# Activation of counter-regulatory mechanisms in a rat renal acute rejection model

**DOI:** 10.1186/1471-2164-9-71

**Published:** 2008-02-08

**Authors:** Bayram Edemir, Sunil M Kurian, Martin Eisenacher, Detlef Lang, Carsten Müller-Tidow, Gert Gabriëls, Daniel R Salomon, Eberhard Schlatter

**Affiliations:** 1 Experimentelle Nephrologie, Medizinische Klinik und Poliklinik D, Universitätsklinikum Münster, Germany; 2 Department of Molecular and Experimental Medicine, The Scripps Research Institute, La Jolla, CA, USA; 3 Integrierte Funktionelle Genomik, Interdisziplinäres Zentrum für Klinische Forschung, Universitätsklinikum Münster, Germany; 4 Hämatologie und Onkologie, Medizinische Klinik und Poliklinik A, Universitätsklinikum Münster, Germany

## Abstract

**Background:**

Microarray analysis provides a powerful approach to identify gene expression alterations following transplantation. In patients the heterogeneity of graft specimens, co-morbidity, co-medications and the challenges in sample collection and preparation complicate conclusions regarding the underlying mechanisms of graft injury, rejection and immune regulation.

**Results:**

We used a rat kidney transplantation model with strict transplant and sample preparation procedures to analyze genome wide changes in gene expression four days after syngeneic and allogeneic transplantation. Both interventions were associated with substantial changes in gene expression. After allogeneic transplantation, genes and pathways related to transport and metabolism were predominantly down-regulated consistent with rejection-mediated graft injury and dysfunction. Up-regulated genes were primarily related to the acute immune response including antigen presentation, T-cell receptor signaling, apoptosis, interferon signaling and complement cascades. We observed a cytokine and chemokine expression profile consistent with activation of a Th1-cell response. A novel finding was up-regulation of several regulatory and protective genes after allogeneic transplantation, specifically IL10, Bcl2a1, C4bpa, Ctla4, HO-1 and the SOCS family.

**Conclusion:**

Our data indicate that in parallel with the predicted activation of immune response and tissue injury pathways, there is simultaneous activation of pathways for counter regulatory and protective mechanisms that would balance and limit the ongoing inflammatory/immune responses. The pathophysiological mechanisms behind and the clinical consequences of alterations in expression of these gene classes in acute rejection, injury and dysfunction vs. protection and immunoregulation, prompt further analyses and open new aspects for therapeutic approaches.

## Background

Microarray analyses have been used to link changes at the level of gene expression to different kidney diseases to obtain markers for diagnosis and prognosis. Microarray analyses of human patients have been used to analyze post-transplant events [1-6]. A recent review of microarray-based studies and the search for biomarkers in organ transplantation is given by Kurian et al. [7]. Microarray analysis of kidney transplant biopsies with acute rejection identified gene expression patterns that distinguish three distinct subtypes of acute rejection that, although indistinguishable by histology, were marked by differences in mechanisms of immune activation and cell proliferation [8]. Interestingly, another study of acute rejection biopsies showed no evidence for up-regulation of cytotoxic T-cell effector molecules despite the fact that these have been considered markers for acute renal rejection [9]. In parallel, gene expression signatures of peripheral blood lymphocytes (PBLs) from transplant patients have also been shown to be capable of classifying patients with acute rejection [6]. Surprisingly, there was essentially no overlap in rejection-diagnostic genes up-regulated in PBL and genes up-regulated in the biopsies from the same patients, indicating that the blood must be considered a very different immune compartment in this setting. As a consequence of these initial studies, the hope of developing validated diagnostic biomarkers for transplantation is tempered by results that challenge an existing dogma on immune response mechanisms and demand additional studies to deconvolute.

There are a number of limitations inherent in clinical studies of gene expression in transplantation. Patients typically represent major differences in gender, age, co-morbidities, clinical histories, immunosuppressive regimes, race/ethnicities, and genetics and it is always difficult to collect samples at specific times relative to the procedures. These differences significantly influence gene expression and that operant variability compromises the results obtained by microarray analysis as well as the confidence to identify the principal underlying immune mechanisms. The use of experimental transplantation models in rats or mice is an opportunity to mitigate these variables and work in a controlled system. Several studies have made use of experimental transplantation to study changes in gene expression related to acute rejection after murine heart, kidney and lung transplantation [10-13]. While several studies have been performed using heart transplantation in rats [14,15], to our knowledge, a study of acute kidney transplant rejection in rats has not been reported.

In previous studies [16,17], we showed in well characterized rat renal transplantation models, no evidence of tissue necrosis at day 4 while histological changes consistent with acute rejection including activated lymphocyte infiltration were found consistently. In those studies we showed differences in gene expression for selected sodium and water transporters that were relevant to tissue injury and renal compensation. In the present study, we made use of these transplantation models and continued our studies using day 4 as the measuring point to represent a time at which there is full representation of acute rejection mechanisms but before the widespread renal tissue injury that can confuse the gene signatures. Thus, changes in gene expression related solely to acute rejection in allogeneic transplantation or linked to the surgical procedure in syngeneic transplantation can be evaluated. Native, untreated kidneys were used as the control group. No immunosuppression was used to specifically analyze the influence of acute rejection on gene expression independent of the effects of immunosuppressive regimens. Thereby, maximum homogeneity in procedures, sample preparation, timing, and handling was achieved. In contrast to the design of renal transplantation studies performed in mice [12,13], we used bilaterally nephrectomized recipient rats, such that the animal's transplant was required to maintain the full metabolic and synthetic function of the organism as is the case in the clinical transplant situation.

## Results

### Correlation and clustering of the samples

**Figure 1:**
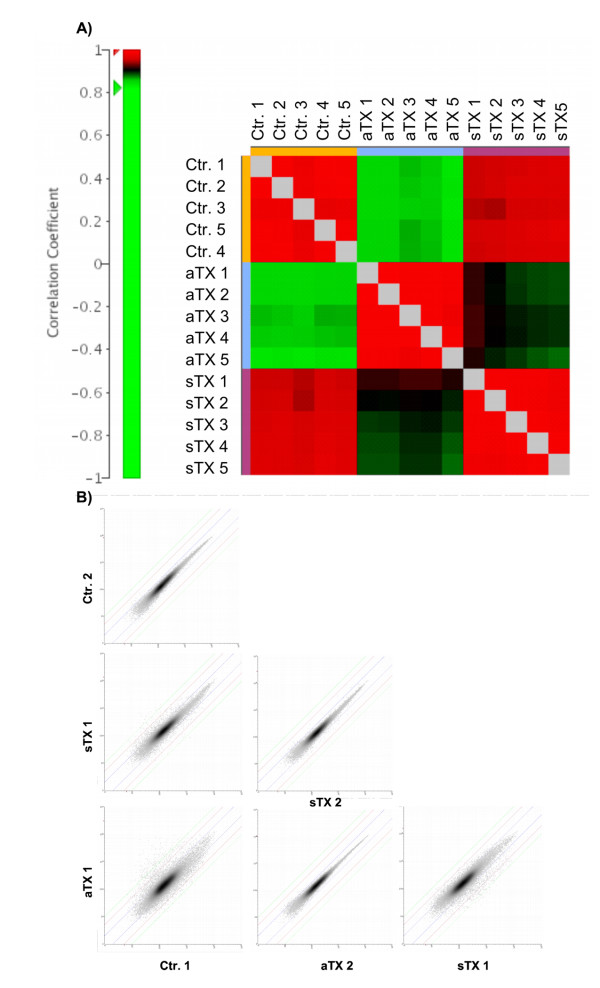
**Correlation and clustering of the samples**. A) Correlation analyses of the samples showed the expected results with higher correlation between control group (ctr) and syngeneic group (sTX) samples compared to acute rejection group (aTX). B) The representative scatter plots of all genes between different samples shows, that the observed correlation is not dominated by a small number of high-expressing genes.

The global experiment structure was inspected utilizing different methods intended to validate whether the groups were homogeneous and whether the within-group variance was smaller than the between-group variance. Furthermore, group distances could be roughly estimated. The correlations within the "acute group" (allogeneic kidney transplant with acute rejection), "syngeneic group" (syngeneic transplant as control for surgical changes) and "control group" (untreated, native kidneys) showed a homogenous correlation of 0.97–0.99 (Fig. 1A). The correlation between the control group and the syngeneic group was 0.95–0.97, between control and the acute group was 0.84–0.87, and between the acute group and the syngeneic group was 0.88–0.92. The representative scatter plots of all genes between different samples showed that the observed correlations are not dominated by a small number of highly expressed genes (Fig. 1B). The syngeneic group was more similar to the control group than the acute group was to the control group. Thus, the processes of acute rejection were the most relevant factors in driving the observed changes in gene expression.

### Identification of differentially expressed genes

**Figure 2:**
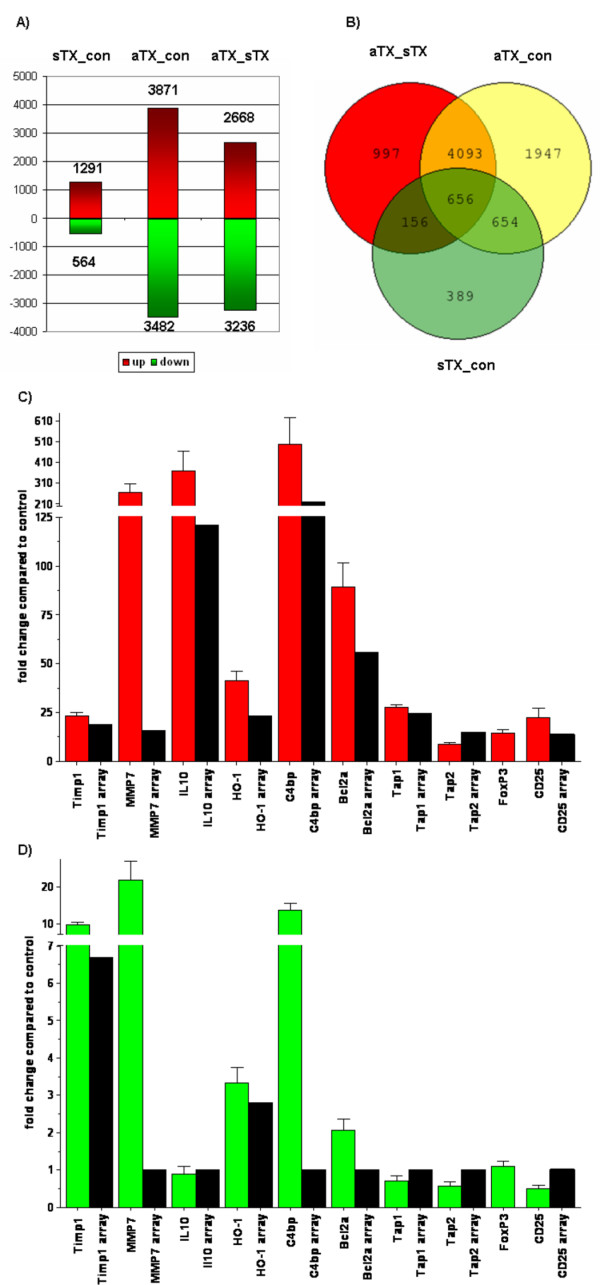
**Differently expressed genes between the control, syngeneic and allogeneic group**. Class comparisons were performed using BRB array tools to identify genes with significant changes in expression levels between the syngeneic group (sTX_con) or the allogeneic group (aTX_con) compared to the control group and between allogeneic and syngeneic group (aTX_sTX; Fig. 2A). Genes with a p-value < 0.001 were classified as significant regulated. The complete lists with the significant regulated genes are provided with this manuscript (see additional file 1). The Venn diagram in Fig. 2B shows the number of overlapping genes. The changes in expression compared to control for selected genes were analyzed by real time PCR using specific primer pairs or TaqMan gene expression assays. Relative changes were evaluated using the 2^-ΔΔ^Ct method. The changes in gene expression after allogeneic transplantation are shown in Fig. 2C and the results for the syngeneic transplantation group in Fig. 2D. The black columns show the corresponding expression levels on the arrays (ratio of mean signal intensities). MMP7. IL10, C4bpa, Tap1 and Tap2 were not differently expressed after syngeneic transplantation on the array. There was no corresponding probe set for FoxP3 on the array.

Significantly regulated and differentially expressed genes were identified using class comparisons as implemented in BRB ArrayTools for all the possible group comparisons (acute to control, acute to syngeneic and syngeneic to control). Genes with a parametric p value < 0.001 were classified as significantly differentially expressed (Fig. 2A). Thus, 3871 genes were up-regulated and 3483 were down-regulated for the acute group compared to the control. Comparison of the acute to the syngeneic group revealed 2668 genes were up- and 3236 genes were down-regulated. In contrast, comparing the control to syngeneic groups for the impact of the transplant surgery revealed 564 genes were down-regulated and 1291 were up-regulated. The Venn diagram in Figure 2B shows the overlap between the lists of significantly altered genes as a function of the three experimental groups. For further analysis we used the gene lists from syngeneic transplants compared to controls and from allogeneic transplants compared to controls. Specifically, we decided not to report on our comparison of the allogeneic transplants to the syngeneic transplants because the results of the functional and pathway analysis were nearly identical with the results obtained using the comparison of allogeneic transplants with controls. There were marginal differences in the number of genes related to a distinct function or pathway. However, the complete output files with all regulated genes are provided as additional file 1.

Genes with protective or regulatory properties up-regulated after allogeneic transplantation like HO-1 (22 fold), Bcl2a1 (59 fold), C4bpa (219 fold), IL10 (120 fold) or members of a gene family that were highly induced like TIMP1 (18 fold), MMP7 (15 fold), TAP1/2 (24/14 fold) were analyzed by real time PCR (Fig. 2C and 2D). FoxP3 and CD25 are expressed by regulatory T-cells [18]. The results for expression of genes that were not significantly regulated after syngeneic transplantation according to the array were set to 1. The PCR results confirmed the up-regulated genes after allogeneic transplantation identified by the expression arrays. The changes in expression varied between 10-fold for Tap2 up to ~500-fold for C4bp. It is important to note that there is no corresponding probe set on the Rat 230 2.0 microarray for FoxP3. Therefore the FoxP3 was also chosen for Real-Time PCR analysis. Interestingly the expression of FoxP3 was highly induced after allogeneic transplantation (~14 fold).

The genes analyzed by real time PCR were not identified to be differently expressed after syngeneic transplantation compared to control. However, the real time PCR results showed up-regulated expression of MMP7 (~21-fold) and C4bp (~13-fold) after syngeneic transplantation.

### Genes involved in immune response

A selection of transcripts determined to be up-regulated as a function of acute rejection is shown in Table 1. The recruitment of cells related to the immune response is indicated by up-regulation of CD8 (229-fold), the marker for cytotoxic T-cells and by the up-regulation of granzyme-A (67-fold), granzyme-B (103-fold) and perforin (25-fold), cytotoxic effector molecules released by CD8+ T-cells. Evidence for the involvement and activation of macrophages is the elevated expression of CD163, the marker for macrophages [19] and ficolin B, which is expressed by activated macrophages [20].

**Table 1:** Selection of significant up-regulated expression of cytokines, cytokine receptors, chemokines, chemokine receptors, regulatory or protective genes after allogeneic transplantation compared to control group.

**Fold change**	**Probe set**	**Description**	**Gene symbol**
**Cytokines**			

121.2	1387711_at	interleukin 10	Il10
84.1	1370790_at	interferon gamma	Ifng
41.6	1391384_at	tumor necrosis factor (TNF superfamily, member 2)	Tnf
15.4	1398256_at	interleukin 1 beta	Il1b
13.1	1368592_at	interleukin 1 alpha	Il1a
8.6	1369665_a_at	interleukin 18	Il18
8.4	1368763_at	interleukin 3	Il3
7.4	1369191_at	interleukin 6	Il6
6.1	1369208_at	interleukin 7	Il7

**Cytokine receptors**			

17	1389092_at	interleukin 2 receptor, gamma	Il2rg
13.6	1387591_at	interleukin 2 receptor, alpha chain	Il2ra
11.8	1369697_at	interleukin 8 receptor, beta	Il8rb
10.1	1387394_at	interleukin 2 receptor, beta chain	Il2rb
3.5	1370728_at	interleukin 13 receptor, alpha 1	Il13ra1
3	1386987_at	interleukin 6 receptor	Il6r

**Chemokines**			

139.7	1382454_at	chemokine (C-X-C motif) ligand 9	Cxcl9
122.8	1387831_at	small inducible cytokine subfamily C, member 1 (lymphotactin)	Xcl1
39.3	1387969_at	chemokine (C-X-C motif) ligand 10	Cxcl10
25.7	1391925_at	chemokine (C-C motif) ligand 19 (predicted)	Ccl19_predicted
21.5	1369815_at	chemokine (C-C motif) ligand 3	Ccl3
17.5	1367973_at	chemokine (C-C motif) ligand 2	Ccl2
16.5	1379365_at	chemokine (C-X-C motif) ligand 11	Cxcl11
15.2	1370832_at	small inducible cytokine A4	Ccl4
6.7	1387316_at	chemokine (C-X-C motif) ligand 1	Cxcl1
5.8	1369983_at	chemokine (C-C motif) ligand 5	Ccl5

**Chemokine receptors**			

55.9	1369290_at	chemokine (C-C) receptor 5	Ccr5
53.5	1370083_at	macrophage inflammatory protein-1 alpha receptor gene	Ccr1
45.9	1393929_at	chemokine (C-C motif) receptor 6 (predicted)	Ccr6_predicted
41.1	1387742_at	chemokine (C-C motif) receptor 2	Ccr2
11.2	1368192_at	chemokine (C-X-C motif) receptor 3	Cxcr3

**Regulatory or protective genes**			

219.364	1369764_at	complement component 4 binding protein, alpha	C4bpa
121.25	1387711_at	interleukin 10	Il10
55.975	1368482_at	B-cell leukemia/lymphoma 2 related protein A1	Bcl2a1
39.519	1371252_at	suppressor of cytokine signaling 1	Socs1
39	1387835_at	interleukin 1 receptor antagonist	Il1rn
36.72	1387608_at	indoleamine 2,3-dioxygenase	Indo
28.885	1382622_at	cystatin F (leukocystatin) (predicted)	Cst7_predicted
23.06	1370080_at	heme oxygenase (decycling) 1	Hmox1
17.922	1370113_at	inhibitor of apoptosis protein 1	Birc3
16.732	1368695_at	complement component 4 binding protein, beta	C4bpb
8.305	1387638_a_at	cytotoxic T-lymphocyte-associated protein 4	Ctla4

**Immune cells related genes**			

229.344	1385414_at	CD8 antigen, alpha chain	Cd8a
144.253	1387378_at	ficolin B	Fcnb
103.106	1370628_at	granzyme B	Gzmb
67.326	1379293_at	granzyme A	Gzma
35.114	1393917_at	CD163 antigen (predicted)	Cd163_predicted
30.581	1387472_at	CD3 antigen delta polypeptide	Cd3d
28.319	1387739_at	CD8 antigen, beta chain	Cd8b
25	1370096_at	perforin 1 (pore forming protein)	Prf1
18.368	1370483_at	CD244 natural killer cell receptor 2B4	Cd244

T-cells and macrophages release factors like cytokines for differentiation and proliferation and chemokines for recruitment of other immunologically active cells. Thus, the expression pattern of cytokines and chemokines like Cxcl9, Xcl1, Cxcl10 or Ccl3 shown in Table 1 is consistent with their well established importance in the signaling mechanisms activated after allogeneic transplantation (for review see [21]).

### Functional annotation using gene ontology terms

To identify activated or suppressed functions, the lists of significantly up- or down-regulated genes after syngeneic transplantation and allogeneic transplantation were analyzed to identify over-represented GO-terms. The third GO-level gives the best compromise between specificity and list coverage [22]. After syngeneic transplantation, 18 GO-terms were enriched in the up-regulated and 1 in the down-regulated group of genes on GO-level 3. We also analyzed over-represented GO-terms without filtering on the third level. Table 2 shows the ten most significant over-represented genes related to biological process (BP), cellular component (CC) and molecular function (MF). Due to the limitation of space the complete lists are also provided with this manuscript (see additional file 2).

**Table 2:** Overrepresented GO categories compared to control within the syngeneic transplantation group. The top 10 significantly overrepresented GO categories on the third level (p < 0.05, Fisher's exact test, with Benjamini and Hochberg correction) related to biological process (BP), cellular component (CC) and molecular function (MF) in the set of genes down- or up-regulated after syngeneic transplantation (sTX) compared to control are shown in Table 2 The numbers indicate the quantity of genes corresponding with the GO-terms.

**sTX_down**			
**Category**	**Term**	**Count**	**p-value**

BP	organic acid metabolism	57	1,1E-10
BP	carboxylic acid metabolism	56	1,5E-10
BP	amino acid and derivative metabolism	41	7,1E-9
BP	amino acid metabolism	31	2,2E-6
BP	nitrogen compound metabolism	40	8,3E-6
BP	amine metabolism	38	9,7E-6
BP	sulfur amino acid metabolism	10	1,8E-5
BP	amino acid derivative metabolism	18	6,0E-5
BP	amino acid catabolism	13	1,1E-4
BP	nitrogen compound catabolism	15	1,2E-4
CC	mitochondrion	59	1,9E-7
CC	microbody	14	4,0E-4
CC	peroxisome	14	4,0E-4
MF	oxidoreductase activity	59	3,2E-9
MF	catalytic activity	195	1,5E-6
MF	lyase activity	20	1,7E-3

**sTX_up**			

BP	mitotic cell cycle	44	5,3E-7
BP	cell division	30	6,1E-7
BP	cell cycle	81	6,8E-7
BP	cell adhesion	70	2,5E-6
BP	mitosis	29	2,6E-6
BP	M phase of mitotic cell cycle	29	2,7E-6
BP	cell proliferation	83	1,1E-5
BP	M phase	33	3,9E-5
BP	regulation of cell cycle	54	7,9E-5
BP	regulation of progression through cell cycle	54	7,9E-5
CC	extracellular matrix	56	3,5E-16
CC	extracellular matrix (sensu Metazoa)	54	2,7E-15
CC	intracellular non-membrane-bound organelle	144	6,0E-13
CC	non-membrane-bound organelle	144	6,0E-13
CC	collagen	16	2,0E-7
CC	ribosome	36	2,4E-5
CC	cytoskeleton	76	4,1E-5
CC	spindle	17	4,1E-5
CC	microtubule cytoskeleton	40	4,2E-5
CC	chromosome	36	4,4E-5
MF	structural molecule activity	75	3,7E-8
MF	extracellular matrix structural constituent	23	9,6E-8
MF	structural constituent of ribosome	32	9,3E-5
MF	collagen binding	8	1,7E-2
MF	pattern binding	17	4,3E-2
MF	enzyme inhibitor activity	29	4,7E-2

Decreased expression of metabolism-related genes after syngeneic transplantation suggests the suppression of BP-related genes. Increased expression of genes related to "cell proliferation" and "cell cycle" within the BP category, "extra cellular matrix" and "collagen" within the CC category and "collagen binding" within the MF category reveal the activation of tissue injury recovery mechanisms related to the transplant surgery itself.

After allogeneic transplantation, 58 GO-terms were significantly enriched in the up-regulated and 29 in the down-regulated genes on GO-level 3. Without the application of any filter, 250 GO-terms were enriched in the up- and 119 in the down-regulated set of genes. Table 3 shows the ten most significant over-represented genes related to BP, CC and MF. As expected, the increased expression of genes related to the BP categories "immune response", "defense response" and the CC category "T-cell receptor complex" and the MF category called "cytokine binding" indicates the activation of the immune response. These GO-terms are consistent with infiltration of the graft by T-cells and describe signaling mechanisms involved in activation, differentiation and recruitment of immune cells. The BP 'cell death' group describes the downstream processes following T-cell activation. The decreased expression of genes related to the BP categories "metabolism", "transport", "excretion" and the MF category "primary active transporter activity" indicates the challenges posed to the graft to maintain renal function. A major part of renal function is mediated by several transporters and transport systems all primarily or secondarily coupled to energy consumption. The down-regulation of genes that are involved in metabolism or transport leads to a decreased tubular function of the kidney.

**Table 3:** Overrepresented GO categories compared to control within the allogeneic transplantation group. The top 10 significantly overrepresented GO categories on the third level (p < 0.05, Fisher's exact test, with Benjamini and Hochberg correction) related to biological process (BP), cellular component (CC) and molecular function (MF) in the set of genes down- or up-regulated after allogeneic transplantation (aTX) compared to control are shown in Table 3. The numbers indicate the quantity of genes corresponding with the GO-terms.

**aTX_down**			
**Category**	**Term**	**Count**	**p-value**

BP	nitrogen compound metabolism	119	8,1E-11
BP	catabolism	130	3,7E-6
BP	cellular metabolism	864	3,9E-4
BP	transport	408	5,1E-4
BP	establishment of localization	459	1,4E-3
BP	excretion	18	5,1E-3
CC	organelle inner membrane	110	1,7E-25
CC	mitochondrial envelope	116	1,1E-23
CC	organelle envelope	128	1,9E-15
CC	cytoplasm	695	5,8E-15
CC	organelle membrane	165	9,3E-15
CC	mitochondrial lumen	41	4,6E-7
CC	membrane fraction	171	8,8E-7
MF	electron carrier activity	45	7,6E-17
MF	oxidoreductase activity, acting on NADH or NADPH	37	1,8E-12
MF	primary active transporter activity	55	2,5E-8
MF	metal ion transporter activity	39	4,3E-7
MF	oxidoreductase activity, acting on the CH-CH group of donors	22	8,8E-6
MF	oxidoreductase activity, acting on CH-OH group of donors	38	1,1E-4
MF	coenzyme binding	21	2,0E-4

**aTX_up**			

BP	immune response	283	1,7E-44
BP	defense response	292	8,1E-43
BP	response to pest, pathogen or parasite	180	6,4E-26
BP	response to other organism	180	2,3E-24
BP	macromolecule metabolism	797	1,4E-23
BP	cell activation	78	2,7E-14
BP	cell death	198	1,9E-13
BP	response to wounding	141	1,8E-11
BP	primary metabolism	1079	8,0E-9
BP	cell cycle	171	2,2E-8
CC	ribonucleoprotein complex	138	1,7E-22
CC	intracellular non-membrane-bound organelle	318	3,9E-18
CC	nucleus	581	3,0E-12
CC	ribosome	81	1,2E-11
CC	cytosolic small ribosomal subunit (sensu Eukaryota)	23	2,2E-8
CC	T cell receptor complex	14	1,5E-6
CC	nuclear envelope	44	2,0E-5
CC	intracellular organelle	1000	2,3E-5
CC	spliceosome complex	25	9,6E-5
CC	proteasome complex (sensu Eukaryota)	25	1,5E-4
MF	RNA binding	178	3,5E-31
MF	cytokine binding	30	8,5E-4
MF	sugar binding	36	6,8E-3
MF	translation factor activity, nucleic acid binding	33	8,6E-3
MF	translation initiation factor activity	22	1,3E-2
MF	protease activator activity	10	4,3E-2

### Pathways affected after syngeneic and allogeneic transplantation

The functional annotation with DAVID identified pathways related to the Kyoto Encyclopedia of Genes and Genomes (KEGG) [23] that were affected after syngeneic or allogeneic transplantation. The pathways affected after syngeneic transplantation are listed in Table 4. The majority of down-regulated genes are related to metabolism pathways. Increased expression of genes involved in "cell cycle" and "extra cellular matrix (ECM)-receptor interaction" are suggestive of active recovery processes following the surgery.

**Table 4:** Pathways with up- or down-regulated genes after syngeneic transplantation or allogeneic transplantation. Pathway charts were obtained using DAVID functional annotation tools (p < 0.05, Fisher's exact test, p-value with Benjamini correction). Pathways with a significantly elevated number of genes down- or up-regulated after syngeneic transplantation (sTX) or allogeneic transplantation (aTX) compared to control are shown Table 4. The numbers indicate the genes affected within the pathways.

**sTX-down**		**sTX-up**	
**Pathway**	**Count**	**Pathway**	**Count**

tryptophan metabolism	13	ribosome	22
methionine metabolism	7	cell cycle	28
valine, leucine and isoleucine degradation	11	ribosome	13
lysine degradation	9	focal adhesion	31
fatty acid metabolism	11	ecm-receptor interaction	16
pyruvate metabolism	9	cell communication	16
beta-alanine metabolism	7		
propanoate metabolism	8		
butanoate metabolism	9		
selenoamino acid metabolism	6		
caprolactam degradation	5		
alkaloid biosynthesis II	5		
limonene and pinene degradation	5		
glycine, serine and threonine metabolism	7		

**aTX-down**		**aTX-up**	

**Pathway**	**Count**	**Pathway**	**Count**

oxidative phosphorylation	48	antigen processing and presentation	46
fatty acid metabolism	29	ribosome	43
valine, leucine and isoleucine degradation	25	cell cycle	50
glutathione metabolism	21	proteasome	22
citrate cycle (tca cycle)	13	t cell receptor signaling pathway	45
pyruvate metabolism	17	type I diabetes mellitus	28
butanoate metabolism	20	hematopoietic cell lineage	33
glycine, serine and threonine metabolism	17	cell adhesion molecules (cams)	50
metabolism of xenobiotics by cytochrome p450	23	cytokine-cytokine receptor interaction	48
tryptophan metabolism	21	toll-like receptor signaling pathway	26
propanoate metabolism	14		
reductive carboxylate cycle (CO2 fixation)	6		

Pathways with enrichment of down- or up-regulated genes after allogeneic transplantation are listed in Table 4. The majority of the pathways with down-regulated genes are also related to metabolism. In all cases the numbers of genes related to metabolism pathways were higher after allogeneic than syngeneic transplantation indicating a stronger suppression of these pathways in the context of acute rejection. But some pathways, however, were affected only after allogeneic transplantation, like 'oxidative phosphorylation', 'citrate cycle' or 'metabolism of xenobiotics by cytochrome p450' indicating an acute rejection mediated down-regulation of genes related to these pathways. The majority of pathways with enrichment in up-regulated genes can be directly or indirectly linked to the immune response.

**Figure 3:**
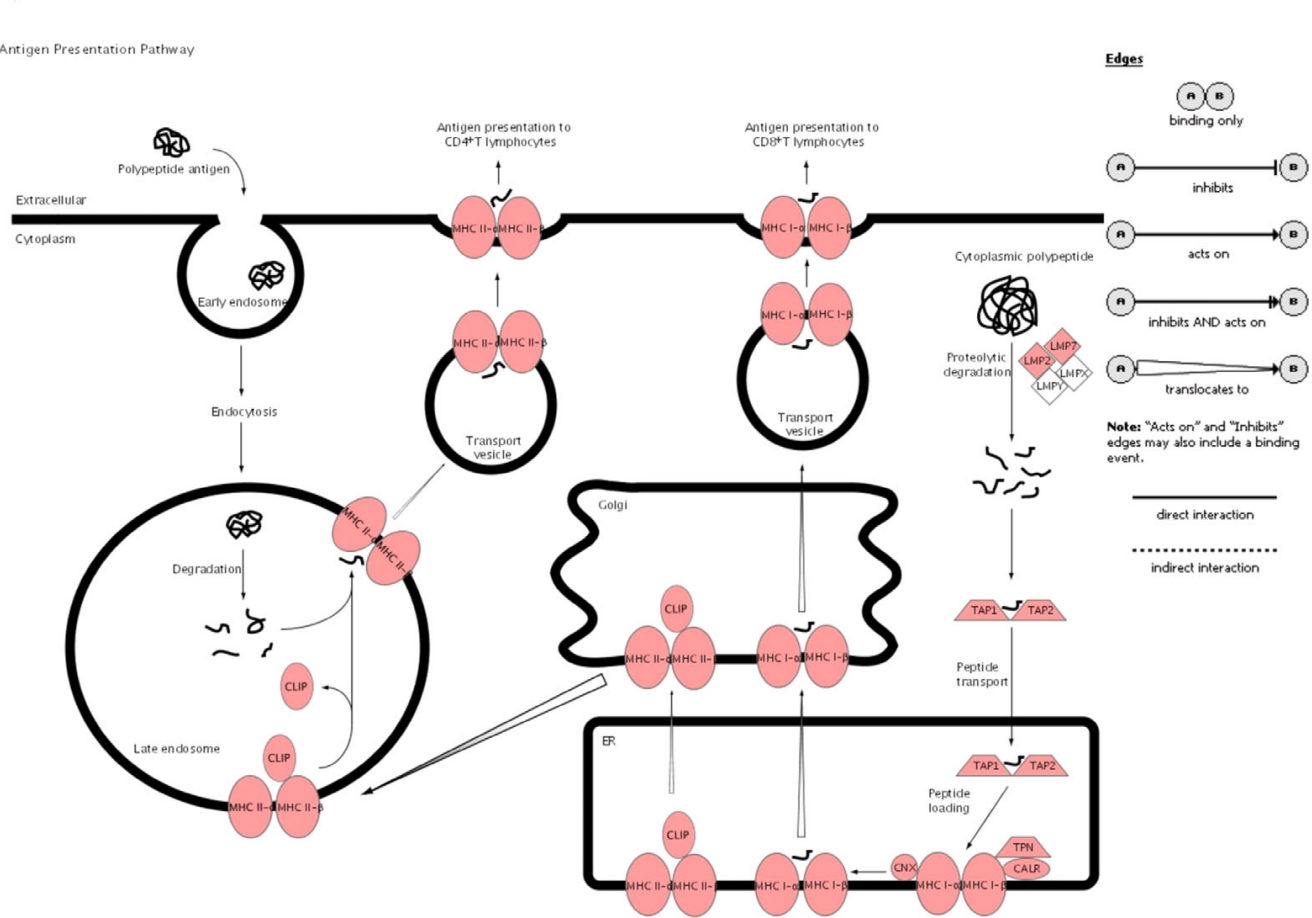
**Antigen presentation pathway**. Ingenuity Pathways Knowledge Base was used to display pathways containing multiple differently expressed genes. Significant up-regulation of genes compared to control was observed in the canonical antigen presentation pathway after allogeneic transplantation. Up-regulated genes are highlighted in red the down-regulated genes are highlighted in green. The legend in Fig 3 applies also to Figs. 4-7.

**Figure 4:**
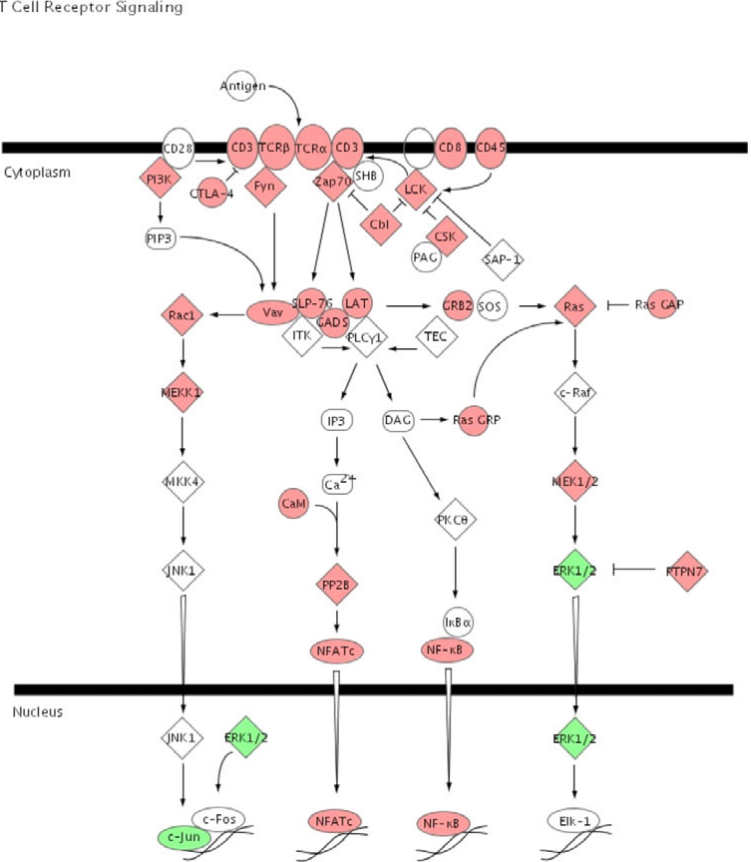
**TCR signaling pathway**. Ingenuity Pathways Knowledge Base was used to display pathways containing multiple differently expressed genes. Significant up-regulation of genes in the canonical TCR signaling pathway was observed after allogeneic transplantation. Up-regulated genes are highlighted in red the down-regulated genes are highlighted in green.

**Figure 5:**
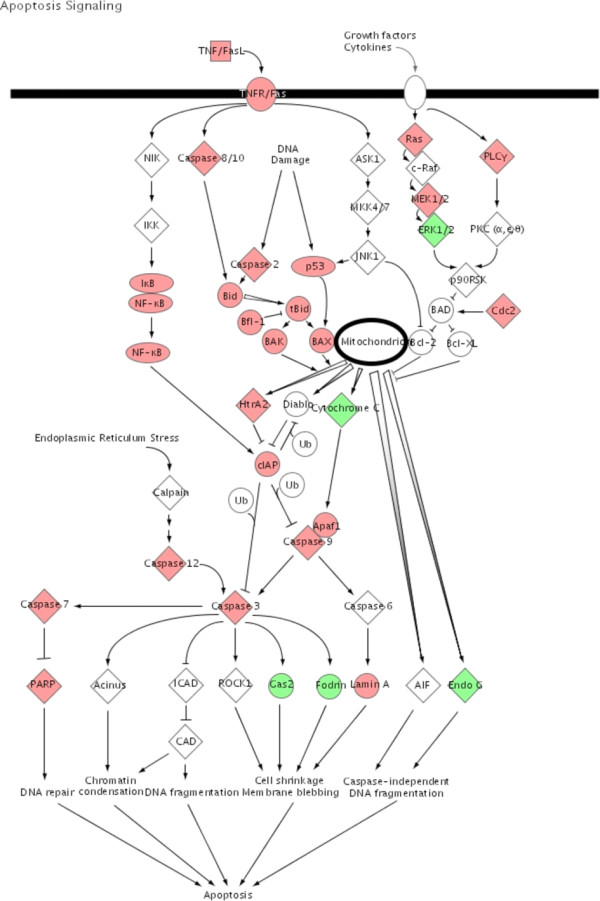
**Apoptosis signaling**. In the same way as described in figure 3 significant regulated genes after allogeneic transplantation was observed in the canonical apoptosis signaling Up-regulated genes are highlighted in red the down-regulated genes are highlighted in green.

**Figure 6:**
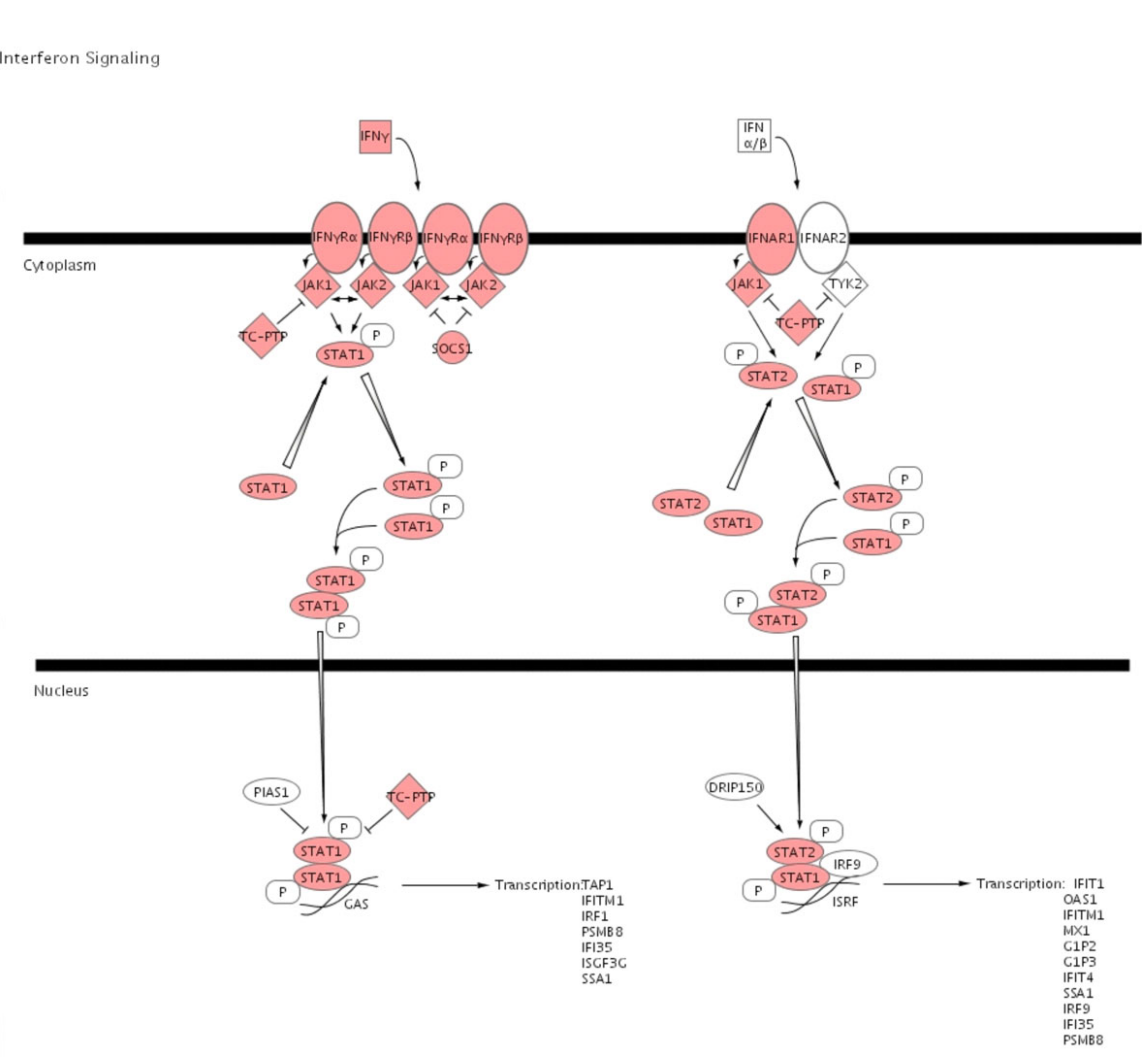
**Interferon signaling**. Significant up-regulation of genes was also observed in the canonical interferon signaling pathway. Up-regulated genes are highlighted in red.

**Figure 7:**
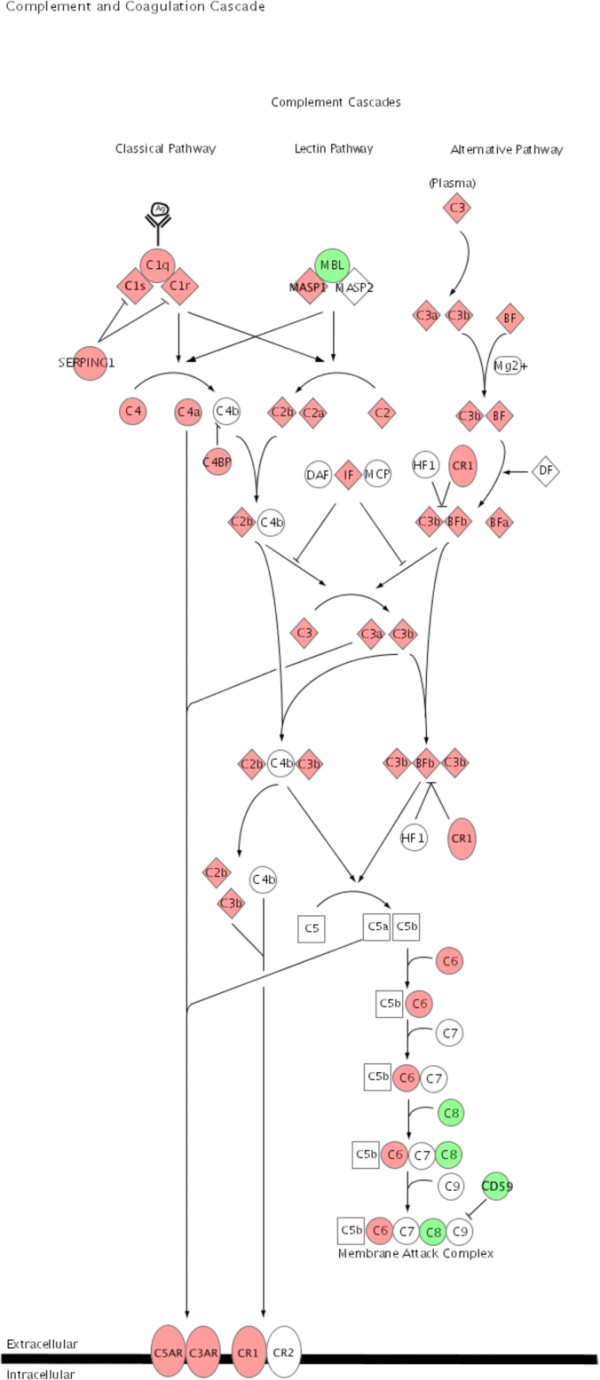
**Complement cascade**. Significant regulated genes after allogeneic transplantation were associated with the canonical complement cascade using the Ingenuity Pathways Knowledge Base to visualize genes differently expressed in the selected pathway. Up regulated genes are highlighted in red the down regulated genes are highlighted in green.

To illustrate the pathways activated by acute rejection related to the immune response, we used IPA to map networks of up- and down-regulated genes after allogeneic transplantation to functional pathways for antigen-presentation (Fig. 3), TCR-signaling (Fig. 4), apoptosis (Fig. 5), IFNγ (Fig. 6) and complement cascades (Fig. 7). Within the antigen processing and presentation pathway nearly all genes were affected (Fig. 3). The immunoproteasome alters the activity of the proteasome by replacement of the β1, β2 and β5 subunits of the 20S core proteasome by LMP2, LMP7 and Psmb10 [24]. The expression of these subunits was strongly induced by acute rejection. The antigens processed by the proteasome are transported by molecules associated with the antigen processing (TAP) complex [25]. Thus, we saw strong induction of TAP1 (24-fold) and TAP2 (15-fold; Fig. 2C).

The recognition of allogeneic MHC by the T-cell's cognate antigen receptor (TCR) will induce activation signals and transcriptional activity (Fig. 4). The gene expression changes indicate an induction of NFATc1 and NF-κB related pathways. Within these pathways, genes with counter regulatory properties like CTLA-4 or cbl were also up-regulated.

Fig. 6 illustrates the signaling pathway for IFNγ. IFNγ acts as a pro-inflammatory cytokine with altered expression after allogeneic transplantation (Table 1). Nearly all the genes within this pathway showed altered expression in the setting of acute rejection. The expression of signal transducer and activator of transcription (STAT) 1 and STAT 2, the downstream effectors of IFNγ signaling, was highly induced. But the increased expression of the suppressor of cytokine signaling (SOCS) 1 and of the protein tyrosine phosphatase non-receptor type 2 (TC-PtP), both negative regulators of IFNγ signaling, indicate the simultaneous activation of regulatory mechanisms to limit the IFNγ signaling.

### Potential novel markers for acute rejection

Several genes in the list of up-regulated genes after allogeneic transplantation might serve as novel markers for acute rejection (Table 5). One candidate is the ETS transcription factor, Spic (SPI-C; 113-fold). SPI-C is expressed during B-lymphocyte development and in activated macrophages [26]. SPI-C also interacts with STAT6 promoting IL4 dependent IgE expression [27]. No elevated expression level of IL4 and only a marginal up-regulation of STAT6 after allogeneic transplantation were found so that the function of SPI-C remains unclear. Phospholipase-A2, group IID (98-fold) and phospholipase-A2, group IIA (75-fold) have been shown to be up-regulated in human broncho-epithelial and nasal epithelial cells after treatment with IFNγ [28]. The authors postulated a possible involvement of both in cytokine-mediated inflammation.

**Table 5:** Potential novel transcriptional markers for acute rejection

**Fold change**	**Probe set**	**Description**	**Gene symbol**
113.071	1378047_at	Spi-C transcription factor (Spi-1/PU.1 related) (predicted)	Spic_predicted
98.594	1392308_at	phospholipase A2, group IID (predicted)	Pla2g2d_predicted
94.648	1392496_at	Trypsin V-A	LOC312273
75.765	1368128_at	phospholipase A2, group IIA (platelets, synovial fluid)	Pla2g2a
46.437	1369766_at	prostaglandin E receptor 2, subtype EP2	Ptger2
39.419	1392171_at	chitinase 3-like 1	Chi3l1
30.24	1370869_at	branched chain aminotransferase 1, cytosolic	Bcat1
26.936	1368413_at	amiloride binding protein 1	Abp1

## Discussion

The MHC antigens are the main barrier for an acceptance of the graft by the host organism and antigen presentation is essential for the activation of T-cells. T-cells are activated when the TCR recognizes allo-MHC class I molecules in the case of CD8+ and MHC class II molecules in the case of CD4+ T-cells [29]. Host T-cells can recognize MHC antigens by either indirect or direct presentation [30]. During indirect presentation, peptides derived from donor MHC class I or MHC class II molecules are processed and presented to CD4+ T-cells by host antigen presenting cells expressing the recipient's MHC class II molecules. The direct antigen presentation pathway is unique to transplantation. In this pathway the T-cell receptors directly recognize an intact MHC molecule expressed on donor cells. In this context, it is important that for our studies with acute rejection, nearly all the genes associated with the antigen processing and presentation pathways were affected (Fig. 3).

Our data also indicates that T-cell cytotoxicity is the main immunological pathway activated in response to acute rejection in allogeneic transplantation. The cytotoxic molecules are perforin/granzyme for CD8+ T-cells and the Fas/Fas-ligand system for CD4+ T-cells [31,32]. In this context, the absolute changes in expression of genes involved in antigen processing and presentation by MHC class I molecules indicates that this part of the antigen presentation is intensely up-regulated. That is also consistent with the up-regulation of the immunoproteasome and TAP1/2 transcripts (Fig. 3) likely to be driven by the up-regulation of interferon γ [33,34], which we have also documented. IFNγ can also induce the expression of the chemokines, CXCL9, CXCL10 and CXCL11 [35]. These data are consistent with other studies showing the changes in gene expression related to IFNγ or cytotoxic T lymphocytes in murine transplantation models [12,13].

The induction of tolerance to the graft would be the treatment of choice after transplantation. Increased expression of protective genes like metallothionein-1 or α2β- crystallin were reported with a mouse cardiac transplant model [36,37]. In this same context, we observed increased expression of a number of genes known to have protective or regulatory properties (Figs. 4, 5, 6, 7) creating a much more comprehensive list than reported in any previous studies. Our study also indicates the endogenous activation of counter regulatory mechanisms within several pathways that have been linked to acute rejection. For example, genes with anti- apoptotic functions include B-cell leukemia/lymphoma 2 related protein A1 (Bfl-1) (Fig. 5) and the Inhibitor of Apoptosis Proteins (IAP) [38,39]. The expression of Heme Oxygenase 1 (HO1; 23-fold) was induced after allogeneic transplantation. The expression of HO1 can suppress graft rejection and leads to long term graft survival in some models [40]. The Cytotoxic T-Lymphocyte-Associated protein 4 (CTLA-4) is a negative regulator of T-cells (Fig. 4) [41]. The function of IL10 is to limit the action of the immune response. IL10 was initially characterized as a cytokine produced by Th2-cells, which inhibited the production of cytokines such as IL2, TNFα and IFNγ [42]. IL10 also modulates the expression of chemokines, chemokine receptors and MHC class II molecules [43]. Thus, the induction of IL10 after allogeneic transplantation indicates a possible feed back mechanism of the immune response (Table 1, Fig. 2).

The complement system is part of the innate immune response. The expression profile we demonstrate in allogeneic transplants for a number of complement components indicates the activation of the complement cascade by acute rejection (Fig. 7). Its major function is the elimination of pathogens and the stimulation of an inflammatory response [44,45]. Local gene expression for complement components has been shown for C3 and C1 during acute rejection in kidney and heart transplantation models, respectively [36,46]. One of the novel genes in our study with the strongest expression after allogeneic transplantation was the C4b binding protein alpha (C4bpa; 219 fold up-regulated). C4bpa is a cofactor for factor 1 in the degradation of C4b in the complement cascade [47]. The degradation of C4b inhibits the formation of the C4b2a complex, a key step in producing an inflammatory response [48].

Recently regulatory T-cells have been recognized as a promising tool for the induction of tolerance [49]. In these studies of our rat renal transplants we also find transcriptional evidence for the presence of regulatory T-cells within the graft. The function of regulatory T-cells is to regulate or suppress the activation, regulation and function of effector T-cells [50]. The development of regulatory T-cells appears to be controlled by FoxP3 [18], which we find is up-regulated after allogeneic transplantation (Fig. 2). The intragraft expression of FoxP3 has also been demonstrated in cardiac allograft patients [51]. The presence of regulatory T-cells within the grafts we studied is further supported by the concomitant increase in expression of CD25 [49] and tryptophan hydroxylase [52].

## Conclusion

We have used a well-defined rat kidney transplantation model to demonstrate global changes in gene expression levels after syngeneic and allogeneic transplantation with the emphasis on early acute rejection in the allogeneic transplants. Using DNA microarrays and real time PCR analysis, we were able to identify major immune effector-related pathways that are activated after allogeneic transplantation and clearly linked by multiple lines of published evidence to acute rejection mechanisms of tissue injury. That we also found a list of genes that are equally highly differentially expressed in acute rejection but not presently linked in the literature represents opportunities for additional studies and discovery. In parallel, we have also identified a number of up-regulated genes that are linked to tissue protective and immune counter-regulation mechanisms. These observations fit with the evolving view of immunity as a balance between effector and regulatory mechanisms.

## Methods

### Kidney transplantation

Male Lewis-Brown-Norway (LBN) and Lewis (LEW) rats (250–300 g, Charles River, Sulzfeld, Germany) with free access to standard rat chow (Ssniff, Soest, Germany) and tap water were used. Experiments were approved by a governmental committee on animal welfare and were performed in accordance with national animal protection guidelines. Renal transplantation was performed as published before [17,53]. For the present study, all recipients were bilaterally nephrectomized immediately before transplantation. In brief, the left kidney including ureter, renal artery, a piece of aorta and renal vein was transplanted into the recipient. For the acute rejection-model, kidneys of LBN-rats (n = 5) were transplanted into LEW-rats and, for the syngeneic transplantation-model, kidneys obtained from LBN-rats (n = 5) were transplanted into LBN-rats. The acute rejection-model leads to marked histological changes typical for acute transplant rejection [53] For the control group, we used native untreated kidneys from LBN-rats (n = 5).

### RNA isolation, labeling, hybridization and scanning

On day four after transplantation, the transplanted kidneys were removed and total RNA was isolated using RNeasy-kit (Qiagen, Hilden, Germany) incubated with 10 U DNase I (Promega, Heidelberg, Germany) to digest genomic DNA. RNA quality was measured using the Agilent 2100 BioAnalyzer (Agilent, Palo Alto, CA, USA). Total RNA was used to prepare biotinylated target RNA. Briefly, 10 μg of total RNA was used to generate first-strand cDNA by using a T7-linked oligo (dT) primer. After second strand synthesis, in vitro transcription was performed with biotinylated UTP and CTP (Enzo Diagnostics, New York, NY, USA).

Target cDNAs generated from each sample were then processed as per manufacturer's recommendation using an Affymetrix GeneChip instrument system. Labeled samples were hybridized to the Rat Genome 230 2.0 Array. Arrays were washed and stained with streptavidin-phycoerythrin before being scanned on an Affymetrix GeneChip scanner. Data were analyzed using Affymetrix GCOS array analysis software. The data discussed in this publication have been deposited in NCBIs Gene Expression Omnibus [54] and are accessible through GEO Series accession number GSE6497.

### Statistical analysis

Experiment reports were inspected to assure equal experiment quality. GeneData Refiner (GeneData, Basel, Switzerland) was used to check quality and import experiments with detection and masking of outliers (the masked area was 0.026 %, 0.01 % and 0.02 % for the control, the syngeneic and allogeneic group respectively). The array defects, assessment using 3'/5' ratios of housekeeping controls, glyseraldehyde-3-phosphate dehydrogenase (GAPDH) and β-Actin, and condensation of intensities to signal values using the "Affymetrix Statistical (MAS5)" method. In GeneData Analyst normalization onto arithmetic mean 400 was performed. Correlation coefficients of all pair wise scatter plots were calculated and summarized in a colored matrix. Clustering was done using the Euclidean distance measure and the complete linkage method. Correlation coefficients and clustering enable a visualization and assessment of the experiment structure based on global expression behavior. Identification of significantly different expressed genes between the control, allogeneic and syngeneic groups were identified using class comparison with the BRB ArrayTools developed by Dr. Richard Simon and Amy Peng [55]. The type of univariate test used was a two-sample T-test. Exact multivariate permutations test was computed based on 126 available permutations. Nominal significance level of each univariate test was set to 0.001. Confidence level of false discovery rate assessment used was 90 % and the maximum allowed numbers of false-positive genes were set to 10.

### Functional annotation and pathway analysis

Up- and down-regulated genes in the allogeneic and syngeneic groups compared to control group were separately analyzed to identify enrichment of different functional pathways and gene ontology (GO) terms [56] using the Database for Annotation, Visualization, and Integrated Discovery (DAVID) [22]. The separate analysis of the down- or up-regulated genes was intended to provide information on suppressed or induced functions, respectively. The enrichment analysis was performed on the third GO level, because this gave the best compromise of specificity and coverage of the gene list. We performed the analysis with and without setting the filter on the third GO level. The Rat 230 2.0 gene chip served as the background list (Fishers exact test with corrected p-value < 0.05, Benjamini & Hochberg [57]). To illustrate the pathways with affected genes in the acute rejection group the data were analyzed through the use of Ingenuity Pathways Analysis (IPA; Ingenuity^® ^Systems, Redwood City, CA, USA).

### Real time PCR

Expression profiles for selected genes were analyzed by real time PCR. Total RNA (10 μg) isolated from the same samples was used for cDNA synthesis with the High Capacity cDNA Reverse Transcription Kit. Real time PCR was performed using SYBR Green PCR Master Mix or TaqMan Universal PCR Master Mix on an ABI PRISM 7700 Sequence Detection System. Specific primer pairs or TaqMan Gene expression assays were used. The PCR efficiency of the primers pairs was tested in dilution series with a cDNA from the allogeneic transplanted group. A list with the corresponding primer sequences or TaqMan assay IDs is provided with this manuscript (additional file 3).

All instruments and reagents were purchased by Applied Biosystems (Darmstadt, Germany). Relative gene expression values were evaluated with the 2^-ΔΔCt ^method using GAPDH or 18s-RNA as housekeeping genes [58].

## Authors' contributions

BE performed the experimental design, data analysis and drafted the manuscript, ME performed data analysis, SK contributed to data acquisition, analysis and drafting the manuscript, DL interpretation of data, CM contributed in data analysis, GG suggested the study, helped with interpretation and drafting of the manuscript, DS data analysis and drafting the manuscript, ES coordinated the study and contributed materials and resources. All authors read and approved the final manuscript.

## Supplementary Material

Additional file 1 Gene lists from the class comparison between the groups analyzed with BRB ArrayTools. Click here for file

Additional file 2 Overrepresented gene ontology annotations in the gene lists up or down regulated after allogeneic transplantation compared to control or after syngeneic transplantation compared to control. Click here for file

Additional file 3 Table with the sequences of the primers and IDs of the TaqMan assays used for real time PCR analysis. Click here for file
