# Supplementary material for: Activation of counter-regulatory mechanisms in a rat renal acute rejection model
Source: BMC Genomics. 2008 Feb 8;9:71. doi: 10.1186/1471-2164-9-71 (PMC2262896; doi:10.1186/1471-2164-9-71)
Supplement: Additional file 1 — Gene lists from the class comparison between the groups analyzed with BRB ArrayTools. [file 1471-2164-9-71-S1.zip › ControlvsAllogeneic/Annotations.html]

   TABLE {font-size: 9pt}    

       Gene Info     


    Gene Ontology   
    Biocarta Pathways         Kegg Pathways     
    Probe set : 1374551_at    Name:   interferon-induced protein 35 (predicted)    GB acc:   BM388891    UniGene:   Rn.44680    Gene Symbol:   Ifi35_predicted    Locus Link:   287719    Chromosome:   10    Cytoband:   Chr:10q32.1    SOURCE    GENECARD       Gene Ontology:   biological process:  immune response   biological process:  response to virus   cellular component:  nucleus   molecular function:  protein binding      BioCarta Pathways:     KEGG Pathways:  
    Probe set : 1387946_at    Name:   lectin, galactoside-binding, soluble, 3 binding protein    GB acc:   AF065438    UniGene:   Rn.3251    Gene Symbol:   Lgals3bp    Locus Link:   245955    Chromosome:   10    Cytoband:   Chr:10q32.3    SOURCE    GENECARD       Gene Ontology:   cellular component:  extracellular space   cellular component:  membrane   molecular function:  scavenger receptor activity   molecular function:  protein binding   molecular function:  isomerase activity      BioCarta Pathways:     KEGG Pathways:  
    Probe set : 1385439_x_at    Name:   Transcribed locus, strongly similar to XP_220978.3 PREDICTED: similar to Probable ATP-dependent helicase LGP2 (Protein D11Lgp2) [Rattus norvegicus]    GB acc:   BF417541    UniGene:   Rn.105654        SOURCE        Gene Ontology:     BioCarta Pathways:     KEGG Pathways:  
    Probe set : 1371537_at    Name:   Transcribed locus    GB acc:   AI179422    UniGene:   Rn.107246        SOURCE        Gene Ontology:     BioCarta Pathways:     KEGG Pathways:  
    Probe set : 1373490_at    Name:   glia maturation factor, gamma    GB acc:   BG666787    UniGene:   Rn.15333    Gene Symbol:   Gmfg    Locus Link:   113940    Chromosome:   1    Cytoband:   Chr:1q21    SOURCE    GENECARD       Gene Ontology:   biological process:  protein amino acid phosphorylation   cellular component:  intracellular      BioCarta Pathways:     KEGG Pathways:  
    Probe set : 1372406_at    Name:   minichromosome maintenance deficient 3 (S. cerevisiae) (predicted) /// similar to DNA replication licensing factor MCM3 (DNA polymerase alpha holoenzyme-associated protein P1) (P1-MCM3)    GB acc:   BM383428    UniGene:   Rn.101621    Gene Symbol:   Mcm3_predicted /// LOC367976    Locus Link:   316273 /// 367976    Chromosome:   9    Cytoband:   Chr:9q13    SOURCE    GENECARD       Gene Ontology:   cellular component:  nucleus   cellular component:  alpha DNA polymerase:primase complex      BioCarta Pathways:     KEGG Pathways:  
    Probe set : 1389210_at    Name:   lymphocyte cytosolic protein 1 (predicted)    GB acc:   BE109711    UniGene:   Rn.14256    Gene Symbol:   Lcp1_predicted    Locus Link:   306071    Chromosome:   15    Cytoband:   Chr:15q11    SOURCE    GENECARD       Gene Ontology:   biological process:  response to wounding   biological process:  actin filament bundle formation      BioCarta Pathways:     KEGG Pathways:  
    Probe set : 1373818_at    Name:   Potassium channel tetramerisation domain containing 12 (predicted)    GB acc:   AI408351    UniGene:   Rn.13832     Locus Link:   364458    Chromosome:   15    Cytoband:   Chr:15q22    SOURCE        Gene Ontology:   biological process:  potassium ion transport   cellular component:  voltage-gated potassium channel complex   cellular component:  membrane   molecular function:  voltage-gated potassium channel activity      BioCarta Pathways:     KEGG Pathways:  
    Probe set : 1367676_at    Name:   high mobility group box 2 /// similar to High mobility group protein 2 (HMG-2) /// similar to High mobility group protein 2 (HMG-2) /// similar to High mobility group protein 2 (HMG-2)    GB acc:   NM_017187    UniGene:   Rn.141159    Gene Symbol:   Hmgb2 /// LOC498072 /// LOC498388 /// LOC498988    Locus Link:   29395 /// 498072 /// 498388 /// 498988    Chromosome:   16    Cytoband:   Chr:16p11 /// Chr:11q12 /// Chr:14q21 /// Chr:1p12    SOURCE    GENECARD       Gene Ontology:   biological process:  DNA replication   biological process:  DNA unwinding during replication   biological process:  DNA repair   biological process:  base-excision repair, DNA ligation   biological process:  DNA packaging   biological process:  establishment and/or maintenance of chromatin architecture   biological process:  nucleosome assembly   biological process:  regulation of transcription, DNA-dependent   biological process:  regulation of transcription from RNA polymerase II promoter   biological process:  nervous system development   biological process:  nervous system development   biological process:  DNA replication   biological process:  DNA unwinding during replication   biological process:  DNA repair   biological process:  base-excision repair, DNA ligation   biological process:  establishment and/or maintenance of chromatin architecture   biological process:  nucleosome assembly   biological process:  regulation of transcription from RNA polymerase II promoter      BioCarta Pathways:     KEGG Pathways:  
    Probe set : 1388164_at    Name:   RT1 class Ib, locus S3    GB acc:   AF029241    UniGene:   Rn.143874    Gene Symbol:   RT1-S3    Locus Link:   294228    Chromosome:   20    Cytoband:   Chr:20p12    SOURCE    GENECARD       Gene Ontology:   biological process:  immune response   biological process:  antigen presentation   cellular component:  MHC class I protein complex   cellular component:  MHC class I protein complex   cellular component:  integral to membrane   molecular function:  peptide binding   molecular function:  MHC class I receptor activity      BioCarta Pathways:     KEGG Pathways:  
    Probe set : 1389873_at    Name:   apoptosis-associated speck-like protein containing a CARD    GB acc:   BI282953    UniGene:   Rn.7817    Gene Symbol:   Pycard    Locus Link:   282817    Chromosome:   1    Cytoband:   Chr:1q36    SOURCE    GENECARD       Gene Ontology:   biological process:  induction of apoptosis   biological process:  regulation of apoptosis   cellular component:  intracellular   molecular function:  protein binding      BioCarta Pathways:     KEGG Pathways:  
    Probe set : 1383564_at     GB acc:   BF411036              Gene Ontology:     BioCarta Pathways:     KEGG Pathways:  
    Probe set : 1373240_at    Name:   dehydrogenase/reductase (SDR family) member 3 (predicted)    GB acc:   BI276935    UniGene:   Rn.12011    Gene Symbol:   Dhrs3_predicted    Locus Link:   313689    Chromosome:   5    Cytoband:   Chr:5q36    SOURCE    GENECARD       Gene Ontology:   biological process:  fatty acid metabolism   biological process:  visual perception   biological process:  metabolism   cellular component:  extracellular space   cellular component:  integral to membrane   molecular function:  nucleotide binding   molecular function:  electron transporter activity   molecular function:  oxidoreductase activity      BioCarta Pathways:     KEGG Pathways:  
    Probe set : 1371209_at    Name:   RT1 class I, CE5    GB acc:   AJ243338    UniGene:   Rn.145027    Gene Symbol:   RT1-CE5    Locus Link:   309607    Chromosome:   20    Cytoband:   Chr:20p12    SOURCE    GENECARD       Gene Ontology:   biological process:  antigen presentation   cellular component:  MHC class I protein complex   molecular function:  MHC class I receptor activity      BioCarta Pathways:     KEGG Pathways:  
    Probe set : 1387770_at    Name:   putative ISG12(a) protein    GB acc:   NM_130743     Gene Symbol:   RGD:1303168    Locus Link:   360415    Chromosome:   6    Cytoband:   Chr:6q32     GENECARD       Gene Ontology:     BioCarta Pathways:     KEGG Pathways:  
    Probe set : 1373575_at    Name:   similar to NADH dehydrogenase (ubiquinone) Fe-S protein 2    GB acc:   BE111722     Gene Symbol:   LOC498279    Locus Link:   498279    Chromosome:   13    Cytoband:   Chr:13q24     GENECARD       Gene Ontology:     BioCarta Pathways:     KEGG Pathways:  
    Probe set : 1372070_at    Name:   interferon gamma inducible protein 30 (predicted)    GB acc:   BM389261    UniGene:   Rn.3370    Gene Symbol:   Ifi30_predicted    Locus Link:   290644    Chromosome:   16    Cytoband:   Chr:16p14    SOURCE    GENECARD       Gene Ontology:   biological process:  immune response   biological process:  antigen processing, exogenous antigen via MHC class II   cellular component:  extracellular region   cellular component:  extracellular space   cellular component:  lysosome   molecular function:  disulfide oxidoreductase activity   molecular function:  oxidoreductase activity      BioCarta Pathways:     KEGG Pathways:  
    Probe set : 1373672_at     GB acc:   BM384419              Gene Ontology:     BioCarta Pathways:     KEGG Pathways:  
    Probe set : 1373025_at    Name:   complement component 1, q subcomponent, gamma polypeptide (predicted)    GB acc:   AI411618    UniGene:   Rn.2393    Gene Symbol:   C1qg_predicted    Locus Link:   362634    Chromosome:   5    Cytoband:   Chr:5q36    SOURCE    GENECARD       Gene Ontology:   cellular component:  extracellular region   cellular component:  extracellular space   cellular component:  cytoplasm      BioCarta Pathways:     KEGG Pathways:  
    Probe set : 1388784_at    Name:   colony stimulating factor 1 receptor    GB acc:   BI285793    UniGene:   Rn.72599    Gene Symbol:   Csf1r    Locus Link:   307403    Chromosome:   18    Cytoband:   Chr:18q12.1    SOURCE    GENECARD       Gene Ontology:     BioCarta Pathways:     KEGG Pathways:  
    Probe set : 1387687_at    Name:   immunoglobulin superfamily, member 6    GB acc:   NM_133542    UniGene:   Rn.16316    Gene Symbol:   Igsf6    Locus Link:   171064    Chromosome:   1    Cytoband:   Chr:1q36    SOURCE    GENECARD       Gene Ontology:   cellular component:  integral to membrane      BioCarta Pathways:     KEGG Pathways:  
    Probe set : 1369186_at    Name:   caspase 1    GB acc:   D85899    UniGene:   Rn.37508    Gene Symbol:   Casp1    Locus Link:   25166    Chromosome:   8    Cytoband:   Chr:8q11    SOURCE    GENECARD       Gene Ontology:   cellular component:  extracellular region   cellular component:  intracellular   cellular component:  cytoplasm      BioCarta Pathways:     KEGG Pathways:  
    Probe set : 1372752_at    Name:   transmembrane 4 superfamily member 7 (predicted)    GB acc:   BF282632    UniGene:   Rn.11531    Gene Symbol:   Tm4sf7_predicted    Locus Link:   293627    Chromosome:   1    Cytoband:   Chr:1q41    SOURCE    GENECARD       Gene Ontology:   biological process:  protein complex assembly   cellular component:  integral to plasma membrane   cellular component:  integral to membrane      BioCarta Pathways:     KEGG Pathways:  
    Probe set : 1373523_at    Name:   Fc fragment of IgG, low affinity IIIa, receptor for (CD16)    GB acc:   AI011757    UniGene:   Rn.23977    Gene Symbol:   Fcgr3a    Locus Link:   304966    Chromosome:   13    Cytoband:   Chr:13q24    SOURCE    GENECARD       Gene Ontology:   cellular component:  integral to membrane   molecular function:  receptor activity      BioCarta Pathways:     KEGG Pathways:  
    Probe set : 1370493_a_at    Name:   leukocyte immunoglobulin-like receptor, subfamily B (with TM and ITIM domains), member 3 (predicted)    GB acc:   AF169637    UniGene:   Rn.143808    Gene Symbol:   Lilrb3_predicted    Locus Link:   361493    Chromosome:   1    Cytoband:   Chr:1q12    SOURCE    GENECARD       Gene Ontology:   biological process:  immune response   cellular component:  integral to membrane   molecular function:  receptor activity      BioCarta Pathways:     KEGG Pathways:  
    Probe set : 1388213_a_at    Name:   RT1 class Ib, locus S3    GB acc:   AJ243973    UniGene:   Rn.143874    Gene Symbol:   RT1-S3    Locus Link:   294228    Chromosome:   20    Cytoband:   Chr:20p12    SOURCE    GENECARD       Gene Ontology:   biological process:  immune response   biological process:  antigen presentation   cellular component:  MHC class I protein complex   cellular component:  MHC class I protein complex   cellular component:  integral to membrane   molecular function:  peptide binding   molecular function:  MHC class I receptor activity      BioCarta Pathways:     KEGG Pathways:  
    Probe set : 1382043_at    Name:   unc-93 homolog B (C. elegans) (predicted)    GB acc:   AI234533    UniGene:   Rn.23633    Gene Symbol:   Unc93b_predicted    Locus Link:   361689    Chromosome:   1    Cytoband:   Chr:1q42    SOURCE    GENECARD       Gene Ontology:     BioCarta Pathways:     KEGG Pathways:  
    Probe set : 1383748_at    Name:   Unknown (protein for MGC:72598)    GB acc:   H31483    UniGene:   Rn.56102    Gene Symbol:   RGD:735063    Locus Link:   298598    Chromosome:   5    Cytoband:   Chr:5q36    SOURCE    GENECARD       Gene Ontology:   cellular component:  coated pit   cellular component:  clathrin vesicle coat   cellular component:  membrane   molecular function:  protein binding      BioCarta Pathways:     KEGG Pathways:  
    Probe set : 1390022_at    Name:   actin related protein 2/3 complex, subunit 5 (predicted)    GB acc:   AA946538    UniGene:   Rn.1639    Gene Symbol:   Arpc5_predicted    Locus Link:   360854    Chromosome:   13    Cytoband:   Chr:13q21    SOURCE    GENECARD       Gene Ontology:   molecular function:  structural constituent of cytoskeleton      BioCarta Pathways:     KEGG Pathways:  
    Probe set : 1379766_at    Name:   Thyroglobulin    GB acc:   AI500952    UniGene:   Rn.10429    Gene Symbol:   Tg    Locus Link:   24826    Chromosome:   7    Cytoband:   Chr:7q33    SOURCE    GENECARD       Gene Ontology:   cellular component:  extracellular region   cellular component:  extracellular space   cellular component:  extracellular space   molecular function:  hormone activity   molecular function:  protein binding   molecular function:  protein binding      BioCarta Pathways:     KEGG Pathways:  
    Probe set : 1374544_at    Name:   CDNA clone MGC:112715 IMAGE:7377326    GB acc:   BM384374    UniGene:   Rn.137475        SOURCE        Gene Ontology:     BioCarta Pathways:     KEGG Pathways:  
    Probe set : 1389114_at    Name:   similar to Hypothetical protein MGC59076 (predicted)    GB acc:   AW523874    UniGene:   Rn.16112    Gene Symbol:   RGD1309144_predicted    Locus Link:   297332    Chromosome:   4    Cytoband:   Chr:4q32    SOURCE    GENECARD       Gene Ontology:   biological process:  threonine biosynthesis   biological process:  metabolism   molecular function:  catalytic activity   molecular function:  threonine synthase activity      BioCarta Pathways:     KEGG Pathways:  
    Probe set : 1398348_at    Name:   Transcribed locus    GB acc:   AA945604    UniGene:   Rn.111929        SOURCE        Gene Ontology:     BioCarta Pathways:     KEGG Pathways:  
    Probe set : 1369204_at    Name:   hemopoietic cell kinase    GB acc:   NM_013185    UniGene:   Rn.10945    Gene Symbol:   Hck    Locus Link:   25734    Chromosome:   3    Cytoband:   Chr:3q41    SOURCE    GENECARD       Gene Ontology:   biological process:  protein amino acid phosphorylation   biological process:  intracellular signaling cascade   biological process:  mesoderm development      BioCarta Pathways:     KEGG Pathways:  
    Probe set : 1368723_at    Name:   linker for activation of T cells    GB acc:   NM_030853    UniGene:   Rn.9773    Gene Symbol:   Lat    Locus Link:   81511    Chromosome:   1    Cytoband:   Chr:1q36    SOURCE    GENECARD       Gene Ontology:   molecular function:  receptor signaling protein activity      BioCarta Pathways:     KEGG Pathways:  
    Probe set : 1391979_at    Name:   Transcribed locus    GB acc:   AA851345    UniGene:   Rn.6731        SOURCE        Gene Ontology:     BioCarta Pathways:     KEGG Pathways:  
    Probe set : 1370383_s_at    Name:   RT1 class II, locus Db1    GB acc:   BI279526    UniGene:   Rn.144821    Gene Symbol:   RT1-Db1    Locus Link:   294270    Chromosome:   20    Cytoband:   Chr:20p12    SOURCE    GENECARD       Gene Ontology:   cellular component:  MHC class II protein complex   cellular component:  membrane   cellular component:  integral to membrane   molecular function:  MHC class II receptor activity      BioCarta Pathways:     KEGG Pathways:  
    Probe set : 1392894_at    Name:   fibrinogen-like 2    GB acc:   AI716194    UniGene:   Rn.64635    Gene Symbol:   Fgl2    Locus Link:   84586    Chromosome:   4    Cytoband:   Chr:4q11    SOURCE    GENECARD       Gene Ontology:   biological process:  parturition   biological process:  cytolysis   cellular component:  extracellular space   molecular function:  peptidase activity      BioCarta Pathways:     KEGG Pathways:  
    Probe set : 1370822_at    Name:   RT1 class II, locus Ba    GB acc:   AF307302    UniGene:   Rn.25717    Gene Symbol:   RT1-Ba    Locus Link:   309621    Chromosome:   20    Cytoband:   Chr:20p12    SOURCE    GENECARD       Gene Ontology:   cellular component:  MHC class II protein complex   cellular component:  membrane   molecular function:  MHC class II receptor activity      BioCarta Pathways:     KEGG Pathways:  
    Probe set : 1379613_at    Name:   Similar to hypothetical protein MGC29390 (predicted)    GB acc:   BM390301    UniGene:   Rn.54182     Locus Link:   303903    Chromosome:   11    Cytoband:   Chr:11q22    SOURCE        Gene Ontology:     BioCarta Pathways:     KEGG Pathways:  
    Probe set : 1372013_at    Name:   interferon induced transmembrane protein 1 (predicted)    GB acc:   BG380285    UniGene:   Rn.22087    Gene Symbol:   Ifitm1_predicted    Locus Link:   293618    Chromosome:   1    Cytoband:   Chr:1q41    SOURCE    GENECARD       Gene Ontology:   cellular component:  plasma membrane   cellular component:  integral to membrane   molecular function:  receptor signaling protein activity      BioCarta Pathways:     KEGG Pathways:  
    Probe set : 1373592_at    Name:   similar to SPI6    GB acc:   AI407094    UniGene:   Rn.95177    Gene Symbol:   MGC94010    Locus Link:   361241    Chromosome:   17    Cytoband:   Chr:17p12    SOURCE    GENECARD       Gene Ontology:   molecular function:  serine-type endopeptidase inhibitor activity      BioCarta Pathways:     KEGG Pathways:  
    Probe set : 1377916_at    Name:   schlafen 2 (predicted)    GB acc:   AW915529    UniGene:   Rn.105052    Gene Symbol:   Slfn2_predicted    Locus Link:   303380    Chromosome:   10    Cytoband:   Chr:10q26    SOURCE    GENECARD       Gene Ontology:   biological process:  negative regulation of cell proliferation      BioCarta Pathways:     KEGG Pathways:  
    Probe set : 1373932_at    Name:   Cytochrome b-245, beta polypeptide    GB acc:   BE098739    UniGene:   Rn.98491    Gene Symbol:   Cybb    Locus Link:   66021    Chromosome:   X    Cytoband:   Chr:Xq13    SOURCE    GENECARD       Gene Ontology:   biological process:  electron transport   cellular component:  intracellular   cellular component:  membrane   cellular component:  integral to membrane      BioCarta Pathways:     KEGG Pathways:  
    Probe set : 1382442_at    Name:   Similar to NF-kappa-B-repressing factor (NFKB-repressing factor) (Transcription factor NRF)    GB acc:   BF288000    UniGene:   Rn.48945     Locus Link:   298316    Chromosome:   X    Cytoband:   Chr:Xq12    SOURCE        Gene Ontology:     BioCarta Pathways:     KEGG Pathways:  
    Probe set : 1387892_at    Name:   tubulin, beta 5    GB acc:   BI285440    UniGene:   Rn.2458    Gene Symbol:   Tubb5    Locus Link:   29214    Chromosome:   20    Cytoband:   Chr:20p12    SOURCE    GENECARD       Gene Ontology:     BioCarta Pathways:     KEGG Pathways:  
    Probe set : 1368010_at    Name:   protein tyrosine phosphatase, non-receptor type 6    GB acc:   NM_053908    UniGene:   Rn.18985    Gene Symbol:   Ptpn6    Locus Link:   116689    Chromosome:   4    Cytoband:   Chr:4q42    SOURCE    GENECARD       Gene Ontology:   cellular component:  cytoplasm   cellular component:  cytoskeleton   cellular component:  membrane      BioCarta Pathways:     KEGG Pathways:  
    Probe set : 1385571_at    Name:   Similar to SP140 nuclear body protein isoform 1    GB acc:   AA923865    UniGene:   Rn.15232     Locus Link:   363269    Chromosome:   9    Cytoband:   Chr:9q35    SOURCE        Gene Ontology:   cellular component:  nucleus      BioCarta Pathways:     KEGG Pathways:  
    Probe set : 1370290_at    Name:   tubulin, beta 5    GB acc:   AB011679    UniGene:   Rn.2458    Gene Symbol:   Tubb5    Locus Link:   29214    Chromosome:   20    Cytoband:   Chr:20p12    SOURCE    GENECARD       Gene Ontology:     BioCarta Pathways:     KEGG Pathways:  
    Probe set : 1374388_at    Name:   EF hand domain containing 2 (predicted)    GB acc:   AI408294    UniGene:   Rn.92713    Gene Symbol:   Efhd2_predicted    Locus Link:   298609    Chromosome:   5    Cytoband:   Chr:5q36    SOURCE    GENECARD       Gene Ontology:   biological process:  muscle development   molecular function:  calcium ion binding      BioCarta Pathways:     KEGG Pathways:  
    Probe set : 1378257_at    Name:   three prime repair exonuclease 1 (predicted)    GB acc:   BG377140    UniGene:   Rn.98617    Gene Symbol:   Trex1_predicted    Locus Link:   301014    Chromosome:   8    Cytoband:   Chr:8q32    SOURCE    GENECARD       Gene Ontology:   cellular component:  nucleus   cellular component:  nuclear membrane   cellular component:  intracellular      BioCarta Pathways:     KEGG Pathways:  
    Probe set : 1370603_a_at    Name:   protein tyrosine phosphatase, receptor type, C    GB acc:   M10072    UniGene:   Rn.90166    Gene Symbol:   Ptprc    Locus Link:   24699    Chromosome:   13    Cytoband:   Chr:13q13    SOURCE    GENECARD       Gene Ontology:     BioCarta Pathways:     KEGG Pathways:  
    Probe set : 1385557_at    Name:   Transcribed locus    GB acc:   AA925349    UniGene:   Rn.128108        SOURCE        Gene Ontology:     BioCarta Pathways:     KEGG Pathways:  
    Probe set : 1370215_at    Name:   complement component 1, q subcomponent, beta polypeptide    GB acc:   AW434057    UniGene:   Rn.6702    Gene Symbol:   C1qb    Locus Link:   29687    Chromosome:   5    Cytoband:   Chr:5q36    SOURCE    GENECARD       Gene Ontology:   cellular component:  complement component C1q complex   cellular component:  extracellular space   cellular component:  cytoplasm      BioCarta Pathways:     KEGG Pathways:  
    Probe set : 1376652_at    Name:   complement component 1, q subcomponent, alpha polypeptide (predicted)    GB acc:   BF418957    UniGene:   Rn.105647    Gene Symbol:   C1qa_predicted    Locus Link:   298566    Chromosome:   5    Cytoband:   Chr:5q36    SOURCE    GENECARD       Gene Ontology:   cellular component:  complement component C1q complex   cellular component:  extracellular space   cellular component:  cytoplasm      BioCarta Pathways:     KEGG Pathways:  
    Probe set : 1395124_at    Name:   tryptophanyl-tRNA synthetase (predicted)    GB acc:   BE101688    UniGene:   Rn.14939    Gene Symbol:   Wars_predicted    Locus Link:   314442    Chromosome:   6    Cytoband:   Chr:6q32    SOURCE    GENECARD       Gene Ontology:   cellular component:  soluble fraction   cellular component:  cytoplasm      BioCarta Pathways:     KEGG Pathways:  
    Probe set : 1370516_at    Name:   peptide/histidine transporter PHT2    GB acc:   AB026665    UniGene:   Rn.17317    Gene Symbol:   Slc15a3    Locus Link:   246239    Chromosome:   1    Cytoband:   Chr:1q43    SOURCE    GENECARD       Gene Ontology:   biological process:  transport   biological process:  oligopeptide transport   biological process:  protein catabolism   cellular component:  lysosomal membrane   cellular component:  membrane   cellular component:  integral to membrane   molecular function:  transporter activity   molecular function:  proton-dependent oligopeptide transporter activity      BioCarta Pathways:     KEGG Pathways:  
    Probe set : 1376845_at    Name:   putative ISG12(b) protein    GB acc:   AA819034    UniGene:   Rn.22509    Gene Symbol:   isg12(b)    Locus Link:   299269    Chromosome:   6    Cytoband:   Chr:6q32    SOURCE    GENECARD       Gene Ontology:     BioCarta Pathways:     KEGG Pathways:  
    Probe set : 1371123_x_at    Name:   RT1 class Ib, locus S3    GB acc:   AJ243973    UniGene:   Rn.143874    Gene Symbol:   RT1-S3    Locus Link:   294228    Chromosome:   20    Cytoband:   Chr:20p12    SOURCE    GENECARD       Gene Ontology:   biological process:  immune response   biological process:  antigen presentation   cellular component:  MHC class I protein complex   cellular component:  MHC class I protein complex   cellular component:  integral to membrane   molecular function:  peptide binding   molecular function:  MHC class I receptor activity      BioCarta Pathways:     KEGG Pathways:  
    Probe set : 1379381_at    Name:   MHC class II transactivator    GB acc:   AI045321    UniGene:   Rn.16740    Gene Symbol:   C2ta    Locus Link:   85483    Chromosome:   10    Cytoband:   Chr:10q11    SOURCE    GENECARD       Gene Ontology:   cellular component:  nucleus      BioCarta Pathways:     KEGG Pathways:  
    Probe set : 1370585_a_at    Name:   protein kinase C, beta 1    GB acc:   X04440    UniGene:   Rn.91118    Gene Symbol:   Prkcb1    Locus Link:   25023    Chromosome:   1    Cytoband:   Chr:1q36    SOURCE    GENECARD       Gene Ontology:   cellular component:  nucleus   cellular component:  cytoplasm   cellular component:  plasma membrane   cellular component:  membrane   molecular function:  protein kinase activity   molecular function:  protein serine/threonine kinase activity   molecular function:  protein kinase CK2 activity   molecular function:  cAMP-dependent protein kinase activity   molecular function:  protein kinase C activity   molecular function:  protein kinase C activity   molecular function:  protein-tyrosine kinase activity   molecular function:  calcium channel regulator activity   molecular function:  calcium ion binding   molecular function:  protein binding   molecular function:  ATP binding   molecular function:  transferase activity   molecular function:  diacylglycerol binding   molecular function:  nucleotide binding   molecular function:  zinc ion binding   molecular function:  kinase activity   molecular function:  metal ion binding      BioCarta Pathways:     KEGG Pathways:  
    Probe set : 1379748_at    Name:   Transcribed locus    GB acc:   AA819629    UniGene:   Rn.16644        SOURCE        Gene Ontology:     BioCarta Pathways:     KEGG Pathways:  
    Probe set : 1382926_s_at    Name:   LOC499533    GB acc:   BF287720    UniGene:   Rn.141366    Gene Symbol:   LOC499533    Locus Link:   499533    Chromosome:   2    Cytoband:   Chr:2q16    SOURCE    GENECARD       Gene Ontology:     BioCarta Pathways:     KEGG Pathways:  
    Probe set : 1372468_at    Name:   CD97 antigen (predicted)    GB acc:   BI296525    UniGene:   Rn.107655    Gene Symbol:   Cd97_predicted    Locus Link:   361383    Chromosome:   19    Cytoband:   Chr:19q11    SOURCE    GENECARD       Gene Ontology:     BioCarta Pathways:     KEGG Pathways:  
    Probe set : 1367974_at    Name:   annexin A3    GB acc:   NM_012823    UniGene:   Rn.6589    Gene Symbol:   Anxa3    Locus Link:   25291    Chromosome:   14    Cytoband:   Chr:14p22    SOURCE    GENECARD       Gene Ontology:   cellular component:  cytoplasm      BioCarta Pathways:     KEGG Pathways:  
    Probe set : 1367816_at    Name:   homeobox only domain    GB acc:   NM_133621    UniGene:   Rn.2989    Gene Symbol:   Hod    Locus Link:   171160    Chromosome:   14    Cytoband:   Chr:14p11    SOURCE    GENECARD       Gene Ontology:   cellular component:  nucleus   molecular function:  DNA binding   molecular function:  transcription factor activity      BioCarta Pathways:     KEGG Pathways:  
    Probe set : 1370904_at    Name:   major histocompatibility complex, class II, DM alpha    GB acc:   BI301490    UniGene:   Rn.35809    Gene Symbol:   RGD:735053    Locus Link:   294274    Chromosome:   20    Cytoband:   Chr:20p12    SOURCE    GENECARD       Gene Ontology:   cellular component:  MHC class II protein complex   cellular component:  membrane   molecular function:  MHC class II receptor activity      BioCarta Pathways:     KEGG Pathways:  
    Probe set : 1379357_at    Name:   Transcribed locus    GB acc:   AI408767    UniGene:   Rn.135304        SOURCE        Gene Ontology:     BioCarta Pathways:     KEGG Pathways:  
    Probe set : 1389006_at    Name:   Macrophage expressed gene 1    GB acc:   AI170394    UniGene:   Rn.4301    Gene Symbol:   Mpeg1    Locus Link:   64552    Chromosome:   1    Cytoband:   Chr:1q43    SOURCE    GENECARD       Gene Ontology:     BioCarta Pathways:     KEGG Pathways:  
    Probe set : 1394940_at    Name:   similar to hypothetical protein FLJ20037 (predicted)    GB acc:   BI294811    UniGene:   Rn.788    Gene Symbol:   RGD1311381_predicted    Locus Link:   300870    Chromosome:   8    Cytoband:   Chr:8q31    SOURCE    GENECARD       Gene Ontology:     BioCarta Pathways:     KEGG Pathways:  
    Probe set : 1382311_at    Name:   TRAF2 binding protein    GB acc:   BM384466    UniGene:   Rn.136924    Gene Symbol:   RGD:1359151    Locus Link:   310877    Chromosome:   2    Cytoband:   Chr:2q42    SOURCE    GENECARD       Gene Ontology:     BioCarta Pathways:     KEGG Pathways:  
    Probe set : 1379496_at    Name:   Transcribed locus    GB acc:   AI029460    UniGene:   Rn.148708        SOURCE        Gene Ontology:     BioCarta Pathways:     KEGG Pathways:  
    Probe set : 1370119_at    Name:   leucocyte specific transcript 1    GB acc:   NM_022634    UniGene:   Rn.91085    Gene Symbol:   Lst1    Locus Link:   64569    Chromosome:   20    Cytoband:   Chr:20p12    SOURCE    GENECARD       Gene Ontology:   biological process:  cellular morphogenesis   biological process:  immune response   cellular component:  integral to membrane   cellular component:  membrane      BioCarta Pathways:     KEGG Pathways:  
    Probe set : 1368518_at    Name:   CD53 antigen    GB acc:   NM_012523    UniGene:   Rn.31988    Gene Symbol:   Cd53    Locus Link:   24251    Chromosome:   2    Cytoband:   Chr:2q34-q41    SOURCE    GENECARD       Gene Ontology:   biological process:  signal transduction   biological process:  antimicrobial humoral response (sensu Vertebrata)   biological process:  regulation of growth      BioCarta Pathways:     KEGG Pathways:  
    Probe set : 1374397_at    Name:   eukaryotic translation initiation factor 2, subunit 2 (beta)    GB acc:   BE107498    UniGene:   Rn.133804    Gene Symbol:   Eif2s2    Locus Link:   296302    Chromosome:   3    Cytoband:   Chr:3q42    SOURCE    GENECARD       Gene Ontology:   biological process:  translational initiation   biological process:  translational initiation   molecular function:  translation initiation factor activity   molecular function:  translation initiation factor activity      BioCarta Pathways:     KEGG Pathways:  
    Probe set : 1388347_at    Name:   similar to lymphocyte antigen 6 complex, locus E    GB acc:   AI233210    UniGene:   Rn.3321    Gene Symbol:   LOC362934    Locus Link:   362934    Chromosome:   7    Cytoband:   Chr:7q34    SOURCE    GENECARD       Gene Ontology:     BioCarta Pathways:     KEGG Pathways:  
    Probe set : 1379344_at    Name:   Cytochrome b-245, beta polypeptide    GB acc:   AI176057    UniGene:   Rn.98491    Gene Symbol:   Cybb    Locus Link:   66021    Chromosome:   X    Cytoband:   Chr:Xq13    SOURCE    GENECARD       Gene Ontology:   biological process:  electron transport   cellular component:  intracellular   cellular component:  membrane   cellular component:  integral to membrane      BioCarta Pathways:     KEGG Pathways:  
    Probe set : 1377239_at    Name:   amyloid beta (A4) precursor protein-binding, family B, member 1 interacting protein (predicted)    GB acc:   BM386169    UniGene:   Rn.38834    Gene Symbol:   Apbb1ip_predicted    Locus Link:   307171    Chromosome:   17    Cytoband:   Chr:17q12.3    SOURCE    GENECARD       Gene Ontology:   biological process:  signal transduction   biological process:  neuropeptide signaling pathway      BioCarta Pathways:     KEGG Pathways:  
    Probe set : 1372011_at    Name:   Guanine deaminase    GB acc:   BI292028    UniGene:   Rn.24783    Gene Symbol:   Gda    Locus Link:   83585    Chromosome:   1    Cytoband:   Chr:1q51    SOURCE    GENECARD       Gene Ontology:   cellular component:  intracellular      BioCarta Pathways:     KEGG Pathways:  
    Probe set : 1374718_at    Name:   Similar to deltex 3-like    GB acc:   AA945915    UniGene:   Rn.1971     Locus Link:   498089      SOURCE        Gene Ontology:     BioCarta Pathways:     KEGG Pathways:  
    Probe set : 1378428_at    Name:   Transcribed locus    GB acc:   AI233552    UniGene:   Rn.23777        SOURCE        Gene Ontology:     BioCarta Pathways:     KEGG Pathways:  
    Probe set : 1370422_at    Name:   receptor-interacting serine-threonine kinase 3    GB acc:   AF036537    UniGene:   Rn.7110    Gene Symbol:   Ripk3    Locus Link:   246240    Chromosome:   15    Cytoband:   Chr:15p13    SOURCE    GENECARD       Gene Ontology:   cellular component:  intracellular      BioCarta Pathways:     KEGG Pathways:  
    Probe set : 1389553_at    Name:   dendritic cell inhibitory receptor 3    GB acc:   BF393825    UniGene:   Rn.137522    Gene Symbol:   RGD:1359528    Locus Link:   362431    Chromosome:   4    Cytoband:   Chr:4q42    SOURCE    GENECARD       Gene Ontology:   molecular function:  receptor activity   molecular function:  sugar binding      BioCarta Pathways:     KEGG Pathways:  
    Probe set : 1371017_at    Name:   T-cell receptor gamma chain    GB acc:   Z27087    UniGene:   Rn.10427    Gene Symbol:   Tcrg    Locus Link:   24821    Chromosome:   17    Cytoband:   Chr:17q11    SOURCE    GENECARD       Gene Ontology:   biological process:  immune response   biological process:  cellular defense response   cellular component:  integral to plasma membrane   cellular component:  gamma-delta T cell receptor complex   molecular function:  transmembrane receptor activity      BioCarta Pathways:     KEGG Pathways:  
    Probe set : 1374578_at    Name:   Transcribed locus    GB acc:   AI409501    UniGene:   Rn.17879        SOURCE        Gene Ontology:     BioCarta Pathways:     KEGG Pathways:  
    Probe set : 1383372_at    Name:   platelet-activating factor receptor    GB acc:   BF407234    UniGene:   Rn.10137    Gene Symbol:   Ptafr    Locus Link:   58949    Chromosome:   5    Cytoband:   Chr:5q36    SOURCE    GENECARD       Gene Ontology:   cellular component:  integral to plasma membrane   cellular component:  integral to membrane      BioCarta Pathways:     KEGG Pathways:  
    Probe set : 1379741_at    Name:   ATPase, H+ transporting, lysosomal V0 subunit A isoform 4 (predicted)    GB acc:   BG377116    UniGene:   Rn.24723    Gene Symbol:   Atp6v0a4_predicted    Locus Link:   296981    Chromosome:   4    Cytoband:   Chr:4q22    SOURCE    GENECARD       Gene Ontology:   cellular component:  membrane   cellular component:  integral to membrane   cellular component:  hydrogen-translocating V-type ATPase complex   molecular function:  hydrogen-exporting ATPase activity, phosphorylative mechanism   molecular function:  hydrogen ion transporter activity      BioCarta Pathways:     KEGG Pathways:  
    Probe set : 1392722_at    Name:   Transcribed locus    GB acc:   AA819134    UniGene:   Rn.16615        SOURCE        Gene Ontology:     BioCarta Pathways:     KEGG Pathways:  
    Probe set : 1390798_at     GB acc:   BF288130              Gene Ontology:     BioCarta Pathways:     KEGG Pathways:  
    Probe set : 1369964_at    Name:   coronin, actin binding protein 1A    GB acc:   NM_130411    UniGene:   Rn.6990    Gene Symbol:   Coro1a    Locus Link:   155151    Chromosome:   1    Cytoband:   Chr:1q36    SOURCE    GENECARD       Gene Ontology:   biological process:  transport   biological process:  cell motility   biological process:  mitosis   biological process:  T cell differentiation   molecular function:  actin binding   molecular function:  actin binding   molecular function:  structural molecule activity      BioCarta Pathways:     KEGG Pathways:  
    Probe set : 1368006_at    Name:   lysosomal-associated protein transmembrane 5    GB acc:   NM_053538    UniGene:   Rn.24799    Gene Symbol:   Laptm5    Locus Link:   89783    Chromosome:   5    Cytoband:   Chr:5q36    SOURCE    GENECARD       Gene Ontology:   cellular component:  lysosome   cellular component:  lysosome   cellular component:  integral to membrane      BioCarta Pathways:     KEGG Pathways:  
    Probe set : 1383420_at    Name:   Similar to lactation elevated 1    GB acc:   BM390554    UniGene:   Rn.24314     Locus Link:   502479      SOURCE        Gene Ontology:     BioCarta Pathways:     KEGG Pathways:  
    Probe set : 1383426_at    Name:   proline-serine-threonine phosphatase-interacting protein 1 (predicted)    GB acc:   BI290159    UniGene:   Rn.6937    Gene Symbol:   Pstpip1_predicted    Locus Link:   300732    Chromosome:   8    Cytoband:   Chr:8q24    SOURCE    GENECARD       Gene Ontology:   cellular component:  stress fiber   cellular component:  contractile ring   molecular function:  actin binding   molecular function:  protein binding   molecular function:  protein phosphatase binding      BioCarta Pathways:     KEGG Pathways:  
    Probe set : 1372757_at    Name:   Signal transducer and activator of transcription 1    GB acc:   BM386875    UniGene:   Rn.33229    Gene Symbol:   Stat1    Locus Link:   25124    Chromosome:   9    Cytoband:   Chr:9q31-q32    SOURCE    GENECARD       Gene Ontology:   cellular component:  nucleus   cellular component:  cytoplasm      BioCarta Pathways:     KEGG Pathways:  
    Probe set : 1385029_at    Name:   Transcribed locus    GB acc:   BE110921    UniGene:   Rn.124278        SOURCE        Gene Ontology:     BioCarta Pathways:     KEGG Pathways:  
    Probe set : 1375955_at    Name:   zinc finger protein 313    GB acc:   BI289415    UniGene:   Rn.49925    Gene Symbol:   RGD:1303139    Locus Link:   362277    Chromosome:   3    Cytoband:   Chr:3q42    SOURCE    GENECARD       Gene Ontology:   cellular component:  ubiquitin ligase complex   cellular component:  nucleus      BioCarta Pathways:     KEGG Pathways:  
    Probe set : 1379425_at    Name:   similar to RIKEN cDNA 3110037I16 (predicted)    GB acc:   AI549208    UniGene:   Rn.35470    Gene Symbol:   RGD1309802_predicted    Locus Link:   313563    Chromosome:   5    Cytoband:   Chr:5q36    SOURCE    GENECARD       Gene Ontology:     BioCarta Pathways:     KEGG Pathways:  
    Probe set : 1367705_at    Name:   glutaredoxin 1 (thioltransferase)    GB acc:   AF319950    UniGene:   Rn.1484    Gene Symbol:   Glrx1    Locus Link:   64045    Chromosome:   2    Cytoband:   Chr:2q11    SOURCE    GENECARD       Gene Ontology:   biological process:  electron transport   biological process:  transport   biological process:  cell redox homeostasis      BioCarta Pathways:     KEGG Pathways:  
    Probe set : 1376144_at    Name:   similar to B aggressive lymphoma (predicted)    GB acc:   AA819679    UniGene:   Rn.2158    Gene Symbol:   RGD1307534_predicted    Locus Link:   303905    Chromosome:   11    Cytoband:   Chr:11q22    SOURCE    GENECARD       Gene Ontology:     BioCarta Pathways:     KEGG Pathways:  
    Probe set : 1387048_at    Name:   nuclear RNA helicase, DECD variant of DEAD box family    GB acc:   NM_053563    UniGene:   Rn.14550    Gene Symbol:   Ddx39    Locus Link:   89827    Chromosome:   19    Cytoband:   Chr:19q11    SOURCE    GENECARD       Gene Ontology:   biological process:  nuclear mRNA splicing, via spliceosome   biological process:  mRNA processing   cellular component:  nucleus      BioCarta Pathways:     KEGG Pathways:  
    Probe set : 1370623_at    Name:   fibrinogen-like 2    GB acc:   AF323608    UniGene:   Rn.64635    Gene Symbol:   Fgl2    Locus Link:   84586    Chromosome:   4    Cytoband:   Chr:4q11    SOURCE    GENECARD       Gene Ontology:   biological process:  parturition   biological process:  cytolysis   cellular component:  extracellular space   molecular function:  peptidase activity      BioCarta Pathways:     KEGG Pathways:  
    Probe set : 1386277_at    Name:   ubiquitin-activating enzyme E1-like (predicted)    GB acc:   AI177396    UniGene:   Rn.98275    Gene Symbol:   Ube1l_predicted    Locus Link:   301000    Chromosome:   8    Cytoband:   Chr:8q32    SOURCE    GENECARD       Gene Ontology:   biological process:  ubiquitin cycle      BioCarta Pathways:     KEGG Pathways:  
    Probe set : 1368835_at    Name:   signal transducer and activator of transcription 1    GB acc:   AW434718    UniGene:   Rn.33229    Gene Symbol:   Stat1    Locus Link:   25124    Chromosome:   9    Cytoband:   Chr:9q31-q32    SOURCE    GENECARD       Gene Ontology:   cellular component:  nucleus   cellular component:  cytoplasm      BioCarta Pathways:     KEGG Pathways:  
    Probe set : 1368558_s_at    Name:   allograft inflammatory factor 1    GB acc:   NM_017196    UniGene:   Rn.32080    Gene Symbol:   Aif1    Locus Link:   29427    Chromosome:   20    Cytoband:   Chr:20p12    SOURCE    GENECARD       Gene Ontology:   molecular function:  calcium ion binding   molecular function:  protein binding      BioCarta Pathways:     KEGG Pathways:  
    Probe set : 1388236_x_at    Name:   RT1 class I, CE12    GB acc:   M24026    UniGene:   Rn.116792    Gene Symbol:   RGD:1359103    Locus Link:   309600    Chromosome:   20    Cytoband:   Chr:20p12    SOURCE    GENECARD       Gene Ontology:   biological process:  antigen presentation   cellular component:  MHC class I protein complex   molecular function:  MHC class I receptor activity      BioCarta Pathways:     KEGG Pathways:  
    Probe set : 1385276_a_at    Name:   similar to hypothetical protein FLJ11354 (predicted)    GB acc:   AA818849    UniGene:   Rn.140601    Gene Symbol:   RGD1310093_predicted    Locus Link:   303538    Chromosome:   10    Cytoband:   Chr:10q32.1    SOURCE    GENECARD       Gene Ontology:     BioCarta Pathways:     KEGG Pathways:  
    Probe set : 1367732_at    Name:   guanine nucleotide binding protein, beta 1    GB acc:   U34958    UniGene:   Rn.126047    Gene Symbol:   Gnb1    Locus Link:   24400    Chromosome:   5    Cytoband:   Chr:5q36    SOURCE    GENECARD       Gene Ontology:   cellular component:  heterotrimeric G-protein complex   cellular component:  heterotrimeric G-protein complex   cellular component:  membrane   molecular function:  GTPase activity   molecular function:  signal transducer activity      BioCarta Pathways:     KEGG Pathways:  
    Probe set : 1368490_at    Name:   CD14 antigen    GB acc:   NM_021744    UniGene:   Rn.42942    Gene Symbol:   Cd14    Locus Link:   60350    Chromosome:   18    Cytoband:   Chr:18p11    SOURCE    GENECARD       Gene Ontology:     BioCarta Pathways:     KEGG Pathways:  
    Probe set : 1371015_at    Name:   myxovirus (influenza virus) resistance 1    GB acc:   X52711    UniGene:   Rn.10373    Gene Symbol:   Mx1    Locus Link:   24575    Chromosome:   11    Cytoband:   Chr:11q12    SOURCE    GENECARD       Gene Ontology:   biological process:  defense response   biological process:  immune response   biological process:  response to virus   cellular component:  nucleus   molecular function:  GTPase activity   molecular function:  GTP binding   molecular function:  nucleotide binding      BioCarta Pathways:     KEGG Pathways:  
    Probe set : 1387202_at    Name:   intercellular adhesion molecule 1    GB acc:   NM_012967    UniGene:   Rn.12    Gene Symbol:   Icam1    Locus Link:   25464    Chromosome:   8    Cytoband:   Chr:8q13    SOURCE    GENECARD       Gene Ontology:   molecular function:  transmembrane receptor activity   molecular function:  protein binding   molecular function:  protein binding      BioCarta Pathways:     KEGG Pathways:  
    Probe set : 1373785_at    Name:   similar to expressed sequence AI132321    GB acc:   AA848738    UniGene:   Rn.6379    Gene Symbol:   LOC362792    Locus Link:   362792    Chromosome:   6    Cytoband:   Chr:6q32    SOURCE    GENECARD       Gene Ontology:     BioCarta Pathways:     KEGG Pathways:  
    Probe set : 1379717_at    Name:   similar to ATP-binding cassette, subfamily G, member 3    GB acc:   BM385804    UniGene:   Rn.114189    Gene Symbol:   LOC498435    Locus Link:   498435      SOURCE    GENECARD       Gene Ontology:     BioCarta Pathways:     KEGG Pathways:  
    Probe set : 1387605_at    Name:   caspase 12    GB acc:   NM_130422    UniGene:   Rn.81078    Gene Symbol:   Casp12    Locus Link:   156117    Chromosome:   8    Cytoband:   Chr:8q11    SOURCE    GENECARD       Gene Ontology:   cellular component:  intracellular   cellular component:  endoplasmic reticulum   cellular component:  endoplasmic reticulum      BioCarta Pathways:     KEGG Pathways:  
    Probe set : 1376151_a_at     GB acc:   AI407953              Gene Ontology:     BioCarta Pathways:     KEGG Pathways:  
    Probe set : 1393280_at    Name:   lymphocyte antigen 86 (predicted)    GB acc:   AA874924    UniGene:   Rn.3176    Gene Symbol:   Ly86_predicted    Locus Link:   291359    Chromosome:   17    Cytoband:   Chr:17p12    SOURCE    GENECARD       Gene Ontology:   cellular component:  extracellular space   cellular component:  plasma membrane   molecular function:  signal transducer activity      BioCarta Pathways:     KEGG Pathways:  
    Probe set : 1367662_at    Name:   hydroxysteroid (17-beta) dehydrogenase 10    GB acc:   NM_031682    UniGene:   Rn.2700    Gene Symbol:   Hsd17b10    Locus Link:   63864    Chromosome:   X    Cytoband:   Chr:Xq21    SOURCE    GENECARD       Gene Ontology:   biological process:  lipid metabolism   biological process:  metabolism   biological process:  steroid metabolism      BioCarta Pathways:     KEGG Pathways:  
    Probe set : 1379568_at    Name:   interferon-induced protein with tetratricopeptide repeats 2 (predicted)    GB acc:   AI009765    UniGene:   Rn.6175    Gene Symbol:   Ifit2_predicted    Locus Link:   294091    Chromosome:   1    Cytoband:   Chr:1q52    SOURCE    GENECARD       Gene Ontology:   biological process:  immune response   molecular function:  binding      BioCarta Pathways:     KEGG Pathways:  
    Probe set : 1382153_at    Name:   C-type (calcium dependent, carbohydrate recognition domain) lectin, superfamily member 6    GB acc:   AI171821    UniGene:   Rn.24032    Gene Symbol:   RGD:1359662    Locus Link:   474143    Chromosome:   4    Cytoband:   Chr:4q42    SOURCE    GENECARD       Gene Ontology:   biological process:  immune response   cellular component:  integral to membrane   molecular function:  sugar binding   molecular function:  receptor activity      BioCarta Pathways:     KEGG Pathways:  
    Probe set : 1395003_at    Name:   dendritic cell inhibitory receptor 4    GB acc:   BI278559    UniGene:   Rn.47351    Gene Symbol:   RGD:1359109    Locus Link:   362430    Chromosome:   4    Cytoband:   Chr:4q42    SOURCE    GENECARD       Gene Ontology:   molecular function:  receptor activity   molecular function:  sugar binding      BioCarta Pathways:     KEGG Pathways:  
    Probe set : 1374033_at    Name:   proteasome (prosome, macropain) subunit, beta type 10 (predicted)    GB acc:   BG373505    UniGene:   Rn.24968    Gene Symbol:   Psmb10_predicted    Locus Link:   291983    Chromosome:   19    Cytoband:   Chr:19q12    SOURCE    GENECARD       Gene Ontology:   biological process:  proteolysis   biological process:  ubiquitin-dependent protein catabolism   biological process:  humoral immune response   cellular component:  cytosol   cellular component:  proteasome core complex (sensu Eukaryota)   cellular component:  protein complex      BioCarta Pathways:     KEGG Pathways:  
    Probe set : 1377826_at    Name:   Transcribed locus, weakly similar to NP_036457.1 microtubule-associated protein, RP/EB family, member 1; adenomatous polyposis coli-binding protein EB1 [Homo sapiens]    GB acc:   AI176721    UniGene:   Rn.17666        SOURCE        Gene Ontology:     BioCarta Pathways:     KEGG Pathways:  
    Probe set : 1388149_at    Name:   transporter 1, ATP-binding cassette, sub-family B (MDR/TAP)    GB acc:   X57523    UniGene:   Rn.10763    Gene Symbol:   Tap1    Locus Link:   24811    Chromosome:   20    Cytoband:   Chr:20p12    SOURCE    GENECARD       Gene Ontology:   biological process:  protein complex assembly   biological process:  transport   biological process:  oligopeptide transport   biological process:  intracellular protein transport   biological process:  defense response   biological process:  immune response   biological process:  antigen processing, endogenous antigen via MHC class I   biological process:  cytosol to ER transport   biological process:  peptide antigen transport   biological process:  antigen presentation, endogenous peptide antigen   biological process:  protein complex assembly   biological process:  intracellular protein transport   biological process:  peptide transport   biological process:  antigen processing, endogenous antigen via MHC class I   biological process:  cytosol to ER transport   biological process:  antigen presentation, endogenous peptide antigen   biological process:  protein transport   molecular function:  nucleotide binding   molecular function:  nucleotide binding   molecular function:  transporter activity   molecular function:  ATP binding   molecular function:  oligopeptide transporter activity   molecular function:  peptide antigen-transporting ATPase activity   molecular function:  peptide antigen-transporting ATPase activity   molecular function:  ATPase activity   molecular function:  nucleoside-triphosphatase activity   molecular function:  MHC class I protein binding   molecular function:  phosphate binding   molecular function:  peptide antigen binding   molecular function:  ATPase activity, coupled to transmembrane movement of substances   molecular function:  protein homodimerization activity   molecular function:  TAP1 binding   molecular function:  TAP2 binding   molecular function:  tapasin binding   molecular function:  protein heterodimerization activity   molecular function:  protein homodimerization activity   molecular function:  TAP1 binding   molecular function:  TAP2 binding   molecular function:  ATP binding   molecular function:  peptide transporter activity   molecular function:  peptide antigen-transporting ATPase activity   molecular function:  MHC class I protein binding   molecular function:  phosphate binding   molecular function:  peptide antigen binding   molecular function:  tapasin binding   molecular function:  protein heterodimerization activity      BioCarta Pathways:     KEGG Pathways:  
    Probe set : 1373401_at    Name:   Transcribed locus    GB acc:   AI176034    UniGene:   Rn.152256        SOURCE        Gene Ontology:     BioCarta Pathways:     KEGG Pathways:  
    Probe set : 1389732_at    Name:   Transcribed locus, strongly similar to NP_082154.2 hypothetical protein LOC71712 [Mus musculus]    GB acc:   BM386323    UniGene:   Rn.1826        SOURCE        Gene Ontology:     BioCarta Pathways:     KEGG Pathways:  
    Probe set : 1385926_at    Name:   Transcribed locus    GB acc:   BF550565    UniGene:   Rn.23216        SOURCE        Gene Ontology:     BioCarta Pathways:     KEGG Pathways:  
    Probe set : 1387134_at    Name:   schlafen 3    GB acc:   NM_053687    UniGene:   Rn.80831    Gene Symbol:   Slfn3    Locus Link:   114247    Chromosome:   10    Cytoband:   Chr:10q26    SOURCE    GENECARD       Gene Ontology:   biological process:  T cell differentiation      BioCarta Pathways:     KEGG Pathways:  
    Probe set : 1370237_at    Name:   L-3-hydroxyacyl-Coenzyme A dehydrogenase, short chain    GB acc:   AA799574    UniGene:   Rn.92789    Gene Symbol:   Hadhsc    Locus Link:   113965    Chromosome:   2    Cytoband:   Chr:2q43    SOURCE    GENECARD       Gene Ontology:   biological process:  fatty acid metabolism   biological process:  lipid metabolism   cellular component:  mitochondrion      BioCarta Pathways:     KEGG Pathways:  
    Probe set : 1387354_at    Name:   signal transducer and activator of transcription 1    GB acc:   NM_032612    UniGene:   Rn.33229    Gene Symbol:   Stat1    Locus Link:   25124    Chromosome:   9    Cytoband:   Chr:9q31-q32    SOURCE    GENECARD       Gene Ontology:   cellular component:  nucleus   cellular component:  cytoplasm      BioCarta Pathways:     KEGG Pathways:  
    Probe set : 1386885_at    Name:   enoyl coenzyme A hydratase 1, peroxisomal    GB acc:   NM_022594    UniGene:   Rn.6148    Gene Symbol:   Ech1    Locus Link:   64526    Chromosome:   1    Cytoband:   Chr:1q21    SOURCE    GENECARD       Gene Ontology:   cellular component:  mitochondrion   cellular component:  peroxisome      BioCarta Pathways:     KEGG Pathways:  
    Probe set : 1384187_at    Name:   similar to Adapter-related protein complex 1 sigma 1B subunit (Sigma-adaptin 1B) (Adaptor protein complex AP-1 sigma-1B subunit) (Golgi adaptor HA1/AP1 adaptin sigma-1B subunit) (Clathrin assembly protein complex 1 sigma-1B small chain) (Sigma 1B s...    GB acc:   BF551686    UniGene:   Rn.17309    Gene Symbol:   LOC302671    Locus Link:   302671    Chromosome:   X    Cytoband:   Chr:Xq21    SOURCE    GENECARD       Gene Ontology:     BioCarta Pathways:     KEGG Pathways:  
    Probe set : 1368227_at    Name:   solute carrier family 28 (sodium-coupled nucleoside transporter), member 2    GB acc:   NM_031664    UniGene:   Rn.10140    Gene Symbol:   Slc28a2    Locus Link:   60423    Chromosome:   3    Cytoband:   Chr:3q35    SOURCE    GENECARD       Gene Ontology:   biological process:  nucleobase, nucleoside, nucleotide and nucleic acid metabolism   biological process:  transport   biological process:  purine nucleoside transport   molecular function:  nucleoside:sodium symporter activity   molecular function:  purine nucleoside transporter activity   molecular function:  nucleoside binding      BioCarta Pathways:     KEGG Pathways:  
    Probe set : 1383448_at    Name:   interferon dependent positive acting transcription factor 3 gamma (predicted)    GB acc:   AI029121    UniGene:   Rn.144965    Gene Symbol:   Isgf3g_predicted    Locus Link:   305896    Chromosome:   15    Cytoband:   Chr:15p13    SOURCE    GENECARD       Gene Ontology:     BioCarta Pathways:     KEGG Pathways:  
    Probe set : 1387893_at    Name:   complement component 1, s subcomponent /// similar to complement component 1, s subcomponent    GB acc:   D88250    UniGene:   Rn.145641    Gene Symbol:   C1s /// LOC500313    Locus Link:   192262 /// 500313    Chromosome:   4    Cytoband:   Chr:4q42    SOURCE    GENECARD       Gene Ontology:   cellular component:  extracellular region   cellular component:  extracellular region   cellular component:  extracellular space      BioCarta Pathways:     KEGG Pathways:  
    Probe set : 1368754_at    Name:   pyrimidinergic receptor P2Y, G-protein coupled, 6    GB acc:   NM_057124    UniGene:   Rn.10671    Gene Symbol:   P2ry6    Locus Link:   117264    Chromosome:   1    Cytoband:   Chr:1q32    SOURCE    GENECARD       Gene Ontology:     BioCarta Pathways:     KEGG Pathways:  
    Probe set : 1383189_at    Name:   Transcribed locus    GB acc:   AA818143    UniGene:   Rn.133346        SOURCE        Gene Ontology:     BioCarta Pathways:     KEGG Pathways:  
    Probe set : 1373026_at    Name:   similar to RIKEN cDNA 2410030K01 (predicted)    GB acc:   BI303598    UniGene:   Rn.18387    Gene Symbol:   RGD1307631_predicted    Locus Link:   363028    Chromosome:   8    Cytoband:   Chr:8q13    SOURCE    GENECARD       Gene Ontology:     BioCarta Pathways:     KEGG Pathways:  
    Probe set : 1369181_at    Name:   cytochrome b-245, beta polypeptide    GB acc:   NM_023965    UniGene:   Rn.98491    Gene Symbol:   Cybb    Locus Link:   66021    Chromosome:   X    Cytoband:   Chr:Xq13    SOURCE    GENECARD       Gene Ontology:   biological process:  electron transport   cellular component:  intracellular   cellular component:  membrane   cellular component:  integral to membrane      BioCarta Pathways:     KEGG Pathways:  
    Probe set : 1378443_at    Name:   SLAM family member 9 (predicted)    GB acc:   BI295964    UniGene:   Rn.43136    Gene Symbol:   Slamf9_predicted    Locus Link:   289235    Chromosome:   13    Cytoband:   Chr:13q24    SOURCE    GENECARD       Gene Ontology:   cellular component:  extracellular space   cellular component:  integral to membrane      BioCarta Pathways:     KEGG Pathways:  
    Probe set : 1388761_at    Name:   histone deacetylase 1 (predicted)    GB acc:   AI180339    UniGene:   Rn.123394    Gene Symbol:   Hdac1_predicted    Locus Link:   297893    Chromosome:   5    Cytoband:   Chr:5q36    SOURCE    GENECARD       Gene Ontology:     BioCarta Pathways:     KEGG Pathways:  
    Probe set : 1386925_at    Name:   actin related protein 2/3 complex, subunit 1B    GB acc:   NM_019289    UniGene:   Rn.2090    Gene Symbol:   Arpc1b    Locus Link:   54227    Chromosome:   12    Cytoband:   Chr:12p11    SOURCE    GENECARD       Gene Ontology:   biological process:  cell motility   cellular component:  Arp2/3 protein complex   cellular component:  Arp2/3 protein complex   molecular function:  structural constituent of cytoskeleton      BioCarta Pathways:     KEGG Pathways:  
    Probe set : 1380129_at    Name:   Transcribed locus    GB acc:   AA818937    UniGene:   Rn.148708        SOURCE        Gene Ontology:     BioCarta Pathways:     KEGG Pathways:  
    Probe set : 1368059_at    Name:   crystallin, mu    GB acc:   NM_053955    UniGene:   Rn.24561    Gene Symbol:   Crym    Locus Link:   117024      SOURCE    GENECARD       Gene Ontology:   biological process:  visual perception      BioCarta Pathways:     KEGG Pathways:  
    Probe set : 1374601_at    Name:   interferon gamma receptor 2 (predicted)    GB acc:   BI274482    UniGene:   Rn.23305    Gene Symbol:   Ifngr2_predicted    Locus Link:   360697    Chromosome:   11    Cytoband:   Chr:11q11    SOURCE    GENECARD       Gene Ontology:   biological process:  cell surface receptor linked signal transduction   biological process:  response to virus   biological process:  response to pathogenic bacteria      BioCarta Pathways:     KEGG Pathways:  
    Probe set : 1388763_at    Name:   Transcribed locus, strongly similar to NP_031751.1 calponin 2 [Mus musculus]    GB acc:   AI230762    UniGene:   Rn.43993        SOURCE        Gene Ontology:     BioCarta Pathways:     KEGG Pathways:  
    Probe set : 1392785_at    Name:   potassium channel tetramerisation domain containing 12 (predicted)    GB acc:   AA800908    UniGene:   Rn.13832    Gene Symbol:   Kctd12_predicted    Locus Link:   364458    Chromosome:   15    Cytoband:   Chr:15q22    SOURCE    GENECARD       Gene Ontology:   biological process:  potassium ion transport   cellular component:  voltage-gated potassium channel complex   cellular component:  membrane   molecular function:  voltage-gated potassium channel activity      BioCarta Pathways:     KEGG Pathways:  
    Probe set : 1372004_at    Name:   heme binding protein 1 (predicted)    GB acc:   AI102065    UniGene:   Rn.6886    Gene Symbol:   Hebp1_predicted    Locus Link:   362454    Chromosome:   4    Cytoband:   Chr:4q43    SOURCE    GENECARD       Gene Ontology:   biological process:  circadian rhythm   biological process:  heme metabolism   cellular component:  cytoplasm   cellular component:  mitochondrion   cellular component:  cytosol   molecular function:  binding   molecular function:  heme binding      BioCarta Pathways:     KEGG Pathways:  
    Probe set : 1396163_at    Name:   interferon gamma induced GTPase (predicted)    GB acc:   BF563316    UniGene:   Rn.113342    Gene Symbol:   Igtp_predicted    Locus Link:   303163    Chromosome:   10    Cytoband:   Chr:10q22    SOURCE    GENECARD       Gene Ontology:   cellular component:  cytosol   molecular function:  GTPase activity      BioCarta Pathways:     KEGG Pathways:  
    Probe set : 1397372_at    Name:   similar to Mob4B protein /// MOB1, Mps One Binder kinase activator-like 1B (yeast) (predicted)    GB acc:   BI289004    UniGene:   Rn.129443    Gene Symbol:   LOC308302 /// Mobk1b_predicted    Locus Link:   308302 /// 362389    Chromosome:   1    Cytoband:   Chr:1q12 /// Chr:4q34    SOURCE    GENECARD       Gene Ontology:     BioCarta Pathways:     KEGG Pathways:  
    Probe set : 1395034_at    Name:   similar to RIKEN cDNA 4933430F08 (predicted)    GB acc:   AI408816    UniGene:   Rn.43613    Gene Symbol:   RGD1311481_predicted    Locus Link:   309178    Chromosome:   1    Cytoband:   Chr:1q43    SOURCE    GENECARD       Gene Ontology:     BioCarta Pathways:     KEGG Pathways:  
    Probe set : 1392547_at    Name:   hypothetical LOC302884    GB acc:   AI716211    UniGene:   Rn.106101    Gene Symbol:   RGD:1359583    Locus Link:   302884    Chromosome:   3    Cytoband:   Chr:3q35    SOURCE    GENECARD       Gene Ontology:     BioCarta Pathways:     KEGG Pathways:  
    Probe set : 1373900_at    Name:   keratin complex 2, basic, gene 7 (predicted)    GB acc:   BI284344    UniGene:   Rn.7913    Gene Symbol:   Krt2-7_predicted    Locus Link:   300242    Chromosome:   7    Cytoband:   Chr:7q36    SOURCE    GENECARD       Gene Ontology:   biological process:  cytoskeleton organization and biogenesis   cellular component:  intermediate filament   molecular function:  structural molecule activity   molecular function:  structural constituent of cytoskeleton      BioCarta Pathways:     KEGG Pathways:  
    Probe set : 1371959_at    Name:   histone 2, H2aa (predicted) /// similar to Hist2h2aa1 protein    GB acc:   AI235236    UniGene:   Rn.8353    Gene Symbol:   Hist2h2aa_predicted /// LOC499676    Locus Link:   365877 /// 499676    Chromosome:   2    Cytoband:   Chr:2q34    SOURCE    GENECARD       Gene Ontology:     BioCarta Pathways:     KEGG Pathways:  
    Probe set : 1383516_at    Name:   fibrinogen-like 2    GB acc:   BG663284    UniGene:   Rn.64635    Gene Symbol:   Fgl2    Locus Link:   84586    Chromosome:   4    Cytoband:   Chr:4q11    SOURCE    GENECARD       Gene Ontology:   biological process:  parturition   biological process:  cytolysis   cellular component:  extracellular space   molecular function:  peptidase activity      BioCarta Pathways:     KEGG Pathways:  
    Probe set : 1371440_at    Name:   Beta-2 microglobulin    GB acc:   AW916647    UniGene:   Rn.1868    Gene Symbol:   B2m    Locus Link:   24223    Chromosome:   3    Cytoband:   Chr:3q35    SOURCE    GENECARD       Gene Ontology:   molecular function:  receptor activity   molecular function:  MHC class I receptor activity      BioCarta Pathways:     KEGG Pathways:  
    Probe set : 1370445_at    Name:   phosphatidylserine-specific phospholipase A1    GB acc:   D88666    UniGene:   Rn.10696    Gene Symbol:   RGD:621261    Locus Link:   85311      SOURCE    GENECARD       Gene Ontology:   biological process:  lipid metabolism   biological process:  lipid catabolism   cellular component:  extracellular space   molecular function:  catalytic activity   molecular function:  hydrolase activity      BioCarta Pathways:     KEGG Pathways:  
    Probe set : 1387711_at    Name:   interleukin 10    GB acc:   L02926    UniGene:   Rn.9868    Gene Symbol:   Il10    Locus Link:   25325    Chromosome:   13    Cytoband:   Chr:13q13    SOURCE    GENECARD       Gene Ontology:   biological process:  negative regulation of cytokine production   biological process:  anti-apoptosis   biological process:  inflammatory response   biological process:  immune response   biological process:  immune response   biological process:  cytoplasmic sequestering of NF-kappaB   biological process:  tyrosine phosphorylation of STAT protein   biological process:  cell-cell signaling   biological process:  hemopoiesis   biological process:  B cell differentiation   biological process:  immune cell chemotaxis   biological process:  T-helper 2 type immune response   biological process:  B cell proliferation   biological process:  negative regulation of T cell proliferation   biological process:  defense response to bacteria   biological process:  negative regulation of nitric oxide biosynthesis   biological process:  negative regulation of interferon-gamma biosynthesis   biological process:  regulation of isotype switching   biological process:  negative regulation of MHC class II biosynthesis   biological process:  positive regulation of MHC class II biosynthesis   biological process:  negative regulation of interferon-alpha biosynthesis   biological process:  anti-apoptosis   biological process:  inflammatory response   biological process:  cytoplasmic sequestering of NF-kappaB   biological process:  hemopoiesis   biological process:  B cell differentiation   biological process:  T-helper 2 type immune response   biological process:  B cell proliferation   biological process:  negative regulation of T cell proliferation   biological process:  negative regulation of nitric oxide biosynthesis   biological process:  negative regulation of interferon-gamma biosynthesis   biological process:  regulation of isotype switching   biological process:  negative regulation of MHC class II biosynthesis      BioCarta Pathways:     KEGG Pathways:  
    Probe set : 1372213_at    Name:   similar to hypothetical protein MGC6835    GB acc:   BM390487    UniGene:   Rn.22047    Gene Symbol:   LOC500300    Locus Link:   500300    Chromosome:   4    Cytoband:   Chr:4q42    SOURCE    GENECARD       Gene Ontology:     BioCarta Pathways:     KEGG Pathways:  
    Probe set : 1374627_at    Name:   Transcribed locus    GB acc:   BF283727    UniGene:   Rn.35673        SOURCE        Gene Ontology:     BioCarta Pathways:     KEGG Pathways:  
    Probe set : 1370202_at    Name:   HRAS like suppressor    GB acc:   BG378637    UniGene:   Rn.11377    Gene Symbol:   Hrasls3    Locus Link:   24913    Chromosome:   1    Cytoband:   Chr:1q43    SOURCE    GENECARD       Gene Ontology:   biological process:  cell cycle   biological process:  negative regulation of progression through cell cycle   cellular component:  cytoplasm   cellular component:  plasma membrane   cellular component:  integral to membrane      BioCarta Pathways:     KEGG Pathways:  
    Probe set : 1368921_a_at    Name:   CD44 antigen    GB acc:   NM_012924    UniGene:   Rn.1120    Gene Symbol:   Cd44    Locus Link:   25406    Chromosome:   3    Cytoband:   Chr:3q31    SOURCE    GENECARD       Gene Ontology:     BioCarta Pathways:     KEGG Pathways:  
    Probe set : 1373881_at    Name:   Rho, GDP dissociation inhibitor (GDI) beta (predicted)    GB acc:   BF285771    UniGene:   Rn.15842    Gene Symbol:   Arhgdib_predicted    Locus Link:   362456    Chromosome:   4    Cytoband:   Chr:4q43    SOURCE    GENECARD       Gene Ontology:   cellular component:  cytoplasm   cellular component:  cytoplasmic membrane-bound vesicle   molecular function:  Rho GDP-dissociation inhibitor activity   molecular function:  GTPase activator activity      BioCarta Pathways:     KEGG Pathways:  
    Probe set : 1375010_at    Name:   CD68 antigen (predicted)    GB acc:   AI177761    UniGene:   Rn.12478    Gene Symbol:   Cd68_predicted    Locus Link:   287435    Chromosome:   10    Cytoband:   Chr:10q24    SOURCE    GENECARD       Gene Ontology:     BioCarta Pathways:     KEGG Pathways:  
    Probe set : 1371078_at    Name:   RT1 class Ib, locus Aw2    GB acc:   AI500830    UniGene:   Rn.116787    Gene Symbol:   RT1-Aw2    Locus Link:   24737    Chromosome:   20    Cytoband:   Chr:20p12    SOURCE    GENECARD       Gene Ontology:   cellular component:  integral to membrane   cellular component:  MHC class I protein complex   molecular function:  MHC class I receptor activity      BioCarta Pathways:     KEGG Pathways:  
    Probe set : 1371152_a_at    Name:   2',5'-oligoadenylate synthetase 1, 40/46kDa    GB acc:   Z18877    UniGene:   Rn.10383    Gene Symbol:   Oas1    Locus Link:   192281    Chromosome:   12    Cytoband:   Chr:12q16    SOURCE    GENECARD       Gene Ontology:   biological process:  immune response   biological process:  response to virus      BioCarta Pathways:     KEGG Pathways:  
    Probe set : 1390659_at    Name:   CD44 antigen    GB acc:   BI302830    UniGene:   Rn.1120    Gene Symbol:   Cd44    Locus Link:   25406    Chromosome:   3    Cytoband:   Chr:3q31    SOURCE    GENECARD       Gene Ontology:     BioCarta Pathways:     KEGG Pathways:  
    Probe set : 1369952_at    Name:   poly(A) binding protein, cytoplasmic 1    GB acc:   NM_134353    UniGene:   Rn.127813    Gene Symbol:   Pabpc1    Locus Link:   171350    Chromosome:   7    Cytoband:   Chr:7q22    SOURCE    GENECARD       Gene Ontology:   biological process:  mRNA polyadenylylation   biological process:  mRNA metabolism   cellular component:  cytoplasm      BioCarta Pathways:     KEGG Pathways:  
    Probe set : 1369766_at    Name:   prostaglandin E receptor 2, subtype EP2    GB acc:   U48858    UniGene:   Rn.10264    Gene Symbol:   Ptger2    Locus Link:   81752    Chromosome:   15    Cytoband:   Chr:15p14    SOURCE    GENECARD       Gene Ontology:   cellular component:  integral to membrane   cellular component:  integral to membrane      BioCarta Pathways:     KEGG Pathways:  
    Probe set : 1384946_at     GB acc:   AI070419              Gene Ontology:     BioCarta Pathways:     KEGG Pathways:  
    Probe set : 1389997_at    Name:   CD3 antigen, epsilon polypeptide (predicted)    GB acc:   AA945909    UniGene:   Rn.96221    Gene Symbol:   Cd3e_predicted    Locus Link:   315609    Chromosome:   8    Cytoband:   Chr:8q22    SOURCE    GENECARD       Gene Ontology:     BioCarta Pathways:     KEGG Pathways:  
    Probe set : 1373658_at    Name:   Rac GTPase-activating protein 1 (predicted)    GB acc:   AI409259    UniGene:   Rn.101301     Locus Link:   315298    Chromosome:   7    Cytoband:   Chr:7q36    SOURCE        Gene Ontology:   biological process:  electron transport   biological process:  transport   biological process:  intracellular signaling cascade   cellular component:  mitochondrion      BioCarta Pathways:     KEGG Pathways:  
    Probe set : 1388212_a_at    Name:   RT1 class Ib, locus S3    GB acc:   AJ243974    UniGene:   Rn.143874    Gene Symbol:   RT1-S3    Locus Link:   294228    Chromosome:   20    Cytoband:   Chr:20p12    SOURCE    GENECARD       Gene Ontology:   biological process:  immune response   biological process:  antigen presentation   cellular component:  MHC class I protein complex   cellular component:  MHC class I protein complex   cellular component:  integral to membrane   molecular function:  peptide binding   molecular function:  MHC class I receptor activity      BioCarta Pathways:     KEGG Pathways:  
    Probe set : 1376795_at    Name:   phosphoinositide-3-kinase adaptor protein 1 (predicted)    GB acc:   AW916092    UniGene:   Rn.15411    Gene Symbol:   Pik3ap1_predicted    Locus Link:   294048    Chromosome:   1    Cytoband:   Chr:1q54    SOURCE    GENECARD       Gene Ontology:   cellular component:  nucleus   cellular component:  cytoplasm   cellular component:  plasma membrane   molecular function:  transmembrane receptor protein tyrosine kinase signaling protein activity   molecular function:  protein binding   molecular function:  kinase activity      BioCarta Pathways:     KEGG Pathways:  
    Probe set : 1369973_at    Name:   xanthine dehydrogenase /// hypothetical gene supported by NM_017154    GB acc:   NM_017154    UniGene:   Rn.129174    Gene Symbol:   Xdh /// LOC497811    Locus Link:   29289 /// 497811    Chromosome:   6    Cytoband:   Chr:6q12 /// Chr:6q13    SOURCE    GENECARD       Gene Ontology:   cellular component:  cytosol      BioCarta Pathways:     KEGG Pathways:  
    Probe set : 1384874_at    Name:   Transcribed locus    GB acc:   BF558516    UniGene:   Rn.96188        SOURCE        Gene Ontology:     BioCarta Pathways:     KEGG Pathways:  
    Probe set : 1377698_at    Name:   tumor necrosis factor receptor superfamily, member 5    GB acc:   AW433947    UniGene:   Rn.25180    Gene Symbol:   RGD:619830    Locus Link:   171369    Chromosome:   3    Cytoband:   Chr:3q42    SOURCE    GENECARD       Gene Ontology:   biological process:  apoptosis   biological process:  immune response   biological process:  signal transduction   cellular component:  membrane   molecular function:  receptor activity   molecular function:  transmembrane receptor activity      BioCarta Pathways:     KEGG Pathways:  
    Probe set : 1390738_at    Name:   bone marrow stromal cell antigen 2    GB acc:   BM385476    UniGene:   Rn.32168    Gene Symbol:   Bst2    Locus Link:   378947    Chromosome:   16    Cytoband:   Chr:16p14    SOURCE    GENECARD       Gene Ontology:   cellular component:  intracellular   cellular component:  apical plasma membrane      BioCarta Pathways:     KEGG Pathways:  
    Probe set : 1370080_at    Name:   heme oxygenase (decycling) 1    GB acc:   NM_012580    UniGene:   Rn.3160    Gene Symbol:   Hmox1    Locus Link:   24451    Chromosome:   19    Cytoband:   Chr:19p11    SOURCE    GENECARD       Gene Ontology:     BioCarta Pathways:     KEGG Pathways:  
    Probe set : 1371210_s_at    Name:   RT1 class Ib, locus Aw2 /// RT1 class I, CE5    GB acc:   AJ276126    UniGene:   Rn.40130    Gene Symbol:   RT1-Aw2 /// RT1-CE5    Locus Link:   24737 /// 309607    Chromosome:   20    Cytoband:   Chr:20p12    SOURCE    GENECARD       Gene Ontology:   cellular component:  integral to membrane   cellular component:  MHC class I protein complex   molecular function:  MHC class I receptor activity      BioCarta Pathways:     KEGG Pathways:  
    Probe set : 1370892_at    Name:   complement component 4a /// complement component 4, gene 2    GB acc:   BI285347    UniGene:   Rn.81052    Gene Symbol:   C4a /// C4-2    Locus Link:   24233 /// 406161    Chromosome:   20    Cytoband:   Chr:20p12    SOURCE    GENECARD       Gene Ontology:   cellular component:  extracellular region   cellular component:  extracellular region   molecular function:  endopeptidase inhibitor activity      BioCarta Pathways:     KEGG Pathways:  
    Probe set : 1398616_at    Name:   similar to Adapter-related protein complex 1 sigma 1B subunit (Sigma-adaptin 1B) (Adaptor protein complex AP-1 sigma-1B subunit) (Golgi adaptor HA1/AP1 adaptin sigma-1B subunit) (Clathrin assembly protein complex 1 sigma-1B small chain) (Sigma 1B s...    GB acc:   AI045228    UniGene:   Rn.17309    Gene Symbol:   LOC302671    Locus Link:   302671    Chromosome:   X    Cytoband:   Chr:Xq21    SOURCE    GENECARD       Gene Ontology:     BioCarta Pathways:     KEGG Pathways:  
    Probe set : 1369663_at    Name:   epoxide hydrolase 2, cytoplasmic    GB acc:   NM_022936    UniGene:   Rn.54495    Gene Symbol:   Ephx2    Locus Link:   65030    Chromosome:   15    Cytoband:   Chr:15p12    SOURCE    GENECARD       Gene Ontology:     BioCarta Pathways:     KEGG Pathways:  
    Probe set : 1373439_at    Name:   Lamin B receptor    GB acc:   AI178491    UniGene:   Rn.6499    Gene Symbol:   Lbr    Locus Link:   89789    Chromosome:   13    Cytoband:   Chr:13q26    SOURCE    GENECARD       Gene Ontology:     BioCarta Pathways:     KEGG Pathways:  
    Probe set : 1382535_at    Name:   Potassium channel tetramerisation domain containing 12 (predicted)    GB acc:   AI030942    UniGene:   Rn.13832     Locus Link:   364458    Chromosome:   15    Cytoband:   Chr:15q22    SOURCE        Gene Ontology:   biological process:  potassium ion transport   cellular component:  voltage-gated potassium channel complex   cellular component:  membrane   molecular function:  voltage-gated potassium channel activity      BioCarta Pathways:     KEGG Pathways:  
    Probe set : 1372112_at    Name:   similar to 9230105E10Rik protein (predicted)    GB acc:   AA945761    UniGene:   Rn.12117    Gene Symbol:   RGD1304579_predicted    Locus Link:   308906    Chromosome:   1    Cytoband:   Chr:1q32    SOURCE    GENECARD       Gene Ontology:   biological process:  regulation of transcription, DNA-dependent   biological process:  protein ubiquitination   cellular component:  nucleus   cellular component:  ubiquitin ligase complex   cellular component:  intracellular   molecular function:  ubiquitin-protein ligase activity   molecular function:  zinc ion binding   molecular function:  metal ion binding      BioCarta Pathways:     KEGG Pathways:  
    Probe set : 1379818_at     GB acc:   BF288109              Gene Ontology:     BioCarta Pathways:     KEGG Pathways:  
    Probe set : 1384164_at    Name:   Transcribed locus    GB acc:   BF290416    UniGene:   Rn.6639        SOURCE        Gene Ontology:     BioCarta Pathways:     KEGG Pathways:  
    Probe set : 1371970_at    Name:   similar to expressed sequence AW413625    GB acc:   AA799328    UniGene:   Rn.112856    Gene Symbol:   LOC499322    Locus Link:   499322    Chromosome:   1    Cytoband:   Chr:1q43    SOURCE    GENECARD       Gene Ontology:     BioCarta Pathways:     KEGG Pathways:  
    Probe set : 1370449_at    Name:   G protein-coupled receptor 105    GB acc:   U76206    UniGene:   Rn.87082    Gene Symbol:   Gpr105    Locus Link:   171108    Chromosome:   2    Cytoband:   Chr:2q31    SOURCE    GENECARD       Gene Ontology:   cellular component:  integral to membrane   cellular component:  integral to membrane   cellular component:  membrane      BioCarta Pathways:     KEGG Pathways:  
    Probe set : 1383585_s_at    Name:   Sorting nexin 10 (predicted)    GB acc:   AI043753    UniGene:   Rn.44192     Locus Link:   297096    Chromosome:   4    Cytoband:   Chr:4q24    SOURCE        Gene Ontology:   molecular function:  protein transporter activity      BioCarta Pathways:     KEGG Pathways:  
    Probe set : 1369586_at    Name:   mast cell protease 8    GB acc:   NM_021598    UniGene:   Rn.55163    Gene Symbol:   Mcpt8    Locus Link:   29269    Chromosome:   15    Cytoband:   Chr:15p12    SOURCE    GENECARD       Gene Ontology:   biological process:  proteolysis      BioCarta Pathways:     KEGG Pathways:  
    Probe set : 1398828_at    Name:   FK506 binding protein 1a    GB acc:   U09386    UniGene:   Rn.80611    Gene Symbol:   Fkbp1a    Locus Link:   25639    Chromosome:   3    Cytoband:   Chr:3q41    SOURCE    GENECARD       Gene Ontology:   biological process:  protein folding   biological process:  transport   biological process:  positive regulation of I-kappaB kinase/NF-kappaB cascade   cellular component:  microsome   cellular component:  membrane      BioCarta Pathways:     KEGG Pathways:  
    Probe set : 1389510_at    Name:   Ly1 antibody reactive clone (predicted)    GB acc:   BE111765    UniGene:   Rn.137862    Gene Symbol:   Lyar_predicted    Locus Link:   289707    Chromosome:   14    Cytoband:   Chr:14q21    SOURCE    GENECARD       Gene Ontology:   cellular component:  nucleus      BioCarta Pathways:     KEGG Pathways:  
    Probe set : 1387227_at    Name:   Wiskott-Aldrich syndrome protein interacting protein    GB acc:   NM_057192    UniGene:   Rn.7914    Gene Symbol:   Waspip    Locus Link:   117538    Chromosome:   3    Cytoband:   Chr:3q23    SOURCE    GENECARD       Gene Ontology:   cellular component:  actin filament   cellular component:  actin cytoskeleton   molecular function:  actin binding   molecular function:  profilin binding      BioCarta Pathways:     KEGG Pathways:  
    Probe set : 1375796_at    Name:   interferon gamma induced GTPase (predicted) /// similar to Ac2-233    GB acc:   BI300770    UniGene:   Rn.113342    Gene Symbol:   Igtp_predicted /// LOC501695    Locus Link:   303163 /// 501695    Chromosome:   10    Cytoband:   Chr:10q22    SOURCE    GENECARD       Gene Ontology:   cellular component:  cytosol   molecular function:  GTPase activity      BioCarta Pathways:     KEGG Pathways:  
    Probe set : 1393038_at    Name:   Fc receptor, IgG, high affinity I (predicted)    GB acc:   BM391631    UniGene:   Rn.16643    Gene Symbol:   Fcgr1_predicted    Locus Link:   295279    Chromosome:   2    Cytoband:   Chr:2q34    SOURCE    GENECARD       Gene Ontology:   biological process:  antibody-dependent cellular cytotoxicity   biological process:  positive regulation of type IIa hypersensitivity   biological process:  positive regulation of type III hypersensitivity   biological process:  receptor mediated endocytosis   biological process:  phagocytosis, recognition   biological process:  phagocytosis, engulfment   biological process:  immune response   biological process:  signal transduction   biological process:  cell surface receptor linked signal transduction   biological process:  antigen presentation, exogenous antigen   biological process:  antigen presentation, exogenous antigen via MHC class I   biological process:  defense response to pathogenic bacteria   biological process:  positive regulation of phagocytosis   biological process:  regulation of immune response      BioCarta Pathways:     KEGG Pathways:  
    Probe set : 1387005_at    Name:   cathepsin S    GB acc:   NM_017320    UniGene:   Rn.11347    Gene Symbol:   Ctss    Locus Link:   50654    Chromosome:   2    Cytoband:   Chr:2q34    SOURCE    GENECARD       Gene Ontology:   biological process:  proteolysis   cellular component:  extracellular space   cellular component:  lysosome   cellular component:  membrane      BioCarta Pathways:     KEGG Pathways:  
    Probe set : 1368565_at    Name:   solute carrier family 1 (glial high affinity glutamate transporter), member 3    GB acc:   NM_019225    UniGene:   Rn.34134    Gene Symbol:   Slc1a3    Locus Link:   29483    Chromosome:   2    Cytoband:   Chr:2q16    SOURCE    GENECARD       Gene Ontology:     BioCarta Pathways:     KEGG Pathways:  
    Probe set : 1387690_at    Name:   caspase 3    GB acc:   U84410    UniGene:   Rn.10562    Gene Symbol:   Casp3    Locus Link:   25402    Chromosome:   16    Cytoband:   Chr:16q11    SOURCE    GENECARD       Gene Ontology:   biological process:  B cell homeostasis   biological process:  proteolysis   biological process:  apoptosis   biological process:  induction of apoptosis   biological process:  induction of apoptosis   biological process:  intracellular signaling cascade   biological process:  intracellular signaling cascade   biological process:  induction of apoptosis via death domain receptors   biological process:  induction of apoptosis by oxidative stress   biological process:  response to wounding   biological process:  neuron differentiation   biological process:  keratinocyte differentiation   biological process:  negative regulation of B cell proliferation   biological process:  T cell homeostasis   biological process:  cell fate commitment   biological process:  negative regulation of cyclin dependent protein kinase activity   biological process:  negative regulation of progression through cell cycle   biological process:  negative regulation of activated T cell proliferation   cellular component:  cytoplasm   cellular component:  cytosol      BioCarta Pathways:     KEGG Pathways:  
    Probe set : 1378047_at    Name:   Spi-C transcription factor (Spi-1/PU.1 related) (predicted)    GB acc:   AA901337    UniGene:   Rn.12056    Gene Symbol:   Spic_predicted    Locus Link:   314711    Chromosome:   7    Cytoband:   Chr:7q13    SOURCE    GENECARD       Gene Ontology:   biological process:  regulation of transcription, DNA-dependent   cellular component:  nucleus   cellular component:  transcription factor complex   molecular function:  DNA binding   molecular function:  transcription factor activity      BioCarta Pathways:     KEGG Pathways:  
    Probe set : 1379957_at    Name:   similar to schlafen 8    GB acc:   BE107457    UniGene:   Rn.137139    Gene Symbol:   RGD:1359139    Locus Link:   303378    Chromosome:   10    Cytoband:   Chr:10q26    SOURCE    GENECARD       Gene Ontology:     BioCarta Pathways:     KEGG Pathways:  
    Probe set : 1374165_at    Name:   similar to Rasa4 protein    GB acc:   AA818264    UniGene:   Rn.1366    Gene Symbol:   LOC288589    Locus Link:   288589    Chromosome:   12    Cytoband:   Chr:12q12    SOURCE    GENECARD       Gene Ontology:     BioCarta Pathways:     KEGG Pathways:  
    Probe set : 1370539_at    Name:   RAB8B, member RAS oncogene family    GB acc:   U53475    UniGene:   Rn.10995    Gene Symbol:   Rab8b    Locus Link:   266688    Chromosome:   8    Cytoband:   Chr:8q24    SOURCE    GENECARD       Gene Ontology:     BioCarta Pathways:     KEGG Pathways:  
    Probe set : 1387678_at    Name:   neuroblastoma RAS viral (v-ras) oncogene homolog    GB acc:   NM_080766    UniGene:   Rn.11271    Gene Symbol:   Nras    Locus Link:   24605    Chromosome:   2    Cytoband:   Chr:2q34    SOURCE    GENECARD       Gene Ontology:   cellular component:  membrane fraction   cellular component:  Golgi stack   cellular component:  membrane      BioCarta Pathways:     KEGG Pathways:  
    Probe set : 1376908_at    Name:   similar to This ORF is capable of encoding 404aa which is homologous to two human interferon-inducible proteins, 54 kDa and 56 kDa proteins; ORF    GB acc:   AW531805    UniGene:   Rn.35775    Gene Symbol:   RGD:1359681    Locus Link:   309526    Chromosome:   1    Cytoband:   Chr:1q52    SOURCE    GENECARD       Gene Ontology:   molecular function:  binding      BioCarta Pathways:     KEGG Pathways:  
    Probe set : 1387878_at    Name:   glutamate dehydrogenase 1    GB acc:   AW916644    UniGene:   Rn.55106    Gene Symbol:   Glud1    Locus Link:   24399    Chromosome:   16    Cytoband:   Chr:16p16    SOURCE    GENECARD       Gene Ontology:   biological process:  amino acid metabolism   biological process:  glutamate catabolism   cellular component:  mitochondrion      BioCarta Pathways:     KEGG Pathways:  
    Probe set : 1372478_at    Name:   similar to LNV    GB acc:   AI137605    UniGene:   Rn.12081    Gene Symbol:   LOC501065    Locus Link:   501065    Chromosome:   8    Cytoband:   Chr:8q32    SOURCE    GENECARD       Gene Ontology:     BioCarta Pathways:     KEGG Pathways:  
    Probe set : 1389488_at    Name:   hypothetical protein LOC498544    GB acc:   AA800750    UniGene:   Rn.132048    Gene Symbol:   LOC498544    Locus Link:   498544    Chromosome:   15    Cytoband:   Chr:15q11    SOURCE    GENECARD       Gene Ontology:     BioCarta Pathways:     KEGG Pathways:  
    Probe set : 1372093_at    Name:   Max interacting protein 1    GB acc:   AI409308    UniGene:   Rn.37510    Gene Symbol:   Mxi1    Locus Link:   25701    Chromosome:   1    Cytoband:   Chr:1q55    SOURCE    GENECARD       Gene Ontology:   cellular component:  nucleus   cellular component:  nucleus      BioCarta Pathways:     KEGG Pathways:  
    Probe set : 1382603_at    Name:   Programmed cell death 1 ligand 2 (predicted)    GB acc:   AW521319    UniGene:   Rn.130112     Locus Link:   309304    Chromosome:   1    Cytoband:   Chr:1q52    SOURCE        Gene Ontology:   biological process:  immune response      BioCarta Pathways:     KEGG Pathways:  
    Probe set : 1371336_at    Name:   hematological and neurological expressed sequence 1    GB acc:   AA942716    UniGene:   Rn.138819    Gene Symbol:   RGD:1359325    Locus Link:   287828    Chromosome:   10    Cytoband:   Chr:10q32.3    SOURCE    GENECARD       Gene Ontology:     BioCarta Pathways:     KEGG Pathways:  
    Probe set : 1384180_at    Name:   interferon-induced protein with tetratricopeptide repeats 2 (predicted)    GB acc:   BE118697    UniGene:   Rn.6175    Gene Symbol:   Ifit2_predicted    Locus Link:   294091    Chromosome:   1    Cytoband:   Chr:1q52    SOURCE    GENECARD       Gene Ontology:   biological process:  immune response   molecular function:  binding      BioCarta Pathways:     KEGG Pathways:  
    Probe set : 1382546_at    Name:   PHD finger protein 11 (predicted)    GB acc:   AW920026    UniGene:   Rn.145792    Gene Symbol:   Phf11_predicted    Locus Link:   361051    Chromosome:   15    Cytoband:   Chr:15p12    SOURCE    GENECARD       Gene Ontology:   biological process:  regulation of transcription, DNA-dependent   molecular function:  DNA binding   molecular function:  protein binding   molecular function:  zinc ion binding      BioCarta Pathways:     KEGG Pathways:  
    Probe set : 1388416_at    Name:   low density lipoprotein receptor-related protein 1 (predicted)    GB acc:   BI282758    UniGene:   Rn.22436    Gene Symbol:   Lrp1_predicted    Locus Link:   299858    Chromosome:   7    Cytoband:   Chr:7q22    SOURCE    GENECARD       Gene Ontology:     BioCarta Pathways:     KEGG Pathways:  
    Probe set : 1367574_at    Name:   vimentin    GB acc:   NM_031140    UniGene:   Rn.2710    Gene Symbol:   Vim    Locus Link:   81818    Chromosome:   17    Cytoband:   Chr:17q12.3    SOURCE    GENECARD       Gene Ontology:   biological process:  intermediate filament-based process      BioCarta Pathways:     KEGG Pathways:  
    Probe set : 1372034_at    Name:   Similar to hypothetical protein MGC29390 (predicted)    GB acc:   BF284106    UniGene:   Rn.54182     Locus Link:   303903    Chromosome:   11    Cytoband:   Chr:11q22    SOURCE        Gene Ontology:     BioCarta Pathways:     KEGG Pathways:  
    Probe set : 1376006_at    Name:   Transcribed locus    GB acc:   BG373799    UniGene:   Rn.46830        SOURCE        Gene Ontology:     BioCarta Pathways:     KEGG Pathways:  
    Probe set : 1378927_at     GB acc:   BE329364              Gene Ontology:     BioCarta Pathways:     KEGG Pathways:  
    Probe set : 1370913_at    Name:   Best5 protein    GB acc:   AI409634    UniGene:   Rn.14882    Gene Symbol:   RGD:620495    Locus Link:   65190    Chromosome:   6    Cytoband:   Chr:6q14    SOURCE    GENECARD       Gene Ontology:   biological process:  ossification   biological process:  regulation of ossification   molecular function:  catalytic activity   molecular function:  iron ion binding      BioCarta Pathways:     KEGG Pathways:  
    Probe set : 1388255_x_at    Name:   RT1 class I, CE5    GB acc:   AJ243338    UniGene:   Rn.145027    Gene Symbol:   RT1-CE5    Locus Link:   309607    Chromosome:   20    Cytoband:   Chr:20p12    SOURCE    GENECARD       Gene Ontology:   biological process:  antigen presentation   cellular component:  MHC class I protein complex   molecular function:  MHC class I receptor activity      BioCarta Pathways:     KEGG Pathways:  
    Probe set : 1371596_at    Name:   ribonucleic acid binding protein S1 (predicted)    GB acc:   AI008971    UniGene:   Rn.7235    Gene Symbol:   Rnps1_predicted    Locus Link:   287113    Chromosome:   10    Cytoband:   Chr:10q12    SOURCE    GENECARD       Gene Ontology:   cellular component:  nucleus      BioCarta Pathways:     KEGG Pathways:  
    Probe set : 1388340_at    Name:   Ns5atp9 protein    GB acc:   BF281153    UniGene:   Rn.7233    Gene Symbol:   RGD:1303041    Locus Link:   300795    Chromosome:   8    Cytoband:   Chr:8q24    SOURCE    GENECARD       Gene Ontology:     BioCarta Pathways:     KEGG Pathways:  
    Probe set : 1372404_at    Name:   RAS-related C3 botulinum substrate 2 (predicted)    GB acc:   AI010476    UniGene:   Rn.2863    Gene Symbol:   Rac2_predicted    Locus Link:   366957    Chromosome:   7    Cytoband:   Chr:7q34    SOURCE    GENECARD       Gene Ontology:   cellular component:  membrane fraction   cellular component:  nuclear membrane   cellular component:  cytoplasm   molecular function:  GTPase activity   molecular function:  GTP binding      BioCarta Pathways:     KEGG Pathways:  
    Probe set : 1385143_at     GB acc:   AI548940              Gene Ontology:     BioCarta Pathways:     KEGG Pathways:  
    Probe set : 1369214_a_at    Name:   MHC class II transactivator    GB acc:   NM_053529    UniGene:   Rn.16740    Gene Symbol:   Mhc2ta    Locus Link:   85483    Chromosome:   10    Cytoband:   Chr:10q11    SOURCE    GENECARD       Gene Ontology:   cellular component:  nucleus      BioCarta Pathways:     KEGG Pathways:  
    Probe set : 1388066_a_at    Name:   G protein-coupled receptor kinase 6    GB acc:   AF040750    UniGene:   Rn.10633    Gene Symbol:   Gprk6    Locus Link:   59076    Chromosome:   17    Cytoband:   Chr:17p14    SOURCE    GENECARD       Gene Ontology:     BioCarta Pathways:     KEGG Pathways:  
    Probe set : 1383578_at    Name:   Transcribed locus    GB acc:   AI071227    UniGene:   Rn.20474        SOURCE        Gene Ontology:     BioCarta Pathways:     KEGG Pathways:  
    Probe set : 1386916_at    Name:   aconitase 1    GB acc:   NM_017321    UniGene:   Rn.35934    Gene Symbol:   Aco1    Locus Link:   50655    Chromosome:   5    Cytoband:   Chr:5q22    SOURCE    GENECARD       Gene Ontology:   cellular component:  cytoplasm   cellular component:  cytoplasm      BioCarta Pathways:     KEGG Pathways:  
    Probe set : 1376943_at     GB acc:   BM386570              Gene Ontology:     BioCarta Pathways:     KEGG Pathways:  
    Probe set : 1377048_at    Name:   similar to cDNA sequence BC021917 (predicted)    GB acc:   H31813    UniGene:   Rn.55630    Gene Symbol:   RGD1311026_predicted    Locus Link:   361730    Chromosome:   1    Cytoband:   Chr:1q43    SOURCE    GENECARD       Gene Ontology:   biological process:  glycerol metabolism   molecular function:  glycerone kinase activity      BioCarta Pathways:     KEGG Pathways:  
    Probe set : 1369726_at    Name:   TAP binding protein    GB acc:   NM_033098    UniGene:   Rn.51023    Gene Symbol:   Tapbp    Locus Link:   25217    Chromosome:   20    Cytoband:   Chr:20p12    SOURCE    GENECARD       Gene Ontology:     BioCarta Pathways:     KEGG Pathways:  
    Probe set : 1382026_at    Name:   Rho GTPase activating protein 9 (predicted)    GB acc:   BE116750    UniGene:   Rn.143729    Gene Symbol:   Arhgap9_predicted    Locus Link:   362893    Chromosome:   7    Cytoband:   Chr:7q22    SOURCE    GENECARD       Gene Ontology:   molecular function:  GTPase activator activity      BioCarta Pathways:     KEGG Pathways:  
    Probe set : 1387984_at    Name:   chemokine-like factor 1    GB acc:   AF253065    UniGene:   Rn.81197    Gene Symbol:   Cklf1    Locus Link:   245978    Chromosome:   19    Cytoband:   Chr:19p14    SOURCE    GENECARD       Gene Ontology:   cellular component:  integral to membrane   cellular component:  extracellular space   cellular component:  membrane   molecular function:  cytokine activity      BioCarta Pathways:     KEGG Pathways:  
    Probe set : 1373504_at    Name:   GLI pathogenesis-related 1 (glioma) (predicted)    GB acc:   BF287967    UniGene:   Rn.65764    Gene Symbol:   Glipr1_predicted    Locus Link:   299783    Chromosome:   7    Cytoband:   Chr:7q21    SOURCE    GENECARD       Gene Ontology:   cellular component:  extracellular region   cellular component:  extracellular space   cellular component:  integral to membrane      BioCarta Pathways:     KEGG Pathways:  
    Probe set : 1395426_at    Name:   similar to ubiquitin-conjugating enzyme E2 variant 1    GB acc:   AW142658     Gene Symbol:   LOC499945    Locus Link:   499945    Chromosome:   3    Cytoband:   Chr:3q42     GENECARD       Gene Ontology:     BioCarta Pathways:     KEGG Pathways:  
    Probe set : 1385925_at    Name:   Transcribed locus    GB acc:   AA963975    UniGene:   Rn.23216        SOURCE        Gene Ontology:     BioCarta Pathways:     KEGG Pathways:  
    Probe set : 1390292_at    Name:   transmembrane protein 8 (five membrane-spanning domains) (predicted)    GB acc:   AW527467    UniGene:   Rn.21890    Gene Symbol:   Tmem8_predicted    Locus Link:   303004    Chromosome:   10    Cytoband:   Chr:10q12    SOURCE    GENECARD       Gene Ontology:   biological process:  cell adhesion   cellular component:  extracellular space   cellular component:  integral to plasma membrane   cellular component:  integral to membrane   molecular function:  protein binding      BioCarta Pathways:     KEGG Pathways:  
    Probe set : 1368975_at    Name:   CD38 antigen    GB acc:   NM_013127    UniGene:   Rn.11414    Gene Symbol:   Cd38    Locus Link:   25668    Chromosome:   14    Cytoband:   Chr:14q21    SOURCE    GENECARD       Gene Ontology:   cellular component:  membrane fraction   cellular component:  nucleus   cellular component:  plasma membrane   cellular component:  integral to membrane      BioCarta Pathways:     KEGG Pathways:  
    Probe set : 1372741_at    Name:   similar to RIKEN cDNA C330023F11 (predicted)    GB acc:   AI412013    UniGene:   Rn.23989    Gene Symbol:   RGD1311440_predicted    Locus Link:   305021    Chromosome:   13    Cytoband:   Chr:13q26    SOURCE    GENECARD       Gene Ontology:   molecular function:  oxidoreductase activity   molecular function:  saccharopine dehydrogenase (NAD+, L-glutamate-forming) activity      BioCarta Pathways:     KEGG Pathways:  
    Probe set : 1382277_at     GB acc:   AA894336              Gene Ontology:     BioCarta Pathways:     KEGG Pathways:  
    Probe set : 1368693_at    Name:   Gardner-Rasheed feline sarcoma viral (Fgr) oncogene homolog    GB acc:   NM_024145    UniGene:   Rn.11309    Gene Symbol:   Fgr    Locus Link:   79113    Chromosome:   5    Cytoband:   Chr:5q36    SOURCE    GENECARD       Gene Ontology:   cellular component:  intracellular      BioCarta Pathways:     KEGG Pathways:  
    Probe set : 1367710_at    Name:   protease (prosome, macropain) 28 subunit, beta    GB acc:   NM_017257    UniGene:   Rn.127779    Gene Symbol:   Psme2    Locus Link:   29614    Chromosome:   15    Cytoband:   Chr:15p13    SOURCE    GENECARD       Gene Ontology:   biological process:  immune response   cellular component:  cytosol   cellular component:  proteasome activator complex   cellular component:  protein complex   molecular function:  proteasome activator activity   molecular function:  proteasome activator activity      BioCarta Pathways:     KEGG Pathways:  
    Probe set : 1381556_at    Name:   Similar to BC013672 protein    GB acc:   BI303853    UniGene:   Rn.73586     Locus Link:   498673      SOURCE        Gene Ontology:     BioCarta Pathways:     KEGG Pathways:  
    Probe set : 1367715_at    Name:   tumor necrosis factor receptor superfamily, member 1a    GB acc:   NM_013091    UniGene:   Rn.11119    Gene Symbol:   Tnfrsf1a    Locus Link:   25625    Chromosome:   4    Cytoband:   Chr:4q42    SOURCE    GENECARD       Gene Ontology:     BioCarta Pathways:     KEGG Pathways:  
    Probe set : 1368808_at    Name:   CAP, adenylate cyclase-associated protein 1 (yeast)    GB acc:   NM_022383    UniGene:   Rn.112585    Gene Symbol:   Cap1    Locus Link:   64185    Chromosome:   5    Cytoband:   Chr:5q36    SOURCE    GENECARD       Gene Ontology:   cellular component:  cytoplasm   cellular component:  membrane   cellular component:  cortical actin cytoskeleton   molecular function:  adenylate cyclase binding   molecular function:  actin binding      BioCarta Pathways:     KEGG Pathways:  
    Probe set : 1368136_at    Name:   thymopoietin    GB acc:   NM_012887    UniGene:   Rn.3364    Gene Symbol:   Tmpo    Locus Link:   25359    Chromosome:   7    Cytoband:   Chr:7q13    SOURCE    GENECARD       Gene Ontology:   biological process:  regulation of transcription      BioCarta Pathways:     KEGG Pathways:  
    Probe set : 1374307_at    Name:   CaM-kinase II inhibitor alpha    GB acc:   AI236027    UniGene:   Rn.12600    Gene Symbol:   LOC287005    Locus Link:   287005    Chromosome:   5    Cytoband:   Chr:5q36    SOURCE    GENECARD       Gene Ontology:   cellular component:  dendrite   cellular component:  cell soma   molecular function:  protein kinase binding   molecular function:  kinase activity      BioCarta Pathways:     KEGG Pathways:  
    Probe set : 1369944_at    Name:   MARCKS-like protein    GB acc:   NM_030862    UniGene:   Rn.2486    Gene Symbol:   Mlp    Locus Link:   81520    Chromosome:   5    Cytoband:   Chr:5q36    SOURCE    GENECARD       Gene Ontology:   biological process:  vesicle-mediated transport   molecular function:  calmodulin binding      BioCarta Pathways:     KEGG Pathways:  
    Probe set : 1390707_at    Name:   regulator of G-protein signalling 10    GB acc:   BI281632    UniGene:   Rn.23957    Gene Symbol:   Rgs10    Locus Link:   54290    Chromosome:   1    Cytoband:   Chr:1q36    SOURCE    GENECARD       Gene Ontology:   molecular function:  signal transducer activity   molecular function:  GTPase activator activity      BioCarta Pathways:     KEGG Pathways:  
    Probe set : 1368043_at    Name:   sorting nexin 1    GB acc:   NM_053411    UniGene:   Rn.144614    Gene Symbol:   Snx1    Locus Link:   84471    Chromosome:   8    Cytoband:   Chr:8q24    SOURCE    GENECARD       Gene Ontology:   cellular component:  early endosome   cellular component:  early endosome   cellular component:  Golgi apparatus   cellular component:  Golgi stack      BioCarta Pathways:     KEGG Pathways:  
    Probe set : 1372097_at    Name:   Interferon consensus sequence binding protein 1 (predicted)    GB acc:   BF284262    UniGene:   Rn.3765    Gene Symbol:   Icsbp1    Locus Link:   292060    Chromosome:   19    Cytoband:   Chr:19q12    SOURCE    GENECARD       Gene Ontology:   cellular component:  nucleus   molecular function:  DNA binding   molecular function:  transcription factor activity      BioCarta Pathways:     KEGG Pathways:  
    Probe set : 1374337_at    Name:   Similar to chromosome 17 open reading frame 27 (predicted)    GB acc:   AI408954    UniGene:   Rn.67402     Locus Link:   303735    Chromosome:   10    Cytoband:   Chr:10q32.3    SOURCE        Gene Ontology:     BioCarta Pathways:     KEGG Pathways:  
    Probe set : 1376109_at    Name:   Transcribed locus, weakly similar to NP_062289.1 DNA binding protein with his-thr domain [Mus musculus]    GB acc:   BM387711    UniGene:   Rn.48053        SOURCE        Gene Ontology:     BioCarta Pathways:     KEGG Pathways:  
    Probe set : 1376880_at    Name:   similar to T2K protein kinase homolog    GB acc:   AI639447    UniGene:   Rn.30683    Gene Symbol:   LOC299827    Locus Link:   299827    Chromosome:   7    Cytoband:   Chr:7q22    SOURCE    GENECARD       Gene Ontology:     BioCarta Pathways:     KEGG Pathways:  
    Probe set : 1384603_at    Name:   ATP-binding cassette, sub-family A (ABC1), member 4 (predicted)    GB acc:   AI602131    UniGene:   Rn.40415    Gene Symbol:   Abca4_predicted    Locus Link:   310836    Chromosome:   2    Cytoband:   Chr:2q41    SOURCE    GENECARD       Gene Ontology:   biological process:  transport   biological process:  visual perception   biological process:  phototransduction, visible light   cellular component:  membrane fraction   cellular component:  integral to plasma membrane   molecular function:  ATP binding   molecular function:  ATPase activity   molecular function:  ATPase activity, coupled to transmembrane movement of substances      BioCarta Pathways:     KEGG Pathways:  
    Probe set : 1372254_at    Name:   serine (or cysteine) peptidase inhibitor, clade G, member 1    GB acc:   AW915763    UniGene:   Rn.100285    Gene Symbol:   Serping1    Locus Link:   295703    Chromosome:   3    Cytoband:   Chr:3q24    SOURCE    GENECARD       Gene Ontology:   biological process:  negative regulation of complement activation, classical pathway   molecular function:  serine-type endopeptidase inhibitor activity   molecular function:  serine-type endopeptidase inhibitor activity      BioCarta Pathways:     KEGG Pathways:  
    Probe set : 1375529_at    Name:   carbonic reductase 4    GB acc:   BF404908    UniGene:   Rn.3599    Gene Symbol:   Cbr4    Locus Link:   359725    Chromosome:   16    Cytoband:   Chr:16p12    SOURCE    GENECARD       Gene Ontology:   biological process:  metabolism   molecular function:  oxidoreductase activity      BioCarta Pathways:     KEGG Pathways:  
    Probe set : 1374284_at    Name:   Ras association (RalGDS/AF-6) domain family 4 (predicted)    GB acc:   AI227769    UniGene:   Rn.11482    Gene Symbol:   Rassf4_predicted    Locus Link:   362423    Chromosome:   4    Cytoband:   Chr:4q42    SOURCE    GENECARD       Gene Ontology:   biological process:  signal transduction   biological process:  neuropeptide signaling pathway   molecular function:  protein binding   molecular function:  oxidoreductase activity      BioCarta Pathways:     KEGG Pathways:  
    Probe set : 1368420_at    Name:   ceruloplasmin    GB acc:   NM_012532    UniGene:   Rn.32777    Gene Symbol:   Cp    Locus Link:   24268    Chromosome:   2    Cytoband:   Chr:2q24    SOURCE    GENECARD       Gene Ontology:     BioCarta Pathways:     KEGG Pathways:  
    Probe set : 1373066_at    Name:   Transcribed locus    GB acc:   AI407797    UniGene:   Rn.40480        SOURCE        Gene Ontology:     BioCarta Pathways:     KEGG Pathways:  
    Probe set : 1367780_at    Name:   pituitary tumor-transforming 1    GB acc:   NM_022391    UniGene:   Rn.271    Gene Symbol:   Pttg1    Locus Link:   64193    Chromosome:   10    Cytoband:   Chr:10q21    SOURCE    GENECARD       Gene Ontology:   biological process:  DNA replication and chromosome cycle   biological process:  regulation of cell growth   biological process:  regulation of cell growth   biological process:  DNA metabolism   biological process:  DNA repair   biological process:  transcription from RNA polymerase II promoter   biological process:  chromosome segregation   biological process:  mitosis   biological process:  spermatogenesis   biological process:  negative regulation of cell proliferation   biological process:  negative regulation of cell proliferation   biological process:  homologous chromosome segregation   biological process:  cellular physiological process   biological process:  response to DNA damage stimulus   biological process:  cell cycle   biological process:  cell division   cellular component:  nucleus   cellular component:  cytoplasm   molecular function:  transcription factor activity   molecular function:  cysteine protease inhibitor activity   molecular function:  protein binding      BioCarta Pathways:     KEGG Pathways:  
    Probe set : 1389092_at    Name:   interleukin 2 receptor, gamma (severe combined immunodeficiency)    GB acc:   AI178808    UniGene:   Rn.146125    Gene Symbol:   Il2rg    Locus Link:   140924    Chromosome:   X    Cytoband:   Chr:Xq31    SOURCE    GENECARD       Gene Ontology:     BioCarta Pathways:     KEGG Pathways:  
    Probe set : 1373992_at    Name:   similar to interferon-inducible GTPase /// similar to MGC108823 protein /// similar to MGC108823 protein    GB acc:   AI408440    UniGene:   Rn.2746    Gene Symbol:   RGD:1359421 /// LOC498871 /// LOC498872    Locus Link:   307414 /// 498871 /// 498872    Chromosome:   18    Cytoband:   Chr:18q12.1    SOURCE    GENECARD       Gene Ontology:     BioCarta Pathways:     KEGG Pathways:  
    Probe set : 1376029_at    Name:   RAB2, member RAS oncogene family-like    GB acc:   BI295991    UniGene:   Rn.107413    Gene Symbol:   RGD:1302985    Locus Link:   294283    Chromosome:   20    Cytoband:   Chr:20p12    SOURCE    GENECARD       Gene Ontology:     BioCarta Pathways:     KEGG Pathways:  
    Probe set : 1373110_at    Name:   Transcribed locus    GB acc:   AI407487    UniGene:   Rn.1452        SOURCE        Gene Ontology:     BioCarta Pathways:     KEGG Pathways:  
    Probe set : 1370882_at    Name:   major histocompatibility complex, class II, DM beta    GB acc:   AI171966    UniGene:   Rn.5892    Gene Symbol:   RGD:735096    Locus Link:   294273    Chromosome:   20    Cytoband:   Chr:20p12    SOURCE    GENECARD       Gene Ontology:   cellular component:  MHC protein complex   cellular component:  membrane   cellular component:  integral to membrane   molecular function:  MHC class II receptor activity      BioCarta Pathways:     KEGG Pathways:  
    Probe set : 1371977_at    Name:   actin related protein 2/3 complex, subunit 3 (predicted)    GB acc:   BG381477    UniGene:   Rn.4233    Gene Symbol:   Arpc3_predicted    Locus Link:   288669    Chromosome:   12    Cytoband:   Chr:12q16    SOURCE    GENECARD       Gene Ontology:   biological process:  cell motility   biological process:  regulation of actin filament polymerization   cellular component:  Arp2/3 protein complex   cellular component:  actin cytoskeleton   cellular component:  lamellipodium   molecular function:  structural constituent of cytoskeleton      BioCarta Pathways:     KEGG Pathways:  
    Probe set : 1369936_at    Name:   calmodulin 1    GB acc:   X13933    UniGene:   Rn.4166    Gene Symbol:   Calm1    Locus Link:   24242    Chromosome:   6    Cytoband:   Chr:6q31-q32    SOURCE    GENECARD       Gene Ontology:     BioCarta Pathways:     KEGG Pathways:  
    Probe set : 1370320_at    Name:   MAWD binding protein    GB acc:   AY083160    UniGene:   Rn.2417    Gene Symbol:   Mawbp    Locus Link:   171564    Chromosome:   20    Cytoband:   Chr:20p11    SOURCE    GENECARD       Gene Ontology:   biological process:  biosynthesis   molecular function:  catalytic activity   molecular function:  oxidoreductase activity      BioCarta Pathways:     KEGG Pathways:  
    Probe set : 1380077_at    Name:   Transcribed locus    GB acc:   AI412131    UniGene:   Rn.43019        SOURCE        Gene Ontology:     BioCarta Pathways:     KEGG Pathways:  
    Probe set : 1388202_at    Name:   RT1 class Ib, locus Aw2    GB acc:   BI395698    UniGene:   Rn.145377    Gene Symbol:   RT1-Aw2    Locus Link:   24737    Chromosome:   20    Cytoband:   Chr:20p12    SOURCE    GENECARD       Gene Ontology:   biological process:  antigen presentation   cellular component:  MHC class I protein complex   molecular function:  MHC class I receptor activity      BioCarta Pathways:     KEGG Pathways:  
    Probe set : 1389387_at    Name:   CDNA clone MGC:93913 IMAGE:7112877    GB acc:   BF561377    UniGene:   Rn.103325        SOURCE        Gene Ontology:   cellular component:  cytosol   cellular component:  protein complex      BioCarta Pathways:     KEGG Pathways:  
    Probe set : 1373037_at    Name:   ubiquitin-conjugating enzyme E2L 6 (predicted)    GB acc:   BI279216    UniGene:   Rn.8609    Gene Symbol:   Ube2l6_predicted    Locus Link:   295704    Chromosome:   3    Cytoband:   Chr:3q24    SOURCE    GENECARD       Gene Ontology:   biological process:  protein modification   biological process:  ubiquitin cycle      BioCarta Pathways:     KEGG Pathways:  
    Probe set : 1369110_x_at    Name:   RT1 class Ib, locus Aw2    GB acc:   NM_012645    UniGene:   Rn.40130    Gene Symbol:   RT1-Aw2    Locus Link:   24737    Chromosome:   20    Cytoband:   Chr:20p12    SOURCE    GENECARD       Gene Ontology:   cellular component:  integral to membrane   cellular component:  MHC class I protein complex   molecular function:  MHC class I receptor activity      BioCarta Pathways:     KEGG Pathways:  
    Probe set : 1391737_at    Name:   similar to p40-phox    GB acc:   AW526210    UniGene:   Rn.46120    Gene Symbol:   LOC500904    Locus Link:   500904    Chromosome:   7    Cytoband:   Chr:7q34    SOURCE    GENECARD       Gene Ontology:     BioCarta Pathways:     KEGG Pathways:  
    Probe set : 1388868_at    Name:   zinc finger protein 216 (predicted)    GB acc:   AI172425    UniGene:   Rn.4050    Gene Symbol:   Zfp216_predicted    Locus Link:   293960    Chromosome:   1    Cytoband:   Chr:1q51    SOURCE    GENECARD       Gene Ontology:   molecular function:  nucleic acid binding   molecular function:  DNA binding   molecular function:  zinc ion binding      BioCarta Pathways:     KEGG Pathways:  
    Probe set : 1392960_at    Name:   similar to RIKEN cDNA 2310004I24 gene (predicted)    GB acc:   AI713860    UniGene:   Rn.32842    Gene Symbol:   RGD1309906_predicted    Locus Link:   287406    Chromosome:   10    Cytoband:   Chr:10q24    SOURCE    GENECARD       Gene Ontology:   molecular function:  hydrolase activity      BioCarta Pathways:     KEGG Pathways:  
    Probe set : 1377669_at    Name:   low Mr GTP-binding protein    GB acc:   BM389524    UniGene:   Rn.37360    Gene Symbol:   Rab27a    Locus Link:   50645    Chromosome:   8    Cytoband:   Chr:8q24    SOURCE    GENECARD       Gene Ontology:   biological process:  small GTPase mediated signal transduction   biological process:  protein transport   molecular function:  GTPase activity   molecular function:  GTP binding   molecular function:  GTP binding      BioCarta Pathways:     KEGG Pathways:  
    Probe set : 1382622_at    Name:   cystatin F (leukocystatin) (predicted)    GB acc:   AI502757    UniGene:   Rn.24282    Gene Symbol:   Cst7_predicted    Locus Link:   296257    Chromosome:   3    Cytoband:   Chr:3q41    SOURCE    GENECARD       Gene Ontology:   cellular component:  extracellular space   molecular function:  endopeptidase inhibitor activity   molecular function:  cysteine protease inhibitor activity      BioCarta Pathways:     KEGG Pathways:  
    Probe set : 1392881_at    Name:   alpha-2,3-sialyltransferase ST3Gal IV    GB acc:   BF289499    UniGene:   Rn.24125    Gene Symbol:   RGD:1303068    Locus Link:   363040    Chromosome:   8    Cytoband:   Chr:8q21    SOURCE    GENECARD       Gene Ontology:   biological process:  protein amino acid glycosylation   cellular component:  Golgi stack   cellular component:  integral to membrane   cellular component:  integral to Golgi membrane   molecular function:  sialyltransferase activity   molecular function:  transferase activity   molecular function:  transferase activity, transferring glycosyl groups      BioCarta Pathways:     KEGG Pathways:  
    Probe set : 1383131_at    Name:   integrin beta 2 (predicted)    GB acc:   AI177292    UniGene:   Rn.42962    Gene Symbol:   Itgb2_predicted    Locus Link:   309684    Chromosome:   20    Cytoband:   Chr:20p12    SOURCE    GENECARD       Gene Ontology:   cellular component:  extracellular space   cellular component:  integrin complex   cellular component:  integral to membrane   molecular function:  receptor activity   molecular function:  protein binding   molecular function:  protein kinase binding      BioCarta Pathways:     KEGG Pathways:  
    Probe set : 1375211_at    Name:   ribonuclease T2 (predicted)    GB acc:   BM391506    UniGene:   Rn.18019    Gene Symbol:   Rnaset2_predicted    Locus Link:   292306    Chromosome:   1    Cytoband:   Chr:1q11    SOURCE    GENECARD       Gene Ontology:   biological process:  RNA catabolism   cellular component:  extracellular region   cellular component:  mitochondrion      BioCarta Pathways:     KEGG Pathways:  
    Probe set : 1389154_at    Name:   lipoic acid synthetase (predicted)    GB acc:   AW524453    UniGene:   Rn.25547    Gene Symbol:   Lias_predicted    Locus Link:   305348    Chromosome:   14    Cytoband:   Chr:14p11    SOURCE    GENECARD       Gene Ontology:   biological process:  lipoic acid biosynthesis   biological process:  lipoate biosynthesis   cellular component:  mitochondrion      BioCarta Pathways:     KEGG Pathways:  
    Probe set : 1398788_at    Name:   glucose regulated protein, 58 kDa    GB acc:   NM_017319    UniGene:   Rn.11527    Gene Symbol:   Grp58    Locus Link:   29468    Chromosome:   3    Cytoband:   Chr:3q35    SOURCE    GENECARD       Gene Ontology:   cellular component:  extracellular space   cellular component:  endoplasmic reticulum      BioCarta Pathways:     KEGG Pathways:  
    Probe set : 1392334_at    Name:   RT1 class II, locus Ba    GB acc:   AW528743    UniGene:   Rn.25717    Gene Symbol:   RT1-Ba    Locus Link:   309621    Chromosome:   20    Cytoband:   Chr:20p12    SOURCE    GENECARD       Gene Ontology:   cellular component:  MHC class II protein complex   cellular component:  membrane   molecular function:  MHC class II receptor activity      BioCarta Pathways:     KEGG Pathways:  
    Probe set : 1387952_a_at    Name:   CD44 antigen    GB acc:   AF065147    UniGene:   Rn.1120    Gene Symbol:   Cd44    Locus Link:   25406    Chromosome:   3    Cytoband:   Chr:3q31    SOURCE    GENECARD       Gene Ontology:     BioCarta Pathways:     KEGG Pathways:  
    Probe set : 1369764_at    Name:   complement component 4 binding protein, alpha    GB acc:   NM_012516    UniGene:   Rn.10408    Gene Symbol:   C4bpa    Locus Link:   24235    Chromosome:   13    Cytoband:   Chr:13q13    SOURCE    GENECARD       Gene Ontology:   cellular component:  extracellular space   molecular function:  complement binding      BioCarta Pathways:     KEGG Pathways:  
    Probe set : 1397892_at    Name:   eukaryotic translation initiation factor 1A (predicted)    GB acc:   BI301150    UniGene:   Rn.3361    Gene Symbol:   Eif1a_predicted    Locus Link:   317163    Chromosome:   18    Cytoband:   Chr:18q11    SOURCE    GENECARD       Gene Ontology:   biological process:  protein biosynthesis   biological process:  translational initiation   cellular component:  eukaryotic translation initiation factor 4F complex   molecular function:  translation factor activity, nucleic acid binding   molecular function:  RNA binding   molecular function:  translation initiation factor activity      BioCarta Pathways:     KEGG Pathways:  
    Probe set : 1368679_a_at    Name:   Yamaguchi sarcoma viral (v-yes-1) oncogene homolog    GB acc:   L14782    UniGene:   Rn.4338    Gene Symbol:   Lyn    Locus Link:   81515    Chromosome:   5    Cytoband:   Chr:5q12    SOURCE    GENECARD       Gene Ontology:   cellular component:  intracellular      BioCarta Pathways:     KEGG Pathways:  
    Probe set : 1388740_at    Name:   similar to cDNA sequence BC032204 (predicted)    GB acc:   AI177645    UniGene:   Rn.105838    Gene Symbol:   RGD1310168_predicted    Locus Link:   309186    Chromosome:   1    Cytoband:   Chr:1q43    SOURCE    GENECARD       Gene Ontology:     BioCarta Pathways:     KEGG Pathways:  
    Probe set : 1371480_at    Name:   LOC499655    GB acc:   AI407985    UniGene:   Rn.128820    Gene Symbol:   LOC499655    Locus Link:   499655    Chromosome:   2    Cytoband:   Chr:2q34    SOURCE    GENECARD       Gene Ontology:     BioCarta Pathways:     KEGG Pathways:  
    Probe set : 1378482_at    Name:   kidney cell line derived transcript 1 (predicted)    GB acc:   AI411065    UniGene:   Rn.16336    Gene Symbol:   Kdt1_predicted    Locus Link:   362325    Chromosome:   4    Cytoband:   Chr:4q21    SOURCE    GENECARD       Gene Ontology:     BioCarta Pathways:     KEGG Pathways:  
    Probe set : 1372475_at    Name:   PTEN induced putative kinase 1 (predicted)    GB acc:   AI410877    UniGene:   Rn.103380    Gene Symbol:   Pink1_predicted    Locus Link:   298575    Chromosome:   5    Cytoband:   Chr:5q36    SOURCE    GENECARD       Gene Ontology:   biological process:  protein amino acid phosphorylation   biological process:  response to stress   biological process:  protein kinase cascade   cellular component:  mitochondrion      BioCarta Pathways:     KEGG Pathways:  
    Probe set : 1370883_at    Name:   RT1 class II, locus Da    GB acc:   Y00480    UniGene:   Rn.103146    Gene Symbol:   RT1-Da    Locus Link:   294269    Chromosome:   20    Cytoband:   Chr:20p12    SOURCE    GENECARD       Gene Ontology:   biological process:  immune response   cellular component:  MHC class II protein complex   cellular component:  membrane      BioCarta Pathways:     KEGG Pathways:  
    Probe set : 1386660_at    Name:   Transcribed locus    GB acc:   BF565021    UniGene:   Rn.146221        SOURCE        Gene Ontology:     BioCarta Pathways:     KEGG Pathways:  
    Probe set : 1389408_at    Name:   ribonucleotide reductase M2    GB acc:   BG379338    UniGene:   Rn.101236    Gene Symbol:   Rrm2    Locus Link:   362720    Chromosome:   6    Cytoband:   Chr:6q16    SOURCE    GENECARD       Gene Ontology:   biological process:  DNA replication   biological process:  deoxyribonucleoside diphosphate metabolism   biological process:  deoxyribonucleotide metabolism   cellular component:  cytoplasm   cellular component:  ribonucleoside-diphosphate reductase complex      BioCarta Pathways:     KEGG Pathways:  
    Probe set : 1391106_at    Name:   Transcribed locus    GB acc:   BM385061    UniGene:   Rn.20457        SOURCE        Gene Ontology:     BioCarta Pathways:     KEGG Pathways:  
    Probe set : 1370885_at    Name:   cathepsin Z    GB acc:   AA849399    UniGene:   Rn.1475    Gene Symbol:   Ctsz    Locus Link:   252929      SOURCE    GENECARD       Gene Ontology:   biological process:  proteolysis   cellular component:  extracellular space      BioCarta Pathways:     KEGG Pathways:  
    Probe set : 1367855_at    Name:   scavenger receptor class B, member 1    GB acc:   AF071495    UniGene:   Rn.88169    Gene Symbol:   Scarb1    Locus Link:   25073    Chromosome:   12    Cytoband:   Chr:12q15-q16    SOURCE    GENECARD       Gene Ontology:   biological process:  cell adhesion   molecular function:  receptor activity      BioCarta Pathways:     KEGG Pathways:  
    Probe set : 1395810_at    Name:   Transcribed locus    GB acc:   BM383094    UniGene:   Rn.146551        SOURCE        Gene Ontology:     BioCarta Pathways:     KEGG Pathways:  
    Probe set : 1390510_at    Name:   membrane-spanning 4-domains, subfamily A, member 6B    GB acc:   BI294706    UniGene:   Rn.17885    Gene Symbol:   RGD:1359381    Locus Link:   293749    Chromosome:   1    Cytoband:   Chr:1q43    SOURCE    GENECARD       Gene Ontology:   biological process:  signal transduction   cellular component:  integral to membrane   molecular function:  receptor activity      BioCarta Pathways:     KEGG Pathways:  
    Probe set : 1390396_at    Name:   Similar to eukaryotic translation initiation factor 4E member 3    GB acc:   AI101338    UniGene:   Rn.22427     Locus Link:   297481    Chromosome:   4    Cytoband:   Chr:4q34    SOURCE        Gene Ontology:     BioCarta Pathways:     KEGG Pathways:  
    Probe set : 1375917_at    Name:   glycoprotein 49b /// similar to GP49B1    GB acc:   BF282961    UniGene:   Rn.24628    Gene Symbol:   RGD:1359090 /// LOC499078    Locus Link:   292594 /// 499078    Chromosome:   1    Cytoband:   Chr:1q12    SOURCE    GENECARD       Gene Ontology:     BioCarta Pathways:     KEGG Pathways:  
    Probe set : 1385051_at    Name:   Transcribed locus    GB acc:   BE108569    UniGene:   Rn.139567        SOURCE        Gene Ontology:     BioCarta Pathways:     KEGG Pathways:  
    Probe set : 1393400_at    Name:   zeta-chain (TCR) associated protein kinase 70kDa    GB acc:   BM387946    UniGene:   Rn.17862    Gene Symbol:   Zap70    Locus Link:   301348    Chromosome:   9    Cytoband:   Chr:9q21    SOURCE    GENECARD       Gene Ontology:   cellular component:  cytosol   cellular component:  T cell receptor complex   cellular component:  cytoplasm      BioCarta Pathways:     KEGG Pathways:  
    Probe set : 1383272_at    Name:   similar to ankyrin repeat and SOCS box-containing protein 11    GB acc:   BI289784    UniGene:   Rn.6582    Gene Symbol:   LOC302666    Locus Link:   302666    Chromosome:   X    Cytoband:   Chr:Xq21    SOURCE    GENECARD       Gene Ontology:     BioCarta Pathways:     KEGG Pathways:  
    Probe set : 1387818_at    Name:   caspase 11    GB acc:   NM_053736    UniGene:   Rn.16195    Gene Symbol:   Casp11    Locus Link:   114555    Chromosome:   8    Cytoband:   Chr:8q11    SOURCE    GENECARD       Gene Ontology:   cellular component:  intracellular      BioCarta Pathways:     KEGG Pathways:  
    Probe set : 1369909_s_at    Name:   fasting-inducible integral membrane protein TM6P1    GB acc:   NM_139107    UniGene:   Rn.144670    Gene Symbol:   Tm6p1    Locus Link:   245966    Chromosome:   4    Cytoband:   Chr:4q33    SOURCE    GENECARD       Gene Ontology:   biological process:  catabolism   cellular component:  integral to membrane      BioCarta Pathways:     KEGG Pathways:  
    Probe set : 1367712_at    Name:   tissue inhibitor of metalloproteinase 1    GB acc:   NM_053819    UniGene:   Rn.25754    Gene Symbol:   Timp1    Locus Link:   116510    Chromosome:   X    Cytoband:   Chr:Xq12    SOURCE    GENECARD       Gene Ontology:   biological process:  C21-steroid hormone biosynthesis   biological process:  erythrocyte maturation   cellular component:  extracellular matrix (sensu Metazoa)   cellular component:  basement membrane   cellular component:  extracellular space   molecular function:  metalloendopeptidase inhibitor activity   molecular function:  metalloendopeptidase inhibitor activity   molecular function:  enzyme inhibitor activity      BioCarta Pathways:     KEGG Pathways:  
    Probe set : 1374180_at    Name:   Transcribed locus    GB acc:   AI236753    UniGene:   Rn.146914        SOURCE        Gene Ontology:     BioCarta Pathways:     KEGG Pathways:  
    Probe set : 1371539_at    Name:   nucleolar protein family A, member 2 (predicted)    GB acc:   AW921151    UniGene:   Rn.4216    Gene Symbol:   Nola2_predicted    Locus Link:   287273    Chromosome:   10    Cytoband:   Chr:10q22    SOURCE    GENECARD       Gene Ontology:   biological process:  nuclear mRNA splicing, via spliceosome   biological process:  mRNA processing   biological process:  protein biosynthesis   molecular function:  RNA binding   molecular function:  structural constituent of ribosome      BioCarta Pathways:     KEGG Pathways:  
    Probe set : 1382533_at    Name:   similar to HIV-1 Rev binding protein    GB acc:   AI012699    UniGene:   Rn.3734    Gene Symbol:   LOC363266    Locus Link:   363266    Chromosome:   9    Cytoband:   Chr:9q34    SOURCE    GENECARD       Gene Ontology:   biological process:  regulation of GTPase activity      BioCarta Pathways:     KEGG Pathways:  
    Probe set : 1374469_at    Name:   Similar to C20orf118    GB acc:   BG372375    UniGene:   Rn.22305     Locus Link:   502689      SOURCE        Gene Ontology:     BioCarta Pathways:     KEGG Pathways:  
    Probe set : 1388332_at    Name:   Ras-related C3 botulinum toxin substrate 1    GB acc:   AA799542    UniGene:   Rn.29157    Gene Symbol:   Rac1    Locus Link:   363875    Chromosome:   12    Cytoband:   Chr:12p11    SOURCE    GENECARD       Gene Ontology:   molecular function:  GTPase activity   molecular function:  GTP binding   molecular function:  nucleotide binding   molecular function:  GTP binding      BioCarta Pathways:     KEGG Pathways:  
    Probe set : 1395184_at     GB acc:   AI411699              Gene Ontology:     BioCarta Pathways:     KEGG Pathways:  
    Probe set : 1388337_at    Name:   nucleoside phosphorylase    GB acc:   AI102495    UniGene:   Rn.2738    Gene Symbol:   Np    Locus Link:   290029    Chromosome:   15    Cytoband:   Chr:15p14    SOURCE    GENECARD       Gene Ontology:     BioCarta Pathways:     KEGG Pathways:  
    Probe set : 1388182_at    Name:   DNA primase, p49 subunit    GB acc:   BE108911    UniGene:   Rn.92497    Gene Symbol:   RGD:621380    Locus Link:   246327    Chromosome:   7    Cytoband:   Chr:7q11    SOURCE    GENECARD       Gene Ontology:   biological process:  DNA replication   biological process:  DNA replication, synthesis of RNA primer      BioCarta Pathways:     KEGG Pathways:  
    Probe set : 1369100_at    Name:   angiotensin/vasopressin receptor    GB acc:   NM_134375    UniGene:   Rn.9669    Gene Symbol:   Nalp6    Locus Link:   171390    Chromosome:   1    Cytoband:   Chr:1q41    SOURCE    GENECARD       Gene Ontology:   cellular component:  integral to membrane      BioCarta Pathways:     KEGG Pathways:  
    Probe set : 1380071_at    Name:   Transcribed locus    GB acc:   BI285978    UniGene:   Rn.147453        SOURCE        Gene Ontology:     BioCarta Pathways:     KEGG Pathways:  
    Probe set : 1390909_at    Name:   Transcribed locus    GB acc:   AI171480    UniGene:   Rn.17796        SOURCE        Gene Ontology:     BioCarta Pathways:     KEGG Pathways:  
    Probe set : 1392655_at    Name:   Similar to SP140 nuclear body protein isoform 1    GB acc:   AA997266    UniGene:   Rn.15232     Locus Link:   363269    Chromosome:   9    Cytoband:   Chr:9q35    SOURCE        Gene Ontology:   cellular component:  nucleus      BioCarta Pathways:     KEGG Pathways:  
    Probe set : 1377125_at    Name:   DnaJ (Hsp40) homolog, subfamily C, member 6 (predicted)    GB acc:   AW523481    UniGene:   Rn.23812    Gene Symbol:   Dnajc6_predicted    Locus Link:   313409    Chromosome:   5    Cytoband:   Chr:5q33    SOURCE    GENECARD       Gene Ontology:     BioCarta Pathways:     KEGG Pathways:  
    Probe set : 1375020_at    Name:   Transcribed locus    GB acc:   AI706777    UniGene:   Rn.147692        SOURCE        Gene Ontology:     BioCarta Pathways:     KEGG Pathways:  
    Probe set : 1386861_at    Name:   H2A histone family, member Z    GB acc:   NM_022674    UniGene:   Rn.112573    Gene Symbol:   H2afz    Locus Link:   58940    Chromosome:   2    Cytoband:   Chr:2q44    SOURCE    GENECARD       Gene Ontology:   molecular function:  DNA binding   molecular function:  DNA binding      BioCarta Pathways:     KEGG Pathways:  
    Probe set : 1369979_at    Name:   src family associated phosphoprotein 2    GB acc:   NM_130413    UniGene:   Rn.12934    Gene Symbol:   Scap2    Locus Link:   155183    Chromosome:   4    Cytoband:   Chr:4q24    SOURCE    GENECARD       Gene Ontology:   biological process:  negative regulation of cell proliferation   cellular component:  cytoplasm   cellular component:  plasma membrane      BioCarta Pathways:     KEGG Pathways:  
    Probe set : 1387884_at    Name:   proteasome (prosome, macropain) subunit, alpha type 5    GB acc:   AI011191    UniGene:   Rn.1276    Gene Symbol:   Psma5    Locus Link:   29672    Chromosome:   2    Cytoband:   Chr:2q34    SOURCE    GENECARD       Gene Ontology:   biological process:  ubiquitin-dependent protein catabolism   cellular component:  cytosol   cellular component:  proteasome core complex (sensu Eukaryota)   cellular component:  protein complex      BioCarta Pathways:     KEGG Pathways:  
    Probe set : 1383392_at    Name:   complement component 2    GB acc:   AI716125    UniGene:   Rn.98333    Gene Symbol:   C2    Locus Link:   24231    Chromosome:   20    Cytoband:   Chr:20p12    SOURCE    GENECARD       Gene Ontology:   biological process:  proteolysis   biological process:  complement activation   biological process:  complement activation, classical pathway   cellular component:  extracellular space   cellular component:  extracellular region      BioCarta Pathways:     KEGG Pathways:  
    Probe set : 1377299_at    Name:   nuclear autoantigenic sperm protein    GB acc:   AI137618    UniGene:   Rn.48344    Gene Symbol:   RGD:1359609    Locus Link:   298441    Chromosome:   5    Cytoband:   Chr:5q36    SOURCE    GENECARD       Gene Ontology:   molecular function:  binding      BioCarta Pathways:     KEGG Pathways:  
    Probe set : 1392407_at    Name:   Similar to RIKEN cDNA 6030405P05 gene    GB acc:   AI012031    UniGene:   Rn.131539     Locus Link:   498282      SOURCE        Gene Ontology:     BioCarta Pathways:     KEGG Pathways:  
    Probe set : 1376038_at    Name:   testis expressed gene 2 (predicted)    GB acc:   AI411054    UniGene:   Rn.94152    Gene Symbol:   Tex2_predicted    Locus Link:   303611    Chromosome:   10    Cytoband:   Chr:10q32.1    SOURCE    GENECARD       Gene Ontology:     BioCarta Pathways:     KEGG Pathways:  
    Probe set : 1370428_x_at    Name:   RT1 class Ib, locus Aw2 /// RT1 class Ia, locus A2 /// RT1 class I, A3    GB acc:   AJ249701    UniGene:   Rn.40130    Gene Symbol:   RT1-Aw2 /// RT1-A2 /// RGD:1359610    Locus Link:   24737 /// 24974 /// 309627    Chromosome:   20    Cytoband:   Chr:20p12    SOURCE    GENECARD       Gene Ontology:   cellular component:  MHC class I protein complex   cellular component:  integral to membrane   cellular component:  MHC class I protein complex   molecular function:  MHC class I receptor activity      BioCarta Pathways:     KEGG Pathways:  
    Probe set : 1391224_at    Name:   similar to 4921517L17Rik protein (predicted)    GB acc:   AI169436    UniGene:   Rn.17118    Gene Symbol:   RGD1311678_predicted    Locus Link:   296311    Chromosome:   3    Cytoband:   Chr:3q42    SOURCE    GENECARD       Gene Ontology:   biological process:  protein biosynthesis   cellular component:  intracellular   cellular component:  ribosome   molecular function:  nucleotide binding   molecular function:  structural constituent of ribosome      BioCarta Pathways:     KEGG Pathways:  
    Probe set : 1369989_at    Name:   pyridoxine 5'-phosphate oxidase    GB acc:   NM_022601    UniGene:   Rn.6299    Gene Symbol:   Pnpo    Locus Link:   64533      SOURCE    GENECARD       Gene Ontology:   biological process:  pyridoxine biosynthesis      BioCarta Pathways:     KEGG Pathways:  
    Probe set : 1370056_at    Name:   Ly6-C antigen    GB acc:   NM_020103    UniGene:   Rn.40119    Gene Symbol:   Ly6c    Locus Link:   56778    Chromosome:   7    Cytoband:   Chr:7q34    SOURCE    GENECARD       Gene Ontology:   biological process:  pathogenesis   cellular component:  extracellular region      BioCarta Pathways:     KEGG Pathways:  
    Probe set : 1370924_at    Name:   similar to T-cell receptor beta-2 chain C region    GB acc:   BG378607    UniGene:   Rn.34871    Gene Symbol:   LOC362350    Locus Link:   362350    Chromosome:   4    Cytoband:   Chr:4q22    SOURCE    GENECARD       Gene Ontology:     BioCarta Pathways:     KEGG Pathways:  
    Probe set : 1387995_a_at    Name:   interferon induced transmembrane protein 3    GB acc:   BI285494    UniGene:   Rn.145191    Gene Symbol:   Ifitm3    Locus Link:   361673    Chromosome:   1    Cytoband:   Chr:1q41    SOURCE    GENECARD       Gene Ontology:     BioCarta Pathways:     KEGG Pathways:  
    Probe set : 1386009_at    Name:   Transcribed locus    GB acc:   AA946110    UniGene:   Rn.134664        SOURCE        Gene Ontology:     BioCarta Pathways:     KEGG Pathways:  
    Probe set : 1375895_at    Name:   Serine racemase    GB acc:   BI275908    UniGene:   Rn.101331    Gene Symbol:   Srr    Locus Link:   303306    Chromosome:   10    Cytoband:   Chr:10q24    SOURCE    GENECARD       Gene Ontology:   biological process:  amino acid metabolism   biological process:  metabolism   molecular function:  serine racemase activity   molecular function:  catalytic activity      BioCarta Pathways:     KEGG Pathways:  
    Probe set : 1380393_at    Name:   crystallin, zeta (predicted)    GB acc:   AI409083    UniGene:   Rn.6746    Gene Symbol:   Cryz_predicted    Locus Link:   362061    Chromosome:   2    Cytoband:   Chr:2q45    SOURCE    GENECARD       Gene Ontology:   biological process:  sensory organ development   biological process:  visual perception   cellular component:  cytoplasm      BioCarta Pathways:     KEGG Pathways:  
    Probe set : 1377576_at    Name:   similar to RIKEN cDNA 1110013G13 (predicted)    GB acc:   AA891800    UniGene:   Rn.37581    Gene Symbol:   RGD1307965_predicted    Locus Link:   310856    Chromosome:   2    Cytoband:   Chr:2q43    SOURCE    GENECARD       Gene Ontology:     BioCarta Pathways:     KEGG Pathways:  
    Probe set : 1368976_at    Name:   CD38 antigen    GB acc:   D30795    UniGene:   Rn.11414    Gene Symbol:   Cd38    Locus Link:   25668    Chromosome:   14    Cytoband:   Chr:14q21    SOURCE    GENECARD       Gene Ontology:   cellular component:  membrane fraction   cellular component:  nucleus   cellular component:  plasma membrane   cellular component:  integral to membrane      BioCarta Pathways:     KEGG Pathways:  
    Probe set : 1371365_at    Name:   similar to Ubiquitin-conjugating enzyme E2S    GB acc:   BI279788    UniGene:   Rn.120398    Gene Symbol:   LOC292588    Locus Link:   292588    Chromosome:   1    Cytoband:   Chr:1q12    SOURCE    GENECARD       Gene Ontology:     BioCarta Pathways:     KEGG Pathways:  
    Probe set : 1374730_at    Name:   Tyro protein tyrosine kinase binding protein    GB acc:   AI102519    UniGene:   Rn.11534    Gene Symbol:   Tyrobp    Locus Link:   361537    Chromosome:   1    Cytoband:   Chr:1q21    SOURCE    GENECARD       Gene Ontology:   cellular component:  integral to membrane      BioCarta Pathways:     KEGG Pathways:  
    Probe set : 1384787_at    Name:   CD3 antigen, gamma polypeptide (predicted)    GB acc:   AI044631    UniGene:   Rn.106603    Gene Symbol:   Cd3g_predicted    Locus Link:   300678    Chromosome:   8    Cytoband:   Chr:8q22    SOURCE    GENECARD       Gene Ontology:   cellular component:  integral to plasma membrane   cellular component:  integral to membrane   cellular component:  T cell receptor complex      BioCarta Pathways:     KEGG Pathways:  
    Probe set : 1396262_at    Name:   pre-B-cell colony enhancing factor 1    GB acc:   BM384211    UniGene:   Rn.7264    Gene Symbol:   Pbef1    Locus Link:   297508    Chromosome:   6    Cytoband:   Chr:6q16    SOURCE    GENECARD       Gene Ontology:   biological process:  cell cycle   biological process:  pyridine nucleotide biosynthesis   cellular component:  nucleus   cellular component:  cytoplasm   cellular component:  nucleus      BioCarta Pathways:     KEGG Pathways:  
    Probe set : 1393848_at    Name:   ribonucleotide reductase M2    GB acc:   AA957260    UniGene:   Rn.101236    Gene Symbol:   Rrm2    Locus Link:   362720    Chromosome:   6    Cytoband:   Chr:6q16    SOURCE    GENECARD       Gene Ontology:   biological process:  DNA replication   biological process:  deoxyribonucleoside diphosphate metabolism   biological process:  deoxyribonucleotide metabolism   cellular component:  cytoplasm   cellular component:  ribonucleoside-diphosphate reductase complex      BioCarta Pathways:     KEGG Pathways:  
    Probe set : 1367663_at    Name:   protease (prosome, macropain) 28 subunit, alpha    GB acc:   NM_017264    UniGene:   Rn.144575    Gene Symbol:   Psme1    Locus Link:   29630    Chromosome:   15    Cytoband:   Chr:15p13    SOURCE    GENECARD       Gene Ontology:   biological process:  immune response   cellular component:  proteasome complex (sensu Eukaryota)   cellular component:  cytosol   cellular component:  proteasome activator complex   molecular function:  proteasome activator activity   molecular function:  proteasome activator activity   molecular function:  peptidase activity      BioCarta Pathways:     KEGG Pathways:  
    Probe set : 1389696_at    Name:   Transcribed locus    GB acc:   BF409820    UniGene:   Rn.62928        SOURCE        Gene Ontology:     BioCarta Pathways:     KEGG Pathways:  
    Probe set : 1395886_at    Name:   ARP3 actin-related protein 3 homolog (yeast)    GB acc:   H31273    UniGene:   Rn.103326    Gene Symbol:   Actr3    Locus Link:   81732    Chromosome:   13    Cytoband:   Chr:13q11    SOURCE    GENECARD       Gene Ontology:   biological process:  cell motility   molecular function:  structural molecule activity   molecular function:  structural constituent of cytoskeleton   molecular function:  protein binding      BioCarta Pathways:     KEGG Pathways:  
    Probe set : 1376976_at    Name:   secreted and transmembrane 1    GB acc:   AI009823    UniGene:   Rn.25030    Gene Symbol:   Sectm1    Locus Link:   287884    Chromosome:   10    Cytoband:   Chr:10q32.3    SOURCE    GENECARD       Gene Ontology:   biological process:  immune response   cellular component:  Golgi apparatus   cellular component:  integral to membrane   cellular component:  integral to membrane      BioCarta Pathways:     KEGG Pathways:  
    Probe set : 1387434_at    Name:   solute carrier family 22 (organic cation transporter), member 4    GB acc:   NM_022270    UniGene:   Rn.54434    Gene Symbol:   Slc22a4    Locus Link:   64037      SOURCE    GENECARD       Gene Ontology:     BioCarta Pathways:     KEGG Pathways:  
    Probe set : 1391417_at    Name:   similar to betaine-homocysteine methyltransferase 2    GB acc:   AA925145    UniGene:   Rn.8494    Gene Symbol:   LOC365972    Locus Link:   365972      SOURCE    GENECARD       Gene Ontology:   molecular function:  homocysteine S-methyltransferase activity      BioCarta Pathways:     KEGG Pathways:  
    Probe set : 1379604_at    Name:   apolipoprotein L, 3 (predicted) /// similar to RIKEN cDNA 2210421G13    GB acc:   BF284937    UniGene:   Rn.145692    Gene Symbol:   Apol3_predicted /// LOC500903    Locus Link:   315108 /// 500903    Chromosome:   7    Cytoband:   Chr:7q34    SOURCE    GENECARD       Gene Ontology:   cellular component:  extracellular region   molecular function:  signal transducer activity   molecular function:  lipid transporter activity   molecular function:  lipid binding      BioCarta Pathways:     KEGG Pathways:  
    Probe set : 1369584_at    Name:   suppressor of cytokine signaling 3    GB acc:   NM_053565    UniGene:   Rn.127801    Gene Symbol:   Socs3    Locus Link:   89829    Chromosome:   10    Cytoband:   Chr:10q32.3    SOURCE    GENECARD       Gene Ontology:   molecular function:  protein binding      BioCarta Pathways:     KEGG Pathways:  
    Probe set : 1376613_at    Name:   similar to hypothetical protein    GB acc:   BI279201    UniGene:   Rn.107832    Gene Symbol:   MGC73022    Locus Link:   309196    Chromosome:   1    Cytoband:   Chr:1q43    SOURCE    GENECARD       Gene Ontology:   molecular function:  binding      BioCarta Pathways:     KEGG Pathways:  
    Probe set : 1389275_at     GB acc:   BM385271              Gene Ontology:     BioCarta Pathways:     KEGG Pathways:  
    Probe set : 1377659_at    Name:   myeloid leukemia factor 1 (predicted)    GB acc:   BI302005    UniGene:   Rn.54860    Gene Symbol:   Mlf1_predicted    Locus Link:   310483    Chromosome:   2    Cytoband:   Chr:2q31    SOURCE    GENECARD       Gene Ontology:   biological process:  hemopoiesis   biological process:  cell differentiation   cellular component:  nucleus   cellular component:  cytoplasm   cellular component:  centrosome   molecular function:  protein binding   molecular function:  protein domain specific binding      BioCarta Pathways:     KEGG Pathways:  
    Probe set : 1383553_at    Name:   similar to CDNA sequence BC034204 (predicted)    GB acc:   BE097591    UniGene:   Rn.12444    Gene Symbol:   RGD1311293_predicted    Locus Link:   315599    Chromosome:   8    Cytoband:   Chr:8q22    SOURCE    GENECARD       Gene Ontology:   molecular function:  ATP binding   molecular function:  nucleotide binding      BioCarta Pathways:     KEGG Pathways:  
    Probe set : 1392544_at    Name:   rcd1 (required for cell differentiation) homolog 1 (S. pombe) (predicted)    GB acc:   AI044879    UniGene:   Rn.18033    Gene Symbol:   Rqcd1_predicted    Locus Link:   301513    Chromosome:   9    Cytoband:   Chr:9q33    SOURCE    GENECARD       Gene Ontology:   biological process:  sex differentiation   biological process:  cytokine and chemokine mediated signaling pathway   molecular function:  binding      BioCarta Pathways:     KEGG Pathways:  
    Probe set : 1393044_at    Name:   thymidylate kinase family LPS-inducible member (predicted)    GB acc:   BF282370    UniGene:   Rn.100440    Gene Symbol:   Tyki_predicted    Locus Link:   314004    Chromosome:   6    Cytoband:   Chr:6q16    SOURCE    GENECARD       Gene Ontology:   biological process:  dTDP biosynthesis   biological process:  dTTP biosynthesis   biological process:  nucleotide biosynthesis      BioCarta Pathways:     KEGG Pathways:  
    Probe set : 1368016_at    Name:   peroxisomal trans-2-enoyl-CoA reductase    GB acc:   NM_133299    UniGene:   Rn.19267    Gene Symbol:   Pecr    Locus Link:   113956    Chromosome:   9    Cytoband:   Chr:9q33    SOURCE    GENECARD       Gene Ontology:   cellular component:  mitochondrion   cellular component:  peroxisome   cellular component:  mitochondrion   molecular function:  oxidoreductase activity   molecular function:  trans-2-enoyl-CoA reductase (NADPH) activity      BioCarta Pathways:     KEGG Pathways:  
    Probe set : 1369197_at    Name:   apoptotic peptidase activating factor 1    GB acc:   NM_023979    UniGene:   Rn.64522    Gene Symbol:   Apaf1    Locus Link:   78963    Chromosome:   7    Cytoband:   Chr:7q13    SOURCE    GENECARD       Gene Ontology:   biological process:  neural tube closure   biological process:  neural tube closure   biological process:  apoptosis   biological process:  induction of apoptosis   biological process:  caspase activation   biological process:  development   biological process:  nervous system development   biological process:  brain development   biological process:  brain development   biological process:  caspase activation via cytochrome c   biological process:  forebrain development   biological process:  forebrain development   biological process:  regulation of apoptosis   biological process:  defense response   biological process:  defense response to pathogen   cellular component:  intracellular   cellular component:  soluble fraction   cellular component:  nucleus   cellular component:  cytosol      BioCarta Pathways:     KEGG Pathways:  
    Probe set : 1368732_at    Name:   transporter 2, ATP-binding cassette, sub-family B (MDR/TAP)    GB acc:   NM_032056    UniGene:   Rn.10372    Gene Symbol:   Tap2    Locus Link:   24812    Chromosome:   20    Cytoband:   Chr:20p12    SOURCE    GENECARD       Gene Ontology:   molecular function:  nucleotide binding   molecular function:  nucleotide binding   molecular function:  DNA binding   molecular function:  transporter activity   molecular function:  ATP binding   molecular function:  ATP binding   molecular function:  oligopeptide transporter activity   molecular function:  peptide antigen-transporting ATPase activity   molecular function:  transcriptional activator activity   molecular function:  ATPase activity   molecular function:  nucleoside-triphosphatase activity   molecular function:  ATPase activity, coupled to transmembrane movement of substances   molecular function:  protein homodimerization activity   molecular function:  TAP1 binding   molecular function:  TAP2 binding   molecular function:  protein homodimerization activity   molecular function:  TAP1 binding   molecular function:  TAP2 binding      BioCarta Pathways:     KEGG Pathways:  
    Probe set : 1384413_at    Name:   Similar to solute carrier family 35, member A5    GB acc:   AA900068    UniGene:   Rn.15016     Locus Link:   498081      SOURCE        Gene Ontology:     BioCarta Pathways:     KEGG Pathways:  
    Probe set : 1374431_at    Name:   ATPase, H+ transporting, V1 subunit A, isoform 1 (predicted)    GB acc:   BE120644    UniGene:   Rn.1431     Locus Link:   360716    Chromosome:   11    Cytoband:   Chr:11q21    SOURCE        Gene Ontology:     BioCarta Pathways:     KEGG Pathways:  
    Probe set : 1373338_at    Name:   Transcribed locus    GB acc:   AI175767    UniGene:   Rn.98471        SOURCE        Gene Ontology:     BioCarta Pathways:     KEGG Pathways:  
    Probe set : 1369677_at    Name:   cannabinoid receptor 1 (brain)    GB acc:   X55812    UniGene:   Rn.89774    Gene Symbol:   Cnr1    Locus Link:   25248    Chromosome:   5    Cytoband:   Chr:5q21    SOURCE    GENECARD       Gene Ontology:   cellular component:  plasma membrane   cellular component:  integral to plasma membrane   cellular component:  integral to membrane      BioCarta Pathways:     KEGG Pathways:  
    Probe set : 1383731_at    Name:   Similar to RIKEN cDNA 2310036D22    GB acc:   AA818065    UniGene:   Rn.106000    Gene Symbol:   MGC94135    Locus Link:   312132    Chromosome:   4    Cytoband:   Chr:4q21    SOURCE    GENECARD       Gene Ontology:     BioCarta Pathways:     KEGG Pathways:  
    Probe set : 1393123_at    Name:   complement component 8, gamma polypeptide (predicted)    GB acc:   BM392153    UniGene:   Rn.17399    Gene Symbol:   C8g_predicted    Locus Link:   296545    Chromosome:   3    Cytoband:   Chr:3p13    SOURCE    GENECARD       Gene Ontology:   cellular component:  membrane attack complex   cellular component:  extracellular space   molecular function:  transporter activity   molecular function:  binding   molecular function:  retinol binding      BioCarta Pathways:     KEGG Pathways:  
    Probe set : 1381593_x_at    Name:   RT1 class II, locus Ba    GB acc:   AW528743    UniGene:   Rn.25717    Gene Symbol:   RT1-Ba    Locus Link:   309621    Chromosome:   20    Cytoband:   Chr:20p12    SOURCE    GENECARD       Gene Ontology:   cellular component:  MHC class II protein complex   cellular component:  membrane   molecular function:  MHC class II receptor activity      BioCarta Pathways:     KEGG Pathways:  
    Probe set : 1388324_at    Name:   nitrilase 1    GB acc:   BG668463    UniGene:   Rn.64425    Gene Symbol:   Nit1    Locus Link:   289222    Chromosome:   13    Cytoband:   Chr:13q24    SOURCE    GENECARD       Gene Ontology:   biological process:  nitrogen compound metabolism      BioCarta Pathways:     KEGG Pathways:  
    Probe set : 1379229_at     GB acc:   AI501127              Gene Ontology:     BioCarta Pathways:     KEGG Pathways:  
    Probe set : 1382590_at    Name:   Similar to RIKEN cDNA 2310015N21    GB acc:   BI277914    UniGene:   Rn.126330     Locus Link:   501095      SOURCE        Gene Ontology:     BioCarta Pathways:     KEGG Pathways:  
    Probe set : 1387867_at    Name:   aldehyde dehydrogenase family 9, subfamily A1    GB acc:   BG672075    UniGene:   Rn.98155    Gene Symbol:   Aldh9a1    Locus Link:   64040    Chromosome:   13    Cytoband:   Chr:13q24    SOURCE    GENECARD       Gene Ontology:   cellular component:  cytosol   cellular component:  cytosol      BioCarta Pathways:     KEGG Pathways:  
    Probe set : 1383241_at    Name:   complement component 1, r subcomponent (predicted)    GB acc:   BI292425    UniGene:   Rn.70397    Gene Symbol:   C1r_predicted    Locus Link:   312705    Chromosome:   4    Cytoband:   Chr:4q42    SOURCE    GENECARD       Gene Ontology:   biological process:  proteolysis   biological process:  immune response   biological process:  complement activation, classical pathway   cellular component:  extracellular region      BioCarta Pathways:     KEGG Pathways:  
    Probe set : 1387566_at    Name:   phospholipase A2, group IVA (cytosolic, calcium-dependent)    GB acc:   NM_133551    UniGene:   Rn.10162    Gene Symbol:   Pla2g4a    Locus Link:   24653    Chromosome:   13    Cytoband:   Chr:13q21    SOURCE    GENECARD       Gene Ontology:   cellular component:  cytosol   cellular component:  integral to membrane      BioCarta Pathways:     KEGG Pathways:  
    Probe set : 1388526_at    Name:   Transcribed locus    GB acc:   AI169075    UniGene:   Rn.100005        SOURCE        Gene Ontology:     BioCarta Pathways:     KEGG Pathways:  
    Probe set : 1388988_at    Name:   similar to RIKEN cDNA 1810013B01    GB acc:   AI175070    UniGene:   Rn.2080    Gene Symbol:   RGD:1359649    Locus Link:   300983    Chromosome:   8    Cytoband:   Chr:8q32    SOURCE    GENECARD       Gene Ontology:   molecular function:  catalytic activity   molecular function:  hydrolase activity      BioCarta Pathways:     KEGG Pathways:  
    Probe set : 1379732_at    Name:   syntaxin 11 (predicted)    GB acc:   AW920037    UniGene:   Rn.52178    Gene Symbol:   Stx11_predicted    Locus Link:   292483    Chromosome:   1    Cytoband:   Chr:1p13    SOURCE    GENECARD       Gene Ontology:   cellular component:  Golgi apparatus   cellular component:  membrane   molecular function:  SNAP receptor activity   molecular function:  protein transporter activity      BioCarta Pathways:     KEGG Pathways:  
    Probe set : 1387859_at    Name:   nitrogen fixation gene 1 (S. cerevisiae)    GB acc:   AI410876    UniGene:   Rn.3151    Gene Symbol:   Nfs1    Locus Link:   84594    Chromosome:   3    Cytoband:   Chr:3q42    SOURCE    GENECARD       Gene Ontology:   biological process:  metabolism   cellular component:  extracellular space   cellular component:  mitochondrion   cellular component:  mitochondrial matrix   molecular function:  transaminase activity   molecular function:  transferase activity   molecular function:  cysteine desulfurase activity      BioCarta Pathways:     KEGG Pathways:  
    Probe set : 1381376_at    Name:   Similar to [Mouse primary response gene B94 mRNA, 3end.], gene product    GB acc:   AI103284    UniGene:   Rn.34387     Locus Link:   299339    Chromosome:   6    Cytoband:   Chr:6q32    SOURCE        Gene Ontology:     BioCarta Pathways:     KEGG Pathways:  
    Probe set : 1373524_at    Name:   BCL2-associated transcription factor 1 (predicted)    GB acc:   AA946482    UniGene:   Rn.15652     Locus Link:   293017    Chromosome:   1    Cytoband:   Chr:1p12    SOURCE        Gene Ontology:   cellular component:  nucleus   molecular function:  DNA binding   molecular function:  transcriptional repressor activity      BioCarta Pathways:     KEGG Pathways:  
    Probe set : 1371776_at    Name:   Phosphatidylinositol 3-kinase, regulatory subunit, polypeptide 1    GB acc:   AA819268    UniGene:   Rn.10599    Gene Symbol:   Pik3r1    Locus Link:   25513    Chromosome:   2    Cytoband:   Chr:2q12    SOURCE    GENECARD       Gene Ontology:   cellular component:  phosphoinositide 3-kinase complex      BioCarta Pathways:     KEGG Pathways:  
    Probe set : 1390134_at    Name:   Transcribed locus    GB acc:   AI411274    UniGene:   Rn.46830        SOURCE        Gene Ontology:     BioCarta Pathways:     KEGG Pathways:  
    Probe set : 1386879_at    Name:   lectin, galactose binding, soluble 3    GB acc:   NM_031832    UniGene:   Rn.764    Gene Symbol:   Lgals3    Locus Link:   83781    Chromosome:   15    Cytoband:   Chr:15p14    SOURCE    GENECARD       Gene Ontology:   biological process:  heterophilic cell adhesion   cellular component:  extracellular region   cellular component:  nucleus   cellular component:  cytoplasm   cellular component:  plasma membrane   molecular function:  protein binding   molecular function:  sugar binding   molecular function:  IgE binding      BioCarta Pathways:     KEGG Pathways:  
    Probe set : 1367850_at    Name:   Fc gamma receptor II beta    GB acc:   NM_053843    UniGene:   Rn.131543    Gene Symbol:   LOC498276    Locus Link:   498276    Chromosome:   13    Cytoband:   Chr:13q24    SOURCE    GENECARD       Gene Ontology:     BioCarta Pathways:     KEGG Pathways:  
    Probe set : 1382325_at    Name:   glycine C-acetyltransferase (2-amino-3-ketobutyrate-coenzyme A ligase) (predicted)    GB acc:   AW525471    UniGene:   Rn.43940    Gene Symbol:   Gcat_predicted    Locus Link:   366959    Chromosome:   7    Cytoband:   Chr:7q34    SOURCE    GENECARD       Gene Ontology:   cellular component:  mitochondrion      BioCarta Pathways:     KEGG Pathways:  
    Probe set : 1392647_at    Name:   Tryptophan hydroxylase    GB acc:   BF282318    UniGene:   Rn.9392    Gene Symbol:   Tph    Locus Link:   24848    Chromosome:   1    Cytoband:   Chr:1q22    SOURCE    GENECARD       Gene Ontology:   cellular component:  extracellular region      BioCarta Pathways:     KEGG Pathways:  
    Probe set : 1383407_at    Name:   Similar to RIKEN cDNA 1200013B08    GB acc:   BM390872    UniGene:   Rn.15328     Locus Link:   317578    Chromosome:   X    Cytoband:   Chr:Xq35    SOURCE        Gene Ontology:     BioCarta Pathways:     KEGG Pathways:  
    Probe set : 1384580_at    Name:   complement component 6    GB acc:   AI045191    UniGene:   Rn.16145    Gene Symbol:   C6    Locus Link:   24237    Chromosome:   2    Cytoband:   Chr:2q16    SOURCE    GENECARD       Gene Ontology:   cellular component:  membrane attack complex   cellular component:  extracellular space   cellular component:  integral to membrane      BioCarta Pathways:     KEGG Pathways:  
    Probe set : 1388243_at    Name:   gene model 1012, (NCBI)    GB acc:   BG663422    UniGene:   Rn.44656    Gene Symbol:   Gm1012    Locus Link:   117257    Chromosome:   3    Cytoband:   Chr:3q35    SOURCE    GENECARD       Gene Ontology:   biological process:  G-protein coupled receptor protein signaling pathway   biological process:  synaptic transmission   biological process:  signal transduction   cellular component:  integral to plasma membrane   cellular component:  integral to membrane      BioCarta Pathways:     KEGG Pathways:  
    Probe set : 1378802_at    Name:   similar to hypothetical protein MGC13047    GB acc:   AI105366    UniGene:   Rn.102445    Gene Symbol:   RGD:1303232    Locus Link:   309381    Chromosome:   1    Cytoband:   Chr:1q54    SOURCE    GENECARD       Gene Ontology:   biological process:  electron transport   biological process:  carotenoid biosynthesis      BioCarta Pathways:     KEGG Pathways:  
    Probe set : 1377759_at    Name:   BH3 interacting domain death agonist    GB acc:   BG666928    UniGene:   Rn.31142    Gene Symbol:   Bid    Locus Link:   64625    Chromosome:   4    Cytoband:   Chr:4q42    SOURCE    GENECARD       Gene Ontology:   biological process:  apoptosis   biological process:  regulation of apoptosis   biological process:  positive regulation of apoptosis   cellular component:  cytoplasm      BioCarta Pathways:     KEGG Pathways:  
    Probe set : 1367946_at    Name:   PDZ and LIM domain 1 (elfin)    GB acc:   NM_017365    UniGene:   Rn.11170    Gene Symbol:   Pdlim1    Locus Link:   54133    Chromosome:   1    Cytoband:   Chr:1q53    SOURCE    GENECARD       Gene Ontology:   cellular component:  transcription factor complex   cellular component:  cytoskeleton      BioCarta Pathways:     KEGG Pathways:  
    Probe set : 1370382_at    Name:   RT1 class II, locus Db1    GB acc:   BI279526    UniGene:   Rn.144821    Gene Symbol:   RT1-Db1    Locus Link:   294270    Chromosome:   20    Cytoband:   Chr:20p12    SOURCE    GENECARD       Gene Ontology:   cellular component:  MHC class II protein complex   cellular component:  membrane   cellular component:  integral to membrane   molecular function:  MHC class II receptor activity      BioCarta Pathways:     KEGG Pathways:  
    Probe set : 1388629_at    Name:   inosine 5-monophosphate dehydrogenase 2    GB acc:   BF281278    UniGene:   Rn.8093    Gene Symbol:   Impdh2    Locus Link:   301005    Chromosome:   8    Cytoband:   Chr:8q32    SOURCE    GENECARD       Gene Ontology:   biological process:  GMP biosynthesis   molecular function:  IMP dehydrogenase activity   molecular function:  catalytic activity   molecular function:  IMP dehydrogenase activity      BioCarta Pathways:     KEGG Pathways:  
    Probe set : 1371239_s_at    Name:   tropomyosin 3, gamma    GB acc:   AF053361    UniGene:   Rn.37575    Gene Symbol:   Tpm3    Locus Link:   117557    Chromosome:   2    Cytoband:   Chr:2q34    SOURCE    GENECARD       Gene Ontology:   biological process:  regulation of muscle contraction   biological process:  muscle development   cellular component:  cytoskeleton   cellular component:  muscle thin filament tropomyosin   molecular function:  actin binding      BioCarta Pathways:     KEGG Pathways:  
    Probe set : 1387022_at    Name:   aldehyde dehydrogenase family 1, member A1    GB acc:   NM_022407    UniGene:   Rn.6132    Gene Symbol:   Aldh1a1    Locus Link:   24188    Chromosome:   1    Cytoband:   Chr:1q51    SOURCE    GENECARD       Gene Ontology:   cellular component:  cytosol      BioCarta Pathways:     KEGG Pathways:  
    Probe set : 1374537_at    Name:   Carbohydrate (chondroitin) synthase 1 (predicted)    GB acc:   AI406533    UniGene:   Rn.13788     Locus Link:   292999    Chromosome:   1    Cytoband:   Chr:1q22    SOURCE        Gene Ontology:   biological process:  chondroitin sulfate biosynthesis   molecular function:  glucuronosyl-N-acetylgalactosaminyl-proteoglycan 4-beta-N-acetylgalactosaminyltransferase activity      BioCarta Pathways:     KEGG Pathways:  
    Probe set : 1379698_at    Name:   Transcribed locus    GB acc:   AI178631    UniGene:   Rn.22530        SOURCE        Gene Ontology:     BioCarta Pathways:     KEGG Pathways:  
    Probe set : 1368092_at    Name:   fumarylacetoacetate hydrolase    GB acc:   NM_017181    UniGene:   Rn.9195    Gene Symbol:   Fah    Locus Link:   29383    Chromosome:   1    Cytoband:   Chr:1q31    SOURCE    GENECARD       Gene Ontology:   cellular component:  nucleus   cellular component:  nucleus      BioCarta Pathways:     KEGG Pathways:  
    Probe set : 1388484_at    Name:   ubiquitin-conjugating enzyme E2C (predicted)    GB acc:   BI296084    UniGene:   Rn.3102    Gene Symbol:   Ube2c_predicted    Locus Link:   296368    Chromosome:   3    Cytoband:   Chr:3q42    SOURCE    GENECARD       Gene Ontology:   molecular function:  ubiquitin conjugating enzyme activity   molecular function:  ubiquitin-protein ligase activity   molecular function:  ligase activity      BioCarta Pathways:     KEGG Pathways:  
    Probe set : 1370897_at    Name:   Branched chain ketoacid dehydrogenase E1, alpha polypeptide    GB acc:   J02827    UniGene:   Rn.49145    Gene Symbol:   Bckdha    Locus Link:   25244    Chromosome:   1    Cytoband:   Chr:1q21    SOURCE    GENECARD       Gene Ontology:   biological process:  metabolism   biological process:  branched chain family amino acid catabolism   biological process:  branched chain family amino acid catabolism      BioCarta Pathways:     KEGG Pathways:  
    Probe set : 1369559_a_at    Name:   CD47 antigen (Rh-related antigen, integrin-associated signal transducer)    GB acc:   NM_019195    UniGene:   Rn.7409    Gene Symbol:   Cd47    Locus Link:   29364    Chromosome:   11    Cytoband:   Chr:11q21    SOURCE    GENECARD       Gene Ontology:   cellular component:  extracellular space   cellular component:  integral to plasma membrane   cellular component:  integral to membrane   molecular function:  protein binding      BioCarta Pathways:     KEGG Pathways:  
    Probe set : 1373024_at    Name:   adaptor-related protein complex 3, sigma 1 subunit (predicted)    GB acc:   BI296511    UniGene:   Rn.3070    Gene Symbol:   Ap3s1_predicted    Locus Link:   302290    Chromosome:   18    Cytoband:   Chr:18q11    SOURCE    GENECARD       Gene Ontology:   molecular function:  transporter activity      BioCarta Pathways:     KEGG Pathways:  
    Probe set : 1377408_at    Name:   phospholipase A2, group VI    GB acc:   AW251242    UniGene:   Rn.44692    Gene Symbol:   Pla2g6    Locus Link:   360426    Chromosome:   7    Cytoband:   Chr:7q34    SOURCE    GENECARD       Gene Ontology:   cellular component:  cytoplasm   cellular component:  membrane      BioCarta Pathways:     KEGG Pathways:  
    Probe set : 1368418_a_at    Name:   ceruloplasmin    GB acc:   AF202115    UniGene:   Rn.32777    Gene Symbol:   Cp    Locus Link:   24268    Chromosome:   2    Cytoband:   Chr:2q24    SOURCE    GENECARD       Gene Ontology:     BioCarta Pathways:     KEGG Pathways:  
    Probe set : 1376056_at    Name:   similar to poly (ADP-ribose) polymerase family, member 10    GB acc:   BF291214    UniGene:   Rn.137552    Gene Symbol:   LOC300043    Locus Link:   300043    Chromosome:   7    Cytoband:   Chr:7q34    SOURCE    GENECARD       Gene Ontology:     BioCarta Pathways:     KEGG Pathways:  
    Probe set : 1381875_at    Name:   N-myc (and STAT) interactor (predicted)    GB acc:   BM386847    UniGene:   Rn.18496    Gene Symbol:   Nmi_predicted    Locus Link:   311021    Chromosome:   3    Cytoband:   Chr:3q12    SOURCE    GENECARD       Gene Ontology:   biological process:  transcription from RNA polymerase II promoter   biological process:  inflammatory response   biological process:  JAK-STAT cascade   cellular component:  cytoplasm   molecular function:  transcription cofactor activity      BioCarta Pathways:     KEGG Pathways:  
    Probe set : 1398246_s_at    Name:   Fc receptor, IgG, low affinity III /// Fc gamma receptor II beta    GB acc:   NM_053843    UniGene:   Rn.6050    Gene Symbol:   Fcgr3 /// LOC498276    Locus Link:   116591 /// 498276    Chromosome:   13    Cytoband:   Chr:13q24    SOURCE    GENECARD       Gene Ontology:   biological process:  antibody-dependent cellular cytotoxicity   biological process:  positive regulation of type IIa hypersensitivity   biological process:  positive regulation of type III hypersensitivity   biological process:  positive regulation of type I hypersensitivity   biological process:  serotonin secretion   biological process:  phagocytosis, recognition   biological process:  phagocytosis, engulfment   biological process:  immune response   biological process:  cell surface receptor linked signal transduction   biological process:  neutrophil chemotaxis   biological process:  cell-mediated immune response   biological process:  cell-mediated immune response   biological process:  positive regulation of tumor necrosis factor-alpha biosynthesis   biological process:  antigen presentation, exogenous antigen via MHC class I   biological process:  mast cell activation   biological process:  positive regulation of phagocytosis   biological process:  regulation of immune response   cellular component:  extracellular space   cellular component:  plasma membrane   cellular component:  integral to membrane      BioCarta Pathways:     KEGG Pathways:  
    Probe set : 1384935_at    Name:   Cleavage stimulation factor, 3' pre-RNA, subunit 3, 77kDa (predicted)    GB acc:   AI070621    UniGene:   Rn.46377     Locus Link:   362178    Chromosome:   3    Cytoband:   Chr:3q32    SOURCE        Gene Ontology:   biological process:  mRNA polyadenylylation   biological process:  mRNA cleavage   cellular component:  nucleus   molecular function:  RNA binding   molecular function:  binding      BioCarta Pathways:     KEGG Pathways:  
    Probe set : 1383320_at    Name:   lymphocyte protein tyrosine kinase    GB acc:   AI137137    UniGene:   Rn.22791    Gene Symbol:   Lck    Locus Link:   313050    Chromosome:   5    Cytoband:   Chr:5q36    SOURCE    GENECARD       Gene Ontology:   biological process:  protein amino acid phosphorylation   biological process:  intracellular signaling cascade   biological process:  T cell activation      BioCarta Pathways:     KEGG Pathways:  
    Probe set : 1374897_at     GB acc:   AI406842              Gene Ontology:     BioCarta Pathways:     KEGG Pathways:  
    Probe set : 1373262_at    Name:   Similar to 2310014H01Rik protein (predicted)    GB acc:   AI102745    UniGene:   Rn.16324     Locus Link:   361790    Chromosome:   20    Cytoband:   Chr:20p12    SOURCE        Gene Ontology:     BioCarta Pathways:     KEGG Pathways:  
    Probe set : 1372199_at    Name:   PTEN induced putative kinase 1 (predicted)    GB acc:   AI233191    UniGene:   Rn.103380    Gene Symbol:   Pink1_predicted    Locus Link:   298575    Chromosome:   5    Cytoband:   Chr:5q36    SOURCE    GENECARD       Gene Ontology:   biological process:  protein amino acid phosphorylation   biological process:  response to stress   biological process:  protein kinase cascade   cellular component:  mitochondrion      BioCarta Pathways:     KEGG Pathways:  
    Probe set : 1367909_at    Name:   dicarbonyl L-xylulose reductase    GB acc:   NM_134387    UniGene:   Rn.19540    Gene Symbol:   Dcxr    Locus Link:   171408      SOURCE    GENECARD       Gene Ontology:   cellular component:  microvillus   cellular component:  brush border   cellular component:  membrane      BioCarta Pathways:     KEGG Pathways:  
    Probe set : 1388574_at    Name:   tryptophanyl-tRNA synthetase (predicted)    GB acc:   BG670966    UniGene:   Rn.14939    Gene Symbol:   Wars_predicted    Locus Link:   314442    Chromosome:   6    Cytoband:   Chr:6q32    SOURCE    GENECARD       Gene Ontology:   cellular component:  soluble fraction   cellular component:  cytoplasm      BioCarta Pathways:     KEGG Pathways:  
    Probe set : 1370200_at    Name:   glutamate dehydrogenase 1    GB acc:   BI284411    UniGene:   Rn.55106    Gene Symbol:   Glud1    Locus Link:   24399    Chromosome:   16    Cytoband:   Chr:16p16    SOURCE    GENECARD       Gene Ontology:   biological process:  amino acid metabolism   biological process:  glutamate catabolism   cellular component:  mitochondrion      BioCarta Pathways:     KEGG Pathways:  
    Probe set : 1392280_at    Name:   toll-like receptor 2    GB acc:   AW526982    UniGene:   Rn.46387    Gene Symbol:   Tlr2    Locus Link:   310553    Chromosome:   2    Cytoband:   Chr:2q34    SOURCE    GENECARD       Gene Ontology:   biological process:  immune response   cellular component:  membrane   molecular function:  receptor activity   molecular function:  receptor activity   molecular function:  transmembrane receptor activity      BioCarta Pathways:     KEGG Pathways:  
    Probe set : 1376693_at    Name:   similar to OEF2    GB acc:   AA998964    UniGene:   Rn.13231    Gene Symbol:   LOC500011    Locus Link:   500011    Chromosome:   4    Cytoband:   Chr:4q13    SOURCE    GENECARD       Gene Ontology:     BioCarta Pathways:     KEGG Pathways:  
    Probe set : 1370910_at    Name:   replication factor C (activator 1) 2    GB acc:   BF283113    UniGene:   Rn.113319    Gene Symbol:   Rfc2    Locus Link:   116468    Chromosome:   12    Cytoband:   Chr:12q12    SOURCE    GENECARD       Gene Ontology:   biological process:  DNA replication   cellular component:  protein complex      BioCarta Pathways:     KEGG Pathways:  
    Probe set : 1380410_at    Name:   Transcribed locus    GB acc:   AI043958    UniGene:   Rn.23216        SOURCE        Gene Ontology:     BioCarta Pathways:     KEGG Pathways:  
    Probe set : 1389071_at    Name:   tangerin    GB acc:   BI287221    UniGene:   Rn.103357    Gene Symbol:   LOC309169    Locus Link:   309169    Chromosome:   1    Cytoband:   Chr:1q43    SOURCE    GENECARD       Gene Ontology:     BioCarta Pathways:     KEGG Pathways:  
    Probe set : 1368207_at    Name:   FXYD domain-containing ion transport regulator 5    GB acc:   NM_021909    UniGene:   Rn.24997    Gene Symbol:   Fxyd5    Locus Link:   60338    Chromosome:   1    Cytoband:   Chr:1q21    SOURCE    GENECARD       Gene Ontology:   biological process:  ion transport   biological process:  transport   cellular component:  extracellular space   cellular component:  membrane   cellular component:  integral to membrane   molecular function:  ion channel activity      BioCarta Pathways:     KEGG Pathways:  
    Probe set : 1384483_at    Name:   Similar to RIKEN cDNA E030034P13 (predicted)    GB acc:   BI274216    UniGene:   Rn.49088     Locus Link:   360488    Chromosome:   10    Cytoband:   Chr:10q12    SOURCE        Gene Ontology:     BioCarta Pathways:     KEGG Pathways:  
    Probe set : 1376281_at    Name:   Similar to hypothetical protein FLJ22349    GB acc:   BM389698    UniGene:   Rn.19890     Locus Link:   500909      SOURCE        Gene Ontology:     BioCarta Pathways:     KEGG Pathways:  
    Probe set : 1388773_at    Name:   similar to [Mouse primary response gene B94 mRNA, 3end.], gene product    GB acc:   BI278479    UniGene:   Rn.34387    Gene Symbol:   LOC299339    Locus Link:   299339    Chromosome:   6    Cytoband:   Chr:6q32    SOURCE    GENECARD       Gene Ontology:     BioCarta Pathways:     KEGG Pathways:  
    Probe set : 1367702_at    Name:   acetyl-Coenzyme A dehydrogenase, medium chain    GB acc:   NM_016986    UniGene:   Rn.6302    Gene Symbol:   Acadm    Locus Link:   24158    Chromosome:   2    Cytoband:   Chr:2q45    SOURCE    GENECARD       Gene Ontology:     BioCarta Pathways:     KEGG Pathways:  
    Probe set : 1389885_at    Name:   similar to RIKEN cDNA 0610025L06 (predicted)    GB acc:   BI294855    UniGene:   Rn.112761    Gene Symbol:   RGD1309967_predicted    Locus Link:   360646    Chromosome:   10    Cytoband:   Chr:10q32.1    SOURCE    GENECARD       Gene Ontology:   molecular function:  zinc ion binding      BioCarta Pathways:     KEGG Pathways:  
    Probe set : 1371694_at     GB acc:   AI317805              Gene Ontology:     BioCarta Pathways:     KEGG Pathways:  
    Probe set : 1378633_at    Name:   leupaxin (predicted)    GB acc:   BE106297    UniGene:   Rn.29200    Gene Symbol:   Lpxn_predicted    Locus Link:   293783    Chromosome:   1    Cytoband:   Chr:1q43    SOURCE    GENECARD       Gene Ontology:   biological process:  protein complex assembly   biological process:  cell adhesion   biological process:  signal transduction   cellular component:  cytoplasm   cellular component:  integral to membrane   molecular function:  zinc ion binding   molecular function:  metal ion binding      BioCarta Pathways:     KEGG Pathways:  
    Probe set : 1383478_at    Name:   Janus kinase 1    GB acc:   BG671504    UniGene:   Rn.90191    Gene Symbol:   Jak1    Locus Link:   84598    Chromosome:   5    Cytoband:   Chr:5q31.3-q35    SOURCE    GENECARD       Gene Ontology:   biological process:  protein amino acid phosphorylation   biological process:  intracellular signaling cascade   biological process:  peptidyl-tyrosine phosphorylation   cellular component:  cytoskeleton      BioCarta Pathways:     KEGG Pathways:  
    Probe set : 1389014_at    Name:   pre-B-cell colony enhancing factor 1    GB acc:   BI297612    UniGene:   Rn.7264    Gene Symbol:   Pbef1    Locus Link:   297508    Chromosome:   6    Cytoband:   Chr:6q16    SOURCE    GENECARD       Gene Ontology:   biological process:  cell cycle   biological process:  pyridine nucleotide biosynthesis   cellular component:  nucleus   cellular component:  cytoplasm   cellular component:  nucleus      BioCarta Pathways:     KEGG Pathways:  
    Probe set : 1388403_at    Name:   similar to NADP+-specific isocitrate dehydrogenase    GB acc:   AI172491    UniGene:   Rn.3490    Gene Symbol:   LOC361596    Locus Link:   361596    Chromosome:   1    Cytoband:   Chr:1q31    SOURCE    GENECARD       Gene Ontology:   biological process:  main pathways of carbohydrate metabolism   biological process:  metabolism   molecular function:  isocitrate dehydrogenase (NADP+) activity   molecular function:  oxidoreductase activity      BioCarta Pathways:     KEGG Pathways:  
    Probe set : 1370693_a_at    Name:   cyclic nucleotide phosphodiesterase 1    GB acc:   M18630    UniGene:   Rn.31762    Gene Symbol:   Cnp1    Locus Link:   25275    Chromosome:   10    Cytoband:   Chr:10q32.1    SOURCE    GENECARD       Gene Ontology:   biological process:  synaptic transmission   biological process:  cyclic nucleotide catabolism   cellular component:  membrane   molecular function:  2',3'-cyclic-nucleotide 3'-phosphodiesterase activity   molecular function:  hydrolase activity      BioCarta Pathways:     KEGG Pathways:  
    Probe set : 1368008_at    Name:   prominin 1    GB acc:   NM_021751    UniGene:   Rn.144589    Gene Symbol:   Prom1    Locus Link:   60357    Chromosome:   14    Cytoband:   Chr:14q21    SOURCE    GENECARD       Gene Ontology:   biological process:  phototransduction   biological process:  glucose homeostasis      BioCarta Pathways:     KEGG Pathways:  
    Probe set : 1373661_a_at    Name:   chemokine (C-X-C motif) receptor 4    GB acc:   AA945737    UniGene:   Rn.44431    Gene Symbol:   Cxcr4    Locus Link:   60628    Chromosome:   13    Cytoband:   Chr:13q12    SOURCE    GENECARD       Gene Ontology:   cellular component:  integral to membrane      BioCarta Pathways:     KEGG Pathways:  
    Probe set : 1367589_at    Name:   aconitase 2, mitochondrial    GB acc:   NM_024398    UniGene:   Rn.43737    Gene Symbol:   Aco2    Locus Link:   79250    Chromosome:   1    Cytoband:   Chr:1q13 or 20q11    SOURCE    GENECARD       Gene Ontology:   cellular component:  mitochondrion      BioCarta Pathways:     KEGG Pathways:  
    Probe set : 1383662_at    Name:   hypothetical protein LOC500956    GB acc:   AI043759    UniGene:   Rn.145694    Gene Symbol:   LOC500956    Locus Link:   500956    Chromosome:   8    Cytoband:   Chr:8q13    SOURCE    GENECARD       Gene Ontology:     BioCarta Pathways:     KEGG Pathways:  
    Probe set : 1379295_at     GB acc:   BI281946              Gene Ontology:     BioCarta Pathways:     KEGG Pathways:  
    Probe set : 1368356_a_at    Name:   type 1 tumor necrosis factor receptor shedding aminopeptidase regulator    GB acc:   NM_030836    UniGene:   Rn.22516    Gene Symbol:   Arts1    Locus Link:   80897    Chromosome:   2    Cytoband:   Chr:2q11    SOURCE    GENECARD       Gene Ontology:   biological process:  regulation of transcription, DNA-dependent   biological process:  proteolysis   biological process:  membrane protein ectodomain proteolysis   biological process:  regulation of blood pressure   biological process:  antigen processing, endogenous antigen via MHC class I   biological process:  antigen processing   biological process:  regulation of innate immune response   biological process:  fat cell differentiation   biological process:  positive regulation of angiogenesis   biological process:  membrane protein ectodomain proteolysis   biological process:  regulation of blood pressure   biological process:  antigen processing, endogenous antigen via MHC class I   biological process:  regulation of innate immune response   biological process:  fat cell differentiation   molecular function:  DNA binding   molecular function:  transcription factor activity   molecular function:  aminopeptidase activity   molecular function:  leucyl aminopeptidase activity   molecular function:  leucyl aminopeptidase activity   molecular function:  membrane alanyl aminopeptidase activity   molecular function:  methionyl aminopeptidase activity   molecular function:  receptor activity   molecular function:  interleukin-6 receptor binding   molecular function:  tumor necrosis factor receptor binding   molecular function:  peptidase activity   molecular function:  metallopeptidase activity   molecular function:  zinc ion binding   molecular function:  zinc ion binding   molecular function:  hydrolase activity   molecular function:  aminopeptidase activity   molecular function:  leucyl aminopeptidase activity   molecular function:  methionyl aminopeptidase activity   molecular function:  interleukin-6 receptor binding   molecular function:  tumor necrosis factor receptor binding   molecular function:  zinc ion binding   molecular function:  metal ion binding      BioCarta Pathways:     KEGG Pathways:  
    Probe set : 1370036_at    Name:   sulfite oxidase    GB acc:   NM_031127    UniGene:   Rn.25720    Gene Symbol:   Suox    Locus Link:   81805    Chromosome:   7    Cytoband:   Chr:7q11    SOURCE    GENECARD       Gene Ontology:   biological process:  electron transport   biological process:  methionine catabolism   cellular component:  mitochondrion      BioCarta Pathways:     KEGG Pathways:  
    Probe set : 1369294_at    Name:   bone marrow stromal cell antigen 1    GB acc:   NM_030848    UniGene:   Rn.10728    Gene Symbol:   Bst1    Locus Link:   81506    Chromosome:   14    Cytoband:   Chr:14q21    SOURCE    GENECARD       Gene Ontology:   biological process:  defense response   cellular component:  extracellular space   cellular component:  plasma membrane   cellular component:  membrane   molecular function:  NAD+ nucleosidase activity   molecular function:  NAD+ nucleosidase activity   molecular function:  hydrolase activity      BioCarta Pathways:     KEGG Pathways:  
    Probe set : 1368274_at    Name:   drebrin-like    GB acc:   NM_031352    UniGene:   Rn.4115    Gene Symbol:   Dbnl    Locus Link:   83527    Chromosome:   14    Cytoband:   Chr:14q21    SOURCE    GENECARD       Gene Ontology:     BioCarta Pathways:     KEGG Pathways:  
    Probe set : 1393187_at    Name:   Similar to Ifi204 protein    GB acc:   BF417625    UniGene:   Rn.141231     Locus Link:   498288      SOURCE        Gene Ontology:     BioCarta Pathways:     KEGG Pathways:  
    Probe set : 1386904_a_at    Name:   cytochrome b-5    GB acc:   AF007107    UniGene:   Rn.1055    Gene Symbol:   Cyb5    Locus Link:   64001    Chromosome:   18    Cytoband:   Chr:18q12.3    SOURCE    GENECARD       Gene Ontology:   biological process:  electron transport   biological process:  fatty acid metabolism   biological process:  transport      BioCarta Pathways:     KEGG Pathways:  
    Probe set : 1383786_at    Name:   Transcribed locus    GB acc:   AW915417    UniGene:   Rn.49241        SOURCE        Gene Ontology:     BioCarta Pathways:     KEGG Pathways:  
    Probe set : 1375006_at    Name:   Similar to RIKEN cDNA B130055L09 (predicted)    GB acc:   BE121050    UniGene:   Rn.23445     Locus Link:   362341    Chromosome:   4    Cytoband:   Chr:4q22    SOURCE        Gene Ontology:     BioCarta Pathways:     KEGG Pathways:  
    Probe set : 1367892_at    Name:   pyruvate dehydrogenase kinase, isoenzyme 2    GB acc:   NM_030872    UniGene:   Rn.88597    Gene Symbol:   Pdk2    Locus Link:   81530    Chromosome:   10    Cytoband:   Chr:10q31    SOURCE    GENECARD       Gene Ontology:   cellular component:  mitochondrion      BioCarta Pathways:     KEGG Pathways:  
    Probe set : 1386934_at    Name:   choline transporter    GB acc:   NM_017348    UniGene:   Rn.10336    Gene Symbol:   Slc6a8    Locus Link:   50690    Chromosome:   X    Cytoband:   Chr:Xq37    SOURCE    GENECARD       Gene Ontology:   cellular component:  integral to plasma membrane   cellular component:  membrane   cellular component:  integral to membrane      BioCarta Pathways:     KEGG Pathways:  
    Probe set : 1387856_at    Name:   calponin 3, acidic    GB acc:   BI274457    UniGene:   Rn.57635    Gene Symbol:   Cnn3    Locus Link:   54321    Chromosome:   2    Cytoband:   Chr:2q41    SOURCE    GENECARD       Gene Ontology:   biological process:  smooth muscle contraction   biological process:  actomyosin structure organization and biogenesis   cellular component:  microtubule   cellular component:  microtubule   cellular component:  actin filament   cellular component:  actin filament      BioCarta Pathways:     KEGG Pathways:  
    Probe set : 1376920_at    Name:   similar to mKIAA2005 protein /// similar to mKIAA2005 protein /// similar to mKIAA2005 protein    GB acc:   BF408536    UniGene:   Rn.118248    Gene Symbol:   LOC500012 /// LOC500014 /// LOC500015    Locus Link:   500012 /// 500014 /// 500015    Chromosome:   4    Cytoband:   Chr:4q13    SOURCE    GENECARD       Gene Ontology:     BioCarta Pathways:     KEGG Pathways:  
    Probe set : 1370188_at    Name:   splicing factor, arginine/serine-rich 10 (transformer 2 homolog, Drosophila)    GB acc:   AW252670    UniGene:   Rn.8538    Gene Symbol:   Sfrs10    Locus Link:   117259    Chromosome:   11    Cytoband:   Chr:11q23    SOURCE    GENECARD       Gene Ontology:   cellular component:  nucleus   cellular component:  nucleus      BioCarta Pathways:     KEGG Pathways:  
    Probe set : 1367908_at    Name:   glycine cleavage system protein H (aminomethyl carrier)    GB acc:   NM_133598    UniGene:   Rn.98456    Gene Symbol:   Gcsh    Locus Link:   171133    Chromosome:   19    Cytoband:   Chr:19q12    SOURCE    GENECARD       Gene Ontology:   biological process:  glycine catabolism   cellular component:  glycine cleavage complex      BioCarta Pathways:     KEGG Pathways:  
    Probe set : 1373072_at    Name:   Transcribed locus    GB acc:   AI170552    UniGene:   Rn.1897        SOURCE        Gene Ontology:     BioCarta Pathways:     KEGG Pathways:  
    Probe set : 1387897_at    Name:   cyclic nucleotide phosphodiesterase 1    GB acc:   L16532    UniGene:   Rn.31762    Gene Symbol:   Cnp1    Locus Link:   25275    Chromosome:   10    Cytoband:   Chr:10q32.1    SOURCE    GENECARD       Gene Ontology:   biological process:  synaptic transmission   biological process:  cyclic nucleotide catabolism   cellular component:  membrane   molecular function:  2',3'-cyclic-nucleotide 3'-phosphodiesterase activity   molecular function:  hydrolase activity      BioCarta Pathways:     KEGG Pathways:  
    Probe set : 1372064_at    Name:   similar to chemokine (C-X-C motif) ligand 16    GB acc:   BI296385    UniGene:   Rn.7070    Gene Symbol:   Cxcl16    Locus Link:   497942    Chromosome:   10    Cytoband:   Chr:10q24    SOURCE    GENECARD       Gene Ontology:     BioCarta Pathways:     KEGG Pathways:  
    Probe set : 1387918_at    Name:   tropomyosin 3, gamma    GB acc:   BI295970    UniGene:   Rn.37575    Gene Symbol:   Tpm3    Locus Link:   117557    Chromosome:   2    Cytoband:   Chr:2q34    SOURCE    GENECARD       Gene Ontology:   biological process:  regulation of muscle contraction   biological process:  muscle development   cellular component:  cytoskeleton   cellular component:  muscle thin filament tropomyosin   molecular function:  actin binding      BioCarta Pathways:     KEGG Pathways:  
    Probe set : 1373258_at    Name:   cathepsin F (predicted)    GB acc:   AI169359    UniGene:   Rn.25087    Gene Symbol:   Ctsf_predicted    Locus Link:   361704    Chromosome:   1    Cytoband:   Chr:1q43    SOURCE    GENECARD       Gene Ontology:   biological process:  proteolysis   cellular component:  extracellular space   cellular component:  lysosome      BioCarta Pathways:     KEGG Pathways:  
    Probe set : 1377603_at    Name:   sorting nexing 24 (predicted)    GB acc:   BF284123    UniGene:   Rn.21066    Gene Symbol:   Snx24_predicted    Locus Link:   361328    Chromosome:   18    Cytoband:   Chr:18q11    SOURCE    GENECARD       Gene Ontology:   biological process:  intracellular signaling cascade   biological process:  protein transport   biological process:  transport      BioCarta Pathways:     KEGG Pathways:  
    Probe set : 1382177_at    Name:   Transcribed locus    GB acc:   BF415064    UniGene:   Rn.15386        SOURCE        Gene Ontology:     BioCarta Pathways:     KEGG Pathways:  
    Probe set : 1368192_at    Name:   chemokine (C-X-C motif) receptor 3    GB acc:   NM_053415    UniGene:   Rn.24787    Gene Symbol:   Cxcr3    Locus Link:   84475    Chromosome:   X    Cytoband:   Chr:Xq31    SOURCE    GENECARD       Gene Ontology:   cellular component:  integral to membrane   cellular component:  integral to membrane      BioCarta Pathways:     KEGG Pathways:  
    Probe set : 1379688_at    Name:   Transcribed locus    GB acc:   BM389813    UniGene:   Rn.147380        SOURCE        Gene Ontology:     BioCarta Pathways:     KEGG Pathways:  
    Probe set : 1370972_x_at     GB acc:   BI300597    UniGene:   Rn.40130    Gene Symbol:   Multiple Gene Symbols    Locus Link:   24737 /// 24973 /// 24974 /// 24977 /// 309600 /// 309603 /// 309607 /// 309627 /// 360231 /// 368153 /// 414779 /// 414784 /// 414789 /// 414819    Chromosome:   20    Cytoband:   Chr:20p12    SOURCE    GENECARD       Gene Ontology:     BioCarta Pathways:     KEGG Pathways:  
    Probe set : 1393114_at    Name:   Transcribed locus    GB acc:   AI136684    UniGene:   Rn.2842        SOURCE        Gene Ontology:     BioCarta Pathways:     KEGG Pathways:  
    Probe set : 1391925_at    Name:   chemokine (C-C motif) ligand 19 (predicted)    GB acc:   AA996885    UniGene:   Rn.12445    Gene Symbol:   Ccl19_predicted    Locus Link:   362506    Chromosome:   5    Cytoband:   Chr:5q22    SOURCE    GENECARD       Gene Ontology:   cellular component:  extracellular region   cellular component:  extracellular space   molecular function:  cytokine activity   molecular function:  chemokine activity      BioCarta Pathways:     KEGG Pathways:  
    Probe set : 1388694_at    Name:   RT1-149 protein    GB acc:   AI233121     Gene Symbol:   RT1-149    Locus Link:   414784    Chromosome:   20    Cytoband:   Chr:20p12     GENECARD       Gene Ontology:     BioCarta Pathways:     KEGG Pathways:  
    Probe set : 1388309_at    Name:   high mobility group AT-hook 1    GB acc:   BG378885    UniGene:   Rn.83614    Gene Symbol:   Hmga1    Locus Link:   117062    Chromosome:   20    Cytoband:   Chr:20p12    SOURCE    GENECARD       Gene Ontology:   biological process:  regulation of transcription, DNA-dependent   biological process:  chromosome organization and biogenesis (sensu Eukaryota)   cellular component:  chromatin   cellular component:  nucleus   molecular function:  DNA binding      BioCarta Pathways:     KEGG Pathways:  
    Probe set : 1368809_at    Name:   CAP, adenylate cyclase-associated protein 1 (yeast)    GB acc:   BG380723    UniGene:   Rn.112585    Gene Symbol:   Cap1    Locus Link:   64185    Chromosome:   5    Cytoband:   Chr:5q36    SOURCE    GENECARD       Gene Ontology:   cellular component:  cytoplasm   cellular component:  membrane   cellular component:  cortical actin cytoskeleton   molecular function:  adenylate cyclase binding   molecular function:  actin binding      BioCarta Pathways:     KEGG Pathways:  
    Probe set : 1388335_at    Name:   transgelin 2 (predicted)    GB acc:   BI285865    UniGene:   Rn.145963    Gene Symbol:   Tagln2_predicted    Locus Link:   304983    Chromosome:   13    Cytoband:   Chr:13q24    SOURCE    GENECARD       Gene Ontology:   biological process:  muscle development      BioCarta Pathways:     KEGG Pathways:  
    Probe set : 1384525_at    Name:   similar to spleen specific 238 kDa protein with PH domain    GB acc:   AI549335    UniGene:   Rn.22563    Gene Symbol:   LOC313438    Locus Link:   313438    Chromosome:   X    Cytoband:   Chr:Xq12    SOURCE    GENECARD       Gene Ontology:     BioCarta Pathways:     KEGG Pathways:  
    Probe set : 1368566_a_at    Name:   MIPP65 protein    GB acc:   AA964381    UniGene:   Rn.6452    Gene Symbol:   RGD:621020    Locus Link:   64539    Chromosome:   20    Cytoband:   Chr:20p12    SOURCE    GENECARD       Gene Ontology:   cellular component:  mitochondrion      BioCarta Pathways:     KEGG Pathways:  
    Probe set : 1368455_at    Name:   natural killer cell group 7 sequence    GB acc:   NM_133540    UniGene:   Rn.20448    Gene Symbol:   Nkg7    Locus Link:   171062    Chromosome:   1    Cytoband:   Chr:1q22    SOURCE    GENECARD       Gene Ontology:     BioCarta Pathways:     KEGG Pathways:  
    Probe set : 1385072_at    Name:   galactose mutarotase (aldose 1-epimerase)    GB acc:   AA997048    UniGene:   Rn.83639    Gene Symbol:   RGD:1359459    Locus Link:   313843    Chromosome:   6    Cytoband:   Chr:6q11    SOURCE    GENECARD       Gene Ontology:   biological process:  carbohydrate metabolism   biological process:  galactose metabolism   molecular function:  aldose 1-epimerase activity   molecular function:  isomerase activity      BioCarta Pathways:     KEGG Pathways:  
    Probe set : 1376723_a_at     GB acc:   AW142765              Gene Ontology:     BioCarta Pathways:     KEGG Pathways:  
    Probe set : 1390956_at    Name:   Transcribed locus    GB acc:   BI294909    UniGene:   Rn.35355        SOURCE        Gene Ontology:     BioCarta Pathways:     KEGG Pathways:  
    Probe set : 1367914_at    Name:   epithelial membrane protein 3    GB acc:   NM_030847    UniGene:   Rn.6782    Gene Symbol:   Emp3    Locus Link:   81505    Chromosome:   1    Cytoband:   Chr:1q22    SOURCE    GENECARD       Gene Ontology:     BioCarta Pathways:     KEGG Pathways:  
    Probe set : 1383783_at    Name:   protocadherin 9 (predicted)    GB acc:   AW521192    UniGene:   Rn.35614    Gene Symbol:   Pcdh9_predicted    Locus Link:   306091    Chromosome:   15    Cytoband:   Chr:15q21    SOURCE    GENECARD       Gene Ontology:   biological process:  cell adhesion   biological process:  homophilic cell adhesion   cellular component:  membrane   cellular component:  integral to membrane   molecular function:  calcium ion binding   molecular function:  protein binding      BioCarta Pathways:     KEGG Pathways:  
    Probe set : 1388140_at    Name:   RAB13, member RAS oncogene family    GB acc:   AW253722    UniGene:   Rn.9819    Gene Symbol:   Rab13    Locus Link:   81756    Chromosome:   2    Cytoband:   Chr:2q34    SOURCE    GENECARD       Gene Ontology:   cellular component:  tight junction      BioCarta Pathways:     KEGG Pathways:  
    Probe set : 1373897_at     GB acc:   AI103106              Gene Ontology:     BioCarta Pathways:     KEGG Pathways:  
    Probe set : 1387277_at    Name:   Yamaguchi sarcoma viral (v-yes-1) oncogene homolog    GB acc:   NM_030857    UniGene:   Rn.4338    Gene Symbol:   Lyn    Locus Link:   81515    Chromosome:   5    Cytoband:   Chr:5q12    SOURCE    GENECARD       Gene Ontology:   cellular component:  intracellular      BioCarta Pathways:     KEGG Pathways:  
    Probe set : 1389571_at    Name:   signal transducer and activator of transcription 2 (predicted)    GB acc:   BG666368    UniGene:   Rn.24237    Gene Symbol:   Stat2_predicted    Locus Link:   288774    Chromosome:   7    Cytoband:   Chr:7q11    SOURCE    GENECARD       Gene Ontology:   cellular component:  nucleus   cellular component:  cytoplasm      BioCarta Pathways:     KEGG Pathways:  
    Probe set : 1397304_at    Name:   interferon gamma induced GTPase (predicted)    GB acc:   AW525366    UniGene:   Rn.113342    Gene Symbol:   Igtp_predicted    Locus Link:   303163    Chromosome:   10    Cytoband:   Chr:10q22    SOURCE    GENECARD       Gene Ontology:   cellular component:  cytosol   molecular function:  GTPase activity      BioCarta Pathways:     KEGG Pathways:  
    Probe set : 1382346_at    Name:   Transcribed locus    GB acc:   BF281960    UniGene:   Rn.13650        SOURCE        Gene Ontology:     BioCarta Pathways:     KEGG Pathways:  
    Probe set : 1384151_at    Name:   Transcribed locus    GB acc:   AI578392    UniGene:   Rn.138244        SOURCE        Gene Ontology:     BioCarta Pathways:     KEGG Pathways:  
    Probe set : 1389470_at    Name:   B-factor, properdin    GB acc:   AI639117    UniGene:   Rn.109148    Gene Symbol:   Bf    Locus Link:   294257    Chromosome:   20    Cytoband:   Chr:20p12    SOURCE    GENECARD       Gene Ontology:   cellular component:  extracellular space   cellular component:  extracellular region      BioCarta Pathways:     KEGG Pathways:  
    Probe set : 1387394_at    Name:   interleukin 2 receptor, beta chain    GB acc:   NM_013195    UniGene:   Rn.5832    Gene Symbol:   Il2rb    Locus Link:   25746      SOURCE    GENECARD       Gene Ontology:     BioCarta Pathways:     KEGG Pathways:  
    Probe set : 1373447_at    Name:   similar to HN1-like protein (predicted)    GB acc:   BF281350    UniGene:   Rn.19357    Gene Symbol:   RGD1305117_predicted    Locus Link:   360492    Chromosome:   10    Cytoband:   Chr:10q12    SOURCE    GENECARD       Gene Ontology:   biological process:  regulation of transcription, DNA-dependent   cellular component:  nucleus   molecular function:  DNA binding      BioCarta Pathways:     KEGG Pathways:  
    Probe set : 1387872_at    Name:   heterogeneous nuclear ribonucleoprotein A1    GB acc:   BF396270    UniGene:   Rn.25771    Gene Symbol:   Hnrpa1    Locus Link:   29578    Chromosome:   7    Cytoband:   Chr:7q36    SOURCE    GENECARD       Gene Ontology:   cellular component:  nucleus   cellular component:  nucleus   cellular component:  ribonucleoprotein complex      BioCarta Pathways:     KEGG Pathways:  
    Probe set : 1377967_at    Name:   Retroviral integration site 2 (predicted)    GB acc:   AI112987    UniGene:   Rn.8884     Locus Link:   292071    Chromosome:   19    Cytoband:   Chr:19q12    SOURCE        Gene Ontology:   biological process:  DNA replication   biological process:  cell cycle   biological process:  regulation of S phase of mitotic cell cycle   cellular component:  nucleus   molecular function:  DNA binding      BioCarta Pathways:     KEGG Pathways:  
    Probe set : 1370090_at    Name:   lymphocyte cytosolic protein 2    GB acc:   NM_130421    UniGene:   Rn.1057    Gene Symbol:   Lcp2    Locus Link:   155918    Chromosome:   10    Cytoband:   Chr:10q12    SOURCE    GENECARD       Gene Ontology:   molecular function:  protein binding      BioCarta Pathways:     KEGG Pathways:  
    Probe set : 1393657_at    Name:   prolylcarboxypeptidase (angiotensinase C) (predicted)    GB acc:   BI285860    UniGene:   Rn.8457    Gene Symbol:   Prcp_predicted    Locus Link:   293118    Chromosome:   1    Cytoband:   Chr:1q32    SOURCE    GENECARD       Gene Ontology:   biological process:  proteolysis   cellular component:  lysosome      BioCarta Pathways:     KEGG Pathways:  
    Probe set : 1388351_at    Name:   similar to chromosome 13 open reading frame 12 (predicted)    GB acc:   BM384128    UniGene:   Rn.28242    Gene Symbol:   RGD1305831_predicted    Locus Link:   288455    Chromosome:   12    Cytoband:   Chr:12p11    SOURCE    GENECARD       Gene Ontology:     BioCarta Pathways:     KEGG Pathways:  
    Probe set : 1388521_at    Name:   pyrroline-5-carboxylate synthetase (glutamate gamma-semialdehyde synthetase) (predicted)    GB acc:   BG380693    UniGene:   Rn.3148    Gene Symbol:   Pycs_predicted    Locus Link:   361755    Chromosome:   1    Cytoband:   Chr:1q54    SOURCE    GENECARD       Gene Ontology:   biological process:  proline biosynthesis   biological process:  amino acid biosynthesis   cellular component:  cytoplasm   cellular component:  mitochondrion   cellular component:  inner membrane      BioCarta Pathways:     KEGG Pathways:  
    Probe set : 1370346_at    Name:   cyclin B1    GB acc:   X64589    UniGene:   Rn.145413    Gene Symbol:   Ccnb1    Locus Link:   25203    Chromosome:   2    Cytoband:   Chr:2q12    SOURCE    GENECARD       Gene Ontology:   cellular component:  nucleus   molecular function:  protein binding   molecular function:  kinase activity   molecular function:  cyclin-dependent protein kinase regulator activity      BioCarta Pathways:     KEGG Pathways:  
    Probe set : 1374574_at    Name:   Similar to hypothetical protein MGC38960 (predicted)    GB acc:   AI547611    UniGene:   Rn.23973     Locus Link:   300836    Chromosome:   8    Cytoband:   Chr:8q24    SOURCE        Gene Ontology:     BioCarta Pathways:     KEGG Pathways:  
    Probe set : 1373876_at     GB acc:   BI295945              Gene Ontology:     BioCarta Pathways:     KEGG Pathways:  
    Probe set : 1377286_at    Name:   GTPase, IMAP family member 4    GB acc:   BM389208    UniGene:   Rn.43169    Gene Symbol:   Gimap4    Locus Link:   286938    Chromosome:   4    Cytoband:   Chr:4q24    SOURCE    GENECARD       Gene Ontology:   molecular function:  GTP binding   molecular function:  nucleotide binding      BioCarta Pathways:     KEGG Pathways:  
    Probe set : 1369716_s_at    Name:   lectin, galactose binding, soluble 5 /// lectin, galactose binding, soluble 9    GB acc:   NM_012976    UniGene:   Rn.64588    Gene Symbol:   Lgals5 /// Lgals9    Locus Link:   25475 /// 25476    Chromosome:   10    Cytoband:   Chr:10q25    SOURCE    GENECARD       Gene Ontology:     BioCarta Pathways:     KEGG Pathways:  
    Probe set : 1377966_at    Name:   Transcribed locus, weakly similar to XP_579465.1 PREDICTED: DNA binding protein N5 [Rattus norvegicus]    GB acc:   BI275560    UniGene:   Rn.22923        SOURCE        Gene Ontology:     BioCarta Pathways:     KEGG Pathways:  
    Probe set : 1368385_a_at    Name:   growth factor receptor bound protein 2    GB acc:   X62853    UniGene:   Rn.3360    Gene Symbol:   Grb2    Locus Link:   81504    Chromosome:   10    Cytoband:   Chr:10q32.3    SOURCE    GENECARD       Gene Ontology:   cellular component:  vesicle membrane      BioCarta Pathways:     KEGG Pathways:  
    Probe set : 1390855_at    Name:   Prolyl endopeptidase    GB acc:   AI235923    UniGene:   Rn.11058    Gene Symbol:   Prep    Locus Link:   83471    Chromosome:   20    Cytoband:   Chr:20q13    SOURCE    GENECARD       Gene Ontology:   biological process:  proteolysis   biological process:  proteolysis   biological process:  protein metabolism   cellular component:  nucleus   cellular component:  cytoplasm   cellular component:  cytosol      BioCarta Pathways:     KEGG Pathways:  
    Probe set : 1370769_a_at    Name:   inducible T-cell co-stimulator    GB acc:   AB023133    UniGene:   Rn.42901    Gene Symbol:   Icos    Locus Link:   64545      SOURCE    GENECARD       Gene Ontology:   biological process:  defense response   cellular component:  extracellular space   cellular component:  integral to plasma membrane   cellular component:  integral to membrane   molecular function:  MHC protein binding   molecular function:  exogenous peptide antigen binding      BioCarta Pathways:     KEGG Pathways:  
    Probe set : 1367590_at    Name:   RAN, member RAS oncogene family    GB acc:   NM_053439    UniGene:   Rn.107698    Gene Symbol:   Ran    Locus Link:   84509    Chromosome:   12    Cytoband:   Chr:12q14    SOURCE    GENECARD       Gene Ontology:   biological process:  regulation of progression through cell cycle   biological process:  microtubule cytoskeleton organization and biogenesis   biological process:  DNA metabolism   biological process:  RNA export from nucleus   biological process:  protein import into nucleus   biological process:  protein export from nucleus   biological process:  protein export from nucleus   biological process:  intracellular protein transport   biological process:  nucleocytoplasmic transport   biological process:  mitotic spindle organization and biogenesis   biological process:  mitosis   biological process:  signal transduction   biological process:  small GTPase mediated signal transduction   biological process:  spermatid development   biological process:  protein transport   biological process:  transport   cellular component:  nucleus   cellular component:  nuclear pore      BioCarta Pathways:     KEGG Pathways:  
    Probe set : 1381228_at    Name:   F-box protein 39 (predicted)    GB acc:   AI073001    UniGene:   Rn.20328     Locus Link:   303287    Chromosome:   10    Cytoband:   Chr:10q24    SOURCE        Gene Ontology:   biological process:  ubiquitin cycle      BioCarta Pathways:     KEGG Pathways:  
    Probe set : 1381014_at    Name:   interferon-induced protein 44 (predicted)    GB acc:   BI274623    UniGene:   Rn.43876    Gene Symbol:   Ifi44_predicted    Locus Link:   310969    Chromosome:   2    Cytoband:   Chr:2q45    SOURCE    GENECARD       Gene Ontology:   biological process:  response to virus   cellular component:  cytoplasm      BioCarta Pathways:     KEGG Pathways:  
    Probe set : 1367837_at    Name:   proteasome (prosome, macropain) subunit, alpha type 4    GB acc:   NM_017281    UniGene:   Rn.11076    Gene Symbol:   Psma4    Locus Link:   29671    Chromosome:   8    Cytoband:   Chr:8q24    SOURCE    GENECARD       Gene Ontology:   biological process:  ubiquitin-dependent protein catabolism   cellular component:  cytosol   cellular component:  proteasome core complex (sensu Eukaryota)   cellular component:  protein complex      BioCarta Pathways:     KEGG Pathways:  
    Probe set : 1373136_at    Name:   hypothetical LOC294390 (predicted)    GB acc:   AI013609    UniGene:   Rn.3372    Gene Symbol:   RGD1307672_predicted    Locus Link:   294390    Chromosome:   20    Cytoband:   Chr:20q11    SOURCE    GENECARD       Gene Ontology:   cellular component:  nucleus   molecular function:  nucleic acid binding   molecular function:  zinc ion binding   molecular function:  metal ion binding      BioCarta Pathways:     KEGG Pathways:  
    Probe set : 1390126_at     GB acc:   BE112913              Gene Ontology:     BioCarta Pathways:     KEGG Pathways:  
    Probe set : 1376700_at    Name:   Transcribed locus, moderately similar to XP_580018.1 PREDICTED: hypothetical protein XP_580018 [Rattus norvegicus]    GB acc:   AI179472    UniGene:   Rn.43951        SOURCE        Gene Ontology:     BioCarta Pathways:     KEGG Pathways:  
    Probe set : 1368474_at    Name:   vascular cell adhesion molecule 1    GB acc:   NM_012889    UniGene:   Rn.11267    Gene Symbol:   Vcam1    Locus Link:   25361    Chromosome:   2    Cytoband:   Chr:2q41    SOURCE    GENECARD       Gene Ontology:   biological process:  cell adhesion   biological process:  cell adhesion   biological process:  cell adhesion   biological process:  cell-cell adhesion   molecular function:  integrin binding   molecular function:  integrin binding   molecular function:  protein binding      BioCarta Pathways:     KEGG Pathways:  
    Probe set : 1388463_at    Name:   putative secreted protein ZSIG11    GB acc:   AW252660    UniGene:   Rn.13429    Gene Symbol:   RGD:1359353    Locus Link:   300988    Chromosome:   8    Cytoband:   Chr:8q32    SOURCE    GENECARD       Gene Ontology:     BioCarta Pathways:     KEGG Pathways:  
    Probe set : 1371447_at    Name:   placenta-specific 8 (predicted)    GB acc:   BG378630    UniGene:   Rn.2649    Gene Symbol:   Plac8_predicted    Locus Link:   360914    Chromosome:   14    Cytoband:   Chr:14p22    SOURCE    GENECARD       Gene Ontology:   biological process:  protein modification   biological process:  ubiquitin cycle   molecular function:  ubiquitin-like activating enzyme activity      BioCarta Pathways:     KEGG Pathways:  
    Probe set : 1368501_s_at    Name:   mast cell protease 8 /// mast cell protease 10 /// mast cell protease 9 /// mast cell protease 8-like 2    GB acc:   NM_019323    UniGene:   Rn.10651    Gene Symbol:   Mcpt8 /// Mcpt10 /// Mcpt9 /// RGD:1302938    Locus Link:   29269 /// 408240 /// 54269 /// 54272    Chromosome:   15    Cytoband:   Chr:15p12 /// Chr:15p13    SOURCE    GENECARD       Gene Ontology:   biological process:  proteolysis   cellular component:  extracellular space      BioCarta Pathways:     KEGG Pathways:  
    Probe set : 1388998_at    Name:   erythrocyte protein band 4.9 (predicted)    GB acc:   AW522835    UniGene:   Rn.9618    Gene Symbol:   Epb4.9_predicted    Locus Link:   361069    Chromosome:   15    Cytoband:   Chr:15p11    SOURCE    GENECARD       Gene Ontology:   biological process:  cellular morphogenesis   biological process:  cytoskeleton organization and biogenesis   biological process:  actin filament bundle formation   cellular component:  plasma membrane   cellular component:  actin cytoskeleton   molecular function:  actin binding   molecular function:  structural constituent of cytoskeleton      BioCarta Pathways:     KEGG Pathways:  
    Probe set : 1389166_at    Name:   calcium and integrin binding family member 2 (predicted)    GB acc:   BF403998    UniGene:   Rn.144898    Gene Symbol:   Cib2_predicted    Locus Link:   300719    Chromosome:   8    Cytoband:   Chr:8q24    SOURCE    GENECARD       Gene Ontology:   biological process:  integrin-mediated signaling pathway   molecular function:  calcium ion binding   molecular function:  protein binding      BioCarta Pathways:     KEGG Pathways:  
    Probe set : 1390133_at    Name:   BCL2/adenovirus E1B 19kDa-interacting protein 1, NIP2 (predicted)    GB acc:   AI178277    UniGene:   Rn.13297    Gene Symbol:   Bnip2_predicted    Locus Link:   300811    Chromosome:   8    Cytoband:   Chr:8q24    SOURCE    GENECARD       Gene Ontology:   biological process:  apoptosis   biological process:  anti-apoptosis   cellular component:  cytoplasm   molecular function:  GTPase activator activity      BioCarta Pathways:     KEGG Pathways:  
    Probe set : 1376785_at    Name:   Synaptonemal complex protein 3    GB acc:   AI104799    UniGene:   Rn.34889    Gene Symbol:   Sycp3    Locus Link:   25561    Chromosome:   7    Cytoband:   Chr:7q13    SOURCE    GENECARD       Gene Ontology:   biological process:  mitosis   biological process:  meiosis   molecular function:  DNA binding   molecular function:  protein binding   molecular function:  protein binding      BioCarta Pathways:     KEGG Pathways:  
    Probe set : 1373830_at    Name:   hypothetical protein LOC619574    GB acc:   AA893539     Gene Symbol:   LOC619574    Locus Link:   619574    Chromosome:   10    Cytoband:   Chr:10q32     GENECARD       Gene Ontology:     BioCarta Pathways:     KEGG Pathways:  
    Probe set : 1397772_at    Name:   Similar to putative membrane steroid receptor (predicted)    GB acc:   BF390195    UniGene:   Rn.17067     Locus Link:   316275    Chromosome:   9    Cytoband:   Chr:9q13    SOURCE        Gene Ontology:   biological process:  G-protein coupled receptor protein signaling pathway   cellular component:  integral to membrane   molecular function:  receptor activity   molecular function:  G-protein coupled receptor activity   molecular function:  steroid binding      BioCarta Pathways:     KEGG Pathways:  
    Probe set : 1381124_at    Name:   Solute carrier family 25 (mitochondrial carrier; ornithine transporter) member 15 (predicted)    GB acc:   AA819672    UniGene:   Rn.66488     Locus Link:   306574    Chromosome:   16    Cytoband:   Chr:16q12.5    SOURCE        Gene Ontology:   molecular function:  L-ornithine transporter activity   molecular function:  transporter activity   molecular function:  binding      BioCarta Pathways:     KEGG Pathways:  
    Probe set : 1372492_at    Name:   claudin 10 (predicted)    GB acc:   AI412937    UniGene:   Rn.99994    Gene Symbol:   Cldn10_predicted    Locus Link:   290485    Chromosome:   15    Cytoband:   Chr:15q24    SOURCE    GENECARD       Gene Ontology:   biological process:  cell adhesion   molecular function:  structural molecule activity      BioCarta Pathways:     KEGG Pathways:  
    Probe set : 1370258_at    Name:   basic leucine zipper and W2 domains 2    GB acc:   BI303268    UniGene:   Rn.13196    Gene Symbol:   Bzw2    Locus Link:   171439    Chromosome:   6    Cytoband:   Chr:6q16    SOURCE    GENECARD       Gene Ontology:   biological process:  regulation of translational initiation   molecular function:  translation initiation factor activity      BioCarta Pathways:     KEGG Pathways:  
    Probe set : 1374478_at    Name:   similar to RIKEN cDNA 2610528J11 (predicted)    GB acc:   AA819329    UniGene:   Rn.1992    Gene Symbol:   RGD1305347_predicted    Locus Link:   362576    Chromosome:   5    Cytoband:   Chr:5q36    SOURCE    GENECARD       Gene Ontology:     BioCarta Pathways:     KEGG Pathways:  
    Probe set : 1374036_at    Name:   minichromosome maintenance deficient 2 mitotin (S. cerevisiae) (predicted)    GB acc:   AI229508    UniGene:   Rn.2715    Gene Symbol:   Mcm2_predicted    Locus Link:   312538    Chromosome:   4    Cytoband:   Chr:4q34    SOURCE    GENECARD       Gene Ontology:   cellular component:  chromatin   cellular component:  nucleus   cellular component:  nuclear origin of replication recognition complex      BioCarta Pathways:     KEGG Pathways:  
    Probe set : 1386956_at    Name:   scavenger receptor class B, member 1    GB acc:   NM_031541    UniGene:   Rn.88169    Gene Symbol:   Scarb1    Locus Link:   25073    Chromosome:   12    Cytoband:   Chr:12q15-q16    SOURCE    GENECARD       Gene Ontology:   biological process:  cell adhesion   molecular function:  receptor activity      BioCarta Pathways:     KEGG Pathways:  
    Probe set : 1370367_at    Name:   solute carrier family 1 (neuronal/epithelial high affinity glutamate transporter, system Xag), member 1    GB acc:   D63772    UniGene:   Rn.6384    Gene Symbol:   Slc1a1    Locus Link:   25550    Chromosome:   1    Cytoband:   Chr:1q52    SOURCE    GENECARD       Gene Ontology:     BioCarta Pathways:     KEGG Pathways:  
    Probe set : 1379891_at    Name:   scotin    GB acc:   AI454858    UniGene:   Rn.102027    Gene Symbol:   RGD:1359661    Locus Link:   301013    Chromosome:   8    Cytoband:   Chr:8q32    SOURCE    GENECARD       Gene Ontology:     BioCarta Pathways:     KEGG Pathways:  
    Probe set : 1371629_at    Name:   CXXC finger 5    GB acc:   AI105444    UniGene:   Rn.105695    Gene Symbol:   RGD:1359466    Locus Link:   291670    Chromosome:   18    Cytoband:   Chr:18p11    SOURCE    GENECARD       Gene Ontology:   molecular function:  DNA binding   molecular function:  zinc ion binding      BioCarta Pathways:     KEGG Pathways:  
    Probe set : 1372236_at    Name:   Similar to Caspase recruitment domain protein 4    GB acc:   BI279079    UniGene:   Rn.53929     Locus Link:   500133      SOURCE        Gene Ontology:     BioCarta Pathways:     KEGG Pathways:  
    Probe set : 1387027_a_at    Name:   lectin, galactose binding, soluble 9    GB acc:   U72741    UniGene:   Rn.10706    Gene Symbol:   Lgals9    Locus Link:   25476    Chromosome:   10    Cytoband:   Chr:10q25    SOURCE    GENECARD       Gene Ontology:   molecular function:  signal transducer activity   molecular function:  sugar binding   molecular function:  galactose binding      BioCarta Pathways:     KEGG Pathways:  
    Probe set : 1389159_at    Name:   Transcribed locus    GB acc:   BM385437    UniGene:   Rn.16626        SOURCE        Gene Ontology:     BioCarta Pathways:     KEGG Pathways:  
    Probe set : 1388458_at    Name:   replication factor C (activator 1) 4 (predicted)    GB acc:   BI291451    UniGene:   Rn.17046    Gene Symbol:   Rfc4_predicted    Locus Link:   288003    Chromosome:   11    Cytoband:   Chr:11q23    SOURCE    GENECARD       Gene Ontology:   biological process:  DNA replication   biological process:  DNA strand elongation   cellular component:  nucleus   cellular component:  delta-DNA polymerase cofactor complex   cellular component:  DNA replication factor C complex      BioCarta Pathways:     KEGG Pathways:  
    Probe set : 1374329_at    Name:   Mcf.2 transforming sequence-like    GB acc:   BI286364    UniGene:   Rn.81062    Gene Symbol:   Mcf2l    Locus Link:   117020      SOURCE    GENECARD       Gene Ontology:   biological process:  electron transport   biological process:  intracellular signaling cascade   biological process:  Rho protein signal transduction   cellular component:  membrane   cellular component:  lamellipodium      BioCarta Pathways:     KEGG Pathways:  
    Probe set : 1379742_at    Name:   similar to SP140 nuclear body protein isoform 1    GB acc:   BI301216     Gene Symbol:   LOC501175    Locus Link:   501175    Chromosome:   9    Cytoband:   Chr:9q35     GENECARD       Gene Ontology:     BioCarta Pathways:     KEGG Pathways:  
    Probe set : 1393108_at    Name:   RT1 class I, A3    GB acc:   BM387813    UniGene:   Rn.116594    Gene Symbol:   RT1-A3    Locus Link:   309627    Chromosome:   20    Cytoband:   Chr:20p12    SOURCE    GENECARD       Gene Ontology:   biological process:  antigen presentation   cellular component:  MHC class I protein complex   molecular function:  MHC class I receptor activity      BioCarta Pathways:     KEGG Pathways:  
    Probe set : 1367973_at    Name:   chemokine (C-C motif) ligand 2    GB acc:   NM_031530    UniGene:   Rn.4772    Gene Symbol:   Ccl2    Locus Link:   24770    Chromosome:   10    Cytoband:   Chr:10q26    SOURCE    GENECARD       Gene Ontology:   biological process:  protein amino acid phosphorylation   biological process:  calcium ion homeostasis   biological process:  anti-apoptosis   biological process:  chemotaxis   biological process:  inflammatory response   biological process:  inflammatory response   biological process:  immune response   biological process:  humoral immune response   biological process:  cell adhesion   biological process:  signal transduction   biological process:  G-protein signaling, coupled to cyclic nucleotide second messenger   biological process:  JAK-STAT cascade   biological process:  cell-cell signaling   biological process:  response to pathogenic bacteria   biological process:  organ morphogenesis   biological process:  viral genome replication   biological process:  sensory perception   cellular component:  extracellular region   cellular component:  extracellular space   cellular component:  cytoplasm   cellular component:  membrane      BioCarta Pathways:     KEGG Pathways:  
    Probe set : 1370621_at    Name:   CD3 antigen, zeta polypeptide    GB acc:   D13555    UniGene:   Rn.9997    Gene Symbol:   Cd3z    Locus Link:   25300    Chromosome:   13    Cytoband:   Chr:13q23    SOURCE    GENECARD       Gene Ontology:   biological process:  cell surface receptor linked signal transduction      BioCarta Pathways:     KEGG Pathways:  
    Probe set : 1367925_at    Name:   major vault protein    GB acc:   NM_022715    UniGene:   Rn.10028    Gene Symbol:   Mvp    Locus Link:   64681    Chromosome:   1    Cytoband:   Chr:1q36    SOURCE    GENECARD       Gene Ontology:   biological process:  response to drug   cellular component:  nucleus   cellular component:  cytoplasm   cellular component:  ribonucleoprotein complex   molecular function:  calcium ion binding      BioCarta Pathways:     KEGG Pathways:  
    Probe set : 1370433_at    Name:   CCA2 protein    GB acc:   AB000199    UniGene:   Rn.11380    Gene Symbol:   Hsd3b7    Locus Link:   246211    Chromosome:   1    Cytoband:   Chr:1q36    SOURCE    GENECARD       Gene Ontology:   cellular component:  endoplasmic reticulum   cellular component:  integral to membrane      BioCarta Pathways:     KEGG Pathways:  
    Probe set : 1372865_at    Name:   zinc finger protein 364 (predicted)    GB acc:   AI171206    UniGene:   Rn.36809    Gene Symbol:   Zfp364_predicted    Locus Link:   362002    Chromosome:   2    Cytoband:   Chr:2q34    SOURCE    GENECARD       Gene Ontology:   biological process:  protein ubiquitination   cellular component:  ubiquitin ligase complex   molecular function:  ubiquitin-protein ligase activity   molecular function:  zinc ion binding      BioCarta Pathways:     KEGG Pathways:  
    Probe set : 1370243_a_at    Name:   prothymosin alpha    GB acc:   AI178772    UniGene:   Rn.817    Gene Symbol:   Ptma    Locus Link:   29222    Chromosome:   9    Cytoband:   Chr:9q35    SOURCE    GENECARD       Gene Ontology:   cellular component:  nucleus      BioCarta Pathways:     KEGG Pathways:  
    Probe set : 1372691_at    Name:   uridine phosphorylase 1 (predicted)    GB acc:   BI292558    UniGene:   Rn.101932    Gene Symbol:   Upp1_predicted    Locus Link:   289801    Chromosome:   14    Cytoband:   Chr:14q21    SOURCE    GENECARD       Gene Ontology:   biological process:  nucleoside metabolism   biological process:  nucleotide catabolism   cellular component:  cytoplasm      BioCarta Pathways:     KEGG Pathways:  
    Probe set : 1398644_at    Name:   Transcribed locus    GB acc:   AI535104    UniGene:   Rn.34740        SOURCE        Gene Ontology:     BioCarta Pathways:     KEGG Pathways:  
    Probe set : 1386871_at    Name:   glutathione peroxidase 4    GB acc:   NM_017165    UniGene:   Rn.3647    Gene Symbol:   Gpx4    Locus Link:   29328    Chromosome:   7    Cytoband:   Chr:7q11    SOURCE    GENECARD       Gene Ontology:     BioCarta Pathways:     KEGG Pathways:  
    Probe set : 1370358_at    Name:   two pore channel 1    GB acc:   AB018253    UniGene:   Rn.24484    Gene Symbol:   Tpcn1    Locus Link:   246215    Chromosome:   12    Cytoband:   Chr:12q16    SOURCE    GENECARD       Gene Ontology:   biological process:  transport   biological process:  ion transport   biological process:  cation transport   cellular component:  integral to membrane   cellular component:  membrane      BioCarta Pathways:     KEGG Pathways:  
    Probe set : 1371662_at    Name:   lysyl-tRNA synthetase    GB acc:   AA892250    UniGene:   Rn.46563    Gene Symbol:   RGD:1359653    Locus Link:   292028    Chromosome:   19    Cytoband:   Chr:19q12    SOURCE    GENECARD       Gene Ontology:   biological process:  tRNA aminoacylation for protein translation   biological process:  lysyl-tRNA aminoacylation   cellular component:  cytoplasm      BioCarta Pathways:     KEGG Pathways:  
    Probe set : 1370096_at    Name:   perforin 1 (pore forming protein)    GB acc:   NM_017330    UniGene:   Rn.11206    Gene Symbol:   Prf1    Locus Link:   50669    Chromosome:   20    Cytoband:   Chr:20q11    SOURCE    GENECARD       Gene Ontology:   biological process:  cytolysis   biological process:  cytolysis   cellular component:  cytosol   cellular component:  integral to membrane   molecular function:  calcium ion binding      BioCarta Pathways:     KEGG Pathways:  
    Probe set : 1367673_at    Name:   selenium binding protein 2    GB acc:   NM_080892    UniGene:   Rn.16617    Gene Symbol:   Selenbp1    Locus Link:   140927    Chromosome:   2    Cytoband:   Chr:2q34    SOURCE    GENECARD       Gene Ontology:   molecular function:  selenium binding      BioCarta Pathways:     KEGG Pathways:  
    Probe set : 1368512_a_at    Name:   glutamyl aminopeptidase    GB acc:   AF214568    UniGene:   Rn.53979    Gene Symbol:   Enpep    Locus Link:   64017    Chromosome:   2    Cytoband:   Chr:2q42    SOURCE    GENECARD       Gene Ontology:   cellular component:  plasma membrane   cellular component:  integral to plasma membrane   cellular component:  integral to membrane      BioCarta Pathways:     KEGG Pathways:  
    Probe set : 1392899_at    Name:   protein regulator of cytokinesis 1 (predicted)    GB acc:   BI284262    UniGene:   Rn.138438    Gene Symbol:   Prc1_predicted    Locus Link:   308761    Chromosome:   1    Cytoband:   Chr:1q31    SOURCE    GENECARD       Gene Ontology:   biological process:  mitotic spindle elongation   biological process:  cytokinesis   cellular component:  nucleus   cellular component:  spindle   cellular component:  spindle microtubule      BioCarta Pathways:     KEGG Pathways:  
    Probe set : 1369665_a_at    Name:   interleukin 18    GB acc:   AJ222813    UniGene:   Rn.11118    Gene Symbol:   Il18    Locus Link:   29197    Chromosome:   8    Cytoband:   Chr:8q23    SOURCE    GENECARD       Gene Ontology:   biological process:  angiogenesis   biological process:  immune response   biological process:  cell-cell signaling   biological process:  induction of apoptosis via death domain receptors   biological process:  regulation of cell adhesion   biological process:  sleep   biological process:  chemokine biosynthesis   biological process:  T-helper 1 type immune response   biological process:  T-helper 2 type immune response   biological process:  interleukin-2 biosynthesis   biological process:  interferon-gamma biosynthesis   biological process:  positive regulation of activated T cell proliferation   biological process:  interleukin-13 biosynthesis   biological process:  granulocyte macrophage colony-stimulating factor biosynthesis   biological process:  regulation of circadian sleep/wake cycle, non-REM sleep   biological process:  angiogenesis   biological process:  induction of apoptosis via death domain receptors   biological process:  regulation of cell adhesion   biological process:  T-helper 1 type immune response   biological process:  interferon-gamma biosynthesis   biological process:  positive regulation of activated T cell proliferation   biological process:  sleep   biological process:  regulation of circadian sleep/wake cycle, non-REM sleep   cellular component:  extracellular region   cellular component:  extracellular region   cellular component:  extracellular space      BioCarta Pathways:     KEGG Pathways:  
    Probe set : 1383086_at    Name:   Transcribed locus    GB acc:   AA818256    UniGene:   Rn.16489        SOURCE        Gene Ontology:     BioCarta Pathways:     KEGG Pathways:  
    Probe set : 1370960_at    Name:   insulin-like growth factor binding protein 5    GB acc:   BE104060    UniGene:   Rn.1593    Gene Symbol:   Igfbp5    Locus Link:   25285    Chromosome:   9    Cytoband:   Chr:9q33    SOURCE    GENECARD       Gene Ontology:   biological process:  regulation of cell growth   biological process:  signal transduction   cellular component:  extracellular region   cellular component:  extracellular space   cellular component:  insulin-like growth factor binding protein complex   molecular function:  insulin-like growth factor binding   molecular function:  growth factor binding      BioCarta Pathways:     KEGG Pathways:  
    Probe set : 1381174_at    Name:   similar to Hypothetical protein KIAA0141    GB acc:   BG381120    UniGene:   Rn.12899    Gene Symbol:   RGD:735029    Locus Link:   307480    Chromosome:   18    Cytoband:   Chr:18p11    SOURCE    GENECARD       Gene Ontology:   molecular function:  binding      BioCarta Pathways:     KEGG Pathways:  
    Probe set : 1397292_at    Name:   LOC500333    GB acc:   BF412804    UniGene:   Rn.94184    Gene Symbol:   LOC500333    Locus Link:   500333    Chromosome:   4    Cytoband:   Chr:4q42    SOURCE    GENECARD       Gene Ontology:     BioCarta Pathways:     KEGG Pathways:  
    Probe set : 1372613_at    Name:   dehydrogenase/reductase (SDR family) member 6 (predicted)    GB acc:   AI232784    UniGene:   Rn.3651    Gene Symbol:   Dhrs6_predicted    Locus Link:   295458    Chromosome:   2    Cytoband:   Chr:2q43    SOURCE    GENECARD       Gene Ontology:   biological process:  metabolism   molecular function:  oxidoreductase activity      BioCarta Pathways:     KEGG Pathways:  
    Probe set : 1384584_at    Name:   Transcribed locus    GB acc:   AA892774    UniGene:   Rn.14792        SOURCE        Gene Ontology:     BioCarta Pathways:     KEGG Pathways:  
    Probe set : 1373629_at    Name:   solute carrier family 7 (cationic amino acid transporter, y+ system), member 6 (predicted)    GB acc:   BE108272    UniGene:   Rn.8066    Gene Symbol:   Slc7a6_predicted    Locus Link:   307811    Chromosome:   19    Cytoband:   Chr:19q12    SOURCE    GENECARD       Gene Ontology:   cellular component:  integral to plasma membrane   cellular component:  integral to membrane   molecular function:  amino acid-polyamine transporter activity      BioCarta Pathways:     KEGG Pathways:  
    Probe set : 1384852_at    Name:   low Mr GTP-binding protein    GB acc:   AI043615    UniGene:   Rn.37360    Gene Symbol:   Rab27a    Locus Link:   50645    Chromosome:   8    Cytoband:   Chr:8q24    SOURCE    GENECARD       Gene Ontology:   biological process:  small GTPase mediated signal transduction   biological process:  protein transport   molecular function:  GTPase activity   molecular function:  GTP binding   molecular function:  GTP binding      BioCarta Pathways:     KEGG Pathways:  
    Probe set : 1389576_at    Name:   U2 small nuclear ribonucleoprotein B (predicted)    GB acc:   AA944322    UniGene:   Rn.7712    Gene Symbol:   Snrpb2_predicted    Locus Link:   362223    Chromosome:   3    Cytoband:   Chr:3q41    SOURCE    GENECARD       Gene Ontology:   biological process:  nuclear mRNA splicing, via spliceosome   biological process:  mRNA processing   biological process:  RNA splicing   molecular function:  RNA binding      BioCarta Pathways:     KEGG Pathways:  
    Probe set : 1375926_at    Name:   Transcribed locus    GB acc:   BG372589    UniGene:   Rn.22507        SOURCE        Gene Ontology:     BioCarta Pathways:     KEGG Pathways:  
    Probe set : 1367825_at    Name:   ral guanine nucleotide dissociation stimulator    GB acc:   NM_019250    UniGene:   Rn.40174    Gene Symbol:   Ralgds    Locus Link:   29622    Chromosome:   3    Cytoband:   Chr:3p12    SOURCE    GENECARD       Gene Ontology:     BioCarta Pathways:     KEGG Pathways:  
    Probe set : 1372604_at    Name:   similar to RIKEN cDNA 2210421G13    GB acc:   BI289459     Gene Symbol:   LOC315106    Locus Link:   315106    Chromosome:   7    Cytoband:   Chr:7q34     GENECARD       Gene Ontology:     BioCarta Pathways:     KEGG Pathways:  
    Probe set : 1374468_at    Name:   myeloid differentiation primary response gene 88    GB acc:   AI236590    UniGene:   Rn.37341    Gene Symbol:   Myd88    Locus Link:   301059    Chromosome:   8    Cytoband:   Chr:8q32    SOURCE    GENECARD       Gene Ontology:   biological process:  Toll signaling pathway   biological process:  positive regulation of I-kappaB kinase/NF-kappaB cascade   biological process:  signal transduction   cellular component:  membrane   molecular function:  Toll binding   molecular function:  transmembrane receptor activity   molecular function:  protein binding      BioCarta Pathways:     KEGG Pathways:  
    Probe set : 1369031_at    Name:   interferon gamma inducing factor binding protein    GB acc:   NM_053374    UniGene:   Rn.20083    Gene Symbol:   Il18bp    Locus Link:   84388    Chromosome:   1    Cytoband:   Chr:1q32    SOURCE    GENECARD       Gene Ontology:     BioCarta Pathways:     KEGG Pathways:  
    Probe set : 1394082_at    Name:   similar to T-Brain-1    GB acc:   BG662631    UniGene:   Rn.8247    Gene Symbol:   LOC311078    Locus Link:   311078    Chromosome:   3    Cytoband:   Chr:3q21    SOURCE    GENECARD       Gene Ontology:     BioCarta Pathways:     KEGG Pathways:  
    Probe set : 1383248_at    Name:   flavin containing monooxygenase 5    GB acc:   AI454611    UniGene:   Rn.7038    Gene Symbol:   Fmo5    Locus Link:   246248    Chromosome:   2    Cytoband:   Chr:2q34    SOURCE    GENECARD       Gene Ontology:   biological process:  electron transport      BioCarta Pathways:     KEGG Pathways:  
    Probe set : 1374565_at    Name:   NIMA (never in mitosis gene a)-related expressed kinase 6    GB acc:   BF282365    UniGene:   Rn.18744    Gene Symbol:   Nek6    Locus Link:   360161    Chromosome:   3    Cytoband:   Chr:3q11    SOURCE    GENECARD       Gene Ontology:   biological process:  protein amino acid phosphorylation      BioCarta Pathways:     KEGG Pathways:  
    Probe set : 1389477_at    Name:   Transcribed locus    GB acc:   BI278855    UniGene:   Rn.35620        SOURCE        Gene Ontology:     BioCarta Pathways:     KEGG Pathways:  
    Probe set : 1390866_at    Name:   Transcribed locus    GB acc:   AI168981    UniGene:   Rn.146252        SOURCE        Gene Ontology:     BioCarta Pathways:     KEGG Pathways:  
    Probe set : 1394479_at    Name:   Transcribed locus    GB acc:   BI288580    UniGene:   Rn.148355        SOURCE        Gene Ontology:     BioCarta Pathways:     KEGG Pathways:  
    Probe set : 1374387_at    Name:   ADP-ribosylation factor-like 6 interacting protein 5    GB acc:   BG662580    UniGene:   Rn.64512    Gene Symbol:   Arl6ip5    Locus Link:   66028    Chromosome:   4    Cytoband:   Chr:4q34    SOURCE    GENECARD       Gene Ontology:   biological process:  L-glutamate transport   biological process:  negative regulation of transport   cellular component:  cytoplasm   cellular component:  endoplasmic reticulum   cellular component:  membrane   cellular component:  integral to membrane   molecular function:  protein binding   molecular function:  protein C-terminus binding      BioCarta Pathways:     KEGG Pathways:  
    Probe set : 1379258_at    Name:   kelch-like 5 (Drosophila) (predicted)    GB acc:   AA998367    UniGene:   Rn.13334    Gene Symbol:   Klhl5_predicted    Locus Link:   305351    Chromosome:   14    Cytoband:   Chr:14p11    SOURCE    GENECARD       Gene Ontology:   biological process:  actin cytoskeleton organization and biogenesis   cellular component:  cytoplasm   cellular component:  cytoskeleton   molecular function:  actin binding   molecular function:  protein binding      BioCarta Pathways:     KEGG Pathways:  
    Probe set : 1389103_at    Name:   similar to RIKEN cDNA 2810037C03 (predicted)    GB acc:   BM391005    UniGene:   Rn.103411    Gene Symbol:   RGD1307397_predicted    Locus Link:   290842    Chromosome:   16    Cytoband:   Chr:16q12.5    SOURCE    GENECARD       Gene Ontology:     BioCarta Pathways:     KEGG Pathways:  
    Probe set : 1367763_at    Name:   acetyl-coenzyme A acetyltransferase 1    GB acc:   D13921    UniGene:   Rn.4054    Gene Symbol:   Acat1    Locus Link:   25014    Chromosome:   8    Cytoband:   Chr:8q24.1    SOURCE    GENECARD       Gene Ontology:   biological process:  lipoprotein biosynthesis   cellular component:  mitochondrion   cellular component:  mitochondrion      BioCarta Pathways:     KEGG Pathways:  
    Probe set : 1368270_at    Name:   apolipoprotein B editing complex 1    GB acc:   NM_012907    UniGene:   Rn.10002    Gene Symbol:   Apobec1    Locus Link:   25383    Chromosome:   4    Cytoband:   Chr:4q42    SOURCE    GENECARD       Gene Ontology:   cellular component:  nucleus   cellular component:  nucleus      BioCarta Pathways:     KEGG Pathways:  
    Probe set : 1370928_at    Name:   LPS-induced TN factor    GB acc:   BI284739    UniGene:   Rn.6940    Gene Symbol:   Litaf    Locus Link:   65161    Chromosome:   10    Cytoband:   Chr:10q11    SOURCE    GENECARD       Gene Ontology:   biological process:  regulation of transcription from RNA polymerase II promoter   biological process:  positive regulation of I-kappaB kinase/NF-kappaB cascade   cellular component:  extracellular region   cellular component:  nucleus   molecular function:  RNA polymerase II transcription factor activity   molecular function:  signal transducer activity   molecular function:  protein binding      BioCarta Pathways:     KEGG Pathways:  
    Probe set : 1392681_at    Name:   RAB2, member RAS oncogene family    GB acc:   AW522578    UniGene:   Rn.44916    Gene Symbol:   Rab2    Locus Link:   65158    Chromosome:   5    Cytoband:   Chr:5q13    SOURCE    GENECARD       Gene Ontology:   cellular component:  endoplasmic reticulum   cellular component:  Golgi apparatus   cellular component:  Golgi stack      BioCarta Pathways:     KEGG Pathways:  
    Probe set : 1368742_at    Name:   complement component 5, receptor 1    GB acc:   NM_053619    UniGene:   Rn.10680    Gene Symbol:   C5r1    Locus Link:   113959    Chromosome:   1    Cytoband:   Chr:1q12    SOURCE    GENECARD       Gene Ontology:   cellular component:  cell surface   cellular component:  cell surface   cellular component:  integral to membrane      BioCarta Pathways:     KEGG Pathways:  
    Probe set : 1377899_at    Name:   similar to RIKEN cDNA 2810025M15 (predicted)    GB acc:   BF419886    UniGene:   Rn.76251    Gene Symbol:   RGD1304982_predicted    Locus Link:   289138    Chromosome:   13    Cytoband:   Chr:13q22    SOURCE    GENECARD       Gene Ontology:     BioCarta Pathways:     KEGG Pathways:  
    Probe set : 1378321_at    Name:   Ras association (RalGDS/AF-6) domain family 4 (predicted)    GB acc:   BG375029    UniGene:   Rn.11482    Gene Symbol:   Rassf4_predicted    Locus Link:   362423    Chromosome:   4    Cytoband:   Chr:4q42    SOURCE    GENECARD       Gene Ontology:   biological process:  signal transduction   biological process:  neuropeptide signaling pathway   molecular function:  protein binding   molecular function:  oxidoreductase activity      BioCarta Pathways:     KEGG Pathways:  
    Probe set : 1391411_at    Name:   Formin-like 2 (predicted)    GB acc:   AI179941    UniGene:   Rn.7075     Locus Link:   311033    Chromosome:   3    Cytoband:   Chr:3q12    SOURCE        Gene Ontology:   cellular component:  nucleus      BioCarta Pathways:     KEGG Pathways:  
    Probe set : 1369549_at    Name:   NKR-P2, ortholog of human NKG2D    GB acc:   NM_133512    UniGene:   Rn.14544    Gene Symbol:   Nkrp2    Locus Link:   24934    Chromosome:   4    Cytoband:   Chr:4q42    SOURCE    GENECARD       Gene Ontology:   cellular component:  integral to membrane      BioCarta Pathways:     KEGG Pathways:  
    Probe set : 1394678_at    Name:   FYVE, RhoGEF and PH domain containing 2 (predicted)    GB acc:   AI411563    UniGene:   Rn.89444     Locus Link:   309653    Chromosome:   20    Cytoband:   Chr:20p12    SOURCE        Gene Ontology:   molecular function:  zinc ion binding      BioCarta Pathways:     KEGG Pathways:  
    Probe set : 1367655_at    Name:   similar to thymosin, beta 10    GB acc:   NM_021261     Gene Symbol:   LOC502600    Locus Link:   502600    Chromosome:   2    Cytoband:   Chr:2q44     GENECARD       Gene Ontology:     BioCarta Pathways:     KEGG Pathways:  
    Probe set : 1367922_at    Name:   a disintegrin and metalloproteinase domain 17 (tumor necrosis factor, alpha, converting enzyme)    GB acc:   NM_020306    UniGene:   Rn.144585    Gene Symbol:   Adam17    Locus Link:   57027    Chromosome:   6    Cytoband:   Chr:6q16    SOURCE    GENECARD       Gene Ontology:     BioCarta Pathways:     KEGG Pathways:  
    Probe set : 1375362_at     GB acc:   BI304125              Gene Ontology:     BioCarta Pathways:     KEGG Pathways:  
    Probe set : 1376688_a_at    Name:   Transcribed locus, moderately similar to NP_705730.1 paired immunoglobin-like type 2 receptor alpha [Mus musculus]    GB acc:   BF288115    UniGene:   Rn.18754        SOURCE        Gene Ontology:     BioCarta Pathways:     KEGG Pathways:  
    Probe set : 1383158_at    Name:   similar to HIV-1 Rev binding protein    GB acc:   AI575747    UniGene:   Rn.3734    Gene Symbol:   LOC363266    Locus Link:   363266    Chromosome:   9    Cytoband:   Chr:9q34    SOURCE    GENECARD       Gene Ontology:   biological process:  regulation of GTPase activity      BioCarta Pathways:     KEGG Pathways:  
    Probe set : 1392527_at    Name:   similar to homolog of yeast TIM14 isoform c    GB acc:   AI137840    UniGene:   Rn.6532    Gene Symbol:   LOC502525    Locus Link:   502525    Chromosome:   2    Cytoband:   Chr:2q25    SOURCE    GENECARD       Gene Ontology:     BioCarta Pathways:     KEGG Pathways:  
    Probe set : 1368081_at    Name:   ATP-binding cassette, sub-family A (ABC1), member 2    GB acc:   NM_024396    UniGene:   Rn.13115    Gene Symbol:   Abca2    Locus Link:   79248    Chromosome:   3    Cytoband:   Chr:3p13    SOURCE    GENECARD       Gene Ontology:     BioCarta Pathways:     KEGG Pathways:  
    Probe set : 1383850_at    Name:   Similar to RIKEN cDNA 1600014C10    GB acc:   BF548042    UniGene:   Rn.2361     Locus Link:   502324      SOURCE        Gene Ontology:     BioCarta Pathways:     KEGG Pathways:  
    Probe set : 1383424_at    Name:   thymidylate kinase family LPS-inducible member (predicted)    GB acc:   BI276216    UniGene:   Rn.100440    Gene Symbol:   Tyki_predicted    Locus Link:   314004    Chromosome:   6    Cytoband:   Chr:6q16    SOURCE    GENECARD       Gene Ontology:   biological process:  dTDP biosynthesis   biological process:  dTTP biosynthesis   biological process:  nucleotide biosynthesis      BioCarta Pathways:     KEGG Pathways:  
    Probe set : 1372328_at    Name:   kinesin-like 8 (predicted)    GB acc:   BM386079    UniGene:   Rn.16800    Gene Symbol:   Knsl8_predicted    Locus Link:   316226    Chromosome:   9    Cytoband:   Chr:9q12    SOURCE    GENECARD       Gene Ontology:   cellular component:  kinesin complex   cellular component:  microtubule   molecular function:  motor activity   molecular function:  microtubule motor activity   molecular function:  binding      BioCarta Pathways:     KEGG Pathways:  
    Probe set : 1398282_at    Name:   kynureninase (L-kynurenine hydrolase)    GB acc:   NM_053902    UniGene:   Rn.10575    Gene Symbol:   Kynu    Locus Link:   116682    Chromosome:   3    Cytoband:   Chr:3q12    SOURCE    GENECARD       Gene Ontology:   cellular component:  cytoplasm      BioCarta Pathways:     KEGG Pathways:  
    Probe set : 1398757_at    Name:   nucleophosmin 1    GB acc:   NM_012992    UniGene:   Rn.112126    Gene Symbol:   Npm1    Locus Link:   25498    Chromosome:   10    Cytoband:   Chr:10q12    SOURCE    GENECARD       Gene Ontology:   biological process:  intracellular protein transport   biological process:  nucleocytoplasmic transport   biological process:  response to stress   biological process:  centrosome cycle   biological process:  cell aging   biological process:  negative regulation of cell proliferation   biological process:  ribosome assembly   biological process:  activation of NF-kappaB transcription factor   biological process:  intracellular protein transport   biological process:  nucleocytoplasmic transport   biological process:  centrosome cycle   biological process:  cell aging   biological process:  negative regulation of cell proliferation      BioCarta Pathways:     KEGG Pathways:  
    Probe set : 1368260_at    Name:   aurora kinase B    GB acc:   NM_053749    UniGene:   Rn.10865    Gene Symbol:   Aurkb    Locus Link:   114592    Chromosome:   10    Cytoband:   Chr:10q24    SOURCE    GENECARD       Gene Ontology:   cellular component:  spindle   cellular component:  spindle   cellular component:  midbody      BioCarta Pathways:     KEGG Pathways:  
    Probe set : 1368409_at    Name:   glutathione S-transferase, theta 2    GB acc:   NM_012796    UniGene:   Rn.87212    Gene Symbol:   Gstt2    Locus Link:   29487    Chromosome:   20    Cytoband:   Chr:20p12    SOURCE    GENECARD       Gene Ontology:   biological process:  glutathione metabolism   biological process:  glutathione metabolism   cellular component:  nucleus      BioCarta Pathways:     KEGG Pathways:  
    Probe set : 1382274_at    Name:   retinoic acid receptor responder (tazarotene induced) 1 (predicted)    GB acc:   AA819288    UniGene:   Rn.16737    Gene Symbol:   Rarres1_predicted    Locus Link:   310486    Chromosome:   2    Cytoband:   Chr:2q31    SOURCE    GENECARD       Gene Ontology:   biological process:  negative regulation of cell proliferation   cellular component:  integral to membrane      BioCarta Pathways:     KEGG Pathways:  
    Probe set : 1378137_at    Name:   similar to ribosome-binding protein p34 - rat (predicted)    GB acc:   BG379788    UniGene:   Rn.2783    Gene Symbol:   RGD1305092_predicted    Locus Link:   287633    Chromosome:   10    Cytoband:   Chr:10q31    SOURCE    GENECARD       Gene Ontology:   cellular component:  integral to membrane      BioCarta Pathways:     KEGG Pathways:  
    Probe set : 1369290_at    Name:   chemokine (C-C) receptor 5    GB acc:   NM_053960    UniGene:   Rn.10736    Gene Symbol:   Ccr5    Locus Link:   117029    Chromosome:   8    Cytoband:   Chr:8q32    SOURCE    GENECARD       Gene Ontology:   cellular component:  endosome   cellular component:  cell surface   cellular component:  integral to membrane      BioCarta Pathways:     KEGG Pathways:  
    Probe set : 1379356_at    Name:   Similar to RIKEN cDNA C230093N12 (predicted)    GB acc:   AI574991    UniGene:   Rn.15953     Locus Link:   365903    Chromosome:   2    Cytoband:   Chr:2q34    SOURCE        Gene Ontology:     BioCarta Pathways:     KEGG Pathways:  
    Probe set : 1379587_at     GB acc:   AI717097              Gene Ontology:     BioCarta Pathways:     KEGG Pathways:  
    Probe set : 1388275_at    Name:   T-cell receptor beta chain    GB acc:   AW919577     Gene Symbol:   Tcrb    Locus Link:   24820    Chromosome:   4    Cytoband:   Chr:4q22     GENECARD       Gene Ontology:   cellular component:  plasma membrane   cellular component:  integral to plasma membrane   cellular component:  external side of plasma membrane   molecular function:  receptor activity   molecular function:  MHC protein binding   molecular function:  peptide antigen binding      BioCarta Pathways:     KEGG Pathways:  
    Probe set : 1372550_at     GB acc:   AI169278              Gene Ontology:     BioCarta Pathways:     KEGG Pathways:  
    Probe set : 1369621_s_at    Name:   FK506 binding protein 1a    GB acc:   NM_053308    UniGene:   Rn.80611    Gene Symbol:   Fkbp1a    Locus Link:   25639    Chromosome:   3    Cytoband:   Chr:3q41    SOURCE    GENECARD       Gene Ontology:   biological process:  protein folding   biological process:  transport   biological process:  positive regulation of I-kappaB kinase/NF-kappaB cascade   cellular component:  microsome   cellular component:  membrane      BioCarta Pathways:     KEGG Pathways:  
    Probe set : 1371074_a_at    Name:   mini chromosome maintenance deficient 6 (S. cerevisiae)    GB acc:   U17565    UniGene:   Rn.33226    Gene Symbol:   Mcmd6    Locus Link:   29685    Chromosome:   13    Cytoband:   Chr:13q12    SOURCE    GENECARD       Gene Ontology:   cellular component:  nucleus      BioCarta Pathways:     KEGG Pathways:  
    Probe set : 1390569_at    Name:   similar to carnosinase 1    GB acc:   AI231438    UniGene:   Rn.15548    Gene Symbol:   RGD:1359493    Locus Link:   307212    Chromosome:   18    Cytoband:   Chr:18q12.3    SOURCE    GENECARD       Gene Ontology:   biological process:  proteolysis   molecular function:  metallopeptidase activity   molecular function:  hydrolase activity   molecular function:  protein dimerization activity      BioCarta Pathways:     KEGG Pathways:  
    Probe set : 1376379_a_at    Name:   similar to RIKEN cDNA 2310004I24 gene (predicted)    GB acc:   AW252141    UniGene:   Rn.32842    Gene Symbol:   RGD1309906_predicted    Locus Link:   287406    Chromosome:   10    Cytoband:   Chr:10q24    SOURCE    GENECARD       Gene Ontology:   molecular function:  hydrolase activity      BioCarta Pathways:     KEGG Pathways:  
    Probe set : 1388330_at    Name:   vitamin K epoxide reductase complex, subunit 1    GB acc:   BM384958    UniGene:   Rn.97942    Gene Symbol:   RGD:1303107    Locus Link:   309004    Chromosome:   1    Cytoband:   Chr:1q36    SOURCE    GENECARD       Gene Ontology:   biological process:  regulation of blood coagulation   biological process:  vitamin K biosynthesis   biological process:  vitamin K metabolism      BioCarta Pathways:     KEGG Pathways:  
    Probe set : 1370400_at    Name:   RT1 class Ib gene, H2-TL-like, grc region (N1) /// RT1 class Ib gene, H2-TL-like, grc region (N3) /// RT1 class Ib gene, H2-TL-like, grc region(N2)    GB acc:   L23128    UniGene:   Rn.107070    Gene Symbol:   RT1-N1 /// RT1-N3 /// RT1-N2    Locus Link:   24748 /// 24750 /// 360323    Chromosome:   20    Cytoband:   Chr:20p12    SOURCE    GENECARD       Gene Ontology:   biological process:  antigen presentation   cellular component:  MHC class I protein complex   cellular component:  MHC class I protein complex   molecular function:  MHC class I receptor activity      BioCarta Pathways:     KEGG Pathways:  
    Probe set : 1397628_at    Name:   similar to RIKEN cDNA 2900092E17 (predicted)    GB acc:   BM390965    UniGene:   Rn.38914    Gene Symbol:   RGD1305592_predicted    Locus Link:   293500    Chromosome:   1    Cytoband:   Chr:1q36    SOURCE    GENECARD       Gene Ontology:     BioCarta Pathways:     KEGG Pathways:  
    Probe set : 1374725_at    Name:   similar to mKIAA1631 protein    GB acc:   AW252811    UniGene:   Rn.13838    Gene Symbol:   LOC310756    Locus Link:   310756    Chromosome:   2    Cytoband:   Chr:2q34    SOURCE    GENECARD       Gene Ontology:     BioCarta Pathways:     KEGG Pathways:  
    Probe set : 1387108_at    Name:   casein kinase 2, beta subunit    GB acc:   BF281311    UniGene:   Rn.11095    Gene Symbol:   Csnk2b    Locus Link:   81650    Chromosome:   20    Cytoband:   Chr:20p12    SOURCE    GENECARD       Gene Ontology:   cellular component:  protein kinase CK2 complex      BioCarta Pathways:     KEGG Pathways:  
    Probe set : 1377832_at    Name:   polo-like kinase 4 (Drosophila) (predicted)    GB acc:   BE109322    UniGene:   Rn.14848    Gene Symbol:   Plk4_predicted    Locus Link:   310344    Chromosome:   2    Cytoband:   Chr:2q26    SOURCE    GENECARD       Gene Ontology:   biological process:  regulation of progression through cell cycle   biological process:  protein amino acid phosphorylation   biological process:  cell cycle      BioCarta Pathways:     KEGG Pathways:  
    Probe set : 1392528_at    Name:   Granulocyte-macrophage colony stimulating receptor alpha (CSFR alpha gene)    GB acc:   AI555854    UniGene:   Rn.128082        SOURCE        Gene Ontology:   cellular component:  integral to membrane   molecular function:  receptor activity      BioCarta Pathways:     KEGG Pathways:  
    Probe set : 1370806_at    Name:   all-trans-13,14-dihydroretinol saturase    GB acc:   AF465614    UniGene:   Rn.55275    Gene Symbol:   Retsat    Locus Link:   246298    Chromosome:   4    Cytoband:   Chr:4q33    SOURCE    GENECARD       Gene Ontology:   biological process:  electron transport   molecular function:  oxidoreductase activity      BioCarta Pathways:     KEGG Pathways:  
    Probe set : 1392737_at    Name:   Transcribed locus    GB acc:   AI044644    UniGene:   Rn.15979        SOURCE        Gene Ontology:     BioCarta Pathways:     KEGG Pathways:  
    Probe set : 1380577_at    Name:   ATP-binding cassette, sub-family G (WHITE), member 2    GB acc:   AI175616    UniGene:   Rn.13131    Gene Symbol:   Abcg2    Locus Link:   312382    Chromosome:   4    Cytoband:   Chr:4q24    SOURCE    GENECARD       Gene Ontology:   biological process:  transport   cellular component:  membrane   cellular component:  integral to membrane      BioCarta Pathways:     KEGG Pathways:  
    Probe set : 1371938_at    Name:   GPI-anchored membrane protein 1 (predicted)    GB acc:   BM390336    UniGene:   Rn.108072     Locus Link:   362173    Chromosome:   3    Cytoband:   Chr:3q32    SOURCE        Gene Ontology:   cellular component:  integral to plasma membrane      BioCarta Pathways:     KEGG Pathways:  
    Probe set : 1373850_at    Name:   sphingomyelin phosphodiesterase, acid-like 3B (predicted)    GB acc:   AW251315     Gene Symbol:   Smpdl3b_predicted    Locus Link:   362619    Chromosome:   5    Cytoband:   Chr:5q36     GENECARD       Gene Ontology:   biological process:  carbohydrate metabolism   cellular component:  extracellular space   molecular function:  hydrolase activity   molecular function:  hydrolase activity, acting on glycosyl bonds      BioCarta Pathways:     KEGG Pathways:  
    Probe set : 1372170_at    Name:   aminoacylase 1    GB acc:   AI411530    UniGene:   Rn.144903    Gene Symbol:   Acy1    Locus Link:   300981    Chromosome:   8    Cytoband:   Chr:8q32    SOURCE    GENECARD       Gene Ontology:   biological process:  proteolysis   biological process:  amino acid metabolism   cellular component:  cytosol   cellular component:  cytoplasm      BioCarta Pathways:     KEGG Pathways:  
    Probe set : 1371968_at    Name:   CGI-119 protein    GB acc:   AI013781    UniGene:   Rn.104707    Gene Symbol:   Cgi119    Locus Link:   362884    Chromosome:   7    Cytoband:   Chr:7q22    SOURCE    GENECARD       Gene Ontology:     BioCarta Pathways:     KEGG Pathways:  
    Probe set : 1390384_at    Name:   similar to Histone H2A.x (H2a/x)    GB acc:   AA875257    UniGene:   Rn.2850    Gene Symbol:   LOC500987    Locus Link:   500987    Chromosome:   8    Cytoband:   Chr:8q22    SOURCE    GENECARD       Gene Ontology:     BioCarta Pathways:     KEGG Pathways:  
    Probe set : 1375989_a_at    Name:   similar to nuclear factor kappa B subunit p100 (predicted)    GB acc:   AI170362    UniGene:   Rn.25549    Gene Symbol:   RGD1307189_predicted    Locus Link:   309452    Chromosome:   1    Cytoband:   Chr:1q54    SOURCE    GENECARD       Gene Ontology:   cellular component:  nucleus      BioCarta Pathways:     KEGG Pathways:  
    Probe set : 1368575_at    Name:   solute carrier family 6 (neurotransmitter transporter), member 18    GB acc:   NM_017163    UniGene:   Rn.9998    Gene Symbol:   Slc6a18    Locus Link:   29323    Chromosome:   1    Cytoband:   Chr:1p11    SOURCE    GENECARD       Gene Ontology:     BioCarta Pathways:     KEGG Pathways:  
    Probe set : 1370340_x_at    Name:   tropomyosin 3, gamma    GB acc:   BI295970    UniGene:   Rn.37575    Gene Symbol:   Tpm3    Locus Link:   117557    Chromosome:   2    Cytoband:   Chr:2q34    SOURCE    GENECARD       Gene Ontology:   biological process:  regulation of muscle contraction   biological process:  muscle development   cellular component:  cytoskeleton   cellular component:  muscle thin filament tropomyosin   molecular function:  actin binding      BioCarta Pathways:     KEGG Pathways:  
    Probe set : 1397655_at     GB acc:   BF415097              Gene Ontology:     BioCarta Pathways:     KEGG Pathways:  
    Probe set : 1368073_at    Name:   interferon regulatory factor 1    GB acc:   NM_012591    UniGene:   Rn.6396    Gene Symbol:   Irf1    Locus Link:   24508    Chromosome:   10    Cytoband:   Chr:10q22    SOURCE    GENECARD       Gene Ontology:   cellular component:  nucleus   molecular function:  DNA binding   molecular function:  transcription factor activity   molecular function:  transcription factor activity      BioCarta Pathways:     KEGG Pathways:  
    Probe set : 1393201_at    Name:   similar to lymphoid-restricted membrane protein    GB acc:   BI283679    UniGene:   Rn.54706    Gene Symbol:   LOC500361    Locus Link:   500361    Chromosome:   4    Cytoband:   Chr:4q44    SOURCE    GENECARD       Gene Ontology:     BioCarta Pathways:     KEGG Pathways:  
    Probe set : 1393666_at    Name:   Transcribed locus    GB acc:   BE101321    UniGene:   Rn.6458        SOURCE        Gene Ontology:     BioCarta Pathways:     KEGG Pathways:  
    Probe set : 1382950_at    Name:   Guanylate nucleotide binding protein 3 (predicted)    GB acc:   AA901350    UniGene:   Rn.15220     Locus Link:   310917    Chromosome:   2    Cytoband:   Chr:2q44    SOURCE        Gene Ontology:   biological process:  immune response   cellular component:  cytosol   molecular function:  GTPase activity   molecular function:  GTP binding      BioCarta Pathways:     KEGG Pathways:  
    Probe set : 1372643_at    Name:   similar to protein 4.1G    GB acc:   BE110569    UniGene:   Rn.1362    Gene Symbol:   LOC309557    Locus Link:   309557    Chromosome:   1    Cytoband:   Chr:1p12    SOURCE    GENECARD       Gene Ontology:     BioCarta Pathways:     KEGG Pathways:  
    Probe set : 1369520_a_at    Name:   branched chain aminotransferase 1, cytosolic    GB acc:   NM_017253    UniGene:   Rn.8273    Gene Symbol:   Bcat1    Locus Link:   29592    Chromosome:   4    Cytoband:   Chr:4q44    SOURCE    GENECARD       Gene Ontology:   cellular component:  cytosol      BioCarta Pathways:     KEGG Pathways:  
    Probe set : 1367672_at    Name:   hydroxysteroid (17-beta) dehydrogenase 4    GB acc:   NM_024392    UniGene:   Rn.2082    Gene Symbol:   Hsd17b4    Locus Link:   79244    Chromosome:   18    Cytoband:   Chr:18q11    SOURCE    GENECARD       Gene Ontology:   cellular component:  mitochondrion   cellular component:  peroxisome      BioCarta Pathways:     KEGG Pathways:  
    Probe set : 1370186_at    Name:   proteosome (prosome, macropain) subunit, beta type 9    GB acc:   AI599350    UniGene:   Rn.13686    Gene Symbol:   Psmb9    Locus Link:   24967    Chromosome:   20    Cytoband:   Chr:20p12    SOURCE    GENECARD       Gene Ontology:   biological process:  proteolysis   biological process:  ubiquitin-dependent protein catabolism   biological process:  immune response      BioCarta Pathways:     KEGG Pathways:  
    Probe set : 1371388_at    Name:   pyruvate dehydrogenase (lipoamide) beta    GB acc:   BM389223    UniGene:   Rn.102424    Gene Symbol:   RGD:1359146    Locus Link:   289950    Chromosome:   15    Cytoband:   Chr:15p14    SOURCE    GENECARD       Gene Ontology:     BioCarta Pathways:     KEGG Pathways:  
    Probe set : 1371033_at    Name:   RT1 class II, locus Bb    GB acc:   AI715202    UniGene:   Rn.20089    Gene Symbol:   RT1-Bb    Locus Link:   309622    Chromosome:   20    Cytoband:   Chr:20p12    SOURCE    GENECARD       Gene Ontology:   cellular component:  MHC class II protein complex   cellular component:  membrane   cellular component:  integral to membrane   molecular function:  MHC class II receptor activity      BioCarta Pathways:     KEGG Pathways:  
    Probe set : 1374799_at    Name:   similar to mKIAA0159 protein    GB acc:   AI407418    UniGene:   Rn.7870    Gene Symbol:   LOC362438    Locus Link:   362438    Chromosome:   4    Cytoband:   Chr:4q42    SOURCE    GENECARD       Gene Ontology:     BioCarta Pathways:     KEGG Pathways:  
    Probe set : 1371106_at    Name:   integrin, beta 7    GB acc:   AF003598    UniGene:   Rn.10767    Gene Symbol:   Itgb7    Locus Link:   25713    Chromosome:   7    Cytoband:   Chr:7q36    SOURCE    GENECARD       Gene Ontology:     BioCarta Pathways:     KEGG Pathways:  
    Probe set : 1367679_at    Name:   CD74 antigen (invariant polpypeptide of major histocompatibility class II antigen-associated)    GB acc:   NM_013069    UniGene:   Rn.33804    Gene Symbol:   Cd74    Locus Link:   25599    Chromosome:   18    Cytoband:   Chr:18q12.1    SOURCE    GENECARD       Gene Ontology:   cellular component:  integral to membrane   cellular component:  membrane   molecular function:  unfolded protein binding   molecular function:  cytokine binding      BioCarta Pathways:     KEGG Pathways:  
    Probe set : 1373997_at    Name:   Transcribed locus    GB acc:   AA925118    UniGene:   Rn.8670        SOURCE        Gene Ontology:     BioCarta Pathways:     KEGG Pathways:  
    Probe set : 1368428_at    Name:   X-prolyl aminopeptidase (aminopeptidase P) 2, membrane-bound    GB acc:   NM_057155    UniGene:   Rn.56142    Gene Symbol:   Xpnpep2    Locus Link:   117522    Chromosome:   X    Cytoband:   Chr:Xq35    SOURCE    GENECARD       Gene Ontology:   biological process:  proteolysis   cellular component:  membrane      BioCarta Pathways:     KEGG Pathways:  
    Probe set : 1373164_at    Name:   Serine/threonine kinase 17b (apoptosis-inducing)    GB acc:   AI012590    UniGene:   Rn.1300    Gene Symbol:   Stk17b    Locus Link:   170904    Chromosome:   9    Cytoband:   Chr:9q31    SOURCE    GENECARD       Gene Ontology:     BioCarta Pathways:     KEGG Pathways:  
    Probe set : 1370890_at    Name:   ARP3 actin-related protein 3 homolog (yeast)    GB acc:   AI012420    UniGene:   Rn.103326    Gene Symbol:   Actr3    Locus Link:   81732    Chromosome:   13    Cytoband:   Chr:13q11    SOURCE    GENECARD       Gene Ontology:   biological process:  cell motility   molecular function:  structural molecule activity   molecular function:  structural constituent of cytoskeleton   molecular function:  protein binding      BioCarta Pathways:     KEGG Pathways:  
    Probe set : 1371782_at    Name:   NIPSNAP-related protein    GB acc:   BF283798    UniGene:   Rn.8287    Gene Symbol:   RGD:1359688    Locus Link:   313211    Chromosome:   5    Cytoband:   Chr:5q24    SOURCE    GENECARD       Gene Ontology:     BioCarta Pathways:     KEGG Pathways:  
    Probe set : 1380019_at    Name:   T-cell, immune regulator 1    GB acc:   BM392376    UniGene:   Rn.12627    Gene Symbol:   Tcirg1    Locus Link:   293650    Chromosome:   1    Cytoband:   Chr:1q42    SOURCE    GENECARD       Gene Ontology:   biological process:  bone resorption   biological process:  proton transport   cellular component:  hydrogen-transporting ATPase V0 domain   cellular component:  membrane   molecular function:  hydrogen-transporting ATPase activity, rotational mechanism      BioCarta Pathways:     KEGG Pathways:  
    Probe set : 1367567_at    Name:   ribosomal protein L6    GB acc:   NM_053971    UniGene:   Rn.2660    Gene Symbol:   Rpl6    Locus Link:   117042    Chromosome:   12    Cytoband:   Chr:12q16    SOURCE    GENECARD       Gene Ontology:   biological process:  regulation of transcription, DNA-dependent   biological process:  protein biosynthesis   biological process:  ribosome biogenesis   molecular function:  DNA binding   molecular function:  RNA binding   molecular function:  structural constituent of ribosome      BioCarta Pathways:     KEGG Pathways:  
    Probe set : 1379452_at    Name:   similar to growth arrest-specific protein 2 - mouse    GB acc:   AI411492    UniGene:   Rn.19800    Gene Symbol:   LOC499156    Locus Link:   499156    Chromosome:   1    Cytoband:   Chr:1q22    SOURCE    GENECARD       Gene Ontology:   biological process:  cell cycle arrest      BioCarta Pathways:     KEGG Pathways:  
    Probe set : 1388455_at    Name:   guanine nucleotide binding protein (G protein), gamma 10    GB acc:   AA799996    UniGene:   Rn.3319    Gene Symbol:   Gng10    Locus Link:   114119    Chromosome:   5    Cytoband:   Chr:5q24    SOURCE    GENECARD       Gene Ontology:   biological process:  G-protein coupled receptor protein signaling pathway   cellular component:  heterotrimeric G-protein complex   cellular component:  heterotrimeric G-protein complex   molecular function:  signal transducer activity      BioCarta Pathways:     KEGG Pathways:  
    Probe set : 1379285_at    Name:   similar to 5830458K16Rik protein (predicted)    GB acc:   AA819788    UniGene:   Rn.4244    Gene Symbol:   RGD1306974_predicted    Locus Link:   360733    Chromosome:   11    Cytoband:   Chr:11q23    SOURCE    GENECARD       Gene Ontology:     BioCarta Pathways:     KEGG Pathways:  
    Probe set : 1376250_at    Name:   nuclear fragile X mental retardation protein interacting protein 1    GB acc:   BI284814    UniGene:   Rn.22071    Gene Symbol:   RGD:1359440    Locus Link:   364430    Chromosome:   15    Cytoband:   Chr:15q11    SOURCE    GENECARD       Gene Ontology:   cellular component:  nucleus   molecular function:  nucleic acid binding   molecular function:  zinc ion binding      BioCarta Pathways:     KEGG Pathways:  
    Probe set : 1367794_at    Name:   alpha-2-macroglobulin    GB acc:   NM_012488    UniGene:   Rn.780    Gene Symbol:   A2m    Locus Link:   24153    Chromosome:   4    Cytoband:   Chr:4q42    SOURCE    GENECARD       Gene Ontology:   cellular component:  extracellular region   cellular component:  extracellular space   cellular component:  extracellular space      BioCarta Pathways:     KEGG Pathways:  
    Probe set : 1381536_at    Name:   Similar to expressed sequence C79127    GB acc:   AI501200    UniGene:   Rn.32751     Locus Link:   292690    Chromosome:   1    Cytoband:   Chr:1q21    SOURCE        Gene Ontology:     BioCarta Pathways:     KEGG Pathways:  
    Probe set : 1388976_at    Name:   similar to BolA domain-containing protein like (11.4 kD) (1P25) (predicted)    GB acc:   AI178272    UniGene:   Rn.13809    Gene Symbol:   RGD1305975_predicted    Locus Link:   297388    Chromosome:   4    Cytoband:   Chr:4q34    SOURCE    GENECARD       Gene Ontology:     BioCarta Pathways:     KEGG Pathways:  
    Probe set : 1367776_at    Name:   cell division cycle 2 homolog A (S. pombe)    GB acc:   NM_019296    UniGene:   Rn.6934    Gene Symbol:   Cdc2a    Locus Link:   54237    Chromosome:   20    Cytoband:   Chr:20p11    SOURCE    GENECARD       Gene Ontology:   cellular component:  nucleus      BioCarta Pathways:     KEGG Pathways:  
    Probe set : 1367628_at    Name:   lectin, galactose binding, soluble 1    GB acc:   NM_019904    UniGene:   Rn.57    Gene Symbol:   Lgals1    Locus Link:   56646    Chromosome:   7    Cytoband:   Chr:7q34    SOURCE    GENECARD       Gene Ontology:   biological process:  heterophilic cell adhesion   biological process:  myoblast differentiation   cellular component:  extracellular space   molecular function:  sugar binding      BioCarta Pathways:     KEGG Pathways:  
    Probe set : 1374628_at    Name:   crystallin, zeta (predicted)    GB acc:   AI232098    UniGene:   Rn.6746    Gene Symbol:   Cryz_predicted    Locus Link:   362061    Chromosome:   2    Cytoband:   Chr:2q45    SOURCE    GENECARD       Gene Ontology:   biological process:  sensory organ development   biological process:  visual perception   cellular component:  cytoplasm      BioCarta Pathways:     KEGG Pathways:  
    Probe set : 1391421_at    Name:   similar to Acyl-CoA dehydrogenase family member 8, mitochondrial precursor (ACAD-8) (Isobutyryl-CoA dehydrogenase)    GB acc:   AI599819    UniGene:   Rn.8978    Gene Symbol:   LOC367046    Locus Link:   367046    Chromosome:   8    Cytoband:   Chr:8q13    SOURCE    GENECARD       Gene Ontology:     BioCarta Pathways:     KEGG Pathways:  
    Probe set : 1388924_at    Name:   angiopoietin-like protein 4    GB acc:   AA818262    UniGene:   Rn.119611    Gene Symbol:   Angptl4    Locus Link:   362850    Chromosome:   7    Cytoband:   Chr:7q12    SOURCE    GENECARD       Gene Ontology:     BioCarta Pathways:     KEGG Pathways:  
    Probe set : 1371795_at     GB acc:   AA848795              Gene Ontology:     BioCarta Pathways:     KEGG Pathways:  
    Probe set : 1377145_at    Name:   similar to very large G-protein coupled receptor 1    GB acc:   BI296089    UniGene:   Rn.16647    Gene Symbol:   LOC294614    Locus Link:   294614    Chromosome:   2    Cytoband:   Chr:2q11    SOURCE    GENECARD       Gene Ontology:     BioCarta Pathways:     KEGG Pathways:  
    Probe set : 1372676_at    Name:   similar to RIKEN cDNA 1110025H10 (predicted)    GB acc:   BI275774    UniGene:   Rn.3918    Gene Symbol:   RGD1304560_predicted    Locus Link:   302980    Chromosome:   10    Cytoband:   Chr:10q12    SOURCE    GENECARD       Gene Ontology:   biological process:  metabolism   cellular component:  mitochondrion      BioCarta Pathways:     KEGG Pathways:  
    Probe set : 1393329_at    Name:   Inducible T-cell co-stimulator    GB acc:   BE099038    UniGene:   Rn.42901    Gene Symbol:   Ailim    Locus Link:   64545      SOURCE    GENECARD       Gene Ontology:   biological process:  defense response   cellular component:  extracellular space   cellular component:  integral to plasma membrane   cellular component:  integral to membrane   molecular function:  MHC protein binding   molecular function:  exogenous peptide antigen binding      BioCarta Pathways:     KEGG Pathways:  
    Probe set : 1382902_at    Name:   potential ubiquitin ligase    GB acc:   AA943147    UniGene:   Rn.107120    Gene Symbol:   Herc6    Locus Link:   362376    Chromosome:   4    Cytoband:   Chr:4q24    SOURCE    GENECARD       Gene Ontology:   biological process:  protein modification   biological process:  ubiquitin cycle   cellular component:  intracellular   molecular function:  ubiquitin-protein ligase activity      BioCarta Pathways:     KEGG Pathways:  
    Probe set : 1380619_at    Name:   similar to RIKEN cDNA 3110001I22 /// similar to RIKEN cDNA 3110001I22    GB acc:   AI177390    UniGene:   Rn.29096    Gene Symbol:   LOC293819 /// LOC361116    Locus Link:   293819 /// 361116    Chromosome:   1    Cytoband:   Chr:1q43 /// Chr:16p14    SOURCE    GENECARD       Gene Ontology:     BioCarta Pathways:     KEGG Pathways:  
    Probe set : 1386773_at    Name:   biotinidase (predicted)    GB acc:   BF524794    UniGene:   Rn.144972    Gene Symbol:   Btd_predicted    Locus Link:   306262    Chromosome:   16    Cytoband:   Chr:16p16    SOURCE    GENECARD       Gene Ontology:   cellular component:  extracellular space      BioCarta Pathways:     KEGG Pathways:  
    Probe set : 1368470_at    Name:   gamma-glutamyl hydrolase    GB acc:   NM_012960    UniGene:   Rn.10260    Gene Symbol:   Ggh    Locus Link:   25455    Chromosome:   5    Cytoband:   Chr:5q21    SOURCE    GENECARD       Gene Ontology:   biological process:  glutamine metabolism   cellular component:  extracellular space   cellular component:  extracellular space   cellular component:  lysosome   cellular component:  lysosome      BioCarta Pathways:     KEGG Pathways:  
    Probe set : 1384298_at    Name:   myosin IF (predicted)    GB acc:   AI764088    UniGene:   Rn.28908    Gene Symbol:   Myo1f_predicted    Locus Link:   314654    Chromosome:   7    Cytoband:   Chr:7q12    SOURCE    GENECARD       Gene Ontology:   cellular component:  myosin   cellular component:  unconventional myosin      BioCarta Pathways:     KEGG Pathways:  
    Probe set : 1389355_at    Name:   immediate early response 5    GB acc:   BF285187    UniGene:   Rn.8590    Gene Symbol:   Ier5    Locus Link:   498256    Chromosome:   13    Cytoband:   Chr:13q21    SOURCE    GENECARD       Gene Ontology:     BioCarta Pathways:     KEGG Pathways:  
    Probe set : 1367600_at    Name:   desmin    GB acc:   NM_022531    UniGene:   Rn.39196    Gene Symbol:   Des    Locus Link:   64362    Chromosome:   9    Cytoband:   Chr:9q33    SOURCE    GENECARD       Gene Ontology:   biological process:  muscle development   biological process:  muscle cell differentiation   cellular component:  kinesin complex   cellular component:  intermediate filament   cellular component:  intermediate filament   molecular function:  structural molecule activity      BioCarta Pathways:     KEGG Pathways:  
    Probe set : 1392171_at    Name:   chitinase 3-like 1    GB acc:   AA945643    UniGene:   Rn.68940    Gene Symbol:   Chi3l1    Locus Link:   89824    Chromosome:   13    Cytoband:   Chr:13q13    SOURCE    GENECARD       Gene Ontology:   biological process:  metabolism   biological process:  carbohydrate metabolism   biological process:  chitin catabolism   cellular component:  extracellular matrix (sensu Metazoa)   cellular component:  extracellular space      BioCarta Pathways:     KEGG Pathways:  
    Probe set : 1367671_at    Name:   proliferating cell nuclear antigen    GB acc:   NM_022381    UniGene:   Rn.223    Gene Symbol:   Pcna    Locus Link:   25737    Chromosome:   3    Cytoband:   Chr:3q36    SOURCE    GENECARD       Gene Ontology:   molecular function:  DNA binding   molecular function:  protein binding   molecular function:  DNA polymerase processivity factor activity      BioCarta Pathways:     KEGG Pathways:  
    Probe set : 1388884_at    Name:   similar to RIKEN cDNA 1810022C23 (predicted)    GB acc:   BM390774    UniGene:   Rn.3194    Gene Symbol:   RGD1310224_predicted    Locus Link:   291076    Chromosome:   17    Cytoband:   Chr:17p12    SOURCE    GENECARD       Gene Ontology:   biological process:  metabolism   cellular component:  peroxisome   molecular function:  catalytic activity   molecular function:  nucleotide binding   molecular function:  RNA binding      BioCarta Pathways:     KEGG Pathways:  
    Probe set : 1385426_at    Name:   similar to hypothetical protein FLJ20647 (predicted)    GB acc:   AI502796    UniGene:   Rn.35486    Gene Symbol:   RGD1305326_predicted    Locus Link:   295462    Chromosome:   2    Cytoband:   Chr:2q43    SOURCE    GENECARD       Gene Ontology:     BioCarta Pathways:     KEGG Pathways:  
    Probe set : 1372516_at    Name:   kinesin family member 22 (predicted)    GB acc:   AI317842    UniGene:   Rn.8601    Gene Symbol:   Kif22_predicted    Locus Link:   293502    Chromosome:   1    Cytoband:   Chr:1q36    SOURCE    GENECARD       Gene Ontology:     BioCarta Pathways:     KEGG Pathways:  
    Probe set : 1373777_at    Name:   Transcribed locus    GB acc:   BF391820    UniGene:   Rn.1141        SOURCE        Gene Ontology:     BioCarta Pathways:     KEGG Pathways:  
    Probe set : 1376100_at    Name:   similar to RIKEN cDNA 2310057H16 (predicted)    GB acc:   BI274903    UniGene:   Rn.98430    Gene Symbol:   RGD1305887_predicted    Locus Link:   307351    Chromosome:   18    Cytoband:   Chr:18q12.1    SOURCE    GENECARD       Gene Ontology:   biological process:  microtubule-based process   biological process:  microtubule-based movement   biological process:  protein polymerization   cellular component:  microtubule   cellular component:  cytoplasm   cellular component:  protein complex      BioCarta Pathways:     KEGG Pathways:  
    Probe set : 1375981_a_at    Name:   similar to Su(var)3-9 homolog    GB acc:   AA997899    UniGene:   Rn.98526    Gene Symbol:   LOC302553    Locus Link:   302553    Chromosome:   X    Cytoband:   Chr:Xq13    SOURCE    GENECARD       Gene Ontology:     BioCarta Pathways:     KEGG Pathways:  
    Probe set : 1368419_at    Name:   ceruloplasmin    GB acc:   AF202115    UniGene:   Rn.32777    Gene Symbol:   Cp    Locus Link:   24268    Chromosome:   2    Cytoband:   Chr:2q24    SOURCE    GENECARD       Gene Ontology:     BioCarta Pathways:     KEGG Pathways:  
    Probe set : 1378418_at    Name:   similar to MGC37193 protein (predicted)    GB acc:   BI285027    UniGene:   Rn.139264    Gene Symbol:   RGD1311681_predicted    Locus Link:   364674    Chromosome:   17    Cytoband:   Chr:17p14    SOURCE    GENECARD       Gene Ontology:     BioCarta Pathways:     KEGG Pathways:  
    Probe set : 1377950_at    Name:   similar to interferon-inducible GTPase (predicted)    GB acc:   AA955213     Gene Symbol:   RGD1309362_predicted    Locus Link:   307415    Chromosome:   18    Cytoband:   Chr:18q12.1     GENECARD       Gene Ontology:   biological process:  cytokine and chemokine mediated signaling pathway      BioCarta Pathways:     KEGG Pathways:  
    Probe set : 1383324_at    Name:   Similar to PALS2-alpha splice variant    GB acc:   AI703599    UniGene:   Rn.49269     Locus Link:   500121      SOURCE        Gene Ontology:     BioCarta Pathways:     KEGG Pathways:  
    Probe set : 1377497_at    Name:   2'-5' oligoadenylate synthetase-like 1 (predicted)    GB acc:   BF419319    UniGene:   Rn.7429    Gene Symbol:   Oasl1_predicted    Locus Link:   304545    Chromosome:   12    Cytoband:   Chr:12q16    SOURCE    GENECARD       Gene Ontology:   biological process:  protein modification   biological process:  immune response   cellular component:  nucleolus   cellular component:  cytoplasm      BioCarta Pathways:     KEGG Pathways:  
    Probe set : 1373854_at    Name:   CDNA clone IMAGE:7324675    GB acc:   AI175795    UniGene:   Rn.3583        SOURCE        Gene Ontology:     BioCarta Pathways:     KEGG Pathways:  
    Probe set : 1367605_at    Name:   profilin 1    GB acc:   NM_022511    UniGene:   Rn.1152    Gene Symbol:   Pfn1    Locus Link:   64303    Chromosome:   10    Cytoband:   Chr:10q24    SOURCE    GENECARD       Gene Ontology:   cellular component:  cytoplasm   cellular component:  cytoplasm   cellular component:  cytoskeleton   cellular component:  actin cytoskeleton      BioCarta Pathways:     KEGG Pathways:  
    Probe set : 1386882_at    Name:   t-complex testis expressed 1    GB acc:   NM_031318    UniGene:   Rn.29901    Gene Symbol:   Tctex1    Locus Link:   83462    Chromosome:   1    Cytoband:   Chr:1q11    SOURCE    GENECARD       Gene Ontology:   biological process:  microtubule-based process   molecular function:  motor activity   molecular function:  microtubule motor activity   molecular function:  protein binding      BioCarta Pathways:     KEGG Pathways:  
    Probe set : 1370172_at    Name:   superoxide dismutase 2, mitochondrial    GB acc:   AA892254    UniGene:   Rn.10488    Gene Symbol:   Sod2    Locus Link:   24787    Chromosome:   1    Cytoband:   Chr:1q21    SOURCE    GENECARD       Gene Ontology:   biological process:  response to superoxide   biological process:  age-dependent response to reactive oxygen species   biological process:  regulation of transcription from RNA polymerase II promoter   biological process:  superoxide metabolism   biological process:  response to oxidative stress   biological process:  cellular defense response (sensu Vertebrata)   biological process:  removal of superoxide radicals   biological process:  removal of superoxide radicals   biological process:  response to superoxide   biological process:  age-dependent response to reactive oxygen species   biological process:  regulation of transcription from RNA polymerase II promoter   biological process:  superoxide metabolism   biological process:  cellular defense response (sensu Vertebrata)   cellular component:  mitochondrion   cellular component:  mitochondrion      BioCarta Pathways:     KEGG Pathways:  
    Probe set : 1390325_at    Name:   CD38 antigen    GB acc:   BI289418    UniGene:   Rn.11414    Gene Symbol:   Cd38    Locus Link:   25668    Chromosome:   14    Cytoband:   Chr:14q21    SOURCE    GENECARD       Gene Ontology:   cellular component:  membrane fraction   cellular component:  nucleus   cellular component:  plasma membrane   cellular component:  integral to membrane      BioCarta Pathways:     KEGG Pathways:  
    Probe set : 1383401_at    Name:   similar to Testis derived transcript    GB acc:   BI288769    UniGene:   Rn.44847    Gene Symbol:   LOC500040    Locus Link:   500040    Chromosome:   4    Cytoband:   Chr:4q21    SOURCE    GENECARD       Gene Ontology:     BioCarta Pathways:     KEGG Pathways:  
    Probe set : 1391489_at    Name:   interferon inducible protein 1 (predicted)    GB acc:   AI407339    UniGene:   Rn.20356    Gene Symbol:   Ifi1_predicted    Locus Link:   303090    Chromosome:   10    Cytoband:   Chr:10q21    SOURCE    GENECARD       Gene Ontology:   biological process:  defense response   cellular component:  endoplasmic reticulum      BioCarta Pathways:     KEGG Pathways:  
    Probe set : 1369427_at    Name:   macrophage expressed gene 1    GB acc:   NM_022617    UniGene:   Rn.4301    Gene Symbol:   Mpeg1    Locus Link:   64552    Chromosome:   1    Cytoband:   Chr:1q43    SOURCE    GENECARD       Gene Ontology:     BioCarta Pathways:     KEGG Pathways:  
    Probe set : 1382003_at    Name:   solute carrier family 16 (monocarboxylic acid transporters), member 13    GB acc:   AI704067    UniGene:   Rn.23260    Gene Symbol:   RGD:1359138    Locus Link:   287451    Chromosome:   10    Cytoband:   Chr:10q24    SOURCE    GENECARD       Gene Ontology:   biological process:  transport   cellular component:  integral to membrane   molecular function:  transporter activity      BioCarta Pathways:     KEGG Pathways:  
    Probe set : 1382314_at    Name:   interferon, alpha-inducible protein (clone IFI-15K) (predicted)    GB acc:   BE096523    UniGene:   Rn.63460    Gene Symbol:   G1p2_predicted    Locus Link:   298693    Chromosome:   5    Cytoband:   Chr:5q36    SOURCE    GENECARD       Gene Ontology:   biological process:  protein modification   biological process:  immune response   biological process:  cell-cell signaling   cellular component:  extracellular space   cellular component:  cytoplasm   molecular function:  protein binding      BioCarta Pathways:     KEGG Pathways:  
    Probe set : 1367805_at    Name:   glutaminase    GB acc:   NM_012569    UniGene:   Rn.5762    Gene Symbol:   Gls    Locus Link:   24398    Chromosome:   9    Cytoband:   Chr:9q31-q32    SOURCE    GENECARD       Gene Ontology:   biological process:  glutamine metabolism   biological process:  glutamine catabolism   biological process:  glutamine catabolism   cellular component:  mitochondrion   cellular component:  mitochondrion   cellular component:  mitochondrial inner membrane   molecular function:  glutaminase activity   molecular function:  glutaminase activity   molecular function:  hydrolase activity      BioCarta Pathways:     KEGG Pathways:  
    Probe set : 1372303_at    Name:   similar to 0910001A06Rik protein (predicted)    GB acc:   BE110633    UniGene:   Rn.105890    Gene Symbol:   RGD1307697_predicted    Locus Link:   299909    Chromosome:   7    Cytoband:   Chr:7q33    SOURCE    GENECARD       Gene Ontology:     BioCarta Pathways:     KEGG Pathways:  
    Probe set : 1372308_at    Name:   CDNA clone IMAGE:7366335    GB acc:   BM386869    UniGene:   Rn.8110        SOURCE        Gene Ontology:     BioCarta Pathways:     KEGG Pathways:  
    Probe set : 1388650_at    Name:   topoisomerase (DNA) 2 alpha    GB acc:   BM385445    UniGene:   Rn.90996    Gene Symbol:   Top2a    Locus Link:   360243    Chromosome:   10    Cytoband:   Chr:10q31    SOURCE    GENECARD       Gene Ontology:     BioCarta Pathways:     KEGG Pathways:  
    Probe set : 1370871_at    Name:   hypothetical gene supported by Y16641; Y16641    GB acc:   BM384165     Gene Symbol:   LOC288146    Locus Link:   288146    Chromosome:   1    Cytoband:   Chr:1p11     GENECARD       Gene Ontology:     BioCarta Pathways:     KEGG Pathways:  
    Probe set : 1372104_at     GB acc:   BF289002              Gene Ontology:     BioCarta Pathways:     KEGG Pathways:  
    Probe set : 1398347_at    Name:   AXL receptor tyrosine kinase (predicted)    GB acc:   AI406520     Gene Symbol:   Axl_predicted    Locus Link:   308444    Chromosome:   1    Cytoband:   Chr:1q21     GENECARD       Gene Ontology:     BioCarta Pathways:     KEGG Pathways:  
    Probe set : 1369630_at    Name:   adenosine kinase    GB acc:   BF283118    UniGene:   Rn.64165    Gene Symbol:   Adk    Locus Link:   25368    Chromosome:   15    Cytoband:   Chr:15p16    SOURCE    GENECARD       Gene Ontology:   biological process:  purine ribonucleoside salvage   biological process:  purine ribonucleoside salvage   biological process:  ribonucleoside monophosphate biosynthesis   cellular component:  nucleus      BioCarta Pathways:     KEGG Pathways:  
    Probe set : 1395268_at    Name:   WW domain containing E3 ubiquitin protein ligase 1 (predicted)    GB acc:   BM383223    UniGene:   Rn.81239    Gene Symbol:   Wwp1_predicted    Locus Link:   297930    Chromosome:   5    Cytoband:   Chr:5q13    SOURCE    GENECARD       Gene Ontology:   cellular component:  ubiquitin ligase complex   cellular component:  intracellular   molecular function:  ubiquitin-protein ligase activity   molecular function:  protein binding   molecular function:  ligase activity      BioCarta Pathways:     KEGG Pathways:  
    Probe set : 1388738_at    Name:   Similar to mKIAA0534 protein    GB acc:   AI411227    UniGene:   Rn.12950     Locus Link:   307992    Chromosome:   1    Cytoband:   Chr:1q55    SOURCE        Gene Ontology:     BioCarta Pathways:     KEGG Pathways:  
    Probe set : 1383395_at    Name:   agmatine ureohydrolase (agmatinase) (predicted)    GB acc:   AI028979    UniGene:   Rn.7880    Gene Symbol:   Agmat_predicted    Locus Link:   298607    Chromosome:   5    Cytoband:   Chr:5q36    SOURCE    GENECARD       Gene Ontology:   cellular component:  extracellular space   cellular component:  mitochondrion      BioCarta Pathways:     KEGG Pathways:  
    Probe set : 1371680_at     GB acc:   BI303631              Gene Ontology:     BioCarta Pathways:     KEGG Pathways:  
    Probe set : 1368000_at    Name:   complement component 3    GB acc:   NM_016994    UniGene:   Rn.11378    Gene Symbol:   C3    Locus Link:   24232    Chromosome:   9    Cytoband:   Chr:9q11    SOURCE    GENECARD       Gene Ontology:   cellular component:  extracellular region   cellular component:  extracellular space      BioCarta Pathways:     KEGG Pathways:  
    Probe set : 1390596_at    Name:   melan-A (predicted)    GB acc:   BG378791    UniGene:   Rn.12340    Gene Symbol:   Mlana_predicted    Locus Link:   293890    Chromosome:   1    Cytoband:   Chr:1q52    SOURCE    GENECARD       Gene Ontology:   cellular component:  integral to plasma membrane      BioCarta Pathways:     KEGG Pathways:  
    Probe set : 1371352_at    Name:   high mobility group protein 17    GB acc:   BM391736    UniGene:   Rn.140742    Gene Symbol:   RGD:620649    Locus Link:   114637    Chromosome:   5    Cytoband:   Chr:5q36    SOURCE    GENECARD       Gene Ontology:   cellular component:  chromosome   cellular component:  chromatin   cellular component:  nucleus   molecular function:  DNA binding      BioCarta Pathways:     KEGG Pathways:  
    Probe set : 1379248_at    Name:   Prolylcarboxypeptidase (angiotensinase C) (predicted)    GB acc:   AA926318    UniGene:   Rn.8457     Locus Link:   293118    Chromosome:   1    Cytoband:   Chr:1q32    SOURCE        Gene Ontology:   biological process:  proteolysis   cellular component:  lysosome      BioCarta Pathways:     KEGG Pathways:  
    Probe set : 1371070_at    Name:   tumor stroma and activated macrophage protein DLM-1    GB acc:   AJ302054    UniGene:   Rn.67419    Gene Symbol:   Zbp1    Locus Link:   171091    Chromosome:   3    Cytoband:   Chr:3q42    SOURCE    GENECARD       Gene Ontology:   biological process:  nuclear mRNA splicing, via spliceosome   biological process:  mRNA processing      BioCarta Pathways:     KEGG Pathways:  
    Probe set : 1370020_at    Name:   solute carrier family 25 (mitochondrial carrier; dicarboxylate transporter), member 10    GB acc:   NM_133418    UniGene:   Rn.3631    Gene Symbol:   RGD:621430    Locus Link:   170943    Chromosome:   10    Cytoband:   Chr:10q32.3    SOURCE    GENECARD       Gene Ontology:   biological process:  transport   biological process:  mitochondrial transport   molecular function:  binding   molecular function:  electrochemical potential-driven transporter activity      BioCarta Pathways:     KEGG Pathways:  
    Probe set : 1393061_at    Name:   similar to cDNA sequence BC021608    GB acc:   AI030103    UniGene:   Rn.107180    Gene Symbol:   RGD:1359544    Locus Link:   300676    Chromosome:   8    Cytoband:   Chr:8q22    SOURCE    GENECARD       Gene Ontology:   molecular function:  binding      BioCarta Pathways:     KEGG Pathways:  
    Probe set : 1392590_at    Name:   Rho GTPase activating protein 24 (predicted)    GB acc:   BG377201    UniGene:   Rn.24657    Gene Symbol:   Arhgap24_predicted    Locus Link:   305156    Chromosome:   14    Cytoband:   Chr:14p22    SOURCE    GENECARD       Gene Ontology:   molecular function:  GTPase activator activity      BioCarta Pathways:     KEGG Pathways:  
    Probe set : 1382078_at    Name:   Transcribed locus    GB acc:   BF283802    UniGene:   Rn.128184        SOURCE        Gene Ontology:     BioCarta Pathways:     KEGG Pathways:  
    Probe set : 1389498_at     GB acc:   BE111203              Gene Ontology:     BioCarta Pathways:     KEGG Pathways:  
    Probe set : 1389034_at    Name:   similar to ubiquitin specific protease UBP43    GB acc:   BI295179    UniGene:   Rn.4165    Gene Symbol:   RGD:1359153    Locus Link:   312688    Chromosome:   4    Cytoband:   Chr:4q42    SOURCE    GENECARD       Gene Ontology:   biological process:  ubiquitin-dependent protein catabolism   molecular function:  cysteine-type endopeptidase activity   molecular function:  ubiquitin thiolesterase activity      BioCarta Pathways:     KEGG Pathways:  
    Probe set : 1374217_at    Name:   similar to chromosome 16 open reading frame 5 (predicted)    GB acc:   BG375463    UniGene:   Rn.101805    Gene Symbol:   RGD1310686_predicted    Locus Link:   360480    Chromosome:   10    Cytoband:   Chr:10q12    SOURCE    GENECARD       Gene Ontology:   cellular component:  nucleus   cellular component:  integral to membrane      BioCarta Pathways:     KEGG Pathways:  
    Probe set : 1387659_at    Name:   guanine deaminase    GB acc:   AF245172    UniGene:   Rn.24783    Gene Symbol:   Gda    Locus Link:   83585    Chromosome:   1    Cytoband:   Chr:1q51    SOURCE    GENECARD       Gene Ontology:   cellular component:  intracellular      BioCarta Pathways:     KEGG Pathways:  
    Probe set : 1371931_at    Name:   general transcription factor II I    GB acc:   BI274753    UniGene:   Rn.27575    Gene Symbol:   Gtf2i    Locus Link:   353256    Chromosome:   12    Cytoband:   Chr:12q12    SOURCE    GENECARD       Gene Ontology:     BioCarta Pathways:     KEGG Pathways:  
    Probe set : 1370628_at    Name:   granzyme B    GB acc:   M34097    UniGene:   Rn.21395    Gene Symbol:   Gzmb    Locus Link:   171528    Chromosome:   15    Cytoband:   Chr:15p12    SOURCE    GENECARD       Gene Ontology:   biological process:  proteolysis   biological process:  apoptosis   biological process:  cytolysis   cellular component:  extracellular space      BioCarta Pathways:     KEGG Pathways:  
    Probe set : 1372875_at    Name:   Transcribed locus    GB acc:   AI408520    UniGene:   Rn.8905        SOURCE        Gene Ontology:     BioCarta Pathways:     KEGG Pathways:  
    Probe set : 1374222_at    Name:   tumor suppressing subtransferable candidate 5    GB acc:   BM391436    UniGene:   Rn.3575    Gene Symbol:   RGD:1303323    Locus Link:   309131    Chromosome:   1    Cytoband:   Chr:1q41    SOURCE    GENECARD       Gene Ontology:   biological process:  transport   biological process:  tetracycline transport   cellular component:  integral to membrane      BioCarta Pathways:     KEGG Pathways:  
    Probe set : 1372585_at    Name:   LOC499318    GB acc:   BM388445    UniGene:   Rn.107400    Gene Symbol:   LOC499318    Locus Link:   499318    Chromosome:   1    Cytoband:   Chr:1q43    SOURCE    GENECARD       Gene Ontology:     BioCarta Pathways:     KEGG Pathways:  
    Probe set : 1369292_at    Name:   hydroxysteroid (17-beta) dehydrogenase 1    GB acc:   NM_012851    UniGene:   Rn.10594    Gene Symbol:   Hsd17b1    Locus Link:   25322    Chromosome:   10    Cytoband:   Chr:10q32.1    SOURCE    GENECARD       Gene Ontology:   cellular component:  cytoplasm   molecular function:  estradiol 17-beta-dehydrogenase activity   molecular function:  estradiol 17-beta-dehydrogenase activity   molecular function:  oxidoreductase activity      BioCarta Pathways:     KEGG Pathways:  
    Probe set : 1393987_s_at    Name:   G protein-coupled receptor kinase 6    GB acc:   BI274742    UniGene:   Rn.10633    Gene Symbol:   Gprk6    Locus Link:   59076    Chromosome:   17    Cytoband:   Chr:17p14    SOURCE    GENECARD       Gene Ontology:     BioCarta Pathways:     KEGG Pathways:  
    Probe set : 1388644_at    Name:   Monoglyceride lipase    GB acc:   AI713204    UniGene:   Rn.40396    Gene Symbol:   Mgll    Locus Link:   29254    Chromosome:   4    Cytoband:   Chr:4q34    SOURCE    GENECARD       Gene Ontology:   cellular component:  synapse      BioCarta Pathways:     KEGG Pathways:  
    Probe set : 1390407_at    Name:   similar to Protein C3orf4 homolog    GB acc:   AA955196    UniGene:   Rn.101823    Gene Symbol:   RGD:1359273    Locus Link:   288182    Chromosome:   11    Cytoband:   Chr:11q12    SOURCE    GENECARD       Gene Ontology:   cellular component:  membrane   cellular component:  integral to membrane      BioCarta Pathways:     KEGG Pathways:  
    Probe set : 1369937_at    Name:   calmodulin 1    GB acc:   BI279113    UniGene:   Rn.4166    Gene Symbol:   Calm1    Locus Link:   24242    Chromosome:   6    Cytoband:   Chr:6q31-q32    SOURCE    GENECARD       Gene Ontology:     BioCarta Pathways:     KEGG Pathways:  
    Probe set : 1388794_at    Name:   RNA binding motif protein, X chromosome retrogene (predicted)    GB acc:   BE107489    UniGene:   Rn.19966    Gene Symbol:   Rbmxrt_predicted    Locus Link:   307779    Chromosome:   19    Cytoband:   Chr:19q11    SOURCE    GENECARD       Gene Ontology:   biological process:  nuclear mRNA splicing, via spliceosome   biological process:  RNA processing   biological process:  mRNA processing   cellular component:  nucleus   cellular component:  ribonucleoprotein complex   molecular function:  RNA binding      BioCarta Pathways:     KEGG Pathways:  
    Probe set : 1372453_at    Name:   down-regulator of transcription 1 (predicted)    GB acc:   AI012637    UniGene:   Rn.6666    Gene Symbol:   Dr1_predicted    Locus Link:   289881    Chromosome:   14    Cytoband:   Chr:14p22    SOURCE    GENECARD       Gene Ontology:   cellular component:  nucleus   molecular function:  DNA binding   molecular function:  transcription corepressor activity   molecular function:  transcription factor binding      BioCarta Pathways:     KEGG Pathways:  
    Probe set : 1389256_at    Name:   Transcribed locus    GB acc:   BG381256    UniGene:   Rn.1630        SOURCE        Gene Ontology:     BioCarta Pathways:     KEGG Pathways:  
    Probe set : 1393144_at    Name:   N-myc (and STAT) interactor (predicted)    GB acc:   BM388202    UniGene:   Rn.18496     Locus Link:   311021    Chromosome:   3    Cytoband:   Chr:3q12    SOURCE        Gene Ontology:   biological process:  transcription from RNA polymerase II promoter   biological process:  inflammatory response   biological process:  JAK-STAT cascade   cellular component:  cytoplasm   molecular function:  transcription cofactor activity      BioCarta Pathways:     KEGG Pathways:  
    Probe set : 1390993_at    Name:   MAWD binding protein    GB acc:   AW532101    UniGene:   Rn.2417    Gene Symbol:   Mawbp    Locus Link:   171564    Chromosome:   20    Cytoband:   Chr:20p11    SOURCE    GENECARD       Gene Ontology:   biological process:  biosynthesis   molecular function:  catalytic activity   molecular function:  oxidoreductase activity      BioCarta Pathways:     KEGG Pathways:  
    Probe set : 1368555_at    Name:   CD37 antigen    GB acc:   NM_017124    UniGene:   Rn.2357    Gene Symbol:   Cd37    Locus Link:   29185    Chromosome:   1    Cytoband:   Chr:1q22    SOURCE    GENECARD       Gene Ontology:   cellular component:  plasma membrane   cellular component:  plasma membrane   cellular component:  integral to membrane      BioCarta Pathways:     KEGG Pathways:  
    Probe set : 1387765_at    Name:   mannose binding lectin 1, protein A    GB acc:   NM_012599    UniGene:   Rn.10273    Gene Symbol:   Mbl1    Locus Link:   24548    Chromosome:   16    Cytoband:   Chr:16p14    SOURCE    GENECARD       Gene Ontology:     BioCarta Pathways:     KEGG Pathways:  
    Probe set : 1367695_at    Name:   quinoid dihydropteridine reductase    GB acc:   NM_022390    UniGene:   Rn.241    Gene Symbol:   Qdpr    Locus Link:   64192    Chromosome:   14    Cytoband:   Chr:14q21    SOURCE    GENECARD       Gene Ontology:     BioCarta Pathways:     KEGG Pathways:  
    Probe set : 1368234_at    Name:   prolyl endopeptidase    GB acc:   NM_031324    UniGene:   Rn.11058    Gene Symbol:   Prep    Locus Link:   83471    Chromosome:   20    Cytoband:   Chr:20q13    SOURCE    GENECARD       Gene Ontology:   biological process:  proteolysis   biological process:  proteolysis   biological process:  protein metabolism   cellular component:  nucleus   cellular component:  cytoplasm   cellular component:  cytosol      BioCarta Pathways:     KEGG Pathways:  
    Probe set : 1372513_at    Name:   ras-related C3 botulinum toxin substrate 1    GB acc:   AI103616    UniGene:   Rn.29157    Gene Symbol:   Rac1    Locus Link:   363875    Chromosome:   12    Cytoband:   Chr:12p11    SOURCE    GENECARD       Gene Ontology:   molecular function:  GTPase activity   molecular function:  GTP binding   molecular function:  nucleotide binding   molecular function:  GTP binding      BioCarta Pathways:     KEGG Pathways:  
    Probe set : 1375674_at    Name:   similar to chromosome 16 open reading frame 5 (predicted)    GB acc:   BI294949    UniGene:   Rn.101805    Gene Symbol:   RGD1310686_predicted    Locus Link:   360480    Chromosome:   10    Cytoband:   Chr:10q12    SOURCE    GENECARD       Gene Ontology:   cellular component:  nucleus   cellular component:  integral to membrane      BioCarta Pathways:     KEGG Pathways:  
    Probe set : 1389592_at    Name:   Transcribed locus    GB acc:   AI317817    UniGene:   Rn.23816        SOURCE        Gene Ontology:     BioCarta Pathways:     KEGG Pathways:  
    Probe set : 1367999_at    Name:   aldehyde dehydrogenase 2    GB acc:   NM_032416    UniGene:   Rn.101781    Gene Symbol:   Aldh2    Locus Link:   29539    Chromosome:   12    Cytoband:   Chr:12q16    SOURCE    GENECARD       Gene Ontology:   biological process:  carbohydrate metabolism   biological process:  alcohol metabolism   biological process:  metabolism   cellular component:  mitochondrion   cellular component:  mitochondrion      BioCarta Pathways:     KEGG Pathways:  
    Probe set : 1373693_at    Name:   G protein-coupled receptor, family C, group 5, member C (predicted)    GB acc:   BF414143    UniGene:   Rn.3658    Gene Symbol:   Gprc5c_predicted    Locus Link:   287805    Chromosome:   10    Cytoband:   Chr:10q32.2    SOURCE    GENECARD       Gene Ontology:   biological process:  G-protein coupled receptor protein signaling pathway   biological process:  visual perception   cellular component:  mitochondrion   cellular component:  membrane   cellular component:  integral to membrane      BioCarta Pathways:     KEGG Pathways:  
    Probe set : 1383421_at    Name:   Transcribed locus    GB acc:   BM386110    UniGene:   Rn.147398        SOURCE        Gene Ontology:     BioCarta Pathways:     KEGG Pathways:  
    Probe set : 1371420_at    Name:   92Aa-Protein    GB acc:   AI412054    UniGene:   Rn.7336    Gene Symbol:   RGD:1359519    Locus Link:   494529      SOURCE    GENECARD       Gene Ontology:     BioCarta Pathways:     KEGG Pathways:  
    Probe set : 1389227_at    Name:   ras homolog gene family, member G (predicted)    GB acc:   BG375902     Gene Symbol:   Rhog_predicted    Locus Link:   308875    Chromosome:   1    Cytoband:   Chr:1q32     GENECARD       Gene Ontology:   molecular function:  GTPase activity   molecular function:  GTP binding      BioCarta Pathways:     KEGG Pathways:  
    Probe set : 1371164_at    Name:   mast cell protease 10    GB acc:   X68657    UniGene:   Rn.48655    Gene Symbol:   Mcpt10    Locus Link:   54269    Chromosome:   15    Cytoband:   Chr:15p12    SOURCE    GENECARD       Gene Ontology:   biological process:  proteolysis   cellular component:  extracellular space      BioCarta Pathways:     KEGG Pathways:  
    Probe set : 1372323_at    Name:   sarcosine dehydrogenase    GB acc:   AI103641    UniGene:   Rn.89832    Gene Symbol:   Sardh    Locus Link:   114123    Chromosome:   3    Cytoband:   Chr:3p12    SOURCE    GENECARD       Gene Ontology:   biological process:  electron transport   biological process:  glycine catabolism   biological process:  choline catabolism      BioCarta Pathways:     KEGG Pathways:  
    Probe set : 1388588_at    Name:   mammary tumor virus receptor 2 (predicted)    GB acc:   AI236101    UniGene:   Rn.6896    Gene Symbol:   Mtvr2_predicted    Locus Link:   309170    Chromosome:   1    Cytoband:   Chr:1q43    SOURCE    GENECARD       Gene Ontology:   molecular function:  receptor activity      BioCarta Pathways:     KEGG Pathways:  
    Probe set : 1372872_at    Name:   Similar to adenylate kinase isozyme 5    GB acc:   BI291271    UniGene:   Rn.16387     Locus Link:   365985    Chromosome:   2    Cytoband:   Chr:2    SOURCE        Gene Ontology:     BioCarta Pathways:     KEGG Pathways:  
    Probe set : 1369262_at    Name:   caspase 8    GB acc:   NM_022277    UniGene:   Rn.54474    Gene Symbol:   Casp8    Locus Link:   64044    Chromosome:   9    Cytoband:   Chr:9q31    SOURCE    GENECARD       Gene Ontology:     BioCarta Pathways:     KEGG Pathways:  
    Probe set : 1367693_at    Name:   tyrosine 3-monooxygenase/tryptophan 5-monooxygenase activation protein, eta polypeptide    GB acc:   NM_013052    UniGene:   Rn.3324    Gene Symbol:   Ywhah    Locus Link:   25576    Chromosome:   14    Cytoband:   Chr:14q21    SOURCE    GENECARD       Gene Ontology:   cellular component:  cytoplasm      BioCarta Pathways:     KEGG Pathways:  
    Probe set : 1374006_at    Name:   kynurenine aminotransferase III    GB acc:   BI295878    UniGene:   Rn.28263    Gene Symbol:   RGD:1359262    Locus Link:   541589      SOURCE    GENECARD       Gene Ontology:   biological process:  biosynthesis      BioCarta Pathways:     KEGG Pathways:  
    Probe set : 1371405_at    Name:   Similar to hypothetical protein MGC52110    GB acc:   BE109143    UniGene:   Rn.3412     Locus Link:   503252      SOURCE        Gene Ontology:     BioCarta Pathways:     KEGG Pathways:  
    Probe set : 1393029_at    Name:   CUG triplet repeat, RNA binding protein 1 (predicted)    GB acc:   BF410771    UniGene:   Rn.22432     Locus Link:   362160    Chromosome:   3    Cytoband:   Chr:3q24    SOURCE        Gene Ontology:   cellular component:  nucleus   cellular component:  cytoplasm   cellular component:  ribonucleoprotein complex      BioCarta Pathways:     KEGG Pathways:  
    Probe set : 1388776_at    Name:   scotin    GB acc:   AI169176    UniGene:   Rn.102027    Gene Symbol:   RGD:1359661    Locus Link:   301013    Chromosome:   8    Cytoband:   Chr:8q32    SOURCE    GENECARD       Gene Ontology:     BioCarta Pathways:     KEGG Pathways:  
    Probe set : 1369688_s_at    Name:   protein tyrosine kinase 2 beta    GB acc:   U69109    UniGene:   Rn.11025    Gene Symbol:   Ptk2b    Locus Link:   50646    Chromosome:   15    Cytoband:   Chr:15p12    SOURCE    GENECARD       Gene Ontology:   biological process:  protein amino acid phosphorylation   cellular component:  cytoskeleton      BioCarta Pathways:     KEGG Pathways:  
    Probe set : 1388460_at    Name:   capping protein (actin filament), gelsolin-like (predicted)    GB acc:   BM384693    UniGene:   Rn.8945    Gene Symbol:   Capg_predicted    Locus Link:   297339    Chromosome:   4    Cytoband:   Chr:4q33    SOURCE    GENECARD       Gene Ontology:   cellular component:  nucleus   cellular component:  F-actin capping protein complex   molecular function:  actin binding      BioCarta Pathways:     KEGG Pathways:  
    Probe set : 1380445_at    Name:   Transcribed locus, strongly similar to XP_515868.1 PREDICTED: sodium channel, voltage-gated, type III, alpha [Pan troglodytes]    GB acc:   BI294409    UniGene:   Rn.37544        SOURCE        Gene Ontology:     BioCarta Pathways:     KEGG Pathways:  
    Probe set : 1368091_at    Name:   5-oxoprolinase (ATP-hydrolysing)    GB acc:   NM_053904    UniGene:   Rn.3066    Gene Symbol:   Oplah    Locus Link:   116684    Chromosome:   7    Cytoband:   Chr:7q34    SOURCE    GENECARD       Gene Ontology:   molecular function:  catalytic activity   molecular function:  hydrolase activity   molecular function:  5-oxoprolinase (ATP-hydrolyzing) activity      BioCarta Pathways:     KEGG Pathways:  
    Probe set : 1368329_at    Name:   solute carrier family 22 (organic anion transporter), member 6    GB acc:   NM_017224    UniGene:   Rn.87849    Gene Symbol:   Slc22a6    Locus Link:   29509    Chromosome:   1    Cytoband:   Chr:1q43    SOURCE    GENECARD       Gene Ontology:     BioCarta Pathways:     KEGG Pathways:  
    Probe set : 1388148_a_at    Name:   low density lipoprotein receptor-related protein associated protein 1    GB acc:   AI232268    UniGene:   Rn.10293    Gene Symbol:   Lrpap1    Locus Link:   116565    Chromosome:   14    Cytoband:   Chr:14q21    SOURCE    GENECARD       Gene Ontology:   cellular component:  extracellular space   cellular component:  endoplasmic reticulum   cellular component:  endoplasmic reticulum      BioCarta Pathways:     KEGG Pathways:  
    Probe set : 1371948_at    Name:   Down syndrome critical region gene 5 (predicted)    GB acc:   BF415030    UniGene:   Rn.53715    Gene Symbol:   Dscr5_predicted    Locus Link:   288238    Chromosome:   11    Cytoband:   Chr:11q11    SOURCE    GENECARD       Gene Ontology:   cellular component:  integral to membrane   molecular function:  transferase activity, transferring glycosyl groups   molecular function:  phosphatidylinositol N-acetylglucosaminyltransferase activity      BioCarta Pathways:     KEGG Pathways:  
    Probe set : 1370113_at    Name:   inhibitor of apoptosis protein 1    GB acc:   NM_023987    UniGene:   Rn.64578    Gene Symbol:   Birc3    Locus Link:   78971    Chromosome:   8    Cytoband:   Chr:8q11    SOURCE    GENECARD       Gene Ontology:   cellular component:  ubiquitin ligase complex   cellular component:  intracellular   molecular function:  ubiquitin-protein ligase activity   molecular function:  protein binding   molecular function:  zinc ion binding      BioCarta Pathways:     KEGG Pathways:  
    Probe set : 1398390_at    Name:   similar to Small inducible cytokine B13 precursor (CXCL13) (B lymphocyte chemoattractant) (CXC chemokine BLC)    GB acc:   AA892854    UniGene:   Rn.6917    Gene Symbol:   LOC498335    Locus Link:   498335    Chromosome:   14    Cytoband:   Chr:14p22    SOURCE    GENECARD       Gene Ontology:   biological process:  immune response   cellular component:  extracellular region   molecular function:  cytokine activity   molecular function:  chemokine activity      BioCarta Pathways:     KEGG Pathways:  
    Probe set : 1385605_at     GB acc:   BF401793              Gene Ontology:     BioCarta Pathways:     KEGG Pathways:  
    Probe set : 1369319_at    Name:   ADP-ribosylation factor-like 6 interacting protein 5    GB acc:   NM_023972    UniGene:   Rn.64512    Gene Symbol:   Arl6ip5    Locus Link:   66028    Chromosome:   4    Cytoband:   Chr:4q34    SOURCE    GENECARD       Gene Ontology:   biological process:  L-glutamate transport   biological process:  negative regulation of transport   cellular component:  cytoplasm   cellular component:  endoplasmic reticulum   cellular component:  membrane   cellular component:  integral to membrane   molecular function:  protein binding   molecular function:  protein C-terminus binding      BioCarta Pathways:     KEGG Pathways:  
    Probe set : 1373940_at    Name:   non-catalytic region of tyrosine kinase adaptor protein 1 (predicted)    GB acc:   BM386507    UniGene:   Rn.8768    Gene Symbol:   Nck1_predicted    Locus Link:   300955    Chromosome:   8    Cytoband:   Chr:8q31    SOURCE    GENECARD       Gene Ontology:   cellular component:  cytoplasm   cellular component:  vesicle membrane      BioCarta Pathways:     KEGG Pathways:  
    Probe set : 1374524_at    Name:   selenocysteine lyase    GB acc:   BM384384    UniGene:   Rn.23954    Gene Symbol:   RGD:1359514    Locus Link:   363285    Chromosome:   9    Cytoband:   Chr:9q36    SOURCE    GENECARD       Gene Ontology:   biological process:  metabolism   molecular function:  transaminase activity   molecular function:  lyase activity      BioCarta Pathways:     KEGG Pathways:  
    Probe set : 1385658_at    Name:   zinc finger protein 313    GB acc:   AI059254    UniGene:   Rn.49925    Gene Symbol:   RGD:1303139    Locus Link:   362277    Chromosome:   3    Cytoband:   Chr:3q42    SOURCE    GENECARD       Gene Ontology:   cellular component:  ubiquitin ligase complex   cellular component:  nucleus      BioCarta Pathways:     KEGG Pathways:  
    Probe set : 1368311_at    Name:   O-6-methylguanine-DNA methyltransferase    GB acc:   NM_012861    UniGene:   Rn.9836    Gene Symbol:   Mgmt    Locus Link:   25332    Chromosome:   1    Cytoband:   Chr:1q41    SOURCE    GENECARD       Gene Ontology:   cellular component:  nucleus      BioCarta Pathways:     KEGG Pathways:  
    Probe set : 1376835_at    Name:   Solute carrier family 35, member B2    GB acc:   BI293600    UniGene:   Rn.129334    Gene Symbol:   Slc35b2    Locus Link:   316241    Chromosome:   9    Cytoband:   Chr:9q12    SOURCE    GENECARD       Gene Ontology:   biological process:  nucleotide-sugar transport   molecular function:  nucleotide-sugar transporter activity      BioCarta Pathways:     KEGG Pathways:  
    Probe set : 1379235_x_at    Name:   similar to CDC45L    GB acc:   AI179539    UniGene:   Rn.98535    Gene Symbol:   LOC287961    Locus Link:   287961    Chromosome:   11    Cytoband:   Chr:11p12    SOURCE    GENECARD       Gene Ontology:     BioCarta Pathways:     KEGG Pathways:  
    Probe set : 1398831_at    Name:   proteasome (prosome, macropain) subunit, beta type 4    GB acc:   NM_031629    UniGene:   Rn.6169    Gene Symbol:   Psmb4    Locus Link:   58854    Chromosome:   2    Cytoband:   Chr:2q34    SOURCE    GENECARD       Gene Ontology:   biological process:  ubiquitin-dependent protein catabolism      BioCarta Pathways:     KEGG Pathways:  
    Probe set : 1381279_at    Name:   receptor (TNFRSF)-interacting serine-threonine kinase 2 (predicted)    GB acc:   BF412519    UniGene:   Rn.139983    Gene Symbol:   Ripk2_predicted    Locus Link:   362491    Chromosome:   5    Cytoband:   Chr:5q13    SOURCE    GENECARD       Gene Ontology:   cellular component:  intracellular      BioCarta Pathways:     KEGG Pathways:  
    Probe set : 1373670_at    Name:   Signal transducer and activator of transcription 2 (predicted)    GB acc:   AA799569    UniGene:   Rn.24237     Locus Link:   288774    Chromosome:   7    Cytoband:   Chr:7q11    SOURCE        Gene Ontology:   cellular component:  nucleus   cellular component:  cytoplasm      BioCarta Pathways:     KEGG Pathways:  
    Probe set : 1372055_at    Name:   Similar to P-Rex1 (predicted)    GB acc:   AI060225    UniGene:   Rn.14163     Locus Link:   311647    Chromosome:   3    Cytoband:   Chr:3q42    SOURCE        Gene Ontology:     BioCarta Pathways:     KEGG Pathways:  
    Probe set : 1379228_at    Name:   similar to flt3 ligand    GB acc:   BF418531    UniGene:   Rn.138312    Gene Symbol:   LOC499148    Locus Link:   499148    Chromosome:   1    Cytoband:   Chr:1q22    SOURCE    GENECARD       Gene Ontology:     BioCarta Pathways:     KEGG Pathways:  
    Probe set : 1368187_at    Name:   glycoprotein (transmembrane) nmb    GB acc:   NM_133298    UniGene:   Rn.13778    Gene Symbol:   Gpnmb    Locus Link:   113955    Chromosome:   4    Cytoband:   Chr:4q24    SOURCE    GENECARD       Gene Ontology:   molecular function:  integrin binding   molecular function:  heparin binding   molecular function:  carbon-nitrogen ligase activity, with glutamine as amido-N-donor      BioCarta Pathways:     KEGG Pathways:  
    Probe set : 1382447_at     GB acc:   AI170441              Gene Ontology:     BioCarta Pathways:     KEGG Pathways:  
    Probe set : 1372256_at     GB acc:   BF550246              Gene Ontology:     BioCarta Pathways:     KEGG Pathways:  
    Probe set : 1384022_at     GB acc:   BI288173              Gene Ontology:     BioCarta Pathways:     KEGG Pathways:  
    Probe set : 1383962_at    Name:   Cd27 binding protein (Hindu God of destruction)    GB acc:   BI275530    UniGene:   Rn.23843    Gene Symbol:   Siva    Locus Link:   362791    Chromosome:   6    Cytoband:   Chr:6q32    SOURCE    GENECARD       Gene Ontology:     BioCarta Pathways:     KEGG Pathways:  
    Probe set : 1367723_a_at    Name:   linker of T-cell receptor pathways    GB acc:   NM_031621    UniGene:   Rn.11228    Gene Symbol:   Lnk    Locus Link:   58838    Chromosome:   12    Cytoband:   Chr:12q16    SOURCE    GENECARD       Gene Ontology:   molecular function:  protein binding      BioCarta Pathways:     KEGG Pathways:  
    Probe set : 1386586_at    Name:   similar to PAK/PLC-interacting protein 1    GB acc:   AA996838    UniGene:   Rn.115052    Gene Symbol:   LOC361232    Locus Link:   361232    Chromosome:   17    Cytoband:   Chr:17p12    SOURCE    GENECARD       Gene Ontology:     BioCarta Pathways:     KEGG Pathways:  
    Probe set : 1373736_at    Name:   Transcribed locus, weakly similar to NP_082606.1 hypothetical protein LOC72704 [Mus musculus]    GB acc:   AI178446    UniGene:   Rn.13717        SOURCE        Gene Ontology:     BioCarta Pathways:     KEGG Pathways:  
    Probe set : 1386897_at    Name:   heterogeneous nuclear ribonucleoproteins methyltransferase-like 2 (S. cerevisiae)    GB acc:   NM_024363    UniGene:   Rn.5870    Gene Symbol:   Hrmt1l2    Locus Link:   60421    Chromosome:   1    Cytoband:   Chr:1q22    SOURCE    GENECARD       Gene Ontology:   molecular function:  methyltransferase activity   molecular function:  N-methyltransferase activity   molecular function:  protein methyltransferase activity   molecular function:  protein methyltransferase activity   molecular function:  histone-arginine N-methyltransferase activity   molecular function:  S-adenosylmethionine-dependent methyltransferase activity   molecular function:  [cytochrome c]-arginine N-methyltransferase activity   molecular function:  transferase activity   molecular function:  protein-arginine omega-N monomethyltransferase activity   molecular function:  protein-arginine omega-N asymmetric methyltransferase activity   molecular function:  protein binding   molecular function:  histone-arginine N-methyltransferase activity   molecular function:  [cytochrome c]-arginine N-methyltransferase activity   molecular function:  protein-arginine omega-N monomethyltransferase activity   molecular function:  protein-arginine omega-N asymmetric methyltransferase activity      BioCarta Pathways:     KEGG Pathways:  
    Probe set : 1385572_at    Name:   Transcribed locus    GB acc:   AI144884    UniGene:   Rn.27718        SOURCE        Gene Ontology:     BioCarta Pathways:     KEGG Pathways:  
    Probe set : 1372461_at    Name:   SET translocation (predicted) /// LOC499767    GB acc:   BG381708    UniGene:   Rn.144530    Gene Symbol:   Set_predicted /// LOC499767    Locus Link:   307947 /// 499767    Chromosome:   19    Cytoband:   Chr:19q12 /// Chr:3p12    SOURCE    GENECARD       Gene Ontology:   cellular component:  nucleus   cellular component:  endoplasmic reticulum   cellular component:  perinuclear region      BioCarta Pathways:     KEGG Pathways:  
    Probe set : 1374606_at    Name:   MYST histone acetyltransferase 1 (predicted)    GB acc:   BF283073    UniGene:   Rn.107364    Gene Symbol:   Prss8    Locus Link:   192107    Chromosome:   1    Cytoband:   Chr:1q36    SOURCE    GENECARD       Gene Ontology:     BioCarta Pathways:     KEGG Pathways:  
    Probe set : 1374512_at    Name:   Transcribed locus, strongly similar to XP_214823.2 PREDICTED: similar to Blvrb protein [Rattus norvegicus]    GB acc:   AI232341    UniGene:   Rn.57038        SOURCE        Gene Ontology:     BioCarta Pathways:     KEGG Pathways:  
    Probe set : 1395840_at    Name:   eukaryotic translation initiation factor 1A (predicted)    GB acc:   AW520679    UniGene:   Rn.3361    Gene Symbol:   Eif1a_predicted    Locus Link:   317163    Chromosome:   18    Cytoband:   Chr:18q11    SOURCE    GENECARD       Gene Ontology:   biological process:  protein biosynthesis   biological process:  translational initiation   cellular component:  eukaryotic translation initiation factor 4F complex   molecular function:  translation factor activity, nucleic acid binding   molecular function:  RNA binding   molecular function:  translation initiation factor activity      BioCarta Pathways:     KEGG Pathways:  
    Probe set : 1377844_at    Name:   zinc binding alcohol dehydrogenase, domain containing 1 (predicted)    GB acc:   AI411520    UniGene:   Rn.135069    Gene Symbol:   Zadh1_predicted    Locus Link:   299194    Chromosome:   6    Cytoband:   Chr:6q31    SOURCE    GENECARD       Gene Ontology:   molecular function:  alcohol dehydrogenase activity, zinc-dependent   molecular function:  zinc ion binding   molecular function:  oxidoreductase activity      BioCarta Pathways:     KEGG Pathways:  
    Probe set : 1371071_at    Name:   guanine nucleotide binding protein beta 4 subunit    GB acc:   BI284800    UniGene:   Rn.10844    Gene Symbol:   RGD:1359321    Locus Link:   294962    Chromosome:   2    Cytoband:   Chr:2q25    SOURCE    GENECARD       Gene Ontology:   biological process:  signal transduction   biological process:  G-protein coupled receptor protein signaling pathway   cellular component:  heterotrimeric G-protein complex   molecular function:  signal transducer activity      BioCarta Pathways:     KEGG Pathways:  
    Probe set : 1368783_at    Name:   inducible T-cell co-stimulator    GB acc:   NM_022610    UniGene:   Rn.42901    Gene Symbol:   Icos    Locus Link:   64545      SOURCE    GENECARD       Gene Ontology:   biological process:  defense response   cellular component:  extracellular space   cellular component:  integral to plasma membrane   cellular component:  integral to membrane   molecular function:  MHC protein binding   molecular function:  exogenous peptide antigen binding      BioCarta Pathways:     KEGG Pathways:  
    Probe set : 1399158_a_at    Name:   nucleophosmin 1    GB acc:   J04943    UniGene:   Rn.54537    Gene Symbol:   Npm1    Locus Link:   25498    Chromosome:   10    Cytoband:   Chr:10q12    SOURCE    GENECARD       Gene Ontology:   biological process:  intracellular protein transport   biological process:  nucleocytoplasmic transport   biological process:  response to stress   biological process:  centrosome cycle   biological process:  cell aging   biological process:  negative regulation of cell proliferation   biological process:  ribosome assembly   biological process:  activation of NF-kappaB transcription factor   biological process:  intracellular protein transport   biological process:  nucleocytoplasmic transport   biological process:  centrosome cycle   biological process:  cell aging   biological process:  negative regulation of cell proliferation      BioCarta Pathways:     KEGG Pathways:  
    Probe set : 1394819_at    Name:   SP110 nuclear body protein (predicted)    GB acc:   AI177957    UniGene:   Rn.6459     Locus Link:   301570    Chromosome:   9    Cytoband:   Chr:9q35    SOURCE        Gene Ontology:   biological process:  regulation of transcription, DNA-dependent   cellular component:  nucleus   molecular function:  transcription factor activity      BioCarta Pathways:     KEGG Pathways:  
    Probe set : 1372076_at    Name:   hepatitis B virus x interacting protein (predicted)    GB acc:   BI294704    UniGene:   Rn.3763    Gene Symbol:   Hbxip_predicted    Locus Link:   295357    Chromosome:   2    Cytoband:   Chr:2q34    SOURCE    GENECARD       Gene Ontology:   biological process:  response to virus   biological process:  viral life cycle   biological process:  viral genome replication      BioCarta Pathways:     KEGG Pathways:  
    Probe set : 1370440_at    Name:   peptide/histidine transporter    GB acc:   AB000280    UniGene:   Rn.10770    Gene Symbol:   Slc15a4    Locus Link:   246280    Chromosome:   12    Cytoband:   Chr:12q14    SOURCE    GENECARD       Gene Ontology:   biological process:  transport   biological process:  oligopeptide transport   biological process:  L-histidine transport   cellular component:  membrane   cellular component:  integral to membrane   cellular component:  integral to membrane   molecular function:  transporter activity   molecular function:  L-histidine transporter activity   molecular function:  copper ion binding      BioCarta Pathways:     KEGG Pathways:  
    Probe set : 1379250_at    Name:   galactose mutarotase (aldose 1-epimerase)    GB acc:   AI012955    UniGene:   Rn.83639    Gene Symbol:   RGD:1359459    Locus Link:   313843    Chromosome:   6    Cytoband:   Chr:6q11    SOURCE    GENECARD       Gene Ontology:   biological process:  carbohydrate metabolism   biological process:  galactose metabolism   molecular function:  aldose 1-epimerase activity   molecular function:  isomerase activity      BioCarta Pathways:     KEGG Pathways:  
    Probe set : 1373775_at    Name:   Hypothetical LOC311956    GB acc:   AI232313    UniGene:   Rn.72440     Locus Link:   311956    Chromosome:   4    Cytoband:   Chr:4q11    SOURCE        Gene Ontology:     BioCarta Pathways:     KEGG Pathways:  
    Probe set : 1379563_at    Name:   interferon regulatory factor 5 (predicted)    GB acc:   AA998071    UniGene:   Rn.17870    Gene Symbol:   Irf5_predicted    Locus Link:   296953    Chromosome:   4    Cytoband:   Chr:4q22    SOURCE    GENECARD       Gene Ontology:   biological process:  regulation of transcription, DNA-dependent   cellular component:  nucleus   molecular function:  DNA binding   molecular function:  transcription factor activity   molecular function:  RNA polymerase III transcription factor activity      BioCarta Pathways:     KEGG Pathways:  
    Probe set : 1385696_at     GB acc:   BI274471              Gene Ontology:     BioCarta Pathways:     KEGG Pathways:  
    Probe set : 1391808_at    Name:   Transcribed locus    GB acc:   AI535140    UniGene:   Rn.14864        SOURCE        Gene Ontology:     BioCarta Pathways:     KEGG Pathways:  
    Probe set : 1398909_at    Name:   similar to QIL1    GB acc:   AA849634    UniGene:   Rn.4199    Gene Symbol:   LOC301124    Locus Link:   301124    Chromosome:   9    Cytoband:   Chr:9q11    SOURCE    GENECARD       Gene Ontology:     BioCarta Pathways:     KEGG Pathways:  
    Probe set : 1390416_at    Name:   solute carrier family 25, member 30    GB acc:   H35736    UniGene:   Rn.19440    Gene Symbol:   RGD:1359702    Locus Link:   361074    Chromosome:   15    Cytoband:   Chr:15q11    SOURCE    GENECARD       Gene Ontology:   biological process:  transport   biological process:  mitochondrial transport   molecular function:  transporter activity   molecular function:  binding      BioCarta Pathways:     KEGG Pathways:  
    Probe set : 1377635_at    Name:   flavin containing monooxygenase 2    GB acc:   BM389350    UniGene:   Rn.3928    Gene Symbol:   Fmo2    Locus Link:   246245    Chromosome:   13    Cytoband:   Chr:13q22    SOURCE    GENECARD       Gene Ontology:   biological process:  electron transport   biological process:  oxygen and reactive oxygen species metabolism      BioCarta Pathways:     KEGG Pathways:  
    Probe set : 1375732_at    Name:   Transcribed locus    GB acc:   BE109509    UniGene:   Rn.2378        SOURCE        Gene Ontology:     BioCarta Pathways:     KEGG Pathways:  
    Probe set : 1371805_at    Name:   similar to RIKEN cDNA C730048E16 (predicted)    GB acc:   AI406508    UniGene:   Rn.11978    Gene Symbol:   RGD1309382_predicted    Locus Link:   299982    Chromosome:   7    Cytoband:   Chr:7q22    SOURCE    GENECARD       Gene Ontology:   biological process:  regulation of transcription, DNA-dependent   molecular function:  DNA binding   molecular function:  transcription factor activity      BioCarta Pathways:     KEGG Pathways:  
    Probe set : 1371295_at    Name:   ribosomal protein S20    GB acc:   AW914097    UniGene:   Rn.144641    Gene Symbol:   Rps20    Locus Link:   122772    Chromosome:   5    Cytoband:   Chr:5q12    SOURCE    GENECARD       Gene Ontology:   biological process:  protein biosynthesis   molecular function:  RNA binding   molecular function:  structural constituent of ribosome      BioCarta Pathways:     KEGG Pathways:  
    Probe set : 1370209_at    Name:   basic transcription element binding protein 1    GB acc:   BE101336    UniGene:   Rn.19481    Gene Symbol:   Bteb1    Locus Link:   117560    Chromosome:   1    Cytoband:   Chr:1q51    SOURCE    GENECARD       Gene Ontology:   cellular component:  nucleus      BioCarta Pathways:     KEGG Pathways:  
    Probe set : 1379287_at    Name:   retinitis pigmentosa 1 (autosomal dominant) (predicted) /// similar to cylindromatosis (turban tumor syndrome) /// similar to cylindromatosis (turban tumor syndrome)    GB acc:   BF391639    UniGene:   Rn.128760    Gene Symbol:   Rp1_predicted /// LOC361373 /// LOC498035    Locus Link:   312937 /// 361373 /// 498035    Chromosome:   5    Cytoband:   Chr:5q12 /// Chr:19p11    SOURCE    GENECARD       Gene Ontology:     BioCarta Pathways:     KEGG Pathways:  
    Probe set : 1367786_at    Name:   proteosome (prosome, macropain) subunit, beta type 8    GB acc:   NM_080767    UniGene:   Rn.29244    Gene Symbol:   Psmb8    Locus Link:   24968    Chromosome:   20    Cytoband:   Chr:20p12    SOURCE    GENECARD       Gene Ontology:   cellular component:  cytosol   cellular component:  proteasome core complex (sensu Eukaryota)   cellular component:  proteasome core complex (sensu Eukaryota)   molecular function:  endopeptidase activity   molecular function:  peptidase activity   molecular function:  hydrolase activity      BioCarta Pathways:     KEGG Pathways:  
    Probe set : 1392731_at    Name:   hypothetical gene supported by NM_130426    GB acc:   BM390522     Gene Symbol:   LOC497808    Locus Link:   497808    Chromosome:   5    Cytoband:   Chr:5q36     GENECARD       Gene Ontology:     BioCarta Pathways:     KEGG Pathways:  
    Probe set : 1388436_at    Name:   small nuclear ribonucleoprotein polypeptide A (predicted)    GB acc:   AI230728    UniGene:   Rn.9347    Gene Symbol:   Snrpa_predicted    Locus Link:   292729    Chromosome:   1    Cytoband:   Chr:1q21    SOURCE    GENECARD       Gene Ontology:   biological process:  nuclear mRNA splicing, via spliceosome   biological process:  mRNA processing   molecular function:  RNA binding   molecular function:  nucleotide binding   molecular function:  nucleic acid binding      BioCarta Pathways:     KEGG Pathways:  
    Probe set : 1397584_at    Name:   Transcribed locus    GB acc:   BF289504    UniGene:   Rn.55786        SOURCE        Gene Ontology:     BioCarta Pathways:     KEGG Pathways:  
    Probe set : 1388071_x_at    Name:   RT1 class Ib, locus Aw2    GB acc:   M24024    UniGene:   Rn.116787    Gene Symbol:   RT1-Aw2    Locus Link:   24737    Chromosome:   20    Cytoband:   Chr:20p12    SOURCE    GENECARD       Gene Ontology:   cellular component:  integral to membrane   cellular component:  MHC class I protein complex   molecular function:  MHC class I receptor activity      BioCarta Pathways:     KEGG Pathways:  
    Probe set : 1371729_at    Name:   Yippee-like 5 (Drosophila) (predicted)    GB acc:   AI169150    UniGene:   Rn.1136     Locus Link:   298792    Chromosome:   6    Cytoband:   Chr:6q13    SOURCE        Gene Ontology:     BioCarta Pathways:     KEGG Pathways:  
    Probe set : 1398602_at    Name:   MAD2 (mitotic arrest deficient, homolog)-like 1 (yeast) (predicted)    GB acc:   AW143296    UniGene:   Rn.19771     Locus Link:   297176    Chromosome:   4    Cytoband:   Chr:4q31    SOURCE        Gene Ontology:     BioCarta Pathways:     KEGG Pathways:  
    Probe set : 1371653_at     GB acc:   BI281955              Gene Ontology:     BioCarta Pathways:     KEGG Pathways:  
    Probe set : 1370463_x_at    Name:   RT1 class I, CE16    GB acc:   U50449     Gene Symbol:   RGD:1359403    Locus Link:   414819       GENECARD       Gene Ontology:     BioCarta Pathways:     KEGG Pathways:  
    Probe set : 1370810_at    Name:   cyclin D2    GB acc:   L09752    UniGene:   Rn.96083    Gene Symbol:   Ccnd2    Locus Link:   64033    Chromosome:   4    Cytoband:   Chr:4q42    SOURCE    GENECARD       Gene Ontology:   cellular component:  nucleus   molecular function:  protein binding   molecular function:  cyclin-dependent protein kinase regulator activity      BioCarta Pathways:     KEGG Pathways:  
    Probe set : 1398853_at    Name:   proteasome (prosome, macropain) subunit, beta type 3    GB acc:   AI169273    UniGene:   Rn.144576    Gene Symbol:   Psmb3    Locus Link:   29676    Chromosome:   10    Cytoband:   Chr:10q31    SOURCE    GENECARD       Gene Ontology:   biological process:  ubiquitin-dependent protein catabolism   biological process:  protein catabolism   cellular component:  cytosol   cellular component:  proteasome core complex (sensu Eukaryota)   cellular component:  protein complex      BioCarta Pathways:     KEGG Pathways:  
    Probe set : 1393460_at    Name:   similar to GARP protein precursor (Garpin) (Glycoprotein A repetitions predominant) (predicted)    GB acc:   BM388665    UniGene:   Rn.138732    Gene Symbol:   RGD1310771_predicted    Locus Link:   303875    Chromosome:   11    Cytoband:   Chr:11q22    SOURCE    GENECARD       Gene Ontology:   cellular component:  extracellular matrix (sensu Metazoa)   cellular component:  integral to membrane      BioCarta Pathways:     KEGG Pathways:  
    Probe set : 1388135_at    Name:   replication protein A2    GB acc:   BM385924    UniGene:   Rn.40389    Gene Symbol:   Rpa2    Locus Link:   59102    Chromosome:   5    Cytoband:   Chr:5q36    SOURCE    GENECARD       Gene Ontology:   biological process:  DNA replication   biological process:  base-excision repair   cellular component:  nucleus   molecular function:  nucleic acid binding   molecular function:  protein N-terminus binding      BioCarta Pathways:     KEGG Pathways:  
    Probe set : 1391803_at    Name:   Transcribed locus    GB acc:   BF557813    UniGene:   Rn.12095        SOURCE        Gene Ontology:     BioCarta Pathways:     KEGG Pathways:  
    Probe set : 1370189_at    Name:   splicing factor, arginine/serine-rich 10 (transformer 2 homolog, Drosophila)    GB acc:   BI290979    UniGene:   Rn.8538    Gene Symbol:   Sfrs10    Locus Link:   117259    Chromosome:   11    Cytoband:   Chr:11q23    SOURCE    GENECARD       Gene Ontology:   cellular component:  nucleus   cellular component:  nucleus      BioCarta Pathways:     KEGG Pathways:  
    Probe set : 1368894_at    Name:   CAP, adenylate cyclase-associated protein, 2 (yeast)    GB acc:   AW919109    UniGene:   Rn.10229    Gene Symbol:   Cap2    Locus Link:   116653      SOURCE    GENECARD       Gene Ontology:   cellular component:  membrane      BioCarta Pathways:     KEGG Pathways:  
    Probe set : 1368467_at    Name:   cytochrome P450, family 4, subfamily F, polypeptide 2    GB acc:   NM_019623    UniGene:   Rn.5722    Gene Symbol:   Cyp4f2    Locus Link:   56266    Chromosome:   7    Cytoband:   Chr:7q11    SOURCE    GENECARD       Gene Ontology:   biological process:  electron transport      BioCarta Pathways:     KEGG Pathways:  
    Probe set : 1372032_at     GB acc:   AA851914              Gene Ontology:     BioCarta Pathways:     KEGG Pathways:  
    Probe set : 1371974_at    Name:   phytanoyl-CoA dioxygenase domain containing 1 (predicted)    GB acc:   BI275763    UniGene:   Rn.32623    Gene Symbol:   Phyhd1_predicted    Locus Link:   296621    Chromosome:   3    Cytoband:   Chr:3p12    SOURCE    GENECARD       Gene Ontology:   molecular function:  oxidoreductase activity, acting on single donors with incorporation of molecular oxygen, incorporation of two atoms of oxygen      BioCarta Pathways:     KEGG Pathways:  
    Probe set : 1387831_at    Name:   small inducible cytokine subfamily C, member 1 (lymphotactin)    GB acc:   NM_134361    UniGene:   Rn.90962    Gene Symbol:   Xcl1    Locus Link:   171371    Chromosome:   13    Cytoband:   Chr:13q23    SOURCE    GENECARD       Gene Ontology:   biological process:  chemotaxis   biological process:  immune response   biological process:  sensory perception   cellular component:  extracellular region   cellular component:  extracellular space   molecular function:  cytokine activity   molecular function:  chemokine activity   molecular function:  chemokine activity      BioCarta Pathways:     KEGG Pathways:  
    Probe set : 1372460_at    Name:   LOC499767    GB acc:   AI172405    UniGene:   Rn.144530    Gene Symbol:   LOC499767    Locus Link:   499767    Chromosome:   3    Cytoband:   Chr:3p12    SOURCE    GENECARD       Gene Ontology:     BioCarta Pathways:     KEGG Pathways:  
    Probe set : 1367614_at    Name:   annexin A1    GB acc:   NM_012904    UniGene:   Rn.1792    Gene Symbol:   Anxa1    Locus Link:   25380    Chromosome:   1    Cytoband:   Chr:1q51    SOURCE    GENECARD       Gene Ontology:   cellular component:  sarcolemma      BioCarta Pathways:     KEGG Pathways:  
    Probe set : 1385001_at    Name:   gasdermin domain containing 1 (predicted)    GB acc:   AI179373    UniGene:   Rn.105659    Gene Symbol:   Gsdmdc1_predicted    Locus Link:   315084    Chromosome:   7    Cytoband:   Chr:7q34    SOURCE    GENECARD       Gene Ontology:     BioCarta Pathways:     KEGG Pathways:  
    Probe set : 1368245_at    Name:   ureidopropionase, beta    GB acc:   NM_053845    UniGene:   Rn.11110    Gene Symbol:   Upb1    Locus Link:   116593    Chromosome:   20    Cytoband:   Chr:20p12    SOURCE    GENECARD       Gene Ontology:   biological process:  nitrogen compound metabolism   biological process:  beta-alanine biosynthesis   biological process:  beta-alanine biosynthesis      BioCarta Pathways:     KEGG Pathways:  
    Probe set : 1375716_at    Name:   interferon gamma receptor 2 (predicted)    GB acc:   AI602715    UniGene:   Rn.23305    Gene Symbol:   Ifngr2_predicted    Locus Link:   360697    Chromosome:   11    Cytoband:   Chr:11q11    SOURCE    GENECARD       Gene Ontology:   biological process:  cell surface receptor linked signal transduction   biological process:  response to virus   biological process:  response to pathogenic bacteria      BioCarta Pathways:     KEGG Pathways:  
    Probe set : 1372562_at    Name:   similar to RIKEN cDNA C030006K11    GB acc:   AI236816    UniGene:   Rn.140135    Gene Symbol:   RGD:1359663    Locus Link:   362946    Chromosome:   7    Cytoband:   Chr:7q34    SOURCE    GENECARD       Gene Ontology:     BioCarta Pathways:     KEGG Pathways:  
    Probe set : 1368940_at    Name:   purinergic receptor P2Y, G-protein coupled 2    GB acc:   NM_017255    UniGene:   Rn.11102    Gene Symbol:   P2ry2    Locus Link:   29597    Chromosome:   1    Cytoband:   Chr:1q32    SOURCE    GENECARD       Gene Ontology:   biological process:  G-protein coupled receptor protein signaling pathway   biological process:  signal transduction      BioCarta Pathways:     KEGG Pathways:  
    Probe set : 1375638_at    Name:   serum deprivation response protein    GB acc:   AI009714    UniGene:   Rn.2472    Gene Symbol:   RGD:1359345    Locus Link:   316384    Chromosome:   9    Cytoband:   Chr:9q22    SOURCE    GENECARD       Gene Ontology:     BioCarta Pathways:     KEGG Pathways:  
    Probe set : 1374038_at    Name:   Transcribed locus    GB acc:   AA799964    UniGene:   Rn.6261        SOURCE        Gene Ontology:     BioCarta Pathways:     KEGG Pathways:  
    Probe set : 1386908_at    Name:   glutaredoxin 1 (thioltransferase)    GB acc:   NM_022278    UniGene:   Rn.1484    Gene Symbol:   Glrx1    Locus Link:   64045    Chromosome:   2    Cytoband:   Chr:2q11    SOURCE    GENECARD       Gene Ontology:   biological process:  electron transport   biological process:  transport   biological process:  cell redox homeostasis      BioCarta Pathways:     KEGG Pathways:  
    Probe set : 1387956_s_at    Name:   chemokine-like factor 1    GB acc:   AF253064    UniGene:   Rn.81197    Gene Symbol:   Cklf1    Locus Link:   245978    Chromosome:   19    Cytoband:   Chr:19p14    SOURCE    GENECARD       Gene Ontology:   cellular component:  integral to membrane   cellular component:  extracellular space   cellular component:  membrane   molecular function:  cytokine activity      BioCarta Pathways:     KEGG Pathways:  
    Probe set : 1381323_at    Name:   retinoblastoma binding protein 5 (predicted)    GB acc:   BG381396    UniGene:   Rn.49131    Gene Symbol:   Rbbp5_predicted    Locus Link:   304794    Chromosome:   13    Cytoband:   Chr:13q13    SOURCE    GENECARD       Gene Ontology:   cellular component:  nucleus      BioCarta Pathways:     KEGG Pathways:  
    Probe set : 1382275_at    Name:   similar to PAK/PLC-interacting protein 1    GB acc:   AI236989    UniGene:   Rn.115052    Gene Symbol:   LOC361232    Locus Link:   361232    Chromosome:   17    Cytoband:   Chr:17p12    SOURCE    GENECARD       Gene Ontology:     BioCarta Pathways:     KEGG Pathways:  
    Probe set : 1370415_at    Name:   Ras association (RalGDS/AF-6) domain family 5    GB acc:   AF002251    UniGene:   Rn.36106    Gene Symbol:   Rassf5    Locus Link:   54355    Chromosome:   13    Cytoband:   Chr:13q13    SOURCE    GENECARD       Gene Ontology:   cellular component:  nucleus   molecular function:  protein binding   molecular function:  diacylglycerol binding      BioCarta Pathways:     KEGG Pathways:  
    Probe set : 1383368_at    Name:   Unknown (protein for MGC:72610)    GB acc:   BF550060    UniGene:   Rn.101402    Gene Symbol:   MGC72610    Locus Link:   304881    Chromosome:   13    Cytoband:   Chr:13q21    SOURCE    GENECARD       Gene Ontology:     BioCarta Pathways:     KEGG Pathways:  
    Probe set : 1370218_at    Name:   lactate dehydrogenase B    GB acc:   AA848319    UniGene:   Rn.1785    Gene Symbol:   Ldhb    Locus Link:   24534    Chromosome:   4    Cytoband:   Chr:4q44    SOURCE    GENECARD       Gene Ontology:   biological process:  glycolysis   biological process:  tricarboxylic acid cycle intermediate metabolism   biological process:  anaerobic glycolysis   cellular component:  cytoplasm      BioCarta Pathways:     KEGG Pathways:  
    Probe set : 1377086_at    Name:   C1q and tumor necrosis factor related protein 3 (predicted)    GB acc:   AI233530    UniGene:   Rn.16507    Gene Symbol:   C1qtnf3_predicted    Locus Link:   294806    Chromosome:   2    Cytoband:   Chr:2q16    SOURCE    GENECARD       Gene Ontology:   biological process:  phosphate transport   cellular component:  extracellular region   cellular component:  extracellular space   cellular component:  cytoplasm      BioCarta Pathways:     KEGG Pathways:  
    Probe set : 1367777_at    Name:   2,4-dienoyl CoA reductase 1, mitochondrial    GB acc:   NM_057197    UniGene:   Rn.2854    Gene Symbol:   Decr1    Locus Link:   117543    Chromosome:   5    Cytoband:   Chr:5q13    SOURCE    GENECARD       Gene Ontology:   biological process:  metabolism   cellular component:  mitochondrion   molecular function:  2,4-dienoyl-CoA reductase (NADPH) activity   molecular function:  2,4-dienoyl-CoA reductase (NADPH) activity   molecular function:  oxidoreductase activity      BioCarta Pathways:     KEGG Pathways:  
    Probe set : 1389181_at    Name:   Rap guanine nucleotide exchange factor (GEF) 6 (predicted)    GB acc:   BM389650    UniGene:   Rn.143915    Gene Symbol:   Rapgef6_predicted    Locus Link:   303141    Chromosome:   10    Cytoband:   Chr:10q22    SOURCE    GENECARD       Gene Ontology:   biological process:  small GTPase mediated signal transduction   biological process:  Ras protein signal transduction   biological process:  regulation of GTPase activity   cellular component:  plasma membrane      BioCarta Pathways:     KEGG Pathways:  
    Probe set : 1380297_at     GB acc:   AW915533              Gene Ontology:     BioCarta Pathways:     KEGG Pathways:  
    Probe set : 1390797_at    Name:   lymphocyte cytosolic protein 2    GB acc:   BF282471    UniGene:   Rn.1057    Gene Symbol:   Lcp2    Locus Link:   155918    Chromosome:   10    Cytoband:   Chr:10q12    SOURCE    GENECARD       Gene Ontology:   molecular function:  protein binding      BioCarta Pathways:     KEGG Pathways:  
    Probe set : 1383391_a_at    Name:   complement component 2    GB acc:   AI716125    UniGene:   Rn.98333    Gene Symbol:   C2    Locus Link:   24231    Chromosome:   20    Cytoband:   Chr:20p12    SOURCE    GENECARD       Gene Ontology:   biological process:  proteolysis   biological process:  complement activation   biological process:  complement activation, classical pathway   cellular component:  extracellular space   cellular component:  extracellular region      BioCarta Pathways:     KEGG Pathways:  
    Probe set : 1390285_at    Name:   similar to BC026645 protein    GB acc:   AI178948    UniGene:   Rn.13220    Gene Symbol:   LOC309112    Locus Link:   309112    Chromosome:   1    Cytoband:   Chr:1q41    SOURCE    GENECARD       Gene Ontology:     BioCarta Pathways:     KEGG Pathways:  
    Probe set : 1368645_at    Name:   protein tyrosine phosphatase, non-receptor type 1    GB acc:   NM_012637    UniGene:   Rn.11317    Gene Symbol:   Ptpn1    Locus Link:   24697    Chromosome:   3    Cytoband:   Chr:3q42    SOURCE    GENECARD       Gene Ontology:   biological process:  protein amino acid dephosphorylation   biological process:  protein amino acid dephosphorylation   cellular component:  integral to membrane   cellular component:  endoplasmic reticulum      BioCarta Pathways:     KEGG Pathways:  
    Probe set : 1368339_at    Name:   calbindin 3, (vitamin D-dependent calcium binding protein)    GB acc:   NM_012521    UniGene:   Rn.9974    Gene Symbol:   Calb3    Locus Link:   24249    Chromosome:   X    Cytoband:   Chr:Xq21    SOURCE    GENECARD       Gene Ontology:   cellular component:  basolateral plasma membrane   cellular component:  apical plasma membrane   molecular function:  calcium ion binding   molecular function:  calcium ion binding   molecular function:  vitamin D binding      BioCarta Pathways:     KEGG Pathways:  
    Probe set : 1386946_at    Name:   carnitine palmitoyltransferase 1, liver    GB acc:   NM_031559    UniGene:   Rn.2856    Gene Symbol:   Cpt1a    Locus Link:   25757    Chromosome:   1    Cytoband:   Chr:1q43    SOURCE    GENECARD       Gene Ontology:     BioCarta Pathways:     KEGG Pathways:  
    Probe set : 1382569_at    Name:   Transcribed locus    GB acc:   BF386242    UniGene:   Rn.64169        SOURCE        Gene Ontology:     BioCarta Pathways:     KEGG Pathways:  
    Probe set : 1393163_at    Name:   Transcribed locus    GB acc:   AI112184    UniGene:   Rn.146411        SOURCE        Gene Ontology:     BioCarta Pathways:     KEGG Pathways:  
    Probe set : 1392914_at     GB acc:   BI275467              Gene Ontology:     BioCarta Pathways:     KEGG Pathways:  
    Probe set : 1385711_at    Name:   similar to RIKEN cDNA 1500031M22 (predicted)    GB acc:   AW507178    UniGene:   Rn.25178    Gene Symbol:   RGD1311752_predicted    Locus Link:   294718    Chromosome:   2    Cytoband:   Chr:2q14    SOURCE    GENECARD       Gene Ontology:     BioCarta Pathways:     KEGG Pathways:  
    Probe set : 1378457_at    Name:   Transcribed locus    GB acc:   AI179450    UniGene:   Rn.34019        SOURCE        Gene Ontology:     BioCarta Pathways:     KEGG Pathways:  
    Probe set : 1378315_at    Name:   Transcribed locus    GB acc:   AI045116    UniGene:   Rn.147582        SOURCE        Gene Ontology:     BioCarta Pathways:     KEGG Pathways:  
    Probe set : 1384103_at    Name:   Similar to novel protein (HT036)    GB acc:   BE106350    UniGene:   Rn.40997     Locus Link:   500536      SOURCE        Gene Ontology:     BioCarta Pathways:     KEGG Pathways:  
    Probe set : 1367901_at    Name:   glucuronidase, beta    GB acc:   NM_017015    UniGene:   Rn.3692    Gene Symbol:   Gusb    Locus Link:   24434    Chromosome:   12    Cytoband:   Chr:12q13    SOURCE    GENECARD       Gene Ontology:   cellular component:  extracellular space   cellular component:  lysosome   cellular component:  lysosome      BioCarta Pathways:     KEGG Pathways:  
    Probe set : 1370309_a_at    Name:   heterogeneous nuclear ribonucleoprotein A/B    GB acc:   AJ238854    UniGene:   Rn.1427    Gene Symbol:   Hnrpab    Locus Link:   83498    Chromosome:   10    Cytoband:   Chr:10q22    SOURCE    GENECARD       Gene Ontology:   cellular component:  nucleus   cellular component:  viral nucleocapsid   cellular component:  ribonucleoprotein complex      BioCarta Pathways:     KEGG Pathways:  
    Probe set : 1393831_at    Name:   Transcribed locus    GB acc:   BM383212    UniGene:   Rn.149113        SOURCE        Gene Ontology:     BioCarta Pathways:     KEGG Pathways:  
    Probe set : 1387198_at    Name:   inositol polyphosphate-5-phosphatase D    GB acc:   NM_019311    UniGene:   Rn.10659    Gene Symbol:   Inpp5d    Locus Link:   54259    Chromosome:   9    Cytoband:   Chr:9q35    SOURCE    GENECARD       Gene Ontology:   biological process:  intracellular signaling cascade   molecular function:  inositol-4,5-bisphosphate 5-phosphatase activity   molecular function:  inositol or phosphatidylinositol phosphatase activity      BioCarta Pathways:     KEGG Pathways:  
    Probe set : 1379582_a_at    Name:   cyclin A2    GB acc:   AA998516    UniGene:   Rn.13094    Gene Symbol:   Ccna2    Locus Link:   114494    Chromosome:   2    Cytoband:   Chr:2q25    SOURCE    GENECARD       Gene Ontology:   biological process:  regulation of progression through cell cycle   biological process:  cell cycle   biological process:  cell division   cellular component:  nucleus      BioCarta Pathways:     KEGG Pathways:  
    Probe set : 1369801_at    Name:   selectin, lymphocyte    GB acc:   D10831    UniGene:   Rn.10461    Gene Symbol:   Sell    Locus Link:   29259    Chromosome:   13    Cytoband:   Chr:13q22    SOURCE    GENECARD       Gene Ontology:   biological process:  defense response   biological process:  cell adhesion   biological process:  heterophilic cell adhesion   molecular function:  protein binding   molecular function:  sugar binding      BioCarta Pathways:     KEGG Pathways:  
    Probe set : 1370232_at    Name:   isovaleryl coenzyme A dehydrogenase    GB acc:   AI102838    UniGene:   Rn.147    Gene Symbol:   Ivd    Locus Link:   24513    Chromosome:   3    Cytoband:   Chr:3q35    SOURCE    GENECARD       Gene Ontology:   biological process:  electron transport   cellular component:  mitochondrion   cellular component:  mitochondrial matrix   cellular component:  mitochondrial matrix      BioCarta Pathways:     KEGG Pathways:  
    Probe set : 1379482_at    Name:   transmembrane 6 superfamily member 1 (predicted)    GB acc:   BI283829    UniGene:   Rn.130366    Gene Symbol:   Tm6sf1_predicted    Locus Link:   361600    Chromosome:   1    Cytoband:   Chr:1q31    SOURCE    GENECARD       Gene Ontology:   cellular component:  integral to membrane      BioCarta Pathways:     KEGG Pathways:  
    Probe set : 1386982_at    Name:   Alpha-1,6-mannosyl-glycoprotein 2-beta-N-acetylglucosaminyltransferase    GB acc:   NM_053604    UniGene:   Rn.2342    Gene Symbol:   Mgat2    Locus Link:   94273    Chromosome:   6    Cytoband:   Chr:6q24    SOURCE    GENECARD       Gene Ontology:   biological process:  protein amino acid N-linked glycosylation   biological process:  oligosaccharide biosynthesis   biological process:  methane metabolism      BioCarta Pathways:     KEGG Pathways:  
    Probe set : 1391637_at    Name:   similar to RIKEN cDNA 5830480G12    GB acc:   AI178168    UniGene:   Rn.15607    Gene Symbol:   RGD:1359304    Locus Link:   295635    Chromosome:   3    Cytoband:   Chr:3q12    SOURCE    GENECARD       Gene Ontology:   molecular function:  GTPase activator activity      BioCarta Pathways:     KEGG Pathways:  
    Probe set : 1390704_at    Name:   SDA1 domain containing 1    GB acc:   BF394953    UniGene:   Rn.12755    Gene Symbol:   RGD:1359112    Locus Link:   289504    Chromosome:   14    Cytoband:   Chr:14p22    SOURCE    GENECARD       Gene Ontology:     BioCarta Pathways:     KEGG Pathways:  
    Probe set : 1377018_at    Name:   Similar to E430002G05Rik protein (predicted)    GB acc:   BF284124    UniGene:   Rn.33642     Locus Link:   311252    Chromosome:   3    Cytoband:   Chr:3q31    SOURCE        Gene Ontology:     BioCarta Pathways:     KEGG Pathways:  
    Probe set : 1371701_at    Name:   NADH dehydrogenase (ubiquinone) 1 beta subcomplex, 9 (predicted)    GB acc:   BG666921    UniGene:   Rn.22045    Gene Symbol:   Ndufb9_predicted    Locus Link:   299954    Chromosome:   7    Cytoband:   Chr:7q33    SOURCE    GENECARD       Gene Ontology:   biological process:  electron transport   biological process:  mitochondrial electron transport, NADH to ubiquinone   biological process:  sensory perception of sound   cellular component:  mitochondrion   cellular component:  mitochondrial inner membrane      BioCarta Pathways:     KEGG Pathways:  
    Probe set : 1368990_at    Name:   cytochrome P450, family 1, subfamily b, polypeptide 1    GB acc:   NM_012940    UniGene:   Rn.10125    Gene Symbol:   Cyp1b1    Locus Link:   25426    Chromosome:   6    Cytoband:   Chr:6q11    SOURCE    GENECARD       Gene Ontology:     BioCarta Pathways:     KEGG Pathways:  
    Probe set : 1384842_s_at    Name:   Tumor necrosis factor receptor superfamily, member 6    GB acc:   AI231531    UniGene:   Rn.144672    Gene Symbol:   RGD:619831    Locus Link:   246097    Chromosome:   1    Cytoband:   Chr:1q52    SOURCE    GENECARD       Gene Ontology:   cellular component:  caveola   cellular component:  membrane   cellular component:  integral to membrane      BioCarta Pathways:     KEGG Pathways:  
    Probe set : 1378193_at    Name:   membrane-spanning 4-domains, subfamily A, member 7 (predicted)    GB acc:   AI408286    UniGene:   Rn.44037    Gene Symbol:   Ms4a7_predicted    Locus Link:   293744    Chromosome:   1    Cytoband:   Chr:1q43    SOURCE    GENECARD       Gene Ontology:   biological process:  signal transduction   cellular component:  integral to membrane   molecular function:  receptor activity      BioCarta Pathways:     KEGG Pathways:  
    Probe set : 1372341_at    Name:   Transcribed locus, strongly similar to XP_484968.2 PREDICTED: similar to RIKEN cDNA C330005L02 [Mus musculus]    GB acc:   AI233213    UniGene:   Rn.17305        SOURCE        Gene Ontology:     BioCarta Pathways:     KEGG Pathways:  
    Probe set : 1390421_at    Name:   Similar to RIKEN cDNA 0610010D20 (predicted)    GB acc:   AI232524    UniGene:   Rn.13905     Locus Link:   293949    Chromosome:   1    Cytoband:   Chr:1q54    SOURCE        Gene Ontology:     BioCarta Pathways:     KEGG Pathways:  
    Probe set : 1372042_at    Name:   chemokine-like factor super family 3 (predicted)    GB acc:   BI294844    UniGene:   Rn.15332    Gene Symbol:   Cklfsf3_predicted    Locus Link:   291813    Chromosome:   19    Cytoband:   Chr:19p14    SOURCE    GENECARD       Gene Ontology:   biological process:  chemotaxis   cellular component:  membrane   cellular component:  integral to membrane   molecular function:  cytokine activity      BioCarta Pathways:     KEGG Pathways:  
    Probe set : 1380546_at    Name:   similar to hypothetical protein FLJ10986 /// similar to hypothetical protein FLJ10986    GB acc:   AA926048    UniGene:   Rn.8436    Gene Symbol:   LOC298250 /// LOC298251    Locus Link:   298250 /// 298251    Chromosome:   5    Cytoband:   Chr:5q33    SOURCE    GENECARD       Gene Ontology:   biological process:  carbohydrate metabolism      BioCarta Pathways:     KEGG Pathways:  
    Probe set : 1369625_at    Name:   aquaporin 1    GB acc:   AA891661    UniGene:   Rn.1618    Gene Symbol:   Aqp1    Locus Link:   25240    Chromosome:   4    Cytoband:   Chr:4q24    SOURCE    GENECARD       Gene Ontology:   biological process:  transport   biological process:  water transport   biological process:  excretion      BioCarta Pathways:     KEGG Pathways:  
    Probe set : 1372930_at    Name:   SP110 nuclear body protein (predicted)    GB acc:   AI411381    UniGene:   Rn.6459     Locus Link:   301570    Chromosome:   9    Cytoband:   Chr:9q35    SOURCE        Gene Ontology:   biological process:  regulation of transcription, DNA-dependent   cellular component:  nucleus   molecular function:  transcription factor activity      BioCarta Pathways:     KEGG Pathways:  
    Probe set : 1373283_at    Name:   Thymopoietin    GB acc:   BE113158    UniGene:   Rn.3364    Gene Symbol:   Tmpo    Locus Link:   25359    Chromosome:   7    Cytoband:   Chr:7q13    SOURCE    GENECARD       Gene Ontology:   biological process:  regulation of transcription      BioCarta Pathways:     KEGG Pathways:  
    Probe set : 1378150_at    Name:   caspase recruitment domain family, member 11 (predicted)    GB acc:   AA945727    UniGene:   Rn.7904    Gene Symbol:   Card11_predicted    Locus Link:   304314    Chromosome:   12    Cytoband:   Chr:12q11    SOURCE    GENECARD       Gene Ontology:   cellular component:  plasma membrane   molecular function:  protein binding      BioCarta Pathways:     KEGG Pathways:  
    Probe set : 1388203_x_at    Name:   RT1 class Ib, locus Aw2 /// RT1 class I, CE5 /// RT1 class I, CE2 /// RT1 class I, CE15 /// RT1 class I, CE10    GB acc:   BI395698    UniGene:   Rn.40130    Gene Symbol:   RT1-Aw2 /// RT1-CE5 /// RT1-CE2 /// RGD:1359630 /// RGD:1359292    Locus Link:   24737 /// 309607 /// 414779 /// 414789 /// 414792    Chromosome:   20    Cytoband:   Chr:20p12    SOURCE    GENECARD       Gene Ontology:   cellular component:  integral to membrane   cellular component:  MHC class I protein complex   molecular function:  MHC class I receptor activity      BioCarta Pathways:     KEGG Pathways:  
    Probe set : 1387742_at    Name:   chemokine (C-C motif) receptor 2    GB acc:   NM_021866    UniGene:   Rn.138119    Gene Symbol:   Ccr2    Locus Link:   60463    Chromosome:   8    Cytoband:   Chr:8q32    SOURCE    GENECARD       Gene Ontology:   biological process:  inflammatory response   biological process:  inflammatory response   biological process:  G-protein coupled receptor protein signaling pathway   cellular component:  integral to membrane      BioCarta Pathways:     KEGG Pathways:  
    Probe set : 1368464_at    Name:   macrophage galactose N-acetyl-galactosamine specific lectin 1    GB acc:   NM_022393    UniGene:   Rn.779    Gene Symbol:   Mgl1    Locus Link:   64195    Chromosome:   10    Cytoband:   Chr:10q24    SOURCE    GENECARD       Gene Ontology:   cellular component:  membrane   cellular component:  integral to membrane   molecular function:  sugar binding   molecular function:  sugar binding   molecular function:  calcium ion binding      BioCarta Pathways:     KEGG Pathways:  
    Probe set : 1389907_at    Name:   similar to archease    GB acc:   AI169317    UniGene:   Rn.144416    Gene Symbol:   LOC297885    Locus Link:   297885    Chromosome:   5    Cytoband:   Chr:5q36    SOURCE    GENECARD       Gene Ontology:     BioCarta Pathways:     KEGG Pathways:  
    Probe set : 1379395_a_at    Name:   histidine triad nucleotide binding protein 2 (predicted)    GB acc:   BI280018    UniGene:   Rn.11657    Gene Symbol:   Hint2_predicted    Locus Link:   313491    Chromosome:   5    Cytoband:   Chr:5q22    SOURCE    GENECARD       Gene Ontology:   cellular component:  extracellular space   cellular component:  mitochondrion   molecular function:  hydrolase activity      BioCarta Pathways:     KEGG Pathways:  
    Probe set : 1384280_at    Name:   Nucleolar and spindle associated protein 1 (predicted)    GB acc:   AA964152    UniGene:   Rn.106933     Locus Link:   311336    Chromosome:   3    Cytoband:   Chr:3q35    SOURCE        Gene Ontology:   cellular component:  nucleus   cellular component:  nucleolus   cellular component:  spindle microtubule   molecular function:  microtubule binding      BioCarta Pathways:     KEGG Pathways:  
    Probe set : 1387806_at    Name:   RAS related protein 1b    GB acc:   NM_134346    UniGene:   Rn.95071    Gene Symbol:   Rap1b    Locus Link:   171337    Chromosome:   7    Cytoband:   Chr:7q22    SOURCE    GENECARD       Gene Ontology:   biological process:  intracellular protein transport   biological process:  small GTPase mediated signal transduction   cellular component:  membrane   molecular function:  GTPase activity   molecular function:  GTP binding   molecular function:  GTP binding   molecular function:  nucleotide binding      BioCarta Pathways:     KEGG Pathways:  
    Probe set : 1394419_at    Name:   similar to RIKEN cDNA 6530401L14 gene (predicted)    GB acc:   BE109109    UniGene:   Rn.51673    Gene Symbol:   RGD1309107_predicted    Locus Link:   296060    Chromosome:   3    Cytoband:   Chr:3q34-q35    SOURCE    GENECARD       Gene Ontology:   molecular function:  GTPase activator activity      BioCarta Pathways:     KEGG Pathways:  
    Probe set : 1389556_at    Name:   kinesin-associated protein 3 (predicted)    GB acc:   BI286000    UniGene:   Rn.24266    Gene Symbol:   Kifap3_predicted    Locus Link:   289168    Chromosome:   13    Cytoband:   Chr:13q22    SOURCE    GENECARD       Gene Ontology:   cellular component:  endoplasmic reticulum   cellular component:  kinesin complex   cellular component:  kinesin II complex      BioCarta Pathways:     KEGG Pathways:  
    Probe set : 1384837_at    Name:   CD69 antigen    GB acc:   AI137672    UniGene:   Rn.23779    Gene Symbol:   Cd69    Locus Link:   29187    Chromosome:   4    Cytoband:   Chr:4q42    SOURCE    GENECARD       Gene Ontology:   cellular component:  external side of plasma membrane   cellular component:  integral to membrane   molecular function:  calcium ion binding   molecular function:  sugar binding      BioCarta Pathways:     KEGG Pathways:  
    Probe set : 1382014_at    Name:   similar to CGI-121 protein (predicted)    GB acc:   AW521951    UniGene:   Rn.19429    Gene Symbol:   RGD1309786_predicted    Locus Link:   297411    Chromosome:   4    Cytoband:   Chr:4q34    SOURCE    GENECARD       Gene Ontology:   molecular function:  protein kinase binding      BioCarta Pathways:     KEGG Pathways:  
    Probe set : 1374651_at    Name:   similar to KIAA0933 protein (predicted)    GB acc:   BM386987    UniGene:   Rn.52409    Gene Symbol:   RGD1306097_predicted    Locus Link:   304077    Chromosome:   11    Cytoband:   Chr:11q11    SOURCE    GENECARD       Gene Ontology:     BioCarta Pathways:     KEGG Pathways:  
    Probe set : 1367829_at    Name:   enoyl Coenzyme A hydratase, short chain, 1, mitochondrial    GB acc:   NM_078623    UniGene:   Rn.6847    Gene Symbol:   Echs1    Locus Link:   140547    Chromosome:   1    Cytoband:   Chr:1q41    SOURCE    GENECARD       Gene Ontology:   cellular component:  mitochondrion   cellular component:  mitochondrion      BioCarta Pathways:     KEGG Pathways:  
    Probe set : 1367952_at    Name:   low density lipoprotein receptor-related protein 2    GB acc:   NM_030827    UniGene:   Rn.26430    Gene Symbol:   Lrp2    Locus Link:   29216    Chromosome:   3    Cytoband:   Chr:3q21    SOURCE    GENECARD       Gene Ontology:     BioCarta Pathways:     KEGG Pathways:  
    Probe set : 1391214_at    Name:   hypothetical protein LOC294430    GB acc:   BI289378    UniGene:   Rn.12813    Gene Symbol:   RGD1304835_predicted    Locus Link:   294430    Chromosome:   20    Cytoband:   Chr:20q11    SOURCE    GENECARD       Gene Ontology:     BioCarta Pathways:     KEGG Pathways:  
    Probe set : 1388389_at    Name:   septin 2    GB acc:   BF282620    UniGene:   Rn.98570    Gene Symbol:   Sept2    Locus Link:   117515    Chromosome:   9    Cytoband:   Chr:9q36    SOURCE    GENECARD       Gene Ontology:   biological process:  cell cycle   molecular function:  nucleotide binding   molecular function:  GTP binding      BioCarta Pathways:     KEGG Pathways:  
    Probe set : 1370067_at    Name:   malic enzyme 1    GB acc:   NM_012600    UniGene:   Rn.3519    Gene Symbol:   Me1    Locus Link:   24552    Chromosome:   8    Cytoband:   Chr:8q31    SOURCE    GENECARD       Gene Ontology:   biological process:  carbohydrate metabolism   biological process:  tricarboxylic acid cycle   biological process:  malate metabolism   cellular component:  soluble fraction   cellular component:  cytosol      BioCarta Pathways:     KEGG Pathways:  
    Probe set : 1367683_at    Name:   karyopherin (importin) alpha 2    GB acc:   NM_053483    UniGene:   Rn.2949    Gene Symbol:   Kpna2    Locus Link:   85245    Chromosome:   10    Cytoband:   Chr:10q32.1    SOURCE    GENECARD       Gene Ontology:   cellular component:  nucleus   cellular component:  cytoskeleton      BioCarta Pathways:     KEGG Pathways:  
    Probe set : 1371171_at    Name:   RT1 class Ib, locus Aw2    GB acc:   M10094    UniGene:   Rn.116787    Gene Symbol:   RT1-Aw2    Locus Link:   24737    Chromosome:   20    Cytoband:   Chr:20p12    SOURCE    GENECARD       Gene Ontology:   cellular component:  integral to membrane   cellular component:  MHC class I protein complex   molecular function:  MHC class I receptor activity      BioCarta Pathways:     KEGG Pathways:  
    Probe set : 1392900_at    Name:   Similar to mKIAA1631 protein    GB acc:   AW888327    UniGene:   Rn.13838     Locus Link:   310756    Chromosome:   2    Cytoband:   Chr:2q34    SOURCE        Gene Ontology:     BioCarta Pathways:     KEGG Pathways:  
    Probe set : 1373557_at    Name:   mini chromosome maintenance deficient 4 homolog (S. cerevisiae) /// similar to mcdc21 protein    GB acc:   AI145081    UniGene:   Rn.8341    Gene Symbol:   Mcmd4 /// LOC310429    Locus Link:   29728 /// 310429    Chromosome:   2    Cytoband:   Chr:2q31    SOURCE    GENECARD       Gene Ontology:   cellular component:  nucleus      BioCarta Pathways:     KEGG Pathways:  
    Probe set : 1370249_at    Name:   benzodiazepin receptor    GB acc:   AI008680    UniGene:   Rn.1820    Gene Symbol:   Bzrp    Locus Link:   24230    Chromosome:   7    Cytoband:   Chr:7q34    SOURCE    GENECARD       Gene Ontology:   biological process:  protein targeting to mitochondrion   biological process:  ion transport   biological process:  signal transduction   molecular function:  receptor activity   molecular function:  benzodiazepine receptor activity   molecular function:  benzodiazepine receptor activity      BioCarta Pathways:     KEGG Pathways:  
    Probe set : 1398326_at    Name:   similar to Nur77 downstream protein 2    GB acc:   BI282332    UniGene:   Rn.2281    Gene Symbol:   RGD:1359417    Locus Link:   361824    Chromosome:   20    Cytoband:   Chr:20p12    SOURCE    GENECARD       Gene Ontology:     BioCarta Pathways:     KEGG Pathways:  
    Probe set : 1372568_at    Name:   EPM2A (laforin) interacting protein 1 (predicted)    GB acc:   AI411270    UniGene:   Rn.2561     Locus Link:   316021    Chromosome:   8    Cytoband:   Chr:8q32    SOURCE        Gene Ontology:   cellular component:  endoplasmic reticulum   molecular function:  protein binding      BioCarta Pathways:     KEGG Pathways:  
    Probe set : 1382215_at    Name:   Similar to Transcription initiation factor TFIID 105 kDa subunit (TAFII-105) (TAFII105)    GB acc:   AA875331    UniGene:   Rn.2882     Locus Link:   291773    Chromosome:   18    Cytoband:   Chr:18p13    SOURCE        Gene Ontology:     BioCarta Pathways:     KEGG Pathways:  
    Probe set : 1394352_at     GB acc:   BF543871              Gene Ontology:     BioCarta Pathways:     KEGG Pathways:  
    Probe set : 1368774_a_at    Name:   espin    GB acc:   NM_019622    UniGene:   Rn.91373    Gene Symbol:   Espn    Locus Link:   56227    Chromosome:   5    Cytoband:   Chr:5q36    SOURCE    GENECARD       Gene Ontology:   biological process:  parallel actin filament bundle formation   biological process:  actin filament bundle formation   biological process:  actin filament bundle formation   cellular component:  brush border   cellular component:  cilium   cellular component:  actin cytoskeleton   cellular component:  actin cytoskeleton   molecular function:  ATP binding   molecular function:  actin filament binding      BioCarta Pathways:     KEGG Pathways:  
    Probe set : 1377945_at    Name:   similar to RIKEN cDNA 2310005B10    GB acc:   AI111512    UniGene:   Rn.26598    Gene Symbol:   MGC95311    Locus Link:   308490    Chromosome:   13    Cytoband:   Chr:13q11    SOURCE    GENECARD       Gene Ontology:     BioCarta Pathways:     KEGG Pathways:  
    Probe set : 1367716_at    Name:   T-cell immunomodulatory protein    GB acc:   NM_133557    UniGene:   Rn.3622    Gene Symbol:   Cda08    Locus Link:   171083    Chromosome:   19    Cytoband:   Chr:19q11    SOURCE    GENECARD       Gene Ontology:   biological process:  cell adhesion   biological process:  cell-matrix adhesion   cellular component:  extracellular space   cellular component:  integrin complex   cellular component:  integral to membrane      BioCarta Pathways:     KEGG Pathways:  
    Probe set : 1394020_at    Name:   Transcribed locus    GB acc:   AI412164    UniGene:   Rn.44086        SOURCE        Gene Ontology:     BioCarta Pathways:     KEGG Pathways:  
    Probe set : 1370987_at    Name:   sialophorin    GB acc:   BF550890    UniGene:   Rn.11144    Gene Symbol:   Spn    Locus Link:   24796    Chromosome:   1    Cytoband:   Chr:1q36    SOURCE    GENECARD       Gene Ontology:   biological process:  response to protozoa   biological process:  negative regulation of type IV hypersensitivity   biological process:  chemotaxis   biological process:  defense response   biological process:  cellular defense response   biological process:  negative regulation of cell adhesion   biological process:  establishment and/or maintenance of cell polarity   biological process:  signal transduction   biological process:  induction of apoptosis by extracellular signals   biological process:  positive regulation of T cell proliferation   biological process:  negative regulation of T cell proliferation   biological process:  positive regulation of tumor necrosis factor-alpha biosynthesis   biological process:  defense response to bacteria   biological process:  negative thymic T cell selection   biological process:  regulation of antiviral response   biological process:  regulation of immune response   biological process:  negative regulation of T cell activation      BioCarta Pathways:     KEGG Pathways:  
    Probe set : 1370060_at    Name:   solute carrier family 25 (mitochondrial carrier; oxoglutarate carrier), member 11    GB acc:   NM_022398    UniGene:   Rn.853    Gene Symbol:   Slc25a11    Locus Link:   64201    Chromosome:   10    Cytoband:   Chr:10q24    SOURCE    GENECARD       Gene Ontology:   biological process:  transport      BioCarta Pathways:     KEGG Pathways:  
    Probe set : 1383712_at    Name:   U87HG mRNA, complete sequence    GB acc:   BF524281    UniGene:   Rn.25244        SOURCE        Gene Ontology:     BioCarta Pathways:     KEGG Pathways:  
    Probe set : 1376858_at     GB acc:   BI281836              Gene Ontology:     BioCarta Pathways:     KEGG Pathways:  
    Probe set : 1367793_at    Name:   D-dopachrome tautomerase    GB acc:   NM_024131    UniGene:   Rn.3464    Gene Symbol:   Ddt    Locus Link:   29318    Chromosome:   20    Cytoband:   Chr:20p12    SOURCE    GENECARD       Gene Ontology:   biological process:  melanin biosynthesis from tyrosine   biological process:  inflammatory response   molecular function:  dopachrome isomerase activity   molecular function:  cytokine activity   molecular function:  isomerase activity      BioCarta Pathways:     KEGG Pathways:  
    Probe set : 1368253_at    Name:   guanidinoacetate methyltransferase    GB acc:   NM_012793    UniGene:   Rn.33890    Gene Symbol:   Gamt    Locus Link:   25257    Chromosome:   7    Cytoband:   Chr:7q11    SOURCE    GENECARD       Gene Ontology:     BioCarta Pathways:     KEGG Pathways:  
    Probe set : 1384231_at    Name:   Transcribed locus    GB acc:   BE108276    UniGene:   Rn.88359        SOURCE        Gene Ontology:     BioCarta Pathways:     KEGG Pathways:  
    Probe set : 1387871_at    Name:   cofilin 1    GB acc:   BM389673    UniGene:   Rn.11675    Gene Symbol:   Cfl1    Locus Link:   29271    Chromosome:   1    Cytoband:   Chr:1q43    SOURCE    GENECARD       Gene Ontology:   biological process:  protein import into nucleus   biological process:  Rho protein signal transduction   biological process:  actin cytoskeleton organization and biogenesis   molecular function:  actin binding   molecular function:  actin binding      BioCarta Pathways:     KEGG Pathways:  
    Probe set : 1398378_at    Name:   glutathione S-transferase kappa 1    GB acc:   AI231779    UniGene:   Rn.109452    Gene Symbol:   Gstk1    Locus Link:   297029    Chromosome:   4    Cytoband:   Chr:4q23    SOURCE    GENECARD       Gene Ontology:   biological process:  protein targeting   biological process:  glutathione metabolism   cellular component:  mitochondrion   cellular component:  mitochondrial matrix   cellular component:  periplasmic space (sensu Gram-negative Bacteria)      BioCarta Pathways:     KEGG Pathways:  
    Probe set : 1371775_at    Name:   Acyl-Coenzyme A dehydrogenase, short/branched chain    GB acc:   AI172057    UniGene:   Rn.44423    Gene Symbol:   Acadsb    Locus Link:   25618    Chromosome:   1    Cytoband:   Chr:1q41    SOURCE    GENECARD       Gene Ontology:   cellular component:  mitochondrion   cellular component:  mitochondrion      BioCarta Pathways:     KEGG Pathways:  
    Probe set : 1396268_at    Name:   SP110 nuclear body protein (predicted)    GB acc:   BF523991    UniGene:   Rn.6459     Locus Link:   301570    Chromosome:   9    Cytoband:   Chr:9q35    SOURCE        Gene Ontology:   biological process:  regulation of transcription, DNA-dependent   cellular component:  nucleus   molecular function:  transcription factor activity      BioCarta Pathways:     KEGG Pathways:  
    Probe set : 1370826_at    Name:   nucleosome assembly protein 1-like 1    GB acc:   AF062594    UniGene:   Rn.18480    Gene Symbol:   Nap1l1    Locus Link:   89825    Chromosome:   7    Cytoband:   Chr:7q21    SOURCE    GENECARD       Gene Ontology:   biological process:  DNA replication   biological process:  nucleosome assembly   biological process:  positive regulation of cell proliferation   cellular component:  nucleus   cellular component:  chromatin assembly complex      BioCarta Pathways:     KEGG Pathways:  
    Probe set : 1378171_at    Name:   Neuropilin 2    GB acc:   BF409060    UniGene:   Rn.10816    Gene Symbol:   Nrp2    Locus Link:   81527    Chromosome:   9    Cytoband:   Chr:9q31    SOURCE    GENECARD       Gene Ontology:   cellular component:  membrane fraction   cellular component:  membrane   cellular component:  integral to membrane      BioCarta Pathways:     KEGG Pathways:  
    Probe set : 1377034_at    Name:   serine (or cysteine) proteinase inhibitor, clade B, member 1a (predicted)    GB acc:   BF411331    UniGene:   Rn.103402    Gene Symbol:   Serpinb1a_predicted    Locus Link:   291091    Chromosome:   17    Cytoband:   Chr:17p12    SOURCE    GENECARD       Gene Ontology:   biological process:  regulation of protein catabolism   cellular component:  cytoplasm   molecular function:  endopeptidase inhibitor activity   molecular function:  serine-type endopeptidase inhibitor activity   molecular function:  peptidase activity      BioCarta Pathways:     KEGG Pathways:  
    Probe set : 1388391_at    Name:   similar to NADH dehydrogenase    GB acc:   AA996544    UniGene:   Rn.3640    Gene Symbol:   LOC363441    Locus Link:   363441    Chromosome:   X    Cytoband:   Chr:Xq11    SOURCE    GENECARD       Gene Ontology:     BioCarta Pathways:     KEGG Pathways:  
    Probe set : 1389654_at    Name:   Transcribed locus    GB acc:   BM392070    UniGene:   Rn.18668        SOURCE        Gene Ontology:     BioCarta Pathways:     KEGG Pathways:  
    Probe set : 1368655_at    Name:   proteoglycan peptide core protein    GB acc:   NM_020074    UniGene:   Rn.40122    Gene Symbol:   RGD:619969    Locus Link:   56782    Chromosome:   20    Cytoband:   Chr:20q11    SOURCE    GENECARD       Gene Ontology:   cellular component:  extracellular space      BioCarta Pathways:     KEGG Pathways:  
    Probe set : 1383986_at    Name:   tropomyosin 3, gamma    GB acc:   AI071098    UniGene:   Rn.37575    Gene Symbol:   Tpm3    Locus Link:   117557    Chromosome:   2    Cytoband:   Chr:2q34    SOURCE    GENECARD       Gene Ontology:   biological process:  regulation of muscle contraction   biological process:  muscle development   cellular component:  cytoskeleton   cellular component:  muscle thin filament tropomyosin   molecular function:  actin binding      BioCarta Pathways:     KEGG Pathways:  
    Probe set : 1389250_at    Name:   Transcribed locus    GB acc:   AW915115    UniGene:   Rn.65477        SOURCE        Gene Ontology:     BioCarta Pathways:     KEGG Pathways:  
    Probe set : 1368528_at    Name:   MIC2 like 1    GB acc:   NM_134459    UniGene:   Rn.20204    Gene Symbol:   RGD:620896    Locus Link:   171485    Chromosome:   15    Cytoband:   Chr:15p16    SOURCE    GENECARD       Gene Ontology:   cellular component:  extracellular space   cellular component:  integral to membrane   cellular component:  integral to membrane      BioCarta Pathways:     KEGG Pathways:  
    Probe set : 1376197_at    Name:   transcription factor 7, T-cell specific (predicted)    GB acc:   AW251860    UniGene:   Rn.106335    Gene Symbol:   Tcf7_predicted    Locus Link:   363595    Chromosome:   10    Cytoband:   Chr:10q22    SOURCE    GENECARD       Gene Ontology:   cellular component:  nucleus   cellular component:  transcription factor complex   molecular function:  DNA binding   molecular function:  transcription factor activity   molecular function:  RNA polymerase II transcription factor activity      BioCarta Pathways:     KEGG Pathways:  
    Probe set : 1389039_at    Name:   Transcribed locus    GB acc:   BM386520    UniGene:   Rn.7736        SOURCE        Gene Ontology:     BioCarta Pathways:     KEGG Pathways:  
    Probe set : 1388537_at    Name:   4-nitrophenylphosphatase domain and non-neuronal SNAP25-like protein homolog 1 (C. elegans) (predicted)    GB acc:   BF554891    UniGene:   Rn.103205    Gene Symbol:   Nipsnap1_predicted    Locus Link:   360971    Chromosome:   14    Cytoband:   Chr:14q21    SOURCE    GENECARD       Gene Ontology:   cellular component:  mitochondrion      BioCarta Pathways:     KEGG Pathways:  
    Probe set : 1398750_at    Name:   calreticulin    GB acc:   NM_022399    UniGene:   Rn.974    Gene Symbol:   Calr    Locus Link:   64202    Chromosome:   19    Cytoband:   Chr:19q11    SOURCE    GENECARD       Gene Ontology:     BioCarta Pathways:     KEGG Pathways:  
    Probe set : 1370144_at    Name:   G protein-binding protein CRFG /// similar to GTP-binding protein NGB /// similar to GTP-binding protein NGB    GB acc:   NM_053689    UniGene:   Rn.106958    Gene Symbol:   Gtpbp4 /// LOC364763 /// LOC498786    Locus Link:   114300 /// 364763 /// 498786    Chromosome:   17    Cytoband:   Chr:17q12.1    SOURCE    GENECARD       Gene Ontology:   biological process:  ribosome biogenesis   biological process:  small GTPase mediated signal transduction   cellular component:  nucleus   cellular component:  nucleus   molecular function:  GTP binding   molecular function:  GTP binding   molecular function:  nucleotide binding      BioCarta Pathways:     KEGG Pathways:  
    Probe set : 1383879_at    Name:   Transcribed locus    GB acc:   BG377332    UniGene:   Rn.146446        SOURCE        Gene Ontology:     BioCarta Pathways:     KEGG Pathways:  
    Probe set : 1385590_at    Name:   Transcribed locus    GB acc:   BI296811    UniGene:   Rn.79620        SOURCE        Gene Ontology:     BioCarta Pathways:     KEGG Pathways:  
    Probe set : 1370127_at    Name:   polymerase (DNA directed), delta 1, catalytic subunit    GB acc:   NM_021662    UniGene:   Rn.88690    Gene Symbol:   Pold1    Locus Link:   59294    Chromosome:   1    Cytoband:   Chr:1q22    SOURCE    GENECARD       Gene Ontology:   cellular component:  nucleus   cellular component:  delta DNA polymerase complex   molecular function:  nucleotide binding   molecular function:  DNA binding   molecular function:  DNA-directed DNA polymerase activity   molecular function:  alpha DNA polymerase activity   molecular function:  beta DNA polymerase activity   molecular function:  delta DNA polymerase activity   molecular function:  epsilon DNA polymerase activity   molecular function:  zeta DNA polymerase activity   molecular function:  gamma DNA-directed DNA polymerase activity   molecular function:  exonuclease activity   molecular function:  3'-5' exonuclease activity   molecular function:  eta DNA polymerase activity   molecular function:  iota DNA polymerase activity   molecular function:  mu DNA polymerase activity   molecular function:  lambda DNA polymerase activity   molecular function:  kappa DNA polymerase activity   molecular function:  nu DNA polymerase activity   molecular function:  theta DNA polymerase activity   molecular function:  transferase activity   molecular function:  hydrolase activity   molecular function:  sigma DNA polymerase activity   molecular function:  protein binding   molecular function:  nuclease activity   molecular function:  zinc ion binding   molecular function:  nucleotidyltransferase activity   molecular function:  metal ion binding      BioCarta Pathways:     KEGG Pathways:  
    Probe set : 1385869_at    Name:   Zinc finger protein 281 (predicted)    GB acc:   AI408878    UniGene:   Rn.22691     Locus Link:   305083    Chromosome:   13    Cytoband:   Chr:13q13    SOURCE        Gene Ontology:   biological process:  negative regulation of transcription from RNA polymerase II promoter   biological process:  regulation of transcription, DNA-dependent   cellular component:  nucleus   cellular component:  DNA-directed RNA polymerase II, core complex      BioCarta Pathways:     KEGG Pathways:  
    Probe set : 1396217_at    Name:   Similar to Adapter-related protein complex 1 sigma 1B subunit (Sigma-adaptin 1B) (Adaptor protein complex AP-1 sigma-1B subunit) (Golgi adaptor HA1/AP1 adaptin sigma-1B subunit) (Clathrin assembly protein complex 1 sigma-1B small chain) (Sigma 1B s...    GB acc:   BF542447    UniGene:   Rn.17309     Locus Link:   302671    Chromosome:   X    Cytoband:   Chr:Xq21    SOURCE        Gene Ontology:     BioCarta Pathways:     KEGG Pathways:  
    Probe set : 1372681_at    Name:   Transcribed locus    GB acc:   BI294716    UniGene:   Rn.11962        SOURCE        Gene Ontology:     BioCarta Pathways:     KEGG Pathways:  
    Probe set : 1368509_at    Name:   Bardet-Biedl syndrome 2 (human)    GB acc:   NM_053618    UniGene:   Rn.15987    Gene Symbol:   Bbs2    Locus Link:   113948    Chromosome:   19    Cytoband:   Chr:19p12    SOURCE    GENECARD       Gene Ontology:   cellular component:  integrin complex      BioCarta Pathways:     KEGG Pathways:  
    Probe set : 1398356_at    Name:   cleavage and polyadenylation specific factor 5 (predicted)    GB acc:   BF420718     Gene Symbol:   Cpsf5_predicted    Locus Link:   291877    Chromosome:   19    Cytoband:   Chr:19p12     GENECARD       Gene Ontology:   biological process:  mRNA cleavage   biological process:  mRNA processing   cellular component:  nucleus   molecular function:  RNA binding      BioCarta Pathways:     KEGG Pathways:  
    Probe set : 1393256_at    Name:   selenophosphate synthetase 1 (predicted)    GB acc:   AI137711    UniGene:   Rn.100803    Gene Symbol:   Sephs1_predicted    Locus Link:   291314    Chromosome:   17    Cytoband:   Chr:17q12.3    SOURCE    GENECARD       Gene Ontology:   biological process:  protein modification      BioCarta Pathways:     KEGG Pathways:  
    Probe set : 1372333_at    Name:   small nuclear ribonucleoprotein E (predicted) /// similar to small nuclear ribonucleoprotein E    GB acc:   BF555033    UniGene:   Rn.145709    Gene Symbol:   Snrpe_predicted /// LOC501075    Locus Link:   360844 /// 501075    Chromosome:   X    Cytoband:   Chr:Xq36 /// Chr:8q32    SOURCE    GENECARD       Gene Ontology:   biological process:  nuclear mRNA splicing, via spliceosome   biological process:  mRNA processing   molecular function:  RNA binding      BioCarta Pathways:     KEGG Pathways:  
    Probe set : 1371912_at    Name:   NADH dehydrogenase (ubiquinone) Fe-S protein 7 (predicted)    GB acc:   AI231358    UniGene:   Rn.2855    Gene Symbol:   Ndufs7_predicted    Locus Link:   362837    Chromosome:   7    Cytoband:   Chr:7q11    SOURCE    GENECARD       Gene Ontology:   biological process:  mitochondrial electron transport, NADH to ubiquinone   cellular component:  extracellular space   cellular component:  mitochondrion   molecular function:  NADH dehydrogenase activity   molecular function:  NADH dehydrogenase (ubiquinone) activity   molecular function:  oxidoreductase activity      BioCarta Pathways:     KEGG Pathways:  
    Probe set : 1373209_at    Name:   Transcribed locus    GB acc:   AI599284    UniGene:   Rn.16374        SOURCE        Gene Ontology:     BioCarta Pathways:     KEGG Pathways:  
    Probe set : 1387621_at    Name:   tumor necrosis factor receptor superfamily, member 4    GB acc:   NM_013049    UniGene:   Rn.48883    Gene Symbol:   Tnfrsf4    Locus Link:   25572    Chromosome:   5    Cytoband:   Chr:5q36    SOURCE    GENECARD       Gene Ontology:   biological process:  immune response   biological process:  cellular defense response   biological process:  cellular defense response   molecular function:  receptor activity   molecular function:  tumor necrosis factor receptor activity   molecular function:  tumor necrosis factor receptor activity      BioCarta Pathways:     KEGG Pathways:  
    Probe set : 1373422_at     GB acc:   BE108280              Gene Ontology:     BioCarta Pathways:     KEGG Pathways:  
    Probe set : 1390240_at    Name:   Transcribed locus    GB acc:   BM389611    UniGene:   Rn.25146        SOURCE        Gene Ontology:     BioCarta Pathways:     KEGG Pathways:  
    Probe set : 1384917_at    Name:   Similar to uracil-DNA glycosylase 2    GB acc:   BF547287    UniGene:   Rn.61269     Locus Link:   499528      SOURCE        Gene Ontology:     BioCarta Pathways:     KEGG Pathways:  
    Probe set : 1375277_at    Name:   similar to hypothetical protein FLJ20433    GB acc:   AA800680    UniGene:   Rn.22790    Gene Symbol:   LOC499745    Locus Link:   499745    Chromosome:   3    Cytoband:   Chr:3p13    SOURCE    GENECARD       Gene Ontology:     BioCarta Pathways:     KEGG Pathways:  
    Probe set : 1388429_at    Name:   similar to gem (nuclear organelle) associated protein 7    GB acc:   BM384085    UniGene:   Rn.13265    Gene Symbol:   LOC499391    Locus Link:   499391      SOURCE    GENECARD       Gene Ontology:     BioCarta Pathways:     KEGG Pathways:  
    Probe set : 1382592_at     GB acc:   AI059054              Gene Ontology:     BioCarta Pathways:     KEGG Pathways:  
    Probe set : 1395508_at    Name:   chaperonin subunit 5 (epsilon)    GB acc:   BG665309    UniGene:   Rn.92645    Gene Symbol:   Cct5    Locus Link:   294864    Chromosome:   2    Cytoband:   Chr:2q22    SOURCE    GENECARD       Gene Ontology:   biological process:  protein folding   biological process:  protein folding   biological process:  cellular protein metabolism      BioCarta Pathways:     KEGG Pathways:  
    Probe set : 1371111_at    Name:   RT1 class I, A3    GB acc:   X82669    UniGene:   Rn.116594    Gene Symbol:   RT1-A3    Locus Link:   309627    Chromosome:   20    Cytoband:   Chr:20p12    SOURCE    GENECARD       Gene Ontology:   biological process:  antigen presentation   cellular component:  MHC class I protein complex   molecular function:  MHC class I receptor activity      BioCarta Pathways:     KEGG Pathways:  
    Probe set : 1391527_at    Name:   Ngfi-A binding protein 2 (predicted)    GB acc:   BE109296    UniGene:   Rn.6880    Gene Symbol:   Nab2    Locus Link:   314910    Chromosome:   7    Cytoband:   Chr:7q22    SOURCE    GENECARD       Gene Ontology:   cellular component:  nucleus   molecular function:  transcription corepressor activity   molecular function:  transcriptional repressor activity      BioCarta Pathways:     KEGG Pathways:  
    Probe set : 1384900_at    Name:   MOB1, Mps One Binder kinase activator-like 2B (yeast) (predicted)    GB acc:   AW530806    UniGene:   Rn.9147    Gene Symbol:   Mobkl2b_predicted    Locus Link:   362833    Chromosome:   7    Cytoband:   Chr:7q11    SOURCE    GENECARD       Gene Ontology:     BioCarta Pathways:     KEGG Pathways:  
    Probe set : 1385502_at    Name:   Tripartite motif protein 21 (predicted)    GB acc:   AI071868    UniGene:   Rn.107862     Locus Link:   308901    Chromosome:   1    Cytoband:   Chr:1q32    SOURCE        Gene Ontology:   biological process:  regulation of transcription, DNA-dependent   biological process:  protein ubiquitination      BioCarta Pathways:     KEGG Pathways:  
    Probe set : 1382189_at    Name:   syndecan 2    GB acc:   AI574779    UniGene:   Rn.11127    Gene Symbol:   Sdc2    Locus Link:   25615    Chromosome:   7    Cytoband:   Chr:7q22    SOURCE    GENECARD       Gene Ontology:   cellular component:  integral to plasma membrane   cellular component:  membrane   cellular component:  integral to membrane   molecular function:  cytoskeletal protein binding      BioCarta Pathways:     KEGG Pathways:  
    Probe set : 1388352_at    Name:   N-acetyltransferase 5 (ARD1 homolog, S. cerevisiae) (predicted)    GB acc:   BI296716    UniGene:   Rn.30327    Gene Symbol:   Nat5_predicted    Locus Link:   362228    Chromosome:   3    Cytoband:   Chr:3q41    SOURCE    GENECARD       Gene Ontology:   molecular function:  N-acetyltransferase activity   molecular function:  acyltransferase activity   molecular function:  transferase activity      BioCarta Pathways:     KEGG Pathways:  
    Probe set : 1388688_at    Name:   Transcribed locus    GB acc:   BI296599    UniGene:   Rn.115416        SOURCE        Gene Ontology:     BioCarta Pathways:     KEGG Pathways:  
    Probe set : 1371213_at    Name:   RT1 class I, A3    GB acc:   AJ005023    UniGene:   Rn.116594    Gene Symbol:   RGD:1359610    Locus Link:   309627    Chromosome:   20    Cytoband:   Chr:20p12    SOURCE    GENECARD       Gene Ontology:   biological process:  antigen presentation   cellular component:  MHC class I protein complex   molecular function:  MHC class I receptor activity      BioCarta Pathways:     KEGG Pathways:  
    Probe set : 1367680_at    Name:   acyl-Coenzyme A oxidase 1, palmitoyl    GB acc:   NM_017340    UniGene:   Rn.31796    Gene Symbol:   Acox1    Locus Link:   50681    Chromosome:   10    Cytoband:   Chr:10q32.3    SOURCE    GENECARD       Gene Ontology:   cellular component:  mitochondrion   cellular component:  peroxisome      BioCarta Pathways:     KEGG Pathways:  
    Probe set : 1388778_at    Name:   Transcribed locus    GB acc:   BF284876    UniGene:   Rn.17629        SOURCE        Gene Ontology:     BioCarta Pathways:     KEGG Pathways:  
    Probe set : 1378914_a_at    Name:   hypothetical protein LOC308990    GB acc:   AI059468    UniGene:   Rn.98391    Gene Symbol:   LOC308990    Locus Link:   308990    Chromosome:   1    Cytoband:   Chr:1q36    SOURCE    GENECARD       Gene Ontology:     BioCarta Pathways:     KEGG Pathways:  
    Probe set : 1372790_at    Name:   Malate dehydrogenase 1, NAD (soluble)    GB acc:   BG671530    UniGene:   Rn.13492    Gene Symbol:   Mdh1    Locus Link:   24551    Chromosome:   14    Cytoband:   Chr:14q22    SOURCE    GENECARD       Gene Ontology:   cellular component:  cytosol      BioCarta Pathways:     KEGG Pathways:  
    Probe set : 1377664_at    Name:   KARP-1 binding protein 1    GB acc:   AI101205    UniGene:   Rn.6424    Gene Symbol:   Kab    Locus Link:   171457    Chromosome:   13    Cytoband:   Chr:13q24    SOURCE    GENECARD       Gene Ontology:     BioCarta Pathways:     KEGG Pathways:  
    Probe set : 1368877_at    Name:   zinc finger protein 354A    GB acc:   NM_052798    UniGene:   Rn.11049    Gene Symbol:   Znf354a    Locus Link:   24522    Chromosome:   10    Cytoband:   Chr:10q21    SOURCE    GENECARD       Gene Ontology:   biological process:  regulation of transcription, DNA-dependent   biological process:  development   biological process:  transcription   cellular component:  intracellular   cellular component:  nucleus   cellular component:  transcription factor complex      BioCarta Pathways:     KEGG Pathways:  
    Probe set : 1387691_at    Name:   tumor necrosis factor superfamily, member 2    GB acc:   NM_012675    UniGene:   Rn.2275    Gene Symbol:   Tnf    Locus Link:   24835    Chromosome:   20    Cytoband:   Chr:20p12    SOURCE    GENECARD       Gene Ontology:   biological process:  regulation of protein amino acid phosphorylation   biological process:  glucose metabolism   biological process:  apoptosis   biological process:  apoptosis   biological process:  induction of apoptosis   biological process:  induction of apoptosis   biological process:  programmed cell death, transformed cells   biological process:  defense response   biological process:  inflammatory response   biological process:  inflammatory response   biological process:  immune response   biological process:  humoral immune response   biological process:  cellular defense response   biological process:  signal transduction   biological process:  signal transduction   biological process:  development   biological process:  cell proliferation   biological process:  positive regulation of cell proliferation   biological process:  induction of apoptosis via death domain receptors   biological process:  organ morphogenesis   biological process:  cytokine and chemokine mediated signaling pathway   biological process:  regulation of cell proliferation   biological process:  defense response to bacteria   biological process:  positive regulation of I-kappaB kinase/NF-kappaB cascade   biological process:  cellular extravasation   biological process:  regulation of osteoclast differentiation   biological process:  positive regulation of osteoclast differentiation   biological process:  negative regulation of glucose import   biological process:  cellular physiological process   biological process:  regulation of immunoglobulin secretion      BioCarta Pathways:     KEGG Pathways:  
    Probe set : 1390849_at    Name:   Transcribed locus    GB acc:   AW520584    UniGene:   Rn.20519        SOURCE        Gene Ontology:     BioCarta Pathways:     KEGG Pathways:  
    Probe set : 1375987_at    Name:   ceramide kinase (predicted)    GB acc:   AW525194    UniGene:   Rn.99537    Gene Symbol:   Cerk_predicted    Locus Link:   300129    Chromosome:   7    Cytoband:   Chr:7q34    SOURCE    GENECARD       Gene Ontology:   biological process:  ceramide metabolism   biological process:  protein kinase C activation   cellular component:  integral to membrane of membrane fraction   cellular component:  membrane fraction      BioCarta Pathways:     KEGG Pathways:  
    Probe set : 1375560_at    Name:   similar to RIKEN cDNA 0610010D20 (predicted)    GB acc:   AA945579    UniGene:   Rn.13905    Gene Symbol:   RGD1310475_predicted    Locus Link:   293949    Chromosome:   1    Cytoband:   Chr:1q54    SOURCE    GENECARD       Gene Ontology:     BioCarta Pathways:     KEGG Pathways:  
    Probe set : 1368066_at    Name:   BCL2-antagonist/killer 1    GB acc:   NM_053812    UniGene:   Rn.14598    Gene Symbol:   Bak1    Locus Link:   116502    Chromosome:   20    Cytoband:   Chr:20p12    SOURCE    GENECARD       Gene Ontology:   biological process:  regulation of apoptosis      BioCarta Pathways:     KEGG Pathways:  
    Probe set : 1383658_at    Name:   lysosomal-associated protein transmembrane 5    GB acc:   BF406601    UniGene:   Rn.24799    Gene Symbol:   Laptm5    Locus Link:   89783    Chromosome:   5    Cytoband:   Chr:5q36    SOURCE    GENECARD       Gene Ontology:   cellular component:  lysosome   cellular component:  lysosome   cellular component:  integral to membrane      BioCarta Pathways:     KEGG Pathways:  
    Probe set : 1394306_at    Name:   zeta-chain (TCR) associated protein kinase 70kDa    GB acc:   BF545972    UniGene:   Rn.17862    Gene Symbol:   Zap70    Locus Link:   301348    Chromosome:   9    Cytoband:   Chr:9q21    SOURCE    GENECARD       Gene Ontology:   cellular component:  cytosol   cellular component:  T cell receptor complex   cellular component:  cytoplasm      BioCarta Pathways:     KEGG Pathways:  
    Probe set : 1373514_at    Name:   similar to chromosome 17 open reading frame 27 (predicted)    GB acc:   AA899109    UniGene:   Rn.67402    Gene Symbol:   RGD1308168_predicted    Locus Link:   303735    Chromosome:   10    Cytoband:   Chr:10q32.3    SOURCE    GENECARD       Gene Ontology:     BioCarta Pathways:     KEGG Pathways:  
    Probe set : 1383073_at    Name:   ubiquitin specific protease 14 (predicted)    GB acc:   BG666028    UniGene:   Rn.11790    Gene Symbol:   Usp14_predicted    Locus Link:   291796    Chromosome:   18    Cytoband:   Chr:18p13    SOURCE    GENECARD       Gene Ontology:   cellular component:  soluble fraction   cellular component:  synaptosome      BioCarta Pathways:     KEGG Pathways:  
    Probe set : 1378296_at     GB acc:   AI012651              Gene Ontology:     BioCarta Pathways:     KEGG Pathways:  
    Probe set : 1390050_at    Name:   golgi phosphoprotein 2 (predicted)    GB acc:   BI288898    UniGene:   Rn.78688    Gene Symbol:   Golph2_predicted    Locus Link:   306725    Chromosome:   17    Cytoband:   Chr:17p14    SOURCE    GENECARD       Gene Ontology:   cellular component:  Golgi apparatus   cellular component:  integral to membrane      BioCarta Pathways:     KEGG Pathways:  
    Probe set : 1376007_at    Name:   Transcribed locus, moderately similar to XP_286747.1 PREDICTED: hypothetical protein XP_286747 [Mus musculus]    GB acc:   AI411772    UniGene:   Rn.139862        SOURCE        Gene Ontology:     BioCarta Pathways:     KEGG Pathways:  
    Probe set : 1373280_at    Name:   RuvB-like 2 (E. coli) (predicted)    GB acc:   AA848324    UniGene:   Rn.146207    Gene Symbol:   Ruvbl2_predicted    Locus Link:   292907    Chromosome:   1    Cytoband:   Chr:1q22    SOURCE    GENECARD       Gene Ontology:   cellular component:  nucleus   cellular component:  cytosol   cellular component:  TIP60 histone acetyltransferase complex      BioCarta Pathways:     KEGG Pathways:  
    Probe set : 1387314_at    Name:   sulfotransferase family 1B, member 1    GB acc:   NM_022513    UniGene:   Rn.1183    Gene Symbol:   Sult1b1    Locus Link:   64305    Chromosome:   14    Cytoband:   Chr:14p21    SOURCE    GENECARD       Gene Ontology:   biological process:  steroid metabolism   biological process:  lipid metabolism   cellular component:  cytosol   cellular component:  cytosol      BioCarta Pathways:     KEGG Pathways:  
    Probe set : 1367897_at    Name:   acyl-Coenzyme A dehydrogenase, very long chain    GB acc:   NM_012891    UniGene:   Rn.33319    Gene Symbol:   Acadvl    Locus Link:   25363    Chromosome:   10    Cytoband:   Chr:10q24    SOURCE    GENECARD       Gene Ontology:   cellular component:  mitochondrion   cellular component:  mitochondrion      BioCarta Pathways:     KEGG Pathways:  
    Probe set : 1380521_at    Name:   hematopoietic cell specific Lyn substrate 1 (predicted)    GB acc:   BF556183    UniGene:   Rn.15578    Gene Symbol:   Hcls1_predicted    Locus Link:   288077    Chromosome:   11    Cytoband:   Chr:11q22    SOURCE    GENECARD       Gene Ontology:     BioCarta Pathways:     KEGG Pathways:  
    Probe set : 1379676_a_at    Name:   similar to RIKEN cDNA 2310005K03    GB acc:   AW527320    UniGene:   Rn.23986    Gene Symbol:   RGD:1359588    Locus Link:   363522    Chromosome:   X    Cytoband:   Chr:Xq37    SOURCE    GENECARD       Gene Ontology:   biological process:  DNA catabolism   molecular function:  deoxyribonuclease activity      BioCarta Pathways:     KEGG Pathways:  
    Probe set : 1374019_at    Name:   LOC499758    GB acc:   BF284939    UniGene:   Rn.7256     Locus Link:   499758      SOURCE        Gene Ontology:     BioCarta Pathways:     KEGG Pathways:  
    Probe set : 1370891_at    Name:   CD48 antigen    GB acc:   X13016    UniGene:   Rn.3705    Gene Symbol:   Cd48    Locus Link:   245962    Chromosome:   13    Cytoband:   Chr:13q24    SOURCE    GENECARD       Gene Ontology:   biological process:  mast cell activation      BioCarta Pathways:     KEGG Pathways:  
    Probe set : 1370973_at    Name:   sodium channel, voltage-gated, type 6, alpha polypeptide    GB acc:   BF285019    UniGene:   Rn.54541    Gene Symbol:   RGD:61922    Locus Link:   64155    Chromosome:   3    Cytoband:   Chr:3q21    SOURCE    GENECARD       Gene Ontology:     BioCarta Pathways:     KEGG Pathways:  
    Probe set : 1386637_at    Name:   fibrinogen-like 2    GB acc:   BF561454    UniGene:   Rn.64635    Gene Symbol:   Fgl2    Locus Link:   84586    Chromosome:   4    Cytoband:   Chr:4q11    SOURCE    GENECARD       Gene Ontology:   biological process:  parturition   biological process:  cytolysis   cellular component:  extracellular space   molecular function:  peptidase activity      BioCarta Pathways:     KEGG Pathways:  
    Probe set : 1374903_at    Name:   glucosaminyl (N-acetyl) transferase 2, I-branching enzyme /// hypothetical gene supported by NM_001001511    GB acc:   AI234819    UniGene:   Rn.8807    Gene Symbol:   Gcnt2 /// LOC497712    Locus Link:   306860 /// 497712    Chromosome:   17    Cytoband:   Chr:17p12    SOURCE    GENECARD       Gene Ontology:   cellular component:  membrane   molecular function:  acetylglucosaminyltransferase activity   molecular function:  transferase activity      BioCarta Pathways:     KEGG Pathways:  
    Probe set : 1387205_at    Name:   RT1 class Ib, locus M3    GB acc:   NM_022921    UniGene:   Rn.92606    Gene Symbol:   RT1-M3    Locus Link:   24747    Chromosome:   20    Cytoband:   Chr:20p12    SOURCE    GENECARD       Gene Ontology:   biological process:  defense response   biological process:  immune response   biological process:  antigen presentation   molecular function:  MHC class I receptor activity      BioCarta Pathways:     KEGG Pathways:  
    Probe set : 1368224_at    Name:   Serine protease inhibitor    GB acc:   NM_031531    UniGene:   Rn.128    Gene Symbol:   Spin2c    Locus Link:   24795    Chromosome:   6    Cytoband:   Chr:6q32    SOURCE    GENECARD       Gene Ontology:   cellular component:  extracellular space   molecular function:  endopeptidase inhibitor activity   molecular function:  serine-type endopeptidase inhibitor activity      BioCarta Pathways:     KEGG Pathways:  
    Probe set : 1373346_at    Name:   similar to hypothetical protein CL25084 (predicted)    GB acc:   AI009492    UniGene:   Rn.102123    Gene Symbol:   RGD1306508_predicted    Locus Link:   289874    Chromosome:   14    Cytoband:   Chr:14q22    SOURCE    GENECARD       Gene Ontology:     BioCarta Pathways:     KEGG Pathways:  
    Probe set : 1370347_at    Name:   PDZ and LIM domain 7    GB acc:   AF095585    UniGene:   Rn.7274    Gene Symbol:   Pdlim7    Locus Link:   286908    Chromosome:   17    Cytoband:   Chr:17p14    SOURCE    GENECARD       Gene Ontology:   biological process:  positive regulation of osteoblast differentiation   molecular function:  protein binding   molecular function:  zinc ion binding   molecular function:  metal ion binding      BioCarta Pathways:     KEGG Pathways:  
    Probe set : 1386943_at    Name:   transmembrane 4 superfamily member 11    GB acc:   NM_022533    UniGene:   Rn.1721    Gene Symbol:   Tm4sf11    Locus Link:   64364    Chromosome:   19    Cytoband:   Chr:19p12    SOURCE    GENECARD       Gene Ontology:   cellular component:  membrane   cellular component:  integral to membrane   molecular function:  ion channel activity      BioCarta Pathways:     KEGG Pathways:  
    Probe set : 1383917_at    Name:   ABI gene family, member 3 (predicted)    GB acc:   BE110730    UniGene:   Rn.95169    Gene Symbol:   Abi3_predicted    Locus Link:   303476    Chromosome:   10    Cytoband:   Chr:10q31    SOURCE    GENECARD       Gene Ontology:   biological process:  cell motility   biological process:  regulation of cell migration   cellular component:  cytoplasm   molecular function:  protein binding      BioCarta Pathways:     KEGG Pathways:  
    Probe set : 1384188_at     GB acc:   AW142766              Gene Ontology:     BioCarta Pathways:     KEGG Pathways:  
    Probe set : 1376390_at    Name:   membrane-spanning 4-domains, subfamily A, member 11 (predicted)    GB acc:   BF395317    UniGene:   Rn.101878    Gene Symbol:   Ms4a11_predicted    Locus Link:   361735    Chromosome:   1    Cytoband:   Chr:1q43    SOURCE    GENECARD       Gene Ontology:     BioCarta Pathways:     KEGG Pathways:  
    Probe set : 1395534_at    Name:   Transcribed locus    GB acc:   AW919370    UniGene:   Rn.8804        SOURCE        Gene Ontology:     BioCarta Pathways:     KEGG Pathways:  
    Probe set : 1371073_at    Name:   UDP-Gal:betaGlcNAc beta 1,4- galactosyltransferase, polypeptide 1    GB acc:   BI286141    UniGene:   Rn.17157    Gene Symbol:   B4galt1    Locus Link:   24390    Chromosome:   5    Cytoband:   Chr:5q22    SOURCE    GENECARD       Gene Ontology:   cellular component:  Golgi apparatus   cellular component:  plasma membrane   cellular component:  integral to membrane      BioCarta Pathways:     KEGG Pathways:  
    Probe set : 1389744_at    Name:   LOC499898    GB acc:   AW527194    UniGene:   Rn.43161    Gene Symbol:   LOC499898    Locus Link:   499898    Chromosome:   3    Cytoband:   Chr:3q41    SOURCE    GENECARD       Gene Ontology:     BioCarta Pathways:     KEGG Pathways:  
    Probe set : 1398303_s_at    Name:   tropomyosin 3, gamma    GB acc:   BI295970    UniGene:   Rn.37575    Gene Symbol:   Tpm3    Locus Link:   117557    Chromosome:   2    Cytoband:   Chr:2q34    SOURCE    GENECARD       Gene Ontology:   biological process:  regulation of muscle contraction   biological process:  muscle development   cellular component:  cytoskeleton   cellular component:  muscle thin filament tropomyosin   molecular function:  actin binding      BioCarta Pathways:     KEGG Pathways:  
    Probe set : 1370573_at    Name:   sarcosine dehydrogenase    GB acc:   AF067650    UniGene:   Rn.89832    Gene Symbol:   Sardh    Locus Link:   114123    Chromosome:   3    Cytoband:   Chr:3p12    SOURCE    GENECARD       Gene Ontology:   biological process:  electron transport   biological process:  glycine catabolism   biological process:  choline catabolism      BioCarta Pathways:     KEGG Pathways:  
    Probe set : 1377646_at    Name:   Transcribed locus    GB acc:   AI177360    UniGene:   Rn.3093        SOURCE        Gene Ontology:     BioCarta Pathways:     KEGG Pathways:  
    Probe set : 1382680_at     GB acc:   BG673602              Gene Ontology:     BioCarta Pathways:     KEGG Pathways:  
    Probe set : 1389365_at    Name:   Similar to RIKEN cDNA 1600014C10    GB acc:   AI228291    UniGene:   Rn.2361     Locus Link:   502324      SOURCE        Gene Ontology:     BioCarta Pathways:     KEGG Pathways:  
    Probe set : 1375137_at    Name:   actin related protein 2/3 complex, subunit 2 (predicted)    GB acc:   AI070323    UniGene:   Rn.93317    Gene Symbol:   Arpc2_predicted    Locus Link:   301511    Chromosome:   9    Cytoband:   Chr:9q33    SOURCE    GENECARD       Gene Ontology:   biological process:  cell motility   biological process:  regulation of actin filament polymerization   cellular component:  cytoskeleton   cellular component:  Arp2/3 protein complex   cellular component:  focal adhesion   molecular function:  structural constituent of cytoskeleton      BioCarta Pathways:     KEGG Pathways:  
    Probe set : 1369208_at    Name:   interleukin 7    GB acc:   AF367210    UniGene:   Rn.10793    Gene Symbol:   Il7    Locus Link:   25647    Chromosome:   2    Cytoband:   Chr:2q23    SOURCE    GENECARD       Gene Ontology:   cellular component:  extracellular region   cellular component:  extracellular space      BioCarta Pathways:     KEGG Pathways:  
    Probe set : 1386669_at    Name:   Transcribed locus    GB acc:   BF565188    UniGene:   Rn.153874        SOURCE        Gene Ontology:     BioCarta Pathways:     KEGG Pathways:  
    Probe set : 1367597_at    Name:   ribosomal protein S8    GB acc:   NM_031706    UniGene:   Rn.3198    Gene Symbol:   Rps8    Locus Link:   65136    Chromosome:   5    Cytoband:   Chr:5q36    SOURCE    GENECARD       Gene Ontology:   biological process:  protein biosynthesis   biological process:  ribosome biogenesis   molecular function:  RNA binding   molecular function:  structural constituent of ribosome      BioCarta Pathways:     KEGG Pathways:  
    Probe set : 1382683_a_at     GB acc:   AA891943              Gene Ontology:     BioCarta Pathways:     KEGG Pathways:  
    Probe set : 1368461_at    Name:   solute carrier family 22 (organic anion transporter), member 8    GB acc:   NM_031332    UniGene:   Rn.23348    Gene Symbol:   Slc22a8    Locus Link:   83500    Chromosome:   1    Cytoband:   Chr:1q43    SOURCE    GENECARD       Gene Ontology:     BioCarta Pathways:     KEGG Pathways:  
    Probe set : 1369998_at    Name:   ADP-ribosylation factor 6    GB acc:   NM_024152    UniGene:   Rn.6993    Gene Symbol:   Arf6    Locus Link:   79121    Chromosome:   6    Cytoband:   Chr:6q24    SOURCE    GENECARD       Gene Ontology:     BioCarta Pathways:     KEGG Pathways:  
    Probe set : 1372685_at    Name:   cyclin-dependent kinase inhibitor 3 (predicted)    GB acc:   BE113362    UniGene:   Rn.107220    Gene Symbol:   Cdkn3_predicted    Locus Link:   289993    Chromosome:   15    Cytoband:   Chr:15p14    SOURCE    GENECARD       Gene Ontology:     BioCarta Pathways:     KEGG Pathways:  
    Probe set : 1391856_at    Name:   similar to semaphorin sem2 (predicted)    GB acc:   BE108859    UniGene:   Rn.32183    Gene Symbol:   RGD1306079_predicted    Locus Link:   290562    Chromosome:   16    Cytoband:   Chr:16p16    SOURCE    GENECARD       Gene Ontology:     BioCarta Pathways:     KEGG Pathways:  
    Probe set : 1375414_at    Name:   TAF9 RNA polymerase II, TATA box binding protein (TBP)-associated factor    GB acc:   AA944572    UniGene:   Rn.8139    Gene Symbol:   Taf9    Locus Link:   373541    Chromosome:   2    Cytoband:   Chr:2q12    SOURCE    GENECARD       Gene Ontology:   biological process:  negative regulation of transcription from RNA polymerase II promoter   biological process:  transcription initiation   cellular component:  transcription factor TFIID complex   cellular component:  nucleus   cellular component:  transcription factor TFIID complex   molecular function:  TATA-binding protein binding   molecular function:  DNA binding      BioCarta Pathways:     KEGG Pathways:  
    Probe set : 1389622_at    Name:   similar to citrin    GB acc:   AI232643    UniGene:   Rn.14686    Gene Symbol:   LOC362322    Locus Link:   362322    Chromosome:   4    Cytoband:   Chr:4q13    SOURCE    GENECARD       Gene Ontology:     BioCarta Pathways:     KEGG Pathways:  
    Probe set : 1389008_at    Name:   Sprouty-related, EVH1 domain containing 2 (predicted)    GB acc:   AI409218    UniGene:   Rn.7582     Locus Link:   305539    Chromosome:   14    Cytoband:   Chr:14q22    SOURCE        Gene Ontology:   biological process:  inactivation of MAPK activity   biological process:  development   biological process:  regulation of signal transduction   cellular component:  plasma membrane   cellular component:  membrane   molecular function:  stem cell factor receptor binding   molecular function:  protein binding      BioCarta Pathways:     KEGG Pathways:  
    Probe set : 1370918_a_at    Name:   ATP synthase, H+ transporting, mitochondrial F1 complex, gamma polypeptide 1    GB acc:   BI275939    UniGene:   Rn.63959    Gene Symbol:   Atp5c1    Locus Link:   116550    Chromosome:   17    Cytoband:   Chr:17q12.3    SOURCE    GENECARD       Gene Ontology:     BioCarta Pathways:     KEGG Pathways:  
    Probe set : 1398296_at    Name:   membrane interacting protein of RGS16    GB acc:   NM_032615    UniGene:   Rn.3731    Gene Symbol:   Mir16    Locus Link:   60418      SOURCE    GENECARD       Gene Ontology:     BioCarta Pathways:     KEGG Pathways:  
    Probe set : 1387624_at    Name:   upstream transcription factor 1    GB acc:   NM_031777    UniGene:   Rn.10845    Gene Symbol:   Usf1    Locus Link:   83586    Chromosome:   13    Cytoband:   Chr:13q24    SOURCE    GENECARD       Gene Ontology:   cellular component:  nucleus      BioCarta Pathways:     KEGG Pathways:  
    Probe set : 1371366_at    Name:   Rho GDP dissociation inhibitor (GDI) alpha    GB acc:   AI408677    UniGene:   Rn.1801    Gene Symbol:   RGD:1359547    Locus Link:   360678    Chromosome:   10    Cytoband:   Chr:10q32.3    SOURCE    GENECARD       Gene Ontology:   cellular component:  cytoplasm   molecular function:  Rho GDP-dissociation inhibitor activity      BioCarta Pathways:     KEGG Pathways:  
    Probe set : 1394051_at    Name:   Transcribed locus    GB acc:   AI009791    UniGene:   Rn.17487        SOURCE        Gene Ontology:     BioCarta Pathways:     KEGG Pathways:  
    Probe set : 1390576_at    Name:   Transcribed locus    GB acc:   BE116370    UniGene:   Rn.28047        SOURCE        Gene Ontology:     BioCarta Pathways:     KEGG Pathways:  
    Probe set : 1373247_at    Name:   Beta-site APP cleaving enzyme    GB acc:   AW435407    UniGene:   Rn.48895    Gene Symbol:   Bace    Locus Link:   29392    Chromosome:   8    Cytoband:   Chr:8q22    SOURCE    GENECARD       Gene Ontology:     BioCarta Pathways:     KEGG Pathways:  
    Probe set : 1368482_at    Name:   B-cell leukemia/lymphoma 2 related protein A1    GB acc:   NM_133416    UniGene:   Rn.19770    Gene Symbol:   Bcl2a1    Locus Link:   170929    Chromosome:   8    Cytoband:   Chr:8q31    SOURCE    GENECARD       Gene Ontology:   cellular component:  intracellular      BioCarta Pathways:     KEGG Pathways:  
    Probe set : 1374667_at    Name:   CDNA clone IMAGE:7302289    GB acc:   AW433942    UniGene:   Rn.23465        SOURCE        Gene Ontology:   biological process:  cell organization and biogenesis   biological process:  actin cytoskeleton organization and biogenesis   molecular function:  actin binding      BioCarta Pathways:     KEGG Pathways:  
    Probe set : 1390918_at    Name:   GH regulated TBC protein 1 (predicted)    GB acc:   BE099056    UniGene:   Rn.19589    Gene Symbol:   Grtp1_predicted    Locus Link:   361180    Chromosome:   16    Cytoband:   Chr:16q12.5    SOURCE    GENECARD       Gene Ontology:   cellular component:  extracellular region   molecular function:  hormone activity      BioCarta Pathways:     KEGG Pathways:  
    Probe set : 1370547_at    Name:   pregnancy-zone protein    GB acc:   M84000    UniGene:   Rn.11295    Gene Symbol:   Pzp    Locus Link:   252922    Chromosome:   4    Cytoband:   Chr:4q42    SOURCE    GENECARD       Gene Ontology:   biological process:  pregnancy   cellular component:  extracellular region   cellular component:  extracellular space      BioCarta Pathways:     KEGG Pathways:  
    Probe set : 1373852_at    Name:   similar to SRD5A2L (predicted)    GB acc:   AA859922    UniGene:   Rn.819    Gene Symbol:   RGD1308828_predicted    Locus Link:   305291    Chromosome:   14    Cytoband:   Chr:14p11    SOURCE    GENECARD       Gene Ontology:     BioCarta Pathways:     KEGG Pathways:  
    Probe set : 1388556_at    Name:   syntaxin 6    GB acc:   BG381589    UniGene:   Rn.100217    Gene Symbol:   Stx6    Locus Link:   60562    Chromosome:   13    Cytoband:   Chr:13q21    SOURCE    GENECARD       Gene Ontology:     BioCarta Pathways:     KEGG Pathways:  
    Probe set : 1377190_at    Name:   Similar to mKIAA0386 protein (predicted)    GB acc:   AI010237    UniGene:   Rn.95357     Locus Link:   306934    Chromosome:   17    Cytoband:   Chr:17p11    SOURCE        Gene Ontology:   biological process:  iron ion transport   biological process:  iron ion homeostasis   molecular function:  binding   molecular function:  ferric iron binding      BioCarta Pathways:     KEGG Pathways:  
    Probe set : 1388397_at    Name:   EBNA1 binding protein 2 (predicted)    GB acc:   BI281160    UniGene:   Rn.9598    Gene Symbol:   Ebna1bp2_predicted    Locus Link:   114021    Chromosome:   5    Cytoband:   Chr:5q36    SOURCE    GENECARD       Gene Ontology:   biological process:  rRNA processing   biological process:  ribosome biogenesis   biological process:  chromosome segregation   cellular component:  membrane fraction   cellular component:  nucleus   cellular component:  nucleolus   molecular function:  protein binding      BioCarta Pathways:     KEGG Pathways:  
    Probe set : 1371763_at    Name:   similar to RIKEN cDNA 4931406C07 (predicted)    GB acc:   BI274533    UniGene:   Rn.3085    Gene Symbol:   RGD1309534_predicted    Locus Link:   363016    Chromosome:   8    Cytoband:   Chr:8q12    SOURCE    GENECARD       Gene Ontology:     BioCarta Pathways:     KEGG Pathways:  
    Probe set : 1375972_at    Name:   Transcribed locus    GB acc:   AA851327    UniGene:   Rn.146380        SOURCE        Gene Ontology:     BioCarta Pathways:     KEGG Pathways:  
    Probe set : 1387819_at    Name:   elastase 1, pancreatic    GB acc:   NM_012552    UniGene:   Rn.6044    Gene Symbol:   Ela1    Locus Link:   24331    Chromosome:   7    Cytoband:   Chr:7q36    SOURCE    GENECARD       Gene Ontology:   biological process:  proteolysis      BioCarta Pathways:     KEGG Pathways:  
    Probe set : 1385656_at    Name:   similar to hypothetical protein MGC13034    GB acc:   AI043676    UniGene:   Rn.122955    Gene Symbol:   LOC499516    Locus Link:   499516    Chromosome:   2    Cytoband:   Chr:2q12    SOURCE    GENECARD       Gene Ontology:     BioCarta Pathways:     KEGG Pathways:  
    Probe set : 1391347_at    Name:   RAB8B, member RAS oncogene family    GB acc:   BF398743    UniGene:   Rn.10995    Gene Symbol:   Rab8b    Locus Link:   266688    Chromosome:   8    Cytoband:   Chr:8q24    SOURCE    GENECARD       Gene Ontology:     BioCarta Pathways:     KEGG Pathways:  
    Probe set : 1368223_at    Name:   a disintegrin-like and metalloprotease (reprolysin type) with thrombospondin type 1 motif, 1    GB acc:   NM_024400    UniGene:   Rn.7897    Gene Symbol:   Adamts1    Locus Link:   79252      SOURCE    GENECARD       Gene Ontology:   biological process:  proteolysis   biological process:  integrin-mediated signaling pathway   biological process:  negative regulation of cell proliferation   cellular component:  extracellular matrix (sensu Metazoa)   cellular component:  plasma membrane   cellular component:  extracellular matrix      BioCarta Pathways:     KEGG Pathways:  
    Probe set : 1368924_at    Name:   growth hormone receptor    GB acc:   NM_017094    UniGene:   Rn.2178    Gene Symbol:   Ghr    Locus Link:   25235    Chromosome:   2    Cytoband:   Chr:2q16    SOURCE    GENECARD       Gene Ontology:     BioCarta Pathways:     KEGG Pathways:  
    Probe set : 1386880_at    Name:   acetyl-Coenzyme A acyltransferase 2 (mitochondrial 3-oxoacyl-Coenzyme A thiolase)    GB acc:   NM_130433    UniGene:   Rn.3786    Gene Symbol:   Acaa2    Locus Link:   170465    Chromosome:   18    Cytoband:   Chr:18q12.3    SOURCE    GENECARD       Gene Ontology:   biological process:  fatty acid metabolism   biological process:  lipid metabolism   cellular component:  mitochondrion   cellular component:  mitochondrial matrix   cellular component:  mitochondrial matrix      BioCarta Pathways:     KEGG Pathways:  
    Probe set : 1390348_at    Name:   folate receptor 2 (fetal) (predicted)    GB acc:   BI274054    UniGene:   Rn.8801    Gene Symbol:   Folr2_predicted    Locus Link:   293154    Chromosome:   1    Cytoband:   Chr:1q32    SOURCE    GENECARD       Gene Ontology:   biological process:  posttranslational protein targeting to membrane   biological process:  folic acid transport   cellular component:  extracellular space   cellular component:  membrane fraction   cellular component:  membrane   molecular function:  receptor activity   molecular function:  folic acid binding   molecular function:  folic acid transporter activity      BioCarta Pathways:     KEGG Pathways:  
    Probe set : 1398256_at    Name:   interleukin 1 beta    GB acc:   NM_031512    UniGene:   Rn.9869    Gene Symbol:   Il1b    Locus Link:   24494    Chromosome:   3    Cytoband:   Chr:3q36    SOURCE    GENECARD       Gene Ontology:   biological process:  regulation of progression through cell cycle   biological process:  angiogenesis   biological process:  apoptosis   biological process:  inflammatory response   biological process:  inflammatory response   biological process:  immune response   biological process:  signal transduction   biological process:  cell-cell signaling   biological process:  cell proliferation   biological process:  negative regulation of cell proliferation   biological process:  cytokine and chemokine mediated signaling pathway   biological process:  cytokine and chemokine mediated signaling pathway   biological process:  antimicrobial humoral response (sensu Vertebrata)   biological process:  neutrophil chemotaxis   biological process:  positive regulation of chemokine biosynthesis   biological process:  positive regulation of interleukin-6 biosynthesis   biological process:  fever   cellular component:  extracellular region   cellular component:  extracellular region   cellular component:  extracellular space      BioCarta Pathways:     KEGG Pathways:  
    Probe set : 1388064_a_at    Name:   solute carrier family 1 (glial high affinity glutamate transporter), member 3    GB acc:   AF265360    UniGene:   Rn.34134    Gene Symbol:   Slc1a3    Locus Link:   29483    Chromosome:   2    Cytoband:   Chr:2q16    SOURCE    GENECARD       Gene Ontology:     BioCarta Pathways:     KEGG Pathways:  
    Probe set : 1369103_at    Name:   fyn proto-oncogene    GB acc:   NM_012755    UniGene:   Rn.19361    Gene Symbol:   Fyn    Locus Link:   25150    Chromosome:   20    Cytoband:   Chr:20q13    SOURCE    GENECARD       Gene Ontology:   cellular component:  intracellular   cellular component:  mitochondrial membrane      BioCarta Pathways:     KEGG Pathways:  
    Probe set : 1383716_at    Name:   Transcribed locus, strongly similar to NP_777569.1 hypothetical protein MGC23909 [Homo sapiens]    GB acc:   BF556301    UniGene:   Rn.137995        SOURCE        Gene Ontology:     BioCarta Pathways:     KEGG Pathways:  
    Probe set : 1370870_at    Name:   malic enzyme 1    GB acc:   M30596    UniGene:   Rn.3519    Gene Symbol:   Me1    Locus Link:   24552    Chromosome:   8    Cytoband:   Chr:8q31    SOURCE    GENECARD       Gene Ontology:   biological process:  carbohydrate metabolism   biological process:  tricarboxylic acid cycle   biological process:  malate metabolism   cellular component:  soluble fraction   cellular component:  cytosol      BioCarta Pathways:     KEGG Pathways:  
    Probe set : 1386890_at    Name:   S100 calcium binding protein A10 (calpactin)    GB acc:   NM_031114    UniGene:   Rn.4083    Gene Symbol:   S100a10    Locus Link:   81778      SOURCE    GENECARD       Gene Ontology:   biological process:  regulation of cell growth   biological process:  regulation of cell differentiation   molecular function:  calcium ion binding      BioCarta Pathways:     KEGG Pathways:  
    Probe set : 1383785_at    Name:   Lymphoid enhancer binding factor 1    GB acc:   BE115061    UniGene:   Rn.21926    Gene Symbol:   Lef1    Locus Link:   161452    Chromosome:   2    Cytoband:   Chr:2q34-q45    SOURCE    GENECARD       Gene Ontology:   cellular component:  nucleus   cellular component:  transcription factor complex   cellular component:  cytoplasm   molecular function:  DNA binding   molecular function:  transcription factor activity   molecular function:  protein binding      BioCarta Pathways:     KEGG Pathways:  
    Probe set : 1373473_a_at    Name:   nucleosome assembly protein 1-like 1    GB acc:   BM386384    UniGene:   Rn.18480    Gene Symbol:   Nap1l1    Locus Link:   89825    Chromosome:   7    Cytoband:   Chr:7q21    SOURCE    GENECARD       Gene Ontology:   biological process:  DNA replication   biological process:  nucleosome assembly   biological process:  positive regulation of cell proliferation   cellular component:  nucleus   cellular component:  chromatin assembly complex      BioCarta Pathways:     KEGG Pathways:  
    Probe set : 1380079_at    Name:   similar to mKIAA2005 protein    GB acc:   AW533923    UniGene:   Rn.118248    Gene Symbol:   LOC500015    Locus Link:   500015    Chromosome:   4    Cytoband:   Chr:4q13    SOURCE    GENECARD       Gene Ontology:     BioCarta Pathways:     KEGG Pathways:  
    Probe set : 1372135_at    Name:   protein tyrosine kinase 9-like (A6-related protein) (predicted)    GB acc:   AI171452     Gene Symbol:   Ptk9l_predicted    Locus Link:   294420    Chromosome:   20    Cytoband:   Chr:20q11     GENECARD       Gene Ontology:   biological process:  intracellular signaling cascade   cellular component:  intracellular   cellular component:  cytoplasm      BioCarta Pathways:     KEGG Pathways:  
    Probe set : 1371511_at    Name:   actin related protein 2/3 complex, subunit 2 (predicted)    GB acc:   AI009775    UniGene:   Rn.93317    Gene Symbol:   Arpc2_predicted    Locus Link:   301511    Chromosome:   9    Cytoband:   Chr:9q33    SOURCE    GENECARD       Gene Ontology:   biological process:  cell motility   biological process:  regulation of actin filament polymerization   cellular component:  cytoskeleton   cellular component:  Arp2/3 protein complex   cellular component:  focal adhesion   molecular function:  structural constituent of cytoskeleton      BioCarta Pathways:     KEGG Pathways:  
    Probe set : 1370294_a_at    Name:   cell division cycle 20 homolog (S. cerevisiae)    GB acc:   U05341    UniGene:   Rn.9262    Gene Symbol:   Cdc20    Locus Link:   64515    Chromosome:   5    Cytoband:   Chr:5q36    SOURCE    GENECARD       Gene Ontology:   cellular component:  spindle   molecular function:  protein binding      BioCarta Pathways:     KEGG Pathways:  
    Probe set : 1382050_at    Name:   Transcribed locus    GB acc:   AI410262    UniGene:   Rn.7315        SOURCE        Gene Ontology:     BioCarta Pathways:     KEGG Pathways:  
    Probe set : 1375585_at    Name:   Transcribed locus    GB acc:   BE111820    UniGene:   Rn.38969        SOURCE        Gene Ontology:     BioCarta Pathways:     KEGG Pathways:  
    Probe set : 1371998_at    Name:   Similar to Mob4B protein    GB acc:   AI176611    UniGene:   Rn.24123     Locus Link:   297387    Chromosome:   4    Cytoband:   Chr:4q34    SOURCE        Gene Ontology:     BioCarta Pathways:     KEGG Pathways:  
    Probe set : 1369940_at    Name:   transaldolase 1    GB acc:   NM_031811    UniGene:   Rn.3136    Gene Symbol:   Taldo1    Locus Link:   83688    Chromosome:   1    Cytoband:   Chr:1q41    SOURCE    GENECARD       Gene Ontology:   biological process:  carbohydrate metabolism   biological process:  pentose-phosphate shunt   cellular component:  cytoplasm   molecular function:  transaldolase activity   molecular function:  transferase activity      BioCarta Pathways:     KEGG Pathways:  
    Probe set : 1375041_at    Name:   Similar to F-box only protein 27    GB acc:   AI599362    UniGene:   Rn.105566     Locus Link:   499114      SOURCE        Gene Ontology:   biological process:  protein catabolism   molecular function:  protein binding      BioCarta Pathways:     KEGG Pathways:  
    Probe set : 1376039_at    Name:   serine/threonine kinase 6    GB acc:   AA996882    UniGene:   Rn.1135    Gene Symbol:   Stk6    Locus Link:   261730    Chromosome:   3    Cytoband:   Chr:3q42    SOURCE    GENECARD       Gene Ontology:   cellular component:  centrosome   cellular component:  centrosome   cellular component:  spindle   cellular component:  spindle      BioCarta Pathways:     KEGG Pathways:  
    Probe set : 1372306_at    Name:   ethylmalonic encephalopathy 1 (predicted)    GB acc:   BM384018    UniGene:   Rn.14691    Gene Symbol:   Ethe1_predicted    Locus Link:   292710    Chromosome:   1    Cytoband:   Chr:1q21    SOURCE    GENECARD       Gene Ontology:   cellular component:  mitochondrion      BioCarta Pathways:     KEGG Pathways:  
    Probe set : 1390042_at    Name:   similar to 1110007F12Rik protein    GB acc:   AI071166    UniGene:   Rn.8661    Gene Symbol:   MGC109491    Locus Link:   362334    Chromosome:   4    Cytoband:   Chr:4q22    SOURCE    GENECARD       Gene Ontology:     BioCarta Pathways:     KEGG Pathways:  
    Probe set : 1382115_at    Name:   similar to RIKEN cDNA 4930521E07 (predicted)    GB acc:   BE099174    UniGene:   Rn.22713    Gene Symbol:   RGD1305166_predicted    Locus Link:   309486    Chromosome:   1    Cytoband:   Chr:1q54    SOURCE    GENECARD       Gene Ontology:     BioCarta Pathways:     KEGG Pathways:  
    Probe set : 1370166_at    Name:   syndecan 2    GB acc:   AI169682    UniGene:   Rn.11127    Gene Symbol:   Sdc2    Locus Link:   25615    Chromosome:   7    Cytoband:   Chr:7q22    SOURCE    GENECARD       Gene Ontology:   cellular component:  integral to plasma membrane   cellular component:  membrane   cellular component:  integral to membrane   molecular function:  cytoskeletal protein binding      BioCarta Pathways:     KEGG Pathways:  
    Probe set : 1388600_at    Name:   Similar to putative membrane steroid receptor (predicted)    GB acc:   AI011713    UniGene:   Rn.17067     Locus Link:   316275    Chromosome:   9    Cytoband:   Chr:9q13    SOURCE        Gene Ontology:   biological process:  G-protein coupled receptor protein signaling pathway   cellular component:  integral to membrane   molecular function:  receptor activity   molecular function:  G-protein coupled receptor activity   molecular function:  steroid binding      BioCarta Pathways:     KEGG Pathways:  
    Probe set : 1371922_at    Name:   Transcribed locus    GB acc:   AI169140    UniGene:   Rn.18638        SOURCE        Gene Ontology:     BioCarta Pathways:     KEGG Pathways:  
    Probe set : 1370948_a_at    Name:   myristoylated alanine rich protein kinase C substrate    GB acc:   M59859    UniGene:   Rn.9560    Gene Symbol:   Marcks    Locus Link:   25603      SOURCE    GENECARD       Gene Ontology:   biological process:  cell motility   cellular component:  plasma membrane   cellular component:  actin cytoskeleton   cellular component:  membrane      BioCarta Pathways:     KEGG Pathways:  
    Probe set : 1398829_at    Name:   FK506 binding protein 1a    GB acc:   AI598372    UniGene:   Rn.80611    Gene Symbol:   Fkbp1a    Locus Link:   25639    Chromosome:   3    Cytoband:   Chr:3q41    SOURCE    GENECARD       Gene Ontology:   biological process:  protein folding   biological process:  transport   biological process:  positive regulation of I-kappaB kinase/NF-kappaB cascade   cellular component:  microsome   cellular component:  membrane      BioCarta Pathways:     KEGG Pathways:  
    Probe set : 1392969_at    Name:   Transcribed locus    GB acc:   BI282309    UniGene:   Rn.12238        SOURCE        Gene Ontology:     BioCarta Pathways:     KEGG Pathways:  
    Probe set : 1398286_at    Name:   cysteine sulfinic acid decarboxylase    GB acc:   M64755    UniGene:   Rn.43232    Gene Symbol:   Csad    Locus Link:   60356    Chromosome:   7    Cytoband:   Chr:7q36    SOURCE    GENECARD       Gene Ontology:     BioCarta Pathways:     KEGG Pathways:  
    Probe set : 1390807_at    Name:   carbonic anhydrase VB, mitochondrial    GB acc:   BG375352    UniGene:   Rn.22441    Gene Symbol:   Ca5b    Locus Link:   302669    Chromosome:   X    Cytoband:   Chr:Xq21    SOURCE    GENECARD       Gene Ontology:   biological process:  one-carbon compound metabolism   cellular component:  mitochondrion      BioCarta Pathways:     KEGG Pathways:  
    Probe set : 1373198_at    Name:   similar to RIKEN cDNA 2810451A06 (predicted)    GB acc:   BF283018    UniGene:   Rn.19564    Gene Symbol:   RGD1311098_predicted    Locus Link:   362998    Chromosome:   7    Cytoband:   Chr:7q36    SOURCE    GENECARD       Gene Ontology:   biological process:  protein folding   cellular component:  integral to membrane   molecular function:  unfolded protein binding   molecular function:  heat shock protein binding      BioCarta Pathways:     KEGG Pathways:  
    Probe set : 1371544_at    Name:   similar to Enhancer of rudimentary homolog    GB acc:   BE109666    UniGene:   Rn.7881    Gene Symbol:   LOC503047    Locus Link:   503047    Chromosome:   6    Cytoband:   Chr:6q24    SOURCE    GENECARD       Gene Ontology:     BioCarta Pathways:     KEGG Pathways:  
    Probe set : 1372103_at    Name:   DnaJ (Hsp40) homolog, subfamily A, member 4 (predicted)    GB acc:   BM386931    UniGene:   Rn.27927     Locus Link:   300721    Chromosome:   8    Cytoband:   Chr:8q24    SOURCE        Gene Ontology:   biological process:  protein folding   molecular function:  heat shock protein binding   molecular function:  unfolded protein binding      BioCarta Pathways:     KEGG Pathways:  
    Probe set : 1389688_at    Name:   Transcribed locus    GB acc:   BM384373    UniGene:   Rn.143891        SOURCE        Gene Ontology:     BioCarta Pathways:     KEGG Pathways:  
    Probe set : 1388166_at    Name:   similar to Igh-6 protein /// similar to Ig H-chain V-region precursor /// similar to single chain Fv antibody fragment scFv 7-10A /// similar to Ig heavy chain V region MC101 precursor    GB acc:   AI411693    UniGene:   Rn.10956    Gene Symbol:   RGD:1359202 /// LOC299458 /// LOC314509 /// LOC366747    Locus Link:   299357 /// 299458 /// 314509 /// 366747    Chromosome:   6    Cytoband:   Chr:6q32 /// Chr:6q33    SOURCE    GENECARD       Gene Ontology:   molecular function:  antigen binding      BioCarta Pathways:     KEGG Pathways:  
    Probe set : 1388528_at    Name:   fibrillarin (predicted)    GB acc:   AW433875    UniGene:   Rn.98803    Gene Symbol:   Fbl_predicted    Locus Link:   292747    Chromosome:   1    Cytoband:   Chr:1q21    SOURCE    GENECARD       Gene Ontology:   biological process:  rRNA processing   biological process:  snoRNA metabolism   biological process:  RNA processing   molecular function:  RNA binding   molecular function:  snoRNA binding   molecular function:  RNA binding      BioCarta Pathways:     KEGG Pathways:  
    Probe set : 1389082_at     GB acc:   BF398612              Gene Ontology:     BioCarta Pathways:     KEGG Pathways:  
    Probe set : 1387793_at    Name:   ERM-binding phosphoprotein    GB acc:   NM_021594    UniGene:   Rn.35142    Gene Symbol:   Slc9a3r1    Locus Link:   59114    Chromosome:   10    Cytoband:   Chr:10q32.2    SOURCE    GENECARD       Gene Ontology:   biological process:  Wnt receptor signaling pathway   biological process:  cAMP-mediated signaling   biological process:  phosphate ion homeostasis   cellular component:  apical plasma membrane   cellular component:  apical part of cell   molecular function:  protein binding      BioCarta Pathways:     KEGG Pathways:  
    Probe set : 1388622_at    Name:   nucleolar protein 5A (predicted)    GB acc:   AW535890    UniGene:   Rn.145204    Gene Symbol:   Nol5a_predicted    Locus Link:   362214    Chromosome:   3    Cytoband:   Chr:3q36    SOURCE    GENECARD       Gene Ontology:   biological process:  rRNA processing   biological process:  ribosome biogenesis   cellular component:  nucleus   cellular component:  nucleolus   molecular function:  RNA binding      BioCarta Pathways:     KEGG Pathways:  
    Probe set : 1388365_at    Name:   ATPase, H+ transporting, V0 subunit D isoform 1 (predicted)    GB acc:   BI276424    UniGene:   Rn.94752    Gene Symbol:   Atp6v0d1_predicted    Locus Link:   291969    Chromosome:   19    Cytoband:   Chr:19q11    SOURCE    GENECARD       Gene Ontology:   biological process:  ATP biosynthesis   biological process:  ATP synthesis coupled proton transport   biological process:  proton transport   cellular component:  proton-transporting two-sector ATPase complex   cellular component:  hydrogen-translocating V-type ATPase complex      BioCarta Pathways:     KEGG Pathways:  
    Probe set : 1381221_at    Name:   similar to T cell receptor V delta 6 (predicted)    GB acc:   AI012548    UniGene:   Rn.145274    Gene Symbol:   RGD1311300_predicted    Locus Link:   364378    Chromosome:   15    Cytoband:   Chr:15p13    SOURCE    GENECARD       Gene Ontology:     BioCarta Pathways:     KEGG Pathways:  
    Probe set : 1388948_at    Name:   START domain containing 10 (predicted)    GB acc:   AI104759    UniGene:   Rn.143765    Gene Symbol:   Stard10_predicted    Locus Link:   293150    Chromosome:   1    Cytoband:   Chr:1q32    SOURCE    GENECARD       Gene Ontology:     BioCarta Pathways:     KEGG Pathways:  
    Probe set : 1373216_at     GB acc:   AI236521              Gene Ontology:     BioCarta Pathways:     KEGG Pathways:  
    Probe set : 1367720_at    Name:   aminolevulinate, delta-, dehydratase    GB acc:   NM_012899    UniGene:   Rn.3941    Gene Symbol:   Alad    Locus Link:   25374      SOURCE    GENECARD       Gene Ontology:   biological process:  porphyrin biosynthesis   biological process:  heme biosynthesis      BioCarta Pathways:     KEGG Pathways:  
    Probe set : 1369969_at    Name:   ADP-ribosyltransferase 1    GB acc:   NM_013063    UniGene:   Rn.11327    Gene Symbol:   Adprt    Locus Link:   25591    Chromosome:   13    Cytoband:   Chr:13q26    SOURCE    GENECARD       Gene Ontology:   cellular component:  intracellular   cellular component:  nucleus   cellular component:  nucleus      BioCarta Pathways:     KEGG Pathways:  
    Probe set : 1371496_at     GB acc:   AI178804              Gene Ontology:     BioCarta Pathways:     KEGG Pathways:  
    Probe set : 1372976_at    Name:   similar to Dorz1 (predicted)    GB acc:   BI294842    UniGene:   Rn.41351    Gene Symbol:   RGD1309721_predicted    Locus Link:   300982    Chromosome:   8    Cytoband:   Chr:8q32    SOURCE    GENECARD       Gene Ontology:   biological process:  aromatic compound metabolism   molecular function:  catalytic activity   molecular function:  hydrolase activity      BioCarta Pathways:     KEGG Pathways:  
    Probe set : 1371916_at    Name:   CDNA clone MGC:105753 IMAGE:7316479    GB acc:   AI409380    UniGene:   Rn.11960        SOURCE        Gene Ontology:     BioCarta Pathways:     KEGG Pathways:  
    Probe set : 1386695_at    Name:   Transcribed locus    GB acc:   BF565756    UniGene:   Rn.47315        SOURCE        Gene Ontology:     BioCarta Pathways:     KEGG Pathways:  
    Probe set : 1395375_at    Name:   similar to hypothetical protein    GB acc:   BG672758    UniGene:   Rn.107832    Gene Symbol:   MGC73022    Locus Link:   309196    Chromosome:   1    Cytoband:   Chr:1q43    SOURCE    GENECARD       Gene Ontology:   molecular function:  binding      BioCarta Pathways:     KEGG Pathways:  
    Probe set : 1391817_at    Name:   Transcribed locus    GB acc:   AA901178    UniGene:   Rn.15144        SOURCE        Gene Ontology:     BioCarta Pathways:     KEGG Pathways:  
    Probe set : 1377183_at    Name:   Transcribed locus    GB acc:   BE098555    UniGene:   Rn.25482        SOURCE        Gene Ontology:     BioCarta Pathways:     KEGG Pathways:  
    Probe set : 1373290_at    Name:   similar to Ezh2 protein    GB acc:   AI012781    UniGene:   Rn.9027    Gene Symbol:   LOC312299    Locus Link:   312299    Chromosome:   4    Cytoband:   Chr:4q24    SOURCE    GENECARD       Gene Ontology:     BioCarta Pathways:     KEGG Pathways:  
    Probe set : 1380110_at     GB acc:   AI229643              Gene Ontology:     BioCarta Pathways:     KEGG Pathways:  
    Probe set : 1371696_at    Name:   G protein-coupled receptor 56    GB acc:   AI412938    UniGene:   Rn.1677    Gene Symbol:   Gpr56    Locus Link:   260326    Chromosome:   19    Cytoband:   Chr:19p13    SOURCE    GENECARD       Gene Ontology:     BioCarta Pathways:     KEGG Pathways:  
    Probe set : 1371577_at    Name:   NADH dehydrogenase (ubiquinone) Fe-S protein 1, 75kDa    GB acc:   AI171362    UniGene:   Rn.1467    Gene Symbol:   RGD:1359670    Locus Link:   301458    Chromosome:   9    Cytoband:   Chr:9q31    SOURCE    GENECARD       Gene Ontology:   biological process:  electron transport   biological process:  mitochondrial electron transport, NADH to ubiquinone      BioCarta Pathways:     KEGG Pathways:  
    Probe set : 1388915_at    Name:   Transcribed locus    GB acc:   BI295753    UniGene:   Rn.834        SOURCE        Gene Ontology:     BioCarta Pathways:     KEGG Pathways:  
    Probe set : 1398500_at    Name:   G protein-coupled receptor 116    GB acc:   BI295477    UniGene:   Rn.25073    Gene Symbol:   Gpr116    Locus Link:   245977    Chromosome:   9    Cytoband:   Chr:9q12    SOURCE    GENECARD       Gene Ontology:   biological process:  signal transduction   biological process:  G-protein coupled receptor protein signaling pathway   biological process:  neuropeptide signaling pathway   cellular component:  membrane   cellular component:  integral to membrane      BioCarta Pathways:     KEGG Pathways:  
    Probe set : 1396984_at    Name:   UDP-glucose ceramide glucosyltransferase-like 1    GB acc:   BM390248    UniGene:   Rn.13004    Gene Symbol:   Ugcgl1    Locus Link:   171129    Chromosome:   9    Cytoband:   Chr:9q21    SOURCE    GENECARD       Gene Ontology:   biological process:  protein folding   biological process:  protein amino acid glycosylation   biological process:  posttranslational protein folding      BioCarta Pathways:     KEGG Pathways:  
    Probe set : 1371691_at     GB acc:   BI282993              Gene Ontology:     BioCarta Pathways:     KEGG Pathways:  
    Probe set : 1388762_at     GB acc:   BF284125              Gene Ontology:     BioCarta Pathways:     KEGG Pathways:  
    Probe set : 1368177_at    Name:   acyl-CoA synthetase long-chain family member 3    GB acc:   NM_057107    UniGene:   Rn.54820    Gene Symbol:   Acsl3    Locus Link:   114024    Chromosome:   9    Cytoband:   Chr:9q33    SOURCE    GENECARD       Gene Ontology:   cellular component:  integral to membrane      BioCarta Pathways:     KEGG Pathways:  
    Probe set : 1369986_at    Name:   hydroxyacyl glutathione hydrolase    GB acc:   NM_033349    UniGene:   Rn.11048    Gene Symbol:   Hagh    Locus Link:   24439    Chromosome:   10    Cytoband:   Chr:10q12    SOURCE    GENECARD       Gene Ontology:   biological process:  spermatogenesis   biological process:  spermatogenesis   molecular function:  hydroxyacylglutathione hydrolase activity   molecular function:  hydrolase activity      BioCarta Pathways:     KEGG Pathways:  
    Probe set : 1370907_at    Name:   sialyltransferase 1    GB acc:   M83143    UniGene:   Rn.54567    Gene Symbol:   Siat1    Locus Link:   25197    Chromosome:   11    Cytoband:   Chr:11q23    SOURCE    GENECARD       Gene Ontology:   biological process:  protein amino acid glycosylation      BioCarta Pathways:     KEGG Pathways:  
    Probe set : 1384330_at    Name:   similar to hypothetical protein FLJ20546    GB acc:   AI763467    UniGene:   Rn.137167    Gene Symbol:   RGD:1359513    Locus Link:   303067    Chromosome:   10    Cytoband:   Chr:10q21    SOURCE    GENECARD       Gene Ontology:     BioCarta Pathways:     KEGG Pathways:  
    Probe set : 1374254_a_at    Name:   hypothetical LOC300441    GB acc:   BM386220    UniGene:   Rn.13486    Gene Symbol:   LOC300441    Locus Link:   300441    Chromosome:   8    Cytoband:   Chr:8q13    SOURCE    GENECARD       Gene Ontology:     BioCarta Pathways:     KEGG Pathways:  
    Probe set : 1389504_at    Name:   Similar to Deltex3    GB acc:   BI297929    UniGene:   Rn.50909     Locus Link:   500847      SOURCE        Gene Ontology:     BioCarta Pathways:     KEGG Pathways:  
    Probe set : 1383429_at    Name:   similar to T-Brain-1    GB acc:   BI289160    UniGene:   Rn.8247    Gene Symbol:   LOC311078    Locus Link:   311078    Chromosome:   3    Cytoband:   Chr:3q21    SOURCE    GENECARD       Gene Ontology:     BioCarta Pathways:     KEGG Pathways:  
    Probe set : 1372459_at    Name:   vasodilator-stimulated phosphoprotein (predicted)    GB acc:   AW520792    UniGene:   Rn.98750    Gene Symbol:   Vasp_predicted    Locus Link:   361517    Chromosome:   1    Cytoband:   Chr:1q21    SOURCE    GENECARD       Gene Ontology:   molecular function:  actin binding   molecular function:  protein binding      BioCarta Pathways:     KEGG Pathways:  
    Probe set : 1373908_at    Name:   Transcribed locus    GB acc:   AI407002    UniGene:   Rn.114474        SOURCE        Gene Ontology:     BioCarta Pathways:     KEGG Pathways:  
    Probe set : 1369752_a_at    Name:   calcium/calmodulin-dependent protein kinase IV    GB acc:   J04446    UniGene:   Rn.11046    Gene Symbol:   Camk4    Locus Link:   25050    Chromosome:   18    Cytoband:   Chr:18p12    SOURCE    GENECARD       Gene Ontology:   cellular component:  nucleus   cellular component:  cytoplasm   cellular component:  calcium- and calmodulin-dependent protein kinase complex   molecular function:  protein kinase activity   molecular function:  protein serine/threonine kinase activity   molecular function:  calmodulin regulated protein kinase activity   molecular function:  calmodulin-dependent protein kinase I activity   molecular function:  calcium- and calmodulin-dependent protein kinase activity   molecular function:  calcium- and calmodulin-dependent protein kinase activity   molecular function:  calmodulin binding   molecular function:  ATP binding   molecular function:  kinase activity   molecular function:  transcriptional activator activity   molecular function:  transferase activity   molecular function:  nucleotide binding   molecular function:  calcium ion binding      BioCarta Pathways:     KEGG Pathways:  
    Probe set : 1373057_at    Name:   ProSAPiP1 protein    GB acc:   AI229142    UniGene:   Rn.7646    Gene Symbol:   ProSAPiP1    Locus Link:   280670    Chromosome:   3    Cytoband:   Chr:3q36    SOURCE    GENECARD       Gene Ontology:   cellular component:  cytoplasm   cellular component:  membrane      BioCarta Pathways:     KEGG Pathways:  
    Probe set : 1398891_at    Name:   mitochondrial ribosomal protein L15 (predicted)    GB acc:   AI103129    UniGene:   Rn.1122    Gene Symbol:   Mrpl15_predicted    Locus Link:   297799    Chromosome:   5    Cytoband:   Chr:5q12    SOURCE    GENECARD       Gene Ontology:   biological process:  mitochondrial genome maintenance   biological process:  protein biosynthesis   molecular function:  structural constituent of ribosome      BioCarta Pathways:     KEGG Pathways:  
    Probe set : 1371445_at    Name:   similar to ribosome-binding protein p34 - rat (predicted)    GB acc:   BF285649    UniGene:   Rn.2783    Gene Symbol:   RGD1305092_predicted    Locus Link:   287633    Chromosome:   10    Cytoband:   Chr:10q31    SOURCE    GENECARD       Gene Ontology:   cellular component:  integral to membrane      BioCarta Pathways:     KEGG Pathways:  
    Probe set : 1388788_at    Name:   glutaryl-Coenzyme A dehydrogenase (predicted)    GB acc:   BG664131    UniGene:   Rn.99039    Gene Symbol:   Gcdh_predicted    Locus Link:   364975    Chromosome:   19    Cytoband:   Chr:19q11    SOURCE    GENECARD       Gene Ontology:   biological process:  electron transport   biological process:  regulation of transcription, DNA-dependent   cellular component:  mitochondrion      BioCarta Pathways:     KEGG Pathways:  
    Probe set : 1368173_at    Name:   nucleolar protein 5    GB acc:   NM_021754    UniGene:   Rn.43930    Gene Symbol:   Nol5    Locus Link:   60373    Chromosome:   9    Cytoband:   Chr:9q31    SOURCE    GENECARD       Gene Ontology:     BioCarta Pathways:     KEGG Pathways:  
    Probe set : 1390383_at    Name:   adipose differentiation-related protein    GB acc:   BI285616    UniGene:   Rn.101967    Gene Symbol:   RGD:1359288    Locus Link:   298199    Chromosome:   5    Cytoband:   Chr:5q32    SOURCE    GENECARD       Gene Ontology:     BioCarta Pathways:     KEGG Pathways:  
    Probe set : 1398373_at    Name:   UDP-Gal:betaGlcNAc beta 1,3-galactosyltransferase, polypeptide 3 (predicted)    GB acc:   AA799400    UniGene:   Rn.3666    Gene Symbol:   B3galt3_predicted    Locus Link:   310508    Chromosome:   2    Cytoband:   Chr:2q32    SOURCE    GENECARD       Gene Ontology:   biological process:  protein amino acid glycosylation   biological process:  oligosaccharide biosynthesis      BioCarta Pathways:     KEGG Pathways:  
    Probe set : 1388822_at    Name:   short coiled-coil protein (predicted) /// similar to short coiled-coil protein    GB acc:   AI409077    UniGene:   Rn.18315    Gene Symbol:   Scoc_predicted /// LOC498931    Locus Link:   364981 /// 498931    Chromosome:   19    Cytoband:   Chr:19q11    SOURCE    GENECARD       Gene Ontology:     BioCarta Pathways:     KEGG Pathways:  
    Probe set : 1369956_at    Name:   interferon gamma receptor 1    GB acc:   NM_053783    UniGene:   Rn.19927    Gene Symbol:   Ifngr    Locus Link:   116465    Chromosome:   1    Cytoband:   Chr:1p12    SOURCE    GENECARD       Gene Ontology:   cellular component:  extracellular space   cellular component:  membrane   cellular component:  integral to membrane      BioCarta Pathways:     KEGG Pathways:  
    Probe set : 1385791_at    Name:   similar to villin-like protein (predicted)    GB acc:   BE112294    UniGene:   Rn.52066    Gene Symbol:   RGD1304843_predicted    Locus Link:   301057    Chromosome:   8    Cytoband:   Chr:8q32    SOURCE    GENECARD       Gene Ontology:     BioCarta Pathways:     KEGG Pathways:  
    Probe set : 1378240_at    Name:   Fanconi anemia D2 protein    GB acc:   AI031007    UniGene:   Rn.32370    Gene Symbol:   RGD:1303172    Locus Link:   312641    Chromosome:   4    Cytoband:   Chr:4q42    SOURCE    GENECARD       Gene Ontology:   biological process:  DNA repair   biological process:  response to DNA damage stimulus   biological process:  cell cycle   cellular component:  nucleus   cellular component:  chromosome      BioCarta Pathways:     KEGG Pathways:  
    Probe set : 1389246_at    Name:   Transcribed locus    GB acc:   BF282414    UniGene:   Rn.8685        SOURCE        Gene Ontology:     BioCarta Pathways:     KEGG Pathways:  
    Probe set : 1395313_s_at     GB acc:   AI179982              Gene Ontology:     BioCarta Pathways:     KEGG Pathways:  
    Probe set : 1378307_at    Name:   Pleckstrin homology domain containing, family H (with MyTH4 domain) member 1 (predicted)    GB acc:   AI412126    UniGene:   Rn.35024     Locus Link:   314262    Chromosome:   6    Cytoband:   Chr:6q24    SOURCE        Gene Ontology:   biological process:  cell adhesion   cellular component:  cytoskeleton      BioCarta Pathways:     KEGG Pathways:  
    Probe set : 1369200_at    Name:   5 nucleotidase    GB acc:   NM_021576    UniGene:   Rn.40132    Gene Symbol:   Nt5    Locus Link:   58813    Chromosome:   8    Cytoband:   Chr:8q31    SOURCE    GENECARD       Gene Ontology:   biological process:  DNA metabolism   biological process:  nucleotide catabolism      BioCarta Pathways:     KEGG Pathways:  
    Probe set : 1388565_at    Name:   acid cluster protein 33    GB acc:   BI289536    UniGene:   Rn.19459    Gene Symbol:   RGD:1359141    Locus Link:   300791    Chromosome:   8    Cytoband:   Chr:8q24    SOURCE    GENECARD       Gene Ontology:   molecular function:  catalytic activity      BioCarta Pathways:     KEGG Pathways:  
    Probe set : 1372110_at    Name:   Similar to hypothetical protein (predicted)    GB acc:   BE113148    UniGene:   Rn.9179     Locus Link:   305332    Chromosome:   14    Cytoband:   Chr:14p11    SOURCE        Gene Ontology:     BioCarta Pathways:     KEGG Pathways:  
    Probe set : 1370345_at    Name:   cyclin B1    GB acc:   L11995    UniGene:   Rn.145413    Gene Symbol:   Ccnb1    Locus Link:   25203    Chromosome:   2    Cytoband:   Chr:2q12    SOURCE    GENECARD       Gene Ontology:   cellular component:  nucleus   molecular function:  protein binding   molecular function:  kinase activity   molecular function:  cyclin-dependent protein kinase regulator activity      BioCarta Pathways:     KEGG Pathways:  
    Probe set : 1378700_at    Name:   Transcribed locus    GB acc:   BF403674    UniGene:   Rn.147829        SOURCE        Gene Ontology:     BioCarta Pathways:     KEGG Pathways:  
    Probe set : 1373757_at    Name:   FLN29 gene product    GB acc:   AW529298    UniGene:   Rn.16272    Gene Symbol:   Fln29    Locus Link:   114635    Chromosome:   12    Cytoband:   Chr:12q16    SOURCE    GENECARD       Gene Ontology:     BioCarta Pathways:     KEGG Pathways:  
    Probe set : 1384457_at    Name:   similar to Fbxw17 protein    GB acc:   AI030755    UniGene:   Rn.100575    Gene Symbol:   LOC498713    Locus Link:   498713    Chromosome:   17    Cytoband:   Chr:17p14    SOURCE    GENECARD       Gene Ontology:     BioCarta Pathways:     KEGG Pathways:  
    Probe set : 1368431_at    Name:   hepsin    GB acc:   NM_017112    UniGene:   Rn.11139    Gene Symbol:   Hpn    Locus Link:   29135    Chromosome:   1    Cytoband:   Chr:1q21    SOURCE    GENECARD       Gene Ontology:   biological process:  proteolysis      BioCarta Pathways:     KEGG Pathways:  
    Probe set : 1375684_at    Name:   neuraminidase 1    GB acc:   BE111846    UniGene:   Rn.128560    Gene Symbol:   Neu1    Locus Link:   24591    Chromosome:   20    Cytoband:   Chr:20p12    SOURCE    GENECARD       Gene Ontology:   biological process:  carbohydrate metabolism   cellular component:  lysosome      BioCarta Pathways:     KEGG Pathways:  
    Probe set : 1371266_at    Name:   afamin    GB acc:   X76456    UniGene:   Rn.126589    Gene Symbol:   Afm    Locus Link:   282708    Chromosome:   14    Cytoband:   Chr:14p22    SOURCE    GENECARD       Gene Ontology:   biological process:  transport   cellular component:  extracellular space   cellular component:  extracellular space   molecular function:  carrier activity      BioCarta Pathways:     KEGG Pathways:  
    Probe set : 1398511_at    Name:   sushi domain containing 2 (predicted)    GB acc:   AA901176    UniGene:   Rn.16299    Gene Symbol:   Susd2_predicted    Locus Link:   294335    Chromosome:   20    Cytoband:   Chr:20p12    SOURCE    GENECARD       Gene Ontology:   cellular component:  extracellular space   cellular component:  integral to membrane      BioCarta Pathways:     KEGG Pathways:  
    Probe set : 1379293_at    Name:   granzyme A    GB acc:   AI104238    UniGene:   Rn.22283    Gene Symbol:   Gzma    Locus Link:   266708    Chromosome:   2    Cytoband:   Chr:2q14    SOURCE    GENECARD       Gene Ontology:   cellular component:  extracellular space   cellular component:  immunological synapse   cellular component:  nucleus      BioCarta Pathways:     KEGG Pathways:  
    Probe set : 1388753_at    Name:   sulfatase 2 (predicted)    GB acc:   AA963797    UniGene:   Rn.4228    Gene Symbol:   Sulf2_predicted    Locus Link:   311642    Chromosome:   3    Cytoband:   Chr:3q42    SOURCE    GENECARD       Gene Ontology:     BioCarta Pathways:     KEGG Pathways:  
    Probe set : 1378033_at     GB acc:   AI029178              Gene Ontology:     BioCarta Pathways:     KEGG Pathways:  
    Probe set : 1385019_at     GB acc:   BM386036              Gene Ontology:     BioCarta Pathways:     KEGG Pathways:  
    Probe set : 1398756_at    Name:   nucleophosmin 1 /// similar to Nucleophosmin (NPM) (Nucleolar phosphoprotein B23) (Numatrin) (Nucleolar protein NO38)    GB acc:   J04943    UniGene:   Rn.54537    Gene Symbol:   Npm1 /// LOC300303    Locus Link:   25498 /// 300303    Chromosome:   10    Cytoband:   Chr:10q12 /// Chr:Xq14    SOURCE    GENECARD       Gene Ontology:   biological process:  intracellular protein transport   biological process:  nucleocytoplasmic transport   biological process:  response to stress   biological process:  centrosome cycle   biological process:  cell aging   biological process:  negative regulation of cell proliferation   biological process:  ribosome assembly   biological process:  activation of NF-kappaB transcription factor   biological process:  intracellular protein transport   biological process:  nucleocytoplasmic transport   biological process:  centrosome cycle   biological process:  cell aging   biological process:  negative regulation of cell proliferation      BioCarta Pathways:     KEGG Pathways:  
    Probe set : 1367769_at    Name:   polymerase (RNA) II (DNA directed) polypeptide G    GB acc:   NM_053948    UniGene:   Rn.106248    Gene Symbol:   Polr2g    Locus Link:   117017    Chromosome:   1    Cytoband:   Chr:1q43    SOURCE    GENECARD       Gene Ontology:   cellular component:  nucleus   cellular component:  DNA-directed RNA polymerase II, core complex      BioCarta Pathways:     KEGG Pathways:  
    Probe set : 1376808_at    Name:   Transcribed locus    GB acc:   BG672572    UniGene:   Rn.21871        SOURCE        Gene Ontology:     BioCarta Pathways:     KEGG Pathways:  
    Probe set : 1390141_at    Name:   formyltetrahydrofolate synthetase domain containing 1 (predicted)    GB acc:   BF281848    UniGene:   Rn.18065    Gene Symbol:   Fthfsdc1_predicted    Locus Link:   361472    Chromosome:   1    Cytoband:   Chr:1p11    SOURCE    GENECARD       Gene Ontology:   biological process:  10-formyltetrahydrofolate biosynthesis   biological process:  folic acid and derivative biosynthesis   cellular component:  mitochondrion      BioCarta Pathways:     KEGG Pathways:  
    Probe set : 1399012_at    Name:   similar to RIKEN cDNA 1110001M20 (predicted)    GB acc:   BI285976    UniGene:   Rn.105907    Gene Symbol:   RGD1310313_predicted    Locus Link:   298308    Chromosome:   5    Cytoband:   Chr:5q34    SOURCE    GENECARD       Gene Ontology:     BioCarta Pathways:     KEGG Pathways:  
    Probe set : 1370369_at    Name:   granzyme M (lymphocyte met-ase 1)    GB acc:   L05175    UniGene:   Rn.9838    Gene Symbol:   Gzmm    Locus Link:   29252    Chromosome:   7    Cytoband:   Chr:7q11    SOURCE    GENECARD       Gene Ontology:   biological process:  proteolysis   biological process:  cytolysis   cellular component:  extracellular space      BioCarta Pathways:     KEGG Pathways:  
    Probe set : 1379885_at    Name:   flavin containing monooxygenase 4    GB acc:   BF282998    UniGene:   Rn.6404    Gene Symbol:   Fmo4    Locus Link:   246247    Chromosome:   13    Cytoband:   Chr:13q22    SOURCE    GENECARD       Gene Ontology:   biological process:  electron transport   biological process:  xenobiotic metabolism      BioCarta Pathways:     KEGG Pathways:  
    Probe set : 1371936_at    Name:   eukaryotic translation initiation factor 4A1    GB acc:   BI284436    UniGene:   Rn.91523    Gene Symbol:   Eif4a1    Locus Link:   287436    Chromosome:   10    Cytoband:   Chr:10q24    SOURCE    GENECARD       Gene Ontology:   biological process:  translational initiation      BioCarta Pathways:     KEGG Pathways:  
    Probe set : 1393249_at    Name:   Friend leukemia integration 1 (predicted)    GB acc:   BF407924    UniGene:   Rn.65827     Locus Link:   315532    Chromosome:   8    Cytoband:   Chr:8q21    SOURCE        Gene Ontology:   cellular component:  nucleus   molecular function:  DNA binding   molecular function:  transcription factor activity   molecular function:  transcriptional activator activity      BioCarta Pathways:     KEGG Pathways:  
    Probe set : 1387502_at    Name:   serine/threonine kinase 17b (apoptosis-inducing)    GB acc:   NM_133392    UniGene:   Rn.1300    Gene Symbol:   Stk17b    Locus Link:   170904    Chromosome:   9    Cytoband:   Chr:9q31    SOURCE    GENECARD       Gene Ontology:     BioCarta Pathways:     KEGG Pathways:  
    Probe set : 1371737_at    Name:   tripartite motif protein 27 (predicted)    GB acc:   BI281720    UniGene:   Rn.8393    Gene Symbol:   Trim27_predicted    Locus Link:   291171    Chromosome:   17    Cytoband:   Chr:17q11    SOURCE    GENECARD       Gene Ontology:     BioCarta Pathways:     KEGG Pathways:  
    Probe set : 1379653_a_at    Name:   similar to SH2 domain protein 1A (Signaling lymphocyte activation molecule-associated protein) (SLAM-associated protein) (T cell signal transduction molecule SAP)    GB acc:   AI176327    UniGene:   Rn.12605    Gene Symbol:   LOC501502    Locus Link:   501502    Chromosome:   X    Cytoband:   Chr:Xq11    SOURCE    GENECARD       Gene Ontology:     BioCarta Pathways:     KEGG Pathways:  
    Probe set : 1373286_at    Name:   CSX-associated LIM    GB acc:   AA875261    UniGene:   Rn.2851    Gene Symbol:   RGD:1359551    Locus Link:   362650    Chromosome:   5    Cytoband:   Chr:5q36    SOURCE    GENECARD       Gene Ontology:   molecular function:  zinc ion binding   molecular function:  metal ion binding      BioCarta Pathways:     KEGG Pathways:  
    Probe set : 1379794_at    Name:   granzyme B    GB acc:   AI029386    UniGene:   Rn.21395    Gene Symbol:   Gzmb    Locus Link:   171528    Chromosome:   15    Cytoband:   Chr:15p12    SOURCE    GENECARD       Gene Ontology:   biological process:  proteolysis   biological process:  apoptosis   biological process:  cytolysis   cellular component:  extracellular space      BioCarta Pathways:     KEGG Pathways:  
    Probe set : 1368609_at    Name:   solute carrier family 10 (sodium/bile acid cotransporter family), member 1    GB acc:   NM_017047    UniGene:   Rn.9913    Gene Symbol:   Slc10a1    Locus Link:   24777    Chromosome:   6    Cytoband:   Chr:6q24    SOURCE    GENECARD       Gene Ontology:   cellular component:  integral to plasma membrane   cellular component:  membrane   cellular component:  integral to membrane      BioCarta Pathways:     KEGG Pathways:  
    Probe set : 1368143_at    Name:   annexin A7    GB acc:   AI013902    UniGene:   Rn.106184    Gene Symbol:   Anxa7    Locus Link:   155423    Chromosome:   15    Cytoband:   Chr:15p16    SOURCE    GENECARD       Gene Ontology:   biological process:  calcium ion homeostasis   biological process:  cell proliferation   biological process:  negative regulation of coagulation   cellular component:  nuclear membrane   cellular component:  plasma membrane      BioCarta Pathways:     KEGG Pathways:  
    Probe set : 1373754_at    Name:   Transcribed locus    GB acc:   AI599232    UniGene:   Rn.1545        SOURCE        Gene Ontology:     BioCarta Pathways:     KEGG Pathways:  
    Probe set : 1371079_at    Name:   Fc receptor, IgG, low affinity IIb    GB acc:   X73371    UniGene:   Rn.33323    Gene Symbol:   Fcgr2b    Locus Link:   289211    Chromosome:   13    Cytoband:   Chr:13q24    SOURCE    GENECARD       Gene Ontology:   cellular component:  integral to plasma membrane   cellular component:  external side of plasma membrane   cellular component:  integral to membrane   molecular function:  receptor activity   molecular function:  receptor signaling protein activity   molecular function:  IgG binding      BioCarta Pathways:     KEGG Pathways:  
    Probe set : 1384173_at    Name:   src family associated phosphoprotein 1    GB acc:   BI290909    UniGene:   Rn.35285    Gene Symbol:   Scap1    Locus Link:   286975    Chromosome:   10    Cytoband:   Chr:10q31    SOURCE    GENECARD       Gene Ontology:   molecular function:  protein domain specific binding      BioCarta Pathways:     KEGG Pathways:  
    Probe set : 1387778_at    Name:   stromal cell derived factor 4    GB acc:   NM_130412    UniGene:   Rn.12964    Gene Symbol:   Sdf4    Locus Link:   155173    Chromosome:   5    Cytoband:   Chr:5q36    SOURCE    GENECARD       Gene Ontology:   cellular component:  extracellular space   cellular component:  Golgi apparatus   cellular component:  Golgi stack   molecular function:  calcium ion binding      BioCarta Pathways:     KEGG Pathways:  
    Probe set : 1371640_at    Name:   hypothetical protein LOC500855    GB acc:   BF289183    UniGene:   Rn.40403    Gene Symbol:   LOC500855    Locus Link:   500855    Chromosome:   7    Cytoband:   Chr:7q22    SOURCE    GENECARD       Gene Ontology:     BioCarta Pathways:     KEGG Pathways:  
    Probe set : 1367738_at    Name:   UNC-119 homolog (C. elegans)    GB acc:   NM_017188    UniGene:   Rn.1609    Gene Symbol:   Unc119    Locus Link:   29402    Chromosome:   10    Cytoband:   Chr:10q25    SOURCE    GENECARD       Gene Ontology:   biological process:  microtubule-based movement   biological process:  visual perception   biological process:  sensory perception   cellular component:  microtubule   molecular function:  structural molecule activity      BioCarta Pathways:     KEGG Pathways:  
    Probe set : 1372310_at    Name:   similar to tumor-related protein (predicted)    GB acc:   AI227941    UniGene:   Rn.1166    Gene Symbol:   RGD1307632_predicted    Locus Link:   364879    Chromosome:   18    Cytoband:   Chr:18q12.1    SOURCE    GENECARD       Gene Ontology:   biological process:  metabolism   molecular function:  catalytic activity      BioCarta Pathways:     KEGG Pathways:  
    Probe set : 1398769_at    Name:   coronin, actin-binding protein, 1B    GB acc:   NM_019222    UniGene:   Rn.2819    Gene Symbol:   Coro1b    Locus Link:   29474    Chromosome:   1    Cytoband:   Chr:1q42    SOURCE    GENECARD       Gene Ontology:   cellular component:  kinesin complex   molecular function:  actin binding      BioCarta Pathways:     KEGG Pathways:  
    Probe set : 1371184_x_at    Name:   Tropomyosin 3, splice variant 1    GB acc:   AF053359    UniGene:   Rn.37575    Gene Symbol:   Tpm3    Locus Link:   117557    Chromosome:   2    Cytoband:   Chr:2q34    SOURCE    GENECARD       Gene Ontology:   biological process:  regulation of muscle contraction   biological process:  muscle development   cellular component:  cytoskeleton   cellular component:  muscle thin filament tropomyosin   molecular function:  actin binding      BioCarta Pathways:     KEGG Pathways:  
    Probe set : 1398430_at    Name:   Transcribed locus    GB acc:   AW524711    UniGene:   Rn.8814        SOURCE        Gene Ontology:     BioCarta Pathways:     KEGG Pathways:  
    Probe set : 1371483_at    Name:   nicotinamide nucleotide transhydrogenase    GB acc:   AA891872    UniGene:   Rn.3128    Gene Symbol:   Nnt    Locus Link:   310378    Chromosome:   2    Cytoband:   Chr:2q16    SOURCE    GENECARD       Gene Ontology:   biological process:  electron transport   biological process:  proton transport   cellular component:  mitochondrion   cellular component:  integral to membrane      BioCarta Pathways:     KEGG Pathways:  
    Probe set : 1370814_at    Name:   dehydrogenase/reductase (SDR family) member 4    GB acc:   AB062758    UniGene:   Rn.12783    Gene Symbol:   Dhrs4    Locus Link:   266686    Chromosome:   15    Cytoband:   Chr:15p13    SOURCE    GENECARD       Gene Ontology:   biological process:  metabolism      BioCarta Pathways:     KEGG Pathways:  
    Probe set : 1372765_a_at    Name:   similar to Peci protein /// similar to RIKEN cDNA 1810022C23 (predicted)    GB acc:   BM390774    UniGene:   Rn.3194    Gene Symbol:   RGD:1359427 /// RGD1310224_predicted    Locus Link:   291075 /// 291076    Chromosome:   17    Cytoband:   Chr:17p12    SOURCE    GENECARD       Gene Ontology:   biological process:  metabolism   cellular component:  peroxisome      BioCarta Pathways:     KEGG Pathways:  
    Probe set : 1392819_at    Name:   membrane-spanning 4-domains, subfamily A, member 11 (predicted)    GB acc:   AW921478    UniGene:   Rn.101878    Gene Symbol:   Ms4a11_predicted    Locus Link:   361735    Chromosome:   1    Cytoband:   Chr:1q43    SOURCE    GENECARD       Gene Ontology:     BioCarta Pathways:     KEGG Pathways:  
    Probe set : 1398255_at    Name:   solute carrier family 15 (H+/peptide transporter), member 2    GB acc:   NM_031672    UniGene:   Rn.89268    Gene Symbol:   Slc15a2    Locus Link:   60577    Chromosome:   11    Cytoband:   Chr:11q22    SOURCE    GENECARD       Gene Ontology:   biological process:  transport   biological process:  oligopeptide transport   biological process:  protein transport      BioCarta Pathways:     KEGG Pathways:  
    Probe set : 1385704_at     GB acc:   AI071148              Gene Ontology:     BioCarta Pathways:     KEGG Pathways:  
    Probe set : 1390226_at    Name:   similar to hypothetical protein LOC340061    GB acc:   AI408343    UniGene:   Rn.41472    Gene Symbol:   LOC498840    Locus Link:   498840    Chromosome:   18    Cytoband:   Chr:18p11    SOURCE    GENECARD       Gene Ontology:     BioCarta Pathways:     KEGG Pathways:  
    Probe set : 1380063_at    Name:   cholesterol 25-hydroxylase (predicted)    GB acc:   AI169398    UniGene:   Rn.28018    Gene Symbol:   Ch25h_predicted    Locus Link:   309527    Chromosome:   1    Cytoband:   Chr:1q52    SOURCE    GENECARD       Gene Ontology:   biological process:  lipid metabolism   biological process:  metabolism   biological process:  cholesterol metabolism   cellular component:  membrane fraction   cellular component:  integral to membrane   molecular function:  catalytic activity   molecular function:  steroid hydroxylase activity   molecular function:  oxidoreductase activity      BioCarta Pathways:     KEGG Pathways:  
    Probe set : 1387221_at    Name:   GTP cyclohydrolase 1    GB acc:   NM_024356    UniGene:   Rn.28195    Gene Symbol:   Gch    Locus Link:   29244    Chromosome:   15    Cytoband:   Chr:15p14    SOURCE    GENECARD       Gene Ontology:   molecular function:  catalytic activity   molecular function:  GTP cyclohydrolase I activity   molecular function:  hydrolase activity      BioCarta Pathways:     KEGG Pathways:  
    Probe set : 1387947_at    Name:   v-maf musculoaponeurotic fibrosarcoma oncogene family, protein B (avian)    GB acc:   U56241    UniGene:   Rn.10725    Gene Symbol:   Mafb    Locus Link:   54264    Chromosome:   3    Cytoband:   Chr:3q42    SOURCE    GENECARD       Gene Ontology:   cellular component:  nucleus   cellular component:  transcription factor complex   molecular function:  DNA binding   molecular function:  protein binding      BioCarta Pathways:     KEGG Pathways:  
    Probe set : 1384215_at     GB acc:   AI144991              Gene Ontology:     BioCarta Pathways:     KEGG Pathways:  
    Probe set : 1393330_at     GB acc:   AI137898              Gene Ontology:     BioCarta Pathways:     KEGG Pathways:  
    Probe set : 1389107_at    Name:   Transcribed locus, weakly similar to XP_236385.3 PREDICTED: similar to junction-associated coiled-coil protein [Rattus norvegicus]    GB acc:   AI008974    UniGene:   Rn.120904        SOURCE        Gene Ontology:     BioCarta Pathways:     KEGG Pathways:  
    Probe set : 1393531_at    Name:   Transcribed locus    GB acc:   AA964815    UniGene:   Rn.23234        SOURCE        Gene Ontology:     BioCarta Pathways:     KEGG Pathways:  
    Probe set : 1399161_a_at    Name:   type 1 tumor necrosis factor receptor shedding aminopeptidase regulator    GB acc:   AF148323    UniGene:   Rn.22516    Gene Symbol:   Arts1    Locus Link:   80897    Chromosome:   2    Cytoband:   Chr:2q11    SOURCE    GENECARD       Gene Ontology:   biological process:  regulation of transcription, DNA-dependent   biological process:  proteolysis   biological process:  membrane protein ectodomain proteolysis   biological process:  regulation of blood pressure   biological process:  antigen processing, endogenous antigen via MHC class I   biological process:  antigen processing   biological process:  regulation of innate immune response   biological process:  fat cell differentiation   biological process:  positive regulation of angiogenesis   biological process:  membrane protein ectodomain proteolysis   biological process:  regulation of blood pressure   biological process:  antigen processing, endogenous antigen via MHC class I   biological process:  regulation of innate immune response   biological process:  fat cell differentiation   molecular function:  DNA binding   molecular function:  transcription factor activity   molecular function:  aminopeptidase activity   molecular function:  leucyl aminopeptidase activity   molecular function:  leucyl aminopeptidase activity   molecular function:  membrane alanyl aminopeptidase activity   molecular function:  methionyl aminopeptidase activity   molecular function:  receptor activity   molecular function:  interleukin-6 receptor binding   molecular function:  tumor necrosis factor receptor binding   molecular function:  peptidase activity   molecular function:  metallopeptidase activity   molecular function:  zinc ion binding   molecular function:  zinc ion binding   molecular function:  hydrolase activity   molecular function:  aminopeptidase activity   molecular function:  leucyl aminopeptidase activity   molecular function:  methionyl aminopeptidase activity   molecular function:  interleukin-6 receptor binding   molecular function:  tumor necrosis factor receptor binding   molecular function:  zinc ion binding   molecular function:  metal ion binding      BioCarta Pathways:     KEGG Pathways:  
    Probe set : 1397611_at    Name:   LOC501124    GB acc:   AW525619    UniGene:   Rn.45049    Gene Symbol:   LOC501124    Locus Link:   501124    Chromosome:   9    Cytoband:   Chr:9q21    SOURCE    GENECARD       Gene Ontology:     BioCarta Pathways:     KEGG Pathways:  
    Probe set : 1373949_at    Name:   similar to RIKEN cDNA 1110012E06 (predicted)    GB acc:   AI070113    UniGene:   Rn.19659    Gene Symbol:   RGD1307977_predicted    Locus Link:   362645    Chromosome:   5    Cytoband:   Chr:5q36    SOURCE    GENECARD       Gene Ontology:     BioCarta Pathways:     KEGG Pathways:  
    Probe set : 1390508_at    Name:   Similar to hypothetical protein MGC27648 (predicted)    GB acc:   BI284393    UniGene:   Rn.1589     Locus Link:   312490    Chromosome:   4    Cytoband:   Chr:4q34    SOURCE        Gene Ontology:     BioCarta Pathways:     KEGG Pathways:  
    Probe set : 1379301_at    Name:   Transcribed locus    GB acc:   BM384812    UniGene:   Rn.28015        SOURCE        Gene Ontology:     BioCarta Pathways:     KEGG Pathways:  
    Probe set : 1370566_at    Name:   retinol dehydrogenase 2    GB acc:   U33500    UniGene:   Rn.94108    Gene Symbol:   Rdh2    Locus Link:   299511    Chromosome:   7    Cytoband:   Chr:7q22    SOURCE    GENECARD       Gene Ontology:   biological process:  retinol metabolism   biological process:  retinoic acid metabolism   biological process:  metabolism   cellular component:  endoplasmic reticulum   cellular component:  microsome   molecular function:  retinol dehydrogenase activity   molecular function:  retinol dehydrogenase activity   molecular function:  oxidoreductase activity      BioCarta Pathways:     KEGG Pathways:  
    Probe set : 1380009_at    Name:   vaccinia related kinase 1 (predicted)    GB acc:   BM392230    UniGene:   Rn.92083    Gene Symbol:   Vrk1_predicted    Locus Link:   362779    Chromosome:   6    Cytoband:   Chr:6q32    SOURCE    GENECARD       Gene Ontology:   biological process:  protein amino acid phosphorylation      BioCarta Pathways:     KEGG Pathways:  
    Probe set : 1392612_at    Name:   Prolactin receptor    GB acc:   AW142962    UniGene:   Rn.9757    Gene Symbol:   Prlr    Locus Link:   24684    Chromosome:   2    Cytoband:   Chr:2q16    SOURCE    GENECARD       Gene Ontology:   biological process:  steroid biosynthesis   biological process:  anti-apoptosis   biological process:  cell surface receptor linked signal transduction   biological process:  transmembrane receptor protein tyrosine kinase activation (dimerization)   biological process:  embryo implantation   biological process:  lactation   biological process:  regulation of cell adhesion   biological process:  regulation of epithelial cell differentiation   biological process:  T cell activation   biological process:  tyrosine phosphorylation of JAK2 protein   biological process:  steroid biosynthesis   biological process:  anti-apoptosis   biological process:  cell surface receptor linked signal transduction   biological process:  transmembrane receptor protein tyrosine kinase activation (dimerization)   biological process:  lactation   biological process:  T cell activation   biological process:  tyrosine phosphorylation of JAK2 protein      BioCarta Pathways:     KEGG Pathways:  
    Probe set : 1376051_at    Name:   crystallin, lamda 1    GB acc:   BI293393    UniGene:   Rn.57632    Gene Symbol:   Cryl1    Locus Link:   290277    Chromosome:   15    Cytoband:   Chr:15p12    SOURCE    GENECARD       Gene Ontology:   biological process:  lipid metabolism   biological process:  fatty acid metabolism   cellular component:  mitochondrion   molecular function:  oxidoreductase activity      BioCarta Pathways:     KEGG Pathways:  
    Probe set : 1396492_at    Name:   similar to NFAT activation molecule 1 (predicted)    GB acc:   BF399099    UniGene:   Rn.140126    Gene Symbol:   RGD1308316_predicted    Locus Link:   362966    Chromosome:   7    Cytoband:   Chr:7q34    SOURCE    GENECARD       Gene Ontology:     BioCarta Pathways:     KEGG Pathways:  
    Probe set : 1373841_at    Name:   Rac/cdc42 guanine nucleotide exchange factor 6    GB acc:   AI177431    UniGene:   Rn.3700    Gene Symbol:   arhgef6    Locus Link:   363509    Chromosome:   X    Cytoband:   Chr:Xq36    SOURCE    GENECARD       Gene Ontology:   molecular function:  guanyl-nucleotide exchange factor activity      BioCarta Pathways:     KEGG Pathways:  
    Probe set : 1392794_at    Name:   Transcribed locus    GB acc:   AA893579    UniGene:   Rn.148291        SOURCE        Gene Ontology:     BioCarta Pathways:     KEGG Pathways:  
    Probe set : 1367835_at    Name:   proprotein convertase subtilisin/kexin type 1 inhibitor    GB acc:   NM_019279    UniGene:   Rn.25745    Gene Symbol:   Pcsk1n    Locus Link:   246333    Chromosome:   X    Cytoband:   Chr:Xq13    SOURCE    GENECARD       Gene Ontology:   biological process:  peptide hormone processing   biological process:  peptide hormone processing      BioCarta Pathways:     KEGG Pathways:  
    Probe set : 1371329_at     GB acc:   BI283681              Gene Ontology:     BioCarta Pathways:     KEGG Pathways:  
    Probe set : 1369672_at    Name:   arachidonate 5-lipoxygenase activating protein    GB acc:   NM_017260    UniGene:   Rn.18399    Gene Symbol:   Alox5ap    Locus Link:   29624    Chromosome:   12    Cytoband:   Chr:12p11    SOURCE    GENECARD       Gene Ontology:   cellular component:  extracellular space   cellular component:  membrane fraction   cellular component:  integral to membrane   molecular function:  binding   molecular function:  enzyme activator activity   molecular function:  enzyme activator activity      BioCarta Pathways:     KEGG Pathways:  
    Probe set : 1383862_at    Name:   Similar to osteoclast inhibitory lectin    GB acc:   BF404050    UniGene:   Rn.95653     Locus Link:   362447    Chromosome:   4    Cytoband:   Chr:4q42    SOURCE        Gene Ontology:     BioCarta Pathways:     KEGG Pathways:  
    Probe set : 1383465_at    Name:   F-box and leucine-rich repeat protein 14 (predicted)    GB acc:   AI058747    UniGene:   Rn.18972    Gene Symbol:   Fbxl14_predicted    Locus Link:   312675    Chromosome:   4    Cytoband:   Chr:4q42    SOURCE    GENECARD       Gene Ontology:   biological process:  ubiquitin cycle      BioCarta Pathways:     KEGG Pathways:  
    Probe set : 1389363_at    Name:   aci-reductone dioxygenase (ARD)-like protein 1    GB acc:   AA997430    UniGene:   Rn.12694    Gene Symbol:   Alp1    Locus Link:   298934    Chromosome:   6    Cytoband:   Chr:6q16    SOURCE    GENECARD       Gene Ontology:   biological process:  methionine salvage   biological process:  amino acid biosynthesis   biological process:  methionine biosynthesis   cellular component:  nucleus   cellular component:  membrane      BioCarta Pathways:     KEGG Pathways:  
    Probe set : 1380537_at    Name:   hematopoietic protein 1 (predicted)    GB acc:   BI293796    UniGene:   Rn.19580    Gene Symbol:   Hem1_predicted    Locus Link:   315348    Chromosome:   7    Cytoband:   Chr:7q36    SOURCE    GENECARD       Gene Ontology:   cellular component:  membrane fraction   cellular component:  integral to plasma membrane      BioCarta Pathways:     KEGG Pathways:  
    Probe set : 1373803_a_at    Name:   growth hormone receptor    GB acc:   AI170771    UniGene:   Rn.2178    Gene Symbol:   Ghr    Locus Link:   25235    Chromosome:   2    Cytoband:   Chr:2q16    SOURCE    GENECARD       Gene Ontology:     BioCarta Pathways:     KEGG Pathways:  
    Probe set : 1377060_at    Name:   methylcrotonoyl-Coenzyme A carboxylase 2 (beta) (predicted)    GB acc:   AI410674    UniGene:   Rn.33635    Gene Symbol:   Mccc2_predicted    Locus Link:   361884    Chromosome:   2    Cytoband:   Chr:2q12    SOURCE    GENECARD       Gene Ontology:   cellular component:  mitochondrion   molecular function:  ligase activity      BioCarta Pathways:     KEGG Pathways:  
    Probe set : 1388176_at    Name:   camello-like 5    GB acc:   AI717047     Gene Symbol:   Cml5    Locus Link:   114020    Chromosome:   4    Cytoband:   Chr:4q34     GENECARD       Gene Ontology:   biological process:  negative regulation of cell adhesion   molecular function:  N-acetyltransferase activity   molecular function:  N-acetyltransferase activity   molecular function:  transferase activity      BioCarta Pathways:     KEGG Pathways:  
    Probe set : 1387932_at    Name:   solute carrier family 1 (neuronal/epithelial high affinity glutamate transporter, system Xag), member 1    GB acc:   U39555    UniGene:   Rn.6384    Gene Symbol:   Slc1a1    Locus Link:   25550    Chromosome:   1    Cytoband:   Chr:1q52    SOURCE    GENECARD       Gene Ontology:     BioCarta Pathways:     KEGG Pathways:  
    Probe set : 1383906_at    Name:   similar to lung inducible neuralized-related C3HC4 RING finger protein    GB acc:   BF416847    UniGene:   Rn.17894    Gene Symbol:   RGD:1359633    Locus Link:   316326    Chromosome:   9    Cytoband:   Chr:9q21    SOURCE    GENECARD       Gene Ontology:   biological process:  protein ubiquitination   cellular component:  ubiquitin ligase complex   molecular function:  ubiquitin-protein ligase activity   molecular function:  zinc ion binding   molecular function:  metal ion binding      BioCarta Pathways:     KEGG Pathways:  
    Probe set : 1389448_at    Name:   peanut-like 2 (Drosophila) (predicted)    GB acc:   AA800004    UniGene:   Rn.6269    Gene Symbol:   Pnutl2_predicted    Locus Link:   287606    Chromosome:   10    Cytoband:   Chr:10q26    SOURCE    GENECARD       Gene Ontology:   biological process:  cytokinesis   biological process:  cell cycle   biological process:  regulation of apoptosis   cellular component:  nucleus   cellular component:  mitochondrion      BioCarta Pathways:     KEGG Pathways:  
    Probe set : 1371575_at    Name:   Moesin    GB acc:   BF281185    UniGene:   Rn.2762    Gene Symbol:   Msn    Locus Link:   81521    Chromosome:   X    Cytoband:   Chr:Xq31    SOURCE    GENECARD       Gene Ontology:   biological process:  cell motility      BioCarta Pathways:     KEGG Pathways:  
    Probe set : 1379316_at    Name:   Transcribed locus    GB acc:   BG373832    UniGene:   Rn.146349        SOURCE        Gene Ontology:     BioCarta Pathways:     KEGG Pathways:  
    Probe set : 1370130_at    Name:   ras homolog gene family, member A    GB acc:   NM_057132    UniGene:   Rn.107401    Gene Symbol:   RGD:619921    Locus Link:   117273    Chromosome:   16    Cytoband:   Chr:16q12.5    SOURCE    GENECARD       Gene Ontology:   biological process:  cell adhesion   biological process:  cell-matrix adhesion   biological process:  integrin-mediated signaling pathway   biological process:  small GTPase mediated signal transduction   biological process:  Rho protein signal transduction   biological process:  striated muscle development   biological process:  protein transport   biological process:  actin cytoskeleton organization and biogenesis   biological process:  cell differentiation   biological process:  positive regulation of NF-kappaB import into nucleus   biological process:  positive regulation of I-kappaB kinase/NF-kappaB cascade   biological process:  stress fiber formation   biological process:  Rho protein signal transduction   biological process:  actin cytoskeleton organization and biogenesis   biological process:  positive regulation of NF-kappaB import into nucleus   biological process:  positive regulation of I-kappaB kinase/NF-kappaB cascade   cellular component:  cytosol   cellular component:  cytoskeleton   cellular component:  membrane      BioCarta Pathways:     KEGG Pathways:  
    Probe set : 1392939_at    Name:   CDNA clone MGC:124984 IMAGE:7134770    GB acc:   BM388933    UniGene:   Rn.137498        SOURCE        Gene Ontology:   biological process:  cation transport   molecular function:  cation transporter activity      BioCarta Pathways:     KEGG Pathways:  
    Probe set : 1367495_at    Name:   similar to prefoldin 4    GB acc:   AI412457     Gene Symbol:   LOC364186    Locus Link:   364186    Chromosome:   14    Cytoband:   Chr:14q21     GENECARD       Gene Ontology:     BioCarta Pathways:     KEGG Pathways:  
    Probe set : 1383654_a_at    Name:   similar to fructosamine-3-kinase    GB acc:   BE112523    UniGene:   Rn.14724    Gene Symbol:   Fnsk    Locus Link:   498034    Chromosome:   10    Cytoband:   Chr:10q32.3    SOURCE    GENECARD       Gene Ontology:     BioCarta Pathways:     KEGG Pathways:  
    Probe set : 1386865_at    Name:   SPARC-like 1 (mast9, hevin)    GB acc:   NM_012946    UniGene:   Rn.3091    Gene Symbol:   Sparcl1    Locus Link:   25434    Chromosome:   14    Cytoband:   Chr:14p22    SOURCE    GENECARD       Gene Ontology:   cellular component:  extracellular matrix (sensu Metazoa)   cellular component:  extracellular space   molecular function:  calcium ion binding   molecular function:  structural molecule activity      BioCarta Pathways:     KEGG Pathways:  
    Probe set : 1389425_at    Name:   similar to 5(3)-deoxyribonucleotidase    GB acc:   AI408674    UniGene:   Rn.12084    Gene Symbol:   LOC501746    Locus Link:   501746    Chromosome:   10    Cytoband:   Chr:10q32.3    SOURCE    GENECARD       Gene Ontology:     BioCarta Pathways:     KEGG Pathways:  
    Probe set : 1385382_at    Name:   Transcribed locus    GB acc:   BI294158    UniGene:   Rn.113277        SOURCE        Gene Ontology:     BioCarta Pathways:     KEGG Pathways:  
    Probe set : 1398647_at    Name:   Similar to hypothetical protein FLJ40283 (predicted)    GB acc:   BI288939    UniGene:   Rn.22645     Locus Link:   361275    Chromosome:   17    Cytoband:   Chr:17q12.3    SOURCE        Gene Ontology:     BioCarta Pathways:     KEGG Pathways:  
    Probe set : 1390835_at    Name:   similar to 1300013J15Rik protein (predicted)    GB acc:   AI013568    UniGene:   Rn.17240    Gene Symbol:   RGD1311123_predicted    Locus Link:   360539    Chromosome:   10    Cytoband:   Chr:10q22-q23    SOURCE    GENECARD       Gene Ontology:   biological process:  multidrug transport   cellular component:  integral to membrane   cellular component:  membrane   molecular function:  drug transporter activity   molecular function:  antiporter activity      BioCarta Pathways:     KEGG Pathways:  
    Probe set : 1389310_at    Name:   Transcribed locus    GB acc:   BF400694    UniGene:   Rn.7568        SOURCE        Gene Ontology:     BioCarta Pathways:     KEGG Pathways:  
    Probe set : 1398591_at    Name:   chemokine (C-C motif) receptor-like 2 (predicted)    GB acc:   AI707080    UniGene:   Rn.67232    Gene Symbol:   Ccrl2_predicted    Locus Link:   316019    Chromosome:   8    Cytoband:   Chr:8q32    SOURCE    GENECARD       Gene Ontology:   cellular component:  integral to plasma membrane   cellular component:  integral to membrane      BioCarta Pathways:     KEGG Pathways:  
    Probe set : 1382727_at     GB acc:   AI071140              Gene Ontology:     BioCarta Pathways:     KEGG Pathways:  
    Probe set : 1375961_at    Name:   frizzled-related protein (predicted)    GB acc:   AI072892    UniGene:   Rn.12034    Gene Symbol:   Frzb_predicted    Locus Link:   295691    Chromosome:   3    Cytoband:   Chr:3q24    SOURCE    GENECARD       Gene Ontology:   cellular component:  extracellular region   cellular component:  extracellular space   cellular component:  membrane   molecular function:  transmembrane receptor activity   molecular function:  Wnt-protein binding      BioCarta Pathways:     KEGG Pathways:  
    Probe set : 1368283_at    Name:   enoyl-Coenzyme A, hydratase/3-hydroxyacyl Coenzyme A dehydrogenase    GB acc:   NM_133606    UniGene:   Rn.3671    Gene Symbol:   Ehhadh    Locus Link:   171142    Chromosome:   11    Cytoband:   Chr:11q23    SOURCE    GENECARD       Gene Ontology:   cellular component:  mitochondrion   cellular component:  peroxisome      BioCarta Pathways:     KEGG Pathways:  
    Probe set : 1382490_at     GB acc:   BF284288              Gene Ontology:     BioCarta Pathways:     KEGG Pathways:  
    Probe set : 1373912_at    Name:   Transcribed locus, weakly similar to XP_488820.1 PREDICTED: hypothetical protein XP_488820 [Mus musculus]    GB acc:   AI170859    UniGene:   Rn.7802        SOURCE        Gene Ontology:     BioCarta Pathways:     KEGG Pathways:  
    Probe set : 1379598_at    Name:   Adenylate cyclase 7    GB acc:   AI071649    UniGene:   Rn.19450    Gene Symbol:   Adcy7    Locus Link:   84420      SOURCE    GENECARD       Gene Ontology:   cellular component:  plasma membrane   cellular component:  integral to plasma membrane   cellular component:  integral to membrane      BioCarta Pathways:     KEGG Pathways:  
    Probe set : 1389413_at    Name:   Oligodendrocyte-myelin glycoprotein    GB acc:   BI279325    UniGene:   Rn.11850    Gene Symbol:   Omg    Locus Link:   450224      SOURCE    GENECARD       Gene Ontology:   cellular component:  integral to membrane      BioCarta Pathways:     KEGG Pathways:  
    Probe set : 1375856_at    Name:   Transcribed locus    GB acc:   AI102258    UniGene:   Rn.7450        SOURCE        Gene Ontology:     BioCarta Pathways:     KEGG Pathways:  
    Probe set : 1374419_at    Name:   Adenylate cyclase 9 (predicted)    GB acc:   AI044435    UniGene:   Rn.18792     Locus Link:   302950    Chromosome:   10    Cytoband:   Chr:10q12    SOURCE        Gene Ontology:     BioCarta Pathways:     KEGG Pathways:  
    Probe set : 1382069_at    Name:   ATPase, class VI, type 11A (predicted)    GB acc:   AA946031    UniGene:   Rn.98540     Locus Link:   306600    Chromosome:   16    Cytoband:   Chr:16q12.5    SOURCE        Gene Ontology:   biological process:  cation transport   biological process:  metabolism   cellular component:  membrane   cellular component:  integral to membrane      BioCarta Pathways:     KEGG Pathways:  
    Probe set : 1376944_at    Name:   Prolactin receptor    GB acc:   AI407163    UniGene:   Rn.9757    Gene Symbol:   Prlr    Locus Link:   24684    Chromosome:   2    Cytoband:   Chr:2q16    SOURCE    GENECARD       Gene Ontology:   biological process:  steroid biosynthesis   biological process:  anti-apoptosis   biological process:  cell surface receptor linked signal transduction   biological process:  transmembrane receptor protein tyrosine kinase activation (dimerization)   biological process:  embryo implantation   biological process:  lactation   biological process:  regulation of cell adhesion   biological process:  regulation of epithelial cell differentiation   biological process:  T cell activation   biological process:  tyrosine phosphorylation of JAK2 protein   biological process:  steroid biosynthesis   biological process:  anti-apoptosis   biological process:  cell surface receptor linked signal transduction   biological process:  transmembrane receptor protein tyrosine kinase activation (dimerization)   biological process:  lactation   biological process:  T cell activation   biological process:  tyrosine phosphorylation of JAK2 protein      BioCarta Pathways:     KEGG Pathways:  
    Probe set : 1389548_at    Name:   alcohol dehydrogenase, iron containing, 1 (predicted)    GB acc:   BF288683    UniGene:   Rn.17173    Gene Symbol:   Adhfe1_predicted    Locus Link:   362474    Chromosome:   5    Cytoband:   Chr:5q11    SOURCE    GENECARD       Gene Ontology:   biological process:  metabolism   molecular function:  iron ion binding   molecular function:  oxidoreductase activity      BioCarta Pathways:     KEGG Pathways:  
    Probe set : 1368085_at    Name:   GTP cyclohydrolase I feedback regulator    GB acc:   NM_133595    UniGene:   Rn.11137    Gene Symbol:   Gchfr    Locus Link:   171128    Chromosome:   3    Cytoband:   Chr:3q35    SOURCE    GENECARD       Gene Ontology:   biological process:  negative regulation of GTP cyclohydrolase I activity   biological process:  regulation of nitric oxide biosynthesis   molecular function:  enzyme inhibitor activity   molecular function:  enzyme binding   molecular function:  GTP cyclohydrolase I binding      BioCarta Pathways:     KEGG Pathways:  
    Probe set : 1385213_at    Name:   Similar to epithelial stromal interaction 1 isoform a    GB acc:   AW524454    UniGene:   Rn.45852     Locus Link:   498547      SOURCE        Gene Ontology:     BioCarta Pathways:     KEGG Pathways:  
    Probe set : 1389140_at    Name:   similar to RIKEN cDNA 5730406I15 (predicted)    GB acc:   AI172437    UniGene:   Rn.7939    Gene Symbol:   RGD1311253_predicted    Locus Link:   293142    Chromosome:   1    Cytoband:   Chr:1q32    SOURCE    GENECARD       Gene Ontology:     BioCarta Pathways:     KEGG Pathways:  
    Probe set : 1394577_at    Name:   Transcribed locus    GB acc:   BI279384    UniGene:   Rn.146573        SOURCE        Gene Ontology:     BioCarta Pathways:     KEGG Pathways:  
    Probe set : 1390386_at    Name:   caspase 3    GB acc:   BM387008    UniGene:   Rn.10562    Gene Symbol:   Casp3    Locus Link:   25402    Chromosome:   16    Cytoband:   Chr:16q11    SOURCE    GENECARD       Gene Ontology:   biological process:  B cell homeostasis   biological process:  proteolysis   biological process:  apoptosis   biological process:  induction of apoptosis   biological process:  induction of apoptosis   biological process:  intracellular signaling cascade   biological process:  intracellular signaling cascade   biological process:  induction of apoptosis via death domain receptors   biological process:  induction of apoptosis by oxidative stress   biological process:  response to wounding   biological process:  neuron differentiation   biological process:  keratinocyte differentiation   biological process:  negative regulation of B cell proliferation   biological process:  T cell homeostasis   biological process:  cell fate commitment   biological process:  negative regulation of cyclin dependent protein kinase activity   biological process:  negative regulation of progression through cell cycle   biological process:  negative regulation of activated T cell proliferation   cellular component:  cytoplasm   cellular component:  cytosol      BioCarta Pathways:     KEGG Pathways:  
    Probe set : 1374857_at    Name:   similar to nucleolar protein family A, member 1    GB acc:   AI579023    UniGene:   Rn.15522    Gene Symbol:   LOC499709    Locus Link:   499709    Chromosome:   2    Cytoband:   Chr:2q43    SOURCE    GENECARD       Gene Ontology:   biological process:  rRNA processing   biological process:  ribosome biogenesis   cellular component:  nucleus   cellular component:  small nucleolar ribonucleoprotein complex   cellular component:  ribonucleoprotein complex   molecular function:  RNA binding   molecular function:  rRNA binding      BioCarta Pathways:     KEGG Pathways:  
    Probe set : 1371487_at    Name:   SH3 domain binding glutamic acid-rich protein-like 3 (predicted)    GB acc:   BG666075    UniGene:   Rn.18564    Gene Symbol:   Sh3bgrl3_predicted    Locus Link:   298544    Chromosome:   5    Cytoband:   Chr:5q36    SOURCE    GENECARD       Gene Ontology:   cellular component:  nucleus      BioCarta Pathways:     KEGG Pathways:  
    Probe set : 1370699_a_at    Name:   epidermal growth factor receptor /// peptidase D    GB acc:   AF187818    UniGene:   Rn.37227    Gene Symbol:   Egfr /// Pepd    Locus Link:   24329 /// 292808    Chromosome:   14    Cytoband:   Chr:14q22 /// Chr:1q21    SOURCE    GENECARD       Gene Ontology:   biological process:  regulation of progression through cell cycle   biological process:  cellular morphogenesis   biological process:  electron transport   biological process:  protein biosynthesis   biological process:  protein amino acid phosphorylation   biological process:  signal transduction   biological process:  signal transduction   biological process:  transmembrane receptor protein tyrosine kinase signaling pathway   biological process:  epidermal growth factor receptor signaling pathway   biological process:  epidermal growth factor receptor signaling pathway   biological process:  protein kinase cascade   biological process:  cell proliferation   biological process:  negative regulation of progression through cell cycle   biological process:  regulation of peptidyl-tyrosine phosphorylation   biological process:  cellular physiological process   biological process:  proteolysis   biological process:  collagen catabolism   molecular function:  protein kinase activity   molecular function:  protein serine/threonine kinase activity   molecular function:  protein-tyrosine kinase activity   molecular function:  transmembrane receptor protein tyrosine kinase activity   molecular function:  signal transducer activity   molecular function:  receptor activity   molecular function:  epidermal growth factor receptor activity   molecular function:  epidermal growth factor receptor activity   molecular function:  epidermal growth factor receptor activity   molecular function:  electron transporter activity   molecular function:  protein binding   molecular function:  ATP binding   molecular function:  kinase activity   molecular function:  transferase activity   molecular function:  X-Pro dipeptidase activity   molecular function:  peptidase activity   molecular function:  metalloexopeptidase activity   molecular function:  metallopeptidase activity   molecular function:  hydrolase activity   molecular function:  dipeptidase activity   molecular function:  manganese ion binding   molecular function:  iron ion binding      BioCarta Pathways:     KEGG Pathways:  
    Probe set : 1391948_at    Name:   B-cell leukemia/lymphoma 11B (predicted)    GB acc:   BM390227    UniGene:   Rn.52748    Gene Symbol:   Bcl11b_predicted    Locus Link:   314423    Chromosome:   6    Cytoband:   Chr:6q32    SOURCE    GENECARD       Gene Ontology:   biological process:  regulation of transcription, DNA-dependent   biological process:  negative regulation of cell proliferation   cellular component:  nucleus   molecular function:  nucleic acid binding   molecular function:  zinc ion binding      BioCarta Pathways:     KEGG Pathways:  
    Probe set : 1374959_at    Name:   NAD(P)H dehydrogenase, quinone 2    GB acc:   AA945624    UniGene:   Rn.7882    Gene Symbol:   RGD:1303320    Locus Link:   291084    Chromosome:   17    Cytoband:   Chr:17p12    SOURCE    GENECARD       Gene Ontology:   biological process:  myeloid cell differentiation   biological process:  electron transport      BioCarta Pathways:     KEGG Pathways:  
    Probe set : 1391040_at    Name:   similar to Retinoblastoma-binding protein 8 (RBBP-8) (CtBP interacting protein) (CtIP) (Retinoblastoma-interacting protein and myosin-like) (RIM) (predicted)    GB acc:   AA997052    UniGene:   Rn.128724    Gene Symbol:   RGD1308872_predicted    Locus Link:   291787    Chromosome:   18    Cytoband:   Chr:18p13    SOURCE    GENECARD       Gene Ontology:     BioCarta Pathways:     KEGG Pathways:  
    Probe set : 1394228_at    Name:   Transcribed locus    GB acc:   AA893147    UniGene:   Rn.13478        SOURCE        Gene Ontology:     BioCarta Pathways:     KEGG Pathways:  
    Probe set : 1382783_at    Name:   Bloom syndrome (predicted)    GB acc:   BF408187    UniGene:   Rn.61121    Gene Symbol:   Blm_predicted    Locus Link:   308755    Chromosome:   1    Cytoband:   Chr:1q31    SOURCE    GENECARD       Gene Ontology:   cellular component:  intracellular   cellular component:  nucleus      BioCarta Pathways:     KEGG Pathways:  
    Probe set : 1373185_at    Name:   signal sequence receptor, beta (predicted)    GB acc:   BE329347    UniGene:   Rn.17458    Gene Symbol:   Ssr2_predicted    Locus Link:   295235    Chromosome:   2    Cytoband:   Chr:2q34    SOURCE    GENECARD       Gene Ontology:   biological process:  cotranslational protein targeting to membrane   cellular component:  extracellular space   cellular component:  endoplasmic reticulum   cellular component:  integral to membrane   molecular function:  receptor activity   molecular function:  signal sequence binding      BioCarta Pathways:     KEGG Pathways:  
    Probe set : 1373772_at    Name:   DNA (cytosine-5-)-methyltransferase 1    GB acc:   AI179516    UniGene:   Rn.6955    Gene Symbol:   Dnmt1    Locus Link:   84350    Chromosome:   8    Cytoband:   Chr:8q13    SOURCE    GENECARD       Gene Ontology:   cellular component:  nucleus      BioCarta Pathways:     KEGG Pathways:  
    Probe set : 1379318_at    Name:   Transcribed locus    GB acc:   BI296812    UniGene:   Rn.146765        SOURCE        Gene Ontology:     BioCarta Pathways:     KEGG Pathways:  
    Probe set : 1367685_at    Name:   ribosomal protein S27a    GB acc:   NM_031113    UniGene:   Rn.106034    Gene Symbol:   Rps27a    Locus Link:   81777    Chromosome:   14    Cytoband:   Chr:14q22    SOURCE    GENECARD       Gene Ontology:   biological process:  protein biosynthesis   biological process:  protein modification   molecular function:  structural constituent of ribosome   molecular function:  zinc ion binding   molecular function:  metal ion binding      BioCarta Pathways:     KEGG Pathways:  
    Probe set : 1372587_at    Name:   endomucin    GB acc:   AI172271    UniGene:   Rn.8542    Gene Symbol:   Emcn    Locus Link:   295490    Chromosome:   2    Cytoband:   Chr:2q43    SOURCE    GENECARD       Gene Ontology:     BioCarta Pathways:     KEGG Pathways:  
    Probe set : 1375522_at    Name:   N-myristoyltransferase 1    GB acc:   AI010491    UniGene:   Rn.830    Gene Symbol:   Nmt1    Locus Link:   259274    Chromosome:   10    Cytoband:   Chr:10q32.1    SOURCE    GENECARD       Gene Ontology:   biological process:  N-terminal protein myristoylation   biological process:  N-terminal peptidyl-glycine N-myristoylation   cellular component:  cytoplasm      BioCarta Pathways:     KEGG Pathways:  
    Probe set : 1382181_at    Name:   similar to RIKEN cDNA A530088I07 gene    GB acc:   AI555069    UniGene:   Rn.17968    Gene Symbol:   LOC311984    Locus Link:   311984    Chromosome:   4    Cytoband:   Chr:4q11    SOURCE    GENECARD       Gene Ontology:     BioCarta Pathways:     KEGG Pathways:  
    Probe set : 1392145_at     GB acc:   BI281801              Gene Ontology:     BioCarta Pathways:     KEGG Pathways:  
    Probe set : 1367838_at    Name:   CTL target antigen    GB acc:   NM_017074    UniGene:   Rn.3881    Gene Symbol:   Cth    Locus Link:   24962    Chromosome:   2    Cytoband:   Chr:2q45    SOURCE    GENECARD       Gene Ontology:   biological process:  amino acid metabolism   biological process:  cysteine biosynthesis   biological process:  amino acid biosynthesis   molecular function:  cystathionine gamma-lyase activity   molecular function:  cystathionine gamma-lyase activity   molecular function:  lyase activity      BioCarta Pathways:     KEGG Pathways:  
    Probe set : 1373961_at    Name:   similar to 4930453N24Rik protein    GB acc:   AI170410    UniGene:   Rn.19101    Gene Symbol:   RGD:1359320    Locus Link:   304176    Chromosome:   11    Cytoband:   Chr:11p12    SOURCE    GENECARD       Gene Ontology:   biological process:  proteolysis   molecular function:  cysteine-type endopeptidase activity      BioCarta Pathways:     KEGG Pathways:  
    Probe set : 1393587_a_at     GB acc:   AI578061              Gene Ontology:     BioCarta Pathways:     KEGG Pathways:  
    Probe set : 1370072_at    Name:   membrane metallo endopeptidase    GB acc:   NM_012608    UniGene:   Rn.33598    Gene Symbol:   Mme    Locus Link:   24590    Chromosome:   2    Cytoband:   Chr:2q31    SOURCE    GENECARD       Gene Ontology:   biological process:  proteolysis   biological process:  cell-cell signaling   cellular component:  integral to plasma membrane   cellular component:  membrane   cellular component:  integral to membrane      BioCarta Pathways:     KEGG Pathways:  
    Probe set : 1388673_at    Name:   lymphocyte specific 1 (predicted)    GB acc:   AI012109    UniGene:   Rn.101608    Gene Symbol:   Lsp1_predicted    Locus Link:   361680    Chromosome:   1    Cytoband:   Chr:1q41    SOURCE    GENECARD       Gene Ontology:   cellular component:  actin cytoskeleton      BioCarta Pathways:     KEGG Pathways:  
    Probe set : 1393757_at    Name:   Similar to OTTHUMP00000018227    GB acc:   BE105343    UniGene:   Rn.113220     Locus Link:   498131      SOURCE        Gene Ontology:     BioCarta Pathways:     KEGG Pathways:  
    Probe set : 1377689_at    Name:   kinetochore associated 1 (predicted)    GB acc:   BF564273    UniGene:   Rn.47918    Gene Symbol:   Kntc1_predicted    Locus Link:   304477    Chromosome:   12    Cytoband:   Chr:12q15-q16    SOURCE    GENECARD       Gene Ontology:   cellular component:  spindle pole   cellular component:  nucleus   cellular component:  kinetochore microtubule   molecular function:  DNA binding   molecular function:  protein binding      BioCarta Pathways:     KEGG Pathways:  
    Probe set : 1372493_at    Name:   similar to RIKEN cDNA 2410005K17 (predicted)    GB acc:   AI102862    UniGene:   Rn.2954    Gene Symbol:   RGD1311232_predicted    Locus Link:   362578    Chromosome:   5    Cytoband:   Chr:5q36    SOURCE    GENECARD       Gene Ontology:     BioCarta Pathways:     KEGG Pathways:  
    Probe set : 1389858_at    Name:   Thymidine kinase 1    GB acc:   BM389006    UniGene:   Rn.4191    Gene Symbol:   Tk1    Locus Link:   24834    Chromosome:   10    Cytoband:   Chr:10    SOURCE    GENECARD       Gene Ontology:   biological process:  DNA metabolism   biological process:  DNA replication   cellular component:  cytoplasm      BioCarta Pathways:     KEGG Pathways:  
    Probe set : 1370030_at    Name:   glutamate cysteine ligase, modifier subunit    GB acc:   NM_017305    UniGene:   Rn.2460    Gene Symbol:   Gclm    Locus Link:   29739    Chromosome:   2    Cytoband:   Chr:2q41    SOURCE    GENECARD       Gene Ontology:   biological process:  cysteine metabolism   biological process:  glutathione biosynthesis      BioCarta Pathways:     KEGG Pathways:  
    Probe set : 1392037_at    Name:   Transcribed locus    GB acc:   BM383048    UniGene:   Rn.43297        SOURCE        Gene Ontology:     BioCarta Pathways:     KEGG Pathways:  
    Probe set : 1375267_at    Name:   peptidylprolyl isomerase C    GB acc:   BI291292    UniGene:   Rn.144176    Gene Symbol:   RGD:1303221    Locus Link:   291463    Chromosome:   18    Cytoband:   Chr:18q11    SOURCE    GENECARD       Gene Ontology:   biological process:  protein folding   molecular function:  peptidyl-prolyl cis-trans isomerase activity   molecular function:  isomerase activity      BioCarta Pathways:     KEGG Pathways:  
    Probe set : 1377737_at    Name:   Similar to hypothetical protein MGC5466 (predicted)    GB acc:   BE098874    UniGene:   Rn.2757     Locus Link:   308667    Chromosome:   1    Cytoband:   Chr:1q22    SOURCE        Gene Ontology:     BioCarta Pathways:     KEGG Pathways:  
    Probe set : 1372009_at    Name:   tyrosyl-tRNA synthetase (predicted)    GB acc:   AI407490    UniGene:   Rn.41002    Gene Symbol:   Yars_predicted    Locus Link:   313047    Chromosome:   5    Cytoband:   Chr:5q36    SOURCE    GENECARD       Gene Ontology:   cellular component:  extracellular space   cellular component:  soluble fraction   cellular component:  cytoplasm      BioCarta Pathways:     KEGG Pathways:  
    Probe set : 1385574_at    Name:   similar to cDNA sequence AF397014 (predicted)    GB acc:   AI501346    UniGene:   Rn.105406    Gene Symbol:   RGD1308114_predicted    Locus Link:   361253    Chromosome:   17    Cytoband:   Chr:17q12.1    SOURCE    GENECARD       Gene Ontology:   biological process:  metabolism   molecular function:  transferase activity      BioCarta Pathways:     KEGG Pathways:  
    Probe set : 1392777_at    Name:   Transcribed locus    GB acc:   BF287676    UniGene:   Rn.14830        SOURCE        Gene Ontology:     BioCarta Pathways:     KEGG Pathways:  
    Probe set : 1390815_at    Name:   Cyclin M1 (predicted)    GB acc:   BF282870    UniGene:   Rn.87733     Locus Link:   309387    Chromosome:   1    Cytoband:   Chr:1q54    SOURCE        Gene Ontology:   biological process:  fatty acid biosynthesis   cellular component:  plasma membrane      BioCarta Pathways:     KEGG Pathways:  
    Probe set : 1372158_at    Name:   LRP16 protein    GB acc:   BI295768    UniGene:   Rn.98178    Gene Symbol:   Lrp16    Locus Link:   246233    Chromosome:   1    Cytoband:   Chr:1q43    SOURCE    GENECARD       Gene Ontology:   cellular component:  mitochondrion      BioCarta Pathways:     KEGG Pathways:  
    Probe set : 1387263_at    Name:   pyruvate kinase, liver and RBC    GB acc:   NM_012624    UniGene:   Rn.48821    Gene Symbol:   Pklr    Locus Link:   24651    Chromosome:   2    Cytoband:   Chr:2q34    SOURCE    GENECARD       Gene Ontology:   biological process:  glycolysis   biological process:  response to pathogen      BioCarta Pathways:     KEGG Pathways:  
    Probe set : 1371509_at    Name:   transforming growth factor beta regulated gene 1 (predicted)    GB acc:   BG666773    UniGene:   Rn.129282    Gene Symbol:   Tbrg1_predicted    Locus Link:   300521    Chromosome:   8    Cytoband:   Chr:8q21    SOURCE    GENECARD       Gene Ontology:   cellular component:  nucleus   molecular function:  DNA binding      BioCarta Pathways:     KEGG Pathways:  
    Probe set : 1368045_at    Name:   solute carrier family 31 (copper transporters), member 1    GB acc:   NM_133600    UniGene:   Rn.2789    Gene Symbol:   RGD:620059    Locus Link:   171135    Chromosome:   5    Cytoband:   Chr:5q24    SOURCE    GENECARD       Gene Ontology:   biological process:  transport   biological process:  copper ion transport   biological process:  ion transport   cellular component:  integral to plasma membrane   cellular component:  integral to membrane   molecular function:  copper ion transporter activity   molecular function:  carrier activity   molecular function:  copper ion binding      BioCarta Pathways:     KEGG Pathways:  
    Probe set : 1371712_at    Name:   similar to snRNP core protein SMX5d    GB acc:   AI176503     Gene Symbol:   LOC292030    Locus Link:   292030    Chromosome:   19    Cytoband:   Chr:19q12     GENECARD       Gene Ontology:     BioCarta Pathways:     KEGG Pathways:  
    Probe set : 1376055_at    Name:   Transcribed locus    GB acc:   AA859768    UniGene:   Rn.123        SOURCE        Gene Ontology:     BioCarta Pathways:     KEGG Pathways:  
    Probe set : 1395574_at    Name:   ROD1 regulator of differentiation 1 (S. pombe)    GB acc:   BE118358    UniGene:   Rn.51103    Gene Symbol:   Rod1    Locus Link:   83515    Chromosome:   5    Cytoband:   Chr:5q24    SOURCE    GENECARD       Gene Ontology:   cellular component:  nucleus      BioCarta Pathways:     KEGG Pathways:  
    Probe set : 1371973_at    Name:   eukaryotic translation initiation factor 3, subunit 6 (predicted)    GB acc:   AI237620    UniGene:   Rn.98547    Gene Symbol:   Eif3s6_predicted    Locus Link:   299872    Chromosome:   7    Cytoband:   Chr:7q31    SOURCE    GENECARD       Gene Ontology:   biological process:  protein biosynthesis   biological process:  regulation of translational initiation   biological process:  cellular physiological process   cellular component:  eukaryotic translation initiation factor 3 complex   molecular function:  translation initiation factor activity      BioCarta Pathways:     KEGG Pathways:  
    Probe set : 1398482_at    Name:   B-cell leukemia/lymphoma 3 (predicted)    GB acc:   AI411774    UniGene:   Rn.68084    Gene Symbol:   Bcl3_predicted    Locus Link:   292700    Chromosome:   1    Cytoband:   Chr:1q21    SOURCE    GENECARD       Gene Ontology:   cellular component:  nucleus   molecular function:  DNA binding   molecular function:  transcription factor activity      BioCarta Pathways:     KEGG Pathways:  
    Probe set : 1390692_at    Name:   cytidine 5'-triphosphate synthase (predicted)    GB acc:   BE105721    UniGene:   Rn.46765    Gene Symbol:   Ctps_predicted    Locus Link:   313560    Chromosome:   5    Cytoband:   Chr:5q36    SOURCE    GENECARD       Gene Ontology:   molecular function:  catalytic activity   molecular function:  CTP synthase activity   molecular function:  ligase activity      BioCarta Pathways:     KEGG Pathways:  
    Probe set : 1374156_at    Name:   membrane protein, palmitoylated 5 (MAGUK p55 subfamily member 5) (predicted)    GB acc:   BE108018    UniGene:   Rn.8181    Gene Symbol:   Mpp5_predicted    Locus Link:   314259    Chromosome:   6    Cytoband:   Chr:6q24    SOURCE    GENECARD       Gene Ontology:   cellular component:  plasma membrane   cellular component:  membrane   cellular component:  integral to membrane   molecular function:  guanylate kinase activity   molecular function:  protein binding      BioCarta Pathways:     KEGG Pathways:  
    Probe set : 1368973_at    Name:   adenosine deaminase, RNA-specific    GB acc:   NM_031006    UniGene:   Rn.10056    Gene Symbol:   Adar    Locus Link:   81635    Chromosome:   2    Cytoband:   Chr:2q34    SOURCE    GENECARD       Gene Ontology:   cellular component:  intracellular   cellular component:  nucleus   cellular component:  nucleus      BioCarta Pathways:     KEGG Pathways:  
    Probe set : 1370936_at    Name:   dimethylglycine dehydrogenase precursor    GB acc:   AA892345    UniGene:   Rn.3646    Gene Symbol:   Dmgdh    Locus Link:   245961    Chromosome:   2    Cytoband:   Chr:2q12    SOURCE    GENECARD       Gene Ontology:   cellular component:  mitochondrion   cellular component:  mitochondrion   cellular component:  cytoplasm      BioCarta Pathways:     KEGG Pathways:  
    Probe set : 1369930_at     GB acc:   NM_017283              Gene Ontology:     BioCarta Pathways:     KEGG Pathways:  
    Probe set : 1372260_at    Name:   leucine zipper domain protein    GB acc:   BI296304    UniGene:   Rn.995    Gene Symbol:   MGC116147    Locus Link:   287061    Chromosome:   10    Cytoband:   Chr:10q12    SOURCE    GENECARD       Gene Ontology:     BioCarta Pathways:     KEGG Pathways:  
    Probe set : 1387839_at    Name:   RT1 class Ib gene, H2-TL-like, grc region (N1) /// RT1 class Ib gene, H2-TL-like, grc region (N3) /// RT1 class Ib gene, H2-TL-like, grc region(N2)    GB acc:   NM_012646    UniGene:   Rn.107070    Gene Symbol:   RT1-N1 /// RT1-N3 /// RT1-N2    Locus Link:   24748 /// 24750 /// 360323    Chromosome:   20    Cytoband:   Chr:20p12    SOURCE    GENECARD       Gene Ontology:   biological process:  antigen presentation   cellular component:  MHC class I protein complex   cellular component:  MHC class I protein complex   molecular function:  MHC class I receptor activity      BioCarta Pathways:     KEGG Pathways:  
    Probe set : 1372117_at    Name:   similar to BB128963 protein (predicted)    GB acc:   BE329026    UniGene:   Rn.3510    Gene Symbol:   RGD1305958_predicted    Locus Link:   309255    Chromosome:   1    Cytoband:   Chr:1q22    SOURCE    GENECARD       Gene Ontology:     BioCarta Pathways:     KEGG Pathways:  
    Probe set : 1398751_at    Name:   similar to ribosomal protein S7    GB acc:   NM_031570     Gene Symbol:   LOC497813    Locus Link:   497813    Chromosome:   6    Cytoband:   Chr:6q16     GENECARD       Gene Ontology:     BioCarta Pathways:     KEGG Pathways:  
    Probe set : 1373053_at     GB acc:   AI169620              Gene Ontology:     BioCarta Pathways:     KEGG Pathways:  
    Probe set : 1369421_at    Name:   topoisomerase (DNA) I    GB acc:   NM_022615    UniGene:   Rn.91572    Gene Symbol:   Top1    Locus Link:   64550    Chromosome:   3    Cytoband:   Chr:3q42    SOURCE    GENECARD       Gene Ontology:     BioCarta Pathways:     KEGG Pathways:  
    Probe set : 1371965_at    Name:   similar to RIKEN cDNA 2010311D03    GB acc:   BF283269    UniGene:   Rn.3269    Gene Symbol:   RGD:1303272    Locus Link:   362134    Chromosome:   3    Cytoband:   Chr:3q12    SOURCE    GENECARD       Gene Ontology:   cellular component:  mitochondrion      BioCarta Pathways:     KEGG Pathways:  
    Probe set : 1368266_at    Name:   arginase 1    GB acc:   NM_017134    UniGene:   Rn.9857    Gene Symbol:   Arg1    Locus Link:   29221    Chromosome:   1    Cytoband:   Chr:1p12    SOURCE    GENECARD       Gene Ontology:     BioCarta Pathways:     KEGG Pathways:  
    Probe set : 1383015_at    Name:   Similar to DKFZP434P1750 protein (predicted)    GB acc:   BF406973    UniGene:   Rn.61004     Locus Link:   361697    Chromosome:   1    Cytoband:   Chr:1q42    SOURCE        Gene Ontology:     BioCarta Pathways:     KEGG Pathways:  
    Probe set : 1372748_at    Name:   similar to KIAA1055 protein (predicted)    GB acc:   BE113335     Gene Symbol:   RGD1307436_predicted    Locus Link:   315880    Chromosome:   8    Cytoband:   Chr:8q31     GENECARD       Gene Ontology:     BioCarta Pathways:     KEGG Pathways:  
    Probe set : 1375523_at    Name:   Similar to Myristoylated alanine-rich C-kinase substrate (MARCKS) (ACAMP-81)    GB acc:   BE108178    UniGene:   Rn.9560    Gene Symbol:   Marcks    Locus Link:   25603    Chromosome:   6    Cytoband:   Chr:6    SOURCE    GENECARD       Gene Ontology:   biological process:  cell motility   cellular component:  plasma membrane   cellular component:  actin cytoskeleton   cellular component:  membrane      BioCarta Pathways:     KEGG Pathways:  
    Probe set : 1369318_at    Name:   fragile histidine triad gene    GB acc:   NM_021774    UniGene:   Rn.145980    Gene Symbol:   Fhit    Locus Link:   60398    Chromosome:   15    Cytoband:   Chr:15p15-p14    SOURCE    GENECARD       Gene Ontology:   biological process:  DNA replication and chromosome cycle   molecular function:  magnesium ion binding   molecular function:  hydrolase activity   molecular function:  manganese ion binding      BioCarta Pathways:     KEGG Pathways:  
    Probe set : 1383655_at    Name:   TNFAIP3 interacting protein 2 (predicted)    GB acc:   BF545192    UniGene:   Rn.17607    Gene Symbol:   Tnip2_predicted    Locus Link:   305451    Chromosome:   14    Cytoband:   Chr:14q21    SOURCE    GENECARD       Gene Ontology:   biological process:  I-kappaB kinase/NF-kappaB cascade   cellular component:  cytoplasm   molecular function:  protein binding      BioCarta Pathways:     KEGG Pathways:  
    Probe set : 1389469_at    Name:   chromodomain helicase DNA binding protein 1-like (predicted)    GB acc:   AA858645    UniGene:   Rn.1478    Gene Symbol:   Chd1l_predicted    Locus Link:   310707    Chromosome:   2    Cytoband:   Chr:2q34    SOURCE    GENECARD       Gene Ontology:   cellular component:  nucleus      BioCarta Pathways:     KEGG Pathways:  
    Probe set : 1374938_at    Name:   ZW10 homolog, centromere/kinetochore protein (Drosophila) (predicted)    GB acc:   BI295067    UniGene:   Rn.25242    Gene Symbol:   Zw10_predicted    Locus Link:   363059    Chromosome:   8    Cytoband:   Chr:8q23    SOURCE    GENECARD       Gene Ontology:   molecular function:  protein binding   molecular function:  centromeric DNA binding      BioCarta Pathways:     KEGG Pathways:  
    Probe set : 1367687_a_at    Name:   peptidylglycine alpha-amidating monooxygenase    GB acc:   M25719    UniGene:   Rn.1121    Gene Symbol:   Pam    Locus Link:   25508    Chromosome:   9    Cytoband:   Chr:9q36    SOURCE    GENECARD       Gene Ontology:   biological process:  peptide amidation   biological process:  protein modification   biological process:  peptide metabolism      BioCarta Pathways:     KEGG Pathways:  
    Probe set : 1388992_at    Name:   E1A binding protein p400 (predicted)    GB acc:   AI169305    UniGene:   Rn.76053    Gene Symbol:   Ep400_predicted    Locus Link:   304569    Chromosome:   12    Cytoband:   Chr:12q16    SOURCE    GENECARD       Gene Ontology:   biological process:  chromatin modification   cellular component:  nucleus      BioCarta Pathways:     KEGG Pathways:  
    Probe set : 1388930_at    Name:   similar to RIKEN cDNA 2310075C12 (predicted)    GB acc:   AI171651    UniGene:   Rn.12767    Gene Symbol:   RGD1305625_predicted    Locus Link:   363013    Chromosome:   8    Cytoband:   Chr:8q11    SOURCE    GENECARD       Gene Ontology:   cellular component:  extracellular space   cellular component:  integral to membrane   molecular function:  receptor activity      BioCarta Pathways:     KEGG Pathways:  
    Probe set : 1368199_at    Name:   nucleoporin 88    GB acc:   NM_053616    UniGene:   Rn.11042    Gene Symbol:   Nup88    Locus Link:   113929    Chromosome:   10    Cytoband:   Chr:10q24    SOURCE    GENECARD       Gene Ontology:   biological process:  transport   biological process:  protein transport   cellular component:  nucleus   cellular component:  nuclear pore   cellular component:  nuclear pore   molecular function:  transporter activity      BioCarta Pathways:     KEGG Pathways:  
    Probe set : 1373889_at    Name:   similar to immunoglobulin superfamily, member 7    GB acc:   BF387360    UniGene:   Rn.145433    Gene Symbol:   LOC498022    Locus Link:   498022    Chromosome:   10    Cytoband:   Chr:10q32.2    SOURCE    GENECARD       Gene Ontology:     BioCarta Pathways:     KEGG Pathways:  
    Probe set : 1381973_at    Name:   solute carrier family 25, member 30    GB acc:   AW530647    UniGene:   Rn.19440    Gene Symbol:   RGD:1359702    Locus Link:   361074    Chromosome:   15    Cytoband:   Chr:15q11    SOURCE    GENECARD       Gene Ontology:   biological process:  transport   biological process:  mitochondrial transport   molecular function:  transporter activity   molecular function:  binding      BioCarta Pathways:     KEGG Pathways:  
    Probe set : 1391409_at    Name:   amnionless (predicted)    GB acc:   BI276097    UniGene:   Rn.7787    Gene Symbol:   Amn_predicted    Locus Link:   314459    Chromosome:   6    Cytoband:   Chr:6q32    SOURCE    GENECARD       Gene Ontology:   biological process:  development   cellular component:  extracellular space   cellular component:  integral to membrane      BioCarta Pathways:     KEGG Pathways:  
    Probe set : 1384628_at    Name:   similar to RIKEN cDNA 0610009A07 (predicted)    GB acc:   AI549292    UniGene:   Rn.34836    Gene Symbol:   RGD1309288_predicted    Locus Link:   308129    Chromosome:   1    Cytoband:   Chr:1p11    SOURCE    GENECARD       Gene Ontology:   biological process:  electron transport   molecular function:  oxidoreductase activity      BioCarta Pathways:     KEGG Pathways:  
    Probe set : 1383369_at    Name:   tripartite motif protein 26    GB acc:   AI028875    UniGene:   Rn.38919    Gene Symbol:   RGD:1359126    Locus Link:   309586    Chromosome:   20    Cytoband:   Chr:20p12    SOURCE    GENECARD       Gene Ontology:     BioCarta Pathways:     KEGG Pathways:  
    Probe set : 1368834_at    Name:   calcium/calmodulin-dependent protein kinase II, delta    GB acc:   AA894330    UniGene:   Rn.87208    Gene Symbol:   Camk2d    Locus Link:   24246    Chromosome:   2    Cytoband:   Chr:2q42-q43    SOURCE    GENECARD       Gene Ontology:   cellular component:  calcium- and calmodulin-dependent protein kinase complex      BioCarta Pathways:     KEGG Pathways:  
    Probe set : 1371807_at    Name:   similar to apoptosis related protein APR-3; p18 protein (predicted)    GB acc:   BE110607    UniGene:   Rn.3206    Gene Symbol:   RGD1311605_predicted    Locus Link:   298841    Chromosome:   6    Cytoband:   Chr:6q14    SOURCE    GENECARD       Gene Ontology:     BioCarta Pathways:     KEGG Pathways:  
    Probe set : 1371505_at    Name:   heterogeneous nuclear ribonucleoprotein C (predicted)    GB acc:   BG381750    UniGene:   Rn.101929    Gene Symbol:   Hnrpc_predicted    Locus Link:   290046    Chromosome:   15    Cytoband:   Chr:15p14    SOURCE    GENECARD       Gene Ontology:   biological process:  nuclear mRNA splicing, via spliceosome   biological process:  mRNA processing   biological process:  RNA splicing   molecular function:  RNA binding   molecular function:  nucleotide binding   molecular function:  nucleic acid binding      BioCarta Pathways:     KEGG Pathways:  
    Probe set : 1375146_at    Name:   similar to RIKEN cDNA 3010027G13 (predicted)    GB acc:   BI282168    UniGene:   Rn.55537    Gene Symbol:   RGD1306193_predicted    Locus Link:   360636    Chromosome:   10    Cytoband:   Chr:10q32.1    SOURCE    GENECARD       Gene Ontology:   biological process:  transport   cellular component:  membrane   cellular component:  integral to membrane   molecular function:  binding      BioCarta Pathways:     KEGG Pathways:  
    Probe set : 1374393_at    Name:   protein tyrosine phosphatase-like (proline instead of catalytic arginine), member b (predicted)    GB acc:   AI169080    UniGene:   Rn.108344    Gene Symbol:   Ptplb_predicted    Locus Link:   288058    Chromosome:   11    Cytoband:   Chr:11q22    SOURCE    GENECARD       Gene Ontology:   biological process:  protein amino acid dephosphorylation   cellular component:  integral to membrane   molecular function:  phosphoprotein phosphatase activity      BioCarta Pathways:     KEGG Pathways:  
    Probe set : 1383625_a_at    Name:   similar to zinc finger protein    GB acc:   BF288164    UniGene:   Rn.102011    Gene Symbol:   LOC500989    Locus Link:   500989    Chromosome:   8    Cytoband:   Chr:8q23    SOURCE    GENECARD       Gene Ontology:     BioCarta Pathways:     KEGG Pathways:  
    Probe set : 1388114_at    Name:   myosin light chain, regulatory B    GB acc:   X05566    UniGene:   Rn.103179    Gene Symbol:   Mrlcb    Locus Link:   50685    Chromosome:   9    Cytoband:   Chr:9q38    SOURCE    GENECARD       Gene Ontology:   biological process:  cytoskeleton organization and biogenesis   biological process:  muscle development   cellular component:  cytoskeleton   cellular component:  apical part of cell   cellular component:  myosin   molecular function:  motor activity   molecular function:  calcium ion binding      BioCarta Pathways:     KEGG Pathways:  
    Probe set : 1374505_at    Name:   similar to growth hormone-inducible soluble protein    GB acc:   AI411031    UniGene:   Rn.23953    Gene Symbol:   LOC502922    Locus Link:   502922    Chromosome:   4    Cytoband:   Chr:4q44    SOURCE    GENECARD       Gene Ontology:     BioCarta Pathways:     KEGG Pathways:  
    Probe set : 1370790_at    Name:   interferon gamma    GB acc:   AF010466    UniGene:   Rn.10795    Gene Symbol:   Ifng    Locus Link:   25712    Chromosome:   7    Cytoband:   Chr:7q22    SOURCE    GENECARD       Gene Ontology:   biological process:  regulation of cell growth   biological process:  programmed cell death, neutrophils   biological process:  programmed cell death, inflammatory cells   biological process:  cell motility   biological process:  defense response   biological process:  immune response   biological process:  cell surface receptor linked signal transduction   biological process:  cell-cell signaling   biological process:  negative regulation of cell proliferation   biological process:  neutrophil chemotaxis   biological process:  defense response to pathogenic bacteria   biological process:  positive regulation of chemokine biosynthesis   biological process:  positive regulation of interleukin-12 biosynthesis   biological process:  positive regulation of MHC class II biosynthesis   biological process:  positive regulation of interleukin-6 biosynthesis   biological process:  regulation of transcription   biological process:  positive regulation of transcription, DNA-dependent   biological process:  positive regulation of isotype switching to IgG isotypes   biological process:  positive regulation of interleukin-1 beta secretion   biological process:  regulation of immune response   biological process:  response to virus   cellular component:  extracellular region   cellular component:  extracellular region   cellular component:  extracellular space      BioCarta Pathways:     KEGG Pathways:  
    Probe set : 1395167_at    Name:   LOC362424 (predicted)    GB acc:   BG672109    UniGene:   Rn.60747    Gene Symbol:   RGD1308850_predicted    Locus Link:   362424    Chromosome:   4    Cytoband:   Chr:4q42    SOURCE    GENECARD       Gene Ontology:     BioCarta Pathways:     KEGG Pathways:  
    Probe set : 1375297_at    Name:   similar to RIKEN cDNA 0610008C08    GB acc:   AI168968    UniGene:   Rn.2579    Gene Symbol:   LOC363474    Locus Link:   363474    Chromosome:   X    Cytoband:   Chr:Xq31    SOURCE    GENECARD       Gene Ontology:     BioCarta Pathways:     KEGG Pathways:  
    Probe set : 1389446_at    Name:   small nuclear ribonucleoprotein polypeptide A' (predicted)    GB acc:   AI576586    UniGene:   Rn.38265    Gene Symbol:   Snrpa1_predicted    Locus Link:   292997    Chromosome:   1    Cytoband:   Chr:1q22    SOURCE    GENECARD       Gene Ontology:   biological process:  RNA splicing   cellular component:  nucleus   cellular component:  snRNP U2   cellular component:  ribonucleoprotein complex   molecular function:  RNA binding      BioCarta Pathways:     KEGG Pathways:  
    Probe set : 1386915_at    Name:   acidic nuclear phosphoprotein 32 family, member B    GB acc:   NM_131911    UniGene:   Rn.20465    Gene Symbol:   Anp32b    Locus Link:   170724    Chromosome:   5    Cytoband:   Chr:5q22    SOURCE    GENECARD       Gene Ontology:   biological process:  S phase of mitotic cell cycle   biological process:  S phase of mitotic cell cycle   biological process:  regulation of cell proliferation   cellular component:  nucleus      BioCarta Pathways:     KEGG Pathways:  
    Probe set : 1377756_at    Name:   similar to RIKEN cDNA 5830446M03 (predicted)    GB acc:   AI232228    UniGene:   Rn.145210    Gene Symbol:   RGD1307688_predicted    Locus Link:   362394    Chromosome:   4    Cytoband:   Chr:4q34    SOURCE    GENECARD       Gene Ontology:     BioCarta Pathways:     KEGG Pathways:  
    Probe set : 1390687_at    Name:   pleckstrin (predicted)    GB acc:   AI556803    UniGene:   Rn.6178    Gene Symbol:   Plek_predicted    Locus Link:   364206    Chromosome:   14    Cytoband:   Chr:14q22    SOURCE    GENECARD       Gene Ontology:   biological process:  intracellular signaling cascade   molecular function:  calcium ion binding      BioCarta Pathways:     KEGG Pathways:  
    Probe set : 1382601_at    Name:   Similar to scavenger receptor type A SR-A    GB acc:   AI237698    UniGene:   Rn.25444     Locus Link:   498638      SOURCE        Gene Ontology:     BioCarta Pathways:     KEGG Pathways:  
    Probe set : 1376605_at    Name:   solute carrier family 17 (anion/sugar transporter), member 5 (predicted)    GB acc:   AA900983    UniGene:   Rn.74591    Gene Symbol:   Slc17a5_predicted    Locus Link:   363103    Chromosome:   8    Cytoband:   Chr:8q31    SOURCE    GENECARD       Gene Ontology:   biological process:  transport   biological process:  sialic acid transport      BioCarta Pathways:     KEGG Pathways:  
    Probe set : 1386989_at    Name:   endothelial differentiation, sphingolipid G-protein-coupled receptor, 5    GB acc:   NM_017192    UniGene:   Rn.2491    Gene Symbol:   Edg5    Locus Link:   29415    Chromosome:   8    Cytoband:   Chr:8q13    SOURCE    GENECARD       Gene Ontology:   biological process:  G-protein coupled receptor protein signaling pathway   biological process:  behavior   biological process:  signal transduction   cellular component:  integral to plasma membrane   cellular component:  integral to membrane      BioCarta Pathways:     KEGG Pathways:  
    Probe set : 1371016_at    Name:   similar to T-cell receptor alpha chain precursor V and C regions (TRA29) - rat (fragment)    GB acc:   BM386010    UniGene:   Rn.9949    Gene Symbol:   RGD:1359684    Locus Link:   364379    Chromosome:   15    Cytoband:   Chr:15p13    SOURCE    GENECARD       Gene Ontology:     BioCarta Pathways:     KEGG Pathways:  
    Probe set : 1374137_at    Name:   E74-like factor 1    GB acc:   AA818055    UniGene:   Rn.98640    Gene Symbol:   Elf1    Locus Link:   85424    Chromosome:   15    Cytoband:   Chr:15q12    SOURCE    GENECARD       Gene Ontology:   cellular component:  nucleus      BioCarta Pathways:     KEGG Pathways:  
    Probe set : 1374449_at    Name:   cell division cycle associated 3 /// similar to cell division cycle associated 3    GB acc:   BF417638    UniGene:   Rn.3246    Gene Symbol:   RGD:1359093 /// LOC501134    Locus Link:   297594 /// 501134    Chromosome:   4    Cytoband:   Chr:4q42 /// Chr:9q22    SOURCE    GENECARD       Gene Ontology:   biological process:  cell division      BioCarta Pathways:     KEGG Pathways:  
    Probe set : 1371908_at    Name:   NTF2-related export protein 1 (predicted)    GB acc:   AA891920    UniGene:   Rn.8541    Gene Symbol:   Nxt1_predicted    Locus Link:   296219    Chromosome:   3    Cytoband:   Chr:3q41    SOURCE    GENECARD       Gene Ontology:   cellular component:  intracellular   cellular component:  nucleus   cellular component:  nuclear pore   cellular component:  cytoplasm   molecular function:  protein binding   molecular function:  Ran GTPase binding   molecular function:  protein transporter activity      BioCarta Pathways:     KEGG Pathways:  
    Probe set : 1390839_at    Name:   similar to RIKEN cDNA E030024M05 (predicted)    GB acc:   AW915795    UniGene:   Rn.12743    Gene Symbol:   RGD1306818_predicted    Locus Link:   298906    Chromosome:   6    Cytoband:   Chr:6q15    SOURCE    GENECARD       Gene Ontology:     BioCarta Pathways:     KEGG Pathways:  
    Probe set : 1398970_at    Name:   Translocation protein 1 (predicted)    GB acc:   BI303004    UniGene:   Rn.98327     Locus Link:   294912    Chromosome:   2    Cytoband:   Chr:2q24    SOURCE        Gene Ontology:   biological process:  cotranslational protein targeting to membrane   biological process:  protein transport   cellular component:  endoplasmic reticulum   cellular component:  integral to membrane   molecular function:  receptor activity   molecular function:  protein transporter activity      BioCarta Pathways:     KEGG Pathways:  
    Probe set : 1389668_at    Name:   similar to AD024 protein (predicted)    GB acc:   AI408269    UniGene:   Rn.16268    Gene Symbol:   RGD1307811_predicted    Locus Link:   295661    Chromosome:   3    Cytoband:   Chr:3q21    SOURCE    GENECARD       Gene Ontology:     BioCarta Pathways:     KEGG Pathways:  
    Probe set : 1371256_at    Name:   protein tyrosine phosphatase, non-receptor type 18 (predicted)    GB acc:   BM386565    UniGene:   Rn.138086    Gene Symbol:   Ptpn18_predicted    Locus Link:   301333    Chromosome:   9    Cytoband:   Chr:9q21    SOURCE    GENECARD       Gene Ontology:   biological process:  protein amino acid dephosphorylation   cellular component:  nucleus   cellular component:  cytoplasm      BioCarta Pathways:     KEGG Pathways:  
    Probe set : 1393777_at    Name:   Transcribed locus    GB acc:   AA998104    UniGene:   Rn.92969        SOURCE        Gene Ontology:     BioCarta Pathways:     KEGG Pathways:  
    Probe set : 1370282_at    Name:   cysteine and glycine-rich protein 2    GB acc:   U44948    UniGene:   Rn.94754    Gene Symbol:   RGD:61950    Locus Link:   29317    Chromosome:   7    Cytoband:   Chr:7q13    SOURCE    GENECARD       Gene Ontology:   biological process:  development   biological process:  cell differentiation   biological process:  myoblast differentiation   cellular component:  nucleus   cellular component:  nucleus   molecular function:  zinc ion binding   molecular function:  metal ion binding      BioCarta Pathways:     KEGG Pathways:  
    Probe set : 1398936_at    Name:   eukaryotic translation initiation factor 2, subunit 3, structural gene X-linked    GB acc:   AI598546    UniGene:   Rn.87025    Gene Symbol:   Eif2s3x    Locus Link:   299027    Chromosome:   X    Cytoband:   Chr:Xq31    SOURCE    GENECARD       Gene Ontology:   biological process:  protein biosynthesis   molecular function:  nucleotide binding   molecular function:  GTP binding      BioCarta Pathways:     KEGG Pathways:  
    Probe set : 1382389_at    Name:   Transcribed locus    GB acc:   AI179755    UniGene:   Rn.148837        SOURCE        Gene Ontology:     BioCarta Pathways:     KEGG Pathways:  
    Probe set : 1368118_at    Name:   B-cell CLL/lymphoma 10    GB acc:   NM_031328    UniGene:   Rn.13007    Gene Symbol:   Bcl10    Locus Link:   83477    Chromosome:   2    Cytoband:   Chr:2q44    SOURCE    GENECARD       Gene Ontology:   cellular component:  intracellular   cellular component:  cytoplasm   cellular component:  lipid raft   molecular function:  protein binding   molecular function:  caspase activator activity      BioCarta Pathways:     KEGG Pathways:  
    Probe set : 1367750_at    Name:   phosphoribosyl pyrophosphate synthetase-associated protein 1    GB acc:   NM_022545    UniGene:   Rn.2290    Gene Symbol:   Prpsap1    Locus Link:   64390    Chromosome:   10    Cytoband:   Chr:10q32.3    SOURCE    GENECARD       Gene Ontology:   biological process:  nucleoside metabolism   biological process:  nucleotide biosynthesis   biological process:  nucleotide biosynthesis   cellular component:  extracellular space      BioCarta Pathways:     KEGG Pathways:  
    Probe set : 1372536_at    Name:   chaperone, ABC1 activity of bc1 complex like (S. pombe) (predicted)    GB acc:   AI105042     Gene Symbol:   Cabc1_predicted    Locus Link:   360887    Chromosome:   13    Cytoband:   Chr:13q26     GENECARD       Gene Ontology:   biological process:  protein folding   cellular component:  mitochondrion   molecular function:  unfolded protein binding      BioCarta Pathways:     KEGG Pathways:  
    Probe set : 1369499_at    Name:   thymidylate synthase    GB acc:   NM_019179    UniGene:   Rn.53975    Gene Symbol:   Tyms    Locus Link:   29261    Chromosome:   9    Cytoband:   Chr:9q38    SOURCE    GENECARD       Gene Ontology:     BioCarta Pathways:     KEGG Pathways:  
    Probe set : 1388617_at    Name:   biphenyl hydrolase-like (serine hydrolase, breast epithelial mucin-associated antigen) (predicted)    GB acc:   AW914746    UniGene:   Rn.22050    Gene Symbol:   Bphl_predicted    Locus Link:   361239    Chromosome:   17    Cytoband:   Chr:17p12    SOURCE    GENECARD       Gene Ontology:   biological process:  aromatic compound metabolism   cellular component:  mitochondrion   molecular function:  catalytic activity   molecular function:  hydrolase activity      BioCarta Pathways:     KEGG Pathways:  
    Probe set : 1374438_at     GB acc:   AI103962              Gene Ontology:     BioCarta Pathways:     KEGG Pathways:  
    Probe set : 1388751_at    Name:   RNA binding motif protein 24 (predicted)    GB acc:   AW433787     Gene Symbol:   Rbm24_predicted    Locus Link:   291031    Chromosome:   17    Cytoband:   Chr:17p13     GENECARD       Gene Ontology:   biological process:  nuclear mRNA splicing, via spliceosome   molecular function:  nucleic acid binding      BioCarta Pathways:     KEGG Pathways:  
    Probe set : 1389457_at    Name:   myeloblastosis oncogene-like 2 (predicted)    GB acc:   BF281184    UniGene:   Rn.20824    Gene Symbol:   Mybl2_predicted    Locus Link:   296344    Chromosome:   3    Cytoband:   Chr:3q42    SOURCE    GENECARD       Gene Ontology:   cellular component:  chromatin   cellular component:  intracellular   cellular component:  nucleus   molecular function:  DNA binding   molecular function:  transcription factor activity      BioCarta Pathways:     KEGG Pathways:  
    Probe set : 1387178_a_at    Name:   cystathionine beta synthase    GB acc:   NM_012522    UniGene:   Rn.87853    Gene Symbol:   Cbs    Locus Link:   24250    Chromosome:   20    Cytoband:   Chr:20p12    SOURCE    GENECARD       Gene Ontology:   cellular component:  cytoplasm      BioCarta Pathways:     KEGG Pathways:  
    Probe set : 1380768_at    Name:   Transcribed locus    GB acc:   BM389322    UniGene:   Rn.121802        SOURCE        Gene Ontology:     BioCarta Pathways:     KEGG Pathways:  
    Probe set : 1389882_at    Name:   immediate early response 5    GB acc:   AI180270    UniGene:   Rn.8590    Gene Symbol:   Ier5    Locus Link:   498256    Chromosome:   13    Cytoband:   Chr:13q21    SOURCE    GENECARD       Gene Ontology:     BioCarta Pathways:     KEGG Pathways:  
    Probe set : 1372186_a_at    Name:   topoisomerase (DNA) 2 alpha    GB acc:   BM385445    UniGene:   Rn.90996    Gene Symbol:   Top2a    Locus Link:   360243    Chromosome:   10    Cytoband:   Chr:10q31    SOURCE    GENECARD       Gene Ontology:     BioCarta Pathways:     KEGG Pathways:  
    Probe set : 1380775_at    Name:   M-phase phosphoprotein 1 (predicted)    GB acc:   BE110723    UniGene:   Rn.138477    Gene Symbol:   Mphosph1_predicted    Locus Link:   309523    Chromosome:   1    Cytoband:   Chr:1q52    SOURCE    GENECARD       Gene Ontology:   biological process:  microtubule-based movement   biological process:  cell cycle arrest   biological process:  regulation of mitosis      BioCarta Pathways:     KEGG Pathways:  
    Probe set : 1379464_at    Name:   Transcribed locus    GB acc:   AI233253    UniGene:   Rn.148930        SOURCE        Gene Ontology:     BioCarta Pathways:     KEGG Pathways:  
    Probe set : 1391387_s_at    Name:   Transcribed locus    GB acc:   AI072956    UniGene:   Rn.20311        SOURCE        Gene Ontology:     BioCarta Pathways:     KEGG Pathways:  
    Probe set : 1395744_at    Name:   Transcribed locus, moderately similar to XP_579465.1 PREDICTED: DNA binding protein N5 [Rattus norvegicus]    GB acc:   BF291140    UniGene:   Rn.24481        SOURCE        Gene Ontology:     BioCarta Pathways:     KEGG Pathways:  
    Probe set : 1372056_at    Name:   Chemokine-like factor super family 6 (predicted)    GB acc:   AI406687    UniGene:   Rn.98431    Gene Symbol:   Cklfsf6    Locus Link:   316035    Chromosome:   8    Cytoband:   Chr:8q32    SOURCE    GENECARD       Gene Ontology:   biological process:  chemotaxis   cellular component:  membrane   cellular component:  integral to membrane   molecular function:  cytokine activity      BioCarta Pathways:     KEGG Pathways:  
    Probe set : 1392597_at     GB acc:   BG670559              Gene Ontology:     BioCarta Pathways:     KEGG Pathways:  
    Probe set : 1382108_at    Name:   V-maf musculoaponeurotic fibrosarcoma oncogene family, protein B (avian)    GB acc:   AA900536    UniGene:   Rn.10725    Gene Symbol:   Mafb    Locus Link:   54264    Chromosome:   3    Cytoband:   Chr:3q42    SOURCE    GENECARD       Gene Ontology:   cellular component:  nucleus   cellular component:  transcription factor complex   molecular function:  DNA binding   molecular function:  protein binding      BioCarta Pathways:     KEGG Pathways:  
    Probe set : 1383171_at    Name:   transcription factor B2, mitochondrial (predicted)    GB acc:   BG373674    UniGene:   Rn.96104    Gene Symbol:   Tfb2m_predicted    Locus Link:   289307    Chromosome:   13    Cytoband:   Chr:13q26    SOURCE    GENECARD       Gene Ontology:   biological process:  rRNA modification   cellular component:  extracellular space   molecular function:  rRNA (adenine-N6,N6-)-dimethyltransferase activity   molecular function:  rRNA methyltransferase activity      BioCarta Pathways:     KEGG Pathways:  
    Probe set : 1373462_at    Name:   embryonic ectoderm development (predicted)    GB acc:   AI170325    UniGene:   Rn.3939    Gene Symbol:   Eed_predicted    Locus Link:   293104    Chromosome:   1    Cytoband:   Chr:1q32    SOURCE    GENECARD       Gene Ontology:   biological process:  imprinting   biological process:  negative regulation of transcription   cellular component:  nucleus   cellular component:  pronucleus   molecular function:  protein binding   molecular function:  transcriptional repressor activity      BioCarta Pathways:     KEGG Pathways:  
    Probe set : 1391662_at    Name:   similar to RIKEN cDNA 9430015G10 (predicted) /// similar to RIKEN cDNA 9430015G10    GB acc:   AI104367    UniGene:   Rn.83717    Gene Symbol:   RGD1311517_predicted /// LOC500599    Locus Link:   313775 /// 500599    Chromosome:   5    Cytoband:   Chr:5q36    SOURCE    GENECARD       Gene Ontology:     BioCarta Pathways:     KEGG Pathways:  
    Probe set : 1371967_at    Name:   mitochondrial ribosomal protein L16    GB acc:   AI410349    UniGene:   Rn.101972    Gene Symbol:   Mrpl16    Locus Link:   293754    Chromosome:   1    Cytoband:   Chr:1q43    SOURCE    GENECARD       Gene Ontology:   biological process:  protein biosynthesis   cellular component:  intracellular   cellular component:  ribosome   molecular function:  structural constituent of ribosome      BioCarta Pathways:     KEGG Pathways:  
    Probe set : 1377818_at    Name:   Similar to junction-mediating and regulatory protein    GB acc:   AW532426    UniGene:   Rn.21208     Locus Link:   293057    Chromosome:   1    Cytoband:   Chr:1q31    SOURCE        Gene Ontology:     BioCarta Pathways:     KEGG Pathways:  
    Probe set : 1382642_at    Name:   zinc finger protein 281 (predicted)    GB acc:   BI291753    UniGene:   Rn.22691    Gene Symbol:   Zfp281_predicted    Locus Link:   305083    Chromosome:   13    Cytoband:   Chr:13q13    SOURCE    GENECARD       Gene Ontology:   biological process:  negative regulation of transcription from RNA polymerase II promoter   biological process:  regulation of transcription, DNA-dependent   cellular component:  nucleus   cellular component:  DNA-directed RNA polymerase II, core complex      BioCarta Pathways:     KEGG Pathways:  
    Probe set : 1373933_at    Name:   Rap guanine nucleotide exchange factor (GEF) 2 (predicted)    GB acc:   BF407276    UniGene:   Rn.23121    Gene Symbol:   Rapgef2_predicted    Locus Link:   310533    Chromosome:   2    Cytoband:   Chr:2q32    SOURCE    GENECARD       Gene Ontology:   cellular component:  integral to plasma membrane   cellular component:  membrane      BioCarta Pathways:     KEGG Pathways:  
    Probe set : 1390507_at    Name:   interferon-stimulated protein (predicted)    GB acc:   BI296097    UniGene:   Rn.16103    Gene Symbol:   Isg20_predicted    Locus Link:   293052    Chromosome:   1    Cytoband:   Chr:1q31    SOURCE    GENECARD       Gene Ontology:   cellular component:  intracellular   cellular component:  nucleus      BioCarta Pathways:     KEGG Pathways:  
    Probe set : 1368391_at    Name:   solute carrier family 7 (cationic amino acid transporter, y+ system), member 1    GB acc:   NM_013111    UniGene:   Rn.9439    Gene Symbol:   Slc7a1    Locus Link:   25648    Chromosome:   12    Cytoband:   Chr:12p11    SOURCE    GENECARD       Gene Ontology:   cellular component:  integral to plasma membrane   cellular component:  membrane   cellular component:  integral to membrane      BioCarta Pathways:     KEGG Pathways:  
    Probe set : 1379665_at    Name:   similar to RIKEN cDNA 4632422M10 (predicted)    GB acc:   AI177835    UniGene:   Rn.144258    Gene Symbol:   RGD1310204_predicted    Locus Link:   294711    Chromosome:   2    Cytoband:   Chr:2q13    SOURCE    GENECARD       Gene Ontology:     BioCarta Pathways:     KEGG Pathways:  
    Probe set : 1386967_at    Name:   ras homolog gene family, member Q    GB acc:   NM_053522    UniGene:   Rn.4169    Gene Symbol:   Rhoq    Locus Link:   85428    Chromosome:   6    Cytoband:   Chr:6q12    SOURCE    GENECARD       Gene Ontology:   cellular component:  plasma membrane      BioCarta Pathways:     KEGG Pathways:  
    Probe set : 1388581_at    Name:   hematological and neurological expressed sequence 1    GB acc:   AI317868    UniGene:   Rn.138819    Gene Symbol:   RGD:1359325    Locus Link:   287828    Chromosome:   10    Cytoband:   Chr:10q32.3    SOURCE    GENECARD       Gene Ontology:     BioCarta Pathways:     KEGG Pathways:  
    Probe set : 1388734_at    Name:   Transcribed locus    GB acc:   AA894193    UniGene:   Rn.11542        SOURCE        Gene Ontology:     BioCarta Pathways:     KEGG Pathways:  
    Probe set : 1398315_at    Name:   ribosomal protein L15    GB acc:   AA800007    UniGene:   Rn.27505    Gene Symbol:   Rpl15    Locus Link:   245981    Chromosome:   15    Cytoband:   Chr:15p16    SOURCE    GENECARD       Gene Ontology:   biological process:  protein biosynthesis   cellular component:  intracellular   cellular component:  ribosome   cellular component:  ribonucleoprotein complex   molecular function:  RNA binding   molecular function:  structural constituent of ribosome   molecular function:  structural constituent of ribosome      BioCarta Pathways:     KEGG Pathways:  
    Probe set : 1392638_at    Name:   Similar to myeloid leukemia factor 1 interacting protein    GB acc:   AI599308    UniGene:   Rn.128609     Locus Link:   306464    Chromosome:   16    Cytoband:   Chr:16q11    SOURCE        Gene Ontology:     BioCarta Pathways:     KEGG Pathways:  
    Probe set : 1383742_at    Name:   sorting nexin 7 (predicted)    GB acc:   AA997003    UniGene:   Rn.29845    Gene Symbol:   Snx7_predicted    Locus Link:   310815    Chromosome:   2    Cytoband:   Chr:2q41    SOURCE    GENECARD       Gene Ontology:   biological process:  transport   biological process:  intracellular signaling cascade   biological process:  protein transport   cellular component:  nucleus   molecular function:  DNA binding      BioCarta Pathways:     KEGG Pathways:  
    Probe set : 1371467_at    Name:   similar to RIKEN cDNA 0610007P06    GB acc:   AA891221    UniGene:   Rn.96145    Gene Symbol:   RGD:1359455    Locus Link:   293103    Chromosome:   1    Cytoband:   Chr:1q32    SOURCE    GENECARD       Gene Ontology:     BioCarta Pathways:     KEGG Pathways:  
    Probe set : 1383181_at    Name:   DnaJ (Hsp40) homolog, subfamily C, member 9 (predicted)    GB acc:   AI704947    UniGene:   Rn.16186    Gene Symbol:   Dnajc9_predicted    Locus Link:   364240    Chromosome:   15    Cytoband:   Chr:15p16    SOURCE    GENECARD       Gene Ontology:   biological process:  protein folding   molecular function:  heat shock protein binding   molecular function:  unfolded protein binding      BioCarta Pathways:     KEGG Pathways:  
    Probe set : 1371604_at    Name:   mitochondrial ribosomal protein L34    GB acc:   AA858969    UniGene:   Rn.2509    Gene Symbol:   RGD:1359397    Locus Link:   290632    Chromosome:   16    Cytoband:   Chr:16p14    SOURCE    GENECARD       Gene Ontology:   biological process:  protein biosynthesis   cellular component:  intracellular   cellular component:  ribosome   cellular component:  ribonucleoprotein complex   molecular function:  structural constituent of ribosome      BioCarta Pathways:     KEGG Pathways:  
    Probe set : 1370682_at    Name:   paired-Ig-like receptor A2 (predicted) /// similar to PIRA5    GB acc:   AF169636    UniGene:   Rn.145293    Gene Symbol:   Pira2_predicted /// LOC365169    Locus Link:   361492 /// 365169    Chromosome:   1    Cytoband:   Chr:1q12    SOURCE    GENECARD       Gene Ontology:     BioCarta Pathways:     KEGG Pathways:  
    Probe set : 1383814_at     GB acc:   BF556870              Gene Ontology:     BioCarta Pathways:     KEGG Pathways:  
    Probe set : 1373040_at    Name:   eukaryotic translation initiation factor 3, subunit 5 (epsilon) (predicted)    GB acc:   BI281752    UniGene:   Rn.41724    Gene Symbol:   Eif3s5_predicted    Locus Link:   293427    Chromosome:   1    Cytoband:   Chr:1q33    SOURCE    GENECARD       Gene Ontology:   biological process:  protein biosynthesis   biological process:  regulation of translational initiation   cellular component:  eukaryotic translation initiation factor 3 complex   molecular function:  translation initiation factor activity      BioCarta Pathways:     KEGG Pathways:  
    Probe set : 1384765_at     GB acc:   BI289371              Gene Ontology:     BioCarta Pathways:     KEGG Pathways:  
    Probe set : 1390598_at    Name:   Transcribed locus    GB acc:   BI294871    UniGene:   Rn.127872        SOURCE        Gene Ontology:     BioCarta Pathways:     KEGG Pathways:  
    Probe set : 1393887_at    Name:   similar to choline phosphotransferase 1; cholinephosphotransferase 1 alpha; cholinephosphotransferase 1    GB acc:   AI511041    UniGene:   Rn.16261    Gene Symbol:   RGD:1359283    Locus Link:   362866    Chromosome:   7    Cytoband:   Chr:7q13    SOURCE    GENECARD       Gene Ontology:   biological process:  phospholipid biosynthesis   molecular function:  transferase activity      BioCarta Pathways:     KEGG Pathways:  
    Probe set : 1383519_at    Name:   Hexokinase 2    GB acc:   BI294137    UniGene:   Rn.91375    Gene Symbol:   Hk2    Locus Link:   25059    Chromosome:   4    Cytoband:   Chr:4q34    SOURCE    GENECARD       Gene Ontology:   biological process:  regulation of progression through cell cycle   biological process:  glucose catabolism   biological process:  glycolysis   cellular component:  mitochondrial outer membrane   cellular component:  mitochondrial outer membrane   cellular component:  membrane      BioCarta Pathways:     KEGG Pathways:  
    Probe set : 1374200_at    Name:   solute carrier family 29 (nucleoside transporters), member 3    GB acc:   BM392280    UniGene:   Rn.15870    Gene Symbol:   Slc29a3    Locus Link:   353307    Chromosome:   20    Cytoband:   Chr:20q11    SOURCE    GENECARD       Gene Ontology:   biological process:  nucleoside transport   biological process:  transport   cellular component:  membrane   cellular component:  integral to membrane   molecular function:  nucleoside transporter activity   molecular function:  nucleoside transporter activity      BioCarta Pathways:     KEGG Pathways:  
    Probe set : 1367593_at    Name:   selenoprotein W, muscle 1    GB acc:   NM_013027    UniGene:   Rn.37192    Gene Symbol:   Sepw1    Locus Link:   25545    Chromosome:   1    Cytoband:   Chr:1q21    SOURCE    GENECARD       Gene Ontology:   biological process:  cell redox homeostasis   molecular function:  selenium binding   molecular function:  oxidoreductase activity   molecular function:  oxidoreductase activity      BioCarta Pathways:     KEGG Pathways:  
    Probe set : 1392461_at    Name:   PRP4 pre-mRNA processing factor 4 homolog (yeast) (predicted)    GB acc:   BI291012    UniGene:   Rn.25206    Gene Symbol:   Prpf4_predicted    Locus Link:   298095    Chromosome:   5    Cytoband:   Chr:5q24    SOURCE    GENECARD       Gene Ontology:   biological process:  nuclear mRNA splicing, via spliceosome   biological process:  RNA splicing   cellular component:  spliceosome complex   cellular component:  ribonucleoprotein complex   cellular component:  U4/U6 x U5 tri-snRNP complex      BioCarta Pathways:     KEGG Pathways:  
    Probe set : 1393050_at    Name:   Strain Wistar Rap2a mRNA, 5' UTR    GB acc:   BG664069    UniGene:   Rn.11727        SOURCE        Gene Ontology:     BioCarta Pathways:     KEGG Pathways:  
    Probe set : 1370177_at    Name:   poliovirus receptor    GB acc:   AI548856    UniGene:   Rn.10677    Gene Symbol:   PVR    Locus Link:   25066    Chromosome:   1    Cytoband:   Chr:1q22    SOURCE    GENECARD       Gene Ontology:   molecular function:  viral receptor activity   molecular function:  receptor activity   molecular function:  protein binding      BioCarta Pathways:     KEGG Pathways:  
    Probe set : 1378440_at    Name:   Transcribed locus    GB acc:   BM385137    UniGene:   Rn.40654        SOURCE        Gene Ontology:     BioCarta Pathways:     KEGG Pathways:  
    Probe set : 1367768_at    Name:   latexin    GB acc:   NM_031655    UniGene:   Rn.11404    Gene Symbol:   Lxn    Locus Link:   59073    Chromosome:   2    Cytoband:   Chr:2q31    SOURCE    GENECARD       Gene Ontology:   molecular function:  enzyme inhibitor activity   molecular function:  metalloendopeptidase inhibitor activity      BioCarta Pathways:     KEGG Pathways:  
    Probe set : 1397526_at    Name:   glutaryl-Coenzyme A dehydrogenase (predicted)    GB acc:   AI412068    UniGene:   Rn.99039    Gene Symbol:   Gcdh_predicted    Locus Link:   364975    Chromosome:   19    Cytoband:   Chr:19q11    SOURCE    GENECARD       Gene Ontology:   biological process:  electron transport   biological process:  regulation of transcription, DNA-dependent   cellular component:  mitochondrion      BioCarta Pathways:     KEGG Pathways:  
    Probe set : 1381993_at    Name:   chloride intracellular channel 2 (predicted)    GB acc:   BI293548    UniGene:   Rn.103254    Gene Symbol:   Clic2_predicted    Locus Link:   294141    Chromosome:   20    Cytoband:   Chr:20p12    SOURCE    GENECARD       Gene Ontology:   biological process:  ion transport   biological process:  chloride transport   biological process:  transport   cellular component:  membrane fraction   cellular component:  membrane   molecular function:  voltage-gated chloride channel activity   molecular function:  ion channel activity   molecular function:  voltage-gated ion channel activity      BioCarta Pathways:     KEGG Pathways:  
    Probe set : 1369029_at    Name:   phospholipid scramblase 1    GB acc:   NM_057194    UniGene:   Rn.96807    Gene Symbol:   Plscr1    Locus Link:   117540    Chromosome:   8    Cytoband:   Chr:8q31    SOURCE    GENECARD       Gene Ontology:   cellular component:  integral to membrane   molecular function:  calcium ion binding   molecular function:  phospholipid scramblase activity      BioCarta Pathways:     KEGG Pathways:  
    Probe set : 1392958_at    Name:   similar to cylindromatosis (turban tumor syndrome)    GB acc:   AI547426     Gene Symbol:   LOC498035    Locus Link:   498035       GENECARD       Gene Ontology:     BioCarta Pathways:     KEGG Pathways:  
    Probe set : 1387382_at    Name:   histamine N-methyltransferase    GB acc:   NM_031044    UniGene:   Rn.13145    Gene Symbol:   Hnmt    Locus Link:   81676    Chromosome:   3    Cytoband:   Chr:3p13    SOURCE    GENECARD       Gene Ontology:   biological process:  regulation of neurotransmitter levels   biological process:  respiratory gaseous exchange      BioCarta Pathways:     KEGG Pathways:  
    Probe set : 1367706_at    Name:   voltage-dependent anion channel 1    GB acc:   NM_031353    UniGene:   Rn.54594    Gene Symbol:   Vdac1    Locus Link:   83529    Chromosome:   10    Cytoband:   Chr:10q22    SOURCE    GENECARD       Gene Ontology:     BioCarta Pathways:     KEGG Pathways:  
    Probe set : 1370379_at    Name:   protease, serine, 8 (prostasin)    GB acc:   AF202076    UniGene:   Rn.107364    Gene Symbol:   Prss8    Locus Link:   192107    Chromosome:   1    Cytoband:   Chr:1q36    SOURCE    GENECARD       Gene Ontology:     BioCarta Pathways:     KEGG Pathways:  
    Probe set : 1387941_s_at    Name:   phospholipase A2, group VI    GB acc:   U51898    UniGene:   Rn.44692    Gene Symbol:   Pla2g6    Locus Link:   360426    Chromosome:   7    Cytoband:   Chr:7q34    SOURCE    GENECARD       Gene Ontology:   cellular component:  cytoplasm   cellular component:  membrane      BioCarta Pathways:     KEGG Pathways:  
    Probe set : 1383684_at    Name:   ASF1 anti-silencing function 1 homolog B (S. cerevisiae) (predicted)    GB acc:   BI288761    UniGene:   Rn.899    Gene Symbol:   Asf1b_predicted    Locus Link:   304648    Chromosome:   19    Cytoband:   Chr:19q11    SOURCE    GENECARD       Gene Ontology:   biological process:  nucleosome assembly   cellular component:  chromatin   molecular function:  histone binding      BioCarta Pathways:     KEGG Pathways:  
    Probe set : 1381357_at    Name:   protein tyrosine phosphatase, non-receptor type 8 (predicted)    GB acc:   AI176755    UniGene:   Rn.144838    Gene Symbol:   Ptpn8_predicted    Locus Link:   295338    Chromosome:   2    Cytoband:   Chr:2q34    SOURCE    GENECARD       Gene Ontology:   biological process:  protein amino acid dephosphorylation      BioCarta Pathways:     KEGG Pathways:  
    Probe set : 1371363_at    Name:   glycerol-3-phosphate dehydrogenase 1 (soluble)    GB acc:   BI277042    UniGene:   Rn.44452    Gene Symbol:   Gpd1    Locus Link:   60666    Chromosome:   7    Cytoband:   Chr:7q36    SOURCE    GENECARD       Gene Ontology:   biological process:  carbohydrate metabolism   biological process:  glycerol-3-phosphate metabolism   biological process:  glycerol-3-phosphate catabolism   cellular component:  glycerol-3-phosphate dehydrogenase complex   cellular component:  cytoplasm      BioCarta Pathways:     KEGG Pathways:  
    Probe set : 1370259_a_at    Name:   parathyroid hormone receptor 1    GB acc:   BE101119    UniGene:   Rn.48025    Gene Symbol:   Pthr1    Locus Link:   56813    Chromosome:   8    Cytoband:   Chr:8q32    SOURCE    GENECARD       Gene Ontology:     BioCarta Pathways:     KEGG Pathways:  
    Probe set : 1370825_a_at    Name:   cell division cycle 42 homolog (S. cerevisiae)    GB acc:   AA925473    UniGene:   Rn.60067    Gene Symbol:   Cdc42    Locus Link:   64465    Chromosome:   5    Cytoband:   Chr:5q36    SOURCE    GENECARD       Gene Ontology:   biological process:  regulation of progression through cell cycle   biological process:  cytokinesis   biological process:  regulation of cell growth   biological process:  actin filament organization   biological process:  establishment and/or maintenance of cell polarity   biological process:  small GTPase mediated signal transduction   biological process:  Rho protein signal transduction   biological process:  protein transport   biological process:  establishment of cell polarity   biological process:  macrophage differentiation   biological process:  filopodium formation   biological process:  cell division   biological process:  response to glucose stimulus   biological process:  regulation of exocytosis   biological process:  establishment and/or maintenance of cell polarity   biological process:  endosome transport   biological process:  cell-cell adhesion   cellular component:  cytoplasm   cellular component:  filopodium   cellular component:  plasma membrane   cellular component:  secretory granule      BioCarta Pathways:     KEGG Pathways:  
    Probe set : 1368379_at    Name:   CD36 antigen (collagen type I receptor, thrombospondin receptor)-like 2    GB acc:   NM_054001    UniGene:   Rn.3957    Gene Symbol:   Scarb2    Locus Link:   117106    Chromosome:   14    Cytoband:   Chr:14p22    SOURCE    GENECARD       Gene Ontology:   biological process:  cell adhesion   biological process:  cell adhesion   molecular function:  receptor activity      BioCarta Pathways:     KEGG Pathways:  
    Probe set : 1387807_at    Name:   platelet-activating factor acetylhydrolase, isoform Ib, alpha subunit 45kDa    GB acc:   NM_031763    UniGene:   Rn.5827    Gene Symbol:   Pafah1b1    Locus Link:   83572    Chromosome:   10    Cytoband:   Chr:10q24    SOURCE    GENECARD       Gene Ontology:   cellular component:  cytoskeleton   cellular component:  microtubule      BioCarta Pathways:     KEGG Pathways:  
    Probe set : 1395875_at    Name:   similar to KIAA0372 gene product (predicted)    GB acc:   BG379050    UniGene:   Rn.49948    Gene Symbol:   RGD1306062_predicted    Locus Link:   294595    Chromosome:   2    Cytoband:   Chr:2q11    SOURCE    GENECARD       Gene Ontology:     BioCarta Pathways:     KEGG Pathways:  
    Probe set : 1388026_at    Name:   CD3 antigen, zeta polypeptide    GB acc:   L08447    UniGene:   Rn.9997    Gene Symbol:   Cd3z    Locus Link:   25300    Chromosome:   13    Cytoband:   Chr:13q23    SOURCE    GENECARD       Gene Ontology:   biological process:  cell surface receptor linked signal transduction      BioCarta Pathways:     KEGG Pathways:  
    Probe set : 1378546_at    Name:   Transcribed locus    GB acc:   AI235245    UniGene:   Rn.147491        SOURCE        Gene Ontology:     BioCarta Pathways:     KEGG Pathways:  
    Probe set : 1390838_at    Name:   similar to KIAA1034-like DNA binding protein    GB acc:   BE115823    UniGene:   Rn.22624    Gene Symbol:   LOC501145    Locus Link:   501145    Chromosome:   9    Cytoband:   Chr:9q31    SOURCE    GENECARD       Gene Ontology:     BioCarta Pathways:     KEGG Pathways:  
    Probe set : 1382924_at    Name:   pantothenate kinase 1 (predicted)    GB acc:   AA850195    UniGene:   Rn.6581    Gene Symbol:   Pank1_predicted    Locus Link:   294088    Chromosome:   1    Cytoband:   Chr:1q52    SOURCE    GENECARD       Gene Ontology:   biological process:  coenzyme A biosynthesis      BioCarta Pathways:     KEGG Pathways:  
    Probe set : 1375525_at    Name:   Microtubule-associated protein, RP/EB family, member 1    GB acc:   BI294783    UniGene:   Rn.7652    Gene Symbol:   Mapre1    Locus Link:   114764    Chromosome:   3    Cytoband:   Chr:3q41    SOURCE    GENECARD       Gene Ontology:   biological process:  cell cycle   biological process:  mitosis   biological process:  cell division   cellular component:  microtubule   molecular function:  microtubule binding      BioCarta Pathways:     KEGG Pathways:  
    Probe set : 1373767_at    Name:   similar to arsenite inducible RNA associated protein (predicted)    GB acc:   AI406908    UniGene:   Rn.19157    Gene Symbol:   RGD1310991_predicted    Locus Link:   360772    Chromosome:   12    Cytoband:   Chr:12q11    SOURCE    GENECARD       Gene Ontology:     BioCarta Pathways:     KEGG Pathways:  
    Probe set : 1377725_at    Name:   Transcribed locus    GB acc:   AI575322    UniGene:   Rn.144053        SOURCE        Gene Ontology:     BioCarta Pathways:     KEGG Pathways:  
    Probe set : 1377774_at     GB acc:   AI029275              Gene Ontology:     BioCarta Pathways:     KEGG Pathways:  
    Probe set : 1383940_at    Name:   cell division cycle associated 1 (predicted)    GB acc:   BG375704    UniGene:   Rn.41428    Gene Symbol:   Cdca1_predicted    Locus Link:   304951    Chromosome:   13    Cytoband:   Chr:13q24    SOURCE    GENECARD       Gene Ontology:   biological process:  chromosome segregation   biological process:  attachment of spindle microtubules to kinetochore   biological process:  cell division   cellular component:  chromosome, pericentric region   cellular component:  kinetochore      BioCarta Pathways:     KEGG Pathways:  
    Probe set : 1374527_at    Name:   similar to hypothetical protein D4Ertd765e (predicted)    GB acc:   AI172274    UniGene:   Rn.1408    Gene Symbol:   RGD1308525_predicted    Locus Link:   298381    Chromosome:   5    Cytoband:   Chr:5q35    SOURCE    GENECARD       Gene Ontology:     BioCarta Pathways:     KEGG Pathways:  
    Probe set : 1398923_at    Name:   similar to D11Bwg0434e protein (predicted)    GB acc:   AI101122    UniGene:   Rn.1025    Gene Symbol:   RGD1305687_predicted    Locus Link:   287453    Chromosome:   10    Cytoband:   Chr:10q24    SOURCE    GENECARD       Gene Ontology:     BioCarta Pathways:     KEGG Pathways:  
    Probe set : 1388333_at    Name:   Ring-box 1 (predicted)    GB acc:   BF291171    UniGene:   Rn.8068     Locus Link:   300084    Chromosome:   7    Cytoband:   Chr:7q34    SOURCE        Gene Ontology:   biological process:  ubiquitin cycle   biological process:  protein ubiquitination   cellular component:  ubiquitin ligase complex   cellular component:  nucleus   cellular component:  SCF ubiquitin ligase complex   molecular function:  ubiquitin-protein ligase activity   molecular function:  protein binding   molecular function:  zinc ion binding      BioCarta Pathways:     KEGG Pathways:  
    Probe set : 1370339_at    Name:   Tropomyosin 3, splice variant 1    GB acc:   L24775    UniGene:   Rn.37575    Gene Symbol:   Tpm3    Locus Link:   117557    Chromosome:   2    Cytoband:   Chr:2q34    SOURCE    GENECARD       Gene Ontology:   biological process:  regulation of muscle contraction   biological process:  muscle development   cellular component:  cytoskeleton   cellular component:  muscle thin filament tropomyosin   molecular function:  actin binding      BioCarta Pathways:     KEGG Pathways:  
    Probe set : 1392479_at    Name:   FK506-binding protein 1a    GB acc:   AA925694    UniGene:   Rn.80611    Gene Symbol:   Fkbp1a    Locus Link:   25639    Chromosome:   3    Cytoband:   Chr:3q41    SOURCE    GENECARD       Gene Ontology:   biological process:  protein folding   biological process:  transport   biological process:  positive regulation of I-kappaB kinase/NF-kappaB cascade   cellular component:  microsome   cellular component:  membrane      BioCarta Pathways:     KEGG Pathways:  
    Probe set : 1374749_at     GB acc:   BM392169              Gene Ontology:     BioCarta Pathways:     KEGG Pathways:  
    Probe set : 1399030_at    Name:   similar to DNA segment, Chr X, Immunex 38, expressed    GB acc:   AA800682    UniGene:   Rn.25900    Gene Symbol:   RGD:1359718    Locus Link:   302559    Chromosome:   X    Cytoband:   Chr:Xq13    SOURCE    GENECARD       Gene Ontology:     BioCarta Pathways:     KEGG Pathways:  
    Probe set : 1389520_at    Name:   similar to Wdr1 protein    GB acc:   BI285874    UniGene:   Rn.3168    Gene Symbol:   LOC360950    Locus Link:   360950    Chromosome:   14    Cytoband:   Chr:14q21    SOURCE    GENECARD       Gene Ontology:     BioCarta Pathways:     KEGG Pathways:  
    Probe set : 1367584_at    Name:   annexin A2    GB acc:   NM_019905    UniGene:   Rn.90546    Gene Symbol:   Anxa2    Locus Link:   56611    Chromosome:   8    Cytoband:   Chr:8q24    SOURCE    GENECARD       Gene Ontology:     BioCarta Pathways:     KEGG Pathways:  
    Probe set : 1371631_at    Name:   similar to 2010003J03Rik protein (predicted)    GB acc:   BF420639    UniGene:   Rn.4163    Gene Symbol:   RGD1304686_predicted    Locus Link:   293673    Chromosome:   1    Cytoband:   Chr:1q43    SOURCE    GENECARD       Gene Ontology:   biological process:  protein biosynthesis   biological process:  translational initiation   molecular function:  translation initiation factor activity   molecular function:  RNA binding      BioCarta Pathways:     KEGG Pathways:  
    Probe set : 1398341_at    Name:   hypothetical LOC287661    GB acc:   BI295767    UniGene:   Rn.107812    Gene Symbol:   LOC287661    Locus Link:   287661    Chromosome:   10    Cytoband:   Chr:10q31    SOURCE    GENECARD       Gene Ontology:     BioCarta Pathways:     KEGG Pathways:  
    Probe set : 1371381_at    Name:   similar to TBC1 domain family member 4    GB acc:   AI007981    UniGene:   Rn.141267    Gene Symbol:   LOC306117    Locus Link:   306117    Chromosome:   15    Cytoband:   Chr:15q21    SOURCE    GENECARD       Gene Ontology:     BioCarta Pathways:     KEGG Pathways:  
    Probe set : 1368236_at    Name:   meprin 1 alpha    GB acc:   NM_013143    UniGene:   Rn.16265    Gene Symbol:   Mep1a    Locus Link:   25684    Chromosome:   9    Cytoband:   Chr:9q12    SOURCE    GENECARD       Gene Ontology:   biological process:  proteolysis   biological process:  proteolysis   biological process:  digestion      BioCarta Pathways:     KEGG Pathways:  
    Probe set : 1386968_at    Name:   protein phosphatase 1, regulatory (inhibitor) subunit 1A    GB acc:   NM_022676    UniGene:   Rn.9756    Gene Symbol:   Ppp1r1a    Locus Link:   58977    Chromosome:   7    Cytoband:   Chr:7q36    SOURCE    GENECARD       Gene Ontology:   biological process:  glycogen metabolism   biological process:  signal transduction   biological process:  carbohydrate metabolism   molecular function:  protein phosphatase inhibitor activity   molecular function:  type 1 serine/threonine specific protein phosphatase inhibitor activity      BioCarta Pathways:     KEGG Pathways:  
    Probe set : 1378746_at     GB acc:   AI013683              Gene Ontology:     BioCarta Pathways:     KEGG Pathways:  
    Probe set : 1388408_at    Name:   similar to RIKEN cDNA 1110020C13 (predicted)    GB acc:   AA800199    UniGene:   Rn.2990    Gene Symbol:   RGD1307129_predicted    Locus Link:   363004    Chromosome:   7    Cytoband:   Chr:7q36    SOURCE    GENECARD       Gene Ontology:     BioCarta Pathways:     KEGG Pathways:  
    Probe set : 1387061_at    Name:   junction plakoglobin    GB acc:   NM_031047    UniGene:   Rn.11255    Gene Symbol:   Jup    Locus Link:   81679    Chromosome:   10    Cytoband:   Chr:10q32.1    SOURCE    GENECARD       Gene Ontology:   biological process:  cell adhesion      BioCarta Pathways:     KEGG Pathways:  
    Probe set : 1376077_at     GB acc:   BG374774              Gene Ontology:     BioCarta Pathways:     KEGG Pathways:  
    Probe set : 1386346_at    Name:   Unknown (protein for MGC:72591)    GB acc:   BI275715    UniGene:   Rn.131677    Gene Symbol:   Tmem19    Locus Link:   299800    Chromosome:   7    Cytoband:   Chr:7q22    SOURCE    GENECARD       Gene Ontology:     BioCarta Pathways:     KEGG Pathways:  
    Probe set : 1368332_at    Name:   guanylate nucleotide binding protein 2    GB acc:   NM_133624    UniGene:   Rn.25736    Gene Symbol:   Gbp2    Locus Link:   171164    Chromosome:   2    Cytoband:   Chr:2q44    SOURCE    GENECARD       Gene Ontology:   biological process:  immune response   biological process:  regulation of transcription, DNA-dependent      BioCarta Pathways:     KEGG Pathways:  
    Probe set : 1393085_at    Name:   similar to hypothetical protein BC018453 (predicted)    GB acc:   BI290341    UniGene:   Rn.92704    Gene Symbol:   RGD1307700_predicted    Locus Link:   363219    Chromosome:   9    Cytoband:   Chr:9q21    SOURCE    GENECARD       Gene Ontology:     BioCarta Pathways:     KEGG Pathways:  
    Probe set : 1391905_at     GB acc:   BE108910              Gene Ontology:     BioCarta Pathways:     KEGG Pathways:  
    Probe set : 1375901_at    Name:   DEAD (Asp-Glu-Ala-Asp) box polypeptide 21b (predicted)    GB acc:   AI178527    UniGene:   Rn.56595    Gene Symbol:   Ddx21b_predicted    Locus Link:   361847    Chromosome:   20    Cytoband:   Chr:20q11    SOURCE    GENECARD       Gene Ontology:   cellular component:  nucleolus      BioCarta Pathways:     KEGG Pathways:  
    Probe set : 1376796_at    Name:   Transcribed locus    GB acc:   BG372452    UniGene:   Rn.147371        SOURCE        Gene Ontology:     BioCarta Pathways:     KEGG Pathways:  
    Probe set : 1371356_at    Name:   tensin like C1 domain containing phosphatase (predicted)    GB acc:   BI294836    UniGene:   Rn.105953    Gene Symbol:   Tenc1_predicted    Locus Link:   315326    Chromosome:   7    Cytoband:   Chr:7q36    SOURCE    GENECARD       Gene Ontology:   biological process:  intracellular signaling cascade   cellular component:  cytoskeleton   molecular function:  actin binding      BioCarta Pathways:     KEGG Pathways:  
    Probe set : 1388118_at    Name:   3-hydroxyisobutyrate dehydrogenase    GB acc:   AI176586    UniGene:   Rn.73    Gene Symbol:   Hibadh    Locus Link:   63938    Chromosome:   4    Cytoband:   Chr:4q24    SOURCE    GENECARD       Gene Ontology:   biological process:  pentose-phosphate shunt   biological process:  valine metabolism   cellular component:  mitochondrion      BioCarta Pathways:     KEGG Pathways:  
    Probe set : 1384292_at    Name:   docking protein 1 (predicted)    GB acc:   BI291018    UniGene:   Rn.117974    Gene Symbol:   Dok1_predicted    Locus Link:   312477    Chromosome:   4    Cytoband:   Chr:4q34    SOURCE    GENECARD       Gene Ontology:     BioCarta Pathways:     KEGG Pathways:  
    Probe set : 1386252_at    Name:   Transcribed locus    GB acc:   BE349825    UniGene:   Rn.54041        SOURCE        Gene Ontology:     BioCarta Pathways:     KEGG Pathways:  
    Probe set : 1380909_at    Name:   Transcribed locus    GB acc:   BM385894    UniGene:   Rn.81275        SOURCE        Gene Ontology:     BioCarta Pathways:     KEGG Pathways:  
    Probe set : 1390161_at    Name:   similar to cysteine and tyrosine-rich protein 1    GB acc:   AA818900    UniGene:   Rn.1528    Gene Symbol:   RGD:1359578    Locus Link:   304138    Chromosome:   11    Cytoband:   Chr:11q11    SOURCE    GENECARD       Gene Ontology:     BioCarta Pathways:     KEGG Pathways:  
    Probe set : 1369617_at    Name:   ubiquitin-conjugating enzyme E2N    GB acc:   NM_053928    UniGene:   Rn.144637    Gene Symbol:   Ube2n    Locus Link:   116725    Chromosome:   4    Cytoband:   Chr:4q12    SOURCE    GENECARD       Gene Ontology:     BioCarta Pathways:     KEGG Pathways:  
    Probe set : 1388137_a_at    Name:   zygin-related protein    GB acc:   AA955230     Gene Symbol:   RGD:628711    Locus Link:   286909       GENECARD       Gene Ontology:     BioCarta Pathways:     KEGG Pathways:  
    Probe set : 1389866_at     GB acc:   AI599296              Gene Ontology:     BioCarta Pathways:     KEGG Pathways:  
    Probe set : 1379302_at    Name:   Transcribed locus    GB acc:   AA848916    UniGene:   Rn.3958        SOURCE        Gene Ontology:     BioCarta Pathways:     KEGG Pathways:  
    Probe set : 1387925_at    Name:   asparagine synthetase    GB acc:   U07202    UniGene:   Rn.11172    Gene Symbol:   Asns    Locus Link:   25612      SOURCE    GENECARD       Gene Ontology:   molecular function:  asparagine synthase (glutamine-hydrolyzing) activity   molecular function:  ligase activity      BioCarta Pathways:     KEGG Pathways:  
    Probe set : 1384638_at    Name:   Similar to RIKEN cDNA 1700013H19 (predicted)    GB acc:   AI030545    UniGene:   Rn.145286     Locus Link:   364976    Chromosome:   19    Cytoband:   Chr:19q11    SOURCE        Gene Ontology:     BioCarta Pathways:     KEGG Pathways:  
    Probe set : 1389392_at    Name:   Transcribed locus    GB acc:   BF399468    UniGene:   Rn.7226        SOURCE        Gene Ontology:     BioCarta Pathways:     KEGG Pathways:  
    Probe set : 1368794_at    Name:   3-hydroxyanthranilate 3,4-dioxygenase    GB acc:   NM_020076    UniGene:   Rn.48675    Gene Symbol:   Haao    Locus Link:   56823    Chromosome:   6    Cytoband:   Chr:6q12    SOURCE    GENECARD       Gene Ontology:   biological process:  regulation of transcription, DNA-dependent   biological process:  metabolism      BioCarta Pathways:     KEGG Pathways:  
    Probe set : 1370930_at    Name:   kinesin family member 1C    GB acc:   BF417285    UniGene:   Rn.89698    Gene Symbol:   Kif1c    Locus Link:   113886    Chromosome:   10    Cytoband:   Chr:10q24    SOURCE    GENECARD       Gene Ontology:     BioCarta Pathways:     KEGG Pathways:  
    Probe set : 1376784_at    Name:   LOC499775    GB acc:   BI274481    UniGene:   Rn.107371    Gene Symbol:   LOC499775    Locus Link:   499775    Chromosome:   3    Cytoband:   Chr:3p12    SOURCE    GENECARD       Gene Ontology:     BioCarta Pathways:     KEGG Pathways:  
    Probe set : 1388711_at    Name:   interleukin 13 receptor, alpha 1    GB acc:   BF282650    UniGene:   Rn.81210    Gene Symbol:   Il13ra1    Locus Link:   252963    Chromosome:   X    Cytoband:   Chr:Xq12    SOURCE    GENECARD       Gene Ontology:   biological process:  cell surface receptor linked signal transduction      BioCarta Pathways:     KEGG Pathways:  
    Probe set : 1367943_at    Name:   nuclear factor of kappa light chain gene enhancer in B-cells inhibitor, beta    GB acc:   NM_030867    UniGene:   Rn.8395    Gene Symbol:   Nfkbib    Locus Link:   81525    Chromosome:   1    Cytoband:   Chr:1q21    SOURCE    GENECARD       Gene Ontology:   biological process:  transcription   biological process:  signal transduction   biological process:  signal transduction   cellular component:  nucleus   molecular function:  transcription coactivator activity   molecular function:  signal transducer activity   molecular function:  receptor activity      BioCarta Pathways:     KEGG Pathways:  
    Probe set : 1374462_at    Name:   kinesin-associated protein 3 (predicted)    GB acc:   AA850935    UniGene:   Rn.24266    Gene Symbol:   Kifap3_predicted    Locus Link:   289168    Chromosome:   13    Cytoband:   Chr:13q22    SOURCE    GENECARD       Gene Ontology:   cellular component:  endoplasmic reticulum   cellular component:  kinesin complex   cellular component:  kinesin II complex      BioCarta Pathways:     KEGG Pathways:  
    Probe set : 1373667_at    Name:   cysteine conjugate-beta lyase (predicted)    GB acc:   BF400674    UniGene:   Rn.110564    Gene Symbol:   Ccbl1_predicted    Locus Link:   311844    Chromosome:   3    Cytoband:   Chr:3p12    SOURCE    GENECARD       Gene Ontology:   biological process:  amino acid derivative metabolism   biological process:  biosynthesis   cellular component:  cytosol   cellular component:  mitochondrion      BioCarta Pathways:     KEGG Pathways:  
    Probe set : 1376624_at    Name:   Transcribed locus    GB acc:   BF288208    UniGene:   Rn.13170        SOURCE        Gene Ontology:     BioCarta Pathways:     KEGG Pathways:  
    Probe set : 1387076_at    Name:   hypoxia inducible factor 1, alpha subunit    GB acc:   NM_024359    UniGene:   Rn.10852    Gene Symbol:   Hif1a    Locus Link:   29560    Chromosome:   6    Cytoband:   Chr:6q24    SOURCE    GENECARD       Gene Ontology:   cellular component:  nucleus   cellular component:  nucleus   cellular component:  flagellum (sensu Eukaryota)      BioCarta Pathways:     KEGG Pathways:  
    Probe set : 1378197_at    Name:   kinesin family member C2    GB acc:   AW433953    UniGene:   Rn.100970    Gene Symbol:   KIFC2    Locus Link:   300053    Chromosome:   7    Cytoband:   Chr:7q34    SOURCE    GENECARD       Gene Ontology:   biological process:  microtubule-based process   biological process:  microtubule-based movement      BioCarta Pathways:     KEGG Pathways:  
    Probe set : 1384614_at     GB acc:   AI169347              Gene Ontology:     BioCarta Pathways:     KEGG Pathways:  
    Probe set : 1367936_at    Name:   serine/threonine kinase 10    GB acc:   NM_019206    UniGene:   Rn.4190    Gene Symbol:   Stk10    Locus Link:   29398      SOURCE    GENECARD       Gene Ontology:   biological process:  protein amino acid phosphorylation      BioCarta Pathways:     KEGG Pathways:  
    Probe set : 1373030_at    Name:   LOC501594    GB acc:   AJ225654    UniGene:   Rn.13504    Gene Symbol:   LOC501594    Locus Link:   501594    Chromosome:   X    Cytoband:   Chr:Xq31    SOURCE    GENECARD       Gene Ontology:     BioCarta Pathways:     KEGG Pathways:  
    Probe set : 1380728_at    Name:   Similar to collapsin response mediator protein-2A    GB acc:   BE118404    UniGene:   Rn.2889     Locus Link:   498539      SOURCE        Gene Ontology:     BioCarta Pathways:     KEGG Pathways:  
    Probe set : 1374169_at    Name:   similar to chromosome 16 open reading frame 5 (predicted)    GB acc:   AI236128    UniGene:   Rn.101805    Gene Symbol:   RGD1310686_predicted    Locus Link:   360480    Chromosome:   10    Cytoband:   Chr:10q12    SOURCE    GENECARD       Gene Ontology:   cellular component:  nucleus   cellular component:  integral to membrane      BioCarta Pathways:     KEGG Pathways:  
    Probe set : 1376649_at    Name:   Protein phosphatase 2 (formerly 2A), regulatory subunit A (PR 65), beta isoform (predicted)    GB acc:   AI111965    UniGene:   Rn.19709     Locus Link:   315648    Chromosome:   8    Cytoband:   Chr:8q23-q24    SOURCE        Gene Ontology:   biological process:  regulation of progression through cell cycle   biological process:  inactivation of MAPK activity   biological process:  regulation of DNA replication   biological process:  regulation of translation   biological process:  protein complex assembly   biological process:  protein amino acid dephosphorylation   biological process:  ceramide metabolism   biological process:  induction of apoptosis   biological process:  RNA splicing   biological process:  response to organic substance   biological process:  second-messenger-mediated signaling   biological process:  regulation of Wnt receptor signaling pathway   biological process:  regulation of cell adhesion   biological process:  negative regulation of cell growth   biological process:  regulation of growth   biological process:  negative regulation of tyrosine phosphorylation of Stat3 protein   biological process:  regulation of transcription   biological process:  regulation of cell differentiation   biological process:  protein amino acid phosphorylation      BioCarta Pathways:     KEGG Pathways:  
    Probe set : 1392940_at    Name:   Transcribed locus    GB acc:   BE095915    UniGene:   Rn.37568        SOURCE        Gene Ontology:     BioCarta Pathways:     KEGG Pathways:  
    Probe set : 1369471_at    Name:   rapostlin    GB acc:   NM_138914    UniGene:   Rn.20445    Gene Symbol:   Fnbp1    Locus Link:   192348    Chromosome:   3    Cytoband:   Chr:3p12    SOURCE    GENECARD       Gene Ontology:   biological process:  nervous system development   biological process:  endocytosis   molecular function:  protein binding      BioCarta Pathways:     KEGG Pathways:  
    Probe set : 1370844_at    Name:   heterogeneous nuclear ribonucleoprotein F    GB acc:   BM387425    UniGene:   Rn.144596    Gene Symbol:   Hnrpf    Locus Link:   64200    Chromosome:   4    Cytoband:   Chr:4q42    SOURCE    GENECARD       Gene Ontology:   biological process:  nuclear mRNA splicing, via spliceosome   biological process:  RNA processing   biological process:  mRNA processing   cellular component:  nucleus   cellular component:  nucleus   cellular component:  viral nucleocapsid   cellular component:  ribonucleoprotein complex      BioCarta Pathways:     KEGG Pathways:  
    Probe set : 1373955_at    Name:   karyopherin (importin) beta 3 (predicted)    GB acc:   BI280339    UniGene:   Rn.79893    Gene Symbol:   Kpnb3_predicted    Locus Link:   306182    Chromosome:   15    Cytoband:   Chr:15q24    SOURCE    GENECARD       Gene Ontology:   cellular component:  nucleus   cellular component:  nuclear pore   cellular component:  cytoplasm      BioCarta Pathways:     KEGG Pathways:  
    Probe set : 1369669_at    Name:   neurolysin (metallopeptidase M3 family)    GB acc:   NM_053970    UniGene:   Rn.11029    Gene Symbol:   Nln    Locus Link:   117041    Chromosome:   2    Cytoband:   Chr:2q13    SOURCE    GENECARD       Gene Ontology:   biological process:  RNA processing   biological process:  proteolysis   cellular component:  mitochondrion      BioCarta Pathways:     KEGG Pathways:  
    Probe set : 1374272_at    Name:   Transcribed locus    GB acc:   BI280268    UniGene:   Rn.138406        SOURCE        Gene Ontology:     BioCarta Pathways:     KEGG Pathways:  
    Probe set : 1392908_at    Name:   hypothetical protein LOC595134    GB acc:   BG381166    UniGene:   Rn.3674    Gene Symbol:   LOC595134    Locus Link:   595134    Chromosome:   5    Cytoband:   Chr:5q36    SOURCE    GENECARD       Gene Ontology:     BioCarta Pathways:     KEGG Pathways:  
    Probe set : 1368096_at    Name:   Ras-related GTP-binding protein Rab29    GB acc:   NM_133590    UniGene:   Rn.4323    Gene Symbol:   Rab7l1    Locus Link:   171122      SOURCE    GENECARD       Gene Ontology:   biological process:  intracellular protein transport   biological process:  small GTPase mediated signal transduction   biological process:  protein transport      BioCarta Pathways:     KEGG Pathways:  
    Probe set : 1380794_at    Name:   Aquarius (predicted)    GB acc:   BE106342    UniGene:   Rn.37299     Locus Link:   366163    Chromosome:   3    Cytoband:   Chr:3q35    SOURCE        Gene Ontology:     BioCarta Pathways:     KEGG Pathways:  
    Probe set : 1389468_at    Name:   ribose 5-phosphate isomerase A (predicted)    GB acc:   BF393902    UniGene:   Rn.12446    Gene Symbol:   Rpia_predicted    Locus Link:   362383    Chromosome:   4    Cytoband:   Chr:4q32    SOURCE    GENECARD       Gene Ontology:   biological process:  pentose-phosphate shunt, non-oxidative branch   cellular component:  intracellular   molecular function:  ribose-5-phosphate isomerase activity   molecular function:  isomerase activity      BioCarta Pathways:     KEGG Pathways:  
    Probe set : 1368191_a_at    Name:   solute carrier family 22 (organic cation transporter), member 1    GB acc:   U76379    UniGene:   Rn.11186    Gene Symbol:   Slc22a1    Locus Link:   24904    Chromosome:   1    Cytoband:   Chr:1q11-q12    SOURCE    GENECARD       Gene Ontology:     BioCarta Pathways:     KEGG Pathways:  
    Probe set : 1380377_at    Name:   Bruton agammaglobulinemia tyrosine kinase    GB acc:   AI113328    UniGene:   Rn.26996    Gene Symbol:   RGD:1359683    Locus Link:   367901    Chromosome:   X    Cytoband:   Chr:Xq34    SOURCE    GENECARD       Gene Ontology:   biological process:  protein amino acid phosphorylation   biological process:  intracellular signaling cascade      BioCarta Pathways:     KEGG Pathways:  
    Probe set : 1391629_at    Name:   hypothetical LOC362677    GB acc:   BI290794    UniGene:   Rn.40729    Gene Symbol:   LOC362677    Locus Link:   362677    Chromosome:   5    Cytoband:   Chr:5q36    SOURCE    GENECARD       Gene Ontology:     BioCarta Pathways:     KEGG Pathways:  
    Probe set : 1373838_at    Name:   fucosyltransferase 4    GB acc:   AI237192    UniGene:   Rn.44467    Gene Symbol:   Fut4    Locus Link:   60670    Chromosome:   8    Cytoband:   Chr:8q11    SOURCE    GENECARD       Gene Ontology:   biological process:  protein amino acid glycosylation      BioCarta Pathways:     KEGG Pathways:  
    Probe set : 1368915_at    Name:   kynurenine 3-monooxygenase (kynurenine 3-hydroxylase)    GB acc:   NM_021593    UniGene:   Rn.35029    Gene Symbol:   Kmo    Locus Link:   59113    Chromosome:   13    Cytoband:   Chr:13q25    SOURCE    GENECARD       Gene Ontology:   biological process:  electron transport   biological process:  aromatic compound metabolism   biological process:  metabolism   cellular component:  mitochondrion   cellular component:  integral to membrane      BioCarta Pathways:     KEGG Pathways:  
    Probe set : 1398612_at    Name:   aldo-keto reductase family 1, member C12 (predicted)    GB acc:   BM383010     Gene Symbol:   Akr1c12_predicted    Locus Link:   361266    Chromosome:   17    Cytoband:   Chr:17q12.3     GENECARD       Gene Ontology:   biological process:  xenobiotic metabolism   molecular function:  aldo-keto reductase activity   molecular function:  oxidoreductase activity      BioCarta Pathways:     KEGG Pathways:  
    Probe set : 1382515_at    Name:   cleavage and polyadenylation specific factor 2 (predicted)    GB acc:   AI059408    UniGene:   Rn.8038    Gene Symbol:   Cpsf2_predicted    Locus Link:   299256    Chromosome:   6    Cytoband:   Chr:6q32    SOURCE    GENECARD       Gene Ontology:   biological process:  mRNA processing   cellular component:  nucleus   molecular function:  RNA binding      BioCarta Pathways:     KEGG Pathways:  
    Probe set : 1376675_at    Name:   similar to mast cell-derived paired immunoglobulin-like receptor1    GB acc:   BI284779    UniGene:   Rn.11988    Gene Symbol:   LOC501736    Locus Link:   501736    Chromosome:   10    Cytoband:   Chr:10q32.2    SOURCE    GENECARD       Gene Ontology:     BioCarta Pathways:     KEGG Pathways:  
    Probe set : 1370064_at    Name:   presenilin 2    GB acc:   AB004454    UniGene:   Rn.11045    Gene Symbol:   Psen2    Locus Link:   81751    Chromosome:   13    Cytoband:   Chr:13q26    SOURCE    GENECARD       Gene Ontology:   biological process:  response to hypoxia   biological process:  cell fate specification   biological process:  protein amino acid N-linked glycosylation   biological process:  membrane protein ectodomain proteolysis   biological process:  membrane protein ectodomain proteolysis   biological process:  chromosome organization and biogenesis (sensu Eukaryota)   biological process:  chromosome organization and biogenesis (sensu Eukaryota)   biological process:  chromosome segregation   biological process:  chromosome segregation   biological process:  Notch receptor processing   biological process:  Notch receptor processing   biological process:  intracellular signaling cascade   biological process:  apoptotic program   biological process:  apoptotic program   biological process:  protein processing   biological process:  protein processing   biological process:  anagen   biological process:  amyloid precursor protein catabolism   biological process:  amyloid precursor protein catabolism   biological process:  positive regulation of enzyme activity   biological process:  positive regulation of enzyme activity   biological process:  Notch signaling pathway   molecular function:  protein binding   molecular function:  protein binding      BioCarta Pathways:     KEGG Pathways:  
    Probe set : 1388509_at     GB acc:   BI274303              Gene Ontology:     BioCarta Pathways:     KEGG Pathways:  
    Probe set : 1371563_at    Name:   similar to CG9135-PA (predicted)    GB acc:   BE109154    UniGene:   Rn.98282    Gene Symbol:   RGD1309986_predicted    Locus Link:   298594    Chromosome:   5    Cytoband:   Chr:5q36    SOURCE    GENECARD       Gene Ontology:     BioCarta Pathways:     KEGG Pathways:  
    Probe set : 1372953_at    Name:   neurocalcin delta    GB acc:   AI101330    UniGene:   Rn.13744    Gene Symbol:   Ncald    Locus Link:   553106    Chromosome:   7    Cytoband:   Chr:7q22    SOURCE    GENECARD       Gene Ontology:   molecular function:  calcium ion binding      BioCarta Pathways:     KEGG Pathways:  
    Probe set : 1370968_at    Name:   nuclear factor of kappa light chain gene enhancer in B-cells 1, p105    GB acc:   AA858801    UniGene:   Rn.2411    Gene Symbol:   Nfkb1    Locus Link:   81736    Chromosome:   2    Cytoband:   Chr:2q43    SOURCE    GENECARD       Gene Ontology:   biological process:  regulation of transcription, DNA-dependent   biological process:  transcription from RNA polymerase II promoter   biological process:  apoptosis   biological process:  anti-apoptosis   biological process:  inflammatory response   biological process:  response to oxidative stress   biological process:  signal transduction   biological process:  response to pathogenic bacteria   biological process:  antibacterial humoral response (sensu Vertebrata)   biological process:  negative regulation of interleukin-12 biosynthesis   biological process:  negative regulation of transcription, DNA-dependent   biological process:  positive regulation of transcription from RNA polymerase II promoter   biological process:  transcription   cellular component:  nucleus   cellular component:  nucleus   cellular component:  cytoplasm   cellular component:  cytoplasm      BioCarta Pathways:     KEGG Pathways:  
    Probe set : 1393917_at    Name:   CD163 antigen (predicted)    GB acc:   BF555488    UniGene:   Rn.8158    Gene Symbol:   Cd163_predicted    Locus Link:   312701    Chromosome:   4    Cytoband:   Chr:4q42    SOURCE    GENECARD       Gene Ontology:   biological process:  antimicrobial humoral response (sensu Vertebrata)   molecular function:  receptor activity   molecular function:  scavenger receptor activity      BioCarta Pathways:     KEGG Pathways:  
    Probe set : 1399033_at    Name:   core binding factor beta    GB acc:   AA945869    UniGene:   Rn.7308    Gene Symbol:   Cbfb    Locus Link:   361391    Chromosome:   19    Cytoband:   Chr:19q11    SOURCE    GENECARD       Gene Ontology:   cellular component:  nucleus   molecular function:  transcription coactivator activity      BioCarta Pathways:     KEGG Pathways:  
    Probe set : 1385706_at    Name:   Similar to Testis derived transcript    GB acc:   BI292758    UniGene:   Rn.44847     Locus Link:   500040      SOURCE        Gene Ontology:     BioCarta Pathways:     KEGG Pathways:  
    Probe set : 1382918_at    Name:   Transcribed locus    GB acc:   AA956561    UniGene:   Rn.147475        SOURCE        Gene Ontology:     BioCarta Pathways:     KEGG Pathways:  
    Probe set : 1380321_at    Name:   mitochondrial tumor suppressor 1    GB acc:   BI287786    UniGene:   Rn.95344    Gene Symbol:   Mtus1    Locus Link:   306487    Chromosome:   16    Cytoband:   Chr:16q12.1    SOURCE    GENECARD       Gene Ontology:   molecular function:  receptor activity      BioCarta Pathways:     KEGG Pathways:  
    Probe set : 1374539_at    Name:   Similar to type IV putative aminophospholipid transporting ATPase    GB acc:   AA850290    UniGene:   Rn.8188     Locus Link:   360932    Chromosome:   14    Cytoband:   Chr:14p11    SOURCE        Gene Ontology:     BioCarta Pathways:     KEGG Pathways:  
    Probe set : 1370082_at    Name:   transforming growth factor, beta 1    GB acc:   NM_021578    UniGene:   Rn.40136    Gene Symbol:   Tgfb1    Locus Link:   59086      SOURCE    GENECARD       Gene Ontology:   biological process:  regulation of progression through cell cycle   biological process:  skeletal development   biological process:  regulation of cell growth   biological process:  regulation of cell growth   biological process:  protein amino acid phosphorylation   biological process:  anti-apoptosis   biological process:  defense response   biological process:  inflammatory response   biological process:  transforming growth factor beta receptor signaling pathway   biological process:  cell-cell signaling   biological process:  striated muscle development   biological process:  cell death   biological process:  cell proliferation   biological process:  negative regulation of cell proliferation   biological process:  organ morphogenesis   biological process:  cell growth   biological process:  regulation of striated muscle development   biological process:  lung development   biological process:  lung development   biological process:  growth   biological process:  regulation of cell proliferation   biological process:  regulation of cell proliferation   biological process:  regulation of protein import into nucleus   biological process:  positive regulation of isotype switching to IgA isotypes   cellular component:  extracellular matrix (sensu Metazoa)   cellular component:  extracellular space      BioCarta Pathways:     KEGG Pathways:  
    Probe set : 1373312_at    Name:   Transcribed locus, strongly similar to NP_064383.1 brain protein 17 [Mus musculus]    GB acc:   BI295064    UniGene:   Rn.128152        SOURCE        Gene Ontology:     BioCarta Pathways:     KEGG Pathways:  
    Probe set : 1381919_at    Name:   Hermansky-Pudlak syndrome 6    GB acc:   BI290986    UniGene:   Rn.69203    Gene Symbol:   Hps6    Locus Link:   309446    Chromosome:   1    Cytoband:   Chr:1q54    SOURCE    GENECARD       Gene Ontology:     BioCarta Pathways:     KEGG Pathways:  
    Probe set : 1377153_a_at    Name:   kelch-like 6 (Drosophila) (predicted)    GB acc:   AI178384    UniGene:   Rn.137232    Gene Symbol:   Klhl6_predicted    Locus Link:   287974    Chromosome:   11    Cytoband:   Chr:11q23    SOURCE    GENECARD       Gene Ontology:   molecular function:  protein binding      BioCarta Pathways:     KEGG Pathways:  
    Probe set : 1384964_at    Name:   similar to Solute carrier family 6 (neurotransmitter transporter), member 20    GB acc:   BI281911    UniGene:   Rn.138076    Gene Symbol:   LOC301083    Locus Link:   301083    Chromosome:   8    Cytoband:   Chr:8q32    SOURCE    GENECARD       Gene Ontology:     BioCarta Pathways:     KEGG Pathways:  
    Probe set : 1374370_at     GB acc:   BF403383              Gene Ontology:     BioCarta Pathways:     KEGG Pathways:  
    Probe set : 1367919_at    Name:   nuclear pore membrane glycoprotein 210    GB acc:   NM_053322    UniGene:   Rn.3189    Gene Symbol:   Pom210    Locus Link:   58958    Chromosome:   4    Cytoband:   Chr:4q34    SOURCE    GENECARD       Gene Ontology:     BioCarta Pathways:     KEGG Pathways:  
    Probe set : 1369728_at    Name:   histone 1, H4m (predicted)    GB acc:   NM_022686     Gene Symbol:   Hist1h4m_predicted    Locus Link:   291152    Chromosome:   17    Cytoband:   Chr:17q11     GENECARD       Gene Ontology:     BioCarta Pathways:     KEGG Pathways:  
    Probe set : 1393067_at     GB acc:   AI710931              Gene Ontology:     BioCarta Pathways:     KEGG Pathways:  
    Probe set : 1376599_at    Name:   ATPase family, AAA domain containing 2 (predicted)    GB acc:   AW915567    UniGene:   Rn.6690     Locus Link:   314993    Chromosome:   7    Cytoband:   Chr:7q33    SOURCE        Gene Ontology:   molecular function:  nucleotide binding   molecular function:  ATP binding   molecular function:  nucleoside-triphosphatase activity      BioCarta Pathways:     KEGG Pathways:  
    Probe set : 1368838_at    Name:   tropomyosin 4    GB acc:   NM_012678    UniGene:   Rn.108199    Gene Symbol:   Tpm4    Locus Link:   24852    Chromosome:   16    Cytoband:   Chr:16p14    SOURCE    GENECARD       Gene Ontology:   biological process:  muscle contraction   biological process:  muscle contraction   biological process:  muscle development   cellular component:  cytoskeleton   cellular component:  kinesin complex   molecular function:  actin binding      BioCarta Pathways:     KEGG Pathways:  
    Probe set : 1398786_at    Name:   proteasome (prosome, macropain) subunit, beta type 2    GB acc:   NM_017284    UniGene:   Rn.1981    Gene Symbol:   Psmb2    Locus Link:   29675    Chromosome:   5    Cytoband:   Chr:5q36    SOURCE    GENECARD       Gene Ontology:   biological process:  ubiquitin-dependent protein catabolism   cellular component:  cytosol   cellular component:  proteasome core complex (sensu Eukaryota)   cellular component:  protein complex      BioCarta Pathways:     KEGG Pathways:  
    Probe set : 1388046_at    Name:   integrin alpha M    GB acc:   AF268593    UniGene:   Rn.54465    Gene Symbol:   Itgam    Locus Link:   25021    Chromosome:   1    Cytoband:   Chr:1q36    SOURCE    GENECARD       Gene Ontology:   cellular component:  integrin complex   cellular component:  external side of plasma membrane   cellular component:  integral to membrane   molecular function:  opsonin binding   molecular function:  receptor activity   molecular function:  protein binding      BioCarta Pathways:     KEGG Pathways:  
    Probe set : 1371863_at    Name:   similar to phosphatidylglycerophosphate synthase (predicted)    GB acc:   BM387059    UniGene:   Rn.50013    Gene Symbol:   RGD1305052_predicted    Locus Link:   303698    Chromosome:   10    Cytoband:   Chr:10q32.3    SOURCE    GENECARD       Gene Ontology:     BioCarta Pathways:     KEGG Pathways:  
    Probe set : 1375346_at    Name:   similar to hypothetical protein FLJ20010    GB acc:   BI290002    UniGene:   Rn.12131    Gene Symbol:   LOC500993    Locus Link:   500993    Chromosome:   8    Cytoband:   Chr:8q23    SOURCE    GENECARD       Gene Ontology:     BioCarta Pathways:     KEGG Pathways:  
    Probe set : 1389980_at    Name:   similar to Protein HSPC163    GB acc:   AI412591    UniGene:   Rn.24859    Gene Symbol:   LOC289324    Locus Link:   289324    Chromosome:   13    Cytoband:   Chr:13q26    SOURCE    GENECARD       Gene Ontology:     BioCarta Pathways:     KEGG Pathways:  
    Probe set : 1398626_s_at    Name:   ARP2 actin-related protein 2 homolog (yeast) (predicted)    GB acc:   AW920881    UniGene:   Rn.102249    Gene Symbol:   Actr2_predicted    Locus Link:   289820    Chromosome:   14    Cytoband:   Chr:14q22    SOURCE    GENECARD       Gene Ontology:   biological process:  cell motility   cellular component:  cytoskeleton   cellular component:  Arp2/3 protein complex   cellular component:  actin cytoskeleton   molecular function:  structural molecule activity   molecular function:  structural constituent of cytoskeleton   molecular function:  protein binding      BioCarta Pathways:     KEGG Pathways:  
    Probe set : 1378592_at    Name:   Similar to RIKEN cDNA 2310035M22 (predicted)    GB acc:   BF396210    UniGene:   Rn.145316     Locus Link:   365813    Chromosome:   2    Cytoband:   Chr:2q31    SOURCE        Gene Ontology:     BioCarta Pathways:     KEGG Pathways:  
    Probe set : 1367989_at    Name:   solute carrier family 2 (facilitated glucose transporter), member 4    GB acc:   NM_012751    UniGene:   Rn.1314    Gene Symbol:   Slc2a4    Locus Link:   25139    Chromosome:   10    Cytoband:   Chr:10q24    SOURCE    GENECARD       Gene Ontology:     BioCarta Pathways:     KEGG Pathways:  
    Probe set : 1372884_at    Name:   Transcribed locus    GB acc:   BG381486    UniGene:   Rn.24338        SOURCE        Gene Ontology:     BioCarta Pathways:     KEGG Pathways:  
    Probe set : 1373786_at    Name:   Transcribed locus    GB acc:   AA924756    UniGene:   Rn.15384        SOURCE        Gene Ontology:     BioCarta Pathways:     KEGG Pathways:  
    Probe set : 1394745_at    Name:   Similar to hypothetical protein FLJ20037 (predicted)    GB acc:   AA850950    UniGene:   Rn.788     Locus Link:   300870    Chromosome:   8    Cytoband:   Chr:8q31    SOURCE        Gene Ontology:     BioCarta Pathways:     KEGG Pathways:  
    Probe set : 1377016_at    Name:   similar to RIKEN cDNA 5730592L21 (predicted)    GB acc:   BE106888    UniGene:   Rn.19198    Gene Symbol:   RGD1310614_predicted    Locus Link:   362978    Chromosome:   7    Cytoband:   Chr:7q34    SOURCE    GENECARD       Gene Ontology:   cellular component:  extracellular space   molecular function:  structural molecule activity   molecular function:  calcium ion binding      BioCarta Pathways:     KEGG Pathways:  
    Probe set : 1379631_at    Name:   colony stimulating factor 1 (macrophage)    GB acc:   AI071665    UniGene:   Rn.83632    Gene Symbol:   Csf1    Locus Link:   78965    Chromosome:   2    Cytoband:   Chr:2q34    SOURCE    GENECARD       Gene Ontology:   biological process:  ossification   biological process:  ossification   biological process:  development   biological process:  development   biological process:  cell proliferation   biological process:  positive regulation of cell proliferation   biological process:  hemopoiesis   biological process:  cell differentiation   biological process:  macrophage differentiation   biological process:  macrophage differentiation   biological process:  regulation of ossification   biological process:  osteoclast differentiation   biological process:  osteoclast differentiation   biological process:  osteoclast differentiation   biological process:  positive regulation of body size   biological process:  odontogenesis   biological process:  odontogenesis   biological process:  positive regulation of odontogenesis (sensu Vertebrata)   biological process:  regulation of ossification   biological process:  positive regulation of body size   biological process:  positive regulation of odontogenesis (sensu Vertebrata)   biological process:  positive regulation of osteoclast differentiation   molecular function:  cytokine activity   molecular function:  macrophage colony stimulating factor receptor binding   molecular function:  growth factor activity      BioCarta Pathways:     KEGG Pathways:  
    Probe set : 1379418_at    Name:   LOC501008    GB acc:   AW916753    UniGene:   Rn.145699    Gene Symbol:   LOC501008    Locus Link:   501008    Chromosome:   8    Cytoband:   Chr:8q24    SOURCE    GENECARD       Gene Ontology:     BioCarta Pathways:     KEGG Pathways:  
    Probe set : 1384331_at    Name:   Neoplastic progression 3 (predicted)    GB acc:   H31896    UniGene:   Rn.2835     Locus Link:   296271    Chromosome:   3    Cytoband:   Chr:3q41    SOURCE        Gene Ontology:   cellular component:  extrachromosomal circular DNA   molecular function:  DNA binding      BioCarta Pathways:     KEGG Pathways:  
    Probe set : 1371576_at    Name:   mitochondrial ribosomal protein S36 (predicted)    GB acc:   AW142276    UniGene:   Rn.3406    Gene Symbol:   Mrps36_predicted    Locus Link:   294696    Chromosome:   2    Cytoband:   Chr:2q12    SOURCE    GENECARD       Gene Ontology:   biological process:  protein biosynthesis   cellular component:  mitochondrion   cellular component:  mitochondrial small ribosomal subunit   molecular function:  structural constituent of ribosome      BioCarta Pathways:     KEGG Pathways:  
    Probe set : 1368139_s_at    Name:   alkaline phosphatase, tissue-nonspecific    GB acc:   Y00714    UniGene:   Rn.82764    Gene Symbol:   Alpl    Locus Link:   25586    Chromosome:   5    Cytoband:   Chr:5q36    SOURCE    GENECARD       Gene Ontology:   biological process:  metabolism   cellular component:  extracellular space   cellular component:  membrane   cellular component:  integral to membrane      BioCarta Pathways:     KEGG Pathways:  
    Probe set : 1382210_at    Name:   Strain Wistar Rap2a mRNA, 5' UTR    GB acc:   BE117392    UniGene:   Rn.11727        SOURCE        Gene Ontology:     BioCarta Pathways:     KEGG Pathways:  
    Probe set : 1397214_at    Name:   Transcribed locus    GB acc:   BF288533    UniGene:   Rn.6731        SOURCE        Gene Ontology:     BioCarta Pathways:     KEGG Pathways:  
    Probe set : 1389555_at    Name:   transcription factor 19    GB acc:   BM387190    UniGene:   Rn.145385    Gene Symbol:   RGD:1302974    Locus Link:   406195    Chromosome:   20    Cytoband:   Chr:20p12    SOURCE    GENECARD       Gene Ontology:   molecular function:  transcription factor activity      BioCarta Pathways:     KEGG Pathways:  
    Probe set : 1370680_at    Name:   staufen, RNA binding protein, homolog 2 (Drosophila)    GB acc:   AF483620    UniGene:   Rn.73714    Gene Symbol:   Stau2    Locus Link:   171500    Chromosome:   5    Cytoband:   Chr:5q11    SOURCE    GENECARD       Gene Ontology:   biological process:  transport   cellular component:  intracellular   cellular component:  nucleus   cellular component:  endoplasmic reticulum   cellular component:  microtubule   molecular function:  RNA binding   molecular function:  double-stranded RNA binding      BioCarta Pathways:     KEGG Pathways:  
    Probe set : 1372672_at    Name:   quinolinate phosphoribosyltransferase (predicted)    GB acc:   AI407095    UniGene:   Rn.9220    Gene Symbol:   Qprt_predicted    Locus Link:   293504    Chromosome:   1    Cytoband:   Chr:1q36    SOURCE    GENECARD       Gene Ontology:   cellular component:  extracellular space      BioCarta Pathways:     KEGG Pathways:  
    Probe set : 1373709_at    Name:   hypothetical LOC289900    GB acc:   BI281706    UniGene:   Rn.28239    Gene Symbol:   RGD:1359592    Locus Link:   289900    Chromosome:   15    Cytoband:   Chr:15p16    SOURCE    GENECARD       Gene Ontology:     BioCarta Pathways:     KEGG Pathways:  
    Probe set : 1372543_at    Name:   similar to RIKEN cDNA 2610029G23    GB acc:   BI282107    UniGene:   Rn.12007    Gene Symbol:   LOC363485    Locus Link:   363485    Chromosome:   X    Cytoband:   Chr:Xq31    SOURCE    GENECARD       Gene Ontology:     BioCarta Pathways:     KEGG Pathways:  
    Probe set : 1387242_at    Name:   Protein kinase, interferon-inducible double stranded RNA dependent    GB acc:   NM_019335    UniGene:   Rn.10022    Gene Symbol:   Prkr    Locus Link:   54287    Chromosome:   6    Cytoband:   Chr:6q11    SOURCE    GENECARD       Gene Ontology:   biological process:  protein amino acid phosphorylation   cellular component:  intracellular      BioCarta Pathways:     KEGG Pathways:  
    Probe set : 1398877_at    Name:   stress-induced-phosphoprotein 1    GB acc:   BI283691    UniGene:   Rn.22614    Gene Symbol:   Stip1    Locus Link:   192277    Chromosome:   1    Cytoband:   Chr:1q43    SOURCE    GENECARD       Gene Ontology:   biological process:  response to stress   cellular component:  nucleus   cellular component:  Golgi apparatus   molecular function:  binding      BioCarta Pathways:     KEGG Pathways:  
    Probe set : 1373043_at     GB acc:   BI275923              Gene Ontology:     BioCarta Pathways:     KEGG Pathways:  
    Probe set : 1376697_at    Name:   carbohydrate sulfotransferase 12 (predicted)    GB acc:   BI275885    UniGene:   Rn.78863    Gene Symbol:   Chst12_predicted    Locus Link:   304322    Chromosome:   12    Cytoband:   Chr:12q11    SOURCE    GENECARD       Gene Ontology:   biological process:  dermatan sulfate biosynthesis   cellular component:  integral to membrane   cellular component:  integral to Golgi membrane      BioCarta Pathways:     KEGG Pathways:  
    Probe set : 1386978_at    Name:   BCL2/adenovirus E1B 19 kDa-interacting protein 3-like    GB acc:   NM_080888    UniGene:   Rn.827    Gene Symbol:   Bnip3l    Locus Link:   140923    Chromosome:   15    Cytoband:   Chr:15p12    SOURCE    GENECARD       Gene Ontology:   biological process:  positive regulation of apoptosis   cellular component:  mitochondrial membrane   cellular component:  integral to membrane      BioCarta Pathways:     KEGG Pathways:  
    Probe set : 1386953_at    Name:   hydroxysteroid 11-beta dehydrogenase 1    GB acc:   NM_017080    UniGene:   Rn.888    Gene Symbol:   Hsd11b1    Locus Link:   25116    Chromosome:   13    Cytoband:   Chr:13q27    SOURCE    GENECARD       Gene Ontology:     BioCarta Pathways:     KEGG Pathways:  
    Probe set : 1373345_at    Name:   amphoterin induced gene 2    GB acc:   AW523747    UniGene:   Rn.98123    Gene Symbol:   Amigo2    Locus Link:   300186    Chromosome:   7    Cytoband:   Chr:7q35    SOURCE    GENECARD       Gene Ontology:   cellular component:  extracellular space   cellular component:  integral to membrane   cellular component:  integral to plasma membrane   molecular function:  protein binding   molecular function:  protein binding      BioCarta Pathways:     KEGG Pathways:  
    Probe set : 1367569_at    Name:   laminin receptor 1 (67kD, ribosomal protein SA)    GB acc:   NM_017138    UniGene:   Rn.999    Gene Symbol:   Lamr1    Locus Link:   29236    Chromosome:   8    Cytoband:   Chr:8q32    SOURCE    GENECARD       Gene Ontology:   biological process:  protein biosynthesis      BioCarta Pathways:     KEGG Pathways:  
    Probe set : 1371012_at    Name:   2-hydroxyphytanoyl-Coenzyme A lyase    GB acc:   AJ245707    UniGene:   Rn.21425    Gene Symbol:   Hpcl2    Locus Link:   85255    Chromosome:   16    Cytoband:   Chr:16p16    SOURCE    GENECARD       Gene Ontology:     BioCarta Pathways:     KEGG Pathways:  
    Probe set : 1371529_at    Name:   Receptor (TNFRSF)-interacting serine-threonine kinase 1 (predicted)    GB acc:   BF289001    UniGene:   Rn.7572     Locus Link:   306886    Chromosome:   17    Cytoband:   Chr:17p12    SOURCE        Gene Ontology:     BioCarta Pathways:     KEGG Pathways:  
    Probe set : 1369070_at    Name:   peroxisomal biogenesis factor 12    GB acc:   NM_053921    UniGene:   Rn.144636    Gene Symbol:   Pex12    Locus Link:   116718    Chromosome:   10    Cytoband:   Chr:10q26    SOURCE    GENECARD       Gene Ontology:   biological process:  peroxisome organization and biogenesis   biological process:  peroxisome organization and biogenesis   biological process:  protein ubiquitination      BioCarta Pathways:     KEGG Pathways:  
    Probe set : 1369158_at    Name:   calcium-sensing receptor    GB acc:   NM_016996    UniGene:   Rn.10019    Gene Symbol:   Casr    Locus Link:   24247    Chromosome:   11    Cytoband:   Chr:11q22    SOURCE    GENECARD       Gene Ontology:     BioCarta Pathways:     KEGG Pathways:  
    Probe set : 1371954_at     GB acc:   BF290193              Gene Ontology:     BioCarta Pathways:     KEGG Pathways:  
    Probe set : 1368304_at    Name:   Flavin containing monooxygenase 3    GB acc:   NM_053433    UniGene:   Rn.28357    Gene Symbol:   Fmo3    Locus Link:   84493    Chromosome:   13    Cytoband:   Chr:13q22    SOURCE    GENECARD       Gene Ontology:   biological process:  electron transport      BioCarta Pathways:     KEGG Pathways:  
    Probe set : 1368220_at    Name:   general transcription factor IIB    GB acc:   NM_031041    UniGene:   Rn.6109    Gene Symbol:   Gtf2b    Locus Link:   81673    Chromosome:   2    Cytoband:   Chr:2q44    SOURCE    GENECARD       Gene Ontology:   cellular component:  nucleus   cellular component:  transcription factor complex   cellular component:  transcription factor complex      BioCarta Pathways:     KEGG Pathways:  
    Probe set : 1372733_at    Name:   Similar to intracellular membrane-associated calcium-independent phospholipase A2 gamma (predicted)    GB acc:   BM391852    UniGene:   Rn.23073     Locus Link:   314075    Chromosome:   6    Cytoband:   Chr:6q21    SOURCE        Gene Ontology:     BioCarta Pathways:     KEGG Pathways:  
    Probe set : 1381570_at    Name:   similar to T-cell activation NFKB-like protein (predicted)    GB acc:   BI293518    UniGene:   Rn.144998    Gene Symbol:   RGD1308055_predicted    Locus Link:   308496    Chromosome:   1    Cytoband:   Chr:1q21    SOURCE    GENECARD       Gene Ontology:     BioCarta Pathways:     KEGG Pathways:  
    Probe set : 1374196_at    Name:   lanC (bacterial lantibiotic synthetase component C)-like 1    GB acc:   AI710389    UniGene:   Rn.38972    Gene Symbol:   Lancl1    Locus Link:   114515    Chromosome:   9    Cytoband:   Chr:9q32    SOURCE    GENECARD       Gene Ontology:   biological process:  G-protein coupled receptor protein signaling pathway   cellular component:  integral to plasma membrane   cellular component:  integral to membrane   molecular function:  G-protein coupled receptor activity      BioCarta Pathways:     KEGG Pathways:  
    Probe set : 1367641_at    Name:   superoxide dismutase 1    GB acc:   NM_017050    UniGene:   Rn.6059    Gene Symbol:   Sod1    Locus Link:   24786    Chromosome:   11    Cytoband:   Chr:11q11    SOURCE    GENECARD       Gene Ontology:   cellular component:  cytoplasm   cellular component:  mitochondrion      BioCarta Pathways:     KEGG Pathways:  
    Probe set : 1371728_at    Name:   Tripartite motif-containing 35    GB acc:   BF281133    UniGene:   Rn.105225     Locus Link:   498538      SOURCE        Gene Ontology:   biological process:  protein ubiquitination   cellular component:  ubiquitin ligase complex   cellular component:  intracellular   molecular function:  ubiquitin-protein ligase activity   molecular function:  zinc ion binding   molecular function:  metal ion binding      BioCarta Pathways:     KEGG Pathways:  
    Probe set : 1370385_at    Name:   phospholipase A2, group VI    GB acc:   U51898    UniGene:   Rn.44692    Gene Symbol:   Pla2g6    Locus Link:   360426    Chromosome:   7    Cytoband:   Chr:7q34    SOURCE    GENECARD       Gene Ontology:   cellular component:  cytoplasm   cellular component:  membrane      BioCarta Pathways:     KEGG Pathways:  
    Probe set : 1375436_at    Name:   nicotinate phosphoribosyltransferase-like protein    GB acc:   BF416417    UniGene:   Rn.22665    Gene Symbol:   RGD:1302945    Locus Link:   315085    Chromosome:   7    Cytoband:   Chr:7q34    SOURCE    GENECARD       Gene Ontology:   biological process:  pyridine nucleotide biosynthesis      BioCarta Pathways:     KEGG Pathways:  
    Probe set : 1372090_at    Name:   similar to RIKEN cDNA 1700108M19    GB acc:   AI231566     Gene Symbol:   LOC298963    Locus Link:   298963    Chromosome:   6    Cytoband:   Chr:6q16     GENECARD       Gene Ontology:     BioCarta Pathways:     KEGG Pathways:  
    Probe set : 1388552_at    Name:   sphingomyelin phosphodiesterase 1, acid lysosomal    GB acc:   BI283738    UniGene:   Rn.18277    Gene Symbol:   Smpd1    Locus Link:   308909    Chromosome:   1    Cytoband:   Chr:1q33    SOURCE    GENECARD       Gene Ontology:   biological process:  sphingomyelin catabolism   molecular function:  sphingomyelin phosphodiesterase activity   molecular function:  integrase activity   molecular function:  hydrolase activity      BioCarta Pathways:     KEGG Pathways:  
    Probe set : 1372850_at    Name:   Similar to Expressed sequence AU019823    GB acc:   AA799464    UniGene:   Rn.135880     Locus Link:   500994      SOURCE        Gene Ontology:     BioCarta Pathways:     KEGG Pathways:  
    Probe set : 1373440_at    Name:   caspase 8 associated protein 2 (predicted)    GB acc:   AI175525    UniGene:   Rn.9052    Gene Symbol:   Casp8ap2_predicted    Locus Link:   313128    Chromosome:   5    Cytoband:   Chr:5q21    SOURCE    GENECARD       Gene Ontology:   biological process:  caspase activation   biological process:  induction of apoptosis via death domain receptors      BioCarta Pathways:     KEGG Pathways:  
    Probe set : 1385641_at    Name:   Transcribed locus, strongly similar to NP_780521.1 hypothetical protein LOC101148 [Mus musculus]    GB acc:   AI070558    UniGene:   Rn.34492        SOURCE        Gene Ontology:     BioCarta Pathways:     KEGG Pathways:  
    Probe set : 1367784_a_at    Name:   clusterin    GB acc:   AF314657    UniGene:   Rn.1780    Gene Symbol:   Clu    Locus Link:   24854    Chromosome:   15    Cytoband:   Chr:15p12    SOURCE    GENECARD       Gene Ontology:   cellular component:  extracellular region   cellular component:  extracellular space      BioCarta Pathways:     KEGG Pathways:  
    Probe set : 1392637_at    Name:   Similar to interferon-inducible GTPase (predicted)    GB acc:   AA923988    UniGene:   Rn.2746     Locus Link:   307414    Chromosome:   18    Cytoband:   Chr:18q12.1    SOURCE        Gene Ontology:     BioCarta Pathways:     KEGG Pathways:  
    Probe set : 1378668_at    Name:   CDNA clone IMAGE:7317308    GB acc:   BG672426    UniGene:   Rn.21474        SOURCE        Gene Ontology:     BioCarta Pathways:     KEGG Pathways:  
    Probe set : 1387018_at    Name:   Arg/Abl-interacting protein ArgBP2    GB acc:   NM_053770    UniGene:   Rn.24612    Gene Symbol:   Argbp2    Locus Link:   114901    Chromosome:   16    Cytoband:   Chr:16q11    SOURCE    GENECARD       Gene Ontology:   biological process:  intracellular signaling cascade   biological process:  transport   cellular component:  nucleus   cellular component:  cytoskeleton   cellular component:  actin cytoskeleton   cellular component:  Z disc      BioCarta Pathways:     KEGG Pathways:  
    Probe set : 1377310_at    Name:   Transcribed locus, weakly similar to XP_575554.1 PREDICTED: similar to RNA binding motif and ELMO domain 1 [Rattus norvegicus]    GB acc:   BG374304    UniGene:   Rn.22341        SOURCE        Gene Ontology:     BioCarta Pathways:     KEGG Pathways:  
    Probe set : 1387913_at    Name:   cytochrome P450, family 2, subfamily d, polypeptide 22    GB acc:   U48220    UniGene:   Rn.26060    Gene Symbol:   Cyp2d22    Locus Link:   171522    Chromosome:   7    Cytoband:   Chr:7q34    SOURCE    GENECARD       Gene Ontology:   biological process:  electron transport   biological process:  drug metabolism      BioCarta Pathways:     KEGG Pathways:  
    Probe set : 1389466_at    Name:   Ankyrin 3, epithelial isoform g    GB acc:   BG374280    UniGene:   Rn.48866     Locus Link:   361833    Chromosome:   20    Cytoband:   Chr:20p11    SOURCE        Gene Ontology:   biological process:  signal transduction   biological process:  positive regulation of apoptosis   cellular component:  cytoskeleton   cellular component:  synapse      BioCarta Pathways:     KEGG Pathways:  
    Probe set : 1379778_at    Name:   similar to HSPC043 protein (predicted)    GB acc:   AI713278    UniGene:   Rn.40728    Gene Symbol:   RGD1307983_predicted    Locus Link:   298032    Chromosome:   5    Cytoband:   Chr:5q24    SOURCE    GENECARD       Gene Ontology:     BioCarta Pathways:     KEGG Pathways:  
    Probe set : 1372360_at    Name:   Transcribed locus    GB acc:   AA817862    UniGene:   Rn.785        SOURCE        Gene Ontology:     BioCarta Pathways:     KEGG Pathways:  
    Probe set : 1388119_at    Name:   hypothetical gene supported by Y16641 /// heterogeneous nuclear ribonucleoprotein A3 /// similar to Hnrpa3 protein /// similar to Hnrpa3 protein    GB acc:   BM392140    UniGene:   Rn.99947    Gene Symbol:   LOC292273 /// Hnrpa3 /// LOC363971 /// LOC499592    Locus Link:   292273 /// 362152 /// 363971 /// 499592    Chromosome:   2    Cytoband:   Chr:2q45 /// Chr:3q23 /// Chr:13q11 /// Chr:2q25    SOURCE    GENECARD       Gene Ontology:   biological process:  mRNA transport      BioCarta Pathways:     KEGG Pathways:  
    Probe set : 1370047_at    Name:   ectonucleotide pyrophosphatase/phosphodiesterase 1    GB acc:   NM_053535    UniGene:   Rn.1199    Gene Symbol:   Enpp1    Locus Link:   85496    Chromosome:   1    Cytoband:   Chr:1p12    SOURCE    GENECARD       Gene Ontology:   biological process:  skeletal development   biological process:  nucleotide metabolism   biological process:  negative regulation of ossification   cellular component:  plasma membrane   cellular component:  integral to membrane      BioCarta Pathways:     KEGG Pathways:  
    Probe set : 1398784_at    Name:   complement component 1, q subcomponent binding protein    GB acc:   NM_019259    UniGene:   Rn.2765    Gene Symbol:   C1qbp    Locus Link:   29681    Chromosome:   10    Cytoband:   Chr:10q24    SOURCE    GENECARD       Gene Ontology:   biological process:  immune response   biological process:  complement activation   cellular component:  mitochondrion   cellular component:  mitochondrial matrix   molecular function:  complement component C1q binding      BioCarta Pathways:     KEGG Pathways:  
    Probe set : 1389566_at    Name:   cyclin B2 (predicted)    GB acc:   AW253821    UniGene:   Rn.6743    Gene Symbol:   Ccnb2_predicted    Locus Link:   363088    Chromosome:   8    Cytoband:   Chr:8q24    SOURCE    GENECARD       Gene Ontology:   cellular component:  nucleus   molecular function:  cyclin-dependent protein kinase regulator activity      BioCarta Pathways:     KEGG Pathways:  
    Probe set : 1392913_at    Name:   similar to zinc finger protein 366 (predicted)    GB acc:   AA964151    UniGene:   Rn.64318    Gene Symbol:   RGD1306491_predicted    Locus Link:   293044    Chromosome:   1    Cytoband:   Chr:1q31    SOURCE    GENECARD       Gene Ontology:     BioCarta Pathways:     KEGG Pathways:  
    Probe set : 1390172_at    Name:   dehydrogenase E1 and transketolase domain containing 1 (predicted)    GB acc:   AI409946    UniGene:   Rn.13539    Gene Symbol:   Dhtkd1_predicted    Locus Link:   361272    Chromosome:   17    Cytoband:   Chr:17q12.3    SOURCE    GENECARD       Gene Ontology:   biological process:  metabolism   biological process:  glycolysis   biological process:  electron transport      BioCarta Pathways:     KEGG Pathways:  
    Probe set : 1375120_at    Name:   inhibitor of DNA binding 4    GB acc:   AI412150    UniGene:   Rn.145407    Gene Symbol:   Idb4    Locus Link:   291023    Chromosome:   17    Cytoband:   Chr:17p14    SOURCE    GENECARD       Gene Ontology:   biological process:  regulation of transcription from RNA polymerase II promoter   cellular component:  nucleus   molecular function:  transcription corepressor activity      BioCarta Pathways:     KEGG Pathways:  
    Probe set : 1384239_at    Name:   Ribosomal protein L31    GB acc:   AA818474    UniGene:   Rn.1101    Gene Symbol:   Rpl31    Locus Link:   64298    Chromosome:   9    Cytoband:   Chr:9q21    SOURCE    GENECARD       Gene Ontology:   biological process:  protein biosynthesis   molecular function:  RNA binding   molecular function:  structural constituent of ribosome      BioCarta Pathways:     KEGG Pathways:  
    Probe set : 1371923_at    Name:   similar to hypothetical protein FLJ20481 (predicted)    GB acc:   BG380281    UniGene:   Rn.16547    Gene Symbol:   RGD1311599_predicted    Locus Link:   361467    Chromosome:   1    Cytoband:   Chr:1p11    SOURCE    GENECARD       Gene Ontology:     BioCarta Pathways:     KEGG Pathways:  
    Probe set : 1368811_at    Name:   lamin B1    GB acc:   NM_053905    UniGene:   Rn.11362    Gene Symbol:   Lmnb1    Locus Link:   116685    Chromosome:   18    Cytoband:   Chr:18p12    SOURCE    GENECARD       Gene Ontology:   molecular function:  structural molecule activity      BioCarta Pathways:     KEGG Pathways:  
    Probe set : 1384327_at    Name:   differentially expressed in FDCP 8 (predicted)    GB acc:   BI291728    UniGene:   Rn.146005    Gene Symbol:   Def8_predicted    Locus Link:   307973    Chromosome:   19    Cytoband:   Chr:19q12    SOURCE    GENECARD       Gene Ontology:   biological process:  intracellular signaling cascade      BioCarta Pathways:     KEGG Pathways:  
    Probe set : 1372517_at    Name:   peptidylprolyl isomerase (cyclophilin)-like 1 (predicted)    GB acc:   BI283851    UniGene:   Rn.2196    Gene Symbol:   Ppil1_predicted    Locus Link:   309651    Chromosome:   20    Cytoband:   Chr:20p12    SOURCE    GENECARD       Gene Ontology:   biological process:  protein folding   biological process:  transport   biological process:  neurotransmitter transport   cellular component:  integral to plasma membrane   cellular component:  membrane   cellular component:  integral to membrane      BioCarta Pathways:     KEGG Pathways:  
    Probe set : 1382105_at    Name:   guanine nucleotide binding protein, beta 5    GB acc:   AI603217    UniGene:   Rn.23042    Gene Symbol:   Gnb5    Locus Link:   83579    Chromosome:   8    Cytoband:   Chr:8q24    SOURCE    GENECARD       Gene Ontology:   cellular component:  heterotrimeric G-protein complex   cellular component:  heterotrimeric G-protein complex   molecular function:  GTPase activity   molecular function:  signal transducer activity   molecular function:  small GTPase regulator activity      BioCarta Pathways:     KEGG Pathways:  
    Probe set : 1368637_at    Name:   caspase recruitment domain family, member 9    GB acc:   NM_022303    UniGene:   Rn.64486    Gene Symbol:   Card9    Locus Link:   64171    Chromosome:   3    Cytoband:   Chr:3p13    SOURCE    GENECARD       Gene Ontology:   biological process:  I-kappaB kinase/NF-kappaB cascade   biological process:  regulation of apoptosis   cellular component:  intracellular   molecular function:  protein binding   molecular function:  protein domain specific binding      BioCarta Pathways:     KEGG Pathways:  
    Probe set : 1369552_at    Name:   SAM domain, SH3 domain and nuclear localisation signals, 1    GB acc:   NM_130821    UniGene:   Rn.81187    Gene Symbol:   Samsn1    Locus Link:   170637    Chromosome:   11    Cytoband:   Chr:11p11    SOURCE    GENECARD       Gene Ontology:     BioCarta Pathways:     KEGG Pathways:  
    Probe set : 1387966_at    Name:   asparaginase-like sperm autoantigen    GB acc:   AF329099    UniGene:   Rn.22774    Gene Symbol:   Asrgl1    Locus Link:   246307    Chromosome:   1    Cytoband:   Chr:1q43    SOURCE    GENECARD       Gene Ontology:   biological process:  glycoprotein catabolism   cellular component:  cytoplasm   molecular function:  N4-(beta-N-acetylglucosaminyl)-L-asparaginase activity   molecular function:  asparaginase activity      BioCarta Pathways:     KEGG Pathways:  
    Probe set : 1383603_at    Name:   Transcribed locus, moderately similar to XP_579465.1 PREDICTED: DNA binding protein N5 [Rattus norvegicus]    GB acc:   BM385552    UniGene:   Rn.24481        SOURCE        Gene Ontology:     BioCarta Pathways:     KEGG Pathways:  
    Probe set : 1370909_at    Name:   nucleoporin 62    GB acc:   BI286040    UniGene:   Rn.54450    Gene Symbol:   Nup62    Locus Link:   65274    Chromosome:   1    Cytoband:   Chr:1q22    SOURCE    GENECARD       Gene Ontology:   biological process:  transport   cellular component:  nucleus   cellular component:  nuclear pore   cellular component:  kinesin complex   molecular function:  structural constituent of nuclear pore   molecular function:  protein binding      BioCarta Pathways:     KEGG Pathways:  
    Probe set : 1367844_at    Name:   guanine nucleotide binding protein, alpha inhibiting 2    GB acc:   M12672    UniGene:   Rn.3036    Gene Symbol:   Gnai2    Locus Link:   81664    Chromosome:   8    Cytoband:   Chr:8q32    SOURCE    GENECARD       Gene Ontology:     BioCarta Pathways:     KEGG Pathways:  
    Probe set : 1372847_at    Name:   ACN9 homolog (S. cerevisiae) (predicted)    GB acc:   AW524458    UniGene:   Rn.101965    Gene Symbol:   Acn9_predicted    Locus Link:   362323    Chromosome:   4    Cytoband:   Chr:4q21    SOURCE    GENECARD       Gene Ontology:   cellular component:  mitochondrion      BioCarta Pathways:     KEGG Pathways:  
    Probe set : 1374756_at    Name:   similar to 1500031N24Rik protein (predicted) /// similar to 1500031N24Rik protein    GB acc:   BI295129    UniGene:   Rn.23996    Gene Symbol:   RGD1311188_predicted /// LOC362936    Locus Link:   315088 /// 362936    Chromosome:   7    Cytoband:   Chr:7q34    SOURCE    GENECARD       Gene Ontology:     BioCarta Pathways:     KEGG Pathways:  
    Probe set : 1380327_at    Name:   Transcribed locus, moderately similar to NP_536694.1 helicase (DNA) B [Mus musculus]    GB acc:   AI408747    UniGene:   Rn.43813        SOURCE        Gene Ontology:     BioCarta Pathways:     KEGG Pathways:  
    Probe set : 1388533_at    Name:   CTD (carboxy-terminal domain, RNA polymerase II, polypeptide A) small phosphatase 1 (predicted)    GB acc:   BM390302    UniGene:   Rn.101881    Gene Symbol:   Ctdsp1_predicted    Locus Link:   363249    Chromosome:   9    Cytoband:   Chr:9q33    SOURCE    GENECARD       Gene Ontology:   cellular component:  nucleus   molecular function:  phosphoprotein phosphatase activity   molecular function:  hydrolase activity      BioCarta Pathways:     KEGG Pathways:  
    Probe set : 1369387_at    Name:   vav 1 oncogene    GB acc:   NM_012759    UniGene:   Rn.48861    Gene Symbol:   Vav1    Locus Link:   25156    Chromosome:   9    Cytoband:   Chr:9q11-q12    SOURCE    GENECARD       Gene Ontology:   cellular component:  intracellular   cellular component:  nucleus      BioCarta Pathways:     KEGG Pathways:  
    Probe set : 1392670_at    Name:   Transcribed locus    GB acc:   BF291168    UniGene:   Rn.65172        SOURCE        Gene Ontology:     BioCarta Pathways:     KEGG Pathways:  
    Probe set : 1389093_at     GB acc:   BI281735              Gene Ontology:     BioCarta Pathways:     KEGG Pathways:  
    Probe set : 1375633_at    Name:   chloride intracellular channel 1    GB acc:   AI012221    UniGene:   Rn.116507    Gene Symbol:   RGD:1303043    Locus Link:   406864    Chromosome:   20    Cytoband:   Chr:20p12    SOURCE    GENECARD       Gene Ontology:   biological process:  transport   biological process:  ion transport   biological process:  chloride transport   cellular component:  membrane   molecular function:  ion channel activity   molecular function:  voltage-gated ion channel activity   molecular function:  voltage-gated chloride channel activity      BioCarta Pathways:     KEGG Pathways:  
    Probe set : 1382571_at    Name:   Transcribed locus    GB acc:   AA925335    UniGene:   Rn.54842        SOURCE        Gene Ontology:     BioCarta Pathways:     KEGG Pathways:  
    Probe set : 1375910_at    Name:   CDC42 effector protein (Rho GTPase binding) 3 (predicted)    GB acc:   AA874943    UniGene:   Rn.4040    Gene Symbol:   Cdc42ep3_predicted    Locus Link:   313838    Chromosome:   6    Cytoband:   Chr:6q11    SOURCE    GENECARD       Gene Ontology:   biological process:  signal transduction   molecular function:  protein binding   molecular function:  cytoskeletal regulatory protein binding      BioCarta Pathways:     KEGG Pathways:  
    Probe set : 1368027_at    Name:   thromboxane A synthase 1    GB acc:   NM_012687    UniGene:   Rn.16283    Gene Symbol:   Tbxas1    Locus Link:   24886    Chromosome:   4    Cytoband:   Chr:4q21-q22    SOURCE    GENECARD       Gene Ontology:   cellular component:  integral to membrane      BioCarta Pathways:     KEGG Pathways:  
    Probe set : 1386980_at    Name:   apolipoprotein M    GB acc:   NM_019373    UniGene:   Rn.262    Gene Symbol:   Apom    Locus Link:   55939    Chromosome:   20    Cytoband:   Chr:20p12    SOURCE    GENECARD       Gene Ontology:   biological process:  transport   biological process:  lipid transport   molecular function:  lipid transporter activity   molecular function:  binding      BioCarta Pathways:     KEGG Pathways:  
    Probe set : 1370154_at    Name:   lysozyme    GB acc:   L12458    UniGene:   Rn.2283    Gene Symbol:   Lyz    Locus Link:   25211    Chromosome:   7    Cytoband:   Chr:7q22    SOURCE    GENECARD       Gene Ontology:   cellular component:  extracellular region   cellular component:  extracellular space   cellular component:  mitochondrion      BioCarta Pathways:     KEGG Pathways:  
    Probe set : 1389085_at    Name:   Transcribed locus    GB acc:   BI296359    UniGene:   Rn.129487        SOURCE        Gene Ontology:     BioCarta Pathways:     KEGG Pathways:  
    Probe set : 1382086_at    Name:   Transcribed locus    GB acc:   AI235883    UniGene:   Rn.19680        SOURCE        Gene Ontology:     BioCarta Pathways:     KEGG Pathways:  
    Probe set : 1383349_at    Name:   CDNA clone IMAGE:7304849    GB acc:   BE115594    UniGene:   Rn.19941        SOURCE        Gene Ontology:     BioCarta Pathways:     KEGG Pathways:  
    Probe set : 1368317_at    Name:   aquaporin 7    GB acc:   NM_019157    UniGene:   Rn.11111    Gene Symbol:   Aqp7    Locus Link:   29171    Chromosome:   5    Cytoband:   Chr:5q22    SOURCE    GENECARD       Gene Ontology:   cellular component:  integral to plasma membrane   cellular component:  membrane   cellular component:  integral to membrane      BioCarta Pathways:     KEGG Pathways:  
    Probe set : 1375030_at    Name:   Transcribed locus    GB acc:   AI639285    UniGene:   Rn.28077        SOURCE        Gene Ontology:     BioCarta Pathways:     KEGG Pathways:  
    Probe set : 1372815_at    Name:   mago-nashi homolog, proliferation-associated (Drosophila) (predicted)    GB acc:   BG673028    UniGene:   Rn.8332    Gene Symbol:   Magoh_predicted    Locus Link:   298385    Chromosome:   5    Cytoband:   Chr:5q35    SOURCE    GENECARD       Gene Ontology:   cellular component:  nucleus   molecular function:  RNA binding      BioCarta Pathways:     KEGG Pathways:  
    Probe set : 1385269_s_at    Name:   hypothetical protein LOC304743    GB acc:   BI289167    UniGene:   Rn.79004    Gene Symbol:   LOC304743    Locus Link:   304743    Chromosome:   13    Cytoband:   Chr:13q11    SOURCE    GENECARD       Gene Ontology:     BioCarta Pathways:     KEGG Pathways:  
    Probe set : 1372079_at    Name:   Transcribed locus    GB acc:   AI410099    UniGene:   Rn.19220        SOURCE        Gene Ontology:     BioCarta Pathways:     KEGG Pathways:  
    Probe set : 1369636_at    Name:   sorbitol dehydrogenase    GB acc:   BI285149    UniGene:   Rn.11334    Gene Symbol:   Sord    Locus Link:   24788    Chromosome:   3    Cytoband:   Chr:3q35    SOURCE    GENECARD       Gene Ontology:   biological process:  sorbitol metabolism   biological process:  visual perception      BioCarta Pathways:     KEGG Pathways:  
    Probe set : 1391652_at    Name:   glycine-N-acyltransferase (predicted)    GB acc:   AI411140    UniGene:   Rn.21872    Gene Symbol:   Glyat_predicted    Locus Link:   293779    Chromosome:   1    Cytoband:   Chr:1q43    SOURCE    GENECARD       Gene Ontology:   biological process:  acyl-CoA metabolism   biological process:  transport   biological process:  response to toxin   cellular component:  mitochondrion   cellular component:  membrane      BioCarta Pathways:     KEGG Pathways:  
    Probe set : 1388908_at    Name:   similar to Peci protein    GB acc:   BI278268    UniGene:   Rn.108029    Gene Symbol:   RGD:1359427    Locus Link:   291075    Chromosome:   17    Cytoband:   Chr:17p12    SOURCE    GENECARD       Gene Ontology:   biological process:  metabolism   molecular function:  acyl-CoA binding   molecular function:  catalytic activity      BioCarta Pathways:     KEGG Pathways:  
    Probe set : 1379386_at    Name:   similar to B99 protein    GB acc:   BE107238    UniGene:   Rn.51142    Gene Symbol:   LOC300126    Locus Link:   300126    Chromosome:   7    Cytoband:   Chr:7q34    SOURCE    GENECARD       Gene Ontology:     BioCarta Pathways:     KEGG Pathways:  
    Probe set : 1389785_at    Name:   similar to RIKEN cDNA 0610006H10 gene (predicted)    GB acc:   AA892933    UniGene:   Rn.22552    Gene Symbol:   RGD1305394_predicted    Locus Link:   293653    Chromosome:   1    Cytoband:   Chr:1q42    SOURCE    GENECARD       Gene Ontology:   biological process:  metabolism   molecular function:  hydrolase activity   molecular function:  hydrolase activity, acting on ester bonds      BioCarta Pathways:     KEGG Pathways:  
    Probe set : 1381968_at    Name:   cellular repressor of E1A-stimulated genes (predicted)    GB acc:   AI029175    UniGene:   Rn.8051    Gene Symbol:   Creg_predicted    Locus Link:   289185    Chromosome:   13    Cytoband:   Chr:13q23    SOURCE    GENECARD       Gene Ontology:   molecular function:  RNA polymerase II transcription factor activity   molecular function:  transcription corepressor activity      BioCarta Pathways:     KEGG Pathways:  
    Probe set : 1393956_at    Name:   serine/threonine kinase 17b (apoptosis-inducing)    GB acc:   BI294556    UniGene:   Rn.1300    Gene Symbol:   Stk17b    Locus Link:   170904    Chromosome:   9    Cytoband:   Chr:9q31    SOURCE    GENECARD       Gene Ontology:     BioCarta Pathways:     KEGG Pathways:  
    Probe set : 1398886_at    Name:   similar to Selenoprotein H    GB acc:   BI285703     Gene Symbol:   LOC502642    Locus Link:   502642    Chromosome:   3    Cytoband:   Chr:3q24     GENECARD       Gene Ontology:     BioCarta Pathways:     KEGG Pathways:  
    Probe set : 1373831_at    Name:   hypothetical protein LOC619574    GB acc:   AW920524     Gene Symbol:   LOC619574    Locus Link:   619574    Chromosome:   10    Cytoband:   Chr:10q32     GENECARD       Gene Ontology:     BioCarta Pathways:     KEGG Pathways:  
    Probe set : 1373200_at    Name:   eukaryotic translation elongation factor 1 epsilon 1 (predicted)    GB acc:   AI600237    UniGene:   Rn.98308    Gene Symbol:   Eef1e1_predicted    Locus Link:   291057    Chromosome:   17    Cytoband:   Chr:17p12    SOURCE    GENECARD       Gene Ontology:   biological process:  protein biosynthesis   molecular function:  translation elongation factor activity      BioCarta Pathways:     KEGG Pathways:  
    Probe set : 1393673_at    Name:   similar to HCDI protein (predicted)    GB acc:   BE120005    UniGene:   Rn.38645    Gene Symbol:   RGD1309307_predicted    Locus Link:   361044    Chromosome:   15    Cytoband:   Chr:15p13    SOURCE    GENECARD       Gene Ontology:     BioCarta Pathways:     KEGG Pathways:  
    Probe set : 1370991_at    Name:   camello-like 3    GB acc:   AF187814    UniGene:   Rn.43582    Gene Symbol:   Cml3    Locus Link:   113892    Chromosome:   4    Cytoband:   Chr:4q34    SOURCE    GENECARD       Gene Ontology:   biological process:  regulation of transcription, DNA-dependent   biological process:  negative regulation of cell adhesion      BioCarta Pathways:     KEGG Pathways:  
    Probe set : 1379073_at    Name:   Similar to RIKEN cDNA 2310067G05    GB acc:   AA859663    UniGene:   Rn.41     Locus Link:   301113    Chromosome:   9    Cytoband:   Chr:9q11    SOURCE        Gene Ontology:     BioCarta Pathways:     KEGG Pathways:  
    Probe set : 1387982_at    Name:   toll-like receptor 4    GB acc:   AF057025    UniGene:   Rn.14534    Gene Symbol:   Tlr4    Locus Link:   29260    Chromosome:   5    Cytoband:   Chr:5q24    SOURCE    GENECARD       Gene Ontology:   biological process:  inflammatory response   biological process:  immune response   biological process:  signal transduction   biological process:  I-kappaB kinase/NF-kappaB cascade   biological process:  activation of NF-kappaB-inducing kinase   biological process:  Toll signaling pathway   biological process:  metabolism   biological process:  detection of pathogenic bacteria   biological process:  detection of fungi   biological process:  T-helper 1 type immune response   biological process:  macrophage activation   biological process:  positive regulation of interleukin-12 biosynthesis   biological process:  innate immune response   biological process:  positive regulation of interleukin-1 biosynthesis   biological process:  positive regulation of interleukin-13 biosynthesis   biological process:  positive regulation of interleukin-6 biosynthesis   biological process:  mast cell activation   biological process:  negative regulation of osteoclast differentiation   biological process:  innate immune response      BioCarta Pathways:     KEGG Pathways:  
    Probe set : 1368992_a_at    Name:   splicing factor, arginine/serine-rich 5    GB acc:   AI104005    UniGene:   Rn.54448    Gene Symbol:   Sfrs5    Locus Link:   29667    Chromosome:   6    Cytoband:   Chr:6q24    SOURCE    GENECARD       Gene Ontology:   cellular component:  nucleus   cellular component:  spliceosome complex   molecular function:  nucleic acid binding   molecular function:  RNA binding   molecular function:  nucleotide binding      BioCarta Pathways:     KEGG Pathways:  
    Probe set : 1395030_at    Name:   Basic transcription element binding protein 1    GB acc:   BM390366    UniGene:   Rn.19481    Gene Symbol:   Bteb1    Locus Link:   117560    Chromosome:   1    Cytoband:   Chr:1q51    SOURCE    GENECARD       Gene Ontology:   cellular component:  nucleus      BioCarta Pathways:     KEGG Pathways:  
    Probe set : 1373055_at    Name:   Transcribed locus    GB acc:   AI407547    UniGene:   Rn.13039        SOURCE        Gene Ontology:     BioCarta Pathways:     KEGG Pathways:  
    Probe set : 1384294_at    Name:   Transcribed locus    GB acc:   BF414285    UniGene:   Rn.16262        SOURCE        Gene Ontology:     BioCarta Pathways:     KEGG Pathways:  
    Probe set : 1367929_at    Name:   CD59 antigen    GB acc:   NM_012925    UniGene:   Rn.1231    Gene Symbol:   Cd59    Locus Link:   25407    Chromosome:   3    Cytoband:   Chr:3q32    SOURCE    GENECARD       Gene Ontology:     BioCarta Pathways:     KEGG Pathways:  
    Probe set : 1384275_at    Name:   Transcribed locus    GB acc:   BI300393    UniGene:   Rn.143977        SOURCE        Gene Ontology:     BioCarta Pathways:     KEGG Pathways:  
    Probe set : 1374113_at    Name:   Bromodomain adjacent to zinc finger domain, 1A (predicted)    GB acc:   BM387384    UniGene:   Rn.1864     Locus Link:   314126    Chromosome:   6    Cytoband:   Chr:6q23    SOURCE        Gene Ontology:   biological process:  regulation of transcription, DNA-dependent   biological process:  protein ubiquitination   cellular component:  ubiquitin ligase complex   cellular component:  nucleus   molecular function:  DNA binding   molecular function:  ubiquitin-protein ligase activity   molecular function:  zinc ion binding      BioCarta Pathways:     KEGG Pathways:  
    Probe set : 1390454_at    Name:   4-nitrophenylphosphatase domain and non-neuronal SNAP25-like protein homolog 1 (C. elegans) (predicted)    GB acc:   BI289807    UniGene:   Rn.103205    Gene Symbol:   Nipsnap1_predicted    Locus Link:   360971    Chromosome:   14    Cytoband:   Chr:14q21    SOURCE    GENECARD       Gene Ontology:   cellular component:  mitochondrion      BioCarta Pathways:     KEGG Pathways:  
    Probe set : 1372886_at    Name:   transforming acidic coiled coil 3    GB acc:   AI556917    UniGene:   Rn.58838    Gene Symbol:   RGD:1302948    Locus Link:   360962    Chromosome:   14    Cytoband:   Chr:14q21    SOURCE    GENECARD       Gene Ontology:     BioCarta Pathways:     KEGG Pathways:  
    Probe set : 1387672_at    Name:   glycine N-methyltransferase    GB acc:   NM_017084    UniGene:   Rn.11142    Gene Symbol:   Gnmt    Locus Link:   25134    Chromosome:   9    Cytoband:   Chr:9q12    SOURCE    GENECARD       Gene Ontology:   biological process:  protein modification   biological process:  S-adenosylmethionine metabolism   biological process:  folic acid metabolism      BioCarta Pathways:     KEGG Pathways:  
    Probe set : 1386274_at    Name:   solute carrier family 17 (sodium phosphate), member 2 (predicted)    GB acc:   AI169925    UniGene:   Rn.28363    Gene Symbol:   Slc17a2_predicted    Locus Link:   306950    Chromosome:   17    Cytoband:   Chr:17p11    SOURCE    GENECARD       Gene Ontology:   biological process:  phosphate metabolism   biological process:  transport   biological process:  sodium ion transport   molecular function:  transporter activity   molecular function:  sodium:phosphate symporter activity   molecular function:  symporter activity      BioCarta Pathways:     KEGG Pathways:  
    Probe set : 1377993_at    Name:   similar to hypothetical protein FLJ12681 (predicted)    GB acc:   AI454466    UniGene:   Rn.59746    Gene Symbol:   RGD1310180_predicted    Locus Link:   360495    Chromosome:   10    Cytoband:   Chr:10q12    SOURCE    GENECARD       Gene Ontology:   cellular component:  integral to membrane      BioCarta Pathways:     KEGG Pathways:  
    Probe set : 1391331_at    Name:   Transcribed locus    GB acc:   BE106891    UniGene:   Rn.45499        SOURCE        Gene Ontology:     BioCarta Pathways:     KEGG Pathways:  
    Probe set : 1387053_at    Name:   flavin containing monooxygenase 1    GB acc:   NM_012792    UniGene:   Rn.867    Gene Symbol:   Fmo1    Locus Link:   25256    Chromosome:   13    Cytoband:   Chr:13q22    SOURCE    GENECARD       Gene Ontology:   biological process:  electron transport   biological process:  transport      BioCarta Pathways:     KEGG Pathways:  
    Probe set : 1370134_at    Name:   acetyl-CoA transporter    GB acc:   NM_022252    UniGene:   Rn.144592    Gene Symbol:   Slc33a1    Locus Link:   64018    Chromosome:   2    Cytoband:   Chr:2q31    SOURCE    GENECARD       Gene Ontology:   biological process:  transport   biological process:  central nervous system development   molecular function:  acetyl-CoA transporter activity   molecular function:  acetyl-CoA transporter activity      BioCarta Pathways:     KEGG Pathways:  
    Probe set : 1370184_at    Name:   cofilin 1    GB acc:   BG666614    UniGene:   Rn.11675    Gene Symbol:   Cfl1    Locus Link:   29271    Chromosome:   1    Cytoband:   Chr:1q43    SOURCE    GENECARD       Gene Ontology:   biological process:  protein import into nucleus   biological process:  Rho protein signal transduction   biological process:  actin cytoskeleton organization and biogenesis   molecular function:  actin binding   molecular function:  actin binding      BioCarta Pathways:     KEGG Pathways:  
    Probe set : 1393745_at    Name:   origin recognition complex, subunit 3-like (S. cerevisiae) (predicted)    GB acc:   BE118179    UniGene:   Rn.139979    Gene Symbol:   Orc3l_predicted    Locus Link:   313138    Chromosome:   5    Cytoband:   Chr:5q21    SOURCE    GENECARD       Gene Ontology:   biological process:  DNA replication   cellular component:  nucleus   cellular component:  nuclear origin of replication recognition complex   molecular function:  DNA binding   molecular function:  DNA replication origin binding      BioCarta Pathways:     KEGG Pathways:  
    Probe set : 1372485_at    Name:   pterin 4 alpha carbinolamine dehydratase/dimerization cofactor of hepatocyte nuclear factor 1 alpha (TCF1) 1    GB acc:   BF281220    UniGene:   Rn.81180    Gene Symbol:   Pcbd1    Locus Link:   29700    Chromosome:   20    Cytoband:   Chr:20q11    SOURCE    GENECARD       Gene Ontology:   biological process:  L-phenylalanine metabolism   biological process:  tetrahydrobiopterin biosynthesis   cellular component:  nucleus   cellular component:  cytoplasm   molecular function:  transcription coactivator activity   molecular function:  4-alpha-hydroxytetrahydrobiopterin dehydratase activity   molecular function:  lyase activity      BioCarta Pathways:     KEGG Pathways:  
    Probe set : 1391690_at    Name:   Transcribed locus    GB acc:   AI171008    UniGene:   Rn.148568        SOURCE        Gene Ontology:     BioCarta Pathways:     KEGG Pathways:  
    Probe set : 1374584_at    Name:   similar to Serine/threonine protein kinase 24    GB acc:   BM388112    UniGene:   Rn.105397    Gene Symbol:   LOC361092    Locus Link:   361092    Chromosome:   15    Cytoband:   Chr:15q25    SOURCE    GENECARD       Gene Ontology:     BioCarta Pathways:     KEGG Pathways:  
    Probe set : 1387083_at    Name:   cardiotrophin 1    GB acc:   NM_017129    UniGene:   Rn.10253    Gene Symbol:   Ctf1    Locus Link:   29201    Chromosome:   1    Cytoband:   Chr:1q36    SOURCE    GENECARD       Gene Ontology:   biological process:  nervous system development   biological process:  nervous system development   cellular component:  extracellular space   molecular function:  cytokine activity   molecular function:  cytokine activity      BioCarta Pathways:     KEGG Pathways:  
    Probe set : 1373975_at    Name:   similar to thioether S-methyltransferase    GB acc:   AI232716    UniGene:   Rn.19133    Gene Symbol:   LOC368066    Locus Link:   368066    Chromosome:   4    Cytoband:   Chr:4q24    SOURCE    GENECARD       Gene Ontology:     BioCarta Pathways:     KEGG Pathways:  
    Probe set : 1383557_at    Name:   Transcribed locus    GB acc:   BG665587    UniGene:   Rn.61749        SOURCE        Gene Ontology:     BioCarta Pathways:     KEGG Pathways:  
    Probe set : 1389162_at    Name:   histone cell cycle regulation defective interacting protein 5 (predicted)    GB acc:   BI277328    UniGene:   Rn.48614    Gene Symbol:   Hirip5_predicted    Locus Link:   297416    Chromosome:   4    Cytoband:   Chr:4q34    SOURCE    GENECARD       Gene Ontology:   cellular component:  cytosol   molecular function:  protein binding      BioCarta Pathways:     KEGG Pathways:  
    Probe set : 1393221_at    Name:   similar to 20-alpha-hydroxysteroid dehydrogenase    GB acc:   AA866264    UniGene:   Rn.14713    Gene Symbol:   LOC498789    Locus Link:   498789    Chromosome:   17    Cytoband:   Chr:17q12.3    SOURCE    GENECARD       Gene Ontology:     BioCarta Pathways:     KEGG Pathways:  
    Probe set : 1371409_at    Name:   similar to cDNA sequence BC031181    GB acc:   AI231196    UniGene:   Rn.3311    Gene Symbol:   LOC498886    Locus Link:   498886    Chromosome:   18    Cytoband:   Chr:18q12.3    SOURCE    GENECARD       Gene Ontology:     BioCarta Pathways:     KEGG Pathways:  
    Probe set : 1376951_at    Name:   MAD2 (mitotic arrest deficient, homolog)-like 1 (yeast) (predicted)    GB acc:   AI101906    UniGene:   Rn.19771     Locus Link:   297176    Chromosome:   4    Cytoband:   Chr:4q31    SOURCE        Gene Ontology:     BioCarta Pathways:     KEGG Pathways:  
    Probe set : 1393929_at    Name:   chemokine (C-C motif) receptor 6 (predicted)    GB acc:   AI045155    UniGene:   Rn.36521    Gene Symbol:   Ccr6_predicted    Locus Link:   308163    Chromosome:   1    Cytoband:   Chr:1q11    SOURCE    GENECARD       Gene Ontology:   cellular component:  integral to plasma membrane   cellular component:  integral to membrane      BioCarta Pathways:     KEGG Pathways:  
    Probe set : 1392975_at    Name:   Transcribed locus, strongly similar to XP_525786.1 PREDICTED: similar to chromosome 2 open reading frame 7 [Pan troglodytes]    GB acc:   AI058938    UniGene:   Rn.153870        SOURCE        Gene Ontology:     BioCarta Pathways:     KEGG Pathways:  
    Probe set : 1376226_at    Name:   Similar to Ab2-076    GB acc:   BG377636    UniGene:   Rn.98393     Locus Link:   365096    Chromosome:   1    Cytoband:   Chr:1q11    SOURCE        Gene Ontology:     BioCarta Pathways:     KEGG Pathways:  
    Probe set : 1387300_at    Name:   crooked neck protein    GB acc:   NM_053797    UniGene:   Rn.127803    Gene Symbol:   Crnkl1    Locus Link:   116481    Chromosome:   3    Cytoband:   Chr:3q41    SOURCE    GENECARD       Gene Ontology:     BioCarta Pathways:     KEGG Pathways:  
    Probe set : 1386867_at    Name:   brain protein 44-like    GB acc:   NM_133561    UniGene:   Rn.3718    Gene Symbol:   Brp44l    Locus Link:   171087    Chromosome:   1    Cytoband:   Chr:1q11    SOURCE    GENECARD       Gene Ontology:     BioCarta Pathways:     KEGG Pathways:  
    Probe set : 1398507_at     GB acc:   BE108415              Gene Ontology:     BioCarta Pathways:     KEGG Pathways:  
    Probe set : 1371732_at    Name:   dermatopontin (predicted)    GB acc:   BI285485    UniGene:   Rn.8396    Gene Symbol:   Dpt_predicted    Locus Link:   289178    Chromosome:   13    Cytoband:   Chr:13q23    SOURCE    GENECARD       Gene Ontology:   biological process:  cell adhesion   cellular component:  extracellular matrix (sensu Metazoa)   cellular component:  extracellular space   molecular function:  protein binding      BioCarta Pathways:     KEGG Pathways:  
    Probe set : 1383640_at    Name:   Phosphatidylinositol glycan, class N (predicted)    GB acc:   BI294569    UniGene:   Rn.131689     Locus Link:   309051    Chromosome:   13    Cytoband:   Chr:13p13    SOURCE        Gene Ontology:   cellular component:  extracellular space   cellular component:  integral to membrane      BioCarta Pathways:     KEGG Pathways:  
    Probe set : 1388628_at    Name:   integral type I protein    GB acc:   BI284430    UniGene:   Rn.98226    Gene Symbol:   RGD:1303327    Locus Link:   300888    Chromosome:   8    Cytoband:   Chr:8q31    SOURCE    GENECARD       Gene Ontology:   biological process:  transport   biological process:  intracellular protein transport   biological process:  protein transport   cellular component:  Golgi stack   cellular component:  membrane   cellular component:  integral to membrane   molecular function:  protein carrier activity      BioCarta Pathways:     KEGG Pathways:  
    Probe set : 1388892_at    Name:   RAB2B, member RAS oncogene family (predicted)    GB acc:   BF395178    UniGene:   Rn.85120    Gene Symbol:   Rab2b_predicted    Locus Link:   305853    Chromosome:   15    Cytoband:   Chr:15p14    SOURCE    GENECARD       Gene Ontology:   biological process:  transport   biological process:  small GTPase mediated signal transduction   biological process:  protein transport   cellular component:  endoplasmic reticulum   cellular component:  Golgi apparatus   molecular function:  GTP binding      BioCarta Pathways:     KEGG Pathways:  
    Probe set : 1372167_at     GB acc:   AW525656              Gene Ontology:     BioCarta Pathways:     KEGG Pathways:  
    Probe set : 1371491_at    Name:   Notch gene homolog 1 (Drosophila)    GB acc:   BM390614    UniGene:   Rn.25046    Gene Symbol:   Notch1    Locus Link:   25496    Chromosome:   3    Cytoband:   Chr:3p13    SOURCE    GENECARD       Gene Ontology:   biological process:  cell fate specification   biological process:  epithelial to mesenchymal transition   biological process:  regulation of transcription, DNA-dependent   biological process:  immune response   biological process:  Notch signaling pathway   biological process:  development   biological process:  determination of left/right symmetry   biological process:  compartment specification   biological process:  endoderm development   biological process:  heart development   biological process:  epidermis development   biological process:  embryonic development   biological process:  cell differentiation   biological process:  keratinocyte differentiation   biological process:  hair follicle morphogenesis   biological process:  positive regulation of apoptosis   biological process:  positive regulation of transcription from RNA polymerase II promoter   biological process:  somatic stem cell division   biological process:  regulation of development   cellular component:  extracellular space   cellular component:  membrane   cellular component:  integral to membrane      BioCarta Pathways:     KEGG Pathways:  
    Probe set : 1387062_a_at    Name:   checkpoint kinase 1 homolog (S. pombe)    GB acc:   NM_080400    UniGene:   Rn.33267    Gene Symbol:   Chek1    Locus Link:   140583    Chromosome:   8    Cytoband:   Chr:8q21    SOURCE    GENECARD       Gene Ontology:   cellular component:  condensed nuclear chromosome   cellular component:  nucleus      BioCarta Pathways:     KEGG Pathways:  
    Probe set : 1368038_at    Name:   synaptojanin 2 binding protein    GB acc:   AF260258    UniGene:   Rn.6258    Gene Symbol:   Synj2bp    Locus Link:   64531    Chromosome:   6    Cytoband:   Chr:6q24    SOURCE    GENECARD       Gene Ontology:   molecular function:  receptor activity   molecular function:  protein binding      BioCarta Pathways:     KEGG Pathways:  
    Probe set : 1377867_at    Name:   similar to Glutaminyl-peptide cyclotransferase precursor (QC) (Glutaminyl-tRNA cyclotransferase) (Glutaminyl cyclase)    GB acc:   BM390001    UniGene:   Rn.7474    Gene Symbol:   LOC313837    Locus Link:   313837    Chromosome:   6    Cytoband:   Chr:6q11    SOURCE    GENECARD       Gene Ontology:     BioCarta Pathways:     KEGG Pathways:  
    Probe set : 1398354_at    Name:   catenin alpha-like 1 (predicted)    GB acc:   AI555457    UniGene:   Rn.33021    Gene Symbol:   Catnal1_predicted    Locus Link:   298019    Chromosome:   5    Cytoband:   Chr:5q24    SOURCE    GENECARD       Gene Ontology:   biological process:  Rho protein signal transduction   cellular component:  cytosol      BioCarta Pathways:     KEGG Pathways:  
    Probe set : 1375270_at    Name:   Transcribed locus    GB acc:   BM384026    UniGene:   Rn.22376        SOURCE        Gene Ontology:     BioCarta Pathways:     KEGG Pathways:  
    Probe set : 1382464_at    Name:   similar to ENSANGP00000021391 (predicted)    GB acc:   BF547020    UniGene:   Rn.29863    Gene Symbol:   RGD1309779_predicted    Locus Link:   363074    Chromosome:   8    Cytoband:   Chr:8q24    SOURCE    GENECARD       Gene Ontology:     BioCarta Pathways:     KEGG Pathways:  
    Probe set : 1388345_at     GB acc:   AI599882              Gene Ontology:     BioCarta Pathways:     KEGG Pathways:  
    Probe set : 1371794_at    Name:   Transcribed locus, strongly similar to NP_035873.1 SH3-domain binding protein 3 [Mus musculus]    GB acc:   BM391449    UniGene:   Rn.6467        SOURCE        Gene Ontology:     BioCarta Pathways:     KEGG Pathways:  
    Probe set : 1369491_at    Name:   D-amino acid oxidase    GB acc:   NM_053626    UniGene:   Rn.81058    Gene Symbol:   Dao1    Locus Link:   114027    Chromosome:   12    Cytoband:   Chr:12q16    SOURCE    GENECARD       Gene Ontology:   biological process:  electron transport   biological process:  tRNA aminoacylation for protein translation   cellular component:  extracellular space   cellular component:  peroxisome      BioCarta Pathways:     KEGG Pathways:  
    Probe set : 1371489_at    Name:   Ring finger protein 4    GB acc:   AI011748    UniGene:   Rn.30015    Gene Symbol:   Rnf4    Locus Link:   29274    Chromosome:   14    Cytoband:   Chr:14q21    SOURCE    GENECARD       Gene Ontology:   cellular component:  ubiquitin ligase complex   cellular component:  nucleus   cellular component:  nucleus      BioCarta Pathways:     KEGG Pathways:  
    Probe set : 1380507_at    Name:   Similar to hypothetical protein MGC33630    GB acc:   BF283420    UniGene:   Rn.43626     Locus Link:   304498    Chromosome:   12    Cytoband:   Chr:12q16    SOURCE        Gene Ontology:     BioCarta Pathways:     KEGG Pathways:  
    Probe set : 1389326_at    Name:   replication factor C (activator 1) 3 (predicted)    GB acc:   AI103093    UniGene:   Rn.3711    Gene Symbol:   Rfc3_predicted    Locus Link:   288414    Chromosome:   12    Cytoband:   Chr:12p12    SOURCE    GENECARD       Gene Ontology:   biological process:  DNA replication   biological process:  DNA strand elongation      BioCarta Pathways:     KEGG Pathways:  
    Probe set : 1396301_x_at    Name:   Transcribed locus    GB acc:   BI277442    UniGene:   Rn.148355        SOURCE        Gene Ontology:     BioCarta Pathways:     KEGG Pathways:  
    Probe set : 1367849_at    Name:   syndecan 1    GB acc:   NM_013026    UniGene:   Rn.11176    Gene Symbol:   Sdc1    Locus Link:   25216    Chromosome:   6    Cytoband:   Chr:6q14    SOURCE    GENECARD       Gene Ontology:   molecular function:  cytoskeletal protein binding      BioCarta Pathways:     KEGG Pathways:  
    Probe set : 1372248_at    Name:   sestrin 1 (predicted)    GB acc:   AI411742    UniGene:   Rn.22395    Gene Symbol:   Sesn1_predicted    Locus Link:   294518    Chromosome:   20    Cytoband:   Chr:20q13    SOURCE    GENECARD       Gene Ontology:   biological process:  response to DNA damage stimulus   biological process:  cell cycle arrest   biological process:  negative regulation of cell proliferation   cellular component:  nucleus      BioCarta Pathways:     KEGG Pathways:  
    Probe set : 1371941_at    Name:   similar to RIKEN cDNA 5230400G24 (predicted)    GB acc:   AI411390    UniGene:   Rn.93479    Gene Symbol:   RGD1310230_predicted    Locus Link:   301563    Chromosome:   9    Cytoband:   Chr:9q34    SOURCE    GENECARD       Gene Ontology:     BioCarta Pathways:     KEGG Pathways:  
    Probe set : 1387011_at    Name:   lipocalin 2    GB acc:   NM_130741    UniGene:   Rn.11303    Gene Symbol:   Lcn2    Locus Link:   170496    Chromosome:   3    Cytoband:   Chr:3p11    SOURCE    GENECARD       Gene Ontology:   biological process:  transport   biological process:  apoptosis   cellular component:  extracellular space   cellular component:  soluble fraction   cellular component:  cytoplasm   molecular function:  transporter activity   molecular function:  binding      BioCarta Pathways:     KEGG Pathways:  
    Probe set : 1379626_at    Name:   Special AT-rich sequence binding protein 1 (predicted)    GB acc:   AA946353    UniGene:   Rn.21462     Locus Link:   316164    Chromosome:   9    Cytoband:   Chr:9q11    SOURCE        Gene Ontology:   cellular component:  nucleus   cellular component:  nuclear heterochromatin   molecular function:  DNA binding   molecular function:  double-stranded DNA binding   molecular function:  transcription factor activity      BioCarta Pathways:     KEGG Pathways:  
    Probe set : 1391572_at    Name:   cysteinyl-tRNA synthetase (predicted)    GB acc:   BE109168    UniGene:   Rn.14865    Gene Symbol:   Cars_predicted    Locus Link:   293638    Chromosome:   1    Cytoband:   Chr:1q41    SOURCE    GENECARD       Gene Ontology:   biological process:  protein biosynthesis   biological process:  cysteinyl-tRNA aminoacylation   cellular component:  soluble fraction   cellular component:  cytoplasm      BioCarta Pathways:     KEGG Pathways:  
    Probe set : 1375504_at    Name:   Polymerase (DNA directed), gamma 2, accessory subunit (predicted)    GB acc:   BM390747    UniGene:   Rn.25075     Locus Link:   303612    Chromosome:   10    Cytoband:   Chr:10q32.1    SOURCE        Gene Ontology:   cellular component:  mitochondrion      BioCarta Pathways:     KEGG Pathways:  
    Probe set : 1395396_at    Name:   similar to CG9135-PA (predicted)    GB acc:   AA848942    UniGene:   Rn.98282    Gene Symbol:   RGD1309986_predicted    Locus Link:   298594    Chromosome:   5    Cytoband:   Chr:5q36    SOURCE    GENECARD       Gene Ontology:     BioCarta Pathways:     KEGG Pathways:  
    Probe set : 1387038_at    Name:   copper chaperone for superoxide dismutase    GB acc:   NM_053425    UniGene:   Rn.12311    Gene Symbol:   Ccs    Locus Link:   84485    Chromosome:   1    Cytoband:   Chr:1q43    SOURCE    GENECARD       Gene Ontology:   cellular component:  soluble fraction   cellular component:  cytoplasm      BioCarta Pathways:     KEGG Pathways:  
    Probe set : 1381976_at    Name:   kinesin family member 21A (predicted)    GB acc:   BE103004    UniGene:   Rn.52470    Gene Symbol:   Kif21a_predicted    Locus Link:   300158    Chromosome:   7    Cytoband:   Chr:7q35    SOURCE    GENECARD       Gene Ontology:   biological process:  microtubule-based process   biological process:  microtubule-based movement   cellular component:  kinesin complex   cellular component:  microtubule associated complex   molecular function:  motor activity   molecular function:  microtubule motor activity   molecular function:  ATP binding      BioCarta Pathways:     KEGG Pathways:  
    Probe set : 1383844_at    Name:   Transcribed locus    GB acc:   BI296898    UniGene:   Rn.26985        SOURCE        Gene Ontology:     BioCarta Pathways:     KEGG Pathways:  
    Probe set : 1380617_at    Name:   GTPase, IMAP family member 5    GB acc:   BI294743    UniGene:   Rn.83549    Gene Symbol:   Gimap5    Locus Link:   246774    Chromosome:   4    Cytoband:   Chr:4q24    SOURCE    GENECARD       Gene Ontology:   molecular function:  GTP binding   molecular function:  nucleotide binding      BioCarta Pathways:     KEGG Pathways:  
    Probe set : 1392947_at    Name:   CDNA clone IMAGE:7379585    GB acc:   AI030349    UniGene:   Rn.6374        SOURCE        Gene Ontology:     BioCarta Pathways:     KEGG Pathways:  
    Probe set : 1368563_at    Name:   aspartoacylase    GB acc:   NM_024399    UniGene:   Rn.21677    Gene Symbol:   Aspa    Locus Link:   79251      SOURCE    GENECARD       Gene Ontology:   biological process:  acetate metabolism   biological process:  aspartate catabolism   biological process:  metabolism      BioCarta Pathways:     KEGG Pathways:  
    Probe set : 1382221_at    Name:   Transcribed locus    GB acc:   AI176944    UniGene:   Rn.15880        SOURCE        Gene Ontology:     BioCarta Pathways:     KEGG Pathways:  
    Probe set : 1370443_at    Name:   deoxyribonuclease II    GB acc:   AF178975    UniGene:   Rn.9509    Gene Symbol:   Dnase2    Locus Link:   171575    Chromosome:   19    Cytoband:   Chr:19q11    SOURCE    GENECARD       Gene Ontology:   cellular component:  extracellular space   cellular component:  lysosome      BioCarta Pathways:     KEGG Pathways:  
    Probe set : 1370462_at    Name:   hyaluronan mediated motility receptor    GB acc:   AI171185    UniGene:   Rn.92304    Gene Symbol:   Hmmr    Locus Link:   25460    Chromosome:   10    Cytoband:   Chr:10q12    SOURCE    GENECARD       Gene Ontology:   biological process:  cell motility   biological process:  cytoskeleton organization and biogenesis   biological process:  cell-cell signaling   molecular function:  hyaluronic acid binding   molecular function:  hyaluronic acid binding   molecular function:  receptor activity      BioCarta Pathways:     KEGG Pathways:  
    Probe set : 1367581_a_at    Name:   secreted phosphoprotein 1    GB acc:   AB001382    UniGene:   Rn.8871    Gene Symbol:   Spp1    Locus Link:   25353    Chromosome:   14    Cytoband:   Chr:14p22    SOURCE    GENECARD       Gene Ontology:   biological process:  ossification   biological process:  anti-apoptosis   biological process:  inflammatory response   biological process:  cell adhesion   biological process:  cell adhesion   biological process:  cell-matrix adhesion   biological process:  cell-cell signaling   biological process:  negative regulation of bone mineralization   biological process:  immune cell chemotaxis   biological process:  T-helper 1 type immune response   biological process:  positive regulation of T cell proliferation   biological process:  regulation of myeloid cell differentiation   biological process:  induction of positive chemotaxis   biological process:  anti-apoptosis   biological process:  inflammatory response   biological process:  cell-matrix adhesion   biological process:  cell-cell signaling   biological process:  negative regulation of bone mineralization   biological process:  immune cell chemotaxis   biological process:  T-helper 1 type immune response   biological process:  positive regulation of T cell proliferation   biological process:  regulation of myeloid cell differentiation   biological process:  induction of positive chemotaxis      BioCarta Pathways:     KEGG Pathways:  
    Probe set : 1380115_at     GB acc:   AA850751              Gene Ontology:     BioCarta Pathways:     KEGG Pathways:  
    Probe set : 1370228_at    Name:   Transferrin /// signal recognition particle receptor, B subunit (predicted)    GB acc:   AA945178    UniGene:   Rn.91296    Gene Symbol:   Tf /// Srprb_predicted    Locus Link:   24825 /// 300965    Chromosome:   8    Cytoband:   Chr:8q32    SOURCE    GENECARD       Gene Ontology:     BioCarta Pathways:     KEGG Pathways:  
    Probe set : 1387975_at    Name:   UDP-glucose ceramide glucosyltransferase    GB acc:   AF047707    UniGene:   Rn.24091    Gene Symbol:   Ugcg    Locus Link:   83626    Chromosome:   5    Cytoband:   Chr:5q24    SOURCE    GENECARD       Gene Ontology:   cellular component:  endoplasmic reticulum   cellular component:  integral to membrane      BioCarta Pathways:     KEGG Pathways:  
    Probe set : 1383323_at    Name:   similar to RIKEN cDNA 1700022L09    GB acc:   AW435234    UniGene:   Rn.117385    Gene Symbol:   LOC499933    Locus Link:   499933    Chromosome:   3    Cytoband:   Chr:3q42    SOURCE    GENECARD       Gene Ontology:     BioCarta Pathways:     KEGG Pathways:  
    Probe set : 1389645_at    Name:   proline dehydrogenase (oxidase) 2 (predicted)    GB acc:   AI233266    UniGene:   Rn.4247    Gene Symbol:   Prodh2_predicted    Locus Link:   361538    Chromosome:   1    Cytoband:   Chr:1q21    SOURCE    GENECARD       Gene Ontology:   biological process:  glutamate biosynthesis   biological process:  proline catabolism   molecular function:  proline dehydrogenase activity      BioCarta Pathways:     KEGG Pathways:  
    Probe set : 1372457_at    Name:   mitochondrial tumor suppressor 1    GB acc:   BF284182    UniGene:   Rn.95344    Gene Symbol:   Mtus1    Locus Link:   306487    Chromosome:   16    Cytoband:   Chr:16q12.1    SOURCE    GENECARD       Gene Ontology:   molecular function:  receptor activity      BioCarta Pathways:     KEGG Pathways:  
    Probe set : 1388809_at    Name:   acid sphingomyelinase-like phosphodiesterase 3A    GB acc:   BM389498    UniGene:   Rn.8142    Gene Symbol:   RGD:1359277    Locus Link:   294422    Chromosome:   20    Cytoband:   Chr:20q11    SOURCE    GENECARD       Gene Ontology:   biological process:  carbohydrate metabolism   molecular function:  hydrolase activity   molecular function:  hydrolase activity, acting on glycosyl bonds      BioCarta Pathways:     KEGG Pathways:  
    Probe set : 1393793_at    Name:   Transcribed locus    GB acc:   AW524669    UniGene:   Rn.125399        SOURCE        Gene Ontology:     BioCarta Pathways:     KEGG Pathways:  
    Probe set : 1368377_at    Name:   granzyme C    GB acc:   NM_134332    UniGene:   Rn.88335    Gene Symbol:   Gzmc    Locus Link:   171290    Chromosome:   15    Cytoband:   Chr:15p12    SOURCE    GENECARD       Gene Ontology:   biological process:  proteolysis   biological process:  cytolysis   cellular component:  extracellular space      BioCarta Pathways:     KEGG Pathways:  
    Probe set : 1368361_a_at    Name:   protein tyrosine phosphatase, non-receptor type 2    GB acc:   NM_053990    UniGene:   Rn.33497    Gene Symbol:   Ptpn2    Locus Link:   117063    Chromosome:   18    Cytoband:   Chr:18q12.1    SOURCE    GENECARD       Gene Ontology:   biological process:  protein amino acid dephosphorylation      BioCarta Pathways:     KEGG Pathways:  
    Probe set : 1378292_at    Name:   Transcribed locus    GB acc:   BE096367    UniGene:   Rn.24358        SOURCE        Gene Ontology:     BioCarta Pathways:     KEGG Pathways:  
    Probe set : 1379275_at    Name:   sorting nexin 10 (predicted)    GB acc:   AI007889    UniGene:   Rn.44192    Gene Symbol:   Snx10_predicted    Locus Link:   297096    Chromosome:   4    Cytoband:   Chr:4q24    SOURCE    GENECARD       Gene Ontology:   molecular function:  protein transporter activity      BioCarta Pathways:     KEGG Pathways:  
    Probe set : 1386901_at    Name:   similar to fatty acid translocase/CD36    GB acc:   NM_031561    UniGene:   Rn.3790    Gene Symbol:   LOC499984    Locus Link:   499984    Chromosome:   4    Cytoband:   Chr:4q11    SOURCE    GENECARD       Gene Ontology:     BioCarta Pathways:     KEGG Pathways:  
    Probe set : 1368573_at    Name:   karyopherin (importin) beta 1    GB acc:   NM_017063    UniGene:   Rn.11061    Gene Symbol:   Kpnb1    Locus Link:   24917    Chromosome:   10    Cytoband:   Chr:10q32.1    SOURCE    GENECARD       Gene Ontology:   cellular component:  nucleus   cellular component:  nuclear pore   cellular component:  cytoplasm      BioCarta Pathways:     KEGG Pathways:  
    Probe set : 1383255_at    Name:   Transcribed locus    GB acc:   BE116202    UniGene:   Rn.147981        SOURCE        Gene Ontology:     BioCarta Pathways:     KEGG Pathways:  
    Probe set : 1382175_at    Name:   Similar to Wilms tumor 1-associating protein (WT1-associated protein) (Putative pre-mRNA splicing regulator female-lethal(2D) homolog)    GB acc:   AW534129    UniGene:   Rn.22883     Locus Link:   499020      SOURCE        Gene Ontology:     BioCarta Pathways:     KEGG Pathways:  
    Probe set : 1389066_at    Name:   Down syndrome critical region gene 1-like 1    GB acc:   BI274408    UniGene:   Rn.24428    Gene Symbol:   Dscr1l1    Locus Link:   140666    Chromosome:   9    Cytoband:   Chr:9q12    SOURCE    GENECARD       Gene Ontology:   biological process:  central nervous system development   biological process:  calcium-mediated signaling   molecular function:  protein phosphatase 2B binding   molecular function:  nucleotide binding      BioCarta Pathways:     KEGG Pathways:  
    Probe set : 1378415_at    Name:   Protein kinase, cAMP-dependent, regulatory, type 2, alpha    GB acc:   BM386879    UniGene:   Rn.9742    Gene Symbol:   Prkar2a    Locus Link:   29699    Chromosome:   8    Cytoband:   Chr:8q32    SOURCE    GENECARD       Gene Ontology:     BioCarta Pathways:     KEGG Pathways:  
    Probe set : 1379419_at    Name:   DNA sequence AY228474    GB acc:   AA962979    UniGene:   Rn.12069    Gene Symbol:   AY228474    Locus Link:   291946    Chromosome:   19    Cytoband:   Chr:19q11    SOURCE    GENECARD       Gene Ontology:     BioCarta Pathways:     KEGG Pathways:  
    Probe set : 1377223_at    Name:   Transcribed locus    GB acc:   AI599621    UniGene:   Rn.147389        SOURCE        Gene Ontology:     BioCarta Pathways:     KEGG Pathways:  
    Probe set : 1373499_at    Name:   growth arrest specific 5    GB acc:   BF287008    UniGene:   Rn.145965    Gene Symbol:   Gas5    Locus Link:   81714    Chromosome:   13    Cytoband:   Chr:13q22    SOURCE    GENECARD       Gene Ontology:   biological process:  rRNA processing      BioCarta Pathways:     KEGG Pathways:  
    Probe set : 1388791_at    Name:   similar to 2810022L02Rik protein (predicted)    GB acc:   BI275911    UniGene:   Rn.14294    Gene Symbol:   RGD1309930_predicted    Locus Link:   316426    Chromosome:   9    Cytoband:   Chr:9q31    SOURCE    GENECARD       Gene Ontology:     BioCarta Pathways:     KEGG Pathways:  
    Probe set : 1380583_s_at    Name:   colony stimulating factor 1 (macrophage)    GB acc:   AW535553    UniGene:   Rn.83632    Gene Symbol:   Csf1    Locus Link:   78965    Chromosome:   2    Cytoband:   Chr:2q34    SOURCE    GENECARD       Gene Ontology:   biological process:  ossification   biological process:  ossification   biological process:  development   biological process:  development   biological process:  cell proliferation   biological process:  positive regulation of cell proliferation   biological process:  hemopoiesis   biological process:  cell differentiation   biological process:  macrophage differentiation   biological process:  macrophage differentiation   biological process:  regulation of ossification   biological process:  osteoclast differentiation   biological process:  osteoclast differentiation   biological process:  osteoclast differentiation   biological process:  positive regulation of body size   biological process:  odontogenesis   biological process:  odontogenesis   biological process:  positive regulation of odontogenesis (sensu Vertebrata)   biological process:  regulation of ossification   biological process:  positive regulation of body size   biological process:  positive regulation of odontogenesis (sensu Vertebrata)   biological process:  positive regulation of osteoclast differentiation   molecular function:  cytokine activity   molecular function:  macrophage colony stimulating factor receptor binding   molecular function:  growth factor activity      BioCarta Pathways:     KEGG Pathways:  
    Probe set : 1389222_at    Name:   Tryptophan hydroxylase    GB acc:   BI282847    UniGene:   Rn.9392    Gene Symbol:   Tph    Locus Link:   24848    Chromosome:   1    Cytoband:   Chr:1q22    SOURCE    GENECARD       Gene Ontology:   cellular component:  extracellular region      BioCarta Pathways:     KEGG Pathways:  
    Probe set : 1370483_at    Name:   CD244 natural killer cell receptor 2B4    GB acc:   AF209406    UniGene:   Rn.53994    Gene Symbol:   Cd244    Locus Link:   64025    Chromosome:   13    Cytoband:   Chr:13q24    SOURCE    GENECARD       Gene Ontology:   biological process:  lymphocyte activation   cellular component:  integral to membrane   molecular function:  receptor activity      BioCarta Pathways:     KEGG Pathways:  
    Probe set : 1369169_at    Name:   solute carrier family 23 (nucleobase transporters), member 1    GB acc:   NM_017315    UniGene:   Rn.144579    Gene Symbol:   Slc23a1    Locus Link:   50621    Chromosome:   18    Cytoband:   Chr:18p11    SOURCE    GENECARD       Gene Ontology:   biological process:  transport   biological process:  sodium ion transport   biological process:  ion transport      BioCarta Pathways:     KEGG Pathways:  
    Probe set : 1385832_s_at    Name:   Similar to RIKEN cDNA 1200013B08    GB acc:   AA924459    UniGene:   Rn.15328     Locus Link:   317578    Chromosome:   X    Cytoband:   Chr:Xq35    SOURCE        Gene Ontology:     BioCarta Pathways:     KEGG Pathways:  
    Probe set : 1372671_at    Name:   similar to RIKEN cDNA 0610038L10 gene /// similar to riboflavin kinase /// similar to riboflavin kinase    GB acc:   BI284293    UniGene:   Rn.13360    Gene Symbol:   LOC317214 /// LOC499328 /// LOC501601    Locus Link:   317214 /// 499328 /// 501601    Chromosome:   X    Cytoband:   Chr:Xq31 /// Chr:1q43    SOURCE    GENECARD       Gene Ontology:   biological process:  riboflavin biosynthesis   molecular function:  riboflavin kinase activity      BioCarta Pathways:     KEGG Pathways:  
    Probe set : 1372069_at    Name:   ankyrin repeat domain 15 (predicted)    GB acc:   BF284716    UniGene:   Rn.86207    Gene Symbol:   Ankrd15_predicted    Locus Link:   309429    Chromosome:   1    Cytoband:   Chr:1q51    SOURCE    GENECARD       Gene Ontology:   biological process:  immune response   biological process:  negative regulation of progression through cell cycle      BioCarta Pathways:     KEGG Pathways:  
    Probe set : 1389176_at    Name:   Inositol polyphosphate-5-phosphatase F (predicted)    GB acc:   BF282674    UniGene:   Rn.60475     Locus Link:   309008    Chromosome:   1    Cytoband:   Chr:1q36-q37    SOURCE        Gene Ontology:     BioCarta Pathways:     KEGG Pathways:  
    Probe set : 1370289_x_at    Name:   kallikrein, submaxillary gland S3    GB acc:   M11566    UniGene:   Rn.34527    Gene Symbol:   Klks3    Locus Link:   292868    Chromosome:   1    Cytoband:   Chr:1q22    SOURCE    GENECARD       Gene Ontology:   biological process:  proteolysis   biological process:  positive regulation of vasoconstriction      BioCarta Pathways:     KEGG Pathways:  
    Probe set : 1388974_at    Name:   signal recognition particle 19 (predicted)    GB acc:   AI101527    UniGene:   Rn.116072    Gene Symbol:   Srp19_predicted    Locus Link:   291685    Chromosome:   18    Cytoband:   Chr:18p12    SOURCE    GENECARD       Gene Ontology:   biological process:  protein targeting   biological process:  cotranslational protein targeting to membrane   cellular component:  signal recognition particle (sensu Eukaryota)   cellular component:  ribonucleoprotein complex   molecular function:  RNA binding   molecular function:  7S RNA binding      BioCarta Pathways:     KEGG Pathways:  
    Probe set : 1379642_at    Name:   Transcribed locus    GB acc:   BM386116    UniGene:   Rn.148999        SOURCE        Gene Ontology:     BioCarta Pathways:     KEGG Pathways:  
    Probe set : 1371925_at    Name:   ATPase type 13A (predicted)    GB acc:   AA893621    UniGene:   Rn.3697    Gene Symbol:   Atp13a_predicted    Locus Link:   290673    Chromosome:   16    Cytoband:   Chr:16p14    SOURCE    GENECARD       Gene Ontology:   biological process:  cation transport   biological process:  metabolism   cellular component:  membrane   cellular component:  integral to membrane      BioCarta Pathways:     KEGG Pathways:  
    Probe set : 1370832_at    Name:   small inducible cytokine A4    GB acc:   U06434    UniGene:   Rn.37880    Gene Symbol:   Ccl4    Locus Link:   116637    Chromosome:   10    Cytoband:   Chr:10q26    SOURCE    GENECARD       Gene Ontology:   cellular component:  extracellular region   cellular component:  extracellular space   molecular function:  chemokine activity   molecular function:  cytokine activity      BioCarta Pathways:     KEGG Pathways:  
    Probe set : 1383548_at    Name:   Similar to hypothetical protein FLJ20189 (predicted)    GB acc:   BI290887    UniGene:   Rn.15268     Locus Link:   315434    Chromosome:   8    Cytoband:   Chr:8q11    SOURCE        Gene Ontology:     BioCarta Pathways:     KEGG Pathways:  
    Probe set : 1367983_at    Name:   flap structure-specific endonuclease 1    GB acc:   NM_053430    UniGene:   Rn.16664    Gene Symbol:   Fen1    Locus Link:   84490    Chromosome:   1    Cytoband:   Chr:1q43    SOURCE    GENECARD       Gene Ontology:   cellular component:  nucleus      BioCarta Pathways:     KEGG Pathways:  
    Probe set : 1390142_at    Name:   Zinc finger, CW-type with coiled-coil domain 3 (predicted)    GB acc:   BE111888    UniGene:   Rn.17121     Locus Link:   304074    Chromosome:   11    Cytoband:   Chr:11q11    SOURCE        Gene Ontology:   molecular function:  ATP binding      BioCarta Pathways:     KEGG Pathways:  
    Probe set : 1374499_at    Name:   TATA box binding protein (Tbp)-associated factor, RNA polymerase I, A (predicted)    GB acc:   BM383402    UniGene:   Rn.104371     Locus Link:   360893    Chromosome:   13    Cytoband:   Chr:13q26    SOURCE        Gene Ontology:   biological process:  transcription from RNA polymerase I promoter   biological process:  transcription from RNA polymerase II promoter   biological process:  transport   cellular component:  RNA polymerase I transcription factor complex   cellular component:  membrane      BioCarta Pathways:     KEGG Pathways:  
    Probe set : 1389403_at    Name:   bone morphogenetic protein 7    GB acc:   AI013715    UniGene:   Rn.18030    Gene Symbol:   Bmp7    Locus Link:   85272    Chromosome:   3    Cytoband:   Chr:3q42    SOURCE    GENECARD       Gene Ontology:   biological process:  BMP signaling pathway   biological process:  regulation of phosphorylation   biological process:  growth   cellular component:  extracellular region   molecular function:  heparin binding   molecular function:  growth factor activity      BioCarta Pathways:     KEGG Pathways:  
    Probe set : 1373017_at    Name:   succinate-Coenzyme A ligase, GDP-forming, beta subunit (predicted)    GB acc:   AI237518    UniGene:   Rn.4098    Gene Symbol:   Suclg2_predicted    Locus Link:   362404    Chromosome:   4    Cytoband:   Chr:4q34    SOURCE    GENECARD       Gene Ontology:   biological process:  tricarboxylic acid cycle   biological process:  succinyl-CoA metabolism   biological process:  metabolism   cellular component:  mitochondrion      BioCarta Pathways:     KEGG Pathways:  
    Probe set : 1384060_at    Name:   Janus kinase 1    GB acc:   BG663208    UniGene:   Rn.90191    Gene Symbol:   Jak1    Locus Link:   84598    Chromosome:   5    Cytoband:   Chr:5q31.3-q35    SOURCE    GENECARD       Gene Ontology:   biological process:  protein amino acid phosphorylation   biological process:  intracellular signaling cascade   biological process:  peptidyl-tyrosine phosphorylation   cellular component:  cytoskeleton      BioCarta Pathways:     KEGG Pathways:  
    Probe set : 1368147_at    Name:   dual specificity phosphatase 1    GB acc:   BE110108    UniGene:   Rn.98260    Gene Symbol:   Dusp1    Locus Link:   114856    Chromosome:   10    Cytoband:   Chr:10q12    SOURCE    GENECARD       Gene Ontology:     BioCarta Pathways:     KEGG Pathways:  
    Probe set : 1388831_at    Name:   Solute carrier family 9 (sodium/hydrogen exchanger), isoform 3 regulator 2    GB acc:   BI277485    UniGene:   Rn.39351    Gene Symbol:   Slc9a3r2    Locus Link:   116501    Chromosome:   10    Cytoband:   Chr:10q12    SOURCE    GENECARD       Gene Ontology:   biological process:  intracellular signaling cascade   cellular component:  nucleus   cellular component:  apical plasma membrane   molecular function:  protein binding      BioCarta Pathways:     KEGG Pathways:  
    Probe set : 1373920_at    Name:   Similar to 106 kDa O-GlcNAc transferase-interacting protein (predicted)    GB acc:   AI175534    UniGene:   Rn.3090     Locus Link:   316085    Chromosome:   8    Cytoband:   Chr:8q32    SOURCE        Gene Ontology:     BioCarta Pathways:     KEGG Pathways:  
    Probe set : 1376160_at    Name:   similar to Avian reticuloendotheliosis viral (v-rel) oncogene related B    GB acc:   BF408850     Gene Symbol:   LOC365510    Locus Link:   365510       GENECARD       Gene Ontology:     BioCarta Pathways:     KEGG Pathways:  
    Probe set : 1368534_at    Name:   adrenergic receptor, alpha 1d    GB acc:   NM_024483    UniGene:   Rn.11314    Gene Symbol:   Adra1d    Locus Link:   29413    Chromosome:   3    Cytoband:   Chr:3q36    SOURCE    GENECARD       Gene Ontology:   cellular component:  membrane fraction   cellular component:  integral to plasma membrane   cellular component:  integral to membrane      BioCarta Pathways:     KEGG Pathways:  
    Probe set : 1388341_at    Name:   RAN GTPase activating protein 1 (predicted)    GB acc:   BI285671    UniGene:   Rn.107099    Gene Symbol:   Rangap1_predicted    Locus Link:   362965    Chromosome:   7    Cytoband:   Chr:7q34    SOURCE    GENECARD       Gene Ontology:   biological process:  signal transduction   cellular component:  soluble fraction   cellular component:  nuclear pore   cellular component:  cytoplasm   molecular function:  GTPase activator activity   molecular function:  Ran GTPase activator activity      BioCarta Pathways:     KEGG Pathways:  
    Probe set : 1384008_at    Name:   Similar to hypothetical protein FLJ40283 (predicted)    GB acc:   AI555224    UniGene:   Rn.22645     Locus Link:   361275    Chromosome:   17    Cytoband:   Chr:17q12.3    SOURCE        Gene Ontology:     BioCarta Pathways:     KEGG Pathways:  
    Probe set : 1372667_at    Name:   similar to RIKEN cDNA 1110059E24    GB acc:   AI170752    UniGene:   Rn.105582    Gene Symbol:   RGD:1359158    Locus Link:   361740    Chromosome:   1    Cytoband:   Chr:1q51    SOURCE    GENECARD       Gene Ontology:     BioCarta Pathways:     KEGG Pathways:  
    Probe set : 1382158_at    Name:   Transcribed locus    GB acc:   BF557889    UniGene:   Rn.12986        SOURCE        Gene Ontology:     BioCarta Pathways:     KEGG Pathways:  
    Probe set : 1398805_at    Name:   APG3 autophagy 3-like (S. cerevisiae)    GB acc:   NM_134394    UniGene:   Rn.3084    Gene Symbol:   Apg3l    Locus Link:   171415    Chromosome:   11    Cytoband:   Chr:11q21    SOURCE    GENECARD       Gene Ontology:   cellular component:  cytoplasmic ubiquitin ligase complex   molecular function:  ubiquitin-like conjugating enzyme activity      BioCarta Pathways:     KEGG Pathways:  
    Probe set : 1376955_at    Name:   procollagen, type IV, alpha 4 (predicted)    GB acc:   AI412936    UniGene:   Rn.22075    Gene Symbol:   Col4a4_predicted    Locus Link:   301562    Chromosome:   9    Cytoband:   Chr:9q34    SOURCE    GENECARD       Gene Ontology:   biological process:  phosphate transport   biological process:  cell adhesion   biological process:  long-term strengthening of neuromuscular junction      BioCarta Pathways:     KEGG Pathways:  
    Probe set : 1368146_at    Name:   dual specificity phosphatase 1    GB acc:   U02553    UniGene:   Rn.98260    Gene Symbol:   Dusp1    Locus Link:   114856    Chromosome:   10    Cytoband:   Chr:10q12    SOURCE    GENECARD       Gene Ontology:     BioCarta Pathways:     KEGG Pathways:  
    Probe set : 1369935_at    Name:   cyclin D3    GB acc:   NM_012766    UniGene:   Rn.3483    Gene Symbol:   Ccnd3    Locus Link:   25193      SOURCE    GENECARD       Gene Ontology:   cellular component:  nucleus      BioCarta Pathways:     KEGG Pathways:  
    Probe set : 1393863_at    Name:   CDNA clone MGC:124654 IMAGE:5622000    GB acc:   AA999005    UniGene:   Rn.148663        SOURCE        Gene Ontology:     BioCarta Pathways:     KEGG Pathways:  
    Probe set : 1395635_at    Name:   Tumor necrosis factor receptor superfamily, member 26 (predicted)    GB acc:   BE098317    UniGene:   Rn.137656     Locus Link:   361685    Chromosome:   1    Cytoband:   Chr:1q41    SOURCE        Gene Ontology:   cellular component:  integral to membrane   molecular function:  receptor activity      BioCarta Pathways:     KEGG Pathways:  
    Probe set : 1376336_at    Name:   Transcribed locus    GB acc:   BM392257    UniGene:   Rn.148006        SOURCE        Gene Ontology:     BioCarta Pathways:     KEGG Pathways:  
    Probe set : 1395579_at    Name:   DEAH (Asp-Glu-Ala-His) box polypeptide 32 (predicted)    GB acc:   BG666347    UniGene:   Rn.105937    Gene Symbol:   Dhx32_predicted    Locus Link:   361667    Chromosome:   1    Cytoband:   Chr:1q41    SOURCE    GENECARD       Gene Ontology:     BioCarta Pathways:     KEGG Pathways:  
    Probe set : 1373093_at    Name:   similar to Mitogen-inducible gene 6 protein homolog (Mig-6) (Gene 33 polypeptide) (predicted)    GB acc:   AI169756    UniGene:   Rn.100336    Gene Symbol:   RGD1307599_predicted    Locus Link:   313729    Chromosome:   5    Cytoband:   Chr:5q36    SOURCE    GENECARD       Gene Ontology:     BioCarta Pathways:     KEGG Pathways:  
    Probe set : 1381173_at    Name:   Transcribed locus    GB acc:   BG375010    UniGene:   Rn.66275        SOURCE        Gene Ontology:     BioCarta Pathways:     KEGG Pathways:  
    Probe set : 1369895_s_at    Name:   podocalyxin-like    GB acc:   AF109393    UniGene:   Rn.13805    Gene Symbol:   Podxl    Locus Link:   192181    Chromosome:   4    Cytoband:   Chr:4q22    SOURCE    GENECARD       Gene Ontology:   biological process:  negative regulation of cell adhesion   biological process:  negative regulation of cell adhesion   biological process:  cell adhesion      BioCarta Pathways:     KEGG Pathways:  
    Probe set : 1371833_at    Name:   brain protein I3 (predicted)    GB acc:   AI230753    UniGene:   Rn.7700    Gene Symbol:   Bri3_predicted    Locus Link:   304284    Chromosome:   12    Cytoband:   Chr:12p11    SOURCE    GENECARD       Gene Ontology:   cellular component:  integral to membrane      BioCarta Pathways:     KEGG Pathways:  
    Probe set : 1385434_at    Name:   Prostaglandin E receptor 2, subtype EP2    GB acc:   AI705087    UniGene:   Rn.10264    Gene Symbol:   Ptger2    Locus Link:   81752    Chromosome:   15    Cytoband:   Chr:15p14    SOURCE    GENECARD       Gene Ontology:   cellular component:  integral to membrane   cellular component:  integral to membrane      BioCarta Pathways:     KEGG Pathways:  
    Probe set : 1371944_at    Name:   ubiquitin-conjugating enzyme E2L 3 (predicted)    GB acc:   BF282658    UniGene:   Rn.131117    Gene Symbol:   Ube2l3_predicted    Locus Link:   363836    Chromosome:   11    Cytoband:   Chr:11q23    SOURCE    GENECARD       Gene Ontology:   biological process:  ubiquitin-dependent protein catabolism   biological process:  ubiquitin cycle   cellular component:  ubiquitin ligase complex      BioCarta Pathways:     KEGG Pathways:  
    Probe set : 1392627_x_at     GB acc:   BI282114              Gene Ontology:     BioCarta Pathways:     KEGG Pathways:  
    Probe set : 1372273_at    Name:   glycophorin C (Gerbich blood group) (predicted) /// similar to glycophorin C    GB acc:   AA944212    UniGene:   Rn.7693    Gene Symbol:   Gypc_predicted /// LOC498835    Locus Link:   364837 /// 498835    Chromosome:   18    Cytoband:   Chr:18p12    SOURCE    GENECARD       Gene Ontology:   biological process:  protein amino acid N-linked glycosylation   biological process:  protein amino acid O-linked glycosylation   biological process:  organ morphogenesis   cellular component:  integral to plasma membrane      BioCarta Pathways:     KEGG Pathways:  
    Probe set : 1390541_at    Name:   harmonin a1    GB acc:   BI291932    UniGene:   Rn.24615    Gene Symbol:   RGD:1303329    Locus Link:   308596    Chromosome:   1    Cytoband:   Chr:1q22    SOURCE    GENECARD       Gene Ontology:   biological process:  regulation of synapse structure and function   cellular component:  synapse   molecular function:  myosin binding   molecular function:  cadherin binding   molecular function:  protein binding      BioCarta Pathways:     KEGG Pathways:  
    Probe set : 1372890_at    Name:   Sphingosine phosphate lyase 1    GB acc:   BM389696    UniGene:   Rn.26953    Gene Symbol:   Spgl1    Locus Link:   286896    Chromosome:   20    Cytoband:   Chr:20q11    SOURCE    GENECARD       Gene Ontology:   cellular component:  integral to endoplasmic reticulum membrane   cellular component:  endoplasmic reticulum   cellular component:  integral to membrane      BioCarta Pathways:     KEGG Pathways:  
    Probe set : 1389197_at    Name:   similar to RIKEN cDNA 9630046K23 (predicted)    GB acc:   BI290535    UniGene:   Rn.7051    Gene Symbol:   RGD1306248_predicted    Locus Link:   288091    Chromosome:   11    Cytoband:   Chr:11q21    SOURCE    GENECARD       Gene Ontology:   cellular component:  extracellular space      BioCarta Pathways:     KEGG Pathways:  
    Probe set : 1379972_at    Name:   similar to 1700060H10Rik protein (predicted)    GB acc:   AI070438    UniGene:   Rn.33054    Gene Symbol:   RGD1308699_predicted    Locus Link:   309790    Chromosome:   20    Cytoband:   Chr:20q11    SOURCE    GENECARD       Gene Ontology:   cellular component:  nucleus      BioCarta Pathways:     KEGG Pathways:  
    Probe set : 1385128_at    Name:   Similar to MGC68837 protein    GB acc:   BE108716    UniGene:   Rn.58294     Locus Link:   498356      SOURCE        Gene Ontology:     BioCarta Pathways:     KEGG Pathways:  
    Probe set : 1387495_at    Name:   transducin-like enhancer of split 4, E(spl) homolog (Drosophila)    GB acc:   NM_019141    UniGene:   Rn.53972    Gene Symbol:   Tle4    Locus Link:   25565    Chromosome:   1    Cytoband:   Chr:1q43    SOURCE    GENECARD       Gene Ontology:   cellular component:  nucleus   cellular component:  nucleus   molecular function:  transcription corepressor activity   molecular function:  transcription corepressor activity   molecular function:  protein binding      BioCarta Pathways:     KEGG Pathways:  
    Probe set : 1397704_at    Name:   similar to establishment of cohesion 1 homolog 2    GB acc:   BF407449     Gene Symbol:   LOC498536    Locus Link:   498536    Chromosome:   15    Cytoband:   Chr:15p12     GENECARD       Gene Ontology:     BioCarta Pathways:     KEGG Pathways:  
    Probe set : 1384907_at     GB acc:   AI411835              Gene Ontology:     BioCarta Pathways:     KEGG Pathways:  
    Probe set : 1393643_at    Name:   reticulocalbin (predicted)    GB acc:   AI112962    UniGene:   Rn.103345    Gene Symbol:   Rcn_predicted    Locus Link:   362182    Chromosome:   3    Cytoband:   Chr:3q32    SOURCE    GENECARD       Gene Ontology:   cellular component:  extracellular space   cellular component:  endoplasmic reticulum   cellular component:  endoplasmic reticulum lumen   molecular function:  calcium ion binding      BioCarta Pathways:     KEGG Pathways:  
    Probe set : 1388355_at    Name:   RNA binding motif protein 17 (predicted)    GB acc:   BF396644    UniGene:   Rn.20694    Gene Symbol:   Rbm17_predicted    Locus Link:   291295    Chromosome:   17    Cytoband:   Chr:17q12.3    SOURCE    GENECARD       Gene Ontology:   biological process:  nuclear mRNA splicing, via spliceosome   biological process:  mRNA processing   cellular component:  intracellular   cellular component:  nucleus   cellular component:  spliceosome complex   molecular function:  nucleic acid binding   molecular function:  RNA binding   molecular function:  nucleotide binding      BioCarta Pathways:     KEGG Pathways:  
    Probe set : 1376709_at    Name:   solute carrier family 39 (metal ion transporter), member 8 (predicted)    GB acc:   BM388442    UniGene:   Rn.28321    Gene Symbol:   Slc39a8_predicted    Locus Link:   295455    Chromosome:   2    Cytoband:   Chr:2q43    SOURCE    GENECARD       Gene Ontology:   biological process:  transport   biological process:  metal ion transport   cellular component:  extracellular space   cellular component:  membrane   cellular component:  integral to membrane   molecular function:  metal ion transporter activity      BioCarta Pathways:     KEGG Pathways:  
    Probe set : 1371839_at    Name:   similar to splicing factor, arginine/serine-rich 2    GB acc:   AA819369    UniGene:   Rn.136644    Gene Symbol:   RGD:1359422    Locus Link:   494445    Chromosome:   10    Cytoband:   Chr:10q32.3    SOURCE    GENECARD       Gene Ontology:   biological process:  nuclear mRNA splicing, via spliceosome   biological process:  mRNA processing   cellular component:  nucleus   molecular function:  nucleotide binding   molecular function:  nucleic acid binding   molecular function:  RNA binding      BioCarta Pathways:     KEGG Pathways:  
    Probe set : 1371498_at    Name:   similar to Jtv1-pending protein    GB acc:   AI412685    UniGene:   Rn.3333    Gene Symbol:   LOC288480    Locus Link:   288480    Chromosome:   12    Cytoband:   Chr:12p11    SOURCE    GENECARD       Gene Ontology:     BioCarta Pathways:     KEGG Pathways:  
    Probe set : 1386866_at    Name:   tyrosine 3-monooxgenase/tryptophan 5-monooxgenase activation protein, gamma polypeptide    GB acc:   NM_019376    UniGene:   Rn.29936    Gene Symbol:   Ywhag    Locus Link:   56010    Chromosome:   12    Cytoband:   Chr:12q12    SOURCE    GENECARD       Gene Ontology:   biological process:  negative regulation of protein kinase activity   biological process:  protein targeting   biological process:  cytoskeleton organization and biogenesis   biological process:  regulation of mitosis   biological process:  signal transduction   biological process:  signal transduction   biological process:  intracellular signaling cascade   biological process:  regulation of signal transduction   biological process:  negative regulation of apoptosis   biological process:  regulation of neuron differentiation   biological process:  regulation of synaptic plasticity   biological process:  negative regulation of protein kinase activity   biological process:  cytoskeleton organization and biogenesis   biological process:  regulation of mitosis   biological process:  regulation of signal transduction   biological process:  negative regulation of apoptosis   biological process:  regulation of neuron differentiation   biological process:  regulation of synaptic plasticity   cellular component:  cytoplasm   cellular component:  cytoplasm   molecular function:  actin binding   molecular function:  protein kinase C binding   molecular function:  insulin-like growth factor receptor binding   molecular function:  enzyme activator activity   molecular function:  protein kinase C inhibitor activity   molecular function:  protein domain specific binding   molecular function:  enzyme regulator activity   molecular function:  protein homodimerization activity   molecular function:  actin binding   molecular function:  protein kinase C binding   molecular function:  insulin-like growth factor receptor binding   molecular function:  protein binding   molecular function:  protein kinase C inhibitor activity   molecular function:  protein homodimerization activity      BioCarta Pathways:     KEGG Pathways:  
    Probe set : 1389442_at    Name:   Transcribed locus    GB acc:   AI406804    UniGene:   Rn.21790        SOURCE        Gene Ontology:     BioCarta Pathways:     KEGG Pathways:  
    Probe set : 1390221_at    Name:   Transcribed locus    GB acc:   BM385216    UniGene:   Rn.8012        SOURCE        Gene Ontology:     BioCarta Pathways:     KEGG Pathways:  
    Probe set : 1374519_at     GB acc:   AI231782              Gene Ontology:     BioCarta Pathways:     KEGG Pathways:  
    Probe set : 1398561_at    Name:   Transcribed locus    GB acc:   BI302791    UniGene:   Rn.148405        SOURCE        Gene Ontology:     BioCarta Pathways:     KEGG Pathways:  
    Probe set : 1388493_at    Name:   Similar to vomeronasal V1r-type receptor V1rc41    GB acc:   AI600030    UniGene:   Rn.129062     Locus Link:   362374    Chromosome:   4    Cytoband:   Chr:4q24    SOURCE        Gene Ontology:     BioCarta Pathways:     KEGG Pathways:  
    Probe set : 1385781_at    Name:   similar to SNF2/RAD54 family protein    GB acc:   BF397986    UniGene:   Rn.72855    Gene Symbol:   LOC317252    Locus Link:   317252    Chromosome:   X    Cytoband:   Chr:Xq31    SOURCE    GENECARD       Gene Ontology:     BioCarta Pathways:     KEGG Pathways:  
    Probe set : 1368773_at    Name:   follicle stimulating hormone primary response gene 1    GB acc:   NM_012955    UniGene:   Rn.10411    Gene Symbol:   Fshprh1    Locus Link:   25448    Chromosome:   X    Cytoband:   Chr:Xq34    SOURCE    GENECARD       Gene Ontology:   biological process:  spermatogenesis   biological process:  female gamete generation   biological process:  sex differentiation      BioCarta Pathways:     KEGG Pathways:  
    Probe set : 1388799_at    Name:   kelch-like 7 (Drosophila) (predicted)    GB acc:   BG374559    UniGene:   Rn.8854    Gene Symbol:   Klhl7_predicted    Locus Link:   362303    Chromosome:   4    Cytoband:   Chr:4q11    SOURCE    GENECARD       Gene Ontology:   molecular function:  protein binding      BioCarta Pathways:     KEGG Pathways:  
    Probe set : 1374838_at    Name:   SP140 nuclear body protein (predicted)    GB acc:   BI293504    UniGene:   Rn.27561    Gene Symbol:   Sp140_predicted    Locus Link:   316580    Chromosome:   9    Cytoband:   Chr:9q35    SOURCE    GENECARD       Gene Ontology:   biological process:  regulation of transcription, DNA-dependent   biological process:  defense response   cellular component:  nucleus   cellular component:  nuclear membrane   cellular component:  nucleoplasm      BioCarta Pathways:     KEGG Pathways:  
    Probe set : 1380293_at    Name:   similar to chromosome 18 open reading frame 54    GB acc:   BF412754    UniGene:   Rn.31250    Gene Symbol:   LOC361346    Locus Link:   361346    Chromosome:   18    Cytoband:   Chr:18q12.1    SOURCE    GENECARD       Gene Ontology:     BioCarta Pathways:     KEGG Pathways:  
    Probe set : 1397672_at    Name:   Transcribed locus    GB acc:   AT006319    UniGene:   Rn.51837        SOURCE        Gene Ontology:     BioCarta Pathways:     KEGG Pathways:  
    Probe set : 1397227_at    Name:   spinal cord expression protein 4    GB acc:   BE120895    UniGene:   Rn.45854    Gene Symbol:   RGD:1303093    Locus Link:   308134    Chromosome:   X    Cytoband:   Chr:Xq34    SOURCE    GENECARD       Gene Ontology:   cellular component:  integral to membrane      BioCarta Pathways:     KEGG Pathways:  
    Probe set : 1382861_at    Name:   similar to transcription elongation factor IIS - mouse    GB acc:   AA997486     Gene Symbol:   LOC498453    Locus Link:   498453    Chromosome:   15    Cytoband:   Chr:15p16     GENECARD       Gene Ontology:     BioCarta Pathways:     KEGG Pathways:  
    Probe set : 1373927_at    Name:   Transcribed locus, strongly similar to XP_226315.3 PREDICTED: similar to High mobility group box 2-like 1 [Rattus norvegicus]    GB acc:   BI283489    UniGene:   Rn.2796        SOURCE        Gene Ontology:     BioCarta Pathways:     KEGG Pathways:  
    Probe set : 1373397_at     GB acc:   AI180161              Gene Ontology:     BioCarta Pathways:     KEGG Pathways:  
    Probe set : 1370376_a_at    Name:   cold shock domain protein A    GB acc:   AA866458    UniGene:   Rn.3306    Gene Symbol:   Csda    Locus Link:   83807    Chromosome:   4    Cytoband:   Chr:4q42    SOURCE    GENECARD       Gene Ontology:   cellular component:  nucleus   cellular component:  nucleus   cellular component:  cytoplasm      BioCarta Pathways:     KEGG Pathways:  
    Probe set : 1393070_at    Name:   secernin 2 (predicted)    GB acc:   BE118406    UniGene:   Rn.15274    Gene Symbol:   Scrn2_predicted    Locus Link:   360612    Chromosome:   10    Cytoband:   Chr:10q31    SOURCE    GENECARD       Gene Ontology:   biological process:  proteolysis   molecular function:  dipeptidase activity      BioCarta Pathways:     KEGG Pathways:  
    Probe set : 1380357_a_at    Name:   Transcribed locus    GB acc:   BF396423    UniGene:   Rn.98569        SOURCE        Gene Ontology:     BioCarta Pathways:     KEGG Pathways:  
    Probe set : 1378247_at    Name:   ELL associated factor 2    GB acc:   BI285131    UniGene:   Rn.20681    Gene Symbol:   Eaf2    Locus Link:   266787    Chromosome:   11    Cytoband:   Chr:11q22    SOURCE    GENECARD       Gene Ontology:   cellular component:  nucleus   cellular component:  nucleoplasm   molecular function:  protein binding   molecular function:  transcriptional activator activity      BioCarta Pathways:     KEGG Pathways:  
    Probe set : 1371462_at    Name:   insulin-like growth factor binding protein 4    GB acc:   BE108969    UniGene:   Rn.112691    Gene Symbol:   Igfbp4    Locus Link:   360622    Chromosome:   10    Cytoband:   Chr:10q31    SOURCE    GENECARD       Gene Ontology:   cellular component:  extracellular region   cellular component:  extracellular space   molecular function:  insulin-like growth factor binding   molecular function:  growth factor binding      BioCarta Pathways:     KEGG Pathways:  
    Probe set : 1399148_s_at    Name:   similar to hypothetical protein MGC38524 (predicted)    GB acc:   AI013567    UniGene:   Rn.105830    Gene Symbol:   RGD1309002_predicted    Locus Link:   290637    Chromosome:   16    Cytoband:   Chr:16p14    SOURCE    GENECARD       Gene Ontology:     BioCarta Pathways:     KEGG Pathways:  
    Probe set : 1370939_at    Name:   acyl-CoA synthetase long-chain family member 1    GB acc:   D90109    UniGene:   Rn.6215    Gene Symbol:   Acsl1    Locus Link:   25288    Chromosome:   16    Cytoband:   Chr:16q11    SOURCE    GENECARD       Gene Ontology:   cellular component:  mitochondrion   cellular component:  integral to membrane      BioCarta Pathways:     KEGG Pathways:  
    Probe set : 1391702_at    Name:   Similar to hypothetical protein FLJ20626 (predicted)    GB acc:   BM389392    UniGene:   Rn.66269     Locus Link:   308365    Chromosome:   1    Cytoband:   Chr:1q21    SOURCE        Gene Ontology:     BioCarta Pathways:     KEGG Pathways:  
    Probe set : 1387851_at    Name:   phosphotriesterase related    GB acc:   NM_022224    UniGene:   Rn.44484    Gene Symbol:   Pter    Locus Link:   63852    Chromosome:   17    Cytoband:   Chr:17q12.3    SOURCE    GENECARD       Gene Ontology:   biological process:  catabolism   biological process:  organophosphate metabolism      BioCarta Pathways:     KEGG Pathways:  
    Probe set : 1374657_at    Name:   Transcribed locus    GB acc:   AA848437    UniGene:   Rn.136572        SOURCE        Gene Ontology:     BioCarta Pathways:     KEGG Pathways:  
    Probe set : 1375266_at     GB acc:   BG380633              Gene Ontology:     BioCarta Pathways:     KEGG Pathways:  
    Probe set : 1367900_at    Name:   glycogenin 1    GB acc:   NM_031043    UniGene:   Rn.3661    Gene Symbol:   Gyg1    Locus Link:   81675    Chromosome:   2    Cytoband:   Chr:2q24    SOURCE    GENECARD       Gene Ontology:   biological process:  glycogen biosynthesis   biological process:  glycogen biosynthesis   biological process:  carbohydrate biosynthesis      BioCarta Pathways:     KEGG Pathways:  
    Probe set : 1379651_at    Name:   Forkhead box P1 (predicted)    GB acc:   BE109884    UniGene:   Rn.33321     Locus Link:   297480    Chromosome:   4    Cytoband:   Chr:4q34    SOURCE        Gene Ontology:   biological process:  regulation of transcription, DNA-dependent   biological process:  negative regulation of transcription   cellular component:  nucleus      BioCarta Pathways:     KEGG Pathways:  
    Probe set : 1368041_at    Name:   synaptojanin 2 binding protein    GB acc:   BF284821    UniGene:   Rn.6258    Gene Symbol:   Synj2bp    Locus Link:   64531    Chromosome:   6    Cytoband:   Chr:6q24    SOURCE    GENECARD       Gene Ontology:   molecular function:  receptor activity   molecular function:  protein binding      BioCarta Pathways:     KEGG Pathways:  
    Probe set : 1367725_at    Name:   serine/threonine-protein kinase pim-3    GB acc:   NM_022602    UniGene:   Rn.6343    Gene Symbol:   Pim3    Locus Link:   64534    Chromosome:   7    Cytoband:   Chr:7q34    SOURCE    GENECARD       Gene Ontology:     BioCarta Pathways:     KEGG Pathways:  
    Probe set : 1369799_at    Name:   4-aminobutyrate aminotransferase    GB acc:   U29701    UniGene:   Rn.10090    Gene Symbol:   Abat    Locus Link:   81632    Chromosome:   10    Cytoband:   Chr:10q12    SOURCE    GENECARD       Gene Ontology:   biological process:  synaptic transmission   biological process:  gamma-aminobutyric acid metabolism   biological process:  neurotransmitter catabolism   cellular component:  mitochondrion   cellular component:  mitochondrial matrix      BioCarta Pathways:     KEGG Pathways:  
    Probe set : 1379760_at    Name:   similar to RIKEN cDNA 1810019A08 (predicted)    GB acc:   AI454285    UniGene:   Rn.145043    Gene Symbol:   RGD1310519_predicted    Locus Link:   310663    Chromosome:   2    Cytoband:   Chr:2q34    SOURCE    GENECARD       Gene Ontology:   biological process:  protein targeting      BioCarta Pathways:     KEGG Pathways:  
    Probe set : 1375423_at     GB acc:   BE098181              Gene Ontology:     BioCarta Pathways:     KEGG Pathways:  
    Probe set : 1374805_at    Name:   similar to hypothetical protein MGC5528    GB acc:   AW251849    UniGene:   Rn.8559    Gene Symbol:   LOC299933    Locus Link:   299933    Chromosome:   7    Cytoband:   Chr:7q31    SOURCE    GENECARD       Gene Ontology:     BioCarta Pathways:     KEGG Pathways:  
    Probe set : 1372297_at    Name:   glutathione S-transferase, alpha 4 (predicted)    GB acc:   AI234527    UniGene:   Rn.57528    Gene Symbol:   Gsta4_predicted    Locus Link:   300850    Chromosome:   8    Cytoband:   Chr:8q31    SOURCE    GENECARD       Gene Ontology:   biological process:  response to stress   biological process:  metabolism   molecular function:  glutathione transferase activity   molecular function:  transferase activity      BioCarta Pathways:     KEGG Pathways:  
    Probe set : 1371494_at    Name:   Transcribed locus    GB acc:   BE110614    UniGene:   Rn.14639        SOURCE        Gene Ontology:     BioCarta Pathways:     KEGG Pathways:  
    Probe set : 1372577_at    Name:   actin related protein 2/3 complex, subunit 4 (predicted)    GB acc:   AI411582    UniGene:   Rn.102266    Gene Symbol:   Arpc4_predicted    Locus Link:   297518    Chromosome:   4    Cytoband:   Chr:4q42    SOURCE    GENECARD       Gene Ontology:   biological process:  actin filament polymerization   cellular component:  cytoskeleton   cellular component:  Arp2/3 protein complex      BioCarta Pathways:     KEGG Pathways:  
    Probe set : 1388534_at    Name:   Solute carrier family 31 (copper transporters), member 1    GB acc:   AA851369    UniGene:   Rn.2789    Gene Symbol:   Slc31a1    Locus Link:   171135    Chromosome:   5    Cytoband:   Chr:5q24    SOURCE    GENECARD       Gene Ontology:   biological process:  transport   biological process:  copper ion transport   biological process:  ion transport   cellular component:  integral to plasma membrane   cellular component:  integral to membrane   molecular function:  copper ion transporter activity   molecular function:  carrier activity   molecular function:  copper ion binding      BioCarta Pathways:     KEGG Pathways:  
    Probe set : 1391109_x_at     GB acc:   BE108349              Gene Ontology:     BioCarta Pathways:     KEGG Pathways:  
    Probe set : 1369081_at    Name:   neuraminidase 1    GB acc:   NM_031522    UniGene:   Rn.128560    Gene Symbol:   Neu1    Locus Link:   24591    Chromosome:   20    Cytoband:   Chr:20p12    SOURCE    GENECARD       Gene Ontology:   biological process:  carbohydrate metabolism   cellular component:  lysosome      BioCarta Pathways:     KEGG Pathways:  
    Probe set : 1389353_at    Name:   Sema domain, transmembrane domain (TM), and cytoplasmic domain, (semaphorin) 6D (predicted)    GB acc:   BM386525    UniGene:   Rn.8257     Locus Link:   311384    Chromosome:   3    Cytoband:   Chr:3q35    SOURCE        Gene Ontology:   biological process:  development   biological process:  nervous system development   cellular component:  integral to membrane      BioCarta Pathways:     KEGG Pathways:  
    Probe set : 1371811_at    Name:   Bernardinelli-Seip congenital lipodystrophy 2 homolog (human)    GB acc:   BI278232    UniGene:   Rn.1686    Gene Symbol:   Bscl2    Locus Link:   361722    Chromosome:   1    Cytoband:   Chr:1q43    SOURCE    GENECARD       Gene Ontology:   cellular component:  integral to membrane   cellular component:  integral to endoplasmic reticulum membrane   cellular component:  endoplasmic reticulum      BioCarta Pathways:     KEGG Pathways:  
    Probe set : 1392001_at    Name:   Pannexin 1    GB acc:   AI232305    UniGene:   Rn.45097    Gene Symbol:   Panx1    Locus Link:   315435    Chromosome:   8    Cytoband:   Chr:8q11-q12    SOURCE    GENECARD       Gene Ontology:   biological process:  cell-cell signaling   cellular component:  gap junction   cellular component:  gap junction   cellular component:  integral to membrane   molecular function:  gap-junction forming channel activity   molecular function:  protein heterodimerization activity      BioCarta Pathways:     KEGG Pathways:  
    Probe set : 1370265_at    Name:   arrestin, beta 2    GB acc:   BF285345    UniGene:   Rn.32973    Gene Symbol:   Arrb2    Locus Link:   25388    Chromosome:   10    Cytoband:   Chr:10q24    SOURCE    GENECARD       Gene Ontology:     BioCarta Pathways:     KEGG Pathways:  
    Probe set : 1371380_at    Name:   pyruvate dehydrogenase E1 alpha 1    GB acc:   AI411413    UniGene:   Rn.3655    Gene Symbol:   Pdha1    Locus Link:   29554    Chromosome:   X    Cytoband:   Chr:Xq21    SOURCE    GENECARD       Gene Ontology:   biological process:  acetyl-CoA metabolism   biological process:  glycolysis   biological process:  metabolism      BioCarta Pathways:     KEGG Pathways:  
    Probe set : 1382843_at    Name:   sphingosine phosphate lyase 1    GB acc:   AI113219    UniGene:   Rn.26953    Gene Symbol:   Sgpl1    Locus Link:   286896    Chromosome:   20    Cytoband:   Chr:20q11    SOURCE    GENECARD       Gene Ontology:   cellular component:  integral to endoplasmic reticulum membrane   cellular component:  endoplasmic reticulum   cellular component:  integral to membrane      BioCarta Pathways:     KEGG Pathways:  
    Probe set : 1392497_at    Name:   Transcribed locus    GB acc:   BE104938    UniGene:   Rn.49273        SOURCE        Gene Ontology:     BioCarta Pathways:     KEGG Pathways:  
    Probe set : 1372907_at    Name:   ATPase, H+ transporting, V0 subunit E isoform 2    GB acc:   BI285242    UniGene:   Rn.11803    Gene Symbol:   Atp6v0e2    Locus Link:   436582    Chromosome:   4    Cytoband:   Chr:4q24    SOURCE    GENECARD       Gene Ontology:   biological process:  ATP synthesis coupled proton transport   cellular component:  integral to membrane   cellular component:  proton-transporting two-sector ATPase complex   molecular function:  hydrogen-transporting ATP synthase activity, rotational mechanism   molecular function:  hydrogen-transporting ATPase activity, rotational mechanism      BioCarta Pathways:     KEGG Pathways:  
    Probe set : 1374157_at     GB acc:   AA858930              Gene Ontology:     BioCarta Pathways:     KEGG Pathways:  
    Probe set : 1385856_at    Name:   RIO kinase 1 (yeast) (predicted)    GB acc:   BE096718    UniGene:   Rn.132553    Gene Symbol:   Riok1_predicted    Locus Link:   291061    Chromosome:   17    Cytoband:   Chr:17p12    SOURCE    GENECARD       Gene Ontology:   molecular function:  protein serine/threonine kinase activity   molecular function:  ATP binding   molecular function:  transferase activity      BioCarta Pathways:     KEGG Pathways:  
    Probe set : 1389695_at     GB acc:   AI410976              Gene Ontology:     BioCarta Pathways:     KEGG Pathways:  
    Probe set : 1382661_at    Name:   nucleoporin 160 (predicted)    GB acc:   AI230642    UniGene:   Rn.21145    Gene Symbol:   Nup160_predicted    Locus Link:   311182    Chromosome:   3    Cytoband:   Chr:3q24    SOURCE    GENECARD       Gene Ontology:   biological process:  mRNA export from nucleus   biological process:  transport   cellular component:  nucleus   cellular component:  nuclear pore   molecular function:  acid phosphatase activity   molecular function:  nucleocytoplasmic transporter activity      BioCarta Pathways:     KEGG Pathways:  
    Probe set : 1369976_at    Name:   dynein, cytoplasmic, light chain 1    GB acc:   NM_053319    UniGene:   Rn.35769    Gene Symbol:   Dnclc1    Locus Link:   58945    Chromosome:   12    Cytoband:   Chr:12q16    SOURCE    GENECARD       Gene Ontology:     BioCarta Pathways:     KEGG Pathways:  
    Probe set : 1375452_at    Name:   meningioma expressed antigen 6 (coiled-coil proline-rich) (predicted)    GB acc:   AI177180    UniGene:   Rn.58607    Gene Symbol:   Mgea6_predicted    Locus Link:   299078    Chromosome:   6    Cytoband:   Chr:6q23    SOURCE    GENECARD       Gene Ontology:   biological process:  RNA processing   cellular component:  integral to membrane      BioCarta Pathways:     KEGG Pathways:  
    Probe set : 1390228_at    Name:   Transcribed locus    GB acc:   BM384446    UniGene:   Rn.31695        SOURCE        Gene Ontology:     BioCarta Pathways:     KEGG Pathways:  
    Probe set : 1370802_at    Name:   similar to integrin beta-5    GB acc:   AF336872     Gene Symbol:   LOC498091    Locus Link:   498091    Chromosome:   11    Cytoband:   Chr:11q22     GENECARD       Gene Ontology:     BioCarta Pathways:     KEGG Pathways:  
    Probe set : 1383148_at    Name:   Transcribed locus    GB acc:   BF559410    UniGene:   Rn.138441        SOURCE        Gene Ontology:     BioCarta Pathways:     KEGG Pathways:  
    Probe set : 1388297_at    Name:   eukaryotic translation elongation factor 1 gamma    GB acc:   BM391203    UniGene:   Rn.38212    Gene Symbol:   RGD:1302939    Locus Link:   293725    Chromosome:   1    Cytoband:   Chr:1q43    SOURCE    GENECARD       Gene Ontology:   biological process:  protein biosynthesis   biological process:  translational elongation   cellular component:  eukaryotic translation elongation factor 1 complex   molecular function:  translation elongation factor activity      BioCarta Pathways:     KEGG Pathways:  
    Probe set : 1398773_at    Name:   src associated in mitosis, 68 kDa    GB acc:   NM_130405    UniGene:   Rn.108177    Gene Symbol:   Khdrbs1    Locus Link:   117268    Chromosome:   5    Cytoband:   Chr:5q36    SOURCE    GENECARD       Gene Ontology:   biological process:  G1/S transition of mitotic cell cycle   biological process:  mRNA processing   biological process:  transport   biological process:  cell cycle arrest   biological process:  signal transduction   biological process:  Ras protein signal transduction   biological process:  cell proliferation   biological process:  negative regulation of transcription   biological process:  regulation of RNA export from nucleus   biological process:  G2/M transition of mitotic cell cycle   biological process:  negative regulation of transcription   biological process:  regulation of RNA export from nucleus   biological process:  cell surface receptor linked signal transduction   biological process:  transcription   biological process:  regulation of transcription, DNA-dependent   biological process:  cell cycle      BioCarta Pathways:     KEGG Pathways:  
    Probe set : 1372840_at    Name:   Similar to Zbed4 protein    GB acc:   AI170289    UniGene:   Rn.3708     Locus Link:   310330    Chromosome:   2    Cytoband:   Chr:2q25    SOURCE        Gene Ontology:     BioCarta Pathways:     KEGG Pathways:  
    Probe set : 1390154_at    Name:   Transcribed locus    GB acc:   BI299824    UniGene:   Rn.144406        SOURCE        Gene Ontology:     BioCarta Pathways:     KEGG Pathways:  
    Probe set : 1374836_at    Name:   RNA, U3 small nucleolar interacting protein 2 (predicted)    GB acc:   AA964554    UniGene:   Rn.11840    Gene Symbol:   Rnu3ip2_predicted    Locus Link:   363134    Chromosome:   8    Cytoband:   Chr:8q32    SOURCE    GENECARD       Gene Ontology:   biological process:  rRNA processing   cellular component:  small nucleolar ribonucleoprotein complex   cellular component:  small nuclear ribonucleoprotein complex   molecular function:  RNA binding      BioCarta Pathways:     KEGG Pathways:  
    Probe set : 1370613_s_at     GB acc:   AF461738    UniGene:   Rn.26489    Gene Symbol:   Ugt1a1 /// Ugt1a6 /// Ugt1a7 /// Ugt1a8 /// Ugt1a2 /// Ugt1a4 /// Ugt1a11 /// Ugt1a5    Locus Link:   113992 /// 154516 /// 24861 /// 301595 /// 396527 /// 396551 /// 396552 /// 574523    Chromosome:   9    Cytoband:   Chr:9q35-q36 /// Chr:9q35    SOURCE    GENECARD       Gene Ontology:     BioCarta Pathways:     KEGG Pathways:  
    Probe set : 1388134_at    Name:   eukaryotic translation elongation factor 1 delta (guanine nucleotide exchange protein)    GB acc:   BG380216    UniGene:   Rn.146086    Gene Symbol:   Eef1d    Locus Link:   300033    Chromosome:   7    Cytoband:   Chr:7q34    SOURCE    GENECARD       Gene Ontology:   biological process:  protein biosynthesis   biological process:  translational elongation   cellular component:  eukaryotic translation elongation factor 1 complex   molecular function:  translation elongation factor activity      BioCarta Pathways:     KEGG Pathways:  
    Probe set : 1386973_a_at    Name:   mitogen activated protein kinase 8 interacting protein    GB acc:   NM_053777    UniGene:   Rn.44266    Gene Symbol:   Mapk8ip    Locus Link:   116457    Chromosome:   3    Cytoband:   Chr:3q24    SOURCE    GENECARD       Gene Ontology:   cellular component:  cytoplasm   cellular component:  cytoplasm      BioCarta Pathways:     KEGG Pathways:  
    Probe set : 1374540_at    Name:   cell division cycle associated 7 (predicted)    GB acc:   AA859235    UniGene:   Rn.16408    Gene Symbol:   Cdca7_predicted    Locus Link:   311742    Chromosome:   3    Cytoband:   Chr:3q22    SOURCE    GENECARD       Gene Ontology:   biological process:  cytokinesis   biological process:  regulation of cell proliferation   biological process:  cell division   cellular component:  nucleus      BioCarta Pathways:     KEGG Pathways:  
    Probe set : 1387969_at    Name:   chemokine (C-X-C motif) ligand 10    GB acc:   U22520    UniGene:   Rn.10584    Gene Symbol:   Cxcl10    Locus Link:   245920    Chromosome:   14    Cytoband:   Chr:14p22    SOURCE    GENECARD       Gene Ontology:   cellular component:  extracellular region   cellular component:  extracellular region   cellular component:  extracellular space   molecular function:  cytokine activity   molecular function:  cytokine activity   molecular function:  chemokine activity   molecular function:  chemokine activity      BioCarta Pathways:     KEGG Pathways:  
    Probe set : 1383152_at    Name:   similar to Cc1-9 (predicted)    GB acc:   BF544427    UniGene:   Rn.72019    Gene Symbol:   RGD1308955_predicted    Locus Link:   293628    Chromosome:   1    Cytoband:   Chr:1q41    SOURCE    GENECARD       Gene Ontology:   biological process:  carbohydrate metabolism   biological process:  chitin catabolism   cellular component:  extracellular space   molecular function:  hydrolase activity, hydrolyzing O-glycosyl compounds   molecular function:  chitinase activity      BioCarta Pathways:     KEGG Pathways:  
    Probe set : 1372904_at    Name:   MOB1, Mps One Binder kinase activator-like 2B (yeast) (predicted)    GB acc:   BI285628    UniGene:   Rn.9147    Gene Symbol:   Mobkl2b_predicted    Locus Link:   362833    Chromosome:   7    Cytoband:   Chr:7q11    SOURCE    GENECARD       Gene Ontology:     BioCarta Pathways:     KEGG Pathways:  
    Probe set : 1377830_a_at    Name:   similar to RIKEN cDNA 1810014F10 gene (predicted)    GB acc:   BF281728    UniGene:   Rn.22205    Gene Symbol:   RGD1311186_predicted    Locus Link:   293587    Chromosome:   1    Cytoband:   Chr:1q41    SOURCE    GENECARD       Gene Ontology:     BioCarta Pathways:     KEGG Pathways:  
    Probe set : 1387064_at    Name:   peroxisomal membrane protein 3    GB acc:   NM_017234    UniGene:   Rn.4065    Gene Symbol:   Pxmp3    Locus Link:   29534    Chromosome:   2    Cytoband:   Chr:2q24    SOURCE    GENECARD       Gene Ontology:   molecular function:  ubiquitin-protein ligase activity   molecular function:  zinc ion binding   molecular function:  metal ion binding      BioCarta Pathways:     KEGG Pathways:  
    Probe set : 1367726_at    Name:   thyroid hormone receptor alpha    GB acc:   NM_031134    UniGene:   Rn.11307    Gene Symbol:   Thra    Locus Link:   81812    Chromosome:   10    Cytoband:   Chr:10q31    SOURCE    GENECARD       Gene Ontology:   cellular component:  nucleus      BioCarta Pathways:     KEGG Pathways:  
    Probe set : 1382921_at    Name:   SDA1 domain containing 1    GB acc:   AA997613    UniGene:   Rn.12755    Gene Symbol:   RGD:1359112    Locus Link:   289504    Chromosome:   14    Cytoband:   Chr:14p22    SOURCE    GENECARD       Gene Ontology:     BioCarta Pathways:     KEGG Pathways:  
    Probe set : 1367890_at    Name:   caspase 2    GB acc:   NM_022522    UniGene:   Rn.1438    Gene Symbol:   Casp2    Locus Link:   64314    Chromosome:   4    Cytoband:   Chr:4q23    SOURCE    GENECARD       Gene Ontology:   cellular component:  intracellular   cellular component:  nucleus      BioCarta Pathways:     KEGG Pathways:  
    Probe set : 1371754_at    Name:   solute carrier family 25 (mitochondrial carrier, phosphate carrier), member 25    GB acc:   AI177358    UniGene:   Rn.17644    Gene Symbol:   Slc25a25    Locus Link:   246771    Chromosome:   3    Cytoband:   Chr:3p11    SOURCE    GENECARD       Gene Ontology:   biological process:  transport   molecular function:  binding   molecular function:  calcium ion binding      BioCarta Pathways:     KEGG Pathways:  
    Probe set : 1376102_at    Name:   similar to RECS1    GB acc:   BI292351    UniGene:   Rn.50364    Gene Symbol:   RGD:1359409    Locus Link:   316516    Chromosome:   9    Cytoband:   Chr:9q33    SOURCE    GENECARD       Gene Ontology:     BioCarta Pathways:     KEGG Pathways:  
    Probe set : 1373264_at    Name:   zinc finger, BED domain containing 3 (predicted)    GB acc:   BE110658    UniGene:   Rn.103511    Gene Symbol:   Zbed3_predicted    Locus Link:   361881    Chromosome:   2    Cytoband:   Chr:2q12    SOURCE    GENECARD       Gene Ontology:   molecular function:  DNA binding      BioCarta Pathways:     KEGG Pathways:  
    Probe set : 1371253_at    Name:   electron transferring flavoprotein, alpha polypeptide    GB acc:   AA942749    UniGene:   Rn.32496    Gene Symbol:   Etfa    Locus Link:   300726    Chromosome:   8    Cytoband:   Chr:8q24    SOURCE    GENECARD       Gene Ontology:   biological process:  electron transport   cellular component:  mitochondrion   cellular component:  electron transfer flavoprotein complex (sensu Eukaryota)   molecular function:  electron transporter activity   molecular function:  electron carrier activity      BioCarta Pathways:     KEGG Pathways:  
    Probe set : 1396077_at    Name:   Transcribed locus    GB acc:   BF554876    UniGene:   Rn.834        SOURCE        Gene Ontology:     BioCarta Pathways:     KEGG Pathways:  
    Probe set : 1371933_at     GB acc:   AI228039              Gene Ontology:     BioCarta Pathways:     KEGG Pathways:  
    Probe set : 1398294_at    Name:   actinin, alpha 1    GB acc:   NM_031005    UniGene:   Rn.6401    Gene Symbol:   Actn1    Locus Link:   81634    Chromosome:   6    Cytoband:   Chr:6q24    SOURCE    GENECARD       Gene Ontology:   cellular component:  cytoskeleton   cellular component:  actin cytoskeleton      BioCarta Pathways:     KEGG Pathways:  
    Probe set : 1377685_at    Name:   similar to establishment of cohesion 1 homolog 1    GB acc:   AW435161    UniGene:   Rn.140694    Gene Symbol:   LOC498820    Locus Link:   498820    Chromosome:   18    Cytoband:   Chr:18p13    SOURCE    GENECARD       Gene Ontology:     BioCarta Pathways:     KEGG Pathways:  
    Probe set : 1367599_at    Name:   ATP synthase, H+ transporting, mitochondrial F0 complex, subunit c (subunit 9), isoform 1    GB acc:   NM_017311    UniGene:   Rn.3357    Gene Symbol:   Atp5g1    Locus Link:   29754    Chromosome:   10    Cytoband:   Chr:10q31    SOURCE    GENECARD       Gene Ontology:     BioCarta Pathways:     KEGG Pathways:  
    Probe set : 1377336_at    Name:   sema domain, immunoglobulin domain (Ig), short basic domain, secreted, (semaphorin) 3B (predicted)    GB acc:   BI275485    UniGene:   Rn.26859    Gene Symbol:   Sema3b_predicted    Locus Link:   363142    Chromosome:   8    Cytoband:   Chr:8q32    SOURCE    GENECARD       Gene Ontology:   cellular component:  extracellular space   cellular component:  endoplasmic reticulum   cellular component:  membrane   molecular function:  receptor activity      BioCarta Pathways:     KEGG Pathways:  
    Probe set : 1386881_at    Name:   insulin-like growth factor binding protein 3    GB acc:   NM_012588    UniGene:   Rn.26369    Gene Symbol:   Igfbp3    Locus Link:   24484    Chromosome:   14    Cytoband:   Chr:14q21    SOURCE    GENECARD       Gene Ontology:   cellular component:  extracellular region   cellular component:  extracellular space   cellular component:  extracellular region      BioCarta Pathways:     KEGG Pathways:  
    Probe set : 1370609_a_at    Name:   solute carrier family 16 (monocarboxylic acid transporters), member 7 /// hypothetical gene supported by NM_017302    GB acc:   X97445    UniGene:   Rn.10524    Gene Symbol:   Slc16a7 /// LOC497820    Locus Link:   29735 /// 497820    Chromosome:   7    Cytoband:   Chr:7q22    SOURCE    GENECARD       Gene Ontology:   biological process:  transport   biological process:  organic anion transport   biological process:  monocarboxylic acid transport      BioCarta Pathways:     KEGG Pathways:  
    Probe set : 1384944_at    Name:   B-cell leukemia/lymphoma 11B (predicted)    GB acc:   BE116855    UniGene:   Rn.52748    Gene Symbol:   Bcl11b_predicted    Locus Link:   314423    Chromosome:   6    Cytoband:   Chr:6q32    SOURCE    GENECARD       Gene Ontology:   biological process:  regulation of transcription, DNA-dependent   biological process:  negative regulation of cell proliferation   cellular component:  nucleus   molecular function:  nucleic acid binding   molecular function:  zinc ion binding      BioCarta Pathways:     KEGG Pathways:  
    Probe set : 1372734_at    Name:   small cell adhesion glycoprotein    GB acc:   AI408095    UniGene:   Rn.144437    Gene Symbol:   Smagp    Locus Link:   300236    Chromosome:   7    Cytoband:   Chr:7q36    SOURCE    GENECARD       Gene Ontology:     BioCarta Pathways:     KEGG Pathways:  
    Probe set : 1384507_at    Name:   Similar to hypothetical protein FLJ11127    GB acc:   AA817708    UniGene:   Rn.16375     Locus Link:   310190    Chromosome:   2    Cytoband:   Chr:2q22    SOURCE        Gene Ontology:     BioCarta Pathways:     KEGG Pathways:  
    Probe set : 1372208_at    Name:   protein phosphatase 1, regulatory (inhibitor) subunit 1B    GB acc:   AA942959    UniGene:   Rn.70366    Gene Symbol:   Ppp1r1b    Locus Link:   360616    Chromosome:   10    Cytoband:   Chr:10q31    SOURCE    GENECARD       Gene Ontology:   cellular component:  cytoplasm      BioCarta Pathways:     KEGG Pathways:  
    Probe set : 1374300_at     GB acc:   AA997139              Gene Ontology:     BioCarta Pathways:     KEGG Pathways:  
    Probe set : 1383711_at    Name:   N-myristoyltransferase 1    GB acc:   BE126380    UniGene:   Rn.830    Gene Symbol:   Nmt1    Locus Link:   259274    Chromosome:   10    Cytoband:   Chr:10q32.1    SOURCE    GENECARD       Gene Ontology:   biological process:  N-terminal protein myristoylation   biological process:  N-terminal peptidyl-glycine N-myristoylation   cellular component:  cytoplasm      BioCarta Pathways:     KEGG Pathways:  
    Probe set : 1368460_at    Name:   solute carrier family 2, member 5    GB acc:   NM_031741    UniGene:   Rn.10900    Gene Symbol:   Slc2a5    Locus Link:   65197    Chromosome:   5    Cytoband:   Chr:5q36    SOURCE    GENECARD       Gene Ontology:     BioCarta Pathways:     KEGG Pathways:  
    Probe set : 1392107_at    Name:   similar to KIAA0963 protein (predicted)    GB acc:   BI302669    UniGene:   Rn.67287    Gene Symbol:   RGD1307194_predicted    Locus Link:   314619    Chromosome:   7    Cytoband:   Chr:7q11    SOURCE    GENECARD       Gene Ontology:     BioCarta Pathways:     KEGG Pathways:  
    Probe set : 1372825_at    Name:   LOC499775    GB acc:   BI290551    UniGene:   Rn.107371    Gene Symbol:   LOC499775    Locus Link:   499775    Chromosome:   3    Cytoband:   Chr:3p12    SOURCE    GENECARD       Gene Ontology:     BioCarta Pathways:     KEGG Pathways:  
    Probe set : 1368040_at    Name:   synaptojanin 2 binding protein    GB acc:   BI285174    UniGene:   Rn.6258    Gene Symbol:   Synj2bp    Locus Link:   64531    Chromosome:   6    Cytoband:   Chr:6q24    SOURCE    GENECARD       Gene Ontology:   molecular function:  receptor activity   molecular function:  protein binding      BioCarta Pathways:     KEGG Pathways:  
    Probe set : 1367883_at    Name:   survival of motor neuron 1, telomeric    GB acc:   NM_022509    UniGene:   Rn.1119    Gene Symbol:   SMN1    Locus Link:   64301    Chromosome:   2    Cytoband:   Chr:2q12    SOURCE    GENECARD       Gene Ontology:     BioCarta Pathways:     KEGG Pathways:  
    Probe set : 1377369_at    Name:   cytochrome b reductase 1 (predicted)    GB acc:   BF419070    UniGene:   Rn.13660    Gene Symbol:   Cybrd1_predicted    Locus Link:   295669    Chromosome:   3    Cytoband:   Chr:3q21    SOURCE    GENECARD       Gene Ontology:   biological process:  electron transport   cellular component:  integral to membrane   molecular function:  transferase activity      BioCarta Pathways:     KEGG Pathways:  
    Probe set : 1382031_at    Name:   Transcribed locus    GB acc:   AA859079    UniGene:   Rn.2564        SOURCE        Gene Ontology:     BioCarta Pathways:     KEGG Pathways:  
    Probe set : 1376801_at    Name:   hypothetical LOC294291    GB acc:   AW915035    UniGene:   Rn.12781    Gene Symbol:   LOC294291    Locus Link:   294291    Chromosome:   20    Cytoband:   Chr:20p12    SOURCE    GENECARD       Gene Ontology:     BioCarta Pathways:     KEGG Pathways:  
    Probe set : 1389213_at    Name:   Transcribed locus    GB acc:   AI408955    UniGene:   Rn.35462        SOURCE        Gene Ontology:     BioCarta Pathways:     KEGG Pathways:  
    Probe set : 1389063_at    Name:   exportin 6 (predicted)    GB acc:   BG378160    UniGene:   Rn.43943    Gene Symbol:   Xpo6_predicted    Locus Link:   293476    Chromosome:   1    Cytoband:   Chr:1q36    SOURCE    GENECARD       Gene Ontology:   biological process:  protein import into nucleus, docking   biological process:  protein export from nucleus   cellular component:  nucleus   cellular component:  nuclear pore   cellular component:  cytoplasm   molecular function:  protein binding   molecular function:  protein transporter activity   molecular function:  binding      BioCarta Pathways:     KEGG Pathways:  
    Probe set : 1367977_at    Name:   synuclein, alpha    GB acc:   NM_019169    UniGene:   Rn.1827    Gene Symbol:   Snca    Locus Link:   29219    Chromosome:   4    Cytoband:   Chr:4q24    SOURCE    GENECARD       Gene Ontology:   biological process:  anti-apoptosis   biological process:  central nervous system development   biological process:  synaptic vesicle transport   cellular component:  cytoplasm   cellular component:  cytoplasm   molecular function:  protein N-terminus binding      BioCarta Pathways:     KEGG Pathways:  
    Probe set : 1386893_at    Name:   granulin    GB acc:   NM_017113    UniGene:   Rn.5820    Gene Symbol:   Grn    Locus Link:   29143    Chromosome:   10    Cytoband:   Chr:10q32.1    SOURCE    GENECARD       Gene Ontology:   biological process:  lipid catabolism   cellular component:  extracellular space   cellular component:  mitochondrion   molecular function:  phospholipase A2 activity   molecular function:  cytokine activity   molecular function:  calcium ion binding      BioCarta Pathways:     KEGG Pathways:  
    Probe set : 1379445_at    Name:   LOC500609    GB acc:   BF283378    UniGene:   Rn.96238     Locus Link:   500609      SOURCE        Gene Ontology:     BioCarta Pathways:     KEGG Pathways:  
    Probe set : 1382074_at    Name:   IBR domain containing 3 (predicted)    GB acc:   AI113237    UniGene:   Rn.18272    Gene Symbol:   Ibrdc3_predicted    Locus Link:   313806    Chromosome:   5    Cytoband:   Chr:5q36    SOURCE    GENECARD       Gene Ontology:     BioCarta Pathways:     KEGG Pathways:  
    Probe set : 1394724_at    Name:   transcription elongation factor A (SII) 1 (predicted)    GB acc:   BE109084    UniGene:   Rn.129120    Gene Symbol:   Tcea1_predicted    Locus Link:   362479    Chromosome:   5    Cytoband:   Chr:5q12    SOURCE    GENECARD       Gene Ontology:   cellular component:  nucleus   cellular component:  nucleoplasm      BioCarta Pathways:     KEGG Pathways:  
    Probe set : 1373226_at    Name:   Similar to 60S ribosomal protein L23    GB acc:   BF400995    UniGene:   Rn.14609     Locus Link:   366003    Chromosome:   3    Cytoband:   Chr:3p13    SOURCE        Gene Ontology:     BioCarta Pathways:     KEGG Pathways:  
    Probe set : 1394673_at    Name:   similar to m33-A isoform    GB acc:   AI454014    UniGene:   Rn.34564    Gene Symbol:   LOC499142    Locus Link:   499142    Chromosome:   1    Cytoband:   Chr:1q22    SOURCE    GENECARD       Gene Ontology:     BioCarta Pathways:     KEGG Pathways:  
    Probe set : 1370963_at    Name:   growth arrest specific 7    GB acc:   AJ131902    UniGene:   Rn.17160    Gene Symbol:   Gas7    Locus Link:   85246    Chromosome:   10    Cytoband:   Chr:10q24    SOURCE    GENECARD       Gene Ontology:   cellular component:  kinesin complex      BioCarta Pathways:     KEGG Pathways:  
    Probe set : 1392905_at    Name:   Guanine nucleotide binding protein, gamma 2    GB acc:   BF552733    UniGene:   Rn.33178    Gene Symbol:   Gng2    Locus Link:   80850      SOURCE    GENECARD       Gene Ontology:   biological process:  signal transduction   biological process:  G-protein coupled receptor protein signaling pathway   cellular component:  heterotrimeric G-protein complex   cellular component:  heterotrimeric G-protein complex   molecular function:  GTPase activity   molecular function:  signal transducer activity   molecular function:  small GTPase regulator activity      BioCarta Pathways:     KEGG Pathways:  
    Probe set : 1370859_at    Name:   thioredoxin domain containing 7    GB acc:   BI284965    UniGene:   Rn.2685    Gene Symbol:   Txndc7    Locus Link:   286906      SOURCE    GENECARD       Gene Ontology:   biological process:  electron transport   biological process:  protein folding   cellular component:  extracellular space   cellular component:  endoplasmic reticulum      BioCarta Pathways:     KEGG Pathways:  
    Probe set : 1371755_at    Name:   Similar to RIKEN cDNA B230219D22    GB acc:   AA799607    UniGene:   Rn.3327     Locus Link:   498701      SOURCE        Gene Ontology:     BioCarta Pathways:     KEGG Pathways:  
    Probe set : 1368178_at    Name:   PDZ domain containing 1    GB acc:   NM_031712    UniGene:   Rn.19842    Gene Symbol:   Pdzk1    Locus Link:   65144    Chromosome:   2    Cytoband:   Chr:2q34    SOURCE    GENECARD       Gene Ontology:   molecular function:  scavenger receptor binding   molecular function:  transporter activity   molecular function:  protein binding      BioCarta Pathways:     KEGG Pathways:  
    Probe set : 1379361_at    Name:   peroxisomal biogenesis factor 11A    GB acc:   BI273703    UniGene:   Rn.14519    Gene Symbol:   Pex11a    Locus Link:   85249    Chromosome:   1    Cytoband:   Chr:1q31    SOURCE    GENECARD       Gene Ontology:   biological process:  peroxisome organization and biogenesis   biological process:  peroxisome membrane biogenesis   biological process:  peroxisome fission      BioCarta Pathways:     KEGG Pathways:  
    Probe set : 1398473_at    Name:   biogenesis of lysosome-related organelles complex-1, subunit 2 (predicted)    GB acc:   BI290651    UniGene:   Rn.19119    Gene Symbol:   Bloc1s2_predicted    Locus Link:   293938    Chromosome:   1    Cytoband:   Chr:1q54    SOURCE    GENECARD       Gene Ontology:   cellular component:  cytosol   molecular function:  protein binding      BioCarta Pathways:     KEGG Pathways:  
    Probe set : 1382692_at    Name:   Transcribed locus    GB acc:   AI045955    UniGene:   Rn.15743        SOURCE        Gene Ontology:     BioCarta Pathways:     KEGG Pathways:  
    Probe set : 1369190_at    Name:   CD2 antigen /// hypothetical gene supported by NM_012830    GB acc:   NM_012830    UniGene:   Rn.10328    Gene Symbol:   Cd2 /// LOC497761    Locus Link:   25299 /// 497761    Chromosome:   2    Cytoband:   Chr:2q34    SOURCE    GENECARD       Gene Ontology:   biological process:  lipid raft polarization   biological process:  protein amino acid phosphorylation   biological process:  induction of apoptosis   biological process:  defense response   biological process:  cell adhesion   biological process:  cell surface receptor linked signal transduction   biological process:  cell-cell adhesion   biological process:  natural killer cell activation   biological process:  positive regulation of dendritic cell activation   biological process:  T cell activation   biological process:  T cell activation   biological process:  regulation of T cell differentiation   biological process:  induction of apoptosis   biological process:  cell surface receptor linked signal transduction   biological process:  cell-cell adhesion   biological process:  T cell activation      BioCarta Pathways:     KEGG Pathways:  
    Probe set : 1387835_at    Name:   interleukin 1 receptor antagonist    GB acc:   NM_022194    UniGene:   Rn.85806    Gene Symbol:   Il1rn    Locus Link:   60582    Chromosome:   3    Cytoband:   Chr:3p13    SOURCE    GENECARD       Gene Ontology:   cellular component:  extracellular region   cellular component:  extracellular region   cellular component:  integral to plasma membrane      BioCarta Pathways:     KEGG Pathways:  
    Probe set : 1376765_at    Name:   similar to maestro    GB acc:   BF283408    UniGene:   Rn.16360    Gene Symbol:   LOC361348    Locus Link:   361348    Chromosome:   18    Cytoband:   Chr:18q12.2    SOURCE    GENECARD       Gene Ontology:     BioCarta Pathways:     KEGG Pathways:  
    Probe set : 1388470_at    Name:   f-box only protein 9 (predicted)    GB acc:   AA892772    UniGene:   Rn.144900    Gene Symbol:   Fbxo9_predicted    Locus Link:   300849    Chromosome:   8    Cytoband:   Chr:8q31    SOURCE    GENECARD       Gene Ontology:   biological process:  protein modification   biological process:  ubiquitin cycle   biological process:  protein ubiquitination   cellular component:  ubiquitin ligase complex   molecular function:  ubiquitin-protein ligase activity   molecular function:  binding      BioCarta Pathways:     KEGG Pathways:  
    Probe set : 1393183_at    Name:   similar to StAR-related protein 1-4E    GB acc:   H33503    UniGene:   Rn.62294    Gene Symbol:   LOC502348    Locus Link:   502348    Chromosome:   1    Cytoband:   Chr:1q31    SOURCE    GENECARD       Gene Ontology:     BioCarta Pathways:     KEGG Pathways:  
    Probe set : 1387759_s_at     GB acc:   J02612    UniGene:   Rn.26489    Gene Symbol:   Ugt1a1 /// Ugt1a6 /// Ugt1a7 /// Ugt1a8 /// Ugt1a2 /// Ugt1a4 /// Ugt1a11 /// Ugt1a5    Locus Link:   113992 /// 154516 /// 24861 /// 301595 /// 396527 /// 396551 /// 396552 /// 574523    Chromosome:   9    Cytoband:   Chr:9q35-q36 /// Chr:9q35    SOURCE    GENECARD       Gene Ontology:     BioCarta Pathways:     KEGG Pathways:  
    Probe set : 1370234_at    Name:   fibronectin 1    GB acc:   AA893484    UniGene:   Rn.1604    Gene Symbol:   Fn1    Locus Link:   25661    Chromosome:   9    Cytoband:   Chr:9q33    SOURCE    GENECARD       Gene Ontology:     BioCarta Pathways:     KEGG Pathways:  
    Probe set : 1381161_a_at    Name:   Similar to hypothetical protein FLJ21127    GB acc:   BI301117    UniGene:   Rn.12905     Locus Link:   304486    Chromosome:   12    Cytoband:   Chr:12q16    SOURCE        Gene Ontology:     BioCarta Pathways:     KEGG Pathways:  
    Probe set : 1369660_at    Name:   defensin beta 1    GB acc:   AF068860    UniGene:   Rn.31800    Gene Symbol:   Defb1    Locus Link:   83687    Chromosome:   16    Cytoband:   Chr:16p    SOURCE    GENECARD       Gene Ontology:   cellular component:  extracellular region   cellular component:  extracellular space      BioCarta Pathways:     KEGG Pathways:  
    Probe set : 1377940_at    Name:   hypothetical LOC287534    GB acc:   BF398271    UniGene:   Rn.13078    Gene Symbol:   RGD:1359691    Locus Link:   287534    Chromosome:   10    Cytoband:   Chr:10q24    SOURCE    GENECARD       Gene Ontology:     BioCarta Pathways:     KEGG Pathways:  
    Probe set : 1371359_at    Name:   myeloid leukemia factor 2 (predicted)    GB acc:   BG381670    UniGene:   Rn.3678    Gene Symbol:   Mlf2_predicted    Locus Link:   312709    Chromosome:   4    Cytoband:   Chr:4q42    SOURCE    GENECARD       Gene Ontology:   cellular component:  nucleus      BioCarta Pathways:     KEGG Pathways:  
    Probe set : 1393729_at    Name:   neuropilin (NRP) and tolloid (TLL)-like 2 (predicted)    GB acc:   BE112114    UniGene:   Rn.96405    Gene Symbol:   Neto2_predicted    Locus Link:   307757    Chromosome:   19    Cytoband:   Chr:19q11    SOURCE    GENECARD       Gene Ontology:   biological process:  endocytosis   cellular component:  coated pit   cellular component:  integral to membrane   molecular function:  receptor activity      BioCarta Pathways:     KEGG Pathways:  
    Probe set : 1371850_at    Name:   3'-phosphoadenosine 5'-phosphosulfate synthase 1 (predicted)    GB acc:   AA892829    UniGene:   Rn.3507    Gene Symbol:   Papss1_predicted    Locus Link:   295443    Chromosome:   2    Cytoband:   Chr:2q43    SOURCE    GENECARD       Gene Ontology:   cellular component:  intracellular   cellular component:  sulfate adenylyltransferase complex (ATP)      BioCarta Pathways:     KEGG Pathways:  
    Probe set : 1391593_at    Name:   Ras association (RalGDS/AF-6) domain family 4 (predicted)    GB acc:   BI286015    UniGene:   Rn.11482    Gene Symbol:   Rassf4_predicted    Locus Link:   362423    Chromosome:   4    Cytoband:   Chr:4q42    SOURCE    GENECARD       Gene Ontology:   biological process:  signal transduction   biological process:  neuropeptide signaling pathway   molecular function:  protein binding   molecular function:  oxidoreductase activity      BioCarta Pathways:     KEGG Pathways:  
    Probe set : 1380726_at    Name:   Similar to asporin precursor    GB acc:   BI290633    UniGene:   Rn.45067     Locus Link:   306805    Chromosome:   17    Cytoband:   Chr:17p14    SOURCE        Gene Ontology:     BioCarta Pathways:     KEGG Pathways:  
    Probe set : 1370384_a_at    Name:   prolactin receptor    GB acc:   M57668    UniGene:   Rn.9757    Gene Symbol:   Prlr    Locus Link:   24684    Chromosome:   2    Cytoband:   Chr:2q16    SOURCE    GENECARD       Gene Ontology:   biological process:  steroid biosynthesis   biological process:  anti-apoptosis   biological process:  cell surface receptor linked signal transduction   biological process:  transmembrane receptor protein tyrosine kinase activation (dimerization)   biological process:  embryo implantation   biological process:  lactation   biological process:  regulation of cell adhesion   biological process:  regulation of epithelial cell differentiation   biological process:  T cell activation   biological process:  tyrosine phosphorylation of JAK2 protein   biological process:  steroid biosynthesis   biological process:  anti-apoptosis   biological process:  cell surface receptor linked signal transduction   biological process:  transmembrane receptor protein tyrosine kinase activation (dimerization)   biological process:  lactation   biological process:  T cell activation   biological process:  tyrosine phosphorylation of JAK2 protein      BioCarta Pathways:     KEGG Pathways:  
    Probe set : 1382255_at     GB acc:   BE110785              Gene Ontology:     BioCarta Pathways:     KEGG Pathways:  
    Probe set : 1389867_at    Name:   CUG triplet repeat,RNA-binding protein 2    GB acc:   BI281086    UniGene:   Rn.30004    Gene Symbol:   Cugbp2    Locus Link:   29428    Chromosome:   17    Cytoband:   Chr:17q12.3    SOURCE    GENECARD       Gene Ontology:   cellular component:  nucleus   molecular function:  RNA binding   molecular function:  RNA binding   molecular function:  nucleotide binding   molecular function:  nucleic acid binding      BioCarta Pathways:     KEGG Pathways:  
    Probe set : 1398863_at    Name:   guanine nucleotide binding protein, beta polypeptide 2    GB acc:   AF277892    UniGene:   Rn.1155    Gene Symbol:   Gnb2    Locus Link:   81667    Chromosome:   12    Cytoband:   Chr:12q12    SOURCE    GENECARD       Gene Ontology:   biological process:  signal transduction   biological process:  G-protein coupled receptor protein signaling pathway   cellular component:  heterotrimeric G-protein complex   molecular function:  GTPase activity   molecular function:  signal transducer activity   molecular function:  calcium channel regulator activity      BioCarta Pathways:     KEGG Pathways:  
    Probe set : 1370308_at    Name:   RS21-C6 protein    GB acc:   AY029335    UniGene:   Rn.109806    Gene Symbol:   Rs21c6    Locus Link:   192252    Chromosome:   1    Cytoband:   Chr:1q36    SOURCE    GENECARD       Gene Ontology:     BioCarta Pathways:     KEGG Pathways:  
    Probe set : 1371613_at    Name:   engulfment and cell motility 2, ced-12 homolog (C. elegans) (predicted)    GB acc:   BM386781    UniGene:   Rn.2097    Gene Symbol:   Elmo2_predicted    Locus Link:   362271    Chromosome:   3    Cytoband:   Chr:3q42    SOURCE    GENECARD       Gene Ontology:   biological process:  phagocytosis   biological process:  apoptosis   cellular component:  cytoskeleton   cellular component:  membrane      BioCarta Pathways:     KEGG Pathways:  
    Probe set : 1367839_at    Name:   farnesyl diphosphate farnesyl transferase 1    GB acc:   NM_019238    UniGene:   Rn.21071    Gene Symbol:   Fdft1    Locus Link:   29580    Chromosome:   15    Cytoband:   Chr:15p12    SOURCE    GENECARD       Gene Ontology:   cellular component:  endoplasmic reticulum   cellular component:  integral to membrane      BioCarta Pathways:     KEGG Pathways:  
    Probe set : 1370789_a_at    Name:   prolactin receptor    GB acc:   L48060    UniGene:   Rn.9757    Gene Symbol:   Prlr    Locus Link:   24684    Chromosome:   2    Cytoband:   Chr:2q16    SOURCE    GENECARD       Gene Ontology:   biological process:  steroid biosynthesis   biological process:  anti-apoptosis   biological process:  cell surface receptor linked signal transduction   biological process:  transmembrane receptor protein tyrosine kinase activation (dimerization)   biological process:  embryo implantation   biological process:  lactation   biological process:  regulation of cell adhesion   biological process:  regulation of epithelial cell differentiation   biological process:  T cell activation   biological process:  tyrosine phosphorylation of JAK2 protein   biological process:  steroid biosynthesis   biological process:  anti-apoptosis   biological process:  cell surface receptor linked signal transduction   biological process:  transmembrane receptor protein tyrosine kinase activation (dimerization)   biological process:  lactation   biological process:  T cell activation   biological process:  tyrosine phosphorylation of JAK2 protein      BioCarta Pathways:     KEGG Pathways:  
    Probe set : 1388175_at    Name:   chloride channel Kb    GB acc:   Z30663    UniGene:   Rn.10339    Gene Symbol:   Clcnkb    Locus Link:   79430    Chromosome:   5    Cytoband:   Chr:5q36    SOURCE    GENECARD       Gene Ontology:     BioCarta Pathways:     KEGG Pathways:  
    Probe set : 1391199_at    Name:   Fas (TNFRSF6)-associated via death domain    GB acc:   BE100715    UniGene:   Rn.16183    Gene Symbol:   Fadd    Locus Link:   266610    Chromosome:   1    Cytoband:   Chr:1q42    SOURCE    GENECARD       Gene Ontology:   molecular function:  protein binding      BioCarta Pathways:     KEGG Pathways:  
    Probe set : 1369286_at    Name:   protein C    GB acc:   NM_012803    UniGene:   Rn.91064    Gene Symbol:   Proc    Locus Link:   25268    Chromosome:   18    Cytoband:   Chr:18p12    SOURCE    GENECARD       Gene Ontology:   cellular component:  extracellular region   cellular component:  extracellular space      BioCarta Pathways:     KEGG Pathways:  
    Probe set : 1384145_at    Name:   Similar to KIAA1919 protein (predicted)    GB acc:   AI511200    UniGene:   Rn.24001     Locus Link:   309809    Chromosome:   20    Cytoband:   Chr:20q12    SOURCE        Gene Ontology:     BioCarta Pathways:     KEGG Pathways:  
    Probe set : 1368079_at    Name:   pyruvate dehydrogenase kinase 1    GB acc:   NM_053826    UniGene:   Rn.11185    Gene Symbol:   Pdk1    Locus Link:   116551    Chromosome:   3    Cytoband:   Chr:3q22    SOURCE    GENECARD       Gene Ontology:   cellular component:  mitochondrion   cellular component:  mitochondrion      BioCarta Pathways:     KEGG Pathways:  
    Probe set : 1376702_at    Name:   Megalencephalic leukoencephalopathy with subcortical cysts 1 (predicted)    GB acc:   AI228307    UniGene:   Rn.98819     Locus Link:   315215    Chromosome:   7    Cytoband:   Chr:7q34    SOURCE        Gene Ontology:   biological process:  transport   cellular component:  integral to membrane of membrane fraction   cellular component:  integral to membrane   molecular function:  ion channel activity      BioCarta Pathways:     KEGG Pathways:  
    Probe set : 1388374_at     GB acc:   AI412011              Gene Ontology:     BioCarta Pathways:     KEGG Pathways:  
    Probe set : 1372279_at    Name:   BCL2-like 13 (apoptosis facilitator) (predicted)    GB acc:   AA892271    UniGene:   Rn.103700    Gene Symbol:   Bcl2l13_predicted    Locus Link:   312682    Chromosome:   4    Cytoband:   Chr:4q42    SOURCE    GENECARD       Gene Ontology:   cellular component:  mitochondrion   cellular component:  integral to membrane   molecular function:  caspase activator activity      BioCarta Pathways:     KEGG Pathways:  
    Probe set : 1373304_at    Name:   alpha-N-acetylglucosaminidase    GB acc:   BM392002    UniGene:   Rn.36938    Gene Symbol:   Naglu    Locus Link:   287711    Chromosome:   10    Cytoband:   Chr:10q32.1    SOURCE    GENECARD       Gene Ontology:   biological process:  biosynthesis   molecular function:  ATP binding   molecular function:  nucleotidyltransferase activity      BioCarta Pathways:     KEGG Pathways:  
    Probe set : 1367917_at    Name:   cytochrome P450, family 2, subfamily d, polypeptide 26    GB acc:   NM_012730    UniGene:   Rn.91355    Gene Symbol:   Cyp2d26    Locus Link:   25053    Chromosome:   7    Cytoband:   Chr:7q34    SOURCE    GENECARD       Gene Ontology:   biological process:  electron transport   cellular component:  endoplasmic reticulum   cellular component:  microsome   cellular component:  membrane      BioCarta Pathways:     KEGG Pathways:  
    Probe set : 1371466_at    Name:   similar to RIKEN cDNA 0610009B22 (predicted) /// similar to Sedlin (Trafficking protein particle complex protein 2)    GB acc:   AI177397    UniGene:   Rn.8274    Gene Symbol:   RGD1306925_predicted /// LOC501550    Locus Link:   287274 /// 501550    Chromosome:   10    Cytoband:   Chr:10q22 /// Chr:Xq21    SOURCE    GENECARD       Gene Ontology:   cellular component:  endoplasmic reticulum   cellular component:  Golgi apparatus   cellular component:  intracellular   molecular function:  intracellular transporter activity      BioCarta Pathways:     KEGG Pathways:  
    Probe set : 1372990_at    Name:   cAMP responsive element binding protein 3 (predicted)    GB acc:   AI179264    UniGene:   Rn.144871    Gene Symbol:   Creb3_predicted    Locus Link:   298400    Chromosome:   5    Cytoband:   Chr:5q22    SOURCE    GENECARD       Gene Ontology:   cellular component:  nucleus   cellular component:  endoplasmic reticulum   cellular component:  integral to membrane   molecular function:  DNA binding   molecular function:  translation activator activity      BioCarta Pathways:     KEGG Pathways:  
    Probe set : 1379448_at    Name:   Ttk protein kinase (predicted)    GB acc:   BE110883    UniGene:   Rn.107533    Gene Symbol:   Ttk_predicted    Locus Link:   315852    Chromosome:   8    Cytoband:   Chr:8q31    SOURCE    GENECARD       Gene Ontology:   cellular component:  spindle      BioCarta Pathways:     KEGG Pathways:  
    Probe set : 1368568_at    Name:   aquaporin 2    GB acc:   D13906    UniGene:   Rn.90076    Gene Symbol:   Aqp2    Locus Link:   25386    Chromosome:   7    Cytoband:   Chr:7q36    SOURCE    GENECARD       Gene Ontology:     BioCarta Pathways:     KEGG Pathways:  
    Probe set : 1367995_at    Name:   catalase    GB acc:   NM_012520    UniGene:   Rn.3001    Gene Symbol:   Cat    Locus Link:   24248    Chromosome:   3    Cytoband:   Chr:3q32-q34    SOURCE    GENECARD       Gene Ontology:   cellular component:  mitochondrion   cellular component:  peroxisome      BioCarta Pathways:     KEGG Pathways:  
    Probe set : 1373595_at    Name:   similar to RIKEN cDNA 1200015A22    GB acc:   BM385463    UniGene:   Rn.15201    Gene Symbol:   MGC94413    Locus Link:   362401    Chromosome:   4    Cytoband:   Chr:4q34    SOURCE    GENECARD       Gene Ontology:     BioCarta Pathways:     KEGG Pathways:  
    Probe set : 1367648_at    Name:   insulin-like growth factor binding protein 2    GB acc:   NM_013122    UniGene:   Rn.6813    Gene Symbol:   Igfbp2    Locus Link:   25662    Chromosome:   9    Cytoband:   Chr:9q33    SOURCE    GENECARD       Gene Ontology:   biological process:  regulation of cell growth   cellular component:  extracellular region   cellular component:  extracellular space   molecular function:  insulin-like growth factor binding   molecular function:  growth factor binding      BioCarta Pathways:     KEGG Pathways:  
    Probe set : 1386376_at    Name:   Transcribed locus    GB acc:   AA892173    UniGene:   Rn.134302        SOURCE        Gene Ontology:     BioCarta Pathways:     KEGG Pathways:  
    Probe set : 1369991_at    Name:   signal peptidase complex 18kD    GB acc:   NM_031723    UniGene:   Rn.13070    Gene Symbol:   Spc18    Locus Link:   65166    Chromosome:   1    Cytoband:   Chr:1q31    SOURCE    GENECARD       Gene Ontology:   biological process:  signal peptide processing   biological process:  proteolysis      BioCarta Pathways:     KEGG Pathways:  
    Probe set : 1374777_at    Name:   hypothetical protein LOC502894    GB acc:   BG380800    UniGene:   Rn.15200    Gene Symbol:   LOC502894    Locus Link:   502894    Chromosome:   4    Cytoband:   Chr:4q42    SOURCE    GENECARD       Gene Ontology:     BioCarta Pathways:     KEGG Pathways:  
    Probe set : 1372670_at    Name:   similar to hypothetical protein FLJ21827 (predicted)    GB acc:   BI281686    UniGene:   Rn.13688    Gene Symbol:   RGD1307682_predicted    Locus Link:   300675    Chromosome:   8    Cytoband:   Chr:8q22    SOURCE    GENECARD       Gene Ontology:     BioCarta Pathways:     KEGG Pathways:  
    Probe set : 1387234_at    Name:   alpha-2-glycoprotein 1, zinc    GB acc:   NM_012826    UniGene:   Rn.5007    Gene Symbol:   Azgp1    Locus Link:   25294    Chromosome:   12    Cytoband:   Chr:12q11    SOURCE    GENECARD       Gene Ontology:     BioCarta Pathways:     KEGG Pathways:  
    Probe set : 1375973_at    Name:   ariadne ubiquitin-conjugating enzyme E2 binding protein homolog 1 (Drosophila) (predicted)    GB acc:   AI600108    UniGene:   Rn.29865    Gene Symbol:   Arih1_predicted    Locus Link:   300756    Chromosome:   8    Cytoband:   Chr:8q24    SOURCE    GENECARD       Gene Ontology:   biological process:  ubiquitin-dependent protein catabolism   biological process:  ubiquitin cycle   biological process:  protein ubiquitination   cellular component:  ubiquitin ligase complex   cellular component:  nucleus      BioCarta Pathways:     KEGG Pathways:  
    Probe set : 1387889_at    Name:   folate receptor 1 (adult)    GB acc:   AI233882    UniGene:   Rn.6912    Gene Symbol:   Folr1    Locus Link:   171049    Chromosome:   1    Cytoband:   Chr:1q32    SOURCE    GENECARD       Gene Ontology:   biological process:  posttranslational protein targeting to membrane   biological process:  folic acid metabolism   cellular component:  extracellular space   cellular component:  membrane fraction   cellular component:  membrane      BioCarta Pathways:     KEGG Pathways:  
    Probe set : 1380425_at    Name:   Ribonuclease L (2',5'-oligoisoadenylate synthetase-dependent)    GB acc:   AI012859    UniGene:   Rn.107928    Gene Symbol:   Rnasel    Locus Link:   359726    Chromosome:   13    Cytoband:   Chr:13q21    SOURCE    GENECARD       Gene Ontology:   biological process:  mRNA processing   biological process:  protein amino acid phosphorylation   molecular function:  protein kinase activity   molecular function:  ATP binding   molecular function:  endoribonuclease activity, producing 5'-phosphomonoesters      BioCarta Pathways:     KEGG Pathways:  
    Probe set : 1398343_at    Name:   DnaJ (Hsp40) homolog, subfamily A, member 4 (predicted)    GB acc:   AI104324    UniGene:   Rn.27927     Locus Link:   300721    Chromosome:   8    Cytoband:   Chr:8q24    SOURCE        Gene Ontology:   biological process:  protein folding   molecular function:  heat shock protein binding   molecular function:  unfolded protein binding      BioCarta Pathways:     KEGG Pathways:  
    Probe set : 1370361_at    Name:   cell growth regulator with EF hand domain 1    GB acc:   U66470    UniGene:   Rn.31842    Gene Symbol:   Cgref1    Locus Link:   245918    Chromosome:   6    Cytoband:   Chr:6q14    SOURCE    GENECARD       Gene Ontology:   cellular component:  extracellular space   molecular function:  calcium ion binding      BioCarta Pathways:     KEGG Pathways:  
    Probe set : 1392648_at    Name:   mannose receptor, C type 1 (predicted)    GB acc:   BI294018    UniGene:   Rn.9197    Gene Symbol:   Mrc1_predicted    Locus Link:   291327    Chromosome:   17    Cytoband:   Chr:17q12.3    SOURCE    GENECARD       Gene Ontology:   biological process:  endocytosis   biological process:  receptor mediated endocytosis   biological process:  pinocytosis      BioCarta Pathways:     KEGG Pathways:  
    Probe set : 1387866_at    Name:   myosin IXb    GB acc:   AI178160    UniGene:   Rn.33782    Gene Symbol:   Myo9b    Locus Link:   25486    Chromosome:   16    Cytoband:   Chr:16p14    SOURCE    GENECARD       Gene Ontology:   cellular component:  cytoskeleton   cellular component:  kinesin complex   cellular component:  myosin   molecular function:  microfilament motor activity   molecular function:  motor activity   molecular function:  actin binding   molecular function:  actin binding   molecular function:  GTPase activator activity   molecular function:  GTPase activator activity   molecular function:  Rho GTPase activator activity   molecular function:  calmodulin binding   molecular function:  ATP binding   molecular function:  zinc ion binding   molecular function:  diacylglycerol binding   molecular function:  ATPase activity, coupled   molecular function:  ATP-dependent protein binding   molecular function:  nucleotide binding   molecular function:  zinc ion binding   molecular function:  metal ion binding      BioCarta Pathways:     KEGG Pathways:  
    Probe set : 1377092_at    Name:   Transcribed locus    GB acc:   BF389682    UniGene:   Rn.153630        SOURCE        Gene Ontology:     BioCarta Pathways:     KEGG Pathways:  
    Probe set : 1374111_at    Name:   similar to pinin    GB acc:   BI301101    UniGene:   Rn.38987    Gene Symbol:   LOC368070    Locus Link:   368070    Chromosome:   6    Cytoband:   Chr:6q23    SOURCE    GENECARD       Gene Ontology:     BioCarta Pathways:     KEGG Pathways:  
    Probe set : 1370824_at    Name:   solute carrier family 38, member 3    GB acc:   AF273025    UniGene:   Rn.32067    Gene Symbol:   Slc38a3    Locus Link:   252919    Chromosome:   8    Cytoband:   Chr:8q32    SOURCE    GENECARD       Gene Ontology:     BioCarta Pathways:     KEGG Pathways:  
    Probe set : 1393342_at    Name:   Solute carrier family 25 (mitochondrial carrier; ornithine transporter) member 15 (predicted)    GB acc:   BG380998    UniGene:   Rn.66488     Locus Link:   306574    Chromosome:   16    Cytoband:   Chr:16q12.5    SOURCE        Gene Ontology:   molecular function:  L-ornithine transporter activity   molecular function:  transporter activity   molecular function:  binding      BioCarta Pathways:     KEGG Pathways:  
    Probe set : 1375554_at    Name:   DEAD (Asp-Glu-Ala-Asp) box polypeptide 47 (predicted)    GB acc:   BM390927    UniGene:   Rn.73790    Gene Symbol:   Ddx47_predicted    Locus Link:   297685    Chromosome:   4    Cytoband:   Chr:4q43    SOURCE    GENECARD       Gene Ontology:   biological process:  RNA metabolism   cellular component:  nucleus   cellular component:  cytoplasm      BioCarta Pathways:     KEGG Pathways:  
    Probe set : 1374626_at    Name:   similar to leucine-rich alpha-2-glycoprotein    GB acc:   BG371585    UniGene:   Rn.17847    Gene Symbol:   RGD:1359464    Locus Link:   367455      SOURCE    GENECARD       Gene Ontology:     BioCarta Pathways:     KEGG Pathways:  
    Probe set : 1368188_at    Name:   4-hydroxyphenylpyruvic acid dioxygenase    GB acc:   NM_017233    UniGene:   Rn.3664    Gene Symbol:   Hpd    Locus Link:   29531    Chromosome:   12    Cytoband:   Chr:12q16    SOURCE    GENECARD       Gene Ontology:   cellular component:  Golgi membrane   cellular component:  endoplasmic reticulum membrane      BioCarta Pathways:     KEGG Pathways:  
    Probe set : 1377607_at    Name:   mitogen activated protein kinase kinase kinase 3 (predicted)    GB acc:   BM385631    UniGene:   Rn.72680    Gene Symbol:   Map3k3_predicted    Locus Link:   303604    Chromosome:   10    Cytoband:   Chr:10q32.1    SOURCE    GENECARD       Gene Ontology:   biological process:  MAPKKK cascade   biological process:  protein amino acid phosphorylation   biological process:  positive regulation of I-kappaB kinase/NF-kappaB cascade      BioCarta Pathways:     KEGG Pathways:  
    Probe set : 1380199_at    Name:   WD repeat domain 22 (predicted)    GB acc:   BI281105    UniGene:   Rn.47944    Gene Symbol:   Wdr22    Locus Link:   314273    Chromosome:   6    Cytoband:   Chr:6q24    SOURCE    GENECARD       Gene Ontology:     BioCarta Pathways:     KEGG Pathways:  
    Probe set : 1382270_at    Name:   Transcribed locus    GB acc:   BM384387    UniGene:   Rn.147463        SOURCE        Gene Ontology:     BioCarta Pathways:     KEGG Pathways:  
    Probe set : 1383936_at    Name:   Elastin microfibril interfacer 2 (predicted)    GB acc:   BM386842    UniGene:   Rn.15970     Locus Link:   316736    Chromosome:   9    Cytoband:   Chr:9q38    SOURCE        Gene Ontology:   biological process:  cell adhesion   cellular component:  extracellular matrix (sensu Metazoa)   molecular function:  protein binding   molecular function:  extracellular matrix constituent conferring elasticity      BioCarta Pathways:     KEGG Pathways:  
    Probe set : 1368790_at    Name:   serine (or cysteine) proteinase inhibitor, clade A (alpha-1 antiproteinase, antitrypsin), member 10    GB acc:   NM_133617    UniGene:   Rn.10502    Gene Symbol:   Serpina10    Locus Link:   171154    Chromosome:   6    Cytoband:   Chr:6q32    SOURCE    GENECARD       Gene Ontology:   biological process:  transport   cellular component:  extracellular space   cellular component:  nucleus      BioCarta Pathways:     KEGG Pathways:  
    Probe set : 1372019_at    Name:   similar to DNA segment, Chr 13, Wayne State University 50, expressed (predicted)    GB acc:   AI231789    UniGene:   Rn.1773    Gene Symbol:   RGD1310128_predicted    Locus Link:   290939    Chromosome:   17    Cytoband:   Chr:17p14    SOURCE    GENECARD       Gene Ontology:     BioCarta Pathways:     KEGG Pathways:  
    Probe set : 1393451_at    Name:   similar to RIKEN cDNA 2610510J17 (predicted)    GB acc:   BI295149    UniGene:   Rn.65502    Gene Symbol:   RGD1310953_predicted    Locus Link:   361416    Chromosome:   19    Cytoband:   Chr:19q12    SOURCE    GENECARD       Gene Ontology:     BioCarta Pathways:     KEGG Pathways:  
    Probe set : 1371800_at    Name:   polyhomeotic-like 2 (Drosophila) (predicted)    GB acc:   AI230538    UniGene:   Rn.2428    Gene Symbol:   Phc2_predicted    Locus Link:   313038    Chromosome:   5    Cytoband:   Chr:5q36    SOURCE    GENECARD       Gene Ontology:   cellular component:  nucleus   molecular function:  protein binding      BioCarta Pathways:     KEGG Pathways:  
    Probe set : 1367920_at    Name:   endothelial differentiation, sphingolipid G-protein-coupled receptor, 5    GB acc:   AB016931    UniGene:   Rn.2491    Gene Symbol:   Edg5    Locus Link:   29415    Chromosome:   8    Cytoband:   Chr:8q13    SOURCE    GENECARD       Gene Ontology:   biological process:  G-protein coupled receptor protein signaling pathway   biological process:  behavior   biological process:  signal transduction   cellular component:  integral to plasma membrane   cellular component:  integral to membrane      BioCarta Pathways:     KEGG Pathways:  
    Probe set : 1389581_at    Name:   similar to RIKEN cDNA 9230117N10 (predicted)    GB acc:   BF390510    UniGene:   Rn.106849    Gene Symbol:   RGD1311155_predicted    Locus Link:   361749    Chromosome:   1    Cytoband:   Chr:1q52    SOURCE    GENECARD       Gene Ontology:   cellular component:  nucleus      BioCarta Pathways:     KEGG Pathways:  
    Probe set : 1368289_at    Name:   group specific component    GB acc:   AA944965    UniGene:   Rn.1437    Gene Symbol:   Gc    Locus Link:   24384    Chromosome:   14    Cytoband:   Chr:14p22    SOURCE    GENECARD       Gene Ontology:   biological process:  transport   biological process:  vitamin D metabolism   biological process:  vitamin transport   cellular component:  extracellular space      BioCarta Pathways:     KEGG Pathways:  
    Probe set : 1386555_at    Name:   casein kinase II, alpha 2, polypeptide (predicted)    GB acc:   BF563960    UniGene:   Rn.40308    Gene Symbol:   Csnk2a2_predicted    Locus Link:   307641    Chromosome:   19    Cytoband:   Chr:19p13    SOURCE    GENECARD       Gene Ontology:   cellular component:  protein kinase CK2 complex      BioCarta Pathways:     KEGG Pathways:  
    Probe set : 1377979_at    Name:   Similar to RIKEN cDNA 2610022G08    GB acc:   AW434228    UniGene:   Rn.20423     Locus Link:   502782      SOURCE        Gene Ontology:     BioCarta Pathways:     KEGG Pathways:  
    Probe set : 1372654_at    Name:   EPS8-like 2 (predicted)    GB acc:   BI285918    UniGene:   Rn.23793    Gene Symbol:   Eps8l2_predicted    Locus Link:   361674    Chromosome:   1    Cytoband:   Chr:1q41    SOURCE    GENECARD       Gene Ontology:   molecular function:  receptor activity      BioCarta Pathways:     KEGG Pathways:  
    Probe set : 1387375_at    Name:   ketohexokinase    GB acc:   NM_031855    UniGene:   Rn.33324    Gene Symbol:   Khk    Locus Link:   25659      SOURCE    GENECARD       Gene Ontology:   biological process:  carbohydrate metabolism   biological process:  carbohydrate catabolism   biological process:  carbohydrate catabolism      BioCarta Pathways:     KEGG Pathways:  
    Probe set : 1373597_at    Name:   processing of precursor 7, ribonuclease P family, (S. cerevisiae) (predicted)    GB acc:   AI102689    UniGene:   Rn.16344    Gene Symbol:   Pop7_predicted    Locus Link:   288564    Chromosome:   12    Cytoband:   Chr:12q12    SOURCE    GENECARD       Gene Ontology:   biological process:  tRNA processing   cellular component:  nucleus   cellular component:  nucleolar ribonuclease P complex   molecular function:  ribonuclease P activity   molecular function:  hydrolase activity      BioCarta Pathways:     KEGG Pathways:  
    Probe set : 1383205_at    Name:   dapper homolog 2, antagonist of beta-catenin (xenopus) (predicted)    GB acc:   BI288833    UniGene:   Rn.100050    Gene Symbol:   Dact2_predicted    Locus Link:   308212    Chromosome:   1    Cytoband:   Chr:1q12    SOURCE    GENECARD       Gene Ontology:     BioCarta Pathways:     KEGG Pathways:  
    Probe set : 1375767_at    Name:   similar to common-site lymphoma leukemia guanine nucleotide exchange factor like (5N754) (predicted)    GB acc:   BI287681    UniGene:   Rn.19912    Gene Symbol:   RGD1308089_predicted    Locus Link:   314249    Chromosome:   6    Cytoband:   Chr:6q24    SOURCE    GENECARD       Gene Ontology:     BioCarta Pathways:     KEGG Pathways:  
    Probe set : 1387348_at    Name:   insulin-like growth factor binding protein 5    GB acc:   BE113270    UniGene:   Rn.1593    Gene Symbol:   Igfbp5    Locus Link:   25285    Chromosome:   9    Cytoband:   Chr:9q33    SOURCE    GENECARD       Gene Ontology:   biological process:  regulation of cell growth   biological process:  signal transduction   cellular component:  extracellular region   cellular component:  extracellular space   cellular component:  insulin-like growth factor binding protein complex   molecular function:  insulin-like growth factor binding   molecular function:  growth factor binding      BioCarta Pathways:     KEGG Pathways:  
    Probe set : 1379317_a_at    Name:   programmed cell death protein 11 (predicted)    GB acc:   BI280308    UniGene:   Rn.136636    Gene Symbol:   Pdcd11_predicted    Locus Link:   309458    Chromosome:   1    Cytoband:   Chr:1q54    SOURCE    GENECARD       Gene Ontology:   biological process:  regulation of transcription, DNA-dependent   biological process:  mRNA processing   biological process:  apoptosis   cellular component:  cytosol      BioCarta Pathways:     KEGG Pathways:  
    Probe set : 1373392_at    Name:   TPA regulated locus (predicted)    GB acc:   AW523924    UniGene:   Rn.2779    Gene Symbol:   Tparl_predicted    Locus Link:   364137    Chromosome:   14    Cytoband:   Chr:14p11    SOURCE    GENECARD       Gene Ontology:   cellular component:  extracellular space   cellular component:  membrane   cellular component:  integral to membrane      BioCarta Pathways:     KEGG Pathways:  
    Probe set : 1376775_at    Name:   similar to RIKEN cDNA 2600010E01 (predicted)    GB acc:   AI059078    UniGene:   Rn.19060    Gene Symbol:   RGD1309969_predicted    Locus Link:   362171    Chromosome:   3    Cytoband:   Chr:3q31    SOURCE    GENECARD       Gene Ontology:     BioCarta Pathways:     KEGG Pathways:  
    Probe set : 1385124_at    Name:   similar to Tribbles homolog 2    GB acc:   BE114077    UniGene:   Rn.17572    Gene Symbol:   LOC313974    Locus Link:   313974    Chromosome:   6    Cytoband:   Chr:6q15    SOURCE    GENECARD       Gene Ontology:     BioCarta Pathways:     KEGG Pathways:  
    Probe set : 1386858_at    Name:   ribosomal protein L13    GB acc:   NM_031101    UniGene:   Rn.908    Gene Symbol:   Rpl13    Locus Link:   81765    Chromosome:   19    Cytoband:   Chr:19q12    SOURCE    GENECARD       Gene Ontology:   biological process:  protein biosynthesis   biological process:  ribosome biogenesis   biological process:  protein biosynthesis      BioCarta Pathways:     KEGG Pathways:  
    Probe set : 1388361_at     GB acc:   BI303856              Gene Ontology:     BioCarta Pathways:     KEGG Pathways:  
    Probe set : 1370818_at    Name:   2-4-dienoyl-Coenzyme A reductase 2, peroxisomal    GB acc:   AF044574    UniGene:   Rn.144598    Gene Symbol:   Decr2    Locus Link:   64461    Chromosome:   10    Cytoband:   Chr:10q12    SOURCE    GENECARD       Gene Ontology:   biological process:  peroxisome organization and biogenesis   biological process:  metabolism   cellular component:  peroxisome   molecular function:  2,4-dienoyl-CoA reductase (NADPH) activity   molecular function:  oxidoreductase activity   molecular function:  2,4-dienoyl-CoA reductase (NADPH) activity      BioCarta Pathways:     KEGG Pathways:  
    Probe set : 1388867_at    Name:   similar to transcription factor    GB acc:   AI227852    UniGene:   Rn.13772    Gene Symbol:   MGC112830    Locus Link:   361178    Chromosome:   16    Cytoband:   Chr:16q12.5    SOURCE    GENECARD       Gene Ontology:   biological process:  regulation of progression through cell cycle   biological process:  regulation of transcription, DNA-dependent   cellular component:  transcription factor complex   molecular function:  transcription factor activity      BioCarta Pathways:     KEGG Pathways:  
    Probe set : 1370281_at    Name:   fatty acid binding protein 5, epidermal    GB acc:   U13253    UniGene:   Rn.98269    Gene Symbol:   Fabp5    Locus Link:   140868      SOURCE    GENECARD       Gene Ontology:   cellular component:  cytoplasm      BioCarta Pathways:     KEGG Pathways:  
    Probe set : 1374221_at    Name:   Solute carrier family 29 (nucleoside transporters), member 3    GB acc:   BM385468    UniGene:   Rn.15870    Gene Symbol:   Slc29a3    Locus Link:   353307    Chromosome:   20    Cytoband:   Chr:20q11    SOURCE    GENECARD       Gene Ontology:   biological process:  nucleoside transport   biological process:  transport   cellular component:  membrane   cellular component:  integral to membrane   molecular function:  nucleoside transporter activity   molecular function:  nucleoside transporter activity      BioCarta Pathways:     KEGG Pathways:  
    Probe set : 1375074_at    Name:   ProSAPiP2 protein    GB acc:   BE113756    UniGene:   Rn.33571    Gene Symbol:   Prosapip2    Locus Link:   266764    Chromosome:   10    Cytoband:   Chr:10q31    SOURCE    GENECARD       Gene Ontology:     BioCarta Pathways:     KEGG Pathways:  
    Probe set : 1367958_at    Name:   abl-interactor 1    GB acc:   NM_024397    UniGene:   Rn.43675    Gene Symbol:   Abi1    Locus Link:   79249    Chromosome:   17    Cytoband:   Chr:17q12.3    SOURCE    GENECARD       Gene Ontology:   biological process:  cellular physiological process   cellular component:  nucleus   cellular component:  lamellipodium   cellular component:  cytoskeleton   molecular function:  protein binding   molecular function:  protein tyrosine kinase activator activity      BioCarta Pathways:     KEGG Pathways:  
    Probe set : 1399089_at    Name:   solute carrier family 38, member 6 (predicted)    GB acc:   BF401591    UniGene:   Rn.55589    Gene Symbol:   Slc38a6_predicted    Locus Link:   299139    Chromosome:   6    Cytoband:   Chr:6q24    SOURCE    GENECARD       Gene Ontology:   biological process:  transport   biological process:  amino acid transport   cellular component:  integral to membrane   cellular component:  membrane   molecular function:  amino acid-polyamine transporter activity      BioCarta Pathways:     KEGG Pathways:  
    Probe set : 1376570_at    Name:   chaperonin subunit 5 (epsilon)    GB acc:   BF417274    UniGene:   Rn.92645    Gene Symbol:   Cct5    Locus Link:   294864    Chromosome:   2    Cytoband:   Chr:2q22    SOURCE    GENECARD       Gene Ontology:   biological process:  protein folding   biological process:  protein folding   biological process:  cellular protein metabolism      BioCarta Pathways:     KEGG Pathways:  
    Probe set : 1390391_at    Name:   Transcribed locus    GB acc:   BF283381    UniGene:   Rn.25327        SOURCE        Gene Ontology:     BioCarta Pathways:     KEGG Pathways:  
    Probe set : 1392893_a_at    Name:   Transcribed locus    GB acc:   AA926239    UniGene:   Rn.105786        SOURCE        Gene Ontology:     BioCarta Pathways:     KEGG Pathways:  
    Probe set : 1370180_at    Name:   nudix (nucleoside diphosphate linked moiety X)-type motif 4    GB acc:   AA891213    UniGene:   Rn.8426    Gene Symbol:   Nudt4    Locus Link:   94267    Chromosome:   7    Cytoband:   Chr:7q13    SOURCE    GENECARD       Gene Ontology:   biological process:  intracellular signaling cascade   biological process:  cyclic nucleotide metabolism   biological process:  calcium-mediated signaling   biological process:  cyclic-nucleotide-mediated signaling   biological process:  phosphoinositide metabolism   biological process:  regulation of RNA export from nucleus   biological process:  intracellular transport   biological process:  intracellular signaling cascade   biological process:  calcium-mediated signaling   biological process:  cyclic-nucleotide-mediated signaling   biological process:  regulation of RNA export from nucleus   biological process:  intracellular transport   cellular component:  intracellular   cellular component:  intracellular      BioCarta Pathways:     KEGG Pathways:  
    Probe set : 1392990_at    Name:   SRY-box containing gene 17 (predicted)    GB acc:   AI031004    UniGene:   Rn.7884    Gene Symbol:   Sox17_predicted    Locus Link:   312936    Chromosome:   5    Cytoband:   Chr:5q12    SOURCE    GENECARD       Gene Ontology:   biological process:  regulation of transcription, DNA-dependent   cellular component:  nucleus   cellular component:  transcription factor complex   molecular function:  DNA binding   molecular function:  transcription factor activity      BioCarta Pathways:     KEGG Pathways:  
    Probe set : 1371781_at    Name:   signal transducer and activator of transcription 3    GB acc:   BI285863    UniGene:   Rn.10247    Gene Symbol:   Stat3    Locus Link:   25125    Chromosome:   10    Cytoband:   Chr:10q32.1    SOURCE    GENECARD       Gene Ontology:   biological process:  negative regulation of transcription from RNA polymerase II promoter   biological process:  eye photoreceptor cell differentiation   biological process:  regulation of transcription, DNA-dependent   biological process:  regulation of transcription from RNA polymerase II promoter   biological process:  transcription from RNA polymerase II promoter   biological process:  cell motility   biological process:  acute-phase response   biological process:  acute-phase response   biological process:  signal transduction   biological process:  intracellular signaling cascade   biological process:  JAK-STAT cascade   biological process:  JAK-STAT cascade   biological process:  nervous system development   biological process:  transcription   cellular component:  nucleus   cellular component:  cytoplasm   cellular component:  plasma membrane      BioCarta Pathways:     KEGG Pathways:  
    Probe set : 1378819_at    Name:   similar to RIKEN cDNA 0610012D14 (predicted)    GB acc:   AW435096    UniGene:   Rn.54118    Gene Symbol:   RGD1310111_predicted    Locus Link:   292875    Chromosome:   1    Cytoband:   Chr:1q22    SOURCE    GENECARD       Gene Ontology:   biological process:  lysine biosynthesis   biological process:  diaminopimelate biosynthesis   biological process:  pyridine nucleotide biosynthesis   molecular function:  oxidoreductase activity      BioCarta Pathways:     KEGG Pathways:  
    Probe set : 1374902_at    Name:   IQ motif containing GTPase activating protein 3 (predicted)    GB acc:   AI071398    UniGene:   Rn.17539    Gene Symbol:   Iqgap3_predicted    Locus Link:   310621    Chromosome:   2    Cytoband:   Chr:2q34    SOURCE    GENECARD       Gene Ontology:   biological process:  small GTPase mediated signal transduction   molecular function:  Ras GTPase activator activity   molecular function:  calmodulin binding      BioCarta Pathways:     KEGG Pathways:  
    Probe set : 1375870_a_at    Name:   RNA binding motif, single stranded interacting protein 1 (predicted)    GB acc:   BF420140    UniGene:   Rn.145202    Gene Symbol:   Rbms1_predicted    Locus Link:   362138    Chromosome:   3    Cytoband:   Chr:3q21    SOURCE    GENECARD       Gene Ontology:   cellular component:  nucleus      BioCarta Pathways:     KEGG Pathways:  
    Probe set : 1377085_at    Name:   carbonic anhydrase 7 (predicted)    GB acc:   BF386665    UniGene:   Rn.16248    Gene Symbol:   Car7_predicted    Locus Link:   291819    Chromosome:   19    Cytoband:   Chr:19p14    SOURCE    GENECARD       Gene Ontology:   biological process:  one-carbon compound metabolism   molecular function:  carbonate dehydratase activity   molecular function:  zinc ion binding   molecular function:  lyase activity      BioCarta Pathways:     KEGG Pathways:  
    Probe set : 1387283_at    Name:   myxovirus (influenza virus) resistance 2    GB acc:   NM_134350    UniGene:   Rn.10374    Gene Symbol:   Mx2    Locus Link:   286918    Chromosome:   11    Cytoband:   Chr:11q21    SOURCE    GENECARD       Gene Ontology:   biological process:  defense response   biological process:  immune response   biological process:  response to virus   molecular function:  GTPase activity   molecular function:  GTP binding   molecular function:  nucleotide binding      BioCarta Pathways:     KEGG Pathways:  
    Probe set : 1367881_at    Name:   protein tyrosine phosphatase, non-receptor type substrate 1    GB acc:   NM_013016    UniGene:   Rn.53971    Gene Symbol:   Ptpns1    Locus Link:   25528    Chromosome:   3    Cytoband:   Chr:3q36    SOURCE    GENECARD       Gene Ontology:   cellular component:  extracellular space   cellular component:  integral to plasma membrane   cellular component:  integral to membrane   molecular function:  protein binding   molecular function:  protein phosphorylated amino acid binding      BioCarta Pathways:     KEGG Pathways:  
    Probe set : 1382084_at    Name:   Similar to EHM2    GB acc:   AA850264    UniGene:   Rn.21106     Locus Link:   500464      SOURCE        Gene Ontology:     BioCarta Pathways:     KEGG Pathways:  
    Probe set : 1376722_at    Name:   nucleoporin 205kDa (predicted)    GB acc:   AI409108    UniGene:   Rn.23694    Gene Symbol:   Nup205_predicted    Locus Link:   362335    Chromosome:   4    Cytoband:   Chr:4q22    SOURCE    GENECARD       Gene Ontology:   biological process:  protein import into nucleus, docking   biological process:  transport   biological process:  nucleocytoplasmic transport   cellular component:  nucleus   cellular component:  nuclear pore   molecular function:  protein binding      BioCarta Pathways:     KEGG Pathways:  
    Probe set : 1391384_at    Name:   G4 protein    GB acc:   AA819227    UniGene:   Rn.2275    Gene Symbol:   Tnf    Locus Link:   24835    Chromosome:   20    Cytoband:   Chr:20p12    SOURCE    GENECARD       Gene Ontology:   biological process:  regulation of protein amino acid phosphorylation   biological process:  glucose metabolism   biological process:  apoptosis   biological process:  apoptosis   biological process:  induction of apoptosis   biological process:  induction of apoptosis   biological process:  programmed cell death, transformed cells   biological process:  defense response   biological process:  inflammatory response   biological process:  inflammatory response   biological process:  immune response   biological process:  humoral immune response   biological process:  cellular defense response   biological process:  signal transduction   biological process:  signal transduction   biological process:  development   biological process:  cell proliferation   biological process:  positive regulation of cell proliferation   biological process:  induction of apoptosis via death domain receptors   biological process:  organ morphogenesis   biological process:  cytokine and chemokine mediated signaling pathway   biological process:  regulation of cell proliferation   biological process:  defense response to bacteria   biological process:  positive regulation of I-kappaB kinase/NF-kappaB cascade   biological process:  cellular extravasation   biological process:  regulation of osteoclast differentiation   biological process:  positive regulation of osteoclast differentiation   biological process:  negative regulation of glucose import   biological process:  cellular physiological process   biological process:  regulation of immunoglobulin secretion      BioCarta Pathways:     KEGG Pathways:  
    Probe set : 1369682_at    Name:   transcription factor 2    GB acc:   NM_013103    UniGene:   Rn.11342    Gene Symbol:   Tcf2    Locus Link:   25640    Chromosome:   10    Cytoband:   Chr:10q26    SOURCE    GENECARD       Gene Ontology:   cellular component:  nucleus   cellular component:  nucleus   cellular component:  transcription factor complex      BioCarta Pathways:     KEGG Pathways:  
    Probe set : 1368251_at    Name:   Janus kinase 3    GB acc:   NM_012855    UniGene:   Rn.11159    Gene Symbol:   Jak3    Locus Link:   25326    Chromosome:   16    Cytoband:   Chr:16p14    SOURCE    GENECARD       Gene Ontology:   cellular component:  cytoskeleton      BioCarta Pathways:     KEGG Pathways:  
    Probe set : 1380245_at     GB acc:   AI411847              Gene Ontology:     BioCarta Pathways:     KEGG Pathways:  
    Probe set : 1382709_at     GB acc:   AI178618              Gene Ontology:     BioCarta Pathways:     KEGG Pathways:  
    Probe set : 1374437_at    Name:   asparaginyl-tRNA synthetase (predicted)    GB acc:   BF419336    UniGene:   Rn.48209    Gene Symbol:   Nars_predicted    Locus Link:   291556    Chromosome:   18    Cytoband:   Chr:18q12.1    SOURCE    GENECARD       Gene Ontology:   cellular component:  intracellular   cellular component:  soluble fraction   cellular component:  cytoplasm      BioCarta Pathways:     KEGG Pathways:  
    Probe set : 1375247_at    Name:   Monoglyceride lipase    GB acc:   BG372713    UniGene:   Rn.40396    Gene Symbol:   Mgll    Locus Link:   29254    Chromosome:   4    Cytoband:   Chr:4q34    SOURCE    GENECARD       Gene Ontology:   cellular component:  synapse      BioCarta Pathways:     KEGG Pathways:  
    Probe set : 1383422_at    Name:   similar to RIKEN cDNA 2310026E23    GB acc:   AW252401    UniGene:   Rn.87596    Gene Symbol:   LOC362564    Locus Link:   362564    Chromosome:   5    Cytoband:   Chr:5q35    SOURCE    GENECARD       Gene Ontology:     BioCarta Pathways:     KEGG Pathways:  
    Probe set : 1368882_at    Name:   sialyltransferase 7c    GB acc:   NM_019123    UniGene:   Rn.88072    Gene Symbol:   Siat7c    Locus Link:   29758    Chromosome:   2    Cytoband:   Chr:2q45    SOURCE    GENECARD       Gene Ontology:   biological process:  protein amino acid glycosylation   molecular function:  sialyltransferase activity   molecular function:  transferase activity   molecular function:  transferase activity, transferring glycosyl groups      BioCarta Pathways:     KEGG Pathways:  
    Probe set : 1368179_at    Name:   thymic stromal-derived lymphopoietin, receptor    GB acc:   NM_134465    UniGene:   Rn.22704    Gene Symbol:   Tslpr    Locus Link:   171499      SOURCE    GENECARD       Gene Ontology:   cellular component:  extracellular space   cellular component:  membrane   cellular component:  integral to membrane      BioCarta Pathways:     KEGG Pathways:  
    Probe set : 1369794_a_at    Name:   6-phosphofructo-2-kinase/fructose-2,6-biphosphatase 3    GB acc:   D87247    UniGene:   Rn.10791    Gene Symbol:   Pfkfb3    Locus Link:   117276    Chromosome:   17    Cytoband:   Chr:17q12.3    SOURCE    GENECARD       Gene Ontology:     BioCarta Pathways:     KEGG Pathways:  
    Probe set : 1377375_at    Name:   aminoadipate-semialdehyde synthase (predicted)    GB acc:   AA944898    UniGene:   Rn.3169    Gene Symbol:   Aass_predicted    Locus Link:   296925    Chromosome:   4    Cytoband:   Chr:4q22    SOURCE    GENECARD       Gene Ontology:   biological process:  electron transport   biological process:  lysine catabolism   biological process:  protein tetramerization   cellular component:  mitochondrion      BioCarta Pathways:     KEGG Pathways:  
    Probe set : 1379298_at    Name:   interferon (alpha and beta) receptor 1 (predicted)    GB acc:   AA799711    UniGene:   Rn.105738    Gene Symbol:   Ifnar1_predicted    Locus Link:   288264    Chromosome:   11    Cytoband:   Chr:11q11    SOURCE    GENECARD       Gene Ontology:   biological process:  cell surface receptor linked signal transduction   biological process:  JAK-STAT cascade   biological process:  response to virus      BioCarta Pathways:     KEGG Pathways:  
    Probe set : 1374739_at    Name:   similar to hematopoietic progenitor protein    GB acc:   AI236771    UniGene:   Rn.32426    Gene Symbol:   LOC500727    Locus Link:   500727    Chromosome:   6    Cytoband:   Chr:6q32    SOURCE    GENECARD       Gene Ontology:     BioCarta Pathways:     KEGG Pathways:  
    Probe set : 1389569_at    Name:   similar to BRIX (predicted)    GB acc:   AW917092    UniGene:   Rn.17880    Gene Symbol:   RGD1308508_predicted    Locus Link:   294799    Chromosome:   2    Cytoband:   Chr:2q16    SOURCE    GENECARD       Gene Ontology:   biological process:  ribosome biogenesis   cellular component:  nucleus      BioCarta Pathways:     KEGG Pathways:  
    Probe set : 1383863_at     GB acc:   BF288303              Gene Ontology:     BioCarta Pathways:     KEGG Pathways:  
    Probe set : 1391262_at    Name:   SUMO/sentrin specific protease 5 (predicted) /// similar to SUMO/sentrin specific protease 5    GB acc:   BE105606    UniGene:   Rn.141357    Gene Symbol:   Senp5_predicted /// LOC499574    Locus Link:   303874 /// 499574    Chromosome:   11    Cytoband:   Chr:11q22 /// Chr:2q24    SOURCE    GENECARD       Gene Ontology:   biological process:  proteolysis   biological process:  ubiquitin cycle   molecular function:  peptidase activity   molecular function:  cysteine-type peptidase activity      BioCarta Pathways:     KEGG Pathways:  
    Probe set : 1372123_at    Name:   succinate dehydrogenase complex, subunit B, iron sulfur (Ip) (predicted)    GB acc:   AI172320    UniGene:   Rn.3902    Gene Symbol:   Sdhb_predicted    Locus Link:   298596    Chromosome:   5    Cytoband:   Chr:5q36    SOURCE    GENECARD       Gene Ontology:   biological process:  tricarboxylic acid cycle   biological process:  electron transport   biological process:  aerobic respiration   cellular component:  mitochondrion   cellular component:  membrane      BioCarta Pathways:     KEGG Pathways:  
    Probe set : 1386454_at    Name:   solute carrier family 23 (nucleobase transporters), member 3 (predicted)    GB acc:   AI639159    UniGene:   Rn.44148    Gene Symbol:   Slc23a3_predicted    Locus Link:   367298    Chromosome:   9    Cytoband:   Chr:9q33    SOURCE    GENECARD       Gene Ontology:     BioCarta Pathways:     KEGG Pathways:  
    Probe set : 1385627_at    Name:   B-cell leukemia/lymphoma 3 (predicted)    GB acc:   BF407019    UniGene:   Rn.68084    Gene Symbol:   Bcl3_predicted    Locus Link:   292700    Chromosome:   1    Cytoband:   Chr:1q21    SOURCE    GENECARD       Gene Ontology:   cellular component:  nucleus   molecular function:  DNA binding   molecular function:  transcription factor activity      BioCarta Pathways:     KEGG Pathways:  
    Probe set : 1383350_at    Name:   solute carrier family 35, member C2 (predicted)    GB acc:   AA963524    UniGene:   Rn.12123    Gene Symbol:   Slc35c2_predicted    Locus Link:   311637    Chromosome:   3    Cytoband:   Chr:3q42    SOURCE    GENECARD       Gene Ontology:   biological process:  transport   cellular component:  integral to membrane      BioCarta Pathways:     KEGG Pathways:  
    Probe set : 1386886_at    Name:   CD164 antigen    GB acc:   NM_031812    UniGene:   Rn.3733    Gene Symbol:   Cd164    Locus Link:   83689    Chromosome:   20    Cytoband:   Chr:20q12    SOURCE    GENECARD       Gene Ontology:     BioCarta Pathways:     KEGG Pathways:  
    Probe set : 1367749_at    Name:   lumican    GB acc:   NM_031050    UniGene:   Rn.3087    Gene Symbol:   Lum    Locus Link:   81682    Chromosome:   7    Cytoband:   Chr:7q13    SOURCE    GENECARD       Gene Ontology:   cellular component:  extracellular matrix (sensu Metazoa)   cellular component:  extracellular space      BioCarta Pathways:     KEGG Pathways:  
    Probe set : 1367775_at    Name:   alpha-methylacyl-CoA racemase    GB acc:   NM_012816    UniGene:   Rn.2590    Gene Symbol:   Amacr    Locus Link:   25284    Chromosome:   2    Cytoband:   Chr:2q16    SOURCE    GENECARD       Gene Ontology:   cellular component:  mitochondrion   cellular component:  peroxisome      BioCarta Pathways:     KEGG Pathways:  
    Probe set : 1384244_at    Name:   similar to RIKEN cDNA 2610207I16 (predicted)    GB acc:   AA964752    UniGene:   Rn.134797    Gene Symbol:   RGD1305387_predicted    Locus Link:   313200    Chromosome:   5    Cytoband:   Chr:5q24    SOURCE    GENECARD       Gene Ontology:   biological process:  metabolism   cellular component:  mitochondrion   molecular function:  sterol carrier activity   molecular function:  oxidoreductase activity      BioCarta Pathways:     KEGG Pathways:  
    Probe set : 1381771_at    Name:   Transcribed locus    GB acc:   AI178422    UniGene:   Rn.20519        SOURCE        Gene Ontology:     BioCarta Pathways:     KEGG Pathways:  
    Probe set : 1371495_at    Name:   cation-dependent mannose-6-phosphate receptor    GB acc:   AA943990    UniGene:   Rn.91427    Gene Symbol:   RGD:1359355    Locus Link:   312689    Chromosome:   4    Cytoband:   Chr:4q42    SOURCE    GENECARD       Gene Ontology:   biological process:  mannose transport   cellular component:  lytic vacuole   cellular component:  integral to membrane   molecular function:  receptor activity   molecular function:  mannose binding   molecular function:  mannose transporter activity      BioCarta Pathways:     KEGG Pathways:  
    Probe set : 1394160_at    Name:   Transmembrane protein 2 (predicted)    GB acc:   AI101945    UniGene:   Rn.107821     Locus Link:   309400    Chromosome:   1    Cytoband:   Chr:1q51    SOURCE        Gene Ontology:   cellular component:  integral to membrane      BioCarta Pathways:     KEGG Pathways:  
    Probe set : 1370187_at    Name:   propionyl coenzyme A carboxylase, beta polypeptide    GB acc:   AI502661    UniGene:   Rn.9732    Gene Symbol:   Pccb    Locus Link:   24624    Chromosome:   8    Cytoband:   Chr:8q31    SOURCE    GENECARD       Gene Ontology:   biological process:  fatty acid catabolism   biological process:  fatty acid biosynthesis      BioCarta Pathways:     KEGG Pathways:  
    Probe set : 1368766_at    Name:   matrix metalloproteinase 7    GB acc:   NM_012864    UniGene:   Rn.10282    Gene Symbol:   Mmp7    Locus Link:   25335    Chromosome:   8    Cytoband:   Chr:8q11    SOURCE    GENECARD       Gene Ontology:   biological process:  proteolysis   biological process:  collagen catabolism   biological process:  peptidoglycan metabolism   cellular component:  extracellular matrix (sensu Metazoa)   cellular component:  extracellular space   cellular component:  nucleus      BioCarta Pathways:     KEGG Pathways:  
    Probe set : 1368294_at    Name:   deoxyribonuclease I-like 3    GB acc:   NM_053907    UniGene:   Rn.29996    Gene Symbol:   Dnase1l3    Locus Link:   116687    Chromosome:   15    Cytoband:   Chr:15p14    SOURCE    GENECARD       Gene Ontology:   cellular component:  extracellular space   cellular component:  nucleus   cellular component:  nucleus      BioCarta Pathways:     KEGG Pathways:  
    Probe set : 1388353_at    Name:   proliferation-associated 2G4, 38kDa    GB acc:   BI296168    UniGene:   Rn.9485    Gene Symbol:   RGD:1302994    Locus Link:   288778    Chromosome:   7    Cytoband:   Chr:7q11    SOURCE    GENECARD       Gene Ontology:   cellular component:  nucleus      BioCarta Pathways:     KEGG Pathways:  
    Probe set : 1396035_at    Name:   similar to RPE-spondin    GB acc:   BF407272    UniGene:   Rn.144410    Gene Symbol:   LOC297757    Locus Link:   297757    Chromosome:   5    Cytoband:   Chr:5q11    SOURCE    GENECARD       Gene Ontology:     BioCarta Pathways:     KEGG Pathways:  
    Probe set : 1371350_at    Name:   similar to S-adenosylmethionine synthetase gamma form (Methionine adenosyltransferase) (AdoMet synthetase) (MAT-II)    GB acc:   AI412159     Gene Symbol:   LOC498211    Locus Link:   498211    Chromosome:   13    Cytoband:   Chr:13p13     GENECARD       Gene Ontology:     BioCarta Pathways:     KEGG Pathways:  
    Probe set : 1399138_at    Name:   similar to expressed sequence AA408877 (predicted)    GB acc:   BG672805     Gene Symbol:   RGD1308513_predicted    Locus Link:   364050    Chromosome:   13    Cytoband:   Chr:13q24     GENECARD       Gene Ontology:   biological process:  protein transport      BioCarta Pathways:     KEGG Pathways:  
    Probe set : 1380621_at    Name:   Similar to tyrosine kinase Fps/Fes    GB acc:   BI289400    UniGene:   Rn.27239     Locus Link:   361597    Chromosome:   1    Cytoband:   Chr:1q31    SOURCE        Gene Ontology:     BioCarta Pathways:     KEGG Pathways:  
    Probe set : 1386162_at    Name:   TXK tyrosine kinase (predicted)    GB acc:   AI009944    UniGene:   Rn.17505    Gene Symbol:   Txk_predicted    Locus Link:   305311    Chromosome:   14    Cytoband:   Chr:14p11    SOURCE    GENECARD       Gene Ontology:   biological process:  protein amino acid phosphorylation   biological process:  intracellular signaling cascade   cellular component:  cytoplasm      BioCarta Pathways:     KEGG Pathways:  
    Probe set : 1371377_at    Name:   ribosomal protein S19    GB acc:   BG668512    UniGene:   Rn.127775    Gene Symbol:   Rps19    Locus Link:   29287    Chromosome:   1    Cytoband:   Chr:1q21    SOURCE    GENECARD       Gene Ontology:   biological process:  protein biosynthesis   biological process:  ribosome biogenesis   biological process:  hemocyte development   molecular function:  RNA binding   molecular function:  structural constituent of ribosome      BioCarta Pathways:     KEGG Pathways:  
    Probe set : 1376692_at    Name:   homeodomain interacting protein kinase 2 (predicted)    GB acc:   BM392321    UniGene:   Rn.8042    Gene Symbol:   Hipk2_predicted    Locus Link:   362342    Chromosome:   4    Cytoband:   Chr:4q22    SOURCE    GENECARD       Gene Ontology:   cellular component:  nucleus   cellular component:  cytoplasm   cellular component:  nuclear body   cellular component:  PML body      BioCarta Pathways:     KEGG Pathways:  
    Probe set : 1391356_at    Name:   Similar to hypothetical protein FLJ10006 (predicted)    GB acc:   BI282688    UniGene:   Rn.26585     Locus Link:   291705    Chromosome:   18    Cytoband:   Chr:18p12    SOURCE        Gene Ontology:   biological process:  electron transport   biological process:  regulation of transcription, DNA-dependent   cellular component:  nucleus   molecular function:  acyl-CoA dehydrogenase activity      BioCarta Pathways:     KEGG Pathways:  
    Probe set : 1372502_at    Name:   Transcribed locus, moderately similar to NP_035028.2 neurofibromatosis 2 [Mus musculus]    GB acc:   AI579645    UniGene:   Rn.7773        SOURCE        Gene Ontology:     BioCarta Pathways:     KEGG Pathways:  
    Probe set : 1379592_at    Name:   similar to citrin    GB acc:   AI045151    UniGene:   Rn.14686    Gene Symbol:   LOC362322    Locus Link:   362322    Chromosome:   4    Cytoband:   Chr:4q13    SOURCE    GENECARD       Gene Ontology:     BioCarta Pathways:     KEGG Pathways:  
    Probe set : 1368856_at    Name:   Janus kinase 2    GB acc:   NM_031514    UniGene:   Rn.18909    Gene Symbol:   Jak2    Locus Link:   24514    Chromosome:   1    Cytoband:   Chr:1q51-q53    SOURCE    GENECARD       Gene Ontology:   biological process:  regulation of progression through cell cycle   biological process:  protein amino acid phosphorylation   biological process:  apoptosis   biological process:  cell motility   biological process:  intracellular signaling cascade   biological process:  JAK-STAT cascade   biological process:  tyrosine phosphorylation of STAT protein   biological process:  STAT protein nuclear translocation   biological process:  mesoderm development   biological process:  negative regulation of cell proliferation   biological process:  peptidyl-tyrosine phosphorylation   biological process:  cytokine and chemokine mediated signaling pathway   biological process:  myeloid cell differentiation   biological process:  cell differentiation   cellular component:  cytoplasm   cellular component:  cytoskeleton      BioCarta Pathways:     KEGG Pathways:  
    Probe set : 1371493_at    Name:   Transcribed locus    GB acc:   BI285640    UniGene:   Rn.146315        SOURCE        Gene Ontology:     BioCarta Pathways:     KEGG Pathways:  
    Probe set : 1378028_at    Name:   MAD2 (mitotic arrest deficient, homolog)-like 1 (yeast) (predicted)    GB acc:   BM384425    UniGene:   Rn.19771    Gene Symbol:   Mad2l1_predicted    Locus Link:   297176    Chromosome:   4    Cytoband:   Chr:4q31    SOURCE    GENECARD       Gene Ontology:     BioCarta Pathways:     KEGG Pathways:  
    Probe set : 1371913_at    Name:   transforming growth factor, beta induced    GB acc:   BG379319    UniGene:   Rn.1046    Gene Symbol:   Tgfbi    Locus Link:   116487    Chromosome:   17    Cytoband:   Chr:17p14    SOURCE    GENECARD       Gene Ontology:   cellular component:  extracellular matrix (sensu Metazoa)      BioCarta Pathways:     KEGG Pathways:  
    Probe set : 1372809_at    Name:   hypothetical gene supported by AF152002    GB acc:   AI716200    UniGene:   Rn.103148    Gene Symbol:   LOC290595    Locus Link:   290595    Chromosome:   16    Cytoband:   Chr:16p14    SOURCE    GENECARD       Gene Ontology:     BioCarta Pathways:     KEGG Pathways:  
    Probe set : 1382005_at    Name:   ROD1 regulator of differentiation 1 (S. pombe)    GB acc:   BI280285    UniGene:   Rn.51103    Gene Symbol:   Rod1    Locus Link:   83515    Chromosome:   5    Cytoband:   Chr:5q24    SOURCE    GENECARD       Gene Ontology:   cellular component:  nucleus      BioCarta Pathways:     KEGG Pathways:  
    Probe set : 1371567_at    Name:   aldehyde dehydrogenase family 7, member A1 (predicted)    GB acc:   AI411279    UniGene:   Rn.7330    Gene Symbol:   Aldh7a1_predicted    Locus Link:   291450    Chromosome:   18    Cytoband:   Chr:18q12.1    SOURCE    GENECARD       Gene Ontology:   biological process:  aldehyde metabolism   biological process:  sensory perception of sound   biological process:  metabolism   cellular component:  mitochondrion   molecular function:  aldehyde dehydrogenase (NAD) activity   molecular function:  oxidoreductase activity      BioCarta Pathways:     KEGG Pathways:  
    Probe set : 1390440_at    Name:   similar to mKIAA0267 protein    GB acc:   BG380512    UniGene:   Rn.25009    Gene Symbol:   LOC302863    Locus Link:   302863    Chromosome:   X    Cytoband:   Chr:Xq36    SOURCE    GENECARD       Gene Ontology:     BioCarta Pathways:     KEGG Pathways:  
    Probe set : 1379687_at    Name:   ARP2 actin-related protein 2 homolog (yeast) (predicted)    GB acc:   AI501579    UniGene:   Rn.102249    Gene Symbol:   Actr2_predicted    Locus Link:   289820    Chromosome:   14    Cytoband:   Chr:14q22    SOURCE    GENECARD       Gene Ontology:   biological process:  cell motility   cellular component:  cytoskeleton   cellular component:  Arp2/3 protein complex   cellular component:  actin cytoskeleton   molecular function:  structural molecule activity   molecular function:  structural constituent of cytoskeleton   molecular function:  protein binding      BioCarta Pathways:     KEGG Pathways:  
    Probe set : 1373273_at    Name:   similar to hypothetical protein FLJ14936 (predicted)    GB acc:   BM384975    UniGene:   Rn.18104    Gene Symbol:   RGD1311129_predicted    Locus Link:   298374    Chromosome:   5    Cytoband:   Chr:5q35    SOURCE    GENECARD       Gene Ontology:     BioCarta Pathways:     KEGG Pathways:  
    Probe set : 1375920_at    Name:   similar to TNF intracellular domain-interacting protein (predicted)    GB acc:   AI406305    UniGene:   Rn.21037    Gene Symbol:   RGD1311487_predicted    Locus Link:   310674    Chromosome:   2    Cytoband:   Chr:2q34    SOURCE    GENECARD       Gene Ontology:     BioCarta Pathways:     KEGG Pathways:  
    Probe set : 1373305_at    Name:   sorting nexin 4 (predicted)    GB acc:   BI280349    UniGene:   Rn.2645    Gene Symbol:   Snx4_predicted    Locus Link:   360725    Chromosome:   11    Cytoband:   Chr:11q22    SOURCE    GENECARD       Gene Ontology:   molecular function:  protein transporter activity      BioCarta Pathways:     KEGG Pathways:  
    Probe set : 1369073_at    Name:   nuclear receptor subfamily 1, group H, member 4    GB acc:   NM_021745    UniGene:   Rn.42943    Gene Symbol:   Nr1h4    Locus Link:   60351    Chromosome:   7    Cytoband:   Chr:7q13    SOURCE    GENECARD       Gene Ontology:   biological process:  regulation of transcription, DNA-dependent   biological process:  bile acid metabolism   biological process:  transcription   cellular component:  nucleus      BioCarta Pathways:     KEGG Pathways:  
    Probe set : 1379538_at    Name:   centromere protein J (predicted)    GB acc:   BE104961    UniGene:   Rn.19524    Gene Symbol:   Cenpj_predicted    Locus Link:   305909    Chromosome:   15    Cytoband:   Chr:15p12    SOURCE    GENECARD       Gene Ontology:   biological process:  microtubule nucleation   biological process:  astral microtubule nucleation   biological process:  positive regulation of JAK-STAT cascade   cellular component:  cytoplasm   cellular component:  centrosome   cellular component:  gamma-tubulin small complex   molecular function:  transcription coactivator activity   molecular function:  protein binding   molecular function:  gamma-tubulin binding      BioCarta Pathways:     KEGG Pathways:  
    Probe set : 1368840_at    Name:   LR8 protein    GB acc:   NM_134390    UniGene:   Rn.26876    Gene Symbol:   Lr8    Locus Link:   171411    Chromosome:   4    Cytoband:   Chr:4q24    SOURCE    GENECARD       Gene Ontology:   cellular component:  integral to membrane      BioCarta Pathways:     KEGG Pathways:  
    Probe set : 1390021_at    Name:   histone 1, H2bh (predicted)    GB acc:   BM391206     Gene Symbol:   Hist1h2bh_predicted    Locus Link:   306945    Chromosome:   17    Cytoband:   Chr:17p11     GENECARD       Gene Ontology:     BioCarta Pathways:     KEGG Pathways:  
    Probe set : 1367591_at    Name:   peroxiredoxin 3    GB acc:   NM_022540    UniGene:   Rn.2011    Gene Symbol:   Prdx3    Locus Link:   64371    Chromosome:   1    Cytoband:   Chr:1q55    SOURCE    GENECARD       Gene Ontology:   cellular component:  extracellular space   cellular component:  mitochondrion      BioCarta Pathways:     KEGG Pathways:  
    Probe set : 1389084_at     GB acc:   AI177159              Gene Ontology:     BioCarta Pathways:     KEGG Pathways:  
    Probe set : 1398806_at    Name:   phosphatidylinositol transfer protein    GB acc:   NM_017231    UniGene:   Rn.9771    Gene Symbol:   Pitpn    Locus Link:   29525    Chromosome:   10    Cytoband:   Chr:10q24    SOURCE    GENECARD       Gene Ontology:   biological process:  transport   biological process:  phospholipid transport   cellular component:  intracellular   cellular component:  integral to membrane      BioCarta Pathways:     KEGG Pathways:  
    Probe set : 1372350_at    Name:   suppressor of var1, 3-like 1 (S. cerevisiae) (predicted)    GB acc:   BI297676    UniGene:   Rn.19830    Gene Symbol:   Supv3l1_predicted    Locus Link:   294385    Chromosome:   20    Cytoband:   Chr:20q11    SOURCE    GENECARD       Gene Ontology:   cellular component:  mitochondrion      BioCarta Pathways:     KEGG Pathways:  
    Probe set : 1373218_at    Name:   Similar to 6430514L14Rik protein (predicted)    GB acc:   BG381051    UniGene:   Rn.23904     Locus Link:   315789    Chromosome:   8    Cytoband:   Chr:8q24    SOURCE        Gene Ontology:     BioCarta Pathways:     KEGG Pathways:  
    Probe set : 1377812_a_at    Name:   transmembrane protein 24 (predicted)    GB acc:   BG378195    UniGene:   Rn.57410    Gene Symbol:   Tmem24_predicted    Locus Link:   300666    Chromosome:   8    Cytoband:   Chr:8q22    SOURCE    GENECARD       Gene Ontology:   cellular component:  integral to membrane      BioCarta Pathways:     KEGG Pathways:  
    Probe set : 1382964_at     GB acc:   BI282553              Gene Ontology:     BioCarta Pathways:     KEGG Pathways:  
    Probe set : 1378213_at    Name:   Transcribed locus    GB acc:   AI177969    UniGene:   Rn.145397        SOURCE        Gene Ontology:     BioCarta Pathways:     KEGG Pathways:  
    Probe set : 1374659_at    Name:   similar to Arpp21 protein    GB acc:   AI227963    UniGene:   Rn.7511    Gene Symbol:   LOC363153    Locus Link:   363153    Chromosome:   8    Cytoband:   Chr:8q32    SOURCE    GENECARD       Gene Ontology:     BioCarta Pathways:     KEGG Pathways:  
    Probe set : 1368186_a_at    Name:   spleen tyrosine kinase    GB acc:   U21683    UniGene:   Rn.87407    Gene Symbol:   Syk    Locus Link:   25155    Chromosome:   17    Cytoband:   Chr:17p14    SOURCE    GENECARD       Gene Ontology:   biological process:  activation of MAPK activity   biological process:  protein complex assembly   biological process:  protein amino acid phosphorylation   biological process:  leukocyte adhesion   biological process:  enzyme linked receptor protein signaling pathway   biological process:  G-protein coupled receptor protein signaling pathway   biological process:  integrin-mediated signaling pathway   biological process:  intracellular signaling cascade   biological process:  cell proliferation   biological process:  organ morphogenesis   biological process:  peptidyl-tyrosine phosphorylation   biological process:  neutrophil chemotaxis   biological process:  positive regulation of B cell differentiation   biological process:  positive regulation of gamma-delta T cell differentiation   biological process:  protein amino acid autophosphorylation   biological process:  regulation of immune response   biological process:  regulation of immune response   biological process:  B cell receptor signaling pathway   biological process:  signal transduction   cellular component:  B cell receptor complex   cellular component:  T cell receptor complex   cellular component:  cytoplasm      BioCarta Pathways:     KEGG Pathways:  
    Probe set : 1378245_at    Name:   similar to 6430514L14Rik protein (predicted)    GB acc:   BF563206    UniGene:   Rn.23904    Gene Symbol:   RGD1311958_predicted    Locus Link:   315789    Chromosome:   8    Cytoband:   Chr:8q24    SOURCE    GENECARD       Gene Ontology:     BioCarta Pathways:     KEGG Pathways:  
    Probe set : 1373947_at    Name:   dermatopontin (predicted)    GB acc:   BI278545    UniGene:   Rn.8396    Gene Symbol:   Dpt_predicted    Locus Link:   289178    Chromosome:   13    Cytoband:   Chr:13q23    SOURCE    GENECARD       Gene Ontology:   biological process:  cell adhesion   cellular component:  extracellular matrix (sensu Metazoa)   cellular component:  extracellular space   molecular function:  protein binding      BioCarta Pathways:     KEGG Pathways:  
    Probe set : 1388603_a_at    Name:   HesB protein    GB acc:   BM384289    UniGene:   Rn.1180    Gene Symbol:   Hbld2    Locus Link:   290985    Chromosome:   17    Cytoband:   Chr:17p14    SOURCE    GENECARD       Gene Ontology:   biological process:  endocrine physiological process   cellular component:  mitochondrion   molecular function:  iron ion binding   molecular function:  metal ion binding   molecular function:  iron-sulfur cluster binding      BioCarta Pathways:     KEGG Pathways:  
    Probe set : 1368137_at    Name:   microtubule-associated protein tau /// hypothetical gene supported by NM_017212    GB acc:   NM_017212    UniGene:   Rn.2455    Gene Symbol:   Mapt /// LOC497674    Locus Link:   29477 /// 497674    Chromosome:   10    Cytoband:   Chr:10q32.1    SOURCE    GENECARD       Gene Ontology:     BioCarta Pathways:     KEGG Pathways:  
    Probe set : 1373618_at    Name:   cAMP responsive element binding protein-like 2 (predicted)    GB acc:   BE113210    UniGene:   Rn.107553    Gene Symbol:   Crebl2_predicted    Locus Link:   362453    Chromosome:   4    Cytoband:   Chr:4q43    SOURCE    GENECARD       Gene Ontology:   biological process:  transcription   biological process:  signal transduction   biological process:  regulation of transcription, DNA-dependent   cellular component:  nucleus   molecular function:  transcription factor activity   molecular function:  DNA binding      BioCarta Pathways:     KEGG Pathways:  
    Probe set : 1387071_a_at    Name:   microtubule-associated protein tau /// hypothetical gene supported by NM_017212    GB acc:   BE107978    UniGene:   Rn.2455    Gene Symbol:   Mapt /// LOC497674    Locus Link:   29477 /// 497674    Chromosome:   10    Cytoband:   Chr:10q32.1    SOURCE    GENECARD       Gene Ontology:     BioCarta Pathways:     KEGG Pathways:  
    Probe set : 1370136_at    Name:   lamin B receptor    GB acc:   NM_134453    UniGene:   Rn.6499    Gene Symbol:   Lbr    Locus Link:   89789    Chromosome:   13    Cytoband:   Chr:13q26    SOURCE    GENECARD       Gene Ontology:     BioCarta Pathways:     KEGG Pathways:  
    Probe set : 1374076_at    Name:   similar to hypothetical protein FLJ34389 (predicted)    GB acc:   AI408984    UniGene:   Rn.105677    Gene Symbol:   RGD1305243_predicted    Locus Link:   361409    Chromosome:   19    Cytoband:   Chr:19q12    SOURCE    GENECARD       Gene Ontology:     BioCarta Pathways:     KEGG Pathways:  
    Probe set : 1381504_at    Name:   similar to asporin precursor    GB acc:   AI639412    UniGene:   Rn.45067    Gene Symbol:   LOC306805    Locus Link:   306805    Chromosome:   17    Cytoband:   Chr:17p14    SOURCE    GENECARD       Gene Ontology:     BioCarta Pathways:     KEGG Pathways:  
    Probe set : 1385414_at    Name:   CD8 antigen, alpha chain    GB acc:   BE117044    UniGene:   Rn.10306    Gene Symbol:   Cd8a    Locus Link:   24930    Chromosome:   4    Cytoband:   Chr:4q33    SOURCE    GENECARD       Gene Ontology:     BioCarta Pathways:     KEGG Pathways:  
    Probe set : 1377666_at    Name:   choline dehydrogenase    GB acc:   BI293004    UniGene:   Rn.22857    Gene Symbol:   Chdh    Locus Link:   290551    Chromosome:   16    Cytoband:   Chr:16p16    SOURCE    GENECARD       Gene Ontology:   biological process:  electron transport   biological process:  alcohol metabolism   cellular component:  mitochondrial inner membrane      BioCarta Pathways:     KEGG Pathways:  
    Probe set : 1374298_at    Name:   Transcribed locus, moderately similar to XP_580018.1 PREDICTED: hypothetical protein XP_580018 [Rattus norvegicus]    GB acc:   BG373455    UniGene:   Rn.62411        SOURCE        Gene Ontology:     BioCarta Pathways:     KEGG Pathways:  
    Probe set : 1377023_at    Name:   dual specificity phosphatase 2 (predicted)    GB acc:   AI408580    UniGene:   Rn.136933    Gene Symbol:   Dusp2_predicted    Locus Link:   311406    Chromosome:   3    Cytoband:   Chr:3q36    SOURCE    GENECARD       Gene Ontology:   biological process:  inactivation of MAPK activity   biological process:  protein amino acid dephosphorylation   cellular component:  nucleus      BioCarta Pathways:     KEGG Pathways:  
    Probe set : 1393230_s_at    Name:   Dead end homolog 1 (zebrafish) (predicted)    GB acc:   AI136714    UniGene:   Rn.12947     Locus Link:   307492    Chromosome:   18    Cytoband:   Chr:18p11    SOURCE        Gene Ontology:   cellular component:  cytoplasm   molecular function:  tRNA ligase activity   molecular function:  histidine-tRNA ligase activity   molecular function:  ATP binding      BioCarta Pathways:     KEGG Pathways:  
    Probe set : 1373851_at    Name:   Transcribed locus    GB acc:   AI171998    UniGene:   Rn.102002        SOURCE        Gene Ontology:     BioCarta Pathways:     KEGG Pathways:  
    Probe set : 1383107_at    Name:   small nuclear ribonucleoprotein D1 (predicted)    GB acc:   BG670306    UniGene:   Rn.18606    Gene Symbol:   Snrpd1_predicted    Locus Link:   291794    Chromosome:   18    Cytoband:   Chr:18p13    SOURCE    GENECARD       Gene Ontology:   biological process:  spliceosome assembly   biological process:  nuclear mRNA splicing, via spliceosome   biological process:  mRNA processing   molecular function:  RNA binding      BioCarta Pathways:     KEGG Pathways:  
    Probe set : 1375985_at    Name:   RAB2B, member RAS oncogene family (predicted)    GB acc:   BF282984    UniGene:   Rn.85120    Gene Symbol:   Rab2b_predicted    Locus Link:   305853    Chromosome:   15    Cytoband:   Chr:15p14    SOURCE    GENECARD       Gene Ontology:   biological process:  transport   biological process:  small GTPase mediated signal transduction   biological process:  protein transport   cellular component:  endoplasmic reticulum   cellular component:  Golgi apparatus   molecular function:  GTP binding      BioCarta Pathways:     KEGG Pathways:  
    Probe set : 1395788_at    Name:   Transcribed locus    GB acc:   BF392117    UniGene:   Rn.57702        SOURCE        Gene Ontology:     BioCarta Pathways:     KEGG Pathways:  
    Probe set : 1376871_at     GB acc:   AA891475    UniGene:   Rn.145774    Gene Symbol:   LOC288549 /// LOC363890 /// LOC363894 /// LOC363899 /// LOC498168 /// LOC498169 /// LOC498199 /// LOC501818 /// LOC501821 /// LOC501828    Locus Link:   288549 /// 363890 /// 363894 /// 363899 /// 498168 /// 498169 /// 498199 /// 501818 /// 501821 /// 501828    Chromosome:   12    Cytoband:   Chr:12q12    SOURCE    GENECARD       Gene Ontology:     BioCarta Pathways:     KEGG Pathways:  
    Probe set : 1371398_at     GB acc:   BF281400              Gene Ontology:     BioCarta Pathways:     KEGG Pathways:  
    Probe set : 1385739_at    Name:   Transcribed locus    GB acc:   BE120816    UniGene:   Rn.53919        SOURCE        Gene Ontology:     BioCarta Pathways:     KEGG Pathways:  
    Probe set : 1387145_at    Name:   gap junction membrane channel protein beta 1    GB acc:   NM_017251    UniGene:   Rn.10444    Gene Symbol:   Gjb1    Locus Link:   29584    Chromosome:   X    Cytoband:   Chr:Xq31    SOURCE    GENECARD       Gene Ontology:   molecular function:  gap-junction forming channel activity   molecular function:  gap-junction forming channel activity   molecular function:  connexon channel activity      BioCarta Pathways:     KEGG Pathways:  
    Probe set : 1371864_at    Name:   Basic transcription element binding protein 1    GB acc:   AW524563    UniGene:   Rn.19481    Gene Symbol:   Bteb1    Locus Link:   117560    Chromosome:   1    Cytoband:   Chr:1q51    SOURCE    GENECARD       Gene Ontology:   cellular component:  nucleus      BioCarta Pathways:     KEGG Pathways:  
    Probe set : 1384028_at    Name:   Transcribed locus    GB acc:   AI072042    UniGene:   Rn.153605        SOURCE        Gene Ontology:     BioCarta Pathways:     KEGG Pathways:  
    Probe set : 1382868_at    Name:   sema domain, transmembrane domain (TM), and cytoplasmic domain, (semaphorin) 6A (predicted)    GB acc:   BM387083    UniGene:   Rn.24818    Gene Symbol:   Sema6a_predicted    Locus Link:   361324    Chromosome:   18    Cytoband:   Chr:18q11    SOURCE    GENECARD       Gene Ontology:   cellular component:  membrane   cellular component:  integral to membrane   cellular component:  axon   molecular function:  receptor activity   molecular function:  protein binding      BioCarta Pathways:     KEGG Pathways:  
    Probe set : 1367588_a_at    Name:   ribosomal protein L13A    GB acc:   NM_022179    UniGene:   Rn.92211    Gene Symbol:   Rpl13a    Locus Link:   317646    Chromosome:   1    Cytoband:   Chr:1q22    SOURCE    GENECARD       Gene Ontology:   biological process:  protein biosynthesis   molecular function:  structural constituent of ribosome   molecular function:  structural constituent of ribosome      BioCarta Pathways:     KEGG Pathways:  
    Probe set : 1396187_at    Name:   Hypothetical protein LOC606294    GB acc:   AI136551    UniGene:   Rn.100975     Locus Link:   606294      SOURCE        Gene Ontology:     BioCarta Pathways:     KEGG Pathways:  
    Probe set : 1375307_at    Name:   chromobox homolog 6 (predicted)    GB acc:   BI275772    UniGene:   Rn.2924    Gene Symbol:   Cbx6_predicted    Locus Link:   315136    Chromosome:   7    Cytoband:   Chr:7q34    SOURCE    GENECARD       Gene Ontology:   biological process:  chromatin assembly or disassembly   biological process:  regulation of transcription, DNA-dependent   biological process:  chromatin modification   cellular component:  chromatin   cellular component:  nucleus   molecular function:  chromatin binding      BioCarta Pathways:     KEGG Pathways:  
    Probe set : 1372535_at    Name:   similar to RIKEN cDNA 2210012G02    GB acc:   AI410203    UniGene:   Rn.17312    Gene Symbol:   LOC366431    Locus Link:   366431    Chromosome:   5    Cytoband:   Chr:5q34    SOURCE    GENECARD       Gene Ontology:     BioCarta Pathways:     KEGG Pathways:  
    Probe set : 1368610_at    Name:   mast cell antigen 32    GB acc:   NM_021585    UniGene:   Rn.40569    Gene Symbol:   Mca32    Locus Link:   59105    Chromosome:   10    Cytoband:   Chr:10q32.1    SOURCE    GENECARD       Gene Ontology:   cellular component:  integral to plasma membrane      BioCarta Pathways:     KEGG Pathways:  
    Probe set : 1388195_at    Name:   CUG triplet repeat,RNA-binding protein 2    GB acc:   AW140475    UniGene:   Rn.30004    Gene Symbol:   Cugbp2    Locus Link:   29428    Chromosome:   17    Cytoband:   Chr:17q12.3    SOURCE    GENECARD       Gene Ontology:   cellular component:  nucleus   molecular function:  RNA binding   molecular function:  RNA binding   molecular function:  nucleotide binding   molecular function:  nucleic acid binding      BioCarta Pathways:     KEGG Pathways:  
    Probe set : 1391849_at    Name:   Similar to MBD2 (methyl-CpG-binding protein)-interacting zinc finger protein (predicted)    GB acc:   BF547270    UniGene:   Rn.38367     Locus Link:   300665    Chromosome:   8    Cytoband:   Chr:8q22    SOURCE        Gene Ontology:     BioCarta Pathways:     KEGG Pathways:  
    Probe set : 1392554_a_at     GB acc:   AW915435              Gene Ontology:     BioCarta Pathways:     KEGG Pathways:  
    Probe set : 1368885_at    Name:   ectonucleoside triphosphate diphosphohydrolase 1    GB acc:   AI009808    UniGene:   Rn.17491    Gene Symbol:   Entpd1    Locus Link:   64519    Chromosome:   1    Cytoband:   Chr:1q54    SOURCE    GENECARD       Gene Ontology:     BioCarta Pathways:     KEGG Pathways:  
    Probe set : 1395364_at    Name:   interferon (alpha and beta) receptor 1 (predicted)    GB acc:   BE099982    UniGene:   Rn.105738    Gene Symbol:   Ifnar1_predicted    Locus Link:   288264    Chromosome:   11    Cytoband:   Chr:11q11    SOURCE    GENECARD       Gene Ontology:   biological process:  cell surface receptor linked signal transduction   biological process:  JAK-STAT cascade   biological process:  response to virus      BioCarta Pathways:     KEGG Pathways:  
    Probe set : 1372415_at    Name:   general transcription factor IIH, polypeptide 3 (predicted)    GB acc:   BM385649    UniGene:   Rn.107170    Gene Symbol:   Gtf2h3_predicted    Locus Link:   288651    Chromosome:   12    Cytoband:   Chr:12q15    SOURCE    GENECARD       Gene Ontology:   biological process:  DNA repair   biological process:  nucleotide-excision repair   biological process:  regulation of transcription, DNA-dependent   cellular component:  nucleus   cellular component:  transcription factor TFIIH complex   molecular function:  damaged DNA binding   molecular function:  transcription factor activity   molecular function:  translation factor activity, nucleic acid binding      BioCarta Pathways:     KEGG Pathways:  
    Probe set : 1374871_at    Name:   asparaginase-like sperm autoantigen    GB acc:   AI233343    UniGene:   Rn.22774    Gene Symbol:   Asrgl1    Locus Link:   246307    Chromosome:   1    Cytoband:   Chr:1q43    SOURCE    GENECARD       Gene Ontology:   biological process:  glycoprotein catabolism   cellular component:  cytoplasm   molecular function:  N4-(beta-N-acetylglucosaminyl)-L-asparaginase activity   molecular function:  asparaginase activity      BioCarta Pathways:     KEGG Pathways:  
    Probe set : 1372546_at    Name:   mitogen-activated protein kinase-activated protein kinase 3 (predicted)    GB acc:   BM390276    UniGene:   Rn.8789    Gene Symbol:   Mapkapk3_predicted    Locus Link:   315994    Chromosome:   8    Cytoband:   Chr:8q32    SOURCE    GENECARD       Gene Ontology:   biological process:  protein amino acid phosphorylation   biological process:  response to stress   biological process:  signal transduction   cellular component:  nucleus      BioCarta Pathways:     KEGG Pathways:  
    Probe set : 1376914_at    Name:   DEP domain containing 1 (predicted)    GB acc:   AW253690    UniGene:   Rn.27037    Gene Symbol:   Depdc1_predicted    Locus Link:   295538    Chromosome:   2    Cytoband:   Chr:2q45    SOURCE    GENECARD       Gene Ontology:   biological process:  intracellular signaling cascade   molecular function:  GTPase activator activity      BioCarta Pathways:     KEGG Pathways:  
    Probe set : 1368358_a_at    Name:   protein tyrosine phosphatase, receptor type, R    GB acc:   NM_053594    UniGene:   Rn.6277    Gene Symbol:   Ptprr    Locus Link:   94202    Chromosome:   7    Cytoband:   Chr:7q22    SOURCE    GENECARD       Gene Ontology:     BioCarta Pathways:     KEGG Pathways:  
    Probe set : 1398807_at    Name:   protein phosphatase 1B, magnesium dependent, beta isoform    GB acc:   NM_033096    UniGene:   Rn.4143    Gene Symbol:   Ppm1b    Locus Link:   24667    Chromosome:   6    Cytoband:   Chr:6q12    SOURCE    GENECARD       Gene Ontology:   biological process:  protein amino acid dephosphorylation   cellular component:  protein serine/threonine phosphatase complex      BioCarta Pathways:     KEGG Pathways:  
    Probe set : 1382154_at    Name:   Transcribed locus, strongly similar to NP_035333.2 protein tyrosine phosphatase, non-receptor type 12 [Mus musculus]    GB acc:   BI289625    UniGene:   Rn.18249        SOURCE        Gene Ontology:     BioCarta Pathways:     KEGG Pathways:  
    Probe set : 1382138_at    Name:   similar to hypothetical protein FLJ20433    GB acc:   BG380221    UniGene:   Rn.22790    Gene Symbol:   LOC499745    Locus Link:   499745    Chromosome:   3    Cytoband:   Chr:3p13    SOURCE    GENECARD       Gene Ontology:     BioCarta Pathways:     KEGG Pathways:  
    Probe set : 1389337_at    Name:   Transcribed locus    GB acc:   AA946032    UniGene:   Rn.7972        SOURCE        Gene Ontology:     BioCarta Pathways:     KEGG Pathways:  
    Probe set : 1368171_at    Name:   lysyl oxidase    GB acc:   NM_017061    UniGene:   Rn.11372    Gene Symbol:   Lox    Locus Link:   24914    Chromosome:   18    Cytoband:   Chr:18q11    SOURCE    GENECARD       Gene Ontology:   biological process:  protein modification   cellular component:  extracellular matrix (sensu Metazoa)   cellular component:  extracellular space      BioCarta Pathways:     KEGG Pathways:  
    Probe set : 1374052_at    Name:   LOC361774 (predicted)    GB acc:   AI599270    UniGene:   Rn.17036     Locus Link:   361774    Chromosome:   1    Cytoband:   Chr:1q55    SOURCE        Gene Ontology:     BioCarta Pathways:     KEGG Pathways:  
    Probe set : 1397949_at     GB acc:   BE117532              Gene Ontology:     BioCarta Pathways:     KEGG Pathways:  
    Probe set : 1388874_at    Name:   Transcribed locus    GB acc:   BE113032    UniGene:   Rn.12182        SOURCE        Gene Ontology:     BioCarta Pathways:     KEGG Pathways:  
    Probe set : 1387357_at    Name:   trimethyllysine hydroxylase, epsilon    GB acc:   NM_133387    UniGene:   Rn.3607    Gene Symbol:   Tmlhe    Locus Link:   170898    Chromosome:   20    Cytoband:   Chr:20p12    SOURCE    GENECARD       Gene Ontology:   biological process:  carnitine biosynthesis   biological process:  carnitine biosynthesis   cellular component:  mitochondrion   cellular component:  mitochondrion      BioCarta Pathways:     KEGG Pathways:  
    Probe set : 1373605_at    Name:   similar to 106 kDa O-GlcNAc transferase-interacting protein (predicted)    GB acc:   AI176540    UniGene:   Rn.3090    Gene Symbol:   RGD1307844_predicted    Locus Link:   316085    Chromosome:   8    Cytoband:   Chr:8q32    SOURCE    GENECARD       Gene Ontology:     BioCarta Pathways:     KEGG Pathways:  
    Probe set : 1391661_at    Name:   Hypothetical gene supported by NM_024381    GB acc:   AA945076    UniGene:   Rn.44654    Gene Symbol:   Gyk    Locus Link:   79223    Chromosome:   X    Cytoband:   Chr:Xq22    SOURCE    GENECARD       Gene Ontology:   biological process:  carbohydrate metabolism   biological process:  glycerol metabolism   biological process:  glycerol-3-phosphate metabolism      BioCarta Pathways:     KEGG Pathways:  
    Probe set : 1381525_at     GB acc:   BF419130              Gene Ontology:     BioCarta Pathways:     KEGG Pathways:  
    Probe set : 1387669_a_at    Name:   epoxide hydrolase 1    GB acc:   NM_012844    UniGene:   Rn.3603    Gene Symbol:   Ephx1    Locus Link:   25315    Chromosome:   13    Cytoband:   Chr:13q26    SOURCE    GENECARD       Gene Ontology:     BioCarta Pathways:     KEGG Pathways:  
    Probe set : 1387100_at    Name:   aquaporin 3    GB acc:   NM_031703    UniGene:   Rn.11109    Gene Symbol:   Aqp3    Locus Link:   65133    Chromosome:   5    Cytoband:   Chr:5q22    SOURCE    GENECARD       Gene Ontology:   biological process:  transport   biological process:  water transport   biological process:  water transport   biological process:  excretion      BioCarta Pathways:     KEGG Pathways:  
    Probe set : 1394390_at    Name:   Transcribed locus    GB acc:   BF394739    UniGene:   Rn.51029        SOURCE        Gene Ontology:     BioCarta Pathways:     KEGG Pathways:  
    Probe set : 1392521_at    Name:   Transcribed locus    GB acc:   AI030119    UniGene:   Rn.18407        SOURCE        Gene Ontology:     BioCarta Pathways:     KEGG Pathways:  
    Probe set : 1375940_a_at    Name:   similar to hypothetical protein FLJ12242 (predicted)    GB acc:   BF396656    UniGene:   Rn.25415    Gene Symbol:   RGD1311154_predicted    Locus Link:   300317    Chromosome:   7    Cytoband:   Chr:7q34    SOURCE    GENECARD       Gene Ontology:     BioCarta Pathways:     KEGG Pathways:  
    Probe set : 1372437_at    Name:   S-phase kinase-associated protein 1A    GB acc:   BM390921    UniGene:   Rn.3477    Gene Symbol:   RGD:1359648    Locus Link:   287280    Chromosome:   10    Cytoband:   Chr:10q22    SOURCE    GENECARD       Gene Ontology:   biological process:  ubiquitin cycle      BioCarta Pathways:     KEGG Pathways:  
    Probe set : 1374429_at    Name:   Transcribed locus, weakly similar to XP_518443.1 PREDICTED: pim-1 oncogene [Pan troglodytes]    GB acc:   AI598401    UniGene:   Rn.129615        SOURCE        Gene Ontology:     BioCarta Pathways:     KEGG Pathways:  
    Probe set : 1387898_at    Name:   heat shock protein, alpha-crystallin-related, B6    GB acc:   D29960    UniGene:   Rn.3201    Gene Symbol:   Hspb6    Locus Link:   192245    Chromosome:   1    Cytoband:   Chr:1q21    SOURCE    GENECARD       Gene Ontology:   biological process:  regulation of muscle contraction   biological process:  protein folding   biological process:  response to unfolded protein   molecular function:  structural constituent of eye lens      BioCarta Pathways:     KEGG Pathways:  
    Probe set : 1393161_at    Name:   small nuclear RNA activating complex, polypeptide 3 (predicted)    GB acc:   AW916962    UniGene:   Rn.47086    Gene Symbol:   Snapc3_predicted    Locus Link:   362537    Chromosome:   5    Cytoband:   Chr:5q31    SOURCE    GENECARD       Gene Ontology:   cellular component:  nucleus   molecular function:  DNA binding      BioCarta Pathways:     KEGG Pathways:  
    Probe set : 1382521_at    Name:   glutaminase    GB acc:   AA925755    UniGene:   Rn.5762    Gene Symbol:   Gls    Locus Link:   24398    Chromosome:   9    Cytoband:   Chr:9q31-q32    SOURCE    GENECARD       Gene Ontology:   biological process:  glutamine metabolism   biological process:  glutamine catabolism   biological process:  glutamine catabolism   cellular component:  mitochondrion   cellular component:  mitochondrion   cellular component:  mitochondrial inner membrane   molecular function:  glutaminase activity   molecular function:  glutaminase activity   molecular function:  hydrolase activity      BioCarta Pathways:     KEGG Pathways:  
    Probe set : 1374800_at    Name:   Solute carrier family 25 (mitochondrial carrier; ornithine transporter) member 15 (predicted)    GB acc:   BI303627    UniGene:   Rn.66488     Locus Link:   306574    Chromosome:   16    Cytoband:   Chr:16q12.5    SOURCE        Gene Ontology:   molecular function:  L-ornithine transporter activity   molecular function:  transporter activity   molecular function:  binding      BioCarta Pathways:     KEGG Pathways:  
    Probe set : 1389540_at    Name:   TG interacting factor (predicted)    GB acc:   AI236141    UniGene:   Rn.144418    Gene Symbol:   Tfig    Locus Link:   316742    Chromosome:   9    Cytoband:   Chr:9q38    SOURCE    GENECARD       Gene Ontology:   cellular component:  nucleus   cellular component:  transcription factor complex   molecular function:  DNA binding   molecular function:  transcription factor activity   molecular function:  transcription corepressor activity      BioCarta Pathways:     KEGG Pathways:  
    Probe set : 1389151_at    Name:   Transcribed locus    GB acc:   AI103440    UniGene:   Rn.113277        SOURCE        Gene Ontology:     BioCarta Pathways:     KEGG Pathways:  
    Probe set : 1384968_at    Name:   SH3-domain binding protein 2 (predicted)    GB acc:   BM384145    UniGene:   Rn.138976    Gene Symbol:   Sh3bp2_predicted    Locus Link:   305450    Chromosome:   14    Cytoband:   Chr:14q21    SOURCE    GENECARD       Gene Ontology:   biological process:  intracellular signaling cascade   molecular function:  SH3/SH2 adaptor activity      BioCarta Pathways:     KEGG Pathways:  
    Probe set : 1377192_a_at    Name:   caseinolytic protease X (E.coli) (predicted)    GB acc:   BM384629    UniGene:   Rn.66864    Gene Symbol:   Clpx_predicted    Locus Link:   300786    Chromosome:   8    Cytoband:   Chr:8q24    SOURCE    GENECARD       Gene Ontology:   biological process:  protein folding   biological process:  protein transport   cellular component:  mitochondrion      BioCarta Pathways:     KEGG Pathways:  
    Probe set : 1372646_at    Name:   similar to RIKEN cDNA 1500015O10 (predicted)    GB acc:   BF418649    UniGene:   Rn.16593    Gene Symbol:   RGD1305645_predicted    Locus Link:   363225    Chromosome:   9    Cytoband:   Chr:9q22    SOURCE    GENECARD       Gene Ontology:     BioCarta Pathways:     KEGG Pathways:  
    Probe set : 1388846_at     GB acc:   AI227978              Gene Ontology:     BioCarta Pathways:     KEGG Pathways:  
    Probe set : 1374638_at    Name:   peroxisomal biogenesis factor 13 (predicted)    GB acc:   AI012951    UniGene:   Rn.144057    Gene Symbol:   Pex13_predicted    Locus Link:   305581    Chromosome:   14    Cytoband:   Chr:14q22    SOURCE    GENECARD       Gene Ontology:   molecular function:  protein transporter activity      BioCarta Pathways:     KEGG Pathways:  
    Probe set : 1395730_at    Name:   glycyl-tRNA synthetase (predicted) /// similar to Glycyl-tRNA synthetase    GB acc:   AW507352    UniGene:   Rn.145629    Gene Symbol:   Gars_predicted /// LOC500135    Locus Link:   297113 /// 500135    Chromosome:   4    Cytoband:   Chr:4q24    SOURCE    GENECARD       Gene Ontology:   cellular component:  soluble fraction   cellular component:  cytoplasm   cellular component:  secretory granule      BioCarta Pathways:     KEGG Pathways:  
    Probe set : 1391955_at    Name:   similar to RAB19, member RAS oncogene family    GB acc:   BI291339    UniGene:   Rn.53184    Gene Symbol:   LOC500088    Locus Link:   500088    Chromosome:   4    Cytoband:   Chr:4q22    SOURCE    GENECARD       Gene Ontology:   biological process:  small GTPase mediated signal transduction   biological process:  protein transport   molecular function:  GTP binding      BioCarta Pathways:     KEGG Pathways:  
    Probe set : 1398903_at    Name:   Esterase D/formylglutathione hydrolase    GB acc:   AI411425    UniGene:   Rn.3682    Gene Symbol:   Esd    Locus Link:   290401    Chromosome:   15    Cytoband:   Chr:15q11    SOURCE    GENECARD       Gene Ontology:   cellular component:  cytoplasmic membrane-bound vesicle      BioCarta Pathways:     KEGG Pathways:  
    Probe set : 1376465_at    Name:   Transcribed locus    GB acc:   BI295240    UniGene:   Rn.24047        SOURCE        Gene Ontology:     BioCarta Pathways:     KEGG Pathways:  
    Probe set : 1369512_at    Name:   carbohydrate (chondroitin 6/keratan) sulfotransferase 3    GB acc:   NM_053408    UniGene:   Rn.64646    Gene Symbol:   Chst3    Locus Link:   84468    Chromosome:   20    Cytoband:   Chr:20q11    SOURCE    GENECARD       Gene Ontology:   biological process:  carbohydrate metabolism   cellular component:  integral to membrane   cellular component:  Golgi stack   molecular function:  transferase activity   molecular function:  sulfotransferase activity   molecular function:  chondroitin 6-sulfotransferase activity      BioCarta Pathways:     KEGG Pathways:  
    Probe set : 1372398_at    Name:   similar to Potassium channel tetramerisation domain containing 15    GB acc:   BG373314    UniGene:   Rn.23171    Gene Symbol:   LOC499129    Locus Link:   499129    Chromosome:   1    Cytoband:   Chr:1q21    SOURCE    GENECARD       Gene Ontology:     BioCarta Pathways:     KEGG Pathways:  
    Probe set : 1368374_a_at    Name:   gamma-glutamyltransferase 1    GB acc:   NM_053840    UniGene:   Rn.10010    Gene Symbol:   Ggt1    Locus Link:   116568      SOURCE    GENECARD       Gene Ontology:   cellular component:  integral to plasma membrane   cellular component:  integral to membrane      BioCarta Pathways:     KEGG Pathways:  
    Probe set : 1368847_at    Name:   RAB10, member RAS oncogene family    GB acc:   NM_017359    UniGene:   Rn.65864    Gene Symbol:   Rab10    Locus Link:   50993    Chromosome:   6    Cytoband:   Chr:6q12    SOURCE    GENECARD       Gene Ontology:   cellular component:  intracellular   cellular component:  Golgi apparatus   cellular component:  Golgi apparatus      BioCarta Pathways:     KEGG Pathways:  
    Probe set : 1398991_at    Name:   Similar to expressed sequence AA408877 (predicted)    GB acc:   BM389704    UniGene:   Rn.7282     Locus Link:   364052    Chromosome:   13    Cytoband:   Chr:13q24    SOURCE        Gene Ontology:   cellular component:  membrane fraction   cellular component:  cytosol   cellular component:  microtubule associated complex   molecular function:  protein kinase C binding   molecular function:  sugar porter activity   molecular function:  protein binding      BioCarta Pathways:     KEGG Pathways:  
    Probe set : 1375106_at    Name:   amnionless (predicted)    GB acc:   AI103368    UniGene:   Rn.7787    Gene Symbol:   Amn_predicted    Locus Link:   314459    Chromosome:   6    Cytoband:   Chr:6q32    SOURCE    GENECARD       Gene Ontology:   biological process:  development   cellular component:  extracellular space   cellular component:  integral to membrane      BioCarta Pathways:     KEGG Pathways:  
    Probe set : 1384147_at    Name:   eukaryotic translation initiation factor 1A (predicted)    GB acc:   AA955540    UniGene:   Rn.3361    Gene Symbol:   Eif1a_predicted    Locus Link:   317163    Chromosome:   18    Cytoband:   Chr:18q11    SOURCE    GENECARD       Gene Ontology:   biological process:  protein biosynthesis   biological process:  translational initiation   cellular component:  eukaryotic translation initiation factor 4F complex   molecular function:  translation factor activity, nucleic acid binding   molecular function:  RNA binding   molecular function:  translation initiation factor activity      BioCarta Pathways:     KEGG Pathways:  
    Probe set : 1380610_at    Name:   Transcribed locus    GB acc:   AI406521    UniGene:   Rn.148591        SOURCE        Gene Ontology:     BioCarta Pathways:     KEGG Pathways:  
    Probe set : 1394881_at    Name:   similar to 6430514L14Rik protein (predicted)    GB acc:   AI578410    UniGene:   Rn.23904    Gene Symbol:   RGD1311958_predicted    Locus Link:   315789    Chromosome:   8    Cytoband:   Chr:8q24    SOURCE    GENECARD       Gene Ontology:     BioCarta Pathways:     KEGG Pathways:  
    Probe set : 1372497_at    Name:   similar to Nbr1 (predicted)    GB acc:   AI104094     Gene Symbol:   RGD1311421_predicted    Locus Link:   303554    Chromosome:   10    Cytoband:   Chr:10q32.1     GENECARD       Gene Ontology:   molecular function:  zinc ion binding      BioCarta Pathways:     KEGG Pathways:  
    Probe set : 1368571_at    Name:   cytoplasmic linker 2    GB acc:   NM_021997    UniGene:   Rn.10893    Gene Symbol:   Cyln2    Locus Link:   29264    Chromosome:   12    Cytoband:   Chr:12q12    SOURCE    GENECARD       Gene Ontology:   biological process:  regulation of transcription, DNA-dependent   biological process:  transcription initiation from RNA polymerase II promoter   molecular function:  RNA polymerase II transcription factor activity   molecular function:  protein binding   molecular function:  microtubule binding      BioCarta Pathways:     KEGG Pathways:  
    Probe set : 1388109_at    Name:   G protein-coupled receptor 116    GB acc:   BI290677    UniGene:   Rn.25073    Gene Symbol:   Gpr116    Locus Link:   245977    Chromosome:   9    Cytoband:   Chr:9q12    SOURCE    GENECARD       Gene Ontology:   biological process:  signal transduction   biological process:  G-protein coupled receptor protein signaling pathway   biological process:  neuropeptide signaling pathway   cellular component:  membrane   cellular component:  integral to membrane      BioCarta Pathways:     KEGG Pathways:  
    Probe set : 1393997_at    Name:   similar to hypothetical protein FLJ32825    GB acc:   AI556333    UniGene:   Rn.38213    Gene Symbol:   LOC500595    Locus Link:   500595    Chromosome:   5    Cytoband:   Chr:5q36    SOURCE    GENECARD       Gene Ontology:     BioCarta Pathways:     KEGG Pathways:  
    Probe set : 1391754_at    Name:   2 ' -5 ' oligoadenylate synthetase 1I    GB acc:   BM391818    UniGene:   Rn.144943    Gene Symbol:   RGD:1359379    Locus Link:   304507    Chromosome:   12    Cytoband:   Chr:12q16    SOURCE    GENECARD       Gene Ontology:   biological process:  immune response      BioCarta Pathways:     KEGG Pathways:  
    Probe set : 1371609_at    Name:   homolog of zebrafish ES1    GB acc:   AI232270    UniGene:   Rn.11498    Gene Symbol:   RGD:1303003    Locus Link:   294326    Chromosome:   20    Cytoband:   Chr:20p12    SOURCE    GENECARD       Gene Ontology:     BioCarta Pathways:     KEGG Pathways:  
    Probe set : 1379967_at    Name:   zinc finger protein 367 (predicted)    GB acc:   AI501039    UniGene:   Rn.35247    Gene Symbol:   Zfp367_predicted    Locus Link:   306695    Chromosome:   17    Cytoband:   Chr:17p14    SOURCE    GENECARD       Gene Ontology:   biological process:  regulation of transcription from RNA polymerase II promoter   cellular component:  nucleus      BioCarta Pathways:     KEGG Pathways:  
    Probe set : 1382436_at    Name:   similar to RIKEN cDNA 2610002M06    GB acc:   BM386497    UniGene:   Rn.8881    Gene Symbol:   LOC363487    Locus Link:   363487    Chromosome:   X    Cytoband:   Chr:Xq31    SOURCE    GENECARD       Gene Ontology:     BioCarta Pathways:     KEGG Pathways:  
    Probe set : 1368204_at    Name:   ligase I, DNA, ATP-dependent /// ligase I, DNA, ATP-dependent (predicted)    GB acc:   NM_030855    UniGene:   Rn.20467    Gene Symbol:   Lig1 /// Lig1_predicted    Locus Link:   360304 /// 81513    Chromosome:   1    Cytoband:   Chr:1q12 /// Chr:1q21    SOURCE    GENECARD       Gene Ontology:   cellular component:  nucleus      BioCarta Pathways:     KEGG Pathways:  
    Probe set : 1390177_at    Name:   Transcribed locus    GB acc:   AI233857    UniGene:   Rn.6397        SOURCE        Gene Ontology:     BioCarta Pathways:     KEGG Pathways:  
    Probe set : 1374293_at     GB acc:   AI137495              Gene Ontology:     BioCarta Pathways:     KEGG Pathways:  
    Probe set : 1374484_at    Name:   similar to RIKEN cDNA 2610033C09 (predicted)    GB acc:   AI230318    UniGene:   Rn.13663    Gene Symbol:   RGD1306421_predicted    Locus Link:   288092    Chromosome:   11    Cytoband:   Chr:11q21    SOURCE    GENECARD       Gene Ontology:     BioCarta Pathways:     KEGG Pathways:  
    Probe set : 1382061_at    Name:   lactate dehydrogenase D (predicted)    GB acc:   AI501131    UniGene:   Rn.137462    Gene Symbol:   Ldhd_predicted    Locus Link:   307858    Chromosome:   19    Cytoband:   Chr:19q12    SOURCE    GENECARD       Gene Ontology:   cellular component:  mitochondrion   cellular component:  mitochondrion      BioCarta Pathways:     KEGG Pathways:  
    Probe set : 1371407_at    Name:   NCK-associated protein 1    GB acc:   AI178285    UniGene:   Rn.2611    Gene Symbol:   Nckap1    Locus Link:   58823    Chromosome:   3    Cytoband:   Chr:3q24    SOURCE    GENECARD       Gene Ontology:   biological process:  apoptosis   biological process:  central nervous system development   biological process:  lamellipodium biogenesis   cellular component:  integral to membrane   cellular component:  lamellipodium   molecular function:  protein binding   molecular function:  protein binding      BioCarta Pathways:     KEGG Pathways:  
    Probe set : 1376895_at    Name:   interleukin 16    GB acc:   AI137163    UniGene:   Rn.104665    Gene Symbol:   Il16    Locus Link:   116996    Chromosome:   1    Cytoband:   Chr:1q31    SOURCE    GENECARD       Gene Ontology:     BioCarta Pathways:     KEGG Pathways:  
    Probe set : 1368375_a_at    Name:   interleukin 15    GB acc:   AF015718    UniGene:   Rn.2490    Gene Symbol:   Il15    Locus Link:   25670    Chromosome:   19    Cytoband:   Chr:19q11    SOURCE    GENECARD       Gene Ontology:     BioCarta Pathways:     KEGG Pathways:  
    Probe set : 1383493_at    Name:   Transcribed locus    GB acc:   BE102202    UniGene:   Rn.7923        SOURCE        Gene Ontology:     BioCarta Pathways:     KEGG Pathways:  
    Probe set : 1387531_at    Name:   methionine sulfoxide reductase A    GB acc:   NM_053307    UniGene:   Rn.41704    Gene Symbol:   Msra    Locus Link:   29447    Chromosome:   15    Cytoband:   Chr:15p12    SOURCE    GENECARD       Gene Ontology:     BioCarta Pathways:     KEGG Pathways:  
    Probe set : 1371391_at    Name:   thioredoxin domain containing 5 (predicted)    GB acc:   BM390196    UniGene:   Rn.128652    Gene Symbol:   Txndc5_predicted    Locus Link:   306869    Chromosome:   17    Cytoband:   Chr:17p12    SOURCE    GENECARD       Gene Ontology:   biological process:  electron transport   cellular component:  endoplasmic reticulum   molecular function:  electron transporter activity   molecular function:  isomerase activity      BioCarta Pathways:     KEGG Pathways:  
    Probe set : 1389632_at    Name:   Transcribed locus, weakly similar to XP_228131.3 PREDICTED: similar to Rho-related BTB domain-containing protein 1 [Rattus norvegicus]    GB acc:   AA799294    UniGene:   Rn.3782        SOURCE        Gene Ontology:     BioCarta Pathways:     KEGG Pathways:  
    Probe set : 1387436_at    Name:   septin 7    GB acc:   NM_022616    UniGene:   Rn.48701    Gene Symbol:   Sept7    Locus Link:   64551    Chromosome:   8    Cytoband:   Chr:8q13    SOURCE    GENECARD       Gene Ontology:   cellular component:  membrane fraction   cellular component:  membrane fraction   molecular function:  GTP binding   molecular function:  nucleotide binding      BioCarta Pathways:     KEGG Pathways:  
    Probe set : 1388836_at    Name:   protein kinase C, eta    GB acc:   AA799981    UniGene:   Rn.10398    Gene Symbol:   Prkch    Locus Link:   81749    Chromosome:   6    Cytoband:   Chr:6q24    SOURCE    GENECARD       Gene Ontology:   biological process:  protein amino acid phosphorylation   biological process:  protein amino acid phosphorylation   biological process:  intracellular signaling cascade      BioCarta Pathways:     KEGG Pathways:  
    Probe set : 1393015_at    Name:   similar to hypothetical protein FLJ14146 (predicted)    GB acc:   BG375354    UniGene:   Rn.39189    Gene Symbol:   RGD1310587_predicted    Locus Link:   360894    Chromosome:   13    Cytoband:   Chr:13q26    SOURCE    GENECARD       Gene Ontology:     BioCarta Pathways:     KEGG Pathways:  
    Probe set : 1370952_at    Name:   glutathione S-transferase, mu 2    GB acc:   AI169331    UniGene:   Rn.625    Gene Symbol:   Gstm2    Locus Link:   24424    Chromosome:   2    Cytoband:   Chr:2q34    SOURCE    GENECARD       Gene Ontology:   molecular function:  glutathione transferase activity   molecular function:  glutathione transferase activity   molecular function:  transferase activity      BioCarta Pathways:     KEGG Pathways:  
    Probe set : 1367485_at    Name:   transcription elongation factor A (SII) 1 (predicted) /// similar to transcription elongation factor IIS - mouse    GB acc:   BM388871    UniGene:   Rn.129120    Gene Symbol:   Tcea1_predicted /// LOC498453    Locus Link:   362479 /// 498453    Chromosome:   5    Cytoband:   Chr:5q12 /// Chr:15p16    SOURCE    GENECARD       Gene Ontology:   cellular component:  nucleus   cellular component:  nucleoplasm      BioCarta Pathways:     KEGG Pathways:  
    Probe set : 1391167_at    Name:   Transcribed locus    GB acc:   AW532567    UniGene:   Rn.47980        SOURCE        Gene Ontology:     BioCarta Pathways:     KEGG Pathways:  
    Probe set : 1383719_at    Name:   microtubule-associated protein 7 (predicted)    GB acc:   AW531489    UniGene:   Rn.23807    Gene Symbol:   Mtap7_predicted    Locus Link:   293016    Chromosome:   1    Cytoband:   Chr:1p12    SOURCE    GENECARD       Gene Ontology:   cellular component:  microtubule associated complex   molecular function:  structural molecule activity   molecular function:  cytoskeletal regulatory protein binding      BioCarta Pathways:     KEGG Pathways:  
    Probe set : 1368552_at    Name:   GrpE-like 1, mitochondrial    GB acc:   NM_024487    UniGene:   Rn.10630    Gene Symbol:   Grpel1    Locus Link:   79563    Chromosome:   14    Cytoband:   Chr:14q21    SOURCE    GENECARD       Gene Ontology:   biological process:  protein folding   cellular component:  extracellular space   cellular component:  mitochondrion      BioCarta Pathways:     KEGG Pathways:  
    Probe set : 1387800_at    Name:   Fas death domain-associated protein    GB acc:   NM_080891    UniGene:   Rn.870    Gene Symbol:   Daxx    Locus Link:   140926    Chromosome:   20    Cytoband:   Chr:20p12    SOURCE    GENECARD       Gene Ontology:   cellular component:  nucleus   cellular component:  kinesin complex   cellular component:  PML body   molecular function:  protein binding   molecular function:  transcriptional repressor activity   molecular function:  protein domain specific binding      BioCarta Pathways:     KEGG Pathways:  
    Probe set : 1377116_at    Name:   Ribonuclease L (2',5'-oligoisoadenylate synthetase-dependent)    GB acc:   BI301478    UniGene:   Rn.107928    Gene Symbol:   Rnasel    Locus Link:   359726    Chromosome:   13    Cytoband:   Chr:13q21    SOURCE    GENECARD       Gene Ontology:   biological process:  mRNA processing   biological process:  protein amino acid phosphorylation   molecular function:  protein kinase activity   molecular function:  ATP binding   molecular function:  endoribonuclease activity, producing 5'-phosphomonoesters      BioCarta Pathways:     KEGG Pathways:  
    Probe set : 1388733_at    Name:   bifunctional apoptosis regulator (predicted)    GB acc:   AI177631    UniGene:   Rn.95242    Gene Symbol:   Bfar_predicted    Locus Link:   304709    Chromosome:   10    Cytoband:   Chr:10q11    SOURCE    GENECARD       Gene Ontology:   biological process:  anti-apoptosis   biological process:  protein ubiquitination   cellular component:  ubiquitin ligase complex   cellular component:  membrane fraction   cellular component:  integral to plasma membrane      BioCarta Pathways:     KEGG Pathways:  
    Probe set : 1373191_at    Name:   similar to hypothetical protein    GB acc:   BG375890     Gene Symbol:   LOC500897    Locus Link:   500897    Chromosome:   7    Cytoband:   Chr:7q34     GENECARD       Gene Ontology:     BioCarta Pathways:     KEGG Pathways:  
    Probe set : 1390917_at     GB acc:   AI227828              Gene Ontology:     BioCarta Pathways:     KEGG Pathways:  
    Probe set : 1372409_at    Name:   MAD2L1 binding protein (predicted)    GB acc:   BI286562    UniGene:   Rn.9596    Gene Symbol:   Mad2l1bp_predicted    Locus Link:   316237    Chromosome:   9    Cytoband:   Chr:9q12    SOURCE    GENECARD       Gene Ontology:   biological process:  mitotic spindle checkpoint   biological process:  regulation of exit from mitosis   cellular component:  nucleus   molecular function:  protein binding      BioCarta Pathways:     KEGG Pathways:  
    Probe set : 1372744_at    Name:   plakophilin 4 (predicted)    GB acc:   BE113437    UniGene:   Rn.17389    Gene Symbol:   Pkp4_predicted    Locus Link:   295625    Chromosome:   3    Cytoband:   Chr:3q21    SOURCE    GENECARD       Gene Ontology:   biological process:  cell adhesion   cellular component:  cytoskeleton   cellular component:  intercellular junction   molecular function:  structural molecule activity   molecular function:  protein binding      BioCarta Pathways:     KEGG Pathways:  
    Probe set : 1380867_a_at    Name:   pleckstrin homology domain containing, family M (with RUN domain) member 1 (predicted)    GB acc:   BM383086    UniGene:   Rn.101335    Gene Symbol:   Plekhm1_predicted    Locus Link:   303584    Chromosome:   10    Cytoband:   Chr:10q32.1    SOURCE    GENECARD       Gene Ontology:   biological process:  intracellular signaling cascade      BioCarta Pathways:     KEGG Pathways:  
    Probe set : 1369948_at    Name:   nerve growth factor receptor (TNFRSF16) associated protein 1    GB acc:   NM_053401    UniGene:   Rn.3126    Gene Symbol:   Ngfrap1    Locus Link:   117089    Chromosome:   X    Cytoband:   Chr:Xq35    SOURCE    GENECARD       Gene Ontology:   cellular component:  cytosol   molecular function:  receptor activity   molecular function:  death receptor binding      BioCarta Pathways:     KEGG Pathways:  
    Probe set : 1390020_at     GB acc:   BI277513              Gene Ontology:     BioCarta Pathways:     KEGG Pathways:  
    Probe set : 1369557_at    Name:   caspase 7    GB acc:   NM_022260    UniGene:   Rn.53995    Gene Symbol:   Casp7    Locus Link:   64026    Chromosome:   1    Cytoband:   Chr:1q55    SOURCE    GENECARD       Gene Ontology:   biological process:  proteolysis   biological process:  apoptosis   biological process:  apoptotic program   cellular component:  cytoplasm      BioCarta Pathways:     KEGG Pathways:  
    Probe set : 1387976_at    Name:   solute carrier family 9 (sodium/hydrogen exchanger), isoform 3 regulator 2    GB acc:   AF294257    UniGene:   Rn.39351    Gene Symbol:   Slc9a3r2    Locus Link:   116501    Chromosome:   10    Cytoband:   Chr:10q12    SOURCE    GENECARD       Gene Ontology:   biological process:  intracellular signaling cascade   cellular component:  nucleus   cellular component:  apical plasma membrane   molecular function:  protein binding      BioCarta Pathways:     KEGG Pathways:  
    Probe set : 1380962_at    Name:   Angiotensin I converting enzyme (peptidyl-dipeptidase A) 2    GB acc:   BE098785    UniGene:   Rn.129779    Gene Symbol:   Ace2    Locus Link:   302668    Chromosome:   X    Cytoband:   Chr:Xq21    SOURCE    GENECARD       Gene Ontology:   biological process:  muscle contraction   biological process:  proteolysis   cellular component:  membrane   cellular component:  integral to membrane      BioCarta Pathways:     KEGG Pathways:  
    Probe set : 1391390_at     GB acc:   AA894192              Gene Ontology:     BioCarta Pathways:     KEGG Pathways:  
    Probe set : 1382161_at    Name:   M-phase phosphoprotein 10 (U3 small nucleolar ribonucleoprotein) (predicted)    GB acc:   AA817668    UniGene:   Rn.103228    Gene Symbol:   Mphosph10_predicted    Locus Link:   293828    Chromosome:   1    Cytoband:   Chr:1q22    SOURCE    GENECARD       Gene Ontology:   biological process:  rRNA processing   cellular component:  small nucleolar ribonucleoprotein complex      BioCarta Pathways:     KEGG Pathways:  
    Probe set : 1369830_at    Name:   protein kinase C, eta    GB acc:   NM_031085    UniGene:   Rn.10398    Gene Symbol:   Prkch    Locus Link:   81749    Chromosome:   6    Cytoband:   Chr:6q24    SOURCE    GENECARD       Gene Ontology:   biological process:  protein amino acid phosphorylation   biological process:  protein amino acid phosphorylation   biological process:  intracellular signaling cascade      BioCarta Pathways:     KEGG Pathways:  
    Probe set : 1371573_at    Name:   ribosomal protein L36a (predicted)    GB acc:   AW142090    UniGene:   Rn.144788    Gene Symbol:   Rpl36a_predicted    Locus Link:   292964    Chromosome:   X    Cytoband:   Chr:Xq34    SOURCE    GENECARD       Gene Ontology:   biological process:  protein biosynthesis   biological process:  ribosome biogenesis   cellular component:  intracellular   cellular component:  cytosolic ribosome (sensu Eukaryota)   cellular component:  ribosome   molecular function:  structural constituent of ribosome      BioCarta Pathways:     KEGG Pathways:  
    Probe set : 1374840_at     GB acc:   BG377716    UniGene:   Rn.41174        SOURCE        Gene Ontology:     BioCarta Pathways:     KEGG Pathways:  
    Probe set : 1388611_at    Name:   transcription elongation factor A (SII), 3 (predicted)    GB acc:   AW252817    UniGene:   Rn.11547    Gene Symbol:   Tcea3_predicted    Locus Link:   298559    Chromosome:   5    Cytoband:   Chr:5q36    SOURCE    GENECARD       Gene Ontology:   cellular component:  nucleus   molecular function:  DNA binding   molecular function:  transcription factor activity   molecular function:  translation elongation factor activity      BioCarta Pathways:     KEGG Pathways:  
    Probe set : 1374592_at     GB acc:   BI282671              Gene Ontology:     BioCarta Pathways:     KEGG Pathways:  
    Probe set : 1377626_at     GB acc:   AA924350              Gene Ontology:     BioCarta Pathways:     KEGG Pathways:  
    Probe set : 1398389_at    Name:   Platelet-activating factor acetylhydrolase, isoform Ib, alpha subunit 45kDa    GB acc:   AI236136    UniGene:   Rn.5827    Gene Symbol:   Pafah1b1    Locus Link:   83572    Chromosome:   10    Cytoband:   Chr:10q24    SOURCE    GENECARD       Gene Ontology:   cellular component:  cytoskeleton   cellular component:  microtubule      BioCarta Pathways:     KEGG Pathways:  
    Probe set : 1368071_at    Name:   Mg87 protein    GB acc:   NM_134410    UniGene:   Rn.11802    Gene Symbol:   Mg87    Locus Link:   171451    Chromosome:   13    Cytoband:   Chr:13q26    SOURCE    GENECARD       Gene Ontology:   biological process:  protein biosynthesis   molecular function:  structural constituent of ribosome      BioCarta Pathways:     KEGG Pathways:  
    Probe set : 1373680_at    Name:   Transcribed locus    GB acc:   AA800708    UniGene:   Rn.3886        SOURCE        Gene Ontology:     BioCarta Pathways:     KEGG Pathways:  
    Probe set : 1373204_at    Name:   hypothetical LOC297077 (predicted)    GB acc:   BM388911    UniGene:   Rn.17071    Gene Symbol:   RGD1310725_predicted    Locus Link:   297077    Chromosome:   4    Cytoband:   Chr:4q24    SOURCE    GENECARD       Gene Ontology:   cellular component:  integral to membrane      BioCarta Pathways:     KEGG Pathways:  
    Probe set : 1396246_at    Name:   Ubiquitin-conjugating enzyme E2D 1, UBC4/5 homolog (yeast) (predicted)    GB acc:   AA965093    UniGene:   Rn.24937     Locus Link:   361831    Chromosome:   20    Cytoband:   Chr:20p11    SOURCE        Gene Ontology:   biological process:  ubiquitin-dependent protein catabolism   biological process:  ubiquitin cycle   molecular function:  ubiquitin conjugating enzyme activity   molecular function:  ubiquitin-protein ligase activity   molecular function:  ligase activity      BioCarta Pathways:     KEGG Pathways:  
    Probe set : 1390415_at    Name:   Thyroid hormone receptor interactor 13 (predicted)    GB acc:   BM392319    UniGene:   Rn.11751     Locus Link:   292206    Chromosome:   1    Cytoband:   Chr:1p11    SOURCE        Gene Ontology:   biological process:  transcription from RNA polymerase II promoter   cellular component:  nucleus      BioCarta Pathways:     KEGG Pathways:  
    Probe set : 1368158_at    Name:   sec1 family domain containing 1    GB acc:   NM_019364    UniGene:   Rn.10761    Gene Symbol:   Scfd1    Locus Link:   54350    Chromosome:   6    Cytoband:   Chr:6q22    SOURCE    GENECARD       Gene Ontology:   cellular component:  endoplasmic reticulum   cellular component:  Golgi apparatus   cellular component:  Golgi vesicle   cellular component:  Golgi stack   molecular function:  syntaxin binding      BioCarta Pathways:     KEGG Pathways:  
    Probe set : 1390819_at    Name:   Thyrotroph embryonic factor    GB acc:   BF419374    UniGene:   Rn.59871    Gene Symbol:   Tef    Locus Link:   29362    Chromosome:   7    Cytoband:   Chr:7q34    SOURCE    GENECARD       Gene Ontology:   cellular component:  nucleus   cellular component:  nucleus      BioCarta Pathways:     KEGG Pathways:  
    Probe set : 1388883_at    Name:   polymerase (DNA-directed), delta 4 (predicted)    GB acc:   BF281976    UniGene:   Rn.104646    Gene Symbol:   Pold4_predicted    Locus Link:   361698    Chromosome:   1    Cytoband:   Chr:1q42    SOURCE    GENECARD       Gene Ontology:   biological process:  DNA replication   cellular component:  nucleus   molecular function:  DNA-directed DNA polymerase activity   molecular function:  delta DNA polymerase activity      BioCarta Pathways:     KEGG Pathways:  
    Probe set : 1383434_at    Name:   Transcribed locus    GB acc:   AA997640    UniGene:   Rn.12530        SOURCE        Gene Ontology:     BioCarta Pathways:     KEGG Pathways:  
    Probe set : 1370170_at    Name:   heterogeneous nuclear ribonucleoprotein U    GB acc:   AI177494    UniGene:   Rn.4328    Gene Symbol:   Hnrpu    Locus Link:   117280    Chromosome:   13    Cytoband:   Chr:13q26    SOURCE    GENECARD       Gene Ontology:   biological process:  RNA processing   molecular function:  DNA binding   molecular function:  RNA binding   molecular function:  transporter activity   molecular function:  ATP binding      BioCarta Pathways:     KEGG Pathways:  
    Probe set : 1372661_at    Name:   transducin (beta)-like 3 (predicted)    GB acc:   AI406314    UniGene:   Rn.32013    Gene Symbol:   Tbl3_predicted    Locus Link:   287120    Chromosome:   10    Cytoband:   Chr:10q12    SOURCE    GENECARD       Gene Ontology:   biological process:  G-protein signaling, coupled to cGMP nucleotide second messenger   molecular function:  receptor signaling protein activity      BioCarta Pathways:     KEGG Pathways:  
    Probe set : 1374011_at    Name:   excision repair cross-complementing rodent repair deficiency, complementation group 3 (predicted)    GB acc:   BM388758    UniGene:   Rn.44012    Gene Symbol:   Ercc3_predicted    Locus Link:   291703    Chromosome:   18    Cytoband:   Chr:18p12    SOURCE    GENECARD       Gene Ontology:   cellular component:  nucleus   cellular component:  transcription factor TFIIH complex      BioCarta Pathways:     KEGG Pathways:  
    Probe set : 1388132_at    Name:   NonO/p54nrb homolog    GB acc:   BE107056    UniGene:   Rn.54645    Gene Symbol:   Sfpq    Locus Link:   252855    Chromosome:   5    Cytoband:   Chr:5q36    SOURCE    GENECARD       Gene Ontology:   molecular function:  nucleic acid binding      BioCarta Pathways:     KEGG Pathways:  
    Probe set : 1381969_at    Name:   Transcribed locus    GB acc:   AA998296    UniGene:   Rn.3958        SOURCE        Gene Ontology:     BioCarta Pathways:     KEGG Pathways:  
    Probe set : 1392755_at    Name:   similar to hypothetical protein AD158 (predicted)    GB acc:   AW920702    UniGene:   Rn.25285    Gene Symbol:   RGD1306585_predicted    Locus Link:   289443    Chromosome:   14    Cytoband:   Chr:14p22    SOURCE    GENECARD       Gene Ontology:   cellular component:  integral to membrane      BioCarta Pathways:     KEGG Pathways:  
    Probe set : 1373740_at    Name:   Transcribed locus, weakly similar to XP_418630.1 PREDICTED: similar to ORF2 [Gallus gallus]    GB acc:   AA851385    UniGene:   Rn.104582        SOURCE        Gene Ontology:     BioCarta Pathways:     KEGG Pathways:  
    Probe set : 1389655_at    Name:   similar to A230072I16Rik protein (predicted)    GB acc:   AA892330    UniGene:   Rn.13456    Gene Symbol:   RGD1306819_predicted    Locus Link:   287593    Chromosome:   10    Cytoband:   Chr:10q26    SOURCE    GENECARD       Gene Ontology:   biological process:  apoptosis   cellular component:  mitochondrion   molecular function:  hydrolase activity      BioCarta Pathways:     KEGG Pathways:  
    Probe set : 1374395_at    Name:   Transcribed locus    GB acc:   AI102825    UniGene:   Rn.114978        SOURCE        Gene Ontology:     BioCarta Pathways:     KEGG Pathways:  
    Probe set : 1392514_at    Name:   brix domain containing 1 (predicted)    GB acc:   BE095565    UniGene:   Rn.25279    Gene Symbol:   Bxdc1_predicted    Locus Link:   294436    Chromosome:   20    Cytoband:   Chr:20q12    SOURCE    GENECARD       Gene Ontology:   cellular component:  nucleus      BioCarta Pathways:     KEGG Pathways:  
    Probe set : 1382100_at    Name:   similar to RIKEN cDNA 2310047O13 (predicted)    GB acc:   AA997169    UniGene:   Rn.106046    Gene Symbol:   RGD1309605_predicted    Locus Link:   291320    Chromosome:   17    Cytoband:   Chr:17q12.3    SOURCE    GENECARD       Gene Ontology:     BioCarta Pathways:     KEGG Pathways:  
    Probe set : 1387651_at    Name:   aquaporin 1    GB acc:   L07268    UniGene:   Rn.1618    Gene Symbol:   Aqp1    Locus Link:   25240    Chromosome:   4    Cytoband:   Chr:4q24    SOURCE    GENECARD       Gene Ontology:   biological process:  transport   biological process:  water transport   biological process:  excretion      BioCarta Pathways:     KEGG Pathways:  
    Probe set : 1388917_at    Name:   myosin IC    GB acc:   BI279786    UniGene:   Rn.10376    Gene Symbol:   Myo1c    Locus Link:   25485    Chromosome:   10    Cytoband:   Chr:10q26    SOURCE    GENECARD       Gene Ontology:   biological process:  transport   biological process:  cytoskeleton organization and biogenesis   cellular component:  myosin   cellular component:  myosin   cellular component:  unconventional myosin   cellular component:  myosin I      BioCarta Pathways:     KEGG Pathways:  
    Probe set : 1370138_at    Name:   lymphoid enhancer binding factor 1    GB acc:   NM_130429    UniGene:   Rn.21926    Gene Symbol:   Lef1    Locus Link:   161452    Chromosome:   2    Cytoband:   Chr:2q34-q45    SOURCE    GENECARD       Gene Ontology:   cellular component:  nucleus   cellular component:  transcription factor complex   cellular component:  cytoplasm   molecular function:  DNA binding   molecular function:  transcription factor activity   molecular function:  protein binding      BioCarta Pathways:     KEGG Pathways:  
    Probe set : 1367452_at    Name:   SMT3 suppressor of mif two 3 homolog 2 (yeast)    GB acc:   NM_133594    UniGene:   Rn.5958    Gene Symbol:   Sumo2    Locus Link:   171127    Chromosome:   5    Cytoband:   Chr:5q11    SOURCE    GENECARD       Gene Ontology:   biological process:  protein modification   biological process:  ubiquitin cycle      BioCarta Pathways:     KEGG Pathways:  
    Probe set : 1384273_at    Name:   Carbohydrate kinase-like (predicted)    GB acc:   BF419310    UniGene:   Rn.19663     Locus Link:   287479    Chromosome:   10    Cytoband:   Chr:10q24    SOURCE        Gene Ontology:   biological process:  carbohydrate metabolism   molecular function:  kinase activity   molecular function:  transferase activity      BioCarta Pathways:     KEGG Pathways:  
    Probe set : 1396292_at    Name:   Similar to peptide N-glycanase    GB acc:   BG379069    UniGene:   Rn.108552     Locus Link:   361014    Chromosome:   15    Cytoband:   Chr:15p16    SOURCE        Gene Ontology:     BioCarta Pathways:     KEGG Pathways:  
    Probe set : 1372157_at    Name:   similar to CGI-143 protein (predicted)    GB acc:   AI232807    UniGene:   Rn.13864    Gene Symbol:   RGD1310586_predicted    Locus Link:   365875    Chromosome:   2    Cytoband:   Chr:2q34    SOURCE    GENECARD       Gene Ontology:     BioCarta Pathways:     KEGG Pathways:  
    Probe set : 1389440_at    Name:   Transcribed locus    GB acc:   AI178476    UniGene:   Rn.16546        SOURCE        Gene Ontology:     BioCarta Pathways:     KEGG Pathways:  
    Probe set : 1370313_at    Name:   brain acyl-CoA hydrolase    GB acc:   D88891    UniGene:   Rn.6024    Gene Symbol:   Bach    Locus Link:   26759    Chromosome:   5    Cytoband:   Chr:5q36    SOURCE    GENECARD       Gene Ontology:   biological process:  lipid metabolism   biological process:  fatty acid metabolism   biological process:  fatty acid catabolism   cellular component:  cytoplasm   cellular component:  cytosol   cellular component:  cytosol      BioCarta Pathways:     KEGG Pathways:  
    Probe set : 1383117_at    Name:   peroxisomal membrane protein 4    GB acc:   AI232414    UniGene:   Rn.7752    Gene Symbol:   Pxmp4    Locus Link:   282634    Chromosome:   3    Cytoband:   Chr:3q41    SOURCE    GENECARD       Gene Ontology:   cellular component:  peroxisome   cellular component:  integral to membrane      BioCarta Pathways:     KEGG Pathways:  
    Probe set : 1390531_at    Name:   Similar to Peroxisomal proliferator-activated receptor A interacting complex 285 kDa protein (PPAR-alpha interacting complex protein 285) (predicted)    GB acc:   BE098021    UniGene:   Rn.47450     Locus Link:   296474    Chromosome:   3    Cytoband:   Chr:3q43    SOURCE        Gene Ontology:     BioCarta Pathways:     KEGG Pathways:  
    Probe set : 1389111_at    Name:   Transcribed locus    GB acc:   BF396316    UniGene:   Rn.17462        SOURCE        Gene Ontology:     BioCarta Pathways:     KEGG Pathways:  
    Probe set : 1382616_at    Name:   glutaminase    GB acc:   AI177367    UniGene:   Rn.5762    Gene Symbol:   Gls    Locus Link:   24398    Chromosome:   9    Cytoband:   Chr:9q31-q32    SOURCE    GENECARD       Gene Ontology:   biological process:  glutamine metabolism   biological process:  glutamine catabolism   biological process:  glutamine catabolism   cellular component:  mitochondrion   cellular component:  mitochondrion   cellular component:  mitochondrial inner membrane   molecular function:  glutaminase activity   molecular function:  glutaminase activity   molecular function:  hydrolase activity      BioCarta Pathways:     KEGG Pathways:  
    Probe set : 1368507_at    Name:   proteasome (prosome, macropain) subunit, alpha type 3    GB acc:   NM_017280    UniGene:   Rn.3997    Gene Symbol:   Psma3    Locus Link:   29670    Chromosome:   6    Cytoband:   Chr:6q24    SOURCE    GENECARD       Gene Ontology:   biological process:  ubiquitin-dependent protein catabolism   cellular component:  cytosol   cellular component:  proteasome core complex (sensu Eukaryota)   cellular component:  protein complex      BioCarta Pathways:     KEGG Pathways:  
    Probe set : 1379499_at    Name:   lymphotoxin B    GB acc:   BF282228    UniGene:   Rn.16681    Gene Symbol:   RGD:1303162    Locus Link:   361795    Chromosome:   20    Cytoband:   Chr:20p12    SOURCE    GENECARD       Gene Ontology:   biological process:  immune response   cellular component:  membrane   molecular function:  tumor necrosis factor receptor binding      BioCarta Pathways:     KEGG Pathways:  
    Probe set : 1388756_at    Name:   similar to RIKEN cDNA 6330579B17 gene (predicted)    GB acc:   AI231606    UniGene:   Rn.15017    Gene Symbol:   RGD1311399_predicted    Locus Link:   298490    Chromosome:   5    Cytoband:   Chr:5q36    SOURCE    GENECARD       Gene Ontology:   molecular function:  ligase activity      BioCarta Pathways:     KEGG Pathways:  
    Probe set : 1370071_at    Name:   adenosine deaminase    GB acc:   NM_130399    UniGene:   Rn.12689    Gene Symbol:   Ada    Locus Link:   24165    Chromosome:   3    Cytoband:   Chr:3q42    SOURCE    GENECARD       Gene Ontology:   cellular component:  cytoplasm      BioCarta Pathways:     KEGG Pathways:  
    Probe set : 1368910_at    Name:   protein phosphatase 2C, magnesium dependent, catalytic subunit    GB acc:   NM_019372    UniGene:   Rn.31799    Gene Symbol:   Ppm2c    Locus Link:   54705    Chromosome:   5    Cytoband:   Chr:5q13    SOURCE    GENECARD       Gene Ontology:   biological process:  protein amino acid dephosphorylation   cellular component:  mitochondrion   cellular component:  mitochondrial matrix   cellular component:  protein serine/threonine phosphatase complex      BioCarta Pathways:     KEGG Pathways:  
    Probe set : 1389903_at    Name:   pituitary tumor-transforming 1 interacting protein (predicted)    GB acc:   AA799616    UniGene:   Rn.4248    Gene Symbol:   Pttg1ip_predicted    Locus Link:   365548    Chromosome:   20    Cytoband:   Chr:20p12    SOURCE    GENECARD       Gene Ontology:   biological process:  protein import into nucleus   biological process:  development      BioCarta Pathways:     KEGG Pathways:  
    Probe set : 1376110_at    Name:   ribonuclease P 25 subunit (human) (predicted)    GB acc:   AI009623    UniGene:   Rn.13924    Gene Symbol:   Rpp25_predicted    Locus Link:   315705    Chromosome:   8    Cytoband:   Chr:8q24    SOURCE    GENECARD       Gene Ontology:   molecular function:  nucleic acid binding      BioCarta Pathways:     KEGG Pathways:  
    Probe set : 1370167_at    Name:   syndecan 2    GB acc:   BG668421    UniGene:   Rn.11127    Gene Symbol:   Sdc2    Locus Link:   25615    Chromosome:   7    Cytoband:   Chr:7q22    SOURCE    GENECARD       Gene Ontology:   cellular component:  integral to plasma membrane   cellular component:  membrane   cellular component:  integral to membrane   molecular function:  cytoskeletal protein binding      BioCarta Pathways:     KEGG Pathways:  
    Probe set : 1387158_at    Name:   meprin 1 beta    GB acc:   NM_013183    UniGene:   Rn.3520    Gene Symbol:   Mep1b    Locus Link:   25727      SOURCE    GENECARD       Gene Ontology:   biological process:  proteolysis   biological process:  proteolysis   biological process:  digestion      BioCarta Pathways:     KEGG Pathways:  
    Probe set : 1368548_at    Name:   solute carrier family 12, member 1    GB acc:   NM_019134    UniGene:   Rn.14799    Gene Symbol:   Slc12a1    Locus Link:   25065    Chromosome:   3    Cytoband:   Chr:3q36    SOURCE    GENECARD       Gene Ontology:     BioCarta Pathways:     KEGG Pathways:  
    Probe set : 1373807_at    Name:   Vascular endothelial growth factor A    GB acc:   AI175732    UniGene:   Rn.1923    Gene Symbol:   Vegf    Locus Link:   83785    Chromosome:   9    Cytoband:   Chr:9q12    SOURCE    GENECARD       Gene Ontology:   biological process:  regulation of progression through cell cycle   biological process:  angiogenesis   biological process:  angiogenesis   biological process:  vasculogenesis   biological process:  anti-apoptosis   biological process:  response to stress   biological process:  signal transduction   biological process:  nervous system development   biological process:  nervous system development   biological process:  mesoderm development   biological process:  cell proliferation   biological process:  positive regulation of cell proliferation   biological process:  positive regulation of cell proliferation   biological process:  positive regulation of signal transduction   biological process:  cell migration   biological process:  positive regulation of vascular endothelial growth factor receptor signaling pathway   biological process:  negative regulation of apoptosis   biological process:  induction of positive chemotaxis   biological process:  development   biological process:  cell differentiation   biological process:  nervous system development      BioCarta Pathways:     KEGG Pathways:  
    Probe set : 1369686_at    Name:   double cortin and calcium/calmodulin-dependent protein kinase-like 1    GB acc:   U78857     Gene Symbol:   Dcamkl1    Locus Link:   83825    Chromosome:   2    Cytoband:   Chr:2q26     GENECARD       Gene Ontology:   cellular component:  integral to plasma membrane      BioCarta Pathways:     KEGG Pathways:  
    Probe set : 1378629_at    Name:   special AT-rich sequence binding protein 1 (predicted)    GB acc:   AI227638    UniGene:   Rn.21462    Gene Symbol:   Satb1_predicted    Locus Link:   316164    Chromosome:   9    Cytoband:   Chr:9q11    SOURCE    GENECARD       Gene Ontology:   cellular component:  nucleus   cellular component:  nuclear heterochromatin   molecular function:  DNA binding   molecular function:  double-stranded DNA binding   molecular function:  transcription factor activity      BioCarta Pathways:     KEGG Pathways:  
    Probe set : 1389833_at    Name:   Sulfatase modifying factor 1 (predicted)    GB acc:   BF285924    UniGene:   Rn.17376     Locus Link:   362409    Chromosome:   4    Cytoband:   Chr:4q41    SOURCE        Gene Ontology:   biological process:  glycosaminoglycan metabolism   cellular component:  endoplasmic reticulum      BioCarta Pathways:     KEGG Pathways:  
    Probe set : 1399154_at    Name:   F-box and leucine-rich repeat protein 11 (predicted)    GB acc:   BF389273    UniGene:   Rn.12228     Locus Link:   361700    Chromosome:   1    Cytoband:   Chr:1q42    SOURCE        Gene Ontology:   biological process:  regulation of transcription, DNA-dependent   biological process:  ubiquitin cycle   molecular function:  DNA binding   molecular function:  zinc ion binding      BioCarta Pathways:     KEGG Pathways:  
    Probe set : 1379561_at    Name:   ATPase family, AAA domain containing 1 (predicted)    GB acc:   AW433567    UniGene:   Rn.54918    Gene Symbol:   Atad1_predicted    Locus Link:   309532    Chromosome:   1    Cytoband:   Chr:1q52    SOURCE    GENECARD       Gene Ontology:   molecular function:  nucleotide binding   molecular function:  ATP binding   molecular function:  nucleoside-triphosphatase activity      BioCarta Pathways:     KEGG Pathways:  
    Probe set : 1388916_at    Name:   Transcribed locus    GB acc:   BE109616    UniGene:   Rn.17586        SOURCE        Gene Ontology:     BioCarta Pathways:     KEGG Pathways:  
    Probe set : 1388027_a_at    Name:   reticulon 4    GB acc:   AF051335    UniGene:   Rn.1348    Gene Symbol:   Rtn4    Locus Link:   83765    Chromosome:   14    Cytoband:   Chr:14q22    SOURCE    GENECARD       Gene Ontology:   molecular function:  protein binding   molecular function:  protein binding      BioCarta Pathways:     KEGG Pathways:  
    Probe set : 1371484_at     GB acc:   BM390252              Gene Ontology:     BioCarta Pathways:     KEGG Pathways:  
    Probe set : 1389902_at    Name:   histocompatibility 13 (predicted)    GB acc:   BI285090    UniGene:   Rn.12102    Gene Symbol:   H13_predicted    Locus Link:   311545    Chromosome:   3    Cytoband:   Chr:3q41    SOURCE    GENECARD       Gene Ontology:   cellular component:  endoplasmic reticulum   cellular component:  endoplasmic reticulum membrane   cellular component:  integral to membrane   molecular function:  peptidase activity   molecular function:  D-alanyl-D-alanine endopeptidase activity   molecular function:  hydrolase activity      BioCarta Pathways:     KEGG Pathways:  
    Probe set : 1388641_at    Name:   phosphoribosylglycinamide formyltransferase (predicted)    GB acc:   AI410319    UniGene:   Rn.55532    Gene Symbol:   Gart_predicted    Locus Link:   288259    Chromosome:   11    Cytoband:   Chr:11q11    SOURCE    GENECARD       Gene Ontology:   cellular component:  cytoplasm      BioCarta Pathways:     KEGG Pathways:  
    Probe set : 1389189_at    Name:   actinin, alpha 1    GB acc:   BF555956    UniGene:   Rn.6401    Gene Symbol:   Actn1    Locus Link:   81634    Chromosome:   6    Cytoband:   Chr:6q24    SOURCE    GENECARD       Gene Ontology:   cellular component:  cytoskeleton   cellular component:  actin cytoskeleton      BioCarta Pathways:     KEGG Pathways:  
    Probe set : 1387232_at    Name:   bone morphogenetic protein 4    GB acc:   NM_012827    UniGene:   Rn.10318    Gene Symbol:   Bmp4    Locus Link:   25296    Chromosome:   15    Cytoband:   Chr:15p14    SOURCE    GENECARD       Gene Ontology:   biological process:  skeletal development   biological process:  angiogenesis   biological process:  metanephros development   biological process:  ureteric bud development   biological process:  eye development (sensu Mammalia)   biological process:  germ cell development   biological process:  mesoderm development   biological process:  mesoderm development   biological process:  mesodermal cell fate determination   biological process:  heart development   biological process:  cell proliferation   biological process:  tissue development   biological process:  BMP signaling pathway   biological process:  forebrain development   biological process:  growth   biological process:  odontogenesis (sensu Vertebrata)   biological process:  cell fate commitment   biological process:  regulation of cell differentiation   biological process:  regulation of cell differentiation   biological process:  positive regulation of cell differentiation   biological process:  ossification   biological process:  development   biological process:  cell differentiation   biological process:  cartilage development   cellular component:  extracellular space      BioCarta Pathways:     KEGG Pathways:  
    Probe set : 1389032_at    Name:   mucolipin 1 (predicted)    GB acc:   AI408501    UniGene:   Rn.106454    Gene Symbol:   Mcoln1_predicted    Locus Link:   288371    Chromosome:   12    Cytoband:   Chr:12p12    SOURCE    GENECARD       Gene Ontology:   cellular component:  lysosome   cellular component:  late endosome   cellular component:  membrane   cellular component:  integral to membrane   molecular function:  ion channel activity   molecular function:  cation channel activity      BioCarta Pathways:     KEGG Pathways:  
    Probe set : 1383747_at    Name:   Ect2 oncogene (predicted)    GB acc:   AI578135    UniGene:   Rn.27564     Locus Link:   361921    Chromosome:   2    Cytoband:   Chr:2q24    SOURCE        Gene Ontology:   cellular component:  intracellular   cellular component:  nucleus   molecular function:  signal transducer activity   molecular function:  guanyl-nucleotide exchange factor activity      BioCarta Pathways:     KEGG Pathways:  
    Probe set : 1376005_at    Name:   Kinesin family member 1B    GB acc:   BE109334    UniGene:   Rn.6526    Gene Symbol:   Kif1b    Locus Link:   117548    Chromosome:   5    Cytoband:   Chr:5q36    SOURCE    GENECARD       Gene Ontology:   biological process:  microtubule-based process   biological process:  microtubule-based movement   biological process:  nerve-nerve synaptic transmission   biological process:  neuromuscular synaptic transmission   biological process:  anterograde axon cargo transport   biological process:  embryonic development   biological process:  microtubule-based movement   biological process:  nerve-nerve synaptic transmission   biological process:  neuromuscular synaptic transmission   biological process:  anterograde axon cargo transport   biological process:  embryonic development   biological process:  cytoskeleton-dependent intracellular transport      BioCarta Pathways:     KEGG Pathways:  
    Probe set : 1375197_at     GB acc:   BG665384              Gene Ontology:     BioCarta Pathways:     KEGG Pathways:  
    Probe set : 1390947_at    Name:   Similar to RIKEN cDNA D930036F22 gene    GB acc:   AA891255    UniGene:   Rn.7269     Locus Link:   362737    Chromosome:   6    Cytoband:   Chr:6q22    SOURCE        Gene Ontology:     BioCarta Pathways:     KEGG Pathways:  
    Probe set : 1390951_at    Name:   Transcribed locus    GB acc:   BE107735    UniGene:   Rn.9566        SOURCE        Gene Ontology:     BioCarta Pathways:     KEGG Pathways:  
    Probe set : 1383220_at    Name:   Transcribed locus    GB acc:   BE114231    UniGene:   Rn.22955        SOURCE        Gene Ontology:     BioCarta Pathways:     KEGG Pathways:  
    Probe set : 1367843_at    Name:   aldo-keto reductase family 7, member A2 (aflatoxin aldehyde reductase)    GB acc:   NM_134407    UniGene:   Rn.8548    Gene Symbol:   RGD:620311    Locus Link:   171445    Chromosome:   5    Cytoband:   Chr:5q36    SOURCE    GENECARD       Gene Ontology:   biological process:  carbohydrate metabolism   biological process:  aldehyde metabolism   cellular component:  mitochondrion   cellular component:  Golgi apparatus   cellular component:  Golgi apparatus   cellular component:  Golgi stack      BioCarta Pathways:     KEGG Pathways:  
    Probe set : 1371403_at    Name:   chaperonin subunit 3 (gamma)    GB acc:   AA799545    UniGene:   Rn.102261    Gene Symbol:   Cct3    Locus Link:   295230    Chromosome:   2    Cytoband:   Chr:2q34    SOURCE    GENECARD       Gene Ontology:   biological process:  chaperonin-mediated tubulin folding   biological process:  protein folding   biological process:  cellular protein metabolism      BioCarta Pathways:     KEGG Pathways:  
    Probe set : 1371986_at    Name:   Transcribed locus    GB acc:   AI576652    UniGene:   Rn.144053        SOURCE        Gene Ontology:     BioCarta Pathways:     KEGG Pathways:  
    Probe set : 1378365_at    Name:   Transcribed locus    GB acc:   AW529483    UniGene:   Rn.138007        SOURCE        Gene Ontology:     BioCarta Pathways:     KEGG Pathways:  
    Probe set : 1394484_at     GB acc:   AI170114              Gene Ontology:     BioCarta Pathways:     KEGG Pathways:  
    Probe set : 1369629_at    Name:   adenosine kinase    GB acc:   U90340    UniGene:   Rn.64165    Gene Symbol:   Adk    Locus Link:   25368    Chromosome:   15    Cytoband:   Chr:15p16    SOURCE    GENECARD       Gene Ontology:   biological process:  purine ribonucleoside salvage   biological process:  purine ribonucleoside salvage   biological process:  ribonucleoside monophosphate biosynthesis   cellular component:  nucleus      BioCarta Pathways:     KEGG Pathways:  
    Probe set : 1391538_at    Name:   cell death-inducing DNA fragmentation factor, alpha subunit-like effector B (predicted)    GB acc:   AI137938    UniGene:   Rn.145275    Gene Symbol:   Cideb_predicted    Locus Link:   364388    Chromosome:   15    Cytoband:   Chr:15p13    SOURCE    GENECARD       Gene Ontology:   cellular component:  intracellular   cellular component:  cytosol   molecular function:  protein binding      BioCarta Pathways:     KEGG Pathways:  
    Probe set : 1368934_at    Name:   cytochrome P450, family 4, subfamily a, polypeptide 10 /// cytochrome P450, family 4, subfamily A, polypeptide 22    GB acc:   NM_016999    UniGene:   Rn.92401    Gene Symbol:   Cyp4a10 /// Cyp4a22    Locus Link:   170544 /// 50549    Chromosome:   5    Cytoband:   Chr:5q36    SOURCE    GENECARD       Gene Ontology:   biological process:  electron transport      BioCarta Pathways:     KEGG Pathways:  
    Probe set : 1391855_at    Name:   B-cell CLL/lymphoma 7A (predicted)    GB acc:   AW534002    UniGene:   Rn.9084     Locus Link:   360809    Chromosome:   12    Cytoband:   Chr:12q16    SOURCE        Gene Ontology:   molecular function:  actin binding      BioCarta Pathways:     KEGG Pathways:  
    Probe set : 1371318_at    Name:   ribosomal protein S16    GB acc:   BG666872    UniGene:   Rn.144646    Gene Symbol:   Rps16    Locus Link:   140655    Chromosome:   1    Cytoband:   Chr:1q21    SOURCE    GENECARD       Gene Ontology:   biological process:  protein biosynthesis   biological process:  ribosome biogenesis   molecular function:  RNA binding   molecular function:  structural constituent of ribosome      BioCarta Pathways:     KEGG Pathways:  
    Probe set : 1392515_at     GB acc:   AI177403    UniGene:   Rn.129033    Gene Symbol:   RGD:1359643 /// RGD:1359487 /// RGD:1359240 /// RGD:1359669 /// RGD:1359210 /// LOC502904 /// LOC502905 /// LOC502906 /// LOC502907    Locus Link:   494205 /// 494206 /// 494207 /// 494209 /// 494211 /// 502904 /// 502905 /// 502906 /// 502907    Chromosome:   4    Cytoband:   Chr:4q42    SOURCE    GENECARD       Gene Ontology:   molecular function:  sugar binding   molecular function:  receptor activity      BioCarta Pathways:     KEGG Pathways:  
    Probe set : 1398765_at    Name:   adaptor-related protein complex 2, mu 1 subunit    GB acc:   NM_053837    UniGene:   Rn.3172    Gene Symbol:   Ap2m1    Locus Link:   116563    Chromosome:   11    Cytoband:   Chr:11q23    SOURCE    GENECARD       Gene Ontology:     BioCarta Pathways:     KEGG Pathways:  
    Probe set : 1378520_at    Name:   B-cell leukemia/lymphoma 11B (predicted)    GB acc:   BF398531    UniGene:   Rn.52748    Gene Symbol:   Bcl11b_predicted    Locus Link:   314423    Chromosome:   6    Cytoband:   Chr:6q32    SOURCE    GENECARD       Gene Ontology:   biological process:  regulation of transcription, DNA-dependent   biological process:  negative regulation of cell proliferation   cellular component:  nucleus   molecular function:  nucleic acid binding   molecular function:  zinc ion binding      BioCarta Pathways:     KEGG Pathways:  
    Probe set : 1373909_at     GB acc:   AI175507              Gene Ontology:     BioCarta Pathways:     KEGG Pathways:  
    Probe set : 1391240_at     GB acc:   AI013954              Gene Ontology:     BioCarta Pathways:     KEGG Pathways:  
    Probe set : 1367741_at    Name:   homocysteine-inducible, endoplasmic reticulum stress-inducible, ubiquitin-like domain member 1    GB acc:   NM_053523    UniGene:   Rn.4028    Gene Symbol:   Herpud1    Locus Link:   85430    Chromosome:   19    Cytoband:   Chr:19p12    SOURCE    GENECARD       Gene Ontology:   biological process:  protein modification   biological process:  response to stress   biological process:  response to unfolded protein   cellular component:  endoplasmic reticulum   cellular component:  endoplasmic reticulum membrane   cellular component:  integral to membrane      BioCarta Pathways:     KEGG Pathways:  
    Probe set : 1368190_at    Name:   renin 1    GB acc:   J02941    UniGene:   Rn.9831    Gene Symbol:   Ren1    Locus Link:   24715    Chromosome:   13    Cytoband:   Chr:13q13    SOURCE    GENECARD       Gene Ontology:   cellular component:  extracellular space      BioCarta Pathways:     KEGG Pathways:  
    Probe set : 1375411_at    Name:   NADH dehydrogenase (ubiquinone) 1 alpha subcomplex, 7 (B14.5a) (predicted)    GB acc:   BI277002    UniGene:   Rn.3422    Gene Symbol:   Ndufa7_predicted    Locus Link:   299643    Chromosome:   7    Cytoband:   Chr:7q12    SOURCE    GENECARD       Gene Ontology:   biological process:  ATP synthesis coupled electron transport   molecular function:  NADH dehydrogenase activity   molecular function:  NADH dehydrogenase (ubiquinone) activity   molecular function:  oxidoreductase activity      BioCarta Pathways:     KEGG Pathways:  
    Probe set : 1378293_at    Name:   tripartite motif protein 26    GB acc:   AI556246    UniGene:   Rn.38919    Gene Symbol:   RGD:1359126    Locus Link:   309586    Chromosome:   20    Cytoband:   Chr:20p12    SOURCE    GENECARD       Gene Ontology:     BioCarta Pathways:     KEGG Pathways:  
    Probe set : 1395697_at    Name:   similar to Ezh2 protein    GB acc:   BF561171    UniGene:   Rn.9027    Gene Symbol:   LOC312299    Locus Link:   312299    Chromosome:   4    Cytoband:   Chr:4q24    SOURCE    GENECARD       Gene Ontology:     BioCarta Pathways:     KEGG Pathways:  
    Probe set : 1371939_at    Name:   GPI-anchored membrane protein 1 (predicted)    GB acc:   AI171369    UniGene:   Rn.108072    Gene Symbol:   Gpiap1_predicted    Locus Link:   362173    Chromosome:   3    Cytoband:   Chr:3q32    SOURCE    GENECARD       Gene Ontology:   cellular component:  integral to plasma membrane      BioCarta Pathways:     KEGG Pathways:  
    Probe set : 1372949_at    Name:   Septin 6 (predicted)    GB acc:   BG663067    UniGene:   Rn.27798     Locus Link:   305227    Chromosome:   14    Cytoband:   Chr:14p22    SOURCE        Gene Ontology:   biological process:  cytokinesis   biological process:  cell cycle   molecular function:  protein binding   molecular function:  GTP binding      BioCarta Pathways:     KEGG Pathways:  
    Probe set : 1372071_at    Name:   similar to 8D6 antigen (predicted)    GB acc:   AI102320    UniGene:   Rn.797    Gene Symbol:   RGD1305860_predicted    Locus Link:   362851    Chromosome:   7    Cytoband:   Chr:7q12    SOURCE    GENECARD       Gene Ontology:   biological process:  development   cellular component:  integral to membrane      BioCarta Pathways:     KEGG Pathways:  
    Probe set : 1392531_at    Name:   Transcribed locus    GB acc:   AI178693    UniGene:   Rn.106867        SOURCE        Gene Ontology:     BioCarta Pathways:     KEGG Pathways:  
    Probe set : 1376263_at    Name:   similar to 1810034B16Rik protein (predicted)    GB acc:   BG378672    UniGene:   Rn.50391    Gene Symbol:   RGD1306222_predicted    Locus Link:   287151    Chromosome:   10    Cytoband:   Chr:10q12    SOURCE    GENECARD       Gene Ontology:   biological process:  nervous system development   biological process:  glial cell differentiation   biological process:  positive regulation of axonogenesis   cellular component:  extracellular space      BioCarta Pathways:     KEGG Pathways:  
    Probe set : 1372869_at    Name:   similar to GTP-binding protein NGB    GB acc:   BE109202     Gene Symbol:   LOC498786    Locus Link:   498786    Chromosome:   17    Cytoband:   Chr:17q12.1     GENECARD       Gene Ontology:     BioCarta Pathways:     KEGG Pathways:  
    Probe set : 1371599_at    Name:   LRRGT00097    GB acc:   AI228587    UniGene:   Rn.116613     Locus Link:   499407      SOURCE        Gene Ontology:     BioCarta Pathways:     KEGG Pathways:  
    Probe set : 1383960_at    Name:   peroxisome biogenesis factor 16 (predicted)    GB acc:   BG376773    UniGene:   Rn.98695    Gene Symbol:   Pex16_predicted    Locus Link:   311203    Chromosome:   3    Cytoband:   Chr:3q24    SOURCE    GENECARD       Gene Ontology:   biological process:  peroxisome organization and biogenesis   cellular component:  peroxisome   cellular component:  integral to peroxisomal membrane      BioCarta Pathways:     KEGG Pathways:  
    Probe set : 1374208_at    Name:   zinc finger CCCH type domain containing 7 (predicted)    GB acc:   BE116569    UniGene:   Rn.15835    Gene Symbol:   Zc3hdc7_predicted    Locus Link:   360466    Chromosome:   10    Cytoband:   Chr:10q11    SOURCE    GENECARD       Gene Ontology:   cellular component:  nucleus   molecular function:  nucleic acid binding   molecular function:  zinc ion binding      BioCarta Pathways:     KEGG Pathways:  
    Probe set : 1371581_at    Name:   Vascular endothelial zinc finger 1 (predicted)    GB acc:   BM388899    UniGene:   Rn.84878     Locus Link:   287615    Chromosome:   10    Cytoband:   Chr:10q26    SOURCE        Gene Ontology:   biological process:  regulation of transcription from RNA polymerase II promoter   biological process:  cellular defense response   cellular component:  nucleus      BioCarta Pathways:     KEGG Pathways:  
    Probe set : 1369950_at    Name:   cyclin-dependent kinase 4    GB acc:   NM_053593    UniGene:   Rn.6115    Gene Symbol:   Cdk4    Locus Link:   94201    Chromosome:   7    Cytoband:   Chr:7q22    SOURCE    GENECARD       Gene Ontology:   cellular component:  cyclin-dependent protein kinase holoenzyme complex   cellular component:  nucleus   cellular component:  transcription factor complex      BioCarta Pathways:     KEGG Pathways:  
    Probe set : 1399095_at    Name:   SMT3 suppressor of mif two 3 homolog 1 (yeast) (predicted)    GB acc:   AI407918    UniGene:   Rn.1221    Gene Symbol:   Sumo1_predicted    Locus Link:   301442    Chromosome:   9    Cytoband:   Chr:9q31    SOURCE    GENECARD       Gene Ontology:   biological process:  DNA repair   biological process:  protein modification   biological process:  ubiquitin cycle   cellular component:  nucleus   cellular component:  nuclear pore   cellular component:  PML body   molecular function:  protein binding      BioCarta Pathways:     KEGG Pathways:  
    Probe set : 1397535_at    Name:   membrane protein, palmitoylated 5 (MAGUK p55 subfamily member 5) (predicted)    GB acc:   BF555544    UniGene:   Rn.8181    Gene Symbol:   Mpp5_predicted    Locus Link:   314259    Chromosome:   6    Cytoband:   Chr:6q24    SOURCE    GENECARD       Gene Ontology:   cellular component:  plasma membrane   cellular component:  membrane   cellular component:  integral to membrane   molecular function:  guanylate kinase activity   molecular function:  protein binding      BioCarta Pathways:     KEGG Pathways:  
    Probe set : 1379801_at    Name:   LOC499790    GB acc:   AI137954    UniGene:   Rn.23792     Locus Link:   499790      SOURCE        Gene Ontology:     BioCarta Pathways:     KEGG Pathways:  
    Probe set : 1367586_at    Name:   lactate dehydrogenase A    GB acc:   NM_017025    UniGene:   Rn.107896    Gene Symbol:   Ldha    Locus Link:   24533    Chromosome:   1    Cytoband:   Chr:1q22    SOURCE    GENECARD       Gene Ontology:   biological process:  glycolysis   biological process:  tricarboxylic acid cycle intermediate metabolism   biological process:  anaerobic glycolysis   cellular component:  cytosol   cellular component:  cytoplasm   molecular function:  lactate dehydrogenase activity   molecular function:  L-lactate dehydrogenase activity   molecular function:  oxidoreductase activity      BioCarta Pathways:     KEGG Pathways:  
    Probe set : 1392486_at    Name:   similar to RIKEN cDNA 2310036D22    GB acc:   AW433977    UniGene:   Rn.106000    Gene Symbol:   RGD:1303037    Locus Link:   312132    Chromosome:   4    Cytoband:   Chr:4q21    SOURCE    GENECARD       Gene Ontology:     BioCarta Pathways:     KEGG Pathways:  
    Probe set : 1373533_at     GB acc:   AI412460              Gene Ontology:     BioCarta Pathways:     KEGG Pathways:  
    Probe set : 1377898_at    Name:   solute carrier family 39 (metal ion transporter), member 5 (predicted)    GB acc:   AI169113    UniGene:   Rn.23867    Gene Symbol:   Slc39a5_predicted    Locus Link:   362812    Chromosome:   7    Cytoband:   Chr:7q11    SOURCE    GENECARD       Gene Ontology:   biological process:  metal ion transport   cellular component:  integral to membrane   molecular function:  metal ion transporter activity      BioCarta Pathways:     KEGG Pathways:  
    Probe set : 1368265_at    Name:   cytochrome P450 monooxygenase CYP2T1    GB acc:   NM_134369    UniGene:   Rn.144661    Gene Symbol:   RGD:620086    Locus Link:   171380    Chromosome:   1    Cytoband:   Chr:1q21    SOURCE    GENECARD       Gene Ontology:   biological process:  electron transport   cellular component:  membrane      BioCarta Pathways:     KEGG Pathways:  
    Probe set : 1372173_at    Name:   similar to U5 snRNP-specific protein (Prp8-binding) (predicted)    GB acc:   AI407945    UniGene:   Rn.15351    Gene Symbol:   RGD1309198_predicted    Locus Link:   313056    Chromosome:   5    Cytoband:   Chr:5q36    SOURCE    GENECARD       Gene Ontology:     BioCarta Pathways:     KEGG Pathways:  
    Probe set : 1386895_at    Name:   melanoma antigen, family D, 1    GB acc:   NM_053409    UniGene:   Rn.54596    Gene Symbol:   Maged1    Locus Link:   84469    Chromosome:   X    Cytoband:   Chr:Xq31    SOURCE    GENECARD       Gene Ontology:   biological process:  regulation of transcription from RNA polymerase II promoter   molecular function:  transcription coactivator activity   molecular function:  protein binding      BioCarta Pathways:     KEGG Pathways:  
    Probe set : 1374646_at    Name:   Casein kinase 1, alpha 1    GB acc:   BM387173    UniGene:   Rn.23810    Gene Symbol:   Csnk1a1    Locus Link:   113927    Chromosome:   18    Cytoband:   Chr:18q12.1    SOURCE    GENECARD       Gene Ontology:   biological process:  protein amino acid phosphorylation   biological process:  Wnt receptor signaling pathway      BioCarta Pathways:     KEGG Pathways:  
    Probe set : 1371389_at    Name:   hypothetical LOC306766    GB acc:   AI170668    UniGene:   Rn.105613    Gene Symbol:   RGD:1359256    Locus Link:   306766    Chromosome:   17    Cytoband:   Chr:17p14    SOURCE    GENECARD       Gene Ontology:     BioCarta Pathways:     KEGG Pathways:  
    Probe set : 1371478_at    Name:   similar to RIKEN cDNA 1110008F13 (predicted)    GB acc:   AI409756    UniGene:   Rn.11832    Gene Symbol:   RGD1307752_predicted    Locus Link:   296315    Chromosome:   3    Cytoband:   Chr:3q42    SOURCE    GENECARD       Gene Ontology:   cellular component:  integral to membrane      BioCarta Pathways:     KEGG Pathways:  
    Probe set : 1368607_at    Name:   cytochrome P450, 4a12    GB acc:   NM_031605    UniGene:   Rn.144685    Gene Symbol:   RGD:628846    Locus Link:   266674    Chromosome:   5    Cytoband:   Chr:5q36    SOURCE    GENECARD       Gene Ontology:   biological process:  electron transport   cellular component:  endoplasmic reticulum   cellular component:  microsome   cellular component:  membrane      BioCarta Pathways:     KEGG Pathways:  
    Probe set : 1389788_at    Name:   hypothetical protein LOC293950    GB acc:   AW522732    UniGene:   Rn.103625    Gene Symbol:   RGD1307336_predicted    Locus Link:   293950    Chromosome:   1    Cytoband:   Chr:1q54    SOURCE    GENECARD       Gene Ontology:     BioCarta Pathways:     KEGG Pathways:  
    Probe set : 1373715_at    Name:   similar to hypothetical protein DKFZp434K1815    GB acc:   BI274516    UniGene:   Rn.144942    Gene Symbol:   RGD:1359442    Locus Link:   304396    Chromosome:   12    Cytoband:   Chr:12q12    SOURCE    GENECARD       Gene Ontology:     BioCarta Pathways:     KEGG Pathways:  
    Probe set : 1382452_at    Name:   serum deprivation response protein    GB acc:   AI603408    UniGene:   Rn.2472    Gene Symbol:   RGD:1359345    Locus Link:   316384    Chromosome:   9    Cytoband:   Chr:9q22    SOURCE    GENECARD       Gene Ontology:     BioCarta Pathways:     KEGG Pathways:  
    Probe set : 1373337_at    Name:   glyoxylate reductase/hydroxypyruvate reductase (predicted)    GB acc:   AI412065    UniGene:   Rn.7815    Gene Symbol:   Grhpr_predicted    Locus Link:   298085    Chromosome:   5    Cytoband:   Chr:5q22    SOURCE    GENECARD       Gene Ontology:   biological process:  L-serine biosynthesis   biological process:  metabolism      BioCarta Pathways:     KEGG Pathways:  
    Probe set : 1374994_at    Name:   Nei endonuclease VIII-like 1 (E. coli) (predicted)    GB acc:   BF394281    UniGene:   Rn.70614     Locus Link:   367090    Chromosome:   8    Cytoband:   Chr:8q24    SOURCE        Gene Ontology:   biological process:  carbohydrate metabolism   biological process:  DNA repair   biological process:  response to DNA damage stimulus   cellular component:  nucleus      BioCarta Pathways:     KEGG Pathways:  
    Probe set : 1377135_at     GB acc:   AA944958              Gene Ontology:     BioCarta Pathways:     KEGG Pathways:  
    Probe set : 1369412_a_at    Name:   solute carrier family 19, member 1    GB acc:   NM_017299    UniGene:   Rn.9042    Gene Symbol:   Slc19a1    Locus Link:   29723    Chromosome:   20    Cytoband:   Chr:20p12    SOURCE    GENECARD       Gene Ontology:   biological process:  transport   biological process:  folic acid transport      BioCarta Pathways:     KEGG Pathways:  
    Probe set : 1389760_at    Name:   ring finger protein 134 (predicted)    GB acc:   AA858786    UniGene:   Rn.16897    Gene Symbol:   Rnf134_predicted    Locus Link:   309457    Chromosome:   1    Cytoband:   Chr:1q54    SOURCE    GENECARD       Gene Ontology:   cellular component:  nucleus   cellular component:  ubiquitin ligase complex      BioCarta Pathways:     KEGG Pathways:  
    Probe set : 1393203_at    Name:   similar to RIKEN cDNA 2010309E21 (predicted)    GB acc:   BG665886    UniGene:   Rn.2397    Gene Symbol:   RGD1304825_predicted    Locus Link:   297415    Chromosome:   4    Cytoband:   Chr:4q34    SOURCE    GENECARD       Gene Ontology:     BioCarta Pathways:     KEGG Pathways:  
    Probe set : 1373683_at    Name:   fyn proto-oncogene    GB acc:   AI230396    UniGene:   Rn.19361    Gene Symbol:   Fyn    Locus Link:   25150    Chromosome:   20    Cytoband:   Chr:20q13    SOURCE    GENECARD       Gene Ontology:   cellular component:  intracellular   cellular component:  mitochondrial membrane      BioCarta Pathways:     KEGG Pathways:  
    Probe set : 1395007_at    Name:   Transcribed locus    GB acc:   BE120572    UniGene:   Rn.151226        SOURCE        Gene Ontology:     BioCarta Pathways:     KEGG Pathways:  
    Probe set : 1372776_at    Name:   F-box and leucine-rich repeat protein 5 (predicted)    GB acc:   BM392017    UniGene:   Rn.8259    Gene Symbol:   Fbxl5_predicted    Locus Link:   305424    Chromosome:   14    Cytoband:   Chr:14q21    SOURCE    GENECARD       Gene Ontology:   biological process:  ubiquitin cycle   biological process:  transport   molecular function:  oxygen transporter activity   molecular function:  iron ion binding      BioCarta Pathways:     KEGG Pathways:  
    Probe set : 1372592_at    Name:   histone deacetylase 6    GB acc:   AI411391    UniGene:   Rn.13453    Gene Symbol:   Hdac6    Locus Link:   84581    Chromosome:   X    Cytoband:   Chr:Xq13    SOURCE    GENECARD       Gene Ontology:     BioCarta Pathways:     KEGG Pathways:  
    Probe set : 1373335_at    Name:   similar to zinc finger, DHHC domain containing 9    GB acc:   BE349808    UniGene:   Rn.15254    Gene Symbol:   LOC302808    Locus Link:   302808    Chromosome:   X    Cytoband:   Chr:Xq35    SOURCE    GENECARD       Gene Ontology:     BioCarta Pathways:     KEGG Pathways:  
    Probe set : 1383776_at    Name:   Transcribed locus    GB acc:   AW525120    UniGene:   Rn.35353        SOURCE        Gene Ontology:     BioCarta Pathways:     KEGG Pathways:  
    Probe set : 1377890_at    Name:   Transcribed locus    GB acc:   AI411360    UniGene:   Rn.41920        SOURCE        Gene Ontology:     BioCarta Pathways:     KEGG Pathways:  
    Probe set : 1389815_at    Name:   protein phosphatase 1, regulatory (inhibitor) subunit 14B    GB acc:   BF547738    UniGene:   Rn.2773    Gene Symbol:   Ppp1r14b    Locus Link:   259225    Chromosome:   1    Cytoband:   Chr:1q43    SOURCE    GENECARD       Gene Ontology:   molecular function:  protein phosphatase inhibitor activity   molecular function:  protein phosphatase type 1 regulator activity   molecular function:  phosphatase inhibitor activity      BioCarta Pathways:     KEGG Pathways:  
    Probe set : 1399025_at    Name:   Similar to hypothetical protein MGC38950 (predicted)    GB acc:   AW916140    UniGene:   Rn.36542     Locus Link:   313418    Chromosome:   5    Cytoband:   Chr:5q34    SOURCE        Gene Ontology:     BioCarta Pathways:     KEGG Pathways:  
    Probe set : 1391091_at    Name:   retinitis pigmentosa 1 (autosomal dominant) (predicted) /// similar to cylindromatosis (turban tumor syndrome)    GB acc:   AI406286    UniGene:   Rn.128760    Gene Symbol:   Rp1_predicted /// LOC361373    Locus Link:   312937 /// 361373    Chromosome:   5    Cytoband:   Chr:5q12 /// Chr:19p11    SOURCE    GENECARD       Gene Ontology:     BioCarta Pathways:     KEGG Pathways:  
    Probe set : 1376746_at    Name:   lactate dehydrogenase D (predicted)    GB acc:   AI145761    UniGene:   Rn.137462    Gene Symbol:   Ldhd_predicted    Locus Link:   307858    Chromosome:   19    Cytoband:   Chr:19q12    SOURCE    GENECARD       Gene Ontology:   cellular component:  mitochondrion   cellular component:  mitochondrion      BioCarta Pathways:     KEGG Pathways:  
    Probe set : 1394591_at    Name:   zinc finger protein 207 (predicted)    GB acc:   AI411280    UniGene:   Rn.102608    Gene Symbol:   Zfp207_predicted    Locus Link:   303763    Chromosome:   10    Cytoband:   Chr:10q26    SOURCE    GENECARD       Gene Ontology:   biological process:  regulation of transcription, DNA-dependent   cellular component:  nucleus   molecular function:  nucleic acid binding   molecular function:  transcription factor activity   molecular function:  zinc ion binding      BioCarta Pathways:     KEGG Pathways:  
    Probe set : 1388772_at    Name:   LSM8 homolog, U6 small nuclear RNA associated (S. cerevisiae) (predicted)    GB acc:   AI177016    UniGene:   Rn.14059    Gene Symbol:   Lsm8_predicted    Locus Link:   296913    Chromosome:   4    Cytoband:   Chr:4q21    SOURCE    GENECARD       Gene Ontology:   biological process:  nuclear mRNA splicing, via spliceosome   biological process:  mRNA processing   cellular component:  nucleus   cellular component:  small nucleolar ribonucleoprotein complex   cellular component:  ribonucleoprotein complex   molecular function:  RNA binding   molecular function:  U6 snRNA binding      BioCarta Pathways:     KEGG Pathways:  
    Probe set : 1389073_at    Name:   similar to RIKEN cDNA 2700029E10 (predicted)    GB acc:   AA851248    UniGene:   Rn.6988    Gene Symbol:   RGD1306561_predicted    Locus Link:   306014    Chromosome:   15    Cytoband:   Chr:15p11    SOURCE    GENECARD       Gene Ontology:   cellular component:  integral to membrane   molecular function:  receptor activity      BioCarta Pathways:     KEGG Pathways:  
    Probe set : 1377751_at    Name:   Transcribed locus    GB acc:   AI233227    UniGene:   Rn.24708        SOURCE        Gene Ontology:     BioCarta Pathways:     KEGG Pathways:  
    Probe set : 1383502_at    Name:   similar to mKIAA0259 protein    GB acc:   BE113753    UniGene:   Rn.21056    Gene Symbol:   LOC315969    Locus Link:   315969    Chromosome:   8    Cytoband:   Chr:8q32    SOURCE    GENECARD       Gene Ontology:     BioCarta Pathways:     KEGG Pathways:  
    Probe set : 1371963_at    Name:   propionyl-coenzyme A carboxylase, alpha polypeptide    GB acc:   BF395042    UniGene:   Rn.6033    Gene Symbol:   Pcca    Locus Link:   54280    Chromosome:   15    Cytoband:   Chr:15q25    SOURCE    GENECARD       Gene Ontology:   biological process:  fatty acid metabolism   biological process:  biotin metabolism   biological process:  metabolism   cellular component:  mitochondrion   cellular component:  mitochondrion      BioCarta Pathways:     KEGG Pathways:  
    Probe set : 1375246_at     GB acc:   BG371965              Gene Ontology:     BioCarta Pathways:     KEGG Pathways:  
    Probe set : 1388977_at    Name:   similar to RIKEN cDNA 9930118K05    GB acc:   BM390401    UniGene:   Rn.2636    Gene Symbol:   RGD:1359727    Locus Link:   310773    Chromosome:   2    Cytoband:   Chr:2q34    SOURCE    GENECARD       Gene Ontology:   biological process:  phospholipid biosynthesis   molecular function:  transferase activity      BioCarta Pathways:     KEGG Pathways:  
    Probe set : 1380123_at     GB acc:   BG374569              Gene Ontology:     BioCarta Pathways:     KEGG Pathways:  
    Probe set : 1369852_at    Name:   coagulation factor X    GB acc:   NM_017143    UniGene:   Rn.21393    Gene Symbol:   F10    Locus Link:   29243    Chromosome:   16    Cytoband:   Chr:16q12.5    SOURCE    GENECARD       Gene Ontology:   biological process:  proteolysis   biological process:  blood coagulation   cellular component:  extracellular region   cellular component:  extracellular space   cellular component:  extrachromosomal DNA      BioCarta Pathways:     KEGG Pathways:  
    Probe set : 1369148_at    Name:   transcription factor 1    GB acc:   NM_012669    UniGene:   Rn.9660    Gene Symbol:   Tcf1    Locus Link:   24817    Chromosome:   12    Cytoband:   Chr:12q16    SOURCE    GENECARD       Gene Ontology:   cellular component:  nucleus   cellular component:  transcription factor complex      BioCarta Pathways:     KEGG Pathways:  
    Probe set : 1368303_at    Name:   period homolog 2 (Drosophila)    GB acc:   NM_031678    UniGene:   Rn.25935    Gene Symbol:   Per2    Locus Link:   63840    Chromosome:   9    Cytoband:   Chr:9q36    SOURCE    GENECARD       Gene Ontology:   cellular component:  nucleus   cellular component:  nucleus   molecular function:  signal transducer activity   molecular function:  protein binding      BioCarta Pathways:     KEGG Pathways:  
    Probe set : 1376728_at    Name:   Transcribed locus    GB acc:   BM392264    UniGene:   Rn.23529        SOURCE        Gene Ontology:     BioCarta Pathways:     KEGG Pathways:  
    Probe set : 1373065_at    Name:   protein tyrosine phosphatase, non-receptor type 18 (predicted)    GB acc:   AW916153    UniGene:   Rn.138086    Gene Symbol:   Ptpn18_predicted    Locus Link:   301333    Chromosome:   9    Cytoband:   Chr:9q21    SOURCE    GENECARD       Gene Ontology:   biological process:  protein amino acid dephosphorylation   cellular component:  nucleus   cellular component:  cytoplasm      BioCarta Pathways:     KEGG Pathways:  
    Probe set : 1370446_at    Name:   non-metastatic cells 7, protein expressed in    GB acc:   AF202049    UniGene:   Rn.82718    Gene Symbol:   Nme7    Locus Link:   171566    Chromosome:   13    Cytoband:   Chr:13q22-q23    SOURCE    GENECARD       Gene Ontology:     BioCarta Pathways:     KEGG Pathways:  
    Probe set : 1395532_at    Name:   Transcribed locus, strongly similar to NP_035873.1 SH3-domain binding protein 3 [Mus musculus]    GB acc:   AI137456    UniGene:   Rn.6467        SOURCE        Gene Ontology:     BioCarta Pathways:     KEGG Pathways:  
    Probe set : 1389041_at    Name:   vacuole 14 protein (S. cerevisiae)    GB acc:   BF408597    UniGene:   Rn.46366    Gene Symbol:   Vac14    Locus Link:   307842    Chromosome:   19    Cytoband:   Chr:19q12    SOURCE    GENECARD       Gene Ontology:   cellular component:  cytoskeleton   molecular function:  binding      BioCarta Pathways:     KEGG Pathways:  
    Probe set : 1397866_at    Name:   serine (or cysteine) proteinase inhibitor, clade B, member 6b (predicted)    GB acc:   BG380122    UniGene:   Rn.115081    Gene Symbol:   Serpinb6b_predicted    Locus Link:   364705    Chromosome:   17    Cytoband:   Chr:17p12    SOURCE    GENECARD       Gene Ontology:   molecular function:  endopeptidase inhibitor activity   molecular function:  serine-type endopeptidase inhibitor activity   molecular function:  peptidase activity      BioCarta Pathways:     KEGG Pathways:  
    Probe set : 1385933_at    Name:   Transcribed locus    GB acc:   AI709424    UniGene:   Rn.19220        SOURCE        Gene Ontology:     BioCarta Pathways:     KEGG Pathways:  
    Probe set : 1371942_at    Name:   similar to Glutathione S-transferase, theta 3    GB acc:   BM390378    UniGene:   Rn.92581    Gene Symbol:   LOC499422    Locus Link:   499422    Chromosome:   20    Cytoband:   Chr:20p12    SOURCE    GENECARD       Gene Ontology:     BioCarta Pathways:     KEGG Pathways:  
    Probe set : 1373749_at    Name:   syntrophin, acidic 1 (predicted)    GB acc:   BF395599    UniGene:   Rn.139656    Gene Symbol:   Snta1_predicted    Locus Link:   362242    Chromosome:   3    Cytoband:   Chr:3q41    SOURCE    GENECARD       Gene Ontology:   biological process:  muscle contraction   biological process:  neuromuscular junction development   cellular component:  cytoskeleton   cellular component:  membrane   cellular component:  postsynaptic membrane      BioCarta Pathways:     KEGG Pathways:  
    Probe set : 1390437_at    Name:   Transcribed locus    GB acc:   BE116385    UniGene:   Rn.14619        SOURCE        Gene Ontology:     BioCarta Pathways:     KEGG Pathways:  
    Probe set : 1367578_at    Name:   peroxiredoxin 2    GB acc:   NM_017169    UniGene:   Rn.2511    Gene Symbol:   Prdx2    Locus Link:   29338    Chromosome:   19    Cytoband:   Chr:19q11    SOURCE    GENECARD       Gene Ontology:   biological process:  oxygen and reactive oxygen species metabolism   biological process:  anti-apoptosis   biological process:  response to oxidative stress   cellular component:  cytoplasm      BioCarta Pathways:     KEGG Pathways:  
    Probe set : 1388595_at    Name:   DEAH (Asp-Glu-Ala-His) box polypeptide 30 (predicted)    GB acc:   AI102995    UniGene:   Rn.22387    Gene Symbol:   Dhx30_predicted    Locus Link:   367172    Chromosome:   8    Cytoband:   Chr:8q32    SOURCE    GENECARD       Gene Ontology:   cellular component:  intracellular      BioCarta Pathways:     KEGG Pathways:  
    Probe set : 1394470_at    Name:   similar to hypothetical protein DKFZp566A1524 (predicted)    GB acc:   AI502258    UniGene:   Rn.36104    Gene Symbol:   RGD1305961_predicted    Locus Link:   298890    Chromosome:   6    Cytoband:   Chr:6q14    SOURCE    GENECARD       Gene Ontology:     BioCarta Pathways:     KEGG Pathways:  
    Probe set : 1370224_at    Name:   signal transducer and activator of transcription 3    GB acc:   BE113920    UniGene:   Rn.10247    Gene Symbol:   Stat3    Locus Link:   25125    Chromosome:   10    Cytoband:   Chr:10q32.1    SOURCE    GENECARD       Gene Ontology:   biological process:  negative regulation of transcription from RNA polymerase II promoter   biological process:  eye photoreceptor cell differentiation   biological process:  regulation of transcription, DNA-dependent   biological process:  regulation of transcription from RNA polymerase II promoter   biological process:  transcription from RNA polymerase II promoter   biological process:  cell motility   biological process:  acute-phase response   biological process:  acute-phase response   biological process:  signal transduction   biological process:  intracellular signaling cascade   biological process:  JAK-STAT cascade   biological process:  JAK-STAT cascade   biological process:  nervous system development   biological process:  transcription   cellular component:  nucleus   cellular component:  cytoplasm   cellular component:  plasma membrane      BioCarta Pathways:     KEGG Pathways:  
    Probe set : 1373990_at    Name:   solute carrier family 7 (cationic amino acid transporter, y+ system), member 12 (predicted) /// similar to solute carrier family 7 (cationic amino acid transporter, y+ system), member 12    GB acc:   AI411935    UniGene:   Rn.133380    Gene Symbol:   Slc7a12_predicted /// LOC361914    Locus Link:   294881 /// 361914    Chromosome:   2    Cytoband:   Chr:2q23    SOURCE    GENECARD       Gene Ontology:   biological process:  transport   biological process:  amino acid transport   molecular function:  amino acid-polyamine transporter activity   molecular function:  amino acid transporter activity      BioCarta Pathways:     KEGG Pathways:  
    Probe set : 1367698_a_at    Name:   septin 9    GB acc:   NM_031837    UniGene:   Rn.91127    Gene Symbol:   Sept9    Locus Link:   83788    Chromosome:   10    Cytoband:   Chr:10q32.3    SOURCE    GENECARD       Gene Ontology:     BioCarta Pathways:     KEGG Pathways:  
    Probe set : 1378249_x_at     GB acc:   BE110241              Gene Ontology:     BioCarta Pathways:     KEGG Pathways:  
    Probe set : 1385816_at    Name:   Transcribed locus, strongly similar to NP_780591.1 hypothetical protein LOC108900 [Mus musculus]    GB acc:   BI293456    UniGene:   Rn.138931        SOURCE        Gene Ontology:     BioCarta Pathways:     KEGG Pathways:  
    Probe set : 1387084_at    Name:   dipeptidylpeptidase 4    GB acc:   NM_012789    UniGene:   Rn.91364    Gene Symbol:   Dpp4    Locus Link:   25253    Chromosome:   3    Cytoband:   Chr:3q21    SOURCE    GENECARD       Gene Ontology:   biological process:  proteolysis   biological process:  proteolysis   biological process:  immune response      BioCarta Pathways:     KEGG Pathways:  
    Probe set : 1397287_at    Name:   similar to 4931417G12Rik protein    GB acc:   BF291148    UniGene:   Rn.74315    Gene Symbol:   LOC500294    Locus Link:   500294    Chromosome:   4    Cytoband:   Chr:4q42    SOURCE    GENECARD       Gene Ontology:     BioCarta Pathways:     KEGG Pathways:  
    Probe set : 1374558_at    Name:   Similar to B7-like protein GL50-B    GB acc:   AI010316    UniGene:   Rn.17428     Locus Link:   499415      SOURCE        Gene Ontology:     BioCarta Pathways:     KEGG Pathways:  
    Probe set : 1395183_at    Name:   Transcribed locus    GB acc:   BM391096    UniGene:   Rn.73529        SOURCE        Gene Ontology:     BioCarta Pathways:     KEGG Pathways:  
    Probe set : 1374625_at    Name:   hairy and enhancer of split 6 (Drosophila) (predicted)    GB acc:   AI176616    UniGene:   Rn.11980    Gene Symbol:   Hes6_predicted    Locus Link:   316626    Chromosome:   9    Cytoband:   Chr:9q36    SOURCE    GENECARD       Gene Ontology:   cellular component:  nucleus   cellular component:  transcription factor complex      BioCarta Pathways:     KEGG Pathways:  
    Probe set : 1392274_at    Name:   Transcribed locus    GB acc:   BE118653    UniGene:   Rn.18332        SOURCE        Gene Ontology:     BioCarta Pathways:     KEGG Pathways:  
    Probe set : 1392713_a_at     GB acc:   AA892541              Gene Ontology:     BioCarta Pathways:     KEGG Pathways:  
    Probe set : 1371683_at    Name:   LSM4 homolog, U6 small nuclear RNA associated (S. cerevisiae) (predicted)    GB acc:   AI103250    UniGene:   Rn.27306    Gene Symbol:   Lsm4_predicted    Locus Link:   290647    Chromosome:   16    Cytoband:   Chr:16p14    SOURCE    GENECARD       Gene Ontology:   molecular function:  DNA binding   molecular function:  RNA binding      BioCarta Pathways:     KEGG Pathways:  
    Probe set : 1371821_at    Name:   WAS protein family, member 2 (predicted)    GB acc:   BM391330    UniGene:   Rn.3856     Locus Link:   313024    Chromosome:   5    Cytoband:   Chr:5q36    SOURCE        Gene Ontology:   cellular component:  ruffle   cellular component:  early endosome   molecular function:  structural constituent of cell wall      BioCarta Pathways:     KEGG Pathways:  
    Probe set : 1370197_a_at    Name:   protein kinase C, zeta    GB acc:   AW533298    UniGene:   Rn.1109    Gene Symbol:   Prkcz    Locus Link:   25522    Chromosome:   5    Cytoband:   Chr:5q36    SOURCE    GENECARD       Gene Ontology:   cellular component:  membrane fraction   cellular component:  cytoplasm   cellular component:  plasma membrane   molecular function:  protein kinase activity   molecular function:  protein kinase activity   molecular function:  protein serine/threonine kinase activity   molecular function:  protein kinase CK2 activity   molecular function:  cAMP-dependent protein kinase activity   molecular function:  atypical protein kinase C activity   molecular function:  atypical protein kinase C activity   molecular function:  protein-tyrosine kinase activity   molecular function:  ATP binding   molecular function:  kinase activity   molecular function:  transferase activity   molecular function:  diacylglycerol binding   molecular function:  nucleotide binding   molecular function:  zinc ion binding   molecular function:  metal ion binding      BioCarta Pathways:     KEGG Pathways:  
    Probe set : 1371338_at    Name:   similar to RIKEN cDNA 1700023M09 (predicted)    GB acc:   AI232272    UniGene:   Rn.1240    Gene Symbol:   RGD1312037_predicted    Locus Link:   292788    Chromosome:   1    Cytoband:   Chr:1q21    SOURCE    GENECARD       Gene Ontology:   cellular component:  endoplasmic reticulum   cellular component:  Golgi apparatus   cellular component:  integral to membrane      BioCarta Pathways:     KEGG Pathways:  
    Probe set : 1367871_at    Name:   cytochrome P450, family 2, subfamily e, polypeptide 1    GB acc:   NM_031543    UniGene:   Rn.1372    Gene Symbol:   Cyp2e1    Locus Link:   25086      SOURCE    GENECARD       Gene Ontology:   biological process:  electron transport   biological process:  xenobiotic metabolism   biological process:  xenobiotic metabolism      BioCarta Pathways:     KEGG Pathways:  
    Probe set : 1385716_at    Name:   Transcribed locus    GB acc:   AW522166    UniGene:   Rn.45316        SOURCE        Gene Ontology:     BioCarta Pathways:     KEGG Pathways:  
    Probe set : 1370561_at    Name:   iGb3 synthase    GB acc:   AF248543    UniGene:   Rn.37737    Gene Symbol:   RGD:727913    Locus Link:   171553    Chromosome:   5    Cytoband:   Chr:5q36    SOURCE    GENECARD       Gene Ontology:   biological process:  glycosphingolipid biosynthesis   molecular function:  transferase activity, transferring glycosyl groups      BioCarta Pathways:     KEGG Pathways:  
    Probe set : 1387885_at    Name:   Fc receptor, IgG, alpha chain transporter    GB acc:   BE110597    UniGene:   Rn.15845    Gene Symbol:   Fcgrt    Locus Link:   29558    Chromosome:   1    Cytoband:   Chr:1q22    SOURCE    GENECARD       Gene Ontology:     BioCarta Pathways:     KEGG Pathways:  
    Probe set : 1398832_at    Name:   nucleolin    GB acc:   NM_012749    UniGene:   Rn.144561    Gene Symbol:   Ncl    Locus Link:   25135    Chromosome:   9    Cytoband:   Chr:9q35    SOURCE    GENECARD       Gene Ontology:   cellular component:  nucleus      BioCarta Pathways:     KEGG Pathways:  
    Probe set : 1373115_at    Name:   similar to 2310061I09Rik protein (predicted)    GB acc:   BI281909    UniGene:   Rn.17453    Gene Symbol:   RGD1304653_predicted    Locus Link:   363213    Chromosome:   9    Cytoband:   Chr:9q21    SOURCE    GENECARD       Gene Ontology:     BioCarta Pathways:     KEGG Pathways:  
    Probe set : 1381857_at    Name:   RAS p21 protein activator 1    GB acc:   AA963157    UniGene:   Rn.12223    Gene Symbol:   Rasa1    Locus Link:   25676    Chromosome:   2    Cytoband:   Chr:2q11    SOURCE    GENECARD       Gene Ontology:   biological process:  intracellular signaling cascade   biological process:  cellular physiological process   molecular function:  GTPase activator activity   molecular function:  Ras GTPase activator activity      BioCarta Pathways:     KEGG Pathways:  
    Probe set : 1396039_at    Name:   solute carrier family 22 (organic anion/cation transporter), member 12    GB acc:   BG377396    UniGene:   Rn.138222    Gene Symbol:   Slc22a12    Locus Link:   171372    Chromosome:   1    Cytoband:   Chr:1q43    SOURCE    GENECARD       Gene Ontology:   biological process:  transport   cellular component:  integral to membrane   molecular function:  transporter activity      BioCarta Pathways:     KEGG Pathways:  
    Probe set : 1375540_at    Name:   similar to hypothetical protein D11Ertd497e (predicted)    GB acc:   BF400209    UniGene:   Rn.39094    Gene Symbol:   RGD1308696_predicted    Locus Link:   287278    Chromosome:   10    Cytoband:   Chr:10q22    SOURCE    GENECARD       Gene Ontology:     BioCarta Pathways:     KEGG Pathways:  
    Probe set : 1389244_x_at    Name:   chemokine (C-X-C motif) receptor 4    GB acc:   AA945737    UniGene:   Rn.44431    Gene Symbol:   Cxcr4    Locus Link:   60628    Chromosome:   13    Cytoband:   Chr:13q12    SOURCE    GENECARD       Gene Ontology:   cellular component:  integral to membrane      BioCarta Pathways:     KEGG Pathways:  
    Probe set : 1398358_a_at    Name:   similar to integrin beta-5    GB acc:   AW520594     Gene Symbol:   LOC498091    Locus Link:   498091    Chromosome:   11    Cytoband:   Chr:11q22     GENECARD       Gene Ontology:     BioCarta Pathways:     KEGG Pathways:  
    Probe set : 1379805_at    Name:   solute carrier family 41, member 2 (predicted)    GB acc:   BF410740    UniGene:   Rn.61775    Gene Symbol:   Slc41a2_predicted    Locus Link:   362861    Chromosome:   7    Cytoband:   Chr:7q13    SOURCE    GENECARD       Gene Ontology:   biological process:  cation transport   cellular component:  integral to membrane   molecular function:  cation transporter activity      BioCarta Pathways:     KEGG Pathways:  
    Probe set : 1386888_at    Name:   eukaryotic translation initiation factor 4E binding protein 1    GB acc:   NM_053857    UniGene:   Rn.11161    Gene Symbol:   Eif4ebp1    Locus Link:   116636    Chromosome:   16    Cytoband:   Chr:16q12.3    SOURCE    GENECARD       Gene Ontology:     BioCarta Pathways:     KEGG Pathways:  
    Probe set : 1372358_at    Name:   TG interacting factor (predicted)    GB acc:   AI012613    UniGene:   Rn.144418    Gene Symbol:   Tfig    Locus Link:   316742    Chromosome:   9    Cytoband:   Chr:9q38    SOURCE    GENECARD       Gene Ontology:   cellular component:  nucleus   cellular component:  transcription factor complex   molecular function:  DNA binding   molecular function:  transcription factor activity   molecular function:  transcription corepressor activity      BioCarta Pathways:     KEGG Pathways:  
    Probe set : 1391338_at    Name:   Transcribed locus    GB acc:   AI603449    UniGene:   Rn.44722        SOURCE        Gene Ontology:     BioCarta Pathways:     KEGG Pathways:  
    Probe set : 1382997_at    Name:   solute carrier family 22 (organic cation transporter), member 13 (predicted)    GB acc:   AA998643    UniGene:   Rn.99291    Gene Symbol:   Slc22a13_predicted    Locus Link:   316062    Chromosome:   8    Cytoband:   Chr:8q32    SOURCE    GENECARD       Gene Ontology:   biological process:  transport   cellular component:  membrane fraction   cellular component:  integral to plasma membrane   molecular function:  transporter activity   molecular function:  organic cation transporter activity      BioCarta Pathways:     KEGG Pathways:  
    Probe set : 1374686_at    Name:   solute carrier family 30 (zinc transporter), member 7 (predicted)    GB acc:   BF283406    UniGene:   Rn.105204    Gene Symbol:   Slc30a7_predicted    Locus Link:   310801    Chromosome:   2    Cytoband:   Chr:2q41    SOURCE    GENECARD       Gene Ontology:   biological process:  cation transport   biological process:  zinc ion transport   molecular function:  zinc ion transporter activity   molecular function:  zinc ion binding   molecular function:  cation transporter activity      BioCarta Pathways:     KEGG Pathways:  
    Probe set : 1376546_at    Name:   similar to hypothetical protein    GB acc:   BE120498    UniGene:   Rn.77399    Gene Symbol:   LOC499157    Locus Link:   499157    Chromosome:   1    Cytoband:   Chr:1q22    SOURCE    GENECARD       Gene Ontology:     BioCarta Pathways:     KEGG Pathways:  
    Probe set : 1384393_at    Name:   Transcribed locus    GB acc:   BE120370    UniGene:   Rn.148597        SOURCE        Gene Ontology:     BioCarta Pathways:     KEGG Pathways:  
    Probe set : 1372438_at    Name:   similar to Nit protein 2 (predicted)    GB acc:   AI411100    UniGene:   Rn.42859    Gene Symbol:   RGD1310494_predicted    Locus Link:   288174    Chromosome:   11    Cytoband:   Chr:11q12    SOURCE    GENECARD       Gene Ontology:   biological process:  nitrogen compound metabolism   cellular component:  mitochondrion   molecular function:  hydrolase activity   molecular function:  hydrolase activity, acting on carbon-nitrogen (but not peptide) bonds      BioCarta Pathways:     KEGG Pathways:  
    Probe set : 1373225_at    Name:   Myeloid cell leukemia sequence 1    GB acc:   AI102618    UniGene:   Rn.4067    Gene Symbol:   Mcl1    Locus Link:   60430    Chromosome:   2    Cytoband:   Chr:2q34    SOURCE    GENECARD       Gene Ontology:   cellular component:  cytoplasm   cellular component:  mitochondrial outer membrane   cellular component:  integral to membrane      BioCarta Pathways:     KEGG Pathways:  
    Probe set : 1374508_at    Name:   nephronophthisis 1 (juvenile) (predicted)    GB acc:   BG663343    UniGene:   Rn.32745    Gene Symbol:   Nphp1_predicted    Locus Link:   296136    Chromosome:   3    Cytoband:   Chr:3q36    SOURCE    GENECARD       Gene Ontology:   cellular component:  membrane   molecular function:  structural molecule activity   molecular function:  protein domain specific binding      BioCarta Pathways:     KEGG Pathways:  
    Probe set : 1392910_at    Name:   block of proliferation 1 (predicted)    GB acc:   AA859968    UniGene:   Rn.101388    Gene Symbol:   Bop1_predicted    Locus Link:   300050    Chromosome:   7    Cytoband:   Chr:7q34    SOURCE    GENECARD       Gene Ontology:   biological process:  rRNA processing   biological process:  ribosomal large subunit biogenesis   biological process:  ribosome biogenesis   cellular component:  nucleus   cellular component:  nucleolus   molecular function:  ribonucleoprotein binding      BioCarta Pathways:     KEGG Pathways:  
    Probe set : 1376128_at    Name:   Transcribed locus    GB acc:   AI103937    UniGene:   Rn.17851        SOURCE        Gene Ontology:     BioCarta Pathways:     KEGG Pathways:  
    Probe set : 1382287_at    Name:   ribonucleic acid binding protein S1 (predicted)    GB acc:   BG375343    UniGene:   Rn.7235    Gene Symbol:   Rnps1_predicted    Locus Link:   287113    Chromosome:   10    Cytoband:   Chr:10q12    SOURCE    GENECARD       Gene Ontology:   cellular component:  nucleus      BioCarta Pathways:     KEGG Pathways:  
    Probe set : 1399091_at    Name:   F-actin capping protein beta subunit    GB acc:   AI232101    UniGene:   Rn.105999    Gene Symbol:   RGD:1359099    Locus Link:   298584    Chromosome:   5    Cytoband:   Chr:5q36    SOURCE    GENECARD       Gene Ontology:   biological process:  actin cytoskeleton organization and biogenesis   cellular component:  F-actin capping protein complex   molecular function:  actin binding      BioCarta Pathways:     KEGG Pathways:  
    Probe set : 1368100_at    Name:   phosphate cytidylyltransferase 2, ethanolamine    GB acc:   NM_053568    UniGene:   Rn.7291    Gene Symbol:   Pcyt2    Locus Link:   89841    Chromosome:   10    Cytoband:   Chr:10q32.3    SOURCE    GENECARD       Gene Ontology:     BioCarta Pathways:     KEGG Pathways:  
    Probe set : 1372774_at    Name:   Coenzyme Q6 homolog (yeast) (predicted)    GB acc:   AI170570    UniGene:   Rn.9283     Locus Link:   299195    Chromosome:   6    Cytoband:   Chr:6q31    SOURCE        Gene Ontology:   cellular component:  extracellular space      BioCarta Pathways:     KEGG Pathways:  
    Probe set : 1367659_s_at    Name:   dodecenoyl-coenzyme A delta isomerase    GB acc:   NM_017306    UniGene:   Rn.80835    Gene Symbol:   Dci    Locus Link:   29740    Chromosome:   10    Cytoband:   Chr:10q12    SOURCE    GENECARD       Gene Ontology:   cellular component:  mitochondrion   cellular component:  mitochondrion   cellular component:  mitochondrion      BioCarta Pathways:     KEGG Pathways:  
    Probe set : 1391161_at     GB acc:   BE107322              Gene Ontology:     BioCarta Pathways:     KEGG Pathways:  
    Probe set : 1384200_at     GB acc:   BE107328              Gene Ontology:     BioCarta Pathways:     KEGG Pathways:  
    Probe set : 1376683_at    Name:   Transcribed locus    GB acc:   AI175063    UniGene:   Rn.17857        SOURCE        Gene Ontology:     BioCarta Pathways:     KEGG Pathways:  
    Probe set : 1387821_at    Name:   RAB3A interacting protein    GB acc:   NM_017313    UniGene:   Rn.31889    Gene Symbol:   Rab3ip    Locus Link:   29885    Chromosome:   7    Cytoband:   Chr:7q22    SOURCE    GENECARD       Gene Ontology:   biological process:  transport   biological process:  protein transport   cellular component:  nucleus   cellular component:  cytosol   cellular component:  nucleus      BioCarta Pathways:     KEGG Pathways:  
    Probe set : 1374235_at    Name:   Down syndrome critical region gene 1-like 1    GB acc:   AI138048    UniGene:   Rn.24428    Gene Symbol:   Dscr1l1    Locus Link:   140666    Chromosome:   9    Cytoband:   Chr:9q12    SOURCE    GENECARD       Gene Ontology:   biological process:  central nervous system development   biological process:  calcium-mediated signaling   molecular function:  protein phosphatase 2B binding   molecular function:  nucleotide binding      BioCarta Pathways:     KEGG Pathways:  
    Probe set : 1387336_at    Name:   N-acetyltransferase 8 (camello like)    GB acc:   NM_022635    UniGene:   Rn.48757    Gene Symbol:   Nat8    Locus Link:   64570    Chromosome:   4    Cytoband:   Chr:4q34    SOURCE    GENECARD       Gene Ontology:   biological process:  negative regulation of cell adhesion   molecular function:  N-acetyltransferase activity   molecular function:  N-acetyltransferase activity   molecular function:  transferase activity      BioCarta Pathways:     KEGG Pathways:  
    Probe set : 1388497_at    Name:   thioesterase superfamily member 2 (predicted)    GB acc:   AI411426    UniGene:   Rn.1730    Gene Symbol:   Them2_predicted    Locus Link:   291135    Chromosome:   17    Cytoband:   Chr:17p11    SOURCE    GENECARD       Gene Ontology:   cellular component:  mitochondrion   molecular function:  catalytic activity   molecular function:  hydrolase activity      BioCarta Pathways:     KEGG Pathways:  
    Probe set : 1396820_at    Name:   histone deacetylase 1 (predicted)    GB acc:   AW530195    UniGene:   Rn.123394    Gene Symbol:   Hdac1_predicted    Locus Link:   297893    Chromosome:   5    Cytoband:   Chr:5q36    SOURCE    GENECARD       Gene Ontology:     BioCarta Pathways:     KEGG Pathways:  
    Probe set : 1376997_at    Name:   Transcribed locus    GB acc:   BI277619    UniGene:   Rn.18388        SOURCE        Gene Ontology:     BioCarta Pathways:     KEGG Pathways:  
    Probe set : 1389633_at    Name:   E2F transcription factor 4 (predicted)    GB acc:   BI290172    UniGene:   Rn.95251     Locus Link:   307794    Chromosome:   19    Cytoband:   Chr:19q11    SOURCE        Gene Ontology:   cellular component:  nucleus   cellular component:  transcription factor complex   cellular component:  cytoplasm   molecular function:  DNA binding   molecular function:  transcription factor activity   molecular function:  protein binding      BioCarta Pathways:     KEGG Pathways:  
    Probe set : 1371869_at    Name:   proteasome (prosome, macropain) subunit, alpha type 7    GB acc:   BF282262    UniGene:   Rn.105784    Gene Symbol:   Psma7    Locus Link:   29674    Chromosome:   3    Cytoband:   Chr:3q43    SOURCE    GENECARD       Gene Ontology:   biological process:  ubiquitin-dependent protein catabolism   cellular component:  cytosol   cellular component:  proteasome core complex (sensu Eukaryota)   molecular function:  endopeptidase activity   molecular function:  peptidase activity   molecular function:  hydrolase activity      BioCarta Pathways:     KEGG Pathways:  
    Probe set : 1388858_at    Name:   mitogen activated protein kinase kinase 3 (predicted)    GB acc:   BI283843    UniGene:   Rn.100064    Gene Symbol:   Map2k3_predicted    Locus Link:   303200    Chromosome:   10    Cytoband:   Chr:10q22    SOURCE    GENECARD       Gene Ontology:     BioCarta Pathways:     KEGG Pathways:  
    Probe set : 1384301_at    Name:   Similar to Kelch-like protein 3    GB acc:   BE113571    UniGene:   Rn.3665     Locus Link:   498697      SOURCE        Gene Ontology:     BioCarta Pathways:     KEGG Pathways:  
    Probe set : 1384499_at    Name:   Butyryl Coenzyme A synthetase 1 (predicted)    GB acc:   BG373566    UniGene:   Rn.38622     Locus Link:   361638    Chromosome:   1    Cytoband:   Chr:1q35    SOURCE        Gene Ontology:   cellular component:  mitochondrion   cellular component:  mitochondrial matrix      BioCarta Pathways:     KEGG Pathways:  
    Probe set : 1392477_at    Name:   ets variant gene 1 (predicted)    GB acc:   AI059914    UniGene:   Rn.76536    Gene Symbol:   Etv1_predicted    Locus Link:   362733    Chromosome:   6    Cytoband:   Chr:6q21    SOURCE    GENECARD       Gene Ontology:   cellular component:  nucleus   molecular function:  DNA binding   molecular function:  transcription factor activity      BioCarta Pathways:     KEGG Pathways:  
    Probe set : 1377456_at    Name:   Transcribed locus    GB acc:   BE116775    UniGene:   Rn.7661        SOURCE        Gene Ontology:     BioCarta Pathways:     KEGG Pathways:  
    Probe set : 1367812_at    Name:   beta-spectrin 3    GB acc:   NM_019167    UniGene:   Rn.20389    Gene Symbol:   Spnb3    Locus Link:   29211    Chromosome:   1    Cytoband:   Chr:1q42    SOURCE    GENECARD       Gene Ontology:     BioCarta Pathways:     KEGG Pathways:  
    Probe set : 1392603_at    Name:   EGL nine homolog 1 (C. elegans)    GB acc:   AI717425    UniGene:   Rn.44718    Gene Symbol:   Egln1    Locus Link:   308913    Chromosome:   19    Cytoband:   Chr:19q12    SOURCE    GENECARD       Gene Ontology:   biological process:  response to hypoxia   biological process:  protein metabolism   cellular component:  cytosol      BioCarta Pathways:     KEGG Pathways:  
    Probe set : 1377594_at    Name:   Src homology 2 domain-containing transforming protein C1    GB acc:   BF548525    UniGene:   Rn.138818    Gene Symbol:   Shc1    Locus Link:   85385    Chromosome:   2    Cytoband:   Chr:2q34    SOURCE    GENECARD       Gene Ontology:   biological process:  intracellular signaling cascade      BioCarta Pathways:     KEGG Pathways:  
    Probe set : 1374624_at    Name:   UDP-N-acetyl-alpha-D-galactosamine:polypeptide N-acetylgalactosaminyltransferase 11 (GalNAc-T11)    GB acc:   AI179979    UniGene:   Rn.53789    Gene Symbol:   Galnt11    Locus Link:   311952    Chromosome:   4    Cytoband:   Chr:4q11    SOURCE    GENECARD       Gene Ontology:   biological process:  protein amino acid O-linked glycosylation   cellular component:  Golgi stack   cellular component:  integral to membrane      BioCarta Pathways:     KEGG Pathways:  
    Probe set : 1375335_at    Name:   heat shock 90kDa protein 1, beta    GB acc:   BI285700    UniGene:   Rn.98667    Gene Symbol:   RGD:1303075    Locus Link:   301252    Chromosome:   9    Cytoband:   Chr:9q12    SOURCE    GENECARD       Gene Ontology:   biological process:  protein folding   biological process:  response to unfolded protein   molecular function:  nucleotide binding   molecular function:  ATP binding   molecular function:  unfolded protein binding      BioCarta Pathways:     KEGG Pathways:  
    Probe set : 1368268_at    Name:   thymine-DNA glycosylase    GB acc:   NM_053729    UniGene:   Rn.98685    Gene Symbol:   Tdg    Locus Link:   114521    Chromosome:   7    Cytoband:   Chr:7q13    SOURCE    GENECARD       Gene Ontology:   biological process:  negative regulation of transcription from RNA polymerase II promoter   biological process:  base-excision repair   biological process:  DNA repair      BioCarta Pathways:     KEGG Pathways:  
    Probe set : 1387631_at    Name:   15-hydroxyprostaglandin dehydrogenase    GB acc:   NM_024390    UniGene:   Rn.6386    Gene Symbol:   Hpgd    Locus Link:   79242    Chromosome:   16    Cytoband:   Chr:16p11    SOURCE    GENECARD       Gene Ontology:   biological process:  prostaglandin metabolism   biological process:  metabolism      BioCarta Pathways:     KEGG Pathways:  
    Probe set : 1394456_at    Name:   Transcribed locus    GB acc:   AW525722    UniGene:   Rn.34019        SOURCE        Gene Ontology:     BioCarta Pathways:     KEGG Pathways:  
    Probe set : 1368047_at    Name:   solute carrier family 13 (sodium-dependent dicarboxylate transporter), member 3    GB acc:   NM_022866    UniGene:   Rn.3587    Gene Symbol:   Slc13a3    Locus Link:   64846    Chromosome:   3    Cytoband:   Chr:3q42    SOURCE    GENECARD       Gene Ontology:     BioCarta Pathways:     KEGG Pathways:  
    Probe set : 1388771_at    Name:   CGG triplet repeat binding protein 1 (predicted)    GB acc:   BG668182    UniGene:   Rn.101743     Locus Link:   288353    Chromosome:   11    Cytoband:   Chr:11p12    SOURCE        Gene Ontology:   cellular component:  nucleus   molecular function:  double-stranded DNA binding      BioCarta Pathways:     KEGG Pathways:  
    Probe set : 1389804_at    Name:   Transcribed locus    GB acc:   BF392605    UniGene:   Rn.16770        SOURCE        Gene Ontology:     BioCarta Pathways:     KEGG Pathways:  
    Probe set : 1368244_at    Name:   arsenic (+3 oxidation state) methyltransferase    GB acc:   NM_080890    UniGene:   Rn.95453    Gene Symbol:   As3mt    Locus Link:   140925    Chromosome:   1    Cytoband:   Chr:1q54    SOURCE    GENECARD       Gene Ontology:     BioCarta Pathways:     KEGG Pathways:  
    Probe set : 1393217_at    Name:   ATP-binding cassette, sub-family G (WHITE), member 3    GB acc:   BF282804    UniGene:   Rn.55321    Gene Symbol:   RGD:1303294    Locus Link:   289453    Chromosome:   14    Cytoband:   Chr:14p22    SOURCE    GENECARD       Gene Ontology:   biological process:  transport   biological process:  regulation of transcription, DNA-dependent   cellular component:  membrane   cellular component:  intracellular      BioCarta Pathways:     KEGG Pathways:  
    Probe set : 1374912_at     GB acc:   BI274467              Gene Ontology:     BioCarta Pathways:     KEGG Pathways:  
    Probe set : 1376733_at    Name:   immunoglobulin superfamily, member 11 (predicted)    GB acc:   AI407898    UniGene:   Rn.20210    Gene Symbol:   Igsf11_predicted    Locus Link:   303926    Chromosome:   11    Cytoband:   Chr:11q21    SOURCE    GENECARD       Gene Ontology:   cellular component:  plasma membrane      BioCarta Pathways:     KEGG Pathways:  
    Probe set : 1388658_at    Name:   surfeit 2    GB acc:   AA942704    UniGene:   Rn.107863    Gene Symbol:   Surf2    Locus Link:   619345    Chromosome:   3    Cytoband:   Chr:3p12    SOURCE    GENECARD       Gene Ontology:     BioCarta Pathways:     KEGG Pathways:  
    Probe set : 1372006_at    Name:   Transcribed locus    GB acc:   BM391274    UniGene:   Rn.954        SOURCE        Gene Ontology:     BioCarta Pathways:     KEGG Pathways:  
    Probe set : 1374057_at    Name:   Similar to Homeobox protein Hox-D8 (Hox-4.3) (Hox-5.4)    GB acc:   AI177143    UniGene:   Rn.13330     Locus Link:   366083    Chromosome:   3    Cytoband:   Chr:3q23    SOURCE        Gene Ontology:     BioCarta Pathways:     KEGG Pathways:  
    Probe set : 1373400_at    Name:   Protein kinase, cAMP-dependent, regulatory, type 2, alpha    GB acc:   AW918535    UniGene:   Rn.9742    Gene Symbol:   Prkar2a    Locus Link:   29699    Chromosome:   8    Cytoband:   Chr:8q32    SOURCE    GENECARD       Gene Ontology:     BioCarta Pathways:     KEGG Pathways:  
    Probe set : 1371341_at    Name:   small nuclear ribonucleoprotein D2 (predicted)    GB acc:   BG372885    UniGene:   Rn.110465    Gene Symbol:   Snrpd2_predicted    Locus Link:   292688    Chromosome:   1    Cytoband:   Chr:1q21    SOURCE    GENECARD       Gene Ontology:   biological process:  spliceosome assembly   biological process:  nuclear mRNA splicing, via spliceosome   biological process:  mRNA processing      BioCarta Pathways:     KEGG Pathways:  
    Probe set : 1374775_at    Name:   antigen identified by monoclonal antibody Ki-67 (predicted) /// similar to Mki67 protein /// similar to Ki-67    GB acc:   AI714002    UniGene:   Rn.73551    Gene Symbol:   Mki67_predicted /// LOC310382 /// LOC366937    Locus Link:   291234 /// 310382 /// 366937    Chromosome:   1    Cytoband:   Chr:1q41 /// Chr:2q26 /// Chr:7q32    SOURCE    GENECARD       Gene Ontology:   biological process:  regulation of progression through cell cycle   biological process:  meiosis   biological process:  cell proliferation   molecular function:  ATP binding      BioCarta Pathways:     KEGG Pathways:  
    Probe set : 1397644_at     GB acc:   BI278369              Gene Ontology:     BioCarta Pathways:     KEGG Pathways:  
    Probe set : 1382419_at    Name:   SoxLZ/Sox6 leucine zipper binding protein in testis (predicted)    GB acc:   BI276776    UniGene:   Rn.40474    Gene Symbol:   Solt_predicted    Locus Link:   294712    Chromosome:   2    Cytoband:   Chr:2q13    SOURCE    GENECARD       Gene Ontology:     BioCarta Pathways:     KEGG Pathways:  
    Probe set : 1374682_at    Name:   Transcribed locus, weakly similar to NP_035569.1 SRY-box 13 [Mus musculus]    GB acc:   BM383170    UniGene:   Rn.30532        SOURCE        Gene Ontology:     BioCarta Pathways:     KEGG Pathways:  
    Probe set : 1395518_at    Name:   Transcribed locus    GB acc:   BF544403    UniGene:   Rn.153933        SOURCE        Gene Ontology:     BioCarta Pathways:     KEGG Pathways:  
    Probe set : 1392933_a_at    Name:   B-cell CLL/lymphoma 7A (predicted)    GB acc:   BF288704    UniGene:   Rn.9084     Locus Link:   360809    Chromosome:   12    Cytoband:   Chr:12q16    SOURCE        Gene Ontology:   molecular function:  actin binding      BioCarta Pathways:     KEGG Pathways:  
    Probe set : 1378543_at    Name:   Nuclear factor, erythroid derived 2, like 3 (predicted)    GB acc:   BE108222    UniGene:   Rn.4057     Locus Link:   312331    Chromosome:   4    Cytoband:   Chr:4q24    SOURCE        Gene Ontology:   biological process:  regulation of transcription, DNA-dependent   biological process:  transcription from RNA polymerase II promoter   cellular component:  extracellular space   cellular component:  nucleus   molecular function:  DNA binding   molecular function:  transcription factor activity   molecular function:  transcription coactivator activity      BioCarta Pathways:     KEGG Pathways:  
    Probe set : 1367760_at    Name:   mitogen activated protein kinase kinase 1    GB acc:   D13341    UniGene:   Rn.5850    Gene Symbol:   Map2k1    Locus Link:   170851    Chromosome:   8    Cytoband:   Chr:8q24    SOURCE    GENECARD       Gene Ontology:   cellular component:  cytosol      BioCarta Pathways:     KEGG Pathways:  
    Probe set : 1380504_at     GB acc:   BI282488              Gene Ontology:     BioCarta Pathways:     KEGG Pathways:  
    Probe set : 1372343_at    Name:   exosome component 8 (predicted)    GB acc:   BM384065     Gene Symbol:   Exosc8_predicted    Locus Link:   295050    Chromosome:   2    Cytoband:   Chr:2q26     GENECARD       Gene Ontology:   biological process:  rRNA processing   biological process:  RNA processing   cellular component:  exosome (RNase complex)   cellular component:  nucleus      BioCarta Pathways:     KEGG Pathways:  
    Probe set : 1392082_a_at    Name:   CD7 antigen (predicted)    GB acc:   BI290548    UniGene:   Rn.8175    Gene Symbol:   Cd7_predicted    Locus Link:   303747    Chromosome:   10    Cytoband:   Chr:10q32.3    SOURCE    GENECARD       Gene Ontology:   molecular function:  receptor activity      BioCarta Pathways:     KEGG Pathways:  
    Probe set : 1397808_at    Name:   Cathepsin C    GB acc:   AW920064    UniGene:   Rn.122504    Gene Symbol:   Ctsc    Locus Link:   25423    Chromosome:   1    Cytoband:   Chr:1q32    SOURCE    GENECARD       Gene Ontology:   biological process:  proteolysis   biological process:  proteolysis   biological process:  proteolysis   biological process:  immune response   cellular component:  extracellular space   cellular component:  lysosome   cellular component:  lysosome      BioCarta Pathways:     KEGG Pathways:  
    Probe set : 1377761_at    Name:   glutamine-fructose-6-phosphate transaminase 2    GB acc:   BI296057    UniGene:   Rn.16089    Gene Symbol:   RGD:1303097    Locus Link:   360518    Chromosome:   10    Cytoband:   Chr:10q22    SOURCE    GENECARD       Gene Ontology:   cellular component:  cytoplasm      BioCarta Pathways:     KEGG Pathways:  
    Probe set : 1367948_a_at    Name:   kinase insert domain protein receptor    GB acc:   U93307    UniGene:   Rn.88869    Gene Symbol:   Kdr    Locus Link:   25589      SOURCE    GENECARD       Gene Ontology:     BioCarta Pathways:     KEGG Pathways:  
    Probe set : 1370949_at    Name:   myristoylated alanine rich protein kinase C substrate    GB acc:   M59859    UniGene:   Rn.9560    Gene Symbol:   Marcks    Locus Link:   25603      SOURCE    GENECARD       Gene Ontology:   biological process:  cell motility   cellular component:  plasma membrane   cellular component:  actin cytoskeleton   cellular component:  membrane      BioCarta Pathways:     KEGG Pathways:  
    Probe set : 1382227_at    Name:   1-acylglycerol-3-phosphate O-acyltransferase 3 (predicted)    GB acc:   AW533203    UniGene:   Rn.100251     Locus Link:   294324    Chromosome:   20    Cytoband:   Chr:20p12    SOURCE        Gene Ontology:   biological process:  phospholipid metabolism   biological process:  metabolism   biological process:  phospholipid biosynthesis   cellular component:  integral to membrane   molecular function:  1-acylglycerol-3-phosphate O-acyltransferase activity   molecular function:  acyltransferase activity   molecular function:  transferase activity      BioCarta Pathways:     KEGG Pathways:  
    Probe set : 1382531_at    Name:   similar to toll-like receptor 7    GB acc:   AI408425    UniGene:   Rn.14819    Gene Symbol:   LOC317468    Locus Link:   317468    Chromosome:   X    Cytoband:   Chr:Xq21    SOURCE    GENECARD       Gene Ontology:     BioCarta Pathways:     KEGG Pathways:  
    Probe set : 1397419_at    Name:   Similar to PALS2-alpha splice variant    GB acc:   AI101314    UniGene:   Rn.49269     Locus Link:   500121      SOURCE        Gene Ontology:     BioCarta Pathways:     KEGG Pathways:  
    Probe set : 1388384_at    Name:   Dynein, cytoplasmic, light chain 1    GB acc:   AI407618    UniGene:   Rn.35769    Gene Symbol:   Dnclc1    Locus Link:   58945    Chromosome:   12    Cytoband:   Chr:12q16    SOURCE    GENECARD       Gene Ontology:     BioCarta Pathways:     KEGG Pathways:  
    Probe set : 1370088_at    Name:   sperm autoantigenic protein 17    GB acc:   NM_053482    UniGene:   Rn.29090    Gene Symbol:   Spa17    Locus Link:   85244    Chromosome:   8    Cytoband:   Chr:8q21    SOURCE    GENECARD       Gene Ontology:   biological process:  signal transduction   biological process:  binding of sperm to zona pellucida   cellular component:  flagellum (sensu Eukaryota)   cellular component:  membrane   molecular function:  protein binding   molecular function:  cAMP-dependent protein kinase regulator activity      BioCarta Pathways:     KEGG Pathways:  
    Probe set : 1371349_at    Name:   similar to collagen alpha1 type VI-precursor    GB acc:   AI598402    UniGene:   Rn.107165    Gene Symbol:   LOC294337    Locus Link:   294337    Chromosome:   20    Cytoband:   Chr:20p12    SOURCE    GENECARD       Gene Ontology:     BioCarta Pathways:     KEGG Pathways:  
    Probe set : 1372396_at    Name:   Similar to 2310047B19Rik protein (predicted)    GB acc:   BE113316    UniGene:   Rn.21057     Locus Link:   363029    Chromosome:   8    Cytoband:   Chr:8q13    SOURCE        Gene Ontology:     BioCarta Pathways:     KEGG Pathways:  
    Probe set : 1394275_at     GB acc:   BF559096              Gene Ontology:     BioCarta Pathways:     KEGG Pathways:  
    Probe set : 1376353_at    Name:   ARP5 actin-related protein 5 homolog (yeast) (predicted)    GB acc:   BI289883    UniGene:   Rn.51809    Gene Symbol:   Actr5_predicted    Locus Link:   362258    Chromosome:   3    Cytoband:   Chr:3q42    SOURCE    GENECARD       Gene Ontology:   cellular component:  actin cytoskeleton   molecular function:  structural constituent of cytoskeleton      BioCarta Pathways:     KEGG Pathways:  
    Probe set : 1398860_at    Name:   neural precursor cell expressed, developmentally down-regulated gene 8    GB acc:   AF095740    UniGene:   Rn.2079    Gene Symbol:   Nedd8    Locus Link:   25490    Chromosome:   15    Cytoband:   Chr:15p13    SOURCE    GENECARD       Gene Ontology:   cellular component:  nucleus   molecular function:  ubiquitin conjugating enzyme activity   molecular function:  protein binding      BioCarta Pathways:     KEGG Pathways:  
    Probe set : 1372276_at    Name:   Transcribed locus    GB acc:   BG378613    UniGene:   Rn.7332        SOURCE        Gene Ontology:     BioCarta Pathways:     KEGG Pathways:  
    Probe set : 1368393_at    Name:   lymphocyte antigen 68    GB acc:   NM_053383    UniGene:   Rn.17490    Gene Symbol:   C1qr1    Locus Link:   84398    Chromosome:   3    Cytoband:   Chr:3q41    SOURCE    GENECARD       Gene Ontology:     BioCarta Pathways:     KEGG Pathways:  
    Probe set : 1370133_at    Name:   regulator of G-protein signaling 19    GB acc:   NM_021661    UniGene:   Rn.88166    Gene Symbol:   Rgs19    Locus Link:   59293    Chromosome:   3    Cytoband:   Chr:3q43    SOURCE    GENECARD       Gene Ontology:     BioCarta Pathways:     KEGG Pathways:  
    Probe set : 1370386_at    Name:   RuvB-like protein 1    GB acc:   AB002406    UniGene:   Rn.86410    Gene Symbol:   Ruvbl1    Locus Link:   65137    Chromosome:   4    Cytoband:   Chr:4q34    SOURCE    GENECARD       Gene Ontology:   cellular component:  nucleus   cellular component:  TIP60 histone acetyltransferase complex      BioCarta Pathways:     KEGG Pathways:  
    Probe set : 1392668_at    Name:   Transcribed locus    GB acc:   AI235429    UniGene:   Rn.118787        SOURCE        Gene Ontology:     BioCarta Pathways:     KEGG Pathways:  
    Probe set : 1370014_at    Name:   syntaxin 4A (placental)    GB acc:   NM_031125    UniGene:   Rn.33218    Gene Symbol:   Stx4a    Locus Link:   81803    Chromosome:   1    Cytoband:   Chr:1q36    SOURCE    GENECARD       Gene Ontology:   molecular function:  protein binding   molecular function:  protein transporter activity      BioCarta Pathways:     KEGG Pathways:  
    Probe set : 1374953_at    Name:   similar to CG12279-PA    GB acc:   AI599484    UniGene:   Rn.9543    Gene Symbol:   LOC500420    Locus Link:   500420    Chromosome:   5    Cytoband:   Chr:5q21    SOURCE    GENECARD       Gene Ontology:     BioCarta Pathways:     KEGG Pathways:  
    Probe set : 1383574_at    Name:   mannosidase 1, alpha (predicted)    GB acc:   AI502956    UniGene:   Rn.3576    Gene Symbol:   Man1a_predicted    Locus Link:   294567    Chromosome:   20    Cytoband:   Chr:20q11    SOURCE    GENECARD       Gene Ontology:   biological process:  carbohydrate metabolism   biological process:  protein amino acid N-linked glycosylation      BioCarta Pathways:     KEGG Pathways:  
    Probe set : 1389251_at    Name:   nudix (nucleoside diphosphate linked moiety X)-type motif 7 (predicted)    GB acc:   AA944380    UniGene:   Rn.99447    Gene Symbol:   Nudt7_predicted    Locus Link:   361413    Chromosome:   19    Cytoband:   Chr:19q12    SOURCE    GENECARD       Gene Ontology:   biological process:  coenzyme A catabolism   biological process:  acetyl-CoA catabolism   cellular component:  peroxisome   molecular function:  acetyl-CoA hydrolase activity      BioCarta Pathways:     KEGG Pathways:  
    Probe set : 1380187_at    Name:   similar to TNF intracellular domain-interacting protein (predicted)    GB acc:   BI286219    UniGene:   Rn.21037    Gene Symbol:   RGD1311487_predicted    Locus Link:   310674    Chromosome:   2    Cytoband:   Chr:2q34    SOURCE    GENECARD       Gene Ontology:     BioCarta Pathways:     KEGG Pathways:  
    Probe set : 1390312_at    Name:   similar to mKIAA2005 protein /// similar to mKIAA2005 protein /// similar to mKIAA2005 protein    GB acc:   BG670441    UniGene:   Rn.118248    Gene Symbol:   LOC500012 /// LOC500014 /// LOC500015    Locus Link:   500012 /// 500014 /// 500015    Chromosome:   4    Cytoband:   Chr:4q13    SOURCE    GENECARD       Gene Ontology:     BioCarta Pathways:     KEGG Pathways:  
    Probe set : 1392496_at    Name:   Trypsin V-A    GB acc:   AI178581    UniGene:   Rn.13006    Gene Symbol:   LOC312273    Locus Link:   312273    Chromosome:   4    Cytoband:   Chr:4q22    SOURCE    GENECARD       Gene Ontology:     BioCarta Pathways:     KEGG Pathways:  
    Probe set : 1375879_at    Name:   G protein-coupled receptor 48    GB acc:   BE111762    UniGene:   Rn.30054    Gene Symbol:   Gpr48    Locus Link:   286994    Chromosome:   3    Cytoband:   Chr:3q33    SOURCE    GENECARD       Gene Ontology:   biological process:  G-protein coupled receptor protein signaling pathway   biological process:  signal transduction   cellular component:  integral to membrane      BioCarta Pathways:     KEGG Pathways:  
    Probe set : 1371803_at    Name:   GM2 ganglioside activator protein    GB acc:   AI717483    UniGene:   Rn.98783    Gene Symbol:   Gm2a    Locus Link:   282838    Chromosome:   10    Cytoband:   Chr:10q22    SOURCE    GENECARD       Gene Ontology:     BioCarta Pathways:     KEGG Pathways:  
    Probe set : 1375908_at    Name:   epithelial V-like antigen (predicted)    GB acc:   BI282616    UniGene:   Rn.8193    Gene Symbol:   Eva_predicted    Locus Link:   300679    Chromosome:   8    Cytoband:   Chr:8q22    SOURCE    GENECARD       Gene Ontology:   biological process:  cell adhesion   biological process:  homophilic cell adhesion   biological process:  morphogenesis   molecular function:  protein binding      BioCarta Pathways:     KEGG Pathways:  
    Probe set : 1375944_at     GB acc:   AA849497              Gene Ontology:     BioCarta Pathways:     KEGG Pathways:  
    Probe set : 1393661_at    Name:   Transcribed locus    GB acc:   BG381658    UniGene:   Rn.146323        SOURCE        Gene Ontology:     BioCarta Pathways:     KEGG Pathways:  
    Probe set : 1389113_at    Name:   Similar to Hypothetical protein MGC32471 (predicted)    GB acc:   AI598307    UniGene:   Rn.9239     Locus Link:   304785    Chromosome:   13    Cytoband:   Chr:13q13    SOURCE        Gene Ontology:     BioCarta Pathways:     KEGG Pathways:  
    Probe set : 1368354_at    Name:   glutathione S-transferase theta 1    GB acc:   NM_053293    UniGene:   Rn.11122    Gene Symbol:   Gstt1    Locus Link:   25260    Chromosome:   20    Cytoband:   Chr:20p12    SOURCE    GENECARD       Gene Ontology:   biological process:  glutathione metabolism   biological process:  response to stress   cellular component:  nucleus   molecular function:  glutathione transferase activity   molecular function:  transferase activity      BioCarta Pathways:     KEGG Pathways:  
    Probe set : 1373611_at    Name:   interleukin 17 receptor (predicted)    GB acc:   BI279744    UniGene:   Rn.106209    Gene Symbol:   Il17r_predicted    Locus Link:   312679    Chromosome:   4    Cytoband:   Chr:4q42    SOURCE    GENECARD       Gene Ontology:   biological process:  cell surface receptor linked signal transduction   cellular component:  extracellular space   cellular component:  integral to plasma membrane   cellular component:  integral to membrane   molecular function:  receptor activity   molecular function:  interleukin receptor activity   molecular function:  interleukin-17 receptor activity      BioCarta Pathways:     KEGG Pathways:  
    Probe set : 1394612_at     GB acc:   AW535206              Gene Ontology:     BioCarta Pathways:     KEGG Pathways:  
    Probe set : 1371146_at    Name:   transcription factor EC    GB acc:   L08812    UniGene:   Rn.9659    Gene Symbol:   Tfec    Locus Link:   26296    Chromosome:   4    Cytoband:   Chr:4q21    SOURCE    GENECARD       Gene Ontology:   biological process:  regulation of transcription, DNA-dependent   biological process:  regulation of transcription, DNA-dependent   biological process:  development   cellular component:  nucleus   cellular component:  transcription factor complex      BioCarta Pathways:     KEGG Pathways:  
    Probe set : 1388294_at    Name:   succinate dehydrogenase complex, subunit D, integral membrane protein    GB acc:   AI176608    UniGene:   Rn.3040    Gene Symbol:   Sdhd    Locus Link:   363061    Chromosome:   8    Cytoband:   Chr:8q23    SOURCE    GENECARD       Gene Ontology:     BioCarta Pathways:     KEGG Pathways:  
    Probe set : 1398325_at    Name:   transmembrane 4 superfamily member 8    GB acc:   AI172464    UniGene:   Rn.998    Gene Symbol:   RGD:1359444    Locus Link:   300733    Chromosome:   8    Cytoband:   Chr:8q24    SOURCE    GENECARD       Gene Ontology:   cellular component:  integral to membrane      BioCarta Pathways:     KEGG Pathways:  
    Probe set : 1379479_at    Name:   kinesin family member 4    GB acc:   AA859926    UniGene:   Rn.22652    Gene Symbol:   Kif4    Locus Link:   84393    Chromosome:   X    Cytoband:   Chr:Xq31    SOURCE    GENECARD       Gene Ontology:   molecular function:  microtubule motor activity   molecular function:  microtubule binding      BioCarta Pathways:     KEGG Pathways:  
    Probe set : 1383305_at    Name:   Transcribed locus    GB acc:   AW530081    UniGene:   Rn.146454        SOURCE        Gene Ontology:     BioCarta Pathways:     KEGG Pathways:  
    Probe set : 1393152_at     GB acc:   AI007821              Gene Ontology:     BioCarta Pathways:     KEGG Pathways:  
    Probe set : 1382029_at    Name:   similar to CDNA sequence BC017647    GB acc:   BE104800    UniGene:   Rn.19152    Gene Symbol:   LOC497960    Locus Link:   497960    Chromosome:   10    Cytoband:   Chr:10q25    SOURCE    GENECARD       Gene Ontology:     BioCarta Pathways:     KEGG Pathways:  
    Probe set : 1371932_at    Name:   similar to hypothetical protein FLJ38482 (predicted)    GB acc:   AI232287    UniGene:   Rn.5808    Gene Symbol:   RGD1309341_predicted    Locus Link:   361137    Chromosome:   16    Cytoband:   Chr:16p13    SOURCE    GENECARD       Gene Ontology:     BioCarta Pathways:     KEGG Pathways:  
    Probe set : 1389263_at    Name:   retinoic acid induced 14 (predicted)    GB acc:   AI234852    UniGene:   Rn.67714    Gene Symbol:   Rai14_predicted    Locus Link:   294804    Chromosome:   2    Cytoband:   Chr:2q16    SOURCE    GENECARD       Gene Ontology:   biological process:  regulation of transcription, DNA-dependent   cellular component:  mitochondrion   molecular function:  DNA binding   molecular function:  transcription factor activity      BioCarta Pathways:     KEGG Pathways:  
    Probe set : 1374892_at    Name:   spermidine/spermine N1-acetyl transferase 2 (predicted)    GB acc:   AI410221    UniGene:   Rn.23795    Gene Symbol:   Sat2_predicted    Locus Link:   360547    Chromosome:   10    Cytoband:   Chr:10q24    SOURCE    GENECARD       Gene Ontology:   biological process:  spermine metabolism   biological process:  spermidine metabolism      BioCarta Pathways:     KEGG Pathways:  
    Probe set : 1369177_at    Name:   phosphatidylinositol 4-kinase type 2 alpha    GB acc:   NM_053735    UniGene:   Rn.93583    Gene Symbol:   Pi4k2a    Locus Link:   114554    Chromosome:   1    Cytoband:   Chr:1q54    SOURCE    GENECARD       Gene Ontology:     BioCarta Pathways:     KEGG Pathways:  
    Probe set : 1389789_at    Name:   Transcribed locus    GB acc:   AI059662    UniGene:   Rn.147438        SOURCE        Gene Ontology:     BioCarta Pathways:     KEGG Pathways:  
    Probe set : 1383597_at    Name:   DNA cross-link repair 1C, PSO2 homolog (S. cerevisiae)    GB acc:   AI072301     Gene Symbol:   Dclre1c    Locus Link:   259171    Chromosome:   17    Cytoband:   Chr:17q12.3     GENECARD       Gene Ontology:   cellular component:  nucleus      BioCarta Pathways:     KEGG Pathways:  
    Probe set : 1373970_at    Name:   similar to RIKEN cDNA 9230117N10 (predicted)    GB acc:   AI716248    UniGene:   Rn.106849    Gene Symbol:   RGD1311155_predicted    Locus Link:   361749    Chromosome:   1    Cytoband:   Chr:1q52    SOURCE    GENECARD       Gene Ontology:   cellular component:  nucleus      BioCarta Pathways:     KEGG Pathways:  
    Probe set : 1376852_at    Name:   methylcrotonoyl-Coenzyme A carboxylase 1 (alpha) (predicted)    GB acc:   BI274460    UniGene:   Rn.15587    Gene Symbol:   Mccc1_predicted    Locus Link:   294972    Chromosome:   2    Cytoband:   Chr:2q25    SOURCE    GENECARD       Gene Ontology:   biological process:  leucine catabolism   biological process:  biotin metabolism   biological process:  metabolism   cellular component:  mitochondrion   cellular component:  mitochondrial matrix      BioCarta Pathways:     KEGG Pathways:  
    Probe set : 1371323_at     GB acc:   BG666002              Gene Ontology:     BioCarta Pathways:     KEGG Pathways:  
    Probe set : 1377686_at    Name:   Transcribed locus    GB acc:   AA859337    UniGene:   Rn.6889        SOURCE        Gene Ontology:     BioCarta Pathways:     KEGG Pathways:  
    Probe set : 1372721_at     GB acc:   AI175447              Gene Ontology:     BioCarta Pathways:     KEGG Pathways:  
    Probe set : 1367998_at    Name:   secretory leukocyte peptidase inhibitor /// similar to secretory leukocyte protease inhibitor    GB acc:   NM_053372    UniGene:   Rn.144851    Gene Symbol:   Slpi /// LOC296356    Locus Link:   296356 /// 84386    Chromosome:   3    Cytoband:   Chr:3q42    SOURCE    GENECARD       Gene Ontology:   molecular function:  peptidase activity      BioCarta Pathways:     KEGG Pathways:  
    Probe set : 1392971_at    Name:   Transcribed locus    GB acc:   BG377743    UniGene:   Rn.2295        SOURCE        Gene Ontology:     BioCarta Pathways:     KEGG Pathways:  
    Probe set : 1387023_at    Name:   glutathione S-transferase, mu type 3    GB acc:   NM_031154    UniGene:   Rn.6036    Gene Symbol:   Gstm3    Locus Link:   81869    Chromosome:   2    Cytoband:   Chr:2q34    SOURCE    GENECARD       Gene Ontology:   biological process:  metabolism   molecular function:  glutathione transferase activity   molecular function:  glutathione transferase activity   molecular function:  transferase activity      BioCarta Pathways:     KEGG Pathways:  
    Probe set : 1377573_at    Name:   carbonic anhydrase VB, mitochondrial    GB acc:   AI411132    UniGene:   Rn.22441    Gene Symbol:   Ca5b    Locus Link:   302669    Chromosome:   X    Cytoband:   Chr:Xq21    SOURCE    GENECARD       Gene Ontology:   biological process:  one-carbon compound metabolism   cellular component:  mitochondrion      BioCarta Pathways:     KEGG Pathways:  
    Probe set : 1374833_at    Name:   Transcribed locus    GB acc:   BF290953    UniGene:   Rn.24673        SOURCE        Gene Ontology:     BioCarta Pathways:     KEGG Pathways:  
    Probe set : 1372205_at    Name:   zinc finger protein 278 (predicted)    GB acc:   AI713121    UniGene:   Rn.71473    Gene Symbol:   Znf278_predicted    Locus Link:   305471    Chromosome:   14    Cytoband:   Chr:14q21    SOURCE    GENECARD       Gene Ontology:   biological process:  regulation of transcription, DNA-dependent   biological process:  negative regulation of transcription   cellular component:  nucleus      BioCarta Pathways:     KEGG Pathways:  
    Probe set : 1375221_at    Name:   similar to CG5554-PA (predicted)    GB acc:   BE109676    UniGene:   Rn.12542    Gene Symbol:   RGD1305146_predicted    Locus Link:   296182    Chromosome:   3    Cytoband:   Chr:3q36    SOURCE    GENECARD       Gene Ontology:   biological process:  electron transport   cellular component:  integral to membrane   molecular function:  electron transporter activity   molecular function:  arsenate reductase (thioredoxin) activity      BioCarta Pathways:     KEGG Pathways:  
    Probe set : 1370377_at    Name:   cytochrome P450, family 2, subfamily d, polypeptide 9 /// cytochrome P450, family 2, subfamily d, polypeptide 10    GB acc:   M25143    UniGene:   Rn.91133    Gene Symbol:   Cyp2d9 /// Cyp2d10    Locus Link:   266684 /// 286963    Chromosome:   7    Cytoband:   Chr:7q34    SOURCE    GENECARD       Gene Ontology:   biological process:  electron transport   cellular component:  endoplasmic reticulum   cellular component:  microsome   cellular component:  membrane      BioCarta Pathways:     KEGG Pathways:  
    Probe set : 1392530_at    Name:   similar to NADH dehydrogenase (ubiquinone) 1, subcomplex unknown, 1    GB acc:   AI409545    UniGene:   Rn.8096    Gene Symbol:   LOC499612    Locus Link:   499612    Chromosome:   2    Cytoband:   Chr:2q26    SOURCE    GENECARD       Gene Ontology:     BioCarta Pathways:     KEGG Pathways:  
    Probe set : 1374703_at    Name:   Chromobox homolog 6 (predicted)    GB acc:   BI304209    UniGene:   Rn.2924     Locus Link:   315136    Chromosome:   7    Cytoband:   Chr:7q34    SOURCE        Gene Ontology:   biological process:  chromatin assembly or disassembly   biological process:  regulation of transcription, DNA-dependent   biological process:  chromatin modification   cellular component:  chromatin   cellular component:  nucleus   molecular function:  chromatin binding      BioCarta Pathways:     KEGG Pathways:  
    Probe set : 1372553_at    Name:   similar to RIKEN cDNA 2610301B20; EST AI428449    GB acc:   AI233205    UniGene:   Rn.7569    Gene Symbol:   MGC94199    Locus Link:   362483    Chromosome:   5    Cytoband:   Chr:5q13    SOURCE    GENECARD       Gene Ontology:     BioCarta Pathways:     KEGG Pathways:  
    Probe set : 1399059_at    Name:   Enhancer of zeste homolog 1 (Drosophila) (predicted)    GB acc:   BI283868    UniGene:   Rn.12929     Locus Link:   303547    Chromosome:   10    Cytoband:   Chr:10q32.1    SOURCE        Gene Ontology:   biological process:  regulation of transcription, DNA-dependent   biological process:  morphogenesis   cellular component:  nucleus   molecular function:  DNA binding   molecular function:  chromatin binding   molecular function:  protein binding      BioCarta Pathways:     KEGG Pathways:  
    Probe set : 1372292_at    Name:   Similar to Serine/threonine protein kinase 24    GB acc:   AI009732    UniGene:   Rn.105397     Locus Link:   361092    Chromosome:   15    Cytoband:   Chr:15q25    SOURCE        Gene Ontology:     BioCarta Pathways:     KEGG Pathways:  
    Probe set : 1376275_at    Name:   Transcribed locus    GB acc:   BE101108    UniGene:   Rn.32291        SOURCE        Gene Ontology:     BioCarta Pathways:     KEGG Pathways:  
    Probe set : 1373823_at    Name:   similar to Cyclin-dependent kinases regulatory subunit 2 (CKS-2)    GB acc:   AA944180    UniGene:   Rn.147433    Gene Symbol:   LOC498709    Locus Link:   498709    Chromosome:   17    Cytoband:   Chr:17p14    SOURCE    GENECARD       Gene Ontology:     BioCarta Pathways:     KEGG Pathways:  
    Probe set : 1386960_at    Name:   solute carrier family 37 (glycerol-6-phosphate transporter), member 4    GB acc:   NM_031589    UniGene:   Rn.1592    Gene Symbol:   Slc37a4    Locus Link:   29573    Chromosome:   8    Cytoband:   Chr:8q22    SOURCE    GENECARD       Gene Ontology:   molecular function:  transporter activity      BioCarta Pathways:     KEGG Pathways:  
    Probe set : 1389646_at    Name:   CDC23 (cell division cycle 23, yeast, homolog) (predicted)    GB acc:   BE100584    UniGene:   Rn.139314    Gene Symbol:   Cdc23_predicted    Locus Link:   291689    Chromosome:   18    Cytoband:   Chr:18p12    SOURCE    GENECARD       Gene Ontology:   cellular component:  anaphase-promoting complex   molecular function:  ubiquitin-protein ligase activity   molecular function:  binding      BioCarta Pathways:     KEGG Pathways:  
    Probe set : 1387034_at    Name:   phenylalanine hydroxylase    GB acc:   NM_012619    UniGene:   Rn.1652    Gene Symbol:   Pah    Locus Link:   24616    Chromosome:   7    Cytoband:   Chr:7q13    SOURCE    GENECARD       Gene Ontology:     BioCarta Pathways:     KEGG Pathways:  
    Probe set : 1398295_at    Name:   solute carrier family 29 (nucleoside transporters), member 1    GB acc:   NM_031684    UniGene:   Rn.5814    Gene Symbol:   Slc29a1    Locus Link:   63997    Chromosome:   9    Cytoband:   Chr:9q12    SOURCE    GENECARD       Gene Ontology:   molecular function:  nucleoside transporter activity   molecular function:  nucleoside transporter activity      BioCarta Pathways:     KEGG Pathways:  
    Probe set : 1389911_at    Name:   similar to cDNA sequence BC019776    GB acc:   BM389126    UniGene:   Rn.64557    Gene Symbol:   RGD:1359271    Locus Link:   316842      SOURCE    GENECARD       Gene Ontology:     BioCarta Pathways:     KEGG Pathways:  
    Probe set : 1383794_at     GB acc:   AI575277              Gene Ontology:     BioCarta Pathways:     KEGG Pathways:  
    Probe set : 1390543_at    Name:   Transcribed locus    GB acc:   BF396416    UniGene:   Rn.8770        SOURCE        Gene Ontology:     BioCarta Pathways:     KEGG Pathways:  
    Probe set : 1389786_at    Name:   Similar to RIKEN cDNA 2410005O16    GB acc:   AA946272    UniGene:   Rn.51261     Locus Link:   500419      SOURCE        Gene Ontology:     BioCarta Pathways:     KEGG Pathways:  
    Probe set : 1371027_at    Name:   Casitas B-lineage lymphoma b    GB acc:   BF556820    UniGene:   Rn.21799    Gene Symbol:   Cblb    Locus Link:   171136    Chromosome:   11    Cytoband:   Chr:11q21    SOURCE    GENECARD       Gene Ontology:   cellular component:  ubiquitin ligase complex   cellular component:  nucleus      BioCarta Pathways:     KEGG Pathways:  
    Probe set : 1382660_at    Name:   Transcribed locus    GB acc:   AA923866    UniGene:   Rn.8284        SOURCE        Gene Ontology:     BioCarta Pathways:     KEGG Pathways:  
    Probe set : 1389852_at    Name:   similar to proliferation associated nuclear element 1 isoform 1 (predicted)    GB acc:   AI598316    UniGene:   Rn.11654    Gene Symbol:   RGD1307309_predicted    Locus Link:   315164    Chromosome:   7    Cytoband:   Chr:7q34    SOURCE    GENECARD       Gene Ontology:     BioCarta Pathways:     KEGG Pathways:  
    Probe set : 1383313_at    Name:   checkpoint with forkhead and ring finger domains (predicted)    GB acc:   BE120149    UniGene:   Rn.15515    Gene Symbol:   Chfr_predicted    Locus Link:   288734    Chromosome:   12    Cytoband:   Chr:12q16    SOURCE    GENECARD       Gene Ontology:   cellular component:  ubiquitin ligase complex   cellular component:  nucleus      BioCarta Pathways:     KEGG Pathways:  
    Probe set : 1368437_at    Name:   carbonic anhydrase 4    GB acc:   NM_019174    UniGene:   Rn.51389    Gene Symbol:   Ca4    Locus Link:   29242    Chromosome:   10    Cytoband:   Chr:10q26    SOURCE    GENECARD       Gene Ontology:   biological process:  one-carbon compound metabolism   cellular component:  extracellular space   cellular component:  membrane   molecular function:  carbonate dehydratase activity   molecular function:  zinc ion binding   molecular function:  lyase activity      BioCarta Pathways:     KEGG Pathways:  
    Probe set : 1370942_at    Name:   RAS p21 protein activator 3    GB acc:   AI170661    UniGene:   Rn.23055    Gene Symbol:   Rasa3    Locus Link:   29372    Chromosome:   16    Cytoband:   Chr:16q12.5    SOURCE    GENECARD       Gene Ontology:   biological process:  intracellular signaling cascade   molecular function:  GTPase activator activity      BioCarta Pathways:     KEGG Pathways:  
    Probe set : 1373436_at    Name:   similar to RIKEN cDNA 1700040L02 (predicted)    GB acc:   AI171288    UniGene:   Rn.137452    Gene Symbol:   RGD1306739_predicted    Locus Link:   361834    Chromosome:   20    Cytoband:   Chr:20p11    SOURCE    GENECARD       Gene Ontology:     BioCarta Pathways:     KEGG Pathways:  
    Probe set : 1373991_at    Name:   Transcribed locus    GB acc:   AI411366    UniGene:   Rn.146464        SOURCE        Gene Ontology:     BioCarta Pathways:     KEGG Pathways:  
    Probe set : 1373217_at    Name:   Transcribed locus    GB acc:   AI010234    UniGene:   Rn.3740        SOURCE        Gene Ontology:     BioCarta Pathways:     KEGG Pathways:  
    Probe set : 1382331_at    Name:   similar to RIKEN cDNA 0610038L10 gene /// similar to RIKEN cDNA 0610038L10 gene /// similar to riboflavin kinase /// similar to riboflavin kinase    GB acc:   AI180421    UniGene:   Rn.13360    Gene Symbol:   LOC311241 /// LOC317214 /// LOC499328 /// LOC501601    Locus Link:   311241 /// 317214 /// 499328 /// 501601    Chromosome:   3    Cytoband:   Chr:3q31 /// Chr:Xq31 /// Chr:1q43    SOURCE    GENECARD       Gene Ontology:   biological process:  riboflavin biosynthesis   molecular function:  riboflavin kinase activity      BioCarta Pathways:     KEGG Pathways:  
    Probe set : 1382496_at     GB acc:   BI288517              Gene Ontology:     BioCarta Pathways:     KEGG Pathways:  
    Probe set : 1377727_at    Name:   bromodomain adjacent to zinc finger domain, 1A (predicted)    GB acc:   BE109134    UniGene:   Rn.1864    Gene Symbol:   Baz1a_predicted    Locus Link:   314126    Chromosome:   6    Cytoband:   Chr:6q23    SOURCE    GENECARD       Gene Ontology:   biological process:  regulation of transcription, DNA-dependent   biological process:  protein ubiquitination   cellular component:  ubiquitin ligase complex   cellular component:  nucleus   molecular function:  DNA binding   molecular function:  ubiquitin-protein ligase activity   molecular function:  zinc ion binding      BioCarta Pathways:     KEGG Pathways:  
    Probe set : 1376136_at     GB acc:   BI296118              Gene Ontology:     BioCarta Pathways:     KEGG Pathways:  
    Probe set : 1368126_at    Name:   acetoacetyl-CoA synthetase    GB acc:   NM_023104    UniGene:   Rn.9215    Gene Symbol:   Aacs    Locus Link:   65984    Chromosome:   12    Cytoband:   Chr:12q14    SOURCE    GENECARD       Gene Ontology:   cellular component:  cytosol      BioCarta Pathways:     KEGG Pathways:  
    Probe set : 1373027_at     GB acc:   AA944375              Gene Ontology:     BioCarta Pathways:     KEGG Pathways:  
    Probe set : 1376623_at    Name:   similar to hypothetical protein FLJ20898    GB acc:   AI409186    UniGene:   Rn.16780    Gene Symbol:   RGD:1359314    Locus Link:   287129    Chromosome:   10    Cytoband:   Chr:10q12    SOURCE    GENECARD       Gene Ontology:   cellular component:  integral to membrane      BioCarta Pathways:     KEGG Pathways:  
    Probe set : 1398892_at    Name:   Niemann Pick type C2    GB acc:   BI287960    UniGene:   Rn.3491    Gene Symbol:   Npc2    Locus Link:   286898    Chromosome:   6    Cytoband:   Chr:6q31    SOURCE    GENECARD       Gene Ontology:     BioCarta Pathways:     KEGG Pathways:  
    Probe set : 1371346_at    Name:   NADH dehydrogenase (ubiquinone) 1 beta subcomplex, 6, 17kDa (predicted)    GB acc:   AI104528    UniGene:   Rn.104528    Gene Symbol:   Ndufb6_predicted    Locus Link:   297990    Chromosome:   5    Cytoband:   Chr:5q22    SOURCE    GENECARD       Gene Ontology:   biological process:  mitochondrial electron transport, NADH to ubiquinone   cellular component:  mitochondrion   molecular function:  NADH dehydrogenase activity   molecular function:  NADH dehydrogenase (ubiquinone) activity   molecular function:  oxidoreductase activity      BioCarta Pathways:     KEGG Pathways:  
    Probe set : 1388121_at    Name:   amyloid beta (A4) precursor-like protein 2    GB acc:   M31322    UniGene:   Rn.1423    Gene Symbol:   Aplp2    Locus Link:   64312    Chromosome:   8    Cytoband:   Chr:8q13    SOURCE    GENECARD       Gene Ontology:   cellular component:  extracellular space   cellular component:  nucleus   cellular component:  integral to membrane      BioCarta Pathways:     KEGG Pathways:  
    Probe set : 1382211_at    Name:   Transcribed locus    GB acc:   AI602542    UniGene:   Rn.107412        SOURCE        Gene Ontology:     BioCarta Pathways:     KEGG Pathways:  
    Probe set : 1399130_at    Name:   similar to RIKEN cDNA 2610022G08    GB acc:   AW918690    UniGene:   Rn.20423    Gene Symbol:   LOC502782    Locus Link:   502782    Chromosome:   4    Cytoband:   Chr:4q24    SOURCE    GENECARD       Gene Ontology:     BioCarta Pathways:     KEGG Pathways:  
    Probe set : 1367790_at    Name:   staphylococcal nuclease domain containing 1    GB acc:   NM_022694    UniGene:   Rn.36148    Gene Symbol:   Snd1    Locus Link:   64635      SOURCE    GENECARD       Gene Ontology:   biological process:  transcription   biological process:  regulation of transcription, DNA-dependent   cellular component:  nucleus   molecular function:  nucleic acid binding   molecular function:  nuclease activity      BioCarta Pathways:     KEGG Pathways:  
    Probe set : 1379283_at    Name:   Transcribed locus    GB acc:   BE117687    UniGene:   Rn.8634        SOURCE        Gene Ontology:     BioCarta Pathways:     KEGG Pathways:  
    Probe set : 1368077_at    Name:   fructose-1,6- biphosphatase 1    GB acc:   NM_012558    UniGene:   Rn.33703    Gene Symbol:   Fbp1    Locus Link:   24362    Chromosome:   17    Cytoband:   Chr:17p14    SOURCE    GENECARD       Gene Ontology:   biological process:  carbohydrate metabolism   biological process:  fructose metabolism   biological process:  gluconeogenesis      BioCarta Pathways:     KEGG Pathways:  
    Probe set : 1367507_at    Name:   Unknown (protein for MGC:72981)    GB acc:   BI281661    UniGene:   Rn.1934    Gene Symbol:   RGD:735224    Locus Link:   293703    Chromosome:   1    Cytoband:   Chr:1q43    SOURCE    GENECARD       Gene Ontology:     BioCarta Pathways:     KEGG Pathways:  
    Probe set : 1371300_at    Name:   ribosomal protein L3    GB acc:   AA892367    UniGene:   Rn.107726    Gene Symbol:   Rpl3    Locus Link:   300079    Chromosome:   7    Cytoband:   Chr:7q34    SOURCE    GENECARD       Gene Ontology:   biological process:  protein biosynthesis   cellular component:  intracellular   cellular component:  ribosome   cellular component:  ribonucleoprotein complex   molecular function:  structural constituent of ribosome      BioCarta Pathways:     KEGG Pathways:  
    Probe set : 1388703_at    Name:   endothelial cell adhesion molecule    GB acc:   BF285026    UniGene:   Rn.17089    Gene Symbol:   RGD:1303286    Locus Link:   300519    Chromosome:   8    Cytoband:   Chr:8q21    SOURCE    GENECARD       Gene Ontology:   biological process:  cell adhesion   cellular component:  integral to membrane   molecular function:  protein binding      BioCarta Pathways:     KEGG Pathways:  
    Probe set : 1370554_at    Name:   ubiquitin carboxyl-terminal esterase L3 (ubiquitin thiolesterase) /// similar to ubiqutin carboxyl-terminal hydrolase l3    GB acc:   AB043959    UniGene:   Rn.46952    Gene Symbol:   Uchl3 /// LOC498560    Locus Link:   114094 /// 498560    Chromosome:   3    Cytoband:   Chr:3q42 /// Chr:15q21    SOURCE    GENECARD       Gene Ontology:   cellular component:  intracellular   cellular component:  cytoplasm      BioCarta Pathways:     KEGG Pathways:  
    Probe set : 1383191_at    Name:   protein phosphatase 1, regulatory (inhibitor) subunit 1A    GB acc:   BE097583    UniGene:   Rn.9756    Gene Symbol:   Ppp1r1a    Locus Link:   58977    Chromosome:   7    Cytoband:   Chr:7q36    SOURCE    GENECARD       Gene Ontology:   biological process:  glycogen metabolism   biological process:  signal transduction   biological process:  carbohydrate metabolism   molecular function:  protein phosphatase inhibitor activity   molecular function:  type 1 serine/threonine specific protein phosphatase inhibitor activity      BioCarta Pathways:     KEGG Pathways:  
    Probe set : 1398599_at    Name:   similar to integral membrane protein 1    GB acc:   BG664929    UniGene:   Rn.120361    Gene Symbol:   LOC500972    Locus Link:   500972    Chromosome:   8    Cytoband:   Chr:8q21    SOURCE    GENECARD       Gene Ontology:     BioCarta Pathways:     KEGG Pathways:  
    Probe set : 1391201_at    Name:   WD repeat and HMG-box DNA binding protein 1 (predicted)    GB acc:   AI012084    UniGene:   Rn.98955     Locus Link:   305827    Chromosome:   15    Cytoband:   Chr:15p14    SOURCE        Gene Ontology:   biological process:  regulation of transcription, DNA-dependent   cellular component:  nucleus   cellular component:  nucleoplasm   cellular component:  cytoplasm   molecular function:  DNA binding      BioCarta Pathways:     KEGG Pathways:  
    Probe set : 1390300_at     GB acc:   BM383635              Gene Ontology:     BioCarta Pathways:     KEGG Pathways:  
    Probe set : 1379365_at    Name:   chemokine (C-X-C motif) ligand 11    GB acc:   BF281987    UniGene:   Rn.13664    Gene Symbol:   Cxcl11    Locus Link:   305236    Chromosome:   14    Cytoband:   Chr:14p22    SOURCE    GENECARD       Gene Ontology:   biological process:  immune response   biological process:  immune response   cellular component:  extracellular region      BioCarta Pathways:     KEGG Pathways:  
    Probe set : 1374034_at    Name:   cysteinyl-tRNA synthetase (predicted)    GB acc:   BG379410    UniGene:   Rn.14865    Gene Symbol:   Cars_predicted    Locus Link:   293638    Chromosome:   1    Cytoband:   Chr:1q41    SOURCE    GENECARD       Gene Ontology:   biological process:  protein biosynthesis   biological process:  cysteinyl-tRNA aminoacylation   cellular component:  soluble fraction   cellular component:  cytoplasm      BioCarta Pathways:     KEGG Pathways:  
    Probe set : 1368851_at    Name:   v-ets erythroblastosis virus E26 oncogene homolog 1 (avian)    GB acc:   NM_012555    UniGene:   Rn.88756    Gene Symbol:   Ets1    Locus Link:   24356    Chromosome:   8    Cytoband:   Chr:8q21    SOURCE    GENECARD       Gene Ontology:   cellular component:  nucleus      BioCarta Pathways:     KEGG Pathways:  
    Probe set : 1380148_at    Name:   Transcribed locus    GB acc:   BE118688    UniGene:   Rn.146647        SOURCE        Gene Ontology:     BioCarta Pathways:     KEGG Pathways:  
    Probe set : 1384988_at    Name:   F-box only protein 5 (predicted)    GB acc:   BM385403    UniGene:   Rn.81585    Gene Symbol:   Fbxo5_predicted    Locus Link:   292263    Chromosome:   1    Cytoband:   Chr:1p11    SOURCE    GENECARD       Gene Ontology:   biological process:  proteolysis   biological process:  ubiquitin cycle   molecular function:  ubiquitin conjugating enzyme activity   molecular function:  ubiquitin-protein ligase activity      BioCarta Pathways:     KEGG Pathways:  
    Probe set : 1391463_at    Name:   DEAD (Asp-Glu-Ala-Asp) box polypeptide 58 (predicted)    GB acc:   AI575264    UniGene:   Rn.38642     Locus Link:   297989    Chromosome:   5    Cytoband:   Chr:5q22    SOURCE        Gene Ontology:     BioCarta Pathways:     KEGG Pathways:  
    Probe set : 1382776_at    Name:   similar to O-acyltransferase (membrane bound) domain containing 1    GB acc:   AI716936    UniGene:   Rn.14994    Gene Symbol:   LOC498741    Locus Link:   498741    Chromosome:   17    Cytoband:   Chr:17p12    SOURCE    GENECARD       Gene Ontology:     BioCarta Pathways:     KEGG Pathways:  
    Probe set : 1367634_at    Name:   ribosomal protein L31    GB acc:   NM_022506    UniGene:   Rn.1101    Gene Symbol:   Rpl31    Locus Link:   64298    Chromosome:   9    Cytoband:   Chr:9q21    SOURCE    GENECARD       Gene Ontology:   biological process:  protein biosynthesis   molecular function:  RNA binding   molecular function:  structural constituent of ribosome      BioCarta Pathways:     KEGG Pathways:  
    Probe set : 1371818_at    Name:   Exportin, tRNA (nuclear export receptor for tRNAs) (predicted)    GB acc:   AW251204    UniGene:   Rn.2238     Locus Link:   314879    Chromosome:   7    Cytoband:   Chr:7q22    SOURCE        Gene Ontology:   biological process:  transport   cellular component:  nucleus   molecular function:  tRNA binding   molecular function:  binding      BioCarta Pathways:     KEGG Pathways:  
    Probe set : 1378470_at    Name:   similar to i-45 protein (49.8 kD) (4H318) (predicted)    GB acc:   BM384409    UniGene:   Rn.18098    Gene Symbol:   RGD1311259_predicted    Locus Link:   315741    Chromosome:   8    Cytoband:   Chr:8q24    SOURCE    GENECARD       Gene Ontology:   biological process:  G-protein coupled receptor protein signaling pathway   cellular component:  integral to membrane   molecular function:  receptor activity   molecular function:  G-protein coupled receptor activity   molecular function:  steroid binding      BioCarta Pathways:     KEGG Pathways:  
    Probe set : 1386966_a_at    Name:   protein kinase C-binding protein Beta15    GB acc:   NM_021764    UniGene:   Rn.37161    Gene Symbol:   Pkcbpb15    Locus Link:   60383    Chromosome:   3    Cytoband:   Chr:3q41    SOURCE    GENECARD       Gene Ontology:   biological process:  protein modification   biological process:  ubiquitin cycle   biological process:  protein ubiquitination   cellular component:  ubiquitin ligase complex   cellular component:  intracellular      BioCarta Pathways:     KEGG Pathways:  
    Probe set : 1390638_at    Name:   Similar to Eph receptor A4    GB acc:   AI072459    UniGene:   Rn.6202     Locus Link:   316539    Chromosome:   9    Cytoband:   Chr:9q33    SOURCE        Gene Ontology:     BioCarta Pathways:     KEGG Pathways:  
    Probe set : 1382763_at     GB acc:   AW921300              Gene Ontology:     BioCarta Pathways:     KEGG Pathways:  
    Probe set : 1372267_at    Name:   proteasome (prosome, macropain) 26S subunit, non-ATPase, 5 (predicted)    GB acc:   AI233729    UniGene:   Rn.13415    Gene Symbol:   Psmd5_predicted    Locus Link:   296651    Chromosome:   3    Cytoband:   Chr:3p11    SOURCE    GENECARD       Gene Ontology:   cellular component:  proteasome complex (sensu Eukaryota)   cellular component:  cytosol      BioCarta Pathways:     KEGG Pathways:  
    Probe set : 1387070_at    Name:   thymopoietin    GB acc:   AI044110    UniGene:   Rn.3364    Gene Symbol:   Tmpo    Locus Link:   25359    Chromosome:   7    Cytoband:   Chr:7q13    SOURCE    GENECARD       Gene Ontology:   biological process:  regulation of transcription      BioCarta Pathways:     KEGG Pathways:  
    Probe set : 1382995_at    Name:   Neuropilin 2    GB acc:   AA859669    UniGene:   Rn.10816    Gene Symbol:   Nrp2    Locus Link:   81527    Chromosome:   9    Cytoband:   Chr:9q31    SOURCE    GENECARD       Gene Ontology:   cellular component:  membrane fraction   cellular component:  membrane   cellular component:  integral to membrane      BioCarta Pathways:     KEGG Pathways:  
    Probe set : 1374709_at    Name:   Transcribed locus    GB acc:   AI406795    UniGene:   Rn.19878        SOURCE        Gene Ontology:     BioCarta Pathways:     KEGG Pathways:  
    Probe set : 1388364_at    Name:   NADH dehydrogenase (ubiquinone) Fe-S protein 3 (predicted)    GB acc:   BG381650    UniGene:   Rn.1261    Gene Symbol:   Ndufs3_predicted    Locus Link:   295923    Chromosome:   3    Cytoband:   Chr:3q24    SOURCE    GENECARD       Gene Ontology:   biological process:  electron transport   biological process:  mitochondrial electron transport, NADH to ubiquinone      BioCarta Pathways:     KEGG Pathways:  
    Probe set : 1394371_at    Name:   nuclear fragile X mental retardation protein interacting protein 1    GB acc:   BF556765    UniGene:   Rn.22071    Gene Symbol:   RGD:1359440    Locus Link:   364430    Chromosome:   15    Cytoband:   Chr:15q11    SOURCE    GENECARD       Gene Ontology:   cellular component:  nucleus   molecular function:  nucleic acid binding   molecular function:  zinc ion binding      BioCarta Pathways:     KEGG Pathways:  
    Probe set : 1374074_at    Name:   cell division cycle 37 homolog (S. cerevisiae)-like (predicted)    GB acc:   BF285292    UniGene:   Rn.60570    Gene Symbol:   Cdc37l_predicted    Locus Link:   293886    Chromosome:   1    Cytoband:   Chr:1q52    SOURCE    GENECARD       Gene Ontology:   biological process:  regulation of progression through cell cycle   biological process:  cytokinesis   biological process:  cell division   cellular component:  cytoplasm   molecular function:  protein binding      BioCarta Pathways:     KEGG Pathways:  
    Probe set : 1370714_a_at    Name:   sialyltransferase 1    GB acc:   M18769    UniGene:   Rn.54567    Gene Symbol:   Siat1    Locus Link:   25197    Chromosome:   11    Cytoband:   Chr:11q23    SOURCE    GENECARD       Gene Ontology:   biological process:  protein amino acid glycosylation      BioCarta Pathways:     KEGG Pathways:  
    Probe set : 1398535_at    Name:   Hbs1-like (S. cerevisiae) (predicted)    GB acc:   AA998579    UniGene:   Rn.98198    Gene Symbol:   Hbs1l_predicted    Locus Link:   293408    Chromosome:   1    Cytoband:   Chr:1p12    SOURCE    GENECARD       Gene Ontology:   biological process:  protein biosynthesis   biological process:  translational elongation   biological process:  signal transduction   molecular function:  translation elongation factor activity   molecular function:  GTP binding   molecular function:  nucleotide binding      BioCarta Pathways:     KEGG Pathways:  
    Probe set : 1378181_at    Name:   ribonuclease P 40 subunit (human) (predicted)    GB acc:   BI279714    UniGene:   Rn.23660    Gene Symbol:   Rpp40_predicted    Locus Link:   291071    Chromosome:   17    Cytoband:   Chr:17p12    SOURCE    GENECARD       Gene Ontology:   biological process:  tRNA 5'-leader removal   biological process:  tRNA processing   cellular component:  nucleus   cellular component:  nucleolar ribonuclease P complex   molecular function:  ribonuclease P activity   molecular function:  hydrolase activity      BioCarta Pathways:     KEGG Pathways:  
    Probe set : 1368209_at    Name:   membrane-associated protein 17    GB acc:   NM_130401    UniGene:   Rn.3605    Gene Symbol:   Map17    Locus Link:   81916    Chromosome:   5    Cytoband:   Chr:5q36    SOURCE    GENECARD       Gene Ontology:   cellular component:  extracellular region   cellular component:  extracellular space   cellular component:  integral to membrane      BioCarta Pathways:     KEGG Pathways:  
    Probe set : 1374244_at    Name:   Ab2-060    GB acc:   AI411141    UniGene:   Rn.43844     Locus Link:   501038      SOURCE        Gene Ontology:     BioCarta Pathways:     KEGG Pathways:  
    Probe set : 1376601_at    Name:   Sema domain, transmembrane domain (TM), and cytoplasmic domain, (semaphorin) 6A (predicted)    GB acc:   BF397526    UniGene:   Rn.24818     Locus Link:   361324    Chromosome:   18    Cytoband:   Chr:18q11    SOURCE        Gene Ontology:   cellular component:  membrane   cellular component:  integral to membrane   cellular component:  axon   molecular function:  receptor activity   molecular function:  protein binding      BioCarta Pathways:     KEGG Pathways:  
    Probe set : 1376595_at    Name:   similar to peroxisome biogenesis factor 1    GB acc:   BF282695    UniGene:   Rn.39842    Gene Symbol:   LOC500006    Locus Link:   500006    Chromosome:   4    Cytoband:   Chr:4q13    SOURCE    GENECARD       Gene Ontology:     BioCarta Pathways:     KEGG Pathways:  
    Probe set : 1390378_at    Name:   Transcribed locus    GB acc:   BE107173    UniGene:   Rn.146837        SOURCE        Gene Ontology:     BioCarta Pathways:     KEGG Pathways:  
    Probe set : 1385585_at    Name:   Transcribed locus    GB acc:   AI111674    UniGene:   Rn.21134        SOURCE        Gene Ontology:     BioCarta Pathways:     KEGG Pathways:  
    Probe set : 1398856_at    Name:   proteasome (prosome, macropain) subunit, alpha type 2    GB acc:   AA801204    UniGene:   Rn.1617    Gene Symbol:   Psma2    Locus Link:   29669    Chromosome:   17    Cytoband:   Chr:17q12.1    SOURCE    GENECARD       Gene Ontology:   biological process:  ubiquitin-dependent protein catabolism   cellular component:  cytosol   cellular component:  proteasome core complex (sensu Eukaryota)   cellular component:  protein complex      BioCarta Pathways:     KEGG Pathways:  
    Probe set : 1379044_at    Name:   Similar to RIKEN cDNA 1700108L22 (predicted)    GB acc:   AW534064    UniGene:   Rn.7988     Locus Link:   310348    Chromosome:   2    Cytoband:   Chr:2q26    SOURCE        Gene Ontology:     BioCarta Pathways:     KEGG Pathways:  
    Probe set : 1393935_at    Name:   Transcribed locus    GB acc:   AA819254    UniGene:   Rn.147489        SOURCE        Gene Ontology:     BioCarta Pathways:     KEGG Pathways:  
    Probe set : 1392573_at    Name:   similar to ubiquitin-conjugating enzyme HR6A    GB acc:   BF284538    UniGene:   Rn.144869    Gene Symbol:   RGD:1359534    Locus Link:   298317    Chromosome:   X    Cytoband:   Chr:Xq12    SOURCE    GENECARD       Gene Ontology:   biological process:  protein modification   biological process:  ubiquitin cycle   molecular function:  ubiquitin-like activating enzyme activity   molecular function:  ligase activity      BioCarta Pathways:     KEGG Pathways:  
    Probe set : 1379847_at    Name:   dysferlin (predicted)    GB acc:   AW141858    UniGene:   Rn.22869    Gene Symbol:   Dysf_predicted    Locus Link:   312492    Chromosome:   4    Cytoband:   Chr:4q34    SOURCE    GENECARD       Gene Ontology:   biological process:  plasma membrane repair   biological process:  muscle contraction   cellular component:  plasma membrane   cellular component:  integral to membrane   cellular component:  sarcolemma   molecular function:  protein binding      BioCarta Pathways:     KEGG Pathways:  
    Probe set : 1371786_at    Name:   tripartite motif-containing 35    GB acc:   BE111791    UniGene:   Rn.105225    Gene Symbol:   Trim35    Locus Link:   498538    Chromosome:   15    Cytoband:   Chr:15p12    SOURCE    GENECARD       Gene Ontology:   biological process:  protein ubiquitination   cellular component:  ubiquitin ligase complex   cellular component:  intracellular   molecular function:  ubiquitin-protein ligase activity   molecular function:  zinc ion binding   molecular function:  metal ion binding      BioCarta Pathways:     KEGG Pathways:  
    Probe set : 1389780_at    Name:   tissue factor pathway inhibitor    GB acc:   AI009783    UniGene:   Rn.15795    Gene Symbol:   Tfpi    Locus Link:   29436    Chromosome:   3    Cytoband:   Chr:3q24    SOURCE    GENECARD       Gene Ontology:   biological process:  blood coagulation   cellular component:  extracellular region   cellular component:  extracellular space      BioCarta Pathways:     KEGG Pathways:  
    Probe set : 1381445_at    Name:   Transcribed locus    GB acc:   AA996810    UniGene:   Rn.59109        SOURCE        Gene Ontology:     BioCarta Pathways:     KEGG Pathways:  
    Probe set : 1373377_at    Name:   sodium channel modifier 1 (predicted)    GB acc:   BG376162    UniGene:   Rn.40268    Gene Symbol:   Scnm1_predicted    Locus Link:   310662    Chromosome:   2    Cytoband:   Chr:2q34    SOURCE    GENECARD       Gene Ontology:     BioCarta Pathways:     KEGG Pathways:  
    Probe set : 1368098_a_at    Name:   small nuclear ribonucleoprotein N    GB acc:   M29294    UniGene:   Rn.11169    Gene Symbol:   Snrpn    Locus Link:   81781    Chromosome:   1    Cytoband:   Chr:1q22    SOURCE    GENECARD       Gene Ontology:   biological process:  mRNA processing   molecular function:  RNA binding      BioCarta Pathways:     KEGG Pathways:  
    Probe set : 1369026_at    Name:   ADP-ribosylation factor interacting protein 1    GB acc:   NM_021763    UniGene:   Rn.37130    Gene Symbol:   Arfip1    Locus Link:   60382    Chromosome:   2    Cytoband:   Chr:2q34    SOURCE    GENECARD       Gene Ontology:   cellular component:  Golgi membrane      BioCarta Pathways:     KEGG Pathways:  
    Probe set : 1368138_at    Name:   hypothetical gene supported by NM_017212    GB acc:   BI281170     Gene Symbol:   LOC497674    Locus Link:   497674    Chromosome:   10    Cytoband:   Chr:10q32.1     GENECARD       Gene Ontology:     BioCarta Pathways:     KEGG Pathways:  
    Probe set : 1380030_at    Name:   zinc finger protein 593 (predicted)    GB acc:   AW523520    UniGene:   Rn.95330    Gene Symbol:   Znf593_predicted    Locus Link:   298546    Chromosome:   5    Cytoband:   Chr:5q36    SOURCE    GENECARD       Gene Ontology:   biological process:  negative regulation of transcription from RNA polymerase II promoter   biological process:  regulation of transcription, DNA-dependent   cellular component:  nucleus   molecular function:  DNA binding   molecular function:  transcription corepressor activity   molecular function:  zinc ion binding      BioCarta Pathways:     KEGG Pathways:  
    Probe set : 1368164_at    Name:   biliverdin reductase A    GB acc:   NM_053850    UniGene:   Rn.9865    Gene Symbol:   Blvra    Locus Link:   116599    Chromosome:   3    Cytoband:   Chr:3q36    SOURCE    GENECARD       Gene Ontology:   biological process:  electron transport   biological process:  metabolism   biological process:  heme catabolism   cellular component:  soluble fraction      BioCarta Pathways:     KEGG Pathways:  
    Probe set : 1383499_at    Name:   Transcribed locus    GB acc:   BG371995    UniGene:   Rn.14698        SOURCE        Gene Ontology:     BioCarta Pathways:     KEGG Pathways:  
    Probe set : 1397744_at    Name:   Dopamine beta hydroxylase    GB acc:   BI276183    UniGene:   Rn.87166    Gene Symbol:   Dbh    Locus Link:   25699    Chromosome:   3    Cytoband:   Chr:3p12    SOURCE    GENECARD       Gene Ontology:     BioCarta Pathways:     KEGG Pathways:  
    Probe set : 1374101_at    Name:   TBC1 domain family, member 10 (predicted)    GB acc:   BF282437    UniGene:   Rn.105859    Gene Symbol:   Tbc1d10_predicted    Locus Link:   360968    Chromosome:   14    Cytoband:   Chr:14q21    SOURCE    GENECARD       Gene Ontology:   cellular component:  microvillus   molecular function:  PDZ domain binding      BioCarta Pathways:     KEGG Pathways:  
    Probe set : 1372920_at     GB acc:   AI411345              Gene Ontology:     BioCarta Pathways:     KEGG Pathways:  
    Probe set : 1368064_a_at    Name:   dopa decarboxylase    GB acc:   U31884    UniGene:   Rn.11064    Gene Symbol:   Ddc    Locus Link:   24311    Chromosome:   14    Cytoband:   Chr:14q21    SOURCE    GENECARD       Gene Ontology:   cellular component:  intracellular      BioCarta Pathways:     KEGG Pathways:  
    Probe set : 1377654_at    Name:   similar to RIKEN cDNA 1810037C20    GB acc:   BE328936    UniGene:   Rn.12310    Gene Symbol:   LOC501664    Locus Link:   501664    Chromosome:   X    Cytoband:   Chr:Xq37    SOURCE    GENECARD       Gene Ontology:     BioCarta Pathways:     KEGG Pathways:  
    Probe set : 1383472_at    Name:   aldehyde dehydrogenase 1 family, member B1 (predicted)    GB acc:   AA997683    UniGene:   Rn.12547    Gene Symbol:   Aldh1b1_predicted    Locus Link:   298079    Chromosome:   5    Cytoband:   Chr:5q22    SOURCE    GENECARD       Gene Ontology:   biological process:  carbohydrate metabolism   biological process:  metabolism   cellular component:  extracellular space   cellular component:  mitochondrion   molecular function:  aldehyde dehydrogenase activity   molecular function:  aldehyde dehydrogenase (NAD) activity   molecular function:  oxidoreductase activity      BioCarta Pathways:     KEGG Pathways:  
    Probe set : 1382928_at    Name:   Transcribed locus    GB acc:   AI233682    UniGene:   Rn.54119        SOURCE        Gene Ontology:     BioCarta Pathways:     KEGG Pathways:  
    Probe set : 1378640_at    Name:   ubiquitin-like, containing PHD and RING finger domains, 1    GB acc:   BE098732    UniGene:   Rn.54318    Gene Symbol:   Uhrf1    Locus Link:   316129    Chromosome:   9    Cytoband:   Chr:9q11    SOURCE    GENECARD       Gene Ontology:   cellular component:  nucleus   cellular component:  ubiquitin ligase complex      BioCarta Pathways:     KEGG Pathways:  
    Probe set : 1388744_at    Name:   minichromosome maintenance protein 7    GB acc:   AI599678    UniGene:   Rn.113    Gene Symbol:   RGD:1303018    Locus Link:   288532    Chromosome:   12    Cytoband:   Chr:12q11    SOURCE    GENECARD       Gene Ontology:   biological process:  DNA replication initiation   cellular component:  nucleus      BioCarta Pathways:     KEGG Pathways:  
    Probe set : 1374563_at    Name:   Transcribed locus    GB acc:   BI301280    UniGene:   Rn.2943        SOURCE        Gene Ontology:     BioCarta Pathways:     KEGG Pathways:  
    Probe set : 1370332_at    Name:   Munc13-4 protein    GB acc:   AF159356    UniGene:   Rn.32635    Gene Symbol:   Unc13h4    Locus Link:   192177    Chromosome:   10    Cytoband:   Chr:10q32.3    SOURCE    GENECARD       Gene Ontology:   biological process:  secretion   biological process:  exocytosis      BioCarta Pathways:     KEGG Pathways:  
    Probe set : 1398516_at    Name:   similar to RIKEN cDNA 0610012C01    GB acc:   BF283013    UniGene:   Rn.13207    Gene Symbol:   LOC498230    Locus Link:   498230    Chromosome:   13    Cytoband:   Chr:13q13    SOURCE    GENECARD       Gene Ontology:     BioCarta Pathways:     KEGG Pathways:  
    Probe set : 1375432_at    Name:   DD6A4-12(7)-2 mRNA, partial sequence    GB acc:   AI171599    UniGene:   Rn.22355        SOURCE        Gene Ontology:     BioCarta Pathways:     KEGG Pathways:  
    Probe set : 1389191_at    Name:   similar to RIKEN cDNA 2210415M20 (predicted)    GB acc:   AA858649    UniGene:   Rn.12707    Gene Symbol:   RGD1309754_predicted    Locus Link:   290851    Chromosome:   16    Cytoband:   Chr:16q12.5    SOURCE    GENECARD       Gene Ontology:     BioCarta Pathways:     KEGG Pathways:  
    Probe set : 1397500_x_at    Name:   Transcribed locus    GB acc:   AA818718    UniGene:   Rn.153901        SOURCE        Gene Ontology:     BioCarta Pathways:     KEGG Pathways:  
    Probe set : 1372430_at    Name:   similar to RIKEN cDNA 1700037H04 (predicted)    GB acc:   H31887    UniGene:   Rn.144317    Gene Symbol:   RGD1311739_predicted    Locus Link:   311428    Chromosome:   3    Cytoband:   Chr:3q36    SOURCE    GENECARD       Gene Ontology:   biological process:  protein biosynthesis   biological process:  aspartyl-tRNA aminoacylation   cellular component:  cytoplasm   molecular function:  aspartate-tRNA ligase activity   molecular function:  ATP binding   molecular function:  ligase activity      BioCarta Pathways:     KEGG Pathways:  
    Probe set : 1390883_at    Name:   Similar to A-kinase anchor protein 11 (Protein kinase A anchoring protein 11) (PRKA11) (A kinase anchor protein 220 kDa) (AKAP 220) (hAKAP220)    GB acc:   AI501759    UniGene:   Rn.10557    Gene Symbol:   Akap11    Locus Link:   25228    Chromosome:   2    Cytoband:   Chr:2    SOURCE    GENECARD       Gene Ontology:   biological process:  protein kinase cascade   cellular component:  peroxisome   cellular component:  peroxisome      BioCarta Pathways:     KEGG Pathways:  
    Probe set : 1374440_at    Name:   dehydrogenase/reductase (SDR family) member 8    GB acc:   BE098506    UniGene:   Rn.102059    Gene Symbol:   RGD:1303235    Locus Link:   289456    Chromosome:   14    Cytoband:   Chr:14p22    SOURCE    GENECARD       Gene Ontology:   biological process:  metabolism   biological process:  steroid biosynthesis   biological process:  lipid biosynthesis   cellular component:  extracellular space   molecular function:  oxidoreductase activity      BioCarta Pathways:     KEGG Pathways:  
    Probe set : 1388634_at    Name:   phosphoglucomutase 1    GB acc:   BI277505    UniGene:   Rn.9970    Gene Symbol:   Pgm1    Locus Link:   24645    Chromosome:   5    Cytoband:   Chr:5q33    SOURCE    GENECARD       Gene Ontology:   cellular component:  cytoplasm      BioCarta Pathways:     KEGG Pathways:  
    Probe set : 1376483_at    Name:   LSM5 homolog, U6 small nuclear RNA associated (S. cerevisiae) (predicted)    GB acc:   AW251852    UniGene:   Rn.8983    Gene Symbol:   Lsm5_predicted    Locus Link:   306222    Chromosome:   4    Cytoband:   Chr:4q24    SOURCE    GENECARD       Gene Ontology:   biological process:  nuclear mRNA splicing, via spliceosome   cellular component:  nucleus   cellular component:  small nucleolar ribonucleoprotein complex   molecular function:  RNA binding      BioCarta Pathways:     KEGG Pathways:  
    Probe set : 1372255_at    Name:   arginyl-tRNA synthetase (predicted)    GB acc:   BF283284    UniGene:   Rn.18711    Gene Symbol:   Rars_predicted    Locus Link:   287191    Chromosome:   10    Cytoband:   Chr:10q12    SOURCE    GENECARD       Gene Ontology:   biological process:  protein biosynthesis   biological process:  tRNA aminoacylation for protein translation   biological process:  arginyl-tRNA aminoacylation   cellular component:  soluble fraction   cellular component:  cytoplasm      BioCarta Pathways:     KEGG Pathways:  
    Probe set : 1376597_at    Name:   zinc finger, CCHC domain containing 10 (predicted)    GB acc:   AI100951    UniGene:   Rn.53927    Gene Symbol:   Zcchc10_predicted    Locus Link:   360524    Chromosome:   10    Cytoband:   Chr:10q22    SOURCE    GENECARD       Gene Ontology:   molecular function:  nucleic acid binding   molecular function:  zinc ion binding   molecular function:  metal ion binding      BioCarta Pathways:     KEGG Pathways:  
    Probe set : 1373253_at    Name:   acyl-Coenzyme A binding domain containing 4 (predicted)    GB acc:   BE111646    UniGene:   Rn.18284    Gene Symbol:   Acbd4_predicted    Locus Link:   303577    Chromosome:   10    Cytoband:   Chr:10q32.1    SOURCE    GENECARD       Gene Ontology:   molecular function:  acyl-CoA binding      BioCarta Pathways:     KEGG Pathways:  
    Probe set : 1389904_at     GB acc:   AI171529              Gene Ontology:     BioCarta Pathways:     KEGG Pathways:  
    Probe set : 1382611_at    Name:   Transcribed locus    GB acc:   AA945938    UniGene:   Rn.24100        SOURCE        Gene Ontology:     BioCarta Pathways:     KEGG Pathways:  
    Probe set : 1384743_at    Name:   Transcribed locus    GB acc:   BF418132    UniGene:   Rn.61749        SOURCE        Gene Ontology:     BioCarta Pathways:     KEGG Pathways:  
    Probe set : 1393297_at    Name:   Transcribed locus    GB acc:   BG377979    UniGene:   Rn.15298        SOURCE        Gene Ontology:     BioCarta Pathways:     KEGG Pathways:  
    Probe set : 1369407_at    Name:   tumor necrosis factor receptor superfamily, member 11b (osteoprotegerin)    GB acc:   NM_012870    UniGene:   Rn.9792    Gene Symbol:   Tnfrsf11b    Locus Link:   25341    Chromosome:   7    Cytoband:   Chr:7q31    SOURCE    GENECARD       Gene Ontology:   cellular component:  extracellular region   cellular component:  extracellular matrix (sensu Metazoa)   cellular component:  extracellular space   molecular function:  receptor activity   molecular function:  cytokine activity   molecular function:  protein binding      BioCarta Pathways:     KEGG Pathways:  
    Probe set : 1390219_at    Name:   similar to WD repeat membrane protein (predicted)    GB acc:   AI412437    UniGene:   Rn.18220    Gene Symbol:   RGD1306997_predicted    Locus Link:   305349    Chromosome:   14    Cytoband:   Chr:14p11    SOURCE    GENECARD       Gene Ontology:     BioCarta Pathways:     KEGG Pathways:  
    Probe set : 1368109_at    Name:   sialyltransferase 9 (CMP-NeuAc:lactosylceramide alpha-2,3-sialyltransferase)    GB acc:   NM_031337    UniGene:   Rn.22706    Gene Symbol:   Siat9    Locus Link:   83505    Chromosome:   4    Cytoband:   Chr:4q33    SOURCE    GENECARD       Gene Ontology:   biological process:  protein amino acid glycosylation   biological process:  ganglioside biosynthesis      BioCarta Pathways:     KEGG Pathways:  
    Probe set : 1367765_at    Name:   transcobalamin 2    GB acc:   NM_022534    UniGene:   Rn.1829    Gene Symbol:   Tcn2    Locus Link:   64365    Chromosome:   14    Cytoband:   Chr:14q21    SOURCE    GENECARD       Gene Ontology:   cellular component:  extracellular space   molecular function:  binding   molecular function:  cobalt ion transporter activity   molecular function:  cobalt ion binding      BioCarta Pathways:     KEGG Pathways:  
    Probe set : 1373149_at    Name:   similar to yippee-like 3    GB acc:   BF285344    UniGene:   Rn.138455    Gene Symbol:   LOC293491    Locus Link:   293491    Chromosome:   1    Cytoband:   Chr:1q36    SOURCE    GENECARD       Gene Ontology:     BioCarta Pathways:     KEGG Pathways:  
    Probe set : 1390163_at     GB acc:   BF282174              Gene Ontology:     BioCarta Pathways:     KEGG Pathways:  
    Probe set : 1374670_at    Name:   similar to hypothetical protein MGC20700 (predicted)    GB acc:   BM384543    UniGene:   Rn.93047    Gene Symbol:   RGD1307722_predicted    Locus Link:   362824    Chromosome:   7    Cytoband:   Chr:7q11    SOURCE    GENECARD       Gene Ontology:     BioCarta Pathways:     KEGG Pathways:  
    Probe set : 1387971_a_at    Name:   mitogen activated protein kinase 8 interacting protein    GB acc:   AF092450    UniGene:   Rn.44266    Gene Symbol:   Mapk8ip    Locus Link:   116457    Chromosome:   3    Cytoband:   Chr:3q24    SOURCE    GENECARD       Gene Ontology:   cellular component:  cytoplasm   cellular component:  cytoplasm      BioCarta Pathways:     KEGG Pathways:  
    Probe set : 1380772_at    Name:   Transcribed locus    GB acc:   BE105726    UniGene:   Rn.40811        SOURCE        Gene Ontology:     BioCarta Pathways:     KEGG Pathways:  
    Probe set : 1380108_at    Name:   WW domain binding protein 1 (predicted)    GB acc:   BI289053    UniGene:   Rn.139817     Locus Link:   297381    Chromosome:   4    Cytoband:   Chr:4q34    SOURCE        Gene Ontology:   cellular component:  integral to membrane   molecular function:  protein binding      BioCarta Pathways:     KEGG Pathways:  
    Probe set : 1368103_at    Name:   ATP-binding cassette, sub-family G (WHITE), member 1    GB acc:   NM_053502    UniGene:   Rn.8398    Gene Symbol:   Abcg1    Locus Link:   85264    Chromosome:   20    Cytoband:   Chr:20p12    SOURCE    GENECARD       Gene Ontology:   biological process:  transport   biological process:  lipid transport   biological process:  cholesterol transport      BioCarta Pathways:     KEGG Pathways:  
    Probe set : 1388400_at    Name:   cell division cycle 34 homolog (S. cerevisiae) (predicted)    GB acc:   BG375350    UniGene:   Rn.2427    Gene Symbol:   Cdc34_predicted    Locus Link:   299602    Chromosome:   7    Cytoband:   Chr:7q11    SOURCE    GENECARD       Gene Ontology:   cellular component:  nucleus      BioCarta Pathways:     KEGG Pathways:  
    Probe set : 1383085_at    Name:   Similar to Sh3bgrl protein    GB acc:   BG672648    UniGene:   Rn.58623     Locus Link:   302363    Chromosome:   X    Cytoband:   Chr:Xq31    SOURCE        Gene Ontology:     BioCarta Pathways:     KEGG Pathways:  
    Probe set : 1384970_at    Name:   similar to RIKEN cDNA 5330437I02 gene    GB acc:   BM389861    UniGene:   Rn.132740    Gene Symbol:   LOC361340    Locus Link:   361340    Chromosome:   18    Cytoband:   Chr:18q12.1    SOURCE    GENECARD       Gene Ontology:     BioCarta Pathways:     KEGG Pathways:  
    Probe set : 1393906_at    Name:   cytochrome P450, family 4, subfamily v, polypeptide 3 (predicted)    GB acc:   AA962978     Gene Symbol:   Cyp4v3_predicted    Locus Link:   361155    Chromosome:   16    Cytoband:   Chr:16q11     GENECARD       Gene Ontology:   biological process:  electron transport   molecular function:  monooxygenase activity      BioCarta Pathways:     KEGG Pathways:  
    Probe set : 1379542_at    Name:   similar to Small nuclear ribonucleoprotein F (snRNP-F) (Sm protein F) (Sm-F) (SmF) /// small nuclear ribonucleoprotein polypeptide F (predicted)    GB acc:   BF522334    UniGene:   Rn.145343    Gene Symbol:   LOC296377 /// Snrpf_predicted    Locus Link:   296377 /// 366871    Chromosome:   3    Cytoband:   Chr:3q42 /// Chr:7q13    SOURCE    GENECARD       Gene Ontology:   biological process:  mRNA processing   cellular component:  nucleus   cellular component:  small nucleolar ribonucleoprotein complex      BioCarta Pathways:     KEGG Pathways:  
    Probe set : 1389297_at    Name:   ERO1-like (S. cerevisiae)    GB acc:   AI146215    UniGene:   Rn.64648    Gene Symbol:   Ero1l    Locus Link:   171562    Chromosome:   15    Cytoband:   Chr:15p14    SOURCE    GENECARD       Gene Ontology:   cellular component:  endoplasmic reticulum   cellular component:  membrane   cellular component:  integral to endoplasmic reticulum membrane   molecular function:  oxidoreductase activity      BioCarta Pathways:     KEGG Pathways:  
    Probe set : 1368355_at    Name:   myosin 5B    GB acc:   NM_017083    UniGene:   Rn.10640    Gene Symbol:   Myo5b    Locus Link:   25132    Chromosome:   18    Cytoband:   Chr:18q12.2-q12.3    SOURCE    GENECARD       Gene Ontology:   biological process:  cytoskeleton organization and biogenesis   cellular component:  cytoskeleton   cellular component:  kinesin complex   cellular component:  myosin      BioCarta Pathways:     KEGG Pathways:  
    Probe set : 1368233_at    Name:   general transcription factor IIF, polypeptide 2    GB acc:   NM_031042    UniGene:   Rn.11000    Gene Symbol:   Gtf2f2    Locus Link:   81674    Chromosome:   15    Cytoband:   Chr:15q11    SOURCE    GENECARD       Gene Ontology:   cellular component:  nucleus   cellular component:  transcription factor TFIIF complex      BioCarta Pathways:     KEGG Pathways:  
    Probe set : 1375997_at    Name:   Transcribed locus    GB acc:   AA799503    UniGene:   Rn.3768        SOURCE        Gene Ontology:     BioCarta Pathways:     KEGG Pathways:  
    Probe set : 1391547_at    Name:   CDNA clone IMAGE:7320555    GB acc:   AI045669    UniGene:   Rn.92807        SOURCE        Gene Ontology:     BioCarta Pathways:     KEGG Pathways:  
    Probe set : 1371847_at     GB acc:   BI274357              Gene Ontology:     BioCarta Pathways:     KEGG Pathways:  
    Probe set : 1371579_at    Name:   Clone UI-R-FJ0-cpu-m-15-0-UI unknown mRNA    GB acc:   AI012456    UniGene:   Rn.29528        SOURCE        Gene Ontology:     BioCarta Pathways:     KEGG Pathways:  
    Probe set : 1395519_at    Name:   similar to B230212L03Rik protein    GB acc:   BG670960    UniGene:   Rn.92940    Gene Symbol:   MGC95152    Locus Link:   297109    Chromosome:   4    Cytoband:   Chr:4q24    SOURCE    GENECARD       Gene Ontology:     BioCarta Pathways:     KEGG Pathways:  
    Probe set : 1371417_at    Name:   low molecular mass ubiquinone-binding protein    GB acc:   AI233054    UniGene:   Rn.3472    Gene Symbol:   Qpc    Locus Link:   497902    Chromosome:   10    Cytoband:   Chr:10q22    SOURCE    GENECARD       Gene Ontology:   biological process:  electron transport   molecular function:  ubiquinol-cytochrome-c reductase activity      BioCarta Pathways:     KEGG Pathways:  
    Probe set : 1381350_at    Name:   Transcribed locus    GB acc:   AI317824    UniGene:   Rn.22987        SOURCE        Gene Ontology:     BioCarta Pathways:     KEGG Pathways:  
    Probe set : 1382013_at    Name:   RNA binding motif protein 24 (predicted)    GB acc:   AI071118     Gene Symbol:   Rbm24_predicted    Locus Link:   291031    Chromosome:   17    Cytoband:   Chr:17p13     GENECARD       Gene Ontology:   biological process:  nuclear mRNA splicing, via spliceosome   molecular function:  nucleic acid binding      BioCarta Pathways:     KEGG Pathways:  
    Probe set : 1390024_at    Name:   Similar to osteoclast inhibitory lectin    GB acc:   AI237685    UniGene:   Rn.95653     Locus Link:   362447    Chromosome:   4    Cytoband:   Chr:4q42    SOURCE        Gene Ontology:     BioCarta Pathways:     KEGG Pathways:  
    Probe set : 1395274_at    Name:   dystonin (predicted)    GB acc:   AI235468    UniGene:   Rn.79807    Gene Symbol:   Dst_predicted    Locus Link:   316313    Chromosome:   9    Cytoband:   Chr:9q21    SOURCE    GENECARD       Gene Ontology:   biological process:  cell cycle arrest   biological process:  cell adhesion   biological process:  retrograde axon cargo transport   cellular component:  cytoskeleton   cellular component:  cytoplasmic membrane-bound vesicle   cellular component:  hemidesmosome      BioCarta Pathways:     KEGG Pathways:  
    Probe set : 1378168_at    Name:   hypothetical LOC287534    GB acc:   BG377391    UniGene:   Rn.13078    Gene Symbol:   RGD:1359691    Locus Link:   287534    Chromosome:   10    Cytoband:   Chr:10q24    SOURCE    GENECARD       Gene Ontology:     BioCarta Pathways:     KEGG Pathways:  
    Probe set : 1382748_at     GB acc:   AI145481              Gene Ontology:     BioCarta Pathways:     KEGG Pathways:  
    Probe set : 1372652_at    Name:   similar to forkhead protein AFXH    GB acc:   BM386443    UniGene:   Rn.19646    Gene Symbol:   LOC302415    Locus Link:   302415    Chromosome:   X    Cytoband:   Chr:Xq31    SOURCE    GENECARD       Gene Ontology:     BioCarta Pathways:     KEGG Pathways:  
    Probe set : 1377327_at    Name:   Transcribed locus    GB acc:   AI008151    UniGene:   Rn.13785        SOURCE        Gene Ontology:     BioCarta Pathways:     KEGG Pathways:  
    Probe set : 1373170_at    Name:   G protein pathway suppressor 2 (predicted)    GB acc:   BI294756    UniGene:   Rn.100409    Gene Symbol:   Gps2_predicted    Locus Link:   303248    Chromosome:   10    Cytoband:   Chr:10q24    SOURCE    GENECARD       Gene Ontology:   biological process:  inactivation of MAPK activity   biological process:  cell cycle   biological process:  JNK cascade   molecular function:  GTPase inhibitor activity      BioCarta Pathways:     KEGG Pathways:  
    Probe set : 1389678_at    Name:   Transcribed locus    GB acc:   BM389496    UniGene:   Rn.38259        SOURCE        Gene Ontology:     BioCarta Pathways:     KEGG Pathways:  
    Probe set : 1376211_a_at    Name:   potassium channel tetramerisation domain containing 6 (predicted)    GB acc:   AI235964    UniGene:   Rn.105101    Gene Symbol:   Kctd6_predicted    Locus Link:   305792    Chromosome:   15    Cytoband:   Chr:15p14    SOURCE    GENECARD       Gene Ontology:   biological process:  potassium ion transport   cellular component:  voltage-gated potassium channel complex   cellular component:  membrane   molecular function:  ion channel activity   molecular function:  voltage-gated potassium channel activity   molecular function:  protein binding      BioCarta Pathways:     KEGG Pathways:  
    Probe set : 1376687_at    Name:   ubiquitin specific protease 1 (predicted)    GB acc:   AI010241    UniGene:   Rn.125994    Gene Symbol:   Usp1_predicted    Locus Link:   313387    Chromosome:   5    Cytoband:   Chr:5q33    SOURCE    GENECARD       Gene Ontology:   biological process:  ubiquitin-dependent protein catabolism   biological process:  ubiquitin cycle      BioCarta Pathways:     KEGG Pathways:  
    Probe set : 1388323_at    Name:   NADH dehydrogenase (ubiquinone) 1 alpha subcomplex, 9 (predicted)    GB acc:   BG381033    UniGene:   Rn.107963    Gene Symbol:   Ndufa9_predicted    Locus Link:   362440    Chromosome:   4    Cytoband:   Chr:4q42    SOURCE    GENECARD       Gene Ontology:   biological process:  sodium ion transport   biological process:  nucleotide-sugar metabolism   cellular component:  extracellular space   cellular component:  mitochondrion   cellular component:  mitochondrial matrix      BioCarta Pathways:     KEGG Pathways:  
    Probe set : 1377752_at    Name:   epithelial membrane protein 2    GB acc:   BE112998    UniGene:   Rn.21730    Gene Symbol:   RGD:1359629    Locus Link:   360468    Chromosome:   10    Cytoband:   Chr:10q11    SOURCE    GENECARD       Gene Ontology:   cellular component:  membrane   cellular component:  integral to membrane      BioCarta Pathways:     KEGG Pathways:  
    Probe set : 1390776_at    Name:   Iroquois related homeobox 3 (Drosophila) (predicted)    GB acc:   AI030203    UniGene:   Rn.22133    Gene Symbol:   Irx3_predicted    Locus Link:   307721    Chromosome:   19    Cytoband:   Chr:19p11    SOURCE    GENECARD       Gene Ontology:   biological process:  regulation of transcription, DNA-dependent   cellular component:  nucleus   cellular component:  transcription factor complex   molecular function:  DNA binding   molecular function:  transcription factor activity      BioCarta Pathways:     KEGG Pathways:  
    Probe set : 1367773_at    Name:   solute carrier family 25, member 1    GB acc:   NM_017307    UniGene:   Rn.8368    Gene Symbol:   Slc25a1    Locus Link:   29743    Chromosome:   11    Cytoband:   Chr:11q23    SOURCE    GENECARD       Gene Ontology:   biological process:  transport   biological process:  mitochondrial citrate transport   biological process:  mitochondrial citrate transport      BioCarta Pathways:     KEGG Pathways:  
    Probe set : 1398987_at    Name:   suppressor of Ty 4 homolog 2 (S. cerevisiae) (predicted)    GB acc:   AW141261    UniGene:   Rn.130855    Gene Symbol:   Supt4h2_predicted    Locus Link:   287608    Chromosome:   10    Cytoband:   Chr:10q26    SOURCE    GENECARD       Gene Ontology:   cellular component:  nucleus   molecular function:  transcription factor activity   molecular function:  positive transcription elongation factor activity   molecular function:  zinc ion binding      BioCarta Pathways:     KEGG Pathways:  
    Probe set : 1372107_at     GB acc:   BG673187              Gene Ontology:     BioCarta Pathways:     KEGG Pathways:  
    Probe set : 1388511_at    Name:   centaurin, delta 2 (predicted)    GB acc:   AI412079    UniGene:   Rn.41376    Gene Symbol:   Centd2_predicted    Locus Link:   361617    Chromosome:   1    Cytoband:   Chr:1q32    SOURCE    GENECARD       Gene Ontology:   biological process:  signal transduction   biological process:  regulation of GTPase activity   molecular function:  GTPase activator activity      BioCarta Pathways:     KEGG Pathways:  
    Probe set : 1394789_at    Name:   LOC499812    GB acc:   AI178097    UniGene:   Rn.43120    Gene Symbol:   LOC499812    Locus Link:   499812    Chromosome:   3    Cytoband:   Chr:3q23    SOURCE    GENECARD       Gene Ontology:     BioCarta Pathways:     KEGG Pathways:  
    Probe set : 1376130_a_at    Name:   dystrobrevin, beta (predicted)    GB acc:   BM390663    UniGene:   Rn.61275    Gene Symbol:   Dtnb_predicted    Locus Link:   362715    Chromosome:   6    Cytoband:   Chr:6q14    SOURCE    GENECARD       Gene Ontology:   cellular component:  cytoskeleton   cellular component:  synapse      BioCarta Pathways:     KEGG Pathways:  
    Probe set : 1371170_a_at    Name:   interleukin 1 alpha    GB acc:   AJ245643    UniGene:   Rn.12300    Gene Symbol:   Il1a    Locus Link:   24493    Chromosome:   3    Cytoband:   Chr:3q36    SOURCE    GENECARD       Gene Ontology:     BioCarta Pathways:     KEGG Pathways:  
    Probe set : 1392541_at    Name:   similar to RIKEN cDNA A030007L17; EST AA673177 (predicted) /// similar to RIKEN cDNA A030007L17    GB acc:   AW919608    UniGene:   Rn.129060    Gene Symbol:   RGD1304876_predicted /// LOC362370    Locus Link:   362368 /// 362370    Chromosome:   4    Cytoband:   Chr:4q24    SOURCE    GENECARD       Gene Ontology:     BioCarta Pathways:     KEGG Pathways:  
    Probe set : 1368132_at    Name:   transducer of ERBB2, 1    GB acc:   NM_133317    UniGene:   Rn.7819    Gene Symbol:   Tob1    Locus Link:   170842    Chromosome:   10    Cytoband:   Chr:10q26    SOURCE    GENECARD       Gene Ontology:   cellular component:  cytoplasm   molecular function:  transcription corepressor activity   molecular function:  protein binding   molecular function:  SMAD binding      BioCarta Pathways:     KEGG Pathways:  
    Probe set : 1391571_at    Name:   Transcribed locus    GB acc:   BG666079    UniGene:   Rn.45627        SOURCE        Gene Ontology:     BioCarta Pathways:     KEGG Pathways:  
    Probe set : 1372777_at    Name:   similar to dJ1033H22.1 (KIAA0554 protein) (predicted)    GB acc:   BM385774    UniGene:   Rn.33820    Gene Symbol:   RGD1305386_predicted    Locus Link:   310839    Chromosome:   2    Cytoband:   Chr:2q42    SOURCE    GENECARD       Gene Ontology:     BioCarta Pathways:     KEGG Pathways:  
    Probe set : 1393647_at    Name:   histone aminotransferase 1 (predicted)    GB acc:   BI285581    UniGene:   Rn.125727    Gene Symbol:   Hat1_predicted    Locus Link:   296501    Chromosome:   3    Cytoband:   Chr:3q21    SOURCE    GENECARD       Gene Ontology:   biological process:  DNA packaging   biological process:  internal protein amino acid acetylation   cellular component:  nucleus      BioCarta Pathways:     KEGG Pathways:  
    Probe set : 1368378_at    Name:   formyltetrahydrofolate dehydrogenase    GB acc:   NM_022547    UniGene:   Rn.2328    Gene Symbol:   Fthfd    Locus Link:   64392      SOURCE    GENECARD       Gene Ontology:   cellular component:  cytoplasm      BioCarta Pathways:     KEGG Pathways:  
    Probe set : 1386933_at    Name:   glycoprotein 2 (zymogen granule membrane)    GB acc:   NM_134418    UniGene:   Rn.11223    Gene Symbol:   Gp2    Locus Link:   171459    Chromosome:   1    Cytoband:   Chr:1q35    SOURCE    GENECARD       Gene Ontology:   cellular component:  extracellular space   cellular component:  membrane fraction   cellular component:  membrane      BioCarta Pathways:     KEGG Pathways:  
    Probe set : 1383067_a_at    Name:   deoxythymidylate kinase (predicted)    GB acc:   AW141143    UniGene:   Rn.95    Gene Symbol:   Dtymk_predicted    Locus Link:   301622    Chromosome:   9    Cytoband:   Chr:9q36    SOURCE    GENECARD       Gene Ontology:     BioCarta Pathways:     KEGG Pathways:  
    Probe set : 1386891_at    Name:   phosphatidylethanolamine binding protein    GB acc:   NM_017236    UniGene:   Rn.29745    Gene Symbol:   Pbp    Locus Link:   29542    Chromosome:   12    Cytoband:   Chr:12q16    SOURCE    GENECARD       Gene Ontology:     BioCarta Pathways:     KEGG Pathways:  
    Probe set : 1373094_at    Name:   general transcription factor II H, polypeptide 1 (predicted)    GB acc:   AW917124    UniGene:   Rn.24733    Gene Symbol:   Gtf2h1_predicted    Locus Link:   361580    Chromosome:   1    Cytoband:   Chr:1q22    SOURCE    GENECARD       Gene Ontology:   cellular component:  nucleus   cellular component:  transcription factor TFIIH complex      BioCarta Pathways:     KEGG Pathways:  
    Probe set : 1390412_at    Name:   Solute carrier family 39 (iron-regulated transporter), member 1    GB acc:   AI229664    UniGene:   Rn.15324    Gene Symbol:   Slc40a1    Locus Link:   170840    Chromosome:   9    Cytoband:   Chr:9q22    SOURCE    GENECARD       Gene Ontology:   biological process:  transport   biological process:  iron ion transport   cellular component:  mitochondrial inner membrane   cellular component:  synaptic vesicle   cellular component:  integral to membrane   molecular function:  iron ion transporter activity   molecular function:  binding   molecular function:  iron ion binding      BioCarta Pathways:     KEGG Pathways:  
    Probe set : 1392922_at    Name:   Transcribed locus    GB acc:   BE116619    UniGene:   Rn.23625        SOURCE        Gene Ontology:     BioCarta Pathways:     KEGG Pathways:  
    Probe set : 1371372_at    Name:   similar to Sid3177p    GB acc:   AA944161    UniGene:   Rn.2344    Gene Symbol:   LOC362809    Locus Link:   362809    Chromosome:   7    Cytoband:   Chr:7q11    SOURCE    GENECARD       Gene Ontology:     BioCarta Pathways:     KEGG Pathways:  
    Probe set : 1387303_at    Name:   solute carrier family 22 (organic cation transporter), member 2    GB acc:   NM_031584    UniGene:   Rn.10555    Gene Symbol:   Slc22a2    Locus Link:   29503    Chromosome:   1    Cytoband:   Chr:1q11    SOURCE    GENECARD       Gene Ontology:     BioCarta Pathways:     KEGG Pathways:  
    Probe set : 1370934_at    Name:   nucleoporin 153kD    GB acc:   L06821    UniGene:   Rn.1347    Gene Symbol:   Nup153    Locus Link:   25281    Chromosome:   17    Cytoband:   Chr:17p13    SOURCE    GENECARD       Gene Ontology:   biological process:  transport      BioCarta Pathways:     KEGG Pathways:  
    Probe set : 1386912_at    Name:   procollagen C-proteinase enhancer protein    GB acc:   NM_019237    UniGene:   Rn.2910    Gene Symbol:   Pcolce    Locus Link:   29569    Chromosome:   12    Cytoband:   Chr:12q12    SOURCE    GENECARD       Gene Ontology:   biological process:  proteolysis   biological process:  development   cellular component:  extracellular space   molecular function:  nucleic acid binding   molecular function:  collagen binding      BioCarta Pathways:     KEGG Pathways:  
    Probe set : 1374794_at    Name:   kinesin-like 7    GB acc:   BF396282    UniGene:   Rn.45205    Gene Symbol:   Knsl7    Locus Link:   353302    Chromosome:   8    Cytoband:   Chr:8q32    SOURCE    GENECARD       Gene Ontology:   biological process:  microtubule-based movement   cellular component:  microtubule associated complex      BioCarta Pathways:     KEGG Pathways:  
    Probe set : 1379770_at    Name:   similar to choline phosphotransferase 1; cholinephosphotransferase 1 alpha; cholinephosphotransferase 1    GB acc:   BF282951    UniGene:   Rn.16261    Gene Symbol:   RGD:1359283    Locus Link:   362866    Chromosome:   7    Cytoband:   Chr:7q13    SOURCE    GENECARD       Gene Ontology:   biological process:  phospholipid biosynthesis   molecular function:  transferase activity      BioCarta Pathways:     KEGG Pathways:  
    Probe set : 1389325_at    Name:   similar to programmed cell death 10    GB acc:   AA848545    UniGene:   Rn.1176    Gene Symbol:   RGD:1359329    Locus Link:   494345    Chromosome:   2    Cytoband:   Chr:2q32    SOURCE    GENECARD       Gene Ontology:   biological process:  apoptosis      BioCarta Pathways:     KEGG Pathways:  
    Probe set : 1380116_at    Name:   Transcribed locus    GB acc:   AI146098    UniGene:   Rn.147656        SOURCE        Gene Ontology:     BioCarta Pathways:     KEGG Pathways:  
    Probe set : 1391274_at    Name:   Transcribed locus    GB acc:   BI292056    UniGene:   Rn.91765        SOURCE        Gene Ontology:     BioCarta Pathways:     KEGG Pathways:  
    Probe set : 1388479_at    Name:   Dihydropyrimidinase-like 3    GB acc:   BI294841    UniGene:   Rn.93365    Gene Symbol:   Dpysl3    Locus Link:   25418    Chromosome:   18    Cytoband:   Chr:18p11    SOURCE    GENECARD       Gene Ontology:   molecular function:  dihydropyrimidinase activity   molecular function:  hydrolase activity      BioCarta Pathways:     KEGG Pathways:  
    Probe set : 1370057_at    Name:   cysteine and glycine-rich protein 1    GB acc:   NM_017148    UniGene:   Rn.108075    Gene Symbol:   Csrp1    Locus Link:   29276    Chromosome:   13    Cytoband:   Chr:13q13    SOURCE    GENECARD       Gene Ontology:   biological process:  nervous system development   biological process:  actin cytoskeleton organization and biogenesis   cellular component:  nucleus   cellular component:  actin cytoskeleton      BioCarta Pathways:     KEGG Pathways:  
    Probe set : 1389496_at    Name:   A kinase (PRKA) anchor protein 7    GB acc:   BI300893    UniGene:   Rn.145179    Gene Symbol:   RGD:1303071    Locus Link:   361458    Chromosome:   1    Cytoband:   Chr:1p12    SOURCE    GENECARD       Gene Ontology:   molecular function:  kinase activity      BioCarta Pathways:     KEGG Pathways:  
    Probe set : 1380021_at    Name:   similar to 2010308M01Rik protein (predicted)    GB acc:   AI059486    UniGene:   Rn.105855    Gene Symbol:   RGD1305120_predicted    Locus Link:   310772    Chromosome:   2    Cytoband:   Chr:2q34    SOURCE    GENECARD       Gene Ontology:     BioCarta Pathways:     KEGG Pathways:  
    Probe set : 1385690_at    Name:   similar to Mut protein /// similar to Mut protein    GB acc:   BI293631    UniGene:   Rn.54738    Gene Symbol:   LOC301276 /// LOC363201    Locus Link:   301276 /// 363201    Chromosome:   9    Cytoband:   Chr:9q12    SOURCE    GENECARD       Gene Ontology:     BioCarta Pathways:     KEGG Pathways:  
    Probe set : 1377634_at    Name:   BCL2/adenovirus E1B 19kDa-interacting protein 1, NIP2 (predicted)    GB acc:   AI715220    UniGene:   Rn.13297    Gene Symbol:   Bnip2_predicted    Locus Link:   300811    Chromosome:   8    Cytoband:   Chr:8q24    SOURCE    GENECARD       Gene Ontology:   biological process:  apoptosis   biological process:  anti-apoptosis   cellular component:  cytoplasm   molecular function:  GTPase activator activity      BioCarta Pathways:     KEGG Pathways:  
    Probe set : 1399035_at    Name:   polymerase (RNA) III (DNA directed) polypeptide H (predicted)    GB acc:   BE097233    UniGene:   Rn.13037    Gene Symbol:   Polr3h_predicted    Locus Link:   300088    Chromosome:   7    Cytoband:   Chr:7q34    SOURCE    GENECARD       Gene Ontology:   cellular component:  nucleus   cellular component:  DNA-directed RNA polymerase III complex   cellular component:  mitochondrion      BioCarta Pathways:     KEGG Pathways:  
    Probe set : 1373138_at    Name:   nudix-type motif 5    GB acc:   AA858886    UniGene:   Rn.55388    Gene Symbol:   RGD:1359284    Locus Link:   361274    Chromosome:   17    Cytoband:   Chr:17q12.3    SOURCE    GENECARD       Gene Ontology:   molecular function:  hydrolase activity      BioCarta Pathways:     KEGG Pathways:  
    Probe set : 1375696_at    Name:   interferon (alpha and beta) receptor 1 (predicted)    GB acc:   BF420426    UniGene:   Rn.105738    Gene Symbol:   Ifnar1_predicted    Locus Link:   288264    Chromosome:   11    Cytoband:   Chr:11q11    SOURCE    GENECARD       Gene Ontology:   biological process:  cell surface receptor linked signal transduction   biological process:  JAK-STAT cascade   biological process:  response to virus      BioCarta Pathways:     KEGG Pathways:  
    Probe set : 1390914_at    Name:   Friend leukemia integration 1 (predicted)    GB acc:   AI071474    UniGene:   Rn.65827    Gene Symbol:   Fli1_predicted    Locus Link:   315532    Chromosome:   8    Cytoband:   Chr:8q21    SOURCE    GENECARD       Gene Ontology:   cellular component:  nucleus   molecular function:  DNA binding   molecular function:  transcription factor activity   molecular function:  transcriptional activator activity      BioCarta Pathways:     KEGG Pathways:  
    Probe set : 1392952_at    Name:   hypothetical protein LOC619561    GB acc:   BI277131     Gene Symbol:   LOC619561    Locus Link:   619561    Chromosome:   10    Cytoband:   Chr:10q26     GENECARD       Gene Ontology:     BioCarta Pathways:     KEGG Pathways:  
    Probe set : 1373903_at    Name:   similar to Hypothetical protein MGC32399 (predicted)    GB acc:   AI011920    UniGene:   Rn.99138    Gene Symbol:   RGD1304715_predicted    Locus Link:   360872    Chromosome:   13    Cytoband:   Chr:13q23    SOURCE    GENECARD       Gene Ontology:     BioCarta Pathways:     KEGG Pathways:  
    Probe set : 1384529_at    Name:   Transcribed locus    GB acc:   BE104877    UniGene:   Rn.139139        SOURCE        Gene Ontology:     BioCarta Pathways:     KEGG Pathways:  
    Probe set : 1368432_a_at    Name:   v-ros UR2 sarcoma virus oncogene homolog 1 (avian)    GB acc:   NM_012874    UniGene:   Rn.87436    Gene Symbol:   Ros1    Locus Link:   25346    Chromosome:   20    Cytoband:   Chr:20q11    SOURCE    GENECARD       Gene Ontology:     BioCarta Pathways:     KEGG Pathways:  
    Probe set : 1374190_at    Name:   citrate lyase beta like (predicted)    GB acc:   BG380750    UniGene:   Rn.137340    Gene Symbol:   Clybl_predicted    Locus Link:   306198    Chromosome:   15    Cytoband:   Chr:15q25    SOURCE    GENECARD       Gene Ontology:   cellular component:  citrate lyase complex   molecular function:  citrate (pro-3S)-lyase activity   molecular function:  lyase activity      BioCarta Pathways:     KEGG Pathways:  
    Probe set : 1397953_at    Name:   Transcribed locus    GB acc:   AI113261    UniGene:   Rn.147460        SOURCE        Gene Ontology:     BioCarta Pathways:     KEGG Pathways:  
    Probe set : 1387780_at    Name:   DnaJ (Hsp40) homolog, subfamily A, member 2    GB acc:   NM_032079    UniGene:   Rn.3904    Gene Symbol:   Dnaja2    Locus Link:   84026    Chromosome:   19    Cytoband:   Chr:19q11    SOURCE    GENECARD       Gene Ontology:   cellular component:  membrane      BioCarta Pathways:     KEGG Pathways:  
    Probe set : 1372030_at    Name:   zinc finger, FYVE domain containing 21 (predicted)    GB acc:   BI283114    UniGene:   Rn.2468    Gene Symbol:   Zfyve21_predicted    Locus Link:   362789    Chromosome:   6    Cytoband:   Chr:6q32    SOURCE    GENECARD       Gene Ontology:   molecular function:  zinc ion binding      BioCarta Pathways:     KEGG Pathways:  
    Probe set : 1368858_at    Name:   UDP-glucuronosyltransferase 8    GB acc:   L21698    UniGene:   Rn.9744    Gene Symbol:   Ugt8    Locus Link:   50555    Chromosome:   2    Cytoband:   Chr:2q42    SOURCE    GENECARD       Gene Ontology:     BioCarta Pathways:     KEGG Pathways:  
    Probe set : 1369323_at    Name:   leptin receptor overlapping transcript    GB acc:   NM_020099    UniGene:   Rn.144583    Gene Symbol:   Leprot    Locus Link:   56766    Chromosome:   5    Cytoband:   Chr:5q33    SOURCE    GENECARD       Gene Ontology:   cellular component:  integral to membrane   molecular function:  receptor activity      BioCarta Pathways:     KEGG Pathways:  
    Probe set : 1368896_at    Name:   MAD homolog 7 (Drosophila)    GB acc:   NM_030858    UniGene:   Rn.29980    Gene Symbol:   Madh7    Locus Link:   81516    Chromosome:   18    Cytoband:   Chr:18q12.3    SOURCE    GENECARD       Gene Ontology:   cellular component:  intracellular   cellular component:  nucleus      BioCarta Pathways:     KEGG Pathways:  
    Probe set : 1390857_at     GB acc:   BI279844              Gene Ontology:     BioCarta Pathways:     KEGG Pathways:  
    Probe set : 1370007_at    Name:   protein disulfide isomerase related protein (calcium-binding protein, intestinal-related)    GB acc:   NM_053849    UniGene:   Rn.39305    Gene Symbol:   Erp70    Locus Link:   116598    Chromosome:   4    Cytoband:   Chr:4q24    SOURCE    GENECARD       Gene Ontology:   biological process:  electron transport   biological process:  protein secretion   cellular component:  extracellular space   cellular component:  endoplasmic reticulum   cellular component:  endoplasmic reticulum lumen      BioCarta Pathways:     KEGG Pathways:  
    Probe set : 1392308_at    Name:   phospholipase A2, group IID (predicted)    GB acc:   AI044869    UniGene:   Rn.109549    Gene Symbol:   Pla2g2d_predicted    Locus Link:   298579    Chromosome:   5    Cytoband:   Chr:5q36    SOURCE    GENECARD       Gene Ontology:   biological process:  phospholipid metabolism   biological process:  inflammatory response   biological process:  lipid catabolism   cellular component:  extracellular region   cellular component:  extracellular space   molecular function:  phospholipase A2 activity   molecular function:  calcium ion binding   molecular function:  hydrolase activity      BioCarta Pathways:     KEGG Pathways:  
    Probe set : 1397610_at    Name:   Transcribed locus    GB acc:   BI290825    UniGene:   Rn.148648        SOURCE        Gene Ontology:     BioCarta Pathways:     KEGG Pathways:  
    Probe set : 1370253_at    Name:   ribosomal protein L22    GB acc:   AI228150    UniGene:   Rn.21738    Gene Symbol:   Rpl22    Locus Link:   81768    Chromosome:   5    Cytoband:   Chr:5q36    SOURCE    GENECARD       Gene Ontology:   biological process:  protein biosynthesis   biological process:  ribosome biogenesis      BioCarta Pathways:     KEGG Pathways:  
    Probe set : 1397652_at    Name:   Transcribed locus    GB acc:   BE108399    UniGene:   Rn.50044        SOURCE        Gene Ontology:     BioCarta Pathways:     KEGG Pathways:  
    Probe set : 1393706_at    Name:   six transmembrane epithelial antigen of the prostate (predicted)    GB acc:   BE109939    UniGene:   Rn.51773    Gene Symbol:   Steap_predicted    Locus Link:   297738    Chromosome:   4    Cytoband:   Chr:4q12    SOURCE    GENECARD       Gene Ontology:   molecular function:  channel or pore class transporter activity      BioCarta Pathways:     KEGG Pathways:  
    Probe set : 1375848_at    Name:   paraoxonase 2 (predicted)    GB acc:   BG668051    UniGene:   Rn.1100    Gene Symbol:   Pon2_predicted    Locus Link:   296851    Chromosome:   4    Cytoband:   Chr:4q13    SOURCE    GENECARD       Gene Ontology:   cellular component:  extracellular region   cellular component:  membrane   molecular function:  aryldialkylphosphatase activity   molecular function:  arylesterase activity   molecular function:  hydrolase activity      BioCarta Pathways:     KEGG Pathways:  
    Probe set : 1393947_at    Name:   solute carrier family 25 (mitochondrial carrier; ornithine transporter) member 15 (predicted)    GB acc:   BG377383    UniGene:   Rn.66488    Gene Symbol:   Slc25a15_predicted    Locus Link:   306574    Chromosome:   16    Cytoband:   Chr:16q12.5    SOURCE    GENECARD       Gene Ontology:   molecular function:  L-ornithine transporter activity   molecular function:  transporter activity   molecular function:  binding      BioCarta Pathways:     KEGG Pathways:  
    Probe set : 1381396_s_at    Name:   Kruppel-like factor 15    GB acc:   AA998067    UniGene:   Rn.22556    Gene Symbol:   Klf15    Locus Link:   85497    Chromosome:   4    Cytoband:   Chr:4q34    SOURCE    GENECARD       Gene Ontology:   biological process:  regulation of transcription, DNA-dependent   cellular component:  nucleus      BioCarta Pathways:     KEGG Pathways:  
    Probe set : 1374821_at    Name:   RAB3A interacting protein    GB acc:   BF399587    UniGene:   Rn.31889    Gene Symbol:   RABIN3    Locus Link:   29885    Chromosome:   7    Cytoband:   Chr:7q22    SOURCE    GENECARD       Gene Ontology:   biological process:  transport   biological process:  protein transport   cellular component:  nucleus   cellular component:  cytosol   cellular component:  nucleus      BioCarta Pathways:     KEGG Pathways:  
    Probe set : 1395411_at    Name:   nicotinate phosphoribosyltransferase-like protein    GB acc:   AW916787    UniGene:   Rn.22665    Gene Symbol:   RGD:1302945    Locus Link:   315085    Chromosome:   7    Cytoband:   Chr:7q34    SOURCE    GENECARD       Gene Ontology:   biological process:  pyridine nucleotide biosynthesis      BioCarta Pathways:     KEGG Pathways:  
    Probe set : 1379340_at    Name:   lamimin, gamma 2    GB acc:   BM385282    UniGene:   Rn.9278    Gene Symbol:   Lamc2    Locus Link:   192362    Chromosome:   13    Cytoband:   Chr:13q21    SOURCE    GENECARD       Gene Ontology:   biological process:  cell adhesion   cellular component:  extracellular matrix (sensu Metazoa)   cellular component:  laminin-2   cellular component:  extracellular matrix      BioCarta Pathways:     KEGG Pathways:  
    Probe set : 1376377_at    Name:   Transcribed locus    GB acc:   BF398599    UniGene:   Rn.9010        SOURCE        Gene Ontology:     BioCarta Pathways:     KEGG Pathways:  
    Probe set : 1368659_at    Name:   alanine-glyoxylate aminotransferase 2    GB acc:   D38100    UniGene:   Rn.1874    Gene Symbol:   Agxt2    Locus Link:   83784    Chromosome:   2    Cytoband:   Chr:2q16    SOURCE    GENECARD       Gene Ontology:   cellular component:  mitochondrion      BioCarta Pathways:     KEGG Pathways:  
    Probe set : 1393401_at    Name:   Similar to 9530046H09Rik protein (predicted)    GB acc:   BE109323    UniGene:   Rn.145219     Locus Link:   362849    Chromosome:   7    Cytoband:   Chr:7q12    SOURCE        Gene Ontology:   biological process:  protein ubiquitination   cellular component:  ubiquitin ligase complex   cellular component:  nucleus   cellular component:  integral to membrane   molecular function:  ubiquitin-protein ligase activity   molecular function:  zinc ion binding   molecular function:  metal ion binding      BioCarta Pathways:     KEGG Pathways:  
    Probe set : 1386975_at    Name:   pyruvate dehydrogenase kinase, isoenzyme 2    GB acc:   AF237719    UniGene:   Rn.88597    Gene Symbol:   Pdk2    Locus Link:   81530    Chromosome:   10    Cytoband:   Chr:10q31    SOURCE    GENECARD       Gene Ontology:   cellular component:  mitochondrion      BioCarta Pathways:     KEGG Pathways:  
    Probe set : 1379286_at    Name:   Transcribed locus    GB acc:   BM383184    UniGene:   Rn.18141        SOURCE        Gene Ontology:     BioCarta Pathways:     KEGG Pathways:  
    Probe set : 1379679_at    Name:   Transcribed locus    GB acc:   BF290584    UniGene:   Rn.55946        SOURCE        Gene Ontology:     BioCarta Pathways:     KEGG Pathways:  
    Probe set : 1370929_at    Name:   low density lipoprotein receptor-related protein associated protein 1    GB acc:   AI170333    UniGene:   Rn.10293    Gene Symbol:   Lrpap1    Locus Link:   116565    Chromosome:   14    Cytoband:   Chr:14q21    SOURCE    GENECARD       Gene Ontology:   cellular component:  extracellular space   cellular component:  endoplasmic reticulum   cellular component:  endoplasmic reticulum      BioCarta Pathways:     KEGG Pathways:  
    Probe set : 1398572_at    Name:   RNA (guanine-7-) methyltransferase (predicted)    GB acc:   BI294251    UniGene:   Rn.20871    Gene Symbol:   Rnmt_predicted    Locus Link:   291534    Chromosome:   18    Cytoband:   Chr:18q12.1    SOURCE    GENECARD       Gene Ontology:   biological process:  mRNA capping   cellular component:  nucleus   cellular component:  mRNA cap complex      BioCarta Pathways:     KEGG Pathways:  
    Probe set : 1370302_at    Name:   thyroid hormone-response protein-1    GB acc:   AF348365    UniGene:   Rn.16033    Gene Symbol:   RGD:620882    Locus Link:   192219    Chromosome:   X    Cytoband:   Chr:Xq21    SOURCE    GENECARD       Gene Ontology:     BioCarta Pathways:     KEGG Pathways:  
    Probe set : 1375289_at     GB acc:   BM387047              Gene Ontology:     BioCarta Pathways:     KEGG Pathways:  
    Probe set : 1386581_at    Name:   integrin, alpha V (vitronectin receptor, alpha polypeptide, antigen CD51) (predicted)    GB acc:   AW141214    UniGene:   Rn.23339    Gene Symbol:   Itgav_predicted    Locus Link:   296456    Chromosome:   3    Cytoband:   Chr:3q24    SOURCE    GENECARD       Gene Ontology:   biological process:  cell-matrix adhesion   biological process:  integrin-mediated signaling pathway   cellular component:  integrin complex   molecular function:  protein binding      BioCarta Pathways:     KEGG Pathways:  
    Probe set : 1393148_at    Name:   transmembrane 7 superfamily member 1 (predicted)    GB acc:   BF542593    UniGene:   Rn.60453    Gene Symbol:   Tm7sf1_predicted    Locus Link:   289287    Chromosome:   17    Cytoband:   Chr:17q12.1    SOURCE    GENECARD       Gene Ontology:   cellular component:  membrane fraction   cellular component:  integral to plasma membrane   cellular component:  integral to membrane      BioCarta Pathways:     KEGG Pathways:  
    Probe set : 1373544_at    Name:   chemokine (C-X-C motif) ligand 9    GB acc:   AI170387    UniGene:   Rn.7391    Gene Symbol:   Cxcl9    Locus Link:   246759    Chromosome:   14    Cytoband:   Chr:14p22    SOURCE    GENECARD       Gene Ontology:   biological process:  inflammatory response   biological process:  immune response   cellular component:  extracellular region   cellular component:  extracellular space   molecular function:  cytokine activity   molecular function:  chemokine activity      BioCarta Pathways:     KEGG Pathways:  
    Probe set : 1373224_at    Name:   alpha-2,3-sialyltransferase ST3Gal IV    GB acc:   BI295550    UniGene:   Rn.24125    Gene Symbol:   RGD:1303068    Locus Link:   363040    Chromosome:   8    Cytoband:   Chr:8q21    SOURCE    GENECARD       Gene Ontology:   biological process:  protein amino acid glycosylation   cellular component:  Golgi stack   cellular component:  integral to membrane   cellular component:  integral to Golgi membrane   molecular function:  sialyltransferase activity   molecular function:  transferase activity   molecular function:  transferase activity, transferring glycosyl groups      BioCarta Pathways:     KEGG Pathways:  
    Probe set : 1373506_at    Name:   Guanylate nucleotide binding protein 2    GB acc:   AA944568    UniGene:   Rn.25736    Gene Symbol:   Gbp2    Locus Link:   171164    Chromosome:   2    Cytoband:   Chr:2q44    SOURCE    GENECARD       Gene Ontology:   biological process:  immune response   biological process:  regulation of transcription, DNA-dependent      BioCarta Pathways:     KEGG Pathways:  
    Probe set : 1372021_at    Name:   nucleoporin 188 (predicted)    GB acc:   AI102685    UniGene:   Rn.11918    Gene Symbol:   Nup188_predicted    Locus Link:   366016    Chromosome:   3    Cytoband:   Chr:3p12    SOURCE    GENECARD       Gene Ontology:     BioCarta Pathways:     KEGG Pathways:  
    Probe set : 1383601_at    Name:   DUF729 domain containing 1 (predicted)    GB acc:   BI295614    UniGene:   Rn.130657     Locus Link:   308720    Chromosome:   1    Cytoband:   Chr:1p12    SOURCE        Gene Ontology:     BioCarta Pathways:     KEGG Pathways:  
    Probe set : 1370821_at    Name:   Cb1-812    GB acc:   AF120100    UniGene:   Rn.112598        SOURCE        Gene Ontology:   biological process:  metabolism   cellular component:  cytoplasm   molecular function:  thiopurine S-methyltransferase activity      BioCarta Pathways:     KEGG Pathways:  
    Probe set : 1390579_at    Name:   similar to RIKEN cDNA 1810029B16 (predicted)    GB acc:   BI294760    UniGene:   Rn.27406    Gene Symbol:   RGD1305222_predicted    Locus Link:   290686    Chromosome:   16    Cytoband:   Chr:16p14    SOURCE    GENECARD       Gene Ontology:     BioCarta Pathways:     KEGG Pathways:  
    Probe set : 1381080_at    Name:   Transcribed locus    GB acc:   AI169081    UniGene:   Rn.33700        SOURCE        Gene Ontology:     BioCarta Pathways:     KEGG Pathways:  
    Probe set : 1395561_at    Name:   heterogeneous nuclear ribonucleoprotein A2/B1 (predicted)    GB acc:   BG666358    UniGene:   Rn.129934    Gene Symbol:   Hnrpa2b1_predicted    Locus Link:   362361    Chromosome:   4    Cytoband:   Chr:4q24    SOURCE    GENECARD       Gene Ontology:   biological process:  nuclear mRNA splicing, via spliceosome   biological process:  RNA processing   biological process:  mRNA processing   cellular component:  nucleus   cellular component:  ribonucleoprotein complex   cellular component:  heterogeneous nuclear ribonucleoprotein complex   molecular function:  nucleic acid binding   molecular function:  RNA binding      BioCarta Pathways:     KEGG Pathways:  
    Probe set : 1393373_at    Name:   similar to encephalopsin    GB acc:   BI289640    UniGene:   Rn.15626    Gene Symbol:   LOC498289    Locus Link:   498289    Chromosome:   13    Cytoband:   Chr:13q25    SOURCE    GENECARD       Gene Ontology:     BioCarta Pathways:     KEGG Pathways:  
    Probe set : 1371328_at    Name:   Cleavage and polyadenylation specific factor 5 (predicted)    GB acc:   BM986552    UniGene:   Rn.107561     Locus Link:   361367    Chromosome:   19    Cytoband:   Chr:19p12    SOURCE        Gene Ontology:     BioCarta Pathways:     KEGG Pathways:  
    Probe set : 1398775_at    Name:   ribosomal protein S15a    GB acc:   NM_053982    UniGene:   Rn.2890    Gene Symbol:   Rps15a    Locus Link:   117053    Chromosome:   1    Cytoband:   Chr:1q35    SOURCE    GENECARD       Gene Ontology:   biological process:  RNA processing   biological process:  protein biosynthesis      BioCarta Pathways:     KEGG Pathways:  
    Probe set : 1368291_at    Name:   baculoviral IAP repeat-containing 2    GB acc:   NM_021752    UniGene:   Rn.43867    Gene Symbol:   Birc2    Locus Link:   60371    Chromosome:   8    Cytoband:   Chr:8q11    SOURCE    GENECARD       Gene Ontology:   cellular component:  ubiquitin ligase complex   cellular component:  intracellular   molecular function:  ubiquitin-protein ligase activity   molecular function:  protein binding   molecular function:  zinc ion binding      BioCarta Pathways:     KEGG Pathways:  
    Probe set : 1378169_at    Name:   similar to peroxisomal long chain acyl-CoA thioesterase Ia    GB acc:   BI295569    UniGene:   Rn.145093    Gene Symbol:   LOC314304    Locus Link:   314304    Chromosome:   6    Cytoband:   Chr:6q31    SOURCE    GENECARD       Gene Ontology:     BioCarta Pathways:     KEGG Pathways:  
    Probe set : 1382682_at    Name:   Similar to hypothetical protein PRO0971 (predicted)    GB acc:   AI172217    UniGene:   Rn.1713     Locus Link:   362037    Chromosome:   2    Cytoband:   Chr:2q42    SOURCE        Gene Ontology:     BioCarta Pathways:     KEGG Pathways:  
    Probe set : 1393247_at    Name:   Similar to zinc finger protein 560    GB acc:   BI298190    UniGene:   Rn.12638     Locus Link:   367033    Chromosome:   8    Cytoband:   Chr:8q13    SOURCE        Gene Ontology:     BioCarta Pathways:     KEGG Pathways:  
    Probe set : 1372780_at    Name:   similar to RIKEN cDNA 1110038M16 (predicted)    GB acc:   BM391310    UniGene:   Rn.8133    Gene Symbol:   RGD1307066_predicted    Locus Link:   313529    Chromosome:   5    Cytoband:   Chr:5q36    SOURCE    GENECARD       Gene Ontology:     BioCarta Pathways:     KEGG Pathways:  
    Probe set : 1372302_at    Name:   similar to hypothetical protein FLJ10579 (predicted)    GB acc:   BF284897    UniGene:   Rn.18772    Gene Symbol:   RGD1308697_predicted    Locus Link:   311328    Chromosome:   3    Cytoband:   Chr:3q35    SOURCE    GENECARD       Gene Ontology:   molecular function:  binding      BioCarta Pathways:     KEGG Pathways:  
    Probe set : 1373406_at    Name:   Torsin family 1, member B (predicted)    GB acc:   BM384991    UniGene:   Rn.6487     Locus Link:   311854    Chromosome:   3    Cytoband:   Chr:3p12    SOURCE        Gene Ontology:   biological process:  protein folding   biological process:  response to unfolded protein   biological process:  chaperone cofactor dependent protein folding   cellular component:  extracellular space   cellular component:  cytoplasm   cellular component:  endoplasmic reticulum   molecular function:  ATP binding   molecular function:  unfolded protein binding      BioCarta Pathways:     KEGG Pathways:  
    Probe set : 1379832_at    Name:   polymerase (RNA) II (DNA directed) polypeptide D (predicted)    GB acc:   BF544977    UniGene:   Rn.128728    Gene Symbol:   Polr2d_predicted    Locus Link:   364834    Chromosome:   18    Cytoband:   Chr:18p12    SOURCE    GENECARD       Gene Ontology:   biological process:  transcription   biological process:  transcription from RNA polymerase II promoter   cellular component:  nucleus   cellular component:  DNA-directed RNA polymerase II, core complex   molecular function:  DNA-directed RNA polymerase activity   molecular function:  transferase activity      BioCarta Pathways:     KEGG Pathways:  
    Probe set : 1372531_at    Name:   protein tyrosine phosphatase, receptor-type, F interacting protein, binding protein 2    GB acc:   BE106488    UniGene:   Rn.2971    Gene Symbol:   Ppfibp2    Locus Link:   308918    Chromosome:   1    Cytoband:   Chr:1q33    SOURCE    GENECARD       Gene Ontology:   biological process:  DNA integration   molecular function:  DNA binding   molecular function:  integrase activity      BioCarta Pathways:     KEGG Pathways:  
    Probe set : 1390144_at    Name:   similar to RIKEN cDNA 3110043O21 gene    GB acc:   BF411622    UniGene:   Rn.13725    Gene Symbol:   RGD:1359108    Locus Link:   313155    Chromosome:   5    Cytoband:   Chr:5q22    SOURCE    GENECARD       Gene Ontology:     BioCarta Pathways:     KEGG Pathways:  
    Probe set : 1374630_at    Name:   chloride intracellular channel 3 (predicted)    GB acc:   AI234249    UniGene:   Rn.25003    Gene Symbol:   Clic3_predicted    Locus Link:   296566    Chromosome:   3    Cytoband:   Chr:3p13    SOURCE    GENECARD       Gene Ontology:   cellular component:  membrane fraction   cellular component:  nucleus   cellular component:  membrane      BioCarta Pathways:     KEGG Pathways:  
    Probe set : 1372768_at    Name:   Similar to RIKEN cDNA 2010009L17 (predicted)    GB acc:   AW251331    UniGene:   Rn.22240     Locus Link:   310005    Chromosome:   2    Cytoband:   Chr:2q12    SOURCE        Gene Ontology:   biological process:  angiogenesis   cellular component:  intracellular   molecular function:  nucleic acid binding      BioCarta Pathways:     KEGG Pathways:  
    Probe set : 1398826_s_at    Name:   nuclear receptor subfamily 2, group F, member 6    GB acc:   NM_139113    UniGene:   Rn.25840    Gene Symbol:   Nr2f6    Locus Link:   245980    Chromosome:   16    Cytoband:   Chr:16p14    SOURCE    GENECARD       Gene Ontology:   cellular component:  intracellular   cellular component:  nucleus      BioCarta Pathways:     KEGG Pathways:  
    Probe set : 1390863_at    Name:   solute carrier family 19 (thiamine transporter), member 2 (predicted)    GB acc:   BF283298    UniGene:   Rn.19386    Gene Symbol:   Slc19a2_predicted    Locus Link:   289175    Chromosome:   13    Cytoband:   Chr:13q22    SOURCE    GENECARD       Gene Ontology:   cellular component:  plasma membrane   cellular component:  membrane   cellular component:  integral to membrane      BioCarta Pathways:     KEGG Pathways:  
    Probe set : 1372959_at    Name:   similar to mKIAA0665 protein (predicted)    GB acc:   BI289467    UniGene:   Rn.17377    Gene Symbol:   RGD1308952_predicted    Locus Link:   303002    Chromosome:   10    Cytoband:   Chr:10q12    SOURCE    GENECARD       Gene Ontology:   cellular component:  intracellular   cellular component:  ribosome      BioCarta Pathways:     KEGG Pathways:  
    Probe set : 1371018_at     GB acc:   BF411765              Gene Ontology:     BioCarta Pathways:     KEGG Pathways:  
    Probe set : 1371903_at    Name:   STIP1 homology and U-Box containing protein 1 (predicted)    GB acc:   BM384012    UniGene:   Rn.6504     Locus Link:   360498    Chromosome:   10    Cytoband:   Chr:10q12    SOURCE        Gene Ontology:     BioCarta Pathways:     KEGG Pathways:  
    Probe set : 1389919_at    Name:   parvin, beta (predicted)    GB acc:   BI296756    UniGene:   Rn.16709    Gene Symbol:   Parvb_predicted    Locus Link:   362973    Chromosome:   7    Cytoband:   Chr:7q34    SOURCE    GENECARD       Gene Ontology:   biological process:  cell adhesion   cellular component:  cytoskeleton   molecular function:  actin binding   molecular function:  protein binding      BioCarta Pathways:     KEGG Pathways:  
    Probe set : 1378092_at    Name:   similar to magnesium-dependent phosphatase-1 (predicted)    GB acc:   AI408718    UniGene:   Rn.19870    Gene Symbol:   RGD1311147_predicted    Locus Link:   290230    Chromosome:   15    Cytoband:   Chr:15p13    SOURCE    GENECARD       Gene Ontology:     BioCarta Pathways:     KEGG Pathways:  
    Probe set : 1389036_at    Name:   similar to RIKEN cDNA 9030221M09 gene (predicted)    GB acc:   BM385629    UniGene:   Rn.49382    Gene Symbol:   RGD1305469_predicted    Locus Link:   305466    Chromosome:   14    Cytoband:   Chr:14q21    SOURCE    GENECARD       Gene Ontology:     BioCarta Pathways:     KEGG Pathways:  
    Probe set : 1367774_at    Name:   glutathione S-transferase A5    GB acc:   NM_031509    UniGene:   Rn.10460    Gene Symbol:   Gsta5    Locus Link:   24421    Chromosome:   9    Cytoband:   Chr:9q13    SOURCE    GENECARD       Gene Ontology:   biological process:  glutathione metabolism   biological process:  response to drug   molecular function:  glutathione transferase activity   molecular function:  glutathione transferase activity   molecular function:  transferase activity      BioCarta Pathways:     KEGG Pathways:  
    Probe set : 1378049_at    Name:   similar to Cas-associated zinc finger protein (predicted)    GB acc:   AI603565    UniGene:   Rn.25043    Gene Symbol:   RGD1306498_predicted    Locus Link:   297879    Chromosome:   5    Cytoband:   Chr:5q36    SOURCE    GENECARD       Gene Ontology:     BioCarta Pathways:     KEGG Pathways:  
    Probe set : 1375026_at    Name:   ceroid-lipofuscinosis, neuronal 6 (predicted)    GB acc:   AI105369    UniGene:   Rn.14593    Gene Symbol:   Cln6_predicted    Locus Link:   315746    Chromosome:   8    Cytoband:   Chr:8q24    SOURCE    GENECARD       Gene Ontology:   biological process:  lysosome organization and biogenesis   biological process:  neuron differentiation   cellular component:  integral to membrane   cellular component:  integral to membrane      BioCarta Pathways:     KEGG Pathways:  
    Probe set : 1368427_at    Name:   A kinase (PRKA) anchor protein 11    GB acc:   NM_012773    UniGene:   Rn.10557    Gene Symbol:   Akap11    Locus Link:   25228      SOURCE    GENECARD       Gene Ontology:   biological process:  protein kinase cascade   cellular component:  peroxisome   cellular component:  peroxisome      BioCarta Pathways:     KEGG Pathways:  
    Probe set : 1390518_at    Name:   similar to putative emu1 protein    GB acc:   AW915157    UniGene:   Rn.81507    Gene Symbol:   LOC498403    Locus Link:   498403    Chromosome:   14    Cytoband:   Chr:14q21    SOURCE    GENECARD       Gene Ontology:     BioCarta Pathways:     KEGG Pathways:  
    Probe set : 1371736_at    Name:   Similar to 1810013D10Rik protein    GB acc:   AI411283    UniGene:   Rn.3288     Locus Link:   501923      SOURCE        Gene Ontology:     BioCarta Pathways:     KEGG Pathways:  
    Probe set : 1378507_at    Name:   Transcribed locus    GB acc:   BE108914    UniGene:   Rn.88974        SOURCE        Gene Ontology:     BioCarta Pathways:     KEGG Pathways:  
    Probe set : 1388717_at    Name:   protein O-fucosyltransferase 2 (predicted)    GB acc:   BM384163    UniGene:   Rn.11702    Gene Symbol:   Pofut2_predicted    Locus Link:   309686    Chromosome:   20    Cytoband:   Chr:20p12    SOURCE    GENECARD       Gene Ontology:   biological process:  fucose metabolism      BioCarta Pathways:     KEGG Pathways:  
    Probe set : 1398789_at    Name:   ribosomal protein L37    GB acc:   NM_031106    UniGene:   Rn.65053    Gene Symbol:   Rpl37    Locus Link:   81770    Chromosome:   2    Cytoband:   Chr:2q16    SOURCE    GENECARD       Gene Ontology:   biological process:  protein biosynthesis      BioCarta Pathways:     KEGG Pathways:  
    Probe set : 1376732_at    Name:   Calreticulin 3 (predicted)    GB acc:   BM388733    UniGene:   Rn.144131     Locus Link:   364529    Chromosome:   16    Cytoband:   Chr:16p14    SOURCE        Gene Ontology:   biological process:  protein folding   cellular component:  endoplasmic reticulum   molecular function:  calcium ion binding   molecular function:  sugar binding   molecular function:  unfolded protein binding      BioCarta Pathways:     KEGG Pathways:  
    Probe set : 1388909_at    Name:   similar to cDNA sequence BC019806 (predicted)    GB acc:   AA891742    UniGene:   Rn.3785    Gene Symbol:   RGD1311534_predicted    Locus Link:   306270    Chromosome:   16    Cytoband:   Chr:16p16    SOURCE    GENECARD       Gene Ontology:     BioCarta Pathways:     KEGG Pathways:  
    Probe set : 1387429_at    Name:   adrenergic receptor kinase, beta 1    GB acc:   NM_012776    UniGene:   Rn.13010    Gene Symbol:   Adrbk1    Locus Link:   25238    Chromosome:   1    Cytoband:   Chr:1q42    SOURCE    GENECARD       Gene Ontology:   cellular component:  soluble fraction   cellular component:  cytoplasm      BioCarta Pathways:     KEGG Pathways:  
    Probe set : 1376804_at    Name:   similar to Myosin VI    GB acc:   BM386777    UniGene:   Rn.18047    Gene Symbol:   LOC315840    Locus Link:   315840    Chromosome:   8    Cytoband:   Chr:8q31    SOURCE    GENECARD       Gene Ontology:     BioCarta Pathways:     KEGG Pathways:  
    Probe set : 1367579_a_at    Name:   tubulin, alpha 1 /// tubulin, alpha 6 (predicted) /// similar to Tubulin alpha-2 chain (Alpha-tubulin 2)    GB acc:   BI285434    UniGene:   Rn.99661    Gene Symbol:   Tuba1 /// Tuba6_predicted /// LOC500929    Locus Link:   300218 /// 500929 /// 64158    Chromosome:   7    Cytoband:   Chr:7q36    SOURCE    GENECARD       Gene Ontology:     BioCarta Pathways:     KEGG Pathways:  
    Probe set : 1367670_at    Name:   fumarate hydratase 1    GB acc:   NM_017005    UniGene:   Rn.29782    Gene Symbol:   Fh1    Locus Link:   24368    Chromosome:   13    Cytoband:   Chr:13q25    SOURCE    GENECARD       Gene Ontology:   biological process:  tricarboxylic acid cycle   biological process:  fumarate metabolism   biological process:  negative regulation of progression through cell cycle   cellular component:  mitochondrion   cellular component:  tricarboxylic acid cycle enzyme complex   molecular function:  catalytic activity   molecular function:  fumarate hydratase activity   molecular function:  lyase activity      BioCarta Pathways:     KEGG Pathways:  
    Probe set : 1373847_at    Name:   transmembrane 4 superfamily member 1 (predicted)    GB acc:   AW435343    UniGene:   Rn.24712    Gene Symbol:   Tm4sf1_predicted    Locus Link:   295061    Chromosome:   2    Cytoband:   Chr:2q31    SOURCE    GENECARD       Gene Ontology:   cellular component:  integral to plasma membrane   cellular component:  integral to membrane      BioCarta Pathways:     KEGG Pathways:  
    Probe set : 1386938_at    Name:   alanyl (membrane) aminopeptidase    GB acc:   NM_031012    UniGene:   Rn.11132    Gene Symbol:   Anpep    Locus Link:   81641    Chromosome:   1    Cytoband:   Chr:1q31    SOURCE    GENECARD       Gene Ontology:   cellular component:  integral to plasma membrane   cellular component:  integral to membrane      BioCarta Pathways:     KEGG Pathways:  
    Probe set : 1382370_at    Name:   cyclin F    GB acc:   BE113938    UniGene:   Rn.15126    Gene Symbol:   Ccnf    Locus Link:   117524    Chromosome:   10    Cytoband:   Chr:10q12    SOURCE    GENECARD       Gene Ontology:   cellular component:  nucleus   molecular function:  cyclin-dependent protein kinase regulator activity      BioCarta Pathways:     KEGG Pathways:  
    Probe set : 1367824_at    Name:   farnesyltransferase, CAAX box, alpha    GB acc:   NM_012847    UniGene:   Rn.5990    Gene Symbol:   Fnta    Locus Link:   25318    Chromosome:   16    Cytoband:   Chr:16q12.4    SOURCE    GENECARD       Gene Ontology:   cellular component:  cytoplasm      BioCarta Pathways:     KEGG Pathways:  
    Probe set : 1378140_at    Name:   ADP-ribosylation factor-like 11 (predicted)    GB acc:   BI296317    UniGene:   Rn.23090    Gene Symbol:   Arl11_predicted    Locus Link:   364396    Chromosome:   15    Cytoband:   Chr:15p12    SOURCE    GENECARD       Gene Ontology:   biological process:  small GTPase mediated signal transduction   molecular function:  GTP binding   molecular function:  nucleotide binding      BioCarta Pathways:     KEGG Pathways:  
    Probe set : 1393794_at    Name:   Similar to tetratricopeptide repeat domain 21B    GB acc:   BI284634    UniGene:   Rn.78500     Locus Link:   295654    Chromosome:   3    Cytoband:   Chr:3q21    SOURCE        Gene Ontology:     BioCarta Pathways:     KEGG Pathways:  
    Probe set : 1391050_at    Name:   similar to Metal-response element-binding transcription factor 2 (Metal-response element DNA-binding protein M96) (predicted)    GB acc:   BI294718    UniGene:   Rn.20300    Gene Symbol:   RGD1304727_predicted    Locus Link:   360905    Chromosome:   14    Cytoband:   Chr:14p22    SOURCE    GENECARD       Gene Ontology:   biological process:  regulation of transcription, DNA-dependent   cellular component:  nucleus   cellular component:  transcription factor complex      BioCarta Pathways:     KEGG Pathways:  
    Probe set : 1393049_at    Name:   myosin IG (predicted)    GB acc:   BF417582    UniGene:   Rn.2799    Gene Symbol:   Myo1g_predicted    Locus Link:   289785    Chromosome:   14    Cytoband:   Chr:14q21    SOURCE    GENECARD       Gene Ontology:   cellular component:  myosin      BioCarta Pathways:     KEGG Pathways:  
    Probe set : 1368762_at    Name:   ubiquitin D    GB acc:   NM_053299    UniGene:   Rn.18473    Gene Symbol:   Ubd    Locus Link:   29168      SOURCE    GENECARD       Gene Ontology:     BioCarta Pathways:     KEGG Pathways:  
    Probe set : 1383845_at    Name:   Transcribed locus    GB acc:   AI059929    UniGene:   Rn.146395        SOURCE        Gene Ontology:     BioCarta Pathways:     KEGG Pathways:  
    Probe set : 1388956_at     GB acc:   BI285945              Gene Ontology:     BioCarta Pathways:     KEGG Pathways:  
    Probe set : 1397441_at    Name:   tetratricopeptide repeat domain 13 (predicted)    GB acc:   BM390838    UniGene:   Rn.98912    Gene Symbol:   Ttc13_predicted    Locus Link:   292095    Chromosome:   19    Cytoband:   Chr:19q12    SOURCE    GENECARD       Gene Ontology:   molecular function:  binding      BioCarta Pathways:     KEGG Pathways:  
    Probe set : 1367622_at    Name:   ATP synthase, H+ transporting, mitochondrial F0 complex, subunit d    GB acc:   NM_019383    UniGene:   Rn.80    Gene Symbol:   Atp5h    Locus Link:   56080    Chromosome:   5    Cytoband:   Chr:5q36    SOURCE    GENECARD       Gene Ontology:     BioCarta Pathways:     KEGG Pathways:  
    Probe set : 1368669_at    Name:   uncoupling protein 2    GB acc:   NM_019354    UniGene:   Rn.13333    Gene Symbol:   Ucp2    Locus Link:   54315    Chromosome:   1    Cytoband:   Chr:1q32    SOURCE    GENECARD       Gene Ontology:   molecular function:  transporter activity   molecular function:  binding      BioCarta Pathways:     KEGG Pathways:  
    Probe set : 1387791_at    Name:   angiotensin 1 converting enzyme    GB acc:   AF201331    UniGene:   Rn.10149    Gene Symbol:   Ace    Locus Link:   24310    Chromosome:   10    Cytoband:   Chr:10q32.1    SOURCE    GENECARD       Gene Ontology:     BioCarta Pathways:     KEGG Pathways:  
    Probe set : 1370280_at    Name:   hypoxanthine guanine phosphoribosyl transferase    GB acc:   M86443    UniGene:   Rn.47    Gene Symbol:   Hprt    Locus Link:   24465    Chromosome:   X    Cytoband:   Chr:Xq36    SOURCE    GENECARD       Gene Ontology:   cellular component:  cytoplasm      BioCarta Pathways:     KEGG Pathways:  
    Probe set : 1367629_at    Name:   cytochrome c oxidase, subunit 7a 3    GB acc:   NM_022503    UniGene:   Rn.1745    Gene Symbol:   Cox7a3    Locus Link:   29507    Chromosome:   8    Cytoband:   Chr:8q24-q31    SOURCE    GENECARD       Gene Ontology:   biological process:  electron transport   molecular function:  cytochrome-c oxidase activity   molecular function:  electron transporter activity   molecular function:  oxidoreductase activity      BioCarta Pathways:     KEGG Pathways:  
    Probe set : 1374727_at    Name:   similar to DnaJ (Hsp40) homolog, subfamily B, member 12    GB acc:   AF244895    UniGene:   Rn.146024    Gene Symbol:   LOC294513    Locus Link:   294513    Chromosome:   20    Cytoband:   Chr:20q11    SOURCE    GENECARD       Gene Ontology:   biological process:  protein folding   molecular function:  heat shock protein binding   molecular function:  unfolded protein binding      BioCarta Pathways:     KEGG Pathways:  
    Probe set : 1388851_at    Name:   heat shock protein, A (predicted)    GB acc:   BI282281    UniGene:   Rn.7535    Gene Symbol:   Hspa9a_predicted    Locus Link:   291671    Chromosome:   18    Cytoband:   Chr:18p12    SOURCE    GENECARD       Gene Ontology:   cellular component:  cytoplasm   cellular component:  mitochondrion   molecular function:  protein binding   molecular function:  ATP binding   molecular function:  unfolded protein binding      BioCarta Pathways:     KEGG Pathways:  
    Probe set : 1373792_at    Name:   ARP1 actin-related protein 1 homolog B (yeast) (predicted)    GB acc:   AI228348    UniGene:   Rn.24317     Locus Link:   316333    Chromosome:   9    Cytoband:   Chr:9q21    SOURCE        Gene Ontology:     BioCarta Pathways:     KEGG Pathways:  
    Probe set : 1387328_at    Name:   Cytochrome P450, subfamily IIC (mephenytoin 4-hydroxylase)    GB acc:   NM_019184    UniGene:   Rn.10870    Gene Symbol:   Cyp2c    Locus Link:   29277    Chromosome:   1    Cytoband:   Chr:1q53    SOURCE    GENECARD       Gene Ontology:   biological process:  electron transport   biological process:  xenobiotic metabolism   cellular component:  endoplasmic reticulum   cellular component:  microsome   cellular component:  membrane      BioCarta Pathways:     KEGG Pathways:  
    Probe set : 1387078_at    Name:   inositol polyphosphate-4-phosphatase, type 1    GB acc:   NM_031002    UniGene:   Rn.11215    Gene Symbol:   Inpp4a    Locus Link:   80849    Chromosome:   9    Cytoband:   Chr:9q21    SOURCE    GENECARD       Gene Ontology:   biological process:  polyphosphate catabolism   biological process:  signal transduction      BioCarta Pathways:     KEGG Pathways:  
    Probe set : 1395944_at    Name:   Transcribed locus    GB acc:   AI548667    UniGene:   Rn.41999        SOURCE        Gene Ontology:     BioCarta Pathways:     KEGG Pathways:  
    Probe set : 1379264_at    Name:   zinc and ring finger 1 (predicted)    GB acc:   BE109004    UniGene:   Rn.32245    Gene Symbol:   Znrf1_predicted    Locus Link:   362367    Chromosome:   4    Cytoband:   Chr:4q24    SOURCE    GENECARD       Gene Ontology:     BioCarta Pathways:     KEGG Pathways:  
    Probe set : 1368661_at    Name:   solute carrier family 13 (sodium-dependent dicarboxylate transporter), member 2    GB acc:   U51153    UniGene:   Rn.10821    Gene Symbol:   Slc13a2    Locus Link:   65202    Chromosome:   10    Cytoband:   Chr:10q25    SOURCE    GENECARD       Gene Ontology:   biological process:  transport   biological process:  sodium ion transport   biological process:  ion transport      BioCarta Pathways:     KEGG Pathways:  
    Probe set : 1372395_at    Name:   Similar to KIAA0597 protein    GB acc:   AA850978    UniGene:   Rn.2039     Locus Link:   294862    Chromosome:   2    Cytoband:   Chr:2q22    SOURCE        Gene Ontology:     BioCarta Pathways:     KEGG Pathways:  
    Probe set : 1388579_at    Name:   Similar to hypothetical gene MGC19595 (predicted)    GB acc:   AI176841    UniGene:   Rn.17455     Locus Link:   306352    Chromosome:   16    Cytoband:   Chr:16p14    SOURCE        Gene Ontology:     BioCarta Pathways:     KEGG Pathways:  
    Probe set : 1368927_at    Name:   membrane bound C2 domain containing protein    GB acc:   NM_017249    UniGene:   Rn.11636    Gene Symbol:   Mbc2    Locus Link:   29579    Chromosome:   7    Cytoband:   Chr:7q11    SOURCE    GENECARD       Gene Ontology:   cellular component:  integral to membrane      BioCarta Pathways:     KEGG Pathways:  
    Probe set : 1393743_at    Name:   Transcribed locus    GB acc:   AI453930    UniGene:   Rn.34553        SOURCE        Gene Ontology:     BioCarta Pathways:     KEGG Pathways:  
    Probe set : 1388178_at    Name:   nuclear receptor coactivator 3    GB acc:   AF322224    UniGene:   Rn.20691    Gene Symbol:   Ncoa3    Locus Link:   84584    Chromosome:   3    Cytoband:   Chr:3q42    SOURCE    GENECARD       Gene Ontology:   biological process:  regulation of transcription, DNA-dependent   biological process:  signal transduction   biological process:  transcription   cellular component:  nucleus      BioCarta Pathways:     KEGG Pathways:  
    Probe set : 1368032_at    Name:   nucleolar and coiled-body phosphoprotein 1    GB acc:   M94288    UniGene:   Rn.9517    Gene Symbol:   Nolc1    Locus Link:   64896    Chromosome:   1    Cytoband:   Chr:1q54    SOURCE    GENECARD       Gene Ontology:     BioCarta Pathways:     KEGG Pathways:  
    Probe set : 1370505_at    Name:   ADP-ribosylarginine hydrolase    GB acc:   M86341    UniGene:   Rn.13315    Gene Symbol:   Adprh    Locus Link:   25371    Chromosome:   11    Cytoband:   Chr:11q21    SOURCE    GENECARD       Gene Ontology:   biological process:  protein modification   biological process:  protein amino acid ADP-ribosylation      BioCarta Pathways:     KEGG Pathways:  
    Probe set : 1369816_at    Name:   RAB3A, member RAS oncogene family    GB acc:   NM_013018    UniGene:   Rn.44409    Gene Symbol:   Rab3a    Locus Link:   25531    Chromosome:   16    Cytoband:   Chr:16p14    SOURCE    GENECARD       Gene Ontology:   cellular component:  intracellular   cellular component:  synaptic vesicle      BioCarta Pathways:     KEGG Pathways:  
    Probe set : 1386868_at    Name:   ribosomal protein S10    GB acc:   NM_031109    UniGene:   Rn.3359    Gene Symbol:   Rps10    Locus Link:   81773    Chromosome:   20    Cytoband:   Chr:20p12    SOURCE    GENECARD       Gene Ontology:   biological process:  regulation of transcription, DNA-dependent   biological process:  protein biosynthesis   cellular component:  nucleus   cellular component:  cytosolic small ribosomal subunit (sensu Eukaryota)   cellular component:  ribonucleoprotein complex   molecular function:  DNA binding   molecular function:  RNA binding   molecular function:  structural constituent of ribosome      BioCarta Pathways:     KEGG Pathways:  
    Probe set : 1398897_at    Name:   ubiquitin-conjugating enzyme E2 variant 1 (predicted)    GB acc:   AI411988     Gene Symbol:   Ube2v1_predicted    Locus Link:   296390    Chromosome:   2    Cytoband:   Chr:2q12     GENECARD       Gene Ontology:   biological process:  protein modification   biological process:  ubiquitin cycle   cellular component:  nucleus   molecular function:  ubiquitin conjugating enzyme activity   molecular function:  protein binding      BioCarta Pathways:     KEGG Pathways:  
    Probe set : 1389223_at     GB acc:   AI177186              Gene Ontology:     BioCarta Pathways:     KEGG Pathways:  
    Probe set : 1387270_at    Name:   hematopoietically expressed homeobox    GB acc:   NM_024385    UniGene:   Rn.12188    Gene Symbol:   Hhex    Locus Link:   79237    Chromosome:   1    Cytoband:   Chr:1q53    SOURCE    GENECARD       Gene Ontology:   cellular component:  nucleus   cellular component:  nucleus   cellular component:  transcription factor complex   molecular function:  DNA binding   molecular function:  transcription factor activity   molecular function:  general transcriptional repressor activity      BioCarta Pathways:     KEGG Pathways:  
    Probe set : 1393508_at    Name:   Transcribed locus    GB acc:   AI043805    UniGene:   Rn.18728        SOURCE        Gene Ontology:     BioCarta Pathways:     KEGG Pathways:  
    Probe set : 1368651_at    Name:   pyruvate kinase, liver and RBC    GB acc:   M17685    UniGene:   Rn.48821    Gene Symbol:   Pklr    Locus Link:   24651    Chromosome:   2    Cytoband:   Chr:2q34    SOURCE    GENECARD       Gene Ontology:   biological process:  glycolysis   biological process:  response to pathogen      BioCarta Pathways:     KEGG Pathways:  
    Probe set : 1369522_a_at    Name:   CD244 natural killer cell receptor 2B4    GB acc:   NM_022259    UniGene:   Rn.53994    Gene Symbol:   Cd244    Locus Link:   64025    Chromosome:   13    Cytoband:   Chr:13q24    SOURCE    GENECARD       Gene Ontology:   biological process:  lymphocyte activation   cellular component:  integral to membrane   molecular function:  receptor activity      BioCarta Pathways:     KEGG Pathways:  
    Probe set : 1388668_at     GB acc:   BM384972              Gene Ontology:     BioCarta Pathways:     KEGG Pathways:  
    Probe set : 1392338_at    Name:   Transcribed locus    GB acc:   AA892989    UniGene:   Rn.22744        SOURCE        Gene Ontology:     BioCarta Pathways:     KEGG Pathways:  
    Probe set : 1393041_at    Name:   SMC2 structural maintenance of chromosomes 2-like 1 (yeast) (predicted)    GB acc:   AW535052    UniGene:   Rn.94452    Gene Symbol:   Smc2l1_predicted    Locus Link:   362519    Chromosome:   5    Cytoband:   Chr:5q23    SOURCE    GENECARD       Gene Ontology:   cellular component:  nucleus   cellular component:  chromosome   cellular component:  membrane      BioCarta Pathways:     KEGG Pathways:  
    Probe set : 1383315_at    Name:   hepatic protein EIIH    GB acc:   AW434208    UniGene:   Rn.145009    Gene Symbol:   RGD:1359311    Locus Link:   308843    Chromosome:   1    Cytoband:   Chr:1q32    SOURCE    GENECARD       Gene Ontology:     BioCarta Pathways:     KEGG Pathways:  
    Probe set : 1371122_at    Name:   TRAF family member-associated Nf-kappa B activator /// hypothetical gene supported by NM_145788    GB acc:   AW140505    UniGene:   Rn.89906    Gene Symbol:   Tank /// LOC497768    Locus Link:   252961 /// 497768    Chromosome:   3    Cytoband:   Chr:3q21    SOURCE    GENECARD       Gene Ontology:   biological process:  signal transduction   biological process:  I-kappaB kinase/NF-kappaB cascade   cellular component:  nucleus   molecular function:  nucleic acid binding   molecular function:  protein binding   molecular function:  zinc ion binding      BioCarta Pathways:     KEGG Pathways:  
    Probe set : 1393017_at    Name:   rhophilin, Rho GTPase binding protein 1 (predicted)    GB acc:   BG378261    UniGene:   Rn.17241    Gene Symbol:   Rhpn1_predicted    Locus Link:   300030    Chromosome:   7    Cytoband:   Chr:7q34    SOURCE    GENECARD       Gene Ontology:   biological process:  signal transduction   cellular component:  intracellular   molecular function:  small GTPase regulator activity   molecular function:  protein binding      BioCarta Pathways:     KEGG Pathways:  
    Probe set : 1373421_at    Name:   TG interacting factor (predicted)    GB acc:   BM392224    UniGene:   Rn.144418    Gene Symbol:   Tgif_predicted    Locus Link:   316742    Chromosome:   9    Cytoband:   Chr:9q38    SOURCE    GENECARD       Gene Ontology:   cellular component:  nucleus   cellular component:  transcription factor complex   molecular function:  DNA binding   molecular function:  transcription factor activity   molecular function:  transcription corepressor activity      BioCarta Pathways:     KEGG Pathways:  
    Probe set : 1382378_at    Name:   cathepsin W (predicted)    GB acc:   BI279370    UniGene:   Rn.34406    Gene Symbol:   Ctsw_predicted    Locus Link:   293676    Chromosome:   1    Cytoband:   Chr:1q43    SOURCE    GENECARD       Gene Ontology:   biological process:  proteolysis   biological process:  immune response   cellular component:  extracellular space   molecular function:  cysteine-type endopeptidase activity   molecular function:  cysteine-type peptidase activity   molecular function:  hydrolase activity      BioCarta Pathways:     KEGG Pathways:  
    Probe set : 1373134_at    Name:   similar to RIKEN cDNA B430104H02    GB acc:   BE329450    UniGene:   Rn.100583    Gene Symbol:   LOC296131    Locus Link:   296131    Chromosome:   3    Cytoband:   Chr:3q36    SOURCE    GENECARD       Gene Ontology:     BioCarta Pathways:     KEGG Pathways:  
    Probe set : 1384295_at    Name:   heat shock protein hsp70-related protein    GB acc:   AI111363    UniGene:   Rn.137392    Gene Symbol:   RGD:1303296    Locus Link:   307133    Chromosome:   17    Cytoband:   Chr:17q12.3    SOURCE    GENECARD       Gene Ontology:   biological process:  protein folding   biological process:  response to unfolded protein   molecular function:  nucleotide binding   molecular function:  ATP binding      BioCarta Pathways:     KEGG Pathways:  
    Probe set : 1371785_at    Name:   tumor necrosis factor receptor superfamily, member 12a    GB acc:   BI303379    UniGene:   Rn.105040    Gene Symbol:   Tnfrsf12a    Locus Link:   302965    Chromosome:   10    Cytoband:   Chr:10q12    SOURCE    GENECARD       Gene Ontology:   cellular component:  integral to membrane   molecular function:  receptor activity      BioCarta Pathways:     KEGG Pathways:  
    Probe set : 1388940_at    Name:   similar to Rho-GTPase-activating protein 25    GB acc:   AW252115    UniGene:   Rn.22776    Gene Symbol:   LOC500246    Locus Link:   500246    Chromosome:   4    Cytoband:   Chr:4q34    SOURCE    GENECARD       Gene Ontology:     BioCarta Pathways:     KEGG Pathways:  
    Probe set : 1373894_at     GB acc:   BF410946              Gene Ontology:     BioCarta Pathways:     KEGG Pathways:  
    Probe set : 1375269_at    Name:   similar to Deoxyribose-phosphate aldolase-like    GB acc:   BM391516    UniGene:   Rn.17191    Gene Symbol:   LOC500355    Locus Link:   500355    Chromosome:   4    Cytoband:   Chr:4q44    SOURCE    GENECARD       Gene Ontology:     BioCarta Pathways:     KEGG Pathways:  
    Probe set : 1384939_at    Name:   Transcribed locus    GB acc:   BF522954    UniGene:   Rn.149181        SOURCE        Gene Ontology:     BioCarta Pathways:     KEGG Pathways:  
    Probe set : 1384303_s_at    Name:   solute carrier family 6 (neurotransmitter transporter), member 17    GB acc:   AI712791    UniGene:   Rn.144487    Gene Symbol:   Slc6a17    Locus Link:   613226      SOURCE    GENECARD       Gene Ontology:   biological process:  transport   biological process:  neurotransmitter transport   cellular component:  integral to plasma membrane   cellular component:  membrane   cellular component:  integral to membrane   molecular function:  neurotransmitter:sodium symporter activity   molecular function:  symporter activity      BioCarta Pathways:     KEGG Pathways:  
    Probe set : 1398369_at    Name:   Transcribed locus    GB acc:   AI599048    UniGene:   Rn.2027        SOURCE        Gene Ontology:     BioCarta Pathways:     KEGG Pathways:  
    Probe set : 1388850_at    Name:   heat shock protein 1, alpha    GB acc:   BG671521    UniGene:   Rn.106682    Gene Symbol:   Hspca    Locus Link:   299331    Chromosome:   6    Cytoband:   Chr:6q32    SOURCE    GENECARD       Gene Ontology:     BioCarta Pathways:     KEGG Pathways:  
    Probe set : 1388158_at    Name:   HLA-B-associated transcript 1A    GB acc:   BG057565    UniGene:   Rn.3516    Gene Symbol:   Bat1a    Locus Link:   114612    Chromosome:   20    Cytoband:   Chr:20p12    SOURCE    GENECARD       Gene Ontology:   biological process:  nuclear mRNA splicing, via spliceosome   biological process:  mRNA processing   cellular component:  nucleus   cellular component:  spliceosome complex      BioCarta Pathways:     KEGG Pathways:  
    Probe set : 1391502_at    Name:   Transcribed locus    GB acc:   AI170453    UniGene:   Rn.28413        SOURCE        Gene Ontology:     BioCarta Pathways:     KEGG Pathways:  
    Probe set : 1369877_at    Name:   CD8 antigen, alpha chain    GB acc:   NM_031538    UniGene:   Rn.10306    Gene Symbol:   Cd8a    Locus Link:   24930    Chromosome:   4    Cytoband:   Chr:4q33    SOURCE    GENECARD       Gene Ontology:     BioCarta Pathways:     KEGG Pathways:  
    Probe set : 1394438_at    Name:   ARP1 actin-related protein 1 homolog A (yeast) (predicted)    GB acc:   BI294346    UniGene:   Rn.32312    Gene Symbol:   Actr1a_predicted    Locus Link:   294010    Chromosome:   1    Cytoband:   Chr:1q54    SOURCE    GENECARD       Gene Ontology:   biological process:  vesicle-mediated transport   molecular function:  motor activity   molecular function:  structural molecule activity   molecular function:  structural constituent of cytoskeleton      BioCarta Pathways:     KEGG Pathways:  
    Probe set : 1368371_at    Name:   potassium voltage-gated channel, subfamily Q, member 1    GB acc:   NM_032073    UniGene:   Rn.9779    Gene Symbol:   Kcnq1    Locus Link:   84020    Chromosome:   1    Cytoband:   Chr:1q41    SOURCE    GENECARD       Gene Ontology:     BioCarta Pathways:     KEGG Pathways:  
    Probe set : 1385252_at    Name:   Tripartite motif protein 34 (predicted)    GB acc:   BI280655    UniGene:   Rn.144794     Locus Link:   293294    Chromosome:   1    Cytoband:   Chr:1q32    SOURCE        Gene Ontology:   biological process:  protein ubiquitination   cellular component:  ubiquitin ligase complex   cellular component:  intracellular   molecular function:  ubiquitin-protein ligase activity   molecular function:  zinc ion binding      BioCarta Pathways:     KEGG Pathways:  
    Probe set : 1372668_at    Name:   LIM domains containing 1 (predicted)    GB acc:   BI289676    UniGene:   Rn.3966     Locus Link:   316101    Chromosome:   8    Cytoband:   Chr:8q32    SOURCE        Gene Ontology:   biological process:  regulation of transcription, DNA-dependent   biological process:  signal transduction   biological process:  development   molecular function:  protein binding   molecular function:  zinc ion binding      BioCarta Pathways:     KEGG Pathways:  
    Probe set : 1373465_at    Name:   PQ loop repeat containing 1 (predicted)    GB acc:   AI228284    UniGene:   Rn.17290    Gene Symbol:   Pqlc1_predicted    Locus Link:   361352    Chromosome:   18    Cytoband:   Chr:18q12.3    SOURCE    GENECARD       Gene Ontology:   biological process:  electron transport   molecular function:  electron transporter activity      BioCarta Pathways:     KEGG Pathways:  
    Probe set : 1383321_at    Name:   tyrosylprotein sulfotransferase 1 (predicted)    GB acc:   BG376248    UniGene:   Rn.144728    Gene Symbol:   Tpst1_predicted    Locus Link:   288617    Chromosome:   12    Cytoband:   Chr:12q13    SOURCE    GENECARD       Gene Ontology:   biological process:  peptidyl-tyrosine sulfation   biological process:  inflammatory response   molecular function:  sulfotransferase activity   molecular function:  protein-tyrosine sulfotransferase activity   molecular function:  transferase activity      BioCarta Pathways:     KEGG Pathways:  
    Probe set : 1380117_at    Name:   Transcribed locus    GB acc:   BF397842    UniGene:   Rn.148018        SOURCE        Gene Ontology:     BioCarta Pathways:     KEGG Pathways:  
    Probe set : 1384485_at    Name:   protein tyrosine phosphatase, receptor type, U    GB acc:   BE116371    UniGene:   Rn.35625    Gene Symbol:   Ptpru    Locus Link:   116680    Chromosome:   5    Cytoband:   Chr:5q36    SOURCE    GENECARD       Gene Ontology:     BioCarta Pathways:     KEGG Pathways:  
    Probe set : 1374636_at    Name:   PHD finger protein 17 (predicted)    GB acc:   BI298596    UniGene:   Rn.90858     Locus Link:   310352    Chromosome:   2    Cytoband:   Chr:2q26    SOURCE        Gene Ontology:   cellular component:  nucleus   cellular component:  cytoplasm   molecular function:  DNA binding   molecular function:  protein binding      BioCarta Pathways:     KEGG Pathways:  
    Probe set : 1373044_at    Name:   similar to Dendritic cell protein GA17    GB acc:   BI285403    UniGene:   Rn.3390    Gene Symbol:   LOC295975    Locus Link:   295975    Chromosome:   3    Cytoband:   Chr:3q32    SOURCE    GENECARD       Gene Ontology:     BioCarta Pathways:     KEGG Pathways:  
    Probe set : 1388453_at    Name:   myeloid-associated differentiation marker    GB acc:   BI296048    UniGene:   Rn.106161    Gene Symbol:   Myadm    Locus Link:   369016    Chromosome:   1    Cytoband:   Chr:1q12    SOURCE    GENECARD       Gene Ontology:   biological process:  myeloid cell differentiation   cellular component:  membrane      BioCarta Pathways:     KEGG Pathways:  
    Probe set : 1399049_at    Name:   similar to RIKEN cDNA 1200003C05 (predicted)    GB acc:   AI230073    UniGene:   Rn.106021    Gene Symbol:   RGD1308917_predicted    Locus Link:   299127    Chromosome:   6    Cytoband:   Chr:6q24    SOURCE    GENECARD       Gene Ontology:     BioCarta Pathways:     KEGG Pathways:  
    Probe set : 1371824_at    Name:   adenylate kinase 3-like 1    GB acc:   AA891949    UniGene:   Rn.1086    Gene Symbol:   Ak3l1    Locus Link:   29223    Chromosome:   5    Cytoband:   Chr:5q33    SOURCE    GENECARD       Gene Ontology:   biological process:  nucleobase, nucleoside, nucleotide and nucleic acid metabolism   cellular component:  mitochondrion      BioCarta Pathways:     KEGG Pathways:  
    Probe set : 1376341_at    Name:   Transcribed locus    GB acc:   BM388895    UniGene:   Rn.28603        SOURCE        Gene Ontology:     BioCarta Pathways:     KEGG Pathways:  
    Probe set : 1377088_at    Name:   similar to RIKEN cDNA 2310046K01 (predicted)    GB acc:   BI275952    UniGene:   Rn.14715    Gene Symbol:   RGD1304644_predicted    Locus Link:   311536    Chromosome:   3    Cytoband:   Chr:3q41    SOURCE    GENECARD       Gene Ontology:   cellular component:  integral to membrane      BioCarta Pathways:     KEGG Pathways:  
    Probe set : 1373197_at    Name:   Similar to Ab1-133 (predicted)    GB acc:   AI578263    UniGene:   Rn.31947     Locus Link:   296384    Chromosome:   3    Cytoband:   Chr:3q42    SOURCE        Gene Ontology:   biological process:  regulation of transcription, DNA-dependent   cellular component:  nucleus   molecular function:  transcription factor activity      BioCarta Pathways:     KEGG Pathways:  
    Probe set : 1372246_at    Name:   osteoclast stimulating factor 1    GB acc:   BI294750    UniGene:   Rn.61513    Gene Symbol:   Ostf1    Locus Link:   259275    Chromosome:   1    Cytoband:   Chr:1q43    SOURCE    GENECARD       Gene Ontology:   biological process:  ossification   biological process:  regulation of transcription, DNA-dependent   biological process:  signal transduction   cellular component:  intracellular   molecular function:  DNA binding   molecular function:  transcription factor activity   molecular function:  protein binding      BioCarta Pathways:     KEGG Pathways:  
    Probe set : 1372162_at    Name:   acetyl-Coenzyme A synthetase 2 (AMP forming)-like (predicted)    GB acc:   AI176565    UniGene:   Rn.98236    Gene Symbol:   Acas2l_predicted    Locus Link:   296259    Chromosome:   3    Cytoband:   Chr:3q41    SOURCE    GENECARD       Gene Ontology:   biological process:  acetyl-CoA biosynthesis   biological process:  metabolism   cellular component:  mitochondrion   cellular component:  mitochondrial matrix   molecular function:  catalytic activity   molecular function:  acetate-CoA ligase activity   molecular function:  ligase activity      BioCarta Pathways:     KEGG Pathways:  
    Probe set : 1372808_at    Name:   similar to methylenetetrahydrofolate dehydrogenase (NAD) (EC 1.5.1.15) / methenyltetrahydrofolate cyclohydrolase (EC 3.5.4.9) precursor - mouse    GB acc:   AW251324    UniGene:   Rn.139814    Gene Symbol:   LOC313410    Locus Link:   313410    Chromosome:   5    Cytoband:   Chr:5q33    SOURCE    GENECARD       Gene Ontology:     BioCarta Pathways:     KEGG Pathways:  
    Probe set : 1372372_at    Name:   similar to Ab2-225 (predicted)    GB acc:   BG372437    UniGene:   Rn.64439    Gene Symbol:   RGD1306952_predicted    Locus Link:   310201    Chromosome:   2    Cytoband:   Chr:2q22    SOURCE    GENECARD       Gene Ontology:   biological process:  aromatic compound catabolism   molecular function:  catalytic activity   molecular function:  hydrolase activity   molecular function:  serine esterase activity      BioCarta Pathways:     KEGG Pathways:  
    Probe set : 1390164_at    Name:   Transcribed locus    GB acc:   BE099850    UniGene:   Rn.23225        SOURCE        Gene Ontology:     BioCarta Pathways:     KEGG Pathways:  
    Probe set : 1389294_at    Name:   cytoplasmic FMR1 interacting protein 1 (predicted)    GB acc:   AI103084    UniGene:   Rn.143776    Gene Symbol:   Cyfip1_predicted    Locus Link:   308666    Chromosome:   1    Cytoband:   Chr:1q22    SOURCE    GENECARD       Gene Ontology:   biological process:  lamellipodium biogenesis   cellular component:  lamellipodium   molecular function:  profilin binding      BioCarta Pathways:     KEGG Pathways:  
    Probe set : 1371361_at    Name:   similar to tensin    GB acc:   BI278826    UniGene:   Rn.3793    Gene Symbol:   LOC301509    Locus Link:   301509    Chromosome:   9    Cytoband:   Chr:9q33    SOURCE    GENECARD       Gene Ontology:     BioCarta Pathways:     KEGG Pathways:  
    Probe set : 1374501_at     GB acc:   BF550209              Gene Ontology:     BioCarta Pathways:     KEGG Pathways:  
    Probe set : 1388879_at     GB acc:   BG669292              Gene Ontology:     BioCarta Pathways:     KEGG Pathways:  
    Probe set : 1385486_at    Name:   Basonuclin 2 (predicted)    GB acc:   AW534737    UniGene:   Rn.134761    Gene Symbol:   LOC298189    Locus Link:   298189    Chromosome:   5    Cytoband:   Chr:5q31    SOURCE    GENECARD       Gene Ontology:   cellular component:  nucleus   molecular function:  nucleic acid binding   molecular function:  DNA binding   molecular function:  zinc ion binding      BioCarta Pathways:     KEGG Pathways:  
    Probe set : 1370436_at    Name:   kidney-specific protein (KS)    GB acc:   AF062389    UniGene:   Rn.14875    Gene Symbol:   RGD:708383    Locus Link:   246263    Chromosome:   1    Cytoband:   Chr:1q35    SOURCE    GENECARD       Gene Ontology:   biological process:  metabolism   molecular function:  catalytic activity      BioCarta Pathways:     KEGG Pathways:  
    Probe set : 1383331_at    Name:   Transcribed locus    GB acc:   BF548234    UniGene:   Rn.8380        SOURCE        Gene Ontology:     BioCarta Pathways:     KEGG Pathways:  
    Probe set : 1372052_at    Name:   Budding uninhibited by benzimidazoles 3 homolog (S. cerevisiae) (predicted)    GB acc:   BI275918    UniGene:   Rn.6897     Locus Link:   361662    Chromosome:   1    Cytoband:   Chr:1q41    SOURCE        Gene Ontology:   biological process:  mitosis   biological process:  mitotic spindle checkpoint   biological process:  cell proliferation   cellular component:  kinetochore   cellular component:  nucleus      BioCarta Pathways:     KEGG Pathways:  
    Probe set : 1389820_at     GB acc:   BF289041              Gene Ontology:     BioCarta Pathways:     KEGG Pathways:  
    Probe set : 1373555_at    Name:   Dynactin 4    GB acc:   BM392315    UniGene:   Rn.23941    Gene Symbol:   Dctn4    Locus Link:   84428    Chromosome:   18    Cytoband:   Chr:18q12.1    SOURCE    GENECARD       Gene Ontology:   molecular function:  protein binding   molecular function:  protein binding      BioCarta Pathways:     KEGG Pathways:  
    Probe set : 1368653_a_at    Name:   fertility protein SP22    GB acc:   BI281756    UniGene:   Rn.30105    Gene Symbol:   Park7    Locus Link:   117287    Chromosome:   5    Cytoband:   Chr:5q36    SOURCE    GENECARD       Gene Ontology:   biological process:  protein folding   cellular component:  nucleus   molecular function:  unfolded protein binding      BioCarta Pathways:     KEGG Pathways:  
    Probe set : 1389456_at    Name:   Transcribed locus    GB acc:   BI296591    UniGene:   Rn.17276        SOURCE        Gene Ontology:     BioCarta Pathways:     KEGG Pathways:  
    Probe set : 1371551_at    Name:   Tnf receptor associated factor 4 (predicted)    GB acc:   BM383323    UniGene:   Rn.3219    Gene Symbol:   Traf4_predicted    Locus Link:   303285    Chromosome:   10    Cytoband:   Chr:10q25    SOURCE    GENECARD       Gene Ontology:   cellular component:  ubiquitin ligase complex   cellular component:  nucleus   molecular function:  ubiquitin-protein ligase activity   molecular function:  receptor activity   molecular function:  zinc ion binding      BioCarta Pathways:     KEGG Pathways:  
    Probe set : 1373127_at    Name:   Similar to Tripartite motif protein 44 (DIPB protein)    GB acc:   BM383645    UniGene:   Rn.8005     Locus Link:   362172    Chromosome:   3    Cytoband:   Chr:3q31    SOURCE        Gene Ontology:   cellular component:  intracellular   molecular function:  zinc ion binding   molecular function:  metal ion binding      BioCarta Pathways:     KEGG Pathways:  
    Probe set : 1390946_at    Name:   Runt-related transcription factor 3    GB acc:   AI709792    UniGene:   Rn.34220    Gene Symbol:   Runx3    Locus Link:   156726    Chromosome:   5    Cytoband:   Chr:5q36    SOURCE    GENECARD       Gene Ontology:   biological process:  regulation of transcription, DNA-dependent   biological process:  axon guidance   biological process:  cell maturation   cellular component:  nucleus   molecular function:  DNA binding   molecular function:  ATP binding   molecular function:  transcription factor activity      BioCarta Pathways:     KEGG Pathways:  
    Probe set : 1389729_at    Name:   similar to cDNA sequence BC003324    GB acc:   AI408907    UniGene:   Rn.12933    Gene Symbol:   RGD:1303140    Locus Link:   288652    Chromosome:   12    Cytoband:   Chr:12q15    SOURCE    GENECARD       Gene Ontology:     BioCarta Pathways:     KEGG Pathways:  
    Probe set : 1370165_at    Name:   small muscle protein, X-linked    GB acc:   AI406565    UniGene:   Rn.4123    Gene Symbol:   Smpx    Locus Link:   84416    Chromosome:   X    Cytoband:   Chr:Xq21    SOURCE    GENECARD       Gene Ontology:   biological process:  striated muscle contraction   cellular component:  nucleus   cellular component:  cytoplasm      BioCarta Pathways:     KEGG Pathways:  
    Probe set : 1398872_at    Name:   ribosomal protein L13 /// ribosomal protein S13    GB acc:   BE107613    UniGene:   Rn.103229    Gene Symbol:   Rpl13 /// Rps13    Locus Link:   161477 /// 81765    Chromosome:   19    Cytoband:   Chr:19q12 /// Chr:3p12    SOURCE    GENECARD       Gene Ontology:   biological process:  protein biosynthesis   biological process:  ribosome biogenesis   biological process:  protein biosynthesis      BioCarta Pathways:     KEGG Pathways:  
    Probe set : 1374146_at    Name:   MAD2 mitotic arrest deficient-like 2 (yeast) (predicted)    GB acc:   BF419366    UniGene:   Rn.27237    Gene Symbol:   Mad2l2_predicted    Locus Link:   313702    Chromosome:   5    Cytoband:   Chr:5q36    SOURCE    GENECARD       Gene Ontology:   biological process:  cell cycle   biological process:  mitosis   biological process:  mitotic spindle checkpoint      BioCarta Pathways:     KEGG Pathways:  
    Probe set : 1368604_at    Name:   Mediterranean fever    GB acc:   NM_031634    UniGene:   Rn.29105    Gene Symbol:   Mefv    Locus Link:   58923    Chromosome:   10    Cytoband:   Chr:10q12    SOURCE    GENECARD       Gene Ontology:   biological process:  inflammatory response   biological process:  microtubule-based process   biological process:  inflammatory response      BioCarta Pathways:     KEGG Pathways:  
    Probe set : 1395662_at    Name:   Transcribed locus    GB acc:   AW531934    UniGene:   Rn.153738        SOURCE        Gene Ontology:     BioCarta Pathways:     KEGG Pathways:  
    Probe set : 1390529_at    Name:   CD83 antigen (predicted)    GB acc:   AI412355    UniGene:   Rn.20096    Gene Symbol:   Cd83_predicted    Locus Link:   361226    Chromosome:   17    Cytoband:   Chr:17p12    SOURCE    GENECARD       Gene Ontology:   biological process:  defense response   biological process:  humoral immune response   biological process:  signal transduction      BioCarta Pathways:     KEGG Pathways:  
    Probe set : 1396267_at     GB acc:   N55607              Gene Ontology:     BioCarta Pathways:     KEGG Pathways:  
    Probe set : 1387703_a_at    Name:   ubiquitin specific protease 2    GB acc:   AF106659    UniGene:   Rn.144632    Gene Symbol:   Usp2    Locus Link:   115771    Chromosome:   8    Cytoband:   Chr:8q22    SOURCE    GENECARD       Gene Ontology:   biological process:  ubiquitin-dependent protein catabolism   biological process:  ubiquitin cycle   cellular component:  centrosome      BioCarta Pathways:     KEGG Pathways:  
    Probe set : 1380431_at    Name:   similar to KIAA0564 protein (predicted)    GB acc:   AA893000     Gene Symbol:   RGD1308772_predicted    Locus Link:   290381    Chromosome:   15    Cytoband:   Chr:15q11-q12     GENECARD       Gene Ontology:     BioCarta Pathways:     KEGG Pathways:  
    Probe set : 1374778_at    Name:   cathepsin C    GB acc:   AI409046    UniGene:   Rn.122504    Gene Symbol:   Ctsc    Locus Link:   25423    Chromosome:   1    Cytoband:   Chr:1q32    SOURCE    GENECARD       Gene Ontology:   biological process:  proteolysis   biological process:  proteolysis   biological process:  proteolysis   biological process:  immune response   cellular component:  extracellular space   cellular component:  lysosome   cellular component:  lysosome      BioCarta Pathways:     KEGG Pathways:  
    Probe set : 1379450_at    Name:   similar to cDNA sequence BC003236 (predicted)    GB acc:   BM383195    UniGene:   Rn.25049    Gene Symbol:   RGD1310822_predicted    Locus Link:   310760    Chromosome:   2    Cytoband:   Chr:2q34    SOURCE    GENECARD       Gene Ontology:     BioCarta Pathways:     KEGG Pathways:  
    Probe set : 1380366_at    Name:   similar to single chain Fv antibody fragment scFv 7-10A    GB acc:   AA996557    UniGene:   Rn.110660    Gene Symbol:   LOC314509    Locus Link:   314509    Chromosome:   6    Cytoband:   Chr:6q33    SOURCE    GENECARD       Gene Ontology:     BioCarta Pathways:     KEGG Pathways:  
    Probe set : 1388532_at    Name:   similar to hypothetical protein (predicted)    GB acc:   AI599496    UniGene:   Rn.145167    Gene Symbol:   RGD1310571_predicted    Locus Link:   361301    Chromosome:   18    Cytoband:   Chr:18p12    SOURCE    GENECARD       Gene Ontology:     BioCarta Pathways:     KEGG Pathways:  
    Probe set : 1376576_at    Name:   dual specificity phosphatase 11 (RNA/RNP complex 1-interacting) (predicted)    GB acc:   BM387750    UniGene:   Rn.24649    Gene Symbol:   Dusp11_predicted    Locus Link:   297412    Chromosome:   4    Cytoband:   Chr:4q34    SOURCE    GENECARD       Gene Ontology:   biological process:  RNA processing   biological process:  protein amino acid dephosphorylation   cellular component:  nucleus      BioCarta Pathways:     KEGG Pathways:  
    Probe set : 1388982_at     GB acc:   BM389066              Gene Ontology:     BioCarta Pathways:     KEGG Pathways:  
    Probe set : 1387284_at    Name:   dihydropyrimidinase    GB acc:   NM_031705    UniGene:   Rn.10586    Gene Symbol:   Dpys    Locus Link:   65135    Chromosome:   7    Cytoband:   Chr:7q22    SOURCE    GENECARD       Gene Ontology:   biological process:  nucleobase, nucleoside, nucleotide and nucleic acid metabolism   biological process:  response to toxin   molecular function:  dihydropyrimidinase activity   molecular function:  dihydropyrimidinase activity   molecular function:  hydrolase activity      BioCarta Pathways:     KEGG Pathways:  
    Probe set : 1374561_at    Name:   hypothetical LOC305452 (predicted)    GB acc:   AW523899    UniGene:   Rn.7798    Gene Symbol:   RGD1309634_predicted    Locus Link:   305452    Chromosome:   14    Cytoband:   Chr:14q21    SOURCE    GENECARD       Gene Ontology:     BioCarta Pathways:     KEGG Pathways:  
    Probe set : 1390556_at     GB acc:   BF408801              Gene Ontology:     BioCarta Pathways:     KEGG Pathways:  
    Probe set : 1383796_at    Name:   CDNA clone IMAGE:7323916    GB acc:   BF550303    UniGene:   Rn.26676        SOURCE        Gene Ontology:     BioCarta Pathways:     KEGG Pathways:  
    Probe set : 1386884_at    Name:   protease, serine, 11 (Igf binding)    GB acc:   NM_031721    UniGene:   Rn.2782    Gene Symbol:   Prss11    Locus Link:   65164    Chromosome:   1    Cytoband:   Chr:1q37    SOURCE    GENECARD       Gene Ontology:   cellular component:  extracellular region   cellular component:  extracellular space      BioCarta Pathways:     KEGG Pathways:  
    Probe set : 1388306_at    Name:   similar to RIKEN cDNA 1810042K04 (predicted)    GB acc:   BM383758    UniGene:   Rn.2926    Gene Symbol:   RGD1305593_predicted    Locus Link:   288513    Chromosome:   12    Cytoband:   Chr:12q11    SOURCE    GENECARD       Gene Ontology:     BioCarta Pathways:     KEGG Pathways:  
    Probe set : 1380057_at    Name:   Transcribed locus    GB acc:   BE097091    UniGene:   Rn.45104        SOURCE        Gene Ontology:     BioCarta Pathways:     KEGG Pathways:  
    Probe set : 1370201_at    Name:   calbindin 1    GB acc:   X04280    UniGene:   Rn.3908    Gene Symbol:   Calb1    Locus Link:   83839    Chromosome:   5    Cytoband:   Chr:5q13    SOURCE    GENECARD       Gene Ontology:   cellular component:  cytoplasm      BioCarta Pathways:     KEGG Pathways:  
    Probe set : 1398974_at    Name:   chromosome segregation 1-like (S. cerevisiae) (predicted)    GB acc:   AI171615    UniGene:   Rn.36664    Gene Symbol:   Cse1l_predicted    Locus Link:   362273    Chromosome:   3    Cytoband:   Chr:3q42    SOURCE    GENECARD       Gene Ontology:   cellular component:  nucleus   cellular component:  nuclear pore   cellular component:  cytoplasm   molecular function:  binding   molecular function:  importin-alpha export receptor activity   molecular function:  protein transporter activity      BioCarta Pathways:     KEGG Pathways:  
    Probe set : 1393844_at    Name:   similar to myeloid leukemia factor 1 interacting protein    GB acc:   AW525736    UniGene:   Rn.128609    Gene Symbol:   LOC306464    Locus Link:   306464    Chromosome:   16    Cytoband:   Chr:16q11    SOURCE    GENECARD       Gene Ontology:     BioCarta Pathways:     KEGG Pathways:  
    Probe set : 1371330_at    Name:   ribosomal protein L11 (predicted)    GB acc:   BI284252    UniGene:   Rn.14687    Gene Symbol:   Rpl11_predicted    Locus Link:   362631    Chromosome:   5    Cytoband:   Chr:5q36    SOURCE    GENECARD       Gene Ontology:   biological process:  protein biosynthesis   molecular function:  RNA binding   molecular function:  structural constituent of ribosome   molecular function:  rRNA binding      BioCarta Pathways:     KEGG Pathways:  
    Probe set : 1388570_at    Name:   similar to RIKEN cDNA 2310005O14    GB acc:   AI232494    UniGene:   Rn.22044    Gene Symbol:   LOC498909    Locus Link:   498909    Chromosome:   19    Cytoband:   Chr:19p12    SOURCE    GENECARD       Gene Ontology:     BioCarta Pathways:     KEGG Pathways:  
    Probe set : 1388520_at    Name:   Sperm associated antigen 9 (predicted)    GB acc:   BI278854    UniGene:   Rn.24583     Locus Link:   360600    Chromosome:   10    Cytoband:   Chr:10q26    SOURCE        Gene Ontology:   biological process:  spermatogenesis   cellular component:  integral to membrane      BioCarta Pathways:     KEGG Pathways:  
    Probe set : 1369008_a_at    Name:   olfactomedin 1    GB acc:   NM_053573    UniGene:   Rn.11005    Gene Symbol:   Olfm1    Locus Link:   93667    Chromosome:   3    Cytoband:   Chr:3p12    SOURCE    GENECARD       Gene Ontology:   biological process:  development   biological process:  nervous system development   molecular function:  latrotoxin receptor activity      BioCarta Pathways:     KEGG Pathways:  
    Probe set : 1372612_at    Name:   dynein light chain-2    GB acc:   BG372973    UniGene:   Rn.36362    Gene Symbol:   Dlc2    Locus Link:   140734    Chromosome:   10    Cytoband:   Chr:10q26    SOURCE    GENECARD       Gene Ontology:   biological process:  microtubule-based process   biological process:  synaptic target recognition   biological process:  synaptic target recognition   cellular component:  microtubule associated complex   cellular component:  dynein complex   cellular component:  microtubule   molecular function:  motor activity   molecular function:  microtubule motor activity   molecular function:  cytoskeletal protein binding      BioCarta Pathways:     KEGG Pathways:  
    Probe set : 1376153_at    Name:   Transcribed locus    GB acc:   BE102621    UniGene:   Rn.22233        SOURCE        Gene Ontology:     BioCarta Pathways:     KEGG Pathways:  
    Probe set : 1370831_at    Name:   monoglyceride lipase    GB acc:   AY081195    UniGene:   Rn.40396    Gene Symbol:   Mgll    Locus Link:   29254    Chromosome:   4    Cytoband:   Chr:4q34    SOURCE    GENECARD       Gene Ontology:   cellular component:  synapse      BioCarta Pathways:     KEGG Pathways:  
    Probe set : 1369683_at    Name:   BH3 interacting domain death agonist    GB acc:   AF136282    UniGene:   Rn.31142    Gene Symbol:   Bid    Locus Link:   64625    Chromosome:   4    Cytoband:   Chr:4q42    SOURCE    GENECARD       Gene Ontology:   biological process:  apoptosis   biological process:  regulation of apoptosis   biological process:  positive regulation of apoptosis   cellular component:  cytoplasm      BioCarta Pathways:     KEGG Pathways:  
    Probe set : 1388539_at    Name:   plakophilin 2 (predicted)    GB acc:   BE113268    UniGene:   Rn.27944    Gene Symbol:   Pkp2_predicted    Locus Link:   287925    Chromosome:   11    Cytoband:   Chr:11q23    SOURCE    GENECARD       Gene Ontology:   biological process:  heart development   biological process:  cell-cell adhesion   molecular function:  structural molecule activity   molecular function:  protein binding   molecular function:  binding      BioCarta Pathways:     KEGG Pathways:  
    Probe set : 1377755_at    Name:   helicase, ATP binding 1 (predicted)    GB acc:   AI179403    UniGene:   Rn.24209    Gene Symbol:   Helic1_predicted    Locus Link:   309887    Chromosome:   20    Cytoband:   Chr:20q13    SOURCE    GENECARD       Gene Ontology:   biological process:  regulation of transcription, DNA-dependent   cellular component:  nucleus      BioCarta Pathways:     KEGG Pathways:  
    Probe set : 1378138_at    Name:   kinesin family member 16B (predicted)    GB acc:   BI296233    UniGene:   Rn.7029    Gene Symbol:   Kif16b_predicted    Locus Link:   311478    Chromosome:   3    Cytoband:   Chr:3q41    SOURCE    GENECARD       Gene Ontology:   biological process:  microtubule-based process   biological process:  microtubule-based movement   biological process:  intracellular signaling cascade   cellular component:  kinesin complex   cellular component:  microtubule associated complex   molecular function:  motor activity   molecular function:  microtubule motor activity   molecular function:  ATP binding      BioCarta Pathways:     KEGG Pathways:  
    Probe set : 1375034_at    Name:   lysosomal phospholipase A2    GB acc:   AI410383    UniGene:   Rn.93631    Gene Symbol:   RGD:1302982    Locus Link:   361401    Chromosome:   19    Cytoband:   Chr:19q12    SOURCE    GENECARD       Gene Ontology:   biological process:  phospholipid catabolism   biological process:  surfactant homeostasis   biological process:  lipid metabolism   cellular component:  lysosome   molecular function:  calcium-independent phospholipase A2 activity   molecular function:  phosphatidylcholine-sterol O-acyltransferase activity      BioCarta Pathways:     KEGG Pathways:  
    Probe set : 1370066_at    Name:   Kelch-like ECH-associated protein 1    GB acc:   NM_057152    UniGene:   Rn.23467    Gene Symbol:   Keap1    Locus Link:   117519    Chromosome:   8    Cytoband:   Chr:8q13    SOURCE    GENECARD       Gene Ontology:   biological process:  regulation of transcription, DNA-dependent   biological process:  transcription   molecular function:  protein binding      BioCarta Pathways:     KEGG Pathways:  
    Probe set : 1382454_at    Name:   chemokine (C-X-C motif) ligand 9    GB acc:   AI044222    UniGene:   Rn.7391    Gene Symbol:   Cxcl9    Locus Link:   246759    Chromosome:   14    Cytoband:   Chr:14p22    SOURCE    GENECARD       Gene Ontology:   biological process:  inflammatory response   biological process:  immune response   cellular component:  extracellular region   cellular component:  extracellular space   molecular function:  cytokine activity   molecular function:  chemokine activity      BioCarta Pathways:     KEGG Pathways:  
    Probe set : 1396942_at    Name:   hypothetical LOC301267    GB acc:   AI408959    UniGene:   Rn.91400    Gene Symbol:   LOC301267    Locus Link:   301267    Chromosome:   9    Cytoband:   Chr:9q12    SOURCE    GENECARD       Gene Ontology:     BioCarta Pathways:     KEGG Pathways:  
    Probe set : 1384191_at    Name:   Transcribed locus    GB acc:   BF387765    UniGene:   Rn.101899        SOURCE        Gene Ontology:     BioCarta Pathways:     KEGG Pathways:  
    Probe set : 1393261_at    Name:   Similar to protein phosphatase 2A B56 regulatory subunit gamma 3 isoform    GB acc:   AI058850    UniGene:   Rn.2153     Locus Link:   500722      SOURCE        Gene Ontology:     BioCarta Pathways:     KEGG Pathways:  
    Probe set : 1394842_at    Name:   Unknown (protein for MGC:72591)    GB acc:   BF289172    UniGene:   Rn.131677    Gene Symbol:   Tmem19    Locus Link:   299800    Chromosome:   7    Cytoband:   Chr:7q22    SOURCE    GENECARD       Gene Ontology:     BioCarta Pathways:     KEGG Pathways:  
    Probe set : 1389013_at    Name:   similar to RIKEN cDNA 1500016H10 (predicted)    GB acc:   BF420644    UniGene:   Rn.102202    Gene Symbol:   RGD1308469_predicted    Locus Link:   317275    Chromosome:   9    Cytoband:   Chr:9q12    SOURCE    GENECARD       Gene Ontology:     BioCarta Pathways:     KEGG Pathways:  
    Probe set : 1392244_at    Name:   LOC498362    GB acc:   AA818153    UniGene:   Rn.16466    Gene Symbol:   LOC498362    Locus Link:   498362    Chromosome:   14    Cytoband:   Chr:14p11    SOURCE    GENECARD       Gene Ontology:     BioCarta Pathways:     KEGG Pathways:  
    Probe set : 1368799_at    Name:   baculoviral IAP repeat-containing 5    GB acc:   NM_022274    UniGene:   Rn.54471    Gene Symbol:   Birc5    Locus Link:   64041      SOURCE    GENECARD       Gene Ontology:     BioCarta Pathways:     KEGG Pathways:  
    Probe set : 1394786_at    Name:   Transcribed locus    GB acc:   AW526631    UniGene:   Rn.46267        SOURCE        Gene Ontology:     BioCarta Pathways:     KEGG Pathways:  
    Probe set : 1368914_at    Name:   runt related transcription factor 1    GB acc:   NM_017325    UniGene:   Rn.11201    Gene Symbol:   Runx1    Locus Link:   50662    Chromosome:   11    Cytoband:   Chr:11q11    SOURCE    GENECARD       Gene Ontology:   cellular component:  nucleus      BioCarta Pathways:     KEGG Pathways:  
    Probe set : 1370919_at    Name:   heterogeneous nuclear ribonucleoprotein M    GB acc:   AI103467    UniGene:   Rn.10156    Gene Symbol:   Hnrpm    Locus Link:   116655    Chromosome:   7    Cytoband:   Chr:7q12    SOURCE    GENECARD       Gene Ontology:     BioCarta Pathways:     KEGG Pathways:  
    Probe set : 1392090_at    Name:   serine (or cysteine) proteinase inhibitor, clade C (antithrombin), member 1 (predicted)    GB acc:   BG377322    UniGene:   Rn.11698    Gene Symbol:   Serpinc1_predicted    Locus Link:   304917    Chromosome:   13    Cytoband:   Chr:13q22    SOURCE    GENECARD       Gene Ontology:   biological process:  blood coagulation   cellular component:  extracellular region   cellular component:  extracellular space   molecular function:  serine-type endopeptidase inhibitor activity   molecular function:  protein binding   molecular function:  heparin binding      BioCarta Pathways:     KEGG Pathways:  
    Probe set : 1392935_at    Name:   Transcribed locus    GB acc:   BE115639    UniGene:   Rn.147598        SOURCE        Gene Ontology:     BioCarta Pathways:     KEGG Pathways:  
    Probe set : 1384877_at    Name:   aquaporin 11    GB acc:   AI072405    UniGene:   Rn.20144    Gene Symbol:   Aqp11    Locus Link:   286758    Chromosome:   1    Cytoband:   Chr:1q32    SOURCE    GENECARD       Gene Ontology:   biological process:  proteolysis   biological process:  transport   cellular component:  membrane   cellular component:  integral to membrane   molecular function:  cysteine-type endopeptidase activity   molecular function:  transporter activity   molecular function:  peptidase activity      BioCarta Pathways:     KEGG Pathways:  
    Probe set : 1377723_at    Name:   Transcribed locus    GB acc:   AI234810    UniGene:   Rn.19876        SOURCE        Gene Ontology:     BioCarta Pathways:     KEGG Pathways:  
    Probe set : 1393431_at    Name:   Transcribed locus    GB acc:   AI500737    UniGene:   Rn.50804        SOURCE        Gene Ontology:     BioCarta Pathways:     KEGG Pathways:  
    Probe set : 1398882_at    Name:   ribosomal protein S5    GB acc:   BI282255    UniGene:   Rn.2100    Gene Symbol:   Rps5    Locus Link:   25538    Chromosome:   1    Cytoband:   Chr:1q21    SOURCE    GENECARD       Gene Ontology:   biological process:  protein biosynthesis   biological process:  ribosome biogenesis   molecular function:  RNA binding   molecular function:  structural constituent of ribosome      BioCarta Pathways:     KEGG Pathways:  
    Probe set : 1384392_at    Name:   cytochrome P450, family 26, subfamily b, polypeptide 1    GB acc:   BF397093    UniGene:   Rn.19898    Gene Symbol:   Cyp26b1    Locus Link:   312495    Chromosome:   4    Cytoband:   Chr:4q34    SOURCE    GENECARD       Gene Ontology:   cellular component:  endoplasmic reticulum   cellular component:  microsome   cellular component:  membrane      BioCarta Pathways:     KEGG Pathways:  
    Probe set : 1393619_at    Name:   CCR4-NOT transcription complex, subunit 6-like (predicted)    GB acc:   AI045762    UniGene:   Rn.16306    Gene Symbol:   Cnot6l_predicted    Locus Link:   360917    Chromosome:   14    Cytoband:   Chr:14p22    SOURCE    GENECARD       Gene Ontology:   biological process:  regulation of transcription, DNA-dependent   cellular component:  nucleus      BioCarta Pathways:     KEGG Pathways:  
    Probe set : 1385277_x_at    Name:   similar to hypothetical protein FLJ11354 (predicted)    GB acc:   AA818849    UniGene:   Rn.140601    Gene Symbol:   RGD1310093_predicted    Locus Link:   303538    Chromosome:   10    Cytoband:   Chr:10q32.1    SOURCE    GENECARD       Gene Ontology:     BioCarta Pathways:     KEGG Pathways:  
    Probe set : 1374846_at    Name:   cardiac lineage protein 1    GB acc:   AI230884    UniGene:   Rn.113703    Gene Symbol:   Clp1    Locus Link:   498008    Chromosome:   10    Cytoband:   Chr:10q32.1    SOURCE    GENECARD       Gene Ontology:   biological process:  negative regulation of transcription from RNA polymerase II promoter   biological process:  negative regulation of cyclin dependent protein kinase activity   cellular component:  nucleus   cellular component:  cytoplasm      BioCarta Pathways:     KEGG Pathways:  
    Probe set : 1382477_at    Name:   SUMO/sentrin specific protease 5 (predicted)    GB acc:   AW532560    UniGene:   Rn.3336     Locus Link:   303874    Chromosome:   11    Cytoband:   Chr:11q22    SOURCE        Gene Ontology:   biological process:  proteolysis   biological process:  ubiquitin cycle   molecular function:  peptidase activity   molecular function:  cysteine-type peptidase activity      BioCarta Pathways:     KEGG Pathways:  
    Probe set : 1382283_at    Name:   Wiskott-Aldrich syndrome protein interacting protein    GB acc:   BF283711    UniGene:   Rn.7914    Gene Symbol:   Waspip    Locus Link:   117538    Chromosome:   3    Cytoband:   Chr:3q23    SOURCE    GENECARD       Gene Ontology:   cellular component:  actin filament   cellular component:  actin cytoskeleton   molecular function:  actin binding   molecular function:  profilin binding      BioCarta Pathways:     KEGG Pathways:  
    Probe set : 1369153_at    Name:   nephrosis 1 homolog, nephrin (human)    GB acc:   NM_022628    UniGene:   Rn.48745    Gene Symbol:   Nphs1    Locus Link:   64563      SOURCE    GENECARD       Gene Ontology:   biological process:  MAPKKK cascade   biological process:  cell adhesion   biological process:  JNK cascade   biological process:  excretion   molecular function:  protein binding      BioCarta Pathways:     KEGG Pathways:  
    Probe set : 1391656_at     GB acc:   AI101416              Gene Ontology:     BioCarta Pathways:     KEGG Pathways:  
    Probe set : 1391935_at    Name:   similar to eukaryotic translation initiation factor 4E member 3    GB acc:   AI575608    UniGene:   Rn.22427    Gene Symbol:   LOC297481    Locus Link:   297481    Chromosome:   4    Cytoband:   Chr:4q34    SOURCE    GENECARD       Gene Ontology:     BioCarta Pathways:     KEGG Pathways:  
    Probe set : 1392916_at    Name:   microtubule-associated protein 7 (predicted)    GB acc:   BM386695    UniGene:   Rn.23807    Gene Symbol:   Mtap7_predicted    Locus Link:   293016    Chromosome:   1    Cytoband:   Chr:1p12    SOURCE    GENECARD       Gene Ontology:   cellular component:  microtubule associated complex   molecular function:  structural molecule activity   molecular function:  cytoskeletal regulatory protein binding      BioCarta Pathways:     KEGG Pathways:  
    Probe set : 1399121_at    Name:   Cell division cycle 27 homolog (S. cerevisiae) (predicted)    GB acc:   BE111600    UniGene:   Rn.13353     Locus Link:   360643    Chromosome:   10    Cytoband:   Chr:10q32.1    SOURCE        Gene Ontology:   molecular function:  binding      BioCarta Pathways:     KEGG Pathways:  
    Probe set : 1384238_at    Name:   Similar to Tweety homolog 2    GB acc:   BM385249    UniGene:   Rn.16666     Locus Link:   287803    Chromosome:   10    Cytoband:   Chr:10q32.2    SOURCE        Gene Ontology:     BioCarta Pathways:     KEGG Pathways:  
    Probe set : 1371606_at    Name:   Transcribed locus    GB acc:   AA819633    UniGene:   Rn.16646        SOURCE        Gene Ontology:     BioCarta Pathways:     KEGG Pathways:  
    Probe set : 1368122_at    Name:   ring finger protein 103    GB acc:   NM_053438    UniGene:   Rn.45685    Gene Symbol:   Rnf103    Locus Link:   84508    Chromosome:   4    Cytoband:   Chr:4q33    SOURCE    GENECARD       Gene Ontology:   biological process:  protein ubiquitination   cellular component:  ubiquitin ligase complex   cellular component:  integral to membrane      BioCarta Pathways:     KEGG Pathways:  
    Probe set : 1383202_at    Name:   similar to U6 snRNA-associated Sm-like protein LSm6 (Sm protein F)    GB acc:   AA818406     Gene Symbol:   LOC498934    Locus Link:   498934    Chromosome:   19    Cytoband:   Chr:19q11     GENECARD       Gene Ontology:     BioCarta Pathways:     KEGG Pathways:  
    Probe set : 1399084_at    Name:   DEAH (Asp-Glu-Ala-His) box polypeptide 16    GB acc:   BF405883    UniGene:   Rn.6832    Gene Symbol:   RGD:1302963    Locus Link:   294232    Chromosome:   20    Cytoband:   Chr:20p12    SOURCE    GENECARD       Gene Ontology:   biological process:  nuclear mRNA splicing, via spliceosome   biological process:  cell cycle   molecular function:  RNA helicase activity      BioCarta Pathways:     KEGG Pathways:  
    Probe set : 1367582_at    Name:   ribosomal protein L29    GB acc:   NM_017150    UniGene:   Rn.3973    Gene Symbol:   Rpl29    Locus Link:   29283    Chromosome:   8    Cytoband:   Chr:8q32    SOURCE    GENECARD       Gene Ontology:     BioCarta Pathways:     KEGG Pathways:  
    Probe set : 1370869_at    Name:   branched chain aminotransferase 1, cytosolic    GB acc:   AI102790    UniGene:   Rn.8273    Gene Symbol:   Bcat1    Locus Link:   29592    Chromosome:   4    Cytoband:   Chr:4q44    SOURCE    GENECARD       Gene Ontology:   cellular component:  cytosol      BioCarta Pathways:     KEGG Pathways:  
    Probe set : 1387085_at    Name:   phosphoribosyl pyrophosphate synthetase 1    GB acc:   M29392    UniGene:   Rn.9761    Gene Symbol:   Prps1    Locus Link:   29562    Chromosome:   X    Cytoband:   Chr:Xq35    SOURCE    GENECARD       Gene Ontology:     BioCarta Pathways:     KEGG Pathways:  
    Probe set : 1372241_at    Name:   ornithine decarboxylase antizyme 1    GB acc:   AI411388    UniGene:   Rn.266    Gene Symbol:   Oaz1    Locus Link:   25502    Chromosome:   7    Cytoband:   Chr:7q11    SOURCE    GENECARD       Gene Ontology:   biological process:  polyamine metabolism   biological process:  polyamine biosynthesis   molecular function:  enzyme inhibitor activity   molecular function:  ornithine decarboxylase inhibitor activity      BioCarta Pathways:     KEGG Pathways:  
    Probe set : 1377911_at    Name:   Transcribed locus    GB acc:   AI029434    UniGene:   Rn.18226        SOURCE        Gene Ontology:     BioCarta Pathways:     KEGG Pathways:  
    Probe set : 1384892_at    Name:   Transcribed locus    GB acc:   AI029798    UniGene:   Rn.146636        SOURCE        Gene Ontology:     BioCarta Pathways:     KEGG Pathways:  
    Probe set : 1383605_at    Name:   similar to alpha-fetoprotein    GB acc:   BE119842    UniGene:   Rn.30321    Gene Symbol:   LOC360919    Locus Link:   360919    Chromosome:   14    Cytoband:   Chr:14p22    SOURCE    GENECARD       Gene Ontology:   biological process:  transport   cellular component:  extracellular space   molecular function:  carrier activity      BioCarta Pathways:     KEGG Pathways:  
    Probe set : 1376816_at    Name:   Hypothetical gene supported by NM_012514    GB acc:   BF284903    UniGene:   Rn.48840    Gene Symbol:   Brca1    Locus Link:   24227    Chromosome:   10    Cytoband:   Chr:10q32.1    SOURCE    GENECARD       Gene Ontology:   biological process:  DNA replication and chromosome cycle   biological process:  cell cycle checkpoint   biological process:  carbohydrate metabolism   biological process:  DNA repair   biological process:  DNA repair   biological process:  regulation of transcription from RNA polymerase II promoter   biological process:  regulation of transcription from RNA polymerase III promoter   biological process:  DNA damage response, signal transduction by p53 class mediator resulting in transcription of p21 class mediator   biological process:  centrosome cycle   biological process:  DNA damage response, signal transduction resulting in induction of apoptosis   biological process:  dosage compensation, by inactivation of X chromosome   biological process:  protein ubiquitination   biological process:  regulation of cell proliferation   biological process:  regulation of apoptosis   biological process:  positive regulation of DNA repair   biological process:  negative regulation of progression through cell cycle   biological process:  negative regulation of centriole replication   biological process:  response to DNA damage stimulus   biological process:  cell cycle      BioCarta Pathways:     KEGG Pathways:  
    Probe set : 1392961_at    Name:   Similar to Mob4B protein    GB acc:   AW534383    UniGene:   Rn.24123     Locus Link:   297387    Chromosome:   4    Cytoband:   Chr:4q34    SOURCE        Gene Ontology:     BioCarta Pathways:     KEGG Pathways:  
    Probe set : 1389725_at    Name:   transmembrane 7 superfamily member 2 (predicted)    GB acc:   BM390364    UniGene:   Rn.8313    Gene Symbol:   Tm7sf2_predicted    Locus Link:   293688    Chromosome:   1    Cytoband:   Chr:1q43    SOURCE    GENECARD       Gene Ontology:   molecular function:  oxidoreductase activity   molecular function:  delta14-sterol reductase activity      BioCarta Pathways:     KEGG Pathways:  
    Probe set : 1368021_at    Name:   alcohol dehydrogenase 1    GB acc:   NM_130780    UniGene:   Rn.40222    Gene Symbol:   Adh1    Locus Link:   24172    Chromosome:   2    Cytoband:   Chr:2q44    SOURCE    GENECARD       Gene Ontology:   biological process:  retinoid metabolism      BioCarta Pathways:     KEGG Pathways:  
    Probe set : 1381819_at    Name:   Transcribed locus    GB acc:   AI137052    UniGene:   Rn.137151        SOURCE        Gene Ontology:     BioCarta Pathways:     KEGG Pathways:  
    Probe set : 1372270_at    Name:   dolichyl-phosphate (UDP-N-acetylglucosamine) N-acetylglucosaminephosphotransferase 1 (GlcNAc-1-P transferase)    GB acc:   BM391270    UniGene:   Rn.129301    Gene Symbol:   H2afx    Locus Link:   300668    Chromosome:   8    Cytoband:   Chr:8q22    SOURCE    GENECARD       Gene Ontology:   biological process:  lipid metabolism   cellular component:  membrane   molecular function:  catalytic activity   molecular function:  transferase activity      BioCarta Pathways:     KEGG Pathways:  
    Probe set : 1388102_at    Name:   leukotriene B4 12-hydroxydehydrogenase    GB acc:   U66322    UniGene:   Rn.10656    Gene Symbol:   Ltb4dh    Locus Link:   192227    Chromosome:   5    Cytoband:   Chr:5q24    SOURCE    GENECARD       Gene Ontology:   biological process:  leukotriene metabolism   biological process:  response to toxin   cellular component:  cytoplasm      BioCarta Pathways:     KEGG Pathways:  
    Probe set : 1382925_at     GB acc:   BF287720              Gene Ontology:     BioCarta Pathways:     KEGG Pathways:  
    Probe set : 1371762_at    Name:   retinol binding protein 4, plasma    GB acc:   AA858962    UniGene:   Rn.108214    Gene Symbol:   Rbp4    Locus Link:   25703    Chromosome:   1    Cytoband:   Chr:1q53    SOURCE    GENECARD       Gene Ontology:   biological process:  transport   biological process:  transport   biological process:  visual perception   cellular component:  extracellular space      BioCarta Pathways:     KEGG Pathways:  
    Probe set : 1390818_at    Name:   ATPase, Na+/K+ transporting, beta 3 polypeptide    GB acc:   BF288088    UniGene:   Rn.5041    Gene Symbol:   Atp1b3    Locus Link:   25390    Chromosome:   8    Cytoband:   Chr:8q31    SOURCE    GENECARD       Gene Ontology:   biological process:  transport   biological process:  potassium ion transport   biological process:  sodium ion transport      BioCarta Pathways:     KEGG Pathways:  
    Probe set : 1374474_at    Name:   Transcribed locus, strongly similar to NP_080091.1 copine family member [Mus musculus]    GB acc:   BE099085    UniGene:   Rn.11746        SOURCE        Gene Ontology:     BioCarta Pathways:     KEGG Pathways:  
    Probe set : 1377879_at    Name:   Transcribed locus    GB acc:   BE102375    UniGene:   Rn.47404        SOURCE        Gene Ontology:     BioCarta Pathways:     KEGG Pathways:  
    Probe set : 1376180_at     GB acc:   AW915012              Gene Ontology:     BioCarta Pathways:     KEGG Pathways:  
    Probe set : 1392454_at    Name:   similar to hypothetical protein FLJ20729 (predicted)    GB acc:   BI296999    UniGene:   Rn.76645    Gene Symbol:   RGD1308723_predicted    Locus Link:   292160    Chromosome:   2    Cytoband:   Chr:2q44    SOURCE    GENECARD       Gene Ontology:     BioCarta Pathways:     KEGG Pathways:  
    Probe set : 1380692_at    Name:   Transcribed locus    GB acc:   BM387127    UniGene:   Rn.148725        SOURCE        Gene Ontology:     BioCarta Pathways:     KEGG Pathways:  
    Probe set : 1368627_at    Name:   regucalcin    GB acc:   NM_031546    UniGene:   Rn.10006    Gene Symbol:   Rgn    Locus Link:   25106    Chromosome:   X    Cytoband:   Chr:Xq11-q12    SOURCE    GENECARD       Gene Ontology:   cellular component:  nucleus   cellular component:  cytoplasm   molecular function:  calcium ion binding   molecular function:  enzyme regulator activity      BioCarta Pathways:     KEGG Pathways:  
    Probe set : 1379339_at    Name:   similar to tumor-related protein (predicted)    GB acc:   BM390448    UniGene:   Rn.1166    Gene Symbol:   RGD1307632_predicted    Locus Link:   364879    Chromosome:   18    Cytoband:   Chr:18q12.1    SOURCE    GENECARD       Gene Ontology:   biological process:  metabolism   molecular function:  catalytic activity      BioCarta Pathways:     KEGG Pathways:  
    Probe set : 1371503_at    Name:   nucleotide binding protein 1 (predicted)    GB acc:   BI289647    UniGene:   Rn.1758    Gene Symbol:   Nubp1_predicted    Locus Link:   287042    Chromosome:   10    Cytoband:   Chr:10q11    SOURCE    GENECARD       Gene Ontology:   biological process:  septin ring assembly   biological process:  transport   molecular function:  nucleotide binding   molecular function:  ATP binding   molecular function:  nucleoside-triphosphatase activity      BioCarta Pathways:     KEGG Pathways:  
    Probe set : 1398812_at    Name:   proteasome (prosome, macropain) subunit, beta type 1    GB acc:   NM_053590    UniGene:   Rn.6016    Gene Symbol:   Psmb1    Locus Link:   94198    Chromosome:   1    Cytoband:   Chr:1q12    SOURCE    GENECARD       Gene Ontology:   biological process:  ubiquitin-dependent protein catabolism   cellular component:  cytosol   cellular component:  proteasome core complex (sensu Eukaryota)   cellular component:  protein complex      BioCarta Pathways:     KEGG Pathways:  
    Probe set : 1390767_at    Name:   signal sequence receptor, alpha (predicted)    GB acc:   BF391148    UniGene:   Rn.25091    Gene Symbol:   Ssr1_predicted    Locus Link:   361233    Chromosome:   17    Cytoband:   Chr:17p12    SOURCE    GENECARD       Gene Ontology:   biological process:  cotranslational protein targeting to membrane   biological process:  positive regulation of cell proliferation   cellular component:  endoplasmic reticulum   cellular component:  integral to membrane   molecular function:  receptor activity   molecular function:  signal sequence binding   molecular function:  calcium ion binding      BioCarta Pathways:     KEGG Pathways:  
    Probe set : 1370105_at    Name:   lunatic fringe gene homolog (Drosophila)    GB acc:   NM_133393    UniGene:   Rn.127809    Gene Symbol:   Lfng    Locus Link:   170905    Chromosome:   12    Cytoband:   Chr:12q11    SOURCE    GENECARD       Gene Ontology:   biological process:  development   biological process:  compartment specification   biological process:  regulation of Notch signaling pathway      BioCarta Pathways:     KEGG Pathways:  
    Probe set : 1388393_at    Name:   proteolipid protein 2    GB acc:   BM385031    UniGene:   Rn.96974    Gene Symbol:   Plp2    Locus Link:   302562    Chromosome:   X    Cytoband:   Chr:Xq13    SOURCE    GENECARD       Gene Ontology:   cellular component:  integral to membrane   cellular component:  membrane   molecular function:  zinc ion binding   molecular function:  metal ion binding      BioCarta Pathways:     KEGG Pathways:  
    Probe set : 1367931_a_at    Name:   polypyrimidine tract binding protein 1    GB acc:   X60790    UniGene:   Rn.64440    Gene Symbol:   Ptbp1    Locus Link:   29497    Chromosome:   7    Cytoband:   Chr:7q11    SOURCE    GENECARD       Gene Ontology:     BioCarta Pathways:     KEGG Pathways:  
    Probe set : 1372082_at    Name:   Unknown (protein for MGC:72598)    GB acc:   BM391772    UniGene:   Rn.56102    Gene Symbol:   RGD:735063    Locus Link:   298598    Chromosome:   5    Cytoband:   Chr:5q36    SOURCE    GENECARD       Gene Ontology:   cellular component:  coated pit   cellular component:  clathrin vesicle coat   cellular component:  membrane   molecular function:  protein binding      BioCarta Pathways:     KEGG Pathways:  
    Probe set : 1380471_at    Name:   Transcribed locus    GB acc:   H31701    UniGene:   Rn.48830        SOURCE        Gene Ontology:     BioCarta Pathways:     KEGG Pathways:  
    Probe set : 1387805_at    Name:   BCL2/adenovirus E1B 19 kDa-interacting protein 3    GB acc:   NM_053420    UniGene:   Rn.2060    Gene Symbol:   Bnip3    Locus Link:   84480    Chromosome:   1    Cytoband:   Chr:1q41    SOURCE    GENECARD       Gene Ontology:   biological process:  apoptosis   biological process:  regulation of apoptosis   biological process:  positive regulation of apoptosis   cellular component:  mitochondrion   cellular component:  integral to membrane   cellular component:  mitochondrial membrane      BioCarta Pathways:     KEGG Pathways:  
    Probe set : 1385093_at    Name:   similar to hypothetical protein LOC284018 isoform b    GB acc:   BG379949    UniGene:   Rn.143962    Gene Symbol:   LOC498014    Locus Link:   498014    Chromosome:   10    Cytoband:   Chr:10q32.1    SOURCE    GENECARD       Gene Ontology:     BioCarta Pathways:     KEGG Pathways:  
    Probe set : 1391453_at    Name:   Transcribed locus, strongly similar to XP_576700.1 PREDICTED: similar to RIKEN cDNA 2900016D05 [Rattus norvegicus]    GB acc:   BF390736    UniGene:   Rn.14957        SOURCE        Gene Ontology:     BioCarta Pathways:     KEGG Pathways:  
    Probe set : 1387957_a_at    Name:   SH3-domain kinase binding protein 1    GB acc:   AF255888    UniGene:   Rn.107226    Gene Symbol:   Sh3kbp1    Locus Link:   84357    Chromosome:   X    Cytoband:   Chr:Xq21    SOURCE    GENECARD       Gene Ontology:   biological process:  regulation of apoptosis   cellular component:  nucleus   cellular component:  kinesin complex   cellular component:  membrane   molecular function:  protein binding   molecular function:  protein binding   molecular function:  protein binding      BioCarta Pathways:     KEGG Pathways:  
    Probe set : 1383886_at    Name:   Transcribed locus    GB acc:   BI275827    UniGene:   Rn.12229        SOURCE        Gene Ontology:     BioCarta Pathways:     KEGG Pathways:  
    Probe set : 1388160_a_at    Name:   isocitrate dehydrogenase 3 (NAD+) beta    GB acc:   AI171793    UniGene:   Rn.1093    Gene Symbol:   Idh3B    Locus Link:   94173    Chromosome:   3    Cytoband:   Chr:3q36    SOURCE    GENECARD       Gene Ontology:   biological process:  tricarboxylic acid cycle   biological process:  metabolism   cellular component:  mitochondrion   molecular function:  isocitrate dehydrogenase (NAD+) activity   molecular function:  oxidoreductase activity      BioCarta Pathways:     KEGG Pathways:  
    Probe set : 1386716_at    Name:   Similar to putative membrane steroid receptor (predicted)    GB acc:   BF562934    UniGene:   Rn.17067     Locus Link:   316275    Chromosome:   9    Cytoband:   Chr:9q13    SOURCE        Gene Ontology:   biological process:  G-protein coupled receptor protein signaling pathway   cellular component:  integral to membrane   molecular function:  receptor activity   molecular function:  G-protein coupled receptor activity   molecular function:  steroid binding      BioCarta Pathways:     KEGG Pathways:  
    Probe set : 1383291_at     GB acc:   BF282631              Gene Ontology:     BioCarta Pathways:     KEGG Pathways:  
    Probe set : 1367746_a_at    Name:   flotillin 2    GB acc:   NM_031830    UniGene:   Rn.2105    Gene Symbol:   Flot2    Locus Link:   83764    Chromosome:   10    Cytoband:   Chr:10q25    SOURCE    GENECARD       Gene Ontology:   biological process:  cell adhesion   biological process:  epidermis development   molecular function:  protein binding      BioCarta Pathways:     KEGG Pathways:  
    Probe set : 1390664_at     GB acc:   AI410837              Gene Ontology:     BioCarta Pathways:     KEGG Pathways:  
    Probe set : 1374194_at     GB acc:   AI170007              Gene Ontology:     BioCarta Pathways:     KEGG Pathways:  
    Probe set : 1372028_at    Name:   similar to Protein CGI-117 (Protein HSPC111) (predicted)    GB acc:   AA893215    UniGene:   Rn.6090    Gene Symbol:   RGD1305727_predicted    Locus Link:   306768    Chromosome:   17    Cytoband:   Chr:17p14    SOURCE    GENECARD       Gene Ontology:     BioCarta Pathways:     KEGG Pathways:  
    Probe set : 1399062_at    Name:   CDNA clone IMAGE:7317308    GB acc:   BI301008    UniGene:   Rn.21474        SOURCE        Gene Ontology:     BioCarta Pathways:     KEGG Pathways:  
    Probe set : 1373386_at    Name:   gap junction membrane channel protein beta 2    GB acc:   AI179953    UniGene:   Rn.9230    Gene Symbol:   Gjb2    Locus Link:   394266    Chromosome:   15    Cytoband:   Chr:15p12    SOURCE    GENECARD       Gene Ontology:   biological process:  cell-cell signaling   biological process:  male genitalia morphogenesis   biological process:  cell communication   molecular function:  connexon channel activity   molecular function:  connexon channel activity      BioCarta Pathways:     KEGG Pathways:  
    Probe set : 1368144_at    Name:   regulator of G-protein signaling 2    GB acc:   AF321837    UniGene:   Rn.1892    Gene Symbol:   Rgs2    Locus Link:   84583    Chromosome:   13    Cytoband:   Chr:13q21    SOURCE    GENECARD       Gene Ontology:   cellular component:  nucleus   molecular function:  signal transducer activity   molecular function:  GTPase activator activity   molecular function:  calmodulin binding      BioCarta Pathways:     KEGG Pathways:  
    Probe set : 1393165_at    Name:   similar to RIKEN cDNA 2310028N02    GB acc:   BG377684    UniGene:   Rn.18267    Gene Symbol:   RGD:1359339    Locus Link:   305070    Chromosome:   13    Cytoband:   Chr:13q27    SOURCE    GENECARD       Gene Ontology:     BioCarta Pathways:     KEGG Pathways:  
    Probe set : 1376317_at     GB acc:   BI282917              Gene Ontology:     BioCarta Pathways:     KEGG Pathways:  
    Probe set : 1398904_at    Name:   non-POU domain containing, octamer-binding /// similar to Nono protein    GB acc:   AI599066    UniGene:   Rn.8381    Gene Symbol:   Nono /// LOC501589    Locus Link:   317259 /// 501589    Chromosome:   X    Cytoband:   Chr:Xq31    SOURCE    GENECARD       Gene Ontology:   cellular component:  nucleus      BioCarta Pathways:     KEGG Pathways:  
    Probe set : 1377110_at    Name:   similar to ADP-ribosylation-like factor 12 protein    GB acc:   BF420720    UniGene:   Rn.23998    Gene Symbol:   LOC497990    Locus Link:   497990    Chromosome:   10    Cytoband:   Chr:10q31    SOURCE    GENECARD       Gene Ontology:     BioCarta Pathways:     KEGG Pathways:  
    Probe set : 1370112_at    Name:   phosphatase and tensin homolog    GB acc:   NM_031606    UniGene:   Rn.22158    Gene Symbol:   Pten    Locus Link:   50557      SOURCE    GENECARD       Gene Ontology:     BioCarta Pathways:     KEGG Pathways:  
    Probe set : 1367959_a_at    Name:   sodium channel, voltage-gated, type I, beta polypeptide    GB acc:   AF182949    UniGene:   Rn.4958    Gene Symbol:   Scn1b    Locus Link:   29686    Chromosome:   1    Cytoband:   Chr:1q21    SOURCE    GENECARD       Gene Ontology:   biological process:  ion transport   biological process:  transport   biological process:  sodium ion transport   cellular component:  extracellular space   cellular component:  integral to membrane   molecular function:  ion channel activity   molecular function:  voltage-gated ion channel activity   molecular function:  sodium ion binding      BioCarta Pathways:     KEGG Pathways:  
    Probe set : 1391014_at    Name:   zinc finger, MYND domain containing 19    GB acc:   AW530427    UniGene:   Rn.40409    Gene Symbol:   Zmynd19    Locus Link:   311791    Chromosome:   3    Cytoband:   Chr:3p13    SOURCE    GENECARD       Gene Ontology:   cellular component:  cytoplasm   cellular component:  internal side of plasma membrane   molecular function:  protein binding   molecular function:  zinc ion binding   molecular function:  metal ion binding      BioCarta Pathways:     KEGG Pathways:  
    Probe set : 1373491_at    Name:   Similar to Metaxin 1, isoform 2    GB acc:   BI276464    UniGene:   Rn.9148     Locus Link:   295241    Chromosome:   2    Cytoband:   Chr:2q34    SOURCE        Gene Ontology:     BioCarta Pathways:     KEGG Pathways:  
    Probe set : 1375220_at    Name:   RE70703p-like    GB acc:   AA818971    UniGene:   Rn.96380    Gene Symbol:   RGD:1303292    Locus Link:   301123    Chromosome:   9    Cytoband:   Chr:9q11    SOURCE    GENECARD       Gene Ontology:   biological process:  protein transport      BioCarta Pathways:     KEGG Pathways:  
    Probe set : 1390650_at    Name:   pericentrin 1 (predicted)    GB acc:   BI290593    UniGene:   Rn.67936    Gene Symbol:   Pcnt1_predicted    Locus Link:   287830    Chromosome:   10    Cytoband:   Chr:10q32.3    SOURCE    GENECARD       Gene Ontology:   cellular component:  integral to membrane   cellular component:  outer membrane   molecular function:  porin activity      BioCarta Pathways:     KEGG Pathways:  
    Probe set : 1389315_at    Name:   similar to Git2 protein    GB acc:   AI408598    UniGene:   Rn.77521    Gene Symbol:   RGD:1359708    Locus Link:   304546    Chromosome:   12    Cytoband:   Chr:12q16    SOURCE    GENECARD       Gene Ontology:   biological process:  regulation of GTPase activity      BioCarta Pathways:     KEGG Pathways:  
    Probe set : 1368055_a_at    Name:   lamin A    GB acc:   BI282866    UniGene:   Rn.44161    Gene Symbol:   Lmna    Locus Link:   60374    Chromosome:   2    Cytoband:   Chr:2q31-q34    SOURCE    GENECARD       Gene Ontology:   biological process:  spermatogenesis   biological process:  muscle development   molecular function:  structural molecule activity   molecular function:  protein binding      BioCarta Pathways:     KEGG Pathways:  
    Probe set : 1382070_at    Name:   activating transcription factor 7 interacting protein (predicted)    GB acc:   AA819758    UniGene:   Rn.98469    Gene Symbol:   Atf7ip_predicted    Locus Link:   312800    Chromosome:   4    Cytoband:   Chr:4q43    SOURCE    GENECARD       Gene Ontology:   cellular component:  nucleus   molecular function:  protein binding      BioCarta Pathways:     KEGG Pathways:  
    Probe set : 1371371_at    Name:   NADH dehydrogenase (ubiquinone) 1 beta subcomplex, 4, 15kDa (predicted)    GB acc:   AW434041    UniGene:   Rn.144724    Gene Symbol:   Ndufb4_predicted    Locus Link:   288088    Chromosome:   11    Cytoband:   Chr:11q21    SOURCE    GENECARD       Gene Ontology:   biological process:  electron transport   biological process:  mitochondrial electron transport, NADH to ubiquinone   cellular component:  membrane fraction   cellular component:  mitochondrion   molecular function:  NADH dehydrogenase activity   molecular function:  NADH dehydrogenase (ubiquinone) activity   molecular function:  oxidoreductase activity      BioCarta Pathways:     KEGG Pathways:  
    Probe set : 1388672_at    Name:   Transcribed locus    GB acc:   AI101373    UniGene:   Rn.9024        SOURCE        Gene Ontology:     BioCarta Pathways:     KEGG Pathways:  
    Probe set : 1383964_at    Name:   Transcribed locus    GB acc:   AI549551    UniGene:   Rn.7482        SOURCE        Gene Ontology:     BioCarta Pathways:     KEGG Pathways:  
    Probe set : 1380201_at    Name:   similar to hypothetical protein FLJ14075    GB acc:   BF412675    UniGene:   Rn.38298    Gene Symbol:   RGD:1359447    Locus Link:   313981    Chromosome:   6    Cytoband:   Chr:6q16    SOURCE    GENECARD       Gene Ontology:   cellular component:  nucleus      BioCarta Pathways:     KEGG Pathways:  
    Probe set : 1392119_at    Name:   Transcribed locus    GB acc:   BF390340    UniGene:   Rn.132297        SOURCE        Gene Ontology:     BioCarta Pathways:     KEGG Pathways:  
    Probe set : 1388339_at    Name:   phosphoprotein enriched in astrocytes 15 (predicted)    GB acc:   BE112895    UniGene:   Rn.7282    Gene Symbol:   Pea15_predicted    Locus Link:   364052    Chromosome:   13    Cytoband:   Chr:13q24    SOURCE    GENECARD       Gene Ontology:   cellular component:  membrane fraction   cellular component:  cytosol   cellular component:  microtubule associated complex   molecular function:  protein kinase C binding   molecular function:  sugar porter activity   molecular function:  protein binding      BioCarta Pathways:     KEGG Pathways:  
    Probe set : 1389389_at    Name:   DEAD (Asp-Glu-Ala-Asp) box polypeptide 56    GB acc:   BF420168    UniGene:   Rn.39458    Gene Symbol:   RGD:1303195    Locus Link:   289780    Chromosome:   14    Cytoband:   Chr:14q21    SOURCE    GENECARD       Gene Ontology:     BioCarta Pathways:     KEGG Pathways:  
    Probe set : 1388366_at    Name:   mitochondrial ribosomal protein L4 (predicted)    GB acc:   BI275903    UniGene:   Rn.13113    Gene Symbol:   Mrpl4_predicted    Locus Link:   363023    Chromosome:   8    Cytoband:   Chr:8q13    SOURCE    GENECARD       Gene Ontology:   biological process:  protein biosynthesis   cellular component:  intracellular   cellular component:  ribosome   cellular component:  ribonucleoprotein complex   molecular function:  structural constituent of ribosome      BioCarta Pathways:     KEGG Pathways:  
    Probe set : 1386917_at    Name:   Pyruvate carboxylase    GB acc:   NM_012744    UniGene:   Rn.11094    Gene Symbol:   Pc    Locus Link:   25104    Chromosome:   1    Cytoband:   Chr:1q42    SOURCE    GENECARD       Gene Ontology:   biological process:  gluconeogenesis   biological process:  metabolism   biological process:  lipid biosynthesis   cellular component:  cytoplasm   cellular component:  mitochondrion      BioCarta Pathways:     KEGG Pathways:  
    Probe set : 1392929_at    Name:   Solute carrier family 4, sodium bicarbonate transporter-like, member 11 (predicted)    GB acc:   BF416678    UniGene:   Rn.6943     Locus Link:   311423    Chromosome:   3    Cytoband:   Chr:3q36    SOURCE        Gene Ontology:   biological process:  anion transport   cellular component:  integral to membrane   molecular function:  inorganic anion exchanger activity      BioCarta Pathways:     KEGG Pathways:  
    Probe set : 1397233_at    Name:   similar to hypothetical protein C730036H08 (predicted)    GB acc:   AW529479    UniGene:   Rn.93158    Gene Symbol:   RGD1309232_predicted    Locus Link:   316023    Chromosome:   8    Cytoband:   Chr:8q32    SOURCE    GENECARD       Gene Ontology:     BioCarta Pathways:     KEGG Pathways:  
    Probe set : 1373934_at    Name:   LOC500840    GB acc:   AI178214    UniGene:   Rn.3702    Gene Symbol:   LOC500840    Locus Link:   500840    Chromosome:   7    Cytoband:   Chr:7q22    SOURCE    GENECARD       Gene Ontology:     BioCarta Pathways:     KEGG Pathways:  
    Probe set : 1373011_at    Name:   hypothetical protein LOC619558    GB acc:   BE109520     Gene Symbol:   LOC619558    Locus Link:   619558    Chromosome:   2    Cytoband:   Chr:2q22     GENECARD       Gene Ontology:     BioCarta Pathways:     KEGG Pathways:  
    Probe set : 1385005_at     GB acc:   AW530264              Gene Ontology:     BioCarta Pathways:     KEGG Pathways:  
    Probe set : 1378080_at    Name:   similar to SCO cytochrome oxidase deficient homolog 1 (yeast)    GB acc:   BI274498    UniGene:   Rn.13276    Gene Symbol:   LOC497930    Locus Link:   497930    Chromosome:   10    Cytoband:   Chr:10q24    SOURCE    GENECARD       Gene Ontology:     BioCarta Pathways:     KEGG Pathways:  
    Probe set : 1388871_at    Name:   ash2 (absent, small, or homeotic)-like (Drosophila) (predicted)    GB acc:   BG378089    UniGene:   Rn.18018    Gene Symbol:   Ash2l_predicted    Locus Link:   290829    Chromosome:   16    Cytoband:   Chr:16q12.4    SOURCE    GENECARD       Gene Ontology:   biological process:  regulation of transcription, DNA-dependent   biological process:  hemopoiesis   cellular component:  nucleus   molecular function:  DNA binding   molecular function:  transcription regulator activity      BioCarta Pathways:     KEGG Pathways:  
    Probe set : 1386898_at    Name:   heat shock 10 kDa protein 1    GB acc:   NM_012966    UniGene:   Rn.106093    Gene Symbol:   Hspe1    Locus Link:   25462    Chromosome:   9    Cytoband:   Chr:9q31    SOURCE    GENECARD       Gene Ontology:   biological process:  protein folding   biological process:  response to unfolded protein   cellular component:  mitochondrion   molecular function:  ATP binding   molecular function:  unfolded protein binding      BioCarta Pathways:     KEGG Pathways:  
    Probe set : 1398852_at    Name:   ribosomal protein S21    GB acc:   BF410811    UniGene:   Rn.4153    Gene Symbol:   Rps21    Locus Link:   81775    Chromosome:   3    Cytoband:   Chr:3q43    SOURCE    GENECARD       Gene Ontology:   biological process:  protein biosynthesis   cellular component:  intracellular   cellular component:  ribosome   cellular component:  ribonucleoprotein complex   molecular function:  structural constituent of ribosome      BioCarta Pathways:     KEGG Pathways:  
    Probe set : 1367895_at    Name:   heterogeneous nuclear ribonucleoprotein K    GB acc:   AI577321    UniGene:   Rn.11854    Gene Symbol:   Hnrpk    Locus Link:   117282    Chromosome:   17    Cytoband:   Chr:17p14    SOURCE    GENECARD       Gene Ontology:   biological process:  RNA processing      BioCarta Pathways:     KEGG Pathways:  
    Probe set : 1371344_at    Name:   ribosomal protein L27a (predicted)    GB acc:   AI177054    UniGene:   Rn.144797    Gene Symbol:   Rpl27a_predicted    Locus Link:   293418    Chromosome:   1    Cytoband:   Chr:1q33    SOURCE    GENECARD       Gene Ontology:   biological process:  protein biosynthesis   cellular component:  intracellular   cellular component:  ribosome   cellular component:  cytosolic large ribosomal subunit (sensu Eukaryota)   molecular function:  RNA binding   molecular function:  structural constituent of ribosome      BioCarta Pathways:     KEGG Pathways:  
    Probe set : 1385440_at    Name:   hematopoietic cell signal transducer    GB acc:   BF419129    UniGene:   Rn.145403    Gene Symbol:   RGD:1359238    Locus Link:   474146    Chromosome:   1    Cytoband:   Chr:1q21    SOURCE    GENECARD       Gene Ontology:     BioCarta Pathways:     KEGG Pathways:  
    Probe set : 1382475_at    Name:   Similar to pellino 3 alpha (predicted)    GB acc:   AI060133    UniGene:   Rn.79888     Locus Link:   309157    Chromosome:   1    Cytoband:   Chr:1q43    SOURCE        Gene Ontology:     BioCarta Pathways:     KEGG Pathways:  
    Probe set : 1384925_at    Name:   Similar to IL-17D    GB acc:   AI045021    UniGene:   Rn.17127     Locus Link:   502011      SOURCE        Gene Ontology:     BioCarta Pathways:     KEGG Pathways:  
    Probe set : 1384202_at    Name:   similar to Tescalcin    GB acc:   BI287326    UniGene:   Rn.103895    Gene Symbol:   LOC288689    Locus Link:   288689    Chromosome:   12    Cytoband:   Chr:12q16    SOURCE    GENECARD       Gene Ontology:     BioCarta Pathways:     KEGG Pathways:  
    Probe set : 1392411_at    Name:   tubulointerstitial nephritis antigen    GB acc:   AI411848    UniGene:   Rn.43851    Gene Symbol:   RGD:1359482    Locus Link:   300846    Chromosome:   8    Cytoband:   Chr:8q24    SOURCE    GENECARD       Gene Ontology:   biological process:  proteolysis   molecular function:  cysteine-type endopeptidase activity   molecular function:  cysteine-type peptidase activity      BioCarta Pathways:     KEGG Pathways:  
    Probe set : 1387883_a_at    Name:   thymosin, beta 4    GB acc:   BG668902    UniGene:   Rn.129715    Gene Symbol:   Tmsb4x    Locus Link:   81814    Chromosome:   X    Cytoband:   Chr:Xq31    SOURCE    GENECARD       Gene Ontology:   cellular component:  cytoskeleton   cellular component:  cytoplasm   molecular function:  actin binding      BioCarta Pathways:     KEGG Pathways:  
    Probe set : 1398337_at    Name:   Similar to RIKEN cDNA 9430077D24 gene    GB acc:   AI169116    UniGene:   Rn.3259     Locus Link:   290341    Chromosome:   15    Cytoband:   Chr:15p11    SOURCE        Gene Ontology:     BioCarta Pathways:     KEGG Pathways:  
    Probe set : 1384112_at    Name:   5 nucleotidase    GB acc:   BI289470    UniGene:   Rn.40132    Gene Symbol:   Nt5    Locus Link:   58813    Chromosome:   8    Cytoband:   Chr:8q31    SOURCE    GENECARD       Gene Ontology:   biological process:  DNA metabolism   biological process:  nucleotide catabolism      BioCarta Pathways:     KEGG Pathways:  
    Probe set : 1389194_at    Name:   Similar to hypothetical protein    GB acc:   AI406491    UniGene:   Rn.3346     Locus Link:   311323    Chromosome:   3    Cytoband:   Chr:3q35    SOURCE        Gene Ontology:     BioCarta Pathways:     KEGG Pathways:  
    Probe set : 1393335_at    Name:   epidermal growth factor-like protein 6    GB acc:   BF418373    UniGene:   Rn.21946    Gene Symbol:   Egfl6    Locus Link:   317470    Chromosome:   X    Cytoband:   Chr:Xq21    SOURCE    GENECARD       Gene Ontology:     BioCarta Pathways:     KEGG Pathways:  
    Probe set : 1384841_at    Name:   Cathepsin C    GB acc:   AA858815    UniGene:   Rn.122504    Gene Symbol:   Ctsc    Locus Link:   25423    Chromosome:   1    Cytoband:   Chr:1q32    SOURCE    GENECARD       Gene Ontology:   biological process:  proteolysis   biological process:  proteolysis   biological process:  proteolysis   biological process:  immune response   cellular component:  extracellular space   cellular component:  lysosome   cellular component:  lysosome      BioCarta Pathways:     KEGG Pathways:  
    Probe set : 1367630_at    Name:   ribosomal protein S11    GB acc:   NM_031110    UniGene:   Rn.2177    Gene Symbol:   Rps11    Locus Link:   81774    Chromosome:   1    Cytoband:   Chr:1q22    SOURCE    GENECARD       Gene Ontology:   biological process:  protein biosynthesis   cellular component:  intracellular   cellular component:  ribosome   cellular component:  ribonucleoprotein complex      BioCarta Pathways:     KEGG Pathways:  
    Probe set : 1382935_at    Name:   similar to Hypothetical protein KIAA0141    GB acc:   BE113823    UniGene:   Rn.12899    Gene Symbol:   RGD:735029    Locus Link:   307480    Chromosome:   18    Cytoband:   Chr:18p11    SOURCE    GENECARD       Gene Ontology:   molecular function:  binding      BioCarta Pathways:     KEGG Pathways:  
    Probe set : 1377968_at    Name:   Transcribed locus    GB acc:   AI069912    UniGene:   Rn.13003        SOURCE        Gene Ontology:     BioCarta Pathways:     KEGG Pathways:  
    Probe set : 1398342_at    Name:   similar to Fus1 protein    GB acc:   BF549775    UniGene:   Rn.120731    Gene Symbol:   LOC501052    Locus Link:   501052    Chromosome:   8    Cytoband:   Chr:8q32    SOURCE    GENECARD       Gene Ontology:     BioCarta Pathways:     KEGG Pathways:  
    Probe set : 1387372_at    Name:   solute carrier family 6 (neurotransmitter transporter, GABA), member 13    GB acc:   NM_133623    UniGene:   Rn.10527    Gene Symbol:   Slc6a13    Locus Link:   171163    Chromosome:   4    Cytoband:   Chr:4q42    SOURCE    GENECARD       Gene Ontology:     BioCarta Pathways:     KEGG Pathways:  
    Probe set : 1370967_at    Name:   Ig active lambda2-like chain mRNA, 3' end    GB acc:   BG374683    UniGene:   Rn.129        SOURCE        Gene Ontology:     BioCarta Pathways:     KEGG Pathways:  
    Probe set : 1370083_at    Name:   macrophage inflammatory protein-1 alpha receptor gene    GB acc:   NM_020542    UniGene:   Rn.34673    Gene Symbol:   Ccr1    Locus Link:   57301    Chromosome:   8    Cytoband:   Chr:8q32    SOURCE    GENECARD       Gene Ontology:   cellular component:  integral to membrane      BioCarta Pathways:     KEGG Pathways:  
    Probe set : 1383064_at    Name:   CDNA clone IMAGE:7379585    GB acc:   AI137878    UniGene:   Rn.6374        SOURCE        Gene Ontology:     BioCarta Pathways:     KEGG Pathways:  
    Probe set : 1398783_at    Name:   G protein pathway suppressor 1    GB acc:   NM_053969    UniGene:   Rn.16873    Gene Symbol:   Gps1    Locus Link:   117039      SOURCE    GENECARD       Gene Ontology:   cellular component:  nucleus   cellular component:  signalosome complex   molecular function:  GTPase inhibitor activity   molecular function:  protein binding      BioCarta Pathways:     KEGG Pathways:  
    Probe set : 1368930_at    Name:   potassium intermediate/small conductance calcium-activated channel, subfamily N, member 4    GB acc:   NM_023021    UniGene:   Rn.44212    Gene Symbol:   Kcnn4    Locus Link:   65206    Chromosome:   1    Cytoband:   Chr:1q21    SOURCE    GENECARD       Gene Ontology:     BioCarta Pathways:     KEGG Pathways:  
    Probe set : 1395694_at    Name:   f-box only protein 9 (predicted)    GB acc:   BG669135    UniGene:   Rn.144900    Gene Symbol:   Fbxo9_predicted    Locus Link:   300849    Chromosome:   8    Cytoband:   Chr:8q31    SOURCE    GENECARD       Gene Ontology:   biological process:  protein modification   biological process:  ubiquitin cycle   biological process:  protein ubiquitination   cellular component:  ubiquitin ligase complex   molecular function:  ubiquitin-protein ligase activity   molecular function:  binding      BioCarta Pathways:     KEGG Pathways:  
    Probe set : 1384658_at    Name:   Transcribed locus    GB acc:   AW530445    UniGene:   Rn.146788        SOURCE        Gene Ontology:     BioCarta Pathways:     KEGG Pathways:  
    Probe set : 1387907_at    Name:   inositol 1,4,5-triphosphate receptor 1    GB acc:   J05510    UniGene:   Rn.2135    Gene Symbol:   Itpr1    Locus Link:   25262    Chromosome:   4    Cytoband:   Chr:4q41    SOURCE    GENECARD       Gene Ontology:     BioCarta Pathways:     KEGG Pathways:  
    Probe set : 1388636_at    Name:   similar to RIKEN cDNA 5730408C10 (predicted)    GB acc:   BI280272    UniGene:   Rn.26488    Gene Symbol:   RGD1305972_predicted    Locus Link:   360554    Chromosome:   10    Cytoband:   Chr:10q24    SOURCE    GENECARD       Gene Ontology:   biological process:  proteolysis   biological process:  protein ubiquitination   cellular component:  ubiquitin ligase complex   molecular function:  ubiquitin-protein ligase activity   molecular function:  peptidase activity   molecular function:  zinc ion binding      BioCarta Pathways:     KEGG Pathways:  
    Probe set : 1376868_at     GB acc:   BM389293              Gene Ontology:     BioCarta Pathways:     KEGG Pathways:  
    Probe set : 1378755_at    Name:   Transcribed locus    GB acc:   AW523689    UniGene:   Rn.88324        SOURCE        Gene Ontology:     BioCarta Pathways:     KEGG Pathways:  
    Probe set : 1372422_at    Name:   Sulfatase modifying factor 1 (predicted)    GB acc:   AI175506    UniGene:   Rn.17376     Locus Link:   362409    Chromosome:   4    Cytoband:   Chr:4q41    SOURCE        Gene Ontology:   biological process:  glycosaminoglycan metabolism   cellular component:  endoplasmic reticulum      BioCarta Pathways:     KEGG Pathways:  
    Probe set : 1380699_at    Name:   Zinc finger homeobox 1b (predicted)    GB acc:   BE110761    UniGene:   Rn.59710     Locus Link:   311071    Chromosome:   3    Cytoband:   Chr:3q12    SOURCE        Gene Ontology:   cellular component:  nucleus      BioCarta Pathways:     KEGG Pathways:  
    Probe set : 1375701_at    Name:   similar to RIKEN cDNA 2810013M15    GB acc:   BF400575    UniGene:   Rn.63360    Gene Symbol:   LOC360568    Locus Link:   360568    Chromosome:   10    Cytoband:   Chr:10q24    SOURCE    GENECARD       Gene Ontology:     BioCarta Pathways:     KEGG Pathways:  
    Probe set : 1367660_at    Name:   fatty acid binding protein 3    GB acc:   NM_024162    UniGene:   Rn.32566    Gene Symbol:   Fabp3    Locus Link:   79131      SOURCE    GENECARD       Gene Ontology:   cellular component:  soluble fraction   cellular component:  cytoplasm      BioCarta Pathways:     KEGG Pathways:  
    Probe set : 1383280_at    Name:   Transcribed locus    GB acc:   BG669130    UniGene:   Rn.107945        SOURCE        Gene Ontology:     BioCarta Pathways:     KEGG Pathways:  
    Probe set : 1377350_at    Name:   Transcribed locus    GB acc:   AA859898    UniGene:   Rn.809        SOURCE        Gene Ontology:     BioCarta Pathways:     KEGG Pathways:  
    Probe set : 1379277_at    Name:   Utrophin    GB acc:   AI575341    UniGene:   Rn.9901    Gene Symbol:   Utrn    Locus Link:   25600    Chromosome:   1    Cytoband:   Chr:1p13    SOURCE    GENECARD       Gene Ontology:     BioCarta Pathways:     KEGG Pathways:  
    Probe set : 1367747_at    Name:   ADP-ribosylation factor-like 3    GB acc:   NM_022700    UniGene:   Rn.9538    Gene Symbol:   Arl3    Locus Link:   64664    Chromosome:   1    Cytoband:   Chr:1q54    SOURCE    GENECARD       Gene Ontology:   biological process:  G-protein coupled receptor protein signaling pathway   biological process:  small GTPase mediated signal transduction      BioCarta Pathways:     KEGG Pathways:  
    Probe set : 1374376_at    Name:   Rho-guanine nucleotide exchange factor (predicted)    GB acc:   BM386483    UniGene:   Rn.33912    Gene Symbol:   Rgnef_predicted    Locus Link:   361882    Chromosome:   2    Cytoband:   Chr:2q12    SOURCE    GENECARD       Gene Ontology:   biological process:  intracellular signaling cascade   biological process:  visual perception   molecular function:  guanyl-nucleotide exchange factor activity      BioCarta Pathways:     KEGG Pathways:  
    Probe set : 1390478_at    Name:   origin recognition complex, subunit 4    GB acc:   BE103926    UniGene:   Rn.98343    Gene Symbol:   RGD:619987    Locus Link:   252936      SOURCE    GENECARD       Gene Ontology:   cellular component:  nuclear origin of replication recognition complex      BioCarta Pathways:     KEGG Pathways:  
    Probe set : 1385126_at    Name:   Transcribed locus    GB acc:   AI060037    UniGene:   Rn.147472        SOURCE        Gene Ontology:     BioCarta Pathways:     KEGG Pathways:  
    Probe set : 1373468_at    Name:   Transcribed locus    GB acc:   BI295896    UniGene:   Rn.153646        SOURCE        Gene Ontology:     BioCarta Pathways:     KEGG Pathways:  
    Probe set : 1389809_at    Name:   Transcribed locus    GB acc:   AI410144    UniGene:   Rn.28828        SOURCE        Gene Ontology:     BioCarta Pathways:     KEGG Pathways:  
    Probe set : 1374306_at    Name:   Transcribed locus    GB acc:   BF394933    UniGene:   Rn.25422        SOURCE        Gene Ontology:     BioCarta Pathways:     KEGG Pathways:  
    Probe set : 1367783_at    Name:   GABA(A) receptor-associated protein like 2    GB acc:   NM_022706    UniGene:   Rn.64537    Gene Symbol:   Gabarapl2    Locus Link:   64670    Chromosome:   19    Cytoband:   Chr:19q12    SOURCE    GENECARD       Gene Ontology:   cellular component:  Golgi membrane   cellular component:  Golgi apparatus   cellular component:  cytoskeleton   cellular component:  Golgi stack      BioCarta Pathways:     KEGG Pathways:  
    Probe set : 1379909_at    Name:   G kinase anchoring protein 1 (predicted)    GB acc:   AA963315    UniGene:   Rn.2003    Gene Symbol:   Gkap1_predicted    Locus Link:   361202    Chromosome:   17    Cytoband:   Chr:17p14    SOURCE    GENECARD       Gene Ontology:   biological process:  signal transduction   biological process:  G-protein signaling, coupled to cGMP nucleotide second messenger   cellular component:  extracellular space   cellular component:  Golgi apparatus   molecular function:  protein binding   molecular function:  kinase activity      BioCarta Pathways:     KEGG Pathways:  
    Probe set : 1380256_at    Name:   polymerase (RNA) III (DNA directed) polypeptide C (predicted)    GB acc:   BE103946    UniGene:   Rn.35251    Gene Symbol:   Polr3c_predicted    Locus Link:   310685    Chromosome:   2    Cytoband:   Chr:2q34    SOURCE    GENECARD       Gene Ontology:   biological process:  transcription   cellular component:  nucleus   molecular function:  DNA binding   molecular function:  DNA-directed RNA polymerase activity   molecular function:  transferase activity      BioCarta Pathways:     KEGG Pathways:  
    Probe set : 1370159_at    Name:   SWI/SNF related, matrix associated, actin dependent regulator of chromatin, subfamily d, member 2    GB acc:   AB003505    UniGene:   Rn.3053    Gene Symbol:   Smarcd2    Locus Link:   83833    Chromosome:   10    Cytoband:   Chr:10q32.1    SOURCE    GENECARD       Gene Ontology:   cellular component:  nucleus   cellular component:  nucleoplasm   cellular component:  SWI/SNF complex   molecular function:  transcription coactivator activity      BioCarta Pathways:     KEGG Pathways:  
    Probe set : 1388723_at    Name:   brain and reproductive organ-expressed protein    GB acc:   AI171289    UniGene:   Rn.1432    Gene Symbol:   Bre    Locus Link:   362704    Chromosome:   6    Cytoband:   Chr:6q13    SOURCE    GENECARD       Gene Ontology:     BioCarta Pathways:     KEGG Pathways:  
    Probe set : 1373633_at    Name:   Transcribed locus    GB acc:   BF399791    UniGene:   Rn.27327        SOURCE        Gene Ontology:     BioCarta Pathways:     KEGG Pathways:  
    Probe set : 1367550_a_at    Name:   beta-amyloid binding protein precursor (predicted)    GB acc:   BI287742    UniGene:   Rn.22973    Gene Symbol:   Bbp_predicted    Locus Link:   362545    Chromosome:   5    Cytoband:   Chr:5q33    SOURCE    GENECARD       Gene Ontology:   biological process:  induction of apoptosis by extracellular signals   cellular component:  integral to plasma membrane   molecular function:  beta-amyloid binding   molecular function:  G-protein coupled receptor activity      BioCarta Pathways:     KEGG Pathways:  
    Probe set : 1383184_at    Name:   zinc and ring finger 1 (predicted)    GB acc:   AW916773    UniGene:   Rn.32245    Gene Symbol:   Znrf1_predicted    Locus Link:   362367    Chromosome:   4    Cytoband:   Chr:4q24    SOURCE    GENECARD       Gene Ontology:     BioCarta Pathways:     KEGG Pathways:  
    Probe set : 1374705_at    Name:   similar to type IV collagen alpha 5 chain    GB acc:   AA891834    UniGene:   Rn.17094    Gene Symbol:   LOC363457    Locus Link:   363457    Chromosome:   X    Cytoband:   Chr:Xq14    SOURCE    GENECARD       Gene Ontology:     BioCarta Pathways:     KEGG Pathways:  
    Probe set : 1392938_s_at    Name:   similar to C11orf17 protein (predicted)    GB acc:   BF561368    UniGene:   Rn.106344    Gene Symbol:   RGD1306959_predicted    Locus Link:   361624    Chromosome:   1    Cytoband:   Chr:1q33    SOURCE    GENECARD       Gene Ontology:     BioCarta Pathways:     KEGG Pathways:  
    Probe set : 1369531_at    Name:   sulfotransferase family, cytosolic, 1C, member 2    GB acc:   NM_133547    UniGene:   Rn.22471    Gene Symbol:   Sult1c2    Locus Link:   171072    Chromosome:   9    Cytoband:   Chr:9q11    SOURCE    GENECARD       Gene Ontology:   cellular component:  lysosome   molecular function:  sulfotransferase activity   molecular function:  transferase activity      BioCarta Pathways:     KEGG Pathways:  
    Probe set : 1374910_at     GB acc:   BE103601              Gene Ontology:     BioCarta Pathways:     KEGG Pathways:  
    Probe set : 1367834_at    Name:   spermidine synthase    GB acc:   NM_053464    UniGene:   Rn.144616    Gene Symbol:   Srm    Locus Link:   84596    Chromosome:   5    Cytoband:   Chr:5q36    SOURCE    GENECARD       Gene Ontology:   biological process:  spermidine biosynthesis      BioCarta Pathways:     KEGG Pathways:  
    Probe set : 1377424_at    Name:   Transcribed locus    GB acc:   AI712672    UniGene:   Rn.146040        SOURCE        Gene Ontology:     BioCarta Pathways:     KEGG Pathways:  
    Probe set : 1387111_at    Name:   dimethylarginine dimethylaminohydrolase 1    GB acc:   NM_022297    UniGene:   Rn.7398    Gene Symbol:   Ddah1    Locus Link:   64157    Chromosome:   2    Cytoband:   Chr:2q44    SOURCE    GENECARD       Gene Ontology:     BioCarta Pathways:     KEGG Pathways:  
    Probe set : 1372062_at    Name:   similar to cyclin-dependent kinase 2-interacting protein    GB acc:   BF394161    UniGene:   Rn.1799    Gene Symbol:   LOC299334    Locus Link:   299334    Chromosome:   6    Cytoband:   Chr:6q32    SOURCE    GENECARD       Gene Ontology:     BioCarta Pathways:     KEGG Pathways:  
    Probe set : 1380346_at    Name:   serine (or cysteine) proteinase inhibitor, clade B, member 1a (predicted)    GB acc:   BE100150    UniGene:   Rn.103402    Gene Symbol:   Serpinb1a_predicted    Locus Link:   291091    Chromosome:   17    Cytoband:   Chr:17p12    SOURCE    GENECARD       Gene Ontology:   biological process:  regulation of protein catabolism   cellular component:  cytoplasm   molecular function:  endopeptidase inhibitor activity   molecular function:  serine-type endopeptidase inhibitor activity   molecular function:  peptidase activity      BioCarta Pathways:     KEGG Pathways:  
    Probe set : 1387888_at    Name:   ribosomal protein S9    GB acc:   AA799768    UniGene:   Rn.109735    Gene Symbol:   Rps9    Locus Link:   81772    Chromosome:   1    Cytoband:   Chr:1q12    SOURCE    GENECARD       Gene Ontology:   biological process:  protein biosynthesis   molecular function:  RNA binding   molecular function:  structural constituent of ribosome      BioCarta Pathways:     KEGG Pathways:  
    Probe set : 1392128_at    Name:   Transcribed locus    GB acc:   BE104541    UniGene:   Rn.50647        SOURCE        Gene Ontology:     BioCarta Pathways:     KEGG Pathways:  
    Probe set : 1367865_at    Name:   limkain b1    GB acc:   NM_133421    UniGene:   Rn.27366    Gene Symbol:   Lkap    Locus Link:   170946    Chromosome:   10    Cytoband:   Chr:10q11    SOURCE    GENECARD       Gene Ontology:   molecular function:  nucleotide binding   molecular function:  nucleic acid binding   molecular function:  RNA binding      BioCarta Pathways:     KEGG Pathways:  
    Probe set : 1368309_at    Name:   thioredoxin reductase 2    GB acc:   NM_022584    UniGene:   Rn.6300    Gene Symbol:   Txnrd2    Locus Link:   50551      SOURCE    GENECARD       Gene Ontology:   cellular component:  cytoplasm   cellular component:  mitochondrion   cellular component:  mitochondrion   cellular component:  mitochondrion   molecular function:  thioredoxin-disulfide reductase activity   molecular function:  thioredoxin-disulfide reductase activity   molecular function:  ATP binding   molecular function:  electron carrier activity   molecular function:  disulfide oxidoreductase activity   molecular function:  oxidoreductase activity   molecular function:  oxidoreductase activity, acting on NADH or NADPH, disulfide as acceptor   molecular function:  metal ion binding   molecular function:  protein dimerization activity   molecular function:  protein dimerization activity   molecular function:  FAD binding   molecular function:  thioredoxin-disulfide reductase activity   molecular function:  selenium binding      BioCarta Pathways:     KEGG Pathways:  
    Probe set : 1371473_at    Name:   cleft lip and palate associated transmembrane protein 1 (predicted)    GB acc:   BI284472    UniGene:   Rn.8842    Gene Symbol:   Clptm1_predicted    Locus Link:   292696    Chromosome:   1    Cytoband:   Chr:1q21    SOURCE    GENECARD       Gene Ontology:   biological process:  development   cellular component:  integral to plasma membrane   cellular component:  integral to membrane      BioCarta Pathways:     KEGG Pathways:  
    Probe set : 1374241_at    Name:   Transcribed locus    GB acc:   AI406271    UniGene:   Rn.147629        SOURCE        Gene Ontology:     BioCarta Pathways:     KEGG Pathways:  
    Probe set : 1388615_at    Name:   Ras-related protein RAP-1A    GB acc:   AI410112    UniGene:   Rn.106321    Gene Symbol:   RGD:1359694    Locus Link:   295347    Chromosome:   2    Cytoband:   Chr:2q34    SOURCE    GENECARD       Gene Ontology:   cellular component:  membrane   molecular function:  nucleotide binding   molecular function:  GTP binding      BioCarta Pathways:     KEGG Pathways:  
    Probe set : 1395065_at    Name:   WD repeat domain 41 (predicted)    GB acc:   AW521367    UniGene:   Rn.139524    Gene Symbol:   Wdr41_predicted    Locus Link:   361879    Chromosome:   2    Cytoband:   Chr:2q12    SOURCE    GENECARD       Gene Ontology:     BioCarta Pathways:     KEGG Pathways:  
    Probe set : 1385132_at    Name:   myeloblastosis oncogene-like 1 (predicted)    GB acc:   AI044253    UniGene:   Rn.15847    Gene Symbol:   Mybl1_predicted    Locus Link:   297783    Chromosome:   5    Cytoband:   Chr:5q11    SOURCE    GENECARD       Gene Ontology:   cellular component:  intracellular   cellular component:  nucleus   molecular function:  DNA binding   molecular function:  transcriptional activator activity      BioCarta Pathways:     KEGG Pathways:  
    Probe set : 1383453_at    Name:   similar to olfactomedin-like 1 /// olfactomedin-like 1 (predicted)    GB acc:   AA946156    UniGene:   Rn.22729    Gene Symbol:   LOC308917 /// Olfml1_predicted    Locus Link:   308917 /// 361621    Chromosome:   1    Cytoband:   Chr:1q33    SOURCE    GENECARD       Gene Ontology:   cellular component:  membrane   molecular function:  latrotoxin receptor activity      BioCarta Pathways:     KEGG Pathways:  
    Probe set : 1373676_at    Name:   Transcribed locus    GB acc:   AI712626    UniGene:   Rn.20033        SOURCE        Gene Ontology:     BioCarta Pathways:     KEGG Pathways:  
    Probe set : 1373118_at     GB acc:   BM387460              Gene Ontology:     BioCarta Pathways:     KEGG Pathways:  
    Probe set : 1383179_at    Name:   Similar to hypothetical protein HSPC129 (predicted)    GB acc:   BI291413    UniGene:   Rn.15161     Locus Link:   311368    Chromosome:   3    Cytoband:   Chr:3q35    SOURCE        Gene Ontology:   molecular function:  phosphoric monoester hydrolase activity      BioCarta Pathways:     KEGG Pathways:  
    Probe set : 1375640_at    Name:   FK506 binding protein 9    GB acc:   AI236786    UniGene:   Rn.105976    Gene Symbol:   Fkbp9    Locus Link:   297123    Chromosome:   4    Cytoband:   Chr:4q24    SOURCE    GENECARD       Gene Ontology:   biological process:  protein folding   molecular function:  calcium ion binding      BioCarta Pathways:     KEGG Pathways:  
    Probe set : 1367957_at    Name:   regulator of G-protein signalling 3    GB acc:   NM_019340    UniGene:   Rn.53900    Gene Symbol:   Rgs3    Locus Link:   54293    Chromosome:   5    Cytoband:   Chr:5q24    SOURCE    GENECARD       Gene Ontology:   molecular function:  signal transducer activity   molecular function:  GTPase activator activity   molecular function:  protein binding      BioCarta Pathways:     KEGG Pathways:  
    Probe set : 1396112_at    Name:   similar to BB128963 protein (predicted)    GB acc:   BF408519    UniGene:   Rn.3510    Gene Symbol:   RGD1305958_predicted    Locus Link:   309255    Chromosome:   1    Cytoband:   Chr:1q22    SOURCE    GENECARD       Gene Ontology:     BioCarta Pathways:     KEGG Pathways:  
    Probe set : 1374118_at    Name:   leucyl-tRNA synthetase (predicted)    GB acc:   AA944176    UniGene:   Rn.7687    Gene Symbol:   Lars_predicted    Locus Link:   291624    Chromosome:   18    Cytoband:   Chr:18p11    SOURCE    GENECARD       Gene Ontology:   biological process:  protein biosynthesis   biological process:  tRNA aminoacylation for protein translation   biological process:  cysteinyl-tRNA aminoacylation   cellular component:  cytoplasm      BioCarta Pathways:     KEGG Pathways:  
    Probe set : 1393584_at     GB acc:   AI045201              Gene Ontology:     BioCarta Pathways:     KEGG Pathways:  
    Probe set : 1386745_at    Name:   Transcribed locus    GB acc:   BF523839    UniGene:   Rn.124150        SOURCE        Gene Ontology:     BioCarta Pathways:     KEGG Pathways:  
    Probe set : 1382993_at    Name:   Bcl-2 binding component 3    GB acc:   AI236152    UniGene:   Rn.25176    Gene Symbol:   Bbc3    Locus Link:   317673      SOURCE    GENECARD       Gene Ontology:   biological process:  apoptosis   biological process:  induction of apoptosis   cellular component:  mitochondrion      BioCarta Pathways:     KEGG Pathways:  
    Probe set : 1375027_at    Name:   Cell cycle progression 1 (predicted)    GB acc:   AI407365    UniGene:   Rn.43385     Locus Link:   363098    Chromosome:   8    Cytoband:   Chr:8q24    SOURCE        Gene Ontology:     BioCarta Pathways:     KEGG Pathways:  
    Probe set : 1367759_at    Name:   H1 histone family, member 0    GB acc:   NM_012578    UniGene:   Rn.3129    Gene Symbol:   H1f0    Locus Link:   24437    Chromosome:   7    Cytoband:   Chr:7q34    SOURCE    GENECARD       Gene Ontology:   biological process:  nucleosome assembly   biological process:  chromosome organization and biogenesis (sensu Eukaryota)   cellular component:  nucleosome   cellular component:  nucleus   cellular component:  chromosome   molecular function:  DNA binding      BioCarta Pathways:     KEGG Pathways:  
    Probe set : 1374831_at    Name:   Similar to cDNA sequence BC023151 (predicted)    GB acc:   AI172579    UniGene:   Rn.23728     Locus Link:   309103    Chromosome:   1    Cytoband:   Chr:1q41    SOURCE        Gene Ontology:     BioCarta Pathways:     KEGG Pathways:  
    Probe set : 1369971_a_at    Name:   heterogeneous nuclear ribonucleoprotein D    GB acc:   AB046616    UniGene:   Rn.94022    Gene Symbol:   Hnrpd    Locus Link:   79256    Chromosome:   14    Cytoband:   Chr:14p22    SOURCE    GENECARD       Gene Ontology:     BioCarta Pathways:     KEGG Pathways:  
    Probe set : AFFX_Rat_beta-actin_5_at    Name:   actin, beta     UniGene:   Rn.94978    Gene Symbol:   Actb    Locus Link:   81822    Chromosome:   12    Cytoband:   Chr:12p11    SOURCE    GENECARD       Gene Ontology:   biological process:  cell motility   biological process:  cytoskeleton organization and biogenesis   biological process:  axonogenesis      BioCarta Pathways:     KEGG Pathways:  
    Probe set : 1376793_at    Name:   similar to RIKEN cDNA 5730414C17 (predicted)    GB acc:   BF397794    UniGene:   Rn.28902    Gene Symbol:   RGD1308377_predicted    Locus Link:   306687    Chromosome:   17    Cytoband:   Chr:17p14    SOURCE    GENECARD       Gene Ontology:     BioCarta Pathways:     KEGG Pathways:  
    Probe set : 1374265_at    Name:   Similar to arylacetamide deacetylase (esterase) (predicted)    GB acc:   BF395101    UniGene:   Rn.20585     Locus Link:   294930    Chromosome:   2    Cytoband:   Chr:2q24    SOURCE        Gene Ontology:     BioCarta Pathways:     KEGG Pathways:  
    Probe set : 1387656_at    Name:   solute carrier family 4, member 1    GB acc:   BE113640    UniGene:   Rn.32202    Gene Symbol:   Slc4a1    Locus Link:   24779    Chromosome:   10    Cytoband:   Chr:10q32    SOURCE    GENECARD       Gene Ontology:   biological process:  transport   biological process:  anion transport   biological process:  cell ion homeostasis   cellular component:  integral to plasma membrane   cellular component:  membrane   cellular component:  integral to membrane      BioCarta Pathways:     KEGG Pathways:  
    Probe set : 1382343_at    Name:   Transcribed locus    GB acc:   AA999037    UniGene:   Rn.33165        SOURCE        Gene Ontology:     BioCarta Pathways:     KEGG Pathways:  
    Probe set : 1389686_at    Name:   Hypothetical LOC363470    GB acc:   BF408898    UniGene:   Rn.16741     Locus Link:   363470    Chromosome:   X    Cytoband:   Chr:Xq22    SOURCE        Gene Ontology:   biological process:  protein amino acid phosphorylation      BioCarta Pathways:     KEGG Pathways:  
    Probe set : 1384259_at    Name:   Rac GTPase-activating protein 1 (predicted)    GB acc:   BF559151    UniGene:   Rn.101301    Gene Symbol:   Racgap1_predicted    Locus Link:   315298    Chromosome:   7    Cytoband:   Chr:7q36    SOURCE    GENECARD       Gene Ontology:   biological process:  electron transport   biological process:  transport   biological process:  intracellular signaling cascade   cellular component:  mitochondrion      BioCarta Pathways:     KEGG Pathways:  
    Probe set : 1395381_at     GB acc:   BF542239              Gene Ontology:     BioCarta Pathways:     KEGG Pathways:  
    Probe set : 1384042_at    Name:   Transcribed locus    GB acc:   BE108303    UniGene:   Rn.24173        SOURCE        Gene Ontology:     BioCarta Pathways:     KEGG Pathways:  
    Probe set : 1382203_at    Name:   growth differentiation factor 1 (predicted)    GB acc:   AI549010    UniGene:   Rn.144974    Gene Symbol:   Gdf1_predicted    Locus Link:   306351    Chromosome:   16    Cytoband:   Chr:16p14    SOURCE    GENECARD       Gene Ontology:   cellular component:  extracellular region   molecular function:  cytokine activity   molecular function:  growth factor activity      BioCarta Pathways:     KEGG Pathways:  
    Probe set : 1379169_at    Name:   similar to RIKEN cDNA 2310067G05    GB acc:   BF399958    UniGene:   Rn.41    Gene Symbol:   LOC301113    Locus Link:   301113    Chromosome:   9    Cytoband:   Chr:9q11    SOURCE    GENECARD       Gene Ontology:     BioCarta Pathways:     KEGG Pathways:  
    Probe set : 1370876_at    Name:   neurofascin    GB acc:   BI285436    UniGene:   Rn.3048    Gene Symbol:   Nfasc    Locus Link:   116690    Chromosome:   13    Cytoband:   Chr:13q13    SOURCE    GENECARD       Gene Ontology:   biological process:  cell adhesion   biological process:  cell-cell adhesion   cellular component:  integral to membrane   molecular function:  protein binding      BioCarta Pathways:     KEGG Pathways:  
    Probe set : 1398885_at    Name:   ribosomal protein L23    GB acc:   AA925327    UniGene:   Rn.103172    Gene Symbol:   Rpl23    Locus Link:   29282    Chromosome:   10    Cytoband:   Chr:10q31    SOURCE    GENECARD       Gene Ontology:   biological process:  protein biosynthesis   biological process:  ribosomal protein import into nucleus   molecular function:  structural constituent of ribosome   molecular function:  structural constituent of ribosome      BioCarta Pathways:     KEGG Pathways:  
    Probe set : 1372023_at    Name:   LOC498674    GB acc:   BI279581    UniGene:   Rn.7314    Gene Symbol:   LOC498674    Locus Link:   498674      SOURCE    GENECARD       Gene Ontology:     BioCarta Pathways:     KEGG Pathways:  
    Probe set : 1397698_at    Name:   Transcribed locus    GB acc:   AW917627    UniGene:   Rn.7962        SOURCE        Gene Ontology:     BioCarta Pathways:     KEGG Pathways:  
    Probe set : 1390698_at    Name:   Transcribed locus, weakly similar to XP_579465.1 PREDICTED: DNA binding protein N5 [Rattus norvegicus]    GB acc:   BF395822    UniGene:   Rn.22587        SOURCE        Gene Ontology:     BioCarta Pathways:     KEGG Pathways:  
    Probe set : 1376770_at    Name:   similar to EF hand domain containing 1    GB acc:   BM389214    UniGene:   Rn.12811    Gene Symbol:   LOC501181    Locus Link:   501181    Chromosome:   9    Cytoband:   Chr:9q35    SOURCE    GENECARD       Gene Ontology:     BioCarta Pathways:     KEGG Pathways:  
    Probe set : 1369929_at    Name:   prosaposin    GB acc:   NM_013013    UniGene:   Rn.97173    Gene Symbol:   Psap    Locus Link:   25524    Chromosome:   20    Cytoband:   Chr:20q11    SOURCE    GENECARD       Gene Ontology:   molecular function:  enzyme activator activity   molecular function:  lipid binding      BioCarta Pathways:     KEGG Pathways:  
    Probe set : 1387910_at    Name:   erythrocyte protein band 4.1-like 1    GB acc:   AB019256    UniGene:   Rn.20361    Gene Symbol:   Epb4.1l1    Locus Link:   59317    Chromosome:   3    Cytoband:   Chr:3q42    SOURCE    GENECARD       Gene Ontology:   biological process:  cortical actin cytoskeleton organization and biogenesis   cellular component:  cytoplasm   cellular component:  cytoskeleton   cellular component:  membrane      BioCarta Pathways:     KEGG Pathways:  
    Probe set : 1372852_at    Name:   similar to protein tyrosine phosphatase, receptor type, C polypeptide-associated protein    GB acc:   BF417048    UniGene:   Rn.143850    Gene Symbol:   LOC499300    Locus Link:   499300    Chromosome:   1    Cytoband:   Chr:1q42    SOURCE    GENECARD       Gene Ontology:     BioCarta Pathways:     KEGG Pathways:  
    Probe set : 1371657_at    Name:   ubiquitin-like 1 (sentrin) activating enzyme E1B (predicted)    GB acc:   AA799692    UniGene:   Rn.3078    Gene Symbol:   Uble1b_predicted    Locus Link:   308508    Chromosome:   1    Cytoband:   Chr:1q21    SOURCE    GENECARD       Gene Ontology:   biological process:  protein modification   biological process:  ubiquitin cycle      BioCarta Pathways:     KEGG Pathways:  
    Probe set : 1376860_at    Name:   Transcribed locus    GB acc:   BE105713    UniGene:   Rn.45804        SOURCE        Gene Ontology:     BioCarta Pathways:     KEGG Pathways:  
    Probe set : 1399065_at     GB acc:   BM389543              Gene Ontology:     BioCarta Pathways:     KEGG Pathways:  
    Probe set : 1373904_at    Name:   similar to RIKEN cDNA 2210402C18 (predicted)    GB acc:   AI103948    UniGene:   Rn.7432    Gene Symbol:   RGD1304585_predicted    Locus Link:   300839    Chromosome:   8    Cytoband:   Chr:8q24    SOURCE    GENECARD       Gene Ontology:     BioCarta Pathways:     KEGG Pathways:  
    Probe set : 1388441_at     GB acc:   BG379987              Gene Ontology:     BioCarta Pathways:     KEGG Pathways:  
    Probe set : 1379345_at    Name:   procollagen, type XV (predicted)    GB acc:   BM386752    UniGene:   Rn.31832    Gene Symbol:   Col15a1_predicted    Locus Link:   298069    Chromosome:   5    Cytoband:   Chr:5q22    SOURCE    GENECARD       Gene Ontology:   biological process:  phosphate transport   biological process:  cell adhesion      BioCarta Pathways:     KEGG Pathways:  
    Probe set : 1389965_at    Name:   trans-golgi network protein 1    GB acc:   AA799818    UniGene:   Rn.11349    Gene Symbol:   Tgoln2    Locus Link:   192152    Chromosome:   4    Cytoband:   Chr:4q33    SOURCE    GENECARD       Gene Ontology:   biological process:  Golgi to endosome transport   molecular function:  protein binding      BioCarta Pathways:     KEGG Pathways:  
    Probe set : 1390523_at    Name:   Transcribed locus    GB acc:   BG373402    UniGene:   Rn.24008        SOURCE        Gene Ontology:     BioCarta Pathways:     KEGG Pathways:  
    Probe set : 1386940_at    Name:   tissue inhibitor of metalloproteinase 2    GB acc:   NM_021989    UniGene:   Rn.10161    Gene Symbol:   Timp2    Locus Link:   29543    Chromosome:   10    Cytoband:   Chr:10q32.3    SOURCE    GENECARD       Gene Ontology:   biological process:  spermatogenesis   biological process:  spermatogenesis   cellular component:  extracellular matrix (sensu Metazoa)   cellular component:  basement membrane   cellular component:  extracellular space   molecular function:  enzyme activator activity   molecular function:  metalloendopeptidase inhibitor activity   molecular function:  enzyme inhibitor activity      BioCarta Pathways:     KEGG Pathways:  
    Probe set : 1396300_at    Name:   Transcribed locus    GB acc:   BI277442    UniGene:   Rn.148355        SOURCE        Gene Ontology:     BioCarta Pathways:     KEGG Pathways:  
    Probe set : 1370820_at    Name:   F-box only protein 6b    GB acc:   AF393484    UniGene:   Rn.57325    Gene Symbol:   Fbxo6b    Locus Link:   192351    Chromosome:   5    Cytoband:   Chr:5q36    SOURCE    GENECARD       Gene Ontology:   biological process:  ubiquitin cycle   biological process:  protein catabolism   biological process:  ER-associated protein catabolism   molecular function:  ubiquitin-protein ligase activity   molecular function:  protein binding   molecular function:  carbohydrate binding      BioCarta Pathways:     KEGG Pathways:  
    Probe set : 1368884_at    Name:   ectonucleoside triphosphate diphosphohydrolase 1    GB acc:   NM_022587    UniGene:   Rn.17491    Gene Symbol:   Entpd1    Locus Link:   64519    Chromosome:   1    Cytoband:   Chr:1q54    SOURCE    GENECARD       Gene Ontology:     BioCarta Pathways:     KEGG Pathways:  
    Probe set : 1381922_at    Name:   solute carrier family 5 (sodium/glucose cotransporter), member 11    GB acc:   BG377720    UniGene:   Rn.112669    Gene Symbol:   Slc5a11    Locus Link:   252854    Chromosome:   1    Cytoband:   Chr:1q36    SOURCE    GENECARD       Gene Ontology:   biological process:  transport   cellular component:  membrane   molecular function:  transporter activity      BioCarta Pathways:     KEGG Pathways:  
    Probe set : 1373583_at    Name:   Transcribed locus    GB acc:   BF396317    UniGene:   Rn.9556        SOURCE        Gene Ontology:     BioCarta Pathways:     KEGG Pathways:  
    Probe set : 1373222_at    Name:   hexosaminidase A    GB acc:   AI230271    UniGene:   Rn.92939    Gene Symbol:   Hexa    Locus Link:   300757    Chromosome:   8    Cytoband:   Chr:8q24    SOURCE    GENECARD       Gene Ontology:   biological process:  carbohydrate metabolism   cellular component:  lysosome   cellular component:  membrane      BioCarta Pathways:     KEGG Pathways:  
    Probe set : 1370341_at    Name:   enolase 2, gamma    GB acc:   AF019973    UniGene:   Rn.10828    Gene Symbol:   Eno2    Locus Link:   24334    Chromosome:   4    Cytoband:   Chr:4q42    SOURCE    GENECARD       Gene Ontology:   biological process:  glycolysis   cellular component:  phosphopyruvate hydratase complex      BioCarta Pathways:     KEGG Pathways:  
    Probe set : 1399026_at    Name:   similar to polyadenylate-binding protein-interacting protein 2    GB acc:   AI178191    UniGene:   Rn.1073    Gene Symbol:   RGD:1359176    Locus Link:   361309    Chromosome:   18    Cytoband:   Chr:18p11    SOURCE    GENECARD       Gene Ontology:     BioCarta Pathways:     KEGG Pathways:  
    Probe set : 1388614_at    Name:   splicing factor 3b, subunit 2 (predicted)    GB acc:   BM386768    UniGene:   Rn.103341    Gene Symbol:   Sf3b2_predicted    Locus Link:   293671    Chromosome:   1    Cytoband:   Chr:1q43    SOURCE    GENECARD       Gene Ontology:   biological process:  nuclear mRNA splicing, via spliceosome   biological process:  mRNA processing   biological process:  RNA splicing   cellular component:  nucleus   cellular component:  spliceosome complex   molecular function:  DNA binding      BioCarta Pathways:     KEGG Pathways:  
    Probe set : 1370921_at    Name:   secretory carrier membrane protein 3    GB acc:   BI281156    UniGene:   Rn.98518    Gene Symbol:   Scamp3    Locus Link:   65169    Chromosome:   2    Cytoband:   Chr:2q34    SOURCE    GENECARD       Gene Ontology:   cellular component:  Golgi membrane   cellular component:  membrane fraction   cellular component:  integral to membrane   molecular function:  protein transporter activity      BioCarta Pathways:     KEGG Pathways:  
    Probe set : 1371421_at    Name:   Similar to FLJ40243 protein (predicted)    GB acc:   BM391873    UniGene:   Rn.104592     Locus Link:   361902    Chromosome:   2    Cytoband:   Chr:2q16    SOURCE        Gene Ontology:     BioCarta Pathways:     KEGG Pathways:  
    Probe set : 1378322_at    Name:   Transcribed locus, strongly similar to NP_705811.8 APG4-D protein [Mus musculus]    GB acc:   BM385592    UniGene:   Rn.139273        SOURCE        Gene Ontology:     BioCarta Pathways:     KEGG Pathways:  
    Probe set : 1377969_at     GB acc:   AI502538              Gene Ontology:     BioCarta Pathways:     KEGG Pathways:  
    Probe set : 1397341_at    Name:   PDZ binding kinase (predicted)    GB acc:   BG663837    UniGene:   Rn.137333     Locus Link:   290326    Chromosome:   15    Cytoband:   Chr:15p12    SOURCE        Gene Ontology:   biological process:  protein amino acid phosphorylation   biological process:  peptidyl-serine phosphorylation   biological process:  peptidyl-threonine phosphorylation      BioCarta Pathways:     KEGG Pathways:  
    Probe set : 1379327_at    Name:   SWI/SNF related, matrix associated, actin dependent regulator of chromatin, subfamily a, member 5 (predicted)    GB acc:   BI292524    UniGene:   Rn.128772    Gene Symbol:   Smarca5_predicted    Locus Link:   307766    Chromosome:   19    Cytoband:   Chr:19q11    SOURCE    GENECARD       Gene Ontology:   biological process:  chromatin remodeling   biological process:  regulation of transcription from RNA polymerase II promoter   biological process:  chromatin modification   cellular component:  nucleoplasm      BioCarta Pathways:     KEGG Pathways:  
    Probe set : 1373986_at    Name:   Transcribed locus    GB acc:   AI410107    UniGene:   Rn.20036        SOURCE        Gene Ontology:     BioCarta Pathways:     KEGG Pathways:  
    Probe set : 1377680_at    Name:   glial cell line derived neurotrophic factor family receptor alpha 3    GB acc:   BG377887    UniGene:   Rn.15499    Gene Symbol:   Gfra3    Locus Link:   84422    Chromosome:   18    Cytoband:   Chr:18p12    SOURCE    GENECARD       Gene Ontology:   biological process:  signal transduction   biological process:  peripheral nervous system development   cellular component:  extrinsic to membrane   molecular function:  receptor activity   molecular function:  receptor activity   molecular function:  receptor binding      BioCarta Pathways:     KEGG Pathways:  
    Probe set : 1388558_at    Name:   Adenylate kinase 3    GB acc:   AI171802    UniGene:   Rn.60    Gene Symbol:   Ak3    Locus Link:   26956    Chromosome:   1    Cytoband:   Chr:1q43    SOURCE    GENECARD       Gene Ontology:   biological process:  amino acid biosynthesis   biological process:  nucleobase, nucleoside, nucleotide and nucleic acid metabolism   cellular component:  mitochondrion   cellular component:  mitochondrion      BioCarta Pathways:     KEGG Pathways:  
    Probe set : 1398809_at    Name:   nuclear distribution gene E homolog 1 (A nidulans)    GB acc:   NM_053347    UniGene:   Rn.20222    Gene Symbol:   Nde1    Locus Link:   83836    Chromosome:   10    Cytoband:   Chr:10q11    SOURCE    GENECARD       Gene Ontology:   cellular component:  centrosome   cellular component:  spindle   molecular function:  protein binding   molecular function:  microtubule binding   molecular function:  protein self binding      BioCarta Pathways:     KEGG Pathways:  
    Probe set : 1393334_at    Name:   Transcribed locus    GB acc:   AW528448    UniGene:   Rn.17664        SOURCE        Gene Ontology:     BioCarta Pathways:     KEGG Pathways:  
    Probe set : 1389169_at    Name:   progesterone receptor membrane component 2 (predicted)    GB acc:   AA944158    UniGene:   Rn.7685    Gene Symbol:   Pgrmc2_predicted    Locus Link:   361940    Chromosome:   2    Cytoband:   Chr:2q26    SOURCE    GENECARD       Gene Ontology:   cellular component:  microsome   cellular component:  integral to membrane   molecular function:  steroid hormone receptor activity   molecular function:  receptor activity   molecular function:  steroid binding      BioCarta Pathways:     KEGG Pathways:  
    Probe set : 1370574_a_at    Name:   protein tyrosine phosphatase, non-receptor type substrate 1    GB acc:   D38468    UniGene:   Rn.53971    Gene Symbol:   Ptpns1    Locus Link:   25528    Chromosome:   3    Cytoband:   Chr:3q36    SOURCE    GENECARD       Gene Ontology:   cellular component:  extracellular space   cellular component:  integral to plasma membrane   cellular component:  integral to membrane   molecular function:  protein binding   molecular function:  protein phosphorylated amino acid binding      BioCarta Pathways:     KEGG Pathways:  
    Probe set : 1371646_at    Name:   phosphogluconate dehydrogenase    GB acc:   AI008441    UniGene:   Rn.915    Gene Symbol:   Pgd    Locus Link:   362660    Chromosome:   5    Cytoband:   Chr:5q36    SOURCE    GENECARD       Gene Ontology:   biological process:  pentose-phosphate shunt   biological process:  pentose-phosphate shunt, oxidative branch      BioCarta Pathways:     KEGG Pathways:  
    Probe set : 1379326_at    Name:   similar to 9530008L14Rik protein (predicted)    GB acc:   AI169837    UniGene:   Rn.16893    Gene Symbol:   RGD1305679_predicted    Locus Link:   361228    Chromosome:   17    Cytoband:   Chr:17p12    SOURCE    GENECARD       Gene Ontology:   cellular component:  integral to membrane      BioCarta Pathways:     KEGG Pathways:  
    Probe set : 1372500_at    Name:   tropomodulin 3 (predicted)    GB acc:   BG378269    UniGene:   Rn.146101    Gene Symbol:   Tmod3_predicted    Locus Link:   300838    Chromosome:   8    Cytoband:   Chr:8q24    SOURCE    GENECARD       Gene Ontology:   biological process:  actin filament organization   biological process:  barbed-end actin filament capping   cellular component:  cytoskeleton   cellular component:  F-actin capping protein complex   molecular function:  actin binding   molecular function:  tropomyosin binding      BioCarta Pathways:     KEGG Pathways:  
    Probe set : 1387280_a_at    Name:   tumor-associated protein 1    GB acc:   NM_017353    UniGene:   Rn.32261    Gene Symbol:   Slc7a5    Locus Link:   50719    Chromosome:   19    Cytoband:   Chr:19q12    SOURCE    GENECARD       Gene Ontology:   biological process:  transport   biological process:  amino acid transport   cellular component:  integral to plasma membrane   cellular component:  membrane   cellular component:  integral to membrane      BioCarta Pathways:     KEGG Pathways:  
    Probe set : 1379396_at    Name:   engulfment and cell motility 1, ced-12 homolog (C. elegans) (predicted)    GB acc:   AI101913    UniGene:   Rn.24911    Gene Symbol:   Elmo1_predicted    Locus Link:   361251    Chromosome:   17    Cytoband:   Chr:17q11    SOURCE    GENECARD       Gene Ontology:   cellular component:  cytoplasm   cellular component:  cytoskeleton   cellular component:  plasma membrane   cellular component:  membrane   molecular function:  protein binding   molecular function:  SH3 domain binding      BioCarta Pathways:     KEGG Pathways:  
    Probe set : 1373015_at    Name:   ring finger protein 11 (predicted)    GB acc:   BI280348    UniGene:   Rn.129983    Gene Symbol:   Rnf11_predicted    Locus Link:   316552    Chromosome:   9    Cytoband:   Chr:9q34    SOURCE    GENECARD       Gene Ontology:   cellular component:  ubiquitin ligase complex      BioCarta Pathways:     KEGG Pathways:  
    Probe set : 1379740_at    Name:   Similar to chromosome 18 open reading frame 54    GB acc:   AW521797    UniGene:   Rn.31250     Locus Link:   361346    Chromosome:   18    Cytoband:   Chr:18q12.1    SOURCE        Gene Ontology:     BioCarta Pathways:     KEGG Pathways:  
    Probe set : 1390046_at    Name:   a disintegrin and metalloprotease domain 8 (predicted)    GB acc:   BI288110    UniGene:   Rn.138249    Gene Symbol:   Adam8_predicted    Locus Link:   309098    Chromosome:   1    Cytoband:   Chr:1q41    SOURCE    GENECARD       Gene Ontology:     BioCarta Pathways:     KEGG Pathways:  
    Probe set : 1379507_at    Name:   Transcribed locus    GB acc:   AW917933    UniGene:   Rn.28424        SOURCE        Gene Ontology:     BioCarta Pathways:     KEGG Pathways:  
    Probe set : 1371080_at    Name:   kallikrein    GB acc:   AI169562    UniGene:   Rn.21391    Gene Symbol:   RGD:735228    Locus Link:   292861    Chromosome:   1    Cytoband:   Chr:1q22    SOURCE    GENECARD       Gene Ontology:   biological process:  proteolysis   molecular function:  serine-type endopeptidase activity      BioCarta Pathways:     KEGG Pathways:  
    Probe set : 1373585_at    Name:   similar to RIKEN cDNA 1810020G14 (predicted)    GB acc:   BE116774    UniGene:   Rn.19779    Gene Symbol:   RGD1310781_predicted    Locus Link:   299179    Chromosome:   6    Cytoband:   Chr:6q24    SOURCE    GENECARD       Gene Ontology:     BioCarta Pathways:     KEGG Pathways:  
    Probe set : 1384451_at    Name:   Transcribed locus    GB acc:   BE116574    UniGene:   Rn.64046        SOURCE        Gene Ontology:     BioCarta Pathways:     KEGG Pathways:  
    Probe set : 1377744_at    Name:   Transcribed locus, moderately similar to XP_576460.1 PREDICTED: similar to hypothetical protein PB402898.00.0 [Rattus norvegicus]    GB acc:   BF392982    UniGene:   Rn.37275        SOURCE        Gene Ontology:     BioCarta Pathways:     KEGG Pathways:  
    Probe set : 1393803_at    Name:   caspase 8 associated protein 2 (predicted)    GB acc:   BF398167    UniGene:   Rn.9052    Gene Symbol:   Casp8ap2_predicted    Locus Link:   313128    Chromosome:   5    Cytoband:   Chr:5q21    SOURCE    GENECARD       Gene Ontology:   biological process:  caspase activation   biological process:  induction of apoptosis via death domain receptors      BioCarta Pathways:     KEGG Pathways:  
    Probe set : 1388483_at    Name:   cofilin 2, muscle (predicted)    GB acc:   BI296011    UniGene:   Rn.101880    Gene Symbol:   Cfl2_predicted    Locus Link:   366624    Chromosome:   6    Cytoband:   Chr:6q23    SOURCE    GENECARD       Gene Ontology:   cellular component:  intracellular   cellular component:  nucleus   cellular component:  cytoskeleton   molecular function:  actin binding      BioCarta Pathways:     KEGG Pathways:  
    Probe set : 1369931_at    Name:   similar to pyruvate kinase (EC 2.7.1.40) isozyme M2 - rat    GB acc:   NM_053297    UniGene:   Rn.115631    Gene Symbol:   LOC498661    Locus Link:   498661    Chromosome:   16    Cytoband:   Chr:16q12.5    SOURCE    GENECARD       Gene Ontology:     BioCarta Pathways:     KEGG Pathways:  
    Probe set : 1367767_at    Name:   3-hydroxy-3-methylglutaryl-Coenzyme A lyase    GB acc:   NM_024386    UniGene:   Rn.12297    Gene Symbol:   Hmgcl    Locus Link:   79238    Chromosome:   5    Cytoband:   Chr:5q36    SOURCE    GENECARD       Gene Ontology:   biological process:  generation of precursor metabolites and energy   biological process:  amino acid metabolism   cellular component:  extracellular space   cellular component:  mitochondrion   cellular component:  mitochondrial matrix      BioCarta Pathways:     KEGG Pathways:  
    Probe set : 1367820_at    Name:   barrier to autointegration factor 1    GB acc:   NM_053631    UniGene:   Rn.19921    Gene Symbol:   Banf1    Locus Link:   114087    Chromosome:   1    Cytoband:   Chr:1q43    SOURCE    GENECARD       Gene Ontology:   cellular component:  nucleus   cellular component:  chromosome   molecular function:  DNA binding      BioCarta Pathways:     KEGG Pathways:  
    Probe set : 1388555_at    Name:   thioredoxin-like 5 (predicted)    GB acc:   AI104292    UniGene:   Rn.106223    Gene Symbol:   Txnl5_predicted    Locus Link:   287474    Chromosome:   10    Cytoband:   Chr:10q24    SOURCE    GENECARD       Gene Ontology:   biological process:  electron transport   molecular function:  electron transporter activity      BioCarta Pathways:     KEGG Pathways:  
    Probe set : 1382253_at    Name:   similar to hypothetical protein FLJ21103 (predicted)    GB acc:   BF419062    UniGene:   Rn.129284    Gene Symbol:   RGD1307173_predicted    Locus Link:   315549    Chromosome:   8    Cytoband:   Chr:8q21    SOURCE    GENECARD       Gene Ontology:     BioCarta Pathways:     KEGG Pathways:  
    Probe set : 1386529_at    Name:   Transcribed locus    GB acc:   BF564309    UniGene:   Rn.146907        SOURCE        Gene Ontology:     BioCarta Pathways:     KEGG Pathways:  
    Probe set : 1382940_at     GB acc:   BI281084              Gene Ontology:     BioCarta Pathways:     KEGG Pathways:  
    Probe set : 1390356_at    Name:   Transcribed locus    GB acc:   AW535401    UniGene:   Rn.15945        SOURCE        Gene Ontology:     BioCarta Pathways:     KEGG Pathways:  
    Probe set : 1383378_at    Name:   Transcribed locus    GB acc:   BI298932    UniGene:   Rn.14926        SOURCE        Gene Ontology:     BioCarta Pathways:     KEGG Pathways:  
    Probe set : 1367468_at    Name:   SCAN domain-containing 1 (predicted)    GB acc:   BM386654    UniGene:   Rn.1095    Gene Symbol:   Scand1_predicted    Locus Link:   362252    Chromosome:   3    Cytoband:   Chr:3q42    SOURCE    GENECARD       Gene Ontology:   cellular component:  nucleus      BioCarta Pathways:     KEGG Pathways:  
    Probe set : 1375996_at    Name:   septin 1 (predicted)    GB acc:   AA894262    UniGene:   Rn.144800    Gene Symbol:   Sept1_predicted    Locus Link:   293507    Chromosome:   1    Cytoband:   Chr:1q36    SOURCE    GENECARD       Gene Ontology:   biological process:  cytokinesis   biological process:  cell cycle   molecular function:  GTP binding   molecular function:  nucleotide binding      BioCarta Pathways:     KEGG Pathways:  
    Probe set : 1399055_at    Name:   bromodomain containing 7 (predicted)    GB acc:   BI275924    UniGene:   Rn.32686    Gene Symbol:   Brd7_predicted    Locus Link:   361374    Chromosome:   19    Cytoband:   Chr:19p11    SOURCE    GENECARD       Gene Ontology:   biological process:  regulation of transcription from RNA polymerase II promoter   cellular component:  nucleus   cellular component:  cytoplasm   molecular function:  protein binding   molecular function:  transcription factor binding   molecular function:  histone binding      BioCarta Pathways:     KEGG Pathways:  
    Probe set : 1388153_at    Name:   acyl-CoA synthetase long-chain family member 1    GB acc:   BI277523    UniGene:   Rn.6215    Gene Symbol:   Acsl1    Locus Link:   25288    Chromosome:   16    Cytoband:   Chr:16q11    SOURCE    GENECARD       Gene Ontology:   cellular component:  mitochondrion   cellular component:  integral to membrane      BioCarta Pathways:     KEGG Pathways:  
    Probe set : 1389658_at    Name:   similar to CG6133-PA (predicted)    GB acc:   BI283104    UniGene:   Rn.27691    Gene Symbol:   RGD1311954_predicted    Locus Link:   361191    Chromosome:   17    Cytoband:   Chr:17p14    SOURCE    GENECARD       Gene Ontology:     BioCarta Pathways:     KEGG Pathways:  
    Probe set : 1381216_at     GB acc:   BI294178              Gene Ontology:     BioCarta Pathways:     KEGG Pathways:  
    Probe set : 1381282_at    Name:   similar to cDNA sequence BC026432 (predicted)    GB acc:   BF416808    UniGene:   Rn.63148    Gene Symbol:   RGD1308345_predicted    Locus Link:   308996    Chromosome:   1    Cytoband:   Chr:1q36    SOURCE    GENECARD       Gene Ontology:     BioCarta Pathways:     KEGG Pathways:  
    Probe set : 1388392_at    Name:   Tax1 (human T-cell leukemia virus type I) binding protein 3 (predicted)    GB acc:   BI274159    UniGene:   Rn.145939    Gene Symbol:   Tax1bp3_predicted    Locus Link:   360564    Chromosome:   10    Cytoband:   Chr:10q24    SOURCE    GENECARD       Gene Ontology:   biological process:  intracellular signaling cascade   biological process:  negative regulation of cell proliferation   cellular component:  nucleus   cellular component:  cytoplasm   molecular function:  protein binding   molecular function:  beta-catenin binding   molecular function:  transcription regulator activity      BioCarta Pathways:     KEGG Pathways:  
    Probe set : 1377750_at    Name:   Rho guanine nucleotide exchange factor (GEF) 3 (predicted)    GB acc:   AI454536    UniGene:   Rn.11687     Locus Link:   290541    Chromosome:   16    Cytoband:   Chr:16p16    SOURCE        Gene Ontology:   biological process:  Rho protein signal transduction   molecular function:  Rho guanyl-nucleotide exchange factor activity      BioCarta Pathways:     KEGG Pathways:  
    Probe set : 1391255_at    Name:   zinc finger, MYND domain containing 15 (predicted)    GB acc:   BE110035    UniGene:   Rn.105972    Gene Symbol:   Zmynd15_predicted    Locus Link:   287457    Chromosome:   10    Cytoband:   Chr:10q24    SOURCE    GENECARD       Gene Ontology:     BioCarta Pathways:     KEGG Pathways:  
    Probe set : 1374079_at    Name:   Transcribed locus    GB acc:   AW915055    UniGene:   Rn.146656        SOURCE        Gene Ontology:     BioCarta Pathways:     KEGG Pathways:  
    Probe set : 1374124_at    Name:   similar to RIKEN cDNA 2610003J06 (predicted)    GB acc:   AA851397    UniGene:   Rn.6504    Gene Symbol:   RGD1307381_predicted    Locus Link:   360498    Chromosome:   10    Cytoband:   Chr:10q12    SOURCE    GENECARD       Gene Ontology:     BioCarta Pathways:     KEGG Pathways:  
    Probe set : 1370811_at    Name:   mercaptopyruvate sulfurtransferase    GB acc:   D50564    UniGene:   Rn.32263    Gene Symbol:   Mpst    Locus Link:   192172    Chromosome:   7    Cytoband:   Chr:7q34    SOURCE    GENECARD       Gene Ontology:   biological process:  sulfate transport   biological process:  cyanate catabolism   biological process:  response to toxin   cellular component:  extracellular space   cellular component:  mitochondrion   cellular component:  mitochondrial matrix      BioCarta Pathways:     KEGG Pathways:  
    Probe set : 1391500_at    Name:   Transcribed locus    GB acc:   BF282889    UniGene:   Rn.19393        SOURCE        Gene Ontology:     BioCarta Pathways:     KEGG Pathways:  
    Probe set : 1383199_at    Name:   Friend leukemia integration 1 (predicted)    GB acc:   AW140820    UniGene:   Rn.65827     Locus Link:   315532    Chromosome:   8    Cytoband:   Chr:8q21    SOURCE        Gene Ontology:   cellular component:  nucleus   molecular function:  DNA binding   molecular function:  transcription factor activity   molecular function:  transcriptional activator activity      BioCarta Pathways:     KEGG Pathways:  
    Probe set : 1375438_at    Name:   golgi SNAP receptor complex member 2    GB acc:   AA964687    UniGene:   Rn.13518    Gene Symbol:   Gosr2    Locus Link:   64154    Chromosome:   10    Cytoband:   Chr:10q32.1    SOURCE    GENECARD       Gene Ontology:     BioCarta Pathways:     KEGG Pathways:  
    Probe set : 1370363_at    Name:   carboxylesterase 3    GB acc:   L46791    UniGene:   Rn.34885    Gene Symbol:   Ces3    Locus Link:   113902    Chromosome:   19    Cytoband:   Chr:19p11    SOURCE    GENECARD       Gene Ontology:   cellular component:  extracellular space   cellular component:  endoplasmic reticulum   cellular component:  endoplasmic reticulum lumen      BioCarta Pathways:     KEGG Pathways:  
    Probe set : 1372876_at    Name:   selenophosphate synthetase 2 (predicted)    GB acc:   AA799700    UniGene:   Rn.100471    Gene Symbol:   Sephs2_predicted    Locus Link:   308993    Chromosome:   1    Cytoband:   Chr:1q36    SOURCE    GENECARD       Gene Ontology:   biological process:  selenocysteine biosynthesis      BioCarta Pathways:     KEGG Pathways:  
    Probe set : 1385359_at    Name:   Similar to RIKEN cDNA 4931406C07 (predicted)    GB acc:   BF406890    UniGene:   Rn.3085     Locus Link:   363016    Chromosome:   8    Cytoband:   Chr:8q12    SOURCE        Gene Ontology:     BioCarta Pathways:     KEGG Pathways:  
    Probe set : 1371592_at    Name:   c-src tyrosine kinase (predicted)    GB acc:   AI600236    UniGene:   Rn.2759    Gene Symbol:   Csk_predicted    Locus Link:   315707    Chromosome:   8    Cytoband:   Chr:8q24    SOURCE    GENECARD       Gene Ontology:   cellular component:  cytoplasm      BioCarta Pathways:     KEGG Pathways:  
    Probe set : 1396499_at    Name:   similar to HSD-29    GB acc:   BF403327    UniGene:   Rn.23833    Gene Symbol:   LOC300201    Locus Link:   300201    Chromosome:   7    Cytoband:   Chr:7q36    SOURCE    GENECARD       Gene Ontology:     BioCarta Pathways:     KEGG Pathways:  
    Probe set : 1370905_at    Name:   dedicator of cytokinesis 9    GB acc:   BI291229    UniGene:   Rn.10431    Gene Symbol:   Dock9    Locus Link:   259237    Chromosome:   15    Cytoband:   Chr:15q25    SOURCE    GENECARD       Gene Ontology:   cellular component:  membrane   molecular function:  guanyl-nucleotide exchange factor activity   molecular function:  GTP binding   molecular function:  GTPase binding      BioCarta Pathways:     KEGG Pathways:  
    Probe set : 1384378_at    Name:   HCR (a-helix coiled-coil rod homolog)    GB acc:   BI293046    UniGene:   Rn.127116    Gene Symbol:   RGD:1302992    Locus Link:   406196    Chromosome:   20    Cytoband:   Chr:20p12    SOURCE    GENECARD       Gene Ontology:     BioCarta Pathways:     KEGG Pathways:  
    Probe set : 1378191_at    Name:   nuclear cap binding protein subunit 1, 80kDa (predicted)    GB acc:   AI549102    UniGene:   Rn.13482    Gene Symbol:   Ncbp1_predicted    Locus Link:   298075    Chromosome:   5    Cytoband:   Chr:5q22    SOURCE    GENECARD       Gene Ontology:   cellular component:  nucleoplasm   molecular function:  RNA cap binding   molecular function:  RNA binding      BioCarta Pathways:     KEGG Pathways:  
    Probe set : 1386892_at    Name:   parathymosin    GB acc:   NM_031975    UniGene:   Rn.3609    Gene Symbol:   Ptms    Locus Link:   83801    Chromosome:   4    Cytoband:   Chr:4q42    SOURCE    GENECARD       Gene Ontology:   cellular component:  nucleus   molecular function:  enzyme inhibitor activity   molecular function:  zinc ion binding   molecular function:  zinc ion binding      BioCarta Pathways:     KEGG Pathways:  
    Probe set : 1391611_at    Name:   kinesin family member 12 (predicted)    GB acc:   BI296819    UniGene:   Rn.32294    Gene Symbol:   Kif12_predicted    Locus Link:   313254    Chromosome:   5    Cytoband:   Chr:5q24    SOURCE    GENECARD       Gene Ontology:   biological process:  microtubule-based process   biological process:  microtubule-based movement   cellular component:  kinesin complex   cellular component:  microtubule associated complex   molecular function:  motor activity   molecular function:  microtubule motor activity   molecular function:  ATP binding      BioCarta Pathways:     KEGG Pathways:  
    Probe set : 1387230_at    Name:   solute carrier family 12, member 3    GB acc:   NM_019345    UniGene:   Rn.10467    Gene Symbol:   Slc12a3    Locus Link:   54300      SOURCE    GENECARD       Gene Ontology:     BioCarta Pathways:     KEGG Pathways:  
    Probe set : 1372557_at    Name:   ADP-ribosylation factor-like 6 (predicted)    GB acc:   AI409182    UniGene:   Rn.101944    Gene Symbol:   Arl6_predicted    Locus Link:   363760    Chromosome:   11    Cytoband:   Chr:11q12    SOURCE    GENECARD       Gene Ontology:   cellular component:  cytoplasm   cellular component:  membrane   molecular function:  protein binding   molecular function:  GTP binding      BioCarta Pathways:     KEGG Pathways:  
    Probe set : 1367752_at    Name:   breast cancer anti-estrogen resistance 1    GB acc:   NM_012931    UniGene:   Rn.40101    Gene Symbol:   Bcar1    Locus Link:   25414    Chromosome:   19    Cytoband:   Chr:19q12    SOURCE    GENECARD       Gene Ontology:   biological process:  cell adhesion   biological process:  cell proliferation   cellular component:  membrane fraction   cellular component:  cytoplasm   molecular function:  signal transducer activity   molecular function:  protein binding      BioCarta Pathways:     KEGG Pathways:  
    Probe set : 1387877_at    Name:   formiminotransferase cyclodeaminase    GB acc:   BE096729    UniGene:   Rn.20140    Gene Symbol:   Ftcd    Locus Link:   89833    Chromosome:   20    Cytoband:   Chr:20p12    SOURCE    GENECARD       Gene Ontology:   cellular component:  cytoplasm   cellular component:  Golgi apparatus   cellular component:  Golgi apparatus   cellular component:  Golgi stack      BioCarta Pathways:     KEGG Pathways:  
    Probe set : 1371844_at     GB acc:   AW144530              Gene Ontology:     BioCarta Pathways:     KEGG Pathways:  
    Probe set : 1371739_at    Name:   Leucine zipper-EF-hand containing transmembrane protein 1    GB acc:   AI104605    UniGene:   Rn.22461    Gene Symbol:   Letm1    Locus Link:   305457    Chromosome:   14    Cytoband:   Chr:14q21    SOURCE    GENECARD       Gene Ontology:   cellular component:  mitochondrion   cellular component:  integral to membrane   molecular function:  calcium ion binding      BioCarta Pathways:     KEGG Pathways:  
    Probe set : 1368300_at    Name:   adenosine A2a receptor    GB acc:   NM_053294    UniGene:   Rn.11180    Gene Symbol:   Adora2a    Locus Link:   25369    Chromosome:   20    Cytoband:   Chr:20p12    SOURCE    GENECARD       Gene Ontology:   biological process:  electron transport   biological process:  cAMP biosynthesis   biological process:  regulation of transcription, DNA-dependent   biological process:  regulation of transcription, DNA-dependent   biological process:  neurotransmitter transport   biological process:  phagocytosis   biological process:  apoptosis   biological process:  inflammatory response   biological process:  cellular defense response   biological process:  G-protein coupled receptor protein signaling pathway   biological process:  G-protein signaling, coupled to cAMP nucleotide second messenger   biological process:  G-protein signaling, adenylate cyclase activating pathway   biological process:  adenylate cyclase activation   biological process:  cell-cell signaling   biological process:  central nervous system development   biological process:  blood coagulation   biological process:  sensory perception   biological process:  circulation   biological process:  signal transduction      BioCarta Pathways:     KEGG Pathways:  
    Probe set : 1367810_at    Name:   choline transporter    GB acc:   AI412218    UniGene:   Rn.10336    Gene Symbol:   Slc6a8    Locus Link:   50690    Chromosome:   X    Cytoband:   Chr:Xq37    SOURCE    GENECARD       Gene Ontology:   cellular component:  integral to plasma membrane   cellular component:  membrane   cellular component:  integral to membrane      BioCarta Pathways:     KEGG Pathways:  
    Probe set : 1377672_at    Name:   sulfotransferase family, cytosolic, 1C, member 2    GB acc:   BI300997    UniGene:   Rn.22471    Gene Symbol:   Sult1c2    Locus Link:   171072    Chromosome:   9    Cytoband:   Chr:9q11    SOURCE    GENECARD       Gene Ontology:   cellular component:  lysosome   molecular function:  sulfotransferase activity   molecular function:  transferase activity      BioCarta Pathways:     KEGG Pathways:  
    Probe set : 1392548_at    Name:   similar to mKIAA1604 protein    GB acc:   BF394282     Gene Symbol:   LOC362153    Locus Link:   362153    Chromosome:   3    Cytoband:   Chr:3q24     GENECARD       Gene Ontology:     BioCarta Pathways:     KEGG Pathways:  
    Probe set : 1398264_at    Name:   solute carrier family 30 (zinc transporter), member 2    GB acc:   NM_012890    UniGene:   Rn.11135    Gene Symbol:   Slc30a2    Locus Link:   25362    Chromosome:   5    Cytoband:   Chr:5q36    SOURCE    GENECARD       Gene Ontology:   biological process:  transport   biological process:  cation transport   biological process:  zinc ion transport   biological process:  zinc ion transport      BioCarta Pathways:     KEGG Pathways:  
    Probe set : 1379719_at    Name:   Transcribed locus    GB acc:   AI408386    UniGene:   Rn.153810        SOURCE        Gene Ontology:     BioCarta Pathways:     KEGG Pathways:  
    Probe set : 1393105_at    Name:   similar to zinc finger protein    GB acc:   BF288164    UniGene:   Rn.102011    Gene Symbol:   LOC500989    Locus Link:   500989    Chromosome:   8    Cytoband:   Chr:8q23    SOURCE    GENECARD       Gene Ontology:     BioCarta Pathways:     KEGG Pathways:  
    Probe set : 1367982_at    Name:   aminolevulinic acid synthase 1    GB acc:   NM_024484    UniGene:   Rn.97126    Gene Symbol:   Alas1    Locus Link:   65155      SOURCE    GENECARD       Gene Ontology:   biological process:  heme biosynthesis   biological process:  metabolism   biological process:  biosynthesis   cellular component:  mitochondrion      BioCarta Pathways:     KEGG Pathways:  
    Probe set : 1368150_at    Name:   solute carrier family 27 (fatty acid transporter), member 2 /// hypothetical gene supported by NM_031736    GB acc:   NM_031736    UniGene:   Rn.3608    Gene Symbol:   Slc27a2 /// LOC497779    Locus Link:   497779 /// 65192    Chromosome:   3    Cytoband:   Chr:3q36    SOURCE    GENECARD       Gene Ontology:   biological process:  lipid metabolism   biological process:  fatty acid metabolism   biological process:  metabolism      BioCarta Pathways:     KEGG Pathways:  
    Probe set : 1388358_at    Name:   electron-transfer-flavoprotein, beta polypeptide    GB acc:   AW252650    UniGene:   Rn.60152    Gene Symbol:   RGD:1303312    Locus Link:   292845    Chromosome:   1    Cytoband:   Chr:1q22    SOURCE    GENECARD       Gene Ontology:   biological process:  fatty acid beta-oxidation   biological process:  electron transport   molecular function:  electron transporter activity      BioCarta Pathways:     KEGG Pathways:  
    Probe set : 1398514_at    Name:   homogentisate 1, 2-dioxygenase (predicted)    GB acc:   AI232328    UniGene:   Rn.145148    Gene Symbol:   Hgd_predicted    Locus Link:   360719    Chromosome:   11    Cytoband:   Chr:11q21    SOURCE    GENECARD       Gene Ontology:   biological process:  L-phenylalanine catabolism   biological process:  tyrosine metabolism   biological process:  tyrosine catabolism      BioCarta Pathways:     KEGG Pathways:  
    Probe set : 1371125_at    Name:   kinesin heavy chain member 2    GB acc:   BF564995    UniGene:   Rn.17829    Gene Symbol:   Kif2    Locus Link:   84391    Chromosome:   2    Cytoband:   Chr:2q14    SOURCE    GENECARD       Gene Ontology:   biological process:  microtubule-based process   biological process:  microtubule-based movement   cellular component:  cytoskeleton   cellular component:  kinesin complex   cellular component:  microtubule associated complex   molecular function:  motor activity   molecular function:  microtubule motor activity   molecular function:  ATP binding      BioCarta Pathways:     KEGG Pathways:  
    Probe set : 1373907_at    Name:   trafficking protein particle complex 4    GB acc:   BI288890    UniGene:   Rn.9580    Gene Symbol:   RGD:1302991    Locus Link:   367073    Chromosome:   8    Cytoband:   Chr:8q22    SOURCE    GENECARD       Gene Ontology:   biological process:  synaptic vesicle transport   biological process:  ER to Golgi transport   cellular component:  Golgi cis-face   molecular function:  syndecan binding      BioCarta Pathways:     KEGG Pathways:  
    Probe set : 1381947_at    Name:   hypothetical LOC295622    GB acc:   AI169006    UniGene:   Rn.43872    Gene Symbol:   LOC295622    Locus Link:   295622    Chromosome:   3    Cytoband:   Chr:3q21    SOURCE    GENECARD       Gene Ontology:     BioCarta Pathways:     KEGG Pathways:  
    Probe set : 1390498_at    Name:   Transcribed locus    GB acc:   AI230554    UniGene:   Rn.147999        SOURCE        Gene Ontology:     BioCarta Pathways:     KEGG Pathways:  
    Probe set : 1381311_at    Name:   EGF-like module containing, mucin-like, hormone receptor-like sequence 1    GB acc:   BE100625    UniGene:   Rn.43404    Gene Symbol:   RGD:1359214    Locus Link:   316137    Chromosome:   9    Cytoband:   Chr:9q11    SOURCE    GENECARD       Gene Ontology:   biological process:  neuropeptide signaling pathway   cellular component:  membrane   molecular function:  G-protein coupled receptor activity   molecular function:  calcium ion binding      BioCarta Pathways:     KEGG Pathways:  
    Probe set : 1374995_at    Name:   engulfment and cell motility 3, ced-12 homolog (C. elegans) (predicted)    GB acc:   BI291645    UniGene:   Rn.144769    Gene Symbol:   Elmo3_predicted    Locus Link:   291962    Chromosome:   19    Cytoband:   Chr:19q11    SOURCE    GENECARD       Gene Ontology:   biological process:  phagocytosis   biological process:  apoptosis   cellular component:  cytoskeleton   cellular component:  membrane      BioCarta Pathways:     KEGG Pathways:  
    Probe set : 1367798_at    Name:   S-adenosylhomocysteine hydrolase    GB acc:   NM_017201    UniGene:   Rn.5878    Gene Symbol:   Ahcy    Locus Link:   29443    Chromosome:   3    Cytoband:   Chr:3q42    SOURCE    GENECARD       Gene Ontology:   cellular component:  cytoplasm   molecular function:  adenosylhomocysteinase activity   molecular function:  adenosylhomocysteinase activity   molecular function:  hydrolase activity      BioCarta Pathways:     KEGG Pathways:  
    Probe set : 1368288_at    Name:   group specific component    GB acc:   NM_012564    UniGene:   Rn.1437    Gene Symbol:   Gc    Locus Link:   24384    Chromosome:   14    Cytoband:   Chr:14p22    SOURCE    GENECARD       Gene Ontology:   biological process:  transport   biological process:  vitamin D metabolism   biological process:  vitamin transport   cellular component:  extracellular space      BioCarta Pathways:     KEGG Pathways:  
    Probe set : 1371730_at    Name:   similar to RIKEN cDNA 1300002A08 (predicted)    GB acc:   BF418786    UniGene:   Rn.103067    Gene Symbol:   RGD1305466_predicted    Locus Link:   291922    Chromosome:   19    Cytoband:   Chr:19p11    SOURCE    GENECARD       Gene Ontology:   biological process:  proteolysis   biological process:  ATP-dependent proteolysis   cellular component:  mitochondrion      BioCarta Pathways:     KEGG Pathways:  
    Probe set : 1380090_at     GB acc:   BF407443              Gene Ontology:     BioCarta Pathways:     KEGG Pathways:  
    Probe set : 1395036_at    Name:   similar to Ac2-190 (predicted)    GB acc:   BF386788    UniGene:   Rn.129949    Gene Symbol:   RGD1306455_predicted    Locus Link:   312952    Chromosome:   5    Cytoband:   Chr:5q12    SOURCE    GENECARD       Gene Ontology:   molecular function:  hydrolase activity      BioCarta Pathways:     KEGG Pathways:  
    Probe set : 1391573_at    Name:   Tumor necrosis factor receptor superfamily, member 21 (predicted)    GB acc:   AA891842    UniGene:   Rn.98293     Locus Link:   316256    Chromosome:   9    Cytoband:   Chr:9q12    SOURCE        Gene Ontology:   biological process:  apoptosis   biological process:  signal transduction   cellular component:  integral to membrane   molecular function:  receptor activity   molecular function:  protein binding      BioCarta Pathways:     KEGG Pathways:  
    Probe set : 1383643_at    Name:   similar to UPF0197 protein C11orf10 homolog    GB acc:   BI282584    UniGene:   Rn.129769    Gene Symbol:   LOC499317    Locus Link:   499317    Chromosome:   1    Cytoband:   Chr:1q43    SOURCE    GENECARD       Gene Ontology:     BioCarta Pathways:     KEGG Pathways:  
    Probe set : 1374466_at    Name:   Protein kinase C, epsilon    GB acc:   AI715484    UniGene:   Rn.34966    Gene Symbol:   Prkce    Locus Link:   29340    Chromosome:   6    Cytoband:   Chr:6q12    SOURCE    GENECARD       Gene Ontology:   cellular component:  membrane fraction   cellular component:  nucleus   cellular component:  cytoplasm      BioCarta Pathways:     KEGG Pathways:  
    Probe set : 1374104_at    Name:   claudin 5    GB acc:   BI281680    UniGene:   Rn.17420    Gene Symbol:   Cldn5    Locus Link:   65131    Chromosome:   11    Cytoband:   Chr:11p12    SOURCE    GENECARD       Gene Ontology:   biological process:  cell-cell adhesion   biological process:  myelination   biological process:  calcium-independent cell-cell adhesion      BioCarta Pathways:     KEGG Pathways:  
    Probe set : 1368163_at    Name:   dipeptidylpeptidase 4    GB acc:   J02997    UniGene:   Rn.91364    Gene Symbol:   Dpp4    Locus Link:   25253    Chromosome:   3    Cytoband:   Chr:3q21    SOURCE    GENECARD       Gene Ontology:   biological process:  proteolysis   biological process:  proteolysis   biological process:  immune response      BioCarta Pathways:     KEGG Pathways:  
    Probe set : 1388725_at    Name:   leptin receptor overlapping transcript    GB acc:   AA892288    UniGene:   Rn.144583    Gene Symbol:   Leprot    Locus Link:   56766    Chromosome:   5    Cytoband:   Chr:5q33    SOURCE    GENECARD       Gene Ontology:   cellular component:  integral to membrane   molecular function:  receptor activity      BioCarta Pathways:     KEGG Pathways:  
    Probe set : 1378505_at     GB acc:   AI232255              Gene Ontology:     BioCarta Pathways:     KEGG Pathways:  
    Probe set : 1388465_at    Name:   Transcribed locus    GB acc:   AA891733    UniGene:   Rn.8288        SOURCE        Gene Ontology:     BioCarta Pathways:     KEGG Pathways:  
    Probe set : 1374171_at    Name:   Similar to SRY (sex determining region Y)-box 5 isoform a    GB acc:   AI170507    UniGene:   Rn.54961    Gene Symbol:   Abcc9    Locus Link:   25560    Chromosome:   4    Cytoband:   Chr:4q44    SOURCE    GENECARD       Gene Ontology:     BioCarta Pathways:     KEGG Pathways:  
    Probe set : 1376641_at    Name:   THO complex 1 (predicted)    GB acc:   BM384228    UniGene:   Rn.139327    Gene Symbol:   Thoc1_predicted    Locus Link:   291797    Chromosome:   18    Cytoband:   Chr:18p13    SOURCE    GENECARD       Gene Ontology:   cellular component:  nucleus      BioCarta Pathways:     KEGG Pathways:  
    Probe set : 1375071_at    Name:   nucleoporin 133 (predicted)    GB acc:   BE119719    UniGene:   Rn.91990    Gene Symbol:   Nup133_predicted    Locus Link:   292085    Chromosome:   19    Cytoband:   Chr:19q12    SOURCE    GENECARD       Gene Ontology:   molecular function:  transporter activity   molecular function:  nucleocytoplasmic transporter activity   molecular function:  porin activity      BioCarta Pathways:     KEGG Pathways:  
    Probe set : 1372728_at    Name:   Sortilin 1    GB acc:   BE103745    UniGene:   Rn.11286    Gene Symbol:   Sort1    Locus Link:   83576    Chromosome:   2    Cytoband:   Chr:2q34    SOURCE    GENECARD       Gene Ontology:     BioCarta Pathways:     KEGG Pathways:  
    Probe set : 1387950_at    Name:   Saccharomyces cerevisiae Nip7p homolog    GB acc:   AF158186    UniGene:   Rn.127815    Gene Symbol:   RGD:620069    Locus Link:   192180    Chromosome:   19    Cytoband:   Chr:19q12    SOURCE    GENECARD       Gene Ontology:   biological process:  ribosome biogenesis   cellular component:  nucleus   molecular function:  RNA binding      BioCarta Pathways:     KEGG Pathways:  
    Probe set : 1373549_at    Name:   DEAD (Asp-Glu-Ala-Asp) box polypeptide 10 (predicted)    GB acc:   BE105699    UniGene:   Rn.99481    Gene Symbol:   Ddx10_predicted    Locus Link:   300710    Chromosome:   8    Cytoband:   Chr:8q24    SOURCE    GENECARD       Gene Ontology:     BioCarta Pathways:     KEGG Pathways:  
    Probe set : 1399010_at    Name:   COP9 (constitutive photomorphogenic) homolog, subunit 7a (Arabidopsis thaliana) (predicted)    GB acc:   BG381740    UniGene:   Rn.1921    Gene Symbol:   Cops7a_predicted    Locus Link:   312710    Chromosome:   4    Cytoband:   Chr:4q42    SOURCE    GENECARD       Gene Ontology:   cellular component:  nucleus   cellular component:  signalosome complex      BioCarta Pathways:     KEGG Pathways:  
    Probe set : 1367856_at    Name:   glucose-6-phosphate dehydrogenase    GB acc:   NM_017006    UniGene:   Rn.11040    Gene Symbol:   G6pdx    Locus Link:   24377    Chromosome:   X    Cytoband:   Chr:Xq37    SOURCE    GENECARD       Gene Ontology:     BioCarta Pathways:     KEGG Pathways:  
    Probe set : 1371254_at    Name:   ubiquinol-cytochrome c reductase, Rieske iron-sulfur polypeptide 1    GB acc:   AI179382    UniGene:   Rn.2603    Gene Symbol:   Uqcrfs1    Locus Link:   291103    Chromosome:   17    Cytoband:   Chr:17p12    SOURCE    GENECARD       Gene Ontology:   biological process:  electron transport   biological process:  electron transport   biological process:  transport      BioCarta Pathways:     KEGG Pathways:  
    Probe set : 1368476_at    Name:   nuclear receptor subfamily 3, group C, member 2    GB acc:   NM_013131    UniGene:   Rn.9678    Gene Symbol:   Nr3c2    Locus Link:   25672    Chromosome:   19    Cytoband:   Chr:19q11    SOURCE    GENECARD       Gene Ontology:   cellular component:  nucleus      BioCarta Pathways:     KEGG Pathways:  
    Probe set : 1389551_at    Name:   lactamase, beta 2 (predicted)    GB acc:   AI008160    UniGene:   Rn.1263    Gene Symbol:   Lactb2_predicted    Locus Link:   297768    Chromosome:   5    Cytoband:   Chr:5q11    SOURCE    GENECARD       Gene Ontology:   cellular component:  mitochondrion   molecular function:  hydrolase activity      BioCarta Pathways:     KEGG Pathways:  
    Probe set : 1383058_at    Name:   Transcribed locus    GB acc:   AI410438    UniGene:   Rn.4287        SOURCE        Gene Ontology:     BioCarta Pathways:     KEGG Pathways:  
    Probe set : 1378043_at    Name:   similar to OEF2    GB acc:   AW532890    UniGene:   Rn.13231    Gene Symbol:   LOC500011    Locus Link:   500011    Chromosome:   4    Cytoband:   Chr:4q13    SOURCE    GENECARD       Gene Ontology:     BioCarta Pathways:     KEGG Pathways:  
    Probe set : 1369370_s_at    Name:   transient receptor potential cation channel, subfamily V, member 4 /// transient receptor potential cation channel, subfamily V, member 1    GB acc:   NM_023970    UniGene:   Rn.64508    Gene Symbol:   Trpv4 /// Trpv1    Locus Link:   66026 /// 83810    Chromosome:   12    Cytoband:   Chr:12q16 /// Chr:10q24    SOURCE    GENECARD       Gene Ontology:   biological process:  regulation of transcription, DNA-dependent   biological process:  transport   biological process:  ion transport   biological process:  cation transport   biological process:  response to osmotic stress   biological process:  hypotonic response   biological process:  osmosensory signaling pathway   biological process:  vasopressin secretion   biological process:  hyperosmotic salinity response   biological process:  regulation of response to osmotic stress   biological process:  cell surface receptor linked signal transduction   biological process:  chemosensory behavior   biological process:  calcium ion transport   biological process:  calcium ion homeostasis   biological process:  regulation of cell volume   biological process:  osmosensory signaling pathway   biological process:  response to mechanical stimulus   biological process:  cation transport      BioCarta Pathways:     KEGG Pathways:  
    Probe set : 1398997_at    Name:   phosphohistidine phosphatase 1 (predicted)    GB acc:   AI102252    UniGene:   Rn.74071    Gene Symbol:   Phpt1_predicted    Locus Link:   296571    Chromosome:   3    Cytoband:   Chr:3p13    SOURCE    GENECARD       Gene Ontology:   biological process:  dephosphorylation   cellular component:  cytosol   molecular function:  phosphoprotein phosphatase activity   molecular function:  phosphohistidine phosphatase activity   molecular function:  hydrolase activity      BioCarta Pathways:     KEGG Pathways:  
    Probe set : 1393732_at    Name:   Transcribed locus    GB acc:   AI071024    UniGene:   Rn.149688        SOURCE        Gene Ontology:     BioCarta Pathways:     KEGG Pathways:  
    Probe set : 1390122_at    Name:   Similar to triggering receptor expressed on myeloid cells-like 1    GB acc:   BI275740    UniGene:   Rn.12212     Locus Link:   501096      SOURCE        Gene Ontology:     BioCarta Pathways:     KEGG Pathways:  
    Probe set : 1393728_at    Name:   Transcribed locus    GB acc:   AA964541    UniGene:   Rn.11836        SOURCE        Gene Ontology:     BioCarta Pathways:     KEGG Pathways:  
    Probe set : 1372402_at    Name:   N-acetylneuraminic acid synthase (sialic acid synthase) (predicted)    GB acc:   AW435213    UniGene:   Rn.17006    Gene Symbol:   Nans_predicted    Locus Link:   298071    Chromosome:   5    Cytoband:   Chr:5q22    SOURCE    GENECARD       Gene Ontology:   cellular component:  cytoplasm   cellular component:  cytosol      BioCarta Pathways:     KEGG Pathways:  
    Probe set : 1389001_at    Name:   Transcribed locus, strongly similar to XP_422815.1 PREDICTED: similar to Importin alpha-4 subunit (Karyopherin alpha-4 subunit) (Qip1 protein) [Gallus gallus]    GB acc:   AW433944    UniGene:   Rn.8372        SOURCE        Gene Ontology:     BioCarta Pathways:     KEGG Pathways:  
    Probe set : 1391518_at     GB acc:   AI579823              Gene Ontology:     BioCarta Pathways:     KEGG Pathways:  
    Probe set : 1385400_at    Name:   ATPase, H+ transporting, V1 subunit B, isoform 1 (predicted)    GB acc:   BI288461    UniGene:   Rn.103610    Gene Symbol:   Atp6v1b1_predicted    Locus Link:   312488    Chromosome:   4    Cytoband:   Chr:4q34    SOURCE    GENECARD       Gene Ontology:     BioCarta Pathways:     KEGG Pathways:  
    Probe set : 1393317_at    Name:   similar to THUMP domain containing 3    GB acc:   BI303231    UniGene:   Rn.139868    Gene Symbol:   LOC500288    Locus Link:   500288    Chromosome:   4    Cytoband:   Chr:4q42    SOURCE    GENECARD       Gene Ontology:     BioCarta Pathways:     KEGG Pathways:  
    Probe set : 1376797_at    Name:   cysteine and glycine-rich protein 2 binding protein (predicted)    GB acc:   AA997458    UniGene:   Rn.36418    Gene Symbol:   Csrp2bp_predicted    Locus Link:   362224    Chromosome:   3    Cytoband:   Chr:3q41    SOURCE    GENECARD       Gene Ontology:   cellular component:  nucleus   molecular function:  N-acetyltransferase activity      BioCarta Pathways:     KEGG Pathways:  
    Probe set : 1369122_at    Name:   Bcl2-associated X protein    GB acc:   AF235993    UniGene:   Rn.10668    Gene Symbol:   Bax    Locus Link:   24887    Chromosome:   1    Cytoband:   Chr:1q31.2    SOURCE    GENECARD       Gene Ontology:   biological process:  release of cytochrome c from mitochondria   biological process:  protein insertion into mitochondrial membrane during induction of apoptosis   biological process:  apoptosis   biological process:  apoptosis   biological process:  induction of apoptosis   biological process:  response to DNA damage stimulus   biological process:  inner mitochondrial membrane organization and biogenesis   biological process:  inner mitochondrial membrane organization and biogenesis   biological process:  outer mitochondrial membrane organization and biogenesis   biological process:  outer mitochondrial membrane organization and biogenesis   biological process:  germ cell development   biological process:  nervous system development   biological process:  induction of apoptosis by extracellular signals   biological process:  induction of apoptosis by intracellular signals   biological process:  negative regulation of survival gene product activity   biological process:  caspase activation via cytochrome c   biological process:  caspase activation via cytochrome c   biological process:  apoptotic mitochondrial changes   biological process:  response to wounding   biological process:  nuclear fragmentation   biological process:  regulation of apoptosis   biological process:  regulation of caspase activation   biological process:  cellular respiration   biological process:  cellular respiration   biological process:  negative regulation of progression through cell cycle   biological process:  retinal cell programmed cell death   biological process:  negative regulation of fibroblast proliferation   biological process:  protein homooligomerization   cellular component:  mitochondrial outer membrane   cellular component:  cytosol   cellular component:  integral to membrane   molecular function:  protein binding      BioCarta Pathways:     KEGG Pathways:  
    Probe set : 1370469_at    Name:   protein tyrosine phosphatase, non-receptor type 7    GB acc:   U28356    UniGene:   Rn.10160    Gene Symbol:   Ptpn7    Locus Link:   246781    Chromosome:   13    Cytoband:   Chr:13q13    SOURCE    GENECARD       Gene Ontology:   biological process:  protein amino acid dephosphorylation   biological process:  dephosphorylation   cellular component:  cytosol      BioCarta Pathways:     KEGG Pathways:  
    Probe set : 1395248_at    Name:   similar to mKIAA0212 protein    GB acc:   BF418342    UniGene:   Rn.8162    Gene Symbol:   LOC297504    Locus Link:   297504    Chromosome:   4    Cytoband:   Chr:4q41    SOURCE    GENECARD       Gene Ontology:     BioCarta Pathways:     KEGG Pathways:  
    Probe set : 1389012_at    Name:   NADH dehydrogenase (ubiquinone) 1 beta subcomplex, 2 (predicted)    GB acc:   BM386606    UniGene:   Rn.18013    Gene Symbol:   Ndufb2_predicted    Locus Link:   362344    Chromosome:   4    Cytoband:   Chr:4q22    SOURCE    GENECARD       Gene Ontology:   biological process:  generation of precursor metabolites and energy   biological process:  mitochondrial electron transport, NADH to ubiquinone   cellular component:  membrane fraction   cellular component:  mitochondrion   molecular function:  NADH dehydrogenase activity   molecular function:  NADH dehydrogenase (ubiquinone) activity   molecular function:  oxidoreductase activity      BioCarta Pathways:     KEGG Pathways:  
    Probe set : 1379308_at    Name:   Transcribed locus, strongly similar to NP_031761.1 procollagen, type IV, alpha 4 [Mus musculus]    GB acc:   BF283228    UniGene:   Rn.15257        SOURCE        Gene Ontology:     BioCarta Pathways:     KEGG Pathways:  
    Probe set : 1382679_at    Name:   similar to WD-repeat protein 43    GB acc:   BF396190    UniGene:   Rn.101883    Gene Symbol:   LOC362703    Locus Link:   362703    Chromosome:   6    Cytoband:   Chr:6q13    SOURCE    GENECARD       Gene Ontology:     BioCarta Pathways:     KEGG Pathways:  
    Probe set : 1394954_at     GB acc:   BE101666              Gene Ontology:     BioCarta Pathways:     KEGG Pathways:  
    Probe set : 1383354_a_at    Name:   F-box only protein 33 (predicted)    GB acc:   BF566664    UniGene:   Rn.41874    Gene Symbol:   Fbxo33_predicted    Locus Link:   314157    Chromosome:   6    Cytoband:   Chr:6q23    SOURCE    GENECARD       Gene Ontology:   biological process:  ubiquitin cycle      BioCarta Pathways:     KEGG Pathways:  
    Probe set : 1373144_at    Name:   similar to Ac2-125    GB acc:   BM388977    UniGene:   Rn.145586    Gene Symbol:   LOC499654    Locus Link:   499654    Chromosome:   2    Cytoband:   Chr:2q34    SOURCE    GENECARD       Gene Ontology:   biological process:  isoprenoid biosynthesis      BioCarta Pathways:     KEGG Pathways:  
    Probe set : 1377696_at    Name:   mesoderm development candidate 1 (predicted)    GB acc:   AI407755    UniGene:   Rn.32437    Gene Symbol:   Mesdc1_predicted    Locus Link:   308795    Chromosome:   1    Cytoband:   Chr:1q31    SOURCE    GENECARD       Gene Ontology:     BioCarta Pathways:     KEGG Pathways:  
    Probe set : 1379710_at    Name:   Transcribed locus, weakly similar to NP_081541.1 hypothetical protein LOC69933 [Mus musculus]    GB acc:   BE108372    UniGene:   Rn.18354        SOURCE        Gene Ontology:     BioCarta Pathways:     KEGG Pathways:  
    Probe set : 1379243_at    Name:   NADH dehydrogenase (ubiquinone) 1 alpha subcomplex, 6 (B14) (predicted)    GB acc:   AA819547    UniGene:   Rn.4013    Gene Symbol:   Ndufa6_predicted    Locus Link:   315167    Chromosome:   7    Cytoband:   Chr:7q34    SOURCE    GENECARD       Gene Ontology:   biological process:  electron transport   cellular component:  membrane fraction   cellular component:  mitochondrion   cellular component:  mitochondrial inner membrane      BioCarta Pathways:     KEGG Pathways:  
    Probe set : 1372148_at    Name:   COMM domain containing 2 (predicted)    GB acc:   BI281210    UniGene:   Rn.4168    Gene Symbol:   Commd2_predicted    Locus Link:   295064    Chromosome:   2    Cytoband:   Chr:2q31    SOURCE    GENECARD       Gene Ontology:     BioCarta Pathways:     KEGG Pathways:  
    Probe set : 1389234_at    Name:   von Willebrand factor    GB acc:   BI298314    UniGene:   Rn.35561    Gene Symbol:   RGD:621759    Locus Link:   116669    Chromosome:   4    Cytoband:   Chr:4q42    SOURCE    GENECARD       Gene Ontology:   cellular component:  extracellular matrix (sensu Metazoa)   molecular function:  protein binding      BioCarta Pathways:     KEGG Pathways:  
    Probe set : 1393212_at    Name:   Transcribed locus    GB acc:   AA964027    UniGene:   Rn.153566        SOURCE        Gene Ontology:     BioCarta Pathways:     KEGG Pathways:  
    Probe set : 1389102_at    Name:   similar to Dock1 protein    GB acc:   BG378147    UniGene:   Rn.1482    Gene Symbol:   LOC309081    Locus Link:   309081    Chromosome:   1    Cytoband:   Chr:1q41    SOURCE    GENECARD       Gene Ontology:     BioCarta Pathways:     KEGG Pathways:  
    Probe set : 1393791_at    Name:   Transcribed locus    GB acc:   AI044622    UniGene:   Rn.131204        SOURCE        Gene Ontology:     BioCarta Pathways:     KEGG Pathways:  
    Probe set : 1373924_at    Name:   similar to C530044N13Rik protein (predicted)    GB acc:   AI712686    UniGene:   Rn.79124    Gene Symbol:   RGD1306568_predicted    Locus Link:   302890    Chromosome:   10    Cytoband:   Chr:10q11    SOURCE    GENECARD       Gene Ontology:   molecular function:  hydrolase activity      BioCarta Pathways:     KEGG Pathways:  
    Probe set : 1388985_at    Name:   hypothetical protein LOC310926 /// LOC501211    GB acc:   AI012869     Gene Symbol:   LOC310926 /// LOC501211    Locus Link:   310926 /// 501211    Chromosome:   2    Cytoband:   Chr:2q44     GENECARD       Gene Ontology:     BioCarta Pathways:     KEGG Pathways:  
    Probe set : 1376573_at    Name:   RAB34, member of RAS oncogene family (predicted)    GB acc:   BM391604    UniGene:   Rn.98493    Gene Symbol:   Rab34_predicted    Locus Link:   360571    Chromosome:   10    Cytoband:   Chr:10q25    SOURCE    GENECARD       Gene Ontology:   cellular component:  ruffle   cellular component:  early endosome   cellular component:  Golgi apparatus   molecular function:  GTPase activity   molecular function:  GTP binding   molecular function:  guanyl nucleotide binding      BioCarta Pathways:     KEGG Pathways:  
    Probe set : 1373373_at    Name:   Glutamine fructose-6-phosphate transaminase 1    GB acc:   BI295011    UniGene:   Rn.118522    Gene Symbol:   MGC95214    Locus Link:   297417    Chromosome:   4    Cytoband:   Chr:4q34    SOURCE    GENECARD       Gene Ontology:   cellular component:  cytoplasm   cellular component:  periplasmic space (sensu Gram-negative Bacteria)   molecular function:  glutamine-fructose-6-phosphate transaminase (isomerizing) activity   molecular function:  transporter activity   molecular function:  sugar binding      BioCarta Pathways:     KEGG Pathways:  
    Probe set : 1375932_at    Name:   Phosphoribosyl pyrophosphate synthetase 2    GB acc:   BF284677    UniGene:   Rn.11320    Gene Symbol:   Prps2    Locus Link:   24689    Chromosome:   X    Cytoband:   Chr:Xq21    SOURCE    GENECARD       Gene Ontology:   biological process:  nucleoside metabolism   biological process:  ribonucleoside monophosphate biosynthesis   biological process:  nucleotide biosynthesis      BioCarta Pathways:     KEGG Pathways:  
    Probe set : 1391539_at    Name:   Transcribed locus    GB acc:   BI291849    UniGene:   Rn.49247        SOURCE        Gene Ontology:     BioCarta Pathways:     KEGG Pathways:  
    Probe set : 1391222_at    Name:   similar to Nedd4 binding protein 1 (predicted)    GB acc:   AA965076    UniGene:   Rn.137980    Gene Symbol:   RGD1305179_predicted    Locus Link:   291921    Chromosome:   19    Cytoband:   Chr:19p11    SOURCE    GENECARD       Gene Ontology:     BioCarta Pathways:     KEGG Pathways:  
    Probe set : 1378029_at    Name:   Transcribed locus    GB acc:   AI233769    UniGene:   Rn.6440        SOURCE        Gene Ontology:     BioCarta Pathways:     KEGG Pathways:  
    Probe set : 1398523_a_at    Name:   interferon (alpha and beta) receptor 1 (predicted)    GB acc:   AA900618    UniGene:   Rn.105738    Gene Symbol:   Ifnar1_predicted    Locus Link:   288264    Chromosome:   11    Cytoband:   Chr:11q11    SOURCE    GENECARD       Gene Ontology:   biological process:  cell surface receptor linked signal transduction   biological process:  JAK-STAT cascade   biological process:  response to virus      BioCarta Pathways:     KEGG Pathways:  
    Probe set : 1376291_at    Name:   similar to WD repeat domain 4    GB acc:   BI282331    UniGene:   Rn.116634    Gene Symbol:   LOC499412    Locus Link:   499412    Chromosome:   20    Cytoband:   Chr:20p12    SOURCE    GENECARD       Gene Ontology:     BioCarta Pathways:     KEGG Pathways:  
    Probe set : 1396229_at    Name:   Transcribed locus    GB acc:   BG378004    UniGene:   Rn.147179        SOURCE        Gene Ontology:     BioCarta Pathways:     KEGG Pathways:  
    Probe set : 1372637_at     GB acc:   AI169241              Gene Ontology:     BioCarta Pathways:     KEGG Pathways:  
    Probe set : 1388942_at    Name:   Transcribed locus    GB acc:   BM390129    UniGene:   Rn.16794        SOURCE        Gene Ontology:     BioCarta Pathways:     KEGG Pathways:  
    Probe set : 1384041_at    Name:   Transcribed locus    GB acc:   BG671630    UniGene:   Rn.23265        SOURCE        Gene Ontology:     BioCarta Pathways:     KEGG Pathways:  
    Probe set : 1372997_at    Name:   Transcribed locus    GB acc:   AI105243    UniGene:   Rn.137692        SOURCE        Gene Ontology:     BioCarta Pathways:     KEGG Pathways:  
    Probe set : 1374766_at    Name:   Similar to mKIAA0493 protein    GB acc:   BE103616    UniGene:   Rn.8212     Locus Link:   314997    Chromosome:   7    Cytoband:   Chr:7q33    SOURCE        Gene Ontology:     BioCarta Pathways:     KEGG Pathways:  
    Probe set : 1384228_at    Name:   similar to nuclear factor of activated T-cells, cytoplasmic, calcineurin-dependent 1    GB acc:   AI501087    UniGene:   Rn.8800    Gene Symbol:   LOC307231    Locus Link:   307231    Chromosome:   18    Cytoband:   Chr:18q12.3    SOURCE    GENECARD       Gene Ontology:     BioCarta Pathways:     KEGG Pathways:  
    Probe set : 1388342_at    Name:   Transcribed locus    GB acc:   BI291344    UniGene:   Rn.7937        SOURCE        Gene Ontology:     BioCarta Pathways:     KEGG Pathways:  
    Probe set : 1370978_at    Name:   secretory carrier membrane protein 1    GB acc:   BM387205    UniGene:   Rn.20374    Gene Symbol:   Scamp1    Locus Link:   29521    Chromosome:   2    Cytoband:   Chr:2q12    SOURCE    GENECARD       Gene Ontology:   molecular function:  protein transporter activity   molecular function:  protein domain specific binding   molecular function:  protein domain specific binding      BioCarta Pathways:     KEGG Pathways:  
    Probe set : 1389165_at    Name:   similar to myocardial ischemic preconditioning upregulated protein 2    GB acc:   AI178292    UniGene:   Rn.23078    Gene Symbol:   LOC498301    Locus Link:   498301    Chromosome:   13    Cytoband:   Chr:13q26    SOURCE    GENECARD       Gene Ontology:     BioCarta Pathways:     KEGG Pathways:  
    Probe set : 1369013_a_at    Name:   mitochondrial ribosomal protein L17    GB acc:   AI103095    UniGene:   Rn.40354    Gene Symbol:   Mrpl17    Locus Link:   171061    Chromosome:   1    Cytoband:   Chr:1q33    SOURCE    GENECARD       Gene Ontology:   biological process:  mitochondrial genome maintenance   biological process:  protein biosynthesis   molecular function:  structural constituent of ribosome      BioCarta Pathways:     KEGG Pathways:  
    Probe set : 1394705_at    Name:   Transcribed locus, weakly similar to XP_580018.1 PREDICTED: hypothetical protein XP_580018 [Rattus norvegicus]    GB acc:   BE117266    UniGene:   Rn.69463        SOURCE        Gene Ontology:     BioCarta Pathways:     KEGG Pathways:  
    Probe set : 1384304_at    Name:   LRP16 protein    GB acc:   AW524615    UniGene:   Rn.98178    Gene Symbol:   Lrp16    Locus Link:   246233    Chromosome:   1    Cytoband:   Chr:1q43    SOURCE    GENECARD       Gene Ontology:   cellular component:  mitochondrion      BioCarta Pathways:     KEGG Pathways:  
    Probe set : 1373921_at    Name:   CDNA clone MGC:124810 IMAGE:7932594    GB acc:   AI169007    UniGene:   Rn.104504        SOURCE        Gene Ontology:   biological process:  metabolism   molecular function:  catalytic activity      BioCarta Pathways:     KEGG Pathways:  
    Probe set : 1389459_at    Name:   Transcribed locus    GB acc:   BE116947    UniGene:   Rn.8045        SOURCE        Gene Ontology:     BioCarta Pathways:     KEGG Pathways:  
    Probe set : 1389836_a_at    Name:   Synapsin III    GB acc:   AI599265    UniGene:   Rn.98839    Gene Symbol:   Syn3    Locus Link:   29130    Chromosome:   7    Cytoband:   Chr:7q13    SOURCE    GENECARD       Gene Ontology:   biological process:  neurotransmitter secretion   biological process:  neurotransmitter secretion      BioCarta Pathways:     KEGG Pathways:  
    Probe set : 1392732_at    Name:   nucleolar and spindle associated protein 1 (predicted)    GB acc:   AI711233    UniGene:   Rn.106933    Gene Symbol:   Nusap1_predicted    Locus Link:   311336    Chromosome:   3    Cytoband:   Chr:3q35    SOURCE    GENECARD       Gene Ontology:   cellular component:  nucleus   cellular component:  nucleolus   cellular component:  spindle microtubule   molecular function:  microtubule binding      BioCarta Pathways:     KEGG Pathways:  
    Probe set : 1398869_at    Name:   proteasome (prosome, macropain) 26S subunit, ATPase, 4    GB acc:   BG378826    UniGene:   Rn.11341    Gene Symbol:   Psmc4    Locus Link:   117262    Chromosome:   1    Cytoband:   Chr:1q21    SOURCE    GENECARD       Gene Ontology:   biological process:  proteolysis   biological process:  protein catabolism      BioCarta Pathways:     KEGG Pathways:  
    Probe set : 1397767_at    Name:   Transcribed locus    GB acc:   BG377356    UniGene:   Rn.41665        SOURCE        Gene Ontology:     BioCarta Pathways:     KEGG Pathways:  
    Probe set : 1372778_at    Name:   Solute carrier family 39 (zinc transporter), member 1 (predicted)    GB acc:   AA799420    UniGene:   Rn.43952     Locus Link:   361986    Chromosome:   2    Cytoband:   Chr:2q34    SOURCE        Gene Ontology:   biological process:  transport   biological process:  zinc ion transport   biological process:  metal ion transport   cellular component:  integral to membrane   molecular function:  metal ion transporter activity      BioCarta Pathways:     KEGG Pathways:  
    Probe set : 1377173_at    Name:   Transcribed locus, moderately similar to XP_580018.1 PREDICTED: hypothetical protein XP_580018 [Rattus norvegicus]    GB acc:   BE104535    UniGene:   Rn.43539        SOURCE        Gene Ontology:     BioCarta Pathways:     KEGG Pathways:  
    Probe set : 1370638_at    Name:   ankyrin 3, epithelial    GB acc:   AF069525    UniGene:   Rn.48866    Gene Symbol:   RGD:620157    Locus Link:   361833    Chromosome:   20    Cytoband:   Chr:20p11    SOURCE    GENECARD       Gene Ontology:   biological process:  signal transduction   biological process:  positive regulation of apoptosis   cellular component:  cytoskeleton   cellular component:  synapse      BioCarta Pathways:     KEGG Pathways:  
    Probe set : 1371252_at    Name:   suppressor of cytokine signaling 1    GB acc:   BE100794    UniGene:   Rn.82754    Gene Symbol:   Socs1    Locus Link:   252971    Chromosome:   10    Cytoband:   Chr:10q11    SOURCE    GENECARD       Gene Ontology:   molecular function:  protein binding   molecular function:  kinase inhibitor activity      BioCarta Pathways:     KEGG Pathways:  
    Probe set : 1370414_at    Name:   Rab38, member of RAS oncogene family    GB acc:   M94043    UniGene:   Rn.9824    Gene Symbol:   Rab38    Locus Link:   252916    Chromosome:   1    Cytoband:   Chr:1q32    SOURCE    GENECARD       Gene Ontology:   cellular component:  cytoplasm   cellular component:  vesicular fraction      BioCarta Pathways:     KEGG Pathways:  
    Probe set : 1371468_at    Name:   Transcribed locus, strongly similar to NP_001003955.1 RAB11 family interacting protein 5 (class I) isoform 1 [Mus musculus]    GB acc:   BI296378    UniGene:   Rn.3496        SOURCE        Gene Ontology:     BioCarta Pathways:     KEGG Pathways:  
    Probe set : 1373679_at    Name:   Transcribed locus    GB acc:   AA946467    UniGene:   Rn.35869        SOURCE        Gene Ontology:     BioCarta Pathways:     KEGG Pathways:  
    Probe set : 1392888_at    Name:   similar to glypican 4    GB acc:   AI071251    UniGene:   Rn.19945    Gene Symbol:   RGD:1359399    Locus Link:   317322    Chromosome:   X    Cytoband:   Chr:Xq36    SOURCE    GENECARD       Gene Ontology:   cellular component:  extracellular matrix (sensu Metazoa)      BioCarta Pathways:     KEGG Pathways:  
    Probe set : 1373782_a_at    Name:   similar to LOC495800 protein    GB acc:   AW253957    UniGene:   Rn.60976    Gene Symbol:   LOC499770    Locus Link:   499770    Chromosome:   3    Cytoband:   Chr:3p12    SOURCE    GENECARD       Gene Ontology:     BioCarta Pathways:     KEGG Pathways:  
    Probe set : 1374632_at    Name:   phosphatidylserine receptor (predicted)    GB acc:   AI232697    UniGene:   Rn.19923    Gene Symbol:   Ptdsr_predicted    Locus Link:   360665    Chromosome:   10    Cytoband:   Chr:10q32.3    SOURCE    GENECARD       Gene Ontology:   cellular component:  plasma membrane   cellular component:  nucleus   cellular component:  nucleus   molecular function:  receptor activity      BioCarta Pathways:     KEGG Pathways:  
    Probe set : 1372403_at    Name:   similar to Nuclear membrane binding protein NUCLING    GB acc:   BI276339    UniGene:   Rn.120531    Gene Symbol:   LOC315732    Locus Link:   315732    Chromosome:   8    Cytoband:   Chr:8q24    SOURCE    GENECARD       Gene Ontology:     BioCarta Pathways:     KEGG Pathways:  
    Probe set : 1388244_s_at    Name:   laminin receptor 1 (67kD, ribosomal protein SA)    GB acc:   BG153272    UniGene:   Rn.999    Gene Symbol:   Lamr1    Locus Link:   29236    Chromosome:   8    Cytoband:   Chr:8q32    SOURCE    GENECARD       Gene Ontology:   biological process:  protein biosynthesis      BioCarta Pathways:     KEGG Pathways:  
    Probe set : 1382139_at    Name:   similar to hypothetical protein FLJ12994 (predicted)    GB acc:   BE108950    UniGene:   Rn.58758    Gene Symbol:   RGD1308795_predicted    Locus Link:   315804    Chromosome:   8    Cytoband:   Chr:8q24    SOURCE    GENECARD       Gene Ontology:     BioCarta Pathways:     KEGG Pathways:  
    Probe set : 1386928_at    Name:   branched chain aminotransferase 2, mitochondrial    GB acc:   NM_022400    UniGene:   Rn.981    Gene Symbol:   Bcat2    Locus Link:   64203    Chromosome:   1    Cytoband:   Chr:1q22    SOURCE    GENECARD       Gene Ontology:   cellular component:  extracellular space   cellular component:  mitochondrion   cellular component:  mitochondrion      BioCarta Pathways:     KEGG Pathways:  
    Probe set : 1377058_at    Name:   similar to solute carrier family 37 (glycerol-3-phosphate transporter), member 2    GB acc:   BI291872    UniGene:   Rn.41938    Gene Symbol:   LOC500973    Locus Link:   500973    Chromosome:   8    Cytoband:   Chr:8q21    SOURCE    GENECARD       Gene Ontology:     BioCarta Pathways:     KEGG Pathways:  
    Probe set : 1376809_at    Name:   similar to RIKEN cDNA 2410025L10    GB acc:   BI288660    UniGene:   Rn.24267    Gene Symbol:   LOC304572    Locus Link:   304572    Chromosome:   12    Cytoband:   Chr:12q16    SOURCE    GENECARD       Gene Ontology:     BioCarta Pathways:     KEGG Pathways:  
    Probe set : 1385086_at    Name:   budding uninhibited by benzimidazoles 1 homolog (S. cerevisiae) (predicted)    GB acc:   BF388785    UniGene:   Rn.56816    Gene Symbol:   Bub1_predicted    Locus Link:   296137    Chromosome:   3    Cytoband:   Chr:3q36    SOURCE    GENECARD       Gene Ontology:     BioCarta Pathways:     KEGG Pathways:  
    Probe set : 1370264_at    Name:   CPG2 protein    GB acc:   BI288556    UniGene:   Rn.89150    Gene Symbol:   RGD:621597    Locus Link:   499010    Chromosome:   1    Cytoband:   Chr:1p11    SOURCE    GENECARD       Gene Ontology:     BioCarta Pathways:     KEGG Pathways:  
    Probe set : 1369493_at    Name:   prolactin receptor    GB acc:   NM_012630    UniGene:   Rn.9757    Gene Symbol:   Prlr    Locus Link:   24684    Chromosome:   2    Cytoband:   Chr:2q16    SOURCE    GENECARD       Gene Ontology:   biological process:  steroid biosynthesis   biological process:  anti-apoptosis   biological process:  cell surface receptor linked signal transduction   biological process:  transmembrane receptor protein tyrosine kinase activation (dimerization)   biological process:  embryo implantation   biological process:  lactation   biological process:  regulation of cell adhesion   biological process:  regulation of epithelial cell differentiation   biological process:  T cell activation   biological process:  tyrosine phosphorylation of JAK2 protein   biological process:  steroid biosynthesis   biological process:  anti-apoptosis   biological process:  cell surface receptor linked signal transduction   biological process:  transmembrane receptor protein tyrosine kinase activation (dimerization)   biological process:  lactation   biological process:  T cell activation   biological process:  tyrosine phosphorylation of JAK2 protein      BioCarta Pathways:     KEGG Pathways:  
    Probe set : 1370865_at    Name:   isocitrate dehydrogenase 3, gamma    GB acc:   BI277627    UniGene:   Rn.2837    Gene Symbol:   Idh3g    Locus Link:   25179    Chromosome:   X    Cytoband:   Chr:Xq37.1-q37.2    SOURCE    GENECARD       Gene Ontology:   biological process:  carbohydrate metabolism   biological process:  tricarboxylic acid cycle   biological process:  metabolism   cellular component:  mitochondrion   cellular component:  isocitrate dehydrogenase complex (NAD+) (sensu Eukaryota)      BioCarta Pathways:     KEGG Pathways:  
    Probe set : 1367456_at    Name:   ubiquitin-conjugating enzyme E2D 3 (UBC4/5 homolog, yeast)    GB acc:   NM_031237    UniGene:   Rn.2778    Gene Symbol:   Ube2d3    Locus Link:   81920    Chromosome:   2    Cytoband:   Chr:2q43    SOURCE    GENECARD       Gene Ontology:     BioCarta Pathways:     KEGG Pathways:  
    Probe set : 1390268_at    Name:   RAB35, member RAS oncogene family (predicted) /// similar to RAB35, member RAS oncogene family    GB acc:   AI013484    UniGene:   Rn.18008    Gene Symbol:   Rab35_predicted /// LOC498191    Locus Link:   288700 /// 498191    Chromosome:   12    Cytoband:   Chr:12q16    SOURCE    GENECARD       Gene Ontology:     BioCarta Pathways:     KEGG Pathways:  
    Probe set : 1379525_at    Name:   similar to chromosome 20 open reading frame 155 (predicted)    GB acc:   BF559514    UniGene:   Rn.35982    Gene Symbol:   RGD1311037_predicted    Locus Link:   366196    Chromosome:   3    Cytoband:   Chr:3q36    SOURCE    GENECARD       Gene Ontology:   biological process:  phospholipid biosynthesis   cellular component:  integral to membrane   molecular function:  transferase activity      BioCarta Pathways:     KEGG Pathways:  
    Probe set : 1383542_at    Name:   RAS guanyl releasing protein 1    GB acc:   BI282819    UniGene:   Rn.32156    Gene Symbol:   Rasgrp1    Locus Link:   29434      SOURCE    GENECARD       Gene Ontology:   biological process:  intracellular signaling cascade   biological process:  small GTPase mediated signal transduction   biological process:  Ras protein signal transduction   cellular component:  membrane fraction      BioCarta Pathways:     KEGG Pathways:  
    Probe set : 1369954_at    Name:   isocitrate dehydrogenase 1 (NADP+), soluble    GB acc:   NM_031510    UniGene:   Rn.3561    Gene Symbol:   Idh1    Locus Link:   24479    Chromosome:   9    Cytoband:   Chr:9q32    SOURCE    GENECARD       Gene Ontology:   cellular component:  peroxisome   cellular component:  cytosol   molecular function:  isocitrate dehydrogenase (NADP+) activity   molecular function:  isocitrate dehydrogenase (NADP+) activity   molecular function:  oxidoreductase activity      BioCarta Pathways:     KEGG Pathways:  
    Probe set : 1370851_a_at    Name:   kalirin, RhoGEF kinase    GB acc:   AI639313    UniGene:   Rn.87882    Gene Symbol:   Kalrn    Locus Link:   84009    Chromosome:   11    Cytoband:   Chr:11q22    SOURCE    GENECARD       Gene Ontology:   cellular component:  neuron projection   cellular component:  cell soma   cellular component:  perinuclear region      BioCarta Pathways:     KEGG Pathways:  
    Probe set : 1369654_at    Name:   protein kinase, AMP-activated, alpha 2 catalytic subunit    GB acc:   NM_023991    UniGene:   Rn.64583    Gene Symbol:   Prkaa2    Locus Link:   78975    Chromosome:   5    Cytoband:   Chr:5q34    SOURCE    GENECARD       Gene Ontology:   cellular component:  nucleus   cellular component:  nucleus      BioCarta Pathways:     KEGG Pathways:  
    Probe set : 1372569_at    Name:   four and a half LIM domains 3 (predicted)    GB acc:   AI171676    UniGene:   Rn.100732    Gene Symbol:   Fhl3_predicted    Locus Link:   313582    Chromosome:   5    Cytoband:   Chr:5q36    SOURCE    GENECARD       Gene Ontology:   biological process:  muscle development   biological process:  actin cytoskeleton organization and biogenesis   cellular component:  stress fiber   cellular component:  nucleus   cellular component:  Z disc   molecular function:  actin binding   molecular function:  zinc ion binding      BioCarta Pathways:     KEGG Pathways:  
    Probe set : 1376065_at    Name:   RRS1 ribosome biogenesis regulator homolog (S. cerevisiae) (predicted)    GB acc:   AI058889    UniGene:   Rn.15120     Locus Link:   297784    Chromosome:   5    Cytoband:   Chr:5q11    SOURCE        Gene Ontology:   biological process:  ribosome biogenesis   biological process:  ribosome biogenesis and assembly   cellular component:  nucleus      BioCarta Pathways:     KEGG Pathways:  
    Probe set : 1371435_at    Name:   nascent-polypeptide-associated complex alpha polypeptide (predicted)    GB acc:   BI279561    UniGene:   Rn.119939    Gene Symbol:   Naca_predicted    Locus Link:   288770    Chromosome:   7    Cytoband:   Chr:7q11    SOURCE    GENECARD       Gene Ontology:   biological process:  protein biosynthesis   cellular component:  nascent polypeptide-associated complex      BioCarta Pathways:     KEGG Pathways:  
    Probe set : 1372518_at    Name:   fibulin 1 (predicted)    GB acc:   AI176918    UniGene:   Rn.9375    Gene Symbol:   Fbln1_predicted    Locus Link:   315191    Chromosome:   7    Cytoband:   Chr:7q34    SOURCE    GENECARD       Gene Ontology:   biological process:  chitin metabolism   molecular function:  extracellular matrix structural constituent   molecular function:  calcium ion binding   molecular function:  chitin binding      BioCarta Pathways:     KEGG Pathways:  
    Probe set : 1375867_at    Name:   zinc finger and BTB domain containing 4 (predicted)    GB acc:   AW524493    UniGene:   Rn.15836    Gene Symbol:   Zbtb4_predicted    Locus Link:   287441    Chromosome:   10    Cytoband:   Chr:10q24    SOURCE    GENECARD       Gene Ontology:     BioCarta Pathways:     KEGG Pathways:  
    Probe set : 1371479_at    Name:   LOC500939    GB acc:   AA946026    UniGene:   Rn.3341    Gene Symbol:   LOC500939    Locus Link:   500939      SOURCE    GENECARD       Gene Ontology:     BioCarta Pathways:     KEGG Pathways:  
    Probe set : 1382663_at    Name:   basic leucine zipper transcription factor, ATF-like (predicted)    GB acc:   AA819819    UniGene:   Rn.16670    Gene Symbol:   Batf_predicted    Locus Link:   299206    Chromosome:   6    Cytoband:   Chr:6q31    SOURCE    GENECARD       Gene Ontology:   biological process:  regulation of transcription, DNA-dependent   biological process:  antimicrobial humoral response (sensu Vertebrata)   cellular component:  nucleus      BioCarta Pathways:     KEGG Pathways:  
    Probe set : 1392464_at     GB acc:   BM392372              Gene Ontology:     BioCarta Pathways:     KEGG Pathways:  
    Probe set : 1382396_at    Name:   Similar to D7Wsu128e protein (predicted)    GB acc:   BE101337    UniGene:   Rn.144798     Locus Link:   293454    Chromosome:   1    Cytoband:   Chr:1q36    SOURCE        Gene Ontology:   biological process:  protein modification      BioCarta Pathways:     KEGG Pathways:  
    Probe set : 1390674_at    Name:   Transcribed locus    GB acc:   BE097195    UniGene:   Rn.14656        SOURCE        Gene Ontology:     BioCarta Pathways:     KEGG Pathways:  
    Probe set : 1379278_at    Name:   similar to hypothetical protein FLJ10871 (predicted)    GB acc:   BE103919    UniGene:   Rn.16509    Gene Symbol:   RGD1311539_predicted    Locus Link:   290322    Chromosome:   15    Cytoband:   Chr:15p12    SOURCE    GENECARD       Gene Ontology:     BioCarta Pathways:     KEGG Pathways:  
    Probe set : 1374182_at    Name:   SWI/SNF related, matrix associated, actin dependent regulator of chromatin, subfamily c, member 1 (predicted)    GB acc:   AA965063    UniGene:   Rn.94211    Gene Symbol:   Smarcc1_predicted    Locus Link:   301020    Chromosome:   8    Cytoband:   Chr:8q32    SOURCE    GENECARD       Gene Ontology:   molecular function:  DNA binding   molecular function:  chromatin binding   molecular function:  transcription coactivator activity      BioCarta Pathways:     KEGG Pathways:  
    Probe set : 1373952_at    Name:   protein kinase, AMP-activated, gamma 2 non-catalytic subunit    GB acc:   AI409841    UniGene:   Rn.51208    Gene Symbol:   Prkag2    Locus Link:   373545    Chromosome:   4    Cytoband:   Chr:4q11    SOURCE    GENECARD       Gene Ontology:   molecular function:  kinase activity      BioCarta Pathways:     KEGG Pathways:  
    Probe set : 1388926_at    Name:   Ectonucleotide pyrophosphatase/phosphodiesterase 5    GB acc:   AI409048    UniGene:   Rn.17107    Gene Symbol:   Enpp5    Locus Link:   316249    Chromosome:   9    Cytoband:   Chr:9q12    SOURCE    GENECARD       Gene Ontology:   biological process:  nucleotide metabolism   molecular function:  hydrolase activity      BioCarta Pathways:     KEGG Pathways:  
    Probe set : 1374791_at    Name:   Similar to sorting nexin 21 isoform a; sorting nexin L; sorting nexin 21 (predicted)    GB acc:   BF282933    UniGene:   Rn.133773     Locus Link:   311629    Chromosome:   3    Cytoband:   Chr:3q42    SOURCE        Gene Ontology:     BioCarta Pathways:     KEGG Pathways:  
    Probe set : 1397328_at    Name:   Transcribed locus    GB acc:   AI555961    UniGene:   Rn.39181        SOURCE        Gene Ontology:     BioCarta Pathways:     KEGG Pathways:  
    Probe set : 1370899_at    Name:   NonO/p54nrb homolog    GB acc:   AI599699    UniGene:   Rn.54645    Gene Symbol:   Sfpq    Locus Link:   252855    Chromosome:   5    Cytoband:   Chr:5q36    SOURCE    GENECARD       Gene Ontology:   molecular function:  nucleic acid binding      BioCarta Pathways:     KEGG Pathways:  
    Probe set : 1374550_at    Name:   similar to map kinase interacting kinase    GB acc:   BI277482    UniGene:   Rn.7910    Gene Symbol:   MGC112775    Locus Link:   500526    Chromosome:   5    Cytoband:   Chr:5q36    SOURCE    GENECARD       Gene Ontology:   biological process:  protein amino acid phosphorylation   molecular function:  protein kinase activity   molecular function:  protein serine/threonine kinase activity   molecular function:  ATP binding      BioCarta Pathways:     KEGG Pathways:  
    Probe set : 1369105_a_at    Name:   protein kinase (cAMP dependent, catalytic) inhibitor beta    GB acc:   AF413572    UniGene:   Rn.9748    Gene Symbol:   Pkib    Locus Link:   24678    Chromosome:   20    Cytoband:   Chr:20q12-q13    SOURCE    GENECARD       Gene Ontology:   biological process:  negative regulation of protein kinase activity   molecular function:  protein kinase inhibitor activity   molecular function:  cAMP-dependent protein kinase inhibitor activity   molecular function:  kinase activity      BioCarta Pathways:     KEGG Pathways:  
    Probe set : 1370207_at    Name:   ATP synthase, H+ transporting, mitochondrial F0 complex, subunit c (subunit 9), isoform 2 /// similar to ATP synthase, H+ transporting, mitochondrial F0 complex, subunit c (subunit 9), isoform 2    GB acc:   AI170772    UniGene:   Rn.29258    Gene Symbol:   Atp5g2 /// LOC499512    Locus Link:   171082 /// 499512    Chromosome:   7    Cytoband:   Chr:7q36 /// Chr:2q12    SOURCE    GENECARD       Gene Ontology:     BioCarta Pathways:     KEGG Pathways:  
    Probe set : 1398759_at    Name:   transforming growth factor beta 1 induced transcript 4    GB acc:   NM_013043    UniGene:   Rn.3545    Gene Symbol:   Tgfb1i4    Locus Link:   25564    Chromosome:   15    Cytoband:   Chr:15q11    SOURCE    GENECARD       Gene Ontology:   biological process:  regulation of transcription, DNA-dependent   biological process:  transcription from RNA polymerase II promoter   biological process:  transcription   cellular component:  nucleus   molecular function:  transcription factor activity      BioCarta Pathways:     KEGG Pathways:  
    Probe set : 1390434_at    Name:   TNFRSF1A-associated via death domain    GB acc:   BM386846    UniGene:   Rn.18545    Gene Symbol:   Tradd    Locus Link:   246756    Chromosome:   19    Cytoband:   Chr:19q11    SOURCE    GENECARD       Gene Ontology:   biological process:  apoptosis   molecular function:  death receptor binding   molecular function:  receptor activity      BioCarta Pathways:     KEGG Pathways:  
    Probe set : 1389123_at    Name:   chemokine (C-C motif) ligand 6    GB acc:   BE095824    UniGene:   Rn.7857    Gene Symbol:   RGD:1303200    Locus Link:   287910    Chromosome:   10    Cytoband:   Chr:10q26    SOURCE    GENECARD       Gene Ontology:   biological process:  chemotaxis   biological process:  immune response   biological process:  sensory perception   cellular component:  extracellular region   cellular component:  extracellular space   molecular function:  cytokine activity   molecular function:  chemokine activity      BioCarta Pathways:     KEGG Pathways:  
    Probe set : 1368588_at    Name:   ATP-dependent, RNA helicase    GB acc:   NM_053525    UniGene:   Rn.18605    Gene Symbol:   Ddx52    Locus Link:   85432    Chromosome:   10    Cytoband:   Chr:10q26    SOURCE    GENECARD       Gene Ontology:   biological process:  rRNA processing   biological process:  rRNA processing   cellular component:  nucleus      BioCarta Pathways:     KEGG Pathways:  
    Probe set : 1388801_at    Name:   Similar to RIKEN cDNA 9030221M09 gene (predicted)    GB acc:   AA945103    UniGene:   Rn.49382     Locus Link:   305466    Chromosome:   14    Cytoband:   Chr:14q21    SOURCE        Gene Ontology:     BioCarta Pathways:     KEGG Pathways:  
    Probe set : 1374420_at    Name:   similar to RIKEN cDNA 2310001A20 (predicted)    GB acc:   BE117936    UniGene:   Rn.2352    Gene Symbol:   RGD1308874_predicted    Locus Link:   366227    Chromosome:   3    Cytoband:   Chr:3q41    SOURCE    GENECARD       Gene Ontology:   biological process:  biosynthesis   cellular component:  integral to membrane   molecular function:  strictosidine synthase activity      BioCarta Pathways:     KEGG Pathways:  
    Probe set : 1388378_at    Name:   eukaryotic translation initiation factor 3, subunit 8, 110kDa (predicted)    GB acc:   AI412236    UniGene:   Rn.4207    Gene Symbol:   Eif3s8_predicted    Locus Link:   293484    Chromosome:   1    Cytoband:   Chr:1q36    SOURCE    GENECARD       Gene Ontology:   biological process:  protein biosynthesis   biological process:  regulation of translational initiation   biological process:  translational initiation   cellular component:  eukaryotic translation initiation factor 3 complex   molecular function:  translation initiation factor activity      BioCarta Pathways:     KEGG Pathways:  
    Probe set : 1372764_at    Name:   similar to density regulated protein drp1    GB acc:   BI276261    UniGene:   Rn.101864    Gene Symbol:   LOC498180    Locus Link:   498180    Chromosome:   12    Cytoband:   Chr:12q15    SOURCE    GENECARD       Gene Ontology:     BioCarta Pathways:     KEGG Pathways:  
    Probe set : 1369409_at    Name:   Ngfi-A binding protein 1    GB acc:   NM_022856    UniGene:   Rn.10099    Gene Symbol:   Nab1    Locus Link:   64824    Chromosome:   9    Cytoband:   Chr:9q22    SOURCE    GENECARD       Gene Ontology:   biological process:  regulation of transcription, DNA-dependent   biological process:  negative regulation of transcription   biological process:  transcription   cellular component:  nucleus   molecular function:  nucleic acid binding   molecular function:  transcriptional repressor activity      BioCarta Pathways:     KEGG Pathways:  
    Probe set : 1376691_at    Name:   Transcribed locus    GB acc:   AI103213    UniGene:   Rn.8434        SOURCE        Gene Ontology:     BioCarta Pathways:     KEGG Pathways:  
    Probe set : 1373378_at    Name:   ATP/GTP binding protein 1 (predicted)    GB acc:   BF410522    UniGene:   Rn.33035    Gene Symbol:   Agtpbp1_predicted    Locus Link:   290986    Chromosome:   17    Cytoband:   Chr:17p14    SOURCE    GENECARD       Gene Ontology:   biological process:  proteolysis   cellular component:  nucleus   cellular component:  cytoplasm   molecular function:  carboxypeptidase A activity      BioCarta Pathways:     KEGG Pathways:  
    Probe set : 1397852_at    Name:   similar to SPT3-associated factor 42 (predicted)    GB acc:   H32108    UniGene:   Rn.23978    Gene Symbol:   RGD1306774_predicted    Locus Link:   360874    Chromosome:   13    Cytoband:   Chr:13q23    SOURCE    GENECARD       Gene Ontology:     BioCarta Pathways:     KEGG Pathways:  
    Probe set : 1383514_s_at    Name:   NMDA receptor-regulated gene 1 (predicted)    GB acc:   BG671648    UniGene:   Rn.12587    Gene Symbol:   Narg1_predicted    Locus Link:   310399    Chromosome:   2    Cytoband:   Chr:2q26    SOURCE    GENECARD       Gene Ontology:   biological process:  angiogenesis   biological process:  transcription   biological process:  regulation of transcription, DNA-dependent   cellular component:  nucleus   cellular component:  cytoplasm   molecular function:  receptor activity   molecular function:  protein binding   molecular function:  N-acetyltransferase activity      BioCarta Pathways:     KEGG Pathways:  
    Probe set : 1392563_at    Name:   Rho GTPase activating protein 8 (predicted)    GB acc:   AW252517    UniGene:   Rn.30314    Gene Symbol:   Arhgap8_predicted    Locus Link:   315189    Chromosome:   7    Cytoband:   Chr:7q34    SOURCE    GENECARD       Gene Ontology:   biological process:  actin cytoskeleton organization and biogenesis   biological process:  positive regulation of cell migration   molecular function:  GTPase activator activity      BioCarta Pathways:     KEGG Pathways:  
    Probe set : 1388327_at    Name:   similar to DNA segment, Chr 10, ERATO Doi 214, expressed (predicted)    GB acc:   AI232357    UniGene:   Rn.1164    Gene Symbol:   RGD1309529_predicted    Locus Link:   294362    Chromosome:   20    Cytoband:   Chr:20p11    SOURCE    GENECARD       Gene Ontology:   cellular component:  extracellular space   cellular component:  mitochondrion      BioCarta Pathways:     KEGG Pathways:  
    Probe set : 1377878_at    Name:   Transcribed locus    GB acc:   AA800782    UniGene:   Rn.146137        SOURCE        Gene Ontology:     BioCarta Pathways:     KEGG Pathways:  
    Probe set : 1390406_at     GB acc:   AI232065              Gene Ontology:     BioCarta Pathways:     KEGG Pathways:  
    Probe set : 1371549_at    Name:   WD repeat domain 23 (predicted)    GB acc:   AI228630    UniGene:   Rn.16542    Gene Symbol:   Wdr23_predicted    Locus Link:   305895    Chromosome:   15    Cytoband:   Chr:15p13    SOURCE    GENECARD       Gene Ontology:     BioCarta Pathways:     KEGG Pathways:  
    Probe set : 1370499_at    Name:   killer cell lectin-like receptor subfamily B member 1A    GB acc:   M62891    UniGene:   Rn.101826    Gene Symbol:   Klrb1a    Locus Link:   362443    Chromosome:   4    Cytoband:   Chr:4q42    SOURCE    GENECARD       Gene Ontology:   cellular component:  integral to membrane   molecular function:  natural killer cell receptor activity   molecular function:  sugar binding      BioCarta Pathways:     KEGG Pathways:  
    Probe set : 1373703_at    Name:   Transcribed locus    GB acc:   BE113247    UniGene:   Rn.13320        SOURCE        Gene Ontology:     BioCarta Pathways:     KEGG Pathways:  
    Probe set : 1393060_at    Name:   similar to KIAA0605 gene product (predicted)    GB acc:   AI059694    UniGene:   Rn.19250    Gene Symbol:   RGD1305459_predicted    Locus Link:   311827    Chromosome:   3    Cytoband:   Chr:3p12    SOURCE    GENECARD       Gene Ontology:     BioCarta Pathways:     KEGG Pathways:  
    Probe set : 1384449_at     GB acc:   AA964588              Gene Ontology:     BioCarta Pathways:     KEGG Pathways:  
    Probe set : 1375516_at    Name:   NADH dehydrogenase (ubiquinone) 1, subcomplex unknown, 2 (predicted)    GB acc:   AA891171    UniGene:   Rn.62471    Gene Symbol:   Ndufc2_predicted    Locus Link:   293130    Chromosome:   1    Cytoband:   Chr:1q32    SOURCE    GENECARD       Gene Ontology:   biological process:  mitochondrial electron transport, NADH to ubiquinone   cellular component:  membrane fraction   cellular component:  mitochondrion   cellular component:  mitochondrial inner membrane   molecular function:  NADH dehydrogenase activity   molecular function:  NADH dehydrogenase (ubiquinone) activity   molecular function:  oxidoreductase activity      BioCarta Pathways:     KEGG Pathways:  
    Probe set : 1388150_at    Name:   exportin 1, CRM1 homolog (yeast)    GB acc:   AW533924    UniGene:   Rn.34087    Gene Symbol:   Xpo1    Locus Link:   85252    Chromosome:   14    Cytoband:   Chr:14q22    SOURCE    GENECARD       Gene Ontology:   cellular component:  nuclear pore   cellular component:  nucleoplasm   cellular component:  cytoplasm   cellular component:  nucleus   molecular function:  binding   molecular function:  protein binding   molecular function:  protein transporter activity   molecular function:  RNA binding      BioCarta Pathways:     KEGG Pathways:  
    Probe set : 1368390_at    Name:   v-raf oncogene homolog 1 (murine sarcoma 3611 virus)    GB acc:   NM_022532    UniGene:   Rn.1714    Gene Symbol:   Araf1    Locus Link:   64363    Chromosome:   X    Cytoband:   Chr:Xq11.1    SOURCE    GENECARD       Gene Ontology:   cellular component:  intracellular   cellular component:  insoluble fraction   cellular component:  mitochondrion   cellular component:  cytosol      BioCarta Pathways:     KEGG Pathways:  
    Probe set : 1394407_at    Name:   Transcribed locus    GB acc:   AW530287    UniGene:   Rn.28162        SOURCE        Gene Ontology:     BioCarta Pathways:     KEGG Pathways:  
    Probe set : 1376277_at    Name:   Transcribed locus    GB acc:   AI412413    UniGene:   Rn.34120        SOURCE        Gene Ontology:     BioCarta Pathways:     KEGG Pathways:  
    Probe set : 1387010_s_at    Name:   sodium channel, voltage-gated, type I, beta polypeptide    GB acc:   NM_017288    UniGene:   Rn.4958    Gene Symbol:   Scn1b    Locus Link:   29686    Chromosome:   1    Cytoband:   Chr:1q21    SOURCE    GENECARD       Gene Ontology:   biological process:  ion transport   biological process:  transport   biological process:  sodium ion transport   cellular component:  extracellular space   cellular component:  integral to membrane   molecular function:  ion channel activity   molecular function:  voltage-gated ion channel activity   molecular function:  sodium ion binding      BioCarta Pathways:     KEGG Pathways:  
    Probe set : 1393405_at    Name:   Transcribed locus    GB acc:   AA925444    UniGene:   Rn.8224        SOURCE        Gene Ontology:     BioCarta Pathways:     KEGG Pathways:  
    Probe set : 1399085_at    Name:   Zinc metalloproteinase, STE24 homolog (S. cerevisiae) (predicted)    GB acc:   BM384770    UniGene:   Rn.25307     Locus Link:   313564    Chromosome:   5    Cytoband:   Chr:5q36    SOURCE        Gene Ontology:   biological process:  proteolysis   biological process:  prenylated protein catabolism      BioCarta Pathways:     KEGG Pathways:  
    Probe set : 1375315_at    Name:   similar to hypothetical protein BC013035    GB acc:   BE098345    UniGene:   Rn.23248    Gene Symbol:   LOC361172    Locus Link:   361172    Chromosome:   16    Cytoband:   Chr:16q12.5    SOURCE    GENECARD       Gene Ontology:     BioCarta Pathways:     KEGG Pathways:  
    Probe set : 1390728_at     GB acc:   AI501517              Gene Ontology:     BioCarta Pathways:     KEGG Pathways:  
    Probe set : 1398844_at    Name:   thioredoxin 2    GB acc:   AA800180    UniGene:   Rn.55043    Gene Symbol:   Txn2    Locus Link:   79462    Chromosome:   7    Cytoband:   Chr:7q34    SOURCE    GENECARD       Gene Ontology:   biological process:  electron transport   biological process:  transport   cellular component:  mitochondrion      BioCarta Pathways:     KEGG Pathways:  
    Probe set : 1383194_a_at    Name:   CDNA clone IMAGE:7379585    GB acc:   AI030349    UniGene:   Rn.6374        SOURCE        Gene Ontology:     BioCarta Pathways:     KEGG Pathways:  
    Probe set : 1370419_a_at    Name:   SH3-domain kinase binding protein 1    GB acc:   AF230520    UniGene:   Rn.107226    Gene Symbol:   Sh3kbp1    Locus Link:   84357    Chromosome:   X    Cytoband:   Chr:Xq21    SOURCE    GENECARD       Gene Ontology:   biological process:  regulation of apoptosis   cellular component:  nucleus   cellular component:  kinesin complex   cellular component:  membrane   molecular function:  protein binding   molecular function:  protein binding   molecular function:  protein binding      BioCarta Pathways:     KEGG Pathways:  
    Probe set : 1374804_at    Name:   similar to Autoantigen NGP-1 (predicted)    GB acc:   AI060050    UniGene:   Rn.18964    Gene Symbol:   RGD1305006_predicted    Locus Link:   362593    Chromosome:   5    Cytoband:   Chr:5q36    SOURCE    GENECARD       Gene Ontology:   biological process:  ribosome biogenesis   cellular component:  nucleus   molecular function:  GTP binding      BioCarta Pathways:     KEGG Pathways:  
    Probe set : 1382131_at    Name:   Transcribed locus    GB acc:   AW523674    UniGene:   Rn.124214        SOURCE        Gene Ontology:     BioCarta Pathways:     KEGG Pathways:  
    Probe set : 1374738_at    Name:   serologically defined colon cancer antigen 10 (predicted)    GB acc:   BF416387    UniGene:   Rn.153577    Gene Symbol:   Sdccag10_predicted    Locus Link:   361887    Chromosome:   2    Cytoband:   Chr:2q13    SOURCE    GENECARD       Gene Ontology:   biological process:  protein folding   cellular component:  nucleus   cellular component:  cytoplasm   molecular function:  peptidyl-prolyl cis-trans isomerase activity   molecular function:  isomerase activity      BioCarta Pathways:     KEGG Pathways:  
    Probe set : 1387361_s_at    Name:   phosphoglycerate kinase 1    GB acc:   NM_053291    UniGene:   Rn.108127    Gene Symbol:   Pgk1    Locus Link:   24644    Chromosome:   X    Cytoband:   Chr:Xq31    SOURCE    GENECARD       Gene Ontology:   biological process:  glycolysis   biological process:  glycolysis   biological process:  phosphorylation   cellular component:  phosphorylase kinase complex      BioCarta Pathways:     KEGG Pathways:  
    Probe set : 1389288_at    Name:   NADH dehydrogenase (ubiquinone) 1 alpha subcomplex, 2 (predicted)    GB acc:   BI279838    UniGene:   Rn.19077    Gene Symbol:   Ndufa2_predicted    Locus Link:   291660    Chromosome:   18    Cytoband:   Chr:18p11    SOURCE    GENECARD       Gene Ontology:   biological process:  generation of precursor metabolites and energy   cellular component:  membrane fraction   cellular component:  mitochondrion   cellular component:  respiratory chain complex I (sensu Eukaryota)   molecular function:  NADH dehydrogenase activity   molecular function:  NADH dehydrogenase (ubiquinone) activity   molecular function:  oxidoreductase activity      BioCarta Pathways:     KEGG Pathways:  
    Probe set : 1395336_at    Name:   similar to RIKEN cDNA 2810022L02    GB acc:   BE098691    UniGene:   Rn.36675    Gene Symbol:   LOC501207    Locus Link:   501207      SOURCE    GENECARD       Gene Ontology:     BioCarta Pathways:     KEGG Pathways:  
    Probe set : 1368215_at    Name:   ceroid-lipofuscinosis, neuronal 2    GB acc:   NM_031357    UniGene:   Rn.43558    Gene Symbol:   Cln2    Locus Link:   83534    Chromosome:   1    Cytoband:   Chr:1q33    SOURCE    GENECARD       Gene Ontology:   biological process:  proteolysis   biological process:  lipid metabolism   biological process:  nervous system development      BioCarta Pathways:     KEGG Pathways:  
    Probe set : 1378536_at    Name:   Transcribed locus    GB acc:   AI638960    UniGene:   Rn.16596        SOURCE        Gene Ontology:     BioCarta Pathways:     KEGG Pathways:  
    Probe set : 1384910_at    Name:   Similar to putative voltage-gated calcium channel alpha(2)delta-4 subunit    GB acc:   AI454016    UniGene:   Rn.34565     Locus Link:   312668    Chromosome:   4    Cytoband:   Chr:4q42    SOURCE        Gene Ontology:     BioCarta Pathways:     KEGG Pathways:  
    Probe set : 1397313_at    Name:   CDNA clone MGC:93828 IMAGE:7110880    GB acc:   AI716841    UniGene:   Rn.118788        SOURCE        Gene Ontology:   biological process:  transcription   biological process:  regulation of transcription, DNA-dependent   cellular component:  nucleus      BioCarta Pathways:     KEGG Pathways:  
    Probe set : 1394056_at    Name:   CDNA clone MGC:124727 IMAGE:7936966    GB acc:   BF551160    UniGene:   Rn.103733        SOURCE        Gene Ontology:     BioCarta Pathways:     KEGG Pathways:  
    Probe set : 1387407_at    Name:   nucleosome assembly protein 1-like 3    GB acc:   NM_133402    UniGene:   Rn.27765    Gene Symbol:   Nap1l3    Locus Link:   170914    Chromosome:   X    Cytoband:   Chr:Xq32    SOURCE    GENECARD       Gene Ontology:   biological process:  nucleosome assembly   cellular component:  nucleus   molecular function:  DNA binding      BioCarta Pathways:     KEGG Pathways:  
    Probe set : 1382780_at    Name:   Transcribed locus    GB acc:   AI059229    UniGene:   Rn.21060        SOURCE        Gene Ontology:     BioCarta Pathways:     KEGG Pathways:  
    Probe set : 1371202_a_at    Name:   nuclear factor I/B    GB acc:   AB012232    UniGene:   Rn.9909    Gene Symbol:   Nfib    Locus Link:   29227    Chromosome:   5    Cytoband:   Chr:5q31    SOURCE    GENECARD       Gene Ontology:   cellular component:  intracellular   cellular component:  nucleus      BioCarta Pathways:     KEGG Pathways:  
    Probe set : 1394737_at    Name:   similar to CG9752-PA (predicted)    GB acc:   BE108874    UniGene:   Rn.53930    Gene Symbol:   RGD1311345_predicted    Locus Link:   361201    Chromosome:   17    Cytoband:   Chr:17p14    SOURCE    GENECARD       Gene Ontology:     BioCarta Pathways:     KEGG Pathways:  
    Probe set : 1383715_at    Name:   similar to hypothetical protein D4Ertd89e (predicted)    GB acc:   BI284371    UniGene:   Rn.22285    Gene Symbol:   RGD1305703_predicted    Locus Link:   362521    Chromosome:   5    Cytoband:   Chr:5q24    SOURCE    GENECARD       Gene Ontology:   biological process:  nucleosome assembly   biological process:  chromosome organization and biogenesis (sensu Eukaryota)   cellular component:  nucleosome   cellular component:  nucleus   molecular function:  DNA binding      BioCarta Pathways:     KEGG Pathways:  
    Probe set : 1386466_at    Name:   Transcribed locus    GB acc:   H33093    UniGene:   Rn.135353        SOURCE        Gene Ontology:     BioCarta Pathways:     KEGG Pathways:  
    Probe set : 1383001_at    Name:   Transcribed locus    GB acc:   AI178157    UniGene:   Rn.7434        SOURCE        Gene Ontology:     BioCarta Pathways:     KEGG Pathways:  
    Probe set : 1389015_at    Name:   similar to FUN14 domain containing 2    GB acc:   AI171982    UniGene:   Rn.132860    Gene Symbol:   LOC361288    Locus Link:   361288    Chromosome:   18    Cytoband:   Chr:18p13    SOURCE    GENECARD       Gene Ontology:     BioCarta Pathways:     KEGG Pathways:  
    Probe set : 1388414_at    Name:   NADH dehydrogenase (ubiquinone) Fe-S protein 5b, 15kDa (NADH-coenzyme Q reductase) (predicted)    GB acc:   AI410498    UniGene:   Rn.141253    Gene Symbol:   Ndufs5b_predicted    Locus Link:   362588    Chromosome:   5    Cytoband:   Chr:5q36    SOURCE    GENECARD       Gene Ontology:   biological process:  mitochondrial electron transport, NADH to ubiquinone   cellular component:  membrane fraction   cellular component:  mitochondrion   molecular function:  NADH dehydrogenase activity   molecular function:  NADH dehydrogenase (ubiquinone) activity   molecular function:  oxidoreductase activity      BioCarta Pathways:     KEGG Pathways:  
    Probe set : 1376161_at    Name:   similar to RIKEN cDNA 1110014L17 (predicted)    GB acc:   AI235294    UniGene:   Rn.21479    Gene Symbol:   RGD1306319_predicted    Locus Link:   305502    Chromosome:   14    Cytoband:   Chr:14q21    SOURCE    GENECARD       Gene Ontology:     BioCarta Pathways:     KEGG Pathways:  
    Probe set : 1370397_at    Name:   cytochrome P450, family 4, subfamily a, polypeptide 14    GB acc:   M33936    UniGene:   Rn.33492    Gene Symbol:   Cyp4a14    Locus Link:   298423    Chromosome:   5    Cytoband:   Chr:5q36    SOURCE    GENECARD       Gene Ontology:   biological process:  electron transport   cellular component:  endoplasmic reticulum   cellular component:  microsome   cellular component:  membrane      BioCarta Pathways:     KEGG Pathways:  
    Probe set : 1374083_at    Name:   Transcribed locus    GB acc:   AA799740    UniGene:   Rn.3717        SOURCE        Gene Ontology:     BioCarta Pathways:     KEGG Pathways:  
    Probe set : 1381461_at    Name:   Transcribed locus    GB acc:   AI058509    UniGene:   Rn.146295        SOURCE        Gene Ontology:     BioCarta Pathways:     KEGG Pathways:  
    Probe set : 1384302_at    Name:   solute carrier family 6 (neurotransmitter transporter), member 17    GB acc:   AI712791    UniGene:   Rn.144487    Gene Symbol:   Slc6a17    Locus Link:   613226      SOURCE    GENECARD       Gene Ontology:   biological process:  transport   biological process:  neurotransmitter transport   cellular component:  integral to plasma membrane   cellular component:  membrane   cellular component:  integral to membrane   molecular function:  neurotransmitter:sodium symporter activity   molecular function:  symporter activity      BioCarta Pathways:     KEGG Pathways:  
    Probe set : 1390717_at    Name:   similar to chromosome 20 open reading frame 155 (predicted)    GB acc:   BM391963    UniGene:   Rn.35982    Gene Symbol:   RGD1311037_predicted    Locus Link:   366196    Chromosome:   3    Cytoband:   Chr:3q36    SOURCE    GENECARD       Gene Ontology:   biological process:  phospholipid biosynthesis   cellular component:  integral to membrane   molecular function:  transferase activity      BioCarta Pathways:     KEGG Pathways:  
    Probe set : 1374611_at     GB acc:   AW535065              Gene Ontology:     BioCarta Pathways:     KEGG Pathways:  
    Probe set : 1397916_s_at    Name:   protein tyrosine phosphatase, non-receptor type 1    GB acc:   AA924431    UniGene:   Rn.11317    Gene Symbol:   Ptpn1    Locus Link:   24697    Chromosome:   3    Cytoband:   Chr:3q42    SOURCE    GENECARD       Gene Ontology:   biological process:  protein amino acid dephosphorylation   biological process:  protein amino acid dephosphorylation   cellular component:  integral to membrane   cellular component:  endoplasmic reticulum      BioCarta Pathways:     KEGG Pathways:  
    Probe set : 1389238_at    Name:   similar to PES1 protein    GB acc:   BG376577    UniGene:   Rn.9521    Gene Symbol:   LOC289740    Locus Link:   289740    Chromosome:   14    Cytoband:   Chr:14q21    SOURCE    GENECARD       Gene Ontology:     BioCarta Pathways:     KEGG Pathways:  
    Probe set : 1374315_at    Name:   Transcribed locus    GB acc:   AI228079    UniGene:   Rn.38221        SOURCE        Gene Ontology:     BioCarta Pathways:     KEGG Pathways:  
    Probe set : 1386857_at    Name:   stathmin 1    GB acc:   NM_017166    UniGene:   Rn.555    Gene Symbol:   Stmn1    Locus Link:   29332    Chromosome:   5    Cytoband:   Chr:5q36    SOURCE    GENECARD       Gene Ontology:   cellular component:  cytoplasm   cellular component:  cytosol   cellular component:  kinesin complex   cellular component:  microtubule   molecular function:  signal transducer activity   molecular function:  tubulin binding   molecular function:  tubulin binding      BioCarta Pathways:     KEGG Pathways:  
    Probe set : 1374411_at    Name:   Mitochondrial ribosomal protein L52 (predicted)    GB acc:   BF414649    UniGene:   Rn.6660     Locus Link:   361037    Chromosome:   15    Cytoband:   Chr:15p13    SOURCE        Gene Ontology:   molecular function:  structural constituent of ribosome      BioCarta Pathways:     KEGG Pathways:  
    Probe set : 1374418_at    Name:   similar to CG8009-PA (predicted)    GB acc:   BF289188    UniGene:   Rn.101963    Gene Symbol:   RGD1308816_predicted    Locus Link:   291914    Chromosome:   19    Cytoband:   Chr:19p11    SOURCE    GENECARD       Gene Ontology:     BioCarta Pathways:     KEGG Pathways:  
    Probe set : 1369497_at    Name:   Robo-1    GB acc:   NM_031537    UniGene:   Rn.48721    Gene Symbol:   LOC24906    Locus Link:   24906    Chromosome:   10    Cytoband:   Chr:10q22    SOURCE    GENECARD       Gene Ontology:     BioCarta Pathways:     KEGG Pathways:  
    Probe set : 1367609_at    Name:   macrophage migration inhibitory factor    GB acc:   NM_031051    UniGene:   Rn.2661    Gene Symbol:   Mif    Locus Link:   81683    Chromosome:   20    Cytoband:   Chr:20p12    SOURCE    GENECARD       Gene Ontology:   cellular component:  extracellular region   cellular component:  extracellular space      BioCarta Pathways:     KEGG Pathways:  
    Probe set : 1390977_at    Name:   suppression of tumorigenicity 7-like    GB acc:   AW527529    UniGene:   Rn.102337    Gene Symbol:   RGD:1359336    Locus Link:   295344    Chromosome:   2    Cytoband:   Chr:2q34    SOURCE    GENECARD       Gene Ontology:   molecular function:  binding      BioCarta Pathways:     KEGG Pathways:  
    Probe set : 1373515_at    Name:   Similar to RIKEN cDNA 1110067D22 (predicted)    GB acc:   BI275737    UniGene:   Rn.17651     Locus Link:   360983    Chromosome:   14    Cytoband:   Chr:14q22    SOURCE        Gene Ontology:     BioCarta Pathways:     KEGG Pathways:  
    Probe set : 1391328_at     GB acc:   AI575783              Gene Ontology:     BioCarta Pathways:     KEGG Pathways:  
    Probe set : 1380365_at    Name:   Transcribed locus    GB acc:   BF403932    UniGene:   Rn.147925        SOURCE        Gene Ontology:     BioCarta Pathways:     KEGG Pathways:  
    Probe set : 1370785_s_at    Name:   translocase of outer mitochondrial membrane 20 homolog (yeast)    GB acc:   D63411    UniGene:   Rn.2143    Gene Symbol:   Tomm20    Locus Link:   266601    Chromosome:   19    Cytoband:   Chr:19q12    SOURCE    GENECARD       Gene Ontology:   molecular function:  protein translocase activity   molecular function:  protein translocase activity   molecular function:  unfolded protein binding      BioCarta Pathways:     KEGG Pathways:  
    Probe set : 1382127_at     GB acc:   AI716708              Gene Ontology:     BioCarta Pathways:     KEGG Pathways:  
    Probe set : 1371155_at    Name:   killer cell lectin-like receptor subfamily C, member 1    GB acc:   AF021350    UniGene:   Rn.9895    Gene Symbol:   Klrc1    Locus Link:   500338    Chromosome:   4    Cytoband:   Chr:4q42    SOURCE    GENECARD       Gene Ontology:   cellular component:  integral to membrane   molecular function:  receptor activity   molecular function:  sugar binding   molecular function:  MHC class I receptor activity      BioCarta Pathways:     KEGG Pathways:  
    Probe set : 1398608_at    Name:   similar to hypothetical gene supported by BC007071 (predicted)    GB acc:   BG371578    UniGene:   Rn.3322    Gene Symbol:   RGD1311868_predicted    Locus Link:   289150    Chromosome:   13    Cytoband:   Chr:13q22    SOURCE    GENECARD       Gene Ontology:     BioCarta Pathways:     KEGG Pathways:  
    Probe set : 1398449_at    Name:   Transcribed locus    GB acc:   AA849031    UniGene:   Rn.53808        SOURCE        Gene Ontology:     BioCarta Pathways:     KEGG Pathways:  
    Probe set : 1368231_at    Name:   signal transducer and activator of transcription 5A    GB acc:   NM_017064    UniGene:   Rn.54486    Gene Symbol:   Stat5a    Locus Link:   24918    Chromosome:   10    Cytoband:   Chr:10q32.1    SOURCE    GENECARD       Gene Ontology:   biological process:  electron transport   biological process:  regulation of transcription, DNA-dependent   biological process:  regulation of transcription, DNA-dependent   biological process:  regulation of transcription from RNA polymerase II promoter   biological process:  immune response   biological process:  signal transduction   biological process:  intracellular signaling cascade   biological process:  JAK-STAT cascade   biological process:  lactation   biological process:  lactation   biological process:  regulation of cell adhesion   biological process:  regulation of epithelial cell differentiation   biological process:  transcription   biological process:  regulation of transcription, DNA-dependent   cellular component:  nucleus   cellular component:  nucleus   cellular component:  cytoplasm   cellular component:  cytoplasm      BioCarta Pathways:     KEGG Pathways:  
    Probe set : 1399057_at    Name:   mortality factor 4 like 1 (predicted)    GB acc:   BE109080    UniGene:   Rn.110179    Gene Symbol:   Morf4l1_predicted    Locus Link:   300891    Chromosome:   8    Cytoband:   Chr:8q31    SOURCE    GENECARD       Gene Ontology:   cellular component:  chromatin   cellular component:  nucleus   cellular component:  chromatin remodeling complex   molecular function:  chromatin binding   molecular function:  protein binding      BioCarta Pathways:     KEGG Pathways:  
    Probe set : 1376026_at    Name:   downstream neighbor of SON (predicted)    GB acc:   AI178243    UniGene:   Rn.17226    Gene Symbol:   Donson_predicted    Locus Link:   288257    Chromosome:   11    Cytoband:   Chr:11q11    SOURCE    GENECARD       Gene Ontology:     BioCarta Pathways:     KEGG Pathways:  
    Probe set : 1371354_at    Name:   Troponin C, cardiac/slow skeletal (predicted)    GB acc:   AI710682    UniGene:   Rn.15460     Locus Link:   290561    Chromosome:   16    Cytoband:   Chr:16p16    SOURCE        Gene Ontology:   biological process:  regulation of muscle contraction   biological process:  muscle development   cellular component:  troponin complex   molecular function:  structural constituent of cytoskeleton   molecular function:  calcium ion binding      BioCarta Pathways:     KEGG Pathways:  
    Probe set : 1396127_at     GB acc:   BF288545              Gene Ontology:     BioCarta Pathways:     KEGG Pathways:  
    Probe set : 1374629_at    Name:   mediator of RNA polymerase II transcription, subunit 8 homolog (yeast) (predicted)    GB acc:   AI713147    UniGene:   Rn.102063    Gene Symbol:   Med8_predicted    Locus Link:   362575    Chromosome:   5    Cytoband:   Chr:5q36    SOURCE    GENECARD       Gene Ontology:   biological process:  regulation of transcription, DNA-dependent   cellular component:  nucleus   molecular function:  receptor activity      BioCarta Pathways:     KEGG Pathways:  
    Probe set : 1367652_at    Name:   insulin-like growth factor binding protein 3    GB acc:   AI713966    UniGene:   Rn.26369    Gene Symbol:   Igfbp3    Locus Link:   24484    Chromosome:   14    Cytoband:   Chr:14q21    SOURCE    GENECARD       Gene Ontology:   cellular component:  extracellular region   cellular component:  extracellular space   cellular component:  extracellular region      BioCarta Pathways:     KEGG Pathways:  
    Probe set : 1378679_at    Name:   Transcribed locus    GB acc:   BG372484    UniGene:   Rn.26727        SOURCE        Gene Ontology:     BioCarta Pathways:     KEGG Pathways:  
    Probe set : 1387879_a_at    Name:   CUG triplet repeat,RNA-binding protein 2    GB acc:   AF090695    UniGene:   Rn.30004    Gene Symbol:   Cugbp2    Locus Link:   29428    Chromosome:   17    Cytoband:   Chr:17q12.3    SOURCE    GENECARD       Gene Ontology:   cellular component:  nucleus   molecular function:  RNA binding   molecular function:  RNA binding   molecular function:  nucleotide binding   molecular function:  nucleic acid binding      BioCarta Pathways:     KEGG Pathways:  
    Probe set : 1391252_at    Name:   ring finger protein 41 (predicted)    GB acc:   BI290717    UniGene:   Rn.74790    Gene Symbol:   Rnf41_predicted    Locus Link:   362814    Chromosome:   7    Cytoband:   Chr:7q11    SOURCE    GENECARD       Gene Ontology:   biological process:  protein ubiquitination   cellular component:  ubiquitin ligase complex   molecular function:  ubiquitin-protein ligase activity   molecular function:  zinc ion binding      BioCarta Pathways:     KEGG Pathways:  
    Probe set : 1383416_at    Name:   Acetyl-coenzyme A acetyltransferase 1    GB acc:   AA899304    UniGene:   Rn.4054    Gene Symbol:   Acat1    Locus Link:   25014    Chromosome:   8    Cytoband:   Chr:8q24.1    SOURCE    GENECARD       Gene Ontology:   biological process:  lipoprotein biosynthesis   cellular component:  mitochondrion   cellular component:  mitochondrion      BioCarta Pathways:     KEGG Pathways:  
    Probe set : 1373624_at    Name:   Transcribed locus    GB acc:   AA945713    UniGene:   Rn.7899        SOURCE        Gene Ontology:     BioCarta Pathways:     KEGG Pathways:  
    Probe set : 1379322_at    Name:   proteasome (prosome, macropain) 26S subunit, non-ATPase, 7 (predicted)    GB acc:   BI289174    UniGene:   Rn.20659    Gene Symbol:   Psmd7_predicted    Locus Link:   307821    Chromosome:   19    Cytoband:   Chr:19q12    SOURCE    GENECARD       Gene Ontology:   cellular component:  cytosol   cellular component:  proteasome regulatory particle (sensu Eukaryota)      BioCarta Pathways:     KEGG Pathways:  
    Probe set : 1385814_at    Name:   mitogen activated protein kinase kinase kinase 3 (predicted)    GB acc:   H32486    UniGene:   Rn.72680    Gene Symbol:   Map3k3_predicted    Locus Link:   303604    Chromosome:   10    Cytoband:   Chr:10q32.1    SOURCE    GENECARD       Gene Ontology:   biological process:  MAPKKK cascade   biological process:  protein amino acid phosphorylation   biological process:  positive regulation of I-kappaB kinase/NF-kappaB cascade      BioCarta Pathways:     KEGG Pathways:  
    Probe set : 1372386_at    Name:   SUMO/sentrin specific protease 2    GB acc:   AW530777    UniGene:   Rn.9367    Gene Symbol:   Senp2    Locus Link:   78973    Chromosome:   11    Cytoband:   Chr:11q23    SOURCE    GENECARD       Gene Ontology:   cellular component:  nucleus   cellular component:  nuclear pore   cellular component:  nuclear pore      BioCarta Pathways:     KEGG Pathways:  
    Probe set : 1379431_at    Name:   Transcribed locus    GB acc:   AW918368    UniGene:   Rn.8749        SOURCE        Gene Ontology:     BioCarta Pathways:     KEGG Pathways:  
    Probe set : 1376870_at    Name:   zinc finger protein 278 (predicted)    GB acc:   BI288076    UniGene:   Rn.71473    Gene Symbol:   Znf278_predicted    Locus Link:   305471    Chromosome:   14    Cytoband:   Chr:14q21    SOURCE    GENECARD       Gene Ontology:   biological process:  regulation of transcription, DNA-dependent   biological process:  negative regulation of transcription   cellular component:  nucleus      BioCarta Pathways:     KEGG Pathways:  
    Probe set : 1387472_at    Name:   CD3 antigen delta polypeptide    GB acc:   NM_013169    UniGene:   Rn.10329    Gene Symbol:   Cd3d    Locus Link:   25710    Chromosome:   8    Cytoband:   Chr:8q22    SOURCE    GENECARD       Gene Ontology:     BioCarta Pathways:     KEGG Pathways:  
    Probe set : 1377808_at    Name:   Similar to IQ motif and WD repeats 1    GB acc:   BF389856    UniGene:   Rn.3917     Locus Link:   289181    Chromosome:   13    Cytoband:   Chr:13q23    SOURCE        Gene Ontology:     BioCarta Pathways:     KEGG Pathways:  
    Probe set : 1388739_at    Name:   similar to chromosome 14 open reading frame 130    GB acc:   BF398626    UniGene:   Rn.7690    Gene Symbol:   RGD:1359144    Locus Link:   314399    Chromosome:   6    Cytoband:   Chr:6q32    SOURCE    GENECARD       Gene Ontology:   biological process:  ubiquitin cycle   molecular function:  ubiquitin-protein ligase activity      BioCarta Pathways:     KEGG Pathways:  
    Probe set : 1384242_at    Name:   Neurolysin (metallopeptidase M3 family)    GB acc:   BI303239    UniGene:   Rn.11029    Gene Symbol:   Nln    Locus Link:   117041    Chromosome:   2    Cytoband:   Chr:2q13    SOURCE    GENECARD       Gene Ontology:   biological process:  RNA processing   biological process:  proteolysis   cellular component:  mitochondrion      BioCarta Pathways:     KEGG Pathways:  
    Probe set : 1375785_at    Name:   Similar to hypothetical protein D4Ertd89e (predicted)    GB acc:   AI101490    UniGene:   Rn.22285     Locus Link:   362521    Chromosome:   5    Cytoband:   Chr:5q24    SOURCE        Gene Ontology:   biological process:  nucleosome assembly   biological process:  chromosome organization and biogenesis (sensu Eukaryota)   cellular component:  nucleosome   cellular component:  nucleus   molecular function:  DNA binding      BioCarta Pathways:     KEGG Pathways:  
    Probe set : 1389617_at     GB acc:   BM392135              Gene Ontology:     BioCarta Pathways:     KEGG Pathways:  
    Probe set : 1374425_at    Name:   transducin-like enhancer of split 1, homolog of Drosophila E(spl) (predicted)    GB acc:   BG380534    UniGene:   Rn.6875    Gene Symbol:   Tle1_predicted    Locus Link:   362533    Chromosome:   5    Cytoband:   Chr:5q31    SOURCE    GENECARD       Gene Ontology:   cellular component:  nucleus   molecular function:  protein binding      BioCarta Pathways:     KEGG Pathways:  
    Probe set : 1390290_at    Name:   surfeit gene 6 (predicted)    GB acc:   BF396659    UniGene:   Rn.146403    Gene Symbol:   Surf6_predicted    Locus Link:   303076    Chromosome:   3    Cytoband:   Chr:3p12    SOURCE    GENECARD       Gene Ontology:   biological process:  ribosome assembly   cellular component:  granular component   cellular component:  nucleus   molecular function:  DNA binding   molecular function:  RNA binding      BioCarta Pathways:     KEGG Pathways:  
    Probe set : 1382431_at    Name:   Hypothetical gene supported by NM_178095    GB acc:   AI103530    UniGene:   Rn.3724    Gene Symbol:   Abca1    Locus Link:   313210    Chromosome:   5    Cytoband:   Chr:5q24    SOURCE    GENECARD       Gene Ontology:     BioCarta Pathways:     KEGG Pathways:  
    Probe set : 1388814_at    Name:   DEAD (Asp-Glu-Ala-Asp) box polypeptide 19    GB acc:   AI408110    UniGene:   Rn.96192    Gene Symbol:   Ddx19    Locus Link:   292022    Chromosome:   19    Cytoband:   Chr:19q12    SOURCE    GENECARD       Gene Ontology:   biological process:  induction of apoptosis   biological process:  response to zinc ion   cellular component:  nucleus      BioCarta Pathways:     KEGG Pathways:  
    Probe set : 1388481_at    Name:   Ribosomal protein S28    GB acc:   AI407731    UniGene:   Rn.6005    Gene Symbol:   Rps28    Locus Link:   50718      SOURCE    GENECARD       Gene Ontology:   biological process:  protein biosynthesis   cellular component:  intracellular   cellular component:  ribosome   cellular component:  cytosolic small ribosomal subunit (sensu Eukaryota)   molecular function:  RNA binding   molecular function:  structural constituent of ribosome      BioCarta Pathways:     KEGG Pathways:  
    Probe set : 1367951_at    Name:   phosphoglycerate mutase 2    GB acc:   NM_017328    UniGene:   Rn.9738    Gene Symbol:   Pgam2    Locus Link:   24959    Chromosome:   14    Cytoband:   Chr:14q21-q22    SOURCE    GENECARD       Gene Ontology:   biological process:  glycolysis   biological process:  striated muscle contraction   biological process:  metabolism      BioCarta Pathways:     KEGG Pathways:  
    Probe set : 1372829_at    Name:   RNA binding motif protein 8 (predicted)    GB acc:   BE113079    UniGene:   Rn.37716    Gene Symbol:   Rbm8_predicted    Locus Link:   295284    Chromosome:   2    Cytoband:   Chr:2q34    SOURCE    GENECARD       Gene Ontology:   cellular component:  nucleus   cellular component:  cytoplasm   molecular function:  RNA binding   molecular function:  mRNA binding      BioCarta Pathways:     KEGG Pathways:  
    Probe set : 1388918_at    Name:   High density lipoprotein binding protein    GB acc:   BG378074    UniGene:   Rn.8515    Gene Symbol:   Hdlbp    Locus Link:   64474    Chromosome:   9    Cytoband:   Chr:9q36    SOURCE    GENECARD       Gene Ontology:   cellular component:  nucleus   cellular component:  plasma membrane      BioCarta Pathways:     KEGG Pathways:  
    Probe set : 1392203_at    Name:   homeo box B2 (predicted)    GB acc:   AI029834    UniGene:   Rn.18333    Gene Symbol:   Hoxb2_predicted    Locus Link:   303489    Chromosome:   10    Cytoband:   Chr:10q31    SOURCE    GENECARD       Gene Ontology:   cellular component:  nucleus   cellular component:  transcription factor complex   molecular function:  DNA binding   molecular function:  transcription factor activity      BioCarta Pathways:     KEGG Pathways:  
    Probe set : 1381937_at    Name:   similar to 60S ribosomal protein L7a    GB acc:   AI045406    UniGene:   Rn.55975    Gene Symbol:   LOC499344    Locus Link:   499344    Chromosome:   1    Cytoband:   Chr:1q52    SOURCE    GENECARD       Gene Ontology:     BioCarta Pathways:     KEGG Pathways:  
    Probe set : 1397830_at    Name:   Insulin-like growth factor binding protein 5    GB acc:   BF399783    UniGene:   Rn.1593    Gene Symbol:   Igfbp5    Locus Link:   25285    Chromosome:   9    Cytoband:   Chr:9q33    SOURCE    GENECARD       Gene Ontology:   biological process:  regulation of cell growth   biological process:  signal transduction   cellular component:  extracellular region   cellular component:  extracellular space   cellular component:  insulin-like growth factor binding protein complex   molecular function:  insulin-like growth factor binding   molecular function:  growth factor binding      BioCarta Pathways:     KEGG Pathways:  
    Probe set : 1387926_at    Name:   sterol-C5-desaturase (fungal ERG3, delta-5-desaturase) homolog (S. cerevisae)    GB acc:   AB052846    UniGene:   Rn.18741    Gene Symbol:   Sc5d    Locus Link:   114100    Chromosome:   8    Cytoband:   Chr:8q22    SOURCE    GENECARD       Gene Ontology:   biological process:  metabolism   molecular function:  catalytic activity      BioCarta Pathways:     KEGG Pathways:  
    Probe set : 1386045_at    Name:   Transcribed locus    GB acc:   AA893205    UniGene:   Rn.143823        SOURCE        Gene Ontology:     BioCarta Pathways:     KEGG Pathways:  
    Probe set : 1397609_at    Name:   TRAF4 associated factor 1    GB acc:   BF287466    UniGene:   Rn.20235    Gene Symbol:   RGD:1303240    Locus Link:   311325    Chromosome:   3    Cytoband:   Chr:3q35    SOURCE    GENECARD       Gene Ontology:     BioCarta Pathways:     KEGG Pathways:  
    Probe set : 1388626_at    Name:   similar to chromosome 20 open reading frame 116 (predicted)    GB acc:   AI638949    UniGene:   Rn.137483    Gene Symbol:   RGD1309979_predicted    Locus Link:   296162    Chromosome:   3    Cytoband:   Chr:3q36    SOURCE    GENECARD       Gene Ontology:     BioCarta Pathways:     KEGG Pathways:  
    Probe set : 1387763_at    Name:   low Mr GTP-binding protein    GB acc:   NM_017317    UniGene:   Rn.37360    Gene Symbol:   Rab27a    Locus Link:   50645    Chromosome:   8    Cytoband:   Chr:8q24    SOURCE    GENECARD       Gene Ontology:   biological process:  small GTPase mediated signal transduction   biological process:  protein transport   molecular function:  GTPase activity   molecular function:  GTP binding   molecular function:  GTP binding      BioCarta Pathways:     KEGG Pathways:  
    Probe set : 1380450_at    Name:   benzodiazapine receptor associated protein 1    GB acc:   AI044632    UniGene:   Rn.15974    Gene Symbol:   Bzrap1    Locus Link:   287609    Chromosome:   10    Cytoband:   Chr:10q26    SOURCE    GENECARD       Gene Ontology:   cellular component:  cytoplasm   cellular component:  mitochondrion   cellular component:  mitochondrion   molecular function:  benzodiazepine receptor binding   molecular function:  protein binding, bridging      BioCarta Pathways:     KEGG Pathways:  
    Probe set : 1385805_at     GB acc:   AI071958              Gene Ontology:     BioCarta Pathways:     KEGG Pathways:  
    Probe set : 1384385_at    Name:   Similar to solute carrier family 7 (cationic amino acid transporter, y+ system), member 12    GB acc:   AI060176    UniGene:   Rn.133380     Locus Link:   361914    Chromosome:   2    Cytoband:   Chr:2q23    SOURCE        Gene Ontology:   biological process:  transport   biological process:  amino acid transport   cellular component:  membrane   cellular component:  integral to membrane   molecular function:  amino acid-polyamine transporter activity      BioCarta Pathways:     KEGG Pathways:  
    Probe set : 1372509_at    Name:   Transcribed locus    GB acc:   AI172024    UniGene:   Rn.22487        SOURCE        Gene Ontology:     BioCarta Pathways:     KEGG Pathways:  
    Probe set : 1376268_at    Name:   ADP-ribosylation factor 6    GB acc:   BM387435    UniGene:   Rn.6993    Gene Symbol:   Arf6    Locus Link:   79121    Chromosome:   6    Cytoband:   Chr:6q24    SOURCE    GENECARD       Gene Ontology:     BioCarta Pathways:     KEGG Pathways:  
    Probe set : 1393817_at    Name:   Transcribed locus    GB acc:   AI070991    UniGene:   Rn.151901        SOURCE        Gene Ontology:     BioCarta Pathways:     KEGG Pathways:  
    Probe set : 1373074_at    Name:   similar to RIKEN cDNA 2700002I20 (predicted)    GB acc:   AI171367    UniGene:   Rn.16742    Gene Symbol:   RGD1307279_predicted    Locus Link:   307210    Chromosome:   18    Cytoband:   Chr:18q12.3    SOURCE    GENECARD       Gene Ontology:     BioCarta Pathways:     KEGG Pathways:  
    Probe set : 1376239_at    Name:   similar to RIKEN cDNA 1110038G14    GB acc:   BF283053    UniGene:   Rn.145217    Gene Symbol:   RGD:1359430    Locus Link:   362802      SOURCE    GENECARD       Gene Ontology:   biological process:  ATP synthesis coupled proton transport   cellular component:  proton-transporting two-sector ATPase complex   molecular function:  ATP binding   molecular function:  hydrolase activity, acting on acid anhydrides, catalyzing transmembrane movement of substances      BioCarta Pathways:     KEGG Pathways:  
    Probe set : 1398749_at    Name:   ribosomal protein L4    GB acc:   NM_022510    UniGene:   Rn.1133    Gene Symbol:   Rpl4    Locus Link:   64302    Chromosome:   8    Cytoband:   Chr:8q24    SOURCE    GENECARD       Gene Ontology:   biological process:  protein biosynthesis   biological process:  metabolism   molecular function:  RNA binding   molecular function:  structural constituent of ribosome   molecular function:  oxidoreductase activity      BioCarta Pathways:     KEGG Pathways:  
    Probe set : 1398598_at    Name:   dystonin (predicted)    GB acc:   BF549679    UniGene:   Rn.79807    Gene Symbol:   Dst_predicted    Locus Link:   316313    Chromosome:   9    Cytoband:   Chr:9q21    SOURCE    GENECARD       Gene Ontology:   biological process:  cell cycle arrest   biological process:  cell adhesion   biological process:  retrograde axon cargo transport   cellular component:  cytoskeleton   cellular component:  cytoplasmic membrane-bound vesicle   cellular component:  hemidesmosome      BioCarta Pathways:     KEGG Pathways:  
    Probe set : 1393091_at    Name:   similar to RIKEN cDNA D130064H19 (predicted)    GB acc:   H34096    UniGene:   Rn.19926    Gene Symbol:   RGD1311241_predicted    Locus Link:   310086    Chromosome:   2    Cytoband:   Chr:2q14    SOURCE    GENECARD       Gene Ontology:     BioCarta Pathways:     KEGG Pathways:  
    Probe set : 1392984_at    Name:   copine III (predicted)    GB acc:   BG671686    UniGene:   Rn.4034    Gene Symbol:   Cpne3_predicted    Locus Link:   313087    Chromosome:   5    Cytoband:   Chr:5q13    SOURCE    GENECARD       Gene Ontology:   biological process:  lipid metabolism   biological process:  vesicle-mediated transport   cellular component:  cytosol      BioCarta Pathways:     KEGG Pathways:  
    Probe set : 1389522_at    Name:   Transcribed locus    GB acc:   BE109446    UniGene:   Rn.21422        SOURCE        Gene Ontology:     BioCarta Pathways:     KEGG Pathways:  
    Probe set : 1395331_at    Name:   similar to hypothetical protein CL25084 (predicted)    GB acc:   BF548263    UniGene:   Rn.102123    Gene Symbol:   RGD1306508_predicted    Locus Link:   289874    Chromosome:   14    Cytoband:   Chr:14q22    SOURCE    GENECARD       Gene Ontology:     BioCarta Pathways:     KEGG Pathways:  
    Probe set : 1398760_at    Name:   ribosomal protein L35a    GB acc:   NM_021264    UniGene:   Rn.2812    Gene Symbol:   Rpl35a    Locus Link:   57809    Chromosome:   11    Cytoband:   Chr:11q22    SOURCE    GENECARD       Gene Ontology:   biological process:  protein biosynthesis      BioCarta Pathways:     KEGG Pathways:  
    Probe set : 1370542_a_at    Name:   E74-like factor 1    GB acc:   AB030216    UniGene:   Rn.98640    Gene Symbol:   Elf1    Locus Link:   85424    Chromosome:   15    Cytoband:   Chr:15q12    SOURCE    GENECARD       Gene Ontology:   cellular component:  nucleus      BioCarta Pathways:     KEGG Pathways:  
    Probe set : 1383376_at    Name:   similar to Williams-Beuren syndrome critical region protein 18    GB acc:   AI717529    UniGene:   Rn.29127    Gene Symbol:   LOC368190    Locus Link:   368190    Chromosome:   12    Cytoband:   Chr:12q12    SOURCE    GENECARD       Gene Ontology:     BioCarta Pathways:     KEGG Pathways:  
    Probe set : 1374129_at    Name:   similar to product is unknown~seizure-related gene    GB acc:   AI104518    UniGene:   Rn.2946    Gene Symbol:   LOC501454    Locus Link:   501454      SOURCE    GENECARD       Gene Ontology:     BioCarta Pathways:     KEGG Pathways:  
    Probe set : 1387054_at    Name:   ATP-binding cassette, sub-family G (WHITE), member 1    GB acc:   AI712719    UniGene:   Rn.8398    Gene Symbol:   Abcg1    Locus Link:   85264    Chromosome:   20    Cytoband:   Chr:20p12    SOURCE    GENECARD       Gene Ontology:   biological process:  transport   biological process:  lipid transport   biological process:  cholesterol transport      BioCarta Pathways:     KEGG Pathways:  
    Probe set : 1383714_at    Name:   Transcribed locus    GB acc:   H32348    UniGene:   Rn.29775        SOURCE        Gene Ontology:     BioCarta Pathways:     KEGG Pathways:  
    Probe set : 1375882_at    Name:   protein phosphatase 3, catalytic subunit, beta isoform    GB acc:   BG380745    UniGene:   Rn.11063    Gene Symbol:   Ppp3cb    Locus Link:   24675    Chromosome:   15    Cytoband:   Chr:15p16    SOURCE    GENECARD       Gene Ontology:   biological process:  protein amino acid dephosphorylation   biological process:  heart development   biological process:  T cell differentiation   cellular component:  protein serine/threonine phosphatase complex   molecular function:  protein phosphatase type 2A activity   molecular function:  protein phosphatase type 1 activity   molecular function:  phosphoprotein phosphatase activity   molecular function:  protein serine/threonine phosphatase activity   molecular function:  calcium-dependent protein serine/threonine phosphatase activity   molecular function:  magnesium-dependent protein serine/threonine phosphatase activity   molecular function:  calmodulin binding   molecular function:  CTD phosphatase activity   molecular function:  protein phosphatase type 2C activity   molecular function:  hydrolase activity   molecular function:  myosin phosphatase activity   molecular function:  protein phosphatase type 2B activity   molecular function:  iron ion binding   molecular function:  zinc ion binding   molecular function:  metal ion binding      BioCarta Pathways:     KEGG Pathways:  
    Probe set : 1371987_at    Name:   polymerase (DNA directed) sigma (predicted)    GB acc:   BI274697    UniGene:   Rn.22554    Gene Symbol:   Pols_predicted    Locus Link:   306672    Chromosome:   17    Cytoband:   Chr:17p14    SOURCE    GENECARD       Gene Ontology:   biological process:  DNA metabolism   biological process:  mitosis   cellular component:  nucleus   molecular function:  DNA binding   molecular function:  DNA-directed DNA polymerase activity   molecular function:  transferase activity      BioCarta Pathways:     KEGG Pathways:  
    Probe set : 1383224_at    Name:   par-6 (partitioning defective 6) homolog beta (C. elegans) (predicted)    GB acc:   BF525012    UniGene:   Rn.21123    Gene Symbol:   Pard6b_predicted    Locus Link:   362279    Chromosome:   3    Cytoband:   Chr:3q42    SOURCE    GENECARD       Gene Ontology:   cellular component:  plasma membrane   cellular component:  tight junction   cellular component:  membrane   molecular function:  protein binding      BioCarta Pathways:     KEGG Pathways:  
    Probe set : 1372232_at    Name:   WW domain binding protein 1 (predicted)    GB acc:   AI170808    UniGene:   Rn.139817    Gene Symbol:   Wbp1_predicted    Locus Link:   297381    Chromosome:   4    Cytoband:   Chr:4q34    SOURCE    GENECARD       Gene Ontology:   cellular component:  integral to membrane   molecular function:  protein binding      BioCarta Pathways:     KEGG Pathways:  
    Probe set : 1389454_at    Name:   programmed cell death 5 (predicted)    GB acc:   BF408447    UniGene:   Rn.9535    Gene Symbol:   Pdcd5_predicted    Locus Link:   292814    Chromosome:   1    Cytoband:   Chr:1q21    SOURCE    GENECARD       Gene Ontology:   biological process:  apoptosis   biological process:  induction of apoptosis      BioCarta Pathways:     KEGG Pathways:  
    Probe set : 1392043_at    Name:   Transcribed locus    GB acc:   BE116206    UniGene:   Rn.121138        SOURCE        Gene Ontology:     BioCarta Pathways:     KEGG Pathways:  
    Probe set : 1388550_at    Name:   similar to MHR23B    GB acc:   BI295122    UniGene:   Rn.67042    Gene Symbol:   LOC298012    Locus Link:   298012    Chromosome:   5    Cytoband:   Chr:5q24    SOURCE    GENECARD       Gene Ontology:   biological process:  nucleotide-excision repair   biological process:  protein modification   cellular component:  nucleus      BioCarta Pathways:     KEGG Pathways:  
    Probe set : 1372617_at    Name:   similar to BTB (POZ) domain containing 2    GB acc:   BM385989    UniGene:   Rn.137561    Gene Symbol:   LOC500793    Locus Link:   500793    Chromosome:   7    Cytoband:   Chr:7q11    SOURCE    GENECARD       Gene Ontology:     BioCarta Pathways:     KEGG Pathways:  
    Probe set : 1379987_at    Name:   similar to RIKEN cDNA 6330416G13 gene (predicted)    GB acc:   BE100570    UniGene:   Rn.139941    Gene Symbol:   RGD1304595_predicted    Locus Link:   298104    Chromosome:   5    Cytoband:   Chr:5q24    SOURCE    GENECARD       Gene Ontology:     BioCarta Pathways:     KEGG Pathways:  
    Probe set : 1379467_at     GB acc:   BF284065              Gene Ontology:     BioCarta Pathways:     KEGG Pathways:  
    Probe set : 1377480_at    Name:   similar to RIKEN cDNA D030060M11 (predicted)    GB acc:   BF387366    UniGene:   Rn.102777    Gene Symbol:   RGD1308076_predicted    Locus Link:   300687    Chromosome:   8    Cytoband:   Chr:8q23    SOURCE    GENECARD       Gene Ontology:     BioCarta Pathways:     KEGG Pathways:  
    Probe set : 1367689_a_at    Name:   cd36 antigen /// similar to fatty acid translocase/CD36    GB acc:   AF072411    UniGene:   Rn.102418    Gene Symbol:   Cd36 /// LOC499984    Locus Link:   29184 /// 499984    Chromosome:   4    Cytoband:   Chr:4q11    SOURCE    GENECARD       Gene Ontology:     BioCarta Pathways:     KEGG Pathways:  
    Probe set : 1387914_at    Name:   cytochrome P450, family 27, subfamily a, polypeptide 1    GB acc:   M73231    UniGene:   Rn.94956    Gene Symbol:   Cyp27a1    Locus Link:   301517    Chromosome:   9    Cytoband:   Chr:9q33    SOURCE    GENECARD       Gene Ontology:   biological process:  cholesterol metabolism   biological process:  electron transport   cellular component:  mitochondrial membrane   cellular component:  mitochondrion   cellular component:  membrane      BioCarta Pathways:     KEGG Pathways:  
    Probe set : 1382476_x_at    Name:   Similar to pellino 3 alpha (predicted)    GB acc:   AI060133    UniGene:   Rn.79888     Locus Link:   309157    Chromosome:   1    Cytoband:   Chr:1q43    SOURCE        Gene Ontology:     BioCarta Pathways:     KEGG Pathways:  
    Probe set : 1370629_at    Name:   ADP-ribosyltransferase 2b    GB acc:   M85193    UniGene:   Rn.107075    Gene Symbol:   Art2b    Locus Link:   293152    Chromosome:   1    Cytoband:   Chr:1q32    SOURCE    GENECARD       Gene Ontology:   biological process:  cell-mediated immune response   biological process:  protein amino acid ADP-ribosylation   cellular component:  cell surface   cellular component:  membrane      BioCarta Pathways:     KEGG Pathways:  
    Probe set : 1381983_at     GB acc:   AI454019              Gene Ontology:     BioCarta Pathways:     KEGG Pathways:  
    Probe set : 1382792_at    Name:   Transcribed locus    GB acc:   BF412158    UniGene:   Rn.147217        SOURCE        Gene Ontology:     BioCarta Pathways:     KEGG Pathways:  
    Probe set : 1397507_at    Name:   amyloid beta (A4) precursor protein-binding, family B, member 1 interacting protein (predicted)    GB acc:   AI029577    UniGene:   Rn.38834    Gene Symbol:   Apbb1ip_predicted    Locus Link:   307171    Chromosome:   17    Cytoband:   Chr:17q12.3    SOURCE    GENECARD       Gene Ontology:   biological process:  signal transduction   biological process:  neuropeptide signaling pathway      BioCarta Pathways:     KEGG Pathways:  
    Probe set : 1372182_at    Name:   phosphofructokinase, platelet    GB acc:   BM389769    UniGene:   Rn.2278    Gene Symbol:   Pfkp    Locus Link:   60416    Chromosome:   17    Cytoband:   Chr:17q12.2    SOURCE    GENECARD       Gene Ontology:   biological process:  glycolysis   cellular component:  cytoplasm   cellular component:  6-phosphofructokinase complex      BioCarta Pathways:     KEGG Pathways:  
    Probe set : 1393162_at    Name:   solute carrier family 39 (metal ion transporter), member 6 (predicted)    GB acc:   AI502079    UniGene:   Rn.99415    Gene Symbol:   Slc39a6_predicted    Locus Link:   291733    Chromosome:   18    Cytoband:   Chr:18p12    SOURCE    GENECARD       Gene Ontology:   biological process:  development   biological process:  nervous system development   biological process:  metal ion transport   cellular component:  endoplasmic reticulum   cellular component:  membrane   cellular component:  integral to membrane   molecular function:  metal ion transporter activity      BioCarta Pathways:     KEGG Pathways:  
    Probe set : 1369966_a_at    Name:   ribosomal protein S24    GB acc:   BG375811    UniGene:   Rn.979    Gene Symbol:   Rps24    Locus Link:   81776    Chromosome:   16    Cytoband:   Chr:16p16    SOURCE    GENECARD       Gene Ontology:   biological process:  protein biosynthesis   biological process:  ribosome biogenesis   molecular function:  RNA binding   molecular function:  structural constituent of ribosome      BioCarta Pathways:     KEGG Pathways:  
    Probe set : 1390923_a_at    Name:   oxysterol binding protein-like 1A    GB acc:   AI137224    UniGene:   Rn.19665    Gene Symbol:   Osbpl1a    Locus Link:   259221      SOURCE    GENECARD       Gene Ontology:   cellular component:  intracellular   molecular function:  phospholipid binding   molecular function:  bioactive lipid receptor activity      BioCarta Pathways:     KEGG Pathways:  
    Probe set : 1376206_at    Name:   Similar to RIKEN cDNA 9030624J02    GB acc:   BF398576    UniGene:   Rn.6279     Locus Link:   361635    Chromosome:   1    Cytoband:   Chr:1q35    SOURCE        Gene Ontology:     BioCarta Pathways:     KEGG Pathways:  
    Probe set : 1382049_at    Name:   similar to thymus atrophy-related protein (predicted)    GB acc:   AW530828    UniGene:   Rn.854    Gene Symbol:   RGD1304607_predicted    Locus Link:   361727    Chromosome:   1    Cytoband:   Chr:1q43    SOURCE    GENECARD       Gene Ontology:     BioCarta Pathways:     KEGG Pathways:  
    Probe set : 1383633_at    Name:   Transcribed locus    GB acc:   BI289884    UniGene:   Rn.153910        SOURCE        Gene Ontology:     BioCarta Pathways:     KEGG Pathways:  
    Probe set : 1387783_a_at    Name:   acetyl-Coenzyme A acyltransferase 1 (peroxisomal 3-oxoacyl-Coenzyme A thiolase)    GB acc:   NM_012489    UniGene:   Rn.8913    Gene Symbol:   Acaa1    Locus Link:   24157    Chromosome:   8    Cytoband:   Chr:8q32    SOURCE    GENECARD       Gene Ontology:   biological process:  generation of precursor metabolites and energy   biological process:  fatty acid metabolism   biological process:  lipid metabolism   cellular component:  peroxisome      BioCarta Pathways:     KEGG Pathways:  
    Probe set : 1389559_at    Name:   similar to Hypothetical protein MGC31278    GB acc:   AA892872    UniGene:   Rn.3891    Gene Symbol:   LOC362919    Locus Link:   362919    Chromosome:   7    Cytoband:   Chr:7q33    SOURCE    GENECARD       Gene Ontology:     BioCarta Pathways:     KEGG Pathways:  
    Probe set : 1392134_x_at    Name:   similar to KIAA0456 protein    GB acc:   BE099652    UniGene:   Rn.72217    Gene Symbol:   LOC360840    Locus Link:   360840    Chromosome:   13    Cytoband:   Chr:13q13    SOURCE    GENECARD       Gene Ontology:     BioCarta Pathways:     KEGG Pathways:  
    Probe set : 1393992_at    Name:   homeo box A7    GB acc:   AA996507     Gene Symbol:   Hoxa7    Locus Link:   500126    Chromosome:   4    Cytoband:   Chr:4q24     GENECARD       Gene Ontology:     BioCarta Pathways:     KEGG Pathways:  
    Probe set : 1383111_at    Name:   2-amino-3-carboxymuconate-6-semialdehyde decarboxylase    GB acc:   AI058895    UniGene:   Rn.43814    Gene Symbol:   Acmsd    Locus Link:   171385    Chromosome:   13    Cytoband:   Chr:13q12    SOURCE    GENECARD       Gene Ontology:   biological process:  metabolism   biological process:  quinolinate metabolism   cellular component:  soluble fraction   cellular component:  cytosol      BioCarta Pathways:     KEGG Pathways:  
    Probe set : 1385088_at    Name:   hypothetical LOC304650 (predicted)    GB acc:   AA891356    UniGene:   Rn.40355    Gene Symbol:   RGD1310262_predicted    Locus Link:   304650    Chromosome:   19    Cytoband:   Chr:19q11    SOURCE    GENECARD       Gene Ontology:     BioCarta Pathways:     KEGG Pathways:  
    Probe set : 1382746_s_at    Name:   Similar to lymphocyte alpha-kinase (predicted)    GB acc:   BE108581    UniGene:   Rn.17057     Locus Link:   310879    Chromosome:   2    Cytoband:   Chr:2q42    SOURCE        Gene Ontology:     BioCarta Pathways:     KEGG Pathways:  
    Probe set : 1393240_at    Name:   EGF-containing fibulin-like extracellular matrix protein 2    GB acc:   AI112299    UniGene:   Rn.16151    Gene Symbol:   RGD:1359496    Locus Link:   293677    Chromosome:   1    Cytoband:   Chr:1q43    SOURCE    GENECARD       Gene Ontology:   biological process:  homophilic cell adhesion   biological process:  blood coagulation   cellular component:  membrane   molecular function:  transmembrane receptor activity   molecular function:  structural molecule activity   molecular function:  calcium ion binding      BioCarta Pathways:     KEGG Pathways:  
    Probe set : 1372987_at    Name:   Transcribed locus, strongly similar to XP_233741.1 PREDICTED: similar to Non-receptor tyrosine-protein kinase TYK2 [Rattus norvegicus]    GB acc:   BF393934    UniGene:   Rn.38688        SOURCE        Gene Ontology:     BioCarta Pathways:     KEGG Pathways:  
    Probe set : 1367864_at    Name:   phosphofructokinase, muscle    GB acc:   NM_031715    UniGene:   Rn.11004    Gene Symbol:   Pfkm    Locus Link:   65152    Chromosome:   7    Cytoband:   Chr:7q36    SOURCE    GENECARD       Gene Ontology:   cellular component:  cytoplasm   cellular component:  6-phosphofructokinase complex      BioCarta Pathways:     KEGG Pathways:  
    Probe set : 1382293_at     GB acc:   BG377109              Gene Ontology:     BioCarta Pathways:     KEGG Pathways:  
    Probe set : 1395587_at    Name:   Similar to Alkaline phytoceramidase (aPHC) (Alkaline ceramidase)    GB acc:   BF389646    UniGene:   Rn.19122     Locus Link:   499210      SOURCE        Gene Ontology:     BioCarta Pathways:     KEGG Pathways:  
    Probe set : 1372357_at    Name:   Transcribed locus    GB acc:   BI281833    UniGene:   Rn.1185        SOURCE        Gene Ontology:     BioCarta Pathways:     KEGG Pathways:  
    Probe set : 1389650_at    Name:   Similar to zinc finger protein 366 (predicted)    GB acc:   BI295153    UniGene:   Rn.64318     Locus Link:   293044    Chromosome:   1    Cytoband:   Chr:1q31    SOURCE        Gene Ontology:     BioCarta Pathways:     KEGG Pathways:  
    Probe set : 1370393_at    Name:   coiled-coil domain containing 5    GB acc:   BF418951    UniGene:   Rn.29736    Gene Symbol:   Ccdc5    Locus Link:   192228    Chromosome:   18    Cytoband:   Chr:18q12.3    SOURCE    GENECARD       Gene Ontology:   biological process:  cell cycle   biological process:  mitosis   biological process:  cell division   cellular component:  microtubule      BioCarta Pathways:     KEGG Pathways:  
    Probe set : 1398491_at    Name:   Transcribed locus, strongly similar to NP_598870.1 immunoglobulin-like domain containing receptor 1 [Mus musculus]    GB acc:   BM383434    UniGene:   Rn.54040        SOURCE        Gene Ontology:     BioCarta Pathways:     KEGG Pathways:  
    Probe set : 1380037_at    Name:   Hypothetical LOC292764    GB acc:   BI294354    UniGene:   Rn.12517    Gene Symbol:   MGC94040    Locus Link:   292764    Chromosome:   1    Cytoband:   Chr:1q21    SOURCE    GENECARD       Gene Ontology:     BioCarta Pathways:     KEGG Pathways:  
    Probe set : 1378013_at    Name:   hypothetical LOC361199    GB acc:   BF283303    UniGene:   Rn.24746    Gene Symbol:   LOC361199    Locus Link:   361199    Chromosome:   17    Cytoband:   Chr:17p14    SOURCE    GENECARD       Gene Ontology:     BioCarta Pathways:     KEGG Pathways:  
    Probe set : 1398780_at    Name:   Rab acceptor 1 (prenylated)    GB acc:   NM_031774    UniGene:   Rn.25604    Gene Symbol:   Rabac1    Locus Link:   83583    Chromosome:   1    Cytoband:   Chr:1q21    SOURCE    GENECARD       Gene Ontology:   biological process:  vesicle-mediated transport      BioCarta Pathways:     KEGG Pathways:  
    Probe set : 1387413_at    Name:   neutrophil cytosolic factor 1    GB acc:   NM_053734    UniGene:   Rn.38575    Gene Symbol:   Ncf1    Locus Link:   114553    Chromosome:   12    Cytoband:   Chr:12q12    SOURCE    GENECARD       Gene Ontology:   cellular component:  cytosol   molecular function:  superoxide-generating NADPH oxidase activity      BioCarta Pathways:     KEGG Pathways:  
    Probe set : 1375491_at     GB acc:   BI299116              Gene Ontology:     BioCarta Pathways:     KEGG Pathways:  
    Probe set : 1396521_at    Name:   StAR-related lipid transfer (START) domain containing 4 (predicted)    GB acc:   BF396135    UniGene:   Rn.57496    Gene Symbol:   Stard4_predicted    Locus Link:   291699    Chromosome:   18    Cytoband:   Chr:18p12    SOURCE    GENECARD       Gene Ontology:   biological process:  steroid biosynthesis   biological process:  transport   biological process:  lipid transport   cellular component:  mitochondrion   molecular function:  lipid binding   molecular function:  cholesterol binding   molecular function:  cholesterol transporter activity      BioCarta Pathways:     KEGG Pathways:  
    Probe set : 1374885_at    Name:   Solute carrier family 12, member 6 (predicted)    GB acc:   AI172278    UniGene:   Rn.13442     Locus Link:   362189    Chromosome:   3    Cytoband:   Chr:3q34    SOURCE        Gene Ontology:     BioCarta Pathways:     KEGG Pathways:  
    Probe set : 1387223_at    Name:   aminoadipate aminotransferase    GB acc:   NM_017193    UniGene:   Rn.11133    Gene Symbol:   Aadat    Locus Link:   29416    Chromosome:   16    Cytoband:   Chr:16p12    SOURCE    GENECARD       Gene Ontology:   biological process:  biosynthesis   cellular component:  mitochondrion      BioCarta Pathways:     KEGG Pathways:  
    Probe set : 1391946_at    Name:   selectin, platelet    GB acc:   BI296054    UniGene:   Rn.10012    Gene Symbol:   Selp    Locus Link:   25651    Chromosome:   13    Cytoband:   Chr:13q22    SOURCE    GENECARD       Gene Ontology:   molecular function:  protein binding   molecular function:  sugar binding      BioCarta Pathways:     KEGG Pathways:  
    Probe set : 1387737_at    Name:   methionine adenosyltransferase II, alpha    GB acc:   NM_134351    UniGene:   Rn.41420    Gene Symbol:   Mat2a    Locus Link:   171347    Chromosome:   4    Cytoband:   Chr:4q33    SOURCE    GENECARD       Gene Ontology:   biological process:  S-adenosylmethionine biosynthesis   biological process:  one-carbon compound metabolism      BioCarta Pathways:     KEGG Pathways:  
    Probe set : 1389386_at    Name:   similar to hypothetical protein DKFZp313N0621    GB acc:   AI170759    UniGene:   Rn.20696    Gene Symbol:   LOC363169    Locus Link:   363169    Chromosome:   8    Cytoband:   Chr:8q32    SOURCE    GENECARD       Gene Ontology:     BioCarta Pathways:     KEGG Pathways:  
    Probe set : 1385187_at    Name:   similar to hypothetical protein MGC38524 (predicted)    GB acc:   AI555738    UniGene:   Rn.105830    Gene Symbol:   RGD1309002_predicted    Locus Link:   290637    Chromosome:   16    Cytoband:   Chr:16p14    SOURCE    GENECARD       Gene Ontology:     BioCarta Pathways:     KEGG Pathways:  
    Probe set : 1370141_at    Name:   myeloid cell leukemia sequence 1    GB acc:   NM_021846     Gene Symbol:   Mcl1    Locus Link:   60430    Chromosome:   2    Cytoband:   Chr:2q34     GENECARD       Gene Ontology:   cellular component:  cytoplasm   cellular component:  mitochondrial outer membrane   cellular component:  integral to membrane   molecular function:  protein binding   molecular function:  protein channel activity   molecular function:  protein heterodimerization activity      BioCarta Pathways:     KEGG Pathways:  
    Probe set : 1398448_at    Name:   Thymic stromal cotransporter (predicted)    GB acc:   BE108346    UniGene:   Rn.20842     Locus Link:   298034    Chromosome:   5    Cytoband:   Chr:5q24    SOURCE        Gene Ontology:   cellular component:  cell surface   cellular component:  integral to membrane      BioCarta Pathways:     KEGG Pathways:  
    Probe set : 1376218_a_at    Name:   Atpase, class I, type 8B, member 2 (predicted)    GB acc:   BI273855    UniGene:   Rn.20321     Locus Link:   361984    Chromosome:   2    Cytoband:   Chr:2q34    SOURCE        Gene Ontology:   biological process:  cation transport   biological process:  metabolism   cellular component:  membrane   cellular component:  integral to membrane      BioCarta Pathways:     KEGG Pathways:  
    Probe set : 1372538_at    Name:   solute carrier family 25 (mitochondrial carrier, peroxisomal membrane protein), member 17 (predicted)    GB acc:   BM389148    UniGene:   Rn.94733    Gene Symbol:   Slc25a17_predicted    Locus Link:   300083    Chromosome:   7    Cytoband:   Chr:7q34    SOURCE    GENECARD       Gene Ontology:   biological process:  transport   biological process:  mitochondrial transport   molecular function:  transporter activity   molecular function:  carrier activity   molecular function:  binding      BioCarta Pathways:     KEGG Pathways:  
    Probe set : 1387905_at    Name:   J domain protein 1    GB acc:   AB062135     Gene Symbol:   LOC619393    Locus Link:   619393       GENECARD       Gene Ontology:     BioCarta Pathways:     KEGG Pathways:  
    Probe set : 1370399_at    Name:   cytochrome P450, family 4, subfamily b, polypeptide 1    GB acc:   M29853    UniGene:   Rn.86651    Gene Symbol:   Cyp4b1    Locus Link:   24307    Chromosome:   5    Cytoband:   Chr:5q36    SOURCE    GENECARD       Gene Ontology:   biological process:  electron transport      BioCarta Pathways:     KEGG Pathways:  
    Probe set : 1377523_at    Name:   Transcribed locus    GB acc:   BE113698    UniGene:   Rn.14648        SOURCE        Gene Ontology:     BioCarta Pathways:     KEGG Pathways:  
    Probe set : 1390967_a_at    Name:   Similar to RIKEN cDNA 2410006F12 (predicted)    GB acc:   AW919980    UniGene:   Rn.98615     Locus Link:   298688    Chromosome:   5    Cytoband:   Chr:5q36    SOURCE        Gene Ontology:   molecular function:  hydrolase activity      BioCarta Pathways:     KEGG Pathways:  
    Probe set : 1373826_at     GB acc:   AI170754              Gene Ontology:     BioCarta Pathways:     KEGG Pathways:  
    Probe set : 1370861_at    Name:   Cytochrome c oxidase, subunit VIa, polypeptide 1    GB acc:   BI282326    UniGene:   Rn.880    Gene Symbol:   Cox6a1    Locus Link:   25282    Chromosome:   12    Cytoband:   Chr:12q16    SOURCE    GENECARD       Gene Ontology:   biological process:  electron transport   biological process:  electron transport   molecular function:  cytochrome-c oxidase activity   molecular function:  cytochrome-c oxidase activity   molecular function:  oxidoreductase activity      BioCarta Pathways:     KEGG Pathways:  
    Probe set : 1372566_at    Name:   mitochondrial carrier homolog 2 (C. elegans) (predicted)    GB acc:   AI412014    UniGene:   Rn.4182    Gene Symbol:   Mtch2_predicted    Locus Link:   295922    Chromosome:   3    Cytoband:   Chr:3q24    SOURCE    GENECARD       Gene Ontology:   biological process:  transport   cellular component:  mitochondrion   cellular component:  integral to membrane   cellular component:  inner membrane   molecular function:  binding      BioCarta Pathways:     KEGG Pathways:  
    Probe set : 1391450_at    Name:   lysyl oxidase-like 2 (predicted)    GB acc:   AI044651    UniGene:   Rn.19018    Gene Symbol:   Loxl2_predicted    Locus Link:   290350    Chromosome:   15    Cytoband:   Chr:15p11    SOURCE    GENECARD       Gene Ontology:   biological process:  protein modification   biological process:  cell adhesion   biological process:  aging   cellular component:  extracellular space   cellular component:  membrane      BioCarta Pathways:     KEGG Pathways:  
    Probe set : 1371976_at    Name:   similar to RIKEN cDNA 9530058B02    GB acc:   AI102758    UniGene:   Rn.106191    Gene Symbol:   LOC497875    Locus Link:   497875    Chromosome:   10    Cytoband:   Chr:10q12    SOURCE    GENECARD       Gene Ontology:     BioCarta Pathways:     KEGG Pathways:  
    Probe set : 1372740_at    Name:   similar to Map4k6-pending protein    GB acc:   AA925557    UniGene:   Rn.32511    Gene Symbol:   RGD:1359135    Locus Link:   303259    Chromosome:   10    Cytoband:   Chr:10q24    SOURCE    GENECARD       Gene Ontology:   molecular function:  small GTPase regulator activity      BioCarta Pathways:     KEGG Pathways:  
    Probe set : 1385250_at    Name:   chromodomain helicase DNA binding protein 6 (predicted)    GB acc:   BF415132    UniGene:   Rn.43055    Gene Symbol:   Chd6_predicted    Locus Link:   311607    Chromosome:   3    Cytoband:   Chr:3q42    SOURCE    GENECARD       Gene Ontology:   biological process:  chromatin assembly or disassembly   cellular component:  chromatin   cellular component:  nucleus   molecular function:  chromatin binding   molecular function:  ATP binding      BioCarta Pathways:     KEGG Pathways:  
    Probe set : 1396755_at    Name:   Transcribed locus    GB acc:   BF399394    UniGene:   Rn.140447        SOURCE        Gene Ontology:     BioCarta Pathways:     KEGG Pathways:  
    Probe set : 1388815_at    Name:   similar to B430201G11Rik protein (predicted)    GB acc:   BE329438    UniGene:   Rn.138371    Gene Symbol:   RGD1311409_predicted    Locus Link:   361502    Chromosome:   1    Cytoband:   Chr:1q12    SOURCE    GENECARD       Gene Ontology:     BioCarta Pathways:     KEGG Pathways:  
    Probe set : 1376590_at    Name:   Transcribed locus    GB acc:   AA818732    UniGene:   Rn.147460        SOURCE        Gene Ontology:     BioCarta Pathways:     KEGG Pathways:  
    Probe set : 1389202_at     GB acc:   AA944520              Gene Ontology:     BioCarta Pathways:     KEGG Pathways:  
    Probe set : 1390802_at    Name:   similar to RIKEN cDNA 5033414D02 (predicted)    GB acc:   AI170769    UniGene:   Rn.106153    Gene Symbol:   RGD1306839_predicted    Locus Link:   293888    Chromosome:   1    Cytoband:   Chr:1q52    SOURCE    GENECARD       Gene Ontology:     BioCarta Pathways:     KEGG Pathways:  
    Probe set : 1372138_at    Name:   Cut-like 1 (Drosophila)    GB acc:   BM390477    UniGene:   Rn.10455    Gene Symbol:   Cutl1    Locus Link:   116639    Chromosome:   12    Cytoband:   Chr:12q12    SOURCE    GENECARD       Gene Ontology:   cellular component:  nucleus   cellular component:  cytoskeleton   cellular component:  kinesin complex      BioCarta Pathways:     KEGG Pathways:  
    Probe set : 1372794_at    Name:   death-associated kinase 2 (predicted)    GB acc:   BM392203    UniGene:   Rn.98325    Gene Symbol:   Dapk2_predicted    Locus Link:   300799    Chromosome:   8    Cytoband:   Chr:8q24    SOURCE    GENECARD       Gene Ontology:   cellular component:  cytoplasm      BioCarta Pathways:     KEGG Pathways:  
    Probe set : 1387732_at    Name:   mitochondrial transcription termination factor 1    GB acc:   NM_053499    UniGene:   Rn.64629    Gene Symbol:   Mterf    Locus Link:   85261    Chromosome:   4    Cytoband:   Chr:4q13    SOURCE    GENECARD       Gene Ontology:   cellular component:  mitochondrion   molecular function:  double-stranded DNA binding   molecular function:  transcription termination factor activity   molecular function:  DNA binding      BioCarta Pathways:     KEGG Pathways:  
    Probe set : 1386718_at    Name:   similar to aldo-keto reductase family 1, member C12    GB acc:   BF566188    UniGene:   Rn.132470    Gene Symbol:   LOC307096    Locus Link:   307096    Chromosome:   17    Cytoband:   Chr:17q12.3    SOURCE    GENECARD       Gene Ontology:     BioCarta Pathways:     KEGG Pathways:  
    Probe set : 1399027_at    Name:   ras homolog gene family, member A    GB acc:   AI408053    UniGene:   Rn.107401    Gene Symbol:   RGD:619921    Locus Link:   117273    Chromosome:   16    Cytoband:   Chr:16q12.5    SOURCE    GENECARD       Gene Ontology:   biological process:  cell adhesion   biological process:  cell-matrix adhesion   biological process:  integrin-mediated signaling pathway   biological process:  small GTPase mediated signal transduction   biological process:  Rho protein signal transduction   biological process:  striated muscle development   biological process:  protein transport   biological process:  actin cytoskeleton organization and biogenesis   biological process:  cell differentiation   biological process:  positive regulation of NF-kappaB import into nucleus   biological process:  positive regulation of I-kappaB kinase/NF-kappaB cascade   biological process:  stress fiber formation   biological process:  Rho protein signal transduction   biological process:  actin cytoskeleton organization and biogenesis   biological process:  positive regulation of NF-kappaB import into nucleus   biological process:  positive regulation of I-kappaB kinase/NF-kappaB cascade   cellular component:  cytosol   cellular component:  cytoskeleton   cellular component:  membrane      BioCarta Pathways:     KEGG Pathways:  
    Probe set : 1386293_at     GB acc:   AA892593              Gene Ontology:     BioCarta Pathways:     KEGG Pathways:  
    Probe set : 1377037_at    Name:   similar to peroxisomal long chain acyl-CoA thioesterase Ia    GB acc:   AI411493    UniGene:   Rn.145093    Gene Symbol:   LOC314304    Locus Link:   314304    Chromosome:   6    Cytoband:   Chr:6q31    SOURCE    GENECARD       Gene Ontology:     BioCarta Pathways:     KEGG Pathways:  
    Probe set : 1368062_at    Name:   adaptor-related protein complex 3, mu 1 subunit    GB acc:   NM_133593    UniGene:   Rn.10959    Gene Symbol:   Ap3m1    Locus Link:   171126    Chromosome:   15    Cytoband:   Chr:15p16    SOURCE    GENECARD       Gene Ontology:   molecular function:  protein transporter activity      BioCarta Pathways:     KEGG Pathways:  
    Probe set : 1389269_at    Name:   similar to RIKEN cDNA 4933433P14 gene (predicted)    GB acc:   BI285251    UniGene:   Rn.3701    Gene Symbol:   RGD1308470_predicted    Locus Link:   362778    Chromosome:   6    Cytoband:   Chr:6q32    SOURCE    GENECARD       Gene Ontology:     BioCarta Pathways:     KEGG Pathways:  
    Probe set : 1379960_at    Name:   sushi domain containing 3 (predicted)    GB acc:   AI406705    UniGene:   Rn.3005    Gene Symbol:   Susd3_predicted    Locus Link:   306810    Chromosome:   17    Cytoband:   Chr:17p14    SOURCE    GENECARD       Gene Ontology:     BioCarta Pathways:     KEGG Pathways:  
    Probe set : 1390287_at    Name:   bridging integrator 2 (predicted)    GB acc:   BM386830    UniGene:   Rn.47919    Gene Symbol:   Bin2_predicted    Locus Link:   366988    Chromosome:   7    Cytoband:   Chr:7q36    SOURCE    GENECARD       Gene Ontology:   cellular component:  cytoplasm   molecular function:  protein binding      BioCarta Pathways:     KEGG Pathways:  
    Probe set : 1374225_at    Name:   CDNA clone IMAGE:7389359    GB acc:   BE112921    UniGene:   Rn.27063        SOURCE        Gene Ontology:     BioCarta Pathways:     KEGG Pathways:  
    Probe set : 1371587_at    Name:   mitogen-activated protein kinase kinase 1 interacting protein 1 (predicted)    GB acc:   AA817829    UniGene:   Rn.1123    Gene Symbol:   Map2k1ip1_predicted    Locus Link:   362045    Chromosome:   2    Cytoband:   Chr:2q44    SOURCE    GENECARD       Gene Ontology:   molecular function:  kinase activity      BioCarta Pathways:     KEGG Pathways:  
    Probe set : 1393421_at    Name:   peroxisomal membrane protein 4    GB acc:   BG664011    UniGene:   Rn.7752    Gene Symbol:   Pxmp4    Locus Link:   282634    Chromosome:   3    Cytoband:   Chr:3q41    SOURCE    GENECARD       Gene Ontology:   cellular component:  peroxisome   cellular component:  integral to membrane      BioCarta Pathways:     KEGG Pathways:  
    Probe set : 1378328_at    Name:   Transcribed locus    GB acc:   AI169255    UniGene:   Rn.28012        SOURCE        Gene Ontology:     BioCarta Pathways:     KEGG Pathways:  
    Probe set : 1381428_a_at    Name:   Histone 1, H2bh (predicted)    GB acc:   AI177364    UniGene:   Rn.42931    Gene Symbol:   Hfe    Locus Link:   29199    Chromosome:   17    Cytoband:   Chr:17p11    SOURCE    GENECARD       Gene Ontology:   molecular function:  MHC class I receptor activity      BioCarta Pathways:     KEGG Pathways:  
    Probe set : 1390497_at    Name:   Transcribed locus    GB acc:   AI175594    UniGene:   Rn.20866        SOURCE        Gene Ontology:     BioCarta Pathways:     KEGG Pathways:  
    Probe set : 1379581_at    Name:   Transcribed locus, weakly similar to XP_579465.1 PREDICTED: DNA binding protein N5 [Rattus norvegicus]    GB acc:   BI288586    UniGene:   Rn.22587        SOURCE        Gene Ontology:     BioCarta Pathways:     KEGG Pathways:  
    Probe set : 1373375_at    Name:   Rab6 interacting protein 1 (predicted)    GB acc:   BI275835    UniGene:   Rn.107101    Gene Symbol:   Rab6ip1_predicted    Locus Link:   308942    Chromosome:   1    Cytoband:   Chr:1q33    SOURCE    GENECARD       Gene Ontology:   molecular function:  nucleic acid binding   molecular function:  protein binding   molecular function:  Rab GTPase binding      BioCarta Pathways:     KEGG Pathways:  
    Probe set : 1396113_at    Name:   similar to Dorz1 (predicted)    GB acc:   AI716909    UniGene:   Rn.41351    Gene Symbol:   RGD1309721_predicted    Locus Link:   300982    Chromosome:   8    Cytoband:   Chr:8q32    SOURCE    GENECARD       Gene Ontology:   biological process:  aromatic compound metabolism   molecular function:  catalytic activity   molecular function:  hydrolase activity      BioCarta Pathways:     KEGG Pathways:  
    Probe set : 1388786_at    Name:   Transcribed locus    GB acc:   BF407452    UniGene:   Rn.7071        SOURCE        Gene Ontology:     BioCarta Pathways:     KEGG Pathways:  
    Probe set : 1373996_at    Name:   Transcribed locus    GB acc:   AI236940    UniGene:   Rn.11426        SOURCE        Gene Ontology:     BioCarta Pathways:     KEGG Pathways:  
    Probe set : 1383165_at    Name:   similar to KIAA1324 protein (predicted)    GB acc:   BM390462    UniGene:   Rn.22751    Gene Symbol:   RGD1310209_predicted    Locus Link:   362019    Chromosome:   2    Cytoband:   Chr:2q34    SOURCE    GENECARD       Gene Ontology:     BioCarta Pathways:     KEGG Pathways:  
    Probe set : 1367656_at    Name:   proteasome (prosome, macropain) subunit, beta type 7    GB acc:   NM_053532    UniGene:   Rn.3846    Gene Symbol:   Psmb7    Locus Link:   85492    Chromosome:   3    Cytoband:   Chr:3q11    SOURCE    GENECARD       Gene Ontology:   biological process:  ubiquitin-dependent protein catabolism   cellular component:  cytosol   cellular component:  proteasome core complex (sensu Eukaryota)   cellular component:  protein complex      BioCarta Pathways:     KEGG Pathways:  
    Probe set : 1376078_at    Name:   Transcribed locus    GB acc:   BI296274    UniGene:   Rn.22415        SOURCE        Gene Ontology:     BioCarta Pathways:     KEGG Pathways:  
    Probe set : 1367803_at    Name:   nucleoporin 54    GB acc:   NM_017361    UniGene:   Rn.11160    Gene Symbol:   Nup54    Locus Link:   53372    Chromosome:   14    Cytoband:   Chr:14p22    SOURCE    GENECARD       Gene Ontology:   cellular component:  nucleus   cellular component:  nuclear pore   cellular component:  nuclear pore   cellular component:  nuclear pore   molecular function:  nucleocytoplasmic transporter activity   molecular function:  protein binding   molecular function:  nucleocytoplasmic transporter activity      BioCarta Pathways:     KEGG Pathways:  
    Probe set : 1384111_at    Name:   signal transducer and activator of transcription 4 (predicted)    GB acc:   AA955354    UniGene:   Rn.137580    Gene Symbol:   Stat4_predicted    Locus Link:   367264    Chromosome:   9    Cytoband:   Chr:9q22    SOURCE    GENECARD       Gene Ontology:   cellular component:  nucleus      BioCarta Pathways:     KEGG Pathways:  
    Probe set : 1398902_at    Name:   Similar to mKIAA0664 protein (predicted)    GB acc:   BF282978    UniGene:   Rn.92100     Locus Link:   303300    Chromosome:   10    Cytoband:   Chr:10q24    SOURCE        Gene Ontology:     BioCarta Pathways:     KEGG Pathways:  
    Probe set : 1393776_at    Name:   Similar to lymphocyte antigen 6 complex, locus E    GB acc:   AA955277    UniGene:   Rn.3321     Locus Link:   362934    Chromosome:   7    Cytoband:   Chr:7q34    SOURCE        Gene Ontology:     BioCarta Pathways:     KEGG Pathways:  
    Probe set : 1396317_at    Name:   cell cycle progression 1 (predicted)    GB acc:   AI764350    UniGene:   Rn.43385    Gene Symbol:   Ccpg1_predicted    Locus Link:   363098    Chromosome:   8    Cytoband:   Chr:8q24    SOURCE    GENECARD       Gene Ontology:     BioCarta Pathways:     KEGG Pathways:  
    Probe set : 1369113_at    Name:   gremlin 1 homolog, cysteine knot superfamily (Xenopus laevis)    GB acc:   NM_019282    UniGene:   Rn.42929    Gene Symbol:   Grem1    Locus Link:   50566    Chromosome:   3    Cytoband:   Chr:3q34    SOURCE    GENECARD       Gene Ontology:   cellular component:  extracellular space   molecular function:  cytokine activity      BioCarta Pathways:     KEGG Pathways:  
    Probe set : 1376829_at    Name:   Transcribed locus    GB acc:   AI716500    UniGene:   Rn.39005        SOURCE        Gene Ontology:     BioCarta Pathways:     KEGG Pathways:  
    Probe set : 1389634_at    Name:   methionyl aminopeptidase 1 (predicted)    GB acc:   AI101470    UniGene:   Rn.6799    Gene Symbol:   Metap1_predicted    Locus Link:   295500    Chromosome:   2    Cytoband:   Chr:2q44    SOURCE    GENECARD       Gene Ontology:   biological process:  proteolysis      BioCarta Pathways:     KEGG Pathways:  
    Probe set : 1372911_at    Name:   Transcribed locus, moderately similar to XP_580018.1 PREDICTED: hypothetical protein XP_580018 [Rattus norvegicus]    GB acc:   BI281950    UniGene:   Rn.27185        SOURCE        Gene Ontology:     BioCarta Pathways:     KEGG Pathways:  
    Probe set : 1382007_at    Name:   SH3-domain GRB2-like B1 (endophilin) (predicted)    GB acc:   AA964563    UniGene:   Rn.144774    Gene Symbol:   Sh3glb1_predicted    Locus Link:   292156    Chromosome:   2    Cytoband:   Chr:2q44    SOURCE    GENECARD       Gene Ontology:     BioCarta Pathways:     KEGG Pathways:  
    Probe set : 1385190_at    Name:   Transcribed locus    GB acc:   BG376453    UniGene:   Rn.66394        SOURCE        Gene Ontology:     BioCarta Pathways:     KEGG Pathways:  
    Probe set : 1377630_at    Name:   transmembrane 4 superfamily member 13 (predicted) /// similar to transmembrane 4 superfamily member 13    GB acc:   AI408602    UniGene:   Rn.24284    Gene Symbol:   Tm4sf13_predicted /// LOC500643    Locus Link:   366602 /// 500643    Chromosome:   6    Cytoband:   Chr:6q16    SOURCE    GENECARD       Gene Ontology:   cellular component:  membrane fraction   cellular component:  integral to plasma membrane   cellular component:  integral to membrane      BioCarta Pathways:     KEGG Pathways:  
    Probe set : 1371886_at    Name:   carnitine acetyltransferase    GB acc:   AI411979    UniGene:   Rn.6249    Gene Symbol:   RGD:1303031    Locus Link:   311849    Chromosome:   3    Cytoband:   Chr:3p12    SOURCE    GENECARD       Gene Ontology:   biological process:  lipid metabolism   biological process:  fatty acid metabolism   biological process:  transport   cellular component:  mitochondrion   cellular component:  peroxisome   cellular component:  endoplasmic reticulum   molecular function:  carnitine O-acetyltransferase activity   molecular function:  acyltransferase activity   molecular function:  transferase activity      BioCarta Pathways:     KEGG Pathways:  
    Probe set : 1377052_at    Name:   Tripartite motif protein 26    GB acc:   AW533469    UniGene:   Rn.38919    Gene Symbol:   Trim26    Locus Link:   309586    Chromosome:   20    Cytoband:   Chr:20p12    SOURCE    GENECARD       Gene Ontology:     BioCarta Pathways:     KEGG Pathways:  
    Probe set : 1370329_at    Name:   cytochrome P450, family 2, subfamily d, polypeptide 22    GB acc:   U48219    UniGene:   Rn.26060    Gene Symbol:   Cyp2d22    Locus Link:   171522    Chromosome:   7    Cytoband:   Chr:7q34    SOURCE    GENECARD       Gene Ontology:   biological process:  electron transport   biological process:  drug metabolism      BioCarta Pathways:     KEGG Pathways:  
    Probe set : 1368907_at    Name:   secretory carrier membrane protein 5    GB acc:   NM_031726    UniGene:   Rn.24420    Gene Symbol:   Scamp5    Locus Link:   65171    Chromosome:   8    Cytoband:   Chr:8q24    SOURCE    GENECARD       Gene Ontology:   biological process:  transport   biological process:  protein transport   cellular component:  synaptic vesicle   cellular component:  integral to membrane      BioCarta Pathways:     KEGG Pathways:  
    Probe set : 1391649_a_at    Name:   Transcribed locus    GB acc:   AW915522    UniGene:   Rn.128940        SOURCE        Gene Ontology:     BioCarta Pathways:     KEGG Pathways:  
    Probe set : 1371332_at    Name:   Histone 1, H4a (predicted)    GB acc:   AA848821    UniGene:   Rn.3987     Locus Link:   364716    Chromosome:   17    Cytoband:   Chr:17p11    SOURCE        Gene Ontology:     BioCarta Pathways:     KEGG Pathways:  
    Probe set : 1389640_at    Name:   Similar to SH3 domain binding glutamic acid-rich protein like 2    GB acc:   BM391811    UniGene:   Rn.7595     Locus Link:   501026      SOURCE        Gene Ontology:     BioCarta Pathways:     KEGG Pathways:  
    Probe set : 1395146_at    Name:   adiponectin receptor 2 (predicted)    GB acc:   BI290608    UniGene:   Rn.101984    Gene Symbol:   Adipor2_predicted    Locus Link:   312670    Chromosome:   4    Cytoband:   Chr:4q42    SOURCE    GENECARD       Gene Ontology:   cellular component:  integral to membrane      BioCarta Pathways:     KEGG Pathways:  
    Probe set : 1389418_at     GB acc:   AI410452              Gene Ontology:     BioCarta Pathways:     KEGG Pathways:  
    Probe set : 1369743_a_at    Name:   purinergic receptor P2X, ligand-gated ion channel 4    GB acc:   U32497    UniGene:   Rn.7176    Gene Symbol:   P2rx4    Locus Link:   29659    Chromosome:   12    Cytoband:   Chr:12q16    SOURCE    GENECARD       Gene Ontology:     BioCarta Pathways:     KEGG Pathways:  
    Probe set : 1382265_at    Name:   similar to KIAA1128 protein (predicted)    GB acc:   AI070061    UniGene:   Rn.96252    Gene Symbol:   RGD1304626_predicted    Locus Link:   306306    Chromosome:   16    Cytoband:   Chr:16p14    SOURCE    GENECARD       Gene Ontology:     BioCarta Pathways:     KEGG Pathways:  
    Probe set : 1368813_at    Name:   CCAAT/enhancer binding protein (C/EBP), delta    GB acc:   BF419200    UniGene:   Rn.6975    Gene Symbol:   Cebpd    Locus Link:   25695      SOURCE    GENECARD       Gene Ontology:   cellular component:  nucleus   cellular component:  nucleus   cellular component:  Golgi stack      BioCarta Pathways:     KEGG Pathways:  
    Probe set : 1386864_at    Name:   phosphoglycerate mutase 1    GB acc:   NM_053290    UniGene:   Rn.1383    Gene Symbol:   Pgam1    Locus Link:   24642      SOURCE    GENECARD       Gene Ontology:   biological process:  glycolysis   biological process:  metabolism   cellular component:  cytosol      BioCarta Pathways:     KEGG Pathways:  
    Probe set : 1372380_at    Name:   similar to RNA binding protein gene with multiple splicing    GB acc:   AI231308    UniGene:   Rn.8565    Gene Symbol:   LOC498642    Locus Link:   498642    Chromosome:   16    Cytoband:   Chr:16q12.3    SOURCE    GENECARD       Gene Ontology:     BioCarta Pathways:     KEGG Pathways:  
    Probe set : 1368965_at    Name:   monocarboxylate transporter    GB acc:   NM_030834    UniGene:   Rn.10826    Gene Symbol:   Slc16a3    Locus Link:   80878    Chromosome:   10    Cytoband:   Chr:10q32.3    SOURCE    GENECARD       Gene Ontology:   biological process:  transport   biological process:  organic anion transport   biological process:  monocarboxylic acid transport   cellular component:  membrane fraction   cellular component:  integral to plasma membrane   cellular component:  integral to membrane   molecular function:  transporter activity   molecular function:  symporter activity   molecular function:  monocarboxylate porter activity      BioCarta Pathways:     KEGG Pathways:  
    Probe set : 1371040_at    Name:   sodium-dependent neutral amino acid transporter ASCT2    GB acc:   BG372334    UniGene:   Rn.28965    Gene Symbol:   Slc1a5    Locus Link:   292657    Chromosome:   1    Cytoband:   Chr:1q21    SOURCE    GENECARD       Gene Ontology:   biological process:  extracellular amino acid transport   biological process:  dicarboxylic acid transport   cellular component:  membrane   molecular function:  amino acid transporter activity   molecular function:  glutamate binding   molecular function:  sodium:dicarboxylate symporter activity      BioCarta Pathways:     KEGG Pathways:  
    Probe set : 1376902_x_at    Name:   Similar to Hypothetical protein 6330514E13    GB acc:   AA964675    UniGene:   Rn.93288     Locus Link:   315117    Chromosome:   7    Cytoband:   Chr:7q34    SOURCE        Gene Ontology:     BioCarta Pathways:     KEGG Pathways:  
    Probe set : 1373508_at    Name:   similar to Selenoprotein T precursor    GB acc:   AI180004     Gene Symbol:   LOC499625    Locus Link:   499625    Chromosome:   2    Cytoband:   Chr:2q31     GENECARD       Gene Ontology:     BioCarta Pathways:     KEGG Pathways:  
    Probe set : 1391822_at    Name:   Transcribed locus    GB acc:   AW253356    UniGene:   Rn.153833        SOURCE        Gene Ontology:     BioCarta Pathways:     KEGG Pathways:  
    Probe set : 1368366_at    Name:   Camello-like 2    GB acc:   NM_021668    UniGene:   Rn.20565    Gene Symbol:   Cml2    Locus Link:   59300    Chromosome:   4    Cytoband:   Chr:4q34    SOURCE    GENECARD       Gene Ontology:   molecular function:  N-acetyltransferase activity   molecular function:  N-acetyltransferase activity   molecular function:  transferase activity      BioCarta Pathways:     KEGG Pathways:  
    Probe set : 1379384_at    Name:   Sp1 transcription factor    GB acc:   BF288328    UniGene:   Rn.44609    Gene Symbol:   Sp1    Locus Link:   24790    Chromosome:   7    Cytoband:   Chr:7q36    SOURCE    GENECARD       Gene Ontology:   cellular component:  nucleus   cellular component:  transcription factor complex      BioCarta Pathways:     KEGG Pathways:  
    Probe set : 1386926_at    Name:   acyl-CoA synthetase long-chain family member 5    GB acc:   NM_053607    UniGene:   Rn.105862    Gene Symbol:   Acsl5    Locus Link:   94340    Chromosome:   1    Cytoband:   Chr:1q55    SOURCE    GENECARD       Gene Ontology:   cellular component:  mitochondrion   cellular component:  integral to membrane      BioCarta Pathways:     KEGG Pathways:  
    Probe set : 1374954_at    Name:   histone deacetylase 11 (predicted)    GB acc:   AI101322    UniGene:   Rn.28065    Gene Symbol:   Hdac11_predicted    Locus Link:   297453    Chromosome:   4    Cytoband:   Chr:4q34    SOURCE    GENECARD       Gene Ontology:   biological process:  regulation of transcription, DNA-dependent   biological process:  chromatin modification   biological process:  histone deacetylation   cellular component:  histone deacetylase complex   cellular component:  nucleus   molecular function:  histone deacetylase activity   molecular function:  transcription factor binding   molecular function:  hydrolase activity      BioCarta Pathways:     KEGG Pathways:  
    Probe set : 1394122_at    Name:   ras homolog gene family, member H (predicted)    GB acc:   AI012081    UniGene:   Rn.20618    Gene Symbol:   Rhoh_predicted    Locus Link:   305341    Chromosome:   14    Cytoband:   Chr:14p11    SOURCE    GENECARD       Gene Ontology:   cellular component:  cytoplasm      BioCarta Pathways:     KEGG Pathways:  
    Probe set : 1379313_at     GB acc:   BE108519              Gene Ontology:     BioCarta Pathways:     KEGG Pathways:  
    Probe set : 1399043_at    Name:   capping protein (actin filament) muscle Z-line, alpha 2    GB acc:   AI175266    UniGene:   Rn.17868    Gene Symbol:   Capza2    Locus Link:   493810    Chromosome:   4    Cytoband:   Chr:4q21    SOURCE    GENECARD       Gene Ontology:   biological process:  actin cytoskeleton organization and biogenesis   cellular component:  F-actin capping protein complex   molecular function:  actin binding      BioCarta Pathways:     KEGG Pathways:  
    Probe set : 1376219_at    Name:   Atpase, class I, type 8B, member 2 (predicted)    GB acc:   BI273855    UniGene:   Rn.20321     Locus Link:   361984    Chromosome:   2    Cytoband:   Chr:2q34    SOURCE        Gene Ontology:   biological process:  cation transport   biological process:  metabolism   cellular component:  membrane   cellular component:  integral to membrane      BioCarta Pathways:     KEGG Pathways:  
    Probe set : 1385334_at    Name:   Cytochrome P450, family 4, subfamily v, polypeptide 3 (predicted)    GB acc:   AA818166    UniGene:   Rn.8023    Gene Symbol:   Loc266761    Locus Link:   266761      SOURCE    GENECARD       Gene Ontology:   biological process:  electron transport      BioCarta Pathways:     KEGG Pathways:  
    Probe set : 1376703_at    Name:   nucleoporin 37 (predicted)    GB acc:   AI501977    UniGene:   Rn.135562    Gene Symbol:   Nup37_predicted    Locus Link:   299706    Chromosome:   7    Cytoband:   Chr:7q13    SOURCE    GENECARD       Gene Ontology:   biological process:  transport   biological process:  protein transport   cellular component:  nucleus   cellular component:  integral to membrane   cellular component:  outer membrane   molecular function:  porin activity      BioCarta Pathways:     KEGG Pathways:  
    Probe set : 1370005_at    Name:   cytochrome b5, outer mitochondrial membrane isoform    GB acc:   NM_030586    UniGene:   Rn.10249    Gene Symbol:   Cyb5m    Locus Link:   80773    Chromosome:   19    Cytoband:   Chr:19q12    SOURCE    GENECARD       Gene Ontology:   biological process:  electron transport   biological process:  transport      BioCarta Pathways:     KEGG Pathways:  
    Probe set : 1371924_at    Name:   olfactomedin-like 3 (predicted)    GB acc:   BI274355    UniGene:   Rn.106761    Gene Symbol:   Olfml3_predicted    Locus Link:   310743    Chromosome:   2    Cytoband:   Chr:2q34    SOURCE    GENECARD       Gene Ontology:   cellular component:  extracellular space      BioCarta Pathways:     KEGG Pathways:  
    Probe set : 1389274_at    Name:   similar to RIKEN cDNA 6720485C15    GB acc:   AA800803    UniGene:   Rn.102152    Gene Symbol:   RGD:1359211    Locus Link:   360639    Chromosome:   10    Cytoband:   Chr:10q32.1    SOURCE    GENECARD       Gene Ontology:   biological process:  nucleosome assembly   biological process:  chromosome organization and biogenesis (sensu Eukaryota)   cellular component:  nucleosome   cellular component:  nucleus   molecular function:  DNA binding   molecular function:  ATP binding      BioCarta Pathways:     KEGG Pathways:  
    Probe set : 1390463_at    Name:   Transcribed locus    GB acc:   BG378294    UniGene:   Rn.63780        SOURCE        Gene Ontology:     BioCarta Pathways:     KEGG Pathways:  
    Probe set : 1375739_at    Name:   EH-domain containing 4    GB acc:   BF395171    UniGene:   Rn.7379    Gene Symbol:   Ehd4    Locus Link:   192204    Chromosome:   3    Cytoband:   Chr:3q35    SOURCE    GENECARD       Gene Ontology:     BioCarta Pathways:     KEGG Pathways:  
    Probe set : 1384243_at    Name:   Transcribed locus    GB acc:   AI111413    UniGene:   Rn.11581        SOURCE        Gene Ontology:     BioCarta Pathways:     KEGG Pathways:  
    Probe set : 1370880_at    Name:   ribonuclease/angiogenin inhibitor 1    GB acc:   X62528    UniGene:   Rn.3756    Gene Symbol:   Rnh1    Locus Link:   245964    Chromosome:   1    Cytoband:   Chr:1q41    SOURCE    GENECARD       Gene Ontology:   biological process:  RNA catabolism   molecular function:  ribonuclease inhibitor activity   molecular function:  ribonuclease inhibitor activity      BioCarta Pathways:     KEGG Pathways:  
    Probe set : 1382008_at    Name:   similar to hypothetical protein FLJ11218 (predicted)    GB acc:   AI044348    UniGene:   Rn.15885    Gene Symbol:   RGD1309804_predicted    Locus Link:   361751    Chromosome:   1    Cytoband:   Chr:1q52    SOURCE    GENECARD       Gene Ontology:   biological process:  electron transport   biological process:  metabolism   molecular function:  oxidoreductase activity      BioCarta Pathways:     KEGG Pathways:  
    Probe set : 1373432_at    Name:   myristoylated alanine rich protein kinase C substrate    GB acc:   BE111604    UniGene:   Rn.9560    Gene Symbol:   Marcks    Locus Link:   25603      SOURCE    GENECARD       Gene Ontology:   biological process:  cell motility   cellular component:  plasma membrane   cellular component:  actin cytoskeleton   cellular component:  membrane      BioCarta Pathways:     KEGG Pathways:  
    Probe set : 1381364_at    Name:   transportin 1 (predicted)    GB acc:   BE116070    UniGene:   Rn.56083    Gene Symbol:   Tnpo1_predicted    Locus Link:   309126    Chromosome:   2    Cytoband:   Chr:2q12    SOURCE    GENECARD       Gene Ontology:   cellular component:  nucleus   cellular component:  nuclear pore   cellular component:  cytoplasm   molecular function:  binding   molecular function:  nuclear localization sequence binding   molecular function:  protein transporter activity      BioCarta Pathways:     KEGG Pathways:  
    Probe set : 1383928_a_at    Name:   Transcribed locus    GB acc:   AI170737    UniGene:   Rn.153582        SOURCE        Gene Ontology:     BioCarta Pathways:     KEGG Pathways:  
    Probe set : 1374884_at    Name:   protein phosphatase 1D magnesium-dependent, delta isoform (predicted)    GB acc:   AI233712    UniGene:   Rn.15540    Gene Symbol:   Ppm1d_predicted    Locus Link:   287585    Chromosome:   10    Cytoband:   Chr:10q26    SOURCE    GENECARD       Gene Ontology:   cellular component:  nucleus   cellular component:  protein serine/threonine phosphatase complex      BioCarta Pathways:     KEGG Pathways:  
    Probe set : 1383912_at    Name:   CDNA clone MGC:109201 IMAGE:7306744    GB acc:   BM390524    UniGene:   Rn.22702        SOURCE        Gene Ontology:     BioCarta Pathways:     KEGG Pathways:  
    Probe set : 1373233_at    Name:   Transcribed locus    GB acc:   AI013502    UniGene:   Rn.48641        SOURCE        Gene Ontology:     BioCarta Pathways:     KEGG Pathways:  
    Probe set : 1392289_at     GB acc:   BI276179              Gene Ontology:     BioCarta Pathways:     KEGG Pathways:  
    Probe set : 1387823_at    Name:   pleiotropic regulator 1 homolog (Arabidopsis)    GB acc:   NM_021757    UniGene:   Rn.44181    Gene Symbol:   Plrg1    Locus Link:   60376    Chromosome:   2    Cytoband:   Chr:2q34    SOURCE    GENECARD       Gene Ontology:   biological process:  nuclear mRNA splicing, via spliceosome   biological process:  mRNA processing   cellular component:  nucleus   cellular component:  spliceosome complex   molecular function:  transcription corepressor activity   molecular function:  signal transducer activity      BioCarta Pathways:     KEGG Pathways:  
    Probe set : 1380254_at    Name:   Transcribed locus, moderately similar to NP_079928.1 general transcription factor III A [Mus musculus]    GB acc:   AA893529    UniGene:   Rn.1571        SOURCE        Gene Ontology:     BioCarta Pathways:     KEGG Pathways:  
    Probe set : 1374326_at    Name:   peter pan homolog (Drosophila) (predicted)    GB acc:   AI178019    UniGene:   Rn.144881    Gene Symbol:   Ppan_predicted    Locus Link:   298699    Chromosome:   8    Cytoband:   Chr:8q13    SOURCE    GENECARD       Gene Ontology:   biological process:  RNA splicing      BioCarta Pathways:     KEGG Pathways:  
    Probe set : 1373842_at    Name:   Type II keratin Kb1    GB acc:   BM390718    UniGene:   Rn.31789    Gene Symbol:   Kb1    Locus Link:   300250    Chromosome:   7    Cytoband:   Chr:7q36    SOURCE    GENECARD       Gene Ontology:   cellular component:  intermediate filament   molecular function:  structural molecule activity      BioCarta Pathways:     KEGG Pathways:  
    Probe set : 1389704_at    Name:   Transcribed locus    GB acc:   AA850490    UniGene:   Rn.7010        SOURCE        Gene Ontology:     BioCarta Pathways:     KEGG Pathways:  
    Probe set : 1382803_at    Name:   mitogen activated protein kinase kinase kinase kinase 1 (predicted)    GB acc:   AI237423    UniGene:   Rn.137063    Gene Symbol:   Map4k1_predicted    Locus Link:   292763    Chromosome:   1    Cytoband:   Chr:1q21    SOURCE    GENECARD       Gene Ontology:     BioCarta Pathways:     KEGG Pathways:  
    Probe set : 1377970_at    Name:   similar to poly (ADP-ribose) polymerase family, member 8    GB acc:   BE105494    UniGene:   Rn.59516    Gene Symbol:   LOC294762    Locus Link:   294762    Chromosome:   2    Cytoband:   Chr:2q15    SOURCE    GENECARD       Gene Ontology:     BioCarta Pathways:     KEGG Pathways:  
    Probe set : 1374191_at    Name:   rhomboid family 1 (Drosophila) (predicted)    GB acc:   AA956764    UniGene:   Rn.6381    Gene Symbol:   Rhbdf1_predicted    Locus Link:   303008    Chromosome:   10    Cytoband:   Chr:10q12    SOURCE    GENECARD       Gene Ontology:   molecular function:  receptor activity   molecular function:  copper ion binding      BioCarta Pathways:     KEGG Pathways:  
    Probe set : 1387972_at    Name:   mucin and cadherin like    GB acc:   AF221952    UniGene:   Rn.18860    Gene Symbol:   Mucdhl    Locus Link:   171554    Chromosome:   1    Cytoband:   Chr:1q41    SOURCE    GENECARD       Gene Ontology:   biological process:  cell adhesion   biological process:  cell adhesion   biological process:  homophilic cell adhesion   biological process:  cell adhesion   molecular function:  calcium ion binding   molecular function:  protein binding   molecular function:  calcium ion binding      BioCarta Pathways:     KEGG Pathways:  
    Probe set : 1397821_at    Name:   Transcribed locus    GB acc:   BF523355    UniGene:   Rn.148049        SOURCE        Gene Ontology:     BioCarta Pathways:     KEGG Pathways:  
    Probe set : 1391015_at    Name:   Similar to DNA segment, Chr 11, ERATO Doi 461, expressed    GB acc:   AI548779    UniGene:   Rn.138612     Locus Link:   497906      SOURCE        Gene Ontology:     BioCarta Pathways:     KEGG Pathways:  
    Probe set : 1387052_at    Name:   glutamic pyruvic transaminase 1, soluble    GB acc:   NM_031039    UniGene:   Rn.6318    Gene Symbol:   Gpt1    Locus Link:   81670    Chromosome:   7    Cytoband:   Chr:7q34    SOURCE    GENECARD       Gene Ontology:   biological process:  gluconeogenesis   biological process:  nitrogen compound metabolism   biological process:  biosynthesis   cellular component:  soluble fraction   cellular component:  cytoplasm   cellular component:  cytosol      BioCarta Pathways:     KEGG Pathways:  
    Probe set : 1367717_at    Name:   ribosomal protein S27    GB acc:   NM_053597    UniGene:   Rn.144623    Gene Symbol:   Rps27    Locus Link:   94266    Chromosome:   2    Cytoband:   Chr:2q34    SOURCE    GENECARD       Gene Ontology:   biological process:  protein biosynthesis   biological process:  signal transduction   biological process:  cell proliferation      BioCarta Pathways:     KEGG Pathways:  
    Probe set : 1380333_at    Name:   protein tyrosine phosphatase, non-receptor type 8 (predicted)    GB acc:   AW915666    UniGene:   Rn.144838    Gene Symbol:   Ptpn8_predicted    Locus Link:   295338    Chromosome:   2    Cytoband:   Chr:2q34    SOURCE    GENECARD       Gene Ontology:   biological process:  protein amino acid dephosphorylation      BioCarta Pathways:     KEGG Pathways:  
    Probe set : 1373371_a_at    Name:   similar to RIKEN cDNA 1110001J03    GB acc:   AA892572    UniGene:   Rn.65180    Gene Symbol:   MGC112899    Locus Link:   500087    Chromosome:   4    Cytoband:   Chr:4q22    SOURCE    GENECARD       Gene Ontology:     BioCarta Pathways:     KEGG Pathways:  
    Probe set : 1373659_at     GB acc:   AI639350              Gene Ontology:     BioCarta Pathways:     KEGG Pathways:  
    Probe set : 1381816_at    Name:   similar to hypothetical protein FLJ11218 (predicted)    GB acc:   BE119617    UniGene:   Rn.15885    Gene Symbol:   RGD1309804_predicted    Locus Link:   361751    Chromosome:   1    Cytoband:   Chr:1q52    SOURCE    GENECARD       Gene Ontology:   biological process:  electron transport   biological process:  metabolism   molecular function:  oxidoreductase activity      BioCarta Pathways:     KEGG Pathways:  
    Probe set : 1389230_at    Name:   Arrestin domain containing 3    GB acc:   AA818910    UniGene:   Rn.4302    Gene Symbol:   Arrdc3    Locus Link:   309945    Chromosome:   2    Cytoband:   Chr:2q11    SOURCE    GENECARD       Gene Ontology:     BioCarta Pathways:     KEGG Pathways:  
    Probe set : 1391563_at    Name:   Transcribed locus    GB acc:   AA963184    UniGene:   Rn.12231        SOURCE        Gene Ontology:     BioCarta Pathways:     KEGG Pathways:  
    Probe set : 1373516_at    Name:   Similar to RIKEN cDNA 2600005C20 (predicted)    GB acc:   BE099830    UniGene:   Rn.54275     Locus Link:   309673    Chromosome:   20    Cytoband:   Chr:20p12    SOURCE        Gene Ontology:     BioCarta Pathways:     KEGG Pathways:  
    Probe set : 1387870_at    Name:   zinc finger protein 36    GB acc:   AB025017    UniGene:   Rn.82737    Gene Symbol:   Zfp36    Locus Link:   79426    Chromosome:   1    Cytoband:   Chr:1q21    SOURCE    GENECARD       Gene Ontology:   cellular component:  nucleus   cellular component:  nucleus   cellular component:  cytoplasm   cellular component:  cytoplasm      BioCarta Pathways:     KEGG Pathways:  
    Probe set : 1388885_at    Name:   similar to Aph1a-pending protein (predicted)    GB acc:   BI275921    UniGene:   Rn.145319    Gene Symbol:   RGD1311546_predicted    Locus Link:   365872    Chromosome:   2    Cytoband:   Chr:2q34    SOURCE    GENECARD       Gene Ontology:   biological process:  metanephros development   biological process:  protein processing   biological process:  positive regulation of enzyme activity   cellular component:  endoplasmic reticulum   cellular component:  Golgi apparatus   cellular component:  integral to membrane   molecular function:  protein binding   molecular function:  peptidase activity      BioCarta Pathways:     KEGG Pathways:  
    Probe set : 1367994_at    Name:   dihydropyrimidine dehydrogenase    GB acc:   NM_031027    UniGene:   Rn.133148    Gene Symbol:   Dpyd    Locus Link:   81656    Chromosome:   2    Cytoband:   Chr:2q41    SOURCE    GENECARD       Gene Ontology:   cellular component:  cytoplasm   cellular component:  mitochondrion   cellular component:  membrane      BioCarta Pathways:     KEGG Pathways:  
    Probe set : 1367537_at    Name:   similar to Clast4 protein    GB acc:   AI012479    UniGene:   Rn.105097    Gene Symbol:   LOC305468    Locus Link:   305468    Chromosome:   14    Cytoband:   Chr:14q21    SOURCE    GENECARD       Gene Ontology:     BioCarta Pathways:     KEGG Pathways:  
    Probe set : 1372901_at    Name:   Similar to RIKEN cDNA 8030451K01    GB acc:   BM386850    UniGene:   Rn.11681     Locus Link:   310416    Chromosome:   2    Cytoband:   Chr:2q26    SOURCE        Gene Ontology:     BioCarta Pathways:     KEGG Pathways:  
    Probe set : 1368980_at    Name:   phospholipase C, epsilon 1    GB acc:   NM_053758    UniGene:   Rn.64650    Gene Symbol:   Plce1    Locus Link:   114633    Chromosome:   1    Cytoband:   Chr:1q53    SOURCE    GENECARD       Gene Ontology:   biological process:  lipid metabolism   biological process:  signal transduction   biological process:  neuropeptide signaling pathway   biological process:  intracellular signaling cascade   biological process:  lipid catabolism   biological process:  activation of MAPK activity   biological process:  regulation of cell growth   biological process:  diacylglycerol biosynthesis   biological process:  regulation of smooth muscle contraction   biological process:  cytoskeleton organization and biogenesis   biological process:  G-protein signaling, coupled to IP3 second messenger (phospholipase C activating)   biological process:  elevation of cytosolic calcium ion concentration   biological process:  heart development   biological process:  regulation of G-protein coupled receptor protein signaling pathway   biological process:  calcium-mediated signaling   biological process:  regulation of protein kinase activity   biological process:  regulation of Ras protein signal transduction   biological process:  inositol phosphate-mediated signaling   cellular component:  membrane fraction      BioCarta Pathways:     KEGG Pathways:  
    Probe set : 1380243_at    Name:   similar to CG14803-PA (predicted)    GB acc:   BE098614    UniGene:   Rn.139999    Gene Symbol:   RGD1304693_predicted    Locus Link:   313108    Chromosome:   5    Cytoband:   Chr:5q21    SOURCE    GENECARD       Gene Ontology:     BioCarta Pathways:     KEGG Pathways:  
    Probe set : 1382136_at    Name:   Similar to GLUT9a    GB acc:   AA858900    UniGene:   Rn.92551     Locus Link:   501925      SOURCE        Gene Ontology:     BioCarta Pathways:     KEGG Pathways:  
    Probe set : 1367651_at    Name:   cathepsin D    GB acc:   NM_134334    UniGene:   Rn.11085    Gene Symbol:   Ctsd    Locus Link:   171293    Chromosome:   1    Cytoband:   Chr:1q41    SOURCE    GENECARD       Gene Ontology:   biological process:  proteolysis   biological process:  proteolysis   cellular component:  mitochondrion   cellular component:  lysosome   cellular component:  lysosome      BioCarta Pathways:     KEGG Pathways:  
    Probe set : 1370238_at     GB acc:   AA891707              Gene Ontology:     BioCarta Pathways:     KEGG Pathways:  
    Probe set : 1392837_at    Name:   P34 protein    GB acc:   AA859108    UniGene:   Rn.144663    Gene Symbol:   P34    Locus Link:   171435    Chromosome:   8    Cytoband:   Chr:8q24    SOURCE    GENECARD       Gene Ontology:   biological process:  transport   biological process:  protein transport      BioCarta Pathways:     KEGG Pathways:  
    Probe set : 1394627_at     GB acc:   AW527508              Gene Ontology:     BioCarta Pathways:     KEGG Pathways:  
    Probe set : 1380086_at    Name:   Transcribed locus    GB acc:   AI059192    UniGene:   Rn.19094        SOURCE        Gene Ontology:     BioCarta Pathways:     KEGG Pathways:  
    Probe set : 1376394_at    Name:   Transcribed locus    GB acc:   AW915454    UniGene:   Rn.14822        SOURCE        Gene Ontology:     BioCarta Pathways:     KEGG Pathways:  
    Probe set : 1379314_at     GB acc:   BF283985              Gene Ontology:     BioCarta Pathways:     KEGG Pathways:  
    Probe set : 1390207_at    Name:   Large tumor suppressor 2 (predicted)    GB acc:   BM390283    UniGene:   Rn.48618     Locus Link:   305922    Chromosome:   15    Cytoband:   Chr:15p12    SOURCE        Gene Ontology:   cellular component:  spindle pole      BioCarta Pathways:     KEGG Pathways:  
    Probe set : 1374414_at    Name:   similar to RIKEN cDNA 1300010M03 (predicted)    GB acc:   AI412962    UniGene:   Rn.13737    Gene Symbol:   RGD1306844_predicted    Locus Link:   363252    Chromosome:   9    Cytoband:   Chr:9q33    SOURCE    GENECARD       Gene Ontology:     BioCarta Pathways:     KEGG Pathways:  
    Probe set : 1388237_s_at    Name:   ATPase, Ca++ transporting, plasma membrane 3    GB acc:   M96626    UniGene:   Rn.11053    Gene Symbol:   Atp2b3    Locus Link:   29599    Chromosome:   X    Cytoband:   Chr:Xq37    SOURCE    GENECARD       Gene Ontology:   cellular component:  plasma membrane   cellular component:  membrane   cellular component:  integral to membrane   molecular function:  magnesium ion binding   molecular function:  calcium-transporting ATPase activity   molecular function:  calcium ion binding   molecular function:  calmodulin binding   molecular function:  ATP binding   molecular function:  ATPase activity, coupled to transmembrane movement of ions, phosphorylative mechanism   molecular function:  hydrolase activity   molecular function:  hydrolase activity, acting on acid anhydrides, catalyzing transmembrane movement of substances   molecular function:  cation-transporting ATPase activity   molecular function:  nucleotide binding   molecular function:  catalytic activity   molecular function:  calcium ion transporter activity   molecular function:  metal ion binding      BioCarta Pathways:     KEGG Pathways:  
    Probe set : 1373521_at    Name:   similar to RIKEN cDNA D430044G18 (predicted)    GB acc:   BF283122    UniGene:   Rn.24687    Gene Symbol:   RGD1309038_predicted    Locus Link:   307393    Chromosome:   18    Cytoband:   Chr:18q12.1    SOURCE    GENECARD       Gene Ontology:   molecular function:  binding      BioCarta Pathways:     KEGG Pathways:  
    Probe set : 1367649_at    Name:   paralemmin    GB acc:   NM_130829    UniGene:   Rn.18947    Gene Symbol:   Palm    Locus Link:   170673    Chromosome:   7    Cytoband:   Chr:7q11    SOURCE    GENECARD       Gene Ontology:   cellular component:  plasma membrane   cellular component:  internal side of plasma membrane   cellular component:  membrane      BioCarta Pathways:     KEGG Pathways:  
    Probe set : 1396719_at    Name:   Transcribed locus    GB acc:   BF399856    UniGene:   Rn.131671        SOURCE        Gene Ontology:     BioCarta Pathways:     KEGG Pathways:  
    Probe set : 1370959_at    Name:   collagen, type III, alpha 1    GB acc:   BI275716    UniGene:   Rn.3247    Gene Symbol:   Col3a1    Locus Link:   84032    Chromosome:   9    Cytoband:   Chr:9q22    SOURCE    GENECARD       Gene Ontology:     BioCarta Pathways:     KEGG Pathways:  
    Probe set : 1372113_at    Name:   flightless I homolog (Drosophila) (predicted)    GB acc:   AA850662    UniGene:   Rn.144698    Gene Symbol:   Fliih_predicted    Locus Link:   287375    Chromosome:   10    Cytoband:   Chr:10q22    SOURCE    GENECARD       Gene Ontology:   cellular component:  nucleus   cellular component:  cytoskeleton   molecular function:  actin binding   molecular function:  lyase activity      BioCarta Pathways:     KEGG Pathways:  
    Probe set : 1390825_at    Name:   solute carrier family 35, member B3 (predicted)    GB acc:   AI010067    UniGene:   Rn.6323    Gene Symbol:   Slc35b3_predicted    Locus Link:   306866    Chromosome:   17    Cytoband:   Chr:17p12    SOURCE    GENECARD       Gene Ontology:   cellular component:  mitochondrion   cellular component:  integral to membrane      BioCarta Pathways:     KEGG Pathways:  
    Probe set : 1371379_at    Name:   similar to Brain protein 44    GB acc:   AI011738    UniGene:   Rn.2487    Gene Symbol:   LOC289182    Locus Link:   289182    Chromosome:   13    Cytoband:   Chr:13q23    SOURCE    GENECARD       Gene Ontology:     BioCarta Pathways:     KEGG Pathways:  
    Probe set : 1391440_at    Name:   testis expressed gene 10 (predicted)    GB acc:   AW521245    UniGene:   Rn.20483    Gene Symbol:   Tex10_predicted    Locus Link:   298065    Chromosome:   5    Cytoband:   Chr:5q22    SOURCE    GENECARD       Gene Ontology:   biological process:  transport   cellular component:  extracellular space   molecular function:  carrier activity      BioCarta Pathways:     KEGG Pathways:  
    Probe set : 1390483_at    Name:   solute carrier family 25 (mitochondrial carrier, palmitoylcarnitine transporter), member 29 (predicted)    GB acc:   BF555120    UniGene:   Rn.15047    Gene Symbol:   Slc25a29_predicted    Locus Link:   314441    Chromosome:   6    Cytoband:   Chr:6q32    SOURCE    GENECARD       Gene Ontology:   biological process:  transport   biological process:  acyl carnitine transport   molecular function:  transporter activity   molecular function:  binding   molecular function:  acyl carnitine transporter activity      BioCarta Pathways:     KEGG Pathways:  
    Probe set : 1376537_at    Name:   Similar to Tyrosine-protein phosphatase, non-receptor type 3 (Protein-tyrosine phosphatase H1) (PTP-H1)    GB acc:   AW435010    UniGene:   Rn.22271     Locus Link:   362524    Chromosome:   5    Cytoband:   Chr:5q24    SOURCE        Gene Ontology:   biological process:  protein amino acid dephosphorylation   cellular component:  cytoplasm   cellular component:  cytoskeleton   cellular component:  membrane      BioCarta Pathways:     KEGG Pathways:  
    Probe set : 1393000_at     GB acc:   AI045970              Gene Ontology:     BioCarta Pathways:     KEGG Pathways:  
    Probe set : 1373519_at    Name:   Transcribed locus    GB acc:   AI412601    UniGene:   Rn.137855        SOURCE        Gene Ontology:     BioCarta Pathways:     KEGG Pathways:  
    Probe set : 1376661_at    Name:   similar to CG11206-PA /// similar to kazrin isoform A    GB acc:   AI556122    UniGene:   Rn.3753    Gene Symbol:   RGD:1359147 /// LOC500579    Locus Link:   313672 /// 500579    Chromosome:   5    Cytoband:   Chr:5q36    SOURCE    GENECARD       Gene Ontology:     BioCarta Pathways:     KEGG Pathways:  
    Probe set : 1375872_at    Name:   Similar to hypothetical protein D4Ertd89e (predicted)    GB acc:   AI144944    UniGene:   Rn.22285     Locus Link:   362521    Chromosome:   5    Cytoband:   Chr:5q24    SOURCE        Gene Ontology:   biological process:  nucleosome assembly   biological process:  chromosome organization and biogenesis (sensu Eukaryota)   cellular component:  nucleosome   cellular component:  nucleus   molecular function:  DNA binding      BioCarta Pathways:     KEGG Pathways:  
    Probe set : 1375566_at    Name:   angiotensin II receptor-associated protein    GB acc:   AI180275    UniGene:   Rn.8471    Gene Symbol:   RGD:1359346    Locus Link:   298646    Chromosome:   5    Cytoband:   Chr:5q36    SOURCE    GENECARD       Gene Ontology:   cellular component:  endoplasmic reticulum   cellular component:  Golgi stack   cellular component:  integral to membrane      BioCarta Pathways:     KEGG Pathways:  
    Probe set : 1371738_at    Name:   similar to hypothetical protein FLJ10375 (predicted)    GB acc:   BI287983    UniGene:   Rn.104231    Gene Symbol:   RGD1308260_predicted    Locus Link:   301111    Chromosome:   8    Cytoband:   Chr:8q32    SOURCE    GENECARD       Gene Ontology:     BioCarta Pathways:     KEGG Pathways:  
    Probe set : 1372545_at    Name:   Similar to Gene model 609    GB acc:   AI411021    UniGene:   Rn.113934     Locus Link:   498080      SOURCE        Gene Ontology:     BioCarta Pathways:     KEGG Pathways:  
    Probe set : 1393293_at    Name:   checkpoint with forkhead and ring finger domains (predicted)    GB acc:   AI029628    UniGene:   Rn.15515    Gene Symbol:   Chfr_predicted    Locus Link:   288734    Chromosome:   12    Cytoband:   Chr:12q16    SOURCE    GENECARD       Gene Ontology:   cellular component:  ubiquitin ligase complex   cellular component:  nucleus      BioCarta Pathways:     KEGG Pathways:  
    Probe set : 1367815_at    Name:   solute carrier family 5 (sodium-dependent vitamin transporter), member 6    GB acc:   NM_130746    UniGene:   Rn.11105    Gene Symbol:   Slc5a6    Locus Link:   170551    Chromosome:   6    Cytoband:   Chr:6q14    SOURCE    GENECARD       Gene Ontology:     BioCarta Pathways:     KEGG Pathways:  
    Probe set : 1396055_at    Name:   procollagen, type VI, alpha 3 (predicted)    GB acc:   AW530165    UniGene:   Rn.2157    Gene Symbol:   Col6a3_predicted    Locus Link:   367313    Chromosome:   9    Cytoband:   Chr:9q35-q36    SOURCE    GENECARD       Gene Ontology:   biological process:  phosphate transport   biological process:  cell adhesion   biological process:  muscle development      BioCarta Pathways:     KEGG Pathways:  
    Probe set : 1383838_at     GB acc:   BI296294              Gene Ontology:     BioCarta Pathways:     KEGG Pathways:  
    Probe set : 1378216_at    Name:   Transcribed locus, weakly similar to NP_032201.1 granulin [Mus musculus]    GB acc:   BM387900    UniGene:   Rn.21695        SOURCE        Gene Ontology:     BioCarta Pathways:     KEGG Pathways:  
    Probe set : 1373546_at    Name:   ATPase, class VI, type 11A (predicted)    GB acc:   AI409922    UniGene:   Rn.98540     Locus Link:   306600    Chromosome:   16    Cytoband:   Chr:16q12.5    SOURCE        Gene Ontology:   biological process:  cation transport   biological process:  metabolism   cellular component:  membrane   cellular component:  integral to membrane      BioCarta Pathways:     KEGG Pathways:  
    Probe set : 1380334_at    Name:   hypothetical RNA binding protein    GB acc:   AI555044    UniGene:   Rn.38803    Gene Symbol:   RGD:1359713    Locus Link:   305340    Chromosome:   14    Cytoband:   Chr:14p11    SOURCE    GENECARD       Gene Ontology:   molecular function:  nucleotide binding   molecular function:  nucleic acid binding   molecular function:  RNA binding      BioCarta Pathways:     KEGG Pathways:  
    Probe set : 1374576_at    Name:   similar to mKIAA0841 protein    GB acc:   BF283857    UniGene:   Rn.137670    Gene Symbol:   LOC361546    Locus Link:   361546    Chromosome:   1    Cytoband:   Chr:1q21    SOURCE    GENECARD       Gene Ontology:     BioCarta Pathways:     KEGG Pathways:  
    Probe set : 1372925_at     GB acc:   AI233135              Gene Ontology:     BioCarta Pathways:     KEGG Pathways:  
    Probe set : 1391893_at    Name:   similar to ATP/GTP-binding protein (predicted)    GB acc:   AI501557    UniGene:   Rn.144332    Gene Symbol:   RGD1307679_predicted    Locus Link:   311166    Chromosome:   3    Cytoband:   Chr:3q24    SOURCE    GENECARD       Gene Ontology:   biological process:  mRNA processing   cellular component:  nucleus   molecular function:  ATP binding   molecular function:  GTP binding      BioCarta Pathways:     KEGG Pathways:  
    Probe set : 1371390_at    Name:   tubulin, beta, 2    GB acc:   AA892044    UniGene:   Rn.110562    Gene Symbol:   Tubb2    Locus Link:   296554    Chromosome:   3    Cytoband:   Chr:3p13    SOURCE    GENECARD       Gene Ontology:   biological process:  microtubule-based movement   biological process:  protein polymerization   cellular component:  cytoplasm   cellular component:  microtubule   cellular component:  protein complex      BioCarta Pathways:     KEGG Pathways:  
    Probe set : 1371997_at    Name:   aldo-keto reductase family 1, member E1 (predicted)    GB acc:   BM390833    UniGene:   Rn.108718    Gene Symbol:   Akr1e1_predicted    Locus Link:   307091    Chromosome:   17    Cytoband:   Chr:17q12.3    SOURCE    GENECARD       Gene Ontology:   molecular function:  oxidoreductase activity      BioCarta Pathways:     KEGG Pathways:  
    Probe set : 1378005_at     GB acc:   AW527486              Gene Ontology:     BioCarta Pathways:     KEGG Pathways:  
    Probe set : 1398367_at    Name:   speckle-type POZ protein (predicted)    GB acc:   BF283504    UniGene:   Rn.107846    Gene Symbol:   Spop_predicted    Locus Link:   287643    Chromosome:   10    Cytoband:   Chr:10q31    SOURCE    GENECARD       Gene Ontology:   biological process:  mRNA processing   cellular component:  nucleus   molecular function:  protein binding      BioCarta Pathways:     KEGG Pathways:  
    Probe set : 1389304_at     GB acc:   AI176323              Gene Ontology:     BioCarta Pathways:     KEGG Pathways:  
    Probe set : 1376068_at     GB acc:   BF283674              Gene Ontology:     BioCarta Pathways:     KEGG Pathways:  
    Probe set : 1391543_at    Name:   receptor (TNFRSF)-interacting serine-threonine kinase 1 (predicted)    GB acc:   BF406678    UniGene:   Rn.7572    Gene Symbol:   Ripk1_predicted    Locus Link:   306886    Chromosome:   17    Cytoband:   Chr:17p12    SOURCE    GENECARD       Gene Ontology:     BioCarta Pathways:     KEGG Pathways:  
    Probe set : 1399034_at    Name:   pecanex homolog (Drosophila) (predicted)    GB acc:   AI009650    UniGene:   Rn.23381    Gene Symbol:   Pcnx_predicted    Locus Link:   314288    Chromosome:   6    Cytoband:   Chr:6q24    SOURCE    GENECARD       Gene Ontology:   cellular component:  integral to membrane      BioCarta Pathways:     KEGG Pathways:  
    Probe set : 1383287_at    Name:   Similar to semaF cytoplasmic domain associated protein 2    GB acc:   BE329232    UniGene:   Rn.128883     Locus Link:   365960    Chromosome:   2    Cytoband:   Chr:2q45    SOURCE        Gene Ontology:     BioCarta Pathways:     KEGG Pathways:  
    Probe set : 1388448_at    Name:   Rap guanine nucleotide exchange factor (GEF) 6 (predicted)    GB acc:   AI235065    UniGene:   Rn.143915     Locus Link:   303141    Chromosome:   10    Cytoband:   Chr:10q22    SOURCE        Gene Ontology:   biological process:  small GTPase mediated signal transduction   biological process:  Ras protein signal transduction   biological process:  regulation of GTPase activity   cellular component:  plasma membrane      BioCarta Pathways:     KEGG Pathways:  
    Probe set : 1389396_at     GB acc:   BE329273              Gene Ontology:     BioCarta Pathways:     KEGG Pathways:  
    Probe set : 1398732_at    Name:   Transcribed locus    GB acc:   BF553297    UniGene:   Rn.32410        SOURCE        Gene Ontology:     BioCarta Pathways:     KEGG Pathways:  
    Probe set : 1384742_at    Name:   Alpha thalassemia/mental retardation syndrome X-linked homolog (human)    GB acc:   BF389776    UniGene:   Rn.107838    Gene Symbol:   Atrx    Locus Link:   246284    Chromosome:   X    Cytoband:   Chr:Xq31    SOURCE    GENECARD       Gene Ontology:   biological process:  DNA repair   biological process:  response to DNA damage stimulus   cellular component:  nucleus      BioCarta Pathways:     KEGG Pathways:  
    Probe set : 1396383_at    Name:   fatty aldehyde dehydrogenase-like    GB acc:   BF412690    UniGene:   Rn.27730    Gene Symbol:   RGD:1359546    Locus Link:   309147    Chromosome:   1    Cytoband:   Chr:1q42    SOURCE    GENECARD       Gene Ontology:   biological process:  metabolism   molecular function:  aldehyde dehydrogenase [NAD(P)+] activity   molecular function:  oxidoreductase activity      BioCarta Pathways:     KEGG Pathways:  
    Probe set : 1384481_at    Name:   Transcribed locus    GB acc:   BG376273    UniGene:   Rn.20703        SOURCE        Gene Ontology:     BioCarta Pathways:     KEGG Pathways:  
    Probe set : 1383826_at    Name:   Rab40b, member RAS oncogene family (predicted)    GB acc:   AA924620    UniGene:   Rn.8397    Gene Symbol:   Rab40b_predicted    Locus Link:   303754    Chromosome:   10    Cytoband:   Chr:10q32.3    SOURCE    GENECARD       Gene Ontology:   biological process:  intracellular signaling cascade   biological process:  small GTPase mediated signal transduction   biological process:  protein transport   molecular function:  GTP binding      BioCarta Pathways:     KEGG Pathways:  
    Probe set : 1367880_at    Name:   laminin, beta 2    GB acc:   NM_012974    UniGene:   Rn.774    Gene Symbol:   Lamb2    Locus Link:   25473    Chromosome:   16    Cytoband:   Chr:16q12.5    SOURCE    GENECARD       Gene Ontology:     BioCarta Pathways:     KEGG Pathways:  
    Probe set : 1371367_at    Name:   TAR DNA binding protein (predicted)    GB acc:   BE107459    UniGene:   Rn.144879    Gene Symbol:   Tardbp_predicted    Locus Link:   298648    Chromosome:   5    Cytoband:   Chr:5q36    SOURCE    GENECARD       Gene Ontology:   cellular component:  nucleus      BioCarta Pathways:     KEGG Pathways:  
    Probe set : 1379043_at    Name:   GH regulated TBC protein 1 (predicted)    GB acc:   BI284837    UniGene:   Rn.19589     Locus Link:   361180    Chromosome:   16    Cytoband:   Chr:16q12.5    SOURCE        Gene Ontology:   cellular component:  extracellular region   molecular function:  hormone activity      BioCarta Pathways:     KEGG Pathways:  
    Probe set : 1375029_at     GB acc:   AI235912              Gene Ontology:     BioCarta Pathways:     KEGG Pathways:  
    Probe set : 1393364_at    Name:   cation-chloride cotransporter 9    GB acc:   BM385460    UniGene:   Rn.15381    Gene Symbol:   Slc12a8    Locus Link:   266733    Chromosome:   11    Cytoband:   Chr:11q22    SOURCE    GENECARD       Gene Ontology:   biological process:  transport   biological process:  amino acid transport   cellular component:  membrane   cellular component:  integral to membrane   molecular function:  amino acid-polyamine transporter activity      BioCarta Pathways:     KEGG Pathways:  
    Probe set : 1368672_at    Name:   arginase 2    GB acc:   NM_019168    UniGene:   Rn.11055    Gene Symbol:   Arg2    Locus Link:   29215    Chromosome:   6    Cytoband:   Chr:6q24    SOURCE    GENECARD       Gene Ontology:   cellular component:  mitochondrion   cellular component:  mitochondrion      BioCarta Pathways:     KEGG Pathways:  
    Probe set : 1373556_at    Name:   similar to CG14903-PA    GB acc:   AI237593    UniGene:   Rn.8319    Gene Symbol:   LOC298861    Locus Link:   298861    Chromosome:   6    Cytoband:   Chr:6q14    SOURCE    GENECARD       Gene Ontology:     BioCarta Pathways:     KEGG Pathways:  
    Probe set : 1379195_s_at    Name:   ligase I, DNA, ATP-dependent /// ligase I, DNA, ATP-dependent (predicted)    GB acc:   AI556346    UniGene:   Rn.20467    Gene Symbol:   Lig1 /// Lig1_predicted    Locus Link:   360304 /// 81513    Chromosome:   1    Cytoband:   Chr:1q12 /// Chr:1q21    SOURCE    GENECARD       Gene Ontology:   cellular component:  nucleus      BioCarta Pathways:     KEGG Pathways:  
    Probe set : 1387253_at    Name:   guanylate cyclase activator 2b    GB acc:   NM_022284    UniGene:   Rn.97593    Gene Symbol:   Guca2b    Locus Link:   64055    Chromosome:   5    Cytoband:   Chr:5q36    SOURCE    GENECARD       Gene Ontology:   cellular component:  extracellular space   molecular function:  enzyme activator activity   molecular function:  calcium sensitive guanylate cyclase activator activity      BioCarta Pathways:     KEGG Pathways:  
    Probe set : 1397176_at    Name:   similar to RIKEN cDNA 1600013P15    GB acc:   AI104417    UniGene:   Rn.29892    Gene Symbol:   RGD:1359565    Locus Link:   290564    Chromosome:   16    Cytoband:   Chr:16p16    SOURCE    GENECARD       Gene Ontology:   cellular component:  nucleus   molecular function:  methyltransferase activity   molecular function:  S-adenosylmethionine-dependent methyltransferase activity   molecular function:  transferase activity      BioCarta Pathways:     KEGG Pathways:  
    Probe set : 1396815_at    Name:   LOC501008    GB acc:   AW251438    UniGene:   Rn.145699    Gene Symbol:   LOC501008    Locus Link:   501008    Chromosome:   8    Cytoband:   Chr:8q24    SOURCE    GENECARD       Gene Ontology:     BioCarta Pathways:     KEGG Pathways:  
    Probe set : 1386662_at    Name:   Secernin 1    GB acc:   BF553981    UniGene:   Rn.103706     Locus Link:   502776      SOURCE        Gene Ontology:   biological process:  proteolysis   molecular function:  dipeptidase activity      BioCarta Pathways:     KEGG Pathways:  
    Probe set : 1390195_at    Name:   Pleckstrin and Sec7 domain containing 4 (predicted)    GB acc:   BM386736    UniGene:   Rn.18242     Locus Link:   311785    Chromosome:   3    Cytoband:   Chr:3p13    SOURCE        Gene Ontology:     BioCarta Pathways:     KEGG Pathways:  
    Probe set : 1388518_at    Name:   F-box and WD-40 domain protein 5 (predicted)    GB acc:   AA799654    UniGene:   Rn.136952    Gene Symbol:   Fbxw5_predicted    Locus Link:   362081    Chromosome:   3    Cytoband:   Chr:3p13    SOURCE    GENECARD       Gene Ontology:   biological process:  ubiquitin cycle      BioCarta Pathways:     KEGG Pathways:  
    Probe set : 1368208_at    Name:   camello-like 1    GB acc:   NM_133558    UniGene:   Rn.3643    Gene Symbol:   Cml1    Locus Link:   171084    Chromosome:   4    Cytoband:   Chr:4q34    SOURCE    GENECARD       Gene Ontology:   molecular function:  N-acetyltransferase activity   molecular function:  N-acetyltransferase activity   molecular function:  transferase activity      BioCarta Pathways:     KEGG Pathways:  
    Probe set : 1372044_at    Name:   similar to Ser/Thr-rich protein T10 in DGCR region (predicted)    GB acc:   AI410321    UniGene:   Rn.101776    Gene Symbol:   RGD1310348_predicted    Locus Link:   360738    Chromosome:   11    Cytoband:   Chr:11q23    SOURCE    GENECARD       Gene Ontology:     BioCarta Pathways:     KEGG Pathways:  
    Probe set : 1375549_at    Name:   Transcribed locus    GB acc:   AI407719    UniGene:   Rn.92548        SOURCE        Gene Ontology:     BioCarta Pathways:     KEGG Pathways:  
    Probe set : 1396337_at     GB acc:   AW529811              Gene Ontology:     BioCarta Pathways:     KEGG Pathways:  
    Probe set : 1374197_at    Name:   Similar to expressed sequence AA415817    GB acc:   AI177275    UniGene:   Rn.1273     Locus Link:   304721    Chromosome:   10    Cytoband:   Chr:10q11    SOURCE        Gene Ontology:     BioCarta Pathways:     KEGG Pathways:  
    Probe set : 1398761_at    Name:   ribosomal protein L5    GB acc:   NM_031099    UniGene:   Rn.92980    Gene Symbol:   Rpl5    Locus Link:   81763    Chromosome:   14    Cytoband:   Chr:14p22    SOURCE    GENECARD       Gene Ontology:   biological process:  protein biosynthesis   biological process:  ribosome biogenesis      BioCarta Pathways:     KEGG Pathways:  
    Probe set : 1389242_at    Name:   Transcribed locus    GB acc:   BG377726    UniGene:   Rn.128067        SOURCE        Gene Ontology:     BioCarta Pathways:     KEGG Pathways:  
    Probe set : 1387916_at    Name:   cytochrome P450 4F6    GB acc:   U39208    UniGene:   Rn.11269    Gene Symbol:   RGD:708365    Locus Link:   266689    Chromosome:   7    Cytoband:   Chr:7q11    SOURCE    GENECARD       Gene Ontology:   biological process:  electron transport   biological process:  leukotriene metabolism   biological process:  inflammatory response      BioCarta Pathways:     KEGG Pathways:  
    Probe set : 1387378_at    Name:   ficolin B    GB acc:   NM_053634    UniGene:   Rn.48281    Gene Symbol:   Fcnb    Locus Link:   114091    Chromosome:   3    Cytoband:   Chr:3p12    SOURCE    GENECARD       Gene Ontology:   biological process:  phosphate transport   cellular component:  cytoplasm   molecular function:  sugar binding      BioCarta Pathways:     KEGG Pathways:  
    Probe set : 1387895_s_at    Name:   cell division cycle 20 homolog (S. cerevisiae)    GB acc:   U05341    UniGene:   Rn.9262    Gene Symbol:   Cdc20    Locus Link:   64515    Chromosome:   5    Cytoband:   Chr:5q36    SOURCE    GENECARD       Gene Ontology:   cellular component:  spindle   molecular function:  protein binding      BioCarta Pathways:     KEGG Pathways:  
    Probe set : 1386914_at    Name:   guanosine monophosphate reductase    GB acc:   NM_057188    UniGene:   Rn.3862    Gene Symbol:   Gmpr    Locus Link:   117533    Chromosome:   17    Cytoband:   Chr:17p13    SOURCE    GENECARD       Gene Ontology:   biological process:  nucleotide metabolism   biological process:  response to cold   biological process:  response to cold      BioCarta Pathways:     KEGG Pathways:  
    Probe set : 1367657_at    Name:   B-cell translocation gene 1, anti-proliferative    GB acc:   NM_017258    UniGene:   Rn.1000    Gene Symbol:   Btg1    Locus Link:   29618    Chromosome:   7    Cytoband:   Chr:7q13    SOURCE    GENECARD       Gene Ontology:   biological process:  protein amino acid methylation   biological process:  spermatid development   biological process:  cell proliferation   biological process:  negative regulation of cell proliferation   biological process:  cell migration   biological process:  negative regulation of cell growth   biological process:  regulation of apoptosis   biological process:  positive regulation of enzyme activity   biological process:  regulation of transcription   biological process:  positive regulation of endothelial cell differentiation   biological process:  positive regulation of myoblast differentiation   biological process:  positive regulation of angiogenesis   biological process:  negative regulation of cell proliferation   biological process:  cell migration   biological process:  negative regulation of cell growth   biological process:  regulation of apoptosis   biological process:  positive regulation of enzyme activity   biological process:  regulation of transcription   biological process:  positive regulation of endothelial cell differentiation   biological process:  positive regulation of myoblast differentiation   biological process:  positive regulation of angiogenesis   biological process:  spermatid development      BioCarta Pathways:     KEGG Pathways:  
    Probe set : 1372466_at    Name:   Transforming growth factor, beta receptor II    GB acc:   AI408571    UniGene:   Rn.9954    Gene Symbol:   Tgfbr2    Locus Link:   81810    Chromosome:   8    Cytoband:   Chr:8q32    SOURCE    GENECARD       Gene Ontology:   cellular component:  membrane   cellular component:  integral to membrane   molecular function:  protein kinase activity   molecular function:  protein serine/threonine kinase activity   molecular function:  transmembrane receptor protein serine/threonine kinase activity   molecular function:  protein kinase CK2 activity   molecular function:  cAMP-dependent protein kinase activity   molecular function:  receptor signaling protein serine/threonine kinase activity   molecular function:  protein-tyrosine kinase activity   molecular function:  receptor activity   molecular function:  transforming growth factor beta receptor activity   molecular function:  transforming growth factor beta receptor activity, type II   molecular function:  transforming growth factor beta receptor activity, type II   molecular function:  ATP binding   molecular function:  kinase activity   molecular function:  transferase activity   molecular function:  nucleotide binding   molecular function:  magnesium ion binding   molecular function:  manganese ion binding   molecular function:  metal ion binding      BioCarta Pathways:     KEGG Pathways:  
    Probe set : 1380884_at    Name:   Transcribed locus    GB acc:   AW524553    UniGene:   Rn.147998        SOURCE        Gene Ontology:     BioCarta Pathways:     KEGG Pathways:  
    Probe set : 1379443_at    Name:   Hermansky-Pudlak syndrome 3 (predicted)    GB acc:   BE120719    UniGene:   Rn.141355    Gene Symbol:   Hps3_predicted    Locus Link:   310288    Chromosome:   2    Cytoband:   Chr:2q24    SOURCE    GENECARD       Gene Ontology:   biological process:  organelle organization and biogenesis   cellular component:  cytoplasm      BioCarta Pathways:     KEGG Pathways:  
    Probe set : 1374545_at    Name:   ring finger and KH domain containing 2 (predicted)    GB acc:   AA945202    UniGene:   Rn.8138    Gene Symbol:   Rkhd2_predicted    Locus Link:   307271    Chromosome:   18    Cytoband:   Chr:18q12.2    SOURCE    GENECARD       Gene Ontology:   biological process:  protein ubiquitination   cellular component:  ubiquitin ligase complex   molecular function:  ubiquitin-protein ligase activity   molecular function:  zinc ion binding      BioCarta Pathways:     KEGG Pathways:  
    Probe set : 1371126_x_at    Name:   granzyme B    GB acc:   X66693    UniGene:   Rn.21395    Gene Symbol:   Gzmb    Locus Link:   171528    Chromosome:   15    Cytoband:   Chr:15p12    SOURCE    GENECARD       Gene Ontology:   biological process:  proteolysis   biological process:  apoptosis   biological process:  cytolysis   cellular component:  extracellular space      BioCarta Pathways:     KEGG Pathways:  
    Probe set : 1389214_at    Name:   similar to Laminin alpha-4 chain precursor    GB acc:   BF406693    UniGene:   Rn.32891    Gene Symbol:   LOC309816    Locus Link:   309816    Chromosome:   20    Cytoband:   Chr:20q12    SOURCE    GENECARD       Gene Ontology:     BioCarta Pathways:     KEGG Pathways:  
    Probe set : 1375349_at    Name:   Transcribed locus, strongly similar to NP_033192.1 sorbin and SH3 domain containing 1 [Mus musculus]    GB acc:   BI295776    UniGene:   Rn.110441        SOURCE        Gene Ontology:     BioCarta Pathways:     KEGG Pathways:  
    Probe set : 1382173_at    Name:   Transcribed locus    GB acc:   BF283569    UniGene:   Rn.51063        SOURCE        Gene Ontology:     BioCarta Pathways:     KEGG Pathways:  
    Probe set : 1391466_at    Name:   Nuclear ubiquitous casein kinase and cyclin-dependent kinase substrate    GB acc:   AI170347    UniGene:   Rn.21324    Gene Symbol:   Nucks    Locus Link:   64709      SOURCE    GENECARD       Gene Ontology:   biological process:  regulation of progression through cell cycle   cellular component:  nucleus   cellular component:  nucleus   cellular component:  cytoplasm   molecular function:  double-stranded DNA binding   molecular function:  single-stranded DNA binding   molecular function:  kinase activity      BioCarta Pathways:     KEGG Pathways:  
    Probe set : 1384265_at    Name:   polymerase (DNA-directed), delta 3, accessory subunit (predicted)    GB acc:   BI278347    UniGene:   Rn.45023    Gene Symbol:   Pold3_predicted    Locus Link:   293144    Chromosome:   1    Cytoband:   Chr:1q32    SOURCE    GENECARD       Gene Ontology:   biological process:  DNA synthesis during DNA repair   biological process:  DNA replication   biological process:  mismatch repair   cellular component:  nucleus   cellular component:  delta DNA polymerase complex   molecular function:  DNA binding   molecular function:  DNA-directed DNA polymerase activity   molecular function:  delta DNA polymerase activity      BioCarta Pathways:     KEGG Pathways:  
    Probe set : 1377797_at    Name:   gene rich cluster, C2f gene (predicted)    GB acc:   AI071470    UniGene:   Rn.24830    Gene Symbol:   Grcc2f_predicted    Locus Link:   312706    Chromosome:   4    Cytoband:   Chr:4q42    SOURCE    GENECARD       Gene Ontology:     BioCarta Pathways:     KEGG Pathways:  
    Probe set : 1374355_at    Name:   similar to MGC47262 protein    GB acc:   BI289733    UniGene:   Rn.46852    Gene Symbol:   LOC315283    Locus Link:   315283    Chromosome:   7    Cytoband:   Chr:7q35-q36    SOURCE    GENECARD       Gene Ontology:     BioCarta Pathways:     KEGG Pathways:  
    Probe set : 1375406_s_at    Name:   rapostlin    GB acc:   BF419792    UniGene:   Rn.20445    Gene Symbol:   Fnbp1    Locus Link:   192348    Chromosome:   3    Cytoband:   Chr:3p12    SOURCE    GENECARD       Gene Ontology:   biological process:  nervous system development   biological process:  endocytosis   molecular function:  protein binding      BioCarta Pathways:     KEGG Pathways:  
    Probe set : 1367885_at    Name:   peroxisomal membrane protein 2    GB acc:   NM_031587    UniGene:   Rn.10292    Gene Symbol:   Pxmp2    Locus Link:   29533    Chromosome:   12    Cytoband:   Chr:12q16    SOURCE    GENECARD       Gene Ontology:   biological process:  peroxisome organization and biogenesis      BioCarta Pathways:     KEGG Pathways:  
    Probe set : 1388880_at    Name:   Schlafen 5 (predicted)    GB acc:   BI278962    UniGene:   Rn.113556     Locus Link:   303377    Chromosome:   10    Cytoband:   Chr:10q26    SOURCE        Gene Ontology:   molecular function:  hydrolase activity      BioCarta Pathways:     KEGG Pathways:  
    Probe set : 1372631_at    Name:   Thymidine kinase 1    GB acc:   AI170596    UniGene:   Rn.4191    Gene Symbol:   Tk1    Locus Link:   24834    Chromosome:   10    Cytoband:   Chr:10    SOURCE    GENECARD       Gene Ontology:   biological process:  DNA metabolism   biological process:  DNA replication   cellular component:  cytoplasm      BioCarta Pathways:     KEGG Pathways:  
    Probe set : 1390553_at    Name:   Transcribed locus    GB acc:   BE104167    UniGene:   Rn.27127        SOURCE        Gene Ontology:     BioCarta Pathways:     KEGG Pathways:  
    Probe set : 1372003_at    Name:   Transcribed locus, strongly similar to NP_056312.2 DKFZP564G2022 protein [Homo sapiens]    GB acc:   BI296755    UniGene:   Rn.17235        SOURCE        Gene Ontology:     BioCarta Pathways:     KEGG Pathways:  
    Probe set : 1374070_at    Name:   glutathione peroxidase 2    GB acc:   AA800587    UniGene:   Rn.3503    Gene Symbol:   Gpx2    Locus Link:   29326    Chromosome:   6    Cytoband:   Chr:6q24    SOURCE    GENECARD       Gene Ontology:   biological process:  response to oxidative stress   cellular component:  cytosol      BioCarta Pathways:     KEGG Pathways:  
    Probe set : 1388076_at    Name:   PAI-1 mRNA-binding protein    GB acc:   AF388527    UniGene:   Rn.7370    Gene Symbol:   Pairbp1    Locus Link:   246303    Chromosome:   4    Cytoband:   Chr:4q31    SOURCE    GENECARD       Gene Ontology:   cellular component:  nucleus   molecular function:  RNA binding   molecular function:  mRNA 3'-UTR binding      BioCarta Pathways:     KEGG Pathways:  
    Probe set : 1394022_at    Name:   inhibitor of DNA binding 4    GB acc:   BE116009    UniGene:   Rn.145407    Gene Symbol:   Idb4    Locus Link:   291023    Chromosome:   17    Cytoband:   Chr:17p14    SOURCE    GENECARD       Gene Ontology:   biological process:  regulation of transcription from RNA polymerase II promoter   cellular component:  nucleus   molecular function:  transcription corepressor activity      BioCarta Pathways:     KEGG Pathways:  
    Probe set : 1388099_a_at    Name:   TCF3 (E2A) fusion partner    GB acc:   AB029495    UniGene:   Rn.12922    Gene Symbol:   Tfpt    Locus Link:   85423    Chromosome:   1    Cytoband:   Chr:1q12    SOURCE    GENECARD       Gene Ontology:   biological process:  induction of apoptosis   cellular component:  nucleus   molecular function:  protein binding   molecular function:  protein heterodimerization activity      BioCarta Pathways:     KEGG Pathways:  
    Probe set : 1369697_at    Name:   interleukin 8 receptor, beta    GB acc:   NM_017183    UniGene:   Rn.90347    Gene Symbol:   Il8rb    Locus Link:   29385    Chromosome:   9    Cytoband:   Chr:9q33    SOURCE    GENECARD       Gene Ontology:   cellular component:  cytoplasm   cellular component:  integral to plasma membrane   cellular component:  integral to membrane      BioCarta Pathways:     KEGG Pathways:  
    Probe set : 1383080_at    Name:   Lysosomal membrane glycoprotein 2    GB acc:   BI292252    UniGene:   Rn.1722    Gene Symbol:   Lamp2    Locus Link:   24944    Chromosome:   X    Cytoband:   Chr:Xq11    SOURCE    GENECARD       Gene Ontology:   biological process:  tRNA aminoacylation for protein translation   molecular function:  tRNA ligase activity   molecular function:  ATP binding      BioCarta Pathways:     KEGG Pathways:  
    Probe set : 1376867_at    Name:   similar to RIKEN cDNA 1110067D22 (predicted)    GB acc:   BE095833    UniGene:   Rn.17651    Gene Symbol:   RGD1307414_predicted    Locus Link:   360983    Chromosome:   14    Cytoband:   Chr:14q22    SOURCE    GENECARD       Gene Ontology:     BioCarta Pathways:     KEGG Pathways:  
    Probe set : 1388024_s_at    Name:   lamina-associated polypeptide 1B    GB acc:   U20286    UniGene:   Rn.11373    Gene Symbol:   Lap1b    Locus Link:   246314    Chromosome:   13    Cytoband:   Chr:13q21    SOURCE    GENECARD       Gene Ontology:   cellular component:  nuclear membrane   cellular component:  nucleus   cellular component:  integral to membrane   molecular function:  lamin binding      BioCarta Pathways:     KEGG Pathways:  
    Probe set : 1393252_at    Name:   Fibulin 1 (predicted)    GB acc:   AA996804    UniGene:   Rn.9375     Locus Link:   315191    Chromosome:   7    Cytoband:   Chr:7q34    SOURCE        Gene Ontology:   biological process:  chitin metabolism   molecular function:  extracellular matrix structural constituent   molecular function:  calcium ion binding   molecular function:  chitin binding      BioCarta Pathways:     KEGG Pathways:  
    Probe set : 1390786_at    Name:   rho/rac guanine nucleotide exchange factor (GEF) 2 (predicted)    GB acc:   BE099756    UniGene:   Rn.12255    Gene Symbol:   Arhgef2_predicted    Locus Link:   310635    Chromosome:   2    Cytoband:   Chr:2q34    SOURCE    GENECARD       Gene Ontology:   cellular component:  microtubule      BioCarta Pathways:     KEGG Pathways:  
    Probe set : 1371092_at    Name:   preprotrypsinogen IV    GB acc:   X15679    UniGene:   Rn.10387    Gene Symbol:   RGD:708585    Locus Link:   286960    Chromosome:   4    Cytoband:   Chr:4q22    SOURCE    GENECARD       Gene Ontology:   biological process:  proteolysis   biological process:  digestion      BioCarta Pathways:     KEGG Pathways:  
    Probe set : 1374475_at    Name:   abhydrolase domain containing 1 (predicted)    GB acc:   AI105131    UniGene:   Rn.40145    Gene Symbol:   Abhd1_predicted    Locus Link:   313917    Chromosome:   6    Cytoband:   Chr:6q14    SOURCE    GENECARD       Gene Ontology:   molecular function:  hydrolase activity   molecular function:  catalytic activity      BioCarta Pathways:     KEGG Pathways:  
    Probe set : 1398350_at    Name:   brain abundant, membrane attached signal protein 1    GB acc:   BG380454    UniGene:   Rn.37779    Gene Symbol:   Basp1    Locus Link:   64160    Chromosome:   2    Cytoband:   Chr:2q22    SOURCE    GENECARD       Gene Ontology:   biological process:  regulation of transcription, DNA-dependent   cellular component:  nucleus   cellular component:  membrane   molecular function:  DNA binding   molecular function:  calmodulin binding      BioCarta Pathways:     KEGG Pathways:  
    Probe set : 1398254_at    Name:   renin binding protein    GB acc:   NM_031095    UniGene:   Rn.11116    Gene Symbol:   Renbp    Locus Link:   81759    Chromosome:   X    Cytoband:   Chr:Xq37    SOURCE    GENECARD       Gene Ontology:   biological process:  mannose metabolism   biological process:  N-acetylglucosamine metabolism   biological process:  regulation of blood pressure      BioCarta Pathways:     KEGG Pathways:  
    Probe set : 1389485_at    Name:   similar to transformed mouse 3T3 cell double minute 1    GB acc:   BE099784    UniGene:   Rn.15576    Gene Symbol:   LOC314859    Locus Link:   314859    Chromosome:   7    Cytoband:   Chr:7q22    SOURCE    GENECARD       Gene Ontology:     BioCarta Pathways:     KEGG Pathways:  
    Probe set : 1367618_a_at    Name:   discs, large homolog 5 (Drosophila) (predicted)    GB acc:   NM_130734    UniGene:   Rn.110214    Gene Symbol:   Dlg5_predicted    Locus Link:   305645    Chromosome:   15    Cytoband:   Chr:15p16    SOURCE    GENECARD       Gene Ontology:   biological process:  regulation of progression through cell cycle   biological process:  intracellular signaling cascade   biological process:  cell-cell adhesion   cellular component:  plasma membrane   cellular component:  cell-cell adherens junction      BioCarta Pathways:     KEGG Pathways:  
    Probe set : 1390049_at    Name:   four and a half LIM domains 1    GB acc:   BI298356    UniGene:   Rn.54261    Gene Symbol:   Fhl1    Locus Link:   25177    Chromosome:   X    Cytoband:   Chr:Xq36    SOURCE    GENECARD       Gene Ontology:   molecular function:  electron transporter activity   molecular function:  zinc ion binding   molecular function:  metal ion binding      BioCarta Pathways:     KEGG Pathways:  
    Probe set : 1372420_at    Name:   similar to FKSG24 (predicted)    GB acc:   AI408084    UniGene:   Rn.106339    Gene Symbol:   RGD1308064_predicted    Locus Link:   290645    Chromosome:   16    Cytoband:   Chr:16p14    SOURCE    GENECARD       Gene Ontology:     BioCarta Pathways:     KEGG Pathways:  
    Probe set : 1367743_at    Name:   phosphofructokinase, liver, B-type    GB acc:   NM_013190    UniGene:   Rn.4212    Gene Symbol:   Pfkl    Locus Link:   25741    Chromosome:   20    Cytoband:   Chr:20p12    SOURCE    GENECARD       Gene Ontology:   biological process:  fructose 6-phosphate metabolism   biological process:  glycolysis   cellular component:  cytoplasm   cellular component:  6-phosphofructokinase complex      BioCarta Pathways:     KEGG Pathways:  
    Probe set : 1398962_at    Name:   similar to cDNA sequence BC005632    GB acc:   BM388453    UniGene:   Rn.15712    Gene Symbol:   RGD:1359682    Locus Link:   299617    Chromosome:   7    Cytoband:   Chr:7q11    SOURCE    GENECARD       Gene Ontology:   biological process:  lipid metabolism   biological process:  fatty acid metabolism   molecular function:  catalytic activity   molecular function:  hydrolase activity      BioCarta Pathways:     KEGG Pathways:  
    Probe set : 1374399_at    Name:   EH-domain containing 4    GB acc:   AA801107    UniGene:   Rn.7379    Gene Symbol:   Ehd4    Locus Link:   192204    Chromosome:   3    Cytoband:   Chr:3q35    SOURCE    GENECARD       Gene Ontology:     BioCarta Pathways:     KEGG Pathways:  
    Probe set : 1368325_at    Name:   epidermal growth factor    GB acc:   NM_012842    UniGene:   Rn.6075    Gene Symbol:   Egf    Locus Link:   25313    Chromosome:   2    Cytoband:   Chr:2q42-q43    SOURCE    GENECARD       Gene Ontology:     BioCarta Pathways:     KEGG Pathways:  
    Probe set : 1376209_at    Name:   similar to hypothetical protein supported by AL449243 (predicted)    GB acc:   BG669208    UniGene:   Rn.38946    Gene Symbol:   RGD1311310_predicted    Locus Link:   315166    Chromosome:   7    Cytoband:   Chr:7q34    SOURCE    GENECARD       Gene Ontology:     BioCarta Pathways:     KEGG Pathways:  
    Probe set : 1376222_at    Name:   similar to HCDI protein (predicted)    GB acc:   AI710677    UniGene:   Rn.38645    Gene Symbol:   RGD1309307_predicted    Locus Link:   361044    Chromosome:   15    Cytoband:   Chr:15p13    SOURCE    GENECARD       Gene Ontology:     BioCarta Pathways:     KEGG Pathways:  
    Probe set : 1390474_at    Name:   similar to RIKEN cDNA 9330161F08    GB acc:   AA859508    UniGene:   Rn.242    Gene Symbol:   LOC362899    Locus Link:   362899    Chromosome:   7    Cytoband:   Chr:7q22    SOURCE    GENECARD       Gene Ontology:     BioCarta Pathways:     KEGG Pathways:  
    Probe set : 1375921_at    Name:   Reticulocalbin (predicted)    GB acc:   BF281577    UniGene:   Rn.103345     Locus Link:   362182    Chromosome:   3    Cytoband:   Chr:3q32    SOURCE        Gene Ontology:   cellular component:  extracellular space   cellular component:  endoplasmic reticulum   cellular component:  endoplasmic reticulum lumen   molecular function:  calcium ion binding      BioCarta Pathways:     KEGG Pathways:  
    Probe set : 1372519_at    Name:   nucleoporin 93 (predicted)    GB acc:   AI169375    UniGene:   Rn.137413    Gene Symbol:   Nup93_predicted    Locus Link:   291874    Chromosome:   19    Cytoband:   Chr:19p12    SOURCE    GENECARD       Gene Ontology:   biological process:  transport   molecular function:  porin activity      BioCarta Pathways:     KEGG Pathways:  
    Probe set : 1383730_at    Name:   Similar to hypothetical protein    GB acc:   BG668930    UniGene:   Rn.107832    Gene Symbol:   MGC73022    Locus Link:   309196    Chromosome:   1    Cytoband:   Chr:1q43    SOURCE    GENECARD       Gene Ontology:   molecular function:  binding      BioCarta Pathways:     KEGG Pathways:  
    Probe set : 1389803_at    Name:   Transcribed locus    GB acc:   BF415514    UniGene:   Rn.47082        SOURCE        Gene Ontology:     BioCarta Pathways:     KEGG Pathways:  
    Probe set : 1389180_at    Name:   Similar to phosphorylase kinase (EC 2.7.1.38) beta chain, non-muscle - rabbit    GB acc:   AI175035    UniGene:   Rn.6488     Locus Link:   361377    Chromosome:   19    Cytoband:   Chr:19q11    SOURCE        Gene Ontology:   biological process:  glycogen metabolism      BioCarta Pathways:     KEGG Pathways:  
    Probe set : 1396853_at    Name:   Adrenergic receptor, alpha 2b    GB acc:   AI549156    UniGene:   Rn.10296    Gene Symbol:   Adra2b    Locus Link:   24174    Chromosome:   3    Cytoband:   Chr:3q36    SOURCE    GENECARD       Gene Ontology:   cellular component:  integral to plasma membrane   cellular component:  integral to membrane      BioCarta Pathways:     KEGG Pathways:  
    Probe set : 1372770_at     GB acc:   BF281357              Gene Ontology:     BioCarta Pathways:     KEGG Pathways:  
    Probe set : 1383182_at    Name:   serine/arginine-rich protein specific kinase 1 (predicted)    GB acc:   AW144184    UniGene:   Rn.2688    Gene Symbol:   Srpk1_predicted    Locus Link:   361811    Chromosome:   20    Cytoband:   Chr:20p12    SOURCE    GENECARD       Gene Ontology:   biological process:  regulation of progression through cell cycle   biological process:  protein amino acid phosphorylation   biological process:  RNA splicing   cellular component:  nucleus      BioCarta Pathways:     KEGG Pathways:  
    Probe set : 1386986_at    Name:   opioid growth factor receptor    GB acc:   NM_053340    UniGene:   Rn.15740    Gene Symbol:   Ogfr    Locus Link:   83525    Chromosome:   3    Cytoband:   Chr:3q43    SOURCE    GENECARD       Gene Ontology:   biological process:  regulation of cell growth   cellular component:  membrane      BioCarta Pathways:     KEGG Pathways:  
    Probe set : 1381678_at    Name:   Basophilic leukemia expressed sequence 06 (Bles06)    GB acc:   AW525560    UniGene:   Rn.147996        SOURCE        Gene Ontology:   molecular function:  protein binding, bridging   molecular function:  actin filament binding      BioCarta Pathways:     KEGG Pathways:  
    Probe set : 1371744_at    Name:   Transcribed locus, strongly similar to NP_057481.2 TH1-like protein; TH1 drosophila homolog; negative elongation factor proteins C and D; TH1-like (Drosophila homolog) [Homo sapiens]    GB acc:   BF282993    UniGene:   Rn.130684        SOURCE        Gene Ontology:     BioCarta Pathways:     KEGG Pathways:  
    Probe set : 1372169_at    Name:   similar to RIKEN cDNA 4121402D02    GB acc:   BE108809    UniGene:   Rn.22437    Gene Symbol:   LOC303514    Locus Link:   303514    Chromosome:   10    Cytoband:   Chr:10q31    SOURCE    GENECARD       Gene Ontology:     BioCarta Pathways:     KEGG Pathways:  
    Probe set : 1376513_a_at     GB acc:   BE098803              Gene Ontology:     BioCarta Pathways:     KEGG Pathways:  
    Probe set : 1389050_at    Name:   Similar to Dual specificity protein phosphatase 3 (T-DSP11)    GB acc:   AI170797    UniGene:   Rn.8286     Locus Link:   498003      SOURCE        Gene Ontology:     BioCarta Pathways:     KEGG Pathways:  
    Probe set : 1373538_at    Name:   ubiquitin specific protease 1 (predicted)    GB acc:   AW525342    UniGene:   Rn.125994    Gene Symbol:   Usp1_predicted    Locus Link:   313387    Chromosome:   5    Cytoband:   Chr:5q33    SOURCE    GENECARD       Gene Ontology:   biological process:  ubiquitin-dependent protein catabolism   biological process:  ubiquitin cycle      BioCarta Pathways:     KEGG Pathways:  
    Probe set : 1389560_at     GB acc:   BF284702              Gene Ontology:     BioCarta Pathways:     KEGG Pathways:  
    Probe set : 1388919_at    Name:   zinc finger protein 541 (predicted)    GB acc:   AW144669    UniGene:   Rn.9137    Gene Symbol:   Znf541_predicted    Locus Link:   308108    Chromosome:   1    Cytoband:   Chr:1q21    SOURCE    GENECARD       Gene Ontology:   biological process:  regulation of transcription, DNA-dependent   cellular component:  nucleus   molecular function:  nucleic acid binding   molecular function:  DNA binding   molecular function:  zinc ion binding      BioCarta Pathways:     KEGG Pathways:  
    Probe set : 1387295_at    Name:   solute carrier family 6 (neurotransmitter transporter, betaine/GABA), member 12    GB acc:   NM_017335    UniGene:   Rn.11352    Gene Symbol:   Slc6a12    Locus Link:   50676    Chromosome:   4    Cytoband:   Chr:4q42    SOURCE    GENECARD       Gene Ontology:   biological process:  transport   biological process:  neurotransmitter transport   biological process:  neurotransmitter transport      BioCarta Pathways:     KEGG Pathways:  
    Probe set : 1388958_a_at    Name:   solute carrier family 2 (facilitated glucose transporter), member 4    GB acc:   AA799760    UniGene:   Rn.1314    Gene Symbol:   Slc2a4    Locus Link:   25139    Chromosome:   10    Cytoband:   Chr:10q24    SOURCE    GENECARD       Gene Ontology:     BioCarta Pathways:     KEGG Pathways:  
    Probe set : 1370395_at    Name:   Huntington disease gene homolog    GB acc:   U18650    UniGene:   Rn.11193    Gene Symbol:   Hdh    Locus Link:   29424    Chromosome:   14    Cytoband:   Chr:14q21    SOURCE    GENECARD       Gene Ontology:     BioCarta Pathways:     KEGG Pathways:  
    Probe set : 1371311_at    Name:   succinate dehydrogenase complex, subunit C    GB acc:   AI009817    UniGene:   Rn.1698    Gene Symbol:   RGD:1359454    Locus Link:   289217    Chromosome:   13    Cytoband:   Chr:13q24    SOURCE    GENECARD       Gene Ontology:   biological process:  tricarboxylic acid cycle   biological process:  electron transport   cellular component:  membrane   molecular function:  succinate dehydrogenase activity      BioCarta Pathways:     KEGG Pathways:  
    Probe set : 1398799_at    Name:   eukaryotic translation initiation factor 4E    GB acc:   NM_053974    UniGene:   Rn.11275    Gene Symbol:   Eif4e    Locus Link:   117045    Chromosome:   2    Cytoband:   Chr:2q44    SOURCE    GENECARD       Gene Ontology:   cellular component:  cytoplasm   cellular component:  eukaryotic translation initiation factor 4F complex      BioCarta Pathways:     KEGG Pathways:  
    Probe set : 1370150_a_at    Name:   thyroid hormone responsive protein    GB acc:   NM_012703    UniGene:   Rn.81140    Gene Symbol:   Thrsp    Locus Link:   25357    Chromosome:   1    Cytoband:   Chr:1q32    SOURCE    GENECARD       Gene Ontology:   biological process:  regulation of transcription from RNA polymerase II promoter   biological process:  lipid metabolism   cellular component:  nucleus      BioCarta Pathways:     KEGG Pathways:  
    Probe set : 1368908_at    Name:   annexin A4    GB acc:   NM_024155    UniGene:   Rn.19270    Gene Symbol:   Anxa4    Locus Link:   79124    Chromosome:   4    Cytoband:   Chr:4q34    SOURCE    GENECARD       Gene Ontology:   biological process:  kidney development   biological process:  exocytosis   cellular component:  apical plasma membrane   molecular function:  phospholipase inhibitor activity   molecular function:  calcium ion binding   molecular function:  calcium-dependent phospholipid binding      BioCarta Pathways:     KEGG Pathways:  
    Probe set : 1384312_at    Name:   Iroquois related homeobox 1 (Drosophila) (predicted) /// similar to iroquois-class homeobox protein Irx1    GB acc:   BF543574    UniGene:   Rn.46018    Gene Symbol:   Irx1_predicted /// LOC501463    Locus Link:   306659 /// 501463    Chromosome:   17    Cytoband:   Chr:17p14    SOURCE    GENECARD       Gene Ontology:   biological process:  regulation of transcription, DNA-dependent   cellular component:  nucleus   cellular component:  transcription factor complex   molecular function:  DNA binding   molecular function:  transcription factor activity      BioCarta Pathways:     KEGG Pathways:  
    Probe set : 1372454_at    Name:   similar to RIKEN cDNA 2210016L21 gene (predicted)    GB acc:   BG378568    UniGene:   Rn.143995    Gene Symbol:   RGD1311899_predicted    Locus Link:   288704    Chromosome:   12    Cytoband:   Chr:12q16    SOURCE    GENECARD       Gene Ontology:     BioCarta Pathways:     KEGG Pathways:  
    Probe set : 1383679_at    Name:   ring finger protein 31 (predicted)    GB acc:   BF411972    UniGene:   Rn.138034    Gene Symbol:   Rnf31_predicted    Locus Link:   364386    Chromosome:   15    Cytoband:   Chr:15p13    SOURCE    GENECARD       Gene Ontology:   cellular component:  cytosol   cellular component:  internal side of plasma membrane   molecular function:  protein binding   molecular function:  ligase activity      BioCarta Pathways:     KEGG Pathways:  
    Probe set : 1382923_at    Name:   synaptotagmin binding, cytoplasmic RNA interacting protein (predicted)    GB acc:   AI072424    UniGene:   Rn.105760    Gene Symbol:   Syncrip_predicted    Locus Link:   363113    Chromosome:   8    Cytoband:   Chr:8q31    SOURCE    GENECARD       Gene Ontology:   biological process:  nuclear mRNA splicing, via spliceosome   biological process:  RNA splicing   biological process:  mRNA processing   cellular component:  nucleus   cellular component:  endoplasmic reticulum   cellular component:  ribonucleoprotein complex   molecular function:  RNA binding   molecular function:  nucleotide binding   molecular function:  nucleic acid binding      BioCarta Pathways:     KEGG Pathways:  
    Probe set : 1395479_at    Name:   similar to BRIX (predicted)    GB acc:   BG373428    UniGene:   Rn.17880    Gene Symbol:   RGD1308508_predicted    Locus Link:   294799    Chromosome:   2    Cytoband:   Chr:2q16    SOURCE    GENECARD       Gene Ontology:   biological process:  ribosome biogenesis   cellular component:  nucleus      BioCarta Pathways:     KEGG Pathways:  
    Probe set : 1371305_at    Name:   Ribosomal protein L8    GB acc:   AI409193    UniGene:   Rn.127774    Gene Symbol:   Rpl8    Locus Link:   26962    Chromosome:   7    Cytoband:   Chr:7q34    SOURCE    GENECARD       Gene Ontology:   biological process:  protein biosynthesis   cellular component:  intracellular   cellular component:  ribosome   cellular component:  cytosolic large ribosomal subunit (sensu Eukaryota)      BioCarta Pathways:     KEGG Pathways:  
    Probe set : 1371899_at    Name:   protein kinase, interferon inducible double stranded RNA dependent activator (predicted)    GB acc:   AI102009    UniGene:   Rn.3217    Gene Symbol:   Prkra_predicted    Locus Link:   311130    Chromosome:   3    Cytoband:   Chr:3q23    SOURCE    GENECARD       Gene Ontology:   cellular component:  intracellular   cellular component:  nucleus      BioCarta Pathways:     KEGG Pathways:  
    Probe set : 1390350_at    Name:   similar to hypothetical protein AL133206 (predicted)    GB acc:   BE097445    UniGene:   Rn.106443    Gene Symbol:   RGD1308418_predicted    Locus Link:   298500    Chromosome:   5    Cytoband:   Chr:5q36    SOURCE    GENECARD       Gene Ontology:     BioCarta Pathways:     KEGG Pathways:  
    Probe set : 1383150_at    Name:   Transcribed locus    GB acc:   BE114751    UniGene:   Rn.137693        SOURCE        Gene Ontology:     BioCarta Pathways:     KEGG Pathways:  
    Probe set : 1372095_at    Name:   Similar to KIAA1161 protein (predicted)    GB acc:   BF281867    UniGene:   Rn.106115     Locus Link:   366360    Chromosome:   5    Cytoband:   Chr:5q22    SOURCE        Gene Ontology:     BioCarta Pathways:     KEGG Pathways:  
    Probe set : 1370250_at    Name:   ubiquitin-conjugating enzyme E2I /// similar to RIKEN cDNA A930001M12 gene    GB acc:   AA799453    UniGene:   Rn.129485    Gene Symbol:   Ube2i /// LOC500440    Locus Link:   25573 /// 500440    Chromosome:   10    Cytoband:   Chr:10q12 /// Chr:5q22    SOURCE    GENECARD       Gene Ontology:     BioCarta Pathways:     KEGG Pathways:  
    Probe set : 1371296_at    Name:   succinate dehydrogenase complex, subunit D, integral membrane protein    GB acc:   AI410703    UniGene:   Rn.3040    Gene Symbol:   Sdhd    Locus Link:   363061    Chromosome:   8    Cytoband:   Chr:8q23    SOURCE    GENECARD       Gene Ontology:     BioCarta Pathways:     KEGG Pathways:  
    Probe set : 1369983_at    Name:   chemokine (C-C motif) ligand 5    GB acc:   NM_031116    UniGene:   Rn.8019    Gene Symbol:   Ccl5    Locus Link:   81780    Chromosome:   10    Cytoband:   Chr:10q26    SOURCE    GENECARD       Gene Ontology:   cellular component:  extracellular region   cellular component:  extracellular space   molecular function:  cytokine activity   molecular function:  chemokine activity      BioCarta Pathways:     KEGG Pathways:  
    Probe set : 1371431_at    Name:   Transcribed locus, strongly similar to XP_232343.3 PREDICTED: similar to Pex5 protein [Rattus norvegicus]    GB acc:   BI296577    UniGene:   Rn.14316        SOURCE        Gene Ontology:     BioCarta Pathways:     KEGG Pathways:  
    Probe set : 1379900_at    Name:   Transcribed locus    GB acc:   AI043697    UniGene:   Rn.18691        SOURCE        Gene Ontology:     BioCarta Pathways:     KEGG Pathways:  
    Probe set : 1380616_at    Name:   sialyltransferase 8 (alpha-2, 8-sialytransferase) D    GB acc:   BI294748    UniGene:   Rn.63696    Gene Symbol:   Siat8d    Locus Link:   116696    Chromosome:   9    Cytoband:   Chr:9q36    SOURCE    GENECARD       Gene Ontology:   cellular component:  Golgi apparatus   cellular component:  integral to membrane   cellular component:  integral to Golgi membrane   molecular function:  sialyltransferase activity   molecular function:  transferase activity   molecular function:  transferase activity, transferring glycosyl groups      BioCarta Pathways:     KEGG Pathways:  
    Probe set : 1382873_at    Name:   similar to cDNA sequence BC003236 (predicted)    GB acc:   BF414250    UniGene:   Rn.25049    Gene Symbol:   RGD1310822_predicted    Locus Link:   310760    Chromosome:   2    Cytoband:   Chr:2q34    SOURCE    GENECARD       Gene Ontology:     BioCarta Pathways:     KEGG Pathways:  
    Probe set : 1388122_at    Name:   glutathione-S-transferase, pi 1 /// glutathione S-transferase, pi 2    GB acc:   X02904    UniGene:   Rn.87063    Gene Symbol:   Gstp1 /// Gstp2    Locus Link:   24426 /// 29438    Chromosome:   1    Cytoband:   Chr:1q43 /// Chr:1q42    SOURCE    GENECARD       Gene Ontology:   biological process:  xenobiotic metabolism   biological process:  central nervous system development   biological process:  metabolism   molecular function:  glutathione transferase activity   molecular function:  glutathione transferase activity   molecular function:  transferase activity      BioCarta Pathways:     KEGG Pathways:  
    Probe set : 1395145_at    Name:   Ral guanine nucleotide dissociation stimulator,-like 1 (predicted)    GB acc:   BF544481    UniGene:   Rn.28005     Locus Link:   289080    Chromosome:   13    Cytoband:   Chr:13q21    SOURCE        Gene Ontology:   biological process:  signal transduction   biological process:  intracellular signaling cascade   biological process:  small GTPase mediated signal transduction   molecular function:  guanyl-nucleotide exchange factor activity   molecular function:  Ral guanyl-nucleotide exchange factor activity      BioCarta Pathways:     KEGG Pathways:  
    Probe set : 1369336_at    Name:   hairless homolog (mouse)    GB acc:   NM_024364    UniGene:   Rn.41543    Gene Symbol:   hr    Locus Link:   60563    Chromosome:   15    Cytoband:   Chr:15p11    SOURCE    GENECARD       Gene Ontology:   biological process:  transcription   biological process:  regulation of transcription, DNA-dependent   cellular component:  nucleus      BioCarta Pathways:     KEGG Pathways:  
    Probe set : 1378421_at    Name:   Transcribed locus    GB acc:   BG671734    UniGene:   Rn.15594        SOURCE        Gene Ontology:     BioCarta Pathways:     KEGG Pathways:  
    Probe set : 1371706_at    Name:   serologically defined colon cancer antigen 3 (predicted)    GB acc:   AI101371    UniGene:   Rn.54139    Gene Symbol:   Sdccag3_predicted    Locus Link:   306322    Chromosome:   16    Cytoband:   Chr:16p14    SOURCE    GENECARD       Gene Ontology:     BioCarta Pathways:     KEGG Pathways:  
    Probe set : 1372957_at    Name:   Similar to hypothetical protein A730008H23 (predicted)    GB acc:   BE101323    UniGene:   Rn.19499     Locus Link:   316602    Chromosome:   9    Cytoband:   Chr:9q35    SOURCE        Gene Ontology:     BioCarta Pathways:     KEGG Pathways:  
    Probe set : 1381933_at    Name:   Transcribed locus    GB acc:   AW434510    UniGene:   Rn.146519        SOURCE        Gene Ontology:     BioCarta Pathways:     KEGG Pathways:  
    Probe set : 1386641_at    Name:   Transcribed locus    GB acc:   BF546770    UniGene:   Rn.21134        SOURCE        Gene Ontology:     BioCarta Pathways:     KEGG Pathways:  
    Probe set : 1380513_at    Name:   similar to Expressed sequence AW060207 (predicted)    GB acc:   BE095638    UniGene:   Rn.146675    Gene Symbol:   RGD1309034_predicted    Locus Link:   305120    Chromosome:   14    Cytoband:   Chr:14p22    SOURCE    GENECARD       Gene Ontology:     BioCarta Pathways:     KEGG Pathways:  
    Probe set : 1372788_at    Name:   ubiquitin-conjugating enzyme E2Q (putative) (predicted)    GB acc:   AI144725    UniGene:   Rn.19034    Gene Symbol:   Ube2q_predicted    Locus Link:   295252    Chromosome:   2    Cytoband:   Chr:2q34    SOURCE    GENECARD       Gene Ontology:   biological process:  protein modification   biological process:  ubiquitin cycle   molecular function:  ubiquitin conjugating enzyme activity      BioCarta Pathways:     KEGG Pathways:  
    Probe set : 1367953_at    Name:   TYRO3 protein tyrosine kinase 3    GB acc:   NM_017092    UniGene:   Rn.8883    Gene Symbol:   Tyro3    Locus Link:   25232    Chromosome:   3    Cytoband:   Chr:3q36.1    SOURCE    GENECARD       Gene Ontology:   biological process:  protein amino acid phosphorylation   biological process:  cell adhesion   biological process:  signal transduction   cellular component:  extracellular space   cellular component:  integral to plasma membrane   cellular component:  integral to membrane      BioCarta Pathways:     KEGG Pathways:  
    Probe set : 1382751_at     GB acc:   BF549536              Gene Ontology:     BioCarta Pathways:     KEGG Pathways:  
    Probe set : 1373892_at    Name:   Transcribed locus    GB acc:   AA945986    UniGene:   Rn.7962        SOURCE        Gene Ontology:     BioCarta Pathways:     KEGG Pathways:  
    Probe set : 1382758_at    Name:   P7 protein    GB acc:   AA955094    UniGene:   Rn.23101    Gene Symbol:   P7    Locus Link:   291942    Chromosome:   19    Cytoband:   Chr:19q11    SOURCE    GENECARD       Gene Ontology:   biological process:  sodium ion transport   cellular component:  membrane   molecular function:  bile acid:sodium symporter activity      BioCarta Pathways:     KEGG Pathways:  
    Probe set : 1392509_at    Name:   Transcribed locus    GB acc:   AA925469    UniGene:   Rn.92651        SOURCE        Gene Ontology:     BioCarta Pathways:     KEGG Pathways:  
    Probe set : 1388596_at    Name:   coactosin-like 1 (Dictyostelium) (predicted)    GB acc:   AI411057    UniGene:   Rn.98506    Gene Symbol:   Cotl1_predicted    Locus Link:   361422    Chromosome:   19    Cytoband:   Chr:19q12    SOURCE    GENECARD       Gene Ontology:   cellular component:  intracellular   cellular component:  cytoskeleton   molecular function:  actin binding   molecular function:  protein binding   molecular function:  enzyme binding      BioCarta Pathways:     KEGG Pathways:  
    Probe set : 1372772_at     GB acc:   BM385098              Gene Ontology:     BioCarta Pathways:     KEGG Pathways:  
    Probe set : 1379934_at    Name:   Lysosomal trafficking regulator    GB acc:   BI285676    UniGene:   Rn.44274    Gene Symbol:   Lyst    Locus Link:   85419      SOURCE    GENECARD       Gene Ontology:   cellular component:  endosome   cellular component:  microtubule cytoskeleton      BioCarta Pathways:     KEGG Pathways:  
    Probe set : 1377907_at    Name:   similar to RIKEN cDNA 6530403A03 (predicted)    GB acc:   BF287924    UniGene:   Rn.39699    Gene Symbol:   RGD1309020_predicted    Locus Link:   291060    Chromosome:   17    Cytoband:   Chr:17p12    SOURCE    GENECARD       Gene Ontology:     BioCarta Pathways:     KEGG Pathways:  
    Probe set : 1382953_at    Name:   Transcribed locus    GB acc:   AI043711    UniGene:   Rn.150151        SOURCE        Gene Ontology:     BioCarta Pathways:     KEGG Pathways:  
    Probe set : 1392174_at    Name:   carbohydrate sulfotransferase 12 (predicted)    GB acc:   BI288174    UniGene:   Rn.78863    Gene Symbol:   Chst12_predicted    Locus Link:   304322    Chromosome:   12    Cytoband:   Chr:12q11    SOURCE    GENECARD       Gene Ontology:   biological process:  dermatan sulfate biosynthesis   cellular component:  integral to membrane   cellular component:  integral to Golgi membrane      BioCarta Pathways:     KEGG Pathways:  
    Probe set : 1373825_at    Name:   Hypothetical LOC314717 (predicted)    GB acc:   BG373078    UniGene:   Rn.103930     Locus Link:   314717    Chromosome:   7    Cytoband:   Chr:7q13    SOURCE        Gene Ontology:     BioCarta Pathways:     KEGG Pathways:  
    Probe set : 1371761_at    Name:   ribosomal protein L34 (predicted)    GB acc:   AI013910     Gene Symbol:   Rpl34_predicted    Locus Link:   362041    Chromosome:   2    Cytoband:   Chr:2q43     GENECARD       Gene Ontology:   biological process:  protein biosynthesis   cellular component:  intracellular   cellular component:  ribosome   cellular component:  cytosolic large ribosomal subunit (sensu Eukaryota)   molecular function:  RNA binding   molecular function:  structural constituent of ribosome      BioCarta Pathways:     KEGG Pathways:  
    Probe set : 1373776_at    Name:   Transcribed locus    GB acc:   AI406341    UniGene:   Rn.136702        SOURCE        Gene Ontology:     BioCarta Pathways:     KEGG Pathways:  
    Probe set : 1374731_at    Name:   Tripartite motif protein 25    GB acc:   BI275929    UniGene:   Rn.34382    Gene Symbol:   Trim25    Locus Link:   494338    Chromosome:   10    Cytoband:   Chr:10q26    SOURCE    GENECARD       Gene Ontology:   biological process:  protein ubiquitination   cellular component:  ubiquitin ligase complex      BioCarta Pathways:     KEGG Pathways:  
    Probe set : 1392032_at    Name:   GRB2-related adaptor protein (predicted)    GB acc:   BF418025    UniGene:   Rn.145247    Gene Symbol:   Grap_predicted    Locus Link:   363616    Chromosome:   10    Cytoband:   Chr:10q23    SOURCE    GENECARD       Gene Ontology:   biological process:  Ras protein signal transduction   biological process:  cell-cell signaling   biological process:  intracellular signaling cascade   cellular component:  cytoplasm   molecular function:  SH3/SH2 adaptor activity      BioCarta Pathways:     KEGG Pathways:  
    Probe set : 1372749_at    Name:   Transcribed locus    GB acc:   AI009786    UniGene:   Rn.95229        SOURCE        Gene Ontology:     BioCarta Pathways:     KEGG Pathways:  
    Probe set : 1383998_at    Name:   Transcribed locus, moderately similar to XP_421321.1 PREDICTED: similar to hypothetical protein FLJ20707 isoform 1 [Gallus gallus]    GB acc:   BF545601    UniGene:   Rn.123807        SOURCE        Gene Ontology:     BioCarta Pathways:     KEGG Pathways:  
    Probe set : 1386979_at    Name:   developmentally regulated protein TPO1    GB acc:   NM_133395    UniGene:   Rn.4099    Gene Symbol:   Tpo1    Locus Link:   170907    Chromosome:   2    Cytoband:   Chr:2q12    SOURCE    GENECARD       Gene Ontology:   cellular component:  plasma membrane   cellular component:  membrane   molecular function:  protein binding   molecular function:  L-serine transporter activity      BioCarta Pathways:     KEGG Pathways:  
    Probe set : 1383065_at    Name:   CDNA clone MGC:124727 IMAGE:7936966    GB acc:   AW434166    UniGene:   Rn.103733        SOURCE        Gene Ontology:     BioCarta Pathways:     KEGG Pathways:  
    Probe set : 1393411_at    Name:   properdin factor, complement    GB acc:   BI277565    UniGene:   Rn.18940    Gene Symbol:   Pfc    Locus Link:   299314    Chromosome:   X    Cytoband:   Chr:Xq12    SOURCE    GENECARD       Gene Ontology:     BioCarta Pathways:     KEGG Pathways:  
    Probe set : 1385168_at    Name:   similar to receptor-interacting factor 1 (predicted)    GB acc:   BM387883    UniGene:   Rn.82110    Gene Symbol:   RGD1306520_predicted    Locus Link:   310775    Chromosome:   2    Cytoband:   Chr:2q34    SOURCE    GENECARD       Gene Ontology:     BioCarta Pathways:     KEGG Pathways:  
    Probe set : 1393357_at    Name:   GLE1 RNA export mediator-like (yeast (predicted)    GB acc:   AW253109    UniGene:   Rn.144353    Gene Symbol:   Gle1l_predicted    Locus Link:   362098    Chromosome:   3    Cytoband:   Chr:3p12    SOURCE    GENECARD       Gene Ontology:   biological process:  mRNA export from nucleus   cellular component:  nuclear pore      BioCarta Pathways:     KEGG Pathways:  
    Probe set : 1382104_at    Name:   WD repeat domain 33 (predicted)    GB acc:   AI113277    UniGene:   Rn.22892    Gene Symbol:   Wdr33_predicted    Locus Link:   307524    Chromosome:   18    Cytoband:   Chr:18p12    SOURCE    GENECARD       Gene Ontology:   biological process:  postreplication repair   biological process:  phosphate transport   biological process:  spermatogenesis   cellular component:  nucleus   cellular component:  cytoplasm      BioCarta Pathways:     KEGG Pathways:  
    Probe set : 1382218_at    Name:   hypothetical LOC298077 (predicted)    GB acc:   AI012175    UniGene:   Rn.146070    Gene Symbol:   RGD1305807_predicted    Locus Link:   298077    Chromosome:   5    Cytoband:   Chr:5q22    SOURCE    GENECARD       Gene Ontology:     BioCarta Pathways:     KEGG Pathways:  
    Probe set : 1383543_at    Name:   similar to CGI-141 protein; EST AA407874 (predicted)    GB acc:   AW918220    UniGene:   Rn.1895    Gene Symbol:   RGD1310691_predicted    Locus Link:   362460    Chromosome:   4    Cytoband:   Chr:4q44    SOURCE    GENECARD       Gene Ontology:     BioCarta Pathways:     KEGG Pathways:  
    Probe set : 1372951_at    Name:   Transcribed locus    GB acc:   BI274172    UniGene:   Rn.21828        SOURCE        Gene Ontology:     BioCarta Pathways:     KEGG Pathways:  
    Probe set : 1399152_at    Name:   epidermal growth factor receptor pathway substrate 15 (predicted)    GB acc:   BF559107    UniGene:   Rn.22316    Gene Symbol:   Eps15_predicted    Locus Link:   313474    Chromosome:   5    Cytoband:   Chr:5q35    SOURCE    GENECARD       Gene Ontology:   biological process:  epidermal growth factor receptor signaling pathway   biological process:  cell proliferation   biological process:  vesicle organization and biogenesis   cellular component:  coated pit   molecular function:  receptor activity   molecular function:  calcium ion binding      BioCarta Pathways:     KEGG Pathways:  
    Probe set : 1382034_at     GB acc:   AA901075              Gene Ontology:     BioCarta Pathways:     KEGG Pathways:  
    Probe set : 1379059_at    Name:   similar to FLJ23471 protein (predicted)    GB acc:   BG373522    UniGene:   Rn.108004    Gene Symbol:   RGD1307875_predicted    Locus Link:   288515    Chromosome:   12    Cytoband:   Chr:12q11    SOURCE    GENECARD       Gene Ontology:     BioCarta Pathways:     KEGG Pathways:  
    Probe set : 1385258_at    Name:   UDP-GlcNAc:betaGal beta-1,3-N-acetylglucosaminyltransferase 7 (predicted)    GB acc:   BF419239    UniGene:   Rn.39638    Gene Symbol:   B3gnt7_predicted    Locus Link:   316583    Chromosome:   9    Cytoband:   Chr:9q35    SOURCE    GENECARD       Gene Ontology:   biological process:  protein amino acid glycosylation   cellular component:  membrane      BioCarta Pathways:     KEGG Pathways:  
    Probe set : 1376642_at    Name:   similar to hypothetical protein FLJ10307 (predicted)    GB acc:   BF548463    UniGene:   Rn.13596    Gene Symbol:   RGD1305649_predicted    Locus Link:   313033    Chromosome:   5    Cytoband:   Chr:5q36    SOURCE    GENECARD       Gene Ontology:   molecular function:  hydrolase activity      BioCarta Pathways:     KEGG Pathways:  
    Probe set : 1398782_at    Name:   N-ethylmaleimide sensitive fusion protein attachment protein alpha    GB acc:   NM_080585    UniGene:   Rn.31745    Gene Symbol:   Napa    Locus Link:   140673    Chromosome:   1    Cytoband:   Chr:1q21    SOURCE    GENECARD       Gene Ontology:   biological process:  transport   biological process:  intracellular protein transport   biological process:  intracellular protein transport   biological process:  intra-Golgi transport   biological process:  intra-Golgi transport   biological process:  membrane fusion   biological process:  membrane fusion   biological process:  brain development   biological process:  brain development   biological process:  protein transport   biological process:  neuron differentiation   biological process:  neuron differentiation   biological process:  apical protein localization   biological process:  apical protein localization   biological process:  ER to Golgi transport   cellular component:  endoplasmic reticulum   cellular component:  Golgi apparatus      BioCarta Pathways:     KEGG Pathways:  
    Probe set : 1395628_at    Name:   Transcribed locus    GB acc:   AW434310    UniGene:   Rn.147027        SOURCE        Gene Ontology:     BioCarta Pathways:     KEGG Pathways:  
    Probe set : 1371849_at    Name:   similar to hypothetical protein FLJ12442 (predicted)    GB acc:   BI285551    UniGene:   Rn.2956    Gene Symbol:   RGD1305524_predicted    Locus Link:   290558    Chromosome:   16    Cytoband:   Chr:16p16    SOURCE    GENECARD       Gene Ontology:     BioCarta Pathways:     KEGG Pathways:  
    Probe set : 1370855_at    Name:   cystatin C    GB acc:   BG666933    UniGene:   Rn.106351    Gene Symbol:   Cst3    Locus Link:   25307    Chromosome:   3    Cytoband:   Chr:3q41    SOURCE    GENECARD       Gene Ontology:   cellular component:  extracellular space   molecular function:  cysteine protease inhibitor activity   molecular function:  endopeptidase inhibitor activity      BioCarta Pathways:     KEGG Pathways:  
    Probe set : 1391544_at    Name:   Similar to adiponutrin    GB acc:   BI285941    UniGene:   Rn.97779     Locus Link:   362972    Chromosome:   7    Cytoband:   Chr:7q34    SOURCE        Gene Ontology:     BioCarta Pathways:     KEGG Pathways:  
    Probe set : 1383528_at    Name:   similar to mKIAA0215 protein    GB acc:   BI276223    UniGene:   Rn.29348    Gene Symbol:   LOC299305    Locus Link:   299305    Chromosome:   X    Cytoband:   Chr:Xq12    SOURCE    GENECARD       Gene Ontology:     BioCarta Pathways:     KEGG Pathways:  
    Probe set : 1375818_at    Name:   lactamase, beta (predicted)    GB acc:   BI300400    UniGene:   Rn.73451    Gene Symbol:   Lactb_predicted    Locus Link:   300803    Chromosome:   8    Cytoband:   Chr:8q24    SOURCE    GENECARD       Gene Ontology:   biological process:  response to antibiotic   cellular component:  extracellular space   cellular component:  integral to membrane   molecular function:  hydrolase activity      BioCarta Pathways:     KEGG Pathways:  
    Probe set : 1382705_at    Name:   Transcribed locus, weakly similar to XP_282988.2 PREDICTED: similar to novel protein [Mus musculus]    GB acc:   BE116909    UniGene:   Rn.147607        SOURCE        Gene Ontology:     BioCarta Pathways:     KEGG Pathways:  
    Probe set : 1370610_at    Name:   Solute carrier family 34 (sodium phosphate), member 1    GB acc:   AB013453    UniGene:   Rn.92022    Gene Symbol:   Slc34a1    Locus Link:   25548    Chromosome:   17    Cytoband:   Chr:17p14    SOURCE    GENECARD       Gene Ontology:     BioCarta Pathways:     KEGG Pathways:  
    Probe set : 1376567_at    Name:   FtsJ homolog 2 (E. coli) (predicted)    GB acc:   AI044868    UniGene:   Rn.95132     Locus Link:   304323    Chromosome:   12    Cytoband:   Chr:12q11    SOURCE        Gene Ontology:   biological process:  cytokinesis   biological process:  rRNA processing   cellular component:  nucleus   molecular function:  methyltransferase activity   molecular function:  transferase activity      BioCarta Pathways:     KEGG Pathways:  
    Probe set : 1379633_a_at    Name:   ubiquitin-activating enzyme E1-like (predicted)    GB acc:   BE096453    UniGene:   Rn.98275    Gene Symbol:   Ube1l_predicted    Locus Link:   301000    Chromosome:   8    Cytoband:   Chr:8q32    SOURCE    GENECARD       Gene Ontology:   biological process:  ubiquitin cycle      BioCarta Pathways:     KEGG Pathways:  
    Probe set : 1391270_at    Name:   Cyclin M3 (predicted)    GB acc:   BE112177    UniGene:   Rn.50710     Locus Link:   301345    Chromosome:   9    Cytoband:   Chr:9q21    SOURCE        Gene Ontology:   biological process:  transcription   biological process:  fatty acid biosynthesis   cellular component:  nucleus   molecular function:  DNA-directed RNA polymerase activity   molecular function:  transferase activity      BioCarta Pathways:     KEGG Pathways:  
    Probe set : 1374295_at    Name:   polymerase (RNA) mitochondrial (DNA directed) (predicted)    GB acc:   AI233096    UniGene:   Rn.24623    Gene Symbol:   Polrmt_predicted    Locus Link:   299604    Chromosome:   7    Cytoband:   Chr:7q11    SOURCE    GENECARD       Gene Ontology:   biological process:  DNA replication, synthesis of RNA primer   biological process:  transcription   cellular component:  mitochondrion   molecular function:  DNA binding   molecular function:  DNA-directed RNA polymerase activity   molecular function:  transferase activity      BioCarta Pathways:     KEGG Pathways:  
    Probe set : 1368647_at    Name:   G protein-coupled receptor kinase 6    GB acc:   NM_031657    UniGene:   Rn.10633    Gene Symbol:   Gprk6    Locus Link:   59076    Chromosome:   17    Cytoband:   Chr:17p14    SOURCE    GENECARD       Gene Ontology:     BioCarta Pathways:     KEGG Pathways:  
    Probe set : 1373090_at    Name:   signal sequence receptor, alpha (predicted)    GB acc:   AI230697    UniGene:   Rn.25091    Gene Symbol:   Ssr1_predicted    Locus Link:   361233    Chromosome:   17    Cytoband:   Chr:17p12    SOURCE    GENECARD       Gene Ontology:   biological process:  cotranslational protein targeting to membrane   biological process:  positive regulation of cell proliferation   cellular component:  endoplasmic reticulum   cellular component:  integral to membrane   molecular function:  receptor activity   molecular function:  signal sequence binding   molecular function:  calcium ion binding      BioCarta Pathways:     KEGG Pathways:  
    Probe set : 1394340_at    Name:   inositol polyphosphate-1-phosphatase (predicted)    GB acc:   BF523172    UniGene:   Rn.20288    Gene Symbol:   Inpp1_predicted    Locus Link:   316376    Chromosome:   9    Cytoband:   Chr:9q22    SOURCE    GENECARD       Gene Ontology:   biological process:  phosphate metabolism   biological process:  signal transduction      BioCarta Pathways:     KEGG Pathways:  
    Probe set : 1384971_at    Name:   similar to Hypothetical protein MGC19163 (predicted)    GB acc:   BI289108    UniGene:   Rn.97407    Gene Symbol:   RGD1309720_predicted    Locus Link:   295971    Chromosome:   3    Cytoband:   Chr:3q32    SOURCE    GENECARD       Gene Ontology:   biological process:  intracellular signaling cascade      BioCarta Pathways:     KEGG Pathways:  
    Probe set : 1374984_at    Name:   erythrocyte protein band 4.1-like 5 (predicted)    GB acc:   AA858925    UniGene:   Rn.2480    Gene Symbol:   Epb4.1l5_predicted    Locus Link:   304733    Chromosome:   13    Cytoband:   Chr:13q11    SOURCE    GENECARD       Gene Ontology:   cellular component:  cytoplasm   cellular component:  cytoskeleton   cellular component:  membrane   molecular function:  binding   molecular function:  cytoskeletal protein binding      BioCarta Pathways:     KEGG Pathways:  
    Probe set : 1386648_at    Name:   DnaJ (Hsp40) homolog, subfamily C, member 6 (predicted)    GB acc:   AW914996    UniGene:   Rn.23812    Gene Symbol:   Dnajc6_predicted    Locus Link:   313409    Chromosome:   5    Cytoband:   Chr:5q33    SOURCE    GENECARD       Gene Ontology:     BioCarta Pathways:     KEGG Pathways:  
    Probe set : 1381336_at    Name:   Transcribed locus    GB acc:   BF408248    UniGene:   Rn.14788        SOURCE        Gene Ontology:     BioCarta Pathways:     KEGG Pathways:  
    Probe set : 1374122_at    Name:   Transcribed locus    GB acc:   BM386808    UniGene:   Rn.2304        SOURCE        Gene Ontology:     BioCarta Pathways:     KEGG Pathways:  
    Probe set : 1387268_at    Name:   RNA polymerase 1-2    GB acc:   NM_031773    UniGene:   Rn.88734    Gene Symbol:   Rpo1-2    Locus Link:   83582    Chromosome:   3    Cytoband:   Chr:3q36    SOURCE    GENECARD       Gene Ontology:   biological process:  transcription   cellular component:  nucleus   cellular component:  nucleolus   cellular component:  DNA-directed RNA polymerase I complex      BioCarta Pathways:     KEGG Pathways:  
    Probe set : 1377033_at    Name:   serine (or cysteine) proteinase inhibitor, clade F, member 2 (predicted)    GB acc:   BG377997    UniGene:   Rn.15774    Gene Symbol:   Serpinf2_predicted    Locus Link:   287527    Chromosome:   10    Cytoband:   Chr:10q24    SOURCE    GENECARD       Gene Ontology:   biological process:  acute-phase response   cellular component:  extracellular region   cellular component:  extracellular space   cellular component:  Golgi apparatus   molecular function:  serine-type endopeptidase inhibitor activity   molecular function:  protein binding      BioCarta Pathways:     KEGG Pathways:  
    Probe set : 1376050_at    Name:   TAF5 RNA polymerase II, TATA box binding protein (TBP)-associated factor (predicted)    GB acc:   AI177663    UniGene:   Rn.144813    Gene Symbol:   Taf5_predicted    Locus Link:   294018    Chromosome:   1    Cytoband:   Chr:1q54    SOURCE    GENECARD       Gene Ontology:   biological process:  regulation of transcription, DNA-dependent   biological process:  regulation of transcription   cellular component:  nucleus   cellular component:  transcription factor TFIID complex   molecular function:  transcription factor activity   molecular function:  transcription regulator activity      BioCarta Pathways:     KEGG Pathways:  
    Probe set : 1389147_at    Name:   Transcribed locus    GB acc:   BE116020    UniGene:   Rn.16536        SOURCE        Gene Ontology:     BioCarta Pathways:     KEGG Pathways:  
    Probe set : 1372758_at    Name:   Transcribed locus    GB acc:   BI282978    UniGene:   Rn.988        SOURCE        Gene Ontology:     BioCarta Pathways:     KEGG Pathways:  
    Probe set : 1372432_at    Name:   PRP3 pre-mRNA processing factor 3 homolog (yeast) (predicted)    GB acc:   BF395125    UniGene:   Rn.116267    Gene Symbol:   Prpf3_predicted    Locus Link:   361995    Chromosome:   2    Cytoband:   Chr:2q34    SOURCE    GENECARD       Gene Ontology:   biological process:  nuclear mRNA splicing, via spliceosome   biological process:  mRNA processing   biological process:  visual perception   cellular component:  nucleus   cellular component:  spliceosome complex      BioCarta Pathways:     KEGG Pathways:  
    Probe set : 1388921_at    Name:   Transcribed locus    GB acc:   BI279587    UniGene:   Rn.24778        SOURCE        Gene Ontology:     BioCarta Pathways:     KEGG Pathways:  
    Probe set : 1381951_at    Name:   Transcribed locus    GB acc:   BF408414    UniGene:   Rn.128717        SOURCE        Gene Ontology:     BioCarta Pathways:     KEGG Pathways:  
    Probe set : 1389344_at    Name:   ubiquitin specific protease 39 (predicted)    GB acc:   BE109258    UniGene:   Rn.50051    Gene Symbol:   Usp39_predicted    Locus Link:   297336    Chromosome:   4    Cytoband:   Chr:4q33    SOURCE    GENECARD       Gene Ontology:   cellular component:  nucleus      BioCarta Pathways:     KEGG Pathways:  
    Probe set : 1382032_at    Name:   Transcribed locus    GB acc:   AI407730    UniGene:   Rn.7451        SOURCE        Gene Ontology:     BioCarta Pathways:     KEGG Pathways:  
    Probe set : 1389271_at     GB acc:   BI274567              Gene Ontology:     BioCarta Pathways:     KEGG Pathways:  
    Probe set : 1388995_at    Name:   Transcribed locus    GB acc:   BF407666    UniGene:   Rn.34945        SOURCE        Gene Ontology:     BioCarta Pathways:     KEGG Pathways:  
    Probe set : 1397476_at    Name:   Ttk protein kinase (predicted)    GB acc:   BM383386    UniGene:   Rn.107533    Gene Symbol:   Ttk_predicted    Locus Link:   315852    Chromosome:   8    Cytoband:   Chr:8q31    SOURCE    GENECARD       Gene Ontology:   cellular component:  spindle      BioCarta Pathways:     KEGG Pathways:  
    Probe set : 1372769_at    Name:   similar to ligatin    GB acc:   AI105290    UniGene:   Rn.107913    Gene Symbol:   LOC498225    Locus Link:   498225    Chromosome:   13    Cytoband:   Chr:13q13    SOURCE    GENECARD       Gene Ontology:   biological process:  translational initiation   cellular component:  membrane   molecular function:  RNA binding   molecular function:  translation initiation factor activity      BioCarta Pathways:     KEGG Pathways:  
    Probe set : 1370297_at    Name:   polo-like kinase 1 (Drosophila)    GB acc:   U10188    UniGene:   Rn.11034    Gene Symbol:   Plk1    Locus Link:   25515    Chromosome:   1    Cytoband:   Chr:1q36    SOURCE    GENECARD       Gene Ontology:   cellular component:  nucleus      BioCarta Pathways:     KEGG Pathways:  
    Probe set : 1382945_at    Name:   Similar to cDNA sequence BC006662 (predicted)    GB acc:   AI028809    UniGene:   Rn.17263     Locus Link:   290500    Chromosome:   15    Cytoband:   Chr:15q25    SOURCE        Gene Ontology:     BioCarta Pathways:     KEGG Pathways:  
    Probe set : 1368214_at    Name:   MAD homolog 2 (Drosophila)    GB acc:   NM_019191    UniGene:   Rn.2755    Gene Symbol:   Madh2    Locus Link:   29357      SOURCE    GENECARD       Gene Ontology:   biological process:  mesoderm formation   biological process:  regulation of transcription, DNA-dependent   biological process:  signal transduction   biological process:  transforming growth factor beta receptor signaling pathway   biological process:  common-partner SMAD protein phosphorylation   biological process:  SMAD protein heteromerization   biological process:  organ morphogenesis   biological process:  anterior/posterior pattern formation   biological process:  cell fate commitment   biological process:  positive regulation of transcription from RNA polymerase II promoter   biological process:  paraxial mesoderm morphogenesis   biological process:  mesoderm formation   biological process:  transforming growth factor beta receptor signaling pathway   biological process:  intracellular signaling cascade   biological process:  anterior/posterior pattern formation   biological process:  cell fate commitment   biological process:  positive regulation of transcription, DNA-dependent   biological process:  positive regulation of transcription from RNA polymerase II promoter   biological process:  paraxial mesoderm morphogenesis   biological process:  regulation of binding   biological process:  transcription      BioCarta Pathways:     KEGG Pathways:  
    Probe set : 1390731_at    Name:   Transcribed locus    GB acc:   BI288533    UniGene:   Rn.48004        SOURCE        Gene Ontology:     BioCarta Pathways:     KEGG Pathways:  
    Probe set : 1398801_at    Name:   CDK105 protein    GB acc:   NM_134415    UniGene:   Rn.144664    Gene Symbol:   RGD:621335    Locus Link:   171456    Chromosome:   2    Cytoband:   Chr:2q12    SOURCE    GENECARD       Gene Ontology:   biological process:  protein biosynthesis   cellular component:  nucleus   cellular component:  ribosome   cellular component:  intracellular   molecular function:  structural constituent of ribosome      BioCarta Pathways:     KEGG Pathways:  
    Probe set : 1386379_at    Name:   Similar to MGC68837 protein    GB acc:   AI172246    UniGene:   Rn.58294     Locus Link:   498356      SOURCE        Gene Ontology:     BioCarta Pathways:     KEGG Pathways:  
    Probe set : 1390999_at    Name:   Transcribed locus    GB acc:   BE108409    UniGene:   Rn.38287        SOURCE        Gene Ontology:     BioCarta Pathways:     KEGG Pathways:  
    Probe set : 1387402_at    Name:   myosin, heavy polypeptide 9    GB acc:   NM_013194    UniGene:   Rn.11385    Gene Symbol:   Myh9    Locus Link:   25745    Chromosome:   7    Cytoband:   Chr:7q34    SOURCE    GENECARD       Gene Ontology:     BioCarta Pathways:     KEGG Pathways:  
    Probe set : 1384368_at    Name:   DNA2 DNA replication helicase 2-like (yeast) (predicted)    GB acc:   AA900084    UniGene:   Rn.21448     Locus Link:   309762    Chromosome:   20    Cytoband:   Chr:20p11    SOURCE        Gene Ontology:     BioCarta Pathways:     KEGG Pathways:  
    Probe set : 1372853_at    Name:   v-rel reticuloendotheliosis viral oncogene homolog A (avian)    GB acc:   BF283772    UniGene:   Rn.19480    Gene Symbol:   Rela    Locus Link:   309165    Chromosome:   1    Cytoband:   Chr:1q43    SOURCE    GENECARD       Gene Ontology:   biological process:  regulation of transcription, DNA-dependent   biological process:  regulation of transcription   cellular component:  nucleus   molecular function:  transcription factor activity      BioCarta Pathways:     KEGG Pathways:  
    Probe set : 1368180_s_at    Name:   glutathione-S-transferase, alpha type2    GB acc:   NM_017013    UniGene:   Rn.144550    Gene Symbol:   Gsta2    Locus Link:   24422    Chromosome:   8    Cytoband:   Chr:8q31    SOURCE    GENECARD       Gene Ontology:   biological process:  metabolism   molecular function:  glutathione transferase activity   molecular function:  glutathione transferase activity   molecular function:  transferase activity      BioCarta Pathways:     KEGG Pathways:  
    Probe set : 1371322_at    Name:   Laminin, gamma 1    GB acc:   BI275624    UniGene:   Rn.7145    Gene Symbol:   Lamc1    Locus Link:   117036    Chromosome:   13    Cytoband:   Chr:13q21    SOURCE    GENECARD       Gene Ontology:   cellular component:  extracellular matrix (sensu Metazoa)   cellular component:  laminin-1      BioCarta Pathways:     KEGG Pathways:  
    Probe set : 1376501_at    Name:   Rho GTPase activating protein 8    GB acc:   AA945062    UniGene:   Rn.7830    Gene Symbol:   RGD:1303143    Locus Link:   300115    Chromosome:   7    Cytoband:   Chr:7q34    SOURCE    GENECARD       Gene Ontology:     BioCarta Pathways:     KEGG Pathways:  
    Probe set : 1373925_at     GB acc:   BE112950              Gene Ontology:     BioCarta Pathways:     KEGG Pathways:  
    Probe set : 1385505_at    Name:   Transcribed locus    GB acc:   AI072068    UniGene:   Rn.146323        SOURCE        Gene Ontology:     BioCarta Pathways:     KEGG Pathways:  
    Probe set : 1384227_at    Name:   Protein tyrosine phosphatase, receptor type, K, extracellular region    GB acc:   AI044031    UniGene:   Rn.126653     Locus Link:   360302    Chromosome:   1    Cytoband:   Chr:1p12    SOURCE        Gene Ontology:   cellular component:  membrane   molecular function:  receptor activity      BioCarta Pathways:     KEGG Pathways:  
    Probe set : 1395246_at    Name:   Protein kinase, cAMP-dependent, regulatory, type 2, alpha    GB acc:   AI104783    UniGene:   Rn.9742    Gene Symbol:   Prkar2a    Locus Link:   29699    Chromosome:   8    Cytoband:   Chr:8q32    SOURCE    GENECARD       Gene Ontology:     BioCarta Pathways:     KEGG Pathways:  
    Probe set : 1367756_at    Name:   G elongation factor    GB acc:   NM_053625    UniGene:   Rn.10913    Gene Symbol:   Gfm    Locus Link:   114017    Chromosome:   2    Cytoband:   Chr:2q31    SOURCE    GENECARD       Gene Ontology:   biological process:  protein biosynthesis   biological process:  translational elongation   biological process:  translational elongation   cellular component:  mitochondrion   molecular function:  translation elongation factor activity   molecular function:  GTP binding   molecular function:  nucleotide binding      BioCarta Pathways:     KEGG Pathways:  
    Probe set : 1393209_at    Name:   Bartter syndrome, infantile, with sensorineural deafness (Barttin)    GB acc:   AI547423    UniGene:   Rn.22715    Gene Symbol:   bsnd    Locus Link:   192675    Chromosome:   5    Cytoband:   Chr:5q34    SOURCE    GENECARD       Gene Ontology:   molecular function:  chloride channel regulator activity   molecular function:  chloride channel activity   molecular function:  protein binding      BioCarta Pathways:     KEGG Pathways:  
    Probe set : 1373503_at    Name:   leucine-rich repeats and calponin homology (CH) domain containing 4 (predicted)    GB acc:   AA892504    UniGene:   Rn.24664    Gene Symbol:   Lrch4_predicted    Locus Link:   360779    Chromosome:   12    Cytoband:   Chr:12q12    SOURCE    GENECARD       Gene Ontology:   biological process:  nervous system development      BioCarta Pathways:     KEGG Pathways:  
    Probe set : 1389832_at    Name:   glutathione S-transferase omega 1    GB acc:   BE113459    UniGene:   Rn.25166    Gene Symbol:   Gsto1    Locus Link:   114846    Chromosome:   1    Cytoband:   Chr:1q54    SOURCE    GENECARD       Gene Ontology:   biological process:  metabolism   biological process:  L-ascorbic acid biosynthesis   cellular component:  cytoplasm   cellular component:  cytoplasm      BioCarta Pathways:     KEGG Pathways:  
    Probe set : 1388117_at    Name:   Similar to small nuclear ribonucleoprotein B    GB acc:   AI411893    UniGene:   Rn.3437    Gene Symbol:   Snrpb    Locus Link:   171365    Chromosome:   3    Cytoband:   Chr:3q36    SOURCE    GENECARD       Gene Ontology:   biological process:  regulation of transcription, DNA-dependent   biological process:  mRNA processing   biological process:  RNA splicing   molecular function:  DNA binding   molecular function:  RNA binding   molecular function:  protein binding      BioCarta Pathways:     KEGG Pathways:  
    Probe set : 1382009_at    Name:   Transcribed locus    GB acc:   AA955721    UniGene:   Rn.146623        SOURCE        Gene Ontology:     BioCarta Pathways:     KEGG Pathways:  
    Probe set : 1376191_at    Name:   15-hydroxyprostaglandin dehydrogenase    GB acc:   AA848820    UniGene:   Rn.6386    Gene Symbol:   Hpgd    Locus Link:   79242    Chromosome:   16    Cytoband:   Chr:16p11    SOURCE    GENECARD       Gene Ontology:   biological process:  prostaglandin metabolism   biological process:  metabolism      BioCarta Pathways:     KEGG Pathways:  
    Probe set : 1368785_a_at    Name:   paired-like homeodomain transcription factor 2    GB acc:   NM_019334    UniGene:   Rn.17591    Gene Symbol:   Pitx2    Locus Link:   54284    Chromosome:   2    Cytoband:   Chr:2q42    SOURCE    GENECARD       Gene Ontology:   cellular component:  nucleus   cellular component:  transcription factor complex      BioCarta Pathways:     KEGG Pathways:  
    Probe set : 1389521_at    Name:   influenza virus NS1A binding protein (predicted)    GB acc:   AI408553    UniGene:   Rn.36174    Gene Symbol:   Ivns1abp_predicted    Locus Link:   289089    Chromosome:   13    Cytoband:   Chr:13q21    SOURCE    GENECARD       Gene Ontology:   biological process:  transcription from RNA polymerase III promoter   biological process:  RNA splicing   biological process:  response to virus   cellular component:  transcription factor complex   cellular component:  spliceosome complex   molecular function:  protein binding      BioCarta Pathways:     KEGG Pathways:  
    Probe set : 1377007_at     GB acc:   AI409696              Gene Ontology:     BioCarta Pathways:     KEGG Pathways:  
    Probe set : 1393957_at    Name:   Transcribed locus    GB acc:   AI548358    UniGene:   Rn.100053        SOURCE        Gene Ontology:     BioCarta Pathways:     KEGG Pathways:  
    Probe set : 1383121_at    Name:   Ceroid-lipofuscinosis, neuronal 2    GB acc:   BM384218    UniGene:   Rn.43558    Gene Symbol:   Cln2    Locus Link:   83534    Chromosome:   1    Cytoband:   Chr:1q33    SOURCE    GENECARD       Gene Ontology:   biological process:  proteolysis   biological process:  lipid metabolism   biological process:  nervous system development      BioCarta Pathways:     KEGG Pathways:  
    Probe set : 1369941_at    Name:   death-associated protein    GB acc:   NM_022526    UniGene:   Rn.1531    Gene Symbol:   Dap    Locus Link:   64322    Chromosome:   2    Cytoband:   Chr:2q22    SOURCE    GENECARD       Gene Ontology:   biological process:  apoptosis   biological process:  induction of apoptosis by extracellular signals   biological process:  programmed cell death      BioCarta Pathways:     KEGG Pathways:  
    Probe set : 1384073_at    Name:   alcohol dehydrogenase, iron containing, 1 (predicted)    GB acc:   BF543577    UniGene:   Rn.17173    Gene Symbol:   Adhfe1_predicted    Locus Link:   362474    Chromosome:   5    Cytoband:   Chr:5q11    SOURCE    GENECARD       Gene Ontology:   biological process:  metabolism   molecular function:  iron ion binding   molecular function:  oxidoreductase activity      BioCarta Pathways:     KEGG Pathways:  
    Probe set : 1370097_a_at    Name:   chemokine (C-X-C motif) receptor 4    GB acc:   U54791    UniGene:   Rn.44431    Gene Symbol:   Cxcr4    Locus Link:   60628    Chromosome:   13    Cytoband:   Chr:13q12    SOURCE    GENECARD       Gene Ontology:   cellular component:  integral to membrane      BioCarta Pathways:     KEGG Pathways:  
    Probe set : 1368340_at    Name:   inositol polyphosphate multikinase    GB acc:   NM_134417    UniGene:   Rn.15187    Gene Symbol:   Impk    Locus Link:   171458    Chromosome:   20    Cytoband:   Chr:20p11    SOURCE    GENECARD       Gene Ontology:   biological process:  phosphoinositide phosphorylation   cellular component:  nucleus      BioCarta Pathways:     KEGG Pathways:  
    Probe set : 1383665_at    Name:   Transcribed locus    GB acc:   BE096055    UniGene:   Rn.41006        SOURCE        Gene Ontology:     BioCarta Pathways:     KEGG Pathways:  
    Probe set : 1390017_at    Name:   Hyaluronan and proteoglycan link protein 2    GB acc:   BF393931    UniGene:   Rn.76545    Gene Symbol:   Hapln2    Locus Link:   64057    Chromosome:   2    Cytoband:   Chr:2q34    SOURCE    GENECARD       Gene Ontology:   biological process:  cell adhesion   cellular component:  extracellular matrix (sensu Metazoa)   cellular component:  extracellular space   molecular function:  hyaluronic acid binding      BioCarta Pathways:     KEGG Pathways:  
    Probe set : 1371945_at    Name:   ubiquitin-conjugating enzyme E2L 3 (predicted)    GB acc:   AI407788    UniGene:   Rn.131117    Gene Symbol:   Ube2l3_predicted    Locus Link:   363836    Chromosome:   11    Cytoband:   Chr:11q23    SOURCE    GENECARD       Gene Ontology:   biological process:  ubiquitin-dependent protein catabolism   biological process:  ubiquitin cycle   cellular component:  ubiquitin ligase complex      BioCarta Pathways:     KEGG Pathways:  
    Probe set : 1372507_at    Name:   similar to RIKEN cDNA 9130410M22    GB acc:   AA901093    UniGene:   Rn.2916    Gene Symbol:   RGD:1359333    Locus Link:   306587    Chromosome:   16    Cytoband:   Chr:16q12.5    SOURCE    GENECARD       Gene Ontology:     BioCarta Pathways:     KEGG Pathways:  
    Probe set : 1372151_at    Name:   transcription elongation regulator 1 (CA150) (predicted)    GB acc:   AI317851    UniGene:   Rn.18770    Gene Symbol:   Tcerg1_predicted    Locus Link:   307474    Chromosome:   18    Cytoband:   Chr:18p11    SOURCE    GENECARD       Gene Ontology:   biological process:  transcription   biological process:  regulation of transcription, DNA-dependent   biological process:  transcription from RNA polymerase II promoter   cellular component:  nucleus   cellular component:  nuclear matrix      BioCarta Pathways:     KEGG Pathways:  
    Probe set : 1367755_at    Name:   cytosolic cysteine dioxygenase 1    GB acc:   NM_052809    UniGene:   Rn.2589    Gene Symbol:   Cdo1    Locus Link:   81718    Chromosome:   18    Cytoband:   Chr:18q11    SOURCE    GENECARD       Gene Ontology:   cellular component:  cytosol   cellular component:  cytosol      BioCarta Pathways:     KEGG Pathways:  
    Probe set : 1396752_at    Name:   Tumor necrosis factor receptor superfamily, member 21 (predicted)    GB acc:   BF405147    UniGene:   Rn.98293     Locus Link:   316256    Chromosome:   9    Cytoband:   Chr:9q12    SOURCE        Gene Ontology:   biological process:  apoptosis   biological process:  signal transduction   cellular component:  integral to membrane   molecular function:  receptor activity   molecular function:  protein binding      BioCarta Pathways:     KEGG Pathways:  
    Probe set : 1397205_at    Name:   dehydrogenase/reductase (SDR family) member 7 (predicted) /// similar to Down-regulated in nephrectomized rat kidney #3    GB acc:   BI288203    UniGene:   Rn.119024    Gene Symbol:   Dhrs7_predicted /// LOC500672    Locus Link:   299131 /// 500672    Chromosome:   6    Cytoband:   Chr:6q24    SOURCE    GENECARD       Gene Ontology:   biological process:  metabolism   molecular function:  oxidoreductase activity      BioCarta Pathways:     KEGG Pathways:  
    Probe set : 1387542_at    Name:   solute carrier family 9, member 3    GB acc:   NM_012654    UniGene:   Rn.9706    Gene Symbol:   Slc9a3    Locus Link:   24784    Chromosome:   1    Cytoband:   Chr:1p11    SOURCE    GENECARD       Gene Ontology:     BioCarta Pathways:     KEGG Pathways:  
    Probe set : 1393684_at    Name:   TBC1D12: TBC1 domain family, member 12 (predicted)    GB acc:   BF389489    UniGene:   Rn.23113    Gene Symbol:   Tbc1d12_predicted    Locus Link:   294071    Chromosome:   1    Cytoband:   Chr:1q53    SOURCE    GENECARD       Gene Ontology:     BioCarta Pathways:     KEGG Pathways:  
    Probe set : 1375630_at    Name:   similar to NHP2-like protein 1 (High mobility group-like nuclear protein 2 homolog 1) ([U4/U6.U5] tri-snRNP 15.5 kDa protein) (OTK27)    GB acc:   AA859366    UniGene:   Rn.153560    Gene Symbol:   RGD:1303103    Locus Link:   300092    Chromosome:   7    Cytoband:   Chr:7q34    SOURCE    GENECARD       Gene Ontology:   biological process:  nuclear mRNA splicing, via spliceosome   biological process:  mRNA processing   biological process:  ribosome biogenesis and assembly   cellular component:  nucleus   cellular component:  spliceosome complex   cellular component:  ribonucleoprotein complex   molecular function:  RNA binding   molecular function:  structural constituent of ribosome      BioCarta Pathways:     KEGG Pathways:  
    Probe set : 1375685_at    Name:   cytoplasmic linker associated protein 1 (predicted)    GB acc:   BI282257    UniGene:   Rn.105840    Gene Symbol:   Clasp1_predicted    Locus Link:   304740    Chromosome:   13    Cytoband:   Chr:13q11    SOURCE    GENECARD       Gene Ontology:   biological process:  negative regulation of microtubule depolymerization   cellular component:  microtubule associated complex   cellular component:  cytoplasmic microtubule   molecular function:  binding   molecular function:  protein binding   molecular function:  microtubule binding      BioCarta Pathways:     KEGG Pathways:  
    Probe set : 1377333_at    Name:   Transcribed locus    GB acc:   BE114427    UniGene:   Rn.18255        SOURCE        Gene Ontology:     BioCarta Pathways:     KEGG Pathways:  
    Probe set : 1387344_at    Name:   aldehyde dehydrogenase family 6, subfamily A1    GB acc:   AI407458    UniGene:   Rn.2098    Gene Symbol:   Aldh6a1    Locus Link:   81708    Chromosome:   6    Cytoband:   Chr:6q31    SOURCE    GENECARD       Gene Ontology:   biological process:  valine metabolism   biological process:  metabolism   cellular component:  mitochondrion      BioCarta Pathways:     KEGG Pathways:  
    Probe set : 1388669_at    Name:   splicing factor 3a, subunit 3 (predicted)    GB acc:   BM391739    UniGene:   Rn.16732    Gene Symbol:   Sf3a3_predicted    Locus Link:   313583    Chromosome:   5    Cytoband:   Chr:5q36    SOURCE    GENECARD       Gene Ontology:   biological process:  nuclear mRNA splicing, via spliceosome   biological process:  mRNA processing   biological process:  RNA splicing   cellular component:  nucleus   cellular component:  spliceosome complex   molecular function:  nucleic acid binding   molecular function:  zinc ion binding      BioCarta Pathways:     KEGG Pathways:  
    Probe set : 1373035_at    Name:   Transcribed locus    GB acc:   AI031032    UniGene:   Rn.12724        SOURCE        Gene Ontology:     BioCarta Pathways:     KEGG Pathways:  
    Probe set : 1390455_at    Name:   abhydrolase domain containing 2 (predicted)    GB acc:   AI013474    UniGene:   Rn.136611    Gene Symbol:   Abhd2_predicted    Locus Link:   293050    Chromosome:   1    Cytoband:   Chr:1q31    SOURCE    GENECARD       Gene Ontology:   cellular component:  integral to membrane   molecular function:  catalytic activity   molecular function:  hydrolase activity      BioCarta Pathways:     KEGG Pathways:  
    Probe set : 1391227_at    Name:   Transcribed locus    GB acc:   AW530423    UniGene:   Rn.20252        SOURCE        Gene Ontology:     BioCarta Pathways:     KEGG Pathways:  
    Probe set : 1369773_at    Name:   bone morphogenetic protein 3    GB acc:   NM_017105    UniGene:   Rn.53974    Gene Symbol:   Bmp3    Locus Link:   25667    Chromosome:   14    Cytoband:   Chr:14p22    SOURCE    GENECARD       Gene Ontology:   cellular component:  extracellular space      BioCarta Pathways:     KEGG Pathways:  
    Probe set : 1368095_at    Name:   adenylate kinase 3    GB acc:   NM_013218    UniGene:   Rn.60    Gene Symbol:   Ak3    Locus Link:   26956    Chromosome:   1    Cytoband:   Chr:1q52    SOURCE    GENECARD       Gene Ontology:   biological process:  amino acid biosynthesis   biological process:  nucleobase, nucleoside, nucleotide and nucleic acid metabolism   cellular component:  mitochondrion   cellular component:  mitochondrion      BioCarta Pathways:     KEGG Pathways:  
    Probe set : 1389379_at    Name:   DNA cross-link repair 1C, PSO2 homolog (S. cerevisiae)    GB acc:   AI011967    UniGene:   Rn.16133    Gene Symbol:   Mt1a    Locus Link:   24567    Chromosome:   19    Cytoband:   Chr:19p12    SOURCE    GENECARD       Gene Ontology:   cellular component:  cytoplasm   cellular component:  lysosome   cellular component:  cytosol   cellular component:  nucleus      BioCarta Pathways:     KEGG Pathways:  
    Probe set : 1372910_at    Name:   Villin (predicted)    GB acc:   AI408770    UniGene:   Rn.12954     Locus Link:   316521    Chromosome:   9    Cytoband:   Chr:9q33    SOURCE        Gene Ontology:   cellular component:  cytoskeleton   cellular component:  brush border   cellular component:  F-actin capping protein complex   molecular function:  actin binding      BioCarta Pathways:     KEGG Pathways:  
    Probe set : 1367619_at    Name:   progesterone receptor membrane component 1    GB acc:   NM_021766    UniGene:   Rn.102356    Gene Symbol:   Pgrmc1    Locus Link:   291948    Chromosome:   X    Cytoband:   Chr:Xq12    SOURCE    GENECARD       Gene Ontology:   biological process:  axon guidance   biological process:  axon guidance   molecular function:  receptor activity   molecular function:  steroid binding      BioCarta Pathways:     KEGG Pathways:  
    Probe set : 1372903_at    Name:   similar to 3000004C01Rik protein (predicted)    GB acc:   AI410054    UniGene:   Rn.112739    Gene Symbol:   RGD1310360_predicted    Locus Link:   303575    Chromosome:   10    Cytoband:   Chr:10q32.1    SOURCE    GENECARD       Gene Ontology:   biological process:  microtubule-based movement   cellular component:  microtubule associated complex   molecular function:  motor activity   molecular function:  ATP binding   molecular function:  microtubule motor activity      BioCarta Pathways:     KEGG Pathways:  
    Probe set : 1395116_at    Name:   CD8 antigen, alpha chain    GB acc:   BI286453    UniGene:   Rn.10306    Gene Symbol:   Cd8a    Locus Link:   24930    Chromosome:   4    Cytoband:   Chr:4q33    SOURCE    GENECARD       Gene Ontology:     BioCarta Pathways:     KEGG Pathways:  
    Probe set : 1390394_at    Name:   Transcribed locus    GB acc:   AW251900    UniGene:   Rn.93973        SOURCE        Gene Ontology:     BioCarta Pathways:     KEGG Pathways:  
    Probe set : 1368201_at    Name:   natriuretic peptide receptor 1    GB acc:   NM_012613    UniGene:   Rn.10463    Gene Symbol:   Npr1    Locus Link:   24603    Chromosome:   2    Cytoband:   Chr:2q34    SOURCE    GENECARD       Gene Ontology:   biological process:  cGMP biosynthesis   biological process:  protein amino acid phosphorylation   biological process:  transport   biological process:  cell surface receptor linked signal transduction   biological process:  intracellular signaling cascade   biological process:  intracellular signaling cascade   biological process:  fluid secretion   biological process:  regulation of blood pressure   biological process:  negative regulation of angiogenesis   biological process:  diuresis   biological process:  natriuresis   biological process:  negative regulation of cell growth   biological process:  regulation of vasodilation   biological process:  regulation of vascular permeability   biological process:  cyclic nucleotide biosynthesis      BioCarta Pathways:     KEGG Pathways:  
    Probe set : 1376672_at    Name:   Transcribed locus    GB acc:   AW533083    UniGene:   Rn.18056        SOURCE        Gene Ontology:     BioCarta Pathways:     KEGG Pathways:  
    Probe set : 1371889_at    Name:   solute carrier family 22 (organic cation transporter), member 17    GB acc:   AW915448    UniGene:   Rn.7312    Gene Symbol:   Slc22a17    Locus Link:   305886    Chromosome:   15    Cytoband:   Chr:15p13    SOURCE    GENECARD       Gene Ontology:   biological process:  tRNA aminoacylation for protein translation   biological process:  transport   biological process:  ion transport   cellular component:  extracellular space   cellular component:  membrane   cellular component:  integral to membrane      BioCarta Pathways:     KEGG Pathways:  
    Probe set : 1373809_at    Name:   DEAD (Asp-Glu-Ala-Asp) box polypeptide 21b (predicted) /// hypothetical protein LOC606294    GB acc:   BI296325    UniGene:   Rn.56595    Gene Symbol:   Ddx21b_predicted /// LOC606294    Locus Link:   361847 /// 606294    Chromosome:   20    Cytoband:   Chr:20q11    SOURCE    GENECARD       Gene Ontology:   cellular component:  nucleolus      BioCarta Pathways:     KEGG Pathways:  
    Probe set : 1379315_at    Name:   similar to RIKEN cDNA 2400009B11 gene (predicted)    GB acc:   BM385768    UniGene:   Rn.24007    Gene Symbol:   RGD1306244_predicted    Locus Link:   293623    Chromosome:   1    Cytoband:   Chr:1q41    SOURCE    GENECARD       Gene Ontology:     BioCarta Pathways:     KEGG Pathways:  
    Probe set : 1385458_a_at    Name:   similar to C11orf17 protein (predicted)    GB acc:   AA799992    UniGene:   Rn.106344    Gene Symbol:   RGD1306959_predicted    Locus Link:   361624    Chromosome:   1    Cytoband:   Chr:1q33    SOURCE    GENECARD       Gene Ontology:     BioCarta Pathways:     KEGG Pathways:  
    Probe set : 1388631_at    Name:   5-azacytidine induced gene 2 (predicted)    GB acc:   AI177093    UniGene:   Rn.140347    Gene Symbol:   Azi2_predicted    Locus Link:   316051    Chromosome:   8    Cytoband:   Chr:8q32    SOURCE    GENECARD       Gene Ontology:   biological process:  I-kappaB kinase/NF-kappaB cascade   cellular component:  cytoplasm      BioCarta Pathways:     KEGG Pathways:  
    Probe set : 1389757_at    Name:   similar to acetyl-coA dehydrogenase -related (111.6 kD) (5G231) (predicted)    GB acc:   AI011481    UniGene:   Rn.13452    Gene Symbol:   RGD1310159_predicted    Locus Link:   304500    Chromosome:   12    Cytoband:   Chr:12q16    SOURCE    GENECARD       Gene Ontology:     BioCarta Pathways:     KEGG Pathways:  
    Probe set : 1389535_at    Name:   death associated protein 3 (predicted)    GB acc:   BI285645    UniGene:   Rn.144833    Gene Symbol:   Dap3_predicted    Locus Link:   295238    Chromosome:   2    Cytoband:   Chr:2q34    SOURCE    GENECARD       Gene Ontology:   molecular function:  structural constituent of ribosome      BioCarta Pathways:     KEGG Pathways:  
    Probe set : 1379669_at     GB acc:   BI275651              Gene Ontology:     BioCarta Pathways:     KEGG Pathways:  
    Probe set : 1385679_at    Name:   Transcribed locus    GB acc:   BG378431    UniGene:   Rn.151616        SOURCE        Gene Ontology:     BioCarta Pathways:     KEGG Pathways:  
    Probe set : 1387346_at    Name:   integrin beta 1    GB acc:   NM_017022    UniGene:   Rn.25733    Gene Symbol:   Itgb1    Locus Link:   24511    Chromosome:   19    Cytoband:   Chr:19q12    SOURCE    GENECARD       Gene Ontology:     BioCarta Pathways:     KEGG Pathways:  
    Probe set : 1368089_at    Name:   phosphodiesterase 2A, cGMP-stimulated    GB acc:   NM_031079    UniGene:   Rn.10044    Gene Symbol:   Pde2a    Locus Link:   81743    Chromosome:   1    Cytoband:   Chr:1q32    SOURCE    GENECARD       Gene Ontology:   biological process:  signal transduction   cellular component:  membrane      BioCarta Pathways:     KEGG Pathways:  
    Probe set : 1388787_at    Name:   Similar to hypothetical protein FLJ13855 (predicted)    GB acc:   BM383736    UniGene:   Rn.16992     Locus Link:   303478    Chromosome:   10    Cytoband:   Chr:10q31    SOURCE        Gene Ontology:     BioCarta Pathways:     KEGG Pathways:  
    Probe set : 1388857_at    Name:   SEC23B (S. cerevisiae) (predicted)    GB acc:   BF289044    UniGene:   Rn.8735    Gene Symbol:   Sec23b_predicted    Locus Link:   362226    Chromosome:   3    Cytoband:   Chr:3q41    SOURCE    GENECARD       Gene Ontology:   molecular function:  protein binding      BioCarta Pathways:     KEGG Pathways:  
    Probe set : 1392079_at    Name:   A kinase (PRKA) anchor protein 7    GB acc:   AW526264    UniGene:   Rn.145179    Gene Symbol:   RGD:1303071    Locus Link:   361458    Chromosome:   1    Cytoband:   Chr:1p12    SOURCE    GENECARD       Gene Ontology:   molecular function:  kinase activity      BioCarta Pathways:     KEGG Pathways:  
    Probe set : 1369381_a_at    Name:   solute carrier family 15 (oligopeptide transporter), member 1    GB acc:   D50306    UniGene:   Rn.10500    Gene Symbol:   Slc15a1    Locus Link:   117261    Chromosome:   15    Cytoband:   Chr:15q25    SOURCE    GENECARD       Gene Ontology:     BioCarta Pathways:     KEGG Pathways:  
    Probe set : 1372455_at    Name:   transmembrane 4 superfamily member 12 (predicted)    GB acc:   AI410264    UniGene:   Rn.145208    Gene Symbol:   Tm4sf12_predicted    Locus Link:   362326    Chromosome:   4    Cytoband:   Chr:4q22    SOURCE    GENECARD       Gene Ontology:   cellular component:  membrane fraction   cellular component:  integral to membrane      BioCarta Pathways:     KEGG Pathways:  
    Probe set : 1371856_at    Name:   CDNA clone MGC:93828 IMAGE:7110880    GB acc:   AI408941    UniGene:   Rn.118788        SOURCE        Gene Ontology:   biological process:  transcription   biological process:  regulation of transcription, DNA-dependent   cellular component:  nucleus      BioCarta Pathways:     KEGG Pathways:  
    Probe set : 1387992_at    Name:   Leukocyte immunoglobulin-like receptor, subfamily B (with TM and ITIM domains), member 3 (predicted)    GB acc:   AF169637    UniGene:   Rn.143808     Locus Link:   361493    Chromosome:   1    Cytoband:   Chr:1q12    SOURCE        Gene Ontology:   biological process:  immune response   cellular component:  integral to membrane   molecular function:  receptor activity      BioCarta Pathways:     KEGG Pathways:  
    Probe set : 1388732_at    Name:   solute carrier family 35, member F5 (predicted)    GB acc:   AI009820    UniGene:   Rn.17967    Gene Symbol:   Slc35f5_predicted    Locus Link:   288993    Chromosome:   13    Cytoband:   Chr:13q11    SOURCE    GENECARD       Gene Ontology:     BioCarta Pathways:     KEGG Pathways:  
    Probe set : 1370878_at    Name:   uroporphyrinogen decarboxylase    GB acc:   Y00350    UniGene:   Rn.4214    Gene Symbol:   Urod    Locus Link:   29421    Chromosome:   5    Cytoband:   Chr:5q36    SOURCE    GENECARD       Gene Ontology:   biological process:  porphyrin biosynthesis   biological process:  heme biosynthesis      BioCarta Pathways:     KEGG Pathways:  
    Probe set : 1385069_at    Name:   Activin A receptor, type 1    GB acc:   BM389711    UniGene:   Rn.87899    Gene Symbol:   Acvr1    Locus Link:   79558    Chromosome:   3    Cytoband:   Chr:3q21    SOURCE    GENECARD       Gene Ontology:   molecular function:  protein kinase activity   molecular function:  protein serine/threonine kinase activity   molecular function:  transmembrane receptor protein serine/threonine kinase activity   molecular function:  protein kinase CK2 activity   molecular function:  cAMP-dependent protein kinase activity   molecular function:  receptor signaling protein serine/threonine kinase activity   molecular function:  receptor activity   molecular function:  transforming growth factor beta receptor activity   molecular function:  ATP binding   molecular function:  kinase activity   molecular function:  activin receptor activity, type I   molecular function:  activin receptor activity, type I   molecular function:  transferase activity   molecular function:  transforming growth factor beta binding   molecular function:  nucleotide binding   molecular function:  magnesium ion binding   molecular function:  manganese ion binding   molecular function:  metal ion binding      BioCarta Pathways:     KEGG Pathways:  
    Probe set : 1384934_at    Name:   Transcribed locus    GB acc:   AA893454    UniGene:   Rn.7329        SOURCE        Gene Ontology:     BioCarta Pathways:     KEGG Pathways:  
    Probe set : 1377900_at    Name:   similar to RIKEN cDNA 1810013B01    GB acc:   BF409560    UniGene:   Rn.2080    Gene Symbol:   RGD:1359649    Locus Link:   300983    Chromosome:   8    Cytoband:   Chr:8q32    SOURCE    GENECARD       Gene Ontology:   molecular function:  catalytic activity   molecular function:  hydrolase activity      BioCarta Pathways:     KEGG Pathways:  
    Probe set : 1371838_at    Name:   similar to splicing factor, arginine/serine-rich 2    GB acc:   AI411155    UniGene:   Rn.136644    Gene Symbol:   RGD:1359422    Locus Link:   494445    Chromosome:   10    Cytoband:   Chr:10q32.3    SOURCE    GENECARD       Gene Ontology:   biological process:  nuclear mRNA splicing, via spliceosome   biological process:  mRNA processing   cellular component:  nucleus   molecular function:  nucleotide binding   molecular function:  nucleic acid binding   molecular function:  RNA binding      BioCarta Pathways:     KEGG Pathways:  
    Probe set : 1370365_at    Name:   glutathione synthetase    GB acc:   L38615    UniGene:   Rn.1692    Gene Symbol:   Gss    Locus Link:   25458    Chromosome:   3    Cytoband:   Chr:3q42    SOURCE    GENECARD       Gene Ontology:     BioCarta Pathways:     KEGG Pathways:  
    Probe set : 1372841_at    Name:   deleted in polyposis 1-like 1 (predicted)    GB acc:   BG376982    UniGene:   Rn.9353    Gene Symbol:   Dp1l1_predicted    Locus Link:   362835    Chromosome:   7    Cytoband:   Chr:7q11    SOURCE    GENECARD       Gene Ontology:   cellular component:  integral to membrane   molecular function:  receptor activity      BioCarta Pathways:     KEGG Pathways:  
    Probe set : 1389709_at    Name:   similar to Zinc finger protein 133    GB acc:   AI146063    UniGene:   Rn.117293    Gene Symbol:   LOC499900    Locus Link:   499900    Chromosome:   3    Cytoband:   Chr:3q41    SOURCE    GENECARD       Gene Ontology:   biological process:  transcription   biological process:  regulation of transcription, DNA-dependent   cellular component:  intracellular   cellular component:  nucleus   molecular function:  nucleic acid binding   molecular function:  zinc ion binding   molecular function:  metal ion binding      BioCarta Pathways:     KEGG Pathways:  
    Probe set : 1372154_at    Name:   similar to 2010321M09Rik protein    GB acc:   AW521019    UniGene:   Rn.9525    Gene Symbol:   RGD:1359616    Locus Link:   300782    Chromosome:   8    Cytoband:   Chr:8q24    SOURCE    GENECARD       Gene Ontology:     BioCarta Pathways:     KEGG Pathways:  
    Probe set : 1370728_at    Name:   interleukin 13 receptor, alpha 1    GB acc:   AY044251    UniGene:   Rn.81210    Gene Symbol:   Il13ra1    Locus Link:   252963    Chromosome:   X    Cytoband:   Chr:Xq12    SOURCE    GENECARD       Gene Ontology:   biological process:  cell surface receptor linked signal transduction      BioCarta Pathways:     KEGG Pathways:  
    Probe set : 1377673_at    Name:   Similar to cyclin K    GB acc:   BF411339    UniGene:   Rn.23     Locus Link:   500715      SOURCE        Gene Ontology:     BioCarta Pathways:     KEGG Pathways:  
    Probe set : 1387804_at    Name:   tripartite motif-containing 63    GB acc:   NM_080903    UniGene:   Rn.40636    Gene Symbol:   Trim63    Locus Link:   140939    Chromosome:   5    Cytoband:   Chr:5q36    SOURCE    GENECARD       Gene Ontology:   cellular component:  contractile fiber   cellular component:  ubiquitin ligase complex   cellular component:  intracellular   cellular component:  nucleus      BioCarta Pathways:     KEGG Pathways:  
    Probe set : 1393795_at     GB acc:   BG377397              Gene Ontology:     BioCarta Pathways:     KEGG Pathways:  
    Probe set : 1377642_at    Name:   caveolin 2    GB acc:   BE349669    UniGene:   Rn.81070    Gene Symbol:   Cav2    Locus Link:   363425    Chromosome:   4    Cytoband:   Chr:4q21    SOURCE    GENECARD       Gene Ontology:   biological process:  synaptic transmission   cellular component:  integral to membrane   cellular component:  caveolar membrane   molecular function:  SNARE binding   molecular function:  syntaxin binding      BioCarta Pathways:     KEGG Pathways:  
    Probe set : 1377840_at     GB acc:   BF415080              Gene Ontology:     BioCarta Pathways:     KEGG Pathways:  
    Probe set : 1384762_at    Name:   Transcribed locus    GB acc:   BE103873    UniGene:   Rn.147915        SOURCE        Gene Ontology:     BioCarta Pathways:     KEGG Pathways:  
    Probe set : 1397876_at    Name:   Transcribed locus    GB acc:   AI549089    UniGene:   Rn.148047        SOURCE        Gene Ontology:     BioCarta Pathways:     KEGG Pathways:  
    Probe set : 1372828_at    Name:   methionine sulfoxide reductase B (predicted)    GB acc:   BM389609    UniGene:   Rn.23802    Gene Symbol:   Mrsb_predicted    Locus Link:   361286    Chromosome:   17    Cytoband:   Chr:17q12.3    SOURCE    GENECARD       Gene Ontology:   biological process:  protein repair   cellular component:  extracellular space   cellular component:  mitochondrion   molecular function:  protein-methionine-R-oxide reductase activity   molecular function:  oxidoreductase activity      BioCarta Pathways:     KEGG Pathways:  
    Probe set : 1371671_at    Name:   G protein-coupled receptor, family C, group 5, member B (predicted)    GB acc:   AI228513    UniGene:   Rn.47330    Gene Symbol:   Gprc5b_predicted    Locus Link:   293546    Chromosome:   1    Cytoband:   Chr:1q35    SOURCE    GENECARD       Gene Ontology:   biological process:  G-protein coupled receptor protein signaling pathway   biological process:  visual perception   cellular component:  extracellular space   cellular component:  membrane   cellular component:  integral to membrane      BioCarta Pathways:     KEGG Pathways:  
    Probe set : 1393149_at     GB acc:   BG663483    UniGene:   Rn.22308    Gene Symbol:   Multiple Gene Symbols    Locus Link:   116741 /// 116742 /// 116778 /// 116779 /// 116780 /// 116781 /// 393085 /// 393086 /// 393088 /// 393089 /// 393091 /// 393092 /// 394223    Chromosome:   18    Cytoband:   Chr:18p11    SOURCE    GENECARD       Gene Ontology:     BioCarta Pathways:     KEGG Pathways:  
    Probe set : 1368888_a_at    Name:   reticulon 4    GB acc:   AJ242963    UniGene:   Rn.1348    Gene Symbol:   Rtn4    Locus Link:   83765    Chromosome:   14    Cytoband:   Chr:14q22    SOURCE    GENECARD       Gene Ontology:   molecular function:  protein binding   molecular function:  protein binding      BioCarta Pathways:     KEGG Pathways:  
    Probe set : 1371416_at    Name:   NADH dehydrogenase (ubiquinone) flavoprotein 1, 51kDa    GB acc:   AI231746    UniGene:   Rn.3159    Gene Symbol:   RGD:1359247    Locus Link:   293655    Chromosome:   1    Cytoband:   Chr:1q42    SOURCE    GENECARD       Gene Ontology:   biological process:  electron transport   biological process:  mitochondrial electron transport, NADH to ubiquinone      BioCarta Pathways:     KEGG Pathways:  
    Probe set : 1374874_at    Name:   similar to RIKEN cDNA 5730509K17 gene    GB acc:   AI410818    UniGene:   Rn.58328    Gene Symbol:   LOC498386    Locus Link:   498386    Chromosome:   14    Cytoband:   Chr:14q21    SOURCE    GENECARD       Gene Ontology:     BioCarta Pathways:     KEGG Pathways:  
    Probe set : 1374126_at    Name:   Transcribed locus    GB acc:   BG374261    UniGene:   Rn.27662        SOURCE        Gene Ontology:     BioCarta Pathways:     KEGG Pathways:  
    Probe set : 1371641_at    Name:   chaperonin subunit 7 (eta) (predicted)    GB acc:   BM388827    UniGene:   Rn.62267    Gene Symbol:   Cct7_predicted    Locus Link:   297406    Chromosome:   4    Cytoband:   Chr:4q34    SOURCE    GENECARD       Gene Ontology:   biological process:  regulation of progression through cell cycle   biological process:  protein folding   cellular component:  cytoplasm   molecular function:  ATP binding   molecular function:  unfolded protein binding      BioCarta Pathways:     KEGG Pathways:  
    Probe set : 1387591_at    Name:   interleukin 2 receptor, alpha chain    GB acc:   NM_013163    UniGene:   Rn.9872    Gene Symbol:   Il2ra    Locus Link:   25704    Chromosome:   17    Cytoband:   Chr:17q12.3    SOURCE    GENECARD       Gene Ontology:   molecular function:  receptor activity   molecular function:  interleukin receptor activity   molecular function:  interleukin-2 receptor activity      BioCarta Pathways:     KEGG Pathways:  
    Probe set : 1382832_at    Name:   similar to CG9135-PA (predicted)    GB acc:   AI236185    UniGene:   Rn.98282    Gene Symbol:   RGD1309986_predicted    Locus Link:   298594    Chromosome:   5    Cytoband:   Chr:5q36    SOURCE    GENECARD       Gene Ontology:     BioCarta Pathways:     KEGG Pathways:  
    Probe set : 1374641_at    Name:   Transcribed locus    GB acc:   BF397653    UniGene:   Rn.40613        SOURCE        Gene Ontology:     BioCarta Pathways:     KEGG Pathways:  
    Probe set : 1393896_at    Name:   similar to ionized calcium binding adapter molecule 2 (Iba2) (predicted)    GB acc:   AI716974    UniGene:   Rn.40666    Gene Symbol:   RGD1305081_predicted    Locus Link:   362107    Chromosome:   3    Cytoband:   Chr:3p11    SOURCE    GENECARD       Gene Ontology:     BioCarta Pathways:     KEGG Pathways:  
    Probe set : 1394761_at    Name:   similar to Rho-GTPase-activating protein 26 (Oligophrenin-1 like protein) (GTPase regulator associated with focal adhesion kinase)    GB acc:   BE114260    UniGene:   Rn.52342    Gene Symbol:   LOC300350    Locus Link:   300350    Chromosome:   8    Cytoband:   Chr:8q11    SOURCE    GENECARD       Gene Ontology:     BioCarta Pathways:     KEGG Pathways:  
    Probe set : 1383586_at     GB acc:   BI295793              Gene Ontology:     BioCarta Pathways:     KEGG Pathways:  
    Probe set : 1396000_at    Name:   Transcribed locus    GB acc:   AI704583    UniGene:   Rn.147147        SOURCE        Gene Ontology:     BioCarta Pathways:     KEGG Pathways:  
    Probe set : 1372655_at     GB acc:   BG380323              Gene Ontology:     BioCarta Pathways:     KEGG Pathways:  
    Probe set : 1373238_at    Name:   similar to SPT3-associated factor 42 (predicted)    GB acc:   AI234844    UniGene:   Rn.23978    Gene Symbol:   RGD1306774_predicted    Locus Link:   360874    Chromosome:   13    Cytoband:   Chr:13q23    SOURCE    GENECARD       Gene Ontology:     BioCarta Pathways:     KEGG Pathways:  
    Probe set : 1380130_at     GB acc:   AW434319              Gene Ontology:     BioCarta Pathways:     KEGG Pathways:  
    Probe set : 1394968_at    Name:   Transcribed locus    GB acc:   BE117366    UniGene:   Rn.149213        SOURCE        Gene Ontology:     BioCarta Pathways:     KEGG Pathways:  
    Probe set : 1387654_at    Name:   unconventional myosin Myr2 I heavy chain    GB acc:   NM_023092    UniGene:   Rn.55135    Gene Symbol:   RGD:620443    Locus Link:   65261    Chromosome:   10    Cytoband:   Chr:10q24    SOURCE    GENECARD       Gene Ontology:   biological process:  transport   biological process:  cytoskeleton organization and biogenesis   cellular component:  myosin   cellular component:  myosin I   cellular component:  myosin I      BioCarta Pathways:     KEGG Pathways:  
    Probe set : 1372362_at    Name:   similar to N-acetylgalactosamine kinase (GalNAc kinase) (Galactokinase 2)    GB acc:   BE329241    UniGene:   Rn.2937    Gene Symbol:   LOC296117    Locus Link:   296117    Chromosome:   3    Cytoband:   Chr:3q36    SOURCE    GENECARD       Gene Ontology:   cellular component:  cytoplasm      BioCarta Pathways:     KEGG Pathways:  
    Probe set : 1369862_at    Name:   proviral integration site 1    GB acc:   NM_017034    UniGene:   Rn.34888    Gene Symbol:   Pim1    Locus Link:   24649    Chromosome:   20    Cytoband:   Chr:20p12    SOURCE    GENECARD       Gene Ontology:   cellular component:  nucleus   cellular component:  cytoplasm   cellular component:  cytoplasm      BioCarta Pathways:     KEGG Pathways:  
    Probe set : 1396014_at    Name:   Transcribed locus    GB acc:   BM384523    UniGene:   Rn.131975        SOURCE        Gene Ontology:     BioCarta Pathways:     KEGG Pathways:  
    Probe set : 1375016_at    Name:   Transcribed locus    GB acc:   AI406912    UniGene:   Rn.24461        SOURCE        Gene Ontology:     BioCarta Pathways:     KEGG Pathways:  
    Probe set : 1368522_at    Name:   timeless homolog (Drosophila)    GB acc:   NM_031340    UniGene:   Rn.31755    Gene Symbol:   Timeless    Locus Link:   83508    Chromosome:   7    Cytoband:   Chr:7q11    SOURCE    GENECARD       Gene Ontology:   cellular component:  nucleus   cellular component:  nucleus      BioCarta Pathways:     KEGG Pathways:  
    Probe set : 1390205_at    Name:   zinc finger, SWIM domain containing 6 (predicted)    GB acc:   BE108876    UniGene:   Rn.105186    Gene Symbol:   Zswim6_predicted    Locus Link:   310062    Chromosome:   2    Cytoband:   Chr:2q14    SOURCE    GENECARD       Gene Ontology:     BioCarta Pathways:     KEGG Pathways:  
    Probe set : 1399159_a_at    Name:   vesicle-associated membrane protein 3    GB acc:   NM_057097    UniGene:   Rn.8480    Gene Symbol:   Vamp3    Locus Link:   29528    Chromosome:   5    Cytoband:   Chr:5q36    SOURCE    GENECARD       Gene Ontology:     BioCarta Pathways:     KEGG Pathways:  
    Probe set : 1388505_at    Name:   Transcribed locus    GB acc:   BE109642    UniGene:   Rn.2378        SOURCE        Gene Ontology:     BioCarta Pathways:     KEGG Pathways:  
    Probe set : 1388363_at    Name:   hnRNP-associated with lethal yellow (predicted)    GB acc:   BM389226    UniGene:   Rn.17077    Gene Symbol:   Raly_predicted    Locus Link:   296301    Chromosome:   3    Cytoband:   Chr:3q41    SOURCE    GENECARD       Gene Ontology:   cellular component:  nucleus   cellular component:  ribonucleoprotein complex   molecular function:  RNA binding   molecular function:  nucleotide binding   molecular function:  nucleic acid binding      BioCarta Pathways:     KEGG Pathways:  
    Probe set : 1372845_at    Name:   ribonuclease P 21kDa subunit (human)    GB acc:   BG380782    UniGene:   Rn.3720    Gene Symbol:   RGD:1303090    Locus Link:   406230    Chromosome:   20    Cytoband:   Chr:20p12    SOURCE    GENECARD       Gene Ontology:     BioCarta Pathways:     KEGG Pathways:  
    Probe set : 1373211_at    Name:   F-box and WD-40 domain protein 4 (predicted)    GB acc:   BF283421    UniGene:   Rn.12644    Gene Symbol:   Fbxw4_predicted    Locus Link:   309444    Chromosome:   1    Cytoband:   Chr:1q54    SOURCE    GENECARD       Gene Ontology:   cellular component:  ubiquitin ligase complex      BioCarta Pathways:     KEGG Pathways:  
    Probe set : 1392743_at    Name:   myc induced nuclear antigen    GB acc:   BI278157    UniGene:   Rn.77753    Gene Symbol:   Mina    Locus Link:   266670    Chromosome:   11    Cytoband:   Chr:11q12    SOURCE    GENECARD       Gene Ontology:   biological process:  cell proliferation   biological process:  regulation of cell proliferation   molecular function:  DNA binding      BioCarta Pathways:     KEGG Pathways:  
    Probe set : 1387782_at    Name:   dynein light chain-2    GB acc:   NM_080697    UniGene:   Rn.36362    Gene Symbol:   Dlc2    Locus Link:   140734    Chromosome:   10    Cytoband:   Chr:10q26    SOURCE    GENECARD       Gene Ontology:   biological process:  microtubule-based process   biological process:  synaptic target recognition   biological process:  synaptic target recognition   cellular component:  microtubule associated complex   cellular component:  dynein complex   cellular component:  microtubule   molecular function:  motor activity   molecular function:  microtubule motor activity   molecular function:  cytoskeletal protein binding      BioCarta Pathways:     KEGG Pathways:  
    Probe set : 1369289_at    Name:   hepatocyte nuclear factor 4, alpha    GB acc:   NM_022180    UniGene:   Rn.44442    Gene Symbol:   Hnf4a    Locus Link:   25735      SOURCE    GENECARD       Gene Ontology:   cellular component:  nucleus   cellular component:  transcription factor complex   cellular component:  transcription factor complex      BioCarta Pathways:     KEGG Pathways:  
    Probe set : 1387306_a_at    Name:   early growth response 2    GB acc:   NM_053633    UniGene:   Rn.89235    Gene Symbol:   Egr2    Locus Link:   114090    Chromosome:   20    Cytoband:   Chr:20p11    SOURCE    GENECARD       Gene Ontology:   cellular component:  nucleus   cellular component:  nucleus      BioCarta Pathways:     KEGG Pathways:  
    Probe set : 1396305_at    Name:   Transcribed locus    GB acc:   BI274350    UniGene:   Rn.146563        SOURCE        Gene Ontology:     BioCarta Pathways:     KEGG Pathways:  
    Probe set : 1398316_at    Name:   similar to LEYDIG CELL TUMOR 10 KD PROTEIN    GB acc:   X62277    UniGene:   Rn.11148    Gene Symbol:   RGD:735128    Locus Link:   288913    Chromosome:   19    Cytoband:   Chr:19q11    SOURCE    GENECARD       Gene Ontology:     BioCarta Pathways:     KEGG Pathways:  
    Probe set : 1393380_at    Name:   Transcribed locus    GB acc:   AW917818    UniGene:   Rn.105091        SOURCE        Gene Ontology:     BioCarta Pathways:     KEGG Pathways:  
    Probe set : 1393443_a_at    Name:   similar to CGI-112 protein (predicted)    GB acc:   AW434918    UniGene:   Rn.144744    Gene Symbol:   RGD1308113_predicted    Locus Link:   290224    Chromosome:   15    Cytoband:   Chr:15p13    SOURCE    GENECARD       Gene Ontology:     BioCarta Pathways:     KEGG Pathways:  
    Probe set : 1383629_a_at    Name:   CDNA clone IMAGE:7386859    GB acc:   BI289966    UniGene:   Rn.17587        SOURCE        Gene Ontology:   molecular function:  nucleic acid binding      BioCarta Pathways:     KEGG Pathways:  
    Probe set : 1386615_at    Name:   carbohydrate sulfotransferase 2 (predicted)    GB acc:   BG665096    UniGene:   Rn.95986    Gene Symbol:   Chst2_predicted    Locus Link:   367145    Chromosome:   8    Cytoband:   Chr:8q31    SOURCE    GENECARD       Gene Ontology:   biological process:  N-acetylglucosamine metabolism   cellular component:  extracellular space   cellular component:  integral to membrane   molecular function:  sulfotransferase activity   molecular function:  transferase activity      BioCarta Pathways:     KEGG Pathways:  
    Probe set : 1371862_at    Name:   ribonucleotide reductase M1    GB acc:   AI412015    UniGene:   Rn.145914    Gene Symbol:   Rrm1    Locus Link:   365320    Chromosome:   1    Cytoband:   Chr:1q32    SOURCE    GENECARD       Gene Ontology:   biological process:  DNA replication   cellular component:  ribonucleoside-diphosphate reductase complex      BioCarta Pathways:     KEGG Pathways:  
    Probe set : 1390957_at    Name:   Similar to Protein C14orf101 homolog    GB acc:   AI112360    UniGene:   Rn.26760     Locus Link:   361030    Chromosome:   15    Cytoband:   Chr:15p14    SOURCE        Gene Ontology:     BioCarta Pathways:     KEGG Pathways:  
    Probe set : 1374999_at    Name:   Transcribed locus    GB acc:   BF399489    UniGene:   Rn.14884        SOURCE        Gene Ontology:     BioCarta Pathways:     KEGG Pathways:  
    Probe set : 1390777_at    Name:   sterol-C5-desaturase (fungal ERG3, delta-5-desaturase) homolog (S. cerevisae)    GB acc:   AI710051    UniGene:   Rn.18741    Gene Symbol:   Sc5d    Locus Link:   114100    Chromosome:   8    Cytoband:   Chr:8q22    SOURCE    GENECARD       Gene Ontology:   biological process:  metabolism   molecular function:  catalytic activity      BioCarta Pathways:     KEGG Pathways:  
    Probe set : 1374030_at    Name:   Transcribed locus    GB acc:   BE100868    UniGene:   Rn.59795        SOURCE        Gene Ontology:     BioCarta Pathways:     KEGG Pathways:  
    Probe set : 1389301_at    Name:   Muscleblind-like 2 (predicted)    GB acc:   AI176665    UniGene:   Rn.1940     Locus Link:   290494    Chromosome:   15    Cytoband:   Chr:15q24    SOURCE        Gene Ontology:   molecular function:  nucleic acid binding      BioCarta Pathways:     KEGG Pathways:  
    Probe set : 1380905_at    Name:   Transcribed locus, weakly similar to NP_083358.1 hypothetical protein LOC74742 [Mus musculus]    GB acc:   AA893260    UniGene:   Rn.86573        SOURCE        Gene Ontology:     BioCarta Pathways:     KEGG Pathways:  
    Probe set : 1390706_at    Name:   spectrin beta 2    GB acc:   BF392456    UniGene:   Rn.93208    Gene Symbol:   Spnb2    Locus Link:   305614    Chromosome:   14    Cytoband:   Chr:14q22    SOURCE    GENECARD       Gene Ontology:   biological process:  barbed-end actin filament capping   cellular component:  cytoskeleton   molecular function:  actin binding      BioCarta Pathways:     KEGG Pathways:  
    Probe set : 1370296_at    Name:   sterol carrier protein 2    GB acc:   M34728    UniGene:   Rn.31887    Gene Symbol:   Scp2    Locus Link:   25541    Chromosome:   5    Cytoband:   Chr:5q35    SOURCE    GENECARD       Gene Ontology:   cellular component:  mitochondrion   cellular component:  peroxisome   molecular function:  sterol carrier protein X-related thiolase activity   molecular function:  sterol carrier activity   molecular function:  lipid binding      BioCarta Pathways:     KEGG Pathways:  
    Probe set : 1376789_at    Name:   Similar to Myosin light chain kinase 2, skeletal/cardiac muscle (MLCK2) (predicted)    GB acc:   AI410679    UniGene:   Rn.132981     Locus Link:   291926    Chromosome:   19    Cytoband:   Chr:19q11    SOURCE        Gene Ontology:     BioCarta Pathways:     KEGG Pathways:  
    Probe set : 1387567_at    Name:   solute carrier family 21, member 1 /// hypothetical gene supported by NM_017111    GB acc:   NM_017111    UniGene:   Rn.9912    Gene Symbol:   Slc21a1 /// LOC497799    Locus Link:   497799 /// 50572    Chromosome:   4    Cytoband:   Chr:4q44    SOURCE    GENECARD       Gene Ontology:     BioCarta Pathways:     KEGG Pathways:  
    Probe set : 1369173_at    Name:   complement component 3a receptor 1    GB acc:   NM_032060    UniGene:   Rn.9772    Gene Symbol:   C3ar1    Locus Link:   84007    Chromosome:   4    Cytoband:   Chr:4q42    SOURCE    GENECARD       Gene Ontology:   cellular component:  integral to membrane   cellular component:  integral to membrane      BioCarta Pathways:     KEGG Pathways:  
    Probe set : 1382435_at    Name:   fizzy/cell division cycle 20 related 1 (Drosophila) (predicted)    GB acc:   AA996835    UniGene:   Rn.12924    Gene Symbol:   Fzr1_predicted    Locus Link:   314642    Chromosome:   7    Cytoband:   Chr:7q11    SOURCE    GENECARD       Gene Ontology:   biological process:  cytokinesis   biological process:  ubiquitin cycle   biological process:  cell cycle   biological process:  mitosis      BioCarta Pathways:     KEGG Pathways:  
    Probe set : 1384936_at     GB acc:   AI136316              Gene Ontology:     BioCarta Pathways:     KEGG Pathways:  
    Probe set : 1379716_at    Name:   CD163 antigen (predicted)    GB acc:   AA956555    UniGene:   Rn.8158     Locus Link:   312701    Chromosome:   4    Cytoband:   Chr:4q42    SOURCE        Gene Ontology:   biological process:  antimicrobial humoral response (sensu Vertebrata)   molecular function:  receptor activity   molecular function:  scavenger receptor activity      BioCarta Pathways:     KEGG Pathways:  
    Probe set : 1395687_at    Name:   activating transcription factor 7 interacting protein (predicted)    GB acc:   BE113420    UniGene:   Rn.98469    Gene Symbol:   Atf7ip_predicted    Locus Link:   312800    Chromosome:   4    Cytoband:   Chr:4q43    SOURCE    GENECARD       Gene Ontology:   cellular component:  nucleus   molecular function:  protein binding      BioCarta Pathways:     KEGG Pathways:  
    Probe set : 1369632_a_at    Name:   ATP-binding cassette, sub-family C (CFTR/MRP), member 8    GB acc:   AB052294    UniGene:   Rn.11187    Gene Symbol:   Abcc8    Locus Link:   25559    Chromosome:   1    Cytoband:   Chr:1q22    SOURCE    GENECARD       Gene Ontology:   biological process:  carbohydrate metabolism   biological process:  transport   biological process:  potassium ion transport      BioCarta Pathways:     KEGG Pathways:  
    Probe set : 1374947_at    Name:   breast cancer anti-estrogen resistance 3 (predicted)    GB acc:   BI286041    UniGene:   Rn.7383    Gene Symbol:   Bcar3_predicted    Locus Link:   310838    Chromosome:   2    Cytoband:   Chr:2q42    SOURCE    GENECARD       Gene Ontology:   molecular function:  guanyl-nucleotide exchange factor activity      BioCarta Pathways:     KEGG Pathways:  
    Probe set : 1382269_at    Name:   cyclin M2 (predicted)    GB acc:   BM388183    UniGene:   Rn.21307    Gene Symbol:   Cnnm2_predicted    Locus Link:   294014    Chromosome:   1    Cytoband:   Chr:1q54    SOURCE    GENECARD       Gene Ontology:   biological process:  fatty acid biosynthesis   cellular component:  integral to membrane      BioCarta Pathways:     KEGG Pathways:  
    Probe set : 1370034_at    Name:   cell division cycle 25 homolog B (S. cerevisiae)    GB acc:   NM_133572    UniGene:   Rn.11312    Gene Symbol:   Cdc25b    Locus Link:   171103    Chromosome:   3    Cytoband:   Chr:3q36    SOURCE    GENECARD       Gene Ontology:   cellular component:  intracellular      BioCarta Pathways:     KEGG Pathways:  
    Probe set : 1379935_at    Name:   chemokine (C-C motif) ligand 7    GB acc:   BF419899    UniGene:   Rn.26815    Gene Symbol:   RGD:1359152    Locus Link:   287561    Chromosome:   10    Cytoband:   Chr:10q26    SOURCE    GENECARD       Gene Ontology:     BioCarta Pathways:     KEGG Pathways:  
    Probe set : 1388767_at    Name:   programmed cell death 6 (predicted)    GB acc:   BI296393    UniGene:   Rn.104071    Gene Symbol:   Pdcd6_predicted    Locus Link:   308061    Chromosome:   1    Cytoband:   Chr:1p11    SOURCE    GENECARD       Gene Ontology:   cellular component:  soluble fraction   cellular component:  microsome   molecular function:  calcium ion binding   molecular function:  protein binding      BioCarta Pathways:     KEGG Pathways:  
    Probe set : 1367691_at    Name:   protein kinase C, delta binding protein    GB acc:   NM_134449    UniGene:   Rn.12281    Gene Symbol:   Prkcdbp    Locus Link:   85332    Chromosome:   1    Cytoband:   Chr:1q33    SOURCE    GENECARD       Gene Ontology:   biological process:  regulation of cell growth   molecular function:  protein kinase C binding   molecular function:  kinase activity      BioCarta Pathways:     KEGG Pathways:  
    Probe set : 1385729_at    Name:   Transcribed locus    GB acc:   BE108687    UniGene:   Rn.134601        SOURCE        Gene Ontology:     BioCarta Pathways:     KEGG Pathways:  
    Probe set : 1388568_at    Name:   eukaryotic translation initiation factor 3 subunit 7    GB acc:   AA891553    UniGene:   Rn.3463    Gene Symbol:   RGD:1303255    Locus Link:   362952    Chromosome:   7    Cytoband:   Chr:7q34    SOURCE    GENECARD       Gene Ontology:   biological process:  translational initiation   molecular function:  translation initiation factor activity      BioCarta Pathways:     KEGG Pathways:  
    Probe set : 1373388_at    Name:   Similar to Hypothetical protein MGC75937    GB acc:   BI279290    UniGene:   Rn.5977     Locus Link:   360822    Chromosome:   12    Cytoband:   Chr:12q16    SOURCE        Gene Ontology:     BioCarta Pathways:     KEGG Pathways:  
    Probe set : 1384943_at    Name:   similar to DNA segment, Chr 16, ERATO Doi 472, expressed    GB acc:   AI137629    UniGene:   Rn.123758    Gene Symbol:   LOC360692    Locus Link:   360692    Chromosome:   11    Cytoband:   Chr:11q11    SOURCE    GENECARD       Gene Ontology:     BioCarta Pathways:     KEGG Pathways:  
    Probe set : 1384438_at    Name:   Transcribed locus, moderately similar to NP_080232.2 hypothetical protein LOC67082 [Mus musculus]    GB acc:   AA924634    UniGene:   Rn.127937        SOURCE        Gene Ontology:     BioCarta Pathways:     KEGG Pathways:  
    Probe set : 1377417_at    Name:   similar to AW046014 protein    GB acc:   BE099931    UniGene:   Rn.21528    Gene Symbol:   LOC363328    Locus Link:   363328      SOURCE    GENECARD       Gene Ontology:     BioCarta Pathways:     KEGG Pathways:  
    Probe set : 1389200_at    Name:   bystin-like    GB acc:   AA851296    UniGene:   Rn.22879    Gene Symbol:   Bysl    Locus Link:   359727      SOURCE    GENECARD       Gene Ontology:     BioCarta Pathways:     KEGG Pathways:  
    Probe set : 1376830_at    Name:   similar to RIKEN cDNA E430013E20 gene (predicted)    GB acc:   AW520496    UniGene:   Rn.25353    Gene Symbol:   RGD1311132_predicted    Locus Link:   291961    Chromosome:   19    Cytoband:   Chr:19q11    SOURCE    GENECARD       Gene Ontology:     BioCarta Pathways:     KEGG Pathways:  
    Probe set : 1371673_at    Name:   transcription factor-like 1 (predicted)    GB acc:   AI413005    UniGene:   Rn.1986    Gene Symbol:   Tcfl1_predicted    Locus Link:   310661    Chromosome:   2    Cytoband:   Chr:2q34    SOURCE    GENECARD       Gene Ontology:   cellular component:  nucleus   cellular component:  transcription factor complex   molecular function:  DNA binding   molecular function:  transcription factor activity   molecular function:  RNA polymerase II transcription factor activity      BioCarta Pathways:     KEGG Pathways:  
    Probe set : 1380932_at     GB acc:   BG373573              Gene Ontology:     BioCarta Pathways:     KEGG Pathways:  
    Probe set : 1389889_at    Name:   similar to mKIAA1402 protein (predicted)    GB acc:   AI412986    UniGene:   Rn.37536    Gene Symbol:   RGD1306404_predicted    Locus Link:   296733    Chromosome:   4    Cytoband:   Chr:4q11    SOURCE    GENECARD       Gene Ontology:     BioCarta Pathways:     KEGG Pathways:  
    Probe set : 1373895_at     GB acc:   BE118440              Gene Ontology:     BioCarta Pathways:     KEGG Pathways:  
    Probe set : 1369934_at    Name:   peptidylprolyl isomerase B    GB acc:   NM_022536    UniGene:   Rn.1893    Gene Symbol:   Ppib    Locus Link:   64367    Chromosome:   8    Cytoband:   Chr:8q24    SOURCE    GENECARD       Gene Ontology:   biological process:  protein folding   cellular component:  extracellular space   cellular component:  endoplasmic reticulum   cellular component:  endoplasmic reticulum lumen      BioCarta Pathways:     KEGG Pathways:  
    Probe set : 1388848_at    Name:   similar to hypothetical protein MGC13251 (predicted)    GB acc:   AA891760    UniGene:   Rn.2343    Gene Symbol:   RGD1308350_predicted    Locus Link:   362790    Chromosome:   6    Cytoband:   Chr:6q32    SOURCE    GENECARD       Gene Ontology:   biological process:  cell organization and biogenesis   biological process:  actin cytoskeleton organization and biogenesis   molecular function:  actin binding   molecular function:  Rho GTPase binding      BioCarta Pathways:     KEGG Pathways:  
    Probe set : 1389417_at    Name:   Transcribed locus    GB acc:   AA946518    UniGene:   Rn.11774        SOURCE        Gene Ontology:     BioCarta Pathways:     KEGG Pathways:  
    Probe set : 1371308_at    Name:   ribosomal protein S4, X-linked    GB acc:   AA799501    UniGene:   Rn.144572    Gene Symbol:   Rps4x    Locus Link:   29426    Chromosome:   X    Cytoband:   Chr:Xq31    SOURCE    GENECARD       Gene Ontology:   molecular function:  RNA binding   molecular function:  structural constituent of ribosome      BioCarta Pathways:     KEGG Pathways:  
    Probe set : 1376619_at    Name:   Similar to protein tyrosine phosphatase, receptor type, D    GB acc:   AI412803    UniGene:   Rn.99077     Locus Link:   313278    Chromosome:   5    Cytoband:   Chr:5q31    SOURCE        Gene Ontology:     BioCarta Pathways:     KEGG Pathways:  
    Probe set : 1383153_at    Name:   Transcribed locus    GB acc:   AI555284    UniGene:   Rn.23647        SOURCE        Gene Ontology:     BioCarta Pathways:     KEGG Pathways:  
    Probe set : 1390859_at    Name:   similar to Nedd4 binding protein 1 (predicted)    GB acc:   BI290968    UniGene:   Rn.137980    Gene Symbol:   RGD1305179_predicted    Locus Link:   291921    Chromosome:   19    Cytoband:   Chr:19p11    SOURCE    GENECARD       Gene Ontology:     BioCarta Pathways:     KEGG Pathways:  
    Probe set : 1383852_at    Name:   Transcribed locus    GB acc:   AI071395    UniGene:   Rn.91036        SOURCE        Gene Ontology:     BioCarta Pathways:     KEGG Pathways:  
    Probe set : 1398628_at     GB acc:   AI059960              Gene Ontology:     BioCarta Pathways:     KEGG Pathways:  
    Probe set : 1379320_at    Name:   Small nuclear ribonucleoprotein D1 (predicted)    GB acc:   AI102591    UniGene:   Rn.18606     Locus Link:   291794    Chromosome:   18    Cytoband:   Chr:18p13    SOURCE        Gene Ontology:   biological process:  spliceosome assembly   biological process:  nuclear mRNA splicing, via spliceosome   biological process:  mRNA processing   molecular function:  RNA binding      BioCarta Pathways:     KEGG Pathways:  
    Probe set : 1372558_at    Name:   NMDA receptor-regulated gene 1 (predicted)    GB acc:   AI177404    UniGene:   Rn.12587    Gene Symbol:   Narg1_predicted    Locus Link:   310399    Chromosome:   2    Cytoband:   Chr:2q26    SOURCE    GENECARD       Gene Ontology:   biological process:  angiogenesis   biological process:  transcription   biological process:  regulation of transcription, DNA-dependent   cellular component:  nucleus   cellular component:  cytoplasm   molecular function:  receptor activity   molecular function:  protein binding   molecular function:  N-acetyltransferase activity      BioCarta Pathways:     KEGG Pathways:  
    Probe set : 1391604_at    Name:   Similar to mKIAA0056 protein    GB acc:   BF418841    UniGene:   Rn.48187     Locus Link:   315508    Chromosome:   8    Cytoband:   Chr:8q13    SOURCE        Gene Ontology:     BioCarta Pathways:     KEGG Pathways:  
    Probe set : 1374622_at    Name:   Transcribed locus    GB acc:   BE107309    UniGene:   Rn.9546        SOURCE        Gene Ontology:     BioCarta Pathways:     KEGG Pathways:  
    Probe set : 1383258_at    Name:   similar to hypothetical protein FLJ14146 (predicted)    GB acc:   BM383065    UniGene:   Rn.39189    Gene Symbol:   RGD1310587_predicted    Locus Link:   360894    Chromosome:   13    Cytoband:   Chr:13q26    SOURCE    GENECARD       Gene Ontology:     BioCarta Pathways:     KEGG Pathways:  
    Probe set : 1376022_at    Name:   Transcribed locus    GB acc:   BI292196    UniGene:   Rn.129608        SOURCE        Gene Ontology:     BioCarta Pathways:     KEGG Pathways:  
    Probe set : 1368168_at    Name:   solute carrier family 34 (sodium phosphate), member 2    GB acc:   NM_053380    UniGene:   Rn.16933    Gene Symbol:   Slc34a2    Locus Link:   84395    Chromosome:   14    Cytoband:   Chr:14q11    SOURCE    GENECARD       Gene Ontology:   biological process:  phosphate metabolism   biological process:  transport   biological process:  phosphate transport   molecular function:  carrier activity   molecular function:  sodium-dependent phosphate transporter activity      BioCarta Pathways:     KEGG Pathways:  
    Probe set : 1383250_at    Name:   similar to KIAA0266 gene product    GB acc:   BE105819    UniGene:   Rn.16331    Gene Symbol:   RGD:1359313    Locus Link:   317579    Chromosome:   X    Cytoband:   Chr:Xq35    SOURCE    GENECARD       Gene Ontology:     BioCarta Pathways:     KEGG Pathways:  
    Probe set : 1367888_at    Name:   MT-protocadherin    GB acc:   NM_053572    UniGene:   Rn.19780    Gene Symbol:   Pcdh21    Locus Link:   93662    Chromosome:   16    Cytoband:   Chr:16p14    SOURCE    GENECARD       Gene Ontology:   biological process:  cell adhesion   biological process:  homophilic cell adhesion   cellular component:  membrane   molecular function:  calcium ion binding   molecular function:  protein binding      BioCarta Pathways:     KEGG Pathways:  
    Probe set : 1372506_at    Name:   proteaseome (prosome, macropain) 28 subunit, 3 (predicted)    GB acc:   AI233766    UniGene:   Rn.7389    Gene Symbol:   Psme3_predicted    Locus Link:   287716    Chromosome:   10    Cytoband:   Chr:10q32.1    SOURCE    GENECARD       Gene Ontology:   cellular component:  proteasome complex (sensu Eukaryota)   cellular component:  cytosol   cellular component:  proteasome activator complex   molecular function:  proteasome activator activity   molecular function:  peptidase activity      BioCarta Pathways:     KEGG Pathways:  
    Probe set : 1378556_at    Name:   Ectonucleotide pyrophosphatase/phosphodiesterase 1    GB acc:   AI411989    UniGene:   Rn.1199    Gene Symbol:   Enpp1    Locus Link:   85496    Chromosome:   1    Cytoband:   Chr:1p12    SOURCE    GENECARD       Gene Ontology:   biological process:  skeletal development   biological process:  nucleotide metabolism   biological process:  negative regulation of ossification   cellular component:  plasma membrane   cellular component:  integral to membrane      BioCarta Pathways:     KEGG Pathways:  
    Probe set : 1383146_at    Name:   similar to neurobeachin    GB acc:   BM388077    UniGene:   Rn.9376    Gene Symbol:   LOC361948    Locus Link:   361948    Chromosome:   2    Cytoband:   Chr:2q26    SOURCE    GENECARD       Gene Ontology:     BioCarta Pathways:     KEGG Pathways:  
    Probe set : 1379323_at    Name:   similar to hypothetical protein MGC14327 (predicted)    GB acc:   BE109498    UniGene:   Rn.23564    Gene Symbol:   RGD1306304_predicted    Locus Link:   311800    Chromosome:   3    Cytoband:   Chr:3p13    SOURCE    GENECARD       Gene Ontology:     BioCarta Pathways:     KEGG Pathways:  
    Probe set : 1384439_at    Name:   CDNA clone IMAGE:7379980    GB acc:   BM385726    UniGene:   Rn.51079        SOURCE        Gene Ontology:     BioCarta Pathways:     KEGG Pathways:  
    Probe set : 1367455_at    Name:   valosin-containing protein    GB acc:   NM_053864    UniGene:   Rn.98891    Gene Symbol:   Vcp    Locus Link:   116643    Chromosome:   5    Cytoband:   Chr:5q22    SOURCE    GENECARD       Gene Ontology:     BioCarta Pathways:     KEGG Pathways:  
    Probe set : 1373196_at    Name:   EF hand domain family, member A2    GB acc:   BF404514    UniGene:   Rn.23547    Gene Symbol:   Efha2    Locus Link:   364601    Chromosome:   16    Cytoband:   Chr:16q12.1    SOURCE    GENECARD       Gene Ontology:   molecular function:  calcium ion binding      BioCarta Pathways:     KEGG Pathways:  
    Probe set : 1381508_at    Name:   Transcribed locus    GB acc:   BE098143    UniGene:   Rn.49769        SOURCE        Gene Ontology:     BioCarta Pathways:     KEGG Pathways:  
    Probe set : 1387296_at    Name:   cytochrome P450, family 2, subfamily J, polypeptide 4    GB acc:   NM_023025    UniGene:   Rn.44992    Gene Symbol:   Cyp2j4    Locus Link:   65210    Chromosome:   5    Cytoband:   Chr:5q33    SOURCE    GENECARD       Gene Ontology:   biological process:  electron transport   cellular component:  microsome   cellular component:  membrane      BioCarta Pathways:     KEGG Pathways:  
    Probe set : 1373779_at     GB acc:   AI011878              Gene Ontology:     BioCarta Pathways:     KEGG Pathways:  
    Probe set : 1384257_at    Name:   non-POU domain containing, octamer-binding /// similar to Nono protein    GB acc:   BF555394    UniGene:   Rn.8381    Gene Symbol:   Nono /// LOC501589    Locus Link:   317259 /// 501589    Chromosome:   X    Cytoband:   Chr:Xq31    SOURCE    GENECARD       Gene Ontology:   cellular component:  nucleus      BioCarta Pathways:     KEGG Pathways:  
    Probe set : 1384015_at    Name:   cytoplasmic linker associated protein 1 (predicted)    GB acc:   AI044920    UniGene:   Rn.105840    Gene Symbol:   Clasp1_predicted    Locus Link:   304740    Chromosome:   13    Cytoband:   Chr:13q11    SOURCE    GENECARD       Gene Ontology:   biological process:  negative regulation of microtubule depolymerization   cellular component:  microtubule associated complex   cellular component:  cytoplasmic microtubule   molecular function:  binding   molecular function:  protein binding   molecular function:  microtubule binding      BioCarta Pathways:     KEGG Pathways:  
    Probe set : 1370838_s_at    Name:   alpha-spectrin 2 /// hypothetical gene supported by NM_171983    GB acc:   BM387423    UniGene:   Rn.5812    Gene Symbol:   Spna2 /// LOC497766    Locus Link:   497766 /// 64159    Chromosome:   3    Cytoband:   Chr:3p12    SOURCE    GENECARD       Gene Ontology:   biological process:  barbed-end actin filament capping      BioCarta Pathways:     KEGG Pathways:  
    Probe set : 1391897_at    Name:   similar to rab6 GTPase activating protein (GAP and centrosome-associated) (predicted)    GB acc:   BG379295    UniGene:   Rn.17748    Gene Symbol:   RGD1304620_predicted    Locus Link:   304914    Chromosome:   13    Cytoband:   Chr:13q22    SOURCE    GENECARD       Gene Ontology:     BioCarta Pathways:     KEGG Pathways:  
    Probe set : 1390500_at    Name:   similar to RIKEN cDNA 9130023F12 gene (predicted)    GB acc:   BG375508    UniGene:   Rn.105388    Gene Symbol:   RGD1311920_predicted    Locus Link:   305544    Chromosome:   14    Cytoband:   Chr:14q22    SOURCE    GENECARD       Gene Ontology:     BioCarta Pathways:     KEGG Pathways:  
    Probe set : 1371872_at    Name:   Nucleosome assembly protein 1-like 1    GB acc:   BE108905    UniGene:   Rn.18480    Gene Symbol:   Nap1l1    Locus Link:   89825    Chromosome:   7    Cytoband:   Chr:7q21    SOURCE    GENECARD       Gene Ontology:   biological process:  DNA replication   biological process:  nucleosome assembly   biological process:  positive regulation of cell proliferation   cellular component:  nucleus   cellular component:  chromatin assembly complex      BioCarta Pathways:     KEGG Pathways:  
    Probe set : 1387684_at    Name:   peroxisome proliferator activated receptor delta    GB acc:   U75918    UniGene:   Rn.96181    Gene Symbol:   Ppard    Locus Link:   25682    Chromosome:   20    Cytoband:   Chr:20p12    SOURCE    GENECARD       Gene Ontology:   biological process:  negative regulation of transcription from RNA polymerase II promoter   biological process:  generation of precursor metabolites and energy   biological process:  regulation of transcription, DNA-dependent   biological process:  regulation of transcription from RNA polymerase II promoter   biological process:  lipid metabolism   biological process:  lipid metabolism   biological process:  cell proliferation   biological process:  nerve ensheathment   biological process:  epidermis development   biological process:  cellular physiological process   biological process:  fatty acid beta-oxidation   biological process:  fatty acid transport   biological process:  regulation of insulin secretion   biological process:  transcription   biological process:  response to temperature stimulus   biological process:  negative regulation of I-kappaB kinase/NF-kappaB cascade   biological process:  response to pain   cellular component:  nucleus      BioCarta Pathways:     KEGG Pathways:  
    Probe set : 1389584_at    Name:   Transcribed locus    GB acc:   AI101266    UniGene:   Rn.13394        SOURCE        Gene Ontology:     BioCarta Pathways:     KEGG Pathways:  
    Probe set : 1383231_at    Name:   similar to Smad nuclear interacting protein    GB acc:   AA955078    UniGene:   Rn.8811    Gene Symbol:   RGD:1359268    Locus Link:   313588    Chromosome:   5    Cytoband:   Chr:5q36    SOURCE    GENECARD       Gene Ontology:     BioCarta Pathways:     KEGG Pathways:  
    Probe set : 1388336_at    Name:   Similar to hypothetical protein D2Ertd391e (predicted)    GB acc:   AI411514    UniGene:   Rn.98797     Locus Link:   362164    Chromosome:   3    Cytoband:   Chr:3q24    SOURCE        Gene Ontology:     BioCarta Pathways:     KEGG Pathways:  
    Probe set : 1377660_at    Name:   similar to transthyretin (4L369) (predicted)    GB acc:   BM387540    UniGene:   Rn.15414    Gene Symbol:   RGD1309350_predicted    Locus Link:   293613    Chromosome:   1    Cytoband:   Chr:1q41    SOURCE    GENECARD       Gene Ontology:     BioCarta Pathways:     KEGG Pathways:  
    Probe set : 1389332_at    Name:   Transcribed locus    GB acc:   BF283404    UniGene:   Rn.13202        SOURCE        Gene Ontology:     BioCarta Pathways:     KEGG Pathways:  
    Probe set : 1397620_at    Name:   putative scaffolding protein POSH    GB acc:   BG372003    UniGene:   Rn.74468    Gene Symbol:   Sh3md2    Locus Link:   306417    Chromosome:   16    Cytoband:   Chr:16p12    SOURCE    GENECARD       Gene Ontology:   biological process:  induction of apoptosis   biological process:  protein ubiquitination   cellular component:  ubiquitin ligase complex   molecular function:  protein binding   molecular function:  ubiquitin-protein ligase activity   molecular function:  zinc ion binding      BioCarta Pathways:     KEGG Pathways:  
    Probe set : 1372109_at     GB acc:   AI102715              Gene Ontology:     BioCarta Pathways:     KEGG Pathways:  
    Probe set : 1370906_at    Name:   branched chain keto acid dehydrogenase E1, beta polypeptide    GB acc:   BM385109    UniGene:   Rn.15623    Gene Symbol:   Bckdhb    Locus Link:   29711    Chromosome:   8    Cytoband:   Chr:8q31    SOURCE    GENECARD       Gene Ontology:   biological process:  amino acid catabolism   biological process:  branched chain family amino acid catabolism      BioCarta Pathways:     KEGG Pathways:  
    Probe set : 1367658_at    Name:   SH3/ankyrin domain gene 3 /// hypothetical gene supported by NM_021676    GB acc:   NM_021676    UniGene:   Rn.42876    Gene Symbol:   Shank3 /// LOC497826    Locus Link:   497826 /// 59312    Chromosome:   7    Cytoband:   Chr:7q34    SOURCE    GENECARD       Gene Ontology:   molecular function:  DNA binding   molecular function:  transcription factor activity   molecular function:  protein binding      BioCarta Pathways:     KEGG Pathways:  
    Probe set : 1374712_at    Name:   proteasome (prosome, macropain) 26S subunit, non-ATPase, 11 (predicted)    GB acc:   AA946509    UniGene:   Rn.11861    Gene Symbol:   Psmd11_predicted    Locus Link:   303353    Chromosome:   10    Cytoband:   Chr:10q26    SOURCE    GENECARD       Gene Ontology:   cellular component:  cytosol   molecular function:  binding      BioCarta Pathways:     KEGG Pathways:  
    Probe set : 1390851_at     GB acc:   AA799676              Gene Ontology:     BioCarta Pathways:     KEGG Pathways:  
    Probe set : 1368498_a_at    Name:   kidney specific organic anion transporter    GB acc:   NM_030837    UniGene:   Rn.127795    Gene Symbol:   RGD:621387    Locus Link:   80899    Chromosome:   4    Cytoband:   Chr:4q44    SOURCE    GENECARD       Gene Ontology:   cellular component:  membrane   cellular component:  integral to membrane      BioCarta Pathways:     KEGG Pathways:  
    Probe set : 1372877_at    Name:   procollagen-lysine, 2-oxoglutarate 5-dioxygenase 3    GB acc:   BI304070    UniGene:   Rn.90152    Gene Symbol:   Plod3    Locus Link:   288583    Chromosome:   12    Cytoband:   Chr:12q12    SOURCE    GENECARD       Gene Ontology:   biological process:  protein metabolism   cellular component:  endoplasmic reticulum   cellular component:  membrane      BioCarta Pathways:     KEGG Pathways:  
    Probe set : 1386280_at    Name:   similar to RIKEN cDNA 0610006F02 (predicted)    GB acc:   AA892888     Gene Symbol:   RGD1305205_predicted    Locus Link:   366792    Chromosome:   7    Cytoband:   Chr:7q11     GENECARD       Gene Ontology:   molecular function:  methyltransferase activity   molecular function:  S-adenosylmethionine-dependent methyltransferase activity   molecular function:  transferase activity      BioCarta Pathways:     KEGG Pathways:  
    Probe set : 1373277_at    Name:   similar to BBP-like protein 2    GB acc:   BG373457    UniGene:   Rn.15627    Gene Symbol:   LOC292995    Locus Link:   292995    Chromosome:   1    Cytoband:   Chr:1q22    SOURCE    GENECARD       Gene Ontology:     BioCarta Pathways:     KEGG Pathways:  
    Probe set : 1389080_at    Name:   zinc responsive protein ZD7    GB acc:   AI233457    UniGene:   Rn.32555    Gene Symbol:   RGD:1359343    Locus Link:   474154    Chromosome:   1    Cytoband:   Chr:1q42    SOURCE    GENECARD       Gene Ontology:   biological process:  nuclear mRNA splicing, via spliceosome   biological process:  mRNA processing   cellular component:  nucleus      BioCarta Pathways:     KEGG Pathways:  
    Probe set : 1379117_at    Name:   Transcribed locus    GB acc:   BF415415    UniGene:   Rn.52558        SOURCE        Gene Ontology:     BioCarta Pathways:     KEGG Pathways:  
    Probe set : 1388888_at    Name:   Transcribed locus    GB acc:   BI274615    UniGene:   Rn.7228        SOURCE        Gene Ontology:     BioCarta Pathways:     KEGG Pathways:  
    Probe set : 1393267_at    Name:   PC4 and SFRS1 interacting protein 1    GB acc:   BI276726    UniGene:   Rn.93075    Gene Symbol:   Psip1    Locus Link:   313323    Chromosome:   5    Cytoband:   Chr:5q31    SOURCE    GENECARD       Gene Ontology:   biological process:  transcription   biological process:  regulation of transcription, DNA-dependent   cellular component:  nucleus   molecular function:  DNA binding      BioCarta Pathways:     KEGG Pathways:  
    Probe set : 1393172_at    Name:   Ngfi-A binding protein 1    GB acc:   AA848648    UniGene:   Rn.10099    Gene Symbol:   Nab1    Locus Link:   64824    Chromosome:   9    Cytoband:   Chr:9q22    SOURCE    GENECARD       Gene Ontology:   biological process:  regulation of transcription, DNA-dependent   biological process:  negative regulation of transcription   biological process:  transcription   cellular component:  nucleus   molecular function:  nucleic acid binding   molecular function:  transcriptional repressor activity      BioCarta Pathways:     KEGG Pathways:  
    Probe set : 1376427_a_at    Name:   glycine dehydrogenase (decarboxylating; glycine decarboxylase, glycine cleavage system protein P) (predicted)    GB acc:   AI029729    UniGene:   Rn.17101    Gene Symbol:   Gldc_predicted    Locus Link:   309312    Chromosome:   1    Cytoband:   Chr:1q52    SOURCE    GENECARD       Gene Ontology:   biological process:  glycine catabolism   cellular component:  mitochondrion   cellular component:  glycine dehydrogenase complex (decarboxylating)   molecular function:  glycine dehydrogenase (decarboxylating) activity   molecular function:  electron transporter activity   molecular function:  oxidoreductase activity      BioCarta Pathways:     KEGG Pathways:  
    Probe set : 1390585_at    Name:   mannan-binding lectin serine peptidase 1    GB acc:   AI169829    UniGene:   Rn.131140    Gene Symbol:   Masp1    Locus Link:   64023    Chromosome:   11    Cytoband:   Chr:11q23    SOURCE    GENECARD       Gene Ontology:   cellular component:  extracellular region      BioCarta Pathways:     KEGG Pathways:  
    Probe set : 1390187_at    Name:   Transcribed locus    GB acc:   AI060043    UniGene:   Rn.122681        SOURCE        Gene Ontology:     BioCarta Pathways:     KEGG Pathways:  
    Probe set : 1395739_at    Name:   similar to RIKEN cDNA C920006C10 (predicted)    GB acc:   BG672302    UniGene:   Rn.21009    Gene Symbol:   RGD1305976_predicted    Locus Link:   362923    Chromosome:   7    Cytoband:   Chr:7q33    SOURCE    GENECARD       Gene Ontology:     BioCarta Pathways:     KEGG Pathways:  
    Probe set : 1397527_at    Name:   tripartite motif protein 47 (predicted)    GB acc:   AI578294    UniGene:   Rn.91719    Gene Symbol:   Trim47_predicted    Locus Link:   287824    Chromosome:   10    Cytoband:   Chr:10q32.3    SOURCE    GENECARD       Gene Ontology:   biological process:  protein ubiquitination   cellular component:  ubiquitin ligase complex   cellular component:  intracellular   cellular component:  nucleus   molecular function:  ubiquitin-protein ligase activity   molecular function:  zinc ion binding      BioCarta Pathways:     KEGG Pathways:  
    Probe set : 1387977_at    Name:   nibrin    GB acc:   AF218575    UniGene:   Rn.25214    Gene Symbol:   Nbn    Locus Link:   85482    Chromosome:   5    Cytoband:   Chr:5q13    SOURCE    GENECARD       Gene Ontology:   cellular component:  nucleus   cellular component:  replication fork   cellular component:  intracellular   molecular function:  damaged DNA binding      BioCarta Pathways:     KEGG Pathways:  
    Probe set : 1370371_a_at    Name:   CEA-related cell adhesion molecule 1 /// CEA-related cell adhesion molecule 10    GB acc:   U23056    UniGene:   Rn.91235    Gene Symbol:   Ceacam1 /// Ceacam10    Locus Link:   287009 /// 81613    Chromosome:   1    Cytoband:   Chr:1q21-q22 /// Chr:1q21    SOURCE    GENECARD       Gene Ontology:   molecular function:  receptor activity   molecular function:  protein binding      BioCarta Pathways:     KEGG Pathways:  
    Probe set : 1372080_at    Name:   Inner membrane protein, mitochondrial (predicted)    GB acc:   BI287936    UniGene:   Rn.47124     Locus Link:   312444    Chromosome:   4    Cytoband:   Chr:4q33    SOURCE        Gene Ontology:     BioCarta Pathways:     KEGG Pathways:  
    Probe set : 1397549_at    Name:   centaurin, gamma 1    GB acc:   AW526039    UniGene:   Rn.91094    Gene Symbol:   Centg1    Locus Link:   65218    Chromosome:   7    Cytoband:   Chr:7q22    SOURCE    GENECARD       Gene Ontology:   biological process:  small GTPase mediated signal transduction   biological process:  regulation of GTPase activity   cellular component:  nucleus      BioCarta Pathways:     KEGG Pathways:  
    Probe set : 1385066_a_at    Name:   Similar to hypothetical protein FLJ13089 (predicted)    GB acc:   BM390527    UniGene:   Rn.95126     Locus Link:   360811    Chromosome:   12    Cytoband:   Chr:12q16    SOURCE        Gene Ontology:   molecular function:  binding      BioCarta Pathways:     KEGG Pathways:  
    Probe set : 1373645_at    Name:   Transcribed locus    GB acc:   BM386246    UniGene:   Rn.106786        SOURCE        Gene Ontology:     BioCarta Pathways:     KEGG Pathways:  
    Probe set : 1372259_at    Name:   DEK oncogene (DNA binding)    GB acc:   BI295054    UniGene:   Rn.25099    Gene Symbol:   RGD:1303246    Locus Link:   306817    Chromosome:   17    Cytoband:   Chr:17p13    SOURCE    GENECARD       Gene Ontology:   biological process:  SRP-dependent cotranslational protein targeting to membrane   cellular component:  nucleus   cellular component:  signal recognition particle (sensu Eukaryota)   molecular function:  DNA binding   molecular function:  RNA binding   molecular function:  GTP binding      BioCarta Pathways:     KEGG Pathways:  
    Probe set : 1367694_at    Name:   hydroxyacyl-Coenzyme A dehydrogenase/3-ketoacyl-Coenzyme A thiolase/enoyl-Coenzyme A hydratase (trifunctional protein), beta subunit    GB acc:   NM_133618    UniGene:   Rn.11253    Gene Symbol:   Hadhb    Locus Link:   171155    Chromosome:   6    Cytoband:   Chr:6q14    SOURCE    GENECARD       Gene Ontology:   biological process:  fatty acid metabolism   biological process:  fatty acid beta-oxidation   biological process:  lipid metabolism   cellular component:  mitochondrion   cellular component:  mitochondrial membrane      BioCarta Pathways:     KEGG Pathways:  
    Probe set : 1389146_at    Name:   hypothetical protein LOC498796    GB acc:   BF283267    UniGene:   Rn.105966    Gene Symbol:   LOC498796    Locus Link:   498796    Chromosome:   17    Cytoband:   Chr:17q12.3    SOURCE    GENECARD       Gene Ontology:     BioCarta Pathways:     KEGG Pathways:  
    Probe set : 1384474_at    Name:   Transcribed locus    GB acc:   AA860010    UniGene:   Rn.872        SOURCE        Gene Ontology:     BioCarta Pathways:     KEGG Pathways:  
    Probe set : 1378867_at     GB acc:   BG374639              Gene Ontology:     BioCarta Pathways:     KEGG Pathways:  
    Probe set : 1378964_at    Name:   Similar to hypothetical protein    GB acc:   AI709953    UniGene:   Rn.107832    Gene Symbol:   MGC73022    Locus Link:   309196    Chromosome:   1    Cytoband:   Chr:1q43    SOURCE    GENECARD       Gene Ontology:   molecular function:  binding      BioCarta Pathways:     KEGG Pathways:  
    Probe set : 1370432_at    Name:   POU domain, class 3, transcription factor 1    GB acc:   M72711    UniGene:   Rn.82720    Gene Symbol:   Pou3f1    Locus Link:   192110    Chromosome:   5    Cytoband:   Chr:5q36    SOURCE    GENECARD       Gene Ontology:   cellular component:  nucleus   cellular component:  nucleus   cellular component:  transcription factor complex   molecular function:  DNA binding   molecular function:  transcription factor activity   molecular function:  transcription factor activity      BioCarta Pathways:     KEGG Pathways:  
    Probe set : 1393320_at    Name:   CDNA clone IMAGE:7375955    GB acc:   BF396985    UniGene:   Rn.7959        SOURCE        Gene Ontology:     BioCarta Pathways:     KEGG Pathways:  
    Probe set : 1393441_at    Name:   similar to cyclin-dependent kinase inhibitor 2D    GB acc:   BI395605    UniGene:   Rn.122002    Gene Symbol:   Cdkn2d    Locus Link:   494444      SOURCE    GENECARD       Gene Ontology:   molecular function:  kinase activity      BioCarta Pathways:     KEGG Pathways:  
    Probe set : 1390656_at    Name:   Transcribed locus    GB acc:   BF389473    UniGene:   Rn.64376        SOURCE        Gene Ontology:     BioCarta Pathways:     KEGG Pathways:  
    Probe set : 1372704_at    Name:   similar to RIKEN cDNA 2310008M10    GB acc:   BE098941    UniGene:   Rn.1889    Gene Symbol:   LOC362040    Locus Link:   362040    Chromosome:   2    Cytoband:   Chr:2q43    SOURCE    GENECARD       Gene Ontology:     BioCarta Pathways:     KEGG Pathways:  
    Probe set : 1375645_at    Name:   similar to DNA segment, Chr 4, ERATO Doi 22, expressed    GB acc:   AI408842    UniGene:   Rn.22136    Gene Symbol:   LOC500575    Locus Link:   500575    Chromosome:   5    Cytoband:   Chr:5q36    SOURCE    GENECARD       Gene Ontology:     BioCarta Pathways:     KEGG Pathways:  
    Probe set : 1375215_x_at    Name:   pyroglutamyl-peptidase I    GB acc:   BE109558    UniGene:   Rn.98380    Gene Symbol:   RGD:1303133    Locus Link:   290648    Chromosome:   16    Cytoband:   Chr:16p14    SOURCE    GENECARD       Gene Ontology:   biological process:  proteolysis   biological process:  proteolysis   cellular component:  cytosol      BioCarta Pathways:     KEGG Pathways:  
    Probe set : 1371879_at    Name:   similar to RIKEN cDNA A930011F22 gene (predicted)    GB acc:   BE110542    UniGene:   Rn.98323    Gene Symbol:   RGD1308919_predicted    Locus Link:   298309    Chromosome:   5    Cytoband:   Chr:5q34    SOURCE    GENECARD       Gene Ontology:     BioCarta Pathways:     KEGG Pathways:  
    Probe set : 1383706_at    Name:   similar to RIKEN cDNA 2900011O08 (predicted)    GB acc:   BF409331    UniGene:   Rn.27543    Gene Symbol:   RGD1305733_predicted    Locus Link:   360459    Chromosome:   10    Cytoband:   Chr:10q11    SOURCE    GENECARD       Gene Ontology:     BioCarta Pathways:     KEGG Pathways:  
    Probe set : 1368121_at    Name:   aldo-keto reductase family 7, member A3 (aflatoxin aldehyde reductase)    GB acc:   NM_013215    UniGene:   Rn.6043    Gene Symbol:   Akr7a3    Locus Link:   26760    Chromosome:   5    Cytoband:   Chr:5q36    SOURCE    GENECARD       Gene Ontology:   cellular component:  cytosol      BioCarta Pathways:     KEGG Pathways:  
    Probe set : 1372896_at    Name:   Similar to CDNA sequence BC063749    GB acc:   BM390440    UniGene:   Rn.16961     Locus Link:   499370      SOURCE        Gene Ontology:     BioCarta Pathways:     KEGG Pathways:  
    Probe set : 1373619_at    Name:   ankyrin repeat domain 10 (predicted)    GB acc:   AA894306    UniGene:   Rn.30154    Gene Symbol:   Ankrd10_predicted    Locus Link:   361183    Chromosome:   16    Cytoband:   Chr:16q12.5    SOURCE    GENECARD       Gene Ontology:   biological process:  regulation of transcription, DNA-dependent   molecular function:  DNA binding   molecular function:  transcription factor activity      BioCarta Pathways:     KEGG Pathways:  
    Probe set : 1368284_at    Name:   plasmalemma vesicle associated protein    GB acc:   NM_020086    UniGene:   Rn.53915    Gene Symbol:   Plvap    Locus Link:   56765    Chromosome:   16    Cytoband:   Chr:16p14    SOURCE    GENECARD       Gene Ontology:   molecular function:  protein homodimerization activity      BioCarta Pathways:     KEGG Pathways:  
    Probe set : 1372149_at    Name:   AU RNA binding protein/enoyl-coenzyme A hydratase (predicted)    GB acc:   BM389502    UniGene:   Rn.50    Gene Symbol:   Auh_predicted    Locus Link:   361215    Chromosome:   17    Cytoband:   Chr:17p14    SOURCE    GENECARD       Gene Ontology:   cellular component:  mitochondrion      BioCarta Pathways:     KEGG Pathways:  
    Probe set : 1388624_at    Name:   similar to Hypothetical UPF0184 protein C9orf16 homolog    GB acc:   BG669070    UniGene:   Rn.2148    Gene Symbol:   LOC499780    Locus Link:   499780    Chromosome:   3    Cytoband:   Chr:3p11    SOURCE    GENECARD       Gene Ontology:     BioCarta Pathways:     KEGG Pathways:  
    Probe set : 1397392_at     GB acc:   AW535120              Gene Ontology:     BioCarta Pathways:     KEGG Pathways:  
    Probe set : 1374890_at    Name:   Transcribed locus, strongly similar to NP_031990.1 ecotropic viral integration site 5 [Mus musculus]    GB acc:   AW533905    UniGene:   Rn.113868        SOURCE        Gene Ontology:     BioCarta Pathways:     KEGG Pathways:  
    Probe set : 1392481_at    Name:   similar to DNA segment, Chr 1, Brigham & Womens Genetics 0212 expressed    GB acc:   BG057566    UniGene:   Rn.22860    Gene Symbol:   LOC363221    Locus Link:   363221    Chromosome:   9    Cytoband:   Chr:9q21    SOURCE    GENECARD       Gene Ontology:     BioCarta Pathways:     KEGG Pathways:  
    Probe set : 1369415_at    Name:   basic helix-loop-helix domain containing, class B2    GB acc:   NM_053328    UniGene:   Rn.81055    Gene Symbol:   Bhlhb2    Locus Link:   79431    Chromosome:   4    Cytoband:   Chr:4q41    SOURCE    GENECARD       Gene Ontology:   cellular component:  nucleus   cellular component:  nucleus      BioCarta Pathways:     KEGG Pathways:  
    Probe set : 1367677_at    Name:   peroxiredoxin 5    GB acc:   NM_053610    UniGene:   Rn.2944    Gene Symbol:   Prdx5    Locus Link:   113898    Chromosome:   1    Cytoband:   Chr:1q43    SOURCE    GENECARD       Gene Ontology:   biological process:  peroxisome organization and biogenesis   cellular component:  mitochondrion   cellular component:  peroxisome      BioCarta Pathways:     KEGG Pathways:  
    Probe set : 1389324_at    Name:   tyrosine kinase receptor 1    GB acc:   BI289129    UniGene:   Rn.13171    Gene Symbol:   Tie1    Locus Link:   89806    Chromosome:   5    Cytoband:   Chr:5q36    SOURCE    GENECARD       Gene Ontology:   biological process:  vasculogenesis   molecular function:  receptor activity   molecular function:  kinase activity      BioCarta Pathways:     KEGG Pathways:  
    Probe set : 1382459_at     GB acc:   BF287235              Gene Ontology:     BioCarta Pathways:     KEGG Pathways:  
    Probe set : 1388145_at    Name:   tenascin XA    GB acc:   BM390128     Gene Symbol:   Tnxa    Locus Link:   25602       GENECARD       Gene Ontology:   biological process:  muscle development      BioCarta Pathways:     KEGG Pathways:  
    Probe set : 1387964_a_at    Name:   ERO1-like (S. cerevisiae)    GB acc:   AY071924    UniGene:   Rn.64648    Gene Symbol:   Ero1l    Locus Link:   171562    Chromosome:   15    Cytoband:   Chr:15p14    SOURCE    GENECARD       Gene Ontology:   cellular component:  endoplasmic reticulum   cellular component:  membrane   cellular component:  integral to endoplasmic reticulum membrane   molecular function:  oxidoreductase activity      BioCarta Pathways:     KEGG Pathways:  
    Probe set : 1396821_at    Name:   Transcribed locus    GB acc:   AW523396    UniGene:   Rn.45624        SOURCE        Gene Ontology:     BioCarta Pathways:     KEGG Pathways:  
    Probe set : 1374693_at    Name:   hypothetical LOC315760 (predicted)    GB acc:   BM388613    UniGene:   Rn.23423    Gene Symbol:   RGD1306243_predicted    Locus Link:   315760    Chromosome:   8    Cytoband:   Chr:8q24    SOURCE    GENECARD       Gene Ontology:   biological process:  protein amino acid ADP-ribosylation   cellular component:  nucleus   molecular function:  NAD+ ADP-ribosyltransferase activity      BioCarta Pathways:     KEGG Pathways:  
    Probe set : 1393219_at    Name:   complement component 2    GB acc:   BF394142    UniGene:   Rn.98333    Gene Symbol:   C2    Locus Link:   24231    Chromosome:   20    Cytoband:   Chr:20p12    SOURCE    GENECARD       Gene Ontology:   biological process:  proteolysis   biological process:  complement activation   biological process:  complement activation, classical pathway   cellular component:  extracellular space   cellular component:  extracellular region      BioCarta Pathways:     KEGG Pathways:  
    Probe set : 1372014_at    Name:   similar to cofactor required for Sp1 transcriptional activation subunit 8    GB acc:   BM392138    UniGene:   Rn.105472    Gene Symbol:   LOC296612    Locus Link:   296612    Chromosome:   3    Cytoband:   Chr:3p12    SOURCE    GENECARD       Gene Ontology:     BioCarta Pathways:     KEGG Pathways:  
    Probe set : 1389816_at     GB acc:   AA926357              Gene Ontology:     BioCarta Pathways:     KEGG Pathways:  
    Probe set : 1388754_at     GB acc:   AI176839              Gene Ontology:     BioCarta Pathways:     KEGG Pathways:  
    Probe set : 1385828_at    Name:   Transcribed locus, strongly similar to XP_525786.1 PREDICTED: similar to chromosome 2 open reading frame 7 [Pan troglodytes]    GB acc:   BF559433    UniGene:   Rn.153870        SOURCE        Gene Ontology:     BioCarta Pathways:     KEGG Pathways:  
    Probe set : 1373704_at    Name:   aquarius (predicted)    GB acc:   BI294892    UniGene:   Rn.37299    Gene Symbol:   Aqr_predicted    Locus Link:   366163    Chromosome:   3    Cytoband:   Chr:3q35    SOURCE    GENECARD       Gene Ontology:     BioCarta Pathways:     KEGG Pathways:  
    Probe set : 1371448_at    Name:   CDNA clone IMAGE:7303896    GB acc:   AI599295    UniGene:   Rn.96020        SOURCE        Gene Ontology:     BioCarta Pathways:     KEGG Pathways:  
    Probe set : 1372561_at    Name:   similar to hypothetical protein AL133206 (predicted)    GB acc:   AI234095    UniGene:   Rn.106443    Gene Symbol:   RGD1308418_predicted    Locus Link:   298500    Chromosome:   5    Cytoband:   Chr:5q36    SOURCE    GENECARD       Gene Ontology:     BioCarta Pathways:     KEGG Pathways:  
    Probe set : 1369401_at    Name:   solute carrier family 21, member 13    GB acc:   NM_130736     Gene Symbol:   Slc21a13    Locus Link:   84608    Chromosome:   4    Cytoband:   Chr:4q44     GENECARD       Gene Ontology:   biological process:  transport   biological process:  ion transport   cellular component:  extracellular space   cellular component:  membrane   cellular component:  integral to membrane   molecular function:  transporter activity      BioCarta Pathways:     KEGG Pathways:  
    Probe set : 1376810_at    Name:   ADP-ribosyltransferase (NAD+; poly (ADP-ribose) polymerase)-like 1 (predicted)    GB acc:   AI103325    UniGene:   Rn.139095    Gene Symbol:   Adprtl1_predicted    Locus Link:   361046    Chromosome:   15    Cytoband:   Chr:15p12    SOURCE    GENECARD       Gene Ontology:   cellular component:  nucleus   cellular component:  spindle   cellular component:  ribonucleoprotein complex      BioCarta Pathways:     KEGG Pathways:  
    Probe set : 1392918_at    Name:   chaperonin subunit 8 (theta) (predicted)    GB acc:   BE115650    UniGene:   Rn.98524    Gene Symbol:   Cct8_predicted    Locus Link:   288305    Chromosome:   11    Cytoband:   Chr:11q11    SOURCE    GENECARD       Gene Ontology:   biological process:  protein folding   cellular component:  cytosol   molecular function:  ATP binding   molecular function:  ATPase activity, coupled   molecular function:  unfolded protein binding      BioCarta Pathways:     KEGG Pathways:  
    Probe set : 1389322_at    Name:   programmed cell death protein 7 (predicted)    GB acc:   AI013847    UniGene:   Rn.8408    Gene Symbol:   Pdcd7_predicted    Locus Link:   363082    Chromosome:   8    Cytoband:   Chr:8q24    SOURCE    GENECARD       Gene Ontology:   biological process:  regulation of transcription, DNA-dependent   biological process:  apoptosis   cellular component:  nucleus      BioCarta Pathways:     KEGG Pathways:  
    Probe set : 1387209_at    Name:   regucalcin gene promotor region related protein    GB acc:   NM_053571    UniGene:   Rn.21873    Gene Symbol:   Rgpr    Locus Link:   89868    Chromosome:   13    Cytoband:   Chr:13q22    SOURCE    GENECARD       Gene Ontology:   cellular component:  nucleus      BioCarta Pathways:     KEGG Pathways:  
    Probe set : 1390397_at    Name:   similar to chromosome 20 open reading frame 39 (predicted)    GB acc:   BF413152    UniGene:   Rn.44092    Gene Symbol:   RGD1310753_predicted    Locus Link:   362235    Chromosome:   3    Cytoband:   Chr:3q41    SOURCE    GENECARD       Gene Ontology:   cellular component:  integral to membrane      BioCarta Pathways:     KEGG Pathways:  
    Probe set : 1391095_at    Name:   matrix metalloproteinase 19 (predicted)    GB acc:   BI294977    UniGene:   Rn.48520    Gene Symbol:   Mmp19_predicted    Locus Link:   304608    Chromosome:   7    Cytoband:   Chr:7q11    SOURCE    GENECARD       Gene Ontology:   biological process:  angiogenesis   biological process:  proteolysis   biological process:  collagen catabolism   cellular component:  extracellular matrix (sensu Metazoa)   cellular component:  extracellular space      BioCarta Pathways:     KEGG Pathways:  
    Probe set : 1373449_at    Name:   TAF5-like RNA polymerase II, p300/CBP-associated factor (PCAF)-associated factor (predicted)    GB acc:   BG375861    UniGene:   Rn.23853    Gene Symbol:   Taf5l_predicted    Locus Link:   307927    Chromosome:   19    Cytoband:   Chr:19q12    SOURCE    GENECARD       Gene Ontology:   cellular component:  nucleus   molecular function:  transcription factor activity   molecular function:  transcription regulator activity      BioCarta Pathways:     KEGG Pathways:  
    Probe set : 1367678_at    Name:   succinate dehydrogenase complex, subunit A, flavoprotein (Fp)    GB acc:   NM_130428    UniGene:   Rn.101725    Gene Symbol:   Sdha    Locus Link:   157074    Chromosome:   1    Cytoband:   Chr:1p11    SOURCE    GENECARD       Gene Ontology:   cellular component:  mitochondrion   cellular component:  respiratory chain complex II   cellular component:  succinate dehydrogenase complex      BioCarta Pathways:     KEGG Pathways:  
    Probe set : 1376861_at    Name:   similar to RIKEN cDNA 1810018L05 /// similar to motile sperm domain containing 1    GB acc:   AW525240    UniGene:   Rn.114936    Gene Symbol:   RGD:1359486 /// LOC501648    Locus Link:   317312 /// 501648    Chromosome:   14    Cytoband:   Chr:14q21 /// Chr:Xq36    SOURCE    GENECARD       Gene Ontology:   cellular component:  integral to membrane   molecular function:  structural molecule activity      BioCarta Pathways:     KEGG Pathways:  
    Probe set : 1382111_at    Name:   similar to RIKEN cDNA 2010001H14    GB acc:   AI454360    UniGene:   Rn.19978    Gene Symbol:   LOC302554    Locus Link:   302554    Chromosome:   X    Cytoband:   Chr:Xq13    SOURCE    GENECARD       Gene Ontology:     BioCarta Pathways:     KEGG Pathways:  
    Probe set : 1383251_at    Name:   ADP-ribosyltransferase (NAD+, poly(ADP-ribose) polymerase)-like 2 (predicted)    GB acc:   AW524533    UniGene:   Rn.22730    Gene Symbol:   Adprtl2_predicted    Locus Link:   290027    Chromosome:   15    Cytoband:   Chr:15p14    SOURCE    GENECARD       Gene Ontology:   biological process:  DNA repair   biological process:  base-excision repair   biological process:  proteolysis   cellular component:  nucleus      BioCarta Pathways:     KEGG Pathways:  
    Probe set : 1388987_at    Name:   similar to expressed sequence AA960436 (predicted)    GB acc:   BF283646    UniGene:   Rn.95149    Gene Symbol:   RGD1305215_predicted    Locus Link:   307643    Chromosome:   19    Cytoband:   Chr:19p13    SOURCE    GENECARD       Gene Ontology:     BioCarta Pathways:     KEGG Pathways:  
    Probe set : 1375056_at    Name:   similar to OCIA domain containing 2    GB acc:   AA943310    UniGene:   Rn.105660    Gene Symbol:   LOC289589    Locus Link:   289589    Chromosome:   14    Cytoband:   Chr:14p11    SOURCE    GENECARD       Gene Ontology:     BioCarta Pathways:     KEGG Pathways:  
    Probe set : 1380688_at    Name:   hypothetical LOC293181 (predicted)    GB acc:   BI283107    UniGene:   Rn.72445    Gene Symbol:   RGD1309623_predicted    Locus Link:   293181    Chromosome:   1    Cytoband:   Chr:1q33    SOURCE    GENECARD       Gene Ontology:     BioCarta Pathways:     KEGG Pathways:  
    Probe set : 1383059_a_at    Name:   G kinase anchoring protein 1 (predicted)    GB acc:   BE112067    UniGene:   Rn.2003    Gene Symbol:   Gkap1_predicted    Locus Link:   361202    Chromosome:   17    Cytoband:   Chr:17p14    SOURCE    GENECARD       Gene Ontology:   biological process:  signal transduction   biological process:  G-protein signaling, coupled to cGMP nucleotide second messenger   cellular component:  extracellular space   cellular component:  Golgi apparatus   molecular function:  protein binding   molecular function:  kinase activity      BioCarta Pathways:     KEGG Pathways:  
    Probe set : 1387776_at    Name:   transglutaminase 2, C polypeptide    GB acc:   BI275994    UniGene:   Rn.10    Gene Symbol:   Tgm2    Locus Link:   56083    Chromosome:   3    Cytoband:   Chr:3q42    SOURCE    GENECARD       Gene Ontology:     BioCarta Pathways:     KEGG Pathways:  
    Probe set : 1391063_at    Name:   kinesin family member 23 (predicted)    GB acc:   BE113443    UniGene:   Rn.63734    Gene Symbol:   Kif23_predicted    Locus Link:   315740    Chromosome:   8    Cytoband:   Chr:8q24    SOURCE    GENECARD       Gene Ontology:   molecular function:  motor activity   molecular function:  microtubule motor activity   molecular function:  ATP binding      BioCarta Pathways:     KEGG Pathways:  
    Probe set : 1392508_at    Name:   similar to RIKEN cDNA 3110010F15 (predicted)    GB acc:   BE119831    UniGene:   Rn.17469    Gene Symbol:   RGD1308378_predicted    Locus Link:   361159    Chromosome:   16    Cytoband:   Chr:16q12.2    SOURCE    GENECARD       Gene Ontology:   cellular component:  intracellular      BioCarta Pathways:     KEGG Pathways:  
    Probe set : 1375886_at    Name:   Transcribed locus    GB acc:   AI236778    UniGene:   Rn.8314        SOURCE        Gene Ontology:     BioCarta Pathways:     KEGG Pathways:  
    Probe set : 1395572_at    Name:   DnaJ (Hsp40) homolog, subfamily A, member 4 (predicted)    GB acc:   BF565278    UniGene:   Rn.27927    Gene Symbol:   Dnaja4_predicted    Locus Link:   300721    Chromosome:   8    Cytoband:   Chr:8q24    SOURCE    GENECARD       Gene Ontology:   biological process:  protein folding   molecular function:  heat shock protein binding   molecular function:  unfolded protein binding      BioCarta Pathways:     KEGG Pathways:  
    Probe set : 1388557_at    Name:   Transcribed locus    GB acc:   BF284922    UniGene:   Rn.37683        SOURCE        Gene Ontology:     BioCarta Pathways:     KEGG Pathways:  
    Probe set : 1388054_a_at    Name:   chondroitin sulfate proteoglycan 2    GB acc:   AF072892    UniGene:   Rn.35666    Gene Symbol:   Cspg2    Locus Link:   114122    Chromosome:   2    Cytoband:   Chr:2q12    SOURCE    GENECARD       Gene Ontology:   biological process:  cell motility   biological process:  cell adhesion   biological process:  cell adhesion   cellular component:  extracellular matrix (sensu Metazoa)   cellular component:  extracellular matrix      BioCarta Pathways:     KEGG Pathways:  
    Probe set : 1398798_at    Name:   methionine aminopeptidase 2    GB acc:   NM_022539    UniGene:   Rn.127860    Gene Symbol:   Metap2    Locus Link:   64370    Chromosome:   7    Cytoband:   Chr:7q13    SOURCE    GENECARD       Gene Ontology:   biological process:  regulation of translation   biological process:  protein modification   biological process:  proteolysis      BioCarta Pathways:     KEGG Pathways:  
    Probe set : 1381940_at    Name:   Transcribed locus    GB acc:   AW527798    UniGene:   Rn.28619        SOURCE        Gene Ontology:     BioCarta Pathways:     KEGG Pathways:  
    Probe set : 1369044_a_at    Name:   phosphodiesterase 4B    GB acc:   AF202733    UniGene:   Rn.37733    Gene Symbol:   Pde4b    Locus Link:   24626    Chromosome:   5    Cytoband:   Chr:5q32    SOURCE    GENECARD       Gene Ontology:   biological process:  smooth muscle contraction   biological process:  signal transduction   cellular component:  soluble fraction   cellular component:  insoluble fraction   cellular component:  cytosol      BioCarta Pathways:     KEGG Pathways:  
    Probe set : 1398361_at    Name:   similar to Rho-GTPase-activating protein PS-GAP-a    GB acc:   BI295127    UniGene:   Rn.40465    Gene Symbol:   LOC498938    Locus Link:   498938    Chromosome:   19    Cytoband:   Chr:19q11    SOURCE    GENECARD       Gene Ontology:     BioCarta Pathways:     KEGG Pathways:  
    Probe set : 1375536_at    Name:   Numb gene homolog (Drosophila)    GB acc:   BI287975    UniGene:   Rn.102078    Gene Symbol:   Numb    Locus Link:   29419    Chromosome:   6    Cytoband:   Chr:6q31    SOURCE    GENECARD       Gene Ontology:   molecular function:  protein binding      BioCarta Pathways:     KEGG Pathways:  
    Probe set : 1369788_s_at    Name:   v-jun sarcoma virus 17 oncogene homolog (avian)    GB acc:   NM_021835    UniGene:   Rn.93714    Gene Symbol:   Jun    Locus Link:   24516    Chromosome:   5    Cytoband:   Chr:5q34.1    SOURCE    GENECARD       Gene Ontology:     BioCarta Pathways:     KEGG Pathways:  
    Probe set : 1368574_at    Name:   adrenergic receptor, alpha 1b    GB acc:   NM_016991    UniGene:   Rn.10032    Gene Symbol:   Adra1b    Locus Link:   24173    Chromosome:   10    Cytoband:   Chr:10q21    SOURCE    GENECARD       Gene Ontology:   cellular component:  integral to plasma membrane   cellular component:  integral to membrane      BioCarta Pathways:     KEGG Pathways:  
    Probe set : 1372423_at    Name:   PERP, TP53 apoptosis effector (predicted)    GB acc:   BI286396    UniGene:   Rn.101762    Gene Symbol:   Perp_predicted    Locus Link:   292949    Chromosome:   1    Cytoband:   Chr:1p12    SOURCE    GENECARD       Gene Ontology:   biological process:  induction of apoptosis      BioCarta Pathways:     KEGG Pathways:  
    Probe set : 1388745_at    Name:   sema domain, immunoglobulin domain (Ig), transmembrane domain (TM) and short cytoplasmic domain, (semaphorin) 4A (predicted)    GB acc:   AI228417    UniGene:   Rn.54039    Gene Symbol:   Sema4a_predicted    Locus Link:   310630    Chromosome:   2    Cytoband:   Chr:2q34    SOURCE    GENECARD       Gene Ontology:   biological process:  development   biological process:  nervous system development   cellular component:  extracellular space   cellular component:  membrane   cellular component:  integral to membrane   molecular function:  receptor activity      BioCarta Pathways:     KEGG Pathways:  
    Probe set : 1373182_at     GB acc:   BF281899              Gene Ontology:     BioCarta Pathways:     KEGG Pathways:  
    Probe set : 1396211_at    Name:   Similar to RIKEN cDNA 2610207I16 (predicted)    GB acc:   BE100009    UniGene:   Rn.134797     Locus Link:   313200    Chromosome:   5    Cytoband:   Chr:5q24    SOURCE        Gene Ontology:   biological process:  metabolism   cellular component:  mitochondrion   molecular function:  sterol carrier activity   molecular function:  oxidoreductase activity      BioCarta Pathways:     KEGG Pathways:  
    Probe set : 1373079_at    Name:   Transcribed locus, weakly similar to XP_342574.1 PREDICTED: similar to Csr1 [Rattus norvegicus]    GB acc:   BI296427    UniGene:   Rn.8272        SOURCE        Gene Ontology:     BioCarta Pathways:     KEGG Pathways:  
    Probe set : 1394499_at    Name:   Taube nuss (predicted)    GB acc:   AI501768    UniGene:   Rn.35557     Locus Link:   316216    Chromosome:   9    Cytoband:   Chr:9q12    SOURCE        Gene Ontology:   biological process:  regulation of fat cell differentiation   biological process:  positive regulation of transcription, DNA-dependent   cellular component:  nucleus   cellular component:  transcription factor TFIID complex   molecular function:  protein binding   molecular function:  transcriptional activator activity      BioCarta Pathways:     KEGG Pathways:  
    Probe set : 1367745_at    Name:   diacylglycerol kinase zeta    GB acc:   NM_031143    UniGene:   Rn.11208    Gene Symbol:   Dgkz    Locus Link:   81821    Chromosome:   3    Cytoband:   Chr:3q24    SOURCE    GENECARD       Gene Ontology:   biological process:  protein kinase C activation   biological process:  intracellular signaling cascade   cellular component:  nucleus   cellular component:  nucleus      BioCarta Pathways:     KEGG Pathways:  
    Probe set : 1379429_at    Name:   Transcribed locus    GB acc:   AI045807    UniGene:   Rn.16505        SOURCE        Gene Ontology:     BioCarta Pathways:     KEGG Pathways:  
    Probe set : 1385806_at    Name:   similar to PAP-1    GB acc:   AA996771    UniGene:   Rn.6585    Gene Symbol:   LOC363032    Locus Link:   363032    Chromosome:   8    Cytoband:   Chr:8q13    SOURCE    GENECARD       Gene Ontology:     BioCarta Pathways:     KEGG Pathways:  
    Probe set : 1386999_at    Name:   tyrosine 3-monooxygenase/tryptophan 5-monooxygenase activation protein, beta polypeptide    GB acc:   BG380730    UniGene:   Rn.8653    Gene Symbol:   Ywhab    Locus Link:   56011    Chromosome:   3    Cytoband:   Chr:3q42    SOURCE    GENECARD       Gene Ontology:   biological process:  protein targeting   molecular function:  protein binding   molecular function:  protein domain specific binding      BioCarta Pathways:     KEGG Pathways:  
    Probe set : 1393808_at    Name:   Transcribed locus, strongly similar to NP_835187.1 fatty acid 2-hydroxylase [Mus musculus]    GB acc:   AW527783    UniGene:   Rn.67886        SOURCE        Gene Ontology:     BioCarta Pathways:     KEGG Pathways:  
    Probe set : 1380544_at    Name:   Transcribed locus    GB acc:   BF396688    UniGene:   Rn.7508        SOURCE        Gene Ontology:     BioCarta Pathways:     KEGG Pathways:  
    Probe set : 1368735_a_at    Name:   transient receptor potential cation channel, subfamily V, member 2    GB acc:   NM_017207    UniGene:   Rn.30221    Gene Symbol:   Trpv2    Locus Link:   29465    Chromosome:   10    Cytoband:   Chr:10q23    SOURCE    GENECARD       Gene Ontology:     BioCarta Pathways:     KEGG Pathways:  
    Probe set : 1392973_at    Name:   LOC499153    GB acc:   AI073173    UniGene:   Rn.61136    Gene Symbol:   LOC499153    Locus Link:   499153    Chromosome:   1    Cytoband:   Chr:1q22    SOURCE    GENECARD       Gene Ontology:     BioCarta Pathways:     KEGG Pathways:  
    Probe set : 1368592_at    Name:   interleukin 1 alpha    GB acc:   NM_017019    UniGene:   Rn.12300    Gene Symbol:   Il1a    Locus Link:   24493    Chromosome:   3    Cytoband:   Chr:3q36    SOURCE    GENECARD       Gene Ontology:     BioCarta Pathways:     KEGG Pathways:  
    Probe set : 1385873_at    Name:   Transcribed locus    GB acc:   BM388405    UniGene:   Rn.25011        SOURCE        Gene Ontology:     BioCarta Pathways:     KEGG Pathways:  
    Probe set : 1383162_at     GB acc:   AI008646              Gene Ontology:     BioCarta Pathways:     KEGG Pathways:  
    Probe set : 1387811_at    Name:   angiotensinogen    GB acc:   NM_134432    UniGene:   Rn.6319    Gene Symbol:   Agt    Locus Link:   24179    Chromosome:   19    Cytoband:   Chr:19q12    SOURCE    GENECARD       Gene Ontology:   cellular component:  extracellular region   cellular component:  extracellular space   cellular component:  soluble fraction      BioCarta Pathways:     KEGG Pathways:  
    Probe set : 1367806_at    Name:   glutaminase    GB acc:   M22586    UniGene:   Rn.5762    Gene Symbol:   Gls    Locus Link:   24398    Chromosome:   9    Cytoband:   Chr:9q31-q32    SOURCE    GENECARD       Gene Ontology:   biological process:  glutamine metabolism   biological process:  glutamine catabolism   biological process:  glutamine catabolism   cellular component:  mitochondrion   cellular component:  mitochondrion   cellular component:  mitochondrial inner membrane   molecular function:  glutaminase activity   molecular function:  glutaminase activity   molecular function:  hydrolase activity      BioCarta Pathways:     KEGG Pathways:  
    Probe set : 1386128_at    Name:   tetranectin (plasminogen binding protein) (predicted)    GB acc:   BF524010    UniGene:   Rn.23478    Gene Symbol:   Tna_predicted    Locus Link:   316099    Chromosome:   8    Cytoband:   Chr:8q32    SOURCE    GENECARD       Gene Ontology:   biological process:  skeletal development   cellular component:  extracellular region   cellular component:  extracellular matrix (sensu Metazoa)   cellular component:  extracellular space   molecular function:  calcium ion binding   molecular function:  sugar binding      BioCarta Pathways:     KEGG Pathways:  
    Probe set : 1381811_at    Name:   Transcribed locus    GB acc:   BM385405    UniGene:   Rn.92548        SOURCE        Gene Ontology:     BioCarta Pathways:     KEGG Pathways:  
    Probe set : 1379254_at    Name:   similar to RIKEN cDNA 1300007B12; clone MNCb-2755 (predicted)    GB acc:   AA945882     Gene Symbol:   RGD1309892_predicted    Locus Link:   289034    Chromosome:   13    Cytoband:   Chr:13q13     GENECARD       Gene Ontology:   cellular component:  integral to membrane      BioCarta Pathways:     KEGG Pathways:  
    Probe set : 1370881_at    Name:   thiosulfate sulfurtransferase    GB acc:   AI411117    UniGene:   Rn.6360    Gene Symbol:   Tst    Locus Link:   25274    Chromosome:   7    Cytoband:   Chr:7q34    SOURCE    GENECARD       Gene Ontology:   biological process:  sulfate transport   biological process:  cyanate catabolism   biological process:  iron-sulfur cluster assembly   cellular component:  mitochondrion   cellular component:  mitochondrion   cellular component:  mitochondrial matrix      BioCarta Pathways:     KEGG Pathways:  
    Probe set : 1379901_at    Name:   TBC1 domain family, member 17 (predicted)    GB acc:   BG375857    UniGene:   Rn.18482    Gene Symbol:   Tbc1d17_predicted    Locus Link:   292886    Chromosome:   1    Cytoband:   Chr:1q22    SOURCE    GENECARD       Gene Ontology:     BioCarta Pathways:     KEGG Pathways:  
    Probe set : 1373434_at    Name:   Transcribed locus    GB acc:   AA944179    UniGene:   Rn.7318        SOURCE        Gene Ontology:     BioCarta Pathways:     KEGG Pathways:  
    Probe set : 1390127_at    Name:   Transcribed locus    GB acc:   BI296216    UniGene:   Rn.147830        SOURCE        Gene Ontology:     BioCarta Pathways:     KEGG Pathways:  
    Probe set : 1398365_at    Name:   similar to RIKEN cDNA 2700055K07 (predicted)    GB acc:   AI171466    UniGene:   Rn.29476    Gene Symbol:   RGD1305061_predicted    Locus Link:   291966    Chromosome:   19    Cytoband:   Chr:19q11    SOURCE    GENECARD       Gene Ontology:   molecular function:  calcium ion binding      BioCarta Pathways:     KEGG Pathways:  
    Probe set : 1369250_at    Name:   solute carrier family 28 (sodium-coupled nucleoside transporter), member 1    GB acc:   NM_053863    UniGene:   Rn.10517    Gene Symbol:   Slc28a1    Locus Link:   116642    Chromosome:   1    Cytoband:   Chr:1q31    SOURCE    GENECARD       Gene Ontology:     BioCarta Pathways:     KEGG Pathways:  
    Probe set : 1382058_at    Name:   related RAS viral (r-ras) oncogene homolog 2 (predicted)    GB acc:   BI292115    UniGene:   Rn.3271    Gene Symbol:   Rras2_predicted    Locus Link:   365355    Chromosome:   1    Cytoband:   Chr:1q34    SOURCE    GENECARD       Gene Ontology:   cellular component:  intracellular   cellular component:  endoplasmic reticulum   cellular component:  plasma membrane   molecular function:  GTPase activity   molecular function:  GTP binding      BioCarta Pathways:     KEGG Pathways:  
    Probe set : 1371727_at     GB acc:   AA893350              Gene Ontology:     BioCarta Pathways:     KEGG Pathways:  
    Probe set : 1390822_at    Name:   cadherin 16 (predicted)    GB acc:   AI411500    UniGene:   Rn.3478    Gene Symbol:   Cdh16_predicted    Locus Link:   307614    Chromosome:   19    Cytoband:   Chr:19p14    SOURCE    GENECARD       Gene Ontology:   biological process:  tRNA aminoacylation for protein translation   biological process:  cell adhesion   biological process:  homophilic cell adhesion   cellular component:  extracellular space   cellular component:  membrane   cellular component:  integral to membrane      BioCarta Pathways:     KEGG Pathways:  
    Probe set : 1374633_at    Name:   Transcribed locus    GB acc:   BF284700    UniGene:   Rn.23149        SOURCE        Gene Ontology:     BioCarta Pathways:     KEGG Pathways:  
    Probe set : 1387769_a_at    Name:   Inhibitor of DNA binding 3, dominant negative helix-loop-helix protein    GB acc:   AF000942    UniGene:   Rn.2760    Gene Symbol:   Id3    Locus Link:   25585    Chromosome:   5    Cytoband:   Chr:5q36    SOURCE    GENECARD       Gene Ontology:   cellular component:  nucleus      BioCarta Pathways:     KEGG Pathways:  
    Probe set : 1371668_at    Name:   Retinoid X receptor alpha    GB acc:   BI285959    UniGene:   Rn.108206    Gene Symbol:   Rxra    Locus Link:   25271    Chromosome:   3    Cytoband:   Chr:3p12    SOURCE    GENECARD       Gene Ontology:   cellular component:  nucleus      BioCarta Pathways:     KEGG Pathways:  
    Probe set : 1399103_at     GB acc:   BF414041              Gene Ontology:     BioCarta Pathways:     KEGG Pathways:  
    Probe set : 1376242_at    Name:   Transcribed locus    GB acc:   BF402365    UniGene:   Rn.38126        SOURCE        Gene Ontology:     BioCarta Pathways:     KEGG Pathways:  
    Probe set : 1378753_at    Name:   thiopurine methyltransferase    GB acc:   AI638971    UniGene:   Rn.31429    Gene Symbol:   Tpmt    Locus Link:   83497    Chromosome:   2    Cytoband:   Chr:2q12    SOURCE    GENECARD       Gene Ontology:   biological process:  protein complex assembly   biological process:  metabolism      BioCarta Pathways:     KEGG Pathways:  
    Probe set : 1395377_at    Name:   bifunctional apoptosis regulator (predicted)    GB acc:   BE107113    UniGene:   Rn.95242    Gene Symbol:   Bfar_predicted    Locus Link:   304709    Chromosome:   10    Cytoband:   Chr:10q11    SOURCE    GENECARD       Gene Ontology:   biological process:  anti-apoptosis   biological process:  protein ubiquitination   cellular component:  ubiquitin ligase complex   cellular component:  membrane fraction   cellular component:  integral to plasma membrane      BioCarta Pathways:     KEGG Pathways:  
    Probe set : 1368301_at    Name:   adenosine A2a receptor    GB acc:   AF228684    UniGene:   Rn.11180    Gene Symbol:   Adora2a    Locus Link:   25369    Chromosome:   20    Cytoband:   Chr:20p12    SOURCE    GENECARD       Gene Ontology:   biological process:  electron transport   biological process:  cAMP biosynthesis   biological process:  regulation of transcription, DNA-dependent   biological process:  regulation of transcription, DNA-dependent   biological process:  neurotransmitter transport   biological process:  phagocytosis   biological process:  apoptosis   biological process:  inflammatory response   biological process:  cellular defense response   biological process:  G-protein coupled receptor protein signaling pathway   biological process:  G-protein signaling, coupled to cAMP nucleotide second messenger   biological process:  G-protein signaling, adenylate cyclase activating pathway   biological process:  adenylate cyclase activation   biological process:  cell-cell signaling   biological process:  central nervous system development   biological process:  blood coagulation   biological process:  sensory perception   biological process:  circulation   biological process:  signal transduction      BioCarta Pathways:     KEGG Pathways:  
    Probe set : 1384548_at    Name:   ribosomal protein L32    GB acc:   BM388159    UniGene:   Rn.110966    Gene Symbol:   Rpl32    Locus Link:   28298    Chromosome:   6    Cytoband:   Chr:6q33    SOURCE    GENECARD       Gene Ontology:   biological process:  protein biosynthesis   molecular function:  RNA binding   molecular function:  structural constituent of ribosome      BioCarta Pathways:     KEGG Pathways:  
    Probe set : 1387820_at    Name:   kallikrein 7    GB acc:   NM_012593    UniGene:   Rn.144553    Gene Symbol:   Klk7    Locus Link:   24523    Chromosome:   1    Cytoband:   Chr:1q22    SOURCE    GENECARD       Gene Ontology:     BioCarta Pathways:     KEGG Pathways:  
    Probe set : 1375205_at    Name:   p300/CBP-associated factor (predicted)    GB acc:   Z83046     Gene Symbol:   Pcaf_predicted    Locus Link:   301164    Chromosome:   9    Cytoband:   Chr:9q11     GENECARD       Gene Ontology:   cellular component:  histone acetyltransferase complex   cellular component:  kinetochore   cellular component:  nucleus      BioCarta Pathways:     KEGG Pathways:  
    Probe set : 1384005_at    Name:   down-regulator of transcription 1 (predicted)    GB acc:   BG662895    UniGene:   Rn.6666    Gene Symbol:   Dr1_predicted    Locus Link:   289881    Chromosome:   14    Cytoband:   Chr:14p22    SOURCE    GENECARD       Gene Ontology:   cellular component:  nucleus   molecular function:  DNA binding   molecular function:  transcription corepressor activity   molecular function:  transcription factor binding      BioCarta Pathways:     KEGG Pathways:  
    Probe set : 1371583_at    Name:   RNA binding motif (RNP1, RRM) protein 3    GB acc:   AI598399    UniGene:   Rn.18057    Gene Symbol:   Rbm3    Locus Link:   114488    Chromosome:   X    Cytoband:   Chr:Xq13    SOURCE    GENECARD       Gene Ontology:   molecular function:  nucleotide binding   molecular function:  nucleic acid binding   molecular function:  RNA binding      BioCarta Pathways:     KEGG Pathways:  
    Probe set : 1389293_at    Name:   Cleavage and polyadenylation specific factor 2 (predicted)    GB acc:   BM390250    UniGene:   Rn.8038     Locus Link:   299256    Chromosome:   6    Cytoband:   Chr:6q32    SOURCE        Gene Ontology:   biological process:  mRNA processing   cellular component:  nucleus   molecular function:  RNA binding      BioCarta Pathways:     KEGG Pathways:  
    Probe set : 1388679_at    Name:   TBC1 domain family, member 14 (predicted)    GB acc:   AI412244    UniGene:   Rn.6099    Gene Symbol:   Tbc1d14_predicted    Locus Link:   360956    Chromosome:   14    Cytoband:   Chr:14q21    SOURCE    GENECARD       Gene Ontology:     BioCarta Pathways:     KEGG Pathways:  
    Probe set : 1392449_at    Name:   RAD18 homolog (S. cerevisiae) (predicted)    GB acc:   BI282152    UniGene:   Rn.139798    Gene Symbol:   Rad18_predicted    Locus Link:   362412    Chromosome:   4    Cytoband:   Chr:4q42    SOURCE    GENECARD       Gene Ontology:   biological process:  DNA repair   biological process:  response to DNA damage stimulus   biological process:  protein ubiquitination   cellular component:  ubiquitin ligase complex   cellular component:  nucleus      BioCarta Pathways:     KEGG Pathways:  
    Probe set : 1374305_at    Name:   adaptor protein complex AP-1, gamma 2 subunit (predicted)    GB acc:   AI408613    UniGene:   Rn.4261    Gene Symbol:   Ap1g2_predicted    Locus Link:   290215    Chromosome:   15    Cytoband:   Chr:15p13    SOURCE    GENECARD       Gene Ontology:   molecular function:  protein binding      BioCarta Pathways:     KEGG Pathways:  
    Probe set : 1389755_at    Name:   similar to transcription factor RAM2    GB acc:   BM391858     Gene Symbol:   LOC619566    Locus Link:   619566    Chromosome:   6    Cytoband:   Chr:6q33     GENECARD       Gene Ontology:     BioCarta Pathways:     KEGG Pathways:  
    Probe set : 1386149_at    Name:   Transcribed locus, weakly similar to XP_576460.1 PREDICTED: similar to hypothetical protein PB402898.00.0 [Rattus norvegicus]    GB acc:   AI175469    UniGene:   Rn.131718        SOURCE        Gene Ontology:     BioCarta Pathways:     KEGG Pathways:  
    Probe set : 1367898_at    Name:   BCL2/adenovirus E1B 19 kDa-interacting protein 3-like    GB acc:   AF441118    UniGene:   Rn.827    Gene Symbol:   Bnip3l    Locus Link:   140923    Chromosome:   15    Cytoband:   Chr:15p12    SOURCE    GENECARD       Gene Ontology:   biological process:  positive regulation of apoptosis   cellular component:  mitochondrial membrane   cellular component:  integral to membrane      BioCarta Pathways:     KEGG Pathways:  
    Probe set : 1373935_at    Name:   polymerase (DNA directed), delta 2, regulatory subunit (predicted)    GB acc:   AI102751    UniGene:   Rn.22989    Gene Symbol:   Pold2_predicted    Locus Link:   289758    Chromosome:   14    Cytoband:   Chr:14q21    SOURCE    GENECARD       Gene Ontology:   biological process:  DNA replication   cellular component:  nucleus   molecular function:  DNA-directed DNA polymerase activity   molecular function:  transferase activity   molecular function:  DNA binding      BioCarta Pathways:     KEGG Pathways:  
    Probe set : 1371524_at    Name:   gene trap locus 3 (predicted)    GB acc:   AI009608    UniGene:   Rn.6708    Gene Symbol:   Gtl3_predicted    Locus Link:   307642    Chromosome:   19    Cytoband:   Chr:19p13    SOURCE    GENECARD       Gene Ontology:     BioCarta Pathways:     KEGG Pathways:  
    Probe set : 1379076_at    Name:   Transcribed locus    GB acc:   BE328961    UniGene:   Rn.12349        SOURCE        Gene Ontology:     BioCarta Pathways:     KEGG Pathways:  
    Probe set : 1397317_at    Name:   Transcribed locus    GB acc:   BI296984    UniGene:   Rn.146350        SOURCE        Gene Ontology:     BioCarta Pathways:     KEGG Pathways:  
    Probe set : 1391607_at    Name:   Similar to Sestrin 2 (Hi95)    GB acc:   BE109653    UniGene:   Rn.126021     Locus Link:   502988      SOURCE        Gene Ontology:     BioCarta Pathways:     KEGG Pathways:  
    Probe set : 1388506_at    Name:   desmoplakin (predicted)    GB acc:   AW144509    UniGene:   Rn.54711    Gene Symbol:   Dsp_predicted    Locus Link:   306871    Chromosome:   17    Cytoband:   Chr:17p12    SOURCE    GENECARD       Gene Ontology:   biological process:  epidermis development   cellular component:  cytoskeleton   cellular component:  intermediate filament   cellular component:  cell-cell adherens junction   molecular function:  structural constituent of cytoskeleton      BioCarta Pathways:     KEGG Pathways:  
    Probe set : 1373041_at    Name:   NADH dehydrogenase (ubiquinone) 1 beta subcomplex 3 (predicted)    GB acc:   AI555535    UniGene:   Rn.107891    Gene Symbol:   Ndufb3_predicted    Locus Link:   301427    Chromosome:   9    Cytoband:   Chr:9q31    SOURCE    GENECARD       Gene Ontology:   biological process:  mitochondrial electron transport, NADH to ubiquinone   cellular component:  mitochondrion   molecular function:  NADH dehydrogenase activity   molecular function:  NADH dehydrogenase (ubiquinone) activity   molecular function:  oxidoreductase activity      BioCarta Pathways:     KEGG Pathways:  
    Probe set : 1387868_at    Name:   lipopolysaccharide binding protein    GB acc:   BF289368    UniGene:   Rn.48863    Gene Symbol:   Lbp    Locus Link:   29469    Chromosome:   3    Cytoband:   Chr:3q42    SOURCE    GENECARD       Gene Ontology:   cellular component:  extracellular space   cellular component:  integral to membrane   molecular function:  lipopolysaccharide binding   molecular function:  lipid binding      BioCarta Pathways:     KEGG Pathways:  
    Probe set : 1371888_at    Name:   mitochondrial ribosomal protein L24    GB acc:   AA892843    UniGene:   Rn.100135    Gene Symbol:   RGD:1359289    Locus Link:   295224    Chromosome:   2    Cytoband:   Chr:2q34    SOURCE    GENECARD       Gene Ontology:   biological process:  protein biosynthesis   cellular component:  intracellular   cellular component:  ribosome   molecular function:  structural constituent of ribosome      BioCarta Pathways:     KEGG Pathways:  
    Probe set : 1368334_at    Name:   growth factor receptor bound protein 7    GB acc:   NM_053403    UniGene:   Rn.28109    Gene Symbol:   Grb7    Locus Link:   84427    Chromosome:   10    Cytoband:   Chr:10q31    SOURCE    GENECARD       Gene Ontology:   biological process:  signal transduction   biological process:  epidermal growth factor receptor signaling pathway   biological process:  intracellular signaling cascade   cellular component:  cytosol   molecular function:  SH3/SH2 adaptor activity   molecular function:  receptor activity      BioCarta Pathways:     KEGG Pathways:  
    Probe set : 1373461_at    Name:   dermal papilla derived protein 6    GB acc:   AI412649    UniGene:   Rn.9041    Gene Symbol:   RGD:1303303    Locus Link:   287446    Chromosome:   10    Cytoband:   Chr:10q24    SOURCE    GENECARD       Gene Ontology:     BioCarta Pathways:     KEGG Pathways:  
    Probe set : 1370398_at    Name:   spermatogenesis associated 7    GB acc:   AF094609    UniGene:   Rn.55448    Gene Symbol:   Spata7    Locus Link:   192225    Chromosome:   6    Cytoband:   Chr:6q32    SOURCE    GENECARD       Gene Ontology:   biological process:  spermatogenesis   molecular function:  DNA binding      BioCarta Pathways:     KEGG Pathways:  
    Probe set : 1382624_at     GB acc:   AA858768              Gene Ontology:     BioCarta Pathways:     KEGG Pathways:  
    Probe set : 1386820_at    Name:   Transcribed locus    GB acc:   AA851099    UniGene:   Rn.146087        SOURCE        Gene Ontology:     BioCarta Pathways:     KEGG Pathways:  
    Probe set : 1386906_a_at    Name:   septin 9    GB acc:   AF180526    UniGene:   Rn.91127    Gene Symbol:   Sept9    Locus Link:   83788    Chromosome:   10    Cytoband:   Chr:10q32.3    SOURCE    GENECARD       Gene Ontology:     BioCarta Pathways:     KEGG Pathways:  
    Probe set : 1392786_at     GB acc:   BG668812              Gene Ontology:     BioCarta Pathways:     KEGG Pathways:  
    Probe set : 1378569_at    Name:   Transcribed locus    GB acc:   BI287991    UniGene:   Rn.19368        SOURCE        Gene Ontology:     BioCarta Pathways:     KEGG Pathways:  
    Probe set : 1385829_at    Name:   similar to mKIAA0657 protein    GB acc:   BI294746    UniGene:   Rn.43633    Gene Symbol:   LOC363257    Locus Link:   363257    Chromosome:   9    Cytoband:   Chr:9q33    SOURCE    GENECARD       Gene Ontology:     BioCarta Pathways:     KEGG Pathways:  
    Probe set : 1371340_at    Name:   ribosomal protein, large P2 /// similar to 60S acidic ribosomal protein P2    GB acc:   AW919054    UniGene:   Rn.976    Gene Symbol:   Rplp2 /// LOC498363    Locus Link:   140662 /// 498363    Chromosome:   1    Cytoband:   Chr:1q41 /// Chr:14p11    SOURCE    GENECARD       Gene Ontology:   biological process:  protein biosynthesis   biological process:  translational elongation   molecular function:  RNA binding   molecular function:  structural constituent of ribosome   molecular function:  structural constituent of ribosome      BioCarta Pathways:     KEGG Pathways:  
    Probe set : 1395192_at    Name:   origin recognition complex, subunit 3-like (S. cerevisiae) (predicted)    GB acc:   BF392026    UniGene:   Rn.139979    Gene Symbol:   Orc3l_predicted    Locus Link:   313138    Chromosome:   5    Cytoband:   Chr:5q21    SOURCE    GENECARD       Gene Ontology:   biological process:  DNA replication   cellular component:  nucleus   cellular component:  nuclear origin of replication recognition complex   molecular function:  DNA binding   molecular function:  DNA replication origin binding      BioCarta Pathways:     KEGG Pathways:  
    Probe set : 1377197_at    Name:   Transcribed locus    GB acc:   BE108892    UniGene:   Rn.146957        SOURCE        Gene Ontology:     BioCarta Pathways:     KEGG Pathways:  
    Probe set : 1368600_at    Name:   solute carrier family 26 (sulfate transporter), member 1    GB acc:   NM_022287    UniGene:   Rn.10016    Gene Symbol:   Slc26a1    Locus Link:   64076    Chromosome:   14    Cytoband:   Chr:14p22    SOURCE    GENECARD       Gene Ontology:   cellular component:  membrane   cellular component:  integral to membrane      BioCarta Pathways:     KEGG Pathways:  
    Probe set : 1392818_at    Name:   growth arrest specific 5    GB acc:   BM384139    UniGene:   Rn.145965    Gene Symbol:   Gas5    Locus Link:   81714    Chromosome:   13    Cytoband:   Chr:13q22    SOURCE    GENECARD       Gene Ontology:   biological process:  rRNA processing      BioCarta Pathways:     KEGG Pathways:  
    Probe set : 1371461_at    Name:   similar to RIKEN cDNA 2410166I05 (predicted)    GB acc:   BF414239    UniGene:   Rn.3864    Gene Symbol:   RGD1305453_predicted    Locus Link:   298549    Chromosome:   5    Cytoband:   Chr:5q36    SOURCE    GENECARD       Gene Ontology:   biological process:  transport   cellular component:  extracellular space   molecular function:  carrier activity      BioCarta Pathways:     KEGG Pathways:  
    Probe set : 1397530_at    Name:   Treacher Collins Franceschetti syndrome 1, homolog (predicted)    GB acc:   BF550362    UniGene:   Rn.7232    Gene Symbol:   Tcof1_predicted    Locus Link:   291571    Chromosome:   18    Cytoband:   Chr:18q12.1    SOURCE    GENECARD       Gene Ontology:   biological process:  skeletal development   biological process:  transport   biological process:  transcription of nuclear rRNA large RNA polymerase I transcript   cellular component:  nucleus   cellular component:  nucleolus   molecular function:  transporter activity      BioCarta Pathways:     KEGG Pathways:  
    Probe set : 1371519_at    Name:   electron-transferring-flavoprotein dehydrogenase    GB acc:   AA851258    UniGene:   Rn.37277    Gene Symbol:   Etfdh    Locus Link:   295143    Chromosome:   2    Cytoband:   Chr:2q32    SOURCE    GENECARD       Gene Ontology:   biological process:  electron transport   biological process:  electron transport   biological process:  transport      BioCarta Pathways:     KEGG Pathways:  
    Probe set : 1390458_at     GB acc:   BG666849              Gene Ontology:     BioCarta Pathways:     KEGG Pathways:  
    Probe set : 1372469_at    Name:   Transcribed locus    GB acc:   AI178901    UniGene:   Rn.147526        SOURCE        Gene Ontology:     BioCarta Pathways:     KEGG Pathways:  
    Probe set : 1384958_at    Name:   Transcribed locus    GB acc:   BI279112    UniGene:   Rn.77908        SOURCE        Gene Ontology:     BioCarta Pathways:     KEGG Pathways:  
    Probe set : 1375781_at    Name:   Transcribed locus    GB acc:   BF419844    UniGene:   Rn.63660        SOURCE        Gene Ontology:     BioCarta Pathways:     KEGG Pathways:  
    Probe set : 1380828_at    Name:   gamma-aminobutyric acid A receptor, alpha 1    GB acc:   AI145413    UniGene:   Rn.28463    Gene Symbol:   Gabra1    Locus Link:   29705    Chromosome:   10    Cytoband:   Chr:10q21    SOURCE    GENECARD       Gene Ontology:     BioCarta Pathways:     KEGG Pathways:  
    Probe set : 1379498_at    Name:   Transcribed locus, weakly similar to NP_060380.2 hypothetical protein FLJ20628 [Homo sapiens]    GB acc:   AW534136    UniGene:   Rn.48269        SOURCE        Gene Ontology:     BioCarta Pathways:     KEGG Pathways:  
    Probe set : 1369006_at    Name:   hexokinase 2    GB acc:   NM_012735    UniGene:   Rn.91375    Gene Symbol:   Hk2    Locus Link:   25059    Chromosome:   4    Cytoband:   Chr:4q34    SOURCE    GENECARD       Gene Ontology:   biological process:  regulation of progression through cell cycle   biological process:  glucose catabolism   biological process:  glycolysis   cellular component:  mitochondrial outer membrane   cellular component:  mitochondrial outer membrane   cellular component:  membrane      BioCarta Pathways:     KEGG Pathways:  
    Probe set : 1379608_at    Name:   Transcribed locus    GB acc:   AA899113    UniGene:   Rn.151465        SOURCE        Gene Ontology:     BioCarta Pathways:     KEGG Pathways:  
    Probe set : 1389228_at    Name:   Similar to RIKEN cDNA 2010309E21 (predicted)    GB acc:   AI412292    UniGene:   Rn.2397     Locus Link:   297415    Chromosome:   4    Cytoband:   Chr:4q34    SOURCE        Gene Ontology:     BioCarta Pathways:     KEGG Pathways:  
    Probe set : 1370879_at    Name:   dihydrolipoamide S-succinyltransferase (E2 component of 2-oxo-glutarate complex)    GB acc:   D90401    UniGene:   Rn.99702    Gene Symbol:   RGD:1359615    Locus Link:   299201    Chromosome:   6    Cytoband:   Chr:6q31    SOURCE    GENECARD       Gene Ontology:   biological process:  tricarboxylic acid cycle   biological process:  metabolism   cellular component:  mitochondrion   cellular component:  oxoglutarate dehydrogenase complex      BioCarta Pathways:     KEGG Pathways:  
    Probe set : 1385800_at     GB acc:   AW531915              Gene Ontology:     BioCarta Pathways:     KEGG Pathways:  
    Probe set : 1388560_at    Name:   similar to Ac2-269 (predicted)    GB acc:   AI412580    UniGene:   Rn.14128    Gene Symbol:   RGD1310479_predicted    Locus Link:   310769    Chromosome:   2    Cytoband:   Chr:2q34    SOURCE    GENECARD       Gene Ontology:     BioCarta Pathways:     KEGG Pathways:  
    Probe set : 1398883_at    Name:   heterogeneous nuclear ribonucleoprotein A2/B1 (predicted)    GB acc:   BI296284    UniGene:   Rn.129934    Gene Symbol:   Hnrpa2b1_predicted    Locus Link:   362361    Chromosome:   4    Cytoband:   Chr:4q24    SOURCE    GENECARD       Gene Ontology:   biological process:  nuclear mRNA splicing, via spliceosome   biological process:  RNA processing   biological process:  mRNA processing   cellular component:  nucleus   cellular component:  ribonucleoprotein complex   cellular component:  heterogeneous nuclear ribonucleoprotein complex   molecular function:  nucleic acid binding   molecular function:  RNA binding      BioCarta Pathways:     KEGG Pathways:  
    Probe set : 1375017_at    Name:   Transcribed locus    GB acc:   BI296120    UniGene:   Rn.73828        SOURCE        Gene Ontology:     BioCarta Pathways:     KEGG Pathways:  
    Probe set : 1389286_at    Name:   similar to 2900070E19Rik protein (predicted)    GB acc:   AI232948    UniGene:   Rn.104008    Gene Symbol:   RGD1308383_predicted    Locus Link:   362776    Chromosome:   6    Cytoband:   Chr:6q32    SOURCE    GENECARD       Gene Ontology:     BioCarta Pathways:     KEGG Pathways:  
    Probe set : 1388300_at    Name:   microsomal glutathione S-transferase 3 (predicted)    GB acc:   AA892234    UniGene:   Rn.1916    Gene Symbol:   Mgst3_predicted    Locus Link:   289197    Chromosome:   13    Cytoband:   Chr:13q24    SOURCE    GENECARD       Gene Ontology:   biological process:  lipid metabolism   biological process:  signal transduction   biological process:  antimicrobial humoral response (sensu Vertebrata)   cellular component:  membrane fraction   cellular component:  microsome   cellular component:  integral to membrane   molecular function:  glutathione transferase activity   molecular function:  peroxidase activity   molecular function:  transferase activity      BioCarta Pathways:     KEGG Pathways:  
    Probe set : 1372043_at    Name:   similar to ribosomal protein P0-like protein; 60S acidic ribosomal protein PO; ribosomal protein, large, P0-like (predicted)    GB acc:   BI282363    UniGene:   Rn.102515    Gene Symbol:   RGD1311709_predicted    Locus Link:   298586    Chromosome:   5    Cytoband:   Chr:5q36    SOURCE    GENECARD       Gene Ontology:     BioCarta Pathways:     KEGG Pathways:  
    Probe set : 1368683_at    Name:   oxidized low density lipoprotein (lectin-like) receptor 1    GB acc:   NM_133306    UniGene:   Rn.87449    Gene Symbol:   Oldlr1    Locus Link:   140914    Chromosome:   4    Cytoband:   Chr:4q42    SOURCE    GENECARD       Gene Ontology:     BioCarta Pathways:     KEGG Pathways:  
    Probe set : 1367612_at    Name:   microsomal glutathione S-transferase 1    GB acc:   NM_134349    UniGene:   Rn.2580    Gene Symbol:   Mgst1    Locus Link:   171341    Chromosome:   4    Cytoband:   Chr:4q44    SOURCE    GENECARD       Gene Ontology:   biological process:  glutathione metabolism   cellular component:  mitochondrion   cellular component:  microsome   cellular component:  membrane   cellular component:  endoplasmic reticulum   molecular function:  glutathione transferase activity   molecular function:  transferase activity      BioCarta Pathways:     KEGG Pathways:  
    Probe set : 1380120_at    Name:   microtubule-associated protein 7 (predicted)    GB acc:   BG374192    UniGene:   Rn.23807    Gene Symbol:   Mtap7_predicted    Locus Link:   293016    Chromosome:   1    Cytoband:   Chr:1p12    SOURCE    GENECARD       Gene Ontology:   cellular component:  microtubule associated complex   molecular function:  structural molecule activity   molecular function:  cytoskeletal regulatory protein binding      BioCarta Pathways:     KEGG Pathways:  
    Probe set : 1389130_at    Name:   E74-like factor 2 (predicted)    GB acc:   AW525131    UniGene:   Rn.107459     Locus Link:   361944    Chromosome:   2    Cytoband:   Chr:2q26    SOURCE        Gene Ontology:   biological process:  regulation of transcription, DNA-dependent   cellular component:  nucleus   molecular function:  DNA binding   molecular function:  transcription factor activity   molecular function:  transcriptional activator activity      BioCarta Pathways:     KEGG Pathways:  
    Probe set : 1385559_at    Name:   Transcribed locus    GB acc:   BF562977    UniGene:   Rn.148168        SOURCE        Gene Ontology:     BioCarta Pathways:     KEGG Pathways:  
    Probe set : 1394397_at    Name:   Ac1158    GB acc:   BF522734    UniGene:   Rn.24353    Gene Symbol:   LOC302898    Locus Link:   302898    Chromosome:   10    Cytoband:   Chr:10q11    SOURCE    GENECARD       Gene Ontology:   biological process:  protein biosynthesis   cellular component:  intracellular   cellular component:  ribosome   molecular function:  structural constituent of ribosome      BioCarta Pathways:     KEGG Pathways:  
    Probe set : 1367830_a_at    Name:   tumor protein p53    GB acc:   NM_030989    UniGene:   Rn.54443    Gene Symbol:   Tp53    Locus Link:   24842    Chromosome:   10    Cytoband:   Chr:10q24    SOURCE    GENECARD       Gene Ontology:   biological process:  protein import into nucleus, translocation   biological process:  cell cycle checkpoint   biological process:  base-excision repair   biological process:  nucleotide-excision repair   biological process:  DNA recombination   biological process:  transcription   biological process:  regulation of transcription, DNA-dependent   biological process:  regulation of transcription from RNA polymerase II promoter   biological process:  regulation of transcription from RNA polymerase II promoter   biological process:  apoptosis   biological process:  response to DNA damage stimulus   biological process:  cell cycle   biological process:  cell cycle arrest   biological process:  cell aging   biological process:  negative regulation of DNA replication   biological process:  cell proliferation   biological process:  induction of apoptosis by hormones   biological process:  DNA damage response, signal transduction resulting in induction of apoptosis   biological process:  caspase activation via cytochrome c   biological process:  response to UV   biological process:  response to X-ray   biological process:  cell differentiation   biological process:  negative regulation of cell growth   biological process:  negative regulation of cell growth   biological process:  DNA damage response, signal transduction by p53 class mediator   biological process:  regulation of cell proliferation   biological process:  DNA damage response, signal transduction by p53 class mediator resulting in induction of apoptosis   biological process:  negative regulation of apoptosis   biological process:  negative regulation of progression through cell cycle   biological process:  regulation of mitochondrial membrane permeability   biological process:  negative regulation of fibroblast proliferation   biological process:  negative regulation of helicase activity   biological process:  protein tetramerization   biological process:  base-excision repair   biological process:  nucleotide-excision repair   biological process:  apoptosis   biological process:  cell cycle arrest   biological process:  development   biological process:  cell aging   biological process:  cell proliferation   biological process:  caspase activation via cytochrome c   biological process:  cell differentiation   biological process:  negative regulation of cell growth   molecular function:  DNA strand annealing activity   molecular function:  DNA binding   molecular function:  transcription factor activity   molecular function:  RNA polymerase II transcription factor activity   molecular function:  RNA polymerase II transcription factor activity   molecular function:  nuclease activity   molecular function:  copper ion binding   molecular function:  protein binding   molecular function:  ATP binding   molecular function:  zinc ion binding   molecular function:  protein binding   molecular function:  DNA strand annealing activity   molecular function:  DNA binding   molecular function:  copper ion binding   molecular function:  protein binding   molecular function:  ATP binding   molecular function:  metal ion binding      BioCarta Pathways:     KEGG Pathways:  
    Probe set : 1389906_at    Name:   Farnesyl diphosphate farnesyl transferase 1    GB acc:   AW530769    UniGene:   Rn.21071    Gene Symbol:   Fdft1    Locus Link:   29580    Chromosome:   15    Cytoband:   Chr:15p12    SOURCE    GENECARD       Gene Ontology:   cellular component:  endoplasmic reticulum   cellular component:  integral to membrane      BioCarta Pathways:     KEGG Pathways:  
    Probe set : 1387183_at    Name:   carnitine O-octanoyltransferase    GB acc:   J02844    UniGene:   Rn.4896    Gene Symbol:   Crot    Locus Link:   83842    Chromosome:   4    Cytoband:   Chr:4q12    SOURCE    GENECARD       Gene Ontology:   cellular component:  peroxisome      BioCarta Pathways:     KEGG Pathways:  
    Probe set : 1370336_at    Name:   pregnancy-induced growth inhibitor    GB acc:   AY081218    UniGene:   Rn.15599    Gene Symbol:   Okl38    Locus Link:   171493    Chromosome:   19    Cytoband:   Chr:19q12    SOURCE    GENECARD       Gene Ontology:   biological process:  negative regulation of cell growth      BioCarta Pathways:     KEGG Pathways:  
    Probe set : 1373080_at    Name:   poly (A) polymerase alpha (predicted)    GB acc:   BI284422    UniGene:   Rn.3455    Gene Symbol:   Papola_predicted    Locus Link:   314417    Chromosome:   6    Cytoband:   Chr:6q32    SOURCE    GENECARD       Gene Ontology:   biological process:  transcription   biological process:  mRNA polyadenylylation   biological process:  mRNA processing   cellular component:  nucleus   cellular component:  cytoplasm      BioCarta Pathways:     KEGG Pathways:  
    Probe set : 1385606_at    Name:   brain protein 16    GB acc:   BF559626    UniGene:   Rn.17102    Gene Symbol:   RGD:1359226    Locus Link:   315094    Chromosome:   7    Cytoband:   Chr:7q34    SOURCE    GENECARD       Gene Ontology:   molecular function:  binding      BioCarta Pathways:     KEGG Pathways:  
    Probe set : 1379406_at    Name:   similar to chromosome 14 open reading frame 21 (predicted)    GB acc:   AA925559    UniGene:   Rn.91559    Gene Symbol:   RGD1308396_predicted    Locus Link:   290235    Chromosome:   15    Cytoband:   Chr:15p13    SOURCE    GENECARD       Gene Ontology:     BioCarta Pathways:     KEGG Pathways:  
    Probe set : 1381017_at    Name:   Transcribed locus    GB acc:   AA925043    UniGene:   Rn.147191        SOURCE        Gene Ontology:     BioCarta Pathways:     KEGG Pathways:  
    Probe set : 1371472_at    Name:   Transcribed locus    GB acc:   BG380581    UniGene:   Rn.19113        SOURCE        Gene Ontology:     BioCarta Pathways:     KEGG Pathways:  
    Probe set : 1394511_at    Name:   similar to KIAA1915 protein (predicted)    GB acc:   BF419160    UniGene:   Rn.63856    Gene Symbol:   RGD1311787_predicted    Locus Link:   298247    Chromosome:   5    Cytoband:   Chr:5q33    SOURCE    GENECARD       Gene Ontology:     BioCarta Pathways:     KEGG Pathways:  
    Probe set : 1379791_at    Name:   CD3 antigen, epsilon polypeptide (predicted)    GB acc:   AW915948    UniGene:   Rn.96221     Locus Link:   315609    Chromosome:   8    Cytoband:   Chr:8q22    SOURCE        Gene Ontology:     BioCarta Pathways:     KEGG Pathways:  
    Probe set : 1376043_at    Name:   similar to PHD zinc finger containing protein JUNE1    GB acc:   BI292073    UniGene:   Rn.20080    Gene Symbol:   RGD:1302969    Locus Link:   360550    Chromosome:   10    Cytoband:   Chr:10q24    SOURCE    GENECARD       Gene Ontology:   biological process:  regulation of transcription, DNA-dependent   molecular function:  protein binding   molecular function:  zinc ion binding      BioCarta Pathways:     KEGG Pathways:  
    Probe set : 1368205_at    Name:   complement factor I    GB acc:   NM_024157    UniGene:   Rn.7424    Gene Symbol:   Cfi    Locus Link:   79126    Chromosome:   2    Cytoband:   Chr:2q43    SOURCE    GENECARD       Gene Ontology:   cellular component:  extracellular region   cellular component:  extracellular space   cellular component:  membrane      BioCarta Pathways:     KEGG Pathways:  
    Probe set : 1395986_at    Name:   slit homolog 2 (Drosophila)    GB acc:   BF391439    UniGene:   Rn.46211    Gene Symbol:   Slit2    Locus Link:   360272    Chromosome:   14    Cytoband:   Chr:14q11    SOURCE    GENECARD       Gene Ontology:   biological process:  metanephros development   biological process:  ureteric bud development   biological process:  chemotaxis   biological process:  cell adhesion   biological process:  G-protein coupled receptor protein signaling pathway   biological process:  nervous system development   biological process:  axon guidance   biological process:  mesoderm migration   biological process:  sensory perception of smell   biological process:  neuron recognition   biological process:  motor axon guidance   biological process:  glial cell migration   biological process:  positive regulation of axonogenesis   biological process:  induction of negative chemotaxis   cellular component:  extracellular matrix (sensu Metazoa)   cellular component:  extracellular space   cellular component:  integral to membrane      BioCarta Pathways:     KEGG Pathways:  
    Probe set : 1373016_at    Name:   dihydrolipoamide dehydrogenase    GB acc:   AI410493    UniGene:   Rn.86962    Gene Symbol:   Dld    Locus Link:   298942    Chromosome:   6    Cytoband:   Chr:6q16    SOURCE    GENECARD       Gene Ontology:   biological process:  electron transport   cellular component:  cytoplasm      BioCarta Pathways:     KEGG Pathways:  
    Probe set : 1377598_at    Name:   similar to 2310002F18Rik protein    GB acc:   AJ225648    UniGene:   Rn.7463    Gene Symbol:   MGC124824    Locus Link:   498332    Chromosome:   14    Cytoband:   Chr:14p22    SOURCE    GENECARD       Gene Ontology:   biological process:  biosynthesis   cellular component:  integral to membrane   molecular function:  prenyltransferase activity      BioCarta Pathways:     KEGG Pathways:  
    Probe set : 1374513_at     GB acc:   BM389104              Gene Ontology:     BioCarta Pathways:     KEGG Pathways:  
    Probe set : 1368025_at    Name:   DNA-damage-inducible transcript 4    GB acc:   NM_080906    UniGene:   Rn.9775    Gene Symbol:   Ddit4    Locus Link:   140942    Chromosome:   20    Cytoband:   Chr:20q11    SOURCE    GENECARD       Gene Ontology:     BioCarta Pathways:     KEGG Pathways:  
    Probe set : 1374262_at    Name:   Transcribed locus    GB acc:   BF396371    UniGene:   Rn.32615        SOURCE        Gene Ontology:     BioCarta Pathways:     KEGG Pathways:  
    Probe set : 1371393_at     GB acc:   AA851222              Gene Ontology:     BioCarta Pathways:     KEGG Pathways:  
    Probe set : 1367996_a_at    Name:   latrophilin 1    GB acc:   AF111099    UniGene:   Rn.10776    Gene Symbol:   Lphn1    Locus Link:   65096    Chromosome:   19    Cytoband:   Chr:19q11    SOURCE    GENECARD       Gene Ontology:   cellular component:  integral to membrane   cellular component:  membrane      BioCarta Pathways:     KEGG Pathways:  
    Probe set : 1383432_at    Name:   LOC362136 (predicted)    GB acc:   AI716676    UniGene:   Rn.23556    Gene Symbol:   RGD1309610_predicted    Locus Link:   362136    Chromosome:   3    Cytoband:   Chr:3q21    SOURCE    GENECARD       Gene Ontology:     BioCarta Pathways:     KEGG Pathways:  
    Probe set : 1381264_at    Name:   Transcribed locus    GB acc:   BE099664    UniGene:   Rn.45379        SOURCE        Gene Ontology:     BioCarta Pathways:     KEGG Pathways:  
    Probe set : 1389778_a_at    Name:   transcription elongation factor B (SIII), polypeptide 3    GB acc:   AI233115    UniGene:   Rn.37427    Gene Symbol:   Tceb3    Locus Link:   25562    Chromosome:   5    Cytoband:   Chr:5q36    SOURCE    GENECARD       Gene Ontology:     BioCarta Pathways:     KEGG Pathways:  
    Probe set : 1389565_at    Name:   Transcribed locus    GB acc:   BE113491    UniGene:   Rn.22478        SOURCE        Gene Ontology:     BioCarta Pathways:     KEGG Pathways:  
    Probe set : 1367988_at    Name:   cytochrome P450, family 2, subfamily c, polypeptide 23    GB acc:   U04733    UniGene:   Rn.2184    Gene Symbol:   Cyp2c23    Locus Link:   83790    Chromosome:   1    Cytoband:   Chr:1q54    SOURCE    GENECARD       Gene Ontology:   biological process:  electron transport   biological process:  arachidonic acid metabolism   biological process:  arachidonic acid metabolism   cellular component:  endoplasmic reticulum   cellular component:  microsome   cellular component:  microsome   cellular component:  membrane      BioCarta Pathways:     KEGG Pathways:  
    Probe set : 1372391_at    Name:   similar to RIKEN cDNA 1500016H10 (predicted)    GB acc:   AI598937    UniGene:   Rn.102202    Gene Symbol:   RGD1308469_predicted    Locus Link:   317275    Chromosome:   9    Cytoband:   Chr:9q12    SOURCE    GENECARD       Gene Ontology:     BioCarta Pathways:     KEGG Pathways:  
    Probe set : 1373427_at    Name:   Ras-related GTP binding D (predicted)    GB acc:   BI288816    UniGene:   Rn.23439    Gene Symbol:   Rragd_predicted    Locus Link:   297960    Chromosome:   5    Cytoband:   Chr:5q21    SOURCE    GENECARD       Gene Ontology:   biological process:  transport   cellular component:  nucleus   cellular component:  cytoplasm      BioCarta Pathways:     KEGG Pathways:  
    Probe set : 1370236_at    Name:   palmitoyl-protein thioesterase    GB acc:   AI234353    UniGene:   Rn.1574    Gene Symbol:   Ppt    Locus Link:   29411    Chromosome:   5    Cytoband:   Chr:5q36    SOURCE    GENECARD       Gene Ontology:   cellular component:  extracellular space   cellular component:  lysosome   molecular function:  catalytic activity   molecular function:  palmitoyl-(protein) hydrolase activity   molecular function:  hydrolase activity      BioCarta Pathways:     KEGG Pathways:  
    Probe set : 1370979_at    Name:   DEAD/H (Asp-Glu-Ala-Asp/His) box polypeptide 20, 103kD    GB acc:   BF282783    UniGene:   Rn.137424    Gene Symbol:   RGD:621744    Locus Link:   84473    Chromosome:   2    Cytoband:   Chr:2q34    SOURCE    GENECARD       Gene Ontology:   biological process:  negative regulation of transcription   molecular function:  transcriptional repressor activity   molecular function:  protein domain specific binding      BioCarta Pathways:     KEGG Pathways:  
    Probe set : 1384863_at    Name:   copine VII (predicted)    GB acc:   AW534032    UniGene:   Rn.145177    Gene Symbol:   Cpne7_predicted    Locus Link:   361433    Chromosome:   19    Cytoband:   Chr:19q12    SOURCE    GENECARD       Gene Ontology:   biological process:  lipid metabolism   molecular function:  transporter activity      BioCarta Pathways:     KEGG Pathways:  
    Probe set : 1369700_at    Name:   chloride channel 7    GB acc:   NM_031568    UniGene:   Rn.10338    Gene Symbol:   Clcn7    Locus Link:   29233    Chromosome:   10    Cytoband:   Chr:10q12    SOURCE    GENECARD       Gene Ontology:   biological process:  transport   biological process:  ion transport   biological process:  chloride transport   molecular function:  ion channel activity   molecular function:  voltage-gated ion channel activity   molecular function:  voltage-gated chloride channel activity      BioCarta Pathways:     KEGG Pathways:  
    Probe set : 1367802_at    Name:   serum/glucocorticoid regulated kinase    GB acc:   NM_019232    UniGene:   Rn.4636    Gene Symbol:   Sgk    Locus Link:   29517    Chromosome:   1    Cytoband:   Chr:1p12    SOURCE    GENECARD       Gene Ontology:   cellular component:  nucleus      BioCarta Pathways:     KEGG Pathways:  
    Probe set : 1373769_at    Name:   phosphoglucomutase 1 (predicted)    GB acc:   AI176913    UniGene:   Rn.131847    Gene Symbol:   Pgm1_predicted    Locus Link:   289632    Chromosome:   14    Cytoband:   Chr:14p11    SOURCE    GENECARD       Gene Ontology:   biological process:  carbohydrate metabolism   biological process:  transport      BioCarta Pathways:     KEGG Pathways:  
    Probe set : 1388116_at    Name:   collagen, type 1, alpha 1    GB acc:   BI285575    UniGene:   Rn.2953    Gene Symbol:   Col1a1    Locus Link:   29393    Chromosome:   10    Cytoband:   Chr:10q31    SOURCE    GENECARD       Gene Ontology:     BioCarta Pathways:     KEGG Pathways:  
    Probe set : 1388645_at     GB acc:   AI175494              Gene Ontology:     BioCarta Pathways:     KEGG Pathways:  
    Probe set : 1387216_at    Name:   lactate dehydrogenase 3, C chain    GB acc:   NM_017266    UniGene:   Rn.9984    Gene Symbol:   Ldhc    Locus Link:   29634    Chromosome:   1    Cytoband:   Chr:1q22    SOURCE    GENECARD       Gene Ontology:   biological process:  glycolysis   biological process:  tricarboxylic acid cycle intermediate metabolism   biological process:  anaerobic glycolysis   cellular component:  cytoplasm      BioCarta Pathways:     KEGG Pathways:  
    Probe set : 1395557_at    Name:   Core promoter element binding protein    GB acc:   BF565718    UniGene:   Rn.7947    Gene Symbol:   Copeb    Locus Link:   58954    Chromosome:   17    Cytoband:   Chr:17q12.2    SOURCE    GENECARD       Gene Ontology:   cellular component:  nucleus   cellular component:  nucleus   cellular component:  cytoplasm      BioCarta Pathways:     KEGG Pathways:  
    Probe set : 1387772_at    Name:   calmodulin 1    GB acc:   NM_031969    UniGene:   Rn.4166    Gene Symbol:   Calm1    Locus Link:   24242    Chromosome:   6    Cytoband:   Chr:6q31-q32    SOURCE    GENECARD       Gene Ontology:     BioCarta Pathways:     KEGG Pathways:  
    Probe set : 1376684_at    Name:   discs, large homolog 7 (Drosophila) (predicted)    GB acc:   AA874827    UniGene:   Rn.3137    Gene Symbol:   Dlg7_predicted    Locus Link:   289997    Chromosome:   15    Cytoband:   Chr:15p14    SOURCE    GENECARD       Gene Ontology:   biological process:  cell-cell signaling   molecular function:  kinase activity      BioCarta Pathways:     KEGG Pathways:  
    Probe set : 1373497_at    Name:   Homeodomain interacting protein kinase 2 (predicted)    GB acc:   BE109208    UniGene:   Rn.8042     Locus Link:   362342    Chromosome:   4    Cytoband:   Chr:4q22    SOURCE        Gene Ontology:   cellular component:  nucleus   cellular component:  cytoplasm   cellular component:  nuclear body   cellular component:  PML body      BioCarta Pathways:     KEGG Pathways:  
    Probe set : 1370213_at    Name:   nuclease sensitive element binding protein 1    GB acc:   BI282111    UniGene:   Rn.110976    Gene Symbol:   Nsep1    Locus Link:   29206    Chromosome:   10    Cytoband:   Chr:10q22    SOURCE    GENECARD       Gene Ontology:   cellular component:  nucleus      BioCarta Pathways:     KEGG Pathways:  
    Probe set : 1371412_a_at    Name:   Neuronal regeneration related protein    GB acc:   BE107450    UniGene:   Rn.8180    Gene Symbol:   Nrep    Locus Link:   338475    Chromosome:   18    Cytoband:   Chr:18p12    SOURCE    GENECARD       Gene Ontology:     BioCarta Pathways:     KEGG Pathways:  
    Probe set : 1382984_at     GB acc:   AI073272              Gene Ontology:     BioCarta Pathways:     KEGG Pathways:  
    Probe set : 1378825_a_at    Name:   Similar to brain specific protein 4    GB acc:   AI011101    UniGene:   Rn.22411     Locus Link:   361987    Chromosome:   2    Cytoband:   Chr:2q34    SOURCE        Gene Ontology:     BioCarta Pathways:     KEGG Pathways:  
    Probe set : 1378180_at    Name:   similar to 6030466N05Rik protein    GB acc:   BI284767    UniGene:   Rn.105816    Gene Symbol:   RGD:1359671    Locus Link:   290741    Chromosome:   16    Cytoband:   Chr:16q11    SOURCE    GENECARD       Gene Ontology:   biological process:  nucleotide biosynthesis      BioCarta Pathways:     KEGG Pathways:  
    Probe set : 1388709_at    Name:   similar to WD-repeat protein 43    GB acc:   BF284695    UniGene:   Rn.101883    Gene Symbol:   LOC362703    Locus Link:   362703    Chromosome:   6    Cytoband:   Chr:6q13    SOURCE    GENECARD       Gene Ontology:     BioCarta Pathways:     KEGG Pathways:  
    Probe set : 1397495_at    Name:   Similar to Protein C9orf10 (predicted)    GB acc:   BE102358    UniGene:   Rn.64941     Locus Link:   291019    Chromosome:   17    Cytoband:   Chr:17p14    SOURCE        Gene Ontology:     BioCarta Pathways:     KEGG Pathways:  
    Probe set : 1389471_at    Name:   Translocase of outer mitochondrial membrane 34 (predicted)    GB acc:   AI412736    UniGene:   Rn.16749     Locus Link:   311621    Chromosome:   3    Cytoband:   Chr:3q42    SOURCE        Gene Ontology:   biological process:  protein folding   biological process:  protein targeting to mitochondrion   molecular function:  signal sequence binding   molecular function:  binding   molecular function:  unfolded protein binding      BioCarta Pathways:     KEGG Pathways:  
    Probe set : 1388476_at    Name:   Tial1 cytotoxic granule-associated RNA binding protein-like 1    GB acc:   AI101391    UniGene:   Rn.22289    Gene Symbol:   Tial1    Locus Link:   361655    Chromosome:   1    Cytoband:   Chr:1q36    SOURCE    GENECARD       Gene Ontology:   biological process:  programmed cell death, activated T cells   molecular function:  RNA binding   molecular function:  nucleotide binding   molecular function:  nucleic acid binding   molecular function:  RNA binding      BioCarta Pathways:     KEGG Pathways:  
    Probe set : 1369754_a_at    Name:   calpastatin    GB acc:   NM_053295    UniGene:   Rn.17481    Gene Symbol:   Cast    Locus Link:   25403    Chromosome:   2    Cytoband:   Chr:2q11    SOURCE    GENECARD       Gene Ontology:   biological process:  protein catabolism   molecular function:  cysteine protease inhibitor activity   molecular function:  calpain inhibitor activity      BioCarta Pathways:     KEGG Pathways:  
    Probe set : 1379937_at    Name:   Interleukin 4 induced 1 (predicted)    GB acc:   AW526968    UniGene:   Rn.46383     Locus Link:   308575    Chromosome:   1    Cytoband:   Chr:1q22    SOURCE        Gene Ontology:   biological process:  electron transport   molecular function:  oxidoreductase activity      BioCarta Pathways:     KEGG Pathways:  
    Probe set : 1373686_at    Name:   serine (or cysteine) proteinase inhibitor, clade A (alpha-1 antiproteinase, antitrypsin), member 6    GB acc:   AA893495    UniGene:   Rn.2374    Gene Symbol:   Serpina6    Locus Link:   299270    Chromosome:   6    Cytoband:   Chr:6q32    SOURCE    GENECARD       Gene Ontology:   molecular function:  serine-type endopeptidase inhibitor activity      BioCarta Pathways:     KEGG Pathways:  
    Probe set : 1390289_at    Name:   similar to RIKEN cDNA A530089I17 (predicted)    GB acc:   BI283804    UniGene:   Rn.11982    Gene Symbol:   RGD1311375_predicted    Locus Link:   309855    Chromosome:   20    Cytoband:   Chr:20q12    SOURCE    GENECARD       Gene Ontology:     BioCarta Pathways:     KEGG Pathways:  
    Probe set : 1391936_a_at    Name:   Transcribed locus    GB acc:   BI289110    UniGene:   Rn.45026        SOURCE        Gene Ontology:     BioCarta Pathways:     KEGG Pathways:  
    Probe set : 1390380_at    Name:   Similar to Ski protein    GB acc:   BE116633    UniGene:   Rn.8311     Locus Link:   313757    Chromosome:   5    Cytoband:   Chr:5q36    SOURCE        Gene Ontology:     BioCarta Pathways:     KEGG Pathways:  
    Probe set : 1390145_at    Name:   Transcribed locus, strongly similar to XP_510406.1 PREDICTED: similar to rabconnectin-3 [Pan troglodytes]    GB acc:   AW524532    UniGene:   Rn.17861        SOURCE        Gene Ontology:     BioCarta Pathways:     KEGG Pathways:  
    Probe set : 1398932_at    Name:   histidine triad nucleotide binding protein 1 (predicted)    GB acc:   AI227884    UniGene:   Rn.138667    Gene Symbol:   Hint1_predicted    Locus Link:   312393    Chromosome:   10    Cytoband:   Chr:10q22    SOURCE    GENECARD       Gene Ontology:   cellular component:  nucleus   molecular function:  hydrolase activity      BioCarta Pathways:     KEGG Pathways:  
    Probe set : 1374688_at    Name:   Tial1 cytotoxic granule-associated RNA binding protein-like 1    GB acc:   AA945696    UniGene:   Rn.22289    Gene Symbol:   Tial1    Locus Link:   361655    Chromosome:   1    Cytoband:   Chr:1q36    SOURCE    GENECARD       Gene Ontology:   biological process:  programmed cell death, activated T cells   molecular function:  RNA binding   molecular function:  nucleotide binding   molecular function:  nucleic acid binding   molecular function:  RNA binding      BioCarta Pathways:     KEGG Pathways:  
    Probe set : 1368543_at    Name:   NADPH oxidase 4    GB acc:   NM_053524    UniGene:   Rn.14744    Gene Symbol:   Nox4    Locus Link:   85431    Chromosome:   1    Cytoband:   Chr:1q32    SOURCE    GENECARD       Gene Ontology:   biological process:  electron transport   biological process:  superoxide release   biological process:  bone resorption   cellular component:  extracellular space   cellular component:  membrane   cellular component:  integral to membrane      BioCarta Pathways:     KEGG Pathways:  
    Probe set : 1383188_at    Name:   Transcribed locus    GB acc:   BM390716    UniGene:   Rn.22635        SOURCE        Gene Ontology:     BioCarta Pathways:     KEGG Pathways:  
    Probe set : 1373048_at    Name:   ARP10 actin-related protein 10 homolog (S. cerevisiae) (predicted)    GB acc:   AI177051    UniGene:   Rn.36338    Gene Symbol:   Actr10_predicted    Locus Link:   299121    Chromosome:   6    Cytoband:   Chr:6q24    SOURCE    GENECARD       Gene Ontology:   biological process:  microtubule-based movement   cellular component:  cytoskeleton   cellular component:  dynactin complex   cellular component:  actin cytoskeleton   molecular function:  structural molecule activity   molecular function:  structural constituent of cytoskeleton   molecular function:  protein binding      BioCarta Pathways:     KEGG Pathways:  
    Probe set : 1374048_at    Name:   neurturin    GB acc:   AI598581    UniGene:   Rn.21952    Gene Symbol:   Nrtn    Locus Link:   84423    Chromosome:   9    Cytoband:   Chr:9q11    SOURCE    GENECARD       Gene Ontology:   biological process:  MAPKKK cascade   biological process:  transmembrane receptor protein tyrosine kinase signaling pathway   biological process:  nervous system development   cellular component:  extracellular space   molecular function:  growth factor activity   molecular function:  growth factor activity      BioCarta Pathways:     KEGG Pathways:  
    Probe set : 1367877_at    Name:   solute carrier family 11 (proton-coupled divalent metal ion transporters), member 2    GB acc:   NM_013173    UniGene:   Rn.11418    Gene Symbol:   Slc11a2    Locus Link:   25715    Chromosome:   7    Cytoband:   Chr:7q36    SOURCE    GENECARD       Gene Ontology:   molecular function:  transporter activity   molecular function:  copper ion transporter activity   molecular function:  iron ion transporter activity   molecular function:  iron ion transporter activity   molecular function:  manganese ion transporter activity   molecular function:  iron ion binding   molecular function:  copper ion binding   molecular function:  zinc ion binding   molecular function:  cadmium ion transporter activity   molecular function:  cobalt ion transporter activity   molecular function:  cobalt ion transporter activity   molecular function:  lead ion transporter activity   molecular function:  nickel ion transporter activity   molecular function:  nickel ion binding   molecular function:  manganese ion binding   molecular function:  cadmium ion binding   molecular function:  cobalt ion binding   molecular function:  iron ion binding      BioCarta Pathways:     KEGG Pathways:  
    Probe set : 1383104_at    Name:   similar to RIKEN cDNA 1810020D17    GB acc:   BI295116    UniGene:   Rn.145188    Gene Symbol:   LOC361606    Locus Link:   361606    Chromosome:   1    Cytoband:   Chr:1q32    SOURCE    GENECARD       Gene Ontology:     BioCarta Pathways:     KEGG Pathways:  
    Probe set : 1369299_at    Name:   platelet-activating factor receptor    GB acc:   NM_053321    UniGene:   Rn.10137    Gene Symbol:   Ptafr    Locus Link:   58949    Chromosome:   5    Cytoband:   Chr:5q36    SOURCE    GENECARD       Gene Ontology:   cellular component:  integral to plasma membrane   cellular component:  integral to membrane      BioCarta Pathways:     KEGG Pathways:  
    Probe set : 1375621_at     GB acc:   AI575254              Gene Ontology:     BioCarta Pathways:     KEGG Pathways:  
    Probe set : 1397958_at    Name:   Transcribed locus    GB acc:   BE111695    UniGene:   Rn.15124        SOURCE        Gene Ontology:     BioCarta Pathways:     KEGG Pathways:  
    Probe set : 1373528_at    Name:   Phosphatidylinositol 3-kinase catalytic delta polypeptide (predicted)    GB acc:   AI407827    UniGene:   Rn.11530     Locus Link:   366508    Chromosome:   5    Cytoband:   Chr:5q36    SOURCE        Gene Ontology:   cellular component:  phosphoinositide 3-kinase complex      BioCarta Pathways:     KEGG Pathways:  
    Probe set : 1389579_at    Name:   Transcribed locus    GB acc:   BI284372    UniGene:   Rn.17918        SOURCE        Gene Ontology:     BioCarta Pathways:     KEGG Pathways:  
    Probe set : 1393018_at    Name:   Similar to RING finger protein 33    GB acc:   AI071984    UniGene:   Rn.19937     Locus Link:   502072      SOURCE        Gene Ontology:     BioCarta Pathways:     KEGG Pathways:  
    Probe set : 1383497_at    Name:   CDNA clone IMAGE:7300848    GB acc:   AA997772    UniGene:   Rn.113742        SOURCE        Gene Ontology:     BioCarta Pathways:     KEGG Pathways:  
    Probe set : 1375216_at    Name:   poliovirus receptor-related 2 (herpesvirus entry mediator B) (predicted)    GB acc:   AA850909    UniGene:   Rn.7444    Gene Symbol:   Pvrl2_predicted    Locus Link:   308417    Chromosome:   1    Cytoband:   Chr:1q21    SOURCE    GENECARD       Gene Ontology:   cellular component:  plasma membrane   cellular component:  integral to membrane   molecular function:  coreceptor activity   molecular function:  receptor activity      BioCarta Pathways:     KEGG Pathways:  
    Probe set : 1395102_at    Name:   potassium channel subunit (Slack)    GB acc:   AW528917    UniGene:   Rn.44275    Gene Symbol:   RGD:621106    Locus Link:   60444    Chromosome:   3    Cytoband:   Chr:3p13    SOURCE    GENECARD       Gene Ontology:   biological process:  ion transport   biological process:  potassium ion transport   biological process:  transport   cellular component:  integral to membrane   cellular component:  membrane      BioCarta Pathways:     KEGG Pathways:  
    Probe set : 1380062_at    Name:   membrane protein, palmitoylated 6 (MAGUK p55 subfamily member 6) (predicted)    GB acc:   BE097259    UniGene:   Rn.27517    Gene Symbol:   Mpp6_predicted    Locus Link:   362359    Chromosome:   4    Cytoband:   Chr:4q24    SOURCE    GENECARD       Gene Ontology:   biological process:  protein complex assembly   cellular component:  plasma membrane   cellular component:  membrane   cellular component:  integral to membrane   molecular function:  guanylate kinase activity   molecular function:  protein binding      BioCarta Pathways:     KEGG Pathways:  
    Probe set : 1373092_at    Name:   Transcribed locus    GB acc:   BE109587    UniGene:   Rn.8191        SOURCE        Gene Ontology:     BioCarta Pathways:     KEGG Pathways:  
    Probe set : 1397838_at    Name:   LOC499976    GB acc:   BF522792    UniGene:   Rn.8155     Locus Link:   499976      SOURCE        Gene Ontology:     BioCarta Pathways:     KEGG Pathways:  
    Probe set : 1374432_at     GB acc:   BE118251              Gene Ontology:     BioCarta Pathways:     KEGG Pathways:  
    Probe set : 1373056_at    Name:   similar to RIKEN cDNA 1110014L17 (predicted)    GB acc:   AI409861    UniGene:   Rn.21479    Gene Symbol:   RGD1306319_predicted    Locus Link:   305502    Chromosome:   14    Cytoband:   Chr:14q21    SOURCE    GENECARD       Gene Ontology:     BioCarta Pathways:     KEGG Pathways:  
    Probe set : 1370242_at    Name:   ribosomal protein S23    GB acc:   AA848284    UniGene:   Rn.144643    Gene Symbol:   Rps23    Locus Link:   124323    Chromosome:   2    Cytoband:   Chr:2q12    SOURCE    GENECARD       Gene Ontology:   biological process:  protein biosynthesis   molecular function:  nucleic acid binding   molecular function:  RNA binding   molecular function:  structural constituent of ribosome      BioCarta Pathways:     KEGG Pathways:  
    Probe set : 1368389_at    Name:   amyloid beta (A4) precursor protein-binding, family A, member 3    GB acc:   NM_031781    UniGene:   Rn.6924    Gene Symbol:   Apba3    Locus Link:   83611    Chromosome:   7    Cytoband:   Chr:7q11    SOURCE    GENECARD       Gene Ontology:   cellular component:  membrane   molecular function:  protein binding   molecular function:  protein binding   molecular function:  protein transporter activity      BioCarta Pathways:     KEGG Pathways:  
    Probe set : 1367926_at    Name:   prohibitin    GB acc:   NM_031851    UniGene:   Rn.29754    Gene Symbol:   Phb    Locus Link:   25344    Chromosome:   19    Cytoband:   Chr:19p13    SOURCE    GENECARD       Gene Ontology:   molecular function:  transcriptional activator activity   molecular function:  transcriptional repressor activity      BioCarta Pathways:     KEGG Pathways:  
    Probe set : 1377703_at    Name:   similar to RIKEN cDNA 2900009I07 (predicted)    GB acc:   AA819694    UniGene:   Rn.24894    Gene Symbol:   RGD1310316_predicted    Locus Link:   309420    Chromosome:   1    Cytoband:   Chr:1q51    SOURCE    GENECARD       Gene Ontology:     BioCarta Pathways:     KEGG Pathways:  
    Probe set : 1376571_at    Name:   similar to RIKEN cDNA 0610027O18 (predicted)    GB acc:   BI285997    UniGene:   Rn.30223    Gene Symbol:   RGD1307118_predicted    Locus Link:   363171    Chromosome:   8    Cytoband:   Chr:8q32    SOURCE    GENECARD       Gene Ontology:     BioCarta Pathways:     KEGG Pathways:  
    Probe set : 1372452_at    Name:   Glycerol-3-phosphate acyltransferase, mitochondrial    GB acc:   BG666882    UniGene:   Rn.44456    Gene Symbol:   Gpam    Locus Link:   29653    Chromosome:   1    Cytoband:   Chr:1q55    SOURCE    GENECARD       Gene Ontology:   cellular component:  mitochondrion   cellular component:  mitochondrion   cellular component:  integral to membrane   cellular component:  membrane      BioCarta Pathways:     KEGG Pathways:  
    Probe set : 1392864_at    Name:   Rho GTPase activating protein 5 (predicted)    GB acc:   BM384008    UniGene:   Rn.20898    Gene Symbol:   Arhgap5_predicted    Locus Link:   299012    Chromosome:   6    Cytoband:   Chr:6q23    SOURCE    GENECARD       Gene Ontology:   cellular component:  cytoplasm   cellular component:  membrane      BioCarta Pathways:     KEGG Pathways:  
    Probe set : 1372414_at    Name:   Transcribed locus    GB acc:   BI281979    UniGene:   Rn.147163        SOURCE        Gene Ontology:     BioCarta Pathways:     KEGG Pathways:  
    Probe set : 1397669_at    Name:   Chemokine (C-C motif) receptor 6 (predicted)    GB acc:   BG666098    UniGene:   Rn.36521     Locus Link:   308163    Chromosome:   1    Cytoband:   Chr:1q11    SOURCE        Gene Ontology:   cellular component:  integral to plasma membrane   cellular component:  integral to membrane      BioCarta Pathways:     KEGG Pathways:  
    Probe set : 1369167_at    Name:   glial cell line derived neurotrophic factor family receptor alpha 2    GB acc:   NM_012750    UniGene:   Rn.90137    Gene Symbol:   Gfra2    Locus Link:   25136    Chromosome:   15    Cytoband:   Chr:15p11    SOURCE    GENECARD       Gene Ontology:   biological process:  transmembrane receptor protein tyrosine kinase signaling pathway   biological process:  nervous system development   cellular component:  extracellular space   cellular component:  membrane   cellular component:  extrinsic to membrane      BioCarta Pathways:     KEGG Pathways:  
    Probe set : 1376271_at    Name:   AA926063gene    GB acc:   AI178914     Gene Symbol:   RGD:1303072    Locus Link:   294284    Chromosome:   20    Cytoband:   Chr:20p12     GENECARD       Gene Ontology:     BioCarta Pathways:     KEGG Pathways:  
    Probe set : 1397647_at    Name:   solute carrier family 25 (mitochondrial carrier; ornithine transporter) member 15 (predicted)    GB acc:   BF554040    UniGene:   Rn.66488    Gene Symbol:   Slc25a15_predicted    Locus Link:   306574    Chromosome:   16    Cytoband:   Chr:16q12.5    SOURCE    GENECARD       Gene Ontology:   molecular function:  L-ornithine transporter activity   molecular function:  transporter activity   molecular function:  binding      BioCarta Pathways:     KEGG Pathways:  
    Probe set : 1390685_at    Name:   Scm-like with four mbt domains 1    GB acc:   BF395810    UniGene:   Rn.14839    Gene Symbol:   Sfmbt1    Locus Link:   58967    Chromosome:   16    Cytoband:   Chr:16p16    SOURCE    GENECARD       Gene Ontology:   biological process:  regulation of transcription   cellular component:  nucleus      BioCarta Pathways:     KEGG Pathways:  
    Probe set : 1380101_at    Name:   poly(A) polymerase gamma (predicted)    GB acc:   BE109349    UniGene:   Rn.27992    Gene Symbol:   Papolg_predicted    Locus Link:   305586    Chromosome:   14    Cytoband:   Chr:14q22    SOURCE    GENECARD       Gene Ontology:   biological process:  transcription   biological process:  mRNA processing   cellular component:  nucleus      BioCarta Pathways:     KEGG Pathways:  
    Probe set : 1372352_at    Name:   arginine-rich, mutated in early stage tumors (predicted)    GB acc:   AI170666    UniGene:   Rn.42796    Gene Symbol:   Armet_predicted    Locus Link:   315989    Chromosome:   8    Cytoband:   Chr:8q32    SOURCE    GENECARD       Gene Ontology:   cellular component:  extracellular space      BioCarta Pathways:     KEGG Pathways:  
    Probe set : 1370163_at    Name:   ornithine decarboxylase 1    GB acc:   BF281299    UniGene:   Rn.874    Gene Symbol:   Odc1    Locus Link:   24609    Chromosome:   6    Cytoband:   Chr:6q16    SOURCE    GENECARD       Gene Ontology:   biological process:  polyamine biosynthesis      BioCarta Pathways:     KEGG Pathways:  
    Probe set : 1372791_at    Name:   Protein kinase, lysine deficient 1    GB acc:   AI235474    UniGene:   Rn.27409    Gene Symbol:   Prkwnk1    Locus Link:   116477    Chromosome:   4    Cytoband:   Chr:4q42    SOURCE    GENECARD       Gene Ontology:   cellular component:  cytoplasm   cellular component:  cytoplasm      BioCarta Pathways:     KEGG Pathways:  
    Probe set : 1380186_at    Name:   Similar to Zinc finger protein 198 (Fused in myeloproliferative disorders protein) (Rearranged in atypical myeloproliferative disorder protein) (predicted)    GB acc:   AI101272    UniGene:   Rn.20430     Locus Link:   305911    Chromosome:   15    Cytoband:   Chr:15p12    SOURCE        Gene Ontology:     BioCarta Pathways:     KEGG Pathways:  
    Probe set : 1368240_a_at    Name:   protein kinase C, beta 1    GB acc:   M13706    UniGene:   Rn.91118    Gene Symbol:   Prkcb1    Locus Link:   25023    Chromosome:   1    Cytoband:   Chr:1q36    SOURCE    GENECARD       Gene Ontology:   cellular component:  nucleus   cellular component:  cytoplasm   cellular component:  plasma membrane   cellular component:  membrane   molecular function:  protein kinase activity   molecular function:  protein serine/threonine kinase activity   molecular function:  protein kinase CK2 activity   molecular function:  cAMP-dependent protein kinase activity   molecular function:  protein kinase C activity   molecular function:  protein kinase C activity   molecular function:  protein-tyrosine kinase activity   molecular function:  calcium channel regulator activity   molecular function:  calcium ion binding   molecular function:  protein binding   molecular function:  ATP binding   molecular function:  transferase activity   molecular function:  diacylglycerol binding   molecular function:  nucleotide binding   molecular function:  zinc ion binding   molecular function:  kinase activity   molecular function:  metal ion binding      BioCarta Pathways:     KEGG Pathways:  
    Probe set : 1375966_at    Name:   Multimerin 2 (predicted)    GB acc:   BE098266    UniGene:   Rn.7966     Locus Link:   306288    Chromosome:   16    Cytoband:   Chr:16p16    SOURCE        Gene Ontology:   cellular component:  extracellular matrix (sensu Metazoa)      BioCarta Pathways:     KEGG Pathways:  
    Probe set : 1376842_at    Name:   Transcribed locus    GB acc:   BF395964    UniGene:   Rn.13464        SOURCE        Gene Ontology:     BioCarta Pathways:     KEGG Pathways:  
    Probe set : 1388373_at    Name:   adiponectin receptor 1    GB acc:   BI289063    UniGene:   Rn.104556    Gene Symbol:   RGD:1303151    Locus Link:   289036    Chromosome:   13    Cytoband:   Chr:13q13    SOURCE    GENECARD       Gene Ontology:   cellular component:  integral to membrane      BioCarta Pathways:     KEGG Pathways:  
    Probe set : 1368056_at    Name:   tuberous sclerosis 2    GB acc:   U24150    UniGene:   Rn.5875    Gene Symbol:   Tsc2    Locus Link:   24855    Chromosome:   10    Cytoband:   Chr:10q12    SOURCE    GENECARD       Gene Ontology:   cellular component:  membrane fraction   cellular component:  nucleus   cellular component:  cytosol   cellular component:  plasma membrane      BioCarta Pathways:     KEGG Pathways:  
    Probe set : 1395524_at    Name:   Similar to hypothetical protein FLJ13089 (predicted)    GB acc:   AW525958    UniGene:   Rn.95126     Locus Link:   360811    Chromosome:   12    Cytoband:   Chr:12q16    SOURCE        Gene Ontology:   molecular function:  binding      BioCarta Pathways:     KEGG Pathways:  
    Probe set : 1370808_at    Name:   diaphorase 1    GB acc:   J03867    UniGene:   Rn.35994    Gene Symbol:   Dia1    Locus Link:   25035    Chromosome:   7    Cytoband:   Chr:7q34    SOURCE    GENECARD       Gene Ontology:     BioCarta Pathways:     KEGG Pathways:  
    Probe set : 1387098_at    Name:   RNA polymerase 1-4    GB acc:   NM_031772    UniGene:   Rn.24877    Gene Symbol:   Rpo1-4    Locus Link:   83581    Chromosome:   4    Cytoband:   Chr:4q33    SOURCE    GENECARD       Gene Ontology:   biological process:  transcription   cellular component:  nucleus   cellular component:  DNA-directed RNA polymerase I complex      BioCarta Pathways:     KEGG Pathways:  
    Probe set : 1392498_at    Name:   similar to protein phosphatase 2A B56 regulatory subunit gamma 3 isoform    GB acc:   BG670494    UniGene:   Rn.2153    Gene Symbol:   LOC500722    Locus Link:   500722    Chromosome:   6    Cytoband:   Chr:6q32    SOURCE    GENECARD       Gene Ontology:     BioCarta Pathways:     KEGG Pathways:  
    Probe set : 1374861_at    Name:   transducin-like enhancer of split 2, homolog of Drosophila E(spl) (predicted)    GB acc:   AI175907    UniGene:   Rn.38683    Gene Symbol:   Tle2_predicted    Locus Link:   299636    Chromosome:   7    Cytoband:   Chr:7q11    SOURCE    GENECARD       Gene Ontology:   cellular component:  nucleus   molecular function:  protein kinase activity   molecular function:  ATP binding      BioCarta Pathways:     KEGG Pathways:  
    Probe set : 1370285_at    Name:   KIAA1536 protein    GB acc:   AY078385    UniGene:   Rn.63647    Gene Symbol:   RGD:619812    Locus Link:   246047    Chromosome:   7    Cytoband:   Chr:7q36    SOURCE    GENECARD       Gene Ontology:     BioCarta Pathways:     KEGG Pathways:  
    Probe set : 1388240_a_at    Name:   integrin alpha 7    GB acc:   X74294    UniGene:   Rn.54492    Gene Symbol:   Itga7    Locus Link:   81008    Chromosome:   7    Cytoband:   Chr:7q11    SOURCE    GENECARD       Gene Ontology:     BioCarta Pathways:     KEGG Pathways:  
    Probe set : 1393551_at     GB acc:   AI044529              Gene Ontology:     BioCarta Pathways:     KEGG Pathways:  
    Probe set : 1395659_at     GB acc:   AI715999              Gene Ontology:     BioCarta Pathways:     KEGG Pathways:  
    Probe set : 1387265_at    Name:   diacylglycerol kinase, gamma    GB acc:   NM_013126    UniGene:   Rn.10544    Gene Symbol:   Dgkg    Locus Link:   25666    Chromosome:   11    Cytoband:   Chr:11p11    SOURCE    GENECARD       Gene Ontology:   biological process:  protein kinase C activation   biological process:  intracellular signaling cascade   biological process:  muscle development      BioCarta Pathways:     KEGG Pathways:  
    Probe set : 1383757_at    Name:   Transcribed locus    GB acc:   AI044802    UniGene:   Rn.20709        SOURCE        Gene Ontology:     BioCarta Pathways:     KEGG Pathways:  
    Probe set : 1383703_at    Name:   FUS interacting protein (serine-arginine rich) 1 (predicted)    GB acc:   BE109151    UniGene:   Rn.105889     Locus Link:   362630    Chromosome:   5    Cytoband:   Chr:5q36    SOURCE        Gene Ontology:   cellular component:  nucleus   cellular component:  nucleoplasm   cellular component:  cytoplasm      BioCarta Pathways:     KEGG Pathways:  
    Probe set : 1368029_at    Name:   guanine nucleotide binding protein, alpha inhibiting 3    GB acc:   NM_013106    UniGene:   Rn.4368    Gene Symbol:   Gnai3    Locus Link:   25643    Chromosome:   2    Cytoband:   Chr:2q34    SOURCE    GENECARD       Gene Ontology:     BioCarta Pathways:     KEGG Pathways:  
    Probe set : 1378056_at    Name:   geminin (predicted)    GB acc:   BM391984    UniGene:   Rn.15438    Gene Symbol:   Gmnn_predicted    Locus Link:   291137    Chromosome:   17    Cytoband:   Chr:17p11    SOURCE    GENECARD       Gene Ontology:   cellular component:  nucleus      BioCarta Pathways:     KEGG Pathways:  
    Probe set : 1394458_at    Name:   SA rat hypertension-associated homolog    GB acc:   BF291026    UniGene:   Rn.88644    Gene Symbol:   Sah    Locus Link:   24763    Chromosome:   1    Cytoband:   Chr:1    SOURCE    GENECARD       Gene Ontology:   cellular component:  mitochondrion   cellular component:  mitochondrial matrix   molecular function:  catalytic activity   molecular function:  fatty-acid ligase activity      BioCarta Pathways:     KEGG Pathways:  
    Probe set : 1398774_at    Name:   ribosomal protein L30    GB acc:   NM_022699    UniGene:   Rn.36878    Gene Symbol:   Rpl30    Locus Link:   64640    Chromosome:   7    Cytoband:   Chr:7q22    SOURCE    GENECARD       Gene Ontology:   biological process:  protein biosynthesis   molecular function:  RNA binding   molecular function:  structural constituent of ribosome      BioCarta Pathways:     KEGG Pathways:  
    Probe set : 1398793_at    Name:   cell division cycle 5-like (S. pombe)    GB acc:   NM_053527    UniGene:   Rn.54977    Gene Symbol:   Cdc5l    Locus Link:   85434    Chromosome:   9    Cytoband:   Chr:9q12    SOURCE    GENECARD       Gene Ontology:   cellular component:  nucleus   cellular component:  nucleus   cellular component:  nuclear speck   cellular component:  spliceosome complex   molecular function:  DNA binding   molecular function:  protein domain specific binding   molecular function:  RNA binding      BioCarta Pathways:     KEGG Pathways:  
    Probe set : 1375857_at    Name:   similar to Myoferlin (Fer-1 like protein 3)    GB acc:   AW917760    UniGene:   Rn.73651    Gene Symbol:   LOC309499    Locus Link:   309499    Chromosome:   1    Cytoband:   Chr:1q53    SOURCE    GENECARD       Gene Ontology:     BioCarta Pathways:     KEGG Pathways:  
    Probe set : 1372092_at    Name:   Amyotrophic lateral sclerosis 2 (juvenile) chromosome region, candidate 3 homolog (human)    GB acc:   BI278968    UniGene:   Rn.26957    Gene Symbol:   Als2cr3    Locus Link:   171086    Chromosome:   9    Cytoband:   Chr:9q31    SOURCE    GENECARD       Gene Ontology:     BioCarta Pathways:     KEGG Pathways:  
    Probe set : 1371636_at    Name:   Similar to novel protein    GB acc:   BE104321    UniGene:   Rn.100818     Locus Link:   313499    Chromosome:   5    Cytoband:   Chr:5q35    SOURCE        Gene Ontology:     BioCarta Pathways:     KEGG Pathways:  
    Probe set : 1390981_at    Name:   Polycystic kidney and hepatic disease 1-like 1 (predicted)    GB acc:   AI028965    UniGene:   Rn.3365     Locus Link:   314917    Chromosome:   7    Cytoband:   Chr:7q31    SOURCE        Gene Ontology:   cellular component:  extracellular space      BioCarta Pathways:     KEGG Pathways:  
    Probe set : 1371309_at    Name:   testis enhanced gene transcript    GB acc:   BI276999    UniGene:   Rn.107678    Gene Symbol:   Tegt    Locus Link:   24822    Chromosome:   7    Cytoband:   Chr:7q36    SOURCE    GENECARD       Gene Ontology:     BioCarta Pathways:     KEGG Pathways:  
    Probe set : 1372942_at    Name:   exosome component 5 (predicted)    GB acc:   AA943578    UniGene:   Rn.7400    Gene Symbol:   Exosc5_predicted    Locus Link:   308441    Chromosome:   1    Cytoband:   Chr:1q21    SOURCE    GENECARD       Gene Ontology:   biological process:  rRNA processing   cellular component:  exosome (RNase complex)   cellular component:  nucleolus   cellular component:  cytoplasm      BioCarta Pathways:     KEGG Pathways:  
    Probe set : 1372428_at    Name:   DnaJ (Hsp40) homolog, subfamily C, member 4 (predicted)    GB acc:   BG380252    UniGene:   Rn.91398    Gene Symbol:   Dnajc4_predicted    Locus Link:   361717    Chromosome:   1    Cytoband:   Chr:1q43    SOURCE    GENECARD       Gene Ontology:   biological process:  protein folding   cellular component:  extracellular space   cellular component:  integral to membrane   molecular function:  heat shock protein binding   molecular function:  unfolded protein binding      BioCarta Pathways:     KEGG Pathways:  
    Probe set : 1398249_at    Name:   solute carrier family 25 (carnitine/acylcarnitine translocase), member 20    GB acc:   NM_053965    UniGene:   Rn.3289    Gene Symbol:   Slc25a20    Locus Link:   117035    Chromosome:   8    Cytoband:   Chr:8q32    SOURCE    GENECARD       Gene Ontology:   biological process:  transport   biological process:  transport      BioCarta Pathways:     KEGG Pathways:  
    Probe set : 1382769_at    Name:   interferon (alpha and beta) receptor 1 (predicted)    GB acc:   AA996880    UniGene:   Rn.105738    Gene Symbol:   Ifnar1_predicted    Locus Link:   288264    Chromosome:   11    Cytoband:   Chr:11q11    SOURCE    GENECARD       Gene Ontology:   biological process:  cell surface receptor linked signal transduction   biological process:  JAK-STAT cascade   biological process:  response to virus      BioCarta Pathways:     KEGG Pathways:  
    Probe set : 1374891_at    Name:   BRF2, subunit of RNA polymerase III transcription initiation factor, BRF1-like (predicted)    GB acc:   BF406141    UniGene:   Rn.13322    Gene Symbol:   Brf2_predicted    Locus Link:   306542    Chromosome:   16    Cytoband:   Chr:16q12.4    SOURCE    GENECARD       Gene Ontology:   biological process:  cell cycle   molecular function:  translation initiation factor activity      BioCarta Pathways:     KEGG Pathways:  
    Probe set : 1388142_at    Name:   chondroitin sulfate proteoglycan 2    GB acc:   AA850991    UniGene:   Rn.35666    Gene Symbol:   Cspg2    Locus Link:   114122    Chromosome:   2    Cytoband:   Chr:2q12    SOURCE    GENECARD       Gene Ontology:   biological process:  cell motility   biological process:  cell adhesion   biological process:  cell adhesion   cellular component:  extracellular matrix (sensu Metazoa)   cellular component:  extracellular matrix      BioCarta Pathways:     KEGG Pathways:  
    Probe set : 1383581_at     GB acc:   BI290662              Gene Ontology:     BioCarta Pathways:     KEGG Pathways:  
    Probe set : 1390250_x_at    Name:   Atpase, class I, type 8B, member 2 (predicted)    GB acc:   BI273855    UniGene:   Rn.20321     Locus Link:   361984    Chromosome:   2    Cytoband:   Chr:2q34    SOURCE        Gene Ontology:   biological process:  cation transport   biological process:  metabolism   cellular component:  membrane   cellular component:  integral to membrane      BioCarta Pathways:     KEGG Pathways:  
    Probe set : 1369222_at    Name:   potassium channel subunit (Slack)    GB acc:   NM_021853    UniGene:   Rn.44275    Gene Symbol:   RGD:621106    Locus Link:   60444    Chromosome:   3    Cytoband:   Chr:3p13    SOURCE    GENECARD       Gene Ontology:   biological process:  ion transport   biological process:  potassium ion transport   biological process:  transport   cellular component:  integral to membrane   cellular component:  membrane      BioCarta Pathways:     KEGG Pathways:  
    Probe set : 1390785_at    Name:   UBX domain containing 4 (predicted)    GB acc:   AI232339    UniGene:   Rn.25112    Gene Symbol:   Ubxd4_predicted    Locus Link:   362716    Chromosome:   6    Cytoband:   Chr:6q14    SOURCE    GENECARD       Gene Ontology:     BioCarta Pathways:     KEGG Pathways:  
    Probe set : 1368128_at    Name:   phospholipase A2, group IIA (platelets, synovial fluid)    GB acc:   NM_031598    UniGene:   Rn.11346    Gene Symbol:   Pla2g2a    Locus Link:   29692    Chromosome:   5    Cytoband:   Chr:5q36    SOURCE    GENECARD       Gene Ontology:   biological process:  regulation of cell growth   biological process:  lipid catabolism   biological process:  lipid catabolism   cellular component:  extracellular region   cellular component:  extracellular space   cellular component:  membrane      BioCarta Pathways:     KEGG Pathways:  
    Probe set : 1391989_at     GB acc:   AI179410              Gene Ontology:     BioCarta Pathways:     KEGG Pathways:  
    Probe set : 1369946_at    Name:   PDRP protein    GB acc:   NM_024384    UniGene:   Rn.42184    Gene Symbol:   Pdrp    Locus Link:   79227    Chromosome:   10    Cytoband:   Chr:10q12    SOURCE    GENECARD       Gene Ontology:     BioCarta Pathways:     KEGG Pathways:  
    Probe set : 1374916_at    Name:   Transcribed locus    GB acc:   BF401373    UniGene:   Rn.39242        SOURCE        Gene Ontology:     BioCarta Pathways:     KEGG Pathways:  
    Probe set : 1374063_at    Name:   splicing factor, arginine/serine-rich 3 (SRp20) (predicted)    GB acc:   AI579288    UniGene:   Rn.9002    Gene Symbol:   Sfrs3_predicted    Locus Link:   361814    Chromosome:   20    Cytoband:   Chr:20p12    SOURCE    GENECARD       Gene Ontology:   biological process:  nuclear mRNA splicing, via spliceosome   biological process:  mRNA splice site selection   biological process:  mRNA processing   cellular component:  nucleus   cellular component:  spliceosome complex   molecular function:  nucleic acid binding   molecular function:  RNA binding      BioCarta Pathways:     KEGG Pathways:  
    Probe set : 1379558_at    Name:   LOC498681    GB acc:   AA957033    UniGene:   Rn.9167     Locus Link:   498681      SOURCE        Gene Ontology:     BioCarta Pathways:     KEGG Pathways:  
    Probe set : 1370592_at    Name:   kidney expressed gene 1    GB acc:   AB019693    UniGene:   Rn.22745    Gene Symbol:   Keg1    Locus Link:   171179    Chromosome:   1    Cytoband:   Chr:1q43    SOURCE    GENECARD       Gene Ontology:   biological process:  regulation of progression through cell cycle   cellular component:  mitochondrion   cellular component:  centrosome   molecular function:  gamma-tubulin binding      BioCarta Pathways:     KEGG Pathways:  
    Probe set : 1380448_at    Name:   AlkB, alkylation repair homolog (E. coli) (predicted)    GB acc:   AI112926    UniGene:   Rn.21131     Locus Link:   362766    Chromosome:   6    Cytoband:   Chr:6q31    SOURCE        Gene Ontology:   biological process:  DNA repair   biological process:  DNA dealkylation      BioCarta Pathways:     KEGG Pathways:  
    Probe set : 1384960_at    Name:   Transcribed locus    GB acc:   BI281250    UniGene:   Rn.124539        SOURCE        Gene Ontology:     BioCarta Pathways:     KEGG Pathways:  
    Probe set : 1370842_at    Name:   branched chain keto acid dehydrogenase kinase    GB acc:   BI277527    UniGene:   Rn.31976    Gene Symbol:   Bckdk    Locus Link:   29603    Chromosome:   1    Cytoband:   Chr:1q36    SOURCE    GENECARD       Gene Ontology:     BioCarta Pathways:     KEGG Pathways:  
    Probe set : 1388887_at    Name:   Glycoprotein galactosyltransferase alpha 1, 3    GB acc:   AI178222    UniGene:   Rn.916    Gene Symbol:   Ggta1    Locus Link:   246766    Chromosome:   3    Cytoband:   Chr:3p11    SOURCE    GENECARD       Gene Ontology:   biological process:  carbohydrate metabolism   cellular component:  membrane      BioCarta Pathways:     KEGG Pathways:  
    Probe set : 1387891_at    Name:   peroxiredoxin 4    GB acc:   BI282076    UniGene:   Rn.17958    Gene Symbol:   Prdx4    Locus Link:   85274      SOURCE    GENECARD       Gene Ontology:   biological process:  I-kappaB phosphorylation   cellular component:  extracellular space   cellular component:  extracellular space   cellular component:  mitochondrion      BioCarta Pathways:     KEGG Pathways:  
    Probe set : 1371914_at    Name:   SWI/SNF related, matrix associated, actin dependent regulator of chromatin, subfamily b, member 1 (predicted)    GB acc:   AI406553    UniGene:   Rn.139601    Gene Symbol:   Smarcb1_predicted    Locus Link:   361825    Chromosome:   20    Cytoband:   Chr:20p12    SOURCE    GENECARD       Gene Ontology:   cellular component:  nuclear chromosome   cellular component:  nucleus   cellular component:  nucleoplasm   molecular function:  protein binding      BioCarta Pathways:     KEGG Pathways:  
    Probe set : 1371553_at    Name:   mitochondrial ribosomal protein L36 (predicted)    GB acc:   BF288120    UniGene:   Rn.7601    Gene Symbol:   Mrpl36_predicted    Locus Link:   364656    Chromosome:   17    Cytoband:   Chr:17p14    SOURCE    GENECARD       Gene Ontology:   biological process:  protein biosynthesis   molecular function:  structural constituent of ribosome      BioCarta Pathways:     KEGG Pathways:  
    Probe set : 1378566_at     GB acc:   BG377991              Gene Ontology:     BioCarta Pathways:     KEGG Pathways:  
    Probe set : 1379107_at    Name:   Solute carrier family 4, member 4    GB acc:   BF416861    UniGene:   Rn.11114    Gene Symbol:   Slc4a4    Locus Link:   84484    Chromosome:   14    Cytoband:   Chr:14p22    SOURCE    GENECARD       Gene Ontology:     BioCarta Pathways:     KEGG Pathways:  
    Probe set : 1375756_at    Name:   Transcribed locus    GB acc:   BF408518    UniGene:   Rn.17100        SOURCE        Gene Ontology:     BioCarta Pathways:     KEGG Pathways:  
    Probe set : 1369492_at    Name:   arylacetamide deacetylase (esterase)    GB acc:   NM_020538    UniGene:   Rn.48732    Gene Symbol:   Aadac    Locus Link:   57300    Chromosome:   2    Cytoband:   Chr:2q31    SOURCE    GENECARD       Gene Ontology:   biological process:  lipid metabolism   biological process:  metabolism      BioCarta Pathways:     KEGG Pathways:  
    Probe set : 1378288_at    Name:   similar to RIKEN cDNA D630035O19 (predicted)    GB acc:   BI292167    UniGene:   Rn.19056    Gene Symbol:   RGD1311100_predicted    Locus Link:   303879    Chromosome:   11    Cytoband:   Chr:11q22    SOURCE    GENECARD       Gene Ontology:     BioCarta Pathways:     KEGG Pathways:  
    Probe set : 1388456_at    Name:   S100 calcium binding protein A1    GB acc:   AI228548    UniGene:   Rn.11091    Gene Symbol:   S100a1    Locus Link:   295214    Chromosome:   2    Cytoband:   Chr:2q34    SOURCE    GENECARD       Gene Ontology:   biological process:  negative regulation of transcription from RNA polymerase II promoter   cellular component:  sarcoplasmic reticulum   molecular function:  calcium ion binding   molecular function:  calcium ion binding   molecular function:  protein homodimerization activity      BioCarta Pathways:     KEGG Pathways:  
    Probe set : 1368605_at    Name:   adaptor protein with pleckstrin homology and src homology 2 domains    GB acc:   NM_053669    UniGene:   Rn.30041    Gene Symbol:   Aps    Locus Link:   114203    Chromosome:   12    Cytoband:   Chr:12q12    SOURCE    GENECARD       Gene Ontology:   biological process:  B-1 B cell homeostasis   biological process:  intracellular signaling cascade   biological process:  nervous system development   biological process:  insulin receptor signaling pathway   biological process:  cytokine and chemokine mediated signaling pathway   biological process:  regulation of metabolism   biological process:  antimicrobial humoral response (sensu Vertebrata)   biological process:  actin cytoskeleton organization and biogenesis   biological process:  glucose homeostasis   biological process:  negative regulation of glucose import   biological process:  regulation of Ras protein signal transduction   biological process:  regulation of immune response   biological process:  antigen receptor-mediated signaling pathway   cellular component:  cytoplasm   cellular component:  actin filament   cellular component:  plasma membrane   cellular component:  membrane      BioCarta Pathways:     KEGG Pathways:  
    Probe set : 1369129_at    Name:   RAS guanyl releasing protein 1    GB acc:   NM_019211    UniGene:   Rn.32156    Gene Symbol:   Rasgrp1    Locus Link:   29434      SOURCE    GENECARD       Gene Ontology:   biological process:  intracellular signaling cascade   biological process:  small GTPase mediated signal transduction   biological process:  Ras protein signal transduction   cellular component:  membrane fraction      BioCarta Pathways:     KEGG Pathways:  
    Probe set : 1387887_at    Name:   ribosomal protein L14    GB acc:   AA874811    UniGene:   Rn.108039    Gene Symbol:   Rpl14    Locus Link:   65043    Chromosome:   8    Cytoband:   Chr:8q32    SOURCE    GENECARD       Gene Ontology:   biological process:  protein biosynthesis   molecular function:  RNA binding   molecular function:  structural constituent of ribosome   molecular function:  structural constituent of ribosome      BioCarta Pathways:     KEGG Pathways:  
    Probe set : 1382685_at     GB acc:   AI101660              Gene Ontology:     BioCarta Pathways:     KEGG Pathways:  
    Probe set : 1392830_at    Name:   Transcribed locus    GB acc:   BF396625    UniGene:   Rn.146623        SOURCE        Gene Ontology:     BioCarta Pathways:     KEGG Pathways:  
    Probe set : 1380277_at    Name:   RAD51 associated protein 1 (predicted)    GB acc:   AI639142    UniGene:   Rn.42022     Locus Link:   312721    Chromosome:   4    Cytoband:   Chr:4q42    SOURCE        Gene Ontology:   biological process:  DNA recombination   cellular component:  nucleus      BioCarta Pathways:     KEGG Pathways:  
    Probe set : 1370816_at    Name:   nuclear receptor subfamily 1, group D, member 1    GB acc:   M25804    UniGene:   Rn.29848    Gene Symbol:   Nr1d1    Locus Link:   252917    Chromosome:   10    Cytoband:   Chr:10q31    SOURCE    GENECARD       Gene Ontology:   biological process:  transcription   biological process:  regulation of transcription, DNA-dependent   biological process:  circadian rhythm   cellular component:  nucleus      BioCarta Pathways:     KEGG Pathways:  
    Probe set : 1379571_at    Name:   plakophilin 4 (predicted)    GB acc:   AA819847    UniGene:   Rn.17389    Gene Symbol:   Pkp4_predicted    Locus Link:   295625    Chromosome:   3    Cytoband:   Chr:3q21    SOURCE    GENECARD       Gene Ontology:   biological process:  cell adhesion   cellular component:  cytoskeleton   cellular component:  intercellular junction   molecular function:  structural molecule activity   molecular function:  protein binding      BioCarta Pathways:     KEGG Pathways:  
    Probe set : 1390789_at    Name:   similar to 5730439E10Rik protein (predicted)    GB acc:   BI296347    UniGene:   Rn.8651    Gene Symbol:   RGD1306270_predicted    Locus Link:   315973    Chromosome:   8    Cytoband:   Chr:8q32    SOURCE    GENECARD       Gene Ontology:   biological process:  signal transduction   biological process:  G-protein coupled receptor protein signaling pathway   cellular component:  integral to membrane      BioCarta Pathways:     KEGG Pathways:  
    Probe set : 1393414_at    Name:   Transcribed locus    GB acc:   AW142650    UniGene:   Rn.8434        SOURCE        Gene Ontology:     BioCarta Pathways:     KEGG Pathways:  
    Probe set : 1373875_at    Name:   similar to RIKEN cDNA 1190005P17 (predicted)    GB acc:   BG381541     Gene Symbol:   RGD1308261_predicted    Locus Link:   299818    Chromosome:   7    Cytoband:   Chr:7q22     GENECARD       Gene Ontology:     BioCarta Pathways:     KEGG Pathways:  
    Probe set : 1367975_at    Name:   annexin A3    GB acc:   BF283732    UniGene:   Rn.6589    Gene Symbol:   Anxa3    Locus Link:   25291    Chromosome:   14    Cytoband:   Chr:14p22    SOURCE    GENECARD       Gene Ontology:   cellular component:  cytoplasm      BioCarta Pathways:     KEGG Pathways:  
    Probe set : 1367580_at    Name:   ribosomal protein L10A    GB acc:   NM_031065    UniGene:   Rn.2262    Gene Symbol:   Rpl10a    Locus Link:   81729    Chromosome:   20    Cytoband:   Chr:20p12    SOURCE    GENECARD       Gene Ontology:   biological process:  protein biosynthesis   molecular function:  structural constituent of ribosome   molecular function:  structural constituent of ribosome      BioCarta Pathways:     KEGG Pathways:  
    Probe set : 1399056_at    Name:   similar to CG10585-PA    GB acc:   AI716436    UniGene:   Rn.20063    Gene Symbol:   RGD:1359372    Locus Link:   365592    Chromosome:   20    Cytoband:   Chr:20q13    SOURCE    GENECARD       Gene Ontology:     BioCarta Pathways:     KEGG Pathways:  
    Probe set : 1374394_at    Name:   WW domain binding protein 2    GB acc:   AI105305    UniGene:   Rn.70500    Gene Symbol:   Wbp2    Locus Link:   192645    Chromosome:   10    Cytoband:   Chr:10q32.3    SOURCE    GENECARD       Gene Ontology:     BioCarta Pathways:     KEGG Pathways:  
    Probe set : 1368282_at    Name:   dipeptidase 1 (renal)    GB acc:   NM_053591    UniGene:   Rn.6051    Gene Symbol:   Dpep1    Locus Link:   94199    Chromosome:   19    Cytoband:   Chr:19q12    SOURCE    GENECARD       Gene Ontology:   biological process:  proteolysis      BioCarta Pathways:     KEGG Pathways:  
    Probe set : 1394290_at    Name:   Transcribed locus    GB acc:   BG669921    UniGene:   Rn.25146        SOURCE        Gene Ontology:     BioCarta Pathways:     KEGG Pathways:  
    Probe set : 1374610_at    Name:   Similar to hypothetical protein 4933408F15    GB acc:   AI599365    UniGene:   Rn.19608     Locus Link:   305166    Chromosome:   14    Cytoband:   Chr:14p22    SOURCE        Gene Ontology:   biological process:  metabolism   molecular function:  acyltransferase activity      BioCarta Pathways:     KEGG Pathways:  
    Probe set : 1374509_at    Name:   Similar to RIKEN cDNA 1110018O08    GB acc:   BI275722    UniGene:   Rn.16101    Gene Symbol:   MGC94226    Locus Link:   288498    Chromosome:   12    Cytoband:   Chr:12p11    SOURCE    GENECARD       Gene Ontology:   biological process:  nucleosome assembly   biological process:  chromosome organization and biogenesis (sensu Eukaryota)   cellular component:  nucleosome   cellular component:  nucleus   molecular function:  DNA binding      BioCarta Pathways:     KEGG Pathways:  
    Probe set : 1385025_a_at    Name:   transmembrane 7 superfamily member 1 (predicted)    GB acc:   AI235734    UniGene:   Rn.60453    Gene Symbol:   Tm7sf1_predicted    Locus Link:   289287    Chromosome:   17    Cytoband:   Chr:17q12.1    SOURCE    GENECARD       Gene Ontology:   cellular component:  membrane fraction   cellular component:  integral to plasma membrane   cellular component:  integral to membrane      BioCarta Pathways:     KEGG Pathways:  
    Probe set : 1391559_at    Name:   similar to RIKEN cDNA 0610030G03    GB acc:   AW525904    UniGene:   Rn.106119    Gene Symbol:   RGD:1359195    Locus Link:   287472    Chromosome:   10    Cytoband:   Chr:10q25    SOURCE    GENECARD       Gene Ontology:   cellular component:  integral to membrane      BioCarta Pathways:     KEGG Pathways:  
    Probe set : 1387785_at    Name:   myotrophin    GB acc:   U21661    UniGene:   Rn.3239    Gene Symbol:   Mtpn    Locus Link:   79215    Chromosome:   4    Cytoband:   Chr:4q22    SOURCE    GENECARD       Gene Ontology:   biological process:  regulation of transcription, DNA-dependent   biological process:  neuron differentiation   molecular function:  DNA binding   molecular function:  transcription factor activity   molecular function:  protein binding      BioCarta Pathways:     KEGG Pathways:  
    Probe set : 1395758_at     GB acc:   AI548569    UniGene:   Rn.105760    Gene Symbol:   LOC301105 /// Syncrip_predicted    Locus Link:   301105 /// 363113    Chromosome:   8    Cytoband:   Chr:8q31    SOURCE    GENECARD       Gene Ontology:   biological process:  nuclear mRNA splicing, via spliceosome   biological process:  RNA splicing   biological process:  mRNA processing   cellular component:  nucleus   cellular component:  endoplasmic reticulum   cellular component:  ribonucleoprotein complex   molecular function:  RNA binding   molecular function:  nucleotide binding   molecular function:  nucleic acid binding      BioCarta Pathways:     KEGG Pathways:  
    Probe set : 1398409_at    Name:   Suppression of tumorigenicity 13    GB acc:   AA850428    UniGene:   Rn.8796    Gene Symbol:   St13    Locus Link:   81800    Chromosome:   7    Cytoband:   Chr:7q34    SOURCE    GENECARD       Gene Ontology:   biological process:  protein folding   cellular component:  cytoplasm      BioCarta Pathways:     KEGG Pathways:  
    Probe set : 1368812_at    Name:   hypothetical gene supported by NM_053905    GB acc:   AI407364     Gene Symbol:   LOC497722    Locus Link:   497722    Chromosome:   18    Cytoband:   Chr:18q12.1     GENECARD       Gene Ontology:     BioCarta Pathways:     KEGG Pathways:  
    Probe set : 1378493_at    Name:   family with sequence similarity 29, member A (predicted)    GB acc:   BE117901    UniGene:   Rn.47423    Gene Symbol:   Fam29a_predicted    Locus Link:   366403    Chromosome:   5    Cytoband:   Chr:5q32    SOURCE    GENECARD       Gene Ontology:     BioCarta Pathways:     KEGG Pathways:  
    Probe set : 1395761_at    Name:   Sema domain, immunoglobulin domain (Ig), short basic domain, secreted, (semaphorin) 3B (predicted)    GB acc:   AW920270    UniGene:   Rn.26859     Locus Link:   363142    Chromosome:   8    Cytoband:   Chr:8q32    SOURCE        Gene Ontology:   cellular component:  extracellular space   cellular component:  endoplasmic reticulum   cellular component:  membrane   molecular function:  receptor activity      BioCarta Pathways:     KEGG Pathways:  
    Probe set : 1373242_at    Name:   TATA box binding protein-like 1 (predicted)    GB acc:   AW434020    UniGene:   Rn.25148    Gene Symbol:   Tbpl1_predicted    Locus Link:   292173    Chromosome:   1    Cytoband:   Chr:1p12    SOURCE    GENECARD       Gene Ontology:   cellular component:  nucleus   cellular component:  transcription factor TFIID complex   cellular component:  cytoplasm   molecular function:  DNA binding   molecular function:  RNA polymerase II transcription factor activity      BioCarta Pathways:     KEGG Pathways:  
    Probe set : 1389865_at    Name:   target of EGR1, member 1 (nuclear) (predicted)    GB acc:   AW525690    UniGene:   Rn.105772    Gene Symbol:   Toe1_predicted    Locus Link:   298443    Chromosome:   5    Cytoband:   Chr:5q36    SOURCE    GENECARD       Gene Ontology:   biological process:  negative regulation of transcription   cellular component:  nucleus   molecular function:  nucleic acid binding   molecular function:  transcriptional repressor activity      BioCarta Pathways:     KEGG Pathways:  
    Probe set : 1383133_at    Name:   Transcribed locus    GB acc:   BI295765    UniGene:   Rn.18130        SOURCE        Gene Ontology:     BioCarta Pathways:     KEGG Pathways:  
    Probe set : 1381915_at    Name:   Transcribed locus    GB acc:   AI111735    UniGene:   Rn.29775        SOURCE        Gene Ontology:     BioCarta Pathways:     KEGG Pathways:  
    Probe set : 1374982_at    Name:   protein phosphatase 1, regulatory (inhibitor) subunit 16A (predicted)    GB acc:   BF412002    UniGene:   Rn.145223    Gene Symbol:   Ppp1r16a_predicted    Locus Link:   362944    Chromosome:   7    Cytoband:   Chr:7q34    SOURCE    GENECARD       Gene Ontology:   cellular component:  cytosol   cellular component:  membrane      BioCarta Pathways:     KEGG Pathways:  
    Probe set : 1388486_at    Name:   Dipeptidylpeptidase 8 (predicted)    GB acc:   BG671315    UniGene:   Rn.17532     Locus Link:   315758    Chromosome:   8    Cytoband:   Chr:8q24    SOURCE        Gene Ontology:   biological process:  proteolysis   biological process:  immune response   cellular component:  cytoplasm   cellular component:  membrane      BioCarta Pathways:     KEGG Pathways:  
    Probe set : 1376071_at    Name:   Transcribed locus    GB acc:   BM389326    UniGene:   Rn.3101        SOURCE        Gene Ontology:     BioCarta Pathways:     KEGG Pathways:  
    Probe set : 1378958_at     GB acc:   AA998314              Gene Ontology:     BioCarta Pathways:     KEGG Pathways:  
    Probe set : 1371885_at    Name:   cytoskeleton-associated protein 1 (predicted)    GB acc:   BI282366    UniGene:   Rn.9550    Gene Symbol:   Ckap1_predicted    Locus Link:   292777    Chromosome:   1    Cytoband:   Chr:1q21    SOURCE    GENECARD       Gene Ontology:   biological process:  protein folding   biological process:  microtubule-based process   cellular component:  cytoskeleton   cellular component:  microtubule cytoskeleton   molecular function:  unfolded protein binding      BioCarta Pathways:     KEGG Pathways:  
    Probe set : 1386927_at    Name:   carnitine palmitoyltransferase 2    GB acc:   NM_012930    UniGene:   Rn.11389    Gene Symbol:   Cpt2    Locus Link:   25413    Chromosome:   5    Cytoband:   Chr:5q35    SOURCE    GENECARD       Gene Ontology:     BioCarta Pathways:     KEGG Pathways:  
    Probe set : 1370155_at    Name:   procollagen, type I, alpha 2    GB acc:   BM388837    UniGene:   Rn.107239    Gene Symbol:   Col1a2    Locus Link:   84352    Chromosome:   4    Cytoband:   Chr:4q13    SOURCE    GENECARD       Gene Ontology:     BioCarta Pathways:     KEGG Pathways:  
    Probe set : 1387125_at    Name:   S100 calcium binding protein A9 (calgranulin B)    GB acc:   NM_053587    UniGene:   Rn.6703    Gene Symbol:   S100a9    Locus Link:   94195    Chromosome:   2    Cytoband:   Chr:2q34    SOURCE    GENECARD       Gene Ontology:   molecular function:  calcium ion binding      BioCarta Pathways:     KEGG Pathways:  
    Probe set : 1385314_at    Name:   Transcribed locus    GB acc:   AA923974    UniGene:   Rn.147371        SOURCE        Gene Ontology:     BioCarta Pathways:     KEGG Pathways:  
    Probe set : 1381162_at    Name:   Similar to septin 10 isoform 1    GB acc:   AI136863    UniGene:   Rn.27246     Locus Link:   309891    Chromosome:   20    Cytoband:   Chr:20q13    SOURCE        Gene Ontology:   biological process:  cell cycle   biological process:  cell division   cellular component:  nucleus   molecular function:  nucleotide binding   molecular function:  GTP binding      BioCarta Pathways:     KEGG Pathways:  
    Probe set : 1378063_at    Name:   Transcribed locus    GB acc:   AW524795    UniGene:   Rn.147728        SOURCE        Gene Ontology:     BioCarta Pathways:     KEGG Pathways:  
    Probe set : 1369425_at    Name:   cadherin 13    GB acc:   NM_138889    UniGene:   Rn.23806    Gene Symbol:   Cdh13    Locus Link:   192248    Chromosome:   19    Cytoband:   Chr:19q12    SOURCE    GENECARD       Gene Ontology:   cellular component:  plasma membrane   cellular component:  external side of plasma membrane   cellular component:  membrane   molecular function:  calcium ion binding   molecular function:  protein binding   molecular function:  protein homodimerization activity      BioCarta Pathways:     KEGG Pathways:  
    Probe set : 1387587_at    Name:   tumor necrosis factor (ligand) superfamily, member 6    GB acc:   NM_012908    UniGene:   Rn.9725    Gene Symbol:   Tnfsf6    Locus Link:   25385    Chromosome:   13    Cytoband:   Chr:13q22    SOURCE    GENECARD       Gene Ontology:   biological process:  apoptosis   biological process:  immune response   biological process:  signal transduction   molecular function:  cytokine activity   molecular function:  tumor necrosis factor receptor binding      BioCarta Pathways:     KEGG Pathways:  
    Probe set : 1398553_at    Name:   similar to CGI-100-like protein    GB acc:   AI137113    UniGene:   Rn.85410    Gene Symbol:   RGD:1359437    Locus Link:   289883    Chromosome:   14    Cytoband:   Chr:14p22    SOURCE    GENECARD       Gene Ontology:   biological process:  intracellular protein transport   cellular component:  endoplasmic reticulum   cellular component:  membrane   cellular component:  integral to membrane   molecular function:  protein carrier activity      BioCarta Pathways:     KEGG Pathways:  
    Probe set : 1383160_at    Name:   cysteine and histidine-rich domain (CHORD)-containing, zinc-binding protein 1 (predicted)    GB acc:   AA892238    UniGene:   Rn.6136    Gene Symbol:   Chordc1_predicted    Locus Link:   315447    Chromosome:   8    Cytoband:   Chr:8q12    SOURCE    GENECARD       Gene Ontology:   molecular function:  calcium ion binding   molecular function:  zinc ion binding      BioCarta Pathways:     KEGG Pathways:  
    Probe set : 1373174_at    Name:   similar to 1110001K21Rik protein    GB acc:   AW915592    UniGene:   Rn.138647    Gene Symbol:   LOC497867    Locus Link:   497867    Chromosome:   10    Cytoband:   Chr:10q12    SOURCE    GENECARD       Gene Ontology:     BioCarta Pathways:     KEGG Pathways:  
    Probe set : 1380035_at    Name:   Similar to RIKEN cDNA 5830433M19    GB acc:   BF523119    UniGene:   Rn.9502     Locus Link:   500501      SOURCE        Gene Ontology:     BioCarta Pathways:     KEGG Pathways:  
    Probe set : 1386096_at    Name:   similar to Metal-response element-binding transcription factor 2 (Metal-response element DNA-binding protein M96) (predicted)    GB acc:   BG666758    UniGene:   Rn.20300    Gene Symbol:   RGD1304727_predicted    Locus Link:   360905    Chromosome:   14    Cytoband:   Chr:14p22    SOURCE    GENECARD       Gene Ontology:   biological process:  regulation of transcription, DNA-dependent   cellular component:  nucleus   cellular component:  transcription factor complex      BioCarta Pathways:     KEGG Pathways:  
    Probe set : 1388172_at    Name:   integral membrane transport UST1r    GB acc:   Y09945    UniGene:   Rn.31066    Gene Symbol:   Ust1r    Locus Link:   192273    Chromosome:   1    Cytoband:   Chr:1q43    SOURCE    GENECARD       Gene Ontology:   biological process:  transport   cellular component:  integral to membrane   molecular function:  transporter activity   molecular function:  transporter activity      BioCarta Pathways:     KEGG Pathways:  
    Probe set : 1373866_at    Name:   similar to hypothetical protein FLJ13448    GB acc:   AI228596    UniGene:   Rn.101706    Gene Symbol:   RGD:1359509    Locus Link:   301416    Chromosome:   9    Cytoband:   Chr:9q31    SOURCE    GENECARD       Gene Ontology:   biological process:  antibiotic biosynthesis   molecular function:  polyketide synthase activity      BioCarta Pathways:     KEGG Pathways:  
    Probe set : 1385625_at    Name:   EGF-containing fibulin-like extracellular matrix protein 2    GB acc:   BF551426    UniGene:   Rn.16151    Gene Symbol:   RGD:1359496    Locus Link:   293677    Chromosome:   1    Cytoband:   Chr:1q43    SOURCE    GENECARD       Gene Ontology:   biological process:  homophilic cell adhesion   biological process:  blood coagulation   cellular component:  membrane   molecular function:  transmembrane receptor activity   molecular function:  structural molecule activity   molecular function:  calcium ion binding      BioCarta Pathways:     KEGG Pathways:  
    Probe set : 1383143_at    Name:   similar to Ephb2 protein    GB acc:   BF549473    UniGene:   Rn.27233    Gene Symbol:   LOC313633    Locus Link:   313633    Chromosome:   5    Cytoband:   Chr:5q36    SOURCE    GENECARD       Gene Ontology:     BioCarta Pathways:     KEGG Pathways:  
    Probe set : 1372889_at    Name:   Solute carrier organic anion transporter family, member 2a1    GB acc:   AI407489    UniGene:   Rn.9671    Gene Symbol:   Slco2a1    Locus Link:   24546    Chromosome:   8    Cytoband:   Chr:8q32    SOURCE    GENECARD       Gene Ontology:   biological process:  transport   biological process:  lipid transport   biological process:  prostaglandin transport   molecular function:  transporter activity   molecular function:  lipid transporter activity   molecular function:  prostaglandin transporter activity      BioCarta Pathways:     KEGG Pathways:  
    Probe set : 1372813_at    Name:   similar to RIKEN cDNA 1810046J19 (predicted)    GB acc:   AA858862    UniGene:   Rn.143893    Gene Symbol:   RGD1306682_predicted    Locus Link:   360617    Chromosome:   10    Cytoband:   Chr:10q31    SOURCE    GENECARD       Gene Ontology:     BioCarta Pathways:     KEGG Pathways:  
    Probe set : 1388863_at    Name:   similar to ubiquitin-conjugating enzyme HR6A    GB acc:   BE111651    UniGene:   Rn.144869    Gene Symbol:   RGD:1359534    Locus Link:   298317    Chromosome:   X    Cytoband:   Chr:Xq12    SOURCE    GENECARD       Gene Ontology:   biological process:  protein modification   biological process:  ubiquitin cycle   molecular function:  ubiquitin-like activating enzyme activity   molecular function:  ligase activity      BioCarta Pathways:     KEGG Pathways:  
    Probe set : 1373733_at    Name:   Bcl-2-related ovarian killer protein    GB acc:   AI227742    UniGene:   Rn.44461    Gene Symbol:   Bok    Locus Link:   29884    Chromosome:   9    Cytoband:   Chr:9q36    SOURCE    GENECARD       Gene Ontology:   molecular function:  protein dimerization activity      BioCarta Pathways:     KEGG Pathways:  
    Probe set : 1397297_at    Name:   Transcribed locus    GB acc:   AI176747    UniGene:   Rn.129187        SOURCE        Gene Ontology:     BioCarta Pathways:     KEGG Pathways:  
    Probe set : 1378940_at    Name:   similar to hypothetical protein FLJ10539 (predicted)    GB acc:   AI170278    UniGene:   Rn.54034    Gene Symbol:   RGD1310610_predicted    Locus Link:   297483    Chromosome:   4    Cytoband:   Chr:4q34    SOURCE    GENECARD       Gene Ontology:     BioCarta Pathways:     KEGG Pathways:  
    Probe set : 1371475_at    Name:   ribonuclease, RNase A family 4    GB acc:   BI284831    UniGene:   Rn.1742    Gene Symbol:   Rnase4    Locus Link:   56759    Chromosome:   15    Cytoband:   Chr:15p14    SOURCE    GENECARD       Gene Ontology:   biological process:  mRNA cleavage      BioCarta Pathways:     KEGG Pathways:  
    Probe set : 1379932_at    Name:   Putative chloride channel (similar to Mm Clcn4-2)    GB acc:   BG378827    UniGene:   Rn.44406    Gene Symbol:   Clcn4-2    Locus Link:   60586    Chromosome:   X    Cytoband:   Chr:Xq21    SOURCE    GENECARD       Gene Ontology:   biological process:  transport   biological process:  ion transport   biological process:  chloride transport   cellular component:  membrane   cellular component:  integral to membrane      BioCarta Pathways:     KEGG Pathways:  
    Probe set : 1379528_at    Name:   Transcribed locus    GB acc:   BI284017    UniGene:   Rn.129894        SOURCE        Gene Ontology:     BioCarta Pathways:     KEGG Pathways:  
    Probe set : 1390185_at    Name:   mRNA decapping enzyme    GB acc:   BE109709    UniGene:   Rn.144684    Gene Symbol:   Dcps    Locus Link:   266605    Chromosome:   8    Cytoband:   Chr:8q21    SOURCE    GENECARD       Gene Ontology:   biological process:  mRNA catabolism, nonsense-mediated decay   cellular component:  nucleus   molecular function:  hydrolase activity      BioCarta Pathways:     KEGG Pathways:  
    Probe set : 1379362_at    Name:   Transcribed locus    GB acc:   BE111121    UniGene:   Rn.13585        SOURCE        Gene Ontology:     BioCarta Pathways:     KEGG Pathways:  
    Probe set : 1381403_at    Name:   malic enzyme 2, NAD(+)-dependent, mitochondrial (predicted)    GB acc:   AI575071    UniGene:   Rn.38235    Gene Symbol:   Me2_predicted    Locus Link:   307270    Chromosome:   18    Cytoband:   Chr:18q12.2    SOURCE    GENECARD       Gene Ontology:   biological process:  pyruvate metabolism   biological process:  malate metabolism   cellular component:  mitochondrion      BioCarta Pathways:     KEGG Pathways:  
    Probe set : 1370969_at    Name:   homeo box A5    GB acc:   BE107303    UniGene:   Rn.8465    Gene Symbol:   Hoxa5    Locus Link:   79241    Chromosome:   4    Cytoband:   Chr:4q24    SOURCE    GENECARD       Gene Ontology:   cellular component:  nucleus   cellular component:  transcription factor complex   cellular component:  transcription factor complex   molecular function:  DNA binding   molecular function:  transcription factor activity   molecular function:  transcription factor activity      BioCarta Pathways:     KEGG Pathways:  
    Probe set : 1387600_at    Name:   gamma-aminobutyric acid (GABA) A receptor, pi    GB acc:   NM_031029    UniGene:   Rn.21401    Gene Symbol:   Gabrp    Locus Link:   81658    Chromosome:   10    Cytoband:   Chr:10q12    SOURCE    GENECARD       Gene Ontology:     BioCarta Pathways:     KEGG Pathways:  
    Probe set : 1383063_a_at    Name:   CDNA clone IMAGE:7379585    GB acc:   AI137878    UniGene:   Rn.6374        SOURCE        Gene Ontology:     BioCarta Pathways:     KEGG Pathways:  
    Probe set : 1391871_at    Name:   Transcribed locus    GB acc:   AI763550    UniGene:   Rn.26672        SOURCE        Gene Ontology:     BioCarta Pathways:     KEGG Pathways:  
    Probe set : 1387608_at    Name:   indoleamine 2,3-dioxygenase    GB acc:   NM_023973    UniGene:   Rn.64513    Gene Symbol:   Indo    Locus Link:   66029    Chromosome:   16    Cytoband:   Chr:16q12.5    SOURCE    GENECARD       Gene Ontology:   cellular component:  cytosol      BioCarta Pathways:     KEGG Pathways:  
    Probe set : 1391773_at    Name:   Transcribed locus    GB acc:   BM390802    UniGene:   Rn.147680        SOURCE        Gene Ontology:     BioCarta Pathways:     KEGG Pathways:  
    Probe set : 1378260_at    Name:   Alcohol dehydrogenase 1    GB acc:   AA817761    UniGene:   Rn.40222    Gene Symbol:   Adh1    Locus Link:   24172    Chromosome:   2    Cytoband:   Chr:2q44    SOURCE    GENECARD       Gene Ontology:   biological process:  retinoid metabolism      BioCarta Pathways:     KEGG Pathways:  
    Probe set : 1388586_at    Name:   synaptojanin 1    GB acc:   BG372397    UniGene:   Rn.22685    Gene Symbol:   Synj1    Locus Link:   85238    Chromosome:   11    Cytoband:   Chr:11q11    SOURCE    GENECARD       Gene Ontology:   cellular component:  membrane coat   cellular component:  clathrin coat      BioCarta Pathways:     KEGG Pathways:  
    Probe set : 1375223_at     GB acc:   BI294737              Gene Ontology:     BioCarta Pathways:     KEGG Pathways:  
    Probe set : 1374720_at    Name:   Transcribed locus    GB acc:   BE109644    UniGene:   Rn.7100        SOURCE        Gene Ontology:     BioCarta Pathways:     KEGG Pathways:  
    Probe set : 1385563_at    Name:   Similar to Catechol O-methyltransferase    GB acc:   AA900591    UniGene:   Rn.110415     Locus Link:   308868    Chromosome:   1    Cytoband:   Chr:1q32    SOURCE        Gene Ontology:     BioCarta Pathways:     KEGG Pathways:  
    Probe set : 1374704_at    Name:   KDEL (Lys-Asp-Glu-Leu) containing 2    GB acc:   BM384752    UniGene:   Rn.98686    Gene Symbol:   Kdelc2    Locus Link:   315664    Chromosome:   8    Cytoband:   Chr:8q24    SOURCE    GENECARD       Gene Ontology:     BioCarta Pathways:     KEGG Pathways:  
    Probe set : 1381217_at    Name:   similar to CGI-09 protein (predicted)    GB acc:   AI639158    UniGene:   Rn.8742    Gene Symbol:   RGD1308877_predicted    Locus Link:   311441    Chromosome:   3    Cytoband:   Chr:3q36    SOURCE    GENECARD       Gene Ontology:     BioCarta Pathways:     KEGG Pathways:  
    Probe set : 1378377_at    Name:   dual adaptor for phosphotyrosine and 3-phosphoinositides 1 (predicted)    GB acc:   BF552453    UniGene:   Rn.136891    Gene Symbol:   Dapp1_predicted    Locus Link:   362046    Chromosome:   2    Cytoband:   Chr:2q44    SOURCE    GENECARD       Gene Ontology:   biological process:  protein amino acid dephosphorylation   biological process:  intracellular signaling cascade   cellular component:  phosphoinositide 3-kinase complex   molecular function:  protein tyrosine phosphatase activity   molecular function:  phospholipid binding   molecular function:  phosphatidylinositol 3-kinase activity      BioCarta Pathways:     KEGG Pathways:  
    Probe set : 1390027_at    Name:   ubiquitin specific protease 8 (predicted)    GB acc:   AI137190    UniGene:   Rn.52174    Gene Symbol:   Usp8_predicted    Locus Link:   296121    Chromosome:   3    Cytoband:   Chr:3q36    SOURCE    GENECARD       Gene Ontology:   cellular component:  intracellular      BioCarta Pathways:     KEGG Pathways:  
    Probe set : 1393020_at    Name:   similar to ZNF258 protein    GB acc:   BE115368    UniGene:   Rn.118708    Gene Symbol:   LOC500548    Locus Link:   500548    Chromosome:   5    Cytoband:   Chr:5q36    SOURCE    GENECARD       Gene Ontology:     BioCarta Pathways:     KEGG Pathways:  
    Probe set : 1379858_at    Name:   methyltransferase like 2 (predicted)    GB acc:   AA955134    UniGene:   Rn.128311    Gene Symbol:   Mettl2_predicted    Locus Link:   363687    Chromosome:   10    Cytoband:   Chr:10q32.1    SOURCE    GENECARD       Gene Ontology:   molecular function:  methyltransferase activity   molecular function:  S-adenosylmethionine-dependent methyltransferase activity   molecular function:  transferase activity      BioCarta Pathways:     KEGG Pathways:  
    Probe set : 1368211_at    Name:   ribosomal protein S14    GB acc:   NM_022672    UniGene:   Rn.144569    Gene Symbol:   Rps14    Locus Link:   29284    Chromosome:   18    Cytoband:   Chr:18q12.1    SOURCE    GENECARD       Gene Ontology:   biological process:  protein biosynthesis   biological process:  ribosome biogenesis   molecular function:  RNA binding   molecular function:  structural constituent of ribosome   molecular function:  structural constituent of ribosome      BioCarta Pathways:     KEGG Pathways:  
    Probe set : 1368777_at    Name:   BRCA1 associated RING domain 1    GB acc:   NM_022622    UniGene:   Rn.48735    Gene Symbol:   Bard1    Locus Link:   64557    Chromosome:   9    Cytoband:   Chr:9q33    SOURCE    GENECARD       Gene Ontology:   biological process:  regulation of transcription, DNA-dependent   biological process:  protein ubiquitination   biological process:  tissue homeostasis   biological process:  response to DNA damage stimulus   biological process:  cell cycle arrest   biological process:  protein ubiquitination   biological process:  negative regulation of mRNA 3'-end processing   biological process:  regulation of phosphorylation   biological process:  positive regulation of apoptosis   biological process:  negative regulation of apoptosis   biological process:  positive regulation of protein catabolism   biological process:  negative regulation of protein export from nucleus      BioCarta Pathways:     KEGG Pathways:  
    Probe set : 1398576_at    Name:   similar to SIRP beta 1 isoform 1 /// protein tyrosine phosphatase, non-receptor type substrate 1-like 3 (predicted)    GB acc:   AI237410    UniGene:   Rn.90529    Gene Symbol:   LOC310212 /// Ptpns1l3_predicted    Locus Link:   310212 /// 310213    Chromosome:   2    Cytoband:   Chr:2q23    SOURCE    GENECARD       Gene Ontology:   molecular function:  receptor activity      BioCarta Pathways:     KEGG Pathways:  
    Probe set : 1375881_at    Name:   destrin (predicted)    GB acc:   AI170442    UniGene:   Rn.111805    Gene Symbol:   Dstn_predicted    Locus Link:   296197    Chromosome:   20    Cytoband:   Chr:20p12    SOURCE    GENECARD       Gene Ontology:   cellular component:  intracellular   molecular function:  actin binding      BioCarta Pathways:     KEGG Pathways:  
    Probe set : 1367831_at    Name:   tumor protein p53    GB acc:   NM_030989    UniGene:   Rn.54443    Gene Symbol:   Tp53    Locus Link:   24842    Chromosome:   10    Cytoband:   Chr:10q24    SOURCE    GENECARD       Gene Ontology:   biological process:  protein import into nucleus, translocation   biological process:  cell cycle checkpoint   biological process:  base-excision repair   biological process:  nucleotide-excision repair   biological process:  DNA recombination   biological process:  transcription   biological process:  regulation of transcription, DNA-dependent   biological process:  regulation of transcription from RNA polymerase II promoter   biological process:  regulation of transcription from RNA polymerase II promoter   biological process:  apoptosis   biological process:  response to DNA damage stimulus   biological process:  cell cycle   biological process:  cell cycle arrest   biological process:  cell aging   biological process:  negative regulation of DNA replication   biological process:  cell proliferation   biological process:  induction of apoptosis by hormones   biological process:  DNA damage response, signal transduction resulting in induction of apoptosis   biological process:  caspase activation via cytochrome c   biological process:  response to UV   biological process:  response to X-ray   biological process:  cell differentiation   biological process:  negative regulation of cell growth   biological process:  negative regulation of cell growth   biological process:  DNA damage response, signal transduction by p53 class mediator   biological process:  regulation of cell proliferation   biological process:  DNA damage response, signal transduction by p53 class mediator resulting in induction of apoptosis   biological process:  negative regulation of apoptosis   biological process:  negative regulation of progression through cell cycle   biological process:  regulation of mitochondrial membrane permeability   biological process:  negative regulation of fibroblast proliferation   biological process:  negative regulation of helicase activity   biological process:  protein tetramerization   biological process:  base-excision repair   biological process:  nucleotide-excision repair   biological process:  apoptosis   biological process:  cell cycle arrest   biological process:  development   biological process:  cell aging   biological process:  cell proliferation   biological process:  caspase activation via cytochrome c   biological process:  cell differentiation   biological process:  negative regulation of cell growth   molecular function:  DNA strand annealing activity   molecular function:  DNA binding   molecular function:  transcription factor activity   molecular function:  RNA polymerase II transcription factor activity   molecular function:  RNA polymerase II transcription factor activity   molecular function:  nuclease activity   molecular function:  copper ion binding   molecular function:  protein binding   molecular function:  ATP binding   molecular function:  zinc ion binding   molecular function:  protein binding   molecular function:  DNA strand annealing activity   molecular function:  DNA binding   molecular function:  copper ion binding   molecular function:  protein binding   molecular function:  ATP binding   molecular function:  metal ion binding      BioCarta Pathways:     KEGG Pathways:  
    Probe set : 1390389_at     GB acc:   BM385936              Gene Ontology:     BioCarta Pathways:     KEGG Pathways:  
    Probe set : 1395354_at    Name:   lanC (bacterial lantibiotic synthetase component C)-like 1    GB acc:   BF549735    UniGene:   Rn.38972    Gene Symbol:   Lancl1    Locus Link:   114515    Chromosome:   9    Cytoband:   Chr:9q32    SOURCE    GENECARD       Gene Ontology:   biological process:  G-protein coupled receptor protein signaling pathway   cellular component:  integral to plasma membrane   cellular component:  integral to membrane   molecular function:  G-protein coupled receptor activity      BioCarta Pathways:     KEGG Pathways:  
    Probe set : 1389219_at    Name:   Similar to serine/threonine kinase    GB acc:   AI406322    UniGene:   Rn.101953     Locus Link:   498312      SOURCE        Gene Ontology:     BioCarta Pathways:     KEGG Pathways:  
    Probe set : 1375250_at    Name:   UDP-Gal:betaGlcNAc beta 1,4- galactosyltransferase, polypeptide 1    GB acc:   BF549771    UniGene:   Rn.17157    Gene Symbol:   B4galt1    Locus Link:   24390    Chromosome:   5    Cytoband:   Chr:5q22    SOURCE    GENECARD       Gene Ontology:   cellular component:  Golgi apparatus   cellular component:  plasma membrane   cellular component:  integral to membrane      BioCarta Pathways:     KEGG Pathways:  
    Probe set : 1373625_at    Name:   serine hydroxymethyl transferase 1 (soluble) (predicted)    GB acc:   AI412012    UniGene:   Rn.3684    Gene Symbol:   Shmt1_predicted    Locus Link:   287379    Chromosome:   10    Cytoband:   Chr:10q22    SOURCE    GENECARD       Gene Ontology:   cellular component:  mitochondrion   cellular component:  cytosol   molecular function:  glycine hydroxymethyltransferase activity   molecular function:  transferase activity      BioCarta Pathways:     KEGG Pathways:  
    Probe set : 1377457_a_at    Name:   sortilin-related receptor, L(DLR class) A repeats-containing (predicted)    GB acc:   AA850618    UniGene:   Rn.92871    Gene Symbol:   Sorl1_predicted    Locus Link:   300652    Chromosome:   8    Cytoband:   Chr:8q22    SOURCE    GENECARD       Gene Ontology:   biological process:  lipid transport   biological process:  receptor mediated endocytosis   biological process:  cholesterol metabolism   cellular component:  integral to plasma membrane   molecular function:  transmembrane receptor activity   molecular function:  lipid transporter activity      BioCarta Pathways:     KEGG Pathways:  
    Probe set : 1368765_at    Name:   chloride channel K1    GB acc:   NM_053327    UniGene:   Rn.88871    Gene Symbol:   Clcnk1    Locus Link:   79425    Chromosome:   5    Cytoband:   Chr:5q36    SOURCE    GENECARD       Gene Ontology:     BioCarta Pathways:     KEGG Pathways:  
    Probe set : 1381749_at    Name:   Transcribed locus    GB acc:   AI703743    UniGene:   Rn.91466        SOURCE        Gene Ontology:     BioCarta Pathways:     KEGG Pathways:  
    Probe set : 1393245_at    Name:   Phytanoyl-CoA hydroxylase    GB acc:   AI029057    UniGene:   Rn.7279    Gene Symbol:   Phyh    Locus Link:   114209    Chromosome:   17    Cytoband:   Chr:17q12.3    SOURCE    GENECARD       Gene Ontology:   cellular component:  peroxisome   cellular component:  peroxisome      BioCarta Pathways:     KEGG Pathways:  
    Probe set : 1374969_at    Name:   forkhead box D4    GB acc:   AA799832    UniGene:   Rn.108090    Gene Symbol:   Foxd4    Locus Link:   252886    Chromosome:   1    Cytoband:   Chr:1q51    SOURCE    GENECARD       Gene Ontology:   biological process:  regulation of transcription, DNA-dependent   biological process:  transcription   cellular component:  nucleus   molecular function:  DNA binding   molecular function:  transcription factor activity      BioCarta Pathways:     KEGG Pathways:  
    Probe set : 1372216_at    Name:   Transcribed locus    GB acc:   AI010259    UniGene:   Rn.17547        SOURCE        Gene Ontology:     BioCarta Pathways:     KEGG Pathways:  
    Probe set : 1374193_at    Name:   Transcribed locus    GB acc:   BI303636    UniGene:   Rn.2723        SOURCE        Gene Ontology:     BioCarta Pathways:     KEGG Pathways:  
    Probe set : 1372709_at    Name:   B-cell receptor-associated protein BAP29    GB acc:   AI172204    UniGene:   Rn.19451    Gene Symbol:   RGD:1359431    Locus Link:   298943    Chromosome:   6    Cytoband:   Chr:6q16    SOURCE    GENECARD       Gene Ontology:   biological process:  intracellular protein transport   biological process:  apoptosis   cellular component:  endoplasmic reticulum   cellular component:  integral to membrane   molecular function:  receptor activity      BioCarta Pathways:     KEGG Pathways:  
    Probe set : 1387109_at    Name:   P450 (cytochrome) oxidoreductase    GB acc:   NM_031576    UniGene:   Rn.11359    Gene Symbol:   Por    Locus Link:   29441    Chromosome:   12    Cytoband:   Chr:12q12    SOURCE    GENECARD       Gene Ontology:   biological process:  electron transport      BioCarta Pathways:     KEGG Pathways:  
    Probe set : 1371299_at    Name:   ribosomal protein S3    GB acc:   BG665124    UniGene:   Rn.127805    Gene Symbol:   Rps3    Locus Link:   140654    Chromosome:   1    Cytoband:   Chr:1q32    SOURCE    GENECARD       Gene Ontology:   biological process:  protein biosynthesis   molecular function:  nucleic acid binding   molecular function:  RNA binding   molecular function:  structural constituent of ribosome      BioCarta Pathways:     KEGG Pathways:  
    Probe set : 1377002_at    Name:   Transcribed locus    GB acc:   AA946209    UniGene:   Rn.107793        SOURCE        Gene Ontology:     BioCarta Pathways:     KEGG Pathways:  
    Probe set : 1375517_at    Name:   Tumor protein p53 inducible nuclear protein 2    GB acc:   AI171654    UniGene:   Rn.86075    Gene Symbol:   LOC362246    Locus Link:   362246    Chromosome:   3    Cytoband:   Chr:3q42    SOURCE    GENECARD       Gene Ontology:   cellular component:  nucleus      BioCarta Pathways:     KEGG Pathways:  
    Probe set : 1383457_at    Name:   similar to hypothetical protein DKFZp761N1114    GB acc:   AI709793    UniGene:   Rn.24654    Gene Symbol:   LOC498228    Locus Link:   498228    Chromosome:   13    Cytoband:   Chr:13q13    SOURCE    GENECARD       Gene Ontology:     BioCarta Pathways:     KEGG Pathways:  
    Probe set : 1389245_at    Name:   proteasome (prosome, macropain) 26S subunit, non-ATPase, 7 (predicted)    GB acc:   BF542903    UniGene:   Rn.20659    Gene Symbol:   Psmd7_predicted    Locus Link:   307821    Chromosome:   19    Cytoband:   Chr:19q12    SOURCE    GENECARD       Gene Ontology:   cellular component:  cytosol   cellular component:  proteasome regulatory particle (sensu Eukaryota)      BioCarta Pathways:     KEGG Pathways:  
    Probe set : 1368676_at    Name:   dynein, cytoplasmic, heavy polypeptide 2 /// hypothetical gene supported by NM_023024    GB acc:   NM_023024    UniGene:   Rn.44896    Gene Symbol:   Dnch2 /// LOC497827    Locus Link:   497827 /// 65209    Chromosome:   8    Cytoband:   Chr:8q11    SOURCE    GENECARD       Gene Ontology:   biological process:  microtubule-based movement   biological process:  transport   cellular component:  cytoplasmic dynein complex   cellular component:  dynein complex      BioCarta Pathways:     KEGG Pathways:  
    Probe set : 1372381_at    Name:   TSPY-like 4 (predicted)    GB acc:   BM383457    UniGene:   Rn.16382     Locus Link:   309828    Chromosome:   20    Cytoband:   Chr:20q12    SOURCE        Gene Ontology:   biological process:  nucleosome assembly   cellular component:  nucleus      BioCarta Pathways:     KEGG Pathways:  
    Probe set : 1373917_at    Name:   Transcribed locus    GB acc:   BE100459    UniGene:   Rn.98549        SOURCE        Gene Ontology:     BioCarta Pathways:     KEGG Pathways:  
    Probe set : 1384087_at     GB acc:   AA925049              Gene Ontology:     BioCarta Pathways:     KEGG Pathways:  
    Probe set : 1389941_at    Name:   ADP-ribosylation factor-like 2 binding protein    GB acc:   BE109130    UniGene:   Rn.985    Gene Symbol:   Arl2bp    Locus Link:   498910    Chromosome:   19    Cytoband:   Chr:19p12    SOURCE    GENECARD       Gene Ontology:     BioCarta Pathways:     KEGG Pathways:  
    Probe set : 1385904_at    Name:   cold inducible RNA binding protein    GB acc:   BG153342    UniGene:   Rn.28931    Gene Symbol:   Cirbp    Locus Link:   81825    Chromosome:   7    Cytoband:   Chr:7q11    SOURCE    GENECARD       Gene Ontology:   biological process:  response to cold   cellular component:  nucleus   molecular function:  nucleic acid binding   molecular function:  RNA binding   molecular function:  nucleotide binding      BioCarta Pathways:     KEGG Pathways:  
    Probe set : 1389185_at     GB acc:   BI281183              Gene Ontology:     BioCarta Pathways:     KEGG Pathways:  
    Probe set : 1371953_at    Name:   Cyclin G2 (predicted)    GB acc:   AI408309    UniGene:   Rn.3989    Gene Symbol:   Ccng2    Locus Link:   29157    Chromosome:   14    Cytoband:   Chr:14p22    SOURCE    GENECARD       Gene Ontology:   molecular function:  cyclin-dependent protein kinase regulator activity      BioCarta Pathways:     KEGG Pathways:  
    Probe set : 1374199_at    Name:   similar to Disco-interacting protein 2 homolog    GB acc:   BI282130    UniGene:   Rn.137473    Gene Symbol:   LOC309692    Locus Link:   309692    Chromosome:   20    Cytoband:   Chr:20p12    SOURCE    GENECARD       Gene Ontology:     BioCarta Pathways:     KEGG Pathways:  
    Probe set : 1368870_at    Name:   Inhibitor of DNA binding 2, dominant negative helix-loop-helix protein    GB acc:   NM_013060    UniGene:   Rn.3272    Gene Symbol:   Id2    Locus Link:   25587    Chromosome:   6    Cytoband:   Chr:6q16    SOURCE    GENECARD       Gene Ontology:   cellular component:  nucleus   cellular component:  nucleus   cellular component:  cytoplasm   molecular function:  protein binding   molecular function:  protein binding   molecular function:  transcription regulator activity      BioCarta Pathways:     KEGG Pathways:  
    Probe set : 1380655_at    Name:   renal tumor antigen (predicted)    GB acc:   AI317878    UniGene:   Rn.63172    Gene Symbol:   Rage_predicted    Locus Link:   362787    Chromosome:   6    Cytoband:   Chr:6q32    SOURCE    GENECARD       Gene Ontology:   biological process:  protein amino acid phosphorylation   biological process:  signal transduction   cellular component:  cytoplasm      BioCarta Pathways:     KEGG Pathways:  
    Probe set : 1387458_at    Name:   ring finger protein 4    GB acc:   NM_019182    UniGene:   Rn.30015    Gene Symbol:   Rnf4    Locus Link:   29274    Chromosome:   14    Cytoband:   Chr:14q21    SOURCE    GENECARD       Gene Ontology:   cellular component:  ubiquitin ligase complex   cellular component:  nucleus   cellular component:  nucleus      BioCarta Pathways:     KEGG Pathways:  
    Probe set : 1376727_at    Name:   similar to RIKEN cDNA 2310034L04 (predicted)    GB acc:   BF283348    UniGene:   Rn.25533    Gene Symbol:   RGD1310157_predicted    Locus Link:   362699    Chromosome:   6    Cytoband:   Chr:6q12    SOURCE    GENECARD       Gene Ontology:   cellular component:  integral to membrane      BioCarta Pathways:     KEGG Pathways:  
    Probe set : 1374785_at    Name:   similar to CD69 antigen (p60, early T-cell activation antigen)    GB acc:   BG380471    UniGene:   Rn.30526    Gene Symbol:   LOC296985    Locus Link:   296985    Chromosome:   4    Cytoband:   Chr:4q22    SOURCE    GENECARD       Gene Ontology:     BioCarta Pathways:     KEGG Pathways:  
    Probe set : 1374062_x_at    Name:   microtubule-associated protein, RP/EB family, member 3    GB acc:   AA946204    UniGene:   Rn.95256    Gene Symbol:   RGD:1359297    Locus Link:   298848    Chromosome:   6    Cytoband:   Chr:6q14    SOURCE    GENECARD       Gene Ontology:   biological process:  cell cycle   biological process:  mitosis   biological process:  cell division   cellular component:  microtubule   molecular function:  microtubule binding      BioCarta Pathways:     KEGG Pathways:  
    Probe set : 1384056_at    Name:   Fibroblast growth factor receptor 3    GB acc:   BF410980    UniGene:   Rn.23671    Gene Symbol:   Fgfr3    Locus Link:   84489    Chromosome:   14    Cytoband:   Chr:14q21    SOURCE    GENECARD       Gene Ontology:   cellular component:  extracellular space   cellular component:  integral to plasma membrane   cellular component:  integral to membrane      BioCarta Pathways:     KEGG Pathways:  
    Probe set : 1377091_at    Name:   NG5 protein    GB acc:   AW524733    UniGene:   Rn.144293    Gene Symbol:   RGD:1359348    Locus Link:   406167      SOURCE    GENECARD       Gene Ontology:     BioCarta Pathways:     KEGG Pathways:  
    Probe set : 1387082_at    Name:   fetuin beta    GB acc:   NM_053348    UniGene:   Rn.2464    Gene Symbol:   Fetub    Locus Link:   83928    Chromosome:   11    Cytoband:   Chr:11q23    SOURCE    GENECARD       Gene Ontology:   cellular component:  extracellular space   molecular function:  cysteine protease inhibitor activity      BioCarta Pathways:     KEGG Pathways:  
    Probe set : 1372373_at    Name:   Similar to putative WD-repeat protein    GB acc:   AI713206    UniGene:   Rn.107767     Locus Link:   317370    Chromosome:   X    Cytoband:   Chr:Xq13    SOURCE        Gene Ontology:     BioCarta Pathways:     KEGG Pathways:  
    Probe set : 1367955_at    Name:   RAB4B, member RAS oncogene family    GB acc:   NM_017355    UniGene:   Rn.7181    Gene Symbol:   Rab4b    Locus Link:   50866    Chromosome:   1    Cytoband:   Chr:1q21    SOURCE    GENECARD       Gene Ontology:   cellular component:  intracellular      BioCarta Pathways:     KEGG Pathways:  
    Probe set : 1368141_at    Name:   cellular nucleic acid binding protein 1    GB acc:   AF242550    UniGene:   Rn.6187    Gene Symbol:   Cnbp1    Locus Link:   64530    Chromosome:   4    Cytoband:   Chr:4q34    SOURCE    GENECARD       Gene Ontology:   cellular component:  nucleus   cellular component:  endoplasmic reticulum   cellular component:  cytosol      BioCarta Pathways:     KEGG Pathways:  
    Probe set : 1381533_at    Name:   Rho family GTPase 1 (predicted)    GB acc:   AI144754    UniGene:   Rn.2319    Gene Symbol:   Rnd1_predicted    Locus Link:   362993    Chromosome:   7    Cytoband:   Chr:7q36    SOURCE    GENECARD       Gene Ontology:   cellular component:  cytoskeleton   cellular component:  adherens junction   molecular function:  GTPase activity   molecular function:  GTP binding   molecular function:  nucleotide binding      BioCarta Pathways:     KEGG Pathways:  
    Probe set : 1370288_a_at    Name:   tropomyosin 1, alpha    GB acc:   AF372216    UniGene:   Rn.87540    Gene Symbol:   Tpm1    Locus Link:   24851    Chromosome:   8    Cytoband:   Chr:8q24    SOURCE    GENECARD       Gene Ontology:   cellular component:  cytoskeleton   cellular component:  muscle thin filament tropomyosin   cellular component:  kinesin complex      BioCarta Pathways:     KEGG Pathways:  
    Probe set : 1390264_at    Name:   similar to RIKEN cDNA 1200014J11 (predicted)    GB acc:   BF413854    UniGene:   Rn.13279    Gene Symbol:   RGD1308139_predicted    Locus Link:   360563    Chromosome:   10    Cytoband:   Chr:10q24    SOURCE    GENECARD       Gene Ontology:     BioCarta Pathways:     KEGG Pathways:  
    Probe set : 1388959_at    Name:   similar to KIAA0153 protein (predicted)    GB acc:   BI297049    UniGene:   Rn.120058    Gene Symbol:   RGD1305319_predicted    Locus Link:   300105    Chromosome:   7    Cytoband:   Chr:7q34    SOURCE    GENECARD       Gene Ontology:     BioCarta Pathways:     KEGG Pathways:  
    Probe set : 1372747_at    Name:   solute carrier family 20, member 2    GB acc:   AI409899    UniGene:   Rn.13642    Gene Symbol:   Slc20a2    Locus Link:   29502    Chromosome:   16    Cytoband:   Chr:16q12.5    SOURCE    GENECARD       Gene Ontology:   biological process:  transport   biological process:  phosphate transport      BioCarta Pathways:     KEGG Pathways:  
    Probe set : 1372321_at    Name:   lymphoblastic leukemia derived sequence 1    GB acc:   BE111805    UniGene:   Rn.17352    Gene Symbol:   RGD:1359244    Locus Link:   304663    Chromosome:   19    Cytoband:   Chr:19q11    SOURCE    GENECARD       Gene Ontology:   biological process:  regulation of transcription   cellular component:  nucleus   molecular function:  transcription regulator activity      BioCarta Pathways:     KEGG Pathways:  
    Probe set : 1389806_at    Name:   Transcribed locus    GB acc:   BF402920    UniGene:   Rn.51515        SOURCE        Gene Ontology:     BioCarta Pathways:     KEGG Pathways:  
    Probe set : 1391581_at    Name:   Rho GTPase activating protein 5 (predicted)    GB acc:   BE104302    UniGene:   Rn.20898     Locus Link:   299012    Chromosome:   6    Cytoband:   Chr:6q23    SOURCE        Gene Ontology:   cellular component:  cytoplasm   cellular component:  membrane      BioCarta Pathways:     KEGG Pathways:  
    Probe set : 1387965_at    Name:   kidney injury molecule 1    GB acc:   AF035963    UniGene:   Rn.11154    Gene Symbol:   Havcr1    Locus Link:   286934    Chromosome:   10    Cytoband:   Chr:10q21    SOURCE    GENECARD       Gene Ontology:   biological process:  cell adhesion   cellular component:  integral to membrane      BioCarta Pathways:     KEGG Pathways:  
    Probe set : 1399142_at    Name:   GLE1 RNA export mediator-like (yeast (predicted)    GB acc:   BF398045    UniGene:   Rn.144353     Locus Link:   362098    Chromosome:   3    Cytoband:   Chr:3p12    SOURCE        Gene Ontology:   biological process:  mRNA export from nucleus   cellular component:  nuclear pore      BioCarta Pathways:     KEGG Pathways:  
    Probe set : 1369685_at    Name:   twist homolog 2 (Drosophila)    GB acc:   NM_021691    UniGene:   Rn.16279    Gene Symbol:   Twist2    Locus Link:   59327    Chromosome:   9    Cytoband:   Chr:9q36    SOURCE    GENECARD       Gene Ontology:   biological process:  mesoderm formation   biological process:  regulation of transcription, DNA-dependent   biological process:  regulation of transcription from RNA polymerase II promoter   biological process:  transcription from RNA polymerase II promoter   biological process:  anti-apoptosis   biological process:  anti-apoptosis   biological process:  development   biological process:  cell differentiation   biological process:  negative regulation of osteoblast differentiation   biological process:  negative regulation of transcription, DNA-dependent   biological process:  negative regulation of osteoblast differentiation   biological process:  negative regulation of transcription, DNA-dependent   biological process:  transcription   biological process:  regulation of transcription      BioCarta Pathways:     KEGG Pathways:  
    Probe set : 1396152_s_at    Name:   insulin-like growth factor binding protein 5    GB acc:   BF399783    UniGene:   Rn.1593    Gene Symbol:   Igfbp5    Locus Link:   25285    Chromosome:   9    Cytoband:   Chr:9q33    SOURCE    GENECARD       Gene Ontology:   biological process:  regulation of cell growth   biological process:  signal transduction   cellular component:  extracellular region   cellular component:  extracellular space   cellular component:  insulin-like growth factor binding protein complex   molecular function:  insulin-like growth factor binding   molecular function:  growth factor binding      BioCarta Pathways:     KEGG Pathways:  
    Probe set : 1372392_at    Name:   Transcribed locus    GB acc:   AI599125    UniGene:   Rn.15997        SOURCE        Gene Ontology:     BioCarta Pathways:     KEGG Pathways:  
    Probe set : 1388489_at     GB acc:   AA875107              Gene Ontology:     BioCarta Pathways:     KEGG Pathways:  
    Probe set : 1386935_at    Name:   nuclear receptor subfamily 4, group A, member 1    GB acc:   NM_024388    UniGene:   Rn.10000    Gene Symbol:   Nr4a1    Locus Link:   79240    Chromosome:   7    Cytoband:   Chr:7q36    SOURCE    GENECARD       Gene Ontology:   cellular component:  nucleus      BioCarta Pathways:     KEGG Pathways:  
    Probe set : 1383355_at    Name:   ATP-binding cassette, sub-family A (ABC1), member 1 /// hypothetical gene supported by NM_178095    GB acc:   AW918387    UniGene:   Rn.3724    Gene Symbol:   Abca1 /// LOC497803    Locus Link:   313210 /// 497803    Chromosome:   5    Cytoband:   Chr:5q24    SOURCE    GENECARD       Gene Ontology:     BioCarta Pathways:     KEGG Pathways:  
    Probe set : 1372615_at    Name:   amine oxidase, copper containing 3    GB acc:   AI070137    UniGene:   Rn.56603    Gene Symbol:   Aoc3    Locus Link:   29473    Chromosome:   10    Cytoband:   Chr:10q32.1    SOURCE    GENECARD       Gene Ontology:   biological process:  cell adhesion      BioCarta Pathways:     KEGG Pathways:  
    Probe set : 1368608_at    Name:   cytochrome P450, family 2, subfamily f, polypeptide 2    GB acc:   NM_019303    UniGene:   Rn.10817    Gene Symbol:   Cyp2f2    Locus Link:   54246    Chromosome:   1    Cytoband:   Chr:1q21    SOURCE    GENECARD       Gene Ontology:   biological process:  electron transport      BioCarta Pathways:     KEGG Pathways:  
    Probe set : 1380485_at    Name:   Similar to RIKEN cDNA 2600016J21    GB acc:   BE106894    UniGene:   Rn.140756     Locus Link:   498678      SOURCE        Gene Ontology:     BioCarta Pathways:     KEGG Pathways:  
    Probe set : 1376754_at    Name:   cysteinyl-tRNA synthetase (predicted)    GB acc:   AI176610    UniGene:   Rn.14865    Gene Symbol:   Cars_predicted    Locus Link:   293638    Chromosome:   1    Cytoband:   Chr:1q41    SOURCE    GENECARD       Gene Ontology:   biological process:  protein biosynthesis   biological process:  cysteinyl-tRNA aminoacylation   cellular component:  soluble fraction   cellular component:  cytoplasm      BioCarta Pathways:     KEGG Pathways:  
    Probe set : 1389409_at    Name:   similar to Testis derived transcript    GB acc:   AI177869    UniGene:   Rn.44847    Gene Symbol:   LOC500040    Locus Link:   500040    Chromosome:   4    Cytoband:   Chr:4q21    SOURCE    GENECARD       Gene Ontology:     BioCarta Pathways:     KEGG Pathways:  
    Probe set : 1390124_at    Name:   similar to hypothetical protein FLJ38426    GB acc:   AA801136    UniGene:   Rn.24187    Gene Symbol:   LOC499866    Locus Link:   499866    Chromosome:   3    Cytoband:   Chr:3q35    SOURCE    GENECARD       Gene Ontology:     BioCarta Pathways:     KEGG Pathways:  
    Probe set : 1373865_at    Name:   synaptosomal-associated protein, 91kDa homolog (mouse)    GB acc:   BF281248    UniGene:   Rn.11022    Gene Symbol:   Snap91    Locus Link:   65178    Chromosome:   8    Cytoband:   Chr:8q31    SOURCE    GENECARD       Gene Ontology:   cellular component:  coated pit   cellular component:  presynaptic membrane   cellular component:  presynaptic membrane   molecular function:  phospholipid binding      BioCarta Pathways:     KEGG Pathways:  
    Probe set : 1374888_at    Name:   Similar to RIKEN cDNA 1300013D05 (predicted)    GB acc:   AI317837    UniGene:   Rn.6626     Locus Link:   360613    Chromosome:   10    Cytoband:   Chr:10q31    SOURCE        Gene Ontology:     BioCarta Pathways:     KEGG Pathways:  
    Probe set : 1388757_at    Name:   Transcribed locus    GB acc:   AI716801    UniGene:   Rn.145906        SOURCE        Gene Ontology:     BioCarta Pathways:     KEGG Pathways:  
    Probe set : 1398931_at     GB acc:   BI282246              Gene Ontology:     BioCarta Pathways:     KEGG Pathways:  
    Probe set : 1377302_a_at    Name:   methylmalonic aciduria (cobalamin deficiency) type A (predicted)    GB acc:   AI145027    UniGene:   Rn.97639    Gene Symbol:   Mmaa_predicted    Locus Link:   291939    Chromosome:   19    Cytoband:   Chr:19q11    SOURCE    GENECARD       Gene Ontology:   cellular component:  mitochondrion   molecular function:  nucleotide binding   molecular function:  ATP binding      BioCarta Pathways:     KEGG Pathways:  
    Probe set : 1372156_at    Name:   similar to RIKEN cDNA 1810014L12 (predicted)    GB acc:   BF414018    UniGene:   Rn.20057    Gene Symbol:   RGD1307423_predicted    Locus Link:   303330    Chromosome:   10    Cytoband:   Chr:10q25    SOURCE    GENECARD       Gene Ontology:   cellular component:  extracellular space   cellular component:  integral to membrane      BioCarta Pathways:     KEGG Pathways:  
    Probe set : 1380210_at     GB acc:   AI136325              Gene Ontology:     BioCarta Pathways:     KEGG Pathways:  
    Probe set : 1375181_at    Name:   similar to 60S ribosomal protein L12    GB acc:   AI170643    UniGene:   Rn.128979    Gene Symbol:   LOC499782    Locus Link:   499782    Chromosome:   3    Cytoband:   Chr:3p11    SOURCE    GENECARD       Gene Ontology:     BioCarta Pathways:     KEGG Pathways:  
    Probe set : 1368386_at    Name:   growth factor receptor bound protein 2    GB acc:   NM_030846    UniGene:   Rn.3360    Gene Symbol:   Grb2    Locus Link:   81504    Chromosome:   10    Cytoband:   Chr:10q32.3    SOURCE    GENECARD       Gene Ontology:   cellular component:  vesicle membrane      BioCarta Pathways:     KEGG Pathways:  
    Probe set : 1368570_at    Name:   lecithin-retinol acyltransferase /// hypothetical gene supported by NM_022280    GB acc:   NM_022280    UniGene:   Rn.137431    Gene Symbol:   Lrat /// LOC497758    Locus Link:   497758 /// 64047    Chromosome:   2    Cytoband:   Chr:2q33 /// Chr:2q34    SOURCE    GENECARD       Gene Ontology:   cellular component:  endoplasmic reticulum   cellular component:  integral to membrane      BioCarta Pathways:     KEGG Pathways:  
    Probe set : 1371244_at    Name:   adenine phosphoribosyl transferase (predicted)    GB acc:   AI412934    UniGene:   Rn.2498    Gene Symbol:   Aprt_predicted    Locus Link:   292072    Chromosome:   19    Cytoband:   Chr:19q12    SOURCE    GENECARD       Gene Ontology:   biological process:  purine ribonucleoside salvage   biological process:  adenine salvage   biological process:  nucleoside metabolism      BioCarta Pathways:     KEGG Pathways:  
    Probe set : 1370941_at    Name:   platelet derived growth factor receptor, alpha polypeptide    GB acc:   AI232379    UniGene:   Rn.55127    Gene Symbol:   Pdgfra    Locus Link:   25267    Chromosome:   14    Cytoband:   Chr:14p11    SOURCE    GENECARD       Gene Ontology:   molecular function:  protein kinase activity   molecular function:  protein serine/threonine kinase activity   molecular function:  protein-tyrosine kinase activity   molecular function:  transmembrane receptor protein tyrosine kinase activity   molecular function:  receptor activity   molecular function:  platelet-derived growth factor alpha-receptor activity   molecular function:  platelet-derived growth factor alpha-receptor activity   molecular function:  vascular endothelial growth factor receptor activity   molecular function:  ATP binding   molecular function:  kinase activity   molecular function:  transferase activity   molecular function:  protein dimerization activity   molecular function:  protein dimerization activity   molecular function:  platelet-derived growth factor binding   molecular function:  nucleotide binding      BioCarta Pathways:     KEGG Pathways:  
    Probe set : AFFX_Rat_beta-actin_M_at    Name:   actin, beta     UniGene:   Rn.94978    Gene Symbol:   Actb    Locus Link:   81822    Chromosome:   12    Cytoband:   Chr:12p11    SOURCE    GENECARD       Gene Ontology:   biological process:  cell motility   biological process:  cytoskeleton organization and biogenesis   biological process:  axonogenesis      BioCarta Pathways:     KEGG Pathways:  
    Probe set : 1388128_at    Name:   ARP3 actin-related protein 3 homolog (yeast)    GB acc:   BI292004    UniGene:   Rn.103326    Gene Symbol:   Actr3    Locus Link:   81732    Chromosome:   13    Cytoband:   Chr:13q11    SOURCE    GENECARD       Gene Ontology:   biological process:  cell motility   molecular function:  structural molecule activity   molecular function:  structural constituent of cytoskeleton   molecular function:  protein binding      BioCarta Pathways:     KEGG Pathways:  
    Probe set : 1374815_at    Name:   STARD3 N-terminal like (predicted)    GB acc:   AA997695    UniGene:   Rn.86009    Gene Symbol:   Stard3nl_predicted    Locus Link:   291182    Chromosome:   17    Cytoband:   Chr:17q11    SOURCE    GENECARD       Gene Ontology:   cellular component:  integral to membrane      BioCarta Pathways:     KEGG Pathways:  
    Probe set : 1377743_at    Name:   Sprouty protein with EVH-1 domain 1, related sequence (predicted)    GB acc:   BI292687    UniGene:   Rn.22990     Locus Link:   296072    Chromosome:   3    Cytoband:   Chr:3q35    SOURCE        Gene Ontology:   biological process:  inactivation of MAPK activity   cellular component:  membrane   molecular function:  stem cell factor receptor binding   molecular function:  protein binding      BioCarta Pathways:     KEGG Pathways:  
    Probe set : 1374139_at    Name:   similar to cerebellar degeneration-related 2 (predicted)    GB acc:   BG671589    UniGene:   Rn.3537    Gene Symbol:   RGD1310578_predicted    Locus Link:   308958    Chromosome:   1    Cytoband:   Chr:1q35-q36    SOURCE    GENECARD       Gene Ontology:   cellular component:  cytoplasm      BioCarta Pathways:     KEGG Pathways:  
    Probe set : 1390987_at    Name:   Transcribed locus    GB acc:   AI406858    UniGene:   Rn.41998        SOURCE        Gene Ontology:     BioCarta Pathways:     KEGG Pathways:  
    Probe set : 1369393_at    Name:   mitogen-activated protein kinase kinase kinase 8    GB acc:   NM_053847    UniGene:   Rn.9939    Gene Symbol:   Map3k8    Locus Link:   116596    Chromosome:   17    Cytoband:   Chr:17q12    SOURCE    GENECARD       Gene Ontology:   cellular component:  intracellular   cellular component:  cytosol      BioCarta Pathways:     KEGG Pathways:  
    Probe set : 1372620_at    Name:   acidic (leucine-rich) nuclear phosphoprotein 32 family, member E (predicted)    GB acc:   AI008642    UniGene:   Rn.146014    Gene Symbol:   Anp32e_predicted    Locus Link:   361999    Chromosome:   2    Cytoband:   Chr:2q34    SOURCE    GENECARD       Gene Ontology:   cellular component:  nucleus   cellular component:  cytoplasm   cellular component:  cytoplasmic membrane-bound vesicle   molecular function:  phosphatase inhibitor activity      BioCarta Pathways:     KEGG Pathways:  
    Probe set : 1374359_at    Name:   Similar to RIKEN cDNA 2810013E07 (predicted)    GB acc:   BM383329    UniGene:   Rn.98992     Locus Link:   297823    Chromosome:   5    Cytoband:   Chr:5q13    SOURCE        Gene Ontology:     BioCarta Pathways:     KEGG Pathways:  
    Probe set : 1373326_at    Name:   Transcribed locus    GB acc:   AI716512    UniGene:   Rn.15193        SOURCE        Gene Ontology:     BioCarta Pathways:     KEGG Pathways:  
    Probe set : 1388847_at    Name:   Positive cofactor 2, multiprotein complex, glutamine/Q-rich-associated protein (predicted)    GB acc:   AI177083    UniGene:   Rn.6342     Locus Link:   360743    Chromosome:   11    Cytoband:   Chr:11q23    SOURCE        Gene Ontology:   biological process:  regulation of transcription, DNA-dependent   cellular component:  nucleus      BioCarta Pathways:     KEGG Pathways:  
    Probe set : 1383175_a_at    Name:   similar to C11orf17 protein (predicted)    GB acc:   BF561368    UniGene:   Rn.106344    Gene Symbol:   RGD1306959_predicted    Locus Link:   361624    Chromosome:   1    Cytoband:   Chr:1q33    SOURCE    GENECARD       Gene Ontology:     BioCarta Pathways:     KEGG Pathways:  
    Probe set : 1367472_at    Name:   similar to ubiquitin-protein ligase (EC 6.3.2.19) E1 - mouse    GB acc:   BG153295    UniGene:   Rn.11800    Gene Symbol:   RGD:1359327    Locus Link:   314432    Chromosome:   X    Cytoband:   Chr:Xq12    SOURCE    GENECARD       Gene Ontology:   biological process:  protein modification   biological process:  ubiquitin cycle      BioCarta Pathways:     KEGG Pathways:  
    Probe set : 1383084_at    Name:   Similar to hypothetical protein FLJ23033    GB acc:   BG671922    UniGene:   Rn.139571     Locus Link:   310982    Chromosome:   2    Cytoband:   Chr:2q44    SOURCE        Gene Ontology:     BioCarta Pathways:     KEGG Pathways:  
    Probe set : 1377785_at    Name:   DEAH (Asp-Glu-Ala-His) box polypeptide 40    GB acc:   BI275330    UniGene:   Rn.29182    Gene Symbol:   RGD:1359618    Locus Link:   287595    Chromosome:   10    Cytoband:   Chr:10q26    SOURCE    GENECARD       Gene Ontology:     BioCarta Pathways:     KEGG Pathways:  
    Probe set : 1390845_at    Name:   Transcribed locus    GB acc:   AI179288    UniGene:   Rn.12856        SOURCE        Gene Ontology:     BioCarta Pathways:     KEGG Pathways:  
    Probe set : 1377232_at    Name:   similar to hypothetical protein (predicted)    GB acc:   BF406608    UniGene:   Rn.9179    Gene Symbol:   RGD1305269_predicted    Locus Link:   305332    Chromosome:   14    Cytoband:   Chr:14p11    SOURCE    GENECARD       Gene Ontology:     BioCarta Pathways:     KEGG Pathways:  
    Probe set : 1385883_at    Name:   Transcribed locus    GB acc:   AI144842    UniGene:   Rn.27701        SOURCE        Gene Ontology:     BioCarta Pathways:     KEGG Pathways:  
    Probe set : 1374613_at    Name:   Transcribed locus    GB acc:   BE100609    UniGene:   Rn.13909        SOURCE        Gene Ontology:     BioCarta Pathways:     KEGG Pathways:  
    Probe set : 1370474_at    Name:   thyroid hormone receptor beta    GB acc:   J03819    UniGene:   Rn.88692    Gene Symbol:   Thrb    Locus Link:   24831    Chromosome:   15    Cytoband:   Chr:15p16    SOURCE    GENECARD       Gene Ontology:   cellular component:  nucleus   cellular component:  nucleus      BioCarta Pathways:     KEGG Pathways:  
    Probe set : 1398768_at    Name:   retinoblastoma binding protein 7    GB acc:   NM_031816    UniGene:   Rn.3600    Gene Symbol:   Rbbp7    Locus Link:   83712    Chromosome:   X    Cytoband:   Chr:Xq21    SOURCE    GENECARD       Gene Ontology:   cellular component:  nucleus   cellular component:  NuRD complex   molecular function:  protein binding   molecular function:  transcriptional repressor activity      BioCarta Pathways:     KEGG Pathways:  
    Probe set : 1370999_at    Name:   sperm associated antigen 5    GB acc:   AF111111    UniGene:   Rn.35359    Gene Symbol:   Spag5    Locus Link:   252918    Chromosome:   10    Cytoband:   Chr:10q25    SOURCE    GENECARD       Gene Ontology:   biological process:  cytokinesis   biological process:  proteolysis   biological process:  cell cycle   biological process:  mitosis   cellular component:  kinetochore   cellular component:  spindle   cellular component:  spindle microtubule   cellular component:  membrane   molecular function:  microtubule binding   molecular function:  peptidase activity   molecular function:  metallopeptidase activity      BioCarta Pathways:     KEGG Pathways:  
    Probe set : 1392928_at    Name:   similar to RIKEN cDNA 2810055F11 (predicted)    GB acc:   AA891693    UniGene:   Rn.105727    Gene Symbol:   RGD1305721_predicted    Locus Link:   314214    Chromosome:   6    Cytoband:   Chr:6q24    SOURCE    GENECARD       Gene Ontology:     BioCarta Pathways:     KEGG Pathways:  
    Probe set : 1393184_at    Name:   P21 (CDKN1A)-activated kinase 2    GB acc:   BF420805    UniGene:   Rn.3840    Gene Symbol:   Pak2    Locus Link:   29432    Chromosome:   11    Cytoband:   Chr:11q22    SOURCE    GENECARD       Gene Ontology:   biological process:  protein amino acid phosphorylation   biological process:  negative regulation of protein kinase activity   biological process:  signal transduction      BioCarta Pathways:     KEGG Pathways:  
    Probe set : 1377887_at    Name:   Acyl-Coenzyme A oxidase 3, pristanoyl    GB acc:   AI013044    UniGene:   Rn.10546    Gene Symbol:   Acox3    Locus Link:   83522    Chromosome:   14    Cytoband:   Chr:14q21    SOURCE    GENECARD       Gene Ontology:   cellular component:  peroxisome   cellular component:  peroxisome   cellular component:  peroxisomal matrix      BioCarta Pathways:     KEGG Pathways:  
    Probe set : 1376651_at    Name:   ADP-ribosylation factor guanine nucleotide-exchange factor 2 (brefeldin A-inhibited)    GB acc:   AW528774    UniGene:   Rn.7868    Gene Symbol:   Arfgef2    Locus Link:   296380    Chromosome:   3    Cytoband:   Chr:3q42    SOURCE    GENECARD       Gene Ontology:   biological process:  exocytosis   biological process:  intracellular signaling cascade   cellular component:  Golgi membrane   cellular component:  cytosol   cellular component:  membrane   molecular function:  guanyl-nucleotide exchange factor activity   molecular function:  binding      BioCarta Pathways:     KEGG Pathways:  
    Probe set : 1383338_at    Name:   Transcribed locus    GB acc:   AA875190    UniGene:   Rn.146915        SOURCE        Gene Ontology:     BioCarta Pathways:     KEGG Pathways:  
    Probe set : 1379281_at    Name:   uterine sensitization-associated gene 1 protein    GB acc:   AA892798    UniGene:   Rn.2415    Gene Symbol:   Sostdc1    Locus Link:   266803    Chromosome:   6    Cytoband:   Chr:6q16    SOURCE    GENECARD       Gene Ontology:   biological process:  embryo implantation   biological process:  Wnt receptor signaling pathway      BioCarta Pathways:     KEGG Pathways:  
    Probe set : 1377850_at    Name:   Zinc and ring finger 1 (predicted)    GB acc:   AI178231    UniGene:   Rn.32245     Locus Link:   362367    Chromosome:   4    Cytoband:   Chr:4q24    SOURCE        Gene Ontology:     BioCarta Pathways:     KEGG Pathways:  
    Probe set : 1382587_at    Name:   Family with sequence similarity 33, member A (predicted)    GB acc:   AA901257    UniGene:   Rn.40571    Gene Symbol:   MGC109093    Locus Link:   287598    Chromosome:   10    Cytoband:   Chr:10q26    SOURCE    GENECARD       Gene Ontology:     BioCarta Pathways:     KEGG Pathways:  
    Probe set : 1387503_at    Name:   carboxypeptidase N, polypeptide 1, 50kD    GB acc:   NM_053526    UniGene:   Rn.145303    Gene Symbol:   Cpn1    Locus Link:   365466    Chromosome:   1    Cytoband:   Chr:1q54    SOURCE    GENECARD       Gene Ontology:   biological process:  proteolysis   biological process:  proteolysis   cellular component:  extracellular space      BioCarta Pathways:     KEGG Pathways:  
    Probe set : 1373300_at     GB acc:   BI296249              Gene Ontology:     BioCarta Pathways:     KEGG Pathways:  
    Probe set : 1387603_at    Name:   killer cell lectin-like receptor subfamily C, member 2    GB acc:   NM_019261    UniGene:   Rn.9894    Gene Symbol:   Klrc2    Locus Link:   29684    Chromosome:   4    Cytoband:   Chr:4q42    SOURCE    GENECARD       Gene Ontology:   molecular function:  MHC class I receptor activity   molecular function:  sugar binding      BioCarta Pathways:     KEGG Pathways:  
    Probe set : 1387505_at    Name:   guanine nucleotide binding protein, alpha inhibiting 1    GB acc:   NM_013145    UniGene:   Rn.11391    Gene Symbol:   Gnai1    Locus Link:   25686    Chromosome:   4    Cytoband:   Chr:4q11    SOURCE    GENECARD       Gene Ontology:   cellular component:  heterotrimeric G-protein complex      BioCarta Pathways:     KEGG Pathways:  
    Probe set : 1373321_at    Name:   Transcribed locus, strongly similar to XP_574622.1 PREDICTED: similar to UPF0197 protein C11orf10 homolog [Rattus norvegicus]    GB acc:   BE111682    UniGene:   Rn.96        SOURCE        Gene Ontology:     BioCarta Pathways:     KEGG Pathways:  
    Probe set : 1368544_a_at    Name:   nucleolar protein 3 (apoptosis repressor with CARD domain)    GB acc:   NM_053516    UniGene:   Rn.86956    Gene Symbol:   Nol3    Locus Link:   85383    Chromosome:   19    Cytoband:   Chr:19q11    SOURCE    GENECARD       Gene Ontology:   cellular component:  nucleus   cellular component:  intracellular   molecular function:  protein binding      BioCarta Pathways:     KEGG Pathways:  
    Probe set : 1376585_at    Name:   mitochondrial ribosomal protein L50 (predicted)    GB acc:   BF395602    UniGene:   Rn.1787    Gene Symbol:   Mrpl50_predicted    Locus Link:   362517    Chromosome:   5    Cytoband:   Chr:5q22    SOURCE    GENECARD       Gene Ontology:   cellular component:  mitochondrion   molecular function:  structural constituent of ribosome   molecular function:  oxidoreductase activity      BioCarta Pathways:     KEGG Pathways:  
    Probe set : 1394070_at    Name:   Transcribed locus    GB acc:   AA893087    UniGene:   Rn.135903        SOURCE        Gene Ontology:     BioCarta Pathways:     KEGG Pathways:  
    Probe set : 1398270_at    Name:   bone morphogenetic protein 2    GB acc:   AA944827    UniGene:   Rn.90931    Gene Symbol:   Bmp2    Locus Link:   29373    Chromosome:   3    Cytoband:   Chr:3q36    SOURCE    GENECARD       Gene Ontology:   biological process:  skeletal development   biological process:  epithelial to mesenchymal transition   biological process:  transforming growth factor beta receptor signaling pathway   biological process:  cell-cell signaling   biological process:  embryonic development   biological process:  organ morphogenesis   biological process:  BMP signaling pathway   biological process:  BMP signaling pathway   biological process:  cardiac cell differentiation   biological process:  growth   biological process:  odontogenesis (sensu Vertebrata)   biological process:  cell fate commitment   biological process:  positive regulation of cell differentiation   biological process:  ossification   biological process:  development   biological process:  cell differentiation   biological process:  cartilage development   cellular component:  extracellular region   cellular component:  extracellular space      BioCarta Pathways:     KEGG Pathways:  
    Probe set : 1367961_at    Name:   nerve growth factor, gamma    GB acc:   NM_031523    UniGene:   Rn.11331    Gene Symbol:   Ngfg    Locus Link:   24594    Chromosome:   1    Cytoband:   Chr:1q22    SOURCE    GENECARD       Gene Ontology:   biological process:  proteolysis      BioCarta Pathways:     KEGG Pathways:  
    Probe set : 1398925_at    Name:   similar to RIKEN cDNA 1300018I05 (predicted)    GB acc:   BI274664    UniGene:   Rn.17113    Gene Symbol:   RGD1307801_predicted    Locus Link:   309656    Chromosome:   20    Cytoband:   Chr:20p12    SOURCE    GENECARD       Gene Ontology:   biological process:  cytokinesis   cellular component:  intracellular   molecular function:  nucleic acid binding      BioCarta Pathways:     KEGG Pathways:  
    Probe set : 1377137_at    Name:   tyrosyl-DNA phosphodiesterase 1 (predicted)    GB acc:   BG379860    UniGene:   Rn.12661    Gene Symbol:   Tdp1_predicted    Locus Link:   314380    Chromosome:   6    Cytoband:   Chr:6q32    SOURCE    GENECARD       Gene Ontology:   biological process:  DNA repair   cellular component:  nucleus   molecular function:  phosphoric diester hydrolase activity   molecular function:  hydrolase activity      BioCarta Pathways:     KEGG Pathways:  
    Probe set : 1386987_at    Name:   interleukin 6 receptor    GB acc:   NM_017020    UniGene:   Rn.1716    Gene Symbol:   Il6r    Locus Link:   24499    Chromosome:   2    Cytoband:   Chr:2q34    SOURCE    GENECARD       Gene Ontology:     BioCarta Pathways:     KEGG Pathways:  
    Probe set : 1368948_at    Name:   moesin    GB acc:   NM_030863    UniGene:   Rn.2762    Gene Symbol:   Msn    Locus Link:   81521    Chromosome:   X    Cytoband:   Chr:Xq31    SOURCE    GENECARD       Gene Ontology:   biological process:  cell motility      BioCarta Pathways:     KEGG Pathways:  
    Probe set : 1388152_at    Name:   microtubule-associated protein 2    GB acc:   BG374290    UniGene:   Rn.10484    Gene Symbol:   Mtap2    Locus Link:   25595    Chromosome:   9    Cytoband:   Chr:9q32    SOURCE    GENECARD       Gene Ontology:   biological process:  microtubule bundle formation   biological process:  negative regulation of microtubule depolymerization   biological process:  dendrite morphogenesis   cellular component:  cytoskeleton   cellular component:  microtubule associated complex   cellular component:  microtubule   molecular function:  structural molecule activity   molecular function:  calmodulin binding   molecular function:  cytoskeletal regulatory protein binding      BioCarta Pathways:     KEGG Pathways:  
    Probe set : 1383262_at    Name:   Unknown (protein for MGC:72591)    GB acc:   BG378159    UniGene:   Rn.131677    Gene Symbol:   Tmem19    Locus Link:   299800    Chromosome:   7    Cytoband:   Chr:7q22    SOURCE    GENECARD       Gene Ontology:     BioCarta Pathways:     KEGG Pathways:  
    Probe set : 1388681_at    Name:   SAR1a gene homolog 2 (S. cerevisiae) (predicted)    GB acc:   AI105294    UniGene:   Rn.36748    Gene Symbol:   Sara2_predicted    Locus Link:   287276    Chromosome:   10    Cytoband:   Chr:10q22    SOURCE    GENECARD       Gene Ontology:   cellular component:  endoplasmic reticulum   cellular component:  Golgi apparatus   cellular component:  membrane   molecular function:  magnesium ion binding   molecular function:  GTP binding      BioCarta Pathways:     KEGG Pathways:  
    Probe set : 1388089_a_at    Name:   ring finger protein 4    GB acc:   AY050655    UniGene:   Rn.30015    Gene Symbol:   Rnf4    Locus Link:   29274    Chromosome:   14    Cytoband:   Chr:14q21    SOURCE    GENECARD       Gene Ontology:   cellular component:  ubiquitin ligase complex   cellular component:  nucleus   cellular component:  nucleus      BioCarta Pathways:     KEGG Pathways:  
    Probe set : 1376824_at    Name:   Similar to RIKEN cDNA 1700027M01 (predicted)    GB acc:   AI408131    UniGene:   Rn.105992     Locus Link:   313668    Chromosome:   5    Cytoband:   Chr:5q36    SOURCE        Gene Ontology:   biological process:  protein modification      BioCarta Pathways:     KEGG Pathways:  
    Probe set : 1394527_at    Name:   Transcribed locus    GB acc:   AI501679    UniGene:   Rn.148928        SOURCE        Gene Ontology:     BioCarta Pathways:     KEGG Pathways:  
    Probe set : 1380421_s_at    Name:   growth factor independent 1    GB acc:   AW532194    UniGene:   Rn.10935    Gene Symbol:   Gfi1    Locus Link:   24388    Chromosome:   14    Cytoband:   Chr:14p22    SOURCE    GENECARD       Gene Ontology:   cellular component:  nucleus      BioCarta Pathways:     KEGG Pathways:  
    Probe set : 1387803_at    Name:   protein phosphatase 2 (formerly 2A), regulatory subunit B (PR 52), beta isoform    GB acc:   NM_022209    UniGene:   Rn.44437    Gene Symbol:   Ppp2r2b    Locus Link:   60660    Chromosome:   18    Cytoband:   Chr:18p11    SOURCE    GENECARD       Gene Ontology:   biological process:  spermatid development   biological process:  signal transduction   cellular component:  protein phosphatase type 2A complex      BioCarta Pathways:     KEGG Pathways:  
    Probe set : 1390032_at    Name:   RNA binding motif, single stranded interacting protein 2 (predicted)    GB acc:   BG374052    UniGene:   Rn.12972    Gene Symbol:   Rbms2_predicted    Locus Link:   288771    Chromosome:   7    Cytoband:   Chr:7q11    SOURCE    GENECARD       Gene Ontology:   biological process:  RNA processing   biological process:  regulation of translation   cellular component:  nucleus   molecular function:  RNA binding   molecular function:  nucleic acid binding      BioCarta Pathways:     KEGG Pathways:  
    Probe set : 1374303_at    Name:   similar to prostate cancer antigen-1 (predicted)    GB acc:   BI288146    UniGene:   Rn.107582    Gene Symbol:   RGD1306377_predicted    Locus Link:   304578    Chromosome:   12    Cytoband:   Chr:12q16    SOURCE    GENECARD       Gene Ontology:     BioCarta Pathways:     KEGG Pathways:  
    Probe set : 1368413_at    Name:   amiloride binding protein 1    GB acc:   NM_022935    UniGene:   Rn.54493    Gene Symbol:   Abp1    Locus Link:   65029    Chromosome:   4    Cytoband:   Chr:4q24    SOURCE    GENECARD       Gene Ontology:   biological process:  transport   biological process:  metabolism   biological process:  amiloride transport   cellular component:  extracellular space   cellular component:  peroxisome   cellular component:  membrane      BioCarta Pathways:     KEGG Pathways:  
    Probe set : 1387364_at    Name:   folate hydrolase    GB acc:   AF040256    UniGene:   Rn.89278    Gene Symbol:   Folh1    Locus Link:   85309    Chromosome:   1    Cytoband:   Chr:1q32    SOURCE    GENECARD       Gene Ontology:   biological process:  proteolysis   biological process:  proteolysis   biological process:  folic acid and derivative metabolism   molecular function:  catalytic activity   molecular function:  aminopeptidase activity   molecular function:  carboxypeptidase activity   molecular function:  membrane dipeptidase activity   molecular function:  serine-type endopeptidase activity   molecular function:  peptidase activity   molecular function:  metallopeptidase activity   molecular function:  gamma-glutamyl hydrolase activity   molecular function:  glutamate carboxypeptidase activity   molecular function:  glutamate carboxypeptidase activity   molecular function:  hydrolase activity   molecular function:  dipeptidase activity   molecular function:  glutamate carboxypeptidase II activity   molecular function:  zinc ion binding   molecular function:  metal ion binding      BioCarta Pathways:     KEGG Pathways:  
    Probe set : 1387271_at    Name:   phytanoyl-CoA hydroxylase    GB acc:   NM_053674    UniGene:   Rn.7279    Gene Symbol:   Phyh    Locus Link:   114209    Chromosome:   17    Cytoband:   Chr:17q12.3    SOURCE    GENECARD       Gene Ontology:   cellular component:  peroxisome   cellular component:  peroxisome      BioCarta Pathways:     KEGG Pathways:  
    Probe set : 1371557_at    Name:   THAP domain containing 4    GB acc:   BI275281    UniGene:   Rn.13958    Gene Symbol:   RGD:1359473    Locus Link:   363291    Chromosome:   9    Cytoband:   Chr:9q36    SOURCE    GENECARD       Gene Ontology:   molecular function:  nucleic acid binding      BioCarta Pathways:     KEGG Pathways:  
    Probe set : 1387812_at    Name:   Subtilisin - like endoprotease    GB acc:   NM_012999    UniGene:   Rn.950    Gene Symbol:   Pace4    Locus Link:   25507    Chromosome:   1    Cytoband:   Chr:1q22    SOURCE    GENECARD       Gene Ontology:   cellular component:  nucleus   cellular component:  membrane      BioCarta Pathways:     KEGG Pathways:  
    Probe set : 1373254_at    Name:   keratin 10    GB acc:   BI282479    UniGene:   Rn.145401    Gene Symbol:   RGD:1359092    Locus Link:   450225    Chromosome:   10    Cytoband:   Chr:10q32.1    SOURCE    GENECARD       Gene Ontology:     BioCarta Pathways:     KEGG Pathways:  
    Probe set : 1383638_at    Name:   CDNA clone IMAGE:7380769    GB acc:   AW524125    UniGene:   Rn.24671        SOURCE        Gene Ontology:     BioCarta Pathways:     KEGG Pathways:  
    Probe set : 1388130_at    Name:   zyxin    GB acc:   AA943537    UniGene:   Rn.107363    Gene Symbol:   Zyx    Locus Link:   114636    Chromosome:   4    Cytoband:   Chr:4q23    SOURCE    GENECARD       Gene Ontology:   biological process:  regulation of inflammatory response   molecular function:  zinc ion binding   molecular function:  metal ion binding      BioCarta Pathways:     KEGG Pathways:  
    Probe set : 1378611_at    Name:   Similar to encephalopsin    GB acc:   BE112744    UniGene:   Rn.15626     Locus Link:   498289      SOURCE        Gene Ontology:     BioCarta Pathways:     KEGG Pathways:  
    Probe set : 1371793_at    Name:   Transcribed locus, strongly similar to NP_081632.1 hypothetical protein LOC70240 [Mus musculus]    GB acc:   AI008697    UniGene:   Rn.105879        SOURCE        Gene Ontology:     BioCarta Pathways:     KEGG Pathways:  
    Probe set : 1390449_at    Name:   CDNA clone IMAGE:7309127    GB acc:   BI289132    UniGene:   Rn.32626        SOURCE        Gene Ontology:     BioCarta Pathways:     KEGG Pathways:  
    Probe set : 1368614_at    Name:   Williams-Beuren syndrome chromosome region 14 homolog (human)    GB acc:   NM_133552    UniGene:   Rn.144656    Gene Symbol:   Wbscr14    Locus Link:   171078      SOURCE    GENECARD       Gene Ontology:   cellular component:  transcription factor complex   cellular component:  nucleus   molecular function:  transcription factor activity   molecular function:  transcription regulator activity      BioCarta Pathways:     KEGG Pathways:  
    Probe set : 1373978_at    Name:   nuclear cap binding protein subunit 1, 80kDa (predicted)    GB acc:   AI410277    UniGene:   Rn.13482    Gene Symbol:   Ncbp1_predicted    Locus Link:   298075    Chromosome:   5    Cytoband:   Chr:5q22    SOURCE    GENECARD       Gene Ontology:   cellular component:  nucleoplasm   molecular function:  RNA cap binding   molecular function:  RNA binding      BioCarta Pathways:     KEGG Pathways:  
    Probe set : 1375182_at    Name:   Similar to RIKEN cDNA D030028O16 (predicted)    GB acc:   BE109671    UniGene:   Rn.103273    Gene Symbol:   MGC105586    Locus Link:   298771    Chromosome:   6    Cytoband:   Chr:6q12    SOURCE    GENECARD       Gene Ontology:   biological process:  proteolysis      BioCarta Pathways:     KEGG Pathways:  
    Probe set : 1373529_at    Name:   Transcribed locus    GB acc:   BE117883    UniGene:   Rn.124162        SOURCE        Gene Ontology:     BioCarta Pathways:     KEGG Pathways:  
    Probe set : 1392992_at    Name:   Transcribed locus    GB acc:   BF395999    UniGene:   Rn.128193        SOURCE        Gene Ontology:     BioCarta Pathways:     KEGG Pathways:  
    Probe set : 1377404_at    Name:   stanniocalcin 1    GB acc:   BM386683    UniGene:   Rn.10647    Gene Symbol:   Stc1    Locus Link:   81801    Chromosome:   15    Cytoband:   Chr:15p11    SOURCE    GENECARD       Gene Ontology:   cellular component:  extracellular region   cellular component:  extracellular space   molecular function:  protein kinase activity   molecular function:  hormone activity   molecular function:  ATP binding      BioCarta Pathways:     KEGG Pathways:  
    Probe set : 1384488_at    Name:   similar to cyclin-dependent kinase inhibitor 2D    GB acc:   BI290067    UniGene:   Rn.122002    Gene Symbol:   Cdkn2d    Locus Link:   494444      SOURCE    GENECARD       Gene Ontology:   molecular function:  kinase activity      BioCarta Pathways:     KEGG Pathways:  
    Probe set : 1375790_at     GB acc:   BI300458              Gene Ontology:     BioCarta Pathways:     KEGG Pathways:  
    Probe set : 1380813_at     GB acc:   AA891239              Gene Ontology:     BioCarta Pathways:     KEGG Pathways:  
    Probe set : 1374547_at    Name:   Similar to RIKEN cDNA A130038L21    GB acc:   BI285509    UniGene:   Rn.109370     Locus Link:   311358    Chromosome:   3    Cytoband:   Chr:3q35    SOURCE        Gene Ontology:     BioCarta Pathways:     KEGG Pathways:  
    Probe set : 1375628_at    Name:   NADH dehydrogenase (ubiquinone) 1 beta subcomplex 8 (predicted)    GB acc:   AI059654    UniGene:   Rn.3383    Gene Symbol:   Ndufb8_predicted    Locus Link:   293991    Chromosome:   1    Cytoband:   Chr:1q54    SOURCE    GENECARD       Gene Ontology:   biological process:  electron transport   biological process:  mitochondrial electron transport, NADH to ubiquinone   molecular function:  NADH dehydrogenase activity   molecular function:  NADH dehydrogenase (ubiquinone) activity   molecular function:  oxidoreductase activity      BioCarta Pathways:     KEGG Pathways:  
    Probe set : 1392644_s_at    Name:   similar to RIKEN cDNA 2610019F03    GB acc:   AA964146    UniGene:   Rn.11716    Gene Symbol:   LOC498662    Locus Link:   498662    Chromosome:   16    Cytoband:   Chr:16q12.5    SOURCE    GENECARD       Gene Ontology:     BioCarta Pathways:     KEGG Pathways:  
    Probe set : 1389652_at    Name:   Transcribed locus    GB acc:   AI171655    UniGene:   Rn.12269        SOURCE        Gene Ontology:     BioCarta Pathways:     KEGG Pathways:  
    Probe set : 1396013_at    Name:   leucine zipper-EF-hand containing transmembrane protein 1    GB acc:   BI293141    UniGene:   Rn.22461    Gene Symbol:   RGD:1359678    Locus Link:   305457    Chromosome:   14    Cytoband:   Chr:14q21    SOURCE    GENECARD       Gene Ontology:   cellular component:  mitochondrion   cellular component:  integral to membrane   molecular function:  calcium ion binding      BioCarta Pathways:     KEGG Pathways:  
    Probe set : 1372689_at    Name:   similar to hypothetical protein FLJ20211 (predicted)    GB acc:   BG381479    UniGene:   Rn.36164    Gene Symbol:   RGD1305478_predicted    Locus Link:   363150    Chromosome:   8    Cytoband:   Chr:8q32    SOURCE    GENECARD       Gene Ontology:     BioCarta Pathways:     KEGG Pathways:  
    Probe set : 1387007_at    Name:   glial cell line derived neurotrophic factor family receptor alpha 1    GB acc:   NM_012959    UniGene:   Rn.88489    Gene Symbol:   Gfra1    Locus Link:   25454    Chromosome:   1    Cytoband:   Chr:1q55    SOURCE    GENECARD       Gene Ontology:   molecular function:  receptor activity   molecular function:  receptor activity      BioCarta Pathways:     KEGG Pathways:  
    Probe set : 1393955_at    Name:   WD repeat domain 44    GB acc:   BI293101    UniGene:   Rn.79380    Gene Symbol:   Wdr44    Locus Link:   246152    Chromosome:   X    Cytoband:   Chr:Xq12    SOURCE    GENECARD       Gene Ontology:   biological process:  regulation of cell migration   cellular component:  microtubule   cellular component:  perinuclear region   molecular function:  Rab GTPase binding      BioCarta Pathways:     KEGG Pathways:  
    Probe set : 1368159_at    Name:   ATP-binding cassette, sub-family B (MDR/TAP), member 6    GB acc:   NM_080582    UniGene:   Rn.54408    Gene Symbol:   Abcb6    Locus Link:   140669    Chromosome:   9    Cytoband:   Chr:9q33    SOURCE    GENECARD       Gene Ontology:   biological process:  transport   biological process:  iron ion homeostasis      BioCarta Pathways:     KEGG Pathways:  
    Probe set : 1374708_at    Name:   similar to Rho guanine nucleotide exchange factor (GEF) 10    GB acc:   BF408594    UniGene:   Rn.18942    Gene Symbol:   LOC306618    Locus Link:   306618    Chromosome:   16    Cytoband:   Chr:16q12.5    SOURCE    GENECARD       Gene Ontology:     BioCarta Pathways:     KEGG Pathways:  
    Probe set : 1373475_at    Name:   hypothetical LOC288065    GB acc:   BM390469    UniGene:   Rn.7523    Gene Symbol:   LOC288065    Locus Link:   288065    Chromosome:   11    Cytoband:   Chr:11q22    SOURCE    GENECARD       Gene Ontology:     BioCarta Pathways:     KEGG Pathways:  
    Probe set : 1375612_at    Name:   Transcribed locus    GB acc:   AA965147    UniGene:   Rn.151553        SOURCE        Gene Ontology:     BioCarta Pathways:     KEGG Pathways:  
    Probe set : 1382363_at    Name:   membrane protein, palmitoylated 5 (MAGUK p55 subfamily member 5) (predicted)    GB acc:   AI072027    UniGene:   Rn.8181    Gene Symbol:   Mpp5_predicted    Locus Link:   314259    Chromosome:   6    Cytoband:   Chr:6q24    SOURCE    GENECARD       Gene Ontology:   cellular component:  plasma membrane   cellular component:  membrane   cellular component:  integral to membrane   molecular function:  guanylate kinase activity   molecular function:  protein binding      BioCarta Pathways:     KEGG Pathways:  
    Probe set : 1372711_at    Name:   similar to cDNA sequence BC005537    GB acc:   AI409292    UniGene:   Rn.23910    Gene Symbol:   LOC498750    Locus Link:   498750    Chromosome:   17    Cytoband:   Chr:17p11    SOURCE    GENECARD       Gene Ontology:     BioCarta Pathways:     KEGG Pathways:  
    Probe set : 1383685_at    Name:   similar to BAP28 protein    GB acc:   BI276972    UniGene:   Rn.47600    Gene Symbol:   LOC361262    Locus Link:   361262    Chromosome:   17    Cytoband:   Chr:17q12.1    SOURCE    GENECARD       Gene Ontology:     BioCarta Pathways:     KEGG Pathways:  
    Probe set : 1376327_at    Name:   tumor necrosis factor receptor superfamily, member 14 (herpesvirus entry mediator) (predicted)    GB acc:   AI169601    UniGene:   Rn.14734    Gene Symbol:   Tnfrsf14_predicted    Locus Link:   366518    Chromosome:   5    Cytoband:   Chr:5q36    SOURCE    GENECARD       Gene Ontology:   cellular component:  integral to plasma membrane   cellular component:  membrane   molecular function:  receptor activity   molecular function:  transmembrane receptor activity   molecular function:  tumor necrosis factor receptor activity      BioCarta Pathways:     KEGG Pathways:  
    Probe set : 1387498_a_at    Name:   Fibroblast growth factor receptor 1    GB acc:   S54008    UniGene:   Rn.9797    Gene Symbol:   Fgfr1    Locus Link:   79114    Chromosome:   16    Cytoband:   Chr:16q12.4    SOURCE    GENECARD       Gene Ontology:   biological process:  MAPKKK cascade   biological process:  skeletal development   biological process:  induction of an organ   biological process:  branching morphogenesis   biological process:  protein amino acid phosphorylation   biological process:  signal transduction   biological process:  brain development   biological process:  brain development   biological process:  salivary gland morphogenesis   biological process:  fibroblast growth factor receptor signaling pathway   biological process:  lung development   biological process:  neurite morphogenesis   biological process:  regulation of cell proliferation   biological process:  regulation of cell proliferation   biological process:  inner ear morphogenesis   cellular component:  extracellular space   cellular component:  integral to plasma membrane   cellular component:  integral to membrane      BioCarta Pathways:     KEGG Pathways:  
    Probe set : 1370168_at    Name:   tyrosine 3-monooxygenase/tryptophan 5-monooxygenase activation protein, theta polypeptide    GB acc:   BI277793    UniGene:   Rn.2502    Gene Symbol:   Ywhaq    Locus Link:   25577    Chromosome:   6    Cytoband:   Chr:6q16    SOURCE    GENECARD       Gene Ontology:   molecular function:  protein kinase C inhibitor activity   molecular function:  protein domain specific binding      BioCarta Pathways:     KEGG Pathways:  
    Probe set : 1389421_at    Name:   polymerase (DNA directed), epsilon    GB acc:   BE101140    UniGene:   Rn.40977    Gene Symbol:   Pole    Locus Link:   304573    Chromosome:   12    Cytoband:   Chr:12q16    SOURCE    GENECARD       Gene Ontology:   biological process:  DNA replication   biological process:  DNA repair   cellular component:  nucleus      BioCarta Pathways:     KEGG Pathways:  
    Probe set : 1398245_at    Name:   synuclein, gamma    GB acc:   NM_031688    UniGene:   Rn.2883    Gene Symbol:   Sncg    Locus Link:   64347    Chromosome:   16    Cytoband:   Chr:16p16    SOURCE    GENECARD       Gene Ontology:   biological process:  peripheral nervous system development   biological process:  peripheral nervous system development   cellular component:  cytoplasm   cellular component:  axon   cellular component:  cell soma      BioCarta Pathways:     KEGG Pathways:  
    Probe set : 1388376_at    Name:   similar to RIKEN cDNA 0610038D11 (predicted)    GB acc:   AI235498    UniGene:   Rn.2190    Gene Symbol:   RGD1309710_predicted    Locus Link:   293700    Chromosome:   1    Cytoband:   Chr:1q43    SOURCE    GENECARD       Gene Ontology:     BioCarta Pathways:     KEGG Pathways:  
    Probe set : 1373859_at    Name:   Transcribed locus    GB acc:   AA819884    UniGene:   Rn.2285        SOURCE        Gene Ontology:     BioCarta Pathways:     KEGG Pathways:  
    Probe set : 1378876_at    Name:   Transcribed locus    GB acc:   BE114024    UniGene:   Rn.46619        SOURCE        Gene Ontology:     BioCarta Pathways:     KEGG Pathways:  
    Probe set : 1374288_at    Name:   FtsJ homolog 3 (E. coli) (predicted)    GB acc:   BG374267    UniGene:   Rn.144933    Gene Symbol:   Ftsj3_predicted    Locus Link:   303608    Chromosome:   10    Cytoband:   Chr:10q32.1    SOURCE    GENECARD       Gene Ontology:   biological process:  cytokinesis   biological process:  rRNA processing   cellular component:  nucleus   molecular function:  structural constituent of chorion (sensu Insecta)   molecular function:  methyltransferase activity   molecular function:  transferase activity      BioCarta Pathways:     KEGG Pathways:  
    Probe set : 1368597_at    Name:   SNF1-like kinase    GB acc:   AB020480    UniGene:   Rn.42905    Gene Symbol:   Snf1lk    Locus Link:   59329    Chromosome:   20    Cytoband:   Chr:20p12    SOURCE    GENECARD       Gene Ontology:   biological process:  negative regulation of transcription from RNA polymerase II promoter   biological process:  protein amino acid phosphorylation   biological process:  protein amino acid phosphorylation   biological process:  cell cycle   biological process:  protein kinase cascade   biological process:  regulation of progression through mitotic cell cycle   biological process:  regulation of cell differentiation   biological process:  protein amino acid phosphorylation   biological process:  protein kinase cascade   biological process:  regulation of progression through mitotic cell cycle   biological process:  regulation of cell differentiation   biological process:  development   biological process:  cell differentiation   cellular component:  nucleus   cellular component:  cytoplasm   cellular component:  cytosol   cellular component:  cytoplasm   molecular function:  magnesium ion binding   molecular function:  protein kinase activity   molecular function:  protein serine/threonine kinase activity   molecular function:  protein serine/threonine kinase activity   molecular function:  protein-tyrosine kinase activity   molecular function:  ATP binding   molecular function:  kinase activity   molecular function:  transcriptional repressor activity   molecular function:  transferase activity   molecular function:  magnesium ion binding   molecular function:  protein serine/threonine kinase activity   molecular function:  ATP binding   molecular function:  nucleotide binding   molecular function:  metal ion binding      BioCarta Pathways:     KEGG Pathways:  
    Probe set : 1374580_at    Name:   SUMO/sentrin specific protease 6 (predicted)    GB acc:   AW535691    UniGene:   Rn.98553    Gene Symbol:   Senp6_predicted    Locus Link:   300860    Chromosome:   8    Cytoband:   Chr:8q31    SOURCE    GENECARD       Gene Ontology:   biological process:  proteolysis   biological process:  ubiquitin cycle   molecular function:  peptidase activity   molecular function:  cysteine-type peptidase activity      BioCarta Pathways:     KEGG Pathways:  
    Probe set : 1374252_at    Name:   centrosomal protein 1 (predicted)    GB acc:   BE108165    UniGene:   Rn.60629    Gene Symbol:   Cep1_predicted    Locus Link:   311886    Chromosome:   3    Cytoband:   Chr:3p11    SOURCE    GENECARD       Gene Ontology:   cellular component:  nucleus   molecular function:  RNA polymerase II transcription factor activity      BioCarta Pathways:     KEGG Pathways:  
    Probe set : 1390944_at    Name:   Snf7 homologue associated with Alix 3    GB acc:   BM384937    UniGene:   Rn.65511    Gene Symbol:   MGC108776    Locus Link:   361916    Chromosome:   2    Cytoband:   Chr:2q23    SOURCE    GENECARD       Gene Ontology:   biological process:  transport   biological process:  protein transport      BioCarta Pathways:     KEGG Pathways:  
    Probe set : 1380465_at    Name:   Transcribed locus    GB acc:   AA965023    UniGene:   Rn.153574        SOURCE        Gene Ontology:     BioCarta Pathways:     KEGG Pathways:  
    Probe set : 1379514_at    Name:   Transcribed locus    GB acc:   AA926014    UniGene:   Rn.15638        SOURCE        Gene Ontology:     BioCarta Pathways:     KEGG Pathways:  
    Probe set : 1376847_at    Name:   CDNA clone IMAGE:7323916    GB acc:   BI302283    UniGene:   Rn.26676        SOURCE        Gene Ontology:     BioCarta Pathways:     KEGG Pathways:  
    Probe set : 1370325_at    Name:   golgi reassembly stacking protein 2    GB acc:   AF110267    UniGene:   Rn.145137    Gene Symbol:   Gorasp2    Locus Link:   113961    Chromosome:   3    Cytoband:   Chr:3q21    SOURCE    GENECARD       Gene Ontology:   biological process:  Golgi organization and biogenesis   molecular function:  protein binding      BioCarta Pathways:     KEGG Pathways:  
    Probe set : 1372200_at    Name:   G-protein signalling modulator 3 (AGS3-like, C. elegans)    GB acc:   BI273727    UniGene:   Rn.7475    Gene Symbol:   RGD:1303014    Locus Link:   406163    Chromosome:   20    Cytoband:   Chr:20p12    SOURCE    GENECARD       Gene Ontology:   biological process:  signal transduction   molecular function:  GTPase activator activity      BioCarta Pathways:     KEGG Pathways:  
    Probe set : 1388488_at    Name:   LSM3 homolog, U6 small nuclear RNA associated (S. cerevisiae) (predicted)    GB acc:   AA943742    UniGene:   Rn.4333    Gene Symbol:   Lsm3_predicted    Locus Link:   297455    Chromosome:   4    Cytoband:   Chr:4q34    SOURCE    GENECARD       Gene Ontology:   biological process:  nuclear mRNA splicing, via spliceosome   biological process:  nuclear mRNA splicing via U2-type spliceosome   biological process:  mRNA processing   molecular function:  RNA binding   molecular function:  U6 snRNA 3'-end binding      BioCarta Pathways:     KEGG Pathways:  
    Probe set : 1373810_at    Name:   phospholipase A2, group XIIA (predicted)    GB acc:   BF284175    UniGene:   Rn.137418    Gene Symbol:   Pla2g12a_predicted    Locus Link:   362039    Chromosome:   2    Cytoband:   Chr:2q43    SOURCE    GENECARD       Gene Ontology:   biological process:  lipid catabolism      BioCarta Pathways:     KEGG Pathways:  
    Probe set : 1398333_at    Name:   Endothelial PAS domain protein 1    GB acc:   AI598434    UniGene:   Rn.55138    Gene Symbol:   Epas1    Locus Link:   29452    Chromosome:   6    Cytoband:   Chr:6q12    SOURCE    GENECARD       Gene Ontology:   cellular component:  nucleus      BioCarta Pathways:     KEGG Pathways:  
    Probe set : 1376947_at    Name:   retinoblastoma binding protein 6 (predicted)    GB acc:   AI178158    UniGene:   Rn.95305    Gene Symbol:   Rbbp6_predicted    Locus Link:   308968    Chromosome:   1    Cytoband:   Chr:1q36    SOURCE    GENECARD       Gene Ontology:   biological process:  regulation of progression through cell cycle   biological process:  protein ubiquitination   cellular component:  ubiquitin ligase complex   cellular component:  nucleus      BioCarta Pathways:     KEGG Pathways:  
    Probe set : 1398366_at    Name:   LOC497968    GB acc:   BF282644    UniGene:   Rn.29982    Gene Symbol:   LOC497968    Locus Link:   497968    Chromosome:   10    Cytoband:   Chr:10q26    SOURCE    GENECARD       Gene Ontology:     BioCarta Pathways:     KEGG Pathways:  
    Probe set : 1392937_at    Name:   cyclin I (predicted)    GB acc:   BF557327    UniGene:   Rn.6670    Gene Symbol:   Ccni_predicted    Locus Link:   289500    Chromosome:   14    Cytoband:   Chr:14p22    SOURCE    GENECARD       Gene Ontology:   molecular function:  cyclin-dependent protein kinase regulator activity      BioCarta Pathways:     KEGG Pathways:  
    Probe set : 1389038_at    Name:   eukaryotic translation termination factor 1 (predicted)    GB acc:   BF285301    UniGene:   Rn.15900    Gene Symbol:   Etf1_predicted    Locus Link:   307503    Chromosome:   18    Cytoband:   Chr:18p12    SOURCE    GENECARD       Gene Ontology:   biological process:  protein biosynthesis   biological process:  translational termination   cellular component:  cytoplasm      BioCarta Pathways:     KEGG Pathways:  
    Probe set : 1377697_at    Name:   Similar to amyloid beta (A4) precursor protein-binding, family B, member 2    GB acc:   BI275738    UniGene:   Rn.24283     Locus Link:   305338    Chromosome:   14    Cytoband:   Chr:14p11    SOURCE        Gene Ontology:     BioCarta Pathways:     KEGG Pathways:  
    Probe set : 1395852_at    Name:   ATP-binding cassette, sub-family A (ABC1), member 3 (predicted)    GB acc:   BF546340    UniGene:   Rn.38406    Gene Symbol:   Abca3_predicted    Locus Link:   302973    Chromosome:   10    Cytoband:   Chr:10q12    SOURCE    GENECARD       Gene Ontology:   biological process:  transport   biological process:  response to drug      BioCarta Pathways:     KEGG Pathways:  
    Probe set : 1388196_at    Name:   NCK-associated protein 1    GB acc:   BM383722    UniGene:   Rn.2611    Gene Symbol:   Nckap1    Locus Link:   58823    Chromosome:   3    Cytoband:   Chr:3q24    SOURCE    GENECARD       Gene Ontology:   biological process:  apoptosis   biological process:  central nervous system development   biological process:  lamellipodium biogenesis   cellular component:  integral to membrane   cellular component:  lamellipodium   molecular function:  protein binding   molecular function:  protein binding      BioCarta Pathways:     KEGG Pathways:  
    Probe set : 1387798_a_at    Name:   complement receptor related protein    GB acc:   D42115    UniGene:   Rn.5825    Gene Symbol:   Crry    Locus Link:   54243    Chromosome:   13    Cytoband:   Chr:13q27    SOURCE    GENECARD       Gene Ontology:   biological process:  regulation of complement activation   biological process:  negative regulation of complement activation   molecular function:  receptor activity      BioCarta Pathways:     KEGG Pathways:  
    Probe set : 1371232_a_at    Name:   chondroitin sulfate proteoglycan 2    GB acc:   AF084544    UniGene:   Rn.35666    Gene Symbol:   Cspg2    Locus Link:   114122    Chromosome:   2    Cytoband:   Chr:2q12    SOURCE    GENECARD       Gene Ontology:   biological process:  cell motility   biological process:  cell adhesion   biological process:  cell adhesion   cellular component:  extracellular matrix (sensu Metazoa)   cellular component:  extracellular matrix      BioCarta Pathways:     KEGG Pathways:  
    Probe set : 1374736_at    Name:   PR domain containing 4    GB acc:   AI172004    UniGene:   Rn.48726    Gene Symbol:   Prdm4    Locus Link:   170820    Chromosome:   7    Cytoband:   Chr:7q13    SOURCE    GENECARD       Gene Ontology:   cellular component:  nucleus      BioCarta Pathways:     KEGG Pathways:  
    Probe set : 1376751_at    Name:   serine/threonine kinase 19    GB acc:   AI044250    UniGene:   Rn.17283    Gene Symbol:   RGD:1359601    Locus Link:   361800    Chromosome:   20    Cytoband:   Chr:20p12    SOURCE    GENECARD       Gene Ontology:   molecular function:  kinase activity      BioCarta Pathways:     KEGG Pathways:  
    Probe set : 1386410_at    Name:   Transcribed locus    GB acc:   AW531135    UniGene:   Rn.151988        SOURCE        Gene Ontology:     BioCarta Pathways:     KEGG Pathways:  
    Probe set : 1376496_at    Name:   hypothetical protein LOC503164    GB acc:   AI717736    UniGene:   Rn.2438    Gene Symbol:   LOC503164    Locus Link:   503164    Chromosome:   7    Cytoband:   Chr:7q34    SOURCE    GENECARD       Gene Ontology:   biological process:  lipid transport   biological process:  lipoprotein metabolism   cellular component:  extracellular region   molecular function:  lipid binding      BioCarta Pathways:     KEGG Pathways:  
    Probe set : 1396065_at    Name:   Transcribed locus    GB acc:   AI548204    UniGene:   Rn.148371        SOURCE        Gene Ontology:     BioCarta Pathways:     KEGG Pathways:  
    Probe set : 1397622_at     GB acc:   AI578003              Gene Ontology:     BioCarta Pathways:     KEGG Pathways:  
    Probe set : 1389730_at    Name:   Transcribed locus    GB acc:   AI176502    UniGene:   Rn.20365        SOURCE        Gene Ontology:     BioCarta Pathways:     KEGG Pathways:  
    Probe set : 1373114_at    Name:   Transcribed locus    GB acc:   AI408442    UniGene:   Rn.18597        SOURCE        Gene Ontology:     BioCarta Pathways:     KEGG Pathways:  
    Probe set : 1379469_at    Name:   similar to transducin (beta)-like 1 X-linked    GB acc:   AA955760    UniGene:   Rn.101383    Gene Symbol:   LOC302711    Locus Link:   302711    Chromosome:   X    Cytoband:   Chr:Xq22    SOURCE    GENECARD       Gene Ontology:     BioCarta Pathways:     KEGG Pathways:  
    Probe set : 1394694_at     GB acc:   AI454646              Gene Ontology:     BioCarta Pathways:     KEGG Pathways:  
    Probe set : 1389790_at    Name:   Transcribed locus    GB acc:   AI410565    UniGene:   Rn.40857        SOURCE        Gene Ontology:     BioCarta Pathways:     KEGG Pathways:  
    Probe set : 1374848_at    Name:   Similar to RIKEN cDNA 2410016F19 (predicted)    GB acc:   AI599867    UniGene:   Rn.9497     Locus Link:   361858    Chromosome:   20    Cytoband:   Chr:20q12    SOURCE        Gene Ontology:     BioCarta Pathways:     KEGG Pathways:  
    Probe set : 1392662_at    Name:   similar to RIKEN cDNA 1500009M05    GB acc:   AI229905    UniGene:   Rn.144840    Gene Symbol:   LOC295457    Locus Link:   295457    Chromosome:   2    Cytoband:   Chr:2q43    SOURCE    GENECARD       Gene Ontology:     BioCarta Pathways:     KEGG Pathways:  
    Probe set : 1369679_a_at    Name:   nuclear factor I/A    GB acc:   AB060652    UniGene:   Rn.10550    Gene Symbol:   Nfia    Locus Link:   25492      SOURCE    GENECARD       Gene Ontology:   cellular component:  intracellular   cellular component:  nucleus   molecular function:  DNA binding   molecular function:  transcription factor activity   molecular function:  heme binding      BioCarta Pathways:     KEGG Pathways:  
    Probe set : 1370752_a_at    Name:   tumor protein p53    GB acc:   AY009504    UniGene:   Rn.54443    Gene Symbol:   Tp53    Locus Link:   24842    Chromosome:   10    Cytoband:   Chr:10q24    SOURCE    GENECARD       Gene Ontology:   biological process:  protein import into nucleus, translocation   biological process:  cell cycle checkpoint   biological process:  base-excision repair   biological process:  nucleotide-excision repair   biological process:  DNA recombination   biological process:  transcription   biological process:  regulation of transcription, DNA-dependent   biological process:  regulation of transcription from RNA polymerase II promoter   biological process:  regulation of transcription from RNA polymerase II promoter   biological process:  apoptosis   biological process:  response to DNA damage stimulus   biological process:  cell cycle   biological process:  cell cycle arrest   biological process:  cell aging   biological process:  negative regulation of DNA replication   biological process:  cell proliferation   biological process:  induction of apoptosis by hormones   biological process:  DNA damage response, signal transduction resulting in induction of apoptosis   biological process:  caspase activation via cytochrome c   biological process:  response to UV   biological process:  response to X-ray   biological process:  cell differentiation   biological process:  negative regulation of cell growth   biological process:  negative regulation of cell growth   biological process:  DNA damage response, signal transduction by p53 class mediator   biological process:  regulation of cell proliferation   biological process:  DNA damage response, signal transduction by p53 class mediator resulting in induction of apoptosis   biological process:  negative regulation of apoptosis   biological process:  negative regulation of progression through cell cycle   biological process:  regulation of mitochondrial membrane permeability   biological process:  negative regulation of fibroblast proliferation   biological process:  negative regulation of helicase activity   biological process:  protein tetramerization   biological process:  base-excision repair   biological process:  nucleotide-excision repair   biological process:  apoptosis   biological process:  cell cycle arrest   biological process:  development   biological process:  cell aging   biological process:  cell proliferation   biological process:  caspase activation via cytochrome c   biological process:  cell differentiation   biological process:  negative regulation of cell growth   molecular function:  DNA strand annealing activity   molecular function:  DNA binding   molecular function:  transcription factor activity   molecular function:  RNA polymerase II transcription factor activity   molecular function:  RNA polymerase II transcription factor activity   molecular function:  nuclease activity   molecular function:  copper ion binding   molecular function:  protein binding   molecular function:  ATP binding   molecular function:  zinc ion binding   molecular function:  protein binding   molecular function:  DNA strand annealing activity   molecular function:  DNA binding   molecular function:  copper ion binding   molecular function:  protein binding   molecular function:  ATP binding   molecular function:  metal ion binding      BioCarta Pathways:     KEGG Pathways:  
    Probe set : 1398386_at    Name:   Transcribed locus, strongly similar to NP_705766.2 zinc finger, CCHC domain containing 6 [Mus musculus]    GB acc:   AW915951    UniGene:   Rn.16458        SOURCE        Gene Ontology:     BioCarta Pathways:     KEGG Pathways:  
    Probe set : 1376885_at    Name:   Similar to chemokine-like factor super family 4    GB acc:   BE095660    UniGene:   Rn.24070     Locus Link:   498902      SOURCE        Gene Ontology:     BioCarta Pathways:     KEGG Pathways:  
    Probe set : 1384831_at    Name:   solute carrier family 7, (cationic amino acid transporter, y+ system) member 13 (predicted)    GB acc:   AI233155    UniGene:   Rn.22708    Gene Symbol:   Slc7a13_predicted    Locus Link:   313089    Chromosome:   5    Cytoband:   Chr:5q13    SOURCE    GENECARD       Gene Ontology:   biological process:  transport   biological process:  amino acid transport   cellular component:  membrane   cellular component:  integral to membrane   molecular function:  amino acid-polyamine transporter activity      BioCarta Pathways:     KEGG Pathways:  
    Probe set : 1375657_at    Name:   Transcribed locus    GB acc:   BE107438    UniGene:   Rn.144180        SOURCE        Gene Ontology:     BioCarta Pathways:     KEGG Pathways:  
    Probe set : 1373494_at    Name:   breakpoint cluster region (predicted)    GB acc:   BE120831    UniGene:   Rn.12782    Gene Symbol:   Bcr_predicted    Locus Link:   309696    Chromosome:   20    Cytoband:   Chr:20p12    SOURCE    GENECARD       Gene Ontology:   biological process:  protein amino acid phosphorylation   biological process:  intracellular signaling cascade      BioCarta Pathways:     KEGG Pathways:  
    Probe set : 1383945_at    Name:   uridine monophosphate kinase (predicted)    GB acc:   AA859827    UniGene:   Rn.24811    Gene Symbol:   Umpk_predicted    Locus Link:   304944    Chromosome:   13    Cytoband:   Chr:13q23    SOURCE    GENECARD       Gene Ontology:   biological process:  biosynthesis      BioCarta Pathways:     KEGG Pathways:  
    Probe set : 1370006_at    Name:   NADH dehydrogenase (ubiquinone) Fe-S protein 6    GB acc:   NM_019223    UniGene:   Rn.127777    Gene Symbol:   Ndufs6    Locus Link:   29478    Chromosome:   2    Cytoband:   Chr:2q12    SOURCE    GENECARD       Gene Ontology:   biological process:  mitochondrial electron transport, NADH to ubiquinone   cellular component:  mitochondrion   cellular component:  respiratory chain complex I (sensu Eukaryota)      BioCarta Pathways:     KEGG Pathways:  
    Probe set : 1384027_a_at    Name:   Transcribed locus    GB acc:   AI072042    UniGene:   Rn.153605        SOURCE        Gene Ontology:     BioCarta Pathways:     KEGG Pathways:  
    Probe set : 1390990_at    Name:   Protein phosphatase 1, catalytic subunit, beta isoform    GB acc:   AI409862    UniGene:   Rn.39034    Gene Symbol:   Ppp1cb    Locus Link:   25594    Chromosome:   6    Cytoband:   Chr:6q13    SOURCE    GENECARD       Gene Ontology:   cellular component:  protein serine/threonine phosphatase complex   molecular function:  protein phosphatase type 2A activity   molecular function:  protein phosphatase type 1 activity   molecular function:  phosphoprotein phosphatase activity   molecular function:  protein serine/threonine phosphatase activity   molecular function:  calcium-dependent protein serine/threonine phosphatase activity   molecular function:  magnesium-dependent protein serine/threonine phosphatase activity   molecular function:  protein binding   molecular function:  CTD phosphatase activity   molecular function:  protein phosphatase type 2C activity   molecular function:  hydrolase activity   molecular function:  myosin phosphatase activity   molecular function:  manganese ion binding   molecular function:  protein phosphatase type 2B activity   molecular function:  iron ion binding   molecular function:  metal ion binding      BioCarta Pathways:     KEGG Pathways:  
    Probe set : 1378433_at    Name:   similar to RIKEN cDNA 5730557B15    GB acc:   BI296242    UniGene:   Rn.60642    Gene Symbol:   LOC310200    Locus Link:   310200    Chromosome:   2    Cytoband:   Chr:2q22    SOURCE    GENECARD       Gene Ontology:     BioCarta Pathways:     KEGG Pathways:  
    Probe set : 1370932_at    Name:   low density lipoprotein receptor-related protein 4    GB acc:   AI070976    UniGene:   Rn.21381    Gene Symbol:   Lrp4    Locus Link:   83469    Chromosome:   3    Cytoband:   Chr:3q24    SOURCE    GENECARD       Gene Ontology:   biological process:  endocytosis   cellular component:  extracellular space   cellular component:  membrane   cellular component:  integral to membrane   molecular function:  receptor activity   molecular function:  calcium ion binding      BioCarta Pathways:     KEGG Pathways:  
    Probe set : 1384340_a_at    Name:   similar to N-acetyltransferase ARD1    GB acc:   AA997134    UniGene:   Rn.12061    Gene Symbol:   LOC363518    Locus Link:   363518    Chromosome:   X    Cytoband:   Chr:Xq37    SOURCE    GENECARD       Gene Ontology:     BioCarta Pathways:     KEGG Pathways:  
    Probe set : 1388666_at    Name:   ectodermal-neural cortex 1    GB acc:   AI179988    UniGene:   Rn.3039    Gene Symbol:   RGD:1303152    Locus Link:   294674    Chromosome:   2    Cytoband:   Chr:2q12    SOURCE    GENECARD       Gene Ontology:   molecular function:  actin binding   molecular function:  protein binding      BioCarta Pathways:     KEGG Pathways:  
    Probe set : 1372433_at    Name:   similar to CG11030-PA (predicted)    GB acc:   AI104715    UniGene:   Rn.98692    Gene Symbol:   RGD1310211_predicted    Locus Link:   305887    Chromosome:   15    Cytoband:   Chr:15p13    SOURCE    GENECARD       Gene Ontology:     BioCarta Pathways:     KEGG Pathways:  
    Probe set : 1398847_at    Name:   nudix (nucleoside diphosphate linked moiety X)-type motif 4    GB acc:   BG376935    UniGene:   Rn.8426    Gene Symbol:   Nudt4    Locus Link:   94267    Chromosome:   7    Cytoband:   Chr:7q13    SOURCE    GENECARD       Gene Ontology:   biological process:  intracellular signaling cascade   biological process:  cyclic nucleotide metabolism   biological process:  calcium-mediated signaling   biological process:  cyclic-nucleotide-mediated signaling   biological process:  phosphoinositide metabolism   biological process:  regulation of RNA export from nucleus   biological process:  intracellular transport   biological process:  intracellular signaling cascade   biological process:  calcium-mediated signaling   biological process:  cyclic-nucleotide-mediated signaling   biological process:  regulation of RNA export from nucleus   biological process:  intracellular transport   cellular component:  intracellular   cellular component:  intracellular      BioCarta Pathways:     KEGG Pathways:  
    Probe set : 1395190_at    Name:   aldo-keto reductase family 1, member B10 (aldose reductase) (predicted)    GB acc:   AI408099    UniGene:   Rn.8475    Gene Symbol:   Akr1b10_predicted    Locus Link:   296972    Chromosome:   4    Cytoband:   Chr:4q22    SOURCE    GENECARD       Gene Ontology:   molecular function:  aldo-keto reductase activity   molecular function:  oxidoreductase activity      BioCarta Pathways:     KEGG Pathways:  
    Probe set : 1393088_at    Name:   RNA (guanine-9-) methyltransferase domain containing 1 (predicted)    GB acc:   BF554013    UniGene:   Rn.47359    Gene Symbol:   Rg9mtd1_predicted    Locus Link:   304012    Chromosome:   11    Cytoband:   Chr:11q12    SOURCE    GENECARD       Gene Ontology:   biological process:  tRNA processing      BioCarta Pathways:     KEGG Pathways:  
    Probe set : 1371237_a_at    Name:   Metallothionein    GB acc:   AF411318    UniGene:   Rn.16133    Gene Symbol:   Mt1a    Locus Link:   24567    Chromosome:   19    Cytoband:   Chr:19p12    SOURCE    GENECARD       Gene Ontology:   cellular component:  cytoplasm   cellular component:  lysosome   cellular component:  cytosol   cellular component:  nucleus      BioCarta Pathways:     KEGG Pathways:  
    Probe set : 1368537_at    Name:   dynactin 4    GB acc:   AI045623    UniGene:   Rn.23941    Gene Symbol:   Dctn4    Locus Link:   84428    Chromosome:   18    Cytoband:   Chr:18q12.1    SOURCE    GENECARD       Gene Ontology:   molecular function:  protein binding   molecular function:  protein binding      BioCarta Pathways:     KEGG Pathways:  
    Probe set : 1375819_at    Name:   similar to RIKEN cDNA 1200011I18 (predicted)    GB acc:   BI299678     Gene Symbol:   RGD1307254_predicted    Locus Link:   298712    Chromosome:   15    Cytoband:   Chr:15q11     GENECARD       Gene Ontology:     BioCarta Pathways:     KEGG Pathways:  
    Probe set : 1373943_at    Name:   CDNA clone IMAGE:7365681    GB acc:   AI237047    UniGene:   Rn.7796        SOURCE        Gene Ontology:     BioCarta Pathways:     KEGG Pathways:  
    Probe set : 1398360_at    Name:   CDNA clone MGC:93791 IMAGE:7109632    GB acc:   AI009805    UniGene:   Rn.29724        SOURCE        Gene Ontology:   cellular component:  integral to membrane      BioCarta Pathways:     KEGG Pathways:  
    Probe set : 1371085_at    Name:   achaete-scute complex homolog-like 3 (Drosophila)    GB acc:   AB046449    UniGene:   Rn.19594    Gene Symbol:   Ascl3    Locus Link:   246301    Chromosome:   1    Cytoband:   Chr:1q33    SOURCE    GENECARD       Gene Ontology:   biological process:  regulation of transcription from RNA polymerase II promoter   biological process:  regulation of transcription   cellular component:  nucleus   cellular component:  transcription factor complex      BioCarta Pathways:     KEGG Pathways:  
    Probe set : 1385634_at     GB acc:   AA817959              Gene Ontology:     BioCarta Pathways:     KEGG Pathways:  
    Probe set : 1369000_at    Name:   neurotrophic tyrosine kinase, receptor, type 1    GB acc:   NM_021589    UniGene:   Rn.39098    Gene Symbol:   Ntrk1    Locus Link:   59109    Chromosome:   2    Cytoband:   Chr:2q34    SOURCE    GENECARD       Gene Ontology:     BioCarta Pathways:     KEGG Pathways:  
    Probe set : 1373977_at    Name:   Kinesin family member 5C (predicted)    GB acc:   BE108253    UniGene:   Rn.50843     Locus Link:   311024    Chromosome:   3    Cytoband:   Chr:3q12    SOURCE        Gene Ontology:   cellular component:  kinesin complex   cellular component:  microtubule associated complex      BioCarta Pathways:     KEGG Pathways:  
    Probe set : 1393730_at    Name:   Transcribed locus    GB acc:   BI277836    UniGene:   Rn.150301        SOURCE        Gene Ontology:     BioCarta Pathways:     KEGG Pathways:  
    Probe set : 1377745_at    Name:   similar to hypothetical protein FLJ20331    GB acc:   BE349673    UniGene:   Rn.13631    Gene Symbol:   LOC310946    Locus Link:   310946    Chromosome:   2    Cytoband:   Chr:2q45    SOURCE    GENECARD       Gene Ontology:     BioCarta Pathways:     KEGG Pathways:  
    Probe set : 1374572_at    Name:   similar to opposite strand transcription unit to Stag3; Gats protein (predicted)    GB acc:   BI294229    UniGene:   Rn.12177    Gene Symbol:   RGD1304774_predicted    Locus Link:   360969    Chromosome:   14    Cytoband:   Chr:14q21    SOURCE    GENECARD       Gene Ontology:     BioCarta Pathways:     KEGG Pathways:  
    Probe set : 1387911_at    Name:   Rab geranylgeranyl transferase componenet, subunit beta    GB acc:   L10416    UniGene:   Rn.107818    Gene Symbol:   Rabggtb    Locus Link:   25533    Chromosome:   2    Cytoband:   Chr:2q45    SOURCE    GENECARD       Gene Ontology:   biological process:  protein modification   biological process:  visual perception   biological process:  protein amino acid geranylgeranylation      BioCarta Pathways:     KEGG Pathways:  
    Probe set : 1397851_at    Name:   Similar to QUAKING isoform 6    GB acc:   BE102226    UniGene:   Rn.85462    Gene Symbol:   Qki    Locus Link:   266718      SOURCE    GENECARD       Gene Ontology:   molecular function:  nucleic acid binding      BioCarta Pathways:     KEGG Pathways:  
    Probe set : 1379862_at     GB acc:   BF400663              Gene Ontology:     BioCarta Pathways:     KEGG Pathways:  
    Probe set : 1383352_at    Name:   Transcribed locus    GB acc:   AI029580    UniGene:   Rn.18262        SOURCE        Gene Ontology:     BioCarta Pathways:     KEGG Pathways:  
    Probe set : 1372627_at     GB acc:   BI299197              Gene Ontology:     BioCarta Pathways:     KEGG Pathways:  
    Probe set : 1389455_at    Name:   SEC24 related gene family, member B (S. cerevisiae) (predicted)    GB acc:   BG380421    UniGene:   Rn.102003    Gene Symbol:   Sec24b_predicted    Locus Link:   295461    Chromosome:   2    Cytoband:   Chr:2q43    SOURCE    GENECARD       Gene Ontology:   biological process:  intracellular protein transport   biological process:  ER to Golgi transport   biological process:  vesicle-mediated transport   molecular function:  transporter activity   molecular function:  protein binding      BioCarta Pathways:     KEGG Pathways:  
    Probe set : 1383300_at    Name:   Dre1 protein    GB acc:   BF550390    UniGene:   Rn.22224    Gene Symbol:   Dre1    Locus Link:   303803    Chromosome:   11    Cytoband:   Chr:11q23    SOURCE    GENECARD       Gene Ontology:   molecular function:  protein binding      BioCarta Pathways:     KEGG Pathways:  
    Probe set : 1383212_at    Name:   CDNA clone IMAGE:7462850    GB acc:   AA925526    UniGene:   Rn.115473        SOURCE        Gene Ontology:     BioCarta Pathways:     KEGG Pathways:  
    Probe set : 1388103_at    Name:   protein distantly related to to the gamma subunit family    GB acc:   AF361355    UniGene:   Rn.92180    Gene Symbol:   Pr1    Locus Link:   245953      SOURCE    GENECARD       Gene Ontology:   biological process:  ion transport   cellular component:  voltage-gated calcium channel complex   cellular component:  integral to membrane   molecular function:  ion channel activity   molecular function:  voltage-gated ion channel activity   molecular function:  calcium channel activity      BioCarta Pathways:     KEGG Pathways:  
    Probe set : 1377437_at    Name:   similar to Veph-A (predicted)    GB acc:   AI013299    UniGene:   Rn.17986    Gene Symbol:   RGD1308217_predicted    Locus Link:   361954    Chromosome:   2    Cytoband:   Chr:2q31    SOURCE    GENECARD       Gene Ontology:     BioCarta Pathways:     KEGG Pathways:  
    Probe set : 1380002_at    Name:   spire homolog 1 (Drosophila) (predicted)    GB acc:   H31747    UniGene:   Rn.132731    Gene Symbol:   Spire1_predicted    Locus Link:   307348    Chromosome:   18    Cytoband:   Chr:18q12.1    SOURCE    GENECARD       Gene Ontology:     BioCarta Pathways:     KEGG Pathways:  
    Probe set : 1388749_at    Name:   Similar to hypothetical protein FLJ13910 (predicted)    GB acc:   BI278823    UniGene:   Rn.11791     Locus Link:   312439    Chromosome:   4    Cytoband:   Chr:4q33    SOURCE        Gene Ontology:     BioCarta Pathways:     KEGG Pathways:  
    Probe set : 1383176_at    Name:   similar to C11orf17 protein (predicted)    GB acc:   BF561368    UniGene:   Rn.106344    Gene Symbol:   RGD1306959_predicted    Locus Link:   361624    Chromosome:   1    Cytoband:   Chr:1q33    SOURCE    GENECARD       Gene Ontology:     BioCarta Pathways:     KEGG Pathways:  
    Probe set : 1375463_at    Name:   Similar to Ski protein    GB acc:   BG380847    UniGene:   Rn.8311     Locus Link:   313757    Chromosome:   5    Cytoband:   Chr:5q36    SOURCE        Gene Ontology:     BioCarta Pathways:     KEGG Pathways:  
    Probe set : 1387059_at    Name:   serine/threonine kinase 39, STE20/SPS1 homolog (yeast)    GB acc:   NM_019362    UniGene:   Rn.34414    Gene Symbol:   Stk39    Locus Link:   54348    Chromosome:   3    Cytoband:   Chr:3q21    SOURCE    GENECARD       Gene Ontology:   biological process:  protein amino acid phosphorylation   biological process:  protein amino acid phosphorylation   biological process:  response to stress      BioCarta Pathways:     KEGG Pathways:  
    Probe set : 1384889_at    Name:   Transcribed locus    GB acc:   AI535467    UniGene:   Rn.124216        SOURCE        Gene Ontology:     BioCarta Pathways:     KEGG Pathways:  
    Probe set : 1385660_at    Name:   similar to KIAA2010 protein (predicted)    GB acc:   AA924079    UniGene:   Rn.22873    Gene Symbol:   RGD1309059_predicted    Locus Link:   314388    Chromosome:   6    Cytoband:   Chr:6q32    SOURCE    GENECARD       Gene Ontology:     BioCarta Pathways:     KEGG Pathways:  
    Probe set : 1376243_at    Name:   similar to serine (or cysteine) proteinase inhibitor, clade B (ovalbumin), member 12    GB acc:   BI278780    UniGene:   Rn.34266    Gene Symbol:   LOC304692    Locus Link:   304692    Chromosome:   13    Cytoband:   Chr:13p13    SOURCE    GENECARD       Gene Ontology:     BioCarta Pathways:     KEGG Pathways:  
    Probe set : 1373116_at    Name:   Karyopherin (importin) alpha 3 (predicted)    GB acc:   AA945320    UniGene:   Rn.18504     Locus Link:   361055    Chromosome:   15    Cytoband:   Chr:15p12    SOURCE        Gene Ontology:   cellular component:  nucleus   cellular component:  nuclear pore   molecular function:  binding   molecular function:  nuclear localization sequence binding   molecular function:  protein transporter activity      BioCarta Pathways:     KEGG Pathways:  
    Probe set : 1398935_at    Name:   Transcribed locus, strongly similar to XP_533460.1 PREDICTED: similar to Sorting nexin-9 [Canis familiaris]    GB acc:   AI009819    UniGene:   Rn.8320        SOURCE        Gene Ontology:     BioCarta Pathways:     KEGG Pathways:  
    Probe set : 1372257_at    Name:   Transcribed locus    GB acc:   BG662579    UniGene:   Rn.7356        SOURCE        Gene Ontology:     BioCarta Pathways:     KEGG Pathways:  
    Probe set : 1377013_at    Name:   Transcribed locus    GB acc:   AI639108    UniGene:   Rn.13077        SOURCE        Gene Ontology:     BioCarta Pathways:     KEGG Pathways:  
    Probe set : 1389613_at    Name:   Similar to MGC68837 protein    GB acc:   AI234712    UniGene:   Rn.58294     Locus Link:   498356      SOURCE        Gene Ontology:     BioCarta Pathways:     KEGG Pathways:  
    Probe set : 1372471_at    Name:   UDP-glucose ceramide glucosyltransferase-like 1    GB acc:   BI283728    UniGene:   Rn.13004    Gene Symbol:   Ugcgl1    Locus Link:   171129    Chromosome:   9    Cytoband:   Chr:9q21    SOURCE    GENECARD       Gene Ontology:   biological process:  protein folding   biological process:  protein amino acid glycosylation   biological process:  posttranslational protein folding      BioCarta Pathways:     KEGG Pathways:  
    Probe set : 1373250_at     GB acc:   AI229404              Gene Ontology:     BioCarta Pathways:     KEGG Pathways:  
    Probe set : 1377893_at    Name:   Transcribed locus    GB acc:   BM390475    UniGene:   Rn.33593        SOURCE        Gene Ontology:     BioCarta Pathways:     KEGG Pathways:  
    Probe set : 1386321_s_at    Name:   tribbles homolog 3 (Drosophila)    GB acc:   H31287    UniGene:   Rn.22325    Gene Symbol:   Trib3    Locus Link:   246273    Chromosome:   3    Cytoband:   Chr:3q41    SOURCE    GENECARD       Gene Ontology:   cellular component:  nucleus      BioCarta Pathways:     KEGG Pathways:  
    Probe set : 1374430_at    Name:   similar to RIKEN cDNA 2700085E05 (predicted)    GB acc:   BI274617    UniGene:   Rn.24630    Gene Symbol:   RGD1307010_predicted    Locus Link:   363644    Chromosome:   10    Cytoband:   Chr:10q24    SOURCE    GENECARD       Gene Ontology:   molecular function:  lyase activity      BioCarta Pathways:     KEGG Pathways:  
    Probe set : 1391628_at    Name:   synovial sarcoma translocation, Chromosome 18 (predicted)    GB acc:   BF418596    UniGene:   Rn.1014    Gene Symbol:   Ss18_predicted    Locus Link:   361295    Chromosome:   18    Cytoband:   Chr:18p13    SOURCE    GENECARD       Gene Ontology:   cellular component:  nucleus      BioCarta Pathways:     KEGG Pathways:  
    Probe set : 1379905_at    Name:   GTP binding protein 1 (predicted)    GB acc:   BM386324    UniGene:   Rn.97210    Gene Symbol:   Gtpbp1_predicted    Locus Link:   300077    Chromosome:   7    Cytoband:   Chr:7q34    SOURCE    GENECARD       Gene Ontology:   molecular function:  electron transporter activity   molecular function:  GTP binding      BioCarta Pathways:     KEGG Pathways:  
    Probe set : 1387377_a_at    Name:   p21 (CDKN1A)-activated kinase 1    GB acc:   U23443    UniGene:   Rn.9149    Gene Symbol:   Pak1    Locus Link:   29431    Chromosome:   1    Cytoband:   Chr:1q32    SOURCE    GENECARD       Gene Ontology:     BioCarta Pathways:     KEGG Pathways:  
    Probe set : 1395960_at    Name:   Transcribed locus, moderately similar to NP_862904.1 epidermodysplasia verruciformis 2 [Mus musculus]    GB acc:   BI301495    UniGene:   Rn.123757        SOURCE        Gene Ontology:     BioCarta Pathways:     KEGG Pathways:  
    Probe set : 1382350_at    Name:   signal transducer and activator of transcription 6 (predicted)    GB acc:   BG377827    UniGene:   Rn.129241    Gene Symbol:   Stat6_predicted    Locus Link:   362896    Chromosome:   7    Cytoband:   Chr:7q22    SOURCE    GENECARD       Gene Ontology:   cellular component:  nucleus   cellular component:  cytoplasm      BioCarta Pathways:     KEGG Pathways:  
    Probe set : 1388446_at    Name:   similar to heterogeneous nuclear ribonucleoprotein A0    GB acc:   BF397834    UniGene:   Rn.98368    Gene Symbol:   LOC498696    Locus Link:   498696    Chromosome:   17    Cytoband:   Chr:17p14    SOURCE    GENECARD       Gene Ontology:     BioCarta Pathways:     KEGG Pathways:  
    Probe set : 1370009_at    Name:   apolipoprotein C-III    GB acc:   NM_012501    UniGene:   Rn.36813    Gene Symbol:   Apoc3    Locus Link:   24207    Chromosome:   8    Cytoband:   Chr:8q23-q24    SOURCE    GENECARD       Gene Ontology:   biological process:  lipid transport   biological process:  lipid catabolism   biological process:  lipoprotein metabolism   cellular component:  extracellular region   cellular component:  extracellular space   molecular function:  lipid transporter activity   molecular function:  lipid binding      BioCarta Pathways:     KEGG Pathways:  
    Probe set : 1384747_at    Name:   transmembrane 7 superfamily member 1 (predicted)    GB acc:   BF404188    UniGene:   Rn.60453    Gene Symbol:   Tm7sf1_predicted    Locus Link:   289287    Chromosome:   17    Cytoband:   Chr:17q12.1    SOURCE    GENECARD       Gene Ontology:   cellular component:  membrane fraction   cellular component:  integral to plasma membrane   cellular component:  integral to membrane      BioCarta Pathways:     KEGG Pathways:  
    Probe set : 1380854_at    Name:   vascular endothelial growth factor B    GB acc:   BF396482    UniGene:   Rn.144620    Gene Symbol:   Vegfb    Locus Link:   89811    Chromosome:   1    Cytoband:   Chr:1q43    SOURCE    GENECARD       Gene Ontology:   biological process:  regulation of progression through cell cycle   biological process:  cell proliferation   cellular component:  membrane   molecular function:  growth factor activity   molecular function:  heparin binding      BioCarta Pathways:     KEGG Pathways:  
    Probe set : 1368984_at    Name:   septin 2    GB acc:   NM_057148    UniGene:   Rn.98570    Gene Symbol:   Sept2    Locus Link:   117515    Chromosome:   9    Cytoband:   Chr:9q36    SOURCE    GENECARD       Gene Ontology:   biological process:  cell cycle   molecular function:  nucleotide binding   molecular function:  GTP binding      BioCarta Pathways:     KEGG Pathways:  
    Probe set : 1371747_at    Name:   similar to RIKEN cDNA 2700038C09 (predicted)    GB acc:   AI406304    UniGene:   Rn.1389    Gene Symbol:   RGD1310660_predicted    Locus Link:   296470    Chromosome:   3    Cytoband:   Chr:3q43    SOURCE    GENECARD       Gene Ontology:     BioCarta Pathways:     KEGG Pathways:  
    Probe set : 1372729_at    Name:   protein C receptor, endothelial (predicted)    GB acc:   AI137406    UniGene:   Rn.144312    Gene Symbol:   Procr_predicted    Locus Link:   362248    Chromosome:   3    Cytoband:   Chr:3q42    SOURCE    GENECARD       Gene Ontology:   biological process:  inflammatory response   biological process:  blood coagulation   biological process:  negative regulation of coagulation   molecular function:  receptor activity   molecular function:  protein C-terminus binding      BioCarta Pathways:     KEGG Pathways:  
    Probe set : 1382046_at    Name:   mitogen activated protein kinase kinase kinase 3 (predicted)    GB acc:   AA963495    UniGene:   Rn.72680    Gene Symbol:   Map3k3_predicted    Locus Link:   303604    Chromosome:   10    Cytoband:   Chr:10q32.1    SOURCE    GENECARD       Gene Ontology:   biological process:  MAPKKK cascade   biological process:  protein amino acid phosphorylation   biological process:  positive regulation of I-kappaB kinase/NF-kappaB cascade      BioCarta Pathways:     KEGG Pathways:  
    Probe set : 1388896_at    Name:   ubiquitin specific protease 33 (predicted)    GB acc:   BF404603    UniGene:   Rn.25188    Gene Symbol:   Usp33_predicted    Locus Link:   310960    Chromosome:   2    Cytoband:   Chr:2q45    SOURCE    GENECARD       Gene Ontology:   biological process:  ubiquitin-dependent protein catabolism   biological process:  ubiquitin cycle   biological process:  protein deubiquitination   cellular component:  cytoplasm   cellular component:  VCB complex      BioCarta Pathways:     KEGG Pathways:  
    Probe set : 1398259_at    Name:   nucleoporin 155    GB acc:   NM_053952    UniGene:   Rn.11328    Gene Symbol:   Nup155    Locus Link:   117021    Chromosome:   2    Cytoband:   Chr:2q16    SOURCE    GENECARD       Gene Ontology:   biological process:  transport   biological process:  nucleocytoplasmic transport      BioCarta Pathways:     KEGG Pathways:  
    Probe set : 1390333_at    Name:   protein phosphatase 4 (formerly X), catalytic subunit    GB acc:   AA957050    UniGene:   Rn.9173    Gene Symbol:   RGD:621225    Locus Link:   171366    Chromosome:   1    Cytoband:   Chr:1q36    SOURCE    GENECARD       Gene Ontology:   cellular component:  protein phosphatase 4 complex   molecular function:  protein phosphatase type 4 activity   molecular function:  hydrolase activity   molecular function:  iron ion binding      BioCarta Pathways:     KEGG Pathways:  
    Probe set : 1399125_at    Name:   inositol polyphosphate-1-phosphatase (predicted)    GB acc:   BI275516    UniGene:   Rn.20288    Gene Symbol:   Inpp1_predicted    Locus Link:   316376    Chromosome:   9    Cytoband:   Chr:9q22    SOURCE    GENECARD       Gene Ontology:   biological process:  phosphate metabolism   biological process:  signal transduction      BioCarta Pathways:     KEGG Pathways:  
    Probe set : 1369962_at    Name:   5-aminoimidazole-4-carboxamide ribonucleotide formyltransferase/IMP cyclohydrolase    GB acc:   NM_031014    UniGene:   Rn.15114    Gene Symbol:   Atic    Locus Link:   81643    Chromosome:   9    Cytoband:   Chr:9q33    SOURCE    GENECARD       Gene Ontology:     BioCarta Pathways:     KEGG Pathways:  
    Probe set : 1367912_at    Name:   latent transforming growth factor beta binding protein 1    GB acc:   NM_021587    UniGene:   Rn.40942    Gene Symbol:   Ltbp1    Locus Link:   59107    Chromosome:   6    Cytoband:   Chr:6q12    SOURCE    GENECARD       Gene Ontology:   biological process:  transforming growth factor beta receptor signaling pathway   cellular component:  extracellular matrix (sensu Metazoa)   cellular component:  extracellular space   molecular function:  transforming growth factor beta receptor activity   molecular function:  calcium ion binding   molecular function:  growth factor binding      BioCarta Pathways:     KEGG Pathways:  
    Probe set : 1382126_at     GB acc:   AW528959              Gene Ontology:     BioCarta Pathways:     KEGG Pathways:  
    Probe set : 1371189_x_at    Name:   laminin receptor 1 (67kD, ribosomal protein SA) /// similar to 40S ribosomal protein SA (p40) (34/67 kDa laminin receptor) /// similar to Ribosomal protein SA    GB acc:   BG153272    UniGene:   Rn.999    Gene Symbol:   Lamr1 /// LOC367030 /// LOC367303    Locus Link:   29236 /// 367030 /// 367303    Chromosome:   8    Cytoband:   Chr:8q32 /// Chr:8q13 /// Chr:9q34    SOURCE    GENECARD       Gene Ontology:   biological process:  protein biosynthesis      BioCarta Pathways:     KEGG Pathways:  
    Probe set : 1381118_at    Name:   similar to RIKEN cDNA 2700007P21 (predicted)    GB acc:   AW526652    UniGene:   Rn.40865    Gene Symbol:   RGD1311463_predicted    Locus Link:   311279    Chromosome:   3    Cytoband:   Chr:3q33    SOURCE    GENECARD       Gene Ontology:     BioCarta Pathways:     KEGG Pathways:  
    Probe set : 1398999_at    Name:   similar to es 64 /// similar to hypothetical protein    GB acc:   BF283508    UniGene:   Rn.145429    Gene Symbol:   LOC363675 /// LOC497993    Locus Link:   363675 /// 497993    Chromosome:   10    Cytoband:   Chr:10q31    SOURCE    GENECARD       Gene Ontology:   biological process:  steroid biosynthesis   molecular function:  cholesterol binding   molecular function:  cholesterol transporter activity      BioCarta Pathways:     KEGG Pathways:  
    Probe set : 1389582_at    Name:   Transcribed locus    GB acc:   AI407405    UniGene:   Rn.125129        SOURCE        Gene Ontology:     BioCarta Pathways:     KEGG Pathways:  
    Probe set : 1368814_at    Name:   aldehyde dehydrogenase family 6, subfamily A1    GB acc:   NM_031057    UniGene:   Rn.2098    Gene Symbol:   Aldh6a1    Locus Link:   81708    Chromosome:   6    Cytoband:   Chr:6q31    SOURCE    GENECARD       Gene Ontology:   biological process:  valine metabolism   biological process:  metabolism   cellular component:  mitochondrion      BioCarta Pathways:     KEGG Pathways:  
    Probe set : 1380152_at    Name:   Similar to RIKEN cDNA 2700059L22 (predicted)    GB acc:   BF407782    UniGene:   Rn.105880     Locus Link:   315645    Chromosome:   8    Cytoband:   Chr:8q23    SOURCE        Gene Ontology:     BioCarta Pathways:     KEGG Pathways:  
    Probe set : 1376001_at    Name:   similar to RNA polymerase I associated factor (PAF53)    GB acc:   BG380440    UniGene:   Rn.11499    Gene Symbol:   LOC313245    Locus Link:   313245    Chromosome:   5    Cytoband:   Chr:5q22    SOURCE    GENECARD       Gene Ontology:     BioCarta Pathways:     KEGG Pathways:  
    Probe set : 1388313_at    Name:   ribosomal protein s25    GB acc:   BM389289    UniGene:   Rn.144642    Gene Symbol:   Rps25    Locus Link:   122799    Chromosome:   8    Cytoband:   Chr:8q22    SOURCE    GENECARD       Gene Ontology:   biological process:  protein biosynthesis   cellular component:  cytosolic small ribosomal subunit (sensu Eukaryota)   molecular function:  RNA binding   molecular function:  structural constituent of ribosome      BioCarta Pathways:     KEGG Pathways:  
    Probe set : 1390514_at    Name:   similar to hypothetical protein from BCRA2 region    GB acc:   BI295160    UniGene:   Rn.19041    Gene Symbol:   RGD:1359603    Locus Link:   288416    Chromosome:   12    Cytoband:   Chr:12p12    SOURCE    GENECARD       Gene Ontology:     BioCarta Pathways:     KEGG Pathways:  
    Probe set : 1387036_at    Name:   hairy and enhancer of split 1 (Drosophila)    GB acc:   NM_024360    UniGene:   Rn.19727    Gene Symbol:   Hes1    Locus Link:   29577    Chromosome:   11    Cytoband:   Chr:11q22    SOURCE    GENECARD       Gene Ontology:   cellular component:  nucleus      BioCarta Pathways:     KEGG Pathways:  
    Probe set : 1371615_at    Name:   diacylglycerol O-acyltransferase homolog 2 (mouse)    GB acc:   BI279069    UniGene:   Rn.9523    Gene Symbol:   Dgat2    Locus Link:   252900    Chromosome:   1    Cytoband:   Chr:1q32    SOURCE    GENECARD       Gene Ontology:   cellular component:  microsome      BioCarta Pathways:     KEGG Pathways:  
    Probe set : 1380053_at    Name:   similar to transmembrane protein TM9SF3    GB acc:   BF397371    UniGene:   Rn.17150    Gene Symbol:   LOC309475    Locus Link:   309475    Chromosome:   1    Cytoband:   Chr:1q54    SOURCE    GENECARD       Gene Ontology:     BioCarta Pathways:     KEGG Pathways:  
    Probe set : 1376253_at    Name:   lymphocyte antigen 6 complex, locus H (predicted)    GB acc:   AW434178    UniGene:   Rn.7534    Gene Symbol:   Ly6h_predicted    Locus Link:   300025    Chromosome:   7    Cytoband:   Chr:7q34    SOURCE    GENECARD       Gene Ontology:   biological process:  cellular defense response   biological process:  nervous system development   cellular component:  extracellular space   cellular component:  integral to plasma membrane   cellular component:  membrane      BioCarta Pathways:     KEGG Pathways:  
    Probe set : 1369209_at    Name:   p34 protein    GB acc:   NM_134398    UniGene:   Rn.144663    Gene Symbol:   RGD:621601    Locus Link:   171435    Chromosome:   8    Cytoband:   Chr:8q24    SOURCE    GENECARD       Gene Ontology:   biological process:  transport   biological process:  protein transport      BioCarta Pathways:     KEGG Pathways:  
    Probe set : 1391730_at    Name:   Myeloid/lymphoid or mixed-lineage leukemia 2 (predicted)    GB acc:   AW433989    UniGene:   Rn.8013     Locus Link:   362996    Chromosome:   7    Cytoband:   Chr:7q36    SOURCE        Gene Ontology:     BioCarta Pathways:     KEGG Pathways:  
    Probe set : 1387809_at    Name:   mitogen-activated protein kinase kinase 6    GB acc:   NM_053703    UniGene:   Rn.17256    Gene Symbol:   Map2k6    Locus Link:   114495    Chromosome:   10    Cytoband:   Chr:10q32.1    SOURCE    GENECARD       Gene Ontology:     BioCarta Pathways:     KEGG Pathways:  
    Probe set : 1382047_at    Name:   similar to AI325464 protein (predicted)    GB acc:   AA899888    UniGene:   Rn.49263    Gene Symbol:   RGD1307976_predicted    Locus Link:   301624    Chromosome:   9    Cytoband:   Chr:9q36    SOURCE    GENECARD       Gene Ontology:     BioCarta Pathways:     KEGG Pathways:  
    Probe set : 1394450_at    Name:   Transcribed locus, moderately similar to XP_484812.1 PREDICTED: hypothetical protein XP_484812 [Mus musculus]    GB acc:   AI228600    UniGene:   Rn.40570        SOURCE        Gene Ontology:     BioCarta Pathways:     KEGG Pathways:  
    Probe set : 1387675_at    Name:   Plasminogen activator, urokinase    GB acc:   X63434    UniGene:   Rn.6064    Gene Symbol:   Plau    Locus Link:   25619    Chromosome:   15    Cytoband:   Chr:15p16    SOURCE    GENECARD       Gene Ontology:   cellular component:  extracellular space      BioCarta Pathways:     KEGG Pathways:  
    Probe set : 1386394_at     GB acc:   AA956971              Gene Ontology:     BioCarta Pathways:     KEGG Pathways:  
    Probe set : 1387039_at    Name:   glypican 1    GB acc:   NM_030828    UniGene:   Rn.7044    Gene Symbol:   Gpc1    Locus Link:   58920      SOURCE    GENECARD       Gene Ontology:   molecular function:  transmembrane receptor activity      BioCarta Pathways:     KEGG Pathways:  
    Probe set : 1392534_at    Name:   transmembrane, prostate androgen induced RNA (predicted)    GB acc:   BI274101    UniGene:   Rn.108034    Gene Symbol:   Tmepai_predicted    Locus Link:   311676    Chromosome:   3    Cytoband:   Chr:3q42    SOURCE    GENECARD       Gene Ontology:   biological process:  androgen receptor signaling pathway   cellular component:  integral to membrane   molecular function:  protein binding      BioCarta Pathways:     KEGG Pathways:  
    Probe set : 1388296_at    Name:   ribosomal protein S18    GB acc:   BF281388    UniGene:   Rn.42766    Gene Symbol:   Rps18    Locus Link:   294282    Chromosome:   20    Cytoband:   Chr:20p12    SOURCE    GENECARD       Gene Ontology:   biological process:  regulation of translation   biological process:  ribosome biogenesis   cellular component:  cytosolic ribosome (sensu Eukaryota)   cellular component:  small ribosomal subunit   molecular function:  structural constituent of ribosome   molecular function:  structural constituent of ribosome      BioCarta Pathways:     KEGG Pathways:  
    Probe set : 1391837_at    Name:   similar to chr2 synaptotagmin    GB acc:   AA955351    UniGene:   Rn.91770    Gene Symbol:   LOC299488    Locus Link:   299488    Chromosome:   6    Cytoband:   Chr:6q33    SOURCE    GENECARD       Gene Ontology:     BioCarta Pathways:     KEGG Pathways:  
    Probe set : 1397788_at    Name:   RIM binding protein 2    GB acc:   AW521711    UniGene:   Rn.125162    Gene Symbol:   LOC266780    Locus Link:   266780    Chromosome:   12    Cytoband:   Chr:12q14    SOURCE    GENECARD       Gene Ontology:   cellular component:  membrane   molecular function:  protein binding   molecular function:  SH3 domain binding      BioCarta Pathways:     KEGG Pathways:  
    Probe set : 1369927_at    Name:   malate dehydrogenase, mitochondrial    GB acc:   NM_031151    UniGene:   Rn.1011    Gene Symbol:   Mor1    Locus Link:   81829    Chromosome:   12    Cytoband:   Chr:12q12    SOURCE    GENECARD       Gene Ontology:   cellular component:  mitochondrion   cellular component:  mitochondrion   cellular component:  mitochondrial matrix      BioCarta Pathways:     KEGG Pathways:  
    Probe set : 1383504_at    Name:   Transcribed locus    GB acc:   AW530604    UniGene:   Rn.23021        SOURCE        Gene Ontology:     BioCarta Pathways:     KEGG Pathways:  
    Probe set : 1376416_at     GB acc:   BI296287              Gene Ontology:     BioCarta Pathways:     KEGG Pathways:  
    Probe set : 1371915_at    Name:   poly(rC) binding protein 4 (predicted)    GB acc:   AW523679    UniGene:   Rn.40405    Gene Symbol:   Pcbp4_predicted    Locus Link:   363133    Chromosome:   8    Cytoband:   Chr:8q32    SOURCE    GENECARD       Gene Ontology:   cellular component:  nucleus   cellular component:  ribonucleoprotein complex   molecular function:  nucleic acid binding   molecular function:  DNA binding   molecular function:  RNA binding      BioCarta Pathways:     KEGG Pathways:  
    Probe set : 1398431_at    Name:   carbonic anhydrase 8 (predicted)    GB acc:   BI294910    UniGene:   Rn.22066    Gene Symbol:   Car8_predicted    Locus Link:   297814    Chromosome:   5    Cytoband:   Chr:5q13    SOURCE    GENECARD       Gene Ontology:   biological process:  one-carbon compound metabolism   biological process:  phosphoinositide-mediated signaling   cellular component:  cytoplasm      BioCarta Pathways:     KEGG Pathways:  
    Probe set : 1376579_at    Name:   leucine aminopeptidase 3 (predicted)    GB acc:   AA945172    UniGene:   Rn.99790    Gene Symbol:   Lap3_predicted    Locus Link:   289668    Chromosome:   14    Cytoband:   Chr:14q21    SOURCE    GENECARD       Gene Ontology:   biological process:  proteolysis   biological process:  protein metabolism   cellular component:  intracellular   cellular component:  cytoplasm   cellular component:  mitochondrion      BioCarta Pathways:     KEGG Pathways:  
    Probe set : 1393629_at    Name:   H2.0-like homeo box 1 (Drosophila) (predicted)    GB acc:   AI501027    UniGene:   Rn.35243    Gene Symbol:   Hlx1_predicted    Locus Link:   364069    Chromosome:   13    Cytoband:   Chr:13q26    SOURCE    GENECARD       Gene Ontology:   biological process:  regulation of transcription, DNA-dependent   biological process:  transcription from RNA polymerase II promoter   biological process:  development   cellular component:  nucleus   cellular component:  transcription factor complex   molecular function:  DNA binding   molecular function:  transcription factor activity      BioCarta Pathways:     KEGG Pathways:  
    Probe set : 1374458_at    Name:   Transcribed locus, strongly similar to NP_573483.1 serum/glucocorticoid regulated kinase 3 [Mus musculus]    GB acc:   AI600031    UniGene:   Rn.17432        SOURCE        Gene Ontology:     BioCarta Pathways:     KEGG Pathways:  
    Probe set : 1374832_at    Name:   Histone 1, H2bh (predicted)    GB acc:   BI299921    UniGene:   Rn.42931    Gene Symbol:   Hfe    Locus Link:   29199    Chromosome:   17    Cytoband:   Chr:17p11    SOURCE    GENECARD       Gene Ontology:   molecular function:  MHC class I receptor activity      BioCarta Pathways:     KEGG Pathways:  
    Probe set : 1381208_at    Name:   histone 2a    GB acc:   BE104595    UniGene:   Rn.39174    Gene Symbol:   RGD:621437    Locus Link:   64646    Chromosome:   17    Cytoband:   Chr:17q11    SOURCE    GENECARD       Gene Ontology:   biological process:  nucleosome assembly   biological process:  chromosome organization and biogenesis (sensu Eukaryota)   cellular component:  nucleosome   cellular component:  nucleus   molecular function:  DNA binding      BioCarta Pathways:     KEGG Pathways:  
    Probe set : 1374615_at     GB acc:   AI175668              Gene Ontology:     BioCarta Pathways:     KEGG Pathways:  
    Probe set : 1379234_a_at    Name:   similar to CDC45L    GB acc:   AI179539    UniGene:   Rn.98535    Gene Symbol:   LOC287961    Locus Link:   287961    Chromosome:   11    Cytoband:   Chr:11p12    SOURCE    GENECARD       Gene Ontology:     BioCarta Pathways:     KEGG Pathways:  
    Probe set : 1370688_at    Name:   glutamate-cysteine ligase, catalytic subunit    GB acc:   J05181    UniGene:   Rn.8365    Gene Symbol:   Gclc    Locus Link:   25283    Chromosome:   8    Cytoband:   Chr:8q31    SOURCE    GENECARD       Gene Ontology:     BioCarta Pathways:     KEGG Pathways:  
    Probe set : 1389128_at    Name:   WD repeat and FYVE domain containing 3 (predicted)    GB acc:   AI146179    UniGene:   Rn.57981     Locus Link:   305164    Chromosome:   14    Cytoband:   Chr:14p22    SOURCE        Gene Ontology:   cellular component:  nuclear membrane   cellular component:  cytoplasm   molecular function:  beta-N-acetylglucosaminylglycopeptide beta-1,4-galactosyltransferase activity   molecular function:  binding   molecular function:  zinc ion binding      BioCarta Pathways:     KEGG Pathways:  
    Probe set : 1372401_at    Name:   N-acetylneuraminic acid synthase (sialic acid synthase) (predicted)    GB acc:   AA859401    UniGene:   Rn.17006    Gene Symbol:   Nans_predicted    Locus Link:   298071    Chromosome:   5    Cytoband:   Chr:5q22    SOURCE    GENECARD       Gene Ontology:   cellular component:  cytoplasm   cellular component:  cytosol      BioCarta Pathways:     KEGG Pathways:  
    Probe set : 1374961_at     GB acc:   BI274511              Gene Ontology:     BioCarta Pathways:     KEGG Pathways:  
    Probe set : 1375562_at    Name:   Transcribed locus    GB acc:   BF408460    UniGene:   Rn.34184        SOURCE        Gene Ontology:     BioCarta Pathways:     KEGG Pathways:  
    Probe set : 1390937_at    Name:   similar to chromosome 14 open reading frame 50 (predicted)    GB acc:   AW523875    UniGene:   Rn.3331    Gene Symbol:   RGD1309051_predicted    Locus Link:   299153    Chromosome:   6    Cytoband:   Chr:6q24    SOURCE    GENECARD       Gene Ontology:     BioCarta Pathways:     KEGG Pathways:  
    Probe set : 1379300_at    Name:   carbohydrate sulfotransferase 2 (predicted)    GB acc:   AW520944    UniGene:   Rn.95986    Gene Symbol:   Chst2_predicted    Locus Link:   367145    Chromosome:   8    Cytoband:   Chr:8q31    SOURCE    GENECARD       Gene Ontology:   biological process:  N-acetylglucosamine metabolism   cellular component:  extracellular space   cellular component:  integral to membrane   molecular function:  sulfotransferase activity   molecular function:  transferase activity      BioCarta Pathways:     KEGG Pathways:  
    Probe set : 1375273_at    Name:   tripartite motif protein 47 (predicted)    GB acc:   AW251625    UniGene:   Rn.91719    Gene Symbol:   Trim47_predicted    Locus Link:   287824    Chromosome:   10    Cytoband:   Chr:10q32.3    SOURCE    GENECARD       Gene Ontology:   biological process:  protein ubiquitination   cellular component:  ubiquitin ligase complex   cellular component:  intracellular   cellular component:  nucleus   molecular function:  ubiquitin-protein ligase activity   molecular function:  zinc ion binding      BioCarta Pathways:     KEGG Pathways:  
    Probe set : 1383397_at    Name:   receptor-associated protein of the synapse (predicted)    GB acc:   BI296106    UniGene:   Rn.17149    Gene Symbol:   Rapsn_predicted    Locus Link:   362161    Chromosome:   3    Cytoband:   Chr:3q24    SOURCE    GENECARD       Gene Ontology:   biological process:  synaptic transmission   biological process:  protein ubiquitination   molecular function:  ubiquitin-protein ligase activity   molecular function:  binding   molecular function:  zinc ion binding      BioCarta Pathways:     KEGG Pathways:  
    Probe set : 1374313_at    Name:   BPY2 interacting protein 1 (predicted)    GB acc:   BI294383    UniGene:   Rn.38446    Gene Symbol:   Bpy2ip1_predicted    Locus Link:   290640    Chromosome:   16    Cytoband:   Chr:16p14    SOURCE    GENECARD       Gene Ontology:   molecular function:  hydrolase activity      BioCarta Pathways:     KEGG Pathways:  
    Probe set : 1372626_at    Name:   Similar to tumor protein D53    GB acc:   AI231999    UniGene:   Rn.24825     Locus Link:   498994      SOURCE        Gene Ontology:     BioCarta Pathways:     KEGG Pathways:  
    Probe set : 1389526_at    Name:   similar to RIKEN cDNA C920006C10 (predicted)    GB acc:   BG373853    UniGene:   Rn.21009    Gene Symbol:   RGD1305976_predicted    Locus Link:   362923    Chromosome:   7    Cytoband:   Chr:7q33    SOURCE    GENECARD       Gene Ontology:     BioCarta Pathways:     KEGG Pathways:  
    Probe set : 1393352_at    Name:   similar to hypothetical protein MGC38960 (predicted)    GB acc:   BI293882    UniGene:   Rn.23973    Gene Symbol:   RGD1310552_predicted    Locus Link:   300836    Chromosome:   8    Cytoband:   Chr:8q24    SOURCE    GENECARD       Gene Ontology:     BioCarta Pathways:     KEGG Pathways:  
    Probe set : 1376047_at    Name:   3'-phosphoadenosine 5'-phosphosulfate synthase 2 (predicted)    GB acc:   BI285321    UniGene:   Rn.79315     Locus Link:   294103    Chromosome:   1    Cytoband:   Chr:1q52    SOURCE        Gene Ontology:   biological process:  sulfate assimilation   biological process:  skeletal development   biological process:  nucleobase, nucleoside, nucleotide and nucleic acid metabolism   cellular component:  intracellular      BioCarta Pathways:     KEGG Pathways:  
    Probe set : 1376792_at    Name:   Transcribed locus    GB acc:   AW251313    UniGene:   Rn.828        SOURCE        Gene Ontology:     BioCarta Pathways:     KEGG Pathways:  
    Probe set : 1387060_at    Name:   core promoter element binding protein    GB acc:   NM_031642    UniGene:   Rn.7947    Gene Symbol:   Copeb    Locus Link:   58954    Chromosome:   17    Cytoband:   Chr:17q12.2    SOURCE    GENECARD       Gene Ontology:   cellular component:  nucleus   cellular component:  nucleus   cellular component:  cytoplasm      BioCarta Pathways:     KEGG Pathways:  
    Probe set : 1388934_at    Name:   vacuolar protein sorting 16    GB acc:   BI285570    UniGene:   Rn.14968    Gene Symbol:   RGD:1359414    Locus Link:   296159    Chromosome:   3    Cytoband:   Chr:3q36    SOURCE    GENECARD       Gene Ontology:   biological process:  intracellular protein transport   cellular component:  cytoplasm      BioCarta Pathways:     KEGG Pathways:  
    Probe set : 1376656_at    Name:   ubiquitin specific protease 20 (predicted)    GB acc:   BI274796    UniGene:   Rn.27074    Gene Symbol:   Usp20_predicted    Locus Link:   311856    Chromosome:   3    Cytoband:   Chr:3p12    SOURCE    GENECARD       Gene Ontology:     BioCarta Pathways:     KEGG Pathways:  
    Probe set : 1397579_x_at    Name:   Similar to RIKEN cDNA 2310011J03    GB acc:   BI294552    UniGene:   Rn.39052    Gene Symbol:   MGC94198    Locus Link:   299612    Chromosome:   7    Cytoband:   Chr:7q11    SOURCE    GENECARD       Gene Ontology:     BioCarta Pathways:     KEGG Pathways:  
    Probe set : 1390790_a_at    Name:   hypothetical LOC361349    GB acc:   AA964219     Gene Symbol:   LOC361349    Locus Link:   361349    Chromosome:   18    Cytoband:   Chr:18q12.3     GENECARD       Gene Ontology:     BioCarta Pathways:     KEGG Pathways:  
    Probe set : 1383336_at    Name:   similar to pinin    GB acc:   BF549763    UniGene:   Rn.38987    Gene Symbol:   LOC368070    Locus Link:   368070    Chromosome:   6    Cytoband:   Chr:6q23    SOURCE    GENECARD       Gene Ontology:     BioCarta Pathways:     KEGG Pathways:  
    Probe set : 1374331_at    Name:   rcd1 (required for cell differentiation) homolog 1 (S. pombe) (predicted)    GB acc:   AI178068    UniGene:   Rn.18033    Gene Symbol:   Rqcd1_predicted    Locus Link:   301513    Chromosome:   9    Cytoband:   Chr:9q33    SOURCE    GENECARD       Gene Ontology:   biological process:  sex differentiation   biological process:  cytokine and chemokine mediated signaling pathway   molecular function:  binding      BioCarta Pathways:     KEGG Pathways:  
    Probe set : 1389450_at    Name:   similar to Putative methyltransferase HUSSY-03 (Williams-Beuren syndrome chromosome region 22 protein homolog) /// similar to Putative methyltransferase HUSSY-03 (Williams-Beuren syndrome chromosome region 22 protein homolog)    GB acc:   BF392737    UniGene:   Rn.19568    Gene Symbol:   LOC360830 /// LOC368084    Locus Link:   360830 /// 368084    Chromosome:   12    Cytoband:   Chr:12q12    SOURCE    GENECARD       Gene Ontology:     BioCarta Pathways:     KEGG Pathways:  
    Probe set : 1389511_s_at     GB acc:   BF403383              Gene Ontology:     BioCarta Pathways:     KEGG Pathways:  
    Probe set : 1391441_at    Name:   Transcribed locus    GB acc:   AA899943    UniGene:   Rn.8955        SOURCE        Gene Ontology:     BioCarta Pathways:     KEGG Pathways:  
    Probe set : 1397385_at    Name:   Transcribed locus    GB acc:   BM387110    UniGene:   Rn.95229        SOURCE        Gene Ontology:     BioCarta Pathways:     KEGG Pathways:  
    Probe set : 1374112_at    Name:   similar to MAPK-interacting and spindle-stabilizing protein    GB acc:   BI296251    UniGene:   Rn.118217    Gene Symbol:   LOC499280    Locus Link:   499280    Chromosome:   1    Cytoband:   Chr:1q41    SOURCE    GENECARD       Gene Ontology:     BioCarta Pathways:     KEGG Pathways:  
    Probe set : 1387117_at    Name:   zinc finger protein 265    GB acc:   AF013965    UniGene:   Rn.11361    Gene Symbol:   Zfp265    Locus Link:   58821    Chromosome:   2    Cytoband:   Chr:2q45    SOURCE    GENECARD       Gene Ontology:   biological process:  RNA splicing   biological process:  RNA splicing   cellular component:  nucleus      BioCarta Pathways:     KEGG Pathways:  
    Probe set : 1396238_at    Name:   UDP-N-acetyl-alpha-D-galactosamine:polypeptide N-acetylgalactosaminyltransferase 14 (predicted)    GB acc:   AI763990    UniGene:   Rn.24642    Gene Symbol:   Galnt14_predicted    Locus Link:   313878    Chromosome:   6    Cytoband:   Chr:6q13    SOURCE    GENECARD       Gene Ontology:   cellular component:  Golgi apparatus   cellular component:  integral to membrane      BioCarta Pathways:     KEGG Pathways:  
    Probe set : 1368174_at    Name:   hypothetical gene supported by NM_019371    GB acc:   NM_019371     Gene Symbol:   LOC497816    Locus Link:   497816    Chromosome:   6    Cytoband:   Chr:6q23     GENECARD       Gene Ontology:     BioCarta Pathways:     KEGG Pathways:  
    Probe set : 1380379_at    Name:   Similar to : F-box protein 44    GB acc:   AI228000    UniGene:   Rn.24824     Locus Link:   500587      SOURCE        Gene Ontology:     BioCarta Pathways:     KEGG Pathways:  
    Probe set : 1391241_at    Name:   Transcribed locus    GB acc:   BF394755    UniGene:   Rn.124211        SOURCE        Gene Ontology:     BioCarta Pathways:     KEGG Pathways:  
    Probe set : 1389774_at    Name:   Transcribed locus    GB acc:   AI008371    UniGene:   Rn.6474        SOURCE        Gene Ontology:     BioCarta Pathways:     KEGG Pathways:  
    Probe set : 1388978_at    Name:   Similar to RIKEN cDNA 2010200I23    GB acc:   BF283704    UniGene:   Rn.95176    Gene Symbol:   MGC94262    Locus Link:   300857    Chromosome:   8    Cytoband:   Chr:8q31    SOURCE    GENECARD       Gene Ontology:   cellular component:  membrane      BioCarta Pathways:     KEGG Pathways:  
    Probe set : 1371891_at    Name:   similar to 1700123O20Rik protein (predicted)    GB acc:   BG371476    UniGene:   Rn.2583    Gene Symbol:   RGD1308430_predicted    Locus Link:   361038    Chromosome:   15    Cytoband:   Chr:15p13    SOURCE    GENECARD       Gene Ontology:     BioCarta Pathways:     KEGG Pathways:  
    Probe set : 1393143_at    Name:   Transcribed locus    GB acc:   AI045866    UniGene:   Rn.16334        SOURCE        Gene Ontology:     BioCarta Pathways:     KEGG Pathways:  
    Probe set : 1372501_at    Name:   splicing factor 3b, subunit 3 (predicted)    GB acc:   BI285007    UniGene:   Rn.7554    Gene Symbol:   Sf3b3_predicted    Locus Link:   292019    Chromosome:   19    Cytoband:   Chr:19q12    SOURCE    GENECARD       Gene Ontology:   biological process:  nuclear mRNA splicing, via spliceosome   biological process:  protein complex assembly   biological process:  RNA splicing   cellular component:  spliceosome complex   cellular component:  small nuclear ribonucleoprotein complex   molecular function:  nucleic acid binding      BioCarta Pathways:     KEGG Pathways:  
    Probe set : 1388151_at    Name:   coronin 7    GB acc:   AI008369    UniGene:   Rn.13808    Gene Symbol:   Coro7    Locus Link:   192276    Chromosome:   10    Cytoband:   Chr:10q12    SOURCE    GENECARD       Gene Ontology:     BioCarta Pathways:     KEGG Pathways:  
    Probe set : 1389388_at    Name:   Transcribed locus    GB acc:   BF291161    UniGene:   Rn.88168        SOURCE        Gene Ontology:     BioCarta Pathways:     KEGG Pathways:  
    Probe set : 1382318_at     GB acc:   AA964856              Gene Ontology:     BioCarta Pathways:     KEGG Pathways:  
    Probe set : 1373564_at    Name:   3-hydroxyisobutyryl-Coenzyme A hydrolase (predicted)    GB acc:   AI410548    UniGene:   Rn.8745    Gene Symbol:   Hibch_predicted    Locus Link:   301384    Chromosome:   9    Cytoband:   Chr:9q22    SOURCE    GENECARD       Gene Ontology:   biological process:  metabolism   molecular function:  catalytic activity   molecular function:  3-hydroxyisobutyryl-CoA hydrolase activity   molecular function:  hydrolase activity      BioCarta Pathways:     KEGG Pathways:  
    Probe set : 1395560_at    Name:   Transcribed locus    GB acc:   BF522151    UniGene:   Rn.24173        SOURCE        Gene Ontology:     BioCarta Pathways:     KEGG Pathways:  
    Probe set : 1388370_at    Name:   cyclin I (predicted)    GB acc:   AA945706    UniGene:   Rn.6670    Gene Symbol:   Ccni_predicted    Locus Link:   289500    Chromosome:   14    Cytoband:   Chr:14p22    SOURCE    GENECARD       Gene Ontology:   molecular function:  cyclin-dependent protein kinase regulator activity      BioCarta Pathways:     KEGG Pathways:  
    Probe set : 1376663_at    Name:   similar to hypothetical protein 6720484B16 (predicted)    GB acc:   BI274245    UniGene:   Rn.136885    Gene Symbol:   RGD1304881_predicted    Locus Link:   304653    Chromosome:   19    Cytoband:   Chr:19q11    SOURCE    GENECARD       Gene Ontology:     BioCarta Pathways:     KEGG Pathways:  
    Probe set : 1369560_at    Name:   glycerol-3-phosphate dehydrogenase 1 (soluble)    GB acc:   NM_022215    UniGene:   Rn.44452    Gene Symbol:   Gpd1    Locus Link:   60666    Chromosome:   7    Cytoband:   Chr:7q36    SOURCE    GENECARD       Gene Ontology:   biological process:  carbohydrate metabolism   biological process:  glycerol-3-phosphate metabolism   biological process:  glycerol-3-phosphate catabolism   cellular component:  glycerol-3-phosphate dehydrogenase complex   cellular component:  cytoplasm      BioCarta Pathways:     KEGG Pathways:  
    Probe set : 1371382_at    Name:   similar to Filamin A (Alpha-filamin) (Filamin 1) (Endothelial actin-binding protein) (Actin-binding protein 280) (ABP-280) (Nonmuscle filamin)    GB acc:   BI283060    UniGene:   Rn.4213    Gene Symbol:   LOC293860    Locus Link:   293860    Chromosome:   X    Cytoband:   Chr:Xq37    SOURCE    GENECARD       Gene Ontology:     BioCarta Pathways:     KEGG Pathways:  
    Probe set : 1374722_at    Name:   C1q and tumor necrosis factor related protein 1    GB acc:   BI296396    UniGene:   Rn.82211    Gene Symbol:   RGD:1359204    Locus Link:   303701    Chromosome:   10    Cytoband:   Chr:10q32.3    SOURCE    GENECARD       Gene Ontology:   biological process:  phosphate transport   cellular component:  cytoplasm      BioCarta Pathways:     KEGG Pathways:  
    Probe set : 1367860_a_at    Name:   matrix metalloproteinase 14 (membrane-inserted)    GB acc:   X83537    UniGene:   Rn.10371    Gene Symbol:   Mmp14    Locus Link:   81707    Chromosome:   15    Cytoband:   Chr:15p13    SOURCE    GENECARD       Gene Ontology:   biological process:  proteolysis   biological process:  collagen catabolism   biological process:  peptidoglycan metabolism      BioCarta Pathways:     KEGG Pathways:  
    Probe set : 1378954_at     GB acc:   AI711116              Gene Ontology:     BioCarta Pathways:     KEGG Pathways:  
    Probe set : 1375440_at    Name:   peptidylprolyl isomerase (cyclophilin)-like 2 (predicted)    GB acc:   BI283709    UniGene:   Rn.145947    Gene Symbol:   Ppil2_predicted    Locus Link:   360746    Chromosome:   11    Cytoband:   Chr:11q23    SOURCE    GENECARD       Gene Ontology:   biological process:  protein folding   biological process:  protein ubiquitination   cellular component:  nucleus   cellular component:  ubiquitin ligase complex   molecular function:  peptidyl-prolyl cis-trans isomerase activity   molecular function:  isomerase activity   molecular function:  ubiquitin-protein ligase activity      BioCarta Pathways:     KEGG Pathways:  
    Probe set : 1377009_at    Name:   Transcribed locus    GB acc:   BI274233    UniGene:   Rn.24077        SOURCE        Gene Ontology:     BioCarta Pathways:     KEGG Pathways:  
    Probe set : 1378467_at    Name:   Transcribed locus    GB acc:   AA851361    UniGene:   Rn.6735        SOURCE        Gene Ontology:     BioCarta Pathways:     KEGG Pathways:  
    Probe set : 1379449_at    Name:   RB1-inducible coiled-coil 1 (predicted)    GB acc:   BG670920    UniGene:   Rn.33137    Gene Symbol:   Rb1cc1_predicted    Locus Link:   312927    Chromosome:   5    Cytoband:   Chr:5q12    SOURCE    GENECARD       Gene Ontology:   cellular component:  nucleus   cellular component:  cytoplasm   molecular function:  kinase activity      BioCarta Pathways:     KEGG Pathways:  
    Probe set : 1383733_at    Name:   Adenylate kinase 3    GB acc:   BF555825    UniGene:   Rn.60    Gene Symbol:   Ak3    Locus Link:   26956    Chromosome:   1    Cytoband:   Chr:1q43    SOURCE    GENECARD       Gene Ontology:   biological process:  amino acid biosynthesis   biological process:  nucleobase, nucleoside, nucleotide and nucleic acid metabolism   cellular component:  mitochondrion   cellular component:  mitochondrion      BioCarta Pathways:     KEGG Pathways:  
    Probe set : 1372363_at    Name:   similar to polymerase (RNA) II (DNA directed) polypeptide H /// similar to polymerase (RNA) II (DNA directed) polypeptide H    GB acc:   BF404414     Gene Symbol:   LOC291795 /// LOC498109    Locus Link:   291795 /// 498109    Chromosome:   18    Cytoband:   Chr:18p13 /// Chr:11q23     GENECARD       Gene Ontology:     BioCarta Pathways:     KEGG Pathways:  
    Probe set : 1373161_at    Name:   similar to RIKEN cDNA 6530411B15    GB acc:   BM386009    UniGene:   Rn.24570    Gene Symbol:   RGD:1359736    Locus Link:   303356    Chromosome:   10    Cytoband:   Chr:10q26    SOURCE    GENECARD       Gene Ontology:     BioCarta Pathways:     KEGG Pathways:  
    Probe set : 1371004_at    Name:   sortilin 1    GB acc:   AI070124    UniGene:   Rn.11286    Gene Symbol:   Sort1    Locus Link:   83576    Chromosome:   2    Cytoband:   Chr:2q34    SOURCE    GENECARD       Gene Ontology:     BioCarta Pathways:     KEGG Pathways:  
    Probe set : 1367644_at     GB acc:   L01115              Gene Ontology:     BioCarta Pathways:     KEGG Pathways:  
    Probe set : 1398413_at    Name:   zinc finger protein 444 (predicted)    GB acc:   AW525177     Gene Symbol:   Znf444_predicted    Locus Link:   292569    Chromosome:   1    Cytoband:   Chr:1q12     GENECARD       Gene Ontology:   biological process:  regulation of transcription, DNA-dependent   cellular component:  nucleus   molecular function:  transcription factor activity   molecular function:  zinc ion binding      BioCarta Pathways:     KEGG Pathways:  
    Probe set : 1376202_at    Name:   UDP-GlcNAc:betaGal beta-1,3-N-acetylglucosaminyltransferase 6 (predicted)    GB acc:   BF416236    UniGene:   Rn.129793    Gene Symbol:   B3gnt6_predicted    Locus Link:   293667    Chromosome:   1    Cytoband:   Chr:1q43    SOURCE    GENECARD       Gene Ontology:   biological process:  poly-N-acetyllactosamine biosynthesis   cellular component:  extracellular space   cellular component:  Golgi apparatus   cellular component:  integral to Golgi membrane      BioCarta Pathways:     KEGG Pathways:  
    Probe set : 1390942_at    Name:   Transcribed locus    GB acc:   AI043817    UniGene:   Rn.17373        SOURCE        Gene Ontology:     BioCarta Pathways:     KEGG Pathways:  
    Probe set : 1387550_a_at    Name:   solute carrier family 14 (urea transporter), member 2    GB acc:   NM_019347    UniGene:   Rn.10157    Gene Symbol:   Slc14a2    Locus Link:   54302    Chromosome:   18    Cytoband:   Chr:18q12.3    SOURCE    GENECARD       Gene Ontology:   cellular component:  membrane fraction   cellular component:  integral to membrane   molecular function:  copper ion binding   molecular function:  urea transporter activity   molecular function:  urea transporter activity      BioCarta Pathways:     KEGG Pathways:  
    Probe set : 1387160_at    Name:   potassium voltage-gated channel, Isk-related subfamily, member 3    GB acc:   NM_022235    UniGene:   Rn.44843    Gene Symbol:   Kcne3    Locus Link:   63883    Chromosome:   1    Cytoband:   Chr:1q32    SOURCE    GENECARD       Gene Ontology:   biological process:  transport   biological process:  ion transport   biological process:  potassium ion transport   cellular component:  voltage-gated potassium channel complex   cellular component:  membrane   cellular component:  integral to membrane      BioCarta Pathways:     KEGG Pathways:  
    Probe set : 1371764_at    Name:   similar to Ubc6p homolog    GB acc:   AI411771    UniGene:   Rn.106299    Gene Symbol:   RGD:1359523    Locus Link:   298689    Chromosome:   5    Cytoband:   Chr:5q36    SOURCE    GENECARD       Gene Ontology:   biological process:  protein modification   biological process:  ubiquitin cycle   molecular function:  ubiquitin-like activating enzyme activity      BioCarta Pathways:     KEGG Pathways:  
    Probe set : 1390173_at    Name:   Transcribed locus    GB acc:   BG377886    UniGene:   Rn.9561        SOURCE        Gene Ontology:     BioCarta Pathways:     KEGG Pathways:  
    Probe set : 1383593_at    Name:   Transcribed locus    GB acc:   BE113211    UniGene:   Rn.107945        SOURCE        Gene Ontology:     BioCarta Pathways:     KEGG Pathways:  
    Probe set : 1369864_a_at    Name:   serine dehydratase    GB acc:   NM_053962    UniGene:   Rn.9918    Gene Symbol:   Sds    Locus Link:   25044      SOURCE    GENECARD       Gene Ontology:     BioCarta Pathways:     KEGG Pathways:  
    Probe set : 1377354_at    Name:   Transcribed locus    GB acc:   BI290660    UniGene:   Rn.21190        SOURCE        Gene Ontology:     BioCarta Pathways:     KEGG Pathways:  
    Probe set : 1368194_at    Name:   1-acylglycerol-3-phosphate O-acyltransferase 4    GB acc:   NM_133406    UniGene:   Rn.41595    Gene Symbol:   Agpat4    Locus Link:   170919    Chromosome:   1    Cytoband:   Chr:1q11    SOURCE    GENECARD       Gene Ontology:   biological process:  phospholipid metabolism   biological process:  metabolism   biological process:  phospholipid biosynthesis   cellular component:  integral to membrane   molecular function:  1-acylglycerol-3-phosphate O-acyltransferase activity   molecular function:  acyltransferase activity   molecular function:  transferase activity      BioCarta Pathways:     KEGG Pathways:  
    Probe set : 1389913_at     GB acc:   BI276990              Gene Ontology:     BioCarta Pathways:     KEGG Pathways:  
    Probe set : 1379491_at    Name:   similar to Fat cell-specific low molecular weight protein (Fat tissue-specific low MW protein) (predicted)    GB acc:   AW524012    UniGene:   Rn.45756    Gene Symbol:   RGD1310648_predicted    Locus Link:   288271    Chromosome:   11    Cytoband:   Chr:11q11    SOURCE    GENECARD       Gene Ontology:     BioCarta Pathways:     KEGG Pathways:  
    Probe set : 1379672_at    Name:   Transcribed locus    GB acc:   AI103938    UniGene:   Rn.27953        SOURCE        Gene Ontology:     BioCarta Pathways:     KEGG Pathways:  
    Probe set : 1390249_at    Name:   similar to DKFZP434H132 protein (predicted)    GB acc:   BF558075    UniGene:   Rn.59239    Gene Symbol:   RGD1305464_predicted    Locus Link:   315702    Chromosome:   8    Cytoband:   Chr:8q24    SOURCE    GENECARD       Gene Ontology:     BioCarta Pathways:     KEGG Pathways:  
    Probe set : 1388764_at    Name:   IQ motif containing GTPase activating protein 1 (predicted)    GB acc:   BM387072    UniGene:   Rn.106939     Locus Link:   361598    Chromosome:   1    Cytoband:   Chr:1q31    SOURCE        Gene Ontology:   biological process:  signal transduction   biological process:  small GTPase mediated signal transduction   cellular component:  cytoplasm   cellular component:  actin filament   molecular function:  GTPase inhibitor activity   molecular function:  Ras GTPase activator activity   molecular function:  calmodulin binding      BioCarta Pathways:     KEGG Pathways:  
    Probe set : 1377905_at    Name:   similar to RIKEN cDNA 2700023B17 (predicted)    GB acc:   BF417956    UniGene:   Rn.2370    Gene Symbol:   RGD1305695_predicted    Locus Link:   361554    Chromosome:   1    Cytoband:   Chr:1q21    SOURCE    GENECARD       Gene Ontology:     BioCarta Pathways:     KEGG Pathways:  
    Probe set : 1373833_at    Name:   similar to RIKEN cDNA 3110040N11 (predicted)    GB acc:   BI288945    UniGene:   Rn.8483    Gene Symbol:   RGD1305713_predicted    Locus Link:   293059    Chromosome:   1    Cytoband:   Chr:1q31    SOURCE    GENECARD       Gene Ontology:     BioCarta Pathways:     KEGG Pathways:  
    Probe set : 1381779_s_at    Name:   mannosidase, alpha, class 2C, member 1    GB acc:   BG380027    UniGene:   Rn.11301    Gene Symbol:   Man2c1    Locus Link:   246136    Chromosome:   8    Cytoband:   Chr:8q24    SOURCE    GENECARD       Gene Ontology:   biological process:  carbohydrate metabolism   biological process:  mannose metabolism   cellular component:  endoplasmic reticulum   cellular component:  endoplasmic reticulum      BioCarta Pathways:     KEGG Pathways:  
    Probe set : 1384634_at     GB acc:   AI235340              Gene Ontology:     BioCarta Pathways:     KEGG Pathways:  
    Probe set : 1367596_at    Name:   ribosomal protein S26    GB acc:   NM_013224    UniGene:   Rn.2388    Gene Symbol:   Rps26    Locus Link:   27139    Chromosome:   7    Cytoband:   Chr:7q11    SOURCE    GENECARD       Gene Ontology:   biological process:  protein biosynthesis   biological process:  tRNA aminoacylation for protein translation   biological process:  aspartyl-tRNA aminoacylation      BioCarta Pathways:     KEGG Pathways:  
    Probe set : 1378104_at    Name:   similar to RIKEN cDNA 8430406I07 (predicted)    GB acc:   AI105459    UniGene:   Rn.23965    Gene Symbol:   RGD1307465_predicted    Locus Link:   296200    Chromosome:   3    Cytoband:   Chr:3q41    SOURCE    GENECARD       Gene Ontology:     BioCarta Pathways:     KEGG Pathways:  
    Probe set : 1391509_at    Name:   tumor-associated calcium signal transducer 2    GB acc:   AA891826    UniGene:   Rn.24809    Gene Symbol:   RGD:1359498    Locus Link:   494343    Chromosome:   4    Cytoband:   Chr:4q31    SOURCE    GENECARD       Gene Ontology:     BioCarta Pathways:     KEGG Pathways:  
    Probe set : 1372846_at    Name:   similar to hypothetical protein MGC20446 (predicted)    GB acc:   AI179227    UniGene:   Rn.13816    Gene Symbol:   RGD1304670_predicted    Locus Link:   361729    Chromosome:   1    Cytoband:   Chr:1q43    SOURCE    GENECARD       Gene Ontology:   biological process:  electron transport   cellular component:  integral to membrane      BioCarta Pathways:     KEGG Pathways:  
    Probe set : 1372638_at    Name:   Transcribed locus    GB acc:   BG371454    UniGene:   Rn.146763        SOURCE        Gene Ontology:     BioCarta Pathways:     KEGG Pathways:  
    Probe set : 1374444_at    Name:   Plexin B1 (predicted)    GB acc:   BI288540    UniGene:   Rn.26773    Gene Symbol:   LOC316009    Locus Link:   316009    Chromosome:   8    Cytoband:   Chr:8q32    SOURCE    GENECARD       Gene Ontology:   biological process:  signal transduction   biological process:  development   cellular component:  integral to plasma membrane   cellular component:  membrane   cellular component:  integral to membrane   molecular function:  receptor activity      BioCarta Pathways:     KEGG Pathways:  
    Probe set : 1371619_at    Name:   similar to HTGN29 protein; keratinocytes associated transmembrane protein 2 (predicted)    GB acc:   BI296718    UniGene:   Rn.106999    Gene Symbol:   RGD1310352_predicted    Locus Link:   303122    Chromosome:   10    Cytoband:   Chr:10q22    SOURCE    GENECARD       Gene Ontology:   cellular component:  integral to membrane      BioCarta Pathways:     KEGG Pathways:  
    Probe set : 1396286_at    Name:   Transcribed locus    GB acc:   BG372956    UniGene:   Rn.50257        SOURCE        Gene Ontology:     BioCarta Pathways:     KEGG Pathways:  
    Probe set : 1375484_at    Name:   Transcribed locus    GB acc:   BG376458    UniGene:   Rn.146323        SOURCE        Gene Ontology:     BioCarta Pathways:     KEGG Pathways:  
    Probe set : 1393101_at    Name:   F-box and leucine-rich repeat protein 10 (predicted)    GB acc:   BM385838    UniGene:   Rn.32641    Gene Symbol:   Fbxl10_predicted    Locus Link:   304495    Chromosome:   12    Cytoband:   Chr:12q16    SOURCE    GENECARD       Gene Ontology:   biological process:  regulation of transcription, DNA-dependent   biological process:  ubiquitin cycle   molecular function:  DNA binding   molecular function:  zinc ion binding      BioCarta Pathways:     KEGG Pathways:  
    Probe set : 1370456_at    Name:   FAT tumor suppressor homolog 3 (Drosophila)    GB acc:   AB076401    UniGene:   Rn.20707    Gene Symbol:   Fat3    Locus Link:   191571    Chromosome:   8    Cytoband:   Chr:8q12    SOURCE    GENECARD       Gene Ontology:   biological process:  homophilic cell adhesion   cellular component:  membrane   molecular function:  calcium ion binding      BioCarta Pathways:     KEGG Pathways:  
    Probe set : 1387810_at    Name:   Kelch-like ECH-associated protein 1    GB acc:   BF414210    UniGene:   Rn.23467    Gene Symbol:   Keap1    Locus Link:   117519    Chromosome:   8    Cytoband:   Chr:8q13    SOURCE    GENECARD       Gene Ontology:   biological process:  regulation of transcription, DNA-dependent   biological process:  transcription   molecular function:  protein binding      BioCarta Pathways:     KEGG Pathways:  
    Probe set : 1389000_at    Name:   Transcribed locus    GB acc:   AA964998    UniGene:   Rn.124985        SOURCE        Gene Ontology:     BioCarta Pathways:     KEGG Pathways:  
    Probe set : 1369018_at    Name:   forkhead box M1    GB acc:   NM_031633    UniGene:   Rn.10665    Gene Symbol:   Foxm1    Locus Link:   58921    Chromosome:   4    Cytoband:   Chr:4q42    SOURCE    GENECARD       Gene Ontology:   biological process:  regulation of transcription, DNA-dependent   biological process:  transcription   cellular component:  nucleus   cellular component:  transcription factor complex   molecular function:  DNA binding   molecular function:  transcription factor activity      BioCarta Pathways:     KEGG Pathways:  
    Probe set : 1370062_at    Name:   hypoxia induced gene 1    GB acc:   NM_080902    UniGene:   Rn.2084    Gene Symbol:   RGD:620215    Locus Link:   140937    Chromosome:   8    Cytoband:   Chr:8q32    SOURCE    GENECARD       Gene Ontology:   cellular component:  integral to membrane      BioCarta Pathways:     KEGG Pathways:  
    Probe set : 1389585_at     GB acc:   BG377510              Gene Ontology:     BioCarta Pathways:     KEGG Pathways:  
    Probe set : 1392487_at    Name:   Transcribed locus    GB acc:   BI303896    UniGene:   Rn.25888        SOURCE        Gene Ontology:     BioCarta Pathways:     KEGG Pathways:  
    Probe set : 1369650_at    Name:   p21 (CDKN1A)-activated kinase 2    GB acc:   U19967    UniGene:   Rn.3840    Gene Symbol:   Pak2    Locus Link:   29432    Chromosome:   11    Cytoband:   Chr:11q22    SOURCE    GENECARD       Gene Ontology:   biological process:  protein amino acid phosphorylation   biological process:  negative regulation of protein kinase activity   biological process:  signal transduction      BioCarta Pathways:     KEGG Pathways:  
    Probe set : 1375726_at    Name:   LIM domain only protein 7    GB acc:   BI284480    UniGene:   Rn.98417    Gene Symbol:   RGD:1302984    Locus Link:   361084    Chromosome:   15    Cytoband:   Chr:15q21    SOURCE    GENECARD       Gene Ontology:   biological process:  cell-cell adhesion   biological process:  actomyosin structure organization and biogenesis   cellular component:  cell-cell adherens junction   cellular component:  apical plasma membrane   molecular function:  actinin binding   molecular function:  protein binding   molecular function:  actin binding   molecular function:  zinc ion binding      BioCarta Pathways:     KEGG Pathways:  
    Probe set : 1392510_at    Name:   similar to hypothetical protein B230314O19 (predicted)    GB acc:   BF283618    UniGene:   Rn.23539    Gene Symbol:   RGD1308013_predicted    Locus Link:   362336    Chromosome:   4    Cytoband:   Chr:4q22    SOURCE    GENECARD       Gene Ontology:     BioCarta Pathways:     KEGG Pathways:  
    Probe set : 1370675_at    Name:   transient receptor potential cation channel, subfamily V, member 1    GB acc:   AF158248    UniGene:   Rn.3073    Gene Symbol:   Trpv1    Locus Link:   83810    Chromosome:   10    Cytoband:   Chr:10q24    SOURCE    GENECARD       Gene Ontology:     BioCarta Pathways:     KEGG Pathways:  
    Probe set : 1398287_at    Name:   plasminogen activator, urokinase    GB acc:   NM_013085    UniGene:   Rn.6064    Gene Symbol:   Plau    Locus Link:   25619    Chromosome:   15    Cytoband:   Chr:15p16    SOURCE    GENECARD       Gene Ontology:   cellular component:  extracellular space      BioCarta Pathways:     KEGG Pathways:  
    Probe set : 1379780_at    Name:   Similar to mKIAA0386 protein (predicted)    GB acc:   BE103199    UniGene:   Rn.95357     Locus Link:   306934    Chromosome:   17    Cytoband:   Chr:17p11    SOURCE        Gene Ontology:   biological process:  iron ion transport   biological process:  iron ion homeostasis   molecular function:  binding   molecular function:  ferric iron binding      BioCarta Pathways:     KEGG Pathways:  
    Probe set : 1370943_at    Name:   sulfotransferase family, cytosolic, 1C, member 1 (predicted)    GB acc:   AI168953     Gene Symbol:   Sult1c1_predicted    Locus Link:   316153    Chromosome:   9    Cytoband:   Chr:9q11     GENECARD       Gene Ontology:   biological process:  amine metabolism   cellular component:  cytoplasm   cellular component:  lysosome   molecular function:  sulfotransferase activity   molecular function:  transferase activity      BioCarta Pathways:     KEGG Pathways:  
    Probe set : 1383795_at    Name:   CDNA clone IMAGE:7379585    GB acc:   BF521753    UniGene:   Rn.6374        SOURCE        Gene Ontology:     BioCarta Pathways:     KEGG Pathways:  
    Probe set : 1378327_at    Name:   doublesex and mab-3 related transcription factor 2 (predicted)    GB acc:   AA963348    UniGene:   Rn.11448    Gene Symbol:   Dmrt2_predicted    Locus Link:   309430    Chromosome:   1    Cytoband:   Chr:1q51    SOURCE    GENECARD       Gene Ontology:   cellular component:  nucleus   molecular function:  DNA binding   molecular function:  transcription factor activity      BioCarta Pathways:     KEGG Pathways:  
    Probe set : 1375647_at    Name:   Transcribed locus    GB acc:   BG671943    UniGene:   Rn.95253        SOURCE        Gene Ontology:     BioCarta Pathways:     KEGG Pathways:  
    Probe set : 1391596_at    Name:   Transcribed locus    GB acc:   AI043603    UniGene:   Rn.15765        SOURCE        Gene Ontology:     BioCarta Pathways:     KEGG Pathways:  
    Probe set : 1389529_at    Name:   Transcribed locus    GB acc:   AI409595    UniGene:   Rn.137163        SOURCE        Gene Ontology:     BioCarta Pathways:     KEGG Pathways:  
    Probe set : 1388507_at    Name:   integrin beta 4 binding protein (predicted)    GB acc:   AI104279    UniGene:   Rn.7553    Gene Symbol:   Itgb4bp_predicted    Locus Link:   305506    Chromosome:   14    Cytoband:   Chr:14q21    SOURCE    GENECARD       Gene Ontology:   cellular component:  nucleus   cellular component:  lamin filament   cellular component:  intermediate filament   molecular function:  translation initiation factor activity   molecular function:  oxidoreductase activity      BioCarta Pathways:     KEGG Pathways:  
    Probe set : 1377679_at    Name:   G protein-coupled receptor 125 (predicted)    GB acc:   BI295085    UniGene:   Rn.22146    Gene Symbol:   Gpr125_predicted    Locus Link:   305408    Chromosome:   14    Cytoband:   Chr:14q11    SOURCE    GENECARD       Gene Ontology:   biological process:  G-protein coupled receptor protein signaling pathway   biological process:  neuropeptide signaling pathway   cellular component:  membrane   cellular component:  integral to membrane   molecular function:  receptor activity   molecular function:  G-protein coupled receptor activity      BioCarta Pathways:     KEGG Pathways:  
    Probe set : 1375945_at    Name:   plexin domain containing 2 (predicted)    GB acc:   BG375285    UniGene:   Rn.36200    Gene Symbol:   Plxdc2_predicted    Locus Link:   361282    Chromosome:   17    Cytoband:   Chr:17q12.3    SOURCE    GENECARD       Gene Ontology:   biological process:  development   biological process:  nervous system development   molecular function:  receptor activity      BioCarta Pathways:     KEGG Pathways:  
    Probe set : 1397167_at     GB acc:   AW534046              Gene Ontology:     BioCarta Pathways:     KEGG Pathways:  
    Probe set : 1384096_at    Name:   Transcribed locus    GB acc:   BI279347    UniGene:   Rn.148741        SOURCE        Gene Ontology:     BioCarta Pathways:     KEGG Pathways:  
    Probe set : 1371656_at    Name:   chaperonin subunit 4 (delta)    GB acc:   BI283657    UniGene:   Rn.97889    Gene Symbol:   Cct4    Locus Link:   29374    Chromosome:   14    Cytoband:   Chr:14q22    SOURCE    GENECARD       Gene Ontology:     BioCarta Pathways:     KEGG Pathways:  
    Probe set : 1390681_at    Name:   Similar to TGFB-induced factor 2    GB acc:   AA964074    UniGene:   Rn.11697     Locus Link:   499930      SOURCE        Gene Ontology:     BioCarta Pathways:     KEGG Pathways:  
    Probe set : 1385853_at    Name:   bromodomain adjacent to zinc finger domain, 1A (predicted)    GB acc:   BF560180    UniGene:   Rn.1864    Gene Symbol:   Baz1a_predicted    Locus Link:   314126    Chromosome:   6    Cytoband:   Chr:6q23    SOURCE    GENECARD       Gene Ontology:   biological process:  regulation of transcription, DNA-dependent   biological process:  protein ubiquitination   cellular component:  ubiquitin ligase complex   cellular component:  nucleus   molecular function:  DNA binding   molecular function:  ubiquitin-protein ligase activity   molecular function:  zinc ion binding      BioCarta Pathways:     KEGG Pathways:  
    Probe set : 1372665_at    Name:   phosphoserine aminotransferase 1    GB acc:   AI230228    UniGene:   Rn.144808    Gene Symbol:   Psat1    Locus Link:   293820    Chromosome:   1    Cytoband:   Chr:1q43    SOURCE    GENECARD       Gene Ontology:   biological process:  L-serine biosynthesis   biological process:  L-serine biosynthesis   biological process:  metabolism   molecular function:  phosphoserine transaminase activity   molecular function:  transaminase activity   molecular function:  transferase activity      BioCarta Pathways:     KEGG Pathways:  
    Probe set : 1392788_at    Name:   Sprouty protein with EVH-1 domain 1, related sequence (predicted)    GB acc:   AA944323    UniGene:   Rn.22990     Locus Link:   296072    Chromosome:   3    Cytoband:   Chr:3q35    SOURCE        Gene Ontology:   biological process:  inactivation of MAPK activity   cellular component:  membrane   molecular function:  stem cell factor receptor binding   molecular function:  protein binding      BioCarta Pathways:     KEGG Pathways:  
    Probe set : 1367610_at    Name:   ribosomal protein L19    GB acc:   NM_031103    UniGene:   Rn.3384    Gene Symbol:   Rpl19    Locus Link:   81767    Chromosome:   10    Cytoband:   Chr:10q31    SOURCE    GENECARD       Gene Ontology:   biological process:  protein biosynthesis   molecular function:  RNA binding   molecular function:  structural constituent of ribosome   molecular function:  structural constituent of ribosome      BioCarta Pathways:     KEGG Pathways:  
    Probe set : 1371342_at    Name:   cytochrome c-1 (predicted)    GB acc:   BI277021    UniGene:   Rn.1413    Gene Symbol:   Cyc1_predicted    Locus Link:   300047    Chromosome:   7    Cytoband:   Chr:7q34    SOURCE    GENECARD       Gene Ontology:   biological process:  electron transport   biological process:  oxidative phosphorylation   cellular component:  mitochondrion   cellular component:  mitochondrial electron transport chain   cellular component:  integral to membrane      BioCarta Pathways:     KEGG Pathways:  
    Probe set : 1380474_at     GB acc:   AI013470              Gene Ontology:     BioCarta Pathways:     KEGG Pathways:  
    Probe set : 1389801_at    Name:   fibroblast growth factor receptor 4    GB acc:   BF391339    UniGene:   Rn.24104    Gene Symbol:   Fgfr4    Locus Link:   25114    Chromosome:   17    Cytoband:   Chr:17p14    SOURCE    GENECARD       Gene Ontology:     BioCarta Pathways:     KEGG Pathways:  
    Probe set : 1380559_at    Name:   Transcribed locus    GB acc:   AI030465    UniGene:   Rn.128277        SOURCE        Gene Ontology:     BioCarta Pathways:     KEGG Pathways:  
    Probe set : 1389649_at    Name:   Transcribed locus    GB acc:   AI411897    UniGene:   Rn.12537        SOURCE        Gene Ontology:     BioCarta Pathways:     KEGG Pathways:  
    Probe set : 1369027_at    Name:   alpha 1,4-galactosyltransferase    GB acc:   NM_022240    UniGene:   Rn.48584    Gene Symbol:   A4galt    Locus Link:   63888    Chromosome:   7    Cytoband:   Chr:7q34    SOURCE    GENECARD       Gene Ontology:   biological process:  glycosphingolipid biosynthesis   biological process:  lipid biosynthesis   biological process:  glycosphingolipid biosynthesis      BioCarta Pathways:     KEGG Pathways:  
    Probe set : 1393436_at    Name:   similar to potential ligand-binding protein    GB acc:   AI549033    UniGene:   Rn.41707    Gene Symbol:   LOC309100    Locus Link:   309100    Chromosome:   1    Cytoband:   Chr:1q41    SOURCE    GENECARD       Gene Ontology:     BioCarta Pathways:     KEGG Pathways:  
    Probe set : 1375655_at    Name:   Transcribed locus    GB acc:   BE107208    UniGene:   Rn.146928        SOURCE        Gene Ontology:     BioCarta Pathways:     KEGG Pathways:  
    Probe set : 1389171_at    Name:   similar to RIKEN cDNA 1110001E17 (predicted)    GB acc:   AI104326    UniGene:   Rn.66154    Gene Symbol:   RGD1307901_predicted    Locus Link:   306327    Chromosome:   16    Cytoband:   Chr:16p14    SOURCE    GENECARD       Gene Ontology:     BioCarta Pathways:     KEGG Pathways:  
    Probe set : 1378179_a_at    Name:   Transcribed locus    GB acc:   AW524864    UniGene:   Rn.48641        SOURCE        Gene Ontology:     BioCarta Pathways:     KEGG Pathways:  
    Probe set : 1376086_at    Name:   similar to RIKEN cDNA 1810047C23 (predicted)    GB acc:   AW528625    UniGene:   Rn.14630    Gene Symbol:   RGD1311161_predicted    Locus Link:   361151    Chromosome:   16    Cytoband:   Chr:16q11    SOURCE    GENECARD       Gene Ontology:     BioCarta Pathways:     KEGG Pathways:  
    Probe set : 1368953_at    Name:   UDP-glucose ceramide glucosyltransferase-like 1    GB acc:   NM_133596    UniGene:   Rn.13004    Gene Symbol:   Ugcgl1    Locus Link:   171129    Chromosome:   9    Cytoband:   Chr:9q21    SOURCE    GENECARD       Gene Ontology:   biological process:  protein folding   biological process:  protein amino acid glycosylation   biological process:  posttranslational protein folding      BioCarta Pathways:     KEGG Pathways:  
    Probe set : 1389052_at    Name:   tetratricopeptide repeat domain 13 (predicted)    GB acc:   BI278584    UniGene:   Rn.98912    Gene Symbol:   Ttc13_predicted    Locus Link:   292095    Chromosome:   19    Cytoband:   Chr:19q12    SOURCE    GENECARD       Gene Ontology:   molecular function:  binding      BioCarta Pathways:     KEGG Pathways:  
    Probe set : 1379724_at     GB acc:   BE103244              Gene Ontology:     BioCarta Pathways:     KEGG Pathways:  
    Probe set : 1393338_at     GB acc:   AW528719              Gene Ontology:     BioCarta Pathways:     KEGG Pathways:  
    Probe set : 1380562_at    Name:   similar to hypothetical protein MGC29784 (predicted)    GB acc:   AW523351    UniGene:   Rn.45622    Gene Symbol:   RGD1307582_predicted    Locus Link:   362416    Chromosome:   4    Cytoband:   Chr:4q42    SOURCE    GENECARD       Gene Ontology:     BioCarta Pathways:     KEGG Pathways:  
    Probe set : 1379473_at    Name:   similar to RIKEN cDNA 1700108L22 (predicted)    GB acc:   BI284815    UniGene:   Rn.7988    Gene Symbol:   RGD1307509_predicted    Locus Link:   310348    Chromosome:   2    Cytoband:   Chr:2q26    SOURCE    GENECARD       Gene Ontology:     BioCarta Pathways:     KEGG Pathways:  
    Probe set : 1379889_at    Name:   lamimin, gamma 2    GB acc:   AW527269    UniGene:   Rn.9278    Gene Symbol:   Lamc2    Locus Link:   192362    Chromosome:   13    Cytoband:   Chr:13q21    SOURCE    GENECARD       Gene Ontology:   biological process:  cell adhesion   cellular component:  extracellular matrix (sensu Metazoa)   cellular component:  laminin-2   cellular component:  extracellular matrix      BioCarta Pathways:     KEGG Pathways:  
    Probe set : 1383644_at    Name:   Transcribed locus    GB acc:   BM385202    UniGene:   Rn.21471        SOURCE        Gene Ontology:     BioCarta Pathways:     KEGG Pathways:  
    Probe set : 1368963_at    Name:   Max interacting protein 1    GB acc:   NM_013160    UniGene:   Rn.37510    Gene Symbol:   Mxi1    Locus Link:   25701    Chromosome:   1    Cytoband:   Chr:1q55    SOURCE    GENECARD       Gene Ontology:   cellular component:  nucleus   cellular component:  nucleus      BioCarta Pathways:     KEGG Pathways:  
    Probe set : 1372065_at    Name:   ADP-ribosyltransferase 3 (predicted)    GB acc:   AI104393    UniGene:   Rn.4077    Gene Symbol:   Art3_predicted    Locus Link:   305235    Chromosome:   14    Cytoband:   Chr:14p22    SOURCE    GENECARD       Gene Ontology:   biological process:  protein amino acid ADP-ribosylation   cellular component:  extracellular space   cellular component:  integral to plasma membrane      BioCarta Pathways:     KEGG Pathways:  
    Probe set : 1390602_a_at    Name:   similar to RIKEN cDNA C430004E15    GB acc:   BG381320    UniGene:   Rn.116819    Gene Symbol:   LOC499749    Locus Link:   499749    Chromosome:   3    Cytoband:   Chr:3p13    SOURCE    GENECARD       Gene Ontology:     BioCarta Pathways:     KEGG Pathways:  
    Probe set : 1391169_at    Name:   similar to RIKEN cDNA B930062P21 gene    GB acc:   BE120139    UniGene:   Rn.88857    Gene Symbol:   LOC500921    Locus Link:   500921    Chromosome:   7    Cytoband:   Chr:7q35    SOURCE    GENECARD       Gene Ontology:     BioCarta Pathways:     KEGG Pathways:  
    Probe set : 1370098_at    Name:   synaptobrevin-like 1    GB acc:   NM_053531    UniGene:   Rn.34151    Gene Symbol:   Sybl1    Locus Link:   85491    Chromosome:   12    Cytoband:   Chr:12q11    SOURCE    GENECARD       Gene Ontology:   biological process:  vesicle-mediated transport   cellular component:  integral to membrane      BioCarta Pathways:     KEGG Pathways:  
    Probe set : 1393119_at    Name:   Transcribed locus    GB acc:   BM388725    UniGene:   Rn.12711        SOURCE        Gene Ontology:     BioCarta Pathways:     KEGG Pathways:  
    Probe set : 1373437_at    Name:   Protein phosphatase 1, regulatory (inhibitor) subunit 8 (predicted)    GB acc:   BI286565    UniGene:   Rn.16580     Locus Link:   313030    Chromosome:   5    Cytoband:   Chr:5q36    SOURCE        Gene Ontology:   biological process:  nuclear mRNA splicing, via spliceosome   biological process:  RNA catabolism   biological process:  cell proliferation   cellular component:  nucleus      BioCarta Pathways:     KEGG Pathways:  
    Probe set : 1387665_at    Name:   betaine-homocysteine methyltransferase    GB acc:   U96133    UniGene:   Rn.11406    Gene Symbol:   Bhmt    Locus Link:   81508    Chromosome:   2    Cytoband:   Chr:2q12    SOURCE    GENECARD       Gene Ontology:     BioCarta Pathways:     KEGG Pathways:  
    Probe set : 1397782_at    Name:   Similar to oxoglutarate dehydrogenase (lipoamide)    GB acc:   BE103050    UniGene:   Rn.45991     Locus Link:   360975    Chromosome:   14    Cytoband:   Chr:14q21    SOURCE        Gene Ontology:   biological process:  glycolysis   biological process:  metabolism      BioCarta Pathways:     KEGG Pathways:  
    Probe set : 1377639_at    Name:   adipocyte-specific adhesion molecule    GB acc:   BI275896    UniGene:   Rn.40141    Gene Symbol:   Asam    Locus Link:   286939    Chromosome:   8    Cytoband:   Chr:8q22    SOURCE    GENECARD       Gene Ontology:   biological process:  cell-cell adhesion   cellular component:  tight junction   cellular component:  integral to membrane      BioCarta Pathways:     KEGG Pathways:  
    Probe set : 1386876_at    Name:   adenylate cyclase 6    GB acc:   NM_012821    UniGene:   Rn.3313    Gene Symbol:   Adcy6    Locus Link:   25289    Chromosome:   7    Cytoband:   Chr:7q36    SOURCE    GENECARD       Gene Ontology:   cellular component:  plasma membrane   cellular component:  integral to membrane      BioCarta Pathways:     KEGG Pathways:  
    Probe set : 1383555_at    Name:   similar to RIKEN cDNA 4930579G22    GB acc:   AW525704    UniGene:   Rn.145152    Gene Symbol:   LOC360799    Locus Link:   360799    Chromosome:   12    Cytoband:   Chr:12q13    SOURCE    GENECARD       Gene Ontology:     BioCarta Pathways:     KEGG Pathways:  
    Probe set : 1395510_at    Name:   abhydrolase domain containing 1 (predicted)    GB acc:   AW916573    UniGene:   Rn.40145    Gene Symbol:   Abhd1_predicted    Locus Link:   313917    Chromosome:   6    Cytoband:   Chr:6q14    SOURCE    GENECARD       Gene Ontology:   molecular function:  hydrolase activity   molecular function:  catalytic activity      BioCarta Pathways:     KEGG Pathways:  
    Probe set : 1385570_at     GB acc:   AI717105              Gene Ontology:     BioCarta Pathways:     KEGG Pathways:  
    Probe set : 1385783_at    Name:   similar to BH3-only member B protein    GB acc:   BF418697    UniGene:   Rn.114731    Gene Symbol:   LOC498630    Locus Link:   498630    Chromosome:   16    Cytoband:   Chr:16q11    SOURCE    GENECARD       Gene Ontology:     BioCarta Pathways:     KEGG Pathways:  
    Probe set : 1381903_at    Name:   F-box only protein 33 (predicted)    GB acc:   AW533341    UniGene:   Rn.41874    Gene Symbol:   Fbxo33_predicted    Locus Link:   314157    Chromosome:   6    Cytoband:   Chr:6q23    SOURCE    GENECARD       Gene Ontology:   biological process:  ubiquitin cycle      BioCarta Pathways:     KEGG Pathways:  
    Probe set : 1379410_at    Name:   Lin-10 protein homolog (C. elegans)    GB acc:   AA925019    UniGene:   Rn.9778    Gene Symbol:   Lin10    Locus Link:   207123    Chromosome:   19    Cytoband:   Chr:19q11    SOURCE    GENECARD       Gene Ontology:   biological process:  Golgi to plasma membrane protein transport   cellular component:  membrane fraction   cellular component:  Golgi trans face   cellular component:  dendrite   cellular component:  synaptic vesicle membrane   molecular function:  receptor binding      BioCarta Pathways:     KEGG Pathways:  
    Probe set : 1370461_at    Name:   hyaluronan mediated motility receptor    GB acc:   AF336825    UniGene:   Rn.92304    Gene Symbol:   Hmmr    Locus Link:   25460    Chromosome:   10    Cytoband:   Chr:10q12    SOURCE    GENECARD       Gene Ontology:   biological process:  cell motility   biological process:  cytoskeleton organization and biogenesis   biological process:  cell-cell signaling   molecular function:  hyaluronic acid binding   molecular function:  hyaluronic acid binding   molecular function:  receptor activity      BioCarta Pathways:     KEGG Pathways:  
    Probe set : 1374403_at    Name:   ephrin B1    GB acc:   BF412072    UniGene:   Rn.44398    Gene Symbol:   Efnb1    Locus Link:   25186    Chromosome:   X    Cytoband:   Chr:Xq22.1    SOURCE    GENECARD       Gene Ontology:     BioCarta Pathways:     KEGG Pathways:  
    Probe set : 1395400_at     GB acc:   AI178515              Gene Ontology:     BioCarta Pathways:     KEGG Pathways:  
    Probe set : 1385423_at    Name:   inner membrane protein, mitochondrial (predicted)    GB acc:   AW521399    UniGene:   Rn.47124    Gene Symbol:   Immt_predicted    Locus Link:   312444    Chromosome:   4    Cytoband:   Chr:4q33    SOURCE    GENECARD       Gene Ontology:     BioCarta Pathways:     KEGG Pathways:  
    Probe set : 1389518_at    Name:   F-box only protein 10 (predicted)    GB acc:   AI009763    UniGene:   Rn.7385    Gene Symbol:   Fbxo10_predicted    Locus Link:   362511    Chromosome:   5    Cytoband:   Chr:5q22    SOURCE    GENECARD       Gene Ontology:   biological process:  ubiquitin cycle   biological process:  protein ubiquitination   cellular component:  ubiquitin ligase complex   cellular component:  ruffle   cellular component:  cytoplasm   molecular function:  ubiquitin-protein ligase activity   molecular function:  protein binding      BioCarta Pathways:     KEGG Pathways:  
    Probe set : 1374834_at    Name:   Splicing factor 3b, subunit 4 (predicted)    GB acc:   AA943520    UniGene:   Rn.116275     Locus Link:   295270    Chromosome:   2    Cytoband:   Chr:2q34    SOURCE        Gene Ontology:   biological process:  nuclear mRNA splicing, via spliceosome   biological process:  mRNA processing   biological process:  RNA splicing   cellular component:  nucleus   cellular component:  spliceosome complex   molecular function:  nucleic acid binding   molecular function:  RNA binding   molecular function:  nucleotide binding      BioCarta Pathways:     KEGG Pathways:  
    Probe set : 1389158_at    Name:   Transcribed locus    GB acc:   BE329196    UniGene:   Rn.22482        SOURCE        Gene Ontology:     BioCarta Pathways:     KEGG Pathways:  
    Probe set : 1373351_at    Name:   Transcribed locus    GB acc:   BI286775    UniGene:   Rn.26749        SOURCE        Gene Ontology:     BioCarta Pathways:     KEGG Pathways:  
    Probe set : 1369708_a_at    Name:   cAMP responsive element binding protein 1    GB acc:   NM_031017    UniGene:   Rn.90061    Gene Symbol:   Creb1    Locus Link:   81646    Chromosome:   9    Cytoband:   Chr:9q32    SOURCE    GENECARD       Gene Ontology:   cellular component:  nucleus   cellular component:  nucleus   cellular component:  transcription factor complex   cellular component:  mitochondrion      BioCarta Pathways:     KEGG Pathways:  
    Probe set : 1372390_at    Name:   Transcribed locus    GB acc:   AI710604    UniGene:   Rn.49740        SOURCE        Gene Ontology:     BioCarta Pathways:     KEGG Pathways:  
    Probe set : 1391090_at    Name:   Transcribed locus    GB acc:   BG380796    UniGene:   Rn.147032        SOURCE        Gene Ontology:     BioCarta Pathways:     KEGG Pathways:  
    Probe set : 1392739_a_at    Name:   similar to RIKEN cDNA 2310005P05 (predicted)    GB acc:   AI454794    UniGene:   Rn.110716    Gene Symbol:   RGD1307401_predicted    Locus Link:   315500    Chromosome:   8    Cytoband:   Chr:8q13    SOURCE    GENECARD       Gene Ontology:   cellular component:  intracellular   cellular component:  nucleus   molecular function:  DNA binding      BioCarta Pathways:     KEGG Pathways:  
    Probe set : 1389373_at    Name:   SMAD, mothers against DPP homolog 1 (Drosophila)    GB acc:   AI029555    UniGene:   Rn.10635    Gene Symbol:   Smad1    Locus Link:   25671    Chromosome:   19    Cytoband:   Chr:19q11    SOURCE    GENECARD       Gene Ontology:   biological process:  MAPKKK cascade   biological process:  regulation of transcription, DNA-dependent   biological process:  regulation of transcription from RNA polymerase II promoter   biological process:  common-partner SMAD protein phosphorylation   biological process:  SMAD protein heteromerization   biological process:  gametogenesis   biological process:  organ morphogenesis   biological process:  BMP signaling pathway   biological process:  homeostasis   biological process:  regulation of transcription, DNA-dependent   biological process:  signal transduction   biological process:  transforming growth factor beta receptor signaling pathway   biological process:  embryonic pattern specification   biological process:  BMP signaling pathway   biological process:  transcription      BioCarta Pathways:     KEGG Pathways:  
    Probe set : 1374089_at    Name:   Transcribed locus    GB acc:   BI289692    UniGene:   Rn.15749        SOURCE        Gene Ontology:     BioCarta Pathways:     KEGG Pathways:  
    Probe set : 1380383_at    Name:   ADP-ribosylation factor 4-like (predicted)    GB acc:   AI030650    UniGene:   Rn.98418     Locus Link:   303559    Chromosome:   10    Cytoband:   Chr:10q32.1    SOURCE        Gene Ontology:   cellular component:  nucleus   molecular function:  GTPase activity   molecular function:  transporter activity   molecular function:  GTP binding      BioCarta Pathways:     KEGG Pathways:  
    Probe set : 1388986_at    Name:   Core promoter element binding protein    GB acc:   AI598339    UniGene:   Rn.7947    Gene Symbol:   Copeb    Locus Link:   58954    Chromosome:   17    Cytoband:   Chr:17q12.2    SOURCE    GENECARD       Gene Ontology:   cellular component:  nucleus   cellular component:  nucleus   cellular component:  cytoplasm      BioCarta Pathways:     KEGG Pathways:  
    Probe set : 1383056_a_at    Name:   LOC499072    GB acc:   AI548910    UniGene:   Rn.12584    Gene Symbol:   LOC499072    Locus Link:   499072    Chromosome:   1    Cytoband:   Chr:1q12    SOURCE    GENECARD       Gene Ontology:     BioCarta Pathways:     KEGG Pathways:  
    Probe set : 1372007_at    Name:   similar to AOF2 protein    GB acc:   BE099064    UniGene:   Rn.6607    Gene Symbol:   LOC500569    Locus Link:   500569    Chromosome:   5    Cytoband:   Chr:5q36    SOURCE    GENECARD       Gene Ontology:     BioCarta Pathways:     KEGG Pathways:  
    Probe set : 1389334_at    Name:   NADH dehydrogenase (ubiquinone) 1 alpha subcomplex 10 /// NADH dehydrogenase 1 alpha subcomplex 10-like protein    GB acc:   BI277594    UniGene:   Rn.102304    Gene Symbol:   Ndufa10 /// LOC316632    Locus Link:   314071 /// 316632    Chromosome:   6    Cytoband:   Chr:6q21    SOURCE    GENECARD       Gene Ontology:   biological process:  nucleobase, nucleoside, nucleotide and nucleic acid metabolism   molecular function:  ATP binding   molecular function:  phosphotransferase activity, alcohol group as acceptor      BioCarta Pathways:     KEGG Pathways:  
    Probe set : 1392835_at    Name:   zinc finger protein 68 (predicted)    GB acc:   AI705060    UniGene:   Rn.47413    Gene Symbol:   Zfp68_predicted    Locus Link:   304337    Chromosome:   12    Cytoband:   Chr:12q11    SOURCE    GENECARD       Gene Ontology:   biological process:  regulation of transcription, DNA-dependent   cellular component:  intracellular   cellular component:  nucleus   molecular function:  nucleic acid binding   molecular function:  DNA binding      BioCarta Pathways:     KEGG Pathways:  
    Probe set : 1370408_at    Name:   putative small membrane protein NID67    GB acc:   AF313411    UniGene:   Rn.8865    Gene Symbol:   Nid67    Locus Link:   286910    Chromosome:   18    Cytoband:   Chr:18q12.1    SOURCE    GENECARD       Gene Ontology:   biological process:  nervous system development   cellular component:  integral to membrane   molecular function:  ion channel activity      BioCarta Pathways:     KEGG Pathways:  
    Probe set : 1387081_at    Name:   reticulocalbin 2    GB acc:   NM_017132    UniGene:   Rn.6133    Gene Symbol:   Rcn2    Locus Link:   29218    Chromosome:   8    Cytoband:   Chr:8q24    SOURCE    GENECARD       Gene Ontology:   biological process:  muscle development   cellular component:  extracellular space   cellular component:  endoplasmic reticulum   molecular function:  calcium ion binding   molecular function:  calcium ion binding   molecular function:  protein binding      BioCarta Pathways:     KEGG Pathways:  
    Probe set : 1376030_at    Name:   centaurin, beta 1 (predicted)    GB acc:   BI275873    UniGene:   Rn.2165    Gene Symbol:   Centb1_predicted    Locus Link:   287443    Chromosome:   10    Cytoband:   Chr:10q24    SOURCE    GENECARD       Gene Ontology:   biological process:  intracellular signaling cascade   biological process:  regulation of GTPase activity   molecular function:  phospholipase C activity   molecular function:  GTPase activator activity      BioCarta Pathways:     KEGG Pathways:  
    Probe set : 1377079_a_at    Name:   protoporphyrinogen oxidase (predicted)    GB acc:   BF386283    UniGene:   Rn.24738    Gene Symbol:   Ppox_predicted    Locus Link:   289219    Chromosome:   13    Cytoband:   Chr:13q24    SOURCE    GENECARD       Gene Ontology:   biological process:  electron transport   biological process:  porphyrin biosynthesis   biological process:  heme biosynthesis   cellular component:  extracellular space   cellular component:  mitochondrion   molecular function:  protoporphyrinogen oxidase activity   molecular function:  electron transporter activity   molecular function:  oxidoreductase activity      BioCarta Pathways:     KEGG Pathways:  
    Probe set : 1377392_at    Name:   Transcribed locus, weakly similar to XP_236909.3 PREDICTED: similar to dishevelled associated activator of morphogenesis 2 [Rattus norvegicus]    GB acc:   BF389722    UniGene:   Rn.102398        SOURCE        Gene Ontology:     BioCarta Pathways:     KEGG Pathways:  
    Probe set : 1389062_at    Name:   RED protein    GB acc:   BE109152    UniGene:   Rn.19642    Gene Symbol:   RGD:1359352    Locus Link:   291659    Chromosome:   18    Cytoband:   Chr:18p11    SOURCE    GENECARD       Gene Ontology:     BioCarta Pathways:     KEGG Pathways:  
    Probe set : 1383168_at    Name:   Transcribed locus, strongly similar to NP_081141.1 hypothetical protein LOC73635 [Mus musculus]    GB acc:   BE102209    UniGene:   Rn.137315        SOURCE        Gene Ontology:     BioCarta Pathways:     KEGG Pathways:  
    Probe set : 1381798_at    Name:   LIM domain only protein 7    GB acc:   BE114958    UniGene:   Rn.98417    Gene Symbol:   RGD:1302984    Locus Link:   361084    Chromosome:   15    Cytoband:   Chr:15q21    SOURCE    GENECARD       Gene Ontology:   biological process:  cell-cell adhesion   biological process:  actomyosin structure organization and biogenesis   cellular component:  cell-cell adherens junction   cellular component:  apical plasma membrane   molecular function:  actinin binding   molecular function:  protein binding   molecular function:  actin binding   molecular function:  zinc ion binding      BioCarta Pathways:     KEGG Pathways:  
    Probe set : 1368049_at    Name:   t-complex protein 1    GB acc:   NM_012670    UniGene:   Rn.7102    Gene Symbol:   Tcp1    Locus Link:   24818    Chromosome:   1    Cytoband:   Chr:1q12    SOURCE    GENECARD       Gene Ontology:     BioCarta Pathways:     KEGG Pathways:  
    Probe set : 1390892_at    Name:   Transcribed locus    GB acc:   AA899893    UniGene:   Rn.14993        SOURCE        Gene Ontology:     BioCarta Pathways:     KEGG Pathways:  
    Probe set : 1372188_at    Name:   Similar to hypothetical protein D15Ertd785e    GB acc:   BM392171    UniGene:   Rn.4227     Locus Link:   300149    Chromosome:   7    Cytoband:   Chr:7q34    SOURCE        Gene Ontology:   biological process:  electron transport      BioCarta Pathways:     KEGG Pathways:  
    Probe set : 1370001_at    Name:   RAB8A, member RAS oncogene family    GB acc:   NM_053998    UniGene:   Rn.16064    Gene Symbol:   Rab8a    Locus Link:   117103    Chromosome:   16    Cytoband:   Chr:16p14    SOURCE    GENECARD       Gene Ontology:   biological process:  small GTPase mediated signal transduction   biological process:  protein transport   biological process:  transport   molecular function:  GTP binding   molecular function:  nucleotide binding      BioCarta Pathways:     KEGG Pathways:  
    Probe set : 1373282_at    Name:   similar to CG7744-PA (predicted)    GB acc:   AI406494    UniGene:   Rn.8763    Gene Symbol:   RGD1306651_predicted    Locus Link:   313720    Chromosome:   5    Cytoband:   Chr:5q36    SOURCE    GENECARD       Gene Ontology:     BioCarta Pathways:     KEGG Pathways:  
    Probe set : 1395821_at    Name:   core binding factor beta    GB acc:   BF524120    UniGene:   Rn.7308    Gene Symbol:   Cbfb    Locus Link:   361391    Chromosome:   19    Cytoband:   Chr:19q11    SOURCE    GENECARD       Gene Ontology:   cellular component:  nucleus   molecular function:  transcription coactivator activity      BioCarta Pathways:     KEGG Pathways:  
    Probe set : 1370337_at    Name:   CCCTC-binding factor    GB acc:   AF133731    UniGene:   Rn.18612    Gene Symbol:   Ctcf    Locus Link:   83726    Chromosome:   19    Cytoband:   Chr:19q12    SOURCE    GENECARD       Gene Ontology:   cellular component:  nucleus      BioCarta Pathways:     KEGG Pathways:  
    Probe set : 1392322_at    Name:   GTPase, IMAP family member 7    GB acc:   BM388319    UniGene:   Rn.28767    Gene Symbol:   Gimap7    Locus Link:   500113    Chromosome:   4    Cytoband:   Chr:4q24    SOURCE    GENECARD       Gene Ontology:   molecular function:  GTP binding      BioCarta Pathways:     KEGG Pathways:  
    Probe set : 1384588_at    Name:   Transcribed locus    GB acc:   BI295602    UniGene:   Rn.79724        SOURCE        Gene Ontology:     BioCarta Pathways:     KEGG Pathways:  
    Probe set : 1395636_at     GB acc:   AI576368              Gene Ontology:     BioCarta Pathways:     KEGG Pathways:  
    Probe set : 1372175_at    Name:   Transcribed locus, weakly similar to XP_342574.1 PREDICTED: similar to Csr1 [Rattus norvegicus]    GB acc:   BE112899    UniGene:   Rn.1132        SOURCE        Gene Ontology:     BioCarta Pathways:     KEGG Pathways:  
    Probe set : 1374660_at    Name:   similar to hypothetical protein FLJ20003 (predicted)    GB acc:   BM385193    UniGene:   Rn.146174    Gene Symbol:   RGD1309025_predicted    Locus Link:   293528    Chromosome:   1    Cytoband:   Chr:1q37    SOURCE    GENECARD       Gene Ontology:     BioCarta Pathways:     KEGG Pathways:  
    Probe set : 1378445_at    Name:   similar to putative repair and recombination helicase RAD26L    GB acc:   BM388759    UniGene:   Rn.12539    Gene Symbol:   LOC361197    Locus Link:   361197    Chromosome:   17    Cytoband:   Chr:17p14    SOURCE    GENECARD       Gene Ontology:     BioCarta Pathways:     KEGG Pathways:  
    Probe set : 1393655_at    Name:   RAD54 like (S. cerevisiae) (predicted)    GB acc:   BF397746    UniGene:   Rn.134695    Gene Symbol:   Rad54l_predicted    Locus Link:   298429    Chromosome:   5    Cytoband:   Chr:5q36    SOURCE    GENECARD       Gene Ontology:   biological process:  DNA repair   biological process:  DNA recombination   biological process:  meiosis   cellular component:  nucleus      BioCarta Pathways:     KEGG Pathways:  
    Probe set : 1368504_at    Name:   lysosomal membrane glycoprotein 1    GB acc:   NM_012857    UniGene:   Rn.40177    Gene Symbol:   Lamp1    Locus Link:   25328    Chromosome:   16    Cytoband:   Chr:16q12.5    SOURCE    GENECARD       Gene Ontology:     BioCarta Pathways:     KEGG Pathways:  
    Probe set : 1371956_at    Name:   CDNA clone MGC:93760 IMAGE:7108525    GB acc:   BG372686    UniGene:   Rn.49463        SOURCE        Gene Ontology:   biological process:  protein ubiquitination   cellular component:  ubiquitin ligase complex   molecular function:  ubiquitin-protein ligase activity   molecular function:  zinc ion binding   molecular function:  metal ion binding      BioCarta Pathways:     KEGG Pathways:  
    Probe set : 1392978_at     GB acc:   BE117086              Gene Ontology:     BioCarta Pathways:     KEGG Pathways:  
    Probe set : 1371926_at    Name:   Interleukin 6 signal transducer    GB acc:   AI171807    UniGene:   Rn.12138    Gene Symbol:   Il6st    Locus Link:   25205    Chromosome:   2    Cytoband:   Chr:2q14-q16    SOURCE    GENECARD       Gene Ontology:     BioCarta Pathways:     KEGG Pathways:  
    Probe set : 1372001_at     GB acc:   BF554893              Gene Ontology:     BioCarta Pathways:     KEGG Pathways:  
    Probe set : 1375083_at    Name:   Transcribed locus    GB acc:   BM387126    UniGene:   Rn.42082        SOURCE        Gene Ontology:     BioCarta Pathways:     KEGG Pathways:  
    Probe set : 1375170_at    Name:   S100 calcium binding protein A11 (calizzarin) (predicted)    GB acc:   BG378926    UniGene:   Rn.145400    Gene Symbol:   S100a11_predicted    Locus Link:   445415    Chromosome:   2    Cytoband:   Chr:2q34    SOURCE    GENECARD       Gene Ontology:   biological process:  negative regulation of DNA replication   biological process:  negative regulation of cell proliferation   cellular component:  nucleus   cellular component:  cytoplasm   cellular component:  extracellular space   molecular function:  cytokine activity   molecular function:  calcium ion binding      BioCarta Pathways:     KEGG Pathways:  
    Probe set : 1374920_at    Name:   Transcribed locus    GB acc:   AI228955    UniGene:   Rn.19095        SOURCE        Gene Ontology:     BioCarta Pathways:     KEGG Pathways:  
    Probe set : 1369077_at    Name:   N-acylsphingosine amidohydrolase 1    GB acc:   NM_053407    UniGene:   Rn.127798    Gene Symbol:   Asah1    Locus Link:   84431    Chromosome:   16    Cytoband:   Chr:16q12.1    SOURCE    GENECARD       Gene Ontology:   biological process:  fatty acid metabolism   biological process:  ceramide metabolism   biological process:  metabolism   cellular component:  lysosome   molecular function:  hydrolase activity   molecular function:  lyase activity   molecular function:  ceramidase activity      BioCarta Pathways:     KEGG Pathways:  
    Probe set : 1367853_at    Name:   hypothetical gene supported by NM_031798    GB acc:   NM_031798     Gene Symbol:   LOC497723    Locus Link:   497723    Chromosome:   18    Cytoband:   Chr:18q12.1     GENECARD       Gene Ontology:     BioCarta Pathways:     KEGG Pathways:  
    Probe set : 1390741_at    Name:   similar to KIAA1086 protein (predicted)    GB acc:   BI294610    UniGene:   Rn.41117    Gene Symbol:   RGD1304737_predicted    Locus Link:   314639    Chromosome:   7    Cytoband:   Chr:7q11    SOURCE    GENECARD       Gene Ontology:     BioCarta Pathways:     KEGG Pathways:  
    Probe set : 1388530_at    Name:   Similar to hypothetical protein    GB acc:   BM389459    UniGene:   Rn.111901     Locus Link:   499672      SOURCE        Gene Ontology:     BioCarta Pathways:     KEGG Pathways:  
    Probe set : 1373176_at    Name:   Polymerase (DNA-directed), delta 3, accessory subunit (predicted)    GB acc:   AA943208    UniGene:   Rn.45023     Locus Link:   293144    Chromosome:   1    Cytoband:   Chr:1q32    SOURCE        Gene Ontology:   biological process:  DNA synthesis during DNA repair   biological process:  DNA replication   biological process:  mismatch repair   cellular component:  nucleus   cellular component:  delta DNA polymerase complex   molecular function:  DNA binding   molecular function:  DNA-directed DNA polymerase activity   molecular function:  delta DNA polymerase activity      BioCarta Pathways:     KEGG Pathways:  
    Probe set : 1371451_at    Name:   ribonuclease H2, large subunit (predicted)    GB acc:   AI010940    UniGene:   Rn.3693    Gene Symbol:   Rnaseh2a_predicted    Locus Link:   364974    Chromosome:   19    Cytoband:   Chr:19q11    SOURCE    GENECARD       Gene Ontology:   biological process:  DNA replication   biological process:  RNA catabolism   biological process:  RNA metabolism      BioCarta Pathways:     KEGG Pathways:  
    Probe set : 1372498_at    Name:   similar to RIKEN cDNA 2810413N20    GB acc:   BF285068    UniGene:   Rn.1274    Gene Symbol:   MGC94686    Locus Link:   307649    Chromosome:   19    Cytoband:   Chr:19p12    SOURCE    GENECARD       Gene Ontology:   molecular function:  methyltransferase activity      BioCarta Pathways:     KEGG Pathways:  
    Probe set : 1370278_at    Name:   ATP synthase, H+ transporting, mitochondrial F1 complex, delta subunit    GB acc:   U00926    UniGene:   Rn.3879    Gene Symbol:   Atp5d    Locus Link:   245965    Chromosome:   7    Cytoband:   Chr:7q11    SOURCE    GENECARD       Gene Ontology:     BioCarta Pathways:     KEGG Pathways:  
    Probe set : 1371313_at    Name:   similar to 60S ribosomal protein L23a    GB acc:   AI172199     Gene Symbol:   LOC501709    Locus Link:   501709    Chromosome:   10    Cytoband:   Chr:10q25     GENECARD       Gene Ontology:     BioCarta Pathways:     KEGG Pathways:  
    Probe set : 1380345_at    Name:   Transcribed locus    GB acc:   AW529036    UniGene:   Rn.47079        SOURCE        Gene Ontology:     BioCarta Pathways:     KEGG Pathways:  
    Probe set : 1367627_at    Name:   glycine amidinotransferase (L-arginine:glycine amidinotransferase)    GB acc:   NM_031031    UniGene:   Rn.17661    Gene Symbol:   Gatm    Locus Link:   81660    Chromosome:   3    Cytoband:   Chr:3q35    SOURCE    GENECARD       Gene Ontology:     BioCarta Pathways:     KEGG Pathways:  
    Probe set : 1374345_at    Name:   Transcribed locus    GB acc:   AI111707    UniGene:   Rn.15962        SOURCE        Gene Ontology:     BioCarta Pathways:     KEGG Pathways:  
    Probe set : 1390854_at    Name:   transmembrane protein 24 (predicted)    GB acc:   BG378195    UniGene:   Rn.57410    Gene Symbol:   Tmem24_predicted    Locus Link:   300666    Chromosome:   8    Cytoband:   Chr:8q22    SOURCE    GENECARD       Gene Ontology:   cellular component:  integral to membrane      BioCarta Pathways:     KEGG Pathways:  
    Probe set : 1387882_at    Name:   basic transcription element binding protein 1    GB acc:   AI071565    UniGene:   Rn.19481    Gene Symbol:   Bteb1    Locus Link:   117560    Chromosome:   1    Cytoband:   Chr:1q51    SOURCE    GENECARD       Gene Ontology:   cellular component:  nucleus      BioCarta Pathways:     KEGG Pathways:  
    Probe set : 1372510_at    Name:   Neoplastic progression 3 (predicted)    GB acc:   AI172302    UniGene:   Rn.2835     Locus Link:   296271    Chromosome:   3    Cytoband:   Chr:3q41    SOURCE        Gene Ontology:   cellular component:  extrachromosomal circular DNA   molecular function:  DNA binding      BioCarta Pathways:     KEGG Pathways:  
    Probe set : 1398268_at    Name:   nuclear transcription factor-Y gamma    GB acc:   BI296061    UniGene:   Rn.1457    Gene Symbol:   Nfyc    Locus Link:   25337    Chromosome:   5    Cytoband:   Chr:5q36    SOURCE    GENECARD       Gene Ontology:   biological process:  regulation of transcription, DNA-dependent   biological process:  transcription   cellular component:  nucleus   cellular component:  transcription factor complex   cellular component:  CCAAT-binding factor complex   molecular function:  DNA binding   molecular function:  transcription factor activity      BioCarta Pathways:     KEGG Pathways:  
    Probe set : 1371988_at    Name:   mannosidase 1, alpha (predicted)    GB acc:   AA892549    UniGene:   Rn.3576    Gene Symbol:   Man1a_predicted    Locus Link:   294567    Chromosome:   20    Cytoband:   Chr:20q11    SOURCE    GENECARD       Gene Ontology:   biological process:  carbohydrate metabolism   biological process:  protein amino acid N-linked glycosylation      BioCarta Pathways:     KEGG Pathways:  
    Probe set : 1367826_at    Name:   nuclear factor, erythroid derived 2, like 2    GB acc:   NM_031789    UniGene:   Rn.10867    Gene Symbol:   Nfe2l2    Locus Link:   83619    Chromosome:   3    Cytoband:   Chr:3q23    SOURCE    GENECARD       Gene Ontology:   biological process:  regulation of transcription, DNA-dependent   biological process:  transcription from RNA polymerase II promoter   biological process:  transcription   cellular component:  nucleus   molecular function:  DNA binding   molecular function:  transcription factor activity      BioCarta Pathways:     KEGG Pathways:  
    Probe set : 1372735_at    Name:   eukaryotic translation initiation factor 3 subunit k (predicted)    GB acc:   BI283109    UniGene:   Rn.1706    Gene Symbol:   eIF3k_predicted    Locus Link:   292762    Chromosome:   1    Cytoband:   Chr:1q21    SOURCE    GENECARD       Gene Ontology:   biological process:  protein biosynthesis   biological process:  translational initiation   cellular component:  nucleus   molecular function:  translation initiation factor activity      BioCarta Pathways:     KEGG Pathways:  
    Probe set : 1375553_at    Name:   similar to RIKEN cDNA 5133400G04 (predicted)    GB acc:   BE096042    UniGene:   Rn.136857    Gene Symbol:   RGD1311742_predicted    Locus Link:   291676    Chromosome:   18    Cytoband:   Chr:18p11    SOURCE    GENECARD       Gene Ontology:     BioCarta Pathways:     KEGG Pathways:  
    Probe set : 1382537_at    Name:   Ras-related GTP binding C (predicted)    GB acc:   AA899991    UniGene:   Rn.6455    Gene Symbol:   Rragc_predicted    Locus Link:   298514    Chromosome:   5    Cytoband:   Chr:5q36    SOURCE    GENECARD       Gene Ontology:   cellular component:  nucleus   cellular component:  cytoplasm   cellular component:  heterotrimeric G-protein complex      BioCarta Pathways:     KEGG Pathways:  
    Probe set : 1387745_at    Name:   antigen identified by monoclonal antibody MRC OX-2 receptor    GB acc:   NM_023953    UniGene:   Rn.124439    Gene Symbol:   Mox2r    Locus Link:   64357    Chromosome:   11    Cytoband:   Chr:11q21    SOURCE    GENECARD       Gene Ontology:   biological process:  G-protein coupled receptor protein signaling pathway   cellular component:  integral to membrane      BioCarta Pathways:     KEGG Pathways:  
    Probe set : 1398966_at    Name:   Transcribed locus, strongly similar to NP_081070.1 differentially expressed in B16F10 1 [Mus musculus]    GB acc:   AI169154    UniGene:   Rn.101076        SOURCE        Gene Ontology:     BioCarta Pathways:     KEGG Pathways:  
    Probe set : 1378601_at    Name:   Transcribed locus    GB acc:   AI413056    UniGene:   Rn.147647        SOURCE        Gene Ontology:     BioCarta Pathways:     KEGG Pathways:  
    Probe set : 1380013_at    Name:   Similar to adiponutrin    GB acc:   BI273908    UniGene:   Rn.97779     Locus Link:   362972    Chromosome:   7    Cytoband:   Chr:7q34    SOURCE        Gene Ontology:     BioCarta Pathways:     KEGG Pathways:  
    Probe set : 1396297_at    Name:   Transcribed locus    GB acc:   BG374359    UniGene:   Rn.66210        SOURCE        Gene Ontology:     BioCarta Pathways:     KEGG Pathways:  
    Probe set : 1374564_at    Name:   deltex 2 homolog (Drosophila)    GB acc:   BE099401    UniGene:   Rn.145894    Gene Symbol:   Dtx2    Locus Link:   304591    Chromosome:   12    Cytoband:   Chr:12q12    SOURCE    GENECARD       Gene Ontology:     BioCarta Pathways:     KEGG Pathways:  
    Probe set : 1378076_at    Name:   Transcribed locus    GB acc:   AA963273    UniGene:   Rn.11429        SOURCE        Gene Ontology:     BioCarta Pathways:     KEGG Pathways:  
    Probe set : 1397634_at     GB acc:   BE098186              Gene Ontology:     BioCarta Pathways:     KEGG Pathways:  
    Probe set : 1373069_at    Name:   mitochondrial ribosomal protein S30 (predicted)    GB acc:   BG378230    UniGene:   Rn.9312    Gene Symbol:   Mrps30_predicted    Locus Link:   294767    Chromosome:   2    Cytoband:   Chr:2q15    SOURCE    GENECARD       Gene Ontology:   biological process:  protein biosynthesis   biological process:  apoptosis   cellular component:  mitochondrion   cellular component:  ribosome   molecular function:  structural constituent of ribosome      BioCarta Pathways:     KEGG Pathways:  
    Probe set : 1367664_at    Name:   ankyrin repeat domain 1 (cardiac muscle)    GB acc:   NM_013220    UniGene:   Rn.3789    Gene Symbol:   Ankrd1    Locus Link:   27064    Chromosome:   1    Cytoband:   Chr:1q53    SOURCE    GENECARD       Gene Ontology:     BioCarta Pathways:     KEGG Pathways:  
    Probe set : 1382160_at    Name:   Casein kinase II, alpha 2, polypeptide (predicted)    GB acc:   AI236331    UniGene:   Rn.40308     Locus Link:   307641    Chromosome:   19    Cytoband:   Chr:19p13    SOURCE        Gene Ontology:   cellular component:  protein kinase CK2 complex      BioCarta Pathways:     KEGG Pathways:  
    Probe set : 1389955_at     GB acc:   BE104483              Gene Ontology:     BioCarta Pathways:     KEGG Pathways:  
    Probe set : 1395710_at    Name:   similar to mesoderm induction early response 1 (MI-ER1)    GB acc:   BI275003    UniGene:   Rn.36542    Gene Symbol:   LOC313418    Locus Link:   313418    Chromosome:   5    Cytoband:   Chr:5q34    SOURCE    GENECARD       Gene Ontology:     BioCarta Pathways:     KEGG Pathways:  
    Probe set : 1367832_at    Name:   lysophospholipase 1    GB acc:   NM_013006    UniGene:   Rn.3594    Gene Symbol:   Lypla1    Locus Link:   25514    Chromosome:   5    Cytoband:   Chr:5q12    SOURCE    GENECARD       Gene Ontology:   biological process:  lipid metabolism   biological process:  fatty acid metabolism   cellular component:  membrane fraction   cellular component:  mitochondrion      BioCarta Pathways:     KEGG Pathways:  
    Probe set : 1373245_at    Name:   procollagen, type IV, alpha 1 (predicted)    GB acc:   BE111752    UniGene:   Rn.53801    Gene Symbol:   Col4a1_predicted    Locus Link:   290905    Chromosome:   16    Cytoband:   Chr:16q12.5    SOURCE    GENECARD       Gene Ontology:   biological process:  phosphate transport   biological process:  cell adhesion      BioCarta Pathways:     KEGG Pathways:  
    Probe set : 1392012_at    Name:   Siah binding protein 1; FBP interacting repressor; pyrimidine tract binding splicing factor; Ro ribonucleoprotein-binding protein 1    GB acc:   BE100353    UniGene:   Rn.96174    Gene Symbol:   Siahbp1    Locus Link:   84401    Chromosome:   7    Cytoband:   Chr:7q34    SOURCE    GENECARD       Gene Ontology:   cellular component:  ribonucleoprotein complex   molecular function:  nucleic acid binding   molecular function:  nucleotide binding      BioCarta Pathways:     KEGG Pathways:  
    Probe set : 1395499_at    Name:   Transcribed locus    GB acc:   BF392443    UniGene:   Rn.30310        SOURCE        Gene Ontology:     BioCarta Pathways:     KEGG Pathways:  
    Probe set : 1372864_at    Name:   Adenylate cyclase 5    GB acc:   AI008095    UniGene:   Rn.6278    Gene Symbol:   Adcy5    Locus Link:   64532    Chromosome:   11    Cytoband:   Chr:11q22    SOURCE    GENECARD       Gene Ontology:   cellular component:  plasma membrane   cellular component:  integral to membrane      BioCarta Pathways:     KEGG Pathways:  
    Probe set : 1384389_at    Name:   Reticulon 4 receptor-like 2    GB acc:   AI112040    UniGene:   Rn.26706    Gene Symbol:   Rtn4rl2    Locus Link:   311169    Chromosome:   3    Cytoband:   Chr:3q24    SOURCE    GENECARD       Gene Ontology:   biological process:  cell surface receptor linked signal transduction   cellular component:  external side of plasma membrane   cellular component:  lipid raft   molecular function:  receptor activity      BioCarta Pathways:     KEGG Pathways:  
    Probe set : 1382739_at    Name:   G protein-coupled receptor 19    GB acc:   AI045718    UniGene:   Rn.99370    Gene Symbol:   Gpr19    Locus Link:   312787    Chromosome:   4    Cytoband:   Chr:4q43    SOURCE    GENECARD       Gene Ontology:   cellular component:  integral to plasma membrane   cellular component:  integral to plasma membrane   cellular component:  integral to membrane      BioCarta Pathways:     KEGG Pathways:  
    Probe set : 1368589_at    Name:   protein tyrosine phosphatase, receptor type, J    GB acc:   NM_017269    UniGene:   Rn.10278    Gene Symbol:   Ptprj    Locus Link:   29645      SOURCE    GENECARD       Gene Ontology:     BioCarta Pathways:     KEGG Pathways:  
    Probe set : 1389739_at    Name:   neuralized-like 2 (Drosophila) (predicted)    GB acc:   AA800025    UniGene:   Rn.98792    Gene Symbol:   Neurl2_predicted    Locus Link:   311633    Chromosome:   3    Cytoband:   Chr:3q42    SOURCE    GENECARD       Gene Ontology:   biological process:  intracellular signaling cascade      BioCarta Pathways:     KEGG Pathways:  
    Probe set : 1377098_at    Name:   Transcribed locus    GB acc:   BF283365    UniGene:   Rn.16632        SOURCE        Gene Ontology:     BioCarta Pathways:     KEGG Pathways:  
    Probe set : 1373822_at    Name:   similar to RIKEN cDNA 1110025L05    GB acc:   BI275708    UniGene:   Rn.140867    Gene Symbol:   LOC502367    Locus Link:   502367    Chromosome:   1    Cytoband:   Chr:1q36    SOURCE    GENECARD       Gene Ontology:     BioCarta Pathways:     KEGG Pathways:  
    Probe set : 1374457_at    Name:   similar to serine C-palmitoyltransferase    GB acc:   AI180194    UniGene:   Rn.88163    Gene Symbol:   LOC503105    Locus Link:   503105      SOURCE    GENECARD       Gene Ontology:     BioCarta Pathways:     KEGG Pathways:  
    Probe set : 1385038_at    Name:   similar to hedgehog-interacting protein    GB acc:   BF390009    UniGene:   Rn.137981    Gene Symbol:   LOC291936    Locus Link:   291936    Chromosome:   19    Cytoband:   Chr:19q11    SOURCE    GENECARD       Gene Ontology:     BioCarta Pathways:     KEGG Pathways:  
    Probe set : 1369990_at    Name:   topoisomerase (DNA) 2 alpha    GB acc:   NM_022183    UniGene:   Rn.90996    Gene Symbol:   Top2a    Locus Link:   360243    Chromosome:   10    Cytoband:   Chr:10q31    SOURCE    GENECARD       Gene Ontology:     BioCarta Pathways:     KEGG Pathways:  
    Probe set : 1380170_at    Name:   GABA(A) receptor-associated protein like 2    GB acc:   BI303388    UniGene:   Rn.64537    Gene Symbol:   Gabarapl2    Locus Link:   64670    Chromosome:   19    Cytoband:   Chr:19q12    SOURCE    GENECARD       Gene Ontology:   cellular component:  Golgi membrane   cellular component:  Golgi apparatus   cellular component:  cytoskeleton   cellular component:  Golgi stack      BioCarta Pathways:     KEGG Pathways:  
    Probe set : 1389464_at    Name:   ligand of numb-protein X 1 (predicted)    GB acc:   BE120953    UniGene:   Rn.25032    Gene Symbol:   Lnx1_predicted    Locus Link:   360926    Chromosome:   14    Cytoband:   Chr:14p11    SOURCE    GENECARD       Gene Ontology:   cellular component:  ubiquitin ligase complex   cellular component:  extracellular space   cellular component:  cytoplasm      BioCarta Pathways:     KEGG Pathways:  
    Probe set : 1390255_at    Name:   similar to mKIAA0843 protein    GB acc:   BG381583    UniGene:   Rn.39037    Gene Symbol:   LOC307395    Locus Link:   307395    Chromosome:   18    Cytoband:   Chr:18q12.1    SOURCE    GENECARD       Gene Ontology:     BioCarta Pathways:     KEGG Pathways:  
    Probe set : 1382777_at    Name:   Neurolysin (metallopeptidase M3 family)    GB acc:   BE108796    UniGene:   Rn.11029    Gene Symbol:   Nln    Locus Link:   117041    Chromosome:   2    Cytoband:   Chr:2q13    SOURCE    GENECARD       Gene Ontology:   biological process:  RNA processing   biological process:  proteolysis   cellular component:  mitochondrion      BioCarta Pathways:     KEGG Pathways:  
    Probe set : 1370147_at    Name:   2-amino-3-carboxymuconate-6-semialdehyde decarboxylase    GB acc:   NM_134372    UniGene:   Rn.43814    Gene Symbol:   Acmsd    Locus Link:   171385    Chromosome:   13    Cytoband:   Chr:13q12    SOURCE    GENECARD       Gene Ontology:   biological process:  metabolism   biological process:  quinolinate metabolism   cellular component:  soluble fraction   cellular component:  cytosol      BioCarta Pathways:     KEGG Pathways:  
    Probe set : 1369635_at    Name:   sorbitol dehydrogenase    GB acc:   NM_017052    UniGene:   Rn.11334    Gene Symbol:   Sord    Locus Link:   24788    Chromosome:   3    Cytoband:   Chr:3q35    SOURCE    GENECARD       Gene Ontology:   biological process:  sorbitol metabolism   biological process:  visual perception      BioCarta Pathways:     KEGG Pathways:  
    Probe set : 1382509_at    Name:   similar to CCR4 (predicted)    GB acc:   AA956256    UniGene:   Rn.122212    Gene Symbol:   RGD1310783_predicted    Locus Link:   287249    Chromosome:   10    Cytoband:   Chr:10q22    SOURCE    GENECARD       Gene Ontology:   biological process:  regulation of transcription, DNA-dependent   cellular component:  nucleus      BioCarta Pathways:     KEGG Pathways:  
    Probe set : 1389170_at    Name:   caspase 7    GB acc:   BF283754    UniGene:   Rn.53995    Gene Symbol:   Casp7    Locus Link:   64026    Chromosome:   1    Cytoband:   Chr:1q55    SOURCE    GENECARD       Gene Ontology:   biological process:  proteolysis   biological process:  apoptosis   biological process:  apoptotic program   cellular component:  cytoplasm      BioCarta Pathways:     KEGG Pathways:  
    Probe set : 1393832_at    Name:   Similar to RIKEN cDNA 0610033I05 (predicted)    GB acc:   AI136337    UniGene:   Rn.117592     Locus Link:   311020    Chromosome:   3    Cytoband:   Chr:3q12    SOURCE        Gene Ontology:     BioCarta Pathways:     KEGG Pathways:  
    Probe set : 1369949_at    Name:   Lutheran blood group (Auberger b antigen included)    GB acc:   NM_031752    UniGene:   Rn.7003    Gene Symbol:   Lu    Locus Link:   78958    Chromosome:   1    Cytoband:   Chr:1q21    SOURCE    GENECARD       Gene Ontology:   biological process:  cell adhesion   biological process:  cell adhesion   biological process:  signal transduction   cellular component:  plasma membrane   cellular component:  integral to plasma membrane   molecular function:  transmembrane receptor activity   molecular function:  laminin receptor activity   molecular function:  laminin receptor activity      BioCarta Pathways:     KEGG Pathways:  
    Probe set : 1371724_at    Name:   small fragment nuclease (predicted)    GB acc:   AA799631    UniGene:   Rn.11496    Gene Symbol:   Smfn_predicted    Locus Link:   300689    Chromosome:   8    Cytoband:   Chr:8q23    SOURCE    GENECARD       Gene Ontology:   biological process:  nucleobase, nucleoside, nucleotide and nucleic acid metabolism   biological process:  nucleotide metabolism   cellular component:  nucleus   cellular component:  mitochondrion   cellular component:  intracellular      BioCarta Pathways:     KEGG Pathways:  
    Probe set : 1376187_at    Name:   Transcribed locus    GB acc:   AI177887    UniGene:   Rn.19332        SOURCE        Gene Ontology:     BioCarta Pathways:     KEGG Pathways:  
    Probe set : 1379201_at     GB acc:   AA851653              Gene Ontology:     BioCarta Pathways:     KEGG Pathways:  
    Probe set : 1390149_at    Name:   transforming acidic coiled coil 2    GB acc:   BI286164    UniGene:   Rn.13989    Gene Symbol:   RGD:1303004    Locus Link:   309025    Chromosome:   1    Cytoband:   Chr:1q37    SOURCE    GENECARD       Gene Ontology:   molecular function:  retinoid X receptor binding      BioCarta Pathways:     KEGG Pathways:  
    Probe set : 1389354_at    Name:   similar to semaF cytoplasmic domain associated protein 2    GB acc:   BF285490    UniGene:   Rn.20103    Gene Symbol:   LOC362057    Locus Link:   362057    Chromosome:   2    Cytoband:   Chr:2q45    SOURCE    GENECARD       Gene Ontology:   molecular function:  protein binding      BioCarta Pathways:     KEGG Pathways:  
    Probe set : 1391045_at    Name:   diacylglycerol O-acyltransferase homolog 2 (mouse)    GB acc:   BI277463    UniGene:   Rn.9523    Gene Symbol:   Dgat2    Locus Link:   252900    Chromosome:   1    Cytoband:   Chr:1q32    SOURCE    GENECARD       Gene Ontology:   cellular component:  microsome      BioCarta Pathways:     KEGG Pathways:  
    Probe set : 1392989_at    Name:   Transcribed locus    GB acc:   BI295986    UniGene:   Rn.25467        SOURCE        Gene Ontology:     BioCarta Pathways:     KEGG Pathways:  
    Probe set : 1373078_at    Name:   Transcribed locus    GB acc:   AI409271    UniGene:   Rn.8222        SOURCE        Gene Ontology:     BioCarta Pathways:     KEGG Pathways:  
    Probe set : 1377442_at    Name:   Similar to Probable serine protease HTRA4 precursor (predicted)    GB acc:   BF409997    UniGene:   Rn.45278     Locus Link:   306564    Chromosome:   16    Cytoband:   Chr:16q12.4    SOURCE        Gene Ontology:     BioCarta Pathways:     KEGG Pathways:  
    Probe set : 1374724_at    Name:   Transcribed locus    GB acc:   BG378232    UniGene:   Rn.11731        SOURCE        Gene Ontology:     BioCarta Pathways:     KEGG Pathways:  
    Probe set : 1388304_at    Name:   NADH dehydrogenase (ubiquinone) 1 beta subcomplex, 5 (predicted)    GB acc:   BI281307    UniGene:   Rn.3367    Gene Symbol:   Ndufb5_predicted    Locus Link:   294964    Chromosome:   2    Cytoband:   Chr:2q25    SOURCE    GENECARD       Gene Ontology:   biological process:  mitochondrial electron transport, NADH to ubiquinone   cellular component:  mitochondrion   molecular function:  NADH dehydrogenase activity   molecular function:  NADH dehydrogenase (ubiquinone) activity   molecular function:  oxidoreductase activity      BioCarta Pathways:     KEGG Pathways:  
    Probe set : 1379312_at    Name:   peroxisome proliferative activated receptor, gamma, coactivator-related 1 (predicted)    GB acc:   AI706508    UniGene:   Rn.9484    Gene Symbol:   Pprc1_predicted    Locus Link:   294007    Chromosome:   1    Cytoband:   Chr:1q54    SOURCE    GENECARD       Gene Ontology:   biological process:  nuclear mRNA splicing, via spliceosome   biological process:  mRNA processing   molecular function:  nucleic acid binding      BioCarta Pathways:     KEGG Pathways:  
    Probe set : 1392296_at    Name:   Transcribed locus    GB acc:   AI502798    UniGene:   Rn.131200        SOURCE        Gene Ontology:     BioCarta Pathways:     KEGG Pathways:  
    Probe set : 1389053_at    Name:   Similar to scruin like at the midline CG5186-PA (predicted)    GB acc:   AI171280    UniGene:   Rn.137393     Locus Link:   312199    Chromosome:   4    Cytoband:   Chr:4q22    SOURCE        Gene Ontology:     BioCarta Pathways:     KEGG Pathways:  
    Probe set : 1377908_at    Name:   CD4 antigen    GB acc:   AI408225    UniGene:   Rn.10748    Gene Symbol:   Cd4    Locus Link:   24932    Chromosome:   4    Cytoband:   Chr:4q42    SOURCE    GENECARD       Gene Ontology:   biological process:  defense response   biological process:  immune response   biological process:  cell adhesion   biological process:  transmembrane receptor protein tyrosine kinase signaling pathway   biological process:  T cell differentiation   biological process:  T cell selection   biological process:  positive regulation of interleukin-2 biosynthesis   biological process:  immune response   biological process:  transmembrane receptor protein tyrosine kinase signaling pathway   biological process:  T cell differentiation   biological process:  T cell selection   biological process:  positive regulation of interleukin-2 biosynthesis      BioCarta Pathways:     KEGG Pathways:  
    Probe set : 1387844_at    Name:   LIM and SH3 protein 1    GB acc:   NM_032613    UniGene:   Rn.94195    Gene Symbol:   Lasp1    Locus Link:   29278    Chromosome:   10    Cytoband:   Chr:10q31    SOURCE    GENECARD       Gene Ontology:     BioCarta Pathways:     KEGG Pathways:  
    Probe set : 1372143_at    Name:   ubiquitin-conjugating enzyme E2 variant 2    GB acc:   AA849958    UniGene:   Rn.3933    Gene Symbol:   Ube2v2    Locus Link:   287927    Chromosome:   11    Cytoband:   Chr:11q23    SOURCE    GENECARD       Gene Ontology:   biological process:  sensory perception of smell   biological process:  protein modification   biological process:  ubiquitin cycle   cellular component:  integral to membrane   molecular function:  receptor activity   molecular function:  odorant binding   molecular function:  ubiquitin-like activating enzyme activity      BioCarta Pathways:     KEGG Pathways:  
    Probe set : 1368671_at    Name:   sushi-repeat-containing protein    GB acc:   NM_022524    UniGene:   Rn.1489    Gene Symbol:   Srpx    Locus Link:   64316    Chromosome:   X    Cytoband:   Chr:Xq13    SOURCE    GENECARD       Gene Ontology:   biological process:  cell adhesion   cellular component:  extracellular space   cellular component:  membrane   molecular function:  protein binding      BioCarta Pathways:     KEGG Pathways:  
    Probe set : 1381056_at    Name:   plakophilin 3 (predicted)    GB acc:   BI286045    UniGene:   Rn.144803    Gene Symbol:   Pkp3_predicted    Locus Link:   293619    Chromosome:   1    Cytoband:   Chr:1q41    SOURCE    GENECARD       Gene Ontology:   biological process:  cell adhesion   molecular function:  structural molecule activity   molecular function:  protein binding      BioCarta Pathways:     KEGG Pathways:  
    Probe set : 1383792_at    Name:   synaptotagmin-like 1 (predicted)    GB acc:   BG378274    UniGene:   Rn.16862    Gene Symbol:   Sytl1_predicted    Locus Link:   297872    Chromosome:   5    Cytoband:   Chr:5q36    SOURCE    GENECARD       Gene Ontology:     BioCarta Pathways:     KEGG Pathways:  
    Probe set : 1380320_at    Name:   similar to hypothetical protein (predicted)    GB acc:   AI176284    UniGene:   Rn.24111    Gene Symbol:   RGD1311361_predicted    Locus Link:   305171    Chromosome:   14    Cytoband:   Chr:14p22    SOURCE    GENECARD       Gene Ontology:     BioCarta Pathways:     KEGG Pathways:  
    Probe set : 1382144_at    Name:   mitochondrial ribosomal protein L47 (predicted)    GB acc:   AW520527    UniGene:   Rn.144828    Gene Symbol:   Mrpl47_predicted    Locus Link:   294963    Chromosome:   2    Cytoband:   Chr:2q25    SOURCE    GENECARD       Gene Ontology:   biological process:  protein biosynthesis   cellular component:  mitochondrion   cellular component:  mitochondrial ribosome   molecular function:  structural constituent of ribosome      BioCarta Pathways:     KEGG Pathways:  
    Probe set : 1397904_at    Name:   Similar to osteoclast inhibitory lectin    GB acc:   BI292042    UniGene:   Rn.95653     Locus Link:   362447    Chromosome:   4    Cytoband:   Chr:4q42    SOURCE        Gene Ontology:     BioCarta Pathways:     KEGG Pathways:  
    Probe set : 1371633_at    Name:   beta catenin-like 1    GB acc:   BI285669    UniGene:   Rn.98091    Gene Symbol:   Ctnnbl1    Locus Link:   296320    Chromosome:   3    Cytoband:   Chr:3q42    SOURCE    GENECARD       Gene Ontology:   molecular function:  binding      BioCarta Pathways:     KEGG Pathways:  
    Probe set : 1392489_at    Name:   Transcribed locus    GB acc:   BE108956    UniGene:   Rn.8458        SOURCE        Gene Ontology:     BioCarta Pathways:     KEGG Pathways:  
    Probe set : 1368200_at    Name:   chemokine (C-X3-C motif) ligand 1    GB acc:   NM_134455    UniGene:   Rn.107266    Gene Symbol:   Cx3cl1    Locus Link:   89808    Chromosome:   19    Cytoband:   Chr:19p12    SOURCE    GENECARD       Gene Ontology:     BioCarta Pathways:     KEGG Pathways:  
    Probe set : 1393563_at    Name:   interleukin 1 receptor accessory protein    GB acc:   BF391914    UniGene:   Rn.10511    Gene Symbol:   Il1rap    Locus Link:   25466    Chromosome:   11    Cytoband:   Chr:11q22    SOURCE    GENECARD       Gene Ontology:   biological process:  cytokine and chemokine mediated signaling pathway   biological process:  interleukin-2 biosynthesis   cellular component:  membrane   cellular component:  integral to membrane      BioCarta Pathways:     KEGG Pathways:  
    Probe set : 1367636_at    Name:   insulin-like growth factor 2 receptor    GB acc:   NM_012756    UniGene:   Rn.270    Gene Symbol:   Igf2r    Locus Link:   25151    Chromosome:   1    Cytoband:   Chr:1q11    SOURCE    GENECARD       Gene Ontology:     BioCarta Pathways:     KEGG Pathways:  
    Probe set : 1392503_at    Name:   RNA binding motif protein 22 (predicted)    GB acc:   BE100371    UniGene:   Rn.105130    Gene Symbol:   Rbm22_predicted    Locus Link:   307410    Chromosome:   18    Cytoband:   Chr:18q12.1    SOURCE    GENECARD       Gene Ontology:   biological process:  nuclear mRNA splicing, via spliceosome   biological process:  mRNA processing   biological process:  cell cycle   cellular component:  ribonucleoprotein complex   cellular component:  spliceosome complex      BioCarta Pathways:     KEGG Pathways:  
    Probe set : 1398811_at    Name:   jumping translocation breakpoint    GB acc:   NM_019213    UniGene:   Rn.91803    Gene Symbol:   Jtb    Locus Link:   29439    Chromosome:   2    Cytoband:   Chr:2q34    SOURCE    GENECARD       Gene Ontology:   cellular component:  membrane fraction   cellular component:  integral to plasma membrane   cellular component:  integral to membrane      BioCarta Pathways:     KEGG Pathways:  
    Probe set : 1376519_at    Name:   brain protein 16    GB acc:   BI280846    UniGene:   Rn.17102    Gene Symbol:   RGD:1359226    Locus Link:   315094    Chromosome:   7    Cytoband:   Chr:7q34    SOURCE    GENECARD       Gene Ontology:   molecular function:  binding      BioCarta Pathways:     KEGG Pathways:  
    Probe set : 1373165_at    Name:   Similar to zinc finger, matrin type 1    GB acc:   BF404539    UniGene:   Rn.8351     Locus Link:   308654    Chromosome:   X    Cytoband:   Chr:Xq34-q35    SOURCE        Gene Ontology:     BioCarta Pathways:     KEGG Pathways:  
    Probe set : 1382443_at    Name:   similar to Pabpc4_predicted protein /// poly A binding protein, cytoplasmic 4 (predicted)    GB acc:   BG376950    UniGene:   Rn.2995    Gene Symbol:   LOC288398 /// Pabpc4_predicted    Locus Link:   288398 /// 298510    Chromosome:   12    Cytoband:   Chr:12p12 /// Chr:5q36    SOURCE    GENECARD       Gene Ontology:   cellular component:  cytoplasm      BioCarta Pathways:     KEGG Pathways:  
    Probe set : 1383926_at    Name:   budding uninhibited by benzimidazoles 1 homolog, beta (S. cerevisiae)    GB acc:   BF557145    UniGene:   Rn.49845    Gene Symbol:   Bub1b    Locus Link:   171576    Chromosome:   3    Cytoband:   Chr:3q35    SOURCE    GENECARD       Gene Ontology:   molecular function:  kinase activity      BioCarta Pathways:     KEGG Pathways:  
    Probe set : 1370203_at    Name:   FGF receptor activating protein 1    GB acc:   AI408972    UniGene:   Rn.94200    Gene Symbol:   Frag1    Locus Link:   116675    Chromosome:   1    Cytoband:   Chr:1q32    SOURCE    GENECARD       Gene Ontology:   cellular component:  integral to membrane   molecular function:  receptor activity      BioCarta Pathways:     KEGG Pathways:  
    Probe set : 1379352_at    Name:   phosphatidylinositol-4-phosphate 5-kinase, type 1 alpha (predicted)    GB acc:   BF406008    UniGene:   Rn.146140    Gene Symbol:   Pip5k1a_predicted    Locus Link:   309419    Chromosome:   1    Cytoband:   Chr:1q51    SOURCE    GENECARD       Gene Ontology:   biological process:  phosphorylation   biological process:  phosphatidylinositol metabolism   cellular component:  membrane   molecular function:  kinase activity   molecular function:  1-phosphatidylinositol-4-phosphate 5-kinase activity   molecular function:  transferase activity      BioCarta Pathways:     KEGG Pathways:  
    Probe set : 1367904_at    Name:   regulated endocrine-specific protein 18    GB acc:   NM_019278    UniGene:   Rn.66430    Gene Symbol:   Resp18    Locus Link:   50561    Chromosome:   9    Cytoband:   Chr:9q33    SOURCE    GENECARD       Gene Ontology:     BioCarta Pathways:     KEGG Pathways:  
    Probe set : 1376907_at    Name:   Microtubule-associated protein 6    GB acc:   AW521452    UniGene:   Rn.37490    Gene Symbol:   Mtap6    Locus Link:   29457    Chromosome:   1    Cytoband:   Chr:1q32    SOURCE    GENECARD       Gene Ontology:   biological process:  microtubule-based process   cellular component:  microtubule associated complex   molecular function:  structural molecule activity   molecular function:  cytoskeletal regulatory protein binding      BioCarta Pathways:     KEGG Pathways:  
    Probe set : 1373096_at    Name:   ARP8 actin-related protein 8 homolog (S. cerevisiae) (predicted)    GB acc:   AI407406    UniGene:   Rn.14756    Gene Symbol:   Actr8_predicted    Locus Link:   361107    Chromosome:   16    Cytoband:   Chr:16p16    SOURCE    GENECARD       Gene Ontology:   cellular component:  cytoskeleton   cellular component:  actin cytoskeleton   molecular function:  structural molecule activity   molecular function:  structural constituent of cytoskeleton      BioCarta Pathways:     KEGG Pathways:  
    Probe set : 1383221_at    Name:   hypothetical LOC360478    GB acc:   BG378463    UniGene:   Rn.137175    Gene Symbol:   LOC360478    Locus Link:   360478    Chromosome:   10    Cytoband:   Chr:10q12    SOURCE    GENECARD       Gene Ontology:     BioCarta Pathways:     KEGG Pathways:  
    Probe set : 1374351_at     GB acc:   AI044117              Gene Ontology:     BioCarta Pathways:     KEGG Pathways:  
    Probe set : 1370085_at    Name:   RAS p21 protein activator 1    GB acc:   NM_013135    UniGene:   Rn.12223    Gene Symbol:   Rasa1    Locus Link:   25676    Chromosome:   2    Cytoband:   Chr:2q11    SOURCE    GENECARD       Gene Ontology:   biological process:  intracellular signaling cascade   biological process:  cellular physiological process   molecular function:  GTPase activator activity   molecular function:  Ras GTPase activator activity      BioCarta Pathways:     KEGG Pathways:  
    Probe set : 1393638_at    Name:   Prostaglandin E receptor 4 (subtype EP4)    GB acc:   AI549199    UniGene:   Rn.16062    Gene Symbol:   Ptger4    Locus Link:   84023    Chromosome:   2    Cytoband:   Chr:2q16    SOURCE    GENECARD       Gene Ontology:   cellular component:  integral to membrane      BioCarta Pathways:     KEGG Pathways:  
    Probe set : 1392725_at    Name:   similar to cDNA sequence BC006662 (predicted)    GB acc:   AA924588    UniGene:   Rn.17263    Gene Symbol:   RGD1304748_predicted    Locus Link:   290500    Chromosome:   15    Cytoband:   Chr:15q25    SOURCE    GENECARD       Gene Ontology:     BioCarta Pathways:     KEGG Pathways:  
    Probe set : 1396086_at    Name:   similar to RIKEN cDNA 1810014L12 (predicted)    GB acc:   H31931    UniGene:   Rn.20057    Gene Symbol:   RGD1307423_predicted    Locus Link:   303330    Chromosome:   10    Cytoband:   Chr:10q25    SOURCE    GENECARD       Gene Ontology:   cellular component:  extracellular space   cellular component:  integral to membrane      BioCarta Pathways:     KEGG Pathways:  
    Probe set : 1383573_at    Name:   Serologically defined colon cancer antigen 33 (predicted)    GB acc:   BE117444    UniGene:   Rn.23119     Locus Link:   307217    Chromosome:   18    Cytoband:   Chr:18q12.3    SOURCE        Gene Ontology:   biological process:  regulation of transcription, DNA-dependent   cellular component:  nucleus   molecular function:  DNA binding   molecular function:  transcription factor activity   molecular function:  zinc ion binding      BioCarta Pathways:     KEGG Pathways:  
    Probe set : 1372684_at    Name:   smoothelin (predicted)    GB acc:   AW435036    UniGene:   Rn.40766    Gene Symbol:   Smtn_predicted    Locus Link:   289734    Chromosome:   14    Cytoband:   Chr:14q21    SOURCE    GENECARD       Gene Ontology:   biological process:  smooth muscle contraction   biological process:  muscle development   cellular component:  actin cytoskeleton   molecular function:  actin binding   molecular function:  structural constituent of muscle      BioCarta Pathways:     KEGG Pathways:  
    Probe set : 1385702_at    Name:   myeloid cell nuclear differentiation antigen (predicted)    GB acc:   AA901151    UniGene:   Rn.15194    Gene Symbol:   Mnda_predicted    Locus Link:   304988    Chromosome:   13    Cytoband:   Chr:13q24    SOURCE    GENECARD       Gene Ontology:   biological process:  regulation of transcription, DNA-dependent   biological process:  cellular defense response   cellular component:  nucleus   molecular function:  DNA binding      BioCarta Pathways:     KEGG Pathways:  
    Probe set : 1397913_at    Name:   Transcribed locus    GB acc:   AW526277    UniGene:   Rn.17276        SOURCE        Gene Ontology:     BioCarta Pathways:     KEGG Pathways:  
    Probe set : 1399039_at    Name:   Similar to Dnajb10 protein    GB acc:   BM383865    UniGene:   Rn.145719     Locus Link:   501165      SOURCE        Gene Ontology:     BioCarta Pathways:     KEGG Pathways:  
    Probe set : 1375869_at    Name:   Unc-51 like kinase 1 (C. elegans)    GB acc:   AI603439    UniGene:   Rn.24509    Gene Symbol:   Ulk1    Locus Link:   360827    Chromosome:   12    Cytoband:   Chr:12q16    SOURCE    GENECARD       Gene Ontology:     BioCarta Pathways:     KEGG Pathways:  
    Probe set : 1371516_at    Name:   exportin 7 (predicted)    GB acc:   BM391834    UniGene:   Rn.32535    Gene Symbol:   Xpo7_predicted    Locus Link:   361070    Chromosome:   15    Cytoband:   Chr:15p11    SOURCE    GENECARD       Gene Ontology:   cellular component:  nucleus   cellular component:  nuclear pore   cellular component:  cytoplasm      BioCarta Pathways:     KEGG Pathways:  
    Probe set : 1373530_at    Name:   cyclin E    GB acc:   AW913890    UniGene:   Rn.15455    Gene Symbol:   Ccne1    Locus Link:   25729    Chromosome:   1    Cytoband:   Chr:1q22    SOURCE    GENECARD       Gene Ontology:   cellular component:  cyclin-dependent protein kinase holoenzyme complex   cellular component:  nucleus   molecular function:  protein binding   molecular function:  kinase activity   molecular function:  cyclin-dependent protein kinase regulator activity      BioCarta Pathways:     KEGG Pathways:  
    Probe set : 1371773_at    Name:   Transcribed locus    GB acc:   AI179413    UniGene:   Rn.22153        SOURCE        Gene Ontology:     BioCarta Pathways:     KEGG Pathways:  
    Probe set : 1387157_at    Name:   polyamine modulated factor 1 binding protein 1    GB acc:   NM_134393    UniGene:   Rn.51502    Gene Symbol:   Pmfbp1    Locus Link:   171414    Chromosome:   19    Cytoband:   Chr:19q12    SOURCE    GENECARD       Gene Ontology:   biological process:  cytoskeleton organization and biogenesis   cellular component:  outer dense fiber   cellular component:  cytoplasm      BioCarta Pathways:     KEGG Pathways:  
    Probe set : 1382258_at    Name:   Rho-guanine nucleotide exchange factor (predicted)    GB acc:   AW521384    UniGene:   Rn.33912    Gene Symbol:   Rgnef_predicted    Locus Link:   361882    Chromosome:   2    Cytoband:   Chr:2q12    SOURCE    GENECARD       Gene Ontology:   biological process:  intracellular signaling cascade   biological process:  visual perception   molecular function:  guanyl-nucleotide exchange factor activity      BioCarta Pathways:     KEGG Pathways:  
    Probe set : 1373607_at    Name:   Transcribed locus    GB acc:   BF418530    UniGene:   Rn.33467        SOURCE        Gene Ontology:     BioCarta Pathways:     KEGG Pathways:  
    Probe set : 1370671_at    Name:   guanylate cyclase 2g    GB acc:   U33847    UniGene:   Rn.10198    Gene Symbol:   Gucy2g    Locus Link:   245708    Chromosome:   1    Cytoband:   Chr:1q55    SOURCE    GENECARD       Gene Ontology:   cellular component:  membrane   cellular component:  integral to membrane      BioCarta Pathways:     KEGG Pathways:  
    Probe set : 1388480_at    Name:   similar to Glycolipid transfer protein    GB acc:   AI412863    UniGene:   Rn.4111    Gene Symbol:   LOC288707    Locus Link:   288707    Chromosome:   12    Cytoband:   Chr:12q16    SOURCE    GENECARD       Gene Ontology:     BioCarta Pathways:     KEGG Pathways:  
    Probe set : 1387135_at    Name:   a disintegrin and metalloproteinase domain 15 (metargidin)    GB acc:   NM_020308    UniGene:   Rn.98788    Gene Symbol:   Adam15    Locus Link:   57025    Chromosome:   2    Cytoband:   Chr:2q34    SOURCE    GENECARD       Gene Ontology:     BioCarta Pathways:     KEGG Pathways:  
    Probe set : 1383576_at    Name:   Protein phosphatase 1, regulatory (inhibitor) subunit 12A    GB acc:   AW917643    UniGene:   Rn.51134    Gene Symbol:   Ppp1r12a    Locus Link:   116670    Chromosome:   7    Cytoband:   Chr:7q21    SOURCE    GENECARD       Gene Ontology:   molecular function:  phosphoprotein phosphatase activity      BioCarta Pathways:     KEGG Pathways:  
    Probe set : 1394850_at     GB acc:   AI232392              Gene Ontology:     BioCarta Pathways:     KEGG Pathways:  
    Probe set : 1378945_at    Name:   Aconitase 2, mitochondrial    GB acc:   AI638990    UniGene:   Rn.43737    Gene Symbol:   Aco2    Locus Link:   79250    Chromosome:   1    Cytoband:   Chr:1q13 or 20q11    SOURCE    GENECARD       Gene Ontology:   cellular component:  mitochondrion      BioCarta Pathways:     KEGG Pathways:  
    Probe set : 1381977_at    Name:   Transcribed locus    GB acc:   BI299008    UniGene:   Rn.146248        SOURCE        Gene Ontology:     BioCarta Pathways:     KEGG Pathways:  
    Probe set : 1373867_at    Name:   Transcribed locus    GB acc:   BI289360    UniGene:   Rn.105803        SOURCE        Gene Ontology:     BioCarta Pathways:     KEGG Pathways:  
    Probe set : 1396309_at    Name:   Similar to RIKEN cDNA 2810453K03 (predicted)    GB acc:   BI292839    UniGene:   Rn.46324     Locus Link:   289657    Chromosome:   14    Cytoband:   Chr:14q11    SOURCE        Gene Ontology:     BioCarta Pathways:     KEGG Pathways:  
    Probe set : 1370792_at    Name:   microtubule-associated protein, RP/EB family, member 1    GB acc:   U75920    UniGene:   Rn.7652    Gene Symbol:   Mapre1    Locus Link:   114764    Chromosome:   3    Cytoband:   Chr:3q41    SOURCE    GENECARD       Gene Ontology:   biological process:  cell cycle   biological process:  mitosis   biological process:  cell division   cellular component:  microtubule   molecular function:  microtubule binding      BioCarta Pathways:     KEGG Pathways:  
    Probe set : 1374212_at    Name:   Transcribed locus    GB acc:   AI012753    UniGene:   Rn.14344        SOURCE        Gene Ontology:     BioCarta Pathways:     KEGG Pathways:  
    Probe set : 1379536_at    Name:   Transcribed locus    GB acc:   BF547014    UniGene:   Rn.21422        SOURCE        Gene Ontology:     BioCarta Pathways:     KEGG Pathways:  
    Probe set : 1378763_at    Name:   hypothetical gene supported by BC088439    GB acc:   BF419627    UniGene:   Rn.64447    Gene Symbol:   LOC500947    Locus Link:   500947    Chromosome:   8    Cytoband:   Chr:8q12    SOURCE    GENECARD       Gene Ontology:     BioCarta Pathways:     KEGG Pathways:  
    Probe set : 1372827_at    Name:   peptidylprolyl isomerase D    GB acc:   AA957342    UniGene:   Rn.136915    Gene Symbol:   RGD:1303174    Locus Link:   361967    Chromosome:   2    Cytoband:   Chr:2q32    SOURCE    GENECARD       Gene Ontology:   biological process:  protein folding      BioCarta Pathways:     KEGG Pathways:  
    Probe set : 1376320_at    Name:   Transcribed locus    GB acc:   BF393051    UniGene:   Rn.8449        SOURCE        Gene Ontology:     BioCarta Pathways:     KEGG Pathways:  
    Probe set : 1384770_at    Name:   Transcribed locus    GB acc:   AI112057    UniGene:   Rn.147581        SOURCE        Gene Ontology:     BioCarta Pathways:     KEGG Pathways:  
    Probe set : 1383228_at     GB acc:   AA875288              Gene Ontology:     BioCarta Pathways:     KEGG Pathways:  
    Probe set : 1385103_at    Name:   fused toes (predicted)    GB acc:   BF390999    UniGene:   Rn.136882    Gene Symbol:   Fts_predicted    Locus Link:   291906    Chromosome:   19    Cytoband:   Chr:19p11    SOURCE    GENECARD       Gene Ontology:   biological process:  ubiquitin cycle   biological process:  apoptosis   biological process:  protein modification   cellular component:  membrane   molecular function:  ubiquitin conjugating enzyme activity   molecular function:  ubiquitin-like activating enzyme activity      BioCarta Pathways:     KEGG Pathways:  
    Probe set : 1382137_at    Name:   abhydrolase domain containing 3 (predicted)    GB acc:   BG662490    UniGene:   Rn.64445    Gene Symbol:   Abhd3_predicted    Locus Link:   291793    Chromosome:   18    Cytoband:   Chr:18p13    SOURCE    GENECARD       Gene Ontology:   cellular component:  integral to membrane   molecular function:  catalytic activity   molecular function:  hydrolase activity      BioCarta Pathways:     KEGG Pathways:  
    Probe set : 1371041_at    Name:   NADH dehydrogenase (ubiquinone) flavoprotein 2    GB acc:   M22756    UniGene:   Rn.28882    Gene Symbol:   Ndufv2    Locus Link:   81728    Chromosome:   9    Cytoband:   Chr:9q37    SOURCE    GENECARD       Gene Ontology:   biological process:  mitochondrial electron transport, NADH to ubiquinone   biological process:  nervous system development   cellular component:  membrane fraction   cellular component:  mitochondrion      BioCarta Pathways:     KEGG Pathways:  
    Probe set : 1382021_at    Name:   Similar to polycystic kidney disease 2    GB acc:   AA850650    UniGene:   Rn.123828     Locus Link:   498328      SOURCE        Gene Ontology:     BioCarta Pathways:     KEGG Pathways:  
    Probe set : 1369943_at    Name:   transglutaminase 2, C polypeptide    GB acc:   NM_019386    UniGene:   Rn.10    Gene Symbol:   Tgm2    Locus Link:   56083    Chromosome:   3    Cytoband:   Chr:3q42    SOURCE    GENECARD       Gene Ontology:     BioCarta Pathways:     KEGG Pathways:  
    Probe set : 1372995_at    Name:   similar to protein kinase D2 (predicted)    GB acc:   AI703715    UniGene:   Rn.15035    Gene Symbol:   RGD1308054_predicted    Locus Link:   292658    Chromosome:   1    Cytoband:   Chr:1q21    SOURCE    GENECARD       Gene Ontology:   biological process:  protein amino acid phosphorylation   biological process:  intracellular signaling cascade      BioCarta Pathways:     KEGG Pathways:  
    Probe set : 1391981_at    Name:   Transcribed locus    GB acc:   AI639181    UniGene:   Rn.131203        SOURCE        Gene Ontology:     BioCarta Pathways:     KEGG Pathways:  
    Probe set : 1388623_at    Name:   EH-domain containing 1 (predicted)    GB acc:   BI277003    UniGene:   Rn.8141     Locus Link:   293692    Chromosome:   1    Cytoband:   Chr:1q43    SOURCE        Gene Ontology:   biological process:  endosome transport   cellular component:  early endosome   cellular component:  endocytic vesicle      BioCarta Pathways:     KEGG Pathways:  
    Probe set : 1376337_at    Name:   SWI/SNF related, matrix associated, actin dependent regulator of chromatin, subfamily a, member 2    GB acc:   AI170385    UniGene:   Rn.94939    Gene Symbol:   RGD:1302988    Locus Link:   361745    Chromosome:   1    Cytoband:   Chr:1q51    SOURCE    GENECARD       Gene Ontology:   biological process:  regulation of cell proliferation      BioCarta Pathways:     KEGG Pathways:  
    Probe set : 1367857_at    Name:   fatty acid desaturase 1    GB acc:   NM_053445    UniGene:   Rn.28161    Gene Symbol:   Fads1    Locus Link:   84575    Chromosome:   1    Cytoband:   Chr:1q43    SOURCE    GENECARD       Gene Ontology:   cellular component:  membrane      BioCarta Pathways:     KEGG Pathways:  
    Probe set : 1368700_at    Name:   phospholipase C-like 1    GB acc:   NM_053456    UniGene:   Rn.10684    Gene Symbol:   Plcl1    Locus Link:   84587    Chromosome:   9    Cytoband:   Chr:9q31    SOURCE    GENECARD       Gene Ontology:   biological process:  lipid metabolism   biological process:  intracellular signaling cascade   biological process:  signal transduction      BioCarta Pathways:     KEGG Pathways:  
    Probe set : 1367481_at    Name:   vacuolar protein sorting 28 (yeast) (predicted)    GB acc:   BI278301    UniGene:   Rn.36912    Gene Symbol:   Vps28_predicted    Locus Link:   300052    Chromosome:   7    Cytoband:   Chr:7q34    SOURCE    GENECARD       Gene Ontology:   biological process:  protein transport      BioCarta Pathways:     KEGG Pathways:  
    Probe set : 1375336_at    Name:   heat shock 90kDa protein 1, beta    GB acc:   AI237389    UniGene:   Rn.98667    Gene Symbol:   RGD:1303075    Locus Link:   301252    Chromosome:   9    Cytoband:   Chr:9q12    SOURCE    GENECARD       Gene Ontology:   biological process:  protein folding   biological process:  response to unfolded protein   molecular function:  nucleotide binding   molecular function:  ATP binding   molecular function:  unfolded protein binding      BioCarta Pathways:     KEGG Pathways:  
    Probe set : 1367754_s_at    Name:   argininosuccinate lyase /// heterogeneous nuclear ribonucleoprotein A/B    GB acc:   NM_031330    UniGene:   Rn.64591    Gene Symbol:   Asl /// Hnrpab    Locus Link:   59085 /// 83498    Chromosome:   12    Cytoband:   Chr:12q13 /// Chr:10q22    SOURCE    GENECARD       Gene Ontology:     BioCarta Pathways:     KEGG Pathways:  
    Probe set : 1383486_at    Name:   Transcribed locus    GB acc:   AA926109    UniGene:   Rn.35842        SOURCE        Gene Ontology:     BioCarta Pathways:     KEGG Pathways:  
    Probe set : 1368039_at    Name:   synaptojanin 2 binding protein    GB acc:   NM_022599    UniGene:   Rn.6258    Gene Symbol:   Synj2bp    Locus Link:   64531    Chromosome:   6    Cytoband:   Chr:6q24    SOURCE    GENECARD       Gene Ontology:   molecular function:  receptor activity   molecular function:  protein binding      BioCarta Pathways:     KEGG Pathways:  
    Probe set : 1368508_at    Name:   proteasome (prosome, macropain) subunit, alpha type 3    GB acc:   BI294912    UniGene:   Rn.3997    Gene Symbol:   Psma3    Locus Link:   29670    Chromosome:   6    Cytoband:   Chr:6q24    SOURCE    GENECARD       Gene Ontology:   biological process:  ubiquitin-dependent protein catabolism   cellular component:  cytosol   cellular component:  proteasome core complex (sensu Eukaryota)   cellular component:  protein complex      BioCarta Pathways:     KEGG Pathways:  
    Probe set : 1383536_at    Name:   Transcribed locus    GB acc:   AI712541    UniGene:   Rn.24681        SOURCE        Gene Ontology:     BioCarta Pathways:     KEGG Pathways:  
    Probe set : 1374482_at     GB acc:   AW142993              Gene Ontology:     BioCarta Pathways:     KEGG Pathways:  
    Probe set : 1372762_at    Name:   similar to RIKEN cDNA 1110033O09 (predicted)    GB acc:   AA849729    UniGene:   Rn.17508    Gene Symbol:   RGD1311839_predicted    Locus Link:   308758    Chromosome:   1    Cytoband:   Chr:1q31    SOURCE    GENECARD       Gene Ontology:     BioCarta Pathways:     KEGG Pathways:  
    Probe set : 1388541_at    Name:   Similar to Hypothetical protein KIAA0152 (predicted)    GB acc:   BE116383    UniGene:   Rn.12649     Locus Link:   304543    Chromosome:   12    Cytoband:   Chr:12q16    SOURCE        Gene Ontology:   biological process:  protein targeting   cellular component:  integral to membrane      BioCarta Pathways:     KEGG Pathways:  
    Probe set : 1374434_at    Name:   similar to specifically androgen-regulated protein    GB acc:   BI291703    UniGene:   Rn.28408    Gene Symbol:   LOC498222    Locus Link:   498222    Chromosome:   13    Cytoband:   Chr:13q13    SOURCE    GENECARD       Gene Ontology:     BioCarta Pathways:     KEGG Pathways:  
    Probe set : 1387072_at    Name:   protein kinase, lysine deficient 1    GB acc:   NM_053794    UniGene:   Rn.27409    Gene Symbol:   Prkwnk1    Locus Link:   116477    Chromosome:   4    Cytoband:   Chr:4q42    SOURCE    GENECARD       Gene Ontology:   cellular component:  cytoplasm   cellular component:  cytoplasm      BioCarta Pathways:     KEGG Pathways:  
    Probe set : 1388895_at     GB acc:   AI175791              Gene Ontology:     BioCarta Pathways:     KEGG Pathways:  
    Probe set : 1377051_at    Name:   Mpv17 transgene, kidney disease mutant-like (predicted)    GB acc:   AA849966    UniGene:   Rn.22842    Gene Symbol:   Mpv17l_predicted    Locus Link:   360463    Chromosome:   10    Cytoband:   Chr:10q11    SOURCE    GENECARD       Gene Ontology:     BioCarta Pathways:     KEGG Pathways:  
    Probe set : 1368546_at    Name:   human immunodeficiency virus type I enhancer binding protein 2    GB acc:   NM_024137    UniGene:   Rn.9802    Gene Symbol:   Hivep2    Locus Link:   29721    Chromosome:   1    Cytoband:   Chr:1p13    SOURCE    GENECARD       Gene Ontology:   biological process:  transcription   biological process:  regulation of transcription, DNA-dependent   biological process:  signal transduction   cellular component:  nucleus   cellular component:  transcription factor complex      BioCarta Pathways:     KEGG Pathways:  
    Probe set : 1392810_at     GB acc:   AI178143              Gene Ontology:     BioCarta Pathways:     KEGG Pathways:  
    Probe set : 1368859_at    Name:   protein phosphatase 1A, magnesium dependent, alpha isoform    GB acc:   NM_017038    UniGene:   Rn.37403    Gene Symbol:   Ppm1a    Locus Link:   24666    Chromosome:   6    Cytoband:   Chr:6q24    SOURCE    GENECARD       Gene Ontology:   biological process:  protein amino acid dephosphorylation   biological process:  positive regulation of I-kappaB kinase/NF-kappaB cascade   cellular component:  protein serine/threonine phosphatase complex   molecular function:  protein phosphatase type 2A activity   molecular function:  protein phosphatase type 1 activity   molecular function:  magnesium ion binding   molecular function:  catalytic activity   molecular function:  phosphoprotein phosphatase activity   molecular function:  protein serine/threonine phosphatase activity   molecular function:  calcium-dependent protein serine/threonine phosphatase activity   molecular function:  magnesium-dependent protein serine/threonine phosphatase activity   molecular function:  signal transducer activity   molecular function:  CTD phosphatase activity   molecular function:  protein phosphatase type 2C activity   molecular function:  hydrolase activity   molecular function:  myosin phosphatase activity   molecular function:  manganese ion binding   molecular function:  protein phosphatase type 2B activity   molecular function:  metal ion binding      BioCarta Pathways:     KEGG Pathways:  
    Probe set : 1385728_at    Name:   AE binding protein 2 (predicted)    GB acc:   BE108006    UniGene:   Rn.1477     Locus Link:   297705    Chromosome:   4    Cytoband:   Chr:4q44    SOURCE        Gene Ontology:   biological process:  regulation of transcription, DNA-dependent   cellular component:  nucleus      BioCarta Pathways:     KEGG Pathways:  
    Probe set : 1388945_at    Name:   similar to 1300014I06Rik protein (predicted)    GB acc:   BM385779    UniGene:   Rn.6535    Gene Symbol:   RGD1311307_predicted    Locus Link:   361238    Chromosome:   17    Cytoband:   Chr:17p12    SOURCE    GENECARD       Gene Ontology:   biological process:  intracellular signaling cascade      BioCarta Pathways:     KEGG Pathways:  
    Probe set : 1370896_a_at    Name:   myosin heavy chain 11    GB acc:   X16262    UniGene:   Rn.94969    Gene Symbol:   Myh11    Locus Link:   24582    Chromosome:   10    Cytoband:   Chr:10q11-q12    SOURCE    GENECARD       Gene Ontology:     BioCarta Pathways:     KEGG Pathways:  
    Probe set : 1392532_at    Name:   Kinesin heavy chain member 2    GB acc:   AI145043    UniGene:   Rn.17829    Gene Symbol:   Kif2    Locus Link:   84391    Chromosome:   2    Cytoband:   Chr:2q12    SOURCE    GENECARD       Gene Ontology:   biological process:  microtubule-based process   biological process:  microtubule-based movement   cellular component:  cytoskeleton   cellular component:  kinesin complex   cellular component:  microtubule associated complex   molecular function:  motor activity   molecular function:  microtubule motor activity   molecular function:  ATP binding      BioCarta Pathways:     KEGG Pathways:  
    Probe set : 1390890_at    Name:   Transcribed locus    GB acc:   AI406854    UniGene:   Rn.13163        SOURCE        Gene Ontology:     BioCarta Pathways:     KEGG Pathways:  
    Probe set : 1387896_at    Name:   sterol carrier protein 2    GB acc:   M62763    UniGene:   Rn.31887    Gene Symbol:   Scp2    Locus Link:   25541    Chromosome:   5    Cytoband:   Chr:5q35    SOURCE    GENECARD       Gene Ontology:   cellular component:  mitochondrion   cellular component:  peroxisome   molecular function:  sterol carrier protein X-related thiolase activity   molecular function:  sterol carrier activity   molecular function:  lipid binding      BioCarta Pathways:     KEGG Pathways:  
    Probe set : 1369450_at    Name:   integral membrane transport protein UST5r    GB acc:   NM_134380    UniGene:   Rn.81214    Gene Symbol:   UST5r    Locus Link:   171398    Chromosome:   1    Cytoband:   Chr:1q43    SOURCE    GENECARD       Gene Ontology:   biological process:  transport   cellular component:  integral to membrane   molecular function:  transporter activity      BioCarta Pathways:     KEGG Pathways:  
    Probe set : 1373147_at    Name:   F-box and leucine-rich repeat protein 3a (predicted)    GB acc:   AI145387    UniGene:   Rn.36356    Gene Symbol:   Fbxl3a_predicted    Locus Link:   306129    Chromosome:   15    Cytoband:   Chr:15q22    SOURCE    GENECARD       Gene Ontology:     BioCarta Pathways:     KEGG Pathways:  
    Probe set : 1367753_at    Name:   SEC31-like 1 (S. cerevisiae)    GB acc:   NM_033021    UniGene:   Rn.6322    Gene Symbol:   Sec31l1    Locus Link:   93646    Chromosome:   14    Cytoband:   Chr:14p22    SOURCE    GENECARD       Gene Ontology:   cellular component:  endosome      BioCarta Pathways:     KEGG Pathways:  
    Probe set : 1379550_a_at    Name:   general transcription factor II I repeat domain-containing 1    GB acc:   BF290483    UniGene:   Rn.84872    Gene Symbol:   Gtf2ird1    Locus Link:   246770    Chromosome:   12    Cytoband:   Chr:12q12    SOURCE    GENECARD       Gene Ontology:   biological process:  regulation of transcription, DNA-dependent   biological process:  development   cellular component:  nucleus      BioCarta Pathways:     KEGG Pathways:  
    Probe set : 1393128_at    Name:   similar to RIKEN cDNA 2610029K21 (predicted)    GB acc:   BI288424    UniGene:   Rn.20166    Gene Symbol:   RGD1311086_predicted    Locus Link:   295228    Chromosome:   2    Cytoband:   Chr:2q34    SOURCE    GENECARD       Gene Ontology:   cellular component:  intracellular   molecular function:  nucleic acid binding      BioCarta Pathways:     KEGG Pathways:  
    Probe set : 1393079_at    Name:   similar to sialic-acid O-acetylesterase (predicted)    GB acc:   AI111605    UniGene:   Rn.21775    Gene Symbol:   RGD1310431_predicted    Locus Link:   363045    Chromosome:   8    Cytoband:   Chr:8q21    SOURCE    GENECARD       Gene Ontology:   cellular component:  extracellular space   cellular component:  lysosome   molecular function:  serine esterase activity   molecular function:  hydrolase activity      BioCarta Pathways:     KEGG Pathways:  
    Probe set : 1385561_at    Name:   similar to peroxisome biogenesis factor 1    GB acc:   BF561031    UniGene:   Rn.39842    Gene Symbol:   LOC500006    Locus Link:   500006    Chromosome:   4    Cytoband:   Chr:4q13    SOURCE    GENECARD       Gene Ontology:     BioCarta Pathways:     KEGG Pathways:  
    Probe set : 1378389_at    Name:   Similar to nuclear factor of activated T-cells, cytoplasmic, calcineurin-dependent 1    GB acc:   BM385157    UniGene:   Rn.8800     Locus Link:   307231    Chromosome:   18    Cytoband:   Chr:18q12.3    SOURCE        Gene Ontology:     BioCarta Pathways:     KEGG Pathways:  
    Probe set : 1367778_at    Name:   hypothetical gene supported by NM_019331    GB acc:   NM_019331     Gene Symbol:   LOC497733    Locus Link:   497733    Chromosome:   1    Cytoband:   Chr:1q31     GENECARD       Gene Ontology:     BioCarta Pathways:     KEGG Pathways:  
    Probe set : 1377198_at    Name:   Src family associated phosphoprotein 2    GB acc:   AA891732    UniGene:   Rn.12934    Gene Symbol:   Scap2    Locus Link:   155183    Chromosome:   4    Cytoband:   Chr:4q24    SOURCE    GENECARD       Gene Ontology:   biological process:  negative regulation of cell proliferation   cellular component:  cytoplasm   cellular component:  plasma membrane      BioCarta Pathways:     KEGG Pathways:  
    Probe set : 1371564_at    Name:   ATPase, H+ transporting, V1 subunit E isoform 1    GB acc:   AI169159    UniGene:   Rn.103171    Gene Symbol:   Atp6v1e1    Locus Link:   297566    Chromosome:   4    Cytoband:   Chr:4q42    SOURCE    GENECARD       Gene Ontology:   biological process:  ATP synthesis coupled proton transport   cellular component:  proton-transporting two-sector ATPase complex   molecular function:  hydrogen-transporting ATP synthase activity, rotational mechanism   molecular function:  hydrogen-transporting ATPase activity, rotational mechanism      BioCarta Pathways:     KEGG Pathways:  
    Probe set : 1386543_at    Name:   similar to F-box and leucine-rich repeat protein 8    GB acc:   BF559650    UniGene:   Rn.11668    Gene Symbol:   LOC498941    Locus Link:   498941    Chromosome:   19    Cytoband:   Chr:19q11    SOURCE    GENECARD       Gene Ontology:     BioCarta Pathways:     KEGG Pathways:  
    Probe set : 1373980_at    Name:   similar to XPA binding protein 1    GB acc:   AW916138    UniGene:   Rn.16253    Gene Symbol:   LOC500619    Locus Link:   500619    Chromosome:   6    Cytoband:   Chr:6q13    SOURCE    GENECARD       Gene Ontology:     BioCarta Pathways:     KEGG Pathways:  
    Probe set : 1392858_at    Name:   nuclear cap binding protein subunit 2 (predicted)    GB acc:   BM387770    UniGene:   Rn.63881    Gene Symbol:   Ncbp2_predicted    Locus Link:   288040    Chromosome:   11    Cytoband:   Chr:11q22    SOURCE    GENECARD       Gene Ontology:   cellular component:  nucleus   molecular function:  RNA cap binding   molecular function:  nucleic acid binding   molecular function:  RNA binding      BioCarta Pathways:     KEGG Pathways:  
    Probe set : 1369285_at    Name:   protein geranylgeranyltransferase type I, beta subunit    GB acc:   NM_031082    UniGene:   Rn.10031    Gene Symbol:   Pggt1b    Locus Link:   81746    Chromosome:   18    Cytoband:   Chr:18q11    SOURCE    GENECARD       Gene Ontology:   biological process:  protein amino acid geranylgeranylation   biological process:  protein amino acid geranylgeranylation   cellular component:  CAAX-protein geranylgeranyltransferase complex   cellular component:  CAAX-protein geranylgeranyltransferase complex      BioCarta Pathways:     KEGG Pathways:  
    Probe set : 1370577_at    Name:   zinc finger protein 455    GB acc:   U67083    UniGene:   Rn.10664    Gene Symbol:   Zfp455    Locus Link:   286979    Chromosome:   2    Cytoband:   Chr:2q23    SOURCE    GENECARD       Gene Ontology:   biological process:  regulation of transcription, DNA-dependent   cellular component:  intracellular   cellular component:  nucleus      BioCarta Pathways:     KEGG Pathways:  
    Probe set : 1383100_at    Name:   family with sequence similarity 18, member B (predicted)    GB acc:   AI070897    UniGene:   Rn.112772    Gene Symbol:   Fam18b_predicted    Locus Link:   309146    Chromosome:   1    Cytoband:   Chr:1q42    SOURCE    GENECARD       Gene Ontology:   cellular component:  integral to membrane      BioCarta Pathways:     KEGG Pathways:  
    Probe set : 1373837_at    Name:   fused toes (predicted)    GB acc:   BI296633    UniGene:   Rn.136882    Gene Symbol:   Fts_predicted    Locus Link:   291906    Chromosome:   19    Cytoband:   Chr:19p11    SOURCE    GENECARD       Gene Ontology:   biological process:  ubiquitin cycle   biological process:  apoptosis   biological process:  protein modification   cellular component:  membrane   molecular function:  ubiquitin conjugating enzyme activity   molecular function:  ubiquitin-like activating enzyme activity      BioCarta Pathways:     KEGG Pathways:  
    Probe set : 1373088_at     GB acc:   BI295811              Gene Ontology:     BioCarta Pathways:     KEGG Pathways:  
    Probe set : 1395404_at    Name:   Transcribed locus    GB acc:   BE110529    UniGene:   Rn.130706        SOURCE        Gene Ontology:     BioCarta Pathways:     KEGG Pathways:
[truncated: 984,742 more chars]
