# Supplementary material for: Activation of counter-regulatory mechanisms in a rat renal acute rejection model
Source: BMC Genomics. 2008 Feb 8;9:71. doi: 10.1186/1471-2164-9-71 (PMC2262896; doi:10.1186/1471-2164-9-71)
Supplement: Additional file 1 — Gene lists from the class comparison between the groups analyzed with BRB ArrayTools. [file 1471-2164-9-71-S1.zip › ControlvsAllogeneic/ControlvsAllogeneic.html]

 

 
 
 Class Comparison Results 
 
P {font-size: 9pt}
TABLE {font-size: 9pt}
 
 
 
 
function popUp(text) {
   var prop =
   "location=no,scrollbars=yes,menubars=no,toolbars=no,resizable=yes";
   popup = window.open("","mywin",prop);
   popup.document.open();
   popup.document.write(text);
   popup.document.close();
   popup.focus();
   }
    
 HELP   
   Description of the problem:   
 
Number of classes: 2
 
Number of genes that passed filtering criteria: 31099
 
Type of univariate test used: Two-sample T-test (with random variance model)
 
 
Column of the Experiment Descriptors sheet that defines class variable : Control vs allogeneic
 
 
WARNING: Distribution assumptions underlying the univariate test random variance model are not satisfied. Random variance model was not used
 
 
Exact Multivariate Permutations test was computed based on 126 available permutations
 
 
 
Nominal significance level of each univariate test: 0.001
 
 
Confidence level of false discovery rate assessment: 90 %
 
Maximum allowed number of false-positive genes: 10
 
Maximum allowed proportion of false-positive genes: 0.1
 
 
   Summary of Results:   
 
 
Number of genes significant at 0.001 level of the univariate test: 7354
 
 
Probability of getting at least 7354 genes significant by chance (at the 0.001 level) if there are no real differences between the classes: 0.00794
 
 
   Genes which discriminate among classes:   
  Table 1  - Sorted by p-value of the univariate test.
 
The first 7354 genes are significant at the nominal 0.001 level of the univariate test
 
With probability of 90 % the first 5086 genes contain no more than 10 false discoveries.
 
 
With probability of 90% the first 8087 genes contain no more than 10% of false discoveries. 
Further extension of the list was halted because the list would contain more than 100 false discoveries
 
 
  
    Parametric  p-value   Geom mean of  intensities  in class  1 :  allogeneic  Geom mean of  intensities  in class  2 :  control  Fold difference of geom means   Probe set Description Anno- tations Gene symbol
   1 p  1283.8 246.1 5.217  1374551_at  interferon-induced protein 35 (predicted)  Info   Ifi35_predicted 
   2 p  2672.5 372.2 7.18  1387946_at  lectin, galactoside-binding, soluble, 3 binding protein  Info   Lgals3bp 
   3 p  443.8 25 17.752  1385439_x_at  Transcribed locus, strongly similar to XP_220978.3 PREDICTED: similar to Probable ATP-dependent helicase LGP2 (Protein D11Lgp2) [Rattus norvegicus]  Info    
   4 p  863.7 147.5 5.856  1371537_at  Transcribed locus  Info    
   5 p  1203.3 186.6 6.449  1373490_at  glia maturation factor, gamma  Info   Gmfg 
   6 p  342.5 24.7 13.866  1372406_at  minichromosome maintenance deficient 3 (S. cerevisiae) (predicted) /// similar to DNA replication licensing factor MCM3 (DNA polymerase alpha holoenzyme-associated protein P1) (P1-MCM3)  Info   Mcm3_predicted /// LOC367976 
   7 p  2789.4 350.9 7.949  1389210_at  lymphocyte cytosolic protein 1 (predicted)  Info   Lcp1_predicted 
   8 p  1508.1 297.1 5.076  1373818_at  Potassium channel tetramerisation domain containing 12 (predicted)  Info    
   9 p  1843.1 192.9 9.555  1367676_at  high mobility group box 2 /// similar to High mobility group protein 2 (HMG-2) /// similar to High mobility group protein 2 (HMG-2) /// similar to High mobility group protein 2 (HMG-2)  Info   Hmgb2 /// LOC498072 /// LOC498388 /// LOC498988 
   10 p  1583.5 185.7 8.527  1388164_at  RT1 class Ib, locus S3  Info   RT1-S3 
   11 p  548.1 67.2 8.156  1389873_at  apoptosis-associated speck-like protein containing a CARD  Info   Pycard 
   12 p  2446.9 315.8 7.748  1383564_at    Info    
   13 p  311.8 797.4 0.391  1373240_at  dehydrogenase/reductase (SDR family) member 3 (predicted)  Info   Dhrs3_predicted 
   14 p  3055.9 265.7 11.501  1371209_at  RT1 class I, CE5  Info   RT1-CE5 
   15 p  4916.5 1007 4.882  1387770_at  putative ISG12(a) protein  Info   RGD:1303168 
   16 p  5116.3 760.1 6.731  1373575_at  similar to NADH dehydrogenase (ubiquinone) Fe-S protein 2  Info   LOC498279 
   17 p  3153.5 1203.3 2.621  1372070_at  interferon gamma inducible protein 30 (predicted)  Info   Ifi30_predicted 
   18 p  1535.7 231.5 6.634  1373672_at    Info    
   19 p  2608.2 270.6 9.639  1373025_at  complement component 1, q subcomponent, gamma polypeptide (predicted)  Info   C1qg_predicted 
   20 p  927.8 204.9 4.528  1388784_at  colony stimulating factor 1 receptor  Info   Csf1r 
   21 p  525.1 21.7 24.198  1387687_at  immunoglobulin superfamily, member 6  Info   Igsf6 
   22 p  929.1 73.5 12.641  1369186_at  caspase 1  Info   Casp1 
   23 p  868.1 478 1.816  1372752_at  transmembrane 4 superfamily member 7 (predicted)  Info   Tm4sf7_predicted 
   24 p  4552.8 279 16.318  1373523_at  Fc fragment of IgG, low affinity IIIa, receptor for (CD16)  Info   Fcgr3a 
   25 p  347.2 24.5 14.171  1370493_a_at  leukocyte immunoglobulin-like receptor, subfamily B (with TM and ITIM domains), member 3 (predicted)  Info   Lilrb3_predicted 
   26 p  2370.9 210.8 11.247  1388213_a_at  RT1 class Ib, locus S3  Info   RT1-S3 
   27 p  1735.7 220.3 7.879  1382043_at  unc-93 homolog B (C. elegans) (predicted)  Info   Unc93b_predicted 
   28 p  304.6 162 1.88  1383748_at  Unknown (protein for MGC:72598)  Info   RGD:735063 
   29 p  3482.4 1508.3 2.309  1390022_at  actin related protein 2/3 complex, subunit 5 (predicted)  Info   Arpc5_predicted 
   30 p  643.7 36.7 17.54  1379766_at  Thyroglobulin  Info   Tg 
   31 p  370 82 4.512  1374544_at  CDNA clone MGC:112715 IMAGE:7377326  Info    
   32 p  555.3 1617.4 0.343  1389114_at  similar to Hypothetical protein MGC59076 (predicted)  Info   RGD1309144_predicted 
   33 p  1205.9 3257.8 0.37  1398348_at  Transcribed locus  Info    
   34 p  1212.4 55 22.044  1369204_at  hemopoietic cell kinase  Info   Hck 
   35 p  433.8 23.1 18.779  1368723_at  linker for activation of T cells  Info   Lat 
   36 p  678.4 51.7 13.122  1391979_at  Transcribed locus  Info    
   37 p  3509.1 588.8 5.96  1370383_s_at  RT1 class II, locus Db1  Info   RT1-Db1 
   38 p  1030.1 193.5 5.324  1392894_at  fibrinogen-like 2  Info   Fgl2 
   39 p  5182.1 799.5 6.482  1370822_at  RT1 class II, locus Ba  Info   RT1-Ba 
   40 p  832.2 64.4 12.922  1379613_at  Similar to hypothetical protein MGC29390 (predicted)  Info    
   41 p  2994 568.6 5.266  1372013_at  interferon induced transmembrane protein 1 (predicted)  Info   Ifitm1_predicted 
   42 p  1362.5 200.3 6.802  1373592_at  similar to SPI6  Info   MGC94010 
   43 p  1789.9 154.6 11.578  1377916_at  schlafen 2 (predicted)  Info   Slfn2_predicted 
   44 p  1164.8 107.1 10.876  1373932_at  Cytochrome b-245, beta polypeptide  Info   Cybb 
   45 p  489.8 28.6 17.126  1382442_at  Similar to NF-kappa-B-repressing factor (NFKB-repressing factor) (Transcription factor NRF)  Info    
   46 p  2245.4 460.3 4.878  1387892_at  tubulin, beta 5  Info   Tubb5 
   47 p  831.5 121.3 6.855  1368010_at  protein tyrosine phosphatase, non-receptor type 6  Info   Ptpn6 
   48 p  810.5 39.2 20.676  1385571_at  Similar to SP140 nuclear body protein isoform 1  Info    
   49 p  1882.7 265.9 7.08  1370290_at  tubulin, beta 5  Info   Tubb5 
   50 p  1394.9 272.3 5.123  1374388_at  EF hand domain containing 2 (predicted)  Info   Efhd2_predicted 
   51 p  1124 152.8 7.356  1378257_at  three prime repair exonuclease 1 (predicted)  Info   Trex1_predicted 
   52 p  555.4 29.4 18.891  1370603_a_at  protein tyrosine phosphatase, receptor type, C  Info   Ptprc 
   53 p  205.4 29.5 6.963  1385557_at  Transcribed locus  Info    
   54 p  2018.3 138.4 14.583  1370215_at  complement component 1, q subcomponent, beta polypeptide  Info   C1qb 
   55 p  3071.3 270 11.375  1376652_at  complement component 1, q subcomponent, alpha polypeptide (predicted)  Info   C1qa_predicted 
   56 p  1331.6 164.4 8.1  1395124_at  tryptophanyl-tRNA synthetase (predicted)  Info   Wars_predicted 
   57 p  736.2 79.3 9.284  1370516_at  peptide/histidine transporter PHT2  Info   Slc15a3 
   58 p  2761.1 305.3 9.044  1376845_at  putative ISG12(b) protein  Info   isg12(b) 
   59 p  3288.8 336 9.788  1371123_x_at  RT1 class Ib, locus S3  Info   RT1-S3 
   60 p  875.2 37.3 23.464  1379381_at  MHC class II transactivator  Info   C2ta 
   61 p  374.7 50.2 7.464  1370585_a_at  protein kinase C, beta 1  Info   Prkcb1 
   62 p  1240.8 158.4 7.833  1379748_at  Transcribed locus  Info    
   63 p  473.5 26.7 17.734  1382926_s_at  LOC499533  Info   LOC499533 
   64 p  470.7 113.5 4.147  1372468_at  CD97 antigen (predicted)  Info   Cd97_predicted 
   65 p  2176 226.1 9.624  1367974_at  annexin A3  Info   Anxa3 
   66 p  1048.2 231.9 4.52  1367816_at  homeobox only domain  Info   Hod 
   67 p  1582.5 210.9 7.504  1370904_at  major histocompatibility complex, class II, DM alpha  Info   RGD:735053 
   68 p  493.7 29.6 16.679  1379357_at  Transcribed locus  Info    
   69 p  3461.3 265.8 13.022  1389006_at  Macrophage expressed gene 1  Info   Mpeg1 
   70 p  439 63.4 6.924  1394940_at  similar to hypothetical protein FLJ20037 (predicted)  Info   RGD1311381_predicted 
   71 p  1069.9 64.1 16.691  1382311_at  TRAF2 binding protein  Info   RGD:1359151 
   72 p  402 49.7 8.089  1379496_at  Transcribed locus  Info    
   73 p  892.5 46.1 19.36  1370119_at  leucocyte specific transcript 1  Info   Lst1 
   74 p  2414.9 172.7 13.983  1368518_at  CD53 antigen  Info   Cd53 
   75 p  1866.3 1045 1.786  1374397_at  eukaryotic translation initiation factor 2, subunit 2 (beta)  Info   Eif2s2 
   76 p  4785.5 1366.4 3.502  1388347_at  similar to lymphocyte antigen 6 complex, locus E  Info   LOC362934 
   77 p  3853.1 389.6 9.89  1379344_at  Cytochrome b-245, beta polypeptide  Info   Cybb 
   78 p  587.1 59.1 9.934  1377239_at  amyloid beta (A4) precursor protein-binding, family B, member 1 interacting protein (predicted)  Info   Apbb1ip_predicted 
   79 p  2166.1 260.9 8.302  1372011_at  Guanine deaminase  Info   Gda 
   80 p  1774.2 119.3 14.872  1374718_at  Similar to deltex 3-like  Info    
   81 p  1023.8 337.8 3.031  1378428_at  Transcribed locus  Info    
   82 p  886.9 50.5 17.562  1370422_at  receptor-interacting serine-threonine kinase 3  Info   Ripk3 
   83 p  1936.9 120 16.141  1389553_at  dendritic cell inhibitory receptor 3  Info   RGD:1359528 
   84 p  141.2 20.3 6.956  1371017_at  T-cell receptor gamma chain  Info   Tcrg 
   85 p  632 114.9 5.5  1374578_at  Transcribed locus  Info    
   86 p  530.1 134.6 3.938  1383372_at  platelet-activating factor receptor  Info   Ptafr 
   87 p  797.5 2314.8 0.345  1379741_at  ATPase, H+ transporting, lysosomal V0 subunit A isoform 4 (predicted)  Info   Atp6v0a4_predicted 
   88 p  233.7 17.9 13.056  1392722_at  Transcribed locus  Info    
   89 p  1022.2 68.6 14.901  1390798_at    Info    
   90 p  2702.6 225 12.012  1369964_at  coronin, actin binding protein 1A  Info   Coro1a 
   91 p  987.9 115.2 8.576  1368006_at  lysosomal-associated protein transmembrane 5  Info   Laptm5 
   92 p  131.7 457.1 0.288  1383420_at  Similar to lactation elevated 1  Info    
   93 p  288.9 42.6 6.782  1383426_at  proline-serine-threonine phosphatase-interacting protein 1 (predicted)  Info   Pstpip1_predicted 
   94 p  3589.3 492.2 7.292  1372757_at  Signal transducer and activator of transcription 1  Info   Stat1 
   95 p  377.4 37.2 10.145  1385029_at  Transcribed locus  Info    
   96 p  781.8 225.8 3.462  1375955_at  zinc finger protein 313  Info   RGD:1303139 
   97 p  301.1 23.3 12.923  1379425_at  similar to RIKEN cDNA 3110037I16 (predicted)  Info   RGD1309802_predicted 
   98 p  1068.7 121.3 8.81  1367705_at  glutaredoxin 1 (thioltransferase)  Info   Glrx1 
   99 p  1947.1 177.2 10.988  1376144_at  similar to B aggressive lymphoma (predicted)  Info   RGD1307534_predicted 
   100 p  949.9 126.8 7.491  1387048_at  nuclear RNA helicase, DECD variant of DEAD box family  Info   Ddx39 
   101 p  331.5 52.7 6.29  1370623_at  fibrinogen-like 2  Info   Fgl2 
   102 p  375.7 65.7 5.718  1386277_at  ubiquitin-activating enzyme E1-like (predicted)  Info   Ube1l_predicted 
   103 p  5358.6 555.8 9.641  1368835_at  signal transducer and activator of transcription 1  Info   Stat1 
   104 p  1478.7 122.8 12.042  1368558_s_at  allograft inflammatory factor 1  Info   Aif1 
   105 p  1095.1 259.3 4.223  1388236_x_at  RT1 class I, CE12  Info   RGD:1359103 
   106 p  509.4 29.4 17.327  1385276_a_at  similar to hypothetical protein FLJ11354 (predicted)  Info   RGD1310093_predicted 
   107 p  878.5 313.9 2.799  1367732_at  guanine nucleotide binding protein, beta 1  Info   Gnb1 
   108 p  1384.8 101.8 13.603  1368490_at  CD14 antigen  Info   Cd14 
   109 p  1440.2 96.1 14.986  1371015_at  myxovirus (influenza virus) resistance 1  Info   Mx1 
   110 p  1558.4 206.4 7.55  1387202_at  intercellular adhesion molecule 1  Info   Icam1 
   111 p  390 97.3 4.008  1373785_at  similar to expressed sequence AI132321  Info   LOC362792 
   112 p  1290.6 195.7 6.595  1379717_at  similar to ATP-binding cassette, subfamily G, member 3  Info   LOC498435 
   113 p  271.4 27.4 9.905  1387605_at  caspase 12  Info   Casp12 
   114 p  4681.9 417.3 11.22  1376151_a_at    Info    
   115 p  1070.8 106.2 10.083  1393280_at  lymphocyte antigen 86 (predicted)  Info   Ly86_predicted 
   116 p  1674.9 3082.8 0.543  1367662_at  hydroxysteroid (17-beta) dehydrogenase 10  Info   Hsd17b10 
   117 p  757.1 109.3 6.927  1379568_at  interferon-induced protein with tetratricopeptide repeats 2 (predicted)  Info   Ifit2_predicted 
   118 p  238.3 21.8 10.931  1382153_at  C-type (calcium dependent, carbohydrate recognition domain) lectin, superfamily member 6  Info   RGD:1359662 
   119 p  483.8 60.9 7.944  1395003_at  dendritic cell inhibitory receptor 4  Info   RGD:1359109 
   120 p  2721.2 135.4 20.097  1374033_at  proteasome (prosome, macropain) subunit, beta type 10 (predicted)  Info   Psmb10_predicted 
   121 p  608.4 76.9 7.912  1377826_at  Transcribed locus, weakly similar to NP_036457.1 microtubule-associated protein, RP/EB family, member 1; adenomatous polyposis coli-binding protein EB1 [Homo sapiens]  Info    
   122 p  1888.4 77.8 24.272  1388149_at  transporter 1, ATP-binding cassette, sub-family B (MDR/TAP)  Info   Tap1 
   123 p  355.7 50.1 7.1  1373401_at  Transcribed locus  Info    
   124 p  1632.2 283.5 5.757  1389732_at  Transcribed locus, strongly similar to NP_082154.2 hypothetical protein LOC71712 [Mus musculus]  Info    
   125 p  440 68.8 6.395  1385926_at  Transcribed locus  Info    
   126 p  1700.9 126.5 13.446  1387134_at  schlafen 3  Info   Slfn3 
   127 p  1260.2 2962 0.425  1370237_at  L-3-hydroxyacyl-Coenzyme A dehydrogenase, short chain  Info   Hadhsc 
   128 p  5985.4 480.5 12.457  1387354_at  signal transducer and activator of transcription 1  Info   Stat1 
   129 p  931.4 2323.4 0.401  1386885_at  enoyl coenzyme A hydratase 1, peroxisomal  Info   Ech1 
   130 p  741.8 59.8 12.405  1384187_at  similar to Adapter-related protein complex 1 sigma 1B subunit (Sigma-adaptin 1B) (Adaptor protein complex AP-1 sigma-1B subunit) (Golgi adaptor HA1/AP1 adaptin sigma-1B subunit) (Clathrin assembly protein complex 1 sigma-1B small chain) (Sigma 1B s...  Info   LOC302671 
   131 p  417.8 81.2 5.145  1368227_at  solute carrier family 28 (sodium-coupled nucleoside transporter), member 2  Info   Slc28a2 
   132 p  526.2 93.4 5.634  1383448_at  interferon dependent positive acting transcription factor 3 gamma (predicted)  Info   Isgf3g_predicted 
   133 p  2868.2 526 5.453  1387893_at  complement component 1, s subcomponent /// similar to complement component 1, s subcomponent  Info   C1s /// LOC500313 
   134 p  346.2 74 4.678  1368754_at  pyrimidinergic receptor P2Y, G-protein coupled, 6  Info   P2ry6 
   135 p  1650.1 166.6 9.905  1383189_at  Transcribed locus  Info    
   136 p  249.6 34.4 7.256  1373026_at  similar to RIKEN cDNA 2410030K01 (predicted)  Info   RGD1307631_predicted 
   137 p  1232 97.4 12.649  1369181_at  cytochrome b-245, beta polypeptide  Info   Cybb 
   138 p  330.8 64 5.169  1378443_at  SLAM family member 9 (predicted)  Info   Slamf9_predicted 
   139 p  587.8 228.5 2.572  1388761_at  histone deacetylase 1 (predicted)  Info   Hdac1_predicted 
   140 p  3159.2 720.9 4.382  1386925_at  actin related protein 2/3 complex, subunit 1B  Info   Arpc1b 
   141 p  505 49.1 10.285  1380129_at  Transcribed locus  Info    
   142 p  1015 2759.4 0.368  1368059_at  crystallin, mu  Info   Crym 
   143 p  801.9 203 3.95  1374601_at  interferon gamma receptor 2 (predicted)  Info   Ifngr2_predicted 
   144 p  1635.3 465.4 3.514  1388763_at  Transcribed locus, strongly similar to NP_031751.1 calponin 2 [Mus musculus]  Info    
   145 p  543.6 109.5 4.964  1392785_at  potassium channel tetramerisation domain containing 12 (predicted)  Info   Kctd12_predicted 
   146 p  536.8 80.6 6.66  1372004_at  heme binding protein 1 (predicted)  Info   Hebp1_predicted 
   147 p  2868.1 82.8 34.639  1396163_at  interferon gamma induced GTPase (predicted)  Info   Igtp_predicted 
   148 p  113.2 45.3 2.499  1397372_at  similar to Mob4B protein /// MOB1, Mps One Binder kinase activator-like 1B (yeast) (predicted)  Info   LOC308302 /// Mobk1b_predicted 
   149 p  901.3 18.4 48.984  1395034_at  similar to RIKEN cDNA 4933430F08 (predicted)  Info   RGD1311481_predicted 
   150 p  1234 37.5 32.907  1392547_at  hypothetical LOC302884  Info   RGD:1359583 
   151 p  542.6 1335.8 0.406  1373900_at  keratin complex 2, basic, gene 7 (predicted)  Info   Krt2-7_predicted 
   152 p  179.9 595.4 0.302  1371959_at  histone 2, H2aa (predicted) /// similar to Hist2h2aa1 protein  Info   Hist2h2aa_predicted /// LOC499676 
   153 p  363.3 61.5 5.907  1383516_at  fibrinogen-like 2  Info   Fgl2 
   154 p  5997.3 2129.4 2.816  1371440_at  Beta-2 microglobulin  Info   B2m 
   155 p  814.1 86.6 9.401  1370445_at  phosphatidylserine-specific phospholipase A1  Info   RGD:621261 
   156 p  145.5 1.2 121.25  1387711_at  interleukin 10  Info   Il10 
   157 p  89 634.5 0.14  1372213_at  similar to hypothetical protein MGC6835  Info   LOC500300 
   158 p  315.6 79.5 3.97  1374627_at  Transcribed locus  Info    
   159 p  3933.1 686.8 5.727  1370202_at  HRAS like suppressor  Info   Hrasls3 
   160 p  713.3 58.5 12.193  1368921_a_at  CD44 antigen  Info   Cd44 
   161 p  2358.1 613 3.847  1373881_at  Rho, GDP dissociation inhibitor (GDI) beta (predicted)  Info   Arhgdib_predicted 
   162 p  1456.1 94.9 15.344  1375010_at  CD68 antigen (predicted)  Info   Cd68_predicted 
   163 p  927.4 94.6 9.803  1371078_at  RT1 class Ib, locus Aw2  Info   RT1-Aw2 
   164 p  579.7 55.9 10.37  1371152_a_at  2',5'-oligoadenylate synthetase 1, 40/46kDa  Info   Oas1 
   165 p  893.5 75.4 11.85  1390659_at  CD44 antigen  Info   Cd44 
   166 p  5140.4 2543.4 2.021  1369952_at  poly(A) binding protein, cytoplasmic 1  Info   Pabpc1 
   167 p  74.3 1.6 46.437  1369766_at  prostaglandin E receptor 2, subtype EP2  Info   Ptger2 
   168 p  634.3 75.2 8.435  1384946_at    Info    
   169 p  523.2 24.3 21.531  1389997_at  CD3 antigen, epsilon polypeptide (predicted)  Info   Cd3e_predicted 
   170 p  248.2 42.5 5.84  1373658_at  Rac GTPase-activating protein 1 (predicted)  Info    
   171 p  3365.3 369.9 9.098  1388212_a_at  RT1 class Ib, locus S3  Info   RT1-S3 
   172 p  877 121.7 7.206  1376795_at  phosphoinositide-3-kinase adaptor protein 1 (predicted)  Info   Pik3ap1_predicted 
   173 p  4422.3 1354.9 3.264  1369973_at  xanthine dehydrogenase /// hypothetical gene supported by NM_017154  Info   Xdh /// LOC497811 
   174 p  33 261.6 0.126  1384874_at  Transcribed locus  Info    
   175 p  542.2 30.3 17.894  1377698_at  tumor necrosis factor receptor superfamily, member 5  Info   RGD:619830 
   176 p  3360.3 582.1 5.773  1390738_at  bone marrow stromal cell antigen 2  Info   Bst2 
   177 p  1238.3 53.7 23.06  1370080_at  heme oxygenase (decycling) 1  Info   Hmox1 
   178 p  974.2 93.6 10.408  1371210_s_at  RT1 class Ib, locus Aw2 /// RT1 class I, CE5  Info   RT1-Aw2 /// RT1-CE5 
   179 p  3837.6 525.8 7.299  1370892_at  complement component 4a /// complement component 4, gene 2  Info   C4a /// C4-2 
   180 p  794.8 80.7 9.849  1398616_at  similar to Adapter-related protein complex 1 sigma 1B subunit (Sigma-adaptin 1B) (Adaptor protein complex AP-1 sigma-1B subunit) (Golgi adaptor HA1/AP1 adaptin sigma-1B subunit) (Clathrin assembly protein complex 1 sigma-1B small chain) (Sigma 1B s...  Info   LOC302671 
   181 p  424.7 1156.2 0.367  1369663_at  epoxide hydrolase 2, cytoplasmic  Info   Ephx2 
   182 p  765.9 281.9 2.717  1373439_at  Lamin B receptor  Info   Lbr 
   183 p  267.7 67.1 3.99  1382535_at  Potassium channel tetramerisation domain containing 12 (predicted)  Info    
   184 p  1154.7 221.4 5.215  1372112_at  similar to 9230105E10Rik protein (predicted)  Info   RGD1304579_predicted 
   185 p  387 30 12.9  1379818_at    Info    
   186 p  142.1 589.6 0.241  1384164_at  Transcribed locus  Info    
   187 p  204.8 14.4 14.222  1371970_at  similar to expressed sequence AW413625  Info   LOC499322 
   188 p  593.3 109.6 5.413  1370449_at  G protein-coupled receptor 105  Info   Gpr105 
   189 p  635.8 90.9 6.994  1383585_s_at  Sorting nexin 10 (predicted)  Info    
   190 p  14.5 1 14.5  1369586_at  mast cell protease 8  Info   Mcpt8 
   191 p  1951.9 959.5 2.034  1398828_at  FK506 binding protein 1a  Info   Fkbp1a 
   192 p  332.1 84.7 3.921  1389510_at  Ly1 antibody reactive clone (predicted)  Info   Lyar_predicted 
   193 p  914.5 287.9 3.176  1387227_at  Wiskott-Aldrich syndrome protein interacting protein  Info   Waspip 
   194 p  944.3 34.7 27.213  1375796_at  interferon gamma induced GTPase (predicted) /// similar to Ac2-233  Info   Igtp_predicted /// LOC501695 
   195 p  907.7 93.2 9.739  1393038_at  Fc receptor, IgG, high affinity I (predicted)  Info   Fcgr1_predicted 
   196 p  2946.4 405.4 7.268  1387005_at  cathepsin S  Info   Ctss 
   197 p  427.2 30.8 13.87  1368565_at  solute carrier family 1 (glial high affinity glutamate transporter), member 3  Info   Slc1a3 
   198 p  264 46.7 5.653  1387690_at  caspase 3  Info   Casp3 
   199 p  158.3 1.4 113.071  1378047_at  Spi-C transcription factor (Spi-1/PU.1 related) (predicted)  Info   Spic_predicted 
   200 p  244.5 38 6.434  1379957_at  similar to schlafen 8  Info   RGD:1359139 
   201 p  201.3 49.1 4.1  1374165_at  similar to Rasa4 protein  Info   LOC288589 
   202 p  306.8 57.3 5.354  1370539_at  RAB8B, member RAS oncogene family  Info   Rab8b 
   203 p  275.7 86.5 3.187  1387678_at  neuroblastoma RAS viral (v-ras) oncogene homolog  Info   Nras 
   204 p  779 51.7 15.068  1376908_at  similar to This ORF is capable of encoding 404aa which is homologous to two human interferon-inducible proteins, 54 kDa and 56 kDa proteins; ORF  Info   RGD:1359681 
   205 p  1810.5 3390.1 0.534  1387878_at  glutamate dehydrogenase 1  Info   Glud1 
   206 p  965.2 177.1 5.45  1372478_at  similar to LNV  Info   LOC501065 
   207 p  1181.7 2376.8 0.497  1389488_at  hypothetical protein LOC498544  Info   LOC498544 
   208 p  503.5 1291.7 0.39  1372093_at  Max interacting protein 1  Info   Mxi1 
   209 p  861.2 40.3 21.37  1382603_at  Programmed cell death 1 ligand 2 (predicted)  Info    
   210 p  1375.8 388.9 3.538  1371336_at  hematological and neurological expressed sequence 1  Info   RGD:1359325 
   211 p  1064.8 145.1 7.338  1384180_at  interferon-induced protein with tetratricopeptide repeats 2 (predicted)  Info   Ifit2_predicted 
   212 p  797.6 54.5 14.635  1382546_at  PHD finger protein 11 (predicted)  Info   Phf11_predicted 
   213 p  744.2 182.2 4.085  1388416_at  low density lipoprotein receptor-related protein 1 (predicted)  Info   Lrp1_predicted 
   214 p  4099.3 635.5 6.451  1367574_at  vimentin  Info   Vim 
   215 p  1788.8 156.4 11.437  1372034_at  Similar to hypothetical protein MGC29390 (predicted)  Info    
   216 p  989.2 347.5 2.847  1376006_at  Transcribed locus  Info    
   217 p  1002.6 63.1 15.889  1378927_at    Info    
   218 p  1632 160.7 10.156  1370913_at  Best5 protein  Info   RGD:620495 
   219 p  1390.3 173.8 7.999  1388255_x_at  RT1 class I, CE5  Info   RT1-CE5 
   220 p  1135.6 643.3 1.765  1371596_at  ribonucleic acid binding protein S1 (predicted)  Info   Rnps1_predicted 
   221 p  854.1 33.2 25.726  1388340_at  Ns5atp9 protein  Info   RGD:1303041 
   222 p  1907.7 221.4 8.617  1372404_at  RAS-related C3 botulinum substrate 2 (predicted)  Info   Rac2_predicted 
   223 p  247 52.8 4.678  1385143_at    Info    
   224 p  559.4 28 19.979  1369214_a_at  MHC class II transactivator  Info   Mhc2ta 
   225 p  126.2 46.4 2.72  1388066_a_at  G protein-coupled receptor kinase 6  Info   Gprk6 
   226 p  264.4 25.8 10.248  1383578_at  Transcribed locus  Info    
   227 p  683.2 1972.5 0.346  1386916_at  aconitase 1  Info   Aco1 
   228 p  322.9 23.4 13.799  1376943_at    Info    
   229 p  488.9 1826.5 0.268  1377048_at  similar to cDNA sequence BC021917 (predicted)  Info   RGD1311026_predicted 
   230 p  1300 186.8 6.959  1369726_at  TAP binding protein  Info   Tapbp 
   231 p  557.1 63.5 8.773  1382026_at  Rho GTPase activating protein 9 (predicted)  Info   Arhgap9_predicted 
   232 p  383 79.5 4.818  1387984_at  chemokine-like factor 1  Info   Cklf1 
   233 p  428.2 97.8 4.378  1373504_at  GLI pathogenesis-related 1 (glioma) (predicted)  Info   Glipr1_predicted 
   234 p  1678.7 904.3 1.856  1395426_at  similar to ubiquitin-conjugating enzyme E2 variant 1  Info   LOC499945 
   235 p  411.5 49.9 8.246  1385925_at  Transcribed locus  Info    
   236 p  366.1 752.6 0.486  1390292_at  transmembrane protein 8 (five membrane-spanning domains) (predicted)  Info   Tmem8_predicted 
   237 p  746.9 18.9 39.519  1368975_at  CD38 antigen  Info   Cd38 
   238 p  672.2 1341.5 0.501  1372741_at  similar to RIKEN cDNA C330023F11 (predicted)  Info   RGD1311440_predicted 
   239 p  363.7 75.4 4.824  1382277_at    Info    
   240 p  908.1 52.4 17.33  1368693_at  Gardner-Rasheed feline sarcoma viral (Fgr) oncogene homolog  Info   Fgr 
   241 p  4730.6 671.6 7.044  1367710_at  protease (prosome, macropain) 28 subunit, beta  Info   Psme2 
   242 p  217.7 14.5 15.014  1381556_at  Similar to BC013672 protein  Info    
   243 p  1140.3 389.8 2.925  1367715_at  tumor necrosis factor receptor superfamily, member 1a  Info   Tnfrsf1a 
   244 p  1970 625.2 3.151  1368808_at  CAP, adenylate cyclase-associated protein 1 (yeast)  Info   Cap1 
   245 p  599.4 270.2 2.218  1368136_at  thymopoietin  Info   Tmpo 
   246 p  227.7 982 0.232  1374307_at  CaM-kinase II inhibitor alpha  Info   LOC287005 
   247 p  1098.8 271.4 4.049  1369944_at  MARCKS-like protein  Info   Mlp 
   248 p  428.8 90.5 4.738  1390707_at  regulator of G-protein signalling 10  Info   Rgs10 
   249 p  876.5 551.9 1.588  1368043_at  sorting nexin 1  Info   Snx1 
   250 p  1236.6 67.9 18.212  1372097_at  Interferon consensus sequence binding protein 1 (predicted)  Info   Icsbp1 
   251 p  874 75.2 11.622  1374337_at  Similar to chromosome 17 open reading frame 27 (predicted)  Info    
   252 p  469.2 78.5 5.977  1376109_at  Transcribed locus, weakly similar to NP_062289.1 DNA binding protein with his-thr domain [Mus musculus]  Info    
   253 p  540.6 249 2.171  1376880_at  similar to T2K protein kinase homolog  Info   LOC299827 
   254 p  46.5 244.5 0.19  1384603_at  ATP-binding cassette, sub-family A (ABC1), member 4 (predicted)  Info   Abca4_predicted 
   255 p  5187.2 1669.8 3.106  1372254_at  serine (or cysteine) peptidase inhibitor, clade G, member 1  Info   Serping1 
   256 p  224.3 429.8 0.522  1375529_at  carbonic reductase 4  Info   Cbr4 
   257 p  270 28.5 9.474  1374284_at  Ras association (RalGDS/AF-6) domain family 4 (predicted)  Info   Rassf4_predicted 
   258 p  1528 148.8 10.269  1368420_at  ceruloplasmin  Info   Cp 
   259 p  366.3 46.6 7.861  1373066_at  Transcribed locus  Info    
   260 p  502 58.8 8.537  1367780_at  pituitary tumor-transforming 1  Info   Pttg1 
   261 p  2053.4 120.7 17.012  1389092_at  interleukin 2 receptor, gamma (severe combined immunodeficiency)  Info   Il2rg 
   262 p  3916 71.5 54.769  1373992_at  similar to interferon-inducible GTPase /// similar to MGC108823 protein /// similar to MGC108823 protein  Info   RGD:1359421 /// LOC498871 /// LOC498872 
   263 p  4028.6 622.5 6.472  1376029_at  RAB2, member RAS oncogene family-like  Info   RGD:1302985 
   264 p  190 623.4 0.305  1373110_at  Transcribed locus  Info    
   265 p  3295.5 557.7 5.909  1370882_at  major histocompatibility complex, class II, DM beta  Info   RGD:735096 
   266 p  2929.2 1315.9 2.226  1371977_at  actin related protein 2/3 complex, subunit 3 (predicted)  Info   Arpc3_predicted 
   267 p  3973.3 1911.9 2.078  1369936_at  calmodulin 1  Info   Calm1 
   268 p  1130.2 2278.6 0.496  1370320_at  MAWD binding protein  Info   Mawbp 
   269 p  364.4 87.7 4.155  1380077_at  Transcribed locus  Info    
   270 p  1004.1 93.6 10.728  1388202_at  RT1 class Ib, locus Aw2  Info   RT1-Aw2 
   271 p  1084.6 398.5 2.722  1389387_at  CDNA clone MGC:93913 IMAGE:7112877  Info    
   272 p  748.9 68.3 10.965  1373037_at  ubiquitin-conjugating enzyme E2L 6 (predicted)  Info   Ube2l6_predicted 
   273 p  1770.7 128.8 13.748  1369110_x_at  RT1 class Ib, locus Aw2  Info   RT1-Aw2 
   274 p  511.5 40 12.787  1391737_at  similar to p40-phox  Info   LOC500904 
   275 p  1235.8 818.7 1.509  1388868_at  zinc finger protein 216 (predicted)  Info   Zfp216_predicted 
   276 p  495 157.6 3.141  1392960_at  similar to RIKEN cDNA 2310004I24 gene (predicted)  Info   RGD1309906_predicted 
   277 p  274 50 5.48  1377669_at  low Mr GTP-binding protein  Info   Rab27a 
   278 p  1178.5 40.8 28.885  1382622_at  cystatin F (leukocystatin) (predicted)  Info   Cst7_predicted 
   279 p  450.3 51.8 8.693  1392881_at  alpha-2,3-sialyltransferase ST3Gal IV  Info   RGD:1303068 
   280 p  656.8 134.3 4.891  1383131_at  integrin beta 2 (predicted)  Info   Itgb2_predicted 
   281 p  4308.4 1604.2 2.686  1375211_at  ribonuclease T2 (predicted)  Info   Rnaset2_predicted 
   282 p  362.3 592.4 0.612  1389154_at  lipoic acid synthetase (predicted)  Info   Lias_predicted 
   283 p  3778.5 1952 1.936  1398788_at  glucose regulated protein, 58 kDa  Info   Grp58 
   284 p  4024.4 1220.3 3.298  1392334_at  RT1 class II, locus Ba  Info   RT1-Ba 
   285 p  934.1 48.4 19.3  1387952_a_at  CD44 antigen  Info   Cd44 
   286 p  241.3 1.1 219.364  1369764_at  complement component 4 binding protein, alpha  Info   C4bpa 
   287 p  464.2 176.4 2.632  1397892_at  eukaryotic translation initiation factor 1A (predicted)  Info   Eif1a_predicted 
   288 p  713.3 97.1 7.346  1368679_a_at  Yamaguchi sarcoma viral (v-yes-1) oncogene homolog  Info   Lyn 
   289 p  575.1 40.1 14.342  1388740_at  similar to cDNA sequence BC032204 (predicted)  Info   RGD1310168_predicted 
   290 p  336.3 88.7 3.791  1371480_at  LOC499655  Info   LOC499655 
   291 p  229 82.7 2.769  1378482_at  kidney cell line derived transcript 1 (predicted)  Info   Kdt1_predicted 
   292 p  409.4 1451.2 0.282  1372475_at  PTEN induced putative kinase 1 (predicted)  Info   Pink1_predicted 
   293 p  6846.9 1391 4.922  1370883_at  RT1 class II, locus Da  Info   RT1-Da 
   294 p  71 13.9 5.108  1386660_at  Transcribed locus  Info    
   295 p  1362 92.7 14.693  1389408_at  ribonucleotide reductase M2  Info   Rrm2 
   296 p  616.9 143.2 4.308  1391106_at  Transcribed locus  Info    
   297 p  2864.2 120.4 23.789  1370885_at  cathepsin Z  Info   Ctsz 
   298 p  142.4 45.6 3.123  1367855_at  scavenger receptor class B, member 1  Info   Scarb1 
   299 p  105 478.5 0.219  1395810_at  Transcribed locus  Info    
   300 p  1822.6 62.8 29.022  1390510_at  membrane-spanning 4-domains, subfamily A, member 6B  Info   RGD:1359381 
   301 p  277.4 54.6 5.081  1390396_at  Similar to eukaryotic translation initiation factor 4E member 3  Info    
   302 p  724.7 62.2 11.651  1375917_at  glycoprotein 49b /// similar to GP49B1  Info   RGD:1359090 /// LOC499078 
   303 p  938.2 21.2 44.255  1385051_at  Transcribed locus  Info    
   304 p  594.2 27.1 21.926  1393400_at  zeta-chain (TCR) associated protein kinase 70kDa  Info   Zap70 
   305 p  31.5 3.6 8.75  1383272_at  similar to ankyrin repeat and SOCS box-containing protein 11  Info   LOC302666 
   306 p  536.1 66 8.123  1387818_at  caspase 11  Info   Casp11 
   307 p  318.9 1089.9 0.293  1369909_s_at  fasting-inducible integral membrane protein TM6P1  Info   Tm6p1 
   308 p  1464.8 78.1 18.755  1367712_at  tissue inhibitor of metalloproteinase 1  Info   Timp1 
   309 p  673.7 273.8 2.461  1374180_at  Transcribed locus  Info    
   310 p  882.9 340.7 2.591  1371539_at  nucleolar protein family A, member 2 (predicted)  Info   Nola2_predicted 
   311 p  401.7 139.2 2.886  1382533_at  similar to HIV-1 Rev binding protein  Info   LOC363266 
   312 p  463.3 36.9 12.556  1374469_at  Similar to C20orf118  Info    
   313 p  2527.6 1625.4 1.555  1388332_at  Ras-related C3 botulinum toxin substrate 1  Info   Rac1 
   314 p  516.7 36.6 14.117  1395184_at    Info    
   315 p  5437.3 1716.3 3.168  1388337_at  nucleoside phosphorylase  Info   Np 
   316 p  385.6 137.7 2.8  1388182_at  DNA primase, p49 subunit  Info   RGD:621380 
   317 p  109.5 461.4 0.237  1369100_at  angiotensin/vasopressin receptor  Info   Nalp6 
   318 p  891.2 156.2 5.706  1380071_at  Transcribed locus  Info    
   319 p  532 39.7 13.401  1390909_at  Transcribed locus  Info    
   320 p  513 107.6 4.768  1392655_at  Similar to SP140 nuclear body protein isoform 1  Info    
   321 p  68.6 194.5 0.353  1377125_at  DnaJ (Hsp40) homolog, subfamily C, member 6 (predicted)  Info   Dnajc6_predicted 
   322 p  431.6 137.4 3.141  1375020_at  Transcribed locus  Info    
   323 p  4892.6 2114.5 2.314  1386861_at  H2A histone family, member Z  Info   H2afz 
   324 p  1468.8 592 2.481  1369979_at  src family associated phosphoprotein 2  Info   Scap2 
   325 p  2346.2 858.7 2.732  1387884_at  proteasome (prosome, macropain) subunit, alpha type 5  Info   Psma5 
   326 p  1756.1 69.6 25.231  1383392_at  complement component 2  Info   C2 
   327 p  331.8 83.8 3.959  1377299_at  nuclear autoantigenic sperm protein  Info   RGD:1359609 
   328 p  608.8 35.5 17.149  1392407_at  Similar to RIKEN cDNA 6030405P05 gene  Info    
   329 p  288.3 587.1 0.491  1376038_at  testis expressed gene 2 (predicted)  Info   Tex2_predicted 
   330 p  3927.4 315.7 12.44  1370428_x_at  RT1 class Ib, locus Aw2 /// RT1 class Ia, locus A2 /// RT1 class I, A3  Info   RT1-Aw2 /// RT1-A2 /// RGD:1359610 
   331 p  54.9 128.8 0.426  1391224_at  similar to 4921517L17Rik protein (predicted)  Info   RGD1311678_predicted 
   332 p  332.2 682.8 0.487  1369989_at  pyridoxine 5'-phosphate oxidase  Info   Pnpo 
   333 p  4413.7 841.1 5.248  1370056_at  Ly6-C antigen  Info   Ly6c 
   334 p  1233.4 32.4 38.068  1370924_at  similar to T-cell receptor beta-2 chain C region  Info   LOC362350 
   335 p  3038.8 1119.1 2.715  1387995_a_at  interferon induced transmembrane protein 3  Info   Ifitm3 
   336 p  248.3 13.9 17.863  1386009_at  Transcribed locus  Info    
   337 p  477.3 162.9 2.93  1375895_at  Serine racemase  Info   Srr 
   338 p  850.2 2464 0.345  1380393_at  crystallin, zeta (predicted)  Info   Cryz_predicted 
   339 p  772.6 1500.4 0.515  1377576_at  similar to RIKEN cDNA 1110013G13 (predicted)  Info   RGD1307965_predicted 
   340 p  337.3 15.6 21.622  1368976_at  CD38 antigen  Info   Cd38 
   341 p  1261.3 593.3 2.126  1371365_at  similar to Ubiquitin-conjugating enzyme E2S  Info   LOC292588 
   342 p  1324.8 108.8 12.176  1374730_at  Tyro protein tyrosine kinase binding protein  Info   Tyrobp 
   343 p  766.5 33.7 22.745  1384787_at  CD3 antigen, gamma polypeptide (predicted)  Info   Cd3g_predicted 
   344 p  735.8 58.8 12.514  1396262_at  pre-B-cell colony enhancing factor 1  Info   Pbef1 
   345 p  313.8 20.7 15.159  1393848_at  ribonucleotide reductase M2  Info   Rrm2 
   346 p  6068.1 1976.5 3.07  1367663_at  protease (prosome, macropain) 28 subunit, alpha  Info   Psme1 
   347 p  515.3 20.8 24.774  1389696_at  Transcribed locus  Info    
   348 p  922.3 273.9 3.367  1395886_at  ARP3 actin-related protein 3 homolog (yeast)  Info   Actr3 
   349 p  1096.2 328.2 3.34  1376976_at  secreted and transmembrane 1  Info   Sectm1 
   350 p  80.8 205.8 0.393  1387434_at  solute carrier family 22 (organic cation transporter), member 4  Info   Slc22a4 
   351 p  1928 4548.6 0.424  1391417_at  similar to betaine-homocysteine methyltransferase 2  Info   LOC365972 
   352 p  494.9 107.1 4.621  1379604_at  apolipoprotein L, 3 (predicted) /// similar to RIKEN cDNA 2210421G13  Info   Apol3_predicted /// LOC500903 
   353 p  230 31.2 7.372  1369584_at  suppressor of cytokine signaling 3  Info   Socs3 
   354 p  420 186.9 2.247  1376613_at  similar to hypothetical protein  Info   MGC73022 
   355 p  170.9 12.8 13.352  1389275_at    Info    
   356 p  13.8 1.1 12.545  1377659_at  myeloid leukemia factor 1 (predicted)  Info   Mlf1_predicted 
   357 p  98.4 219.4 0.448  1383553_at  similar to CDNA sequence BC034204 (predicted)  Info   RGD1311293_predicted 
   358 p  406.5 123.6 3.289  1392544_at  rcd1 (required for cell differentiation) homolog 1 (S. pombe) (predicted)  Info   Rqcd1_predicted 
   359 p  357.2 40.3 8.864  1393044_at  thymidylate kinase family LPS-inducible member (predicted)  Info   Tyki_predicted 
   360 p  2082.8 4044.6 0.515  1368016_at  peroxisomal trans-2-enoyl-CoA reductase  Info   Pecr 
   361 p  237.3 50.4 4.708  1369197_at  apoptotic peptidase activating factor 1  Info   Apaf1 
   362 p  1070.8 72.4 14.79  1368732_at  transporter 2, ATP-binding cassette, sub-family B (MDR/TAP)  Info   Tap2 
   363 p  427.4 154.8 2.761  1384413_at  Similar to solute carrier family 35, member A5  Info    
   364 p  1792.9 2439.9 0.735  1374431_at  ATPase, H+ transporting, V1 subunit A, isoform 1 (predicted)  Info    
   365 p  187.6 502.6 0.373  1373338_at  Transcribed locus  Info    
   366 p  13.4 61.2 0.219  1369677_at  cannabinoid receptor 1 (brain)  Info   Cnr1 
   367 p  263.4 496.6 0.53  1383731_at  Similar to RIKEN cDNA 2310036D22  Info   MGC94135 
   368 p  65.7 180 0.365  1393123_at  complement component 8, gamma polypeptide (predicted)  Info   C8g_predicted 
   369 p  4129.9 1214.4 3.401  1381593_x_at  RT1 class II, locus Ba  Info   RT1-Ba 
   370 p  1371 2785.6 0.492  1388324_at  nitrilase 1  Info   Nit1 
   371 p  215.6 30.7 7.023  1379229_at    Info    
   372 p  227.1 52 4.367  1382590_at  Similar to RIKEN cDNA 2310015N21  Info    
   373 p  908.7 2160.1 0.421  1387867_at  aldehyde dehydrogenase family 9, subfamily A1  Info   Aldh9a1 
   374 p  3122.1 551.9 5.657  1383241_at  complement component 1, r subcomponent (predicted)  Info   C1r_predicted 
   375 p  757.5 192.9 3.927  1387566_at  phospholipase A2, group IVA (cytosolic, calcium-dependent)  Info   Pla2g4a 
   376 p  280.1 817.2 0.343  1388526_at  Transcribed locus  Info    
   377 p  1230.5 3293.7 0.374  1388988_at  similar to RIKEN cDNA 1810013B01  Info   RGD:1359649 
   378 p  140.1 16.9 8.29  1379732_at  syntaxin 11 (predicted)  Info   Stx11_predicted 
   379 p  1058.4 2103.1 0.503  1387859_at  nitrogen fixation gene 1 (S. cerevisiae)  Info   Nfs1 
   380 p  288.1 54.6 5.277  1381376_at  Similar to [Mouse primary response gene B94 mRNA, 3end.], gene product  Info    
   381 p  204.3 480.4 0.425  1373524_at  BCL2-associated transcription factor 1 (predicted)  Info    
   382 p  494.3 184.3 2.682  1371776_at  Phosphatidylinositol 3-kinase, regulatory subunit, polypeptide 1  Info   Pik3r1 
   383 p  324.2 169.7 1.91  1390134_at  Transcribed locus  Info    
   384 p  3345.1 598.3 5.591  1386879_at  lectin, galactose binding, soluble 3  Info   Lgals3 
   385 p  2932.5 116.4 25.193  1367850_at  Fc gamma receptor II beta  Info   LOC498276 
   386 p  167 463.4 0.36  1382325_at  glycine C-acetyltransferase (2-amino-3-ketobutyrate-coenzyme A ligase) (predicted)  Info   Gcat_predicted 
   387 p  1353.3 32.4 41.769  1392647_at  Tryptophan hydroxylase  Info   Tph 
   388 p  228.6 18 12.7  1383407_at  Similar to RIKEN cDNA 1200013B08  Info    
   389 p  143 11.8 12.119  1384580_at  complement component 6  Info   C6 
   390 p  139 71.3 1.95  1388243_at  gene model 1012, (NCBI)  Info   Gm1012 
   391 p  503.7 1685.4 0.299  1378802_at  similar to hypothetical protein MGC13047  Info   RGD:1303232 
   392 p  750.6 223.3 3.361  1377759_at  BH3 interacting domain death agonist  Info   Bid 
   393 p  376.6 160.1 2.352  1367946_at  PDZ and LIM domain 1 (elfin)  Info   Pdlim1 
   394 p  1213.9 96.4 12.592  1370382_at  RT1 class II, locus Db1  Info   RT1-Db1 
   395 p  1118.7 355.7 3.145  1388629_at  inosine 5-monophosphate dehydrogenase 2  Info   Impdh2 
   396 p  1958.5 644.2 3.04  1371239_s_at  tropomyosin 3, gamma  Info   Tpm3 
   397 p  1736.6 4011.9 0.433  1387022_at  aldehyde dehydrogenase family 1, member A1  Info   Aldh1a1 
   398 p  428.6 129.9 3.299  1374537_at  Carbohydrate (chondroitin) synthase 1 (predicted)  Info    
   399 p  695.4 23.1 30.104  1379698_at  Transcribed locus  Info    
   400 p  1055.2 2559.4 0.412  1368092_at  fumarylacetoacetate hydrolase  Info   Fah 
   401 p  439.1 50.8 8.644  1388484_at  ubiquitin-conjugating enzyme E2C (predicted)  Info   Ube2c_predicted 
   402 p  303.3 786.6 0.386  1370897_at  Branched chain ketoacid dehydrogenase E1, alpha polypeptide  Info   Bckdha 
   403 p  1457.9 584.7 2.493  1369559_a_at  CD47 antigen (Rh-related antigen, integrin-associated signal transducer)  Info   Cd47 
   404 p  654.8 270 2.425  1373024_at  adaptor-related protein complex 3, sigma 1 subunit (predicted)  Info   Ap3s1_predicted 
   405 p  102.9 399 0.258  1377408_at  phospholipase A2, group VI  Info   Pla2g6 
   406 p  1790.8 357.4 5.011  1368418_a_at  ceruloplasmin  Info   Cp 
   407 p  529.1 103.3 5.122  1376056_at  similar to poly (ADP-ribose) polymerase family, member 10  Info   LOC300043 
   408 p  354.9 50.9 6.972  1381875_at  N-myc (and STAT) interactor (predicted)  Info   Nmi_predicted 
   409 p  3775.2 185 20.406  1398246_s_at  Fc receptor, IgG, low affinity III /// Fc gamma receptor II beta  Info   Fcgr3 /// LOC498276 
   410 p  334.8 144 2.325  1384935_at  Cleavage stimulation factor, 3' pre-RNA, subunit 3, 77kDa (predicted)  Info    
   411 p  923.6 78.1 11.826  1383320_at  lymphocyte protein tyrosine kinase  Info   Lck 
   412 p  785.8 290.1 2.709  1374897_at    Info    
   413 p  390.8 61.7 6.334  1373262_at  Similar to 2310014H01Rik protein (predicted)  Info    
   414 p  152.7 534.5 0.286  1372199_at  PTEN induced putative kinase 1 (predicted)  Info   Pink1_predicted 
   415 p  898.6 2933.3 0.306  1367909_at  dicarbonyl L-xylulose reductase  Info   Dcxr 
   416 p  2905.3 454 6.399  1388574_at  tryptophanyl-tRNA synthetase (predicted)  Info   Wars_predicted 
   417 p  1207.1 2507.2 0.481  1370200_at  glutamate dehydrogenase 1  Info   Glud1 
   418 p  466.1 33.2 14.039  1392280_at  toll-like receptor 2  Info   Tlr2 
   419 p  1481.6 58.7 25.24  1376693_at  similar to OEF2  Info   LOC500011 
   420 p  313.7 143.4 2.188  1370910_at  replication factor C (activator 1) 2  Info   Rfc2 
   421 p  609.1 91.2 6.679  1380410_at  Transcribed locus  Info    
   422 p  244.1 61.4 3.976  1389071_at  tangerin  Info   LOC309169 
   423 p  727.6 133.4 5.454  1368207_at  FXYD domain-containing ion transport regulator 5  Info   Fxyd5 
   424 p  302.4 44.6 6.78  1384483_at  Similar to RIKEN cDNA E030034P13 (predicted)  Info    
   425 p  124.1 47.8 2.596  1376281_at  Similar to hypothetical protein FLJ22349  Info    
   426 p  662.3 111.3 5.951  1388773_at  similar to [Mouse primary response gene B94 mRNA, 3end.], gene product  Info   LOC299339 
   427 p  2245 4494.7 0.499  1367702_at  acetyl-Coenzyme A dehydrogenase, medium chain  Info   Acadm 
   428 p  892.6 112.1 7.963  1389885_at  similar to RIKEN cDNA 0610025L06 (predicted)  Info   RGD1309967_predicted 
   429 p  1319.7 518.6 2.545  1371694_at    Info    
   430 p  381.3 79.9 4.772  1378633_at  leupaxin (predicted)  Info   Lpxn_predicted 
   431 p  2085.5 1234.1 1.69  1383478_at  Janus kinase 1  Info   Jak1 
   432 p  2625.9 341.4 7.692  1389014_at  pre-B-cell colony enhancing factor 1  Info   Pbef1 
   433 p  2713.7 4413.7 0.615  1388403_at  similar to NADP+-specific isocitrate dehydrogenase  Info   LOC361596 
   434 p  252.3 35.8 7.047  1370693_a_at  cyclic nucleotide phosphodiesterase 1  Info   Cnp1 
   435 p  580.6 1461.4 0.397  1368008_at  prominin 1  Info   Prom1 
   436 p  123.5 52.1 2.37  1373661_a_at  chemokine (C-X-C motif) receptor 4  Info   Cxcr4 
   437 p  2399.2 3991.4 0.601  1367589_at  aconitase 2, mitochondrial  Info   Aco2 
   438 p  601.7 82.1 7.329  1383662_at  hypothetical protein LOC500956  Info   LOC500956 
   439 p  465.6 98.2 4.741  1379295_at    Info    
   440 p  2441.5 508.9 4.798  1368356_a_at  type 1 tumor necrosis factor receptor shedding aminopeptidase regulator  Info   Arts1 
   441 p  198.3 706.3 0.281  1370036_at  sulfite oxidase  Info   Suox 
   442 p  543.9 83.2 6.537  1369294_at  bone marrow stromal cell antigen 1  Info   Bst1 
   443 p  1302.3 404.6 3.219  1368274_at  drebrin-like  Info   Dbnl 
   444 p  592.9 21.7 27.323  1393187_at  Similar to Ifi204 protein  Info    
   445 p  1630 3113.5 0.524  1386904_a_at  cytochrome b-5  Info   Cyb5 
   446 p  754.6 184.6 4.088  1383786_at  Transcribed locus  Info    
   447 p  752.1 235.1 3.199  1375006_at  Similar to RIKEN cDNA B130055L09 (predicted)  Info    
   448 p  825.9 2137.5 0.386  1367892_at  pyruvate dehydrogenase kinase, isoenzyme 2  Info   Pdk2 
   449 p  1015.3 2423.3 0.419  1386934_at  choline transporter  Info   Slc6a8 
   450 p  4470.8 2510.4 1.781  1387856_at  calponin 3, acidic  Info   Cnn3 
   451 p  465 81.7 5.692  1376920_at  similar to mKIAA2005 protein /// similar to mKIAA2005 protein /// similar to mKIAA2005 protein  Info   LOC500012 /// LOC500014 /// LOC500015 
   452 p  1457.8 800.2 1.822  1370188_at  splicing factor, arginine/serine-rich 10 (transformer 2 homolog, Drosophila)  Info   Sfrs10 
   453 p  1051.8 2803.5 0.375  1367908_at  glycine cleavage system protein H (aminomethyl carrier)  Info   Gcsh 
   454 p  1026.3 434.3 2.363  1373072_at  Transcribed locus  Info    
   455 p  758 98 7.735  1387897_at  cyclic nucleotide phosphodiesterase 1  Info   Cnp1 
   456 p  2337.1 1019.3 2.293  1372064_at  similar to chemokine (C-X-C motif) ligand 16  Info   Cxcl16 
   457 p  2007.8 497.5 4.036  1387918_at  tropomyosin 3, gamma  Info   Tpm3 
   458 p  179.9 292.5 0.615  1373258_at  cathepsin F (predicted)  Info   Ctsf_predicted 
   459 p  315.2 143.7 2.193  1377603_at  sorting nexing 24 (predicted)  Info   Snx24_predicted 
   460 p  197.4 51.5 3.833  1382177_at  Transcribed locus  Info    
   461 p  309 27.4 11.277  1368192_at  chemokine (C-X-C motif) receptor 3  Info   Cxcr3 
   462 p  303.3 37.1 8.175  1379688_at  Transcribed locus  Info    
   463 p  1280.8 223 5.743  1370972_x_at    Info   Multiple Gene Symbols 
   464 p  374.4 195.5 1.915  1393114_at  Transcribed locus  Info    
   465 p  1811.2 70.3 25.764  1391925_at  chemokine (C-C motif) ligand 19 (predicted)  Info   Ccl19_predicted 
   466 p  934 155.9 5.991  1388694_at  RT1-149 protein  Info   RT1-149 
   467 p  334.1 108.6 3.076  1388309_at  high mobility group AT-hook 1  Info   Hmga1 
   468 p  2456.9 900.9 2.727  1368809_at  CAP, adenylate cyclase-associated protein 1 (yeast)  Info   Cap1 
   469 p  2436.4 1232.6 1.977  1388335_at  transgelin 2 (predicted)  Info   Tagln2_predicted 
   470 p  475.4 98.5 4.826  1384525_at  similar to spleen specific 238 kDa protein with PH domain  Info   LOC313438 
   471 p  1108.1 2210 0.501  1368566_a_at  MIPP65 protein  Info   RGD:621020 
   472 p  611.1 32.1 19.037  1368455_at  natural killer cell group 7 sequence  Info   Nkg7 
   473 p  637.8 1894.7 0.337  1385072_at  galactose mutarotase (aldose 1-epimerase)  Info   RGD:1359459 
   474 p  428 947.9 0.452  1376723_a_at    Info    
   475 p  1197.3 39 30.7  1390956_at  Transcribed locus  Info    
   476 p  577.5 109.1 5.293  1367914_at  epithelial membrane protein 3  Info   Emp3 
   477 p  16.7 194.7 0.086  1383783_at  protocadherin 9 (predicted)  Info   Pcdh9_predicted 
   478 p  452.5 152.1 2.975  1388140_at  RAB13, member RAS oncogene family  Info   Rab13 
   479 p  881.5 70.7 12.468  1373897_at    Info    
   480 p  134.2 27.7 4.845  1387277_at  Yamaguchi sarcoma viral (v-yes-1) oncogene homolog  Info   Lyn 
   481 p  970.3 148.2 6.547  1389571_at  signal transducer and activator of transcription 2 (predicted)  Info   Stat2_predicted 
   482 p  4795.7 211.5 22.675  1397304_at  interferon gamma induced GTPase (predicted)  Info   Igtp_predicted 
   483 p  305.5 59.7 5.117  1382346_at  Transcribed locus  Info    
   484 p  76.4 41.5 1.841  1384151_at  Transcribed locus  Info    
   485 p  598.1 42.9 13.942  1389470_at  B-factor, properdin  Info   Bf 
   486 p  427.7 42 10.183  1387394_at  interleukin 2 receptor, beta chain  Info   Il2rb 
   487 p  542.3 235.4 2.304  1373447_at  similar to HN1-like protein (predicted)  Info   RGD1305117_predicted 
   488 p  1120.3 303.7 3.689  1387872_at  heterogeneous nuclear ribonucleoprotein A1  Info   Hnrpa1 
   489 p  200 17.1 11.696  1377967_at  Retroviral integration site 2 (predicted)  Info    
   490 p  541.8 27.9 19.419  1370090_at  lymphocyte cytosolic protein 2  Info   Lcp2 
   491 p  156.4 67.3 2.324  1393657_at  prolylcarboxypeptidase (angiotensinase C) (predicted)  Info   Prcp_predicted 
   492 p  3324.8 2029.7 1.638  1388351_at  similar to chromosome 13 open reading frame 12 (predicted)  Info   RGD1305831_predicted 
   493 p  294.6 93.7 3.144  1388521_at  pyrroline-5-carboxylate synthetase (glutamate gamma-semialdehyde synthetase) (predicted)  Info   Pycs_predicted 
   494 p  293.7 32 9.178  1370346_at  cyclin B1  Info   Ccnb1 
   495 p  52.2 169.7 0.308  1374574_at  Similar to hypothetical protein MGC38960 (predicted)  Info    
   496 p  727.8 332.9 2.186  1373876_at    Info    
   497 p  388.1 94.4 4.111  1377286_at  GTPase, IMAP family member 4  Info   Gimap4 
   498 p  439 125.4 3.501  1369716_s_at  lectin, galactose binding, soluble 5 /// lectin, galactose binding, soluble 9  Info   Lgals5 /// Lgals9 
   499 p  301.5 1486.5 0.203  1377966_at  Transcribed locus, weakly similar to XP_579465.1 PREDICTED: DNA binding protein N5 [Rattus norvegicus]  Info    
   500 p  894.9 415.9 2.152  1368385_a_at  growth factor receptor bound protein 2  Info   Grb2 
   501 p  89 291.3 0.306  1390855_at  Prolyl endopeptidase  Info   Prep 
   502 p  162.5 18.4 8.832  1370769_a_at  inducible T-cell co-stimulator  Info   Icos 
   503 p  4156.5 2182.5 1.904  1367590_at  RAN, member RAS oncogene family  Info   Ran 
   504 p  838 77.5 10.813  1381228_at  F-box protein 39 (predicted)  Info    
   505 p  290.7 43.6 6.667  1381014_at  interferon-induced protein 44 (predicted)  Info   Ifi44_predicted 
   506 p  1173.9 486.1 2.415  1367837_at  proteasome (prosome, macropain) subunit, alpha type 4  Info   Psma4 
   507 p  286.1 116.7 2.452  1373136_at  hypothetical LOC294390 (predicted)  Info   RGD1307672_predicted 
   508 p  107.9 403.6 0.267  1390126_at    Info    
   509 p  789 309.8 2.547  1376700_at  Transcribed locus, moderately similar to XP_580018.1 PREDICTED: hypothetical protein XP_580018 [Rattus norvegicus]  Info    
   510 p  1939.6 275.1 7.051  1368474_at  vascular cell adhesion molecule 1  Info   Vcam1 
   511 p  710.3 1707.4 0.416  1388463_at  putative secreted protein ZSIG11  Info   RGD:1359353 
   512 p  6188.1 1272.3 4.864  1371447_at  placenta-specific 8 (predicted)  Info   Plac8_predicted 
   513 p  94.1 2.5 37.64  1368501_s_at  mast cell protease 8 /// mast cell protease 10 /// mast cell protease 9 /// mast cell protease 8-like 2  Info   Mcpt8 /// Mcpt10 /// Mcpt9 /// RGD:1302938 
   514 p  328.5 926 0.355  1388998_at  erythrocyte protein band 4.9 (predicted)  Info   Epb4.9_predicted 
   515 p  72.8 241.8 0.301  1389166_at  calcium and integrin binding family member 2 (predicted)  Info   Cib2_predicted 
   516 p  389.8 166.8 2.337  1390133_at  BCL2/adenovirus E1B 19kDa-interacting protein 1, NIP2 (predicted)  Info   Bnip2_predicted 
   517 p  282.7 799.8 0.353  1376785_at  Synaptonemal complex protein 3  Info   Sycp3 
   518 p  508.2 115.5 4.4  1373830_at  hypothetical protein LOC619574  Info   LOC619574 
   519 p  231.4 34.2 6.766  1397772_at  Similar to putative membrane steroid receptor (predicted)  Info    
   520 p  191.3 486.4 0.393  1381124_at  Solute carrier family 25 (mitochondrial carrier; ornithine transporter) member 15 (predicted)  Info    
   521 p  1084 2787.6 0.389  1372492_at  claudin 10 (predicted)  Info   Cldn10_predicted 
   522 p  347.4 100.7 3.45  1370258_at  basic leucine zipper and W2 domains 2  Info   Bzw2 
   523 p  489.7 1376.5 0.356  1374478_at  similar to RIKEN cDNA 2610528J11 (predicted)  Info   RGD1305347_predicted 
   524 p  442.9 96.3 4.599  1374036_at  minichromosome maintenance deficient 2 mitotin (S. cerevisiae) (predicted)  Info   Mcm2_predicted 
   525 p  122.3 38.7 3.16  1386956_at  scavenger receptor class B, member 1  Info   Scarb1 
   526 p  423.8 1531.1 0.277  1370367_at  solute carrier family 1 (neuronal/epithelial high affinity glutamate transporter, system Xag), member 1  Info   Slc1a1 
   527 p  1095.9 549.9 1.993  1379891_at  scotin  Info   RGD:1359661 
   528 p  560.2 1355.2 0.413  1371629_at  CXXC finger 5  Info   RGD:1359466 
   529 p  502.9 122.5 4.105  1372236_at  Similar to Caspase recruitment domain protein 4  Info    
   530 p  609.7 124.8 4.885  1387027_a_at  lectin, galactose binding, soluble 9  Info   Lgals9 
   531 p  137.1 361.5 0.379  1389159_at  Transcribed locus  Info    
   532 p  252.7 124.4 2.031  1388458_at  replication factor C (activator 1) 4 (predicted)  Info   Rfc4_predicted 
   533 p  50.2 169.6 0.296  1374329_at  Mcf.2 transforming sequence-like  Info   Mcf2l 
   534 p  194 46.6 4.163  1379742_at  similar to SP140 nuclear body protein isoform 1  Info   LOC501175 
   535 p  4732 779.6 6.07  1393108_at  RT1 class I, A3  Info   RT1-A3 
   536 p  3079 175.6 17.534  1367973_at  chemokine (C-C motif) ligand 2  Info   Ccl2 
   537 p  229.2 25.6 8.953  1370621_at  CD3 antigen, zeta polypeptide  Info   Cd3z 
   538 p  1066.2 381.3 2.796  1367925_at  major vault protein  Info   Mvp 
   539 p  479.4 122.9 3.901  1370433_at  CCA2 protein  Info   Hsd3b7 
   540 1e-07 1364.8 560.8 2.434  1372865_at  zinc finger protein 364 (predicted)  Info   Zfp364_predicted 
   541 1e-07 4950.6 2832.4 1.748  1370243_a_at  prothymosin alpha  Info   Ptma 
   542 1e-07 1422.1 102.9 13.82  1372691_at  uridine phosphorylase 1 (predicted)  Info   Upp1_predicted 
   543 1e-07 100.3 1.9 52.789  1398644_at  Transcribed locus  Info    
   544 1e-07 2532.5 4131 0.613  1386871_at  glutathione peroxidase 4  Info   Gpx4 
   545 1e-07 494.4 1040.2 0.475  1370358_at  two pore channel 1  Info   Tpcn1 
   546 1e-07 2267.9 688.8 3.293  1371662_at  lysyl-tRNA synthetase  Info   RGD:1359653 
   547 1e-07 647.5 25.9 25  1370096_at  perforin 1 (pore forming protein)  Info   Prf1 
   548 1e-07 470.4 1118.2 0.421  1367673_at  selenium binding protein 2  Info   Selenbp1 
   549 1e-07 1444.7 2611.8 0.553  1368512_a_at  glutamyl aminopeptidase  Info   Enpep 
   550 1e-07 322.4 48.2 6.689  1392899_at  protein regulator of cytokinesis 1 (predicted)  Info   Prc1_predicted 
   551 1e-07 645.7 74.4 8.679  1369665_a_at  interleukin 18  Info   Il18 
   552 1e-07 115.6 349.9 0.33  1383086_at  Transcribed locus  Info    
   553 1e-07 1097.4 3389.2 0.324  1370960_at  insulin-like growth factor binding protein 5  Info   Igfbp5 
   554 1e-07 166.2 242.5 0.685  1381174_at  similar to Hypothetical protein KIAA0141  Info   RGD:735029 
   555 1e-07 105.3 4.2 25.071  1397292_at  LOC500333  Info   LOC500333 
   556 1e-07 625.7 1914.8 0.327  1372613_at  dehydrogenase/reductase (SDR family) member 6 (predicted)  Info   Dhrs6_predicted 
   557 1e-07 204.7 86.1 2.377  1384584_at  Transcribed locus  Info    
   558 1e-07 276 69.8 3.954  1373629_at  solute carrier family 7 (cationic amino acid transporter, y+ system), member 6 (predicted)  Info   Slc7a6_predicted 
   559 1e-07 174.8 26.7 6.547  1384852_at  low Mr GTP-binding protein  Info   Rab27a 
   560 1e-07 340.6 190 1.793  1389576_at  U2 small nuclear ribonucleoprotein B (predicted)  Info   Snrpb2_predicted 
   561 1e-07 183.1 893.4 0.205  1375926_at  Transcribed locus  Info    
   562 1e-07 386.7 46.5 8.316  1367825_at  ral guanine nucleotide dissociation stimulator  Info   Ralgds 
   563 1e-07 440.9 58.3 7.563  1372604_at  similar to RIKEN cDNA 2210421G13  Info   LOC315106 
   564 1e-07 956.5 335 2.855  1374468_at  myeloid differentiation primary response gene 88  Info   Myd88 
   565 1e-07 1965.4 62.4 31.497  1369031_at  interferon gamma inducing factor binding protein  Info   Il18bp 
   566 1e-07 765.6 326.1 2.348  1394082_at  similar to T-Brain-1  Info   LOC311078 
   567 1e-07 241.6 592.1 0.408  1383248_at  flavin containing monooxygenase 5  Info   Fmo5 
   568 1e-07 315 89.1 3.535  1374565_at  NIMA (never in mitosis gene a)-related expressed kinase 6  Info   Nek6 
   569 1e-07 196.3 58.3 3.367  1389477_at  Transcribed locus  Info    
   570 1e-07 1493.1 345.3 4.324  1390866_at  Transcribed locus  Info    
   571 1e-07 217.1 639.2 0.34  1394479_at  Transcribed locus  Info    
   572 1e-07 1742.8 814.3 2.14  1374387_at  ADP-ribosylation factor-like 6 interacting protein 5  Info   Arl6ip5 
   573 1e-07 437 178.8 2.444  1379258_at  kelch-like 5 (Drosophila) (predicted)  Info   Klhl5_predicted 
   574 1e-07 170.1 47.8 3.559  1389103_at  similar to RIKEN cDNA 2810037C03 (predicted)  Info   RGD1307397_predicted 
   575 1e-07 1753.1 2895.5 0.605  1367763_at  acetyl-coenzyme A acetyltransferase 1  Info   Acat1 
   576 1e-07 211.2 37.5 5.632  1368270_at  apolipoprotein B editing complex 1  Info   Apobec1 
   577 1e-07 3793 1790.6 2.118  1370928_at  LPS-induced TN factor  Info   Litaf 
   578 1e-07 1632.9 2204.4 0.741  1392681_at  RAB2, member RAS oncogene family  Info   Rab2 
   579 1e-07 601.8 70.4 8.548  1368742_at  complement component 5, receptor 1  Info   C5r1 
   580 1e-07 570.7 1167.7 0.489  1377899_at  similar to RIKEN cDNA 2810025M15 (predicted)  Info   RGD1304982_predicted 
   581 1e-07 287 26.7 10.749  1378321_at  Ras association (RalGDS/AF-6) domain family 4 (predicted)  Info   Rassf4_predicted 
   582 1e-07 1169.9 475.1 2.462  1391411_at  Formin-like 2 (predicted)  Info    
   583 1e-07 518.7 37.5 13.832  1369549_at  NKR-P2, ortholog of human NKG2D  Info   Nkrp2 
   584 1e-07 207.4 36.4 5.698  1394678_at  FYVE, RhoGEF and PH domain containing 2 (predicted)  Info    
   585 1e-07 2669.7 264.9 10.078  1367655_at  similar to thymosin, beta 10  Info   LOC502600 
   586 1e-07 821.8 264.4 3.108  1367922_at  a disintegrin and metalloproteinase domain 17 (tumor necrosis factor, alpha, converting enzyme)  Info   Adam17 
   587 1e-07 347.7 139.7 2.489  1375362_at    Info    
   588 1e-07 178.9 27.1 6.601  1376688_a_at  Transcribed locus, moderately similar to NP_705730.1 paired immunoglobin-like type 2 receptor alpha [Mus musculus]  Info    
   589 1e-07 592.1 186.4 3.177  1383158_at  similar to HIV-1 Rev binding protein  Info   LOC363266 
   590 1e-07 951.4 1531.3 0.621  1392527_at  similar to homolog of yeast TIM14 isoform c  Info   LOC502525 
   591 1e-07 126.9 414 0.307  1368081_at  ATP-binding cassette, sub-family A (ABC1), member 2  Info   Abca2 
   592 1e-07 142.9 349.4 0.409  1383850_at  Similar to RIKEN cDNA 1600014C10  Info    
   593 1e-07 708.2 79.5 8.908  1383424_at  thymidylate kinase family LPS-inducible member (predicted)  Info   Tyki_predicted 
   594 1e-07 341.6 856.4 0.399  1372328_at  kinesin-like 8 (predicted)  Info   Knsl8_predicted 
   595 1e-07 252.7 709.3 0.356  1398282_at  kynureninase (L-kynurenine hydrolase)  Info   Kynu 
   596 1e-07 3363.4 1552.1 2.167  1398757_at  nucleophosmin 1  Info   Npm1 
   597 1e-07 274.4 20.9 13.129  1368260_at  aurora kinase B  Info   Aurkb 
   598 1e-07 147.8 339.6 0.435  1368409_at  glutathione S-transferase, theta 2  Info   Gstt2 
   599 1e-07 1397.5 476.2 2.935  1382274_at  retinoic acid receptor responder (tazarotene induced) 1 (predicted)  Info   Rarres1_predicted 
   600 1e-07 596.7 238.3 2.504  1378137_at  similar to ribosome-binding protein p34 - rat (predicted)  Info   RGD1305092_predicted 
   601 1e-07 783 14 55.929  1369290_at  chemokine (C-C) receptor 5  Info   Ccr5 
   602 1e-07 385.3 102.7 3.752  1379356_at  Similar to RIKEN cDNA C230093N12 (predicted)  Info    
   603 1e-07 53.5 193.6 0.276  1379587_at    Info    
   604 1e-07 1055 41.9 25.179  1388275_at  T-cell receptor beta chain  Info   Tcrb 
   605 1e-07 550.1 201.3 2.733  1372550_at    Info    
   606 1e-07 1138 483.3 2.355  1369621_s_at  FK506 binding protein 1a  Info   Fkbp1a 
   607 1e-07 422.9 47 8.998  1371074_a_at  mini chromosome maintenance deficient 6 (S. cerevisiae)  Info   Mcmd6 
   608 1e-07 299.2 993.6 0.301  1390569_at  similar to carnosinase 1  Info   RGD:1359493 
   609 1e-07 368.4 104.3 3.532  1376379_a_at  similar to RIKEN cDNA 2310004I24 gene (predicted)  Info   RGD1309906_predicted 
   610 1e-07 699.8 1601.2 0.437  1388330_at  vitamin K epoxide reductase complex, subunit 1  Info   RGD:1303107 
   611 1e-07 379 37.8 10.026  1370400_at  RT1 class Ib gene, H2-TL-like, grc region (N1) /// RT1 class Ib gene, H2-TL-like, grc region (N3) /// RT1 class Ib gene, H2-TL-like, grc region(N2)  Info   RT1-N1 /// RT1-N3 /// RT1-N2 
   612 1e-07 401.4 122.4 3.279  1397628_at  similar to RIKEN cDNA 2900092E17 (predicted)  Info   RGD1305592_predicted 
   613 1e-07 256.2 77.2 3.319  1374725_at  similar to mKIAA1631 protein  Info   LOC310756 
   614 1e-07 1629.5 1225.6 1.33  1387108_at  casein kinase 2, beta subunit  Info   Csnk2b 
   615 1e-07 160 9.7 16.495  1377832_at  polo-like kinase 4 (Drosophila) (predicted)  Info   Plk4_predicted 
   616 1e-07 277.2 102.2 2.712  1392528_at  Granulocyte-macrophage colony stimulating receptor alpha (CSFR alpha gene)  Info    
   617 1e-07 1654 3315.8 0.499  1370806_at  all-trans-13,14-dihydroretinol saturase  Info   Retsat 
   618 1e-07 748.3 27.7 27.014  1392737_at  Transcribed locus  Info    
   619 1e-07 585.1 1419.9 0.412  1380577_at  ATP-binding cassette, sub-family G (WHITE), member 2  Info   Abcg2 
   620 1e-07 2594.6 1780 1.458  1371938_at  GPI-anchored membrane protein 1 (predicted)  Info    
   621 1e-07 283.9 122.9 2.31  1373850_at  sphingomyelin phosphodiesterase, acid-like 3B (predicted)  Info   Smpdl3b_predicted 
   622 1e-07 3196.8 6211.1 0.515  1372170_at  aminoacylase 1  Info   Acy1 
   623 1e-07 2435.3 967.4 2.517  1371968_at  CGI-119 protein  Info   Cgi119 
   624 1e-07 308.6 94.8 3.255  1390384_at  similar to Histone H2A.x (H2a/x)  Info   LOC500987 
   625 1e-07 261.3 63.1 4.141  1375989_a_at  similar to nuclear factor kappa B subunit p100 (predicted)  Info   RGD1307189_predicted 
   626 1e-07 703.1 1972.8 0.356  1368575_at  solute carrier family 6 (neurotransmitter transporter), member 18  Info   Slc6a18 
   627 1e-07 2013.2 584.3 3.445  1370340_x_at  tropomyosin 3, gamma  Info   Tpm3 
   628 1e-07 114.6 21.5 5.33  1397655_at    Info    
   629 1e-07 4389.9 142.3 30.85  1368073_at  interferon regulatory factor 1  Info   Irf1 
   630 1e-07 130.2 33.3 3.91  1393201_at  similar to lymphoid-restricted membrane protein  Info   LOC500361 
   631 1e-07 79.1 235 0.337  1393666_at  Transcribed locus  Info    
   632 1e-07 4902.4 205.6 23.844  1382950_at  Guanylate nucleotide binding protein 3 (predicted)  Info    
   633 1e-07 619 182.5 3.392  1372643_at  similar to protein 4.1G  Info   LOC309557 
   634 1e-07 399.2 35.5 11.245  1369520_a_at  branched chain aminotransferase 1, cytosolic  Info   Bcat1 
   635 1e-07 1258 2855.8 0.441  1367672_at  hydroxysteroid (17-beta) dehydrogenase 4  Info   Hsd17b4 
   636 1e-07 5654.3 226.5 24.964  1370186_at  proteosome (prosome, macropain) subunit, beta type 9  Info   Psmb9 
   637 1e-07 1273.2 2011.3 0.633  1371388_at  pyruvate dehydrogenase (lipoamide) beta  Info   RGD:1359146 
   638 1e-07 6345.7 1497.6 4.237  1371033_at  RT1 class II, locus Bb  Info   RT1-Bb 
   639 1e-07 119 30.8 3.864  1374799_at  similar to mKIAA0159 protein  Info   LOC362438 
   640 1e-07 156.8 24.1 6.506  1371106_at  integrin, beta 7  Info   Itgb7 
   641 1e-07 8350.3 2896.6 2.883  1367679_at  CD74 antigen (invariant polpypeptide of major histocompatibility class II antigen-associated)  Info   Cd74 
   642 1e-07 225.6 97.9 2.304  1373997_at  Transcribed locus  Info    
   643 1e-07 361.3 1163.5 0.311  1368428_at  X-prolyl aminopeptidase (aminopeptidase P) 2, membrane-bound  Info   Xpnpep2 
   644 1e-07 661.4 179.7 3.681  1373164_at  Serine/threonine kinase 17b (apoptosis-inducing)  Info   Stk17b 
   645 1e-07 2256.3 989 2.281  1370890_at  ARP3 actin-related protein 3 homolog (yeast)  Info   Actr3 
   646 1e-07 347.4 64 5.428  1371782_at  NIPSNAP-related protein  Info   RGD:1359688 
   647 1e-07 312.1 82.7 3.774  1380019_at  T-cell, immune regulator 1  Info   Tcirg1 
   648 1e-07 4899.5 3351.8 1.462  1367567_at  ribosomal protein L6  Info   Rpl6 
   649 1e-07 120.7 753.3 0.16  1379452_at  similar to growth arrest-specific protein 2 - mouse  Info   LOC499156 
   650 1e-07 1085.2 342.2 3.171  1388455_at  guanine nucleotide binding protein (G protein), gamma 10  Info   Gng10 
   651 1e-07 1257.9 87.4 14.392  1379285_at  similar to 5830458K16Rik protein (predicted)  Info   RGD1306974_predicted 
   652 1e-07 492.8 255.8 1.927  1376250_at  nuclear fragile X mental retardation protein interacting protein 1  Info   RGD:1359440 
   653 1e-07 256.6 18.4 13.946  1367794_at  alpha-2-macroglobulin  Info   A2m 
   654 1e-07 22.4 1.5 14.933  1381536_at  Similar to expressed sequence C79127  Info    
   655 1e-07 438.6 772.2 0.568  1388976_at  similar to BolA domain-containing protein like (11.4 kD) (1P25) (predicted)  Info   RGD1305975_predicted 
   656 1e-07 414.1 50.2 8.249  1367776_at  cell division cycle 2 homolog A (S. pombe)  Info   Cdc2a 
   657 1e-07 1417.8 555.3 2.553  1367628_at  lectin, galactose binding, soluble 1  Info   Lgals1 
   658 1e-07 1114.9 2953.4 0.377  1374628_at  crystallin, zeta (predicted)  Info   Cryz_predicted 
   659 1e-07 164.9 293.7 0.561  1391421_at  similar to Acyl-CoA dehydrogenase family member 8, mitochondrial precursor (ACAD-8) (Isobutyryl-CoA dehydrogenase)  Info   LOC367046 
   660 1e-07 438 69.7 6.284  1388924_at  angiopoietin-like protein 4  Info   Angptl4 
   661 1e-07 446.2 1092.7 0.408  1371795_at    Info    
   662 1e-07 234 814.7 0.287  1377145_at  similar to very large G-protein coupled receptor 1  Info   LOC294614 
   663 1e-07 731.3 1868.9 0.391  1372676_at  similar to RIKEN cDNA 1110025H10 (predicted)  Info   RGD1304560_predicted 
   664 1e-07 306.6 31.1 9.859  1393329_at  Inducible T-cell co-stimulator  Info   Ailim 
   665 1e-07 328.3 39.1 8.396  1382902_at  potential ubiquitin ligase  Info   Herc6 
   666 1e-07 206.3 80.7 2.556  1380619_at  similar to RIKEN cDNA 3110001I22 /// similar to RIKEN cDNA 3110001I22  Info   LOC293819 /// LOC361116 
   667 1e-07 295.6 694.5 0.426  1386773_at  biotinidase (predicted)  Info   Btd_predicted 
   668 1e-07 1585.1 2920.5 0.543  1368470_at  gamma-glutamyl hydrolase  Info   Ggh 
   669 1e-07 401.1 26.3 15.251  1384298_at  myosin IF (predicted)  Info   Myo1f_predicted 
   670 1e-07 551.5 120.2 4.588  1389355_at  immediate early response 5  Info   Ier5 
   671 1e-07 239.6 77.3 3.1  1367600_at  desmin  Info   Des 
   672 1e-07 611 15.5 39.419  1392171_at  chitinase 3-like 1  Info   Chi3l1 
   673 1e-07 2524.4 1088.4 2.319  1367671_at  proliferating cell nuclear antigen  Info   Pcna 
   674 1e-07 1392.6 3099.1 0.449  1388884_at  similar to RIKEN cDNA 1810022C23 (predicted)  Info   RGD1310224_predicted 
   675 1e-07 125.4 12.7 9.874  1385426_at  similar to hypothetical protein FLJ20647 (predicted)  Info   RGD1305326_predicted 
   676 1e-07 204.6 18.7 10.941  1372516_at  kinesin family member 22 (predicted)  Info   Kif22_predicted 
   677 1e-07 230.3 20.4 11.289  1373777_at  Transcribed locus  Info    
   678 1e-07 620 25.3 24.506  1376100_at  similar to RIKEN cDNA 2310057H16 (predicted)  Info   RGD1305887_predicted 
   679 1e-07 315.5 91.1 3.463  1375981_a_at  similar to Su(var)3-9 homolog  Info   LOC302553 
   680 1e-07 1384.4 401.5 3.448  1368419_at  ceruloplasmin  Info   Cp 
   681 1e-07 345.1 13.8 25.007  1378418_at  similar to MGC37193 protein (predicted)  Info   RGD1311681_predicted 
   682 1e-07 2644.1 80 33.051  1377950_at  similar to interferon-inducible GTPase (predicted)  Info   RGD1309362_predicted 
   683 1e-07 246.9 553.2 0.446  1383324_at  Similar to PALS2-alpha splice variant  Info    
   684 1e-07 336.5 64.9 5.185  1377497_at  2'-5' oligoadenylate synthetase-like 1 (predicted)  Info   Oasl1_predicted 
   685 1e-07 359.3 712.6 0.504  1373854_at  CDNA clone IMAGE:7324675  Info    
   686 1e-07 5835 2333.1 2.501  1367605_at  profilin 1  Info   Pfn1 
   687 1e-07 1020.8 291.2 3.505  1386882_at  t-complex testis expressed 1  Info   Tctex1 
   688 1e-07 3935.4 1503.5 2.617  1370172_at  superoxide dismutase 2, mitochondrial  Info   Sod2 
   689 1e-07 683.4 43 15.893  1390325_at  CD38 antigen  Info   Cd38 
   690 1e-07 881.5 306.7 2.874  1383401_at  similar to Testis derived transcript  Info   LOC500040 
   691 1e-07 3281 70.9 46.276  1391489_at  interferon inducible protein 1 (predicted)  Info   Ifi1_predicted 
   692 1e-07 631.4 25.4 24.858  1369427_at  macrophage expressed gene 1  Info   Mpeg1 
   693 1e-07 152.1 499.2 0.305  1382003_at  solute carrier family 16 (monocarboxylic acid transporters), member 13  Info   RGD:1359138 
   694 1e-07 2750.8 115.9 23.734  1382314_at  interferon, alpha-inducible protein (clone IFI-15K) (predicted)  Info   G1p2_predicted 
   695 1e-07 147.2 755.7 0.195  1367805_at  glutaminase  Info   Gls 
   696 1e-07 1188.6 221.8 5.359  1372303_at  similar to 0910001A06Rik protein (predicted)  Info   RGD1307697_predicted 
   697 1e-07 158.5 78.1 2.029  1372308_at  CDNA clone IMAGE:7366335  Info    
   698 1e-07 284.4 44.5 6.391  1388650_at  topoisomerase (DNA) 2 alpha  Info   Top2a 
   699 1e-07 2966.6 1468.7 2.02  1370871_at  hypothetical gene supported by Y16641; Y16641  Info   LOC288146 
   700 1e-07 358.3 784 0.457  1372104_at    Info    
   701 1e-07 444.2 200.2 2.219  1398347_at  AXL receptor tyrosine kinase (predicted)  Info   Axl_predicted 
   702 1e-07 1580 3002.6 0.526  1369630_at  adenosine kinase  Info   Adk 
   703 1e-07 157.5 512.6 0.307  1395268_at  WW domain containing E3 ubiquitin protein ligase 1 (predicted)  Info   Wwp1_predicted 
   704 1e-07 463.7 1451.7 0.319  1388738_at  Similar to mKIAA0534 protein  Info    
   705 1e-07 585.1 1773.5 0.33  1383395_at  agmatine ureohydrolase (agmatinase) (predicted)  Info   Agmat_predicted 
   706 1e-07 1120.1 2199.8 0.509  1371680_at    Info    
   707 1e-07 2488.5 129.9 19.157  1368000_at  complement component 3  Info   C3 
   708 1e-07 1.4 65.3 0.021  1390596_at  melan-A (predicted)  Info   Mlana_predicted 
   709 1e-07 3230 1700 1.9  1371352_at  high mobility group protein 17  Info   RGD:620649 
   710 1e-07 234 68.5 3.416  1379248_at  Prolylcarboxypeptidase (angiotensinase C) (predicted)  Info    
   711 1e-07 464 28.5 16.281  1371070_at  tumor stroma and activated macrophage protein DLM-1  Info   Zbp1 
   712 1e-07 604.9 1908.6 0.317  1370020_at  solute carrier family 25 (mitochondrial carrier; dicarboxylate transporter), member 10  Info   RGD:621430 
   713 1e-07 280.4 1113.4 0.252  1393061_at  similar to cDNA sequence BC021608  Info   RGD:1359544 
   714 1e-07 1131.1 2278.5 0.496  1392590_at  Rho GTPase activating protein 24 (predicted)  Info   Arhgap24_predicted 
   715 1e-07 132.6 30.4 4.362  1382078_at  Transcribed locus  Info    
   716 1e-07 206.6 400 0.516  1389498_at    Info    
   717 1e-07 810.3 68.4 11.846  1389034_at  similar to ubiquitin specific protease UBP43  Info   RGD:1359153 
   718 1e-07 124.3 467.9 0.266  1374217_at  similar to chromosome 16 open reading frame 5 (predicted)  Info   RGD1310686_predicted 
   719 1e-07 1436.6 182.8 7.859  1387659_at  guanine deaminase  Info   Gda 
   720 1e-07 368.6 760.2 0.485  1371931_at  general transcription factor II I  Info   Gtf2i 
   721 1e-07 2526.1 24.5 103.106  1370628_at  granzyme B  Info   Gzmb 
   722 1e-07 315.5 154.3 2.045  1372875_at  Transcribed locus  Info    
   723 1e-07 1075.9 2110.8 0.51  1374222_at  tumor suppressing subtransferable candidate 5  Info   RGD:1303323 
   724 1e-07 9150.7 1279 7.155  1372585_at  LOC499318  Info   LOC499318 
   725 1e-07 27.4 99.1 0.276  1369292_at  hydroxysteroid (17-beta) dehydrogenase 1  Info   Hsd17b1 
   726 1e-07 1257.7 476 2.642  1393987_s_at  G protein-coupled receptor kinase 6  Info   Gprk6 
   727 1e-07 407.3 1546.8 0.263  1388644_at  Monoglyceride lipase  Info   Mgll 
   728 1e-07 872.3 499.4 1.747  1390407_at  similar to Protein C3orf4 homolog  Info   RGD:1359273 
   729 1e-07 5370.3 2753.9 1.95  1369937_at  calmodulin 1  Info   Calm1 
   730 1e-07 683.2 283.8 2.407  1388794_at  RNA binding motif protein, X chromosome retrogene (predicted)  Info   Rbmxrt_predicted 
   731 1e-07 480.1 260.7 1.842  1372453_at  down-regulator of transcription 1 (predicted)  Info   Dr1_predicted 
   732 1e-07 78.4 224.4 0.349  1389256_at  Transcribed locus  Info    
   733 1e-07 695.5 88.4 7.868  1393144_at  N-myc (and STAT) interactor (predicted)  Info    
   734 1e-07 214.9 467.8 0.459  1390993_at  MAWD binding protein  Info   Mawbp 
   735 1e-07 213 61.7 3.452  1368555_at  CD37 antigen  Info   Cd37 
   736 1e-07 75.2 219.7 0.342  1387765_at  mannose binding lectin 1, protein A  Info   Mbl1 
   737 1e-07 1543 3258.4 0.474  1367695_at  quinoid dihydropteridine reductase  Info   Qdpr 
   738 1e-07 406.7 1301.7 0.312  1368234_at  prolyl endopeptidase  Info   Prep 
   739 1e-07 2131.9 992.7 2.148  1372513_at  ras-related C3 botulinum toxin substrate 1  Info   Rac1 
   740 1e-07 148.7 406.8 0.366  1375674_at  similar to chromosome 16 open reading frame 5 (predicted)  Info   RGD1310686_predicted 
   741 1e-07 174.5 410.5 0.425  1389592_at  Transcribed locus  Info    
   742 1e-07 1265.1 2823.8 0.448  1367999_at  aldehyde dehydrogenase 2  Info   Aldh2 
   743 1e-07 372.6 972.1 0.383  1373693_at  G protein-coupled receptor, family C, group 5, member C (predicted)  Info   Gprc5c_predicted 
   744 1e-07 153.9 342.2 0.45  1383421_at  Transcribed locus  Info    
   745 1e-07 202.9 600.5 0.338  1371420_at  92Aa-Protein  Info   RGD:1359519 
   746 1e-07 937.8 378.7 2.476  1389227_at  ras homolog gene family, member G (predicted)  Info   Rhog_predicted 
   747 1e-07 10.4 1 10.4  1371164_at  mast cell protease 10  Info   Mcpt10 
   748 1e-07 158.9 640.4 0.248  1372323_at  sarcosine dehydrogenase  Info   Sardh 
   749 1e-07 488.9 273.3 1.789  1388588_at  mammary tumor virus receptor 2 (predicted)  Info   Mtvr2_predicted 
   750 1e-07 69.6 173.9 0.4  1372872_at  Similar to adenylate kinase isozyme 5  Info    
   751 1e-07 348.1 64.9 5.364  1369262_at  caspase 8  Info   Casp8 
   752 1e-07 1636.4 748.9 2.185  1367693_at  tyrosine 3-monooxygenase/tryptophan 5-monooxygenase activation protein, eta polypeptide  Info   Ywhah 
   753 1e-07 1223.1 303.6 4.029  1374006_at  kynurenine aminotransferase III  Info   RGD:1359262 
   754 1e-07 1142.8 2124.6 0.538  1371405_at  Similar to hypothetical protein MGC52110  Info    
   755 1e-07 380.3 129.1 2.946  1393029_at  CUG triplet repeat, RNA binding protein 1 (predicted)  Info    
   756 1e-07 1785.6 710 2.515  1388776_at  scotin  Info   RGD:1359661 
   757 1e-07 262.2 65.1 4.028  1369688_s_at  protein tyrosine kinase 2 beta  Info   Ptk2b 
   758 1e-07 1406.8 258.7 5.438  1388460_at  capping protein (actin filament), gelsolin-like (predicted)  Info   Capg_predicted 
   759 1e-07 256.8 74.3 3.456  1380445_at  Transcribed locus, strongly similar to XP_515868.1 PREDICTED: sodium channel, voltage-gated, type III, alpha [Pan troglodytes]  Info    
   760 1e-07 695.3 1807.9 0.385  1368091_at  5-oxoprolinase (ATP-hydrolysing)  Info   Oplah 
   761 1e-07 592.3 2621.1 0.226  1368329_at  solute carrier family 22 (organic anion transporter), member 6  Info   Slc22a6 
   762 1e-07 1260.4 2381.9 0.529  1388148_a_at  low density lipoprotein receptor-related protein associated protein 1  Info   Lrpap1 
   763 1e-07 825.4 1711.2 0.482  1371948_at  Down syndrome critical region gene 5 (predicted)  Info   Dscr5_predicted 
   764 1e-07 663.1 37 17.922  1370113_at  inhibitor of apoptosis protein 1  Info   Birc3 
   765 1e-07 1273.5 139.3 9.142  1398390_at  similar to Small inducible cytokine B13 precursor (CXCL13) (B lymphocyte chemoattractant) (CXC chemokine BLC)  Info   LOC498335 
   766 1e-07 99.7 3.8 26.237  1385605_at    Info    
   767 1e-07 1436.7 623.1 2.306  1369319_at  ADP-ribosylation factor-like 6 interacting protein 5  Info   Arl6ip5 
   768 1e-07 560.8 302.7 1.853  1373940_at  non-catalytic region of tyrosine kinase adaptor protein 1 (predicted)  Info   Nck1_predicted 
   769 1e-07 826.5 1909.3 0.433  1374524_at  selenocysteine lyase  Info   RGD:1359514 
   770 1e-07 659.2 163.1 4.042  1385658_at  zinc finger protein 313  Info   RGD:1303139 
   771 1e-07 241.2 101 2.388  1368311_at  O-6-methylguanine-DNA methyltransferase  Info   Mgmt 
   772 1e-07 466.1 93.5 4.985  1376835_at  Solute carrier family 35, member B2  Info   Slc35b2 
   773 1e-07 174.1 35.6 4.89  1379235_x_at  similar to CDC45L  Info   LOC287961 
   774 1e-07 3908.7 2332.8 1.676  1398831_at  proteasome (prosome, macropain) subunit, beta type 4  Info   Psmb4 
   775 1e-07 402.3 48.7 8.261  1381279_at  receptor (TNFRSF)-interacting serine-threonine kinase 2 (predicted)  Info   Ripk2_predicted 
   776 1e-07 557.3 114.2 4.88  1373670_at  Signal transducer and activator of transcription 2 (predicted)  Info    
   777 1e-07 440.6 83.9 5.251  1372055_at  Similar to P-Rex1 (predicted)  Info    
   778 1e-07 73.2 35.1 2.085  1379228_at  similar to flt3 ligand  Info   LOC499148 
   779 1e-07 3061 83.3 36.747  1368187_at  glycoprotein (transmembrane) nmb  Info   Gpnmb 
   780 1e-07 92.4 375.7 0.246  1382447_at    Info    
   781 1e-07 1214.4 213.1 5.699  1372256_at    Info    
   782 1e-07 524.5 217.9 2.407  1384022_at    Info    
   783 1e-07 312.5 82.1 3.806  1383962_at  Cd27 binding protein (Hindu God of destruction)  Info   Siva 
   784 1e-07 297.7 190.8 1.56  1367723_a_at  linker of T-cell receptor pathways  Info   Lnk 
   785 1e-07 305.2 63.7 4.791  1386586_at  similar to PAK/PLC-interacting protein 1  Info   LOC361232 
   786 1e-07 37.3 178.5 0.209  1373736_at  Transcribed locus, weakly similar to NP_082606.1 hypothetical protein LOC72704 [Mus musculus]  Info    
   787 1e-07 1124.5 520.1 2.162  1386897_at  heterogeneous nuclear ribonucleoproteins methyltransferase-like 2 (S. cerevisiae)  Info   Hrmt1l2 
   788 1e-07 249.7 46.4 5.381  1385572_at  Transcribed locus  Info    
   789 1e-07 1311.9 562.4 2.333  1372461_at  SET translocation (predicted) /// LOC499767  Info   Set_predicted /// LOC499767 
   790 1e-07 51.1 84.4 0.605  1374606_at  MYST histone acetyltransferase 1 (predicted)  Info   Prss8 
   791 1e-07 297.1 784 0.379  1374512_at  Transcribed locus, strongly similar to XP_214823.2 PREDICTED: similar to Blvrb protein [Rattus norvegicus]  Info    
   792 1e-07 348.5 127.8 2.727  1395840_at  eukaryotic translation initiation factor 1A (predicted)  Info   Eif1a_predicted 
   793 1e-07 359.4 703.9 0.511  1377844_at  zinc binding alcohol dehydrogenase, domain containing 1 (predicted)  Info   Zadh1_predicted 
   794 1e-07 232.9 46.5 5.009  1371071_at  guanine nucleotide binding protein beta 4 subunit  Info   RGD:1359321 
   795 1e-07 365.3 20.1 18.174  1368783_at  inducible T-cell co-stimulator  Info   Icos 
   796 1e-07 3173.8 1333.4 2.38  1399158_a_at  nucleophosmin 1  Info   Npm1 
   797 1e-07 369.9 53.7 6.888  1394819_at  SP110 nuclear body protein (predicted)  Info    
   798 1e-07 1757.8 2782.2 0.632  1372076_at  hepatitis B virus x interacting protein (predicted)  Info   Hbxip_predicted 
   799 2e-07 426.8 136 3.138  1370440_at  peptide/histidine transporter  Info   Slc15a4 
   800 2e-07 410.2 1342.5 0.306  1379250_at  galactose mutarotase (aldose 1-epimerase)  Info   RGD:1359459 
   801 2e-07 1017.8 349.8 2.91  1373775_at  Hypothetical LOC311956  Info    
   802 2e-07 234.8 57.8 4.062  1379563_at  interferon regulatory factor 5 (predicted)  Info   Irf5_predicted 
   803 2e-07 372.2 26.8 13.888  1385696_at    Info    
   804 2e-07 216 51.1 4.227  1391808_at  Transcribed locus  Info    
   805 2e-07 1376.1 2212.2 0.622  1398909_at  similar to QIL1  Info   LOC301124 
   806 2e-07 652.4 1850 0.353  1390416_at  solute carrier family 25, member 30  Info   RGD:1359702 
   807 2e-07 59.4 341.3 0.174  1377635_at  flavin containing monooxygenase 2  Info   Fmo2 
   808 2e-07 175.4 69.1 2.538  1375732_at  Transcribed locus  Info    
   809 2e-07 289.6 532.6 0.544  1371805_at  similar to RIKEN cDNA C730048E16 (predicted)  Info   RGD1309382_predicted 
   810 2e-07 5141.2 3613.5 1.423  1371295_at  ribosomal protein S20  Info   Rps20 
   811 2e-07 784.4 2372.8 0.331  1370209_at  basic transcription element binding protein 1  Info   Bteb1 
   812 2e-07 326.7 125.9 2.595  1379287_at  retinitis pigmentosa 1 (autosomal dominant) (predicted) /// similar to cylindromatosis (turban tumor syndrome) /// similar to cylindromatosis (turban tumor syndrome)  Info   Rp1_predicted /// LOC361373 /// LOC498035 
   813 2e-07 3726.4 121.5 30.67  1367786_at  proteosome (prosome, macropain) subunit, beta type 8  Info   Psmb8 
   814 2e-07 144.2 2.5 57.68  1392731_at  hypothetical gene supported by NM_130426  Info   LOC497808 
   815 2e-07 307.7 74.5 4.13  1388436_at  small nuclear ribonucleoprotein polypeptide A (predicted)  Info   Snrpa_predicted 
   816 2e-07 170.2 475.5 0.358  1397584_at  Transcribed locus  Info    
   817 2e-07 7156.7 3282.5 2.18  1388071_x_at  RT1 class Ib, locus Aw2  Info   RT1-Aw2 
   818 2e-07 469.1 935.5 0.501  1371729_at  Yippee-like 5 (Drosophila) (predicted)  Info    
   819 2e-07 245.4 31.2 7.865  1398602_at  MAD2 (mitotic arrest deficient, homolog)-like 1 (yeast) (predicted)  Info    
   820 2e-07 2679.6 1244 2.154  1371653_at    Info    
   821 2e-07 1014 350.6 2.892  1370463_x_at  RT1 class I, CE16  Info   RGD:1359403 
   822 2e-07 164.5 27.7 5.939  1370810_at  cyclin D2  Info   Ccnd2 
   823 2e-07 2420.3 1425 1.698  1398853_at  proteasome (prosome, macropain) subunit, beta type 3  Info   Psmb3 
   824 2e-07 490.5 81.2 6.041  1393460_at  similar to GARP protein precursor (Garpin) (Glycoprotein A repetitions predominant) (predicted)  Info   RGD1310771_predicted 
   825 2e-07 277.6 72.9 3.808  1388135_at  replication protein A2  Info   Rpa2 
   826 2e-07 135.1 24 5.629  1391803_at  Transcribed locus  Info    
   827 2e-07 1309.1 858.2 1.525  1370189_at  splicing factor, arginine/serine-rich 10 (transformer 2 homolog, Drosophila)  Info   Sfrs10 
   828 2e-07 2.7 57.2 0.047  1368894_at  CAP, adenylate cyclase-associated protein, 2 (yeast)  Info   Cap2 
   829 2e-07 906.4 3106.8 0.292  1368467_at  cytochrome P450, family 4, subfamily F, polypeptide 2  Info   Cyp4f2 
   830 2e-07 669.5 143.3 4.672  1372032_at    Info    
   831 2e-07 1360.2 2084.1 0.653  1371974_at  phytanoyl-CoA dioxygenase domain containing 1 (predicted)  Info   Phyhd1_predicted 
   832 2e-07 958.4 7.8 122.872  1387831_at  small inducible cytokine subfamily C, member 1 (lymphotactin)  Info   Xcl1 
   833 2e-07 1119.3 631.5 1.772  1372460_at  LOC499767  Info   LOC499767 
   834 2e-07 2466.9 1022.4 2.413  1367614_at  annexin A1  Info   Anxa1 
   835 2e-07 170.3 42.1 4.045  1385001_at  gasdermin domain containing 1 (predicted)  Info   Gsdmdc1_predicted 
   836 2e-07 457.1 2200 0.208  1368245_at  ureidopropionase, beta  Info   Upb1 
   837 2e-07 316.7 58.8 5.386  1375716_at  interferon gamma receptor 2 (predicted)  Info   Ifngr2_predicted 
   838 2e-07 393.2 795.6 0.494  1372562_at  similar to RIKEN cDNA C030006K11  Info   RGD:1359663 
   839 2e-07 138.6 36.9 3.756  1368940_at  purinergic receptor P2Y, G-protein coupled 2  Info   P2ry2 
   840 2e-07 131 362.8 0.361  1375638_at  serum deprivation response protein  Info   RGD:1359345 
   841 2e-07 196.9 503.7 0.391  1374038_at  Transcribed locus  Info    
   842 2e-07 1095.5 147.9 7.407  1386908_at  glutaredoxin 1 (thioltransferase)  Info   Glrx1 
   843 2e-07 203 41.3 4.915  1387956_s_at  chemokine-like factor 1  Info   Cklf1 
   844 2e-07 203 110.8 1.832  1381323_at  retinoblastoma binding protein 5 (predicted)  Info   Rbbp5_predicted 
   845 2e-07 287.7 50.3 5.72  1382275_at  similar to PAK/PLC-interacting protein 1  Info   LOC361232 
   846 2e-07 339.5 91.1 3.727  1370415_at  Ras association (RalGDS/AF-6) domain family 5  Info   Rassf5 
   847 2e-07 696.5 415.9 1.675  1383368_at  Unknown (protein for MGC:72610)  Info   MGC72610 
   848 2e-07 3907 5251.4 0.744  1370218_at  lactate dehydrogenase B  Info   Ldhb 
   849 2e-07 75.8 334.2 0.227  1377086_at  C1q and tumor necrosis factor related protein 3 (predicted)  Info   C1qtnf3_predicted 
   850 2e-07 1157.3 1956.8 0.591  1367777_at  2,4-dienoyl CoA reductase 1, mitochondrial  Info   Decr1 
   851 2e-07 534.3 177.8 3.005  1389181_at  Rap guanine nucleotide exchange factor (GEF) 6 (predicted)  Info   Rapgef6_predicted 
   852 2e-07 287 81.4 3.526  1380297_at    Info    
   853 2e-07 246.5 19 12.974  1390797_at  lymphocyte cytosolic protein 2  Info   Lcp2 
   854 2e-07 2108.6 42.2 49.967  1383391_a_at  complement component 2  Info   C2 
   855 2e-07 85.7 399.6 0.214  1390285_at  similar to BC026645 protein  Info   LOC309112 
   856 2e-07 274.4 80.4 3.413  1368645_at  protein tyrosine phosphatase, non-receptor type 1  Info   Ptpn1 
   857 2e-07 16.7 96 0.174  1368339_at  calbindin 3, (vitamin D-dependent calcium binding protein)  Info   Calb3 
   858 2e-07 937.7 1753.1 0.535  1386946_at  carnitine palmitoyltransferase 1, liver  Info   Cpt1a 
   859 2e-07 47.4 196.8 0.241  1382569_at  Transcribed locus  Info    
   860 2e-07 28.3 85.4 0.331  1393163_at  Transcribed locus  Info    
   861 2e-07 81.9 276.8 0.296  1392914_at    Info    
   862 2e-07 107.8 68.8 1.567  1385711_at  similar to RIKEN cDNA 1500031M22 (predicted)  Info   RGD1311752_predicted 
   863 2e-07 142.8 387.2 0.369  1378457_at  Transcribed locus  Info    
   864 2e-07 25 136.4 0.183  1378315_at  Transcribed locus  Info    
   865 2e-07 165.7 356.3 0.465  1384103_at  Similar to novel protein (HT036)  Info    
   866 2e-07 526.5 215 2.449  1367901_at  glucuronidase, beta  Info   Gusb 
   867 2e-07 2425.7 1401.5 1.731  1370309_a_at  heterogeneous nuclear ribonucleoprotein A/B  Info   Hnrpab 
   868 2e-07 494.2 1789.3 0.276  1393831_at  Transcribed locus  Info    
   869 2e-07 284.1 50.7 5.604  1387198_at  inositol polyphosphate-5-phosphatase D  Info   Inpp5d 
   870 2e-07 241.3 11.4 21.167  1379582_a_at  cyclin A2  Info   Ccna2 
   871 2e-07 354.4 19.1 18.555  1369801_at  selectin, lymphocyte  Info   Sell 
   872 2e-07 884.3 1610.1 0.549  1370232_at  isovaleryl coenzyme A dehydrogenase  Info   Ivd 
   873 2e-07 157.9 40.1 3.938  1379482_at  transmembrane 6 superfamily member 1 (predicted)  Info   Tm6sf1_predicted 
   874 2e-07 397.8 207.5 1.917  1386982_at  Alpha-1,6-mannosyl-glycoprotein 2-beta-N-acetylglucosaminyltransferase  Info   Mgat2 
   875 2e-07 299.5 47.6 6.292  1391637_at  similar to RIKEN cDNA 5830480G12  Info   RGD:1359304 
   876 2e-07 88.2 36.8 2.397  1390704_at  SDA1 domain containing 1  Info   RGD:1359112 
   877 2e-07 53.7 399.4 0.134  1377018_at  Similar to E430002G05Rik protein (predicted)  Info    
   878 2e-07 3401.5 4585.5 0.742  1371701_at  NADH dehydrogenase (ubiquinone) 1 beta subcomplex, 9 (predicted)  Info   Ndufb9_predicted 
   879 2e-07 281.9 43 6.556  1368990_at  cytochrome P450, family 1, subfamily b, polypeptide 1  Info   Cyp1b1 
   880 2e-07 215.6 21.1 10.218  1384842_s_at  Tumor necrosis factor receptor superfamily, member 6  Info   RGD:619831 
   881 2e-07 1437 35.9 40.028  1378193_at  membrane-spanning 4-domains, subfamily A, member 7 (predicted)  Info   Ms4a7_predicted 
   882 2e-07 409.9 995.1 0.412  1372341_at  Transcribed locus, strongly similar to XP_484968.2 PREDICTED: similar to RIKEN cDNA C330005L02 [Mus musculus]  Info    
   883 2e-07 1192.9 2578.9 0.463  1390421_at  Similar to RIKEN cDNA 0610010D20 (predicted)  Info    
   884 2e-07 360.3 81.1 4.443  1372042_at  chemokine-like factor super family 3 (predicted)  Info   Cklfsf3_predicted 
   885 2e-07 383.7 775.1 0.495  1380546_at  similar to hypothetical protein FLJ10986 /// similar to hypothetical protein FLJ10986  Info   LOC298250 /// LOC298251 
   886 2e-07 2239.9 4948.8 0.453  1369625_at  aquaporin 1  Info   Aqp1 
   887 2e-07 944.3 179.9 5.249  1372930_at  SP110 nuclear body protein (predicted)  Info    
   888 2e-07 442.2 178.7 2.475  1373283_at  Thymopoietin  Info   Tmpo 
   889 2e-07 253.9 35.9 7.072  1378150_at  caspase recruitment domain family, member 11 (predicted)  Info   Card11_predicted 
   890 2e-07 1177.3 166.5 7.071  1388203_x_at  RT1 class Ib, locus Aw2 /// RT1 class I, CE5 /// RT1 class I, CE2 /// RT1 class I, CE15 /// RT1 class I, CE10  Info   RT1-Aw2 /// RT1-CE5 /// RT1-CE2 /// RGD:1359630 /// RGD:1359292 
   891 2e-07 259.5 6.3 41.19  1387742_at  chemokine (C-C motif) receptor 2  Info   Ccr2 
   892 2e-07 467.5 33.8 13.831  1368464_at  macrophage galactose N-acetyl-galactosamine specific lectin 1  Info   Mgl1 
   893 2e-07 688.5 897.1 0.767  1389907_at  similar to archease  Info   LOC297885 
   894 2e-07 1161.7 1870.4 0.621  1379395_a_at  histidine triad nucleotide binding protein 2 (predicted)  Info   Hint2_predicted 
   895 2e-07 253.5 43.7 5.801  1384280_at  Nucleolar and spindle associated protein 1 (predicted)  Info    
   896 2e-07 2801.5 1037 2.702  1387806_at  RAS related protein 1b  Info   Rap1b 
   897 2e-07 363.6 32.2 11.292  1394419_at  similar to RIKEN cDNA 6530401L14 gene (predicted)  Info   RGD1309107_predicted 
   898 2e-07 186 531.5 0.35  1389556_at  kinesin-associated protein 3 (predicted)  Info   Kifap3_predicted 
   899 2e-07 418.5 24.3 17.222  1384837_at  CD69 antigen  Info   Cd69 
   900 2e-07 178 405.4 0.439  1382014_at  similar to CGI-121 protein (predicted)  Info   RGD1309786_predicted 
   901 2e-07 119.2 45.1 2.643  1374651_at  similar to KIAA0933 protein (predicted)  Info   RGD1306097_predicted 
   902 2e-07 931.8 1854.1 0.503  1367829_at  enoyl Coenzyme A hydratase, short chain, 1, mitochondrial  Info   Echs1 
   903 2e-07 1136 4150.8 0.274  1367952_at  low density lipoprotein receptor-related protein 2  Info   Lrp2 
   904 2e-07 376.3 19.1 19.702  1391214_at  hypothetical protein LOC294430  Info   RGD1304835_predicted 
   905 2e-07 1288.6 754.7 1.707  1388389_at  septin 2  Info   Sept2 
   906 2e-07 197.7 830.2 0.238  1370067_at  malic enzyme 1  Info   Me1 
   907 2e-07 1121.1 420.3 2.667  1367683_at  karyopherin (importin) alpha 2  Info   Kpna2 
   908 2e-07 228.3 53.9 4.236  1371171_at  RT1 class Ib, locus Aw2  Info   RT1-Aw2 
   909 2e-07 1460.3 519.5 2.811  1392900_at  Similar to mKIAA1631 protein  Info    
   910 2e-07 272.1 43.5 6.255  1373557_at  mini chromosome maintenance deficient 4 homolog (S. cerevisiae) /// similar to mcdc21 protein  Info   Mcmd4 /// LOC310429 
   911 2e-07 2920.7 557.4 5.24  1370249_at  benzodiazepin receptor  Info   Bzrp 
   912 2e-07 3987.4 5872.9 0.679  1398326_at  similar to Nur77 downstream protein 2  Info   RGD:1359417 
   913 2e-07 250.2 639.3 0.391  1372568_at  EPM2A (laforin) interacting protein 1 (predicted)  Info    
   914 2e-07 140.3 242.8 0.578  1382215_at  Similar to Transcription initiation factor TFIID 105 kDa subunit (TAFII-105) (TAFII105)  Info    
   915 2e-07 137.9 26.4 5.223  1394352_at    Info    
   916 2e-07 188.9 658.5 0.287  1368774_a_at  espin  Info   Espn 
   917 2e-07 310.3 149.5 2.076  1377945_at  similar to RIKEN cDNA 2310005B10  Info   MGC95311 
   918 2e-07 414.8 775.6 0.535  1367716_at  T-cell immunomodulatory protein  Info   Cda08 
   919 2e-07 268.1 1061.1 0.253  1394020_at  Transcribed locus  Info    
   920 2e-07 557.2 105.4 5.287  1370987_at  sialophorin  Info   Spn 
   921 2e-07 1253.2 1968.5 0.637  1370060_at  solute carrier family 25 (mitochondrial carrier; oxoglutarate carrier), member 11  Info   Slc25a11 
   922 2e-07 256.1 153.1 1.673  1383712_at  U87HG mRNA, complete sequence  Info    
   923 2e-07 97.2 309.1 0.314  1376858_at    Info    
   924 2e-07 1521.5 3313.8 0.459  1367793_at  D-dopachrome tautomerase  Info   Ddt 
   925 2e-07 341.8 1046.8 0.327  1368253_at  guanidinoacetate methyltransferase  Info   Gamt 
   926 2e-07 101.8 9.5 10.716  1384231_at  Transcribed locus  Info    
   927 2e-07 311.6 112.6 2.767  1387871_at  cofilin 1  Info   Cfl1 
   928 2e-07 391.9 1054.1 0.372  1398378_at  glutathione S-transferase kappa 1  Info   Gstk1 
   929 2e-07 985.3 2393.5 0.412  1371775_at  Acyl-Coenzyme A dehydrogenase, short/branched chain  Info   Acadsb 
   930 2e-07 384.1 78.2 4.912  1396268_at  SP110 nuclear body protein (predicted)  Info    
   931 2e-07 1245.2 647.5 1.923  1370826_at  nucleosome assembly protein 1-like 1  Info   Nap1l1 
   932 2e-07 255.4 37.9 6.739  1378171_at  Neuropilin 2  Info   Nrp2 
   933 2e-07 1785.8 721.8 2.474  1377034_at  serine (or cysteine) proteinase inhibitor, clade B, member 1a (predicted)  Info   Serpinb1a_predicted 
   934 2e-07 3155.2 4639.6 0.68  1388391_at  similar to NADH dehydrogenase  Info   LOC363441 
   935 2e-07 357.3 755.9 0.473  1389654_at  Transcribed locus  Info    
   936 2e-07 2276.1 573 3.972  1368655_at  proteoglycan peptide core protein  Info   RGD:619969 
   937 2e-07 2391 1070.6 2.233  1383986_at  tropomyosin 3, gamma  Info   Tpm3 
   938 2e-07 333.5 51.8 6.438  1389250_at  Transcribed locus  Info    
   939 2e-07 142.7 348.1 0.41  1368528_at  MIC2 like 1  Info   RGD:620896 
   940 2e-07 226.1 34.6 6.535  1376197_at  transcription factor 7, T-cell specific (predicted)  Info   Tcf7_predicted 
   941 2e-07 324 88.7 3.653  1389039_at  Transcribed locus  Info    
   942 2e-07 430.7 1116.1 0.386  1388537_at  4-nitrophenylphosphatase domain and non-neuronal SNAP25-like protein homolog 1 (C. elegans) (predicted)  Info   Nipsnap1_predicted 
   943 2e-07 3833.4 2366.1 1.62  1398750_at  calreticulin  Info   Calr 
   944 2e-07 408.3 2697.6 0.151  1370144_at  G protein-binding protein CRFG /// similar to GTP-binding protein NGB /// similar to GTP-binding protein NGB  Info   Gtpbp4 /// LOC364763 /// LOC498786 
   945 2e-07 378.5 818 0.463  1383879_at  Transcribed locus  Info    
   946 2e-07 148 8.3 17.831  1385590_at  Transcribed locus  Info    
   947 2e-07 132.9 38.7 3.434  1370127_at  polymerase (DNA directed), delta 1, catalytic subunit  Info   Pold1 
   948 2e-07 396.9 133.1 2.982  1385869_at  Zinc finger protein 281 (predicted)  Info    
   949 2e-07 102.6 12.1 8.479  1396217_at  Similar to Adapter-related protein complex 1 sigma 1B subunit (Sigma-adaptin 1B) (Adaptor protein complex AP-1 sigma-1B subunit) (Golgi adaptor HA1/AP1 adaptin sigma-1B subunit) (Clathrin assembly protein complex 1 sigma-1B small chain) (Sigma 1B s...  Info    
   950 2e-07 135.4 270.1 0.501  1372681_at  Transcribed locus  Info    
   951 2e-07 137.7 543.9 0.253  1368509_at  Bardet-Biedl syndrome 2 (human)  Info   Bbs2 
   952 2e-07 1037.8 491.9 2.11  1398356_at  cleavage and polyadenylation specific factor 5 (predicted)  Info   Cpsf5_predicted 
   953 2e-07 130.4 231.1 0.564  1393256_at  selenophosphate synthetase 1 (predicted)  Info   Sephs1_predicted 
   954 2e-07 2092.1 1217.8 1.718  1372333_at  small nuclear ribonucleoprotein E (predicted) /// similar to small nuclear ribonucleoprotein E  Info   Snrpe_predicted /// LOC501075 
   955 2e-07 1923.8 2956.1 0.651  1371912_at  NADH dehydrogenase (ubiquinone) Fe-S protein 7 (predicted)  Info   Ndufs7_predicted 
   956 2e-07 310.5 190 1.634  1373209_at  Transcribed locus  Info    
   957 2e-07 86.3 14.2 6.077  1387621_at  tumor necrosis factor receptor superfamily, member 4  Info   Tnfrsf4 
   958 2e-07 968.9 491.4 1.972  1373422_at    Info    
   959 2e-07 101 28.1 3.594  1390240_at  Transcribed locus  Info    
   960 2e-07 1.1 45 0.024  1384917_at  Similar to uracil-DNA glycosylase 2  Info    
   961 2e-07 140.1 38.1 3.677  1375277_at  similar to hypothetical protein FLJ20433  Info   LOC499745 
   962 2e-07 276.6 119.2 2.32  1388429_at  similar to gem (nuclear organelle) associated protein 7  Info   LOC499391 
   963 2e-07 136.4 37.4 3.647  1382592_at    Info    
   964 2e-07 1343.9 711.1 1.89  1395508_at  chaperonin subunit 5 (epsilon)  Info   Cct5 
   965 2e-07 217.6 68.9 3.158  1371111_at  RT1 class I, A3  Info   RT1-A3 
   966 2e-07 425.8 204 2.087  1391527_at  Ngfi-A binding protein 2 (predicted)  Info   Nab2 
   967 2e-07 98.3 14.7 6.687  1384900_at  MOB1, Mps One Binder kinase activator-like 2B (yeast) (predicted)  Info   Mobkl2b_predicted 
   968 2e-07 112.4 23.5 4.783  1385502_at  Tripartite motif protein 21 (predicted)  Info    
   969 2e-07 480.5 1572.9 0.305  1382189_at  syndecan 2  Info   Sdc2 
   970 3e-07 1180.3 721.5 1.636  1388352_at  N-acetyltransferase 5 (ARD1 homolog, S. cerevisiae) (predicted)  Info   Nat5_predicted 
   971 3e-07 203.7 478.7 0.426  1388688_at  Transcribed locus  Info    
   972 3e-07 233.5 36 6.486  1371213_at  RT1 class I, A3  Info   RGD:1359610 
   973 3e-07 1253.1 3550 0.353  1367680_at  acyl-Coenzyme A oxidase 1, palmitoyl  Info   Acox1 
   974 3e-07 283.7 1160.4 0.244  1388778_at  Transcribed locus  Info    
   975 3e-07 165.1 24.2 6.822  1378914_a_at  hypothetical protein LOC308990  Info   LOC308990 
   976 3e-07 509.8 1493.4 0.341  1372790_at  Malate dehydrogenase 1, NAD (soluble)  Info   Mdh1 
   977 3e-07 173.2 49.3 3.513  1377664_at  KARP-1 binding protein 1  Info   Kab 
   978 3e-07 230.3 1214.9 0.19  1368877_at  zinc finger protein 354A  Info   Znf354a 
   979 3e-07 68.2 3.3 20.667  1387691_at  tumor necrosis factor superfamily, member 2  Info   Tnf 
   980 3e-07 140.8 3.9 36.103  1390849_at  Transcribed locus  Info    
   981 3e-07 258.4 86.9 2.974  1375987_at  ceramide kinase (predicted)  Info   Cerk_predicted 
   982 3e-07 444.2 1007.8 0.441  1375560_at  similar to RIKEN cDNA 0610010D20 (predicted)  Info   RGD1310475_predicted 
   983 3e-07 304.9 51.3 5.943  1368066_at  BCL2-antagonist/killer 1  Info   Bak1 
   984 3e-07 682.4 91.3 7.474  1383658_at  lysosomal-associated protein transmembrane 5  Info   Laptm5 
   985 3e-07 232.7 4.4 52.886  1394306_at  zeta-chain (TCR) associated protein kinase 70kDa  Info   Zap70 
   986 3e-07 1309.6 121.7 10.761  1373514_at  similar to chromosome 17 open reading frame 27 (predicted)  Info   RGD1308168_predicted 
   987 3e-07 1382.6 799.8 1.729  1383073_at  ubiquitin specific protease 14 (predicted)  Info   Usp14_predicted 
   988 3e-07 189.9 67.5 2.813  1378296_at    Info    
   989 3e-07 161.3 372.1 0.433  1390050_at  golgi phosphoprotein 2 (predicted)  Info   Golph2_predicted 
   990 3e-07 136.4 335.7 0.406  1376007_at  Transcribed locus, moderately similar to XP_286747.1 PREDICTED: hypothetical protein XP_286747 [Mus musculus]  Info    
   991 3e-07 583 236.1 2.469  1373280_at  RuvB-like 2 (E. coli) (predicted)  Info   Ruvbl2_predicted 
   992 3e-07 575.1 1189 0.484  1387314_at  sulfotransferase family 1B, member 1  Info   Sult1b1 
   993 3e-07 626.5 1178.4 0.532  1367897_at  acyl-Coenzyme A dehydrogenase, very long chain  Info   Acadvl 
   994 3e-07 871.6 81.2 10.734  1380521_at  hematopoietic cell specific Lyn substrate 1 (predicted)  Info   Hcls1_predicted 
   995 3e-07 515.9 249.3 2.069  1379676_a_at  similar to RIKEN cDNA 2310005K03  Info   RGD:1359588 
   996 3e-07 272.3 475.9 0.572  1374019_at  LOC499758  Info    
   997 3e-07 1412.6 388.2 3.639  1370891_at  CD48 antigen  Info   Cd48 
   998 3e-07 5.1 31.5 0.162  1370973_at  sodium channel, voltage-gated, type 6, alpha polypeptide  Info   RGD:61922 
   999 3e-07 241.1 39.8 6.058  1386637_at  fibrinogen-like 2  Info   Fgl2 
   1000 3e-07 403.2 785.6 0.513  1374903_at  glucosaminyl (N-acetyl) transferase 2, I-branching enzyme /// hypothetical gene supported by NM_001001511  Info   Gcnt2 /// LOC497712 
   1001 3e-07 1068.7 98 10.905  1387205_at  RT1 class Ib, locus M3  Info   RT1-M3 
   1002 3e-07 240 20 12  1368224_at  Serine protease inhibitor  Info   Spin2c 
   1003 3e-07 648.4 1207.4 0.537  1373346_at  similar to hypothetical protein CL25084 (predicted)  Info   RGD1306508_predicted 
   1004 3e-07 241.5 99.4 2.43  1370347_at  PDZ and LIM domain 7  Info   Pdlim7 
   1005 3e-07 477.8 1260.5 0.379  1386943_at  transmembrane 4 superfamily member 11  Info   Tm4sf11 
   1006 3e-07 256.6 69.1 3.713  1383917_at  ABI gene family, member 3 (predicted)  Info   Abi3_predicted 
   1007 3e-07 186.9 357 0.524  1384188_at    Info    
   1008 3e-07 1844.3 19.8 93.146  1376390_at  membrane-spanning 4-domains, subfamily A, member 11 (predicted)  Info   Ms4a11_predicted 
   1009 3e-07 36.8 79.8 0.461  1395534_at  Transcribed locus  Info    
   1010 3e-07 417.2 105.8 3.943  1371073_at  UDP-Gal:betaGlcNAc beta 1,4- galactosyltransferase, polypeptide 1  Info   B4galt1 
   1011 3e-07 126.1 440 0.287  1389744_at  LOC499898  Info   LOC499898 
   1012 3e-07 1798 665.4 2.702  1398303_s_at  tropomyosin 3, gamma  Info   Tpm3 
   1013 3e-07 81.4 227.3 0.358  1370573_at  sarcosine dehydrogenase  Info   Sardh 
   1014 3e-07 969.6 3035.6 0.319  1377646_at  Transcribed locus  Info    
   1015 3e-07 345 32.7 10.55  1382680_at    Info    
   1016 3e-07 595.8 226.3 2.633  1389365_at  Similar to RIKEN cDNA 1600014C10  Info    
   1017 3e-07 1974 750.8 2.629  1375137_at  actin related protein 2/3 complex, subunit 2 (predicted)  Info   Arpc2_predicted 
   1018 3e-07 141.4 23.1 6.121  1369208_at  interleukin 7  Info   Il7 
   1019 3e-07 60.8 20.8 2.923  1386669_at  Transcribed locus  Info    
   1020 3e-07 5752.9 3855.8 1.492  1367597_at  ribosomal protein S8  Info   Rps8 
   1021 3e-07 74 539.6 0.137  1382683_a_at    Info    
   1022 3e-07 1069.2 3809.6 0.281  1368461_at  solute carrier family 22 (organic anion transporter), member 8  Info   Slc22a8 
   1023 3e-07 1013.3 519.3 1.951  1369998_at  ADP-ribosylation factor 6  Info   Arf6 
   1024 3e-07 396.5 37.3 10.63  1372685_at  cyclin-dependent kinase inhibitor 3 (predicted)  Info   Cdkn3_predicted 
   1025 3e-07 585.2 1288.5 0.454  1391856_at  similar to semaphorin sem2 (predicted)  Info   RGD1306079_predicted 
   1026 3e-07 1057.6 664.4 1.592  1375414_at  TAF9 RNA polymerase II, TATA box binding protein (TBP)-associated factor  Info   Taf9 
   1027 3e-07 626.4 1625.1 0.385  1389622_at  similar to citrin  Info   LOC362322 
   1028 3e-07 333.6 557.6 0.598  1389008_at  Sprouty-related, EVH1 domain containing 2 (predicted)  Info    
   1029 3e-07 4191.2 5872.5 0.714  1370918_a_at  ATP synthase, H+ transporting, mitochondrial F1 complex, gamma polypeptide 1  Info   Atp5c1 
   1030 3e-07 1285.5 2507.8 0.513  1398296_at  membrane interacting protein of RGS16  Info   Mir16 
   1031 3e-07 335.5 150.5 2.229  1387624_at  upstream transcription factor 1  Info   Usf1 
   1032 3e-07 2082.1 1222.3 1.703  1371366_at  Rho GDP dissociation inhibitor (GDI) alpha  Info   RGD:1359547 
   1033 3e-07 67.7 6.4 10.578  1394051_at  Transcribed locus  Info    
   1034 3e-07 321.7 171.4 1.877  1390576_at  Transcribed locus  Info    
   1035 3e-07 70.2 165.4 0.424  1373247_at  Beta-site APP cleaving enzyme  Info   Bace 
   1036 3e-07 1802.4 32.2 55.975  1368482_at  B-cell leukemia/lymphoma 2 related protein A1  Info   Bcl2a1 
   1037 3e-07 103.1 276.1 0.373  1374667_at  CDNA clone IMAGE:7302289  Info    
   1038 3e-07 371.7 904.4 0.411  1390918_at  GH regulated TBC protein 1 (predicted)  Info   Grtp1_predicted 
   1039 3e-07 415.1 1371.2 0.303  1370547_at  pregnancy-zone protein  Info   Pzp 
   1040 3e-07 340.1 112.9 3.012  1373852_at  similar to SRD5A2L (predicted)  Info   RGD1308828_predicted 
   1041 3e-07 330.7 183 1.807  1388556_at  syntaxin 6  Info   Stx6 
   1042 3e-07 96.2 26 3.7  1377190_at  Similar to mKIAA0386 protein (predicted)  Info    
   1043 3e-07 559.5 237.7 2.354  1388397_at  EBNA1 binding protein 2 (predicted)  Info   Ebna1bp2_predicted 
   1044 3e-07 901.1 3638.7 0.248  1371763_at  similar to RIKEN cDNA 4931406C07 (predicted)  Info   RGD1309534_predicted 
   1045 3e-07 89.1 26.5 3.362  1375972_at  Transcribed locus  Info    
   1046 3e-07 527.9 1389.6 0.38  1387819_at  elastase 1, pancreatic  Info   Ela1 
   1047 3e-07 1236.7 2235 0.553  1385656_at  similar to hypothetical protein MGC13034  Info   LOC499516 
   1048 3e-07 700.3 105.3 6.651  1391347_at  RAB8B, member RAS oncogene family  Info   Rab8b 
   1049 3e-07 753 138.8 5.425  1368223_at  a disintegrin-like and metalloprotease (reprolysin type) with thrombospondin type 1 motif, 1  Info   Adamts1 
   1050 3e-07 77.4 339.5 0.228  1368924_at  growth hormone receptor  Info   Ghr 
   1051 3e-07 733.6 1450.7 0.506  1386880_at  acetyl-Coenzyme A acyltransferase 2 (mitochondrial 3-oxoacyl-Coenzyme A thiolase)  Info   Acaa2 
   1052 3e-07 575 53.9 10.668  1390348_at  folate receptor 2 (fetal) (predicted)  Info   Folr2_predicted 
   1053 3e-07 571.2 36.9 15.48  1398256_at  interleukin 1 beta  Info   Il1b 
   1054 3e-07 99.7 19.7 5.061  1388064_a_at  solute carrier family 1 (glial high affinity glutamate transporter), member 3  Info   Slc1a3 
   1055 3e-07 269.7 61.5 4.385  1369103_at  fyn proto-oncogene  Info   Fyn 
   1056 3e-07 137.9 62.4 2.21  1383716_at  Transcribed locus, strongly similar to NP_777569.1 hypothetical protein MGC23909 [Homo sapiens]  Info    
   1057 3e-07 222.8 938.1 0.238  1370870_at  malic enzyme 1  Info   Me1 
   1058 3e-07 2669.2 874.2 3.053  1386890_at  S100 calcium binding protein A10 (calpactin)  Info   S100a10 
   1059 3e-07 84 17.3 4.855  1383785_at  Lymphoid enhancer binding factor 1  Info   Lef1 
   1060 3e-07 470 162.7 2.889  1373473_a_at  nucleosome assembly protein 1-like 1  Info   Nap1l1 
   1061 3e-07 273.8 32.5 8.425  1380079_at  similar to mKIAA2005 protein  Info   LOC500015 
   1062 3e-07 426 69.4 6.138  1372135_at  protein tyrosine kinase 9-like (A6-related protein) (predicted)  Info   Ptk9l_predicted 
   1063 3e-07 3745.7 2239.6 1.672  1371511_at  actin related protein 2/3 complex, subunit 2 (predicted)  Info   Arpc2_predicted 
   1064 3e-07 264.3 57 4.637  1370294_a_at  cell division cycle 20 homolog (S. cerevisiae)  Info   Cdc20 
   1065 3e-07 344.4 1630.2 0.211  1382050_at  Transcribed locus  Info    
   1066 3e-07 327.4 107.2 3.054  1375585_at  Transcribed locus  Info    
   1067 3e-07 1013.7 566.5 1.789  1371998_at  Similar to Mob4B protein  Info    
   1068 3e-07 2316.6 1707 1.357  1369940_at  transaldolase 1  Info   Taldo1 
   1069 3e-07 349.3 26 13.435  1375041_at  Similar to F-box only protein 27  Info    
   1070 3e-07 124.9 24.8 5.036  1376039_at  serine/threonine kinase 6  Info   Stk6 
   1071 3e-07 1162 2626.5 0.442  1372306_at  ethylmalonic encephalopathy 1 (predicted)  Info   Ethe1_predicted 
   1072 3e-07 1886.2 686 2.75  1390042_at  similar to 1110007F12Rik protein  Info   MGC109491 
   1073 3e-07 94 178.7 0.526  1382115_at  similar to RIKEN cDNA 4930521E07 (predicted)  Info   RGD1305166_predicted 
   1074 3e-07 476.8 1928.9 0.247  1370166_at  syndecan 2  Info   Sdc2 
   1075 3e-07 502.9 66.4 7.574  1388600_at  Similar to putative membrane steroid receptor (predicted)  Info    
   1076 3e-07 1785.7 3802.8 0.47  1371922_at  Transcribed locus  Info    
   1077 3e-07 435.2 65.1 6.685  1370948_a_at  myristoylated alanine rich protein kinase C substrate  Info   Marcks 
   1078 3e-07 1603.9 1021.5 1.57  1398829_at  FK506 binding protein 1a  Info   Fkbp1a 
   1079 3e-07 1431.3 3208.4 0.446  1392969_at  Transcribed locus  Info    
   1080 3e-07 193.4 981.3 0.197  1398286_at  cysteine sulfinic acid decarboxylase  Info   Csad 
   1081 3e-07 722.5 1417.9 0.51  1390807_at  carbonic anhydrase VB, mitochondrial  Info   Ca5b 
   1082 3e-07 240.5 529 0.455  1373198_at  similar to RIKEN cDNA 2810451A06 (predicted)  Info   RGD1311098_predicted 
   1083 3e-07 1868.1 947.1 1.972  1371544_at  similar to Enhancer of rudimentary homolog  Info   LOC503047 
   1084 3e-07 125.4 313.3 0.4  1372103_at  DnaJ (Hsp40) homolog, subfamily A, member 4 (predicted)  Info    
   1085 3e-07 218.9 532.7 0.411  1389688_at  Transcribed locus  Info    
   1086 3e-07 400.2 88.2 4.537  1388166_at  similar to Igh-6 protein /// similar to Ig H-chain V-region precursor /// similar to single chain Fv antibody fragment scFv 7-10A /// similar to Ig heavy chain V region MC101 precursor  Info   RGD:1359202 /// LOC299458 /// LOC314509 /// LOC366747 
   1087 3e-07 996.5 340.9 2.923  1388528_at  fibrillarin (predicted)  Info   Fbl_predicted 
   1088 3e-07 125.8 240.3 0.524  1389082_at    Info    
   1089 3e-07 2051.6 3341.4 0.614  1387793_at  ERM-binding phosphoprotein  Info   Slc9a3r1 
   1090 3e-07 594.3 251.9 2.359  1388622_at  nucleolar protein 5A (predicted)  Info   Nol5a_predicted 
   1091 3e-07 2174.6 3251.3 0.669  1388365_at  ATPase, H+ transporting, V0 subunit D isoform 1 (predicted)  Info   Atp6v0d1_predicted 
   1092 3e-07 68.9 14.6 4.719  1381221_at  similar to T cell receptor V delta 6 (predicted)  Info   RGD1311300_predicted 
   1093 3e-07 594.8 1663.8 0.357  1388948_at  START domain containing 10 (predicted)  Info   Stard10_predicted 
   1094 3e-07 163.7 333.3 0.491  1373216_at    Info    
   1095 3e-07 565.3 1130.9 0.5  1367720_at  aminolevulinate, delta-, dehydratase  Info   Alad 
   1096 4e-07 1093 443.7 2.463  1369969_at  ADP-ribosyltransferase 1  Info   Adprt 
   1097 4e-07 653.6 2082 0.314  1371496_at    Info    
   1098 4e-07 145.1 632.6 0.229  1372976_at  similar to Dorz1 (predicted)  Info   RGD1309721_predicted 
   1099 4e-07 1825 3913.3 0.466  1371916_at  CDNA clone MGC:105753 IMAGE:7316479  Info    
   1100 4e-07 180.3 12.5 14.424  1386695_at  Transcribed locus  Info    
   1101 4e-07 487.9 233.5 2.09  1395375_at  similar to hypothetical protein  Info   MGC73022 
   1102 4e-07 357.8 132.1 2.709  1391817_at  Transcribed locus  Info    
   1103 4e-07 58.5 119.1 0.491  1377183_at  Transcribed locus  Info    
   1104 4e-07 193.6 35.1 5.516  1373290_at  similar to Ezh2 protein  Info   LOC312299 
   1105 4e-07 527.7 165.1 3.196  1380110_at    Info    
   1106 4e-07 931.4 1798.6 0.518  1371696_at  G protein-coupled receptor 56  Info   Gpr56 
   1107 4e-07 1552.9 2345.2 0.662  1371577_at  NADH dehydrogenase (ubiquinone) Fe-S protein 1, 75kDa  Info   RGD:1359670 
   1108 4e-07 352.7 133.5 2.642  1388915_at  Transcribed locus  Info    
   1109 4e-07 129.8 370.4 0.35  1398500_at  G protein-coupled receptor 116  Info   Gpr116 
   1110 4e-07 312.3 153 2.041  1396984_at  UDP-glucose ceramide glucosyltransferase-like 1  Info   Ugcgl1 
   1111 4e-07 2655.7 628 4.229  1371691_at    Info    
   1112 4e-07 758 354.4 2.139  1388762_at    Info    
   1113 4e-07 134.8 366.6 0.368  1368177_at  acyl-CoA synthetase long-chain family member 3  Info   Acsl3 
   1114 4e-07 739.8 2040.8 0.363  1369986_at  hydroxyacyl glutathione hydrolase  Info   Hagh 
   1115 4e-07 801.7 2107.1 0.38  1370907_at  sialyltransferase 1  Info   Siat1 
   1116 4e-07 148.5 63.9 2.324  1384330_at  similar to hypothetical protein FLJ20546  Info   RGD:1359513 
   1117 4e-07 496.3 1124.4 0.441  1374254_a_at  hypothetical LOC300441  Info   LOC300441 
   1118 4e-07 162.4 370.1 0.439  1389504_at  Similar to Deltex3  Info    
   1119 4e-07 1052.4 483.8 2.175  1383429_at  similar to T-Brain-1  Info   LOC311078 
   1120 4e-07 1424.5 373.6 3.813  1372459_at  vasodilator-stimulated phosphoprotein (predicted)  Info   Vasp_predicted 
   1121 4e-07 361.1 89.2 4.048  1373908_at  Transcribed locus  Info    
   1122 4e-07 25.1 2.8 8.964  1369752_a_at  calcium/calmodulin-dependent protein kinase IV  Info   Camk4 
   1123 4e-07 224.2 704.1 0.318  1373057_at  ProSAPiP1 protein  Info   ProSAPiP1 
   1124 4e-07 1261.4 1844.2 0.684  1398891_at  mitochondrial ribosomal protein L15 (predicted)  Info   Mrpl15_predicted 
   1125 4e-07 1421.1 687.1 2.068  1371445_at  similar to ribosome-binding protein p34 - rat (predicted)  Info   RGD1305092_predicted 
   1126 4e-07 1546.9 3542.1 0.437  1388788_at  glutaryl-Coenzyme A dehydrogenase (predicted)  Info   Gcdh_predicted 
   1127 4e-07 628.1 254.2 2.471  1368173_at  nucleolar protein 5  Info   Nol5 
   1128 4e-07 1942.4 929.1 2.091  1390383_at  adipose differentiation-related protein  Info   RGD:1359288 
   1129 4e-07 1155.9 243.1 4.755  1398373_at  UDP-Gal:betaGlcNAc beta 1,3-galactosyltransferase, polypeptide 3 (predicted)  Info   B3galt3_predicted 
   1130 4e-07 1082.6 1450.4 0.746  1388822_at  short coiled-coil protein (predicted) /// similar to short coiled-coil protein  Info   Scoc_predicted /// LOC498931 
   1131 4e-07 1092.2 541.6 2.017  1369956_at  interferon gamma receptor 1  Info   Ifngr 
   1132 4e-07 115 466.2 0.247  1385791_at  similar to villin-like protein (predicted)  Info   RGD1304843_predicted 
   1133 4e-07 79.1 27.4 2.887  1378240_at  Fanconi anemia D2 protein  Info   RGD:1303172 
   1134 4e-07 440.2 184.7 2.383  1389246_at  Transcribed locus  Info    
   1135 4e-07 227.9 46.1 4.944  1395313_s_at    Info    
   1136 4e-07 105.9 500.8 0.211  1378307_at  Pleckstrin homology domain containing, family H (with MyTH4 domain) member 1 (predicted)  Info    
   1137 4e-07 282.2 759 0.372  1369200_at  5 nucleotidase  Info   Nt5 
   1138 4e-07 966.5 547.7 1.765  1388565_at  acid cluster protein 33  Info   RGD:1359141 
   1139 4e-07 168.2 459.2 0.366  1372110_at  Similar to hypothetical protein (predicted)  Info    
   1140 4e-07 320.1 22.3 14.354  1370345_at  cyclin B1  Info   Ccnb1 
   1141 4e-07 198.5 38 5.224  1378700_at  Transcribed locus  Info    
   1142 4e-07 586.3 144.5 4.057  1373757_at  FLN29 gene product  Info   Fln29 
   1143 4e-07 238.2 91.2 2.612  1384457_at  similar to Fbxw17 protein  Info   LOC498713 
   1144 4e-07 679.4 1310.7 0.518  1368431_at  hepsin  Info   Hpn 
   1145 4e-07 87.6 203.1 0.431  1375684_at  neuraminidase 1  Info   Neu1 
   1146 4e-07 186.7 852.8 0.219  1371266_at  afamin  Info   Afm 
   1147 4e-07 373.6 1462.2 0.256  1398511_at  sushi domain containing 2 (predicted)  Info   Susd2_predicted 
   1148 4e-07 868.5 12.9 67.326  1379293_at  granzyme A  Info   Gzma 
   1149 4e-07 971.8 364.3 2.668  1388753_at  sulfatase 2 (predicted)  Info   Sulf2_predicted 
   1150 4e-07 114.9 21.5 5.344  1378033_at    Info    
   1151 4e-07 64.1 13.7 4.679  1385019_at    Info    
   1152 4e-07 488 131.2 3.72  1398756_at  nucleophosmin 1 /// similar to Nucleophosmin (NPM) (Nucleolar phosphoprotein B23) (Numatrin) (Nucleolar protein NO38)  Info   Npm1 /// LOC300303 
   1153 4e-07 1029.6 650.6 1.583  1367769_at  polymerase (RNA) II (DNA directed) polypeptide G  Info   Polr2g 
   1154 4e-07 99 536.6 0.184  1376808_at  Transcribed locus  Info    
   1155 4e-07 67.3 7.2 9.347  1390141_at  formyltetrahydrofolate synthetase domain containing 1 (predicted)  Info   Fthfsdc1_predicted 
   1156 4e-07 2665.1 3621.7 0.736  1399012_at  similar to RIKEN cDNA 1110001M20 (predicted)  Info   RGD1310313_predicted 
   1157 4e-07 150.2 4.2 35.762  1370369_at  granzyme M (lymphocyte met-ase 1)  Info   Gzmm 
   1158 4e-07 392 1515.2 0.259  1379885_at  flavin containing monooxygenase 4  Info   Fmo4 
   1159 4e-07 3333.9 1497.7 2.226  1371936_at  eukaryotic translation initiation factor 4A1  Info   Eif4a1 
   1160 4e-07 252.2 116.8 2.159  1393249_at  Friend leukemia integration 1 (predicted)  Info    
   1161 4e-07 431 128.4 3.357  1387502_at  serine/threonine kinase 17b (apoptosis-inducing)  Info   Stk17b 
   1162 4e-07 497.1 264.5 1.879  1371737_at  tripartite motif protein 27 (predicted)  Info   Trim27_predicted 
   1163 4e-07 70.4 5.8 12.138  1379653_a_at  similar to SH2 domain protein 1A (Signaling lymphocyte activation molecule-associated protein) (SLAM-associated protein) (T cell signal transduction molecule SAP)  Info   LOC501502 
   1164 4e-07 153.1 52.4 2.922  1373286_at  CSX-associated LIM  Info   RGD:1359551 
   1165 4e-07 1042.7 15.1 69.053  1379794_at  granzyme B  Info   Gzmb 
   1166 4e-07 7.9 27 0.293  1368609_at  solute carrier family 10 (sodium/bile acid cotransporter family), member 1  Info   Slc10a1 
   1167 4e-07 878 396 2.217  1368143_at  annexin A7  Info   Anxa7 
   1168 4e-07 198.2 419.7 0.472  1373754_at  Transcribed locus  Info    
   1169 4e-07 483.7 17 28.453  1371079_at  Fc receptor, IgG, low affinity IIb  Info   Fcgr2b 
   1170 4e-07 140.5 49.2 2.856  1384173_at  src family associated phosphoprotein 1  Info   Scap1 
   1171 4e-07 1641 1149 1.428  1387778_at  stromal cell derived factor 4  Info   Sdf4 
   1172 4e-07 895.5 1422.4 0.63  1371640_at  hypothetical protein LOC500855  Info   LOC500855 
   1173 4e-07 328.8 109.7 2.997  1367738_at  UNC-119 homolog (C. elegans)  Info   Unc119 
   1174 4e-07 492.7 1040.1 0.474  1372310_at  similar to tumor-related protein (predicted)  Info   RGD1307632_predicted 
   1175 4e-07 1062.4 423.6 2.508  1398769_at  coronin, actin-binding protein, 1B  Info   Coro1b 
   1176 4e-07 257.3 72 3.574  1371184_x_at  Tropomyosin 3, splice variant 1  Info   Tpm3 
   1177 4e-07 197.8 510.5 0.387  1398430_at  Transcribed locus  Info    
   1178 4e-07 1378.8 2541.5 0.543  1371483_at  nicotinamide nucleotide transhydrogenase  Info   Nnt 
   1179 4e-07 719.6 1273.2 0.565  1370814_at  dehydrogenase/reductase (SDR family) member 4  Info   Dhrs4 
   1180 4e-07 1043.8 2298.2 0.454  1372765_a_at  similar to Peci protein /// similar to RIKEN cDNA 1810022C23 (predicted)  Info   RGD:1359427 /// RGD1310224_predicted 
   1181 4e-07 507.7 25.5 19.91  1392819_at  membrane-spanning 4-domains, subfamily A, member 11 (predicted)  Info   Ms4a11_predicted 
   1182 4e-07 268.4 1199.4 0.224  1398255_at  solute carrier family 15 (H+/peptide transporter), member 2  Info   Slc15a2 
   1183 4e-07 207.7 645.9 0.322  1385704_at    Info    
   1184 4e-07 326.7 45.6 7.164  1390226_at  similar to hypothetical protein LOC340061  Info   LOC498840 
   1185 4e-07 817.9 73.5 11.128  1380063_at  cholesterol 25-hydroxylase (predicted)  Info   Ch25h_predicted 
   1186 4e-07 296.4 18.2 16.286  1387221_at  GTP cyclohydrolase 1  Info   Gch 
   1187 4e-07 261.7 47 5.568  1387947_at  v-maf musculoaponeurotic fibrosarcoma oncogene family, protein B (avian)  Info   Mafb 
   1188 5e-07 39.7 176.5 0.225  1384215_at    Info    
   1189 5e-07 84.8 211.1 0.402  1393330_at    Info    
   1190 5e-07 928.8 2248.7 0.413  1389107_at  Transcribed locus, weakly similar to XP_236385.3 PREDICTED: similar to junction-associated coiled-coil protein [Rattus norvegicus]  Info    
   1191 5e-07 95.7 332.3 0.288  1393531_at  Transcribed locus  Info    
   1192 5e-07 1565.9 494 3.17  1399161_a_at  type 1 tumor necrosis factor receptor shedding aminopeptidase regulator  Info   Arts1 
   1193 5e-07 114.2 25.2 4.532  1397611_at  LOC501124  Info   LOC501124 
   1194 5e-07 412.6 112.7 3.661  1373949_at  similar to RIKEN cDNA 1110012E06 (predicted)  Info   RGD1307977_predicted 
   1195 5e-07 182.4 282.6 0.645  1390508_at  Similar to hypothetical protein MGC27648 (predicted)  Info    
   1196 5e-07 26.8 69.7 0.385  1379301_at  Transcribed locus  Info    
   1197 5e-07 117.4 595.6 0.197  1370566_at  retinol dehydrogenase 2  Info   Rdh2 
   1198 5e-07 227.7 88.4 2.576  1380009_at  vaccinia related kinase 1 (predicted)  Info   Vrk1_predicted 
   1199 5e-07 91.8 466.1 0.197  1392612_at  Prolactin receptor  Info   Prlr 
   1200 5e-07 940.7 2310.6 0.407  1376051_at  crystallin, lamda 1  Info   Cryl1 
   1201 5e-07 166.2 3.9 42.615  1396492_at  similar to NFAT activation molecule 1 (predicted)  Info   RGD1308316_predicted 
   1202 5e-07 355.7 136.6 2.604  1373841_at  Rac/cdc42 guanine nucleotide exchange factor 6  Info   arhgef6 
   1203 5e-07 153.3 19.8 7.742  1392794_at  Transcribed locus  Info    
   1204 5e-07 83.1 292.9 0.284  1367835_at  proprotein convertase subtilisin/kexin type 1 inhibitor  Info   Pcsk1n 
   1205 5e-07 2987 2243.9 1.331  1371329_at    Info    
   1206 5e-07 325.8 52 6.265  1369672_at  arachidonate 5-lipoxygenase activating protein  Info   Alox5ap 
   1207 5e-07 238.7 98.6 2.421  1383862_at  Similar to osteoclast inhibitory lectin  Info    
   1208 5e-07 212.2 114.3 1.857  1383465_at  F-box and leucine-rich repeat protein 14 (predicted)  Info   Fbxl14_predicted 
   1209 5e-07 577.3 959.6 0.602  1389363_at  aci-reductone dioxygenase (ARD)-like protein 1  Info   Alp1 
   1210 5e-07 184.2 15.9 11.585  1380537_at  hematopoietic protein 1 (predicted)  Info   Hem1_predicted 
   1211 5e-07 148.7 507.8 0.293  1373803_a_at  growth hormone receptor  Info   Ghr 
   1212 5e-07 674.4 1552.5 0.434  1377060_at  methylcrotonoyl-Coenzyme A carboxylase 2 (beta) (predicted)  Info   Mccc2_predicted 
   1213 5e-07 156.4 572.4 0.273  1388176_at  camello-like 5  Info   Cml5 
   1214 5e-07 231.1 718.5 0.322  1387932_at  solute carrier family 1 (neuronal/epithelial high affinity glutamate transporter, system Xag), member 1  Info   Slc1a1 
   1215 5e-07 2429.9 348.7 6.968  1383906_at  similar to lung inducible neuralized-related C3HC4 RING finger protein  Info   RGD:1359633 
   1216 5e-07 217.1 709.6 0.306  1389448_at  peanut-like 2 (Drosophila) (predicted)  Info   Pnutl2_predicted 
   1217 5e-07 3006.4 1933.8 1.555  1371575_at  Moesin  Info   Msn 
   1218 5e-07 304.9 83.2 3.665  1379316_at  Transcribed locus  Info    
   1219 5e-07 1426.9 875.3 1.63  1370130_at  ras homolog gene family, member A  Info   RGD:619921 
   1220 5e-07 49.1 29 1.693  1392939_at  CDNA clone MGC:124984 IMAGE:7134770  Info    
   1221 5e-07 672.5 422.7 1.591  1367495_at  similar to prefoldin 4  Info   LOC364186 
   1222 5e-07 82.5 390.7 0.211  1383654_a_at  similar to fructosamine-3-kinase  Info   Fnsk 
   1223 5e-07 95.8 297.3 0.322  1386865_at  SPARC-like 1 (mast9, hevin)  Info   Sparcl1 
   1224 5e-07 229.2 61.2 3.745  1389425_at  similar to 5(3)-deoxyribonucleotidase  Info   LOC501746 
   1225 5e-07 79.1 16.8 4.708  1385382_at  Transcribed locus  Info    
   1226 5e-07 56 167.9 0.334  1398647_at  Similar to hypothetical protein FLJ40283 (predicted)  Info    
   1227 5e-07 1111.8 2517.8 0.442  1390835_at  similar to 1300013J15Rik protein (predicted)  Info   RGD1311123_predicted 
   1228 5e-07 113.8 291.4 0.391  1389310_at  Transcribed locus  Info    
   1229 5e-07 128 27.3 4.689  1398591_at  chemokine (C-C motif) receptor-like 2 (predicted)  Info   Ccrl2_predicted 
   1230 5e-07 176.5 41.4 4.263  1382727_at    Info    
   1231 5e-07 176.2 477.4 0.369  1375961_at  frizzled-related protein (predicted)  Info   Frzb_predicted 
   1232 5e-07 467.7 1402.3 0.334  1368283_at  enoyl-Coenzyme A, hydratase/3-hydroxyacyl Coenzyme A dehydrogenase  Info   Ehhadh 
   1233 5e-07 23.2 70.3 0.33  1382490_at    Info    
   1234 5e-07 513.2 235.2 2.182  1373912_at  Transcribed locus, weakly similar to XP_488820.1 PREDICTED: hypothetical protein XP_488820 [Mus musculus]  Info    
   1235 5e-07 170.8 17.9 9.542  1379598_at  Adenylate cyclase 7  Info   Adcy7 
   1236 5e-07 246.8 25 9.872  1389413_at  Oligodendrocyte-myelin glycoprotein  Info   Omg 
   1237 5e-07 196.4 609.2 0.322  1375856_at  Transcribed locus  Info    
   1238 5e-07 76.3 164.4 0.464  1374419_at  Adenylate cyclase 9 (predicted)  Info    
   1239 5e-07 186.1 383.8 0.485  1382069_at  ATPase, class VI, type 11A (predicted)  Info    
   1240 5e-07 146.9 679.7 0.216  1376944_at  Prolactin receptor  Info   Prlr 
   1241 5e-07 386.1 1328.9 0.291  1389548_at  alcohol dehydrogenase, iron containing, 1 (predicted)  Info   Adhfe1_predicted 
   1242 5e-07 352.6 842.1 0.419  1368085_at  GTP cyclohydrolase I feedback regulator  Info   Gchfr 
   1243 5e-07 209.1 16.1 12.988  1385213_at  Similar to epithelial stromal interaction 1 isoform a  Info    
   1244 5e-07 1061 653.8 1.623  1389140_at  similar to RIKEN cDNA 5730406I15 (predicted)  Info   RGD1311253_predicted 
   1245 5e-07 290.8 646.6 0.45  1394577_at  Transcribed locus  Info    
   1246 5e-07 414.8 93.4 4.441  1390386_at  caspase 3  Info   Casp3 
   1247 5e-07 268.2 112.2 2.39  1374857_at  similar to nucleolar protein family A, member 1  Info   LOC499709 
   1248 5e-07 1236.6 526.7 2.348  1371487_at  SH3 domain binding glutamic acid-rich protein-like 3 (predicted)  Info   Sh3bgrl3_predicted 
   1249 5e-07 1520.6 2841 0.535  1370699_a_at  epidermal growth factor receptor /// peptidase D  Info   Egfr /// Pepd 
   1250 5e-07 54.7 1.6 34.188  1391948_at  B-cell leukemia/lymphoma 11B (predicted)  Info   Bcl11b_predicted 
   1251 5e-07 462.6 1083.3 0.427  1374959_at  NAD(P)H dehydrogenase, quinone 2  Info   RGD:1303320 
   1252 5e-07 199.9 117.1 1.707  1391040_at  similar to Retinoblastoma-binding protein 8 (RBBP-8) (CtBP interacting protein) (CtIP) (Retinoblastoma-interacting protein and myosin-like) (RIM) (predicted)  Info   RGD1308872_predicted 
   1253 5e-07 343.7 847.1 0.406  1394228_at  Transcribed locus  Info    
   1254 5e-07 81.3 36.8 2.209  1382783_at  Bloom syndrome (predicted)  Info   Blm_predicted 
   1255 5e-07 968.5 581.6 1.665  1373185_at  signal sequence receptor, beta (predicted)  Info   Ssr2_predicted 
   1256 5e-07 275.9 94.5 2.92  1373772_at  DNA (cytosine-5-)-methyltransferase 1  Info   Dnmt1 
   1257 5e-07 222.2 518.2 0.429  1379318_at  Transcribed locus  Info    
   1258 5e-07 4846.7 3463.5 1.399  1367685_at  ribosomal protein S27a  Info   Rps27a 
   1259 5e-07 502.1 1211.2 0.415  1372587_at  endomucin  Info   Emcn 
   1260 5e-07 773.6 411 1.882  1375522_at  N-myristoyltransferase 1  Info   Nmt1 
   1261 5e-07 602.1 184.9 3.256  1382181_at  similar to RIKEN cDNA A530088I07 gene  Info   LOC311984 
   1262 5e-07 158.9 363 0.438  1392145_at    Info    
   1263 5e-07 412.3 1737.4 0.237  1367838_at  CTL target antigen  Info   Cth 
   1264 5e-07 440.2 163.4 2.694  1373961_at  similar to 4930453N24Rik protein  Info   RGD:1359320 
   1265 5e-07 349.1 731.9 0.477  1393587_a_at    Info    
   1266 5e-07 641.2 1736.5 0.369  1370072_at  membrane metallo endopeptidase  Info   Mme 
   1267 5e-07 364.6 41 8.893  1388673_at  lymphocyte specific 1 (predicted)  Info   Lsp1_predicted 
   1268 5e-07 128.5 19.7 6.523  1393757_at  Similar to OTTHUMP00000018227  Info    
   1269 6e-07 74.2 18.4 4.033  1377689_at  kinetochore associated 1 (predicted)  Info   Kntc1_predicted 
   1270 6e-07 301.4 95.4 3.159  1372493_at  similar to RIKEN cDNA 2410005K17 (predicted)  Info   RGD1311232_predicted 
   1271 6e-07 307.6 109.9 2.799  1389858_at  Thymidine kinase 1  Info   Tk1 
   1272 6e-07 1673.6 3236.6 0.517  1370030_at  glutamate cysteine ligase, modifier subunit  Info   Gclm 
   1273 6e-07 112.9 19.5 5.79  1392037_at  Transcribed locus  Info    
   1274 6e-07 609.5 2011.8 0.303  1375267_at  peptidylprolyl isomerase C  Info   RGD:1303221 
   1275 6e-07 708.9 336 2.11  1377737_at  Similar to hypothetical protein MGC5466 (predicted)  Info    
   1276 6e-07 978.4 371.6 2.633  1372009_at  tyrosyl-tRNA synthetase (predicted)  Info   Yars_predicted 
   1277 6e-07 188.4 506.6 0.372  1385574_at  similar to cDNA sequence AF397014 (predicted)  Info   RGD1308114_predicted 
   1278 6e-07 115.7 17.6 6.574  1392777_at  Transcribed locus  Info    
   1279 6e-07 13.7 48 0.285  1390815_at  Cyclin M1 (predicted)  Info    
   1280 6e-07 183.9 756.8 0.243  1372158_at  LRP16 protein  Info   Lrp16 
   1281 6e-07 48.5 193.6 0.251  1387263_at  pyruvate kinase, liver and RBC  Info   Pklr 
   1282 6e-07 1334.5 744 1.794  1371509_at  transforming growth factor beta regulated gene 1 (predicted)  Info   Tbrg1_predicted 
   1283 6e-07 406 951.5 0.427  1368045_at  solute carrier family 31 (copper transporters), member 1  Info   RGD:620059 
   1284 6e-07 375.3 192.4 1.951  1371712_at  similar to snRNP core protein SMX5d  Info   LOC292030 
   1285 6e-07 313.8 46.3 6.778  1376055_at  Transcribed locus  Info    
   1286 6e-07 406.8 225.3 1.806  1395574_at  ROD1 regulator of differentiation 1 (S. pombe)  Info   Rod1 
   1287 6e-07 2624.8 1592.9 1.648  1371973_at  eukaryotic translation initiation factor 3, subunit 6 (predicted)  Info   Eif3s6_predicted 
   1288 6e-07 272.9 31.6 8.636  1398482_at  B-cell leukemia/lymphoma 3 (predicted)  Info   Bcl3_predicted 
   1289 6e-07 160.8 55.6 2.892  1390692_at  cytidine 5'-triphosphate synthase (predicted)  Info   Ctps_predicted 
   1290 6e-07 275 534.9 0.514  1374156_at  membrane protein, palmitoylated 5 (MAGUK p55 subfamily member 5) (predicted)  Info   Mpp5_predicted 
   1291 6e-07 260.9 74.3 3.511  1368973_at  adenosine deaminase, RNA-specific  Info   Adar 
   1292 6e-07 231.1 788.3 0.293  1370936_at  dimethylglycine dehydrogenase precursor  Info   Dmgdh 
   1293 6e-07 3364 2078.3 1.619  1369930_at    Info    
   1294 6e-07 509.5 1078.7 0.472  1372260_at  leucine zipper domain protein  Info   MGC116147 
   1295 6e-07 77.3 18.8 4.112  1387839_at  RT1 class Ib gene, H2-TL-like, grc region (N1) /// RT1 class Ib gene, H2-TL-like, grc region (N3) /// RT1 class Ib gene, H2-TL-like, grc region(N2)  Info   RT1-N1 /// RT1-N3 /// RT1-N2 
   1296 6e-07 207.6 379.6 0.547  1372117_at  similar to BB128963 protein (predicted)  Info   RGD1305958_predicted 
   1297 6e-07 4401.2 2901.7 1.517  1398751_at  similar to ribosomal protein S7  Info   LOC497813 
   1298 6e-07 133 359.3 0.37  1373053_at    Info    
   1299 6e-07 174.8 96.5 1.811  1369421_at  topoisomerase (DNA) I  Info   Top1 
   1300 6e-07 565.9 886 0.639  1371965_at  similar to RIKEN cDNA 2010311D03  Info   RGD:1303272 
   1301 6e-07 295.7 36.4 8.124  1368266_at  arginase 1  Info   Arg1 
   1302 6e-07 126 13.4 9.403  1383015_at  Similar to DKFZP434P1750 protein (predicted)  Info    
   1303 6e-07 357 168.5 2.119  1372748_at  similar to KIAA1055 protein (predicted)  Info   RGD1307436_predicted 
   1304 6e-07 953.4 275.7 3.458  1375523_at  Similar to Myristoylated alanine-rich C-kinase substrate (MARCKS) (ACAMP-81)  Info   Marcks 
   1305 6e-07 63.3 215.6 0.294  1369318_at  fragile histidine triad gene  Info   Fhit 
   1306 6e-07 307.9 63.6 4.841  1383655_at  TNFAIP3 interacting protein 2 (predicted)  Info   Tnip2_predicted 
   1307 6e-07 373.4 1163.8 0.321  1389469_at  chromodomain helicase DNA binding protein 1-like (predicted)  Info   Chd1l_predicted 
   1308 6e-07 228.7 116.3 1.966  1374938_at  ZW10 homolog, centromere/kinetochore protein (Drosophila) (predicted)  Info   Zw10_predicted 
   1309 6e-07 696.1 1223 0.569  1367687_a_at  peptidylglycine alpha-amidating monooxygenase  Info   Pam 
   1310 6e-07 256.1 133.2 1.923  1388992_at  E1A binding protein p400 (predicted)  Info   Ep400_predicted 
   1311 6e-07 1917.6 1135.1 1.689  1388930_at  similar to RIKEN cDNA 2310075C12 (predicted)  Info   RGD1305625_predicted 
   1312 6e-07 536.5 241.6 2.221  1368199_at  nucleoporin 88  Info   Nup88 
   1313 6e-07 922.3 40.9 22.55  1373889_at  similar to immunoglobulin superfamily, member 7  Info   LOC498022 
   1314 6e-07 206.6 762.8 0.271  1381973_at  solute carrier family 25, member 30  Info   RGD:1359702 
   1315 6e-07 1042.2 2362.5 0.441  1391409_at  amnionless (predicted)  Info   Amn_predicted 
   1316 6e-07 417.5 1106.1 0.377  1384628_at  similar to RIKEN cDNA 0610009A07 (predicted)  Info   RGD1309288_predicted 
   1317 6e-07 301.3 130 2.318  1383369_at  tripartite motif protein 26  Info   RGD:1359126 
   1318 6e-07 436.8 314.3 1.39  1368834_at  calcium/calmodulin-dependent protein kinase II, delta  Info   Camk2d 
   1319 6e-07 324.7 506.1 0.642  1371807_at  similar to apoptosis related protein APR-3; p18 protein (predicted)  Info   RGD1311605_predicted 
   1320 6e-07 1886.4 1105.1 1.707  1371505_at  heterogeneous nuclear ribonucleoprotein C (predicted)  Info   Hnrpc_predicted 
   1321 6e-07 1686.6 2825.3 0.597  1375146_at  similar to RIKEN cDNA 3010027G13 (predicted)  Info   RGD1306193_predicted 
   1322 6e-07 508.5 260.1 1.955  1374393_at  protein tyrosine phosphatase-like (proline instead of catalytic arginine), member b (predicted)  Info   Ptplb_predicted 
   1323 6e-07 746.7 346.2 2.157  1383625_a_at  similar to zinc finger protein  Info   LOC500989 
   1324 6e-07 2830.1 1578.4 1.793  1388114_at  myosin light chain, regulatory B  Info   Mrlcb 
   1325 6e-07 1292.4 780.2 1.656  1374505_at  similar to growth hormone-inducible soluble protein  Info   LOC502922 
   1326 6e-07 479.6 5.7 84.14  1370790_at  interferon gamma  Info   Ifng 
   1327 6e-07 170.2 540 0.315  1395167_at  LOC362424 (predicted)  Info   RGD1308850_predicted 
   1328 6e-07 801.9 1257.9 0.637  1375297_at  similar to RIKEN cDNA 0610008C08  Info   LOC363474 
   1329 6e-07 321.4 115.1 2.792  1389446_at  small nuclear ribonucleoprotein polypeptide A' (predicted)  Info   Snrpa1_predicted 
   1330 6e-07 1350.8 667.8 2.023  1386915_at  acidic nuclear phosphoprotein 32 family, member B  Info   Anp32b 
   1331 6e-07 322.9 145.5 2.219  1377756_at  similar to RIKEN cDNA 5830446M03 (predicted)  Info   RGD1307688_predicted 
   1332 6e-07 1486.8 64.1 23.195  1390687_at  pleckstrin (predicted)  Info   Plek_predicted 
   1333 6e-07 491 14.1 34.823  1382601_at  Similar to scavenger receptor type A SR-A  Info    
   1334 6e-07 251.9 737.2 0.342  1376605_at  solute carrier family 17 (anion/sugar transporter), member 5 (predicted)  Info   Slc17a5_predicted 
   1335 6e-07 140 45 3.111  1386989_at  endothelial differentiation, sphingolipid G-protein-coupled receptor, 5  Info   Edg5 
   1336 6e-07 1244.2 192 6.48  1371016_at  similar to T-cell receptor alpha chain precursor V and C regions (TRA29) - rat (fragment)  Info   RGD:1359684 
   1337 7e-07 534.8 272.6 1.962  1374137_at  E74-like factor 1  Info   Elf1 
   1338 7e-07 278.1 42.9 6.483  1374449_at  cell division cycle associated 3 /// similar to cell division cycle associated 3  Info   RGD:1359093 /// LOC501134 
   1339 7e-07 321 147.4 2.178  1371908_at  NTF2-related export protein 1 (predicted)  Info   Nxt1_predicted 
   1340 7e-07 402.3 109.1 3.687  1390839_at  similar to RIKEN cDNA E030024M05 (predicted)  Info   RGD1306818_predicted 
   1341 7e-07 669.9 1096.7 0.611  1398970_at  Translocation protein 1 (predicted)  Info    
   1342 7e-07 237 30.1 7.874  1389668_at  similar to AD024 protein (predicted)  Info   RGD1307811_predicted 
   1343 7e-07 621.1 124.9 4.973  1371256_at  protein tyrosine phosphatase, non-receptor type 18 (predicted)  Info   Ptpn18_predicted 
   1344 7e-07 308.4 759.9 0.406  1393777_at  Transcribed locus  Info    
   1345 7e-07 664 1272.7 0.522  1370282_at  cysteine and glycine-rich protein 2  Info   RGD:61950 
   1346 7e-07 429.6 297.5 1.444  1398936_at  eukaryotic translation initiation factor 2, subunit 3, structural gene X-linked  Info   Eif2s3x 
   1347 7e-07 99.7 161.7 0.617  1382389_at  Transcribed locus  Info    
   1348 7e-07 809.7 433 1.87  1368118_at  B-cell CLL/lymphoma 10  Info   Bcl10 
   1349 7e-07 326.3 544.6 0.599  1367750_at  phosphoribosyl pyrophosphate synthetase-associated protein 1  Info   Prpsap1 
   1350 7e-07 117.7 495.8 0.237  1372536_at  chaperone, ABC1 activity of bc1 complex like (S. pombe) (predicted)  Info   Cabc1_predicted 
   1351 7e-07 107.1 32.4 3.306  1369499_at  thymidylate synthase  Info   Tyms 
   1352 7e-07 627 1292.1 0.485  1388617_at  biphenyl hydrolase-like (serine hydrolase, breast epithelial mucin-associated antigen) (predicted)  Info   Bphl_predicted 
   1353 7e-07 475 233.3 2.036  1374438_at    Info    
   1354 7e-07 490.4 1041.5 0.471  1388751_at  RNA binding motif protein 24 (predicted)  Info   Rbm24_predicted 
   1355 7e-07 62.9 18.3 3.437  1389457_at  myeloblastosis oncogene-like 2 (predicted)  Info   Mybl2_predicted 
   1356 7e-07 298 968.6 0.308  1387178_a_at  cystathionine beta synthase  Info   Cbs 
   1357 7e-07 126.8 40.8 3.108  1380768_at  Transcribed locus  Info    
   1358 7e-07 46.7 2.9 16.103  1389882_at  immediate early response 5  Info   Ier5 
   1359 7e-07 157.9 14.2 11.12  1372186_a_at  topoisomerase (DNA) 2 alpha  Info   Top2a 
   1360 7e-07 90.8 35.9 2.529  1380775_at  M-phase phosphoprotein 1 (predicted)  Info   Mphosph1_predicted 
   1361 7e-07 504.2 1700.9 0.296  1379464_at  Transcribed locus  Info    
   1362 7e-07 157.8 58 2.721  1391387_s_at  Transcribed locus  Info    
   1363 7e-07 206 51.2 4.023  1395744_at  Transcribed locus, moderately similar to XP_579465.1 PREDICTED: DNA binding protein N5 [Rattus norvegicus]  Info    
   1364 7e-07 1276.9 526.7 2.424  1372056_at  Chemokine-like factor super family 6 (predicted)  Info   Cklfsf6 
   1365 7e-07 1617.9 3202.1 0.505  1392597_at    Info    
   1366 7e-07 878.3 221.2 3.971  1382108_at  V-maf musculoaponeurotic fibrosarcoma oncogene family, protein B (avian)  Info   Mafb 
   1367 7e-07 227.2 400.3 0.568  1383171_at  transcription factor B2, mitochondrial (predicted)  Info   Tfb2m_predicted 
   1368 7e-07 392.7 155.4 2.527  1373462_at  embryonic ectoderm development (predicted)  Info   Eed_predicted 
   1369 7e-07 64.9 16.3 3.982  1391662_at  similar to RIKEN cDNA 9430015G10 (predicted) /// similar to RIKEN cDNA 9430015G10  Info   RGD1311517_predicted /// LOC500599 
   1370 7e-07 702.8 1095.3 0.642  1371967_at  mitochondrial ribosomal protein L16  Info   Mrpl16 
   1371 7e-07 52.1 165.1 0.316  1377818_at  Similar to junction-mediating and regulatory protein  Info    
   1372 7e-07 296.9 137.8 2.155  1382642_at  zinc finger protein 281 (predicted)  Info   Zfp281_predicted 
   1373 7e-07 850.4 552.5 1.539  1373933_at  Rap guanine nucleotide exchange factor (GEF) 2 (predicted)  Info   Rapgef2_predicted 
   1374 7e-07 139.3 22.9 6.083  1390507_at  interferon-stimulated protein (predicted)  Info   Isg20_predicted 
   1375 7e-07 238.9 78.9 3.028  1368391_at  solute carrier family 7 (cationic amino acid transporter, y+ system), member 1  Info   Slc7a1 
   1376 7e-07 136.1 43.5 3.129  1379665_at  similar to RIKEN cDNA 4632422M10 (predicted)  Info   RGD1310204_predicted 
   1377 7e-07 585.7 323.7 1.809  1386967_at  ras homolog gene family, member Q  Info   Rhoq 
   1378 7e-07 842.7 319 2.642  1388581_at  hematological and neurological expressed sequence 1  Info   RGD:1359325 
   1379 7e-07 282.7 591.5 0.478  1388734_at  Transcribed locus  Info    
   1380 7e-07 4987.6 2898.8 1.721  1398315_at  ribosomal protein L15  Info   Rpl15 
   1381 7e-07 112.1 32.7 3.428  1392638_at  Similar to myeloid leukemia factor 1 interacting protein  Info    
   1382 7e-07 396.7 853.3 0.465  1383742_at  sorting nexin 7 (predicted)  Info   Snx7_predicted 
   1383 7e-07 848.4 554.5 1.53  1371467_at  similar to RIKEN cDNA 0610007P06  Info   RGD:1359455 
   1384 7e-07 562.1 258 2.179  1383181_at  DnaJ (Hsp40) homolog, subfamily C, member 9 (predicted)  Info   Dnajc9_predicted 
   1385 7e-07 339.8 576.3 0.59  1371604_at  mitochondrial ribosomal protein L34  Info   RGD:1359397 
   1386 7e-07 318.1 37.4 8.505  1370682_at  paired-Ig-like receptor A2 (predicted) /// similar to PIRA5  Info   Pira2_predicted /// LOC365169 
   1387 7e-07 47.9 176.9 0.271  1383814_at    Info    
   1388 7e-07 2271.2 1353.6 1.678  1373040_at  eukaryotic translation initiation factor 3, subunit 5 (epsilon) (predicted)  Info   Eif3s5_predicted 
   1389 7e-07 183.9 21.7 8.475  1384765_at    Info    
   1390 8e-07 30.3 137.1 0.221  1390598_at  Transcribed locus  Info    
   1391 8e-07 1241.1 2711.5 0.458  1393887_at  similar to choline phosphotransferase 1; cholinephosphotransferase 1 alpha; cholinephosphotransferase 1  Info   RGD:1359283 
   1392 8e-07 286.3 46.2 6.197  1383519_at  Hexokinase 2  Info   Hk2 
   1393 8e-07 379.2 800.7 0.474  1374200_at  solute carrier family 29 (nucleoside transporters), member 3  Info   Slc29a3 
   1394 8e-07 395.5 165.4 2.391  1367593_at  selenoprotein W, muscle 1  Info   Sepw1 
   1395 8e-07 360.8 228.1 1.582  1392461_at  PRP4 pre-mRNA processing factor 4 homolog (yeast) (predicted)  Info   Prpf4_predicted 
   1396 8e-07 254.7 75.3 3.382  1393050_at  Strain Wistar Rap2a mRNA, 5' UTR  Info    
   1397 8e-07 349.7 51.2 6.83  1370177_at  poliovirus receptor  Info   PVR 
   1398 8e-07 168.4 81 2.079  1378440_at  Transcribed locus  Info    
   1399 8e-07 326.3 90 3.626  1367768_at  latexin  Info   Lxn 
   1400 8e-07 495.7 1158.6 0.428  1397526_at  glutaryl-Coenzyme A dehydrogenase (predicted)  Info   Gcdh_predicted 
   1401 8e-07 687.7 346 1.988  1381993_at  chloride intracellular channel 2 (predicted)  Info   Clic2_predicted 
   1402 8e-07 576.5 222.5 2.591  1369029_at  phospholipid scramblase 1  Info   Plscr1 
   1403 8e-07 463.5 190.4 2.434  1392958_at  similar to cylindromatosis (turban tumor syndrome)  Info   LOC498035 
   1404 8e-07 318.2 2280.4 0.14  1387382_at  histamine N-methyltransferase  Info   Hnmt 
   1405 8e-07 3119.9 4952.4 0.63  1367706_at  voltage-dependent anion channel 1  Info   Vdac1 
   1406 8e-07 524.5 1474.5 0.356  1370379_at  protease, serine, 8 (prostasin)  Info   Prss8 
   1407 8e-07 81.5 309.1 0.264  1387941_s_at  phospholipase A2, group VI  Info   Pla2g6 
   1408 8e-07 116.6 9.4 12.404  1383684_at  ASF1 anti-silencing function 1 homolog B (S. cerevisiae) (predicted)  Info   Asf1b_predicted 
   1409 8e-07 203.3 7.5 27.107  1381357_at  protein tyrosine phosphatase, non-receptor type 8 (predicted)  Info   Ptpn8_predicted 
   1410 8e-07 509.2 1861.9 0.273  1371363_at  glycerol-3-phosphate dehydrogenase 1 (soluble)  Info   Gpd1 
   1411 8e-07 491.2 1698.1 0.289  1370259_a_at  parathyroid hormone receptor 1  Info   Pthr1 
   1412 8e-07 2855.1 1804.5 1.582  1370825_a_at  cell division cycle 42 homolog (S. cerevisiae)  Info   Cdc42 
   1413 8e-07 258.5 81.6 3.168  1368379_at  CD36 antigen (collagen type I receptor, thrombospondin receptor)-like 2  Info   Scarb2 
   1414 8e-07 683.9 471.6 1.45  1387807_at  platelet-activating factor acetylhydrolase, isoform Ib, alpha subunit 45kDa  Info   Pafah1b1 
   1415 8e-07 210.5 119.7 1.759  1395875_at  similar to KIAA0372 gene product (predicted)  Info   RGD1306062_predicted 
   1416 8e-07 119.1 21.3 5.592  1388026_at  CD3 antigen, zeta polypeptide  Info   Cd3z 
   1417 8e-07 139 53 2.623  1378546_at  Transcribed locus  Info    
   1418 8e-07 81.2 254.8 0.319  1390838_at  similar to KIAA1034-like DNA binding protein  Info   LOC501145 
   1419 8e-07 334.1 1087.4 0.307  1382924_at  pantothenate kinase 1 (predicted)  Info   Pank1_predicted 
   1420 8e-07 678.5 439.3 1.545  1375525_at  Microtubule-associated protein, RP/EB family, member 1  Info   Mapre1 
   1421 8e-07 159.5 79.2 2.014  1373767_at  similar to arsenite inducible RNA associated protein (predicted)  Info   RGD1310991_predicted 
   1422 8e-07 289.4 614 0.471  1377725_at  Transcribed locus  Info    
   1423 8e-07 64.9 134.8 0.481  1377774_at    Info    
   1424 8e-07 158.7 24.5 6.478  1383940_at  cell division cycle associated 1 (predicted)  Info   Cdca1_predicted 
   1425 8e-07 141.4 310.1 0.456  1374527_at  similar to hypothetical protein D4Ertd765e (predicted)  Info   RGD1308525_predicted 
   1426 8e-07 1448.5 2420.3 0.598  1398923_at  similar to D11Bwg0434e protein (predicted)  Info   RGD1305687_predicted 
   1427 8e-07 2410.3 1436.9 1.677  1388333_at  Ring-box 1 (predicted)  Info    
   1428 8e-07 3474 1461.7 2.377  1370339_at  Tropomyosin 3, splice variant 1  Info   Tpm3 
   1429 8e-07 148.4 87.3 1.7  1392479_at  FK506-binding protein 1a  Info   Fkbp1a 
   1430 8e-07 201 438.1 0.459  1374749_at    Info    
   1431 8e-07 277.2 434.1 0.639  1399030_at  similar to DNA segment, Chr X, Immunex 38, expressed  Info   RGD:1359718 
   1432 8e-07 1783.3 1096.7 1.626  1389520_at  similar to Wdr1 protein  Info   LOC360950 
   1433 8e-07 3301.4 978 3.376  1367584_at  annexin A2  Info   Anxa2 
   1434 9e-07 421.6 205.7 2.05  1371631_at  similar to 2010003J03Rik protein (predicted)  Info   RGD1304686_predicted 
   1435 9e-07 435.1 1188.3 0.366  1398341_at  hypothetical LOC287661  Info   LOC287661 
   1436 9e-07 3767.7 4780.7 0.788  1371381_at  similar to TBC1 domain family member 4  Info   LOC306117 
   1437 9e-07 1283 3590 0.357  1368236_at  meprin 1 alpha  Info   Mep1a 
   1438 9e-07 473.7 2274.8 0.208  1386968_at  protein phosphatase 1, regulatory (inhibitor) subunit 1A  Info   Ppp1r1a 
   1439 9e-07 104.4 50.4 2.071  1378746_at    Info    
   1440 9e-07 1864.5 803.8 2.32  1388408_at  similar to RIKEN cDNA 1110020C13 (predicted)  Info   RGD1307129_predicted 
   1441 9e-07 288.1 562.7 0.512  1387061_at  junction plakoglobin  Info   Jup 
   1442 9e-07 172.6 69.2 2.494  1376077_at    Info    
   1443 9e-07 220 126.4 1.741  1386346_at  Unknown (protein for MGC:72591)  Info   Tmem19 
   1444 9e-07 6633.9 159.3 41.644  1368332_at  guanylate nucleotide binding protein 2  Info   Gbp2 
   1445 9e-07 272.8 46.3 5.892  1393085_at  similar to hypothetical protein BC018453 (predicted)  Info   RGD1307700_predicted 
   1446 9e-07 196.3 72.9 2.693  1391905_at    Info    
   1447 9e-07 500.9 195.2 2.566  1375901_at  DEAD (Asp-Glu-Ala-Asp) box polypeptide 21b (predicted)  Info   Ddx21b_predicted 
   1448 9e-07 444.7 329.5 1.35  1376796_at  Transcribed locus  Info    
   1449 9e-07 95.3 232.7 0.41  1371356_at  tensin like C1 domain containing phosphatase (predicted)  Info   Tenc1_predicted 
   1450 9e-07 2512.7 4363.9 0.576  1388118_at  3-hydroxyisobutyrate dehydrogenase  Info   Hibadh 
   1451 9e-07 194.9 31.3 6.227  1384292_at  docking protein 1 (predicted)  Info   Dok1_predicted 
   1452 9e-07 207 51.7 4.004  1386252_at  Transcribed locus  Info    
   1453 9e-07 463.1 124.8 3.711  1380909_at  Transcribed locus  Info    
   1454 9e-07 66 159.6 0.414  1390161_at  similar to cysteine and tyrosine-rich protein 1  Info   RGD:1359578 
   1455 9e-07 561.6 251.8 2.23  1369617_at  ubiquitin-conjugating enzyme E2N  Info   Ube2n 
   1456 9e-07 622.5 364.5 1.708  1388137_a_at  zygin-related protein  Info   RGD:628711 
   1457 9e-07 1399.3 2029.8 0.689  1389866_at    Info    
   1458 9e-07 314.4 115.6 2.72  1379302_at  Transcribed locus  Info    
   1459 9e-07 357.3 141.8 2.52  1387925_at  asparagine synthetase  Info   Asns 
   1460 9e-07 457.3 1459 0.313  1384638_at  Similar to RIKEN cDNA 1700013H19 (predicted)  Info    
   1461 9e-07 142.3 588 0.242  1389392_at  Transcribed locus  Info    
   1462 9e-07 507.6 1813.2 0.28  1368794_at  3-hydroxyanthranilate 3,4-dioxygenase  Info   Haao 
   1463 9e-07 155.6 365.2 0.426  1370930_at  kinesin family member 1C  Info   Kif1c 
   1464 9e-07 103 32.8 3.14  1376784_at  LOC499775  Info   LOC499775 
   1465 9e-07 740.3 294.5 2.514  1388711_at  interleukin 13 receptor, alpha 1  Info   Il13ra1 
   1466 9e-07 426.4 251.3 1.697  1367943_at  nuclear factor of kappa light chain gene enhancer in B-cells inhibitor, beta  Info   Nfkbib 
   1467 9e-07 180.3 351.1 0.514  1374462_at  kinesin-associated protein 3 (predicted)  Info   Kifap3_predicted 
   1468 9e-07 1328.5 2291.2 0.58  1373667_at  cysteine conjugate-beta lyase (predicted)  Info   Ccbl1_predicted 
   1469 9e-07 191.8 44.8 4.281  1376624_at  Transcribed locus  Info    
   1470 9e-07 3642.4 1305.1 2.791  1387076_at  hypoxia inducible factor 1, alpha subunit  Info   Hif1a 
   1471 9e-07 98 333.1 0.294  1378197_at  kinesin family member C2  Info   KIFC2 
   1472 9e-07 71.7 431 0.166  1384614_at    Info    
   1473 9e-07 374.4 97.8 3.828  1367936_at  serine/threonine kinase 10  Info   Stk10 
   1474 9e-07 594.6 389.1 1.528  1373030_at  LOC501594  Info   LOC501594 
   1475 9e-07 185.6 63.2 2.937  1380728_at  Similar to collapsin response mediator protein-2A  Info    
   1476 9e-07 194.7 490.8 0.397  1374169_at  similar to chromosome 16 open reading frame 5 (predicted)  Info   RGD1310686_predicted 
   1477 9e-07 275.4 448.5 0.614  1376649_at  Protein phosphatase 2 (formerly 2A), regulatory subunit A (PR 65), beta isoform (predicted)  Info    
   1478 9e-07 107.7 269.7 0.399  1392940_at  Transcribed locus  Info    
   1479 9e-07 85.4 42.3 2.019  1369471_at  rapostlin  Info   Fnbp1 
   1480 9e-07 1328.9 734.5 1.809  1370844_at  heterogeneous nuclear ribonucleoprotein F  Info   Hnrpf 
   1481 9e-07 579.8 300.1 1.932  1373955_at  karyopherin (importin) beta 3 (predicted)  Info   Kpnb3_predicted 
   1482 9e-07 406.8 109.5 3.715  1369669_at  neurolysin (metallopeptidase M3 family)  Info   Nln 
   1483 9e-07 174.4 345.8 0.504  1374272_at  Transcribed locus  Info    
   1484 9e-07 484.3 279 1.736  1392908_at  hypothetical protein LOC595134  Info   LOC595134 
   1485 9e-07 807.3 2480.9 0.325  1368096_at  Ras-related GTP-binding protein Rab29  Info   Rab7l1 
   1486 9e-07 215.2 89.8 2.396  1380794_at  Aquarius (predicted)  Info    
   1487 9e-07 513.5 234.3 2.192  1389468_at  ribose 5-phosphate isomerase A (predicted)  Info   Rpia_predicted 
   1488 9e-07 681.8 1777.3 0.384  1368191_a_at  solute carrier family 22 (organic cation transporter), member 1  Info   Slc22a1 
   1489 9e-07 200.4 18.8 10.66  1380377_at  Bruton agammaglobulinemia tyrosine kinase  Info   RGD:1359683 
   1490 9e-07 64.6 221.5 0.292  1391629_at  hypothetical LOC362677  Info   LOC362677 
   1491 9e-07 116.8 42.1 2.774  1373838_at  fucosyltransferase 4  Info   Fut4 
   1492 9e-07 459.7 1419.7 0.324  1368915_at  kynurenine 3-monooxygenase (kynurenine 3-hydroxylase)  Info   Kmo 
   1493 1e-06 612.7 2819.7 0.217  1398612_at  aldo-keto reductase family 1, member C12 (predicted)  Info   Akr1c12_predicted 
   1494 1e-06 160.4 71.3 2.25  1382515_at  cleavage and polyadenylation specific factor 2 (predicted)  Info   Cpsf2_predicted 
   1495 1e-06 220.7 28.3 7.799  1376675_at  similar to mast cell-derived paired immunoglobulin-like receptor1  Info   LOC501736 
   1496 1e-06 259.1 67.6 3.833  1370064_at  presenilin 2  Info   Psen2 
   1497 1e-06 387 212.7 1.819  1388509_at    Info    
   1498 1e-06 341 154.2 2.211  1371563_at  similar to CG9135-PA (predicted)  Info   RGD1309986_predicted 
   1499 1e-06 124.4 279.9 0.444  1372953_at  neurocalcin delta  Info   Ncald 
   1500 1e-06 678.1 257.1 2.637  1370968_at  nuclear factor of kappa light chain gene enhancer in B-cells 1, p105  Info   Nfkb1 
   1501 1e-06 122.9 3.5 35.114  1393917_at  CD163 antigen (predicted)  Info   Cd163_predicted 
   1502 1e-06 1045 529.4 1.974  1399033_at  core binding factor beta  Info   Cbfb 
   1503 1e-06 72.1 25.5 2.827  1385706_at  Similar to Testis derived transcript  Info    
   1504 1e-06 23.9 114.7 0.208  1382918_at  Transcribed locus  Info    
   1505 1e-06 101.3 215.6 0.47  1380321_at  mitochondrial tumor suppressor 1  Info   Mtus1 
   1506 1e-06 305.1 93.3 3.27  1374539_at  Similar to type IV putative aminophospholipid transporting ATPase  Info    
   1507 1e-06 391 112.8 3.466  1370082_at  transforming growth factor, beta 1  Info   Tgfb1 
   1508 1e-06 393.1 875.4 0.449  1373312_at  Transcribed locus, strongly similar to NP_064383.1 brain protein 17 [Mus musculus]  Info    
   1509 1e-06 101.3 39.4 2.571  1381919_at  Hermansky-Pudlak syndrome 6  Info   Hps6 
   1510 1e-06 169.1 35.7 4.737  1377153_a_at  kelch-like 6 (Drosophila) (predicted)  Info   Klhl6_predicted 
   1511 1e-06 382.5 916.3 0.417  1384964_at  similar to Solute carrier family 6 (neurotransmitter transporter), member 20  Info   LOC301083 
   1512 1e-06 43.8 123.4 0.355  1374370_at    Info    
   1513 1e-06 212.6 62.8 3.385  1367919_at  nuclear pore membrane glycoprotein 210  Info   Pom210 
   1514 1e-06 36.2 131.9 0.274  1369728_at  histone 1, H4m (predicted)  Info   Hist1h4m_predicted 
   1515 1e-06 239.2 575.9 0.415  1393067_at    Info    
   1516 1e-06 226.4 62.3 3.634  1376599_at  ATPase family, AAA domain containing 2 (predicted)  Info    
   1517 1e-06 1197.1 418.4 2.861  1368838_at  tropomyosin 4  Info   Tpm4 
   1518 1e-06 2973 1411.5 2.106  1398786_at  proteasome (prosome, macropain) subunit, beta type 2  Info   Psmb2 
   1519 1e-06 234.7 3.5 67.057  1388046_at  integrin alpha M  Info   Itgam 
   1520 1e-06 379.4 153.3 2.475  1371863_at  similar to phosphatidylglycerophosphate synthase (predicted)  Info   RGD1305052_predicted 
   1521 1e-06 308.8 642.6 0.481  1375346_at  similar to hypothetical protein FLJ20010  Info   LOC500993 
   1522 1e-06 448 220.7 2.03  1389980_at  similar to Protein HSPC163  Info   LOC289324 
   1523 1e-06 3650.7 2268.4 1.609  1398626_s_at  ARP2 actin-related protein 2 homolog (yeast) (predicted)  Info   Actr2_predicted 
   1524 1e-06 305.9 64.6 4.735  1378592_at  Similar to RIKEN cDNA 2310035M22 (predicted)  Info    
   1525 1e-06 25.3 135.7 0.186  1367989_at  solute carrier family 2 (facilitated glucose transporter), member 4  Info   Slc2a4 
   1526 1e-06 91.5 206.7 0.443  1372884_at  Transcribed locus  Info    
   1527 1e-06 66.6 180.2 0.37  1373786_at  Transcribed locus  Info    
   1528 1e-06 55.2 8 6.9  1394745_at  Similar to hypothetical protein FLJ20037 (predicted)  Info    
   1529 1e-06 786.6 295.9 2.658  1377016_at  similar to RIKEN cDNA 5730592L21 (predicted)  Info   RGD1310614_predicted 
   1530 1e-06 280.1 41.5 6.749  1379631_at  colony stimulating factor 1 (macrophage)  Info   Csf1 
   1531 1e-06 99.3 339.2 0.293  1379418_at  LOC501008  Info   LOC501008 
   1532 1e-06 115.3 51.6 2.234  1384331_at  Neoplastic progression 3 (predicted)  Info    
   1533 1.1e-06 1091.1 1619.1 0.674  1371576_at  mitochondrial ribosomal protein S36 (predicted)  Info   Mrps36_predicted 
   1534 1.1e-06 160.9 737.7 0.218  1368139_s_at  alkaline phosphatase, tissue-nonspecific  Info   Alpl 
   1535 1.1e-06 72.7 19.2 3.786  1382210_at  Strain Wistar Rap2a mRNA, 5' UTR  Info    
   1536 1.1e-06 237.3 25.6 9.27  1397214_at  Transcribed locus  Info    
   1537 1.1e-06 314.7 121.7 2.586  1389555_at  transcription factor 19  Info   RGD:1302974 
   1538 1.1e-06 76.9 136.1 0.565  1370680_at  staufen, RNA binding protein, homolog 2 (Drosophila)  Info   Stau2 
   1539 1.1e-06 559 1441.7 0.388  1372672_at  quinolinate phosphoribosyltransferase (predicted)  Info   Qprt_predicted 
   1540 1.1e-06 161.1 282.7 0.57  1373709_at  hypothetical LOC289900  Info   RGD:1359592 
   1541 1.1e-06 388.6 138.6 2.804  1372543_at  similar to RIKEN cDNA 2610029G23  Info   LOC363485 
   1542 1.1e-06 339.4 69 4.919  1387242_at  Protein kinase, interferon-inducible double stranded RNA dependent  Info   Prkr 
   1543 1.1e-06 1096 639.6 1.714  1398877_at  stress-induced-phosphoprotein 1  Info   Stip1 
   1544 1.1e-06 1046.6 325.3 3.217  1373043_at    Info    
   1545 1.1e-06 170.7 40.9 4.174  1376697_at  carbohydrate sulfotransferase 12 (predicted)  Info   Chst12_predicted 
   1546 1.1e-06 772.7 1066.9 0.724  1386978_at  BCL2/adenovirus E1B 19 kDa-interacting protein 3-like  Info   Bnip3l 
   1547 1.1e-06 2839 4277.5 0.664  1386953_at  hydroxysteroid 11-beta dehydrogenase 1  Info   Hsd11b1 
   1548 1.1e-06 234.9 71 3.308  1373345_at  amphoterin induced gene 2  Info   Amigo2 
   1549 1.1e-06 5021.7 3696.4 1.359  1367569_at  laminin receptor 1 (67kD, ribosomal protein SA)  Info   Lamr1 
   1550 1.1e-06 186.9 783.6 0.239  1371012_at  2-hydroxyphytanoyl-Coenzyme A lyase  Info   Hpcl2 
   1551 1.1e-06 358.2 158 2.267  1371529_at  Receptor (TNFRSF)-interacting serine-threonine kinase 1 (predicted)  Info    
   1552 1.1e-06 127.4 222.8 0.572  1369070_at  peroxisomal biogenesis factor 12  Info   Pex12 
   1553 1.1e-06 157.1 468.2 0.336  1369158_at  calcium-sensing receptor  Info   Casr 
   1554 1.1e-06 1130.1 2680.4 0.422  1371954_at    Info    
   1555 1.1e-06 749.7 3629.6 0.207  1368304_at  Flavin containing monooxygenase 3  Info   Fmo3 
   1556 1.1e-06 602.8 374.7 1.609  1368220_at  general transcription factor IIB  Info   Gtf2b 
   1557 1.1e-06 309.7 504 0.614  1372733_at  Similar to intracellular membrane-associated calcium-independent phospholipase A2 gamma (predicted)  Info    
   1558 1.1e-06 81.5 5.7 14.298  1381570_at  similar to T-cell activation NFKB-like protein (predicted)  Info   RGD1308055_predicted 
   1559 1.1e-06 253.4 389.5 0.651  1374196_at  lanC (bacterial lantibiotic synthetase component C)-like 1  Info   Lancl1 
   1560 1.1e-06 4384 5953 0.736  1367641_at  superoxide dismutase 1  Info   Sod1 
   1561 1.1e-06 185.8 315.6 0.589  1371728_at  Tripartite motif-containing 35  Info    
   1562 1.1e-06 80 306.3 0.261  1370385_at  phospholipase A2, group VI  Info   Pla2g6 
   1563 1.1e-06 1390.3 2752.4 0.505  1375436_at  nicotinate phosphoribosyltransferase-like protein  Info   RGD:1302945 
   1564 1.1e-06 1000.6 462.7 2.163  1372090_at  similar to RIKEN cDNA 1700108M19  Info   LOC298963 
   1565 1.1e-06 839.2 1230.1 0.682  1388552_at  sphingomyelin phosphodiesterase 1, acid lysosomal  Info   Smpd1 
   1566 1.1e-06 324.6 130.5 2.487  1372850_at  Similar to Expressed sequence AU019823  Info    
   1567 1.1e-06 343 414.4 0.828  1373440_at  caspase 8 associated protein 2 (predicted)  Info   Casp8ap2_predicted 
   1568 1.1e-06 595.4 137.5 4.33  1385641_at  Transcribed locus, strongly similar to NP_780521.1 hypothetical protein LOC101148 [Mus musculus]  Info    
   1569 1.2e-06 4702.2 871.1 5.398  1367784_a_at  clusterin  Info   Clu 
   1570 1.2e-06 2642.1 39.4 67.058  1392637_at  Similar to interferon-inducible GTPase (predicted)  Info    
   1571 1.2e-06 262.6 370.9 0.708  1378668_at  CDNA clone IMAGE:7317308  Info    
   1572 1.2e-06 109.7 252.1 0.435  1387018_at  Arg/Abl-interacting protein ArgBP2  Info   Argbp2 
   1573 1.2e-06 272.9 137.1 1.991  1377310_at  Transcribed locus, weakly similar to XP_575554.1 PREDICTED: similar to RNA binding motif and ELMO domain 1 [Rattus norvegicus]  Info    
   1574 1.2e-06 25.2 101.3 0.249  1387913_at  cytochrome P450, family 2, subfamily d, polypeptide 22  Info   Cyp2d22 
   1575 1.2e-06 1358.3 3033 0.448  1389466_at  Ankyrin 3, epithelial isoform g  Info    
   1576 1.2e-06 219.2 94.1 2.329  1379778_at  similar to HSPC043 protein (predicted)  Info   RGD1307983_predicted 
   1577 1.2e-06 735.8 362.2 2.031  1372360_at  Transcribed locus  Info    
   1578 1.2e-06 2120.7 1387.7 1.528  1388119_at  hypothetical gene supported by Y16641 /// heterogeneous nuclear ribonucleoprotein A3 /// similar to Hnrpa3 protein /// similar to Hnrpa3 protein  Info   LOC292273 /// Hnrpa3 /// LOC363971 /// LOC499592 
   1579 1.2e-06 70.7 179.8 0.393  1370047_at  ectonucleotide pyrophosphatase/phosphodiesterase 1  Info   Enpp1 
   1580 1.2e-06 2353.9 1437.3 1.638  1398784_at  complement component 1, q subcomponent binding protein  Info   C1qbp 
   1581 1.2e-06 338.6 46.4 7.297  1389566_at  cyclin B2 (predicted)  Info   Ccnb2_predicted 
   1582 1.2e-06 210.1 95 2.212  1392913_at  similar to zinc finger protein 366 (predicted)  Info   RGD1306491_predicted 
   1583 1.2e-06 404.4 1373.7 0.294  1390172_at  dehydrogenase E1 and transketolase domain containing 1 (predicted)  Info   Dhtkd1_predicted 
   1584 1.2e-06 140.3 366.4 0.383  1375120_at  inhibitor of DNA binding 4  Info   Idb4 
   1585 1.2e-06 265.2 153.9 1.723  1384239_at  Ribosomal protein L31  Info   Rpl31 
   1586 1.2e-06 160 43.4 3.687  1371923_at  similar to hypothetical protein FLJ20481 (predicted)  Info   RGD1311599_predicted 
   1587 1.2e-06 205.6 25.9 7.938  1368811_at  lamin B1  Info   Lmnb1 
   1588 1.2e-06 378 678.7 0.557  1384327_at  differentially expressed in FDCP 8 (predicted)  Info   Def8_predicted 
   1589 1.2e-06 369 113.5 3.251  1372517_at  peptidylprolyl isomerase (cyclophilin)-like 1 (predicted)  Info   Ppil1_predicted 
   1590 1.2e-06 80.6 179.2 0.45  1382105_at  guanine nucleotide binding protein, beta 5  Info   Gnb5 
   1591 1.2e-06 41.9 2.9 14.448  1368637_at  caspase recruitment domain family, member 9  Info   Card9 
   1592 1.2e-06 173.4 28.3 6.127  1369552_at  SAM domain, SH3 domain and nuclear localisation signals, 1  Info   Samsn1 
   1593 1.2e-06 367.6 1148 0.32  1387966_at  asparaginase-like sperm autoantigen  Info   Asrgl1 
   1594 1.2e-06 388.4 194.9 1.993  1383603_at  Transcribed locus, moderately similar to XP_579465.1 PREDICTED: DNA binding protein N5 [Rattus norvegicus]  Info    
   1595 1.2e-06 379.7 155.6 2.44  1370909_at  nucleoporin 62  Info   Nup62 
   1596 1.2e-06 831.4 380.1 2.187  1367844_at  guanine nucleotide binding protein, alpha inhibiting 2  Info   Gnai2 
   1597 1.2e-06 207.5 514 0.404  1372847_at  ACN9 homolog (S. cerevisiae) (predicted)  Info   Acn9_predicted 
   1598 1.2e-06 100.1 24.8 4.036  1374756_at  similar to 1500031N24Rik protein (predicted) /// similar to 1500031N24Rik protein  Info   RGD1311188_predicted /// LOC362936 
   1599 1.2e-06 148.3 65.9 2.25  1380327_at  Transcribed locus, moderately similar to NP_536694.1 helicase (DNA) B [Mus musculus]  Info    
   1600 1.2e-06 613.4 1077.8 0.569  1388533_at  CTD (carboxy-terminal domain, RNA polymerase II, polypeptide A) small phosphatase 1 (predicted)  Info   Ctdsp1_predicted 
   1601 1.2e-06 265.3 26.7 9.936  1369387_at  vav 1 oncogene  Info   Vav1 
   1602 1.2e-06 227.9 589.7 0.386  1392670_at  Transcribed locus  Info    
   1603 1.2e-06 597.1 264.8 2.255  1389093_at    Info    
   1604 1.2e-06 4853.6 2408.8 2.015  1375633_at  chloride intracellular channel 1  Info   RGD:1303043 
   1605 1.3e-06 156.7 408.8 0.383  1382571_at  Transcribed locus  Info    
   1606 1.3e-06 198 89.9 2.202  1375910_at  CDC42 effector protein (Rho GTPase binding) 3 (predicted)  Info   Cdc42ep3_predicted 
   1607 1.3e-06 315.6 68.8 4.587  1368027_at  thromboxane A synthase 1  Info   Tbxas1 
   1608 1.3e-06 1603.1 3593 0.446  1386980_at  apolipoprotein M  Info   Apom 
   1609 1.3e-06 7894.1 2871 2.75  1370154_at  lysozyme  Info   Lyz 
   1610 1.3e-06 211.7 642.7 0.329  1389085_at  Transcribed locus  Info    
   1611 1.3e-06 177 31.5 5.619  1382086_at  Transcribed locus  Info    
   1612 1.3e-06 418.9 179.7 2.331  1383349_at  CDNA clone IMAGE:7304849  Info    
   1613 1.3e-06 1268.2 1930 0.657  1368317_at  aquaporin 7  Info   Aqp7 
   1614 1.3e-06 79.1 390.8 0.202  1375030_at  Transcribed locus  Info    
   1615 1.3e-06 983.7 484.1 2.032  1372815_at  mago-nashi homolog, proliferation-associated (Drosophila) (predicted)  Info   Magoh_predicted 
   1616 1.3e-06 80.9 34.3 2.359  1385269_s_at  hypothetical protein LOC304743  Info   LOC304743 
   1617 1.3e-06 1894 1137.4 1.665  1372079_at  Transcribed locus  Info    
   1618 1.3e-06 1534.1 3329.2 0.461  1369636_at  sorbitol dehydrogenase  Info   Sord 
   1619 1.3e-06 779.3 2425 0.321  1391652_at  glycine-N-acyltransferase (predicted)  Info   Glyat_predicted 
   1620 1.3e-06 620 1389.3 0.446  1388908_at  similar to Peci protein  Info   RGD:1359427 
   1621 1.3e-06 86.9 8.4 10.345  1379386_at  similar to B99 protein  Info   LOC300126 
   1622 1.3e-06 1562.5 4244.5 0.368  1389785_at  similar to RIKEN cDNA 0610006H10 gene (predicted)  Info   RGD1305394_predicted 
   1623 1.3e-06 1407.1 723.5 1.945  1381968_at  cellular repressor of E1A-stimulated genes (predicted)  Info   Creg_predicted 
   1624 1.3e-06 480.6 241.9 1.987  1393956_at  serine/threonine kinase 17b (apoptosis-inducing)  Info   Stk17b 
   1625 1.3e-06 1273.8 730.2 1.744  1398886_at  similar to Selenoprotein H  Info   LOC502642 
   1626 1.3e-06 496.6 118.1 4.205  1373831_at  hypothetical protein LOC619574  Info   LOC619574 
   1627 1.3e-06 728.2 443.1 1.643  1373200_at  eukaryotic translation elongation factor 1 epsilon 1 (predicted)  Info   Eef1e1_predicted 
   1628 1.3e-06 13.8 77.2 0.179  1393673_at  similar to HCDI protein (predicted)  Info   RGD1309307_predicted 
   1629 1.3e-06 182.6 1231.3 0.148  1370991_at  camello-like 3  Info   Cml3 
   1630 1.3e-06 153.9 681.9 0.226  1379073_at  Similar to RIKEN cDNA 2310067G05  Info    
   1631 1.3e-06 272.1 29.3 9.287  1387982_at  toll-like receptor 4  Info   Tlr4 
   1632 1.3e-06 776.4 1668.8 0.465  1368992_a_at  splicing factor, arginine/serine-rich 5  Info   Sfrs5 
   1633 1.3e-06 2.2 88.8 0.025  1395030_at  Basic transcription element binding protein 1  Info   Bteb1 
   1634 1.4e-06 229.2 407.3 0.563  1373055_at  Transcribed locus  Info    
   1635 1.4e-06 85.3 20.1 4.244  1384294_at  Transcribed locus  Info    
   1636 1.4e-06 1447.4 2568 0.564  1367929_at  CD59 antigen  Info   Cd59 
   1637 1.4e-06 29.2 118 0.247  1384275_at  Transcribed locus  Info    
   1638 1.4e-06 263.9 93.5 2.822  1374113_at  Bromodomain adjacent to zinc finger domain, 1A (predicted)  Info    
   1639 1.4e-06 793.4 1724 0.46  1390454_at  4-nitrophenylphosphatase domain and non-neuronal SNAP25-like protein homolog 1 (C. elegans) (predicted)  Info   Nipsnap1_predicted 
   1640 1.4e-06 232.9 18.5 12.589  1372886_at  transforming acidic coiled coil 3  Info   RGD:1302948 
   1641 1.4e-06 88.1 555 0.159  1387672_at  glycine N-methyltransferase  Info   Gnmt 
   1642 1.4e-06 44.4 177.2 0.251  1386274_at  solute carrier family 17 (sodium phosphate), member 2 (predicted)  Info   Slc17a2_predicted 
   1643 1.4e-06 61.8 313.5 0.197  1377993_at  similar to hypothetical protein FLJ12681 (predicted)  Info   RGD1310180_predicted 
   1644 1.4e-06 100.9 370.2 0.273  1391331_at  Transcribed locus  Info    
   1645 1.4e-06 935.3 3514.1 0.266  1387053_at  flavin containing monooxygenase 1  Info   Fmo1 
   1646 1.4e-06 295.1 439.7 0.671  1370134_at  acetyl-CoA transporter  Info   Slc33a1 
   1647 1.4e-06 3664.4 2380.5 1.539  1370184_at  cofilin 1  Info   Cfl1 
   1648 1.4e-06 182.2 69.2 2.633  1393745_at  origin recognition complex, subunit 3-like (S. cerevisiae) (predicted)  Info   Orc3l_predicted 
   1649 1.4e-06 936.6 2332.1 0.402  1372485_at  pterin 4 alpha carbinolamine dehydratase/dimerization cofactor of hepatocyte nuclear factor 1 alpha (TCF1) 1  Info   Pcbd1 
   1650 1.4e-06 107.5 21 5.119  1391690_at  Transcribed locus  Info    
   1651 1.4e-06 486.9 965.4 0.504  1374584_at  similar to Serine/threonine protein kinase 24  Info   LOC361092 
   1652 1.4e-06 74.3 199.1 0.373  1387083_at  cardiotrophin 1  Info   Ctf1 
   1653 1.4e-06 206.6 1638.4 0.126  1373975_at  similar to thioether S-methyltransferase  Info   LOC368066 
   1654 1.4e-06 258.2 582.2 0.443  1383557_at  Transcribed locus  Info    
   1655 1.4e-06 426.3 189.2 2.253  1389162_at  histone cell cycle regulation defective interacting protein 5 (predicted)  Info   Hirip5_predicted 
   1656 1.4e-06 1794 3221.2 0.557  1393221_at  similar to 20-alpha-hydroxysteroid dehydrogenase  Info   LOC498789 
   1657 1.4e-06 930.3 1264.3 0.736  1371409_at  similar to cDNA sequence BC031181  Info   LOC498886 
   1658 1.4e-06 78.6 21 3.743  1376951_at  MAD2 (mitotic arrest deficient, homolog)-like 1 (yeast) (predicted)  Info    
   1659 1.4e-06 87.3 1.9 45.947  1393929_at  chemokine (C-C motif) receptor 6 (predicted)  Info   Ccr6_predicted 
   1660 1.4e-06 121.8 223.9 0.544  1392975_at  Transcribed locus, strongly similar to XP_525786.1 PREDICTED: similar to chromosome 2 open reading frame 7 [Pan troglodytes]  Info    
   1661 1.4e-06 45.6 126.1 0.362  1376226_at  Similar to Ab2-076  Info    
   1662 1.4e-06 184.1 103.6 1.777  1387300_at  crooked neck protein  Info   Crnkl1 
   1663 1.4e-06 2502.3 4334.4 0.577  1386867_at  brain protein 44-like  Info   Brp44l 
   1664 1.4e-06 32.9 137.1 0.24  1398507_at    Info    
   1665 1.4e-06 1890.9 1025.1 1.845  1371732_at  dermatopontin (predicted)  Info   Dpt_predicted 
   1666 1.4e-06 145.4 366.2 0.397  1383640_at  Phosphatidylinositol glycan, class N (predicted)  Info    
   1667 1.5e-06 430.6 123.1 3.498  1388628_at  integral type I protein  Info   RGD:1303327 
   1668 1.5e-06 289.3 434.9 0.665  1388892_at  RAB2B, member RAS oncogene family (predicted)  Info   Rab2b_predicted 
   1669 1.5e-06 1438.5 634.6 2.267  1372167_at    Info    
   1670 1.5e-06 213.8 108.7 1.967  1371491_at  Notch gene homolog 1 (Drosophila)  Info   Notch1 
   1671 1.5e-06 112.2 21.4 5.243  1387062_a_at  checkpoint kinase 1 homolog (S. pombe)  Info   Chek1 
   1672 1.5e-06 362.8 907.1 0.4  1368038_at  synaptojanin 2 binding protein  Info   Synj2bp 
   1673 1.5e-06 65.5 13.9 4.712  1377867_at  similar to Glutaminyl-peptide cyclotransferase precursor (QC) (Glutaminyl-tRNA cyclotransferase) (Glutaminyl cyclase)  Info   LOC313837 
   1674 1.5e-06 108 193.4 0.558  1398354_at  catenin alpha-like 1 (predicted)  Info   Catnal1_predicted 
   1675 1.5e-06 115.8 212.3 0.545  1375270_at  Transcribed locus  Info    
   1676 1.5e-06 371.6 792.3 0.469  1382464_at  similar to ENSANGP00000021391 (predicted)  Info   RGD1309779_predicted 
   1677 1.5e-06 945.1 514.6 1.837  1388345_at    Info    
   1678 1.5e-06 269 645 0.417  1371794_at  Transcribed locus, strongly similar to NP_035873.1 SH3-domain binding protein 3 [Mus musculus]  Info    
   1679 1.5e-06 2082.7 4519.7 0.461  1369491_at  D-amino acid oxidase  Info   Dao1 
   1680 1.5e-06 802.9 408.7 1.965  1371489_at  Ring finger protein 4  Info   Rnf4 
   1681 1.5e-06 35.2 94.4 0.373  1380507_at  Similar to hypothetical protein MGC33630  Info    
   1682 1.5e-06 175.4 62.4 2.811  1389326_at  replication factor C (activator 1) 3 (predicted)  Info   Rfc3_predicted 
   1683 1.5e-06 183 518.9 0.353  1396301_x_at  Transcribed locus  Info    
   1684 1.5e-06 241.2 116.1 2.078  1367849_at  syndecan 1  Info   Sdc1 
   1685 1.5e-06 140.4 328.3 0.428  1372248_at  sestrin 1 (predicted)  Info   Sesn1_predicted 
   1686 1.5e-06 724.3 1068.2 0.678  1371941_at  similar to RIKEN cDNA 5230400G24 (predicted)  Info   RGD1310230_predicted 
   1687 1.5e-06 3469.8 108.5 31.98  1387011_at  lipocalin 2  Info   Lcn2 
   1688 1.5e-06 127.2 69.7 1.825  1379626_at  Special AT-rich sequence binding protein 1 (predicted)  Info    
   1689 1.5e-06 143 83.3 1.717  1391572_at  cysteinyl-tRNA synthetase (predicted)  Info   Cars_predicted 
   1690 1.5e-06 129.4 316.9 0.408  1375504_at  Polymerase (DNA directed), gamma 2, accessory subunit (predicted)  Info    
   1691 1.5e-06 143.3 63.9 2.243  1395396_at  similar to CG9135-PA (predicted)  Info   RGD1309986_predicted 
   1692 1.5e-06 332.5 576.9 0.576  1387038_at  copper chaperone for superoxide dismutase  Info   Ccs 
   1693 1.5e-06 161.7 391.4 0.413  1381976_at  kinesin family member 21A (predicted)  Info   Kif21a_predicted 
   1694 1.5e-06 55.9 178.3 0.314  1383844_at  Transcribed locus  Info    
   1695 1.5e-06 388.6 98 3.965  1380617_at  GTPase, IMAP family member 5  Info   Gimap5 
   1696 1.5e-06 38.5 99.7 0.386  1392947_at  CDNA clone IMAGE:7379585  Info    
   1697 1.5e-06 608.7 1602.9 0.38  1368563_at  aspartoacylase  Info   Aspa 
   1698 1.5e-06 73.5 259.9 0.283  1382221_at  Transcribed locus  Info    
   1699 1.5e-06 255.6 98.3 2.6  1370443_at  deoxyribonuclease II  Info   Dnase2 
   1700 1.5e-06 96.1 28.4 3.384  1370462_at  hyaluronan mediated motility receptor  Info   Hmmr 
   1701 1.5e-06 8095.6 2964.7 2.731  1367581_a_at  secreted phosphoprotein 1  Info   Spp1 
   1702 1.5e-06 16.2 121.7 0.133  1380115_at    Info    
   1703 1.5e-06 949.8 224.4 4.233  1370228_at  Transferrin /// signal recognition particle receptor, B subunit (predicted)  Info   Tf /// Srprb_predicted 
   1704 1.5e-06 133.9 50.3 2.662  1387975_at  UDP-glucose ceramide glucosyltransferase  Info   Ugcg 
   1705 1.5e-06 69.9 13 5.377  1383323_at  similar to RIKEN cDNA 1700022L09  Info   LOC499933 
   1706 1.5e-06 352.8 987.8 0.357  1389645_at  proline dehydrogenase (oxidase) 2 (predicted)  Info   Prodh2_predicted 
   1707 1.5e-06 232.9 438.8 0.531  1372457_at  mitochondrial tumor suppressor 1  Info   Mtus1 
   1708 1.5e-06 1553.6 568.4 2.733  1388809_at  acid sphingomyelinase-like phosphodiesterase 3A  Info   RGD:1359277 
   1709 1.5e-06 349.7 1041.4 0.336  1393793_at  Transcribed locus  Info    
   1710 1.5e-06 171.4 24.6 6.967  1368377_at  granzyme C  Info   Gzmc 
   1711 1.6e-06 490.6 206.2 2.379  1368361_a_at  protein tyrosine phosphatase, non-receptor type 2  Info   Ptpn2 
   1712 1.6e-06 91.6 239.8 0.382  1378292_at  Transcribed locus  Info    
   1713 1.6e-06 343 66.4 5.166  1379275_at  sorting nexin 10 (predicted)  Info   Snx10_predicted 
   1714 1.6e-06 196.1 426.1 0.46  1386901_at  similar to fatty acid translocase/CD36  Info   LOC499984 
   1715 1.6e-06 349.9 103 3.397  1368573_at  karyopherin (importin) beta 1  Info   Kpnb1 
   1716 1.6e-06 285.3 124.7 2.288  1383255_at  Transcribed locus  Info    
   1717 1.6e-06 475.5 274.6 1.732  1382175_at  Similar to Wilms tumor 1-associating protein (WT1-associated protein) (Putative pre-mRNA splicing regulator female-lethal(2D) homolog)  Info    
   1718 1.6e-06 109.3 491.7 0.222  1389066_at  Down syndrome critical region gene 1-like 1  Info   Dscr1l1 
   1719 1.6e-06 143.8 289.4 0.497  1378415_at  Protein kinase, cAMP-dependent, regulatory, type 2, alpha  Info   Prkar2a 
   1720 1.6e-06 154.1 411.4 0.375  1379419_at  DNA sequence AY228474  Info   AY228474 
   1721 1.6e-06 55 114.5 0.48  1377223_at  Transcribed locus  Info    
   1722 1.6e-06 510.6 206.5 2.473  1373499_at  growth arrest specific 5  Info   Gas5 
   1723 1.6e-06 384.9 167.3 2.301  1388791_at  similar to 2810022L02Rik protein (predicted)  Info   RGD1309930_predicted 
   1724 1.6e-06 249.8 84.2 2.967  1380583_s_at  colony stimulating factor 1 (macrophage)  Info   Csf1 
   1725 1.6e-06 200.8 124.9 1.608  1389222_at  Tryptophan hydroxylase  Info   Tph 
   1726 1.6e-06 174.5 9.5 18.368  1370483_at  CD244 natural killer cell receptor 2B4  Info   Cd244 
   1727 1.6e-06 311.1 1037 0.3  1369169_at  solute carrier family 23 (nucleobase transporters), member 1  Info   Slc23a1 
   1728 1.6e-06 401.1 58.7 6.833  1385832_s_at  Similar to RIKEN cDNA 1200013B08  Info    
   1729 1.6e-06 1125.3 1903.1 0.591  1372671_at  similar to RIKEN cDNA 0610038L10 gene /// similar to riboflavin kinase /// similar to riboflavin kinase  Info   LOC317214 /// LOC499328 /// LOC501601 
   1730 1.6e-06 274.5 562 0.488  1372069_at  ankyrin repeat domain 15 (predicted)  Info   Ankrd15_predicted 
   1731 1.6e-06 196.5 433.6 0.453  1389176_at  Inositol polyphosphate-5-phosphatase F (predicted)  Info    
   1732 1.6e-06 24.4 377.9 0.065  1370289_x_at  kallikrein, submaxillary gland S3  Info   Klks3 
   1733 1.6e-06 483.5 266.8 1.812  1388974_at  signal recognition particle 19 (predicted)  Info   Srp19_predicted 
   1734 1.6e-06 108.2 28.8 3.757  1379642_at  Transcribed locus  Info    
   1735 1.6e-06 493.9 278.3 1.775  1371925_at  ATPase type 13A (predicted)  Info   Atp13a_predicted 
   1736 1.6e-06 476.6 31.2 15.276  1370832_at  small inducible cytokine A4  Info   Ccl4 
   1737 1.6e-06 213.4 94.9 2.249  1383548_at  Similar to hypothetical protein FLJ20189 (predicted)  Info    
   1738 1.6e-06 165 46.7 3.533  1367983_at  flap structure-specific endonuclease 1  Info   Fen1 
   1739 1.6e-06 324.6 134.3 2.417  1390142_at  Zinc finger, CW-type with coiled-coil domain 3 (predicted)  Info    
   1740 1.6e-06 697 182.2 3.825  1374499_at  TATA box binding protein (Tbp)-associated factor, RNA polymerase I, A (predicted)  Info    
   1741 1.7e-06 347.3 728.6 0.477  1389403_at  bone morphogenetic protein 7  Info   Bmp7 
   1742 1.7e-06 2443.8 3739.2 0.654  1373017_at  succinate-Coenzyme A ligase, GDP-forming, beta subunit (predicted)  Info   Suclg2_predicted 
   1743 1.7e-06 656.3 342 1.919  1384060_at  Janus kinase 1  Info   Jak1 
   1744 1.7e-06 71.9 463.6 0.155  1368147_at  dual specificity phosphatase 1  Info   Dusp1 
   1745 1.7e-06 79.7 203.5 0.392  1388831_at  Solute carrier family 9 (sodium/hydrogen exchanger), isoform 3 regulator 2  Info   Slc9a3r2 
   1746 1.7e-06 25 70.7 0.354  1373920_at  Similar to 106 kDa O-GlcNAc transferase-interacting protein (predicted)  Info    
   1747 1.7e-06 212.3 69.9 3.037  1376160_at  similar to Avian reticuloendotheliosis viral (v-rel) oncogene related B  Info   LOC365510 
   1748 1.7e-06 117 7.8 15  1368534_at  adrenergic receptor, alpha 1d  Info   Adra1d 
   1749 1.7e-06 606.8 295.9 2.051  1388341_at  RAN GTPase activating protein 1 (predicted)  Info   Rangap1_predicted 
   1750 1.7e-06 183.1 517.3 0.354  1384008_at  Similar to hypothetical protein FLJ40283 (predicted)  Info    
   1751 1.7e-06 435.9 246.6 1.768  1372667_at  similar to RIKEN cDNA 1110059E24  Info   RGD:1359158 
   1752 1.7e-06 123 45.6 2.697  1382158_at  Transcribed locus  Info    
   1753 1.7e-06 1430.8 679.9 2.104  1398805_at  APG3 autophagy 3-like (S. cerevisiae)  Info   Apg3l 
   1754 1.7e-06 33 138.3 0.239  1376955_at  procollagen, type IV, alpha 4 (predicted)  Info   Col4a4_predicted 
   1755 1.7e-06 409.9 2158.2 0.19  1368146_at  dual specificity phosphatase 1  Info   Dusp1 
   1756 1.7e-06 487.3 303 1.608  1369935_at  cyclin D3  Info   Ccnd3 
   1757 1.7e-06 244.2 24.3 10.049  1393863_at  CDNA clone MGC:124654 IMAGE:5622000  Info    
   1758 1.7e-06 88.8 18.4 4.826  1395635_at  Tumor necrosis factor receptor superfamily, member 26 (predicted)  Info    
   1759 1.7e-06 202 105.5 1.915  1376336_at  Transcribed locus  Info    
   1760 1.7e-06 133 196 0.679  1395579_at  DEAH (Asp-Glu-Ala-His) box polypeptide 32 (predicted)  Info   Dhx32_predicted 
   1761 1.7e-06 1042.9 3308.3 0.315  1373093_at  similar to Mitogen-inducible gene 6 protein homolog (Mig-6) (Gene 33 polypeptide) (predicted)  Info   RGD1307599_predicted 
   1762 1.7e-06 56.5 14.9 3.792  1381173_at  Transcribed locus  Info    
   1763 1.7e-06 254.1 542.6 0.468  1369895_s_at  podocalyxin-like  Info   Podxl 
   1764 1.7e-06 1174.7 1921.3 0.611  1371833_at  brain protein I3 (predicted)  Info   Bri3_predicted 
   1765 1.7e-06 254.9 24.3 10.49  1385434_at  Prostaglandin E receptor 2, subtype EP2  Info   Ptger2 
   1766 1.7e-06 1841.2 1237.6 1.488  1371944_at  ubiquitin-conjugating enzyme E2L 3 (predicted)  Info   Ube2l3_predicted 
   1767 1.7e-06 564 1119.8 0.504  1392627_x_at    Info    
   1768 1.7e-06 386.2 130.2 2.966  1372273_at  glycophorin C (Gerbich blood group) (predicted) /// similar to glycophorin C  Info   Gypc_predicted /// LOC498835 
   1769 1.7e-06 261.2 568.4 0.46  1390541_at  harmonin a1  Info   RGD:1303329 
   1770 1.7e-06 646.1 260.2 2.483  1372890_at  Sphingosine phosphate lyase 1  Info   Spgl1 
   1771 1.8e-06 295.6 179.9 1.643  1389197_at  similar to RIKEN cDNA 9630046K23 (predicted)  Info   RGD1306248_predicted 
   1772 1.8e-06 65.7 17.9 3.67  1379972_at  similar to 1700060H10Rik protein (predicted)  Info   RGD1308699_predicted 
   1773 1.8e-06 291.1 137.2 2.122  1385128_at  Similar to MGC68837 protein  Info    
   1774 1.8e-06 148.5 71.6 2.074  1387495_at  transducin-like enhancer of split 4, E(spl) homolog (Drosophila)  Info   Tle4 
   1775 1.8e-06 86.4 12.9 6.698  1397704_at  similar to establishment of cohesion 1 homolog 2  Info   LOC498536 
   1776 1.8e-06 182.1 476.7 0.382  1384907_at    Info    
   1777 1.8e-06 523.3 146.1 3.582  1393643_at  reticulocalbin (predicted)  Info   Rcn_predicted 
   1778 1.8e-06 495.7 259.3 1.912  1388355_at  RNA binding motif protein 17 (predicted)  Info   Rbm17_predicted 
   1779 1.8e-06 266 771.2 0.345  1376709_at  solute carrier family 39 (metal ion transporter), member 8 (predicted)  Info   Slc39a8_predicted 
   1780 1.8e-06 706.3 383.6 1.841  1371839_at  similar to splicing factor, arginine/serine-rich 2  Info   RGD:1359422 
   1781 1.8e-06 707.5 455.5 1.553  1371498_at  similar to Jtv1-pending protein  Info   LOC288480 
   1782 1.8e-06 1783 899.7 1.982  1386866_at  tyrosine 3-monooxgenase/tryptophan 5-monooxgenase activation protein, gamma polypeptide  Info   Ywhag 
   1783 1.8e-06 133.5 215.8 0.619  1389442_at  Transcribed locus  Info    
   1784 1.8e-06 153.4 32.8 4.677  1390221_at  Transcribed locus  Info    
   1785 1.8e-06 233.1 97.5 2.391  1374519_at    Info    
   1786 1.8e-06 284 117.6 2.415  1398561_at  Transcribed locus  Info    
   1787 1.8e-06 372.3 114.9 3.24  1388493_at  Similar to vomeronasal V1r-type receptor V1rc41  Info    
   1788 1.8e-06 42.1 9.4 4.479  1385781_at  similar to SNF2/RAD54 family protein  Info   LOC317252 
   1789 1.8e-06 85.4 13.4 6.373  1368773_at  follicle stimulating hormone primary response gene 1  Info   Fshprh1 
   1790 1.8e-06 181.4 357.4 0.508  1388799_at  kelch-like 7 (Drosophila) (predicted)  Info   Klhl7_predicted 
   1791 1.8e-06 556.5 107 5.201  1374838_at  SP140 nuclear body protein (predicted)  Info   Sp140_predicted 
   1792 1.8e-06 104 34.8 2.989  1380293_at  similar to chromosome 18 open reading frame 54  Info   LOC361346 
   1793 1.8e-06 15.2 53.7 0.283  1397672_at  Transcribed locus  Info    
   1794 1.8e-06 9.1 25.2 0.361  1397227_at  spinal cord expression protein 4  Info   RGD:1303093 
   1795 1.8e-06 1246 724.9 1.719  1382861_at  similar to transcription elongation factor IIS - mouse  Info   LOC498453 
   1796 1.8e-06 98.3 52 1.89  1373927_at  Transcribed locus, strongly similar to XP_226315.3 PREDICTED: similar to High mobility group box 2-like 1 [Rattus norvegicus]  Info    
   1797 1.8e-06 1104.5 724.6 1.524  1373397_at    Info    
   1798 1.8e-06 770.8 221.6 3.478  1370376_a_at  cold shock domain protein A  Info   Csda 
   1799 1.8e-06 72.7 228.5 0.318  1393070_at  secernin 2 (predicted)  Info   Scrn2_predicted 
   1800 1.8e-06 340.9 108.5 3.142  1380357_a_at  Transcribed locus  Info    
   1801 1.8e-06 17.6 91.4 0.193  1378247_at  ELL associated factor 2  Info   Eaf2 
   1802 1.8e-06 95.5 211 0.453  1371462_at  insulin-like growth factor binding protein 4  Info   Igfbp4 
   1803 1.8e-06 155.2 77.3 2.008  1399148_s_at  similar to hypothetical protein MGC38524 (predicted)  Info   RGD1309002_predicted 
   1804 1.8e-06 1067.9 1660.2 0.643  1370939_at  acyl-CoA synthetase long-chain family member 1  Info   Acsl1 
   1805 1.8e-06 43.9 71.3 0.616  1391702_at  Similar to hypothetical protein FLJ20626 (predicted)  Info    
   1806 1.8e-06 1244.4 2234.5 0.557  1387851_at  phosphotriesterase related  Info   Pter 
   1807 1.8e-06 203.9 394.9 0.516  1374657_at  Transcribed locus  Info    
   1808 1.8e-06 613.1 197.8 3.1  1375266_at    Info    
   1809 1.8e-06 903.1 490.2 1.842  1367900_at  glycogenin 1  Info   Gyg1 
   1810 1.8e-06 121.4 60.8 1.997  1379651_at  Forkhead box P1 (predicted)  Info    
   1811 1.8e-06 600.5 1081.4 0.555  1368041_at  synaptojanin 2 binding protein  Info   Synj2bp 
   1812 1.9e-06 192.6 1188.6 0.162  1367725_at  serine/threonine-protein kinase pim-3  Info   Pim3 
   1813 1.9e-06 218.1 503.8 0.433  1369799_at  4-aminobutyrate aminotransferase  Info   Abat 
   1814 1.9e-06 325.3 38.2 8.516  1379760_at  similar to RIKEN cDNA 1810019A08 (predicted)  Info   RGD1310519_predicted 
   1815 1.9e-06 167.1 295.4 0.566  1375423_at    Info    
   1816 1.9e-06 82.2 16 5.138  1374805_at  similar to hypothetical protein MGC5528  Info   LOC299933 
   1817 1.9e-06 1695.2 3016.3 0.562  1372297_at  glutathione S-transferase, alpha 4 (predicted)  Info   Gsta4_predicted 
   1818 1.9e-06 159.7 411 0.389  1371494_at  Transcribed locus  Info    
   1819 1.9e-06 1501.3 665.6 2.256  1372577_at  actin related protein 2/3 complex, subunit 4 (predicted)  Info   Arpc4_predicted 
   1820 1.9e-06 635 1206.7 0.526  1388534_at  Solute carrier family 31 (copper transporters), member 1  Info   Slc31a1 
   1821 1.9e-06 49.7 264.9 0.188  1391109_x_at    Info    
   1822 1.9e-06 298.4 753.6 0.396  1369081_at  neuraminidase 1  Info   Neu1 
   1823 1.9e-06 113.2 318 0.356  1389353_at  Sema domain, transmembrane domain (TM), and cytoplasmic domain, (semaphorin) 6D (predicted)  Info    
   1824 1.9e-06 569.7 956.8 0.595  1371811_at  Bernardinelli-Seip congenital lipodystrophy 2 homolog (human)  Info   Bscl2 
   1825 1.9e-06 34.3 141.3 0.243  1392001_at  Pannexin 1  Info   Panx1 
   1826 1.9e-06 376.2 63.8 5.897  1370265_at  arrestin, beta 2  Info   Arrb2 
   1827 1.9e-06 2060.1 3263.1 0.631  1371380_at  pyruvate dehydrogenase E1 alpha 1  Info   Pdha1 
   1828 1.9e-06 218.2 68 3.209  1382843_at  sphingosine phosphate lyase 1  Info   Sgpl1 
   1829 1.9e-06 229.6 116 1.979  1392497_at  Transcribed locus  Info    
   1830 1.9e-06 768.2 1382 0.556  1372907_at  ATPase, H+ transporting, V0 subunit E isoform 2  Info   Atp6v0e2 
   1831 1.9e-06 526.3 172.6 3.049  1374157_at    Info    
   1832 1.9e-06 167.9 81.3 2.065  1385856_at  RIO kinase 1 (yeast) (predicted)  Info   Riok1_predicted 
   1833 2e-06 501.5 792.4 0.633  1389695_at    Info    
   1834 2e-06 180.9 103.4 1.75  1382661_at  nucleoporin 160 (predicted)  Info   Nup160_predicted 
   1835 2e-06 2489.2 1242.6 2.003  1369976_at  dynein, cytoplasmic, light chain 1  Info   Dnclc1 
   1836 2e-06 601.5 1012.1 0.594  1375452_at  meningioma expressed antigen 6 (coiled-coil proline-rich) (predicted)  Info   Mgea6_predicted 
   1837 2e-06 247.7 464.4 0.533  1390228_at  Transcribed locus  Info    
   1838 2e-06 80.2 189.5 0.423  1370802_at  similar to integrin beta-5  Info   LOC498091 
   1839 2e-06 113.1 239.7 0.472  1383148_at  Transcribed locus  Info    
   1840 2e-06 4808.2 2965.8 1.621  1388297_at  eukaryotic translation elongation factor 1 gamma  Info   RGD:1302939 
   1841 2e-06 1269.2 808.1 1.571  1398773_at  src associated in mitosis, 68 kDa  Info   Khdrbs1 
   1842 2e-06 197 115.6 1.704  1372840_at  Similar to Zbed4 protein  Info    
   1843 2e-06 305.1 104.4 2.922  1390154_at  Transcribed locus  Info    
   1844 2e-06 232.3 108 2.151  1374836_at  RNA, U3 small nucleolar interacting protein 2 (predicted)  Info   Rnu3ip2_predicted 
   1845 2e-06 2098.4 3364.1 0.624  1370613_s_at    Info   Ugt1a1 /// Ugt1a6 /// Ugt1a7 /// Ugt1a8 /// Ugt1a2 /// Ugt1a4 /// Ugt1a11 /// Ugt1a5 
   1846 2e-06 1700.5 1044.1 1.629  1388134_at  eukaryotic translation elongation factor 1 delta (guanine nucleotide exchange protein)  Info   Eef1d 
   1847 2e-06 243.6 623 0.391  1386973_a_at  mitogen activated protein kinase 8 interacting protein  Info   Mapk8ip 
   1848 2e-06 241 5.1 47.255  1374540_at  cell division cycle associated 7 (predicted)  Info   Cdca7_predicted 
   1849 2e-06 6033.3 153.2 39.382  1387969_at  chemokine (C-X-C motif) ligand 10  Info   Cxcl10 
   1850 2e-06 227.5 96.6 2.355  1383152_at  similar to Cc1-9 (predicted)  Info   RGD1308955_predicted 
   1851 2e-06 378.3 85.4 4.43  1372904_at  MOB1, Mps One Binder kinase activator-like 2B (yeast) (predicted)  Info   Mobkl2b_predicted 
   1852 2e-06 201.4 385.6 0.522  1377830_a_at  similar to RIKEN cDNA 1810014F10 gene (predicted)  Info   RGD1311186_predicted 
   1853 2e-06 266.2 154.2 1.726  1387064_at  peroxisomal membrane protein 3  Info   Pxmp3 
   1854 2e-06 65.8 177.4 0.371  1367726_at  thyroid hormone receptor alpha  Info   Thra 
   1855 2e-06 152.9 52.2 2.929  1382921_at  SDA1 domain containing 1  Info   RGD:1359112 
   1856 2e-06 392.9 186.5 2.107  1367890_at  caspase 2  Info   Casp2 
   1857 2.1e-06 138.8 1092.5 0.127  1371754_at  solute carrier family 25 (mitochondrial carrier, phosphate carrier), member 25  Info   Slc25a25 
   1858 2.1e-06 662.6 267.2 2.48  1376102_at  similar to RECS1  Info   RGD:1359409 
   1859 2.1e-06 116.4 212.2 0.549  1373264_at  zinc finger, BED domain containing 3 (predicted)  Info   Zbed3_predicted 
   1860 2.1e-06 1376.7 1965.3 0.701  1371253_at  electron transferring flavoprotein, alpha polypeptide  Info   Etfa 
   1861 2.1e-06 166.4 61.6 2.701  1396077_at  Transcribed locus  Info    
   1862 2.1e-06 86.1 269.2 0.32  1371933_at    Info    
   1863 2.1e-06 335.4 165.2 2.03  1398294_at  actinin, alpha 1  Info   Actn1 
   1864 2.1e-06 368.5 245.6 1.5  1377685_at  similar to establishment of cohesion 1 homolog 1  Info   LOC498820 
   1865 2.1e-06 3093.7 3944.7 0.784  1367599_at  ATP synthase, H+ transporting, mitochondrial F0 complex, subunit c (subunit 9), isoform 1  Info   Atp5g1 
   1866 2.1e-06 84.8 237.7 0.357  1377336_at  sema domain, immunoglobulin domain (Ig), short basic domain, secreted, (semaphorin) 3B (predicted)  Info   Sema3b_predicted 
   1867 2.1e-06 170.9 802.2 0.213  1386881_at  insulin-like growth factor binding protein 3  Info   Igfbp3 
   1868 2.1e-06 389.4 1225.1 0.318  1370609_a_at  solute carrier family 16 (monocarboxylic acid transporters), member 7 /// hypothetical gene supported by NM_017302  Info   Slc16a7 /// LOC497820 
   1869 2.1e-06 62.3 8.1 7.691  1384944_at  B-cell leukemia/lymphoma 11B (predicted)  Info   Bcl11b_predicted 
   1870 2.1e-06 849.4 1526.3 0.557  1372734_at  small cell adhesion glycoprotein  Info   Smagp 
   1871 2.1e-06 177.7 45.1 3.94  1384507_at  Similar to hypothetical protein FLJ11127  Info    
   1872 2.1e-06 54.4 293.1 0.186  1372208_at  protein phosphatase 1, regulatory (inhibitor) subunit 1B  Info   Ppp1r1b 
   1873 2.1e-06 319.4 567.7 0.563  1374300_at    Info    
   1874 2.1e-06 398.3 140.2 2.841  1383711_at  N-myristoyltransferase 1  Info   Nmt1 
   1875 2.1e-06 207.3 877.9 0.236  1368460_at  solute carrier family 2, member 5  Info   Slc2a5 
   1876 2.1e-06 426.6 123.2 3.463  1392107_at  similar to KIAA0963 protein (predicted)  Info   RGD1307194_predicted 
   1877 2.1e-06 344.8 141.4 2.438  1372825_at  LOC499775  Info   LOC499775 
   1878 2.1e-06 202.4 426 0.475  1368040_at  synaptojanin 2 binding protein  Info   Synj2bp 
   1879 2.1e-06 367.4 151.5 2.425  1367883_at  survival of motor neuron 1, telomeric  Info   SMN1 
   1880 2.1e-06 92 33.9 2.714  1377369_at  cytochrome b reductase 1 (predicted)  Info   Cybrd1_predicted 
   1881 2.1e-06 26 353.4 0.074  1382031_at  Transcribed locus  Info    
   1882 2.1e-06 204.1 104 1.962  1376801_at  hypothetical LOC294291  Info   LOC294291 
   1883 2.1e-06 191.5 90.3 2.121  1389213_at  Transcribed locus  Info    
   1884 2.1e-06 743.5 405.2 1.835  1389063_at  exportin 6 (predicted)  Info   Xpo6_predicted 
   1885 2.1e-06 18 266.9 0.067  1367977_at  synuclein, alpha  Info   Snca 
   1886 2.1e-06 3306.3 1551.3 2.131  1386893_at  granulin  Info   Grn 
   1887 2.1e-06 29.1 191.9 0.152  1379445_at  LOC500609  Info    
   1888 2.1e-06 469.9 111 4.233  1382074_at  IBR domain containing 3 (predicted)  Info   Ibrdc3_predicted 
   1889 2.1e-06 405 191.1 2.119  1394724_at  transcription elongation factor A (SII) 1 (predicted)  Info   Tcea1_predicted 
   1890 2.2e-06 163 57.7 2.825  1373226_at  Similar to 60S ribosomal protein L23  Info    
   1891 2.2e-06 328.5 19.1 17.199  1394673_at  similar to m33-A isoform  Info   LOC499142 
   1892 2.2e-06 191.5 34.3 5.583  1370963_at  growth arrest specific 7  Info   Gas7 
   1893 2.2e-06 832 32.3 25.759  1392905_at  Guanine nucleotide binding protein, gamma 2  Info   Gng2 
   1894 2.2e-06 2439.7 1187 2.055  1370859_at  thioredoxin domain containing 7  Info   Txndc7 
   1895 2.2e-06 977.3 1317.1 0.742  1371755_at  Similar to RIKEN cDNA B230219D22  Info    
   1896 2.2e-06 1522.4 2893.6 0.526  1368178_at  PDZ domain containing 1  Info   Pdzk1 
   1897 2.2e-06 150.9 466.6 0.323  1379361_at  peroxisomal biogenesis factor 11A  Info   Pex11a 
   1898 2.2e-06 560 244.9 2.287  1398473_at  biogenesis of lysosome-related organelles complex-1, subunit 2 (predicted)  Info   Bloc1s2_predicted 
   1899 2.2e-06 394.8 110.6 3.57  1382692_at  Transcribed locus  Info    
   1900 2.2e-06 182.9 7.7 23.753  1369190_at  CD2 antigen /// hypothetical gene supported by NM_012830  Info   Cd2 /// LOC497761 
   1901 2.2e-06 97.5 2.5 39  1387835_at  interleukin 1 receptor antagonist  Info   Il1rn 
   1902 2.2e-06 647.6 2187.8 0.296  1376765_at  similar to maestro  Info   LOC361348 
   1903 2.2e-06 238.7 526 0.454  1388470_at  f-box only protein 9 (predicted)  Info   Fbxo9_predicted 
   1904 2.2e-06 312.4 131.6 2.374  1393183_at  similar to StAR-related protein 1-4E  Info   LOC502348 
   1905 2.2e-06 1459 2392.3 0.61  1387759_s_at    Info   Ugt1a1 /// Ugt1a6 /// Ugt1a7 /// Ugt1a8 /// Ugt1a2 /// Ugt1a4 /// Ugt1a11 /// Ugt1a5 
   1906 2.2e-06 1598.2 658.2 2.428  1370234_at  fibronectin 1  Info   Fn1 
   1907 2.2e-06 123.6 14.5 8.524  1381161_a_at  Similar to hypothetical protein FLJ21127  Info    
   1908 2.2e-06 1979.4 4251.3 0.466  1369660_at  defensin beta 1  Info   Defb1 
   1909 2.2e-06 110.8 358.3 0.309  1377940_at  hypothetical LOC287534  Info   RGD:1359691 
   1910 2.2e-06 811.5 1197 0.678  1371359_at  myeloid leukemia factor 2 (predicted)  Info   Mlf2_predicted 
   1911 2.2e-06 60.3 129 0.467  1393729_at  neuropilin (NRP) and tolloid (TLL)-like 2 (predicted)  Info   Neto2_predicted 
   1912 2.2e-06 640.8 1086.8 0.59  1371850_at  3'-phosphoadenosine 5'-phosphosulfate synthase 1 (predicted)  Info   Papss1_predicted 
   1913 2.2e-06 224.1 9 24.9  1391593_at  Ras association (RalGDS/AF-6) domain family 4 (predicted)  Info   Rassf4_predicted 
   1914 2.2e-06 12.4 55.2 0.225  1380726_at  Similar to asporin precursor  Info    
   1915 2.2e-06 192.6 969.4 0.199  1370384_a_at  prolactin receptor  Info   Prlr 
   1916 2.2e-06 1517 290.4 5.224  1382255_at    Info    
   1917 2.3e-06 367 202.9 1.809  1389867_at  CUG triplet repeat,RNA-binding protein 2  Info   Cugbp2 
   1918 2.3e-06 1629 1031.7 1.579  1398863_at  guanine nucleotide binding protein, beta polypeptide 2  Info   Gnb2 
   1919 2.3e-06 333.2 127.5 2.613  1370308_at  RS21-C6 protein  Info   Rs21c6 
   1920 2.3e-06 479.4 255.9 1.873  1371613_at  engulfment and cell motility 2, ced-12 homolog (C. elegans) (predicted)  Info   Elmo2_predicted 
   1921 2.3e-06 347.9 828.9 0.42  1367839_at  farnesyl diphosphate farnesyl transferase 1  Info   Fdft1 
   1922 2.3e-06 167.7 574.6 0.292  1370789_a_at  prolactin receptor  Info   Prlr 
   1923 2.3e-06 1119.5 2502.1 0.447  1388175_at  chloride channel Kb  Info   Clcnkb 
   1924 2.3e-06 175.7 74.2 2.368  1391199_at  Fas (TNFRSF6)-associated via death domain  Info   Fadd 
   1925 2.3e-06 140 468.4 0.299  1369286_at  protein C  Info   Proc 
   1926 2.3e-06 680.1 1217.6 0.559  1384145_at  Similar to KIAA1919 protein (predicted)  Info    
   1927 2.3e-06 161.3 56.4 2.86  1368079_at  pyruvate dehydrogenase kinase 1  Info   Pdk1 
   1928 2.3e-06 16 69.6 0.23  1376702_at  Megalencephalic leukoencephalopathy with subcortical cysts 1 (predicted)  Info    
   1929 2.3e-06 469.8 847.1 0.555  1388374_at    Info    
   1930 2.3e-06 196.9 112.6 1.749  1372279_at  BCL2-like 13 (apoptosis facilitator) (predicted)  Info   Bcl2l13_predicted 
   1931 2.3e-06 157.6 335.6 0.47  1373304_at  alpha-N-acetylglucosaminidase  Info   Naglu 
   1932 2.3e-06 893.7 2934.9 0.305  1367917_at  cytochrome P450, family 2, subfamily d, polypeptide 26  Info   Cyp2d26 
   1933 2.3e-06 169.4 277.4 0.611  1371466_at  similar to RIKEN cDNA 0610009B22 (predicted) /// similar to Sedlin (Trafficking protein particle complex protein 2)  Info   RGD1306925_predicted /// LOC501550 
   1934 2.3e-06 364.7 181.6 2.008  1372990_at  cAMP responsive element binding protein 3 (predicted)  Info   Creb3_predicted 
   1935 2.3e-06 109.8 8.3 13.229  1379448_at  Ttk protein kinase (predicted)  Info   Ttk_predicted 
   1936 2.3e-06 1583.4 3256.9 0.486  1368568_at  aquaporin 2  Info   Aqp2 
   1937 2.3e-06 2473.9 4882.7 0.507  1367995_at  catalase  Info   Cat 
   1938 2.3e-06 637.8 341.1 1.87  1373595_at  similar to RIKEN cDNA 1200015A22  Info   MGC94413 
   1939 2.3e-06 192.7 394.8 0.488  1367648_at  insulin-like growth factor binding protein 2  Info   Igfbp2 
   1940 2.3e-06 216.8 732.8 0.296  1386376_at  Transcribed locus  Info    
   1941 2.3e-06 1161 580.9 1.999  1369991_at  signal peptidase complex 18kD  Info   Spc18 
   1942 2.3e-06 100.1 46 2.176  1374777_at  hypothetical protein LOC502894  Info   LOC502894 
   1943 2.3e-06 187.4 368.2 0.509  1372670_at  similar to hypothetical protein FLJ21827 (predicted)  Info   RGD1307682_predicted 
   1944 2.3e-06 52.9 183.5 0.288  1387234_at  alpha-2-glycoprotein 1, zinc  Info   Azgp1 
   1945 2.4e-06 196.2 124.8 1.572  1375973_at  ariadne ubiquitin-conjugating enzyme E2 binding protein homolog 1 (Drosophila) (predicted)  Info   Arih1_predicted 
   1946 2.4e-06 1919.2 3615.2 0.531  1387889_at  folate receptor 1 (adult)  Info   Folr1 
   1947 2.4e-06 302.1 39.4 7.668  1380425_at  Ribonuclease L (2',5'-oligoisoadenylate synthetase-dependent)  Info   Rnasel 
   1948 2.4e-06 299.2 663.7 0.451  1398343_at  DnaJ (Hsp40) homolog, subfamily A, member 4 (predicted)  Info    
   1949 2.4e-06 61.3 199.9 0.307  1370361_at  cell growth regulator with EF hand domain 1  Info   Cgref1 
   1950 2.4e-06 167.4 23.7 7.063  1392648_at  mannose receptor, C type 1 (predicted)  Info   Mrc1_predicted 
   1951 2.4e-06 236.6 112.8 2.098  1387866_at  myosin IXb  Info   Myo9b 
   1952 2.4e-06 575.7 40.3 14.285  1377092_at  Transcribed locus  Info    
   1953 2.4e-06 301.7 128.2 2.353  1374111_at  similar to pinin  Info   LOC368070 
   1954 2.4e-06 111 592.5 0.187  1370824_at  solute carrier family 38, member 3  Info   Slc38a3 
   1955 2.4e-06 281.4 746.8 0.377  1393342_at  Solute carrier family 25 (mitochondrial carrier; ornithine transporter) member 15 (predicted)  Info    
   1956 2.4e-06 403.4 254.4 1.586  1375554_at  DEAD (Asp-Glu-Ala-Asp) box polypeptide 47 (predicted)  Info   Ddx47_predicted 
   1957 2.4e-06 161.1 69.8 2.308  1374626_at  similar to leucine-rich alpha-2-glycoprotein  Info   RGD:1359464 
   1958 2.4e-06 3173.8 5718.5 0.555  1368188_at  4-hydroxyphenylpyruvic acid dioxygenase  Info   Hpd 
   1959 2.4e-06 174.4 105.7 1.65  1377607_at  mitogen activated protein kinase kinase kinase 3 (predicted)  Info   Map3k3_predicted 
   1960 2.4e-06 23.2 55.9 0.415  1380199_at  WD repeat domain 22 (predicted)  Info   Wdr22 
   1961 2.4e-06 61.2 154 0.397  1382270_at  Transcribed locus  Info    
   1962 2.4e-06 222.4 26.8 8.299  1383936_at  Elastin microfibril interfacer 2 (predicted)  Info    
   1963 2.4e-06 370 104.2 3.551  1368790_at  serine (or cysteine) proteinase inhibitor, clade A (alpha-1 antiproteinase, antitrypsin), member 10  Info   Serpina10 
   1964 2.4e-06 474.5 225.6 2.103  1372019_at  similar to DNA segment, Chr 13, Wayne State University 50, expressed (predicted)  Info   RGD1310128_predicted 
   1965 2.4e-06 172.4 76.8 2.245  1393451_at  similar to RIKEN cDNA 2610510J17 (predicted)  Info   RGD1310953_predicted 
   1966 2.4e-06 604 331.1 1.824  1371800_at  polyhomeotic-like 2 (Drosophila) (predicted)  Info   Phc2_predicted 
   1967 2.5e-06 62.3 15.7 3.968  1367920_at  endothelial differentiation, sphingolipid G-protein-coupled receptor, 5  Info   Edg5 
   1968 2.5e-06 291.4 537.3 0.542  1389581_at  similar to RIKEN cDNA 9230117N10 (predicted)  Info   RGD1311155_predicted 
   1969 2.5e-06 14 82.2 0.17  1368289_at  group specific component  Info   Gc 
   1970 2.5e-06 72.8 24.6 2.959  1386555_at  casein kinase II, alpha 2, polypeptide (predicted)  Info   Csnk2a2_predicted 
   1971 2.5e-06 41.8 102 0.41  1377979_at  Similar to RIKEN cDNA 2610022G08  Info    
   1972 2.5e-06 753.2 1912 0.394  1372654_at  EPS8-like 2 (predicted)  Info   Eps8l2_predicted 
   1973 2.5e-06 538 2319 0.232  1387375_at  ketohexokinase  Info   Khk 
   1974 2.5e-06 407.7 234.1 1.742  1373597_at  processing of precursor 7, ribonuclease P family, (S. cerevisiae) (predicted)  Info   Pop7_predicted 
   1975 2.5e-06 172 434.3 0.396  1383205_at  dapper homolog 2, antagonist of beta-catenin (xenopus) (predicted)  Info   Dact2_predicted 
   1976 2.5e-06 145.2 427.5 0.34  1375767_at  similar to common-site lymphoma leukemia guanine nucleotide exchange factor like (5N754) (predicted)  Info   RGD1308089_predicted 
   1977 2.5e-06 227.4 818.2 0.278  1387348_at  insulin-like growth factor binding protein 5  Info   Igfbp5 
   1978 2.5e-06 226 109.6 2.062  1379317_a_at  programmed cell death protein 11 (predicted)  Info   Pdcd11_predicted 
   1979 2.5e-06 410.9 231.8 1.773  1373392_at  TPA regulated locus (predicted)  Info   Tparl_predicted 
   1980 2.5e-06 87.6 22.4 3.911  1376775_at  similar to RIKEN cDNA 2600010E01 (predicted)  Info   RGD1309969_predicted 
   1981 2.5e-06 74.7 163.6 0.457  1385124_at  similar to Tribbles homolog 2  Info   LOC313974 
   1982 2.5e-06 4528.1 2781.7 1.628  1386858_at  ribosomal protein L13  Info   Rpl13 
   1983 2.5e-06 1171.8 1745 0.672  1388361_at    Info    
   1984 2.5e-06 782 2371.5 0.33  1370818_at  2-4-dienoyl-Coenzyme A reductase 2, peroxisomal  Info   Decr2 
   1985 2.5e-06 679.5 305.8 2.222  1388867_at  similar to transcription factor  Info   MGC112830 
   1986 2.5e-06 2476.5 710 3.488  1370281_at  fatty acid binding protein 5, epidermal  Info   Fabp5 
   1987 2.5e-06 258.4 537.6 0.481  1374221_at  Solute carrier family 29 (nucleoside transporters), member 3  Info   Slc29a3 
   1988 2.5e-06 113.6 36.7 3.095  1375074_at  ProSAPiP2 protein  Info   Prosapip2 
   1989 2.5e-06 316.2 142.8 2.214  1367958_at  abl-interactor 1  Info   Abi1 
   1990 2.5e-06 157.4 65.3 2.41  1399089_at  solute carrier family 38, member 6 (predicted)  Info   Slc38a6_predicted 
   1991 2.6e-06 2862.7 1737.9 1.647  1376570_at  chaperonin subunit 5 (epsilon)  Info   Cct5 
   1992 2.6e-06 521.6 86.5 6.03  1390391_at  Transcribed locus  Info    
   1993 2.6e-06 133 237.1 0.561  1392893_a_at  Transcribed locus  Info    
   1994 2.6e-06 2586.9 3743.4 0.691  1370180_at  nudix (nucleoside diphosphate linked moiety X)-type motif 4  Info   Nudt4 
   1995 2.6e-06 49.8 169 0.295  1392990_at  SRY-box containing gene 17 (predicted)  Info   Sox17_predicted 
   1996 2.6e-06 1049.7 377.4 2.781  1371781_at  signal transducer and activator of transcription 3  Info   Stat3 
   1997 2.6e-06 147.3 614 0.24  1378819_at  similar to RIKEN cDNA 0610012D14 (predicted)  Info   RGD1310111_predicted 
   1998 2.6e-06 108.1 6.6 16.379  1374902_at  IQ motif containing GTPase activating protein 3 (predicted)  Info   Iqgap3_predicted 
   1999 2.6e-06 429.1 209.2 2.051  1375870_a_at  RNA binding motif, single stranded interacting protein 1 (predicted)  Info   Rbms1_predicted 
   2000 2.6e-06 77.3 161.4 0.479  1377085_at  carbonic anhydrase 7 (predicted)  Info   Car7_predicted 
   2001 2.6e-06 495.8 63.6 7.796  1387283_at  myxovirus (influenza virus) resistance 2  Info   Mx2 
   2002 2.6e-06 402.1 164.4 2.446  1367881_at  protein tyrosine phosphatase, non-receptor type substrate 1  Info   Ptpns1 
   2003 2.6e-06 110.2 372.8 0.296  1382084_at  Similar to EHM2  Info    
   2004 2.6e-06 501.3 179.7 2.79  1376722_at  nucleoporin 205kDa (predicted)  Info   Nup205_predicted 
   2005 2.6e-06 179 4.3 41.628  1391384_at  G4 protein  Info   Tnf 
   2006 2.6e-06 325.2 1077.8 0.302  1369682_at  transcription factor 2  Info   Tcf2 
   2007 2.6e-06 189.6 64.6 2.935  1368251_at  Janus kinase 3  Info   Jak3 
   2008 2.6e-06 199.7 401.2 0.498  1380245_at    Info    
   2009 2.6e-06 111.8 26.7 4.187  1382709_at    Info    
   2010 2.6e-06 765.3 448 1.708  1374437_at  asparaginyl-tRNA synthetase (predicted)  Info   Nars_predicted 
   2011 2.7e-06 653.9 2134 0.306  1375247_at  Monoglyceride lipase  Info   Mgll 
   2012 2.7e-06 84.9 222.6 0.381  1383422_at  similar to RIKEN cDNA 2310026E23  Info   LOC362564 
   2013 2.7e-06 191.3 476.5 0.401  1368882_at  sialyltransferase 7c  Info   Siat7c 
   2014 2.7e-06 354.6 167.2 2.121  1368179_at  thymic stromal-derived lymphopoietin, receptor  Info   Tslpr 
   2015 2.7e-06 205.2 46.8 4.385  1369794_a_at  6-phosphofructo-2-kinase/fructose-2,6-biphosphatase 3  Info   Pfkfb3 
   2016 2.7e-06 1388.1 2760.6 0.503  1377375_at  aminoadipate-semialdehyde synthase (predicted)  Info   Aass_predicted 
   2017 2.7e-06 217.9 101.1 2.155  1379298_at  interferon (alpha and beta) receptor 1 (predicted)  Info   Ifnar1_predicted 
   2018 2.7e-06 335.6 90 3.729  1374739_at  similar to hematopoietic progenitor protein  Info   LOC500727 
   2019 2.7e-06 442.5 238.2 1.858  1389569_at  similar to BRIX (predicted)  Info   RGD1308508_predicted 
   2020 2.7e-06 429.4 163.6 2.625  1383863_at    Info    
   2021 2.7e-06 155 39.4 3.934  1391262_at  SUMO/sentrin specific protease 5 (predicted) /// similar to SUMO/sentrin specific protease 5  Info   Senp5_predicted /// LOC499574 
   2022 2.7e-06 2856.2 3652.8 0.782  1372123_at  succinate dehydrogenase complex, subunit B, iron sulfur (Ip) (predicted)  Info   Sdhb_predicted 
   2023 2.7e-06 128.7 472 0.273  1386454_at  solute carrier family 23 (nucleobase transporters), member 3 (predicted)  Info   Slc23a3_predicted 
   2024 2.7e-06 165.2 33.7 4.902  1385627_at  B-cell leukemia/lymphoma 3 (predicted)  Info   Bcl3_predicted 
   2025 2.7e-06 134.8 238.5 0.565  1383350_at  solute carrier family 35, member C2 (predicted)  Info   Slc35c2_predicted 
   2026 2.7e-06 3318.1 4212.8 0.788  1386886_at  CD164 antigen  Info   Cd164 
   2027 2.7e-06 248.9 47.4 5.251  1367749_at  lumican  Info   Lum 
   2028 2.7e-06 824.9 3132.2 0.263  1367775_at  alpha-methylacyl-CoA racemase  Info   Amacr 
   2029 2.7e-06 998.5 1576.5 0.633  1384244_at  similar to RIKEN cDNA 2610207I16 (predicted)  Info   RGD1305387_predicted 
   2030 2.7e-06 163.7 2.9 56.448  1381771_at  Transcribed locus  Info    
   2031 2.7e-06 1801.6 1358 1.327  1371495_at  cation-dependent mannose-6-phosphate receptor  Info   RGD:1359355 
   2032 2.7e-06 125.9 46.9 2.684  1394160_at  Transmembrane protein 2 (predicted)  Info    
   2033 2.7e-06 1305.9 2294.8 0.569  1370187_at  propionyl coenzyme A carboxylase, beta polypeptide  Info   Pccb 
   2034 2.7e-06 22.1 1.4 15.786  1368766_at  matrix metalloproteinase 7  Info   Mmp7 
   2035 2.7e-06 88 27.1 3.247  1368294_at  deoxyribonuclease I-like 3  Info   Dnase1l3 
   2036 2.7e-06 702.5 337.8 2.08  1388353_at  proliferation-associated 2G4, 38kDa  Info   RGD:1302994 
   2037 2.8e-06 90.7 464.5 0.195  1396035_at  similar to RPE-spondin  Info   LOC297757 
   2038 2.8e-06 1387 3058.6 0.453  1371350_at  similar to S-adenosylmethionine synthetase gamma form (Methionine adenosyltransferase) (AdoMet synthetase) (MAT-II)  Info   LOC498211 
   2039 2.8e-06 141.8 220.4 0.643  1399138_at  similar to expressed sequence AA408877 (predicted)  Info   RGD1308513_predicted 
   2040 2.8e-06 256.8 31.7 8.101  1380621_at  Similar to tyrosine kinase Fps/Fes  Info    
   2041 2.8e-06 69.3 25.3 2.739  1386162_at  TXK tyrosine kinase (predicted)  Info   Txk_predicted 
   2042 2.8e-06 6323.7 3585.9 1.763  1371377_at  ribosomal protein S19  Info   Rps19 
   2043 2.8e-06 129.3 331.4 0.39  1376692_at  homeodomain interacting protein kinase 2 (predicted)  Info   Hipk2_predicted 
   2044 2.8e-06 408.3 193.2 2.113  1391356_at  Similar to hypothetical protein FLJ10006 (predicted)  Info    
   2045 2.8e-06 206.1 455.8 0.452  1372502_at  Transcribed locus, moderately similar to NP_035028.2 neurofibromatosis 2 [Mus musculus]  Info    
   2046 2.8e-06 336.6 843.5 0.399  1379592_at  similar to citrin  Info   LOC362322 
   2047 2.8e-06 264.7 61.3 4.318  1368856_at  Janus kinase 2  Info   Jak2 
   2048 2.8e-06 376.5 691.9 0.544  1371493_at  Transcribed locus  Info    
   2049 2.8e-06 94.4 19.9 4.744  1378028_at  MAD2 (mitotic arrest deficient, homolog)-like 1 (yeast) (predicted)  Info   Mad2l1_predicted 
   2050 2.8e-06 2403.4 666.5 3.606  1371913_at  transforming growth factor, beta induced  Info   Tgfbi 
   2051 2.8e-06 48.5 7.8 6.218  1372809_at  hypothetical gene supported by AF152002  Info   LOC290595 
   2052 2.8e-06 1462.6 916.4 1.596  1382005_at  ROD1 regulator of differentiation 1 (S. pombe)  Info   Rod1 
   2053 2.8e-06 2603.8 4427.5 0.588  1371567_at  aldehyde dehydrogenase family 7, member A1 (predicted)  Info   Aldh7a1_predicted 
   2054 2.8e-06 150.3 321.2 0.468  1390440_at  similar to mKIAA0267 protein  Info   LOC302863 
   2055 2.8e-06 545.6 261.7 2.085  1379687_at  ARP2 actin-related protein 2 homolog (yeast) (predicted)  Info   Actr2_predicted 
   2056 2.8e-06 285.7 147.9 1.932  1373273_at  similar to hypothetical protein FLJ14936 (predicted)  Info   RGD1311129_predicted 
   2057 2.8e-06 212.9 35.6 5.98  1375920_at  similar to TNF intracellular domain-interacting protein (predicted)  Info   RGD1311487_predicted 
   2058 2.8e-06 322.2 197.4 1.632  1373305_at  sorting nexin 4 (predicted)  Info   Snx4_predicted 
   2059 2.8e-06 242.1 610.6 0.396  1369073_at  nuclear receptor subfamily 1, group H, member 4  Info   Nr1h4 
   2060 2.8e-06 127.3 51 2.496  1379538_at  centromere protein J (predicted)  Info   Cenpj_predicted 
   2061 2.8e-06 2709.1 1097.7 2.468  1368840_at  LR8 protein  Info   Lr8 
   2062 2.8e-06 954.8 1436.5 0.665  1390021_at  histone 1, H2bh (predicted)  Info   Hist1h2bh_predicted 
   2063 2.8e-06 1618.6 2013.3 0.804  1367591_at  peroxiredoxin 3  Info   Prdx3 
   2064 2.8e-06 58.4 251.1 0.233  1389084_at    Info    
   2065 2.9e-06 886 544.2 1.628  1398806_at  phosphatidylinositol transfer protein  Info   Pitpn 
   2066 2.9e-06 305.8 395.3 0.774  1372350_at  suppressor of var1, 3-like 1 (S. cerevisiae) (predicted)  Info   Supv3l1_predicted 
   2067 2.9e-06 79.4 270 0.294  1373218_at  Similar to 6430514L14Rik protein (predicted)  Info    
   2068 2.9e-06 219.2 498.7 0.44  1377812_a_at  transmembrane protein 24 (predicted)  Info   Tmem24_predicted 
   2069 2.9e-06 127.3 279.9 0.455  1382964_at    Info    
   2070 2.9e-06 37 5.3 6.981  1378213_at  Transcribed locus  Info    
   2071 2.9e-06 12.1 1.4 8.643  1374659_at  similar to Arpp21 protein  Info   LOC363153 
   2072 2.9e-06 127 56.9 2.232  1368186_a_at  spleen tyrosine kinase  Info   Syk 
   2073 2.9e-06 65.7 185.2 0.355  1378245_at  similar to 6430514L14Rik protein (predicted)  Info   RGD1311958_predicted 
   2074 2.9e-06 2145.3 687.5 3.12  1373947_at  dermatopontin (predicted)  Info   Dpt_predicted 
   2075 2.9e-06 728.7 1038.1 0.702  1388603_a_at  HesB protein  Info   Hbld2 
   2076 2.9e-06 205.9 500.2 0.412  1368137_at  microtubule-associated protein tau /// hypothetical gene supported by NM_017212  Info   Mapt /// LOC497674 
   2077 2.9e-06 93 193.4 0.481  1373618_at  cAMP responsive element binding protein-like 2 (predicted)  Info   Crebl2_predicted 
   2078 2.9e-06 219.6 671.8 0.327  1387071_a_at  microtubule-associated protein tau /// hypothetical gene supported by NM_017212  Info   Mapt /// LOC497674 
   2079 2.9e-06 210.8 100.7 2.093  1370136_at  lamin B receptor  Info   Lbr 
   2080 2.9e-06 131.6 74.3 1.771  1374076_at  similar to hypothetical protein FLJ34389 (predicted)  Info   RGD1305243_predicted 
   2081 2.9e-06 9.9 43.2 0.229  1381504_at  similar to asporin precursor  Info   LOC306805 
   2082 2.9e-06 733.9 3.2 229.344  1385414_at  CD8 antigen, alpha chain  Info   Cd8a 
   2083 2.9e-06 363.5 925.4 0.393  1377666_at  choline dehydrogenase  Info   Chdh 
   2084 2.9e-06 278.3 68.8 4.045  1374298_at  Transcribed locus, moderately similar to XP_580018.1 PREDICTED: hypothetical protein XP_580018 [Rattus norvegicus]  Info    
   2085 2.9e-06 335.1 68.1 4.921  1377023_at  dual specificity phosphatase 2 (predicted)  Info   Dusp2_predicted 
   2086 2.9e-06 151 369.8 0.408  1393230_s_at  Dead end homolog 1 (zebrafish) (predicted)  Info    
   2087 3e-06 160 300.2 0.533  1373851_at  Transcribed locus  Info    
   2088 3e-06 243.5 107.7 2.261  1383107_at  small nuclear ribonucleoprotein D1 (predicted)  Info   Snrpd1_predicted 
   2089 3e-06 171.8 311.9 0.551  1375985_at  RAB2B, member RAS oncogene family (predicted)  Info   Rab2b_predicted 
   2090 3e-06 84.8 1.8 47.111  1395788_at  Transcribed locus  Info    
   2091 3e-06 103.3 28.9 3.574  1376871_at    Info   LOC288549 /// LOC363890 /// LOC363894 /// LOC363899 /// LOC498168 /// LOC498169 /// LOC498199 /// LOC501818 /// LOC501821 /// LOC501828 
   2092 3e-06 3261.2 4638.4 0.703  1371398_at    Info    
   2093 3e-06 549.5 1054.1 0.521  1385739_at  Transcribed locus  Info    
   2094 3e-06 61.7 295.6 0.209  1387145_at  gap junction membrane channel protein beta 1  Info   Gjb1 
   2095 3e-06 905.3 2387.5 0.379  1371864_at  Basic transcription element binding protein 1  Info   Bteb1 
   2096 3e-06 607.2 1152.1 0.527  1384028_at  Transcribed locus  Info    
   2097 3e-06 80.3 229.7 0.35  1382868_at  sema domain, transmembrane domain (TM), and cytoplasmic domain, (semaphorin) 6A (predicted)  Info   Sema6a_predicted 
   2098 3e-06 4194.7 2838.5 1.478  1367588_a_at  ribosomal protein L13A  Info   Rpl13a 
   2099 3e-06 245.7 100 2.457  1396187_at  Hypothetical protein LOC606294  Info    
   2100 3e-06 178.3 365.4 0.488  1375307_at  chromobox homolog 6 (predicted)  Info   Cbx6_predicted 
   2101 3e-06 270.9 115.7 2.341  1372535_at  similar to RIKEN cDNA 2210012G02  Info   LOC366431 
   2102 3.1e-06 185 14.8 12.5  1368610_at  mast cell antigen 32  Info   Mca32 
   2103 3.1e-06 240.8 95.9 2.511  1388195_at  CUG triplet repeat,RNA-binding protein 2  Info   Cugbp2 
   2104 3.1e-06 102.7 43.4 2.366  1391849_at  Similar to MBD2 (methyl-CpG-binding protein)-interacting zinc finger protein (predicted)  Info    
   2105 3.1e-06 157.6 38.6 4.083  1392554_a_at    Info    
   2106 3.1e-06 185.2 77.5 2.39  1368885_at  ectonucleoside triphosphate diphosphohydrolase 1  Info   Entpd1 
   2107 3.1e-06 236.3 106.1 2.227  1395364_at  interferon (alpha and beta) receptor 1 (predicted)  Info   Ifnar1_predicted 
   2108 3.1e-06 150.6 84.8 1.776  1372415_at  general transcription factor IIH, polypeptide 3 (predicted)  Info   Gtf2h3_predicted 
   2109 3.1e-06 490.3 1532.2 0.32  1374871_at  asparaginase-like sperm autoantigen  Info   Asrgl1 
   2110 3.1e-06 178.7 95.2 1.877  1372546_at  mitogen-activated protein kinase-activated protein kinase 3 (predicted)  Info   Mapkapk3_predicted 
   2111 3.1e-06 92.5 8 11.562  1376914_at  DEP domain containing 1 (predicted)  Info   Depdc1_predicted 
   2112 3.1e-06 110.9 279.3 0.397  1368358_a_at  protein tyrosine phosphatase, receptor type, R  Info   Ptprr 
   2113 3.1e-06 720.5 1269.9 0.567  1398807_at  protein phosphatase 1B, magnesium dependent, beta isoform  Info   Ppm1b 
   2114 3.2e-06 957 656.7 1.457  1382154_at  Transcribed locus, strongly similar to NP_035333.2 protein tyrosine phosphatase, non-receptor type 12 [Mus musculus]  Info    
   2115 3.2e-06 109.3 18.4 5.94  1382138_at  similar to hypothetical protein FLJ20433  Info   LOC499745 
   2116 3.2e-06 368.1 160.7 2.291  1389337_at  Transcribed locus  Info    
   2117 3.2e-06 221.7 69.7 3.181  1368171_at  lysyl oxidase  Info   Lox 
   2118 3.2e-06 222.1 146.3 1.518  1374052_at  LOC361774 (predicted)  Info    
   2119 3.2e-06 64.9 110.9 0.585  1397949_at    Info    
   2120 3.2e-06 655.6 1410.5 0.465  1388874_at  Transcribed locus  Info    
   2121 3.2e-06 406.1 1406.4 0.289  1387357_at  trimethyllysine hydroxylase, epsilon  Info   Tmlhe 
   2122 3.2e-06 246.2 598.9 0.411  1373605_at  similar to 106 kDa O-GlcNAc transferase-interacting protein (predicted)  Info   RGD1307844_predicted 
   2123 3.2e-06 463.4 1315.2 0.352  1391661_at  Hypothetical gene supported by NM_024381  Info   Gyk 
   2124 3.2e-06 189.2 77.8 2.432  1381525_at    Info    
   2125 3.2e-06 774.2 1347.1 0.575  1387669_a_at  epoxide hydrolase 1  Info   Ephx1 
   2126 3.2e-06 532.5 1254.7 0.424  1387100_at  aquaporin 3  Info   Aqp3 
   2127 3.3e-06 63.1 25.7 2.455  1394390_at  Transcribed locus  Info    
   2128 3.3e-06 28.8 68.6 0.42  1392521_at  Transcribed locus  Info    
   2129 3.3e-06 91.3 187.4 0.487  1375940_a_at  similar to hypothetical protein FLJ12242 (predicted)  Info   RGD1311154_predicted 
   2130 3.3e-06 3437.5 4409.7 0.78  1372437_at  S-phase kinase-associated protein 1A  Info   RGD:1359648 
   2131 3.3e-06 267.5 60.5 4.421  1374429_at  Transcribed locus, weakly similar to XP_518443.1 PREDICTED: pim-1 oncogene [Pan troglodytes]  Info    
   2132 3.3e-06 105.2 312.9 0.336  1387898_at  heat shock protein, alpha-crystallin-related, B6  Info   Hspb6 
   2133 3.3e-06 178.9 67.6 2.646  1393161_at  small nuclear RNA activating complex, polypeptide 3 (predicted)  Info   Snapc3_predicted 
   2134 3.3e-06 994.3 424.7 2.341  1382521_at  glutaminase  Info   Gls 
   2135 3.3e-06 365.6 890.1 0.411  1374800_at  Solute carrier family 25 (mitochondrial carrier; ornithine transporter) member 15 (predicted)  Info    
   2136 3.3e-06 442.6 1408.4 0.314  1389540_at  TG interacting factor (predicted)  Info   Tfig 
   2137 3.3e-06 311.3 63.1 4.933  1389151_at  Transcribed locus  Info    
   2138 3.3e-06 177.1 36.9 4.799  1384968_at  SH3-domain binding protein 2 (predicted)  Info   Sh3bp2_predicted 
   2139 3.4e-06 551.6 1197 0.461  1377192_a_at  caseinolytic protease X (E.coli) (predicted)  Info   Clpx_predicted 
   2140 3.4e-06 285.5 842.4 0.339  1372646_at  similar to RIKEN cDNA 1500015O10 (predicted)  Info   RGD1305645_predicted 
   2141 3.4e-06 173.3 70.3 2.465  1388846_at    Info    
   2142 3.4e-06 570.4 876.6 0.651  1374638_at  peroxisomal biogenesis factor 13 (predicted)  Info   Pex13_predicted 
   2143 3.4e-06 231.6 131.5 1.761  1395730_at  glycyl-tRNA synthetase (predicted) /// similar to Glycyl-tRNA synthetase  Info   Gars_predicted /// LOC500135 
   2144 3.4e-06 92 14.4 6.389  1391955_at  similar to RAB19, member RAS oncogene family  Info   LOC500088 
   2145 3.4e-06 1505 2276.6 0.661  1398903_at  Esterase D/formylglutathione hydrolase  Info   Esd 
   2146 3.4e-06 66.9 121.7 0.55  1376465_at  Transcribed locus  Info    
   2147 3.4e-06 9 1.4 6.429  1369512_at  carbohydrate (chondroitin 6/keratan) sulfotransferase 3  Info   Chst3 
   2148 3.4e-06 99.6 210.6 0.473  1372398_at  similar to Potassium channel tetramerisation domain containing 15  Info   LOC499129 
   2149 3.4e-06 3499.5 5541.4 0.632  1368374_a_at  gamma-glutamyltransferase 1  Info   Ggt1 
   2150 3.4e-06 810.9 374.4 2.166  1368847_at  RAB10, member RAS oncogene family  Info   Rab10 
   2151 3.4e-06 247.3 527.9 0.468  1398991_at  Similar to expressed sequence AA408877 (predicted)  Info    
   2152 3.4e-06 444.4 1071.9 0.415  1375106_at  amnionless (predicted)  Info   Amn_predicted 
   2153 3.4e-06 179.1 58.4 3.067  1384147_at  eukaryotic translation initiation factor 1A (predicted)  Info   Eif1a_predicted 
   2154 3.4e-06 91.9 312.3 0.294  1380610_at  Transcribed locus  Info    
   2155 3.5e-06 57.9 215.9 0.268  1394881_at  similar to 6430514L14Rik protein (predicted)  Info   RGD1311958_predicted 
   2156 3.5e-06 558.3 875.7 0.638  1372497_at  similar to Nbr1 (predicted)  Info   RGD1311421_predicted 
   2157 3.5e-06 355.2 107.1 3.317  1368571_at  cytoplasmic linker 2  Info   Cyln2 
   2158 3.5e-06 206.3 339.9 0.607  1388109_at  G protein-coupled receptor 116  Info   Gpr116 
   2159 3.5e-06 189.4 448.7 0.422  1393997_at  similar to hypothetical protein FLJ32825  Info   LOC500595 
   2160 3.5e-06 127.1 21.8 5.83  1391754_at  2 ' -5 ' oligoadenylate synthetase 1I  Info   RGD:1359379 
   2161 3.5e-06 614.9 988.6 0.622  1371609_at  homolog of zebrafish ES1  Info   RGD:1303003 
   2162 3.5e-06 120 41.2 2.913  1379967_at  zinc finger protein 367 (predicted)  Info   Zfp367_predicted 
   2163 3.5e-06 112.8 50 2.256  1382436_at  similar to RIKEN cDNA 2610002M06  Info   LOC363487 
   2164 3.5e-06 233.2 120.6 1.934  1368204_at  ligase I, DNA, ATP-dependent /// ligase I, DNA, ATP-dependent (predicted)  Info   Lig1 /// Lig1_predicted 
   2165 3.5e-06 257.8 140 1.841  1390177_at  Transcribed locus  Info    
   2166 3.5e-06 351.8 669.6 0.525  1374293_at    Info    
   2167 3.5e-06 150.6 98.5 1.529  1374484_at  similar to RIKEN cDNA 2610033C09 (predicted)  Info   RGD1306421_predicted 
   2168 3.5e-06 406.4 810.5 0.501  1382061_at  lactate dehydrogenase D (predicted)  Info   Ldhd_predicted 
   2169 3.5e-06 2118.5 2937.1 0.721  1371407_at  NCK-associated protein 1  Info   Nckap1 
   2170 3.5e-06 62.6 21.3 2.939  1376895_at  interleukin 16  Info   Il16 
   2171 3.5e-06 223.7 89.6 2.497  1368375_a_at  interleukin 15  Info   Il15 
   2172 3.5e-06 81.7 24.1 3.39  1383493_at  Transcribed locus  Info    
   2173 3.6e-06 286.2 945.5 0.303  1387531_at  methionine sulfoxide reductase A  Info   Msra 
   2174 3.6e-06 649.3 392.1 1.656  1371391_at  thioredoxin domain containing 5 (predicted)  Info   Txndc5_predicted 
   2175 3.6e-06 286.8 713.4 0.402  1389632_at  Transcribed locus, weakly similar to XP_228131.3 PREDICTED: similar to Rho-related BTB domain-containing protein 1 [Rattus norvegicus]  Info    
   2176 3.6e-06 391.8 233.5 1.678  1387436_at  septin 7  Info   Sept7 
   2177 3.6e-06 358.7 110.6 3.243  1388836_at  protein kinase C, eta  Info   Prkch 
   2178 3.6e-06 354.1 843.3 0.42  1393015_at  similar to hypothetical protein FLJ14146 (predicted)  Info   RGD1310587_predicted 
   2179 3.6e-06 204.9 644.4 0.318  1370952_at  glutathione S-transferase, mu 2  Info   Gstm2 
   2180 3.6e-06 1509 800.6 1.885  1367485_at  transcription elongation factor A (SII) 1 (predicted) /// similar to transcription elongation factor IIS - mouse  Info   Tcea1_predicted /// LOC498453 
   2181 3.6e-06 265.1 123.9 2.14  1391167_at  Transcribed locus  Info    
   2182 3.6e-06 143.9 304.4 0.473  1383719_at  microtubule-associated protein 7 (predicted)  Info   Mtap7_predicted 
   2183 3.6e-06 853.1 1285.5 0.664  1368552_at  GrpE-like 1, mitochondrial  Info   Grpel1 
   2184 3.6e-06 267.4 104.8 2.552  1387800_at  Fas death domain-associated protein  Info   Daxx 
   2185 3.6e-06 326.6 65.1 5.017  1377116_at  Ribonuclease L (2',5'-oligoisoadenylate synthetase-dependent)  Info   Rnasel 
   2186 3.6e-06 256.4 149.6 1.714  1388733_at  bifunctional apoptosis regulator (predicted)  Info   Bfar_predicted 
   2187 3.6e-06 299.7 508.3 0.59  1373191_at  similar to hypothetical protein  Info   LOC500897 
   2188 3.7e-06 107.7 52.8 2.04  1390917_at    Info    
   2189 3.7e-06 203.7 97.2 2.096  1372409_at  MAD2L1 binding protein (predicted)  Info   Mad2l1bp_predicted 
   2190 3.7e-06 488.2 1136.9 0.429  1372744_at  plakophilin 4 (predicted)  Info   Pkp4_predicted 
   2191 3.7e-06 271.5 131.7 2.062  1380867_a_at  pleckstrin homology domain containing, family M (with RUN domain) member 1 (predicted)  Info   Plekhm1_predicted 
   2192 3.7e-06 554.8 1128.6 0.492  1369948_at  nerve growth factor receptor (TNFRSF16) associated protein 1  Info   Ngfrap1 
   2193 3.7e-06 1644.1 3067.8 0.536  1390020_at    Info    
   2194 3.7e-06 294 106.8 2.753  1369557_at  caspase 7  Info   Casp7 
   2195 3.7e-06 96 182.4 0.526  1387976_at  solute carrier family 9 (sodium/hydrogen exchanger), isoform 3 regulator 2  Info   Slc9a3r2 
   2196 3.7e-06 74.2 280.9 0.264  1380962_at  Angiotensin I converting enzyme (peptidyl-dipeptidase A) 2  Info   Ace2 
   2197 3.7e-06 222 650.1 0.341  1391390_at    Info    
   2198 3.7e-06 143.9 82.4 1.746  1382161_at  M-phase phosphoprotein 10 (U3 small nucleolar ribonucleoprotein) (predicted)  Info   Mphosph10_predicted 
   2199 3.8e-06 173.7 37.5 4.632  1369830_at  protein kinase C, eta  Info   Prkch 
   2200 3.8e-06 2563.4 1535.8 1.669  1371573_at  ribosomal protein L36a (predicted)  Info   Rpl36a_predicted 
   2201 3.8e-06 252.8 141.4 1.788  1374840_at    Info    
   2202 3.8e-06 131.9 433.9 0.304  1388611_at  transcription elongation factor A (SII), 3 (predicted)  Info   Tcea3_predicted 
   2203 3.8e-06 256.5 435.6 0.589  1374592_at    Info    
   2204 3.8e-06 7434.8 108.7 68.397  1377626_at    Info    
   2205 3.8e-06 460.5 680.6 0.677  1398389_at  Platelet-activating factor acetylhydrolase, isoform Ib, alpha subunit 45kDa  Info   Pafah1b1 
   2206 3.8e-06 649.5 1291.7 0.503  1368071_at  Mg87 protein  Info   Mg87 
   2207 3.8e-06 343.3 807.3 0.425  1373680_at  Transcribed locus  Info    
   2208 3.8e-06 1290.6 593.9 2.173  1373204_at  hypothetical LOC297077 (predicted)  Info   RGD1310725_predicted 
   2209 3.8e-06 23.7 67.4 0.352  1396246_at  Ubiquitin-conjugating enzyme E2D 1, UBC4/5 homolog (yeast) (predicted)  Info    
   2210 3.8e-06 120.1 18.9 6.354  1390415_at  Thyroid hormone receptor interactor 13 (predicted)  Info    
   2211 3.8e-06 697.3 438.6 1.59  1368158_at  sec1 family domain containing 1  Info   Scfd1 
   2212 3.8e-06 159.6 266.9 0.598  1390819_at  Thyrotroph embryonic factor  Info   Tef 
   2213 3.8e-06 304.5 661.6 0.46  1388883_at  polymerase (DNA-directed), delta 4 (predicted)  Info   Pold4_predicted 
   2214 3.8e-06 21.7 1.8 12.056  1383434_at  Transcribed locus  Info    
   2215 3.8e-06 1686.4 1038.2 1.624  1370170_at  heterogeneous nuclear ribonucleoprotein U  Info   Hnrpu 
   2216 3.8e-06 195.1 115.5 1.689  1372661_at  transducin (beta)-like 3 (predicted)  Info   Tbl3_predicted 
   2217 3.8e-06 401.2 277.2 1.447  1374011_at  excision repair cross-complementing rodent repair deficiency, complementation group 3 (predicted)  Info   Ercc3_predicted 
   2218 3.8e-06 503.9 219.9 2.291  1388132_at  NonO/p54nrb homolog  Info   Sfpq 
   2219 3.9e-06 695.1 356.6 1.949  1381969_at  Transcribed locus  Info    
   2220 3.9e-06 202.2 55.9 3.617  1392755_at  similar to hypothetical protein AD158 (predicted)  Info   RGD1306585_predicted 
   2221 3.9e-06 444.7 1165.4 0.382  1373740_at  Transcribed locus, weakly similar to XP_418630.1 PREDICTED: similar to ORF2 [Gallus gallus]  Info    
   2222 3.9e-06 320.5 154.8 2.07  1389655_at  similar to A230072I16Rik protein (predicted)  Info   RGD1306819_predicted 
   2223 3.9e-06 550.9 293.4 1.878  1374395_at  Transcribed locus  Info    
   2224 3.9e-06 157.6 58.9 2.676  1392514_at  brix domain containing 1 (predicted)  Info   Bxdc1_predicted 
   2225 3.9e-06 208.9 373.3 0.56  1382100_at  similar to RIKEN cDNA 2310047O13 (predicted)  Info   RGD1309605_predicted 
   2226 3.9e-06 1739.2 4088.6 0.425  1387651_at  aquaporin 1  Info   Aqp1 
   2227 3.9e-06 435.1 743.6 0.585  1388917_at  myosin IC  Info   Myo1c 
   2228 3.9e-06 41.7 3.9 10.692  1370138_at  lymphoid enhancer binding factor 1  Info   Lef1 
   2229 3.9e-06 2370.3 1625.8 1.458  1367452_at  SMT3 suppressor of mif two 3 homolog 2 (yeast)  Info   Sumo2 
   2230 3.9e-06 110.5 270.5 0.409  1384273_at  Carbohydrate kinase-like (predicted)  Info    
   2231 3.9e-06 19.6 35.8 0.547  1396292_at  Similar to peptide N-glycanase  Info    
   2232 3.9e-06 253.7 448.5 0.566  1372157_at  similar to CGI-143 protein (predicted)  Info   RGD1310586_predicted 
   2233 3.9e-06 166 17.3 9.595  1389440_at  Transcribed locus  Info    
   2234 3.9e-06 445.7 210.4 2.118  1370313_at  brain acyl-CoA hydrolase  Info   Bach 
   2235 3.9e-06 260.1 698 0.373  1383117_at  peroxisomal membrane protein 4  Info   Pxmp4 
   2236 3.9e-06 209.6 72 2.911  1390531_at  Similar to Peroxisomal proliferator-activated receptor A interacting complex 285 kDa protein (PPAR-alpha interacting complex protein 285) (predicted)  Info    
   2237 3.9e-06 145.6 284.8 0.511  1389111_at  Transcribed locus  Info    
   2238 4e-06 181.4 57.6 3.149  1382616_at  glutaminase  Info   Gls 
   2239 4e-06 2675.7 1393.5 1.92  1368507_at  proteasome (prosome, macropain) subunit, alpha type 3  Info   Psma3 
   2240 4e-06 251.5 34.3 7.332  1379499_at  lymphotoxin B  Info   RGD:1303162 
   2241 4e-06 284.3 588.8 0.483  1388756_at  similar to RIKEN cDNA 6330579B17 gene (predicted)  Info   RGD1311399_predicted 
   2242 4e-06 387.5 168.8 2.296  1370071_at  adenosine deaminase  Info   Ada 
   2243 4e-06 159.4 79.4 2.008  1368910_at  protein phosphatase 2C, magnesium dependent, catalytic subunit  Info   Ppm2c 
   2244 4e-06 1564.3 2342.5 0.668  1389903_at  pituitary tumor-transforming 1 interacting protein (predicted)  Info   Pttg1ip_predicted 
   2245 4e-06 51 271 0.188  1376110_at  ribonuclease P 25 subunit (human) (predicted)  Info   Rpp25_predicted 
   2246 4e-06 420.7 1730.7 0.243  1370167_at  syndecan 2  Info   Sdc2 
   2247 4e-06 235.6 1397.6 0.169  1387158_at  meprin 1 beta  Info   Mep1b 
   2248 4e-06 1024.7 2068.5 0.495  1368548_at  solute carrier family 12, member 1  Info   Slc12a1 
   2249 4e-06 346.8 895.5 0.387  1373807_at  Vascular endothelial growth factor A  Info   Vegf 
   2250 4e-06 56.3 21.5 2.619  1369686_at  double cortin and calcium/calmodulin-dependent protein kinase-like 1  Info   Dcamkl1 
   2251 4.1e-06 108.7 43.8 2.482  1378629_at  special AT-rich sequence binding protein 1 (predicted)  Info   Satb1_predicted 
   2252 4.1e-06 411.9 788.6 0.522  1389833_at  Sulfatase modifying factor 1 (predicted)  Info    
   2253 4.1e-06 297.8 161.3 1.846  1399154_at  F-box and leucine-rich repeat protein 11 (predicted)  Info    
   2254 4.1e-06 1068.5 549.9 1.943  1379561_at  ATPase family, AAA domain containing 1 (predicted)  Info   Atad1_predicted 
   2255 4.1e-06 378.3 981.2 0.386  1388916_at  Transcribed locus  Info    
   2256 4.1e-06 1225.2 759.2 1.614  1388027_a_at  reticulon 4  Info   Rtn4 
   2257 4.1e-06 486.9 301.7 1.614  1371484_at    Info    
   2258 4.1e-06 1007.2 624.6 1.613  1389902_at  histocompatibility 13 (predicted)  Info   H13_predicted 
   2259 4.1e-06 284 192.4 1.476  1388641_at  phosphoribosylglycinamide formyltransferase (predicted)  Info   Gart_predicted 
   2260 4.1e-06 712.5 368.7 1.932  1389189_at  actinin, alpha 1  Info   Actn1 
   2261 4.1e-06 122.5 510.5 0.24  1387232_at  bone morphogenetic protein 4  Info   Bmp4 
   2262 4.1e-06 404.9 706.8 0.573  1389032_at  mucolipin 1 (predicted)  Info   Mcoln1_predicted 
   2263 4.1e-06 339.8 43.9 7.74  1383747_at  Ect2 oncogene (predicted)  Info    
   2264 4.1e-06 150.8 63.8 2.364  1376005_at  Kinesin family member 1B  Info   Kif1b 
   2265 4.2e-06 2224.4 3413.2 0.652  1375197_at    Info    
   2266 4.2e-06 204.1 117.8 1.733  1390947_at  Similar to RIKEN cDNA D930036F22 gene  Info    
   2267 4.2e-06 7.6 54.9 0.138  1390951_at  Transcribed locus  Info    
   2268 4.2e-06 48.2 121.6 0.396  1383220_at  Transcribed locus  Info    
   2269 4.2e-06 1518.4 3953.7 0.384  1367843_at  aldo-keto reductase family 7, member A2 (aflatoxin aldehyde reductase)  Info   RGD:620311 
   2270 4.2e-06 1770.5 1197.8 1.478  1371403_at  chaperonin subunit 3 (gamma)  Info   Cct3 
   2271 4.2e-06 513.2 826.5 0.621  1371986_at  Transcribed locus  Info    
   2272 4.2e-06 15.1 71.6 0.211  1378365_at  Transcribed locus  Info    
   2273 4.2e-06 2.5 43.8 0.057  1394484_at    Info    
   2274 4.2e-06 611.8 1192.5 0.513  1369629_at  adenosine kinase  Info   Adk 
   2275 4.2e-06 160.7 395.9 0.406  1391538_at  cell death-inducing DNA fragmentation factor, alpha subunit-like effector B (predicted)  Info   Cideb_predicted 
   2276 4.2e-06 443.2 979.4 0.453  1368934_at  cytochrome P450, family 4, subfamily a, polypeptide 10 /// cytochrome P450, family 4, subfamily A, polypeptide 22  Info   Cyp4a10 /// Cyp4a22 
   2277 4.2e-06 34.4 81.3 0.423  1391855_at  B-cell CLL/lymphoma 7A (predicted)  Info    
   2278 4.3e-06 5813 4094.3 1.42  1371318_at  ribosomal protein S16  Info   Rps16 
   2279 4.3e-06 255 31.4 8.121  1392515_at    Info   RGD:1359643 /// RGD:1359487 /// RGD:1359240 /// RGD:1359669 /// RGD:1359210 /// LOC502904 /// LOC502905 /// LOC502906 /// LOC502907 
   2280 4.3e-06 3140.5 2328.6 1.349  1398765_at  adaptor-related protein complex 2, mu 1 subunit  Info   Ap2m1 
   2281 4.3e-06 12.2 1.3 9.385  1378520_at  B-cell leukemia/lymphoma 11B (predicted)  Info   Bcl11b_predicted 
   2282 4.3e-06 111.5 277.5 0.402  1373909_at    Info    
   2283 4.3e-06 22.6 75.9 0.298  1391240_at    Info    
   2284 4.3e-06 756.5 1715.6 0.441  1367741_at  homocysteine-inducible, endoplasmic reticulum stress-inducible, ubiquitin-like domain member 1  Info   Herpud1 
   2285 4.3e-06 147.9 313.2 0.472  1368190_at  renin 1  Info   Ren1 
   2286 4.3e-06 1201 1713.8 0.701  1375411_at  NADH dehydrogenase (ubiquinone) 1 alpha subcomplex, 7 (B14.5a) (predicted)  Info   Ndufa7_predicted 
   2287 4.3e-06 199.1 110.2 1.807  1378293_at  tripartite motif protein 26  Info   RGD:1359126 
   2288 4.3e-06 195.1 27.4 7.12  1395697_at  similar to Ezh2 protein  Info   LOC312299 
   2289 4.3e-06 2008 1501 1.338  1371939_at  GPI-anchored membrane protein 1 (predicted)  Info   Gpiap1_predicted 
   2290 4.3e-06 654.1 399.7 1.636  1372949_at  Septin 6 (predicted)  Info    
   2291 4.3e-06 324.5 573.8 0.566  1372071_at  similar to 8D6 antigen (predicted)  Info   RGD1305860_predicted 
   2292 4.3e-06 150.2 65.7 2.286  1392531_at  Transcribed locus  Info    
   2293 4.3e-06 88.9 276.4 0.322  1376263_at  similar to 1810034B16Rik protein (predicted)  Info   RGD1306222_predicted 
   2294 4.3e-06 463.9 2892.2 0.16  1372869_at  similar to GTP-binding protein NGB  Info   LOC498786 
   2295 4.3e-06 884 1429.1 0.619  1371599_at  LRRGT00097  Info    
   2296 4.3e-06 166.4 327.1 0.509  1383960_at  peroxisome biogenesis factor 16 (predicted)  Info   Pex16_predicted 
   2297 4.3e-06 336.8 142.7 2.36  1374208_at  zinc finger CCCH type domain containing 7 (predicted)  Info   Zc3hdc7_predicted 
   2298 4.4e-06 1323.2 744.2 1.778  1371581_at  Vascular endothelial zinc finger 1 (predicted)  Info    
   2299 4.4e-06 910.6 474.2 1.92  1369950_at  cyclin-dependent kinase 4  Info   Cdk4 
   2300 4.4e-06 1305.5 1070.3 1.22  1399095_at  SMT3 suppressor of mif two 3 homolog 1 (yeast) (predicted)  Info   Sumo1_predicted 
   2301 4.4e-06 295.4 557.6 0.53  1397535_at  membrane protein, palmitoylated 5 (MAGUK p55 subfamily member 5) (predicted)  Info   Mpp5_predicted 
   2302 4.4e-06 38.2 101.1 0.378  1379801_at  LOC499790  Info    
   2303 4.4e-06 4252.5 1803.4 2.358  1367586_at  lactate dehydrogenase A  Info   Ldha 
   2304 4.4e-06 419.7 755.8 0.555  1392486_at  similar to RIKEN cDNA 2310036D22  Info   RGD:1303037 
   2305 4.4e-06 141.8 301.9 0.47  1373533_at    Info    
   2306 4.4e-06 4.9 51.8 0.095  1377898_at  solute carrier family 39 (metal ion transporter), member 5 (predicted)  Info   Slc39a5_predicted 
   2307 4.4e-06 75.9 159 0.477  1368265_at  cytochrome P450 monooxygenase CYP2T1  Info   RGD:620086 
   2308 4.5e-06 262.8 133.2 1.973  1372173_at  similar to U5 snRNP-specific protein (Prp8-binding) (predicted)  Info   RGD1309198_predicted 
   2309 4.5e-06 481.2 756 0.637  1386895_at  melanoma antigen, family D, 1  Info   Maged1 
   2310 4.5e-06 1225.7 898.5 1.364  1374646_at  Casein kinase 1, alpha 1  Info   Csnk1a1 
   2311 4.5e-06 1301.9 4205.3 0.31  1371389_at  hypothetical LOC306766  Info   RGD:1359256 
   2312 4.5e-06 2102.6 1384.7 1.518  1371478_at  similar to RIKEN cDNA 1110008F13 (predicted)  Info   RGD1307752_predicted 
   2313 4.5e-06 1858.9 2842.4 0.654  1368607_at  cytochrome P450, 4a12  Info   RGD:628846 
   2314 4.5e-06 82 156.6 0.524  1389788_at  hypothetical protein LOC293950  Info   RGD1307336_predicted 
   2315 4.5e-06 136.1 72.1 1.888  1373715_at  similar to hypothetical protein DKFZp434K1815  Info   RGD:1359442 
   2316 4.5e-06 46.6 129.3 0.36  1382452_at  serum deprivation response protein  Info   RGD:1359345 
   2317 4.5e-06 1715.7 4095.3 0.419  1373337_at  glyoxylate reductase/hydroxypyruvate reductase (predicted)  Info   Grhpr_predicted 
   2318 4.5e-06 27.9 74.3 0.376  1374994_at  Nei endonuclease VIII-like 1 (E. coli) (predicted)  Info    
   2319 4.6e-06 67 381.6 0.176  1377135_at    Info    
   2320 4.6e-06 501.4 998.5 0.502  1369412_a_at  solute carrier family 19, member 1  Info   Slc19a1 
   2321 4.6e-06 161.6 77.7 2.08  1389760_at  ring finger protein 134 (predicted)  Info   Rnf134_predicted 
   2322 4.6e-06 157.2 92.9 1.692  1393203_at  similar to RIKEN cDNA 2010309E21 (predicted)  Info   RGD1304825_predicted 
   2323 4.6e-06 255.3 66 3.868  1373683_at  fyn proto-oncogene  Info   Fyn 
   2324 4.6e-06 24 50.6 0.474  1395007_at  Transcribed locus  Info    
   2325 4.6e-06 1104.5 531.8 2.077  1372776_at  F-box and leucine-rich repeat protein 5 (predicted)  Info   Fbxl5_predicted 
   2326 4.6e-06 335.7 816.5 0.411  1372592_at  histone deacetylase 6  Info   Hdac6 
   2327 4.6e-06 299.3 594 0.504  1373335_at  similar to zinc finger, DHHC domain containing 9  Info   LOC302808 
   2328 4.6e-06 235.2 405.5 0.58  1383776_at  Transcribed locus  Info    
   2329 4.6e-06 69.7 185.7 0.375  1377890_at  Transcribed locus  Info    
   2330 4.7e-06 1392.8 639.5 2.178  1389815_at  protein phosphatase 1, regulatory (inhibitor) subunit 14B  Info   Ppp1r14b 
   2331 4.7e-06 752.5 476.3 1.58  1399025_at  Similar to hypothetical protein MGC38950 (predicted)  Info    
   2332 4.7e-06 128.6 49.2 2.614  1391091_at  retinitis pigmentosa 1 (autosomal dominant) (predicted) /// similar to cylindromatosis (turban tumor syndrome)  Info   Rp1_predicted /// LOC361373 
   2333 4.7e-06 224.8 638 0.352  1376746_at  lactate dehydrogenase D (predicted)  Info   Ldhd_predicted 
   2334 4.7e-06 506.5 321.5 1.575  1394591_at  zinc finger protein 207 (predicted)  Info   Zfp207_predicted 
   2335 4.7e-06 342.6 142.5 2.404  1388772_at  LSM8 homolog, U6 small nuclear RNA associated (S. cerevisiae) (predicted)  Info   Lsm8_predicted 
   2336 4.7e-06 258.5 128.9 2.005  1389073_at  similar to RIKEN cDNA 2700029E10 (predicted)  Info   RGD1306561_predicted 
   2337 4.7e-06 179.6 358.5 0.501  1377751_at  Transcribed locus  Info    
   2338 4.7e-06 201.6 70.8 2.847  1383502_at  similar to mKIAA0259 protein  Info   LOC315969 
   2339 4.7e-06 404.3 829.5 0.487  1371963_at  propionyl-coenzyme A carboxylase, alpha polypeptide  Info   Pcca 
   2340 4.7e-06 2276.5 1718.3 1.325  1375246_at    Info    
   2341 4.7e-06 921.9 615.6 1.498  1388977_at  similar to RIKEN cDNA 9930118K05  Info   RGD:1359727 
   2342 4.7e-06 138.2 314 0.44  1380123_at    Info    
   2343 4.7e-06 81.7 5.4 15.13  1369852_at  coagulation factor X  Info   F10 
   2344 4.8e-06 91.3 232.9 0.392  1369148_at  transcription factor 1  Info   Tcf1 
   2345 4.8e-06 100.2 232.9 0.43  1368303_at  period homolog 2 (Drosophila)  Info   Per2 
   2346 4.8e-06 874.1 1650.6 0.53  1376728_at  Transcribed locus  Info    
   2347 4.8e-06 964.3 293.6 3.284  1373065_at  protein tyrosine phosphatase, non-receptor type 18 (predicted)  Info   Ptpn18_predicted 
   2348 4.8e-06 234 559.9 0.418  1370446_at  non-metastatic cells 7, protein expressed in  Info   Nme7 
   2349 4.8e-06 379.1 738.9 0.513  1395532_at  Transcribed locus, strongly similar to NP_035873.1 SH3-domain binding protein 3 [Mus musculus]  Info    
   2350 4.8e-06 375 250.2 1.499  1389041_at  vacuole 14 protein (S. cerevisiae)  Info   Vac14 
   2351 4.8e-06 201.4 29.2 6.897  1397866_at  serine (or cysteine) proteinase inhibitor, clade B, member 6b (predicted)  Info   Serpinb6b_predicted 
   2352 4.8e-06 644.7 281.2 2.293  1385933_at  Transcribed locus  Info    
   2353 4.8e-06 3153 5149.8 0.612  1371942_at  similar to Glutathione S-transferase, theta 3  Info   LOC499422 
   2354 4.8e-06 123.9 505.4 0.245  1373749_at  syntrophin, acidic 1 (predicted)  Info   Snta1_predicted 
   2355 4.8e-06 91.5 242.5 0.377  1390437_at  Transcribed locus  Info    
   2356 4.8e-06 3224.3 2297 1.404  1367578_at  peroxiredoxin 2  Info   Prdx2 
   2357 4.8e-06 253.7 383.1 0.662  1388595_at  DEAH (Asp-Glu-Ala-His) box polypeptide 30 (predicted)  Info   Dhx30_predicted 
   2358 4.8e-06 107.1 9.6 11.156  1394470_at  similar to hypothetical protein DKFZp566A1524 (predicted)  Info   RGD1305961_predicted 
   2359 4.8e-06 1442.2 573.5 2.515  1370224_at  signal transducer and activator of transcription 3  Info   Stat3 
   2360 4.8e-06 89.6 1890.3 0.047  1373990_at  solute carrier family 7 (cationic amino acid transporter, y+ system), member 12 (predicted) /// similar to solute carrier family 7 (cationic amino acid transporter, y+ system), member 12  Info   Slc7a12_predicted /// LOC361914 
   2361 4.8e-06 854.1 424.6 2.012  1367698_a_at  septin 9  Info   Sept9 
   2362 4.9e-06 227.2 751.8 0.302  1378249_x_at    Info    
   2363 4.9e-06 46.2 10 4.62  1385816_at  Transcribed locus, strongly similar to NP_780591.1 hypothetical protein LOC108900 [Mus musculus]  Info    
   2364 4.9e-06 791.7 1715.9 0.461  1387084_at  dipeptidylpeptidase 4  Info   Dpp4 
   2365 4.9e-06 60 133.4 0.45  1397287_at  similar to 4931417G12Rik protein  Info   LOC500294 
   2366 4.9e-06 240.7 501.3 0.48  1374558_at  Similar to B7-like protein GL50-B  Info    
   2367 4.9e-06 126.4 45 2.809  1395183_at  Transcribed locus  Info    
   2368 4.9e-06 296.9 765.5 0.388  1374625_at  hairy and enhancer of split 6 (Drosophila) (predicted)  Info   Hes6_predicted 
   2369 4.9e-06 266.7 473 0.564  1392274_at  Transcribed locus  Info    
   2370 4.9e-06 217.8 545.6 0.399  1392713_a_at    Info    
   2371 4.9e-06 611.3 359.6 1.7  1371683_at  LSM4 homolog, U6 small nuclear RNA associated (S. cerevisiae) (predicted)  Info   Lsm4_predicted 
   2372 4.9e-06 1137.5 793.7 1.433  1371821_at  WAS protein family, member 2 (predicted)  Info    
   2373 4.9e-06 189.4 372.2 0.509  1370197_a_at  protein kinase C, zeta  Info   Prkcz 
   2374 5e-06 688.5 1098.5 0.627  1371338_at  similar to RIKEN cDNA 1700023M09 (predicted)  Info   RGD1312037_predicted 
   2375 5e-06 736.8 2560.4 0.288  1367871_at  cytochrome P450, family 2, subfamily e, polypeptide 1  Info   Cyp2e1 
   2376 5e-06 174.4 446.7 0.39  1385716_at  Transcribed locus  Info    
   2377 5e-06 246.6 49.8 4.952  1370561_at  iGb3 synthase  Info   RGD:727913 
   2378 5e-06 529.6 980.2 0.54  1387885_at  Fc receptor, IgG, alpha chain transporter  Info   Fcgrt 
   2379 5e-06 1678.3 1013.6 1.656  1398832_at  nucleolin  Info   Ncl 
   2380 5e-06 264.4 142.2 1.859  1373115_at  similar to 2310061I09Rik protein (predicted)  Info   RGD1304653_predicted 
   2381 5e-06 397.1 294.3 1.349  1381857_at  RAS p21 protein activator 1  Info   Rasa1 
   2382 5e-06 303.7 1297.2 0.234  1396039_at  solute carrier family 22 (organic anion/cation transporter), member 12  Info   Slc22a12 
   2383 5e-06 314.5 192.1 1.637  1375540_at  similar to hypothetical protein D11Ertd497e (predicted)  Info   RGD1308696_predicted 
   2384 5.1e-06 151.7 56.3 2.694  1389244_x_at  chemokine (C-X-C motif) receptor 4  Info   Cxcr4 
   2385 5.1e-06 313.2 640.9 0.489  1398358_a_at  similar to integrin beta-5  Info   LOC498091 
   2386 5.1e-06 76.9 17.2 4.471  1379805_at  solute carrier family 41, member 2 (predicted)  Info   Slc41a2_predicted 
   2387 5.1e-06 499.5 96.5 5.176  1386888_at  eukaryotic translation initiation factor 4E binding protein 1  Info   Eif4ebp1 
   2388 5.1e-06 116.1 265.1 0.438  1372358_at  TG interacting factor (predicted)  Info   Tfig 
   2389 5.1e-06 34.7 74.9 0.463  1391338_at  Transcribed locus  Info    
   2390 5.1e-06 54.7 337.2 0.162  1382997_at  solute carrier family 22 (organic cation transporter), member 13 (predicted)  Info   Slc22a13_predicted 
   2391 5.1e-06 126.5 44.9 2.817  1374686_at  solute carrier family 30 (zinc transporter), member 7 (predicted)  Info   Slc30a7_predicted 
   2392 5.1e-06 80.9 182.7 0.443  1376546_at  similar to hypothetical protein  Info   LOC499157 
   2393 5.2e-06 109.8 11 9.982  1384393_at  Transcribed locus  Info    
   2394 5.2e-06 1542.3 2881.6 0.535  1372438_at  similar to Nit protein 2 (predicted)  Info   RGD1310494_predicted 
   2395 5.2e-06 1727.7 1012.6 1.706  1373225_at  Myeloid cell leukemia sequence 1  Info   Mcl1 
   2396 5.2e-06 405.3 599.1 0.677  1374508_at  nephronophthisis 1 (juvenile) (predicted)  Info   Nphp1_predicted 
   2397 5.2e-06 257.4 144.8 1.778  1392910_at  block of proliferation 1 (predicted)  Info   Bop1_predicted 
   2398 5.2e-06 286.6 557.4 0.514  1376128_at  Transcribed locus  Info    
   2399 5.2e-06 401.2 175.7 2.283  1382287_at  ribonucleic acid binding protein S1 (predicted)  Info   Rnps1_predicted 
   2400 5.2e-06 2398.1 1596.2 1.502  1399091_at  F-actin capping protein beta subunit  Info   RGD:1359099 
   2401 5.2e-06 319.4 651.2 0.49  1368100_at  phosphate cytidylyltransferase 2, ethanolamine  Info   Pcyt2 
   2402 5.3e-06 654.9 1869.2 0.35  1372774_at  Coenzyme Q6 homolog (yeast) (predicted)  Info    
   2403 5.3e-06 1028.2 1842 0.558  1367659_s_at  dodecenoyl-coenzyme A delta isomerase  Info   Dci 
   2404 5.3e-06 123.7 50.8 2.435  1391161_at    Info    
   2405 5.3e-06 301.6 97.2 3.103  1384200_at    Info    
   2406 5.3e-06 296.3 519.5 0.57  1376683_at  Transcribed locus  Info    
   2407 5.3e-06 195.8 380.4 0.515  1387821_at  RAB3A interacting protein  Info   Rab3ip 
   2408 5.3e-06 65.7 194.8 0.337  1374235_at  Down syndrome critical region gene 1-like 1  Info   Dscr1l1 
   2409 5.3e-06 569.1 2893 0.197  1387336_at  N-acetyltransferase 8 (camello like)  Info   Nat8 
   2410 5.3e-06 190.3 433.7 0.439  1388497_at  thioesterase superfamily member 2 (predicted)  Info   Them2_predicted 
   2411 5.3e-06 626.3 270.4 2.316  1396820_at  histone deacetylase 1 (predicted)  Info   Hdac1_predicted 
   2412 5.3e-06 273 572.8 0.477  1376997_at  Transcribed locus  Info    
   2413 5.3e-06 428.5 226.3 1.894  1389633_at  E2F transcription factor 4 (predicted)  Info    
   2414 5.3e-06 3299.5 1698.8 1.942  1371869_at  proteasome (prosome, macropain) subunit, alpha type 7  Info   Psma7 
   2415 5.3e-06 496.7 347.4 1.43  1388858_at  mitogen activated protein kinase kinase 3 (predicted)  Info   Map2k3_predicted 
   2416 5.4e-06 66.4 234.8 0.283  1384301_at  Similar to Kelch-like protein 3  Info    
   2417 5.4e-06 243.6 606.4 0.402  1384499_at  Butyryl Coenzyme A synthetase 1 (predicted)  Info    
   2418 5.4e-06 242.1 567.5 0.427  1392477_at  ets variant gene 1 (predicted)  Info   Etv1_predicted 
   2419 5.4e-06 40.9 101.8 0.402  1377456_at  Transcribed locus  Info    
   2420 5.4e-06 88.9 272.8 0.326  1367812_at  beta-spectrin 3  Info   Spnb3 
   2421 5.4e-06 279.4 409.3 0.683  1392603_at  EGL nine homolog 1 (C. elegans)  Info   Egln1 
   2422 5.4e-06 629.1 279.1 2.254  1377594_at  Src homology 2 domain-containing transforming protein C1  Info   Shc1 
   2423 5.4e-06 515.3 1868 0.276  1374624_at  UDP-N-acetyl-alpha-D-galactosamine:polypeptide N-acetylgalactosaminyltransferase 11 (GalNAc-T11)  Info   Galnt11 
   2424 5.4e-06 3464.9 2091.8 1.656  1375335_at  heat shock 90kDa protein 1, beta  Info   RGD:1303075 
   2425 5.4e-06 309.2 166.9 1.853  1368268_at  thymine-DNA glycosylase  Info   Tdg 
   2426 5.4e-06 86.5 252.8 0.342  1387631_at  15-hydroxyprostaglandin dehydrogenase  Info   Hpgd 
   2427 5.5e-06 301.4 680.7 0.443  1394456_at  Transcribed locus  Info    
   2428 5.5e-06 2049.3 4126.1 0.497  1368047_at  solute carrier family 13 (sodium-dependent dicarboxylate transporter), member 3  Info   Slc13a3 
   2429 5.5e-06 724.5 415.7 1.743  1388771_at  CGG triplet repeat binding protein 1 (predicted)  Info    
   2430 5.5e-06 134.4 50 2.688  1389804_at  Transcribed locus  Info    
   2431 5.5e-06 144.4 259.3 0.557  1368244_at  arsenic (+3 oxidation state) methyltransferase  Info   As3mt 
   2432 5.5e-06 397 166.9 2.379  1393217_at  ATP-binding cassette, sub-family G (WHITE), member 3  Info   RGD:1303294 
   2433 5.5e-06 96.2 10.1 9.525  1374912_at    Info    
   2434 5.5e-06 410 1233.6 0.332  1376733_at  immunoglobulin superfamily, member 11 (predicted)  Info   Igsf11_predicted 
   2435 5.5e-06 264.4 363.4 0.728  1388658_at  surfeit 2  Info   Surf2 
   2436 5.5e-06 495.1 177.1 2.796  1372006_at  Transcribed locus  Info    
   2437 5.5e-06 928.6 1662.3 0.559  1374057_at  Similar to Homeobox protein Hox-D8 (Hox-4.3) (Hox-5.4)  Info    
   2438 5.5e-06 572 1137 0.503  1373400_at  Protein kinase, cAMP-dependent, regulatory, type 2, alpha  Info   Prkar2a 
   2439 5.5e-06 1932.7 1064.1 1.816  1371341_at  small nuclear ribonucleoprotein D2 (predicted)  Info   Snrpd2_predicted 
   2440 5.5e-06 318.3 31.7 10.041  1374775_at  antigen identified by monoclonal antibody Ki-67 (predicted) /// similar to Mki67 protein /// similar to Ki-67  Info   Mki67_predicted /// LOC310382 /// LOC366937 
   2441 5.5e-06 214.2 82.2 2.606  1397644_at    Info    
   2442 5.5e-06 148.7 24.8 5.996  1382419_at  SoxLZ/Sox6 leucine zipper binding protein in testis (predicted)  Info   Solt_predicted 
   2443 5.5e-06 128.9 306.2 0.421  1374682_at  Transcribed locus, weakly similar to NP_035569.1 SRY-box 13 [Mus musculus]  Info    
   2444 5.5e-06 329.5 624.7 0.527  1395518_at  Transcribed locus  Info    
   2445 5.5e-06 109.2 252.5 0.432  1392933_a_at  B-cell CLL/lymphoma 7A (predicted)  Info    
   2446 5.6e-06 179.8 292.4 0.615  1378543_at  Nuclear factor, erythroid derived 2, like 3 (predicted)  Info    
   2447 5.6e-06 666.3 396.7 1.68  1367760_at  mitogen activated protein kinase kinase 1  Info   Map2k1 
   2448 5.6e-06 11.5 61 0.189  1380504_at    Info    
   2449 5.6e-06 227.2 123.7 1.837  1372343_at  exosome component 8 (predicted)  Info   Exosc8_predicted 
   2450 5.6e-06 174.3 39.1 4.458  1392082_a_at  CD7 antigen (predicted)  Info   Cd7_predicted 
   2451 5.6e-06 1188.1 288 4.125  1397808_at  Cathepsin C  Info   Ctsc 
   2452 5.6e-06 109 50.3 2.167  1377761_at  glutamine-fructose-6-phosphate transaminase 2  Info   RGD:1303097 
   2453 5.6e-06 285.9 677.9 0.422  1367948_a_at  kinase insert domain protein receptor  Info   Kdr 
   2454 5.6e-06 277.9 84.5 3.289  1370949_at  myristoylated alanine rich protein kinase C substrate  Info   Marcks 
   2455 5.6e-06 124.4 250.2 0.497  1382227_at  1-acylglycerol-3-phosphate O-acyltransferase 3 (predicted)  Info    
   2456 5.6e-06 110 9.3 11.828  1382531_at  similar to toll-like receptor 7  Info   LOC317468 
   2457 5.6e-06 126.1 234.6 0.538  1397419_at  Similar to PALS2-alpha splice variant  Info    
   2458 5.6e-06 28.5 66 0.432  1388384_at  Dynein, cytoplasmic, light chain 1  Info   Dnclc1 
   2459 5.6e-06 89.5 176.1 0.508  1370088_at  sperm autoantigenic protein 17  Info   Spa17 
   2460 5.7e-06 698.8 229.5 3.045  1371349_at  similar to collagen alpha1 type VI-precursor  Info   LOC294337 
   2461 5.7e-06 119.6 195.1 0.613  1372396_at  Similar to 2310047B19Rik protein (predicted)  Info    
   2462 5.7e-06 295.1 110.6 2.668  1394275_at    Info    
   2463 5.7e-06 88.3 38.6 2.288  1376353_at  ARP5 actin-related protein 5 homolog (yeast) (predicted)  Info   Actr5_predicted 
   2464 5.7e-06 3084.5 2291 1.346  1398860_at  neural precursor cell expressed, developmentally down-regulated gene 8  Info   Nedd8 
   2465 5.7e-06 153.1 304.9 0.502  1372276_at  Transcribed locus  Info    
   2466 5.7e-06 245.5 154.1 1.593  1368393_at  lymphocyte antigen 68  Info   C1qr1 
   2467 5.7e-06 188.4 15.7 12  1370133_at  regulator of G-protein signaling 19  Info   Rgs19 
   2468 5.7e-06 264 161.1 1.639  1370386_at  RuvB-like protein 1  Info   Ruvbl1 
   2469 5.7e-06 179 61.7 2.901  1392668_at  Transcribed locus  Info    
   2470 5.7e-06 798.5 394.9 2.022  1370014_at  syntaxin 4A (placental)  Info   Stx4a 
   2471 5.7e-06 343.7 189.7 1.812  1374953_at  similar to CG12279-PA  Info   LOC500420 
   2472 5.7e-06 600.3 295.1 2.034  1383574_at  mannosidase 1, alpha (predicted)  Info   Man1a_predicted 
   2473 5.7e-06 233.2 405.6 0.575  1389251_at  nudix (nucleoside diphosphate linked moiety X)-type motif 7 (predicted)  Info   Nudt7_predicted 
   2474 5.8e-06 166.2 64.5 2.577  1380187_at  similar to TNF intracellular domain-interacting protein (predicted)  Info   RGD1311487_predicted 
   2475 5.8e-06 488.5 137.4 3.555  1390312_at  similar to mKIAA2005 protein /// similar to mKIAA2005 protein /// similar to mKIAA2005 protein  Info   LOC500012 /// LOC500014 /// LOC500015 
   2476 5.8e-06 511.1 5.4 94.648  1392496_at  Trypsin V-A  Info   LOC312273 
   2477 5.8e-06 369 637.6 0.579  1375879_at  G protein-coupled receptor 48  Info   Gpr48 
   2478 5.8e-06 772.6 386.4 1.999  1371803_at  GM2 ganglioside activator protein  Info   Gm2a 
   2479 5.8e-06 189.8 372.3 0.51  1375908_at  epithelial V-like antigen (predicted)  Info   Eva_predicted 
   2480 5.8e-06 1583.1 4007.9 0.395  1375944_at    Info    
   2481 5.8e-06 87.8 191 0.46  1393661_at  Transcribed locus  Info    
   2482 5.8e-06 73.4 232.4 0.316  1389113_at  Similar to Hypothetical protein MGC32471 (predicted)  Info    
   2483 5.8e-06 764 1089.9 0.701  1368354_at  glutathione S-transferase theta 1  Info   Gstt1 
   2484 5.8e-06 424 176.3 2.405  1373611_at  interleukin 17 receptor (predicted)  Info   Il17r_predicted 
   2485 5.8e-06 99.6 191.8 0.519  1394612_at    Info    
   2486 5.8e-06 372 104.6 3.556  1371146_at  transcription factor EC  Info   Tfec 
   2487 5.9e-06 2272.5 3161.6 0.719  1388294_at  succinate dehydrogenase complex, subunit D, integral membrane protein  Info   Sdhd 
   2488 5.9e-06 1090.4 1481.4 0.736  1398325_at  transmembrane 4 superfamily member 8  Info   RGD:1359444 
   2489 5.9e-06 78.7 14.6 5.39  1379479_at  kinesin family member 4  Info   Kif4 
   2490 5.9e-06 152.7 279.8 0.546  1383305_at  Transcribed locus  Info    
   2491 5.9e-06 327.6 622.3 0.526  1393152_at    Info    
   2492 5.9e-06 114.8 82.7 1.388  1382029_at  similar to CDNA sequence BC017647  Info   LOC497960 
   2493 5.9e-06 429.7 269.9 1.592  1371932_at  similar to hypothetical protein FLJ38482 (predicted)  Info   RGD1309341_predicted 
   2494 5.9e-06 387.5 215.1 1.801  1389263_at  retinoic acid induced 14 (predicted)  Info   Rai14_predicted 
   2495 6e-06 297.2 820.3 0.362  1374892_at  spermidine/spermine N1-acetyl transferase 2 (predicted)  Info   Sat2_predicted 
   2496 6e-06 158.5 79.2 2.001  1369177_at  phosphatidylinositol 4-kinase type 2 alpha  Info   Pi4k2a 
   2497 6e-06 295.4 126.3 2.339  1389789_at  Transcribed locus  Info    
   2498 6e-06 63.5 25.2 2.52  1383597_at  DNA cross-link repair 1C, PSO2 homolog (S. cerevisiae)  Info   Dclre1c 
   2499 6e-06 596.7 1190.9 0.501  1373970_at  similar to RIKEN cDNA 9230117N10 (predicted)  Info   RGD1311155_predicted 
   2500 6e-06 483.2 1242.2 0.389  1376852_at  methylcrotonoyl-Coenzyme A carboxylase 1 (alpha) (predicted)  Info   Mccc1_predicted 
   2501 6e-06 3844.4 5232 0.735  1371323_at    Info    
   2502 6e-06 123.6 32.9 3.757  1377686_at  Transcribed locus  Info    
   2503 6e-06 969 591.5 1.638  1372721_at    Info    
   2504 6e-06 3621.2 55.7 65.013  1367998_at  secretory leukocyte peptidase inhibitor /// similar to secretory leukocyte protease inhibitor  Info   Slpi /// LOC296356 
   2505 6e-06 224.5 474.7 0.473  1392971_at  Transcribed locus  Info    
   2506 6e-06 40.9 113.5 0.36  1387023_at  glutathione S-transferase, mu type 3  Info   Gstm3 
   2507 6e-06 529.9 910.7 0.582  1377573_at  carbonic anhydrase VB, mitochondrial  Info   Ca5b 
   2508 6e-06 45.6 136.9 0.333  1374833_at  Transcribed locus  Info    
   2509 6.1e-06 111.4 170.3 0.654  1372205_at  zinc finger protein 278 (predicted)  Info   Znf278_predicted 
   2510 6.1e-06 387.5 629.1 0.616  1375221_at  similar to CG5554-PA (predicted)  Info   RGD1305146_predicted 
   2511 6.1e-06 481.2 1817.4 0.265  1370377_at  cytochrome P450, family 2, subfamily d, polypeptide 9 /// cytochrome P450, family 2, subfamily d, polypeptide 10  Info   Cyp2d9 /// Cyp2d10 
   2512 6.1e-06 1323.2 1873.8 0.706  1392530_at  similar to NADH dehydrogenase (ubiquinone) 1, subcomplex unknown, 1  Info   LOC499612 
   2513 6.1e-06 221.2 493.7 0.448  1374703_at  Chromobox homolog 6 (predicted)  Info    
   2514 6.1e-06 168.3 255.5 0.659  1372553_at  similar to RIKEN cDNA 2610301B20; EST AI428449  Info   MGC94199 
   2515 6.1e-06 328.6 584.2 0.562  1399059_at  Enhancer of zeste homolog 1 (Drosophila) (predicted)  Info    
   2516 6.1e-06 261.3 467.5 0.559  1372292_at  Similar to Serine/threonine protein kinase 24  Info    
   2517 6.1e-06 237.9 122.2 1.947  1376275_at  Transcribed locus  Info    
   2518 6.1e-06 650.1 155 4.194  1373823_at  similar to Cyclin-dependent kinases regulatory subunit 2 (CKS-2)  Info   LOC498709 
   2519 6.1e-06 575.6 1896.1 0.304  1386960_at  solute carrier family 37 (glycerol-6-phosphate transporter), member 4  Info   Slc37a4 
   2520 6.1e-06 154.3 86.4 1.786  1389646_at  CDC23 (cell division cycle 23, yeast, homolog) (predicted)  Info   Cdc23_predicted 
   2521 6.1e-06 2262.5 4969.7 0.455  1387034_at  phenylalanine hydroxylase  Info   Pah 
   2522 6.1e-06 50.2 154.3 0.325  1398295_at  solute carrier family 29 (nucleoside transporters), member 1  Info   Slc29a1 
   2523 6.2e-06 506.1 236.7 2.138  1389911_at  similar to cDNA sequence BC019776  Info   RGD:1359271 
   2524 6.2e-06 64.2 19.7 3.259  1383794_at    Info    
   2525 6.2e-06 234.6 785.1 0.299  1390543_at  Transcribed locus  Info    
   2526 6.2e-06 45.6 167.4 0.272  1389786_at  Similar to RIKEN cDNA 2410005O16  Info    
   2527 6.2e-06 208.1 76.7 2.713  1371027_at  Casitas B-lineage lymphoma b  Info   Cblb 
   2528 6.2e-06 142.8 288.5 0.495  1382660_at  Transcribed locus  Info    
   2529 6.2e-06 89.3 16.3 5.479  1389852_at  similar to proliferation associated nuclear element 1 isoform 1 (predicted)  Info   RGD1307309_predicted 
   2530 6.2e-06 216 122.6 1.762  1383313_at  checkpoint with forkhead and ring finger domains (predicted)  Info   Chfr_predicted 
   2531 6.3e-06 356.5 697.7 0.511  1368437_at  carbonic anhydrase 4  Info   Ca4 
   2532 6.3e-06 184.3 93 1.982  1370942_at  RAS p21 protein activator 3  Info   Rasa3 
   2533 6.3e-06 84.8 171.4 0.495  1373436_at  similar to RIKEN cDNA 1700040L02 (predicted)  Info   RGD1306739_predicted 
   2534 6.3e-06 1068.1 2655.4 0.402  1373991_at  Transcribed locus  Info    
   2535 6.3e-06 83.4 175.6 0.475  1373217_at  Transcribed locus  Info    
   2536 6.3e-06 707.7 1323.7 0.535  1382331_at  similar to RIKEN cDNA 0610038L10 gene /// similar to RIKEN cDNA 0610038L10 gene /// similar to riboflavin kinase /// similar to riboflavin kinase  Info   LOC311241 /// LOC317214 /// LOC499328 /// LOC501601 
   2537 6.3e-06 182.5 514 0.355  1382496_at    Info    
   2538 6.3e-06 172.2 43 4.005  1377727_at  bromodomain adjacent to zinc finger domain, 1A (predicted)  Info   Baz1a_predicted 
   2539 6.3e-06 97.4 194.3 0.501  1376136_at    Info    
   2540 6.3e-06 110.5 50.1 2.206  1368126_at  acetoacetyl-CoA synthetase  Info   Aacs 
   2541 6.3e-06 432.3 869.3 0.497  1373027_at    Info    
   2542 6.4e-06 94 290.5 0.324  1376623_at  similar to hypothetical protein FLJ20898  Info   RGD:1359314 
   2543 6.4e-06 2151.4 1553.8 1.385  1398892_at  Niemann Pick type C2  Info   Npc2 
   2544 6.4e-06 1749 2650.9 0.66  1371346_at  NADH dehydrogenase (ubiquinone) 1 beta subcomplex, 6, 17kDa (predicted)  Info   Ndufb6_predicted 
   2545 6.4e-06 875 1446.2 0.605  1388121_at  amyloid beta (A4) precursor-like protein 2  Info   Aplp2 
   2546 6.4e-06 249 668.4 0.373  1382211_at  Transcribed locus  Info    
   2547 6.4e-06 359.7 581.1 0.619  1399130_at  similar to RIKEN cDNA 2610022G08  Info   LOC502782 
   2548 6.4e-06 870.4 443.8 1.961  1367790_at  staphylococcal nuclease domain containing 1  Info   Snd1 
   2549 6.5e-06 115.1 168.6 0.683  1379283_at  Transcribed locus  Info    
   2550 6.5e-06 3245.7 6435.7 0.504  1368077_at  fructose-1,6- biphosphatase 1  Info   Fbp1 
   2551 6.5e-06 351.8 188 1.871  1367507_at  Unknown (protein for MGC:72981)  Info   RGD:735224 
   2552 6.5e-06 6420.4 4406.5 1.457  1371300_at  ribosomal protein L3  Info   Rpl3 
   2553 6.5e-06 286.7 488.3 0.587  1388703_at  endothelial cell adhesion molecule  Info   RGD:1303286 
   2554 6.5e-06 698 440.9 1.583  1370554_at  ubiquitin carboxyl-terminal esterase L3 (ubiquitin thiolesterase) /// similar to ubiqutin carboxyl-terminal hydrolase l3  Info   Uchl3 /// LOC498560 
   2555 6.5e-06 128.5 575.1 0.223  1383191_at  protein phosphatase 1, regulatory (inhibitor) subunit 1A  Info   Ppp1r1a 
   2556 6.5e-06 168.8 120 1.407  1398599_at  similar to integral membrane protein 1  Info   LOC500972 
   2557 6.5e-06 49.8 2.7 18.444  1391201_at  WD repeat and HMG-box DNA binding protein 1 (predicted)  Info    
   2558 6.5e-06 21.8 55.8 0.391  1390300_at    Info    
   2559 6.5e-06 7481.8 453.4 16.502  1379365_at  chemokine (C-X-C motif) ligand 11  Info   Cxcl11 
   2560 6.5e-06 230.6 129.4 1.782  1374034_at  cysteinyl-tRNA synthetase (predicted)  Info   Cars_predicted 
   2561 6.5e-06 595.5 297.8 2  1368851_at  v-ets erythroblastosis virus E26 oncogene homolog 1 (avian)  Info   Ets1 
   2562 6.6e-06 207.2 85.5 2.423  1380148_at  Transcribed locus  Info    
   2563 6.6e-06 81.5 20.1 4.055  1384988_at  F-box only protein 5 (predicted)  Info   Fbxo5_predicted 
   2564 6.6e-06 656.8 290.3 2.262  1391463_at  DEAD (Asp-Glu-Ala-Asp) box polypeptide 58 (predicted)  Info    
   2565 6.6e-06 262.1 120.1 2.182  1382776_at  similar to O-acyltransferase (membrane bound) domain containing 1  Info   LOC498741 
   2566 6.6e-06 6396.6 4826.3 1.325  1367634_at  ribosomal protein L31  Info   Rpl31 
   2567 6.6e-06 682.8 1174.6 0.581  1371818_at  Exportin, tRNA (nuclear export receptor for tRNAs) (predicted)  Info    
   2568 6.6e-06 430.4 1130 0.381  1378470_at  similar to i-45 protein (49.8 kD) (4H318) (predicted)  Info   RGD1311259_predicted 
   2569 6.6e-06 331 198.9 1.664  1386966_a_at  protein kinase C-binding protein Beta15  Info   Pkcbpb15 
   2570 6.6e-06 41.9 116.2 0.361  1390638_at  Similar to Eph receptor A4  Info    
   2571 6.6e-06 126.5 186.1 0.68  1382763_at    Info    
   2572 6.6e-06 688.8 466 1.478  1372267_at  proteasome (prosome, macropain) 26S subunit, non-ATPase, 5 (predicted)  Info   Psmd5_predicted 
   2573 6.6e-06 78.8 32.8 2.402  1387070_at  thymopoietin  Info   Tmpo 
   2574 6.6e-06 251 60.5 4.149  1382995_at  Neuropilin 2  Info   Nrp2 
   2575 6.6e-06 49.2 166.5 0.295  1374709_at  Transcribed locus  Info    
   2576 6.6e-06 1510.8 2405.7 0.628  1388364_at  NADH dehydrogenase (ubiquinone) Fe-S protein 3 (predicted)  Info   Ndufs3_predicted 
   2577 6.6e-06 143.8 85.3 1.686  1394371_at  nuclear fragile X mental retardation protein interacting protein 1  Info   RGD:1359440 
   2578 6.7e-06 125.9 201 0.626  1374074_at  cell division cycle 37 homolog (S. cerevisiae)-like (predicted)  Info   Cdc37l_predicted 
   2579 6.7e-06 225.2 719.2 0.313  1370714_a_at  sialyltransferase 1  Info   Siat1 
   2580 6.7e-06 90.7 164.4 0.552  1398535_at  Hbs1-like (S. cerevisiae) (predicted)  Info   Hbs1l_predicted 
   2581 6.7e-06 273 114.6 2.382  1378181_at  ribonuclease P 40 subunit (human) (predicted)  Info   Rpp40_predicted 
   2582 6.8e-06 1390.9 2410.9 0.577  1368209_at  membrane-associated protein 17  Info   Map17 
   2583 6.8e-06 117.4 721.3 0.163  1374244_at  Ab2-060  Info    
   2584 6.8e-06 53.4 113 0.473  1376601_at  Sema domain, transmembrane domain (TM), and cytoplasmic domain, (semaphorin) 6A (predicted)  Info    
   2585 6.9e-06 147.9 245.6 0.602  1376595_at  similar to peroxisome biogenesis factor 1  Info   LOC500006 
   2586 6.9e-06 90.3 180.5 0.5  1390378_at  Transcribed locus  Info    
   2587 6.9e-06 98.5 292.9 0.336  1385585_at  Transcribed locus  Info    
   2588 6.9e-06 2411 1382.2 1.744  1398856_at  proteasome (prosome, macropain) subunit, alpha type 2  Info   Psma2 
   2589 6.9e-06 57.4 144.3 0.398  1379044_at  Similar to RIKEN cDNA 1700108L22 (predicted)  Info    
   2590 6.9e-06 273 599.6 0.455  1393935_at  Transcribed locus  Info    
   2591 6.9e-06 459.4 331.1 1.387  1392573_at  similar to ubiquitin-conjugating enzyme HR6A  Info   RGD:1359534 
   2592 6.9e-06 48 97.3 0.493  1379847_at  dysferlin (predicted)  Info   Dysf_predicted 
   2593 6.9e-06 790.4 1519.2 0.52  1371786_at  tripartite motif-containing 35  Info   Trim35 
   2594 6.9e-06 43.6 80.2 0.544  1389780_at  tissue factor pathway inhibitor  Info   Tfpi 
   2595 6.9e-06 114.2 248.6 0.459  1381445_at  Transcribed locus  Info    
   2596 7e-06 140.7 383.7 0.367  1373377_at  sodium channel modifier 1 (predicted)  Info   Scnm1_predicted 
   2597 7e-06 159.3 254.5 0.626  1368098_a_at  small nuclear ribonucleoprotein N  Info   Snrpn 
   2598 7e-06 146.9 101.1 1.453  1369026_at  ADP-ribosylation factor interacting protein 1  Info   Arfip1 
   2599 7e-06 154.8 453.2 0.342  1368138_at  hypothetical gene supported by NM_017212  Info   LOC497674 
   2600 7e-06 105 51.4 2.043  1380030_at  zinc finger protein 593 (predicted)  Info   Znf593_predicted 
   2601 7e-06 373.8 851.6 0.439  1368164_at  biliverdin reductase A  Info   Blvra 
   2602 7e-06 45.4 81.5 0.557  1383499_at  Transcribed locus  Info    
   2603 7e-06 29.2 118.1 0.247  1397744_at  Dopamine beta hydroxylase  Info   Dbh 
   2604 7.1e-06 290 521.8 0.556  1374101_at  TBC1 domain family, member 10 (predicted)  Info   Tbc1d10_predicted 
   2605 7.1e-06 119.3 373 0.32  1372920_at    Info    
   2606 7.1e-06 300.8 1138.2 0.264  1368064_a_at  dopa decarboxylase  Info   Ddc 
   2607 7.1e-06 208.4 418.8 0.498  1377654_at  similar to RIKEN cDNA 1810037C20  Info   LOC501664 
   2608 7.1e-06 29.3 127 0.231  1383472_at  aldehyde dehydrogenase 1 family, member B1 (predicted)  Info   Aldh1b1_predicted 
   2609 7.1e-06 144.6 380.9 0.38  1382928_at  Transcribed locus  Info    
   2610 7.1e-06 67.4 12 5.617  1378640_at  ubiquitin-like, containing PHD and RING finger domains, 1  Info   Uhrf1 
   2611 7.1e-06 520.4 222.7 2.337  1388744_at  minichromosome maintenance protein 7  Info   RGD:1303018 
   2612 7.1e-06 74.2 24.2 3.066  1374563_at  Transcribed locus  Info    
   2613 7.2e-06 26.3 2.2 11.955  1370332_at  Munc13-4 protein  Info   Unc13h4 
   2614 7.2e-06 72.3 170.3 0.425  1398516_at  similar to RIKEN cDNA 0610012C01  Info   LOC498230 
   2615 7.2e-06 250.8 163.2 1.537  1375432_at  DD6A4-12(7)-2 mRNA, partial sequence  Info    
   2616 7.2e-06 242.2 135.5 1.787  1389191_at  similar to RIKEN cDNA 2210415M20 (predicted)  Info   RGD1309754_predicted 
   2617 7.2e-06 72.4 134.8 0.537  1397500_x_at  Transcribed locus  Info    
   2618 7.2e-06 291.9 129.5 2.254  1372430_at  similar to RIKEN cDNA 1700037H04 (predicted)  Info   RGD1311739_predicted 
   2619 7.2e-06 118.1 227.7 0.519  1390883_at  Similar to A-kinase anchor protein 11 (Protein kinase A anchoring protein 11) (PRKA11) (A kinase anchor protein 220 kDa) (AKAP 220) (hAKAP220)  Info   Akap11 
   2620 7.2e-06 960.3 1450.7 0.662  1374440_at  dehydrogenase/reductase (SDR family) member 8  Info   RGD:1303235 
   2621 7.3e-06 406.6 680.8 0.597  1388634_at  phosphoglucomutase 1  Info   Pgm1 
   2622 7.3e-06 383 177 2.164  1376483_at  LSM5 homolog, U6 small nuclear RNA associated (S. cerevisiae) (predicted)  Info   Lsm5_predicted 
   2623 7.3e-06 1283.6 453.5 2.83  1372255_at  arginyl-tRNA synthetase (predicted)  Info   Rars_predicted 
   2624 7.3e-06 155.6 90.7 1.716  1376597_at  zinc finger, CCHC domain containing 10 (predicted)  Info   Zcchc10_predicted 
   2625 7.3e-06 230.6 626.9 0.368  1373253_at  acyl-Coenzyme A binding domain containing 4 (predicted)  Info   Acbd4_predicted 
   2626 7.3e-06 2988.5 1745.2 1.712  1389904_at    Info    
   2627 7.3e-06 95.4 454 0.21  1382611_at  Transcribed locus  Info    
   2628 7.3e-06 407.8 883.8 0.461  1384743_at  Transcribed locus  Info    
   2629 7.3e-06 100.9 8.5 11.871  1393297_at  Transcribed locus  Info    
   2630 7.3e-06 76.1 286.9 0.265  1369407_at  tumor necrosis factor receptor superfamily, member 11b (osteoprotegerin)  Info   Tnfrsf11b 
   2631 7.4e-06 62.1 128.4 0.484  1390219_at  similar to WD repeat membrane protein (predicted)  Info   RGD1306997_predicted 
   2632 7.4e-06 263.7 436.3 0.604  1368109_at  sialyltransferase 9 (CMP-NeuAc:lactosylceramide alpha-2,3-sialyltransferase)  Info   Siat9 
   2633 7.4e-06 833.2 1882.1 0.443  1367765_at  transcobalamin 2  Info   Tcn2 
   2634 7.4e-06 219.2 458.9 0.478  1373149_at  similar to yippee-like 3  Info   LOC293491 
   2635 7.4e-06 119.2 61.5 1.938  1390163_at    Info    
   2636 7.4e-06 418.4 100.7 4.155  1374670_at  similar to hypothetical protein MGC20700 (predicted)  Info   RGD1307722_predicted 
   2637 7.4e-06 179.2 368.4 0.486  1387971_a_at  mitogen activated protein kinase 8 interacting protein  Info   Mapk8ip 
   2638 7.4e-06 76.6 25.9 2.958  1380772_at  Transcribed locus  Info    
   2639 7.4e-06 65.7 132 0.498  1380108_at  WW domain binding protein 1 (predicted)  Info    
   2640 7.4e-06 576.6 193.6 2.978  1368103_at  ATP-binding cassette, sub-family G (WHITE), member 1  Info   Abcg1 
   2641 7.5e-06 509.5 309.1 1.648  1388400_at  cell division cycle 34 homolog (S. cerevisiae) (predicted)  Info   Cdc34_predicted 
   2642 7.5e-06 556.1 323.1 1.721  1383085_at  Similar to Sh3bgrl protein  Info    
   2643 7.5e-06 23.3 65.1 0.358  1384970_at  similar to RIKEN cDNA 5330437I02 gene  Info   LOC361340 
   2644 7.5e-06 113.8 269.1 0.423  1393906_at  cytochrome P450, family 4, subfamily v, polypeptide 3 (predicted)  Info   Cyp4v3_predicted 
   2645 7.5e-06 1089.4 382.3 2.85  1379542_at  similar to Small nuclear ribonucleoprotein F (snRNP-F) (Sm protein F) (Sm-F) (SmF) /// small nuclear ribonucleoprotein polypeptide F (predicted)  Info   LOC296377 /// Snrpf_predicted 
   2646 7.5e-06 266.2 122.9 2.166  1389297_at  ERO1-like (S. cerevisiae)  Info   Ero1l 
   2647 7.5e-06 221 432.3 0.511  1368355_at  myosin 5B  Info   Myo5b 
   2648 7.5e-06 214.9 106.2 2.024  1368233_at  general transcription factor IIF, polypeptide 2  Info   Gtf2f2 
   2649 7.5e-06 198.5 613.3 0.324  1375997_at  Transcribed locus  Info    
   2650 7.6e-06 34 117.7 0.289  1391547_at  CDNA clone IMAGE:7320555  Info    
   2651 7.6e-06 694.9 1539.4 0.451  1371847_at    Info    
   2652 7.6e-06 1616.4 1122.2 1.44  1371579_at  Clone UI-R-FJ0-cpu-m-15-0-UI unknown mRNA  Info    
   2653 7.7e-06 61.5 202.4 0.304  1395519_at  similar to B230212L03Rik protein  Info   MGC95152 
   2654 7.7e-06 3011.1 4249.9 0.709  1371417_at  low molecular mass ubiquinone-binding protein  Info   Qpc 
   2655 7.7e-06 63.5 190.5 0.333  1381350_at  Transcribed locus  Info    
   2656 7.7e-06 282.3 518.4 0.545  1382013_at  RNA binding motif protein 24 (predicted)  Info   Rbm24_predicted 
   2657 7.7e-06 3257.7 1665.4 1.956  1390024_at  Similar to osteoclast inhibitory lectin  Info    
   2658 7.7e-06 129.7 231.7 0.56  1395274_at  dystonin (predicted)  Info   Dst_predicted 
   2659 7.7e-06 80.5 263.4 0.306  1378168_at  hypothetical LOC287534  Info   RGD:1359691 
   2660 7.8e-06 367.8 667.1 0.551  1382748_at    Info    
   2661 7.8e-06 88.5 179.2 0.494  1372652_at  similar to forkhead protein AFXH  Info   LOC302415 
   2662 7.8e-06 176.1 83.8 2.101  1377327_at  Transcribed locus  Info    
   2663 7.8e-06 163.3 301.2 0.542  1373170_at  G protein pathway suppressor 2 (predicted)  Info   Gps2_predicted 
   2664 7.8e-06 120.2 349.1 0.344  1389678_at  Transcribed locus  Info    
   2665 7.8e-06 282.1 481.8 0.586  1376211_a_at  potassium channel tetramerisation domain containing 6 (predicted)  Info   Kctd6_predicted 
   2666 7.8e-06 409.5 219.3 1.867  1376687_at  ubiquitin specific protease 1 (predicted)  Info   Usp1_predicted 
   2667 7.9e-06 1726.6 2177.3 0.793  1388323_at  NADH dehydrogenase (ubiquinone) 1 alpha subcomplex, 9 (predicted)  Info   Ndufa9_predicted 
   2668 7.9e-06 68.7 22.7 3.026  1377752_at  epithelial membrane protein 2  Info   RGD:1359629 
   2669 7.9e-06 276.6 623.5 0.444  1390776_at  Iroquois related homeobox 3 (Drosophila) (predicted)  Info   Irx3_predicted 
   2670 7.9e-06 483.3 775.1 0.624  1367773_at  solute carrier family 25, member 1  Info   Slc25a1 
   2671 7.9e-06 593.4 441.4 1.344  1398987_at  suppressor of Ty 4 homolog 2 (S. cerevisiae) (predicted)  Info   Supt4h2_predicted 
   2672 7.9e-06 334.5 624.1 0.536  1372107_at    Info    
   2673 7.9e-06 236.3 119.4 1.979  1388511_at  centaurin, delta 2 (predicted)  Info   Centd2_predicted 
   2674 7.9e-06 49.2 99.4 0.495  1394789_at  LOC499812  Info   LOC499812 
   2675 7.9e-06 330.1 136.2 2.424  1376130_a_at  dystrobrevin, beta (predicted)  Info   Dtnb_predicted 
   2676 7.9e-06 44.4 13.3 3.338  1371170_a_at  interleukin 1 alpha  Info   Il1a 
   2677 8e-06 1043.6 2127 0.491  1392541_at  similar to RIKEN cDNA A030007L17; EST AA673177 (predicted) /// similar to RIKEN cDNA A030007L17  Info   RGD1304876_predicted /// LOC362370 
   2678 8e-06 751.7 1501.2 0.501  1368132_at  transducer of ERBB2, 1  Info   Tob1 
   2679 8e-06 37.8 65.4 0.578  1391571_at  Transcribed locus  Info    
   2680 8e-06 581.1 848 0.685  1372777_at  similar to dJ1033H22.1 (KIAA0554 protein) (predicted)  Info   RGD1305386_predicted 
   2681 8e-06 930.5 486.8 1.911  1393647_at  histone aminotransferase 1 (predicted)  Info   Hat1_predicted 
   2682 8e-06 627.4 1268.9 0.494  1368378_at  formyltetrahydrofolate dehydrogenase  Info   Fthfd 
   2683 8e-06 106.6 928.2 0.115  1386933_at  glycoprotein 2 (zymogen granule membrane)  Info   Gp2 
   2684 8e-06 454.9 255.4 1.781  1383067_a_at  deoxythymidylate kinase (predicted)  Info   Dtymk_predicted 
   2685 8e-06 2913.4 3975.7 0.733  1386891_at  phosphatidylethanolamine binding protein  Info   Pbp 
   2686 8e-06 262.5 137.1 1.915  1373094_at  general transcription factor II H, polypeptide 1 (predicted)  Info   Gtf2h1_predicted 
   2687 8e-06 198.8 414.8 0.479  1390412_at  Solute carrier family 39 (iron-regulated transporter), member 1  Info   Slc40a1 
   2688 8e-06 301.3 107 2.816  1392922_at  Transcribed locus  Info    
   2689 8e-06 2656 2053.6 1.293  1371372_at  similar to Sid3177p  Info   LOC362809 
   2690 8.1e-06 862.9 3631 0.238  1387303_at  solute carrier family 22 (organic cation transporter), member 2  Info   Slc22a2 
   2691 8.1e-06 633.5 317.4 1.996  1370934_at  nucleoporin 153kD  Info   Nup153 
   2692 8.1e-06 734.1 254.8 2.881  1386912_at  procollagen C-proteinase enhancer protein  Info   Pcolce 
   2693 8.1e-06 99.1 17.4 5.695  1374794_at  kinesin-like 7  Info   Knsl7 
   2694 8.1e-06 907.5 1949 0.466  1379770_at  similar to choline phosphotransferase 1; cholinephosphotransferase 1 alpha; cholinephosphotransferase 1  Info   RGD:1359283 
   2695 8.1e-06 1307.9 824 1.587  1389325_at  similar to programmed cell death 10  Info   RGD:1359329 
   2696 8.1e-06 399.5 235.6 1.696  1380116_at  Transcribed locus  Info    
   2697 8.1e-06 95.5 192.5 0.496  1391274_at  Transcribed locus  Info    
   2698 8.1e-06 208.3 68.1 3.059  1388479_at  Dihydropyrimidinase-like 3  Info   Dpysl3 
   2699 8.2e-06 1307.5 619.5 2.111  1370057_at  cysteine and glycine-rich protein 1  Info   Csrp1 
   2700 8.2e-06 317.1 142.1 2.232  1389496_at  A kinase (PRKA) anchor protein 7  Info   RGD:1303071 
   2701 8.2e-06 375.6 132.8 2.828  1380021_at  similar to 2010308M01Rik protein (predicted)  Info   RGD1305120_predicted 
   2702 8.2e-06 261.3 525.9 0.497  1385690_at  similar to Mut protein /// similar to Mut protein  Info   LOC301276 /// LOC363201 
   2703 8.2e-06 332.3 102.9 3.229  1377634_at  BCL2/adenovirus E1B 19kDa-interacting protein 1, NIP2 (predicted)  Info   Bnip2_predicted 
   2704 8.2e-06 245.1 123.4 1.986  1399035_at  polymerase (RNA) III (DNA directed) polypeptide H (predicted)  Info   Polr3h_predicted 
   2705 8.3e-06 201.4 98.8 2.038  1373138_at  nudix-type motif 5  Info   RGD:1359284 
   2706 8.3e-06 189.1 65.6 2.883  1375696_at  interferon (alpha and beta) receptor 1 (predicted)  Info   Ifnar1_predicted 
   2707 8.3e-06 210.2 96.4 2.18  1390914_at  Friend leukemia integration 1 (predicted)  Info   Fli1_predicted 
   2708 8.3e-06 171.3 434.6 0.394  1392952_at  hypothetical protein LOC619561  Info   LOC619561 
   2709 8.3e-06 199.2 114.8 1.735  1373903_at  similar to Hypothetical protein MGC32399 (predicted)  Info   RGD1304715_predicted 
   2710 8.4e-06 33.5 94.2 0.356  1384529_at  Transcribed locus  Info    
   2711 8.4e-06 32 158.6 0.202  1368432_a_at  v-ros UR2 sarcoma virus oncogene homolog 1 (avian)  Info   Ros1 
   2712 8.4e-06 229.6 404.8 0.567  1374190_at  citrate lyase beta like (predicted)  Info   Clybl_predicted 
   2713 8.4e-06 190.3 48 3.965  1397953_at  Transcribed locus  Info    
   2714 8.4e-06 1324.6 1040.1 1.274  1387780_at  DnaJ (Hsp40) homolog, subfamily A, member 2  Info   Dnaja2 
   2715 8.4e-06 446.7 730.3 0.612  1372030_at  zinc finger, FYVE domain containing 21 (predicted)  Info   Zfyve21_predicted 
   2716 8.4e-06 28.3 88.9 0.318  1368858_at  UDP-glucuronosyltransferase 8  Info   Ugt8 
   2717 8.5e-06 305.3 162 1.885  1369323_at  leptin receptor overlapping transcript  Info   Leprot 
   2718 8.5e-06 101.2 261.3 0.387  1368896_at  MAD homolog 7 (Drosophila)  Info   Madh7 
   2719 8.5e-06 577.7 1374.4 0.42  1390857_at    Info    
   2720 8.5e-06 864 365 2.367  1370007_at  protein disulfide isomerase related protein (calcium-binding protein, intestinal-related)  Info   Erp70 
   2721 8.5e-06 315.5 3.2 98.594  1392308_at  phospholipase A2, group IID (predicted)  Info   Pla2g2d_predicted 
   2722 8.5e-06 16.4 47 0.349  1397610_at  Transcribed locus  Info    
   2723 8.5e-06 4383.4 3083.2 1.422  1370253_at  ribosomal protein L22  Info   Rpl22 
   2724 8.5e-06 124.9 268.2 0.466  1397652_at  Transcribed locus  Info    
   2725 8.6e-06 421.5 118.8 3.548  1393706_at  six transmembrane epithelial antigen of the prostate (predicted)  Info   Steap_predicted 
   2726 8.6e-06 888.2 641.5 1.385  1375848_at  paraoxonase 2 (predicted)  Info   Pon2_predicted 
   2727 8.6e-06 323.2 837.1 0.386  1393947_at  solute carrier family 25 (mitochondrial carrier; ornithine transporter) member 15 (predicted)  Info   Slc25a15_predicted 
   2728 8.6e-06 139.2 446.5 0.312  1381396_s_at  Kruppel-like factor 15  Info   Klf15 
   2729 8.6e-06 28.6 80.2 0.357  1374821_at  RAB3A interacting protein  Info   RABIN3 
   2730 8.6e-06 422.9 882.3 0.479  1395411_at  nicotinate phosphoribosyltransferase-like protein  Info   RGD:1302945 
   2731 8.7e-06 122.9 32.1 3.829  1379340_at  lamimin, gamma 2  Info   Lamc2 
   2732 8.7e-06 48.7 12 4.058  1376377_at  Transcribed locus  Info    
   2733 8.7e-06 2454.3 3777.1 0.65  1368659_at  alanine-glyoxylate aminotransferase 2  Info   Agxt2 
   2734 8.7e-06 257.7 98.8 2.608  1393401_at  Similar to 9530046H09Rik protein (predicted)  Info    
   2735 8.7e-06 356.4 915.1 0.389  1386975_at  pyruvate dehydrogenase kinase, isoenzyme 2  Info   Pdk2 
   2736 8.7e-06 41.4 124.6 0.332  1379286_at  Transcribed locus  Info    
   2737 8.7e-06 62 176.3 0.352  1379679_at  Transcribed locus  Info    
   2738 8.8e-06 483 1325.1 0.365  1370929_at  low density lipoprotein receptor-related protein associated protein 1  Info   Lrpap1 
   2739 8.8e-06 432.4 230.2 1.878  1398572_at  RNA (guanine-7-) methyltransferase (predicted)  Info   Rnmt_predicted 
   2740 8.8e-06 161.3 313.7 0.514  1370302_at  thyroid hormone-response protein-1  Info   RGD:620882 
   2741 8.8e-06 527.7 845.8 0.624  1375289_at    Info    
   2742 8.8e-06 152.3 108.7 1.401  1386581_at  integrin, alpha V (vitronectin receptor, alpha polypeptide, antigen CD51) (predicted)  Info   Itgav_predicted 
   2743 8.8e-06 386.1 764.4 0.505  1393148_at  transmembrane 7 superfamily member 1 (predicted)  Info   Tm7sf1_predicted 
   2744 8.8e-06 5318.8 57.8 92.021  1373544_at  chemokine (C-X-C motif) ligand 9  Info   Cxcl9 
   2745 8.8e-06 367.3 12.9 28.473  1373224_at  alpha-2,3-sialyltransferase ST3Gal IV  Info   RGD:1303068 
   2746 8.8e-06 252.1 392.6 0.642  1373506_at  Guanylate nucleotide binding protein 2  Info   Gbp2 
   2747 8.8e-06 178.7 106.2 1.683  1372021_at  nucleoporin 188 (predicted)  Info   Nup188_predicted 
   2748 8.9e-06 179.2 8.5 21.082  1383601_at  DUF729 domain containing 1 (predicted)  Info    
   2749 8.9e-06 926.3 2568.7 0.361  1370821_at  Cb1-812  Info    
   2750 8.9e-06 161.1 41.6 3.873  1390579_at  similar to RIKEN cDNA 1810029B16 (predicted)  Info   RGD1305222_predicted 
   2751 9e-06 94 635.2 0.148  1381080_at  Transcribed locus  Info    
   2752 9e-06 1486.4 799.8 1.858  1395561_at  heterogeneous nuclear ribonucleoprotein A2/B1 (predicted)  Info   Hnrpa2b1_predicted 
   2753 9e-06 233.9 55.6 4.207  1393373_at  similar to encephalopsin  Info   LOC498289 
   2754 9e-06 1192.5 1795.6 0.664  1371328_at  Cleavage and polyadenylation specific factor 5 (predicted)  Info    
   2755 9e-06 5464.8 3385.7 1.614  1398775_at  ribosomal protein S15a  Info   Rps15a 
   2756 9e-06 470.1 198.1 2.373  1368291_at  baculoviral IAP repeat-containing 2  Info   Birc2 
   2757 9e-06 124.4 237.4 0.524  1378169_at  similar to peroxisomal long chain acyl-CoA thioesterase Ia  Info   LOC314304 
   2758 9.1e-06 142.7 63 2.265  1382682_at  Similar to hypothetical protein PRO0971 (predicted)  Info    
   2759 9.1e-06 58.1 27.5 2.113  1393247_at  Similar to zinc finger protein 560  Info    
   2760 9.1e-06 133.7 224.7 0.595  1372780_at  similar to RIKEN cDNA 1110038M16 (predicted)  Info   RGD1307066_predicted 
   2761 9.1e-06 635.3 345.5 1.839  1372302_at  similar to hypothetical protein FLJ10579 (predicted)  Info   RGD1308697_predicted 
   2762 9.1e-06 234.9 135.5 1.734  1373406_at  Torsin family 1, member B (predicted)  Info    
   2763 9.1e-06 907.3 594.1 1.527  1379832_at  polymerase (RNA) II (DNA directed) polypeptide D (predicted)  Info   Polr2d_predicted 
   2764 9.1e-06 172 272.6 0.631  1372531_at  protein tyrosine phosphatase, receptor-type, F interacting protein, binding protein 2  Info   Ppfibp2 
   2765 9.1e-06 192.1 79 2.432  1390144_at  similar to RIKEN cDNA 3110043O21 gene  Info   RGD:1359108 
   2766 9.2e-06 59.8 233 0.257  1374630_at  chloride intracellular channel 3 (predicted)  Info   Clic3_predicted 
   2767 9.2e-06 274.9 196.2 1.401  1372768_at  Similar to RIKEN cDNA 2010009L17 (predicted)  Info    
   2768 9.2e-06 357.9 589.6 0.607  1398826_s_at  nuclear receptor subfamily 2, group F, member 6  Info   Nr2f6 
   2769 9.2e-06 193.5 373 0.519  1390863_at  solute carrier family 19 (thiamine transporter), member 2 (predicted)  Info   Slc19a2_predicted 
   2770 9.2e-06 63.2 229.8 0.275  1372959_at  similar to mKIAA0665 protein (predicted)  Info   RGD1308952_predicted 
   2771 9.2e-06 46.7 158 0.296  1371018_at    Info    
   2772 9.2e-06 145.4 264.3 0.55  1371903_at  STIP1 homology and U-Box containing protein 1 (predicted)  Info    
   2773 9.2e-06 94.4 55 1.716  1389919_at  parvin, beta (predicted)  Info   Parvb_predicted 
   2774 9.2e-06 231.6 122.9 1.884  1378092_at  similar to magnesium-dependent phosphatase-1 (predicted)  Info   RGD1311147_predicted 
   2775 9.2e-06 365.1 237.3 1.539  1389036_at  similar to RIKEN cDNA 9030221M09 gene (predicted)  Info   RGD1305469_predicted 
   2776 9.2e-06 1732.3 3922.5 0.442  1367774_at  glutathione S-transferase A5  Info   Gsta5 
   2777 9.3e-06 340.4 710.3 0.479  1378049_at  similar to Cas-associated zinc finger protein (predicted)  Info   RGD1306498_predicted 
   2778 9.3e-06 995.3 1988.6 0.501  1375026_at  ceroid-lipofuscinosis, neuronal 6 (predicted)  Info   Cln6_predicted 
   2779 9.3e-06 176.9 305.7 0.579  1368427_at  A kinase (PRKA) anchor protein 11  Info   Akap11 
   2780 9.3e-06 16.9 64.3 0.263  1390518_at  similar to putative emu1 protein  Info   LOC498403 
   2781 9.3e-06 866.9 1364.9 0.635  1371736_at  Similar to 1810013D10Rik protein  Info    
   2782 9.3e-06 78.3 13.3 5.887  1378507_at  Transcribed locus  Info    
   2783 9.3e-06 189 132.1 1.431  1388717_at  protein O-fucosyltransferase 2 (predicted)  Info   Pofut2_predicted 
   2784 9.4e-06 4924.3 3609.2 1.364  1398789_at  ribosomal protein L37  Info   Rpl37 
   2785 9.4e-06 30.3 71.2 0.426  1376732_at  Calreticulin 3 (predicted)  Info    
   2786 9.4e-06 219.8 489.6 0.449  1388909_at  similar to cDNA sequence BC019806 (predicted)  Info   RGD1311534_predicted 
   2787 9.4e-06 227.1 87 2.61  1387429_at  adrenergic receptor kinase, beta 1  Info   Adrbk1 
   2788 9.4e-06 451.8 845 0.535  1376804_at  similar to Myosin VI  Info   LOC315840 
   2789 9.4e-06 6582.5 4056.6 1.623  1367579_a_at  tubulin, alpha 1 /// tubulin, alpha 6 (predicted) /// similar to Tubulin alpha-2 chain (Alpha-tubulin 2)  Info   Tuba1 /// Tuba6_predicted /// LOC500929 
   2790 9.4e-06 1788.4 2333.3 0.766  1367670_at  fumarate hydratase 1  Info   Fh1 
   2791 9.4e-06 689.2 322.7 2.136  1373847_at  transmembrane 4 superfamily member 1 (predicted)  Info   Tm4sf1_predicted 
   2792 9.4e-06 1580.9 3409.7 0.464  1386938_at  alanyl (membrane) aminopeptidase  Info   Anpep 
   2793 9.4e-06 133.3 24.5 5.441  1382370_at  cyclin F  Info   Ccnf 
   2794 9.5e-06 755.1 491.9 1.535  1367824_at  farnesyltransferase, CAAX box, alpha  Info   Fnta 
   2795 9.5e-06 98.6 17.9 5.508  1378140_at  ADP-ribosylation factor-like 11 (predicted)  Info   Arl11_predicted 
   2796 9.5e-06 176.5 570.2 0.31  1393794_at  Similar to tetratricopeptide repeat domain 21B  Info    
   2797 9.5e-06 156 76.4 2.042  1391050_at  similar to Metal-response element-binding transcription factor 2 (Metal-response element DNA-binding protein M96) (predicted)  Info   RGD1304727_predicted 
   2798 9.5e-06 327.8 44.2 7.416  1393049_at  myosin IG (predicted)  Info   Myo1g_predicted 
   2799 9.5e-06 5535.1 47.1 117.518  1368762_at  ubiquitin D  Info   Ubd 
   2800 9.5e-06 156.4 241.3 0.648  1383845_at  Transcribed locus  Info    
   2801 9.6e-06 121.4 64.9 1.871  1388956_at    Info    
   2802 9.6e-06 232.3 109.8 2.116  1397441_at  tetratricopeptide repeat domain 13 (predicted)  Info   Ttc13_predicted 
   2803 9.6e-06 3514.9 4385.4 0.802  1367622_at  ATP synthase, H+ transporting, mitochondrial F0 complex, subunit d  Info   Atp5h 
   2804 9.6e-06 568.4 233 2.439  1368669_at  uncoupling protein 2  Info   Ucp2 
   2805 9.6e-06 57.7 94.4 0.611  1387791_at  angiotensin 1 converting enzyme  Info   Ace 
   2806 9.6e-06 1709.7 1009.1 1.694  1370280_at  hypoxanthine guanine phosphoribosyl transferase  Info   Hprt 
   2807 9.7e-06 4518.4 5680.8 0.795  1367629_at  cytochrome c oxidase, subunit 7a 3  Info   Cox7a3 
   2808 9.7e-06 73.1 124.2 0.589  1374727_at  similar to DnaJ (Hsp40) homolog, subfamily B, member 12  Info   LOC294513 
   2809 9.7e-06 1420.8 2051.7 0.692  1388851_at  heat shock protein, A (predicted)  Info   Hspa9a_predicted 
   2810 9.7e-06 272.1 443 0.614  1373792_at  ARP1 actin-related protein 1 homolog B (yeast) (predicted)  Info    
   2811 9.7e-06 97.3 1726.1 0.056  1387328_at  Cytochrome P450, subfamily IIC (mephenytoin 4-hydroxylase)  Info   Cyp2c 
   2812 9.7e-06 258.9 140.1 1.848  1387078_at  inositol polyphosphate-4-phosphatase, type 1  Info   Inpp4a 
   2813 9.7e-06 120.2 59.6 2.017  1395944_at  Transcribed locus  Info    
   2814 9.7e-06 667.9 1110.3 0.602  1379264_at  zinc and ring finger 1 (predicted)  Info   Znrf1_predicted 
   2815 9.7e-06 218.2 895.4 0.244  1368661_at  solute carrier family 13 (sodium-dependent dicarboxylate transporter), member 2  Info   Slc13a2 
   2816 9.7e-06 593.9 1077.2 0.551  1372395_at  Similar to KIAA0597 protein  Info    
   2817 9.8e-06 59.5 190.9 0.312  1388579_at  Similar to hypothetical gene MGC19595 (predicted)  Info    
   2818 9.8e-06 214.7 74.5 2.882  1368927_at  membrane bound C2 domain containing protein  Info   Mbc2 
   2819 9.8e-06 256.9 487.6 0.527  1393743_at  Transcribed locus  Info    
   2820 9.8e-06 152.7 77.6 1.968  1388178_at  nuclear receptor coactivator 3  Info   Ncoa3 
   2821 9.8e-06 368.1 181.9 2.024  1368032_at  nucleolar and coiled-body phosphoprotein 1  Info   Nolc1 
   2822 9.9e-06 231.6 130.2 1.779  1370505_at  ADP-ribosylarginine hydrolase  Info   Adprh 
   2823 9.9e-06 54.8 112 0.489  1369816_at  RAB3A, member RAS oncogene family  Info   Rab3a 
   2824 9.9e-06 3878.8 2691.9 1.441  1386868_at  ribosomal protein S10  Info   Rps10 
   2825 9.9e-06 1817.9 1116.8 1.628  1398897_at  ubiquitin-conjugating enzyme E2 variant 1 (predicted)  Info   Ube2v1_predicted 
   2826 9.9e-06 20.9 58.5 0.357  1389223_at    Info    
   2827 1e-05 140.3 29.7 4.724  1387270_at  hematopoietically expressed homeobox  Info   Hhex 
   2828 1e-05 212.4 1692.1 0.126  1393508_at  Transcribed locus  Info    
   2829 1e-05 131.6 470.9 0.279  1368651_at  pyruvate kinase, liver and RBC  Info   Pklr 
   2830 1.01e-05 56.3 8.4 6.702  1369522_a_at  CD244 natural killer cell receptor 2B4  Info   Cd244 
   2831 1.01e-05 188.6 114.2 1.651  1388668_at    Info    
   2832 1.01e-05 4.2 51.1 0.082  1392338_at  Transcribed locus  Info    
   2833 1.01e-05 167.8 48.9 3.431  1393041_at  SMC2 structural maintenance of chromosomes 2-like 1 (yeast) (predicted)  Info   Smc2l1_predicted 
   2834 1.01e-05 59.3 312.5 0.19  1383315_at  hepatic protein EIIH  Info   RGD:1359311 
   2835 1.01e-05 188.6 60.2 3.133  1371122_at  TRAF family member-associated Nf-kappa B activator /// hypothetical gene supported by NM_145788  Info   Tank /// LOC497768 
   2836 1.01e-05 133.3 434.7 0.307  1393017_at  rhophilin, Rho GTPase binding protein 1 (predicted)  Info   Rhpn1_predicted 
   2837 1.01e-05 891.5 304.1 2.932  1373421_at  TG interacting factor (predicted)  Info   Tgif_predicted 
   2838 1.01e-05 121.5 5 24.3  1382378_at  cathepsin W (predicted)  Info   Ctsw_predicted 
   2839 1.01e-05 357.2 640.6 0.558  1373134_at  similar to RIKEN cDNA B430104H02  Info   LOC296131 
   2840 1.01e-05 370.1 190.8 1.94  1384295_at  heat shock protein hsp70-related protein  Info   RGD:1303296 
   2841 1.01e-05 601.5 202.4 2.972  1371785_at  tumor necrosis factor receptor superfamily, member 12a  Info   Tnfrsf12a 
   2842 1.01e-05 109.4 12.8 8.547  1388940_at  similar to Rho-GTPase-activating protein 25  Info   LOC500246 
   2843 1.01e-05 292.9 156.4 1.873  1373894_at    Info    
   2844 1.02e-05 926.5 1291.8 0.717  1375269_at  similar to Deoxyribose-phosphate aldolase-like  Info   LOC500355 
   2845 1.02e-05 76.9 9.2 8.359  1384939_at  Transcribed locus  Info    
   2846 1.02e-05 33.2 94.7 0.351  1384303_s_at  solute carrier family 6 (neurotransmitter transporter), member 17  Info   Slc6a17 
   2847 1.02e-05 528 330.1 1.6  1398369_at  Transcribed locus  Info    
   2848 1.02e-05 3934 2500.8 1.573  1388850_at  heat shock protein 1, alpha  Info   Hspca 
   2849 1.02e-05 1453.7 826.7 1.758  1388158_at  HLA-B-associated transcript 1A  Info   Bat1a 
   2850 1.02e-05 82.5 18.8 4.388  1391502_at  Transcribed locus  Info    
   2851 1.02e-05 578.7 11.3 51.212  1369877_at  CD8 antigen, alpha chain  Info   Cd8a 
   2852 1.03e-05 374.3 251.6 1.488  1394438_at  ARP1 actin-related protein 1 homolog A (yeast) (predicted)  Info   Actr1a_predicted 
   2853 1.03e-05 111.4 222.5 0.501  1368371_at  potassium voltage-gated channel, subfamily Q, member 1  Info   Kcnq1 
   2854 1.03e-05 381.7 133.2 2.866  1385252_at  Tripartite motif protein 34 (predicted)  Info    
   2855 1.03e-05 236.3 146.5 1.613  1372668_at  LIM domains containing 1 (predicted)  Info    
   2856 1.03e-05 241.7 484.6 0.499  1373465_at  PQ loop repeat containing 1 (predicted)  Info   Pqlc1_predicted 
   2857 1.03e-05 206.5 101.9 2.026  1383321_at  tyrosylprotein sulfotransferase 1 (predicted)  Info   Tpst1_predicted 
   2858 1.03e-05 70.6 210.4 0.336  1380117_at  Transcribed locus  Info    
   2859 1.03e-05 27.1 75.5 0.359  1384485_at  protein tyrosine phosphatase, receptor type, U  Info   Ptpru 
   2860 1.04e-05 270 444 0.608  1374636_at  PHD finger protein 17 (predicted)  Info    
   2861 1.04e-05 1931.5 1463.4 1.32  1373044_at  similar to Dendritic cell protein GA17  Info   LOC295975 
   2862 1.04e-05 640.2 296.7 2.158  1388453_at  myeloid-associated differentiation marker  Info   Myadm 
   2863 1.04e-05 534.8 695.8 0.769  1399049_at  similar to RIKEN cDNA 1200003C05 (predicted)  Info   RGD1308917_predicted 
   2864 1.05e-05 913.4 2566.2 0.356  1371824_at  adenylate kinase 3-like 1  Info   Ak3l1 
   2865 1.05e-05 31.9 72.2 0.442  1376341_at  Transcribed locus  Info    
   2866 1.05e-05 41.1 113.1 0.363  1377088_at  similar to RIKEN cDNA 2310046K01 (predicted)  Info   RGD1304644_predicted 
   2867 1.05e-05 693.2 195.7 3.542  1373197_at  Similar to Ab1-133 (predicted)  Info    
   2868 1.05e-05 1283.3 752.8 1.705  1372246_at  osteoclast stimulating factor 1  Info   Ostf1 
   2869 1.05e-05 1019.4 1870.7 0.545  1372162_at  acetyl-Coenzyme A synthetase 2 (AMP forming)-like (predicted)  Info   Acas2l_predicted 
   2870 1.06e-05 208.2 10.9 19.101  1372808_at  similar to methylenetetrahydrofolate dehydrogenase (NAD) (EC 1.5.1.15) / methenyltetrahydrofolate cyclohydrolase (EC 3.5.4.9) precursor - mouse  Info   LOC313410 
   2871 1.06e-05 1692.3 4032.1 0.42  1372372_at  similar to Ab2-225 (predicted)  Info   RGD1306952_predicted 
   2872 1.06e-05 41.1 201.6 0.204  1390164_at  Transcribed locus  Info    
   2873 1.06e-05 929.4 540.8 1.719  1389294_at  cytoplasmic FMR1 interacting protein 1 (predicted)  Info   Cyfip1_predicted 
   2874 1.06e-05 448.5 1014.5 0.442  1371361_at  similar to tensin  Info   LOC301509 
   2875 1.06e-05 654.5 434.5 1.506  1374501_at    Info    
   2876 1.06e-05 102.8 40.7 2.526  1388879_at    Info    
   2877 1.07e-05 163.7 393.7 0.416  1385486_at  Basonuclin 2 (predicted)  Info   LOC298189 
   2878 1.07e-05 2679.2 5219.5 0.513  1370436_at  kidney-specific protein (KS)  Info   RGD:708383 
   2879 1.07e-05 28.6 55.2 0.518  1383331_at  Transcribed locus  Info    
   2880 1.07e-05 1013.9 734.3 1.381  1372052_at  Budding uninhibited by benzimidazoles 3 homolog (S. cerevisiae) (predicted)  Info    
   2881 1.07e-05 2801.9 3561.8 0.787  1389820_at    Info    
   2882 1.07e-05 481.7 286.3 1.683  1373555_at  Dynactin 4  Info   Dctn4 
   2883 1.07e-05 1989.4 2650.1 0.751  1368653_a_at  fertility protein SP22  Info   Park7 
   2884 1.07e-05 89.2 186.8 0.478  1389456_at  Transcribed locus  Info    
   2885 1.07e-05 261.7 414.8 0.631  1371551_at  Tnf receptor associated factor 4 (predicted)  Info   Traf4_predicted 
   2886 1.07e-05 606 839.2 0.722  1373127_at  Similar to Tripartite motif protein 44 (DIPB protein)  Info    
   2887 1.08e-05 198.2 39.9 4.967  1390946_at  Runt-related transcription factor 3  Info   Runx3 
   2888 1.08e-05 723.4 393.8 1.837  1389729_at  similar to cDNA sequence BC003324  Info   RGD:1303140 
   2889 1.08e-05 47.6 266.1 0.179  1370165_at  small muscle protein, X-linked  Info   Smpx 
   2890 1.08e-05 4795.1 3409.7 1.406  1398872_at  ribosomal protein L13 /// ribosomal protein S13  Info   Rpl13 /// Rps13 
   2891 1.08e-05 236.4 136.5 1.732  1374146_at  MAD2 mitotic arrest deficient-like 2 (yeast) (predicted)  Info   Mad2l2_predicted 
   2892 1.08e-05 136.1 32.8 4.149  1368604_at  Mediterranean fever  Info   Mefv 
   2893 1.08e-05 96 42.5 2.259  1395662_at  Transcribed locus  Info    
   2894 1.08e-05 254.5 111 2.293  1390529_at  CD83 antigen (predicted)  Info   Cd83_predicted 
   2895 1.08e-05 216.4 78.9 2.743  1396267_at    Info    
   2896 1.09e-05 101.7 378.6 0.269  1387703_a_at  ubiquitin specific protease 2  Info   Usp2 
   2897 1.09e-05 113 244.7 0.462  1380431_at  similar to KIAA0564 protein (predicted)  Info   RGD1308772_predicted 
   2898 1.09e-05 1717.7 943.2 1.821  1374778_at  cathepsin C  Info   Ctsc 
   2899 1.09e-05 606.1 351 1.727  1379450_at  similar to cDNA sequence BC003236 (predicted)  Info   RGD1310822_predicted 
   2900 1.09e-05 151.6 51.3 2.955  1380366_at  similar to single chain Fv antibody fragment scFv 7-10A  Info   LOC314509 
   2901 1.09e-05 281.7 214 1.316  1388532_at  similar to hypothetical protein (predicted)  Info   RGD1310571_predicted 
   2902 1.09e-05 370.7 214.7 1.727  1376576_at  dual specificity phosphatase 11 (RNA/RNP complex 1-interacting) (predicted)  Info   Dusp11_predicted 
   2903 1.09e-05 219.2 353.7 0.62  1388982_at    Info    
   2904 1.09e-05 211.3 653.8 0.323  1387284_at  dihydropyrimidinase  Info   Dpys 
   2905 1.09e-05 143.7 91.2 1.576  1374561_at  hypothetical LOC305452 (predicted)  Info   RGD1309634_predicted 
   2906 1.09e-05 85.7 165.5 0.518  1390556_at    Info    
   2907 1.1e-05 59.8 129.3 0.462  1383796_at  CDNA clone IMAGE:7323916  Info    
   2908 1.1e-05 1120.6 708.8 1.581  1386884_at  protease, serine, 11 (Igf binding)  Info   Prss11 
   2909 1.1e-05 232.3 151 1.538  1388306_at  similar to RIKEN cDNA 1810042K04 (predicted)  Info   RGD1305593_predicted 
   2910 1.11e-05 96.7 428.2 0.226  1380057_at  Transcribed locus  Info    
   2911 1.11e-05 1365.7 3130.2 0.436  1370201_at  calbindin 1  Info   Calb1 
   2912 1.11e-05 594.9 383 1.553  1398974_at  chromosome segregation 1-like (S. cerevisiae) (predicted)  Info   Cse1l_predicted 
   2913 1.12e-05 45.7 21.5 2.126  1393844_at  similar to myeloid leukemia factor 1 interacting protein  Info   LOC306464 
   2914 1.12e-05 5234.8 4094.5 1.278  1371330_at  ribosomal protein L11 (predicted)  Info   Rpl11_predicted 
   2915 1.12e-05 758.2 1324.1 0.573  1388570_at  similar to RIKEN cDNA 2310005O14  Info   LOC498909 
   2916 1.12e-05 551.6 807.8 0.683  1388520_at  Sperm associated antigen 9 (predicted)  Info    
   2917 1.13e-05 245.7 153.5 1.601  1369008_a_at  olfactomedin 1  Info   Olfm1 
   2918 1.13e-05 993.8 1772.1 0.561  1372612_at  dynein light chain-2  Info   Dlc2 
   2919 1.13e-05 340.4 134.8 2.525  1376153_at  Transcribed locus  Info    
   2920 1.13e-05 573.6 1732.5 0.331  1370831_at  monoglyceride lipase  Info   Mgll 
   2921 1.14e-05 319.6 84.9 3.764  1369683_at  BH3 interacting domain death agonist  Info   Bid 
   2922 1.14e-05 107.8 145.2 0.742  1388539_at  plakophilin 2 (predicted)  Info   Pkp2_predicted 
   2923 1.14e-05 234.6 138.2 1.698  1377755_at  helicase, ATP binding 1 (predicted)  Info   Helic1_predicted 
   2924 1.14e-05 76.7 190.9 0.402  1378138_at  kinesin family member 16B (predicted)  Info   Kif16b_predicted 
   2925 1.14e-05 445.2 218.8 2.035  1375034_at  lysosomal phospholipase A2  Info   RGD:1302982 
   2926 1.14e-05 281.5 162.6 1.731  1370066_at  Kelch-like ECH-associated protein 1  Info   Keap1 
   2927 1.14e-05 5715.5 40.9 139.743  1382454_at  chemokine (C-X-C motif) ligand 9  Info   Cxcl9 
   2928 1.15e-05 34.4 152.9 0.225  1396942_at  hypothetical LOC301267  Info   LOC301267 
   2929 1.15e-05 263.2 9.2 28.609  1384191_at  Transcribed locus  Info    
   2930 1.15e-05 38.6 84.2 0.458  1393261_at  Similar to protein phosphatase 2A B56 regulatory subunit gamma 3 isoform  Info    
   2931 1.15e-05 286.3 129.2 2.216  1394842_at  Unknown (protein for MGC:72591)  Info   Tmem19 
   2932 1.15e-05 764.7 537.1 1.424  1389013_at  similar to RIKEN cDNA 1500016H10 (predicted)  Info   RGD1308469_predicted 
   2933 1.15e-05 58 125 0.464  1392244_at  LOC498362  Info   LOC498362 
   2934 1.15e-05 170 35.1 4.843  1368799_at  baculoviral IAP repeat-containing 5  Info   Birc5 
   2935 1.16e-05 559.4 1277.2 0.438  1394786_at  Transcribed locus  Info    
   2936 1.16e-05 218 51.6 4.225  1368914_at  runt related transcription factor 1  Info   Runx1 
   2937 1.16e-05 604.6 348.5 1.735  1370919_at  heterogeneous nuclear ribonucleoprotein M  Info   Hnrpm 
   2938 1.16e-05 736.1 1612.8 0.456  1392090_at  serine (or cysteine) proteinase inhibitor, clade C (antithrombin), member 1 (predicted)  Info   Serpinc1_predicted 
   2939 1.16e-05 63.1 139.6 0.452  1392935_at  Transcribed locus  Info    
   2940 1.17e-05 137.4 322.4 0.426  1384877_at  aquaporin 11  Info   Aqp11 
   2941 1.17e-05 357 533.8 0.669  1377723_at  Transcribed locus  Info    
   2942 1.17e-05 788.7 357.9 2.204  1393431_at  Transcribed locus  Info    
   2943 1.17e-05 5021.9 3566.6 1.408  1398882_at  ribosomal protein S5  Info   Rps5 
   2944 1.18e-05 17.3 96.2 0.18  1384392_at  cytochrome P450, family 26, subfamily b, polypeptide 1  Info   Cyp26b1 
   2945 1.18e-05 160.1 91.7 1.746  1393619_at  CCR4-NOT transcription complex, subunit 6-like (predicted)  Info   Cnot6l_predicted 
   2946 1.18e-05 402.2 15.6 25.782  1385277_x_at  similar to hypothetical protein FLJ11354 (predicted)  Info   RGD1310093_predicted 
   2947 1.18e-05 738.4 1076.3 0.686  1374846_at  cardiac lineage protein 1  Info   Clp1 
   2948 1.19e-05 92.8 37.2 2.495  1382477_at  SUMO/sentrin specific protease 5 (predicted)  Info    
   2949 1.19e-05 410.1 120.4 3.406  1382283_at  Wiskott-Aldrich syndrome protein interacting protein  Info   Waspip 
   2950 1.19e-05 47.8 122.6 0.39  1369153_at  nephrosis 1 homolog, nephrin (human)  Info   Nphs1 
   2951 1.19e-05 86.2 200.9 0.429  1391656_at    Info    
   2952 1.19e-05 189.3 33.6 5.634  1391935_at  similar to eukaryotic translation initiation factor 4E member 3  Info   LOC297481 
   2953 1.19e-05 463.5 991.1 0.468  1392916_at  microtubule-associated protein 7 (predicted)  Info   Mtap7_predicted 
   2954 1.19e-05 287.3 181.9 1.579  1399121_at  Cell division cycle 27 homolog (S. cerevisiae) (predicted)  Info    
   2955 1.19e-05 229.8 359.9 0.639  1384238_at  Similar to Tweety homolog 2  Info    
   2956 1.2e-05 128.6 249.2 0.516  1371606_at  Transcribed locus  Info    
   2957 1.2e-05 285.1 430.7 0.662  1368122_at  ring finger protein 103  Info   Rnf103 
   2958 1.2e-05 690.6 410.7 1.682  1383202_at  similar to U6 snRNA-associated Sm-like protein LSm6 (Sm protein F)  Info   LOC498934 
   2959 1.2e-05 307.6 211.3 1.456  1399084_at  DEAH (Asp-Glu-Ala-His) box polypeptide 16  Info   RGD:1302963 
   2960 1.2e-05 4266.3 3241.3 1.316  1367582_at  ribosomal protein L29  Info   Rpl29 
   2961 1.2e-05 756 25 30.24  1370869_at  branched chain aminotransferase 1, cytosolic  Info   Bcat1 
   2962 1.2e-05 444.6 278.9 1.594  1387085_at  phosphoribosyl pyrophosphate synthetase 1  Info   Prps1 
   2963 1.2e-05 4544.5 3769.6 1.206  1372241_at  ornithine decarboxylase antizyme 1  Info   Oaz1 
   2964 1.2e-05 470.4 1027.4 0.458  1377911_at  Transcribed locus  Info    
   2965 1.2e-05 92.2 34.6 2.665  1384892_at  Transcribed locus  Info    
   2966 1.21e-05 224.6 861.5 0.261  1383605_at  similar to alpha-fetoprotein  Info   LOC360919 
   2967 1.21e-05 49.8 9.8 5.082  1376816_at  Hypothetical gene supported by NM_012514  Info   Brca1 
   2968 1.21e-05 316.5 148.9 2.126  1392961_at  Similar to Mob4B protein  Info    
   2969 1.21e-05 100.8 282.4 0.357  1389725_at  transmembrane 7 superfamily member 2 (predicted)  Info   Tm7sf2_predicted 
   2970 1.21e-05 430.1 2462 0.175  1368021_at  alcohol dehydrogenase 1  Info   Adh1 
   2971 1.22e-05 133.8 51.4 2.603  1381819_at  Transcribed locus  Info    
   2972 1.22e-05 613.3 313.9 1.954  1372270_at  dolichyl-phosphate (UDP-N-acetylglucosamine) N-acetylglucosaminephosphotransferase 1 (GlcNAc-1-P transferase)  Info   H2afx 
   2973 1.22e-05 247.1 421 0.587  1388102_at  leukotriene B4 12-hydroxydehydrogenase  Info   Ltb4dh 
   2974 1.22e-05 162.5 10 16.25  1382925_at    Info    
   2975 1.22e-05 115.1 1011.7 0.114  1371762_at  retinol binding protein 4, plasma  Info   Rbp4 
   2976 1.22e-05 114.7 264.1 0.434  1390818_at  ATPase, Na+/K+ transporting, beta 3 polypeptide  Info   Atp1b3 
   2977 1.22e-05 286.9 145 1.979  1374474_at  Transcribed locus, strongly similar to NP_080091.1 copine family member [Mus musculus]  Info    
   2978 1.23e-05 67.6 182.5 0.37  1377879_at  Transcribed locus  Info    
   2979 1.23e-05 73.1 151.1 0.484  1376180_at    Info    
   2980 1.23e-05 210.8 105.2 2.004  1392454_at  similar to hypothetical protein FLJ20729 (predicted)  Info   RGD1308723_predicted 
   2981 1.23e-05 231.8 60.9 3.806  1380692_at  Transcribed locus  Info    
   2982 1.23e-05 495.6 2919.7 0.17  1368627_at  regucalcin  Info   Rgn 
   2983 1.23e-05 255.9 461.8 0.554  1379339_at  similar to tumor-related protein (predicted)  Info   RGD1307632_predicted 
   2984 1.24e-05 761.9 540.4 1.41  1371503_at  nucleotide binding protein 1 (predicted)  Info   Nubp1_predicted 
   2985 1.24e-05 3033.9 2038.3 1.488  1398812_at  proteasome (prosome, macropain) subunit, beta type 1  Info   Psmb1 
   2986 1.24e-05 286.8 171.7 1.67  1390767_at  signal sequence receptor, alpha (predicted)  Info   Ssr1_predicted 
   2987 1.24e-05 116 35.6 3.258  1370105_at  lunatic fringe gene homolog (Drosophila)  Info   Lfng 
   2988 1.24e-05 942.3 422.7 2.229  1388393_at  proteolipid protein 2  Info   Plp2 
   2989 1.25e-05 1485 1011.7 1.468  1367931_a_at  polypyrimidine tract binding protein 1  Info   Ptbp1 
   2990 1.25e-05 733.6 452.8 1.62  1372082_at  Unknown (protein for MGC:72598)  Info   RGD:735063 
   2991 1.25e-05 196.2 557.5 0.352  1380471_at  Transcribed locus  Info    
   2992 1.25e-05 1035.2 1810 0.572  1387805_at  BCL2/adenovirus E1B 19 kDa-interacting protein 3  Info   Bnip3 
   2993 1.25e-05 42.5 86.2 0.493  1385093_at  similar to hypothetical protein LOC284018 isoform b  Info   LOC498014 
   2994 1.25e-05 80.4 25.4 3.165  1391453_at  Transcribed locus, strongly similar to XP_576700.1 PREDICTED: similar to RIKEN cDNA 2900016D05 [Rattus norvegicus]  Info    
   2995 1.25e-05 275.5 115.2 2.391  1387957_a_at  SH3-domain kinase binding protein 1  Info   Sh3kbp1 
   2996 1.26e-05 49.9 111.3 0.448  1383886_at  Transcribed locus  Info    
   2997 1.26e-05 1522 2096.4 0.726  1388160_a_at  isocitrate dehydrogenase 3 (NAD+) beta  Info   Idh3B 
   2998 1.26e-05 98.3 25.1 3.916  1386716_at  Similar to putative membrane steroid receptor (predicted)  Info    
   2999 1.26e-05 3153.8 883.9 3.568  1383291_at    Info    
   3000 1.26e-05 831.4 426.1 1.951  1367746_a_at  flotillin 2  Info   Flot2 
   3001 1.26e-05 54.5 138.4 0.394  1390664_at    Info    
   3002 1.27e-05 309.5 181.9 1.701  1374194_at    Info    
   3003 1.27e-05 647.2 361.7 1.789  1372028_at  similar to Protein CGI-117 (Protein HSPC111) (predicted)  Info   RGD1305727_predicted 
   3004 1.27e-05 578.1 395.6 1.461  1399062_at  CDNA clone IMAGE:7317308  Info    
   3005 1.27e-05 722.1 1607.8 0.449  1373386_at  gap junction membrane channel protein beta 2  Info   Gjb2 
   3006 1.27e-05 371.9 131.6 2.826  1368144_at  regulator of G-protein signaling 2  Info   Rgs2 
   3007 1.27e-05 136.4 265.7 0.513  1393165_at  similar to RIKEN cDNA 2310028N02  Info   RGD:1359339 
   3008 1.27e-05 115.8 36.8 3.147  1376317_at    Info    
   3009 1.27e-05 1454.4 1048.3 1.387  1398904_at  non-POU domain containing, octamer-binding /// similar to Nono protein  Info   Nono /// LOC501589 
   3010 1.27e-05 156.1 11.7 13.342  1377110_at  similar to ADP-ribosylation-like factor 12 protein  Info   LOC497990 
   3011 1.27e-05 422.1 301.1 1.402  1370112_at  phosphatase and tensin homolog  Info   Pten 
   3012 1.27e-05 67.2 251 0.268  1367959_a_at  sodium channel, voltage-gated, type I, beta polypeptide  Info   Scn1b 
   3013 1.28e-05 106.6 59 1.807  1391014_at  zinc finger, MYND domain containing 19  Info   Zmynd19 
   3014 1.28e-05 407.8 291.2 1.4  1373491_at  Similar to Metaxin 1, isoform 2  Info    
   3015 1.28e-05 1132.3 1686.5 0.671  1375220_at  RE70703p-like  Info   RGD:1303292 
   3016 1.28e-05 157.8 63.2 2.497  1390650_at  pericentrin 1 (predicted)  Info   Pcnt1_predicted 
   3017 1.28e-05 254 152.1 1.67  1389315_at  similar to Git2 protein  Info   RGD:1359708 
   3018 1.28e-05 496.2 281.8 1.761  1368055_a_at  lamin A  Info   Lmna 
   3019 1.28e-05 216.5 130.8 1.655  1382070_at  activating transcription factor 7 interacting protein (predicted)  Info   Atf7ip_predicted 
   3020 1.29e-05 2871.8 4122.4 0.697  1371371_at  NADH dehydrogenase (ubiquinone) 1 beta subcomplex, 4, 15kDa (predicted)  Info   Ndufb4_predicted 
   3021 1.29e-05 201.3 113.1 1.78  1388672_at  Transcribed locus  Info    
   3022 1.29e-05 66.6 105.6 0.631  1383964_at  Transcribed locus  Info    
   3023 1.29e-05 213.5 113.9 1.874  1380201_at  similar to hypothetical protein FLJ14075  Info   RGD:1359447 
   3024 1.3e-05 127.6 243.9 0.523  1392119_at  Transcribed locus  Info    
   3025 1.3e-05 358.4 214.4 1.672  1388339_at  phosphoprotein enriched in astrocytes 15 (predicted)  Info   Pea15_predicted 
   3026 1.3e-05 141.5 73.5 1.925  1389389_at  DEAD (Asp-Glu-Ala-Asp) box polypeptide 56  Info   RGD:1303195 
   3027 1.3e-05 812 968.9 0.838  1388366_at  mitochondrial ribosomal protein L4 (predicted)  Info   Mrpl4_predicted 
   3028 1.31e-05 350.6 1050.5 0.334  1386917_at  Pyruvate carboxylase  Info   Pc 
   3029 1.31e-05 252.2 508.5 0.496  1392929_at  Solute carrier family 4, sodium bicarbonate transporter-like, member 11 (predicted)  Info    
   3030 1.31e-05 25.4 102.4 0.248  1397233_at  similar to hypothetical protein C730036H08 (predicted)  Info   RGD1309232_predicted 
   3031 1.31e-05 155.1 287.2 0.54  1373934_at  LOC500840  Info   LOC500840 
   3032 1.32e-05 621.4 1626.7 0.382  1373011_at  hypothetical protein LOC619558  Info   LOC619558 
   3033 1.32e-05 45.8 246.2 0.186  1385005_at    Info    
   3034 1.33e-05 325.9 198.2 1.644  1378080_at  similar to SCO cytochrome oxidase deficient homolog 1 (yeast)  Info   LOC497930 
   3035 1.33e-05 443.1 261.8 1.693  1388871_at  ash2 (absent, small, or homeotic)-like (Drosophila) (predicted)  Info   Ash2l_predicted 
   3036 1.33e-05 3270.9 3904 0.838  1386898_at  heat shock 10 kDa protein 1  Info   Hspe1 
   3037 1.33e-05 5195.9 3560.4 1.459  1398852_at  ribosomal protein S21  Info   Rps21 
   3038 1.33e-05 2981.1 2121.2 1.405  1367895_at  heterogeneous nuclear ribonucleoprotein K  Info   Hnrpk 
   3039 1.33e-05 6475.9 4338.1 1.493  1371344_at  ribosomal protein L27a (predicted)  Info   Rpl27a_predicted 
   3040 1.34e-05 171.6 22.7 7.559  1385440_at  hematopoietic cell signal transducer  Info   RGD:1359238 
   3041 1.34e-05 31.8 86.9 0.366  1382475_at  Similar to pellino 3 alpha (predicted)  Info    
   3042 1.34e-05 42.9 94.9 0.452  1384925_at  Similar to IL-17D  Info    
   3043 1.34e-05 64.2 220.9 0.291  1384202_at  similar to Tescalcin  Info   LOC288689 
   3044 1.34e-05 317.9 870.7 0.365  1392411_at  tubulointerstitial nephritis antigen  Info   RGD:1359482 
   3045 1.34e-05 9467 5495 1.723  1387883_a_at  thymosin, beta 4  Info   Tmsb4x 
   3046 1.35e-05 519.7 266.3 1.952  1398337_at  Similar to RIKEN cDNA 9430077D24 gene  Info    
   3047 1.35e-05 167.7 378.4 0.443  1384112_at  5 nucleotidase  Info   Nt5 
   3048 1.36e-05 259.7 358.2 0.725  1389194_at  Similar to hypothetical protein  Info    
   3049 1.36e-05 34.4 91.6 0.376  1393335_at  epidermal growth factor-like protein 6  Info   Egfl6 
   3050 1.36e-05 152.8 51.1 2.99  1384841_at  Cathepsin C  Info   Ctsc 
   3051 1.36e-05 5414.5 3879.7 1.396  1367630_at  ribosomal protein S11  Info   Rps11 
   3052 1.36e-05 204.4 405.9 0.504  1382935_at  similar to Hypothetical protein KIAA0141  Info   RGD:735029 
   3053 1.36e-05 116.9 53.5 2.185  1377968_at  Transcribed locus  Info    
   3054 1.36e-05 156.5 247.2 0.633  1398342_at  similar to Fus1 protein  Info   LOC501052 
   3055 1.36e-05 91.5 514.8 0.178  1387372_at  solute carrier family 6 (neurotransmitter transporter, GABA), member 13  Info   Slc6a13 
   3056 1.37e-05 83.8 35 2.394  1370967_at  Ig active lambda2-like chain mRNA, 3' end  Info    
   3057 1.37e-05 155.2 2.9 53.517  1370083_at  macrophage inflammatory protein-1 alpha receptor gene  Info   Ccr1 
   3058 1.37e-05 271.9 559.9 0.486  1383064_at  CDNA clone IMAGE:7379585  Info    
   3059 1.38e-05 955.7 1375.7 0.695  1398783_at  G protein pathway suppressor 1  Info   Gps1 
   3060 1.38e-05 187.7 7.8 24.064  1368930_at  potassium intermediate/small conductance calcium-activated channel, subfamily N, member 4  Info   Kcnn4 
   3061 1.38e-05 221.2 474.4 0.466  1395694_at  f-box only protein 9 (predicted)  Info   Fbxo9_predicted 
   3062 1.38e-05 204.7 2.7 75.815  1384658_at  Transcribed locus  Info    
   3063 1.39e-05 514.2 796.1 0.646  1387907_at  inositol 1,4,5-triphosphate receptor 1  Info   Itpr1 
   3064 1.39e-05 246.9 449.7 0.549  1388636_at  similar to RIKEN cDNA 5730408C10 (predicted)  Info   RGD1305972_predicted 
   3065 1.39e-05 465.5 977.2 0.476  1376868_at    Info    
   3066 1.39e-05 60.4 141.3 0.427  1378755_at  Transcribed locus  Info    
   3067 1.39e-05 171.1 308.8 0.554  1372422_at  Sulfatase modifying factor 1 (predicted)  Info    
   3068 1.39e-05 265.2 83 3.195  1380699_at  Zinc finger homeobox 1b (predicted)  Info    
   3069 1.39e-05 171 104.2 1.641  1375701_at  similar to RIKEN cDNA 2810013M15  Info   LOC360568 
   3070 1.4e-05 927.3 2080.5 0.446  1367660_at  fatty acid binding protein 3  Info   Fabp3 
   3071 1.4e-05 59.9 180.8 0.331  1383280_at  Transcribed locus  Info    
   3072 1.4e-05 77.5 26.5 2.925  1377350_at  Transcribed locus  Info    
   3073 1.4e-05 214.7 395.5 0.543  1379277_at  Utrophin  Info   Utrn 
   3074 1.4e-05 275.1 483.5 0.569  1367747_at  ADP-ribosylation factor-like 3  Info   Arl3 
   3075 1.4e-05 131.6 289.4 0.455  1374376_at  Rho-guanine nucleotide exchange factor (predicted)  Info   Rgnef_predicted 
   3076 1.4e-05 134.7 213.1 0.632  1390478_at  origin recognition complex, subunit 4  Info   RGD:619987 
   3077 1.4e-05 25.4 60.5 0.42  1385126_at  Transcribed locus  Info    
   3078 1.41e-05 50.8 4.2 12.095  1373468_at  Transcribed locus  Info    
   3079 1.41e-05 228.8 542.6 0.422  1389809_at  Transcribed locus  Info    
   3080 1.41e-05 168.6 59.3 2.843  1374306_at  Transcribed locus  Info    
   3081 1.41e-05 1710.7 2213.3 0.773  1367783_at  GABA(A) receptor-associated protein like 2  Info   Gabarapl2 
   3082 1.41e-05 108.5 236.4 0.459  1379909_at  G kinase anchoring protein 1 (predicted)  Info   Gkap1_predicted 
   3083 1.41e-05 232.2 143.1 1.623  1380256_at  polymerase (RNA) III (DNA directed) polypeptide C (predicted)  Info   Polr3c_predicted 
   3084 1.42e-05 227 131.4 1.728  1370159_at  SWI/SNF related, matrix associated, actin dependent regulator of chromatin, subfamily d, member 2  Info   Smarcd2 
   3085 1.42e-05 271.7 395.5 0.687  1388723_at  brain and reproductive organ-expressed protein  Info   Bre 
   3086 1.42e-05 84.5 32.1 2.632  1373633_at  Transcribed locus  Info    
   3087 1.42e-05 396 599.7 0.66  1367550_a_at  beta-amyloid binding protein precursor (predicted)  Info   Bbp_predicted 
   3088 1.42e-05 74.2 122.5 0.606  1383184_at  zinc and ring finger 1 (predicted)  Info   Znrf1_predicted 
   3089 1.42e-05 533.1 874.3 0.61  1374705_at  similar to type IV collagen alpha 5 chain  Info   LOC363457 
   3090 1.42e-05 343 159.3 2.153  1392938_s_at  similar to C11orf17 protein (predicted)  Info   RGD1306959_predicted 
   3091 1.43e-05 355.4 1704.8 0.208  1369531_at  sulfotransferase family, cytosolic, 1C, member 2  Info   Sult1c2 
   3092 1.43e-05 87 275.1 0.316  1374910_at    Info    
   3093 1.44e-05 333 181.5 1.835  1367834_at  spermidine synthase  Info   Srm 
   3094 1.44e-05 92.7 163.3 0.568  1377424_at  Transcribed locus  Info    
   3095 1.44e-05 1477.8 3550.9 0.416  1387111_at  dimethylarginine dimethylaminohydrolase 1  Info   Ddah1 
   3096 1.44e-05 213.7 121.8 1.755  1372062_at  similar to cyclin-dependent kinase 2-interacting protein  Info   LOC299334 
   3097 1.45e-05 634.3 276.9 2.291  1380346_at  serine (or cysteine) proteinase inhibitor, clade B, member 1a (predicted)  Info   Serpinb1a_predicted 
   3098 1.45e-05 4471.7 3211.3 1.392  1387888_at  ribosomal protein S9  Info   Rps9 
   3099 1.45e-05 56.3 13.8 4.08  1392128_at  Transcribed locus  Info    
   3100 1.45e-05 228.5 156.1 1.464  1367865_at  limkain b1  Info   Lkap 
   3101 1.45e-05 140.4 201.2 0.698  1368309_at  thioredoxin reductase 2  Info   Txnrd2 
   3102 1.45e-05 681.8 1037.7 0.657  1371473_at  cleft lip and palate associated transmembrane protein 1 (predicted)  Info   Clptm1_predicted 
   3103 1.45e-05 285.3 751.4 0.38  1374241_at  Transcribed locus  Info    
   3104 1.46e-05 1685.3 1197.1 1.408  1388615_at  Ras-related protein RAP-1A  Info   RGD:1359694 
   3105 1.46e-05 111.9 58.5 1.913  1395065_at  WD repeat domain 41 (predicted)  Info   Wdr41_predicted 
   3106 1.46e-05 163.2 482 0.339  1385132_at  myeloblastosis oncogene-like 1 (predicted)  Info   Mybl1_predicted 
   3107 1.46e-05 298.9 610.4 0.49  1383453_at  similar to olfactomedin-like 1 /// olfactomedin-like 1 (predicted)  Info   LOC308917 /// Olfml1_predicted 
   3108 1.46e-05 109.5 220.3 0.497  1373676_at  Transcribed locus  Info    
   3109 1.46e-05 68.3 144.8 0.472  1373118_at    Info    
   3110 1.46e-05 279.4 146.8 1.903  1383179_at  Similar to hypothetical protein HSPC129 (predicted)  Info    
   3111 1.47e-05 452.2 996.4 0.454  1375640_at  FK506 binding protein 9  Info   Fkbp9 
   3112 1.47e-05 333 138.7 2.401  1367957_at  regulator of G-protein signalling 3  Info   Rgs3 
   3113 1.47e-05 160.7 303.5 0.529  1396112_at  similar to BB128963 protein (predicted)  Info   RGD1305958_predicted 
   3114 1.47e-05 513.6 334.5 1.535  1374118_at  leucyl-tRNA synthetase (predicted)  Info   Lars_predicted 
   3115 1.47e-05 335.4 732.8 0.458  1393584_at    Info    
   3116 1.47e-05 151 66.5 2.271  1386745_at  Transcribed locus  Info    
   3117 1.47e-05 187 74.6 2.507  1382993_at  Bcl-2 binding component 3  Info   Bbc3 
   3118 1.47e-05 395.8 568.4 0.696  1375027_at  Cell cycle progression 1 (predicted)  Info    
   3119 1.47e-05 577.4 1465 0.394  1367759_at  H1 histone family, member 0  Info   H1f0 
   3120 1.48e-05 135.2 84.4 1.602  1374831_at  Similar to cDNA sequence BC023151 (predicted)  Info    
   3121 1.48e-05 760.3 351.4 2.164  1369971_a_at  heterogeneous nuclear ribonucleoprotein D  Info   Hnrpd 
   3122 1.48e-05 6991.6 3504.5 1.995  AFFX_Rat_beta-actin_5_at  actin, beta  Info   Actb 
   3123 1.48e-05 791.7 558.7 1.417  1376793_at  similar to RIKEN cDNA 5730414C17 (predicted)  Info   RGD1308377_predicted 
   3124 1.49e-05 445.1 178.4 2.495  1374265_at  Similar to arylacetamide deacetylase (esterase) (predicted)  Info    
   3125 1.49e-05 97.4 395.2 0.246  1387656_at  solute carrier family 4, member 1  Info   Slc4a1 
   3126 1.49e-05 93.2 40.7 2.29  1382343_at  Transcribed locus  Info    
   3127 1.49e-05 359.9 197.5 1.822  1389686_at  Hypothetical LOC363470  Info    
   3128 1.49e-05 71.4 17.3 4.127  1384259_at  Rac GTPase-activating protein 1 (predicted)  Info   Racgap1_predicted 
   3129 1.5e-05 108.2 238 0.455  1395381_at    Info    
   3130 1.5e-05 73.2 41.2 1.777  1384042_at  Transcribed locus  Info    
   3131 1.5e-05 275.3 227.5 1.21  1382203_at  growth differentiation factor 1 (predicted)  Info   Gdf1_predicted 
   3132 1.51e-05 5.8 60.6 0.096  1379169_at  similar to RIKEN cDNA 2310067G05  Info   LOC301113 
   3133 1.51e-05 257.8 108.3 2.38  1370876_at  neurofascin  Info   Nfasc 
   3134 1.51e-05 5286.6 4048.7 1.306  1398885_at  ribosomal protein L23  Info   Rpl23 
   3135 1.51e-05 170.3 326.1 0.522  1372023_at  LOC498674  Info   LOC498674 
   3136 1.51e-05 144.8 270.1 0.536  1397698_at  Transcribed locus  Info    
   3137 1.51e-05 303.3 128.3 2.364  1390698_at  Transcribed locus, weakly similar to XP_579465.1 PREDICTED: DNA binding protein N5 [Rattus norvegicus]  Info    
   3138 1.51e-05 476.2 1037.5 0.459  1376770_at  similar to EF hand domain containing 1  Info   LOC501181 
   3139 1.52e-05 4742 2875.5 1.649  1369929_at  prosaposin  Info   Psap 
   3140 1.52e-05 158.6 350.8 0.452  1387910_at  erythrocyte protein band 4.1-like 1  Info   Epb4.1l1 
   3141 1.52e-05 418.7 16.1 26.006  1372852_at  similar to protein tyrosine phosphatase, receptor type, C polypeptide-associated protein  Info   LOC499300 
   3142 1.52e-05 854.4 534 1.6  1371657_at  ubiquitin-like 1 (sentrin) activating enzyme E1B (predicted)  Info   Uble1b_predicted 
   3143 1.52e-05 112.4 318.2 0.353  1376860_at  Transcribed locus  Info    
   3144 1.53e-05 390.2 763.6 0.511  1399065_at    Info    
   3145 1.53e-05 160.4 70 2.291  1373904_at  similar to RIKEN cDNA 2210402C18 (predicted)  Info   RGD1304585_predicted 
   3146 1.53e-05 958 1822.6 0.526  1388441_at    Info    
   3147 1.53e-05 131 46.5 2.817  1379345_at  procollagen, type XV (predicted)  Info   Col15a1_predicted 
   3148 1.54e-05 1491.3 2018.1 0.739  1389965_at  trans-golgi network protein 1  Info   Tgoln2 
   3149 1.54e-05 49.9 123.1 0.405  1390523_at  Transcribed locus  Info    
   3150 1.54e-05 865.7 507.2 1.707  1386940_at  tissue inhibitor of metalloproteinase 2  Info   Timp2 
   3151 1.54e-05 142.6 422.7 0.337  1396300_at  Transcribed locus  Info    
   3152 1.54e-05 1050 734.5 1.43  1370820_at  F-box only protein 6b  Info   Fbxo6b 
   3153 1.54e-05 113.3 25.4 4.461  1368884_at  ectonucleoside triphosphate diphosphohydrolase 1  Info   Entpd1 
   3154 1.54e-05 256.9 469.1 0.548  1381922_at  solute carrier family 5 (sodium/glucose cotransporter), member 11  Info   Slc5a11 
   3155 1.54e-05 145.6 300.4 0.485  1373583_at  Transcribed locus  Info    
   3156 1.55e-05 1271.7 1733.2 0.734  1373222_at  hexosaminidase A  Info   Hexa 
   3157 1.55e-05 82.9 26.7 3.105  1370341_at  enolase 2, gamma  Info   Eno2 
   3158 1.55e-05 734.2 1006.1 0.73  1399026_at  similar to polyadenylate-binding protein-interacting protein 2  Info   RGD:1359176 
   3159 1.55e-05 714.1 469.3 1.522  1388614_at  splicing factor 3b, subunit 2 (predicted)  Info   Sf3b2_predicted 
   3160 1.55e-05 603.8 1009.4 0.598  1370921_at  secretory carrier membrane protein 3  Info   Scamp3 
   3161 1.55e-05 2317.6 4118.2 0.563  1371421_at  Similar to FLJ40243 protein (predicted)  Info    
   3162 1.55e-05 82.8 43.4 1.908  1378322_at  Transcribed locus, strongly similar to NP_705811.8 APG4-D protein [Mus musculus]  Info    
   3163 1.56e-05 72.5 282.4 0.257  1377969_at    Info    
   3164 1.56e-05 123.1 17 7.241  1397341_at  PDZ binding kinase (predicted)  Info    
   3165 1.56e-05 347.2 185.3 1.874  1379327_at  SWI/SNF related, matrix associated, actin dependent regulator of chromatin, subfamily a, member 5 (predicted)  Info   Smarca5_predicted 
   3166 1.56e-05 814.4 1470.6 0.554  1373986_at  Transcribed locus  Info    
   3167 1.56e-05 121.2 403 0.301  1377680_at  glial cell line derived neurotrophic factor family receptor alpha 3  Info   Gfra3 
   3168 1.57e-05 1648.2 2351.4 0.701  1388558_at  Adenylate kinase 3  Info   Ak3 
   3169 1.57e-05 256.2 145.3 1.763  1398809_at  nuclear distribution gene E homolog 1 (A nidulans)  Info   Nde1 
   3170 1.57e-05 86.2 35.9 2.401  1393334_at  Transcribed locus  Info    
   3171 1.57e-05 178.3 293.4 0.608  1389169_at  progesterone receptor membrane component 2 (predicted)  Info   Pgrmc2_predicted 
   3172 1.58e-05 140.2 57.5 2.438  1370574_a_at  protein tyrosine phosphatase, non-receptor type substrate 1  Info   Ptpns1 
   3173 1.58e-05 1140.7 597.9 1.908  1371646_at  phosphogluconate dehydrogenase  Info   Pgd 
   3174 1.58e-05 187.8 568.1 0.331  1379326_at  similar to 9530008L14Rik protein (predicted)  Info   RGD1305679_predicted 
   3175 1.59e-05 598 265.9 2.249  1372500_at  tropomodulin 3 (predicted)  Info   Tmod3_predicted 
   3176 1.59e-05 118.5 44.1 2.687  1387280_a_at  tumor-associated protein 1  Info   Slc7a5 
   3177 1.59e-05 126.4 39.6 3.192  1379396_at  engulfment and cell motility 1, ced-12 homolog (C. elegans) (predicted)  Info   Elmo1_predicted 
   3178 1.6e-05 368.7 643.2 0.573  1373015_at  ring finger protein 11 (predicted)  Info   Rnf11_predicted 
   3179 1.6e-05 62.7 16.6 3.777  1379740_at  Similar to chromosome 18 open reading frame 54  Info    
   3180 1.6e-05 238.2 138 1.726  1390046_at  a disintegrin and metalloprotease domain 8 (predicted)  Info   Adam8_predicted 
   3181 1.6e-05 111.7 2 55.85  1379507_at  Transcribed locus  Info    
   3182 1.61e-05 31.4 829 0.038  1371080_at  kallikrein  Info   RGD:735228 
   3183 1.61e-05 955 1553.1 0.615  1373585_at  similar to RIKEN cDNA 1810020G14 (predicted)  Info   RGD1310781_predicted 
   3184 1.61e-05 180.4 95.9 1.881  1384451_at  Transcribed locus  Info    
   3185 1.61e-05 171.7 58.7 2.925  1377744_at  Transcribed locus, moderately similar to XP_576460.1 PREDICTED: similar to hypothetical protein PB402898.00.0 [Rattus norvegicus]  Info    
   3186 1.61e-05 108.3 44.5 2.434  1393803_at  caspase 8 associated protein 2 (predicted)  Info   Casp8ap2_predicted 
   3187 1.61e-05 622.3 898.4 0.693  1388483_at  cofilin 2, muscle (predicted)  Info   Cfl2_predicted 
   3188 1.61e-05 3145.1 1147.2 2.742  1369931_at  similar to pyruvate kinase (EC 2.7.1.40) isozyme M2 - rat  Info   LOC498661 
   3189 1.61e-05 606.3 817.3 0.742  1367767_at  3-hydroxy-3-methylglutaryl-Coenzyme A lyase  Info   Hmgcl 
   3190 1.62e-05 1400.2 1037.1 1.35  1367820_at  barrier to autointegration factor 1  Info   Banf1 
   3191 1.62e-05 1126.3 2113.2 0.533  1388555_at  thioredoxin-like 5 (predicted)  Info   Txnl5_predicted 
   3192 1.62e-05 173.6 88.9 1.953  1382253_at  similar to hypothetical protein FLJ21103 (predicted)  Info   RGD1307173_predicted 
   3193 1.63e-05 51.8 92.4 0.561  1386529_at  Transcribed locus  Info    
   3194 1.63e-05 64.6 125 0.517  1382940_at    Info    
   3195 1.63e-05 1356.4 1012.1 1.34  1390356_at  Transcribed locus  Info    
   3196 1.63e-05 150.1 265.2 0.566  1383378_at  Transcribed locus  Info    
   3197 1.64e-05 821.9 1286 0.639  1367468_at  SCAN domain-containing 1 (predicted)  Info   Scand1_predicted 
   3198 1.64e-05 159.5 60.1 2.654  1375996_at  septin 1 (predicted)  Info   Sept1_predicted 
   3199 1.64e-05 432.3 234.9 1.84  1399055_at  bromodomain containing 7 (predicted)  Info   Brd7_predicted 
   3200 1.64e-05 606.4 906.5 0.669  1388153_at  acyl-CoA synthetase long-chain family member 1  Info   Acsl1 
   3201 1.64e-05 577.2 278.2 2.075  1389658_at  similar to CG6133-PA (predicted)  Info   RGD1311954_predicted 
   3202 1.64e-05 95.4 11.9 8.017  1381216_at    Info    
   3203 1.64e-05 45.8 74.3 0.616  1381282_at  similar to cDNA sequence BC026432 (predicted)  Info   RGD1308345_predicted 
   3204 1.65e-05 934.3 529 1.766  1388392_at  Tax1 (human T-cell leukemia virus type I) binding protein 3 (predicted)  Info   Tax1bp3_predicted 
   3205 1.65e-05 430.3 232.5 1.851  1377750_at  Rho guanine nucleotide exchange factor (GEF) 3 (predicted)  Info    
   3206 1.65e-05 103.9 40.3 2.578  1391255_at  zinc finger, MYND domain containing 15 (predicted)  Info   Zmynd15_predicted 
   3207 1.65e-05 133.9 235.8 0.568  1374079_at  Transcribed locus  Info    
   3208 1.65e-05 134.2 244 0.55  1374124_at  similar to RIKEN cDNA 2610003J06 (predicted)  Info   RGD1307381_predicted 
   3209 1.66e-05 873.8 1520 0.575  1370811_at  mercaptopyruvate sulfurtransferase  Info   Mpst 
   3210 1.66e-05 285.2 424.2 0.672  1391500_at  Transcribed locus  Info    
   3211 1.67e-05 174.6 66.4 2.63  1383199_at  Friend leukemia integration 1 (predicted)  Info    
   3212 1.67e-05 580.5 1047.3 0.554  1375438_at  golgi SNAP receptor complex member 2  Info   Gosr2 
   3213 1.67e-05 72 305.7 0.236  1370363_at  carboxylesterase 3  Info   Ces3 
   3214 1.67e-05 873.5 1426.1 0.613  1372876_at  selenophosphate synthetase 2 (predicted)  Info   Sephs2_predicted 
   3215 1.67e-05 57 16.8 3.393  1385359_at  Similar to RIKEN cDNA 4931406C07 (predicted)  Info    
   3216 1.68e-05 581.6 280.7 2.072  1371592_at  c-src tyrosine kinase (predicted)  Info   Csk_predicted 
   3217 1.68e-05 1.2 7 0.171  1396499_at  similar to HSD-29  Info   LOC300201 
   3218 1.69e-05 151.8 242.7 0.625  1370905_at  dedicator of cytokinesis 9  Info   Dock9 
   3219 1.69e-05 105.8 56.6 1.869  1384378_at  HCR (a-helix coiled-coil rod homolog)  Info   RGD:1302992 
   3220 1.69e-05 241.3 676.3 0.357  1378191_at  nuclear cap binding protein subunit 1, 80kDa (predicted)  Info   Ncbp1_predicted 
   3221 1.69e-05 750.6 1178.4 0.637  1386892_at  parathymosin  Info   Ptms 
   3222 1.69e-05 306.9 448.9 0.684  1391611_at  kinesin family member 12 (predicted)  Info   Kif12_predicted 
   3223 1.7e-05 411.4 836.4 0.492  1387230_at  solute carrier family 12, member 3  Info   Slc12a3 
   3224 1.7e-05 224.4 327.3 0.686  1372557_at  ADP-ribosylation factor-like 6 (predicted)  Info   Arl6_predicted 
   3225 1.7e-05 209.3 398.3 0.525  1367752_at  breast cancer anti-estrogen resistance 1  Info   Bcar1 
   3226 1.7e-05 41.6 170.4 0.244  1387877_at  formiminotransferase cyclodeaminase  Info   Ftcd 
   3227 1.7e-05 930.3 1263.7 0.736  1371844_at    Info    
   3228 1.7e-05 505.6 914 0.553  1371739_at  Leucine zipper-EF-hand containing transmembrane protein 1  Info   Letm1 
   3229 1.71e-05 145.3 48.9 2.971  1368300_at  adenosine A2a receptor  Info   Adora2a 
   3230 1.71e-05 568.6 1112.5 0.511  1367810_at  choline transporter  Info   Slc6a8 
   3231 1.71e-05 685.7 2547 0.269  1377672_at  sulfotransferase family, cytosolic, 1C, member 2  Info   Sult1c2 
   3232 1.71e-05 88.9 35.7 2.49  1392548_at  similar to mKIAA1604 protein  Info   LOC362153 
   3233 1.71e-05 68.7 191.4 0.359  1398264_at  solute carrier family 30 (zinc transporter), member 2  Info   Slc30a2 
   3234 1.72e-05 100.8 207.3 0.486  1379719_at  Transcribed locus  Info    
   3235 1.72e-05 162 54.4 2.978  1393105_at  similar to zinc finger protein  Info   LOC500989 
   3236 1.72e-05 552.2 1018.3 0.542  1367982_at  aminolevulinic acid synthase 1  Info   Alas1 
   3237 1.72e-05 2032.8 4456.9 0.456  1368150_at  solute carrier family 27 (fatty acid transporter), member 2 /// hypothetical gene supported by NM_031736  Info   Slc27a2 /// LOC497779 
   3238 1.72e-05 1086.5 2194.3 0.495  1388358_at  electron-transfer-flavoprotein, beta polypeptide  Info   RGD:1303312 
   3239 1.72e-05 1203.5 2876.3 0.418  1398514_at  homogentisate 1, 2-dioxygenase (predicted)  Info   Hgd_predicted 
   3240 1.73e-05 202.7 114.4 1.772  1371125_at  kinesin heavy chain member 2  Info   Kif2 
   3241 1.73e-05 437.7 331.8 1.319  1373907_at  trafficking protein particle complex 4  Info   RGD:1302991 
   3242 1.73e-05 21.1 71.8 0.294  1381947_at  hypothetical LOC295622  Info   LOC295622 
   3243 1.74e-05 204.2 380 0.537  1390498_at  Transcribed locus  Info    
   3244 1.74e-05 133.4 71.4 1.868  1381311_at  EGF-like module containing, mucin-like, hormone receptor-like sequence 1  Info   RGD:1359214 
   3245 1.74e-05 161.3 263.1 0.613  1374995_at  engulfment and cell motility 3, ced-12 homolog (C. elegans) (predicted)  Info   Elmo3_predicted 
   3246 1.74e-05 1152.9 1736.2 0.664  1367798_at  S-adenosylhomocysteine hydrolase  Info   Ahcy 
   3247 1.74e-05 193.2 1624 0.119  1368288_at  group specific component  Info   Gc 
   3248 1.74e-05 777.9 1338.1 0.581  1371730_at  similar to RIKEN cDNA 1300002A08 (predicted)  Info   RGD1305466_predicted 
   3249 1.74e-05 29.4 66.4 0.443  1380090_at    Info    
   3250 1.74e-05 103.8 47.9 2.167  1395036_at  similar to Ac2-190 (predicted)  Info   RGD1306455_predicted 
   3251 1.75e-05 235.7 542.5 0.434  1391573_at  Tumor necrosis factor receptor superfamily, member 21 (predicted)  Info    
   3252 1.76e-05 1025.1 673 1.523  1383643_at  similar to UPF0197 protein C11orf10 homolog  Info   LOC499317 
   3253 1.77e-05 117.3 245.8 0.477  1374466_at  Protein kinase C, epsilon  Info   Prkce 
   3254 1.77e-05 92.5 200.2 0.462  1374104_at  claudin 5  Info   Cldn5 
   3255 1.77e-05 2041.7 3416.2 0.598  1368163_at  dipeptidylpeptidase 4  Info   Dpp4 
   3256 1.77e-05 838.2 480.2 1.746  1388725_at  leptin receptor overlapping transcript  Info   Leprot 
   3257 1.78e-05 21.9 78.2 0.28  1378505_at    Info    
   3258 1.78e-05 186.5 351.2 0.531  1388465_at  Transcribed locus  Info    
   3259 1.78e-05 63.8 169.7 0.376  1374171_at  Similar to SRY (sex determining region Y)-box 5 isoform a  Info   Abcc9 
   3260 1.79e-05 273.2 146.3 1.867  1376641_at  THO complex 1 (predicted)  Info   Thoc1_predicted 
   3261 1.79e-05 91.1 29 3.141  1375071_at  nucleoporin 133 (predicted)  Info   Nup133_predicted 
   3262 1.79e-05 193 488.4 0.395  1372728_at  Sortilin 1  Info   Sort1 
   3263 1.79e-05 291.2 190.5 1.529  1387950_at  Saccharomyces cerevisiae Nip7p homolog  Info   RGD:620069 
   3264 1.79e-05 141.1 81.8 1.725  1373549_at  DEAD (Asp-Glu-Ala-Asp) box polypeptide 10 (predicted)  Info   Ddx10_predicted 
   3265 1.8e-05 450.7 612.6 0.736  1399010_at  COP9 (constitutive photomorphogenic) homolog, subunit 7a (Arabidopsis thaliana) (predicted)  Info   Cops7a_predicted 
   3266 1.8e-05 1130.1 506.1 2.233  1367856_at  glucose-6-phosphate dehydrogenase  Info   G6pdx 
   3267 1.8e-05 3532.9 4417.9 0.8  1371254_at  ubiquinol-cytochrome c reductase, Rieske iron-sulfur polypeptide 1  Info   Uqcrfs1 
   3268 1.8e-05 78.4 188.4 0.416  1368476_at  nuclear receptor subfamily 3, group C, member 2  Info   Nr3c2 
   3269 1.8e-05 1271.6 3063.4 0.415  1389551_at  lactamase, beta 2 (predicted)  Info   Lactb2_predicted 
   3270 1.8e-05 300.3 957.5 0.314  1383058_at  Transcribed locus  Info    
   3271 1.81e-05 171.3 3 57.1  1378043_at  similar to OEF2  Info   LOC500011 
   3272 1.81e-05 133.9 244.7 0.547  1369370_s_at  transient receptor potential cation channel, subfamily V, member 4 /// transient receptor potential cation channel, subfamily V, member 1  Info   Trpv4 /// Trpv1 
   3273 1.81e-05 605.5 954.1 0.635  1398997_at  phosphohistidine phosphatase 1 (predicted)  Info   Phpt1_predicted 
   3274 1.81e-05 75.5 357.6 0.211  1393732_at  Transcribed locus  Info    
   3275 1.81e-05 179.5 68.2 2.632  1390122_at  Similar to triggering receptor expressed on myeloid cells-like 1  Info    
   3276 1.81e-05 103.1 27.2 3.79  1393728_at  Transcribed locus  Info    
   3277 1.81e-05 509.1 287.3 1.772  1372402_at  N-acetylneuraminic acid synthase (sialic acid synthase) (predicted)  Info   Nans_predicted 
   3278 1.81e-05 635.6 375.6 1.692  1389001_at  Transcribed locus, strongly similar to XP_422815.1 PREDICTED: similar to Importin alpha-4 subunit (Karyopherin alpha-4 subunit) (Qip1 protein) [Gallus gallus]  Info    
   3279 1.81e-05 84.1 19.6 4.291  1391518_at    Info    
   3280 1.82e-05 158.8 428.9 0.37  1385400_at  ATPase, H+ transporting, V1 subunit B, isoform 1 (predicted)  Info   Atp6v1b1_predicted 
   3281 1.82e-05 462.3 289.3 1.598  1393317_at  similar to THUMP domain containing 3  Info   LOC500288 
   3282 1.82e-05 118.8 239.7 0.496  1376797_at  cysteine and glycine-rich protein 2 binding protein (predicted)  Info   Csrp2bp_predicted 
   3283 1.83e-05 440.2 233.3 1.887  1369122_at  Bcl2-associated X protein  Info   Bax 
   3284 1.83e-05 261.3 14.9 17.537  1370469_at  protein tyrosine phosphatase, non-receptor type 7  Info   Ptpn7 
   3285 1.83e-05 278.2 141.4 1.967  1395248_at  similar to mKIAA0212 protein  Info   LOC297504 
   3286 1.83e-05 1860.9 2504.4 0.743  1389012_at  NADH dehydrogenase (ubiquinone) 1 beta subcomplex, 2 (predicted)  Info   Ndufb2_predicted 
   3287 1.84e-05 144.5 488.6 0.296  1379308_at  Transcribed locus, strongly similar to NP_031761.1 procollagen, type IV, alpha 4 [Mus musculus]  Info    
   3288 1.84e-05 124.1 74 1.677  1382679_at  similar to WD-repeat protein 43  Info   LOC362703 
   3289 1.84e-05 352.7 211.3 1.669  1394954_at    Info    
   3290 1.84e-05 150.2 91.1 1.649  1383354_a_at  F-box only protein 33 (predicted)  Info   Fbxo33_predicted 
   3291 1.84e-05 118.5 172.7 0.686  1373144_at  similar to Ac2-125  Info   LOC499654 
   3292 1.84e-05 231.9 114.9 2.018  1377696_at  mesoderm development candidate 1 (predicted)  Info   Mesdc1_predicted 
   3293 1.85e-05 28.9 83.2 0.347  1379710_at  Transcribed locus, weakly similar to NP_081541.1 hypothetical protein LOC69933 [Mus musculus]  Info    
   3294 1.85e-05 1337.7 2577.5 0.519  1379243_at  NADH dehydrogenase (ubiquinone) 1 alpha subcomplex, 6 (B14) (predicted)  Info   Ndufa6_predicted 
   3295 1.85e-05 277 186.7 1.484  1372148_at  COMM domain containing 2 (predicted)  Info   Commd2_predicted 
   3296 1.85e-05 105.5 393.8 0.268  1389234_at  von Willebrand factor  Info   RGD:621759 
   3297 1.85e-05 27.6 80.3 0.344  1393212_at  Transcribed locus  Info    
   3298 1.86e-05 260.8 452.4 0.576  1389102_at  similar to Dock1 protein  Info   LOC309081 
   3299 1.86e-05 25.6 1.7 15.059  1393791_at  Transcribed locus  Info    
   3300 1.86e-05 326.3 515.2 0.633  1373924_at  similar to C530044N13Rik protein (predicted)  Info   RGD1306568_predicted 
   3301 1.87e-05 1028.6 413.1 2.49  1388985_at  hypothetical protein LOC310926 /// LOC501211  Info   LOC310926 /// LOC501211 
   3302 1.87e-05 381 699.1 0.545  1376573_at  RAB34, member of RAS oncogene family (predicted)  Info   Rab34_predicted 
   3303 1.87e-05 613.1 332.7 1.843  1373373_at  Glutamine fructose-6-phosphate transaminase 1  Info   MGC95214 
   3304 1.87e-05 577.9 1073.1 0.539  1375932_at  Phosphoribosyl pyrophosphate synthetase 2  Info   Prps2 
   3305 1.87e-05 237.6 854 0.278  1391539_at  Transcribed locus  Info    
   3306 1.87e-05 126.3 28.8 4.385  1391222_at  similar to Nedd4 binding protein 1 (predicted)  Info   RGD1305179_predicted 
   3307 1.88e-05 55.8 152 0.367  1378029_at  Transcribed locus  Info    
   3308 1.88e-05 94.4 40.8 2.314  1398523_a_at  interferon (alpha and beta) receptor 1 (predicted)  Info   Ifnar1_predicted 
   3309 1.88e-05 78.4 24 3.267  1376291_at  similar to WD repeat domain 4  Info   LOC499412 
   3310 1.89e-05 35.1 103.8 0.338  1396229_at  Transcribed locus  Info    
   3311 1.89e-05 18 73.5 0.245  1372637_at    Info    
   3312 1.89e-05 388.6 601.6 0.646  1388942_at  Transcribed locus  Info    
   3313 1.89e-05 118.8 44.2 2.688  1384041_at  Transcribed locus  Info    
   3314 1.89e-05 532.8 890.6 0.598  1372997_at  Transcribed locus  Info    
   3315 1.89e-05 395.1 637.6 0.62  1374766_at  Similar to mKIAA0493 protein  Info    
   3316 1.9e-05 129.5 21.4 6.051  1384228_at  similar to nuclear factor of activated T-cells, cytoplasmic, calcineurin-dependent 1  Info   LOC307231 
   3317 1.9e-05 254.9 137.4 1.855  1388342_at  Transcribed locus  Info    
   3318 1.91e-05 612.3 348.7 1.756  1370978_at  secretory carrier membrane protein 1  Info   Scamp1 
   3319 1.91e-05 796.5 485.3 1.641  1389165_at  similar to myocardial ischemic preconditioning upregulated protein 2  Info   LOC498301 
   3320 1.91e-05 2598.8 1837.9 1.414  1369013_a_at  mitochondrial ribosomal protein L17  Info   Mrpl17 
   3321 1.91e-05 420 681.8 0.616  1394705_at  Transcribed locus, weakly similar to XP_580018.1 PREDICTED: hypothetical protein XP_580018 [Rattus norvegicus]  Info    
   3322 1.92e-05 46.5 145.2 0.32  1384304_at  LRP16 protein  Info   Lrp16 
   3323 1.92e-05 166.6 423.1 0.394  1373921_at  CDNA clone MGC:124810 IMAGE:7932594  Info    
   3324 1.92e-05 309.7 426.3 0.726  1389459_at  Transcribed locus  Info    
   3325 1.93e-05 1872.1 3306 0.566  1389836_a_at  Synapsin III  Info   Syn3 
   3326 1.93e-05 260.2 22.5 11.564  1392732_at  nucleolar and spindle associated protein 1 (predicted)  Info   Nusap1_predicted 
   3327 1.93e-05 1266.6 896.9 1.412  1398869_at  proteasome (prosome, macropain) 26S subunit, ATPase, 4  Info   Psmc4 
   3328 1.93e-05 76.9 31.1 2.473  1397767_at  Transcribed locus  Info    
   3329 1.94e-05 1062 626.9 1.694  1372778_at  Solute carrier family 39 (zinc transporter), member 1 (predicted)  Info    
   3330 1.94e-05 143.5 72.4 1.982  1377173_at  Transcribed locus, moderately similar to XP_580018.1 PREDICTED: hypothetical protein XP_580018 [Rattus norvegicus]  Info    
   3331 1.94e-05 199.9 478.3 0.418  1370638_at  ankyrin 3, epithelial  Info   RGD:620157 
   3332 1.94e-05 418.9 10.6 39.519  1371252_at  suppressor of cytokine signaling 1  Info   Socs1 
   3333 1.95e-05 106.2 44 2.414  1370414_at  Rab38, member of RAS oncogene family  Info   Rab38 
   3334 1.95e-05 354.2 693.4 0.511  1371468_at  Transcribed locus, strongly similar to NP_001003955.1 RAB11 family interacting protein 5 (class I) isoform 1 [Mus musculus]  Info    
   3335 1.95e-05 186.1 98.7 1.886  1373679_at  Transcribed locus  Info    
   3336 1.96e-05 801.7 1458.7 0.55  1392888_at  similar to glypican 4  Info   RGD:1359399 
   3337 1.96e-05 96.6 33.4 2.892  1373782_a_at  similar to LOC495800 protein  Info   LOC499770 
   3338 1.96e-05 161.6 92.3 1.751  1374632_at  phosphatidylserine receptor (predicted)  Info   Ptdsr_predicted 
   3339 1.97e-05 99.5 212.8 0.468  1372403_at  similar to Nuclear membrane binding protein NUCLING  Info   LOC315732 
   3340 1.97e-05 3544.4 2567 1.381  1388244_s_at  laminin receptor 1 (67kD, ribosomal protein SA)  Info   Lamr1 
   3341 1.97e-05 283.8 197.7 1.436  1382139_at  similar to hypothetical protein FLJ12994 (predicted)  Info   RGD1308795_predicted 
   3342 1.97e-05 338.6 638.4 0.53  1386928_at  branched chain aminotransferase 2, mitochondrial  Info   Bcat2 
   3343 1.98e-05 89.4 26.9 3.323  1377058_at  similar to solute carrier family 37 (glycerol-3-phosphate transporter), member 2  Info   LOC500973 
   3344 1.98e-05 79.1 138.1 0.573  1376809_at  similar to RIKEN cDNA 2410025L10  Info   LOC304572 
   3345 1.99e-05 143.4 8.8 16.295  1385086_at  budding uninhibited by benzimidazoles 1 homolog (S. cerevisiae) (predicted)  Info   Bub1_predicted 
   3346 1.99e-05 128.8 235.3 0.547  1370264_at  CPG2 protein  Info   RGD:621597 
   3347 1.99e-05 132.9 513.4 0.259  1369493_at  prolactin receptor  Info   Prlr 
   3348 1.99e-05 2287.9 3438.2 0.665  1370865_at  isocitrate dehydrogenase 3, gamma  Info   Idh3g 
   3349 2e-05 4247.2 2714.1 1.565  1367456_at  ubiquitin-conjugating enzyme E2D 3 (UBC4/5 homolog, yeast)  Info   Ube2d3 
   3350 2e-05 304.1 158.2 1.922  1390268_at  RAB35, member RAS oncogene family (predicted) /// similar to RAB35, member RAS oncogene family  Info   Rab35_predicted /// LOC498191 
   3351 2e-05 362.3 697.8 0.519  1379525_at  similar to chromosome 20 open reading frame 155 (predicted)  Info   RGD1311037_predicted 
   3352 2.01e-05 331.6 82 4.044  1383542_at  RAS guanyl releasing protein 1  Info   Rasgrp1 
   3353 2.01e-05 2168.7 4403.3 0.493  1369954_at  isocitrate dehydrogenase 1 (NADP+), soluble  Info   Idh1 
   3354 2.02e-05 23.6 53.2 0.444  1370851_a_at  kalirin, RhoGEF kinase  Info   Kalrn 
   3355 2.02e-05 162.9 443.6 0.367  1369654_at  protein kinase, AMP-activated, alpha 2 catalytic subunit  Info   Prkaa2 
   3356 2.02e-05 228.2 113.2 2.016  1372569_at  four and a half LIM domains 3 (predicted)  Info   Fhl3_predicted 
   3357 2.02e-05 163.9 88.4 1.854  1376065_at  RRS1 ribosome biogenesis regulator homolog (S. cerevisiae) (predicted)  Info    
   3358 2.02e-05 3999.2 2808.3 1.424  1371435_at  nascent-polypeptide-associated complex alpha polypeptide (predicted)  Info   Naca_predicted 
   3359 2.03e-05 137 31.6 4.335  1372518_at  fibulin 1 (predicted)  Info   Fbln1_predicted 
   3360 2.03e-05 205.4 291.6 0.704  1375867_at  zinc finger and BTB domain containing 4 (predicted)  Info   Zbtb4_predicted 
   3361 2.03e-05 273 420.2 0.65  1371479_at  LOC500939  Info   LOC500939 
   3362 2.03e-05 286 11.1 25.766  1382663_at  basic leucine zipper transcription factor, ATF-like (predicted)  Info   Batf_predicted 
   3363 2.03e-05 317.6 203.2 1.563  1392464_at    Info    
   3364 2.03e-05 263.7 161.4 1.634  1382396_at  Similar to D7Wsu128e protein (predicted)  Info    
   3365 2.03e-05 141.6 14.1 10.043  1390674_at  Transcribed locus  Info    
   3366 2.03e-05 280.9 167.7 1.675  1379278_at  similar to hypothetical protein FLJ10871 (predicted)  Info   RGD1311539_predicted 
   3367 2.03e-05 152.6 85.8 1.779  1374182_at  SWI/SNF related, matrix associated, actin dependent regulator of chromatin, subfamily c, member 1 (predicted)  Info   Smarcc1_predicted 
   3368 2.03e-05 99.4 148.2 0.671  1373952_at  protein kinase, AMP-activated, gamma 2 non-catalytic subunit  Info   Prkag2 
   3369 2.04e-05 921.1 1429.4 0.644  1388926_at  Ectonucleotide pyrophosphatase/phosphodiesterase 5  Info   Enpp5 
   3370 2.04e-05 43 98.7 0.436  1374791_at  Similar to sorting nexin 21 isoform a; sorting nexin L; sorting nexin 21 (predicted)  Info    
   3371 2.04e-05 136.8 60.4 2.265  1397328_at  Transcribed locus  Info    
   3372 2.04e-05 353.9 160.2 2.209  1370899_at  NonO/p54nrb homolog  Info   Sfpq 
   3373 2.05e-05 242.2 106.3 2.278  1374550_at  similar to map kinase interacting kinase  Info   MGC112775 
   3374 2.05e-05 25.5 4.5 5.667  1369105_a_at  protein kinase (cAMP dependent, catalytic) inhibitor beta  Info   Pkib 
   3375 2.06e-05 2071 3237 0.64  1370207_at  ATP synthase, H+ transporting, mitochondrial F0 complex, subunit c (subunit 9), isoform 2 /// similar to ATP synthase, H+ transporting, mitochondrial F0 complex, subunit c (subunit 9), isoform 2  Info   Atp5g2 /// LOC499512 
   3376 2.06e-05 5064.1 4205.8 1.204  1398759_at  transforming growth factor beta 1 induced transcript 4  Info   Tgfb1i4 
   3377 2.06e-05 186.1 93.6 1.988  1390434_at  TNFRSF1A-associated via death domain  Info   Tradd 
   3378 2.06e-05 256.1 114.4 2.239  1389123_at  chemokine (C-C motif) ligand 6  Info   RGD:1303200 
   3379 2.06e-05 166.9 97.2 1.717  1368588_at  ATP-dependent, RNA helicase  Info   Ddx52 
   3380 2.06e-05 270.7 180 1.504  1388801_at  Similar to RIKEN cDNA 9030221M09 gene (predicted)  Info    
   3381 2.07e-05 1482.1 2703.4 0.548  1374420_at  similar to RIKEN cDNA 2310001A20 (predicted)  Info   RGD1308874_predicted 
   3382 2.07e-05 2151.3 1690 1.273  1388378_at  eukaryotic translation initiation factor 3, subunit 8, 110kDa (predicted)  Info   Eif3s8_predicted 
   3383 2.07e-05 448.4 265.5 1.689  1372764_at  similar to density regulated protein drp1  Info   LOC498180 
   3384 2.08e-05 312.9 149.6 2.092  1369409_at  Ngfi-A binding protein 1  Info   Nab1 
   3385 2.08e-05 253.9 691.4 0.367  1376691_at  Transcribed locus  Info    
   3386 2.08e-05 159 319.5 0.498  1373378_at  ATP/GTP binding protein 1 (predicted)  Info   Agtpbp1_predicted 
   3387 2.09e-05 133.9 65.3 2.051  1397852_at  similar to SPT3-associated factor 42 (predicted)  Info   RGD1306774_predicted 
   3388 2.09e-05 239.5 128.3 1.867  1383514_s_at  NMDA receptor-regulated gene 1 (predicted)  Info   Narg1_predicted 
   3389 2.09e-05 124.7 262.9 0.474  1392563_at  Rho GTPase activating protein 8 (predicted)  Info   Arhgap8_predicted 
   3390 2.09e-05 3732.4 5568 0.67  1388327_at  similar to DNA segment, Chr 10, ERATO Doi 214, expressed (predicted)  Info   RGD1309529_predicted 
   3391 2.1e-05 38.4 77.5 0.495  1377878_at  Transcribed locus  Info    
   3392 2.1e-05 307.6 585.1 0.526  1390406_at    Info    
   3393 2.1e-05 696.6 1049.5 0.664  1371549_at  WD repeat domain 23 (predicted)  Info   Wdr23_predicted 
   3394 2.11e-05 86.1 14.2 6.063  1370499_at  killer cell lectin-like receptor subfamily B member 1A  Info   Klrb1a 
   3395 2.11e-05 269.7 96.5 2.795  1373703_at  Transcribed locus  Info    
   3396 2.11e-05 35.8 131.6 0.272  1393060_at  similar to KIAA0605 gene product (predicted)  Info   RGD1305459_predicted 
   3397 2.11e-05 72.9 15.6 4.673  1384449_at    Info    
   3398 2.12e-05 1663.6 2329.4 0.714  1375516_at  NADH dehydrogenase (ubiquinone) 1, subcomplex unknown, 2 (predicted)  Info   Ndufc2_predicted 
   3399 2.12e-05 692 380.7 1.818  1388150_at  exportin 1, CRM1 homolog (yeast)  Info   Xpo1 
   3400 2.13e-05 245.2 395.3 0.62  1368390_at  v-raf oncogene homolog 1 (murine sarcoma 3611 virus)  Info   Araf1 
   3401 2.13e-05 141.8 255.6 0.555  1394407_at  Transcribed locus  Info    
   3402 2.13e-05 85.7 126 0.68  1376277_at  Transcribed locus  Info    
   3403 2.13e-05 105.9 348.6 0.304  1387010_s_at  sodium channel, voltage-gated, type I, beta polypeptide  Info   Scn1b 
   3404 2.14e-05 45.7 18.9 2.418  1393405_at  Transcribed locus  Info    
   3405 2.14e-05 543.1 937 0.58  1399085_at  Zinc metalloproteinase, STE24 homolog (S. cerevisiae) (predicted)  Info    
   3406 2.14e-05 156 303.1 0.515  1375315_at  similar to hypothetical protein BC013035  Info   LOC361172 
   3407 2.14e-05 204.6 99.3 2.06  1390728_at    Info    
   3408 2.15e-05 1063.5 1485.7 0.716  1398844_at  thioredoxin 2  Info   Txn2 
   3409 2.15e-05 43.3 106.9 0.405  1383194_a_at  CDNA clone IMAGE:7379585  Info    
   3410 2.15e-05 168.9 61.7 2.737  1370419_a_at  SH3-domain kinase binding protein 1  Info   Sh3kbp1 
   3411 2.15e-05 164.1 94.5 1.737  1374804_at  similar to Autoantigen NGP-1 (predicted)  Info   RGD1305006_predicted 
   3412 2.15e-05 157.3 319.3 0.493  1382131_at  Transcribed locus  Info    
   3413 2.16e-05 122.7 268.5 0.457  1374738_at  serologically defined colon cancer antigen 10 (predicted)  Info   Sdccag10_predicted 
   3414 2.16e-05 3628.5 2661.4 1.363  1387361_s_at  phosphoglycerate kinase 1  Info   Pgk1 
   3415 2.16e-05 1094.2 1672.1 0.654  1389288_at  NADH dehydrogenase (ubiquinone) 1 alpha subcomplex, 2 (predicted)  Info   Ndufa2_predicted 
   3416 2.17e-05 98.8 36.7 2.692  1395336_at  similar to RIKEN cDNA 2810022L02  Info   LOC501207 
   3417 2.17e-05 894.1 1195.2 0.748  1368215_at  ceroid-lipofuscinosis, neuronal 2  Info   Cln2 
   3418 2.17e-05 219.4 459.7 0.477  1378536_at  Transcribed locus  Info    
   3419 2.17e-05 2.6 23.1 0.113  1384910_at  Similar to putative voltage-gated calcium channel alpha(2)delta-4 subunit  Info    
   3420 2.17e-05 376.4 189.7 1.984  1397313_at  CDNA clone MGC:93828 IMAGE:7110880  Info    
   3421 2.17e-05 63.2 111.9 0.565  1394056_at  CDNA clone MGC:124727 IMAGE:7936966  Info    
   3422 2.18e-05 83.9 142 0.591  1387407_at  nucleosome assembly protein 1-like 3  Info   Nap1l3 
   3423 2.18e-05 96 224.1 0.428  1382780_at  Transcribed locus  Info    
   3424 2.18e-05 274.4 489.1 0.561  1371202_a_at  nuclear factor I/B  Info   Nfib 
   3425 2.18e-05 459.3 732.3 0.627  1394737_at  similar to CG9752-PA (predicted)  Info   RGD1311345_predicted 
   3426 2.18e-05 182.6 254.4 0.718  1383715_at  similar to hypothetical protein D4Ertd89e (predicted)  Info   RGD1305703_predicted 
   3427 2.19e-05 110.7 228.5 0.484  1386466_at  Transcribed locus  Info    
   3428 2.19e-05 101.6 32.1 3.165  1383001_at  Transcribed locus  Info    
   3429 2.19e-05 320.1 535.2 0.598  1389015_at  similar to FUN14 domain containing 2  Info   LOC361288 
   3430 2.21e-05 1828.4 2603.1 0.702  1388414_at  NADH dehydrogenase (ubiquinone) Fe-S protein 5b, 15kDa (NADH-coenzyme Q reductase) (predicted)  Info   Ndufs5b_predicted 
   3431 2.21e-05 346.5 502.7 0.689  1376161_at  similar to RIKEN cDNA 1110014L17 (predicted)  Info   RGD1306319_predicted 
   3432 2.21e-05 1871.6 4421.1 0.423  1370397_at  cytochrome P450, family 4, subfamily a, polypeptide 14  Info   Cyp4a14 
   3433 2.21e-05 58.9 115.8 0.509  1374083_at  Transcribed locus  Info    
   3434 2.21e-05 496.1 228.6 2.17  1381461_at  Transcribed locus  Info    
   3435 2.22e-05 158.8 596.8 0.266  1384302_at  solute carrier family 6 (neurotransmitter transporter), member 17  Info   Slc6a17 
   3436 2.22e-05 276.2 522.1 0.529  1390717_at  similar to chromosome 20 open reading frame 155 (predicted)  Info   RGD1311037_predicted 
   3437 2.23e-05 317.8 233.7 1.36  1374611_at    Info    
   3438 2.23e-05 231.1 96.9 2.385  1397916_s_at  protein tyrosine phosphatase, non-receptor type 1  Info   Ptpn1 
   3439 2.23e-05 278.3 145.5 1.913  1389238_at  similar to PES1 protein  Info   LOC289740 
   3440 2.23e-05 27.6 67.4 0.409  1374315_at  Transcribed locus  Info    
   3441 2.23e-05 2037.4 1396.9 1.459  1386857_at  stathmin 1  Info   Stmn1 
   3442 2.24e-05 549.6 372.7 1.475  1374411_at  Mitochondrial ribosomal protein L52 (predicted)  Info    
   3443 2.24e-05 511.2 326.2 1.567  1374418_at  similar to CG8009-PA (predicted)  Info   RGD1308816_predicted 
   3444 2.24e-05 229.8 8.9 25.82  1369497_at  Robo-1  Info   LOC24906 
   3445 2.24e-05 2816.9 3839.5 0.734  1367609_at  macrophage migration inhibitory factor  Info   Mif 
   3446 2.25e-05 93.6 143.5 0.652  1390977_at  suppression of tumorigenicity 7-like  Info   RGD:1359336 
   3447 2.25e-05 261.6 469.1 0.558  1373515_at  Similar to RIKEN cDNA 1110067D22 (predicted)  Info    
   3448 2.25e-05 28.9 2.1 13.762  1391328_at    Info    
   3449 2.25e-05 29.4 82.5 0.356  1380365_at  Transcribed locus  Info    
   3450 2.25e-05 1281.9 1763.3 0.727  1370785_s_at  translocase of outer mitochondrial membrane 20 homolog (yeast)  Info   Tomm20 
   3451 2.26e-05 514.9 1270.8 0.405  1382127_at    Info    
   3452 2.26e-05 39.4 3.2 12.312  1371155_at  killer cell lectin-like receptor subfamily C, member 1  Info   Klrc1 
   3453 2.26e-05 195.3 91 2.146  1398608_at  similar to hypothetical gene supported by BC007071 (predicted)  Info   RGD1311868_predicted 
   3454 2.26e-05 169.6 6.1 27.803  1398449_at  Transcribed locus  Info    
   3455 2.27e-05 172.9 100 1.729  1368231_at  signal transducer and activator of transcription 5A  Info   Stat5a 
   3456 2.27e-05 2204.7 1781.6 1.237  1399057_at  mortality factor 4 like 1 (predicted)  Info   Morf4l1_predicted 
   3457 2.27e-05 120.8 29.8 4.054  1376026_at  downstream neighbor of SON (predicted)  Info   Donson_predicted 
   3458 2.27e-05 95 214.7 0.442  1371354_at  Troponin C, cardiac/slow skeletal (predicted)  Info    
   3459 2.28e-05 23.9 129.2 0.185  1396127_at    Info    
   3460 2.28e-05 133.9 63 2.125  1374629_at  mediator of RNA polymerase II transcription, subunit 8 homolog (yeast) (predicted)  Info   Med8_predicted 
   3461 2.28e-05 290.2 1201.1 0.242  1367652_at  insulin-like growth factor binding protein 3  Info   Igfbp3 
   3462 2.29e-05 1651.3 527.8 3.129  1378679_at  Transcribed locus  Info    
   3463 2.29e-05 140.3 41.3 3.397  1387879_a_at  CUG triplet repeat,RNA-binding protein 2  Info   Cugbp2 
   3464 2.29e-05 196.9 127.9 1.539  1391252_at  ring finger protein 41 (predicted)  Info   Rnf41_predicted 
   3465 2.29e-05 128.2 318.6 0.402  1383416_at  Acetyl-coenzyme A acetyltransferase 1  Info   Acat1 
   3466 2.29e-05 357.6 681.2 0.525  1373624_at  Transcribed locus  Info    
   3467 2.29e-05 488.6 333 1.467  1379322_at  proteasome (prosome, macropain) 26S subunit, non-ATPase, 7 (predicted)  Info   Psmd7_predicted 
   3468 2.3e-05 69.7 34.6 2.014  1385814_at  mitogen activated protein kinase kinase kinase 3 (predicted)  Info   Map3k3_predicted 
   3469 2.3e-05 187.5 123.3 1.521  1372386_at  SUMO/sentrin specific protease 2  Info   Senp2 
   3470 2.3e-05 80.5 174.6 0.461  1379431_at  Transcribed locus  Info    
   3471 2.3e-05 46.8 114.4 0.409  1376870_at  zinc finger protein 278 (predicted)  Info   Znf278_predicted 
   3472 2.3e-05 379.2 12.4 30.581  1387472_at  CD3 antigen delta polypeptide  Info   Cd3d 
   3473 2.32e-05 28.4 65.6 0.433  1377808_at  Similar to IQ motif and WD repeats 1  Info    
   3474 2.32e-05 369.5 229.6 1.609  1388739_at  similar to chromosome 14 open reading frame 130  Info   RGD:1359144 
   3475 2.32e-05 168.2 76.2 2.207  1384242_at  Neurolysin (metallopeptidase M3 family)  Info   Nln 
   3476 2.32e-05 175 336 0.521  1375785_at  Similar to hypothetical protein D4Ertd89e (predicted)  Info    
   3477 2.32e-05 425.2 254.4 1.671  1389617_at    Info    
   3478 2.32e-05 19 46.6 0.408  1374425_at  transducin-like enhancer of split 1, homolog of Drosophila E(spl) (predicted)  Info   Tle1_predicted 
   3479 2.32e-05 215.2 110.2 1.953  1390290_at  surfeit gene 6 (predicted)  Info   Surf6_predicted 
   3480 2.32e-05 295.6 135.4 2.183  1382431_at  Hypothetical gene supported by NM_178095  Info   Abca1 
   3481 2.32e-05 787.1 2002.7 0.393  1388814_at  DEAD (Asp-Glu-Ala-Asp) box polypeptide 19  Info   Ddx19 
   3482 2.33e-05 4840.6 3540.3 1.367  1388481_at  Ribosomal protein S28  Info   Rps28 
   3483 2.33e-05 2.5 45.9 0.054  1367951_at  phosphoglycerate mutase 2  Info   Pgam2 
   3484 2.33e-05 895.9 491.6 1.822  1372829_at  RNA binding motif protein 8 (predicted)  Info   Rbm8_predicted 
   3485 2.33e-05 375 657.1 0.571  1388918_at  High density lipoprotein binding protein  Info   Hdlbp 
   3486 2.34e-05 215.6 468.4 0.46  1392203_at  homeo box B2 (predicted)  Info   Hoxb2_predicted 
   3487 2.35e-05 78.5 189.6 0.414  1381937_at  similar to 60S ribosomal protein L7a  Info   LOC499344 
   3488 2.35e-05 154 437.6 0.352  1397830_at  Insulin-like growth factor binding protein 5  Info   Igfbp5 
   3489 2.35e-05 280.1 508.4 0.551  1387926_at  sterol-C5-desaturase (fungal ERG3, delta-5-desaturase) homolog (S. cerevisae)  Info   Sc5d 
   3490 2.36e-05 13 84.2 0.154  1386045_at  Transcribed locus  Info    
   3491 2.36e-05 58.9 2.2 26.773  1397609_at  TRAF4 associated factor 1  Info   RGD:1303240 
   3492 2.36e-05 417.7 646.3 0.646  1388626_at  similar to chromosome 20 open reading frame 116 (predicted)  Info   RGD1309979_predicted 
   3493 2.36e-05 88.6 11.7 7.573  1387763_at  low Mr GTP-binding protein  Info   Rab27a 
   3494 2.36e-05 111 28.6 3.881  1380450_at  benzodiazapine receptor associated protein 1  Info   Bzrap1 
   3495 2.36e-05 259.9 435 0.597  1385805_at    Info    
   3496 2.36e-05 1.9 65.6 0.029  1384385_at  Similar to solute carrier family 7 (cationic amino acid transporter, y+ system), member 12  Info    
   3497 2.37e-05 181.9 366 0.497  1372509_at  Transcribed locus  Info    
   3498 2.38e-05 739.6 308.3 2.399  1376268_at  ADP-ribosylation factor 6  Info   Arf6 
   3499 2.38e-05 65.7 26.7 2.461  1393817_at  Transcribed locus  Info    
   3500 2.38e-05 214.1 397.8 0.538  1373074_at  similar to RIKEN cDNA 2700002I20 (predicted)  Info   RGD1307279_predicted 
   3501 2.38e-05 76.1 263.3 0.289  1376239_at  similar to RIKEN cDNA 1110038G14  Info   RGD:1359430 
   3502 2.39e-05 4285.5 3085.9 1.389  1398749_at  ribosomal protein L4  Info   Rpl4 
   3503 2.39e-05 557.7 976.4 0.571  1398598_at  dystonin (predicted)  Info   Dst_predicted 
   3504 2.39e-05 203.4 138.4 1.47  1393091_at  similar to RIKEN cDNA D130064H19 (predicted)  Info   RGD1311241_predicted 
   3505 2.39e-05 191.9 135 1.421  1392984_at  copine III (predicted)  Info   Cpne3_predicted 
   3506 2.39e-05 200.2 366.6 0.546  1389522_at  Transcribed locus  Info    
   3507 2.39e-05 354.6 593.1 0.598  1395331_at  similar to hypothetical protein CL25084 (predicted)  Info   RGD1306508_predicted 
   3508 2.4e-05 4544.7 3269.5 1.39  1398760_at  ribosomal protein L35a  Info   Rpl35a 
   3509 2.4e-05 248.8 101.3 2.456  1370542_a_at  E74-like factor 1  Info   Elf1 
   3510 2.4e-05 223.9 412 0.543  1383376_at  similar to Williams-Beuren syndrome critical region protein 18  Info   LOC368190 
   3511 2.41e-05 286.3 430.5 0.665  1374129_at  similar to product is unknown~seizure-related gene  Info   LOC501454 
   3512 2.41e-05 99.6 48.4 2.058  1387054_at  ATP-binding cassette, sub-family G (WHITE), member 1  Info   Abcg1 
   3513 2.41e-05 226.2 375.5 0.602  1383714_at  Transcribed locus  Info    
   3514 2.41e-05 388.1 216.6 1.792  1375882_at  protein phosphatase 3, catalytic subunit, beta isoform  Info   Ppp3cb 
   3515 2.41e-05 198.8 132.1 1.505  1371987_at  polymerase (DNA directed) sigma (predicted)  Info   Pols_predicted 
   3516 2.42e-05 451.7 771.3 0.586  1383224_at  par-6 (partitioning defective 6) homolog beta (C. elegans) (predicted)  Info   Pard6b_predicted 
   3517 2.43e-05 286.3 508.1 0.563  1372232_at  WW domain binding protein 1 (predicted)  Info   Wbp1_predicted 
   3518 2.43e-05 925.7 530.2 1.746  1389454_at  programmed cell death 5 (predicted)  Info   Pdcd5_predicted 
   3519 2.43e-05 64.4 121.1 0.532  1392043_at  Transcribed locus  Info    
   3520 2.43e-05 1599.3 1187.8 1.346  1388550_at  similar to MHR23B  Info   LOC298012 
   3521 2.43e-05 242.3 411 0.59  1372617_at  similar to BTB (POZ) domain containing 2  Info   LOC500793 
   3522 2.43e-05 138.9 76.8 1.809  1379987_at  similar to RIKEN cDNA 6330416G13 gene (predicted)  Info   RGD1304595_predicted 
   3523 2.44e-05 25.2 157.5 0.16  1379467_at    Info    
   3524 2.44e-05 141.3 100.5 1.406  1377480_at  similar to RIKEN cDNA D030060M11 (predicted)  Info   RGD1308076_predicted 
   3525 2.45e-05 128.7 308.3 0.417  1367689_a_at  cd36 antigen /// similar to fatty acid translocase/CD36  Info   Cd36 /// LOC499984 
   3526 2.45e-05 430.8 143.4 3.004  1387914_at  cytochrome P450, family 27, subfamily a, polypeptide 1  Info   Cyp27a1 
   3527 2.45e-05 31 74.6 0.416  1382476_x_at  Similar to pellino 3 alpha (predicted)  Info    
   3528 2.46e-05 20.9 6.5 3.215  1370629_at  ADP-ribosyltransferase 2b  Info   Art2b 
   3529 2.46e-05 93.2 153 0.609  1381983_at    Info    
   3530 2.46e-05 56.1 17.2 3.262  1382792_at  Transcribed locus  Info    
   3531 2.48e-05 43.4 1.7 25.529  1397507_at  amyloid beta (A4) precursor protein-binding, family B, member 1 interacting protein (predicted)  Info   Apbb1ip_predicted 
   3532 2.49e-05 1371.4 438.2 3.13  1372182_at  phosphofructokinase, platelet  Info   Pfkp 
   3533 2.49e-05 139.5 49 2.847  1393162_at  solute carrier family 39 (metal ion transporter), member 6 (predicted)  Info   Slc39a6_predicted 
   3534 2.49e-05 5802.5 3791.7 1.53  1369966_a_at  ribosomal protein S24  Info   Rps24 
   3535 2.5e-05 231.8 443.5 0.523  1390923_a_at  oxysterol binding protein-like 1A  Info   Osbpl1a 
   3536 2.51e-05 182.6 99.8 1.83  1376206_at  Similar to RIKEN cDNA 9030624J02  Info    
   3537 2.51e-05 68.3 120.9 0.565  1382049_at  similar to thymus atrophy-related protein (predicted)  Info   RGD1304607_predicted 
   3538 2.51e-05 661.3 1221.7 0.541  1383633_at  Transcribed locus  Info    
   3539 2.52e-05 632.7 1772 0.357  1387783_a_at  acetyl-Coenzyme A acyltransferase 1 (peroxisomal 3-oxoacyl-Coenzyme A thiolase)  Info   Acaa1 
   3540 2.52e-05 628.2 429.7 1.462  1389559_at  similar to Hypothetical protein MGC31278  Info   LOC362919 
   3541 2.53e-05 105.9 43.1 2.457  1392134_x_at  similar to KIAA0456 protein  Info   LOC360840 
   3542 2.53e-05 45.9 141 0.326  1393992_at  homeo box A7  Info   Hoxa7 
   3543 2.53e-05 917.9 3269.1 0.281  1383111_at  2-amino-3-carboxymuconate-6-semialdehyde decarboxylase  Info   Acmsd 
   3544 2.53e-05 132 214.2 0.616  1385088_at  hypothetical LOC304650 (predicted)  Info   RGD1310262_predicted 
   3545 2.55e-05 80.9 30.9 2.618  1382746_s_at  Similar to lymphocyte alpha-kinase (predicted)  Info    
   3546 2.55e-05 243.2 103.3 2.354  1393240_at  EGF-containing fibulin-like extracellular matrix protein 2  Info   RGD:1359496 
   3547 2.55e-05 210.7 122 1.727  1372987_at  Transcribed locus, strongly similar to XP_233741.1 PREDICTED: similar to Non-receptor tyrosine-protein kinase TYK2 [Rattus norvegicus]  Info    
   3548 2.55e-05 216.3 443.2 0.488  1367864_at  phosphofructokinase, muscle  Info   Pfkm 
   3549 2.55e-05 494 1241.8 0.398  1382293_at    Info    
   3550 2.56e-05 135.5 66.8 2.028  1395587_at  Similar to Alkaline phytoceramidase (aPHC) (Alkaline ceramidase)  Info    
   3551 2.56e-05 413.7 271.8 1.522  1372357_at  Transcribed locus  Info    
   3552 2.56e-05 82.1 168.3 0.488  1389650_at  Similar to zinc finger protein 366 (predicted)  Info    
   3553 2.56e-05 113.7 44 2.584  1370393_at  coiled-coil domain containing 5  Info   Ccdc5 
   3554 2.56e-05 140 267.3 0.524  1398491_at  Transcribed locus, strongly similar to NP_598870.1 immunoglobulin-like domain containing receptor 1 [Mus musculus]  Info    
   3555 2.56e-05 67.5 16.1 4.193  1380037_at  Hypothetical LOC292764  Info   MGC94040 
   3556 2.57e-05 83.4 606.4 0.138  1378013_at  hypothetical LOC361199  Info   LOC361199 
   3557 2.58e-05 841.8 1372.4 0.613  1398780_at  Rab acceptor 1 (prenylated)  Info   Rabac1 
   3558 2.58e-05 192.8 22.5 8.569  1387413_at  neutrophil cytosolic factor 1  Info   Ncf1 
   3559 2.59e-05 2.4 20 0.12  1375491_at    Info    
   3560 2.59e-05 94.5 39 2.423  1396521_at  StAR-related lipid transfer (START) domain containing 4 (predicted)  Info   Stard4_predicted 
   3561 2.59e-05 163.5 312.5 0.523  1374885_at  Solute carrier family 12, member 6 (predicted)  Info    
   3562 2.59e-05 3873.2 6035.9 0.642  1387223_at  aminoadipate aminotransferase  Info   Aadat 
   3563 2.59e-05 62.2 12.3 5.057  1391946_at  selectin, platelet  Info   Selp 
   3564 2.59e-05 578.3 1496 0.387  1387737_at  methionine adenosyltransferase II, alpha  Info   Mat2a 
   3565 2.59e-05 423.3 643.6 0.658  1389386_at  similar to hypothetical protein DKFZp313N0621  Info   LOC363169 
   3566 2.59e-05 171.2 99.1 1.728  1385187_at  similar to hypothetical protein MGC38524 (predicted)  Info   RGD1309002_predicted 
   3567 2.6e-05 921 560.3 1.644  1370141_at  myeloid cell leukemia sequence 1  Info   Mcl1 
   3568 2.61e-05 152.2 257.1 0.592  1398448_at  Thymic stromal cotransporter (predicted)  Info    
   3569 2.61e-05 39.7 16.6 2.392  1376218_a_at  Atpase, class I, type 8B, member 2 (predicted)  Info    
   3570 2.61e-05 393.5 532.4 0.739  1372538_at  solute carrier family 25 (mitochondrial carrier, peroxisomal membrane protein), member 17 (predicted)  Info   Slc25a17_predicted 
   3571 2.62e-05 276.9 521.3 0.531  1387905_at  J domain protein 1  Info   LOC619393 
   3572 2.62e-05 73.3 177.6 0.413  1370399_at  cytochrome P450, family 4, subfamily b, polypeptide 1  Info   Cyp4b1 
   3573 2.62e-05 71.5 134.4 0.532  1377523_at  Transcribed locus  Info    
   3574 2.62e-05 194.9 129.7 1.503  1390967_a_at  Similar to RIKEN cDNA 2410006F12 (predicted)  Info    
   3575 2.63e-05 599.9 998.1 0.601  1373826_at    Info    
   3576 2.63e-05 3831.6 4629.9 0.828  1370861_at  Cytochrome c oxidase, subunit VIa, polypeptide 1  Info   Cox6a1 
   3577 2.63e-05 324.5 473.2 0.686  1372566_at  mitochondrial carrier homolog 2 (C. elegans) (predicted)  Info   Mtch2_predicted 
   3578 2.64e-05 134.7 44.2 3.048  1391450_at  lysyl oxidase-like 2 (predicted)  Info   Loxl2_predicted 
   3579 2.64e-05 492.6 920 0.535  1371976_at  similar to RIKEN cDNA 9530058B02  Info   LOC497875 
   3580 2.64e-05 392.5 618.8 0.634  1372740_at  similar to Map4k6-pending protein  Info   RGD:1359135 
   3581 2.64e-05 6.1 47.8 0.128  1385250_at  chromodomain helicase DNA binding protein 6 (predicted)  Info   Chd6_predicted 
   3582 2.65e-05 132.2 74 1.786  1396755_at  Transcribed locus  Info    
   3583 2.65e-05 635.2 417 1.523  1388815_at  similar to B430201G11Rik protein (predicted)  Info   RGD1311409_predicted 
   3584 2.65e-05 62.5 115.8 0.54  1376590_at  Transcribed locus  Info    
   3585 2.65e-05 366.5 162 2.262  1389202_at    Info    
   3586 2.65e-05 279.4 185.5 1.506  1390802_at  similar to RIKEN cDNA 5033414D02 (predicted)  Info   RGD1306839_predicted 
   3587 2.65e-05 533.8 1042.3 0.512  1372138_at  Cut-like 1 (Drosophila)  Info   Cutl1 
   3588 2.65e-05 206.2 501.4 0.411  1372794_at  death-associated kinase 2 (predicted)  Info   Dapk2_predicted 
   3589 2.66e-05 368.2 238.8 1.542  1387732_at  mitochondrial transcription termination factor 1  Info   Mterf 
   3590 2.67e-05 144.3 339.7 0.425  1386718_at  similar to aldo-keto reductase family 1, member C12  Info   LOC307096 
   3591 2.67e-05 2312 1581.4 1.462  1399027_at  ras homolog gene family, member A  Info   RGD:619921 
   3592 2.68e-05 59.6 112.8 0.528  1386293_at    Info    
   3593 2.68e-05 180.4 504.2 0.358  1377037_at  similar to peroxisomal long chain acyl-CoA thioesterase Ia  Info   LOC314304 
   3594 2.68e-05 605.8 525.1 1.154  1368062_at  adaptor-related protein complex 3, mu 1 subunit  Info   Ap3m1 
   3595 2.69e-05 166.4 76 2.189  1389269_at  similar to RIKEN cDNA 4933433P14 gene (predicted)  Info   RGD1308470_predicted 
   3596 2.69e-05 39.1 110.6 0.354  1379960_at  sushi domain containing 3 (predicted)  Info   Susd3_predicted 
   3597 2.7e-05 421.8 158.7 2.658  1390287_at  bridging integrator 2 (predicted)  Info   Bin2_predicted 
   3598 2.7e-05 119.8 37.1 3.229  1374225_at  CDNA clone IMAGE:7389359  Info    
   3599 2.7e-05 740.9 503.6 1.471  1371587_at  mitogen-activated protein kinase kinase 1 interacting protein 1 (predicted)  Info   Map2k1ip1_predicted 
   3600 2.7e-05 115.4 389.3 0.296  1393421_at  peroxisomal membrane protein 4  Info   Pxmp4 
   3601 2.71e-05 3.8 67.3 0.056  1378328_at  Transcribed locus  Info    
   3602 2.71e-05 1019.8 2487 0.41  1381428_a_at  Histone 1, H2bh (predicted)  Info   Hfe 
   3603 2.72e-05 109.6 172.9 0.634  1390497_at  Transcribed locus  Info    
   3604 2.72e-05 191.3 97.2 1.968  1379581_at  Transcribed locus, weakly similar to XP_579465.1 PREDICTED: DNA binding protein N5 [Rattus norvegicus]  Info    
   3605 2.72e-05 402.4 270.2 1.489  1373375_at  Rab6 interacting protein 1 (predicted)  Info   Rab6ip1_predicted 
   3606 2.72e-05 100 293.8 0.34  1396113_at  similar to Dorz1 (predicted)  Info   RGD1309721_predicted 
   3607 2.72e-05 299.9 502.9 0.596  1388786_at  Transcribed locus  Info    
   3608 2.73e-05 153.5 207 0.742  1373996_at  Transcribed locus  Info    
   3609 2.73e-05 19.9 61.5 0.324  1383165_at  similar to KIAA1324 protein (predicted)  Info   RGD1310209_predicted 
   3610 2.73e-05 1708.8 1257.6 1.359  1367656_at  proteasome (prosome, macropain) subunit, beta type 7  Info   Psmb7 
   3611 2.74e-05 188.3 661.5 0.285  1376078_at  Transcribed locus  Info    
   3612 2.74e-05 279.8 143.4 1.951  1367803_at  nucleoporin 54  Info   Nup54 
   3613 2.74e-05 68 17.7 3.842  1384111_at  signal transducer and activator of transcription 4 (predicted)  Info   Stat4_predicted 
   3614 2.74e-05 747.6 1277.7 0.585  1398902_at  Similar to mKIAA0664 protein (predicted)  Info    
   3615 2.74e-05 188.5 61.7 3.055  1393776_at  Similar to lymphocyte antigen 6 complex, locus E  Info    
   3616 2.75e-05 117 266.1 0.44  1396317_at  cell cycle progression 1 (predicted)  Info   Ccpg1_predicted 
   3617 2.75e-05 108.5 308.4 0.352  1369113_at  gremlin 1 homolog, cysteine knot superfamily (Xenopus laevis)  Info   Grem1 
   3618 2.75e-05 6.6 24.3 0.272  1376829_at  Transcribed locus  Info    
   3619 2.75e-05 337.6 227 1.487  1389634_at  methionyl aminopeptidase 1 (predicted)  Info   Metap1_predicted 
   3620 2.75e-05 83.9 534.6 0.157  1372911_at  Transcribed locus, moderately similar to XP_580018.1 PREDICTED: hypothetical protein XP_580018 [Rattus norvegicus]  Info    
   3621 2.75e-05 277.4 153.2 1.811  1382007_at  SH3-domain GRB2-like B1 (endophilin) (predicted)  Info   Sh3glb1_predicted 
   3622 2.75e-05 176.5 84.7 2.084  1385190_at  Transcribed locus  Info    
   3623 2.75e-05 324.4 199.1 1.629  1377630_at  transmembrane 4 superfamily member 13 (predicted) /// similar to transmembrane 4 superfamily member 13  Info   Tm4sf13_predicted /// LOC500643 
   3624 2.76e-05 503.8 1031.7 0.488  1371886_at  carnitine acetyltransferase  Info   RGD:1303031 
   3625 2.76e-05 116.2 45.6 2.548  1377052_at  Tripartite motif protein 26  Info   Trim26 
   3626 2.76e-05 90.6 296.6 0.305  1370329_at  cytochrome P450, family 2, subfamily d, polypeptide 22  Info   Cyp2d22 
   3627 2.76e-05 23.1 46.5 0.497  1368907_at  secretory carrier membrane protein 5  Info   Scamp5 
   3628 2.76e-05 137.2 62.8 2.185  1391649_a_at  Transcribed locus  Info    
   3629 2.77e-05 1330.8 2710.9 0.491  1371332_at  Histone 1, H4a (predicted)  Info    
   3630 2.78e-05 289.1 467.5 0.618  1389640_at  Similar to SH3 domain binding glutamic acid-rich protein like 2  Info    
   3631 2.78e-05 289.2 164.9 1.754  1395146_at  adiponectin receptor 2 (predicted)  Info   Adipor2_predicted 
   3632 2.79e-05 121.4 219.8 0.552  1389418_at    Info    
   3633 2.8e-05 383.3 193.9 1.977  1369743_a_at  purinergic receptor P2X, ligand-gated ion channel 4  Info   P2rx4 
   3634 2.8e-05 141.1 294.7 0.479  1382265_at  similar to KIAA1128 protein (predicted)  Info   RGD1304626_predicted 
   3635 2.8e-05 376.2 185.9 2.024  1368813_at  CCAAT/enhancer binding protein (C/EBP), delta  Info   Cebpd 
   3636 2.81e-05 5175.7 3809.1 1.359  1386864_at  phosphoglycerate mutase 1  Info   Pgam1 
   3637 2.81e-05 364.9 543.9 0.671  1372380_at  similar to RNA binding protein gene with multiple splicing  Info   LOC498642 
   3638 2.81e-05 310.2 43.4 7.147  1368965_at  monocarboxylate transporter  Info   Slc16a3 
   3639 2.81e-05 322.7 122.2 2.641  1371040_at  sodium-dependent neutral amino acid transporter ASCT2  Info   Slc1a5 
   3640 2.81e-05 14.7 3 4.9  1376902_x_at  Similar to Hypothetical protein 6330514E13  Info    
   3641 2.81e-05 1190.9 909.2 1.31  1373508_at  similar to Selenoprotein T precursor  Info   LOC499625 
   3642 2.82e-05 28.7 63.7 0.451  1391822_at  Transcribed locus  Info    
   3643 2.83e-05 390.5 2025.7 0.193  1368366_at  Camello-like 2  Info   Cml2 
   3644 2.84e-05 95.6 55.7 1.716  1379384_at  Sp1 transcription factor  Info   Sp1 
   3645 2.84e-05 888.9 314.8 2.824  1386926_at  acyl-CoA synthetase long-chain family member 5  Info   Acsl5 
   3646 2.84e-05 55.3 148.6 0.372  1374954_at  histone deacetylase 11 (predicted)  Info   Hdac11_predicted 
   3647 2.85e-05 343.7 13.5 25.459  1394122_at  ras homolog gene family, member H (predicted)  Info   Rhoh_predicted 
   3648 2.85e-05 39 17.1 2.281  1379313_at    Info    
   3649 2.86e-05 1466 975.3 1.503  1399043_at  capping protein (actin filament) muscle Z-line, alpha 2  Info   Capza2 
   3650 2.86e-05 180.4 92 1.961  1376219_at  Atpase, class I, type 8B, member 2 (predicted)  Info    
   3651 2.86e-05 189 322.9 0.585  1385334_at  Cytochrome P450, family 4, subfamily v, polypeptide 3 (predicted)  Info   Loc266761 
   3652 2.87e-05 298.2 169.6 1.758  1376703_at  nucleoporin 37 (predicted)  Info   Nup37_predicted 
   3653 2.87e-05 492.7 743.3 0.663  1370005_at  cytochrome b5, outer mitochondrial membrane isoform  Info   Cyb5m 
   3654 2.87e-05 917.2 314.8 2.914  1371924_at  olfactomedin-like 3 (predicted)  Info   Olfml3_predicted 
   3655 2.87e-05 127.5 223.5 0.57  1389274_at  similar to RIKEN cDNA 6720485C15  Info   RGD:1359211 
   3656 2.87e-05 73.9 140.3 0.527  1390463_at  Transcribed locus  Info    
   3657 2.88e-05 680 393.9 1.726  1375739_at  EH-domain containing 4  Info   Ehd4 
   3658 2.88e-05 46.2 101.6 0.455  1384243_at  Transcribed locus  Info    
   3659 2.89e-05 1137.8 594.8 1.913  1370880_at  ribonuclease/angiogenin inhibitor 1  Info   Rnh1 
   3660 2.89e-05 406.2 543.1 0.748  1382008_at  similar to hypothetical protein FLJ11218 (predicted)  Info   RGD1309804_predicted 
   3661 2.89e-05 798.4 336 2.376  1373432_at  myristoylated alanine rich protein kinase C substrate  Info   Marcks 
   3662 2.9e-05 183.6 103.4 1.776  1381364_at  transportin 1 (predicted)  Info   Tnpo1_predicted 
   3663 2.9e-05 67.3 124.7 0.54  1383928_a_at  Transcribed locus  Info    
   3664 2.9e-05 112 166.3 0.673  1374884_at  protein phosphatase 1D magnesium-dependent, delta isoform (predicted)  Info   Ppm1d_predicted 
   3665 2.91e-05 451.8 212.4 2.127  1383912_at  CDNA clone MGC:109201 IMAGE:7306744  Info    
   3666 2.91e-05 326.7 157.2 2.078  1373233_at  Transcribed locus  Info    
   3667 2.91e-05 250.8 107.1 2.342  1392289_at    Info    
   3668 2.93e-05 628.2 436.8 1.438  1387823_at  pleiotropic regulator 1 homolog (Arabidopsis)  Info   Plrg1 
   3669 2.94e-05 22 63.6 0.346  1380254_at  Transcribed locus, moderately similar to NP_079928.1 general transcription factor III A [Mus musculus]  Info    
   3670 2.94e-05 266.1 139.2 1.912  1374326_at  peter pan homolog (Drosophila) (predicted)  Info   Ppan_predicted 
   3671 2.95e-05 475.6 773.7 0.615  1373842_at  Type II keratin Kb1  Info   Kb1 
   3672 2.95e-05 29 96.5 0.301  1389704_at  Transcribed locus  Info    
   3673 2.95e-05 154.2 27.2 5.669  1382803_at  mitogen activated protein kinase kinase kinase kinase 1 (predicted)  Info   Map4k1_predicted 
   3674 2.95e-05 190.8 71.7 2.661  1377970_at  similar to poly (ADP-ribose) polymerase family, member 8  Info   LOC294762 
   3675 2.97e-05 162.4 282.6 0.575  1374191_at  rhomboid family 1 (Drosophila) (predicted)  Info   Rhbdf1_predicted 
   3676 2.98e-05 957.4 1327.9 0.721  1387972_at  mucin and cadherin like  Info   Mucdhl 
   3677 2.99e-05 48 100.5 0.478  1397821_at  Transcribed locus  Info    
   3678 3e-05 60.8 21.2 2.868  1391015_at  Similar to DNA segment, Chr 11, ERATO Doi 461, expressed  Info    
   3679 3e-05 30.3 103.4 0.293  1387052_at  glutamic pyruvic transaminase 1, soluble  Info   Gpt1 
   3680 3e-05 5721.1 4490.8 1.274  1367717_at  ribosomal protein S27  Info   Rps27 
   3681 3e-05 71.3 3.5 20.371  1380333_at  protein tyrosine phosphatase, non-receptor type 8 (predicted)  Info   Ptpn8_predicted 
   3682 3e-05 366 688.3 0.532  1373371_a_at  similar to RIKEN cDNA 1110001J03  Info   MGC112899 
   3683 3.01e-05 19.6 100.8 0.194  1373659_at    Info    
   3684 3.01e-05 97.7 163.1 0.599  1381816_at  similar to hypothetical protein FLJ11218 (predicted)  Info   RGD1309804_predicted 
   3685 3.03e-05 484.3 870.7 0.556  1389230_at  Arrestin domain containing 3  Info   Arrdc3 
   3686 3.03e-05 9.7 32.6 0.298  1391563_at  Transcribed locus  Info    
   3687 3.03e-05 236.6 135.3 1.749  1373516_at  Similar to RIKEN cDNA 2600005C20 (predicted)  Info    
   3688 3.03e-05 1094.3 2400.2 0.456  1387870_at  zinc finger protein 36  Info   Zfp36 
   3689 3.04e-05 878.2 627.6 1.399  1388885_at  similar to Aph1a-pending protein (predicted)  Info   RGD1311546_predicted 
   3690 3.05e-05 104.4 169.6 0.616  1367994_at  dihydropyrimidine dehydrogenase  Info   Dpyd 
   3691 3.06e-05 265.4 197.9 1.341  1367537_at  similar to Clast4 protein  Info   LOC305468 
   3692 3.07e-05 251.8 171.1 1.472  1372901_at  Similar to RIKEN cDNA 8030451K01  Info    
   3693 3.07e-05 18.4 47.7 0.386  1368980_at  phospholipase C, epsilon 1  Info   Plce1 
   3694 3.07e-05 43.4 9.3 4.667  1380243_at  similar to CG14803-PA (predicted)  Info   RGD1304693_predicted 
   3695 3.07e-05 101.1 61.6 1.641  1382136_at  Similar to GLUT9a  Info    
   3696 3.07e-05 3161 2367.2 1.335  1367651_at  cathepsin D  Info   Ctsd 
   3697 3.07e-05 4010.6 5040.2 0.796  1370238_at    Info    
   3698 3.07e-05 221.3 127.7 1.733  1392837_at  P34 protein  Info   P34 
   3699 3.07e-05 134 184.8 0.725  1394627_at    Info    
   3700 3.08e-05 29.9 5.8 5.155  1380086_at  Transcribed locus  Info    
   3701 3.08e-05 49 7.4 6.622  1376394_at  Transcribed locus  Info    
   3702 3.08e-05 239.6 340.5 0.704  1379314_at    Info    
   3703 3.08e-05 478.8 697.6 0.686  1390207_at  Large tumor suppressor 2 (predicted)  Info    
   3704 3.09e-05 217.7 358.5 0.607  1374414_at  similar to RIKEN cDNA 1300010M03 (predicted)  Info   RGD1306844_predicted 
   3705 3.09e-05 1.4 12.4 0.113  1388237_s_at  ATPase, Ca++ transporting, plasma membrane 3  Info   Atp2b3 
   3706 3.09e-05 83.1 238.2 0.349  1373521_at  similar to RIKEN cDNA D430044G18 (predicted)  Info   RGD1309038_predicted 
   3707 3.09e-05 200.9 411.4 0.488  1367649_at  paralemmin  Info   Palm 
   3708 3.1e-05 23.6 44.1 0.535  1396719_at  Transcribed locus  Info    
   3709 3.1e-05 2138.2 755.7 2.829  1370959_at  collagen, type III, alpha 1  Info   Col3a1 
   3710 3.1e-05 533.7 327 1.632  1372113_at  flightless I homolog (Drosophila) (predicted)  Info   Fliih_predicted 
   3711 3.1e-05 351.4 641.6 0.548  1390825_at  solute carrier family 35, member B3 (predicted)  Info   Slc35b3_predicted 
   3712 3.1e-05 2234.1 3582.2 0.624  1371379_at  similar to Brain protein 44  Info   LOC289182 
   3713 3.11e-05 159 72.4 2.196  1391440_at  testis expressed gene 10 (predicted)  Info   Tex10_predicted 
   3714 3.11e-05 93 182.7 0.509  1390483_at  solute carrier family 25 (mitochondrial carrier, palmitoylcarnitine transporter), member 29 (predicted)  Info   Slc25a29_predicted 
   3715 3.11e-05 66.1 118.5 0.558  1376537_at  Similar to Tyrosine-protein phosphatase, non-receptor type 3 (Protein-tyrosine phosphatase H1) (PTP-H1)  Info    
   3716 3.12e-05 75.6 165.2 0.458  1393000_at    Info    
   3717 3.12e-05 131.4 229.1 0.574  1373519_at  Transcribed locus  Info    
   3718 3.13e-05 152.3 294.5 0.517  1376661_at  similar to CG11206-PA /// similar to kazrin isoform A  Info   RGD:1359147 /// LOC500579 
   3719 3.13e-05 77 168 0.458  1375872_at  Similar to hypothetical protein D4Ertd89e (predicted)  Info    
   3720 3.13e-05 484.1 744.6 0.65  1375566_at  angiotensin II receptor-associated protein  Info   RGD:1359346 
   3721 3.13e-05 138.2 234.3 0.59  1371738_at  similar to hypothetical protein FLJ10375 (predicted)  Info   RGD1308260_predicted 
   3722 3.14e-05 193.2 305.1 0.633  1372545_at  Similar to Gene model 609  Info    
   3723 3.14e-05 96 55.7 1.724  1393293_at  checkpoint with forkhead and ring finger domains (predicted)  Info   Chfr_predicted 
   3724 3.14e-05 273.2 594.7 0.459  1367815_at  solute carrier family 5 (sodium-dependent vitamin transporter), member 6  Info   Slc5a6 
   3725 3.15e-05 126.7 51.6 2.455  1396055_at  procollagen, type VI, alpha 3 (predicted)  Info   Col6a3_predicted 
   3726 3.15e-05 116.1 261.2 0.444  1383838_at    Info    
   3727 3.15e-05 14.5 43.2 0.336  1378216_at  Transcribed locus, weakly similar to NP_032201.1 granulin [Mus musculus]  Info    
   3728 3.16e-05 1082.1 1780.6 0.608  1373546_at  ATPase, class VI, type 11A (predicted)  Info    
   3729 3.16e-05 761.2 1367.2 0.557  1380334_at  hypothetical RNA binding protein  Info   RGD:1359713 
   3730 3.16e-05 138.9 65 2.137  1374576_at  similar to mKIAA0841 protein  Info   LOC361546 
   3731 3.16e-05 161.7 280.8 0.576  1372925_at    Info    
   3732 3.17e-05 102.1 41.8 2.443  1391893_at  similar to ATP/GTP-binding protein (predicted)  Info   RGD1307679_predicted 
   3733 3.18e-05 2286.6 1531.4 1.493  1371390_at  tubulin, beta, 2  Info   Tubb2 
   3734 3.18e-05 470.1 884.1 0.532  1371997_at  aldo-keto reductase family 1, member E1 (predicted)  Info   Akr1e1_predicted 
   3735 3.19e-05 35 83 0.422  1378005_at    Info    
   3736 3.19e-05 439.4 260.6 1.686  1398367_at  speckle-type POZ protein (predicted)  Info   Spop_predicted 
   3737 3.19e-05 364.7 264.2 1.38  1389304_at    Info    
   3738 3.19e-05 58.3 130.9 0.445  1376068_at    Info    
   3739 3.2e-05 160.3 49.9 3.212  1391543_at  receptor (TNFRSF)-interacting serine-threonine kinase 1 (predicted)  Info   Ripk1_predicted 
   3740 3.2e-05 201.7 338.9 0.595  1399034_at  pecanex homolog (Drosophila) (predicted)  Info   Pcnx_predicted 
   3741 3.2e-05 449.2 222.4 2.02  1383287_at  Similar to semaF cytoplasmic domain associated protein 2  Info    
   3742 3.21e-05 508.4 380.1 1.338  1388448_at  Rap guanine nucleotide exchange factor (GEF) 6 (predicted)  Info    
   3743 3.22e-05 78.4 197.1 0.398  1389396_at    Info    
   3744 3.22e-05 52.5 110.4 0.476  1398732_at  Transcribed locus  Info    
   3745 3.23e-05 90.5 30.1 3.007  1384742_at  Alpha thalassemia/mental retardation syndrome X-linked homolog (human)  Info   Atrx 
   3746 3.23e-05 119.4 38.4 3.109  1396383_at  fatty aldehyde dehydrogenase-like  Info   RGD:1359546 
   3747 3.23e-05 31.7 90.6 0.35  1384481_at  Transcribed locus  Info    
   3748 3.24e-05 45.8 108 0.424  1383826_at  Rab40b, member RAS oncogene family (predicted)  Info   Rab40b_predicted 
   3749 3.24e-05 138.6 330.8 0.419  1367880_at  laminin, beta 2  Info   Lamb2 
   3750 3.24e-05 812.5 477.3 1.702  1371367_at  TAR DNA binding protein (predicted)  Info   Tardbp_predicted 
   3751 3.26e-05 77 148 0.52  1379043_at  GH regulated TBC protein 1 (predicted)  Info    
   3752 3.27e-05 217 152.3 1.425  1375029_at    Info    
   3753 3.27e-05 115.2 302.2 0.381  1393364_at  cation-chloride cotransporter 9  Info   Slc12a8 
   3754 3.27e-05 205.7 537.1 0.383  1368672_at  arginase 2  Info   Arg2 
   3755 3.27e-05 162.7 104.4 1.558  1373556_at  similar to CG14903-PA  Info   LOC298861 
   3756 3.28e-05 98.7 38.8 2.544  1379195_s_at  ligase I, DNA, ATP-dependent /// ligase I, DNA, ATP-dependent (predicted)  Info   Lig1 /// Lig1_predicted 
   3757 3.28e-05 17.7 41.4 0.428  1387253_at  guanylate cyclase activator 2b  Info   Guca2b 
   3758 3.29e-05 125.1 53.2 2.352  1397176_at  similar to RIKEN cDNA 1600013P15  Info   RGD:1359565 
   3759 3.29e-05 16.4 47.8 0.343  1396815_at  LOC501008  Info   LOC501008 
   3760 3.29e-05 118 221.5 0.533  1386662_at  Secernin 1  Info    
   3761 3.29e-05 119.4 33.5 3.564  1390195_at  Pleckstrin and Sec7 domain containing 4 (predicted)  Info    
   3762 3.3e-05 465.4 680 0.684  1388518_at  F-box and WD-40 domain protein 5 (predicted)  Info   Fbxw5_predicted 
   3763 3.31e-05 323 1774.4 0.182  1368208_at  camello-like 1  Info   Cml1 
   3764 3.31e-05 185.4 392.7 0.472  1372044_at  similar to Ser/Thr-rich protein T10 in DGCR region (predicted)  Info   RGD1310348_predicted 
   3765 3.31e-05 45.8 207.1 0.221  1375549_at  Transcribed locus  Info    
   3766 3.31e-05 14.7 32.5 0.452  1396337_at    Info    
   3767 3.32e-05 360.8 579.1 0.623  1374197_at  Similar to expressed sequence AA415817  Info    
   3768 3.32e-05 5269.3 4323.5 1.219  1398761_at  ribosomal protein L5  Info   Rpl5 
   3769 3.32e-05 117 231.7 0.505  1389242_at  Transcribed locus  Info    
   3770 3.32e-05 122.9 245 0.502  1387916_at  cytochrome P450 4F6  Info   RGD:708365 
   3771 3.32e-05 1081.9 7.5 144.253  1387378_at  ficolin B  Info   Fcnb 
   3772 3.33e-05 236.6 34.6 6.838  1387895_s_at  cell division cycle 20 homolog (S. cerevisiae)  Info   Cdc20 
   3773 3.33e-05 53.9 125.5 0.429  1386914_at  guanosine monophosphate reductase  Info   Gmpr 
   3774 3.33e-05 3223.3 2291.3 1.407  1367657_at  B-cell translocation gene 1, anti-proliferative  Info   Btg1 
   3775 3.34e-05 1889.7 1270.3 1.488  1372466_at  Transforming growth factor, beta receptor II  Info   Tgfbr2 
   3776 3.34e-05 18.2 58.1 0.313  1380884_at  Transcribed locus  Info    
   3777 3.34e-05 171.6 136 1.262  1379443_at  Hermansky-Pudlak syndrome 3 (predicted)  Info   Hps3_predicted 
   3778 3.34e-05 461.6 246.8 1.87  1374545_at  ring finger and KH domain containing 2 (predicted)  Info   Rkhd2_predicted 
   3779 3.34e-05 17.3 2 8.65  1371126_x_at  granzyme B  Info   Gzmb 
   3780 3.35e-05 174.8 56.1 3.116  1389214_at  similar to Laminin alpha-4 chain precursor  Info   LOC309816 
   3781 3.35e-05 293 476.3 0.615  1375349_at  Transcribed locus, strongly similar to NP_033192.1 sorbin and SH3 domain containing 1 [Mus musculus]  Info    
   3782 3.35e-05 18.7 64.9 0.288  1382173_at  Transcribed locus  Info    
   3783 3.36e-05 118.4 65.4 1.81  1391466_at  Nuclear ubiquitous casein kinase and cyclin-dependent kinase substrate  Info   Nucks 
   3784 3.36e-05 131.5 75.1 1.751  1384265_at  polymerase (DNA-directed), delta 3, accessory subunit (predicted)  Info   Pold3_predicted 
   3785 3.36e-05 161.6 62.7 2.577  1377797_at  gene rich cluster, C2f gene (predicted)  Info   Grcc2f_predicted 
   3786 3.36e-05 129.6 49.5 2.618  1374355_at  similar to MGC47262 protein  Info   LOC315283 
   3787 3.37e-05 73.6 37.2 1.978  1375406_s_at  rapostlin  Info   Fnbp1 
   3788 3.37e-05 358.9 732.6 0.49  1367885_at  peroxisomal membrane protein 2  Info   Pxmp2 
   3789 3.37e-05 647.8 408.5 1.586  1388880_at  Schlafen 5 (predicted)  Info    
   3790 3.38e-05 210.9 83.8 2.517  1372631_at  Thymidine kinase 1  Info   Tk1 
   3791 3.38e-05 52.1 18.7 2.786  1390553_at  Transcribed locus  Info    
   3792 3.38e-05 487.7 233 2.093  1372003_at  Transcribed locus, strongly similar to NP_056312.2 DKFZP564G2022 protein [Homo sapiens]  Info    
   3793 3.38e-05 782.4 255.8 3.059  1374070_at  glutathione peroxidase 2  Info   Gpx2 
   3794 3.38e-05 2937.3 1877.9 1.564  1388076_at  PAI-1 mRNA-binding protein  Info   Pairbp1 
   3795 3.38e-05 323.2 1056.9 0.306  1394022_at  inhibitor of DNA binding 4  Info   Idb4 
   3796 3.39e-05 104.8 51.4 2.039  1388099_a_at  TCF3 (E2A) fusion partner  Info   Tfpt 
   3797 3.4e-05 11.8 1 11.8  1369697_at  interleukin 8 receptor, beta  Info   Il8rb 
   3798 3.4e-05 1943.6 1186.5 1.638  1383080_at  Lysosomal membrane glycoprotein 2  Info   Lamp2 
   3799 3.4e-05 109.6 298.1 0.368  1376867_at  similar to RIKEN cDNA 1110067D22 (predicted)  Info   RGD1307414_predicted 
   3800 3.4e-05 619.1 378.1 1.637  1388024_s_at  lamina-associated polypeptide 1B  Info   Lap1b 
   3801 3.4e-05 242.2 83.6 2.897  1393252_at  Fibulin 1 (predicted)  Info    
   3802 3.4e-05 353.8 203.2 1.741  1390786_at  rho/rac guanine nucleotide exchange factor (GEF) 2 (predicted)  Info   Arhgef2_predicted 
   3803 3.4e-05 133.7 9.3 14.376  1371092_at  preprotrypsinogen IV  Info   RGD:708585 
   3804 3.41e-05 156.6 291.6 0.537  1374475_at  abhydrolase domain containing 1 (predicted)  Info   Abhd1_predicted 
   3805 3.41e-05 1001.1 1514.6 0.661  1398350_at  brain abundant, membrane attached signal protein 1  Info   Basp1 
   3806 3.41e-05 278.7 453.5 0.615  1398254_at  renin binding protein  Info   Renbp 
   3807 3.41e-05 91.2 56.4 1.617  1389485_at  similar to transformed mouse 3T3 cell double minute 1  Info   LOC314859 
   3808 3.41e-05 4610.2 3121 1.477  1367618_a_at  discs, large homolog 5 (Drosophila) (predicted)  Info   Dlg5_predicted 
   3809 3.42e-05 74.5 140.4 0.531  1390049_at  four and a half LIM domains 1  Info   Fhl1 
   3810 3.43e-05 481.1 377.8 1.273  1372420_at  similar to FKSG24 (predicted)  Info   RGD1308064_predicted 
   3811 3.43e-05 526 301.6 1.744  1367743_at  phosphofructokinase, liver, B-type  Info   Pfkl 
   3812 3.43e-05 701.7 1002.2 0.7  1398962_at  similar to cDNA sequence BC005632  Info   RGD:1359682 
   3813 3.43e-05 207.1 106.2 1.95  1374399_at  EH-domain containing 4  Info   Ehd4 
   3814 3.44e-05 997.4 3872.3 0.258  1368325_at  epidermal growth factor  Info   Egf 
   3815 3.45e-05 1583.4 2683.9 0.59  1376209_at  similar to hypothetical protein supported by AL449243 (predicted)  Info   RGD1311310_predicted 
   3816 3.46e-05 44.4 148.8 0.298  1376222_at  similar to HCDI protein (predicted)  Info   RGD1309307_predicted 
   3817 3.46e-05 131.3 240.4 0.546  1390474_at  similar to RIKEN cDNA 9330161F08  Info   LOC362899 
   3818 3.48e-05 62.4 21.9 2.849  1375921_at  Reticulocalbin (predicted)  Info    
   3819 3.48e-05 369.6 137.5 2.688  1372519_at  nucleoporin 93 (predicted)  Info   Nup93_predicted 
   3820 3.49e-05 188.5 115.4 1.633  1383730_at  Similar to hypothetical protein  Info   MGC73022 
   3821 3.5e-05 30.6 92.4 0.331  1389803_at  Transcribed locus  Info    
   3822 3.5e-05 158 298.8 0.529  1389180_at  Similar to phosphorylase kinase (EC 2.7.1.38) beta chain, non-muscle - rabbit  Info    
   3823 3.5e-05 178.8 279.6 0.639  1396853_at  Adrenergic receptor, alpha 2b  Info   Adra2b 
   3824 3.51e-05 1091.9 640.3 1.705  1372770_at    Info    
   3825 3.51e-05 152 86.3 1.761  1383182_at  serine/arginine-rich protein specific kinase 1 (predicted)  Info   Srpk1_predicted 
   3826 3.52e-05 407.8 164.4 2.481  1386986_at  opioid growth factor receptor  Info   Ogfr 
   3827 3.52e-05 69 23.7 2.911  1381678_at  Basophilic leukemia expressed sequence 06 (Bles06)  Info    
   3828 3.53e-05 140.7 186.8 0.753  1371744_at  Transcribed locus, strongly similar to NP_057481.2 TH1-like protein; TH1 drosophila homolog; negative elongation factor proteins C and D; TH1-like (Drosophila homolog) [Homo sapiens]  Info    
   3829 3.53e-05 334 216.5 1.543  1372169_at  similar to RIKEN cDNA 4121402D02  Info   LOC303514 
   3830 3.54e-05 108 28.1 3.843  1376513_a_at    Info    
   3831 3.55e-05 635.9 928.2 0.685  1389050_at  Similar to Dual specificity protein phosphatase 3 (T-DSP11)  Info    
   3832 3.55e-05 222.8 107.3 2.076  1373538_at  ubiquitin specific protease 1 (predicted)  Info   Usp1_predicted 
   3833 3.55e-05 409.3 581.4 0.704  1389560_at    Info    
   3834 3.56e-05 158 243.2 0.65  1388919_at  zinc finger protein 541 (predicted)  Info   Znf541_predicted 
   3835 3.56e-05 42.9 126.1 0.34  1387295_at  solute carrier family 6 (neurotransmitter transporter, betaine/GABA), member 12  Info   Slc6a12 
   3836 3.57e-05 26.1 67.3 0.388  1388958_a_at  solute carrier family 2 (facilitated glucose transporter), member 4  Info   Slc2a4 
   3837 3.57e-05 102.8 57.7 1.782  1370395_at  Huntington disease gene homolog  Info   Hdh 
   3838 3.59e-05 3381.8 4308.8 0.785  1371311_at  succinate dehydrogenase complex, subunit C  Info   RGD:1359454 
   3839 3.59e-05 1585.7 1137.1 1.395  1398799_at  eukaryotic translation initiation factor 4E  Info   Eif4e 
   3840 3.6e-05 15.8 127.2 0.124  1370150_a_at  thyroid hormone responsive protein  Info   Thrsp 
   3841 3.6e-05 883.6 450.7 1.961  1368908_at  annexin A4  Info   Anxa4 
   3842 3.6e-05 157.3 332 0.474  1384312_at  Iroquois related homeobox 1 (Drosophila) (predicted) /// similar to iroquois-class homeobox protein Irx1  Info   Irx1_predicted /// LOC501463 
   3843 3.61e-05 275.2 381.6 0.721  1372454_at  similar to RIKEN cDNA 2210016L21 gene (predicted)  Info   RGD1311899_predicted 
   3844 3.61e-05 229.4 136.9 1.676  1383679_at  ring finger protein 31 (predicted)  Info   Rnf31_predicted 
   3845 3.61e-05 275.2 134.1 2.052  1382923_at  synaptotagmin binding, cytoplasmic RNA interacting protein (predicted)  Info   Syncrip_predicted 
   3846 3.61e-05 134.2 67.7 1.982  1395479_at  similar to BRIX (predicted)  Info   RGD1308508_predicted 
   3847 3.62e-05 4738.8 3259.5 1.454  1371305_at  Ribosomal protein L8  Info   Rpl8 
   3848 3.63e-05 291.4 412 0.707  1371899_at  protein kinase, interferon inducible double stranded RNA dependent activator (predicted)  Info   Prkra_predicted 
   3849 3.63e-05 130.7 65 2.011  1390350_at  similar to hypothetical protein AL133206 (predicted)  Info   RGD1308418_predicted 
   3850 3.64e-05 175.9 113.1 1.555  1383150_at  Transcribed locus  Info    
   3851 3.64e-05 91.3 144.4 0.632  1372095_at  Similar to KIAA1161 protein (predicted)  Info    
   3852 3.64e-05 1108.2 723.8 1.531  1370250_at  ubiquitin-conjugating enzyme E2I /// similar to RIKEN cDNA A930001M12 gene  Info   Ube2i /// LOC500440 
   3853 3.64e-05 1590.2 2246.2 0.708  1371296_at  succinate dehydrogenase complex, subunit D, integral membrane protein  Info   Sdhd 
   3854 3.64e-05 412.3 70.8 5.823  1369983_at  chemokine (C-C motif) ligand 5  Info   Ccl5 
   3855 3.64e-05 236.1 480.3 0.492  1371431_at  Transcribed locus, strongly similar to XP_232343.3 PREDICTED: similar to Pex5 protein [Rattus norvegicus]  Info    
   3856 3.66e-05 53.6 84.8 0.632  1379900_at  Transcribed locus  Info    
   3857 3.67e-05 67.6 4.3 15.721  1380616_at  sialyltransferase 8 (alpha-2, 8-sialytransferase) D  Info   Siat8d 
   3858 3.68e-05 196.2 124.2 1.58  1382873_at  similar to cDNA sequence BC003236 (predicted)  Info   RGD1310822_predicted 
   3859 3.69e-05 2183.1 3444.7 0.634  1388122_at  glutathione-S-transferase, pi 1 /// glutathione S-transferase, pi 2  Info   Gstp1 /// Gstp2 
   3860 3.69e-05 308.4 557.3 0.553  1395145_at  Ral guanine nucleotide dissociation stimulator,-like 1 (predicted)  Info    
   3861 3.69e-05 24.7 36.2 0.682  1369336_at  hairless homolog (mouse)  Info   hr 
   3862 3.7e-05 18.1 39.7 0.456  1378421_at  Transcribed locus  Info    
   3863 3.7e-05 326.3 437.6 0.746  1371706_at  serologically defined colon cancer antigen 3 (predicted)  Info   Sdccag3_predicted 
   3864 3.71e-05 71 6.7 10.597  1372957_at  Similar to hypothetical protein A730008H23 (predicted)  Info    
   3865 3.71e-05 165.4 115.1 1.437  1381933_at  Transcribed locus  Info    
   3866 3.71e-05 60.4 235.3 0.257  1386641_at  Transcribed locus  Info    
   3867 3.71e-05 316.3 167.4 1.889  1380513_at  similar to Expressed sequence AW060207 (predicted)  Info   RGD1309034_predicted 
   3868 3.71e-05 133.1 86.9 1.532  1372788_at  ubiquitin-conjugating enzyme E2Q (putative) (predicted)  Info   Ube2q_predicted 
   3869 3.71e-05 62.1 143.1 0.434  1367953_at  TYRO3 protein tyrosine kinase 3  Info   Tyro3 
   3870 3.71e-05 74.6 20.2 3.693  1382751_at    Info    
   3871 3.72e-05 565.9 980.4 0.577  1373892_at  Transcribed locus  Info    
   3872 3.72e-05 49.7 23 2.161  1382758_at  P7 protein  Info   P7 
   3873 3.73e-05 146.4 72.5 2.019  1392509_at  Transcribed locus  Info    
   3874 3.74e-05 1353.8 911.2 1.486  1388596_at  coactosin-like 1 (Dictyostelium) (predicted)  Info   Cotl1_predicted 
   3875 3.74e-05 260.1 393.8 0.66  1372772_at    Info    
   3876 3.74e-05 207.8 104.6 1.987  1379934_at  Lysosomal trafficking regulator  Info   Lyst 
   3877 3.75e-05 24 9.3 2.581  1377907_at  similar to RIKEN cDNA 6530403A03 (predicted)  Info   RGD1309020_predicted 
   3878 3.76e-05 22.8 55.6 0.41  1382953_at  Transcribed locus  Info    
   3879 3.77e-05 76.3 29.8 2.56  1392174_at  carbohydrate sulfotransferase 12 (predicted)  Info   Chst12_predicted 
   3880 3.77e-05 365.1 544.5 0.671  1373825_at  Hypothetical LOC314717 (predicted)  Info    
   3881 3.77e-05 4589.5 3785 1.213  1371761_at  ribosomal protein L34 (predicted)  Info   Rpl34_predicted 
   3882 3.77e-05 160.7 95.7 1.679  1373776_at  Transcribed locus  Info    
   3883 3.77e-05 884.9 425.8 2.078  1374731_at  Tripartite motif protein 25  Info   Trim25 
   3884 3.77e-05 105.5 27.2 3.879  1392032_at  GRB2-related adaptor protein (predicted)  Info   Grap_predicted 
   3885 3.77e-05 349.6 246.9 1.416  1372749_at  Transcribed locus  Info    
   3886 3.78e-05 667.7 383.2 1.742  1383998_at  Transcribed locus, moderately similar to XP_421321.1 PREDICTED: similar to hypothetical protein FLJ20707 isoform 1 [Gallus gallus]  Info    
   3887 3.78e-05 73.9 111.7 0.662  1386979_at  developmentally regulated protein TPO1  Info   Tpo1 
   3888 3.78e-05 142 251.2 0.565  1383065_at  CDNA clone MGC:124727 IMAGE:7936966  Info    
   3889 3.78e-05 62.7 4.4 14.25  1393411_at  properdin factor, complement  Info   Pfc 
   3890 3.78e-05 112.6 38 2.963  1385168_at  similar to receptor-interacting factor 1 (predicted)  Info   RGD1306520_predicted 
   3891 3.79e-05 105.7 43 2.458  1393357_at  GLE1 RNA export mediator-like (yeast (predicted)  Info   Gle1l_predicted 
   3892 3.8e-05 87.9 137.5 0.639  1382104_at  WD repeat domain 33 (predicted)  Info   Wdr33_predicted 
   3893 3.8e-05 131.5 49 2.684  1382218_at  hypothetical LOC298077 (predicted)  Info   RGD1305807_predicted 
   3894 3.8e-05 138.4 69.1 2.003  1383543_at  similar to CGI-141 protein; EST AA407874 (predicted)  Info   RGD1310691_predicted 
   3895 3.8e-05 174.3 96.3 1.81  1372951_at  Transcribed locus  Info    
   3896 3.81e-05 431.6 205.1 2.104  1399152_at  epidermal growth factor receptor pathway substrate 15 (predicted)  Info   Eps15_predicted 
   3897 3.81e-05 26.3 98.5 0.267  1382034_at    Info    
   3898 3.81e-05 65.8 23.4 2.812  1379059_at  similar to FLJ23471 protein (predicted)  Info   RGD1307875_predicted 
   3899 3.81e-05 69.9 36.8 1.899  1385258_at  UDP-GlcNAc:betaGal beta-1,3-N-acetylglucosaminyltransferase 7 (predicted)  Info   B3gnt7_predicted 
   3900 3.82e-05 63.5 20.7 3.068  1376642_at  similar to hypothetical protein FLJ10307 (predicted)  Info   RGD1305649_predicted 
   3901 3.82e-05 804.1 1028.4 0.782  1398782_at  N-ethylmaleimide sensitive fusion protein attachment protein alpha  Info   Napa 
   3902 3.83e-05 21.8 63.6 0.343  1395628_at  Transcribed locus  Info    
   3903 3.84e-05 54.5 104.2 0.523  1371849_at  similar to hypothetical protein FLJ12442 (predicted)  Info   RGD1305524_predicted 
   3904 3.84e-05 2061.4 2778.9 0.742  1370855_at  cystatin C  Info   Cst3 
   3905 3.85e-05 19 76.3 0.249  1391544_at  Similar to adiponutrin  Info    
   3906 3.85e-05 99.6 83.5 1.193  1383528_at  similar to mKIAA0215 protein  Info   LOC299305 
   3907 3.85e-05 443.5 235.4 1.884  1375818_at  lactamase, beta (predicted)  Info   Lactb_predicted 
   3908 3.86e-05 192.3 360 0.534  1382705_at  Transcribed locus, weakly similar to XP_282988.2 PREDICTED: similar to novel protein [Mus musculus]  Info    
   3909 3.86e-05 195.8 1089.9 0.18  1370610_at  Solute carrier family 34 (sodium phosphate), member 1  Info   Slc34a1 
   3910 3.86e-05 116.8 77.2 1.513  1376567_at  FtsJ homolog 2 (E. coli) (predicted)  Info    
   3911 3.87e-05 324.1 10.4 31.163  1379633_a_at  ubiquitin-activating enzyme E1-like (predicted)  Info   Ube1l_predicted 
   3912 3.87e-05 198.8 430.7 0.462  1391270_at  Cyclin M3 (predicted)  Info    
   3913 3.87e-05 104.9 175 0.599  1374295_at  polymerase (RNA) mitochondrial (DNA directed) (predicted)  Info   Polrmt_predicted 
   3914 3.88e-05 277.4 157.2 1.765  1368647_at  G protein-coupled receptor kinase 6  Info   Gprk6 
   3915 3.88e-05 1026.9 731.1 1.405  1373090_at  signal sequence receptor, alpha (predicted)  Info   Ssr1_predicted 
   3916 3.88e-05 282.8 131.3 2.154  1394340_at  inositol polyphosphate-1-phosphatase (predicted)  Info   Inpp1_predicted 
   3917 3.88e-05 169.7 367.1 0.462  1384971_at  similar to Hypothetical protein MGC19163 (predicted)  Info   RGD1309720_predicted 
   3918 3.89e-05 63.9 123.5 0.517  1374984_at  erythrocyte protein band 4.1-like 5 (predicted)  Info   Epb4.1l5_predicted 
   3919 3.9e-05 15.1 53 0.285  1386648_at  DnaJ (Hsp40) homolog, subfamily C, member 6 (predicted)  Info   Dnajc6_predicted 
   3920 3.9e-05 117 233.8 0.5  1381336_at  Transcribed locus  Info    
   3921 3.9e-05 47.2 130.5 0.362  1374122_at  Transcribed locus  Info    
   3922 3.9e-05 85.7 50 1.714  1387268_at  RNA polymerase 1-2  Info   Rpo1-2 
   3923 3.91e-05 649 1159.9 0.56  1377033_at  serine (or cysteine) proteinase inhibitor, clade F, member 2 (predicted)  Info   Serpinf2_predicted 
   3924 3.91e-05 110 35.3 3.116  1376050_at  TAF5 RNA polymerase II, TATA box binding protein (TBP)-associated factor (predicted)  Info   Taf5_predicted 
   3925 3.92e-05 788.8 1342 0.588  1389147_at  Transcribed locus  Info    
   3926 3.92e-05 685.6 906.5 0.756  1372758_at  Transcribed locus  Info    
   3927 3.93e-05 245.8 149.9 1.64  1372432_at  PRP3 pre-mRNA processing factor 3 homolog (yeast) (predicted)  Info   Prpf3_predicted 
   3928 3.94e-05 56 146.3 0.383  1388921_at  Transcribed locus  Info    
   3929 3.95e-05 11.7 52.4 0.223  1381951_at  Transcribed locus  Info    
   3930 3.96e-05 325.9 197.1 1.653  1389344_at  ubiquitin specific protease 39 (predicted)  Info   Usp39_predicted 
   3931 3.96e-05 149.7 236.9 0.632  1382032_at  Transcribed locus  Info    
   3932 3.96e-05 92 178.9 0.514  1389271_at    Info    
   3933 3.96e-05 592.6 851.9 0.696  1388995_at  Transcribed locus  Info    
   3934 3.96e-05 35 3.3 10.606  1397476_at  Ttk protein kinase (predicted)  Info   Ttk_predicted 
   3935 3.96e-05 402.5 276.6 1.455  1372769_at  similar to ligatin  Info   LOC498225 
   3936 3.97e-05 199 12.6 15.794  1370297_at  polo-like kinase 1 (Drosophila)  Info   Plk1 
   3937 3.97e-05 129.5 193.7 0.669  1382945_at  Similar to cDNA sequence BC006662 (predicted)  Info    
   3938 3.97e-05 364.6 218.1 1.672  1368214_at  MAD homolog 2 (Drosophila)  Info   Madh2 
   3939 3.97e-05 444.7 232.5 1.913  1390731_at  Transcribed locus  Info    
   3940 3.98e-05 1394.2 872.4 1.598  1398801_at  CDK105 protein  Info   RGD:621335 
   3941 3.98e-05 168.4 71.7 2.349  1386379_at  Similar to MGC68837 protein  Info    
   3942 3.98e-05 9.7 1.2 8.083  1390999_at  Transcribed locus  Info    
   3943 3.98e-05 319.6 173.2 1.845  1387402_at  myosin, heavy polypeptide 9  Info   Myh9 
   3944 4.01e-05 179.9 293.4 0.613  1384368_at  DNA2 DNA replication helicase 2-like (yeast) (predicted)  Info    
   3945 4.01e-05 371.5 276.9 1.342  1372853_at  v-rel reticuloendotheliosis viral oncogene homolog A (avian)  Info   Rela 
   3946 4.01e-05 2214.5 5783 0.383  1368180_s_at  glutathione-S-transferase, alpha type2  Info   Gsta2 
   3947 4.01e-05 574.6 385.6 1.49  1371322_at  Laminin, gamma 1  Info   Lamc1 
   3948 4.02e-05 231.3 119.4 1.937  1376501_at  Rho GTPase activating protein 8  Info   RGD:1303143 
   3949 4.02e-05 190.5 127.1 1.499  1373925_at    Info    
   3950 4.02e-05 127 222 0.572  1385505_at  Transcribed locus  Info    
   3951 4.02e-05 147.3 228.3 0.645  1384227_at  Protein tyrosine phosphatase, receptor type, K, extracellular region  Info    
   3952 4.02e-05 126.1 252.4 0.5  1395246_at  Protein kinase, cAMP-dependent, regulatory, type 2, alpha  Info   Prkar2a 
   3953 4.03e-05 418.1 617.3 0.677  1367756_at  G elongation factor  Info   Gfm 
   3954 4.03e-05 198 496.1 0.399  1393209_at  Bartter syndrome, infantile, with sensorineural deafness (Barttin)  Info   bsnd 
   3955 4.04e-05 61.2 151 0.405  1373503_at  leucine-rich repeats and calponin homology (CH) domain containing 4 (predicted)  Info   Lrch4_predicted 
   3956 4.04e-05 1995.5 2891.9 0.69  1389832_at  glutathione S-transferase omega 1  Info   Gsto1 
   3957 4.05e-05 1730.6 1299 1.332  1388117_at  Similar to small nuclear ribonucleoprotein B  Info   Snrpb 
   3958 4.05e-05 231.7 104.2 2.224  1382009_at  Transcribed locus  Info    
   3959 4.05e-05 123.6 287.7 0.43  1376191_at  15-hydroxyprostaglandin dehydrogenase  Info   Hpgd 
   3960 4.06e-05 82.7 159.2 0.519  1368785_a_at  paired-like homeodomain transcription factor 2  Info   Pitx2 
   3961 4.06e-05 1138.9 1895.9 0.601  1389521_at  influenza virus NS1A binding protein (predicted)  Info   Ivns1abp_predicted 
   3962 4.07e-05 96.3 145.6 0.661  1377007_at    Info    
   3963 4.08e-05 141.4 14 10.1  1393957_at  Transcribed locus  Info    
   3964 4.09e-05 414.5 722.1 0.574  1383121_at  Ceroid-lipofuscinosis, neuronal 2  Info   Cln2 
   3965 4.1e-05 1363.2 891.5 1.529  1369941_at  death-associated protein  Info   Dap 
   3966 4.1e-05 152.4 819.2 0.186  1384073_at  alcohol dehydrogenase, iron containing, 1 (predicted)  Info   Adhfe1_predicted 
   3967 4.1e-05 114 48.5 2.351  1370097_a_at  chemokine (C-X-C motif) receptor 4  Info   Cxcr4 
   3968 4.1e-05 585.9 1200.4 0.488  1368340_at  inositol polyphosphate multikinase  Info   Impk 
   3969 4.11e-05 286.5 137.7 2.081  1383665_at  Transcribed locus  Info    
   3970 4.11e-05 29.1 16 1.819  1390017_at  Hyaluronan and proteoglycan link protein 2  Info   Hapln2 
   3971 4.12e-05 829.5 690.1 1.202  1371945_at  ubiquitin-conjugating enzyme E2L 3 (predicted)  Info   Ube2l3_predicted 
   3972 4.12e-05 473.8 748.5 0.633  1372507_at  similar to RIKEN cDNA 9130410M22  Info   RGD:1359333 
   3973 4.12e-05 291.5 168.2 1.733  1372151_at  transcription elongation regulator 1 (CA150) (predicted)  Info   Tcerg1_predicted 
   3974 4.12e-05 1260.6 2521.5 0.5  1367755_at  cytosolic cysteine dioxygenase 1  Info   Cdo1 
   3975 4.13e-05 12.3 54.1 0.227  1396752_at  Tumor necrosis factor receptor superfamily, member 21 (predicted)  Info    
   3976 4.13e-05 970.9 4031.7 0.241  1397205_at  dehydrogenase/reductase (SDR family) member 7 (predicted) /// similar to Down-regulated in nephrectomized rat kidney #3  Info   Dhrs7_predicted /// LOC500672 
   3977 4.14e-05 346.8 939.8 0.369  1387542_at  solute carrier family 9, member 3  Info   Slc9a3 
   3978 4.14e-05 67.9 15.3 4.438  1393684_at  TBC1D12: TBC1 domain family, member 12 (predicted)  Info   Tbc1d12_predicted 
   3979 4.14e-05 1279 797.1 1.605  1375630_at  similar to NHP2-like protein 1 (High mobility group-like nuclear protein 2 homolog 1) ([U4/U6.U5] tri-snRNP 15.5 kDa protein) (OTK27)  Info   RGD:1303103 
   3980 4.15e-05 363.1 181.9 1.996  1375685_at  cytoplasmic linker associated protein 1 (predicted)  Info   Clasp1_predicted 
   3981 4.15e-05 66.6 367.8 0.181  1377333_at  Transcribed locus  Info    
   3982 4.16e-05 2199 3861 0.57  1387344_at  aldehyde dehydrogenase family 6, subfamily A1  Info   Aldh6a1 
   3983 4.17e-05 396.4 188.3 2.105  1388669_at  splicing factor 3a, subunit 3 (predicted)  Info   Sf3a3_predicted 
   3984 4.17e-05 407.4 182.3 2.235  1373035_at  Transcribed locus  Info    
   3985 4.17e-05 218.3 120.8 1.807  1390455_at  abhydrolase domain containing 2 (predicted)  Info   Abhd2_predicted 
   3986 4.17e-05 122.5 16.7 7.335  1391227_at  Transcribed locus  Info    
   3987 4.18e-05 33.6 69.2 0.486  1369773_at  bone morphogenetic protein 3  Info   Bmp3 
   3988 4.18e-05 1255.7 1894.5 0.663  1368095_at  adenylate kinase 3  Info   Ak3 
   3989 4.2e-05 101.7 36.7 2.771  1389379_at  DNA cross-link repair 1C, PSO2 homolog (S. cerevisiae)  Info   Mt1a 
   3990 4.21e-05 175.8 98 1.794  1372910_at  Villin (predicted)  Info    
   3991 4.21e-05 1825.3 3081 0.592  1367619_at  progesterone receptor membrane component 1  Info   Pgrmc1 
   3992 4.21e-05 78.3 5.5 14.236  1372903_at  similar to 3000004C01Rik protein (predicted)  Info   RGD1310360_predicted 
   3993 4.22e-05 120.8 11.9 10.151  1395116_at  CD8 antigen, alpha chain  Info   Cd8a 
   3994 4.23e-05 224.5 83.9 2.676  1390394_at  Transcribed locus  Info    
   3995 4.23e-05 124.3 77.2 1.61  1368201_at  natriuretic peptide receptor 1  Info   Npr1 
   3996 4.23e-05 58 140.4 0.413  1376672_at  Transcribed locus  Info    
   3997 4.24e-05 95.7 195.9 0.489  1371889_at  solute carrier family 22 (organic cation transporter), member 17  Info   Slc22a17 
   3998 4.24e-05 398.6 675.5 0.59  1373809_at  DEAD (Asp-Glu-Ala-Asp) box polypeptide 21b (predicted) /// hypothetical protein LOC606294  Info   Ddx21b_predicted /// LOC606294 
   3999 4.25e-05 174 268.9 0.647  1379315_at  similar to RIKEN cDNA 2400009B11 gene (predicted)  Info   RGD1306244_predicted 
   4000 4.26e-05 246.8 119.5 2.065  1385458_a_at  similar to C11orf17 protein (predicted)  Info   RGD1306959_predicted 
   4001 4.26e-05 275.5 177.2 1.555  1388631_at  5-azacytidine induced gene 2 (predicted)  Info   Azi2_predicted 
   4002 4.27e-05 121 282.3 0.429  1389757_at  similar to acetyl-coA dehydrogenase -related (111.6 kD) (5G231) (predicted)  Info   RGD1310159_predicted 
   4003 4.28e-05 378.9 245.2 1.545  1389535_at  death associated protein 3 (predicted)  Info   Dap3_predicted 
   4004 4.28e-05 149.7 261.8 0.572  1379669_at    Info    
   4005 4.3e-05 528.8 911.2 0.58  1385679_at  Transcribed locus  Info    
   4006 4.3e-05 1010.4 701.9 1.44  1387346_at  integrin beta 1  Info   Itgb1 
   4007 4.3e-05 149.6 310.9 0.481  1368089_at  phosphodiesterase 2A, cGMP-stimulated  Info   Pde2a 
   4008 4.3e-05 477.3 316.9 1.506  1388787_at  Similar to hypothetical protein FLJ13855 (predicted)  Info    
   4009 4.32e-05 795.1 539 1.475  1388857_at  SEC23B (S. cerevisiae) (predicted)  Info   Sec23b_predicted 
   4010 4.32e-05 90.4 31.6 2.861  1392079_at  A kinase (PRKA) anchor protein 7  Info   RGD:1303071 
   4011 4.32e-05 42.9 143.5 0.299  1369381_a_at  solute carrier family 15 (oligopeptide transporter), member 1  Info   Slc15a1 
   4012 4.33e-05 327.7 635.5 0.516  1372455_at  transmembrane 4 superfamily member 12 (predicted)  Info   Tm4sf12_predicted 
   4013 4.34e-05 1318 945.9 1.393  1371856_at  CDNA clone MGC:93828 IMAGE:7110880  Info    
   4014 4.34e-05 168.8 14.2 11.887  1387992_at  Leukocyte immunoglobulin-like receptor, subfamily B (with TM and ITIM domains), member 3 (predicted)  Info    
   4015 4.34e-05 211.8 139.3 1.52  1388732_at  solute carrier family 35, member F5 (predicted)  Info   Slc35f5_predicted 
   4016 4.36e-05 662.5 871.3 0.76  1370878_at  uroporphyrinogen decarboxylase  Info   Urod 
   4017 4.36e-05 143.6 237 0.606  1385069_at  Activin A receptor, type 1  Info   Acvr1 
   4018 4.36e-05 174.4 51.9 3.36  1384934_at  Transcribed locus  Info    
   4019 4.37e-05 921.9 2395.5 0.385  1377900_at  similar to RIKEN cDNA 1810013B01  Info   RGD:1359649 
   4020 4.38e-05 1905.8 1186.2 1.607  1371838_at  similar to splicing factor, arginine/serine-rich 2  Info   RGD:1359422 
   4021 4.38e-05 972.2 2709.5 0.359  1370365_at  glutathione synthetase  Info   Gss 
   4022 4.39e-05 143.9 438.6 0.328  1372841_at  deleted in polyposis 1-like 1 (predicted)  Info   Dp1l1_predicted 
   4023 4.39e-05 99.4 52 1.912  1389709_at  similar to Zinc finger protein 133  Info   LOC499900 
   4024 4.39e-05 440.2 298.8 1.473  1372154_at  similar to 2010321M09Rik protein  Info   RGD:1359616 
   4025 4.39e-05 364.4 104.1 3.5  1370728_at  interleukin 13 receptor, alpha 1  Info   Il13ra1 
   4026 4.4e-05 253.7 501.7 0.506  1377673_at  Similar to cyclin K  Info    
   4027 4.4e-05 6.2 49.2 0.126  1387804_at  tripartite motif-containing 63  Info   Trim63 
   4028 4.4e-05 125.9 49.7 2.533  1393795_at    Info    
   4029 4.41e-05 280.3 378.3 0.741  1377642_at  caveolin 2  Info   Cav2 
   4030 4.43e-05 20.6 34.6 0.595  1377840_at    Info    
   4031 4.44e-05 74.2 7.1 10.451  1384762_at  Transcribed locus  Info    
   4032 4.44e-05 24.2 68.1 0.355  1397876_at  Transcribed locus  Info    
   4033 4.44e-05 315.5 492.2 0.641  1372828_at  methionine sulfoxide reductase B (predicted)  Info   Mrsb_predicted 
   4034 4.45e-05 46.5 82.3 0.565  1371671_at  G protein-coupled receptor, family C, group 5, member B (predicted)  Info   Gprc5b_predicted 
   4035 4.45e-05 71.5 20.5 3.488  1393149_at    Info   Multiple Gene Symbols 
   4036 4.47e-05 1734.9 1157.6 1.499  1368888_a_at  reticulon 4  Info   Rtn4 
   4037 4.48e-05 1627.8 2147.4 0.758  1371416_at  NADH dehydrogenase (ubiquinone) flavoprotein 1, 51kDa  Info   RGD:1359247 
   4038 4.48e-05 49.4 116.8 0.423  1374874_at  similar to RIKEN cDNA 5730509K17 gene  Info   LOC498386 
   4039 4.48e-05 243.7 460.1 0.53  1374126_at  Transcribed locus  Info    
   4040 4.48e-05 1427.6 985.9 1.448  1371641_at  chaperonin subunit 7 (eta) (predicted)  Info   Cct7_predicted 
   4041 4.48e-05 103.5 7.6 13.618  1387591_at  interleukin 2 receptor, alpha chain  Info   Il2ra 
   4042 4.48e-05 227 108 2.102  1382832_at  similar to CG9135-PA (predicted)  Info   RGD1309986_predicted 
   4043 4.49e-05 447.7 784.2 0.571  1374641_at  Transcribed locus  Info    
   4044 4.49e-05 102.1 204.1 0.5  1393896_at  similar to ionized calcium binding adapter molecule 2 (Iba2) (predicted)  Info   RGD1305081_predicted 
   4045 4.5e-05 159.6 242.2 0.659  1394761_at  similar to Rho-GTPase-activating protein 26 (Oligophrenin-1 like protein) (GTPase regulator associated with focal adhesion kinase)  Info   LOC300350 
   4046 4.5e-05 487.8 400.1 1.219  1383586_at    Info    
   4047 4.5e-05 141.8 416.7 0.34  1396000_at  Transcribed locus  Info    
   4048 4.5e-05 479.1 849.1 0.564  1372655_at    Info    
   4049 4.51e-05 200.5 102.8 1.95  1373238_at  similar to SPT3-associated factor 42 (predicted)  Info   RGD1306774_predicted 
   4050 4.52e-05 67.8 21 3.229  1380130_at    Info    
   4051 4.52e-05 127 70.4 1.804  1394968_at  Transcribed locus  Info    
   4052 4.52e-05 228.2 298 0.766  1387654_at  unconventional myosin Myr2 I heavy chain  Info   RGD:620443 
   4053 4.52e-05 367.3 557.7 0.659  1372362_at  similar to N-acetylgalactosamine kinase (GalNAc kinase) (Galactokinase 2)  Info   LOC296117 
   4054 4.53e-05 112.4 25.4 4.425  1369862_at  proviral integration site 1  Info   Pim1 
   4055 4.54e-05 19 98 0.194  1396014_at  Transcribed locus  Info    
   4056 4.54e-05 63 111.6 0.565  1375016_at  Transcribed locus  Info    
   4057 4.55e-05 70.6 20.1 3.512  1368522_at  timeless homolog (Drosophila)  Info   Timeless 
   4058 4.55e-05 303.3 188 1.613  1390205_at  zinc finger, SWIM domain containing 6 (predicted)  Info   Zswim6_predicted 
   4059 4.57e-05 1050.4 723.6 1.452  1399159_a_at  vesicle-associated membrane protein 3  Info   Vamp3 
   4060 4.57e-05 425.2 199.4 2.132  1388505_at  Transcribed locus  Info    
   4061 4.57e-05 413.9 192.4 2.151  1388363_at  hnRNP-associated with lethal yellow (predicted)  Info   Raly_predicted 
   4062 4.58e-05 799.7 303.4 2.636  1372845_at  ribonuclease P 21kDa subunit (human)  Info   RGD:1303090 
   4063 4.58e-05 303.3 134.7 2.252  1373211_at  F-box and WD-40 domain protein 4 (predicted)  Info   Fbxw4_predicted 
   4064 4.6e-05 134.3 63.9 2.102  1392743_at  myc induced nuclear antigen  Info   Mina 
   4065 4.6e-05 375.2 608.4 0.617  1387782_at  dynein light chain-2  Info   Dlc2 
   4066 4.6e-05 280.5 757.2 0.37  1369289_at  hepatocyte nuclear factor 4, alpha  Info   Hnf4a 
   4067 4.6e-05 246.6 60 4.11  1387306_a_at  early growth response 2  Info   Egr2 
   4068 4.61e-05 20.5 45.5 0.451  1396305_at  Transcribed locus  Info    
   4069 4.61e-05 406.5 541.2 0.751  1398316_at  similar to LEYDIG CELL TUMOR 10 KD PROTEIN  Info   RGD:735128 
   4070 4.61e-05 129.6 200.8 0.645  1393380_at  Transcribed locus  Info    
   4071 4.61e-05 4.4 40.1 0.11  1393443_a_at  similar to CGI-112 protein (predicted)  Info   RGD1308113_predicted 
   4072 4.63e-05 144.8 81.3 1.781  1383629_a_at  CDNA clone IMAGE:7386859  Info    
   4073 4.63e-05 120.3 242.3 0.496  1386615_at  carbohydrate sulfotransferase 2 (predicted)  Info   Chst2_predicted 
   4074 4.65e-05 440 186.9 2.354  1371862_at  ribonucleotide reductase M1  Info   Rrm1 
   4075 4.65e-05 69.8 130.2 0.536  1390957_at  Similar to Protein C14orf101 homolog  Info    
   4076 4.66e-05 19.3 40 0.483  1374999_at  Transcribed locus  Info    
   4077 4.67e-05 249 520.1 0.479  1390777_at  sterol-C5-desaturase (fungal ERG3, delta-5-desaturase) homolog (S. cerevisae)  Info   Sc5d 
   4078 4.68e-05 215.2 319.2 0.674  1374030_at  Transcribed locus  Info    
   4079 4.68e-05 932.6 1286.3 0.725  1389301_at  Muscleblind-like 2 (predicted)  Info    
   4080 4.68e-05 188.5 475.9 0.396  1380905_at  Transcribed locus, weakly similar to NP_083358.1 hypothetical protein LOC74742 [Mus musculus]  Info    
   4081 4.68e-05 370.3 518.8 0.714  1390706_at  spectrin beta 2  Info   Spnb2 
   4082 4.68e-05 3146 3595.4 0.875  1370296_at  sterol carrier protein 2  Info   Scp2 
   4083 4.69e-05 7.4 76.1 0.097  1376789_at  Similar to Myosin light chain kinase 2, skeletal/cardiac muscle (MLCK2) (predicted)  Info    
   4084 4.7e-05 176.4 1544.8 0.114  1387567_at  solute carrier family 21, member 1 /// hypothetical gene supported by NM_017111  Info   Slc21a1 /// LOC497799 
   4085 4.7e-05 56.9 13.1 4.344  1369173_at  complement component 3a receptor 1  Info   C3ar1 
   4086 4.7e-05 173.9 114.3 1.521  1382435_at  fizzy/cell division cycle 20 related 1 (Drosophila) (predicted)  Info   Fzr1_predicted 
   4087 4.71e-05 21.8 52.4 0.416  1384936_at    Info    
   4088 4.71e-05 76.5 10.4 7.356  1379716_at  CD163 antigen (predicted)  Info    
   4089 4.72e-05 59 33.2 1.777  1395687_at  activating transcription factor 7 interacting protein (predicted)  Info   Atf7ip_predicted 
   4090 4.73e-05 20.5 49.5 0.414  1369632_a_at  ATP-binding cassette, sub-family C (CFTR/MRP), member 8  Info   Abcc8 
   4091 4.73e-05 34.8 90.4 0.385  1374947_at  breast cancer anti-estrogen resistance 3 (predicted)  Info   Bcar3_predicted 
   4092 4.73e-05 89.4 190.5 0.469  1382269_at  cyclin M2 (predicted)  Info   Cnnm2_predicted 
   4093 4.74e-05 92.9 33.6 2.765  1370034_at  cell division cycle 25 homolog B (S. cerevisiae)  Info   Cdc25b 
   4094 4.74e-05 1150.3 8 143.787  1379935_at  chemokine (C-C motif) ligand 7  Info   RGD:1359152 
   4095 4.74e-05 1377.5 1028.9 1.339  1388767_at  programmed cell death 6 (predicted)  Info   Pdcd6_predicted 
   4096 4.74e-05 554.6 352.5 1.573  1367691_at  protein kinase C, delta binding protein  Info   Prkcdbp 
   4097 4.74e-05 139.9 336.2 0.416  1385729_at  Transcribed locus  Info    
   4098 4.75e-05 1034 637.2 1.623  1388568_at  eukaryotic translation initiation factor 3 subunit 7  Info   RGD:1303255 
   4099 4.75e-05 332 489.8 0.678  1373388_at  Similar to Hypothetical protein MGC75937  Info    
   4100 4.77e-05 53.9 17.6 3.062  1384943_at  similar to DNA segment, Chr 16, ERATO Doi 472, expressed  Info   LOC360692 
   4101 4.77e-05 124.7 371.5 0.336  1384438_at  Transcribed locus, moderately similar to NP_080232.2 hypothetical protein LOC67082 [Mus musculus]  Info    
   4102 4.78e-05 69.9 32 2.184  1377417_at  similar to AW046014 protein  Info   LOC363328 
   4103 4.79e-05 192.8 107.4 1.795  1389200_at  bystin-like  Info   Bysl 
   4104 4.82e-05 22.2 33.1 0.671  1376830_at  similar to RIKEN cDNA E430013E20 gene (predicted)  Info   RGD1311132_predicted 
   4105 4.82e-05 352.1 240.7 1.463  1371673_at  transcription factor-like 1 (predicted)  Info   Tcfl1_predicted 
   4106 4.82e-05 26.5 69.5 0.381  1380932_at    Info    
   4107 4.82e-05 67.1 44.4 1.511  1389889_at  similar to mKIAA1402 protein (predicted)  Info   RGD1306404_predicted 
   4108 4.82e-05 144.4 332 0.435  1373895_at    Info    
   4109 4.82e-05 3003.4 2193.1 1.369  1369934_at  peptidylprolyl isomerase B  Info   Ppib 
   4110 4.83e-05 140.3 29.8 4.708  1388848_at  similar to hypothetical protein MGC13251 (predicted)  Info   RGD1308350_predicted 
   4111 4.84e-05 111 76.8 1.445  1389417_at  Transcribed locus  Info    
   4112 4.85e-05 6061.6 4429.5 1.368  1371308_at  ribosomal protein S4, X-linked  Info   Rps4x 
   4113 4.85e-05 312 516 0.605  1376619_at  Similar to protein tyrosine phosphatase, receptor type, D  Info    
   4114 4.85e-05 20.6 70.2 0.293  1383153_at  Transcribed locus  Info    
   4115 4.86e-05 154.3 60 2.572  1390859_at  similar to Nedd4 binding protein 1 (predicted)  Info   RGD1305179_predicted 
   4116 4.86e-05 213 446.2 0.477  1383852_at  Transcribed locus  Info    
   4117 4.87e-05 457.4 1277.6 0.358  1398628_at    Info    
   4118 4.87e-05 43.5 13.9 3.129  1379320_at  Small nuclear ribonucleoprotein D1 (predicted)  Info    
   4119 4.88e-05 533.7 338.9 1.575  1372558_at  NMDA receptor-regulated gene 1 (predicted)  Info   Narg1_predicted 
   4120 4.88e-05 181.1 98 1.848  1391604_at  Similar to mKIAA0056 protein  Info    
   4121 4.89e-05 177.3 342.2 0.518  1374622_at  Transcribed locus  Info    
   4122 4.89e-05 32.3 84 0.385  1383258_at  similar to hypothetical protein FLJ14146 (predicted)  Info   RGD1310587_predicted 
   4123 4.89e-05 239.9 128.5 1.867  1376022_at  Transcribed locus  Info    
   4124 4.89e-05 1239.4 241 5.143  1368168_at  solute carrier family 34 (sodium phosphate), member 2  Info   Slc34a2 
   4125 4.89e-05 192.9 127.3 1.515  1383250_at  similar to KIAA0266 gene product  Info   RGD:1359313 
   4126 4.9e-05 60.3 14.2 4.246  1367888_at  MT-protocadherin  Info   Pcdh21 
   4127 4.9e-05 720.9 448 1.609  1372506_at  proteaseome (prosome, macropain) 28 subunit, 3 (predicted)  Info   Psme3_predicted 
   4128 4.91e-05 101.8 269.5 0.378  1378556_at  Ectonucleotide pyrophosphatase/phosphodiesterase 1  Info   Enpp1 
   4129 4.92e-05 81 135 0.6  1383146_at  similar to neurobeachin  Info   LOC361948 
   4130 4.92e-05 174.2 272.4 0.64  1379323_at  similar to hypothetical protein MGC14327 (predicted)  Info   RGD1306304_predicted 
   4131 4.92e-05 107.6 54.4 1.978  1384439_at  CDNA clone IMAGE:7379980  Info    
   4132 4.92e-05 2308.1 1793 1.287  1367455_at  valosin-containing protein  Info   Vcp 
   4133 4.92e-05 56.7 137.4 0.413  1373196_at  EF hand domain family, member A2  Info   Efha2 
   4134 4.93e-05 82.7 129.2 0.64  1381508_at  Transcribed locus  Info    
   4135 4.94e-05 81.5 158.4 0.515  1387296_at  cytochrome P450, family 2, subfamily J, polypeptide 4  Info   Cyp2j4 
   4136 4.94e-05 154.1 228.1 0.676  1373779_at    Info    
   4137 4.95e-05 729.9 510.5 1.43  1384257_at  non-POU domain containing, octamer-binding /// similar to Nono protein  Info   Nono /// LOC501589 
   4138 4.95e-05 138.7 67.7 2.049  1384015_at  cytoplasmic linker associated protein 1 (predicted)  Info   Clasp1_predicted 
   4139 4.97e-05 1217.3 2041.7 0.596  1370838_s_at  alpha-spectrin 2 /// hypothetical gene supported by NM_171983  Info   Spna2 /// LOC497766 
   4140 4.98e-05 107.7 45.4 2.372  1391897_at  similar to rab6 GTPase activating protein (GAP and centrosome-associated) (predicted)  Info   RGD1304620_predicted 
   4141 4.98e-05 401.1 564.9 0.71  1390500_at  similar to RIKEN cDNA 9130023F12 gene (predicted)  Info   RGD1311920_predicted 
   4142 4.98e-05 652.1 398.6 1.636  1371872_at  Nucleosome assembly protein 1-like 1  Info   Nap1l1 
   4143 4.99e-05 19.5 45.1 0.432  1387684_at  peroxisome proliferator activated receptor delta  Info   Ppard 
   4144 5e-05 8.6 28.5 0.302  1389584_at  Transcribed locus  Info    
   4145 5.01e-05 240.7 137.1 1.756  1383231_at  similar to Smad nuclear interacting protein  Info   RGD:1359268 
   4146 5.01e-05 227.5 337.9 0.673  1388336_at  Similar to hypothetical protein D2Ertd391e (predicted)  Info    
   4147 5.02e-05 56.5 174.4 0.324  1377660_at  similar to transthyretin (4L369) (predicted)  Info   RGD1309350_predicted 
   4148 5.02e-05 176.4 388.4 0.454  1389332_at  Transcribed locus  Info    
   4149 5.02e-05 64.9 184.5 0.352  1397620_at  putative scaffolding protein POSH  Info   Sh3md2 
   4150 5.04e-05 468 699.6 0.669  1372109_at    Info    
   4151 5.04e-05 976.7 1774 0.551  1370906_at  branched chain keto acid dehydrogenase E1, beta polypeptide  Info   Bckdhb 
   4152 5.04e-05 111.2 182.6 0.609  1367658_at  SH3/ankyrin domain gene 3 /// hypothetical gene supported by NM_021676  Info   Shank3 /// LOC497826 
   4153 5.05e-05 999 677.8 1.474  1374712_at  proteasome (prosome, macropain) 26S subunit, non-ATPase, 11 (predicted)  Info   Psmd11_predicted 
   4154 5.05e-05 1446.9 3918.8 0.369  1390851_at    Info    
   4155 5.05e-05 461.8 1359.2 0.34  1368498_a_at  kidney specific organic anion transporter  Info   RGD:621387 
   4156 5.06e-05 664.7 458.6 1.449  1372877_at  procollagen-lysine, 2-oxoglutarate 5-dioxygenase 3  Info   Plod3 
   4157 5.06e-05 367.7 1036.2 0.355  1386280_at  similar to RIKEN cDNA 0610006F02 (predicted)  Info   RGD1305205_predicted 
   4158 5.06e-05 498.9 699.7 0.713  1373277_at  similar to BBP-like protein 2  Info   LOC292995 
   4159 5.07e-05 531.8 422 1.26  1389080_at  zinc responsive protein ZD7  Info   RGD:1359343 
   4160 5.07e-05 7 25.5 0.275  1379117_at  Transcribed locus  Info    
   4161 5.07e-05 143.7 221.1 0.65  1388888_at  Transcribed locus  Info    
   4162 5.08e-05 220.2 120.6 1.826  1393267_at  PC4 and SFRS1 interacting protein 1  Info   Psip1 
   4163 5.09e-05 340 230.5 1.475  1393172_at  Ngfi-A binding protein 1  Info   Nab1 
   4164 5.09e-05 148.2 330.9 0.448  1376427_a_at  glycine dehydrogenase (decarboxylating; glycine decarboxylase, glycine cleavage system protein P) (predicted)  Info   Gldc_predicted 
   4165 5.09e-05 99.8 55.8 1.789  1390585_at  mannan-binding lectin serine peptidase 1  Info   Masp1 
   4166 5.1e-05 162.3 112.5 1.443  1390187_at  Transcribed locus  Info    
   4167 5.1e-05 310.1 180.9 1.714  1395739_at  similar to RIKEN cDNA C920006C10 (predicted)  Info   RGD1305976_predicted 
   4168 5.1e-05 63.2 32 1.975  1397527_at  tripartite motif protein 47 (predicted)  Info   Trim47_predicted 
   4169 5.11e-05 356 233.1 1.527  1387977_at  nibrin  Info   Nbn 
   4170 5.11e-05 113.7 35.4 3.212  1370371_a_at  CEA-related cell adhesion molecule 1 /// CEA-related cell adhesion molecule 10  Info   Ceacam1 /// Ceacam10 
   4171 5.12e-05 1758.7 2345.3 0.75  1372080_at  Inner membrane protein, mitochondrial (predicted)  Info    
   4172 5.14e-05 25 3.1 8.065  1397549_at  centaurin, gamma 1  Info   Centg1 
   4173 5.15e-05 114.1 44 2.593  1385066_a_at  Similar to hypothetical protein FLJ13089 (predicted)  Info    
   4174 5.15e-05 177.1 312.5 0.567  1373645_at  Transcribed locus  Info    
   4175 5.16e-05 1490.9 1150.4 1.296  1372259_at  DEK oncogene (DNA binding)  Info   RGD:1303246 
   4176 5.17e-05 2295.2 2968.1 0.773  1367694_at  hydroxyacyl-Coenzyme A dehydrogenase/3-ketoacyl-Coenzyme A thiolase/enoyl-Coenzyme A hydratase (trifunctional protein), beta subunit  Info   Hadhb 
   4177 5.17e-05 1002.5 1861.8 0.538  1389146_at  hypothetical protein LOC498796  Info   LOC498796 
   4178 5.18e-05 23.1 91.7 0.252  1384474_at  Transcribed locus  Info    
   4179 5.19e-05 39.2 8.8 4.455  1378867_at    Info    
   4180 5.21e-05 453.3 277.9 1.631  1378964_at  Similar to hypothetical protein  Info   MGC73022 
   4181 5.23e-05 18.7 150.6 0.124  1370432_at  POU domain, class 3, transcription factor 1  Info   Pou3f1 
   4182 5.23e-05 208.8 108.5 1.924  1393320_at  CDNA clone IMAGE:7375955  Info    
   4183 5.24e-05 149.5 63.4 2.358  1393441_at  similar to cyclin-dependent kinase inhibitor 2D  Info   Cdkn2d 
   4184 5.25e-05 49.6 155.1 0.32  1390656_at  Transcribed locus  Info    
   4185 5.26e-05 1060.9 679.2 1.562  1372704_at  similar to RIKEN cDNA 2310008M10  Info   LOC362040 
   4186 5.27e-05 640.8 445.8 1.437  1375645_at  similar to DNA segment, Chr 4, ERATO Doi 22, expressed  Info   LOC500575 
   4187 5.29e-05 1268.6 2563.8 0.495  1375215_x_at  pyroglutamyl-peptidase I  Info   RGD:1303133 
   4188 5.29e-05 295.3 155.4 1.9  1371879_at  similar to RIKEN cDNA A930011F22 gene (predicted)  Info   RGD1308919_predicted 
   4189 5.29e-05 38.1 77.1 0.494  1383706_at  similar to RIKEN cDNA 2900011O08 (predicted)  Info   RGD1305733_predicted 
   4190 5.3e-05 1878.7 5458.9 0.344  1368121_at  aldo-keto reductase family 7, member A3 (aflatoxin aldehyde reductase)  Info   Akr7a3 
   4191 5.32e-05 198.4 98.4 2.016  1372896_at  Similar to CDNA sequence BC063749  Info    
   4192 5.32e-05 126.4 222.9 0.567  1373619_at  ankyrin repeat domain 10 (predicted)  Info   Ankrd10_predicted 
   4193 5.33e-05 476 1155.8 0.412  1368284_at  plasmalemma vesicle associated protein  Info   Plvap 
   4194 5.35e-05 396.7 810.1 0.49  1372149_at  AU RNA binding protein/enoyl-coenzyme A hydratase (predicted)  Info   Auh_predicted 
   4195 5.36e-05 242.8 421.1 0.577  1388624_at  similar to Hypothetical UPF0184 protein C9orf16 homolog  Info   LOC499780 
   4196 5.36e-05 2.5 39.7 0.063  1397392_at    Info    
   4197 5.37e-05 272.1 482.3 0.564  1374890_at  Transcribed locus, strongly similar to NP_031990.1 ecotropic viral integration site 5 [Mus musculus]  Info    
   4198 5.37e-05 260.8 177.5 1.469  1392481_at  similar to DNA segment, Chr 1, Brigham & Womens Genetics 0212 expressed  Info   LOC363221 
   4199 5.38e-05 181.8 413.2 0.44  1369415_at  basic helix-loop-helix domain containing, class B2  Info   Bhlhb2 
   4200 5.38e-05 3460.4 2821.3 1.227  1367677_at  peroxiredoxin 5  Info   Prdx5 
   4201 5.38e-05 87 166.7 0.522  1389324_at  tyrosine kinase receptor 1  Info   Tie1 
   4202 5.38e-05 57.2 2.2 26  1382459_at    Info    
   4203 5.4e-05 62.9 162.2 0.388  1388145_at  tenascin XA  Info   Tnxa 
   4204 5.4e-05 85.8 43.1 1.991  1387964_a_at  ERO1-like (S. cerevisiae)  Info   Ero1l 
   4205 5.41e-05 12.9 49.2 0.262  1396821_at  Transcribed locus  Info    
   4206 5.42e-05 104.7 187.1 0.56  1374693_at  hypothetical LOC315760 (predicted)  Info   RGD1306243_predicted 
   4207 5.42e-05 2199 13.6 161.691  1393219_at  complement component 2  Info   C2 
   4208 5.43e-05 212.1 99.6 2.13  1372014_at  similar to cofactor required for Sp1 transcriptional activation subunit 8  Info   LOC296612 
   4209 5.43e-05 128.1 198.9 0.644  1389816_at    Info    
   4210 5.43e-05 939 590.8 1.589  1388754_at    Info    
   4211 5.43e-05 59.4 116.8 0.509  1385828_at  Transcribed locus, strongly similar to XP_525786.1 PREDICTED: similar to chromosome 2 open reading frame 7 [Pan troglodytes]  Info    
   4212 5.44e-05 340.1 222.9 1.526  1373704_at  aquarius (predicted)  Info   Aqr_predicted 
   4213 5.44e-05 629 1079 0.583  1371448_at  CDNA clone IMAGE:7303896  Info    
   4214 5.44e-05 301.5 211.8 1.424  1372561_at  similar to hypothetical protein AL133206 (predicted)  Info   RGD1308418_predicted 
   4215 5.45e-05 265 2161.6 0.123  1369401_at  solute carrier family 21, member 13  Info   Slc21a13 
   4216 5.46e-05 175.3 110.2 1.591  1376810_at  ADP-ribosyltransferase (NAD+; poly (ADP-ribose) polymerase)-like 1 (predicted)  Info   Adprtl1_predicted 
   4217 5.47e-05 1195.9 694.3 1.722  1392918_at  chaperonin subunit 8 (theta) (predicted)  Info   Cct8_predicted 
   4218 5.47e-05 205.1 346.9 0.591  1389322_at  programmed cell death protein 7 (predicted)  Info   Pdcd7_predicted 
   4219 5.47e-05 23.8 59.9 0.397  1387209_at  regucalcin gene promotor region related protein  Info   Rgpr 
   4220 5.47e-05 48.6 12.6 3.857  1390397_at  similar to chromosome 20 open reading frame 39 (predicted)  Info   RGD1310753_predicted 
   4221 5.48e-05 56.9 17.3 3.289  1391095_at  matrix metalloproteinase 19 (predicted)  Info   Mmp19_predicted 
   4222 5.49e-05 140.4 101.1 1.389  1373449_at  TAF5-like RNA polymerase II, p300/CBP-associated factor (PCAF)-associated factor (predicted)  Info   Taf5l_predicted 
   4223 5.5e-05 2598.4 3407.7 0.763  1367678_at  succinate dehydrogenase complex, subunit A, flavoprotein (Fp)  Info   Sdha 
   4224 5.5e-05 147.2 225.5 0.653  1376861_at  similar to RIKEN cDNA 1810018L05 /// similar to motile sperm domain containing 1  Info   RGD:1359486 /// LOC501648 
   4225 5.51e-05 454.8 1016.8 0.447  1382111_at  similar to RIKEN cDNA 2010001H14  Info   LOC302554 
   4226 5.52e-05 216.7 127.8 1.696  1383251_at  ADP-ribosyltransferase (NAD+, poly(ADP-ribose) polymerase)-like 2 (predicted)  Info   Adprtl2_predicted 
   4227 5.53e-05 99.6 46.9 2.124  1388987_at  similar to expressed sequence AA960436 (predicted)  Info   RGD1305215_predicted 
   4228 5.55e-05 523.7 321.5 1.629  1375056_at  similar to OCIA domain containing 2  Info   LOC289589 
   4229 5.57e-05 69.4 169 0.411  1380688_at  hypothetical LOC293181 (predicted)  Info   RGD1309623_predicted 
   4230 5.57e-05 38.3 87.4 0.438  1383059_a_at  G kinase anchoring protein 1 (predicted)  Info   Gkap1_predicted 
   4231 5.59e-05 1434.3 494.9 2.898  1387776_at  transglutaminase 2, C polypeptide  Info   Tgm2 
   4232 5.59e-05 124.7 28 4.454  1391063_at  kinesin family member 23 (predicted)  Info   Kif23_predicted 
   4233 5.59e-05 101.7 61.6 1.651  1392508_at  similar to RIKEN cDNA 3110010F15 (predicted)  Info   RGD1308378_predicted 
   4234 5.61e-05 409.5 779 0.526  1375886_at  Transcribed locus  Info    
   4235 5.62e-05 77.2 153.2 0.504  1395572_at  DnaJ (Hsp40) homolog, subfamily A, member 4 (predicted)  Info   Dnaja4_predicted 
   4236 5.63e-05 3459.1 1383.1 2.501  1388557_at  Transcribed locus  Info    
   4237 5.63e-05 205.9 44.5 4.627  1388054_a_at  chondroitin sulfate proteoglycan 2  Info   Cspg2 
   4238 5.63e-05 1309.1 938.1 1.395  1398798_at  methionine aminopeptidase 2  Info   Metap2 
   4239 5.64e-05 37.3 149.1 0.25  1381940_at  Transcribed locus  Info    
   4240 5.64e-05 153.5 39.5 3.886  1369044_a_at  phosphodiesterase 4B  Info   Pde4b 
   4241 5.64e-05 119.8 212.4 0.564  1398361_at  similar to Rho-GTPase-activating protein PS-GAP-a  Info   LOC498938 
   4242 5.64e-05 328.4 511.1 0.643  1375536_at  Numb gene homolog (Drosophila)  Info   Numb 
   4243 5.65e-05 588 1684.4 0.349  1369788_s_at  v-jun sarcoma virus 17 oncogene homolog (avian)  Info   Jun 
   4244 5.65e-05 42.4 85.7 0.495  1368574_at  adrenergic receptor, alpha 1b  Info   Adra1b 
   4245 5.66e-05 489.8 965.7 0.507  1372423_at  PERP, TP53 apoptosis effector (predicted)  Info   Perp_predicted 
   4246 5.66e-05 935 600.8 1.556  1388745_at  sema domain, immunoglobulin domain (Ig), transmembrane domain (TM) and short cytoplasmic domain, (semaphorin) 4A (predicted)  Info   Sema4a_predicted 
   4247 5.67e-05 182.6 335.6 0.544  1373182_at    Info    
   4248 5.68e-05 6.3 20.9 0.301  1396211_at  Similar to RIKEN cDNA 2610207I16 (predicted)  Info    
   4249 5.68e-05 274.4 440.4 0.623  1373079_at  Transcribed locus, weakly similar to XP_342574.1 PREDICTED: similar to Csr1 [Rattus norvegicus]  Info    
   4250 5.69e-05 88.1 48.7 1.809  1394499_at  Taube nuss (predicted)  Info    
   4251 5.7e-05 337 173.3 1.945  1367745_at  diacylglycerol kinase zeta  Info   Dgkz 
   4252 5.7e-05 662.7 402.9 1.645  1379429_at  Transcribed locus  Info    
   4253 5.71e-05 269.7 126.2 2.137  1385806_at  similar to PAP-1  Info   LOC363032 
   4254 5.72e-05 1294.6 694 1.865  1386999_at  tyrosine 3-monooxygenase/tryptophan 5-monooxygenase activation protein, beta polypeptide  Info   Ywhab 
   4255 5.72e-05 79.4 180.2 0.441  1393808_at  Transcribed locus, strongly similar to NP_835187.1 fatty acid 2-hydroxylase [Mus musculus]  Info    
   4256 5.73e-05 128.9 65.4 1.971  1380544_at  Transcribed locus  Info    
   4257 5.73e-05 76.2 17.8 4.281  1368735_a_at  transient receptor potential cation channel, subfamily V, member 2  Info   Trpv2 
   4258 5.73e-05 93.6 255.1 0.367  1392973_at  LOC499153  Info   LOC499153 
   4259 5.73e-05 49.8 3.8 13.105  1368592_at  interleukin 1 alpha  Info   Il1a 
   4260 5.74e-05 210.9 102.9 2.05  1385873_at  Transcribed locus  Info    
   4261 5.74e-05 964.1 1650.5 0.584  1383162_at    Info    
   4262 5.74e-05 65.1 250.2 0.26  1387811_at  angiotensinogen  Info   Agt 
   4263 5.75e-05 98.2 209.8 0.468  1367806_at  glutaminase  Info   Gls 
   4264 5.75e-05 131.4 34 3.865  1386128_at  tetranectin (plasminogen binding protein) (predicted)  Info   Tna_predicted 
   4265 5.76e-05 55.6 183.6 0.303  1381811_at  Transcribed locus  Info    
   4266 5.76e-05 740.6 1223.4 0.605  1379254_at  similar to RIKEN cDNA 1300007B12; clone MNCb-2755 (predicted)  Info   RGD1309892_predicted 
   4267 5.76e-05 1978.5 3233.3 0.612  1370881_at  thiosulfate sulfurtransferase  Info   Tst 
   4268 5.77e-05 122.8 195.4 0.628  1379901_at  TBC1 domain family, member 17 (predicted)  Info   Tbc1d17_predicted 
   4269 5.77e-05 158.8 239.1 0.664  1373434_at  Transcribed locus  Info    
   4270 5.78e-05 23.9 62.9 0.38  1390127_at  Transcribed locus  Info    
   4271 5.78e-05 48.2 7.4 6.514  1398365_at  similar to RIKEN cDNA 2700055K07 (predicted)  Info   RGD1305061_predicted 
   4272 5.79e-05 40.8 101.6 0.402  1369250_at  solute carrier family 28 (sodium-coupled nucleoside transporter), member 1  Info   Slc28a1 
   4273 5.81e-05 528.7 406.7 1.3  1382058_at  related RAS viral (r-ras) oncogene homolog 2 (predicted)  Info   Rras2_predicted 
   4274 5.81e-05 1383 2276.7 0.607  1371727_at    Info    
   4275 5.81e-05 1498.6 2543 0.589  1390822_at  cadherin 16 (predicted)  Info   Cdh16_predicted 
   4276 5.81e-05 110.1 205.6 0.536  1374633_at  Transcribed locus  Info    
   4277 5.82e-05 1256 568.5 2.209  1387769_a_at  Inhibitor of DNA binding 3, dominant negative helix-loop-helix protein  Info   Id3 
   4278 5.82e-05 298.9 477.7 0.626  1371668_at  Retinoid X receptor alpha  Info   Rxra 
   4279 5.82e-05 138.6 226.7 0.611  1399103_at    Info    
   4280 5.83e-05 68 121 0.562  1376242_at  Transcribed locus  Info    
   4281 5.84e-05 507.7 926 0.548  1378753_at  thiopurine methyltransferase  Info   Tpmt 
   4282 5.86e-05 125.3 73.4 1.707  1395377_at  bifunctional apoptosis regulator (predicted)  Info   Bfar_predicted 
   4283 5.86e-05 84.3 13 6.485  1368301_at  adenosine A2a receptor  Info   Adora2a 
   4284 5.86e-05 6448.4 5061.9 1.274  1384548_at  ribosomal protein L32  Info   Rpl32 
   4285 5.86e-05 420.9 3478.3 0.121  1387820_at  kallikrein 7  Info   Klk7 
   4286 5.88e-05 881.1 684.6 1.287  1375205_at  p300/CBP-associated factor (predicted)  Info   Pcaf_predicted 
   4287 5.88e-05 149.1 112.6 1.324  1384005_at  down-regulator of transcription 1 (predicted)  Info   Dr1_predicted 
   4288 5.89e-05 1274.8 589.4 2.163  1371583_at  RNA binding motif (RNP1, RRM) protein 3  Info   Rbm3 
   4289 5.89e-05 317.7 233.1 1.363  1389293_at  Cleavage and polyadenylation specific factor 2 (predicted)  Info    
   4290 5.9e-05 505.4 785.1 0.644  1388679_at  TBC1 domain family, member 14 (predicted)  Info   Tbc1d14_predicted 
   4291 5.9e-05 86.3 10.3 8.379  1392449_at  RAD18 homolog (S. cerevisiae) (predicted)  Info   Rad18_predicted 
   4292 5.92e-05 152.5 83.4 1.829  1374305_at  adaptor protein complex AP-1, gamma 2 subunit (predicted)  Info   Ap1g2_predicted 
   4293 5.93e-05 105.9 54.1 1.957  1389755_at  similar to transcription factor RAM2  Info   LOC619566 
   4294 5.93e-05 12.5 36.4 0.343  1386149_at  Transcribed locus, weakly similar to XP_576460.1 PREDICTED: similar to hypothetical protein PB402898.00.0 [Rattus norvegicus]  Info    
   4295 5.94e-05 896.3 1145 0.783  1367898_at  BCL2/adenovirus E1B 19 kDa-interacting protein 3-like  Info   Bnip3l 
   4296 5.95e-05 246.7 159 1.552  1373935_at  polymerase (DNA directed), delta 2, regulatory subunit (predicted)  Info   Pold2_predicted 
   4297 5.95e-05 353.7 220.1 1.607  1371524_at  gene trap locus 3 (predicted)  Info   Gtl3_predicted 
   4298 5.95e-05 6.4 29.9 0.214  1379076_at  Transcribed locus  Info    
   4299 5.96e-05 78.5 24.3 3.23  1397317_at  Transcribed locus  Info    
   4300 5.96e-05 365.2 699.7 0.522  1391607_at  Similar to Sestrin 2 (Hi95)  Info    
   4301 5.96e-05 441.3 713.8 0.618  1388506_at  desmoplakin (predicted)  Info   Dsp_predicted 
   4302 5.97e-05 2386.3 3263.2 0.731  1373041_at  NADH dehydrogenase (ubiquinone) 1 beta subcomplex 3 (predicted)  Info   Ndufb3_predicted 
   4303 5.97e-05 125.3 47.2 2.655  1387868_at  lipopolysaccharide binding protein  Info   Lbp 
   4304 5.98e-05 1046.4 671.7 1.558  1371888_at  mitochondrial ribosomal protein L24  Info   RGD:1359289 
   4305 5.98e-05 82.5 178.5 0.462  1368334_at  growth factor receptor bound protein 7  Info   Grb7 
   4306 5.98e-05 393.7 245.4 1.604  1373461_at  dermal papilla derived protein 6  Info   RGD:1303303 
   4307 5.99e-05 98.7 160.4 0.615  1370398_at  spermatogenesis associated 7  Info   Spata7 
   4308 5.99e-05 67.4 24.2 2.785  1382624_at    Info    
   4309 6e-05 107 173.5 0.617  1386820_at  Transcribed locus  Info    
   4310 6e-05 623.1 331.4 1.88  1386906_a_at  septin 9  Info   Sept9 
   4311 6.01e-05 63.8 132.8 0.48  1392786_at    Info    
   4312 6.01e-05 18.4 50.4 0.365  1378569_at  Transcribed locus  Info    
   4313 6.01e-05 45.7 112.3 0.407  1385829_at  similar to mKIAA0657 protein  Info   LOC363257 
   4314 6.02e-05 4845.4 3659 1.324  1371340_at  ribosomal protein, large P2 /// similar to 60S acidic ribosomal protein P2  Info   Rplp2 /// LOC498363 
   4315 6.03e-05 135.3 50.4 2.685  1395192_at  origin recognition complex, subunit 3-like (S. cerevisiae) (predicted)  Info   Orc3l_predicted 
   4316 6.03e-05 48.9 75.5 0.648  1377197_at  Transcribed locus  Info    
   4317 6.03e-05 487.6 1003 0.486  1368600_at  solute carrier family 26 (sulfate transporter), member 1  Info   Slc26a1 
   4318 6.03e-05 130.3 35.3 3.691  1392818_at  growth arrest specific 5  Info   Gas5 
   4319 6.03e-05 963.8 1136.4 0.848  1371461_at  similar to RIKEN cDNA 2410166I05 (predicted)  Info   RGD1305453_predicted 
   4320 6.03e-05 50.9 25.6 1.988  1397530_at  Treacher Collins Franceschetti syndrome 1, homolog (predicted)  Info   Tcof1_predicted 
   4321 6.04e-05 553.7 832.1 0.665  1371519_at  electron-transferring-flavoprotein dehydrogenase  Info   Etfdh 
   4322 6.04e-05 47.3 71.8 0.659  1390458_at    Info    
   4323 6.05e-05 157.5 280.6 0.561  1372469_at  Transcribed locus  Info    
   4324 6.06e-05 497.6 361.3 1.377  1384958_at  Transcribed locus  Info    
   4325 6.07e-05 27.1 7.3 3.712  1375781_at  Transcribed locus  Info    
   4326 6.07e-05 7 22.1 0.317  1380828_at  gamma-aminobutyric acid A receptor, alpha 1  Info   Gabra1 
   4327 6.09e-05 40.7 93.6 0.435  1379498_at  Transcribed locus, weakly similar to NP_060380.2 hypothetical protein FLJ20628 [Homo sapiens]  Info    
   4328 6.1e-05 129.9 13.5 9.622  1369006_at  hexokinase 2  Info   Hk2 
   4329 6.1e-05 88.9 22.9 3.882  1379608_at  Transcribed locus  Info    
   4330 6.1e-05 378.5 255.8 1.48  1389228_at  Similar to RIKEN cDNA 2010309E21 (predicted)  Info    
   4331 6.11e-05 1016.1 1507.7 0.674  1370879_at  dihydrolipoamide S-succinyltransferase (E2 component of 2-oxo-glutarate complex)  Info   RGD:1359615 
   4332 6.12e-05 184.3 83.6 2.205  1385800_at    Info    
   4333 6.13e-05 563.1 351.1 1.604  1388560_at  similar to Ac2-269 (predicted)  Info   RGD1310479_predicted 
   4334 6.13e-05 1478 939.8 1.573  1398883_at  heterogeneous nuclear ribonucleoprotein A2/B1 (predicted)  Info   Hnrpa2b1_predicted 
   4335 6.14e-05 37.1 135.9 0.273  1375017_at  Transcribed locus  Info    
   4336 6.14e-05 1327.9 2012.6 0.66  1389286_at  similar to 2900070E19Rik protein (predicted)  Info   RGD1308383_predicted 
   4337 6.15e-05 2922.1 5076.9 0.576  1388300_at  microsomal glutathione S-transferase 3 (predicted)  Info   Mgst3_predicted 
   4338 6.15e-05 299.6 165.6 1.809  1372043_at  similar to ribosomal protein P0-like protein; 60S acidic ribosomal protein PO; ribosomal protein, large, P0-like (predicted)  Info   RGD1311709_predicted 
   4339 6.15e-05 212.2 5.6 37.893  1368683_at  oxidized low density lipoprotein (lectin-like) receptor 1  Info   Oldlr1 
   4340 6.16e-05 3299.6 3565.4 0.925  1367612_at  microsomal glutathione S-transferase 1  Info   Mgst1 
   4341 6.17e-05 28.4 84 0.338  1380120_at  microtubule-associated protein 7 (predicted)  Info   Mtap7_predicted 
   4342 6.17e-05 238.2 148.6 1.603  1389130_at  E74-like factor 2 (predicted)  Info    
   4343 6.17e-05 34.7 20.8 1.668  1385559_at  Transcribed locus  Info    
   4344 6.17e-05 422 271.2 1.556  1394397_at  Ac1158  Info   LOC302898 
   4345 6.18e-05 271.3 142.5 1.904  1367830_a_at  tumor protein p53  Info   Tp53 
   4346 6.18e-05 143.6 277.7 0.517  1389906_at  Farnesyl diphosphate farnesyl transferase 1  Info   Fdft1 
   4347 6.19e-05 240.1 397.4 0.604  1387183_at  carnitine O-octanoyltransferase  Info   Crot 
   4348 6.19e-05 334.3 1509.9 0.221  1370336_at  pregnancy-induced growth inhibitor  Info   Okl38 
   4349 6.19e-05 703.2 488 1.441  1373080_at  poly (A) polymerase alpha (predicted)  Info   Papola_predicted 
   4350 6.2e-05 94.3 55.1 1.711  1385606_at  brain protein 16  Info   RGD:1359226 
   4351 6.2e-05 157.5 101 1.559  1379406_at  similar to chromosome 14 open reading frame 21 (predicted)  Info   RGD1308396_predicted 
   4352 6.2e-05 131.3 59.5 2.207  1381017_at  Transcribed locus  Info    
   4353 6.21e-05 111.3 300.9 0.37  1371472_at  Transcribed locus  Info    
   4354 6.21e-05 61.4 33 1.861  1394511_at  similar to KIAA1915 protein (predicted)  Info   RGD1311787_predicted 
   4355 6.21e-05 777.5 8.4 92.56  1379791_at  CD3 antigen, epsilon polypeptide (predicted)  Info    
   4356 6.23e-05 469.6 232.2 2.022  1376043_at  similar to PHD zinc finger containing protein JUNE1  Info   RGD:1302969 
   4357 6.23e-05 113.8 52.5 2.168  1368205_at  complement factor I  Info   Cfi 
   4358 6.23e-05 47.9 118.3 0.405  1395986_at  slit homolog 2 (Drosophila)  Info   Slit2 
   4359 6.24e-05 751.8 1251.4 0.601  1373016_at  dihydrolipoamide dehydrogenase  Info   Dld 
   4360 6.24e-05 138.9 206.2 0.674  1377598_at  similar to 2310002F18Rik protein  Info   MGC124824 
   4361 6.24e-05 1950.9 1425.4 1.369  1374513_at    Info    
   4362 6.26e-05 235.8 699.1 0.337  1368025_at  DNA-damage-inducible transcript 4  Info   Ddit4 
   4363 6.27e-05 27.2 78.7 0.346  1374262_at  Transcribed locus  Info    
   4364 6.28e-05 618.3 910.9 0.679  1371393_at    Info    
   4365 6.29e-05 36.9 129.1 0.286  1367996_a_at  latrophilin 1  Info   Lphn1 
   4366 6.29e-05 33.3 100.6 0.331  1383432_at  LOC362136 (predicted)  Info   RGD1309610_predicted 
   4367 6.29e-05 21.5 56 0.384  1381264_at  Transcribed locus  Info    
   4368 6.29e-05 336.7 188.9 1.782  1389778_a_at  transcription elongation factor B (SIII), polypeptide 3  Info   Tceb3 
   4369 6.3e-05 125.1 51.7 2.42  1389565_at  Transcribed locus  Info    
   4370 6.3e-05 3829.3 6651.9 0.576  1367988_at  cytochrome P450, family 2, subfamily c, polypeptide 23  Info   Cyp2c23 
   4371 6.31e-05 494.7 359.2 1.377  1372391_at  similar to RIKEN cDNA 1500016H10 (predicted)  Info   RGD1308469_predicted 
   4372 6.31e-05 124.2 216 0.575  1373427_at  Ras-related GTP binding D (predicted)  Info   Rragd_predicted 
   4373 6.32e-05 829.1 567.2 1.462  1370236_at  palmitoyl-protein thioesterase  Info   Ppt 
   4374 6.32e-05 144.9 92.9 1.56  1370979_at  DEAD/H (Asp-Glu-Ala-Asp/His) box polypeptide 20, 103kD  Info   RGD:621744 
   4375 6.33e-05 81.8 153.6 0.533  1384863_at  copine VII (predicted)  Info   Cpne7_predicted 
   4376 6.33e-05 140.8 62.1 2.267  1369700_at  chloride channel 7  Info   Clcn7 
   4377 6.33e-05 1917.9 4761.9 0.403  1367802_at  serum/glucocorticoid regulated kinase  Info   Sgk 
   4378 6.33e-05 359.6 196.2 1.833  1373769_at  phosphoglucomutase 1 (predicted)  Info   Pgm1_predicted 
   4379 6.34e-05 1014 253.3 4.003  1388116_at  collagen, type 1, alpha 1  Info   Col1a1 
   4380 6.34e-05 1353.8 837.4 1.617  1388645_at    Info    
   4381 6.34e-05 54.9 28.1 1.954  1387216_at  lactate dehydrogenase 3, C chain  Info   Ldhc 
   4382 6.35e-05 245.8 93.8 2.62  1395557_at  Core promoter element binding protein  Info   Copeb 
   4383 6.35e-05 2432.8 1694.8 1.435  1387772_at  calmodulin 1  Info   Calm1 
   4384 6.35e-05 123.6 13.1 9.435  1376684_at  discs, large homolog 7 (Drosophila) (predicted)  Info   Dlg7_predicted 
   4385 6.35e-05 168.7 326.5 0.517  1373497_at  Homeodomain interacting protein kinase 2 (predicted)  Info    
   4386 6.36e-05 4125 3050.9 1.352  1370213_at  nuclease sensitive element binding protein 1  Info   Nsep1 
   4387 6.37e-05 458.5 1674.2 0.274  1371412_a_at  Neuronal regeneration related protein  Info   Nrep 
   4388 6.37e-05 68.4 33.8 2.024  1382984_at    Info    
   4389 6.38e-05 153 86.5 1.769  1378825_a_at  Similar to brain specific protein 4  Info    
   4390 6.39e-05 78.9 45.1 1.749  1378180_at  similar to 6030466N05Rik protein  Info   RGD:1359671 
   4391 6.39e-05 191.2 109.3 1.749  1388709_at  similar to WD-repeat protein 43  Info   LOC362703 
   4392 6.39e-05 33.8 117.3 0.288  1397495_at  Similar to Protein C9orf10 (predicted)  Info    
   4393 6.4e-05 230 114.3 2.012  1389471_at  Translocase of outer mitochondrial membrane 34 (predicted)  Info    
   4394 6.41e-05 474.5 307.5 1.543  1388476_at  Tial1 cytotoxic granule-associated RNA binding protein-like 1  Info   Tial1 
   4395 6.42e-05 232.8 130.7 1.781  1369754_a_at  calpastatin  Info   Cast 
   4396 6.42e-05 16.1 34.5 0.467  1379937_at  Interleukin 4 induced 1 (predicted)  Info    
   4397 6.43e-05 1.5 12.5 0.12  1373686_at  serine (or cysteine) proteinase inhibitor, clade A (alpha-1 antiproteinase, antitrypsin), member 6  Info   Serpina6 
   4398 6.43e-05 134.4 206.1 0.652  1390289_at  similar to RIKEN cDNA A530089I17 (predicted)  Info   RGD1311375_predicted 
   4399 6.44e-05 573.1 1053 0.544  1391936_a_at  Transcribed locus  Info    
   4400 6.45e-05 321.7 549.2 0.586  1390380_at  Similar to Ski protein  Info    
   4401 6.46e-05 209 368.9 0.567  1390145_at  Transcribed locus, strongly similar to XP_510406.1 PREDICTED: similar to rabconnectin-3 [Pan troglodytes]  Info    
   4402 6.47e-05 2470.6 3123.1 0.791  1398932_at  histidine triad nucleotide binding protein 1 (predicted)  Info   Hint1_predicted 
   4403 6.47e-05 146.8 238.3 0.616  1374688_at  Tial1 cytotoxic granule-associated RNA binding protein-like 1  Info   Tial1 
   4404 6.47e-05 115.1 624.6 0.184  1368543_at  NADPH oxidase 4  Info   Nox4 
   4405 6.48e-05 54.2 186.4 0.291  1383188_at  Transcribed locus  Info    
   4406 6.48e-05 691.1 600 1.152  1373048_at  ARP10 actin-related protein 10 homolog (S. cerevisiae) (predicted)  Info   Actr10_predicted 
   4407 6.48e-05 35.7 92.4 0.386  1374048_at  neurturin  Info   Nrtn 
   4408 6.48e-05 485.5 913.1 0.532  1367877_at  solute carrier family 11 (proton-coupled divalent metal ion transporters), member 2  Info   Slc11a2 
   4409 6.48e-05 112.4 220.2 0.51  1383104_at  similar to RIKEN cDNA 1810020D17  Info   LOC361606 
   4410 6.51e-05 121.6 23.9 5.088  1369299_at  platelet-activating factor receptor  Info   Ptafr 
   4411 6.51e-05 47.5 73.4 0.647  1375621_at    Info    
   4412 6.51e-05 302.2 13.1 23.069  1397958_at  Transcribed locus  Info    
   4413 6.53e-05 310.1 49.1 6.316  1373528_at  Phosphatidylinositol 3-kinase catalytic delta polypeptide (predicted)  Info    
   4414 6.54e-05 11 33.2 0.331  1389579_at  Transcribed locus  Info    
   4415 6.54e-05 230.3 615.7 0.374  1393018_at  Similar to RING finger protein 33  Info    
   4416 6.56e-05 86.2 200.2 0.431  1383497_at  CDNA clone IMAGE:7300848  Info    
   4417 6.58e-05 237.1 96.1 2.467  1375216_at  poliovirus receptor-related 2 (herpesvirus entry mediator B) (predicted)  Info   Pvrl2_predicted 
   4418 6.58e-05 31.2 91.5 0.341  1395102_at  potassium channel subunit (Slack)  Info   RGD:621106 
   4419 6.58e-05 262.2 659.6 0.398  1380062_at  membrane protein, palmitoylated 6 (MAGUK p55 subfamily member 6) (predicted)  Info   Mpp6_predicted 
   4420 6.6e-05 298.7 496.2 0.602  1373092_at  Transcribed locus  Info    
   4421 6.61e-05 34.9 103.7 0.337  1397838_at  LOC499976  Info    
   4422 6.61e-05 118.4 263.4 0.45  1374432_at    Info    
   4423 6.62e-05 270.2 425.3 0.635  1373056_at  similar to RIKEN cDNA 1110014L17 (predicted)  Info   RGD1306319_predicted 
   4424 6.63e-05 7141.6 5094.8 1.402  1370242_at  ribosomal protein S23  Info   Rps23 
   4425 6.64e-05 187.5 278.6 0.673  1368389_at  amyloid beta (A4) precursor protein-binding, family A, member 3  Info   Apba3 
   4426 6.64e-05 216.5 325.1 0.666  1367926_at  prohibitin  Info   Phb 
   4427 6.65e-05 149.4 225.3 0.663  1377703_at  similar to RIKEN cDNA 2900009I07 (predicted)  Info   RGD1310316_predicted 
   4428 6.65e-05 197.9 307.8 0.643  1376571_at  similar to RIKEN cDNA 0610027O18 (predicted)  Info   RGD1307118_predicted 
   4429 6.66e-05 247.9 464.5 0.534  1372452_at  Glycerol-3-phosphate acyltransferase, mitochondrial  Info   Gpam 
   4430 6.67e-05 76.6 189.4 0.404  1392864_at  Rho GTPase activating protein 5 (predicted)  Info   Arhgap5_predicted 
   4431 6.68e-05 178.9 296 0.604  1372414_at  Transcribed locus  Info    
   4432 6.68e-05 57.7 25.6 2.254  1397669_at  Chemokine (C-C motif) receptor 6 (predicted)  Info    
   4433 6.68e-05 20 79.5 0.252  1369167_at  glial cell line derived neurotrophic factor family receptor alpha 2  Info   Gfra2 
   4434 6.68e-05 22.1 1.9 11.632  1376271_at  AA926063gene  Info   RGD:1303072 
   4435 6.69e-05 248 537.7 0.461  1397647_at  solute carrier family 25 (mitochondrial carrier; ornithine transporter) member 15 (predicted)  Info   Slc25a15_predicted 
   4436 6.7e-05 60.6 30.6 1.98  1390685_at  Scm-like with four mbt domains 1  Info   Sfmbt1 
   4437 6.7e-05 86.3 50.8 1.699  1380101_at  poly(A) polymerase gamma (predicted)  Info   Papolg_predicted 
   4438 6.71e-05 1496.4 658.5 2.272  1372352_at  arginine-rich, mutated in early stage tumors (predicted)  Info   Armet_predicted 
   4439 6.73e-05 1558.7 2872.6 0.543  1370163_at  ornithine decarboxylase 1  Info   Odc1 
   4440 6.73e-05 1057.2 1644.8 0.643  1372791_at  Protein kinase, lysine deficient 1  Info   Prkwnk1 
   4441 6.74e-05 152.4 99.1 1.538  1380186_at  Similar to Zinc finger protein 198 (Fused in myeloproliferative disorders protein) (Rearranged in atypical myeloproliferative disorder protein) (predicted)  Info    
   4442 6.74e-05 128.7 5.8 22.19  1368240_a_at  protein kinase C, beta 1  Info   Prkcb1 
   4443 6.74e-05 85.2 138.4 0.616  1375966_at  Multimerin 2 (predicted)  Info    
   4444 6.74e-05 106.6 55.4 1.924  1376842_at  Transcribed locus  Info    
   4445 6.75e-05 1304.7 1864.2 0.7  1388373_at  adiponectin receptor 1  Info   RGD:1303151 
   4446 6.75e-05 203.1 281.4 0.722  1368056_at  tuberous sclerosis 2  Info   Tsc2 
   4447 6.76e-05 61.6 27.7 2.224  1395524_at  Similar to hypothetical protein FLJ13089 (predicted)  Info    
   4448 6.77e-05 599.3 1022.2 0.586  1370808_at  diaphorase 1  Info   Dia1 
   4449 6.77e-05 138.9 73.9 1.88  1387098_at  RNA polymerase 1-4  Info   Rpo1-4 
   4450 6.77e-05 306.8 209.3 1.466  1392498_at  similar to protein phosphatase 2A B56 regulatory subunit gamma 3 isoform  Info   LOC500722 
   4451 6.78e-05 36.6 131.4 0.279  1374861_at  transducin-like enhancer of split 2, homolog of Drosophila E(spl) (predicted)  Info   Tle2_predicted 
   4452 6.8e-05 124.6 248.1 0.502  1370285_at  KIAA1536 protein  Info   RGD:619812 
   4453 6.8e-05 16.4 48.4 0.339  1388240_a_at  integrin alpha 7  Info   Itga7 
   4454 6.81e-05 332.2 646 0.514  1393551_at    Info    
   4455 6.81e-05 42.9 87.5 0.49  1395659_at    Info    
   4456 6.82e-05 46.3 291.7 0.159  1387265_at  diacylglycerol kinase, gamma  Info   Dgkg 
   4457 6.82e-05 120.1 222.6 0.54  1383757_at  Transcribed locus  Info    
   4458 6.84e-05 188.6 133.1 1.417  1383703_at  FUS interacting protein (serine-arginine rich) 1 (predicted)  Info    
   4459 6.84e-05 1281.8 1092.9 1.173  1368029_at  guanine nucleotide binding protein, alpha inhibiting 3  Info   Gnai3 
   4460 6.85e-05 185.1 29.5 6.275  1378056_at  geminin (predicted)  Info   Gmnn_predicted 
   4461 6.86e-05 68 137.7 0.494  1394458_at  SA rat hypertension-associated homolog  Info   Sah 
   4462 6.87e-05 4507.7 3383.6 1.332  1398774_at  ribosomal protein L30  Info   Rpl30 
   4463 6.88e-05 570.2 404 1.411  1398793_at  cell division cycle 5-like (S. pombe)  Info   Cdc5l 
   4464 6.89e-05 390.4 264.6 1.475  1375857_at  similar to Myoferlin (Fer-1 like protein 3)  Info   LOC309499 
   4465 6.9e-05 283.1 420.4 0.673  1372092_at  Amyotrophic lateral sclerosis 2 (juvenile) chromosome region, candidate 3 homolog (human)  Info   Als2cr3 
   4466 6.9e-05 1405 1991.6 0.705  1371636_at  Similar to novel protein  Info    
   4467 6.9e-05 31.9 91.8 0.347  1390981_at  Polycystic kidney and hepatic disease 1-like 1 (predicted)  Info    
   4468 6.91e-05 3881.3 5070.3 0.765  1371309_at  testis enhanced gene transcript  Info   Tegt 
   4469 6.92e-05 272.1 380.6 0.715  1372942_at  exosome component 5 (predicted)  Info   Exosc5_predicted 
   4470 6.94e-05 81.9 148.5 0.552  1372428_at  DnaJ (Hsp40) homolog, subfamily C, member 4 (predicted)  Info   Dnajc4_predicted 
   4471 6.94e-05 397 562.6 0.706  1398249_at  solute carrier family 25 (carnitine/acylcarnitine translocase), member 20  Info   Slc25a20 
   4472 6.95e-05 439.4 256.1 1.716  1382769_at  interferon (alpha and beta) receptor 1 (predicted)  Info   Ifnar1_predicted 
   4473 6.95e-05 75.8 43.7 1.735  1374891_at  BRF2, subunit of RNA polymerase III transcription initiation factor, BRF1-like (predicted)  Info   Brf2_predicted 
   4474 6.97e-05 152 40.3 3.772  1388142_at  chondroitin sulfate proteoglycan 2  Info   Cspg2 
   4475 6.97e-05 120 12.8 9.375  1383581_at    Info    
   4476 6.98e-05 112 54.5 2.055  1390250_x_at  Atpase, class I, type 8B, member 2 (predicted)  Info    
   4477 6.99e-05 39 105.3 0.37  1369222_at  potassium channel subunit (Slack)  Info   RGD:621106 
   4478 6.99e-05 440.9 638.2 0.691  1390785_at  UBX domain containing 4 (predicted)  Info   Ubxd4_predicted 
   4479 7e-05 644 8.5 75.765  1368128_at  phospholipase A2, group IIA (platelets, synovial fluid)  Info   Pla2g2a 
   4480 7e-05 16 2.3 6.957  1391989_at    Info    
   4481 7e-05 218.4 110.4 1.978  1369946_at  PDRP protein  Info   Pdrp 
   4482 7.01e-05 91.4 59.9 1.526  1374916_at  Transcribed locus  Info    
   4483 7.02e-05 1787.8 1099.8 1.626  1374063_at  splicing factor, arginine/serine-rich 3 (SRp20) (predicted)  Info   Sfrs3_predicted 
   4484 7.03e-05 68.1 96.3 0.707  1379558_at  LOC498681  Info    
   4485 7.04e-05 88.3 221.3 0.399  1370592_at  kidney expressed gene 1  Info   Keg1 
   4486 7.05e-05 246.1 131.2 1.876  1380448_at  AlkB, alkylation repair homolog (E. coli) (predicted)  Info    
   4487 7.05e-05 16.8 81.9 0.205  1384960_at  Transcribed locus  Info    
   4488 7.06e-05 730.2 957 0.763  1370842_at  branched chain keto acid dehydrogenase kinase  Info   Bckdk 
   4489 7.06e-05 494.5 948.3 0.521  1388887_at  Glycoprotein galactosyltransferase alpha 1, 3  Info   Ggta1 
   4490 7.06e-05 604 363.1 1.663  1387891_at  peroxiredoxin 4  Info   Prdx4 
   4491 7.07e-05 454.8 316.3 1.438  1371914_at  SWI/SNF related, matrix associated, actin dependent regulator of chromatin, subfamily b, member 1 (predicted)  Info   Smarcb1_predicted 
   4492 7.07e-05 625.9 798.5 0.784  1371553_at  mitochondrial ribosomal protein L36 (predicted)  Info   Mrpl36_predicted 
   4493 7.09e-05 10.6 34.7 0.305  1378566_at    Info    
   4494 7.1e-05 13.6 62.4 0.218  1379107_at  Solute carrier family 4, member 4  Info   Slc4a4 
   4495 7.1e-05 250.2 483 0.518  1375756_at  Transcribed locus  Info    
   4496 7.12e-05 36.2 106.3 0.341  1369492_at  arylacetamide deacetylase (esterase)  Info   Aadac 
   4497 7.13e-05 159.3 316 0.504  1378288_at  similar to RIKEN cDNA D630035O19 (predicted)  Info   RGD1311100_predicted 
   4498 7.14e-05 271.5 573.3 0.474  1388456_at  S100 calcium binding protein A1  Info   S100a1 
   4499 7.15e-05 52.3 23.4 2.235  1368605_at  adaptor protein with pleckstrin homology and src homology 2 domains  Info   Aps 
   4500 7.15e-05 42.7 15.7 2.72  1369129_at  RAS guanyl releasing protein 1  Info   Rasgrp1 
   4501 7.16e-05 3563.7 2552.1 1.396  1387887_at  ribosomal protein L14  Info   Rpl14 
   4502 7.16e-05 46.7 144.7 0.323  1382685_at    Info    
   4503 7.16e-05 125.8 67.5 1.864  1392830_at  Transcribed locus  Info    
   4504 7.17e-05 33.2 8.3 4  1380277_at  RAD51 associated protein 1 (predicted)  Info    
   4505 7.17e-05 45.6 224.9 0.203  1370816_at  nuclear receptor subfamily 1, group D, member 1  Info   Nr1d1 
   4506 7.18e-05 104.8 238 0.44  1379571_at  plakophilin 4 (predicted)  Info   Pkp4_predicted 
   4507 7.2e-05 158.8 263.9 0.602  1390789_at  similar to 5730439E10Rik protein (predicted)  Info   RGD1306270_predicted 
   4508 7.2e-05 144.8 389.2 0.372  1393414_at  Transcribed locus  Info    
   4509 7.21e-05 210.5 93.7 2.247  1373875_at  similar to RIKEN cDNA 1190005P17 (predicted)  Info   RGD1308261_predicted 
   4510 7.22e-05 182.6 10.1 18.079  1367975_at  annexin A3  Info   Anxa3 
   4511 7.23e-05 4545.6 2795 1.626  1367580_at  ribosomal protein L10A  Info   Rpl10a 
   4512 7.23e-05 52.6 130.4 0.403  1399056_at  similar to CG10585-PA  Info   RGD:1359372 
   4513 7.23e-05 265.3 410.5 0.646  1374394_at  WW domain binding protein 2  Info   Wbp2 
   4514 7.25e-05 82.6 183.4 0.45  1368282_at  dipeptidase 1 (renal)  Info   Dpep1 
   4515 7.25e-05 47.1 15.1 3.119  1394290_at  Transcribed locus  Info    
   4516 7.26e-05 350.6 630 0.557  1374610_at  Similar to hypothetical protein 4933408F15  Info    
   4517 7.27e-05 97.9 170.6 0.574  1374509_at  Similar to RIKEN cDNA 1110018O08  Info   MGC94226 
   4518 7.27e-05 210.7 382.9 0.55  1385025_a_at  transmembrane 7 superfamily member 1 (predicted)  Info   Tm7sf1_predicted 
   4519 7.28e-05 81.8 151.4 0.54  1391559_at  similar to RIKEN cDNA 0610030G03  Info   RGD:1359195 
   4520 7.29e-05 455 288.7 1.576  1387785_at  myotrophin  Info   Mtpn 
   4521 7.29e-05 50.6 20 2.53  1395758_at    Info   LOC301105 /// Syncrip_predicted 
   4522 7.29e-05 306.1 473.3 0.647  1398409_at  Suppression of tumorigenicity 13  Info   St13 
   4523 7.29e-05 42.7 2.5 17.08  1368812_at  hypothetical gene supported by NM_053905  Info   LOC497722 
   4524 7.3e-05 116.1 38 3.055  1378493_at  family with sequence similarity 29, member A (predicted)  Info   Fam29a_predicted 
   4525 7.31e-05 54.1 108.6 0.498  1395761_at  Sema domain, immunoglobulin domain (Ig), short basic domain, secreted, (semaphorin) 3B (predicted)  Info    
   4526 7.31e-05 393.2 217.6 1.807  1373242_at  TATA box binding protein-like 1 (predicted)  Info   Tbpl1_predicted 
   4527 7.33e-05 99.7 57.4 1.737  1389865_at  target of EGR1, member 1 (nuclear) (predicted)  Info   Toe1_predicted 
   4528 7.33e-05 201.6 123.9 1.627  1383133_at  Transcribed locus  Info    
   4529 7.34e-05 243.2 360.4 0.675  1381915_at  Transcribed locus  Info    
   4530 7.34e-05 108.1 227.8 0.475  1374982_at  protein phosphatase 1, regulatory (inhibitor) subunit 16A (predicted)  Info   Ppp1r16a_predicted 
   4531 7.34e-05 214.9 345.1 0.623  1388486_at  Dipeptidylpeptidase 8 (predicted)  Info    
   4532 7.35e-05 99.7 157.8 0.632  1376071_at  Transcribed locus  Info    
   4533 7.36e-05 133.8 63.8 2.097  1378958_at    Info    
   4534 7.36e-05 679.9 492.1 1.382  1371885_at  cytoskeleton-associated protein 1 (predicted)  Info   Ckap1_predicted 
   4535 7.39e-05 324.9 418.8 0.776  1386927_at  carnitine palmitoyltransferase 2  Info   Cpt2 
   4536 7.4e-05 1606.3 584.3 2.749  1370155_at  procollagen, type I, alpha 2  Info   Col1a2 
   4537 7.4e-05 221.5 9.3 23.817  1387125_at  S100 calcium binding protein A9 (calgranulin B)  Info   S100a9 
   4538 7.41e-05 191.8 118.8 1.614  1385314_at  Transcribed locus  Info    
   4539 7.43e-05 336.6 192.8 1.746  1381162_at  Similar to septin 10 isoform 1  Info    
   4540 7.44e-05 12.5 29.2 0.428  1378063_at  Transcribed locus  Info    
   4541 7.45e-05 11.1 26.8 0.414  1369425_at  cadherin 13  Info   Cdh13 
   4542 7.46e-05 91.3 20.1 4.542  1387587_at  tumor necrosis factor (ligand) superfamily, member 6  Info   Tnfsf6 
   4543 7.46e-05 117.6 40.5 2.904  1398553_at  similar to CGI-100-like protein  Info   RGD:1359437 
   4544 7.47e-05 580.7 369.2 1.573  1383160_at  cysteine and histidine-rich domain (CHORD)-containing, zinc-binding protein 1 (predicted)  Info   Chordc1_predicted 
   4545 7.47e-05 378.9 594.2 0.638  1373174_at  similar to 1110001K21Rik protein  Info   LOC497867 
   4546 7.47e-05 204.1 124.8 1.635  1380035_at  Similar to RIKEN cDNA 5830433M19  Info    
   4547 7.48e-05 193.5 78.4 2.468  1386096_at  similar to Metal-response element-binding transcription factor 2 (Metal-response element DNA-binding protein M96) (predicted)  Info   RGD1304727_predicted 
   4548 7.48e-05 518.1 2116.1 0.245  1388172_at  integral membrane transport UST1r  Info   Ust1r 
   4549 7.48e-05 445.4 742 0.6  1373866_at  similar to hypothetical protein FLJ13448  Info   RGD:1359509 
   4550 7.49e-05 203.1 96.4 2.107  1385625_at  EGF-containing fibulin-like extracellular matrix protein 2  Info   RGD:1359496 
   4551 7.5e-05 32.2 64 0.503  1383143_at  similar to Ephb2 protein  Info   LOC313633 
   4552 7.51e-05 160.7 315 0.51  1372889_at  Solute carrier organic anion transporter family, member 2a1  Info   Slco2a1 
   4553 7.51e-05 587.9 747.8 0.786  1372813_at  similar to RIKEN cDNA 1810046J19 (predicted)  Info   RGD1306682_predicted 
   4554 7.53e-05 525.7 318.6 1.65  1388863_at  similar to ubiquitin-conjugating enzyme HR6A  Info   RGD:1359534 
   4555 7.55e-05 175.6 434.3 0.404  1373733_at  Bcl-2-related ovarian killer protein  Info   Bok 
   4556 7.55e-05 116.3 36.8 3.16  1397297_at  Transcribed locus  Info    
   4557 7.56e-05 63.6 23.3 2.73  1378940_at  similar to hypothetical protein FLJ10539 (predicted)  Info   RGD1310610_predicted 
   4558 7.57e-05 472.6 201.3 2.348  1371475_at  ribonuclease, RNase A family 4  Info   Rnase4 
   4559 7.58e-05 146.6 342.6 0.428  1379932_at  Putative chloride channel (similar to Mm Clcn4-2)  Info   Clcn4-2 
   4560 7.59e-05 414.8 610.9 0.679  1379528_at  Transcribed locus  Info    
   4561 7.59e-05 355 200.3 1.772  1390185_at  mRNA decapping enzyme  Info   Dcps 
   4562 7.6e-05 53.5 122 0.439  1379362_at  Transcribed locus  Info    
   4563 7.62e-05 222.6 96.5 2.307  1381403_at  malic enzyme 2, NAD(+)-dependent, mitochondrial (predicted)  Info   Me2_predicted 
   4564 7.63e-05 111.4 240.6 0.463  1370969_at  homeo box A5  Info   Hoxa5 
   4565 7.64e-05 81.3 26.8 3.034  1387600_at  gamma-aminobutyric acid (GABA) A receptor, pi  Info   Gabrp 
   4566 7.64e-05 186.9 393 0.476  1383063_a_at  CDNA clone IMAGE:7379585  Info    
   4567 7.64e-05 76.2 146.3 0.521  1391871_at  Transcribed locus  Info    
   4568 7.64e-05 183.6 5 36.72  1387608_at  indoleamine 2,3-dioxygenase  Info   Indo 
   4569 7.66e-05 108.2 144.2 0.75  1391773_at  Transcribed locus  Info    
   4570 7.67e-05 27.4 105.6 0.259  1378260_at  Alcohol dehydrogenase 1  Info   Adh1 
   4571 7.67e-05 459.1 349.4 1.314  1388586_at  synaptojanin 1  Info   Synj1 
   4572 7.68e-05 81.8 169.4 0.483  1375223_at    Info    
   4573 7.7e-05 79.2 24.7 3.206  1374720_at  Transcribed locus  Info    
   4574 7.71e-05 9.6 20.8 0.462  1385563_at  Similar to Catechol O-methyltransferase  Info    
   4575 7.72e-05 185.5 95.7 1.938  1374704_at  KDEL (Lys-Asp-Glu-Leu) containing 2  Info   Kdelc2 
   4576 7.72e-05 93.3 55.9 1.669  1381217_at  similar to CGI-09 protein (predicted)  Info   RGD1308877_predicted 
   4577 7.72e-05 187.8 10.4 18.058  1378377_at  dual adaptor for phosphotyrosine and 3-phosphoinositides 1 (predicted)  Info   Dapp1_predicted 
   4578 7.72e-05 576.9 827.1 0.697  1390027_at  ubiquitin specific protease 8 (predicted)  Info   Usp8_predicted 
   4579 7.73e-05 92.4 14 6.6  1393020_at  similar to ZNF258 protein  Info   LOC500548 
   4580 7.74e-05 124.3 67.3 1.847  1379858_at  methyltransferase like 2 (predicted)  Info   Mettl2_predicted 
   4581 7.74e-05 6401.6 4792 1.336  1368211_at  ribosomal protein S14  Info   Rps14 
   4582 7.75e-05 27.3 2.6 10.5  1368777_at  BRCA1 associated RING domain 1  Info   Bard1 
   4583 7.76e-05 70.6 7.3 9.671  1398576_at  similar to SIRP beta 1 isoform 1 /// protein tyrosine phosphatase, non-receptor type substrate 1-like 3 (predicted)  Info   LOC310212 /// Ptpns1l3_predicted 
   4584 7.76e-05 3006.9 2376.9 1.265  1375881_at  destrin (predicted)  Info   Dstn_predicted 
   4585 7.76e-05 620.1 319 1.944  1367831_at  tumor protein p53  Info   Tp53 
   4586 7.77e-05 247.8 131.3 1.887  1390389_at    Info    
   4587 7.78e-05 101.1 155.6 0.65  1395354_at  lanC (bacterial lantibiotic synthetase component C)-like 1  Info   Lancl1 
   4588 7.79e-05 119.5 227.4 0.526  1389219_at  Similar to serine/threonine kinase  Info    
   4589 7.8e-05 95.4 46.6 2.047  1375250_at  UDP-Gal:betaGlcNAc beta 1,4- galactosyltransferase, polypeptide 1  Info   B4galt1 
   4590 7.8e-05 653.1 976 0.669  1373625_at  serine hydroxymethyl transferase 1 (soluble) (predicted)  Info   Shmt1_predicted 
   4591 7.81e-05 446.7 839.1 0.532  1377457_a_at  sortilin-related receptor, L(DLR class) A repeats-containing (predicted)  Info   Sorl1_predicted 
   4592 7.81e-05 161.3 354.7 0.455  1368765_at  chloride channel K1  Info   Clcnk1 
   4593 7.81e-05 180.2 427.3 0.422  1381749_at  Transcribed locus  Info    
   4594 7.82e-05 1385.4 1980.4 0.7  1393245_at  Phytanoyl-CoA hydroxylase  Info   Phyh 
   4595 7.82e-05 90.4 171.3 0.528  1374969_at  forkhead box D4  Info   Foxd4 
   4596 7.83e-05 99.2 67.8 1.463  1372216_at  Transcribed locus  Info    
   4597 7.83e-05 196 117 1.675  1374193_at  Transcribed locus  Info    
   4598 7.83e-05 519.7 331.8 1.566  1372709_at  B-cell receptor-associated protein BAP29  Info   RGD:1359431 
   4599 7.84e-05 379 540.7 0.701  1387109_at  P450 (cytochrome) oxidoreductase  Info   Por 
   4600 7.84e-05 5392.1 3846.9 1.402  1371299_at  ribosomal protein S3  Info   Rps3 
   4601 7.84e-05 90.3 25.9 3.486  1377002_at  Transcribed locus  Info    
   4602 7.85e-05 547.2 954 0.574  1375517_at  Tumor protein p53 inducible nuclear protein 2  Info   LOC362246 
   4603 7.85e-05 222.1 635 0.35  1383457_at  similar to hypothetical protein DKFZp761N1114  Info   LOC498228 
   4604 7.87e-05 877 600.5 1.46  1389245_at  proteasome (prosome, macropain) 26S subunit, non-ATPase, 7 (predicted)  Info   Psmd7_predicted 
   4605 7.87e-05 83.8 177.5 0.472  1368676_at  dynein, cytoplasmic, heavy polypeptide 2 /// hypothetical gene supported by NM_023024  Info   Dnch2 /// LOC497827 
   4606 7.88e-05 81.4 182.7 0.446  1372381_at  TSPY-like 4 (predicted)  Info    
   4607 7.88e-05 694.5 517.2 1.343  1373917_at  Transcribed locus  Info    
   4608 7.88e-05 60.8 17.5 3.474  1384087_at    Info    
   4609 7.88e-05 556.7 1012 0.55  1389941_at  ADP-ribosylation factor-like 2 binding protein  Info   Arl2bp 
   4610 7.89e-05 106.1 216.6 0.49  1385904_at  cold inducible RNA binding protein  Info   Cirbp 
   4611 7.9e-05 92.3 189.4 0.487  1389185_at    Info    
   4612 7.9e-05 536.9 321.5 1.67  1371953_at  Cyclin G2 (predicted)  Info   Ccng2 
   4613 7.91e-05 92.3 137.3 0.672  1374199_at  similar to Disco-interacting protein 2 homolog  Info   LOC309692 
   4614 7.91e-05 796.5 1258.8 0.633  1368870_at  Inhibitor of DNA binding 2, dominant negative helix-loop-helix protein  Info   Id2 
   4615 7.92e-05 41.8 75.7 0.552  1380655_at  renal tumor antigen (predicted)  Info   Rage_predicted 
   4616 7.92e-05 687.5 357.6 1.923  1387458_at  ring finger protein 4  Info   Rnf4 
   4617 7.93e-05 421.2 615.8 0.684  1376727_at  similar to RIKEN cDNA 2310034L04 (predicted)  Info   RGD1310157_predicted 
   4618 7.93e-05 38.6 70 0.551  1374785_at  similar to CD69 antigen (p60, early T-cell activation antigen)  Info   LOC296985 
   4619 7.94e-05 34 81.9 0.415  1374062_x_at  microtubule-associated protein, RP/EB family, member 3  Info   RGD:1359297 
   4620 7.95e-05 83 141.7 0.586  1384056_at  Fibroblast growth factor receptor 3  Info   Fgfr3 
   4621 7.95e-05 31.4 74.6 0.421  1377091_at  NG5 protein  Info   RGD:1359348 
   4622 7.96e-05 63.9 125 0.511  1387082_at  fetuin beta  Info   Fetub 
   4623 7.97e-05 160.8 260.6 0.617  1372373_at  Similar to putative WD-repeat protein  Info    
   4624 7.98e-05 264.2 155.6 1.698  1367955_at  RAB4B, member RAS oncogene family  Info   Rab4b 
   4625 8e-05 3027.8 2239.2 1.352  1368141_at  cellular nucleic acid binding protein 1  Info   Cnbp1 
   4626 8e-05 97.5 11.9 8.193  1381533_at  Rho family GTPase 1 (predicted)  Info   Rnd1_predicted 
   4627 8e-05 524.9 272.9 1.923  1370288_a_at  tropomyosin 1, alpha  Info   Tpm1 
   4628 8.01e-05 228.3 153.8 1.484  1390264_at  similar to RIKEN cDNA 1200014J11 (predicted)  Info   RGD1308139_predicted 
   4629 8.03e-05 260.6 117 2.227  1388959_at  similar to KIAA0153 protein (predicted)  Info   RGD1305319_predicted 
   4630 8.03e-05 107.8 164.9 0.654  1372747_at  solute carrier family 20, member 2  Info   Slc20a2 
   4631 8.03e-05 79.7 24.4 3.266  1372321_at  lymphoblastic leukemia derived sequence 1  Info   RGD:1359244 
   4632 8.04e-05 14.1 38.4 0.367  1389806_at  Transcribed locus  Info    
   4633 8.04e-05 159.5 270.3 0.59  1391581_at  Rho GTPase activating protein 5 (predicted)  Info    
   4634 8.04e-05 743 14.7 50.544  1387965_at  kidney injury molecule 1  Info   Havcr1 
   4635 8.05e-05 261 157.5 1.657  1399142_at  GLE1 RNA export mediator-like (yeast (predicted)  Info    
   4636 8.05e-05 7.5 1 7.5  1369685_at  twist homolog 2 (Drosophila)  Info   Twist2 
   4637 8.05e-05 271.7 734.6 0.37  1396152_s_at  insulin-like growth factor binding protein 5  Info   Igfbp5 
   4638 8.06e-05 153.2 304.8 0.503  1372392_at  Transcribed locus  Info    
   4639 8.07e-05 2165.6 2705.4 0.8  1388489_at    Info    
   4640 8.09e-05 177.4 916.4 0.194  1386935_at  nuclear receptor subfamily 4, group A, member 1  Info   Nr4a1 
   4641 8.1e-05 125.1 69.8 1.792  1383355_at  ATP-binding cassette, sub-family A (ABC1), member 1 /// hypothetical gene supported by NM_178095  Info   Abca1 /// LOC497803 
   4642 8.12e-05 33.8 84 0.402  1372615_at  amine oxidase, copper containing 3  Info   Aoc3 
   4643 8.12e-05 15 59 0.254  1368608_at  cytochrome P450, family 2, subfamily f, polypeptide 2  Info   Cyp2f2 
   4644 8.14e-05 29.9 73.1 0.409  1380485_at  Similar to RIKEN cDNA 2600016J21  Info    
   4645 8.14e-05 171.9 114 1.508  1376754_at  cysteinyl-tRNA synthetase (predicted)  Info   Cars_predicted 
   4646 8.15e-05 365.7 145.3 2.517  1389409_at  similar to Testis derived transcript  Info   LOC500040 
   4647 8.15e-05 230.6 124.1 1.858  1390124_at  similar to hypothetical protein FLJ38426  Info   LOC499866 
   4648 8.15e-05 26.5 97 0.273  1373865_at  synaptosomal-associated protein, 91kDa homolog (mouse)  Info   Snap91 
   4649 8.16e-05 94.7 50 1.894  1374888_at  Similar to RIKEN cDNA 1300013D05 (predicted)  Info    
   4650 8.16e-05 172.8 83 2.082  1388757_at  Transcribed locus  Info    
   4651 8.18e-05 627.8 788.5 0.796  1398931_at    Info    
   4652 8.23e-05 49.3 83.9 0.588  1377302_a_at  methylmalonic aciduria (cobalamin deficiency) type A (predicted)  Info   Mmaa_predicted 
   4653 8.24e-05 428.9 281.7 1.523  1372156_at  similar to RIKEN cDNA 1810014L12 (predicted)  Info   RGD1307423_predicted 
   4654 8.24e-05 82.2 28.3 2.905  1380210_at    Info    
   4655 8.25e-05 6041.4 3928.6 1.538  1375181_at  similar to 60S ribosomal protein L12  Info   LOC499782 
   4656 8.27e-05 835.9 581 1.439  1368386_at  growth factor receptor bound protein 2  Info   Grb2 
   4657 8.27e-05 51.9 11.5 4.513  1368570_at  lecithin-retinol acyltransferase /// hypothetical gene supported by NM_022280  Info   Lrat /// LOC497758 
   4658 8.28e-05 2132.2 1501.5 1.42  1371244_at  adenine phosphoribosyl transferase (predicted)  Info   Aprt_predicted 
   4659 8.31e-05 1098.9 594 1.85  1370941_at  platelet derived growth factor receptor, alpha polypeptide  Info   Pdgfra 
   4660 8.32e-05 8224.7 5225.9 1.574  AFFX_Rat_beta-actin_M_at  actin, beta  Info   Actb 
   4661 8.32e-05 231.1 94.1 2.456  1388128_at  ARP3 actin-related protein 3 homolog (yeast)  Info   Actr3 
   4662 8.33e-05 410.1 251.9 1.628  1374815_at  STARD3 N-terminal like (predicted)  Info   Stard3nl_predicted 
   4663 8.33e-05 309.3 114.7 2.697  1377743_at  Sprouty protein with EVH-1 domain 1, related sequence (predicted)  Info    
   4664 8.33e-05 262.6 465.5 0.564  1374139_at  similar to cerebellar degeneration-related 2 (predicted)  Info   RGD1310578_predicted 
   4665 8.35e-05 99.1 152.1 0.652  1390987_at  Transcribed locus  Info    
   4666 8.37e-05 81.6 30.1 2.711  1369393_at  mitogen-activated protein kinase kinase kinase 8  Info   Map3k8 
   4667 8.37e-05 620.5 375.7 1.652  1372620_at  acidic (leucine-rich) nuclear phosphoprotein 32 family, member E (predicted)  Info   Anp32e_predicted 
   4668 8.38e-05 96.3 35 2.751  1374359_at  Similar to RIKEN cDNA 2810013E07 (predicted)  Info    
   4669 8.39e-05 7.2 25.7 0.28  1373326_at  Transcribed locus  Info    
   4670 8.4e-05 218 152 1.434  1388847_at  Positive cofactor 2, multiprotein complex, glutamine/Q-rich-associated protein (predicted)  Info    
   4671 8.41e-05 195.4 109.8 1.78  1383175_a_at  similar to C11orf17 protein (predicted)  Info   RGD1306959_predicted 
   4672 8.42e-05 1714.8 1160.9 1.477  1367472_at  similar to ubiquitin-protein ligase (EC 6.3.2.19) E1 - mouse  Info   RGD:1359327 
   4673 8.42e-05 16.2 34.1 0.475  1383084_at  Similar to hypothetical protein FLJ23033  Info    
   4674 8.43e-05 191.4 250.3 0.765  1377785_at  DEAH (Asp-Glu-Ala-His) box polypeptide 40  Info   RGD:1359618 
   4675 8.43e-05 452.6 676.1 0.669  1390845_at  Transcribed locus  Info    
   4676 8.43e-05 153.1 297.6 0.514  1377232_at  similar to hypothetical protein (predicted)  Info   RGD1305269_predicted 
   4677 8.43e-05 226.3 140.5 1.611  1385883_at  Transcribed locus  Info    
   4678 8.43e-05 141.5 256.1 0.553  1374613_at  Transcribed locus  Info    
   4679 8.43e-05 48.8 128.3 0.38  1370474_at  thyroid hormone receptor beta  Info   Thrb 
   4680 8.43e-05 2001.3 1468.1 1.363  1398768_at  retinoblastoma binding protein 7  Info   Rbbp7 
   4681 8.44e-05 78.1 5.3 14.736  1370999_at  sperm associated antigen 5  Info   Spag5 
   4682 8.45e-05 346.4 723.3 0.479  1392928_at  similar to RIKEN cDNA 2810055F11 (predicted)  Info   RGD1305721_predicted 
   4683 8.46e-05 134 78.7 1.703  1393184_at  P21 (CDKN1A)-activated kinase 2  Info   Pak2 
   4684 8.47e-05 239.9 323.7 0.741  1377887_at  Acyl-Coenzyme A oxidase 3, pristanoyl  Info   Acox3 
   4685 8.47e-05 188.6 290.5 0.649  1376651_at  ADP-ribosylation factor guanine nucleotide-exchange factor 2 (brefeldin A-inhibited)  Info   Arfgef2 
   4686 8.47e-05 104.4 58.8 1.776  1383338_at  Transcribed locus  Info    
   4687 8.47e-05 770.2 1787.3 0.431  1379281_at  uterine sensitization-associated gene 1 protein  Info   Sostdc1 
   4688 8.48e-05 146.7 226.1 0.649  1377850_at  Zinc and ring finger 1 (predicted)  Info    
   4689 8.49e-05 32.6 18.1 1.801  1382587_at  Family with sequence similarity 33, member A (predicted)  Info   MGC109093 
   4690 8.49e-05 90.4 180.3 0.501  1387503_at  carboxypeptidase N, polypeptide 1, 50kD  Info   Cpn1 
   4691 8.51e-05 93.6 236.9 0.395  1373300_at    Info    
   4692 8.51e-05 34.3 2.1 16.333  1387603_at  killer cell lectin-like receptor subfamily C, member 2  Info   Klrc2 
   4693 8.52e-05 136.6 315.7 0.433  1387505_at  guanine nucleotide binding protein, alpha inhibiting 1  Info   Gnai1 
   4694 8.53e-05 128.6 280.2 0.459  1373321_at  Transcribed locus, strongly similar to XP_574622.1 PREDICTED: similar to UPF0197 protein C11orf10 homolog [Rattus norvegicus]  Info    
   4695 8.54e-05 65.7 17.5 3.754  1368544_a_at  nucleolar protein 3 (apoptosis repressor with CARD domain)  Info   Nol3 
   4696 8.55e-05 466.7 676.3 0.69  1376585_at  mitochondrial ribosomal protein L50 (predicted)  Info   Mrpl50_predicted 
   4697 8.6e-05 94.4 250.6 0.377  1394070_at  Transcribed locus  Info    
   4698 8.6e-05 58.5 30.5 1.918  1398270_at  bone morphogenetic protein 2  Info   Bmp2 
   4699 8.6e-05 704.9 3552 0.198  1367961_at  nerve growth factor, gamma  Info   Ngfg 
   4700 8.61e-05 834.4 485.9 1.717  1398925_at  similar to RIKEN cDNA 1300018I05 (predicted)  Info   RGD1307801_predicted 
   4701 8.67e-05 189.9 76 2.499  1377137_at  tyrosyl-DNA phosphodiesterase 1 (predicted)  Info   Tdp1_predicted 
   4702 8.67e-05 86 28.1 3.06  1386987_at  interleukin 6 receptor  Info   Il6r 
   4703 8.68e-05 770.2 387 1.99  1368948_at  moesin  Info   Msn 
   4704 8.7e-05 27.9 69.3 0.403  1388152_at  microtubule-associated protein 2  Info   Mtap2 
   4705 8.71e-05 487.8 355.8 1.371  1383262_at  Unknown (protein for MGC:72591)  Info   Tmem19 
   4706 8.71e-05 1015.7 1296.6 0.783  1388681_at  SAR1a gene homolog 2 (S. cerevisiae) (predicted)  Info   Sara2_predicted 
   4707 8.73e-05 487.7 229.9 2.121  1388089_a_at  ring finger protein 4  Info   Rnf4 
   4708 8.73e-05 120.2 210.4 0.571  1376824_at  Similar to RIKEN cDNA 1700027M01 (predicted)  Info    
   4709 8.74e-05 50.8 12.2 4.164  1394527_at  Transcribed locus  Info    
   4710 8.74e-05 13.7 1.4 9.786  1380421_s_at  growth factor independent 1  Info   Gfi1 
   4711 8.74e-05 38.7 90.8 0.426  1387803_at  protein phosphatase 2 (formerly 2A), regulatory subunit B (PR 52), beta isoform  Info   Ppp2r2b 
   4712 8.77e-05 308.2 174.8 1.763  1390032_at  RNA binding motif, single stranded interacting protein 2 (predicted)  Info   Rbms2_predicted 
   4713 8.8e-05 118.8 186.3 0.638  1374303_at  similar to prostate cancer antigen-1 (predicted)  Info   RGD1306377_predicted 
   4714 8.83e-05 126.6 4.7 26.936  1368413_at  amiloride binding protein 1  Info   Abp1 
   4715 8.83e-05 40.9 188.8 0.217  1387364_at  folate hydrolase  Info   Folh1 
   4716 8.83e-05 1876.9 2483.1 0.756  1387271_at  phytanoyl-CoA hydroxylase  Info   Phyh 
   4717 8.83e-05 938 1367.8 0.686  1371557_at  THAP domain containing 4  Info   RGD:1359473 
   4718 8.84e-05 51.4 190.9 0.269  1387812_at  Subtilisin - like endoprotease  Info   Pace4 
   4719 8.86e-05 36.8 74.3 0.495  1373254_at  keratin 10  Info   RGD:1359092 
   4720 8.86e-05 101.3 80.3 1.262  1383638_at  CDNA clone IMAGE:7380769  Info    
   4721 8.86e-05 824.7 435.3 1.895  1388130_at  zyxin  Info   Zyx 
   4722 8.87e-05 73.9 43.6 1.695  1378611_at  Similar to encephalopsin  Info    
   4723 8.89e-05 88.5 180.2 0.491  1371793_at  Transcribed locus, strongly similar to NP_081632.1 hypothetical protein LOC70240 [Mus musculus]  Info    
   4724 8.9e-05 80.1 209.8 0.382  1390449_at  CDNA clone IMAGE:7309127  Info    
   4725 8.9e-05 60.4 131 0.461  1368614_at  Williams-Beuren syndrome chromosome region 14 homolog (human)  Info   Wbscr14 
   4726 8.9e-05 687.6 1562.1 0.44  1373978_at  nuclear cap binding protein subunit 1, 80kDa (predicted)  Info   Ncbp1_predicted 
   4727 8.97e-05 106.4 176.2 0.604  1375182_at  Similar to RIKEN cDNA D030028O16 (predicted)  Info   MGC105586 
   4728 8.97e-05 837.9 1256.6 0.667  1373529_at  Transcribed locus  Info    
   4729 8.97e-05 92.5 156.8 0.59  1392992_at  Transcribed locus  Info    
   4730 8.97e-05 101.3 225.1 0.45  1377404_at  stanniocalcin 1  Info   Stc1 
   4731 8.98e-05 250.8 83.6 3  1384488_at  similar to cyclin-dependent kinase inhibitor 2D  Info   Cdkn2d 
   4732 8.98e-05 22.3 48.9 0.456  1375790_at    Info    
   4733 9e-05 217 301.5 0.72  1380813_at    Info    
   4734 9e-05 265.1 396.4 0.669  1374547_at  Similar to RIKEN cDNA A130038L21  Info    
   4735 9.01e-05 296.6 510.6 0.581  1375628_at  NADH dehydrogenase (ubiquinone) 1 beta subcomplex 8 (predicted)  Info   Ndufb8_predicted 
   4736 9.02e-05 163 304 0.536  1392644_s_at  similar to RIKEN cDNA 2610019F03  Info   LOC498662 
   4737 9.03e-05 302.9 493.4 0.614  1389652_at  Transcribed locus  Info    
   4738 9.04e-05 249.6 457 0.546  1396013_at  leucine zipper-EF-hand containing transmembrane protein 1  Info   RGD:1359678 
   4739 9.06e-05 209 138.4 1.51  1372689_at  similar to hypothetical protein FLJ20211 (predicted)  Info   RGD1305478_predicted 
   4740 9.06e-05 54.8 88.4 0.62  1387007_at  glial cell line derived neurotrophic factor family receptor alpha 1  Info   Gfra1 
   4741 9.07e-05 127.8 61.7 2.071  1393955_at  WD repeat domain 44  Info   Wdr44 
   4742 9.1e-05 55.5 164.8 0.337  1368159_at  ATP-binding cassette, sub-family B (MDR/TAP), member 6  Info   Abcb6 
   4743 9.1e-05 94.8 140 0.677  1374708_at  similar to Rho guanine nucleotide exchange factor (GEF) 10  Info   LOC306618 
   4744 9.1e-05 395.1 265.9 1.486  1373475_at  hypothetical LOC288065  Info   LOC288065 
   4745 9.11e-05 128 64.6 1.981  1375612_at  Transcribed locus  Info    
   4746 9.11e-05 92.4 184.7 0.5  1382363_at  membrane protein, palmitoylated 5 (MAGUK p55 subfamily member 5) (predicted)  Info   Mpp5_predicted 
   4747 9.12e-05 1359.8 907 1.499  1372711_at  similar to cDNA sequence BC005537  Info   LOC498750 
   4748 9.12e-05 110.9 41.4 2.679  1383685_at  similar to BAP28 protein  Info   LOC361262 
   4749 9.13e-05 746.4 270.6 2.758  1376327_at  tumor necrosis factor receptor superfamily, member 14 (herpesvirus entry mediator) (predicted)  Info   Tnfrsf14_predicted 
   4750 9.14e-05 131.8 244.4 0.539  1387498_a_at  Fibroblast growth factor receptor 1  Info   Fgfr1 
   4751 9.14e-05 2452.5 1868.3 1.313  1370168_at  tyrosine 3-monooxygenase/tryptophan 5-monooxygenase activation protein, theta polypeptide  Info   Ywhaq 
   4752 9.15e-05 64.1 19.4 3.304  1389421_at  polymerase (DNA directed), epsilon  Info   Pole 
   4753 9.15e-05 71.5 169.1 0.423  1398245_at  synuclein, gamma  Info   Sncg 
   4754 9.16e-05 1085.1 810.9 1.338  1388376_at  similar to RIKEN cDNA 0610038D11 (predicted)  Info   RGD1309710_predicted 
   4755 9.16e-05 138.5 249.3 0.556  1373859_at  Transcribed locus  Info    
   4756 9.16e-05 5 57.3 0.087  1378876_at  Transcribed locus  Info    
   4757 9.18e-05 169.8 93.8 1.81  1374288_at  FtsJ homolog 3 (E. coli) (predicted)  Info   Ftsj3_predicted 
   4758 9.18e-05 45.4 182.5 0.249  1368597_at  SNF1-like kinase  Info   Snf1lk 
   4759 9.18e-05 220.4 325.1 0.678  1374580_at  SUMO/sentrin specific protease 6 (predicted)  Info   Senp6_predicted 
   4760 9.18e-05 103.3 56.5 1.828  1374252_at  centrosomal protein 1 (predicted)  Info   Cep1_predicted 
   4761 9.18e-05 112.4 242 0.464  1390944_at  Snf7 homologue associated with Alix 3  Info   MGC108776 
   4762 9.2e-05 287.3 609.3 0.472  1380465_at  Transcribed locus  Info    
   4763 9.21e-05 97 53.2 1.823  1379514_at  Transcribed locus  Info    
   4764 9.24e-05 77.9 188.9 0.412  1376847_at  CDNA clone IMAGE:7323916  Info    
   4765 9.25e-05 408.5 298.7 1.368  1370325_at  golgi reassembly stacking protein 2  Info   Gorasp2 
   4766 9.27e-05 216.3 78.7 2.748  1372200_at  G-protein signalling modulator 3 (AGS3-like, C. elegans)  Info   RGD:1303014 
   4767 9.29e-05 731.9 469 1.561  1388488_at  LSM3 homolog, U6 small nuclear RNA associated (S. cerevisiae) (predicted)  Info   Lsm3_predicted 
   4768 9.31e-05 115.1 178 0.647  1373810_at  phospholipase A2, group XIIA (predicted)  Info   Pla2g12a_predicted 
   4769 9.32e-05 1133.1 1852.6 0.612  1398333_at  Endothelial PAS domain protein 1  Info   Epas1 
   4770 9.33e-05 86.1 50.4 1.708  1376947_at  retinoblastoma binding protein 6 (predicted)  Info   Rbbp6_predicted 
   4771 9.34e-05 65.2 109.7 0.594  1398366_at  LOC497968  Info   LOC497968 
   4772 9.34e-05 541.3 686.1 0.789  1392937_at  cyclin I (predicted)  Info   Ccni_predicted 
   4773 9.35e-05 628.5 408.4 1.539  1389038_at  eukaryotic translation termination factor 1 (predicted)  Info   Etf1_predicted 
   4774 9.35e-05 135.9 208.8 0.651  1377697_at  Similar to amyloid beta (A4) precursor protein-binding, family B, member 2  Info    
   4775 9.37e-05 152.4 228.7 0.666  1395852_at  ATP-binding cassette, sub-family A (ABC1), member 3 (predicted)  Info   Abca3_predicted 
   4776 9.37e-05 1169.4 1673.2 0.699  1388196_at  NCK-associated protein 1  Info   Nckap1 
   4777 9.38e-05 885.2 660.7 1.34  1387798_a_at  complement receptor related protein  Info   Crry 
   4778 9.38e-05 199.1 46.2 4.31  1371232_a_at  chondroitin sulfate proteoglycan 2  Info   Cspg2 
   4779 9.38e-05 174.2 107.6 1.619  1374736_at  PR domain containing 4  Info   Prdm4 
   4780 9.4e-05 155.9 75.2 2.073  1376751_at  serine/threonine kinase 19  Info   RGD:1359601 
   4781 9.4e-05 169.5 77.8 2.179  1386410_at  Transcribed locus  Info    
   4782 9.4e-05 157.4 10 15.74  1376496_at  hypothetical protein LOC503164  Info   LOC503164 
   4783 9.43e-05 17.1 1.5 11.4  1396065_at  Transcribed locus  Info    
   4784 9.43e-05 38.4 76.9 0.499  1397622_at    Info    
   4785 9.43e-05 219.9 429.1 0.512  1389730_at  Transcribed locus  Info    
   4786 9.44e-05 236.9 126.2 1.877  1373114_at  Transcribed locus  Info    
   4787 9.44e-05 27.8 3.2 8.688  1379469_at  similar to transducin (beta)-like 1 X-linked  Info   LOC302711 
   4788 9.45e-05 28 51.2 0.547  1394694_at    Info    
   4789 9.46e-05 145 86.9 1.669  1389790_at  Transcribed locus  Info    
   4790 9.46e-05 182.3 153.9 1.185  1374848_at  Similar to RIKEN cDNA 2410016F19 (predicted)  Info    
   4791 9.46e-05 377.9 298.3 1.267  1392662_at  similar to RIKEN cDNA 1500009M05  Info   LOC295457 
   4792 9.47e-05 205.2 467.9 0.439  1369679_a_at  nuclear factor I/A  Info   Nfia 
   4793 9.49e-05 237.9 122.3 1.945  1370752_a_at  tumor protein p53  Info   Tp53 
   4794 9.51e-05 280.1 209.3 1.338  1398386_at  Transcribed locus, strongly similar to NP_705766.2 zinc finger, CCHC domain containing 6 [Mus musculus]  Info    
   4795 9.51e-05 345.4 649.4 0.532  1376885_at  Similar to chemokine-like factor super family 4  Info    
   4796 9.52e-05 2180.6 5696.6 0.383  1384831_at  solute carrier family 7, (cationic amino acid transporter, y+ system) member 13 (predicted)  Info   Slc7a13_predicted 
   4797 9.53e-05 252.3 474 0.532  1375657_at  Transcribed locus  Info    
   4798 9.55e-05 372 276.1 1.347  1373494_at  breakpoint cluster region (predicted)  Info   Bcr_predicted 
   4799 9.55e-05 151.1 60.5 2.498  1383945_at  uridine monophosphate kinase (predicted)  Info   Umpk_predicted 
   4800 9.57e-05 1876 2509 0.748  1370006_at  NADH dehydrogenase (ubiquinone) Fe-S protein 6  Info   Ndufs6 
   4801 9.58e-05 554.3 955.3 0.58  1384027_a_at  Transcribed locus  Info    
   4802 9.58e-05 92.9 169.7 0.547  1390990_at  Protein phosphatase 1, catalytic subunit, beta isoform  Info   Ppp1cb 
   4803 9.58e-05 67.4 107.6 0.626  1378433_at  similar to RIKEN cDNA 5730557B15  Info   LOC310200 
   4804 9.6e-05 48.4 116.4 0.416  1370932_at  low density lipoprotein receptor-related protein 4  Info   Lrp4 
   4805 9.6e-05 150.4 83.5 1.801  1384340_a_at  similar to N-acetyltransferase ARD1  Info   LOC363518 
   4806 9.6e-05 174.4 84.4 2.066  1388666_at  ectodermal-neural cortex 1  Info   RGD:1303152 
   4807 9.63e-05 276.2 196.5 1.406  1372433_at  similar to CG11030-PA (predicted)  Info   RGD1310211_predicted 
   4808 9.63e-05 864.8 1563.6 0.553  1398847_at  nudix (nucleoside diphosphate linked moiety X)-type motif 4  Info   Nudt4 
   4809 9.63e-05 47.4 159.8 0.297  1395190_at  aldo-keto reductase family 1, member B10 (aldose reductase) (predicted)  Info   Akr1b10_predicted 
   4810 9.66e-05 161.6 107 1.51  1393088_at  RNA (guanine-9-) methyltransferase domain containing 1 (predicted)  Info   Rg9mtd1_predicted 
   4811 9.68e-05 4173.7 6811.4 0.613  1371237_a_at  Metallothionein  Info   Mt1a 
   4812 9.68e-05 133.9 85.2 1.572  1368537_at  dynactin 4  Info   Dctn4 
   4813 9.69e-05 105.2 62.7 1.678  1375819_at  similar to RIKEN cDNA 1200011I18 (predicted)  Info   RGD1307254_predicted 
   4814 9.7e-05 314.3 183.5 1.713  1373943_at  CDNA clone IMAGE:7365681  Info    
   4815 9.7e-05 933.2 1448.5 0.644  1398360_at  CDNA clone MGC:93791 IMAGE:7109632  Info    
   4816 9.7e-05 31.7 52.8 0.6  1371085_at  achaete-scute complex homolog-like 3 (Drosophila)  Info   Ascl3 
   4817 9.71e-05 108.9 52 2.094  1385634_at    Info    
   4818 9.72e-05 16.7 39 0.428  1369000_at  neurotrophic tyrosine kinase, receptor, type 1  Info   Ntrk1 
   4819 9.74e-05 55.9 135 0.414  1373977_at  Kinesin family member 5C (predicted)  Info    
   4820 9.74e-05 112.4 42.8 2.626  1393730_at  Transcribed locus  Info    
   4821 9.75e-05 239.7 159.7 1.501  1377745_at  similar to hypothetical protein FLJ20331  Info   LOC310946 
   4822 9.75e-05 166.1 241.5 0.688  1374572_at  similar to opposite strand transcription unit to Stag3; Gats protein (predicted)  Info   RGD1304774_predicted 
   4823 9.75e-05 940.4 638.7 1.472  1387911_at  Rab geranylgeranyl transferase componenet, subunit beta  Info   Rabggtb 
   4824 9.77e-05 83.3 37.8 2.204  1397851_at  Similar to QUAKING isoform 6  Info   Qki 
   4825 9.79e-05 198.6 98.2 2.022  1379862_at    Info    
   4826 9.79e-05 89.7 53.5 1.677  1383352_at  Transcribed locus  Info    
   4827 9.8e-05 650.2 368.6 1.764  1372627_at    Info    
   4828 9.81e-05 188.1 160.1 1.175  1389455_at  SEC24 related gene family, member B (S. cerevisiae) (predicted)  Info   Sec24b_predicted 
   4829 9.82e-05 172.4 317.5 0.543  1383300_at  Dre1 protein  Info   Dre1 
   4830 9.84e-05 44.9 78.2 0.574  1383212_at  CDNA clone IMAGE:7462850  Info    
   4831 9.84e-05 592.8 1240 0.478  1388103_at  protein distantly related to to the gamma subunit family  Info   Pr1 
   4832 9.85e-05 35.7 91.3 0.391  1377437_at  similar to Veph-A (predicted)  Info   RGD1308217_predicted 
   4833 9.86e-05 95.7 140.3 0.682  1380002_at  spire homolog 1 (Drosophila) (predicted)  Info   Spire1_predicted 
   4834 9.87e-05 338.7 582 0.582  1388749_at  Similar to hypothetical protein FLJ13910 (predicted)  Info    
   4835 9.87e-05 110 57.8 1.903  1383176_at  similar to C11orf17 protein (predicted)  Info   RGD1306959_predicted 
   4836 9.88e-05 134.9 222.8 0.605  1375463_at  Similar to Ski protein  Info    
   4837 9.89e-05 240.1 473.4 0.507  1387059_at  serine/threonine kinase 39, STE20/SPS1 homolog (yeast)  Info   Stk39 
   4838 9.9e-05 90 43.3 2.079  1384889_at  Transcribed locus  Info    
   4839 9.91e-05 463.6 339.7 1.365  1385660_at  similar to KIAA2010 protein (predicted)  Info   RGD1309059_predicted 
   4840 9.93e-05 18.8 38.8 0.485  1376243_at  similar to serine (or cysteine) proteinase inhibitor, clade B (ovalbumin), member 12  Info   LOC304692 
   4841 0.0001001 384.1 279.3 1.375  1373116_at  Karyopherin (importin) alpha 3 (predicted)  Info    
   4842 0.0001001 391.2 620.3 0.631  1398935_at  Transcribed locus, strongly similar to XP_533460.1 PREDICTED: similar to Sorting nexin-9 [Canis familiaris]  Info    
   4843 0.0001004 385 680.4 0.566  1372257_at  Transcribed locus  Info    
   4844 0.0001005 46.1 199.1 0.232  1377013_at  Transcribed locus  Info    
   4845 0.0001007 220.6 148.2 1.489  1389613_at  Similar to MGC68837 protein  Info    
   4846 0.0001009 577.4 342.9 1.684  1372471_at  UDP-glucose ceramide glucosyltransferase-like 1  Info   Ugcgl1 
   4847 0.0001009 100.1 31 3.229  1373250_at    Info    
   4848 0.000101 127.4 78.9 1.615  1377893_at  Transcribed locus  Info    
   4849 0.0001013 55.4 25.7 2.156  1386321_s_at  tribbles homolog 3 (Drosophila)  Info   Trib3 
   4850 0.0001014 682.2 414.9 1.644  1374430_at  similar to RIKEN cDNA 2700085E05 (predicted)  Info   RGD1307010_predicted 
   4851 0.0001017 326.9 251 1.302  1391628_at  synovial sarcoma translocation, Chromosome 18 (predicted)  Info   Ss18_predicted 
   4852 0.000102 94.4 58.2 1.622  1379905_at  GTP binding protein 1 (predicted)  Info   Gtpbp1_predicted 
   4853 0.0001021 200.1 64.6 3.098  1387377_a_at  p21 (CDKN1A)-activated kinase 1  Info   Pak1 
   4854 0.0001021 30.2 2.2 13.727  1395960_at  Transcribed locus, moderately similar to NP_862904.1 epidermodysplasia verruciformis 2 [Mus musculus]  Info    
   4855 0.0001021 106.1 61.3 1.731  1382350_at  signal transducer and activator of transcription 6 (predicted)  Info   Stat6_predicted 
   4856 0.0001022 1855.1 1457.5 1.273  1388446_at  similar to heterogeneous nuclear ribonucleoprotein A0  Info   LOC498696 
   4857 0.0001027 274.8 589.1 0.466  1370009_at  apolipoprotein C-III  Info   Apoc3 
   4858 0.000103 50.7 103.7 0.489  1384747_at  transmembrane 7 superfamily member 1 (predicted)  Info   Tm7sf1_predicted 
   4859 0.000103 112.5 198.8 0.566  1380854_at  vascular endothelial growth factor B  Info   Vegfb 
   4860 0.0001034 405.1 173.4 2.336  1368984_at  septin 2  Info   Sept2 
   4861 0.0001035 454.4 768.4 0.591  1371747_at  similar to RIKEN cDNA 2700038C09 (predicted)  Info   RGD1310660_predicted 
   4862 0.0001036 72.7 35.1 2.071  1372729_at  protein C receptor, endothelial (predicted)  Info   Procr_predicted 
   4863 0.0001036 429.3 283.1 1.516  1382046_at  mitogen activated protein kinase kinase kinase 3 (predicted)  Info   Map3k3_predicted 
   4864 0.0001039 340.9 491.1 0.694  1388896_at  ubiquitin specific protease 33 (predicted)  Info   Usp33_predicted 
   4865 0.0001039 195.5 124.1 1.575  1398259_at  nucleoporin 155  Info   Nup155 
   4866 0.0001039 314.4 147 2.139  1390333_at  protein phosphatase 4 (formerly X), catalytic subunit  Info   RGD:621225 
   4867 0.000104 537.4 293.4 1.832  1399125_at  inositol polyphosphate-1-phosphatase (predicted)  Info   Inpp1_predicted 
   4868 0.0001041 681.7 481.3 1.416  1369962_at  5-aminoimidazole-4-carboxamide ribonucleotide formyltransferase/IMP cyclohydrolase  Info   Atic 
   4869 0.0001044 79.8 180.7 0.442  1367912_at  latent transforming growth factor beta binding protein 1  Info   Ltbp1 
   4870 0.0001045 285.6 442.8 0.645  1382126_at    Info    
   4871 0.0001046 987.4 684.5 1.443  1371189_x_at  laminin receptor 1 (67kD, ribosomal protein SA) /// similar to 40S ribosomal protein SA (p40) (34/67 kDa laminin receptor) /// similar to Ribosomal protein SA  Info   Lamr1 /// LOC367030 /// LOC367303 
   4872 0.0001047 50.5 28.4 1.778  1381118_at  similar to RIKEN cDNA 2700007P21 (predicted)  Info   RGD1311463_predicted 
   4873 0.0001049 352.2 233 1.512  1398999_at  similar to es 64 /// similar to hypothetical protein  Info   LOC363675 /// LOC497993 
   4874 0.0001051 203.5 131.8 1.544  1389582_at  Transcribed locus  Info    
   4875 0.0001051 889 2121.6 0.419  1368814_at  aldehyde dehydrogenase family 6, subfamily A1  Info   Aldh6a1 
   4876 0.0001052 120.9 276.6 0.437  1380152_at  Similar to RIKEN cDNA 2700059L22 (predicted)  Info    
   4877 0.0001054 119 58 2.052  1376001_at  similar to RNA polymerase I associated factor (PAF53)  Info   LOC313245 
   4878 0.0001054 1787.7 1160.4 1.541  1388313_at  ribosomal protein s25  Info   Rps25 
   4879 0.0001055 689.5 583.5 1.182  1390514_at  similar to hypothetical protein from BCRA2 region  Info   RGD:1359603 
   4880 0.0001055 110.2 255.5 0.431  1387036_at  hairy and enhancer of split 1 (Drosophila)  Info   Hes1 
   4881 0.0001056 190.7 423.9 0.45  1371615_at  diacylglycerol O-acyltransferase homolog 2 (mouse)  Info   Dgat2 
   4882 0.0001056 76.5 128.3 0.596  1380053_at  similar to transmembrane protein TM9SF3  Info   LOC309475 
   4883 0.0001058 80.6 38.3 2.104  1376253_at  lymphocyte antigen 6 complex, locus H (predicted)  Info   Ly6h_predicted 
   4884 0.0001062 160.4 98.5 1.628  1369209_at  p34 protein  Info   RGD:621601 
   4885 0.0001062 28.1 59.3 0.474  1391730_at  Myeloid/lymphoid or mixed-lineage leukemia 2 (predicted)  Info    
   4886 0.0001062 37.4 88.3 0.424  1387809_at  mitogen-activated protein kinase kinase 6  Info   Map2k6 
   4887 0.0001064 64.6 103.7 0.623  1382047_at  similar to AI325464 protein (predicted)  Info   RGD1307976_predicted 
   4888 0.0001064 53.7 103.9 0.517  1394450_at  Transcribed locus, moderately similar to XP_484812.1 PREDICTED: hypothetical protein XP_484812 [Mus musculus]  Info    
   4889 0.0001065 3115.4 4169.9 0.747  1387675_at  Plasminogen activator, urokinase  Info   Plau 
   4890 0.0001067 385.7 238.2 1.619  1386394_at    Info    
   4891 0.0001067 93.9 149.6 0.628  1387039_at  glypican 1  Info   Gpc1 
   4892 0.0001068 288.8 605.8 0.477  1392534_at  transmembrane, prostate androgen induced RNA (predicted)  Info   Tmepai_predicted 
   4893 0.0001068 6055.3 5053.7 1.198  1388296_at  ribosomal protein S18  Info   Rps18 
   4894 0.000107 146.1 73.3 1.993  1391837_at  similar to chr2 synaptotagmin  Info   LOC299488 
   4895 0.000107 142.5 288.9 0.493  1397788_at  RIM binding protein 2  Info   LOC266780 
   4896 0.0001072 1985.8 2656.6 0.747  1369927_at  malate dehydrogenase, mitochondrial  Info   Mor1 
   4897 0.0001075 111.8 139.6 0.801  1383504_at  Transcribed locus  Info    
   4898 0.0001076 106.5 158.5 0.672  1376416_at    Info    
   4899 0.0001077 159.2 313.9 0.507  1371915_at  poly(rC) binding protein 4 (predicted)  Info   Pcbp4_predicted 
   4900 0.000108 48 121.9 0.394  1398431_at  carbonic anhydrase 8 (predicted)  Info   Car8_predicted 
   4901 0.0001081 811.1 488.7 1.66  1376579_at  leucine aminopeptidase 3 (predicted)  Info   Lap3_predicted 
   4902 0.0001082 99 15.2 6.513  1393629_at  H2.0-like homeo box 1 (Drosophila) (predicted)  Info   Hlx1_predicted 
   4903 0.0001083 365 188.5 1.936  1374458_at  Transcribed locus, strongly similar to NP_573483.1 serum/glucocorticoid regulated kinase 3 [Mus musculus]  Info    
   4904 0.0001084 8 35.4 0.226  1374832_at  Histone 1, H2bh (predicted)  Info   Hfe 
   4905 0.0001088 23.2 57.3 0.405  1381208_at  histone 2a  Info   RGD:621437 
   4906 0.0001089 213.5 150.8 1.416  1374615_at    Info    
   4907 0.000109 123.4 52.4 2.355  1379234_a_at  similar to CDC45L  Info   LOC287961 
   4908 0.000109 3767.8 7912.2 0.476  1370688_at  glutamate-cysteine ligase, catalytic subunit  Info   Gclc 
   4909 0.0001091 619.2 925 0.669  1389128_at  WD repeat and FYVE domain containing 3 (predicted)  Info    
   4910 0.0001093 717.1 443.2 1.618  1372401_at  N-acetylneuraminic acid synthase (sialic acid synthase) (predicted)  Info   Nans_predicted 
   4911 0.0001094 154.8 77.2 2.005  1374961_at    Info    
   4912 0.0001098 45.5 81.5 0.558  1375562_at  Transcribed locus  Info    
   4913 0.00011 280.8 473.6 0.593  1390937_at  similar to chromosome 14 open reading frame 50 (predicted)  Info   RGD1309051_predicted 
   4914 0.00011 66.1 181.4 0.364  1379300_at  carbohydrate sulfotransferase 2 (predicted)  Info   Chst2_predicted 
   4915 0.0001101 96.9 54.5 1.778  1375273_at  tripartite motif protein 47 (predicted)  Info   Trim47_predicted 
   4916 0.0001101 40.4 93.9 0.43  1383397_at  receptor-associated protein of the synapse (predicted)  Info   Rapsn_predicted 
   4917 0.0001101 120.6 53.3 2.263  1374313_at  BPY2 interacting protein 1 (predicted)  Info   Bpy2ip1_predicted 
   4918 0.0001102 82.8 224 0.37  1372626_at  Similar to tumor protein D53  Info    
   4919 0.0001103 724.3 488.3 1.483  1389526_at  similar to RIKEN cDNA C920006C10 (predicted)  Info   RGD1305976_predicted 
   4920 0.0001104 97.7 226 0.432  1393352_at  similar to hypothetical protein MGC38960 (predicted)  Info   RGD1310552_predicted 
   4921 0.0001104 335.3 569 0.589  1376047_at  3'-phosphoadenosine 5'-phosphosulfate synthase 2 (predicted)  Info    
   4922 0.0001105 224.9 374.4 0.601  1376792_at  Transcribed locus  Info    
   4923 0.0001105 600.8 224.4 2.677  1387060_at  core promoter element binding protein  Info   Copeb 
   4924 0.0001106 191.2 133.7 1.43  1388934_at  vacuolar protein sorting 16  Info   RGD:1359414 
   4925 0.0001106 203.9 346.4 0.589  1376656_at  ubiquitin specific protease 20 (predicted)  Info   Usp20_predicted 
   4926 0.0001106 41.2 112.3 0.367  1397579_x_at  Similar to RIKEN cDNA 2310011J03  Info   MGC94198 
   4927 0.0001108 112.4 52.6 2.137  1390790_a_at  hypothetical LOC361349  Info   LOC361349 
   4928 0.0001109 274.1 135.5 2.023  1383336_at  similar to pinin  Info   LOC368070 
   4929 0.0001109 220.1 130 1.693  1374331_at  rcd1 (required for cell differentiation) homolog 1 (S. pombe) (predicted)  Info   Rqcd1_predicted 
   4930 0.0001109 323.8 199.4 1.624  1389450_at  similar to Putative methyltransferase HUSSY-03 (Williams-Beuren syndrome chromosome region 22 protein homolog) /// similar to Putative methyltransferase HUSSY-03 (Williams-Beuren syndrome chromosome region 22 protein homolog)  Info   LOC360830 /// LOC368084 
   4931 0.000111 212.5 375.9 0.565  1389511_s_at    Info    
   4932 0.0001111 57.9 30.8 1.88  1391441_at  Transcribed locus  Info    
   4933 0.0001111 152.4 89.8 1.697  1397385_at  Transcribed locus  Info    
   4934 0.0001112 113 182.5 0.619  1374112_at  similar to MAPK-interacting and spindle-stabilizing protein  Info   LOC499280 
   4935 0.0001112 265 212.3 1.248  1387117_at  zinc finger protein 265  Info   Zfp265 
   4936 0.0001113 49.9 193.8 0.257  1396238_at  UDP-N-acetyl-alpha-D-galactosamine:polypeptide N-acetylgalactosaminyltransferase 14 (predicted)  Info   Galnt14_predicted 
   4937 0.0001113 47.8 113.5 0.421  1368174_at  hypothetical gene supported by NM_019371  Info   LOC497816 
   4938 0.0001114 96.7 153.3 0.631  1380379_at  Similar to : F-box protein 44  Info    
   4939 0.0001115 37 120.6 0.307  1391241_at  Transcribed locus  Info    
   4940 0.0001115 43.5 89 0.489  1389774_at  Transcribed locus  Info    
   4941 0.0001115 621.3 891.2 0.697  1388978_at  Similar to RIKEN cDNA 2010200I23  Info   MGC94262 
   4942 0.0001117 441.2 325.7 1.355  1371891_at  similar to 1700123O20Rik protein (predicted)  Info   RGD1308430_predicted 
   4943 0.0001117 255.6 489.7 0.522  1393143_at  Transcribed locus  Info    
   4944 0.0001118 507.3 305.4 1.661  1372501_at  splicing factor 3b, subunit 3 (predicted)  Info   Sf3b3_predicted 
   4945 0.000112 297.1 214.7 1.384  1388151_at  coronin 7  Info   Coro7 
   4946 0.000112 179.1 258 0.694  1389388_at  Transcribed locus  Info    
   4947 0.0001123 116 195.7 0.593  1382318_at    Info    
   4948 0.0001123 275.1 415.5 0.662  1373564_at  3-hydroxyisobutyryl-Coenzyme A hydrolase (predicted)  Info   Hibch_predicted 
   4949 0.0001124 92.1 48.7 1.891  1395560_at  Transcribed locus  Info    
   4950 0.0001125 2025.9 2575.7 0.787  1388370_at  cyclin I (predicted)  Info   Ccni_predicted 
   4951 0.0001127 134 87.7 1.528  1376663_at  similar to hypothetical protein 6720484B16 (predicted)  Info   RGD1304881_predicted 
   4952 0.0001128 137.3 328.7 0.418  1369560_at  glycerol-3-phosphate dehydrogenase 1 (soluble)  Info   Gpd1 
   4953 0.0001128 907.8 478.2 1.898  1371382_at  similar to Filamin A (Alpha-filamin) (Filamin 1) (Endothelial actin-binding protein) (Actin-binding protein 280) (ABP-280) (Nonmuscle filamin)  Info   LOC293860 
   4954 0.000113 118.7 28.5 4.165  1374722_at  C1q and tumor necrosis factor related protein 1  Info   RGD:1359204 
   4955 0.0001131 551.9 265.1 2.082  1367860_a_at  matrix metalloproteinase 14 (membrane-inserted)  Info   Mmp14 
   4956 0.0001132 26.8 60.6 0.442  1378954_at    Info    
   4957 0.0001132 380.5 261.7 1.454  1375440_at  peptidylprolyl isomerase (cyclophilin)-like 2 (predicted)  Info   Ppil2_predicted 
   4958 0.0001134 64.4 145 0.444  1377009_at  Transcribed locus  Info    
   4959 0.0001136 114.3 74.1 1.543  1378467_at  Transcribed locus  Info    
   4960 0.0001137 200.7 294.3 0.682  1379449_at  RB1-inducible coiled-coil 1 (predicted)  Info   Rb1cc1_predicted 
   4961 0.0001138 411 616 0.667  1383733_at  Adenylate kinase 3  Info   Ak3 
   4962 0.000114 310.7 162.2 1.916  1372363_at  similar to polymerase (RNA) II (DNA directed) polypeptide H /// similar to polymerase (RNA) II (DNA directed) polypeptide H  Info   LOC291795 /// LOC498109 
   4963 0.000114 511.3 727 0.703  1373161_at  similar to RIKEN cDNA 6530411B15  Info   RGD:1359736 
   4964 0.000114 43.3 76.4 0.567  1371004_at  sortilin 1  Info   Sort1 
   4965 0.0001142 158.9 319.2 0.498  1367644_at    Info    
   4966 0.0001144 93 134.6 0.691  1398413_at  zinc finger protein 444 (predicted)  Info   Znf444_predicted 
   4967 0.0001144 190.5 296 0.644  1376202_at  UDP-GlcNAc:betaGal beta-1,3-N-acetylglucosaminyltransferase 6 (predicted)  Info   B3gnt6_predicted 
   4968 0.0001145 93.8 180 0.521  1390942_at  Transcribed locus  Info    
   4969 0.0001146 62.7 216.3 0.29  1387550_a_at  solute carrier family 14 (urea transporter), member 2  Info   Slc14a2 
   4970 0.0001146 36 8.2 4.39  1387160_at  potassium voltage-gated channel, Isk-related subfamily, member 3  Info   Kcne3 
   4971 0.0001148 599.3 428.2 1.4  1371764_at  similar to Ubc6p homolog  Info   RGD:1359523 
   4972 0.000115 228.3 364.9 0.626  1390173_at  Transcribed locus  Info    
   4973 0.0001152 82 179.3 0.457  1383593_at  Transcribed locus  Info    
   4974 0.0001152 21.8 115.9 0.188  1369864_a_at  serine dehydratase  Info   Sds 
   4975 0.0001152 19.3 50.6 0.381  1377354_at  Transcribed locus  Info    
   4976 0.0001152 199.3 86.3 2.309  1368194_at  1-acylglycerol-3-phosphate O-acyltransferase 4  Info   Agpat4 
   4977 0.0001159 45.2 78.1 0.579  1389913_at    Info    
   4978 0.0001159 30.8 153 0.201  1379491_at  similar to Fat cell-specific low molecular weight protein (Fat tissue-specific low MW protein) (predicted)  Info   RGD1310648_predicted 
   4979 0.000116 24.2 68 0.356  1379672_at  Transcribed locus  Info    
   4980 0.0001163 105.9 37.9 2.794  1390249_at  similar to DKFZP434H132 protein (predicted)  Info   RGD1305464_predicted 
   4981 0.0001166 1288.2 800.6 1.609  1388764_at  IQ motif containing GTPase activating protein 1 (predicted)  Info    
   4982 0.0001167 264.9 173.8 1.524  1377905_at  similar to RIKEN cDNA 2700023B17 (predicted)  Info   RGD1305695_predicted 
   4983 0.0001169 124 72.1 1.72  1373833_at  similar to RIKEN cDNA 3110040N11 (predicted)  Info   RGD1305713_predicted 
   4984 0.0001176 211.8 303.3 0.698  1381779_s_at  mannosidase, alpha, class 2C, member 1  Info   Man2c1 
   4985 0.0001176 16.7 49.3 0.339  1384634_at    Info    
   4986 0.0001178 5265.2 3637.1 1.448  1367596_at  ribosomal protein S26  Info   Rps26 
   4987 0.0001178 130.2 95.7 1.361  1378104_at  similar to RIKEN cDNA 8430406I07 (predicted)  Info   RGD1307465_predicted 
   4988 0.000118 132.4 297.8 0.445  1391509_at  tumor-associated calcium signal transducer 2  Info   RGD:1359498 
   4989 0.000118 203.8 151 1.35  1372846_at  similar to hypothetical protein MGC20446 (predicted)  Info   RGD1304670_predicted 
   4990 0.000118 132 197.4 0.669  1372638_at  Transcribed locus  Info    
   4991 0.0001181 45.7 101.9 0.448  1374444_at  Plexin B1 (predicted)  Info   LOC316009 
   4992 0.0001186 859.6 1115 0.771  1371619_at  similar to HTGN29 protein; keratinocytes associated transmembrane protein 2 (predicted)  Info   RGD1310352_predicted 
   4993 0.0001187 111.2 48.6 2.288  1396286_at  Transcribed locus  Info    
   4994 0.0001188 106.2 231.6 0.459  1375484_at  Transcribed locus  Info    
   4995 0.0001188 317.3 231.7 1.369  1393101_at  F-box and leucine-rich repeat protein 10 (predicted)  Info   Fbxl10_predicted 
   4996 0.0001188 23.1 55.4 0.417  1370456_at  FAT tumor suppressor homolog 3 (Drosophila)  Info   Fat3 
   4997 0.000119 121.1 75.1 1.613  1387810_at  Kelch-like ECH-associated protein 1  Info   Keap1 
   4998 0.0001191 291.8 227.4 1.283  1389000_at  Transcribed locus  Info    
   4999 0.0001191 67.2 20.8 3.231  1369018_at  forkhead box M1  Info   Foxm1 
   5000 0.0001192 1644.6 1076.7 1.527  1370062_at  hypoxia induced gene 1  Info   RGD:620215 
   5001 0.0001193 99.4 178.7 0.556  1389585_at    Info    
   5002 0.0001195 207.5 126.5 1.64  1392487_at  Transcribed locus  Info    
   5003 0.0001196 248.2 111.7 2.222  1369650_at  p21 (CDKN1A)-activated kinase 2  Info   Pak2 
   5004 0.0001196 216.4 394.7 0.548  1375726_at  LIM domain only protein 7  Info   RGD:1302984 
   5005 0.0001196 33 125.8 0.262  1392510_at  similar to hypothetical protein B230314O19 (predicted)  Info   RGD1308013_predicted 
   5006 0.0001197 165.7 397.4 0.417  1370675_at  transient receptor potential cation channel, subfamily V, member 1  Info   Trpv1 
   5007 0.0001198 1890 2555 0.74  1398287_at  plasminogen activator, urokinase  Info   Plau 
   5008 0.0001199 52.4 17.8 2.944  1379780_at  Similar to mKIAA0386 protein (predicted)  Info    
   5009 0.0001201 3284.8 6097.7 0.539  1370943_at  sulfotransferase family, cytosolic, 1C, member 1 (predicted)  Info   Sult1c1_predicted 
   5010 0.0001202 238.1 481.8 0.494  1383795_at  CDNA clone IMAGE:7379585  Info    
   5011 0.0001203 48 107.5 0.447  1378327_at  doublesex and mab-3 related transcription factor 2 (predicted)  Info   Dmrt2_predicted 
   5012 0.0001203 102.1 264.9 0.385  1375647_at  Transcribed locus  Info    
   5013 0.0001203 5.9 1.3 4.538  1391596_at  Transcribed locus  Info    
   5014 0.0001203 158.8 222.8 0.713  1389529_at  Transcribed locus  Info    
   5015 0.0001204 1794.3 1368.9 1.311  1388507_at  integrin beta 4 binding protein (predicted)  Info   Itgb4bp_predicted 
   5016 0.0001205 96.8 158.7 0.61  1377679_at  G protein-coupled receptor 125 (predicted)  Info   Gpr125_predicted 
   5017 0.0001206 148.2 221.4 0.669  1375945_at  plexin domain containing 2 (predicted)  Info   Plxdc2_predicted 
   5018 0.0001206 293.1 20 14.655  1397167_at    Info    
   5019 0.0001206 17.9 34.8 0.514  1384096_at  Transcribed locus  Info    
   5020 0.0001207 2325 1627.6 1.428  1371656_at  chaperonin subunit 4 (delta)  Info   Cct4 
   5021 0.0001211 42.3 19.5 2.169  1390681_at  Similar to TGFB-induced factor 2  Info    
   5022 0.0001212 86.3 20.9 4.129  1385853_at  bromodomain adjacent to zinc finger domain, 1A (predicted)  Info   Baz1a_predicted 
   5023 0.0001214 1883.6 3265.2 0.577  1372665_at  phosphoserine aminotransferase 1  Info   Psat1 
   5024 0.0001214 28.1 11.4 2.465  1392788_at  Sprouty protein with EVH-1 domain 1, related sequence (predicted)  Info    
   5025 0.0001215 5895.9 4629.9 1.273  1367610_at  ribosomal protein L19  Info   Rpl19 
   5026 0.0001216 2639.1 3360.5 0.785  1371342_at  cytochrome c-1 (predicted)  Info   Cyc1_predicted 
   5027 0.0001216 118.5 44.2 2.681  1380474_at    Info    
   5028 0.0001218 131.9 238.8 0.552  1389801_at  fibroblast growth factor receptor 4  Info   Fgfr4 
   5029 0.0001219 42.9 6.3 6.81  1380559_at  Transcribed locus  Info    
   5030 0.0001219 1017.5 1853.4 0.549  1389649_at  Transcribed locus  Info    
   5031 0.0001221 83.3 123.4 0.675  1369027_at  alpha 1,4-galactosyltransferase  Info   A4galt 
   5032 0.0001222 2.3 26.3 0.087  1393436_at  similar to potential ligand-binding protein  Info   LOC309100 
   5033 0.0001224 135.8 102.7 1.322  1375655_at  Transcribed locus  Info    
   5034 0.0001225 58.5 132.3 0.442  1389171_at  similar to RIKEN cDNA 1110001E17 (predicted)  Info   RGD1307901_predicted 
   5035 0.0001225 211.6 74.9 2.825  1378179_a_at  Transcribed locus  Info    
   5036 0.0001225 481.3 658.8 0.731  1376086_at  similar to RIKEN cDNA 1810047C23 (predicted)  Info   RGD1311161_predicted 
   5037 0.0001225 215.3 116.7 1.845  1368953_at  UDP-glucose ceramide glucosyltransferase-like 1  Info   Ugcgl1 
   5038 0.0001227 265.5 147.9 1.795  1389052_at  tetratricopeptide repeat domain 13 (predicted)  Info   Ttc13_predicted 
   5039 0.0001228 200 394 0.508  1379724_at    Info    
   5040 0.0001231 42.3 92.7 0.456  1393338_at    Info    
   5041 0.0001231 114.9 216.9 0.53  1380562_at  similar to hypothetical protein MGC29784 (predicted)  Info   RGD1307582_predicted 
   5042 0.0001232 83 175.4 0.473  1379473_at  similar to RIKEN cDNA 1700108L22 (predicted)  Info   RGD1307509_predicted 
   5043 0.0001232 34 9.6 3.542  1379889_at  lamimin, gamma 2  Info   Lamc2 
   5044 0.0001232 34 76.8 0.443  1383644_at  Transcribed locus  Info    
   5045 0.0001232 119.3 311.4 0.383  1368963_at  Max interacting protein 1  Info   Mxi1 
   5046 0.0001233 95.2 59.6 1.597  1372065_at  ADP-ribosyltransferase 3 (predicted)  Info   Art3_predicted 
   5047 0.0001233 274.8 419.4 0.655  1390602_a_at  similar to RIKEN cDNA C430004E15  Info   LOC499749 
   5048 0.0001233 72.7 113.9 0.638  1391169_at  similar to RIKEN cDNA B930062P21 gene  Info   LOC500921 
   5049 0.0001233 624.4 819.8 0.762  1370098_at  synaptobrevin-like 1  Info   Sybl1 
   5050 0.0001234 103.4 34.1 3.032  1393119_at  Transcribed locus  Info    
   5051 0.0001236 334.7 225.7 1.483  1373437_at  Protein phosphatase 1, regulatory (inhibitor) subunit 8 (predicted)  Info    
   5052 0.0001239 108.4 214.3 0.506  1387665_at  betaine-homocysteine methyltransferase  Info   Bhmt 
   5053 0.0001239 7.8 89.1 0.088  1397782_at  Similar to oxoglutarate dehydrogenase (lipoamide)  Info    
   5054 0.0001242 69.7 38.1 1.829  1377639_at  adipocyte-specific adhesion molecule  Info   Asam 
   5055 0.0001243 45.6 96.8 0.471  1386876_at  adenylate cyclase 6  Info   Adcy6 
   5056 0.0001245 138.8 113.7 1.221  1383555_at  similar to RIKEN cDNA 4930579G22  Info   LOC360799 
   5057 0.0001245 68.8 94.4 0.729  1395510_at  abhydrolase domain containing 1 (predicted)  Info   Abhd1_predicted 
   5058 0.0001247 3.3 25.9 0.127  1385570_at    Info    
   5059 0.0001249 211.3 392.2 0.539  1385783_at  similar to BH3-only member B protein  Info   LOC498630 
   5060 0.0001251 280.8 149.1 1.883  1381903_at  F-box only protein 33 (predicted)  Info   Fbxo33_predicted 
   5061 0.0001252 203.1 307.5 0.66  1379410_at  Lin-10 protein homolog (C. elegans)  Info   Lin10 
   5062 0.0001253 79.6 22.4 3.554  1370461_at  hyaluronan mediated motility receptor  Info   Hmmr 
   5063 0.0001254 132 210 0.629  1374403_at  ephrin B1  Info   Efnb1 
   5064 0.0001256 91.9 60.9 1.509  1395400_at    Info    
   5065 0.0001258 214.1 300.7 0.712  1385423_at  inner membrane protein, mitochondrial (predicted)  Info   Immt_predicted 
   5066 0.0001262 711.9 398.7 1.786  1389518_at  F-box only protein 10 (predicted)  Info   Fbxo10_predicted 
   5067 0.0001262 703.9 528.6 1.332  1374834_at  Splicing factor 3b, subunit 4 (predicted)  Info    
   5068 0.0001263 124.6 56.9 2.19  1389158_at  Transcribed locus  Info    
   5069 0.0001263 48.4 91.8 0.527  1373351_at  Transcribed locus  Info    
   5070 0.0001263 76.6 40.5 1.891  1369708_a_at  cAMP responsive element binding protein 1  Info   Creb1 
   5071 0.0001264 49.4 134.5 0.367  1372390_at  Transcribed locus  Info    
   5072 0.0001265 11.9 30.6 0.389  1391090_at  Transcribed locus  Info    
   5073 0.0001265 47.3 115.7 0.409  1392739_a_at  similar to RIKEN cDNA 2310005P05 (predicted)  Info   RGD1307401_predicted 
   5074 0.0001266 550 324.5 1.695  1389373_at  SMAD, mothers against DPP homolog 1 (Drosophila)  Info   Smad1 
   5075 0.0001266 67.7 174.5 0.388  1374089_at  Transcribed locus  Info    
   5076 0.0001266 105.9 230.8 0.459  1380383_at  ADP-ribosylation factor 4-like (predicted)  Info    
   5077 0.0001266 487.4 180.9 2.694  1388986_at  Core promoter element binding protein  Info   Copeb 
   5078 0.0001267 199.1 365.2 0.545  1383056_a_at  LOC499072  Info   LOC499072 
   5079 0.0001267 267.9 146.1 1.834  1372007_at  similar to AOF2 protein  Info   LOC500569 
   5080 0.0001268 667.3 905.5 0.737  1389334_at  NADH dehydrogenase (ubiquinone) 1 alpha subcomplex 10 /// NADH dehydrogenase 1 alpha subcomplex 10-like protein  Info   Ndufa10 /// LOC316632 
   5081 0.0001269 177.5 221.7 0.801  1392835_at  zinc finger protein 68 (predicted)  Info   Zfp68_predicted 
   5082 0.0001273 119.1 76.4 1.559  1370408_at  putative small membrane protein NID67  Info   Nid67 
   5083 0.0001275 395.2 249.2 1.586  1387081_at  reticulocalbin 2  Info   Rcn2 
   5084 0.0001275 107.3 10.8 9.935  1376030_at  centaurin, beta 1 (predicted)  Info   Centb1_predicted 
   5085 0.0001276 118.5 216.8 0.547  1377079_a_at  protoporphyrinogen oxidase (predicted)  Info   Ppox_predicted 
   5086 0.0001276 38.8 96.6 0.402  1377392_at  Transcribed locus, weakly similar to XP_236909.3 PREDICTED: similar to dishevelled associated activator of morphogenesis 2 [Rattus norvegicus]  Info    
   5087 0.0001279 578.6 328.3 1.762  1389062_at  RED protein  Info   RGD:1359352 
   5088 0.0001282 171.6 100.3 1.711  1383168_at  Transcribed locus, strongly similar to NP_081141.1 hypothetical protein LOC73635 [Mus musculus]  Info    
   5089 0.0001289 21.2 50.4 0.421  1381798_at  LIM domain only protein 7  Info   RGD:1302984 
   5090 0.0001291 2159.4 1568.7 1.377  1368049_at  t-complex protein 1  Info   Tcp1 
   5091 0.0001291 72 22.3 3.229  1390892_at  Transcribed locus  Info    
   5092 0.0001291 192.7 126.6 1.522  1372188_at  Similar to hypothetical protein D15Ertd785e  Info    
   5093 0.0001292 846.9 684.8 1.237  1370001_at  RAB8A, member RAS oncogene family  Info   Rab8a 
   5094 0.0001292 76 162 0.469  1373282_at  similar to CG7744-PA (predicted)  Info   RGD1306651_predicted 
   5095 0.0001292 188.2 71.1 2.647  1395821_at  core binding factor beta  Info   Cbfb 
   5096 0.0001292 401.2 328.9 1.22  1370337_at  CCCTC-binding factor  Info   Ctcf 
   5097 0.0001292 122.9 8.3 14.807  1392322_at  GTPase, IMAP family member 7  Info   Gimap7 
   5098 0.0001294 160.1 110.2 1.453  1384588_at  Transcribed locus  Info    
   5099 0.0001295 24 50.4 0.476  1395636_at    Info    
   5100 0.0001295 803.5 1297.1 0.619  1372175_at  Transcribed locus, weakly similar to XP_342574.1 PREDICTED: similar to Csr1 [Rattus norvegicus]  Info    
   5101 0.0001295 814.1 609.1 1.337  1374660_at  similar to hypothetical protein FLJ20003 (predicted)  Info   RGD1309025_predicted 
   5102 0.0001297 120.8 187.6 0.644  1378445_at  similar to putative repair and recombination helicase RAD26L  Info   LOC361197 
   5103 0.0001298 70.4 6.3 11.175  1393655_at  RAD54 like (S. cerevisiae) (predicted)  Info   Rad54l_predicted 
   5104 0.0001299 2875.1 3633.9 0.791  1368504_at  lysosomal membrane glycoprotein 1  Info   Lamp1 
   5105 0.0001299 499.8 784.1 0.637  1371956_at  CDNA clone MGC:93760 IMAGE:7108525  Info    
   5106 0.00013 461 277.6 1.661  1392978_at    Info    
   5107 0.0001302 735.3 461.7 1.593  1371926_at  Interleukin 6 signal transducer  Info   Il6st 
   5108 0.0001304 446.2 320.5 1.392  1372001_at    Info    
   5109 0.0001304 108.7 253.3 0.429  1375083_at  Transcribed locus  Info    
   5110 0.0001305 3380.9 1895.8 1.783  1375170_at  S100 calcium binding protein A11 (calizzarin) (predicted)  Info   S100a11_predicted 
   5111 0.0001306 205.2 340.4 0.603  1374920_at  Transcribed locus  Info    
   5112 0.0001308 683.1 481.8 1.418  1369077_at  N-acylsphingosine amidohydrolase 1  Info   Asah1 
   5113 0.0001309 252.7 396 0.638  1367853_at  hypothetical gene supported by NM_031798  Info   LOC497723 
   5114 0.000131 43.7 56.6 0.772  1390741_at  similar to KIAA1086 protein (predicted)  Info   RGD1304737_predicted 
   5115 0.0001311 703 404.9 1.736  1388530_at  Similar to hypothetical protein  Info    
   5116 0.0001311 182.2 94.9 1.92  1373176_at  Polymerase (DNA-directed), delta 3, accessory subunit (predicted)  Info    
   5117 0.0001315 487.4 265.1 1.839  1371451_at  ribonuclease H2, large subunit (predicted)  Info   Rnaseh2a_predicted 
   5118 0.0001315 692.6 993.5 0.697  1372498_at  similar to RIKEN cDNA 2810413N20  Info   MGC94686 
   5119 0.0001315 2885.5 3791.6 0.761  1370278_at  ATP synthase, H+ transporting, mitochondrial F1 complex, delta subunit  Info   Atp5d 
   5120 0.0001316 3528.2 2711.5 1.301  1371313_at  similar to 60S ribosomal protein L23a  Info   LOC501709 
   5121 0.0001316 28.5 50.7 0.562  1380345_at  Transcribed locus  Info    
   5122 0.0001317 2854.6 6020.1 0.474  1367627_at  glycine amidinotransferase (L-arginine:glycine amidinotransferase)  Info   Gatm 
   5123 0.0001318 60.7 151 0.402  1374345_at  Transcribed locus  Info    
   5124 0.0001318 125 246.7 0.507  1390854_at  transmembrane protein 24 (predicted)  Info   Tmem24_predicted 
   5125 0.0001319 107.6 346.8 0.31  1387882_at  basic transcription element binding protein 1  Info   Bteb1 
   5126 0.000132 215.8 108.1 1.996  1372510_at  Neoplastic progression 3 (predicted)  Info    
   5127 0.000132 552.1 763.5 0.723  1398268_at  nuclear transcription factor-Y gamma  Info   Nfyc 
   5128 0.0001321 641 420.3 1.525  1371988_at  mannosidase 1, alpha (predicted)  Info   Man1a_predicted 
   5129 0.0001322 1417.5 971.1 1.46  1367826_at  nuclear factor, erythroid derived 2, like 2  Info   Nfe2l2 
   5130 0.0001323 961.5 638.7 1.505  1372735_at  eukaryotic translation initiation factor 3 subunit k (predicted)  Info   eIF3k_predicted 
   5131 0.0001323 46.5 109.2 0.426  1375553_at  similar to RIKEN cDNA 5133400G04 (predicted)  Info   RGD1311742_predicted 
   5132 0.0001324 714.2 534.4 1.336  1382537_at  Ras-related GTP binding C (predicted)  Info   Rragc_predicted 
   5133 0.0001325 40.5 14.5 2.793  1387745_at  antigen identified by monoclonal antibody MRC OX-2 receptor  Info   Mox2r 
   5134 0.0001325 484.2 615.3 0.787  1398966_at  Transcribed locus, strongly similar to NP_081070.1 differentially expressed in B16F10 1 [Mus musculus]  Info    
   5135 0.0001325 44.6 91.9 0.485  1378601_at  Transcribed locus  Info    
   5136 0.0001325 42.5 110.1 0.386  1380013_at  Similar to adiponutrin  Info    
   5137 0.0001326 37.6 7.8 4.821  1396297_at  Transcribed locus  Info    
   5138 0.0001328 111.8 54.3 2.059  1374564_at  deltex 2 homolog (Drosophila)  Info   Dtx2 
   5139 0.0001328 58 6 9.667  1378076_at  Transcribed locus  Info    
   5140 0.000133 257.8 421.4 0.612  1397634_at    Info    
   5141 0.000133 372.2 469.7 0.792  1373069_at  mitochondrial ribosomal protein S30 (predicted)  Info   Mrps30_predicted 
   5142 0.0001331 11 25.6 0.43  1367664_at  ankyrin repeat domain 1 (cardiac muscle)  Info   Ankrd1 
   5143 0.0001332 79.9 30.3 2.637  1382160_at  Casein kinase II, alpha 2, polypeptide (predicted)  Info    
   5144 0.0001333 9.1 21.8 0.417  1389955_at    Info    
   5145 0.0001334 61.1 25.5 2.396  1395710_at  similar to mesoderm induction early response 1 (MI-ER1)  Info   LOC313418 
   5146 0.0001335 1229.1 1925.5 0.638  1367832_at  lysophospholipase 1  Info   Lypla1 
   5147 0.0001336 2078.8 1210.6 1.717  1373245_at  procollagen, type IV, alpha 1 (predicted)  Info   Col4a1_predicted 
   5148 0.0001336 69 21.8 3.165  1392012_at  Siah binding protein 1; FBP interacting repressor; pyrimidine tract binding splicing factor; Ro ribonucleoprotein-binding protein 1  Info   Siahbp1 
   5149 0.0001337 148.7 218.9 0.679  1395499_at  Transcribed locus  Info    
   5150 0.000134 19.8 42.8 0.463  1372864_at  Adenylate cyclase 5  Info   Adcy5 
   5151 0.000134 12.8 2.6 4.923  1384389_at  Reticulon 4 receptor-like 2  Info   Rtn4rl2 
   5152 0.0001345 39.7 11.9 3.336  1382739_at  G protein-coupled receptor 19  Info   Gpr19 
   5153 0.0001346 299.4 150.6 1.988  1368589_at  protein tyrosine phosphatase, receptor type, J  Info   Ptprj 
   5154 0.0001349 63.1 194.1 0.325  1389739_at  neuralized-like 2 (Drosophila) (predicted)  Info   Neurl2_predicted 
   5155 0.000135 212 804.7 0.263  1377098_at  Transcribed locus  Info    
   5156 0.000135 1130.4 743.8 1.52  1373822_at  similar to RIKEN cDNA 1110025L05  Info   LOC502367 
   5157 0.0001352 570.4 383.7 1.487  1374457_at  similar to serine C-palmitoyltransferase  Info   LOC503105 
   5158 0.0001352 24.7 91.4 0.27  1385038_at  similar to hedgehog-interacting protein  Info   LOC291936 
   5159 0.0001355 549.9 676 0.813  1369990_at  topoisomerase (DNA) 2 alpha  Info   Top2a 
   5160 0.0001357 208.5 318.8 0.654  1380170_at  GABA(A) receptor-associated protein like 2  Info   Gabarapl2 
   5161 0.0001357 37 91.6 0.404  1389464_at  ligand of numb-protein X 1 (predicted)  Info   Lnx1_predicted 
   5162 0.0001357 156.3 303 0.516  1390255_at  similar to mKIAA0843 protein  Info   LOC307395 
   5163 0.0001358 139.3 72 1.935  1382777_at  Neurolysin (metallopeptidase M3 family)  Info   Nln 
   5164 0.0001358 346.3 1412.8 0.245  1370147_at  2-amino-3-carboxymuconate-6-semialdehyde decarboxylase  Info   Acmsd 
   5165 0.0001359 1352.5 2414.7 0.56  1369635_at  sorbitol dehydrogenase  Info   Sord 
   5166 0.000136 562.6 354.4 1.587  1382509_at  similar to CCR4 (predicted)  Info   RGD1310783_predicted 
   5167 0.000136 750.5 310.7 2.416  1389170_at  caspase 7  Info   Casp7 
   5168 0.0001361 136 18.4 7.391  1393832_at  Similar to RIKEN cDNA 0610033I05 (predicted)  Info    
   5169 0.0001364 379.6 772.6 0.491  1369949_at  Lutheran blood group (Auberger b antigen included)  Info   Lu 
   5170 0.0001367 1017.6 578.7 1.758  1371724_at  small fragment nuclease (predicted)  Info   Smfn_predicted 
   5171 0.0001368 266.4 407.9 0.653  1376187_at  Transcribed locus  Info    
   5172 0.000137 85.4 53.4 1.599  1379201_at    Info    
   5173 0.000137 450.7 804.9 0.56  1390149_at  transforming acidic coiled coil 2  Info   RGD:1303004 
   5174 0.0001371 613.1 797.9 0.768  1389354_at  similar to semaF cytoplasmic domain associated protein 2  Info   LOC362057 
   5175 0.0001372 148.7 296.3 0.502  1391045_at  diacylglycerol O-acyltransferase homolog 2 (mouse)  Info   Dgat2 
   5176 0.0001372 195.9 333.9 0.587  1392989_at  Transcribed locus  Info    
   5177 0.0001373 549.8 1307.7 0.42  1373078_at  Transcribed locus  Info    
   5178 0.0001373 21.7 45.1 0.481  1377442_at  Similar to Probable serine protease HTRA4 precursor (predicted)  Info    
   5179 0.0001374 68.7 225.7 0.304  1374724_at  Transcribed locus  Info    
   5180 0.0001374 2056.5 3047.7 0.675  1388304_at  NADH dehydrogenase (ubiquinone) 1 beta subcomplex, 5 (predicted)  Info   Ndufb5_predicted 
   5181 0.0001376 123.7 42 2.945  1379312_at  peroxisome proliferative activated receptor, gamma, coactivator-related 1 (predicted)  Info   Pprc1_predicted 
   5182 0.0001377 45.1 17.8 2.534  1392296_at  Transcribed locus  Info    
   5183 0.0001379 469.4 332.2 1.413  1389053_at  Similar to scruin like at the midline CG5186-PA (predicted)  Info    
   5184 0.0001379 106.5 62.8 1.696  1377908_at  CD4 antigen  Info   Cd4 
   5185 0.000138 266.4 154.5 1.724  1387844_at  LIM and SH3 protein 1  Info   Lasp1 
   5186 0.000138 332 242.1 1.371  1372143_at  ubiquitin-conjugating enzyme E2 variant 2  Info   Ube2v2 
   5187 0.0001383 111.5 67.3 1.657  1368671_at  sushi-repeat-containing protein  Info   Srpx 
   5188 0.0001386 50.2 134.6 0.373  1381056_at  plakophilin 3 (predicted)  Info   Pkp3_predicted 
   5189 0.0001388 65.5 36.5 1.795  1383792_at  synaptotagmin-like 1 (predicted)  Info   Sytl1_predicted 
   5190 0.0001392 129.9 94.9 1.369  1380320_at  similar to hypothetical protein (predicted)  Info   RGD1311361_predicted 
   5191 0.0001392 312.2 235.2 1.327  1382144_at  mitochondrial ribosomal protein L47 (predicted)  Info   Mrpl47_predicted 
   5192 0.0001392 166.5 93.4 1.783  1397904_at  Similar to osteoclast inhibitory lectin  Info    
   5193 0.0001392 383.3 254.3 1.507  1371633_at  beta catenin-like 1  Info   Ctnnbl1 
   5194 0.00014 58.9 30.1 1.957  1392489_at  Transcribed locus  Info    
   5195 0.0001401 1144.8 752.5 1.521  1368200_at  chemokine (C-X3-C motif) ligand 1  Info   Cx3cl1 
   5196 0.0001401 112.4 58.5 1.921  1393563_at  interleukin 1 receptor accessory protein  Info   Il1rap 
   5197 0.0001402 1224.1 2708.8 0.452  1367636_at  insulin-like growth factor 2 receptor  Info   Igf2r 
   5198 0.0001402 222.9 127.4 1.75  1392503_at  RNA binding motif protein 22 (predicted)  Info   Rbm22_predicted 
   5199 0.0001405 641.3 413.6 1.551  1398811_at  jumping translocation breakpoint  Info   Jtb 
   5200 0.0001409 161.7 116.1 1.393  1376519_at  brain protein 16  Info   RGD:1359226 
   5201 0.0001411 75.7 140.4 0.539  1373165_at  Similar to zinc finger, matrin type 1  Info    
   5202 0.0001412 293 203.9 1.437  1382443_at  similar to Pabpc4_predicted protein /// poly A binding protein, cytoplasmic 4 (predicted)  Info   LOC288398 /// Pabpc4_predicted 
   5203 0.0001413 83.3 4 20.825  1383926_at  budding uninhibited by benzimidazoles 1 homolog, beta (S. cerevisiae)  Info   Bub1b 
   5204 0.0001415 202.1 142.6 1.417  1370203_at  FGF receptor activating protein 1  Info   Frag1 
   5205 0.0001417 62 102.2 0.607  1379352_at  phosphatidylinositol-4-phosphate 5-kinase, type 1 alpha (predicted)  Info   Pip5k1a_predicted 
   5206 0.0001417 440.4 751.7 0.586  1367904_at  regulated endocrine-specific protein 18  Info   Resp18 
   5207 0.0001417 104.4 44.1 2.367  1376907_at  Microtubule-associated protein 6  Info   Mtap6 
   5208 0.0001418 294.1 358.4 0.821  1373096_at  ARP8 actin-related protein 8 homolog (S. cerevisiae) (predicted)  Info   Actr8_predicted 
   5209 0.000142 554.2 880.4 0.629  1383221_at  hypothetical LOC360478  Info   LOC360478 
   5210 0.0001421 126.2 79.5 1.587  1374351_at    Info    
   5211 0.0001422 403.2 252.8 1.595  1370085_at  RAS p21 protein activator 1  Info   Rasa1 
   5212 0.0001422 241.3 129.8 1.859  1393638_at  Prostaglandin E receptor 4 (subtype EP4)  Info   Ptger4 
   5213 0.0001423 81.3 169.5 0.48  1392725_at  similar to cDNA sequence BC006662 (predicted)  Info   RGD1304748_predicted 
   5214 0.0001427 264.5 160.5 1.648  1396086_at  similar to RIKEN cDNA 1810014L12 (predicted)  Info   RGD1307423_predicted 
   5215 0.0001431 248.9 412.3 0.604  1383573_at  Serologically defined colon cancer antigen 33 (predicted)  Info    
   5216 0.0001432 79.2 225.6 0.351  1372684_at  smoothelin (predicted)  Info   Smtn_predicted 
   5217 0.0001432 362.4 9.8 36.98  1385702_at  myeloid cell nuclear differentiation antigen (predicted)  Info   Mnda_predicted 
   5218 0.0001434 18.5 30.3 0.611  1397913_at  Transcribed locus  Info    
   5219 0.0001435 284.9 543 0.525  1399039_at  Similar to Dnajb10 protein  Info    
   5220 0.0001436 101.9 172.8 0.59  1375869_at  Unc-51 like kinase 1 (C. elegans)  Info   Ulk1 
   5221 0.0001437 283.3 410.3 0.69  1371516_at  exportin 7 (predicted)  Info   Xpo7_predicted 
   5222 0.0001437 80.1 6.3 12.714  1373530_at  cyclin E  Info   Ccne1 
   5223 0.0001437 427.3 801.1 0.533  1371773_at  Transcribed locus  Info    
   5224 0.0001438 43.6 82.7 0.527  1387157_at  polyamine modulated factor 1 binding protein 1  Info   Pmfbp1 
   5225 0.0001442 91.7 188.6 0.486  1382258_at  Rho-guanine nucleotide exchange factor (predicted)  Info   Rgnef_predicted 
   5226 0.0001444 133.7 214.2 0.624  1373607_at  Transcribed locus  Info    
   5227 0.0001447 12.2 36 0.339  1370671_at  guanylate cyclase 2g  Info   Gucy2g 
   5228 0.0001447 522.5 307.2 1.701  1388480_at  similar to Glycolipid transfer protein  Info   LOC288707 
   5229 0.0001448 486.6 244.5 1.99  1387135_at  a disintegrin and metalloproteinase domain 15 (metargidin)  Info   Adam15 
   5230 0.0001448 249.8 151.5 1.649  1383576_at  Protein phosphatase 1, regulatory (inhibitor) subunit 12A  Info   Ppp1r12a 
   5231 0.0001449 51.2 143.2 0.358  1394850_at    Info    
   5232 0.0001451 6.9 25.4 0.272  1378945_at  Aconitase 2, mitochondrial  Info   Aco2 
   5233 0.0001452 111.2 69.2 1.607  1381977_at  Transcribed locus  Info    
   5234 0.0001452 289.3 181.8 1.591  1373867_at  Transcribed locus  Info    
   5235 0.0001455 51.7 92.1 0.561  1396309_at  Similar to RIKEN cDNA 2810453K03 (predicted)  Info    
   5236 0.0001456 622.5 374.1 1.664  1370792_at  microtubule-associated protein, RP/EB family, member 1  Info   Mapre1 
   5237 0.0001457 131.3 65.3 2.011  1374212_at  Transcribed locus  Info    
   5238 0.0001458 124.8 210.4 0.593  1379536_at  Transcribed locus  Info    
   5239 0.0001458 29.9 69.3 0.431  1378763_at  hypothetical gene supported by BC088439  Info   LOC500947 
   5240 0.0001459 828.7 654.8 1.266  1372827_at  peptidylprolyl isomerase D  Info   RGD:1303174 
   5241 0.0001459 11.3 45.7 0.247  1376320_at  Transcribed locus  Info    
   5242 0.0001459 17.4 172.7 0.101  1384770_at  Transcribed locus  Info    
   5243 0.000146 165.9 269.4 0.616  1383228_at    Info    
   5244 0.0001461 344.4 612.9 0.562  1385103_at  fused toes (predicted)  Info   Fts_predicted 
   5245 0.0001461 85.4 171.3 0.499  1382137_at  abhydrolase domain containing 3 (predicted)  Info   Abhd3_predicted 
   5246 0.0001462 1995.2 2876.6 0.694  1371041_at  NADH dehydrogenase (ubiquinone) flavoprotein 2  Info   Ndufv2 
   5247 0.0001464 277.7 441.3 0.629  1382021_at  Similar to polycystic kidney disease 2  Info    
   5248 0.0001466 3498.8 1607.3 2.177  1369943_at  transglutaminase 2, C polypeptide  Info   Tgm2 
   5249 0.0001466 113.8 54.6 2.084  1372995_at  similar to protein kinase D2 (predicted)  Info   RGD1308054_predicted 
   5250 0.0001466 62.1 109.6 0.567  1391981_at  Transcribed locus  Info    
   5251 0.0001467 280.6 427.5 0.656  1388623_at  EH-domain containing 1 (predicted)  Info    
   5252 0.0001468 281.8 510.9 0.552  1376337_at  SWI/SNF related, matrix associated, actin dependent regulator of chromatin, subfamily a, member 2  Info   RGD:1302988 
   5253 0.0001469 446.6 823.9 0.542  1367857_at  fatty acid desaturase 1  Info   Fads1 
   5254 0.0001472 150.3 325.3 0.462  1368700_at  phospholipase C-like 1  Info   Plcl1 
   5255 0.0001472 1055 807.2 1.307  1367481_at  vacuolar protein sorting 28 (yeast) (predicted)  Info   Vps28_predicted 
   5256 0.0001476 4984.1 3417.1 1.459  1375336_at  heat shock 90kDa protein 1, beta  Info   RGD:1303075 
   5257 0.0001476 724.9 1473.9 0.492  1367754_s_at  argininosuccinate lyase /// heterogeneous nuclear ribonucleoprotein A/B  Info   Asl /// Hnrpab 
   5258 0.0001477 46.6 2.7 17.259  1383486_at  Transcribed locus  Info    
   5259 0.000148 61.2 172.6 0.355  1368039_at  synaptojanin 2 binding protein  Info   Synj2bp 
   5260 0.000148 380.5 303.5 1.254  1368508_at  proteasome (prosome, macropain) subunit, alpha type 3  Info   Psma3 
   5261 0.0001485 224.3 325.4 0.689  1383536_at  Transcribed locus  Info    
   5262 0.0001486 104 154.4 0.674  1374482_at    Info    
   5263 0.0001487 274.1 457.4 0.599  1372762_at  similar to RIKEN cDNA 1110033O09 (predicted)  Info   RGD1311839_predicted 
   5264 0.0001489 509.1 825.6 0.617  1388541_at  Similar to Hypothetical protein KIAA0152 (predicted)  Info    
   5265 0.000149 64.7 115.3 0.561  1374434_at  similar to specifically androgen-regulated protein  Info   LOC498222 
   5266 0.0001491 239.8 449 0.534  1387072_at  protein kinase, lysine deficient 1  Info   Prkwnk1 
   5267 0.0001492 325.1 250.8 1.296  1388895_at    Info    
   5268 0.0001494 599.9 1348.7 0.445  1377051_at  Mpv17 transgene, kidney disease mutant-like (predicted)  Info   Mpv17l_predicted 
   5269 0.0001496 166.1 86.2 1.927  1368546_at  human immunodeficiency virus type I enhancer binding protein 2  Info   Hivep2 
   5270 0.0001497 79 11.3 6.991  1392810_at    Info    
   5271 0.0001498 211.6 272.2 0.777  1368859_at  protein phosphatase 1A, magnesium dependent, alpha isoform  Info   Ppm1a 
   5272 0.0001498 37.5 21.5 1.744  1385728_at  AE binding protein 2 (predicted)  Info    
   5273 0.0001498 143.8 264.7 0.543  1388945_at  similar to 1300014I06Rik protein (predicted)  Info   RGD1311307_predicted 
   5274 0.0001499 67 183.5 0.365  1370896_a_at  myosin heavy chain 11  Info   Myh11 
   5275 0.0001501 104 47.6 2.185  1392532_at  Kinesin heavy chain member 2  Info   Kif2 
   5276 0.0001503 46.2 3 15.4  1390890_at  Transcribed locus  Info    
   5277 0.0001503 1060.5 1383.6 0.766  1387896_at  sterol carrier protein 2  Info   Scp2 
   5278 0.0001503 228.5 438.2 0.521  1369450_at  integral membrane transport protein UST5r  Info   UST5r 
   5279 0.0001506 161.6 228.9 0.706  1373147_at  F-box and leucine-rich repeat protein 3a (predicted)  Info   Fbxl3a_predicted 
   5280 0.0001507 221.3 334.8 0.661  1367753_at  SEC31-like 1 (S. cerevisiae)  Info   Sec31l1 
   5281 0.000151 152.8 227.9 0.67  1379550_a_at  general transcription factor II I repeat domain-containing 1  Info   Gtf2ird1 
   5282 0.000151 67.9 20 3.395  1393128_at  similar to RIKEN cDNA 2610029K21 (predicted)  Info   RGD1311086_predicted 
   5283 0.0001512 113.6 187.2 0.607  1393079_at  similar to sialic-acid O-acetylesterase (predicted)  Info   RGD1310431_predicted 
   5284 0.0001512 127.5 237.4 0.537  1385561_at  similar to peroxisome biogenesis factor 1  Info   LOC500006 
   5285 0.0001513 105 44.5 2.36  1378389_at  Similar to nuclear factor of activated T-cells, cytoplasmic, calcineurin-dependent 1  Info    
   5286 0.0001519 275.7 543.6 0.507  1367778_at  hypothetical gene supported by NM_019331  Info   LOC497733 
   5287 0.0001522 315.7 203.7 1.55  1377198_at  Src family associated phosphoprotein 2  Info   Scap2 
   5288 0.0001524 1314.2 1812 0.725  1371564_at  ATPase, H+ transporting, V1 subunit E isoform 1  Info   Atp6v1e1 
   5289 0.0001525 35.9 19 1.889  1386543_at  similar to F-box and leucine-rich repeat protein 8  Info   LOC498941 
   5290 0.0001527 168.5 127.8 1.318  1373980_at  similar to XPA binding protein 1  Info   LOC500619 
   5291 0.0001529 447 348.4 1.283  1392858_at  nuclear cap binding protein subunit 2 (predicted)  Info   Ncbp2_predicted 
   5292 0.0001529 129.5 68.4 1.893  1369285_at  protein geranylgeranyltransferase type I, beta subunit  Info   Pggt1b 
   5293 0.0001531 114.2 44.6 2.561  1370577_at  zinc finger protein 455  Info   Zfp455 
   5294 0.0001532 584.9 878.7 0.666  1383100_at  family with sequence similarity 18, member B (predicted)  Info   Fam18b_predicted 
   5295 0.0001533 349.5 514.1 0.68  1373837_at  fused toes (predicted)  Info   Fts_predicted 
   5296 0.0001533 905.1 1447.5 0.625  1373088_at    Info    
   5297 0.0001538 267.2 391 0.683  1395404_at  Transcribed locus  Info    
   5298 0.0001544 225.6 345.5 0.653  1377806_at  vascular endothelial zinc finger 1 (predicted)  Info   Vezf1_predicted 
   5299 0.0001549 145.1 92.2 1.574  1367521_at    Info    
   5300 0.0001549 306.3 452.4 0.677  1367718_at  choline kinase beta  Info   Chkb 
   5301 0.0001553 59.9 29.8 2.01  1377889_at  similar to human mRNA for KIAA0539 protein  Info   LOC498058 
   5302 0.0001556 207.3 350.9 0.591  1383830_a_at  oxysterol binding protein-like 1A  Info   Osbpl1a 
   5303 0.0001557 91 44.5 2.045  1375187_at  Transcribed locus  Info    
   5304 0.0001557 185.7 271.9 0.683  1372650_at  similar to Dynamin binding protein (Scaffold protein Tuba)  Info   LOC309362 
   5305 0.0001558 69.3 124.5 0.557  1376767_at  G protein-coupled receptor 116  Info   Gpr116 
   5306 0.0001559 104.1 192 0.542  1391021_at  similar to KIAA1749 protein (predicted)  Info   RGD1304623_predicted 
   5307 0.0001559 1541.6 1086.5 1.419  1398300_at  ATPase, Na+/K+ transporting, beta 3 polypeptide  Info   Atp1b3 
   5308 0.0001565 137.1 70.2 1.953  1390472_at    Info    
   5309 0.0001566 318.2 173.2 1.837  1372050_at  Similar to hypothetical protein MGC38524 (predicted)  Info    
   5310 0.0001566 59.3 117.4 0.505  1382811_at  Mitochondrial ribosomal protein L16  Info   Mrpl16 
   5311 0.0001566 224.6 323.3 0.695  1377076_at  Similar to sialic-acid O-acetylesterase (predicted)  Info    
   5312 0.0001567 208.5 119.5 1.745  1377760_at  similar to hypothetical protein MGC28606 (predicted)  Info   RGD1310661_predicted 
   5313 0.0001568 2.3 21.2 0.108  1371083_at  Serine protease inhibitor  Info   Spin2a 
   5314 0.000157 275 153 1.797  1373840_at  nudix -type motif 9  Info   RGD:1359522 
   5315 0.0001573 279.8 458.8 0.61  1371513_at  LOC500067  Info   LOC500067 
   5316 0.0001574 264.4 391.6 0.675  1373420_at  evolutionarily conserved signaling intermediate in Toll pathway  Info   RGD:1359488 
   5317 0.0001575 108.9 21.5 5.065  1384350_at  Transcribed locus  Info    
   5318 0.0001575 115.9 157.1 0.738  1394041_at  CDNA clone IMAGE:7131734  Info    
   5319 0.000158 148.4 224.1 0.662  1376057_at    Info    
   5320 0.000158 569.3 868.7 0.655  1372720_at  BTB (POZ) domain containing 1 (predicted)  Info   Btbd1_predicted 
   5321 0.000158 120.9 63 1.919  1378399_at  similar to RIKEN cDNA 2810453I06  Info   LOC498145 
   5322 0.0001582 106.3 191.9 0.554  1388104_at  G protein-coupled receptor 48  Info   Gpr48 
   5323 0.0001583 23.9 9.9 2.414  1398402_at  forkhead box P4 (predicted)  Info   Foxp4_predicted 
   5324 0.0001584 384 259.4 1.48  1382135_at  similar to CG9346-PA (predicted)  Info   RGD1307882_predicted 
   5325 0.0001589 1002.7 735.4 1.363  1373033_at  similar to chromosome 6 open reading frame 83; similar to RIKEN cDNA 5630401J11  Info   RGD:1359119 
   5326 0.0001589 133.3 223.6 0.596  1382219_at  similar to RIKEN cDNA 1700108L22 (predicted)  Info   RGD1307509_predicted 
   5327 0.0001591 15 52.6 0.285  1379363_at  Maltase-glucoamylase (predicted)  Info    
   5328 0.0001592 203.4 292.2 0.696  1372882_at  similar to CG12379-PA (predicted)  Info   RGD1308635_predicted 
   5329 0.0001592 289.1 173.6 1.665  1368405_at  v-ral simian leukemia viral oncogene homolog A (ras related)  Info   Rala 
   5330 0.0001593 73.2 139.3 0.525  1379405_at    Info    
   5331 0.0001593 132.2 21.8 6.064  1382493_at  Transcribed locus, strongly similar to XP_223060.3 PREDICTED: similar to LEK1 [Rattus norvegicus]  Info    
   5332 0.0001594 326 454.7 0.717  1372839_at  mitochondrial ribosomal protein S35 (predicted)  Info   Mrps35_predicted 
   5333 0.0001596 557 267.1 2.085  1372542_at  Similar to QUAKING isoform 6  Info   Qki 
   5334 0.0001603 367.8 254.8 1.443  1387206_at  UDP-Gal:betaGlcNAc beta 1,4-galactosyltransferase, polypeptide 6  Info   B4galt6 
   5335 0.0001604 387.5 286.8 1.351  1374523_at  6-phosphogluconolactonase (predicted)  Info   Pgls_predicted 
   5336 0.0001605 74.4 49.8 1.494  1379488_at  TP53 regulating kinase (predicted)  Info   Trp53rk_predicted 
   5337 0.0001606 181.4 131.9 1.375  1368652_at  caspase 9  Info   Casp9 
   5338 0.0001607 19.1 52 0.367  1381808_at  Heterogeneous nuclear ribonucleoprotein A/B  Info   Hnrpab 
   5339 0.0001608 130.7 79.5 1.644  1378016_at  Echinoderm microtubule associated protein like 4 (predicted)  Info    
   5340 0.0001609 308.9 462.8 0.667  1377524_at  similar to CG18661-PA (predicted)  Info   RGD1307155_predicted 
   5341 0.0001609 37.6 22.7 1.656  1394599_at  similar to cDNA sequence BC022133  Info   LOC362399 
   5342 0.0001611 105.4 325.8 0.324  1386720_at  transmembrane protease, serine 8 (intestinal)  Info   Tmprss8 
   5343 0.0001612 482.2 1264.8 0.381  1384838_at  solute carrier family 34 (sodium phosphate), member 3  Info   Slc34a3 
   5344 0.0001614 88 142.6 0.617  1372172_at  nephronophthisis 1 (juvenile) (predicted)  Info   Nphp1_predicted 
   5345 0.0001619 15.7 55.2 0.284  1380923_at  N-acylsphingosine amidohydrolase 3-like (predicted)  Info    
   5346 0.0001622 992 1499 0.662  1389023_at  Similar to Oligosaccharyl transferase 3 CG7748-PA (predicted)  Info    
   5347 0.0001623 113.7 214.1 0.531  1373872_at  Transcribed locus, strongly similar to XP_574462.1 PREDICTED: similar to hypothetical protein C230069C04 [Rattus norvegicus]  Info    
   5348 0.0001623 173 375.2 0.461  1373631_at  RAP1, GTPase activating protein 1 (predicted)  Info   Rap1ga1_predicted 
   5349 0.0001624 96.4 254.8 0.378  1383319_at  solute carrier family 4, sodium bicarbonate transporter-like, member 11 (predicted)  Info   Slc4a11_predicted 
   5350 0.0001628 12.4 34.6 0.358  1384183_at    Info    
   5351 0.0001628 1231 1621.3 0.759  1371798_at  Guanine nucleotide binding protein, alpha 12  Info   Gna12 
   5352 0.0001629 158 18.9 8.36  1383709_at  Transcribed locus  Info    
   5353 0.0001629 1814.8 2569.3 0.706  1389964_at  NADH dehydrogenase (ubiquinone) 1, alpha/beta subcomplex, 1 (predicted)  Info   Ndufab1_predicted 
   5354 0.000163 159.8 22.3 7.166  1393830_at  SH2 domain protein 2A  Info   RGD:1303076 
   5355 0.0001632 838.5 1163 0.721  1370690_at  heat shock protein, A (predicted)  Info   Hspa9a_predicted 
   5356 0.0001632 102.9 138.6 0.742  1382039_at  RAB, member of RAS oncogene family-like 2A (predicted)  Info   Rabl2a_predicted 
   5357 0.0001633 2535.8 3055.2 0.83  1371642_at  eukaryotic translation initiation factor 4A2 (predicted)  Info   Eif4a2_predicted 
   5358 0.0001635 229.6 458.4 0.501  1377209_at  similar to hypothetical protein FLJ12587 (predicted)  Info   RGD1310815_predicted 
   5359 0.0001635 150.7 240.1 0.628  1382330_at  Transcribed locus  Info    
   5360 0.0001636 32.3 3.1 10.419  1369411_at  growth factor independent 1  Info   Gfi1 
   5361 0.0001637 102.4 229.7 0.446  1390727_at  Transcribed locus  Info    
   5362 0.0001638 51.8 105.2 0.492  1368897_at  MAD homolog 7 (Drosophila)  Info   Madh7 
   5363 0.0001639 113 205.8 0.549  1379495_at  Transcribed locus  Info    
   5364 0.0001639 29.5 65.2 0.452  1379573_at    Info    
   5365 0.0001639 63.1 34.6 1.824  1373223_at  Transcribed locus  Info    
   5366 0.0001641 186.1 105.9 1.757  1389621_at  FIP1 like 1 (S. cerevisiae) (predicted)  Info   Fip1l1_predicted 
   5367 0.0001644 524.3 725.3 0.723  1375898_at  similar to RNA binding protein gene with multiple splicing  Info   LOC498642 
   5368 0.0001645 268.3 76.5 3.507  1378032_at    Info    
   5369 0.0001649 464.8 334.3 1.39  1388953_at  guanine nucleotide binding protein-like 3 (nucleolar)  Info   Gnl3 
   5370 0.0001649 121.6 43.9 2.77  1397548_at  similar to PTPL1-associated RhoGAP 1 (predicted)  Info   RGD1308662_predicted 
   5371 0.000165 41.8 16.7 2.503  1379945_at  similar to T-cell activation kelch repeat protein  Info   LOC500262 
   5372 0.000165 609.6 1930.2 0.316  1387974_a_at  kidney specific organic anion transporter  Info   RGD:621387 
   5373 0.0001652 53 161.1 0.329  1390119_at  secreted frizzled-related protein 2  Info   Sfrp2 
   5374 0.0001653 4.5 52.7 0.085  1387044_at  glycoprotein hormone alpha 2  Info   Gpha2 
   5375 0.0001656 73.2 123.9 0.591  1390998_at  GLIS family zinc finger 1 (predicted)  Info   Glis1_predicted 
   5376 0.0001657 147.4 55.2 2.67  1374784_at  phosphoribosyl transferase domain containing 1 (predicted)  Info   Prtfdc1_predicted 
   5377 0.0001658 74.9 10.9 6.872  1369306_at  killer cell lectin-like receptor, subfamily D, member 1  Info   Klrd1 
   5378 0.0001663 24.7 51 0.484  1396512_at  Transcribed locus  Info    
   5379 0.0001663 1077.5 814.7 1.323  1372607_at  nucleotide binding protein 2 (predicted)  Info   Nubp2_predicted 
   5380 0.0001665 229.3 386.1 0.594  1388643_at  fucosyltransferase 8 (alpha (1,6) fucosyltransferase)  Info   RGD:1303096 
   5381 0.0001668 899.9 1233.2 0.73  1398938_at  acid phosphatase 1, soluble  Info   Acp1 
   5382 0.0001668 546 342.7 1.593  1373049_at  similar to D1Ertd622e protein  Info   LOC501195 
   5383 0.0001671 352.5 568.7 0.62  1372287_at  Myotubularin related protein 6 (predicted)  Info    
   5384 0.0001674 929.2 716.5 1.297  1372100_at  similar to Small membrane protein 1 (predicted)  Info   RGD1305638_predicted 
   5385 0.0001675 98.8 193.4 0.511  1374060_at  Transcribed locus  Info    
   5386 0.0001675 468.5 299.2 1.566  1393336_at  SWAP complex protein (predicted)  Info   Swap70_predicted 
   5387 0.0001676 98.8 62.3 1.586  1375067_at  Transcribed locus  Info    
   5388 0.0001682 108.3 184.3 0.588  1379673_at    Info    
   5389 0.0001684 233 359.5 0.648  1382500_at  Secernin 1  Info    
   5390 0.0001685 4595.5 3461.6 1.328  1398762_at  syndecan binding protein  Info   Sdcbp 
   5391 0.0001686 293.8 485 0.606  1369065_a_at  ATPase, Ca++ transporting, cardiac muscle, slow twitch 2  Info   Atp2a2 
   5392 0.0001691 238.1 380.9 0.625  1376705_a_at  RAB24, member RAS oncogene family (predicted)  Info   Rab24_predicted 
   5393 0.0001692 3.2 34.4 0.093  1379517_at  regulator of G-protein signaling 6  Info   Rgs6 
   5394 0.0001692 49.8 78.2 0.637  1392636_at  Transcribed locus  Info    
   5395 0.0001693 20 43.4 0.461  1385578_at  Transcribed locus  Info    
   5396 0.0001693 143.4 411.7 0.348  1374022_at  Transcribed locus  Info    
   5397 0.0001693 85.5 240.1 0.356  1385614_at  melanophilin (predicted)  Info   Mlph_predicted 
   5398 0.0001693 60.7 100.6 0.603  1396202_at  Transcribed locus  Info    
   5399 0.0001693 148.5 242 0.614  1382124_at  Cyclin M3 (predicted)  Info    
   5400 0.0001694 13.6 4.9 2.776  1391514_at  Transcribed locus  Info    
   5401 0.0001694 135.9 280.3 0.485  1397729_x_at  similar to RIKEN cDNA 1600029D21  Info   RGD:1359217 
   5402 0.0001697 181.5 23.8 7.626  1373722_at  kinesin family member 20A (predicted)  Info   Kif20a_predicted 
   5403 0.0001703 556.6 1004.4 0.554  1376640_at    Info    
   5404 0.0001705 1527.1 1232 1.24  1398794_at  transcription elongation factor B (SIII), polypeptide 1  Info   Tceb1 
   5405 0.0001707 187.4 445.8 0.42  1390403_at  similar to CG8312-PA (predicted)  Info   RGD1304790_predicted 
   5406 0.0001715 44.5 194.9 0.228  1376248_at  sulfotransferase family, cytosolic, 2B, member 1 (predicted)  Info   Sult2b1_predicted 
   5407 0.0001718 91.3 324.2 0.282  1384774_at  transmembrane protease, serine 8 (intestinal)  Info   Tmprss8 
   5408 0.0001719 13.7 26.9 0.509  1369361_at  Klotho  Info   Kl 
   5409 0.0001721 222.4 346 0.643  1391282_at  Transcribed locus, moderately similar to XP_484071.1 PREDICTED: similar to Als2 protein [Mus musculus]  Info    
   5410 0.0001731 1.6 5.6 0.286  1382764_at  Similar to hypothetical protein MGC38960 (predicted)  Info    
   5411 0.0001734 74.1 44.4 1.669  1395513_at  similar to cDNA clone MGC:36552 (predicted)  Info   RGD1306898_predicted 
   5412 0.0001738 67.7 100.3 0.675  1394590_at  NFKB inhibitor interacting Ras-like protein 1 (predicted)  Info   Nkiras1_predicted 
   5413 0.0001739 150.9 69.5 2.171  1399118_at  similar to RIKEN cDNA 4121402D02  Info   LOC303514 
   5414 0.000174 87.1 138.8 0.628  1368928_at  tripartite motif protein 3  Info   Trim3 
   5415 0.0001742 413.6 324.1 1.276  1369642_at  platelet-activating factor acetylhydrolase, isoform 1b, alpha2 subunit  Info   Pafah1b2 
   5416 0.0001744 106.9 228.8 0.467  1393626_at  Transcribed locus  Info    
   5417 0.0001749 140.4 81.4 1.725  1382052_at  Transcribed locus  Info    
   5418 0.0001751 1113.6 839.9 1.326  1377668_at  YY1 transcription factor  Info   Yy1 
   5419 0.0001754 218 143.6 1.518  1377020_at  CDNA clone IMAGE:7375616  Info    
   5420 0.0001755 239.8 167.6 1.431  1398344_at  cofactor required for Sp1 transcriptional activation, subunit 6 (predicted)  Info   Crsp6_predicted 
   5421 0.0001761 166.3 81.9 2.031  1372125_at  glutathione peroxidase 7 (predicted)  Info   Gpx7_predicted 
   5422 0.0001763 26.3 42.1 0.625  1382844_at  Transcribed locus  Info    
   5423 0.0001764 179.1 104.9 1.707  1373285_at  Transcribed locus  Info    
   5424 0.0001768 660.6 328.2 2.013  1377726_at  tripartite motif protein 25  Info   Trim25 
   5425 0.0001772 1860.2 2288.3 0.813  1367479_at    Info    
   5426 0.0001781 1006.9 691.6 1.456  1387636_a_at  P11 protein  Info   RGD:621692 
   5427 0.0001782 136.1 231.1 0.589  1373883_at    Info    
   5428 0.0001783 123.2 10.3 11.961  1384555_at  similar to RIKEN cDNA 4432409M07  Info   LOC363326 
   5429 0.0001783 127.8 186.5 0.685  1377949_s_at    Info    
   5430 0.0001784 366 568.2 0.644  1372467_at  Transcribed locus  Info    
   5431 0.0001784 372.7 450 0.828  1371614_at  autophagy 12-like (S. cerevisiae) (predicted)  Info   Apg12l_predicted 
   5432 0.0001785 36.8 104.9 0.351  1375729_at  similar to Eph receptor A4  Info   LOC316539 
   5433 0.0001785 100.8 13.5 7.467  1393982_at  polymerase (DNA directed), epsilon 2 (p59 subunit) (predicted)  Info   Pole2_predicted 
   5434 0.0001788 39.1 88.7 0.441  1391556_at  Transcribed locus  Info    
   5435 0.0001789 137.9 83.2 1.657  1370923_at  expressed in non-metastatic cells 6, protein (nucleoside diphosphate kinase)  Info   Nme6 
   5436 0.000179 6.7 52.6 0.127  1374384_at  Crystallin, gamma C  Info   Crygc 
   5437 0.0001792 193.8 268.5 0.722  1372098_at    Info    
   5438 0.0001793 128.6 60.8 2.115  1383626_at    Info    
   5439 0.0001795 68.2 34.7 1.965  1393919_at  similar to RIKEN cDNA 4933405A16 (predicted)  Info   RGD1305778_predicted 
   5440 0.0001795 54 138.6 0.39  1389370_at  PHD finger protein 1  Info   RGD:1303205 
   5441 0.0001796 382.4 240.8 1.588  1377042_at    Info    
   5442 0.0001796 76.6 137 0.559  1380250_at  sushi, nidogen and EGF-like domains 1  Info   Sned1 
   5443 0.0001799 98.5 188 0.524  1372754_at    Info    
   5444 0.00018 88.9 43.6 2.039  1395349_at  Transcribed locus  Info    
   5445 0.0001805 63.7 17.7 3.599  1385099_at    Info    
   5446 0.0001807 61.4 19.3 3.181  1373751_at  Transcribed locus  Info    
   5447 0.0001809 412.7 708.5 0.582  1398262_at  phosphoribosyl pyrophosphate synthetase 2  Info   Prps2 
   5448 0.0001811 4383.8 3714.3 1.18  1367603_at  Similar to Tpi1 protein  Info   Tpi1 
   5449 0.0001812 62.1 144.6 0.429  1385773_at  Transcribed locus, weakly similar to XP_576460.1 PREDICTED: similar to hypothetical protein PB402898.00.0 [Rattus norvegicus]  Info    
   5450 0.0001812 70.8 14.3 4.951  1371115_at  protein tyrosine phosphatase, receptor type, E  Info   Ptpre 
   5451 0.0001815 55.8 187.8 0.297  1369634_at  solute carrier family 4, member 1  Info   Slc4a1 
   5452 0.0001816 411.7 213.7 1.927  1388590_at  zinc ribbon domain containing, 1  Info   RGD:1303114 
   5453 0.0001816 11 53.1 0.207  1388605_at  WAP four-disulfide core domain 3 (predicted)  Info   Wfdc3_predicted 
   5454 0.0001816 359.7 109.4 3.288  1370864_at  collagen, type 1, alpha 1  Info   Col1a1 
   5455 0.0001819 18.1 52.8 0.343  1376710_at  Similar to PYM protein  Info    
   5456 0.0001821 590.9 314.6 1.878  1370004_at  H2A histone family, member Y  Info   H2afy 
   5457 0.0001823 58.4 102.1 0.572  1374131_at  Transcribed locus  Info    
   5458 0.0001826 543.2 713.1 0.762  1382689_at  similar to RGD1310870_predicted protein  Info   LOC499242 
   5459 0.0001827 32.5 133.3 0.244  1373778_at  Acetyl-Coenzyme A carboxylase beta  Info   Acacb 
   5460 0.0001827 3192.5 5492.4 0.581  1368826_at  catechol-O-methyltransferase  Info   Comt 
   5461 0.0001828 328.8 145.7 2.257  1368871_at  mitogen activated protein kinase kinase kinase 1  Info   Map3k1 
   5462 0.0001831 71.4 36.7 1.946  1391107_at  similar to 5-3 exonuclease (predicted)  Info   RGD1309713_predicted 
   5463 0.0001832 208.3 409.6 0.509  1373615_at  frizzled-related protein (predicted)  Info   Frzb_predicted 
   5464 0.0001834 24.8 10.9 2.275  1398621_at  adenylate kinase 7 (predicted)  Info   Ak7_predicted 
   5465 0.0001838 223.8 364.7 0.614  1382381_at  Transcribed locus  Info    
   5466 0.0001839 137.8 54.6 2.524  1373091_at  Transcribed locus  Info    
   5467 0.0001841 1906 2934.4 0.65  1371131_a_at  upregulated by 1,25-dihydroxyvitamin D-3  Info   Txnip 
   5468 0.0001848 840 1581 0.531  1383893_at  ATPase, H+ transporting, V1 subunit G, isoform 3 (predicted)  Info   Atp6v1g3_predicted 
   5469 0.0001849 422.8 283.1 1.493  1371535_at  phosphomannomutase 2 (predicted)  Info   Pmm2_predicted 
   5470 0.0001855 60.4 99.3 0.608  1391478_at  zinc finger protein 532 (predicted)  Info   Znf532_predicted 
   5471 0.0001856 430.2 739.6 0.582  1373634_at  Transcribed locus  Info    
   5472 0.0001866 247.4 460.4 0.537  1367940_at  chemokine orphan receptor 1  Info   Cmkor1 
   5473 0.0001866 46 86.6 0.531  1396215_at  Similar to RIKEN cDNA 2610022G08  Info    
   5474 0.0001867 321.7 262.2 1.227  1371981_at  Transcribed locus, strongly similar to NP_031860.1 Down syndrome critical region protein A [Mus musculus]  Info    
   5475 0.0001868 138.4 6.7 20.657  1390137_at  TRAF4 associated factor 1  Info   RGD:1303240 
   5476 0.0001868 85.7 141.9 0.604  1389491_at  Sideroflexin 5  Info   Sfxn5 
   5477 0.0001869 790.4 580.5 1.362  1388828_at  hypothetical LOC315617 (predicted)  Info   RGD1308311_predicted 
   5478 0.0001869 624.1 393.9 1.584  1371868_at  breast carcinoma amplified sequence 2 (predicted)  Info   Bcas2_predicted 
   5479 0.0001872 368.6 206.2 1.788  1393248_at  similar to expressed sequence C87860 (predicted)  Info   RGD1308468_predicted 
   5480 0.0001872 760.1 466.1 1.631  1375635_at  Transcribed locus  Info    
   5481 0.0001873 13.2 30 0.44  1392795_at  Sema domain, transmembrane domain (TM), and cytoplasmic domain, (semaphorin) 6A (predicted)  Info    
   5482 0.0001874 20.5 46.1 0.445  1376213_at  Transcribed locus  Info    
   5483 0.0001874 276.2 12.9 21.411  1369025_at  CD5 antigen  Info   Cd5 
   5484 0.0001876 87.9 54 1.628  1392038_at  similar to PI-3-kinase-related kinase SMG-1 isoform 2  Info   LOC499250 
   5485 0.0001879 718.8 526.2 1.366  1374061_at  similar to RIKEN cDNA 1110055L24 (predicted)  Info   RGD1307606_predicted 
   5486 0.0001879 548.8 858.8 0.639  1371531_at  Hypothetical LOC299944 (predicted)  Info    
   5487 0.0001882 1134.3 841.5 1.348  1367491_at  apoptosis inhibitor 5 (predicted)  Info   Api5_predicted 
   5488 0.0001885 82.5 169.9 0.486  1390404_at  laminin, alpha 2 (predicted)  Info   Lama2_predicted 
   5489 0.0001886 665.8 875.7 0.76  1375173_at  RDCR-0918-3 protein  Info   RGD:708461 
   5490 0.0001887 58.3 9.5 6.137  1397769_at  Similar to RIKEN cDNA 6330512M04 gene  Info    
   5491 0.0001888 86.5 177.2 0.488  1373291_at    Info    
   5492 0.000189 31.5 68.7 0.459  1371137_at  acyl-Coenzyme A oxidase 2, branched chain  Info   Acox2 
   5493 0.000189 332.7 222.5 1.495  1388897_at  WD repeat domain 5 (predicted)  Info   Wdr5_predicted 
   5494 0.0001893 87.8 60.1 1.461  1383292_at  inner centromere protein antigens 135/155kDa (predicted)  Info   Incenp_predicted 
   5495 0.0001893 112.4 12.2 9.213  1379557_at  Similar to Gamma-parvin  Info    
   5496 0.0001894 358.2 277 1.293  1373077_at  similar to hypothetical protein D11Ertd497e (predicted)  Info   RGD1308696_predicted 
   5497 0.0001895 89.3 29.3 3.048  1394743_at  similar to novel protein  Info   LOC362010 
   5498 0.0001896 328.6 534.9 0.614  1374650_at  neural precursor cell expressed, developmentally down-regulated gene 9 (predicted)  Info   Nedd9_predicted 
   5499 0.00019 1079.4 615.6 1.753  1388960_at  pyrophosphatase  Info   Pp 
   5500 0.00019 211.7 149.2 1.419  1383289_at  Ngg1 interacting factor 3-like 1 (S. pombe) (predicted)  Info   Nif3l1_predicted 
   5501 0.0001902 450.3 332.7 1.353  1375272_at  Phosphatidylinositol 4-kinase type 2 alpha  Info   Pi4KII 
   5502 0.0001902 2416.5 4329 0.558  1372261_at  Transcribed locus  Info    
   5503 0.0001905 120.3 301.6 0.399  1381776_at  similar to RIKEN cDNA 1810015P03 (predicted)  Info   RGD1305260_predicted 
   5504 0.0001909 33.7 2.4 14.042  1373257_at  similar to protein phosphatase 1, regulatory (inhibitory) subunit 1C; thymocyte ARPP; DNA segment, Chr 9, Brigham & Womens Genetics 1012 expressed (predicted)  Info   RGD1307215_predicted 
   5505 0.0001912 799.9 1170.4 0.683  1374188_at  Translocation protein 1 (predicted)  Info    
   5506 0.0001913 95.1 55.1 1.726  1378079_at  golgi autoantigen, golgin subfamily a, 3 (predicted) /// similar to male-enhanced antigen-2  Info   Golga3_predicted /// LOC500010 
   5507 0.0001918 31.3 64.2 0.488  1397836_at  sirtuin (silent mating type information regulation 2 homolog) 4 (S. cerevisiae) (predicted)  Info   Sirt4_predicted 
   5508 0.0001918 177.2 420.1 0.422  1380975_at  Transcribed locus  Info    
   5509 0.0001919 152.8 101.6 1.504  1374186_at  Similar to KIAA1185 protein (predicted)  Info    
   5510 0.0001921 1262.6 4808.8 0.263  1368084_at  deoxyribonuclease I  Info   Dnase1 
   5511 0.0001922 101.6 196.7 0.517  1387004_at  neuroblastoma, suppression of tumorigenicity 1  Info   Nbl1 
   5512 0.0001922 68 142.9 0.476  1370853_at  CaM-kinase II inhibitor alpha  Info   RGD:708430 
   5513 0.0001928 7 22.6 0.31  1384616_at  similar to mKIAA0674 protein (predicted)  Info   RGD1304816_predicted 
   5514 0.0001936 319 1086.7 0.294  1387987_at  solute carrier family 22 (organic anion transporter), member 19  Info   Slc22a19 
   5515 0.0001938 178.1 109.9 1.621  1373768_at  BMS1-like, ribosome assembly protein (yeast) (predicted)  Info   Bms1l_predicted 
   5516 0.0001941 60.2 19.2 3.135  1395442_at  Transcribed locus  Info    
   5517 0.0001942 5339.3 4173.6 1.279  1398830_at  ribosomal protein L28  Info   Rpl28 
   5518 0.0001943 68 147.6 0.461  1391669_at  protein tyrosine phosphatase, receptor type, B (predicted)  Info   Ptprb_predicted 
   5519 0.0001947 2.2 26.2 0.084  1376667_at  cytochrome P450, family 26, subfamily b, polypeptide 1  Info   Cyp26b1 
   5520 0.0001947 659.2 378.4 1.742  1371836_at  RAB5C, member RAS oncogene family (predicted)  Info   Rab5c_predicted 
   5521 0.0001947 617.3 495.7 1.245  1375663_at  similar to RIKEN cDNA 2510010F15 (predicted)  Info   RGD1307608_predicted 
   5522 0.0001947 5697.6 9184.1 0.62  1368440_at  solute carrier family 3, member 1  Info   Slc3a1 
   5523 0.0001958 80.4 122.3 0.657  1386962_at  phospholipase C, beta 4  Info   Plcb4 
   5524 0.0001958 90.9 61.8 1.471  1383660_at  Transcribed locus  Info    
   5525 0.0001959 164.3 87.7 1.873  1390118_at  similar to ATP/GTP-binding protein (predicted)  Info   RGD1307679_predicted 
   5526 0.000196 21.7 40.7 0.533  1377963_at  Transcribed locus  Info    
   5527 0.0001967 250.4 182.3 1.374  1388059_a_at  solute carrier family 11 (proton-coupled divalent metal ion transporters), member 2  Info   Slc11a2 
   5528 0.0001967 239.3 4.3 55.651  1387667_at  nitric oxide synthase 2, inducible  Info   Nos2 
   5529 0.0001969 138.6 315.9 0.439  1395373_at  Transcribed locus  Info    
   5530 0.0001969 651.9 427.2 1.526  1367551_a_at  similar to CG14980-PB (predicted)  Info   RGD1306410_predicted 
   5531 0.000197 76.4 124.6 0.613  1391570_at  similar to mKIAA0843 protein  Info   LOC307395 
   5532 0.0001972 51.9 151.2 0.343  1387339_at  selenoprotein P, plasma, 1  Info   Sepp1 
   5533 0.0001975 1100.2 962.5 1.143  1371687_at  calnexin  Info   Canx 
   5534 0.0001976 623 994.2 0.627  1383240_at  Integrin, alpha 6  Info   Itga6 
   5535 0.0001978 103.9 67 1.551  1390924_at  TAF3 RNA polymerase II, TATA box binding protein (TBP)-associated factor (predicted)  Info    
   5536 0.000198 352.3 517.1 0.681  1388423_at  hypothetical protein MGC15854  Info   RGD:1302996 
   5537 0.000198 361 434.6 0.831  1372894_at  similar to PL6 protein (predicted)  Info   RGD1310540_predicted 
   5538 0.0001983 41.5 14.3 2.902  1370450_at  tropomyosin 3, gamma  Info   Tpm3 
   5539 0.0001985 180.6 376.1 0.48  1372190_at  Aquaporin 4  Info   Aqp4 
   5540 0.0001986 31.4 62.4 0.503  1376918_at  Serine (or cysteine) proteinase inhibitor, clade C (antithrombin), member 1 (predicted)  Info    
   5541 0.000199 35.6 83.9 0.424  1390456_at  Transcribed locus  Info    
   5542 0.0001993 249.4 149.3 1.67  1388864_at  similar to ATP-dependent RNA-helicase  Info   LOC360815 
   5543 0.0001994 49.9 140.2 0.356  1379057_at  Transcribed locus, strongly similar to NP_659132.1 hypothetical protein LOC227545 [Mus musculus]  Info    
   5544 0.0001995 39.5 65.4 0.604  1383642_at    Info    
   5545 0.0001998 94.5 54.3 1.74  1373353_at    Info    
   5546 0.0001998 112.2 197.8 0.567  1390000_at  jumonji domain containing 3 (predicted)  Info   Jmjd3_predicted 
   5547 2e-04 167.8 98.5 1.704  1387201_at  ring finger protein 138  Info   Rnf138 
   5548 2e-04 89.6 156.7 0.572  1373857_at  Family with sequence similarity 36, member A (predicted)  Info    
   5549 0.0002002 143.8 277.9 0.517  1379336_at  Transcribed locus  Info    
   5550 0.0002003 62.9 158.2 0.398  1382462_at  Transcribed locus  Info    
   5551 0.0002003 890.8 1158.2 0.769  1371964_at  G-rich RNA sequence binding factor 1 (predicted)  Info   Grsf1_predicted 
   5552 0.000201 482.1 763.8 0.631  1398950_at    Info    
   5553 0.0002011 133.2 225.7 0.59  1376169_at  Transcribed locus  Info    
   5554 0.0002012 231.6 134.9 1.717  1368026_at  hepatoma-derived growth factor, related protein 2  Info   Hdgfrp2 
   5555 0.0002012 856.3 1252 0.684  1389549_at  proline synthetase co-transcribed (predicted)  Info   Prosc_predicted 
   5556 0.0002015 594.2 315.9 1.881  1397674_at  eukaryotic translation initiation factor 3, subunit 8, 110kDa (predicted)  Info   Eif3s8_predicted 
   5557 0.0002015 279.3 163.7 1.706  1395533_at  dead end homolog 1 (zebrafish) (predicted)  Info   Dnd1_predicted 
   5558 0.0002019 753.4 416 1.811  1373140_at  Interleukin 6 signal transducer  Info   Il6st 
   5559 0.0002019 36.4 16 2.275  1397737_at  Transcribed locus  Info    
   5560 0.000202 32.6 18.7 1.743  1397515_at  similar to hypothetical protein FLJ40362 (predicted)  Info   RGD1310052_predicted 
   5561 0.000202 76.6 24.1 3.178  1382027_at  LOC498010  Info   Itgb3 
   5562 0.0002021 107.8 180.8 0.596  1387127_at  attractin  Info   Atrn 
   5563 0.0002021 1489.3 2154.9 0.691  1367808_at  translocase of inner mitochondrial membrane 8 homolog b (yeast)  Info   Timm8b 
   5564 0.0002026 3733.3 2664.3 1.401  1398854_at  ribosomal protein L24  Info   Rpl24 
   5565 0.0002028 356 797.3 0.447  1370248_at  FXYD domain-containing ion transport regulator 6  Info   Fxyd6 
   5566 0.000203 344.7 435.3 0.792  1379441_at  zinc finger protein 294 (predicted)  Info   Zfp294_predicted 
   5567 0.0002033 56.2 22.6 2.487  1370711_a_at  nucleoporin like 1  Info   Nupl1 
   5568 0.0002034 105.6 158.7 0.665  1370330_at  signal-induced proliferation-associated 1 like 1  Info   Sipa1l1 
   5569 0.0002035 17.1 41.8 0.409  1380370_at    Info    
   5570 0.0002036 98.9 56.6 1.747  1369568_at  syntaxin 6  Info   Stx6 
   5571 0.0002037 314.7 1002.7 0.314  1375231_a_at  CXXC finger 5  Info   RGD:1359466 
   5572 0.000204 2.2 11.4 0.193  1398743_at  Transcribed locus  Info    
   5573 0.0002042 25.9 73.1 0.354  1392013_at  DEP domain containing 2 (predicted)  Info    
   5574 0.0002042 459.7 612 0.751  1389333_at    Info    
   5575 0.0002047 401.8 656.2 0.612  1387294_at  SH3-domain binding protein 5 (BTK-associated)  Info   Sh3bp5 
   5576 0.0002048 234.1 350.8 0.667  1383077_at  similar to RIKEN cDNA 1110063F24 (predicted)  Info   RGD1311622_predicted 
   5577 0.0002051 216.8 502.7 0.431  1367847_at  nuclear protein 1  Info   Nupr1 
   5578 0.0002051 152.5 114.4 1.333  1384821_at  Transcribed locus  Info    
   5579 0.0002053 174.2 99 1.76  1373761_at  Transcribed locus  Info    
   5580 0.0002055 204.1 381.1 0.536  1398281_at  thiopurine methyltransferase  Info   Tpmt 
   5581 0.0002061 17.8 36.5 0.488  1397637_at  Transcribed locus  Info    
   5582 0.0002061 43.3 22.9 1.891  1377782_at  Phosphoribosylformylglycinamidine synthase (FGAR amidotransferase) (predicted)  Info    
   5583 0.0002067 38.5 121 0.318  1389350_at  apolipoprotein H (predicted)  Info   Apoh_predicted 
   5584 0.0002074 117.4 169.9 0.691  1390213_at  Transcribed locus  Info    
   5585 0.0002075 171.2 380.8 0.45  1368078_at  endothelial cell-specific molecule 1  Info   Esm1 
   5586 0.0002077 155.7 87.3 1.784  1373868_at  BCL2-associated transcription factor 1 (predicted)  Info   Bclaf1_predicted 
   5587 0.0002079 319.8 216 1.481  1384254_at  similar to OTU domain containing 1  Info   LOC498803 
   5588 0.0002089 68.6 33.4 2.054  1385765_at  similar to lin-9 homolog (C. elegans)  Info   LOC360888 
   5589 0.000209 188.4 410 0.46  1373309_at  Similar to RIKEN cDNA 1810054O13 (predicted)  Info    
   5590 0.0002091 121.7 73.9 1.647  1376827_at  Echinoderm microtubule associated protein like 4 (predicted)  Info    
   5591 0.0002091 123.4 76.9 1.605  1377252_at  similar to RIKEN cDNA 1200011M11 (predicted)  Info   RGD1306862_predicted 
   5592 0.0002091 87.3 8 10.912  1368324_at  breast cancer 2  Info   Brca2 
   5593 0.0002092 336.3 193.4 1.739  1371663_at  similar to expressed sequence AW556797 (predicted)  Info   RGD1305138_predicted 
   5594 0.0002095 89.4 52.2 1.713  1390441_at  Development and differentiation enhancing (predicted)  Info    
   5595 0.0002096 21.1 55.8 0.378  1373985_at  Transcribed locus  Info    
   5596 0.0002099 16.2 60.6 0.267  1377422_at  myotubularin related protein 1 (predicted)  Info   Mtmr1_predicted 
   5597 0.0002105 20.5 40.7 0.504  1380133_at  odd-skipped related 2 (Drosophila) (predicted)  Info   Osr2_predicted 
   5598 0.0002108 100.2 40.7 2.462  1388439_at  similar to 65kDa FK506-binding protein  Info   LOC360627 
   5599 0.000211 2191.9 1624 1.35  1367711_at  proteasome (prosome, macropain) 26S subunit, ATPase 2  Info   Psmc2 
   5600 0.0002119 350.8 543.6 0.645  1398537_at  autocrine motility factor receptor (predicted)  Info   Amfr_predicted 
   5601 0.0002121 3797.1 5457.1 0.696  1387799_at  FXYD domain-containing ion transport regulator 2  Info   Fxyd2 
   5602 0.0002123 52.4 27.1 1.934  1376062_at  syndecan 1  Info   Sdc1 
   5603 0.0002124 225.1 408.4 0.551  1367894_at  insulin induced gene 1  Info   Insig1 
   5604 0.0002124 1390.8 1749.2 0.795  1388417_at  anaphase-promoting complex subunit 5 (predicted)  Info   Anapc5_predicted 
   5605 0.0002127 113.9 195.4 0.583  1372985_at  zinc finger protein 444 (predicted)  Info   Znf444_predicted 
   5606 0.0002129 228.5 144 1.587  1384016_at  similar to R31449_3  Info   LOC500790 
   5607 0.000213 221.6 341.8 0.648  1398450_at  Transcribed locus  Info    
   5608 0.0002132 148.9 46 3.237  1372868_at  torsin family 3, member A (predicted)  Info   Tor3a_predicted 
   5609 0.0002135 258 130.1 1.983  1388939_at  procollagen, type XV (predicted)  Info   Col15a1_predicted 
   5610 0.0002136 1099.1 2521.3 0.436  1374251_at  Transcribed locus  Info    
   5611 0.0002138 1143.6 770.9 1.483  1371949_at  basic leucine zipper and W2 domains 1  Info   Bzw1 
   5612 0.0002142 380.3 301 1.263  1371622_at  similar to candidate tumor suppressor OVCA2  Info   LOC497954 
   5613 0.0002143 46.6 123.9 0.376  1394216_at  similar to RIKEN cDNA 2810055F11 (predicted)  Info   RGD1305721_predicted 
   5614 0.0002146 259.8 388.5 0.669  1382818_at  similar to hypothetical protein D630010C10 (predicted)  Info   RGD1309752_predicted 
   5615 0.0002149 87.1 25.7 3.389  1392927_at  progestin and adipoQ receptor family member IV (predicted)  Info   Paqr4_predicted 
   5616 0.0002149 26.3 56.8 0.463  1377459_at  lymphocyte antigen 6 complex, locus G6E  Info   RGD:1303112 
   5617 0.0002151 68 32.9 2.067  1387264_at  potassium channel, subfamily K, member 6 /// hypothetical gene supported by NM_053806  Info   Kcnk6 /// LOC497732 
   5618 0.0002151 36.4 145.6 0.25  1380579_at  matrilin 1, cartilage matrix protein  Info   RGD:1359410 
   5619 0.0002151 6.1 29.5 0.207  1380763_at    Info    
   5620 0.0002154 271.7 519 0.524  1372012_at  24-dehydrocholesterol reductase (predicted)  Info   Dhcr24_predicted 
   5621 0.0002154 322.8 642.9 0.502  1384394_at    Info    
   5622 0.0002155 362 273 1.326  1367499_at  solute carrier family 35, member C1 (predicted)  Info   Slc35c1_predicted 
   5623 0.0002156 82.3 34.7 2.372  1391505_x_at    Info    
   5624 0.000216 33 61.5 0.537  1387050_s_at  kininogen 1 /// similar to alpha-1 major acute phase protein prepeptide  Info   Kng1 /// RGD:1359376 
   5625 0.000216 302.2 452.8 0.667  1393983_at  exportin, tRNA (nuclear export receptor for tRNAs) (predicted)  Info   Xpot_predicted 
   5626 0.0002162 77.3 237.3 0.326  1393474_at  sulfotransferase family, cytosolic, 2B, member 1 (predicted)  Info   Sult2b1_predicted 
   5627 0.0002162 4110.5 6651.1 0.618  1368680_a_at  solute carrier family 34 (sodium phosphate), member 1  Info   Slc34a1 
   5628 0.0002163 359.2 237.6 1.512  1372329_at  similar to hypothetical protein PRO0971 (predicted)  Info   RGD1311435_predicted 
   5629 0.0002165 678.6 980.2 0.692  1387855_at  guanosine diphosphate dissociation inhibitor 1  Info   Gdi1 
   5630 0.0002167 1005 1483.5 0.677  1375933_at    Info    
   5631 0.0002169 83.1 31 2.681  1394315_at  Basophilic leukemia expressed sequence 06 (Bles06)  Info    
   5632 0.0002169 228 356.1 0.64  1372367_at    Info    
   5633 0.0002171 241.7 368.1 0.657  1369304_at  6-pyruvoyl-tetrahydropterin synthase  Info   Pts 
   5634 0.0002171 147.2 80.7 1.824  1382633_at  Transcribed locus  Info    
   5635 0.0002174 227.3 295.2 0.77  1389672_at  similar to mKIAA0701 protein  Info   LOC363009 
   5636 0.0002175 164 109.5 1.498  1375447_at  GTP binding protein 1 (predicted)  Info   Gtpbp1_predicted 
   5637 0.0002175 198.3 395.8 0.501  1373122_at  ajuba homolog (Xenopus laevis)  Info   Jub 
   5638 0.0002177 96.2 34.3 2.805  1370780_at  RAB31, member RAS oncogene family  Info   Rab31 
   5639 0.0002183 334.9 801.9 0.418  1387092_at  FXYD domain-containing ion transport regulator 4  Info   Fxyd4 
   5640 0.0002188 85.4 162.2 0.527  1374243_at  Transcribed locus  Info    
   5641 0.0002193 87.6 157.8 0.555  1393654_at  nei endonuclease VIII-like 1 (E. coli) (predicted)  Info   Neil1_predicted 
   5642 0.0002193 17.9 53.9 0.332  1382083_at  coagulation factor C homolog (Limulus polyphemus) (predicted)  Info   Coch_predicted 
   5643 0.0002196 85.1 41.4 2.056  1382355_at  UDP-N-acetyl-alpha-D-galactosamine:polypeptide N-acetylgalactosaminyltransferase 4 (GalNAc-T4)  Info   Galnt4 
   5644 0.00022 68.5 122.4 0.56  1390972_at  similar to CGI-121 protein (predicted)  Info   RGD1309786_predicted 
   5645 0.0002202 218.8 144.8 1.511  1398985_at  GTP binding protein 1 (predicted)  Info    
   5646 0.0002204 269.1 170.4 1.579  1390109_at    Info    
   5647 0.0002206 60.6 35 1.731  1382187_at  similar to RIKEN cDNA 2610029K21 (predicted)  Info   RGD1311086_predicted 
   5648 0.0002206 428.1 732.3 0.585  1376082_at  ecotropic viral integration site 1 (predicted)  Info   Evi1_predicted 
   5649 0.0002208 97.9 26.3 3.722  1387276_at  activity and neurotransmitter-induced early gene protein 4 (ania-4)  Info   RGD:621187 
   5650 0.0002209 83.1 177.9 0.467  1392483_at  Similar to choline phosphotransferase 1; cholinephosphotransferase 1 alpha; cholinephosphotransferase 1  Info   MGC95241 
   5651 0.0002211 184.7 279.3 0.661  1376009_at  meningioma expressed antigen 6 (coiled-coil proline-rich) (predicted)  Info   Mgea6_predicted 
   5652 0.0002211 329.6 579.4 0.569  1376487_at  Rhesus blood group-associated C glycoprotein  Info   Rhcg 
   5653 0.0002217 414.8 665.8 0.623  1391433_at  mitochondrial acyl-CoA thioesterase 1  Info   Mte1 
   5654 0.0002217 39.6 77.9 0.508  1382072_at  Similar to cis-Golgi matrix protein GM130  Info    
   5655 0.0002217 146.1 228.3 0.64  1398447_at  Transcribed locus  Info    
   5656 0.0002217 49.9 119.9 0.416  1385670_at  serine dehydratase-like (predicted)  Info   Sdsl_predicted 
   5657 0.0002218 41.1 13 3.162  1380696_at  Similar to RIKEN cDNA C230093N12 (predicted)  Info    
   5658 0.0002221 195.4 6.9 28.319  1387739_at  CD8 antigen, beta chain  Info   Cd8b 
   5659 0.0002225 35.7 10.3 3.466  1397798_at  Transcribed locus  Info    
   5660 0.0002225 61 2.4 25.417  1385733_at  exonuclease 1 (predicted)  Info   Exo1_predicted 
   5661 0.0002226 2435.5 1850.9 1.316  1388989_at  similar to RIKEN cDNA 3110001N18 (predicted)  Info   RGD1309517_predicted 
   5662 0.0002226 1.4 5.9 0.237  1381158_at  speedy homolog 1 (Drosophila)  Info   Spdy1 
   5663 0.0002232 121.2 8 15.15  1368494_at  S100 calcium binding protein A8 (calgranulin A)  Info   S100a8 
   5664 0.0002239 393.7 259.9 1.515  1383705_at  CDNA clone IMAGE:7317308  Info    
   5665 0.0002239 169.6 296.5 0.572  1391438_at  Transcribed locus, strongly similar to NP_031990.1 ecotropic viral integration site 5 [Mus musculus]  Info    
   5666 0.0002239 233.8 394.8 0.592  1379402_at  ATP-binding cassette, sub-family C (CFTR/MRP), member 4  Info   Abcc4 
   5667 0.0002245 1690 2540 0.665  1375631_at  pleckstrin homology domain containing, family B (evectins) member 2 (predicted)  Info   Plekhb2_predicted 
   5668 0.000225 117 54.1 2.163  1385649_at  integrin alpha 5  Info   Itga5 
   5669 0.000225 56.7 28.3 2.004  1385751_at    Info    
   5670 0.0002253 46.9 84.5 0.555  1379590_at  similar to U2 small nuclear ribonucleoprotein auxiliary factor 35 kDa subunit related-protein 1 (U2(RNU2) small nuclear RNA auxillary factor 1-like 1) (SP2)  Info   LOC498425 
   5671 0.0002254 62.3 105.4 0.591  1383399_at  synaptic vesicle glycoprotein 2a  Info   Sv2a 
   5672 0.0002254 274.4 166.8 1.645  1376025_at  HMT1 hnRNP methyltransferase-like 1 (S. cerevisiae)  Info   Hrmt1l1 
   5673 0.0002255 63.8 37.6 1.697  1392688_at  Ras association domain family 1  Info   RGD:1359383 
   5674 0.0002255 439.3 632.2 0.695  1369562_at  neural visinin-like Ca2+-binding protein type 3  Info   Hpcal1 
   5675 0.0002255 105.3 3.9 27  1371618_s_at  tubulin, beta 3  Info   Tubb3 
   5676 0.0002259 447.9 324.6 1.38  1374135_at  importin 4 (predicted)  Info   Ipo4_predicted 
   5677 0.0002259 1108.6 907 1.222  1377608_a_at  TAR DNA binding protein (predicted)  Info   Tardbp_predicted 
   5678 0.0002263 334.6 612.6 0.546  1389253_at  vanin 1 (predicted)  Info   Vnn1_predicted 
   5679 0.0002264 567.4 826.8 0.686  1373507_at  acyl-Coenzyme A binding domain containing 5 (predicted)  Info   Acbd5_predicted 
   5680 0.0002268 74 22.4 3.304  1395231_at  similar to hematopoietic progenitor protein  Info   LOC500727 
   5681 0.0002269 180 278.1 0.647  1372583_at  Transcribed locus  Info    
   5682 0.0002275 296 174.7 1.694  1376839_at  Transcribed locus  Info    
   5683 0.0002278 221.7 91.2 2.431  1387646_a_at  Max protein  Info   Max 
   5684 0.0002278 188.9 135.9 1.39  1379377_at  ganglioside-induced differentiation-associated-protein 2 (predicted)  Info   Gdap2_predicted 
   5685 0.0002281 3868.1 5990.8 0.646  1368060_at  heat-responsive protein 12  Info   Hrsp12 
   5686 0.0002282 42.6 14.7 2.898  1390823_at  Transcribed locus, weakly similar to NP_116026.1 S-phase kinase-associated protein 2 isoform 2; CDK2/cyclin A-associated protein p45 [Homo sapiens]  Info    
   5687 0.0002284 112.8 176 0.641  1380017_at  Transcribed locus  Info    
   5688 0.0002288 523.5 273.8 1.912  1385553_at  nuclear cap binding protein subunit 2 (predicted)  Info   Ncbp2_predicted 
   5689 0.0002291 59.7 90.8 0.657  1374760_at  Transcribed locus  Info    
   5690 0.0002296 155.1 61.6 2.518  1376076_at    Info    
   5691 0.0002297 32.5 52.5 0.619  1393811_at  similar to putative repair and recombination helicase RAD26L  Info   LOC361197 
   5692 0.0002298 485.7 850.9 0.571  1367771_at  delta sleep inducing peptide, immunoreactor  Info   Dsipi 
   5693 0.0002299 1084.5 764.1 1.419  1367484_at  ubiquitin-conjugating enzyme E2E 2 (UBC4/5 homolog, yeast) (predicted) /// similar to Ubiquitin-conjugating enzyme E2 E1 (Ubiquitin-protein ligase E1) (Ubiquitin carrier protein E1) (UbcM3)  Info   Ube2e2_predicted /// LOC498449 
   5694 0.0002299 228.3 134.8 1.694  1388707_at  Transcribed locus  Info    
   5695 0.0002301 185.2 66.9 2.768  1378282_at  casein kinase II, alpha 2, polypeptide (predicted)  Info   Csnk2a2_predicted 
   5696 0.0002304 1121.2 968.8 1.157  1387777_at  integrin linked kinase  Info   Ilk 
   5697 0.0002309 589.5 366.6 1.608  1384714_x_at  similar to 60S ribosomal protein L8 /// similar to mitochondrial tryptophanyl tRNA synthetase 2  Info   LOC499374 /// LOC499679 
   5698 0.0002315 108.6 48.6 2.235  1392417_s_at  TP53 regulating kinase (predicted)  Info   Trp53rk_predicted 
   5699 0.0002318 67.4 18 3.744  1393581_at  calmodulin binding protein 1 (predicted)  Info   Calmbp1_predicted 
   5700 0.0002318 67 3.8 17.632  1381833_at  cell division cycle associated 2 (predicted)  Info   Cdca2_predicted 
   5701 0.0002321 1760.8 1165.1 1.511  1386954_at  adenylate kinase 2  Info   Ak2 
   5702 0.0002323 73.7 153.6 0.48  1396101_at  stanniocalcin 1  Info   Stc1 
   5703 0.0002323 715.4 2682.6 0.267  1367631_at  connective tissue growth factor  Info   Ctgf 
   5704 0.0002325 1.2 6 0.2  1397922_at  Transcribed locus  Info    
   5705 0.0002326 97.8 33.3 2.937  1379348_at  exosome component 2 (predicted)  Info   Exosc2_predicted 
   5706 0.0002327 69 167.2 0.413  1367845_at  neurofilament 3, medium  Info   Nef3 
   5707 0.0002327 74.5 206.7 0.36  1367742_at  carnitine palmitoyltransferase 1b  Info   Cpt1b 
   5708 0.0002329 67.1 94.3 0.712  1398818_at  guanine nucleotide binding protein (G protein), gamma 5 subunit  Info   Gng5 
   5709 0.0002329 131.4 213.7 0.615  1388911_at  DNA primase, p58 subunit  Info   Prim2 
   5710 0.0002333 360.8 288.3 1.251  1372366_at  HIV-1 tat interactive protein, homolog (human)  Info   Htatip 
   5711 0.0002335 727.3 1492.1 0.487  1373431_at  leucine-rich repeat-containing 5 (predicted)  Info   Lrrc5_predicted 
   5712 0.0002335 309.3 195.6 1.581  1372619_at  mitochondrial ribosomal protein L49 (predicted)  Info   Mrpl49_predicted 
   5713 0.0002337 19.7 50.1 0.393  1378929_at  Transcribed locus, weakly similar to XP_580238.1 PREDICTED: hypothetical protein XP_580238 [Rattus norvegicus]  Info    
   5714 0.0002337 819.2 519.8 1.576  1387262_at  Sjogren syndrome antigen B  Info   Ssb 
   5715 0.0002338 220.3 346.2 0.636  1375916_at  Similar to RIKEN cDNA 5330414D10 (predicted)  Info    
   5716 0.0002338 57.7 91.6 0.63  1390590_at  protein O-fucosyltransferase 2 (predicted)  Info   Pofut2_predicted 
   5717 0.0002338 126 237.9 0.53  1387104_at  sodium channel, nonvoltage-gated, type I, alpha polypeptide  Info   Scnn1a 
   5718 0.000234 85.4 193.6 0.441  1384887_at    Info    
   5719 0.0002342 273.7 213.4 1.283  1373495_at  ubiquitin-conjugating enzyme E2, J1 (predicted)  Info   Ube2j1_predicted 
   5720 0.0002346 292.1 180.3 1.62  1375695_at  YTH domain family 2 (predicted)  Info   Ythdf2_predicted 
   5721 0.0002348 160.2 77.9 2.056  1368404_at  drebrin 1  Info   Dbn1 
   5722 0.000235 1741.5 2766 0.63  1377720_x_at    Info    
   5723 0.000235 213.2 136.1 1.566  1370993_at  laminin, gamma 1  Info   Lamc1 
   5724 0.0002354 1613.6 2569.2 0.628  1386909_a_at  voltage-dependent anion channel 1  Info   Vdac1 
   5725 0.000236 644.7 840.2 0.767  1372150_at  Similar to mKIAA0190 protein  Info    
   5726 0.0002363 257.6 724.9 0.355  1368080_at  response gene to complement 32  Info   Rgc32 
   5727 0.0002365 93.2 151.3 0.616  1373141_at    Info    
   5728 0.0002369 183.3 283.1 0.647  1380171_at  Adrenergic receptor, alpha 2b  Info   Adra2b 
   5729 0.000237 403.8 603.5 0.669  1372344_at  Hbs1-like (S. cerevisiae) (predicted)  Info   Hbs1l_predicted 
   5730 0.0002372 345.2 192.7 1.791  1367874_at  ras homolog gene family, member Q  Info   Rhoq 
   5731 0.0002373 514.8 370.2 1.391  1377676_at  nuclear ubiquitous casein kinase and cyclin-dependent kinase substrate  Info   Nucks 
   5732 0.0002378 277 159.5 1.737  1372106_at  EH-domain containing 4  Info   Ehd4 
   5733 0.0002378 495.3 291.2 1.701  1380582_at  colony stimulating factor 1 (macrophage)  Info   Csf1 
   5734 0.0002382 358.2 467.1 0.767  1371789_at  similar to ClpP protease  Info   LOC301117 
   5735 0.0002383 930.2 648.3 1.435  1389010_at  leukotriene A4 hydrolase (predicted)  Info   Lta4h_predicted 
   5736 0.0002384 47.9 101.9 0.47  1396408_at  Transcribed locus  Info    
   5737 0.0002387 396.4 296.2 1.338  1373099_at  phosphatidylinositol glycan, class S  Info   RGD:1359436 
   5738 0.000239 212.2 140.2 1.514  1387236_at  dynactin 4  Info   Dctn4 
   5739 0.0002391 376.7 256 1.471  1389598_at  ceroid-lipofuscinosis, neuronal 6 (predicted)  Info   Cln6_predicted 
   5740 0.0002395 48.6 14.9 3.262  1395665_at    Info    
   5741 0.0002395 60.8 35.5 1.713  1392716_at  Transcribed locus  Info    
   5742 0.0002396 144.2 235.8 0.612  1374747_at  PFTAIRE protein kinase 1 (predicted)  Info    
   5743 0.0002399 374.5 701.1 0.534  1391864_at    Info    
   5744 0.00024 2.4 15.7 0.153  1398604_at  Oxysterol binding protein 2 (predicted)  Info    
   5745 0.0002404 703.9 514.6 1.368  1388425_at  similar to RIKEN cDNA D130038B21 (predicted)  Info   RGD1305890_predicted 
   5746 0.0002404 43.1 15.6 2.763  1374015_at  Transcribed locus  Info    
   5747 0.0002405 36.3 2.7 13.444  1392754_at  Similar to cysteine-rich glycoprotein  Info    
   5748 0.0002405 63.5 103 0.617  1374883_at  myotubularin related protein 7 (predicted)  Info   Mtmr7_predicted 
   5749 0.0002407 93.8 67.4 1.392  1373120_at  Spermatogenesis associated 2  Info   Spata2 
   5750 0.0002409 168.2 110 1.529  1367967_at  leprecan 1  Info   Lepre1 
   5751 0.000241 33.6 64.2 0.523  1372293_at  Toll-like receptor 5  Info   Tlr5 
   5752 0.0002412 22.4 84.8 0.264  1393898_at  Similar to KIAA0460 protein (predicted)  Info    
   5753 0.0002417 495.7 694.7 0.714  1393314_at  Fibroblast growth factor 1  Info   Fgf1 
   5754 0.0002418 194.6 114.6 1.698  1376129_at  Similar to vacuolar protein sorting 13C protein  Info    
   5755 0.0002427 43.5 115.6 0.376  1392149_at  Eukaryotic translation initiation factor 4B (predicted)  Info   MGC94923 
   5756 0.0002428 49.4 90.9 0.543  1376635_at  Transketolase  Info   Tkt 
   5757 0.0002429 1098.1 1568.1 0.7  1388380_at  similar to Protein CGI-51  Info   RGD:1303219 
   5758 0.0002431 78.9 9.5 8.305  1387638_a_at  cytotoxic T-lymphocyte-associated protein 4  Info   Ctla4 
   5759 0.0002433 2124.5 7248.5 0.293  1387218_at  trefoil factor 3  Info   Tff3 
   5760 0.0002438 568.3 1315.8 0.432  1396103_at  similar to Ab2-225 (predicted)  Info   RGD1306952_predicted 
   5761 0.000244 409.4 230.1 1.779  1373657_at  Solute carrier family 31, member 2 (predicted)  Info    
   5762 0.0002451 313 218.2 1.434  1373467_at  Similar to TBP-associated factor 172 (TAF-172) (TAF(II)170)  Info    
   5763 0.0002452 625 412.9 1.514  1374526_at    Info    
   5764 0.0002457 428.6 555.4 0.772  1373160_at  similar to HTPAP protein  Info   LOC498653 
   5765 0.0002468 1769.5 1451.2 1.219  1399050_at  adenylosuccinate synthetase 2, non muscle (predicted)  Info   Adss2_predicted 
   5766 0.0002469 92.6 169.5 0.546  1392529_at  Transcribed locus  Info    
   5767 0.000247 128.4 45.2 2.841  1389651_at  apelin, AGTRL1 ligand  Info   Apln 
   5768 0.000247 143.2 80.8 1.772  1372939_at  hypothetical LOC287199 (predicted)  Info   RGD1307203_predicted 
   5769 0.0002476 20.6 57 0.361  1385724_at    Info    
   5770 0.0002482 2984.9 1890.7 1.579  1399160_a_at  ubiquitin-conjugating enzyme E2D 3 (UBC4/5 homolog, yeast)  Info   Ube2d3 
   5771 0.0002486 14.7 39.5 0.372  1379646_at  Similar to CDNA sequence BC024479  Info    
   5772 0.0002489 758.3 584.3 1.298  1373563_at  similar to putative nucleic acid binding protein RY-1; EST AI449063 (predicted)  Info   RGD1310925_predicted 
   5773 0.0002492 83.3 31.8 2.619  1376018_at  similar to Lmnb2 protein  Info   LOC299625 
   5774 0.0002495 16.8 79 0.213  1393263_at  Kin of IRRE like 3 (Drosophila) (predicted)  Info    
   5775 0.0002497 1430.7 2646.2 0.541  1386872_at  insulin-like growth factor 2 receptor  Info   Igf2r 
   5776 0.0002501 147.5 275.3 0.536  1377738_a_at  cysteine and histidine rich 1  Info   Cyhr1 
   5777 0.0002504 49.2 110.4 0.446  1393409_at  Transcribed locus  Info    
   5778 0.0002507 31.8 15 2.12  1388010_at  Pro-melanin-concentrating hormone  Info   Pmch 
   5779 0.0002508 2.5 43.3 0.058  1367739_at  Cytochrom c oxidase subunit VIII-H (heart/muscle)  Info   Cox8h 
   5780 0.0002508 24.9 72.6 0.343  1369795_at  potassium inwardly-rectifying channel, subfamily J, member 12  Info   Kcnj12 
   5781 0.0002511 108.6 189.1 0.574  1376494_at  similar to DNA polymerase epsilon p17 subunit (DNA polymerase epsilon subunit 3) (Chromatin accessibility complex 17) (HuCHRAC17) (CHRAC-17)  Info   RGD:1359475 
   5782 0.0002518 130 226.5 0.574  1380182_at  similar to KIAA1217  Info   LOC291356 
   5783 0.0002518 98.8 64.4 1.534  1385395_at    Info    
   5784 0.0002519 84 30 2.8  1373085_at  carbonyl reductase 3 (predicted)  Info   Cbr3_predicted 
   5785 0.0002523 3267.4 4005.2 0.816  1371321_at    Info    
   5786 0.0002524 135.9 70.6 1.925  1381237_at  Transcribed locus  Info    
   5787 0.0002526 1.6 11.3 0.142  1394668_at  immunoglobulin superfamily, member 4B (predicted)  Info   Igsf4b_predicted 
   5788 0.0002528 483.6 317.7 1.522  1376869_at  Transducin-like enhancer of split 4, E(spl) homolog (Drosophila)  Info   Tle4 
   5789 0.0002534 157.5 261.3 0.603  1398942_at  CDNA clone MGC:95041 IMAGE:7123253  Info    
   5790 0.0002538 57.4 115.2 0.498  1379580_at  chromodomain helicase DNA binding protein 6 (predicted)  Info   Chd6_predicted 
   5791 0.000254 743.5 393.1 1.891  1382094_at  Transcribed locus  Info    
   5792 0.0002542 31.6 10 3.16  1370442_at  thymosin beta-like protein 1  Info   Tmsbl1 
   5793 0.0002542 422.7 280.9 1.505  1374279_at  small inducible cytokine subfamily E, member 1  Info   Scye1 
   5794 0.0002544 12.6 32.7 0.385  1374542_at  Similar to RIKEN cDNA 1200015N20 (predicted)  Info    
   5795 0.000255 83.4 142.3 0.586  1373301_at    Info    
   5796 0.0002551 39.9 95.7 0.417  1391506_at  PHD finger protein 17 (predicted)  Info    
   5797 0.0002554 7.9 2 3.95  1376369_at  trophinin associated protein (tastin) (predicted)  Info   Troap_predicted 
   5798 0.0002556 146.4 205.5 0.712  1372010_at  similar to MK-5 type 2  Info   MGC116327 
   5799 0.0002558 74.6 40.4 1.847  1394589_at  Transcribed locus  Info    
   5800 0.0002559 286.9 635.4 0.452  1368446_at  serine protease inhibitor, Kazal type 1  Info   Spink1 
   5801 0.0002561 124.4 79.8 1.559  1374958_at  similar to RIKEN cDNA 2610017G09 (predicted)  Info   RGD1306103_predicted 
   5802 0.0002561 66.3 2.4 27.625  1392628_at  Transcribed locus  Info    
   5803 0.0002567 318.2 573.7 0.555  1371990_at  CDNA clone IMAGE:7930963  Info    
   5804 0.000257 339.1 648.1 0.523  1393933_at  sortilin-related receptor, L(DLR class) A repeats-containing (predicted)  Info   Sorl1_predicted 
   5805 0.0002572 115.8 271 0.427  1370747_at  fibroblast growth factor 9  Info   Fgf9 
   5806 0.0002575 332.8 495 0.672  1373201_at  Dihydrolipoamide branched chain transacylase E2  Info   Dbt 
   5807 0.0002584 109.6 180.5 0.607  1389561_at  similar to RIKEN cDNA 1810021J13 (predicted)  Info   RGD1311364_predicted 
   5808 0.0002584 122.7 245.6 0.5  1384639_at  deleted in polyposis 1-like 1 (predicted)  Info   Dp1l1_predicted 
   5809 0.0002586 180 92.5 1.946  1384352_at  PAP associated domain containing 4 (predicted)  Info   Papd4_predicted 
   5810 0.0002589 136 66.7 2.039  1376198_at  adipocyte-specific adhesion molecule  Info   Asam 
   5811 0.0002593 4655 3330.2 1.398  1367568_a_at  matrix Gla protein  Info   Mgp 
   5812 0.0002594 469.2 735.3 0.638  1367836_at  carnitine palmitoyltransferase 1, liver  Info   Cpt1a 
   5813 0.0002594 365.4 526.5 0.694  1373366_at  alpha glucosidase 2, alpha neutral subunit (predicted)  Info   G2an_predicted 
   5814 0.0002598 112.5 44 2.557  1382730_at  Similar to osteoclast inhibitory lectin  Info    
   5815 0.0002604 548.4 382.5 1.434  1371704_at  Rho family GTPase 1 (predicted)  Info   Rnd1 
   5816 0.0002607 84.5 59.9 1.411  1377836_at  Similar to suppressor of hairy wing homolog 4 isoform 1  Info    
   5817 0.0002614 203.2 110.7 1.836  1374716_at  similar to RIKEN cDNA 2810430M08 (predicted)  Info   RGD1306106_predicted 
   5818 0.0002615 256.3 468.2 0.547  1368193_at  solute carrier family 26, member 4  Info   Slc26a4 
   5819 0.0002616 379.6 535.7 0.709  1371702_at  transmembrane 4 superfamily member 2  Info   Tm4sf2 
   5820 0.0002618 905.8 623.8 1.452  1373100_at  similar to RIKEN cDNA 2900010J23  Info   LOC499779 
   5821 0.0002619 66.8 31.2 2.141  1378342_at  LOC498937  Info   LOC498937 
   5822 0.0002621 3862.4 4738.2 0.815  1370284_at  ATP synthase, H+ transporting, mitochondrial F1 complex, epsilon subunit  Info   Atp5e 
   5823 0.0002622 95.1 46.4 2.05  1368167_at  cathepsin E  Info   Ctse 
   5824 0.0002624 177.8 267.3 0.665  1372849_at  DnaJ (Hsp40) homolog, subfamily A, member 2  Info   Dnaja2 
   5825 0.0002628 801.1 474 1.69  1390989_at  similar to Mospd2 protein  Info   LOC363463 
   5826 0.0002636 41 85.6 0.479  1381262_at  Transcribed locus  Info    
   5827 0.0002637 737 285.7 2.58  1387074_at  regulator of G-protein signaling 2  Info   Rgs2 
   5828 0.0002641 273.3 367.7 0.743  1377141_at  Similar to RIKEN cDNA 1110018J18  Info    
   5829 0.0002644 111.5 45.4 2.456  1377380_at  Transcribed locus  Info    
   5830 0.0002645 256.4 425 0.603  1383523_at  Rho/rac guanine nucleotide exchange factor (GEF) 18 (predicted)  Info    
   5831 0.0002648 314.7 233.4 1.348  1378110_at  Metadherin  Info   Lyric 
   5832 0.0002649 38.4 95.6 0.402  1388274_at  brain expressed myelocytomatosis oncogene  Info   Bmyc 
   5833 0.000265 73.2 123.4 0.593  1391148_at  Transcribed locus  Info    
   5834 0.000265 521.7 230.7 2.261  1392842_at  Schlafen 5 (predicted)  Info    
   5835 0.000265 14.4 33.2 0.434  1375073_at  similar to sterile alpha motif domain containing 11  Info   LOC500601 
   5836 0.0002651 5.9 51.7 0.114  1379848_at    Info    
   5837 0.0002651 202.7 254.2 0.797  1376894_at  pallidin (predicted)  Info   Pldn_predicted 
   5838 0.0002652 33.8 15.2 2.224  1370518_a_at  syntaxin binding protein 1  Info   Stxbp1 
   5839 0.0002653 20.8 110.3 0.189  1391485_at    Info    
   5840 0.0002654 175.8 247.3 0.711  1383531_at    Info    
   5841 0.0002656 117 323.6 0.362  1378739_at  similar to cell death-inducing DFFA-like effector c  Info   LOC500292 
   5842 0.0002658 105.8 168.4 0.628  1370317_at  potassium channel regulator 1  Info   RGD:708500 
   5843 0.0002658 133.4 83.3 1.601  1376834_at  Ataxia telangiectasia mutated homolog (human)  Info   Atm 
   5844 0.0002664 397.8 609.5 0.653  1373560_at  similar to N-acetylglucosaminyltrasnferase IVb  Info   LOC303100 
   5845 0.0002666 132 90.5 1.459  1392829_at  Transcribed locus, strongly similar to XP_529072.1 PREDICTED: similar to cleavage stimulation factor subunit 2 [Pan troglodytes]  Info    
   5846 0.0002669 129.9 76.1 1.707  1372989_at  Transcribed locus  Info    
   5847 0.000267 99.5 52.3 1.902  1375442_at  M-phase phosphoprotein 10 (U3 small nucleolar ribonucleoprotein) (predicted)  Info   Mphosph10_predicted 
   5848 0.000267 350.3 219.5 1.596  1371695_at  translocated promoter region (predicted)  Info   Tpr_predicted 
   5849 0.0002677 651.3 559.3 1.164  1371860_at  similar to RIKEN cDNA 1110059P08 (predicted)  Info   RGD1305031_predicted 
   5850 0.0002677 152.6 264.2 0.578  1387558_at  transient receptor potential cation channel, subfamily V, member 4  Info   Trpv4 
   5851 0.0002681 626.8 279 2.247  1367905_at  ectonucleotide pyrophosphatase/phosphodiesterase 3  Info   Enpp3 
   5852 0.0002686 259 24.8 10.444  1377185_at    Info    
   5853 0.0002688 2783 2438.7 1.141  1398851_at  tyrosine 3-monooxygenase/tryptophan 5-monooxygenase activation protein, epsilon polypeptide  Info   Ywhae 
   5854 0.0002689 670.1 359.1 1.866  1388129_at  structure specific recognition protein 1  Info   Ssrp1 
   5855 0.0002689 231.3 411.3 0.562  1373480_at  Transcribed locus  Info    
   5856 0.000269 208.8 9.7 21.526  1369815_at  chemokine (C-C motif) ligand 3  Info   Ccl3 
   5857 0.0002691 50.6 32.9 1.538  1392844_at  Transcribed locus  Info    
   5858 0.0002694 319.5 415.8 0.768  1388677_at  ubiquitin-associated protein 1 (predicted)  Info   Ubap1_predicted 
   5859 0.0002698 45.9 24.5 1.873  1385580_at  Transcribed locus  Info    
   5860 0.0002699 174.7 113.6 1.538  1381822_x_at  TP53 regulating kinase (predicted) /// TP53 regulating kinase (predicted)  Info   Trp53rk_predicted /// Tp53rk_predicted 
   5861 0.00027 46 3.4 13.529  1392440_at  Transcribed locus  Info    
   5862 0.0002704 55 193.3 0.285  1393037_at    Info    
   5863 0.0002709 72.8 119.7 0.608  1376679_at  Transcribed locus  Info    
   5864 0.0002713 3075.1 1872.2 1.643  1388331_at  tumor rejection antigen gp96 (predicted)  Info   Tra1_predicted 
   5865 0.0002717 433 724.7 0.597  1388821_at  similar to Tribbles homolog 2  Info   LOC313974 
   5866 0.0002718 67.9 239.4 0.284  1368583_a_at  histidine-rich glycoprotein  Info   Hrg 
   5867 0.0002718 24.2 1.9 12.737  1370387_at  cytochrome P450, family 3, subfamily a, polypeptide 13  Info   Cyp3a13 
   5868 0.000272 126 45.9 2.745  1368172_a_at  lysyl oxidase  Info   Lox 
   5869 0.0002722 349.3 309.9 1.127  1389509_at  Similar to hypothetical protein 2610304F09 (predicted)  Info    
   5870 0.0002722 366.7 265.9 1.379  1371326_at  casein kinase II, alpha 2, polypeptide (predicted)  Info   Csnk2a2_predicted 
   5871 0.0002731 404 627.5 0.644  1393388_at  Zinc finger, DHHC domain containing 3 (predicted)  Info   Zdhhc3 
   5872 0.0002733 198.4 269.9 0.735  1372054_at  Similar to sex-determination protein homolog Fem1a  Info    
   5873 0.0002734 25.3 109.3 0.231  1385994_at  sporulation protein, meiosis-specific, SPO11 homolog (S. cerevisiae) (predicted)  Info   Spo11_predicted 
   5874 0.0002735 61.9 24 2.579  1373771_at  Transcribed locus  Info    
   5875 0.0002736 83.5 30.8 2.711  1395165_at  PC4 and SFRS1 interacting protein 1  Info   Psip1 
   5876 0.0002741 44.8 88.1 0.509  1397356_at  Rho GTPase activating protein 1 (predicted)  Info    
   5877 0.0002742 80.1 51.6 1.552  1369236_at  PR domain containing 4  Info   Prdm4 
   5878 0.0002744 206 321 0.642  1384046_at    Info    
   5879 0.0002744 70.6 40.2 1.756  1392706_at  Cadherin 23 (otocadherin)  Info   Cdh23 
   5880 0.0002745 56.6 150.1 0.377  1378896_at    Info    
   5881 0.0002746 136.7 83.1 1.645  1385052_at  RAB3A interacting protein (rabin3)-like 1  Info   Rab3il1 
   5882 0.0002746 317.2 193.3 1.641  1374014_at  neutral sphingomyelinase (N-SMase) activation associated factor /// hypothetical gene supported by NM_181389  Info   Nsmaf /// LOC497801 
   5883 0.0002747 147 243.4 0.604  1378241_at  Transcribed locus  Info    
   5884 0.0002752 69.7 33.8 2.062  1394278_at  LOC500640  Info   LOC500640 
   5885 0.0002754 53.4 5 10.68  1384752_at  similar to osteoclast inhibitory lectin  Info   LOC362446 
   5886 0.0002756 348.3 755 0.461  1388792_at  growth arrest and DNA-damage-inducible 45 gamma (predicted)  Info   Gadd45g_predicted 
   5887 0.0002757 408.5 547.5 0.746  1388692_at  Transcribed locus, strongly similar to NP_079814.1 spermatogenesis associated 11 [Mus musculus]  Info    
   5888 0.0002761 5.6 8.8 0.636  1389120_at  potassium voltage gated channel, Shaw-related subfamily, member 3  Info   Kcnc3 
   5889 0.0002762 5611.8 4930.8 1.138  1371301_at  ribosomal protein L9  Info   Rpl9 
   5890 0.0002764 408.4 310.1 1.317  1371684_at  pelota homolog  Info   RGD:1359591 
   5891 0.0002765 418.3 207.6 2.015  1372325_at  elastin microfibril interfacer 1 (predicted)  Info   Emilin1_predicted 
   5892 0.0002771 155.4 70.8 2.195  1385157_at  chromobox homolog 1 (Drosophila HP1 beta) (predicted)  Info   Cbx1_predicted 
   5893 0.0002779 1252.7 985.2 1.272  1367870_at  thioredoxin-like 2  Info   Txnl2 
   5894 0.000278 30.1 80.5 0.374  1386691_at  F-box only protein 36 (predicted)  Info   Fbxo36_predicted 
   5895 0.0002799 581.7 442.1 1.316  1373610_at    Info    
   5896 0.0002811 89.3 30.9 2.89  1383690_at  similar to ornithine decarboxylase-like protein  Info   LOC366473 
   5897 0.0002813 265.5 388.9 0.683  1368863_at  non-metastatic cells 3, protein expressed in  Info   Nme3 
   5898 0.0002826 13.1 25.3 0.518  1395963_at  Mitogen activated protein kinase kinase kinase 12  Info   Map3k12 
   5899 0.0002829 174.1 91.9 1.894  1376990_at  Similar to HCDI protein (predicted)  Info    
   5900 0.0002829 101.3 153.2 0.661  1369972_at  serine (or cysteine) proteinase inhibitor, clade B, member 5  Info   Serpinb5 
   5901 0.000283 361 244.5 1.476  1368165_at  phosphoribosyl pyrophosphate synthetase 1  Info   Prps1 
   5902 0.0002833 1106.5 862.3 1.283  1371410_at  similar to cDNA sequence BC056474  Info   LOC288925 
   5903 0.0002835 39.3 70.2 0.56  1373699_at  Similar to RIKEN cDNA 1810059A23  Info    
   5904 0.0002835 95.1 47.9 1.985  1393683_at  similar to KIAA0368 (predicted)  Info   RGD1306148_predicted 
   5905 0.0002838 57.6 124 0.465  1385899_at  Transcribed locus  Info    
   5906 0.000284 22.7 3.4 6.676  1378331_at  Mesoderm development candiate 2 (predicted)  Info   Mesdc2 
   5907 0.0002847 78 42.2 1.848  1368279_at  myeloid/lymphoid or mixed-lineage leukemia (trithorax (Drosophila) homolog); translocated to, 3  Info   RGD:620940 
   5908 0.0002848 488.4 617.1 0.791  1368444_at  small glutamine-rich tetratricopeptide repeat (TPR)-containing, alpha  Info   Sgta 
   5909 0.0002854 206.6 97.1 2.128  1387795_at  polymerase (DNA directed), alpha 2  Info   Pola2 
   5910 0.0002862 81.2 54.9 1.479  1397362_at  Transcribed locus  Info    
   5911 0.0002865 91 175.9 0.517  1391534_at  elongation of very long chain fatty acids (FEN1/Elo2, SUR4/Elo3, yeast)-like 2 (predicted)  Info   Elovl2_predicted 
   5912 0.0002866 854.3 1094.7 0.78  1372252_at  trafficking protein particle complex 1 (predicted)  Info   Trappc1_predicted 
   5913 0.0002867 270.9 167.2 1.62  1378178_at  SMC6 structural maintenance of chromosomes 6-like 1 (yeast) (predicted)  Info   Smc6l1_predicted 
   5914 0.0002867 117.5 69.8 1.683  1380265_at  small nuclear RNA activating complex, polypeptide 1 (predicted)  Info   Snapc1_predicted 
   5915 0.0002869 89.4 150.1 0.596  1389908_at  Nuclear factor I/A  Info   Nfia 
   5916 0.0002872 229.3 304.9 0.752  1367528_at  F-box and WD-40 domain protein 8 (predicted)  Info    
   5917 0.0002872 150.3 117.8 1.276  1377359_at  similar to protein P3  Info   LOC501665 
   5918 0.0002877 543.9 356.9 1.524  1372863_at  pam, highwire, rpm 1 (predicted)  Info   Phr1_predicted 
   5919 0.0002877 26.3 53.9 0.488  1380852_at  hypothetical LOC294883 (predicted)  Info   RGD1305844_predicted 
   5920 0.000288 74 47.3 1.564  1384538_at    Info    
   5921 0.000288 64.8 110.5 0.586  1395965_at  Retinoblastoma binding protein 6 (predicted)  Info    
   5922 0.0002883 37.7 75.3 0.501  1393344_at  Transcribed locus  Info    
   5923 0.0002883 153.9 27.8 5.536  1376255_at  mitogen activated protein kinase kinase kinase kinase 1 (predicted)  Info   Map4k1_predicted 
   5924 0.0002884 276.6 217.8 1.27  1367515_at  CCR4-NOT transcription complex, subunit 7 (predicted)  Info   Cnot7_predicted 
   5925 0.000289 101.4 243.8 0.416  1369526_at  acyl-Coenzyme A dehydrogenase, short/branched chain  Info   Acadsb 
   5926 0.0002891 5093.6 6610.1 0.771  1388113_at  cytochrome c oxidase, subunit VIIIa  Info   Cox8a 
   5927 0.0002893 93 254.9 0.365  1394594_at  Transcribed locus  Info    
   5928 0.0002899 475.3 265.5 1.79  1375673_at  Mitogen activated protein kinase kinase kinase 1  Info   Map3k1 
   5929 0.0002904 610.1 1029.9 0.592  1398250_at  cytosolic acyl-CoA thioesterase 1  Info   Cte1 
   5930 0.0002913 35.2 87 0.405  1382838_at    Info    
   5931 0.0002914 391.9 622.5 0.63  1374591_at  Similar to protein tyrosine phosphatase, receptor type, D  Info    
   5932 0.0002917 163.1 61.7 2.643  1378264_at  Nuclear autoantigenic sperm protein  Info   Nasp 
   5933 0.0002917 290.8 184.6 1.575  1388803_at  deoxyhypusine synthase  Info   RGD:1303326 
   5934 0.0002918 403 1024.4 0.393  1368249_at  Kruppel-like factor 15  Info   Klf15 
   5935 0.0002918 62.1 88.9 0.699  1381467_at  Transcribed locus, weakly similar to XP_373633.2 PREDICTED: hypothetical protein XP_373633 [Homo sapiens]  Info    
   5936 0.0002922 342.8 207.5 1.652  1372145_at  threonyl-tRNA synthetase  Info   RGD:1359527 
   5937 0.0002923 54.9 32.2 1.705  1382273_at  similar to Apoptosis facilitator Bcl-2-like protein 14  Info   LOC500348 
   5938 0.0002924 6673.8 4491 1.486  1367640_at  ribosomal protein S12  Info   Rps12 
   5939 0.0002929 3 11.6 0.259  1393850_at  similar to CRYPTIC  Info   LOC501121 
   5940 0.000293 24.2 4.4 5.5  1392285_at  similar to scotin (predicted)  Info   RGD1312041_predicted 
   5941 0.0002931 65.7 45.9 1.431  1376247_at  phosphoenolpyruvate carboxykinase 2 (mitochondrial) (predicted)  Info   Pck2_predicted 
   5942 0.0002937 307.7 493.9 0.623  1371014_at  phospholipase C, beta 1  Info   Plcb1 
   5943 0.0002937 112.5 173.4 0.649  1390281_a_at  similar to Hypothetical protein FLJ32452  Info   LOC362810 
   5944 0.0002937 322.6 508.9 0.634  1388618_at  nidogen 2 (predicted)  Info   Nid2_predicted 
   5945 0.0002938 94.7 154 0.615  1393100_at  similar to 1110008L16Rik protein (predicted)  Info   RGD1305089_predicted 
   5946 0.000294 145.3 58.8 2.471  1383596_at  similar to RIKEN cDNA 5830446M03 (predicted)  Info   RGD1307688_predicted 
   5947 0.0002941 70.9 8.7 8.149  1378199_at  Transcribed locus  Info    
   5948 0.0002943 288.7 419.6 0.688  1372085_at  ADP-ribosylation factor-like 6 interacting protein 2 (predicted)  Info   Arl6ip2_predicted 
   5949 0.0002944 174.5 328.1 0.532  1382835_at  Transcribed locus  Info    
   5950 0.0002948 85.6 24.5 3.494  1371267_at  RT1 class Ib, locus Aw2 /// RT1-149 protein  Info   RT1-Aw2 /// RT1-149 
   5951 0.000295 56.6 110.8 0.511  1376279_at  processing of precursor 1, ribonuclease P/MRP family, (S. cerevisiae) (predicted)  Info   Pop1_predicted 
   5952 0.000295 572.3 368.6 1.553  1373150_at  catechol-O-methyltransferase domain containing 1 (predicted)  Info   Comtd1_predicted 
   5953 0.0002954 202.8 359.5 0.564  1390117_at  Transcribed locus  Info    
   5954 0.0002956 222.5 351.3 0.633  1388825_at  Transcribed locus  Info    
   5955 0.0002959 1502.1 2065.2 0.727  1373193_at    Info    
   5956 0.000296 870.3 1586.2 0.549  1377719_a_at    Info    
   5957 0.0002962 40.1 58.1 0.69  1376912_at  hypothetical LOC287388 (predicted)  Info   RGD1310324_predicted 
   5958 0.0002967 60 163.4 0.367  1377789_at  Transcribed locus  Info    
   5959 0.000297 329.7 541.9 0.608  1368206_at  peroxisomal acyl-CoA thioesterase 1  Info   Pte1 
   5960 0.0002971 265.8 390.4 0.681  1375846_at  Xenotropic and polytropic retrovirus receptor 1 (predicted)  Info    
   5961 0.0002974 92.1 33.9 2.717  1389671_at  transient receptor potential cation channel, subfamily C, member 2  Info   Trpc2 
   5962 0.000298 30.6 50.6 0.605  1381088_at    Info    
   5963 0.0002987 234.7 914.8 0.257  1386994_at  B-cell translocation gene 2, anti-proliferative  Info   Btg2 
   5964 0.0002992 2.5 17.5 0.143  1398169_at  Transcribed locus  Info    
   5965 0.0002993 152.3 101.6 1.499  1391190_at    Info    
   5966 0.0002997 2475.3 1869.2 1.324  1374385_at  similar to Poly(rC)-binding protein 1 (Alpha-CP1) (hnRNP-E1)  Info   LOC500242 
   5967 0.0002998 59.9 138.6 0.432  1391434_at  RAB3A, member RAS oncogene family  Info   Rab3a 
   5968 0.0002999 284.8 116.6 2.443  1370990_at  similar to cytokine receptor related protein 4  Info   RGD:621517 
   5969 3e-04 54.9 26.4 2.08  1380259_at  similar to RIKEN cDNA 2410017P07 (predicted)  Info   RGD1311358_predicted 
   5970 0.0003001 793 526.3 1.507  1372810_at    Info    
   5971 0.0003004 77.1 179.2 0.43  1371436_at  dimethylarginine dimethylaminohydrolase 2  Info   RGD:1302955 
   5972 0.0003004 395.3 538.4 0.734  1398878_at  similar to CPSF4 protein  Info   MGC108785 
   5973 0.0003005 80 41.5 1.928  1375060_at  similar to Cc2-27 (predicted)  Info   RGD1310244_predicted 
   5974 0.0003006 119.5 80.4 1.486  1378008_at  Transcribed locus  Info    
   5975 0.000301 21 53.9 0.39  1376945_at  Transcribed locus  Info    
   5976 0.0003011 86.2 26.2 3.29  1385639_at    Info    
   5977 0.0003013 6.7 16.3 0.411  1380326_at  Transcribed locus  Info    
   5978 0.0003015 1689.3 4621.1 0.366  1371030_at  secreted phosphoprotein 2  Info   Spp2 
   5979 0.0003019 364.3 618 0.589  1379736_at  Similar to hypothetical protein MGC28931 (predicted)  Info    
   5980 0.0003026 571.8 1043.3 0.548  1388395_at  G0/G1 switch gene 2 (predicted)  Info   G0s2_predicted 
   5981 0.0003032 2290 1886.5 1.214  1371434_at  CDNA clone MGC:112638 IMAGE:7376647  Info    
   5982 0.0003037 151.9 399.4 0.38  1380389_at  Prostaglandin E receptor 3 (subtype EP3)  Info   Ptger3 
   5983 0.0003037 151.4 77.9 1.944  1384553_at  similar to hypothetical protein FLJ13089 (predicted)  Info   RGD1305685_predicted 
   5984 0.0003043 316.4 432.5 0.732  1374586_at  Similar to ADP-ribosylation factor related protein 2  Info    
   5985 0.0003044 69.1 45.5 1.519  1375953_at    Info    
   5986 0.0003056 43.4 88.6 0.49  1383694_at  similar to RIKEN cDNA 1110061N23  Info   LOC499748 
   5987 0.0003062 100.6 47.6 2.113  1373787_at  Solute carrier family 6 (neurotransmitter transporter, glycine), member 9  Info   Slc6a9 
   5988 0.0003067 396.4 500.8 0.792  1372879_at  AKT1 substrate 1 (proline-rich) (predicted)  Info   Akt1s1_predicted 
   5989 0.0003071 230.3 299.1 0.77  1390501_at  Transcribed locus  Info    
   5990 0.0003072 28.3 5.5 5.145  1370550_at  limbic system-associated membrane protein  Info   Lsamp 
   5991 0.0003073 102 193.1 0.528  1381871_at  BAI1-associated protein 1 (predicted)  Info   Baiap1_predicted 
   5992 0.0003079 447.3 999 0.448  1383559_at  DEAD (Asp-Glu-Ala-Asp) box polypeptide 19  Info   Ddx19 
   5993 0.0003079 139.4 336.4 0.414  1383744_at  Transcribed locus  Info    
   5994 0.0003081 48.8 63.9 0.764  1385632_at  similar to RIKEN cDNA A430093J20 gene  Info   LOC367113 
   5995 0.0003082 64.4 49.1 1.312  1378990_at  Transcribed locus  Info    
   5996 0.0003083 739.2 580 1.274  1389623_at  activating transcription factor 1 (predicted)  Info   Atf1_predicted 
   5997 0.0003084 362.9 688.8 0.527  1393294_at  cytoplasmic polyadenylation element binding protein 4 (predicted)  Info   Cpeb4_predicted 
   5998 0.0003084 121.7 219.6 0.554  1385098_at  Transcribed locus  Info    
   5999 0.0003084 19 54.9 0.346  1374865_at  similar to RIKEN cDNA 6030419C18 gene  Info   LOC501007 
   6000 0.0003085 42.8 11.9 3.597  1369380_at  kinesin-related protein 2  Info   Kif2c 
   6001 0.0003098 36.6 69.7 0.525  1391085_at  Transcribed locus  Info    
   6002 0.0003102 46.6 11 4.236  1376485_at  TRAF-interacting protein (predicted)  Info   Traip_predicted 
   6003 0.0003106 249.8 358.5 0.697  1368314_at  gamma-glutamyl carboxylase  Info   Ggcx 
   6004 0.0003108 60 27 2.222  1382167_at  similar to SHP2-interacting transmembrane adaptor protein  Info   LOC500449 
   6005 0.000311 23.8 150.9 0.158  1371400_at  thyroid hormone responsive protein  Info   Thrsp 
   6006 0.000311 95.7 134.9 0.709  1380695_at  LOC500518  Info    
   6007 0.0003122 215.7 350.2 0.616  1374582_at  potassium channel tetramerisation domain containing 9 (predicted)  Info   Kctd9_predicted 
   6008 0.0003123 57.4 98.1 0.585  1394820_at  Transcribed locus  Info    
   6009 0.0003124 40.8 100.6 0.406  1370937_a_at  integrin alpha 7  Info   Itga7 
   6010 0.0003125 712.6 451.5 1.578  1387786_at  myotrophin  Info   Mtpn 
   6011 0.0003126 51.7 82.7 0.625  1398597_at  Transcribed locus  Info    
   6012 0.0003126 160.7 105.8 1.519  1378166_at  Gene trap locus 7 (predicted)  Info    
   6013 0.0003131 84.1 40 2.103  1385268_at  hypothetical protein LOC304743  Info   LOC304743 
   6014 0.0003134 105.3 275 0.383  1387908_at  RAS, dexamethasone-induced 1  Info   Rasd1 
   6015 0.0003135 31.7 73.5 0.431  1382267_at  four jointed box 1 (Drosophila) (predicted)  Info   Fjx1_predicted 
   6016 0.0003135 80.8 232.8 0.347  1371091_at  unknown protein  Info   RGD:708546 
   6017 0.0003138 294.4 462.8 0.636  1393039_a_at    Info    
   6018 0.0003139 1971 1205.6 1.635  1368280_at  cathepsin C  Info   Ctsc 
   6019 0.0003143 353.8 220.1 1.607  1392542_at  similar to CDC42 small effector 2 /// similar to CDC42 small effector 2  Info   LOC501691 /// LOC502327 
   6020 0.0003143 972.8 787.7 1.235  1375892_at  ELAV (embryonic lethal, abnormal vision, Drosophila)-like 1 (Hu antigen R) (predicted)  Info   Elavl1_predicted 
   6021 0.0003144 18.3 3 6.1  1393253_at  zinc finger protein 365  Info   Zfp365 
   6022 0.0003145 96.1 48.4 1.986  1392717_at  Kinesin family member 1B  Info   Kif1b 
   6023 0.0003146 4.5 1.1 4.091  1391345_at  Transcribed locus  Info    
   6024 0.0003146 39.8 126 0.316  1382487_at  testis expressed gene 14 (predicted)  Info   Tex14_predicted 
   6025 0.0003154 259.7 430.1 0.604  1377643_at  homeo box D10 (predicted)  Info   Hoxd10_predicted 
   6026 0.0003166 31.9 9.4 3.394  1393975_a_at  similar to RIKEN cDNA 1110035L05 (predicted)  Info   RGD1307059_predicted 
   6027 0.0003167 208.1 337.9 0.616  1382455_at  Transcribed locus  Info    
   6028 0.0003169 27.3 61.1 0.447  1380964_at  Similar to dystrobrevin alpha isoform 1  Info    
   6029 0.0003171 15.6 44.6 0.35  1384431_at  Similar to RIKEN cDNA 4930431B11 gene (predicted)  Info    
   6030 0.0003178 24.7 14.8 1.669  1397692_at  cytotoxic granule-associated RNA binding protein 1 (predicted)  Info   Tia1_predicted 
   6031 0.000318 482.4 388.2 1.243  1374711_at  cleavage and polyadenylation specificity factor 3 (predicted)  Info   Cpsf3_predicted 
   6032 0.0003181 26.2 6.7 3.91  1386052_at  Transcribed locus  Info    
   6033 0.0003187 377.1 277.5 1.359  1397675_at  eukaryotic translation initiation factor 4H  Info   RGD:1359222 
   6034 0.0003189 644.2 403.2 1.598  1370867_at  guanine nucleotide binding protein, beta polypeptide 2  Info   Gnb2 
   6035 0.0003192 3940.2 4611.9 0.854  1386887_at  cytochrome c oxidase subunit Vb  Info   Cox5b 
   6036 0.0003197 325.2 174.9 1.859  1372830_at  similar to PI-3-kinase-related kinase SMG-1 isoform 2  Info   LOC499250 
   6037 0.0003201 957.8 1436.3 0.667  1383698_at  pyruvate dehydrogenase E1 alpha 1  Info   Pdha1 
   6038 0.0003203 431.7 300.3 1.438  1372719_at  Transcribed locus  Info    
   6039 0.0003206 83.2 113.1 0.736  1376802_at  Similar to hypothetical protein MGC10854 (predicted)  Info    
   6040 0.0003206 465.8 387.8 1.201  1388990_at  PREDICTED: Rattus norvegicus spermatogenesis-related protein (Srp) (Mki67ip), mRNA  Info    
   6041 0.0003208 212.1 340.7 0.623  1383558_at  similar to MJ0495-like protein SelB  Info   LOC500255 
   6042 0.000321 129.7 224.1 0.579  1373643_at  Transcribed locus  Info    
   6043 0.0003223 224.9 355.5 0.633  1372111_at    Info    
   6044 0.0003223 121.6 215.7 0.564  1395316_at  DEAD (Asp-Glu-Ala-Asp) box polypeptide 21a (predicted) /// melanoma antigen, family H, 1  Info   Ddx21a_predicted /// RGD:1359316 
   6045 0.0003224 292.8 138.7 2.111  1368002_at  mutS homolog 2 (E. coli)  Info   Msh2 
   6046 0.0003234 140.7 225.2 0.625  1390114_at  myelin protein zero-like 1  Info   RGD:1359140 
   6047 0.0003235 98 58.6 1.672  1376201_at  similar to RIKEN cDNA 2810013M15  Info   LOC360568 
   6048 0.0003237 21.1 59.5 0.355  1392809_at  Transcribed locus  Info    
   6049 0.0003239 3081 2547.7 1.209  1387862_at  tyrosine 3-monooxygenase/tryptophan 5-monooxygenase activation protein, theta polypeptide  Info   Ywhaq 
   6050 0.000324 11.1 25.1 0.442  1391018_at  myosin VC (predicted)  Info   Myo5c_predicted 
   6051 0.0003241 32.9 15.1 2.179  1396363_at  Transcribed locus  Info    
   6052 0.0003246 84.4 138.2 0.611  1376278_at  Transcribed locus  Info    
   6053 0.0003248 52 123.2 0.422  1374417_at  Siah binding protein 1; FBP interacting repressor; pyrimidine tract binding splicing factor; Ro ribonucleoprotein-binding protein 1  Info   Siahbp1 
   6054 0.0003249 154.8 262.3 0.59  1390592_at  Transcribed locus  Info    
   6055 0.0003252 134.3 64.3 2.089  1376811_a_at  cleavage and polyadenylation specific factor 6, 68kDa (predicted)  Info   Cpsf6_predicted 
   6056 0.0003255 78.7 21.8 3.61  1372153_at  type I keratin KA15  Info   RGD:1303044 
   6057 0.0003256 98.7 214.1 0.461  1385432_at  CDNA clone MGC:95146 IMAGE:7129361  Info    
   6058 0.0003256 16.6 39.4 0.421  1381515_at  CDNA clone IMAGE:7123062  Info    
   6059 0.0003262 51.9 99.5 0.522  1382673_at  N-arginine dibasic convertase 1  Info   Nrd1 
   6060 0.0003267 229.8 148.2 1.551  1394318_at  cAMP-regulated phosphoprotein 19  Info   RGD:71054 
   6061 0.0003274 180 232 0.776  1374094_at  Calcium binding protein 39-like (predicted)  Info    
   6062 0.0003277 8.3 153.3 0.054  1370522_at  glucagon receptor  Info   Gcgr 
   6063 0.0003281 3.5 14.2 0.246  1378192_at  Similar to RIKEN cDNA 0610027O18 (predicted)  Info    
   6064 0.0003285 223.1 129.1 1.728  1384246_at  SWI/SNF related, matrix associated, actin dependent regulator of chromatin, subfamily a, member 5 (predicted)  Info   Smarca5_predicted 
   6065 0.0003287 251.2 312.9 0.803  1388697_at  inositol polyphosphate-5-phosphatase A (predicted)  Info   Inpp5a_predicted 
   6066 0.0003295 81.9 156 0.525  1382412_at  Transcribed locus  Info    
   6067 0.0003297 17.8 51 0.349  1374187_at  Transcribed locus  Info    
   6068 0.0003298 5.3 23.9 0.222  1397266_at  Transcription factor CP2-like 2 (predicted)  Info    
   6069 0.0003307 222.7 443.8 0.502  1374939_at  Cytoplasmic FMR1 interacting protein 2 (predicted)  Info    
   6070 0.0003311 1158.9 812.2 1.427  1379671_at  similar to RIKEN cDNA 1200003O06 (predicted)  Info   RGD1305751_predicted 
   6071 0.0003316 206.1 146.5 1.407  1368341_at  polymerase (DNA directed), beta  Info   Polb 
   6072 0.0003319 101.1 231.9 0.436  1392324_at  EPS8-like 2 (predicted)  Info   Eps8l2_predicted 
   6073 0.0003322 59.2 174 0.34  1373689_at  Transcribed locus  Info    
   6074 0.0003325 286.7 127.1 2.256  1375709_at  signal-induced proliferation-associated gene 1  Info   RGD:1303184 
   6075 0.0003325 310 628.7 0.493  1370333_a_at  insulin-like growth factor 1  Info   Igf1 
   6076 0.0003331 41.9 68.1 0.615  1374218_at  Similar to KIAA0522 protein  Info    
   6077 0.0003332 4185.3 7875.1 0.531  1368333_at  uromodulin  Info   Umod 
   6078 0.0003335 375.5 291.6 1.288  1388105_at  D123 gene product  Info   RGD:619766 
   6079 0.0003341 2293.3 1688.6 1.358  1398817_at  ADP-ribosylation factor 1  Info   Arf1 
   6080 0.0003344 435.7 330 1.32  1384467_at  CDNA clone MGC:109551 IMAGE:7325365  Info    
   6081 0.0003352 174.3 142 1.227  1377102_at  similar to cDNA sequence BC014795 (predicted)  Info   RGD1306829_predicted 
   6082 0.0003353 1943 1181.7 1.644  1375424_at  ARP2 actin-related protein 2 homolog (yeast) (predicted)  Info   Actr2_predicted 
   6083 0.0003356 140.6 266.3 0.528  1374963_s_at    Info    
   6084 0.0003356 221.7 362.2 0.612  1372018_at  Cdc42-binding protein kinase beta  Info   Cdc42bpb 
   6085 0.0003362 247.5 183.8 1.347  1374150_at  Similar to EWS/FLI1 activated transcript 2 (EAT-2)  Info    
   6086 0.0003364 239.4 191.8 1.248  1385941_at  Wolf-Hirschhorn syndrome candidate 1-like 1 (predicted)  Info    
   6087 0.0003369 16.7 30.2 0.553  1386577_at  Similar to RIKEN cDNA 4930429H24 (predicted)  Info    
   6088 0.000337 643.9 916.7 0.702  1368426_at  carnitine O-octanoyltransferase  Info   Crot 
   6089 0.0003379 149 331.3 0.45  1390036_at  solute carrier family 16 (monocarboxylic acid transporters), member 6  Info   Slc16a6 
   6090 0.0003386 2462.9 5413 0.455  1372264_at  phosphoenolpyruvate carboxykinase 1  Info   Pck1 
   6091 0.0003386 633 1146.2 0.552  1393894_at  cytochrome P450, 4a12  Info   RGD:628846 
   6092 0.000339 379.8 303.8 1.25  1389125_at  mitochondrial ribosomal protein L1 (predicted)  Info   Mrpl1_predicted 
   6093 0.0003393 112 148.7 0.753  1382010_at    Info    
   6094 0.0003394 143.3 224.6 0.638  1370335_at  disabled homolog 2 (Drosophila) interacting protein  Info   Dab2ip 
   6095 0.0003395 119.2 59.3 2.01  1379696_at  Transcribed locus, weakly similar to NP_001001306.1 hypothetical protein LOC329695 [Mus musculus]  Info    
   6096 0.0003397 50.7 119.4 0.425  1374276_at  Similar to SPT3-associated factor 42 (predicted)  Info    
   6097 0.00034 81.6 143.9 0.567  1394535_at  kinesin family member 16B (predicted)  Info   Kif16b_predicted 
   6098 0.0003404 178.7 125.5 1.424  1393153_at  Zinc finger protein 161  Info   Zfp161 
   6099 0.0003404 356 207.4 1.716  1376407_a_at  LSM7 homolog, U6 small nuclear RNA associated (S. cerevisiae) (predicted)  Info   Lsm7_predicted 
   6100 0.0003406 112.4 175.3 0.641  1373763_at  zinc finger protein 297B (predicted)  Info   Zfp297b_predicted 
   6101 0.000341 136.4 230.5 0.592  1373137_at  Transcribed locus  Info    
   6102 0.000341 942.1 738.6 1.276  1375974_at  Similar to RNA-binding protein isoform G3BP-2a (predicted)  Info    
   6103 0.0003411 90.6 48.8 1.857  1386392_at  anaphase promoting complex subunit 10 (predicted)  Info   Anapc10_predicted 
   6104 0.0003417 263.7 363.8 0.725  1368117_at  gephyrin  Info   Gphn 
   6105 0.0003419 215.4 128.8 1.672  1391719_at    Info    
   6106 0.000342 548.2 344.3 1.592  1374844_at  Transcribed locus, strongly similar to NP_006319.1 RNA binding motif protein 14; coactivator activator; SYT interacting protein [Homo sapiens]  Info    
   6107 0.0003421 483.7 355.2 1.362  1377577_at  guanine monphosphate synthetase (predicted)  Info   Gmps_predicted 
   6108 0.0003422 35.3 51.1 0.691  1393438_at  Zinc finger protein 316 (predicted)  Info    
   6109 0.0003427 31.7 13.3 2.383  1396132_at  similar to FCRL (predicted)  Info   RGD1306176_predicted 
   6110 0.0003429 25.2 2.2 11.455  1391301_at  histone 1, H2bp (predicted)  Info   Hist1h2bp_predicted 
   6111 0.000343 134.7 29.6 4.551  1379387_at  Transcribed locus  Info    
   6112 0.0003434 306.7 476.5 0.644  1367638_at  malonyl-CoA decarboxylase  Info   Mlycd 
   6113 0.0003438 141.4 95.8 1.476  1394320_at  cyclin dependent kinase 2  Info   Cdk2 
   6114 0.0003438 89.8 147.1 0.61  1374100_at    Info    
   6115 0.000344 719.9 529 1.361  1370208_at  nuclear receptor subfamily 1, group H, member 2  Info   Nr1h2 
   6116 0.0003445 196.8 128.2 1.535  1389580_at  LOC499580  Info   LOC499580 
   6117 0.0003446 22.3 57.3 0.389  1393279_at    Info    
   6118 0.000345 257.6 387.2 0.665  1399166_a_at  Similar to Bcl7b protein  Info    
   6119 0.0003452 326.8 508.8 0.642  1371251_at  galactose-1-phosphate uridyl transferase (predicted)  Info   Galt_predicted 
   6120 0.0003453 375.4 187.6 2.001  1372417_at  SERTA domain containing 1  Info   Sertad1 
   6121 0.0003455 199.4 338.8 0.589  1383041_x_at    Info    
   6122 0.0003457 131.8 24.2 5.446  1384981_at  CD6 antigen  Info   Cd6 
   6123 0.0003458 62.6 100.5 0.623  1382384_at  similar to Ras association (RalGDS/AF-6) domain family 6  Info   MGC114410 
   6124 0.000346 210.1 294.4 0.714  1379668_at  similar to hypothetical protein B230397C21  Info   LOC361174 
   6125 0.000346 97.4 1202.7 0.081  1381233_at  Transcribed locus  Info    
   6126 0.000346 80.4 133 0.605  1391397_at  Transcribed locus  Info    
   6127 0.0003466 72.8 182.4 0.399  1369347_s_at  prominin 2  Info   Prom2 
   6128 0.0003467 78.6 43.6 1.803  1381189_at  fizzy/cell division cycle 20 related 1 (Drosophila) (predicted)  Info   Fzr1_predicted 
   6129 0.0003468 1929.8 1334.5 1.446  1372743_at  sorting nexin 5 (predicted)  Info   Snx5_predicted 
   6130 0.0003469 101.8 153.5 0.663  1392538_at  similar to Hypothetical protein MGC75937  Info   LOC360822 
   6131 0.0003473 181.2 52.3 3.465  1376098_a_at  myosin IG (predicted)  Info   Myo1g_predicted 
   6132 0.0003473 867.9 1592.3 0.545  1370076_at  potassium inwardly-rectifying channel, subfamily J, member 16  Info   Kcnj16 
   6133 0.0003474 11.4 39.1 0.292  1377788_at    Info    
   6134 0.0003475 40.5 57.7 0.702  1392784_at  growth arrest specific 6  Info   Gas6 
   6135 0.0003479 160.9 111.5 1.443  1376887_at  Similar to zinc finger protein 445  Info    
   6136 0.000348 131.7 200.8 0.656  1369069_at  A kinase (PRKA) anchor protein 1  Info   Akap1 
   6137 0.0003483 58.8 83.3 0.706  1383894_at  Transcribed locus  Info    
   6138 0.0003493 9.4 35.3 0.266  1378135_at  Transcribed locus, moderately similar to NP_001001306.1 hypothetical protein LOC329695 [Mus musculus]  Info    
   6139 0.0003493 74.5 171.2 0.435  1395027_at  Transcribed locus  Info    
   6140 0.0003501 161.9 85.3 1.898  1399048_at  coactivator-associated arginine methyltransferase 1 (predicted)  Info   Carm1_predicted 
   6141 0.0003502 179.9 125.7 1.431  1399087_at  similar to hypothetical protein FLJ31528 (predicted)  Info   RGD1307410_predicted 
   6142 0.0003503 291.8 162.1 1.8  1393881_at  NMDA receptor-regulated gene 1 (predicted)  Info   Narg1_predicted 
   6143 0.0003507 36.4 72.8 0.5  1390915_at  Transcribed locus, strongly similar to XP_521209.1 PREDICTED: hypothetical protein XP_521209 [Pan troglodytes]  Info    
   6144 0.0003507 59.3 7.4 8.014  1386275_at  Transcribed locus  Info    
   6145 0.000351 4034.4 4907.7 0.822  1374426_at  ubiquinol-cytochrome c reductase binding protein (predicted)  Info   Uqcrb_predicted 
   6146 0.000351 274.7 487.8 0.563  1370032_at  ERM-binding phosphoprotein  Info   Slc9a3r1 
   6147 0.0003515 22.3 50.3 0.443  1395398_at  Acidic (leucine-rich) nuclear phosphoprotein 32 family, member A  Info   Anp32a 
   6148 0.0003517 381.9 246.8 1.547  1384427_at  transformed mouse 3T3 cell double minute 2 (predicted)  Info   Mdm2_predicted 
   6149 0.0003535 109.5 178.7 0.613  1376498_at  similar to 2900002H16Rik protein (predicted)  Info   RGD1307973_predicted 
   6150 0.0003536 145.3 225.2 0.645  1383278_at  centaurin, gamma 2 (predicted)  Info   Centg2_predicted 
   6151 0.0003539 96.8 299.2 0.324  1395026_at  flavin containing monooxygenase 4  Info   Fmo4 
   6152 0.0003539 87.1 133.9 0.65  1393197_at  abhydrolase domain containing 8 (predicted)  Info   Abhd8_predicted 
   6153 0.0003545 63.4 281.5 0.225  1369259_at  deiodinase, iodothyronine, type I  Info   Dio1 
   6154 0.0003553 27.4 49.2 0.557  1378487_at  E1A binding protein p300  Info   Ep300 
   6155 0.0003553 963.8 1227.2 0.785  1398327_at  pleckstrin homology domain containing, family C (with FERM domain) member 1 (predicted)  Info   Plekhc1_predicted 
   6156 0.0003558 182.1 274.6 0.663  1388856_at  kit ligand  Info   Kitl 
   6157 0.0003559 1267.2 1754.9 0.722  1388487_at  Adducin 1 (alpha)  Info   Add1 
   6158 0.0003561 168.5 254.9 0.661  1385753_at  Similar to mKIAA0089 protein  Info    
   6159 0.0003565 579.2 723.2 0.801  1390111_at    Info    
   6160 0.0003571 404.9 463.1 0.874  1392719_at  5,10-methenyltetrahydrofolate synthetase (5-formyltetrahydrofolate cyclo-ligase) (predicted)  Info   Mthfs_predicted 
   6161 0.0003573 55.9 2.6 21.5  1368503_at  GTP cyclohydrolase 1  Info   Gch 
   6162 0.0003574 108.4 67.6 1.604  1382306_at  Ariadne ubiquitin-conjugating enzyme E2 binding protein homolog 1 (Drosophila) (predicted)  Info    
   6163 0.0003578 86.9 49.4 1.759  1383246_at  Similar to Hypothetical protein A630084C13 (predicted)  Info    
   6164 0.0003583 85.4 155.1 0.551  1368372_at  steroid sulfatase  Info   Sts 
   6165 0.0003584 96.7 53.2 1.818  1370519_at  syntaxin binding protein 1  Info   Stxbp1 
   6166 0.0003585 80.3 149.5 0.537  1383939_at  Transcribed locus, moderately similar to XP_580018.1 PREDICTED: hypothetical protein XP_580018 [Rattus norvegicus]  Info    
   6167 0.0003595 160.9 112.9 1.425  1378977_at  Transcribed locus  Info    
   6168 0.0003596 30.9 8.3 3.723  1368210_at  interleukin 24  Info   Il24 
   6169 0.0003603 110.5 64.2 1.721  1390272_at  similar to RIKEN cDNA 2410012M04 (predicted)  Info   RGD1307867_predicted 
   6170 0.0003604 182.8 102.1 1.79  1384428_at  similar to OTTHUMP00000040081  Info   LOC502228 
   6171 0.0003608 213.6 132 1.618  1376468_at  dead end homolog 1 (zebrafish) (predicted)  Info   Dnd1_predicted 
   6172 0.000361 560.8 441.1 1.271  1373052_at  phosducin-like 3 (predicted)  Info   Pdcl3_predicted 
   6173 0.0003611 83.6 174.9 0.478  1393559_at  Transcribed locus  Info    
   6174 0.0003613 176.1 96.1 1.832  1393035_at  Transcribed locus  Info    
   6175 0.0003614 230.6 155.3 1.485  1383091_at  amyloid beta precursor protein (cytoplasmic tail) binding protein 2 (predicted)  Info   Appbp2_predicted 
   6176 0.0003616 45.1 85.7 0.526  1385924_at  Transcribed locus  Info    
   6177 0.0003619 155.1 237.5 0.653  1375011_at  Transcribed locus  Info    
   6178 0.0003624 328.6 520.4 0.631  1369061_at  glutathione reductase  Info   Gsr 
   6179 0.000363 315.7 557.4 0.566  1383047_at  growth arrest specific 6  Info   Gas6 
   6180 0.000363 501.1 1216.6 0.412  1382434_at  ectonucleoside triphosphate diphosphohydrolase 5  Info   Entpd5 
   6181 0.0003631 2504.3 3172.2 0.789  1388320_at  serine protease inhibitor, Kunitz type 2  Info   Spint2 
   6182 0.0003633 328.7 182.2 1.804  1390116_at  Polymerase I and transcript release factor (predicted)  Info    
   6183 0.0003638 182.7 101.7 1.796  1369963_at  platelet-activating factor acetylhydrolase, isoform 1b, alpha1 subunit  Info   RGD:620333 
   6184 0.000365 142.6 221.3 0.644  1385043_at  Similar to channel-interacting PDZ domain protein isoform 1  Info    
   6185 0.0003657 558.1 379.6 1.47  1370070_at  synaptojanin 1  Info   Synj1 
   6186 0.000366 306 376 0.814  1370300_at  prolactin regulatory element binding  Info   Preb 
   6187 0.0003664 521.8 422.5 1.235  1372058_at  Transcribed locus, strongly similar to NP_776109.1 NudC domain containing 3 [Mus musculus]  Info    
   6188 0.0003664 248.5 151.3 1.642  1389279_at  RAN, member RAS oncogene family  Info   Ran 
   6189 0.0003668 626.5 898.4 0.697  1372262_at  LOC500344  Info    
   6190 0.0003668 111.6 173.1 0.645  1380811_at  Transcribed locus  Info    
   6191 0.0003672 16.7 33.3 0.502  1393382_at  Similar to Hypothetical protein A430033K04  Info    
   6192 0.0003673 340.6 184.6 1.845  1385487_at    Info    
   6193 0.0003678 576.3 738 0.781  1371637_at  heterochromatin protein 1, binding protein 3  Info   Hp1bp3 
   6194 0.0003683 450.3 601 0.749  1372504_at    Info   LOC502017 /// LOC502540 
   6195 0.0003686 185.3 110.4 1.678  1375998_at  anaphase promoting complex subunit 7 (predicted)  Info   Anapc7_predicted 
   6196 0.0003687 25.3 5.2 4.865  1396708_at  similar to hypothetical protein MGC33486 (predicted)  Info   RGD1310680_predicted 
   6197 0.0003697 513.7 344.3 1.492  1389144_at  similar to OTTHUMP00000018508  Info   LOC498559 
   6198 0.0003703 39.3 25.3 1.553  1382353_at  DNA methyltransferase 3A (predicted)  Info   Dnmt3a_predicted 
   6199 0.0003721 313.4 653.7 0.479  1383332_at  Transcribed locus, weakly similar to NP_001002778.1 hypothetical protein LOC442814 [Mus musculus]  Info    
   6200 0.0003725 406 252.1 1.61  1371658_at  neighbor of Cox4 (predicted)  Info   Noc4_predicted 
   6201 0.0003727 116.4 76.5 1.522  1383485_at  transformed mouse 3T3 cell double minute 2 (predicted)  Info   Mdm2_predicted 
   6202 0.0003742 70 101.5 0.69  1387415_a_at  syntaxin binding protein 5 (tomosyn)  Info   Stxbp5 
   6203 0.0003747 23.1 3.4 6.794  1380635_at  Transcribed locus  Info    
   6204 0.000375 27 53.5 0.505  1371045_at  amiloride-sensitive cation channel 2, neuronal  Info   Accn2 
   6205 0.000375 90.9 158.4 0.574  1369678_a_at  nuclear factor I/A  Info   Nfia 
   6206 0.0003751 307.8 413.5 0.744  1372074_at  nudix (nucleotide diphosphate linked moiety X)-type motif 3 (predicted)  Info   Nudt3_predicted 
   6207 0.0003752 79.2 47.6 1.664  1378508_at  Transcribed locus  Info    
   6208 0.0003754 95.6 148.7 0.643  1368318_at  homer homolog 1 (Drosophila)  Info   Homer1 
   6209 0.0003761 46 5.9 7.797  1388056_at  2-5 oligoadenylate synthetase 1B  Info   Oas1b 
   6210 0.0003762 115.8 342.2 0.338  1383943_at  dynein, axonemal, heavy polypeptide 7  Info   Dnah7 
   6211 0.0003764 122.9 220.8 0.557  1384283_at  similar to ankyrin repeat and SOCS box-containing protein 9  Info   LOC367785 
   6212 0.0003765 193.3 97 1.993  1372202_at  similar to expressed sequence AI597479 (predicted)  Info   RGD1310553_predicted 
   6213 0.0003771 37.2 64.1 0.58  1372708_at  Transcribed locus  Info    
   6214 0.0003801 48.7 72.6 0.671  1376630_at    Info    
   6215 0.0003803 291.5 444.3 0.656  1371560_at  interferon regulatory factor 3  Info   Irf3 
   6216 0.0003809 131.5 82.5 1.594  1383880_at  integrin, alpha V (vitronectin receptor, alpha polypeptide, antigen CD51) (predicted)  Info   Itgav_predicted 
   6217 0.000381 76.2 145.4 0.524  1370724_a_at  nuclear factor I/A  Info   Nfia 
   6218 0.0003814 917.2 571.4 1.605  1370173_at  superoxide dismutase 2, mitochondrial  Info   Sod2 
   6219 0.0003817 6.5 31.2 0.208  1383620_at    Info    
   6220 0.0003822 119.6 76.9 1.555  1380349_at  Transcribed locus  Info    
   6221 0.0003831 122.2 179.2 0.682  1387417_a_at  SH2-B PH domain containing signaling mediator 1  Info   Sh2bpsm1 
   6222 0.0003834 699.3 528.2 1.324  1375652_at  signal sequence receptor, gamma  Info   Ssr3 
   6223 0.0003844 149.2 103 1.449  1375551_at  similar to hypothetical protein MGC2574  Info   RGD:1359280 
   6224 0.0003848 434.3 1009.2 0.43  1393516_at  Transcribed locus  Info    
   6225 0.0003848 45.6 23.2 1.966  1394643_at  Transcribed locus  Info    
   6226 0.0003857 737.1 1037.7 0.71  1399098_at  glyoxylase 1  Info   Glo1 
   6227 0.0003862 438.1 286.5 1.529  1387114_at  protein kinase C, delta  Info   Prkcd 
   6228 0.0003863 217.5 134.1 1.622  1383621_at  Transcribed locus  Info    
   6229 0.0003864 42.6 72.7 0.586  1374957_at  similar to ribosomal protein L27a  Info   LOC292474 
   6230 0.0003866 253.5 132.1 1.919  1395465_at    Info    
   6231 0.0003866 906.5 507.6 1.786  1367713_at  eukaryotic translation initiation factor 2, subunit 1 alpha  Info   Eif2s1 
   6232 0.0003873 235.3 342.7 0.687  1383963_at  Transcribed locus  Info    
   6233 0.0003875 521.1 689.5 0.756  1372441_at  chromodomain helicase DNA binding protein 4  Info   Chd4 
   6234 0.0003878 120.4 192 0.627  1383423_at  Transcribed locus  Info    
   6235 0.0003878 39.9 8.8 4.534  1391260_at  Transcribed locus  Info    
   6236 0.0003879 773 470.8 1.642  1368436_at  nuclear distribution gene C homolog (Aspergillus)  Info   Nudc 
   6237 0.000388 1349.7 1051.4 1.284  1371418_at  chaperonin containing TCP1, subunit 2 (beta)  Info   RGD:1359143 
   6238 0.000388 15.3 34.2 0.447  1368648_at  cytochrome c oxidase subunit IV isoform 2  Info   Cox4i2 
   6239 0.000388 203.8 286.6 0.711  1387920_at  mannosidase, alpha, class 2C, member 1  Info   Man2c1 
   6240 0.0003889 126.3 96.2 1.313  1380815_at  similar to hypothetical protein FLJ11752  Info   LOC304923 
   6241 0.0003893 763.3 1159 0.659  1371643_at  cyclin D1  Info   Ccnd1 
   6242 0.0003896 56.3 75.9 0.742  1376042_at  Similar to Faciogenital dysplasia homolog  Info    
   6243 0.0003901 48.9 90.8 0.539  1392944_at  similar to talin 2  Info   LOC315776 
   6244 0.0003902 264.6 372.9 0.71  1367504_at    Info    
   6245 0.0003906 94.2 156.7 0.601  1378088_at  similar to Zdhhc1 protein  Info   LOC291967 
   6246 0.000391 936.6 724.7 1.292  1398905_at  ATPase, H+ transporting, V1 subunit G isoform 1 (predicted)  Info   Atp6v1g1_predicted 
   6247 0.0003913 361.3 636.3 0.568  1372490_at  Similar to mKIAA0089 protein  Info    
   6248 0.0003919 241.5 158 1.528  1390788_a_at  WD repeat domain 36 (predicted)  Info   Wdr36_predicted 
   6249 0.0003922 371.3 566.6 0.655  1376593_at  Transcribed locus  Info    
   6250 0.0003923 124.7 75.1 1.66  1382972_at  Transcribed locus, strongly similar to XP_226713.2 PREDICTED: similar to Src-associated protein SAW [Rattus norvegicus]  Info    
   6251 0.0003923 121.5 86.5 1.405  1368593_at  CD1d1 antigen  Info   Cd1d1 
   6252 0.0003924 106.5 49.4 2.156  1377918_at  similar to Stomatin-like 1  Info   LOC300748 
   6253 0.0003926 132 12.8 10.312  1384778_at  chymotrypsin C (caldecrin) (predicted)  Info   Ctrc_predicted 
   6254 0.0003927 40.8 71 0.575  1385087_at  Transcribed locus  Info    
   6255 0.0003929 793.4 1485.9 0.534  1367950_at  solute carrier family 22 (organic cation transporter), member 5  Info   Slc22a5 
   6256 0.0003937 164.2 112.7 1.457  1372436_at  tumor protein D52-like 2  Info   Tpd52l2 
   6257 0.0003942 190.6 310.8 0.613  1386166_at  ATPase, H+ transporting, V1 subunit G, isoform 3 (predicted)  Info   Atp6v1g3_predicted 
   6258 0.0003952 1442.1 1027.5 1.404  1372247_at  dolichyl-di-phosphooligosaccharide-protein glycotransferase (predicted)  Info   Ddost_predicted 
   6259 0.0003952 10.6 36 0.294  1383571_at  hypothetical protein LOC303515  Info   LOC303515 
   6260 0.000396 2888.6 2283.4 1.265  1386996_at  myosin light chain, regulatory B  Info   Mrlcb 
   6261 0.0003963 99.2 164.3 0.604  1376058_at  mitochondrial tumor suppressor 1  Info   Mtus1 
   6262 0.0003967 500.6 683.5 0.732  1372814_at  similar to RIKEN cDNA 2010005O13 (predicted)  Info   RGD1310623_predicted 
   6263 0.0003969 341.4 226.9 1.505  1390515_at  Transcribed locus  Info    
   6264 0.000397 62.6 27.6 2.268  1398143_at  Hypothetical LOC363470  Info    
   6265 0.0003973 125.1 27 4.633  1375806_at  similar to [Mouse primary response gene B94 mRNA, 3end.], gene product  Info   LOC299339 
   6266 0.000398 52.9 136.8 0.387  1378484_at  Transcribed locus  Info    
   6267 0.0003981 99.5 173 0.575  1376230_at    Info    
   6268 0.0003981 335.8 217.1 1.547  1370223_at  ADP-ribosylation factor related protein 1  Info   Arfrp1 
   6269 0.0003982 13.1 42.5 0.308  1381729_at  similar to RIKEN cDNA 1700102P08  Info   LOC498675 
   6270 0.0003994 139 19.7 7.056  1371928_at  cell division cycle associated 8  Info   Cdca8 
   6271 0.0003997 1051.2 1298.1 0.81  1388410_at  UDP-glucose pyrophosphorylase 2 (predicted)  Info   Ugp2_predicted 
   6272 0.0004007 320.2 215 1.489  1393641_at  B-cell linker  Info   Blnk 
   6273 0.0004011 135.1 88.6 1.525  1377627_at  similar to down-regulated in metastasis  Info   LOC314713 
   6274 0.0004014 4463.8 6068.1 0.736  1387773_at  cytochrome c, somatic  Info   Cycs 
   6275 0.0004015 525.5 673.2 0.781  1381982_at  UDP-N-acteylglucosamine pyrophosphorylase 1-like 1 (predicted)  Info   Uap1l1_predicted 
   6276 0.0004023 441.6 544.8 0.811  1377872_at  similar to chromosome 7 open reading frame 30 (predicted)  Info   RGD1306936_predicted 
   6277 0.0004025 3385.9 5249 0.645  1387013_at  kidney-specific membrane protein  Info   Tmem27 
   6278 0.0004026 445.8 938.4 0.475  1367811_at  3-phosphoglycerate dehydrogenase  Info   Phgdh 
   6279 0.0004027 222.3 151.7 1.465  1389742_at  Transcribed locus  Info    
   6280 0.0004028 12.7 24 0.529  1371708_at  carbonic anhydrase 6 (predicted)  Info   Car6_predicted 
   6281 0.000403 220.8 133.9 1.649  1373263_at  similar to SUMO/sentrin specific protease 5  Info   LOC499312 
   6282 0.000403 68.6 4.1 16.732  1368695_at  complement component 4 binding protein, beta  Info   C4bpb 
   6283 0.0004031 320.3 403.1 0.795  1382699_s_at  Hermansky-Pudlak syndrome 1 homolog (human)  Info   Hps1 
   6284 0.0004031 699.3 258.3 2.707  1387854_at  procollagen, type I, alpha 2  Info   Col1a2 
   6285 0.0004033 70.7 145 0.488  1369768_at  H1 histone family, member 4  Info   H1f4 
   6286 0.0004043 203.2 139.8 1.454  1395355_at  Transcribed locus  Info    
   6287 0.0004046 139 75.4 1.844  1384617_at  Unknown (protein for MGC:72614)  Info   RGD:735075 
   6288 0.0004061 21.9 2.6 8.423  1368763_at  interleukin 3  Info   Il3 
   6289 0.0004066 198 139.9 1.415  1370662_a_at  adaptor-related protein complex 2, beta 1 subunit  Info   Ap2b1 
   6290 0.0004068 177.8 122.1 1.456  1399120_at  similar to RIKEN cDNA 2610027O18 (predicted)  Info   RGD1306567_predicted 
   6291 0.0004073 297.8 1623.7 0.183  1381251_at  similar to RIKEN cDNA 4921520P21; DMRTC1  Info   RGD:1359563 
   6292 0.0004081 117.8 72.6 1.623  1373082_at  Protein kinase inhibitor, alpha  Info   Pkia 
   6293 0.0004089 92.3 42 2.198  1369718_at  signal sequence receptor, gamma  Info   Ssr3 
   6294 0.000409 24.8 11 2.255  1391082_at  Similar to PR-domain protein 11 (predicted)  Info    
   6295 0.0004097 81.9 107.1 0.765  1384119_at  Transcribed locus  Info    
   6296 0.0004099 24.1 52.1 0.463  1380226_at  Similar to Sterile alpha motif domain containing 10  Info    
   6297 0.0004102 18.6 45.8 0.406  1383211_at  Transcribed locus  Info    
   6298 0.0004107 55.8 5.3 10.528  1385336_at  docking protein 2 (predicted)  Info   Dok2_predicted 
   6299 0.0004111 229.8 372.6 0.617  1395410_at  protein phosphatase 2 (formerly 2A), regulatory subunit B'', alpha (predicted)  Info   Ppp2r3a_predicted 
   6300 0.000412 14.4 42.6 0.338  1392652_at  Transcribed locus  Info    
   6301 0.0004124 1.1 8.3 0.133  1390262_a_at    Info    
   6302 0.0004128 256.1 134.3 1.907  1379420_at  similar to Dehydrogenase/reductase SDR family member 7 precursor (Retinal short-chain dehydrogenase/reductase 4)  Info   LOC299135 
   6303 0.0004129 100.9 158.8 0.635  1381470_at  V-kit Hardy-Zuckerman 4 feline sarcoma viral oncogene homolog  Info   Kit 
   6304 0.0004129 371.8 253.2 1.468  1368412_a_at  protein tyrosine phosphatase, receptor type, O  Info   Ptpro 
   6305 0.0004136 152.3 88.6 1.719  1374733_at  symplekin (predicted)  Info   Sympk_predicted 
   6306 0.0004148 113.8 177 0.643  1376155_at  similar to hypothetical protein 2BE2121 - Chinese hamster (fragment)  Info   LOC499507 
   6307 0.0004153 46.7 12.4 3.766  1385865_at  similar to RIKEN cDNA 2510040D07  Info   LOC501048 
   6308 0.0004153 50 94.3 0.53  1393695_at  Tudor domain containing 3 (predicted)  Info    
   6309 0.0004153 454.2 348.9 1.302  1374798_at  Unknown (protein for MGC:72610)  Info   MGC72610 
   6310 0.0004165 31.4 11.6 2.707  1381572_at  similar to RIKEN cDNA 1810065E05  Info   LOC308429 
   6311 0.0004173 3381.9 2945.3 1.148  1367635_at  prolyl 4-hydroxylase, beta polypeptide  Info   P4hb 
   6312 0.0004177 183.3 369.6 0.496  1374594_at  similar to RIKEN cDNA 1600029D21  Info   RGD:1359217 
   6313 0.0004186 418 265.7 1.573  1378638_a_at  similar to 2700029M09Rik protein (predicted)  Info   RGD1311747_predicted 
   6314 0.0004189 26 104.9 0.248  1394112_at  Hydroxyacid oxidase 1  Info   Hao1 
   6315 0.0004189 644.3 485.6 1.327  1399015_at  Transmembrane trafficking protein 21  Info   Tmp21 
   6316 0.000419 341.4 545.7 0.626  1376163_at  similar to Expressed sequence AI649392 (predicted)  Info   RGD1305043_predicted 
   6317 0.0004195 765.5 512.7 1.493  1388492_at  TNFAIP3 interacting protein 1 (predicted)  Info   Tnip1_predicted 
   6318 0.0004197 38.5 82.8 0.465  1382853_at  Transcribed locus  Info    
   6319 0.0004199 391.2 263.6 1.484  1382615_at  SEC61, alpha subunit (S. cerevisiae)  Info   Sec61a1 
   6320 0.0004202 208.3 338.6 0.615  1369310_at  brain abundant, membrane attached signal protein 1  Info   Basp1 
   6321 0.0004203 343.2 436.8 0.786  1398983_at  mitochondrial ribosomal protein L30 (predicted)  Info   Mrpl30_predicted 
   6322 0.000422 27.6 69.7 0.396  1397450_at  Transcribed locus  Info    
   6323 0.000422 28.7 12.2 2.352  1382791_at  Transcribed locus  Info    
   6324 0.0004222 467.3 294.5 1.587  1388647_at    Info    
   6325 0.0004222 688.5 932.8 0.738  1389265_at  glucan (1,4-alpha-), branching enzyme 1 (predicted)  Info   Gbe1_predicted 
   6326 0.000423 59.1 82 0.721  1384398_at  LOC498947  Info   LOC498947 
   6327 0.0004233 1000.9 1846.8 0.542  1387037_at  cubilin (intrinsic factor-cobalamin receptor)  Info   Cubn 
   6328 0.0004234 215.9 270.4 0.798  1386959_a_at  mitogen activated protein kinase kinase 5  Info   Map2k5 
   6329 0.0004241 85.5 8.3 10.301  1397437_at  AT rich interactive domain 5A (Mrf1 like) (predicted)  Info    
   6330 0.0004242 61.9 102.8 0.602  1376975_at  Transcribed locus  Info    
   6331 0.0004245 224.7 346.8 0.648  1393096_at  similar to ribosomal protein L27a  Info   LOC292474 
   6332 0.000425 537.8 2039.6 0.264  1379411_at  similar to ENSANGP00000020885  Info   LOC306366 
   6333 0.0004275 54.4 106.8 0.509  1383947_at  Transcribed locus  Info    
   6334 0.0004276 1389.8 1172.8 1.185  1392465_at  similar to SAP18  Info   LOC498002 
   6335 0.000429 87.3 226.3 0.386  1395601_at  Transcribed locus  Info    
   6336 0.0004291 38.3 82.3 0.465  1391131_at  Transcribed locus  Info    
   6337 0.0004294 140.8 60.2 2.339  1385798_at  Transcribed locus  Info    
   6338 0.0004297 4.8 35.3 0.136  1390355_at  ryanodine receptor 1, skeletal muscle  Info   Ryr1 
   6339 0.0004299 120.8 167.5 0.721  1372465_at    Info    
   6340 0.0004302 21.8 64.9 0.336  1370132_at  FK506 binding protein 1b  Info   Fkbp1b 
   6341 0.000432 959 648.9 1.478  1386900_at  ribosome associated membrane protein 4  Info   RAMP4 
   6342 0.0004321 325.5 502.8 0.647  1372449_at  Transcribed locus  Info    
   6343 0.0004323 50.3 11.3 4.451  1376316_at  Origin recognition complex, subunit 6-like (S. cerevisiae) (predicted)  Info    
   6344 0.0004328 64.8 45 1.44  1393914_at  similar to Ftsj homolog  Info   LOC363450 
   6345 0.0004335 76.8 129.3 0.594  1395045_at  NADH dehydrogenase (ubiquinone) 1 alpha subcomplex, 7 (B14.5a) (predicted)  Info   Ndufa7_predicted 
   6346 0.0004336 343.1 220.1 1.559  1377675_at  LOC498261  Info   LOC498261 
   6347 0.0004346 292.9 373.2 0.785  1389713_at  Similar to RIKEN cDNA 5830434P21  Info    
   6348 0.0004347 463 378.1 1.225  1387485_a_at  protein phosphatase 2 (formerly 2A), regulatory subunit B (PR 52), alpha isoform  Info   Ppp2r2a 
   6349 0.0004357 1279.5 957.3 1.337  1371848_at    Info    
   6350 0.0004361 87.9 131.3 0.669  1389687_at  Abelson helper integration site 1  Info   Ahi1 
   6351 0.0004362 208.6 139.9 1.491  1385070_at  Transcribed locus, moderately similar to XP_488956.1 PREDICTED: hypothetical protein LOC66602 [Mus musculus]  Info    
   6352 0.0004362 516.7 222 2.327  1368829_at  fibrillin 1  Info   Fbn1 
   6353 0.0004363 524.9 386.7 1.357  1372015_at  transforming, acidic coiled-coil containing protein 1a  Info   RGD:1303120 
   6354 0.0004367 122.5 87.8 1.395  1384281_at  Transcribed locus  Info    
   6355 0.0004373 422.3 236.4 1.786  1388413_at  ribosome binding protein 1 homolog 180kDa (dog) (predicted)  Info   Rrbp1_predicted 
   6356 0.0004377 115.6 198.7 0.582  1391574_at    Info    
   6357 0.0004382 24.9 81.2 0.307  1392921_at  Transcribed locus  Info    
   6358 0.0004384 450.8 275.8 1.635  1384388_at  similar to gp25L2 protein (predicted)  Info   RGD1307627_predicted 
   6359 0.0004384 85.5 166.9 0.512  1374278_at  Transcribed locus  Info    
   6360 0.0004386 141.4 93.8 1.507  1373706_at  Swi/SNF related matrix associated, actin dependent regulator of chromatin, subfamily a-like 1 (predicted)  Info   Smarcal1_predicted 
   6361 0.0004387 102.6 52.3 1.962  1382934_at  Pannexin 1  Info   Panx1 
   6362 0.0004396 171.8 120.3 1.428  1384017_at  leucine-rich repeat kinase 1 (predicted)  Info   Lrrk1_predicted 
   6363 0.0004398 26.3 59.2 0.444  1392607_at  Similar to erythrocyte membrane protein band 4.1 (elliptocytosis 1, RH-linked) isoform 1  Info    
   6364 0.0004399 253.3 392.8 0.645  1378605_at  similar to single Ig IL-1 receptor related protein (predicted)  Info   RGD1306732_predicted 
   6365 0.0004412 604.1 759.9 0.795  1372258_at  Transcribed locus, moderately similar to XP_529507.1 PREDICTED: hypothetical protein XP_529507 [Pan troglodytes]  Info    
   6366 0.0004413 328.8 118 2.786  1368308_at  myelocytomatosis viral oncogene homolog (avian)  Info   Myc 
   6367 0.0004414 204.5 323.7 0.632  1373578_at  Transcribed locus, strongly similar to NP_032717.1 neurofilament 3, medium [Mus musculus]  Info    
   6368 0.0004415 17.5 28.3 0.618  1377337_at  C-myc binding protein (predicted)  Info    
   6369 0.0004426 546.5 318.6 1.715  1383616_at  similar to class II cytokine receptor 4  Info   LOC304091 
   6370 0.0004429 148.6 205.7 0.722  1377837_at  zinc finger protein 183 (RING finger, C3HC4 type)  Info   RGD:1303128 
   6371 0.0004431 101.5 174.9 0.58  1391212_at  transcription elongation factor A (SII)-like 1  Info   Tceal1 
   6372 0.0004432 128 231.7 0.552  1368453_at  fatty acid desaturase 2  Info   Fads2 
   6373 0.0004434 82.8 53.3 1.553  1387824_at  splicing factor, arginine/serine-rich 12  Info   Sfrs12 
   6374 0.0004437 37.6 4 9.4  1398439_a_at    Info    
   6375 0.0004448 217.3 168.7 1.288  1374255_at  Phenylalanine-tRNA synthetase-like, alpha subunit (predicted)  Info    
   6376 0.0004458 240.2 335.2 0.717  1399083_at  Williams Beuren syndrome chromosome region 21 (predicted) /// similar to Williams-Beuren syndrome critical region protein 21  Info   Wbscr21_predicted /// LOC368083 
   6377 0.0004459 102 312.9 0.326  1384775_s_at  transmembrane protease, serine 8 (intestinal)  Info   Tmprss8 
   6378 0.0004462 47.4 69.4 0.683  1378652_at  Transcribed locus  Info    
   6379 0.0004464 73.1 147.7 0.495  1378085_at  epsin 3 (predicted)  Info   Epn3_predicted 
   6380 0.0004465 492.7 261.8 1.882  1387343_at  CCAAT/enhancer binding protein (C/EBP), delta  Info   Cebpd 
   6381 0.0004467 355.9 472.2 0.754  1385838_a_at  beta-amyloid binding protein precursor (predicted)  Info   Bbp_predicted 
   6382 0.0004467 242.9 156.4 1.553  1373869_at  sterol O-acyltransferase 1  Info   Soat1 
   6383 0.000447 121.9 61.5 1.982  1390259_at  Ubiquitin-conjugating enzyme E2D 1, UBC4/5 homolog (yeast) (predicted)  Info    
   6384 0.0004479 306.6 203.1 1.51  1371676_at  longevity assurance homolog 5 (S. cerevisiae) (predicted)  Info   Lass5_predicted 
   6385 0.0004479 98 157 0.624  1383931_at  Protein tyrosine phosphatase, receptor type, K, extracellular region  Info    
   6386 0.0004483 1277.9 1497 0.854  1373013_at  similar to polyadenylate-binding protein-interacting protein 2  Info   RGD:1359176 
   6387 0.0004484 73.3 32.9 2.228  1383874_at  LOC500640  Info   LOC500640 
   6388 0.0004489 167.3 104.6 1.599  1388002_at  TAO kinase 1  Info   Taok1 
   6389 0.0004491 3983.6 6774.8 0.588  1372523_at  glutamate-cysteine ligase, catalytic subunit  Info   Gclc 
   6390 0.0004492 21.9 49.4 0.443  1397049_at  Transcribed locus, strongly similar to NP_081963.1 calcium-binding tyrosine-phosphorylation regulated protein [Mus musculus]  Info    
   6391 0.0004493 38 74.2 0.512  1389430_at  Transcribed locus  Info    
   6392 0.0004496 98 12.3 7.967  1374735_at  Rho GTPase activating protein 4  Info   Arhgap4 
   6393 0.0004496 100.9 64.8 1.557  1394213_at  similar to RIKEN cDNA 1200014J11 (predicted)  Info   RGD1308139_predicted 
   6394 0.0004501 77.7 138.5 0.561  1374103_at  Frequenin homolog (Drosophila)  Info   Freq 
   6395 0.0004509 355.3 517.2 0.687  1374758_at  Transcribed locus  Info    
   6396 0.0004509 624.2 860.8 0.725  1373020_at  similar to mitochondria-associated granulocyte macrophage CSF signaling molecule  Info   LOC287065 
   6397 0.0004513 1042.4 523 1.993  1371527_at  epithelial membrane protein 1  Info   Emp1 
   6398 0.0004521 315.8 501.8 0.629  1372860_at  similar to phospholysine phosphohistidine inorganic pyrophosphate phosphata (5M590)  Info   RGD:1359187 
   6399 0.0004525 18.7 52.2 0.358  1385558_at  Similar to hypothetical protein D930024E11  Info    
   6400 0.0004525 21.6 43.7 0.494  1396460_at  Transcribed locus, strongly similar to NP_780301.1 ras homolog gene family, member f [Mus musculus]  Info    
   6401 0.0004535 28.5 71.6 0.398  1382244_at  quinolinate phosphoribosyltransferase (predicted)  Info   Qprt_predicted 
   6402 0.0004538 91.5 186.9 0.49  1374822_at  Similar to 106 kDa O-GlcNAc transferase-interacting protein (predicted)  Info    
   6403 0.000454 1214.6 1559 0.779  1373901_at    Info    
   6404 0.0004545 680.1 904.7 0.752  1372624_at  Transmembrane protein 16F (predicted)  Info    
   6405 0.000455 392.2 266.2 1.473  1373426_at    Info    
   6406 0.0004551 41.6 105.4 0.395  1374546_at  Protocadherin 17 (predicted)  Info    
   6407 0.0004554 67 122.3 0.548  1376240_at  Transcribed locus, strongly similar to NP_036462.1 MYST histone acetyltransferase (monocytic leukemia) 4; histone acetyltransferase; monocytic leukemia zinc finger protein-related factor [Homo sapiens]  Info    
   6408 0.0004555 313.1 533.7 0.587  1373518_at  Similar to RIKEN cDNA 1300007B12; clone MNCb-2755 (predicted)  Info    
   6409 0.0004571 274.5 404 0.679  1371655_at  mannose-P-dolichol utilization defect 1 (predicted)  Info   Mpdu1_predicted 
   6410 0.0004575 338.8 534.8 0.634  1372196_at  nuclear receptor co-repressor 2 (predicted)  Info   Ncor2_predicted 
   6411 0.0004576 188.6 529 0.357  1383933_at  Dead end homolog 1 (zebrafish) (predicted)  Info    
   6412 0.0004583 161.7 109 1.483  1376937_at  similar to 4631422O05Rik protein  Info   LOC304020 
   6413 0.0004583 32.1 9.9 3.242  1377718_at  similar to CDNA sequence BC022692  Info   LOC501552 
   6414 0.0004584 151.7 243.1 0.624  1395347_at  Arylsulfatase A (predicted)  Info    
   6415 0.000459 29.9 10.5 2.848  1377637_at  Transcribed locus  Info    
   6416 0.0004596 307.7 198.6 1.549  1395409_at  protein phosphatase 2A, regulatory subunit B' (PR 53) (predicted)  Info   Ppp2r4_predicted 
   6417 0.0004613 93.1 144.5 0.644  1378152_at  Transcribed locus  Info    
   6418 0.0004614 566.1 348.3 1.625  1373488_at  Transcribed locus  Info    
   6419 0.0004621 299.7 362.6 0.827  1388317_at  Transcription factor Pur-beta  Info    
   6420 0.0004625 50 94.6 0.529  1377180_at  Transcribed locus, moderately similar to NP_998724.1 superkiller viralicidic activity 2-like [Rattus norvegicus]  Info    
   6421 0.0004633 47.6 21.1 2.256  1383163_at  immunoglobulin joining chain (predicted)  Info   Igj_predicted 
   6422 0.0004636 1695.9 2157.6 0.786  1398870_at  translocase of outer mitochondrial membrane 20 homolog (yeast)  Info   Tomm20 
   6423 0.0004638 130.3 258.4 0.504  1390546_at    Info    
   6424 0.000464 2.9 23.3 0.124  1379899_at  Transcribed locus  Info    
   6425 0.0004648 44 107.4 0.41  1376184_at  Ly6/neurotoxin 1 (predicted)  Info   Lynx1_predicted 
   6426 0.0004653 123.5 182.7 0.676  1373627_at  Similar to putative phosphoinositide 5-phosphatase type II; C62  Info    
   6427 0.0004655 693.8 869.4 0.798  1389481_at  similar to GI:13385412-like protein splice form I  Info   RGD:735065 
   6428 0.0004659 95.7 46.3 2.067  1382252_at  Transcribed locus  Info    
   6429 0.000466 85.8 127.4 0.673  1376678_at  similar to amyloid beta (A4) precursor protein-binding, family B, member 2  Info   LOC305338 
   6430 0.000466 214.8 275.9 0.779  1378037_at  similar to RIKEN cDNA 0610009J22 (predicted)  Info   RGD1310738_predicted 
   6431 0.0004661 35.9 70.3 0.511  1379321_at  Hypothetical LOC305960 (predicted)  Info    
   6432 0.0004663 295.6 463.2 0.638  1372579_at  Endoglin  Info    
   6433 0.0004663 194 23.4 8.291  1375900_at  similar to T-cell antigen 4-1BB precursor - mouse  Info   LOC500590 
   6434 0.000467 231.4 128.3 1.804  1375341_at  Kua homolog (predicted)  Info   Kua_predicted 
   6435 0.000468 60.1 94.1 0.639  1389689_at  valyl-tRNA synthetase 2-like  Info   RGD:1303122 
   6436 0.000469 135.3 74.6 1.814  1376497_a_at  similar to FLJ20859 protein  Info   LOC305845 
   6437 0.0004695 48.8 82.9 0.589  1378021_at  Transcribed locus  Info    
   6438 0.0004701 23.4 121.1 0.193  1393139_at  similar to Apolipoprotein C2  Info   LOC292697 
   6439 0.0004704 114.1 173.9 0.656  1385517_at  general transcription factor II I  Info   Gtf2i 
   6440 0.0004706 1770.7 3572.4 0.496  1370026_at  crystallin, alpha B  Info   Cryab 
   6441 0.0004718 79.3 29.2 2.716  1377117_at  Transcribed locus  Info    
   6442 0.0004718 126.7 6.6 19.197  1381441_at    Info    
   6443 0.0004733 193.6 241.5 0.802  1374461_at  zinc finger, DHHC domain containing 4 (predicted)  Info   Zdhhc4_predicted 
   6444 0.0004736 47 75.2 0.625  1379781_at  Similar to BAF53a (predicted)  Info    
   6445 0.0004737 14.5 69.3 0.209  1379950_at  Camello-like 2  Info   Cml2 
   6446 0.0004738 313.2 211.1 1.484  1368668_at  phospholipase A2, activating protein  Info   Plaa 
   6447 0.000474 101.4 22.4 4.527  1385797_at  Actin alpha cardiac 1  Info   Actc1 
   6448 0.0004745 83.9 22.6 3.712  1378733_at  Transcribed locus, moderately similar to XP_580018.1 PREDICTED: hypothetical protein XP_580018 [Rattus norvegicus]  Info    
   6449 0.0004747 1236.4 1613.6 0.766  1388445_at  annexin A11 (predicted)  Info   Anxa11_predicted 
   6450 0.0004764 417 593.6 0.702  1388680_at  similar to contains transmembrane (TM) region and ATP binding region (predicted)  Info   RGD1311230_predicted 
   6451 0.0004766 40.6 3.2 12.688  1391077_at  Claspin homolog (Xenopus laevis) (predicted)  Info    
   6452 0.0004769 101.7 187.8 0.542  1373266_at  similar to downregulated in renal cell carcinoma (predicted)  Info   RGD1306327_predicted 
   6453 0.0004773 448.4 318.5 1.408  1377194_a_at  similar to RIKEN cDNA 2310015N07 (predicted)  Info   RGD1308637_predicted 
   6454 0.0004775 87.7 36.4 2.409  1397917_at  Transcribed locus  Info    
   6455 0.0004777 94.6 60.4 1.566  1378345_at  Transcribed locus  Info    
   6456 0.0004783 122.7 232.9 0.527  1379755_at  Transcribed locus  Info    
   6457 0.0004785 66.2 39 1.697  1377077_at  plexin A3  Info   Plxna3 
   6458 0.0004786 242.2 133.9 1.809  1393173_at  RAS p21 protein activator 1  Info   Rasa1 
   6459 0.000479 330.3 230.9 1.43  1383951_at  similar to hypothetical protein FLJ31951 (predicted)  Info   RGD1309561_predicted 
   6460 0.000479 435.9 630.7 0.691  1398424_at  WD SOCS-box protein 2  Info   Wsb2 
   6461 0.0004791 319.9 491.2 0.651  1392044_at  Transcribed locus  Info    
   6462 0.00048 689.8 429.5 1.606  1376272_s_at  AA926063gene  Info   RGD:1303072 
   6463 0.0004809 40.5 76.3 0.531  1395604_at  MMS19-like (MET18 homolog, S. cerevisiae)  Info   LOC171124 
   6464 0.0004812 18.6 223.4 0.083  1368779_a_at  guanylate cyclase 1, soluble, beta 2  Info   Gucy1b2 
   6465 0.0004818 350.7 239.2 1.466  1383074_at    Info    
   6466 0.0004833 92.2 42.6 2.164  1367891_a_at  caspase 2  Info   Casp2 
   6467 0.0004836 8563 6077.7 1.409  AFFX_Rat_beta-actin_3_at  actin, beta  Info   Actb 
   6468 0.000484 335.6 661 0.508  1378156_at  Transcribed locus  Info    
   6469 0.000484 38.4 59.8 0.642  1388174_at  wingless-type MMTV integration site family, member 2B  Info   Wnt2b 
   6470 0.000484 47.3 77.4 0.611  1395132_at    Info    
   6471 0.0004842 54.8 68.8 0.797  1385462_at  mitogen activated protein kinase kinase kinase kinase 2 (predicted)  Info   Map4k2_predicted 
   6472 0.0004847 34.6 56 0.618  1376217_at  cancer susceptibility candidate 1 (predicted)  Info   Casc1_predicted 
   6473 0.0004849 226.1 153.4 1.474  1370460_at  ubiquitin specific protease 15  Info   Usp15 
   6474 0.0004855 93.8 70 1.34  1390351_at  Transcribed locus  Info    
   6475 0.0004857 413.7 282.7 1.463  1388478_at  Transcribed locus  Info    
   6476 0.000486 38.5 81.2 0.474  1380741_at  Transcribed locus  Info    
   6477 0.000487 66.6 40.6 1.64  1392847_at  Casitas B-lineage lymphoma-like 1 (predicted)  Info   Cbll1_predicted 
   6478 0.0004877 6 43.4 0.138  1373567_at  similar to Tle6 protein  Info   LOC299637 
   6479 0.0004881 154 214 0.72  1382602_at  similar to zinc finger protein 650  Info   LOC311115 
   6480 0.0004882 39.8 107.3 0.371  1370334_at  evectin-1  Info   Plekhb1 
   6481 0.0004885 12 31.3 0.383  1381070_at    Info    
   6482 0.0004885 47.7 93.9 0.508  1390969_at  Transcribed locus  Info    
   6483 0.0004895 202.2 299.7 0.675  1389478_at  similar to hypothetical protein FLJ14360 (predicted)  Info   RGD1306288_predicted 
   6484 0.00049 804.6 1188.5 0.677  1398933_at  similar to RIKEN cDNA 2010110M21 (predicted)  Info   RGD1309691_predicted 
   6485 0.0004902 48.3 21.5 2.247  1383958_at  cell division cycle associated 2 (predicted)  Info   Cdca2_predicted 
   6486 0.0004905 36.2 18.9 1.915  1385564_at  similar to LIM domain containing preferred translocation partner in lipoma; LIM domain-containing preferred translocation partner in lipoma; Lipoma-preferred-partner gene  Info   LOC288010 
   6487 0.0004907 395.4 683.6 0.578  1397185_at  Transcribed locus  Info    
   6488 0.0004924 54.3 96 0.566  1377011_at  Similar to hypothetical protein CG003 (predicted)  Info    
   6489 0.0004929 34.4 69.2 0.497  1380773_at  6-phosphogluconolactonase (predicted)  Info    
   6490 0.0004933 76.7 9.1 8.429  1387269_s_at  plasminogen activator, urokinase receptor  Info   Plaur 
   6491 0.0004935 387.9 616.1 0.63  1367828_at  acyl-coenzyme A dehydrogenase, short chain  Info   Acads 
   6492 0.0004943 165.9 236.1 0.703  1368712_at  zinc finger protein 386 (Kruppel-like)  Info   Znf386 
   6493 0.0004953 55.7 99.8 0.558  1385369_at  similar to hypothetical protein FLJ32844 (predicted)  Info   RGD1311430_predicted 
   6494 0.0004964 113.3 172.4 0.657  1383069_at  Similar to RNA binding motif, single stranded interacting protein 3  Info    
   6495 0.0004973 73.2 45.2 1.619  1385504_at  fring  Info   Rffl 
   6496 0.0004973 45.4 90.6 0.501  1374745_at  similar to sorting nexin 21 isoform a; sorting nexin L; sorting nexin 21 (predicted)  Info   RGD1311169_predicted 
   6497 0.000498 2369.8 2993.2 0.792  1370230_at  ATP synthase, H+ transporting, mitochondrial F0 complex, subunit F6  Info   Atp5j 
   6498 0.0004981 34.4 57.6 0.597  1376464_at  Thymocyte protein thy28  Info   MGC72984 
   6499 0.0004981 46.3 14.7 3.15  1368489_at  fos-like antigen 1  Info   Fosl1 
   6500 0.0004982 276 203.9 1.354  1382643_at  sorting nexin 16  Info   Snx16 
   6501 0.0004983 343.5 280.7 1.224  1389717_at  similar to KIAA0157 gene product is novel. (predicted)  Info   RGD1308918_predicted 
   6502 0.0004995 39.5 80.8 0.489  1398312_s_at  solute carrier family 14 (urea transporter), member 2  Info   Slc14a2 
   6503 0.0004996 39.5 60.8 0.65  1396302_at  Transcribed locus  Info    
   6504 0.0004998 26.6 17.8 1.494  1391621_at  E2F transcription factor 7 (predicted)  Info    
   6505 5e-04 2746.3 3335.1 0.823  1388301_at  ubiquinol-cytochrome c reductase core protein I  Info   RGD:1303314 
   6506 0.0005001 1264.6 996.8 1.269  1371337_at  cytochrome c oxidase subunit VIIa polypeptide 2 like (predicted)  Info   Cox7a2l_predicted 
   6507 0.0005006 27.7 71.4 0.388  1384487_at  doublesex and mab-3 related transcription factor 2 (predicted)  Info   Dmrt2_predicted 
   6508 0.0005014 2.7 18.8 0.144  1380127_at  Transcribed locus  Info    
   6509 0.0005016 56.9 5.1 11.157  1390481_a_at  similar to RIKEN cDNA 2700084L22 (predicted)  Info   RGD1310816_predicted 
   6510 0.0005017 60.8 5.5 11.055  1371196_at  MBP13 Vbeta14 gene  Info    
   6511 0.0005019 194.7 282.7 0.689  1374085_at  Max dimerization protein 4 (predicted)  Info   Mxd4_predicted 
   6512 0.000502 104.5 71.2 1.468  1391195_at    Info    
   6513 0.0005022 24.6 3.3 7.455  1375978_at  FCH domain only 1 (predicted)  Info   Fcho1_predicted 
   6514 0.0005037 459.3 367.2 1.251  1372680_at  Similar to malignant cell expression-enhanced gene/tumor progression-enhanc (predicted)  Info    
   6515 0.0005037 349.9 429.8 0.814  1375337_at  a disintegrin and metalloproteinase domain 9 (meltrin gamma) (predicted)  Info   Adam9_predicted 
   6516 0.0005038 169.3 112.1 1.51  1397540_at  Transcribed locus  Info    
   6517 0.0005052 45.3 5.6 8.089  1381474_at  similar to mCHCR  Info   LOC302492 
   6518 0.0005052 1332.5 823 1.619  1390113_a_at  Similar to KIAA0731 protein (predicted)  Info    
   6519 0.0005055 159.2 95.1 1.674  1368129_at  Scm-like with four mbt domains 1  Info   Sfmbt1 
   6520 0.0005059 345.5 500.5 0.69  1388383_at  Transcribed locus  Info    
   6521 0.0005059 380.5 255.4 1.49  1374072_at  Vascular endothelial zinc finger 1 (predicted)  Info    
   6522 0.000506 49.8 117 0.426  1385668_at  Transcribed locus  Info    
   6523 0.0005063 932.1 647.8 1.439  1368331_at  chitobiase, di-N-acetyl-  Info   Ctbs 
   6524 0.0005092 198.3 136.7 1.451  1377100_at  androgen-induced proliferation inhibitor (predicted)  Info   Aprin_predicted 
   6525 0.0005094 72.7 144.2 0.504  1377776_at  similar to hypothetical protein FLJ36175  Info   RGD:1359296 
   6526 0.0005096 67.6 9.9 6.828  1388069_at  killer cell lectin-like receptor subfamily B member 1B  Info   Klrb1b 
   6527 0.0005097 869.1 1156.5 0.751  1398845_at  eukaryotic translation initiation factor 5  Info   Eif5 
   6528 0.0005105 299.9 220.5 1.36  1389220_at    Info    
   6529 0.0005107 34 60 0.567  1391553_at  Transcribed locus  Info    
   6530 0.0005119 46.9 4.9 9.571  1385831_at  Similar to RIKEN cDNA 1200013B08  Info    
   6531 0.0005129 388.2 314 1.236  1373576_at    Info    
   6532 0.000513 686.1 506.1 1.356  1371463_at  transcription factor INI  Info   Phf5a 
   6533 0.0005139 27.3 2 13.65  1385522_at  origin recognition complex, subunit 1-like (S.cereviaiae)  Info   Orc1l 
   6534 0.0005143 371.2 523.6 0.709  1391158_a_at  spinocerebellar ataxia 2 (olivopontocerebellar ataxia 2, autosomal dominant, ataxin 2) (predicted)  Info   Sca2_predicted 
   6535 0.0005152 28.9 81 0.357  1395052_at  Transcribed locus  Info    
   6536 0.0005156 313.4 470.6 0.666  1395725_at  similar to intracellular membrane-associated calcium-independent phospholipase A2 gamma (predicted)  Info   RGD1311444_predicted 
   6537 0.0005157 112.6 57.4 1.962  1377390_at  beta-site APP-cleaving enzyme 2  Info   RGD:1303241 
   6538 0.0005158 24.8 2.1 11.81  1398519_at    Info    
   6539 0.0005161 57.5 4.3 13.372  1396231_at  similar to Ras GTPase-activating protein nGAP (RAS protein activator like 1) (predicted)  Info   RGD1309019_predicted 
   6540 0.0005162 24.2 44.5 0.544  1382356_at  similar to EF-hand domain (C-terminal) containing 2  Info   LOC302507 
   6541 0.0005167 42.8 17 2.518  1391388_at  Transcribed locus  Info    
   6542 0.0005176 166.9 36.1 4.623  1387391_at  cyclin-dependent kinase inhibitor 1A  Info   Cdkn1a 
   6543 0.0005178 28.3 96.6 0.293  1379518_at  Ras responsive element binding protein 1 (predicted)  Info    
   6544 0.0005185 73.6 34.9 2.109  1368346_at  UDP-Gal:betaGlcNAc beta 1,3-galactosyltransferase, polypeptide 4  Info   B3galt4 
   6545 0.0005189 71.3 130.7 0.546  1369468_at  frizzled homolog 4 (Drosophila)  Info   Fzd4 
   6546 0.0005191 95.1 8.9 10.685  1373489_at  similar to chromatin assembly factor-I p150 subunit  Info   LOC363333 
   6547 0.0005191 489.9 894.7 0.548  1389412_at    Info    
   6548 0.0005194 24.7 14.7 1.68  1379878_at  Transcribed locus  Info    
   6549 0.0005196 91.1 30.6 2.977  1385220_at    Info    
   6550 0.0005199 218.3 139.2 1.568  1385727_at  wound inducible transcript 3.0  Info   RGD:1303233 
   6551 0.0005203 185 120 1.542  1371534_at  similar to RIKEN cDNA 1300006L01 (predicted)  Info   RGD1307826_predicted 
   6552 0.0005205 3 2.1 1.429  1370210_at  Ras-induced senescence 1  Info   Ris1 
   6553 0.0005213 640.2 1105.3 0.579  1388870_at  Transcribed locus, strongly similar to NP_620412.1 musashi 2 isoform a [Homo sapiens]  Info    
   6554 0.0005216 80.6 29.9 2.696  1378540_at    Info    
   6555 0.0005222 47.2 78.5 0.601  1397569_at  Similar to teratocyte-specific carboxylesterase  Info    
   6556 0.0005227 197 358.5 0.55  1374839_at  spastic paraplegia 7 homolog (human)  Info   Spg7 
   6557 0.0005228 15.1 46.3 0.326  1380387_at  Transcribed locus  Info    
   6558 0.0005235 148.3 13 11.408  1375028_at  Transcribed locus  Info    
   6559 0.0005235 48.1 92.3 0.521  1397268_at    Info    
   6560 0.0005235 534.9 726.3 0.736  1388467_at  small glutamine-rich tetratricopeptide repeat (TPR)-containing, alpha  Info   Sgta 
   6561 0.000525 238.7 371.4 0.643  1373162_at  NYGGF3  Info    
   6562 0.0005252 1418.2 1164.6 1.218  1371395_at  chromobox homolog 3 (HP1 gamma homolog, Drosophila)  Info   Cbx3 
   6563 0.0005255 2518.1 2188.8 1.15  1367475_at  cell division cycle 42 homolog (S. cerevisiae)  Info   Cdc42 
   6564 0.0005257 1945.8 1668.8 1.166  1388390_at  eukaryotic translation initiation factor 3, subunit 3 gamma  Info   Eif3s3 
   6565 0.0005263 870.7 599.1 1.453  1375904_at  Transcribed locus  Info    
   6566 0.0005269 70.5 31.7 2.224  1378198_at  Transcribed locus  Info    
   6567 0.0005272 201.3 309.4 0.651  1376848_at  Transcribed locus  Info    
   6568 0.0005276 76.5 42.9 1.783  1377387_a_at  similar to RIKEN cDNA 1810009K13  Info   LOC498110 
   6569 0.0005278 19.2 45.5 0.422  1369494_a_at  growth hormone releasing hormone receptor  Info   Ghrhr 
   6570 0.0005281 202.1 103.8 1.947  1369443_at  angiopoietin-like 2  Info   Angptl2 
   6571 0.0005284 383.7 1630.8 0.235  1368321_at  early growth response 1  Info   Egr1 
   6572 0.0005285 110.6 57.7 1.917  1395039_at  Transcribed locus  Info    
   6573 0.0005285 137.6 53.5 2.572  1369382_at  c-mer proto-oncogene tyrosine kinase  Info   Mertk 
   6574 0.0005286 151.8 105.7 1.436  1391086_at  katanin p60 subunit A 1  Info   RGD:1303062 
   6575 0.0005294 157.6 96.1 1.64  1376185_at  kinesin family member C1  Info   RGD:1359118 
   6576 0.0005296 279.4 168.7 1.656  1388405_at  similar to colonic and hepatic tumor over-expressed protein isoform a  Info   LOC311191 
   6577 0.00053 129.9 195.2 0.665  1385113_at  similar to Homeobox protein Hox-C10 (Hox-3.6) (predicted)  Info   RGD1307250_predicted 
   6578 0.0005301 85.5 176.3 0.485  1377029_at  RAR-related orphan receptor alpha (predicted)  Info   Rora_predicted 
   6579 0.0005302 123.7 81 1.527  1378040_at    Info    
   6580 0.0005305 26.9 54.2 0.496  1391479_at  Transcribed locus  Info    
   6581 0.0005305 150.2 109.7 1.369  1376664_at  liver regeneration-related protein  Info   RGD:708394 
   6582 0.0005305 135 90 1.5  1395445_at  similar to regulatory factor X-associated protein  Info   LOC499617 
   6583 0.0005306 773.2 965 0.801  1373501_at  Transcribed locus  Info    
   6584 0.0005314 1128 1263.6 0.893  1371612_at  tRNA splicing endonuclease 34 homolog  Info   RGD:1359564 
   6585 0.0005326 41.5 18.2 2.28  1374616_at  platelet-derived growth factor receptor-like (predicted)  Info   Pdgfrl_predicted 
   6586 0.000533 9467.7 6366.4 1.487  1367595_s_at  beta-2 microglobulin  Info   B2m 
   6587 0.0005332 1334.1 1051.8 1.268  1371688_at  translocating chain-associating membrane protein  Info   RGD:1359100 
   6588 0.0005333 212.1 364.8 0.581  1382134_at  similar to olfactomedin-like 1 /// olfactomedin-like 1 (predicted)  Info   LOC308917 /// Olfml1_predicted 
   6589 0.0005341 118.3 195.3 0.606  1374783_at  Similar to suppressor of Ty 3 homolog  Info    
   6590 0.0005345 481.4 334.4 1.44  1373481_at  R3H domain (binds single-stranded nucleic acids) containing (predicted)  Info   R3hdm_predicted 
   6591 0.0005346 205.7 312.6 0.658  1389027_at  Lectin, mannose-binding 2-like (predicted)  Info    
   6592 0.0005348 44.5 82.5 0.539  1374160_at  Similar to RIKEN cDNA 1110063G11 (predicted)  Info    
   6593 0.0005351 27.4 65.1 0.421  1384346_at  Transcribed locus  Info    
   6594 0.0005354 414.6 576 0.72  1398981_at    Info    
   6595 0.0005357 65.2 42.2 1.545  1382077_at  GLI-Kruppel family member GLI3  Info   Gli3 
   6596 0.0005364 6.7 100.3 0.067  1387033_at  uncoupling protein 1  Info   Ucp1 
   6597 0.0005366 130.9 206.4 0.634  1376800_at  Chimerin (chimaerin) 2  Info   Chn2 
   6598 0.0005367 1661.4 2438.8 0.681  1393138_at  Jun D proto-oncogene  Info   Jund 
   6599 0.0005372 173.5 244.5 0.71  1388475_at  Similar to prion protein interacting protein 1  Info    
   6600 0.0005375 198 110.8 1.787  1385020_at  similar to DNA segment, Chr 19, Brigham & Womens Genetics 1357 expressed (predicted) /// hypothetical protein LOC499339  Info   RGD1308750_predicted /// LOC499339 
   6601 0.0005378 65.3 17.9 3.648  1394953_at  sprouty protein with EVH-1 domain 1, related sequence (predicted)  Info   Spred1_predicted 
   6602 0.0005378 102.6 56.6 1.813  1395329_at  similar to GDP-mannose pyrophosphorylase B  Info   LOC363145 
   6603 0.0005392 11 49.9 0.22  1371731_at  Phosphofructokinase, liver, B-type  Info   Pfkl 
   6604 0.0005403 772 629.2 1.227  1387041_at  ubiquilin 1  Info   Ubqln1 
   6605 0.0005403 431.9 762.6 0.566  1379578_at  similar to Zbtb20 protein  Info   LOC288105 
   6606 0.0005407 20.8 41.7 0.499  1370615_at  UDP-glucuronosyltransferase  Info   RGD:708417 
   6607 0.0005421 247.2 334 0.74  1378287_at  radixin  Info   RGD:1359472 
   6608 0.0005432 636.8 796.8 0.799  1371465_at    Info    
   6609 0.0005432 2483.8 2026.5 1.226  1386004_s_at  mortality factor 4 like 1 (predicted)  Info   Morf4l1_predicted 
   6610 0.0005433 452.9 620.5 0.73  1371374_at  similar to homolog of yeast MAF1  Info   RGD:1359315 
   6611 0.0005444 639.1 516.5 1.237  1367935_at  smu-1 suppressor of mec-8 and unc-52 homolog (C. elegans)  Info   Smu1 
   6612 0.0005446 606 397.5 1.525  1383089_at  RAB21, member RAS oncogene family  Info   RGD:1303150 
   6613 0.0005452 76.5 124.5 0.614  1398277_at  activin A receptor, type 1  Info   Acvr1 
   6614 0.0005465 59.1 93.9 0.629  1390323_at  Transcribed locus  Info    
   6615 0.0005467 89.5 6.4 13.984  1378915_x_at  hypothetical protein LOC308990  Info   LOC308990 
   6616 0.0005471 47.6 8 5.95  1398719_at  chromodomain protein, Y chromosome-like 2 (predicted)  Info   Cdyl2_predicted 
   6617 0.0005475 230.1 142.5 1.615  1389302_at  similar to RIKEN cDNA D030070L09 (predicted)  Info   RGD1310199_predicted 
   6618 0.0005476 1252.4 2441 0.513  1390530_at  Transcribed locus  Info    
   6619 0.0005477 104.9 4.5 23.311  1391317_at  similar to RIKEN cDNA 2810433K01 (predicted)  Info   RGD1310784_predicted 
   6620 0.0005479 161.4 104.5 1.544  1373293_at  similar to DNA segment, Chr 16, Brigham & Womens Genetics 1547 expressed (predicted)  Info   RGD1309089_predicted 
   6621 0.0005482 53.1 180.4 0.294  1370310_at  3-hydroxy-3-methylglutaryl-Coenzyme A synthase 2  Info   Hmgcs2 
   6622 0.0005486 10.3 2.5 4.12  1385759_at  serine (or cysteine) proteinase inhibitor, clade B (ovalbumin), member 10  Info   Serpinb10 
   6623 0.0005507 81.7 35.7 2.289  1398062_at  similar to hypothetical protein AL133206 (predicted)  Info   RGD1308418_predicted 
   6624 0.0005509 106 46.2 2.294  1379603_at  similar to CBL E3 ubiquitin protein ligase (Signal transduction protein CBL) (Proto-oncogene c-CBL) (Casitas B-lineage lymphoma proto-oncogene)  Info   LOC500985 
   6625 0.0005513 337.2 482.4 0.699  1399052_at  Toll interacting protein (predicted)  Info    
   6626 0.0005516 55 101.6 0.541  1391813_at  ankyrin repeat and SOCS box-containing 13 (predicted)  Info   Asb13_predicted 
   6627 0.0005517 149.4 220.7 0.677  1383019_at  FMS-like tyrosine kinase 1  Info   Flt1 
   6628 0.0005525 1260.6 1740.6 0.724  1388975_at  succinate-Coenzyme A ligase, ADP-forming, beta subunit (predicted)  Info   Sucla2_predicted 
   6629 0.0005531 108 63.5 1.701  1383922_a_at  similar to N-acetyltransferase ARD1  Info   LOC363518 
   6630 0.0005547 24 60.8 0.395  1380136_at  Transcribed locus  Info    
   6631 0.000555 69.3 38.5 1.8  1384285_at  similar to Hypothetical protein MGC48332  Info   RGD:1359308 
   6632 0.000555 38.8 17.5 2.217  1398266_a_at  early growth response 2  Info   Egr2 
   6633 0.0005561 127.9 71.2 1.796  1395363_at  methionine-tRNA synthetase (predicted)  Info   Mars_predicted 
   6634 0.0005565 36.7 107.5 0.341  1377877_at    Info    
   6635 0.0005567 138.6 228.1 0.608  1373267_at  Sh3 domain YSC-like 1 (predicted)  Info   Sh3yl1_predicted 
   6636 0.0005574 3343.8 4304.8 0.777  1388315_at  similar to neuronal protein 15.6  Info   LOC299310 
   6637 0.0005575 67.5 27.9 2.419  1373669_at  glucosamine-6-phosphate deaminase 2 (predicted)  Info   Gnpda2_predicted 
   6638 0.0005576 29.8 71.1 0.419  1386530_at  Transcribed locus  Info    
   6639 0.0005585 59.2 87.7 0.675  1367813_at  protein phosphatase 1, regulatory (inhibitor) subunit 14A  Info   Ppp1r14a 
   6640 0.0005585 163.1 111.9 1.458  1393046_at  similar to Protein C20orf20 (predicted)  Info   RGD1308612_predicted 
   6641 0.0005592 8558.6 6045.9 1.416  1398836_s_at  actin, beta  Info   Actb 
   6642 0.0005597 48.8 17.2 2.837  1390816_at    Info    
   6643 0.0005597 423 235.1 1.799  1390832_at  Transcribed locus  Info    
   6644 0.0005607 262.4 351 0.748  1373194_at  caspase 8 associated protein 2 (predicted)  Info   Casp8ap2_predicted 
   6645 0.0005615 65.3 7.5 8.707  1390623_at    Info    
   6646 0.0005618 362.7 738.9 0.491  1373329_at  transmembrane protease, serine 2  Info   Tmprss2 
   6647 0.0005622 145.9 75 1.945  1375015_at  Transcribed locus  Info    
   6648 0.0005622 312.2 459.6 0.679  1373808_at  CDNA clone IMAGE:7193711  Info    
   6649 0.0005636 61.8 141.7 0.436  1381201_at  MARVEL (membrane-associating) domain containing 2 (predicted)  Info   Mrvldc2_predicted 
   6650 0.0005648 85.4 122 0.7  1383790_at  Transcribed locus  Info    
   6651 0.0005652 82 129.9 0.631  1387046_at  selective LIM binding factor, rat homolog  Info   RGD:620744 
   6652 0.0005654 111.1 184.4 0.602  1399109_at  Transcribed locus  Info    
   6653 0.0005657 118.6 182.3 0.651  1373119_at  potassium channel tetramerisation domain containing 3 (predicted)  Info   Kctd3_predicted 
   6654 0.0005658 1217.5 1774.1 0.686  1367969_at  peroxiredoxin 6  Info   Prdx6 
   6655 0.0005661 96.3 63.1 1.526  1382148_at  Transcribed locus, moderately similar to XP_576460.1 PREDICTED: similar to hypothetical protein PB402898.00.0 [Rattus norvegicus]  Info    
   6656 0.0005666 35.3 4.2 8.405  1391009_a_at    Info    
   6657 0.0005677 426.9 573.2 0.745  1388401_at    Info    
   6658 0.0005683 68.8 40.6 1.695  1394710_at  5-azacytidine induced gene 2 (predicted)  Info   Azi2_predicted 
   6659 0.0005688 18.3 40.2 0.455  1378471_at    Info    
   6660 0.0005688 142.8 239.4 0.596  1394948_at  Arrestin domain containing 3  Info   Arrdc3 
   6661 0.0005689 287.4 197 1.459  1391220_at  Transcribed locus  Info    
   6662 0.0005692 21.2 60.3 0.352  1383293_at  Transcribed locus  Info    
   6663 0.0005693 24.7 46.5 0.531  1388876_at  Phospholamban  Info   Pln 
   6664 0.0005705 96.5 55 1.755  1390820_at  zinc finger, CCHC domain containing 11 (predicted)  Info   Zcchc11_predicted 
   6665 0.0005706 55.1 118.8 0.464  1389805_at  Transcribed locus  Info    
   6666 0.0005707 841.5 415.2 2.027  1389966_at  procollagen, type VI, alpha 3 (predicted)  Info   Col6a3_predicted 
   6667 0.0005708 141.7 220.8 0.642  1371635_at  transmembrane domain protein regulated in adipocytes  Info   Tpra40 
   6668 0.000571 34.7 96.1 0.361  1385626_at  similar to B230212L03Rik protein  Info   MGC95152 
   6669 0.0005711 107.6 46.1 2.334  1389537_at  Treacher Collins Franceschetti syndrome 1, homolog (predicted)  Info   Tcof1_predicted 
   6670 0.0005713 65.8 98.9 0.665  1384621_at  Transcribed locus, weakly similar to XP_519783.1 PREDICTED: similar to hypothetical protein MGC34646 [Pan troglodytes]  Info    
   6671 0.0005729 1012.9 1260.1 0.804  1399132_at  cullin 3 (predicted)  Info   Cul3_predicted 
   6672 0.0005734 36.4 81.7 0.446  1393545_at  transcription factor CP2-like 2 (predicted)  Info   Tcfcp2l2_predicted 
   6673 0.000574 111.9 171.7 0.652  1397363_at  poliovirus receptor-related 3 (predicted)  Info   Pvrl3_predicted 
   6674 0.0005741 280.8 95.9 2.928  1368943_at  ribonuclease, RNase A family 4  Info   Rnase4 
   6675 0.0005751 36.6 67.7 0.541  1393910_at  similar to Fam13a1 protein (predicted)  Info   RGD1309807_predicted 
   6676 0.0005751 13.1 45.7 0.287  1385320_at  similar to Pdz-containing protein  Info   LOC317379 
   6677 0.0005751 222.3 147.3 1.509  1391491_a_at  similar to MHR23B  Info   LOC298012 
   6678 0.0005755 304.5 428.1 0.711  1372221_at  Transcribed locus, strongly similar to NP_003335.1 ubiquitin-conjugating enzyme E2H isoform 1; ubiquitin-protein ligase H; ubiquitin carrier protein H; ubiquitin-conjugating enzyme E2H (homologous to yeast UBC8) [Homo sapiens]  Info    
   6679 0.0005755 14.3 38.7 0.37  1379078_at  Transcribed locus  Info    
   6680 0.000576 7.1 36.2 0.196  1389206_at  Transcribed locus  Info    
   6681 0.000577 94.1 130.3 0.722  1390202_at  PR domain containing 2, with ZNF domain  Info    
   6682 0.0005771 1329.5 773.6 1.719  1371310_s_at  serine (or cysteine) proteinase inhibitor, clade H, member 1  Info   Serpinh1 
   6683 0.0005774 78.4 152.9 0.513  1390101_at  similar to hypothetical protein MGC31967  Info   LOC313496 
   6684 0.0005774 1.5 11 0.136  1393387_at  Transcribed locus  Info    
   6685 0.0005779 10.3 33.8 0.305  1369090_at  protein kinase, cGMP-dependent, type II  Info   Prkg2 
   6686 0.0005787 27.8 69.1 0.402  1377926_at  Transcribed locus  Info    
   6687 0.0005789 297.1 428.5 0.693  1388430_at  prostate tumor over expressed gene 1 (predicted)  Info   Ptov1_predicted 
   6688 0.0005793 130.3 162.4 0.802  1376159_at  Transcribed locus  Info    
   6689 0.0005796 89.2 7.8 11.436  1397411_at  FYVE, RhoGEF and PH domain containing 2 (predicted)  Info   Fgd2_predicted 
   6690 0.0005801 797 1644.6 0.485  1368947_at  growth arrest and DNA-damage-inducible 45 alpha  Info   Gadd45a 
   6691 0.0005801 276.9 205.3 1.349  1389596_at    Info    
   6692 0.0005804 110 44.6 2.466  1396280_at  similar to G patch domain and KOW motifs  Info   LOC302560 
   6693 0.0005805 266.9 192.6 1.386  1373129_at  Transcribed locus  Info    
   6694 0.0005808 43 101.2 0.425  1387373_at  phosphodiesterase 4D interacting protein (myomegalin)  Info   Pde4dip 
   6695 0.0005819 1.7 9.7 0.175  1387164_at  leukocyte cell derived chemotaxin 1  Info   Lect1 
   6696 0.0005825 22.4 47.7 0.47  1398233_at  Transcribed locus  Info    
   6697 0.0005828 627.9 907.3 0.692  1382048_at  ATPase, H+ transporting, V1 subunit A, isoform 1 (predicted)  Info   Atp6v1a1_predicted 
   6698 0.0005838 723.9 595.8 1.215  1372067_at  thioredoxin domain containing 1 (predicted)  Info   Txndc1_predicted 
   6699 0.0005838 592.3 884.3 0.67  1370286_at  solute carrier family 38, member 2  Info   Slc38a2 
   6700 0.0005843 429.8 307 1.4  1398867_at  neuronal differentiation-related gene  Info   Prp19 
   6701 0.0005852 635.2 381.3 1.666  1398345_at    Info    
   6702 0.0005854 102.2 52.2 1.958  1374764_at  similar to 2610033H07Rik protein (predicted)  Info   RGD1305605_predicted 
   6703 0.0005856 2772.9 3380.7 0.82  1368521_at  napsin A aspartic peptidase  Info   Napsa 
   6704 0.0005871 192.8 138 1.397  1374707_at  similar to 2610301G19Rik protein (predicted)  Info   RGD1309922_predicted 
   6705 0.000589 72.5 134.6 0.539  1389463_at  protein kinase, cAMP dependent regulatory, type I, beta  Info   Prkar1b 
   6706 0.0005892 196.6 295.4 0.666  1378860_at  Similar to hypothetical protein D930020E02  Info    
   6707 0.0005893 50.3 20.3 2.478  1381567_at  hypothetical LOC294390 (predicted)  Info   RGD1307672_predicted 
   6708 0.0005899 26 37.9 0.686  1392726_at  similar to pyruvate dehydrogenase phosphatase regulatory subunit (predicted)  Info   RGD1308307_predicted 
   6709 0.0005903 688.3 476 1.446  1371521_at  Pyruvate dehydrogenase E1 alpha 2  Info   Pdha2 
   6710 0.0005904 87.7 53 1.655  1382745_at  Similar to lymphocyte alpha-kinase (predicted)  Info    
   6711 0.0005913 13.5 24.5 0.551  1396467_at  Similar to macroH2A2  Info    
   6712 0.0005918 109.4 172.5 0.634  1372040_at  calmodulin binding transcription activator 2 (predicted)  Info   Camta2_predicted 
   6713 0.0005919 36.3 60.8 0.597  1378978_a_at  Similar to KIAA1205 protein (predicted)  Info    
   6714 0.0005921 93 58.3 1.595  1387261_at  protein phosphatase 3, catalytic subunit, beta isoform  Info   Ppp3cb 
   6715 0.0005921 36.2 60.3 0.6  1397911_at  Transcribed locus  Info    
   6716 0.0005927 72.9 23.5 3.102  1371058_at    Info    
   6717 0.000593 6394.2 9075.5 0.705  1370299_at  aldolase B  Info   Aldob 
   6718 0.000594 77.3 125.4 0.616  1375085_at  similar to RIKEN cDNA 1810012H11  Info   LOC497933 
   6719 0.0005947 147.8 94.8 1.559  1391078_at  replication factor C 1  Info   Recc1 
   6720 0.0005951 159.4 230.8 0.691  1386755_at  similar to RIKEN cDNA 1810060J02  Info   RGD:1359502 
   6721 0.0005961 51.4 19.1 2.691  1382472_at  Transcribed locus  Info    
   6722 0.0005967 185.9 149.7 1.242  1384002_at  similar to Potential phospholipid-transporting ATPase IF (ATPase class I type 11B) (ATPase IR)  Info   LOC361929 
   6723 0.0005981 301.5 399.7 0.754  1399102_at  similar to hypothetical protein MGC15523 (predicted)  Info   RGD1306356_predicted 
   6724 0.0005984 88.3 32.5 2.717  1387390_at  granzyme K  Info   Gzmk 
   6725 0.0005987 479.1 355.4 1.348  1383288_at  Transformed mouse 3T3 cell double minute 2 (predicted)  Info    
   6726 0.0005989 284.1 135.1 2.103  1387351_at  fibrillin 1  Info   Fbn1 
   6727 0.000599 2915.6 3698.2 0.788  1371419_at  spectrin beta 2  Info   Spnb2 
   6728 0.0005998 91.4 188.2 0.486  1392999_at  Transcribed locus  Info    
   6729 0.0005999 36.3 10.6 3.425  1386797_at  solute carrier family 39 (metal ion transporter), member 6 (predicted)  Info   Slc39a6_predicted 
   6730 6e-04 97.9 51.9 1.886  1384523_at  similar to postmeiotic segregation increased 1  Info   MGC72584 
   6731 0.0006001 203 106.5 1.906  1372578_at  valyl-tRNA synthetase 2  Info   Vars2 
   6732 0.0006012 299.2 199.3 1.501  1370356_at  RNA binding motif protein 10  Info   Rbm10 
   6733 0.0006018 299.1 397.3 0.753  1377809_at    Info    
   6734 0.0006019 163.4 115.8 1.411  1374357_at  CDC91 cell division cycle 91-like 1 (S. cerevisiae)  Info   Cdc91l1 
   6735 0.0006022 129.9 213.4 0.609  1372481_at  Transcribed locus, moderately similar to NP_598415.1 CD34 antigen [Mus musculus]  Info    
   6736 0.0006032 35.8 74.9 0.478  1391516_at  similar to OCTN3  Info   LOC303140 
   6737 0.0006034 503.4 671.2 0.75  1375896_at  similar to polyploidy associated protein kinase PAPK-A  Info   LOC501146 
   6738 0.0006045 178.3 119.8 1.488  1390385_at  Similar to D-glucuronyl C5-epimerase  Info    
   6739 0.0006047 212.7 306 0.695  1376060_at  Protein phosphatase 1H (PP2C domain containing) (predicted)  Info    
   6740 0.0006052 61.4 143.6 0.428  1387740_at  peroxisomal biogenesis factor 11A  Info   Pex11a 
   6741 0.0006054 1002.2 769.9 1.302  1373133_at  similar to ADP-ribosylation factor-like 10C  Info   LOC500282 
   6742 0.0006067 47.9 22.3 2.148  1375936_at  Desmocollin 2 (predicted)  Info    
   6743 0.0006072 11.6 48.1 0.241  1383760_at  Transcribed locus, strongly similar to NP_758458.1 zinc finger, CSL domain containing 2 [Mus musculus]  Info    
   6744 0.0006074 711.4 397.8 1.788  1367913_at  cytoglobin  Info   Cygb 
   6745 0.0006078 3624.1 4547.7 0.797  1367721_at  syndecan 4  Info   Sdc4 
   6746 0.0006093 41.8 73.3 0.57  1391944_at  similar to Hypothetical protein C6orf60 (predicted)  Info   RGD1311294_predicted 
   6747 0.00061 1981.3 1546.1 1.281  1398770_at  large subunit ribosomal protein L36a  Info   RGD:621156 
   6748 0.0006104 27.1 43.1 0.629  1394702_at  Transcribed locus  Info    
   6749 0.0006109 31.5 78.3 0.402  1378998_at  Transcribed locus  Info    
   6750 0.0006113 106 269 0.394  1383469_at  Aldehyde dehydrogenase family 1, subfamily A3  Info   Aldh1a3 
   6751 0.0006118 100.5 126.8 0.793  1382234_at  Hypothetical protein LOC311254  Info    
   6752 0.0006118 101.6 166.1 0.612  1384277_at  Transcribed locus, moderately similar to NP_076356.1 hypothetical protein LOC77547 [Mus musculus]  Info    
   6753 0.000612 481.9 717.2 0.672  1388852_at  similar to pM5 protein; DNA segment, Chr 7, ERATO Doi 156, expressed (predicted)  Info   RGD1305240_predicted 
   6754 0.0006123 394.3 526.2 0.749  1387771_a_at  mitogen activated protein kinase 3  Info   Mapk3 
   6755 0.0006123 142.1 81.5 1.744  1380028_at  Transcribed locus  Info    
   6756 0.0006124 30.8 73.3 0.42  1380089_at  Transcribed locus  Info    
   6757 0.0006127 408.4 540.1 0.756  1388833_at  similar to DNA polymerase epsilon p17 subunit (DNA polymerase epsilon subunit 3) (Chromatin accessibility complex 17) (HuCHRAC17) (CHRAC-17)  Info   RGD:1359475 
   6758 0.0006132 160.5 276.2 0.581  1369212_s_at  erythrocyte protein band 4.1-like 1  Info   Epb4.1l1 
   6759 0.0006137 37 17.3 2.139  1387834_at  megakaryocyte-associated tyrosine kinase  Info   Matk 
   6760 0.0006149 1056.1 744.4 1.419  1368843_at  YME1-like 1 (S. cerevisiae)  Info   Yme1l1 
   6761 0.0006162 211.4 339.1 0.623  1379483_at  Basic helix-loop-helix domain containing, class B2  Info   Bhlhb2 
   6762 0.0006166 198 290.2 0.682  1393719_at  Transcribed locus  Info    
   6763 0.0006181 91.6 147.3 0.622  1393309_at  Transcribed locus  Info    
   6764 0.0006184 48 27.5 1.745  1374270_at  Testis specific, 14  Info    
   6765 0.000619 120.7 75.7 1.594  1385644_at  ring finger protein 121 (predicted)  Info   Rnf121_predicted 
   6766 0.0006194 532.4 318.2 1.673  1370848_at  solute carrier family 2 (facilitated glucose transporter), member 1  Info   Slc2a1 
   6767 0.0006203 173.2 251.1 0.69  1373002_at  mitochondrial ribosomal protein S9 (predicted)  Info   Mrps9_predicted 
   6768 0.0006212 3.4 17.5 0.194  1369364_at  ATPase, Na+/K+ transporting, alpha 4 polypeptide  Info   Atp1a4 
   6769 0.0006212 310.3 647.1 0.48  1389129_at  similar to mKIAA0665 protein (predicted)  Info   RGD1308952_predicted 
   6770 0.0006215 89.6 46.7 1.919  1390364_at  Transcribed locus  Info    
   6771 0.0006219 57.4 125.9 0.456  1394939_at  similar to protein phosphatase type 1B (formely 2C), Mg-dependent, beta isoform (predicted)  Info   RGD1308501_predicted 
   6772 0.0006219 40.5 4.3 9.419  1380313_at  killer cell lectin-like receptor, family E, member 1  Info   Klre1 
   6773 0.0006219 1.5 10.4 0.144  1370534_at  activin receptor-like kinase 7  Info   Acvr1c 
   6774 0.0006241 913.4 645.9 1.414  1387917_at  lamina-associated polypeptide 1B  Info   Lap1b 
   6775 0.0006241 142.6 185 0.771  1388058_at  TAF6 RNA polymerase II, TATA box binding protein (TBP)-associated factor (predicted)  Info   Taf6_predicted 
   6776 0.0006244 214.4 105.2 2.038  1382629_at  similar to male sterility domain containing 2  Info   LOC499247 
   6777 0.0006246 20.7 46.6 0.444  1396057_at  Similar to actin monomer-binding protein (predicted)  Info    
   6778 0.000626 144 224.1 0.643  1389019_at  Golgi autoantigen, golgin subfamily b, macrogolgin 1  Info   LOC192243 
   6779 0.000626 218.1 312.8 0.697  1376882_at    Info    
   6780 0.000626 80.3 7.3 11  1376084_a_at  extra spindle poles like 1 (S. cerevisiae) (predicted)  Info   Espl1_predicted 
   6781 0.0006281 483.2 350.6 1.378  1389372_at  Transcribed locus  Info    
   6782 0.0006282 120.3 227 0.53  1373424_at  DiGeorge syndrome critical region gene 6 (predicted)  Info   Dgcr6_predicted 
   6783 0.0006287 22.3 36.2 0.616  1372296_at  similar to putative SH3BGR protein  Info   LOC498066 
   6784 0.0006288 154.7 122.4 1.264  1394136_at  PAP associated domain containing 1 (predicted)  Info   Papd1_predicted 
   6785 0.0006292 335.7 216.6 1.55  1375686_at  peptidylprolyl isomerase (cyclophilin)-like 3  Info   Ppil3 
   6786 0.0006298 95.9 78.3 1.225  1373394_at    Info    
   6787 0.0006306 87.7 44.9 1.953  1382400_at  rearranged L-myc fusion sequence (predicted)  Info   Rlf_predicted 
   6788 0.0006311 135 72.4 1.865  1391427_at  Similar to RIKEN cDNA 1500031M22 (predicted)  Info    
   6789 0.0006315 84.3 143 0.59  1390399_at  cAMP responsive element binding protein-like 2 (predicted)  Info   Crebl2_predicted 
   6790 0.0006316 37 5.8 6.379  1392040_at  similar to 2810453L12Rik protein (predicted)  Info   RGD1306255_predicted 
   6791 0.0006316 15.5 32.9 0.471  1379943_at  Transcribed locus  Info    
   6792 0.0006317 698.9 454.7 1.537  1367621_at  death-associated protein kinase 3  Info   Dapk3 
   6793 0.0006319 57.9 110 0.526  1377469_at  Transcribed locus  Info    
   6794 0.0006321 41.7 79.8 0.523  1385374_at  Thyrotroph embryonic factor  Info   Tef 
   6795 0.0006331 143.7 78.3 1.835  1382461_at  similar to arginine N-methyltransferase p82 isoform (predicted)  Info   RGD1304869_predicted 
   6796 0.0006331 979.2 1318.9 0.742  1376265_at    Info    
   6797 0.0006332 41.5 5.1 8.137  1377334_at  RT1 class II, locus Ba  Info   RT1-Ba 
   6798 0.0006335 180.8 138.7 1.304  1389423_at  Discoidin domain receptor family, member 2 (predicted)  Info   Ddr2 
   6799 0.0006349 95.6 47 2.034  1390502_at  similar to RIKEN cDNA 1700025G04 gene (predicted)  Info   RGD1309104_predicted 
   6800 0.000635 24.9 5.9 4.22  1377699_at  BTB and CNC homology 1 (predicted)  Info   Bach1_predicted 
   6801 0.0006364 2179.2 1738.2 1.254  1388774_at    Info    
   6802 0.0006368 118.6 90.3 1.313  1390197_at  Similar to hypothetical gene supported by AF226663  Info    
   6803 0.000637 215.9 337.8 0.639  1393098_at  similar to Lethal giant larvae homolog 2  Info   LOC360661 
   6804 0.0006374 137.9 50.4 2.736  1387432_at  ROD1 regulator of differentiation 1 (S. pombe)  Info   Rod1 
   6805 0.0006385 1554.9 1835.7 0.847  1367496_at  transmembrane 9 superfamily member 2  Info   RGD:1359230 
   6806 0.0006386 223.5 127.3 1.756  1368012_at  telomerase associated protein 1  Info   Tep1 
   6807 0.0006389 209.9 117.3 1.789  1389659_at  similar to ctla-2-beta protein (141 AA)  Info   LOC498690 
   6808 0.0006393 21.6 39.1 0.552  1382190_at  MAS-related GPR, member F  Info   Mrgprf 
   6809 0.0006398 102.2 73.4 1.392  1396449_at    Info    
   6810 0.0006407 1511.4 1126.4 1.342  1373862_at  Toll-like receptor adaptor molecule 2 (predicted)  Info   LOC252889 
   6811 0.0006415 102.3 175 0.585  1376049_at  Similar to mKIAA3013 protein  Info    
   6812 0.0006422 38.8 10.6 3.66  1383952_at  NEDD9 interacting protein with calponin homology and LIM domains (predicted)  Info   Nical_predicted 
   6813 0.0006426 25.8 49.4 0.522  1389208_at  similar to AI448196 protein  Info   LOC363495 
   6814 0.000643 119.1 183.7 0.648  1393192_at  Transcribed locus  Info    
   6815 0.0006435 140.7 100.6 1.399  1391921_at  RNA guanylyltransferase and 5'-phosphatase (predicted)  Info   Rngtt_predicted 
   6816 0.0006437 23.9 11.7 2.043  1391511_at  Transcribed locus  Info    
   6817 0.0006441 36 12.4 2.903  1397221_s_at  Tumor necrosis factor receptor superfamily, member 6  Info   RGD:619831 
   6818 0.0006449 8711.1 6071.5 1.435  1398835_at  actin, beta  Info   Actb 
   6819 0.0006461 3.3 34.1 0.097  1382132_at  Transcribed locus  Info    
   6820 0.0006467 196.9 139.5 1.411  1367863_at  BCL2/adenovirus E1B 19kDa-interacting protein 1  Info   Bnip1 
   6821 0.0006475 94.2 64.3 1.465  1394327_at  similar to chromosome 1 open reading frame 50  Info   LOC313551 
   6822 0.0006486 164.8 121.8 1.353  1376274_at  similar to RIKEN cDNA 1110056N09 (predicted)  Info   RGD1306301_predicted 
   6823 0.00065 2487.6 2010.8 1.237  1371428_at    Info    
   6824 0.0006502 54.4 11.3 4.814  1369849_at  cannabinoid receptor 2 (macrophage)  Info   Cnr2 
   6825 0.0006503 839.1 617.9 1.358  1383361_at    Info    
   6826 0.0006504 278.2 488.6 0.569  1372836_at  Transcribed locus  Info    
   6827 0.0006506 73 42.8 1.706  1383849_at  similar to hypothetical protein FLJ12994 (predicted)  Info   RGD1308795_predicted 
   6828 0.0006508 216.7 192.7 1.125  1399029_at  ubiquitin specific protease 48  Info   Usp48 
   6829 0.0006508 36.2 83.7 0.432  1373159_at  Transcribed locus  Info    
   6830 0.000651 63.9 106.3 0.601  1388818_at  Transcribed locus  Info    
   6831 0.0006511 1131.3 1587.3 0.713  1368181_at  methylenetetrahydrofolate dehydrogenase (NADP+ dependent), methenyltetrahydrofolate cyclohydrolase, formyltetrahydrofolate synthase  Info   Mthfd1 
   6832 0.000652 12.6 34.2 0.368  1382433_at  Transcribed locus  Info    
   6833 0.000652 373.3 232.5 1.606  1374976_a_at  Sterol O-acyltransferase 1  Info   Soat1 
   6834 0.0006524 291.7 437.4 0.667  1392492_at  Transcribed locus  Info    
   6835 0.0006527 87.7 49 1.79  1376419_at  Transcribed locus  Info    
   6836 0.0006531 688.7 503 1.369  1371660_at  zinc finger, HIT domain containing 1 (predicted)  Info   Znhit1_predicted 
   6837 0.0006536 1181.4 949.9 1.244  1371709_at  mitochondrial ribosomal protein L3 (predicted)  Info   Mrpl3_predicted 
   6838 0.0006546 18.7 53.8 0.348  1381956_at  Transcribed locus  Info    
   6839 0.000655 1284.8 1049.4 1.224  1372077_at  serine/threonine kinase receptor associated protein (predicted)  Info   Strap_predicted 
   6840 0.0006558 107.7 148.8 0.724  1376698_at  Transcribed locus  Info    
   6841 0.0006559 96.2 154.4 0.623  1373930_at  similar to Disco-interacting protein 2 homolog  Info   LOC309692 
   6842 0.000657 3677.9 3008.1 1.223  1398841_at  RAB1, member RAS oncogene family  Info   Rab1 
   6843 0.0006573 1565.1 1024.6 1.528  1398778_at  proteasome (prosome, macropain) subunit, alpha type 1  Info   Psma1 
   6844 0.0006576 38 3.3 11.515  1385451_at  sialyltransferase 8 (alpha-2, 8-sialytransferase) D  Info   Siat8d 
   6845 0.0006581 266.1 390.1 0.682  1393310_at  Itchy homolog E3 ubiquitin protein ligase  Info   Itch 
   6846 0.0006582 214.9 339.5 0.633  1373542_at  sphingosine kinase 2 (predicted)  Info   Sphk2_predicted 
   6847 0.0006585 83.6 11.8 7.085  1376668_at  similar to RIKEN cDNA 4922503N01 (predicted)  Info   RGD1311126_predicted 
   6848 0.0006586 105.5 182.8 0.577  1389005_at    Info    
   6849 0.0006588 1562.4 2020.6 0.773  1369942_at  actinin alpha 4  Info   Actn4 
   6850 0.0006602 291.9 190.2 1.535  1390409_at  LOC361774 (predicted)  Info   RGD1306116_predicted 
   6851 0.0006602 39.3 75.7 0.519  1394025_at  MAD homolog 6 (Drosophila) (predicted)  Info   Smad6_predicted 
   6852 0.0006607 19.1 49.1 0.389  1384284_at  Transcribed locus, strongly similar to NP_780301.1 ras homolog gene family, member f [Mus musculus]  Info    
   6853 0.0006616 102.6 55.1 1.862  1396185_at  RNA binding motif protein 17 (predicted)  Info   Rbm17_predicted 
   6854 0.0006624 267.5 169.8 1.575  1396866_s_at  peroxisomal membrane protein 3  Info   Pxmp3 
   6855 0.0006647 75.6 6.5 11.631  1388084_at  killer cell lectin-like receptor subfamily A, member 22  Info   Klra22 
   6856 0.0006649 140.5 87 1.615  1389065_at  similar to DNA segment, Chr 8, ERATO Doi 233, expressed (predicted)  Info   RGD1310161_predicted 
   6857 0.0006658 186.4 355.5 0.524  1382544_at  Coenzyme Q6 homolog (yeast) (predicted)  Info    
   6858 0.0006659 4179.1 3683.2 1.135  1375632_at  similar to 60S ribosomal protein L38  Info   LOC502741 
   6859 0.0006669 184 89.8 2.049  1376321_at  family with sequence similarity 38, member A (predicted)  Info   Fam38a_predicted 
   6860 0.000667 171.3 276.4 0.62  1382041_at  1-acylglycerol-3-phosphate O-acyltransferase 3 (predicted)  Info   Agpat3_predicted 
   6861 0.0006677 752.1 592.1 1.27  1388704_at  LOC499834  Info    
   6862 0.0006681 2158.4 1703.2 1.267  1368819_at  integrin beta 1  Info   Itgb1 
   6863 0.0006683 294.2 214.4 1.372  1379725_at  Transcribed locus  Info    
   6864 0.0006683 286.4 196.5 1.458  1390278_at  similar to RIKEN cDNA G430041M01  Info   LOC500116 
   6865 0.0006684 216 151.5 1.426  1390214_a_at  CDKN1A interacting zinc finger protein 1 (predicted)  Info   Ciz1_predicted 
   6866 0.0006685 139 86.5 1.607  1387412_at  phosphatidylinositol-4-phosphate 5-kinase, type II, alpha  Info   Pip5k2a 
   6867 0.0006685 61.9 119.3 0.519  1395809_at  sestrin 1 (predicted)  Info   Sesn1_predicted 
   6868 0.0006693 1130.4 1380.4 0.819  1371962_at  Tu translation elongation factor, mitochondrial (predicted)  Info   Tufm_predicted 
   6869 0.0006695 245.4 327.8 0.749  1376401_at  Transcribed locus  Info    
   6870 0.0006698 30 52.3 0.574  1396847_at  Transcribed locus  Info    
   6871 0.0006698 45.8 78.3 0.585  1384504_at  BAI1-associated protein 1 (predicted)  Info   Baiap1_predicted 
   6872 0.0006698 72.4 27.8 2.604  1387483_at  phospholipase C, gamma 2  Info   Plcg2 
   6873 0.0006701 334.4 259 1.291  1399071_at    Info    
   6874 0.0006705 23 166.9 0.138  1388097_at  calcium channel, voltage-dependent, gamma subunit 5  Info   Cacng5 
   6875 0.000671 68.3 30.2 2.262  1384897_at  Transcribed locus  Info    
   6876 0.0006719 172.5 130.2 1.325  1374502_at  similar to rab3 GTPase-activating protein, non-catalytic subunit (predicted)  Info   RGD1311518_predicted 
   6877 0.000673 69 26.8 2.575  1389500_at  3-alpha-hydroxysteroid dehydrogenase  Info   LOC191574 
   6878 0.0006731 385.8 258.8 1.491  1374695_at  Transcribed locus  Info    
   6879 0.0006734 64.4 46.7 1.379  1389674_at  zinc finger protein 282 (predicted)  Info   Zfp282_predicted 
   6880 0.0006744 525.4 407.1 1.291  1391412_at  5'-3' exoribonuclease 2 (predicted)  Info   Xrn2_predicted 
   6881 0.0006748 112.7 26.1 4.318  1380336_at  Transcribed locus  Info    
   6882 0.0006774 51 93.5 0.545  1382402_at  Unc-51 like kinase 1 (C. elegans)  Info   Ulk1 
   6883 0.0006791 218.3 298.2 0.732  1372152_at  similar to RIKEN cDNA 5430428G01 (predicted) /// similar to RIKEN cDNA 5430428G01  Info   RGD1312003_predicted /// LOC499699 
   6884 0.0006794 5429.4 4411.7 1.231  1370866_at  ribosomal protein L41  Info   Rpl41 
   6885 0.0006805 524.9 684.6 0.767  1373590_at  stomatin (predicted)  Info   Stom_predicted 
   6886 0.0006811 544.5 316.1 1.723  1375428_at  cellular repressor of E1A-stimulated genes (predicted)  Info   Creg_predicted 
   6887 0.0006812 47 88.6 0.53  1388177_at  dendrin  Info   Ddn 
   6888 0.0006831 259.5 430.7 0.603  1389648_at  Receptor-interacting serine-threonine kinase 4 (predicted)  Info    
   6889 0.0006838 36.5 56.3 0.648  1375914_at  similar to KIAA1862 protein (predicted)  Info   RGD1306967_predicted 
   6890 0.0006847 241.8 163.4 1.48  1373452_at  RNA terminal phosphate cyclase-like 1 (predicted)  Info   Rcl1_predicted 
   6891 0.000685 113.9 7.6 14.987  1380414_at  B-cell leukemia/lymphoma 3 (predicted)  Info   Bcl3_predicted 
   6892 0.000685 577.3 809.2 0.713  1371429_at  dystroglycan 1  Info   Dag1 
   6893 0.0006852 53.1 102.3 0.519  1398585_at  F-box only protein 21 (predicted)  Info   Fbxo21_predicted 
   6894 0.0006854 75.2 176.1 0.427  1369213_at  neural cell adhesion molecule L1  Info   RGD:619777 
   6895 0.0006854 300.9 191 1.575  1367540_at  Transcribed locus  Info    
   6896 0.000686 123.4 56.7 2.176  1373215_at  active BCR-related gene (predicted)  Info   Abr_predicted 
   6897 0.0006865 68.5 28.9 2.37  1387658_at  eukaryotic elongation factor-2 kinase  Info   Eef2k 
   6898 0.0006866 14.5 7.7 1.883  1395038_at  Similar to hypothetical protein BC011981 (predicted)  Info    
   6899 0.0006875 83.2 9.1 9.143  1387945_at  Similar to TRGV9 protein  Info    
   6900 0.0006879 7 42.8 0.164  1375001_at  Transcribed locus  Info    
   6901 0.000688 52.1 33.3 1.565  1382319_at  G protein-coupled receptor 68 (predicted)  Info    
   6902 0.0006886 178.7 100.5 1.778  1370114_a_at  phosphatidylinositol 3-kinase, regulatory subunit, polypeptide 1  Info   Pik3r1 
   6903 0.0006894 491 383.2 1.281  1399137_at  methionyl aminopeptidase 1 (predicted)  Info   Metap1_predicted 
   6904 0.0006895 25.2 82.7 0.305  1370691_a_at  thyroid hormone receptor alpha  Info   Thra 
   6905 0.0006904 80.8 50.3 1.606  1391177_at  Transcribed locus  Info    
   6906 0.0006909 53.2 22.1 2.407  1382566_at  Transcribed locus  Info    
   6907 0.0006942 136.1 107.3 1.268  1374151_at  Hepatoma-derived growth factor, related protein 3  Info   Hdgfrp3 
   6908 0.0006943 328.5 210 1.564  1373738_at  Transcribed locus  Info    
   6909 0.0006953 131.9 191 0.691  1377884_at  Transcribed locus  Info    
   6910 0.0006961 94.6 201.6 0.469  1374143_at  Transcribed locus  Info    
   6911 0.0006983 613.3 815.7 0.752  1388542_at  similar to RIKEN cDNA 2010315L10 (predicted) /// similar to 2010315L10Rik protein  Info   RGD1306660_predicted /// LOC292282 
   6912 0.000699 62.3 117.9 0.528  1385929_at  Zinc finger protein 11 (predicted)  Info    
   6913 0.0006997 151.9 87.1 1.744  1376701_a_at  similar to 5930416I19Rik protein  Info   RGD:1359404 
   6914 7e-04 34.3 4 8.575  1384174_at  Transcribed locus  Info    
   6915 0.0007004 50.8 26.7 1.903  1391044_s_at  similar to Lck-interacting transmembrane adaptor protein LIME  Info   LOC362289 
   6916 0.0007008 269.9 345.9 0.78  1372227_at  Muted (predicted)  Info    
   6917 0.0007009 95.5 70.1 1.362  1390928_at  similar to tigger transposable element derived 2  Info   LOC500150 
   6918 0.0007015 178.5 135.9 1.313  1394605_at  similar to CDNA sequence BC017647  Info   LOC497960 
   6919 0.0007021 131.5 94.9 1.386  1377819_at  Similar to mKIAA1111 protein  Info    
   6920 0.0007026 529 298.7 1.771  1389351_at  similar to FLI-LRR associated protein-1  Info   RGD:1359548 
   6921 0.0007062 26.6 63.4 0.42  1391444_at  Similar to teratocyte-specific carboxylesterase  Info    
   6922 0.0007065 49 20.7 2.367  1376866_at  Excision repair cross-complementing rodent repair deficiency, complementation group 1 (predicted)  Info    
   6923 0.0007067 46.5 21.1 2.204  1395086_at  similar to expressed sequence AV312086 (predicted)  Info   RGD1305514_predicted 
   6924 0.0007079 221 131.8 1.677  1373003_at  Squamous cell carcinoma antigen recognized by T-cells 3 (predicted)  Info    
   6925 0.000708 192.9 290.9 0.663  1385887_at  Transcribed locus  Info    
   6926 0.0007082 129.7 325.4 0.399  1387959_at  lysophospholipase  Info   RGD:708388 
   6927 0.0007083 438.5 252.2 1.739  1371546_at  similar to TR4 orphan receptor associated protein TRA16  Info   LOC361128 
   6928 0.0007092 89.8 150.7 0.596  1376125_at  Zinc finger and BTB domain containing 10  Info   Rinzf 
   6929 0.0007098 1353.6 1146.2 1.181  1367494_at  similar to CGI-35 protein (predicted)  Info   RGD1310899_predicted 
   6930 0.0007099 263.8 169.8 1.554  1383156_at  kinesin heavy chain member 2  Info   Kif2 
   6931 0.0007106 1275.7 884.2 1.443  1373195_at  fusion (involved in t(12;16) in malignant liposarcoma) (predicted)  Info   Fus_predicted 
   6932 0.0007108 163.3 113.2 1.443  1395555_at  golgi SNAP receptor complex member 1  Info   Gosr1 
   6933 0.0007109 1344.3 2484.1 0.541  1370352_at  carboxylesterase 1 /// similar to Liver carboxylesterase B-1 precursor (Liver microsomal carboxylesterase)  Info   RGD:621508 /// LOC501232 
   6934 0.0007117 270.1 373.7 0.723  1374597_at  similar to CDNA sequence BC034664  Info   LOC498733 
   6935 0.0007138 364.2 578 0.63  1371940_at  similar to microfilament and actin filament cross-linker protein isoform a  Info   LOC362587 
   6936 0.0007138 682.9 564.8 1.209  1387775_at  general transcription factor IIa 2  Info   Gtf2a2 
   6937 0.0007139 246 160.4 1.534  1383269_at  ring finger protein 2 (predicted)  Info   Rnf2_predicted 
   6938 0.0007149 19.6 45.2 0.434  1381391_at  hypothetical LOC287798  Info   RGD:1359207 
   6939 0.0007168 27.4 38.7 0.708  1383753_at    Info    
   6940 0.0007171 222.7 340.4 0.654  1388905_at  membralin (predicted)  Info   RGD1311136_predicted 
   6941 0.0007185 10 38.8 0.258  1396646_at  similar to RIKEN cDNA 4930431B11 gene (predicted)  Info   RGD1308555_predicted 
   6942 0.0007205 553.4 789.8 0.701  1390392_at  similar to RIKEN cDNA 2500001K11 (predicted)  Info   RGD1309602_predicted 
   6943 0.0007219 105.6 169.6 0.623  1371716_at  Transcribed locus, strongly similar to NP_937803.1 SWI/SNF-related matrix-associated actin-dependent regulator of chromatin c2 [Mus musculus]  Info    
   6944 0.000722 276.7 335.7 0.824  1367518_at  similar to fatso (predicted)  Info   RGD1305121_predicted 
   6945 0.0007231 171.8 138.1 1.244  1383232_at  Transcribed locus  Info    
   6946 0.0007242 324.8 409 0.794  1378098_at  Similar to CG4768-PA (predicted)  Info    
   6947 0.0007243 13.7 32.9 0.416  1387287_a_at  ATP-binding cassette, sub-family C (CFTR/MRP), member 9  Info   Abcc9 
   6948 0.0007251 2067.5 2499.2 0.827  1371894_at  Glucosamine (N-acetyl)-6-sulfatase (predicted)  Info    
   6949 0.0007255 28.5 14 2.036  1383539_at  Transcribed locus  Info    
   6950 0.000726 25.2 10.5 2.4  1391296_at  Similar to COX11 homolog, cytochrome c oxidase assembly protein  Info    
   6951 0.0007274 187.3 236.3 0.793  1399104_at  Transcribed locus  Info    
   6952 0.0007284 36.9 91.6 0.403  1380044_x_at    Info    
   6953 0.000729 198.9 377.3 0.527  1387057_at  solute carrier family 7 (cationic amino acid transporter, y+ system), member 8 /// synaptic Ras GTPase activating protein 1 homolog (rat)  Info   Slc7a8 /// Syngap1 
   6954 0.0007291 870.8 644.1 1.352  1389482_at  similar to EST AA792894 (predicted)  Info   RGD1306495_predicted 
   6955 0.0007298 165.3 102.6 1.611  1374945_at  similar to RIKEN cDNA 6720458F09 gene  Info   RGD:1359191 
   6956 0.0007299 27.3 52 0.525  1367992_at  secretory granule neuroendocrine protein 1  Info   Sgne1 
   6957 0.0007301 232.8 358.1 0.65  1376446_at  similar to RIKEN cDNA D430028G21  Info   RGD:1359371 
   6958 0.0007307 576.1 656.4 0.878  1372473_at  tight junction protein 1 (predicted)  Info   Tjp1_predicted 
   6959 0.0007316 39.1 76.3 0.512  1381296_at  Arginase 2  Info   Arg2 
   6960 0.0007334 165.6 280.8 0.59  1395721_at  3'-phosphoadenosine 5'-phosphosulfate synthase 2 (predicted)  Info   Papss2_predicted 
   6961 0.0007339 304.8 460.2 0.662  1394871_at  Solute carrier family 31 (copper transporters), member 1  Info   Slc31a1 
   6962 0.000734 511.3 630.7 0.811  1367761_at  nudE nuclear distribution gene E homolog like 1 (A. nidulans)  Info   Ndel1 
   6963 0.0007357 64.1 113 0.567  1382004_at  Transcribed locus  Info    
   6964 0.0007366 5.5 78.7 0.07  1376913_at    Info    
   6965 0.000737 1414.2 1738.7 0.813  1371639_at  vacuolar protein sorting 29 (S. pombe) (predicted)  Info   Vps29_predicted 
   6966 0.0007376 29.6 47.2 0.627  1383669_at  Transcribed locus  Info    
   6967 0.0007377 134.3 214 0.628  1374774_at  Transcribed locus  Info    
   6968 0.0007381 295.1 454.6 0.649  1376762_at  Similar to Pleckstrin homology domain-containing protein family A member 1 (Tandem PH domain containing protein-1) (TAPP-1)  Info    
   6969 0.0007385 73.2 102.7 0.713  1373084_at  WD repeat domain 20 (predicted)  Info   Wdr20_predicted 
   6970 0.0007386 83.2 153.9 0.541  1377266_at  Transcribed locus  Info    
   6971 0.0007391 71 97.1 0.731  1375531_at  similar to Leucine-rich and immunoglobulin-like domains 3  Info   LOC299830 
   6972 0.0007391 13.7 31.8 0.431  1392852_at    Info    
   6973 0.0007396 285.7 182.7 1.564  1390208_at  HIV-1 Tat interactive protein 2 (predicted)  Info   Htatip2_predicted 
   6974 0.00074 437.6 344.1 1.272  1367502_at  mitochondrial ribosomal protein L21 (predicted)  Info   Mrpl21_predicted 
   6975 0.0007403 28 4.5 6.222  1368800_at  tumor necrosis factor (ligand) superfamily, member 5  Info   Tnfsf5 
   6976 0.000741 844.5 1159 0.729  1371358_at  glycoprotein, synaptic 2  Info   Gpsn2 
   6977 0.0007411 316.7 244.7 1.294  1398471_at  component of oligomeric golgi complex 6  Info   RGD:1303283 
   6978 0.0007413 1464 1777.5 0.824  1398894_at  Unknown (protein for MGC:72959)  Info   Commd3 
   6979 0.0007421 24.9 76.3 0.326  1396614_at  secreted frizzled-related protein 2  Info   Sfrp2 
   6980 0.0007429 139.9 73.1 1.914  1378172_at  Transcribed locus  Info    
   6981 0.000743 150.7 225 0.67  1391476_at  Transcribed locus  Info    
   6982 0.000744 29.2 5.2 5.615  1387568_at  paired-Ig-like receptor B  Info   Pirb 
   6983 0.0007456 75.8 126.4 0.6  1367787_at  islet cell autoantigen 1  Info   Ica1 
   6984 0.0007472 18.8 30.6 0.614  1380640_at  Transcribed locus  Info    
   6985 0.0007474 115.1 78.1 1.474  1389237_at  similar to Zc3hc1 protein  Info   LOC296957 
   6986 0.0007479 306.2 467.4 0.655  1374144_at  Transcribed locus  Info    
   6987 0.0007484 1210.5 2050.8 0.59  1383161_a_at    Info    
   6988 0.0007489 25.2 4.6 5.478  1369867_at  sialyltransferase 8 A  Info   Siat8a 
   6989 0.0007492 51.1 35.3 1.448  1390442_at  Similar to mKIAA0133 protein (predicted)  Info    
   6990 0.0007493 918.7 2140.6 0.429  1368397_at  UDP-glucuronosyltransferase 2 family, member 5 /// UDP glycosyltransferase 2 family, polypeptide B4  Info   Ugt2b5 /// Ugt2b4 
   6991 0.0007494 79.7 49.1 1.623  1374590_at  phospholipase C-like 2 (predicted)  Info   Plcl2_predicted 
   6992 0.0007497 123.7 73.1 1.692  1369305_at  RAB3A interacting protein (rabin3)-like 1  Info   Rab3il1 
   6993 0.0007502 82.5 52.3 1.577  1389664_at  similar to hypothetical protein (predicted)  Info   RGD1305937_predicted 
   6994 0.0007503 45.7 6.1 7.492  1369191_at  interleukin 6  Info   Il6 
   6995 0.0007505 35.8 17.8 2.011  1382610_at  ATPase family, AAA domain containing 1 (predicted)  Info    
   6996 0.0007514 75.7 44.7 1.694  1379462_at  Transcribed locus, moderately similar to XP_580018.1 PREDICTED: hypothetical protein XP_580018 [Rattus norvegicus]  Info    
   6997 0.0007514 11.3 29 0.39  1378681_at  Transcribed locus  Info    
   6998 0.0007538 175.5 219.1 0.801  1389313_at  Transcribed locus, strongly similar to XP_203601.5 PREDICTED: phosphofurin acidic cluster sorting protein 1-like [Mus musculus]  Info    
   6999 0.000754 146.6 94.6 1.55  1377126_at  COMM domain containing 7 (predicted)  Info   Commd7_predicted 
   7000 0.0007547 443.7 393.8 1.127  1367487_at  similar to UDP-Gal:betaGlcNAc beta 1,4-galactosyltransferase, polypeptide 3  Info   RGD:1359221 
   7001 0.0007555 89.1 7.5 11.88  1378386_at  solute carrier family 2 (facilitated glucose transporter), member 6 (predicted)  Info   Slc2a6_predicted 
   7002 0.0007558 49.1 28 1.754  1390689_at  ribosomal protein S6 kinase, polypeptide 1 (predicted)  Info   Rps6kc1_predicted 
   7003 0.0007561 42.6 21.6 1.972  1386073_at  Similar to T-Brain-1  Info    
   7004 0.0007575 138.7 88.7 1.564  1382288_at  thyroid hormone receptor interactor 4 (predicted)  Info   Trip4_predicted 
   7005 0.0007586 97.8 56.2 1.74  1384107_at  Transcribed locus  Info    
   7006 0.0007588 35.5 67.1 0.529  1376768_at  Transcribed locus  Info    
   7007 0.0007603 34.4 77.6 0.443  1377407_at  similar to Aa2-174 (predicted)  Info   RGD1309578_predicted 
   7008 0.0007603 31.9 2.8 11.393  1391626_at  similar to RIKEN cDNA 2610034E18 gene (predicted)  Info   RGD1309007_predicted 
   7009 0.0007618 342.1 259.5 1.318  1399020_at  similar to family with sequence similarity 40, member A  Info   LOC362012 
   7010 0.0007626 712.5 840.6 0.848  1371634_at  similar to RIKEN cDNA 1810020E01 (predicted)  Info   RGD1305677_predicted 
   7011 0.000763 16.9 40.9 0.413  1393477_at  Transcribed locus  Info    
   7012 0.0007631 232.6 154.1 1.509  1379401_a_at  F-box only protein 33 (predicted)  Info   Fbxo33_predicted 
   7013 0.0007642 143.5 71.9 1.996  1397458_at  phosphomannomutase 2 (predicted)  Info   Pmm2_predicted 
   7014 0.0007655 465.5 1001.9 0.465  1368057_at  ATP-binding cassette, sub-family D (ALD), member 3  Info   Abcd3 
   7015 0.0007667 46.6 268.8 0.173  1374855_at  period homolog 1 (Drosophila)  Info   Per1 
   7016 0.0007684 173.8 264.2 0.658  1390132_at  Transcribed locus  Info    
   7017 0.0007691 57.8 26.4 2.189  1369010_at  protein kinase Chk2  Info   Chek2 
   7018 0.0007704 251.8 139.9 1.8  1395642_at  similar to RIKEN cDNA 4632412I24  Info   LOC313744 
   7019 0.0007708 337.9 241.1 1.401  1372377_at  DEAD (Asp-Glu-Ala-Asp) box polypeptide 41 (predicted)  Info   Ddx41_predicted 
   7020 0.0007724 11 19.6 0.561  1381612_at  Transcribed locus  Info    
   7021 0.0007729 230.7 355 0.65  1383129_at  CG6210-like  Info   RGD:735139 
   7022 0.0007729 104.7 45.4 2.306  1374198_at  B7 homlog 3  Info   B7h3 
   7023 0.0007738 47.5 84.4 0.563  1375206_at  hypothetical LOC287466 (predicted)  Info   RGD1308212_predicted 
   7024 0.0007751 208.9 293.6 0.712  1371919_at  similar to RP2 protein, testosterone-regulated - ricefield mouse (Mus caroli)  Info   RGD:1302935 
   7025 0.0007753 302.4 209.3 1.445  1398481_at  Transcribed locus  Info    
   7026 0.0007759 366.4 557.4 0.657  1389177_at  PERP, TP53 apoptosis effector (predicted)  Info   Perp_predicted 
   7027 0.0007794 74.9 126 0.594  1383607_at  Ac1158  Info   LOC302898 
   7028 0.0007797 401.1 298.2 1.345  1367478_at  protease, serine, 25 (predicted)  Info   Prss25_predicted 
   7029 0.0007797 30.3 52.5 0.577  1369839_at  amphiphysin 1  Info   Amph1 
   7030 0.0007806 59.2 31.2 1.897  1382581_at  ets homologous factor (predicted)  Info   Ehf_predicted 
   7031 0.0007809 5566.5 4338.5 1.283  1398871_at  ribosomal protein L17  Info   RGD:1303019 
   7032 0.0007834 199.4 293.7 0.679  1390199_at  Transcribed locus  Info    
   7033 0.0007836 148.3 98.9 1.499  1391414_at    Info    
   7034 0.0007842 79.7 36.2 2.202  1390232_at  similar to RIKEN cDNA 1810055G02 (predicted)  Info   RGD1311946_predicted 
   7035 0.0007845 641.7 432.1 1.485  1383068_at  deoxythymidylate kinase (predicted)  Info   Dtymk_predicted 
   7036 0.000785 267.4 558.8 0.479  1387121_a_at  N-myc downstream regulated gene 2  Info   Ndrg2 
   7037 0.0007854 16.4 28.9 0.567  1380067_at  myosin VC (predicted)  Info   Myo5c_predicted 
   7038 0.0007858 511.7 334.4 1.53  1372787_at  charged amino acid rich leucine zipper 1 (predicted)  Info   Crlz1_predicted 
   7039 0.0007861 4.1 15.3 0.268  1397841_at  Transcribed locus  Info    
   7040 0.0007873 217.1 125.1 1.735  1383396_at  fibronectin type III domain containing 3 (predicted)  Info   Fndc3_predicted 
   7041 0.0007876 106.2 74.6 1.424  1380824_at  Hook homolog 3  Info   Hook3 
   7042 0.000788 89.9 131 0.686  1376323_at    Info    
   7043 0.0007885 80.3 213.1 0.377  1385912_at  similar to interferon regulatory factor 10  Info   LOC502685 
   7044 0.0007895 384.9 299.1 1.287  1373241_at    Info    
   7045 0.0007904 75.3 136.9 0.55  1390246_at  Transcribed locus, moderately similar to NP_796093.1 hypothetical protein LOC320264 [Mus musculus]  Info    
   7046 0.0007906 436.5 697.2 0.626  1381190_at    Info    
   7047 0.000791 473.4 295.9 1.6  1371632_at  Similar to Coronin, actin binding protein 1C  Info    
   7048 0.0007916 171.7 109.2 1.572  1385090_at  RAD17 homolog (S. pombe) (predicted)  Info   Rad17_predicted 
   7049 0.000792 384 302.8 1.268  1372784_at  similar to Muf1-pending protein (predicted)  Info   RGD1311221_predicted 
   7050 0.0007927 207.3 119.2 1.739  1377263_at  cofactor required for Sp1 transcriptional activation, subunit 9 (predicted)  Info   Crsp9_predicted 
   7051 0.0007928 307.2 572.9 0.536  1398318_at  mucin 1, transmembrane  Info   Muc1 
   7052 0.0007929 390.3 310.9 1.255  1388474_at  Transcribed locus, strongly similar to NP_989596.1 ubiquitin-conjugating enzyme E2I (UBC9 homolog, yeast) [Gallus gallus]  Info    
   7053 0.0007933 94.5 167.4 0.565  1376776_at  similar to hypothetical protein FLJ33084  Info   LOC500528 
   7054 0.0007938 222.1 141.1 1.574  1377250_at  Transcribed locus  Info    
   7055 0.0007947 295 370.7 0.796  1368067_at  zinc finger protein 148  Info   Znf148 
   7056 0.0007953 199.8 311.7 0.641  1374027_at  Zinc finger protein 187  Info   Azf8 
   7057 0.000797 88.6 139.8 0.634  1387549_at  N-deacetylase/N-sulfotransferase (heparan glucosaminyl) 1  Info   Ndst1 
   7058 0.0007972 51.5 32.4 1.59  1368246_at  adaptor-related protein complex 3, mu 2 subunit  Info   Ap3m2 
   7059 0.0007982 217 184.7 1.175  1383700_at  similar to D15Wsu75e protein (predicted)  Info   RGD1305776_predicted 
   7060 0.0007989 1646.8 1425 1.156  1389940_at  coated vesicle membrane protein  Info   Rnp24 
   7061 0.0007995 287.8 150.8 1.908  1388625_at  similar to RIKEN cDNA 4921521J11 (predicted)  Info   RGD1304711_predicted 
   7062 0.0008009 1.8 10.5 0.171  1382204_at    Info    
   7063 0.0008015 75.2 158.9 0.473  1390123_at  similar to cDNA sequence BC018222 (predicted)  Info   RGD1305586_predicted 
   7064 0.0008016 244.4 183.5 1.332  1375042_at  Asparagine-linked glycosylation 8 homolog (yeast, alpha-1,3-glucosyltransferase) (predicted)  Info    
   7065 0.0008023 14.5 36.8 0.394  1384720_at    Info    
   7066 0.0008024 113.8 56.7 2.007  1375224_at  pleckstrin homology-like domain, family A, member 3 (predicted)  Info   Phlda3_predicted 
   7067 0.0008029 217.2 167.6 1.296  1390577_at  similar to KIAA1464 protein (predicted)  Info   RGD1311587_predicted 
   7068 0.0008046 353.1 654.5 0.539  1373566_at  DD6A4-12(7)-1 mRNA, partial sequence  Info    
   7069 0.0008052 161.2 317.8 0.507  1392569_at  Insulin-like growth factor I mRNA, 3' end of mRNA  Info    
   7070 0.0008053 47 75.9 0.619  1377090_at  dedicator of cytokinesis 6 (predicted)  Info   Dock6_predicted 
   7071 0.0008058 1.5 12.2 0.123  1374088_at  Myopalladin (predicted)  Info    
   7072 0.0008089 38.4 66.1 0.581  1375183_at  Transcribed locus  Info    
   7073 0.0008099 152.3 72.4 2.104  1371173_a_at  calpastatin  Info   Cast 
   7074 0.0008121 52.3 25.2 2.075  1375625_at  similar to putative membrane steroid receptor (predicted)  Info   RGD1311710_predicted 
   7075 0.000813 18.1 92 0.197  1372434_at  CASK-interacting protein CIP98  Info   Cip98 
   7076 0.0008131 136.4 176.7 0.772  1382240_at  Transcribed locus, strongly similar to NP_057371.1 HP1-BP74; likely ortholog of mouse heterochromatin protein 1, binding protein 3 [Homo sapiens]  Info    
   7077 0.0008132 64.7 112.5 0.575  1377032_at  Rho GTPase activating protein 15  Info   Arhgap15 
   7078 0.0008135 61.1 32.9 1.857  1368258_at  apelin, AGTRL1 ligand  Info   Apln 
   7079 0.0008137 25.8 49.5 0.521  1397615_at  Transcribed locus  Info    
   7080 0.0008145 274.7 418.1 0.657  1372443_at  low density lipoprotein receptor-related protein 11 (predicted)  Info   Lrp11_predicted 
   7081 0.0008147 104 156.9 0.663  1389536_at  Transcribed locus  Info    
   7082 0.0008153 71.5 44.7 1.6  1394268_at    Info    
   7083 0.0008159 21 73.1 0.287  1375057_at  similar to Hypothetical protein 6720430O15  Info   LOC311210 
   7084 0.0008162 57.8 8.4 6.881  1397859_x_at    Info    
   7085 0.0008167 117.3 71.2 1.647  1383896_at  CDNA clone MGC:105591 IMAGE:7313723  Info    
   7086 0.0008172 90.9 138.8 0.655  1382388_at  Similar to hypothetical protein MGC27699 (predicted)  Info    
   7087 0.0008173 414.2 679.1 0.61  1375894_at  leucine zipper transcription factor-like 1 (predicted)  Info   Lztfl1_predicted 
   7088 0.000818 133.2 54.7 2.435  1372653_at  FK506 binding protein 11 (predicted)  Info   Fkbp11_predicted 
   7089 0.0008182 101.5 64.7 1.569  1376553_at  thymine glycol DNA glycosylase/AP lyase (predicted)  Info   Nthl1_predicted 
   7090 0.000819 940.6 1286.7 0.731  1398928_at  divalent cation tolerant protein CUTA  Info   RGD:1303306 
   7091 0.000819 268.3 197.2 1.361  1373946_at  similar to RIKEN cDNA 2810037C14 (predicted)  Info   RGD1305915_predicted 
   7092 0.0008192 24.8 41.9 0.592  1384484_at  Transcribed locus  Info    
   7093 0.0008198 76.1 41.8 1.821  1387427_at  RAD50 homolog (S. cerevisiae)  Info   Rad50 
   7094 0.0008219 52 75.3 0.691  1393308_at  Similar to BMI1-like protein  Info    
   7095 0.0008221 144.1 109.4 1.317  1369265_at  SUMO/sentrin specific protease 2  Info   Senp2 
   7096 0.0008226 22.2 59 0.376  1383848_at  Adrenergic receptor, beta 1  Info   Adrb1 
   7097 0.0008238 12.8 62.5 0.205  1378560_at  period homolog 2 (Drosophila)  Info   Per2 
   7098 0.0008246 20.1 46.5 0.432  1372858_at    Info    
   7099 0.0008247 192.7 282.7 0.682  1398601_at  spire homolog 1 (Drosophila) (predicted)  Info   Spire1_predicted 
   7100 0.000825 145.8 256.3 0.569  1388038_at  attractin  Info   Atrn 
   7101 0.0008255 10.1 20 0.505  1380770_at  Transcribed locus  Info    
   7102 0.0008257 114.9 61.8 1.859  1376037_at  achalasia, adrenocortical insufficiency, alacrimia (Allgrove, triple-A) (predicted)  Info   Aaas_predicted 
   7103 0.0008261 72.8 43 1.693  1389990_at    Info    
   7104 0.0008263 18.1 44 0.411  1379761_at  Transcribed locus  Info    
   7105 0.0008265 192.8 135.4 1.424  1392986_at  Striatin, calmodulin binding protein  Info   Strn 
   7106 0.0008267 317.4 491.7 0.646  1371043_a_at  POU domain, class 3, transcription factor 3  Info   Pou3f3 
   7107 0.0008268 30.7 65.7 0.467  1392729_at  Transcribed locus  Info    
   7108 0.0008269 15.8 31.4 0.503  1371109_at  complement component 8, beta polypeptide  Info   C8b 
   7109 0.000827 50.3 31.7 1.587  1394209_at  Transcribed locus  Info    
   7110 0.0008272 29.4 17.1 1.719  1377260_at  Transcribed locus  Info    
   7111 0.0008274 1756.5 3780.2 0.465  1386944_a_at  glucose-6-phosphatase, catalytic  Info   G6pc 
   7112 0.0008277 45.7 14.4 3.174  1390469_at  nurim (nuclear envelope membrane protein)  Info   RGD:1302966 
   7113 0.0008278 701.9 552.2 1.271  1375705_at  Guanine nucleotide binding protein, beta 1  Info   Gnb1 
   7114 0.0008282 81.4 57.1 1.426  1393207_at  Similar to 2210403N09Rik protein (predicted)  Info    
   7115 0.0008286 111.4 142.4 0.782  1387948_at  intestinal cell kinase  Info   Ick 
   7116 0.0008287 318.6 255.7 1.246  1389021_at  similar to HCV NS3-transactivated protein 1 (predicted)  Info   RGD1306332_predicted 
   7117 0.0008301 117.1 203.4 0.576  1371545_at  Platelet/endothelial cell adhesion molecule  Info   Pecam 
   7118 0.0008313 141.8 191 0.742  1368643_at  spermatogenesis associated 6  Info   Spata6 
   7119 0.0008314 271.4 398.9 0.68  1373448_at  acylphosphatase 1, erythrocyte (common) type (predicted)  Info   Acyp1_predicted 
   7120 0.0008316 351.9 262.5 1.341  1388585_at  Transcribed locus, weakly similar to XP_580159.1 PREDICTED: hypothetical protein XP_580159 [Rattus norvegicus]  Info    
   7121 0.0008319 189.3 16.6 11.404  1379311_at  AT rich interactive domain 5A (Mrf1 like) (predicted)  Info    
   7122 0.0008325 31.2 136.2 0.229  1368271_a_at  fatty acid binding protein 4, adipocyte  Info   Fabp4 
   7123 0.0008333 48.9 82.7 0.591  1374896_at  Transcribed locus  Info    
   7124 0.0008339 469.2 367.8 1.276  1373454_at  similar to RIKEN cDNA 0610037P05 (predicted)  Info   RGD1305823_predicted 
   7125 0.0008339 259.9 130.3 1.995  1369955_at  Collagen, type V, alpha 1  Info   Col5a1 
   7126 0.0008343 36.9 15.4 2.396  1376258_at  Serine (or cysteine) proteinase inhibitor, clade B, member 1a (predicted)  Info    
   7127 0.0008356 728.9 557 1.309  1371368_at  SEC61, alpha subunit (S. cerevisiae)  Info   Sec61a1 
   7128 0.0008358 2624.1 3245.4 0.809  1371415_at  ubiquinol-cytochrome c reductase hinge protein (predicted)  Info   Uqcrh_predicted 
   7129 0.0008364 129.1 245.5 0.526  1384116_at  Transcribed locus  Info    
   7130 0.0008365 30.4 3.1 9.806  1374352_at  Actin alpha cardiac 1  Info   Actc1 
   7131 0.0008372 10.1 54 0.187  1392803_at    Info    
   7132 0.0008377 507.3 268.4 1.89  1373780_at  tetraspan 1  Info   RGD:1303308 
   7133 0.0008381 337.1 230.9 1.46  1388720_at  Hypothetical LOC361225  Info    
   7134 0.0008392 70.3 46.9 1.499  1385601_at  FSHD region gene 1 (predicted)  Info   Frg1_predicted 
   7135 0.0008407 448 604.5 0.741  1389338_at  hypothetical LOC293114 (predicted)  Info   RGD1308371_predicted 
   7136 0.0008418 202.7 168.7 1.202  1373664_at  phosphatidylinositol glycan, class C (predicted)  Info   Pigc_predicted 
   7137 0.0008419 251.1 191.7 1.31  1390722_at  Unknown sequence  Info    
   7138 0.0008419 2143.7 1304 1.644  1367661_at  S100 calcium binding protein A6 (calcyclin)  Info   S100a6 
   7139 0.0008432 25.2 62.7 0.402  1393454_at  Protocadherin 17 (predicted)  Info    
   7140 0.0008438 5.9 1.2 4.917  1397864_at  Transcribed locus  Info    
   7141 0.0008438 48.3 87.7 0.551  1372521_at  ras homolog gene family, member N (predicted)  Info   Rohn_predicted 
   7142 0.0008449 638.2 446.1 1.431  1371873_at  Transcribed locus  Info    
   7143 0.0008453 1580.8 1209.6 1.307  1398960_at    Info    
   7144 0.0008456 2408.7 1716.2 1.404  1371422_at  MORF-related gene X  Info   RGD:1359471 
   7145 0.0008462 206.1 342.2 0.602  1399041_at  similar to OPA3 protein  Info   LOC502305 
   7146 0.0008466 33.9 16.8 2.018  1377554_at  tumor necrosis factor (ligand) superfamily, member 9  Info   Tnfsf9 
   7147 0.0008474 11.8 89.3 0.132  1398592_at    Info    
   7148 0.0008482 195.3 138.2 1.413  1383377_at  GA repeat binding protein, alpha (predicted)  Info   Gabpa 
   7149 0.0008507 706.5 812.9 0.869  1399019_at  abhydrolase domain containing 4 (predicted)  Info   Abhd4_predicted 
   7150 0.0008519 868.4 475.8 1.825  1370314_at  solute carrier family 20 (phosphate transporter), member 1  Info   Slc20a1 
   7151 0.000852 1193.5 862.4 1.384  1387120_at  proteasome (prosome, macropain) 26S subunit, ATPase 3  Info   Psmc3 
   7152 0.0008523 6742.3 4907 1.374  1367573_at  ribosomal protein S6  Info   Rps6 
   7153 0.0008526 83.8 43.3 1.935  1368941_at  prostaglandin E receptor 4 (subtype EP4)  Info   Ptger4 
   7154 0.000853 722.8 463.6 1.559  1371830_at  ubiquitin-like 1 (sentrin) activating enzyme E1A (predicted)  Info   Uble1a_predicted 
   7155 0.0008537 92.3 61.7 1.496  1380753_at  similar to expressed sequence AV312086 (predicted)  Info   RGD1305514_predicted 
   7156 0.0008545 52.2 5.6 9.321  1387548_at  hyaluronan synthase 2  Info   Has2 
   7157 0.0008559 310.2 209.1 1.484  1388900_at  hypothetical LOC361797  Info   LOC361797 
   7158 0.0008561 71.3 44 1.62  1393140_at    Info    
   7159 0.0008563 100 27.6 3.623  1394039_at  Kruppel-like factor 5  Info   Klf5 
   7160 0.0008567 58.8 26.2 2.244  1377982_at  deltex 4 homolog (Drosophila) (predicted)  Info   Dtx4_predicted 
   7161 0.0008567 10.2 22 0.464  1382954_at  Transcribed locus  Info    
   7162 0.0008567 349.8 240.2 1.456  1379662_a_at  SNF related kinase  Info   Snrk 
   7163 0.0008572 70.1 135.3 0.518  1392072_at  Transcribed locus  Info    
   7164 0.0008587 2.8 13 0.215  1386399_at  Transcribed locus  Info    
   7165 0.0008592 181.8 222.3 0.818  1391443_at  similar to chromosome 14 open reading frame 35  Info   LOC498489 
   7166 0.0008595 164 91.7 1.788  1393653_at  similar to putative protein product of HMFN2073  Info   LOC501174 
   7167 0.0008607 231 146.7 1.575  1399006_at  upstream transcription factor 1  Info   Usf1 
   7168 0.000861 699.4 540.3 1.294  1372126_at  Transcribed locus  Info    
   7169 0.0008613 91.2 45.9 1.987  1393822_at  Transcribed locus  Info    
   7170 0.0008616 528.6 353.7 1.494  1395813_at  similar to EST AA792894 (predicted)  Info   RGD1306495_predicted 
   7171 0.0008628 121.8 234.1 0.52  1369974_at  vesicle-associated membrane protein 2  Info   Vamp2 
   7172 0.0008641 115.3 79.9 1.443  1383961_a_at  DnaJ (Hsp40) homolog, subfamily C, member 11 (predicted)  Info   Dnajc11_predicted 
   7173 0.0008649 142.4 228.2 0.624  1370181_at  RAB4A, member RAS oncogene family  Info   Rab4a 
   7174 0.0008651 345.3 170.1 2.03  1370894_at  claudin 7  Info   Cldn7 
   7175 0.0008652 105.1 174.6 0.602  1399155_at  Similar to RIKEN cDNA 5830434P21  Info    
   7176 0.0008654 125.2 78.1 1.603  1398384_at  exosome component 9 (predicted)  Info   Exosc9_predicted 
   7177 0.0008664 65.8 43.6 1.509  1374856_at  similar to RIKEN cDNA 4930556P03 (predicted)  Info   RGD1306433_predicted 
   7178 0.0008665 205.6 134.9 1.524  1385676_at  CD2 antigen (cytoplasmic tail) binding protein 2 (predicted)  Info   Cd2bp2_predicted 
   7179 0.000867 1356.3 1870.4 0.725  1371482_at  NADH dehydrogenase (ubiquinone) Fe-S protein 2 (predicted)  Info   Ndufs2_predicted 
   7180 0.0008677 115.1 223.2 0.516  1398401_at  tudor domain containing 3 (predicted)  Info   Tdrd3_predicted 
   7181 0.0008681 701.9 428 1.64  1371698_at  similar to 116 kDa U5 small nuclear ribonucleoprotein component (U5 snRNP-specific protein, 116 kDa) (U5-116 kDa)  Info   LOC287739 
   7182 0.0008682 1156.3 1475.5 0.784  1387863_at  upstream of NRAS  Info   Unr 
   7183 0.0008682 191.3 317.1 0.603  1388662_at  Transcribed locus  Info    
   7184 0.0008682 4.6 13.9 0.331  1396628_at  Similar to neuronal interacting factor X 1 (NIX1)  Info    
   7185 0.0008686 156.9 113.4 1.384  1376560_at  Transcribed locus  Info    
   7186 0.0008704 118.3 196.3 0.603  1395460_at  cask-interacting protein 2 (predicted)  Info   Caskin2_predicted 
   7187 0.0008712 135.2 86.8 1.558  1370665_at  hypoxia up-regulated 1  Info   Hyou1 
   7188 0.0008724 200.6 146 1.374  1383227_at  Transcribed locus  Info    
   7189 0.0008746 97 162.1 0.598  1374334_at  Immunoglobulin heavy chain (alpha polypeptide)  Info   Igha 
   7190 0.0008751 52.2 85.8 0.608  1372572_at  sialyltransferase 7F  Info   RGD:1303196 
   7191 0.0008753 1632.7 1310.7 1.246  1371585_at  G1 to S phase transition 1  Info   Gspt1 
   7192 0.0008781 218.6 288.9 0.757  1390684_at  Transcribed locus  Info    
   7193 0.0008792 15.2 301.2 0.05  1387214_at  carboxylesterase 1  Info   RGD:621508 
   7194 0.0008799 164.9 268.3 0.615  1374835_at  Kelch-like 12 (Drosophila)  Info   Klhl12 
   7195 0.0008802 40.3 18 2.239  1385695_at  lysyl oxidase-like 3 (predicted)  Info   Loxl3_predicted 
   7196 0.000881 190.6 598.4 0.319  1383832_at  similar to MGC52019 protein (predicted)  Info   RGD1311652_predicted 
   7197 0.0008812 59.3 20.9 2.837  1396993_at  Transcribed locus  Info    
   7198 0.0008838 24.1 75.1 0.321  1374782_at  Transcribed locus  Info    
   7199 0.0008841 160.9 110.1 1.461  1387020_at  cytochrome P450, subfamily 51  Info   Cyp51 
   7200 0.0008848 274.5 373.8 0.734  1376953_at  Transcribed locus  Info    
   7201 0.0008865 316.6 410.4 0.771  1390128_at    Info    
   7202 0.0008867 36.9 61.1 0.604  1376747_at  Membrane associated guanylate kinase 1 b NT-short isoform  Info    
   7203 0.0008887 1239.5 2435.6 0.509  1387808_at  solute carrier family 7 (cationic amino acid transporter, y+ system), member 7  Info   Slc7a7 
   7204 0.0008891 150.8 223.2 0.676  1396053_at  neural precursor cell expressed, developmentally down-regulated gene 9 (predicted)  Info   Nedd9_predicted 
   7205 0.0008905 482.8 306.7 1.574  1377691_at  SEC22 vesicle trafficking protein-like 1 (S. cerevisiae) (predicted)  Info   Sec22l1_predicted 
   7206 0.000891 54.6 28.9 1.889  1389360_at  FXYD domain-containing ion transport regulator 3  Info   Fxyd3 
   7207 0.0008916 229.6 299.1 0.768  1372491_at  similar to Ski protein  Info   LOC313757 
   7208 0.0008926 73.1 133.5 0.548  1391885_at  Spermatogenesis associated 9 (predicted)  Info    
   7209 0.0008935 13.5 83.5 0.162  1386705_at    Info    
   7210 0.0008942 74.8 38.4 1.948  1374216_at  Transcribed locus  Info    
   7211 0.000898 14.6 34.3 0.426  1371121_at  Sertoli cell protein 1  Info   RGD:620279 
   7212 0.0008991 84.7 126.4 0.67  1372348_at  Histone cell cycle regulation defective interacting protein 5 (predicted)  Info    
   7213 0.0008993 3048.3 2633.4 1.158  1371386_at  similar to Protein C9orf10 (predicted)  Info   RGD1306643_predicted 
   7214 0.0009003 17.8 49.6 0.359  1376124_at  Wiskott-Aldrich syndrome-like (human)  Info   Wasl 
   7215 0.0009018 593.7 476.9 1.245  1367772_at  chloride channel, nucleotide-sensitive, 1A  Info   Clns1a 
   7216 0.0009021 369 265.8 1.388  1376662_at  Protein phosphatase 1, regulatory (inhibitor) subunit 12A  Info   Ppp1r12a 
   7217 0.0009029 252.2 836.2 0.302  1367851_at  prostaglandin D2 synthase  Info   Ptgds 
   7218 0.000903 185 27.3 6.777  1387316_at  chemokine (C-X-C motif) ligand 1  Info   Cxcl1 
   7219 0.0009042 97.9 57.2 1.712  1393972_at  Transcribed locus  Info    
   7220 0.000905 81.4 150.5 0.541  1385556_at    Info    
   7221 0.0009059 206.4 135.8 1.52  1377262_at    Info    
   7222 0.0009075 32.3 16.5 1.958  1384099_at  solute carrier family 39 (metal ion transporter), member 6 (predicted)  Info   Slc39a6_predicted 
   7223 0.0009079 7476.1 5921.8 1.262  1371297_at  ribosomal protein L7a (predicted)  Info   Rpl7a_predicted 
   7224 0.0009093 851.5 1150.2 0.74  1371444_at    Info    
   7225 0.0009098 388.9 505.4 0.769  1373061_at  sorting nexin 17 (predicted)  Info   Snx17_predicted 
   7226 0.0009103 6.8 47.5 0.143  1382239_at  similar to Myosin light chain kinase 2, skeletal/cardiac muscle (MLCK2) (predicted)  Info   RGD1305801_predicted 
   7227 0.0009121 1381.2 836.3 1.652  1399143_at  ubiquitin-conjugating enzyme E2N  Info   Ube2n 
   7228 0.0009124 375.3 612.6 0.613  1376194_at  Transcribed locus  Info    
   7229 0.0009128 109.6 180.7 0.607  1390311_at  Transcribed locus  Info    
   7230 0.0009135 7.4 14.8 0.5  1393761_at  Transcribed locus, moderately similar to XP_221920.3 PREDICTED: similar to RB-associated KRAB repressor [Rattus norvegicus]  Info    
   7231 0.0009141 81.7 42.7 1.913  1395118_at  similar to RNA (guanine-9-) methyltransferase domain containing 2  Info   LOC295496 
   7232 0.0009146 76.1 122.2 0.623  1372470_at  armadillo repeat gene deleted in velo-cardio-facial syndrome (predicted)  Info   Arvcf_predicted 
   7233 0.0009158 386.3 531.6 0.727  1370684_s_at  hypothetical protein LOC501546  Info   LOC501546 
   7234 0.0009163 35.6 22.5 1.582  1397641_at  Transcribed locus  Info    
   7235 0.0009164 46.4 75.5 0.615  1380637_at  similar to MEGF11 protein  Info   LOC363079 
   7236 0.0009167 90.2 48.9 1.845  1385344_at  AE binding protein 2 (predicted)  Info    
   7237 0.000918 60.5 34.6 1.749  1391139_at  Transcribed locus  Info    
   7238 0.000919 1665.1 2169.5 0.768  1368102_at  hydroxysteroid 11-beta dehydrogenase 2  Info   Hsd11b2 
   7239 0.0009191 35.7 72.4 0.493  1370465_at  ATP-binding cassette, sub-family B (MDR/TAP), member 1A  Info   Abcb1a 
   7240 0.0009194 219.4 287.7 0.763  1374109_at  diacylglycerol kinase, theta (predicted)  Info   Dgkq_predicted 
   7241 0.0009205 305.3 222.2 1.374  1374700_at  Sprouty protein with EVH-1 domain 1, related sequence (predicted)  Info    
   7242 0.0009207 147.1 102.8 1.431  1389595_at  similar to hypothetical protein FLJ20399 (predicted)  Info   RGD1305612_predicted 
   7243 0.0009208 115.8 188.2 0.615  1375915_at  interleukin-1 receptor-associated kinase 1 binding protein 1 (predicted)  Info   Irak1bp1_predicted 
   7244 0.000921 120.1 207.6 0.579  1391280_at    Info    
   7245 0.0009223 91.7 142.3 0.644  1390457_at  Transcribed locus  Info    
   7246 0.0009227 195.9 249.2 0.786  1368886_at  mitogen activated protein kinase kinase kinase 12  Info   Map3k12 
   7247 0.0009234 5906.3 4377.4 1.349  1367560_at  acidic ribosomal phosphoprotein P0  Info   Arbp 
   7248 0.0009236 521.3 826.9 0.63  1386538_at  similar to KIAA1919 protein (predicted)  Info   RGD1310495_predicted 
   7249 0.0009238 361.4 255.9 1.412  1370351_at  tudor domain containing 7  Info   Tdrd7 
   7250 0.0009258 60 15.3 3.922  1398706_at    Info    
   7251 0.0009267 296.2 417.8 0.709  1374585_at  enoyl Coenzyme A hydratase domain containing 1  Info   RGD:1359654 
   7252 0.000927 168.5 102.5 1.644  1368967_at  eukaryotic translation initiation factor 2B, subunit 3 gamma  Info   Eif2b3 
   7253 0.0009273 58.7 40.2 1.46  1375943_at  Similar to Hypothetical protein KIAA0419 (predicted)  Info    
   7254 0.0009276 73.2 114.5 0.639  1380193_at  Transcribed locus  Info    
   7255 0.0009284 300.9 510 0.59  1370509_at  pyruvate dehydrogenase phosphatase isoenzyme 2  Info   Pdp2 
   7256 0.0009292 57.1 86.6 0.659  1373789_at  Similar to zinc finger protein 651  Info    
   7257 0.0009297 2.2 10.7 0.206  1388289_s_at  follicle - stimulating hormone subunit beta  Info   Fshb 
   7258 0.0009301 60.3 103.1 0.585  1374566_at  Nischarin (predicted)  Info   Nisch 
   7259 0.0009306 5.3 27.6 0.192  1396249_at  Natriuretic peptide receptor 3  Info   Npr3 
   7260 0.0009312 54.7 14.2 3.852  1392791_at  Transcribed locus  Info    
   7261 0.0009324 596 442 1.348  1386930_at  proteasome (prosome, macropain) 26S subunit, non-ATPase, 4  Info   Psmd4 
   7262 0.0009329 144.3 239.6 0.602  1395928_at  Nuclear receptor subfamily 1, group D, member 1  Info   Nr1d1 
   7263 0.0009342 117.3 70.6 1.661  1376451_at  similar to krev interaction trapped-1A  Info   LOC362317 
   7264 0.0009343 498.9 365.6 1.365  1372723_at  Importin 9 (predicted)  Info    
   7265 0.0009344 330.4 434.6 0.76  1389647_at  Sorting nexin 13 (predicted)  Info    
   7266 0.000935 352.9 598.5 0.59  1383437_at    Info    
   7267 0.0009351 80.2 57 1.407  1379299_at  Cockayne syndrome 1 (classical) (predicted)  Info   Ckn1_predicted 
   7268 0.0009359 65.3 111 0.588  1398440_at  Transcribed locus  Info    
   7269 0.0009379 865 1105 0.783  1369982_at  adaptor protein complex AP-2, alpha 2 subunit  Info   Ap2a2 
   7270 0.0009382 1025 1230.8 0.833  1398779_at  actin related protein 2/3 complex, subunit 1A  Info   Arpc1a 
   7271 0.0009391 29.2 48 0.608  1380437_at  Transcribed locus  Info    
   7272 0.0009394 117.5 163.5 0.719  1372954_at  Transcribed locus  Info    
   7273 0.0009402 286.1 424.1 0.675  1372722_at  Transcribed locus  Info    
   7274 0.0009409 182.7 159.9 1.143  1393214_at  Similar to hypothetical protein A230042K10  Info    
   7275 0.0009409 57.8 10.3 5.612  1368250_at  tektin 1  Info   Tekt1 
   7276 0.000941 20 8.2 2.439  1380066_at  transferrin receptor 2 (predicted)  Info   Trfr2_predicted 
   7277 0.0009417 198.2 135.5 1.463  1378378_at  pantothenate kinase 2 (Hallervorden-Spatz syndrome) (predicted)  Info   Pank2_predicted 
   7278 0.0009421 201.9 295.7 0.683  1388471_at  similar to RIKEN cDNA E430026E19 (predicted)  Info   RGD1307494_predicted 
   7279 0.0009421 804.9 1013.6 0.794  1367911_at  isocitrate dehydrogenase 3 (NAD+) alpha  Info   Idh3a 
   7280 0.0009421 606.7 485.3 1.25  1377735_at  pleckstrin homology domain-containing, family A (phosphoinositide binding specific) member 3 (predicted)  Info   Plekha3_predicted 
   7281 0.0009429 459.5 353.5 1.3  1388386_at    Info    
   7282 0.0009433 128.4 213.6 0.601  1395551_at  Similar to novel protein  Info    
   7283 0.0009436 54 261.4 0.207  1387363_at  folate hydrolase  Info   Folh1 
   7284 0.0009452 251.3 385.9 0.651  1374448_at  reversion-inducing-cysteine-rich protein with kazal motifs (predicted)  Info   Reck_predicted 
   7285 0.0009459 233.4 165.8 1.408  1374051_at  similar to hypothetical protein D15Ertd785e  Info   MGC114417 
   7286 0.0009459 14.8 27 0.548  1368879_a_at  guanine nucleotide binding protein, alpha o  Info   Gnao 
   7287 0.000946 23.5 45 0.522  1387983_at  thyroid hormone receptor beta  Info   Thrb 
   7288 0.0009469 48.7 78 0.624  1383988_at    Info    
   7289 0.0009493 94.4 60.8 1.553  1374003_at  LOC498007  Info   LOC498007 
   7290 0.0009493 21.4 67.2 0.318  1383566_at  T-complex-associated testis expressed 1 (predicted)  Info    
   7291 0.000951 93.8 66.9 1.402  1389681_at  Poliovirus receptor-related 2 (herpesvirus entry mediator B) (predicted)  Info    
   7292 0.0009517 36.2 4.5 8.044  1376799_a_at  cytokine receptor-like factor 1 (predicted)  Info   Crlf1_predicted 
   7293 0.0009533 125.6 160.5 0.783  1371906_at  Transcribed locus  Info    
   7294 0.0009549 25.2 8.7 2.897  1378537_at  Transcribed locus  Info    
   7295 0.0009551 61.7 111.8 0.552  1390097_at  TSPY-like 4 (predicted)  Info   Tspyl4_predicted 
   7296 0.0009554 43.1 96.6 0.446  1384138_at  Potassium channel subunit (Slack)  Info   Slack 
   7297 0.000956 102.6 64.1 1.601  1370040_at  8-oxoguanine DNA-glycosylase 1  Info   Ogg1 
   7298 0.0009566 579.5 461.6 1.255  1398808_at  Inositol (myo)-1(or 4)-monophosphatase 1  Info   Impa1 
   7299 0.0009568 40.1 80.4 0.499  1371020_at  RIM binding protein 2  Info   Rimbp2 
   7300 0.0009569 51.2 32.4 1.58  1393998_at  GA repeat binding protein, beta 1 (predicted) /// similar to GA binding protein beta chain (GABP-beta-2 subunit) (GABP-2) (GABP-beta-1 subunit) (GABPB-1) (Transcription factor E4TF1-53) (Transcription factor E4TF1-47) (Nuclear respiratory factor-2)  Info   Gabpb1_predicted /// LOC499883 
   7301 0.0009577 10.5 2.2 4.773  1371227_at  colony stimulating factor 2 (granulocyte-macrophage)  Info   Csf2 
   7302 0.0009579 17.4 2.1 8.286  1384920_at  Transcribed locus  Info    
   7303 0.0009582 16.1 2.4 6.708  1392582_at  similar to NAG14 protein (predicted)  Info   RGD1311013_predicted 
   7304 0.0009583 715.7 907.2 0.789  1367506_at  mitochondrial ribosomal protein L11  Info   RGD:1359665 
   7305 0.0009588 69 31.4 2.197  1384252_at  similar to hypothetical protein BC015148 (predicted)  Info   RGD1309095_predicted 
   7306 0.0009589 366 606.2 0.604  1371682_at  microtubule-associated protein 1 light chain 3 alpha  Info   Map1lc3a 
   7307 0.0009607 49 20.1 2.438  1382409_at  testis specific, 14  Info   Tsga14 
   7308 0.000962 178.5 98.3 1.816  1379423_at  Transcribed locus, strongly similar to NP_033131.2 Ras suppressor protein 1 [Mus musculus]  Info    
   7309 0.0009623 15.2 32.3 0.471  1397885_at  Disabled homolog 2 (Drosophila) interacting protein  Info   Dab2ip 
   7310 0.0009623 14.1 26 0.542  1396106_at  Similar to dystrobrevin alpha isoform 1  Info    
   7311 0.0009627 65.7 35.4 1.856  1374621_at  similar to TAFI95  Info   LOC361420 
   7312 0.0009639 38 7.1 5.352  1395828_at  kinesin family member 23 (predicted)  Info   Kif23_predicted 
   7313 0.0009662 14.6 35 0.417  1380883_at  Similar to chromosome 13 open reading frame 3 (predicted)  Info    
   7314 0.000968 23.5 56.5 0.416  1394778_at  Nuclear factor I/B  Info   Nfib 
   7315 0.0009686 187.2 227.4 0.823  1369732_a_at  sialyltransferase 4B (beta-galactoside alpha-2,3-sialyltransferase)  Info   Siat4b 
   7316 0.0009698 57.9 87.8 0.659  1390341_at  Transcribed locus  Info    
   7317 0.00097 280.6 198.7 1.412  1373207_at  Transcribed locus  Info    
   7318 0.0009702 501.8 651.7 0.77  1370025_at  phosphatidylinositol-4-phosphate 5-kinase, type II, gamma  Info   Pip5k2c 
   7319 0.0009704 250.6 371.3 0.675  1383813_at  ADP-ribosylation factor-like 2 binding protein  Info   Arl2bp 
   7320 0.0009705 434.9 317.1 1.371  1389312_at  similar to RIKEN cDNA 0610016J10 gene (predicted)  Info   RGD1309929_predicted 
   7321 0.0009714 35.1 19.6 1.791  1369614_at  RAP2B, member of RAS oncogene family  Info   Rap2b 
   7322 0.0009731 373 495.7 0.752  1371717_at    Info    
   7323 0.0009734 612.2 811.3 0.755  1374522_at  Transcribed locus, strongly similar to XP_135706.3 PREDICTED: similar to mKIAA0268 protein [Mus musculus]  Info    
   7324 0.0009737 70.7 5.6 12.625  1375040_at  nuclear factor, erythroid derived 2 (predicted)  Info   Nfe2_predicted 
   7325 0.0009746 563.3 450.4 1.251  1386910_a_at  apurinic/apyrimidinic endonuclease 1  Info   Apex1 
   7326 0.0009755 35.7 78.3 0.456  1370211_at  neurogranin  Info   Nrgn 
   7327 0.0009756 94.3 134.2 0.703  1378248_at  Similar to amyloid beta (A4) precursor protein-binding, family B, member 2  Info    
   7328 0.000976 63.7 121.5 0.524  1371059_at  protein kinase, cAMP-dependent, regulatory, type 2, alpha  Info   Prkar2a 
   7329 0.0009766 198.1 114.5 1.73  1384797_at  similar to RIKEN cDNA 5730596K20 (predicted)  Info   RGD1309871_predicted 
   7330 0.0009786 36.8 62.7 0.587  1391906_at  kinesin family member 1B  Info   Kif1b 
   7331 0.0009786 879.6 694.3 1.267  1377769_at  adaptor protein complex AP-1, sigma 1 (predicted)  Info   Ap1s1_predicted 
   7332 0.0009789 359.3 249.3 1.441  1370974_at  Vps54-like  Info   Vps54 
   7333 0.0009798 339.2 461.1 0.736  1374349_at  CTD (carboxy-terminal domain, RNA polymerase II, polypeptide A) small phosphatase-like (predicted)  Info    
   7334 0.0009803 183 120.1 1.524  1380730_at  CDNA clone IMAGE:7375616  Info    
   7335 0.0009814 1580 1026.2 1.54  1372439_at  procollagen, type IV, alpha 1 (predicted)  Info   Col4a1_predicted 
   7336 0.0009826 545.2 377.2 1.445  1389289_at  Ewing sarcoma breakpoint region 1 (predicted)  Info   Ewsr1_predicted 
   7337 0.000983 75.8 129.2 0.587  1372171_at    Info    
   7338 0.0009842 51.6 27.3 1.89  1384059_at  PC4 and SFRS1 interacting protein 1  Info   Psip1 
   7339 0.0009849 114.7 66.4 1.727  1392920_at  Elongation factor RNA polymerase II-like 3 (predicted)  Info    
   7340 0.0009851 14.6 2.3 6.348  1395480_at  Transcribed locus  Info    
   7341 0.0009871 4.3 23.1 0.186  1368380_at  vitronectin  Info   Vtn 
   7342 0.0009875 1028.8 677.5 1.519  1387101_at  acyl-CoA synthetase long-chain family member 4  Info   Acsl4 
   7343 0.0009894 445.2 607.5 0.733  1388194_at  dihydrolipoamide S-acetyltransferase (E2 component of pyruvate dehydrogenase complex)  Info   Dlat 
   7344 0.0009898 33.2 7.7 4.312  1375379_at  Transporter 1, ATP-binding cassette, sub-family B (MDR/TAP)  Info   Tap1 
   7345 0.0009906 229.2 439.4 0.522  1377351_at  sushi domain containing 3 (predicted)  Info   Susd3_predicted 
   7346 0.0009909 52.3 93.7 0.558  1382057_at  similar to oocyte-testis gene 1  Info   LOC499378 
   7347 0.0009921 73.4 36.2 2.028  1394490_at  Transcribed locus  Info    
   7348 0.0009929 19.8 38.5 0.514  1392340_at  similar to Kelch-like protein 3  Info   LOC498697 
   7349 0.0009939 11.6 2.7 4.296  1397077_at  Similar to RIKEN cDNA 2810409H07 (predicted)  Info    
   7350 0.0009946 192.9 248.5 0.776  1373157_at  ubiquitin specific protease 47 (predicted)  Info   Usp47_predicted 
   7351 0.0009978 70.1 155.7 0.45  1373915_at  dystrophia myotonica kinase, B15 (predicted)  Info   Dm15_predicted 
   7352 0.000998 53.1 24.7 2.15  1379584_at  C-type (calcium dependent, carbohydrate-recognition domain) lectin, superfamily member 2 (activation-induced) (predicted)  Info    
   7353 0.0009981 126.8 20 6.34  1383589_at  Transcribed locus  Info    
   7354 0.0009999 106.9 210.2 0.509  1376267_at  Solute carrier family 16 (monocarboxylic acid transporters), member 6  Info   Slc16a6 
   7355 0.0010001 195.3 139.1 1.404  1376680_at    Info    
   7356 0.0010009 289.8 370.1 0.783  1373502_at  Similar to RIKEN cDNA 4933427L07 (predicted)  Info    
   7357 0.0010013 347.9 569.5 0.611  1386478_at  mitochondrial carrier triple repeat 1 (predicted)  Info   Mcart1_predicted 
   7358 0.0010016 305 462.7 0.659  1390026_at  Bcl2-associated athanogene 3 (predicted)  Info   Bag3_predicted 
   7359 0.0010017 3.4 13.8 0.246  1369058_at  synaptotagmin 3  Info   Syt3 
   7360 0.0010045 200.3 141.4 1.417  1375906_at  Transcribed locus, strongly similar to XP_356361.2 PREDICTED: similar to CXorf44 protein [Mus musculus]  Info    
   7361 0.0010046 43.1 13.5 3.193  1385551_at  RT1 class Ib, locus H2-Q-like, grc region /// similar to class I histocompatibility antigen alpha chain - cotton-top tamarin  Info   RT1-O /// LOC499402 
   7362 0.001005 25.4 53.3 0.477  1374123_at  Transcribed locus  Info    
   7363 0.0010052 327.3 545.2 0.6  1370530_a_at  phospholipase D1  Info   Pld1 
   7364 0.0010053 20.2 60.9 0.332  1387704_at  estrogen receptor 1  Info   Esr1 
   7365 0.0010055 341.1 417.9 0.816  1376067_at  CCR4-NOT transcription complex, subunit 2 (predicted)  Info    
   7366 0.0010056 670.4 537.1 1.248  1387002_at  maternal G10 transcript  Info   G10 
   7367 0.0010059 542.6 789.5 0.687  1388454_at  Similar to RNA binding protein with multiple splicing 2  Info    
   7368 0.001006 459 667.9 0.687  1392301_at  SH3 domain and tetratricopeptide repeats 1 (predicted)  Info   Sh3tc1_predicted 
   7369 0.0010067 169.3 83.7 2.023  1368005_at  inositol 1, 4, 5-triphosphate receptor 3  Info   Itpr3 
   7370 0.0010074 225.4 131.1 1.719  1370261_at  ribosomal protein S6 kinase polypeptide 1  Info   Rps6ka1 
   7371 0.0010078 46.7 15.8 2.956  1377410_at  similar to hypothetical protein FLJ23311 (predicted)  Info   RGD1308091_predicted 
   7372 0.0010082 84.8 131.8 0.643  1374117_at  brain-specific angiogenesis inhibitor 1-associated protein 2  Info   Baiap2 
   7373 0.0010095 84.5 145 0.583  1377874_at  T-box 2 (predicted)  Info   Tbx2 
   7374 0.0010104 209.3 335.5 0.624  1389215_at  selenophosphate synthetase 1 (predicted)  Info   Sephs1_predicted 
   7375 0.0010112 74.8 133.4 0.561  1380600_at    Info    
   7376 0.0010121 131.4 100.8 1.304  1376625_at    Info    
   7377 0.0010123 127.7 65 1.965  1368867_at  GERp95  Info   Eif2c2 
   7378 0.0010133 21 42.3 0.496  1384309_at  Transcribed locus  Info    
   7379 0.0010168 93.4 153.2 0.61  1368133_at  multiple PDZ domain protein  Info   Mpdz 
   7380 0.0010178 420.1 566.4 0.742  1398968_at  Similar to RIKEN cDNA 3930401K13 (predicted)  Info   3930401k13rik 
   7381 0.0010192 1.8 6.2 0.29  1389160_at  erythroid associated factor (predicted)  Info   Eraf_predicted 
   7382 0.0010196 537 713.6 0.753  1372317_at  EH-domain containing 1 (predicted)  Info   Ehd1_predicted 
   7383 0.0010196 511.7 413.4 1.238  1371741_at  ARP1 actin-related protein 1 homolog A (yeast) (predicted)  Info   Actr1a_predicted 
   7384 0.0010209 281.7 207.7 1.356  1388769_at  similar to ring finger and WD repeat domain 1 isoform 1  Info   LOC360491 
   7385 0.001021 385.5 1114.8 0.346  1398463_at  Transcribed locus  Info    
   7386 0.0010212 52.7 99.3 0.531  1398710_at  cytochrome P450, family 2, subfamily u, polypeptide 1 (predicted)  Info   Cyp2u1_predicted 
   7387 0.0010214 52.8 16.7 3.162  1380835_at  similar to hypothetical protein FLJ12671  Info   RGD:1359413 
   7388 0.0010218 18.3 43 0.426  1382281_at  Similar to DAZ associated protein 2  Info    
   7389 0.0010232 136.5 216 0.632  1379519_at  Transcribed locus, moderately similar to NP_001001306.1 hypothetical protein LOC329695 [Mus musculus]  Info    
   7390 0.0010234 81.5 143.1 0.57  1397383_at  similar to empty spiracles homolog 1  Info   LOC500235 
   7391 0.001024 56.2 103.7 0.542  1389307_at  similar to Amyloid beta (A4) precursor-like protein 1  Info   LOC502317 
   7392 0.0010267 3.1 30.5 0.102  1369064_a_at  sodium channel, voltage-gated, type 8, alpha polypeptide  Info   Scn8a 
   7393 0.0010268 60.4 8 7.55  1387648_at  chemokine (C-X-C motif) ligand 5  Info   Cxcl5 
   7394 0.0010271 92 180.3 0.51  1376518_at    Info    
   7395 0.0010281 3600.6 4181.6 0.861  1388199_at  tumor-associated calcium signal transducer 1  Info   Tacstd1 
   7396 0.0010281 2 8.6 0.233  1382785_at  kinesin family member 1A (predicted)  Info   Kif1a_predicted 
   7397 0.0010283 185.7 257.8 0.72  1372450_at    Info    
   7398 0.0010285 212.9 125.8 1.692  1370887_at  transforming growth factor beta 1 induced transcript 1  Info   Tgfb1i1 
   7399 0.0010292 141.7 182.6 0.776  1374860_at    Info    
   7400 0.0010296 16.5 3.9 4.231  1384556_at  ATPase, class V, type 10A (predicted)  Info   Atp10a_predicted 
   7401 0.0010307 15.2 31 0.49  1378055_at  Transcribed locus  Info    
   7402 0.0010308 74.9 53.1 1.411  1383877_a_at  Transcribed locus  Info    
   7403 0.0010309 23.5 48 0.49  1384888_at  suppressor of cytokine signaling 7 (predicted)  Info   Socs7_predicted 
   7404 0.0010312 271 351.4 0.771  1383208_at  similar to DNA polymerase epsilon p17 subunit (DNA polymerase epsilon subunit 3) (Chromatin accessibility complex 17) (HuCHRAC17) (CHRAC-17)  Info   RGD:1359475 
   7405 0.0010331 340 239.3 1.421  1371904_at  SET and MYND domain containing 2  Info   Smyd2 
   7406 0.0010334 57.1 88.2 0.647  1381049_at  zinc finger, FYVE domain containing 9 (predicted)  Info   Zfyve9_predicted 
   7407 0.0010341 17.3 2.7 6.407  1368722_at  lymphotoxin A  Info   Lta 
   7408 0.001037 52.6 92.9 0.566  1383060_at  G kinase anchoring protein 1 (predicted)  Info   Gkap1_predicted 
   7409 0.0010372 131.4 18.5 7.103  1383630_at  docking protein 3 (predicted)  Info   Dok3_predicted 
   7410 0.0010375 717.2 556.8 1.288  1367492_at  DnaJ (Hsp40) homolog, subfamily C, member 8 (predicted)  Info   Dnajc8_predicted 
   7411 0.001038 45.9 68.6 0.669  1372226_at  Transcribed locus, strongly similar to NP_055665.1 SLIT-ROBO Rho GTPase-activating protein 2; SLIT-ROBO Rho GTPase-activating protein 3; WAVE-associated Rac GTPase activating protein [Homo sapiens]  Info    
   7412 0.001038 33.7 83.6 0.403  1385322_at    Info    
   7413 0.0010397 535.7 741.8 0.722  1373087_at  Transcribed locus  Info    
   7414 0.0010398 43 9.7 4.433  1370086_at  fibrinogen, gamma polypeptide  Info   Fgg 
   7415 0.0010399 298.5 417.3 0.715  1388713_at  thiamine triphosphatase  Info   Thtpa 
   7416 0.00104 47.9 18.6 2.575  1394869_at  Transcribed locus  Info    
   7417 0.0010421 608 810.2 0.75  1367520_at  apolipoprotein A-I binding protein (predicted)  Info   Apoa1bp_predicted 
   7418 0.0010433 1294.5 1049.6 1.233  1387035_a_at  nadrin  Info   Arhgap17 
   7419 0.0010435 88.7 56.9 1.559  1376950_at  similar to RIKEN cDNA 2610034H20 (predicted)  Info   RGD1309793_predicted 
   7420 0.0010441 1156.3 809.3 1.429  1388715_at  glycyl-tRNA synthetase (predicted)  Info   Gars_predicted 
   7421 0.0010442 150.9 241.7 0.624  1383884_at  Transcribed locus  Info    
   7422 0.0010457 343.9 230.6 1.491  1375852_at  3-hydroxy-3-methylglutaryl-Coenzyme A reductase  Info   Hmgcr 
   7423 0.0010462 3086.1 2516.8 1.226  1388759_at  Chloride intracellular channel 4  Info   Clic4 
   7424 0.0010466 628.5 809.6 0.776  1387840_at  acid phosphatase 1, soluble  Info   Acp1 
   7425 0.0010501 23.3 75.4 0.309  1375305_at  hypothetical LOC362256  Info   LOC362256 
   7426 0.0010513 153.6 192.8 0.797  1373918_at  retinol dehydrogenase 11 (predicted)  Info   Rdh11_predicted 
   7427 0.0010522 78.6 151.8 0.518  1368119_at  phosphatidylinositol (4,5) bisphosphate 5-phosphatase, A  Info   Pib5pa 
   7428 0.0010524 40.2 66.1 0.608  1378547_at  Transcribed locus  Info    
   7429 0.0010539 103.8 144.8 0.717  1373948_at  similar to CGTHBA protein (-14 gene protein) (predicted)  Info   RGD1308665_predicted 
   7430 0.0010551 368.1 262.3 1.403  1388729_at  Harvey rat sarcoma oncogene, subgroup R (predicted)  Info   Rras_predicted 
   7431 0.0010552 284.2 549.2 0.517  1388422_at  LIM and senescent cell antigen like domains 2 (predicted)  Info   Lims2_predicted 
   7432 0.0010553 5239.7 4125.5 1.27  1367561_at  ribosomal protein L27  Info   Rpl27 
   7433 0.0010556 16.3 48.5 0.336  1377632_at    Info    
   7434 0.0010557 141.2 260 0.543  1380526_at  glutaryl-Coenzyme A dehydrogenase (predicted)  Info   Gcdh_predicted 
   7435 0.0010559 79.3 127.7 0.621  1399064_at  Resistance to inhibitors of cholinesterase 8B (C. elegans)  Info   Ric8b 
   7436 0.001056 296 426.5 0.694  1392763_at  1-acylglycerol-3-phosphate O-acyltransferase 1  Info   RGD:1303287 
   7437 0.0010574 102.3 58.6 1.746  1388966_at  Purinergic receptor P2Y, G-protein coupled 2  Info   P2ry2 
   7438 0.0010575 14.1 4.2 3.357  1391363_at    Info    
   7439 0.0010596 281.5 437.2 0.644  1372160_at  bladder cancer associated protein homolog (human)  Info   Blcap 
   7440 0.0010596 47.9 16.1 2.975  1376381_at  XK-related protein 5  Info   RGD:1359335 
   7441 0.0010608 6.6 46 0.143  1374148_at  LOC498009  Info   LOC498009 
   7442 0.001061 51.7 88.1 0.587  1385624_at  similar to suppressor of Ty 3 homolog  Info   LOC501104 
   7443 0.0010615 55.9 28.1 1.989  1382417_at  transformed mouse 3T3 cell double minute 4 (predicted)  Info   Mdm4_predicted 
   7444 0.0010623 53.8 32.4 1.66  1379637_at  Transcribed locus  Info    
   7445 0.0010628 78.5 123.6 0.635  1393612_a_at  similar to Hypothetical protein MGC19163 (predicted)  Info   RGD1309720_predicted 
   7446 0.0010633 74.9 51 1.469  1390982_at  chromatin assembly factor 1, subunit B (p60) (predicted)  Info   Chaf1b_predicted 
   7447 0.0010645 123.9 91.5 1.354  1383301_at  hypothetical protein LOC498154  Info   LOC498154 
   7448 0.0010664 247.1 110.4 2.238  1370017_at  emerin  Info   Emd 
   7449 0.0010684 48.3 2.9 16.655  1387880_at  CUG triplet repeat,RNA-binding protein 2  Info   Cugbp2 
   7450 0.0010703 89.7 57.1 1.571  1392615_at  Similar to mKIAA1724 protein  Info    
   7451 0.0010708 1415.3 2058.1 0.688  1389393_at  similar to RIKEN cDNA 2210412D01 (predicted)  Info   RGD1308210_predicted 
   7452 0.001071 75.2 38.8 1.938  1395654_at  Similar to RIKEN cDNA 2610528H13 (predicted)  Info    
   7453 0.0010715 237.8 343.4 0.692  1367915_at  diacylglycerol O-acyltransferase 1  Info   Dgat1 
   7454 0.0010738 10.1 2.2 4.591  1396066_at  similar to Laminin alpha-4 chain precursor  Info   LOC309816 
   7455 0.0010741 159.3 311.6 0.511  1388469_at  Insulin-like growth factor I mRNA, 3' end of mRNA  Info    
   7456 0.001076 2.8 43.9 0.064  1397855_at  Similar to cysteine sulfinic acid decarboxylase  Info    
   7457 0.0010778 318.7 431.6 0.738  1379294_at  T-cell receptor rearranged alpha-chain V-region mRNA (V-alpha-3-V-J-C) partial cds, hybridoma KL  Info    
   7458 0.001078 17.5 43.7 0.4  1393507_at  Transcribed locus  Info    
   7459 0.0010783 1080 1566.1 0.69  1368399_a_at  plasma glutamate carboxypeptidase  Info   Pgcp 
   7460 0.0010786 290.2 383.8 0.756  1388817_at  hypothetical protein LOC310665  Info   LOC310665 
   7461 0.0010792 61.3 106.2 0.577  1394459_at  Similar to Protein CXorf17 homolog  Info    
   7462 0.0010793 163.8 11.9 13.765  1396205_at  Transcribed locus, weakly similar to XP_282988.2 PREDICTED: similar to novel protein [Mus musculus]  Info    
   7463 0.0010808 454.7 303.5 1.498  1388354_at  similar to U5 snRNP-specific protein, 200 kDa  Info   LOC296126 
   7464 0.0010814 1330 1029.6 1.292  1398796_at  transmembrane trafficking protein 21  Info   Tmp21 
   7465 0.0010828 191.2 259.3 0.737  1389336_at  processing of precursor 5, ribonuclease P/MRP family (S. cerevisiae) (predicted)  Info   Pop5_predicted 
   7466 0.0010836 208.9 140.8 1.484  1375209_at  Oxysterol binding protein-like 11 (predicted)  Info    
   7467 0.0010846 193.1 252.9 0.764  1374485_at  Transcribed locus  Info    
   7468 0.0010864 417.4 642.5 0.65  1383920_at  similar to aminomethyltransferase  Info   RGD:1359408 
   7469 0.0010879 60.2 33.9 1.776  1394409_at  melanoma associated antigen (mutated) 1 (predicted)  Info   Mum1_predicted 
   7470 0.0010887 71.6 53 1.351  1398683_at  similar to replication protein-binding trans-activator RBT1  Info   MGC108974 
   7471 0.0010895 551.6 797.8 0.691  1398317_at  3'(2'),5'-bisphosphate nucleotidase  Info   Bpnt1 
   7472 0.0010903 8.9 90.7 0.098  1374167_at  similar to autoantigen  Info   LOC361399 
   7473 0.0010925 3065.8 2565.7 1.195  1371384_at  basic transcription factor 3 (predicted)  Info   Btf3_predicted 
   7474 0.001095 13 3.3 3.939  1374192_at  Transcribed locus  Info    
   7475 0.0010958 3.4 1.2 2.833  1392451_at  similar to hypothetical protein FLJ37118  Info   LOC362659 
   7476 0.0010958 33.7 60.4 0.558  1381803_at    Info    
   7477 0.0010967 100.7 71 1.418  1395704_at  Transcribed locus  Info    
   7478 0.0010994 41.3 72 0.574  1392841_at  Transcribed locus, strongly similar to XP_515868.1 PREDICTED: sodium channel, voltage-gated, type III, alpha [Pan troglodytes]  Info    
   7479 0.0010996 111.6 188.5 0.592  1389321_at  Transcribed locus  Info    
   7480 0.0011008 152.4 99.5 1.532  1385761_s_at  Transcribed locus  Info    
   7481 0.0011009 534.8 355.4 1.505  1381972_at  cereblon (predicted)  Info   Crbn_predicted 
   7482 0.001101 295.7 497.3 0.595  1379904_at    Info    
   7483 0.0011024 19.3 39.4 0.49  1382800_at  Transcribed locus  Info    
   7484 0.0011027 402.8 302.6 1.331  1374456_at  Transcribed locus, strongly similar to XP_619138.1 PREDICTED: similar to Ras GTPase-activating-like protein IQGAP2 [Mus musculus]  Info    
   7485 0.001104 129.3 73.2 1.766  1370916_at  tec protein tyrosine kinase  Info   RGD:620973 
   7486 0.0011062 1796.3 1365.8 1.315  1388777_at  signal sequence receptor, gamma  Info   Ssr3 
   7487 0.0011076 1227.2 995.9 1.232  1375338_at  RAB10, member RAS oncogene family  Info   Rab10 
   7488 0.0011089 24.9 49 0.508  1394195_at  Transcribed locus  Info    
   7489 0.0011101 181.3 265.1 0.684  1388698_at  extracellular matrix protein 1  Info   Ecm1 
   7490 0.0011116 5027.8 5992.9 0.839  1370277_at  solute carrier family 25 (mitochondrial carrier; adenine nucleotide translocator), member 3  Info   Slc25a3 
   7491 0.0011117 60.7 89.2 0.68  1378243_at  transmembrane protein 17 (predicted)  Info   Tmem17_predicted 
   7492 0.0011123 93.3 185.8 0.502  1379782_a_at    Info    
   7493 0.0011126 78.6 117.5 0.669  1391183_at  Transcribed locus  Info    
   7494 0.0011131 114.2 82.4 1.386  1371055_at  RAB12, member RAS oncogene family  Info   Rab12 
   7495 0.0011143 117.6 189.3 0.621  1393707_at  Bcl2-like 2  Info   Bcl2l2 
   7496 0.0011145 459.9 332.3 1.384  1379810_at  small inducible cytokine subfamily E, member 1  Info   Scye1 
   7497 0.0011158 118.2 72.1 1.639  1392991_at  Transcribed locus, strongly similar to NP_079855.1 TAF12 RNA polymerase II, TATA box binding protein (TBP)-associated factor [Mus musculus]  Info    
   7498 0.0011163 119.3 92.4 1.291  1376132_at  glycosyltransferase-like protein  Info   LOC362129 
   7499 0.0011174 162.5 266 0.611  1374350_at  Transcribed locus  Info    
   7500 0.0011184 394.8 269.9 1.463  1368134_a_at  interleukin 4 receptor  Info   Il4r 
   7501 0.001121 98.6 129.9 0.759  1383604_at  Phosphatidylinositol glycan, class N (predicted)  Info    
   7502 0.001121 360.2 287.9 1.251  1373845_at  Phosphatidylethanlomine binding protein 2  Info   Pbp2 
   7503 0.0011221 26.9 53.9 0.499  1397768_at  Transcribed locus  Info    
   7504 0.0011229 35.2 68.9 0.511  1381997_at  adipocyte complement related protein of 30 kDa  Info   Acdc 
   7505 0.0011229 205.9 129.9 1.585  1399088_at  tousled-like kinase 2 (Arabidopsis) (predicted)  Info   Tlk2_predicted 
   7506 0.0011236 563.5 414.6 1.359  1373387_at    Info    
   7507 0.0011236 17.7 36.6 0.484  1398662_at  Transcribed locus  Info    
   7508 0.0011239 63.5 45.9 1.383  1384878_at  N-myristoyltransferase 2  Info   Nmt2 
   7509 0.0011241 6.9 21.9 0.315  1373333_at  similar to Microsomal signal peptidase 23 kDa subunit (SPase 22 kDa subunit) (SPC22/23)  Info   MGC109340 
   7510 0.0011245 245.2 339.3 0.723  1379784_at  Peroxisome biogenesis factor 7 (predicted)  Info    
   7511 0.0011254 374.9 271.6 1.38  1368065_at  regulator of G-protein signaling 19 interacting protein 1  Info   Rgs19ip1 
   7512 0.0011255 97.6 62.8 1.554  1384965_at  serine/threonine kinase 2  Info   Slk 
   7513 0.0011272 135 241.6 0.559  1374172_at  Procollagen, type VIII, alpha 2 (predicted)  Info    
   7514 0.0011274 194.1 120.2 1.615  1394093_at  SWI/SNF related, matrix associated, actin dependent regulator of chromatin, subfamily a, member 5 (predicted)  Info   Smarca5_predicted 
   7515 0.0011277 123.9 61.4 2.018  1391026_at  Immediate early response 5-like  Info    
   7516 0.0011289 22 45 0.489  1380786_at  Transcribed locus  Info    
   7517 0.0011289 112.2 215.6 0.52  1398634_at    Info    
   7518 0.0011293 1713.6 1465.3 1.169  1398800_at  tyrosine 3-monooxygenase/tryptophan 5-monooxygenase activation protein, beta polypeptide  Info   Ywhab 
   7519 0.0011298 175.3 116.5 1.505  1383099_at  LOC499526  Info   LOC499526 
   7520 0.0011314 104.1 71.8 1.45  1382062_at  similar to hypothetical protein FLJ13273 (predicted)  Info   RGD1311822_predicted 
   7521 0.0011324 95.9 48.6 1.973  1390848_at  Similar to CKT2  Info    
   7522 0.0011325 39.2 72.9 0.538  1394101_at  Erythrocyte protein band 4.1-like 5 (predicted)  Info    
   7523 0.0011333 27 59.2 0.456  1382403_at  similar to RIKEN cDNA 2610019F03  Info   LOC498662 
   7524 0.0011334 85.4 126.2 0.677  1377162_at  Transcribed locus  Info    
   7525 0.0011337 70.8 132.3 0.535  1372139_at  similar to RIKEN cDNA 5530601I19 (predicted)  Info   RGD1308399_predicted 
   7526 0.0011343 737 958.9 0.769  1388699_at  mannosidase 2, alpha B1  Info   Man2b1 
   7527 0.0011345 1055.1 719.9 1.466  1388369_at  similar to gp25L2 protein (predicted)  Info   RGD1307627_predicted 
   7528 0.0011345 27.6 66.5 0.415  1369485_at  cytosolic acetyl-CoA hydrolase  Info   Cach 
   7529 0.0011349 379.2 243.9 1.555  1372187_at  Protein kinase C, nu (predicted)  Info    
   7530 0.0011358 3050.9 3560.3 0.857  1370235_at  diazepam binding inhibitor  Info   Dbi 
   7531 0.0011367 4157 5305.3 0.784  1367653_a_at  malate dehydrogenase 1, NAD (soluble)  Info   Mdh1 
   7532 0.001137 787.7 1018.6 0.773  1399004_at    Info    
   7533 0.0011395 37.9 22.4 1.692  1383729_at  ankyrin repeat and SOCS box-containing 16 (predicted)  Info   Asb16_predicted 
   7534 0.0011399 421 220.7 1.908  1395173_at  GPI-anchored membrane protein 1 (predicted)  Info   Gpiap1_predicted 
   7535 0.0011416 104.8 57.1 1.835  1381847_at  Similar to BC021442 protein  Info    
   7536 0.0011439 172.2 132.6 1.299  1392744_at  Autophagy 7-like (S. cerevisiae) (predicted)  Info    
   7537 0.0011444 3877.9 5090.4 0.762  1370276_at  ATP synthase, H+ transporting, mitochondrial F1 complex, O subunit  Info   Atp5o 
   7538 0.0011452 287.4 162.4 1.77  1373984_at  Transcribed locus  Info    
   7539 0.0011459 252.9 319.9 0.791  1372600_at  Similar to F-box only protein 31  Info    
   7540 0.0011466 329.7 222.9 1.479  1396145_at  Septin 6 (predicted)  Info    
   7541 0.001147 460.7 632.8 0.728  1371920_at  polymerase (DNA-directed), delta interacting protein 2 (predicted)  Info   Poldip2_predicted 
   7542 0.0011474 7.6 2 3.8  1381725_at  Transcribed locus  Info    
   7543 0.0011482 475.3 344.5 1.38  1388447_at  Transcribed locus, strongly similar to NP_084275.3 limb-bud and heart [Mus musculus]  Info    
   7544 0.0011508 297.5 153 1.944  1382463_at  Transcribed locus  Info    
   7545 0.0011519 36.8 79.7 0.462  1368784_at  apobec-1 complementation factor  Info   Acf 
   7546 0.0011533 313.6 386.9 0.811  1367907_a_at  clathrin, light polypeptide (Lcb)  Info   Cltb 
   7547 0.0011546 59.6 20.2 2.95  1386998_at  aldolase C, fructose-biphosphate  Info   Aldoc 
   7548 0.0011551 213.3 305.5 0.698  1373539_at  Transcribed locus  Info    
   7549 0.0011558 228.2 352 0.648  1377166_at  amyotrophic lateral sclerosis 2 (juvenile) (predicted)  Info   Als2_predicted 
   7550 0.0011564 35.8 88.4 0.405  1393271_at  Transcribed locus  Info    
   7551 0.0011575 1.1 7.4 0.149  1369691_at  sodium channel, voltage-gated, type III, alpha polypeptide /// hypothetical gene supported by NM_013119  Info   Scn3a /// LOC497770 
   7552 0.0011575 129.8 246.3 0.527  1383624_at  similar to hypothetical protein LOC284018 isoform b  Info   LOC498014 
   7553 0.0011582 2267.3 1630.2 1.391  1398764_at  ribosomal protein L21  Info   Rpl21 
   7554 0.0011588 95 161 0.59  1383981_at  transformation related protein 53 binding protein 2 (predicted)  Info   Trp53bp2_predicted 
   7555 0.0011601 69.1 41.1 1.681  1380760_at  Transcribed locus  Info    
   7556 0.0011601 39.5 117.4 0.336  1388604_at    Info    
   7557 0.001161 55.6 78.8 0.706  1369433_at  IP63 protein  Info   RGD:620464 
   7558 0.0011622 53.5 31.9 1.677  1384404_at  PRP3 pre-mRNA processing factor 3 homolog (yeast) (predicted)  Info   Prpf3_predicted 
   7559 0.0011625 1571.9 1892.5 0.831  1388326_at  NADH dehydrogenase (ubiquinone) Fe-S protein 8 (predicted)  Info   Ndufs8_predicted 
   7560 0.0011649 37.5 11.2 3.348  1378887_at  Mesoderm development candidate 1 (predicted)  Info    
   7561 0.0011663 10.8 2.1 5.143  1395838_at  Transcribed locus  Info    
   7562 0.0011673 45.5 24.4 1.865  1386621_at    Info    
   7563 0.0011685 39.2 16.8 2.333  1375649_at  Transcribed locus  Info    
   7564 0.0011691 1.6 8.1 0.198  1376726_at  similar to Carbonic anhydrase-related protein 10 (Carbonic anhydrase-related protein X) (CARP X) (CA-RP X)  Info   LOC360599 
   7565 0.0011699 261.7 423.9 0.617  1367997_at  suppressor of K+ transport defect 3  Info   Skd3 
   7566 0.0011703 333.9 479.3 0.697  1379703_at  hypothetical LOC315055 (predicted)  Info   RGD1311559_predicted 
   7567 0.0011708 87.1 210.9 0.413  1368313_a_at  transient receptor potential cation channel, subfamily V, member 1  Info   Trpv1 
   7568 0.001172 103.4 60.8 1.701  1379704_at  zinc finger protein 143 (predicted)  Info   Zfp143_predicted 
   7569 0.0011729 56.2 6.1 9.213  1398589_at  similar to paired immunoglobin-like type 2 receptor alpha  Info   LOC288568 
   7570 0.001173 20.7 4.7 4.404  1385222_at    Info    
   7571 0.0011736 440.9 295.9 1.49  1375280_at  CGI-146 protein  Info   RGD:1359392 
   7572 0.0011739 11.4 27.7 0.412  1373896_at    Info    
   7573 0.0011743 733.7 1008.8 0.727  1372179_at  neural visinin-like Ca2+-binding protein type 3  Info   Hpcal1 
   7574 0.0011758 322.8 465.2 0.694  1387903_at  praja 2, RING-H2 motif containing  Info   Pja2 
   7575 0.0011773 45.5 72.5 0.628  1387185_at  amyloid beta (A4) precursor protein-binding, family B, member 3  Info   Apbb3 
   7576 0.0011785 355.6 242.2 1.468  1369057_at  syntaxin binding protein 2  Info   Stxbp2 
   7577 0.001179 766.7 989 0.775  1375934_at  similar to RIKEN cDNA D330045A20  Info   LOC315911 
   7578 0.0011791 287.2 391.4 0.734  1390563_at    Info    
   7579 0.0011814 35.1 102.5 0.342  1393422_at  serine dehydratase-like (predicted)  Info   Sdsl_predicted 
   7580 0.0011829 119.6 71.3 1.677  1375188_at  katanin p80 (WD40-containing) subunit B 1 (predicted)  Info   Katnb1_predicted 
   7581 0.0011835 778.5 637.9 1.22  1371471_at  glioma tumor suppressor candidate region gene 2  Info   RGD:1303085 
   7582 0.0011838 551.4 778.3 0.708  1378692_at  Paired box gene 8  Info   Pax8 
   7583 0.0011851 45.4 80.1 0.567  1388485_at  Chemokine (C-X-C motif) ligand 14 (predicted)  Info    
   7584 0.0011854 71.3 100.6 0.709  1391206_at  Transcribed locus  Info    
   7585 0.0011856 70.2 113.6 0.618  1378441_at  Transcribed locus  Info    
   7586 0.0011887 2078.5 2397.1 0.867  1371605_at  similar to RIKEN cDNA 2410011G03 (predicted)  Info   RGD1311462_predicted 
   7587 0.0011906 59.9 7.5 7.987  1384338_at  similar to CDC45L  Info   LOC287961 
   7588 0.0011908 43.7 5.7 7.667  1397663_at  Transcribed locus  Info    
   7589 0.0011908 340.3 480.2 0.709  1373639_at  similar to RIKEN cDNA 2410022L05  Info   LOC290925 
   7590 0.0011913 259.4 189.5 1.369  1373169_at  Similar to 1-acylglycerolphosphate acyltransferase-epsilon (predicted)  Info    
   7591 0.0011921 186.7 279.5 0.668  1376774_at  Putative membrane protein Re9 (member 1)  Info    
   7592 0.0011929 252 211.2 1.193  1372451_at  ubiquitin specific protease 49 (predicted) /// similar to Ubiquitin specific protease homolog 49 (TRF-proximal protein homolog)  Info   Usp49_predicted /// LOC501098 
   7593 0.0011937 121.6 174.9 0.695  1374694_at  Transcribed locus  Info    
   7594 0.0011938 87.4 42.9 2.037  1372590_at  Transcribed locus  Info    
   7595 0.0011943 3357.9 2844.6 1.18  1371375_at    Info    
   7596 0.0011946 99.4 54.7 1.817  1389862_at  similar to hypothetical protein MGC3731 (predicted)  Info   RGD1305327_predicted 
   7597 0.0011971 1580.3 1986.2 0.796  1372706_at  hexosaminidase B (predicted)  Info   Hexb_predicted 
   7598 0.001199 1165.1 1525.2 0.764  1373819_at  hypothetical protein LOC500855  Info   LOC500855 
   7599 0.0011992 28.4 64.2 0.442  1378492_at  FERM, RhoGEF (Arhgef) and pleckstrin domain protein 1 (chondrocyte-derived) (predicted)  Info    
   7600 0.0012015 109.1 167 0.653  1394815_at  Transcribed locus  Info    
   7601 0.0012027 223.5 138.8 1.61  1390098_at  similar to RIKEN cDNA 2510005D08 (predicted)  Info   RGD1306762_predicted 
   7602 0.0012031 27.1 11.8 2.297  1397709_at  similar to PI-3-kinase-related kinase SMG-1 isoform 2  Info   LOC499250 
   7603 0.0012036 368.5 286.7 1.285  1376010_at  PRP4 pre-mRNA processing factor 4 homolog B (yeast) (predicted)  Info   Prpf4b_predicted 
   7604 0.0012039 363.5 564.8 0.644  1390102_at  disrupted in renal carcinoma 2 (predicted)  Info   Dirc2_predicted 
   7605 0.0012043 117.6 69.3 1.697  1380001_at  similar to pinin  Info   LOC368070 
   7606 0.0012068 148 188.1 0.787  1397812_at  SUMO/sentrin specific protease 6 (predicted)  Info   Senp6_predicted 
   7607 0.0012072 16.1 2.4 6.708  1385297_at  RecQ protein-like 4 (predicted)  Info   Recql4_predicted 
   7608 0.0012094 48.1 91.1 0.528  1377865_at  Transcribed locus  Info    
   7609 0.0012097 37.1 4.1 9.049  1389542_at  centromere protein E (predicted)  Info   Cenpe_predicted 
   7610 0.0012107 51.4 34.4 1.494  1369431_at  UDP-N-acetyl-alpha-D-galactosamine:polypeptide N-acetylgalactosaminyltransferase 7 (GalNAc-T7)  Info   Galnt7 
   7611 0.0012108 49.7 33.5 1.484  1369441_at  calpain 5  Info   Capn5 
   7612 0.0012136 1.2 4.8 0.25  1377104_at  Similar to BC028663 protein  Info    
   7613 0.0012138 184.7 135.9 1.359  1374332_at  DEAD (Asp-Glu-Ala-Asp) box polypeptide 49 (predicted)  Info   Ddx49_predicted 
   7614 0.0012142 82 38.7 2.119  1395517_at  LIM domains containing 1 (predicted)  Info   Limd1_predicted 
   7615 0.0012143 45.2 24.2 1.868  1376324_at  Similar to zinc finger protein 21 isoform 2  Info    
   7616 0.0012149 927.4 775.3 1.196  1372155_at  tripartite motif protein 28  Info   Trim28 
   7617 0.0012149 3.8 11.8 0.322  1375376_at  Similar to WAC  Info    
   7618 0.0012151 50.7 116.4 0.436  1387816_at  insulin-like growth factor binding protein, acid labile subunit  Info   Igfals 
   7619 0.0012165 98.2 68.4 1.436  1372898_at  similar to 2700008B19Rik protein  Info   LOC497972 
   7620 0.0012167 168.3 114.2 1.474  1372826_at    Info    
   7621 0.0012174 508.7 378 1.346  1372714_at  similar to RIKEN cDNA 8430437G11 (predicted)  Info   RGD1307778_predicted 
   7622 0.0012184 12.5 37 0.338  1383442_at  similar to hypothetical protein FLJ13941  Info   RGD:1359433 
   7623 0.0012185 198.8 122.6 1.622  1376205_at  Transcribed locus  Info    
   7624 0.0012197 102.6 68.3 1.502  1389314_at  nuclear VCP-like (predicted)  Info   Nvl_predicted 
   7625 0.0012198 286.8 524.1 0.547  1373922_at  TPR domain, ankyrin-repeat and coiled-coil-containing  Info   Tanc 
   7626 0.0012225 73.1 30.2 2.421  1395297_at  similar to hypothetical protein FLJ10652 (predicted)  Info   RGD1309621_predicted 
   7627 0.0012257 397 250.7 1.584  1376737_at  similar to SUMO/sentrin specific protease 5  Info   LOC499312 
   7628 0.0012258 2856.9 2323.1 1.23  1398880_at  RNA polymerase II transcriptional coactivator  Info   Rpo2tc1 
   7629 0.0012259 129.9 160.4 0.81  1395440_at  similar to RIKEN cDNA 5133401N09  Info   LOC498695 
   7630 0.0012264 71.2 39.1 1.821  1368074_at  galactose-4-epimerase, UDP  Info   Gale 
   7631 0.0012265 49.8 83.4 0.597  1376156_at  hypothetical LOC316091 (predicted)  Info   RGD1304827_predicted 
   7632 0.0012267 127.4 77 1.655  1377860_at  similar to bM410K19.2.2 (novel protein similar to extra-cellular matrix proteins and chondroitin sulfate proteoglycans, variant 2) (predicted)  Info   RGD1306981_predicted 
   7633 0.001227 142.2 96.3 1.477  1384390_at  Importin 9 (predicted)  Info    
   7634 0.0012271 108.3 156.9 0.69  1368166_at  ADP-ribosylation factor-like 2  Info   Arl2 
   7635 0.0012283 28 37.2 0.753  1390856_at  Transcribed locus  Info    
   7636 0.0012297 80.1 137.4 0.583  1371132_a_at  ankyrin 3, epithelial  Info   RGD:620157 
   7637 0.0012337 40.2 15.3 2.627  1369484_at  WNT1 inducible signaling pathway protein 2  Info   Wisp2 
   7638 0.0012362 992.2 765.9 1.295  1387031_at  endoplasmic retuclum protein 29  Info   Erp29 
   7639 0.0012375 569.7 394.5 1.444  1371985_a_at  HLA-B associated transcript 5  Info   RGD:1303164 
   7640 0.0012377 4892.5 3387.9 1.444  1372701_at  heat shock protein 1, alpha  Info   Hspca 
   7641 0.0012382 6.7 40.1 0.167  1398567_at  similar to FLJ42986 protein  Info   LOC501127 
   7642 0.0012412 71.4 132.1 0.54  1379397_at  RAR-related orphan receptor alpha (predicted)  Info   Rora_predicted 
   7643 0.0012426 165.1 221.2 0.746  1391410_at  Transcribed locus  Info    
   7644 0.0012429 46 3.9 11.795  1391878_at  similar to Myb proto-oncogene protein (C-myb)  Info   LOC498982 
   7645 0.0012435 310.1 213.6 1.452  1377998_at  coproporphyrinogen oxidase (predicted)  Info   Cpox_predicted 
   7646 0.0012445 73.6 124.4 0.592  1375121_at  MAD homolog 6 (Drosophila) (predicted)  Info   Smad6_predicted 
   7647 0.0012465 258.2 362 0.713  1399036_at  ubiquitin specific protease 47 (predicted)  Info   Usp47_predicted 
   7648 0.0012479 8.2 25.3 0.324  1381936_at  calcium/calmodulin-dependent protein kinase II gamma  Info   Camk2g 
   7649 0.0012488 244 345.9 0.705  1398242_at  protein phosphatase 5, catalytic subunit  Info   Ppp5c 
   7650 0.0012488 106.2 65.8 1.614  1376611_at  Polymerase (DNA directed), alpha 1  Info   Pola1 
   7651 0.0012506 33.2 80.9 0.41  1383439_at  Neuronal PAS domain protein 2 (predicted)  Info    
   7652 0.0012508 53.9 85.7 0.629  1370011_at  adenylate kinase 1  Info   Ak1 
   7653 0.0012542 204.2 322.2 0.634  1378027_at  poliovirus receptor-related 3 (predicted)  Info   Pvrl3_predicted 
   7654 0.0012573 105.1 57 1.844  1372410_at  C1q and tumor necrosis factor related protein 6 (predicted)  Info    
   7655 0.0012588 23.2 35.7 0.65  1382510_at  Transcribed locus  Info    
   7656 0.0012591 99.4 160.1 0.621  1382593_at  Ets variant gene 1 (predicted)  Info    
   7657 0.0012596 696.5 869.5 0.801  1367800_at  plasminogen activator, tissue  Info   Plat 
   7658 0.0012645 72.8 53.6 1.358  1381986_at  Neuron navigator 1 (predicted)  Info    
   7659 0.0012659 2.4 26.2 0.092  1397678_at  Transcribed locus  Info    
   7660 0.0012693 56 111.4 0.503  1383686_at  synaptogyrin 1  Info   Syngr1 
   7661 0.0012701 666.7 496.2 1.344  1372304_at  COMM domain containing 4 (predicted)  Info   Commd4_predicted 
   7662 0.0012702 630.6 375.4 1.68  1383127_at  CDNA clone IMAGE:7320912  Info    
   7663 0.0012703 127.8 168.1 0.76  1373832_at  Sorting nexin 19 (predicted)  Info    
   7664 0.0012708 165 92.4 1.786  1398451_at  Transcribed locus  Info    
   7665 0.0012715 77.9 97.4 0.8  1374032_at  PHD finger protein 12 (predicted)  Info   Phf12_predicted 
   7666 0.001273 1.2 5.8 0.207  1396114_at  F-box only protein 33 (predicted)  Info    
   7667 0.0012748 34.5 64.6 0.534  1382118_at  paternally expressed 3 (predicted)  Info   Peg3_predicted 
   7668 0.0012759 4877.2 5586.7 0.873  1367585_a_at  ATPase, Na+/K+ transporting, alpha 1 polypeptide  Info   Atp1a1 
   7669 0.0012778 12.8 33.3 0.384  1391228_at  testis specific gene A2 (predicted)  Info   Tsga2_predicted 
   7670 0.001279 856.7 2254.7 0.38  1370725_a_at  glucose-6-phosphatase, catalytic  Info   G6pc 
   7671 0.0012803 142.1 94.2 1.508  1372870_at  KDEL (Lys-Asp-Glu-Leu) endoplasmic reticulum protein retention receptor 3 (predicted)  Info   Kdelr3_predicted 
   7672 0.001281 148.7 208.5 0.713  1371064_at  pericentriolar material 1  Info   Pcm1 
   7673 0.0012825 82.5 147.7 0.559  1391315_at    Info    
   7674 0.0012846 80.6 151.7 0.531  1371665_at  SWI/SNF related, matrix associated, actin dependent regulator of chromatin, subfamily d, member 3 (predicted)  Info   Smarcd3_predicted 
   7675 0.0012855 17.5 35.2 0.497  1378783_at  Transcribed locus  Info    
   7676 0.0012859 6.7 17.8 0.376  1382398_at  similar to MGC68837 protein  Info   LOC498356 
   7677 0.0012894 236.8 167 1.418  1398330_at  syntaxin binding protein 1  Info   Stxbp1 
   7678 0.0012896 10.9 46.1 0.236  1379713_at    Info    
   7679 0.0012911 115.7 153.2 0.755  1374116_at  Similar to Metaxin 1, isoform 2  Info    
   7680 0.0012929 143.9 79.1 1.819  1393620_at  sestrin 3 (predicted)  Info   Sesn3_predicted 
   7681 0.0012958 70.8 43.7 1.62  1384602_at  G protein-coupled receptor 107 (predicted)  Info   Gpr107_predicted 
   7682 0.0012966 633.5 429.3 1.476  1374323_at  BRCA2 and CDKN1A interacting protein (predicted)  Info   Bccip_predicted 
   7683 0.0013019 286.4 194.8 1.47  1377713_at  checkpoint suppressor 1 (predicted)  Info   Ches1_predicted 
   7684 0.0013026 192.3 129.5 1.485  1379768_at  CDNA clone IMAGE:7374368  Info    
   7685 0.0013031 246.2 196.2 1.255  1392562_at  similar to RIKEN cDNA 2610001E06 (predicted)  Info   RGD1308126_predicted 
   7686 0.0013032 62.8 217.4 0.289  1368596_at  SNF1-like kinase  Info   Snf1lk 
   7687 0.0013036 3010.8 2597.9 1.159  1371427_at  similar to signal peptidase 12kDa (predicted)  Info   RGD1309125_predicted 
   7688 0.0013037 13.7 27.7 0.495  1382444_at  Transcribed locus  Info    
   7689 0.0013045 10 23.3 0.429  1379148_at  Calcium channel, voltage-dependent, beta 4 subunit  Info   Cacnb4 
   7690 0.0013045 103.2 171.9 0.6  1398610_at  CDNA clone MGC:124727 IMAGE:7936966  Info    
   7691 0.0013058 112.5 131 0.859  1379731_at  Transcribed locus  Info    
   7692 0.0013059 74.2 120.9 0.614  1378002_at  osmotic stress protein 94 kDa (predicted)  Info   OSP94_predicted 
   7693 0.001307 43.1 81.4 0.529  1373580_at  Transcribed locus  Info    
   7694 0.0013084 166.3 292 0.57  1392789_at  Transcribed locus, strongly similar to XP_484968.2 PREDICTED: similar to RIKEN cDNA C330005L02 [Mus musculus]  Info    
   7695 0.0013096 41.6 65.1 0.639  1369439_at  angiotensin II receptor, type-1  Info   RGD:67391 
   7696 0.0013097 3.2 17.4 0.184  1380669_at    Info    
   7697 0.0013137 509.4 244.8 2.081  1370895_at  collagen, type V, alpha 2  Info   Col5a2 
   7698 0.0013145 379.8 285.6 1.33  1398899_at  polymerase (RNA) II (DNA directed) polypeptide C (predicted)  Info   Polr2c_predicted 
   7699 0.0013146 62.5 37.6 1.662  1382865_at  testis specific, 14  Info   Tsga14 
   7700 0.0013152 125.7 186.1 0.675  1379541_at    Info    
   7701 0.0013165 144.4 220.4 0.655  1372736_at  Amylo-1, 6-glucosidase, 4-alpha-glucanotransferase (glycogen debranching enzyme, glycogen storage disease type III) (predicted)  Info    
   7702 0.0013168 252.5 386.1 0.654  1398618_s_at    Info    
   7703 0.0013184 1110 2156.5 0.515  1383798_at  similar to CDNA sequence BC026682  Info   RGD:1359194 
   7704 0.0013194 10.4 39.8 0.261  1380969_at  Similar to mKIAA0940 protein  Info    
   7705 0.0013248 134.2 193.1 0.695  1372193_at  imprinted and ancient  Info   Impact 
   7706 0.0013256 772.1 657.1 1.175  1398813_at  ubiquitin-activating enzyme E1C  Info   Ube1c 
   7707 0.0013279 61.9 28.5 2.172  1390940_at    Info    
   7708 0.0013283 543.7 373 1.458  1392746_x_at  Similar to KIAA0731 protein (predicted)  Info    
   7709 0.0013284 147 196.4 0.748  1371627_at  Angiomotin-like 1 (predicted)  Info    
   7710 0.001329 186.9 240.9 0.776  1378506_at  Transcribed locus  Info    
   7711 0.0013298 1601.7 1964.6 0.815  1367642_at  succinate-CoA ligase, GDP-forming, alpha subunit  Info   Suclg1 
   7712 0.0013311 129.4 82.8 1.563  1379485_at  eukaryotic translation initiation factor 3, subunit 10 (theta) (predicted)  Info   Eif3s10_predicted 
   7713 0.0013327 2.1 15.5 0.135  1393549_at  Transcribed locus  Info    
   7714 0.0013354 72.2 153 0.472  1385074_at  SWI/SNF related, matrix associated, actin dependent regulator of chromatin, subfamily a, member 2  Info   RGD:1302988 
   7715 0.0013357 206.4 250.9 0.823  1373730_at  Similar to hypothetical protein MGC20460 (predicted)  Info    
   7716 0.0013359 2558.3 1417.5 1.805  1370956_at  decorin  Info   Dcn 
   7717 0.0013385 11.1 27.7 0.401  1378459_at    Info    
   7718 0.0013408 352.1 575.4 0.612  1390710_x_at  sortilin-related receptor, L(DLR class) A repeats-containing (predicted)  Info   Sorl1_predicted 
   7719 0.001342 215.4 273.6 0.787  1370176_at  amyotrophic lateral sclerosis 2 (juvenile) chromosome region, candidate 3 homolog (human)  Info   Als2cr3 
   7720 0.0013423 2.1 10.9 0.193  1387679_at  solute carrier family 21, member 10  Info   Slc21a10 
   7721 0.0013444 35.8 87 0.411  1397015_at  Transcribed locus  Info    
   7722 0.0013457 64.1 97.1 0.66  1381825_at  similar to hypothetical protein FLJ34633  Info   RGD:1303271 
   7723 0.0013467 99.8 178.2 0.56  1377015_at  Malic enzyme 3, NADP(+)-dependent, mitochondrial (predicted)  Info    
   7724 0.0013467 80.8 115.6 0.699  1379816_at  Similar to RIKEN cDNA 2410025L10  Info    
   7725 0.0013491 490.5 382 1.284  1384323_at  proteasome (prosome, macropain) 26S subunit, ATPase, 6 (predicted)  Info   Psmc6_predicted 
   7726 0.0013499 76.3 46.5 1.641  1381019_x_at  development and differentiation enhancing (predicted)  Info   Ddef1_predicted 
   7727 0.0013501 93 44.3 2.099  1368033_at  nucleolar and coiled-body phosphoprotein 1  Info   Nolc1 
   7728 0.0013508 4.5 41.8 0.108  1387370_at  tropomodulin 1  Info   Tmod1 
   7729 0.0013513 81.1 152.8 0.531  1393427_s_at  EGF-like domain 7  Info   Egfl7 
   7730 0.0013524 245.4 140 1.753  1370054_at  cyclin-dependent kinase inhibitor 2C (p18, inhibits CDK4)  Info   Cdkn2c 
   7731 0.0013524 121.1 92.7 1.306  1390647_at  Putative homeodomain transcription factor 2 (predicted)  Info    
   7732 0.0013526 160.8 227.3 0.707  1379031_at  Transcribed locus  Info    
   7733 0.0013544 161.3 226.6 0.712  1373270_at  similar to D11Ertd498e protein (predicted)  Info   RGD1307754_predicted 
   7734 0.0013566 138.9 87.4 1.589  1379385_at  tumor suppressing subtransferable candidate 1 (predicted)  Info   Tssc1_predicted 
   7735 0.0013576 218.4 182.5 1.197  1391437_at  similar to BC002216 protein  Info   RGD:1359656 
   7736 0.0013577 43.8 111.1 0.394  1397251_at  Similar to hypothetical protein MGC32132 (predicted)  Info    
   7737 0.0013586 39.1 14.6 2.678  1380822_at  Transcribed locus  Info    
   7738 0.001359 474.7 613.8 0.773  1371689_at  Eukaryotic translation elongation factor 1 alpha 1  Info   Eef1a1 
   7739 0.0013594 123.7 89.5 1.382  1374386_at  Protein kinase C and casein kinase substrate in neurons 1  Info   Pacsin1 
   7740 0.0013599 197.3 124 1.591  1381203_at  SH3-domain GRB2-like B1 (endophilin) (predicted)  Info   Sh3glb1_predicted 
   7741 0.0013611 75.4 99.4 0.759  1373880_at  Similar to mKIAA1632 protein (predicted)  Info    
   7742 0.0013619 91 68 1.338  1381975_at    Info    
   7743 0.0013658 279.2 367 0.761  1387875_at  PTK2 protein tyrosine kinase 2  Info   Ptk2 
   7744 0.0013658 76.8 53.8 1.428  1395418_at  zinc finger protein 131 (predicted)  Info   Zfp131_predicted 
   7745 0.0013659 7.8 54.6 0.143  1383390_at  Similar to hypothetical protein FLJ25477 isoform 2  Info    
   7746 0.0013673 1096.2 718.9 1.525  1370287_a_at  tropomyosin 1, alpha  Info   Tpm1 
   7747 0.0013704 83.6 41.9 1.995  1392886_a_at  similar to FCRL (predicted)  Info   RGD1306176_predicted 
   7748 0.0013709 1.8 11 0.164  1384647_at  Rho guanine nucleotide exchange factor 7  Info   Arhgef7 
   7749 0.0013712 435.8 302.4 1.441  1368241_a_at  flotillin 1  Info   Flot1 
   7750 0.0013715 1.9 9.1 0.209  1384696_at  similar to RIKEN cDNA 1700001E04  Info   MGC116197 
   7751 0.001372 39.4 75 0.525  1374049_at  similar to Myosin light chain 1 slow a  Info   LOC317454 
   7752 0.0013754 44.4 17.6 2.523  1368523_at  Ca  Info   Cadps 
   7753 0.0013759 33 17 1.941  1387507_at  interleukin 1 receptor accessory protein  Info   Il1rap 
   7754 0.0013767 261.8 325.2 0.805  1376790_at  Galactosylceramidase  Info   Galc 
   7755 0.001377 321.5 570.2 0.564  1394472_at  Ral guanine nucleotide dissociation stimulator,-like 1 (predicted)  Info    
   7756 0.0013783 563.2 450.6 1.25  1367482_at  similar to anaphase promoting complex subunit 11 homolog  Info   LOC498030 
   7757 0.0013792 416 1018.4 0.408  1397164_at  Transcribed locus, weakly similar to XP_580018.1 PREDICTED: hypothetical protein XP_580018 [Rattus norvegicus]  Info    
   7758 0.0013802 25.9 44.9 0.577  1383057_at  ASF1 anti-silencing function 1 homolog A (S. cerevisiae) (predicted)  Info   Asf1a_predicted 
   7759 0.0013823 806.9 1053.7 0.766  1374318_at  Transcribed locus  Info    
   7760 0.0013835 193.3 130.8 1.478  1376936_at  similar to sodium/calcium exchanger protein  Info   LOC498185 
   7761 0.0013837 1022.1 719.4 1.421  1388328_at  similar to Eukaryotic translation initiation factor 3 subunit 2 (eIF-3 beta) (eIF3 p36) (eIF3i) (TGF-beta receptor interacting protein 1) (TRIP-1)  Info   LOC499619 
   7762 0.0013853 66.4 41.4 1.604  1373522_at  Transcribed locus  Info    
   7763 0.0013861 39.6 66.8 0.593  1378979_x_at  Similar to KIAA1205 protein (predicted)  Info    
   7764 0.0013868 29.6 45.5 0.651  1372690_at  Transcribed locus  Info    
   7765 0.0013892 6.1 34.3 0.178  1390092_at  Transcribed locus  Info    
   7766 0.0013895 33.7 49.4 0.682  1368901_at  thrombomodulin  Info   Thbd 
   7767 0.0013908 128.6 202.6 0.635  1382371_at  hypothetical protein LOC362011  Info   LOC362011 
   7768 0.0013929 43.6 82.2 0.53  1368599_at  solute carrier family 9, member 2  Info   Slc9a2 
   7769 0.001395 58.6 83.9 0.698  1369680_at  solute carrier family 2 (facilitated glucose transporter), member 13  Info   Slc2a13 
   7770 0.0013962 55 10.3 5.34  1369119_a_at  5-hydroxytryptamine (serotonin) receptor 7  Info   Htr7 
   7771 0.0013963 29.3 12.4 2.363  1375833_at  Protein tyrosine phosphatase, non-receptor type 4  Info   Ptpn4 
   7772 0.0013971 43.3 82 0.528  1380675_s_at  carbonic anhydrase 11  Info   Car11 
   7773 0.0013977 97.9 135.5 0.723  1375951_at  thrombomodulin  Info   Thbd 
   7774 0.0014025 26.7 63.3 0.422  1398265_at  ATP-binding cassette, sub-family C (CFTR/MRP), member 9  Info   Abcc9 
   7775 0.0014041 61.6 27.1 2.273  1367791_at  receptor (calcitonin) activity modifying protein 1  Info   Ramp1 
   7776 0.0014047 154.8 215.4 0.719  1374454_at  Similar to RIKEN cDNA 5330414D10 (predicted)  Info    
   7777 0.0014054 35.1 55.2 0.636  1376107_at  Arginase 2  Info   Arg2 
   7778 0.0014063 51.9 79.2 0.655  1398517_at  Similar to HSPC182 protein (predicted)  Info    
   7779 0.0014065 25.6 3.3 7.758  1388173_at  potassium voltage-gated channel, Shal-related family, member 1  Info   Kcnd1 
   7780 0.0014089 168.2 79.7 2.11  1380596_at  Transcribed locus  Info    
   7781 0.00141 131.2 174.2 0.753  1371593_at  Transcribed locus  Info    
   7782 0.0014101 52.2 28.2 1.851  1392168_at  vaccinia related kinase 1 (predicted)  Info   Vrk1_predicted 
   7783 0.0014109 206.5 262.6 0.786  1374847_at  leucine-zipper-like transcriptional regulator, 1 (predicted)  Info   Lztr1_predicted 
   7784 0.0014122 57 82.7 0.689  1377093_at  Bromodomain and PHD finger containing, 3 (predicted)  Info    
   7785 0.0014125 246 378.8 0.649  1388701_at  Myeloid/lymphoid or mixed lineage-leukemia translocation to 6 homolog (Drosophila) (predicted)  Info    
   7786 0.0014125 109.8 192.4 0.571  1385722_at  Transcribed locus, weakly similar to NP_035507.1 single-minded homolog 2 [Mus musculus]  Info    
   7787 0.0014126 722.7 436.7 1.655  1372002_at  gap junction membrane channel protein alpha 1  Info   Gja1 
   7788 0.0014129 121.5 169.7 0.716  1383216_at  Cohen syndrome homolog 1 (predicted)  Info    
   7789 0.0014129 69.6 118.2 0.589  1380203_at    Info    
   7790 0.0014133 254.1 177.8 1.429  1373879_at  Transcribed locus  Info    
   7791 0.0014133 327.5 456.5 0.717  1377353_a_at  tumor necrosis factor (ligand) superfamily, member 13 (predicted)  Info   Tnfsf13_predicted 
   7792 0.0014136 341.8 186.2 1.836  1389558_at  Transcribed locus  Info    
   7793 0.0014169 157.7 127.1 1.241  1374152_at  WD repeat domain 46  Info   Wdr46 
   7794 0.0014189 29.7 4.4 6.75  1382774_at    Info    
   7795 0.0014217 464.9 388.2 1.198  1398934_at  Mitogen-activated protein kinase kinase kinase 7 interacting protein 2 (predicted)  Info    
   7796 0.001424 73.4 110.8 0.662  1392924_at  Transcribed locus  Info    
   7797 0.0014247 110.5 137.3 0.805  1376177_at  similar to RIKEN cDNA 5730593F17  Info   LOC497983 
   7798 0.0014285 18.1 33.4 0.542  1377208_at  Muscleblind-like 2 (predicted)  Info    
   7799 0.0014298 568.4 450.5 1.262  1371982_at  AIP1  Info   RGD:708575 
   7800 0.0014299 56.8 34.1 1.666  1377943_at    Info    
   7801 0.0014305 15.7 60.7 0.259  1388545_at  Transcribed locus  Info    
   7802 0.0014327 21.5 37.9 0.567  1385808_at  PHD finger protein 7 (predicted)  Info   Phf7_predicted 
   7803 0.0014341 39.5 67.8 0.583  1380869_at  Transcribed locus, moderately similar to NP_001001306.1 hypothetical protein LOC329695 [Mus musculus]  Info    
   7804 0.0014347 154.6 108.9 1.42  1378259_at    Info    
   7805 0.0014351 241.2 154.1 1.565  1373632_at  TAF9 RNA polymerase II, TATA box binding protein (TBP)-associated factor  Info   Taf9 
   7806 0.0014352 16.2 5.2 3.115  1379051_at  Similar to RIKEN cDNA 2310057M21 (predicted)  Info    
   7807 0.0014396 1102.1 844.4 1.305  1371559_at  CDNA clone MGC:109551 IMAGE:7325365  Info    
   7808 0.00144 194.3 254.7 0.763  1398432_at  similar to Ankyrin repeat domain protein 28  Info   LOC306264 
   7809 0.00144 43.7 105.2 0.415  1372892_at    Info    
   7810 0.0014407 78.1 150.7 0.518  1392025_x_at    Info    
   7811 0.0014439 239.6 134.5 1.781  1376436_at  similar to cDNA sequence BC003322 (predicted)  Info   RGD1308805_predicted 
   7812 0.0014447 656 883.8 0.742  1375526_at  similar to novel protein of unknown function (DUF423) family member  Info   LOC287442 
   7813 0.0014491 68.3 121 0.564  1369159_at  androgen receptor  Info   Ar 
   7814 0.0014508 47.7 74.5 0.64  1384479_at  UDP-N-acetyl-alpha-D-galactosamine:polypeptide N-acetylgalactosaminyltransferase 3 (predicted)  Info    
   7815 0.0014509 50 70.2 0.712  1385335_at  tripartite motif protein 39  Info   RGD:1303115 
   7816 0.0014533 41.6 64.3 0.647  1387456_at  staufen, RNA binding protein, homolog 2 (Drosophila)  Info   Stau2 
   7817 0.0014545 32.7 5.2 6.288  1387454_at  niban protein  Info   Niban 
   7818 0.0014552 147.7 95.8 1.542  1389017_at  similar to Antxr2 protein  Info   LOC305633 
   7819 0.0014579 109.6 10.2 10.745  1394359_at  similar to DUF729 domain containing 1  Info   LOC364500 
   7820 0.001458 77.4 130.9 0.591  1395251_at    Info    
   7821 0.0014582 46.5 71 0.655  1374159_at  Similar to RN49018 (predicted)  Info    
   7822 0.0014587 346 408.4 0.847  1374075_at  N-ethylmaleimide-sensitive factor attachment protein, gamma (predicted)  Info   Napg 
   7823 0.0014608 129.2 89.3 1.447  1372924_at  Transcribed locus  Info    
   7824 0.0014623 151.5 205.3 0.738  1384136_at    Info    
   7825 0.0014634 4267.8 3470.9 1.23  1390019_at  H3 histone, family 3B  Info   RGD:621095 
   7826 0.0014679 117.4 174.2 0.674  1389562_at  Transcribed locus  Info    
   7827 0.0014693 64.7 40.6 1.594  1398617_at  Transcribed locus  Info    
   7828 0.00147 172.4 253.7 0.68  1390535_at  CTD (carboxy-terminal domain, RNA polymerase II, polypeptide A) phosphatase, subunit 1 (predicted)  Info    
   7829 0.0014704 376.6 289.3 1.302  1375970_at  Transcribed locus  Info    
   7830 0.0014766 110.2 60.9 1.81  1377041_at  manic fringe homolog (Drosophila)  Info   Mfng 
   7831 0.0014787 155.9 89.4 1.744  1395767_at  similar to HEF-like protein (predicted) /// cleavage stimulation factor, 3' pre-RNA, subunit 1 (predicted)  Info   RGD1310643_predicted /// Cstf1_predicted 
   7832 0.0014791 387.2 266.3 1.454  1373824_at  craniofacial development protein 1  Info   Cfdp1 
   7833 0.0014808 75.8 149.5 0.507  1370531_a_at  phospholipase D1  Info   Pld1 
   7834 0.0014821 585.2 915.7 0.639  1398508_at  similar to polyglutamine-containing protein (predicted)  Info   RGD1310994_predicted 
   7835 0.0014823 1022.1 819.2 1.248  1371768_at  similar to HSPC182 protein (predicted)  Info   RGD1307854_predicted 
   7836 0.001483 15.7 31.5 0.498  1377786_at  Transcribed locus  Info    
   7837 0.0014857 44.3 5.9 7.508  1392061_at  minichromosome maintenance deficient 10 (S. cerevisiae) (predicted)  Info   Mcm10_predicted 
   7838 0.0014862 55.1 26.6 2.071  1396221_at  Transcribed locus  Info    
   7839 0.0014867 287.1 454.4 0.632  1397300_at  Transcribed locus  Info    
   7840 0.0014893 111.5 78.6 1.419  1398611_at  similar to cullin 4B  Info   LOC302502 
   7841 0.0014904 210.1 276.5 0.76  1374054_at  Transcribed locus  Info    
   7842 0.0014909 251.3 341.9 0.735  1389639_at  similar to Shb protein  Info   LOC362513 
   7843 0.0014912 607.1 442.1 1.373  1383126_at  V-akt murine thymoma viral oncogene homolog 1  Info   Akt1 
   7844 0.001492 11.6 35.7 0.325  1393376_at  SRY-box containing gene 6 (predicted)  Info   Sox6_predicted 
   7845 0.0014926 32.3 60.2 0.537  1377736_at  Transcribed locus  Info    
   7846 0.0014928 208.9 135.9 1.537  1395199_at  eukaryotic translation initiation factor 3, subunit 1 alpha (predicted)  Info   Eif3s1_predicted 
   7847 0.001493 98.6 53.1 1.857  1380301_at    Info    
   7848 0.0014935 97.7 47.6 2.053  1376237_at  Chemokine (C-C motif) receptor 6 (predicted)  Info    
   7849 0.0014941 197.2 125.7 1.569  1373417_at  phospholipid scramblase 3 (predicted)  Info   Plscr3_predicted 
   7850 0.0014954 103.1 163.9 0.629  1378309_at  ubiquitin specific protease 20 (predicted)  Info   Usp20_predicted 
   7851 0.0014963 80.9 159.1 0.508  1386817_at    Info    
   7852 0.0014964 3646.9 4178.4 0.873  1398755_at  ATPase, H+ transporting, V0 subunit C  Info   Atp6v0c 
   7853 0.0014971 75.6 143.5 0.527  1374666_at  Similar to mKIAA0824 protein  Info    
   7854 0.0014973 102.1 73.9 1.382  1376288_at  Transcribed locus  Info    
   7855 0.0014981 57.6 34.5 1.67  1395503_at  similar to RIKEN cDNA 3230401I01 (predicted)  Info   RGD1307904_predicted 
   7856 0.0014988 312.7 229.1 1.365  1378100_at  similar to glioma-amplified sequence-41 (predicted)  Info   RGD1305741_predicted 
   7857 0.001499 233.5 386.7 0.604  1387015_at  profilin 2  Info   Pfn2 
   7858 0.0015008 60.5 97.3 0.622  1388305_at  v-raf oncogene homolog 1 (murine sarcoma 3611 virus)  Info   Araf1 
   7859 0.0015049 19.4 84.4 0.23  1386120_at  LOC500416  Info   LOC500416 
   7860 0.0015055 593 477 1.243  1372801_at  COMM domain containing 10  Info   RGD:1303015 
   7861 0.0015064 445.8 759.2 0.587  1384240_at  angiotensin II receptor, type 1 (AT1A)  Info   Agtr1a 
   7862 0.0015074 283.4 222.6 1.273  1374517_at  RecQ protein-like (predicted)  Info   Recql_predicted 
   7863 0.0015075 147 91.9 1.6  1373171_at  similar to hypothetical protein DKFZp434L0117 (predicted)  Info   RGD1311520_predicted 
   7864 0.0015095 200.2 284.3 0.704  1369473_at  phosphoglucomutase 1  Info   Pgm1 
   7865 0.0015109 74.2 40.1 1.85  1379019_at  properdin factor, complement  Info   Pfc 
   7866 0.0015132 459.5 715.7 0.642  1390121_at  GLIS family zinc finger 2 (predicted)  Info    
   7867 0.001515 737.2 579.7 1.272  1367523_at  similar to hypothetical protein, 2-6 (predicted)  Info   RGD1304906_predicted 
   7868 0.0015156 96.1 140 0.686  1383122_at  protein kinase, AMP-activated, gamma 2 non-catalytic subunit  Info   Prkag2 
   7869 0.001517 1579.9 1935.3 0.816  1372484_at  Similar to RIKEN cDNA 4732496G21 gene (predicted)  Info   MGC93965 
   7870 0.0015181 295.1 203.6 1.449  1368862_at  v-akt murine thymoma viral oncogene homolog 1  Info   Akt1 
   7871 0.0015209 90.9 14.8 6.142  1370423_at  guanine nucleotide binding protein, alpha 15  Info   Gna15 
   7872 0.0015217 76.8 15.9 4.83  1380303_at  Transcribed locus  Info    
   7873 0.0015248 43.1 19.8 2.177  1394744_at  Eukaryotic translation elongation factor 1 gamma  Info   Eef1g 
   7874 0.0015282 120.6 194.3 0.621  1372866_at  similar to hypothetical protein MGC18873 (predicted)  Info   RGD1311914_predicted 
   7875 0.0015285 412.2 261.3 1.577  1388837_at  Similar to RIKEN cDNA 1110028E10 (predicted)  Info    
   7876 0.0015308 21.3 79.5 0.268  1385352_at  Solute carrier family 16 (monocarboxylic acid transporters), member 13  Info   MGC93826 
   7877 0.0015311 106.7 132.3 0.807  1397312_at  exportin 7 (predicted)  Info   Xpo7_predicted 
   7878 0.0015346 620.4 469.9 1.32  1388217_a_at  calumenin  Info   Calu 
   7879 0.0015359 69.9 98.5 0.71  1389340_at    Info    
   7880 0.0015403 46.5 71.3 0.652  1391812_at  Similar to RIKEN cDNA E330026B02 (predicted)  Info    
   7881 0.0015419 1441.4 856.4 1.683  1397766_at  Similar to RIKEN cDNA 4933405A16 (predicted)  Info    
   7882 0.0015424 77.1 46.8 1.647  1386701_at  Transcribed locus  Info    
   7883 0.0015443 205.5 153.3 1.341  1388893_at  glycosyltransferase AD-017  Info   RGD:1359293 
   7884 0.0015461 155.9 106.5 1.464  1393459_at  fragile X mental retardation syndrome 1 homolog  Info   Fmr1 
   7885 0.0015466 96.5 140.5 0.687  1377620_at    Info    
   7886 0.001547 171.5 248 0.692  1398252_at  nuclear receptor binding factor 1  Info   Nrbf1 
   7887 0.0015471 14.2 3.4 4.176  1396864_at    Info    
   7888 0.0015485 16.8 5.5 3.055  1377980_at    Info    
   7889 0.0015497 2718.3 3076.7 0.884  1371837_at  ddx5 gene  Info   RGD:619906 
   7890 0.0015532 61.9 104.2 0.594  1372682_at  similar to RIKEN cDNA 2810432L12 (predicted)  Info   RGD1307218_predicted 
   7891 0.0015535 71.2 102.9 0.692  1374266_at  Protocadherin 1 (cadherin-like 1) (predicted)  Info    
   7892 0.0015539 205.9 123 1.674  1388742_at    Info    
   7893 0.0015564 510.6 628.2 0.813  1368075_at  lipase A, lysosomal acid  Info   Lipa 
   7894 0.0015564 54 32.1 1.682  1383677_at  Similar to KIAA0794 protein (predicted)  Info    
   7895 0.001558 225.9 136.5 1.655  1376368_at  CUE domain containing 2 (predicted)  Info   Cuedc2_predicted 
   7896 0.0015587 1522.2 1289.8 1.18  1367473_at  TOM22 protein  Info   RGD:1303260 
   7897 0.0015643 30.3 135.3 0.224  1375707_at    Info    
   7898 0.0015658 61.3 30.4 2.016  1375449_at  Zinc finger homeobox 1b (predicted)  Info    
   7899 0.0015665 633 858 0.738  1372108_at  similar to RIKEN cDNA 2810422B04  Info   LOC500199 
   7900 0.001569 41.5 73.3 0.566  1393064_at  Transcribed locus  Info    
   7901 0.0015717 238.2 329.7 0.722  1382807_at  similar to hypothetical protein (predicted)  Info   RGD1310450_predicted 
   7902 0.001573 85.5 39.8 2.148  1393365_at  Transcribed locus  Info    
   7903 0.0015776 32.4 59.5 0.545  1394779_at  Transcribed locus  Info    
   7904 0.0015816 1.4 9.2 0.152  1397172_at  eukaryotic translation initiation factor 2C, 3 (predicted)  Info   Eif2c3_predicted 
   7905 0.0015846 30.9 17.6 1.756  1369772_at  solute carrier family 6 (neurotransmitter transporter, glycine), member 9  Info   Slc6a9 
   7906 0.0015867 83.1 44.8 1.855  1395135_at  similar to Wbscr27 protein  Info   LOC360794 
   7907 0.0015892 223.6 294.2 0.76  1382085_at  similar to RIKEN cDNA 1810059A23  Info   LOC363289 
   7908 0.0015901 85 34.6 2.457  1379062_at  similar to Expressed sequence AU019823  Info   LOC500994 
   7909 0.0015914 91.8 48 1.912  1375988_at    Info    
   7910 0.0015919 6848.9 5386.6 1.271  1398324_at  similar to 60S ribosomal protein L18a  Info   RGD:1302976 
   7911 0.0015934 160.7 246.6 0.652  1397349_at  Similar to reproduction 8 (predicted)  Info    
   7912 0.0015936 66.5 49.7 1.338  1373656_at  Transcribed locus  Info    
   7913 0.0015941 177.2 153 1.158  1389090_at  Werner helicase interacting protein 1  Info   Wrnip1 
   7914 0.001595 12.6 35.7 0.353  1389449_at  similar to CG10671-like (predicted)  Info   RGD1306911_predicted 
   7915 0.0015955 69.5 114.4 0.608  1382184_at  similar to regulator of Fas-induced apoptosis /// Fas apoptotic inhibitory molecule  Info   LOC498224 /// RGD:1359282 
   7916 0.0015968 12.3 4.2 2.929  1380269_at  FUS interacting protein (serine-arginine rich) 1 (predicted)  Info    
   7917 0.0015986 83.4 51.4 1.623  1392546_at    Info    
   7918 0.0016014 187.2 169.4 1.105  1370183_at  dual-specificity tyrosine-(Y)-phosphorylation regulated kinase 1A  Info   Dyrk1a 
   7919 0.0016037 29.8 9.7 3.072  1393891_at  procollagen, type VIII, alpha 1 (predicted)  Info   Col8a1_predicted 
   7920 0.0016038 58.5 43 1.36  1382722_at  scavenger receptor class F, member 1 (predicted) /// similar to Scavenger receptor class F, member 1  Info   Scarf1_predicted /// LOC499066 
   7921 0.0016044 461 322.9 1.428  1382045_at  TBC1 domain family, member 15 (predicted)  Info   Tbc1d15_predicted 
   7922 0.0016063 597.8 719.7 0.831  1375413_at  sirtuin (silent mating type information regulation 2 homolog) 2 (S. cerevisiae)  Info   Sirt2 
   7923 0.0016067 211.5 157.2 1.345  1376574_at  Lymphocyte antigen 68  Info   C1qr1 
   7924 0.0016069 88.6 50.3 1.761  1372584_at  similar to DKFZP566K1924 protein (predicted)  Info   RGD1308373_predicted 
   7925 0.0016116 248.5 160.9 1.544  1387836_at  Prenylated SNARE protein  Info   Ykt6 
   7926 0.0016151 156.5 255.9 0.612  1382197_at  ras homolog gene family, member D (predicted)  Info   Rhod_predicted 
   7927 0.0016162 303.6 415.8 0.73  1373469_at  Transcribed locus  Info    
   7928 0.0016166 91.1 131.9 0.691  1383799_at    Info    
   7929 0.0016196 67.7 49.4 1.37  1383772_at  Transcribed locus  Info    
   7930 0.0016204 39.6 60.2 0.658  1368830_at  Rous sarcoma oncogene  Info   Src 
   7931 0.0016204 85.3 136.7 0.624  1373814_at  similar to mKIAA1002 protein (predicted)  Info   RGD1310066_predicted 
   7932 0.0016205 8.3 21.5 0.386  1395170_at    Info    
   7933 0.0016206 134.2 262.5 0.511  1375448_at  Transcribed locus  Info    
   7934 0.0016219 146.2 210.5 0.695  1376731_at  Phosphatidylinositol glycan, class N (predicted)  Info    
   7935 0.0016225 3.3 13.9 0.237  1375679_at  similar to RIKEN cDNA 2310035C23 (predicted)  Info   RGD1307235_predicted 
   7936 0.0016228 414.1 283.3 1.462  1370002_at  Rho guanine nucleotide exchange factor (GEF) 1  Info   Arhgef1 
   7937 0.001623 91.3 10.3 8.864  1377340_at  tissue factor pathway inhibitor 2  Info   Tfpi2 
   7938 0.0016236 82.7 6.5 12.723  1387794_at  ficolin A  Info   Fcna 
   7939 0.0016248 144.9 198.2 0.731  1382866_at  similar to Putative protein 15E1.2 (predicted)  Info   RGD1309698_predicted 
   7940 0.0016259 182.5 149.5 1.221  1380106_at  zinc finger protein 162  Info   Zfp162 
   7941 0.0016303 227.3 167.2 1.359  1392180_at  Sp1 transcription factor  Info   Sp1 
   7942 0.0016317 109.4 60.2 1.817  AFFX_Rat_Hexokinase_5_at  hexokinase 1  Info   Hk1 
   7943 0.001632 132.4 68.3 1.939  1377174_at  SET binding factor 1 (predicted)  Info   Sbf1_predicted 
   7944 0.0016333 123.6 71.7 1.724  1376708_at  Transcribed locus, strongly similar to NP_666286.1 hypothetical protein LOC232748 [Mus musculus]  Info    
   7945 0.0016366 17.7 38.8 0.456  1397296_at  Rhomboid, veinlet-like 4 (Drosophila) (predicted)  Info    
   7946 0.0016393 184.3 298.8 0.617  1370043_at  activated leukocyte cell adhesion molecule  Info   Alcam 
   7947 0.0016393 202.4 317.9 0.637  1375760_at  Transducer of ERBB2, 2  Info   Tob2 
   7948 0.0016407 376.2 316.6 1.188  1388912_at    Info    
   7949 0.0016423 13 36.2 0.359  1393437_at  Transcribed locus  Info    
   7950 0.0016438 363.4 261.5 1.39  1374316_at  Transcribed locus  Info    
   7951 0.0016448 217.8 149.1 1.461  1393367_at  polynucleotide kinase 3 ' -phosphatase  Info   RGD:1303331 
   7952 0.0016466 441.2 343 1.286  1373849_at  similar to BAF53a (predicted)  Info   RGD1307747_predicted 
   7953 0.0016478 1193.7 1021.4 1.169  1367480_at  Transcribed locus, strongly similar to NP_055555.1 DEAD (Asp-Glu-Ala-Asp) box polypeptide 48; eukaryotic initiation factor 4A-like NUK-34 [Homo sapiens]  Info    
   7954 0.0016496 39.2 60.5 0.648  1370392_at  transient receptor potential cation channel, subfamily M, member 4  Info   Trpm4 
   7955 0.0016516 122.6 65.4 1.875  1388954_at    Info    
   7956 0.001652 1199.2 1546.9 0.775  1372347_at  Transcribed locus  Info    
   7957 0.0016559 35.4 58.7 0.603  1378291_at  FK506 binding protein 3 (predicted)  Info    
   7958 0.0016568 114.2 91.6 1.247  1383267_at  Similar to RIKEN cDNA 2210404D11 (predicted)  Info    
   7959 0.0016573 46.3 25.7 1.802  1385133_at    Info    
   7960 0.0016587 178.2 120.7 1.476  1373004_at  DnaJ (Hsp40) homolog, subfamily C, member 2  Info   Dnajc2 
   7961 0.0016591 2793.2 2395.4 1.166  1373877_at  Phosphatidylinositol binding clathrin assembly protein  Info   Picalm 
   7962 0.0016614 137 212.2 0.646  1374486_at  Similar to hypothetical protein FLJ32884 (predicted)  Info    
   7963 0.0016622 253.3 432.4 0.586  1376214_at  Similar to RIKEN cDNA 1810037K07 (predicted)  Info    
   7964 0.0016679 261.2 173.7 1.504  1372253_at  CCR4-NOT transcription complex, subunit 8 (predicted)  Info   Cnot8_predicted 
   7965 0.0016691 109 66.9 1.629  1391170_at  similar to mKIAA1757 protein (predicted)  Info   RGD1310433_predicted 
   7966 0.0016695 212.1 297.2 0.714  1399107_at  similar to RIKEN cDNA 1810030N24 (predicted)  Info   RGD1305158_predicted 
   7967 0.0016698 204.9 149.7 1.369  1390769_at  Ribose-phosphate pyrophosphokinase I -like  Info   LOC314140 
   7968 0.00167 116.6 147.5 0.791  1382037_at  cysteine-rich motor neuron 1 (predicted)  Info   Crim1_predicted 
   7969 0.0016709 927.7 711.3 1.304  1367972_at  TBP-interacting protein 120A  Info   Tip120A 
   7970 0.001671 108 196.1 0.551  1374496_at  similar to RIKEN cDNA 1200009O22; EST AI316813 (predicted)  Info   RGD1310827_predicted 
   7971 0.0016742 9.5 69.6 0.136  1387852_at  thyroid hormone responsive protein  Info   Thrsp 
   7972 0.0016754 308.3 252.1 1.223  1382141_at  SET domain, bifurcated 1 (predicted)  Info   Setdb1_predicted 
   7973 0.0016785 56.7 107.8 0.526  1379803_at  LIM domain only 4 (predicted)  Info   Lmo4_predicted 
   7974 0.0016789 77.4 7.4 10.459  1376872_at  Transcribed locus  Info    
   7975 0.0016801 77.7 57.5 1.351  1396320_at  CDC2-related kinase 7  Info   Crk7 
   7976 0.0016829 148.9 314.5 0.473  1392865_at  fibroblast growth factor 9  Info   Fgf9 
   7977 0.0016903 54.6 17.1 3.193  1368350_at  protein tyrosine phosphatase, receptor-type, Z polypeptide 1 /// hypothetical gene supported by NM_013080  Info   Ptprz1 /// LOC497784 
   7978 0.001691 151.7 204.6 0.741  1398579_at  Transcribed locus, moderately similar to XP_580018.1 PREDICTED: hypothetical protein XP_580018 [Rattus norvegicus]  Info    
   7979 0.0016917 1.1 4.6 0.239  1374260_at  Transcribed locus  Info    
   7980 0.0016918 52.1 23.6 2.208  1377851_at  Transcribed locus, weakly similar to NP_997348.1 FLJ45337 protein [Homo sapiens]  Info    
   7981 0.0016933 42.2 13.8 3.058  1376983_at    Info    
   7982 0.0016936 238.9 306.2 0.78  1372412_at  similar to hypothetical protein FLJ10477 (predicted)  Info   RGD1308109_predicted 
   7983 0.0016949 185.1 140.4 1.318  1377151_at  Transcribed locus  Info    
   7984 0.0016951 1.6 6.4 0.25  1378426_at  Similar to 2410024A21Rik protein (predicted)  Info    
   7985 0.0016973 135.5 171.4 0.791  1386942_at  cis-Golgi matrix protein GM130  Info   Golga2 
   7986 0.0016994 10.3 28.8 0.358  1383014_at  Transcribed locus  Info    
   7987 0.0017005 123.4 72.4 1.704  1397255_at  similar to hypothetical protein MGC2574  Info   RGD:1359280 
   7988 0.0017009 24.7 56.7 0.436  1393433_at  similar to Agrin (predicted)  Info   RGD1306592_predicted 
   7989 0.001701 208.6 274.6 0.76  1376303_a_at  membralin (predicted)  Info   RGD1311136_predicted 
   7990 0.0017024 187.6 126.9 1.478  1374141_at  similar to RIKEN cDNA F730014I05 (predicted)  Info   RGD1310800_predicted 
   7991 0.0017037 3295.3 1836.6 1.794  1370219_at  cytochrome b-245, alpha polypeptide  Info   Cyba 
   7992 0.0017072 193.4 260 0.744  1390555_at  similar to Suppressor of cytokine signaling 5  Info   LOC500616 
   7993 0.0017075 695.6 506.1 1.374  1367464_at  siah binding protein 1; FBP interacting repressor; pyrimidine tract binding splicing factor; Ro ribonucleoprotein-binding protein 1  Info   RGD:621674 
   7994 0.0017087 250.4 334.1 0.749  1395362_at  Transcribed locus  Info    
   7995 0.0017125 70.8 154.3 0.459  1373963_at    Info    
   7996 0.0017128 124.9 267.9 0.466  1390847_at  similar to RIKEN cDNA 1810054O13 (predicted)  Info   RGD1305749_predicted 
   7997 0.0017145 70.1 101.6 0.69  1391370_at  Transcribed locus  Info    
   7998 0.0017153 118 86.4 1.366  1389981_at  RNA binding motif protein 27 (predicted)  Info   Rbm27_predicted 
   7999 0.0017169 239.1 316.4 0.756  1372551_at  Fas-activated serine/threonine kinase (predicted)  Info    
   8000 0.0017172 48.6 90 0.54  1368219_at  chloride channel 2  Info   Clcn2 
   8001 0.0017224 59.4 158.4 0.375  1388561_at  Transcribed locus  Info    
   8002 0.0017237 9.2 68.7 0.134  1397688_at  protocadherin 9 (predicted)  Info   Pcdh9_predicted 
   8003 0.0017272 205.1 139.6 1.469  1379249_at  Similar to Wilms tumor 1-associating protein (WT1-associated protein) (Putative pre-mRNA splicing regulator female-lethal(2D) homolog)  Info    
   8004 0.0017274 80.7 36.5 2.211  1396742_at  karyopherin (importin) beta 3 (predicted)  Info   Kpnb3_predicted 
   8005 0.0017282 9.8 33 0.297  1391147_at  Similar to opioid growth factor receptor  Info    
   8006 0.0017306 549.3 661.7 0.83  1373846_at  similar to RIKEN cDNA 5730434I03 gene (predicted)  Info   RGD1311774_predicted 
   8007 0.0017318 127.7 78.5 1.627  1375130_at  zinc finger protein 216 (predicted)  Info   Zfp216_predicted 
   8008 0.0017362 80.8 58.9 1.372  1376266_at  Transcribed locus  Info    
   8009 0.0017365 121.6 167.8 0.725  1383443_at  similar to RIKEN cDNA 1110018J18  Info   LOC498685 
   8010 0.0017412 51.3 87.9 0.584  1392551_at  Transcribed locus  Info    
   8011 0.0017447 17.4 2.6 6.692  1396304_at  Transcribed locus  Info    
   8012 0.0017462 310.7 463.1 0.671  1380598_at  Transcribed locus  Info    
   8013 0.0017464 219.9 12.4 17.734  1370429_at  RT1 class Ib, locus Aw2  Info   RT1-Aw2 
   8014 0.0017469 55.5 91.4 0.607  1390466_at  Transcribed locus  Info    
   8015 0.0017477 79.4 29.3 2.71  1382919_at  Solute carrier family 39 (zinc transporter), member 14 (predicted)  Info    
   8016 0.0017478 84.1 7.5 11.213  1376231_at  similar to Hypothetical UPF0080 protein KIAA0186  Info   LOC499914 
   8017 0.001749 6.9 27.3 0.253  1397322_at  Transcribed locus, weakly similar to XP_341407.2 PREDICTED: similar to RIKEN cDNA 5730469M10 [Rattus norvegicus]  Info    
   8018 0.001749 247.2 175 1.413  1374489_at  GTP binding protein 2 (predicted)  Info   Gtpbp2_predicted 
   8019 0.0017493 217.3 159.2 1.365  1383929_at  Transcribed locus  Info    
   8020 0.0017505 14.1 23.9 0.59  1369086_a_at  calcium channel, voltage-dependent, L type, alpha 1D subunit  Info   Cacna1d 
   8021 0.0017507 16.7 39.4 0.424  1376677_a_at  Transcribed locus  Info    
   8022 0.0017541 285.1 198.7 1.435  1389395_at  Transcribed locus, moderately similar to NP_065184.2 selenoprotein N, 1 isoform 1 precursor; muscular dystrophy with rigid spine 1; rigid spine muscular dystrophy 1 [Homo sapiens]  Info    
   8023 0.0017564 117 74.1 1.579  1380163_at  SEC23 interacting protein (predicted)  Info    
   8024 0.0017574 99.6 35 2.846  1386191_a_at  Transcribed locus  Info    
   8025 0.0017589 65.8 31.6 2.082  1380690_at  Cell division cycle associated 7 (predicted)  Info    
   8026 0.0017591 970.3 1320.9 0.735  1368272_at  glutamate oxaloacetate transaminase 1  Info   Got1 
   8027 0.0017608 104.1 80.2 1.298  1388578_at  similar to membrane targeting translocation system protein like (2A562)  Info   LOC289378 
   8028 0.0017624 265.9 168.1 1.582  1395061_at  D4, zinc and double PHD fingers family 2 (predicted)  Info   Dpf2_predicted 
   8029 0.0017626 83.2 55.8 1.491  1397617_at  Similar to Retinoblastoma-binding protein 2 (RBBP-2)  Info    
   8030 0.0017632 7.4 51.7 0.143  1380172_at  kinesin family member 5C (predicted)  Info   Kif5c_predicted 
   8031 0.0017667 489 707.4 0.691  1393216_at  acetyl-CoA transporter  Info   Slc33a1 
   8032 0.0017681 73.6 113.4 0.649  1377084_at  Similar to hypothetical protein (predicted)  Info    
   8033 0.0017687 86 57.4 1.498  1372792_at  similar to hypothetical protein MGC15677  Info   LOC292780 
   8034 0.0017707 412.7 243.9 1.692  1398362_at  Transcribed locus  Info    
   8035 0.0017723 227.9 143.9 1.584  1384858_a_at  similar to RIKEN cDNA D530033C11 (predicted)  Info   RGD1307179_predicted 
   8036 0.0017743 1051.1 750.7 1.4  1371918_at  CD99  Info    
   8037 0.0017751 21.6 53.4 0.404  1390671_at  Insulin-like growth factor 1 receptor  Info   Igf1r 
   8038 0.0017752 15.5 44.8 0.346  1368898_at  MAD homolog 7 (Drosophila)  Info   Madh7 
   8039 0.0017767 101.1 69.2 1.461  1396692_at    Info    
   8040 0.0017784 126.8 89 1.425  1379831_at  similar to multiple hat domains (predicted)  Info   RGD1311017_predicted 
   8041 0.0017801 78.3 106.3 0.737  1378215_at  similar to RIKEN cDNA 4833422P03 (predicted)  Info   RGD1311568_predicted 
   8042 0.0017807 144.4 95.1 1.518  1381145_at  Transcribed locus  Info    
   8043 0.0017865 75.8 125 0.606  1393142_at  similar to p10-binding protein  Info   LOC367153 
   8044 0.0017876 12 31.6 0.38  1388632_at  RAB6B, member RAS oncogene family (predicted)  Info    
   8045 0.0017879 43.4 68.7 0.632  1369543_s_at  putative regulation protein GS3  Info   RGD:621729 
   8046 0.0017903 201 256.8 0.783  1374664_at  Similar to TTF-I interacting peptide 20; TIP20; Transcription Termination Factor I Interacting Peptide 20 (predicted)  Info    
   8047 0.0017903 139.4 253.3 0.55  1384217_at  Transcribed locus  Info    
   8048 0.0017917 26.5 57.6 0.46  1381618_at  Transcribed locus  Info    
   8049 0.0017919 66.1 36.2 1.826  1395376_at  DEAD/H (Asp-Glu-Ala-Asp/His) box polypeptide 11 (CHL1-like helicase homolog, S. cerevisiae) (predicted)  Info   Ddx11_predicted 
   8050 0.0017926 78.7 13.9 5.662  1384063_at  collagen triple helix repeat containing 1  Info   Cthrc1 
   8051 0.0017928 103.4 147.5 0.701  1378395_at  Transcribed locus  Info    
   8052 0.0017943 483.9 370.6 1.306  1388468_at  Similar to hypothetical protein  Info    
   8053 0.0017945 71.5 27.5 2.6  1375014_at    Info    
   8054 0.001796 91.3 123.7 0.738  1392588_at  receptor interacting protein kinase 5  Info   Ripk5 
   8055 0.0017962 27.4 13.8 1.986  1392099_at  similar to Hypothetical protein MGC57096  Info   LOC310463 
   8056 0.0017994 58 29.1 1.993  1373219_at  snail homolog, (Drosophila)  Info   Snai1 
   8057 0.0018012 102.8 80.3 1.28  1390201_at  Ras-related protein RAP-1A  Info   RGD:1359694 
   8058 0.0018045 227.4 301.3 0.755  1392468_at  similar to KIAA1078 protein (predicted)  Info   RGD1310950_predicted 
   8059 0.0018075 64.7 48.9 1.323  1385691_at    Info    
   8060 0.0018094 2.6 8.8 0.295  1370601_a_at  glutamate receptor, ionotropic, N-methyl-D-aspartate 3A  Info   Grin3a 
   8061 0.0018107 75.9 113.1 0.671  1367970_at  profilin 2  Info   Pfn2 
   8062 0.0018125 364.2 299 1.218  1368083_at  cyclin H  Info   Ccnh 
   8063 0.0018131 12.5 2.9 4.31  1382901_at    Info    
   8064 0.001814 50.3 101.1 0.498  1379814_at  CDNA clone IMAGE:7302159  Info    
   8065 0.0018145 247.7 172.2 1.438  1372931_at  Similar to DXImx39e protein  Info    
   8066 0.0018153 1.9 11.1 0.171  1394765_at  Transcribed locus  Info    
   8067 0.0018175 164.2 93.8 1.751  1383230_at  LOC360664 (predicted)  Info   RGD1311078_predicted 
   8068 0.0018192 574 482.8 1.189  1398875_at  estrous-specific protein, 250 kDa /// similar to RIKEN cDNA 1500004O14  Info   RGD:708426 /// LOC366277 
   8069 0.0018213 62.1 95.4 0.651  1382200_at  similar to Prr6 protein  Info   LOC501702 
   8070 0.0018217 659.4 812.8 0.811  1371433_at  Transcribed locus  Info    
   8071 0.0018245 184.4 342.5 0.538  1368281_at  dipeptidase 1 (renal)  Info   Dpep1 
   8072 0.0018252 3.4 14.9 0.228  1385793_at  Similar to RIKEN cDNA 2310067G05  Info    
   8073 0.0018285 30.9 15.4 2.006  1397336_at  poly(A) polymerase gamma (predicted)  Info   Papolg_predicted 
   8074 0.0018324 32.7 68.6 0.477  1397365_at  Similar to ribosomal protein L27a  Info    
   8075 0.0018334 113.5 193.9 0.585  1378003_at  Transcribed locus  Info    
   8076 0.0018335 424.1 291.8 1.453  1385758_at  similar to B430201G11Rik protein (predicted)  Info   RGD1311409_predicted 
   8077 0.0018341 37 19.4 1.907  1392995_at  Transcribed locus  Info    
   8078 0.0018343 652.8 890.8 0.733  1382971_at  Programmed cell death 6 interacting protein  Info    
   8079 0.0018356 162.9 115.1 1.415  1374620_at  CEA-related cell adhesion molecule 1  Info   Ceacam1 
   8080 0.0018379 54.4 98.3 0.553  1377961_at  similar to AT motif-binding factor  Info   LOC307829 
   8081 0.0018424 3.3 14.5 0.228  1398192_at  Parkin  Info   Park2 
   8082 0.0018425 41.5 21.8 1.904  1390867_at    Info    
   8083 0.0018441 78.3 48.6 1.611  1396150_at  claudin 1  Info   Cldn1 
   8084 0.0018479 27.3 44.2 0.618  1379971_at  zinc finger CCCH type domain containing 6 (predicted)  Info   Zc3hdc6_predicted 
   8085 0.0018494 163.5 287.2 0.569  1380022_at  similar to AL023001 protein (predicted)  Info   RGD1305001_predicted 
   8086 0.0018524 21.6 65.4 0.33  1372240_at  sarcoglycan, alpha (dystrophin-associated glycoprotein) (predicted)  Info   Sgca_predicted 
   8087 0.0018545 826.4 548.1 1.508  1387860_at  calpain 2  Info   Capn2 
 
  
 
    
'Observed v. Expected' table of GO classes and parent classes, in list of 8087 genes shown above:
    
Only GO classes and parent classes with at least 5 observations in the selected subset and with an 'Observed vs. Expected' ratio of at least 2 are shown.  
 Cellular Component 
 
  
  GO id GO classification Observed in selected subset Expected in selected subset Observed/ Expected
  0005732 small nucleolar ribonucleoprotein complex  7 2.13 3.29
  0042613 MHC class II protein complex  9 3.04 2.96
  0005830 cytosolic ribosome (sensu Eukaryota)  8 2.73 2.93
  0042101 T cell receptor complex  5 1.82 2.74
  0005664 nuclear origin of replication recognition complex  5 1.82 2.74
  0005885 Arp2/3 protein complex  9 3.34 2.69
  0001772 immunological synapse  38 14.27 2.66
  0042611 MHC protein complex  32 12.15 2.63
  0042612 MHC class I protein complex  22 8.5 2.59
  0005839 proteasome core complex (sensu Eukaryota)  12 4.86 2.47
  0000808 origin recognition complex  5 2.13 2.35
  0005657 replication fork  9 3.95 2.28
  0030894 replisome  8 3.64 2.2
  0005741 mitochondrial outer membrane  5 2.43 2.06
 
  
 Molecular Function 
 
  
  GO id GO classification Observed in selected subset Expected in selected subset Observed/ Expected
  0042605 peptide antigen binding  6 1.82 3.3
  0000064 L-ornithine transporter activity  5 1.52 3.3
  0042287 MHC protein binding  6 2.12 2.83
  0003891 delta DNA polymerase activity  5 1.82 2.75
  0045012 MHC class II receptor activity  9 3.34 2.7
  0030106 MHC class I receptor activity  29 11.22 2.58
  0016893 endonuclease activity, active with either ribo- or deoxyribonucleic acids and producing 5`-phosphomonoesters  6 2.43 2.47
  0016891 endoribonuclease activity, producing 5`-phosphomonoesters  6 2.43 2.47
  0016805 dipeptidase activity  6 2.43 2.47
  0050136 NADH dehydrogenase (quinone) activity  13 5.46 2.38
  0016655 oxidoreductase activity, acting on NADH or NADPH, quinone or similar compound as acceptor  13 5.46 2.38
  0008137 NADH dehydrogenase (ubiquinone) activity  13 5.46 2.38
  0003954 NADH dehydrogenase activity  13 5.46 2.38
  0004521 endoribonuclease activity  7 3.03 2.31
  0004540 ribonuclease activity  8 3.64 2.2
  0016651 oxidoreductase activity, acting on NADH or NADPH  15 6.98 2.15
  0015081 sodium ion transporter activity  13 6.07 2.14
  0003690 double-stranded DNA binding  9 4.25 2.12
  0015179 L-amino acid transporter activity  7 3.34 2.1
  0004518 nuclease activity  17 8.19 2.08
  0019955 cytokine binding  8 3.94 2.03
  0008642 ubiquitin-like activating enzyme activity  8 3.94 2.03
  0004519 endonuclease activity  8 3.94 2.03
 
  
 Biological Process 
 
  
  GO id GO classification Observed in selected subset Expected in selected subset Observed/ Expected
  0051223 regulation of protein transport  5 1.6 3.12
  0042977 tyrosine phosphorylation of JAK2 protein  5 1.6 3.12
  0042976 activation of JAK protein  5 1.6 3.12
  0009618 response to pathogenic bacteria  7 2.24 3.12
  0007595 lactation  6 1.92 3.12
  0042828 response to pathogen  10 3.52 2.84
  0006740 NADPH regeneration  7 2.56 2.73
  0006739 NADP metabolism  7 2.56 2.73
  0006284 base-excision repair  7 2.56 2.73
  0006098 pentose-phosphate shunt  7 2.56 2.73
  0051017 actin filament bundle formation  6 2.24 2.68
  0030856 regulation of epithelial cell differentiation  6 2.24 2.68
  0050870 positive regulation of T cell activation  5 1.92 2.6
  0043094 metabolic compound salvage  5 1.92 2.6
  0030593 neutrophil chemotaxis  5 1.92 2.6
  0015833 peptide transport  5 1.92 2.6
  0008635 caspase activation via cytochrome c  5 1.92 2.6
  0007171 transmembrane receptor protein tyrosine kinase activation (dimerization)  5 1.92 2.6
  0006857 oligopeptide transport  5 1.92 2.6
  0006760 folic acid and derivative metabolism  5 1.92 2.6
  0006271 DNA strand elongation  5 1.92 2.6
  0006030 chitin metabolism  5 1.92 2.6
  0019882 antigen presentation  19 7.37 2.58
  0046822 regulation of nucleocytoplasmic transport  9 3.52 2.55
  0042773 ATP synthesis coupled electron transport  20 8.01 2.5
  0006084 acetyl-CoA metabolism  16 6.41 2.5
  0042775 ATP synthesis coupled electron transport (sensu Eukaryota)  18 7.37 2.44
  0030595 immune cell chemotaxis  7 2.88 2.43
  0009065 glutamine family amino acid catabolism  7 2.88 2.43
  0006120 mitochondrial electron transport, NADH to ubiquinone  17 7.05 2.41
  0046356 acetyl-CoA catabolism  13 5.45 2.39
  0009109 coenzyme catabolism  13 5.45 2.39
  0050900 immune cell migration  9 3.84 2.34
  0019362 pyridine nucleotide metabolism  12 5.13 2.34
  0009060 aerobic respiration  12 5.13 2.34
  0006099 tricarboxylic acid cycle  12 5.13 2.34
  0006044 N-acetylglucosamine metabolism  9 3.84 2.34
  0009063 amino acid catabolism  20 8.65 2.31
  0045333 cellular respiration  14 6.09 2.3
  0007568 aging  8 3.52 2.27
  0006405 RNA-nucleus export  8 3.52 2.27
  0050672 negative regulation of lymphocyte proliferation  5 2.24 2.23
  0045672 positive regulation of osteoclast differentiation  5 2.24 2.23
  0045657 positive regulation of monocyte differentiation  5 2.24 2.23
  0042130 negative regulation of T cell proliferation  5 2.24 2.23
  0030333 antigen processing  5 2.24 2.23
  0019835 cytolysis  10 4.49 2.23
  0009071 serine family amino acid catabolism  5 2.24 2.23
  0007088 regulation of mitosis  5 2.24 2.23
  0006546 glycine catabolism  5 2.24 2.23
  0009617 response to bacteria  12 5.45 2.2
  0051187 cofactor catabolism  14 6.41 2.19
  0051051 negative regulation of transport  7 3.2 2.19
  0006769 nicotinamide metabolism  7 3.2 2.19
  0008629 induction of apoptosis by intracellular signals  9 4.16 2.16
  0006100 tricarboxylic acid cycle intermediate metabolism  9 4.16 2.16
  0007259 JAK-STAT cascade  20 9.29 2.15
  0009615 response to virus  17 8.01 2.12
  0045670 regulation of osteoclast differentiation  6 2.88 2.08
  0042742 defense response to bacteria  8 3.84 2.08
  0042108 positive regulation of cytokine biosynthesis  6 2.88 2.08
  0030316 osteoclast differentiation  6 2.88 2.08
  0030217 T cell differentiation  8 3.84 2.08
  0016066 cellular defense response (sensu Vertebrata)  14 6.73 2.08
  0008625 induction of apoptosis via death domain receptors  8 3.84 2.08
  0007569 cell aging  6 2.88 2.08
  0007566 embryo implantation  6 2.88 2.08
  0006839 mitochondrial transport  8 3.84 2.08
  0006733 oxidoreduction coenzyme metabolism  12 5.77 2.08
  0001894 tissue homeostasis  8 3.84 2.08
  0001819 positive regulation of cytokine production  6 2.88 2.08
  0001776 immune cell homeostasis  6 2.88 2.08
  0050863 regulation of T cell activation  11 5.45 2.02
  0019221 cytokine and chemokine mediated signaling pathway  11 5.45 2.02
  0050867 positive regulation of cell activation  9 4.49 2.01
  0042088 T-helper 1 type immune response  9 4.49 2.01
  0006041 glucosamine metabolism  9 4.49 2.01
  0006119 oxidative phosphorylation  25 12.49 2
 
  
  
 
  
 
  
 
 
 
 
   Date and time of the analysis:   
Name of the project file: Project.xls
 
Time of the analysis: Fri Apr 07 13:25:43 2006
 
BRB-ArrayTools Version: 3.3
 
 
   Filtering parameters:   
   Spot Filters: OFF
    
    
Normalization: OFF
   
   Gene Filters: OFF
   
   Gene Subsets: OFF
   
      View expression of significant genes.
