# Supplementary material for: Activation of counter-regulatory mechanisms in a rat renal acute rejection model
Source: BMC Genomics. 2008 Feb 8;9:71. doi: 10.1186/1471-2164-9-71 (PMC2262896; doi:10.1186/1471-2164-9-71)
Supplement: Additional file 1 — Gene lists from the class comparison between the groups analyzed with BRB ArrayTools. [file 1471-2164-9-71-S1.zip › ControlvsAllogeneic/ExpressionTable.html]

  
 
 
    Table.  Normalized Log-Transformed Gene Expressions for Significant Genes  
  
    Experiment  Allogeneic1 Allogeneic2 Allogeneic3 Allogeneic4 Allogeneic5 Control1 Control2 Control3 Control4 Control5
    Class  allogeneic allogeneic allogeneic allogeneic allogeneic control control control control control
   1   1374551_at   10.357 10.312 10.297 10.317 10.347 7.927 7.855 8.009 7.96 7.965
   2   1387946_at   11.427 11.407 11.36 11.316 11.411 8.621 8.445 8.522 8.525 8.587
   3   1385439_x_at   8.782 8.757 8.664 8.888 8.878 4.717 4.618 4.635 4.527 4.719
   4   1371537_at   9.81 9.722 9.702 9.735 9.803 7.192 7.3 7.216 7.196 7.12
   5   1373490_at   10.17 10.149 10.305 10.339 10.2 7.508 7.516 7.607 7.593 7.494
   6   1372406_at   8.436 8.465 8.388 8.458 8.354 4.385 4.689 4.709 4.703 4.637
   7   1389210_at   11.317 11.478 11.54 11.503 11.391 8.438 8.401 8.585 8.415 8.436
   8   1373818_at   10.591 10.642 10.544 10.469 10.546 8.319 8.148 8.184 8.178 8.245
   9   1367676_at   10.85 10.853 10.921 10.791 10.825 7.527 7.758 7.641 7.434 7.6
   10   1388164_at   10.594 10.583 10.618 10.659 10.69 7.466 7.516 7.718 7.592 7.391
   11   1389873_at   9.115 9.126 9.097 8.993 9.16 5.914 6.124 6.156 6.182 5.971
   12   1383564_at   11.243 11.238 11.277 11.268 11.258 8.203 8.246 8.186 8.393 8.487
   13   1373240_at   8.34 8.27 8.264 8.261 8.287 9.648 9.578 9.717 9.611 9.641
   14   1371209_at   11.64 11.663 11.616 11.416 11.552 7.934 8.168 7.947 8.014 8.207
   15   1387770_at   12.28 12.263 12.245 12.165 12.365 9.924 9.899 9.937 10.048 10.072
   16   1373575_at   12.328 12.314 12.312 12.261 12.39 9.53 9.523 9.782 9.49 9.526
   17   1372070_at   11.636 11.632 11.622 11.545 11.679 10.238 10.229 10.254 10.279 10.164
   18   1373672_at   10.557 10.528 10.7 10.621 10.518 7.958 7.912 7.688 7.874 7.842
   19   1373025_at   11.406 11.352 11.438 11.274 11.274 8.18 8.038 8.215 7.874 8.094
   20   1388784_at   9.886 9.905 9.849 9.909 9.739 7.758 7.761 7.65 7.615 7.61
   21   1387687_at   8.967 9.008 9.125 9.029 9.055 4.161 4.455 4.751 4.38 4.455
   22   1369186_at   9.793 9.82 9.867 9.807 10.012 6.035 6.254 6.283 6.386 6.043
   23   1372752_at   9.744 9.727 9.792 9.787 9.759 8.896 8.895 8.956 8.889 8.868
   24   1373523_at   12.336 12.163 12.271 11.92 12.073 8.114 7.969 8.3 8.153 8.083
   25   1370493_a_at   8.439 8.405 8.62 8.435 8.299 4.77 4.566 4.786 4.523 4.432
   26   1388213_a_at   11.222 11.231 11.104 11.163 11.335 7.805 7.788 7.895 7.572 7.537
   27   1382043_at   10.764 10.796 10.695 10.729 10.823 7.82 8.018 7.72 7.711 7.648
   28   1383748_at   8.241 8.243 8.242 8.262 8.267 7.37 7.355 7.26 7.374 7.339
   29   1390022_at   11.833 11.754 11.782 11.68 11.781 10.536 10.589 10.598 10.52 10.55
   30   1379766_at   9.195 9.159 9.566 9.439 9.293 5.128 5.319 5.358 5.177 5.002
   31   1374544_at   8.477 8.447 8.571 8.571 8.591 6.375 6.296 6.478 6.225 6.412
   32   1389114_at   9.223 9.061 9.052 9.146 9.103 10.633 10.717 10.609 10.7 10.638
   33   1398348_at   10.291 10.21 10.187 10.288 10.204 11.578 11.726 11.654 11.665 11.725
   34   1369204_at   10.186 10.279 10.294 10.253 10.206 5.594 5.484 6.075 5.94 5.808
   35   1368723_at   8.723 8.618 8.851 8.87 8.743 4.38 4.42 4.896 4.441 4.498
   36   1391979_at   9.101 9.438 9.488 9.498 9.505 5.769 5.75 5.785 5.644 5.513
   37   1370383_s_at   11.797 11.769 11.876 11.744 11.698 9.145 9.024 9.372 9.247 9.222
   38   1392894_at   9.931 9.992 10.011 10.113 9.996 7.72 7.565 7.718 7.522 7.454
   39   1370822_at   12.449 12.377 12.421 12.228 12.222 9.622 9.526 9.634 9.828 9.605
   40   1379613_at   9.722 9.665 9.545 9.795 9.778 5.965 6.101 6.152 6.129 5.702
   41   1372013_at   11.461 11.515 11.565 11.577 11.621 9.191 9.007 9.331 9.16 9.066
   42   1373592_at   10.45 10.399 10.523 10.453 10.235 7.725 7.627 7.534 7.805 7.538
   43   1377916_at   10.922 10.84 10.714 10.663 10.888 7.2 7.211 7.518 7.362 7.071
   44   1373932_at   10.242 10.262 10.252 10.168 10.005 6.702 6.471 6.797 6.913 6.827
   45   1382442_at   8.891 8.898 8.909 9.081 8.901 4.733 4.886 5.196 4.785 4.599
   46   1387892_at   11.149 11.121 11.105 11.137 11.152 8.783 8.974 8.999 8.704 8.772
   47   1368010_at   9.594 9.619 9.715 9.734 9.836 7.038 6.898 7.073 6.832 6.77
   48   1385571_at   9.592 9.868 9.588 9.47 9.795 5.328 5.238 5.393 4.988 5.512
   49   1370290_at   10.895 10.869 10.798 10.898 10.933 8.085 8.126 8.227 8.033 7.801
   50   1374388_at   10.28 10.39 10.513 10.574 10.473 8 8.18 8.143 8.109 8.014
   51   1378257_at   10.088 10.133 10.109 10.151 10.19 7.26 7.249 7.525 7.137 7.106
   52   1370603_a_at   9.098 9.085 9.263 9.179 8.961 4.801 5.238 4.642 4.911 4.788
   53   1385557_at   7.672 7.723 7.735 7.571 7.709 4.794 4.758 4.969 4.775 5.109
   54   1370215_at   11.029 11.005 11.02 10.866 10.975 7.332 6.843 7.125 6.939 7.323
   55   1376652_at   11.6 11.588 11.723 11.507 11.507 8.012 8.045 8.383 7.861 8.082
   56   1395124_at   10.427 10.387 10.267 10.369 10.444 7.459 7.21 7.603 7.318 7.217
   57   1370516_at   9.47 9.37 9.421 9.633 9.727 6.233 6.294 6.478 6.379 6.164
   58   1376845_at   11.333 11.338 11.61 11.301 11.573 8.355 8.342 8.266 8.049 8.259
   59   1371123_x_at   11.731 11.632 11.623 11.639 11.792 8.529 8.379 8.465 8.509 8.078
   60   1379381_at   9.772 9.659 9.697 9.812 9.928 5.274 4.993 5.647 5.126 5.074
   61   1370585_a_at   8.505 8.482 8.706 8.588 8.467 5.75 5.519 5.841 5.496 5.639
   62   1379748_at   10.256 10.289 10.199 10.232 10.409 7.161 7.374 7.539 7.141 7.324
   63   1382926_s_at   8.754 8.868 8.938 8.907 8.968 4.934 4.477 4.934 4.878 4.476
   64   1372468_at   8.856 8.806 8.932 8.95 8.848 6.805 6.911 6.914 6.857 6.645
   65   1367974_at   11.027 10.817 11.217 11.22 11.156 7.945 7.936 7.767 7.706 7.75
   66   1367816_at   10.062 10.084 10.071 9.939 10.013 7.881 7.982 7.799 7.677 7.946
   67   1370904_at   10.662 10.621 10.806 10.616 10.435 7.719 7.545 7.728 7.9 7.71
   68   1379357_at   9.152 9.045 8.904 8.683 8.953 4.916 5.067 5.014 4.862 4.584
   69   1389006_at   11.864 11.737 11.791 11.753 11.64 8.113 8.058 8.247 8.172 7.68
   70   1394940_at   8.841 8.73 8.943 8.719 8.657 5.895 5.993 6.214 5.957 5.869
   71   1382311_at   10.002 10.105 10.044 10.04 10.126 6.098 5.918 6.396 5.858 5.746
   72   1379496_at   8.593 8.652 8.604 8.744 8.662 5.792 5.564 5.725 5.346 5.75
   73   1370119_at   9.794 9.643 9.839 9.861 9.872 5.489 5.251 5.783 5.802 5.314
   74   1368518_at   11.258 11.263 11.403 11.251 11.013 7.382 7.178 7.706 7.538 7.358
   75   1374397_at   10.891 10.833 10.887 10.818 10.901 10.07 10.062 10.026 9.974 10.014
   76   1388347_at   12.303 12.31 12.171 12.105 12.233 10.453 10.395 10.328 10.375 10.53
   77   1379344_at   12.018 12.03 12.033 11.665 11.813 8.685 8.609 8.737 8.383 8.614
   78   1377239_at   9.167 9.188 9.169 9.112 9.352 6.106 5.791 6.059 5.64 5.831
   79   1372011_at   11.079 11.142 11.113 10.874 11.196 8.176 8.131 7.776 8.008 8.045
   80   1374718_at   10.825 10.879 10.734 10.697 10.83 6.58 6.92 7.258 6.867 6.868
   81   1378428_at   10.081 10.075 9.891 9.956 9.996 8.411 8.511 8.36 8.365 8.354
   82   1370422_at   9.784 9.839 9.678 9.765 9.897 5.696 5.508 6.061 5.639 5.381
   83   1389553_at   10.673 10.724 11.048 11.015 11.138 6.827 6.929 7.17 6.855 6.756
   84   1371017_at   7.251 7.08 7.217 7.101 7.059 4.218 4.392 4.18 4.586 4.325
   85   1374578_at   9.246 9.236 9.354 9.342 9.341 6.945 6.725 6.677 7.04 6.836
   86   1383372_at   8.995 9.083 9.074 9.152 8.947 7.151 7.133 7.089 7.094 6.896
   87   1379741_at   9.604 9.634 9.767 9.665 9.527 11.14 11.202 11.206 11.227 11.109
   88   1392722_at   7.962 8.003 7.673 7.889 7.815 4.137 4.163 4.464 4.176 3.884
   89   1390798_at   9.938 9.991 10.148 9.965 9.946 6.091 6.38 6.299 5.921 5.809
   90   1369964_at   11.413 11.429 11.619 11.329 11.21 7.81 7.721 8.047 7.926 7.563
   91   1368006_at   9.931 9.776 10.139 9.969 9.927 6.712 6.889 7.1 6.7 6.838
   92   1383420_at   6.983 7.05 6.992 7.082 7.099 8.889 8.977 8.789 8.839 8.686
   93   1383426_at   8.147 8.065 8.283 8.197 8.182 5.507 5.22 5.646 5.379 5.311
   94   1372757_at   11.878 11.832 11.889 11.665 11.783 9.023 8.923 9.105 9.003 8.661
   95   1385029_at   8.422 8.429 8.402 8.681 8.866 5.184 5.351 5.271 5.121 5.159
   96   1375955_at   9.496 9.593 9.579 9.748 9.637 7.78 7.835 7.949 7.754 7.776
   97   1379425_at   8.255 8.187 8.087 8.257 8.386 4.408 4.893 4.55 4.564 4.297
   98   1367705_at   9.885 10.107 10.207 10.051 10.058 7.003 6.607 6.994 7.049 6.958
   99   1376144_at   10.886 10.911 10.788 10.938 11.113 7.423 7.407 7.822 7.279 7.413
   100   1387048_at   9.941 9.904 9.864 9.806 9.944 6.697 7.22 6.934 7.018 7.061
   101   1370623_at   8.37 8.249 8.424 8.339 8.482 5.767 5.87 5.797 5.449 5.711
   102   1386277_at   8.527 8.494 8.421 8.577 8.747 6.036 5.889 6.125 5.949 6.194
   103   1368835_at   12.448 12.431 12.464 12.23 12.365 9.038 9.12 9.466 9.048 8.921
   104   1368558_s_at   10.397 10.444 10.615 10.504 10.69 7.147 7.048 7.083 6.798 6.624
   105   1388236_x_at   10.039 9.891 10.136 10.198 10.221 8.011 8.023 8.106 7.95 8.002
   106   1385276_a_at   9.034 9.138 8.747 8.876 9.168 4.771 5.052 4.688 5.189 4.695
   107   1367732_at   9.692 9.703 9.781 9.799 9.919 8.333 8.34 8.288 8.213 8.296
   108   1368490_at   10.396 10.459 10.433 10.352 10.537 6.838 6.543 7.012 6.6 6.357
   109   1371015_at   10.642 10.638 10.172 10.341 10.666 6.615 6.443 6.851 6.459 6.564
   110   1387202_at   10.616 10.669 10.36 10.759 10.626 7.822 7.675 7.802 7.69 7.457
   111   1373785_at   8.679 8.677 8.755 8.492 8.434 6.661 6.593 6.561 6.627 6.579
   112   1379717_at   10.332 10.34 10.228 10.228 10.541 7.747 7.748 7.575 7.599 7.393
   113   1387605_at   8.009 8.046 7.927 7.989 8.449 4.631 4.699 4.864 4.798 4.897
   114   1376151_a_at   12.281 12.194 12.192 12.078 12.219 8.429 8.644 9.03 8.856 8.566
   115   1393280_at   9.975 10.051 10.37 9.947 9.979 6.545 6.598 6.949 6.798 6.767
   116   1367662_at   10.747 10.698 10.689 10.674 10.741 11.637 11.622 11.627 11.52 11.544
   117   1379568_at   9.491 9.594 9.337 9.589 9.812 6.881 6.858 6.77 6.639 6.711
   118   1382153_at   7.87 7.92 7.898 7.966 7.829 4.54 4.589 4.437 4.63 4.031
   119   1395003_at   8.821 8.892 8.901 8.943 9.034 5.73 5.783 5.934 6.244 5.954
   120   1374033_at   11.398 11.347 11.392 11.337 11.575 7.168 6.979 7.482 7.109 6.665
   121   1377826_at   9.056 9.3 9.204 9.316 9.369 6.199 6.032 6.469 6.413 6.214
   122   1388149_at   10.894 10.853 10.738 10.898 11.033 6.302 6.138 6.805 5.975 6.185
   123   1373401_at   8.342 8.408 8.578 8.632 8.412 5.783 5.719 5.65 5.715 5.368
   124   1389732_at   10.709 10.682 10.479 10.627 10.866 8.056 8.294 8.003 8.177 8.207
   125   1385926_at   8.692 8.827 8.63 8.847 8.91 6.254 5.916 6.256 6.123 5.972
   126   1387134_at   10.857 10.714 10.928 10.441 10.719 7.028 6.767 7.278 6.819 7.023
   127   1370237_at   10.248 10.243 10.307 10.426 10.273 11.534 11.514 11.482 11.617 11.514
   128   1387354_at   12.674 12.614 12.572 12.378 12.498 8.792 8.8 9.326 8.906 8.719
   129   1386885_at   9.844 9.918 9.961 9.814 9.779 11.247 11.127 11.21 11.221 11.105
   130   1384187_at   9.515 9.513 9.508 9.532 9.606 5.805 6.17 6.167 5.552 5.811
   131   1368227_at   8.692 8.563 8.86 8.735 8.685 6.48 6.346 6.192 6.482 6.214
   132   1383448_at   8.991 8.96 8.984 9.084 9.179 6.38 6.491 6.421 6.714 6.72
   133   1387893_at   11.583 11.586 11.433 11.331 11.497 9.266 9.095 8.902 9.003 8.929
   134   1368754_at   8.496 8.213 8.538 8.472 8.459 6.132 6.23 6.313 6.072 6.303
   135   1383189_at   10.722 10.86 10.417 10.645 10.798 7.106 7.449 7.457 7.57 7.321
   136   1373026_at   7.973 8.002 7.827 7.995 8.021 5.254 5.127 4.985 4.831 5.319
   137   1369181_at   10.25 10.24 10.493 10.181 10.17 6.454 6.616 6.831 6.841 6.288
   138   1378443_at   8.307 8.247 8.41 8.6 8.285 5.977 6.165 6.034 5.917 5.902
   139   1388761_at   9.153 9.255 9.17 9.186 9.232 7.834 7.988 7.83 7.744 7.784
   140   1386925_at   11.593 11.601 11.582 11.66 11.691 9.494 9.541 9.684 9.49 9.259
   141   1380129_at   9.109 8.959 8.792 9.097 8.943 5.729 5.35 5.553 5.924 5.534
   142   1368059_at   9.978 9.907 10.021 10.109 9.92 11.368 11.538 11.461 11.414 11.37
   143   1374601_at   9.555 9.749 9.644 9.608 9.68 7.825 7.65 7.745 7.48 7.625
   144   1388763_at   10.759 10.749 10.783 10.542 10.545 8.927 8.785 8.906 8.806 8.888
   145   1392785_at   9.069 9.099 9.13 8.923 9.211 6.999 6.722 6.776 6.619 6.758
   146   1372004_at   8.985 8.987 9.14 9.108 9.12 6.543 6.082 6.507 6.301 6.231
   147   1396163_at   11.577 11.493 11.66 11.336 11.363 6.118 6.234 6.911 6.561 6.035
   148   1397372_at   6.866 6.798 6.842 6.805 6.801 5.519 5.413 5.634 5.536 5.404
   149   1395034_at   9.791 9.758 9.916 9.695 9.92 3.972 4.285 4.865 4.081 3.788
   150   1392547_at   10.261 10.288 10.312 10.231 10.254 5.147 5.028 5.906 5.049 5.023
   151   1373900_at   9.035 9.1 9.06 9.221 9.004 10.339 10.425 10.372 10.45 10.333
   152   1371959_at   7.465 7.445 7.443 7.517 7.587 9.295 9.157 9.377 9.085 9.176
   153   1383516_at   8.299 8.396 8.681 8.479 8.669 5.952 6.023 6.029 5.927 5.782
   154   1371440_at   12.683 12.587 12.532 12.4 12.549 11.073 11.117 10.98 11.075 11.036
   155   1370445_at   9.581 9.571 9.728 9.816 9.649 6.764 6.466 6.476 6.154 6.323
   156   1387711_at   6.86 7.05 7.511 7.416 7.086 0 0 1.036 0 0.014
   157   1372213_at   6.61 6.506 6.403 6.223 6.635 9.449 9.297 9.425 9.107 9.27
   158   1374627_at   8.175 8.397 8.307 8.309 8.324 6.277 6.236 6.475 6.416 6.16
   159   1370202_at   11.883 11.797 12.047 11.991 11.99 9.305 9.41 9.62 9.557 9.226
   160   1368921_a_at   9.587 9.684 9.362 9.406 9.353 5.893 5.535 6.194 5.909 5.826
   161   1373881_at   11.135 11.204 11.275 11.179 11.225 9.224 9.145 9.495 9.163 9.271
   162   1375010_at   10.436 10.379 10.589 10.556 10.579 6.516 6.386 7.075 6.484 6.383
   163   1371078_at   9.937 9.778 9.875 9.892 9.803 6.365 6.608 6.65 6.908 6.29
   164   1371152_a_at   9.206 9.265 8.832 9.216 9.377 5.777 5.564 5.956 5.951 5.778
   165   1390659_at   9.868 9.914 9.542 9.837 9.855 6.252 6.247 6.581 6.178 5.928
   166   1369952_at   12.386 12.32 12.336 12.217 12.379 11.36 11.286 11.351 11.272 11.293
   167   1369766_at   5.911 6.182 6.679 6.222 6.086 0.978 0.263 1.036 0.687 0.526
   168   1384946_at   9.355 9.329 9.245 9.208 9.409 6.204 6.218 6.613 6.05 6.079
   169   1389997_at   8.957 8.896 9.525 9.078 8.7 4.495 4.607 4.523 4.869 4.508
   170   1373658_at   7.961 7.945 7.901 8.092 7.878 5.23 5.362 5.556 5.264 5.641
   171   1388212_a_at   11.806 11.667 11.722 11.548 11.841 8.801 8.401 8.378 8.334 8.74
   172   1376795_at   9.71 9.753 9.827 9.812 9.78 6.716 7.207 6.787 6.81 7.116
   173   1369973_at   12.17 12.144 12.099 11.979 12.16 10.366 10.523 10.234 10.458 10.439
   174   1384874_at   4.877 4.867 5.11 5.12 5.24 8.294 8.007 8.001 8.035 7.818
   175   1377698_at   9.164 9.116 8.818 9.069 9.247 4.732 4.983 5.337 4.975 4.579
   176   1390738_at   11.653 11.625 11.69 11.762 11.843 8.986 9.068 9.434 9.137 9.3
   177   1370080_at   9.929 9.968 10.614 10.591 10.268 5.605 5.638 5.996 5.754 5.744
   178   1371210_s_at   9.887 9.932 9.984 9.916 9.922 6.253 6.553 6.339 6.923 6.678
   179   1370892_at   11.958 11.846 12.054 11.886 11.785 8.859 8.887 9.357 8.966 9.123
   180   1398616_at   9.744 9.661 9.47 9.522 9.774 6.175 6.201 6.674 6.151 6.469
   181   1369663_at   8.758 8.726 8.652 8.807 8.709 10.211 10.159 10.018 10.296 10.192
   182   1373439_at   9.494 9.629 9.538 9.55 9.695 8.152 8.171 8.116 8.013 8.244
   183   1382535_at   8.105 8.117 8.1 7.947 8.052 6.034 5.978 6.112 6.297 5.924
   184   1372112_at   10.055 10.134 10.185 10.152 10.341 7.839 7.786 8.023 7.721 7.584
   185   1379818_at   8.55 8.614 8.537 8.655 8.624 5.235 4.667 4.822 4.606 5.194
   186   1384164_at   7.243 7.212 7.204 7.162 6.931 9.249 9.28 9.134 9.05 9.305
   187   1371970_at   7.807 7.662 7.474 7.708 7.74 3.635 4.132 3.47 4.043 3.963
   188   1370449_at   9.105 9.211 9.145 9.316 9.286 6.685 6.618 7.074 6.742 6.759
   189   1383585_s_at   9.174 9.273 9.483 9.269 9.364 6.658 6.363 6.286 6.75 6.472
   190   1369586_at   4.08 4.012 3.342 3.838 4.005 0.176 0 0 0 0
   191   1398828_at   10.907 10.897 10.965 10.966 10.919 9.949 9.797 9.931 9.992 9.862
   192   1389510_at   8.36 8.37 8.195 8.406 8.548 6.333 6.351 6.469 6.549 6.322
   193   1387227_at   9.779 9.814 9.921 9.873 9.798 8.341 8.235 8.019 8.139 8.112
   194   1375796_at   9.642 9.69 10.012 9.744 10.328 5 5.079 5.519 5.178 4.8
   195   1393038_at   9.977 9.823 9.761 9.678 9.89 6.54 6.287 6.932 6.402 6.553
   196   1387005_at   11.495 11.49 11.465 11.474 11.7 8.664 8.44 8.856 8.458 8.897
   197   1368565_at   8.759 8.784 8.904 8.698 8.549 5.101 4.707 5.299 4.611 5.016
   198   1387690_at   8.057 7.945 8.179 8.157 7.885 5.525 5.612 5.475 5.773 5.339
   199   1378047_at   7.344 7.277 7.433 7.238 7.24 1.293 0.07 0.895 0.39 0
   200   1379957_at   7.932 8.093 7.747 7.83 8.067 5.497 5.041 5.284 5.217 5.209
   201   1374165_at   7.433 7.631 7.633 7.75 7.819 5.741 5.507 5.611 5.615 5.609
   202   1370539_at   8.161 8.084 8.323 8.315 8.424 5.806 5.98 5.839 5.958 5.614
   203   1387678_at   8.14 8.124 8.026 8.233 8.011 6.489 6.527 6.515 6.342 6.303
   204   1376908_at   9.624 9.611 9.233 9.809 9.75 5.92 5.536 5.888 5.373 5.751
   205   1387878_at   10.861 10.85 10.805 10.869 10.727 11.674 11.802 11.722 11.706 11.731
   206   1372478_at   9.888 9.947 10 9.87 9.869 7.615 7.357 7.738 7.312 7.321
   207   1389488_at   10.213 10.212 10.143 10.293 10.173 11.165 11.218 11.256 11.143 11.293
   208   1372093_at   8.981 8.962 8.952 9.059 8.925 10.349 10.236 10.497 10.264 10.329
   209   1382603_at   9.724 9.768 9.798 9.743 9.718 5.568 5.281 5.828 5.078 4.918
   210   1371336_at   10.354 10.346 10.354 10.509 10.566 8.541 8.493 8.78 8.636 8.566
   211   1384180_at   10.024 10.078 9.912 10.004 10.264 7.279 7.239 7.363 6.839 7.185
   212   1382546_at   9.646 9.615 9.483 9.717 9.737 5.897 5.557 6.252 5.598 5.532
   213   1388416_at   9.449 9.658 9.469 9.563 9.559 7.437 7.535 7.297 7.603 7.675
   214   1367574_at   12.078 12.005 12.008 11.869 12.046 9.396 9.058 9.56 9.128 9.416
   215   1372034_at   10.772 10.786 10.678 10.81 10.978 7.1 7.386 7.731 7.121 7.109
   216   1376006_at   9.97 9.869 9.928 10.038 9.945 8.348 8.625 8.461 8.383 8.386
   217   1378927_at   9.813 9.842 9.984 10.072 10.138 5.696 5.78 6.482 5.986 5.949
   218   1370913_at   10.679 10.672 10.564 10.81 10.637 7.232 7.625 7.6 7.135 7.049
   219   1388255_x_at   10.322 10.472 10.609 10.391 10.412 7.43 7.253 7.289 7.828 7.405
   220   1371596_at   10.216 10.128 10.143 10.115 10.144 9.378 9.372 9.362 9.258 9.277
   221   1388340_at   9.767 9.739 9.878 9.688 9.619 4.829 4.986 5.33 4.579 5.537
   222   1372404_at   10.712 10.939 10.988 10.953 10.895 7.814 7.688 8.168 7.767 7.516
   223   1385143_at   7.886 8.119 7.733 8.006 8 5.619 5.848 5.837 5.589 5.715
   224   1369214_a_at   9.223 9.14 9.16 9.097 9.019 4.777 4.584 5.436 4.654 4.58
   225   1388066_a_at   7.005 6.9 7.059 7.062 6.872 5.667 5.461 5.561 5.461 5.533
   226   1383578_at   8.085 8.238 7.981 8.014 7.914 4.355 4.749 4.785 4.527 5.032
   227   1386916_at   9.33 9.356 9.504 9.524 9.367 10.889 11.058 11.022 10.932 10.828
   228   1376943_at   8.123 8.203 8.508 8.474 8.367 4.433 4.785 4.731 4.674 4.114
   229   1377048_at   8.917 8.872 9.052 9.079 8.747 10.717 10.901 10.929 10.771 10.856
   230   1369726_at   10.491 10.478 10.155 10.193 10.405 7.478 7.712 7.479 7.317 7.741
   231   1382026_at   8.958 9.096 9.317 9.234 9.005 6.029 5.766 6.29 5.773 6.08
   232   1387984_at   8.459 8.5 8.719 8.493 8.734 6.249 6.437 6.189 6.491 6.195
   233   1373504_at   8.677 8.628 8.974 8.638 8.793 6.724 6.605 6.681 6.616 6.432
   234   1395426_at   10.677 10.742 10.673 10.714 10.76 9.93 9.808 9.771 9.828 9.766
   235   1385925_at   8.842 8.653 8.554 8.747 8.628 5.928 5.498 5.315 5.708 5.748
   236   1390292_at   8.499 8.474 8.547 8.553 8.507 9.593 9.59 9.416 9.629 9.551
   237   1368975_at   9.474 9.525 9.606 9.557 9.562 3.663 4.415 4.8 4.434 3.903
   238   1372741_at   9.32 9.298 9.433 9.475 9.437 10.393 10.404 10.374 10.436 10.341
   239   1382277_at   8.378 8.421 8.375 8.576 8.782 6.123 6.285 6.269 6.156 6.345
   240   1368693_at   9.759 9.741 9.851 9.988 9.795 5.315 5.757 6.061 6.025 5.404
   241   1367710_at   12.28 12.237 12.149 12.104 12.269 9.235 9.417 9.772 9.177 9.357
   242   1381556_at   7.646 7.838 7.473 7.756 8.118 3.732 3.907 3.53 3.916 4.185
   243   1367715_at   10.022 10.045 10.169 10.2 10.341 8.62 8.632 8.642 8.597 8.542
   244   1368808_at   10.916 10.887 10.935 10.996 10.986 9.463 9.263 9.164 9.393 9.158
   245   1368136_at   9.205 9.273 9.212 9.271 9.176 8.136 8.193 7.992 7.984 8.084
   246   1374307_at   7.936 7.909 7.985 7.728 7.596 9.925 10.088 9.911 9.873 9.901
   247   1369944_at   10.036 10.12 9.941 10.209 10.204 8.298 8.056 8.05 8.09 7.928
   248   1390707_at   8.682 8.647 8.984 8.836 8.573 6.481 6.386 6.639 6.414 6.577
   249   1368043_at   9.731 9.789 9.813 9.815 9.73 9.107 9.042 9.145 9.133 9.114
   250   1372097_at   10.287 10.332 10.195 10.225 10.322 5.97 6.176 6.671 5.828 5.78
   251   1374337_at   9.76 9.705 9.622 9.876 9.895 6.141 6.241 6.713 5.947 6.119
   252   1376109_at   8.813 8.83 8.654 8.967 9.107 6.344 6.107 6.377 6.469 6.175
   253   1376880_at   8.983 9.08 9.059 9.058 9.213 7.93 8.029 7.914 8.003 7.925
   254   1384603_at   5.58 5.558 5.617 5.391 5.542 8.023 7.903 8.215 7.728 7.8
   255   1372254_at   12.455 12.337 12.452 12.187 12.273 10.704 10.742 10.824 10.661 10.596
   256   1375529_at   7.787 7.764 7.791 7.756 7.947 8.778 8.749 8.728 8.768 8.716
   257   1374284_at   8.022 8.085 7.98 8.332 7.967 4.966 4.97 4.995 4.811 4.415
   258   1368420_at   10.779 10.78 10.193 10.447 10.687 7.437 7.11 7.09 7.13 7.321
   259   1373066_at   8.587 8.707 8.359 8.385 8.548 5.486 5.454 5.611 5.284 5.875
   260   1367780_at   8.707 8.741 9.11 9.064 9.236 5.923 6.109 5.796 5.768 5.794
   261   1389092_at   10.981 10.959 11.195 11.037 10.847 6.973 6.507 7.425 6.936 6.736
   262   1373992_at   11.983 12.011 11.944 11.785 11.954 5.805 5.922 7.027 6.184 5.865
   263   1376029_at   12.023 11.967 11.916 11.907 12.067 9.155 9.206 9.674 9.279 9.096
   264   1373110_at   7.515 7.589 7.689 7.618 7.438 9.338 9.385 9.125 9.192 9.38
   265   1370882_at   11.771 11.72 11.756 11.61 11.574 9.371 9.155 9.24 9.035 8.817
   266   1371977_at   11.524 11.5 11.464 11.472 11.622 10.301 10.464 10.423 10.35 10.271
   267   1369936_at   12.03 12.049 11.859 11.906 11.936 10.934 10.858 10.951 10.918 10.843
   268   1370320_at   10.206 10.14 10.165 10.126 10.076 11.205 11.233 11.054 11.184 11.093
   269   1380077_at   8.531 8.56 8.516 8.514 8.425 6.189 6.626 6.576 6.514 6.371
   270   1388202_at   9.74 9.815 10.181 10.036 10.086 6.611 6.415 6.791 6.729 6.196
   271   1389387_at   10.024 9.975 10.088 10.136 10.192 8.738 8.745 8.588 8.587 8.535
   272   1373037_at   9.541 9.61 9.469 9.358 9.764 6.117 5.695 6.439 6.186 6.03
   273   1369110_x_at   10.791 10.617 10.96 10.817 10.765 6.51 7.258 6.959 7.013 7.306
   274   1391737_at   9.063 9.063 8.896 8.844 9.127 5.081 5.394 5.545 4.931 5.657
   275   1388868_at   10.285 10.286 10.23 10.22 10.336 9.707 9.643 9.66 9.688 9.688
   276   1392960_at   8.959 8.925 8.873 8.932 9.067 7.108 7.296 7.464 7.362 7.272
   277   1377669_at   8.018 8.068 8.285 8.093 8.024 5.663 5.738 5.897 5.402 5.515
   278   1382622_at   10.245 10.195 10.391 10.151 10.031 5.329 5.12 5.973 5.471 4.858
   279   1392881_at   8.789 8.887 8.681 8.732 8.984 5.501 5.39 5.834 5.733 6.023
   280   1383131_at   9.417 9.267 9.558 9.421 9.133 7.137 7.151 7.035 7.164 6.86
   281   1375211_at   12.15 12.111 12.112 11.975 12.016 10.682 10.674 10.786 10.593 10.504
   282   1389154_at   8.505 8.501 8.572 8.506 8.421 9.164 9.255 9.232 9.192 9.21
   283   1398788_at   11.909 11.898 11.825 11.863 11.922 10.945 10.955 11.024 10.918 10.812
   284   1392334_at   12.034 12.113 11.897 11.939 11.89 10.38 10.051 10.315 10.257 10.262
   285   1387952_a_at   10.009 10.033 9.692 9.83 9.772 5.402 5.371 6.229 5.535 5.445
   286   1369764_at   8.383 8.618 7.294 8.097 7.183 0 0 0.66 0 0
   287   1397892_at   8.868 8.687 8.896 8.882 8.96 7.434 7.455 7.372 7.468 7.583
   288   1368679_a_at   9.482 9.471 9.563 9.423 9.453 7.051 6.565 6.429 6.431 6.532
   289   1388740_at   9.047 9.092 9.364 9.212 9.122 5.295 4.822 5.624 5.626 5.26
   290   1371480_at   8.383 8.309 8.247 8.509 8.521 6.349 6.433 6.449 6.692 6.429
   291   1378482_at   7.839 7.831 7.87 7.751 7.905 6.533 6.383 6.268 6.434 6.231
   292   1372475_at   8.714 8.804 8.755 8.72 8.393 10.561 10.49 10.476 10.534 10.453
   293   1370883_at   12.927 12.844 12.777 12.455 12.703 10.469 10.378 10.617 10.427 10.319
   294   1386660_at   5.945 6.086 6.134 6.202 6.386 3.553 3.933 3.837 3.873 3.779
   295   1389408_at   10.422 10.531 10.462 10.404 10.238 6.167 6.682 7.033 6.282 6.504
   296   1391106_at   9.327 9.424 9.083 9.141 9.371 7.083 7.25 7.33 7.012 7.133
   297   1370885_at   11.611 11.579 11.299 11.331 11.599 6.908 6.791 7.574 6.779 6.509
   298   1367855_at   7.116 7.034 7.269 7.18 7.17 5.456 5.461 5.736 5.469 5.431
   299   1395810_at   6.731 6.731 6.828 6.843 6.439 8.892 9.1 8.756 8.892 8.872
   300   1390510_at   10.802 10.909 10.755 10.809 10.885 6.019 5.608 6.73 5.808 5.696
   301   1390396_at   7.929 8.138 8.072 8.168 8.272 6.007 5.791 5.611 5.574 5.868
   302   1375917_at   9.467 9.554 9.97 9.097 9.418 6.012 5.849 5.82 6.065 6.044
   303   1385051_at   10.004 9.897 9.623 9.854 9.992 4.09 4.434 5.199 3.916 4.388
   304   1393400_at   9.205 9.329 9.551 9.132 8.856 4.755 4.674 5.187 4.918 4.279
   305   1383272_at   5.017 4.898 5.142 4.723 5.113 2 2.029 1.872 1.876 1.428
   306   1387818_at   9.164 9.069 8.892 9.07 9.137 6.107 5.89 6.176 6.37 5.681
   307   1369909_s_at   8.253 8.191 8.376 8.56 8.205 10.139 10.038 10.076 10.178 10.02
   308   1367712_at   10.475 10.54 10.517 10.544 10.506 6.498 5.984 6.847 6.262 5.849
   309   1374180_at   9.345 9.431 9.393 9.413 9.398 8.074 8.07 8.261 7.934 8.147
   310   1371539_at   9.816 9.664 9.754 9.819 9.877 8.265 8.526 8.404 8.491 8.375
   311   1382533_at   8.504 8.574 8.727 8.746 8.699 7.245 7.046 7.064 7.214 7.034
   312   1374469_at   8.784 8.788 8.588 8.991 9.13 5.225 5.021 5.674 5.128 4.982
   313   1388332_at   11.318 11.304 11.214 11.304 11.377 10.655 10.645 10.681 10.666 10.686
   314   1395184_at   9.104 9.151 8.954 8.799 9.057 4.986 5.026 5.785 5.149 5.013
   315   1388337_at   12.571 12.462 12.417 12.209 12.384 10.719 10.653 10.893 10.722 10.739
   316   1388182_at   8.586 8.58 8.6 8.611 8.577 7.216 7.171 7.236 6.938 6.969
   317   1369100_at   6.719 6.8 6.746 6.79 6.817 8.909 9.015 9.03 8.708 8.588
   318   1380071_at   9.597 9.691 9.703 9.869 10.138 7.16 7.327 7.395 7.174 7.381
   319   1390909_at   9.201 8.992 8.9 9.036 9.146 5.279 5.216 5.671 5.576 4.811
   320   1392655_at   9.11 9.057 8.835 8.947 9.064 6.863 6.884 6.727 6.436 6.839
   321   1377125_at   6.201 6.091 6.177 6.006 6.029 7.58 7.785 7.466 7.611 7.575
   322   1375020_at   8.651 8.546 8.841 8.862 8.868 7.097 7.035 7.123 7.193 7.065
   323   1386861_at   12.295 12.234 12.257 12.127 12.37 11.07 11.131 11.068 11.036 10.926
   324   1369979_at   10.565 10.587 10.536 10.368 10.546 9.272 9.319 9.198 9.176 9.082
   325   1387884_at   11.089 11.121 11.184 11.247 11.339 9.635 9.82 9.834 9.796 9.645
   326   1383392_at   10.827 10.804 10.781 10.741 10.739 6.481 5.795 6.709 5.935 5.687
   327   1377299_at   8.327 8.3 8.244 8.512 8.487 6.54 6.383 6.524 6.319 6.178
   328   1392407_at   9.06 9.154 9.287 9.393 9.355 4.826 5.085 5.777 5.127 4.928
   329   1376038_at   8.216 8.121 8.203 8.223 8.095 9.258 9.292 9.094 9.152 9.192
   330   1370428_x_at   11.984 11.928 11.852 11.932 12 8.232 8.199 8.912 8.107 8.062
   331   1391224_at   5.85 5.702 5.701 5.863 5.779 7.036 7.089 6.853 7.036 7.031
   332   1369989_at   8.399 8.376 8.457 8.334 8.315 9.426 9.543 9.403 9.309 9.397
   333   1370056_at   12.361 12.145 12.042 11.958 12.032 9.641 9.807 9.548 9.612 9.973
   334   1370924_at   10.165 10.177 10.728 10.418 9.855 5.017 4.969 5.462 4.408 5.238
   335   1387995_a_at   11.555 11.565 11.437 11.587 11.701 10.268 10.084 9.986 10.17 10.132
   336   1386009_at   7.931 7.881 7.837 8.027 8.105 3.644 3.444 4.406 3.535 3.947
   337   1375895_at   8.794 8.939 8.762 9.035 8.964 7.372 7.46 7.41 7.265 7.232
   338   1380393_at   9.715 9.721 9.85 9.85 9.523 11.222 11.237 11.375 11.216 11.284
   339   1377576_at   9.528 9.528 9.623 9.679 9.609 10.495 10.653 10.507 10.516 10.584
   340   1368976_at   8.304 8.231 8.61 8.622 8.223 4.209 4.105 3.463 3.669 4.376
   341   1371365_at   10.233 10.274 10.261 10.395 10.34 9.18 9.249 9.341 9.163 9.13
   342   1374730_at   9.971 10.348 10.682 10.273 10.584 6.966 6.511 7.001 6.749 6.599
   343   1384787_at   9.583 9.553 9.919 9.58 9.276 5.008 4.643 5.684 5.079 4.963
   344   1396262_at   9.501 9.556 9.455 9.301 9.803 6.018 5.922 6.042 6.073 5.338
   345   1393848_at   8.295 8.307 8.557 8.04 8.27 4.133 4.891 4.012 4.406 4.412
   346   1367663_at   12.744 12.644 12.598 12.345 12.504 10.98 10.959 11.008 10.893 10.904
   347   1389696_at   8.918 8.915 8.898 9.025 9.291 4.341 4.312 3.756 4.545 4.928
   348   1395886_at   9.762 9.873 9.929 9.834 9.849 8.364 7.944 8.003 8.073 8.104
   349   1376976_at   10.064 10.058 10.118 10.157 10.095 8.336 8.289 8.4 8.158 8.61
   350   1387434_at   6.403 6.325 6.426 6.307 6.219 7.744 7.777 7.736 7.519 7.65
   351   1391417_at   10.883 10.924 10.982 10.992 10.784 12.199 12.164 11.996 12.19 12.205
   352   1379604_at   8.916 8.953 8.835 8.966 9.085 6.799 6.466 6.647 6.996 6.807
   353   1369584_at   7.641 7.903 8.013 7.947 7.722 5.164 4.578 5.029 5.135 4.918
   354   1376613_at   8.608 8.679 8.804 8.776 8.704 7.434 7.516 7.54 7.667 7.573
   355   1389275_at   7.536 7.382 7.386 7.626 7.155 3.646 3.401 3.37 3.782 4.168
   356   1377659_at   4.02 3.615 3.772 3.844 3.697 0 0 0 0.731 0
   357   1383553_at   6.617 6.47 6.715 6.628 6.676 7.858 7.696 7.802 7.81 7.722
   358   1392544_at   8.576 8.685 8.716 8.649 8.709 7.061 6.984 7.082 6.942 6.681
   359   1393044_at   8.543 8.473 8.306 8.464 8.617 5.317 5.34 5.413 5.707 4.895
   360   1368016_at   11 10.97 10.979 11.025 11.147 11.914 11.917 12.047 12.03 12.001
   361   1369197_at   7.885 7.813 7.791 7.762 8.201 5.49 5.588 5.619 5.784 5.795
   362   1368732_at   9.967 9.932 10.147 10.302 9.974 6.051 5.908 6.8 6.086 6.046
   363   1384413_at   8.781 8.77 8.785 8.684 8.678 7.219 7.172 7.455 7.38 7.146
   364   1374431_at   10.813 10.851 10.802 10.812 10.762 11.233 11.208 11.266 11.274 11.281
   365   1373338_at   7.546 7.618 7.645 7.568 7.382 8.971 9.101 9.038 8.882 8.874
   366   1369677_at   3.944 3.628 3.595 3.824 3.747 5.887 5.935 6.144 5.694 6.012
   367   1383731_at   7.985 7.995 8.09 8.05 8.086 9.029 9.02 8.976 8.908 8.848
   368   1393123_at   6.109 5.88 5.975 6.227 6 7.492 7.488 7.58 7.412 7.487
   369   1381593_x_at   12.123 12.094 11.905 12.021 11.915 10.29 10.057 10.276 10.447 10.161
   370   1388324_at   10.428 10.442 10.511 10.463 10.261 11.409 11.436 11.416 11.511 11.448
   371   1379229_at   7.584 7.577 7.928 7.878 7.794 5.12 5.205 4.626 4.829 4.918
   372   1382590_at   7.79 7.696 7.904 7.987 7.759 5.734 5.769 5.384 5.787 5.826
   373   1387867_at   9.807 9.772 9.8 10.024 9.735 11.046 11.09 11.159 11.079 11.011
   374   1383241_at   11.857 11.743 11.411 11.378 11.652 8.933 9.165 8.989 9.271 9.184
   375   1387566_at   9.54 9.551 9.419 9.49 9.825 7.538 7.705 7.653 7.664 7.396
   376   1388526_at   8.139 7.966 8.119 8.157 8.269 9.849 9.713 9.645 9.591 9.575
   377   1388988_at   10.15 10.205 10.441 10.38 10.149 11.671 11.654 11.644 11.752 11.707
   378   1379732_at   6.97 6.863 7.266 7.172 7.378 4.284 4.27 4.155 3.788 3.899
   379   1387859_at   10.168 10.081 10.012 10.018 9.958 11.048 11.126 10.962 11.012 11.045
   380   1381376_at   8.237 8.38 8.097 8.119 8.018 5.951 5.51 5.619 5.84 5.939
   381   1373524_at   7.713 7.773 7.678 7.696 7.514 8.954 9.002 8.926 8.85 8.807
   382   1371776_at   9.01 8.991 8.822 9.025 8.898 7.581 7.398 7.659 7.581 7.411
   383   1390134_at   8.198 8.366 8.313 8.419 8.406 7.421 7.453 7.4 7.383 7.376
   384   1386879_at   11.72 11.693 11.954 11.646 11.526 9.264 9.085 9.552 9.154 9.069
   385   1367850_at   11.503 11.459 11.567 11.389 11.672 6.704 6.799 7.669 6.546 6.598
   386   1382325_at   7.343 7.436 7.234 7.51 7.394 8.795 8.917 9 8.852 8.718
   387   1392647_at   10.327 10.268 10.901 10.411 10.104 5.088 4.598 5.777 4.846 4.789
   388   1383407_at   7.878 7.833 7.942 7.768 7.762 3.747 4.223 4.45 4.591 3.858
   389   1384580_at   7.158 7.083 7.169 6.928 7.46 3.534 3.358 3.214 4.008 3.72
   390   1388243_at   7.144 7.019 7.191 7.175 7.066 6.242 6.109 6.203 6.087 6.137
   391   1378802_at   8.883 8.86 9.139 9.102 8.898 10.842 10.751 10.696 10.776 10.528
   392   1377759_at   9.519 9.52 9.57 9.629 9.521 7.839 7.986 7.736 7.912 7.542
   393   1367946_at   8.371 8.549 8.579 8.646 8.64 7.349 7.318 7.404 7.242 7.299
   394   1370382_at   10.053 10.405 10.099 10.429 10.241 6.824 6.316 6.968 6.664 6.181
   395   1388629_at   10.121 10.225 10.025 10.11 10.158 8.606 8.479 8.644 8.34 8.303
   396   1371239_s_at   10.9 10.846 10.94 10.939 11.053 9.395 9.459 9.158 9.455 9.19
   397   1387022_at   10.654 10.751 10.91 10.802 10.694 11.877 11.938 11.945 12.041 12.05
   398   1374537_at   8.66 8.575 8.808 8.817 8.857 7.082 7.217 6.899 6.922 6.988
   399   1379698_at   9.372 9.283 9.434 9.595 9.524 4.463 4.345 5.362 4.055 4.436
   400   1368092_at   10.015 10.024 10.152 10.127 9.899 11.283 11.412 11.317 11.393 11.203
   401   1388484_at   8.754 8.842 8.8 8.795 8.701 5.523 5.202 5.968 5.937 5.698
   402   1370897_at   8.224 8.12 8.281 8.267 8.331 9.576 9.683 9.777 9.598 9.464
   403   1369559_a_at   10.452 10.482 10.558 10.487 10.57 9.188 9.154 9.313 9.3 9.003
   404   1373024_at   9.215 9.249 9.408 9.446 9.457 8.061 8.105 8.145 8.102 7.971
   405   1377408_at   6.807 6.618 6.651 6.878 6.471 8.685 8.54 8.836 8.569 8.571
   406   1368418_a_at   10.829 10.877 10.651 10.776 10.899 8.564 8.479 8.106 8.611 8.648
   407   1376056_at   9.004 8.996 9.024 9.053 9.16 6.681 6.503 7.078 6.498 6.693
   408   1381875_at   8.352 8.408 8.254 8.599 8.743 5.719 5.827 5.869 5.595 5.335
   409   1398246_s_at   11.965 11.89 11.856 11.709 11.992 7.414 7.533 8.278 7.246 7.188
   410   1384935_at   8.409 8.313 8.317 8.464 8.433 7.024 7.274 7.142 7.133 7.277
   411   1383320_at   9.757 9.871 10.126 9.952 9.55 6.126 6.057 6.732 6.473 6.051
   412   1374897_at   9.45 9.66 9.513 9.708 9.759 8.135 8.258 8.254 8.16 8.095
   413   1373262_at   8.657 8.594 8.606 8.759 8.435 5.905 5.747 6.183 6.231 5.67
   414   1372199_at   7.119 7.158 7.314 7.533 7.149 9.089 9.16 9.087 9.001 8.973
   415   1367909_at   9.754 9.682 9.891 10.009 9.721 11.478 11.582 11.375 11.681 11.475
   416   1388574_at   11.627 11.577 11.42 11.37 11.528 8.761 8.765 9.264 8.773 8.57
   417   1370200_at   10.106 10.231 10.269 10.384 10.197 11.24 11.337 11.261 11.334 11.287
   418   1392280_at   8.935 8.911 9.002 8.738 8.736 4.649 4.915 5.682 4.997 5.025
   419   1376693_at   10.627 10.579 10.301 10.418 10.739 5.302 5.843 6.48 5.612 6.142
   420   1370910_at   8.172 8.293 8.36 8.387 8.254 7.045 7.25 7.195 7.117 7.211
   421   1380410_at   9.265 9.28 9.081 9.162 9.464 6.268 6.543 6.918 6.364 6.458
   422   1389071_at   7.858 7.819 7.894 8.019 8.067 6.029 6.041 5.87 6.106 5.656
   423   1368207_at   9.296 9.389 9.654 9.612 9.583 7.096 7.09 7.257 7.148 6.71
   424   1384483_at   8.136 8.118 8.203 8.349 8.394 5.272 5.632 5.35 5.869 5.266
   425   1376281_at   7.003 6.754 6.986 7.023 7.009 5.591 5.42 5.631 5.658 5.588
   426   1388773_at   9.356 9.393 9.477 9.456 9.174 6.509 6.753 7.178 6.847 6.709
   427   1367702_at   11.08 11.076 11.166 11.271 11.07 12.068 12.118 12.217 12.176 12.091
   428   1389885_at   9.81 9.824 9.955 9.78 9.641 6.667 6.737 7.189 7.016 6.433
   429   1371694_at   10.31 10.321 10.332 10.325 10.543 9.042 9.177 8.948 8.909 9.015
   430   1378633_at   8.602 8.583 8.811 8.624 8.252 6.463 6.226 6.456 6.252 6.201
   431   1383478_at   11.024 11.007 10.991 11.127 10.981 10.268 10.183 10.308 10.325 10.262
   432   1389014_at   11.464 11.415 11.372 11.175 11.366 8.311 8.486 8.895 8.202 8.183
   433   1388403_at   11.426 11.43 11.406 11.399 11.37 12.047 12.104 12.223 12.115 12.051
   434   1370693_a_at   7.902 8.069 7.849 7.888 8.186 5.362 5.109 5.482 5.052 4.812
   435   1368008_at   9.089 9.142 9.112 9.233 9.331 10.396 10.467 10.672 10.512 10.519
   436   1373661_a_at   6.859 6.886 7.058 7.038 6.901 5.629 5.607 5.705 5.849 5.728
   437   1367589_at   11.258 11.238 11.241 11.25 11.155 11.911 12.058 11.926 12.008 11.911
   438   1383662_at   9.08 9.283 9.1 9.262 9.44 6.166 6.231 6.582 6.105 6.711
   439   1379295_at   8.689 8.847 8.905 9.039 8.835 6.626 6.807 6.273 6.663 6.724
   440   1368356_a_at   11.144 11.281 11.215 11.234 11.395 8.856 9.348 8.964 8.759 9.029
   441   1370036_at   7.757 7.72 7.478 7.738 7.465 9.548 9.649 9.42 9.366 9.337
   442   1369294_at   9.007 8.924 9.218 9.189 9.098 6.127 6.492 6.787 6.245 6.241
   443   1368274_at   10.293 10.242 10.404 10.419 10.376 8.624 8.602 8.755 8.879 8.442
   444   1393187_at   9.12 9.31 9.111 9.233 9.285 3.784 4.483 5.197 4.299 4.429
   445   1386904_a_at   10.666 10.667 10.674 10.738 10.609 11.639 11.72 11.483 11.624 11.554
   446   1383786_at   9.52 9.56 9.524 9.309 9.885 7.461 7.614 7.488 7.601 7.477
   447   1375006_at   9.586 9.717 9.309 9.568 9.594 7.783 8.007 7.917 7.917 7.763
   448   1367892_at   9.572 9.654 9.81 9.861 9.552 11.049 11.067 10.995 11.136 11.062
   449   1386934_at   10.019 10.045 9.977 10.062 9.835 11.344 11.342 11.11 11.19 11.228
   450   1387856_at   12.148 12.122 12.083 12.045 12.233 11.314 11.346 11.227 11.339 11.242
   451   1376920_at   8.898 8.844 8.623 8.978 8.962 6.342 6.569 6.506 6.373 5.973
   452   1370188_at   10.475 10.507 10.474 10.488 10.604 9.774 9.65 9.607 9.612 9.579
   453   1367908_at   10.07 10.059 9.906 10.194 9.965 11.434 11.556 11.283 11.534 11.459
   454   1373072_at   9.981 10.05 10.02 9.947 10.018 8.705 8.868 8.598 8.724 8.917
   455   1387897_at   9.555 9.401 9.605 9.568 9.7 6.186 6.432 6.862 6.905 6.685
   456   1372064_at   11.265 11.249 11.034 11.243 11.161 9.981 10.084 9.85 10.026 10.026
   457   1387918_at   10.974 10.904 10.891 11.018 11.07 9.004 9.056 8.709 9.225 8.8
   458   1373258_at   7.511 7.423 7.458 7.47 7.592 8.204 8.251 8.186 8.137 8.185
   459   1377603_at   8.237 8.309 8.314 8.273 8.366 7.098 7.025 7.326 7.183 7.205
   460   1382177_at   7.577 7.742 7.551 7.633 7.622 5.762 5.948 5.651 5.679 5.399
   461   1368192_at   8.371 8.158 8.649 8.15 8.029 4.577 4.628 5.231 4.914 4.534
   462   1379688_at   8.228 8.2 8.283 8.335 8.176 5.019 5.24 5.772 5.02 5.025
   463   1370972_x_at   10.347 10.271 10.424 10.233 10.339 7.532 7.821 8.215 7.83 7.605
   464   1393114_at   8.556 8.6 8.407 8.526 8.654 7.574 7.649 7.583 7.581 7.669
   465   1391925_at   11.111 10.959 10.711 10.578 10.755 6.771 5.795 6.501 5.754 5.854
   466   1388694_at   10.031 9.898 9.616 9.776 10.016 7.645 7.255 7.136 7.319 7.069
   467   1388309_at   8.273 8.371 8.416 8.521 8.34 6.811 6.794 6.956 6.715 6.54
   468   1368809_at   11.381 11.334 11.18 11.098 11.321 9.757 9.759 9.991 9.734 9.835
   469   1388335_at   11.237 11.159 11.205 11.331 11.321 10.2 10.297 10.296 10.368 10.176
   470   1384525_at   8.776 8.874 8.879 8.94 8.995 6.465 6.87 6.863 6.347 6.565
   471   1368566_a_at   10.139 10.049 10.191 10.159 10.033 11.174 11.214 11.059 11.097 11.004
   472   1368455_at   9.076 9.189 9.663 9.51 8.838 4.808 4.852 5.592 4.858 4.915
   473   1385072_at   9.448 9.425 9.302 9.334 9.076 10.924 11.007 10.765 10.842 10.901
   474   1376723_a_at   8.718 8.632 8.803 8.796 8.759 9.982 9.834 10.002 9.877 9.748
   475   1390956_at   10.314 10.195 9.975 10.349 10.295 4.963 5.074 6.206 5.213 4.976
   476   1367914_at   8.993 9.021 9.21 9.231 9.414 6.464 6.667 6.912 6.86 6.946
   477   1383783_at   4.147 4.186 3.757 4.45 3.776 7.246 7.531 7.943 7.726 7.581
   478   1388140_at   8.78 8.739 8.707 8.854 9.028 7.203 7.268 7.427 7.241 7.104
   479   1373897_at   9.902 9.765 9.511 9.85 9.891 5.874 6.021 6.793 6.031 5.995
   480   1387277_at   7.082 7.035 7.074 7.089 7.06 4.856 4.704 5.2 4.574 4.631
   481   1389571_at   9.811 9.841 9.769 10.078 10.111 7.143 7.095 7.622 6.947 7.25
   482   1397304_at   12.298 12.241 12.328 12.082 12.187 7.279 7.498 8.543 7.77 7.533
   483   1382346_at   8.239 8.14 8.194 8.325 8.377 5.879 5.96 6.236 5.558 5.859
   484   1384151_at   6.212 6.262 6.287 6.321 6.193 5.337 5.38 5.425 5.259 5.472
   485   1389470_at   9.249 9.184 9.07 9.361 9.257 5.158 5.215 6.143 5.263 5.337
   486   1387394_at   8.652 8.672 8.872 8.881 8.624 5.509 5.14 5.912 5.017 5.392
   487   1373447_at   9.119 9.045 9.011 9.094 9.146 7.863 8.079 7.809 7.76 7.883
   488   1387872_at   10.031 10.076 9.985 10.21 10.346 8.313 8.215 8.468 8.137 8.099
   489   1377967_at   7.623 7.816 7.267 7.707 7.805 4.082 4.059 4.644 3.878 3.835
   490   1370090_at   9.027 9.098 9.129 9.151 9.002 4.49 4.302 4.991 5.509 4.722
   491   1393657_at   7.197 7.329 7.211 7.38 7.33 6.106 6.016 6.035 6.244 5.963
   492   1388351_at   11.737 11.676 11.666 11.66 11.756 10.958 10.979 11.09 10.999 10.91
   493   1388521_at   8.23 7.991 8.327 8.205 8.261 6.704 6.687 6.47 6.467 6.423
   494   1370346_at   8.193 8.326 8.103 8.245 8.125 4.423 5.246 5.059 4.994 5.285
   495   1374574_at   5.71 5.828 5.716 5.713 5.562 7.41 7.534 7.547 7.136 7.406
   496   1373876_at   9.479 9.492 9.482 9.55 9.535 8.362 8.378 8.584 8.293 8.279
   497   1377286_at   8.48 8.467 8.912 8.619 8.524 6.635 6.748 6.412 6.458 6.548
   498   1369716_s_at   8.823 8.839 8.605 8.776 8.848 7.108 6.674 7.053 6.946 7.072
   499   1377966_at   8.408 8.267 8.296 8.334 7.875 10.516 10.733 10.609 10.515 10.315
   500   1368385_a_at   9.638 9.753 9.85 9.964 9.823 8.69 8.676 8.752 8.697 8.685
   501   1390855_at   6.322 6.5 6.536 6.593 6.429 8.088 8.281 8.425 8.088 8.048
   502   1370769_a_at   7.282 7.178 7.819 7.335 7.107 3.903 4.434 4.324 4.299 4.057
   503   1367590_at   12.107 12.02 12.085 11.89 12.003 11.13 11.17 11.07 11.079 11.01
   504   1381228_at   9.679 9.715 9.451 9.697 10.012 6.086 6.282 6.777 6.346 5.888
   505   1381014_at   8.098 8.067 7.891 8.381 8.48 5.525 5.328 5.718 5.212 5.452
   506   1367837_at   10.177 10.063 10.139 10.282 10.325 9.057 8.888 8.946 8.942 8.791
   507   1373136_at   8.105 8.206 8.094 8.15 8.246 6.928 6.702 7.004 6.946 6.754
   508   1390126_at   6.735 6.983 6.643 6.881 6.525 8.748 8.702 8.457 8.7 8.676
   509   1376700_at   9.613 9.58 9.49 9.639 9.798 8.267 8.209 8.442 8.172 8.285
   510   1368474_at   11.177 11.057 10.777 10.64 10.957 8.278 8.397 7.851 7.897 8.097
   511   1388463_at   9.45 9.47 9.562 9.598 9.282 10.704 10.745 10.655 10.854 10.731
   512   1371447_at   12.791 12.704 12.582 12.33 12.57 10.048 10.295 10.568 10.257 10.398
   513   1368501_s_at   6.615 6.679 6.708 6.084 6.691 0.872 1.642 1.491 1.864 0.604
   514   1388998_at   8.366 8.248 8.457 8.563 8.164 9.896 9.856 9.923 9.82 9.78
   515   1389166_at   6.028 6.221 6.412 6.154 6.116 8.01 8.102 7.845 7.844 7.786
   516   1390133_at   8.531 8.543 8.584 8.608 8.768 7.334 7.449 7.473 7.228 7.428
   517   1376785_at   8.161 8.275 8.114 8.149 8.017 9.711 9.849 9.629 9.494 9.534
   518   1373830_at   8.959 8.897 8.874 9.002 9.214 6.776 6.61 7.072 7.05 6.753
   519   1397772_at   7.716 7.894 7.936 8.016 7.708 5.135 5.285 5.402 4.68 4.984
   520   1381124_at   7.613 7.655 7.4 7.633 7.598 8.966 9.064 8.76 8.959 8.88
   521   1372492_at   9.967 10.084 10.228 10.204 9.928 11.333 11.403 11.496 11.507 11.485
   522   1370258_at   8.408 8.424 8.364 8.456 8.552 6.681 6.726 6.893 6.6 6.373
   523   1374478_at   8.869 8.777 8.91 9.119 9.004 10.497 10.456 10.526 10.402 10.253
   524   1374036_at   8.724 8.825 8.99 8.72 8.696 6.479 6.411 6.924 6.701 6.435
   525   1386956_at   6.861 6.962 6.971 6.829 7.049 5.53 5.331 5.108 5.249 5.156
   526   1370367_at   8.744 8.644 8.836 8.947 8.465 10.48 10.628 10.733 10.507 10.554
   527   1379891_at   10.014 10.057 10.229 10.164 10.026 9.172 9.005 9.088 9.111 9.14
   528   1371629_at   9.136 8.968 9.196 9.247 9.103 10.458 10.42 10.514 10.378 10.251
   529   1372236_at   8.993 8.879 8.857 9.113 9.028 6.841 6.979 7.23 6.667 6.964
   530   1387027_a_at   9.4 9.413 8.984 9.115 9.348 6.906 6.818 6.994 7.255 6.843
   531   1389159_at   7.019 7.038 6.973 7.204 7.259 8.669 8.451 8.462 8.491 8.417
   532   1388458_at   8.001 8.036 8.024 8 7.846 6.999 7.052 6.863 6.868 7.012
   533   1374329_at   5.815 5.683 5.575 5.599 5.573 7.646 7.35 7.308 7.215 7.513
   534   1379742_at   7.316 7.839 7.59 7.625 7.629 5.464 5.386 5.529 5.767 5.563
   535   1393108_at   12.251 12.242 12.214 12.137 12.197 9.478 9.475 10.131 9.441 9.507
   536   1367973_at   11.683 11.652 11.553 11.494 11.558 6.994 7.551 8.202 7.364 7.167
   537   1370621_at   7.806 7.762 8.105 7.949 7.581 4.381 4.699 5.17 4.487 4.658
   538   1367925_at   10.022 9.936 9.977 10.091 10.266 8.568 8.624 8.617 8.674 8.39
   539   1370433_at   8.863 8.91 8.765 8.92 9.068 6.96 6.832 7.054 7.184 6.676
   540   1372865_at   10.375 10.431 10.413 10.326 10.528 9.191 9.327 9.03 9.064 9.045
   541   1370243_a_at   12.323 12.224 12.223 12.216 12.382 11.477 11.526 11.384 11.453 11.499
   542   1372691_at   10.434 10.48 10.452 10.443 10.561 6.242 6.523 7.159 6.367 7.132
   543   1398644_at   6.54 6.702 6.635 6.705 6.656 1.88 0.39 1.39 0.496 0.604
   544   1386871_at   11.271 11.257 11.415 11.313 11.276 12.037 11.998 12.068 12.024 11.935
   545   1370358_at   8.991 8.887 8.956 9.11 8.804 10.036 10.064 10.014 9.947 10.052
   546   1371662_at   11.089 11.07 11.161 11.061 11.354 9.578 9.538 9.504 9.252 9.268
   547   1370096_at   9.066 9.078 9.882 9.622 9.046 5.032 4.668 4.993 4.68 4.113
   548   1367673_at   8.919 8.871 8.92 8.848 8.831 10.096 10.282 10.163 10.183 9.91
   549   1368512_a_at   10.442 10.393 10.576 10.578 10.494 11.317 11.364 11.341 11.294 11.438
   550   1392899_at   8.198 8.362 8.103 8.606 8.395 5.22 5.548 5.856 5.788 5.537
   551   1369665_a_at   9.369 9.327 9.333 9.267 9.377 6.791 6.023 6.089 6.313 5.872
   552   1383086_at   6.871 6.726 7.081 6.84 6.743 8.321 8.418 8.632 8.391 8.491
   553   1370960_at   10.054 9.973 10.287 10.018 10.167 11.807 11.842 11.501 11.799 11.686
   554   1381174_at   7.372 7.325 7.366 7.42 7.401 7.847 7.969 7.89 7.957 7.947
   555   1397292_at   6.519 6.383 7.208 6.721 6.762 1.7 1.803 2.356 1.766 2.692
   556   1372613_at   9.348 9.315 9.469 9.179 9.136 10.949 10.923 10.694 11.05 10.9
   557   1384584_at   7.54 7.631 7.617 7.726 7.873 6.373 6.477 6.372 6.525 6.393
   558   1373629_at   8.299 8.014 8.005 7.916 8.307 6.067 6.13 6.359 5.978 6.097
   559   1384852_at   7.374 7.351 7.588 7.549 7.385 4.848 4.483 4.731 5.178 4.459
   560   1389576_at   8.535 8.282 8.389 8.435 8.417 7.594 7.56 7.613 7.563 7.518
   561   1375926_at   7.565 7.402 7.757 7.657 7.201 9.858 9.578 9.819 9.769 9.991
   562   1367825_at   8.59 8.576 8.375 8.721 8.713 5.509 5.268 5.934 5.183 5.806
   563   1372604_at   8.869 8.587 8.597 8.826 9.042 6.035 5.79 6.132 5.956 5.418
   564   1374468_at   9.983 9.964 9.741 9.871 9.948 8.581 8.429 8.287 8.439 8.204
   565   1369031_at   10.858 10.888 10.954 11.014 10.989 5.982 5.899 6.818 5.928 5.191
   566   1394082_at   9.518 9.509 9.606 9.552 9.717 8.484 8.36 8.22 8.437 8.244
   567   1383248_at   8.03 7.885 7.888 7.987 7.791 9.277 9.109 9.374 9.198 9.09
   568   1374565_at   8.343 8.33 8.188 8.214 8.422 6.546 6.423 6.77 6.358 6.286
   569   1389477_at   7.52 7.542 7.564 7.869 7.589 6.021 5.845 5.769 6.005 5.684
   570   1390866_at   10.621 10.616 10.475 10.468 10.541 8.07 8.696 8.568 8.372 8.452
   571   1394479_at   7.704 7.76 7.868 7.918 7.559 9.301 9.422 9.359 9.39 9.129
   572   1374387_at   10.714 10.694 10.767 10.794 10.868 9.726 9.582 9.834 9.62 9.586
   573   1379258_at   8.826 8.703 8.617 8.831 8.88 7.465 7.476 7.661 7.386 7.423
   574   1389103_at   7.419 7.326 7.566 7.265 7.477 5.271 5.584 5.714 5.675 5.657
   575   1367763_at   10.85 10.721 10.775 10.777 10.756 11.549 11.558 11.399 11.54 11.452
   576   1368270_at   7.579 7.523 8.186 7.74 7.584 5.066 5.3 5.152 5.299 5.318
   577   1370928_at   11.948 11.906 11.86 11.899 11.833 10.778 10.773 11.016 10.748 10.716
   578   1392681_at   10.662 10.602 10.701 10.716 10.685 11.076 11.091 11.14 11.12 11.104
   579   1368742_at   9.154 9.14 9.125 9.263 9.484 6.076 5.78 6.683 6.129 6.019
   580   1377899_at   9.112 9.195 9.079 9.199 9.198 10.085 10.368 10.129 10.188 10.178
   581   1378321_at   8.287 8.183 8.115 8.118 8.121 4.348 4.659 5.13 5.178 4.381
   582   1391411_at   10.112 10.233 10.252 10.228 10.136 8.954 9.058 8.759 8.734 8.956
   583   1369549_at   9.046 8.927 9.267 8.947 8.907 4.655 5.31 5.826 5.271 5.09
   584   1394678_at   7.575 7.802 7.599 7.766 7.739 5.327 4.902 5.271 4.898 5.534
   585   1367655_at   11.424 11.554 11.822 10.865 11.248 7.996 8.032 8.312 8.06 7.847
   586   1367922_at   9.606 9.731 9.513 9.735 9.828 7.807 8.042 8.201 8.045 8.136
   587   1375362_at   8.391 8.314 8.534 8.401 8.569 7.071 7.197 7.06 7.29 7.013
   588   1376688_a_at   7.415 7.612 7.441 7.309 7.635 5.246 4.532 4.692 4.543 4.78
   589   1383158_at   9.241 9.208 9.166 9.134 9.3 7.563 7.78 7.569 7.255 7.545
   590   1392527_at   9.826 9.839 9.915 9.961 9.928 10.575 10.661 10.532 10.612 10.524
   591   1368081_at   6.93 6.942 6.98 7.229 6.857 8.658 8.741 8.474 8.87 8.724
   592   1383850_at   7.07 7.329 7.155 7.045 7.193 8.396 8.534 8.447 8.305 8.562
   593   1383424_at   9.411 9.512 9.122 9.646 9.649 6.258 6.53 6.509 6.466 5.799
   594   1372328_at   8.303 8.386 8.447 8.597 8.349 9.765 9.781 9.852 9.753 9.558
   595   1398282_at   7.968 7.959 8.049 8.13 7.799 9.349 9.612 9.6 9.43 9.36
   596   1398757_at   11.755 11.707 11.632 11.639 11.845 10.655 10.616 10.713 10.451 10.565
   597   1368260_at   8.153 8.207 8.062 8.195 7.884 4.421 4.596 4.583 3.644 4.669
   598   1368409_at   7.101 7.096 7.307 7.378 7.154 8.481 8.418 8.356 8.455 8.33
   599   1382274_at   10.463 10.44 10.637 10.507 10.196 8.847 8.768 8.984 8.984 8.894
   600   1378137_at   9.203 9.169 9.154 9.308 9.27 8.08 7.954 7.805 7.936 7.708
   601   1369290_at   9.422 9.494 9.536 9.712 9.901 3.903 2.993 4.814 3.803 3.5
   602   1379356_at   8.496 8.578 8.528 8.615 8.732 6.642 7.013 6.628 6.436 6.694
   603   1379587_at   5.756 5.831 5.694 6.014 5.417 7.661 7.598 7.544 7.593 7.59
   604   1388275_at   9.991 9.923 10.457 10.106 9.738 5.225 5.028 6.231 5.091 5.372
   605   1372550_at   9 8.95 9.064 9.277 9.227 7.728 7.714 7.483 7.696 7.647
   606   1369621_s_at   10.017 10.057 10.187 10.176 10.324 8.939 8.839 8.828 9.034 8.944
   607   1371074_a_at   8.755 8.795 8.606 8.688 8.776 5.21 5.735 5.943 5.78 5.107
   608   1390569_at   8.274 8.315 8.255 8.269 8.013 9.758 9.862 10.197 10.042 9.925
   609   1376379_a_at   8.521 8.496 8.515 8.409 8.684 6.684 7.038 6.555 6.665 6.58
   610   1388330_at   9.283 9.33 9.507 9.575 9.559 10.684 10.64 10.703 10.597 10.6
   611   1370400_at   8.55 8.461 8.672 8.376 8.772 5.09 5.053 4.898 5.834 5.331
   612   1397628_at   8.622 8.58 8.408 8.825 8.808 6.941 6.744 6.965 7.027 6.999
   613   1374725_at   8.007 7.979 7.742 8.095 8.183 6.318 6.232 6.091 6.438 6.27
   614   1387108_at   10.649 10.693 10.714 10.663 10.631 10.283 10.256 10.305 10.239 10.212
   615   1377832_at   7.307 7.381 7.465 7.315 7.144 2.508 3.131 3.609 3.508 3.618
   616   1392528_at   8.013 8.075 7.988 8.246 8.252 6.733 6.747 6.795 6.52 6.58
   617   1370806_at   10.714 10.664 10.662 10.863 10.557 11.653 11.641 11.711 11.752 11.72
   618   1392737_at   9.505 9.495 9.57 9.565 9.602 4.343 4.135 5.524 5.157 4.805
   619   1380577_at   9.172 9.072 9.265 9.364 9.089 10.446 10.521 10.326 10.574 10.491
   620   1371938_at   11.404 11.397 11.301 11.336 11.269 10.771 10.832 10.826 10.775 10.785
   621   1373850_at   8.12 8.08 8.323 8.177 8.047 6.993 7.065 6.963 6.838 6.849
   622   1372170_at   11.612 11.587 11.774 11.716 11.524 12.591 12.557 12.562 12.695 12.597
   623   1371968_at   11.197 11.184 11.291 11.229 11.349 9.871 9.846 10.171 9.896 9.806
   624   1390384_at   8.128 8.512 8.086 8.332 8.291 6.673 6.447 6.577 6.683 6.453
   625   1375989_a_at   8.148 8.226 7.691 7.932 8.152 6.118 5.841 6.069 5.97 5.898
   626   1368575_at   9.398 9.371 9.597 9.521 9.402 10.929 10.965 11.191 10.845 10.802
   627   1370340_x_at   10.965 10.961 10.87 10.973 11.107 9.257 9.366 9.134 9.322 8.873
   628   1397655_at   6.579 7.007 6.696 7.132 6.785 4.213 4.488 4.626 4.259 4.559
   629   1368073_at   12.186 12.125 12.057 12.037 12.094 6.738 6.925 8.186 7.119 6.796
   630   1393201_at   6.98 7.085 7.141 7.077 6.84 5.021 5.185 4.936 5.331 4.804
   631   1393666_at   6.277 6.392 6.232 6.242 6.384 7.77 8.102 7.995 7.668 7.845
   632   1382950_at   12.36 12.362 12.345 11.979 12.25 7.508 7.26 8.611 7.496 7.543
   633   1372643_at   9.085 9.193 9.396 9.515 9.18 7.591 7.531 7.652 7.436 7.349
   634   1369520_a_at   8.602 8.514 8.691 8.856 8.541 4.841 5.088 5.832 4.874 5.117
   635   1367672_at   10.268 10.453 10.301 10.355 10.107 11.465 11.528 11.37 11.532 11.504
   636   1370186_at   12.604 12.519 12.466 12.27 12.466 7.516 7.705 8.796 7.602 7.499
   637   1371388_at   10.215 10.309 10.37 10.402 10.275 10.94 11.013 10.951 10.991 10.975
   638   1371033_at   12.782 12.68 12.72 12.472 12.504 10.624 10.325 10.769 10.32 10.705
   639   1374799_at   6.895 6.837 6.924 7.037 6.777 4.758 5.239 5.11 4.828 4.791
   640   1371106_at   7.094 7.167 7.461 7.235 7.505 4.58 4.728 4.321 4.975 4.346
   641   1367679_at   13.217 13.118 13.079 12.8 12.924 11.512 11.43 11.397 11.59 11.571
   642   1373997_at   7.792 7.886 7.884 7.797 7.73 6.668 6.675 6.615 6.718 6.388
   643   1368428_at   8.425 8.326 8.68 8.718 8.337 10.19 10.25 10.116 10.277 10.088
   644   1373164_at   9.294 9.166 9.616 9.458 9.311 7.509 7.471 7.603 7.244 7.621
   645   1370890_at   11.102 11.16 11.199 11.109 11.129 9.907 10.09 10.105 9.839 9.808
   646   1371782_at   8.463 8.451 8.236 8.438 8.614 5.743 5.699 6.308 6.106 6.139
   647   1380019_at   8.295 8.295 8.393 8.016 8.431 6.456 6.073 6.401 6.459 6.464
   648   1367567_at   12.267 12.256 12.277 12.189 12.304 11.749 11.739 11.75 11.68 11.635
   649   1379452_at   7.009 7.11 7.009 6.881 6.57 9.434 9.707 9.187 9.673 9.786
   650   1388455_at   9.985 10.085 10.016 10.056 10.275 8.366 8.563 8.575 8.427 8.162
   651   1379285_at   10.139 10.155 10.229 10.43 10.53 6.112 6.194 7.092 6.132 6.718
   652   1376250_at   8.886 9.064 8.811 8.963 9.001 8.045 8.063 7.984 7.908 7.995
   653   1367794_at   8.122 8.026 7.437 8.031 8.4 4.373 3.866 4.485 3.865 4.401
   654   1381536_at   4.768 4.737 3.708 4.752 4.461 0.444 0.595 0.766 0.74 0.485
   655   1388976_at   8.75 8.657 8.813 8.793 8.871 9.622 9.685 9.558 9.558 9.542
   656   1367776_at   8.706 8.891 8.586 8.742 8.543 5.058 5.774 5.664 5.786 5.962
   657   1367628_at   10.349 10.415 10.435 10.421 10.727 9.21 9.025 9.177 9.096 9.079
   658   1374628_at   10.093 10.12 10.241 10.283 9.876 11.556 11.529 11.522 11.607 11.427
   659   1391421_at   7.331 7.367 7.309 7.389 7.43 8.041 8.201 8.245 8.249 8.254
   660   1388924_at   8.746 8.828 8.316 8.981 9.002 6.015 6.234 6.34 6.115 5.912
   661   1371795_at   8.767 8.733 9.034 8.759 8.716 10.09 10.216 10.127 9.973 10.062
   662   1377145_at   7.878 7.625 8.048 7.949 7.852 9.926 9.606 9.695 9.522 9.601
   663   1372676_at   9.417 9.369 9.675 9.667 9.443 10.819 10.98 10.806 10.807 10.928
   664   1393329_at   8.124 7.918 8.618 8.598 8.043 4.572 5.072 5.066 5.196 4.894
   665   1382902_at   8.114 8.441 8.337 8.613 8.29 5.312 5.084 5.844 5.021 5.184
   666   1380619_at   7.62 7.77 7.829 7.579 7.643 6.278 6.397 6.385 6.473 6.142
   667   1386773_at   8.033 8.21 8.32 8.28 8.195 9.515 9.446 9.536 9.432 9.27
   668   1368470_at   10.53 10.544 10.643 10.71 10.724 11.416 11.514 11.571 11.506 11.553
   669   1384298_at   8.566 8.498 8.909 8.803 8.463 5.102 4.987 4.907 4.589 4.003
   670   1389355_at   9.27 9.166 8.881 9.121 9.099 6.597 7.225 7.004 6.827 6.897
   671   1367600_at   7.837 7.887 7.804 7.971 8.025 6.535 6.178 6.334 6.264 6.054
   672   1392171_at   9.303 9.155 9.415 9.342 9.061 3.23 4.471 4.714 3.928 3.43
   673   1367671_at   11.382 11.308 11.308 11.31 11.201 10.035 10.131 10.295 9.931 10.047
   674   1388884_at   10.482 10.372 10.504 10.582 10.278 11.521 11.659 11.613 11.686 11.509
   675   1385426_at   6.655 6.904 7.13 7.299 6.865 3.616 3.429 4.213 3.648 3.403
   676   1372516_at   7.78 7.645 7.568 7.658 7.732 4.098 4.553 4.361 3.557 4.573
   677   1373777_at   7.771 7.738 7.866 8 7.861 4.296 4.351 4.85 3.698 4.547
   678   1376100_at   9.396 9.118 9.132 9.213 9.523 4.135 4.558 5.241 4.148 5.216
   679   1375981_a_at   8.232 8.267 8.179 8.405 8.424 6.193 6.484 6.715 6.559 6.594
   680   1368419_at   10.491 10.575 10.232 10.321 10.556 8.643 8.706 8.383 8.69 8.823
   681   1378418_at   8.468 8.443 8.334 8.416 8.492 3.331 4.155 3.984 4.411 3.044
   682   1377950_at   11.438 11.413 11.288 11.224 11.48 6.196 6.126 7.329 6.322 5.641
   683   1383324_at   7.86 7.965 7.985 8.1 7.83 9.015 9.167 9.252 9.079 9.045
   684   1377497_at   8.757 8.124 8.559 8.408 8.126 6.13 6.006 6.027 6.089 5.85
   685   1373854_at   8.324 8.517 8.456 8.572 8.575 9.464 9.562 9.39 9.518 9.451
   686   1367605_at   12.588 12.498 12.64 12.358 12.469 11.159 11.112 11.05 11.362 11.258
   687   1386882_at   9.88 9.871 9.994 10.045 10.188 8.145 8.407 8.21 7.905 8.262
   688   1370172_at   12.005 11.891 11.94 11.852 12.023 10.55 10.719 10.652 10.544 10.305
   689   1390325_at   9.364 9.182 9.221 9.68 9.635 5.506 4.988 6.145 5.22 5.278
   690   1383401_at   9.799 9.875 9.666 9.756 9.823 8.202 8.273 8.461 8.004 8.363
   691   1391489_at   11.73 11.741 11.584 11.594 11.751 5.925 5.566 7.291 6.237 5.722
   692   1369427_at   9.316 9.249 9.435 9.191 9.321 4.731 4.565 4.676 5.485 3.876
   693   1382003_at   7.369 7.019 7.416 7.378 7.062 9.053 9.075 8.886 8.94 8.865
   694   1382314_at   11.473 11.455 11.093 11.489 11.618 6.672 6.879 7.75 6.653 6.331
   695   1367805_at   7.271 7.236 7.147 7.564 6.792 9.489 9.669 9.636 9.588 9.427
   696   1372303_at   9.983 10.12 10.332 10.277 10.365 7.832 8.17 7.449 7.773 7.743
   697   1372308_at   7.246 7.159 7.425 7.396 7.318 6.24 6.389 6.321 6.232 6.257
   698   1388650_at   7.978 8.017 8.179 8.373 8.213 5.053 5.535 5.531 5.383 5.869
   699   1370871_at   11.464 11.506 11.602 11.514 11.587 10.469 10.673 10.501 10.583 10.375
   700   1372104_at   8.489 8.476 8.347 8.631 8.481 9.521 9.754 9.529 9.593 9.677
   701   1398347_at   8.711 8.902 8.672 8.864 8.826 7.726 7.634 7.766 7.594 7.504
   702   1369630_at   10.664 10.722 10.674 10.596 10.471 11.557 11.509 11.477 11.574 11.642
   703   1395268_at   7.214 7.246 7.481 7.417 7.139 9.041 9.108 9.125 8.733 9.001
   704   1388738_at   8.752 9.095 8.845 8.783 8.81 10.462 10.743 10.416 10.343 10.554
   705   1383395_at   9.173 9.145 9.232 9.464 8.95 10.76 10.798 10.923 10.714 10.766
   706   1371680_at   10.096 10.128 10.071 10.282 10.07 11.105 11.183 11.182 10.982 11.064
   707   1368000_at   11.62 11.643 10.641 10.98 11.521 6.894 6.768 7.525 6.936 6.982
   708   1390596_at   0.367 0 0.832 1.384 0 6.336 6.078 6.24 6.084 5.412
   709   1371352_at   11.722 11.709 11.66 11.514 11.682 10.76 10.794 10.6 10.795 10.708
   710   1379248_at   7.903 7.738 7.918 7.901 7.893 6.271 6.192 5.771 6.25 6.003
   711   1371070_at   8.645 8.783 8.69 9.026 9.147 4.823 4.188 5.101 5.403 4.66
   712   1370020_at   9.292 9.17 9.24 9.479 9.021 10.865 10.759 11.06 10.993 10.814
   713   1393061_at   8.1 8.14 8.302 8.369 7.747 10.061 10.098 10.205 10.18 10.061
   714   1392590_at   10.032 10.08 10.206 10.274 10.126 11.151 11.29 11.137 11.066 11.125
   715   1382078_at   7.21 6.979 7.147 7.025 6.893 4.77 4.617 4.943 5.118 5.183
   716   1389498_at   7.642 7.754 7.691 7.785 7.584 8.641 8.735 8.699 8.643 8.501
   717   1389034_at   9.6 9.697 9.55 9.673 9.793 5.901 5.638 6.724 5.843 6.375
   718   1374217_at   6.911 7.009 7.015 7.239 6.612 8.857 8.894 8.961 8.905 8.733
   719   1387659_at   10.275 10.265 10.502 10.402 10.998 7.493 7.84 7.413 7.6 7.225
   720   1371931_at   8.438 8.409 8.57 8.63 8.583 9.591 9.605 9.693 9.486 9.476
   721   1370628_at   11.153 11.098 11.632 11.534 11.095 3.436 4.569 5.718 4.68 4.669
   722   1372875_at   8.238 8.264 8.324 8.477 8.205 7.277 7.363 7.29 7.264 7.153
   723   1374222_at   10.023 10.073 9.982 10.191 10.087 10.995 11.125 10.926 11.156 11.015
   724   1372585_at   13.401 13.256 13.221 12.877 13.043 10.069 10.161 10.834 10.27 10.27
   725   1369292_at   4.964 4.885 4.678 4.762 4.587 6.776 6.835 6.549 6.61 6.384
   726   1393987_s_at   10.292 10.284 10.352 10.307 10.248 8.968 8.807 9.164 8.721 8.814
   727   1388644_at   8.812 8.751 8.56 8.785 8.442 10.467 10.638 10.895 10.436 10.539
   728   1390407_at   9.762 9.722 9.79 9.765 9.805 9.005 9.093 8.987 8.887 8.848
   729   1369937_at   12.469 12.349 12.362 12.303 12.47 11.323 11.469 11.555 11.452 11.337
   730   1388794_at   9.422 9.382 9.349 9.355 9.572 8.255 8.323 8.102 8.023 8.041
   731   1372453_at   8.901 8.89 8.83 8.908 9.007 7.98 8.011 7.944 8.013 8.184
   732   1389256_at   6.298 6.27 6.075 6.298 6.526 7.796 7.898 7.932 7.657 7.768
   733   1393144_at   9.373 9.431 9.244 9.555 9.607 6.5 6.586 6.918 5.947 6.379
   734   1390993_at   7.758 7.74 7.836 7.736 7.668 8.94 9.021 8.699 8.914 8.774
   735   1368555_at   7.595 7.613 7.892 7.869 7.704 5.757 5.757 6.158 6.046 6.017
   736   1387765_at   6.238 6.143 6.419 6.125 6.242 7.829 7.825 7.992 7.675 7.575
   737   1367695_at   10.539 10.634 10.699 10.663 10.422 11.737 11.739 11.543 11.689 11.641
   738   1368234_at   8.753 8.694 8.772 8.806 8.314 10.302 10.307 10.48 10.32 10.323
   739   1372513_at   11.001 11.162 11.061 10.977 11.088 10.093 9.835 9.901 10.077 9.871
   740   1375674_at   7.109 7.416 7.208 7.27 7.076 8.731 8.537 8.842 8.68 8.551
   741   1389592_at   7.3 7.367 7.49 7.689 7.39 8.616 8.733 8.714 8.657 8.686
   742   1367999_at   10.319 10.25 10.247 10.521 10.188 11.348 11.456 11.536 11.458 11.519
   743   1373693_at   8.541 8.554 8.705 8.627 8.281 9.852 9.95 10.047 9.867 9.91
   744   1383421_at   7.152 7.23 7.27 7.474 7.201 8.416 8.472 8.484 8.282 8.439
   745   1371420_at   7.581 7.492 7.683 7.926 7.642 9.291 9.23 9.382 9.063 9.184
   746   1389227_at   9.84 9.794 9.71 10.03 9.992 8.616 8.467 8.712 8.547 8.482
   747   1371164_at   3.747 3.223 3.546 2.757 3.636 0 0 0.333 0 0
   748   1372323_at   7.153 7.385 7.442 7.574 7.005 9.333 9.417 9.441 9.283 9.14
   749   1388588_at   8.932 8.935 8.992 8.906 8.902 8.093 8.058 8.212 8.161 7.946
   750   1372872_at   6.128 6.11 6.208 6.227 5.929 7.51 7.543 7.481 7.447 7.231
   751   1369262_at   8.346 8.503 8.315 8.432 8.622 5.581 6.003 6.132 6.373 6.007
   752   1367693_at   10.7 10.613 10.57 10.739 10.759 9.468 9.596 9.73 9.421 9.527
   753   1374006_at   10.12 10.31 10.479 10.351 10.021 8.21 8.331 8.517 8.115 8.059
   754   1371405_at   10.103 10.164 10.147 10.184 10.195 11.064 11.087 10.905 11.005 11.204
   755   1393029_at   8.495 8.57 8.66 8.49 8.64 7.052 6.877 7.315 6.959 6.861
   756   1388776_at   10.629 10.758 10.919 10.85 10.856 9.583 9.519 9.586 9.375 9.296
   757   1369688_s_at   7.956 7.988 8.181 8.163 7.885 6.355 6.078 5.739 5.933 6.013
   758   1388460_at   10.515 10.482 10.406 10.355 10.534 7.943 8.028 8.479 7.619 8.006
   759   1380445_at   7.803 8.008 8.013 8.02 8.178 6.107 6.477 6.337 6.153 6.004
   760   1368091_at   9.48 9.245 9.437 9.631 9.415 10.777 10.836 10.998 10.799 10.69
   761   1368329_at   9.273 9.15 9.511 9.289 8.828 11.33 11.493 11.165 11.389 11.404
   762   1388148_a_at   10.221 10.311 10.265 10.392 10.309 11.234 11.274 11.176 11.336 11.071
   763   1371948_at   9.74 9.673 9.725 9.503 9.803 10.745 10.77 10.809 10.767 10.612
   764   1370113_at   9.434 9.343 8.969 9.493 9.627 4.505 5.07 5.82 5.386 5.262
   765   1398390_at   10.642 10.651 10.364 10.055 9.861 7.261 6.795 7.351 7.183 7.021
   766   1385605_at   6.525 7.016 6.399 6.502 6.751 1.778 1.66 1.978 2.864 1.395
   767   1369319_at   10.451 10.536 10.434 10.411 10.611 9.423 9.304 9.125 9.397 9.168
   768   1373940_at   9.014 9.111 9.158 9.181 9.194 8.292 8.295 8.199 8.319 8.103
   769   1374524_at   9.655 9.602 9.81 9.779 9.608 10.871 10.973 10.694 10.99 10.966
   770   1385658_at   9.209 9.239 9.173 9.631 9.571 7.503 7.195 7.27 7.511 7.268
   771   1368311_at   7.899 7.843 7.946 7.876 8.005 6.565 6.9 6.662 6.662 6.503
   772   1376835_at   8.972 8.851 8.64 8.977 8.884 6.344 6.634 6.964 6.499 6.292
   773   1379235_x_at   7.467 7.344 7.369 7.599 7.439 5.184 5.592 5.098 4.811 5.086
   774   1398831_at   12.02 11.952 11.945 11.787 11.958 11.193 11.251 11.186 11.181 11.127
   775   1381279_at   8.799 8.674 8.333 8.46 8.994 5.869 5.629 5.899 5.188 5.446
   776   1373670_at   9.131 8.956 9.082 9.153 9.289 7.056 6.448 7.094 6.666 6.911
   777   1372055_at   8.604 8.711 8.931 8.935 8.735 6.785 6.574 6.132 6.228 6.233
   778   1379228_at   6.222 6.132 6.173 6.221 6.223 5.169 5.17 4.94 5.082 5.301
   779   1368187_at   11.443 11.524 11.739 11.636 11.558 5.633 6.401 7.457 6.101 6.314
   780   1382447_at   6.573 6.391 6.812 6.539 6.332 8.551 8.748 8.67 8.542 8.255
   781   1372256_at   10.169 10.163 10.317 10.251 10.33 7.826 7.678 8.227 7.569 7.377
   782   1384022_at   8.804 9.037 9.162 9.093 9.078 7.854 7.644 7.799 7.693 7.848
   783   1383962_at   8.284 8.22 8.512 8.154 8.267 6.519 6.45 6.532 6.268 6.025
   784   1367723_a_at   8.21 8.205 8.177 8.261 8.236 7.69 7.617 7.509 7.558 7.506
   785   1386586_at   8.068 8.18 8.236 8.247 8.539 5.916 5.95 6.316 6.114 5.67
   786   1373736_at   5.327 5.328 4.914 5.314 5.224 7.593 7.625 7.718 7.313 7.15
   787   1386897_at   10.165 10.115 10.04 10.104 10.252 8.954 9.106 9.181 9.003 8.869
   788   1385572_at   7.9 8.016 7.904 7.931 8.068 5.744 5.659 5.431 5.044 5.808
   789   1372461_at   10.365 10.243 10.298 10.445 10.437 9.2 9.097 9.337 9.019 9.023
   790   1374606_at   5.736 5.636 5.696 5.674 5.63 6.518 6.38 6.285 6.386 6.427
   791   1374512_at   8.157 8.193 8.312 8.291 8.121 9.692 9.764 9.545 9.713 9.36
   792   1395840_at   8.265 8.416 8.386 8.554 8.605 6.927 6.861 7.14 7.142 6.919
   793   1377844_at   8.476 8.361 8.442 8.542 8.626 9.469 9.519 9.549 9.396 9.363
   794   1371071_at   7.663 7.729 7.781 7.9 8.245 5.686 5.66 5.593 5.187 5.57
   795   1368783_at   8.493 8.478 8.747 8.746 8.1 4.283 3.758 4.794 3.966 4.838
   796   1399158_a_at   11.652 11.736 11.483 11.565 11.723 10.501 10.504 10.282 10.228 10.389
   797   1394819_at   8.44 8.229 8.61 8.487 8.89 5.852 5.473 6.175 5.634 5.601
   798   1372076_at   10.739 10.733 10.779 10.823 10.824 11.359 11.437 11.415 11.565 11.434
   799   1370440_at   8.712 8.614 8.693 8.878 8.789 7.071 7.227 6.979 7.323 6.837
   800   1379250_at   8.503 8.59 8.89 8.912 8.506 10.283 10.421 10.472 10.482 10.296
   801   1373775_at   9.951 9.974 9.828 10.018 10.185 8.458 8.475 8.695 8.31 8.314
   802   1379563_at   8.094 7.954 7.986 7.757 7.587 5.59 5.845 5.805 6.024 6
   803   1385696_at   8.311 8.392 8.662 8.978 8.357 4.449 4.216 5.272 4.948 4.831
   804   1391808_at   7.667 7.709 7.593 7.828 7.978 5.616 5.733 6.018 5.628 5.384
   805   1398909_at   10.481 10.37 10.421 10.37 10.49 11.135 11.18 11.072 11.159 11.01
   806   1390416_at   9.341 9.432 9.367 9.527 9.08 10.839 10.677 10.919 10.958 10.873
   807   1377635_at   5.996 6.05 5.928 5.521 5.965 8.682 8.535 7.993 8.406 8.458
   808   1375732_at   7.277 7.446 7.583 7.615 7.35 5.989 6.265 6.114 6.058 6.129
   809   1371805_at   8.213 8.228 8.099 8.266 8.083 9.108 9.154 9.079 8.955 8.988
   810   1371295_at   12.355 12.259 12.368 12.274 12.384 11.835 11.849 11.767 11.847 11.798
   811   1370209_at   9.498 9.643 9.641 9.754 9.542 11.355 11.226 11.196 10.909 11.376
   812   1379287_at   8.245 8.425 8.237 8.469 8.384 7.01 7.005 7.193 6.823 6.849
   813   1367786_at   11.841 11.837 11.784 11.888 11.967 6.789 6.712 8.07 6.599 6.456
   814   1392731_at   7.275 7.02 7.144 7.055 7.366 2.163 0.411 0.766 2.011 1.333
   815   1388436_at   8.233 8.14 8.169 8.414 8.37 5.933 6.316 6.219 6.568 6.063
   816   1397584_at   7.264 7.198 7.544 7.621 7.431 8.898 8.907 8.992 8.766 8.903
   817   1388071_x_at   12.95 12.847 12.825 12.594 12.81 11.691 11.672 11.683 11.783 11.574
   818   1371729_at   8.827 8.811 8.927 8.908 8.895 9.993 9.948 9.88 9.852 9.675
   819   1398602_at   7.965 7.892 7.901 8.081 7.856 4.499 5.113 4.612 5.352 5.252
   820   1371653_at   11.394 11.393 11.318 11.369 11.466 10.338 10.3 10.435 10.062 10.269
   821   1370463_x_at   9.931 9.993 10.288 9.953 9.764 8.36 8.435 8.452 8.568 8.453
   822   1370810_at   7.178 7.392 7.445 7.563 7.232 4.786 4.467 5.278 4.801 4.62
   823   1398853_at   11.336 11.314 11.267 11.143 11.145 10.445 10.51 10.461 10.535 10.432
   824   1393460_at   8.782 8.946 8.912 8.934 9.117 6.378 5.865 6.781 6.355 6.334
   825   1388135_at   8.067 8.071 8.285 8.213 7.949 6.144 6.173 5.934 6.543 6.145
   826   1391803_at   7.166 7.154 7.117 6.885 7.07 4.901 4.791 4.692 4.388 4.139
   827   1370189_at   10.303 10.413 10.345 10.297 10.414 9.827 9.751 9.694 9.685 9.769
   828   1368894_at   1.104 1.506 1.036 2.098 1.339 5.95 5.657 6.274 6.083 5.224
   829   1368467_at   9.775 9.857 9.839 10.146 9.503 11.577 11.692 11.607 11.526 11.604
   830   1372032_at   9.065 9.249 9.555 9.63 9.436 7.101 7.487 7.006 7.133 7.089
   831   1371974_at   10.416 10.389 10.479 10.308 10.456 11.028 11.036 10.96 11.009 11.094
   832   1387831_at   9.947 9.949 10.03 9.932 9.665 3.269 1.655 2.784 4.2 2.88
   833   1372460_at   10.087 10.168 10.062 10.169 10.156 9.236 9.45 9.227 9.358 9.243
   834   1367614_at   11.228 11.234 11.256 11.278 11.347 9.947 10.175 10.166 9.867 9.833
   835   1385001_at   7.486 7.476 7.101 7.393 7.606 5.5 5.452 5.408 5.553 5.067
   836   1368245_at   8.903 8.869 8.92 9.06 8.429 11.103 11.138 11.354 11.094 10.827
   837   1375716_at   8.449 8.129 8.319 8.292 8.344 5.941 5.465 5.844 6.315 5.829
   838   1372562_at   8.576 8.666 8.552 8.736 8.565 9.727 9.758 9.546 9.645 9.503
   839   1368940_at   6.886 7.164 7.211 7.133 7.179 5.248 5.114 5.485 5.294 4.894
   840   1375638_at   7.012 7.236 6.88 6.982 7.06 8.566 8.711 8.499 8.415 8.324
   841   1374038_at   7.535 7.776 7.42 7.649 7.728 8.933 9.107 8.997 8.815 9.03
   842   1386908_at   10.015 10.012 10.177 10.095 10.189 7.32 7.271 7.69 6.646 7.117
   843   1387956_s_at   7.437 7.517 7.773 7.692 7.908 5.373 5.595 5.254 5.595 5.021
   844   1381323_at   7.522 7.664 7.709 7.73 7.702 6.792 6.838 6.8 6.872 6.655
   845   1382275_at   8.025 8.028 8.253 8.272 8.265 5.826 5.825 5.796 5.719 5.1
   846   1370415_at   8.305 8.345 8.506 8.577 8.303 6.528 6.691 6.647 6.55 6.132
   847   1383368_at   9.538 9.463 9.399 9.423 9.396 8.808 8.634 8.616 8.753 8.69
   848   1370218_at   11.894 11.922 11.971 11.899 11.973 12.389 12.317 12.332 12.418 12.336
   849   1377086_at   6.209 6.189 6.284 6.511 6.024 8.498 8.581 7.997 8.453 8.393
   850   1367777_at   10.132 10.124 10.156 10.251 10.22 10.951 10.915 10.899 11.068 10.838
   851   1389181_at   9.035 8.973 9.258 9.094 8.947 7.446 7.714 7.582 7.315 7.313
   852   1380297_at   8.199 8.205 7.836 8.176 8.408 6.434 6.399 6.412 6.374 6.114
   853   1390797_at   8.005 7.931 7.923 7.975 7.892 4.046 4.022 5.106 3.854 4.223
   854   1383391_a_at   11.008 11.04 11.111 11.001 11.051 4.688 4.995 6.674 5.336 5.301
   855   1390285_at   6.364 6.412 6.413 6.659 6.26 8.667 8.602 9.057 8.345 8.541
   856   1368645_at   7.822 8.093 8.246 8.165 8.174 6.365 6.286 6.375 6.55 6.072
   857   1368339_at   4.511 4.253 3.817 4.043 3.668 6.653 6.589 6.608 6.556 6.515
   858   1386946_at   9.91 9.973 9.798 9.747 9.936 10.726 10.8 10.883 10.78 10.688
   859   1382569_at   5.146 5.659 5.822 5.618 5.583 7.662 7.592 7.513 7.801 7.534
   860   1393163_at   4.814 4.716 4.677 4.868 5.036 6.345 6.407 6.627 6.502 6.197
   861   1392914_at   6.082 6.382 6.567 6.45 6.298 8.139 8.205 8.299 7.997 7.925
   862   1385711_at   6.716 6.689 6.734 6.774 6.848 6.151 6.126 6.157 6.08 6.007
   863   1378457_at   7.052 7.166 7.253 7.308 7.009 8.52 8.669 8.724 8.696 8.375
   864   1378315_at   4.986 4.918 4.503 4.475 4.328 7.029 7.354 7.095 7.039 6.941
   865   1384103_at   7.292 7.338 7.557 7.411 7.264 8.555 8.506 8.337 8.552 8.433
   866   1367901_at   8.914 8.95 9.024 9.119 9.194 7.605 7.624 7.853 7.766 7.892
   867   1370309_a_at   11.205 11.207 11.241 11.278 11.29 10.565 10.556 10.393 10.393 10.357
   868   1393831_at   8.893 8.817 9.094 9.197 8.744 10.807 11.045 10.826 10.587 10.761
   869   1387198_at   7.962 8.239 8.167 8.3 8.085 5.986 5.951 5.462 5.648 5.267
   870   1379582_a_at   7.597 8.195 8.169 7.643 7.97 3.181 4.19 2.856 3.531 3.828
   871   1369801_at   8.42 8.46 8.56 8.398 8.508 4.934 3.43 4.424 4.012 4.494
   872   1370232_at   9.631 9.775 9.878 9.837 9.82 10.57 10.717 10.653 10.723 10.6
   873   1379482_at   7.234 7.296 7.425 7.3 7.263 5.515 5.61 5.174 4.972 5.362
   874   1386982_at   8.6 8.611 8.579 8.595 8.795 7.67 7.85 7.61 7.663 7.693
   875   1391637_at   8.115 8.159 8.181 8.254 8.422 5.639 5.415 5.687 6.02 5.106
   876   1390704_at   6.452 6.426 6.497 6.432 6.507 4.996 5.265 5.426 5.235 5.082
   877   1377018_at   6.076 5.76 5.197 5.639 6.066 8.51 8.8 8.814 8.522 8.561
   878   1371701_at   11.76 11.755 11.745 11.726 11.673 12.155 12.24 12.142 12.163 12.115
   879   1368990_at   8.262 8.242 7.593 8.106 8.493 5.224 5.455 5.635 5.47 5.354
   880   1384842_s_at   7.739 7.832 7.56 7.736 7.895 3.834 4.031 4.654 4.695 4.796
   881   1378193_at   10.36 10.352 10.621 10.543 10.569 4.396 4.632 6.227 5.271 5.297
   882   1372341_at   8.658 8.734 8.846 8.607 8.551 10.143 10.044 9.878 9.833 9.895
   883   1390421_at   10.29 10.219 10.162 10.305 10.125 11.355 11.416 11.109 11.427 11.355
   884   1372042_at   8.486 8.403 8.166 8.637 8.772 6.318 6.1 6.534 6.254 6.506
   885   1380546_at   8.587 8.519 8.515 8.559 8.739 9.584 9.769 9.594 9.552 9.491
   886   1369625_at   11.151 11.075 11.32 11.147 10.953 12.275 12.186 12.201 12.327 12.376
   887   1372930_at   9.901 9.95 9.701 9.917 9.946 7.23 7.378 7.973 7.256 7.618
   888   1373283_at   9.005 8.683 8.699 8.712 8.844 7.364 7.67 7.479 7.467 7.428
   889   1378150_at   7.989 7.902 7.92 8.062 8.069 5.172 5.01 5.608 5.41 4.625
   890   1388203_x_at   10.102 10.08 10.302 10.257 10.266 7.51 7.369 7.539 7.726 6.754
   891   1387742_at   8.082 8.22 7.884 7.858 8.053 3.823 2.287 2.147 2.144 2.878
   892   1368464_at   8.695 8.631 8.677 8.842 9.499 5.002 5.08 5.68 4.981 4.657
   893   1389907_at   9.402 9.436 9.388 9.46 9.452 9.77 9.876 9.814 9.777 9.809
   894   1379395_a_at   10.155 10.121 10.17 10.32 10.144 10.929 10.917 10.849 10.834 10.816
   895   1384280_at   7.912 8.022 7.947 8.098 7.949 4.91 5.314 5.666 5.666 5.689
   896   1387806_at   11.498 11.498 11.471 11.288 11.505 10.109 10.234 9.797 9.895 10.056
   897   1394419_at   8.484 8.48 8.337 8.615 8.614 5.11 5.248 5.475 4.247 4.958
   898   1389556_at   7.451 7.732 7.562 7.658 7.293 8.988 9.231 9.099 8.939 9.013
   899   1384837_at   8.625 8.669 8.859 8.947 8.447 5.116 4.388 5.004 4.704 3.808
   900   1382014_at   7.432 7.528 7.589 7.519 7.312 8.744 8.764 8.632 8.711 8.464
   901   1374651_at   6.98 6.746 6.98 6.783 6.998 5.455 5.342 5.653 5.376 5.65
   902   1367829_at   9.983 9.913 9.953 9.794 9.677 10.894 10.907 10.788 10.871 10.822
   903   1367952_at   10.012 10.009 10.18 10.447 10.1 11.91 12.143 12.01 11.787 12.247
   904   1391214_at   8.7 8.653 8.646 8.488 8.291 3.809 4.578 5.065 4.111 3.704
   905   1388389_at   10.314 10.267 10.293 10.308 10.476 9.603 9.621 9.597 9.471 9.507
   906   1370067_at   7.621 7.698 7.742 7.851 7.225 9.568 9.603 9.819 9.593 9.904
   907   1367683_at   10.216 10.119 10.102 10.109 10.107 8.626 8.949 8.451 8.736 8.814
   908   1371171_at   8.033 7.835 7.882 7.661 7.763 6.09 5.591 5.892 5.744 5.45
   909   1392900_at   10.385 10.571 10.45 10.573 10.582 9.046 9.237 9.152 8.797 8.874
   910   1373557_at   8.05 8.241 8.008 8.096 8.045 4.871 5.823 5.593 5.504 5.427
   911   1370249_at   11.531 11.554 11.565 11.345 11.566 9.058 9.255 9.596 8.91 8.794
   912   1398326_at   11.963 11.899 11.995 11.934 12.014 12.507 12.475 12.45 12.58 12.587
   913   1372568_at   8.109 8.008 8.093 7.808 7.816 9.233 9.219 9.496 9.377 9.276
   914   1382215_at   7.104 7.142 7.15 7.193 7.072 7.956 8.071 7.922 7.822 7.847
   915   1394352_at   7.005 7.016 6.994 7.37 7.153 4.308 4.948 4.665 4.644 5.038
   916   1368774_a_at   7.61 7.478 7.639 7.731 7.35 9.164 9.655 9.473 9.285 9.238
   917   1377945_at   8.34 8.368 8.204 8.296 8.178 7.259 7.322 7.26 7.016 7.264
   918   1367716_at   8.672 8.614 8.816 8.684 8.696 9.59 9.594 9.442 9.705 9.664
   919   1394020_at   8.182 8.223 8.176 7.935 7.817 9.938 10.137 10.325 9.775 10.082
   920   1370987_at   9.112 9.003 9.183 9.342 8.969 6.684 7.208 6.604 6.694 6.407
   921   1370060_at   10.237 10.187 10.373 10.342 10.317 10.919 10.931 10.947 11.02 10.898
   922   1383712_at   7.98 7.927 7.963 8.027 8.107 7.357 7.187 7.25 7.312 7.185
   923   1376858_at   6.818 6.545 6.391 6.481 6.777 8.357 8.369 8.035 8.289 8.31
   924   1367793_at   10.455 10.427 10.739 10.714 10.521 11.641 11.664 11.759 11.746 11.662
   925   1368253_at   8.261 8.495 8.638 8.541 8.149 9.982 9.999 10.195 10.005 9.977
   926   1384231_at   6.633 6.752 6.43 6.714 6.817 3.206 2.965 4.029 3.05 2.962
   927   1387871_at   8.055 8.295 8.352 8.219 8.497 6.861 6.962 6.871 6.71 6.67
   928   1398378_at   8.623 8.539 8.505 8.52 8.883 10.005 10.038 10.244 9.925 9.996
   929   1371775_at   9.848 10.067 10.187 9.85 9.77 11.221 11.213 11.224 11.274 11.192
   930   1396268_at   8.496 8.672 8.584 8.491 8.682 6.465 5.82 6.557 6.46 6.145
   931   1370826_at   10.257 10.24 10.146 10.313 10.455 9.302 9.427 9.352 9.255 9.357
   932   1378171_at   7.945 7.744 7.88 8.214 8.201 5.384 4.968 5.403 5.615 4.848
   933   1377034_at   10.677 10.799 10.81 10.815 10.91 9.368 9.43 9.754 9.55 9.377
   934   1388391_at   11.601 11.566 11.681 11.66 11.61 12.191 12.181 12.259 12.184 12.085
   935   1389654_at   8.392 8.38 8.416 8.616 8.601 9.621 9.518 9.679 9.434 9.558
   936   1368655_at   11.115 11.12 11.345 11.119 11.063 9.106 9.136 8.97 9 9.599
   937   1383986_at   11.224 11.241 11.113 11.207 11.332 9.982 10.126 10.271 9.904 10.038
   938   1389250_at   8.234 8.306 8.417 8.397 8.553 5.583 5.395 5.415 6.242 5.838
   939   1368528_at   7.128 7.039 7.155 7.177 7.286 8.463 8.663 8.454 8.229 8.407
   940   1376197_at   7.91 8.054 7.811 7.759 7.571 4.94 5.336 5.586 4.813 4.896
   941   1389039_at   8.412 8.305 8.082 8.305 8.596 6.247 6.731 6.457 6.376 6.543
   942   1388537_at   8.65 8.692 8.828 8.914 8.669 10.233 10.296 9.967 10.162 9.962
   943   1398750_at   11.938 11.92 11.893 11.836 11.935 11.255 11.265 11.142 11.09 11.29
   944   1370144_at   8.882 8.784 8.805 8.842 8.055 11.526 11.266 11.562 11.415 11.218
   945   1383879_at   8.473 8.454 8.73 8.586 8.578 9.597 9.768 9.752 9.524 9.738
   946   1385590_at   7.053 7.021 7.559 7.334 7.079 3.596 2.7 3.281 3.353 2.293
   947   1370127_at   7.203 7.162 7.055 6.869 6.982 5.503 5.267 5.466 5.058 5.078
   948   1385869_at   8.591 8.672 8.635 8.652 8.613 6.748 7.287 7.096 6.935 7.217
   949   1396217_at   6.59 6.661 7.055 6.462 6.636 3.892 4 3.608 3.395 3.091
   950   1372681_at   7.199 7.23 6.999 6.983 6.996 8.176 7.998 8.108 8.061 8.045
   951   1368509_at   7.362 7.09 7.17 7.235 6.668 9.039 9.17 9.182 9.064 8.98
   952   1398356_at   10.071 10.044 10.06 9.888 10.034 9.069 9.057 8.765 8.963 8.856
   953   1393256_at   7.057 6.96 7.05 7.087 6.981 8.002 7.912 7.789 7.756 7.802
   954   1372333_at   10.914 11.031 11.062 11.042 11.105 10.341 10.271 10.274 10.25 10.114
   955   1371912_at   10.952 10.916 11.02 10.83 10.83 11.482 11.523 11.558 11.553 11.532
   956   1373209_at   8.186 8.315 8.255 8.378 8.258 7.494 7.643 7.643 7.533 7.538
   957   1387621_at   6.453 6.376 6.226 6.552 6.553 3.501 3.993 4.163 3.425 4.055
   958   1373422_at   9.919 9.911 9.822 9.989 9.96 8.819 9.111 8.943 8.831 9
   959   1390240_at   6.72 6.761 6.708 6.655 6.449 4.656 4.778 5.057 5.026 4.534
   960   1384917_at   0 0.239 0.516 0 0.189 5.702 5.876 6.166 4.314 5.402
   961   1375277_at   7.117 7.001 7.216 7.219 7.099 5.185 4.882 5.232 5.462 5.497
   962   1388429_at   8.034 8.118 8.314 7.937 8.154 6.836 7.035 6.917 6.917 6.779
   963   1382592_at   7.009 7.042 7.131 7.226 7.051 5.243 5.433 5.496 4.999 4.946
   964   1395508_at   10.366 10.366 10.326 10.365 10.539 9.554 9.602 9.373 9.419 9.421
   965   1371111_at   7.798 7.643 7.829 7.654 7.905 6.211 5.931 5.916 6.401 6.075
   966   1391527_at   8.64 8.688 8.639 8.858 8.846 7.704 7.694 7.625 7.532 7.808
   967   1384900_at   6.393 6.614 6.883 6.695 6.512 3.515 4.14 3.784 4.312 3.628
   968   1385502_at   6.755 7.123 6.693 6.796 6.696 4.492 4.399 5.023 4.415 4.454
   969   1382189_at   8.86 8.872 9.069 9.151 8.59 10.731 10.706 10.474 10.586 10.599
   970   1388352_at   10.274 10.218 10.187 10.11 10.234 9.597 9.533 9.474 9.383 9.487
   971   1388688_at   7.64 7.526 7.815 7.805 7.567 8.92 8.905 8.792 8.821 9.077
   972   1371213_at   7.876 7.763 7.722 7.93 8.044 5.304 5.131 5.358 5.481 4.569
   973   1367680_at   10.324 10.363 10.442 10.383 9.945 11.736 11.794 11.711 11.899 11.828
   974   1388778_at   8.301 8.223 8.142 8.32 7.755 10.062 10.233 10.446 10.015 10.146
   975   1378914_a_at   7.156 7.368 7.701 7.383 7.227 4.612 4.621 4.906 4.795 4.048
   976   1372790_at   8.891 9.066 9.134 9.12 8.759 10.578 10.589 10.726 10.502 10.326
   977   1377664_at   7.398 7.276 7.305 7.441 7.764 5.796 5.724 5.713 5.451 5.44
   978   1368877_at   7.78 7.777 8.229 7.789 7.662 10.548 10.44 9.884 10.166 10.195
   979   1387691_at   5.5 6.425 6.272 6.168 6.093 2.287 1.333 1.736 1.057 2.094
   980   1390849_at   6.899 7.001 7.225 7.159 7.403 1.339 2.982 1.275 1.903 2.278
   981   1375987_at   7.806 8.011 8.067 8.108 8.075 6.568 6.362 6.663 6.436 6.181
   982   1375560_at   8.78 8.769 8.843 8.834 8.75 10.119 9.973 9.869 10.153 9.771
   983   1368066_at   8.103 8.032 8.37 8.481 8.275 5.392 5.349 5.934 6.051 5.679
   984   1383658_at   9.352 9.264 9.367 9.526 9.563 6.512 6.687 7.067 6.077 6.219
   985   1394306_at   7.639 8.137 8.267 7.876 7.393 1.465 1.516 3.082 1.848 2.711
   986   1373514_at   10.458 10.426 10.216 10.299 10.377 6.957 6.379 7.386 6.513 7.402
   987   1383073_at   10.39 10.422 10.401 10.528 10.425 9.556 9.685 9.782 9.545 9.65
   988   1378296_at   7.653 7.482 7.455 7.602 7.652 6.186 5.843 6.188 5.903 6.263
   989   1390050_at   7.361 7.403 7.28 7.235 7.387 8.401 8.575 8.757 8.589 8.376
   990   1376007_at   6.936 7.113 7.109 7.097 7.206 8.385 8.566 8.523 8.29 8.191
   991   1373280_at   9.291 9.229 9.046 9.237 9.134 7.676 7.901 7.869 7.851 8.119
   992   1387314_at   8.999 9.108 9.293 9.286 9.152 10.238 10.245 10.282 10.242 10.071
   993   1367897_at   9.25 9.365 9.126 9.359 9.356 10.139 10.241 10.303 10.22 10.11
   994   1380521_at   9.577 9.564 9.81 9.972 9.915 6.386 6.369 7.041 6.074 5.846
   995   1379676_a_at   8.864 9.051 9.084 8.918 9.137 7.933 7.944 8.115 7.853 7.963
   996   1374019_at   7.995 8.117 8.027 8.113 8.194 8.939 8.989 8.931 8.805 8.81
   997   1370891_at   10.493 10.49 10.629 10.387 10.32 8.66 8.789 8.189 8.731 8.633
   998   1370973_at   2.844 2.503 2.098 1.989 2.325 4.849 5.034 5.021 4.791 5.184
   999   1386637_at   7.744 7.775 8.175 7.853 8.019 5.519 5.332 4.899 5.114 5.719
   1000   1374903_at   8.665 8.732 8.671 8.708 8.5 9.567 9.683 9.642 9.467 9.729
   1001   1387205_at   10.052 10.035 9.854 10.073 10.295 6.437 6.66 7.348 6.071 6.558
   1002   1368224_at   7.973 8.063 7.577 8.061 7.861 4.111 4.438 5.092 4.041 3.927
   1003   1373346_at   9.347 9.43 9.238 9.331 9.357 10.324 10.165 10.327 10.084 10.288
   1004   1370347_at   7.767 7.734 7.997 8.056 8.027 6.608 6.67 6.793 6.549 6.557
   1005   1386943_at   8.917 8.864 8.843 8.967 8.91 10.473 10.494 10.105 10.337 10.09
   1006   1383917_at   7.876 7.737 7.865 8.324 8.215 5.984 6.097 6.219 6.203 6.056
   1007   1384188_at   7.576 7.502 7.558 7.689 7.405 8.384 8.543 8.566 8.397 8.508
   1008   1376390_at   10.861 10.897 10.838 10.714 10.934 4.409 3.482 5.87 3.949 3.845
   1009   1395534_at   5.023 5.274 5.31 5.287 5.123 6.428 6.395 6.298 6.295 6.175
   1010   1371073_at   8.79 8.682 8.633 8.675 8.742 7.11 6.414 6.563 6.647 6.894
   1011   1389744_at   6.664 6.913 7.142 7.231 6.944 8.715 8.99 8.84 8.686 8.675
   1012   1398303_s_at   10.694 10.82 10.918 10.764 10.865 9.539 9.509 9.205 9.49 9.148
   1013   1370573_at   6.395 6.167 6.632 6.321 6.214 7.853 7.822 7.658 7.955 7.855
   1014   1377646_at   9.97 9.952 10.209 9.845 9.63 11.611 11.724 11.547 11.434 11.522
   1015   1382680_at   8.293 8.367 8.355 8.478 8.658 5.146 5.098 5.714 4.687 4.513
   1016   1389365_at   9.174 9.099 9.073 9.285 9.463 7.707 7.876 7.891 7.957 7.68
   1017   1375137_at   10.939 10.881 11.027 10.882 11.005 9.716 9.526 9.424 9.77 9.327
   1018   1369208_at   7.09 7.14 7.244 7.19 7.056 4.085 4.745 5.026 4.306 4.489
   1019   1386669_at   5.76 5.73 6.003 6.031 6.109 4.265 4.265 4.512 4.553 4.287
   1020   1367597_at   12.497 12.448 12.449 12.457 12.6 11.95 11.93 11.954 11.903 11.827
   1021   1382683_a_at   6.068 6.473 6.342 6.365 5.801 9.221 8.589 9.426 9.04 9.101
   1022   1368461_at   10.021 10.03 10.275 10.152 9.834 11.952 11.909 11.541 12.034 12.041
   1023   1369998_at   9.873 9.979 10.161 9.957 9.954 9.135 8.979 8.983 8.917 9.087
   1024   1372685_at   8.651 8.511 8.748 8.743 8.502 4.588 5.657 5.114 5.02 5.733
   1025   1391856_at   9.25 9.126 9.091 9.377 9.121 10.41 10.418 10.41 10.23 10.189
   1026   1375414_at   10.02 10.093 10.09 10.003 10.027 9.508 9.39 9.353 9.362 9.266
   1027   1389622_at   9.282 9.229 9.345 9.415 9.184 10.668 10.892 10.408 10.732 10.631
   1028   1389008_at   8.326 8.278 8.436 8.342 8.53 9.093 9.183 9.129 9.11 9.101
   1029   1370918_a_at   12.085 12.051 12.043 11.966 12.021 12.492 12.496 12.46 12.594 12.558
   1030   1398296_at   10.367 10.373 10.401 10.337 10.161 11.318 11.404 11.361 11.208 11.169
   1031   1387624_at   8.428 8.325 8.158 8.518 8.523 7.274 7.317 7.161 7.245 7.172
   1032   1371366_at   11.016 11.024 10.984 11.001 11.094 10.267 10.224 10.425 10.201 10.16
   1033   1394051_at   6.061 6.11 6.25 6.201 5.778 2.84 2.063 2.467 3.293 2.729
   1034   1390576_at   8.305 8.425 8.213 8.384 8.321 7.332 7.334 7.496 7.562 7.381
   1035   1373247_at   6.218 6.222 6 5.981 6.25 7.388 7.49 7.481 7.252 7.239
   1036   1368482_at   10.761 10.738 10.922 10.864 10.794 4.228 4.718 6.231 5.479 4.381
   1037   1374667_at   6.585 6.63 6.614 6.791 6.817 8.299 8.239 7.855 8.116 8.037
   1038   1390918_at   8.48 8.426 8.623 8.701 8.46 9.93 9.805 9.585 9.938 9.847
   1039   1370547_at   8.815 8.716 8.777 8.873 8.306 10.306 10.519 10.334 10.408 10.538
   1040   1373852_at   8.205 8.364 8.461 8.444 8.576 6.593 6.894 7.06 6.869 6.676
   1041   1388556_at   8.41 8.348 8.234 8.409 8.445 7.458 7.663 7.458 7.549 7.45
   1042   1377190_at   6.601 6.532 6.705 6.46 6.646 4.741 4.88 4.511 4.992 4.37
   1043   1388397_at   9.106 9.051 8.962 9.167 9.353 7.826 8.032 7.929 7.762 7.916
   1044   1371763_at   9.85 9.788 10.061 10.023 9.355 11.774 11.936 11.881 11.813 11.742
   1045   1375972_at   6.505 6.404 6.306 6.453 6.721 4.441 4.811 4.996 4.679 4.711
   1046   1387819_at   8.944 8.798 9.286 9.103 9.089 10.442 10.43 10.371 10.587 10.373
   1047   1385656_at   10.116 10.255 10.396 10.375 10.22 11.079 11.149 11.077 11.148 11.178
   1048   1391347_at   9.154 9.245 9.65 9.508 9.702 6.764 6.419 6.901 7.122 6.389
   1049   1368223_at   9.628 9.601 8.983 9.743 9.827 7.205 6.934 7.212 7.191 7.043
   1050   1368924_at   6.413 6.486 6.48 5.946 6.042 8.563 8.536 8.132 8.376 8.429
   1051   1386880_at   9.432 9.534 9.481 9.579 9.569 10.458 10.559 10.307 10.65 10.538
   1052   1390348_at   8.783 9 9.578 9.327 9.149 5.606 5.69 6.396 5.748 5.327
   1053   1398256_at   9.234 9.184 9.18 9.089 9.102 5.125 4.924 6.21 4.801 4.975
   1054   1388064_a_at   6.718 6.732 6.76 6.757 6.232 4.181 4.519 4.257 3.961 4.578
   1055   1369103_at   7.914 7.841 8.285 8.262 8.075 6.213 6.149 5.77 5.918 5.658
   1056   1383716_at   7.052 7.291 7.107 6.897 7.188 5.874 6.006 6 6.049 5.893
   1057   1370870_at   7.733 7.711 7.867 8.188 7.5 9.67 10.064 9.985 9.744 9.906
   1058   1386890_at   11.482 11.368 11.204 11.354 11.503 9.72 9.826 10.092 9.57 9.651
   1059   1383785_at   6.648 5.984 6.643 6.279 6.412 3.908 4.307 3.927 4.232 4.19
   1060   1373473_a_at   8.87 8.891 8.631 8.916 9.075 7.295 7.29 7.349 7.19 7.606
   1061   1380079_at   8.316 8.143 8.187 8.212 7.626 5.142 5.032 4.825 5.535 4.58
   1062   1372135_at   8.696 8.554 8.843 8.812 8.767 6.181 6.061 6.282 6.524 5.541
   1063   1371511_at   11.93 11.863 11.877 11.777 11.908 11.05 11.184 11.263 11.063 11.086
   1064   1370294_a_at   8.115 7.89 8.207 8.092 7.925 5.734 6.277 5.775 5.91 5.472
   1065   1382050_at   8.421 8.556 8.567 8.57 8.025 10.712 10.894 10.338 10.552 10.859
   1066   1375585_at   8.251 8.327 8.201 8.469 8.526 6.609 6.921 6.901 6.804 6.486
   1067   1371998_at   10.019 10.02 10.002 9.986 9.9 9.026 9.083 9.307 9.21 9.104
   1068   1369940_at   11.19 11.155 11.147 11.183 11.214 10.783 10.723 10.812 10.689 10.679
   1069   1375041_at   8.362 8.471 8.26 8.556 8.593 4.484 3.871 4.996 4.986 5.172
   1070   1376039_at   7.165 6.998 6.87 6.778 7.013 4.415 4.62 4.902 4.972 4.254
   1071   1372306_at   10.068 10.131 10.232 10.32 10.16 11.441 11.308 11.185 11.306 11.555
   1072   1390042_at   10.811 10.807 10.994 11.015 10.779 9.238 9.554 9.314 9.337 9.668
   1073   1382115_at   6.487 6.563 6.447 6.684 6.595 7.641 7.39 7.496 7.411 7.468
   1074   1370166_at   8.93 8.981 9.184 8.943 8.449 11.034 10.994 10.756 10.826 10.958
   1075   1388600_at   8.909 8.885 9.065 9.044 8.968 5.725 5.851 6.787 5.95 5.954
   1076   1371922_at   10.676 10.701 11.035 10.846 10.754 11.849 11.973 11.807 11.938 11.896
   1077   1370948_a_at   8.783 8.709 8.882 8.502 8.952 6.044 6.364 5.476 6.331 5.906
   1078   1398829_at   10.608 10.594 10.698 10.595 10.742 9.936 9.977 10.111 9.974 9.985
   1079   1392969_at   10.425 10.337 10.631 10.666 10.357 11.595 11.645 11.573 11.667 11.758
   1080   1398286_at   7.469 7.544 7.609 8.013 7.343 9.936 9.999 10.291 9.691 9.775
   1081   1390807_at   9.433 9.485 9.582 9.655 9.329 10.479 10.541 10.418 10.389 10.52
   1082   1373198_at   7.848 7.889 7.805 8.107 7.901 9.092 9.209 8.921 9.078 8.936
   1083   1371544_at   10.892 10.802 10.911 10.796 10.936 9.923 10.017 9.765 9.993 9.739
   1084   1372103_at   6.937 6.872 6.972 7.157 6.915 8.374 8.369 8.26 8.028 8.426
   1085   1389688_at   7.635 7.77 7.77 7.954 7.743 8.858 9.184 9.214 8.963 9.066
   1086   1388166_at   8.7 8.789 8.681 8.604 8.449 6.34 6.15 6.354 6.927 6.54
   1087   1388528_at   9.874 9.907 9.876 10.006 10.14 8.438 8.67 8.523 8.225 8.21
   1088   1389082_at   6.911 7.024 7.043 6.995 6.902 7.881 8.041 8.009 7.876 7.738
   1089   1387793_at   10.937 10.972 11.125 11.073 10.905 11.738 11.732 11.727 11.705 11.629
   1090   1388622_at   9.208 9.173 9.017 9.345 9.333 8.023 8.156 7.827 7.96 7.919
   1091   1388365_at   11.084 11.086 11.177 11.123 10.962 11.689 11.641 11.623 11.697 11.684
   1092   1381221_at   6.274 5.9 6.1 6.097 6.161 3.742 3.63 4.142 4.233 3.587
   1093   1388948_at   9.27 9.232 9.235 9.264 9.079 10.663 10.712 11.04 10.54 10.547
   1094   1373216_at   7.3 7.47 7.18 7.531 7.293 8.386 8.296 8.422 8.398 8.402
   1095   1367720_at   9.076 9.067 9.277 9.183 9.111 10.112 10.346 10.084 10.131 10.042
   1096   1369969_at   10.046 10.032 10.012 10.128 10.253 8.893 9.02 8.669 8.62 8.765
   1097   1371496_at   9.406 9.297 9.536 9.516 9.006 11.15 10.936 10.868 11.112 11.053
   1098   1372976_at   7.181 7.004 7.398 7.409 6.915 9.291 9.253 9.658 9.269 9.056
   1099   1371916_at   10.833 10.804 11.023 10.867 10.642 11.925 11.925 11.801 12.037 11.983
   1100   1386695_at   7.196 7.46 7.294 7.732 7.787 3.134 3.29 4.202 4.17 3.44
   1101   1395375_at   8.846 8.977 9.096 8.841 8.891 7.918 7.782 7.804 8.045 7.787
   1102   1391817_at   8.51 8.409 8.618 8.409 8.467 7.171 7.223 7.04 7.066 6.726
   1103   1377183_at   5.975 5.919 5.933 5.867 5.66 6.892 6.891 7.038 6.806 6.852
   1104   1373290_at   7.496 7.674 7.697 7.566 7.554 5.351 5.15 5.031 5.535 4.603
   1105   1380110_at   9.031 9.026 9.058 8.968 9.135 7.432 7.645 7.479 7.009 7.272
   1106   1371696_at   9.857 9.863 9.848 10.025 9.724 10.888 10.931 10.765 10.728 10.751
   1107   1371577_at   10.599 10.578 10.602 10.642 10.581 11.152 11.288 11.282 11.151 11.104
   1108   1388915_at   8.388 8.466 8.235 8.645 8.576 7.029 7.062 7.276 6.92 7.014
   1109   1398500_at   6.92 6.962 6.825 7.375 7.02 8.578 8.555 8.44 8.468 8.623
   1110   1396984_at   8.182 8.366 8.293 8.293 8.3 7.331 7.382 7.358 7.114 7.101
   1111   1371691_at   11.626 11.565 11.053 11.147 11.485 9.392 9.105 9.181 9.265 9.53
   1112   1388762_at   9.589 9.597 9.518 9.573 9.554 8.661 8.501 8.565 8.263 8.357
   1113   1368177_at   7.003 6.898 7.182 7.3 6.989 8.641 8.646 8.305 8.506 8.494
   1114   1369986_at   9.398 9.391 9.668 9.833 9.365 10.994 11.066 11.031 10.978 10.905
   1115   1370907_at   9.642 9.601 9.819 9.778 9.395 11.028 11.077 11.216 10.88 11.004
   1116   1384330_at   7.138 7.354 7.112 7.255 7.211 5.978 6.18 5.977 5.771 6.086
   1117   1374254_a_at   8.821 8.747 9.074 9.129 9.004 10.03 10.128 10.173 10.167 10.176
   1118   1389504_at   7.411 7.412 7.343 7.391 7.162 8.753 8.468 8.581 8.468 8.388
   1119   1383429_at   9.864 10.008 10.149 10.003 10.174 8.876 9.109 8.889 8.826 8.891
   1120   1372459_at   10.465 10.482 10.531 10.511 10.393 8.693 8.429 8.97 8.309 8.326
   1121   1373908_at   8.38 8.438 8.25 8.495 8.92 6.498 6.595 6.641 6.231 6.428
   1122   1369752_a_at   4.731 4.55 4.494 4.814 4.663 1.438 1.632 1.884 0.731 1.748
   1123   1373057_at   7.894 7.696 7.765 8.002 7.686 9.727 9.603 9.429 9.235 9.304
   1124   1398891_at   10.351 10.187 10.349 10.333 10.284 10.857 10.89 10.886 10.823 10.787
   1125   1371445_at   10.457 10.446 10.455 10.467 10.539 9.503 9.524 9.566 9.214 9.316
   1126   1388788_at   10.701 10.652 10.658 10.654 10.311 11.849 11.76 11.687 11.885 11.77
   1127   1368173_at   9.371 9.267 9.16 9.338 9.339 8.011 8.019 8.242 7.919 7.759
   1128   1390383_at   10.921 10.856 10.889 10.967 10.986 10.048 9.856 9.965 9.722 9.707
   1129   1398373_at   10.006 10.257 10.06 10.186 10.364 7.932 7.921 8.419 7.673 7.682
   1130   1388822_at   10.064 10.043 10.072 10.068 10.154 10.484 10.583 10.472 10.498 10.473
   1131   1369956_at   10.154 10.015 10.2 10.132 9.964 9.154 8.989 8.93 9.185 9.147
   1132   1385791_at   6.808 6.82 7.01 7.159 6.428 9.039 8.861 8.912 8.809 8.703
   1133   1378240_at   6.354 6.365 6.435 6.193 6.183 4.611 4.87 5.081 4.619 4.708
   1134   1389246_at   8.54 8.688 8.854 8.934 8.894 7.57 7.578 7.441 7.43 7.628
   1135   1395313_s_at   7.796 7.807 7.962 7.987 7.609 5.612 5.123 5.48 5.435 5.976
   1136   1378307_at   6.45 6.669 7.033 6.915 6.567 8.975 9.003 9.31 8.88 8.672
   1137   1369200_at   8.022 8.071 8.356 8.326 7.927 9.604 9.691 9.604 9.478 9.463
   1138   1388565_at   9.936 9.919 9.96 9.912 9.857 9.106 9.244 9.087 8.92 9.129
   1139   1372110_at   7.384 7.355 7.432 7.605 7.194 8.83 9.118 8.743 8.789 8.734
   1140   1370345_at   8.569 8.14 8.312 8.289 8.301 3.555 4.656 5.033 4.675 4.471
   1141   1378700_at   7.56 7.62 7.556 7.679 7.748 4.754 5.01 5.516 5.456 5.508
   1142   1373757_at   9.041 9.119 9.143 9.267 9.408 7.296 7.194 7.485 7.14 6.761
   1143   1384457_at   7.876 7.924 7.684 7.962 8.036 6.473 6.304 6.71 6.628 6.441
   1144   1368431_at   9.425 9.402 9.422 9.537 9.253 10.338 10.349 10.517 10.332 10.243
   1145   1375684_at   6.343 6.461 6.616 6.439 6.405 7.665 7.859 7.61 7.744 7.454
   1146   1371266_at   7.578 7.576 7.782 7.74 7.047 9.787 9.653 9.942 9.548 9.751
   1147   1398511_at   8.608 8.584 8.653 8.717 8.164 10.625 10.729 10.213 10.561 10.442
   1148   1379293_at   9.42 9.354 10.516 10.138 9.384 4.4 2.738 4.154 4.09 3.057
   1149   1388753_at   9.999 10.016 9.737 9.792 10.078 8.525 8.515 8.747 8.369 8.389
   1150   1378033_at   6.739 6.772 6.657 7.004 7.047 4.456 4.079 4.772 4.709 4.131
   1151   1385019_at   5.967 6.21 6.29 5.736 5.814 3.623 3.487 3.916 3.762 4.074
   1152   1398756_at   8.809 8.898 8.975 8.885 9.087 6.876 7.017 7.481 6.801 7.004
   1153   1367769_at   10.075 9.973 10.009 9.963 10.02 9.307 9.505 9.308 9.296 9.312
   1154   1376808_at   6.819 6.577 6.567 6.795 6.392 9.015 9.15 9.572 8.842 8.758
   1155   1390141_at   5.561 6.268 6.07 6.369 6.094 2.524 2.818 3.131 2.467 3.316
   1156   1399012_at   11.336 11.392 11.39 11.396 11.387 11.783 11.737 11.893 11.849 11.851
   1157   1370369_at   7.344 7.067 7.429 7.5 6.811 2.057 1.828 1.899 3.285 1.356
   1158   1379885_at   8.497 8.664 8.898 8.822 8.193 10.467 10.513 10.666 10.638 10.544
   1159   1371936_at   11.636 11.641 11.617 11.751 11.869 10.606 10.669 10.635 10.504 10.329
   1160   1393249_at   7.8 7.985 8.084 8.07 7.954 6.936 6.935 6.662 6.957 6.848
   1161   1387502_at   8.751 8.679 8.958 8.76 8.61 7.204 7.002 6.634 7 7.183
   1162   1371737_at   8.899 8.784 9.008 9.089 9.006 7.941 8.085 8.032 8.05 8.127
   1163   1379653_a_at   6.04 6.172 6.297 6.552 5.628 2.558 2.331 2.053 2.478 3.2
   1164   1373286_at   7.355 7.092 7.086 7.339 7.419 5.721 5.566 5.893 5.513 5.87
   1165   1379794_at   9.781 9.846 10.447 10.303 9.753 4.064 3.348 5.295 3.795 3.067
   1166   1368609_at   3.091 3.136 2.687 3.124 2.888 4.63 4.79 4.863 4.98 4.52
   1167   1368143_at   9.703 9.782 9.671 9.86 9.875 8.5 8.609 8.874 8.528 8.635
   1168   1373754_at   7.712 7.561 7.571 7.702 7.609 8.71 8.73 8.927 8.686 8.514
   1169   1371079_at   8.897 8.903 8.927 8.775 9.089 4.27 4.251 4.986 3.946 2.996
   1170   1384173_at   7.14 7.158 7.109 7.226 7.039 5.6 5.284 5.888 5.683 5.653
   1171   1387778_at   10.627 10.627 10.672 10.741 10.735 10.159 10.083 10.197 10.228 10.164
   1172   1371640_at   9.849 9.776 9.831 9.686 9.891 10.535 10.426 10.493 10.525 10.391
   1173   1367738_at   8.326 8.421 8.414 8.3 8.345 6.902 6.692 7.021 6.853 6.417
   1174   1372310_at   8.781 8.934 9.056 9.138 8.815 10.06 10.094 9.992 10.015 9.95
   1175   1398769_at   10.14 9.943 9.861 10.066 10.255 8.782 8.684 8.921 8.624 8.622
   1176   1371184_x_at   7.902 7.89 8.021 8.086 8.138 5.86 6.229 6.436 5.947 6.375
   1177   1398430_at   7.528 7.662 7.624 7.852 7.473 9.064 9.114 9.004 8.742 9.055
   1178   1371483_at   10.484 10.399 10.518 10.433 10.311 11.341 11.423 11.384 11.253 11.157
   1179   1370814_at   9.419 9.364 9.505 9.585 9.583 10.274 10.356 10.413 10.315 10.213
   1180   1372765_a_at   10.099 10.016 10.209 10.023 9.791 11.061 11.262 11.117 11.219 11.173
   1181   1392819_at   8.983 9.107 9.02 8.827 9.003 4.621 3.963 5.719 4.664 4.402
   1182   1398255_at   8.02 8.102 8.216 8.318 7.683 10.175 10.276 10.545 9.929 10.216
   1183   1385704_at   7.814 7.724 7.379 7.811 7.766 9.517 9.446 9.234 9.098 9.381
   1184   1390226_at   8.248 8.235 8.331 8.465 8.481 5.552 5.467 6.164 5.025 5.354
   1185   1380063_at   9.588 9.583 9.701 9.677 9.83 5.749 5.546 6.417 6.667 6.624
   1186   1387221_at   8.041 8.107 8.104 8.271 8.534 4.601 3.982 4.368 3.274 4.697
   1187   1387947_at   7.757 7.69 8.17 8.281 8.26 5.893 5.659 5.579 5.254 5.394
   1188   1384215_at   5.573 5.377 5.382 4.959 5.26 7.116 7.718 7.485 7.635 7.365
   1189   1393330_at   6.175 6.537 6.277 6.495 6.543 7.883 7.722 7.688 7.733 7.584
   1190   1389107_at   9.822 9.755 10.062 9.936 9.721 11.074 11.324 11.2 10.968 11.109
   1191   1393531_at   6.53 6.286 6.744 6.827 6.512 8.491 8.411 8.511 8.385 8.085
   1192   1399161_a_at   10.675 10.612 10.598 10.597 10.583 8.718 8.921 9.318 9.058 8.727
   1193   1397611_at   6.679 7.028 6.636 6.9 6.933 4.413 4.898 4.338 4.637 4.985
   1194   1373949_at   8.613 8.655 8.733 8.635 8.806 6.953 6.599 7.231 6.704 6.593
   1195   1390508_at   7.452 7.604 7.515 7.566 7.419 8.188 8.166 8.155 8.165 8.041
   1196   1379301_at   4.86 4.784 4.975 4.629 4.462 6.062 6.072 6.123 6.222 6.133
   1197   1370566_at   6.471 6.983 7.187 7.045 6.689 9.189 9.108 9.028 9.573 9.193
   1198   1380009_at   7.967 7.998 7.57 7.83 7.79 6.525 6.506 6.614 6.355 6.331
   1199   1392612_at   6.627 6.784 6.561 6.647 5.986 8.707 8.934 8.97 8.648 9.063
   1200   1376051_at   9.891 9.887 10.053 9.973 9.584 11.111 11.284 11.067 11.228 11.181
   1201   1396492_at   7.075 7.551 7.638 7.542 7.077 1.057 2.07 3.128 2.043 1.475
   1202   1373841_at   8.493 8.568 8.494 8.478 8.34 7.348 6.819 7.164 7.022 7.115
   1203   1392794_at   7.35 7.644 6.955 7.143 7.208 4.547 4.353 3.982 4.759 3.883
   1204   1367835_at   6.175 6.31 6.475 6.69 6.229 8.424 8.074 7.992 8.343 8.139
   1205   1371329_at   11.529 11.494 11.578 11.558 11.564 11.139 11.203 11.054 11.146 11.118
   1206   1369672_at   8.211 8.319 8.119 8.346 8.745 6.126 5.56 5.245 5.753 5.816
   1207   1383862_at   7.854 7.831 7.98 8.028 7.802 6.434 6.574 6.525 6.855 6.727
   1208   1383465_at   7.836 7.716 7.566 7.81 7.718 6.779 6.788 6.827 6.988 6.805
   1209   1389363_at   9.087 9.031 9.224 9.238 9.287 9.929 9.919 9.874 9.93 9.88
   1210   1380537_at   7.601 7.676 7.061 7.617 7.669 3.192 4.404 4.271 4.021 4.073
   1211   1373803_a_at   7.22 7.182 7.401 7.34 6.94 9.292 9.052 8.748 8.968 8.88
   1212   1377060_at   9.205 9.457 9.474 9.56 9.292 10.588 10.708 10.723 10.467 10.516
   1213   1388176_at   7.255 7.343 7.525 7.423 6.9 9.056 9.324 9.175 8.954 9.296
   1214   1387932_at   7.646 7.723 8.095 8.122 7.677 9.371 9.539 9.565 9.545 9.424
   1215   1383906_at   11.401 11.349 10.72 11.141 11.622 8.248 8.361 8.778 8.182 8.66
   1216   1389448_at   7.677 7.728 7.451 7.883 8.074 9.533 9.419 9.651 9.364 9.389
   1217   1371575_at   11.581 11.58 11.513 11.544 11.551 10.963 11.015 10.955 10.774 10.88
   1218   1379316_at   8.197 8.232 8.08 8.344 8.407 6.21 6.4 6.812 6.179 6.289
   1219   1370130_at   10.43 10.446 10.528 10.56 10.429 9.874 9.807 9.67 9.827 9.69
   1220   1392939_at   5.541 5.562 5.644 5.671 5.677 4.75 4.967 4.883 4.766 4.933
   1221   1367495_at   9.35 9.392 9.401 9.344 9.48 8.712 8.764 8.764 8.8 8.578
   1222   1383654_a_at   6.179 6.332 6.805 6.285 6.23 8.818 8.772 8.345 8.752 8.362
   1223   1386865_at   6.417 6.419 6.463 6.661 6.951 8.368 8.159 8.229 8.093 8.229
   1224   1389425_at   7.67 7.641 7.822 7.936 8.134 5.631 5.851 6.15 6.117 5.927
   1225   1385382_at   6.342 6.428 6.19 6.464 6.106 4.132 4.434 3.595 4.024 4.187
   1226   1398647_at   5.648 5.784 5.782 5.98 5.841 7.751 7.204 7.268 7.363 7.37
   1227   1390835_at   10.19 10.113 10.182 10.201 9.908 11.298 11.463 11.09 11.334 11.304
   1228   1389310_at   6.963 6.746 6.835 6.844 6.767 8.099 8.097 8.528 8.092 8.12
   1229   1398591_at   6.816 6.973 7.215 6.993 7 5.223 4.841 4.66 4.764 4.358
   1230   1382727_at   7.335 7.516 7.416 7.375 7.677 5.226 5.251 5.217 5.264 5.893
   1231   1375961_at   7.479 7.456 7.19 7.392 7.788 8.888 8.934 8.852 8.848 8.975
   1232   1368283_at   8.98 8.873 8.983 9.042 8.468 10.539 10.387 10.422 10.531 10.388
   1233   1382490_at   4.351 4.595 4.771 4.629 4.338 6.357 6.208 6.106 6.085 5.922
   1234   1373912_at   8.903 8.992 8.912 8.965 9.245 7.873 7.855 8.053 7.788 7.821
   1235   1379598_at   7.176 7.609 7.463 7.509 7.324 4.269 3.617 4.855 3.838 4.225
   1236   1389413_at   7.847 8.151 8.133 7.853 7.752 3.84 4.771 5.096 4.846 4.678
   1237   1375856_at   7.603 7.685 7.835 7.554 7.411 9.319 9.563 9.063 9.172 9.136
   1238   1374419_at   6.363 6.324 6.209 6.209 6.158 7.524 7.224 7.492 7.361 7.203
   1239   1382069_at   7.737 7.642 7.373 7.425 7.523 8.558 8.688 8.546 8.549 8.58
   1240   1376944_at   7.116 7.159 7.231 7.483 7.003 9.105 9.715 9.587 9.088 9.549
   1241   1389548_at   8.545 8.664 8.898 8.619 8.239 10.437 10.45 10.129 10.467 10.397
   1242   1368085_at   8.269 8.414 8.52 8.72 8.386 9.827 9.765 9.749 9.668 9.582
   1243   1385213_at   7.63 7.598 7.785 7.644 7.881 4.076 3.738 4.058 3.328 4.851
   1244   1389140_at   9.988 10.014 10.07 10.082 10.102 9.425 9.358 9.454 9.321 9.207
   1245   1394577_at   8.133 8.132 8.218 8.209 8.227 9.154 9.484 9.55 9.215 9.28
   1246   1390386_at   8.746 8.678 8.73 8.649 8.678 6.552 6.265 6.89 6.854 6.164
   1247   1374857_at   7.921 7.999 8.006 8.228 8.181 6.901 6.761 6.982 6.806 6.601
   1248   1371487_at   10.107 10.157 10.305 10.369 10.423 8.996 9.023 9.275 8.947 8.963
   1249   1370699_a_at   10.561 10.592 10.681 10.647 10.371 11.447 11.525 11.372 11.552 11.465
   1250   1391948_at   5.767 5.133 5.978 6.262 5.727 0.506 0.043 1.664 0.251 1.118
   1251   1374959_at   8.877 8.901 8.984 8.906 8.601 10.075 10.256 9.969 9.968 10.138
   1252   1391040_at   7.587 7.558 7.595 7.81 7.665 6.959 6.881 6.895 6.794 6.827
   1253   1394228_at   8.391 8.344 8.499 8.643 8.248 9.707 9.751 9.925 9.553 9.696
   1254   1382783_at   6.208 6.389 6.344 6.341 6.442 5.246 5.122 5.352 5.312 4.97
   1255   1373185_at   9.883 9.878 9.901 9.91 10.025 9.113 9.263 9.307 9.153 9.083
   1256   1373772_at   8.006 8.239 7.998 8.075 8.223 6.519 6.668 6.857 6.3 6.468
   1257   1379318_at   7.766 7.63 7.738 8.039 7.805 8.92 8.917 9.171 8.97 9.109
   1258   1367685_at   12.283 12.212 12.264 12.172 12.283 11.838 11.731 11.751 11.785 11.685
   1259   1372587_at   8.997 9.069 8.998 8.904 8.893 10.423 10.361 9.962 10.3 10.165
   1260   1375522_at   9.624 9.55 9.542 9.655 9.606 8.707 8.74 8.449 8.781 8.739
   1261   1382181_at   9.319 9.404 8.991 9.114 9.341 7.572 7.682 7.73 7.236 7.434
   1262   1392145_at   7.061 7.225 7.358 7.483 7.431 8.466 8.533 8.612 8.496 8.413
   1263   1367838_at   8.663 8.641 9.033 8.888 8.213 10.781 10.808 10.854 10.622 10.748
   1264   1373961_at   8.752 8.814 8.663 8.784 8.897 7.196 7.429 7.6 7.448 7.09
   1265   1393587_a_at   8.256 8.421 8.5 8.561 8.498 9.532 9.677 9.565 9.416 9.387
   1266   1370072_at   9.336 9.325 9.364 9.518 9.08 10.718 10.848 10.984 10.564 10.697
   1267   1388673_at   8.559 8.558 8.687 8.353 8.395 6.027 5.442 5.376 4.708 5.228
   1268   1393757_at   7.036 6.93 7.092 7.143 6.826 4.082 4.066 4.419 4.954 3.977
   1269   1377689_at   6.104 6.106 6.192 6.232 6.434 3.778 4.296 4.564 4.193 4.196
   1270   1372493_at   8.113 8.247 8.014 8.288 8.517 6.586 6.615 6.475 6.835 6.369
   1271   1389858_at   8.081 8.207 8.183 8.437 8.417 6.812 7.055 6.724 6.715 6.594
   1272   1370030_at   10.716 10.731 10.857 10.733 10.507 11.677 11.683 11.581 11.588 11.771
   1273   1392037_at   6.699 7.072 6.434 6.822 7.064 4.109 4.535 4.614 4.252 3.914
   1274   1375267_at   9.152 9.231 9.159 9.5 9.215 10.952 11.089 11.285 10.864 10.681
   1275   1377737_at   9.491 9.536 9.395 9.4 9.525 8.432 8.629 8.28 8.385 8.235
   1276   1372009_at   9.872 9.862 9.815 10.125 9.998 8.37 8.527 8.671 8.359 8.76
   1277   1385574_at   7.416 7.631 7.597 7.814 7.33 8.828 8.897 9.088 9.088 9.022
   1278   1392777_at   6.601 6.871 6.918 6.972 6.912 3.637 4.116 4.558 4.507 3.864
   1279   1390815_at   3.784 4.036 3.846 3.568 3.648 5.339 5.605 5.413 5.683 5.883
   1280   1372158_at   7.585 7.804 7.163 7.751 7.312 9.756 9.616 9.64 9.437 9.371
   1281   1387263_at   5.536 5.515 5.949 5.662 5.337 7.745 7.624 7.782 7.595 7.239
   1282   1371509_at   10.506 10.445 10.309 10.278 10.372 9.66 9.612 9.509 9.46 9.455
   1283   1368045_at   8.592 8.627 8.845 8.741 8.521 9.896 10.007 9.65 9.988 9.93
   1284   1371712_at   8.413 8.549 8.589 8.672 8.536 7.687 7.463 7.731 7.489 7.573
   1285   1376055_at   8.366 8.253 8.311 8.228 8.31 5.099 5.309 6.207 5.373 5.671
   1286   1395574_at   8.557 8.632 8.582 8.749 8.822 7.89 7.886 7.794 7.784 7.724
   1287   1371973_at   11.409 11.38 11.396 11.219 11.385 10.724 10.703 10.566 10.54 10.655
   1288   1398482_at   8.115 7.84 7.896 8.384 8.227 4.674 5.125 5.123 5.542 4.435
   1289   1390692_at   7.03 7.317 7.288 7.419 7.592 5.689 5.802 5.973 5.856 5.669
   1290   1374156_at   8.167 8.123 8.062 8.085 8.079 9.046 9.258 9.063 8.852 9.097
   1291   1368973_at   7.82 8.083 7.879 8.203 8.153 6.181 6.005 6.287 6.57 6.034
   1292   1370936_at   7.894 8.009 7.865 8.08 7.412 9.711 9.695 9.58 9.651 9.476
   1293   1369930_at   11.827 11.775 11.686 11.59 11.701 10.998 11.106 11.064 10.967 10.971
   1294   1372260_at   8.888 9.055 8.867 9.05 9.105 10.173 10.181 10.14 10.005 9.877
   1295   1387839_at   6.615 6.283 6.006 6.121 6.333 4.618 4.216 4.078 4.146 4.108
   1296   1372117_at   7.688 7.569 7.714 7.771 7.745 8.531 8.766 8.508 8.49 8.547
   1297   1398751_at   12.133 12.124 11.973 12.101 12.189 11.59 11.473 11.476 11.504 11.47
   1298   1373053_at   7.258 7.109 7.163 6.841 6.907 8.283 8.446 8.671 8.539 8.507
   1299   1369421_at   7.486 7.433 7.293 7.532 7.505 6.752 6.525 6.514 6.563 6.608
   1300   1371965_at   9.186 9.09 9.179 9.187 9.079 9.713 9.924 9.83 9.743 9.745
   1301   1368266_at   7.898 7.948 8.146 8.694 8.353 5.087 5.119 5.718 5.242 4.757
   1302   1383015_at   7.115 6.939 7.026 7.065 6.741 3.259 3.858 4.351 3.243 4.033
   1303   1372748_at   8.621 8.383 8.416 8.385 8.594 7.512 7.549 7.303 7.331 7.289
   1304   1375523_at   9.988 10.03 9.716 9.657 10.094 8.08 8.277 7.842 8.337 7.998
   1305   1369318_at   6.168 6.037 5.716 6.005 5.992 7.813 7.97 7.375 7.847 7.755
   1306   1383655_at   8.171 8.09 8.209 8.216 8.644 6.204 6.019 6.288 5.567 5.87
   1307   1389469_at   8.435 8.589 8.486 8.813 8.399 10.247 10.123 10.501 10.032 10.02
   1308   1374938_at   7.697 7.715 7.891 7.904 7.98 6.91 6.851 6.799 6.988 6.759
   1309   1367687_a_at   9.484 9.409 9.26 9.493 9.569 10.187 10.307 10.222 10.254 10.311
   1310   1388992_at   7.972 8.047 7.784 8.157 8.042 6.988 7.122 7.107 7.02 7.049
   1311   1388930_at   10.949 10.95 10.827 10.801 10.999 10.237 10.232 10.1 10.125 10.05
   1312   1368199_at   9.1 9.104 8.95 9.041 9.142 7.871 7.864 8.208 7.849 7.792
   1313   1373889_at   9.707 9.65 10.185 9.986 9.718 5.485 5.025 6.244 5.579 4.433
   1314   1381973_at   7.601 7.693 7.928 7.911 7.319 9.743 9.385 9.59 9.721 9.437
   1315   1391409_at   10.019 9.916 10.046 10.11 10.036 11.255 11.472 11.01 11.173 11.12
   1316   1384628_at   8.623 8.748 8.889 8.895 8.373 10.111 10.168 10.034 10.114 10.13
   1317   1383369_at   8.243 8.225 8.172 8.235 8.3 6.78 7.269 6.997 6.935 7.129
   1318   1368834_at   8.738 8.765 8.769 8.818 8.764 8.326 8.362 8.317 8.18 8.296
   1319   1371807_at   8.291 8.346 8.311 8.303 8.463 8.99 9.078 9.027 8.918 8.904
   1320   1371505_at   10.903 10.893 10.751 10.897 10.963 10.038 10.215 10.152 9.988 10.157
   1321   1375146_at   10.745 10.685 10.862 10.721 10.587 11.518 11.437 11.446 11.538 11.382
   1322   1374393_at   9.014 8.913 8.91 9.029 9.085 7.975 8.082 8.216 7.976 7.864
   1323   1383625_a_at   9.568 9.533 9.477 9.576 9.569 8.341 8.59 8.647 8.332 8.267
   1324   1388114_at   11.535 11.522 11.485 11.305 11.484 10.623 10.678 10.556 10.51 10.753
   1325   1374505_at   10.302 10.348 10.434 10.188 10.407 9.696 9.612 9.56 9.632 9.538
   1326   1370790_at   9.202 9.063 8.904 8.687 8.674 2.278 1.646 3.628 1.57 3.491
   1327   1395167_at   7.44 7.359 7.527 7.506 7.225 8.999 9.049 9.426 8.78 9.13
   1328   1375297_at   9.655 9.668 9.639 9.742 9.533 10.36 10.374 10.292 10.251 10.207
   1329   1389446_at   8.219 8.395 8.155 8.283 8.587 6.948 6.861 6.95 6.912 6.562
   1330   1386915_at   10.346 10.364 10.278 10.487 10.523 9.376 9.408 9.571 9.227 9.333
   1331   1377756_at   8.165 8.153 8.358 8.477 8.52 7.223 7.23 7.243 7.103 7.126
   1332   1390687_at   10.472 10.485 10.556 10.581 10.596 6.047 6.134 6.65 6.391 4.784
   1333   1382601_at   8.813 8.855 8.856 8.951 9.223 4.229 3.775 4.452 2.454 4.165
   1334   1376605_at   7.902 7.956 8.163 8.117 7.746 9.517 9.688 9.727 9.363 9.335
   1335   1386989_at   6.966 7.332 6.906 7.275 7.169 5.53 5.249 5.488 5.435 5.755
   1336   1371016_at   10.156 10.229 10.619 10.425 9.977 7.237 7.497 8.156 7.639 7.396
   1337   1374137_at   9.143 9.188 9.068 8.898 9.017 8.143 8.249 8.042 8.036 7.984
   1338   1374449_at   7.98 8.205 7.902 8.319 8.192 4.905 5.721 5.629 5.092 5.763
   1339   1371908_at   8.337 8.36 8.282 8.422 8.232 7.265 7.31 7.216 7.308 6.918
   1340   1390839_at   8.677 8.64 8.654 8.536 8.753 6.701 6.68 7.073 6.362 7.031
   1341   1398970_at   9.457 9.444 9.366 9.368 9.303 10.113 10.173 10.17 9.94 10.098
   1342   1389668_at   8.129 7.904 7.556 7.891 7.963 4.476 5.235 5.274 4.418 5.166
   1343   1371256_at   9.179 9.377 9.266 9.153 9.419 6.928 6.979 7.53 6.806 6.579
   1344   1393777_at   8.132 8.144 8.375 8.511 8.182 9.565 9.737 9.392 9.542 9.611
   1345   1370282_at   9.308 9.327 9.533 9.47 9.238 10.443 10.313 10.337 10.202 10.274
   1346   1398936_at   8.703 8.7 8.8 8.786 8.745 8.295 8.291 8.192 8.158 8.148
   1347   1382389_at   6.579 6.691 6.642 6.693 6.591 7.313 7.495 7.25 7.268 7.361
   1348   1368118_at   9.609 9.498 9.69 9.809 9.701 8.827 8.632 8.744 8.735 8.853
   1349   1367750_at   8.396 8.25 8.246 8.413 8.444 9.107 9.169 9.133 8.988 9.048
   1350   1372536_at   7 6.896 6.544 7.248 6.709 9.016 9.103 9.091 8.931 8.627
   1351   1369499_at   7.019 6.598 6.791 6.665 6.639 4.873 5.062 5.05 5.343 4.771
   1352   1388617_at   9.206 9.252 9.495 9.372 9.137 10.39 10.385 10.218 10.421 10.263
   1353   1374438_at   8.671 8.917 8.825 9.055 8.991 7.765 7.882 7.91 7.939 7.835
   1354   1388751_at   9.05 9.056 9.055 8.819 8.708 10.021 9.977 10.024 9.974 10.125
   1355   1389457_at   5.968 6.139 5.695 5.964 6.103 4.133 4.307 4.026 3.964 4.525
   1356   1387178_a_at   8.212 8.194 8.405 8.483 7.801 9.915 10.011 9.964 9.879 9.83
   1357   1380768_at   7.026 7.093 7.065 6.927 6.822 5.371 5.144 5.081 5.514 5.642
   1358   1389882_at   5.943 5.687 5.008 5.398 5.699 1.373 1.057 1.214 2.433 1.546
   1359   1372186_a_at   7.343 7.372 7.145 7.506 7.148 3.529 4.525 4.268 3.297 3.498
   1360   1380775_at   6.447 6.796 6.316 6.388 6.578 5.079 5.323 5.161 5.066 5.201
   1361   1379464_at   8.985 9.091 9.266 8.776 8.771 10.805 10.827 10.952 10.577 10.499
   1362   1391387_s_at   7.087 7.381 7.178 7.392 7.471 5.696 5.969 5.808 5.728 6.093
   1363   1395744_at   7.691 7.659 7.626 7.709 7.747 5.794 6.123 5.328 5.735 5.403
   1364   1372056_at   10.293 10.339 10.138 10.287 10.534 8.934 9.246 8.861 9.09 9.073
   1365   1392597_at   10.695 10.62 10.726 10.68 10.579 11.635 11.767 11.819 11.482 11.521
   1366   1382108_at   9.554 9.56 9.814 9.976 9.989 7.738 7.964 8.058 7.751 7.437
   1367   1383171_at   7.857 7.735 7.94 7.931 7.676 8.69 8.681 8.583 8.694 8.578
   1368   1373462_at   8.603 8.617 8.492 8.652 8.723 7.084 7.264 7.343 7.583 7.124
   1369   1391662_at   5.938 6.019 6.202 5.954 5.988 3.556 4.021 4.4 4.131 4.012
   1370   1371967_at   9.483 9.466 9.474 9.49 9.371 10.198 10.153 9.98 10.131 10.025
   1371   1377818_at   5.601 5.98 5.406 5.861 5.671 7.553 7.339 7.487 7.212 7.245
   1372   1382642_at   8.104 8.1 8.098 8.361 8.405 7.19 7.164 6.96 7.107 7.112
   1373   1373933_at   9.692 9.728 9.725 9.786 9.728 9.181 9.178 9.108 8.949 9.132
   1374   1390507_at   6.929 7.109 6.982 7.355 7.238 4.204 4.134 5.098 4.563 4.58
   1375   1368391_at   7.786 7.759 7.863 8.086 8.007 6.199 6.562 6.485 6.229 6.036
   1376   1379665_at   7.221 7.085 7.01 6.989 7.138 5.691 5.733 5.209 5.257 5.328
   1377   1386967_at   9.161 9.156 9.134 9.236 9.282 8.427 8.467 8.173 8.248 8.376
   1378   1388581_at   9.58 9.648 9.639 9.804 9.923 8.239 8.398 8.579 8.261 8.111
   1379   1388734_at   8.029 8.192 8.146 8.223 8.125 9.079 9.327 9.19 9.042 9.404
   1380   1398315_at   12.429 12.334 12.296 12.087 12.275 11.506 11.499 11.498 11.534 11.47
   1381   1392638_at   6.886 6.69 7.086 6.757 6.623 5.397 4.87 4.902 4.893 5.087
   1382   1383742_at   8.667 8.663 8.621 8.685 8.523 9.648 10.001 9.8 9.648 9.588
   1383   1371467_at   9.803 9.712 9.641 9.772 9.715 9.082 9.171 9.22 9.03 9.073
   1384   1383181_at   9.046 9.079 9.094 9.234 9.221 7.857 8.005 8.278 7.992 7.924
   1385   1371604_at   8.388 8.277 8.508 8.451 8.419 9.137 9.176 9.232 9.269 9.04
   1386   1370682_at   8.102 8.26 8.59 8.352 8.264 5.638 4.985 5.795 4.997 4.703
   1387   1383814_at   5.483 5.862 5.746 5.65 5.168 7.555 7.577 7.404 7.559 7.241
   1388   1373040_at   11.271 11.17 11.113 11.049 11.144 10.463 10.48 10.445 10.361 10.265
   1389   1384765_at   7.264 7.403 7.694 7.673 7.58 4.518 4.56 4.67 4.826 3.638
   1390   1390598_at   4.569 4.798 5.172 5.261 4.816 6.979 7.067 7.463 6.969 7.019
   1391   1393887_at   10.364 10.217 10.432 10.371 10.003 11.413 11.492 11.319 11.386 11.414
   1392   1383519_at   8.007 8.274 7.927 8.352 8.248 5.071 5.827 5.908 5.675 5.163
   1393   1374200_at   8.436 8.543 8.75 8.698 8.408 9.638 9.726 9.54 9.735 9.586
   1394   1367593_at   8.517 8.65 8.49 8.594 8.887 7.593 7.277 7.369 7.318 7.291
   1395   1392461_at   8.526 8.541 8.39 8.585 8.433 7.941 7.843 7.847 7.773 7.763
   1396   1393050_at   7.953 8.057 7.702 8.023 8.229 6.131 6.592 6.236 6.038 6.181
   1397   1370177_at   8.479 8.428 8.311 8.66 8.372 5.839 5.319 6.012 5.128 6.097
   1398   1378440_at   7.207 7.411 7.438 7.413 7.51 6.237 6.333 6.336 6.236 6.555
   1399   1367768_at   8.31 8.324 8.105 8.356 8.656 6.287 6.581 6.755 6.619 6.215
   1400   1397526_at   9.005 8.939 9.059 9.072 8.693 10.279 10.137 10.019 10.332 10.124
   1401   1381993_at   9.438 9.537 9.269 9.313 9.571 8.403 8.555 8.319 8.399 8.495
   1402   1369029_at   9.256 9.242 8.932 9.096 9.329 7.854 7.861 7.997 7.676 7.599
   1403   1392958_at   8.889 8.781 8.737 8.899 8.976 7.504 7.651 7.759 7.282 7.667
   1404   1387382_at   8.455 8.368 8.493 8.698 7.557 11.235 11.353 11.151 11.065 10.971
   1405   1367706_at   11.674 11.63 11.71 11.532 11.49 12.241 12.234 12.226 12.322 12.347
   1406   1370379_at   9.01 9.025 9.246 9.169 8.726 10.676 10.582 10.302 10.578 10.491
   1407   1387941_s_at   6.311 6.542 6.088 6.3 6.5 8.191 8.444 8.554 7.886 8.284
   1408   1383684_at   6.85 6.905 6.908 6.833 6.829 2.328 3.184 3.86 3.662 3.126
   1409   1381357_at   7.673 7.397 8.096 7.697 7.475 2.208 3.406 3.194 2.077 3.696
   1410   1371363_at   8.927 8.811 9.357 9.176 8.69 10.927 10.986 10.837 10.935 10.627
   1411   1370259_a_at   8.923 8.73 9.045 9.361 8.641 10.702 10.846 10.686 10.767 10.648
   1412   1370825_a_at   11.507 11.516 11.544 11.345 11.486 10.874 10.893 10.752 10.846 10.723
   1413   1368379_at   7.921 8.032 7.85 8.08 8.188 6.531 6.604 6.416 6.141 6.061
   1414   1387807_at   9.377 9.381 9.36 9.468 9.503 8.956 8.895 8.908 8.793 8.854
   1415   1395875_at   7.657 7.756 7.597 7.826 7.752 6.922 6.955 7.013 6.874 6.751
   1416   1388026_at   6.8 6.709 7.181 7.002 6.79 4.622 4.574 3.811 4.704 4.341
   1417   1378546_at   7.072 6.935 7.303 7.252 7.032 5.767 5.613 5.57 5.693 5.999
   1418   1390838_at   6.141 6.27 6.508 6.524 6.276 7.885 7.913 8.376 7.889 7.904
   1419   1382924_at   8.38 8.598 8.534 8.404 8.005 10.048 10.35 9.941 9.992 10.102
   1420   1375525_at   9.393 9.287 9.421 9.516 9.414 8.846 8.781 8.834 8.733 8.701
   1421   1373767_at   7.123 7.241 7.507 7.342 7.376 6.432 6.251 6.35 6.237 6.266
   1422   1377725_at   8.058 8.17 8.275 8.211 8.171 9.331 9.369 9.425 9.132 9.054
   1423   1377774_at   6.021 5.922 6.099 5.822 6.232 7.16 6.961 7.066 7.081 7.104
   1424   1383940_at   7.268 7.335 7.265 7.331 7.352 4.204 4.834 4.082 4.902 5.059
   1425   1374527_at   6.981 7.003 7.289 7.351 7.094 8.32 8.28 8.361 8.28 8.144
   1426   1398923_at   10.367 10.429 10.516 10.558 10.631 11.279 11.293 11.182 11.162 11.289
   1427   1388333_at   11.295 11.301 11.244 11.059 11.276 10.49 10.541 10.42 10.575 10.418
   1428   1370339_at   11.712 11.712 11.796 11.728 11.864 10.491 10.846 10.428 10.467 10.335
   1429   1392479_at   7.137 7.239 7.246 7.176 7.269 6.551 6.366 6.459 6.562 6.302
   1430   1374749_at   7.6 7.738 7.623 7.688 7.608 8.625 8.89 9.022 8.622 8.717
   1431   1399030_at   8.144 8.017 8.043 8.272 8.098 8.769 8.82 8.746 8.74 8.733
   1432   1389520_at   10.748 10.696 10.887 10.888 10.783 10.098 10.145 10.191 9.982 10.079
   1433   1367584_at   11.735 11.664 11.646 11.688 11.712 9.81 9.964 10.418 9.693 9.784
   1434   1371631_at   8.707 8.565 8.833 8.821 8.672 7.69 7.833 7.782 7.603 7.513
   1435   1398341_at   8.745 8.738 8.932 8.967 8.443 10.192 10.105 10.399 10.25 10.127
   1436   1371381_at   11.895 11.858 11.928 11.819 11.897 12.199 12.193 12.2 12.282 12.241
   1437   1368236_at   10.342 10.393 10.553 10.419 9.921 11.78 11.796 11.771 11.795 11.907
   1438   1386968_at   9.006 8.875 9.048 8.936 8.574 11.082 11.154 11.662 11.097 10.762
   1439   1378746_at   6.816 6.675 6.801 6.577 6.663 5.711 5.67 5.748 5.407 5.739
   1440   1388408_at   10.9 10.936 10.821 10.786 10.881 9.611 9.755 9.923 9.443 9.521
   1441   1387061_at   8.007 8.032 8.176 8.341 8.296 9.16 9.161 9.189 9.12 9.051
   1442   1376077_at   7.408 7.218 7.461 7.562 7.505 6.097 5.873 6.352 6.176 6.062
   1443   1386346_at   7.696 7.75 7.733 7.847 7.882 7.116 6.91 7.053 6.979 6.852
   1444   1368332_at   12.834 12.743 12.595 12.593 12.714 6.801 6.806 8.885 7.063 7.024
   1445   1393085_at   7.992 7.98 7.954 8.164 8.369 5.742 5.344 5.521 6.038 5.023
   1446   1391905_at   7.544 7.636 7.522 7.637 7.744 6.227 6.527 6.154 6.105 5.926
   1447   1375901_at   8.828 9.052 8.815 9.005 9.142 7.612 7.806 7.577 7.341 7.708
   1448   1376796_at   8.751 8.765 8.869 8.783 8.817 8.375 8.448 8.317 8.364 8.316
   1449   1371356_at   6.485 6.74 6.73 6.661 6.258 7.963 7.87 7.832 7.801 7.846
   1450   1388118_at   11.357 11.331 11.389 11.227 11.171 12.141 12.111 11.946 12.064 12.195
   1451   1384292_at   7.584 7.542 7.501 7.629 7.778 5.356 4.871 4.999 5.308 4.302
   1452   1386252_at   7.582 7.736 7.446 7.778 7.927 5.273 5.987 5.884 5.688 5.622
   1453   1380909_at   8.692 8.719 8.653 8.986 9.226 7.122 7.087 7.094 6.68 6.836
   1454   1390161_at   5.819 6.101 5.883 6.246 6.171 7.419 7.432 7.199 7.258 7.282
   1455   1369617_at   9.264 9.152 9.238 9.123 8.891 8.079 7.806 7.924 8.11 7.963
   1456   1388137_a_at   9.181 9.177 9.286 9.318 9.447 8.504 8.492 8.557 8.579 8.417
   1457   1389866_at   10.33 10.423 10.493 10.548 10.458 10.972 10.988 11.039 10.996 10.94
   1458   1379302_at   8.29 8.292 8.375 8.234 8.293 6.896 6.736 6.883 7.193 6.556
   1459   1387925_at   8.375 8.466 8.467 8.551 8.545 6.984 7.073 7.506 7.024 7.15
   1460   1384638_at   8.816 8.882 9.039 9.063 8.385 10.526 10.458 10.592 10.497 10.48
   1461   1389392_at   7.09 7.157 7.117 7.271 7.128 9.538 9.426 8.674 9.203 9.158
   1462   1368794_at   8.951 8.832 9.228 9.192 8.736 10.708 11.005 11.008 10.892 10.508
   1463   1370930_at   7.333 7.177 7.157 7.422 7.319 8.612 8.686 8.573 8.252 8.439
   1464   1376784_at   6.84 6.455 6.783 6.733 6.623 5.165 5.115 4.769 4.823 5.3
   1465   1388711_at   9.581 9.53 9.291 9.54 9.718 8.122 8.467 8.188 8.185 8.048
   1466   1367943_at   8.679 8.703 8.679 8.91 8.708 8.077 7.971 7.856 8.006 7.955
   1467   1374462_at   7.4 7.479 7.416 7.597 7.577 8.33 8.63 8.321 8.528 8.469
   1468   1373667_at   10.476 10.381 10.256 10.351 10.415 11.034 11.157 11.095 11.286 11.238
   1469   1376624_at   7.436 7.765 7.823 7.5 7.392 5.493 5.635 5.753 5.001 5.552
   1470   1387076_at   11.991 11.853 11.935 11.706 11.668 10.23 10.428 10.256 10.168 10.669
   1471   1378197_at   6.611 6.628 6.891 6.687 6.255 8.522 8.516 8.427 8.361 8.073
   1472   1384614_at   5.848 6.172 6.312 6.385 6.1 9.073 8.291 9.053 8.395 8.946
   1473   1367936_at   8.367 8.455 8.631 8.687 8.602 6.701 6.785 6.886 6.131 6.554
   1474   1373030_at   9.128 9.212 9.118 9.312 9.31 8.612 8.594 8.567 8.58 8.669
   1475   1380728_at   7.529 7.466 7.323 7.659 7.703 5.846 6.007 6.294 5.741 6.021
   1476   1374169_at   7.481 7.649 7.631 7.732 7.532 8.933 8.871 9.268 8.729 8.895
   1477   1376649_at   8.037 8.122 8.024 8.206 8.139 8.793 8.941 8.846 8.702 8.763
   1478   1392940_at   6.587 6.947 6.665 6.96 6.596 8.07 8.188 8.176 7.898 8.042
   1479   1369471_at   6.529 6.284 6.558 6.453 6.26 5.521 5.361 5.292 5.492 5.35
   1480   1370844_at   10.347 10.289 10.283 10.492 10.469 9.567 9.677 9.446 9.489 9.424
   1481   1373955_at   9.034 9.142 9.212 9.277 9.233 8.149 8.362 8.371 8.162 8.102
   1482   1369669_at   8.69 8.771 8.462 8.705 8.713 6.843 7.058 6.286 6.918 6.766
   1483   1374272_at   7.362 7.426 7.473 7.63 7.341 8.336 8.628 8.461 8.357 8.387
   1484   1392908_at   8.772 8.948 8.968 8.949 8.962 7.995 8.273 8.16 8.069 8.123
   1485   1368096_at   9.647 9.695 9.775 9.849 9.319 11.218 11.247 11.586 11.2 11.131
   1486   1380794_at   7.571 7.779 7.565 7.859 7.974 6.436 6.672 6.414 6.512 6.406
   1487   1389468_at   8.897 8.849 8.905 9.186 9.185 7.853 7.83 7.88 8.019 7.78
   1488   1368191_a_at   9.446 9.473 9.46 9.452 9.236 10.781 10.788 10.498 11.088 10.822
   1489   1380377_at   7.699 7.541 7.684 7.572 7.738 3.508 4.388 4.931 3.832 4.516
   1490   1391629_at   5.851 5.917 6.217 6.182 5.896 7.762 7.99 8.019 7.77 7.414
   1491   1373838_at   6.589 7.016 6.979 6.902 6.852 5.227 5.24 5.374 5.655 5.489
   1492   1368915_at   8.89 8.856 9.009 9.07 8.398 10.4 10.529 10.471 10.545 10.412
   1493   1398612_at   9.224 9.096 9.654 9.524 8.796 11.357 11.439 11.699 11.367 11.445
   1494   1382515_at   7.134 7.271 7.285 7.523 7.414 6.183 6.192 6.337 6.057 6.01
   1495   1376675_at   7.734 7.725 7.758 7.707 8.005 4.758 4.925 4.049 5.047 5.335
   1496   1370064_at   7.927 7.917 8.185 8.055 8.004 5.852 6.181 6.494 6.155 5.711
   1497   1388509_at   8.627 8.702 8.523 8.557 8.574 7.745 7.855 7.776 7.518 7.769
   1498   1371563_at   8.363 8.469 8.266 8.44 8.529 7.281 7.139 7.308 7.514 7.103
   1499   1372953_at   6.994 6.918 6.842 6.986 7.054 8.156 8.278 8.304 8.033 7.874
   1500   1370968_at   9.392 9.394 9.295 9.494 9.452 8.302 7.745 8.05 7.828 8.104
   1501   1393917_at   6.438 6.61 7.492 7.095 7.07 2.287 2.828 1.057 1.157 1.628
   1502   1399033_at   9.972 10.128 10.171 9.875 10.001 9.075 9.018 8.905 9.026 9.217
   1503   1385706_at   6.34 6.151 6.051 6.248 6.072 4.536 4.426 4.925 4.886 4.586
   1504   1382918_at   4.48 4.725 4.825 4.892 3.963 6.809 6.88 6.834 6.908 6.777
   1505   1380321_at   6.668 6.59 6.845 6.708 6.502 7.928 7.793 7.605 7.642 7.793
   1506   1374539_at   8.285 8.257 8.164 8.188 8.372 6.426 6.471 6.98 6.243 6.602
   1507   1370082_at   8.602 8.351 8.795 8.684 8.622 6.896 6.466 7.012 7.065 6.648
   1508   1373312_at   8.462 8.455 8.608 8.908 8.66 9.765 9.788 9.85 9.682 9.785
   1509   1381919_at   6.651 6.803 6.444 6.632 6.782 5.308 5.567 5.347 5.117 5.155
   1510   1377153_a_at   7.376 7.413 7.567 7.467 7.185 5.139 5.095 4.748 5.719 5.082
   1511   1384964_at   8.44 8.496 8.495 8.781 8.685 9.744 9.945 9.654 9.814 10.04
   1512   1374370_at   5.249 5.534 5.707 5.371 5.404 7.142 6.953 6.908 7.068 6.666
   1513   1367919_at   7.705 7.791 7.662 7.988 7.513 6.069 6.084 6.238 5.621 5.852
   1514   1369728_at   5.542 5.319 4.834 5.149 5.044 6.912 7.017 7.263 6.873 7.153
   1515   1393067_at   7.875 7.92 7.701 7.888 8.126 9.214 9.195 9.341 8.926 9.171
   1516   1376599_at   7.969 7.895 7.673 7.828 7.747 5.728 5.828 6.069 5.753 6.424
   1517   1368838_at   10.11 10.05 10.377 10.298 10.291 9.074 8.55 8.633 8.725 8.562
   1518   1398786_at   11.516 11.456 11.613 11.51 11.594 10.564 10.444 10.437 10.662 10.209
   1519   1388046_at   7.827 7.817 8.107 7.913 7.71 1.506 0.444 3.278 1.816 1.945
   1520   1371863_at   8.56 8.78 8.368 8.518 8.613 7.071 7.29 7.458 7.119 7.361
   1521   1375346_at   8.221 8.269 8.29 8.435 8.138 9.349 9.298 9.525 9.338 9.128
   1522   1389980_at   8.711 8.773 8.833 8.786 8.935 7.735 7.965 7.638 7.929 7.665
   1523   1398626_s_at   11.917 11.764 11.821 11.797 11.871 10.994 11.22 11.237 11.18 11.107
   1524   1378592_at   8.169 8.269 8.213 8.22 8.412 5.978 6.296 5.709 6.47 5.616
   1525   1367989_at   4.771 4.639 4.754 4.904 4.243 7.063 7.265 7.532 6.784 6.779
   1526   1372884_at   6.597 6.51 6.32 6.632 6.52 7.64 7.734 7.876 7.451 7.755
   1527   1373786_at   6.107 5.826 6.382 6.122 5.855 7.436 7.382 7.568 7.581 7.499
   1528   1394745_at   5.981 5.869 5.681 5.621 5.775 2.77 3.32 3.542 2.967 2.403
   1529   1377016_at   9.416 9.492 9.647 9.71 9.832 8.16 8.107 8.502 8.076 8.201
   1530   1379631_at   8.309 8.332 7.703 8.178 8.127 5.356 4.778 5.402 5.871 5.475
   1531   1379418_at   6.414 6.851 6.48 6.652 6.77 8.512 8.69 8.046 8.412 8.369
   1532   1384331_at   6.762 6.767 7.041 6.919 6.757 5.685 5.772 5.754 5.802 5.431
   1533   1371576_at   10.113 10.104 10.188 10.078 9.974 10.679 10.705 10.56 10.689 10.672
   1534   1368139_s_at   7.421 7.338 7.55 7.625 6.717 9.554 9.477 9.593 9.627 9.383
   1535   1382210_at   6.077 5.984 6.149 6.223 6.485 4.515 4.065 4.534 3.937 4.259
   1536   1397214_at   8.021 7.888 7.97 7.924 7.651 4.781 5.042 4.957 3.752 4.86
   1537   1389555_at   8.357 8.422 8.231 8.405 8.075 6.959 6.958 7.161 6.904 6.651
   1538   1370680_at   6.297 6.234 6.277 6.305 6.213 7.196 7.204 7.036 7.128 6.881
   1539   1372672_at   9.095 9.018 9.33 9.294 8.896 10.408 10.617 10.594 10.566 10.284
   1540   1373709_at   7.274 7.194 7.464 7.36 7.368 8.203 8.041 8.24 8.186 8.046
   1541   1372543_at   8.459 8.509 8.641 8.695 8.706 7.058 7.319 7.379 6.85 6.965
   1542   1387242_at   8.343 8.528 8.093 8.484 8.587 6.085 5.834 6.009 6.691 5.926
   1543   1398877_at   10.029 10.096 10.036 10.139 10.19 9.184 9.357 9.456 9.225 9.384
   1544   1373043_at   9.963 9.953 9.906 10.041 10.294 8.21 8.403 8.617 8.003 8.494
   1545   1376697_at   7.109 7.32 7.683 7.429 7.534 5.133 5.491 5.68 5.008 5.462
   1546   1386978_at   9.61 9.589 9.519 9.58 9.67 10.07 10.148 9.994 10.058 10.026
   1547   1386953_at   11.504 11.538 11.541 11.41 11.363 12.125 11.976 12.039 12.056 12.116
   1548   1373345_at   7.798 7.825 7.617 7.787 8.352 6.204 6.109 6.264 6.011 6.159
   1549   1367569_at   12.345 12.318 12.243 12.236 12.328 11.856 11.802 11.941 11.854 11.806
   1550   1371012_at   7.781 7.549 7.782 7.53 7.09 9.588 9.751 9.809 9.656 9.266
   1551   1371529_at   8.391 8.337 8.43 8.676 8.589 7.186 7.315 7.515 7.346 7.158
   1552   1369070_at   6.945 6.952 7.067 7.056 6.946 7.754 7.999 7.821 7.748 7.675
   1553   1369158_at   7.273 7.288 7.468 7.322 7.127 8.691 8.929 9.225 8.608 8.902
   1554   1371954_at   10.03 10.044 10.115 10.309 10.213 11.461 11.283 11.182 11.367 11.647
   1555   1368304_at   9.691 9.639 9.73 9.809 8.882 11.817 11.697 11.819 11.84 11.955
   1556   1368220_at   9.254 9.284 9.216 9.083 9.339 8.641 8.54 8.562 8.552 8.453
   1557   1372733_at   8.153 8.324 8.269 8.36 8.269 8.947 9.11 8.938 8.871 9.021
   1558   1381570_at   6.005 6.817 6.065 6.455 6.401 2.29 1.722 3.24 2.454 2.82
   1559   1374196_at   7.948 7.978 7.941 8.053 8.006 8.6 8.517 8.715 8.69 8.506
   1560   1367641_at   12.219 12.068 12.08 12.095 12.029 12.523 12.547 12.514 12.536 12.576
   1561   1371728_at   7.477 7.451 7.575 7.718 7.467 8.292 8.377 8.223 8.252 8.366
   1562   1370385_at   6.318 6.396 5.904 6.67 6.318 8.341 8.301 8.502 8.118 8.03
   1563   1375436_at   10.315 10.336 10.575 10.624 10.356 11.416 11.55 11.326 11.462 11.379
   1564   1372090_at   9.802 9.785 10.057 10.119 10.07 8.766 8.996 8.928 8.759 8.82
   1565   1388552_at   9.626 9.685 9.839 9.703 9.711 10.281 10.208 10.34 10.277 10.217
   1566   1372850_at   8.218 8.467 8.295 8.303 8.429 7.129 7.29 7.045 6.768 6.907
   1567   1373440_at   8.438 8.447 8.449 8.377 8.4 8.715 8.716 8.726 8.663 8.653
   1568   1385641_at   9.182 9.258 9.138 9.287 9.224 7.325 7.138 7.429 6.507 7.12
   1569   1367784_a_at   12.388 12.373 11.522 12.173 12.539 9.873 9.697 9.871 9.8 9.592
   1570   1392637_at   11.34 11.412 11.277 11.374 11.434 4.718 4.823 7.141 5.109 4.715
   1571   1378668_at   8.095 8.029 8.035 8.045 7.979 8.511 8.597 8.612 8.424 8.531
   1572   1387018_at   6.734 6.805 6.872 6.531 6.943 8.045 8.018 8.124 7.777 7.923
   1573   1377310_at   8.026 8.042 7.946 8.198 8.249 7.22 7.184 7.133 6.962 6.994
   1574   1387913_at   4.56 4.728 4.964 4.715 4.322 6.826 6.707 6.891 6.638 6.25
   1575   1389466_at   10.273 10.328 10.552 10.619 10.266 11.561 11.745 11.462 11.487 11.578
   1576   1379778_at   7.843 7.793 7.642 7.93 7.673 6.322 6.782 6.601 6.612 6.465
   1577   1372360_at   9.489 9.65 9.573 9.45 9.454 8.668 8.548 8.26 8.46 8.568
   1578   1388119_at   10.98 11.035 11.051 11.072 11.114 10.498 10.553 10.45 10.362 10.329
   1579   1370047_at   6.169 6.004 6.34 6.192 6.018 7.747 7.59 7.402 7.455 7.259
   1580   1398784_at   11.188 11.141 11.192 11.182 11.302 10.591 10.583 10.41 10.513 10.349
   1581   1389566_at   8.375 8.574 8.381 8.394 8.295 4.982 5.915 5.685 5.072 6.032
   1582   1392913_at   7.691 7.664 7.742 7.642 7.834 6.823 6.437 6.705 6.453 6.431
   1583   1390172_at   8.721 8.745 8.78 8.856 8.197 10.384 10.643 10.261 10.324 10.508
   1584   1375120_at   6.845 7.209 7.169 7.23 7.209 8.65 8.398 8.641 8.631 8.265
   1585   1384239_at   8.134 7.919 8.073 8.081 8.048 7.364 7.359 7.095 7.251 7.261
   1586   1371923_at   7.152 7.511 7.077 7.389 7.481 5.068 5.27 5.684 5.569 5.6
   1587   1368811_at   7.549 7.559 7.568 7.935 7.809 3.956 4.96 4.533 4.794 5.231
   1588   1384327_at   8.533 8.435 8.574 8.697 8.572 9.364 9.473 9.554 9.381 9.261
   1589   1372517_at   8.342 8.3 8.371 8.852 8.772 6.84 6.777 7 6.875 6.64
   1590   1382105_at   6.171 6.157 6.503 6.425 6.408 7.5 7.652 7.519 7.433 7.323
   1591   1368637_at   5.84 5.591 5.148 4.989 5.376 2.406 0.934 1.176 1.475 1.753
   1592   1369552_at   6.96 7.326 7.701 7.611 7.593 5.114 4.734 5.209 4.392 4.666
   1593   1387966_at   8.37 8.38 8.418 8.923 8.52 10.165 10.27 10.375 10.046 9.97
   1594   1383603_at   8.628 8.503 8.607 8.68 8.589 7.523 7.88 7.613 7.488 7.529
   1595   1370909_at   8.479 8.576 8.364 8.686 8.739 7.063 7.338 7.508 7.274 7.224
   1596   1367844_at   9.489 9.511 9.819 9.842 9.836 8.692 8.512 8.568 8.544 8.535
   1597   1372847_at   7.846 7.537 7.775 7.801 7.527 9.019 9.089 9.112 9.096 8.714
   1598   1374756_at   6.828 6.637 6.381 6.726 6.659 4.116 4.63 4.918 4.788 4.699
   1599   1380327_at   7.175 7.07 7.135 7.406 7.276 5.918 5.974 6.087 6.291 5.939
   1600   1388533_at   9.183 9.232 9.316 9.414 9.159 10.188 10.161 9.983 10.033 10.004
   1601   1369387_at   8.035 8.143 7.967 8.088 8.023 4.955 4.126 5.576 4.319 4.707
   1602   1392670_at   7.765 7.957 7.875 7.918 7.647 9.222 9.477 9.234 8.911 9.177
   1603   1389093_at   9.152 9.221 9.154 9.291 9.291 8.205 8.209 8.009 8.077 7.742
   1604   1375633_at   12.264 12.258 12.226 12.179 12.298 11.133 11.276 11.475 11.263 11.024
   1605   1382571_at   7.583 7.108 7.348 7.286 7.135 8.758 8.872 8.546 8.527 8.672
   1606   1375910_at   7.398 7.599 7.736 7.871 7.541 6.513 6.484 6.4 6.443 6.609
   1607   1368027_at   8.385 8.443 8.467 8.08 8.136 6.43 5.574 6.291 5.991 6.235
   1608   1386980_at   10.718 10.616 10.742 10.733 10.425 11.869 11.807 11.552 11.931 11.896
   1609   1370154_at   13.223 13.131 12.849 12.705 12.825 11.466 11.29 11.623 11.57 11.488
   1610   1389085_at   7.707 7.609 7.896 8.039 7.379 9.449 9.36 9.365 9.146 9.319
   1611   1382086_at   7.697 7.697 7.138 7.334 7.474 4.836 5.403 4.802 4.544 5.296
   1612   1383349_at   8.572 8.687 8.651 8.706 8.937 7.557 7.683 7.559 7.378 7.271
   1613   1368317_at   10.347 10.285 10.339 10.407 10.165 10.867 10.908 10.949 10.986 10.863
   1614   1375030_at   6.464 6.438 6.233 6.506 5.888 8.534 8.91 8.958 8.367 8.282
   1615   1372815_at   9.961 9.896 9.896 9.894 10.064 8.882 9.188 8.94 8.791 8.795
   1616   1385269_s_at   6.485 6.285 6.149 6.331 6.44 5.053 5.38 4.916 5.061 5.081
   1617   1372079_at   10.837 10.851 10.938 10.94 10.871 10.191 10.325 10.061 10.022 10.158
   1618   1369636_at   10.703 10.587 10.717 10.628 10.281 11.733 11.762 11.57 11.676 11.764
   1619   1391652_at   9.683 9.458 9.954 9.66 9.274 11.19 11.297 11.068 11.252 11.411
   1620   1388908_at   9.291 9.273 9.545 9.261 9.011 10.336 10.47 10.481 10.395 10.519
   1621   1379386_at   6.475 6.57 6.301 6.34 6.522 3.57 3.773 2.536 2.98 2.534
   1622   1389785_at   10.511 10.511 10.864 10.846 10.316 12.118 12.03 12.096 12.095 11.917
   1623   1381968_at   10.417 10.401 10.523 10.45 10.502 9.427 9.435 9.783 9.431 9.417
   1624   1393956_at   8.869 8.917 9.113 8.891 8.754 7.993 7.799 8.061 7.928 7.81
   1625   1398886_at   10.264 10.204 10.319 10.324 10.464 9.667 9.563 9.42 9.463 9.448
   1626   1373831_at   8.672 9.266 8.934 8.736 9.173 6.694 6.626 6.798 7.176 7.125
   1627   1373200_at   9.48 9.485 9.431 9.485 9.659 8.844 8.911 8.768 8.673 8.762
   1628   1393673_at   4.033 3.597 4.024 3.944 3.343 6.467 6.447 6.536 6.084 5.817
   1629   1370991_at   7.66 7.636 8.028 7.417 6.821 10.272 10.301 9.962 10.323 10.471
   1630   1379073_at   7.283 7.42 7.195 7.692 6.737 9.623 9.296 9.284 9.44 9.424
   1631   1387982_at   8.247 8.122 8.071 7.83 8.171 4.78 4.073 5.138 5.542 4.821
   1632   1368992_a_at   9.686 9.573 9.628 9.687 9.43 10.753 10.843 10.856 10.502 10.569
   1633   1395030_at   0.595 0.556 0.39 2.06 2.025 6.886 6.434 6.891 5.972 6.177
   1634   1373055_at   7.819 7.704 7.855 8.004 7.821 8.624 8.652 8.811 8.711 8.552
   1635   1384294_at   6.152 6.422 6.357 6.662 6.477 4.526 4.602 4.432 4.266 3.808
   1636   1367929_at   10.426 10.432 10.635 10.458 10.545 11.325 11.296 11.413 11.445 11.154
   1637   1384275_at   5.34 4.741 4.745 4.709 4.808 6.603 7.082 6.795 6.768 7.162
   1638   1374113_at   8.195 8.165 7.996 7.763 8.1 6.815 6.467 6.45 6.677 6.323
   1639   1390454_at   9.685 9.528 9.685 9.666 9.596 10.759 10.944 10.53 10.92 10.606
   1640   1372886_at   7.845 7.905 7.814 7.855 7.899 3.213 4.334 4.17 5 4.313
   1641   1387672_at   6.367 6.536 6.675 6.666 6.06 9.105 9.441 9.45 9.093 8.492
   1642   1386274_at   5.677 5.123 5.859 5.613 5.089 7.475 7.489 7.48 7.376 7.529
   1643   1377993_at   5.82 5.972 5.93 6.328 5.692 8.195 8.401 8.775 8.238 7.852
   1644   1391331_at   6.69 6.59 6.759 6.697 6.551 8.357 8.508 9.089 8.354 8.353
   1645   1387053_at   9.889 10.026 10.089 9.955 9.388 11.845 11.731 11.478 11.945 11.896
   1646   1370134_at   8.154 8.222 8.222 8.206 8.22 8.911 8.723 8.82 8.791 8.656
   1647   1370184_at   11.873 11.838 11.916 11.731 11.839 11.143 11.258 11.114 11.251 11.319
   1648   1393745_at   7.418 7.558 7.599 7.493 7.479 6.377 5.777 6.221 6.207 5.978
   1649   1372485_at   9.692 9.786 10.069 10.059 9.75 11.196 11.328 10.961 11.304 11.149
   1650   1391690_at   7.004 6.786 6.909 6.53 6.513 4.369 3.875 4.699 4.269 4.734
   1651   1374584_at   8.794 8.826 9.023 9.117 8.879 10.065 9.964 9.912 9.789 9.846
   1652   1387083_at   6.264 6.145 6.331 6.351 5.981 7.649 7.819 7.624 7.779 7.315
   1653   1373975_at   7.886 7.74 8.166 7.84 6.822 10.817 10.552 10.807 10.639 10.576
   1654   1383557_at   7.875 8.161 8.097 8.066 7.863 9.288 9.378 9.027 9.029 9.205
   1655   1389162_at   8.602 8.716 8.576 8.793 8.992 7.356 7.559 7.645 7.639 7.617
   1656   1393221_at   10.876 10.853 10.892 10.833 10.591 11.576 11.685 11.566 11.676 11.764
   1657   1371409_at   9.822 9.795 9.891 9.894 9.906 10.3 10.392 10.278 10.323 10.229
   1658   1376951_at   6.103 6.419 6.16 6.34 6.456 4.524 4.331 4.801 3.994 4.317
   1659   1393929_at   6.405 5.777 7.112 6.777 6.169 0.227 1.367 1.05 0 2.004
   1660   1392975_at   6.872 6.765 7.12 7.006 6.88 7.766 7.875 7.891 7.723 7.78
   1661   1376226_at   5.478 5.589 5.365 5.373 5.754 7.243 7.099 6.987 6.785 6.779
   1662   1387300_at   7.59 7.651 7.464 7.351 7.564 6.636 6.676 6.831 6.72 6.609
   1663   1386867_at   11.337 11.326 11.459 11.198 11.125 12.067 12.089 12.026 12.165 12.062
   1664   1398507_at   4.927 5.093 5.385 4.977 4.827 7.091 7.568 7.083 6.759 6.997
   1665   1371732_at   10.916 10.894 10.798 10.813 11.003 10.086 10.057 9.768 10.029 10.069
   1666   1383640_at   7.184 7.016 7.035 7.225 7.458 8.314 8.673 8.67 8.453 8.471
   1667   1388628_at   8.844 8.807 8.62 8.578 8.901 7.018 7.066 7.314 6.588 6.734
   1668   1388892_at   8.099 8.074 8.25 8.272 8.188 8.716 8.851 8.786 8.727 8.742
   1669   1372167_at   10.587 10.637 10.546 10.235 10.447 9.414 9.456 9.119 9.323 9.235
   1670   1371491_at   7.605 7.611 7.678 7.886 7.92 6.814 6.826 6.767 6.624 6.793
   1671   1387062_a_at   6.749 7.076 6.803 6.662 6.764 4.081 4.502 4.997 4.491 4.019
   1672   1368038_at   8.451 8.506 8.606 8.628 8.324 10.021 10.018 9.694 9.824 9.568
   1673   1377867_at   6 6.421 6.084 5.871 5.79 4.34 3.638 3.598 3.63 3.8
   1674   1398354_at   6.71 6.71 6.719 6.754 6.878 7.624 7.666 7.667 7.657 7.363
   1675   1375270_at   6.951 6.743 6.772 6.965 6.844 7.666 7.733 7.912 7.745 7.595
   1676   1382464_at   8.433 8.476 8.56 8.773 8.447 9.646 9.856 9.568 9.536 9.543
   1677   1388345_at   9.82 9.943 9.82 9.892 9.946 8.879 9.177 9.061 8.84 9.079
   1678   1371794_at   8.167 8.154 7.898 8.038 8.1 9.231 9.647 9.391 9.155 9.242
   1679   1369491_at   10.965 10.923 11.271 11.099 10.863 12.119 12.119 11.98 12.28 12.212
   1680   1371489_at   9.596 9.577 9.64 9.668 9.764 8.833 8.757 8.418 8.696 8.671
   1681   1380507_at   5.374 5.051 5.215 5.224 4.819 6.523 6.593 6.783 6.478 6.426
   1682   1389326_at   7.307 7.427 7.299 7.558 7.68 5.818 6.076 6.273 5.873 5.779
   1683   1396301_x_at   7.219 7.468 7.647 7.738 7.507 8.903 9.262 8.899 9.16 8.873
   1684   1367849_at   7.896 7.937 7.984 7.904 7.848 6.866 6.939 7.114 6.665 6.714
   1685   1372248_at   7.016 7.242 7.128 7.131 7.148 8.505 8.538 8.476 8.148 8.129
   1686   1371941_at   9.429 9.449 9.594 9.506 9.525 10.089 10.104 10.148 9.976 9.988
   1687   1387011_at   12.243 12.15 10.731 11.613 12.067 6.77 6.342 7.833 6.602 6.26
   1688   1379626_at   6.934 6.968 7.118 7.106 6.832 6.056 6.285 6.119 6.104 6.046
   1689   1391572_at   7.151 7.234 7.124 7.252 7.037 6.324 6.233 6.379 6.478 6.489
   1690   1375504_at   7.075 6.923 7.226 7.05 6.805 8.279 8.599 8.246 8.224 8.193
   1691   1395396_at   7.321 7.274 6.832 7.213 7.172 6.05 6.101 5.946 5.956 5.932
   1692   1387038_at   8.269 8.272 8.416 8.482 8.447 9.239 9.26 9.154 9.2 9.008
   1693   1381976_at   7.116 7.423 7.344 7.499 7.303 8.611 8.877 8.636 8.396 8.542
   1694   1383844_at   5.949 5.88 5.365 5.963 5.866 7.344 7.622 7.591 7.571 7.264
   1695   1380617_at   8.347 8.464 8.89 8.79 8.519 6.875 6.75 6.165 6.666 6.62
   1696   1392947_at   5.195 5.319 5.104 5.287 5.421 6.565 6.988 6.684 6.513 6.449
   1697   1368563_at   9.166 9.102 9.612 9.331 9.038 10.69 10.792 10.605 10.582 10.564
   1698   1382221_at   6.034 6.205 6.209 6.519 6.035 8.115 8.056 7.621 8.326 7.992
   1699   1370443_at   8 7.753 8.02 8.046 8.171 6.484 6.883 6.769 6.501 6.462
   1700   1370462_at   6.447 6.661 6.389 6.907 6.528 4.634 4.902 4.532 5.043 5.039
   1701   1367581_a_at   13.205 13.093 12.799 12.777 13.04 11.615 11.464 11.799 11.449 11.342
   1702   1380115_at   4.666 3.902 4.008 3.873 3.635 7.065 6.929 6.338 7.214 7.089
   1703   1370228_at   10.192 10.176 9.517 10.05 9.523 7.907 7.797 7.75 7.619 7.979
   1704   1387975_at   6.919 6.983 7.248 7.148 7.028 5.662 5.445 5.993 5.493 5.669
   1705   1383323_at   6.064 6.51 5.924 6.152 5.982 3.284 3.973 4.184 3.561 3.52
   1706   1389645_at   8.495 8.354 8.409 8.618 8.438 10.097 10.254 9.604 9.905 9.88
   1707   1372457_at   7.838 7.88 7.898 7.898 7.806 8.849 8.925 8.531 8.715 8.868
   1708   1388809_at   10.543 10.443 10.939 10.572 10.51 9.224 9.229 9.257 9.197 8.848
   1709   1393793_at   8.37 8.364 8.692 8.664 8.16 10.052 10.126 10.057 9.732 10.155
   1710   1368377_at   7.437 7.55 7.453 7.422 7.246 4.418 4.013 5.32 4.83 4.532
   1711   1368361_a_at   8.94 8.789 8.7 9.047 9.217 7.673 7.695 7.803 7.711 7.558
   1712   1378292_at   6.326 6.568 6.533 6.719 6.443 7.897 8.12 7.59 7.897 8.025
   1713   1379275_at   8.583 8.304 8.312 8.47 8.441 6.14 5.414 6.021 6.537 6.157
   1714   1386901_at   7.688 7.801 7.424 7.498 7.667 8.619 8.824 8.642 8.673 8.917
   1715   1368573_at   8.367 8.522 8.312 8.542 8.511 6.773 6.811 6.577 7.033 6.24
   1716   1383255_at   7.996 8.264 8.039 8.158 8.326 6.886 7.222 7.011 6.847 6.847
   1717   1382175_at   8.932 8.847 8.846 8.843 8.998 8.154 8.22 8.186 7.928 8.017
   1718   1389066_at   6.991 6.749 6.944 6.834 6.345 8.791 8.819 9.448 8.733 8.916
   1719   1378415_at   7.113 7.298 7.165 7.224 7.039 8.294 8.234 8.315 8.076 7.966
   1720   1379419_at   7.132 7.293 7.328 7.095 7.493 8.449 8.528 8.745 8.761 8.938
   1721   1377223_at   5.921 5.616 5.703 5.892 5.776 7.016 6.947 6.766 6.789 6.675
   1722   1373499_at   8.892 8.888 8.982 8.952 9.265 7.834 7.812 7.693 7.398 7.713
   1723   1388791_at   8.493 8.477 8.477 8.842 8.653 7.608 7.216 7.418 7.333 7.357
   1724   1380583_s_at   8.137 7.998 7.86 7.976 7.851 6.397 6.07 6.706 6.573 6.23
   1725   1389222_at   7.729 7.603 7.628 7.618 7.671 6.985 7.031 6.99 7.048 6.771
   1726   1370483_at   7.199 7.377 7.714 7.644 7.302 2.945 4.082 3.625 2.195 3.409
   1727   1369169_at   8.211 8.275 8.567 8.497 7.856 10.012 10.15 10.16 9.897 9.872
   1728   1385832_s_at   8.524 8.632 8.838 8.84 8.405 5.561 5.6 6.672 5.707 5.837
   1729   1372671_at   10.121 10.238 10.039 10.222 10.06 10.839 11.073 10.84 10.847 10.872
   1730   1372069_at   8.058 8.032 8.286 8.222 7.904 9.164 9.209 9.248 9.05 9.003
   1731   1389176_at   7.565 7.721 7.687 7.718 7.4 8.923 8.736 8.655 8.58 8.907
   1732   1370289_x_at   4.964 4.658 4.728 4.656 4.047 8.985 8.999 8.527 8.797 7.501
   1733   1388974_at   8.893 8.868 8.96 8.955 8.912 8.016 8.256 8.048 8.126 7.851
   1734   1379642_at   6.505 6.829 7.026 6.826 6.601 4.991 5.056 5.083 4.617 4.49
   1735   1371925_at   8.878 8.943 9.079 8.916 8.925 8.043 8.141 8.296 8.164 7.96
   1736   1370832_at   8.709 8.462 9.315 9.389 8.609 4.601 4.667 5.61 5.536 4.406
   1737   1383548_at   7.751 7.6 7.684 7.735 7.917 6.53 6.76 6.632 6.627 6.291
   1738   1367983_at   7.23 7.421 7.351 7.487 7.34 5.307 5.834 5.852 5.586 5.151
   1739   1390142_at   8.471 8.142 8.442 8.367 8.291 7.038 6.941 7.26 6.847 7.261
   1740   1374499_at   9.302 9.439 9.283 9.513 9.689 7.755 7.267 7.844 7.132 7.55
   1741   1389403_at   8.414 8.352 8.411 8.668 8.354 9.682 9.568 9.372 9.568 9.354
   1742   1373017_at   11.301 11.281 11.166 11.263 11.264 11.867 11.885 11.705 11.942 11.944
   1743   1384060_at   9.202 9.325 9.454 9.484 9.326 8.589 8.447 8.294 8.466 8.294
   1744   1368147_at   5.917 6.541 6.123 6.054 6.203 9.256 8.656 9.236 8.244 8.892
   1745   1388831_at   6.187 6.065 6.274 6.497 6.563 7.55 7.68 7.813 7.766 7.536
   1746   1373920_at   4.776 4.452 4.671 4.904 4.421 6.098 6.254 6.383 6.033 5.955
   1747   1376160_at   7.481 7.718 7.828 7.83 7.792 5.696 6.18 6.346 6.193 6.215
   1748   1368534_at   6.607 6.978 6.424 7.223 7.122 3.119 3.392 2.674 3.57 2.036
   1749   1388341_at   9.132 9.186 9.162 9.444 9.302 8.043 8.219 8.348 8.103 8.333
   1750   1384008_at   7.402 7.426 7.739 7.769 7.246 8.949 9.145 8.802 9.037 9.143
   1751   1372667_at   8.83 8.823 8.617 8.716 8.854 7.923 8.11 7.837 7.994 7.867
   1752   1382158_at   6.984 7.155 6.69 6.817 7.069 5.595 5.507 5.737 5.455 5.26
   1753   1398805_at   10.34 10.453 10.552 10.506 10.562 9.262 9.292 9.689 9.443 9.361
   1754   1376955_at   4.979 4.791 5.584 5.009 4.849 7.283 7.238 6.778 7.152 7.108
   1755   1368146_at   8.731 8.886 8.768 8.722 8.29 11.199 10.994 11.35 10.473 11.362
   1756   1369935_at   8.939 8.794 8.948 8.984 8.979 8.267 8.157 8.39 8.244 8.157
   1757   1393863_at   7.706 7.823 8.375 7.867 7.889 4.892 4.741 5.252 4.248 3.873
   1758   1395635_at   6.203 6.081 6.585 6.727 6.762 4.273 3.805 4.216 4.551 4.146
   1759   1376336_at   7.517 7.717 7.606 7.608 7.843 6.687 6.837 6.57 6.834 6.678
   1760   1395579_at   7.106 7.061 7.017 7.093 6.999 7.625 7.546 7.613 7.757 7.531
   1761   1373093_at   9.869 9.84 10.34 10.254 9.829 11.777 11.684 11.772 11.403 11.822
   1762   1381173_at   5.83 6.111 5.638 5.834 5.689 4.302 3.777 3.973 3.491 3.927
   1763   1369895_s_at   7.981 7.918 7.97 8.159 7.917 8.941 9.33 9.182 8.925 9.04
   1764   1371833_at   10.128 10.103 10.287 10.343 10.13 11.005 10.835 10.916 10.933 10.851
   1765   1385434_at   7.927 7.887 8.009 8.146 8 5.03 5.089 4.467 4.808 3.606
   1766   1371944_at   10.852 10.813 10.944 10.795 10.827 10.376 10.266 10.159 10.335 10.23
   1767   1392627_x_at   9.012 9.096 9.169 9.123 9.297 10.272 10.284 9.997 10.113 9.979
   1768   1372273_at   8.433 8.508 8.687 8.673 8.664 7.124 7.296 6.745 7.21 6.751
   1769   1390541_at   7.849 7.978 8.095 8.159 8.063 9.106 9.383 9.241 9.064 8.96
   1770   1372890_at   9.15 9.133 9.396 9.571 9.428 8.175 8.025 8.147 7.944 7.827
   1771   1389197_at   8.199 8.105 8.182 8.269 8.283 7.427 7.584 7.61 7.485 7.35
   1772   1379972_at   6.085 5.931 5.979 5.812 6.383 3.73 4.121 4.359 4.383 4.206
   1773   1385128_at   8.202 8.155 8.048 8.207 8.313 7.284 7.286 6.955 6.948 7.026
   1774   1387495_at   7.065 7.245 7.186 7.216 7.357 6.221 6.21 5.957 6.051 6.366
   1775   1397704_at   6.581 6.649 6.47 6.324 6.142 3.917 4.201 2.978 3.64 3.73
   1776   1384907_at   7.556 7.555 7.426 7.477 7.53 8.657 9.189 8.749 8.755 9.135
   1777   1393643_at   8.905 8.965 8.683 9.092 9.514 7.209 7.167 7.404 7.105 7.069
   1778   1388355_at   8.89 8.854 8.808 9.085 9.13 7.915 8.111 8.09 7.935 8.041
   1779   1376709_at   8.127 7.839 8.069 8.286 7.954 9.416 9.649 9.953 9.482 9.454
   1780   1371839_at   9.524 9.471 9.532 9.426 9.367 8.76 8.519 8.646 8.616 8.376
   1781   1371498_at   9.44 9.473 9.497 9.414 9.509 8.851 9.002 8.808 8.786 8.709
   1782   1386866_at   10.714 10.714 10.699 10.937 10.936 9.793 9.968 9.922 9.675 9.709
   1783   1389442_at   7.134 7.103 7 7.079 6.989 7.62 7.873 7.852 7.681 7.744
   1784   1390221_at   7.035 7.315 7.564 7.492 6.898 5.084 4.887 5.294 4.624 5.289
   1785   1374519_at   7.864 8.069 7.697 7.777 7.917 6.729 6.671 6.331 6.775 6.532
   1786   1398561_at   8.194 8.44 8.145 7.884 8.087 6.8 7.009 6.943 6.918 6.721
   1787   1388493_at   8.36 8.504 8.782 8.488 8.566 6.876 6.831 7.261 6.536 6.718
   1788   1385781_at   5.211 5.601 5.338 5.268 5.553 3.362 3.128 3.772 2.87 3.004
   1789   1368773_at   6.483 6.803 6.19 6.143 6.461 3.413 3.521 3.839 4.415 3.529
   1790   1388799_at   7.477 7.556 7.308 7.518 7.656 8.4 8.582 8.647 8.402 8.374
   1791   1374838_at   8.984 9.194 9.12 9.126 9.177 6.784 6.765 7.404 6.467 6.285
   1792   1380293_at   6.789 6.83 6.759 6.558 6.563 5.49 5.211 4.84 4.921 5.147
   1793   1397672_at   3.775 4.28 3.848 4.114 3.612 5.567 6.035 5.78 5.786 5.562
   1794   1397227_at   3.227 3.144 3.434 3.011 3.109 4.893 4.34 4.796 4.695 4.562
   1795   1382861_at   10.425 10.343 10.294 10.183 10.171 9.657 9.492 9.467 9.407 9.486
   1796   1373927_at   6.804 6.574 6.539 6.584 6.594 5.846 5.541 5.673 5.818 5.619
   1797   1373397_at   10.133 10.055 10.05 10.142 10.166 9.519 9.583 9.563 9.503 9.337
   1798   1370376_a_at   9.387 9.447 9.897 9.688 9.531 7.573 7.665 8.181 7.619 7.92
   1799   1393070_at   6.266 5.897 6.287 6.378 6.094 7.945 7.86 8.112 7.782 7.481
   1800   1380357_a_at   8.135 8.634 8.343 8.578 8.376 7.143 6.735 6.613 6.574 6.741
   1801   1378247_at   4.143 4.22 3.782 4.617 3.911 6.616 6.543 6.846 6.523 6.047
   1802   1371462_at   6.5 6.38 6.662 6.55 6.797 7.879 7.811 7.614 7.748 7.555
   1803   1399148_s_at   7.325 7.199 7.157 7.33 7.38 6.347 6.486 6.251 6.217 6.064
   1804   1370939_at   10.126 10.087 10.092 10.076 9.921 10.731 10.768 10.769 10.637 10.582
   1805   1391702_at   5.508 5.383 5.618 5.395 5.369 6.154 6.037 6.169 6.211 6.212
   1806   1387851_at   10.222 10.216 10.259 10.342 10.367 11.015 11.328 11.009 11.204 11.072
   1807   1374657_at   7.743 7.583 7.746 7.656 7.632 8.496 8.884 8.638 8.615 8.495
   1808   1375266_at   9.099 9.105 9.126 9.702 9.266 7.412 7.77 7.776 7.581 7.601
   1809   1367900_at   9.819 9.82 9.826 9.721 9.907 8.893 8.973 9.076 9.039 8.706
   1810   1379651_at   6.974 6.979 6.822 7.054 6.788 6.138 5.81 5.974 5.931 5.783
   1811   1368041_at   9.064 9.112 9.265 9.373 9.335 10.017 10.204 10.043 10.083 10.047
   1812   1367725_at   7.525 7.514 7.791 7.851 7.267 10.517 10.042 10.71 9.641 10.165
   1813   1369799_at   7.797 7.657 7.923 7.94 7.527 9.028 9.121 8.761 8.993 8.981
   1814   1379760_at   8.309 8.131 8.454 8.306 8.528 5.125 4.628 6.094 5.398 5.035
   1815   1375423_at   7.339 7.371 7.409 7.434 7.369 8.215 8.293 8.33 8.236 7.959
   1816   1374805_at   6.416 6.31 6.149 6.464 6.47 4.06 4.172 4.543 3.448 3.8
   1817   1372297_at   10.656 10.524 10.84 10.855 10.761 11.504 11.597 11.488 11.562 11.641
   1818   1371494_at   7.298 7.384 7.146 7.407 7.361 8.523 8.892 8.967 8.536 8.498
   1819   1372577_at   10.489 10.461 10.48 10.663 10.667 9.225 9.399 9.696 9.273 9.3
   1820   1388534_at   9.213 9.312 9.469 9.353 9.206 10.201 10.457 10.232 10.177 10.117
   1821   1391109_x_at   5.805 5.329 5.795 5.825 5.428 7.921 8.134 8.552 7.525 8.114
   1822   1369081_at   8.193 8.015 8.097 8.522 8.278 9.629 9.456 9.553 9.764 9.386
   1823   1389353_at   6.92 6.965 6.495 6.948 6.785 8.356 8.335 8.42 7.986 8.468
   1824   1371811_at   9.069 9.009 9.321 9.195 9.176 9.845 9.982 9.96 9.885 9.839
   1825   1392001_at   5.388 5.243 4.995 4.968 4.903 7.403 7.401 7.18 7.091 6.639
   1826   1370265_at   8.491 8.663 8.674 8.435 8.513 6.551 5.599 6.333 6.005 5.488
   1827   1371380_at   10.972 10.916 11.014 11.182 10.958 11.649 11.656 11.758 11.588 11.708
   1828   1382843_at   7.769 7.652 7.824 7.914 7.69 6.158 5.871 5.867 6.563 5.982
   1829   1392497_at   7.887 7.899 7.763 7.648 8.017 6.994 6.957 6.729 6.792 6.816
   1830   1372907_at   9.532 9.514 9.761 9.616 9.502 10.393 10.472 10.382 10.605 10.311
   1831   1374157_at   9.033 9.024 9.063 9.108 8.971 7.606 7.665 7.49 6.931 7.465
   1832   1385856_at   7.424 7.243 7.245 7.566 7.479 6.54 6.378 6.253 6.341 6.21
   1833   1389695_at   8.887 8.982 9.074 9.038 8.87 9.706 9.686 9.57 9.519 9.67
   1834   1382661_at   7.541 7.605 7.596 7.349 7.403 6.766 6.794 6.616 6.703 6.581
   1835   1369976_at   11.325 11.225 11.194 11.227 11.437 10.142 10.175 10.5 10.195 10.384
   1836   1375452_at   9.188 9.083 9.302 9.409 9.18 10.041 10.026 10.005 9.937 9.907
   1837   1390228_at   7.967 8.024 8.082 7.949 7.74 9.013 8.857 8.898 8.747 8.781
   1838   1370802_at   6.019 6.424 6.336 6.413 6.435 7.452 7.822 7.505 7.546 7.505
   1839   1383148_at   6.724 6.875 6.88 6.948 6.682 7.793 8.154 7.783 7.8 7.995
   1840   1388297_at   12.126 12.157 12.369 12.178 12.325 11.506 11.526 11.654 11.506 11.48
   1841   1398773_at   10.326 10.27 10.255 10.323 10.374 9.641 9.77 9.704 9.479 9.698
   1842   1372840_at   7.477 7.567 7.624 7.715 7.725 6.721 6.882 6.814 6.985 6.861
   1843   1390154_at   8.312 8.258 8.034 8.371 8.291 6.452 6.796 7.068 6.738 6.473
   1844   1374836_at   7.993 7.653 7.894 7.797 7.963 6.684 6.665 7.009 6.645 6.77
   1845   1370613_s_at   11.088 11.021 11.077 10.999 10.989 11.529 11.718 11.764 11.721 11.849
   1846   1388134_at   10.726 10.708 10.634 10.743 10.847 10.119 10.05 10.099 10.017 9.855
   1847   1386973_a_at   7.596 7.868 8.111 8.182 7.885 9.196 9.352 9.405 9.272 9.191
   1848   1374540_at   8.077 7.807 7.869 7.892 7.92 2.281 1.118 3.855 1.799 2.66
   1849   1387969_at   12.744 12.629 12.425 12.383 12.612 6.61 6.552 8.923 7.077 7.134
   1850   1383152_at   7.756 7.727 7.656 7.897 8.114 6.49 6.765 6.706 6.44 6.569
   1851   1372904_at   8.402 8.374 8.563 8.751 8.727 6.462 6.161 7.017 6.203 6.236
   1852   1377830_a_at   7.529 7.73 7.725 7.654 7.628 8.5 8.629 8.763 8.684 8.378
   1853   1387064_at   8.097 8.141 8.132 7.895 8.018 7.253 7.439 7.159 7.23 7.26
   1854   1367726_at   5.822 6.004 6.182 6.005 6.192 7.267 7.433 7.809 7.535 7.31
   1855   1382921_at   7.145 7.383 7.103 7.24 7.41 5.822 5.76 6.031 5.519 5.395
   1856   1367890_at   8.509 8.646 8.744 8.592 8.598 7.467 7.305 7.642 7.78 7.521
   1857   1371754_at   7.329 6.849 7.176 7.117 7.112 10.412 10.234 10.551 9.22 10.051
   1858   1376102_at   9.279 9.45 9.203 9.41 9.518 7.945 7.816 8.345 8.024 8.179
   1859   1373264_at   6.887 7 6.715 6.903 6.812 7.835 7.876 7.623 7.615 7.7
   1860   1371253_at   10.39 10.36 10.521 10.464 10.4 10.999 10.955 10.889 11.01 10.849
   1861   1396077_at   7.415 7.068 7.259 7.483 7.67 5.764 6.008 5.839 6.016 6.095
   1862   1371933_at   6.439 6.549 6.623 6.423 6.101 8.034 8.327 7.764 8.272 7.965
   1863   1398294_at   8.227 8.296 8.306 8.586 8.533 7.551 7.317 7.31 7.349 7.312
   1864   1377685_at   8.464 8.439 8.628 8.538 8.558 7.893 7.993 7.962 8.022 7.831
   1865   1367599_at   11.572 11.604 11.6 11.584 11.616 11.893 12.001 11.952 12.011 11.871
   1866   1377336_at   6.713 6.103 6.428 6.549 6.237 7.907 7.948 7.979 7.97 7.662
   1867   1386881_at   7.603 7.542 7.329 7.273 7.336 10.019 9.876 9.008 9.683 9.654
   1868   1370609_a_at   8.574 8.567 8.883 8.611 8.392 10.219 10.431 10.566 10.162 9.917
   1869   1384944_at   5.733 5.657 6.001 6.075 6.34 2.63 2.884 2.667 3.109 3.789
   1870   1372734_at   9.712 9.694 9.731 9.862 9.652 10.527 10.658 10.77 10.439 10.486
   1871   1384507_at   7.327 7.329 7.445 7.582 7.685 5.612 5.331 6.023 5.204 5.307
   1872   1372208_at   5.561 5.821 5.996 5.831 5.614 8.236 7.797 8.882 8.075 7.986
   1873   1374300_at   8.303 8.179 8.374 8.291 8.45 9.272 9.215 9.209 9.036 9.014
   1874   1383711_at   8.641 8.644 8.774 8.467 8.663 7.565 7.031 6.912 7.153 6.995
   1875   1368460_at   7.552 7.895 7.797 8.119 7.114 9.822 9.776 9.773 9.826 9.693
   1876   1392107_at   8.657 8.621 8.694 8.862 8.85 6.89 6.775 7.498 6.808 6.751
   1877   1372825_at   8.392 8.273 8.468 8.512 8.503 7.248 7.178 7.374 7.125 6.792
   1878   1368040_at   7.414 7.613 7.727 7.786 7.766 8.808 8.788 8.519 8.836 8.723
   1879   1367883_at   8.525 8.44 8.347 8.486 8.807 7.341 7.286 7.42 7.002 7.168
   1880   1377369_at   6.658 6.476 6.325 6.537 6.624 5.333 4.782 5.209 5.201 4.9
   1881   1382031_at   3.95 4.197 4.989 4.883 5.472 8.626 8.579 8.057 8.2 8.864
   1882   1376801_at   7.49 7.826 7.56 7.738 7.75 6.798 6.744 6.747 6.506 6.706
   1883   1389213_at   7.535 7.491 7.492 7.762 7.626 6.668 6.395 6.334 6.403 6.683
   1884   1389063_at   9.365 9.47 9.602 9.643 9.61 8.571 8.855 8.668 8.611 8.607
   1885   1367977_at   4.654 4.637 3.846 4.353 3.374 7.581 7.848 8.826 8.075 7.971
   1886   1386893_at   11.593 11.651 11.633 11.678 11.899 10.456 10.575 10.863 10.629 10.473
   1887   1379445_at   4.816 4.52 5.216 5.458 4.311 7.641 7.699 7.722 7.575 7.285
   1888   1382074_at   8.66 8.82 8.925 9.02 8.956 7.049 6.926 7.068 6.737 6.193
   1889   1394724_at   8.66 8.653 8.658 8.56 8.777 7.808 7.614 7.296 7.639 7.532
   1890   1373226_at   7.513 7.331 7.231 7.289 7.38 5.754 5.963 5.752 6.233 5.554
   1891   1394673_at   8.185 8.344 8.355 8.373 8.542 4.775 3.546 5.082 4.498 3.383
   1892   1370963_at   7.638 7.535 7.446 7.505 7.783 4.511 5.365 5.57 4.762 5.285
   1893   1392905_at   9.719 9.717 9.629 9.591 9.846 5.096 4.37 5.688 3.92 5.987
   1894   1370859_at   11.291 11.25 11.142 11.159 11.422 10.113 10.374 10.325 9.992 10.261
   1895   1371755_at   9.948 10.022 9.858 9.929 9.907 10.377 10.328 10.29 10.402 10.418
   1896   1368178_at   10.465 10.509 10.729 10.712 10.446 11.471 11.655 11.367 11.53 11.47
   1897   1379361_at   7.432 7.278 7.309 7.119 7.052 9.313 8.841 8.651 8.832 8.695
   1898   1398473_at   9.105 9.148 9.16 9.079 9.154 8.088 8.071 8.049 7.91 7.563
   1899   1382692_at   8.604 8.62 8.832 8.808 8.26 7.024 6.917 6.501 6.524 6.98
   1900   1369190_at   7.611 7.414 7.617 7.6 7.334 2.893 2.703 4.387 2.553 2.202
   1901   1387835_at   6.531 6.69 6.819 6.724 6.276 1.575 0.856 0.678 2.907 0.604
   1902   1376765_at   9.343 9.386 9.671 9.496 8.8 11.135 11.12 11.077 11.076 11.067
   1903   1388470_at   7.891 7.855 8.023 8.051 7.675 9.178 9.066 9.175 8.947 8.83
   1904   1393183_at   8.161 8.342 8.202 8.312 8.419 7.216 7.276 7.034 6.881 6.792
   1905   1387759_s_at   10.516 10.443 10.654 10.535 10.406 11.134 11.238 11.13 11.273 11.347
   1906   1370234_at   10.534 10.51 10.474 10.71 10.983 9.305 9.422 9.291 9.261 9.534
   1907   1381161_a_at   6.935 6.992 6.956 7.085 6.782 3.027 4.055 4.099 3.604 4.52
   1908   1369660_at   10.97 10.934 11.039 10.976 10.835 12.154 12.079 12.296 11.945 11.794
   1909   1377940_at   6.557 6.821 7.009 7.105 6.463 8.666 8.313 8.611 8.362 8.473
   1910   1371359_at   9.674 9.652 9.751 9.725 9.52 10.243 10.199 10.221 10.305 10.158
   1911   1393729_at   6.089 6.009 5.65 6.046 5.777 6.919 7.042 7.088 7.067 6.94
   1912   1371850_at   9.243 9.245 9.421 9.377 9.332 10.161 10.141 9.876 10.145 10.106
   1913   1391593_at   7.606 7.894 7.846 7.858 7.837 2.539 2.269 4.294 2.92 3.829
   1914   1380726_at   3.693 3.741 4.163 3.315 3.234 5.713 5.91 5.685 5.992 5.631
   1915   1370384_a_at   7.597 7.306 7.757 8.097 7.188 9.837 10.126 10.124 9.542 9.975
   1916   1382255_at   10.833 10.417 10.406 10.166 11.013 7.918 8.524 7.94 8.087 8.441
   1917   1389867_at   8.483 8.425 8.688 8.523 8.479 7.735 7.839 7.528 7.575 7.644
   1918   1398863_at   10.612 10.599 10.673 10.671 10.794 9.966 9.989 10.182 9.95 9.968
   1919   1370308_at   8.321 8.594 8.319 8.33 8.337 6.92 6.991 7.386 6.889 6.786
   1920   1371613_at   8.943 8.926 9.009 8.783 8.864 8.056 8.122 7.749 7.995 8.073
   1921   1367839_at   8.361 8.293 8.644 8.606 8.309 9.692 9.677 9.968 9.588 9.551
   1922   1370789_a_at   7.36 7.339 7.632 7.662 6.957 9.169 9.157 9.239 9.372 8.894
   1923   1388175_at   10.155 10.167 10.283 10.008 10.031 11.352 11.161 11.563 11.289 11.079
   1924   1391199_at   7.317 7.667 7.345 7.413 7.542 6.069 6.35 6.304 6.373 5.97
   1925   1369286_at   7.269 6.945 7.311 7.395 6.728 8.783 9.152 8.893 8.742 8.788
   1926   1384145_at   9.48 9.399 9.514 9.263 9.392 10.161 10.384 10.081 10.309 10.314
   1927   1368079_at   7.41 7.334 7.182 7.242 7.501 6.018 5.885 6.084 5.557 5.541
   1928   1376702_at   4.095 3.595 4.253 4.343 3.724 6.084 6.08 6.492 6.079 5.873
   1929   1388374_at   8.886 8.837 8.784 8.966 8.907 9.704 9.768 9.952 9.611 9.596
   1930   1372279_at   7.62 7.573 7.51 7.604 7.8 6.8 6.946 6.81 6.863 6.656
   1931   1373304_at   7.282 7.145 7.458 7.456 7.162 8.41 8.405 8.583 8.353 8.201
   1932   1367917_at   9.833 9.94 9.788 10.158 9.3 11.565 11.432 11.528 11.599 11.471
   1933   1371466_at   7.361 7.525 7.375 7.386 7.374 8.187 8.267 8.115 7.987 8.023
   1934   1372990_at   8.325 8.401 8.452 8.713 8.661 7.358 7.494 7.566 7.552 7.552
   1935   1379448_at   7.06 6.648 6.746 6.342 7.099 2.483 3.515 2.934 2.444 3.865
   1936   1368568_at   10.483 10.473 10.779 10.649 10.759 11.695 11.575 11.881 11.639 11.556
   1937   1367995_at   11.314 11.325 11.375 11.375 10.974 12.308 12.277 12.137 12.229 12.316
   1938   1373595_at   9.153 9.208 9.286 9.428 9.51 8.393 8.465 8.517 8.392 8.303
   1939   1367648_at   7.6 7.586 7.597 7.693 7.476 8.705 8.88 8.434 8.621 8.484
   1940   1386376_at   7.807 7.708 7.891 7.933 7.462 9.496 9.522 9.962 9.24 9.366
   1941   1369991_at   10.069 10.098 10.138 10.246 10.355 9.188 9.197 8.964 9.377 9.184
   1942   1374777_at   6.615 6.717 6.569 6.608 6.716 5.601 5.389 5.844 5.379 5.411
   1943   1372670_at   7.619 7.643 7.553 7.575 7.359 8.589 8.542 8.703 8.479 8.31
   1944   1387234_at   5.701 5.907 5.743 5.966 5.308 7.59 7.217 7.795 7.401 7.597
   1945   1375973_at   7.557 7.571 7.598 7.681 7.675 6.993 7.041 6.835 7.084 6.867
   1946   1387889_at   10.954 10.875 10.917 10.902 10.884 11.822 11.811 11.556 12.022 11.889
   1947   1380425_at   8.284 8.163 8.048 8.292 8.408 4.629 5.676 5.88 5.462 4.851
   1948   1398343_at   8.01 8.077 8.309 8.501 8.227 9.409 9.439 9.415 9.203 9.405
   1949   1370361_at   6.15 6.069 5.944 5.582 5.942 7.629 7.735 7.952 7.295 7.606
   1950   1392648_at   7.467 7.252 6.973 7.263 7.981 4.732 4.218 5.151 4.388 4.346
   1951   1387866_at   8 7.697 7.843 7.958 7.933 6.65 6.74 6.878 6.751 7.069
   1952   1377092_at   8.921 9.148 8.984 9.43 9.363 5.208 4.9 6.55 4.98 5.032
   1953   1374111_at   8.097 8.092 8.184 8.308 8.505 7.101 7.2 6.931 6.793 6.985
   1954   1370824_at   6.806 6.858 7.082 7.039 6.189 9.159 9.331 9.616 9.093 8.855
   1955   1393342_at   8.003 8.024 8.314 8.376 7.966 9.441 9.754 9.355 9.731 9.443
   1956   1375554_at   8.723 8.724 8.499 8.554 8.78 8.018 7.96 8.028 7.987 7.962
   1957   1374626_at   7.237 7.268 7.472 7.294 7.389 6.08 5.983 5.896 6.256 6.408
   1958   1368188_at   11.623 11.613 11.657 11.648 11.619 12.557 12.503 12.207 12.523 12.617
   1959   1377607_at   7.555 7.42 7.411 7.445 7.401 6.867 6.666 6.56 6.814 6.715
   1960   1380199_at   4.734 4.624 4.569 4.418 4.322 5.947 5.872 5.852 5.858 5.497
   1961   1382270_at   6.051 5.821 5.747 6.139 5.918 7.352 7.386 7.382 7.287 6.926
   1962   1383936_at   7.612 7.546 7.771 7.894 8.161 4.57 5.06 4.016 5.402 4.681
   1963   1368790_at   8.459 8.614 8.107 8.621 8.856 6.915 6.749 6.881 6.501 6.468
   1964   1372019_at   8.857 8.736 8.753 8.971 9.135 7.738 7.889 7.988 7.763 7.71
   1965   1393451_at   7.363 7.244 7.448 7.566 7.528 6.071 6.363 6.132 6.514 6.233
   1966   1371800_at   9.202 9.083 9.101 9.427 9.379 8.382 8.445 8.357 8.35 8.321
   1967   1367920_at   5.871 5.891 6.005 6.011 6.032 3.876 3.877 3.676 4.619 3.826
   1968   1389581_at   8.152 8.12 8.377 8.14 8.145 9.075 9.204 8.876 9.028 9.164
   1969   1368289_at   4.246 3.966 3.816 3.846 3.18 6.163 6.362 6.827 6.352 6.102
   1970   1386555_at   6.112 6.119 5.798 6.504 6.397 4.555 4.761 4.54 4.715 4.535
   1971   1377979_at   5.468 5.523 5.276 5.426 5.229 6.716 6.79 6.5 6.934 6.424
   1972   1372654_at   9.56 9.627 9.557 9.603 9.437 10.888 10.965 11.277 10.659 10.715
   1973   1387375_at   8.969 8.805 9.439 9.502 8.642 11.069 11.3 11.292 11.172 11.062
   1974   1373597_at   8.499 8.61 8.645 8.811 8.791 7.815 7.834 7.989 7.913 7.806
   1975   1383205_at   7.502 7.6 7.343 7.348 7.336 8.818 8.68 9.062 8.447 8.806
   1976   1375767_at   7.215 7.276 7.341 7.326 6.752 8.717 8.822 8.962 8.679 8.519
   1977   1387348_at   7.388 7.774 8.224 7.77 7.991 9.769 9.53 9.525 9.911 9.647
   1978   1379317_a_at   7.602 7.779 7.768 8.039 7.914 6.587 6.839 6.853 6.767 6.831
   1979   1373392_at   8.524 8.667 8.715 8.76 8.746 7.949 7.901 7.981 7.756 7.696
   1980   1376775_at   6.645 6.316 6.556 6.454 6.294 3.949 4.431 4.878 4.624 4.548
   1981   1385124_at   6.211 6.295 5.954 6.383 6.27 7.196 7.338 7.293 7.579 7.363
   1982   1386858_at   12.234 12.182 12.053 12.036 12.218 11.456 11.465 11.568 11.416 11.304
   1983   1388361_at   10.169 10.2 10.16 10.183 10.26 10.77 10.91 10.798 10.739 10.628
   1984   1370818_at   9.685 9.658 9.866 9.577 9.27 11.281 11.235 10.85 11.413 11.278
   1985   1388867_at   9.54 9.573 9.304 9.268 9.357 8.332 8.514 8.088 8.166 8.181
   1986   1370281_at   11.131 11.127 11.331 11.434 11.349 9.455 9.505 9.978 9.224 9.196
   1987   1374221_at   7.95 7.972 7.943 8.277 7.924 9.049 9.258 9.074 9.095 8.876
   1988   1375074_at   6.719 6.938 6.987 6.869 6.623 4.916 4.986 5.277 5.207 5.6
   1989   1367958_at   8.228 8.249 8.337 8.413 8.297 7.385 6.987 6.912 7.322 7.183
   1990   1399089_at   7.295 7.342 7.304 7.474 7.079 5.999 5.728 6.017 6.192 6.215
   1991   1376570_at   11.499 11.456 11.452 11.433 11.576 10.742 10.764 10.972 10.681 10.657
   1992   1390391_at   8.891 8.901 8.99 9.236 9.115 6.054 6.515 7.173 6.433 5.995
   1993   1392893_a_at   6.93 7.015 7.061 7.224 7.05 8.014 8.016 7.783 7.848 7.786
   1994   1370180_at   11.337 11.354 11.397 11.301 11.295 11.854 11.877 11.985 11.73 11.904
   1995   1392990_at   5.245 5.647 5.999 5.607 5.698 7.65 7.497 7.349 7.103 7.404
   1996   1371781_at   10.217 10.021 9.964 10.023 9.954 8.832 8.476 8.572 8.167 8.753
   1997   1378819_at   7.093 6.918 7.675 7.492 6.838 9.263 9.166 9.433 9.373 9.075
   1998   1374902_at   6.651 6.857 6.736 6.85 6.684 3.5 1.664 2.513 3.438 2.467
   1999   1375870_a_at   8.545 8.73 8.736 8.71 9.004 7.834 7.799 7.612 7.586 7.712
   2000   1377085_at   6.177 6.346 6.136 6.38 6.32 7.245 7.299 7.197 7.633 7.301
   2001   1387283_at   9.072 8.7 8.336 9.23 9.43 6.26 5.74 6.441 5.567 5.946
   2002   1367881_at   8.512 8.679 8.481 8.812 8.773 7.379 7.559 7.281 7.513 7.071
   2003   1382084_at   6.795 6.738 6.888 6.862 6.637 8.721 8.495 8.996 8.265 8.234
   2004   1376722_at   8.964 8.96 8.913 8.979 9.031 7.394 7.825 7.65 7.082 7.495
   2005   1391384_at   7.838 7.555 7.236 7.475 7.313 1.599 2.854 3.259 0.731 2
   2006   1369682_at   8.341 8.233 8.103 8.605 8.443 10.136 10.051 10.479 9.957 9.746
   2007   1368251_at   7.571 7.339 7.668 7.649 7.609 5.594 6.194 6.273 5.944 6.065
   2008   1380245_at   7.636 7.62 7.851 7.678 7.423 8.589 8.78 8.614 8.501 8.756
   2009   1382709_at   6.498 6.961 7.192 6.836 6.54 4.836 4.385 4.994 4.525 4.946
   2010   1374437_at   9.484 9.529 9.6 9.691 9.595 8.899 8.972 8.715 8.671 8.78
   2011   1375247_at   9.299 9.29 9.588 9.4 9.188 10.991 11.364 11.073 11.256 10.613
   2012   1383422_at   6.208 6.364 6.585 6.56 6.324 7.649 8.146 7.858 7.652 7.686
   2013   1368882_at   7.698 7.653 7.641 7.574 7.333 8.949 9.045 8.533 8.999 8.955
   2014   1368179_at   8.345 8.376 8.502 8.609 8.519 7.383 7.298 7.495 7.607 7.147
   2015   1369794_a_at   7.487 7.661 7.946 7.936 7.373 5.891 5.209 5.783 5.223 5.631
   2016   1377375_at   10.371 10.369 10.557 10.495 10.403 11.371 11.638 11.192 11.408 11.545
   2017   1379298_at   7.743 7.929 7.628 7.756 7.781 6.876 6.757 6.567 6.698 6.398
   2018   1374739_at   8.192 8.345 8.402 8.666 8.348 6.628 6.299 6.862 6.628 6.044
   2019   1389569_at   8.75 8.911 8.601 8.81 8.874 7.998 7.763 7.778 8.02 7.922
   2020   1383863_at   8.757 8.649 8.683 8.689 8.954 7.477 7.607 7.456 7.221 7.009
   2021   1391262_at   7.071 7.418 7.091 7.488 7.311 5.637 4.918 5.007 5.595 5.343
   2022   1372123_at   11.502 11.484 11.47 11.421 11.522 11.774 11.834 11.885 11.897 11.784
   2023   1386454_at   6.733 7 7.109 7.332 6.863 8.903 8.992 9.264 8.723 8.531
   2024   1385627_at   7.351 7.438 6.889 7.671 7.491 4.899 4.632 5.234 5.501 5.105
   2025   1383350_at   6.943 7.043 7.153 7.26 6.975 7.955 7.915 7.885 7.979 7.754
   2026   1386886_at   11.786 11.685 11.73 11.624 11.656 12.039 12.035 12.052 12.013 12.064
   2027   1367749_at   8.011 8.031 7.777 7.495 8.484 5.62 5.246 5.958 5.347 5.657
   2028   1367775_at   9.691 9.675 10.017 9.947 9.11 11.588 11.696 11.578 11.721 11.482
   2029   1384244_at   9.918 9.985 9.921 10.105 9.889 10.596 10.699 10.697 10.646 10.475
   2030   1381771_at   7.107 7.535 7.476 7.192 7.463 1.709 2.483 2.609 0.88 0
   2031   1371495_at   10.823 10.762 10.861 10.779 10.851 10.431 10.469 10.298 10.433 10.405
   2032   1394160_at   7.01 6.985 6.799 6.871 7.215 5.776 5.725 5.606 5.243 5.403
   2033   1370187_at   10.366 10.269 10.438 10.414 10.268 11.25 11.168 11.108 11.322 10.972
   2034   1368766_at   5.219 3.849 4.548 4.335 4.383 0.411 0 1.063 1.202 0
   2035   1368294_at   6.598 6.875 6.12 6.351 6.356 4.893 4.783 4.796 4.833 4.487
   2036   1388353_at   9.42 9.229 9.553 9.61 9.471 8.27 8.334 8.343 8.421 8.633
   2037   1396035_at   6.797 6.624 6.754 6.496 5.847 8.901 8.791 9.25 8.723 8.633
   2038   1371350_at   10.56 10.537 10.569 10.312 10.211 11.542 11.653 11.596 11.357 11.746
   2039   1399138_at   7.139 7.218 7.04 7.251 7.089 7.911 7.757 7.79 7.671 7.791
   2040   1380621_at   7.968 7.893 7.984 8.094 8.081 5.176 5.592 5.404 4.483 4.283
   2041   1386162_at   5.984 6.098 6.31 6.308 5.869 4.379 4.649 4.672 4.661 4.945
   2042   1371377_at   12.81 12.7 12.551 12.424 12.648 11.819 11.862 11.799 11.714 11.847
   2043   1376692_at   6.866 6.881 7.01 7.29 7.029 8.319 8.707 8.385 8.226 8.226
   2044   1391356_at   8.349 8.666 8.765 8.8 8.788 7.536 7.609 7.487 7.623 7.716
   2045   1372502_at   7.71 7.522 7.766 7.886 7.552 8.789 9.035 8.961 8.648 8.73
   2046   1379592_at   8.61 8.434 8.379 8.441 8.11 9.788 9.823 9.461 9.913 9.616
   2047   1368856_at   7.919 8.232 7.846 8.023 8.22 6.172 6.184 6.151 5.86 5.326
   2048   1371493_at   8.547 8.491 8.485 8.678 8.582 9.448 9.607 9.525 9.378 9.214
   2049   1378028_at   6.406 6.242 6.764 6.767 6.626 4.272 4.369 4.899 3.926 4.114
   2050   1371913_at   11.162 11.159 11.388 11.315 11.13 9.249 9.408 9.954 9.129 9.161
   2051   1372809_at   5.652 5.598 4.896 5.953 5.904 3.242 2.93 3.113 3.044 2.503
   2052   1382005_at   10.538 10.559 10.559 10.398 10.517 9.79 9.938 9.886 9.668 9.918
   2053   1371567_at   11.375 11.273 11.381 11.464 11.24 12.091 12.12 11.932 12.249 12.17
   2054   1390440_at   7.272 7.382 6.986 7.23 7.289 8.244 8.414 8.426 8.101 8.451
   2055   1379687_at   9.259 9.211 9.037 9.102 8.85 8.234 8.061 7.956 7.906 8.003
   2056   1373273_at   8.003 8.156 8.236 8.119 8.277 7.371 7.332 7.206 7.123 7.009
   2057   1375920_at   7.519 7.84 7.761 7.897 7.652 5.025 5.163 5.913 5.063 4.606
   2058   1373305_at   8.394 8.342 8.154 8.287 8.482 7.518 7.64 7.643 7.666 7.657
   2059   1369073_at   8.133 7.964 8.064 7.831 7.607 9.335 9.347 8.985 9.291 9.313
   2060   1379538_at   6.731 7.066 6.91 7.069 7.186 5.466 5.75 5.931 5.689 5.528
   2061   1368840_at   11.532 11.604 11.101 11.277 11.504 10.197 9.99 9.976 10.297 10.041
   2062   1390021_at   9.872 9.832 9.891 10.042 9.858 10.594 10.378 10.503 10.463 10.504
   2063   1367591_at   10.639 10.66 10.744 10.617 10.643 11.006 11.016 10.972 10.931 10.952
   2064   1389084_at   5.697 5.678 6.027 5.927 6.006 8.084 8.036 8.425 7.914 7.402
   2065   1398806_at   9.706 9.679 9.769 9.916 9.887 9.183 9.095 9.142 8.962 9.057
   2066   1372350_at   8.239 8.277 8.245 8.246 8.275 8.671 8.727 8.571 8.573 8.592
   2067   1373218_at   6.479 5.967 6.473 6.319 6.319 8.055 7.871 8.547 7.958 7.953
   2068   1377812_a_at   7.93 7.828 7.92 7.769 7.434 8.989 9.064 8.823 8.88 9.056
   2069   1382964_at   7.152 6.779 6.875 7.012 7.143 8.205 8.347 8.003 8.077 8.013
   2070   1378213_at   4.887 5.33 5.293 4.969 5.565 2.16 2.698 1.84 3.03 2.353
   2071   1374659_at   2.984 3.761 4.281 3.745 3.231 0.632 0.566 0.496 0.864 0
   2072   1368186_a_at   7.22 6.757 7.113 6.993 6.863 5.784 5.984 5.894 5.647 5.837
   2073   1378245_at   6.134 5.843 5.91 6.008 6.294 7.534 7.553 7.89 7.396 7.291
   2074   1373947_at   11.175 11.109 10.927 10.928 11.195 9.549 9.426 8.939 9.702 9.51
   2075   1388603_a_at   9.45 9.488 9.519 9.541 9.549 10.007 10.106 10.062 9.871 10.052
   2076   1368137_at   7.6 7.546 7.783 7.959 7.541 9.115 9.15 8.944 8.74 8.883
   2077   1373618_at   6.718 6.625 6.395 6.48 6.481 7.502 7.78 7.756 7.48 7.459
   2078   1387071_a_at   7.788 7.735 8.038 7.874 7.459 9.414 9.693 9.183 9.521 9.149
   2079   1370136_at   7.629 7.62 7.849 7.887 7.614 6.832 6.655 6.428 6.759 6.594
   2080   1374076_at   7.107 7.046 6.932 7.076 7.039 6.114 6.258 6.409 6.262 6.033
   2081   1381504_at   3.146 3.602 2.93 3.278 3.546 5.361 5.548 4.937 5.615 5.708
   2082   1385414_at   9.421 9.397 9.837 9.624 9.318 0.367 1.899 3.995 0.263 1.753
   2083   1377666_at   8.567 8.499 8.46 8.609 8.394 9.763 10.249 9.934 9.665 9.658
   2084   1374298_at   8.401 8.321 8.115 7.929 7.836 5.951 6.628 5.845 5.998 6.101
   2085   1377023_at   8.303 8.342 8.614 8.592 8.091 5.897 6.079 6.45 6.472 5.547
   2086   1393230_s_at   7.123 7.128 7.235 7.375 7.33 8.436 8.855 8.632 8.264 8.466
   2087   1373851_at   7.363 7.196 7.388 7.454 7.21 8.108 8.445 8.274 8.163 8.158
   2088   1383107_at   7.99 7.851 7.934 7.9 7.964 6.619 7.07 6.832 6.484 6.752
   2089   1375985_at   7.336 7.529 7.529 7.283 7.445 8.239 8.362 8.44 8.113 8.271
   2090   1395788_at   6.663 6.583 6.344 5.893 6.549 2.284 0 0.239 1.546 0
   2091   1376871_at   6.436 6.474 7.008 6.699 6.836 5.303 4.851 4.648 4.765 4.686
   2092   1371398_at   11.752 11.643 11.682 11.569 11.71 12.174 12.245 12.09 12.253 12.135
   2093   1385739_at   9.046 9.054 9.06 9.25 9.101 10.014 10.306 9.948 9.883 10.059
   2094   1387145_at   5.785 6.144 6.594 5.588 5.629 8.249 8.073 8.078 8.339 8.3
   2095   1371864_at   9.83 9.779 9.901 9.925 9.676 11.349 11.266 10.879 11.075 11.537
   2096   1384028_at   9.096 9.096 9.257 9.39 9.391 10.15 10.32 10.214 10.119 10.046
   2097   1382868_at   6.105 6.251 6.344 6.468 6.466 7.764 7.751 8.228 7.55 7.926
   2098   1367588_a_at   12.069 12.007 12.076 11.918 12.102 11.464 11.559 11.539 11.433 11.359
   2099   1396187_at   7.944 7.775 7.903 8.157 7.923 6.948 6.651 6.488 6.728 6.407
   2100   1375307_at   7.326 7.497 7.413 7.618 7.534 8.424 8.649 8.694 8.517 8.282
   2101   1372535_at   8.249 7.938 8.08 7.899 8.243 6.575 7.039 6.859 6.848 6.95
   2102   1368610_at   7.613 7.553 7.408 7.497 7.585 3.345 3.189 4.336 4.876 3.707
   2103   1388195_at   7.74 7.567 8.186 8.104 7.962 6.627 6.529 6.611 6.584 6.566
   2104   1391849_at   6.606 6.957 6.665 6.668 6.514 5.202 5.329 5.656 5.537 5.469
   2105   1392554_a_at   7.137 7.278 7.646 7.443 6.998 5.599 5.248 5.557 5.004 4.938
   2106   1368885_at   7.473 7.488 7.289 7.743 7.672 6.44 6.244 6.019 6.268 6.415
   2107   1395364_at   8.006 7.96 7.809 7.708 7.94 6.687 6.438 6.724 6.897 6.9
   2108   1372415_at   7.294 7.181 7.159 7.278 7.264 6.301 6.458 6.208 6.483 6.583
   2109   1374871_at   8.952 8.922 9.094 9.236 8.483 10.492 10.518 10.854 10.508 10.535
   2110   1372546_at   7.582 7.239 7.664 7.408 7.515 6.627 6.656 6.535 6.495 6.551
   2111   1376914_at   6.473 6.638 6.608 6.383 6.555 1.832 3.459 3.186 3.493 3.074
   2112   1368358_a_at   6.589 6.954 6.722 6.95 6.748 8.23 8.248 8.273 7.771 8.108
   2113   1398807_at   9.368 9.487 9.562 9.619 9.429 10.276 10.43 10.454 10.191 10.201
   2114   1382154_at   9.961 9.887 9.903 9.892 9.868 9.408 9.503 9.298 9.244 9.343
   2115   1382138_at   6.636 6.96 6.688 6.665 6.914 3.702 3.864 4.923 4.121 4.391
   2116   1389337_at   8.589 8.512 8.368 8.423 8.727 7.393 7.205 7.623 7.263 7.158
   2117   1368171_at   7.705 7.657 7.654 7.675 8.273 6.414 5.965 5.959 6.111 6.169
   2118   1374052_at   7.63 7.848 7.794 7.906 7.798 7.232 7.204 7.228 7.209 7.093
   2119   1397949_at   5.91 6.164 6.039 6.011 5.978 6.847 6.969 6.693 6.778 6.68
   2120   1388874_at   9.198 9.269 9.52 9.591 9.206 10.577 10.492 10.273 10.474 10.495
   2121   1387357_at   8.636 8.773 8.933 8.888 8.097 10.505 10.479 10.586 10.356 10.363
   2122   1373605_at   7.89 7.977 7.865 8.038 7.948 9.404 9.309 9.384 8.812 9.221
   2123   1391661_at   8.934 8.851 9.143 8.894 8.458 10.427 10.613 10.268 10.237 10.26
   2124   1381525_at   7.593 7.467 7.418 7.706 7.636 6.441 6.132 6.522 6.333 5.983
   2125   1387669_a_at   9.532 9.542 9.642 9.698 9.569 10.31 10.45 10.194 10.519 10.507
   2126   1387100_at   8.906 8.959 8.986 9.251 9.18 10.297 10.606 10.251 10.225 10.086
   2127   1394390_at   6.057 6.158 5.916 5.686 6.074 4.689 4.471 4.668 4.621 4.957
   2128   1392521_at   4.915 4.846 4.513 5.018 4.952 5.999 6.062 6.089 6 6.355
   2129   1375940_a_at   6.552 6.476 6.369 6.612 6.552 7.552 7.74 7.596 7.611 7.249
   2130   1372437_at   11.736 11.731 11.806 11.689 11.773 12.139 12.167 12.024 12.117 12.085
   2131   1374429_at   8.218 8.228 7.947 8.072 7.851 5.513 5.871 6.226 5.586 6.401
   2132   1387898_at   6.888 6.873 6.667 6.677 6.477 8.387 8.06 8.664 8.031 8.305
   2133   1393161_at   7.416 7.401 7.556 7.436 7.608 6.079 6.293 5.789 5.851 6.38
   2134   1382521_at   9.889 9.929 10.056 9.899 10.015 8.81 8.951 8.719 8.346 8.827
   2135   1374800_at   8.396 8.501 8.461 8.806 8.407 9.763 10.122 9.72 9.754 9.63
   2136   1389540_at   8.82 8.837 8.936 9.078 8.279 10.509 10.618 10.447 10.264 10.461
   2137   1389151_at   8.048 8.269 8.353 8.376 8.367 6.45 5.843 6.294 5.975 5.339
   2138   1384968_at   7.283 7.352 7.514 7.637 7.557 5.772 5.171 5.418 5.013 4.65
   2139   1377192_a_at   9.024 9.097 9.309 9.275 8.832 10.273 10.383 10.123 10.195 10.152
   2140   1372646_at   8.327 8.173 8.04 8.103 8.144 9.797 9.953 9.967 9.601 9.273
   2141   1388846_at   7.351 7.58 7.442 7.354 7.458 6.379 6.202 6.309 5.991 5.797
   2142   1374638_at   9.094 9.111 9.266 9.138 9.17 9.778 9.93 9.801 9.704 9.666
   2143   1395730_at   7.921 7.84 7.717 7.947 7.852 7.26 7.054 6.908 6.973 6.997
   2144   1391955_at   6.695 6.415 6.524 6.46 6.527 3.24 3.939 4.48 4.184 3.421
   2145   1398903_at   10.435 10.523 10.543 10.655 10.622 11.179 11.173 11.075 11.264 11.072
   2146   1376465_at   6.054 6.045 5.883 6.182 6.158 6.742 7.063 6.874 6.985 6.969
   2147   1369512_at   3.186 2.742 3.474 2.934 3.517 0.791 0 0.029 0.88 0.595
   2148   1372398_at   6.654 6.77 6.682 6.514 6.57 7.766 7.799 7.944 7.439 7.644
   2149   1368374_a_at   11.657 11.813 11.796 11.891 11.707 12.323 12.428 12.537 12.524 12.368
   2150   1368847_at   9.701 9.908 9.499 9.762 9.447 8.692 8.461 8.555 8.617 8.416
   2151   1398991_at   7.891 8.066 7.83 8.004 7.96 8.986 9.205 9.293 8.887 8.85
   2152   1375106_at   8.825 8.709 8.939 8.943 8.563 10.145 10.103 9.83 10.324 9.928
   2153   1384147_at   7.491 7.378 7.456 7.58 7.518 5.382 6.232 5.996 5.825 5.908
   2154   1380610_at   6.41 6.598 6.604 6.722 6.274 8.143 8.244 8.814 8.067 8.166
   2155   1394881_at   5.713 5.547 6.011 6.354 5.651 7.645 7.64 7.958 7.952 7.575
   2156   1372497_at   9.056 9.12 9.035 9.193 9.22 9.743 9.952 9.714 9.739 9.724
   2157   1368571_at   8.825 8.499 8.196 8.529 8.313 6.868 6.823 7.043 6.517 6.462
   2158   1388109_at   7.77 7.601 7.709 7.697 7.665 8.368 8.467 8.56 8.215 8.435
   2159   1393997_at   7.84 7.651 7.496 7.52 7.319 8.849 8.923 8.568 8.947 8.76
   2160   1391754_at   6.716 6.767 6.953 7.19 7.321 4.302 4.748 4.725 4.718 3.754
   2161   1371609_at   9.207 9.133 9.305 9.338 9.339 9.897 10.082 9.993 9.812 9.962
   2162   1379967_at   6.717 7.086 6.81 6.958 6.964 5.027 5.598 5.267 5.249 5.682
   2163   1382436_at   6.755 6.652 7.018 6.788 6.877 5.531 5.65 5.891 5.742 5.401
   2164   1368204_at   7.804 7.813 7.819 8.014 7.88 6.977 7.056 6.75 7.066 6.718
   2165   1390177_at   7.925 7.906 7.912 8.036 8.272 7.012 7.187 7.15 7.209 7.088
   2166   1374293_at   8.339 8.407 8.521 8.646 8.38 9.217 9.431 9.576 9.41 9.301
   2167   1374484_at   7.319 7.153 7.169 7.203 7.328 6.681 6.55 6.526 6.619 6.738
   2168   1382061_at   8.772 8.695 8.605 8.834 8.428 9.618 9.826 9.655 9.712 9.503
   2169   1371407_at   11.065 11.072 10.916 11.105 11.085 11.455 11.575 11.545 11.466 11.56
   2170   1376895_at   5.778 6.202 5.786 6.336 5.739 4.277 4.343 4.547 4.321 4.564
   2171   1368375_a_at   7.936 7.851 7.613 7.75 7.877 6.329 6.724 6.511 6.182 6.68
   2172   1383493_at   6.253 6.367 6.354 6.433 6.354 4.368 4.383 5.193 4.452 4.563
   2173   1387531_at   8.024 8.203 8.63 8.25 7.699 9.94 9.903 9.885 9.89 9.806
   2174   1371391_at   9.34 9.274 9.188 9.356 9.556 8.645 8.672 8.629 8.58 8.55
   2175   1389632_at   7.971 8.003 8.175 8.491 8.179 9.453 9.443 9.733 9.478 9.285
   2176   1387436_at   8.649 8.512 8.702 8.634 8.573 7.938 7.946 7.655 7.965 7.834
   2177   1388836_at   8.369 8.253 8.884 8.715 8.212 7.023 6.743 6.668 6.63 6.88
   2178   1393015_at   8.367 8.331 8.332 8.775 8.535 9.74 9.89 9.859 9.567 9.544
   2179   1370952_at   7.521 7.466 7.707 7.773 7.927 9.64 9.328 9.531 9.206 8.954
   2180   1367485_at   10.608 10.501 10.602 10.54 10.547 9.814 9.834 9.53 9.619 9.429
   2181   1391167_at   8.019 7.964 8.116 7.998 8.156 7.161 7.023 7.068 6.869 6.642
   2182   1383719_at   7.078 7.118 7.355 7.356 6.939 8.3 8.403 8.089 8.231 8.226
   2183   1368552_at   9.706 9.648 9.759 9.796 9.774 10.443 10.396 10.323 10.304 10.175
   2184   1387800_at   8.187 8.088 8.034 8.128 7.877 6.569 6.58 7.124 6.548 6.735
   2185   1377116_at   8.123 8.388 8.09 8.596 8.56 5.709 5.878 6.317 6.567 5.654
   2186   1388733_at   8.025 8.047 7.905 7.935 8.098 7.364 7.361 7.103 7.203 7.094
   2187   1373191_at   8.245 8.274 8.233 8.335 8.049 8.925 9.087 9.095 8.841 9
   2188   1390917_at   6.524 6.822 6.689 6.875 6.846 5.681 5.947 5.666 5.557 5.762
   2189   1372409_at   7.41 7.713 7.624 7.853 7.75 6.513 6.43 6.616 6.759 6.696
   2190   1372744_at   8.915 8.931 8.905 9.133 8.773 10.14 10.285 10.396 9.854 10.081
   2191   1380867_a_at   7.995 8.145 8.202 8.08 8.003 7.314 7.126 6.9 6.84 7.025
   2192   1369948_at   9.011 9.037 9.083 9.248 9.2 10.288 9.848 10.274 10.136 10.156
   2193   1390020_at   10.638 10.707 10.73 10.74 10.601 11.645 11.754 11.401 11.712 11.403
   2194   1369557_at   8.198 8.226 8.33 8.079 8.165 6.903 6.612 6.428 7.132 6.617
   2195   1387976_at   6.507 6.543 6.573 6.708 6.595 7.557 7.685 7.544 7.539 7.228
   2196   1380962_at   6.11 6.354 6.329 6.449 5.822 7.812 8.286 8.531 7.895 8.147
   2197   1391390_at   8.003 7.553 7.738 8.167 7.511 9.529 9.255 9.29 9.234 9.414
   2198   1382161_at   7.166 7.179 7.016 7.202 7.28 6.281 6.43 6.558 6.239 6.315
   2199   1369830_at   7.237 7.033 7.978 7.701 7.255 5.246 5.257 4.864 5.366 5.416
   2200   1371573_at   11.274 11.274 11.258 11.303 11.51 10.629 10.617 10.687 10.58 10.411
   2201   1374840_at   8.15 7.938 7.907 7.977 7.938 7.222 7.28 7.138 7.159 6.92
   2202   1388611_at   7.138 6.545 7.374 7.1 7.059 8.811 8.582 9.013 8.694 8.704
   2203   1374592_at   7.895 8.023 8.022 8.045 8.028 8.827 8.76 8.953 8.727 8.568
   2204   1377626_at   12.948 12.901 12.992 12.783 12.676 6.219 5.906 8.904 6.552 6.238
   2205   1398389_at   8.95 8.881 8.783 8.805 8.816 9.475 9.498 9.323 9.304 9.454
   2206   1368071_at   9.209 9.348 9.386 9.418 9.354 10.383 10.571 10.346 10.314 10.062
   2207   1373680_at   8.432 8.379 8.387 8.746 8.173 9.705 9.628 9.488 9.86 9.603
   2208   1373204_at   10.5 10.423 10.008 10.33 10.408 9.219 9.056 9.318 9.333 9.144
   2209   1396246_at   4.3 4.705 4.635 4.836 4.349 5.808 6.204 6.065 5.983 6.314
   2210   1390415_at   6.949 6.95 6.744 6.834 7.062 3.903 4.49 3.674 4.136 5.003
   2211   1368158_at   9.372 9.466 9.531 9.365 9.495 8.862 8.894 8.811 8.668 8.649
   2212   1390819_at   7.265 7.3 7.283 7.54 7.203 8.041 7.94 8.127 8.087 8.106
   2213   1388883_at   8.053 8.034 8.54 8.378 8.246 9.405 9.312 9.305 9.461 9.366
   2214   1383434_at   4.624 4.31 4.444 3.997 4.828 0.888 0.084 0.926 1.828 0.454
   2215   1370170_at   10.692 10.69 10.609 10.809 10.799 10.032 10.148 10.098 9.863 9.958
   2216   1372661_at   7.579 7.505 7.549 7.71 7.696 6.723 6.897 7.007 6.898 6.731
   2217   1374011_at   8.559 8.747 8.575 8.711 8.65 8.076 8.172 8.192 8.11 8.022
   2218   1388132_at   8.787 8.829 8.913 9.14 9.217 7.534 7.809 7.868 7.783 7.91
   2219   1381969_at   9.273 9.413 9.475 9.545 9.5 8.597 8.649 8.532 8.328 8.286
   2220   1392755_at   7.985 7.535 7.826 7.86 7.093 5.72 5.698 5.836 5.97 5.8
   2221   1373740_at   8.549 8.652 9.019 9.042 8.721 10.25 10.376 10.29 9.978 10.038
   2222   1389655_at   8.319 8.471 8.247 8.233 8.35 7.152 7.435 7.034 7.482 7.268
   2223   1374395_at   9.12 9.067 8.968 9.144 9.23 8.305 8.311 8.131 7.952 8.284
   2224   1392514_at   7.318 7.234 6.993 7.31 7.645 5.863 6.123 5.735 5.953 5.728
   2225   1382100_at   7.566 7.717 7.707 7.84 7.705 8.492 8.732 8.645 8.433 8.419
   2226   1387651_at   10.676 10.631 11.061 10.868 10.584 12.136 11.854 11.816 12.084 12.096
   2227   1388917_at   8.801 8.581 8.76 8.877 8.807 9.468 9.671 9.643 9.448 9.461
   2228   1370138_at   5.101 5.198 5.389 5.586 5.644 2.676 2.524 1.098 1.77 1.669
   2229   1367452_at   11.095 11.185 11.336 11.213 11.225 10.694 10.75 10.618 10.692 10.58
   2230   1384273_at   6.876 7.142 6.652 6.485 6.785 8.177 8.077 8.134 7.962 8.047
   2231   1396292_at   4.101 4.296 4.281 4.459 4.327 5.019 5.286 5.204 5.051 5.241
   2232   1372157_at   7.965 7.949 8.005 8.094 7.922 8.873 8.848 8.895 8.889 8.539
   2233   1389440_at   7.298 7.572 7.317 7.449 7.238 4.418 3.198 4.951 3.956 4.027
   2234   1370313_at   8.676 8.788 8.78 8.909 8.847 7.601 7.594 7.971 7.524 7.893
   2235   1383117_at   7.871 7.786 8.232 8.227 7.998 9.457 9.78 9.287 9.435 9.277
   2236   1390531_at   7.613 7.582 7.509 7.978 7.873 6.455 5.819 6.297 6.12 6.156
   2237   1389111_at   7.12 7.179 7.265 7.313 7.051 8.102 8.441 8.093 8.023 8.11
   2238   1382616_at   7.538 7.538 7.676 7.385 7.377 6.165 5.973 6.033 5.395 5.678
   2239   1368507_at   11.236 11.333 11.425 11.506 11.428 10.384 10.595 10.566 10.478 10.2
   2240   1379499_at   8.316 8.023 7.804 7.897 7.832 5.042 5.302 5.844 4.966 4.353
   2241   1388756_at   8.111 8.245 8.031 8.282 8.087 9.284 9.329 9.378 8.956 9.061
   2242   1370071_at   8.599 8.39 8.598 8.807 8.596 7.315 7.562 7.102 7.544 7.47
   2243   1368910_at   7.267 7.286 7.41 7.19 7.43 6.12 6.437 6.422 6.457 6.115
   2244   1389903_at   10.597 10.593 10.655 10.68 10.531 11.218 11.217 11.018 11.284 11.232
   2245   1376110_at   5.574 5.743 5.567 6.044 5.435 8.371 8.431 7.403 8.282 7.925
   2246   1370167_at   8.828 8.717 9.129 8.875 8.035 10.8 10.732 10.735 10.698 10.821
   2247   1387158_at   7.784 7.791 8.188 8.482 7.156 10.369 10.456 10.694 10.388 10.337
   2248   1368548_at   9.829 9.899 10.003 9.982 10.292 11.04 11.162 11.03 10.881 10.959
   2249   1373807_at   8.351 8.435 8.587 8.504 8.313 9.914 9.772 10.14 9.444 9.762
   2250   1369686_at   5.752 6.165 5.941 5.599 5.618 4.209 4.41 4.402 4.602 4.519
   2251   1378629_at   6.721 6.658 7.012 6.894 6.537 5.538 5.356 5.738 5.268 5.366
   2252   1389833_at   8.594 8.635 8.82 8.841 8.541 9.652 9.503 9.553 9.84 9.569
   2253   1399154_at   8.15 8.194 8.128 8.238 8.38 7.23 7.293 7.257 7.595 7.291
   2254   1379561_at   10.098 10.109 9.923 10.073 10.104 8.981 9.286 9.25 8.868 9.13
   2255   1388916_at   8.459 8.612 8.532 8.81 8.403 9.884 10.269 9.668 9.824 10.046
   2256   1388027_a_at   10.247 10.325 10.201 10.208 10.313 9.728 9.571 9.62 9.544 9.378
   2257   1371484_at   8.981 8.896 8.8 8.986 8.974 8.393 8.172 8.318 8.189 8.113
   2258   1389902_at   9.975 10.009 9.827 9.965 10.104 9.257 9.31 9.444 9.235 9.188
   2259   1388641_at   8.099 8.226 8.087 8.191 8.144 7.626 7.659 7.684 7.459 7.514
   2260   1389189_at   9.504 9.362 9.312 9.628 9.577 8.602 8.34 8.62 8.422 8.648
   2261   1387232_at   6.857 6.93 7.183 6.96 6.751 8.782 8.972 9.631 8.983 8.611
   2262   1389032_at   8.699 8.582 8.584 8.855 8.589 9.272 9.482 9.564 9.504 9.504
   2263   1383747_at   8.466 8.509 8.127 8.419 8.522 4.492 5.921 5.572 5.421 5.873
   2264   1376005_at   7.177 7.078 7.255 7.359 7.312 5.915 5.652 6.032 6.14 6.237
   2265   1375197_at   11.06 10.983 11.166 11.19 11.197 11.763 11.775 11.809 11.745 11.594
   2266   1390947_at   7.728 7.765 7.55 7.585 7.737 6.991 7.039 6.834 6.746 6.793
   2267   1390951_at   2.585 2.472 3.432 3.348 2.793 5.985 6.056 6.111 5.3 5.439
   2268   1383220_at   5.892 5.429 5.451 5.638 5.545 6.968 6.84 7.241 6.855 6.724
   2269   1367843_at   10.526 10.497 10.891 10.655 10.272 12.043 12.014 11.671 12.086 11.93
   2270   1371403_at   10.781 10.738 10.682 10.839 10.91 10.157 10.308 10.288 10.148 10.23
   2271   1371986_at   8.878 9.036 9.079 8.963 9.062 9.861 9.707 9.709 9.596 9.581
   2272   1378365_at   4.105 4.492 3.517 3.679 3.797 6.207 6.556 6.117 5.983 5.945
   2273   1394484_at   1.433 1.975 0.07 1.74 1.485 5.458 5.483 4.879 5.974 5.468
   2274   1369629_at   9.316 9.257 9.377 9.291 9.044 10.323 10.174 9.992 10.376 10.234
   2275   1391538_at   7.483 7.185 7.079 7.677 7.219 8.544 8.772 8.691 8.535 8.603
   2276   1368934_at   8.549 8.635 8.94 8.953 8.883 9.935 10.007 9.725 10.101 9.91
   2277   1391855_at   5.225 5.081 5.174 4.983 5.063 6.581 6.391 5.976 6.284 6.492
   2278   1371318_at   12.601 12.546 12.488 12.346 12.544 11.992 12.054 11.999 11.989 11.963
   2279   1392515_at   8.412 7.944 8.465 7.078 8.073 4.781 4.788 5.399 5.047 4.854
   2280   1398765_at   11.641 11.584 11.519 11.663 11.677 11.129 11.25 11.249 11.148 11.151
   2281   1378520_at   3.436 3.449 4.241 3.285 3.643 0 0 0.816 0.138 1.157
   2282   1373909_at   6.731 6.972 7.079 6.677 6.543 8.047 8.232 7.884 8.263 8.154
   2283   1391240_at   4.833 4.706 4.238 4.457 4.265 6.341 6.574 6.269 6.099 5.945
   2284   1367741_at   9.59 9.515 9.788 9.578 9.344 10.886 10.658 10.79 10.475 10.914
   2285   1368190_at   7.128 7.123 7.401 7.291 7.102 8.396 8.228 8.057 8.25 8.525
   2286   1375411_at   10.228 10.17 10.374 10.187 10.191 10.77 10.761 10.791 10.761 10.631
   2287   1378293_at   7.487 7.573 7.612 7.816 7.698 6.695 6.686 6.973 6.832 6.733
   2288   1395697_at   7.582 7.728 7.635 7.613 7.484 5.245 4.971 3.807 5.108 4.756
   2289   1371939_at   10.971 11.003 10.933 11.005 10.945 10.574 10.64 10.443 10.501 10.601
   2290   1372949_at   9.25 9.318 9.285 9.462 9.452 8.775 8.734 8.526 8.567 8.612
   2291   1372071_at   8.419 8.363 8.392 8.095 8.443 9.173 9.275 9.082 9.227 9.065
   2292   1392531_at   7.326 7.161 7.157 7.294 7.215 6.13 6.226 6.137 6.061 5.639
   2293   1376263_at   6.54 6.109 6.655 6.784 6.283 8.26 7.945 7.868 8.203 8.277
   2294   1372869_at   9.176 9.099 9 9.066 7.947 11.692 11.38 11.669 11.401 11.348
   2295   1371599_at   9.673 9.738 9.858 9.911 9.76 10.453 10.584 10.596 10.363 10.408
   2296   1383960_at   7.131 7.384 7.311 7.587 7.48 8.367 8.426 8.297 8.462 8.216
   2297   1374208_at   8.389 8.325 8.233 8.426 8.606 7.441 7.007 7.319 6.953 7.063
   2298   1371581_at   10.42 10.402 10.217 10.377 10.432 9.487 9.688 9.668 9.523 9.332
   2299   1369950_at   9.728 9.878 9.63 9.963 9.954 8.901 8.978 8.906 8.985 8.677
   2300   1399095_at   10.365 10.33 10.36 10.409 10.287 10.013 10.04 10.096 10.101 10.069
   2301   1397535_at   8.027 8.041 8.303 8.402 8.259 9.141 9.238 9.163 9.018 9.054
   2302   1379801_at   5.089 5.503 5.409 5.113 5.159 6.512 6.54 7.017 6.715 6.513
   2303   1367586_at   12.105 12.034 12.103 12.026 12.003 10.735 10.798 11.232 10.755 10.562
   2304   1392486_at   8.606 8.68 8.931 8.689 8.66 9.608 9.701 9.626 9.463 9.412
   2305   1373533_at   7.338 7.292 6.931 7.05 7.128 8.228 8.364 8.268 7.994 8.336
   2306   1377898_at   2.403 2.428 2.556 2.85 1.227 5.812 5.752 5.511 6.121 5.284
   2307   1368265_at   6.37 6.299 5.917 6.397 6.249 7.423 7.144 7.347 7.339 7.314
   2308   1372173_at   7.95 7.857 8.035 8.186 8.162 7.189 6.888 7.218 7.044 6.946
   2309   1386895_at   8.926 8.934 8.799 8.837 9.055 9.574 9.535 9.512 9.71 9.48
   2310   1374646_at   10.248 10.338 10.257 10.159 10.295 9.868 9.883 9.741 9.807 9.759
   2311   1371389_at   10.517 10.369 10.593 10.477 9.775 12.047 11.869 12.027 12.174 12.074
   2312   1371478_at   10.956 10.978 11.148 11.098 11.01 10.331 10.558 10.503 10.408 10.376
   2313   1368607_at   10.766 10.804 10.977 10.993 10.762 11.516 11.472 11.387 11.506 11.483
   2314   1389788_at   6.31 6.527 6.115 6.453 6.379 7.339 7.444 7.285 7.234 7.153
   2315   1373715_at   7.115 7.179 6.969 7.086 7.092 6.142 6.065 6.42 5.976 6.253
   2316   1382452_at   5.492 5.634 5.214 5.667 5.703 7.125 7.244 7.163 6.78 6.76
   2317   1373337_at   10.799 10.753 10.974 10.876 10.32 11.993 12.045 11.911 12.057 11.992
   2318   1374994_at   4.934 4.574 4.708 5.071 4.729 6.337 5.929 6.286 6.454 6.067
   2319   1377135_at   6.01 5.729 6.405 6.615 5.575 8.537 8.553 9.03 8.444 8.315
   2320   1369412_a_at   9.044 9.011 8.926 8.946 8.923 10.136 10.032 10.143 9.777 9.73
   2321   1389760_at   7.464 7.402 7.225 7.331 7.258 6.398 6.227 5.981 6.302 6.489
   2322   1393203_at   7.226 7.258 7.193 7.49 7.317 6.613 6.36 6.594 6.586 6.538
   2323   1373683_at   7.921 7.854 8.162 8.073 7.972 6.563 6.12 5.523 5.869 6.144
   2324   1395007_at   4.567 4.68 4.53 4.387 4.746 5.646 5.475 5.577 5.671 5.941
   2325   1372776_at   9.918 10.043 10.259 10.093 10.233 9.148 9.285 8.911 9.039 8.89
   2326   1372592_at   8.248 8.269 8.561 8.681 8.197 9.743 9.692 9.85 9.645 9.436
   2327   1373335_at   8.43 8.148 8.165 8.203 8.182 9.054 9.487 9.249 9.122 9.161
   2328   1383776_at   7.919 7.745 7.751 7.982 7.99 8.663 8.742 8.734 8.48 8.699
   2329   1377890_at   5.984 6.194 6.332 6.183 5.921 7.691 7.601 7.804 7.363 7.223
   2330   1389815_at   10.406 10.433 10.315 10.376 10.689 9.374 9.405 9.495 9.311 9.018
   2331   1399025_at   9.68 9.633 9.539 9.497 9.428 8.792 8.867 9.04 8.904 8.874
   2332   1391091_at   6.938 6.958 7.052 7.186 6.901 5.956 5.68 5.739 5.278 5.454
   2333   1376746_at   7.827 7.912 7.97 7.947 7.405 9.224 9.378 9.587 9.364 9.034
   2334   1394591_at   8.989 9.035 8.987 8.882 9.029 8.285 8.518 8.306 8.345 8.188
   2335   1388772_at   8.293 8.513 8.455 8.345 8.495 7.201 7.444 7.236 7.118 6.777
   2336   1389073_at   8.045 7.844 7.977 8.244 7.96 7.065 6.93 7.194 7.05 6.81
   2337   1377751_at   7.462 7.537 7.587 7.392 7.464 8.444 8.736 8.224 8.43 8.594
   2338   1383502_at   7.766 7.645 7.517 7.739 7.61 6.065 6.324 6.493 5.71 6.139
   2339   1371963_at   8.494 8.658 8.87 8.83 8.445 9.654 9.774 9.797 9.697 9.559
   2340   1375246_at   11.184 11.177 11.121 11.105 11.177 10.713 10.826 10.827 10.66 10.708
   2341   1388977_at   9.755 9.895 9.826 9.789 9.977 9.22 9.356 9.339 9.162 9.252
   2342   1380123_at   7.16 7.118 7.097 7.062 7.116 8.512 8.507 8.058 8.381 8.014
   2343   1369852_at   6.62 6.339 6.203 6.397 6.204 2.711 2.787 3.438 1.782 1.506
   2344   1369148_at   6.575 6.554 6.45 6.588 6.398 8.069 8.193 7.685 7.824 7.548
   2345   1368303_at   6.645 6.804 6.417 6.586 6.783 7.998 7.747 8.079 7.591 7.902
   2346   1376728_at   9.63 9.799 9.8 9.902 9.728 10.706 10.809 10.774 10.408 10.746
   2347   1373065_at   9.915 9.824 9.894 9.816 10.118 8.209 8.197 8.67 8.185 7.727
   2348   1370446_at   7.913 7.761 8.044 8.081 7.555 8.952 9.082 9.323 9.222 9.066
   2349   1395532_at   8.57 8.602 8.45 8.762 8.448 9.564 9.731 9.562 9.475 9.314
   2350   1389041_at   8.611 8.445 8.604 8.529 8.564 7.808 7.937 8.012 8.012 8.066
   2351   1397866_at   7.414 7.474 7.853 7.949 7.581 4.524 5.338 5.194 5.17 4.109
   2352   1385933_at   9.27 9.332 9.522 9.253 9.286 8.29 8.376 7.825 8.184 8.004
   2353   1371942_at   11.524 11.603 11.691 11.627 11.667 12.281 12.281 12.162 12.482 12.446
   2354   1373749_at   6.702 6.924 7.361 7.164 6.616 9.121 9.281 8.589 9.124 8.792
   2355   1390437_at   6.617 6.597 6.256 6.301 6.811 7.821 8.073 7.738 7.836 8.141
   2356   1367578_at   11.683 11.666 11.747 11.54 11.639 11.118 11.178 11.276 11.147 11.108
   2357   1388595_at   8.019 7.977 7.902 7.984 8.052 8.423 8.658 8.703 8.532 8.592
   2358   1394470_at   6.859 6.642 6.673 6.581 6.959 4.442 2.846 3.394 2.922 2.733
   2359   1370224_at   10.195 10.288 10.682 10.533 10.772 9.224 9.275 9.235 8.978 9.106
   2360   1373990_at   7.185 7.451 6.126 6.484 5.179 10.886 10.873 11.073 10.687 10.903
   2361   1367698_a_at   9.647 9.65 9.8 9.848 9.746 8.731 8.763 9.024 8.594 8.538
   2362   1378249_x_at   7.822 7.737 7.999 7.963 7.619 9.615 9.259 10.077 9.476 9.344
   2363   1385816_at   5.935 5.369 5.5 5.407 5.442 3.814 3.162 2.805 3.227 3.611
   2364   1387084_at   9.75 9.538 9.356 9.831 9.67 10.776 10.872 10.849 10.535 10.692
   2365   1397287_at   5.821 5.841 5.948 6.145 5.782 7.016 7.123 7.187 7.222 6.751
   2366   1374558_at   7.822 7.836 8.015 8.187 7.695 8.953 9.144 8.908 8.98 8.861
   2367   1395183_at   6.773 6.906 7.119 7.045 7.069 5.471 5.246 5.921 5.557 5.257
   2368   1374625_at   7.821 8.091 8.43 8.417 8.311 9.676 9.597 9.677 9.562 9.389
   2369   1392274_at   7.959 8.088 7.967 8.076 8.205 8.835 9.113 8.841 8.897 8.742
   2370   1392713_a_at   7.927 7.748 7.8 7.888 7.471 8.962 9.448 9.057 9.068 8.924
   2371   1371683_at   9.153 9.153 9.192 9.375 9.405 8.445 8.628 8.505 8.357 8.516
   2372   1371821_at   10.09 10.14 10.091 10.244 10.193 9.632 9.701 9.616 9.501 9.713
   2373   1370197_a_at   7.402 7.552 7.784 7.632 7.456 8.546 8.729 8.434 8.389 8.603
   2374   1371338_at   9.33 9.251 9.545 9.573 9.436 10.074 10.111 10.079 10.14 10.103
   2375   1367871_at   9.486 9.597 9.774 9.745 9.024 11.38 11.418 10.967 11.55 11.296
   2376   1385716_at   7.369 7.099 7.678 7.546 7.541 8.866 8.933 8.972 8.675 8.569
   2377   1370561_at   7.861 7.878 7.795 8.12 8.076 5.735 5.243 5.222 6.348 5.643
   2378   1387885_at   8.956 8.924 8.889 9.306 9.167 9.957 9.929 9.876 9.991 9.932
   2379   1398832_at   10.584 10.683 10.739 10.708 10.851 10.082 10.108 9.998 9.828 9.911
   2380   1373115_at   8.042 8.022 8.041 8.1 8.029 7.322 7.046 6.998 7.38 7.011
   2381   1381857_at   8.61 8.698 8.588 8.606 8.664 8.289 8.141 8.133 8.16 8.282
   2382   1396039_at   8.078 8.037 8.679 8.67 7.768 10.284 10.563 10.298 10.413 10.148
   2383   1375540_at   8.385 8.394 8.138 8.342 8.226 7.465 7.73 7.533 7.611 7.589
   2384   1389244_x_at   7.113 7.043 7.289 7.514 7.264 5.797 5.745 5.82 5.531 6.185
   2385   1398358_a_at   8.51 8.289 8.212 8.32 8.123 9.492 9.36 9.365 9.345 9.057
   2386   1379805_at   6.005 5.933 6.108 6.527 6.754 4.39 4.073 4.374 3.782 3.886
   2387   1386888_at   8.874 8.801 8.848 9.098 9.2 6.37 6.559 7.339 6.602 6.09
   2388   1372358_at   6.979 6.619 6.819 7.064 6.818 7.99 8.317 8.135 7.853 7.955
   2389   1391338_at   5.135 5.196 5.025 5.252 4.976 6.194 6.189 6.506 6.292 5.949
   2390   1382997_at   5.709 5.966 5.394 6.371 5.434 8.388 8.144 9.024 8.321 8.111
   2391   1374686_at   6.907 7.098 6.886 6.855 7.166 5.723 5.81 5.165 5.473 5.265
   2392   1376546_at   6.409 6.507 6.473 6.215 6.088 7.469 7.714 7.437 7.649 7.299
   2393   1384393_at   6.508 6.825 6.814 6.792 6.953 3.409 3.9 3.326 4.22 2.444
   2394   1372438_at   10.603 10.637 10.781 10.484 10.45 11.524 11.552 11.253 11.575 11.559
   2395   1373225_at   10.751 10.673 10.794 10.806 10.749 9.843 9.848 10.025 9.989 10.215
   2396   1374508_at   8.624 8.562 8.773 8.676 8.68 9.148 9.265 9.365 9.183 9.173
   2397   1392910_at   7.911 8.016 7.967 8.164 7.982 7.212 7.206 7.373 6.973 7.123
   2398   1376128_at   8.301 7.948 8.165 8.326 8.076 9.066 9.345 9.05 9.088 9.064
   2399   1382287_at   8.713 8.725 8.628 8.537 8.639 7.68 7.261 7.478 7.694 7.171
   2400   1399091_at   11.08 11.17 11.287 11.277 11.324 10.544 10.72 10.69 10.655 10.593
   2401   1368100_at   8.195 8.331 8.219 8.422 8.43 9.259 9.295 9.676 9.268 9.237
   2402   1372774_at   9.368 9.405 9.602 9.479 8.921 10.742 11.139 10.941 10.665 10.854
   2403   1367659_s_at   10.001 10.005 10.017 9.999 10.007 10.781 10.779 10.758 11.162 10.756
   2404   1391161_at   7.215 7.05 6.864 6.707 6.918 5.954 5.669 5.643 5.424 5.64
   2405   1384200_at   8.19 7.744 8.361 8.465 8.422 6.819 6.509 6.511 6.414 6.757
   2406   1376683_at   8.061 8.215 8.362 8.276 8.142 9.007 9.196 9.028 8.847 9.027
   2407   1387821_at   7.567 7.427 7.649 7.803 7.618 8.505 8.804 8.443 8.63 8.475
   2408   1374235_at   5.738 6.189 5.957 6.197 6.104 7.763 7.497 7.991 7.437 7.34
   2409   1387336_at   9.116 9.044 9.654 9.486 8.463 11.339 11.457 11.543 11.773 11.38
   2410   1388497_at   7.398 7.448 7.616 7.828 7.571 8.79 8.774 8.969 8.808 8.462
   2411   1396820_at   9.19 9.213 9.329 9.301 9.42 8.277 8.044 7.896 8.363 7.814
   2412   1376997_at   8.071 8.048 7.986 8.195 8.164 8.976 9.112 9.487 9.003 9.232
   2413   1389633_at   8.624 8.722 8.877 8.832 8.661 7.676 7.624 7.973 7.917 7.922
   2414   1371869_at   11.711 11.606 11.634 11.661 11.828 10.675 10.753 11.018 10.682 10.523
   2415   1388858_at   8.999 8.886 9.015 8.958 8.923 8.582 8.361 8.437 8.474 8.349
   2416   1384301_at   6.339 6.068 5.873 5.932 6.055 7.853 7.945 8.403 7.524 7.651
   2417   1384499_at   7.778 7.833 7.765 7.994 8.272 9.085 9.506 9.161 9.346 9.122
   2418   1392477_at   7.778 7.796 8.209 7.687 8.128 8.98 9.256 9.14 9.248 9.118
   2419   1377456_at   5.367 5.23 5.595 4.968 5.606 6.628 6.737 6.751 6.588 6.646
   2420   1367812_at   6.236 6.35 6.44 6.794 6.551 8.012 8.062 8.526 7.8 8.06
   2421   1392603_at   8.149 8.057 8.079 8.257 8.088 8.758 8.721 8.722 8.627 8.556
   2422   1377594_at   9.154 9.213 9.245 9.474 9.4 7.927 8.016 8.152 8.465 8.062
   2423   1374624_at   9.042 9.051 9.342 9.091 8.52 10.715 10.843 10.55 11.171 11.057
   2424   1375335_at   11.729 11.7 11.709 11.648 12.007 11.089 11.041 10.997 10.949 11.077
   2425   1368268_at   8.08 8.242 8.557 8.284 8.198 7.439 7.436 7.287 7.363 7.39
   2426   1387631_at   6.591 6.726 6.339 6.584 5.93 7.898 8.066 8.076 7.971 7.898
   2427   1394456_at   8.08 8.13 8.402 8.422 8.142 9.366 9.614 9.593 9.291 9.191
   2428   1368047_at   10.94 10.927 11.25 11.125 10.762 11.948 12.045 11.889 12.142 12.03
   2429   1388771_at   9.482 9.539 9.463 9.508 9.513 8.735 8.924 8.551 8.519 8.768
   2430   1389804_at   6.813 7.047 7.215 7.103 7.176 5.771 5.559 5.553 5.327 6.006
   2431   1368244_at   7.026 7.31 7.051 7.142 7.341 8.074 8.125 7.861 8.056 7.977
   2432   1393217_at   8.568 8.711 8.515 8.546 8.824 7.367 7.403 7.043 7.688 7.412
   2433   1374912_at   6.291 6.51 6.735 6.743 6.659 4.272 3.727 3.206 2.759 2.74
   2434   1376733_at   8.502 8.586 8.749 8.897 8.664 10.42 10.474 9.754 10.433 10.262
   2435   1388658_at   8.097 8.012 8.07 7.995 8.058 8.619 8.377 8.495 8.535 8.502
   2436   1372006_at   8.863 8.746 8.808 9.11 9.232 7.383 7.547 7.122 7.54 7.749
   2437   1374057_at   9.785 9.795 9.879 9.832 10.003 10.694 10.635 10.935 10.514 10.718
   2438   1373400_at   9.181 9.174 9.02 9.305 9.119 10.106 10.293 10.386 9.983 9.988
   2439   1371341_at   11.019 11.041 10.875 10.787 10.859 10.012 10.189 10.212 10.002 9.862
   2440   1374775_at   8.182 8.31 8.388 8.455 8.236 3.925 5.586 5.616 5.029 4.787
   2441   1397644_at   7.798 8.026 7.386 7.592 7.91 6.347 6.184 6.574 6.387 6.311
   2442   1382419_at   7.259 7.349 7.033 7.329 7.111 4.075 4.793 4.955 4.098 5.254
   2443   1374682_at   7.02 6.99 6.922 7.344 6.774 8.133 8.277 8.493 8.087 8.301
   2444   1395518_at   8.271 8.445 8.476 8.369 8.261 9.227 9.406 9.228 9.073 9.502
   2445   1392933_a_at   6.807 6.808 6.76 6.842 6.636 8.195 7.872 7.625 8.002 8.206
   2446   1378543_at   7.485 7.513 7.475 7.531 7.447 8.172 8.349 8.315 8.129 7.994
   2447   1367760_at   9.197 9.31 9.483 9.499 9.41 8.697 8.614 8.755 8.573 8.52
   2448   1380504_at   3.685 3.543 3.569 2.971 3.863 6.137 6.459 5.614 5.529 5.916
   2449   1372343_at   7.732 7.934 7.937 7.653 7.884 6.903 7.141 7.005 6.924 6.78
   2450   1392082_a_at   7.307 7.562 7.96 7.579 6.817 5.521 5.119 5.136 5.409 5.255
   2451   1397808_at   10.166 10.269 10.116 10.232 10.288 7.605 8.335 8.767 8.018 8.124
   2452   1377761_at   6.649 6.654 6.891 6.874 6.775 5.669 5.484 5.424 5.93 5.758
   2453   1367948_a_at   8.282 8.347 7.901 8.141 8.126 9.534 9.659 9.231 9.187 9.414
   2454   1370949_at   7.615 7.979 8.189 8.518 8.29 6.255 6.297 6.537 6.502 6.415
   2455   1382227_at   6.874 6.95 7.049 7.128 6.791 7.918 8.158 8.067 7.972 7.72
   2456   1382531_at   6.752 6.835 6.926 6.495 6.901 3.949 2.281 2.658 3.35 3.858
   2457   1397419_at   6.866 6.989 6.816 7.148 7.072 8.016 7.991 7.745 7.745 7.872
   2458   1388384_at   5.037 5.002 4.525 4.663 4.95 6.068 6.155 5.941 5.905 6.153
   2459   1370088_at   6.326 6.371 6.361 6.653 6.707 7.349 7.392 7.595 7.523 7.442
   2460   1371349_at   9.316 9.377 9.191 9.37 9.99 7.936 7.78 7.793 7.678 8.023
   2461   1372396_at   6.864 6.948 6.775 7.012 6.91 7.709 7.733 7.547 7.441 7.609
   2462   1394275_at   8.345 8.185 8.041 8.049 8.406 6.904 7.125 6.529 6.559 6.832
   2463   1376353_at   6.651 6.41 6.442 6.491 6.327 5.239 4.986 5.397 5.567 5.16
   2464   1398860_at   11.559 11.555 11.507 11.653 11.68 11.177 11.179 11.221 11.16 11.072
   2465   1372276_at   7.442 7.205 7.284 7.236 7.124 8.428 8.429 8.222 8.033 8.148
   2466   1368393_at   7.902 7.982 7.997 7.912 7.906 7.257 7.349 7.037 7.361 7.332
   2467   1370133_at   7.676 7.446 7.512 7.523 7.631 4.221 3.575 3.027 5.048 4.011
   2468   1370386_at   8.082 8.009 7.836 8.161 8.135 7.261 7.251 7.376 7.339 7.432
   2469   1392668_at   7.588 7.419 7.591 7.492 7.331 6.424 6.029 5.811 5.873 5.606
   2470   1370014_at   9.602 9.625 9.501 9.707 9.771 8.772 8.769 8.746 8.452 8.388
   2471   1374953_at   8.473 8.521 8.249 8.307 8.576 7.483 7.739 7.628 7.46 7.529
   2472   1383574_at   9.171 9.234 9.297 9.243 9.203 8.244 8.162 7.929 8.171 8.52
   2473   1389251_at   7.648 7.779 8.022 7.959 7.919 8.633 8.713 8.729 8.703 8.542
   2474   1380187_at   7.424 7.188 7.128 7.651 7.494 5.941 6.039 6.249 6.096 5.731
   2475   1390312_at   8.793 8.94 8.68 9.164 9.084 6.718 6.791 7.374 7.177 7.45
   2476   1392496_at   8.989 8.856 9.253 9.025 8.863 2.496 3.087 4.356 1.39 0.872
   2477   1375879_at   8.44 8.508 8.541 8.552 8.596 9.322 9.561 9.247 9.131 9.321
   2478   1371803_at   9.655 9.54 9.651 9.535 9.587 8.483 8.451 8.935 8.634 8.467
   2479   1375908_at   7.543 7.612 7.702 7.632 7.351 8.577 8.513 8.784 8.466 8.362
   2480   1375944_at   10.713 10.664 10.869 10.736 10.16 11.963 11.965 12.073 11.844 11.999
   2481   1393661_at   6.345 6.329 6.751 6.601 6.255 7.545 7.682 7.71 7.471 7.481
   2482   1389113_at   5.993 6.255 6.456 6.388 5.902 7.769 7.739 8.297 7.638 7.859
   2483   1368354_at   9.609 9.438 9.705 9.512 9.624 10.065 10.045 10.097 10.123 10.12
   2484   1373611_at   8.65 8.607 8.798 8.72 8.864 7.364 7.712 7.743 7.242 7.246
   2485   1394612_at   6.637 6.681 6.796 6.703 6.377 7.526 7.585 7.477 7.534 7.796
   2486   1371146_at   8.608 8.509 8.56 8.443 8.576 7.009 6.883 6.053 6.696 6.906
   2487   1388294_at   11.129 11.126 11.27 11.121 11.104 11.648 11.707 11.624 11.651 11.503
   2488   1398325_at   10.065 10.038 9.999 10.147 10.203 10.554 10.589 10.537 10.473 10.511
   2489   1379479_at   6.488 6.508 6.111 6.254 6.133 3.438 4.233 4.486 3.399 3.8
   2490   1383305_at   7.214 7.245 7.231 7.467 7.113 8.161 8.288 8.061 7.939 8.193
   2491   1393152_at   8.309 8.297 8.453 8.486 8.235 9.396 9.481 9.067 9.282 9.181
   2492   1382029_at   6.821 6.893 6.779 6.931 6.788 6.433 6.391 6.249 6.428 6.348
   2493   1371932_at   8.802 8.676 8.685 8.633 8.94 8.022 8.061 8.12 8.005 8.174
   2494   1389263_at   8.531 8.699 8.471 8.7 8.588 7.764 7.684 7.54 7.814 7.942
   2495   1374892_at   8.088 8.224 8.406 8.38 7.977 9.92 9.851 9.384 9.813 9.431
   2496   1369177_at   7.261 7.205 7.564 7.23 7.282 6.458 6.446 6.078 6.298 6.261
   2497   1389789_at   8.14 8.182 8.058 8.322 8.331 6.674 7.223 6.996 7.183 6.827
   2498   1383597_at   5.953 5.982 6.199 5.814 6.001 4.785 4.341 4.582 5 4.561
   2499   1373970_at   9.388 9.325 9.176 9.305 8.91 10.122 10.145 10.242 10.215 10.365
   2500   1376852_at   8.833 8.888 9.093 9.167 8.601 10.225 10.199 10.607 10.197 10.165
   2501   1371323_at   11.945 11.871 11.97 11.911 11.846 12.328 12.31 12.257 12.427 12.444
   2502   1377686_at   7.048 7.033 7.113 6.633 6.919 4.902 5.02 5.311 5.44 4.522
   2503   1372721_at   9.798 9.876 9.921 9.916 10.092 9.258 9.351 9.059 9.186 9.187
   2504   1367998_at   11.895 11.793 11.786 11.624 12.012 5.657 5.159 7.926 4.544 5.706
   2505   1392971_at   7.588 7.782 7.62 7.985 8.079 8.967 8.931 8.865 8.77 8.921
   2506   1387023_at   4.996 5.42 5.395 5.192 5.776 6.743 6.706 6.801 6.879 7.006
   2507   1377573_at   8.947 9.001 9.145 9.21 8.946 9.92 9.96 9.688 9.844 9.743
   2508   1374833_at   5.273 5.799 5.528 5.695 5.263 7.275 7.22 7.203 6.689 7.1
   2509   1372205_at   6.813 6.821 6.679 6.859 6.826 7.449 7.562 7.438 7.28 7.329
   2510   1375221_at   8.616 8.615 8.596 8.739 8.424 9.296 9.453 9.186 9.298 9.254
   2511   1370377_at   9.079 9.083 9.077 9.102 8.212 10.799 10.726 11.044 10.793 10.777
   2512   1392530_at   10.402 10.244 10.339 10.397 10.466 10.833 10.851 10.969 10.908 10.797
   2513   1374703_at   7.735 7.778 7.484 8.024 7.924 8.788 9.031 9.116 8.827 8.976
   2514   1372553_at   7.491 7.36 7.423 7.332 7.37 8.135 7.909 8.072 8.006 7.863
   2515   1399059_at   8.31 8.387 8.285 8.523 8.297 9.143 9.158 9.45 9.106 9.094
   2516   1372292_at   7.978 7.912 8.183 8.121 7.954 8.941 9.015 8.738 8.942 8.708
   2517   1376275_at   7.877 7.958 7.749 8.04 7.845 7.037 6.676 7.026 7.091 6.835
   2518   1373823_at   9.351 9.428 9.299 9.328 9.316 7.592 7.536 6.922 6.688 7.644
   2519   1386960_at   9.156 9.036 9.496 9.394 8.761 11.085 10.946 10.506 10.999 10.907
   2520   1389646_at   7.311 7.081 7.346 7.255 7.352 6.465 6.413 6.347 6.651 6.286
   2521   1387034_at   11.127 11.141 11.323 11.141 10.987 12.292 12.361 11.911 12.413 12.419
   2522   1398295_at   5.763 5.631 5.89 5.535 5.431 7.59 6.809 7.283 7.448 7.219
   2523   1389911_at   8.851 8.875 8.895 9.066 9.229 7.816 7.854 8.189 7.79 7.786
   2524   1383794_at   5.771 6.024 6.054 6.101 6.073 4.471 4.583 4.291 3.721 4.433
   2525   1390543_at   7.792 7.784 8.188 8.204 7.402 9.673 9.875 9.584 9.442 9.509
   2526   1389786_at   5.686 5.383 5.776 5.606 5.109 7.377 7.564 7.681 6.897 7.415
   2527   1371027_at   7.477 7.548 7.794 7.923 7.764 6.168 6.419 6.61 6.108 6.002
   2528   1382660_at   7.118 7.239 6.918 7.313 7.201 8.117 8.39 8.209 7.959 8.187
   2529   1389852_at   6.32 6.895 6.338 6.464 6.389 3.724 3.963 3.557 4.773 4.114
   2530   1383313_at   7.637 7.768 8.009 7.755 7.607 6.969 6.931 6.847 7.044 6.9
   2531   1368437_at   8.323 8.519 8.294 8.67 8.583 9.27 9.391 9.437 9.601 9.533
   2532   1370942_at   7.445 7.523 7.514 7.373 7.776 6.461 6.446 6.479 6.8 6.51
   2533   1373436_at   6.359 6.344 6.463 6.548 6.317 7.539 7.365 7.654 7.41 7.136
   2534   1373991_at   9.964 9.99 10.389 10.215 9.746 11.339 11.51 11.158 11.444 11.422
   2535   1373217_at   6.462 6.311 6.223 6.331 6.583 7.538 7.668 7.5 7.181 7.393
   2536   1382331_at   9.597 9.345 9.37 9.671 9.352 10.479 10.449 10.306 10.421 10.197
   2537   1382496_at   7.394 7.347 7.883 7.626 7.307 9.162 8.71 8.882 9.23 9.044
   2538   1377727_at   7.216 7.164 7.436 7.383 7.942 5.048 5.719 5.427 5.723 5.221
   2539   1376136_at   6.654 6.39 6.74 6.713 6.534 7.809 7.451 7.507 7.733 7.511
   2540   1368126_at   6.656 6.808 6.756 6.795 6.926 5.911 5.639 5.738 5.294 5.657
   2541   1373027_at   8.627 8.686 8.782 8.974 8.71 9.843 10 9.637 9.569 9.769
   2542   1376623_at   6.786 6.432 6.45 6.644 6.464 8.322 8.306 7.621 8.347 8.315
   2543   1398892_at   11.009 11.09 11.051 11.092 11.113 10.63 10.665 10.69 10.561 10.461
   2544   1371346_at   10.844 10.723 10.916 10.636 10.742 11.442 11.351 11.272 11.433 11.364
   2545   1388121_at   9.945 9.616 9.72 9.805 9.78 10.424 10.47 10.612 10.392 10.593
   2546   1382211_at   7.931 7.8 8.054 8.205 7.81 9.321 9.65 9.609 9.024 9.319
   2547   1399130_at   8.567 8.473 8.332 8.496 8.585 9.17 9.255 9.289 9.194 9.005
   2548   1367790_at   9.721 9.634 9.764 9.844 9.865 8.736 8.835 8.998 8.506 8.895
   2549   1379283_at   6.752 6.978 6.803 6.849 6.854 7.393 7.522 7.411 7.29 7.371
   2550   1368077_at   11.819 11.746 11.735 11.634 11.388 12.64 12.555 12.497 12.804 12.763
   2551   1367507_at   8.624 8.464 8.454 8.42 8.331 7.766 7.436 7.662 7.543 7.363
   2552   1371300_at   12.801 12.669 12.558 12.522 12.693 12.112 12.142 12.119 12.107 12.047
   2553   1388703_at   8.269 8.141 8.037 8.272 8.097 8.932 8.821 9.138 8.94 8.826
   2554   1370554_at   9.534 9.529 9.328 9.324 9.521 8.865 8.861 8.776 8.777 8.642
   2555   1383191_at   7.209 7.187 7.209 6.845 6.581 9.118 9.268 9.747 8.867 8.838
   2556   1398599_at   7.393 7.319 7.534 7.344 7.404 6.883 6.874 7.022 6.857 6.897
   2557   1391201_at   5.902 5.512 5.528 5.578 5.664 1.428 2.905 1.144 0.506 1.111
   2558   1390300_at   4.195 4.375 4.288 4.61 4.759 5.913 6.055 5.688 5.633 5.719
   2559   1379365_at   13.175 13.021 12.632 12.626 12.892 8.438 8.324 10.285 8.295 8.78
   2560   1374034_at   7.811 7.75 7.832 7.998 7.857 6.987 7.165 7.042 6.767 7.117
   2561   1368851_at   9.166 9.374 9.287 9.313 8.95 8.309 8.339 8.007 8.162 8.273
   2562   1380148_at   7.77 7.633 7.701 7.509 7.861 6.216 6.256 6.544 6.783 6.287
   2563   1384988_at   6.576 6.363 6.308 6.135 6.357 4.76 4.529 4.569 3.87 3.92
   2564   1391463_at   9.232 9.282 9.313 9.46 9.509 7.96 8.234 8.522 7.996 8.195
   2565   1382776_at   7.899 7.976 7.898 8.045 8.353 7.043 6.983 7.024 6.795 6.697
   2566   1367634_at   12.732 12.676 12.639 12.53 12.639 12.285 12.231 12.276 12.226 12.165
   2567   1371818_at   9.404 9.45 9.495 9.512 9.215 10.127 10.399 10.19 10.089 10.185
   2568   1378470_at   8.694 8.697 8.971 8.938 8.448 10.225 10.276 10.367 9.985 9.858
   2569   1386966_a_at   8.509 8.301 8.452 8.231 8.361 7.472 7.687 7.738 7.568 7.716
   2570   1390638_at   5.244 5.392 5.172 5.766 5.362 6.97 7.114 6.673 6.959 6.59
   2571   1382763_at   6.919 6.91 7.025 7.16 6.899 7.467 7.555 7.553 7.591 7.533
   2572   1372267_at   9.443 9.386 9.485 9.414 9.411 8.874 8.981 8.95 8.83 8.687
   2573   1387070_at   6.162 6.206 6.24 6.473 6.423 5.297 4.834 5.017 4.779 5.254
   2574   1382995_at   7.995 7.856 7.745 8.068 8.194 6.224 5.454 6.416 5.908 5.591
   2575   1374709_at   5.448 5.748 5.834 5.736 5.344 7.182 7.735 7.709 7.093 7.179
   2576   1388364_at   10.614 10.553 10.679 10.501 10.459 11.257 11.287 11.29 11.299 11.027
   2577   1394371_at   7.198 7.114 7.194 7.327 7.005 6.577 6.365 6.275 6.453 6.402
   2578   1374074_at   6.945 7.022 6.988 7.053 6.874 7.59 7.724 7.829 7.496 7.617
   2579   1370714_a_at   7.932 7.836 8.053 7.944 7.312 9.787 9.223 9.44 9.611 9.39
   2580   1398535_at   6.282 6.469 6.586 6.602 6.577 7.28 7.347 7.584 7.265 7.33
   2581   1378181_at   7.955 8.167 7.955 8.13 8.258 7.208 6.91 6.632 6.812 6.644
   2582   1368209_at   10.485 10.208 10.564 10.526 10.426 11.269 11.343 11.074 11.222 11.268
   2583   1374244_at   6.947 6.977 7.277 7.268 5.91 9.496 9.633 9.412 9.432 9.499
   2584   1376601_at   5.78 5.897 5.716 5.74 5.556 7.005 6.764 7.022 6.77 6.538
   2585   1376595_at   7.221 7.294 7.228 7.157 7.141 7.801 8.145 8.042 7.874 7.839
   2586   1390378_at   6.623 6.424 6.51 6.63 6.295 7.419 7.777 7.47 7.346 7.47
   2587   1385585_at   6.878 6.721 6.608 6.621 6.282 8.065 8.369 8.563 8.036 7.936
   2588   1398856_at   11.305 11.199 11.194 11.191 11.288 10.618 10.38 10.512 10.475 10.18
   2589   1379044_at   5.795 5.525 5.79 6.305 5.794 7.17 7.229 7.229 7.109 7.125
   2590   1393935_at   8.152 8.02 8.234 8.12 7.939 9.181 9.415 9.494 9.038 9.012
   2591   1392573_at   8.893 8.834 8.906 8.765 8.821 8.249 8.461 8.32 8.423 8.402
   2592   1379847_at   5.46 5.475 5.752 5.629 5.612 6.661 6.817 6.661 6.311 6.575
   2593   1371786_at   9.633 9.606 9.62 9.701 9.572 10.808 10.748 10.342 10.482 10.465
   2594   1389780_at   5.562 5.383 5.391 5.411 5.484 6.254 6.377 6.057 6.497 6.446
   2595   1381445_at   6.751 6.715 6.779 6.954 6.975 7.876 8.175 8.196 7.763 7.779
   2596   1373377_at   7.224 6.725 7.414 7.285 7.037 8.701 8.815 8.516 8.465 8.424
   2597   1368098_a_at   7.223 7.279 7.359 7.39 7.329 7.811 7.972 8.181 8.002 7.99
   2598   1369026_at   7.179 7.195 7.198 7.154 7.266 6.786 6.766 6.59 6.54 6.621
   2599   1368138_at   7.031 7.283 7.376 7.597 7.085 8.593 9.104 8.664 8.677 9.083
   2600   1380030_at   6.526 6.812 6.801 6.511 6.925 5.551 5.813 5.821 5.64 5.59
   2601   1368164_at   8.442 8.41 8.641 8.77 8.468 9.65 9.704 10.076 9.728 9.513
   2602   1383499_at   5.326 5.373 5.508 5.78 5.535 6.388 6.391 6.358 6.272 6.335
   2603   1397744_at   4.348 5.363 5.061 4.599 4.959 6.901 6.943 7.115 6.861 6.597
   2604   1374101_at   7.974 8.089 8.378 8.319 8.139 8.911 8.984 9.11 9.088 9.045
   2605   1372920_at   6.942 7.045 7.095 6.807 6.605 8.808 8.511 8.835 8.463 8.1
   2606   1368064_a_at   8.209 8.26 8.259 8.35 8.086 10.367 10.344 9.433 10.399 10.219
   2607   1377654_at   7.681 7.742 7.571 7.789 7.731 8.782 8.597 8.998 8.456 8.718
   2608   1383472_at   5.237 5.183 4.515 4.795 4.636 7.02 6.622 7.517 6.876 6.906
   2609   1382928_at   6.944 6.945 7.448 7.479 7.062 8.572 8.692 8.579 8.693 8.332
   2610   1378640_at   5.829 5.986 5.809 6.311 6.443 3.664 4.003 3.949 2.866 3.421
   2611   1388744_at   8.997 8.942 8.967 9.067 9.145 8.053 7.646 8.094 7.549 7.652
   2612   1374563_at   5.72 6.18 6.4 6.508 6.26 4.769 4.599 4.722 4.307 4.598
   2613   1370332_at   4.633 5.224 5.035 4.307 4.395 1.727 1.417 0.526 1.651 0.251
   2614   1398516_at   6.173 6.327 6.397 6.135 5.845 7.277 7.669 7.416 7.443 7.256
   2615   1375432_at   7.856 8.139 7.89 7.9 8.067 7.4 7.372 7.394 7.284 7.304
   2616   1389191_at   7.738 7.846 7.838 8.086 8.091 7.057 7.075 6.96 7.205 7.117
   2617   1397500_x_at   5.993 6.361 6.312 6.187 6.037 6.995 7.215 7.008 7.175 6.984
   2618   1372430_at   8.336 8.089 8.29 8.039 8.193 6.77 7.118 7.18 6.785 7.234
   2619   1390883_at   6.754 7.029 6.673 6.992 6.974 7.827 7.874 7.936 7.607 7.912
   2620   1374440_at   9.963 9.881 9.97 9.97 9.752 10.519 10.434 10.61 10.391 10.559
   2621   1388634_at   8.572 8.74 8.667 8.748 8.612 9.263 9.447 9.631 9.31 9.404
   2622   1376483_at   8.432 8.578 8.523 8.555 8.817 7.494 7.793 7.394 7.383 7.276
   2623   1372255_at   10.42 10.252 10.031 10.405 10.521 8.562 8.971 9.22 8.708 8.664
   2624   1376597_at   7.135 7.283 7.212 7.309 7.472 6.433 6.635 6.392 6.438 6.621
   2625   1373253_at   7.937 7.622 7.848 8.032 7.807 9.258 9.477 9.625 9.196 8.904
   2626   1389904_at   11.661 11.583 11.617 11.392 11.473 10.701 10.968 10.709 10.643 10.825
   2627   1382611_at   6.426 7.049 6.777 6.777 5.85 9.117 8.71 8.855 8.742 8.708
   2628   1384743_at   8.563 8.559 8.702 8.728 8.806 9.739 10.118 9.68 9.536 9.865
   2629   1393297_at   6.398 6.771 6.76 6.762 6.591 2.592 4.07 2.094 3.265 3.424
   2630   1369407_at   6.373 6.421 6.249 6.005 6.201 8.176 8.242 8.72 8.038 7.647
   2631   1390219_at   6.096 5.989 6.013 6.055 5.633 6.881 7.105 6.921 6.926 7.191
   2632   1368109_at   8.148 7.934 8.055 8.092 7.986 8.725 8.92 8.818 8.561 8.821
   2633   1367765_at   9.623 9.716 9.801 9.682 9.691 10.931 11.104 10.449 10.941 10.965
   2634   1373149_at   7.586 7.704 7.712 7.975 7.904 8.936 8.682 9.079 8.835 8.678
   2635   1390163_at   6.999 6.775 6.83 6.89 6.993 5.799 5.938 5.919 6.254 5.808
   2636   1374670_at   8.803 8.729 8.439 8.683 8.889 6.071 6.377 6.781 7.015 7.023
   2637   1387971_a_at   7.272 7.279 7.745 7.562 7.571 8.483 8.514 8.641 8.598 8.387
   2638   1380772_at   6.239 6.208 6.201 6.547 6.098 4.916 4.354 4.709 5.048 4.434
   2639   1380108_at   6.097 5.756 6.187 6.138 6.015 7.17 6.871 7.041 7.198 6.943
   2640   1368103_at   9.064 9.095 9.022 9.464 9.212 7.335 7.657 8.029 7.663 7.3
   2641   1388400_at   8.935 8.944 9.033 9.199 8.853 8.31 8.205 8.409 8.23 8.206
   2642   1383085_at   9.106 9.149 9.135 8.953 9.252 8.387 8.447 8.161 8.457 8.226
   2643   1384970_at   4.125 4.409 4.821 4.671 4.678 5.968 5.95 6.219 6.184 5.803
   2644   1393906_at   6.933 6.675 6.925 7.06 6.559 8.292 7.949 7.883 8.229 8.007
   2645   1379542_at   9.821 9.834 10.088 10.406 10.298 8.653 8.538 8.884 8.448 8.371
   2646   1389297_at   7.931 8.051 8.05 8.255 7.996 6.848 7.208 7.106 6.678 6.867
   2647   1368355_at   7.682 7.649 7.822 7.998 7.788 8.689 8.962 8.882 8.564 8.682
   2648   1368233_at   7.614 7.794 7.703 7.747 7.882 6.864 6.899 6.862 6.477 6.549
   2649   1375997_at   7.724 7.47 7.96 7.867 7.144 9.324 9.192 9.048 9.36 9.379
   2650   1391547_at   4.931 5.009 5.167 4.963 5.369 6.817 6.993 7.424 6.558 6.604
   2651   1371847_at   9.368 9.384 9.464 9.534 9.453 10.596 10.838 10.191 10.72 10.596
   2652   1371579_at   10.698 10.679 10.54 10.701 10.676 10.235 10.226 10.113 10.039 10.047
   2653   1395519_at   5.555 6.206 6.186 5.953 5.809 7.758 7.653 7.97 7.68 7.245
   2654   1371417_at   11.58 11.607 11.651 11.389 11.553 11.989 12.082 12.103 12.057 12.035
   2655   1381350_at   5.942 6.138 5.935 6.384 5.54 7.513 7.728 7.755 7.489 7.381
   2656   1382013_at   8.138 8.258 8.246 8.018 8.046 9.105 9.08 8.749 9.007 9.149
   2657   1390024_at   11.681 11.707 11.811 11.498 11.652 10.838 10.721 10.455 10.901 10.594
   2658   1395274_at   7.048 6.966 6.979 7.203 6.898 7.831 8.081 7.756 7.898 7.714
   2659   1378168_at   6.064 6.518 6.038 6.643 6.397 8.327 8.035 7.835 8.277 7.73
   2660   1382748_at   8.533 8.497 8.519 8.53 8.536 9.436 9.681 9.225 9.227 9.339
   2661   1372652_at   6.455 6.428 6.315 6.575 6.568 7.463 7.73 7.622 7.39 7.225
   2662   1377327_at   7.309 7.554 7.623 7.344 7.471 6.389 6.088 6.617 6.359 6.488
   2663   1373170_at   7.191 7.525 7.286 7.431 7.323 8.138 8.275 8.472 8.118 8.169
   2664   1389678_at   6.882 6.867 7.043 7.226 6.528 8.463 8.462 8.794 8.208 8.31
   2665   1376211_a_at   8.137 8.036 8.317 8.242 7.968 9.001 9.015 8.796 8.869 8.881
   2666   1376687_at   8.724 8.759 8.722 8.543 8.639 7.636 8.026 7.879 7.587 7.756
   2667   1388323_at   10.778 10.721 10.806 10.714 10.75 11.111 11.118 11.153 11.072 10.987
   2668   1377752_at   5.854 6.108 5.927 6.212 6.405 4.302 4.787 4.614 4.134 4.689
   2669   1390776_at   7.902 8.049 8.02 8.276 8.31 9.247 9.147 9.62 9.228 9.18
   2670   1367773_at   8.978 8.844 8.717 9.08 8.964 9.624 9.632 9.656 9.518 9.56
   2671   1398987_at   9.235 9.154 9.176 9.256 9.244 8.831 8.796 8.895 8.715 8.693
   2672   1372107_at   8.424 8.302 8.275 8.346 8.582 9.341 9.255 9.497 9.065 9.271
   2673   1388511_at   7.761 7.813 7.753 7.989 8.106 7.045 6.904 7.048 6.702 6.799
   2674   1394789_at   5.351 5.619 5.615 5.909 5.611 6.525 6.729 6.721 6.515 6.688
   2675   1376130_a_at   8.389 8.295 8.394 8.432 8.325 6.93 7.159 7.544 6.96 6.857
   2676   1371170_a_at   6.017 5.41 5.12 5.197 5.621 3.786 3.904 3.579 3.609 3.777
   2677   1392541_at   9.933 9.943 10.2 10.296 9.765 11.07 11.12 11.104 10.939 11.039
   2678   1368132_at   9.601 9.613 9.59 9.522 9.444 10.487 10.562 10.903 10.452 10.356
   2679   1391571_at   5.182 5.064 5.306 5.47 5.18 5.983 6.131 6.078 5.918 6.041
   2680   1372777_at   9.143 9.128 9.207 9.308 9.127 9.774 9.761 9.562 9.768 9.775
   2681   1393647_at   9.725 9.761 9.87 10.037 9.917 8.942 8.991 9.156 8.822 8.724
   2682   1368378_at   9.15 9.229 9.348 9.457 9.282 10.138 10.534 10.502 10.212 10.16
   2683   1386933_at   7.473 7.3 6.559 6.462 5.888 9.973 9.728 10.122 9.959 9.509
   2684   1383067_a_at   8.78 8.791 8.742 8.836 8.997 7.914 8.2 8.124 7.905 7.841
   2685   1386891_at   11.508 11.504 11.562 11.466 11.502 11.945 11.886 12.038 12.066 11.851
   2686   1373094_at   8.174 7.942 7.889 8.125 8.052 7.331 7.231 6.985 6.984 6.967
   2687   1390412_at   7.625 7.406 7.83 7.644 7.67 8.745 8.769 8.447 8.598 8.922
   2688   1392922_at   8.103 8.264 8.363 8.267 8.179 6.468 6.464 7.021 7.137 6.62
   2689   1371372_at   11.389 11.392 11.368 11.31 11.416 11.06 11.078 10.908 10.953 11.021
   2690   1387303_at   9.776 9.609 10.113 10.199 9.068 11.844 11.801 11.948 11.708 11.83
   2691   1370934_at   9.302 9.373 9.274 9.224 9.363 8.224 8.588 8.081 8.184 8.476
   2692   1386912_at   9.598 9.47 9.219 9.349 9.963 8.106 7.956 8.223 7.736 7.946
   2693   1374794_at   6.649 6.734 6.513 6.814 6.443 4.733 3.297 4.285 4.326 3.943
   2694   1379770_at   9.778 9.841 10.077 9.958 9.475 11.017 10.956 10.801 11 10.869
   2695   1389325_at   10.4 10.335 10.358 10.237 10.435 9.745 9.848 9.726 9.553 9.56
   2696   1380116_at   8.648 8.698 8.747 8.642 8.477 8.031 7.998 7.764 7.878 7.729
   2697   1391274_at   6.371 6.468 6.66 6.585 6.801 7.649 7.67 7.685 7.618 7.323
   2698   1388479_at   7.665 7.789 7.322 7.607 8.131 6.266 6.213 6.229 5.928 5.814
   2699   1370057_at   10.276 10.291 10.2 10.426 10.571 9.271 9.281 9.512 8.984 9.327
   2700   1389496_at   8.071 8.182 8.397 8.483 8.413 7.134 7.138 7.316 7.313 6.85
   2701   1380021_at   8.525 8.407 8.534 8.685 8.614 6.886 7.333 7.41 6.987 6.648
   2702   1385690_at   8.11 8.03 8.142 8.055 7.81 9.216 9.248 8.834 8.969 8.927
   2703   1377634_at   8.402 8.21 8.333 8.442 8.495 7.073 6.804 6.175 6.904 6.471
   2704   1399035_at   7.863 7.847 7.983 8.1 7.894 6.692 6.812 7.123 7.135 6.976
   2705   1373138_at   7.569 7.419 7.67 7.667 7.943 6.686 6.744 6.67 6.617 6.417
   2706   1375696_at   7.428 7.774 7.75 7.486 7.377 6.289 5.949 5.579 6.15 6.214
   2707   1390914_at   7.634 7.522 7.78 7.862 7.781 6.702 6.645 6.416 6.852 6.339
   2708   1392952_at   7.265 7.375 7.578 7.726 7.158 8.879 8.806 8.475 8.967 8.69
   2709   1373903_at   7.6 7.621 7.686 7.638 7.645 6.712 6.77 6.941 6.691 7.101
   2710   1384529_at   4.725 5.11 5.107 5.359 5.019 6.432 6.76 6.211 6.585 6.801
   2711   1368432_a_at   4.711 5.093 5.048 5.674 4.469 7.405 7.439 6.968 7.164 7.572
   2712   1374190_at   7.978 7.755 7.838 7.715 7.929 8.759 8.746 8.411 8.728 8.662
   2713   1397953_at   7.533 7.709 7.255 7.623 7.739 5.679 5.548 6.209 5.265 5.219
   2714   1387780_at   10.425 10.356 10.323 10.326 10.428 10.032 10.107 10.029 9.995 9.949
   2715   1372030_at   8.745 8.689 8.961 8.814 8.806 9.566 9.58 9.446 9.635 9.334
   2716   1368858_at   4.703 4.918 5.259 4.642 4.58 6.642 6.724 6.573 6.283 6.152
   2717   1369323_at   8.044 8.253 8.232 8.366 8.375 7.506 7.33 7.201 7.486 7.175
   2718   1368896_at   6.575 6.751 6.88 6.639 6.458 8.017 8.269 8.295 7.683 7.883
   2719   1390857_at   8.976 8.979 9.194 9.51 9.211 10.512 10.606 10.528 10.26 10.218
   2720   1370007_at   9.595 9.76 9.524 9.894 10.002 8.4 8.468 8.678 8.274 8.738
   2721   1392308_at   8.264 8.312 8.686 8.542 7.703 0.614 3.645 2.583 0.164 1.305
   2722   1397610_at   4.171 3.645 3.879 4.159 4.34 5.44 5.712 5.669 5.688 5.259
   2723   1370253_at   12.142 12.082 12.087 12.065 12.113 11.549 11.554 11.773 11.591 11.483
   2724   1397652_at   6.836 6.987 6.906 7.002 7.089 7.81 8.314 8.275 7.876 8.06
   2725   1393706_at   8.785 8.861 8.291 8.793 8.868 6.988 7.078 6.335 7.174 6.888
   2726   1375848_at   9.773 9.821 9.73 9.739 9.91 9.343 9.376 9.211 9.296 9.401
   2727   1393947_at   8.372 8.271 8.469 8.544 8.026 9.625 9.917 9.37 9.933 9.701
   2728   1381396_s_at   7.13 7 7.564 7.289 6.625 8.994 8.711 8.65 8.752 8.904
   2729   1374821_at   4.577 5.012 4.722 4.73 5.157 6.631 6.386 6.345 5.983 6.28
   2730   1395411_at   8.636 8.641 8.839 8.859 8.646 9.857 9.822 9.446 10.013 9.788
   2731   1379340_at   7.019 6.991 6.764 7.018 6.918 4.963 4.819 5.519 4.435 5.291
   2732   1376377_at   5.88 5.655 5.202 5.635 5.651 4.104 3.053 3.453 3.632 3.671
   2733   1368659_at   11.306 11.258 11.185 11.271 11.285 11.795 11.767 11.803 11.998 12.052
   2734   1393401_at   7.932 8.005 7.894 8.101 8.115 6.458 6.572 6.964 6.259 6.883
   2735   1386975_at   8.494 8.35 8.607 8.616 8.32 9.979 9.666 9.447 10.096 10.001
   2736   1379286_at   5.716 5.186 5.227 5.202 5.531 7.128 7.336 6.778 6.698 6.863
   2737   1379679_at   6.009 5.994 5.913 6.165 5.695 7.693 7.367 7.269 7.836 7.145
   2738   1370929_at   8.757 8.712 9.216 9.251 8.644 10.436 10.578 10.342 10.317 10.186
   2739   1398572_at   8.667 8.676 8.671 8.765 9.001 8.002 8.01 7.753 7.733 7.735
   2740   1370302_at   7.254 7.087 7.434 7.499 7.394 8.257 8.278 8.399 8.088 8.444
   2741   1375289_at   8.986 8.959 9.111 9.177 8.984 9.666 9.853 9.851 9.6 9.65
   2742   1386581_at   7.226 7.15 7.271 7.316 7.292 6.862 6.655 6.842 6.71 6.749
   2743   1393148_at   8.403 8.476 8.802 8.821 8.462 9.479 9.68 9.6 9.488 9.643
   2744   1373544_at   12.534 12.475 12.331 12.113 12.431 5.259 4.486 8.322 5.462 5.73
   2745   1373224_at   8.6 8.443 8.348 8.487 8.726 2.223 3.864 4.697 4.668 2.998
   2746   1373506_at   7.931 8.007 7.965 8.071 7.914 8.677 8.626 8.791 8.457 8.533
   2747   1372021_at   7.556 7.523 7.353 7.467 7.51 6.916 6.81 6.661 6.743 6.522
   2748   1383601_at   7.376 7.578 7.482 7.511 7.479 1.438 3.692 3.542 3.843 2.95
   2749   1370821_at   9.781 9.911 9.997 10.216 9.371 11.329 11.185 11.46 11.392 11.268
   2750   1390579_at   7.287 7.257 7.172 7.492 7.45 5.483 4.734 5.756 5.212 5.701
   2751   1381080_at   6.468 6.516 7.193 6.72 5.874 9.425 9.757 8.664 9.355 9.355
   2752   1395561_at   10.444 10.37 10.587 10.643 10.643 9.913 9.633 9.529 9.619 9.523
   2753   1393373_at   7.788 7.852 7.716 7.894 8.099 6.231 6.207 5.854 5.449 5.242
   2754   1371328_at   10.126 10.21 10.246 10.277 10.24 10.784 11.016 10.707 10.79 10.754
   2755   1398775_at   12.602 12.495 12.361 12.223 12.399 11.791 11.785 11.643 11.696 11.711
   2756   1368291_at   8.73 8.797 8.869 8.948 9.04 7.885 7.53 7.72 7.775 7.24
   2757   1378169_at   7.033 6.954 6.983 6.714 7.112 7.924 8.043 7.681 7.999 7.81
   2758   1382682_at   6.997 7.295 6.927 7.226 7.338 6.16 6.015 6.054 5.652 6.012
   2759   1393247_at   5.633 5.772 6.031 5.819 6.046 4.888 4.885 4.928 4.559 4.643
   2760   1372780_at   7.039 7.048 7.179 7.065 6.983 7.717 7.766 8.063 7.839 7.675
   2761   1372302_at   9.309 9.253 9.362 9.269 9.363 8.524 8.634 8.517 8.145 8.343
   2762   1373406_at   7.832 7.972 7.718 7.843 8.014 7.145 7.148 7.236 6.944 6.937
   2763   1379832_at   9.901 9.973 9.809 9.762 9.682 9.31 9.242 9.142 9.25 9.129
   2764   1372531_at   7.479 7.419 7.485 7.444 7.306 8.257 8.195 7.937 8.025 8.039
   2765   1390144_at   7.854 7.677 7.567 7.281 7.551 6.204 6.069 6.586 6.245 6.416
   2766   1374630_at   5.924 6.167 6.118 6.133 5.168 7.953 7.821 7.998 7.903 7.646
   2767   1372768_at   8.06 8.007 8.029 8.196 8.224 7.652 7.626 7.661 7.594 7.547
   2768   1398826_s_at   8.347 8.465 8.428 8.589 8.588 9.28 9.376 9.13 9.168 9.065
   2769   1390863_at   7.573 7.709 7.698 7.591 7.409 8.601 8.506 8.808 8.463 8.336
   2770   1372959_at   6.123 5.92 6.265 6.12 5.481 7.903 7.906 7.367 8.157 7.891
   2771   1371018_at   5.3 5.688 6.117 5.397 5.231 7.447 7.471 7.083 7.239 7.28
   2772   1371903_at   7.01 7.179 7.448 7.182 7.1 8.18 8.095 7.932 8.078 7.944
   2773   1389919_at   6.648 6.514 6.485 6.725 6.431 5.888 5.58 5.834 5.868 5.732
   2774   1378092_at   7.784 7.786 7.79 7.881 8.035 6.875 7.162 7.066 6.889 6.712
   2775   1389036_at   8.518 8.607 8.467 8.405 8.562 7.933 7.88 8.064 7.806 7.769
   2776   1367774_at   10.736 10.687 10.904 10.768 10.698 11.956 11.923 11.526 12.124 12.159
   2777   1378049_at   8.16 8.336 8.574 8.578 8.407 9.594 9.617 9.213 9.422 9.516
   2778   1375026_at   9.862 9.825 9.95 10.06 10.099 10.977 11.057 10.676 11.187 10.891
   2779   1368427_at   7.424 7.483 7.364 7.596 7.468 8.355 8.293 8.44 8.066 8.125
   2780   1390518_at   4.365 4.401 3.48 4.288 3.839 5.927 6.155 5.866 5.856 6.228
   2781   1371736_at   9.849 9.769 9.803 9.593 9.784 10.4 10.559 10.495 10.316 10.303
   2782   1378507_at   6.398 6.311 5.935 6.397 6.412 3.048 3.543 4.439 3.539 4.101
   2783   1388717_at   7.607 7.438 7.553 7.556 7.659 7.165 6.975 7.101 6.99 6.995
   2784   1398789_at   12.367 12.259 12.27 12.149 12.284 11.929 11.805 11.769 11.812 11.773
   2785   1376732_at   4.779 5.015 5.206 4.702 4.913 6.319 6.146 6.378 5.919 6.01
   2786   1388909_at   7.791 7.915 7.705 7.941 7.547 8.726 8.969 9.127 8.717 9.138
   2787   1387429_at   7.492 7.629 7.932 8.16 7.924 6.491 6.649 6.496 6.199 6.377
   2788   1376804_at   8.809 8.691 8.594 9.02 8.984 9.738 9.874 9.661 9.656 9.685
   2789   1367579_a_at   12.911 12.721 12.701 12.497 12.593 11.962 11.97 12.041 11.986 11.972
   2790   1367670_at   10.782 10.759 10.746 10.905 10.829 11.169 11.263 11.2 11.205 11.104
   2791   1373847_at   9.282 9.241 9.472 9.603 9.545 8.462 8.505 8.327 8.355 8.021
   2792   1386938_at   10.523 10.45 10.841 10.898 10.42 11.599 11.845 11.85 11.667 11.716
   2793   1382370_at   6.696 7.282 7.238 7.173 6.902 5.009 4.702 5.139 4.248 3.978
   2794   1367824_at   9.533 9.548 9.406 9.59 9.725 8.958 8.945 9.06 8.91 8.838
   2795   1378140_at   6.52 6.491 7.1 6.364 6.64 3.756 4.55 3.988 3.736 4.79
   2796   1393794_at   7.4 7.313 7.243 7.71 7.653 8.787 8.828 9.462 9.398 9.301
   2797   1391050_at   7.195 7.026 7.311 7.437 7.458 6.126 6.489 6.316 6.148 6.202
   2798   1393049_at   8.241 8.388 8.641 8.507 8.006 4.573 5.503 5.837 5.249 6.172
   2799   1368762_at   12.733 12.623 12.216 12.235 12.365 4.693 4.732 8.304 5.119 4.943
   2800   1383845_at   7.276 7.358 7.301 7.339 7.173 8.018 7.96 7.769 7.8 8.026
   2801   1388956_at   6.919 6.827 6.814 7.03 7.026 6.172 5.807 5.849 6.15 6.124
   2802   1397441_at   7.879 7.85 7.618 8.07 7.88 7.076 6.711 6.738 6.57 6.796
   2803   1367622_at   11.838 11.776 11.76 11.694 11.828 12.101 12.1 12.049 12.166 12.076
   2804   1368669_at   8.857 9.049 9.279 9.338 9.232 7.85 7.632 8.19 7.715 7.933
   2805   1387791_at   5.892 5.854 5.802 6.013 5.686 6.559 6.555 6.741 6.464 6.489
   2806   1370280_at   10.647 10.682 10.823 10.757 10.79 9.959 10.071 9.72 10.023 10.121
   2807   1367629_at   12.185 12.116 12.219 12.073 12.115 12.481 12.436 12.479 12.541 12.422
   2808   1374727_at   6.125 6.353 6.238 6.071 6.174 6.94 7.173 6.975 6.815 6.879
   2809   1388851_at   10.431 10.516 10.37 10.63 10.415 11.035 11.016 10.972 11.078 10.912
   2810   1373792_at   8.018 8.072 7.992 8.216 8.143 8.764 8.801 8.952 8.594 8.845
   2811   1387328_at   7.412 7.172 6.958 6.339 5.143 10.615 10.72 11.111 10.883 10.438
   2812   1387078_at   8.122 8.102 8.1 7.921 7.835 7.321 7.108 7.129 7.195 6.898
   2813   1395944_at   7.044 6.599 7.056 6.927 6.923 5.925 5.795 5.973 6.07 5.728
   2814   1379264_at   9.443 9.287 9.331 9.618 9.239 10.084 10.164 10.189 10.011 10.135
   2815   1368661_at   7.722 7.756 8.246 7.985 7.137 9.863 9.987 9.956 9.774 9.452
   2816   1372395_at   9.132 9.144 9.332 9.293 9.169 9.953 10.351 9.951 9.977 10.133
   2817   1388579_at   5.891 6.236 6.127 5.641 5.581 7.65 7.545 7.895 7.597 7.195
   2818   1368927_at   7.624 7.664 7.747 7.968 7.726 6.121 6.238 6.647 5.762 6.325
   2819   1393743_at   8.025 7.956 7.926 8.065 8.054 8.752 9.193 8.992 8.703 9.008
   2820   1388178_at   7.35 6.966 7.201 7.44 7.316 6.285 6.285 6.325 6.421 6.073
   2821   1368032_at   8.41 8.484 8.359 8.604 8.763 7.609 7.714 7.449 7.486 7.278
   2822   1370505_at   7.784 7.858 7.89 7.79 7.955 6.905 7.024 7.276 7.095 6.823
   2823   1369816_at   5.927 6.056 5.721 5.438 5.742 6.781 6.795 6.838 6.803 6.823
   2824   1386868_at   12.041 11.947 11.874 11.779 11.966 11.494 11.43 11.373 11.35 11.325
   2825   1398897_at   10.824 10.734 10.729 10.908 10.947 10.003 10.315 10.188 10.045 10.075
   2826   1389223_at   4.146 4.168 4.667 4.645 4.312 6.047 6.136 5.645 5.647 5.879
   2827   1387270_at   7.035 6.79 7.154 7.309 7.374 4.904 4.128 5.17 5.302 4.951
   2828   1393508_at   8.035 7.934 8.152 7.608 6.923 10.833 10.956 9.901 10.835 11.098
   2829   1368651_at   6.857 6.604 7.385 7.444 6.911 8.849 9.006 9.152 8.808 8.582
   2830   1369522_a_at   5.104 5.699 6.074 6.441 5.752 2.506 3.167 2.93 3.553 3.189
   2831   1388668_at   7.509 7.493 7.523 7.609 7.662 6.773 6.765 7.071 6.687 6.88
   2832   1392338_at   3.44 1.531 1.687 1.996 1.709 5.905 5.505 5.919 5.323 5.719
   2833   1393041_at   7.101 7.545 7.467 7.454 7.386 5.46 6.266 5.418 5.389 5.53
   2834   1383315_at   5.563 5.593 6.446 6.238 5.606 8.573 8.704 7.841 8.081 8.24
   2835   1371122_at   7.517 7.613 7.582 7.562 7.522 6.002 6.112 5.507 6.383 5.555
   2836   1393017_at   6.639 6.755 7.42 7.492 6.987 8.782 8.717 8.87 8.698 8.752
   2837   1373421_at   9.811 9.909 9.522 9.875 9.883 8.422 8.208 8.679 7.85 8.084
   2838   1382378_at   6.7 6.887 7.221 6.887 6.927 2.433 3.959 2.345 1.7 1.227
   2839   1373134_at   8.5 8.391 8.441 8.61 8.461 9.378 9.454 9.419 9.346 9.02
   2840   1384295_at   8.538 8.459 8.388 8.464 8.808 7.584 7.707 7.717 7.497 7.373
   2841   1371785_at   9.081 9.096 9.209 9.533 9.244 7.577 7.501 8.06 7.283 7.886
   2842   1388940_at   6.495 6.604 6.746 7.112 6.912 2.86 4.315 4.341 3.178 3.708
   2843   1373894_at   7.938 8.036 8.279 8.301 8.418 7.28 7.269 7.281 7.384 7.231
   2844   1375269_at   9.797 9.788 9.779 9.935 9.979 10.328 10.434 10.306 10.303 10.304
   2845   1384939_at   5.988 6.271 6.287 6.3 6.483 4.083 2.254 3.172 3.542 2.993
   2846   1384303_s_at   5.2 4.635 4.999 5.246 5.193 6.664 6.795 6.73 6.247 6.387
   2847   1398369_at   8.94 8.955 9.025 9.232 9.069 8.311 8.39 8.478 8.221 8.432
   2848   1388850_at   11.912 11.886 12.023 11.814 12.074 11.156 11.408 11.305 11.206 11.366
   2849   1388158_at   10.497 10.526 10.528 10.446 10.531 9.713 9.79 9.91 9.618 9.425
   2850   1391502_at   6.173 6.415 6.151 6.462 6.625 4.133 5.021 3.952 4.037 4.019
   2851   1369877_at   8.899 8.842 9.53 9.565 9.046 2.29 4.265 4.935 2.07 3.9
   2852   1394438_at   8.533 8.539 8.43 8.608 8.63 8.076 7.963 7.96 8.063 7.813
   2853   1368371_at   6.825 6.843 6.984 6.628 6.72 7.969 7.954 7.864 7.57 7.632
   2854   1385252_at   8.522 8.631 8.621 8.355 8.753 6.941 7.16 7.481 6.617 7.09
   2855   1372668_at   7.738 8.002 7.72 7.955 8.008 7.12 7.274 7.218 7.131 7.229
   2856   1373465_at   7.792 8.014 7.923 8.079 7.777 9.17 9.05 8.701 8.871 8.812
   2857   1383321_at   7.849 7.478 7.547 7.762 7.813 6.737 6.443 6.71 6.589 6.879
   2858   1380117_at   6.482 5.971 6.017 6.392 5.848 7.765 7.587 7.814 7.405 8.013
   2859   1384485_at   4.889 4.593 4.381 5.091 4.851 6.198 5.917 6.306 6.419 6.348
   2860   1374636_at   7.936 7.911 8.18 8.291 8.067 8.751 8.841 8.806 8.773 8.801
   2861   1373044_at   10.941 10.92 10.861 10.847 11.009 10.595 10.463 10.556 10.525 10.437
   2862   1388453_at   9.247 9.169 9.413 9.566 9.216 8.033 8.203 8.439 8.011 8.379
   2863   1399049_at   9.014 8.978 9.087 9.196 9.04 9.462 9.439 9.469 9.426 9.416
   2864   1371824_at   9.841 9.76 9.998 10.201 9.375 11.181 11.542 11.419 11.215 11.27
   2865   1376341_at   4.991 4.81 4.732 5.283 5.157 6.323 6.141 6.206 5.947 6.251
   2866   1377088_at   5.109 5.477 5.454 5.699 5.073 6.651 6.594 7.002 7.053 6.805
   2867   1373197_at   9.592 9.376 9.161 9.384 9.673 7.414 7.154 8.135 7.608 7.752
   2868   1372246_at   10.268 10.264 10.279 10.253 10.564 9.465 9.585 9.726 9.43 9.575
   2869   1372162_at   9.904 9.906 10.14 10.205 9.813 10.793 10.993 10.915 10.722 10.925
   2870   1372808_at   7.554 7.619 7.696 8.064 7.575 3.83 3.798 4.649 2.359 2.566
   2871   1372372_at   10.806 10.842 10.915 10.802 10.259 12.005 11.928 11.807 12.039 12.108
   2872   1390164_at   4.823 5.916 5.297 5.633 5.137 7.762 7.785 7.934 7.675 7.121
   2873   1389294_at   9.815 9.867 9.828 9.883 9.908 9.08 9.373 8.917 9.046 8.978
   2874   1371361_at   8.681 8.753 9.056 8.668 8.887 10.07 10.115 9.634 10.18 9.933
   2875   1374501_at   9.452 9.342 9.275 9.369 9.332 8.849 8.928 8.693 8.643 8.703
   2876   1388879_at   6.824 6.973 6.535 6.688 6.397 5.486 5.303 5.112 5.216 5.626
   2877   1385486_at   7.038 7.386 7.326 7.486 7.537 8.7 8.909 8.352 8.677 8.466
   2878   1370436_at   11.237 11.325 11.488 11.515 11.373 12.428 12.362 12.021 12.452 12.485
   2879   1383331_at   4.823 4.93 5.1 4.733 4.614 5.809 5.662 5.765 5.968 5.732
   2880   1372052_at   9.895 9.999 9.949 10.127 9.959 9.54 9.518 9.58 9.548 9.416
   2881   1389820_at   11.484 11.377 11.471 11.427 11.502 11.726 11.82 11.764 11.89 11.791
   2882   1373555_at   8.898 8.951 8.889 8.816 9.006 8.144 8.304 7.95 8.08 8.328
   2883   1368653_a_at   10.919 10.898 10.942 11.001 11.031 11.38 11.463 11.299 11.428 11.289
   2884   1389456_at   6.488 6.398 6.561 6.553 6.393 7.66 7.832 7.572 7.212 7.449
   2885   1371551_at   7.941 7.951 7.961 8.212 8.093 8.567 8.656 8.783 8.672 8.804
   2886   1373127_at   9.247 9.276 9.372 9.178 9.143 9.815 9.669 9.725 9.674 9.682
   2887   1390946_at   7.017 7.598 7.855 7.872 7.814 5.941 5.28 4.849 5.208 5.31
   2888   1389729_at   9.478 9.528 9.687 9.369 9.43 8.701 8.588 8.42 8.548 8.85
   2889   1370165_at   5.002 6.095 5.202 5.754 5.81 7.979 8.01 8.591 8.084 7.615
   2890   1398872_at   12.302 12.236 12.302 12.066 12.231 11.786 11.78 11.664 11.77 11.677
   2891   1374146_at   7.942 7.774 7.911 7.964 7.835 7.125 7.234 7.262 6.918 6.923
   2892   1368604_at   7.145 7.561 7.301 6.673 6.766 4.968 5.067 5.176 5.388 4.589
   2893   1395662_at   6.532 6.748 6.519 6.499 6.63 5.59 5.531 5.338 5.59 5.001
   2894   1390529_at   8.114 8.222 7.719 8.067 7.835 6.781 6.768 7.002 6.906 6.517
   2895   1396267_at   7.644 7.838 7.818 7.805 7.683 6.347 6.781 5.882 6.315 6.181
   2896   1387703_a_at   6.668 6.731 6.981 6.548 6.416 8.865 8.556 8.83 7.917 8.654
   2897   1380431_at   6.89 7.106 6.701 6.835 6.571 7.727 8.123 7.976 7.819 8.028
   2898   1374778_at   10.769 10.696 10.735 10.63 10.901 9.871 10.172 9.756 9.862 9.745
   2899   1379450_at   9.191 9.203 9.227 9.333 9.262 8.464 8.528 8.594 8.157 8.532
   2900   1380366_at   7.287 7.445 7.359 7.211 6.92 5.191 5.712 6.006 5.802 5.695
   2901   1388532_at   8.111 8.139 8.073 8.157 8.211 7.819 7.767 7.732 7.773 7.617
   2902   1376576_at   8.528 8.363 8.543 8.597 8.64 7.691 7.958 7.839 7.64 7.603
   2903   1388982_at   7.774 7.715 7.687 7.797 7.908 8.546 8.656 8.414 8.315 8.4
   2904   1387284_at   7.652 7.607 8.073 8.078 7.205 9.385 9.491 9.343 9.279 9.265
   2905   1374561_at   7.201 7.163 7.023 7.288 7.16 6.511 6.354 6.585 6.446 6.657
   2906   1390556_at   6.398 6.359 6.461 6.664 6.227 7.634 7.304 7.294 7.349 7.272
   2907   1383796_at   5.849 5.918 5.923 6.191 5.635 6.826 7.244 6.899 7.017 7.086
   2908   1386884_at   10.176 10.165 10.06 10.095 10.156 9.552 9.599 9.239 9.42 9.536
   2909   1388306_at   7.906 7.782 7.842 7.887 7.881 7.374 7.277 7.193 7.321 7.027
   2910   1380057_at   6.578 6.268 6.978 6.744 6.412 8.439 8.965 9.358 8.558 8.39
   2911   1370201_at   10.535 10.489 10.627 10.141 10.285 11.751 11.689 11.757 11.572 11.291
   2912   1398974_at   9.351 9.264 9.206 9.12 9.142 8.607 8.44 8.715 8.491 8.652
   2913   1393844_at   5.639 5.684 5.339 5.666 5.249 4.568 4.2 4.49 4.509 4.351
   2914   1371330_at   12.406 12.337 12.375 12.247 12.404 12.037 12.045 11.99 12.003 11.922
   2915   1388570_at   9.519 9.454 9.574 9.661 9.624 10.394 10.427 10.568 10.358 10.107
   2916   1388520_at   9.147 9.084 9.056 9.167 9.083 9.635 9.868 9.596 9.604 9.586
   2917   1369008_a_at   7.956 7.949 7.746 7.932 8.12 7.225 7.349 7.357 7.203 7.176
   2918   1372612_at   9.801 9.807 10.045 10.055 10.075 10.71 10.889 10.952 10.789 10.617
   2919   1376153_at   8.352 8.431 8.014 8.486 8.771 6.941 7.164 7.265 7.093 6.909
   2920   1370831_at   8.97 8.89 9.381 9.184 9.395 10.736 11.002 10.749 11.009 10.297
   2921   1369683_at   8.235 8.204 8.413 8.38 8.368 6.966 6.27 5.834 6.689 6.283
   2922   1388539_at   6.799 6.752 6.676 6.75 6.782 7.215 7.142 7.314 7.158 7.081
   2923   1377755_at   7.741 7.957 8.01 7.859 7.804 7.113 7.182 7.305 6.977 6.978
   2924   1378138_at   6.239 6.043 6.134 6.63 6.263 7.447 7.926 7.551 7.391 7.568
   2925   1375034_at   8.665 8.667 8.893 9.054 8.713 7.747 7.574 7.923 7.662 7.962
   2926   1370066_at   8.057 8.033 8.036 8.247 8.312 7.416 7.428 7.423 7.128 7.329
   2927   1382454_at   12.69 12.605 12.256 12.304 12.548 4.243 4.31 8.201 4.698 5.319
   2928   1396942_at   4.976 5.018 5.18 5.304 5.048 7.363 7.127 8.021 7.029 6.74
   2929   1384191_at   8.021 8.07 8.106 8.041 7.961 1.47 2.895 3.866 4.435 3.329
   2930   1393261_at   5.05 5.382 5.446 4.952 5.518 6.513 6.365 6.373 6.323 6.402
   2931   1394842_at   8.076 8.076 8.021 8.238 8.396 7.078 7.165 6.95 7.209 6.665
   2932   1389013_at   9.551 9.553 9.533 9.658 9.6 9.183 9.18 8.957 9.044 8.981
   2933   1392244_at   6.108 5.883 5.632 5.883 5.785 6.902 6.982 6.994 7.242 6.71
   2934   1368799_at   7.247 7.279 7.38 7.575 7.564 5.512 5.166 5.728 4.506 4.765
   2935   1394786_at   8.924 8.941 9.238 9.412 9.125 10.148 10.486 10.541 10.145 10.273
   2936   1368914_at   7.711 7.604 7.487 7.804 8.235 5.223 5.991 5.921 5.303 6.005
   2937   1370919_at   9.209 9.132 9.078 9.36 9.42 8.504 8.562 8.498 8.382 8.278
   2938   1392090_at   9.527 9.495 9.64 9.595 9.363 10.654 10.749 10.249 10.883 10.742
   2939   1392935_at   6.065 5.871 5.883 5.854 6.226 6.967 7.332 6.839 7.209 7.28
   2940   1384877_at   7.023 7.123 7.202 7.366 6.796 8.489 8.491 8.34 8.327 8.016
   2941   1377723_at   8.423 8.364 8.489 8.583 8.54 9.066 9.219 8.951 8.989 9.076
   2942   1393431_at   9.74 9.606 9.374 9.468 9.929 8.527 8.641 8.59 8.28 8.38
   2943   1398882_at   12.361 12.333 12.367 12.154 12.254 11.879 11.8 11.72 11.864 11.738
   2944   1384392_at   4.09 3.964 3.577 4.211 4.703 6.341 6.896 6.038 7.055 6.612
   2945   1393619_at   7.234 7.291 7.335 7.377 7.375 6.278 6.723 6.438 6.494 6.659
   2946   1385277_x_at   8.815 8.517 8.372 8.65 8.905 3.444 5.176 3.278 2.86 5.056
   2947   1374846_at   9.538 9.541 9.478 9.596 9.488 10.08 10.229 9.973 10.135 9.941
   2948   1382477_at   6.274 6.56 6.75 6.436 6.663 5.025 5.18 4.963 5.361 5.559
   2949   1382283_at   8.633 8.573 8.708 8.72 8.763 7.188 7.172 6.21 6.919 7.067
   2950   1369153_at   5.708 5.429 5.252 5.788 5.712 6.768 7.219 7.138 6.768 6.795
   2951   1391656_at   6.509 6.492 6.44 6.531 6.18 7.65 7.783 7.947 7.585 7.285
   2952   1391935_at   7.27 7.354 7.665 7.407 8.125 5.069 5.561 5.365 4.328 5.036
   2953   1392916_at   8.698 8.691 8.961 9.168 8.763 10.039 10.155 9.902 9.921 9.747
   2954   1399121_at   8.211 8.308 8.008 8.1 8.206 7.572 7.589 7.57 7.351 7.454
   2955   1384238_at   7.901 7.821 7.744 7.961 7.794 8.459 8.502 8.634 8.56 8.303
   2956   1371606_at   6.764 7.183 7.161 7.07 6.855 7.873 8.122 8.049 7.929 7.832
   2957   1368122_at   8.154 8.103 8.079 8.282 8.159 8.668 8.783 8.911 8.614 8.778
   2958   1383202_at   9.324 9.336 9.407 9.565 9.526 8.718 8.703 8.773 8.773 8.443
   2959   1399084_at   8.198 8.262 8.322 8.309 8.231 7.664 7.902 7.73 7.59 7.731
   2960   1367582_at   12.098 12.034 12.056 11.986 12.119 11.723 11.648 11.631 11.751 11.559
   2961   1370869_at   9.467 9.542 9.508 9.617 9.677 2.971 5.262 6.015 4.204 4.781
   2962   1387085_at   8.839 8.808 8.684 8.856 8.794 8.334 8.132 8.168 7.976 8.009
   2963   1372241_at   12.204 12.15 12.128 12.079 12.188 11.831 11.846 11.898 11.914 11.913
   2964   1377911_at   8.882 8.952 9.001 8.996 8.558 9.972 10.289 9.836 9.844 10.082
   2965   1384892_at   6.192 6.609 6.457 6.683 6.69 5.483 5.229 5.042 5.002 4.802
   2966   1383605_at   7.807 8.045 8.09 7.991 7.124 9.661 9.69 10.084 9.502 9.816
   2967   1376816_at   5.702 5.379 5.481 5.834 5.8 3.913 3.652 2.738 2.818 3.346
   2968   1392961_at   8.526 8.35 8.233 8.148 8.273 7.584 7.115 7.062 7.214 7.119
   2969   1389725_at   6.81 6.532 6.65 6.289 6.999 8.241 8.116 8.441 7.843 8.069
   2970   1368021_at   8.976 8.888 9.193 8.827 7.858 11.285 11.459 10.774 11.334 11.477
   2971   1381819_at   7.068 7.236 6.667 7.134 7.216 6.028 5.594 5.406 5.686 5.706
   2972   1372270_at   9.193 9.231 8.979 9.319 9.581 8.358 8.314 8.339 8.242 8.218
   2973   1388102_at   7.895 8 7.736 7.943 8.168 8.654 8.824 8.801 8.634 8.674
   2974   1382925_at   7.185 7.347 7.332 7.419 7.441 3.653 3.853 4.265 2.945 1.868
   2975   1371762_at   7.075 6.998 6.964 7.454 5.744 9.881 9.931 10.581 9.872 9.647
   2976   1390818_at   6.995 6.98 6.447 6.974 6.812 7.866 7.959 8.118 8.281 8.002
   2977   1374474_at   7.934 8.185 8.011 8.294 8.397 7.129 7.138 7.386 7.201 7.045
   2978   1377879_at   5.987 6.165 5.959 6.365 5.919 7.533 7.836 7.733 7.255 7.202
   2979   1376180_at   5.918 6.194 6.149 6.378 6.319 7.134 7.177 7.526 7.253 7.107
   2980   1392454_at   7.566 7.711 7.545 7.759 8.02 6.716 6.926 6.554 6.743 6.644
   2981   1380692_at   7.686 7.983 8.215 7.607 7.791 5.557 6.565 5.935 5.77 5.816
   2982   1368627_at   9.128 9.165 8.974 9.528 7.97 11.317 11.54 11.7 11.483 11.517
   2983   1379339_at   8.038 7.953 8.194 8.007 7.806 9.056 8.91 8.727 8.854 8.709
   2984   1371503_at   9.553 9.507 9.631 9.606 9.569 9.139 9.121 9.178 9.039 8.911
   2985   1398812_at   11.529 11.588 11.546 11.489 11.683 10.928 11.015 11.106 11.083 10.834
   2986   1390767_at   8.145 8.091 8.169 8.125 8.288 7.631 7.526 7.254 7.412 7.295
   2987   1370105_at   6.516 6.728 7.012 7.004 7.027 5.468 5.403 4.697 4.928 5.274
   2988   1388393_at   9.808 9.821 9.856 10.008 9.906 8.728 8.604 9.171 8.561 8.553
   2989   1367931_a_at   10.485 10.548 10.541 10.548 10.558 9.953 10.178 10.03 9.859 9.892
   2990   1372082_at   9.449 9.498 9.436 9.632 9.579 8.811 8.808 8.679 8.759 9.055
   2991   1380471_at   7.259 7.66 7.479 8.028 7.653 8.987 9.269 9.428 8.997 8.932
   2992   1387805_at   9.933 10.026 10.301 10.005 9.813 10.838 10.862 10.778 10.742 10.889
   2993   1385093_at   5.611 5.39 5.53 5.305 5.203 6.359 6.419 6.189 6.531 6.653
   2994   1391453_at   6.194 6.055 6.663 6.575 6.155 4.392 4.928 4.529 4.482 5.012
   2995   1387957_a_at   7.919 7.892 8.374 8.149 8.196 6.917 6.878 7.1 6.842 6.502
   2996   1383886_at   5.68 5.648 5.823 5.821 5.238 6.906 6.952 6.693 6.786 6.653
   2997   1388160_a_at   10.592 10.542 10.636 10.585 10.504 11.04 11.177 10.929 11.059 10.963
   2998   1386716_at   6.793 6.899 6.643 6.405 6.354 4.824 4.156 5.147 4.786 4.34
   2999   1383291_at   11.768 11.711 11.381 11.452 11.803 10.001 9.92 9.103 10.028 9.887
   3000   1367746_a_at   9.644 9.658 9.483 9.835 9.878 8.665 8.69 9.002 8.573 8.745
   3001   1390664_at   5.573 6.021 5.847 5.767 5.639 7.135 7.066 7.426 7.227 6.709
   3002   1374194_at   8.242 8.202 8.185 8.317 8.424 7.599 7.678 7.562 7.322 7.374
   3003   1372028_at   9.228 9.359 9.23 9.365 9.508 8.559 8.701 8.433 8.531 8.269
   3004   1399062_at   9.174 9.151 9.182 9.178 9.191 8.705 8.727 8.407 8.657 8.644
   3005   1373386_at   9.577 9.58 9.598 9.634 9.091 10.718 10.831 10.694 10.572 10.44
   3006   1368144_at   8.496 8.448 8.532 8.514 8.703 7.246 7.346 7.238 6.55 6.821
   3007   1393165_at   7.036 7.136 7.292 6.936 7.061 8.079 8.339 7.875 8.067 7.908
   3008   1376317_at   6.955 6.858 6.684 6.81 6.972 5.216 5.484 5.264 5.477 4.574
   3009   1398904_at   10.575 10.504 10.414 10.471 10.566 10.061 10.133 10.064 9.892 10.019
   3010   1377110_at   7.277 7.305 7.527 7.096 7.229 3.86 3.483 4.662 3.502 2.257
   3011   1370112_at   8.866 8.81 8.645 8.61 8.675 8.253 8.249 8.182 8.233 8.254
   3012   1367959_a_at   6.055 6.165 6.071 6.316 5.746 8.275 7.727 8.502 7.788 7.566
   3013   1391014_at   6.741 6.553 6.602 6.841 6.941 5.969 5.754 5.889 6.029 5.772
   3014   1373491_at   8.674 8.669 8.625 8.627 8.764 8.08 8.152 8.118 8.259 8.319
   3015   1375220_at   10.103 10.126 10.253 10.105 10.138 10.639 10.874 10.813 10.689 10.584
   3016   1390650_at   7.097 7.385 7.163 7.452 7.412 5.751 6.418 6.05 5.826 5.861
   3017   1389315_at   7.926 8.021 8.043 8.018 7.934 7.392 7.186 6.989 7.304 7.374
   3018   1368055_a_at   8.905 8.848 8.988 8.999 9.034 8.125 8.403 8.116 8.146 7.904
   3019   1382070_at   7.705 7.569 7.84 7.771 7.906 7.008 7.002 6.874 7.095 7.176
   3020   1371371_at   11.458 11.309 11.589 11.49 11.593 11.968 12.038 11.996 12.066 11.978
   3021   1388672_at   7.526 7.647 7.502 7.745 7.847 6.707 7.022 6.697 6.826 6.854
   3022   1383964_at   6.104 6.166 5.921 6.22 5.874 6.715 6.779 6.67 6.737 6.707
   3023   1380201_at   7.692 7.545 7.769 7.993 7.691 6.953 6.901 6.935 6.725 6.645
   3024   1392119_at   6.942 6.829 7.034 7.22 6.953 8.112 8.032 7.875 7.676 7.956
   3025   1388339_at   8.553 8.465 8.393 8.482 8.535 7.925 7.594 7.63 7.919 7.653
   3026   1389389_at   7.091 7.337 7.02 7.021 7.255 6.144 6.17 6.49 6.033 6.157
   3027   1388366_at   9.672 9.67 9.693 9.665 9.627 9.953 9.919 9.973 9.829 9.926
   3028   1386917_at   8.443 8.383 8.692 8.79 7.962 9.922 10.192 9.784 10.039 10.248
   3029   1392929_at   8.062 7.929 8.022 8.154 7.727 9.025 9.192 8.705 9.012 9.017
   3030   1397233_at   4.823 4.596 4.712 3.995 5.206 6.921 6.668 6.729 6.408 6.664
   3031   1373934_at   7.311 7.248 7.079 7.563 7.183 8.09 8.237 8.313 8.051 8.139
   3032   1373011_at   9.244 9.231 9.367 9.33 9.224 10.983 10.731 10.121 10.75 10.754
   3033   1385005_at   5.72 5.244 6.004 5.687 4.936 8.218 8.337 7.331 7.911 7.921
   3034   1378080_at   8.394 8.255 8.305 8.38 8.407 7.579 7.792 7.637 7.75 7.394
   3035   1388871_at   8.652 8.733 8.65 8.894 9.029 8.058 8.042 8.1 7.912 8.049
   3036   1386898_at   11.675 11.685 11.64 11.645 11.732 11.912 12.007 11.926 11.875 11.934
   3037   1398852_at   12.457 12.309 12.341 12.179 12.429 11.78 11.899 11.756 11.828 11.725
   3038   1367895_at   11.523 11.515 11.523 11.494 11.652 11.142 11.133 10.937 11.088 10.954
   3039   1371344_at   12.829 12.732 12.582 12.486 12.675 12.082 12.05 12.122 12.116 12.044
   3040   1385440_at   7.579 7.102 7.816 7.549 7.069 4.093 4.913 4.663 5.171 3.668
   3041   1382475_at   4.884 4.665 5.098 5.413 4.89 6.526 6.47 6.157 6.363 6.693
   3042   1384925_at   5.097 5.396 5.533 5.71 5.385 6.579 6.598 6.802 6.449 6.416
   3043   1384202_at   6.184 5.896 6.292 6.15 5.503 8.08 7.507 8.104 7.62 7.626
   3044   1392411_at   8.07 8.279 8.656 8.422 8.135 9.85 10.019 9.35 9.74 9.871
   3045   1387883_a_at   13.407 13.339 13.196 12.976 13.126 12.46 12.37 12.349 12.533 12.407
   3046   1398337_at   9.124 8.988 8.746 8.995 9.254 8.178 8.074 7.901 8.186 7.946
   3047   1384112_at   7.175 7.147 7.624 7.568 7.435 8.549 8.848 8.388 8.551 8.482
   3048   1389194_at   8.017 8.05 8 8.01 8.025 8.487 8.633 8.448 8.334 8.523
   3049   1393335_at   5.307 4.892 4.977 5.119 5.229 6.318 6.686 6.947 6.329 6.303
   3050   1384841_at   7.18 7.186 7.416 7.24 7.258 5.752 5.613 6.057 5.861 5.092
   3051   1367630_at   12.533 12.455 12.354 12.262 12.409 11.894 11.875 12.008 11.918 11.914
   3052   1382935_at   7.772 7.386 7.75 7.828 7.641 8.71 8.633 8.845 8.718 8.418
   3053   1377968_at   6.955 6.939 6.56 6.88 7.008 5.595 5.546 6.059 5.743 5.766
   3054   1398342_at   7.216 7.406 7.329 7.229 7.271 8.089 8.051 7.999 7.814 7.796
   3055   1387372_at   6.678 6.556 6.946 6.831 5.569 9.289 9.081 8.665 9.006 8.998
   3056   1370967_at   6.394 6.535 6.214 6.343 6.463 5.426 5.401 5.071 4.916 4.826
   3057   1370083_at   7.127 7.201 7.129 7.054 7.881 0 0.774 2.976 2.86 0.963
   3058   1383064_at   7.954 7.791 8.128 8.237 8.324 9.207 9.29 9.066 9.111 8.971
   3059   1398783_at   9.954 9.919 9.983 9.866 9.78 10.492 10.454 10.281 10.521 10.382
   3060   1368930_at   7.641 7.647 7.543 7.586 7.346 1.852 2.021 3.935 2.755 4.24
   3061   1395694_at   7.691 7.929 7.806 7.66 7.86 9.047 9.106 8.527 8.989 8.781
   3062   1384658_at   7.507 7.682 7.828 7.662 7.708 0.722 0.824 3.958 1.531 0.189
   3063   1387907_at   8.952 8.924 8.903 9.132 9.121 9.607 9.803 9.646 9.53 9.599
   3064   1388636_at   7.913 7.925 7.996 8.038 7.867 8.808 8.843 9.1 8.557 8.757
   3065   1376868_at   8.696 8.814 9.103 9.009 8.691 9.918 10.135 9.961 9.655 9.994
   3066   1378755_at   5.887 6.117 5.906 6.055 5.614 7.024 7.021 7.533 7.079 7.057
   3067   1372422_at   7.426 7.319 7.456 7.648 7.245 8.188 8.214 8.444 8.377 8.129
   3068   1380699_at   8.015 8.042 7.858 8.053 8.287 6.96 6.284 5.983 6.461 6.186
   3069   1375701_at   7.426 7.364 7.228 7.442 7.629 6.585 6.78 6.736 6.632 6.783
   3070   1367660_at   9.758 9.815 10.036 10.019 9.655 10.994 11.149 11.316 10.931 10.724
   3071   1383280_at   5.947 6.275 5.802 5.604 5.889 7.294 7.907 7.579 7.148 7.562
   3072   1377350_at   6.069 6.193 6.267 6.725 6.125 4.374 4.727 4.713 4.699 5.114
   3073   1379277_at   7.771 7.573 7.638 7.878 7.869 8.593 8.802 8.782 8.435 8.525
   3074   1367747_at   8.051 7.934 8.099 8.086 8.349 9.089 8.965 8.913 8.859 8.761
   3075   1374376_at   7.043 6.848 6.844 7.3 7.163 8.079 8.254 8.47 8.039 8.043
   3076   1390478_at   7.302 7.055 6.924 7.094 6.992 7.762 7.838 7.659 7.708 7.71
   3077   1385126_at   4.391 4.78 4.989 4.759 4.428 5.99 6.13 5.891 5.705 5.873
   3078   1373468_at   5.723 5.57 6.09 5.745 5.205 2.646 2.7 1.233 2.587 1.137
   3079   1389809_at   7.583 7.716 7.96 8.179 7.752 9.233 9.016 9.269 9.092 8.809
   3080   1374306_at   7.325 7.375 6.929 7.599 7.757 5.604 5.944 6.083 5.939 5.883
   3081   1367783_at   10.774 10.773 10.666 10.668 10.821 11.129 11.161 11.162 11.066 11.042
   3082   1379909_at   7.076 6.775 6.562 6.56 6.832 7.866 8.162 7.77 7.764 7.863
   3083   1380256_at   7.909 7.705 7.748 7.898 8.035 7.127 7.337 7.145 7.109 7.086
   3084   1370159_at   7.719 7.883 7.866 7.782 7.881 7.142 6.955 7.224 6.78 7.088
   3085   1388723_at   8.122 7.968 7.982 8.179 8.178 8.574 8.659 8.586 8.568 8.751
   3086   1373633_at   6.531 6.275 6.241 6.432 6.527 5.188 5.375 4.983 4.566 4.903
   3087   1367550_a_at   8.67 8.55 8.603 8.638 8.687 9.177 9.267 9.441 9.107 9.149
   3088   1383184_at   6.01 6.216 6.301 6.214 6.324 6.836 7.083 6.804 6.924 7.034
   3089   1374705_at   8.974 9.119 9.087 9.019 9.093 9.731 9.876 9.572 9.697 9.983
   3090   1392938_s_at   8.398 8.375 8.18 8.45 8.707 7.232 7.498 7.534 7.149 7.166
   3091   1369531_at   8.242 8.413 8.935 8.852 7.925 10.894 10.864 10.142 10.784 10.993
   3092   1374910_at   6.342 6.444 5.926 6.51 6.989 8.086 8.298 8.108 7.992 8.034
   3093   1367834_at   8.333 8.302 8.434 8.405 8.423 7.724 7.675 7.511 7.25 7.361
   3094   1377424_at   6.541 6.454 6.717 6.579 6.383 7.54 7.472 7.282 7.282 7.18
   3095   1387111_at   10.356 10.387 11.02 10.477 10.406 11.834 11.8 11.585 11.849 11.901
   3096   1372062_at   7.699 7.816 7.705 7.601 7.878 6.963 6.789 7.195 6.848 6.847
   3097   1380346_at   9.303 9.082 9.65 9.228 9.282 7.849 8.001 8.344 8.251 8.121
   3098   1387888_at   12.115 12.071 12.076 12.124 12.247 11.642 11.61 11.733 11.737 11.523
   3099   1392128_at   5.65 5.967 5.843 5.837 5.783 3.703 3.094 4.351 3.689 4.082
   3100   1367865_at   7.932 7.824 7.655 7.933 7.837 7.288 7.37 7.18 7.293 7.3
   3101   1368309_at   7.097 7.056 7.151 7.231 7.134 7.785 7.686 7.704 7.551 7.536
   3102   1371473_at   9.373 9.439 9.399 9.606 9.249 9.95 9.941 10.081 10.056 10.068
   3103   1374241_at   7.937 8.013 8.044 8.642 8.144 9.41 9.734 9.758 9.502 9.362
   3104   1388615_at   10.771 10.751 10.82 10.667 10.586 10.2 10.337 10.248 10.135 10.207
   3105   1395065_at   6.7 6.631 6.667 7.011 7.024 5.904 5.911 5.961 5.906 5.672
   3106   1385132_at   7.418 7.307 7.725 7.413 6.888 8.818 8.665 9.036 8.809 9.235
   3107   1383453_at   8.17 8.352 8.11 8.249 8.238 9.224 9.486 8.877 9.345 9.338
   3108   1373676_at   6.493 6.888 6.623 6.971 6.902 7.746 7.73 7.993 7.654 7.794
   3109   1373118_at   6.168 5.877 6.19 6.164 6.073 7.101 7.183 7.53 6.904 7.169
   3110   1383179_at   7.969 8.158 8.183 8.15 8.173 7.295 7.48 7.01 7.215 6.99
   3111   1375640_at   8.608 8.79 8.579 8.953 9.174 9.923 9.981 10.128 9.964 9.806
   3112   1367957_at   8.352 8.215 8.4 8.551 8.379 6.962 7.189 7.497 7.183 6.75
   3113   1396112_at   7.263 7.279 7.41 7.453 7.238 8.466 8.435 8.007 8.193 8.128
   3114   1374118_at   8.989 8.914 9.001 9.111 9.007 8.386 8.532 8.496 8.295 8.221
   3115   1393584_at   8.32 8.349 8.467 8.324 8.487 9.784 9.454 9.386 9.187 9.776
   3116   1386745_at   7.232 7.346 7.244 7.14 7.228 6.3 6.273 5.614 6.026 6.06
   3117   1382993_at   7.524 7.526 7.327 7.79 7.567 5.923 6.21 6.498 6.495 5.98
   3118   1375027_at   8.553 8.619 8.583 8.609 8.779 9.147 9.258 9.222 9.049 9.078
   3119   1367759_at   9.202 9.161 9.034 9.274 9.197 10.706 10.552 10.889 10.089 10.347
   3120   1374831_at   7.014 7.178 6.972 7.092 7.139 6.451 6.415 6.583 6.345 6.203
   3121   1369971_a_at   9.529 9.627 9.336 9.628 9.732 8.575 8.673 8.596 8.278 8.163
   3122   AFFX_Rat_beta-actin_5_at   12.915 12.776 12.868 12.615 12.683 11.91 11.54 11.638 12.049 11.738
   3123   1376793_at   9.593 9.633 9.574 9.665 9.679 9.039 9.306 9.16 9.09 9.034
   3124   1374265_at   8.999 8.736 8.576 8.768 8.912 7.5 7.652 7.804 7.329 7.107
   3125   1387656_at   6.469 6.298 6.824 6.798 6.644 8.63 8.478 9.331 8.539 8.153
   3126   1382343_at   6.52 6.414 6.529 6.663 6.589 5.643 5.455 5.324 5.414 4.907
   3127   1389686_at   8.485 8.538 8.415 8.534 8.485 7.551 7.833 7.752 7.314 7.678
   3128   1384259_at   6.181 6.397 6.137 6.185 5.894 4.482 3.542 3.709 4.555 4.273
   3129   1395381_at   6.816 6.756 6.814 6.483 6.917 8.13 7.64 8.037 7.985 7.681
   3130   1384042_at   6.022 6.18 6.166 6.336 6.268 5.149 5.433 5.551 5.252 5.44
   3131   1382203_at   8.055 8.167 8.075 8.111 8.115 7.809 7.842 7.788 7.912 7.796
   3132   1379169_at   2.536 1.748 3.604 2.757 2.029 6.504 5.444 6.006 5.737 5.916
   3133   1370876_at   7.982 7.88 7.893 8.076 8.22 7.172 6.507 6.547 6.733 6.835
   3134   1398885_at   12.43 12.397 12.338 12.284 12.392 12.009 11.962 12.093 11.956 11.896
   3135   1372023_at   7.41 7.612 7.316 7.307 7.415 8.522 8.428 8.506 8.124 8.166
   3136   1397698_at   7.001 7.194 7.408 7.193 7.095 7.962 8.265 7.933 8.226 8.001
   3137   1390698_at   8.199 8.106 8.086 8.437 8.396 7.062 7.368 6.667 6.941 6.978
   3138   1376770_at   9.001 8.994 8.876 8.88 8.728 9.998 9.909 10.439 9.959 9.789
   3139   1369929_at   12.272 12.262 12.323 12.104 12.095 11.605 11.538 11.248 11.552 11.503
   3140   1387910_at   7.211 7.046 7.411 7.547 7.332 8.467 8.569 8.703 8.182 8.351
   3141   1372852_at   8.657 8.737 8.839 8.86 8.455 2.06 4.259 4.909 4.575 4.259
   3142   1371657_at   9.648 9.654 9.701 9.81 9.881 9.162 9.159 9.134 8.963 8.886
   3143   1376860_at   6.702 6.862 7.155 6.891 6.452 8.287 8.365 7.903 8.425 8.589
   3144   1399065_at   8.364 8.537 8.597 8.883 8.659 9.474 9.53 9.82 9.541 9.517
   3145   1373904_at   7.117 7.308 7.257 7.343 7.603 6.397 6.053 5.907 6.347 5.94
   3146   1388441_at   9.794 9.62 9.94 10.165 10.001 10.911 10.824 10.926 10.725 10.773
   3147   1379345_at   6.96 6.81 6.864 7.171 7.363 5.693 5.06 5.631 5.538 5.766
   3148   1389965_at   10.592 10.577 10.565 10.564 10.414 10.931 11.08 10.948 10.897 11.037
   3149   1390523_at   5.393 5.442 5.705 6.047 5.624 7.158 7.1 6.866 6.862 6.733
   3150   1386940_at   9.753 9.773 9.855 9.509 9.898 9.063 9.01 9.064 8.998 8.797
   3151   1396300_at   7.191 7.189 7.099 7.326 6.976 8.549 9.297 8.591 8.811 8.369
   3152   1370820_at   9.943 10.069 10.078 10.035 10.056 9.601 9.507 9.547 9.614 9.335
   3153   1368884_at   6.911 6.651 6.852 6.849 6.859 5.115 4.535 4.888 4.972 3.836
   3154   1381922_at   7.756 7.999 8.145 8.198 7.925 8.898 9.012 8.892 8.871 8.695
   3155   1373583_at   6.957 7.086 7.239 7.333 7.312 8.087 8.53 8.095 8.109 8.334
   3156   1373222_at   10.311 10.206 10.255 10.373 10.418 10.664 10.843 10.741 10.758 10.79
   3157   1370341_at   6.286 6.232 6.476 6.28 6.595 4.949 4.213 4.934 5.088 4.51
   3158   1399026_at   9.621 9.573 9.46 9.516 9.431 10.031 10.072 9.902 9.971 9.897
   3159   1388614_at   9.438 9.395 9.385 9.615 9.567 8.845 8.86 9.019 8.736 8.913
   3160   1370921_at   9.127 9.104 9.262 9.36 9.337 10.024 9.862 10.176 9.992 9.842
   3161   1371421_at   11.297 11.128 11.38 11.143 10.944 11.95 12.032 11.848 12.117 12.092
   3162   1378322_at   6.19 6.542 6.32 6.269 6.54 5.633 5.309 5.567 5.422 5.266
   3163   1377969_at   6.12 5.991 6.508 6.484 5.802 8.286 8.066 8.669 7.985 7.702
   3164   1397341_at   6.865 6.862 6.745 7.215 7.03 3.005 4.227 4.793 4.018 4.394
   3165   1379327_at   8.329 8.488 8.427 8.697 8.257 7.718 7.591 7.574 7.413 7.374
   3166   1373986_at   9.573 9.62 9.77 9.88 9.505 10.673 10.519 10.31 10.488 10.62
   3167   1377680_at   6.723 6.725 7.354 7.114 6.693 8.595 8.743 9.121 8.382 8.432
   3168   1388558_at   10.638 10.615 10.721 10.741 10.718 11.213 11.345 11.088 11.091 11.261
   3169   1398809_at   7.898 7.942 8.08 8.071 8.014 7.363 7.387 6.977 7.078 7.112
   3170   1393334_at   6.49 6.352 6.211 6.417 6.677 5.538 5.303 4.957 5.096 4.939
   3171   1389169_at   7.444 7.55 7.411 7.59 7.397 8.317 8.31 8.294 8.009 8.055
   3172   1370574_a_at   7.141 6.948 7.033 7.309 7.224 6.177 5.694 5.77 6.081 5.51
   3173   1371646_at   10.039 10.078 10.076 10.207 10.378 9.049 9.252 9.504 9.223 9.091
   3174   1379326_at   7.319 7.636 8.112 7.229 7.467 9.024 9.197 9.046 9.431 9.053
   3175   1372500_at   9.078 9.064 9.155 9.264 9.56 7.978 8.396 8.038 7.978 7.885
   3176   1387280_a_at   6.811 6.731 6.87 7.107 6.926 5.51 5.22 5.992 5.251 5.338
   3177   1379396_at   6.772 6.576 7.1 7.211 7.249 5.602 5.556 5.315 5.112 4.943
   3178   1373015_at   8.383 8.478 8.691 8.641 8.439 9.484 9.455 9.143 9.243 9.32
   3179   1379740_at   5.924 5.908 5.741 6.063 6.216 3.975 4.554 3.424 4.343 3.961
   3180   1390046_at   7.715 7.832 7.867 7.942 8.122 7.218 7.172 7.101 6.911 7.143
   3181   1379507_at   6.476 6.664 7.444 7.015 6.418 0 2.091 2.78 0.014 0
   3182   1371080_at   6.133 6.007 5.085 3.853 3.775 9.712 9.969 9.76 9.666 9.369
   3183   1373585_at   9.833 9.864 9.986 9.984 9.83 10.645 10.707 10.335 10.685 10.632
   3184   1384451_at   7.39 7.593 7.346 7.577 7.572 6.585 6.824 6.391 6.407 6.71
   3185   1377744_at   7.468 7.423 7.33 7.371 7.525 5.519 6.088 6.396 5.812 5.56
   3186   1393803_at   6.66 6.864 6.563 6.861 6.846 5.078 5.831 5.368 5.608 5.494
   3187   1388483_at   9.284 9.215 9.308 9.405 9.195 9.924 9.884 9.691 9.731 9.826
   3188   1369931_at   11.599 11.6 11.604 11.637 11.655 10.121 10.131 10.764 9.899 9.904
   3189   1367767_at   9.221 9.283 9.275 9.227 9.213 9.665 9.824 9.543 9.684 9.658
   3190   1367820_at   10.422 10.479 10.444 10.505 10.408 10.142 10.103 9.95 9.921 9.976
   3191   1388555_at   10.044 10.028 10.335 10.252 10.027 10.975 11.072 10.849 11.304 11.026
   3192   1382253_at   7.293 7.299 7.602 7.522 7.481 6.709 6.196 6.539 6.527 6.397
   3193   1386529_at   5.592 5.565 5.748 5.799 5.764 6.452 6.461 6.396 6.833 6.511
   3194   1382940_at   6.111 5.862 6.274 5.853 5.964 7.213 6.892 6.978 6.918 6.83
   3195   1390356_at   10.431 10.335 10.357 10.402 10.503 9.895 10.09 10.03 9.925 9.974
   3196   1383378_at   7.323 7.287 7.122 7.254 7.162 8.325 8.111 7.9 7.876 8.042
   3197   1367468_at   9.483 9.737 9.756 9.835 9.604 10.355 10.396 10.309 10.373 10.21
   3198   1375996_at   7.448 7.1 7.464 7.608 6.967 5.947 6.174 6.005 5.816 5.609
   3199   1399055_at   8.741 8.797 8.595 8.866 8.779 7.846 7.921 8.154 7.627 7.83
   3200   1388153_at   9.251 9.166 9.262 9.374 9.167 9.911 9.868 9.913 9.785 9.645
   3201   1389658_at   9.021 9.1 9.123 9.213 9.407 8.259 8.274 8.263 8 7.805
   3202   1381216_at   6.268 6.321 6.876 6.651 6.764 2.508 3.32 4.23 3.986 3.825
   3203   1381282_at   5.474 5.666 5.42 5.61 5.415 6.13 6.319 6.21 6.361 6.058
   3204   1388392_at   9.78 9.866 9.652 9.879 10.161 8.998 9.023 8.98 9.073 9.16
   3205   1377750_at   8.715 8.624 8.705 8.82 8.881 7.838 8.002 8.107 7.676 7.682
   3206   1391255_at   6.68 6.703 6.291 7.021 6.8 5.162 5.619 5.274 5.149 5.464
   3207   1374079_at   6.941 7.129 7.202 7.186 6.87 7.825 7.707 7.883 8.06 7.931
   3208   1374124_at   6.992 6.853 7.007 7.267 7.222 7.979 7.884 8.051 7.996 7.742
   3209   1370811_at   9.776 9.815 9.923 9.811 9.531 10.417 10.714 10.522 10.7 10.498
   3210   1391500_at   8.11 8.062 8.191 8.197 8.22 8.703 8.929 8.745 8.66 8.606
   3211   1383199_at   7.355 7.452 7.435 7.628 7.37 6.397 6.195 5.535 5.991 6.145
   3212   1375438_at   9.084 9.143 9.266 9.326 9.086 10.127 10.068 9.754 10.223 9.989
   3213   1370363_at   6.218 6.325 6.572 6.367 5.371 8.452 8.422 7.942 8.315 8.149
   3214   1372876_at   9.741 9.79 9.941 9.795 9.586 10.581 10.592 10.353 10.509 10.355
   3215   1385359_at   5.638 6 5.925 5.905 5.695 3.517 4.319 3.839 4.539 4.146
   3216   1371592_at   9.012 9.099 9.154 9.359 9.295 7.878 8.109 8.432 8.002 8.244
   3217   1396499_at   0 1.17 0 0.151 0 3.32 2.625 2.907 2.836 2.353
   3218   1370905_at   7.307 7.311 7.162 7.255 7.195 7.939 8.159 7.927 7.752 7.837
   3219   1384378_at   6.509 6.739 6.835 6.842 6.699 5.969 5.707 5.941 5.571 5.926
   3220   1378191_at   7.9 7.964 8.151 8.167 7.39 9.478 9.498 9.092 9.384 9.557
   3221   1386892_at   9.6 9.47 9.54 9.684 9.465 10.167 10.424 10.181 10.165 10.077
   3222   1391611_at   8.237 8.406 8.203 8.107 8.356 8.873 8.826 8.709 8.814 8.829
   3223   1387230_at   8.681 8.504 8.815 8.749 8.673 9.774 9.552 10.072 9.571 9.571
   3224   1372557_at   7.846 7.759 7.738 7.797 7.91 8.449 8.478 8.342 8.195 8.309
   3225   1367752_at   7.675 7.639 7.757 7.946 7.531 8.837 8.801 8.49 8.534 8.526
   3226   1387877_at   5.427 5.652 5.351 5.773 4.688 7.196 7.565 7.814 7.221 7.27
   3227   1371844_at   9.846 9.843 9.794 9.924 9.901 10.327 10.404 10.375 10.179 10.232
   3228   1371739_at   8.96 8.844 9.015 9.03 9.06 9.687 10.01 10.056 9.623 9.804
   3229   1368300_at   7.133 7.339 6.722 7.573 7.148 5.565 5.591 5.77 5.86 5.275
   3230   1367810_at   9.114 9.155 9.226 9.213 9.049 10.271 10.318 9.747 10.088 10.174
   3231   1377672_at   9.437 9.331 9.912 9.505 8.922 11.355 11.621 10.833 11.272 11.491
   3232   1392548_at   6.637 6.21 6.367 6.487 6.667 4.75 5.196 5.18 5.482 5.176
   3233   1398264_at   6.276 5.938 6.165 6.298 5.831 7.756 7.713 7.05 7.717 7.667
   3234   1379719_at   6.693 6.755 6.602 6.648 6.578 7.382 7.754 8.028 7.542 7.773
   3235   1393105_at   7.469 7.197 7.14 7.205 7.69 5.924 5.619 6.231 5.473 5.586
   3236   1367982_at   9.096 9.094 9.181 9.137 9.036 9.908 9.953 10.347 9.963 9.789
   3237   1368150_at   11.133 11.061 11.175 11.068 10.51 12.164 12.049 12.064 12.159 12.172
   3238   1388358_at   10.057 9.889 9.97 10.302 10.209 11.032 11.216 11.236 11.207 10.807
   3239   1398514_at   10.435 10.126 10.604 10.195 9.806 11.445 11.534 11.485 11.54 11.446
   3240   1371125_at   7.639 7.714 7.637 7.783 7.544 6.971 7.051 6.717 6.845 6.604
   3241   1373907_at   8.716 8.667 8.879 8.773 8.836 8.374 8.443 8.392 8.32 8.342
   3242   1381947_at   4.472 4.216 4.759 4.655 3.897 5.901 5.994 6.227 6.574 6.139
   3243   1390498_at   7.691 7.577 7.632 7.938 7.532 8.427 8.733 8.532 8.43 8.729
   3244   1381311_at   7.037 6.959 6.883 7.228 7.193 6.434 6.092 6.178 6.068 6.016
   3245   1374995_at   7.29 7.375 7.324 7.193 7.488 7.987 8.059 8.264 7.966 7.92
   3246   1367798_at   10.135 10.176 10.161 10.212 10.171 10.792 10.828 10.943 10.671 10.575
   3247   1368288_at   8.11 7.894 7.894 7.8 6.272 10.502 10.714 10.81 10.614 10.687
   3248   1371730_at   9.475 9.581 9.456 9.786 9.718 10.317 10.535 10.461 10.405 10.212
   3249   1380090_at   4.835 4.434 5.018 4.889 5.213 6.044 6.106 6.031 6.022 6.06
   3250   1395036_at   6.694 6.801 6.546 6.808 6.643 5.766 5.74 5.504 5.731 5.174
   3251   1391573_at   7.782 7.791 7.991 7.994 7.846 9.308 9.146 8.902 8.703 9.359
   3252   1383643_at   9.888 9.916 9.942 10.08 10.182 9.36 9.4 9.504 9.428 9.28
   3253   1374466_at   6.856 6.811 6.861 6.985 6.855 7.93 8.203 8.12 7.914 7.541
   3254   1374104_at   6.369 6.479 6.238 6.753 6.815 7.532 7.782 7.764 7.608 7.542
   3255   1368163_at   11.126 11.107 10.997 10.86 10.888 11.816 11.88 11.557 11.63 11.806
   3256   1388725_at   9.572 9.632 9.667 9.794 9.89 8.905 9.152 8.876 8.739 8.865
   3257   1378505_at   4.748 3.97 4.317 4.647 4.573 6.391 6.528 5.797 6.601 6.125
   3258   1388465_at   7.59 7.577 7.468 7.594 7.489 8.342 8.425 8.822 8.437 8.254
   3259   1374171_at   5.887 6.051 5.829 5.79 6.416 7.512 7.77 7.257 7.178 7.317
   3260   1376641_at   7.961 8.103 8.134 8.166 8.104 7.226 7.052 7.346 7.424 6.917
   3261   1375071_at   6.525 6.589 6.676 6.328 6.433 4.19 5.082 5.065 4.864 5.091
   3262   1372728_at   7.474 7.481 7.481 7.761 7.767 8.878 8.828 9.446 8.748 8.76
   3263   1387950_at   8.141 8.179 8.09 8.149 8.368 7.526 7.744 7.492 7.612 7.492
   3264   1373549_at   7.096 6.959 7.067 7.319 7.262 6.281 6.212 6.544 6.329 6.406
   3265   1399010_at   8.758 8.72 8.769 8.954 8.88 9.221 9.215 9.285 9.24 9.333
   3266   1367856_at   10.139 10.165 10.125 10.244 10.039 8.827 9.002 9.45 8.892 8.746
   3267   1371254_at   11.843 11.764 11.863 11.743 11.722 12.105 12.103 12.071 12.193 12.073
   3268   1368476_at   6.35 6.237 6.222 6.263 6.388 7.451 7.455 8.096 7.346 7.439
   3269   1389551_at   10.269 10.28 10.726 10.364 9.923 11.565 11.696 11.373 11.595 11.675
   3270   1383058_at   8.163 8.067 8.37 8.421 8.129 9.936 10.066 10.426 9.666 9.422
   3271   1378043_at   7.555 7.464 7.549 7.217 7.315 3.746 2.198 0.299 1.281 0.367
   3272   1369370_s_at   6.914 7.057 7.174 7.308 6.87 8.074 8.032 7.793 7.899 7.877
   3273   1398997_at   9.204 9.147 9.144 9.297 9.417 9.899 9.961 9.986 9.942 9.703
   3274   1393732_at   6.026 6.354 6.065 6.412 6.335 7.923 9.043 8.085 8.331 9.029
   3275   1390122_at   7.459 7.423 7.343 7.377 7.839 5.971 5.67 6.306 6.365 6.149
   3276   1393728_at   6.676 6.34 6.608 7.237 6.578 4.867 4.646 5.093 4.232 4.988
   3277   1372402_at   8.867 9 8.884 9.005 9.203 8.128 8.274 8.301 8.211 7.917
   3278   1389001_at   9.197 9.254 9.266 9.422 9.421 8.499 8.534 8.327 8.709 8.696
   3279   1391518_at   6.453 6.34 6.102 6.455 6.619 3.589 4.406 4.41 4.918 4.126
   3280   1385400_at   7.029 7.311 7.371 7.597 7.246 8.808 8.874 9.121 8.537 8.383
   3281   1393317_at   8.821 8.858 8.873 8.822 8.89 7.917 8.234 8.364 8.224 8.145
   3282   1376797_at   6.781 6.723 6.847 6.999 7.111 8.081 8.015 8.041 7.706 7.683
   3283   1369122_at   8.77 8.619 8.65 8.812 9.059 7.905 8.015 7.95 7.822 7.639
   3284   1370469_at   8.076 7.95 8.292 7.952 7.879 3.471 2.302 4.596 4.648 4.463
   3285   1395248_at   7.972 8.192 8.174 8.159 8.102 7.374 7.117 6.781 7.172 7.273
   3286   1389012_at   10.895 10.878 10.872 10.722 10.94 11.303 11.393 11.254 11.288 11.213
   3287   1379308_at   7.209 7.328 7.237 7.261 6.837 8.837 9.142 8.291 9.134 9.258
   3288   1382679_at   6.965 7.144 6.823 6.89 6.956 6.338 6.354 6.029 6.109 6.216
   3289   1394954_at   8.539 8.373 8.444 8.502 8.452 7.704 7.673 7.937 7.823 7.478
   3290   1383354_a_at   7.027 7.284 7.302 7.258 7.283 6.411 6.7 6.386 6.606 6.441
   3291   1373144_at   6.761 6.991 6.849 6.999 6.845 7.412 7.506 7.539 7.344 7.359
   3292   1377696_at   7.853 8.109 7.857 7.877 7.591 6.816 6.826 6.607 7.089 6.881
   3293   1379710_at   4.825 4.741 4.841 5.002 4.851 6.269 6.284 6.902 6.525 5.908
   3294   1379243_at   10.118 10.231 10.504 10.616 10.459 11.333 11.399 11.4 11.394 11.133
   3295   1372148_at   8.273 8.033 8.165 8.097 8.001 7.667 7.51 7.546 7.579 7.422
   3296   1389234_at   6.834 6.815 6.847 7.173 5.938 8.627 8.601 8.515 8.589 8.774
   3297   1393212_at   4.92 4.594 4.99 4.842 4.584 6.432 6.212 6.839 5.945 6.211
   3298   1389102_at   7.937 8.243 7.886 7.999 8.068 8.726 8.874 9.034 8.798 8.675
   3299   1393791_at   4.53 4.276 5.057 4.798 4.722 0 2.18 0 0.556 1.195
   3300   1373924_at   8.19 8.494 8.378 8.325 8.362 9.122 8.964 9.05 9.091 8.818
   3301   1388985_at   10.004 10.032 9.759 9.772 10.465 8.527 8.777 8.554 8.681 8.912
   3302   1376573_at   8.703 8.692 8.515 8.46 8.498 9.424 9.51 9.141 9.58 9.592
   3303   1373373_at   9.141 9.199 9.2 9.392 9.368 8.21 8.649 8.451 8.19 8.391
   3304   1375932_at   9.22 9.306 9.115 9.138 9.095 10.054 10.212 10.294 9.765 10.012
   3305   1391539_at   7.696 7.919 8.422 8.02 7.406 9.708 10.025 9.958 9.388 9.61
   3306   1391222_at   6.547 6.639 7.196 6.991 7.532 5.162 4.483 4.649 5.265 4.691
   3307   1378029_at   5.555 5.863 6.199 5.739 5.648 7.445 7.601 7.036 7.041 7.115
   3308   1398523_a_at   6.577 6.656 6.524 6.636 6.408 5.736 5.464 5.174 5.382 4.988
   3309   1376291_at   5.853 6.321 6.366 6.416 6.503 4.873 4.157 4.863 4.277 4.748
   3310   1396229_at   5.546 4.71 5.149 5.148 5.112 6.339 6.89 6.98 6.586 6.695
   3311   1372637_at   4.451 4.192 3.905 4.323 3.977 6.053 6.035 6.985 6.094 5.83
   3312   1388942_at   8.414 8.669 8.628 8.684 8.616 9.288 9.349 9.117 9.305 9.105
   3313   1384041_at   7.159 6.606 6.739 7.013 6.942 5.556 5.332 5.478 5.86 5.105
   3314   1372997_at   9.022 9.159 8.975 9.083 9.049 10.029 9.66 9.818 9.605 9.881
   3315   1374766_at   8.536 8.64 8.707 8.677 8.57 9.378 9.529 9.349 9.142 9.185
   3316   1384228_at   6.694 7.142 7.195 7.135 6.919 3.764 5.422 4.44 4.245 4.232
   3317   1388342_at   8.1 8.037 7.926 8.042 7.862 7.043 7.333 6.796 7.169 7.171
   3318   1370978_at   9.28 9.257 9.233 9.299 9.222 8.379 8.585 8.705 8.196 8.364
   3319   1389165_at   9.658 9.637 9.731 9.569 9.592 8.931 9.033 8.634 8.986 9.03
   3320   1369013_a_at   11.366 11.336 11.305 11.193 11.518 10.786 10.833 10.856 10.842 10.901
   3321   1394705_at   8.638 8.776 8.819 8.832 8.506 9.241 9.428 9.466 9.411 9.52
   3322   1384304_at   5.854 5.415 5.903 5.571 4.951 7.378 7.258 7.023 7.084 7.168
   3323   1373921_at   7.327 7.543 7.162 7.451 7.417 8.587 8.617 9.264 8.622 8.534
   3324   1389459_at   8.334 8.258 8.38 8.259 8.141 8.685 8.733 8.813 8.649 8.798
   3325   1389836_a_at   10.851 10.795 11.083 11.037 10.587 11.631 11.735 11.653 11.713 11.722
   3326   1392732_at   7.925 7.965 8.093 8.187 7.947 4.326 5.15 5.18 3.058 4.754
   3327   1398869_at   10.243 10.251 10.252 10.373 10.415 9.809 9.807 9.92 9.846 9.661
   3328   1397767_at   5.987 6.281 6.296 6.185 6.575 5.032 5.261 4.773 5.086 4.647
   3329   1372778_at   10.034 9.993 9.873 10.076 10.287 9.365 9.408 9.116 9.323 9.248
   3330   1377173_at   7.229 6.873 7.273 7.18 7.272 6.279 5.975 6.342 6.305 5.986
   3331   1370638_at   7.408 7.522 7.902 7.888 7.495 8.797 9.2 8.658 8.837 9.017
   3332   1371252_at   8.57 8.521 8.668 8.899 8.894 4.658 2.83 4.931 2.664 1.945
   3333   1370414_at   6.518 6.588 6.668 6.845 7.033 5.412 5.604 5.791 5.194 5.298
   3334   1371468_at   8.544 8.428 8.287 8.581 8.502 9.476 9.596 9.401 9.092 9.623
   3335   1373679_at   7.52 7.419 7.699 7.605 7.457 6.646 6.896 6.496 6.379 6.708
   3336   1392888_at   9.513 9.69 9.617 9.779 9.636 10.598 10.58 10.167 10.591 10.617
   3337   1373782_a_at   6.787 6.877 6.337 6.263 6.706 5.106 4.941 5.122 4.714 5.436
   3338   1374632_at   7.265 7.571 7.158 7.255 7.43 6.658 6.559 6.466 6.359 6.603
   3339   1372403_at   6.672 6.57 6.582 7.036 6.323 7.85 7.789 7.631 7.646 7.751
   3340   1388244_s_at   11.764 11.689 11.763 11.86 11.881 11.331 11.353 11.426 11.33 11.189
   3341   1382139_at   7.998 8.205 8.205 8.206 8.13 7.598 7.676 7.734 7.644 7.483
   3342   1386928_at   8.436 8.387 8.473 8.41 8.311 9.308 9.301 9.677 9.231 9.075
   3343   1377058_at   6.092 6.507 6.645 6.567 6.598 5.189 4.709 4.172 4.785 4.902
   3344   1376809_at   6.187 6.34 6.285 6.314 6.402 7.28 7.091 7.297 6.852 7.028
   3345   1385086_at   7.105 7.193 7.178 7.194 7.148 3.428 4.279 3.208 3.274 1.506
   3346   1370264_at   7.051 6.772 7.169 7.062 6.992 7.817 7.893 8.05 7.64 7.993
   3347   1369493_at   6.942 7.1 7.252 7.578 6.403 8.725 9.089 9.316 8.858 9.031
   3348   1370865_at   11.244 11.233 11.279 11.018 11.025 11.749 11.816 11.624 11.793 11.755
   3349   1367456_at   12.2 12.151 12.114 11.866 11.931 11.467 11.491 11.319 11.379 11.375
   3350   1390268_at   8.036 8.173 8.251 8.272 8.509 7.252 7.582 7.251 7.158 7.286
   3351   1379525_at   8.261 8.461 8.57 8.817 8.397 9.485 9.58 9.315 9.509 9.345
   3352   1383542_at   8.528 8.317 8.344 8.327 8.352 6.052 6.347 7.179 5.881 6.326
   3353   1369954_at   11.109 11.11 11.141 11.161 10.892 12.135 12.07 11.729 12.318 12.27
   3354   1370851_a_at   4.611 4.321 4.932 4.663 4.278 5.646 5.724 5.592 5.813 5.892
   3355   1369654_at   7.276 7.038 7.666 7.412 7.348 8.735 9.175 8.394 8.899 8.763
   3356   1372569_at   7.83 7.777 8.119 7.683 7.76 6.865 6.689 7.054 6.929 6.576
   3357   1376065_at   7.258 7.207 7.274 7.511 7.533 6.459 6.421 6.226 6.663 6.557
   3358   1371435_at   12.04 11.968 11.86 11.838 12.121 11.518 11.486 11.437 11.436 11.4
   3359   1372518_at   7.058 7.3 6.405 6.967 7.762 4.807 4.949 5.27 4.803 5.074
   3360   1375867_at   7.651 7.585 7.704 7.664 7.809 8.177 8.115 8.332 8.227 8.089
   3361   1371479_at   8.101 8.063 8.005 8.131 8.165 8.885 8.652 8.825 8.689 8.523
   3362   1382663_at   7.989 8.094 7.947 8.419 8.351 2.111 2.716 5.116 3.966 3.474
   3363   1392464_at   8.323 8.225 8.313 8.404 8.291 7.703 7.703 7.873 7.481 7.572
   3364   1382396_at   7.982 8.003 7.888 8.126 8.217 7.353 7.304 7.222 7.536 7.256
   3365   1390674_at   6.913 7.2 7.381 7.225 7.01 3.048 4.286 4.453 4.482 2.811
   3366   1379278_at   8.048 8.094 8.094 8.195 8.239 7.402 7.537 7.481 7.427 7.101
   3367   1374182_at   7.206 7.182 7.238 7.372 7.268 6.143 6.608 6.548 6.518 6.3
   3368   1373952_at   6.436 6.627 6.685 6.66 6.769 7.243 7.171 7.33 7.162 7.149
   3369   1388926_at   9.75 9.734 9.97 9.995 9.787 10.554 10.479 10.597 10.429 10.347
   3370   1374791_at   5.458 5.532 5.623 5.027 5.492 6.827 6.632 6.78 6.543 6.346
   3371   1397328_at   6.933 7.183 7.205 6.928 7.23 5.946 5.96 5.697 6.305 5.676
   3372   1370899_at   8.564 8.477 8.214 8.593 8.488 7.344 7.601 7.303 6.935 7.437
   3373   1374550_at   7.664 7.72 7.9 8.158 8.157 6.661 6.581 7.052 6.728 6.639
   3374   1369105_a_at   4.706 4.263 4.586 4.472 5.33 1.736 2.932 1.744 2.23 2.154
   3375   1370207_at   10.938 10.938 10.979 11.112 11.114 11.498 11.532 11.77 11.719 11.783
   3376   1398759_at   12.386 12.333 12.247 12.276 12.289 12.045 12.014 12.075 11.982 12.075
   3377   1390434_at   7.654 7.347 7.557 7.498 7.643 6.599 6.621 6.785 6.54 6.198
   3378   1389123_at   8.23 8.146 8.087 7.788 7.75 6.89 6.925 6.946 6.938 6.489
   3379   1368588_at   7.2 7.336 7.363 7.572 7.442 6.421 6.792 6.596 6.676 6.53
   3380   1388801_at   8.052 8.073 7.941 8.152 8.184 7.414 7.403 7.654 7.418 7.571
   3381   1374420_at   10.457 10.407 10.779 10.683 10.341 11.367 11.55 11.244 11.401 11.44
   3382   1388378_at   11.083 11.01 11.025 11.129 11.107 10.713 10.805 10.784 10.672 10.64
   3383   1372764_at   8.855 8.734 8.749 8.909 8.797 8.044 8.245 8.203 7.95 7.82
   3384   1369409_at   8.335 8.454 8.369 8.382 7.907 7.09 7.045 7.274 7.428 7.29
   3385   1376691_at   7.967 8.002 8.184 8.098 7.688 9.477 9.501 8.933 9.457 9.798
   3386   1373378_at   7.492 7.242 7.182 7.502 7.144 8.446 8.557 8.253 8.073 8.269
   3387   1397852_at   7.043 6.962 7.169 7.119 7.035 6.134 5.851 5.888 6.422 5.853
   3388   1383514_s_at   8.03 8.046 7.77 7.835 7.839 7.173 7.169 6.726 6.906 7.042
   3389   1392563_at   6.912 6.809 7.013 7.3 6.777 7.871 7.885 8.172 8.005 8.258
   3390   1388327_at   11.893 11.848 12.011 11.836 11.742 12.445 12.263 12.489 12.467 12.55
   3391   1377878_at   5.231 5.241 5.309 5.393 5.144 6.452 6.601 6.158 6.119 6.049
   3392   1390406_at   8.196 8.199 8.25 8.332 8.347 9.13 9.221 9.558 9.082 8.972
   3393   1371549_at   9.495 9.404 9.542 9.496 9.284 10.132 10.113 10.098 9.91 9.925
   3394   1370499_at   6.564 6.454 6.829 6.123 6.171 4.592 3.335 3.797 4.215 3.181
   3395   1373703_at   8.265 8.178 7.543 8.104 8.286 6.526 6.631 6.941 6.366 6.499
   3396   1393060_at   4.884 5.221 5.14 5.084 5.482 7.443 7.29 6.459 6.733 7.275
   3397   1384449_at   6.582 6.073 6.279 6.06 5.942 3.152 3.974 4.018 4.525 4.15
   3398   1375516_at   10.665 10.636 10.742 10.789 10.669 11.17 11.206 11.334 11.181 11.038
   3399   1388150_at   9.532 9.54 9.273 9.426 9.404 8.609 8.807 8.458 8.32 8.669
   3400   1368390_at   7.843 7.809 8.152 8.064 7.82 8.707 8.523 8.62 8.678 8.608
   3401   1394407_at   7.145 7.128 7.015 7.277 7.172 8.062 8.311 7.894 7.871 7.851
   3402   1376277_at   6.328 6.295 6.502 6.437 6.546 6.999 7.071 7.006 6.829 6.979
   3403   1387010_s_at   6.507 7.06 6.825 6.917 6.322 8.597 8.368 8.888 8.063 8.31
   3404   1393405_at   5.311 5.797 5.528 5.431 5.504 4.109 4.42 4.276 4.534 3.849
   3405   1399085_at   8.935 9.056 9.173 9.179 9.083 9.793 9.907 10.158 9.741 9.76
   3406   1375315_at   7.302 7.471 7.207 7.124 7.326 8.09 8.119 8.481 8.076 8.452
   3407   1390728_at   7.634 7.731 7.67 7.572 7.777 6.583 6.829 6.583 6.908 6.266
   3408   1398844_at   10.025 10.04 10.131 10.063 10.014 10.5 10.529 10.732 10.452 10.471
   3409   1383194_a_at   5.153 5.633 5.611 5.08 5.708 6.86 6.82 6.71 6.816 6.497
   3410   1370419_a_at   7.259 7.06 7.272 7.911 7.498 6.195 6.032 5.904 5.742 5.865
   3411   1374804_at   7.373 7.469 7.284 7.313 7.354 6.702 6.62 6.23 6.602 6.654
   3412   1382131_at   7.365 7.512 7.105 7.247 7.26 8.332 8.502 8.075 8.139 8.545
   3413   1374738_at   6.921 7.021 6.817 7.074 6.86 8.193 8.342 7.632 8.106 8.071
   3414   1387361_s_at   11.775 11.768 11.91 11.801 11.873 11.328 11.472 11.241 11.451 11.398
   3415   1389288_at   10.023 10.034 10.062 10.202 10.157 10.714 10.79 10.818 10.735 10.481
   3416   1395336_at   6.429 6.438 6.716 6.902 6.65 5.377 5.264 4.756 5.537 5.055
   3417   1368215_at   9.828 9.743 9.887 9.894 9.669 10.217 10.208 10.165 10.246 10.28
   3418   1378536_at   7.658 7.894 7.904 7.809 7.621 8.878 9.24 8.649 8.751 8.704
   3419   1384910_at   0.669 0.757 1.766 1.982 1.744 4.91 4.051 3.966 5.087 4.625
   3420   1397313_at   8.626 8.643 8.54 8.401 8.571 7.823 7.682 7.212 7.489 7.631
   3421   1394056_at   6.185 6.05 5.901 5.903 5.871 6.644 7.023 6.847 6.648 6.87
   3422   1387407_at   6.236 6.335 6.484 6.363 6.534 7.155 7.377 6.998 7.027 7.195
   3423   1382780_at   6.547 6.413 6.958 6.453 6.55 8.044 7.918 7.535 7.61 7.933
   3424   1371202_a_at   8.109 7.98 7.949 8.271 8.191 8.868 9.04 9.149 8.735 8.879
   3425   1394737_at   8.71 8.712 8.764 9.049 8.981 9.571 9.574 9.529 9.456 9.451
   3426   1383715_at   7.519 7.449 7.567 7.6 7.428 8.097 8.019 8.044 7.849 7.945
   3427   1386466_at   6.85 6.899 6.781 6.674 6.749 7.913 8.156 7.833 7.458 7.821
   3428   1383001_at   6.399 6.578 6.564 6.995 6.796 4.857 5.077 4.474 5.335 5.288
   3429   1389015_at   8.172 8.316 8.557 8.364 8.203 9.076 9.243 9.039 9.012 8.949
   3430   1388414_at   10.744 10.697 10.866 10.939 10.936 11.28 11.347 11.432 11.39 11.281
   3431   1376161_at   8.385 8.326 8.612 8.515 8.346 9.003 9.008 8.87 9.016 8.971
   3432   1370397_at   10.794 10.744 11.249 10.976 10.587 11.958 11.985 11.976 12.302 12.331
   3433   1374083_at   5.943 5.793 5.58 6.027 6.058 6.662 6.91 7.012 6.965 6.732
   3434   1381461_at   8.957 8.933 8.759 8.974 9.149 7.836 7.964 8.182 7.6 7.602
   3435   1384302_at   7.209 7.423 7.39 7.554 6.977 9.152 9.412 9.861 8.896 8.784
   3436   1390717_at   7.985 7.944 8.233 8.337 8.051 8.932 9.257 8.826 9.049 9.078
   3437   1374611_at   8.249 8.212 8.444 8.384 8.271 7.963 7.841 7.824 7.879 7.837
   3438   1397916_s_at   7.711 7.773 8.201 7.774 7.803 6.603 6.427 6.944 6.715 6.3
   3439   1389238_at   8.141 7.858 7.928 8.32 8.355 7.125 7.223 7.258 7.075 7.245
   3440   1374315_at   4.828 4.624 5.038 4.881 4.572 6.036 6.002 5.843 5.957 6.532
   3441   1386857_at   11.025 10.979 10.837 11.033 11.087 10.407 10.542 10.29 10.514 10.487
   3442   1374411_at   9.096 9.016 9.065 9.078 9.257 8.576 8.662 8.375 8.595 8.499
   3443   1374418_at   9.065 9.017 8.819 8.935 9.154 8.247 8.459 8.244 8.349 8.451
   3444   1369497_at   7.876 7.904 7.446 7.976 8.02 3.604 3.562 1.189 3.109 4.289
   3445   1367609_at   11.417 11.396 11.493 11.595 11.399 11.932 11.844 11.941 12.001 11.815
   3446   1390977_at   6.535 6.44 6.71 6.443 6.613 7.244 7.076 7.308 7.13 7.066
   3447   1373515_at   8.019 7.998 8.145 8.074 7.92 8.872 9.205 8.822 8.785 8.684
   3448   1391328_at   4.695 5.353 5.535 3.259 5.429 1.31 1.05 1.131 0.807 0.993
   3449   1380365_at   4.859 4.737 4.702 4.908 5.195 6.433 6.528 6.603 5.796 6.473
   3450   1370785_s_at   10.28 10.33 10.403 10.355 10.253 10.815 10.807 10.912 10.751 10.636
   3451   1382127_at   9.009 9.191 9.168 9.206 8.466 10.372 10.422 10.186 10.194 10.385
   3452   1371155_at   5.189 5.157 6.657 5.107 4.394 2.176 1.566 1.036 1.895 1.628
   3453   1398608_at   7.644 7.604 7.763 7.56 7.478 6.463 6.669 6.753 6.571 6.084
   3454   1398449_at   7.451 7.293 7.586 7.61 7.089 1.541 3.173 3.913 3.299 1.137
   3455   1368231_at   7.479 7.265 7.555 7.507 7.365 6.85 6.455 6.758 6.515 6.639
   3456   1399057_at   11.088 11.093 11.105 11.074 11.172 10.801 10.874 10.822 10.811 10.687
   3457   1376026_at   7.069 7.032 6.848 6.851 6.782 4.941 4.951 4.586 4.341 5.665
   3458   1371354_at   6.588 6.665 6.88 6.488 6.227 7.744 7.766 8.033 7.639 7.55
   3459   1396127_at   3.892 5.13 5.045 4.446 4.368 7.274 7.197 6.397 7.127 7.071
   3460   1374629_at   6.987 7.111 6.86 7.017 7.349 5.959 5.753 6.17 6.211 5.794
   3461   1367652_at   8.127 8.052 8.359 8.231 8.136 10.548 10.495 9.42 10.651 10.037
   3462   1378679_at   10.763 10.81 10.582 10.571 10.722 9.231 9.355 8.569 8.642 9.422
   3463   1387879_a_at   6.555 7.507 7.471 7.054 7.075 5.24 5.408 5.07 5.433 5.692
   3464   1391252_at   7.571 7.513 7.646 7.755 7.623 7.169 7.034 6.944 7.034 6.813
   3465   1383416_at   7.197 6.603 7.018 7.325 6.868 8.534 8.402 8.353 8.052 8.238
   3466   1373624_at   8.276 8.511 8.429 8.716 8.479 9.609 9.461 9.527 9.283 9.179
   3467   1379322_at   8.857 8.95 8.848 8.963 9.044 8.274 8.559 8.39 8.393 8.28
   3468   1385814_at   6.278 6.021 6.191 5.973 6.154 5.261 5.012 4.831 5.411 5.043
   3469   1372386_at   7.669 7.47 7.424 7.565 7.628 6.936 7.065 6.811 7.06 6.86
   3470   1379431_at   6.291 6.446 6.656 6.144 6.116 7.578 7.659 7.229 7.319 7.455
   3471   1376870_at   5.468 5.23 5.535 5.933 5.573 6.98 6.81 7.095 6.767 6.541
   3472   1387472_at   8.392 8.373 9.065 8.78 8.224 2.16 2.526 4.879 4.039 4.53
   3473   1377808_at   4.584 5.023 5.062 4.618 4.863 6.283 5.941 6.243 5.895 5.811
   3474   1388739_at   8.451 8.486 8.572 8.581 8.557 7.833 8.06 7.643 7.731 7.948
   3475   1384242_at   7.001 7.684 7.342 7.521 7.42 6.207 6.41 6.382 6.058 6.204
   3476   1375785_at   7.228 7.342 7.805 7.518 7.363 8.357 8.529 8.393 8.276 8.407
   3477   1389617_at   8.634 8.628 8.822 8.831 8.745 8.048 8.228 7.787 7.925 7.968
   3478   1374425_at   4.539 4.117 4.1 4.223 4.277 5.717 5.852 5.641 5.202 5.296
   3479   1390290_at   7.508 7.64 7.593 7.924 8.083 6.711 6.806 6.839 6.764 6.796
   3480   1382431_at   8.129 8.319 8.278 8.424 7.886 7.067 6.965 6.824 7.319 7.232
   3481   1388814_at   9.591 9.547 9.851 9.835 9.278 11.014 11.031 10.528 11.141 11.124
   3482   1388481_at   12.25 12.209 12.182 12.254 12.31 11.814 11.69 11.737 11.96 11.748
   3483   1367951_at   1.7 2.457 1.828 0.604 0 5.823 5.089 6.074 5.227 5.384
   3484   1372829_at   9.774 9.762 9.621 9.879 10.001 9.04 9.036 9.067 8.906 8.657
   3485   1388918_at   8.449 8.392 8.572 8.802 8.539 9.502 9.288 9.483 9.181 9.346
   3486   1392203_at   7.538 7.639 7.864 7.862 7.859 9.001 9.11 8.833 8.936 8.477
   3487   1381937_at   6.207 6.002 6.368 6.104 6.794 7.692 7.651 7.447 7.51 7.536
   3488   1397830_at   7.256 7.155 7.475 7.207 7.242 9.214 8.658 8.354 9.094 8.547
   3489   1387926_at   8.164 8.201 8.328 8.063 7.893 9.092 9.127 8.75 9.022 8.958
   3490   1386045_at   4.403 3.942 4.133 3.2 2.832 6.486 6.243 6.657 6.459 6.136
   3491   1397609_at   5.684 5.606 6.046 5.833 6.227 2.524 0.872 2.202 0 0
   3492   1388626_at   8.71 8.624 8.713 8.822 8.662 9.273 9.458 9.52 9.214 9.214
   3493   1387763_at   6.321 6.359 6.728 6.49 6.448 3.646 2.658 3.011 4.529 3.87
   3494   1380450_at   6.286 6.726 7.423 6.888 6.647 5.257 4.527 4.948 4.648 4.808
   3495   1385805_at   7.849 7.909 8.009 8.245 8.098 8.685 8.696 8.952 8.75 8.741
   3496   1384385_at   0 0 2.646 1.799 0 6.272 6.156 6.542 5.607 5.599
   3497   1372509_at   7.375 7.624 7.624 7.454 7.458 8.6 8.531 8.848 8.361 8.238
   3498   1376268_at   9.328 9.359 9.481 9.665 9.819 8.343 8.616 7.98 8.32 8.083
   3499   1393817_at   6.044 5.972 6.134 6.22 5.824 4.74 5.093 4.267 4.821 4.764
   3500   1373074_at   7.936 7.604 7.641 7.741 7.789 8.681 8.807 8.809 8.494 8.39
   3501   1376239_at   6.627 6.311 6.134 5.777 6.404 8.23 8.003 8.482 7.87 7.618
   3502   1398749_at   12.2 12.103 11.89 12.022 12.111 11.651 11.596 11.578 11.569 11.564
   3503   1398598_at   9.132 9.157 9.063 9.248 9.017 9.67 10.074 10.12 9.811 9.982
   3504   1393091_at   7.604 7.782 7.511 7.602 7.844 7.057 7.139 7.119 7.109 7.141
   3505   1392984_at   7.499 7.527 7.779 7.543 7.573 7.041 7.03 7.114 7.177 7.024
   3506   1389522_at   7.502 7.888 7.811 7.561 7.463 8.594 8.435 8.673 8.505 8.384
   3507   1395331_at   8.354 8.416 8.425 8.55 8.604 9.482 9.234 9.121 9.087 9.136
   3508   1398760_at   12.186 12.05 12.167 12.055 12.291 11.73 11.753 11.63 11.585 11.675
   3509   1370542_a_at   8.188 8.053 8.048 7.766 7.74 6.991 6.851 6.363 6.685 6.423
   3510   1383376_at   7.896 7.974 8.001 7.643 7.52 8.808 8.63 8.706 8.629 8.658
   3511   1374129_at   8.076 8.199 8.243 8.103 8.186 8.889 8.723 8.637 8.891 8.609
   3512   1387054_at   6.711 6.626 6.543 6.785 6.529 5.377 5.587 5.99 5.408 5.625
   3513   1383714_at   7.626 7.778 7.741 8.073 7.89 8.417 8.592 8.544 8.629 8.582
   3514   1375882_at   8.386 8.725 8.456 8.697 8.737 7.749 7.912 7.845 7.544 7.743
   3515   1371987_at   7.723 7.67 7.58 7.467 7.737 7.046 6.872 7.109 7.073 7.128
   3516   1383224_at   8.962 8.903 8.639 8.822 8.77 9.433 9.627 9.839 9.513 9.544
   3517   1372232_at   8.016 8.206 8.141 8.151 8.294 8.939 8.92 9.309 8.813 8.964
   3518   1389454_at   9.775 9.824 9.684 9.902 10.087 9.131 9.187 9.112 8.988 8.835
   3519   1392043_at   6.018 5.967 6.171 6.142 5.744 7.036 7.104 6.956 6.75 6.755
   3520   1388550_at   10.628 10.689 10.58 10.629 10.691 10.216 10.373 10.184 10.097 10.199
   3521   1372617_at   7.674 7.881 7.86 8.14 8.05 8.678 8.579 8.763 8.642 8.754
   3522   1379987_at   7.104 7.133 7.189 7.136 7.027 6.199 6.175 6.538 5.994 6.407
   3523   1379467_at   3.845 4.987 5.317 4.591 4.545 7.999 7.165 6.991 7.198 7.143
   3524   1377480_at   7.154 7.149 7.061 7.059 7.288 6.72 6.648 6.736 6.627 6.521
   3525   1367689_a_at   7.269 7.033 6.786 6.933 7.019 8.402 8.219 7.833 8.562 8.325
   3526   1387914_at   8.988 8.755 8.442 8.677 8.893 7.353 7.156 7.647 6.788 6.876
   3527   1382476_x_at   5.002 5.035 5.167 5.143 4.418 6.211 6.321 6.086 6.139 6.349
   3528   1370629_at   4.834 4.174 4.267 4.535 4.111 2.644 2.941 2.536 3.093 2.31
   3529   1381983_at   6.55 6.454 6.598 6.695 6.41 7.318 7.366 7.13 7.398 7.075
   3530   1382792_at   5.69 5.629 5.984 5.966 5.785 4.315 3.513 4.594 3.914 4.181
   3531   1397507_at   5.876 5.287 5.304 5.444 5.281 2.84 0.164 0.651 0 0.227
   3532   1372182_at   10.351 10.382 10.344 10.529 10.502 8.7 8.808 9.464 8.506 8.4
   3533   1393162_at   7.06 7.159 6.863 7.202 7.335 5.396 6.093 5.549 5.213 5.822
   3534   1369966_a_at   12.631 12.504 12.417 12.374 12.586 11.997 12.022 11.827 11.754 11.843
   3535   1390923_a_at   7.677 7.76 8.112 7.88 7.856 8.869 9.026 8.766 8.767 8.536
   3536   1376206_at   7.491 7.61 7.539 7.537 7.385 6.568 6.919 6.369 6.768 6.578
   3537   1382049_at   6.291 5.891 6.21 6.056 6.02 6.796 7.121 6.803 7.013 6.858
   3538   1383633_at   9.333 9.347 9.53 9.391 9.246 10.186 10.47 10.47 10.028 10.119
   3539   1387783_a_at   9.426 9.513 9.247 9.535 8.805 10.896 10.754 10.398 11.02 10.888
   3540   1389559_at   9.273 9.171 9.253 9.351 9.427 8.684 8.92 8.659 8.723 8.749
   3541   1392134_x_at   6.737 6.751 6.825 6.546 6.772 5.703 5.411 4.907 5.667 5.467
   3542   1393992_at   5.957 5.305 5.276 5.62 5.44 7.025 7.59 7.289 7.034 6.761
   3543   1383111_at   9.986 10.005 10.14 10.05 9.03 11.718 11.756 11.457 11.676 11.766
   3544   1385088_at   6.985 6.95 7.088 7.116 7.083 7.848 7.934 7.793 7.579 7.561
   3545   1382746_s_at   6.301 6.178 6.086 6.556 6.568 4.481 5.135 4.962 5.22 4.956
   3546   1393240_at   8.022 7.826 7.505 7.903 8.373 6.651 6.784 6.72 6.66 6.641
   3547   1372987_at   7.451 7.69 7.794 7.817 7.845 6.771 7.017 7.095 6.897 6.875
   3548   1367864_at   7.601 7.7 7.66 7.948 7.877 8.718 8.92 9.095 8.72 8.506
   3549   1382293_at   9.005 8.964 9.031 8.747 8.995 10.18 10.164 10.822 10.271 9.954
   3550   1395587_at   7.05 6.759 7.289 6.992 7.321 6.247 6.131 6.037 5.918 5.977
   3551   1372357_at   8.714 8.662 8.689 8.668 8.73 8.01 8.338 8.13 7.971 7.981
   3552   1389650_at   6.235 6.271 6.347 6.518 6.425 7.699 7.215 7.576 7.116 7.37
   3553   1370393_at   6.847 6.864 6.513 6.979 6.941 5.395 5.458 5.302 5.968 5.174
   3554   1398491_at   7.03 7.266 7.172 7.162 7.016 8.055 8.013 8.425 7.976 7.841
   3555   1380037_at   5.887 6.196 5.992 6.042 6.272 4.164 3.18 3.931 4.563 4.196
   3556   1378013_at   6.566 6.762 6.722 6.593 5.264 9.245 9.412 8.565 9.553 9.445
   3557   1398780_at   9.574 9.55 9.747 9.841 9.876 10.425 10.413 10.583 10.406 10.287
   3558   1387413_at   7.695 7.717 7.748 7.544 7.253 4.721 3.508 3.912 5.472 4.832
   3559   1375491_at   0.595 1.091 0.714 2.585 1.275 4.269 4.255 4.44 4.276 4.377
   3560   1396521_at   6.449 6.471 6.637 6.629 6.625 5.686 5.329 4.795 5.296 5.329
   3561   1374885_at   7.365 7.573 7.105 7.439 7.286 8.235 8.419 8.08 8.206 8.498
   3562   1387223_at   11.987 11.896 12.111 11.89 11.712 12.544 12.434 12.577 12.596 12.646
   3563   1391946_at   5.848 5.901 5.652 5.731 6.667 4.218 3.847 3.037 3.362 3.648
   3564   1387737_at   9.431 8.899 9.42 9.258 8.87 10.674 10.157 10.575 10.59 10.738
   3565   1389386_at   8.636 8.709 8.755 8.783 8.745 9.451 9.405 9.082 9.394 9.318
   3566   1385187_at   7.425 7.346 7.378 7.479 7.47 6.958 6.621 6.579 6.571 6.426
   3567   1370141_at   9.733 9.81 9.835 10.001 9.856 9.338 9.072 8.913 9.213 9.116
   3568   1398448_at   7.203 7.403 7.153 7.394 7.097 8.086 8.21 7.9 7.9 7.935
   3569   1376218_a_at   5.165 5.076 5.31 5.718 5.294 4.271 3.984 3.975 3.773 4.275
   3570   1372538_at   8.548 8.525 8.705 8.696 8.628 8.999 8.987 9.136 9.147 9.014
   3571   1387905_at   7.953 8.143 8.206 8.232 8.031 8.769 9.309 8.954 9.143 8.955
   3572   1370399_at   6.479 6.265 6.074 5.974 6.19 7.621 7.542 7.015 7.71 7.473
   3573   1377523_at   6.337 6.011 6.245 6.282 5.928 7.137 7.306 6.924 6.968 7.016
   3574   1390967_a_at   7.582 7.45 7.757 7.703 7.541 6.863 7.057 7.087 7.021 7.064
   3575   1373826_at   9.265 9.108 9.319 9.327 9.123 10.112 9.765 9.955 10.13 9.852
   3576   1370861_at   11.983 11.887 11.901 11.83 11.918 12.147 12.178 12.123 12.239 12.196
   3577   1372566_at   8.406 8.288 8.441 8.223 8.355 8.901 9.048 8.919 8.783 8.78
   3578   1391450_at   6.666 6.833 7.05 7.178 7.643 5.555 5.402 5.22 5.428 5.727
   3579   1371976_at   8.843 8.855 9.072 9.052 8.9 9.811 10.006 10.101 9.718 9.592
   3580   1372740_at   8.583 8.451 8.618 8.767 8.664 9.218 9.391 9.424 9.193 9.14
   3581   1385250_at   3.257 2.903 2.828 2.293 1.799 5.498 5.135 5.034 6.178 6.047
   3582   1396755_at   6.996 7.12 6.85 7.199 7.067 6.258 6.241 6.451 5.988 6.11
   3583   1388815_at   9.329 9.244 9.304 9.434 9.243 8.586 8.702 8.938 8.631 8.662
   3584   1376590_at   6.181 6.059 5.765 5.91 5.914 6.755 7.094 6.966 6.774 6.687
   3585   1389202_at   8.212 8.613 8.576 8.682 8.505 7.3 7.732 7.336 7.047 7.284
   3586   1390802_at   8.072 8.271 7.978 8.121 8.188 7.538 7.54 7.69 7.518 7.39
   3587   1372138_at   8.76 9.196 9.309 8.956 9.08 10.118 9.834 9.961 10.04 10.175
   3588   1372794_at   7.596 7.68 8.078 7.545 7.54 8.75 9.282 9.02 9.106 8.692
   3589   1387732_at   8.461 8.557 8.477 8.551 8.575 7.67 7.927 7.955 8.093 7.853
   3590   1386718_at   7.438 7.166 7.016 7.127 7.118 8.425 8.151 8.879 8.262 8.325
   3591   1399027_at   11.081 11.021 11.353 11.265 11.155 10.61 10.669 10.606 10.683 10.567
   3592   1386293_at   5.975 5.956 5.786 5.91 5.859 6.642 7.087 7.044 6.63 6.684
   3593   1377037_at   7.528 7.328 7.94 7.465 7.214 8.996 9.373 8.619 9.011 8.89
   3594   1368062_at   9.217 9.207 9.239 9.238 9.313 9.051 9.066 9.055 9.031 8.979
   3595   1389269_at   7.495 7.423 7.479 7.164 7.331 6.279 6.617 6.09 6.331 5.921
   3596   1379960_at   5.19 4.913 5.727 5.274 5.349 7.018 7.06 6.815 6.553 6.498
   3597   1390287_at   8.671 8.653 8.781 8.898 8.6 7.425 7.38 7.798 7.026 6.923
   3598   1374225_at   6.886 6.757 6.554 7.196 7.127 5.583 5.493 4.704 5.042 5.237
   3599   1371587_at   9.519 9.403 9.648 9.445 9.651 8.953 9.098 8.849 8.969 9.011
   3600   1393421_at   6.683 6.75 7.457 7.091 6.273 8.543 8.733 8.525 8.707 8.517
   3601   1378328_at   3.608 1.618 1.043 1.632 1.651 6.464 6.077 6.606 5.541 5.671
   3602   1381428_a_at   10.198 10.188 10.077 9.983 9.524 11.233 11.114 11.427 11.098 11.529
   3603   1390497_at   6.882 6.823 6.915 6.668 6.594 7.486 7.403 7.27 7.495 7.516
   3604   1379581_at   7.637 7.596 7.37 7.621 7.675 6.535 6.499 6.333 6.921 6.724
   3605   1373375_at   8.625 8.704 8.693 8.634 8.606 8.013 8.13 7.972 7.966 8.307
   3606   1396113_at   6.478 6.85 6.652 7.017 6.221 8.148 8.073 8.507 8.41 7.855
   3607   1388786_at   8.189 8.122 8.15 8.404 8.275 8.925 9.108 9.17 8.868 8.799
   3608   1373996_at   7.305 7.177 7.394 7.309 7.127 7.684 7.648 7.689 7.717 7.73
   3609   1383165_at   3.742 4.396 4.46 4.538 4.43 6.363 5.765 5.682 6.075 5.822
   3610   1367656_at   10.73 10.639 10.716 10.837 10.772 10.281 10.31 10.395 10.342 10.154
   3611   1376078_at   7.526 7.329 8.039 7.883 7.008 9.101 9.674 9.472 9.188 9.412
   3612   1367803_at   8.173 8.138 8.048 7.911 8.372 7.285 7.111 6.926 7.41 7.088
   3613   1384111_at   6.291 6.203 6.305 6.097 5.542 3.82 4.029 4.826 3.911 4.138
   3614   1398902_at   9.352 9.506 9.699 9.638 9.535 10.271 10.46 10.5 10.202 10.163
   3615   1393776_at   7.432 7.519 7.366 7.584 7.891 6.477 6.175 5.587 5.802 5.694
   3616   1396317_at   6.819 6.911 6.525 7.132 6.966 7.776 8.195 8.284 7.887 8.136
   3617   1369113_at   6.616 6.93 6.851 6.878 6.533 8.375 8.524 8.636 7.795 8.013
   3618   1376829_at   3.319 2.973 2.325 2.511 2.493 4.171 4.835 4.812 4.519 4.686
   3619   1389634_at   8.532 8.32 8.342 8.464 8.339 7.893 7.925 7.871 7.817 7.629
   3620   1372911_at   6.579 6.344 6.669 6.411 5.951 9.5 8.962 8.13 9.811 8.91
   3621   1382007_at   8.016 8.06 8.182 8.196 8.126 7.271 7.506 7.19 6.949 7.383
   3622   1385190_at   7.434 7.281 7.23 7.674 7.702 6.305 6.387 6.689 6.239 6.404
   3623   1377630_at   8.193 8.234 8.513 8.436 8.332 7.612 7.689 7.816 7.47 7.599
   3624   1371886_at   9.027 9.072 9.235 8.971 8.577 10.042 10.046 10 10.145 9.821
   3625   1377052_at   6.573 6.942 6.895 7.094 6.801 5.806 5.262 5.234 5.85 5.402
   3626   1370329_at   5.869 6.913 6.482 6.665 6.578 8.213 8.07 8.563 8.246 7.968
   3627   1368907_at   4.427 4.392 4.432 4.558 4.831 5.279 5.418 5.78 5.623 5.588
   3628   1391649_a_at   7.145 6.975 6.833 7.216 7.329 6.073 5.997 6.264 5.682 5.849
   3629   1371332_at   10.422 10.42 10.43 10.166 10.453 11.471 11.519 11.454 10.984 11.595
   3630   1389640_at   8.288 8.087 8.193 8.289 8.021 8.868 9.082 8.73 8.771 8.892
   3631   1395146_at   7.96 8.064 8.127 8.341 8.389 7.51 7.375 7.211 7.328 7.402
   3632   1389418_at   7.125 6.809 6.839 6.94 6.904 7.686 7.877 7.805 7.519 8.013
   3633   1369743_a_at   8.314 8.44 8.57 8.784 8.803 7.683 7.731 7.486 7.694 7.402
   3634   1382265_at   6.974 6.904 7.338 7.294 7.191 8.204 8.45 7.911 8.315 8.134
   3635   1368813_at   8.454 8.407 8.489 8.741 8.686 7.898 7.523 7.481 7.286 7.504
   3636   1386864_at   12.466 12.426 12.339 12.201 12.255 11.918 11.896 11.861 11.936 11.866
   3637   1372380_at   8.433 8.347 8.564 8.685 8.529 9.123 9.061 9.171 9.117 8.963
   3638   1368965_at   7.971 8.3 8.02 8.548 8.547 4.504 5.785 6.293 5.594 5.019
   3639   1371040_at   8.081 8.548 8.42 8.34 8.282 7.026 6.512 7.341 7.078 6.708
   3640   1376902_x_at   3.251 3.66 3.635 4.344 4.503 1.993 1.736 1.566 1.287 1.299
   3641   1373508_at   10.169 10.171 10.32 10.233 10.195 9.897 9.848 9.709 9.9 9.788
   3642   1391822_at   4.719 4.951 4.728 4.595 5.217 6.159 5.747 6.172 5.922 5.97
   3643   1368366_at   8.638 8.637 9.15 9.002 7.619 10.69 10.926 11.092 11.06 11.153
   3644   1379384_at   6.537 6.451 6.531 6.749 6.625 5.924 5.69 5.563 5.845 5.976
   3645   1386926_at   9.849 9.472 9.879 9.903 9.877 8.1 8.289 8.905 8.061 8.137
   3646   1374954_at   5.538 5.8 5.903 5.934 5.775 7.197 7.036 7.786 7.168 6.891
   3647   1394122_at   8.078 8.336 8.346 8.668 8.697 2.772 3.521 5.475 4.41 2.575
   3648   1379313_at   5.573 5.192 5.408 5.347 4.911 3.875 4.398 4.051 4.067 4.093
   3649   1399043_at   10.493 10.515 10.498 10.474 10.608 10.056 10.076 9.957 9.81 9.749
   3650   1376219_at   7.224 7.544 7.547 7.4 7.759 6.657 6.327 6.717 6.495 6.422
   3651   1385334_at   7.502 7.638 7.548 7.473 7.648 8.35 8.645 8.175 8.306 8.199
   3652   1376703_at   8.26 8.21 8.166 8.225 8.239 7.379 7.128 7.557 7.665 7.301
   3653   1370005_at   8.938 8.907 9.07 9.022 8.786 9.711 9.575 9.465 9.428 9.51
   3654   1371924_at   9.831 9.885 9.534 9.779 10.177 8.557 8.224 7.754 8.567 8.388
   3655   1389274_at   6.932 7 6.816 6.985 7.238 7.84 7.865 8 7.677 7.637
   3656   1390463_at   6.199 6.079 6.38 6.351 6.031 7.349 7.232 7.191 6.875 7.016
   3657   1375739_at   9.236 9.156 9.481 9.569 9.606 8.63 8.679 8.638 8.549 8.613
   3658   1384243_at   5.351 5.306 5.495 5.851 5.639 6.507 6.964 6.772 6.605 6.484
   3659   1370880_at   9.998 10.034 10.203 10.322 10.203 9.193 9.377 9.383 9.253 8.874
   3660   1382008_at   8.684 8.691 8.778 8.576 8.602 8.993 9.077 9.205 9.085 9.064
   3661   1373432_at   9.589 9.869 9.869 9.36 9.517 8.371 8.795 8.146 8.344 8.305
   3662   1381364_at   7.619 7.602 7.325 7.523 7.534 6.709 6.91 6.691 6.399 6.748
   3663   1383928_a_at   6.295 6.127 6.031 6.018 5.891 6.966 6.759 7.196 7.074 6.813
   3664   1374884_at   6.923 6.923 6.706 6.792 6.693 7.525 7.377 7.247 7.338 7.401
   3665   1383912_at   8.677 8.776 8.889 8.741 9.014 7.763 7.965 7.334 7.933 7.657
   3666   1373233_at   8.326 8.327 8.543 8.205 8.358 7.139 7.394 7.124 7.696 7.128
   3667   1392289_at   8.031 7.697 7.955 8.073 8.095 6.834 6.945 6.364 7.029 6.538
   3668   1387823_at   9.176 9.308 9.266 9.394 9.332 8.837 8.935 8.704 8.716 8.662
   3669   1380254_at   4.407 4.639 4.603 4.626 4.032 5.561 5.828 6.272 5.99 6.306
   3670   1374326_at   8.028 7.979 7.898 8.077 8.298 7.356 6.968 7.275 7.113 6.894
   3671   1373842_at   8.821 8.91 9.009 8.945 8.784 9.614 9.805 9.623 9.351 9.586
   3672   1389704_at   5.095 4.704 4.513 5.201 4.789 6.839 6.738 6.947 6.096 6.338
   3673   1382803_at   7.34 7.328 7.296 7.405 6.975 5.24 4.992 5.202 4.699 3.681
   3674   1377970_at   7.492 7.453 7.496 7.492 7.946 6.033 6.3 6.637 5.85 5.995
   3675   1374191_at   7.31 7.16 7.277 7.591 7.38 8.185 8.028 8.367 8.046 8.088
   3676   1387972_at   9.938 9.842 9.844 10.069 9.822 10.414 10.423 10.41 10.374 10.253
   3677   1397821_at   5.353 5.382 5.855 5.783 5.547 6.714 6.734 6.549 6.843 6.419
   3678   1391015_at   5.88 6.067 6.026 5.866 5.786 4.796 4.662 4.39 3.791 4.38
   3679   1387052_at   4.747 5.183 5.003 4.444 5.228 6.846 6.639 7.182 6.308 6.486
   3680   1367717_at   12.55 12.483 12.455 12.363 12.56 12.191 12.131 12.159 12.117 12.066
   3681   1380333_at   5.989 6.302 6.928 6.248 5.31 0.454 2.744 1.057 2.409 2.376
   3682   1373371_a_at   8.489 8.495 8.439 8.667 8.489 9.342 9.558 9.708 9.416 9.111
   3683   1373659_at   4.041 4.151 4.345 4.331 4.613 6.697 6.803 7.53 6.021 6.224
   3684   1381816_at   6.613 6.599 6.811 6.458 6.568 7.305 7.326 7.512 7.47 7.136
   3685   1389230_at   8.772 8.813 9.003 9.017 8.993 9.831 9.885 9.984 9.546 9.584
   3686   1391563_at   3.349 3.136 3.152 3.853 2.935 5.412 4.867 5.279 4.877 4.699
   3687   1373516_at   7.881 7.799 7.86 8.032 7.859 6.877 7.01 7.3 6.939 7.276
   3688   1387870_at   10 10.129 10.206 10.1 10.044 11.108 10.987 11.558 10.967 11.525
   3689   1388885_at   9.729 9.636 9.763 9.864 9.901 9.329 9.282 9.378 9.298 9.182
   3690   1367994_at   6.722 6.884 6.71 6.733 6.48 7.262 7.333 7.552 7.492 7.391
   3691   1367537_at   8.008 8.04 8.059 8.074 8.08 7.816 7.597 7.569 7.54 7.619
   3692   1372901_at   8.072 7.962 8.092 7.893 7.862 7.363 7.48 7.411 7.558 7.281
   3693   1368980_at   4.25 4.55 4.171 3.671 4.358 5.396 5.484 5.723 5.774 5.504
   3694   1380243_at   5.791 5.586 5.254 5.233 5.333 3.746 3.838 2.618 2.976 2.943
   3695   1382136_at   6.692 6.506 6.477 6.807 6.819 5.795 5.984 6.062 5.972 5.914
   3696   1367651_at   11.594 11.672 11.595 11.512 11.759 11.223 11.273 11.169 11.253 11.128
   3697   1370238_at   12.068 12.014 11.971 11.864 11.931 12.255 12.317 12.327 12.34 12.257
   3698   1392837_at   7.596 7.714 7.768 7.949 7.924 6.762 6.979 7.051 7.178 7.012
   3699   1394627_at   7.009 7.047 7.098 7.157 7.018 7.505 7.689 7.436 7.583 7.438
   3700   1380086_at   5.056 5.188 4.333 4.83 5.104 2.223 3.47 2.263 2.365 2.302
   3701   1376394_at   5.859 5.557 6.066 5.194 5.398 2.602 3.707 2 3.187 2.905
   3702   1379314_at   7.803 7.888 7.889 7.986 7.956 8.347 8.6 8.407 8.297 8.407
   3703   1390207_at   8.914 8.948 8.723 8.993 8.939 9.455 9.608 9.341 9.418 9.409
   3704   1374414_at   7.755 7.657 7.607 7.922 7.891 8.482 8.59 8.637 8.406 8.313
   3705   1388237_s_at   0 0.111 1.501 0 0.856 3.836 3.877 3.315 4.202 2.932
   3706   1373521_at   6.139 6.495 6.372 6.626 6.247 7.67 8.168 8.384 7.648 7.61
   3707   1367649_at   7.413 7.669 7.635 7.998 7.534 8.68 8.731 8.941 8.531 8.539
   3708   1396719_at   4.672 4.598 4.242 4.828 4.465 5.504 5.365 5.524 5.36 5.56
   3709   1370959_at   11.108 11.085 10.566 10.923 11.629 9.528 9.425 9.627 9.512 9.716
   3710   1372113_at   9.107 9.005 8.829 9.13 9.229 8.367 8.511 8.363 8.197 8.328
   3711   1390825_at   8.451 8.5 8.365 8.351 8.617 9.32 9.364 9.639 9.212 9.092
   3712   1371379_at   11.041 11.053 11.285 11.173 11.074 11.896 11.856 11.629 11.978 11.674
   3713   1391440_at   7.221 7.18 7.39 7.249 7.525 6.4 6.264 6.381 5.754 6.086
   3714   1390483_at   6.647 6.11 6.785 6.604 6.55 7.462 7.476 7.546 7.505 7.577
   3715   1376537_at   6.077 5.956 5.951 6.227 6.016 6.795 6.854 7.231 6.785 6.777
   3716   1393000_at   6.199 6.137 6.126 6.307 6.43 7.207 7.42 7.809 7.3 7.105
   3717   1373519_at   7 6.925 7.149 7.171 6.946 7.644 8.019 8.028 7.685 7.823
   3718   1376661_at   7.438 7.099 7.288 7.355 7.074 8.113 8.233 8.534 8.068 8.064
   3719   1375872_at   6.125 6.422 6.294 6.277 6.216 7.395 7.826 7.445 7.105 7.192
   3720   1375566_at   8.917 8.902 8.798 9.005 8.974 9.513 9.576 9.38 9.767 9.466
   3721   1371738_at   7.201 6.886 7.063 7.282 7.12 7.787 7.918 8.048 7.916 7.691
   3722   1372545_at   7.477 7.393 7.659 7.728 7.714 8.239 8.106 8.327 8.331 8.263
   3723   1393293_at   6.406 6.612 6.864 6.514 6.527 5.933 5.683 5.715 5.924 5.745
   3724   1367815_at   7.98 8.086 8.31 8.411 7.681 9.19 9.322 9.196 9.102 9.271
   3725   1396055_at   6.849 6.84 7.032 7.073 7.136 6.006 5.964 5.226 5.559 5.69
   3726   1383838_at   6.54 6.933 7.072 7.185 6.563 7.856 8.004 8.124 8.086 8.074
   3727   1378216_at   4.007 3.579 3.502 3.959 4.223 5.276 5.746 5.425 5.676 5.034
   3728   1373546_at   10.159 10.172 9.951 10.062 10.054 10.68 11.022 10.853 10.585 10.85
   3729   1380334_at   9.44 9.423 9.753 9.677 9.567 10.377 10.598 10.514 10.142 10.453
   3730   1374576_at   7.193 7.084 7.397 6.97 6.944 5.963 6.286 6.101 6.087 5.672
   3731   1372925_at   7.237 7.266 7.303 7.345 7.536 8.191 8.284 8.19 8.174 7.826
   3732   1391893_at   6.544 6.552 6.605 6.78 6.892 5.61 5.048 5.057 5.674 5.541
   3733   1371390_at   11.102 11.106 11.222 11.244 11.12 10.679 10.767 10.529 10.504 10.425
   3734   1371997_at   8.7 8.885 8.645 9.127 9.028 9.927 9.759 9.917 9.702 9.635
   3735   1378005_at   5.374 5.409 4.922 5.271 4.67 6.42 6.382 6.423 6.219 6.435
   3736   1398367_at   8.76 8.667 8.693 8.871 8.906 8.185 8.137 8.129 7.844 7.835
   3737   1389304_at   8.379 8.491 8.537 8.601 8.543 7.949 8.16 8.031 7.968 8.12
   3738   1376068_at   5.697 5.767 5.998 6.121 5.743 7.038 7.068 7.362 7.039 6.653
   3739   1391543_at   7.495 7.343 7.245 7.376 7.162 5.981 5.844 5.22 5.131 6.031
   3740   1399034_at   7.699 7.405 7.653 7.808 7.714 8.508 8.572 8.248 8.356 8.34
   3741   1383287_at   8.584 8.67 8.84 9.032 8.93 7.694 7.841 7.523 8.054 7.874
   3742   1388448_at   8.957 9.026 8.943 9.002 9.021 8.656 8.64 8.634 8.506 8.414
   3743   1389396_at   6.185 6.231 6.67 6.362 6.014 8.05 7.572 7.469 7.644 7.379
   3744   1398732_at   5.598 5.781 5.442 5.603 6.142 6.903 6.723 6.892 6.733 6.68
   3745   1384742_at   6.403 7.005 6.144 6.233 6.713 4.903 5.314 4.752 4.769 4.818
   3746   1396383_at   7.015 6.488 6.962 7.129 6.903 4.857 5.838 5.284 5.26 5.073
   3747   1384481_at   4.973 4.838 5.206 4.838 5.075 6.492 6.643 6.905 6.574 5.891
   3748   1383826_at   5.08 5.683 5.622 5.692 5.502 6.891 7.046 6.712 6.572 6.551
   3749   1367880_at   6.935 6.993 6.996 7.226 7.422 8.313 8.472 8.779 8.14 8.144
   3750   1371367_at   9.583 9.644 9.478 9.803 9.823 8.803 9.059 9.015 8.711 8.905
   3751   1379043_at   6.047 6.071 6.339 6.598 6.282 7.329 7.039 7.235 7.152 7.291
   3752   1375029_at   7.72 7.676 7.7 7.83 7.882 7.183 7.126 7.399 7.269 7.276
   3753   1393364_at   6.972 6.901 6.853 6.981 6.535 8.265 8.264 7.693 8.508 8.468
   3754   1368672_at   7.79 7.648 8.004 7.715 7.263 9.183 8.942 9.466 8.879 8.875
   3755   1373556_at   7.283 7.173 7.374 7.398 7.501 6.781 6.808 6.703 6.733 6.507
   3756   1379195_s_at   6.804 6.299 6.78 6.768 6.472 5.612 4.927 5.461 5.326 5.058
   3757   1387253_at   3.834 4.144 4.452 4.059 4.231 5.604 5.622 5.365 5.096 5.177
   3758   1397176_at   6.974 6.934 6.962 6.858 7.109 5.708 5.491 6.013 6.091 5.358
   3759   1396815_at   3.987 4.448 3.95 3.53 4.25 5.684 5.869 5.639 5.289 5.416
   3760   1386662_at   6.846 7.088 6.945 7.027 6.506 7.927 7.759 7.701 7.762 7.807
   3761   1390195_at   7.011 6.863 6.866 6.97 6.787 5.242 5.288 5.659 4.498 4.635
   3762   1388518_at   8.816 8.95 8.942 8.827 8.777 9.34 9.502 9.569 9.261 9.376
   3763   1368208_at   8.236 8.326 8.925 8.839 7.349 10.646 10.764 11.119 10.609 10.828
   3764   1372044_at   7.413 7.829 7.77 7.28 7.383 8.701 8.771 8.378 8.559 8.678
   3765   1375549_at   5.779 5.09 5.573 5.881 5.261 7.912 7.486 8.064 6.948 8.062
   3766   1396337_at   4.034 4.24 3.849 3.711 3.555 5.003 5.014 5.085 4.801 5.218
   3767   1374197_at   8.585 8.516 8.497 8.47 8.408 9.335 9.241 8.896 9.146 9.271
   3768   1398761_at   12.448 12.371 12.31 12.322 12.366 12.067 12.095 12.152 12 12.076
   3769   1389242_at   6.926 7.061 6.846 6.97 6.547 7.787 7.8 8.106 7.949 7.637
   3770   1387916_at   6.744 7.218 6.93 6.909 6.908 7.864 7.658 8.222 7.939 8.002
   3771   1387378_at   10.322 10.24 9.787 9.911 10.136 1.536 1.406 5.592 1.687 4.285
   3772   1387895_s_at   7.434 7.87 8.431 8.203 7.492 4.953 5.469 4.256 5.871 5.023
   3773   1386914_at   5.836 5.607 5.781 5.815 5.716 6.982 7.14 7.363 6.846 6.525
   3774   1367657_at   11.583 11.696 11.754 11.597 11.642 11.041 11.13 11.344 11.168 11.127
   3775   1372466_at   10.929 10.967 10.731 10.87 10.923 10.307 10.511 10.181 10.248 10.306
   3776   1380884_at   4.006 4.388 4.593 4.358 3.601 6.007 6.103 5.52 5.801 5.876
   3777   1379443_at   7.533 7.448 7.382 7.415 7.338 7.089 7.165 7.059 7.101 7.026
   3778   1374545_at   8.65 8.859 8.805 8.864 9.073 7.879 7.94 7.749 8.261 7.906
   3779   1371126_x_at   3.89 4.241 4.522 4.411 3.487 0.536 0.642 0.239 1.811 1.727
   3780   1389214_at   7.423 7.497 6.841 7.486 8.001 5.877 5.718 6.037 5.798 5.616
   3781   1375349_at   8.083 8.102 8.206 8.483 8.1 8.909 8.859 8.831 9.04 8.84
   3782   1382173_at   4.177 4.521 3.833 4.264 4.331 6.146 6.186 6.209 5.287 6.27
   3783   1391466_at   6.722 6.778 6.909 7.09 6.937 6.236 6.163 6.064 5.86 5.83
   3784   1384265_at   6.995 7.13 7.149 6.999 6.92 6.312 6.279 6.292 6.379 5.888
   3785   1377797_at   7.118 7.23 7.287 7.497 7.547 6.159 6.042 5.458 6.29 5.906
   3786   1374355_at   6.663 6.925 6.913 7.203 7.386 5.654 5.533 6.044 5.5 5.412
   3787   1375406_s_at   5.894 6.176 6.073 6.592 6.27 5.229 5.207 5.243 5.13 5.277
   3788   1367885_at   8.429 8.417 8.597 8.67 8.325 9.489 9.526 9.878 9.486 9.206
   3789   1388880_at   9.386 9.161 9.271 9.41 9.469 8.679 8.539 8.889 8.645 8.62
   3790   1372631_at   7.822 7.617 7.481 7.763 7.917 6.045 6.581 6.832 6.201 6.286
   3791   1390553_at   5.965 5.527 5.556 5.801 5.666 4.365 4.277 4.174 3.674 4.642
   3792   1372003_at   9.009 9.086 8.807 8.855 8.893 8.116 7.989 8.056 7.604 7.557
   3793   1374070_at   9.701 9.738 9.334 9.525 9.761 7.355 8.431 8.053 8.145 8.01
   3794   1388076_at   11.559 11.506 11.299 11.49 11.748 10.893 10.927 10.895 10.901 10.758
   3795   1394022_at   8.561 8.042 8.476 8.525 8.077 9.909 10.719 9.991 9.812 9.797
   3796   1388099_a_at   6.757 6.557 6.922 6.412 6.906 5.732 5.684 5.927 5.486 5.589
   3797   1369697_at   4.385 4.077 3.053 2.104 4.162 0 0 0 0 0
   3798   1383080_at   11.01 11.027 10.909 10.777 10.9 10.223 10.26 10.033 10.092 10.455
   3799   1376867_at   6.669 6.988 7.09 6.755 6.378 8.449 8.487 8.207 8.142 7.815
   3800   1388024_s_at   9.209 9.119 9.384 9.247 9.411 8.598 8.74 8.57 8.576 8.329
   3801   1393252_at   7.914 8.133 7.593 7.776 8.184 6.726 6.478 5.883 6.24 6.599
   3802   1390786_at   8.53 8.517 8.437 8.484 8.366 7.707 7.606 7.999 7.465 7.557
   3803   1371092_at   6.869 7.14 7.03 7.44 6.838 2.971 1.664 4.014 3.274 4.194
   3804   1374475_at   7.311 6.947 7.275 7.385 7.536 8.055 8.252 8.317 8.215 8.1
   3805   1398350_at   9.922 9.89 9.807 10.038 10.179 10.668 10.587 10.556 10.47 10.543
   3806   1398254_at   8.041 7.985 8.151 8.292 8.142 8.746 8.976 8.687 8.996 8.72
   3807   1389485_at   6.453 6.543 6.383 6.523 6.652 5.889 6.005 5.86 5.591 5.739
   3808   1367618_a_at   12.336 12.229 12.118 11.948 12.223 11.594 11.598 11.636 11.658 11.553
   3809   1390049_at   6.456 6.296 6.327 5.976 6.04 7.345 7.049 7.202 7.079 6.993
   3810   1372420_at   8.842 8.912 8.868 8.972 8.957 8.497 8.524 8.562 8.691 8.532
   3811   1367743_at   8.952 8.982 9.038 9.188 9.035 8.037 8.287 8.548 8.193 8.119
   3812   1398962_at   9.444 9.48 9.397 9.584 9.368 10.113 10.012 9.87 10.004 9.846
   3813   1374399_at   7.847 7.901 7.36 7.56 7.803 6.918 6.761 6.743 6.629 6.598
   3814   1368325_at   10.044 10.113 10.374 10.235 9.045 11.892 11.883 11.938 11.911 11.971
   3815   1376209_at   10.479 10.416 10.835 10.804 10.61 11.402 11.371 11.466 11.455 11.256
   3816   1376222_at   5.442 4.807 5.922 5.811 5.38 7.104 7.32 7.468 7.032 7.164
   3817   1390474_at   7.198 6.854 7.062 6.902 7.169 7.895 7.891 8.21 7.77 7.78
   3818   1375921_at   6.18 6.254 5.6 5.736 6.049 4.01 4.413 4.394 4.768 4.672
   3819   1372519_at   8.447 8.504 8.365 8.589 8.743 7.07 7.043 7.712 6.895 6.796
   3820   1383730_at   7.632 7.429 7.59 7.485 7.656 6.731 6.855 7.119 6.847 6.698
   3821   1389803_at   4.685 4.941 4.993 5.537 4.525 6.283 6.658 6.612 6.367 6.733
   3822   1389180_at   7.615 7.42 7.232 7.11 7.14 8.178 8.441 8.218 8.19 8.088
   3823   1396853_at   7.325 7.408 7.662 7.655 7.361 8.194 8.158 8.154 8.037 8.093
   3824   1372770_at   10.117 10.053 10.004 10.042 10.247 9.425 9.502 9.225 9.416 9.045
   3825   1383182_at   6.998 7.312 7.307 7.377 7.247 6.459 6.692 6.247 6.403 6.358
   3826   1386986_at   8.826 8.347 8.449 8.81 8.925 7.265 7.613 7.629 7.215 7.083
   3827   1381678_at   5.862 5.954 6.346 6.347 6.029 4.702 3.957 4.693 4.867 4.602
   3828   1371744_at   7.19 7.064 7.05 7.096 7.283 7.48 7.54 7.592 7.6 7.514
   3829   1372169_at   8.227 8.326 8.311 8.552 8.501 7.773 7.689 7.739 7.921 7.67
   3830   1376513_a_at   6.601 6.567 7.004 6.916 6.687 5.255 4.005 4.717 4.993 5.084
   3831   1389050_at   9.321 9.166 9.344 9.488 9.245 9.844 9.97 9.746 9.818 9.913
   3832   1373538_at   7.512 7.547 8.056 7.842 8.04 6.768 6.842 6.706 6.85 6.561
   3833   1389560_at   8.686 8.587 8.518 8.802 8.792 9.256 9.141 9.236 9.154 9.131
   3834   1388919_at   7.252 7.291 7.218 7.334 7.423 7.765 7.935 8.127 8.005 7.797
   3835   1387295_at   5.66 5.627 5.285 5.029 5.506 6.905 7.101 7.406 6.987 6.495
   3836   1388958_a_at   4.846 4.58 5.015 4.826 4.258 6.293 6.092 6.289 5.783 5.908
   3837   1370395_at   6.677 6.57 6.769 6.583 6.817 5.986 5.982 5.878 5.508 5.903
   3838   1371311_at   11.737 11.716 11.843 11.669 11.653 12.078 12.048 12.003 12.163 12.073
   3839   1398799_at   10.639 10.62 10.609 10.594 10.692 10.363 10.146 10.047 10.074 10.125
   3840   1370150_a_at   3.569 4.208 4.318 3.794 4.033 6.952 6.741 8.281 6.684 6.296
   3841   1368908_at   9.747 9.709 9.573 9.806 10.102 8.948 8.965 8.897 8.72 8.55
   3842   1384312_at   7.442 7.264 6.996 7.566 7.221 8.433 8.449 8.598 8.072 8.322
   3843   1372454_at   8.02 8.118 8.142 8.191 8.051 8.58 8.719 8.618 8.539 8.423
   3844   1383679_at   7.85 7.606 7.797 7.922 8.033 7.066 6.959 7.284 7.153 7.023
   3845   1382923_at   7.776 8.191 8.048 8.184 8.323 7.232 7.273 6.906 6.842 7.08
   3846   1395479_at   7.111 7.094 6.987 6.989 7.159 6.305 6.26 6.163 6.012 5.667
   3847   1371305_at   12.245 12.275 12.124 12.101 12.307 11.786 11.572 11.565 11.799 11.63
   3848   1371899_at   8.186 8.122 8.29 8.179 8.158 8.685 8.85 8.628 8.743 8.528
   3849   1390350_at   6.855 7.169 6.985 7.216 6.926 6.053 6.013 5.884 6.379 5.785
   3850   1383150_at   7.477 7.34 7.482 7.623 7.371 6.844 6.909 6.883 6.587 6.882
   3851   1372095_at   6.453 6.607 6.257 6.531 6.712 7.261 7.193 7.148 7.132 7.135
   3852   1370250_at   9.967 9.981 10.163 10.231 10.228 9.381 9.416 9.614 9.491 9.596
   3853   1371296_at   10.644 10.53 10.775 10.66 10.565 11.169 11.163 11.154 11.216 10.965
   3854   1369983_at   8.879 8.909 8.432 9.313 7.905 5.511 5.888 6.53 6.288 6.507
   3855   1371431_at   7.826 7.66 7.823 8.351 7.755 8.97 8.952 8.911 8.916 8.79
   3856   1379900_at   5.529 5.888 5.789 5.763 5.745 6.38 6.585 6.442 6.378 6.243
   3857   1380616_at   6.332 6.235 6.116 5.814 5.903 2.362 1.063 1.623 3.819 1.674
   3858   1382873_at   7.499 7.577 7.592 7.779 7.631 6.929 6.746 7.047 6.923 7.136
   3859   1388122_at   11.129 11.106 10.877 11.118 11.231 11.557 11.831 11.765 11.72 11.878
   3860   1395145_at   8.256 8.138 8.38 8.384 8.186 9.263 9.215 8.795 9.052 9.286
   3861   1369336_at   4.47 4.651 4.562 4.839 4.603 5.148 5.163 5.099 5.256 5.232
   3862   1378421_at   3.924 4.142 4.333 4.302 4.202 5.589 5 5.485 5.065 5.412
   3863   1371706_at   8.357 8.281 8.403 8.379 8.331 8.907 8.766 8.852 8.668 8.675
   3864   1372957_at   5.986 5.803 6.139 6.544 6.273 2.365 1.824 2.982 2.365 4.147
   3865   1381933_at   7.315 7.49 7.341 7.306 7.399 6.94 6.866 6.806 6.962 6.661
   3866   1386641_at   5.463 5.999 6.565 6.007 5.546 7.678 8.268 8.143 7.703 7.599
   3867   1380513_at   8.362 8.273 8.122 8.31 8.458 7.582 7.646 7.132 7.27 7.305
   3868   1372788_at   6.927 7.216 7.08 7.131 6.928 6.466 6.305 6.386 6.601 6.449
   3869   1367953_at   5.877 6.012 5.888 5.717 6.292 7.144 7.421 7.384 7.034 6.822
   3870   1382751_at   6.128 6.644 6.312 6.075 5.951 3.851 4.482 4.985 4.324 4.022
   3871   1373892_at   8.897 9.242 9.149 9.166 9.267 9.945 10.128 10.026 9.704 9.884
   3872   1382758_at   5.769 5.721 5.693 5.221 5.768 4.496 4.43 4.268 4.65 4.77
   3873   1392509_at   6.927 7.046 7.341 7.432 7.222 6.087 5.96 6.428 6.13 6.298
   3874   1388596_at   10.38 10.354 10.484 10.436 10.36 9.89 10.056 9.764 9.767 9.681
   3875   1372772_at   7.827 8.012 8.181 8.132 7.961 8.56 8.719 8.669 8.513 8.646
   3876   1379934_at   7.782 7.767 7.437 7.514 7.996 6.623 6.871 6.755 6.493 6.802
   3877   1377907_at   4.467 4.296 4.301 5.052 4.8 3.063 3.355 3.146 3.445 3.103
   3878   1382953_at   4.215 4.32 4.882 4.574 4.565 5.634 5.929 6.04 5.917 5.465
   3879   1392174_at   6.153 6.035 5.949 6.465 6.666 5.165 4.615 4.756 4.958 4.999
   3880   1373825_at   8.676 8.549 8.497 8.53 8.309 9.083 9.227 9.061 8.989 9.084
   3881   1371761_at   12.199 12.138 12.195 12.059 12.229 11.939 11.867 11.905 11.872 11.847
   3882   1373776_at   7.314 7.342 7.187 7.351 7.447 6.51 6.876 6.382 6.547 6.586
   3883   1374731_at   9.457 9.786 9.646 10.074 9.985 8.664 8.81 8.953 8.63 8.612
   3884   1392032_at   6.665 6.679 6.897 6.816 6.549 4.271 4.943 4.16 5.131 5.313
   3885   1372749_at   8.388 8.386 8.315 8.529 8.63 7.905 7.914 8.024 7.915 7.98
   3886   1383998_at   9.375 9.423 9.429 9.261 9.427 8.679 8.759 8.241 8.537 8.694
   3887   1386979_at   6.385 6.256 6.252 6.172 5.973 6.759 6.871 6.731 6.857 6.802
   3888   1383065_at   7.054 7.194 7.167 7.41 6.924 7.988 8.117 7.977 8.029 7.751
   3889   1393411_at   6.278 5.824 5.698 5.984 6.065 3.96 1.496 1.709 1.82 1.692
   3890   1385168_at   6.762 6.796 6.775 6.667 7.076 4.777 5.398 4.898 5.743 5.422
   3891   1393357_at   6.659 6.675 6.677 6.735 6.874 5.791 5.577 5.152 5.635 4.98
   3892   1382104_at   6.213 6.46 6.639 6.57 6.403 7.186 7.066 7.029 7.156 7.079
   3893   1382218_at   7.074 7.045 7.016 7.124 6.935 5.471 5.569 6.143 5.783 5.105
   3894   1383543_at   6.931 7.104 7.277 7.166 7.088 6.374 6.172 5.716 6.203 6.092
   3895   1372951_at   7.675 7.361 7.523 7.337 7.331 6.772 6.682 6.702 6.404 6.386
   3896   1399152_at   8.701 8.691 8.831 8.627 8.917 7.915 7.904 7.264 7.735 7.581
   3897   1382034_at   4.317 4.606 4.73 5.1 4.841 6.777 6.951 5.863 6.691 6.831
   3898   1379059_at   5.803 6.091 5.742 5.993 6.572 4.587 4.685 4.818 4.176 4.487
   3899   1385258_at   6.127 6.196 6.053 6.248 6.017 5.172 5.216 5.075 5.584 4.961
   3900   1376642_at   5.936 6.005 5.623 6.249 6.126 3.898 4.352 4.369 4.948 4.282
   3901   1398782_at   9.679 9.69 9.553 9.584 9.751 10.025 9.916 10.001 10.055 10.033
   3902   1395628_at   4.839 4.377 4.677 4.236 4.091 6.075 6.264 6.08 6.042 5.494
   3903   1371849_at   5.668 5.733 5.935 5.553 5.947 6.872 6.532 6.91 6.716 6.488
   3904   1370855_at   11.008 11.037 10.984 10.946 11.072 11.58 11.454 11.288 11.483 11.396
   3905   1391544_at   4.198 3.899 3.863 4.945 4.346 6.085 6.397 6.755 6.133 5.896
   3906   1383528_at   6.55 6.66 6.705 6.646 6.631 6.374 6.451 6.347 6.393 6.358
   3907   1375818_at   8.604 8.685 8.683 8.888 9.104 7.922 8 7.632 7.868 7.974
   3908   1382705_at   7.554 7.535 7.883 7.426 7.539 8.619 8.483 8.211 8.475 8.672
   3909   1370610_at   7.619 7.65 8.374 7.451 6.974 10.603 9.733 10.356 10.258 9.5
   3910   1376567_at   6.646 6.864 6.925 6.977 6.928 6.422 6.307 6.253 6.207 6.165
   3911   1379633_a_at   8.383 8.287 8.072 8.315 8.644 1.063 3.511 3.605 4.365 4.32
   3912   1391270_at   7.617 7.332 7.856 7.93 7.44 8.794 8.664 8.991 8.761 8.543
   3913   1374295_at   6.67 6.594 6.659 6.853 6.785 7.586 7.293 7.657 7.458 7.263
   3914   1368647_at   8.159 8.076 8.164 8.261 7.919 7.265 7.329 7.494 7.392 7.001
   3915   1373090_at   10.029 10.043 9.951 9.958 10.04 9.555 9.611 9.572 9.292 9.54
   3916   1394340_at   8.106 8.134 8.063 8.3 8.116 7.091 6.982 7.507 6.811 6.791
   3917   1384971_at   7.441 7.426 7.703 7.262 7.202 8.54 8.9 8.422 8.263 8.475
   3918   1374984_at   5.987 6.119 6.208 5.922 5.753 7.067 6.87 7.226 6.822 6.759
   3919   1386648_at   4.668 3.64 3.921 4.006 3.345 5.684 5.754 5.716 5.659 5.828
   3920   1381336_at   6.529 6.757 6.829 7.14 7.096 7.953 7.944 7.936 7.708 7.804
   3921   1374122_at   5.357 5.757 5.578 5.76 5.354 7.161 7.133 7.371 6.443 7.034
   3922   1387268_at   6.356 6.193 6.336 6.532 6.693 5.655 5.685 5.542 5.574 5.771
   3923   1377033_at   9.381 9.267 9.302 9.609 9.151 10.182 10.103 10.433 10.164 10.017
   3924   1376050_at   6.734 6.897 6.699 6.807 6.772 5.074 5.105 5.245 5.769 4.525
   3925   1389147_at   9.474 9.551 9.741 9.706 9.646 10.5 10.61 10.135 10.329 10.378
   3926   1372758_at   9.463 9.399 9.431 9.407 9.406 9.8 9.874 9.717 9.745 9.985
   3927   1372432_at   7.963 7.904 7.703 8.105 8.03 7.23 7.279 7.405 7.127 7.096
   3928   1388921_at   5.66 5.956 5.943 5.821 5.654 7.091 7.13 7.763 6.795 7.184
   3929   1381951_at   3.566 3.333 3.667 4.205 2.956 5.629 5.974 6.032 5.837 5.087
   3930   1389344_at   8.318 8.357 8.336 8.342 8.389 7.549 7.8 7.709 7.311 7.747
   3931   1382032_at   7.081 7.397 7.072 7.33 7.246 7.937 8.007 7.773 7.953 7.769
   3932   1389271_at   6.307 6.584 6.345 6.755 6.623 7.463 7.4 7.805 7.365 7.383
   3933   1388995_at   9.059 9.238 9.232 9.221 9.303 9.717 9.767 9.877 9.565 9.747
   3934   1397476_at   5.302 5.049 5.106 4.941 5.246 1.585 2.954 0.895 0.856 2.39
   3935   1372769_at   8.719 8.469 8.7 8.732 8.646 8.153 8.235 8.085 8.13 7.956
   3936   1370297_at   7.596 7.68 7.57 7.788 7.55 4.461 4.328 2.488 4.569 2.438
   3937   1382945_at   7.119 6.778 7.016 7.037 7.136 7.543 7.552 7.671 7.679 7.542
   3938   1368214_at   8.447 8.569 8.553 8.444 8.538 7.916 7.861 7.926 7.472 7.669
   3939   1390731_at   8.912 8.837 8.501 8.739 8.995 7.718 7.959 7.725 7.783 8.12
   3940   1398801_at   10.735 10.327 10.319 10.399 10.445 9.863 9.75 9.823 9.743 9.665
   3941   1386379_at   7.395 7.463 7.293 7.331 7.496 6.391 6.348 5.677 5.967 6.432
   3942   1390999_at   3.418 2.173 3.405 3.828 3.557 1.208 0 0.151 0 0
   3943   1387402_at   8.211 8.167 8.152 8.626 8.445 7.588 7.37 7.372 7.297 7.554
   3944   1384368_at   7.358 7.415 7.541 7.661 7.482 8.254 8.348 8.316 8.076 7.991
   3945   1372853_at   8.516 8.399 8.631 8.584 8.556 8.245 8.079 8.112 8.047 8.084
   3946   1368180_s_at   11.168 11.089 11.513 11.295 10.498 12.46 12.501 12.441 12.564 12.523
   3947   1371322_at   9.02 9.129 9.096 9.32 9.266 8.452 8.676 8.554 8.574 8.699
   3948   1376501_at   7.816 7.921 7.776 7.764 7.992 6.557 7.161 6.859 7.108 6.812
   3949   1373925_at   7.417 7.566 7.618 7.63 7.637 7.032 6.883 7.081 7.132 6.821
   3950   1385505_at   6.915 6.922 7.035 7.005 7.065 7.628 8.118 7.863 7.587 7.777
   3951   1384227_at   7.325 7.174 7.059 7.222 7.232 7.703 8.04 7.799 7.709 7.924
   3952   1395246_at   7.179 7.01 6.885 6.892 6.923 8.35 7.982 8.06 7.749 7.758
   3953   1367756_at   8.54 8.665 8.661 8.905 8.768 9.24 9.245 9.377 9.305 9.182
   3954   1393209_at   7.546 7.601 7.87 7.591 7.54 8.911 8.947 9.518 8.774 8.623
   3955   1373503_at   5.713 5.937 5.817 6.122 6.083 7.188 7.271 7.154 6.851 7.728
   3956   1389832_at   10.849 10.855 11.028 11.083 10.997 11.59 11.421 11.532 11.587 11.358
   3957   1388117_at   10.731 10.727 10.871 10.697 10.759 10.308 10.431 10.21 10.337 10.43
   3958   1382009_at   7.66 7.678 7.889 7.975 8.077 6.363 6.58 6.668 7.039 6.869
   3959   1376191_at   6.471 6.946 7.074 7.154 7.102 8.188 8.252 8.381 8.158 7.863
   3960   1368785_a_at   6.291 6.179 6.46 6.522 6.401 7.595 7.275 6.99 7.423 7.289
   3961   1389521_at   10.012 9.959 10.224 10.216 10.356 10.864 10.972 11.024 10.874 10.71
   3962   1377007_at   6.498 6.563 6.478 6.644 6.763 7.119 7.307 7.319 7.075 7.11
   3963   1393957_at   7.065 6.99 7.151 7.114 7.399 2.566 3.116 4.545 4.546 4.264
   3964   1383121_at   8.559 8.69 8.664 8.899 8.663 9.471 9.761 9.265 9.418 9.565
   3965   1369941_at   10.451 10.5 10.151 10.435 10.527 9.749 9.9 9.731 9.867 9.753
   3966   1384073_at   7.24 7.452 8.055 7.158 6.351 9.905 9.305 9.497 9.956 9.727
   3967   1370097_a_at   6.869 6.813 6.969 7.008 6.506 5.83 5.792 5.305 5.289 5.787
   3968   1368340_at   8.975 8.937 9.268 9.587 9.205 10.166 10.197 10.431 10.217 10.135
   3969   1383665_at   8.225 8.222 7.84 8.274 8.25 7.41 7.04 7.133 6.773 7.168
   3970   1390017_at   4.79 4.8 5.07 4.688 4.956 3.762 4.031 4.252 4.065 3.901
   3971   1371945_at   9.697 9.67 9.711 9.738 9.664 9.438 9.472 9.389 9.512 9.342
   3972   1372507_at   8.797 8.795 9.069 9.074 8.705 9.514 9.623 9.59 9.551 9.462
   3973   1372151_at   8.034 8.152 8.203 8.305 8.241 7.42 7.555 7.569 7.093 7.331
   3974   1367755_at   10.378 10.506 10.335 10.345 9.935 11.331 11.282 11.576 11.112 11.2
   3975   1396752_at   3.946 2.778 4.155 3.593 3.606 5.904 5.584 6.177 5.533 5.584
   3976   1397205_at   9.893 9.888 10.437 10.334 9.063 11.903 11.81 12.199 11.844 12.13
   3977   1387542_at   8.551 8.77 8.724 8.306 7.839 9.982 9.981 9.729 9.775 9.913
   3978   1393684_at   6.167 5.946 6.042 6.102 6.173 4.121 4.409 4.529 3.266 3.365
   3979   1375630_at   10.146 10.251 10.343 10.43 10.433 9.87 9.525 9.637 9.648 9.512
   3980   1375685_at   8.16 8.399 8.584 8.748 8.631 7.513 7.623 7.691 7.338 7.369
   3981   1377333_at   6.063 5.901 6.508 6.596 5.221 8.291 8.562 9.179 8.468 8.115
   3982   1387344_at   11.143 11.103 11.253 11.012 11.001 11.886 11.851 11.627 12.112 12.098
   3983   1388669_at   8.469 8.627 8.366 8.659 9.032 7.648 7.68 7.474 7.662 7.319
   3984   1373035_at   8.72 8.665 8.362 8.739 8.866 7.538 7.546 7.817 7.091 7.56
   3985   1390455_at   8.029 7.844 7.554 7.677 7.748 6.9 6.787 7.159 6.779 6.957
   3986   1391227_at   6.965 7.152 6.701 6.89 6.975 4.214 4.566 4.962 3.029 3.519
   3987   1369773_at   5.281 5.265 4.714 5.233 4.858 6.006 6.265 6.102 6.198 5.999
   3988   1368095_at   10.342 10.359 10.399 10.224 10.148 10.954 10.999 10.739 10.987 10.76
   3989   1389379_at   6.494 6.757 6.819 6.564 6.709 5.621 5.137 4.593 5.411 5.231
   3990   1372910_at   7.269 7.395 7.471 7.596 7.557 6.623 6.652 6.707 6.803 6.29
   3991   1367619_at   10.879 10.862 10.925 10.819 10.685 11.637 11.627 11.259 11.702 11.721
   3992   1372903_at   6.44 6.231 6.116 6.639 6.027 3.149 0.872 2.325 2.37 3.586
   3993   1395116_at   6.815 6.382 7.32 7.05 7.014 3.824 4.093 4.392 2.183 3.349
   3994   1390394_at   7.732 7.995 7.775 7.72 7.83 6.227 6.244 6.563 6.952 5.966
   3995   1368201_at   6.768 6.956 6.958 7.041 7.067 6.094 6.286 6.506 6.262 6.203
   3996   1376672_at   5.577 5.876 5.865 5.934 6.033 6.882 7.255 7.625 6.902 7.005
   3997   1371889_at   6.615 6.166 6.57 6.609 6.943 7.527 7.621 7.734 7.628 7.558
   3998   1373809_at   8.804 8.755 8.427 8.702 8.504 9.186 9.548 9.425 9.383 9.458
   3999   1379315_at   7.343 7.561 7.392 7.557 7.362 8.111 8.059 8.274 8.01 7.899
   4000   1385458_a_at   7.997 8.027 7.569 7.926 8.216 7.112 6.805 7.051 6.808 6.727
   4001   1388631_at   8.141 7.929 8.052 8.171 8.237 7.35 7.627 7.564 7.479 7.324
   4002   1389757_at   6.94 7 6.895 7.149 6.613 7.884 8.279 8.483 8.233 7.826
   4003   1389535_at   8.579 8.509 8.492 8.564 8.686 7.898 8.154 8.034 7.851 7.752
   4004   1379669_at   7.09 7.075 7.212 7.467 7.287 7.944 8.022 8.295 7.886 8.013
   4005   1385679_at   8.913 8.923 9.072 9.07 9.254 9.845 10.1 9.642 9.819 9.752
   4006   1387346_at   10.007 9.968 9.927 10.085 9.916 9.522 9.582 9.532 9.275 9.364
   4007   1368089_at   6.87 7.167 7.344 7.483 7.258 8.463 8.179 8.025 8.444 8.291
   4008   1388787_at   8.885 8.843 8.748 8.95 9.068 8.122 8.365 8.423 8.32 8.31
   4009   1388857_at   9.516 9.587 9.695 9.782 9.595 9.022 9.206 9.144 8.904 9.094
   4010   1392079_at   6.262 6.319 6.546 6.735 6.629 4.639 5.085 5.245 5.395 4.555
   4011   1369381_a_at   5.516 5.438 5.852 5.202 5.099 7.429 7.438 6.496 7.231 7.228
   4012   1372455_at   8.438 8.424 8.161 8.228 8.53 9.326 9.474 9.562 9.033 9.165
   4013   1371856_at   10.348 10.404 10.328 10.32 10.42 9.926 10.054 9.751 9.77 9.927
   4014   1387992_at   7.457 6.545 7.9 7.427 7.666 4.223 3.905 4.558 4.085 2.356
   4015   1388732_at   7.783 7.521 7.789 7.691 7.849 7.021 7.305 7.127 7.101 7.056
   4016   1370878_at   9.316 9.498 9.332 9.296 9.418 9.737 9.816 9.84 9.782 9.66
   4017   1385069_at   7.144 7.146 6.928 7.257 7.353 8.04 7.703 7.862 7.884 7.956
   4018   1384934_at   7.531 7.61 7.107 7.406 7.577 5.384 5.856 6.394 5.3 5.551
   4019   1377900_at   9.805 9.849 10.234 10.031 9.323 11.276 11.107 11.019 11.498 11.23
   4020   1371838_at   11.001 10.932 10.856 10.806 10.886 10.376 10.264 10.36 10.089 9.971
   4021   1370365_at   10.311 9.787 9.865 10.119 9.544 11.171 11.314 11.901 11.274 11.358
   4022   1372841_at   7.119 6.921 7.326 7.522 6.959 8.488 8.635 9.378 8.883 8.501
   4023   1389709_at   6.789 6.681 6.612 6.367 6.729 5.578 5.735 5.483 5.686 6.021
   4024   1372154_at   8.645 8.645 8.816 8.912 8.892 8.257 8.357 8.181 8.182 8.137
   4025   1370728_at   8.559 8.711 8.482 8.366 8.428 7.208 6.631 6.163 7.198 6.308
   4026   1377673_at   7.969 7.894 8.029 8.132 7.91 9.092 9.282 8.581 8.92 8.978
   4027   1387804_at   3.45 3.23 1.628 2.31 2.548 6.301 5.525 5.567 5.472 5.233
   4028   1393795_at   6.841 6.66 7.04 6.797 7.546 5.713 5.837 5.477 5.629 5.526
   4029   1377642_at   8.093 8.073 8.239 8.092 8.157 8.632 8.671 8.411 8.54 8.563
   4030   1377840_at   4.408 4.584 4.262 4.388 4.195 5.174 5.142 5.232 4.86 5.152
   4031   1384762_at   6.355 6.116 6.188 6.249 6.161 1.614 3.498 3.944 2.202 2.884
   4032   1397876_at   4.668 4.73 4.853 3.979 4.759 6.071 6.082 6.414 6.1 5.779
   4033   1372828_at   8.202 8.307 8.21 8.456 8.331 9.01 9.039 8.846 9.085 8.736
   4034   1371671_at   5.552 5.512 5.802 5.416 5.417 6.574 6.336 6.471 6.138 6.295
   4035   1393149_at   6.292 6.158 5.963 5.777 6.613 4.666 4.614 3.905 3.957 4.652
   4036   1368888_a_at   10.71 10.69 10.692 10.753 10.959 10.19 10.333 10.238 10.078 10.046
   4037   1371416_at   10.594 10.594 10.659 10.791 10.706 11.022 11.11 11.178 11.039 10.993
   4038   1374874_at   5.414 5.897 5.18 5.763 5.878 6.959 6.891 7.021 6.831 6.639
   4039   1374126_at   7.901 7.953 7.891 8.013 7.886 8.797 9.198 8.995 8.596 8.642
   4040   1371641_at   10.447 10.439 10.388 10.555 10.568 9.913 10.021 10.118 9.889 9.786
   4041   1387591_at   7.144 6.4 6.913 6.677 6.331 3.666 3.583 3.512 2.58 1.333
   4042   1382832_at   7.876 7.52 7.682 7.998 8.057 6.787 6.914 6.867 6.8 6.408
   4043   1374641_at   8.685 8.767 8.852 8.958 8.769 9.389 9.856 9.799 9.513 9.518
   4044   1393896_at   6.592 6.503 6.487 6.755 7.034 7.536 7.805 7.481 7.686 7.856
   4045   1394761_at   7.156 7.397 7.223 7.489 7.326 7.913 7.957 8.062 7.775 7.894
   4046   1383586_at   8.907 8.921 8.921 8.975 8.927 8.692 8.642 8.528 8.728 8.631
   4047   1396000_at   7.263 7.076 7.331 7.314 6.753 8.554 9.214 8.218 8.745 8.783
   4048   1372655_at   8.808 8.836 9.054 8.995 8.828 9.678 10.027 9.466 9.697 9.782
   4049   1373238_at   7.442 7.479 7.659 7.808 7.848 6.648 6.592 6.668 7.015 6.492
   4050   1380130_at   5.995 6.404 5.613 6.175 6.227 4.284 4.777 3.809 4.554 4.525
   4051   1394968_at   7.007 7.041 7.039 6.954 6.903 6.432 6.179 5.791 6.199 6.082
   4052   1387654_at   7.844 7.866 7.827 7.847 7.788 8.321 8.164 8.341 8.115 8.156
   4053   1372362_at   8.428 8.439 8.549 8.501 8.688 9.124 9.32 9.109 8.948 9.115
   4054   1369862_at   6.698 6.678 6.622 6.897 7.169 4.386 4.533 5.393 3.974 5.05
   4055   1396014_at   4.676 4.728 3.956 4.32 3.569 6.492 6.807 5.893 7.026 6.857
   4056   1375016_at   6.017 6.06 6.088 5.922 5.797 6.705 7.071 6.861 6.844 6.533
   4057   1368522_at   6.129 6.32 6.347 6.143 5.769 3.688 4.479 4.051 4.697 4.748
   4058   1390205_at   8.055 8.129 8.453 8.287 8.298 7.692 7.645 7.403 7.519 7.512
   4059   1399159_a_at   9.812 10.054 10.047 10.056 10.214 9.467 9.49 9.551 9.543 9.444
   4060   1388505_at   8.589 8.815 8.917 8.759 8.579 7.375 7.662 8.021 7.389 7.752
   4061   1388363_at   8.729 8.688 8.457 8.818 8.775 7.373 7.681 8.023 7.516 7.347
   4062   1372845_at   9.733 9.831 9.699 9.421 9.533 8.53 8.448 7.65 8.42 8.177
   4063   1373211_at   8.211 8.096 8.545 8.322 8.048 7.061 7.214 7.437 6.874 6.783
   4064   1392743_at   6.885 6.914 7.061 7.18 7.307 6.12 6.344 5.879 5.935 5.705
   4065   1387782_at   8.387 8.437 8.573 8.794 8.567 9.189 9.21 9.423 9.301 9.121
   4066   1369289_at   8.079 8.068 8.367 8.231 7.913 9.976 9.189 9.17 9.736 9.752
   4067   1387306_a_at   7.737 7.897 7.978 8.137 7.983 5.814 5.711 6.876 5.482 5.648
   4068   1396305_at   4.492 4.419 4.114 4.204 4.555 5.548 5.716 5.504 5.698 5.067
   4069   1398316_at   8.676 8.649 8.546 8.758 8.708 9.124 9.153 9.078 9.109 8.935
   4070   1393380_at   6.993 7.133 6.986 7.075 6.899 7.617 7.819 7.659 7.412 7.741
   4071   1393443_a_at   2.832 1.986 1.718 3.094 1.104 5.736 5.589 5.339 5.153 4.804
   4072   1383629_a_at   7.335 6.948 7.134 7.155 7.318 6.26 6.195 6.62 6.24 6.412
   4073   1386615_at   6.614 6.924 7.069 7.203 6.742 7.835 8.133 7.872 8.02 7.741
   4074   1371862_at   8.815 8.876 8.624 8.789 8.804 7.221 7.499 7.806 7.235 7.967
   4075   1390957_at   6.036 6.095 5.904 6.246 6.347 6.906 6.937 7.352 6.96 6.971
   4076   1374999_at   4.241 4.437 4.195 4.479 4.014 5.657 5.24 5.416 5.054 5.24
   4077   1390777_at   8.137 8.235 8.053 7.697 7.677 9.121 9.149 8.875 8.835 9.133
   4078   1374030_at   7.672 7.745 7.742 7.876 7.712 8.252 8.564 8.215 8.258 8.302
   4079   1389301_at   9.878 9.946 9.839 9.864 9.799 10.344 10.309 10.261 10.209 10.521
   4080   1380905_at   7.369 7.444 7.853 7.622 7.503 8.86 9.085 8.378 9.244 8.905
   4081   1390706_at   8.464 8.457 8.559 8.656 8.527 9.063 9.092 8.826 9.029 9.085
   4082   1370296_at   11.58 11.613 11.608 11.631 11.664 11.778 11.817 11.789 11.887 11.788
   4083   1376789_at   2.868 3.621 3.259 3.22 1.491 6.044 6.85 5.6 6.364 6.394
   4084   1387567_at   7.593 7.763 8.019 8.013 5.926 10.546 10.668 10.742 10.503 10.508
   4085   1369173_at   5.609 5.611 6.068 5.985 5.874 3.126 3.41 4.14 4.458 3.448
   4086   1382435_at   7.374 7.318 7.499 7.434 7.587 6.822 6.789 6.817 7.06 6.696
   4087   1384936_at   4.498 4.494 4.274 4.223 4.737 5.79 5.932 5.489 6.021 5.332
   4088   1379716_at   5.239 6 6.662 6.649 6.741 3.722 3.434 3.648 2.501 3.567
   4089   1395687_at   5.649 5.746 5.881 6.079 6.055 5.069 4.968 4.982 5.29 4.957
   4090   1369632_a_at   4.415 4.452 4.498 4.356 4.08 5.522 5.669 6.127 5.573 5.253
   4091   1374947_at   5.147 5.326 5.022 5.008 5.104 6.682 6.607 6.952 6.2 6.051
   4092   1382269_at   6.198 6.427 6.297 6.826 6.659 7.507 7.351 7.814 7.581 7.615
   4093   1370034_at   6.503 6.426 6.51 6.608 6.64 5.371 4.664 4.608 5.23 5.476
   4094   1379935_at   10.305 10.173 9.893 10.189 10.278 1.632 4.428 5.373 3.157 0.401
   4095   1388767_at   10.383 10.377 10.359 10.527 10.493 10.013 9.991 10.155 9.911 9.964
   4096   1367691_at   9.037 9.115 8.98 9.081 9.364 8.54 8.591 8.355 8.339 8.481
   4097   1385729_at   7.038 7.165 7.404 7.342 6.694 8.268 8.701 8.489 8.125 8.383
   4098   1388568_at   9.932 9.928 9.918 10.182 10.11 9.378 9.405 9.482 9.107 9.206
   4099   1373388_at   8.347 8.291 8.209 8.57 8.458 8.975 8.911 9.029 8.837 8.928
   4100   1384943_at   5.588 5.57 6.335 5.893 5.37 4.178 4.521 3.862 4.198 3.947
   4101   1384438_at   6.487 6.575 7.28 7.369 7.102 8.511 8.489 8.847 8.474 8.365
   4102   1377417_at   5.945 5.971 6.09 6.287 6.347 5.269 4.802 4.781 5.302 4.84
   4103   1389200_at   7.444 7.523 7.669 7.705 7.614 6.821 6.799 6.597 6.484 7.035
   4104   1376830_at   4.288 4.551 4.584 4.549 4.393 5.075 4.999 5.217 5.005 4.955
   4105   1371673_at   8.41 8.367 8.566 8.389 8.567 7.861 7.911 8.1 7.913 7.771
   4106   1380932_at   4.446 4.84 4.82 4.907 4.615 6.333 6.033 6.491 6.156 5.584
   4107   1389889_at   5.946 6.117 5.92 6.154 6.209 5.552 5.445 5.292 5.542 5.531
   4108   1373895_at   7.232 7.34 7.426 6.817 7.055 8.411 8.494 8.69 8.173 8.107
   4109   1369934_at   11.554 11.536 11.469 11.566 11.638 11.099 11.121 11.249 11.094 10.93
   4110   1388848_at   7.106 6.805 7.428 7.351 6.973 4.275 4.833 5.815 4.98 4.583
   4111   1389417_at   6.836 6.888 6.654 6.862 6.734 6.217 6.337 6.35 6.328 6.081
   4112   1371308_at   12.682 12.57 12.637 12.361 12.577 12.12 12.106 12.054 12.146 12.138
   4113   1376619_at   8.189 8.185 8.368 8.371 8.315 8.956 9.334 8.908 8.971 8.887
   4114   1383153_at   3.779 4.644 3.946 4.553 4.889 6.283 6.181 6.229 6.096 5.876
   4115   1390859_at   7.172 7.091 7.237 7.558 7.29 6.288 6.095 5.792 5.385 5.979
   4116   1383852_at   7.45 7.585 7.836 7.974 7.83 8.79 9.171 8.738 8.622 8.685
   4117   1398628_at   8.796 8.833 9.367 8.818 8.373 10.311 10.566 9.949 10.355 10.416
   4118   1379320_at   5.824 5.703 4.934 5.264 5.494 3.358 3.675 4.053 4.095 3.825
   4119   1372558_at   9.041 9.122 8.984 9.077 9.076 8.393 8.441 8.305 8.204 8.681
   4120   1391604_at   7.35 7.56 7.461 7.628 7.505 6.401 6.728 6.816 6.794 6.335
   4121   1374622_at   7.382 7.362 7.54 7.611 7.456 8.382 8.487 8.403 8.066 8.757
   4122   1383258_at   4.936 4.839 4.869 5.202 5.223 6.104 6.595 6.844 6.412 6.008
   4123   1376022_at   7.859 7.836 8.087 7.899 7.85 7.271 7.115 6.711 7.117 6.812
   4124   1368168_at   10.436 10.45 9.41 10.577 10.504 7.934 7.956 8.619 7.693 7.363
   4125   1383250_at   7.514 7.402 7.69 7.704 7.649 7.018 7.127 6.96 7.028 6.827
   4126   1367888_at   6.065 6.35 5.702 5.702 5.756 2.993 4.283 3.945 3.726 4.209
   4127   1372506_at   9.403 9.46 9.362 9.631 9.613 9.018 8.743 8.778 8.883 8.614
   4128   1378556_at   6.767 7.061 6.41 6.774 6.338 8.333 8.181 8.058 7.629 8.17
   4129   1383146_at   6.237 6.221 6.285 6.394 6.565 6.931 7.046 7.253 6.935 7.217
   4130   1379323_at   7.235 7.399 7.431 7.53 7.628 8.048 8.1 8.117 8.241 7.942
   4131   1384439_at   6.828 6.716 6.77 6.823 6.612 6.069 5.388 5.89 5.859 5.616
   4132   1367455_at   11.148 11.108 11.095 11.241 11.271 10.775 10.877 10.86 10.816 10.713
   4133   1373196_at   5.548 5.64 5.909 6.19 5.845 7.487 6.984 6.87 6.915 7.255
   4134   1381508_at   6.131 6.39 6.369 6.423 6.534 7.087 7.04 6.962 7.123 6.854
   4135   1387296_at   6.416 6.482 6.079 6.507 6.255 7.37 7.006 7.448 7.514 7.2
   4136   1373779_at   7.393 7.345 7.119 7.211 7.271 7.746 7.842 8.003 7.698 7.878
   4137   1384257_at   9.484 9.453 9.422 9.648 9.552 9.152 9.039 8.96 8.993 8.834
   4138   1384015_at   7.007 7.277 6.939 7.236 7.121 6.054 5.961 6.525 5.874 5.994
   4139   1370838_s_at   9.991 10.173 10.388 10.458 10.237 10.909 11.067 11.078 10.855 11.069
   4140   1391897_at   6.903 6.709 6.704 6.831 6.606 5.272 5.833 5.773 5.048 5.589
   4141   1390500_at   8.788 8.641 8.595 8.727 8.489 9.061 9.265 9.149 9.085 9.15
   4142   1371872_at   9.348 9.284 9.102 9.537 9.473 8.658 8.798 8.653 8.58 8.504
   4143   1387684_at   4.122 4.399 4.403 4.267 4.232 5.944 5.673 5.237 5.15 5.476
   4144   1389584_at   3.449 2.299 3.189 3.118 3.428 4.955 4.975 4.59 4.848 4.786
   4145   1383231_at   7.86 7.987 7.716 7.903 8.089 6.917 6.943 7.163 7.372 7.101
   4146   1388336_at   7.785 7.656 7.823 7.936 7.947 8.336 8.417 8.585 8.354 8.312
   4147   1377660_at   6.019 6.08 5.653 5.762 5.583 7.062 7.709 8.016 7.328 7.117
   4148   1389332_at   7.202 7.267 7.34 7.702 7.804 8.628 8.882 8.423 8.479 8.594
   4149   1397620_at   6.007 6.046 5.978 6.116 5.956 8.041 7.679 7.629 6.888 7.399
   4150   1372109_at   8.863 8.863 8.921 8.901 8.804 9.474 9.561 9.595 9.432 9.191
   4151   1370906_at   10.02 9.717 9.931 10.124 9.868 10.944 10.889 10.938 10.507 10.686
   4152   1367658_at   6.732 7.101 6.74 6.828 6.588 7.477 7.428 7.628 7.523 7.505
   4153   1374712_at   9.871 9.78 10.084 10.036 10.051 9.327 9.503 9.497 9.379 9.318
   4154   1390851_at   10.521 10.523 10.913 10.679 9.858 11.875 11.886 11.809 12.003 12.107
   4155   1368498_a_at   8.882 8.812 9.24 9.175 8.148 10.501 10.407 10.275 10.497 10.364
   4156   1372877_at   9.276 9.4 9.229 9.466 9.512 8.716 8.818 8.949 8.807 8.916
   4157   1386280_at   8.593 8.532 8.842 8.837 7.807 10.026 9.943 10.072 10.046 9.998
   4158   1373277_at   8.869 8.848 9.084 9.078 8.934 9.464 9.554 9.486 9.336 9.413
   4159   1389080_at   9.061 9.178 8.964 8.999 9.072 8.675 8.755 8.776 8.666 8.734
   4160   1379117_at   2.26 3.252 2.556 3 2.935 4.598 4.909 5.122 4.163 4.583
   4161   1388888_at   7.076 7.13 7.21 7.356 7.064 7.936 7.728 7.806 7.601 7.871
   4162   1393267_at   7.719 7.985 7.686 7.764 7.76 6.988 7.261 6.833 6.745 6.741
   4163   1393172_at   8.411 8.254 8.502 8.408 8.472 7.901 7.942 7.934 7.834 7.632
   4164   1376427_a_at   7.135 7.137 7.561 7.117 7.105 8.398 8.584 8.129 8.669 8.07
   4165   1390585_at   6.783 6.526 6.713 6.475 6.712 5.733 5.864 5.492 5.998 5.924
   4166   1390187_at   7.225 7.335 7.283 7.374 7.496 6.741 6.889 6.856 6.659 6.925
   4167   1395739_at   8.19 8.305 8.248 8.305 8.335 7.705 7.724 7.349 7.479 7.239
   4168   1397527_at   6.12 5.916 5.727 6.093 6.057 4.941 4.833 5.392 4.985 4.845
   4169   1387977_at   8.301 8.487 8.458 8.551 8.582 7.906 8.067 7.711 7.778 7.862
   4170   1370371_a_at   6.586 6.769 7.068 6.805 6.918 5.402 4.398 5.314 5.521 5.098
   4171   1372080_at   10.757 10.703 10.798 10.785 10.857 11.245 11.256 11.298 11.041 11.138
   4172   1397549_at   4.825 4.641 4.531 4.713 4.497 0.687 0.84 2.101 2.032 2.606
   4173   1385066_a_at   6.828 6.681 6.669 7.002 6.99 4.905 5.614 5.791 5.299 5.682
   4174   1373645_at   7.441 7.299 7.698 7.586 7.317 8.184 8.298 8.523 8.105 8.328
   4175   1372259_at   10.618 10.616 10.469 10.51 10.496 10.171 10.276 10.189 10.051 10.153
   4176   1367694_at   11.182 11.189 11.214 11.089 11.147 11.475 11.526 11.417 11.642 11.616
   4177   1389146_at   9.951 9.925 10.206 10.135 9.631 10.855 10.951 10.751 10.736 11.019
   4178   1384474_at   4.089 4.68 5.068 4.836 3.976 6.067 6.487 6.939 6.599 6.501
   4179   1378867_at   4.916 5.529 5.85 5.082 5.08 3.474 3.372 3.589 2.534 2.696
   4180   1378964_at   8.814 8.901 8.654 8.928 8.824 8.03 7.986 8.394 8.004 8.179
   4181   1370432_at   3.937 4.704 3.68 4.859 3.936 7.629 7.475 7.78 6.062 7.226
   4182   1393320_at   7.579 7.781 7.542 7.869 7.757 6.507 6.892 6.515 6.903 6.991
   4183   1393441_at   7.121 7.228 7.376 7.245 7.151 5.606 6.041 6.355 5.664 6.264
   4184   1390656_at   6.006 5.773 5.677 5.855 4.851 7.097 7.296 7.467 7.277 7.251
   4185   1372704_at   10.108 10.121 9.901 9.969 10.156 9.336 9.491 9.628 9.298 9.286
   4186   1375645_at   9.3 9.253 9.212 9.402 9.452 8.793 8.741 8.972 8.822 8.674
   4187   1375215_x_at   10.005 10.129 10.58 10.363 10.469 11.363 11.579 11.161 11.335 11.183
   4188   1371879_at   8.442 8.237 7.995 8.109 8.248 7.099 7.602 7.362 7.197 7.138
   4189   1383706_at   5.009 5.222 5.609 5.307 5.108 6.39 6.334 6.037 6.459 6.123
   4190   1368121_at   10.85 10.818 11.408 11.067 10.234 12.31 12.4 12.316 12.499 12.547
   4191   1372896_at   7.741 7.646 7.606 7.601 7.566 6.884 6.786 6.346 6.282 6.803
   4192   1373619_at   7.204 6.872 7.204 6.76 6.866 7.961 7.812 7.811 7.656 7.761
   4193   1368284_at   8.688 8.648 9.069 8.827 9.242 10.503 10.056 9.795 10.233 10.286
   4194   1372149_at   8.407 8.846 8.438 8.741 8.729 9.439 9.511 9.979 9.793 9.588
   4195   1388624_at   7.732 7.916 7.908 8.097 7.966 8.582 8.771 9 8.717 8.52
   4196   1397392_at   2.814 2 0 0.566 1.281 5.135 5.586 5.4 4.995 5.434
   4197   1374890_at   7.978 8.03 8.046 8.143 8.242 8.987 9.153 8.585 8.996 8.847
   4198   1392481_at   8.007 7.865 8.077 8.063 8.123 7.368 7.608 7.554 7.314 7.513
   4199   1369415_at   7.51 7.697 7.562 7.497 7.267 9.016 8.7 8.956 8.316 8.465
   4200   1367677_at   11.702 11.715 11.746 11.759 11.862 11.455 11.473 11.516 11.497 11.37
   4201   1389324_at   6.152 6.565 6.441 6.446 6.614 7.58 7.101 7.556 7.398 7.268
   4202   1382459_at   5.301 5.589 5.742 6.465 6.095 2.278 0 0 2.696 0.696
   4203   1388145_at   6.007 5.73 6.018 6.351 5.77 7.397 7.403 7.794 7.068 7.047
   4204   1387964_a_at   6.38 6.102 6.63 6.287 6.715 5.237 5.535 5.328 5.54 5.505
   4205   1396821_at   3.904 2.814 3.888 3.742 4.123 5.917 5.789 5.477 5.495 5.428
   4206   1374693_at   6.805 6.677 6.615 6.693 6.757 7.701 7.771 7.187 7.476 7.605
   4207   1393219_at   11.202 11.201 11.192 10.843 11.075 4.717 4.75 6.25 1.683 1.449
   4208   1372014_at   7.6 7.481 7.673 7.949 7.942 6.6 6.984 6.384 6.482 6.738
   4209   1389816_at   7.023 6.862 7.236 6.859 7.025 7.596 7.701 7.592 7.768 7.522
   4210   1388754_at   9.9 9.83 9.805 9.851 9.99 9.313 9.086 9.462 9.142 9.03
   4211   1385828_at   5.824 5.719 5.83 6.155 5.935 6.959 6.984 7.03 6.899 6.47
   4212   1373704_at   8.435 8.462 8.289 8.516 8.347 7.796 7.944 7.929 7.573 7.76
   4213   1371448_at   9.189 9.236 9.419 9.505 9.135 9.926 10.095 10.13 9.922 10.304
   4214   1372561_at   8.157 8.164 8.223 8.35 8.287 7.824 7.556 7.796 7.818 7.639
   4215   1369401_at   8.115 8.199 8.733 8.622 6.58 11.131 11.027 11.268 10.929 11.035
   4216   1376810_at   7.309 7.383 7.479 7.563 7.535 6.743 7.007 6.747 6.568 6.853
   4217   1392918_at   10.211 10.294 10.009 10.353 10.253 9.33 9.689 9.202 9.469 9.507
   4218   1389322_at   7.688 7.594 7.717 7.823 7.581 8.237 8.666 8.263 8.608 8.419
   4219   1387209_at   5.007 4.467 4.536 4.239 4.625 5.764 5.95 6.118 5.531 6.164
   4220   1390397_at   5.669 5.698 5 5.569 6.085 4.288 3.401 3.29 3.811 3.467
   4221   1391095_at   5.745 5.592 5.843 5.85 6.12 4.205 4.164 3.331 4.529 4.316
   4222   1373449_at   6.943 7.169 7.163 7.144 7.248 6.667 6.704 6.638 6.745 6.545
   4223   1367678_at   11.24 11.294 11.402 11.431 11.351 11.788 11.807 11.624 11.782 11.671
   4224   1376861_at   7.356 7.104 7.363 7.178 7.008 7.828 7.904 7.807 7.865 7.681
   4225   1382111_at   8.677 8.807 9.205 8.897 8.559 10.049 10.132 9.622 10.203 9.943
   4226   1383251_at   7.874 7.807 7.552 7.741 7.824 7.098 7.159 6.982 7.054 6.695
   4227   1388987_at   6.495 6.669 6.577 6.65 6.799 5.611 5.481 5.597 5.941 5.126
   4228   1375056_at   9.046 9.09 8.998 8.961 9.067 8.192 8.532 8.072 8.497 8.352
   4229   1380688_at   6.449 5.839 6.084 6.334 5.877 7.322 7.382 7.826 7.326 7.145
   4230   1383059_a_at   5.499 5.553 4.82 5.078 5.347 6.672 6.441 6.496 6.246 6.396
   4231   1387776_at   10.511 10.542 10.288 10.502 10.588 9.38 8.811 8.297 9.27 8.996
   4232   1391063_at   7.065 6.958 6.642 7.372 6.774 5.588 4.021 4.866 4.747 4.824
   4233   1392508_at   6.618 6.664 6.683 6.669 6.71 5.848 5.974 5.721 6.275 5.905
   4234   1375886_at   8.602 8.592 8.867 8.719 8.608 9.742 9.74 9.19 9.596 9.759
   4235   1395572_at   6.313 6.369 6.556 6.142 5.967 7.475 7.198 7.004 7.37 7.252
   4236   1388557_at   11.915 11.866 11.467 11.555 11.978 10.544 10.663 9.895 10.554 10.513
   4237   1388054_a_at   7.607 7.174 7.623 7.536 8.489 5.527 5.727 5.943 4.848 5.329
   4238   1398798_at   10.336 10.46 10.314 10.324 10.338 9.894 9.963 10.02 9.761 9.73
   4239   1381940_at   4.757 4.537 5.582 5.708 5.518 7.363 7.534 7.142 6.971 7.091
   4240   1369044_a_at   7.148 6.887 7.45 7.402 7.422 5.807 5.572 4.504 5.526 5.104
   4241   1398361_at   6.98 6.796 7.196 6.872 6.678 7.862 7.878 7.585 7.713 7.614
   4242   1375536_at   8.201 8.349 8.308 8.582 8.357 9.023 9.158 8.815 8.993 8.998
   4243   1369788_s_at   9.283 9.409 8.94 9.25 9.115 10.797 10.584 11.083 10.094 11.032
   4244   1368574_at   5.547 5.433 4.934 5.539 5.579 6.541 6.387 6.481 6.457 6.236
   4245   1372423_at   8.806 8.952 8.874 8.99 9.057 9.887 10.366 9.669 9.781 9.874
   4246   1388745_at   9.773 9.883 9.679 10.082 9.927 9.268 9.254 9.291 9.293 9.049
   4247   1373182_at   7.576 7.432 7.499 7.84 7.216 8.204 8.423 8.483 8.365 8.477
   4248   1396211_at   3.405 2.787 2.575 2.077 2.441 4.257 4.36 4.533 4.367 4.413
   4249   1373079_at   8.06 8.039 7.895 8.202 8.303 8.869 8.902 8.836 8.652 8.655
   4250   1394499_at   6.512 6.351 6.537 6.624 6.281 5.713 5.72 5.818 5.359 5.412
   4251   1367745_at   8.438 8.473 8.336 8.334 8.402 7.411 7.455 7.881 7.248 7.19
   4252   1379429_at   9.42 9.436 9.456 9.216 9.334 8.845 8.724 8.388 8.762 8.553
   4253   1385806_at   7.992 8.181 7.933 8.088 8.181 6.487 6.933 7.083 7.146 7.247
   4254   1386999_at   10.513 10.557 10.225 10.066 10.33 9.588 9.623 9.236 9.392 9.354
   4255   1393808_at   5.847 6.532 6.356 6.51 6.312 7.445 7.323 7.799 7.584 7.315
   4256   1380544_at   6.801 7.026 7.238 6.986 7.001 6.141 6.249 6.214 5.806 5.743
   4257   1368735_a_at   6.297 6.344 6.244 6.326 6.052 4.249 3.663 4.747 3.41 4.681
   4258   1392973_at   6.325 6.419 6.695 6.662 6.641 8.358 8.288 7.731 8.138 7.459
   4259   1368592_at   5.34 6.044 5.846 5.529 5.431 0.66 1.084 2.299 2.501 3.144
   4260   1385873_at   7.578 7.86 7.675 7.666 7.822 6.661 6.872 6.215 6.88 6.795
   4261   1383162_at   9.832 9.868 10.146 10.023 9.696 10.557 10.52 10.763 10.849 10.753
   4262   1387811_at   6.174 6.025 6.382 6.372 5.167 7.879 7.772 8.319 7.716 8.15
   4263   1367806_at   6.479 7.007 6.452 6.575 6.575 7.867 7.748 7.751 7.873 7.325
   4264   1386128_at   6.673 6.99 6.545 7.189 7.791 5.318 5.307 4.914 4.684 5.222
   4265   1381811_at   5.603 5.954 5.918 5.768 5.739 7.948 7.415 7.714 6.734 7.79
   4266   1379254_at   9.636 9.751 9.388 9.555 9.333 10.251 10.242 10.42 10.085 10.286
   4267   1370881_at   10.966 10.948 11.174 10.949 10.714 11.576 11.767 11.48 11.715 11.756
   4268   1379901_at   7.149 6.998 6.827 6.935 6.791 7.658 7.618 7.802 7.49 7.484
   4269   1373434_at   7.291 7.379 7.189 7.461 7.233 7.966 8.052 7.963 7.752 7.774
   4270   1390127_at   4.14 4.999 4.458 4.681 4.622 6.198 6.168 6.104 5.779 5.629
   4271   1398365_at   5.187 5.52 5.742 6.125 5.376 2.411 2.809 2.618 2.465 4.102
   4272   1369250_at   5.099 5.563 5.395 5.632 5.066 7.045 6.784 6.292 6.686 6.53
   4273   1382058_at   9.054 9.088 8.918 9.136 9.036 8.718 8.73 8.564 8.712 8.614
   4274   1371727_at   10.4 10.47 10.647 10.424 10.226 11.202 11.279 10.907 11.221 11.154
   4275   1390822_at   10.698 10.561 10.425 10.392 10.671 11.122 11.348 11.584 11.28 11.226
   4276   1374633_at   6.8 6.885 6.924 6.871 6.435 7.73 7.903 7.429 7.665 7.694
   4277   1387769_a_at   10.284 10.191 10.06 10.186 10.752 8.818 9.215 9.196 9.346 9.179
   4278   1371668_at   8.156 8.06 8.202 8.316 8.384 8.959 9.103 8.88 8.862 8.695
   4279   1399103_at   7.001 6.937 7.167 7.163 7.307 7.788 7.689 8.063 7.741 7.84
   4280   1376242_at   5.963 5.867 6.119 6.248 6.243 6.696 7.096 7.064 6.794 6.945
   4281   1378753_at   8.895 8.982 8.995 9.072 8.995 9.72 9.982 10.211 9.589 9.773
   4282   1395377_at   6.823 6.91 6.783 7.032 7.295 6.277 6.308 6.101 6.17 6.138
   4283   1368301_at   6.704 6.238 6.308 6.204 6.534 4.317 2.42 3.681 3.872 4.191
   4284   1384548_at   12.72 12.606 12.707 12.552 12.689 12.367 12.354 12.351 12.237 12.219
   4285   1387820_at   9.146 9.069 9.393 8.735 7.244 11.832 11.894 11.908 11.831 11.356
   4286   1375205_at   9.697 9.799 9.75 9.82 9.85 9.394 9.562 9.33 9.382 9.427
   4287   1384005_at   7.161 7.229 7.387 7.136 7.189 6.9 6.839 6.762 6.742 6.834
   4288   1371583_at   10.097 10.243 10.136 10.392 10.712 9.259 9.509 9.223 9.054 8.971
   4289   1389293_at   8.273 8.254 8.236 8.344 8.45 7.737 7.922 7.99 7.832 7.842
   4290   1388679_at   8.995 9.006 9.034 9.007 8.865 9.782 9.754 9.389 9.484 9.675
   4291   1392449_at   5.938 6.611 6.494 6.687 6.426 3.889 3.883 3.167 1.967 3.924
   4292   1374305_at   7.119 7.293 7.477 7.253 7.12 6.609 6.461 6.494 6.252 6.094
   4293   1389755_at   6.697 6.561 6.697 6.819 6.856 5.549 5.82 6.177 5.67 5.576
   4294   1386149_at   4.116 3.863 3.455 3.013 3.769 4.941 5.35 5.206 5.244 5.194
   4295   1367898_at   9.725 9.855 9.728 9.86 9.872 10.092 10.247 10.226 10.135 10.106
   4296   1373935_at   7.884 7.868 7.893 8.054 8.035 7.501 7.25 7.461 7.123 7.227
   4297   1371524_at   8.338 8.483 8.485 8.442 8.583 7.983 7.883 7.554 7.637 7.853
   4298   1379076_at   2.018 2.602 2.793 3.172 2.784 5.129 4.775 5.026 5.456 4.131
   4299   1397317_at   6.04 6.143 6.11 6.609 6.569 4.902 4.775 4.759 3.878 4.702
   4300   1391607_at   8.283 8.333 8.581 8.686 8.681 9.597 9.699 9.258 9.276 9.423
   4301   1388506_at   8.806 8.862 8.713 8.883 8.663 9.355 9.45 9.729 9.281 9.582
   4302   1373041_at   11.192 11.149 11.186 11.216 11.359 11.587 11.763 11.79 11.665 11.555
   4303   1387868_at   7.174 7.231 6.401 7.111 6.932 5.811 5.67 5.182 5.612 5.535
   4304   1371888_at   10.048 10.119 9.873 9.93 10.186 9.438 9.508 9.483 9.176 9.353
   4305   1368334_at   6.264 6.352 6.292 6.648 6.276 7.424 7.353 7.869 7.63 7.124
   4306   1373461_at   8.426 8.488 8.646 8.825 8.719 7.928 7.877 8.135 7.902 7.852
   4307   1370398_at   6.537 6.693 6.338 6.686 6.873 7.362 7.318 7.376 7.286 7.285
   4308   1382624_at   5.868 6.106 6.151 5.914 6.334 4.698 5 4.467 4.818 3.992
   4309   1386820_at   6.792 6.744 6.886 6.807 6.48 7.507 7.402 7.499 7.561 7.226
   4310   1386906_a_at   9.298 9.24 9.488 9.27 9.121 8.483 8.343 8.708 8.151 8.177
   4311   1392786_at   5.996 5.547 6.141 6.027 6.271 7.092 7.239 6.978 6.857 7.098
   4312   1378569_at   3.944 4.307 4.756 3.756 4.262 5.732 5.897 5.456 5.704 5.493
   4313   1385829_at   5.464 5.76 5.699 5.753 4.888 6.869 6.874 6.881 6.722 6.71
   4314   1371340_at   12.379 12.273 12.082 12.176 12.302 11.853 11.869 11.82 11.844 11.8
   4315   1395192_at   7.451 7.227 6.818 6.873 7.031 6.131 5.563 5.44 5.82 5.319
   4316   1377197_at   5.6 5.432 5.644 5.73 5.65 6.122 6.289 6.152 6.157 6.473
   4317   1368600_at   8.637 8.969 9.042 9.331 8.668 9.954 10.124 9.842 9.955 9.975
   4318   1392818_at   6.891 7.016 7.422 6.697 7.1 5.897 4.597 4.996 5.24 4.984
   4319   1371461_at   9.957 9.885 9.951 9.901 9.869 10.244 10.109 10.151 10.1 10.146
   4320   1397530_at   5.489 5.856 5.832 5.828 5.35 4.959 4.515 4.651 4.571 4.685
   4321   1371519_at   9.017 9.132 9.183 9.192 9.041 9.841 9.661 9.794 9.75 9.456
   4322   1390458_at   5.664 5.4 5.677 5.616 5.463 6.122 6.334 6.241 6.116 6.014
   4323   1372469_at   7.213 7.17 7.122 7.554 7.435 8.057 8.223 8.366 8.019 7.999
   4324   1384958_at   8.935 8.915 8.875 9.083 8.987 8.477 8.503 8.509 8.346 8.651
   4325   1375781_at   4.496 4.89 5.413 4.117 4.888 3.084 2.621 3.203 2.616 2.803
   4326   1380828_at   2.918 2.791 2.816 3.079 2.406 4.271 4.249 4.021 4.729 5.057
   4327   1379498_at   5.068 5.142 5.101 5.775 5.645 6.488 6.693 6.605 6.538 6.421
   4328   1369006_at   6.732 6.863 6.947 7.367 7.196 2.546 4.082 5.023 3.303 3.838
   4329   1379608_at   6.233 7.005 6.664 6.238 6.227 4.769 5.189 4.097 4.279 4.249
   4330   1389228_at   8.495 8.585 8.414 8.613 8.714 7.845 8.14 8.091 7.989 7.929
   4331   1370879_at   9.837 9.887 10.028 10.134 10.059 10.515 10.714 10.616 10.417 10.528
   4332   1385800_at   7.277 7.239 7.816 7.785 7.512 6.286 6.157 6.436 6.369 6.678
   4333   1388560_at   9.048 9.052 8.977 9.344 9.266 8.401 8.513 8.362 8.36 8.643
   4334   1398883_at   10.417 10.499 10.434 10.616 10.682 9.829 10.108 9.945 9.767 9.733
   4335   1375017_at   4.673 5.096 5.82 5.646 4.822 7.211 7.15 7.336 6.954 6.781
   4336   1389286_at   10.291 10.244 10.344 10.543 10.452 10.938 11.117 11.1 10.847 10.871
   4337   1388300_at   11.517 11.466 11.692 11.383 11.506 12.332 12.367 11.958 12.486 12.406
   4338   1372043_at   8.21 8.375 7.885 8.384 8.28 7.368 7.302 7.498 7.521 7.168
   4339   1368683_at   6.996 7.68 8.387 8.326 7.258 0 2.776 3.339 2.939 3.341
   4340   1367612_at   11.717 11.674 11.684 11.649 11.717 11.774 11.803 11.816 11.804 11.802
   4341   1380120_at   4.439 4.893 5.421 4.512 4.881 6.509 6.327 6.724 6.081 6.318
   4342   1389130_at   7.789 7.968 7.781 7.885 8.056 7.005 7.111 7.394 7.344 7.223
   4343   1385559_at   4.977 5.156 5.13 5.195 5.122 4.446 4.663 4.287 4.365 4.119
   4344   1394397_at   8.776 8.722 8.567 8.639 8.9 8.084 8.227 7.984 8.207 7.913
   4345   1367830_a_at   7.708 8.148 8.243 8.309 8.011 7.213 6.932 7.14 7.263 7.229
   4346   1389906_at   7.028 6.94 7.365 7.267 7.228 8.04 8.222 8.426 8.049 7.849
   4347   1387183_at   7.949 8.031 7.841 7.896 7.821 8.884 8.65 8.478 8.754 8.407
   4348   1370336_at   8.494 8.39 8.799 8.786 7.455 10.465 10.425 11.109 10.254 10.548
   4349   1373080_at   9.334 9.403 9.584 9.573 9.395 9.007 9.071 8.814 8.888 8.873
   4350   1385606_at   6.368 6.38 6.634 6.8 6.615 6.012 5.755 5.723 5.663 5.772
   4351   1379406_at   7.158 7.27 7.324 7.376 7.371 6.676 6.426 6.809 6.814 6.568
   4352   1381017_at   7.022 7.01 7.001 7.019 7.131 6.068 6.303 5.979 5.485 5.637
   4353   1371472_at   6.81 6.577 6.408 6.867 7.33 8.333 8.388 8.426 7.851 8.167
   4354   1394511_at   6.06 6.198 5.763 6.085 5.597 4.948 5.114 5.016 5.139 5.015
   4355   1379791_at   9.563 9.586 10.027 9.621 9.216 0.251 2.245 5.188 3.623 4.049
   4356   1376043_at   9.031 9.034 8.854 8.781 8.677 7.976 7.602 7.907 8.199 7.611
   4357   1368205_at   6.87 7.047 6.86 6.521 6.851 6.18 5.657 5.506 5.621 5.602
   4358   1395986_at   5.44 5.386 5.402 5.662 6.024 6.866 7.307 6.931 6.754 6.575
   4359   1373016_at   9.331 9.49 9.585 9.73 9.635 10.438 10.376 10.203 10.367 10.064
   4360   1377598_at   7.181 7.244 6.942 7.013 7.211 7.842 7.657 7.669 7.711 7.561
   4361   1374513_at   10.98 10.948 11.032 10.894 10.796 10.471 10.582 10.379 10.383 10.57
   4362   1368025_at   7.94 7.537 8.14 7.828 7.961 9.524 9.751 9.839 8.832 9.3
   4363   1374262_at   4.492 4.299 4.741 5.106 5.187 6.215 6.398 5.963 6.307 6.609
   4364   1371393_at   9.327 9.09 9.269 9.289 9.385 9.778 9.962 9.962 9.726 9.727
   4365   1367996_a_at   5.098 4.57 5.57 5.421 5.368 6.962 7.49 6.636 7.247 6.727
   4366   1383432_at   5.433 5.209 4.598 5.087 4.961 6.713 6.528 7.14 6.716 6.168
   4367   1381264_at   4.024 4.376 4.826 4.505 4.398 5.82 6.3 5.683 5.607 5.63
   4368   1389778_a_at   8.154 8.354 8.583 8.442 8.443 7.715 7.762 7.371 7.597 7.362
   4369   1389565_at   6.69 6.922 7.369 7.083 6.772 5.9 6.031 5.584 5.518 5.424
   4370   1367988_at   11.929 11.901 12.045 11.765 11.874 12.634 12.585 12.452 12.846 12.981
   4371   1372391_at   8.86 8.909 8.943 8.973 9.068 8.511 8.668 8.379 8.462 8.423
   4372   1373427_at   6.807 6.793 6.976 7.251 6.956 7.925 7.845 7.716 7.541 7.747
   4373   1370236_at   9.843 9.812 9.678 9.624 9.521 9.185 9.195 9.171 8.987 9.201
   4374   1370979_at   7.265 7.149 7.141 7.228 7.113 6.563 6.832 6.407 6.402 6.485
   4375   1384863_at   6.047 6.25 6.686 6.314 6.471 7.409 7.338 7.1 7.244 7.222
   4376   1369700_at   7.044 7.073 7.267 7.154 7.148 6.349 5.947 5.659 6.235 5.592
   4377   1367802_at   10.918 10.856 11.374 10.791 10.588 12.492 12.173 11.811 12.275 12.336
   4378   1373769_at   8.307 8.425 8.506 8.523 8.689 7.866 7.61 7.521 7.769 7.314
   4379   1388116_at   9.677 9.696 9.644 10.069 10.843 7.823 7.924 8.507 7.798 7.871
   4380   1388645_at   10.329 10.313 10.332 10.478 10.562 9.603 9.789 9.957 9.685 9.516
   4381   1387216_at   5.796 5.652 5.642 5.975 5.835 4.392 4.833 4.994 4.995 4.861
   4382   1395557_at   8.085 8.091 7.695 7.906 7.93 6.956 6.205 6.916 6.167 6.512
   4383   1387772_at   11.312 11.287 11.082 11.261 11.3 10.629 10.906 10.755 10.602 10.741
   4384   1376684_at   7.028 7.127 6.783 6.896 6.911 2.376 4.369 4.606 3.101 4.123
   4385   1373497_at   7.286 7.367 7.413 7.674 7.254 8.045 8.417 8.44 8.634 8.219
   4386   1370213_at   12.062 12.011 11.921 11.921 12.136 11.598 11.569 11.69 11.576 11.442
   4387   1371412_a_at   8.721 8.766 8.449 8.944 9.324 10.954 10.951 9.919 10.915 10.807
   4388   1382984_at   6.132 6.057 5.876 6.047 6.366 5.043 4.849 5.084 5.477 4.938
   4389   1378825_a_at   7.452 7.19 7.297 7.244 7.106 6.628 6.262 6.465 6.189 6.634
   4390   1378180_at   6.15 6.189 6.347 6.483 6.338 5.439 5.639 5.18 5.558 5.66
   4391   1388709_at   7.553 7.372 7.468 7.725 7.777 6.834 6.894 6.913 6.513 6.705
   4392   1397495_at   5.423 5.565 5.127 4.87 4.403 7.123 7.146 6.794 6.764 6.543
   4393   1389471_at   7.725 7.745 7.769 8.097 7.893 7.07 6.469 6.91 6.693 7.043
   4394   1388476_at   8.881 8.792 8.881 8.938 8.959 8.309 8.308 8.441 8.289 7.975
   4395   1369754_a_at   7.885 7.867 7.932 7.787 7.843 7.378 7.059 6.749 7.093 6.872
   4396   1379937_at   4.403 4.131 3.931 3.946 3.624 5.228 5.265 5.097 5.073 4.878
   4397   1373686_at   1.546 0 1.189 0 0 3.806 3.658 2.836 4.233 3.678
   4398   1390289_at   6.91 7.131 7.147 7.159 7.004 7.845 7.735 7.687 7.72 7.448
   4399   1391936_a_at   9.224 9.265 9.139 8.876 9.309 9.972 10.112 10.334 9.823 9.961
   4400   1390380_at   8.285 8.276 8.106 8.515 8.466 9.102 9.161 9.305 8.87 9.067
   4401   1390145_at   7.675 7.749 7.555 7.81 7.748 8.494 8.879 8.482 8.264 8.515
   4402   1398932_at   11.299 11.252 11.228 11.25 11.324 11.688 11.59 11.482 11.708 11.575
   4403   1374688_at   7.272 7.233 7.332 7.245 6.908 7.969 7.902 7.921 7.69 8.001
   4404   1368543_at   7.181 7.091 7.128 6.777 6.056 9.499 9.403 8.321 9.628 9.584
   4405   1383188_at   5.278 5.923 6.24 6.194 5.172 7.743 7.595 7.379 7.567 7.426
   4406   1373048_at   9.355 9.453 9.427 9.418 9.512 9.257 9.202 9.228 9.236 9.221
   4407   1374048_at   5.123 5.027 5.381 5.513 4.737 6.69 6.668 6.757 6.105 6.43
   4408   1367877_at   8.916 8.881 8.689 9.072 9.059 9.917 10.033 10.022 9.629 9.572
   4409   1383104_at   6.921 6.895 6.961 6.691 6.597 7.805 7.958 8.013 7.731 7.409
   4410   1369299_at   7.078 7.387 6.898 7.006 6.261 4.996 4.81 4.288 5.058 3.744
   4411   1375621_at   5.316 5.608 5.567 5.689 5.671 6.254 6.161 6.268 6.284 6.023
   4412   1397958_at   8.213 8.123 8.134 8.298 8.429 4.338 4.203 4.567 1.356 4.12
   4413   1373528_at   8.291 8.359 8.307 8.267 8.16 5.915 5.948 6.427 5.424 4.367
   4414   1389579_at   3.894 3.157 3.134 3.949 3.154 4.927 4.983 5.38 5.122 4.854
   4415   1393018_at   7.584 7.844 8.346 8.029 7.434 9.146 9.307 9.623 9.147 9.106
   4416   1383497_at   6.434 6.266 6.236 6.949 6.265 7.665 7.871 7.801 7.428 7.461
   4417   1375216_at   7.877 7.963 7.473 8.072 8.064 6.101 6.826 6.755 6.742 6.509
   4418   1395102_at   4.849 4.889 5.019 5.029 5.026 6.59 6.74 7.112 6.104 6.032
   4419   1380062_at   7.706 7.559 8.178 8.397 8.334 9.332 9.489 9.464 9.298 9.243
   4420   1373092_at   7.983 8.336 8.065 8.371 8.358 8.857 9.048 8.842 8.927 9.099
   4421   1397838_at   4.775 5.12 4.939 5.313 5.47 7.122 7.087 6.456 6.408 6.41
   4422   1374432_at   6.876 7.077 6.622 6.777 7.087 7.947 8.268 8.382 7.706 7.902
   4423   1373056_at   8.039 7.93 7.914 8.342 8.166 8.663 8.704 8.756 8.852 8.685
   4424   1370242_at   12.962 12.827 12.768 12.625 12.828 12.363 12.32 12.216 12.41 12.265
   4425   1368389_at   7.435 7.59 7.522 7.695 7.513 8.062 8.149 8.318 8.141 7.939
   4426   1367926_at   7.608 7.89 7.774 7.72 7.798 8.442 8.357 8.48 8.322 8.122
   4427   1377703_at   7.307 6.964 7.311 7.254 7.282 7.94 7.857 7.797 7.807 7.677
   4428   1376571_at   7.555 7.536 7.623 7.665 7.765 8.28 8.406 8.292 8.365 7.986
   4429   1372452_at   7.835 7.928 7.984 8.227 7.794 8.685 9.028 9.135 8.693 8.756
   4430   1392864_at   6.117 6.285 6.192 6.05 6.654 7.677 8.016 7.411 7.2 7.523
   4431   1372414_at   7.479 7.263 7.606 7.571 7.494 8.187 8.327 8.431 8.025 8.078
   4432   1397669_at   5.846 5.785 5.73 6.011 5.882 4.928 4.64 5.086 4.281 4.452
   4433   1369167_at   4.78 4.408 4.442 3.402 4.584 6.409 6.417 5.881 6.35 6.509
   4434   1376271_at   4.461 4.313 4.4 4.781 4.368 1.411 0 0 2.444 0.74
   4435   1397647_at   7.887 7.969 8.174 8.123 7.617 9.249 9.118 8.759 9.347 8.88
   4436   1390685_at   5.912 5.989 5.571 6.016 6.121 4.642 5.168 5 5.04 4.825
   4437   1380101_at   6.279 6.618 6.362 6.485 6.415 5.565 5.387 5.764 5.82 5.801
   4438   1372352_at   10.412 10.481 10.348 10.537 10.958 9.145 9.291 9.286 9.285 9.808
   4439   1370163_at   10.498 10.592 10.697 10.838 10.405 11.582 11.364 11.768 11.247 11.48
   4440   1372791_at   10.047 10.156 9.969 10.044 10.014 10.614 10.863 10.85 10.442 10.648
   4441   1380186_at   7.074 7.236 7.124 7.5 7.323 6.536 6.593 6.701 6.622 6.704
   4442   1368240_a_at   7.036 7.036 7.022 7.262 6.686 1.828 3.581 1.618 4.277 1.333
   4443   1375966_at   6.263 6.207 6.433 6.593 6.569 6.948 7.145 7.051 7.207 7.212
   4444   1376842_at   6.58 6.678 6.71 6.818 6.897 5.792 5.63 5.554 5.783 6.205
   4445   1388373_at   10.256 10.362 10.308 10.472 10.349 10.793 10.916 11.06 10.831 10.721
   4446   1368056_at   7.629 7.669 7.738 7.728 7.564 8.188 8.114 8.301 7.975 8.104
   4447   1395524_at   6.197 6.164 5.841 5.627 5.893 4.746 4.915 4.674 5.139 4.492
   4448   1370808_at   9.168 9.218 8.999 9.371 9.38 9.97 9.953 10.254 10.016 9.794
   4449   1387098_at   7.143 7.104 7.024 7.362 6.958 6.083 6.361 6.327 6.391 5.874
   4450   1392498_at   8.155 8.194 8.401 8.408 8.147 7.768 7.822 7.584 7.737 7.637
   4451   1374861_at   4.938 4.631 5.378 5.451 5.567 7.186 7.078 7.572 6.603 6.75
   4452   1370285_at   6.728 6.782 6.951 7.184 7.158 7.83 7.719 8.193 8.156 7.875
   4453   1388240_a_at   4.55 3.911 3.812 3.534 4.369 5.889 5.658 5.315 5.556 5.572
   4454   1393551_at   8.266 8.392 8.133 8.421 8.667 9.434 9.451 8.98 9.47 9.341
   4455   1395659_at   5.306 5.569 5.464 5.695 5.084 6.416 6.621 6.609 6.469 6.145
   4456   1387265_at   5.424 4.707 6.262 5.871 5.404 8.459 8.64 7.293 8.422 8.127
   4457   1383757_at   6.992 6.926 6.929 6.955 6.739 7.687 7.937 8.144 7.509 7.715
   4458   1383703_at   7.377 7.61 7.657 7.602 7.55 6.908 7.115 6.992 7.131 7.137
   4459   1368029_at   10.276 10.293 10.275 10.38 10.396 10.051 10.133 10.122 10.069 10.095
   4460   1378056_at   7.596 7.208 7.758 7.487 7.613 5.077 5.117 5.896 4.445 3.879
   4461   1394458_at   6.181 6.138 5.854 6.084 6.185 7.111 7.463 7.234 6.746 6.973
   4462   1398774_at   12.121 12.162 12.157 12.085 12.165 11.674 11.702 11.933 11.659 11.653
   4463   1398793_at   9.189 9.012 9.082 9.274 9.221 8.767 8.734 8.559 8.689 8.542
   4464   1375857_at   8.566 8.609 8.689 8.652 8.527 8.049 8.266 8.099 7.976 7.849
   4465   1372092_at   7.938 8.243 8.098 8.337 8.111 8.713 8.818 8.741 8.611 8.694
   4466   1371636_at   10.508 10.559 10.522 10.367 10.327 10.885 11.007 10.818 10.991 11.097
   4467   1390981_at   4.261 5.153 5.283 5.153 5.13 6.835 6.523 6.454 6.366 6.427
   4468   1371309_at   11.999 11.979 11.883 11.862 11.889 12.353 12.231 12.185 12.42 12.351
   4469   1372942_at   7.924 8.093 7.976 8.208 8.24 8.596 8.609 8.506 8.581 8.569
   4470   1372428_at   6.486 6.273 6.466 6.391 6.162 7.186 7.121 7.592 7.119 7.052
   4471   1398249_at   8.546 8.607 8.658 8.652 8.703 9.087 9.274 9.217 9.179 8.922
   4472   1382769_at   8.93 8.603 8.757 8.75 8.857 7.721 7.875 8.138 8.188 8.081
   4473   1374891_at   6.082 6.33 6.458 6.298 6.053 5.526 5.607 5.4 5.524 5.191
   4474   1388142_at   7.192 7.141 6.828 7.393 7.688 4.935 5.111 6.147 5.311 5.162
   4475   1383581_at   6.728 6.832 6.727 7.182 7.065 3.542 3.349 4.663 4.506 2.356
   4476   1390250_x_at   6.752 6.478 6.804 7.014 6.986 5.516 5.787 5.866 6.075 5.591
   4477   1369222_at   5.278 5.198 5.455 5.26 5.235 6.964 6.628 7.294 6.462 6.243
   4478   1390785_at   8.929 8.787 8.818 8.858 8.531 9.326 9.357 9.35 9.232 9.324
   4479   1368128_at   9.552 9.645 9.036 8.97 9.451 3.632 0.526 4.964 1.93 4.418
   4480   1391989_at   3.101 4.025 4.54 3.945 4.407 1.816 1.546 0.956 1.39 0.214
   4481   1369946_at   7.488 7.534 7.981 7.988 7.864 6.722 6.864 6.722 7.03 6.596
   4482   1374916_at   6.349 6.611 6.482 6.493 6.634 6.015 5.986 5.963 5.666 5.899
   4483   1374063_at   10.818 10.859 10.68 10.745 10.918 10.283 10.037 10.318 9.979 9.898
   4484   1379558_at   6.111 6.191 5.966 6.145 6.038 6.556 6.603 6.788 6.497 6.502
   4485   1370592_at   6.465 6.095 6.685 6.77 6.308 7.892 8.173 7.386 7.76 7.738
   4486   1380448_at   7.944 7.724 8.12 8.072 7.856 7.291 6.891 6.869 7.258 6.869
   4487   1384960_at   4.077 4.544 3.575 4.857 3.296 6.359 6.051 6.582 6.515 6.271
   4488   1370842_at   9.496 9.387 9.636 9.502 9.54 9.95 9.958 9.775 9.9 9.928
   4489   1388887_at   9.149 8.93 9.111 8.96 8.599 9.97 10.022 9.578 9.933 9.943
   4490   1387891_at   9.289 9.224 9.065 9.056 9.559 8.51 8.568 8.516 8.555 8.374
   4491   1371914_at   8.823 8.814 8.913 8.752 8.843 8.443 8.312 8.255 8.429 8.087
   4492   1371553_at   9.253 9.28 9.361 9.304 9.25 9.604 9.662 9.791 9.611 9.537
   4493   1378566_at   3.18 2.846 3.654 3.987 3.392 5.303 5.379 4.756 5.217 4.934
   4494   1379107_at   3.847 3.934 3.708 3.604 3.721 6.948 5.641 6.185 5.246 5.794
   4495   1375756_at   7.746 7.987 8.17 7.938 7.994 9.227 8.781 8.919 9.049 8.603
   4496   1369492_at   5.208 5.536 5.357 5.272 4.525 6.806 6.821 6.275 6.907 6.854
   4497   1378288_at   7.18 7.13 7.46 7.467 7.339 8.336 8.545 8.028 8.547 8.062
   4498   1388456_at   8.11 7.992 8.339 8.074 7.91 9.031 9.057 9.662 9.006 9.06
   4499   1368605_at   5.785 5.614 5.517 5.94 5.687 4.513 4.642 5.018 4.219 4.356
   4500   1369129_at   5.532 5.708 5.482 5.383 4.967 4.074 3.579 4.464 3.945 3.779
   4501   1387887_at   11.835 11.802 11.687 11.725 11.947 11.41 11.335 11.277 11.403 11.162
   4502   1382685_at   5.742 5.789 5.715 5.253 5.227 7.598 7.579 6.877 6.72 7.11
   4503   1392830_at   6.824 6.927 6.951 7.08 7.095 5.681 6.01 6.251 6.277 6.162
   4504   1380277_at   5.197 5.238 4.979 4.746 5.097 2.842 3.597 3.66 2.328 2.874
   4505   1370816_at   5.841 4.885 5.851 5.966 5.02 7.914 7.772 7.495 7.356 8.53
   4506   1379571_at   6.522 6.459 6.958 6.843 6.776 8.383 7.82 7.868 7.711 7.69
   4507   1390789_at   7.149 7.424 7.195 7.534 7.251 8.007 8.034 7.83 8.228 8.121
   4508   1393414_at   7.062 7.215 7.539 7.016 7.055 8.77 8.626 7.962 8.86 8.804
   4509   1373875_at   7.552 7.77 7.724 7.717 7.826 6.815 6.95 6.494 6.372 6.121
   4510   1367975_at   7.373 7.573 7.663 7.367 7.586 3.635 3.986 4.254 1.157 3.652
   4511   1367580_at   12.344 12.263 12.025 11.957 12.164 11.604 11.423 11.419 11.546 11.252
   4512   1399056_at   5.818 5.138 5.897 5.906 5.822 7.229 7.03 7.232 6.938 6.706
   4513   1374394_at   7.991 7.962 8.046 8.277 7.981 8.743 8.882 8.666 8.573 8.542
   4514   1368282_at   6.576 6.529 6.369 6.275 6.09 7.751 7.681 7.033 7.54 7.589
   4515   1394290_at   5.731 5.569 5.617 5.554 5.32 3.309 4.547 3.811 3.741 4.181
   4516   1374610_at   8.515 8.317 8.493 8.642 8.299 9.149 9.369 9.435 9.019 9.523
   4517   1374509_at   6.524 6.558 6.579 6.74 6.662 7.28 7.703 7.519 7.453 7.119
   4518   1385025_a_at   7.577 7.615 7.884 7.899 7.617 8.735 8.527 8.245 8.691 8.705
   4519   1391559_at   6.274 6.325 6.037 6.451 6.684 7.31 7.18 7.149 7.424 7.149
   4520   1387785_at   8.753 8.803 8.906 8.794 8.892 8.332 8.138 7.89 8.349 8.157
   4521   1395758_at   5.576 5.421 5.425 6.037 5.844 4.817 4.227 4.037 4.333 4.198
   4522   1398409_at   8.081 8.301 8.225 8.318 8.365 8.913 9.008 8.78 8.684 9.047
   4523   1368812_at   5.264 5.22 5.257 5.958 5.375 1.856 0.848 0 3.096 0.824
   4524   1378493_at   6.425 6.738 6.908 7.077 7.147 5.3 5.729 5.135 5.402 4.673
   4525   1395761_at   5.471 5.868 5.782 5.978 5.695 6.711 6.87 7.006 6.391 6.837
   4526   1373242_at   8.306 8.551 8.588 8.691 8.959 7.864 7.734 7.871 7.708 7.649
   4527   1389865_at   6.745 6.664 6.644 6.554 6.593 6.117 5.781 5.688 6.04 5.583
   4528   1383133_at   7.505 7.735 7.527 7.65 7.86 6.923 7.108 6.711 6.996 7.028
   4529   1381915_at   7.746 7.923 8.02 8.083 7.856 8.391 8.616 8.452 8.408 8.599
   4530   1374982_at   7.056 6.633 6.865 6.532 6.697 8.136 7.812 7.991 7.733 7.486
   4531   1388486_at   7.75 7.655 7.72 7.985 7.628 8.466 8.585 8.551 8.304 8.249
   4532   1376071_at   6.687 6.855 6.483 6.675 6.5 7.354 7.418 7.093 7.28 7.368
   4533   1378958_at   7.036 7.057 7.077 7.224 6.924 6.213 6.099 5.57 6.295 5.804
   4534   1371885_at   9.376 9.394 9.374 9.291 9.612 8.885 8.996 9.042 8.909 8.882
   4535   1386927_at   8.402 8.417 8.329 8.287 8.283 8.685 8.823 8.635 8.786 8.622
   4536   1370155_at   10.459 10.393 10.365 10.622 11.409 9.201 9.123 9.249 9.224 9.156
   4537   1387125_at   7.643 7.739 6.981 7.868 8.725 2.319 4.209 4.271 3.731 1.521
   4538   1385314_at   7.5 7.618 7.397 7.558 7.843 6.772 6.953 6.81 7.079 6.85
   4539   1381162_at   8.478 8.549 8.075 8.28 8.593 7.616 7.629 7.519 7.452 7.74
   4540   1378063_at   3.29 3.818 3.651 3.776 3.708 4.474 4.97 5.302 4.8 4.798
   4541   1369425_at   3.666 3.557 3.24 3.459 3.458 4.123 4.888 4.87 4.936 4.901
   4542   1387587_at   6.096 6.535 7.293 6.454 6.187 4.435 3.941 4.929 3.804 4.534
   4543   1398553_at   6.57 6.388 7.225 6.872 7.334 5.309 5.543 5 5.307 5.534
   4544   1383160_at   9.062 9.104 9.267 9.129 9.348 8.301 8.664 8.483 8.508 8.685
   4545   1373174_at   8.495 8.441 8.56 8.648 8.684 9.265 9.236 9.096 9.454 9.022
   4546   1380035_at   7.417 7.772 7.574 7.704 7.899 6.916 7.067 6.813 6.952 7.066
   4547   1386096_at   7.375 7.489 7.697 7.613 7.808 6.577 6.328 6.404 6.473 5.68
   4548   1388172_at   9.301 9.104 9.502 9.19 7.988 11.186 11.073 10.8 11.075 11.101
   4549   1373866_at   8.738 8.745 8.934 8.939 8.638 9.711 9.593 9.562 9.235 9.575
   4550   1385625_at   7.751 7.855 7.347 7.494 7.884 6.543 6.729 6.314 6.888 6.48
   4551   1383143_at   4.875 4.816 4.937 5.119 5.306 5.854 5.707 6.253 6.151 6.035
   4552   1372889_at   7.351 7.414 7.287 7.517 7.072 8.254 8.359 8.683 8.09 8.111
   4553   1372813_at   9.156 9.141 9.289 9.211 9.201 9.468 9.673 9.582 9.547 9.464
   4554   1388863_at   8.918 8.928 9.214 8.915 9.215 8.378 8.487 8.386 8.185 8.141
   4555   1373733_at   7.203 7.386 7.269 7.585 7.836 8.834 8.809 9.179 8.627 8.365
   4556   1397297_at   6.869 6.831 6.354 7.041 7.216 5.716 5.284 4.701 5.326 4.974
   4557   1378940_at   5.876 6.069 5.814 5.851 6.343 5.11 4.668 4.351 4.105 4.482
   4558   1371475_at   9 8.804 8.522 8.647 9.449 7.763 7.691 7.544 7.621 7.645
   4559   1379932_at   7.365 7.193 7.22 7.285 6.916 8.583 8.838 7.952 8.414 8.316
   4560   1379528_at   8.669 8.765 8.565 8.842 8.64 9.182 9.257 9.469 9.132 9.235
   4561   1390185_at   8.448 8.474 8.474 8.407 8.555 7.359 7.648 7.959 7.801 7.465
   4562   1379362_at   5.853 5.947 5.47 5.921 5.51 7.03 7.02 7.239 6.498 6.869
   4563   1381403_at   7.875 7.799 7.715 8.068 7.536 6.432 6.552 7.133 6.428 6.416
   4564   1370969_at   7.039 6.984 6.767 6.532 6.678 7.712 7.861 8.291 8.04 7.649
   4565   1387600_at   6.455 6.271 6.25 6.078 6.668 5.169 4.882 4.868 4.769 4.022
   4566   1383063_a_at   7.381 7.442 7.675 7.447 7.786 8.882 8.817 8.191 8.548 8.655
   4567   1391871_at   6.158 6.094 6.427 6.354 6.227 7.094 7.471 7.293 6.816 7.29
   4568   1387608_at   7.28 7.431 7.72 7.706 7.465 1.449 3.079 3.857 0 3.16
   4569   1391773_at   6.669 6.711 6.791 6.875 6.743 7.228 7.312 7.145 7.093 7.082
   4570   1378260_at   4.806 4.512 5.496 4.517 4.55 6.986 6.589 6.055 6.96 7.019
   4571   1388586_at   8.794 8.796 8.802 8.789 9.033 8.416 8.503 8.499 8.378 8.447
   4572   1375223_at   6.278 6.284 6.533 6.251 6.426 7.377 7.36 7.905 7.191 7.189
   4573   1374720_at   6.311 6.294 6.043 6.567 6.32 4.705 4.836 4.74 5.036 3.809
   4574   1385563_at   3.055 3.348 3.32 3.315 3.305 4.485 4.62 4.434 3.83 4.513
   4575   1374704_at   7.335 7.404 7.421 7.659 7.859 6.353 6.435 6.831 6.62 6.663
   4576   1381217_at   6.506 6.563 6.462 6.527 6.66 6.127 5.695 5.903 5.691 5.604
   4577   1378377_at   7.732 7.472 7.624 7.471 7.466 3.782 3.87 4.696 3.211 1.339
   4578   1390027_at   9.031 9.037 9.322 9.181 9.29 9.773 9.686 9.565 9.73 9.706
   4579   1393020_at   6.504 6.248 6.766 6.423 6.706 4.129 4.134 2.472 4.539 3.787
   4580   1379858_at   6.759 6.73 7.02 7.028 7.251 6.149 6.081 6.235 6.089 5.812
   4581   1368211_at   12.797 12.676 12.638 12.463 12.647 12.259 12.218 12.174 12.277 12.204
   4582   1368777_at   4.255 5.001 5.241 4.888 4.466 1.029 1.637 0.189 2.776 1.275
   4583   1398576_at   6.087 6.195 6.267 6.077 6.08 2.141 2.131 2.144 4.067 3.812
   4584   1375881_at   11.6 11.506 11.646 11.447 11.571 11.316 11.209 11.175 11.234 11.141
   4585   1367831_at   9.201 9.283 9.049 9.449 9.4 8.236 8.092 8.713 8.364 8.181
   4586   1390389_at   7.788 8.195 8.05 7.895 7.836 7.006 7.322 6.705 7.095 7.053
   4587   1395354_at   6.759 6.548 6.882 6.474 6.633 7.293 7.277 7.147 7.414 7.278
   4588   1389219_at   6.893 6.765 6.639 7.14 7.067 7.81 8.076 7.65 7.646 7.964
   4589   1375250_at   6.156 6.502 6.716 6.905 6.601 5.621 5.327 5.601 5.689 5.467
   4590   1373625_at   9.378 9.382 9.441 9.402 9.152 9.868 10.067 9.733 10.026 9.96
   4591   1377457_a_at   8.719 8.689 8.904 8.965 8.74 9.404 9.893 9.992 9.523 9.752
   4592   1368765_at   7.086 7.394 7.473 7.395 7.32 8.459 8.468 8.981 8.259 8.186
   4593   1381749_at   7.104 7.546 7.49 7.966 7.361 8.79 8.977 8.821 8.41 8.698
   4594   1393245_at   10.489 10.408 10.346 10.495 10.442 11.002 11.082 11.052 10.893 10.729
   4595   1374969_at   6.378 6.322 6.639 6.526 6.629 7.367 7.526 7.775 7.159 7.274
   4596   1372216_at   6.69 6.634 6.717 6.412 6.711 6.124 5.955 6.089 6.011 6.235
   4597   1374193_at   7.647 7.631 7.535 7.617 7.642 6.599 7.088 6.978 7.017 6.667
   4598   1372709_at   8.951 8.951 9.185 9.034 8.988 8.398 8.52 8.546 8.259 8.147
   4599   1387109_at   8.674 8.64 8.469 8.589 8.458 9.256 9.139 9.025 8.948 9.026
   4600   1371299_at   12.439 12.393 12.412 12.265 12.475 11.96 11.919 12.086 11.778 11.804
   4601   1377002_at   6.333 6.344 6.449 6.812 6.548 4.574 5.459 4.326 4.184 4.919
   4602   1375517_at   9.106 9.203 9.104 9.082 8.985 9.81 10.137 10.15 9.659 9.733
   4603   1383457_at   7.813 7.676 8.055 7.767 7.664 9.397 9.464 9.889 9.055 8.749
   4604   1389245_at   9.692 9.824 9.65 9.839 9.877 9.091 9.33 9.4 9.119 9.21
   4605   1368676_at   6.604 6.5 6.1 6.344 6.395 7.251 7.614 7.862 7.415 7.218
   4606   1372381_at   5.94 6.621 6.117 6.731 6.327 7.396 7.705 7.516 7.527 7.42
   4607   1373917_at   9.417 9.502 9.337 9.45 9.492 9.095 9.151 8.993 8.962 8.872
   4608   1384087_at   6.034 5.767 6.032 5.93 5.869 3.302 4.076 4.079 4.645 4.554
   4609   1389941_at   8.859 8.951 9.306 9.3 9.188 10.087 10.046 10.153 9.764 9.865
   4610   1385904_at   6.468 6.852 6.693 6.857 6.777 7.856 7.938 7.381 8.031 7.587
   4611   1389185_at   6.398 6.699 6.517 6.566 6.46 7.701 7.718 7.875 7.383 7.149
   4612   1371953_at   8.923 8.909 9.279 8.963 9.269 8.355 8.281 8.316 8.179 8.513
   4613   1374199_at   6.52 6.479 6.5 6.424 6.715 7.145 7.169 7.194 7.134 6.864
   4614   1368870_at   9.676 9.578 9.637 9.631 9.665 10.521 10.45 10.326 10.091 10.102
   4615   1380655_at   5.209 5.395 5.373 5.631 5.318 6.235 6.055 6.452 6.455 6.016
   4616   1387458_at   9.389 9.367 9.544 9.442 9.385 8.801 8.423 8.069 8.659 8.46
   4617   1376727_at   8.572 8.608 8.829 8.793 8.79 9.292 9.436 9.122 9.279 9.203
   4618   1374785_at   4.97 5.301 5.331 5.299 5.461 6.077 6.287 6.005 6.355 5.918
   4619   1374062_x_at   4.722 4.887 5.245 5.363 5.227 6.21 6.417 6.808 6.175 6.166
   4620   1384056_at   6.208 6.332 6.313 6.45 6.568 7.222 7.161 7.417 6.98 6.953
   4621   1377091_at   4.759 5.004 5.419 4.725 4.968 5.899 6.481 6.352 6.384 5.992
   4622   1387082_at   5.93 6.163 6.071 6.06 5.761 7.092 6.956 7.282 6.894 6.606
   4623   1372373_at   7.327 7.306 7.289 7.378 7.347 7.996 8.16 8.309 7.848 7.816
   4624   1367955_at   8.045 7.938 7.979 8.148 8.118 7.609 7.198 7.098 7.383 7.12
   4625   1368141_at   11.693 11.628 11.475 11.46 11.564 11.182 11.194 11.195 11.067 11.005
   4626   1381533_at   6.74 6.943 6.079 6.885 6.387 2.491 4.778 3.36 3.313 3.936
   4627   1370288_a_at   8.977 8.91 8.741 9.236 9.315 8.354 7.957 8.074 8.118 7.959
   4628   1390264_at   7.76 7.811 7.634 7.944 8.025 7.22 7.4 7.274 7.229 7.2
   4629   1388959_at   8.041 7.935 7.733 7.998 8.421 6.911 6.794 7.06 6.486 7.101
   4630   1372747_at   6.781 6.844 6.667 6.834 6.637 7.524 7.337 7.54 7.217 7.21
   4631   1372321_at   6.385 6.298 6.458 6.262 6.179 4.431 4.092 4.995 5.28 4.24
   4632   1389806_at   3.981 3.738 3.379 4.475 3.506 5.266 5.368 5.28 5.157 5.25
   4633   1391581_at   7.258 7.368 7.379 7.369 7.211 8.117 8.295 7.759 7.971 8.249
   4634   1387965_at   9.54 9.492 8.536 9.771 10.348 3.769 1.536 5.761 4.854 3.448
   4635   1399142_at   7.96 8.021 8.04 8.213 7.905 7.423 7.202 7.305 7.526 7.042
   4636   1369685_at   3.555 1.836 2.063 3.579 3.524 0 0 0 0 0.07
   4637   1396152_s_at   8.153 8.061 8.282 7.958 7.976 9.844 9.499 8.945 9.987 9.328
   4638   1372392_at   7.067 7.202 7.141 7.568 7.32 8.34 8.409 8.49 7.959 8.059
   4639   1388489_at   10.946 11.044 11.151 11.128 11.134 11.36 11.451 11.453 11.373 11.37
   4640   1386935_at   7.559 7.5 7.762 7.369 7.163 10.257 9.766 10.668 8.845 9.663
   4641   1383355_at   6.992 7.205 7.079 6.862 6.696 6.186 6.031 6.345 6.152 5.907
   4642   1372615_at   4.858 4.776 5.111 5.601 5.039 6.12 6.583 6.609 6.498 6.151
   4643   1368608_at   4.07 4.129 4.163 3.767 3.402 6.576 5.588 5.23 6.082 5.94
   4644   1380485_at   4.868 4.574 4.816 5.121 5.143 6.642 6.383 6.004 6.05 5.877
   4645   1376754_at   7.43 7.324 7.413 7.553 7.409 6.836 6.747 6.738 6.731 7.111
   4646   1389409_at   8.376 8.233 8.556 8.566 8.842 7.146 7.227 7.696 7.078 6.767
   4647   1390124_at   7.895 8.017 7.679 7.72 7.936 6.763 7.269 6.871 6.75 7.126
   4648   1373865_at   4.738 4.448 5.174 4.955 4.316 6.688 6.996 5.835 6.636 6.84
   4649   1374888_at   6.317 6.402 6.789 6.881 6.434 5.633 5.747 5.436 5.697 5.707
   4650   1388757_at   7.067 7.425 7.676 7.553 7.446 6.26 6.433 6.715 6.368 6.096
   4651   1398931_at   9.236 9.404 9.381 9.195 9.254 9.57 9.679 9.616 9.619 9.631
   4652   1377302_a_at   5.573 5.598 5.715 5.749 5.479 6.36 6.605 6.583 6.301 6.107
   4653   1372156_at   8.694 8.754 8.652 8.853 8.769 8.101 8.234 8.351 8.105 7.9
   4654   1380210_at   6.379 6.161 6.645 6.66 5.962 5.017 5.12 4.277 4.639 5.06
   4655   1375181_at   12.765 12.665 12.559 12.312 12.501 11.993 11.948 11.932 12.016 11.81
   4656   1368386_at   9.569 9.536 9.777 9.916 9.738 9.195 9.219 9.207 9.128 9.163
   4657   1368570_at   6.157 5.92 5.074 5.587 5.752 3.872 3.99 2.655 3.621 3.474
   4658   1371244_at   11.023 11.054 10.976 10.996 11.242 10.562 10.626 10.651 10.556 10.366
   4659   1370941_at   10.131 10.225 9.763 9.958 10.432 9.123 9.258 9.343 9.219 9.129
   4660   AFFX_Rat_beta-actin_M_at   13.173 13.075 13.1 12.796 12.885 12.379 12.167 12.332 12.509 12.371
   4661   1388128_at   7.89 7.808 7.814 7.875 7.876 6.791 6.808 6.775 6.528 5.881
   4662   1374815_at   8.675 8.596 8.798 8.744 8.587 7.817 7.885 7.9 8.311 7.97
   4663   1377743_at   8.234 8.402 8.357 8.238 8.134 7.158 6.541 6.35 7.377 6.781
   4664   1374139_at   8.001 7.894 8.074 8.303 7.913 8.807 9.12 8.972 8.618 8.795
   4665   1390987_at   6.763 6.768 6.469 6.538 6.618 7.33 7.215 7.428 7.194 7.078
   4666   1369393_at   6.279 6.428 6.092 6.325 6.628 5.294 5.139 4.984 4.266 4.869
   4667   1372620_at   9.307 9.151 9.245 9.359 9.323 8.643 8.791 8.504 8.235 8.594
   4668   1374359_at   6.593 6.765 6.697 6.49 6.404 4.993 5.396 4.522 5.633 5.104
   4669   1373326_at   3.328 3.377 2.138 2.862 2.519 4.548 4.855 4.455 4.662 4.895
   4670   1388847_at   7.725 7.777 7.918 7.681 7.74 7.306 7.174 7.201 7.113 7.446
   4671   1383175_a_at   7.633 7.684 7.342 7.513 7.882 6.885 6.954 6.81 6.681 6.563
   4672   1367472_at   10.662 10.769 10.649 10.845 10.795 10.159 10.244 10.403 10.02 10.079
   4673   1383084_at   3.951 4.421 3.994 4.013 3.693 4.893 5.413 5.121 4.954 5.077
   4674   1377785_at   7.486 7.608 7.457 7.729 7.621 7.989 8.022 7.913 7.934 7.979
   4675   1390845_at   8.699 8.811 8.682 8.925 8.993 9.374 9.575 9.357 9.43 9.269
   4676   1377232_at   7.249 6.954 7.349 7.422 7.317 8.232 8.593 8.19 7.983 8.087
   4677   1385883_at   7.742 7.957 7.685 7.784 7.944 6.842 7.199 7.141 7.29 7.199
   4678   1374613_at   7.35 7.028 7.129 7.116 7.102 8.051 8.293 8.055 7.956 7.649
   4679   1370474_at   5.264 5.252 5.956 5.629 5.949 7.14 7.167 6.979 7.15 6.581
   4680   1398768_at   11.091 11.013 10.861 10.831 11.037 10.478 10.644 10.526 10.448 10.502
   4681   1370999_at   6.382 6.094 6.184 6.471 6.309 1.48 0.816 3.219 3.086 3.436
   4682   1392928_at   8.7 8.455 8.567 8.343 8.117 9.551 9.722 9.095 9.604 9.52
   4683   1393184_at   7.017 7.203 7.04 6.983 7.087 6.094 6.556 6.241 6.096 6.5
   4684   1377887_at   7.778 7.843 7.943 7.966 8.001 8.292 8.298 8.421 8.454 8.229
   4685   1376651_at   7.518 7.478 7.675 7.81 7.317 8.154 8.216 8.181 8.201 8.159
   4686   1383338_at   6.685 6.986 6.652 6.384 6.825 5.773 5.907 6.062 5.883 5.767
   4687   1379281_at   9.67 9.445 9.868 9.591 9.37 10.529 10.853 11.33 10.623 10.684
   4688   1377850_at   7.105 7.307 7.112 7.301 7.16 7.933 7.972 7.911 7.652 7.636
   4689   1382587_at   5.02 4.937 5.202 5.095 4.881 4.383 4.168 4.383 3.833 4.137
   4690   1387503_at   6.15 6.454 6.657 6.736 6.49 7.769 7.466 7.203 7.578 7.458
   4691   1373300_at   6.393 6.314 6.626 6.87 6.539 7.586 7.861 8.338 7.524 8.132
   4692   1387603_at   4.743 5.249 5.72 5.338 4.454 0.687 1.782 0 2.683 0.239
   4693   1387505_at   6.979 7.19 7.332 7.222 6.747 8.346 8.501 7.798 8.475 8.393
   4694   1373321_at   6.982 6.862 6.905 7.066 7.216 8.187 8.552 7.791 8.274 7.847
   4695   1368544_a_at   5.777 6.169 5.853 6.001 6.392 4.624 4.2 4.333 3.22 4.254
   4696   1376585_at   8.956 8.776 9.02 8.727 8.852 9.433 9.566 9.383 9.361 9.264
   4697   1394070_at   6.368 6.435 6.824 6.618 6.555 7.881 7.898 8.594 7.499 7.976
   4698   1398270_at   5.912 5.861 5.52 6.273 5.785 5.053 4.964 4.891 4.788 4.961
   4699   1367961_at   9.833 9.779 10.131 9.226 8.338 11.826 11.853 11.759 11.918 11.617
   4700   1398925_at   9.643 9.614 9.631 9.903 9.733 9.033 9.186 8.869 8.908 8.626
   4701   1377137_at   7.342 7.67 7.451 7.656 7.726 6.036 6.441 6.536 5.695 6.534
   4702   1386987_at   6.43 6.204 6.488 6.208 6.804 4.888 4.725 5.226 4.122 5.1
   4703   1368948_at   9.522 9.654 9.681 9.657 9.432 8.948 8.357 8.26 8.648 8.769
   4704   1388152_at   4.974 4.457 4.612 5.001 4.961 6.111 6.402 6.395 5.628 6.035
   4705   1383262_at   8.944 8.844 8.87 9.014 8.98 8.588 8.571 8.343 8.349 8.525
   4706   1388681_at   9.929 9.974 10.034 10.098 9.907 10.394 10.355 10.332 10.404 10.217
   4707   1388089_a_at   9.067 8.785 8.857 8.995 8.946 8.154 7.945 7.352 8.036 7.737
   4708   1376824_at   6.83 6.833 6.798 7.026 7.059 7.54 7.806 7.995 7.786 7.46
   4709   1394527_at   5.251 5.684 5.865 5.569 5.961 2.911 4.231 3.282 4.152 3.492
   4710   1380421_s_at   3.323 3.335 4.533 4.015 3.697 0 0 2.021 0.444 0
   4711   1387803_at   5.407 5.231 5.185 5.383 5.174 6.468 6.593 6.896 6.646 5.92
   4712   1390032_at   8.13 8.135 8.294 8.453 8.328 7.485 7.757 7.506 7.254 7.246
   4713   1374303_at   6.833 6.884 6.71 6.981 7.056 7.617 7.663 7.514 7.62 7.294
   4714   1368413_at   7.345 7.278 6.151 6.752 7.396 1.84 3.138 3.225 0 2.956
   4715   1387364_at   5.397 5.795 5.791 4.963 4.819 7.664 7.833 6.663 7.864 7.779
   4716   1387271_at   10.81 10.862 10.855 10.931 10.913 11.304 11.35 11.379 11.271 11.086
   4717   1371557_at   9.878 9.836 9.784 9.926 9.943 10.371 10.536 10.607 10.358 10.217
   4718   1387812_at   5.82 5.713 5.703 5.986 5.193 7.444 7.462 8.441 7.42 7.117
   4719   1373254_at   5.148 5.259 4.758 5.353 5.489 6.274 6.429 6.182 6.059 6.128
   4720   1383638_at   6.641 6.732 6.609 6.655 6.677 6.201 6.301 6.308 6.454 6.369
   4721   1388130_at   9.541 9.474 9.722 9.945 9.758 8.929 8.664 9.057 8.572 8.609
   4722   1378611_at   6.291 6.218 6.135 6.083 6.313 5.481 5.681 5.447 5.1 5.522
   4723   1371793_at   6.339 6.225 6.465 6.56 6.747 7.729 7.591 7.68 7.274 7.191
   4724   1390449_at   5.722 6.254 6.382 6.503 6.756 7.675 7.988 7.46 7.773 7.668
   4725   1368614_at   6.045 6.038 5.979 5.662 5.854 7.107 7.005 7.484 6.931 6.641
   4726   1373978_at   9.372 9.409 9.732 9.524 9.09 10.718 10.74 10.108 10.75 10.73
   4727   1375182_at   6.858 6.559 6.721 6.925 6.604 7.568 7.359 7.653 7.475 7.251
   4728   1373529_at   9.693 9.634 9.697 9.755 9.773 10.142 10.577 10.327 10.186 10.244
   4729   1392992_at   6.652 6.45 6.537 6.579 6.441 7.126 7.553 7.496 7.076 7.215
   4730   1377404_at   6.664 6.477 6.548 6.808 6.817 7.737 7.703 7.643 8.383 7.605
   4731   1384488_at   7.646 7.912 8.063 8.283 7.949 6.527 6.399 6.91 6.378 5.714
   4732   1375790_at   4.229 4.647 4.413 4.272 4.848 5.768 5.259 5.826 5.706 5.504
   4733   1380813_at   7.69 7.839 7.848 7.672 7.759 8.367 8.296 8.245 8.233 8.039
   4734   1374547_at   8.07 8.022 7.855 8.162 8.143 8.655 8.671 8.659 8.409 8.76
   4735   1375628_at   8.257 8.086 8.364 8.058 8.297 8.932 9.326 9.011 8.927 8.784
   4736   1392644_s_at   7.017 7.466 7.36 7.583 7.318 8.005 8.446 8.214 8.17 8.404
   4737   1389652_at   8.008 8.288 8.252 8.454 8.212 9.012 9.139 8.977 8.762 8.843
   4738   1396013_at   7.662 7.95 7.933 8.375 7.897 8.862 8.96 8.748 8.805 8.805
   4739   1372689_at   7.811 7.712 7.837 7.524 7.652 6.888 7.219 7.15 7.203 7.101
   4740   1387007_at   5.563 5.694 5.963 5.852 5.81 6.554 6.431 6.447 6.646 6.25
   4741   1393955_at   7.07 7.205 6.926 6.858 6.928 5.511 6.306 5.883 6.096 5.94
   4742   1368159_at   5.564 5.384 5.536 6.324 6.16 7.132 7.285 7.714 7.171 7.52
   4743   1374708_at   6.446 6.533 6.723 6.485 6.649 7.061 7.077 7.148 7.014 7.345
   4744   1373475_at   8.472 8.575 8.611 8.719 8.753 8.019 8.011 8.245 8.119 7.879
   4745   1375612_at   7.07 7.248 7.182 6.893 6.608 5.817 6.037 5.91 6.042 6.257
   4746   1382363_at   6.605 6.203 6.675 6.695 6.472 7.805 7.664 7.19 7.554 7.432
   4747   1372711_at   10.417 10.311 10.325 10.421 10.572 9.855 10.031 9.876 9.696 9.667
   4748   1383685_at   6.559 6.445 6.682 7.007 7.272 5.22 5.64 5.661 5.334 5.009
   4749   1376327_at   9.371 9.389 9.758 9.557 9.643 8.511 8.26 8.258 7.962 7.409
   4750   1387498_a_at   6.91 7.041 6.92 7.237 7.102 7.831 8.199 8.181 7.68 7.776
   4751   1370168_at   11.328 11.241 11.295 11.162 11.274 10.965 10.964 10.771 10.891 10.746
   4752   1389421_at   5.973 5.856 5.958 6.133 6.086 3.608 4.587 4.892 4.424 3.894
   4753   1398245_at   5.889 6.277 6.387 6.421 5.828 7.302 7.275 7.869 7.251 7.31
   4754   1388376_at   10.005 10.012 10.112 10.165 10.123 9.563 9.618 9.841 9.689 9.606
   4755   1373859_at   6.984 6.974 7.344 7.166 7.103 7.948 8.324 7.905 7.755 7.877
   4756   1378876_at   1.043 3.128 2.459 3.526 1.384 5.627 5.879 6.021 6.055 5.626
   4757   1374288_at   7.248 7.149 7.447 7.547 7.647 6.587 6.812 6.364 6.527 6.465
   4758   1368597_at   5.228 5.646 5.385 5.781 5.486 8.003 7.695 7.997 6.632 7.231
   4759   1374580_at   7.76 7.874 7.703 7.964 7.619 8.482 8.346 8.415 8.243 8.236
   4760   1374252_at   6.643 6.452 6.808 6.737 6.813 5.579 5.659 6.14 5.936 5.789
   4761   1390944_at   6.46 6.871 7.023 7.207 6.5 7.861 8.03 7.982 7.96 7.761
   4762   1380465_at   8.176 8.017 8.563 8.341 7.735 9.236 9.413 9.079 9.254 9.274
   4763   1379514_at   6.237 6.725 6.739 6.703 6.597 5.796 5.809 5.818 5.811 5.435
   4764   1376847_at   6.079 6.317 6.776 6.437 5.805 7.557 7.52 7.451 7.459 7.821
   4765   1370325_at   8.744 8.585 8.691 8.591 8.76 8.26 8.389 8.228 8.103 8.133
   4766   1372200_at   7.442 7.45 7.9 8.13 7.862 5.897 6.231 6.806 6.408 6.151
   4767   1388488_at   9.474 9.564 9.451 9.608 9.48 8.909 8.772 9.134 8.922 8.63
   4768   1373810_at   6.808 6.857 6.983 6.907 6.679 7.658 7.588 7.255 7.484 7.394
   4769   1398333_at   10.116 10.109 10.024 10.167 10.314 10.882 11.101 10.582 10.775 10.936
   4770   1376947_at   6.287 6.543 6.289 6.405 6.614 5.41 5.898 5.536 5.752 5.681
   4771   1398366_at   6.082 5.93 6.037 6.043 6.039 7.023 6.877 6.883 6.664 6.444
   4772   1392937_at   9.025 9.146 9.048 9.112 9.07 9.456 9.536 9.338 9.47 9.311
   4773   1389038_at   9.11 9.195 9.276 9.508 9.39 8.713 8.725 8.756 8.699 8.476
   4774   1377697_at   7.019 7.074 6.984 7.278 7.078 7.64 7.898 7.62 7.532 7.84
   4775   1395852_at   7.186 7.238 7.237 7.446 7.155 7.807 8.07 7.705 7.75 7.855
   4776   1388196_at   10.209 10.244 10.273 10.156 10.076 10.828 10.804 10.48 10.757 10.673
   4777   1387798_a_at   9.697 9.747 9.777 9.832 9.896 9.354 9.513 9.223 9.413 9.336
   4778   1371232_a_at   7.328 7.261 7.335 7.732 8.531 5.245 5.897 5.601 5.872 5.033
   4779   1374736_at   7.43 7.473 7.469 7.519 7.333 6.653 6.605 7.03 6.901 6.56
   4780   1376751_at   6.947 7.392 7.218 7.287 7.581 6.536 6.319 6.224 5.903 6.183
   4781   1386410_at   7.236 7.264 7.446 7.636 7.445 6.455 6.291 6.637 5.809 6.216
   4782   1376496_at   7.371 7.275 6.861 7.407 7.579 4.307 4.262 3.254 1.333 3.469
   4783   1396065_at   3.967 4.212 3.346 4.261 4.681 0.214 2.299 0.506 0 0
   4784   1397622_at   5.32 4.989 5.188 5.339 5.474 6.358 6.515 6.329 6.28 5.841
   4785   1389730_at   7.858 7.747 7.559 7.887 7.853 8.65 8.966 9.057 8.662 8.39
   4786   1373114_at   7.669 7.677 7.84 8.033 8.222 6.912 7.138 6.967 7.112 6.766
   4787   1379469_at   4.722 5.002 4.307 4.814 5.148 1.057 0.475 2.546 1.899 2.508
   4788   1394694_at   4.646 4.985 4.819 4.866 4.715 5.831 5.727 5.431 5.962 5.438
   4789   1389790_at   7.122 7.338 7.129 7.149 7.163 6.38 6.585 6.464 6.114 6.661
   4790   1374848_at   7.446 7.572 7.516 7.475 7.544 7.297 7.291 7.222 7.327 7.191
   4791   1392662_at   8.557 8.597 8.556 8.526 8.573 8.207 8.331 8.177 8.31 8.078
   4792   1369679_a_at   7.247 8.009 7.892 7.614 7.644 9.199 8.97 8.78 8.602 8.8
   4793   1370752_a_at   8.013 7.687 7.874 8.074 7.824 7.18 6.606 6.987 7.164 6.735
   4794   1398386_at   8.094 8.262 8.17 8.049 8.075 7.66 7.652 7.864 7.621 7.749
   4795   1376885_at   8.243 8.218 8.57 8.666 8.463 9.157 9.508 9.607 9.271 9.171
   4796   1384831_at   11.022 11.25 11.243 11.517 10.421 12.435 12.448 12.347 12.463 12.686
   4797   1375657_at   7.671 7.743 8.037 8.323 8.121 8.84 9.039 8.85 8.835 8.88
   4798   1373494_at   8.597 8.524 8.546 8.496 8.533 8.185 8.155 8.18 7.88 8.145
   4799   1383945_at   6.952 7.433 7.13 7.177 7.505 6.029 6.141 5.996 6.115 5.314
   4800   1370006_at   10.831 10.819 10.784 10.96 10.974 11.275 11.331 11.424 11.279 11.155
   4801   1384027_a_at   9.086 8.903 8.963 9.279 9.342 9.81 9.906 10.155 9.864 9.764
   4802   1390990_at   6.41 6.315 6.664 6.6 6.697 7.374 7.666 7.582 7.242 7.168
   4803   1378433_at   5.901 6.093 5.988 6.209 6.178 6.99 6.775 6.794 6.621 6.565
   4804   1370932_at   5.597 5.243 5.435 6.059 5.651 6.983 7.219 6.686 6.571 6.856
   4805   1384340_a_at   7.242 7.473 7.183 6.841 7.422 6.351 6.385 6.524 6.283 6.377
   4806   1388666_at   7.493 7.47 6.935 7.626 7.708 6.207 6.391 6.455 6.399 6.547
   4807   1372433_at   8.055 8.11 8.059 8.222 8.103 7.479 7.65 7.494 7.655 7.815
   4808   1398847_at   9.574 9.791 9.821 9.865 9.729 10.387 10.353 10.934 10.698 10.682
   4809   1395190_at   5.273 5.244 5.845 5.684 5.787 7.64 7.088 6.62 7.76 7.493
   4810   1393088_at   7.236 7.362 7.22 7.346 7.515 6.686 6.939 6.625 6.614 6.841
   4811   1371237_a_at   12.196 12.162 11.989 11.968 11.82 12.813 12.585 12.597 12.71 12.964
   4812   1368537_at   7.101 6.951 7.067 7.166 7.04 6.547 6.375 6.57 6.469 6.107
   4813   1375819_at   6.479 6.636 6.713 6.738 7.018 6.169 6.019 5.916 5.889 5.859
   4814   1373943_at   8.189 8.222 8.439 8.486 8.143 7.577 7.766 7.249 7.481 7.523
   4815   1398360_at   9.8 9.668 9.981 10.011 9.871 10.477 10.613 10.65 10.468 10.293
   4816   1371085_at   5.194 4.988 5.123 4.806 4.832 5.904 5.724 5.632 5.522 5.826
   4817   1385634_at   6.559 6.681 6.674 6.937 6.982 5.701 5.808 5.22 5.861 5.907
   4818   1369000_at   4.245 4.186 3.976 3.929 3.97 5.153 4.744 5.64 5.359 5.537
   4819   1373977_at   6.176 5.767 5.402 6.076 5.6 6.968 7.365 7.266 6.989 6.796
   4820   1393730_at   6.702 6.76 6.796 6.814 6.989 5.484 5.433 4.753 5.501 5.927
   4821   1377745_at   7.922 7.951 7.954 7.783 7.916 7.423 7.407 7.406 7.338 7.022
   4822   1374572_at   7.323 7.291 7.291 7.524 7.449 7.874 8.129 7.777 7.939 7.861
   4823   1387911_at   9.85 9.815 9.712 9.999 10.011 9.448 9.427 9.306 9.252 9.162
   4824   1397851_at   6.263 6.542 6.272 6.435 6.386 5.473 5.109 4.819 5.678 5.128
   4825   1379862_at   7.536 7.494 7.691 7.59 7.859 6.896 6.668 6.726 6.653 6.141
   4826   1383352_at   6.397 6.516 6.482 6.422 6.615 5.652 5.419 5.943 5.777 5.922
   4827   1372627_at   9.176 9.402 9.281 9.443 9.423 8.581 8.738 8.723 8.387 8.201
   4828   1389455_at   7.594 7.549 7.518 7.571 7.545 7.314 7.415 7.251 7.362 7.273
   4829   1383300_at   7.384 7.326 7.384 7.523 7.531 8.136 8.113 8.171 8.726 8.407
   4830   1383212_at   5.551 5.238 5.464 5.601 5.595 6.514 6.331 6.253 5.975 6.375
   4831   1388103_at   8.912 9.12 9.54 9.443 9.042 10.503 10.185 10.001 10.42 10.272
   4832   1377437_at   5.054 4.841 5.661 4.861 5.377 6.248 6.639 6.839 6.483 6.352
   4833   1380002_at   6.504 6.493 6.677 6.692 6.536 7.198 7.043 7.354 7.068 7.001
   4834   1388749_at   8.285 8.341 8.566 8.588 8.238 9.157 9.463 9.254 9.03 9.02
   4835   1383176_at   6.733 6.678 6.821 6.762 6.911 6.21 5.751 6.079 5.577 5.652
   4836   1375463_at   6.864 7.34 7.058 7.194 6.925 7.844 7.839 7.899 7.599 7.818
   4837   1387059_at   7.851 7.829 7.945 8.067 7.847 8.881 8.916 9.338 8.547 8.753
   4838   1384889_at   6.45 6.274 6.506 6.653 6.578 5.481 5.88 5.384 5.383 5.047
   4839   1385660_at   9.038 8.879 8.699 8.856 8.812 8.439 8.491 8.403 8.303 8.406
   4840   1376243_at   4.46 4.337 4.081 3.775 4.51 5.15 5.389 5.143 5.349 5.353
   4841   1373116_at   8.687 8.392 8.603 8.687 8.557 8.112 8.198 8.12 8.007 8.191
   4842   1398935_at   8.644 8.442 8.63 8.82 8.522 9.355 9.488 9.265 9.182 9.094
   4843   1372257_at   8.576 8.477 8.484 8.797 8.609 9.254 9.54 9.643 9.104 9.51
   4844   1377013_at   6.126 5.333 5.662 5.365 5.15 7.953 7.645 8.363 7.144 7.083
   4845   1389613_at   7.841 7.522 7.874 7.953 7.736 7.151 7.321 7.223 7.15 7.211
   4846   1372471_at   9.116 9.214 9.026 9.281 9.229 8.263 8.695 8.602 8.218 8.332
   4847   1373250_at   6.501 6.849 6.344 6.81 6.721 5.084 4.565 5.494 5.291 4.348
   4848   1377893_at   6.995 7.137 6.84 7.053 6.942 6.136 6.241 6.271 6.622 6.236
   4849   1386321_s_at   5.684 5.582 6.02 5.913 5.757 4.658 4.963 4.946 4.223 4.625
   4850   1374430_at   9.393 9.412 9.411 9.249 9.605 8.794 8.97 8.523 8.542 8.655
   4851   1391628_at   8.299 8.317 8.287 8.415 8.445 8.112 7.938 7.98 7.98 7.846
   4852   1379905_at   6.586 6.579 6.441 6.605 6.594 5.999 5.642 5.699 6.145 5.827
   4853   1387377_a_at   7.732 7.95 7.237 7.511 7.793 6.213 6.524 5.439 6.179 5.711
   4854   1395960_at   4.933 4.24 5.119 5.773 4.524 0.31 2.903 1.151 0.536 0.807
   4855   1382350_at   6.678 6.515 6.831 6.804 6.816 5.83 6.138 5.682 6.171 5.865
   4856   1388446_at   10.883 10.893 10.86 10.775 10.877 10.634 10.577 10.407 10.418 10.51
   4857   1370009_at   8.183 7.929 8.273 8.215 7.909 9.282 9.434 8.673 9.338 9.284
   4858   1384747_at   5.978 5.664 5.711 5.458 5.506 6.822 6.95 6.281 6.754 6.675
   4859   1380854_at   6.698 6.886 6.78 6.995 6.709 7.635 7.847 7.731 7.709 7.254
   4860   1368984_at   8.767 8.647 8.583 8.578 8.735 7.94 7.326 6.985 7.688 7.253
   4861   1371747_at   9.05 8.621 8.581 8.966 8.92 9.521 9.546 9.777 9.507 9.577
   4862   1372729_at   6.022 6.133 6.2 5.931 6.63 5.463 5 5 5.091 5.121
   4863   1382046_at   8.561 8.721 8.701 8.982 8.763 8.16 8.032 8.106 8.098 8.329
   4864   1388896_at   8.357 8.431 8.338 8.472 8.468 9.069 9.106 8.813 8.754 8.959
   4865   1398259_at   7.562 7.699 7.529 7.453 7.811 7.029 7.167 6.887 6.771 6.922
   4866   1390333_at   8.274 8.062 8.491 8.56 8.094 7.304 7.366 7.006 6.848 7.476
   4867   1399125_at   9.015 8.999 8.898 9.198 9.24 7.99 8.202 8.544 8.275 7.97
   4868   1369962_at   9.409 9.454 9.257 9.432 9.512 8.966 8.967 9.032 8.704 8.885
   4869   1367912_at   6.117 6.005 6.305 6.321 6.846 7.442 7.749 7.423 7.267 7.607
   4870   1382126_at   8.188 8.203 8.112 8.227 8.058 8.783 9.11 8.749 8.661 8.649
   4871   1371189_x_at   9.817 9.829 9.938 10.063 10.091 9.508 9.474 9.382 9.486 9.246
   4872   1381118_at   5.611 5.453 5.718 5.765 5.742 4.865 5.161 4.541 4.709 4.857
   4873   1398999_at   8.328 8.355 8.384 8.504 8.729 7.954 7.853 7.914 7.887 7.714
   4874   1389582_at   7.686 7.655 7.47 7.756 7.779 6.999 6.988 6.94 6.967 7.318
   4875   1368814_at   9.804 9.88 10.104 9.851 9.342 11.184 10.81 10.705 11.381 11.175
   4876   1380152_at   7.043 7.181 6.898 7.088 6.378 7.97 8.385 8.26 7.904 8.038
   4877   1376001_at   7.025 6.776 6.785 6.715 7.172 6.063 5.749 5.507 6.171 5.801
   4878   1388313_at   10.599 10.628 10.925 10.904 10.963 10.163 10.268 10.273 10.065 10.133
   4879   1390514_at   9.489 9.384 9.405 9.493 9.375 9.241 9.2 9.216 9.109 9.177
   4880   1387036_at   6.879 6.794 6.922 6.783 6.543 7.764 8.144 8.524 7.614 7.939
   4881   1371615_at   7.487 7.575 7.749 7.794 7.271 8.463 8.891 9.17 8.595 8.518
   4882   1380053_at   6.362 6.322 5.971 6.433 6.201 7.061 7.243 6.878 6.931 6.904
   4883   1376253_at   6.306 6.346 6.467 6.511 6.037 5.532 5.431 5.364 5.151 4.813
   4884   1369209_at   7.167 7.46 7.262 7.394 7.345 6.362 6.665 6.526 6.875 6.682
   4885   1391730_at   4.828 4.442 4.535 5.179 5.068 5.963 6.011 5.843 5.713 5.924
   4886   1387809_at   4.895 5.478 4.955 5.36 5.445 6.858 6.415 6.588 6.328 6.131
   4887   1382047_at   5.662 6.044 6.143 6.086 6.128 6.663 6.762 6.564 6.737 6.753
   4888   1394450_at   5.908 5.789 5.441 6.029 5.568 6.946 6.742 6.763 6.549 6.495
   4889   1387675_at   11.617 11.54 11.67 11.658 11.541 11.901 12.005 12.219 12.024 11.98
   4890   1386394_at   8.486 8.407 8.609 8.839 8.615 8.054 7.911 8.009 7.689 7.817
   4891   1387039_at   6.295 6.73 6.679 6.522 6.537 7.163 7.296 7.403 7.066 7.197
   4892   1392534_at   7.913 7.932 8.355 8.461 8.207 9.462 9.172 9.461 9.215 8.903
   4893   1388296_at   12.647 12.561 12.587 12.446 12.579 12.323 12.306 12.274 12.353 12.259
   4894   1391837_at   7.148 7.186 7.202 7.178 7.241 6.384 6.437 6.22 6.284 5.656
   4895   1397788_at   7.037 6.969 7.063 7.152 7.553 8.289 8.236 8.455 7.983 7.909
   4896   1369927_at   10.957 10.906 10.873 11.033 11.009 11.404 11.461 11.278 11.234 11.499
   4897   1383504_at   6.772 6.777 6.893 6.754 6.83 7.198 7.136 6.999 7.09 7.202
   4898   1376416_at   6.715 6.703 6.9 6.85 6.505 7.359 7.234 7.457 7.244 7.249
   4899   1371915_at   7.089 7.342 7.186 7.678 7.277 8.41 8.284 8.577 8.191 8.009
   4900   1398431_at   5.266 5.076 6.073 5.642 5.862 6.89 7.052 7.013 6.76 6.933
   4901   1376579_at   9.565 9.523 9.853 9.715 9.663 9.031 9.175 8.679 8.964 8.816
   4902   1393629_at   6.261 6.783 6.717 6.702 6.687 4.913 3.932 4.43 2.716 3.64
   4903   1374458_at   8.901 8.634 8.309 8.36 8.355 7.595 7.707 7.301 7.693 7.497
   4904   1374832_at   3.198 2.06 2.755 3.406 3.602 4.764 4.912 5.26 5.373 5.427
   4905   1381208_at   4.574 4.453 4.073 4.8 4.768 6.047 6.029 5.878 5.332 5.924
   4906   1374615_at   7.722 7.74 7.682 7.819 7.727 7.095 7.463 7.299 7.111 7.212
   4907   1379234_a_at   7.133 6.967 7.271 6.859 6.502 5.749 5.474 5.795 6.086 5.455
   4908   1370688_at   12.034 11.902 12.139 12.01 11.312 13.015 12.857 12.858 12.968 13.052
   4909   1389128_at   9.194 9.237 9.203 9.397 9.34 9.655 10.085 9.834 9.776 9.916
   4910   1372401_at   9.494 9.438 9.434 9.491 9.573 8.819 8.815 9.119 8.566 8.641
   4911   1374961_at   7.291 7.13 7.14 7.495 7.315 5.8 6.269 6.552 6.371 6.359
   4912   1375562_at   5.427 5.235 5.433 5.794 5.651 6.269 6.626 6.299 6.271 6.277
   4913   1390937_at   8.044 8.054 8.039 8.319 8.212 8.801 8.806 9.251 8.761 8.82
   4914   1379300_at   5.515 5.845 6.447 6.452 5.972 7.378 7.522 7.892 7.362 7.361
   4915   1375273_at   6.665 6.424 6.438 6.739 6.73 5.945 5.651 5.745 6.008 5.485
   4916   1383397_at   5.3 5.209 5.686 5.246 5.236 6.892 6.503 6.801 6.534 6.039
   4917   1374313_at   6.929 7.075 6.88 7.021 6.666 5.577 5.234 5.93 6.109 5.831
   4918   1372626_at   6.325 6.299 6.419 6.365 6.447 7.755 7.868 8.526 7.573 7.314
   4919   1389526_at   9.444 9.524 9.535 9.51 9.489 8.938 9.225 8.777 8.913 8.805
   4920   1393352_at   6.097 6.751 6.396 6.821 6.988 7.63 7.861 7.956 7.724 7.928
   4921   1376047_at   8.464 8.463 8.423 8.372 8.224 9.108 9.36 9.404 8.976 8.913
   4922   1376792_at   7.771 7.727 8.095 7.848 7.625 8.535 8.801 8.54 8.393 8.473
   4923   1387060_at   9.127 9.011 9.113 9.346 9.558 7.939 7.855 8.382 7.406 7.469
   4924   1388934_at   7.446 7.545 7.536 7.603 7.766 6.982 7.191 7.099 7.131 6.91
   4925   1376656_at   7.52 7.579 7.706 8.053 7.501 8.325 8.479 8.551 8.39 8.437
   4926   1397579_x_at   5.242 5.243 4.808 5.697 5.826 6.751 6.708 6.959 6.543 7.094
   4927   1390790_a_at   6.568 6.759 6.825 7.194 6.715 5.555 5.875 6.091 5.464 5.596
   4928   1383336_at   7.834 8.037 8.029 8.179 8.415 7.39 7.214 7.118 6.906 6.782
   4929   1374331_at   7.539 7.664 7.761 8.013 7.934 7.061 6.846 7.234 7.036 6.934
   4930   1389450_at   8.551 8.48 8.29 8.184 8.189 7.48 7.751 7.795 7.493 7.678
   4931   1389511_s_at   7.577 7.645 7.854 7.887 7.693 8.51 8.526 8.941 8.425 8.369
   4932   1391441_at   5.63 5.938 6.116 5.843 5.755 5.179 4.9 4.889 4.618 5.134
   4933   1397385_at   7.167 7.289 6.927 7.433 7.442 6.371 6.669 6.408 6.485 6.512
   4934   1374112_at   6.806 6.629 6.979 6.808 6.879 7.657 7.392 7.726 7.49 7.294
   4935   1387117_at   8.049 7.991 7.971 8.051 8.188 7.745 7.737 7.675 7.814 7.679
   4936   1396238_at   5.255 4.853 6.367 5.878 5.851 7.574 7.841 7.741 7.327 7.508
   4937   1368174_at   5.386 5.872 5.635 5.793 5.205 6.924 6.959 7.16 6.43 6.661
   4938   1380379_at   6.655 6.582 6.623 6.608 6.512 7.131 7.24 7.615 7.199 7.115
   4939   1391241_at   5.265 5.218 4.797 5.079 5.695 6.895 7.187 6.169 7.204 7.118
   4940   1389774_at   5.253 5.836 5.341 5.602 5.176 6.666 6.388 6.687 6.329 6.309
   4941   1388978_at   9.321 9.189 9.388 9.279 9.217 9.82 10.009 9.745 9.607 9.817
   4942   1371891_at   8.719 8.643 8.95 8.843 8.772 8.437 8.402 8.292 8.254 8.351
   4943   1393143_at   7.833 7.793 8.228 8.26 7.875 8.8 8.99 8.826 8.807 9.256
   4944   1372501_at   8.792 8.841 8.938 9.332 9.03 8.133 8.347 8.353 8.221 8.22
   4945   1388151_at   8.193 8.17 8.284 8.324 8.102 7.652 7.827 7.667 7.918 7.668
   4946   1389388_at   7.464 7.304 7.522 7.64 7.493 8.025 8.173 7.871 7.933 8.054
   4947   1382318_at   6.708 6.683 6.992 7.022 6.888 7.76 7.49 7.854 7.448 7.51
   4948   1373564_at   7.98 8.043 8.032 8.17 8.294 8.798 8.516 8.75 8.844 8.585
   4949   1395560_at   6.764 6.627 6.249 6.291 6.692 5.536 5.83 5.39 5.721 5.551
   4950   1388370_at   11 10.992 10.922 10.896 11.112 11.33 11.326 11.393 11.215 11.389
   4951   1376663_at   7.014 6.866 7.078 7.21 7.165 6.375 6.691 6.326 6.423 6.455
   4952   1369560_at   6.852 7.174 7.624 7.098 6.76 8.59 8.272 8.074 8.57 8.298
   4953   1371382_at   9.825 9.746 9.74 9.975 9.845 8.777 8.825 9.368 8.63 8.908
   4954   1374722_at   6.929 7.048 6.407 6.742 7.33 5.337 3.976 5.326 4.845 4.684
   4955   1367860_a_at   8.984 8.945 9.377 9.377 8.857 7.948 7.906 8.291 8.296 7.81
   4956   1378954_at   4.918 4.642 4.729 5.087 4.351 5.638 6.161 6.185 5.904 5.713
   4957   1375440_at   8.444 8.417 8.628 8.762 8.608 7.958 8.105 8.147 8.036 7.913
   4958   1377009_at   5.801 6.167 6.444 5.829 5.804 7.278 7.004 7.218 6.896 7.505
   4959   1378467_at   6.796 6.801 6.726 6.843 7.018 6.32 6.416 5.978 6.186 6.157
   4960   1379449_at   7.628 7.525 7.696 7.793 7.603 8.235 8.282 8.272 7.944 8.273
   4961   1383733_at   8.734 8.786 8.795 8.623 8.478 9.253 9.33 9.058 9.409 9.284
   4962   1372363_at   8.041 8.151 8.37 8.322 8.514 7.656 7.121 7.413 7.426 7.091
   4963   1373161_at   9.129 9.046 8.798 8.984 9.033 9.503 9.6 9.622 9.426 9.379
   4964   1371004_at   5.379 5.398 5.123 5.524 5.755 6.262 6.287 6.446 6.126 6.161
   4965   1367644_at   7.254 7.011 7.265 7.394 7.638 8.3 8.402 8.637 8.23 8.021
   4966   1398413_at   6.491 6.616 6.606 6.486 6.494 6.991 6.916 7.03 7.329 7.093
   4967   1376202_at   7.459 7.462 7.42 7.78 7.748 8.221 8.312 8.308 8.104 8.101
   4968   1390942_at   6.53 6.37 6.598 6.768 6.492 7.778 7.394 7.138 7.727 7.423
   4969   1387550_a_at   5.783 5.42 5.933 6.296 6.422 7.447 7.835 8.281 7.266 7.958
   4970   1387160_at   5.52 4.983 5.19 5.105 5.049 2.373 3.401 3.923 3.048 2.472
   4971   1371764_at   9.175 9.156 9.163 9.308 9.334 8.736 8.748 8.904 8.545 8.779
   4972   1390173_at   7.61 7.745 7.941 8.053 7.824 8.677 8.54 8.504 8.528 8.307
   4973   1383593_at   6.011 6.607 6.607 6.332 6.228 7.228 7.326 7.614 7.396 7.867
   4974   1369864_a_at   4.609 3.722 4.721 4.841 4.346 7.223 6.642 7.769 6.247 6.402
   4975   1377354_at   3.648 4.697 4.22 4.242 4.534 5.423 5.806 5.881 5.696 5.495
   4976   1368194_at   7.51 7.663 7.289 7.713 8.018 6.284 6.847 6.558 6.342 6.13
   4977   1389913_at   5.211 5.369 5.621 5.634 5.657 6.513 6.169 6.388 6.202 6.163
   4978   1379491_at   5.076 5.409 5.329 5.259 3.644 7.385 7.141 7.214 7.28 7.265
   4979   1379672_at   4.454 4.047 5.101 4.586 4.795 5.989 6.28 6.432 5.995 5.737
   4980   1390249_at   6.663 6.74 6.6 6.905 6.726 5.467 5.536 5.531 4.447 5.242
   4981   1388764_at   10.376 10.398 10.29 10.329 10.263 9.622 9.771 9.93 9.388 9.514
   4982   1377905_at   7.913 7.95 8.048 8.258 8.078 7.629 7.459 7.348 7.506 7.266
   4983   1373833_at   6.678 7.036 7.058 6.791 7.208 5.993 6.307 6.113 6.29 6.153
   4984   1381779_s_at   7.677 7.747 7.805 7.64 7.763 8.117 8.151 8.456 8.141 8.357
   4985   1384634_at   3.876 4.307 3.779 4.529 3.814 6.023 5.581 5.966 5.407 5.144
   4986   1367596_at   12.523 12.407 12.415 12.123 12.343 11.93 11.834 11.693 11.853 11.832
   4987   1378104_at   6.895 7.229 7.003 6.919 7.079 6.555 6.611 6.548 6.647 6.538
   4988   1391509_at   6.951 6.916 7.187 7.252 6.936 8.115 8.451 8.687 7.895 7.941
   4989   1372846_at   7.772 7.654 7.615 7.524 7.791 7.23 7.161 7.38 7.21 7.212
   4990   1372638_at   6.883 6.929 7.027 7.164 7.221 7.562 7.699 7.781 7.594 7.487
   4991   1374444_at   5.692 5.546 4.995 5.759 5.574 6.581 6.641 6.982 6.395 6.756
   4992   1371619_at   9.629 9.686 9.747 9.803 9.873 10.117 10.225 10.159 10.031 10.083
   4993   1396286_at   6.473 6.745 6.818 7.022 6.924 6.067 5.222 5.516 5.447 5.758
   4994   1375484_at   6.628 6.415 6.683 7.157 6.771 7.579 7.882 8.193 7.681 7.941
   4995   1393101_at   8.234 8.243 8.234 8.344 8.492 7.975 7.721 7.867 7.827 7.892
   4996   1370456_at   4.698 4.401 4.581 4.844 4.119 5.938 6.146 5.613 5.871 5.391
   4997   1387810_at   6.939 6.681 6.947 6.98 7.052 6.259 6.218 6.159 6.494 6.024
   4998   1389000_at   8.083 8.271 8.254 8.227 8.109 7.903 7.85 7.862 7.831 7.7
   4999   1369018_at   6.025 6.522 5.893 5.992 5.923 3.574 4.788 4.406 4.438 4.696
   5000   1370062_at   10.609 10.598 10.71 10.807 10.693 10.083 10.111 10.326 10.007 9.836
   5001   1389585_at   6.401 6.546 6.902 6.786 6.54 7.669 7.674 7.325 7.3 7.439
   5002   1392487_at   7.469 7.645 7.592 7.983 7.794 6.856 6.887 7.147 7.022 7.004
   5003   1369650_at   8.055 8.094 7.899 8.061 7.667 7.272 6.828 6.407 6.902 6.611
   5004   1375726_at   7.572 7.554 7.765 8.133 7.763 8.505 8.66 8.859 8.633 8.467
   5005   1392510_at   5.272 5.094 5.598 4.796 4.471 7.265 7.188 6.186 7.126 7.111
   5006   1370675_at   7.174 7.26 7.509 7.703 7.214 8.699 8.928 8.053 8.783 8.709
   5007   1398287_at   10.854 10.838 10.911 11.002 10.815 11.363 11.367 11.409 11.346 11.111
   5008   1379780_at   5.702 5.731 6.045 5.686 5.389 3.846 4.275 4.142 4.824 3.668
   5009   1370943_at   11.814 11.769 11.919 11.576 11.33 12.586 12.519 12.343 12.61 12.812
   5010   1383795_at   7.656 8.038 7.804 8.116 7.864 8.691 9.283 9.118 8.699 8.771
   5011   1378327_at   5.525 5.23 5.493 5.616 6.068 6.469 6.882 6.879 6.952 6.561
   5012   1375647_at   6.561 6.569 6.417 7.034 6.786 7.854 8.32 8.555 7.658 7.86
   5013   1391596_at   2.623 2.367 1.744 2.725 3.392 0.651 0 0.239 0 0.872
   5014   1389529_at   7.387 7.281 7.452 7.33 7.105 7.776 7.952 7.781 7.746 7.743
   5015   1388507_at   10.699 10.813 10.774 10.951 10.809 10.324 10.428 10.543 10.447 10.351
   5016   1377679_at   6.549 6.715 6.828 6.52 6.373 7.531 7.287 7.118 7.301 7.314
   5017   1375945_at   7.029 7.121 7.25 7.241 7.416 7.933 7.79 7.874 7.704 7.652
   5018   1397167_at   8.086 8.198 8.173 8.239 8.28 2.331 5 5.266 3.875 5.155
   5019   1384096_at   4.584 3.829 4.123 4.222 4.04 5.003 5.064 5.355 5.101 5.074
   5020   1371656_at   11.252 11.149 11.148 11.068 11.297 10.645 10.774 10.841 10.502 10.581
   5021   1390681_at   5.271 5.471 5.292 5.603 5.375 4.232 3.944 4.722 4 4.52
   5022   1385853_at   5.938 6.419 6.538 6.472 6.794 4.527 4.851 3.422 4.822 4.291
   5023   1372665_at   10.754 10.791 11.1 11.083 10.668 11.725 11.714 11.388 11.774 11.765
   5024   1392788_at   5.126 5.023 5.028 4.654 4.221 3.581 3.806 3.385 3.429 3.348
   5025   1367610_at   12.657 12.56 12.51 12.377 12.524 12.195 12.102 12.162 12.188 12.237
   5026   1371342_at   11.381 11.305 11.295 11.358 11.49 11.779 11.81 11.653 11.712 11.619
   5027   1380474_at   6.908 6.645 6.848 6.771 7.273 5.438 5.197 5.038 5.609 6.052
   5028   1389801_at   7.146 7.055 7.196 6.896 6.925 7.822 7.88 8.271 7.598 7.928
   5029   1380559_at   5.378 5.56 5.116 5.711 5.356 2.263 2.66 1.496 3.057 3.81
   5030   1389649_at   10.025 10.023 9.97 9.91 10.026 10.905 11.118 10.39 10.904 10.963
   5031   1369027_at   6.298 6.32 6.362 6.254 6.663 6.859 6.902 7.065 6.91 6.998
   5032   1393436_at   2.862 0.74 1.39 1.131 0 4.525 4.778 5.234 4.871 4.164
   5033   1375655_at   7.152 6.996 6.981 7.095 7.202 6.781 6.687 6.654 6.737 6.55
   5034   1389171_at   5.65 5.708 6.208 5.654 6.132 7.236 7.14 7.192 7.077 6.591
   5035   1378179_a_at   7.577 7.828 7.631 7.752 7.839 6.676 6.606 5.562 6.349 5.941
   5036   1376086_at   8.907 8.885 8.831 8.916 9.016 9.442 9.396 9.442 9.405 9.134
   5037   1368953_at   7.753 7.679 7.683 7.664 7.973 7.025 7.051 6.493 7.056 6.708
   5038   1389052_at   7.9 7.941 8.025 8.151 8.246 6.877 7.4 7.446 7.218 7.102
   5039   1379724_at   7.381 7.404 7.757 7.845 7.833 8.699 8.767 8.797 8.578 8.269
   5040   1393338_at   5.246 5.629 5.076 5.562 5.494 6.502 6.81 6.828 6.185 6.347
   5041   1380562_at   6.634 6.791 7.108 6.845 6.841 7.915 7.604 8.02 7.843 7.424
   5042   1379473_at   6.088 6.228 6.555 6.668 6.34 7.439 7.599 7.809 7.231 7.195
   5043   1379889_at   5.128 5.029 4.76 5.149 5.365 3.087 3.946 2.599 2.987 3.694
   5044   1383644_at   4.963 5.375 4.8 4.751 5.557 6.151 6.385 6.209 6.158 6.408
   5045   1368963_at   6.997 7.016 7.186 7.069 6.223 8.546 7.932 8.328 8.391 8.215
   5046   1372065_at   6.586 6.658 6.318 6.488 6.813 5.862 6.023 6.013 5.766 5.816
   5047   1390602_a_at   8.058 8.088 8.238 8.179 7.948 8.838 8.893 8.479 8.683 8.668
   5048   1391169_at   6.478 6.03 6.29 6.137 5.983 6.768 6.875 6.878 6.777 6.86
   5049   1370098_at   9.207 9.137 9.399 9.412 9.277 9.695 9.677 9.683 9.73 9.611
   5050   1393119_at   6.534 6.561 7.016 6.884 6.464 5.365 5.163 5.635 4.467 4.824
   5051   1373437_at   8.335 8.302 8.403 8.446 8.448 7.807 8.057 7.687 7.911 7.63
   5052   1387665_at   6.586 6.857 6.707 6.447 7.205 7.654 7.902 7.635 7.865 7.66
   5053   1397782_at   4.113 1.891 2.07 3.614 3.141 6.627 6.84 7.078 5.522 6.322
   5054   1377639_at   5.95 6.337 6.022 6.122 6.179 5.111 5.541 5.034 5.084 5.48
   5055   1386876_at   5.471 5.391 5.503 5.693 5.497 6.626 6.508 7.128 6.507 6.215
   5056   1383555_at   7.152 7.12 7.179 7.119 7.012 6.941 6.817 6.756 6.808 6.823
   5057   1395510_at   6.187 6.214 6.048 6.071 6.005 6.451 6.659 6.442 6.695 6.556
   5058   1385570_at   1.795 0.585 2.157 1.151 2.893 4.223 4.964 5.136 4.688 4.463
   5059   1385783_at   7.576 7.689 7.799 8.091 7.462 8.705 8.727 8.761 8.462 8.422
   5060   1381903_at   8.167 8.079 8.182 8.037 8.202 6.874 7.489 7.035 7.538 7.167
   5061   1379410_at   7.795 7.861 7.406 7.718 7.548 8.19 8.227 8.303 8.281 8.321
   5062   1370461_at   6.606 6.304 6.037 6.273 6.354 3.77 4.32 4.288 5.231 4.817
   5063   1374403_at   6.996 6.863 7.158 7.146 7.06 7.895 7.621 7.743 7.857 7.454
   5064   1395400_at   6.414 6.51 6.546 6.535 6.605 6.198 5.924 5.899 5.927 5.694
   5065   1385423_at   7.739 7.688 7.623 7.784 7.878 8.384 8.196 8.117 8.346 8.117
   5066   1389518_at   9.193 9.28 9.495 9.732 9.679 8.702 8.721 8.769 8.547 8.457
   5067   1374834_at   9.465 9.408 9.424 9.571 9.427 8.96 9.147 9.074 8.892 9.157
   5068   1389158_at   7.016 6.873 6.677 7.117 7.123 6.079 5.748 5.816 6.152 5.355
   5069   1373351_at   5.647 5.392 5.341 5.595 6.011 6.394 6.748 6.45 6.457 6.555
   5070   1369708_a_at   6.289 6.142 6.222 6.429 6.21 5.502 5.195 5.421 5.641 4.933
   5071   1372390_at   5.619 5.642 5.359 5.315 6.198 6.805 7.495 7.09 6.739 7.226
   5072   1391090_at   3.88 3.384 3.88 2.956 3.751 5.098 4.972 5.134 4.766 4.703
   5073   1392739_a_at   5.558 5.185 5.801 6.027 5.249 7.166 6.871 6.743 6.913 6.577
   5074   1389373_at   9.033 9.139 8.889 9.127 9.329 8.487 8.514 8.427 8.113 8.168
   5075   1374089_at   5.919 6.059 6.06 6.26 6.109 7.003 7.868 7.895 7.057 7.413
   5076   1380383_at   6.768 6.831 6.603 6.591 6.84 7.871 7.916 8.357 7.693 7.417
   5077   1388986_at   8.938 9.214 8.647 8.746 9.099 7.507 7.616 8.056 6.955 7.361
   5078   1383056_a_at   7.58 7.455 7.921 7.706 7.525 8.265 8.501 8.858 8.515 8.423
   5079   1372007_at   7.829 7.993 7.847 8.306 8.351 7.091 7.034 7.336 7.165 7.327
   5080   1389334_at   9.387 9.5 9.345 9.317 9.362 9.657 9.914 9.974 9.774 9.794
   5081   1392835_at   7.422 7.459 7.39 7.457 7.627 7.725 7.791 7.859 7.818 7.771
   5082   1370408_at   6.68 6.794 7.047 6.872 7.086 6.215 6.281 6.242 6.108 6.435
   5083   1387081_at   8.664 8.619 8.605 8.448 8.797 8.011 8.222 7.749 7.867 7.956
   5084   1376030_at   6.533 6.811 7.034 6.781 6.567 3.703 3.633 4.959 2.561 2.305
   5085   1377079_a_at   6.686 6.813 7.059 6.966 6.919 7.645 7.992 7.966 7.797 7.403
   5086   1377392_at   4.957 5.379 5.693 5.44 4.927 6.472 6.79 6.97 6.352 6.386
   5087   1389062_at   9.102 9.125 9.005 9.199 9.45 8.379 8.556 8.552 8.088 8.22
   5088   1383168_at   7.176 7.619 7.271 7.518 7.527 6.452 6.574 6.888 6.733 6.595
   5089   1381798_at   4.486 3.916 4.807 4.344 4.469 6.055 5.593 5.694 5.548 5.388
   5090   1368049_at   11.085 11.047 10.909 11.114 11.228 10.61 10.707 10.715 10.499 10.545
   5091   1390892_at   5.831 6.19 6.006 6.408 6.419 4.642 4.255 4.516 3.839 5.159
   5092   1372188_at   7.663 7.556 7.629 7.548 7.555 7.26 7.042 6.902 6.742 6.973
   5093   1370001_at   9.653 9.781 9.752 9.737 9.708 9.38 9.342 9.564 9.434 9.377
   5094   1373282_at   6.175 6.437 6.348 6.354 5.924 7.399 7.441 7.73 6.958 7.173
   5095   1395821_at   7.246 7.391 7.737 7.775 7.633 6.569 6.184 5.51 6.357 6.134
   5096   1370337_at   8.761 8.638 8.571 8.568 8.704 8.329 8.346 8.391 8.325 8.416
   5097   1392322_at   6.683 6.921 7.312 7.119 6.674 4.428 3.816 1.157 2.915 2.987
   5098   1384588_at   7.28 7.307 7.371 7.414 7.241 6.837 6.944 6.884 6.536 6.719
   5099   1395636_at   4.752 4.566 4.654 4.347 4.595 5.934 5.735 5.908 5.174 5.521
   5100   1372175_at   9.832 9.812 9.579 9.553 9.475 10.339 10.504 10.089 10.34 10.434
   5101   1374660_at   9.619 9.608 9.705 9.65 9.764 9.282 9.399 9.316 9.136 9.119
   5102   1378445_at   7.044 6.723 6.974 6.839 7.002 7.72 7.72 7.374 7.476 7.467
   5103   1393655_at   6.129 6.327 5.848 6.342 6.044 1.744 3.72 3.041 3.556 1.22
   5104   1368504_at   11.472 11.506 11.456 11.436 11.577 11.847 11.92 11.666 11.86 11.843
   5105   1371956_at   8.841 8.931 8.843 9.054 9.156 9.69 9.859 9.556 9.46 9.51
   5106   1392978_at   8.825 8.78 8.743 8.88 9.016 8.235 8.161 8.386 7.924 7.878
   5107   1371926_at   9.562 9.711 9.292 9.58 9.465 8.688 8.968 9.044 8.719 8.836
   5108   1372001_at   8.694 8.739 8.809 8.978 8.788 8.292 8.31 8.421 8.437 8.161
   5109   1375083_at   6.801 6.795 6.463 7.114 6.645 7.844 8.097 8.438 7.966 7.577
   5110   1375170_at   11.724 11.711 11.479 11.765 11.937 10.816 10.741 11.273 10.785 10.827
   5111   1374920_at   7.744 7.794 7.695 7.687 7.483 8.398 8.666 8.426 8.092 8.474
   5112   1369077_at   9.393 9.352 9.516 9.39 9.428 9.098 8.962 8.695 8.969 8.838
   5113   1367853_at   7.768 8.117 7.928 8.084 8.009 8.654 8.799 8.71 8.375 8.609
   5114   1390741_at   5.518 5.302 5.472 5.409 5.549 5.791 5.749 5.843 5.796 5.937
   5115   1388530_at   9.147 9.573 9.498 9.435 9.633 8.763 8.693 8.764 8.346 8.74
   5116   1373176_at   7.662 7.588 7.074 7.626 7.596 6.346 6.586 6.415 6.756 6.74
   5117   1371451_at   9.034 8.822 8.797 8.913 9.079 7.845 8.188 8.4 7.762 8.057
   5118   1372498_at   9.398 9.279 9.591 9.621 9.292 9.974 10.022 9.89 9.924 9.972
   5119   1370278_at   11.574 11.53 11.627 11.371 11.371 11.907 11.873 11.855 11.968 11.839
   5120   1371313_at   11.881 11.77 11.813 11.661 11.799 11.463 11.542 11.339 11.36 11.32
   5121   1380345_at   4.707 5.033 4.676 4.984 4.757 5.771 5.381 5.887 5.789 5.492
   5122   1367627_at   11.636 11.626 11.536 11.311 11.285 12.682 12.579 12.021 12.714 12.783
   5123   1374345_at   5.898 6.051 6.473 5.716 5.476 7.142 7.252 7.583 7.191 7.024
   5124   1390854_at   6.931 6.82 7.183 7.154 6.744 8.174 7.912 7.579 8.196 7.872
   5125   1387882_at   6.2 6.632 7.156 6.92 6.841 8.803 8.065 7.909 8.667 8.745
   5126   1372510_at   7.673 7.557 7.897 7.919 7.723 6.707 6.943 7.13 6.39 6.611
   5127   1398268_at   9.055 8.974 9.196 9.243 9.076 9.703 9.565 9.665 9.495 9.454
   5128   1371988_at   9.281 9.342 9.397 9.328 9.273 8.886 8.851 8.4 8.692 8.748
   5129   1367826_at   10.414 10.553 10.572 10.391 10.416 9.902 9.893 9.69 10.088 10.044
   5130   1372735_at   9.872 9.934 9.707 9.931 10.103 9.172 9.408 9.495 9.246 9.272
   5131   1375553_at   5.686 5.711 5.159 5.72 5.416 6.938 6.762 7.145 6.722 6.286
   5132   1382537_at   9.454 9.317 9.547 9.574 9.508 9.158 9.096 8.914 9.046 9.095
   5133   1387745_at   5.249 5.365 5.589 5.135 5.369 4.194 3.197 4.331 3.621 3.924
   5134   1398966_at   8.825 8.886 8.962 8.973 8.951 9.258 9.353 9.328 9.276 9.112
   5135   1378601_at   5.505 5.472 5.752 4.953 5.709 6.557 6.637 6.635 6.413 6.371
   5136   1380013_at   5.256 5.336 5.583 5.895 4.969 7.045 6.764 6.864 6.932 6.311
   5137   1396297_at   5.025 5.291 5.669 5.462 4.711 2.508 3.414 3.696 2.151 3.091
   5138   1374564_at   6.586 6.699 6.952 6.989 6.799 5.898 5.968 5.828 5.241 5.877
   5139   1378076_at   6.028 5.419 5.768 6.313 5.758 2.932 1.934 1.144 3.484 3.426
   5140   1397634_at   7.856 7.952 7.957 8.18 8.105 8.671 8.748 9.03 8.524 8.623
   5141   1373069_at   8.395 8.524 8.55 8.66 8.57 8.873 8.932 8.881 8.904 8.788
   5142   1367664_at   3.341 3.721 3.445 3.623 3.146 4.487 4.887 5.149 4.355 4.515
   5143   1382160_at   6.177 6.452 6.032 6.38 6.556 5.342 4.916 4.982 5.111 4.259
   5144   1389955_at   3.718 3.123 3 3.041 3.06 4.478 4.874 4.093 4.459 4.335
   5145   1395710_at   6.06 6.066 5.846 6.043 5.647 5.103 4.694 4.333 4.964 4.276
   5146   1367832_at   10.246 10.217 10.39 10.231 10.232 11.022 11.128 10.596 10.895 10.914
   5147   1373245_at   10.899 10.976 10.951 11.119 11.162 10.278 10.531 9.897 10.199 10.303
   5148   1392012_at   5.86 6.433 5.628 6.523 6.101 3.87 4.249 4.748 4.555 4.818
   5149   1395499_at   7.068 7.136 7.362 7.346 7.171 7.812 7.744 7.857 7.568 7.889
   5150   1372864_at   4.591 4.095 4.573 4.345 3.937 5.639 5.251 5.543 5.122 5.543
   5151   1384389_at   3.652 4.676 3.611 2.753 3.686 1.646 1.07 1.151 1.208 1.791
   5152   1382739_at   5.33 5.024 5.441 5.838 4.927 4.184 3.418 3.846 3.313 3.082
   5153   1368589_at   8.184 8.062 8.087 8.33 8.466 7.14 7.428 7.584 6.876 7.144
   5154   1389739_at   6.058 5.956 6.15 6.382 5.356 8.234 7.602 7.393 7.445 7.33
   5155   1377098_at   7.745 7.66 8.116 7.846 7.273 9.883 10.176 8.718 9.745 9.74
   5156   1373822_at   10.257 10.226 10.062 9.995 10.172 9.497 9.594 9.784 9.476 9.343
   5157   1374457_at   9.089 9.059 8.993 9.216 9.422 8.583 8.666 8.655 8.543 8.471
   5158   1385038_at   4.529 3.777 5.187 5.219 4.432 6.438 6.699 6.645 6.327 6.461
   5159   1369990_at   9.131 9.138 9.142 9.072 9.033 9.369 9.38 9.5 9.287 9.469
   5160   1380170_at   7.662 7.826 7.582 7.881 7.567 8.346 8.389 8.487 8.242 8.118
   5161   1389464_at   5.344 4.591 5.374 5.193 5.552 6.375 6.508 6.895 6.397 6.409
   5162   1390255_at   7.189 7.428 7.306 7.35 7.166 7.973 8.565 8.174 8.537 7.966
   5163   1382777_at   7.108 7.123 7.117 7.19 7.073 5.822 6.028 6.384 6.027 6.59
   5164   1370147_at   8.623 8.468 9.002 8.474 7.612 10.67 10.647 9.713 10.767 10.525
   5165   1369635_at   10.383 10.44 10.574 10.467 10.143 11.327 11.338 10.854 11.421 11.248
   5166   1382509_at   9.098 9.309 9.01 9.08 9.183 8.545 8.683 8.196 8.541 8.38
   5167   1389170_at   9.383 9.407 9.857 9.633 9.477 8.078 7.904 8.878 8.304 8.233
   5168   1393832_at   7.454 6.762 7.18 6.881 7.164 3.496 3.036 5.223 4.567 4.703
   5169   1369949_at   8.484 8.355 8.7 8.713 8.589 9.437 9.321 9.784 9.398 10.028
   5170   1371724_at   9.973 9.827 9.959 10.036 10.16 9.192 9.285 9.489 8.851 9.067
   5171   1376187_at   7.878 8.09 8.053 8.255 8.011 8.592 8.821 8.844 8.525 8.578
   5172   1379201_at   6.343 6.223 6.733 6.446 6.339 5.7 5.732 5.69 5.928 5.649
   5173   1390149_at   8.592 8.706 8.958 9.181 8.643 9.69 9.766 9.683 9.45 9.674
   5174   1389354_at   9.277 9.21 9.112 9.306 9.395 9.693 9.645 9.529 9.646 9.687
   5175   1391045_at   7.212 7.351 7.406 7.269 6.844 8.409 8.181 8.499 8.025 7.939
   5176   1392989_at   7.573 7.583 7.69 7.644 7.58 8.482 8.712 8.164 8.11 8.449
   5177   1373078_at   8.921 9.02 9.474 9.11 8.989 10.414 10.65 9.754 10.428 10.519
   5178   1377442_at   4.54 4.227 4.093 4.525 4.812 5.839 5.384 5.464 5.333 5.453
   5179   1374724_at   5.935 6.165 6.228 6.257 5.925 8.273 8.336 7.003 7.831 7.648
   5180   1388304_at   10.978 10.911 11.034 11.005 11.102 11.463 11.805 11.678 11.553 11.368
   5181   1379312_at   6.912 6.951 6.68 7.004 7.205 5.973 5.441 5.671 5.13 4.743
   5182   1392296_at   5.339 5.5 6.015 5.584 5.03 3.81 4.472 4.221 4.26 3.995
   5183   1389053_at   8.816 8.852 8.877 8.958 8.869 8.553 8.42 8.369 8.411 8.127
   5184   1377908_at   6.739 6.659 6.707 7.095 6.474 5.873 6.036 5.909 5.915 6.133
   5185   1387844_at   8.024 7.933 8.066 8.094 8.172 7.371 7.202 6.995 7.634 7.155
   5186   1372143_at   8.416 8.326 8.359 8.25 8.525 7.737 8.021 7.925 7.978 7.937
   5187   1368671_at   6.579 6.905 6.745 6.824 6.952 6.244 5.934 5.944 6.313 5.932
   5188   1381056_at   5.995 5.578 5.109 5.891 5.677 7.255 6.883 7.544 6.846 6.834
   5189   1383792_at   5.99 5.723 6.276 6.166 6.017 5.214 5.416 5.23 5.179 4.904
   5190   1380320_at   6.959 7.068 7.075 7.153 6.854 6.534 6.654 6.435 6.659 6.563
   5191   1382144_at   8.283 8.278 8.15 8.376 8.344 7.958 8.014 7.783 7.843 7.793
   5192   1397904_at   7.375 7.346 7.501 7.265 7.413 6.312 6.567 6.962 6.337 6.546
   5193   1371633_at   8.627 8.574 8.415 8.689 8.608 7.964 8.029 8.245 7.915 7.797
   5194   1392489_at   5.845 5.849 5.666 5.993 6.048 5.102 4.807 4.809 5.277 4.553
   5195   1368200_at   10.226 10.303 9.886 10.124 10.266 9.593 9.573 9.52 9.693 9.398
   5196   1393563_at   6.907 6.602 6.824 6.802 6.928 6.136 6.029 5.618 6.053 5.514
   5197   1367636_at   10.443 10.321 10.392 10.367 9.764 11.413 11.46 10.971 11.61 11.563
   5198   1392503_at   7.764 7.749 7.733 7.836 7.92 7.091 7.03 7.243 7.038 6.564
   5199   1398811_at   9.192 9.302 9.224 9.423 9.483 8.841 8.835 8.671 8.682 8.431
   5200   1376519_at   7.184 7.373 7.239 7.533 7.355 6.873 6.878 6.974 6.815 6.758
   5201   1373165_at   5.995 6.211 6.22 6.712 6.076 7.166 7.239 7.146 6.986 7.128
   5202   1382443_at   8.105 8.203 8.097 8.311 8.259 7.83 7.646 7.463 7.785 7.634
   5203   1383926_at   6.465 6.494 6.086 6.5 6.354 1.485 1.757 4.525 1.007 1.299
   5204   1370203_at   7.681 7.629 7.498 7.719 7.768 7.15 6.98 7.303 7.088 7.257
   5205   1379352_at   6.036 5.952 5.786 6.006 5.99 6.616 6.989 6.384 6.677 6.708
   5206   1367904_at   8.653 8.764 8.687 9.065 8.744 9.488 9.37 9.781 9.741 9.39
   5207   1376907_at   6.64 6.657 6.557 6.905 6.768 4.978 5.572 5.595 5.976 5.193
   5208   1373096_at   8.258 8.281 8.112 8.141 8.208 8.528 8.558 8.417 8.488 8.436
   5209   1383221_at   9.007 8.922 8.966 9.375 9.301 9.691 9.814 9.818 9.865 9.722
   5210   1374351_at   6.878 6.999 7.231 6.947 6.844 6.417 6.471 6.14 6.394 6.144
   5211   1370085_at   8.52 8.481 8.867 8.582 8.827 7.996 8.147 7.89 8.06 7.814
   5212   1393638_at   7.768 7.579 8.106 8.15 7.971 6.846 6.909 7.102 7.279 6.965
   5213   1392725_at   5.96 6.495 6.487 6.436 6.348 7.794 7.167 7.513 7.415 7.137
   5214   1396086_at   8.184 7.97 8.116 7.976 7.989 7.587 7.321 7.445 7.274 7.004
   5215   1383573_at   7.987 8.127 7.804 8.083 7.797 8.66 8.825 8.429 8.621 8.903
   5216   1372684_at   6.569 5.979 5.773 6.618 6.598 8.106 7.774 8.021 7.845 7.341
   5217   1385702_at   8.347 8.654 8.501 8.247 8.758 2.403 1.05 5.504 4.287 3.194
   5218   1397913_at   4.161 4.309 4.023 4.522 4.018 4.821 5.061 4.959 4.928 4.824
   5219   1399039_at   8.137 8.142 8.015 8.393 8.083 8.978 9.114 9.543 8.865 8.923
   5220   1375869_at   6.593 6.665 6.531 6.97 6.597 7.403 7.406 7.746 7.284 7.326
   5221   1371516_at   8.13 8.157 8.037 8.3 8.107 8.708 8.839 8.743 8.44 8.672
   5222   1373530_at   6.133 6.29 5.983 6.574 6.634 1.59 2.969 4.5 2.558 1.705
   5223   1371773_at   8.566 9.072 8.754 8.779 8.524 9.526 9.9 9.419 9.828 9.555
   5224   1387157_at   5.454 5.433 5.36 5.379 5.614 6.545 6.204 6.623 6.531 5.942
   5225   1382258_at   6.551 6.455 6.364 6.587 6.639 7.467 7.576 8.091 7.21 7.45
   5226   1373607_at   7.222 7.077 6.986 7.193 6.836 7.619 7.972 7.837 7.695 7.589
   5227   1370671_at   3.409 4.072 3.512 3.104 3.955 5.073 5.428 5.434 5.262 4.648
   5228   1388480_at   8.86 8.908 9.074 9.185 9.12 8.219 8.221 8.596 8.272 8.008
   5229   1387135_at   8.766 8.918 8.9 8.959 9.09 7.792 7.989 8.426 7.851 7.609
   5230   1383576_at   8.084 7.795 8.12 7.959 7.865 7.257 7.153 7.112 7.116 7.575
   5231   1394850_at   5.895 5.954 5.842 5.19 5.514 7.146 7.367 6.533 7.471 7.29
   5232   1378945_at   2.516 2.328 3.381 3.312 2.43 5.037 4.346 5.062 4.371 4.526
   5233   1381977_at   6.838 6.621 6.669 6.92 6.938 6.07 6.065 6.394 6.12 5.917
   5234   1373867_at   8.302 8.189 8.185 8 8.206 7.289 7.753 7.365 7.648 7.476
   5235   1396309_at   5.666 5.324 5.927 5.704 5.839 6.398 6.49 6.782 6.433 6.524
   5236   1370792_at   9.258 9.216 9.355 9.355 9.225 8.819 8.579 8.263 8.714 8.362
   5237   1374212_at   7.102 7.107 6.588 7.204 7.185 6.248 6.141 6.062 5.676 6.016
   5238   1379536_at   6.898 7.08 6.644 7.027 7.169 7.803 7.764 7.883 7.541 7.594
   5239   1378763_at   4.537 5.126 5.353 4.995 4.488 6.085 6.332 6.077 6.146 5.936
   5240   1372827_at   9.615 9.716 9.694 9.812 9.637 9.345 9.402 9.467 9.26 9.3
   5241   1376320_at   3.998 4.258 2.996 3.552 2.7 5.471 5.623 5.661 5.477 5.344
   5242   1384770_at   4.951 4.834 4.498 3.984 2.322 7.423 7.311 7.836 7.199 7.391
   5243   1383228_at   7.103 7.279 7.361 7.643 7.483 8.131 7.923 7.99 8.161 8.163
   5244   1385103_at   8.335 8.254 8.296 8.54 8.715 9.407 9.313 9.464 9.106 9.008
   5245   1382137_at   6.298 6.672 6.191 6.596 6.324 7.371 7.865 7.332 7.173 7.361
   5246   1371041_at   10.889 10.779 10.942 11.086 11.116 11.428 11.536 11.656 11.423 11.408
   5247   1382021_at   7.959 8.057 8.342 8.266 7.964 8.824 8.582 8.783 8.783 8.956
   5248   1369943_at   11.917 11.883 11.715 11.591 11.757 10.976 10.524 10.106 10.765 10.881
   5249   1372995_at   6.918 6.684 6.613 7.037 6.897 5.507 5.726 6.251 5.851 5.524
   5250   1391981_at   5.699 5.816 6.256 5.943 6.067 6.863 7.023 6.625 6.691 6.677
   5251   1388623_at   8.164 8.066 8.177 8.146 8.108 8.77 9.026 8.58 8.792 8.532
   5252   1376337_at   8.211 8.245 7.886 8.176 8.175 8.915 8.945 9.331 8.672 9.121
   5253   1367857_at   8.91 8.886 8.531 8.745 8.942 9.78 9.982 9.322 9.671 9.676
   5254   1368700_at   6.914 7.589 7.296 7.321 7.039 8.315 8.628 7.993 8.224 8.567
   5255   1367481_at   10.059 10.093 9.99 10.027 10.045 9.736 9.709 9.601 9.769 9.469
   5256   1375336_at   12.412 12.362 12.089 12.224 12.329 11.617 11.703 11.77 11.661 11.943
   5257   1367754_s_at   9.505 9.616 9.71 9.39 9.287 10.652 10.756 10.01 10.626 10.584
   5258   1383486_at   5.175 5.53 5.676 5.62 5.716 0 1.17 3.249 2.362 0.475
   5259   1368039_at   5.887 6.05 6.35 6.015 5.382 7.664 7.35 6.942 7.855 7.347
   5260   1368508_at   8.508 8.539 8.625 8.531 8.656 8.156 8.313 8.296 8.146 8.318
   5261   1383536_at   7.601 7.741 7.887 7.86 7.956 8.253 8.485 8.396 8.215 8.38
   5262   1374482_at   6.722 6.722 6.623 6.78 6.653 7.334 7.376 7.477 7.068 7.098
   5263   1372762_at   8.109 7.951 8.198 8.05 8.183 9.078 9.007 8.85 8.734 8.517
   5264   1388541_at   9.105 9.041 8.892 9.016 8.904 9.731 10.043 9.568 9.516 9.587
   5265   1374434_at   5.785 6.19 5.952 6.159 5.989 7.155 6.608 6.922 6.905 6.653
   5266   1387072_at   7.869 8.054 7.704 7.757 8.143 8.891 8.489 9.133 8.733 8.807
   5267   1388895_at   8.294 8.287 8.389 8.437 8.316 8.023 8.08 7.968 7.983 7.798
   5268   1377051_at   8.948 9.42 9.666 9.189 8.919 10.457 10.614 10.08 10.574 10.26
   5269   1368546_at   7.295 7.249 7.314 7.73 7.291 6.414 6.56 6.771 6.217 6.187
   5270   1392810_at   6.246 6.308 6.359 6.074 6.531 3.414 1.956 4.179 4.191 3.734
   5271   1368859_at   7.639 7.769 7.719 7.725 7.775 8.107 8.251 8.062 8.074 7.95
   5272   1385728_at   5.019 5.126 5.335 5.377 5.29 4.403 4.432 4.123 4.746 4.429
   5273   1388945_at   7.234 6.926 7.419 7.214 7.048 8.176 8.069 8.301 7.708 7.985
   5274   1370896_a_at   6.132 5.422 6.282 6.247 6.25 7.476 7.421 8.019 7.143 7.54
   5275   1392532_at   6.826 6.896 6.402 6.603 6.772 5.629 6 5.692 5.165 5.373
   5276   1390890_at   5.259 5.789 5.657 6.051 4.897 2.094 0.454 2.121 0.138 3.065
   5277   1387896_at   10.056 10.06 10.097 10.005 10.034 10.471 10.577 10.502 10.269 10.351
   5278   1369450_at   7.641 7.828 7.676 7.746 8.288 8.73 8.787 9.053 8.712 8.595
   5279   1373147_at   7.136 7.302 7.406 7.41 7.425 7.848 7.887 7.984 7.804 7.671
   5280   1367753_at   7.697 7.831 7.684 7.99 7.746 8.351 8.295 8.631 8.231 8.427
   5281   1379550_a_at   7.234 7.266 7.093 7.329 7.357 7.868 7.58 8.031 7.852 7.83
   5282   1393128_at   6.329 6.31 6.041 5.939 5.802 4.356 4.835 4.875 3.817 3.744
   5283   1393079_at   6.884 6.917 6.765 6.978 6.598 7.539 7.681 7.288 7.768 7.467
   5284   1385561_at   7.028 7.077 7.343 6.866 6.66 8.028 7.967 7.701 8.019 7.743
   5285   1378389_at   6.566 6.813 6.622 6.856 6.716 5.694 5.719 5.86 5.107 4.994
   5286   1367778_at   8.158 8.111 7.962 8.276 8.027 9.029 9.586 9.12 8.828 8.868
   5287   1377198_at   8.096 8.202 8.378 8.487 8.348 7.532 7.859 7.761 7.528 7.672
   5288   1371564_at   10.262 10.327 10.247 10.481 10.483 10.778 10.87 10.968 10.693 10.808
   5289   1386543_at   5.087 5.475 4.967 5.132 5.171 4.31 4.285 4.246 4.53 3.862
   5290   1373980_at   7.318 7.412 7.41 7.363 7.478 7.032 6.975 7.184 6.89 6.905
   5291   1392858_at   8.762 8.744 8.743 8.837 8.934 8.502 8.388 8.325 8.466 8.541
   5292   1369285_at   6.993 6.926 7.086 7.099 6.979 6.285 6.261 5.988 6.327 5.618
   5293   1370577_at   6.745 6.709 7.056 6.603 7.063 5.812 5.689 4.836 5.336 5.714
   5294   1383100_at   9.308 9.136 9.239 9.153 9.124 9.752 9.956 9.958 9.541 9.69
   5295   1373837_at   8.458 8.32 8.391 8.535 8.542 8.98 9.028 9.24 8.99 8.792
   5296   1373088_at   9.715 9.562 9.907 9.99 9.936 10.467 10.566 10.685 10.468 10.31
   5297   1395404_at   7.855 8.056 8.062 8.244 8.091 8.681 8.689 8.663 8.62 8.401
   5298   1377806_at   7.832 7.588 7.794 8.013 7.86 8.46 8.385 8.654 8.374 8.29
   5299   1367521_at   6.991 7.061 7.214 7.342 7.297 6.642 6.419 6.482 6.737 6.356
   5300   1367718_at   8.194 8.306 8.279 8.15 8.366 8.883 8.81 9.07 8.691 8.653
   5301   1377889_at   5.774 5.769 6.017 5.842 6.12 5.065 5.237 4.476 4.977 4.725
   5302   1383830_a_at   7.74 7.63 7.768 7.82 7.52 8.685 8.583 8.574 8.254 8.179
   5303   1375187_at   6.539 6.714 6.404 6.39 6.489 5.38 5.573 5.787 5.665 4.967
   5304   1372650_at   7.622 7.35 7.729 7.453 7.531 7.899 8.106 8.172 8.089 8.168
   5305   1376767_at   5.852 5.936 6.126 6.34 6.324 7.229 6.896 6.748 7.003 6.925
   5306   1391021_at   6.328 6.735 6.843 6.639 6.963 7.771 7.708 7.417 7.636 7.394
   5307   1398300_at   10.518 10.473 10.678 10.572 10.71 10.111 10.182 10.24 9.929 9.965
   5308   1390472_at   7.15 7.023 7.086 7.178 7.06 6.106 6.469 6.392 5.675 6.021
   5309   1372050_at   8.066 8.252 8.288 8.402 8.561 7.138 7.585 7.705 7.277 7.477
   5310   1382811_at   6.262 5.957 5.774 5.853 5.599 7.051 7.093 6.946 6.68 6.605
   5311   1377076_at   7.753 7.805 7.793 8.016 7.692 8.168 8.44 8.456 8.376 8.244
   5312   1377760_at   7.783 7.551 7.745 7.635 7.806 6.856 7.259 6.981 6.827 6.583
   5313   1371083_at   2.011 1.214 1.975 0.856 0 3.717 4.413 5.45 3.957 4.509
   5314   1373840_at   8.185 7.876 7.879 8.292 8.286 7.377 7.343 7.431 6.967 7.169
   5315   1371513_at   8.229 8.088 7.934 8.191 8.201 8.755 9.095 9.014 8.745 8.599
   5316   1373420_at   7.902 7.996 8.156 8.25 7.928 8.61 8.503 8.795 8.631 8.527
   5317   1384350_at   7.029 6.819 6.779 6.669 6.541 5.229 4.111 3.567 5.235 3.979
   5318   1394041_at   7.035 6.963 6.77 6.74 6.776 7.383 7.308 7.212 7.305 7.27
   5319   1376057_at   7.321 7.19 7.099 7.021 7.437 7.848 7.736 7.77 7.71 7.978
   5320   1372720_at   9.082 9.218 9.004 9.184 9.277 9.788 9.836 9.993 9.543 9.653
   5321   1378399_at   6.679 6.82 7.031 7.108 6.95 5.77 5.785 6.337 5.81 6.187
   5322   1388104_at   6.787 6.717 6.422 6.843 6.892 7.653 7.845 7.597 7.582 7.243
   5323   1398402_at   4.236 5.084 4.587 4.586 4.401 3.694 3.023 3.014 3.426 3.346
   5324   1382135_at   8.63 8.609 8.563 8.64 8.482 8.186 8.233 7.953 7.862 7.862
   5325   1373033_at   9.816 9.891 10.127 10.069 9.945 9.623 9.55 9.538 9.492 9.409
   5326   1382219_at   6.846 7.06 7.156 7.299 6.932 7.873 7.825 7.967 7.507 7.853
   5327   1379363_at   4.088 3.545 3.724 4.128 4.051 4.898 6.433 5.75 5.644 5.856
   5328   1372882_at   7.664 7.565 7.81 7.536 7.766 8.39 8.216 8.181 8.05 8.118
   5329   1368405_at   7.989 8.022 8.067 8.363 8.436 7.485 7.353 7.653 7.331 7.377
   5330   1379405_at   6.043 6.066 6.321 6.263 6.277 7.339 7.487 7.056 6.91 6.818
   5331   1382493_at   7.29 6.982 6.841 6.95 7.168 3.432 5.124 3.628 4.833 5.225
   5332   1372839_at   8.24 8.292 8.409 8.371 8.431 8.869 9.056 8.766 8.735 8.719
   5333   1372542_at   9.286 9.219 8.825 8.899 9.379 7.988 8.415 7.887 7.784 8.231
   5334   1387206_at   8.45 8.521 8.362 8.649 8.631 7.767 8.043 8.048 8.013 8.096
   5335   1374523_at   8.631 8.653 8.555 8.496 8.655 7.943 8.23 8.213 8.173 8.262
   5336   1379488_at   6.234 6.141 6.112 6.121 6.479 5.754 5.635 5.578 5.752 5.476
   5337   1368652_at   7.508 7.428 7.347 7.636 7.594 7.094 7.079 7.159 6.976 6.911
   5338   1381808_at   4.576 3.672 4.163 4.672 4.192 5.807 5.963 5.381 5.419 5.936
   5339   1378016_at   6.829 6.949 6.959 7.094 7.317 6.254 6.209 6.487 6.465 6.149
   5340   1377524_at   8.327 8.304 8.254 8.166 8.305 9.005 9.05 8.842 8.786 8.587
   5341   1394599_at   5.254 5.552 5.142 5.017 5.198 4.583 4.277 4.452 4.578 4.629
   5342   1386720_at   6.603 6.772 7.084 7.141 5.998 8.511 8.485 8.603 8.277 7.861
   5343   1384838_at   8.95 8.77 9.103 8.967 8.776 10.532 10.516 9.518 10.583 10.374
   5344   1372172_at   6.34 6.743 6.565 6.322 6.332 7.12 7.39 7.079 7.16 7.031
   5345   1380923_at   3.811 3.69 4.222 3.617 4.52 6.185 6 6.141 5.079 5.528
   5346   1389023_at   9.812 9.833 10.11 9.989 10.027 10.44 10.789 10.583 10.393 10.544
   5347   1373872_at   7.096 6.696 6.881 6.747 6.723 7.634 7.85 8.134 7.46 7.633
   5348   1373631_at   7.426 7.372 7.483 7.463 7.427 8.49 8.507 9.19 8.327 8.244
   5349   1383319_at   6.713 6.316 6.659 7.056 6.214 7.892 7.999 8.529 7.924 7.624
   5350   1384183_at   4.024 4.13 2.975 3.675 3.365 5.263 5.027 5.273 4.989 5.003
   5351   1371798_at   10.313 10.32 10.163 10.207 10.325 10.714 10.825 10.537 10.63 10.609
   5352   1383709_at   7.161 7.294 7.214 7.519 7.329 4.126 2.558 5.159 4.912 4.45
   5353   1389964_at   10.81 10.803 10.949 10.723 10.844 11.341 11.419 11.492 11.279 11.105
   5354   1393830_at   7.002 7.34 7.759 7.637 6.863 5.444 5.041 3.387 4.777 3.751
   5355   1370690_at   9.634 9.673 9.744 9.858 9.649 10.305 10.249 9.987 10.258 10.119
   5356   1382039_at   6.662 6.661 6.561 6.775 6.763 7.008 7.006 7.088 7.248 7.223
   5357   1371642_at   11.284 11.214 11.396 11.323 11.324 11.606 11.582 11.477 11.643 11.577
   5358   1377209_at   7.683 7.745 8.176 7.98 7.632 8.766 9.063 8.456 8.882 9.036
   5359   1382330_at   7.121 7.234 7.038 7.29 7.496 7.999 7.975 8.049 7.712 7.802
   5360   1369411_at   4.621 5.707 4.643 5.306 4.796 1.367 0.669 1.17 1.58 3.385
   5361   1390727_at   6.532 6.313 6.697 7.013 6.838 7.894 7.805 8.3 7.574 7.645
   5362   1368897_at   5.595 5.365 5.97 5.908 5.631 7.102 6.795 6.612 6.495 6.583
   5363   1379495_at   6.559 6.586 7.086 6.992 6.876 7.746 7.956 7.575 7.589 7.559
   5364   1379573_at   4.741 5.385 5.04 4.526 4.713 6.053 6.319 6.046 5.898 5.822
   5365   1373223_at   5.942 6.06 5.688 5.828 6.381 5.185 4.961 5.267 5.144 5.006
   5366   1389621_at   7.642 7.541 7.498 7.399 7.62 6.458 6.698 6.949 6.505 7.027
   5367   1375898_at   9.122 8.931 9.188 8.969 8.962 9.618 9.604 9.475 9.461 9.354
   5368   1378032_at   8.227 8.158 7.601 7.821 8.532 6.086 6.145 7.122 5.918 6.012
   5369   1388953_at   8.859 8.869 8.751 8.898 8.925 8.522 8.305 8.175 8.508 8.415
   5370   1397548_at   7.162 6.894 6.745 6.986 6.843 4.789 5.278 5.858 5.41 5.948
   5371   1379945_at   5.439 5.341 5.317 5.229 5.595 3.978 4.512 3.44 4.4 3.985
   5372   1387974_a_at   9.395 9.369 9.752 9.437 8.306 10.737 10.969 10.929 11.027 10.911
   5373   1390119_at   6.319 5.984 5.147 5.439 5.751 7.475 7.474 7.658 6.999 7.052
   5374   1387044_at   1.22 3.719 2.398 1.521 2.011 5.019 5.639 6.877 5.415 5.653
   5375   1390998_at   6.339 5.929 6.106 6.422 6.173 6.898 7.203 6.994 6.925 6.744
   5376   1374784_at   7.14 7.181 6.819 6.941 7.936 5.84 5.828 5.459 5.812 5.998
   5377   1369306_at   6.207 6.358 6.584 6.189 5.798 3.353 4.106 3.907 3.925 1.922
   5378   1396512_at   4.369 5.024 4.685 4.426 4.619 5.676 5.586 5.969 5.811 5.326
   5379   1372607_at   10.02 10.045 9.979 10.141 10.183 9.625 9.818 9.743 9.602 9.563
   5380   1388643_at   7.908 7.981 7.737 7.788 7.791 8.673 8.93 8.297 8.573 8.491
   5381   1398938_at   9.749 9.771 10.044 9.776 9.728 10.306 10.375 10.195 10.285 10.179
   5382   1373049_at   8.87 9.039 9.114 9.276 9.165 8.456 8.697 8.288 8.29 8.374
   5383   1372287_at   8.191 8.438 8.582 8.533 8.563 9.179 9.424 8.983 9.055 9.117
   5384   1372100_at   9.715 9.914 9.777 9.871 10.021 9.455 9.493 9.549 9.49 9.436
   5385   1374060_at   6.723 6.775 6.189 6.686 6.756 7.327 7.677 7.91 7.548 7.514
   5386   1393336_at   8.802 8.778 8.877 9.061 8.842 8.133 8.311 7.954 8.445 8.283
   5387   1375067_at   6.511 6.876 6.53 6.724 6.494 6.03 6.181 5.788 5.877 5.925
   5388   1379673_at   6.892 6.513 6.479 6.831 7.079 7.575 7.524 7.517 7.538 7.476
   5389   1382500_at   7.929 7.877 7.934 7.891 7.689 8.613 8.592 8.191 8.424 8.629
   5390   1398762_at   12.267 12.138 12.263 12.007 12.156 11.794 11.757 11.607 11.802 11.827
   5391   1369065_a_at   8.139 8.258 8.215 8.27 8.112 8.76 9.041 9.144 8.594 9.071
   5392   1376705_a_at   7.785 7.722 7.94 8.064 7.964 8.683 8.767 8.557 8.576 8.283
   5393   1379517_at   0 2.011 1.384 3.055 1.88 4.758 5.223 5.494 5.328 4.724
   5394   1392636_at   5.646 5.309 5.801 5.631 5.807 6.228 6.215 6.409 6.353 6.238
   5395   1385578_at   4.146 4.279 4.288 4.503 4.379 5.624 5.793 5.206 4.935 5.633
   5396   1374022_at   7.018 7.173 7.572 7.569 6.486 8.665 8.423 8.612 8.625 9.103
   5397   1385614_at   6.216 6.205 6.711 6.62 6.338 8.428 7.992 7.24 8.131 7.747
   5398   1396202_at   6.002 6.081 5.741 5.981 5.817 6.76 6.811 6.548 6.803 6.345
   5399   1382124_at   7.261 7.252 7.358 7.16 7.042 8.083 7.883 8.066 7.986 7.577
   5400   1391514_at   4.027 3.839 3.141 4.049 3.797 1.989 2.046 2.696 2.16 2.625
   5401   1397729_x_at   6.886 6.785 7.364 7.289 7.107 8.146 7.952 8.56 7.99 8.006
   5402   1373722_at   7.595 7.531 7.508 7.661 7.224 4.559 4.407 3.123 4.978 5.811
   5403   1376640_at   8.938 8.954 8.998 9.261 9.451 9.881 10.111 10.212 9.855 9.8
   5404   1398794_at   10.564 10.593 10.478 10.622 10.625 10.312 10.307 10.35 10.231 10.134
   5405   1390403_at   7.605 7.298 7.695 7.712 7.441 8.949 8.767 9.373 8.507 8.405
   5406   1376248_at   6.106 5.833 5.521 5.655 4.262 7.807 7.629 7.555 7.553 7.489
   5407   1384774_at   6.496 6.626 6.949 6.912 5.58 8.443 8.437 8.7 8.015 8.11
   5408   1369361_at   3.874 3.851 3.79 3.916 3.432 5.058 4.772 4.432 4.534 4.964
   5409   1391282_at   7.787 7.899 7.71 7.841 7.748 8.437 8.584 8.683 8.209 8.261
   5410   1382764_at   0.774 0.454 1.043 0 1.007 2.144 2.609 1.915 2.742 2.993
   5411   1395513_at   5.961 6.102 6.423 6.414 6.153 5.683 5.463 5.54 5.365 5.305
   5412   1394590_at   5.918 6.296 6.02 6.133 6.041 6.752 6.791 6.517 6.672 6.51
   5413   1399118_at   7.328 7.125 7.164 7.383 7.185 5.9 6.365 5.85 6.639 5.841
   5414   1368928_at   6.222 6.468 6.557 6.662 6.316 7.275 7.243 7.11 6.942 7.014
   5415   1369642_at   8.684 8.705 8.632 8.706 8.733 8.341 8.381 8.145 8.411 8.423
   5416   1393626_at   6.736 6.411 6.901 7.083 6.572 7.615 8.052 8.157 7.811 7.556
   5417   1382052_at   7.08 6.993 6.943 7.367 7.282 6.514 6.375 6.337 6.482 6.025
   5418   1377668_at   10.086 10.149 10.133 10.125 10.112 9.805 9.776 9.472 9.76 9.757
   5419   1377020_at   7.705 7.796 7.922 7.568 7.848 7.253 7.136 6.967 7.369 7.103
   5420   1398344_at   7.938 7.897 7.938 7.798 7.957 7.456 7.241 7.314 7.645 7.29
   5421   1372125_at   7.151 7.298 7.257 7.249 7.931 6.585 6.314 6.19 6.282 6.407
   5422   1382844_at   4.554 4.963 4.745 4.745 4.589 5.342 5.452 5.621 5.172 5.389
   5423   1373285_at   7.28 7.317 7.357 7.781 7.69 6.65 6.618 6.91 6.63 6.759
   5424   1377726_at   9.401 9.144 9.156 9.438 9.698 8.023 8.392 8.742 8.317 8.318
   5425   1367479_at   10.81 10.777 10.92 10.911 10.889 11.246 11.074 11.195 11.208 11.077
   5426   1387636_a_at   9.802 9.848 9.998 10.053 10.177 9.416 9.535 9.444 9.506 9.269
   5427   1373883_at   6.896 7.127 6.953 7.139 7.324 7.821 7.91 8.151 7.619 7.759
   5428   1384555_at   7.029 7.081 7.155 6.803 6.656 4.159 3.623 4.772 2.395 1.891
   5429   1377949_s_at   6.715 6.922 7.102 7.126 7.123 7.622 7.499 7.533 7.557 7.506
   5430   1372467_at   8.576 8.417 8.611 8.584 8.391 9.224 9.198 9.406 8.98 8.943
   5431   1371614_at   8.489 8.497 8.589 8.648 8.487 8.885 8.736 8.84 8.829 8.779
   5432   1375729_at   5.101 4.794 5.636 5.377 5.1 6.958 6.873 7.096 6.556 6.08
   5433   1393982_at   6.362 6.608 6.883 6.876 6.548 4.521 4.231 4.398 2.214 3.405
   5434   1391556_at   5.22 4.876 5.254 5.233 5.864 6.393 6.544 6.338 6.764 6.314
   5435   1370923_at   7.006 7.211 7.1 7.093 7.129 6.271 6.337 6.143 6.775 6.371
   5436   1374384_at   2.945 3.374 2.868 3.43 1.057 6.102 5.672 5.299 5.597 5.909
   5437   1372098_at   7.61 7.598 7.617 7.635 7.53 8.002 8.242 8.171 8.087 7.842
   5438   1383626_at   6.945 6.641 7.131 6.955 7.362 5.81 6.371 5.916 5.741 5.797
   5439   1393919_at   6.07 6.231 5.914 6.318 5.924 5.456 4.751 5.176 5.273 4.929
   5440   1389370_at   5.065 6.038 5.691 6.01 5.969 7.449 6.85 7.014 7.134 7.125
   5441   1377042_at   8.443 8.345 8.76 8.756 8.59 7.997 7.958 8.049 7.832 7.722
   5442   1380250_at   6.236 6.402 6.163 6.051 6.442 7.307 6.737 7.301 7.126 7.02
   5443   1372754_at   6.821 6.181 6.896 6.56 6.655 7.658 7.534 7.417 7.756 7.407
   5444   1395349_at   6.598 6.564 6.626 6.231 6.349 5.637 5.157 5.736 5.623 5.076
   5445   1385099_at   5.791 5.698 6.111 6.29 6.081 4.941 3.38 4.307 4.269 3.83
   5446   1373751_at   5.771 5.99 6.227 6.002 5.716 3.548 4.628 4.176 4.933 4.06
   5447   1398262_at   8.727 8.637 8.73 8.71 8.641 9.471 9.699 9.749 9.292 9.133
   5448   1367603_at   12.144 12.082 12.09 12.036 12.138 11.869 11.92 11.886 11.879 11.741
   5449   1385773_at   5.767 5.679 6.344 5.939 6.051 7.267 7.414 7.264 7.332 6.603
   5450   1371115_at   6.278 5.785 6.125 6.358 6.181 4.886 3.227 4.123 2.986 3.971
   5451   1369634_at   5.273 6.003 6.086 6.028 5.624 7.772 7.692 8.045 7.502 6.754
   5452   1388590_at   8.47 8.37 8.976 8.87 8.743 7.919 7.804 7.904 7.485 7.586
   5453   1388605_at   3.366 3.925 3.522 4 2.478 5.823 6.106 5.999 4.893 5.831
   5454   1370864_at   8.231 8.328 8.06 8.424 9.409 6.783 6.522 7.023 6.513 7.022
   5455   1376710_at   4.233 3.57 4.491 4.505 4.093 5.855 5.994 5.306 6.098 5.363
   5456   1370004_at   9.215 9.134 8.875 9.374 9.436 8.285 8.388 8.613 8.041 8.16
   5457   1374131_at   5.67 5.836 5.734 6.172 5.922 6.66 6.882 6.356 6.725 6.743
   5458   1382689_at   9.109 8.953 9.142 9.074 9.149 9.425 9.531 9.463 9.343 9.629
   5459   1373778_at   5.19 5.76 4.223 4.924 5.022 6.86 7.079 7.63 6.489 7.236
   5460   1368826_at   11.714 11.692 11.807 11.583 11.408 12.495 12.53 12.031 12.556 12.505
   5461   1368871_at   8.208 8.158 8.281 8.54 8.618 6.99 7.403 7.659 7.101 6.784
   5462   1391107_at   6.046 6.066 6.288 5.899 6.49 5.343 5.279 5.435 5.074 4.851
   5463   1373615_at   7.637 7.622 7.703 7.499 8.05 8.913 8.715 8.229 8.792 8.742
   5464   1398621_at   4.763 4.391 5.081 4.697 4.235 3.642 3.482 3.036 3.583 3.474
   5465   1382381_at   7.757 7.845 7.931 7.964 7.532 8.541 8.787 8.35 8.454 8.421
   5466   1373091_at   6.621 7.008 7.275 7.368 7.261 5.728 5.724 5.884 6.237 5.282
   5467   1371131_a_at   10.977 10.801 10.738 10.962 11.004 11.601 11.282 11.748 11.418 11.545
   5468   1383893_at   9.703 9.573 9.845 9.837 9.613 10.602 10.578 11.11 10.485 10.358
   5469   1371535_at   8.686 8.535 8.618 8.946 8.833 8.091 8.038 8.199 8.31 8.086
   5470   1391478_at   6.025 5.817 5.876 6.125 5.737 6.749 6.664 6.729 6.299 6.725
   5471   1373634_at   8.755 8.831 8.523 8.781 8.855 9.396 9.896 9.5 9.28 9.58
   5472   1367940_at   7.679 7.823 7.999 8.17 8.081 9.051 8.958 8.957 8.454 8.814
   5473   1396215_at   5.632 5.629 5.579 5.734 5.045 6.543 6.393 6.439 6.598 6.204
   5474   1371981_at   8.355 8.35 8.251 8.337 8.354 8.141 7.935 8.117 7.965 8.015
   5475   1390137_at   7.044 7.174 6.845 7.174 7.327 1.239 4.2 4.124 2.996 1.151
   5476   1389491_at   6.115 6.507 6.318 6.557 6.609 7.031 7.315 7.289 7.109 7.001
   5477   1388828_at   9.474 9.638 9.61 9.706 9.704 9.086 9.299 9.236 9.022 9.262
   5478   1371868_at   9.222 9.202 9.208 9.363 9.434 8.753 8.907 8.416 8.515 8.518
   5479   1393248_at   8.582 8.565 8.283 8.537 8.663 7.604 8.114 7.645 7.626 7.453
   5480   1375635_at   9.496 9.633 9.522 9.583 9.616 8.695 8.891 9.218 8.609 8.908
   5481   1392795_at   3.394 4.194 3.977 3.688 3.359 5.193 4.802 4.963 4.855 4.718
   5482   1376213_at   4.643 3.898 4.774 4.397 4.069 5.551 5.437 5.308 5.676 5.659
   5483   1369025_at   8.115 8.092 8.392 8.143 7.806 4.65 3.777 4.626 4.307 1.063
   5484   1392038_at   6.604 6.572 6.322 6.552 6.24 5.516 5.813 5.767 5.682 5.997
   5485   1374061_at   9.448 9.509 9.521 9.332 9.637 9.05 9.215 9.017 8.987 8.929
   5486   1371531_at   8.97 8.855 9.139 9.333 9.205 9.704 9.921 9.796 9.686 9.624
   5487   1367491_at   10.157 10.185 10.191 10.143 10.063 9.743 9.924 9.626 9.733 9.558
   5488   1390404_at   6.207 6.221 6.105 6.346 6.949 7.476 7.558 7.252 7.319 7.437
   5489   1375173_at   9.308 9.328 9.262 9.505 9.492 9.694 9.864 9.85 9.735 9.728
   5490   1397769_at   5.735 5.165 5.634 6.314 6.485 3.96 2.711 2.465 4.056 3.07
   5491   1373291_at   6.47 6.073 6.431 6.735 6.463 7.648 7.565 7.703 7.043 7.387
   5492   1371137_at   4.752 4.83 4.883 5.527 4.886 5.969 6.064 6.503 6.017 5.956
   5493   1388897_at   8.145 8.36 8.469 8.579 8.337 7.897 7.786 7.865 7.839 7.601
   5494   1383292_at   6.28 6.353 6.458 6.654 6.536 5.901 5.804 6.043 6.012 5.787
   5495   1379557_at   6.527 6.747 6.846 6.986 6.955 3.714 3.355 4.918 1.971 4.105
   5496   1373077_at   8.35 8.418 8.437 8.553 8.664 8.13 8.086 8.153 8.12 8.08
   5497   1394743_at   6.618 6.516 6.154 6.897 6.218 5.052 5.081 4.184 5.385 4.659
   5498   1374650_at   8.285 8.294 8.578 8.471 8.173 9.141 9.288 9.104 8.813 8.97
   5499   1388960_at   10.073 10.052 9.963 10.113 10.179 9.22 9.303 9.691 9.146 8.969
   5500   1383289_at   7.664 7.865 7.832 7.572 7.695 7.309 7.384 7.074 7.158 7.181
   5501   1375272_at   8.72 8.973 8.858 8.83 8.692 8.472 8.389 8.46 8.34 8.23
   5502   1372261_at   11.44 11.346 11.469 11.156 10.783 12.056 12.094 12.025 12.04 12.184
   5503   1381776_at   6.674 7.009 6.35 7.127 7.394 8.034 8.017 8.504 8.347 8.28
   5504   1373257_at   4.625 5.276 5.333 5.189 4.952 1.433 0 3.181 1.459 0.111
   5505   1374188_at   9.545 9.539 9.611 9.771 9.753 10.127 10.246 10.426 10.144 10.021
   5506   1378079_at   6.629 6.641 6.498 6.687 6.4 5.718 6.11 5.433 5.811 5.846
   5507   1397836_at   4.508 5.001 5.054 5.422 4.844 5.899 5.919 6.159 5.901 6.142
   5508   1380975_at   7.551 7.198 7.417 7.671 7.506 8.77 9.168 8.161 8.516 8.958
   5509   1374186_at   7.189 7.265 7.156 7.184 7.482 6.497 6.913 6.679 6.604 6.64
   5510   1368084_at   10.605 10.642 10.879 10.156 9.228 12.239 12.269 12.408 12.101 12.14
   5511   1387004_at   6.394 6.672 6.778 6.713 6.775 7.662 7.507 8.097 7.48 7.354
   5512   1370853_at   5.875 5.86 6.279 6.532 5.888 7.191 7.439 6.857 7.211 7.096
   5513   1384616_at   3.294 2.609 2.722 2.854 2.508 3.722 4.736 4.625 4.355 5.055
   5514   1387987_at   8.271 8.12 8.904 8.642 7.651 10.18 9.988 10.675 9.694 9.892
   5515   1373768_at   7.499 7.462 7.297 7.706 7.421 6.922 6.734 6.969 6.785 6.487
   5516   1395442_at   5.653 6.142 5.749 6.056 5.961 4.437 4.983 4.067 3.524 4.289
   5517   1398830_at   12.487 12.396 12.31 12.319 12.399 12.08 11.928 12.163 12.03 11.934
   5518   1391669_at   5.947 6.229 6.156 6.066 6.038 7.088 7.84 7.17 7.055 6.875
   5519   1376667_at   0.31 2.157 0.595 0.176 2.356 5.161 4.169 4.278 5.656 4.297
   5520   1371836_at   9.178 9.242 9.644 9.508 9.251 8.691 8.52 8.775 8.277 8.556
   5521   1375663_at   9.288 9.21 9.281 9.315 9.255 9.018 9.054 8.889 8.808 8.998
   5522   1368440_at   12.61 12.543 12.631 12.461 12.135 13.183 13.025 13.045 13.271 13.3
   5523   1386962_at   6.314 6.467 6.384 6.36 6.123 7.046 6.993 7.112 6.782 6.738
   5524   1383660_at   6.414 6.331 6.616 6.588 6.58 6.104 6.069 5.905 5.927 5.737
   5525   1390118_at   7.296 7.205 7.37 7.534 7.398 6.6 6.131 6.496 6.837 6.211
   5526   1377963_at   4.098 4.682 4.489 4.255 4.67 5.19 5.588 5.444 5.351 5.155
   5527   1388059_a_at   7.976 7.93 7.897 8.068 7.971 7.591 7.342 7.494 7.71 7.414
   5528   1387667_at   7.823 7.781 8.249 8.104 7.556 0 2.807 3.587 0 4.162
   5529   1395373_at   7.132 7.424 7.193 7.204 6.62 8.339 8.624 8.265 8.432 7.856
   5530   1367551_a_at   9.203 9.304 9.448 9.266 9.521 8.752 8.988 8.737 8.688 8.53
   5531   1391570_at   6.151 6.268 6.064 6.416 6.378 7.13 6.902 6.654 7.013 7.106
   5532   1387339_at   5.441 5.414 6.031 6.149 5.46 7.57 7.347 6.555 7.33 7.398
   5533   1371687_at   10.164 10.129 10.08 10.083 10.062 9.936 9.968 9.897 9.923 9.829
   5534   1383240_at   9.203 9.268 9.361 9.398 9.185 9.926 10.325 9.771 9.87 9.895
   5535   1390924_at   6.685 6.61 6.719 6.672 6.808 5.892 6.333 5.841 6.204 6.06
   5536   1388423_at   8.512 8.436 8.373 8.584 8.4 8.937 9.008 9.236 9.108 8.783
   5537   1372894_at   8.409 8.475 8.462 8.615 8.519 8.743 8.714 8.743 8.769 8.848
   5538   1370450_at   5.161 5.381 5.627 5.101 5.611 4.087 4.403 3.906 3.149 3.654
   5539   1372190_at   7.483 7.522 7.421 7.339 7.72 8.508 8.541 8.998 8.06 8.668
   5540   1376918_at   4.891 4.752 4.929 5.254 5.041 5.692 5.979 6.111 5.679 6.354
   5541   1390456_at   5.565 5.219 4.716 4.836 5.442 6.332 6.762 6.248 6.365 6.242
   5542   1388864_at   8.034 7.924 7.627 8.118 8.108 7.228 7.427 7.318 7.047 7.089
   5543   1379057_at   5.313 5.676 5.506 6.06 5.653 6.972 7.22 7.829 6.659 6.977
   5544   1383642_at   5.189 5.018 5.474 5.45 5.381 6.099 6.233 5.943 6.078 5.807
   5545   1373353_at   6.561 6.551 6.468 6.576 6.658 5.566 5.605 5.997 6.109 5.534
   5546   1390000_at   6.793 6.54 6.974 6.83 6.915 8.019 7.61 7.583 7.487 7.441
   5547   1387201_at   7.235 7.222 7.513 7.454 7.532 6.839 6.87 6.373 6.521 6.51
   5548   1373857_at   6.403 6.465 6.303 6.952 6.307 7.168 7.277 7.358 7.31 7.345
   5549   1379336_at   7.072 7.143 7.092 7.408 7.126 8.024 8.16 8.564 7.73 8.114
   5550   1382462_at   5.844 5.91 6.032 6.545 5.548 7.077 7.721 7.473 7.076 7.181
   5551   1371964_at   9.793 9.707 9.797 9.891 9.807 10.21 10.36 10.11 10.069 10.139
   5552   1398950_at   8.641 8.931 9.09 9.144 8.76 9.607 9.617 9.667 9.555 9.439
   5553   1376169_at   7.061 7.017 7.006 7.094 7.11 7.702 8.086 8.09 7.502 7.71
   5554   1368026_at   7.706 7.834 8.015 7.897 7.826 7.233 7.313 7.184 6.935 6.715
   5555   1389549_at   9.679 9.714 9.886 9.833 9.598 10.289 10.409 10.042 10.411 10.299
   5556   1397674_at   9.069 9.076 9.258 9.304 9.368 8.706 8.134 8.085 8.508 8.085
   5557   1395533_at   7.986 8.07 8.016 8.206 8.352 7.64 7.405 7.177 7.459 7.093
   5558   1373140_at   9.615 9.753 9.154 9.448 9.817 8.526 8.844 8.758 8.595 8.777
   5559   1397737_at   4.8 5.588 5.048 5.243 5.249 4.222 4.093 3.632 4.299 3.756
   5560   1397515_at   4.832 4.834 5.062 4.98 5.415 4.271 4.154 4.248 4.41 4.023
   5561   1382027_at   5.504 6.146 6.694 6.5 6.456 4.306 4.279 4.503 4.79 5.088
   5562   1387127_at   6.748 6.716 6.627 6.885 6.783 7.304 7.71 7.453 7.239 7.783
   5563   1367808_at   10.407 10.381 10.636 10.68 10.598 11.045 11.217 11.158 11.058 10.89
   5564   1398854_at   11.905 11.892 11.979 11.715 11.84 11.431 11.483 11.142 11.458 11.385
   5565   1370248_at   8.416 8.363 8.537 8.491 8.572 9.462 9.577 10.306 9.589 9.26
   5566   1379441_at   8.508 8.483 8.393 8.424 8.337 8.667 8.891 8.839 8.711 8.721
   5567   1370711_a_at   5.878 5.574 6.027 6.119 5.469 4.572 4.746 4.065 4.914 4.199
   5568   1370330_at   6.785 6.672 6.865 6.621 6.671 7.052 7.458 7.217 7.343 7.48
   5569   1380370_at   4.302 4.218 3.831 4.493 3.617 5.226 5.286 5.563 5.759 5.089
   5570   1369568_at   6.442 6.662 6.317 6.898 6.821 5.834 5.839 5.659 6.024 5.758
   5571   1375231_a_at   8.259 8.366 8.219 8.372 8.272 9.645 9.684 9.85 9.676 10.993
   5572   1398743_at   1.84 0.454 1.189 1.411 0.632 3.38 3.965 3.83 3.913 2.491
   5573   1392013_at   4.566 5.112 4.507 4.373 4.906 6.037 6.852 5.824 6.358 5.888
   5574   1389333_at   8.66 8.809 8.868 8.948 8.938 9.264 9.385 9.267 9.199 9.172
   5575   1387294_at   8.708 8.694 8.551 8.542 8.756 9.344 9.51 9.124 9.158 9.653
   5576   1383077_at   7.863 8.11 7.876 7.877 7.63 8.621 8.355 8.42 8.511 8.366
   5577   1367847_at   7.525 7.693 7.966 7.842 7.776 8.914 9.265 9.47 8.558 8.661
   5578   1384821_at   7.294 7.206 7.107 7.335 7.322 6.934 6.666 6.9 6.798 6.89
   5579   1373761_at   7.457 7.39 7.354 7.489 7.533 6.269 6.591 6.599 6.644 7.041
   5580   1398281_at   7.533 7.68 7.797 7.569 7.785 8.609 8.801 8.766 8.615 8.079
   5581   1397637_at   4.179 4.505 4.044 3.911 4.133 5.05 5.695 5.092 5.075 5.028
   5582   1377782_at   5.191 5.452 5.502 5.719 5.326 4.857 4.715 4.405 4.313 4.308
   5583   1389350_at   5.194 5.409 5.7 5.656 4.379 6.858 6.98 7.207 6.922 6.629
   5584   1390213_at   6.843 6.617 6.882 7.092 6.942 7.487 7.354 7.346 7.48 7.374
   5585   1368078_at   7.304 7.325 7.709 7.563 7.199 8.624 9.1 8.516 8.474 8.15
   5586   1373868_at   7.322 7.262 7.271 7.238 7.322 6.845 6.588 6.419 6.311 6.079
   5587   1384254_at   8.409 8.286 8.443 8.324 8.144 7.601 7.866 7.976 7.658 7.673
   5588   1385765_at   5.974 6.294 5.794 6.253 6.187 5.23 4.739 4.753 5.206 5.379
   5589   1373309_at   7.573 7.561 7.657 7.709 7.289 8.736 8.968 8.059 8.827 8.807
   5590   1376827_at   6.905 6.727 6.926 6.848 7.233 6.373 6.292 6.217 6.226 5.928
   5591   1377252_at   7.06 6.806 6.832 6.863 7.176 6.561 6.243 6.1 6.199 6.225
   5592   1368324_at   6.328 6.536 6.368 6.357 6.654 1.714 4.725 3.593 2.157 2.807
   5593   1371663_at   8.177 8.124 8.5 8.658 8.511 7.68 7.79 7.59 7.543 7.374
   5594   1390441_at   6.362 6.303 6.621 6.676 6.45 5.501 5.92 5.565 5.963 5.578
   5595   1373985_at   3.963 4.854 3.892 4.403 4.87 5.875 5.893 5.902 5.793 5.547
   5596   1377422_at   3.777 3.242 3.922 4.362 4.774 6.234 6.152 6.05 5.69 5.48
   5597   1380133_at   4.34 4.527 4.351 4.18 4.374 5.569 5.511 4.786 5.516 5.347
   5598   1388439_at   6.689 6.429 6.306 6.458 7.352 5.572 5.436 5.174 5.149 5.399
   5599   1367711_at   11.013 11.064 11.049 11.186 11.177 10.631 10.695 10.847 10.666 10.487
   5600   1398537_at   8.432 8.471 8.364 8.583 8.421 9.291 9.305 8.841 9.038 8.957
   5601   1387799_at   11.999 11.941 12.001 11.789 11.723 12.341 12.278 12.339 12.559 12.552
   5602   1376062_at   6.06 5.57 5.754 5.272 5.895 4.66 4.629 4.963 4.818 4.732
   5603   1367894_at   7.623 7.669 7.973 7.89 7.917 8.66 8.835 9.017 8.434 8.425
   5604   1388417_at   10.538 10.526 10.338 10.409 10.398 10.735 10.803 10.892 10.7 10.732
   5605   1372985_at   6.972 6.893 6.759 6.903 6.634 7.614 7.522 7.939 7.688 7.289
   5606   1384016_at   7.591 7.787 7.797 8.028 7.979 7.037 7.051 7.404 7.11 7.249
   5607   1398450_at   7.79 7.892 7.734 7.892 7.653 8.454 8.529 8.527 8.076 8.498
   5608   1372868_at   6.806 7.261 7.263 7.427 7.334 4.835 5.688 6.074 5.943 5.075
   5609   1388939_at   7.921 7.949 7.662 8.32 8.204 6.793 7.124 6.91 6.914 7.377
   5610   1374251_at   9.994 10.17 10.338 10.428 9.58 11.425 11.56 10.887 11.307 11.321
   5611   1371949_at   10.116 10.201 10.063 10.125 10.291 9.767 9.746 9.37 9.628 9.442
   5612   1371622_at   8.536 8.521 8.599 8.585 8.613 8.259 8.373 8.295 8.124 8.117
   5613   1394216_at   5.338 5.938 5.64 4.984 5.807 6.996 7.116 6.409 7.189 7.053
   5614   1382818_at   7.926 8.014 7.937 8.085 8.144 8.321 8.777 8.57 8.597 8.743
   5615   1392927_at   6.334 6.258 6.167 6.675 6.785 4.783 4.598 4.895 3.807 5.321
   5616   1377459_at   4.322 4.787 5.147 4.942 4.38 5.972 5.707 6.028 5.698 5.739
   5617   1387264_at   6.002 5.831 5.911 6.126 6.566 5.055 4.989 4.686 5.166 5.293
   5618   1380579_at   5.727 5.817 4.269 5.006 5.118 7.486 7.146 7.392 7.234 6.674
   5619   1380763_at   1.727 2.956 3.063 3.239 2.032 4.191 5.109 5.162 5.195 4.753
   5620   1372012_at   7.828 8.027 8.299 8.256 8.018 9.064 9.403 9.036 8.925 8.669
   5621   1384394_at   8.134 8.054 8.485 8.56 8.439 9.463 9.696 9.214 9.28 8.989
   5622   1367499_at   8.407 8.398 8.492 8.672 8.53 7.966 8.031 8.159 8.143 8.165
   5623   1391505_x_at   6.015 6.121 6.721 6.287 6.674 5.115 5.564 5.005 5.152 4.739
   5624   1387050_s_at   5.07 5.241 5.053 5.156 4.697 6.182 6.027 5.94 6.011 5.548
   5625   1393983_at   8.052 8.154 8.324 8.455 8.212 8.909 8.961 8.617 8.783 8.844
   5626   1393474_at   6.36 6.35 6.555 6.546 5.546 8.239 8.075 7.409 8.187 7.544
   5627   1368680_a_at   12.141 12.075 12.128 11.983 11.697 12.687 12.632 12.472 12.834 12.871
   5628   1372329_at   8.337 8.432 8.474 8.584 8.617 7.969 8.15 7.746 7.876 7.722
   5629   1387855_at   9.326 9.373 9.348 9.458 9.526 10.039 9.935 10.156 9.753 9.801
   5630   1375933_at   10.005 10.035 9.846 10.148 9.831 10.563 10.644 10.29 10.533 10.643
   5631   1394315_at   6.308 6.063 6.79 6.492 6.233 5.044 4.699 4.425 5.524 5.074
   5632   1372367_at   8.005 7.834 7.648 7.902 7.775 8.466 8.776 8.444 8.283 8.413
   5633   1369304_at   7.896 7.753 7.887 8.061 7.988 8.282 8.392 8.701 8.644 8.601
   5634   1382633_at   7.074 6.883 7.285 7.262 7.502 6.155 6.321 6.636 6.387 6.174
   5635   1389672_at   7.767 7.831 7.931 7.923 7.69 8.172 8.341 8.227 8.137 8.151
   5636   1375447_at   7.428 7.219 7.351 7.574 7.216 6.767 6.597 6.968 6.834 6.706
   5637   1373122_at   7.628 7.443 7.648 7.989 7.448 8.641 8.54 9.031 8.652 8.278
   5638   1370780_at   6.343 6.505 6.528 6.893 6.67 5.422 5.222 4.249 5.275 5.328
   5639   1387092_at   8.208 8.267 8.637 8.263 8.564 9.682 9.404 10.313 9.319 9.519
   5640   1374243_at   6.275 6.55 6.317 6.384 6.553 7.259 7.705 7.429 6.891 7.427
   5641   1393654_at   6.202 6.38 6.787 6.382 6.516 7.582 7.314 7.064 7.398 7.152
   5642   1382083_at   4.47 4.141 4.082 4.482 3.622 6.065 5.594 6.297 5.617 5.185
   5643   1382355_at   6.26 6.381 6.239 6.547 6.63 5.605 5.794 5.142 5.017 5.298
   5644   1390972_at   6.001 6.093 6.079 6.057 6.26 6.91 7.326 6.592 6.785 7.063
   5645   1398985_at   7.653 7.835 7.681 7.875 7.823 7.293 7.126 7.432 6.969 7.07
   5646   1390109_at   7.959 7.908 8.181 8.111 8.201 7.524 7.413 7.652 7.323 7.153
   5647   1382187_at   5.948 5.632 6.166 5.956 5.903 5.287 5.243 5.045 5.259 4.806
   5648   1376082_at   8.607 8.868 8.58 8.751 8.904 9.408 9.697 9.819 9.28 9.378
   5649   1387276_at   6.575 6.515 6.417 6.504 7.052 5.202 4.527 3.886 4.526 5.445
   5650   1392483_at   6.176 6.155 6.925 6.165 6.459 7.586 7.643 7.27 7.626 7.247
   5651   1376009_at   7.612 7.546 7.508 7.511 7.468 8.286 8.226 7.958 8.298 7.859
   5652   1376487_at   8.416 8.379 8.386 8.321 8.321 9.25 9.032 9.609 9.152 8.848
   5653   1391433_at   8.764 8.731 8.746 8.644 8.598 9.531 9.586 9.071 9.197 9.51
   5654   1382072_at   5.558 5.159 5.179 5.288 5.355 6.614 6.61 5.988 6.14 6.069
   5655   1398447_at   7.163 7.135 6.946 7.466 7.241 7.626 7.911 7.81 7.939 7.887
   5656   1385670_at   6.157 5.339 5.403 5.643 5.662 6.605 6.913 7.379 6.972 6.66
   5657   1380696_at   5.254 5.105 5.935 5.303 5.211 4.174 3.608 2.913 3.832 3.967
   5658   1387739_at   7.451 7.503 8.221 7.699 7.177 0 3.027 4.388 3.134 3.426
   5659   1397798_at   5.03 5.06 5.472 5.473 4.747 2.714 2.986 3.642 4.112 3.374
   5660   1385733_at   5.776 6.203 5.914 5.906 5.856 0 2.809 0 0.287 3.294
   5661   1388989_at   11.099 11.173 11.338 11.28 11.36 10.81 10.968 10.875 10.876 10.741
   5662   1381158_at   0 0.39 0.367 1.299 0.496 2.934 1.744 2.299 2.778 3.072
   5663   1368494_at   7.276 6.497 5.984 6.9 7.948 3.754 1.718 3.636 1.761 4.126
   5664   1383705_at   8.581 8.567 8.711 8.572 8.672 8.193 8.117 7.705 8.15 7.942
   5665   1391438_at   7.157 7.461 7.559 7.292 7.56 8.215 8.433 7.963 8.435 8.015
   5666   1379402_at   7.712 7.681 7.918 8.168 7.865 8.608 8.938 8.518 8.48 8.581
   5667   1375631_at   10.708 10.64 10.906 10.821 10.539 11.341 11.257 11.088 11.389 11.479
   5668   1385649_at   6.782 6.592 7.026 6.936 7.015 5.786 5.394 5.994 6.186 5.423
   5669   1385751_at   5.894 5.837 5.83 5.449 6.116 4.66 5.189 4.614 5.009 4.65
   5670   1379590_at   5.743 5.644 5.629 5.315 5.428 6.525 6.399 6.704 6.045 6.335
   5671   1383399_at   5.834 5.822 5.796 6.106 6.245 6.694 6.837 6.901 6.444 6.72
   5672   1376025_at   7.996 7.966 7.896 8.233 8.41 7.438 7.306 7.561 7.403 7.202
   5673   1392688_at   5.771 5.863 6.084 6.187 6.069 5.491 5.181 5.371 5.168 4.949
   5674   1369562_at   8.591 8.83 8.824 8.952 8.698 9.468 9.192 9.168 9.344 9.349
   5675   1371618_s_at   6.45 6.442 6.681 6.856 7.161 2.669 0.444 3.758 0 2.971
   5676   1374135_at   8.778 8.864 8.701 8.929 8.764 8.236 8.462 8.208 8.292 8.515
   5677   1377608_a_at   10.085 10.16 10.095 10.044 10.188 9.88 9.935 9.804 9.721 9.785
   5678   1389253_at   8.387 8.289 8.46 8.26 8.534 9.391 9.212 8.782 9.5 9.409
   5679   1373507_at   9.04 9.083 9.23 9.131 9.257 9.661 9.971 9.534 9.596 9.694
   5680   1395231_at   6.149 5.942 6.188 6.331 6.438 5.044 3.932 4.547 5.051 3.851
   5681   1372583_at   7.191 7.622 7.598 7.458 7.591 8.171 8.222 8.005 7.958 8.241
   5682   1376839_at   8.216 7.998 8.159 8.333 8.34 7.52 7.405 7.8 7.325 7.196
   5683   1387646_a_at   7.47 7.655 8.145 7.977 7.713 6.779 6.109 6.524 6.954 6.187
   5684   1379377_at   7.424 7.42 7.586 7.758 7.62 7.1 7.173 7.049 6.955 7.158
   5685   1368060_at   11.98 11.961 12.084 11.796 11.766 12.512 12.507 12.288 12.711 12.725
   5686   1390823_at   5.104 5.401 5.494 5.662 5.404 4.178 3.874 3.007 4.08 4.24
   5687   1380017_at   6.689 6.644 6.893 6.956 6.907 7.658 7.645 7.337 7.286 7.37
   5688   1385553_at   8.864 8.975 8.974 8.957 9.389 8.333 8.249 7.698 8.23 7.975
   5689   1374760_at   6.063 5.956 5.772 5.904 5.802 6.193 6.541 6.55 6.642 6.6
   5690   1376076_at   7.123 7.065 7.13 7.341 7.728 6.186 6.306 6.154 5.411 5.669
   5691   1393811_at   4.92 4.982 4.884 5.282 5.035 5.896 5.728 5.75 5.798 5.397
   5692   1367771_at   8.826 8.965 9.147 8.758 8.924 10.028 9.768 9.464 9.505 9.898
   5693   1367484_at   10.157 10.114 10.052 9.896 10.196 9.718 9.71 9.453 9.435 9.571
   5694   1388707_at   7.989 7.895 7.563 7.767 7.962 6.862 6.938 7.159 7.032 7.383
   5695   1378282_at   7.37 7.326 7.556 7.578 7.837 6.123 6.322 6.444 6.191 5.233
   5696   1387777_at   10.18 10.075 10.054 10.197 10.148 9.937 9.927 9.93 9.852 9.953
   5697   1384714_x_at   9.003 9.026 9.235 9.357 9.396 8.549 8.585 8.334 8.734 8.388
   5698   1392417_s_at   7.001 6.673 6.875 6.666 6.601 5.483 5.254 5.474 5.567 6.243
   5699   1393581_at   5.939 6.038 5.885 6.287 6.226 3.511 4.683 4.908 3.503 4.264
   5700   1381833_at   6.317 6.047 6.254 5.617 6.096 1.978 1.454 4.329 1.328 0.516
   5701   1386954_at   10.716 10.697 10.738 10.881 10.878 10.306 10.241 9.889 10.376 10.12
   5702   1396101_at   6.36 5.93 6.11 6.375 6.248 7.405 7.135 6.853 7.727 7.197
   5703   1367631_at   9.374 9.324 10.098 9.77 8.847 11.972 11.652 11.222 10.688 11.412
   5704   1397922_at   0 0 1.546 0 0 1.903 2.674 2.78 3.03 2.566
   5705   1379348_at   6.41 6.465 6.651 6.847 6.686 4.591 5.309 5.328 5.641 4.428
   5706   1367845_at   6.039 5.846 6.037 6.118 6.502 6.962 7.334 7.802 7.753 7.075
   5707   1367742_at   6.16 6.242 6.092 6.356 6.241 7.577 7.781 8.526 7.358 7.216
   5708   1398818_at   5.939 6.11 6.171 5.995 6.127 6.725 6.635 6.62 6.435 6.382
   5709   1388911_at   7.094 7.214 6.976 7.184 6.723 7.795 7.68 7.96 7.701 7.56
   5710   1372366_at   8.522 8.523 8.445 8.504 8.481 8.317 8.189 8.204 8.132 8.016
   5711   1373431_at   9.406 9.599 9.803 9.581 9.144 10.719 10.83 10.12 10.472 10.575
   5712   1372619_at   8.181 8.249 8.115 8.387 8.43 7.572 7.726 7.621 7.825 7.314
   5713   1378929_at   4.423 4.402 3.983 4.823 3.888 5.867 5.546 5.958 5.217 5.644
   5714   1387262_at   9.694 9.594 9.492 9.706 9.905 9.067 9.263 9.078 8.833 8.868
   5715   1375916_at   7.619 7.5 7.951 7.88 7.966 8.428 8.56 8.293 8.453 8.441
   5716   1390590_at   6.037 5.638 5.631 5.983 5.961 6.653 6.552 6.513 6.567 6.304
   5717   1387104_at   6.944 6.91 7.169 7.239 6.623 7.965 7.749 8.147 7.603 8.009
   5718   1384887_at   6.446 6.505 6.533 6.5 6.096 7.169 7.902 8.077 7.444 7.393
   5719   1373495_at   8.072 8.123 8.174 8.096 8.017 7.793 7.76 7.548 7.846 7.743
   5720   1375695_at   7.981 7.926 8.239 8.396 8.41 7.618 7.394 7.557 7.4 7.503
   5721   1368404_at   6.799 7.32 7.492 7.301 7.706 6.218 6.509 6.32 6.087 6.288
   5722   1377720_x_at   10.554 10.681 11 10.951 10.644 11.302 11.3 11.502 11.603 11.461
   5723   1370993_at   7.546 7.768 7.687 7.879 7.8 7.129 7.223 6.754 7.209 7.128
   5724   1386909_a_at   10.526 10.509 10.851 10.785 10.609 11.518 11.242 11.073 11.481 11.322
   5725   1372150_at   9.304 9.353 9.291 9.295 9.419 9.645 9.809 9.877 9.575 9.667
   5726   1368080_at   8.168 8.201 7.995 8.033 7.65 9.704 9.584 8.75 9.407 10.062
   5727   1373141_at   6.565 6.275 6.578 6.637 6.653 7.246 7.338 7.511 7.084 7.027
   5728   1380171_at   7.561 7.456 7.514 7.457 7.6 8.314 8.419 8.096 7.961 7.936
   5729   1372344_at   8.46 8.719 8.707 8.787 8.614 9.145 9.235 9.509 9.213 9.084
   5730   1367874_at   8.456 8.438 8.303 8.529 8.43 7.977 7.641 7.197 7.677 7.46
   5731   1377676_at   8.96 9.01 8.973 9.093 9.002 8.505 8.732 8.659 8.352 8.412
   5732   1372106_at   8.136 8.167 8.013 8.114 8.139 7.467 7.566 7.51 6.948 7.093
   5733   1380582_at   9.005 9.069 8.953 8.954 8.781 8.209 7.848 8.269 8.074 8.529
   5734   1371789_at   8.451 8.463 8.522 8.488 8.498 8.885 8.951 8.857 8.65 8.995
   5735   1389010_at   10.062 9.904 9.693 9.712 9.937 9.36 9.493 9.313 9.316 9.221
   5736   1396408_at   5.296 5.791 5.84 5.619 5.364 7.066 6.886 6.319 6.566 6.517
   5737   1373099_at   8.468 8.58 8.743 8.769 8.594 8.175 8.283 8.218 8.288 8.088
   5738   1387236_at   7.815 7.845 7.612 7.771 7.604 7.24 7.269 7.074 7.236 6.841
   5739   1389598_at   8.4 8.481 8.574 8.665 8.666 8.033 8.108 8.189 7.843 7.826
   5740   1395665_at   5.793 5.793 5.174 5.654 5.598 4.27 3.106 4.547 3.725 3.836
   5741   1392716_at   5.989 5.658 5.838 5.971 6.179 5.138 4.97 5.08 5.488 5.079
   5742   1374747_at   7.218 7.096 6.843 7.424 7.28 7.907 8.087 7.792 7.852 7.769
   5743   1391864_at   8.575 8.456 8.782 8.712 8.221 9.513 9.731 9.121 9.568 9.335
   5744   1398604_at   2.043 1.907 1.029 0.623 0.696 3.299 4.062 4.502 4.773 3.23
   5745   1388425_at   9.461 9.333 9.435 9.403 9.665 9.042 9.144 8.989 9.002 8.859
   5746   1374015_at   5.418 5.067 5.181 5.659 5.818 4.229 4.336 4.233 3.467 3.555
   5747   1392754_at   4.659 5.426 5.718 5.179 4.93 0.848 0.556 3.556 1.669 0.546
   5748   1374883_at   5.873 5.935 5.921 5.957 6.261 6.53 6.583 7.011 6.718 6.589
   5749   1373120_at   6.596 6.644 6.525 6.36 6.634 5.941 6.185 6.027 6.225 5.997
   5750   1367967_at   7.224 7.227 7.412 7.578 7.53 6.9 6.552 6.9 6.764 6.792
   5751   1372293_at   5.135 4.701 5.375 4.993 5.155 6.178 6.064 6.233 5.748 5.795
   5752   1393898_at   3.971 4.151 5.335 4.882 4.075 6.625 6.784 6.382 5.891 6.351
   5753   1393314_at   8.837 8.9 8.92 9.113 8.996 9.411 9.644 9.497 9.283 9.367
   5754   1376129_at   7.668 7.701 7.538 7.612 7.503 6.812 6.823 6.441 7.128 7.001
   5755   1392149_at   5.277 5.73 5.289 5.553 5.371 6.796 6.691 7.655 6.655 6.465
   5756   1376635_at   5.399 5.824 5.574 5.85 5.489 6.632 6.669 6.703 6.138 6.385
   5757   1388380_at   10.03 10.018 10.008 10.303 10.145 10.646 10.627 10.772 10.627 10.402
   5758   1387638_a_at   6.034 6.116 6.988 6.351 6.025 4.274 3.312 3.328 1.585 3.763
   5759   1387218_at   11.409 11.336 11.313 11.272 9.935 12.743 12.748 12.93 12.813 12.883
   5760   1396103_at   9.067 9.212 9.535 9.147 8.791 10.527 10.55 9.77 10.382 10.58
   5761   1373657_at   8.54 8.563 8.619 8.772 8.893 7.848 8.17 7.965 7.772 7.475
   5762   1373467_at   8.207 8.372 8.394 8.439 8.038 7.851 7.728 7.836 7.647 7.787
   5763   1374526_at   9.339 9.382 9.189 9.237 9.292 8.723 8.951 8.785 8.539 8.45
   5764   1373160_at   8.811 8.762 8.825 8.583 8.737 9.221 9.195 8.999 9.059 9.115
   5765   1399050_at   10.852 10.853 10.633 10.85 10.757 10.506 10.483 10.555 10.509 10.462
   5766   1392529_at   6.588 6.1 6.816 6.636 6.528 7.419 7.232 7.661 7.297 7.418
   5767   1389651_at   6.767 7.044 7.391 7.465 6.358 5.101 5.553 5.849 5.35 5.641
   5768   1372939_at   6.898 7.285 7.269 7.261 7.097 6.567 6.221 6.082 6.628 6.182
   5769   1385724_at   4.477 3.927 4.585 4.898 3.919 6.242 5.933 5.679 5.888 5.42
   5770   1399160_a_at   11.639 11.581 11.76 11.346 11.391 11.055 10.883 10.625 10.965 10.896
   5771   1379646_at   3.096 4.18 4.228 3.68 4.192 5.094 5.491 5.347 5.346 5.245
   5772   1373563_at   9.498 9.632 9.459 9.592 9.653 9.014 9.267 9.268 9.219 9.185
   5773   1376018_at   6.484 5.974 6.118 6.62 6.704 4.543 4.674 5.213 5.469 5.062
   5774   1393263_at   4.305 3.611 3.734 4.268 4.448 6.19 6.201 7.518 5.763 5.848
   5775   1386872_at   10.429 10.391 10.765 10.671 10.157 11.386 11.461 11.05 11.616 11.335
   5776   1377738_a_at   7.024 7.17 7.168 7.29 7.371 8.14 8.291 8.464 7.9 7.73
   5777   1393409_at   5.628 6.109 5.488 5.327 5.548 7.079 6.821 6.328 6.698 7.007
   5778   1388010_at   4.722 5.519 4.789 4.822 5.098 3.68 4.009 3.897 4.198 3.748
   5779   1367739_at   0.956 0 1.868 1.151 2.529 5.227 5.096 7.332 5.247 4.274
   5780   1369795_at   4.394 4.777 4.626 4.9 4.499 6.8 6.658 5.702 5.995 5.751
   5781   1376494_at   6.991 6.536 6.878 6.956 6.453 7.613 7.681 7.569 7.321 7.632
   5782   1380182_at   7.05 7.052 6.588 7.133 7.286 7.656 7.97 7.753 7.87 7.868
   5783   1385395_at   6.65 6.717 6.398 6.873 6.497 6.083 6.026 5.807 6.116 6.015
   5784   1373085_at   6.432 6.554 6.126 5.957 6.897 5.473 4.672 4.996 4.948 4.45
   5785   1371321_at   11.628 11.598 11.687 11.668 11.789 11.911 11.944 12.094 11.98 11.91
   5786   1381237_at   7.165 7.049 6.836 7.362 7.021 6.138 5.933 6.403 6.432 5.803
   5787   1394668_at   0.275 0.978 1.057 0.345 0.856 4.195 4.088 2.858 4.176 2.16
   5788   1376869_at   9.054 8.779 8.893 8.916 8.945 8.326 8.384 8.459 7.976 8.414
   5789   1398942_at   7.193 7.405 7.05 7.38 7.467 8.177 8.213 7.713 8.042 8.003
   5790   1379580_at   5.815 6.19 5.57 5.889 5.752 6.516 6.734 6.723 7.046 7.219
   5791   1382094_at   9.426 9.523 9.495 9.566 9.681 8.562 9.105 8.666 8.23 8.531
   5792   1370442_at   4.982 4.83 4.851 5.111 5.139 2.842 3.79 3.79 3.629 2.558
   5793   1374279_at   8.629 8.71 8.568 8.855 8.855 8.116 8.348 8.198 7.886 8.122
   5794   1374542_at   2.878 4.045 3.681 3.919 3.773 5.051 5.257 5.149 4.792 4.916
   5795   1373301_at   6.329 6.35 6.495 6.636 6.099 7.39 7.159 7.287 6.965 6.961
   5796   1391506_at   5.394 4.97 5.064 5.683 5.488 6.463 6.979 6.887 6.162 6.414
   5797   1376369_at   2.387 3.194 3.291 3.681 2.401 0.714 1.202 1.339 1.401 0.465
   5798   1372010_at   7.057 7.204 7.131 7.311 7.264 7.624 7.832 7.842 7.538 7.579
   5799   1394589_at   6.074 5.946 6.315 6.253 6.521 5.466 5.212 5.004 5.41 5.585
   5800   1368446_at   8.265 8.369 8.119 8.084 7.985 9.445 9.606 8.728 9.646 9.132
   5801   1374958_at   6.758 6.963 7.003 7.205 6.869 6.172 6.38 6.259 6.217 6.568
   5802   1392628_at   5.729 6.319 6.049 6.052 6.104 3.157 3.103 0 0 0
   5803   1371990_at   8.193 8.284 8.38 8.397 8.316 9.207 9.08 9.63 9.079 8.825
   5804   1393933_at   8.237 8.319 8.529 8.645 8.298 9.066 9.519 9.732 9.309 9.074
   5805   1370747_at   6.791 6.725 6.854 7.088 6.818 7.741 8.217 8.73 7.698 8.026
   5806   1373201_at   8.069 8.339 8.485 8.524 8.475 8.989 9.084 8.857 8.926 8.9
   5807   1389561_at   6.899 6.482 6.838 6.883 6.78 7.795 7.547 7.339 7.49 7.308
   5808   1384639_at   6.903 6.865 7.137 7.231 6.56 7.711 7.861 8.155 8.247 7.726
   5809   1384352_at   7.419 7.685 7.39 7.404 7.562 6.847 6.224 6.215 6.492 6.876
   5810   1376198_at   7.124 6.878 6.643 7.199 7.591 6.051 6.139 6.045 6.158 5.903
   5811   1367568_a_at   12.154 12.126 12.035 12.21 12.396 11.743 11.594 11.628 11.667 11.874
   5812   1367836_at   8.908 8.975 8.927 8.792 8.769 9.788 9.408 9.411 9.714 9.291
   5813   1373366_at   8.497 8.529 8.523 8.519 8.499 9.154 9.121 9.248 8.842 8.837
   5814   1382730_at   6.815 7.049 6.654 6.841 6.71 5.033 5.263 5.813 5.106 6.085
   5815   1371704_at   9.181 9.08 8.879 9.196 9.16 8.46 8.688 8.757 8.515 8.476
   5816   1377836_at   6.19 6.474 6.444 6.398 6.498 5.819 5.884 5.8 6.125 5.89
   5817   1374716_at   7.41 7.5 7.764 7.964 7.696 6.768 6.441 6.897 7.062 6.786
   5818   1368193_at   7.935 7.745 8.164 8.154 8.01 8.841 8.82 9.28 8.864 8.549
   5819   1371702_at   8.639 8.495 8.41 8.482 8.816 9.096 9.17 9.079 9.019 8.962
   5820   1373100_at   9.769 9.733 9.875 9.914 9.825 9.149 9.298 9.546 9.345 9.087
   5821   1378342_at   6.032 6.207 5.527 6.149 6.395 5.203 4.901 4.866 4.663 5.175
   5822   1370284_at   12.071 11.933 11.879 11.798 11.896 12.209 12.223 12.265 12.179 12.174
   5823   1368167_at   6.65 6.532 6.82 6.605 6.247 5.555 5.567 6.02 5.22 5.32
   5824   1372849_at   7.48 7.407 7.418 7.679 7.388 8.182 8.178 8.197 7.807 7.949
   5825   1390989_at   9.58 9.736 9.809 9.456 9.649 8.961 9.114 8.489 8.899 8.98
   5826   1381262_at   4.855 5.09 5.637 5.587 5.621 6.41 6.427 6.505 6.213 6.544
   5827   1387074_at   9.571 9.484 9.423 9.403 9.747 8.095 8.162 8.916 7.614 8.005
   5828   1377141_at   8.016 8.087 8.013 8.192 8.165 8.611 8.689 8.388 8.525 8.4
   5829   1377380_at   6.554 6.701 6.876 6.944 6.932 5.318 5.304 5.742 6.136 5.017
   5830   1383523_at   7.76 8.062 7.933 8.345 7.911 8.677 8.75 8.976 8.631 8.622
   5831   1378110_at   8.282 8.395 8.188 8.288 8.338 7.838 8.021 7.678 7.821 7.975
   5832   1388274_at   5.185 5.249 4.71 5.482 5.698 6.459 7.051 6.513 6.631 6.244
   5833   1391148_at   5.803 6.085 6.257 6.442 6.376 6.84 7.018 6.978 6.858 7.041
   5834   1392842_at   8.808 8.951 8.86 9.127 9.388 7.717 7.732 8.46 7.803 7.538
   5835   1375073_at   3.329 4.018 3.898 3.918 4.099 5.17 5.276 5.339 4.909 4.581
   5836   1379848_at   1.39 3.279 1.795 3.838 2.511 5.739 6.12 5.309 6.233 5.062
   5837   1376894_at   7.607 7.784 7.626 7.667 7.631 8.02 8.142 7.932 7.915 7.939
   5838   1370518_a_at   5.209 5.013 5.265 4.966 4.939 4.38 4.177 3.623 4.012 3.437
   5839   1391485_at   3.172 4.411 4.773 4.868 4.66 7.15 6.861 7.124 6.918 5.872
   5840   1383531_at   7.379 7.329 7.595 7.592 7.393 8.139 7.966 7.844 7.975 7.826
   5841   1378739_at   6.704 6.973 7.071 7.119 6.482 8.25 8.385 9.081 8.112 7.864
   5842   1370317_at   6.695 6.882 6.464 6.856 6.726 7.254 7.512 7.307 7.257 7.649
   5843   1376834_at   7.19 6.867 7.007 7.053 7.18 6.532 6.587 6.46 6.196 6.126
   5844   1373560_at   8.68 8.359 8.739 8.616 8.785 9.296 9.167 9.193 9.115 9.487
   5845   1392829_at   6.818 7.265 7.152 6.928 7.057 6.497 6.423 6.559 6.612 6.406
   5846   1372989_at   6.595 7.072 7.071 7.059 7.31 6.099 6.278 6.374 6.272 6.231
   5847   1375442_at   6.601 6.542 6.372 6.686 6.983 5.94 5.747 5.309 5.888 5.662
   5848   1371695_at   8.488 8.426 8.204 8.59 8.553 7.594 8.067 7.855 7.63 7.744
   5849   1371860_at   9.299 9.348 9.404 9.38 9.305 9.126 9.156 9.103 9.214 9.037
   5850   1387558_at   7.078 7.224 7.198 7.56 7.207 7.919 8.398 8.025 7.807 8.079
   5851   1367905_at   8.987 9.27 9.219 9.439 9.544 7.846 8.349 8.661 7.87 7.896
   5852   1377185_at   7.833 7.932 7.933 8.116 8.271 3.539 3.118 5.432 5.772 5.313
   5853   1398851_at   11.467 11.419 11.415 11.42 11.491 11.344 11.274 11.201 11.237 11.203
   5854   1388129_at   9.345 9.343 9.286 9.499 9.467 8.526 8.676 8.856 8.041 8.342
   5855   1373480_at   7.905 7.816 7.876 7.946 7.725 8.805 9.124 8.601 8.401 8.489
   5856   1369815_at   7.484 7.611 8 7.895 7.54 4.801 1.891 4.006 4.424 1.275
   5857   1392844_at   5.519 5.815 5.682 5.616 5.668 5.068 4.748 5.146 4.962 5.27
   5858   1388677_at   8.277 8.192 8.285 8.35 8.495 8.735 8.769 8.626 8.764 8.604
   5859   1385580_at   5.718 5.569 5.484 5.382 5.452 4.741 4.66 4.079 4.791 4.793
   5860   1381822_x_at   7.515 7.377 7.25 7.483 7.619 6.923 7.003 6.542 6.799 6.87
   5861   1392440_at   5.218 5.175 5.536 5.992 5.696 0.322 2.084 0.74 3.71 1.891
   5862   1393037_at   5.976 6.65 5.65 5.773 4.855 7.449 7.623 7.596 7.517 7.788
   5863   1376679_at   6.248 5.984 6.286 6.234 6.178 7.066 7.192 6.904 6.702 6.652
   5864   1388331_at   11.702 11.599 11.334 11.463 11.835 10.784 11.075 10.973 10.635 10.885
   5865   1388821_at   8.586 8.692 8.954 8.963 8.596 9.313 9.285 9.584 9.591 9.732
   5866   1368583_a_at   6.077 6.331 6.754 6.129 5.138 8.355 8.006 7.685 7.821 7.649
   5867   1370387_at   5.66 5.596 4.007 4.207 3.507 1.614 0.66 2.138 0.124 0.164
   5868   1368172_a_at   6.977 6.523 7.382 6.506 7.501 5.573 5.824 5.131 5.49 5.589
   5869   1389509_at   8.38 8.475 8.443 8.479 8.466 8.293 8.304 8.307 8.282 8.193
   5870   1371326_at   8.413 8.443 8.637 8.701 8.399 8.077 8.117 7.898 8.132 8.05
   5871   1393388_at   8.902 8.586 8.457 8.622 8.723 9.341 9.359 9.49 9.207 9.07
   5872   1372054_at   7.567 7.47 7.65 7.73 7.745 8.203 8.092 8.166 7.958 7.964
   5873   1385994_at   5.491 4.517 3.491 4.86 4.949 6.887 6.888 6.927 6.693 6.465
   5874   1373771_at   5.861 5.672 5.557 6.259 6.413 4.464 4.931 4.581 4.848 4.098
   5875   1395165_at   6.148 6.492 6.325 6.584 6.369 5.441 5.448 4.698 4.304 4.831
   5876   1397356_at   5.319 5.294 5.573 5.63 5.618 6.493 6.651 6.036 6.286 6.839
   5877   1369236_at   6.327 6.509 6.336 6.203 6.247 5.601 5.772 5.716 5.947 5.412
   5878   1384046_at   7.624 7.731 7.781 7.662 7.634 8.359 8.575 7.965 8.394 8.339
   5879   1392706_at   6.013 6.063 6.087 6.178 6.368 5.452 5.699 5.255 5.229 5.006
   5880   1378896_at   5.845 5.936 6.121 5.95 5.259 7.603 7.32 6.566 7.376 7.285
   5881   1385052_at   7.13 7.086 7.184 6.777 7.295 6.438 6.344 6.316 6.63 6.152
   5882   1374014_at   8.175 8.023 8.399 8.572 8.377 7.503 7.785 7.687 7.59 7.406
   5883   1378241_at   7.06 7.068 7.165 7.296 7.408 7.874 8.089 8.173 7.888 7.613
   5884   1394278_at   5.95 5.884 5.905 6.334 6.542 5.298 5.281 4.931 5.135 4.751
   5885   1384752_at   5.591 5.532 5.978 5.905 5.686 1.144 1.111 3.714 3.44 2.144
   5886   1388792_at   8.479 8.131 8.357 8.668 8.587 9.56 9.323 9.953 9.123 9.842
   5887   1388692_at   8.708 8.635 8.836 8.575 8.616 9.276 9.119 9.05 9.073 8.966
   5888   1389120_at   2.284 2.534 2.536 2.548 2.462 3.189 3.027 2.903 3.082 3.469
   5889   1371301_at   12.514 12.434 12.459 12.387 12.477 12.31 12.208 12.222 12.297 12.302
   5890   1371684_at   8.591 8.638 8.715 8.772 8.652 8.337 8.232 8.421 8.086 8.307
   5891   1372325_at   8.734 8.627 8.376 8.772 9.035 7.974 7.264 7.73 7.902 7.617
   5892   1385157_at   7.14 7.085 7.367 7.6 7.205 5.909 6.028 6.205 6.735 5.846
   5893   1367870_at   10.346 10.324 10.216 10.209 10.36 9.945 10.117 9.925 9.856 9.877
   5894   1386691_at   4.713 4.566 5.294 5.497 4.496 6.319 6.372 6.726 6.098 6.142
   5895   1373610_at   9.085 9.247 9.123 9.274 9.192 8.825 8.927 8.694 8.636 8.86
   5896   1383690_at   6.188 6.813 6.233 6.372 6.795 4.944 4.478 5.154 5.624 4.55
   5897   1368863_at   7.943 7.902 7.959 8.15 8.309 8.626 8.624 8.569 8.741 8.456
   5898   1395963_at   3.726 3.7 4.142 3.625 3.362 4.459 4.605 4.675 4.566 4.996
   5899   1376990_at   7.288 7.298 7.29 7.633 7.71 6.558 6.309 6.958 6.455 6.333
   5900   1369972_at   6.777 6.822 6.427 6.762 6.522 7.265 7.154 7.474 7.246 7.16
   5901   1368165_at   8.271 8.5 8.459 8.619 8.632 8.08 8.066 7.848 7.742 7.932
   5902   1371410_at   10.024 10.04 10.126 10.13 10.24 9.617 9.863 9.752 9.831 9.698
   5903   1373699_at   5.261 5.286 5.073 5.136 5.723 6.074 6.083 6.408 6.154 5.949
   5904   1393683_at   6.56 6.211 6.702 6.491 6.894 5.893 5.65 5.37 5.727 5.268
   5905   1385899_at   5.481 5.799 5.756 6.09 6.114 6.896 7.084 7.366 6.522 6.902
   5906   1378331_at   4.336 5.044 3.352 4.978 4.824 2.583 1.05 2.242 0.949 1.915
   5907   1368279_at   6.497 6.375 6.046 6.211 6.298 5.727 5.615 5.074 5.385 5.187
   5908   1368444_at   8.825 8.876 8.926 9.093 8.938 9.278 9.213 9.388 9.251 9.217
   5909   1387795_at   7.599 7.895 7.631 7.885 7.445 6.596 6.588 7.122 6.143 6.561
   5910   1397362_at   6.349 6.159 6.235 6.468 6.507 5.821 5.993 5.614 5.777 5.686
   5911   1391534_at   6.676 6.574 6.602 6.46 6.227 7.454 7.85 7.037 7.601 7.354
   5912   1372252_at   9.59 9.738 9.681 9.792 9.893 10.071 10.147 10.018 10.177 10.07
   5913   1378178_at   8.124 8.079 7.813 8.074 8.318 7.477 7.504 7.487 7.074 7.384
   5914   1380265_at   6.819 6.934 6.52 7.017 7.094 6.104 6.11 5.881 6.304 6.224
   5915   1389908_at   6.396 6.16 6.475 6.707 6.672 7.342 7.191 7.19 7.42 7.006
   5916   1367528_at   8.031 7.849 7.679 7.834 7.812 8.34 8.313 8.142 8.194 8.273
   5917   1377359_at   7.234 7.189 7.169 7.407 7.161 6.911 6.838 6.94 6.949 6.762
   5918   1372863_at   8.971 9.133 8.974 9.17 9.187 8.325 8.739 8.636 8.311 8.386
   5919   1380852_at   4.372 4.912 4.757 4.819 4.73 5.698 6.208 5.312 5.772 5.773
   5920   1384538_at   6.302 6.12 6.115 6.512 5.996 5.746 5.597 5.524 5.405 5.547
   5921   1395965_at   5.967 6.248 6.156 5.712 6.011 7.089 6.849 6.698 6.583 6.723
   5922   1393344_at   5.504 4.619 5.396 5.387 5.274 6.155 6.223 6.317 6.139 6.34
   5923   1376255_at   7.198 7.177 7.345 7.429 7.179 5.033 5.044 5.456 5.227 3.22
   5924   1367515_at   8.082 8.1 8.072 8.124 8.18 7.865 7.857 7.644 7.631 7.838
   5925   1369526_at   6.699 6.566 7.065 6.541 6.448 8.503 7.777 7.557 8.167 7.645
   5926   1388113_at   12.375 12.293 12.411 12.24 12.253 12.642 12.574 12.61 12.835 12.791
   5927   1394594_at   6.511 6.593 6.619 6.637 6.336 8.014 8.357 7.097 8.188 8.313
   5928   1375673_at   8.94 8.903 8.616 9.063 8.941 7.872 8.122 8.476 7.851 7.941
   5929   1398250_at   9.051 9.119 9.652 9.278 9.164 9.959 10.126 9.793 10.14 10.024
   5930   1382838_at   5.448 5.515 4.528 4.888 5.305 6.121 6.509 6.765 6.449 6.369
   5931   1374591_at   8.532 8.662 8.511 8.788 8.579 9.273 9.535 9.242 8.954 9.405
   5932   1378264_at   7.177 7.555 6.997 7.449 7.57 6.376 6.041 6.181 5.939 5.201
   5933   1388803_at   8.284 8.229 8.197 8.04 8.17 7.642 7.519 7.788 7.506 7.185
   5934   1368249_at   8.474 8.797 9.214 8.794 7.993 10.233 10.019 9.809 9.797 10.145
   5935   1381467_at   6.04 5.816 6.045 5.892 5.986 6.506 6.415 6.667 6.552 6.234
   5936   1372145_at   8.243 8.341 8.349 8.671 8.502 7.422 7.711 7.974 7.784 7.595
   5937   1382273_at   5.524 5.529 5.789 6.144 5.904 4.913 5.044 4.934 4.984 5.17
   5938   1367640_at   12.915 12.807 12.603 12.464 12.733 12.187 12.068 12.269 12.169 11.972
   5939   1393850_at   1.761 1.696 1.618 1.422 1.511 4.326 3.716 3.989 2.711 2.911
   5940   1392285_at   5.221 4.737 3.503 4.839 4.69 2.501 1.791 2.59 2.592 1.157
   5941   1376247_at   6.011 5.895 5.995 6.004 6.282 5.599 5.68 5.362 5.459 5.498
   5942   1371014_at   8.22 8.246 8.341 8.414 8.107 8.939 9.271 8.667 8.854 9.008
   5943   1390281_a_at   6.897 6.58 6.936 6.821 6.837 7.479 7.482 7.59 7.519 7.121
   5944   1388618_at   8.317 8.236 8.114 8.538 8.465 8.807 9.03 9.235 8.851 9.034
   5945   1393100_at   6.624 6.663 6.38 6.55 6.609 7.3 7.291 7.623 7.085 7.033
   5946   1383596_at   7.347 7.172 6.518 7.372 7.508 5.774 5.961 6.187 6.013 5.456
   5947   1378199_at   5.945 6.269 6.444 6.206 5.87 2.333 4.366 2.922 4.119 1.899
   5948   1372085_at   8.232 8.038 8.224 8.346 8.028 8.947 8.693 8.559 8.661 8.706
   5949   1382835_at   7.289 7.135 7.718 7.75 7.344 8.251 8.591 8.541 8.242 8.165
   5950   1371267_at   6.356 6.478 6.443 6.389 6.435 3.566 4.362 5.063 5.161 4.917
   5951   1376279_at   5.905 5.396 5.835 6.22 5.753 6.75 6.89 7.036 6.499 6.783
   5952   1373150_at   9.113 9.103 9.169 9.188 9.229 8.521 8.78 8.636 8.527 8.166
   5953   1390117_at   7.646 7.596 7.497 8.036 7.547 8.379 8.873 8.398 8.386 8.413
   5954   1388825_at   7.716 7.699 7.875 7.873 7.827 8.17 8.754 8.554 8.303 8.502
   5955   1373193_at   10.532 10.408 10.539 10.559 10.726 10.895 11.074 11.134 11.095 10.861
   5956   1377719_a_at   9.531 9.669 9.97 10.089 9.568 10.514 10.399 10.92 10.698 10.625
   5957   1376912_at   5.229 5.446 5.303 5.251 5.392 6.035 6.023 5.655 5.713 5.874
   5958   1377789_at   5.687 5.963 5.991 6.247 5.647 6.947 7.584 8.021 6.881 7.325
   5959   1368206_at   8.352 8.181 8.433 8.562 8.298 8.88 9.103 9.415 9.133 8.878
   5960   1375846_at   7.967 8.059 8.071 8.056 8.119 8.71 8.828 8.3 8.583 8.622
   5961   1389671_at   6.462 6.724 6.101 6.657 6.683 4.525 5.23 4.824 5.761 5.082
   5962   1381088_at   5.119 4.74 5.172 4.765 4.879 5.688 5.89 5.62 5.712 5.399
   5963   1386994_at   7.672 7.808 7.961 8.16 7.773 9.9 9.614 10.871 8.931 9.871
   5964   1398169_at   2.452 0.526 1.48 0.475 1.551 4.545 3.999 4.436 4.605 3.06
   5965   1391190_at   7.083 7.214 7.229 7.275 7.453 6.874 6.745 6.595 6.698 6.426
   5966   1374385_at   11.332 11.374 11.274 11.139 11.248 10.838 10.965 10.868 10.984 10.686
   5967   1391434_at   5.749 5.343 6.006 6.051 6.372 7.27 6.869 7.027 7.426 6.984
   5968   1370990_at   8.08 8.146 8.115 8.053 8.375 7.205 6.946 6.089 7.198 6.89
   5969   1380259_at   5.628 6.042 5.416 5.966 5.847 4.454 4.476 5.18 4.714 4.778
   5970   1372810_at   9.643 9.599 9.749 9.633 9.532 9.247 9.18 9.098 8.746 8.926
   5971   1371436_at   6.388 5.957 5.869 6.366 6.76 7.496 7.473 7.92 7.282 7.255
   5972   1398878_at   8.651 8.775 8.44 8.769 8.499 9.061 9.043 9.17 9.075 9.014
   5973   1375060_at   6.184 6.24 6.31 6.39 6.485 5.24 5.429 5.855 4.955 5.404
   5974   1378008_at   6.917 6.908 6.612 6.892 7.176 6.347 6.364 6.34 6.213 6.382
   5975   1376945_at   4.191 4.681 4.68 4.268 4.155 5.099 5.625 5.795 6.053 6.183
   5976   1385639_at   6.373 6.669 6.105 6.565 6.436 4.776 4.506 5.082 3.815 5.372
   5977   1380326_at   3.131 3.074 2.205 2.491 2.866 4.236 4.302 4.033 3.688 3.88
   5978   1371030_at   10.953 10.848 10.894 11.056 9.861 12.179 12.341 11.797 12.321 12.232
   5979   1379736_at   8.375 8.358 8.768 8.673 8.372 9.439 9.506 9.164 9.236 9.013
   5980   1388395_at   9.114 9.086 9.295 9.273 9.029 9.876 9.772 10.144 10.496 9.848
   5981   1371434_at   11.152 11.156 11.155 11.099 11.243 10.81 11.009 10.933 10.795 10.861
   5982   1380389_at   7.226 7.502 6.935 7.343 7.226 8.503 8.446 9.48 8.357 8.422
   5983   1384553_at   6.978 7.334 7.219 7.302 7.38 5.958 6.413 6.767 6.11 6.175
   5984   1374586_at   8.177 8.237 8.307 8.379 8.427 8.838 8.923 8.732 8.716 8.574
   5985   1375953_at   5.936 6.006 6.319 6.217 6.074 5.648 5.279 5.537 5.652 5.427
   5986   1383694_at   5.351 5.693 5.194 5.573 5.392 6.537 6.577 6.856 6.416 5.962
   5987   1373787_at   6.716 6.361 6.442 7.216 6.525 5.434 5.749 5.592 5.299 5.785
   5988   1372879_at   8.478 8.524 8.762 8.704 8.686 8.984 8.929 8.979 8.973 8.975
   5989   1390501_at   7.84 7.964 7.933 7.797 7.704 8.219 8.084 8.207 8.296 8.317
   5990   1370550_at   4.77 4.766 4.915 4.136 5.526 2.195 3.732 2.236 2.134 1.96
   5991   1381871_at   6.564 6.385 6.55 6.852 7.013 7.513 7.911 7.744 7.404 7.394
   5992   1383559_at   8.702 8.714 9.097 8.895 8.617 10.196 10.015 9.323 10.312 9.975
   5993   1383744_at   7.345 7.153 7.503 6.781 6.834 8.742 8.531 8.632 7.905 8.162
   5994   1385632_at   5.77 5.491 5.553 5.674 5.563 6.118 6.051 5.876 5.978 5.97
   5995   1378990_at   5.914 6.114 5.901 6.016 6.104 5.585 5.65 5.738 5.462 5.66
   5996   1389623_at   9.421 9.508 9.443 9.65 9.627 9.199 9.265 9.235 9.107 9.094
   5997   1393294_at   8.487 8.212 8.811 8.665 8.342 9.507 9.589 9.074 9.295 9.674
   5998   1385098_at   6.872 6.989 6.898 7.015 6.862 7.932 8.219 7.429 7.593 7.722
   5999   1374865_at   4.677 4.584 4.403 4.031 3.542 5.875 5.542 5.358 6.154 5.97
   6000   1369380_at   5.762 5.76 5.175 5.109 5.299 3.373 4.079 3.485 4.206 2.694
   6001   1391085_at   4.991 4.942 5.303 5.418 5.31 6.157 6.196 6.162 6.424 5.68
   6002   1376485_at   5.212 5.621 5.574 5.637 5.673 2.163 3.89 3.931 3.488 3.84
   6003   1368314_at   8.017 7.965 7.746 7.958 8.14 8.56 8.585 8.587 8.395 8.302
   6004   1382167_at   5.747 5.816 6.353 6.043 5.573 4.482 4.631 5.194 4.541 4.938
   6005   1371400_at   4.702 4.549 4.221 4.481 4.902 6.875 7.087 8.85 7.07 6.307
   6006   1380695_at   6.467 6.688 6.471 6.741 6.533 7.015 7.207 6.877 7.104 7.175
   6007   1374582_at   7.481 7.703 8.055 7.965 7.561 8.499 8.538 8.363 8.408 8.452
   6008   1394820_at   5.555 6.042 5.899 5.814 5.901 6.861 6.635 6.386 6.808 6.393
   6009   1370937_a_at   4.962 5.722 5.409 5.751 4.916 6.602 6.516 7.037 6.775 6.331
   6010   1387786_at   9.338 9.387 9.702 9.453 9.504 8.806 9.046 8.5 8.861 8.881
   6011   1398597_at   5.577 5.803 5.433 5.75 5.904 6.562 6.501 6.176 6.376 6.233
   6012   1378166_at   7.36 7.207 7.315 7.415 7.345 6.491 6.863 7.012 6.597 6.663
   6013   1385268_at   6.309 6.728 6.241 6.046 6.645 5.092 5.686 5.391 5.435 4.998
   6014   1387908_at   6.893 6.757 6.024 6.978 6.938 8.48 8.292 8.144 7.622 7.978
   6015   1382267_at   5.017 4.869 5.115 5.325 4.598 6.138 6.007 6.674 6.428 5.754
   6016   1371091_at   6.199 6.21 6.525 6.432 6.317 7.641 7.737 8.829 7.476 7.631
   6017   1393039_a_at   7.992 8.201 8.231 8.329 8.254 8.937 8.965 9.065 8.765 8.539
   6018   1368280_at   11.079 11.146 10.904 10.652 10.942 10.464 10.197 10.034 10.377 10.106
   6019   1392542_at   8.481 8.418 8.644 8.426 8.364 7.988 7.771 7.467 8.019 7.664
   6020   1375892_at   9.922 9.905 9.988 9.944 9.871 9.584 9.621 9.781 9.493 9.629
   6021   1393253_at   4.423 3.506 4.247 4.15 4.624 1.333 0.214 2.305 2.05 2.124
   6022   1392717_at   6.458 6.316 6.338 6.805 7.018 5.386 5.584 5.804 5.788 5.424
   6023   1391345_at   2.356 0.895 2.472 2.592 2.578 0 0 0.566 0 0
   6024   1382487_at   6.049 5.603 4.655 5.039 5.224 7.094 7.02 7.349 6.924 6.5
   6025   1377643_at   8.096 8.005 8.019 7.886 8.097 8.562 8.804 9.168 8.546 8.662
   6026   1393975_a_at   4.761 4.685 5.033 5.118 5.387 2.816 2.832 4.191 3.411 2.876
   6027   1382455_at   7.578 7.624 7.518 7.917 7.869 8.564 8.638 8.317 8.229 8.256
   6028   1380964_at   4.841 5.289 4.58 4.849 4.307 5.924 5.915 6.315 5.759 5.748
   6029   1384431_at   3.864 4.541 4.095 3.843 3.467 4.88 5.689 5.311 5.578 5.936
   6030   1397692_at   4.448 4.695 4.837 4.318 4.821 3.761 3.968 4.044 3.953 3.696
   6031   1374711_at   8.892 8.892 8.826 8.934 9.026 8.636 8.558 8.559 8.741 8.51
   6032   1386052_at   4.727 4.693 4.683 4.846 4.616 2.711 3.513 3.448 1.915 2.18
   6033   1397675_at   8.446 8.392 8.706 8.602 8.649 8.237 8.12 8.037 8.179 8.009
   6034   1370867_at   9.224 9.131 9.359 9.398 9.545 8.721 8.589 8.764 8.851 8.353
   6035   1386887_at   12.017 11.972 11.914 11.83 11.987 12.123 12.194 12.184 12.22 12.134
   6036   1372830_at   8.535 8.556 8.143 8.113 8.379 7.403 7.775 7.576 7.074 7.426
   6037   1383698_at   9.989 9.987 9.889 10.054 9.599 10.677 10.457 10.488 10.481 10.339
   6038   1372719_at   8.617 8.754 8.559 8.91 8.929 8.316 8.217 8.238 8.069 8.311
   6039   1376802_at   6.288 6.294 6.287 6.482 6.541 6.864 6.684 6.98 6.798 6.781
   6040   1388990_at   8.891 8.878 8.882 8.922 8.745 8.597 8.675 8.534 8.525 8.665
   6041   1383558_at   7.914 7.726 7.419 7.946 7.638 8.516 8.514 8.404 8.443 8.185
   6042   1373643_at   6.914 6.763 6.849 7.383 7.186 7.824 7.903 7.973 7.624 7.715
   6043   1372111_at   7.683 7.726 8.095 7.825 7.737 8.425 8.558 8.741 8.268 8.377
   6044   1395316_at   6.674 6.871 6.835 7.012 7.237 7.868 8.043 7.799 7.522 7.533
   6045   1368002_at   8.084 8.678 7.89 8.211 8.105 7.216 7.248 7.449 6.84 6.829
   6046   1390114_at   7.062 7.154 7.039 7.175 7.249 7.745 7.813 8.216 7.71 7.591
   6047   1376201_at   6.558 6.656 6.57 6.525 6.762 5.75 5.813 6.177 6.084 5.535
   6048   1392809_at   4.138 3.955 4.797 4.434 4.688 6.118 6.296 5.178 5.956 5.922
   6049   1387862_at   11.669 11.645 11.594 11.441 11.597 11.28 11.365 11.313 11.252 11.366
   6050   1391018_at   3.307 2.884 3.61 3.66 3.891 4.722 4.673 4.75 4.292 4.802
   6051   1396363_at   4.57 4.78 5.031 5.421 5.403 3.838 4.129 3.732 4.103 3.799
   6052   1376278_at   6.521 6.276 6.31 6.592 6.298 6.975 7.138 7.479 6.921 7.038
   6053   1374417_at   5.698 5.674 5.497 5.628 6.011 7.304 6.789 7.449 6.759 6.422
   6054   1390592_at   7.101 7.113 7.364 7.467 7.327 7.948 8.122 8.382 7.969 7.755
   6055   1376811_a_at   6.676 7.047 6.846 7.62 7.159 5.964 6.079 5.784 5.963 6.241
   6056   1372153_at   6.167 6.214 5.851 6.478 6.785 4.267 3.851 5.394 4.599 4.113
   6057   1385432_at   6.355 6.52 6.845 6.651 6.752 7.419 7.987 8.267 7.468 7.567
   6058   1381515_at   4.158 3.939 3.623 4.504 4.049 5.394 5.756 4.983 4.952 5.415
   6059   1382673_at   5.359 5.559 5.758 5.684 6.128 6.722 6.903 6.698 6.407 6.453
   6060   1394318_at   7.867 7.79 7.777 7.699 8.089 7.317 7.262 6.892 7.342 7.244
   6061   1374094_at   7.446 7.476 7.567 7.406 7.566 7.889 7.944 7.803 7.968 7.684
   6062   1370522_at   4.403 4.972 1.911 1.872 2.067 7.298 7.185 7.959 6.839 7.018
   6063   1378192_at   1.104 1.922 1.367 1.824 2.778 3.742 3.618 3.847 4.485 3.428
   6064   1384246_at   7.781 8.115 7.435 7.801 7.876 7.194 7.163 6.878 6.823 7.003
   6065   1388697_at   8.008 7.881 7.879 8.143 7.954 8.297 8.244 8.357 8.3 8.251
   6066   1382412_at   6.355 6.188 6.251 6.413 6.575 7.539 6.854 7.54 7.439 7.056
   6067   1374187_at   4.395 4.205 3.886 4.111 4.153 5.768 6.207 4.795 5.615 5.978
   6068   1397266_at   2.227 2.403 2.124 1.816 3.441 5.315 4.769 4.622 3.914 4.273
   6069   1374939_at   7.91 7.648 8.132 7.817 7.49 8.855 8.904 9.146 8.661 8.402
   6070   1379671_at   10.164 10.172 10.161 10.22 10.175 9.789 9.73 9.328 9.728 9.753
   6071   1368341_at   7.708 7.722 7.753 7.74 7.513 7.295 7.168 6.935 7.315 7.26
   6072   1392324_at   6.648 6.807 7.094 6.617 6.135 8.187 7.555 8.061 7.895 7.59
   6073   1373689_at   5.39 6.149 6.211 6.072 5.62 7.437 7.432 8.172 6.941 7.233
   6074   1375709_at   8.175 7.994 8.436 8.133 8.079 7.156 7.205 7.42 6.394 6.773
   6075   1370333_a_at   8.254 8.078 8.161 8.021 8.867 9.191 9.406 9.505 9.303 9.075
   6076   1374218_at   5.289 5.114 5.692 5.417 5.435 5.897 6.214 6.27 5.994 6.069
   6077   1368333_at   12.183 12.105 12.311 12.086 11.47 12.904 12.811 12.911 13.053 13.037
   6078   1388105_at   8.539 8.646 8.573 8.504 8.5 8.13 8.347 8.064 8.288 8.109
   6079   1398817_at   11.195 11.083 11.246 11.134 11.159 10.849 10.642 10.557 10.918 10.642
   6080   1384467_at   8.636 8.693 8.861 8.777 8.868 8.497 8.382 8.207 8.426 8.319
   6081   1377102_at   7.413 7.383 7.536 7.485 7.411 7.274 7.186 7.103 7.155 7.029
   6082   1375424_at   10.866 10.876 10.903 10.899 11.076 10.097 10.46 10.488 10.079 9.91
   6083   1374963_s_at   6.927 7.198 7.124 7.27 7.161 7.997 8.307 8.463 7.771 7.745
   6084   1372018_at   7.633 7.589 7.844 8.069 7.83 8.542 8.609 8.704 8.417 8.231
   6085   1374150_at   7.887 7.903 7.99 7.982 7.994 7.606 7.559 7.487 7.279 7.677
   6086   1385941_at   7.922 7.902 7.838 7.833 8.02 7.613 7.687 7.602 7.435 7.579
   6087   1386577_at   3.949 3.841 3.93 4.424 4.168 5.228 4.857 4.769 5.03 4.688
   6088   1368426_at   9.385 9.299 9.211 9.227 9.533 9.847 9.873 10.026 9.816 9.64
   6089   1390036_at   7.385 7.421 7.558 6.971 6.758 8.462 8.774 8.044 8.296 8.285
   6090   1372264_at   11.424 11.427 11.58 11.266 10.634 12.436 12.39 12.105 12.381 12.698
   6091   1393894_at   9.017 9.025 9.372 9.574 9.541 10.04 10.423 10.255 10.015 10.082
   6092   1389125_at   8.598 8.624 8.532 8.542 8.55 8.116 8.354 8.365 8.252 8.149
   6093   1382010_at   6.75 6.818 6.831 6.824 6.813 7.399 7.27 7.15 7.262 6.999
   6094   1370335_at   7.068 7.056 7.225 7.069 7.397 7.912 7.864 7.468 7.909 7.902
   6095   1379696_at   6.694 6.777 6.809 6.846 7.358 6.128 6.17 5.515 5.878 5.758
   6096   1374276_at   5.319 5.846 5.737 5.771 5.645 7.215 7.069 6.193 7.152 6.871
   6097   1394535_at   6.096 6.175 6.697 6.509 6.273 7.216 7.406 7.22 7.087 6.916
   6098   1393153_at   7.654 7.541 7.282 7.505 7.422 6.952 7.091 7.048 6.755 7.01
   6099   1376407_a_at   8.517 8.363 8.572 8.409 8.518 7.895 7.85 7.527 7.924 7.285
   6100   1373763_at   6.707 6.751 6.976 7.042 6.587 7.512 7.679 7.354 7.308 7.416
   6101   1373137_at   6.91 7.021 7.095 7.394 7.037 8.089 7.843 8.034 7.567 7.71
   6102   1375974_at   9.742 9.823 9.899 9.968 9.967 9.466 9.654 9.433 9.579 9.512
   6103   1386392_at   6.375 6.482 6.293 6.785 6.576 5.78 5.677 5.283 5.936 5.369
   6104   1368117_at   7.955 8.021 7.981 8.172 8.083 8.472 8.64 8.675 8.307 8.441
   6105   1391719_at   7.508 7.703 7.904 7.836 7.803 6.665 7.075 7.299 7.071 6.936
   6106   1374844_at   9.315 9.146 8.789 8.966 9.277 8.287 8.518 8.496 8.302 8.534
   6107   1377577_at   8.904 8.945 8.749 9.04 8.95 8.432 8.68 8.507 8.388 8.354
   6108   1393438_at   5.227 5.239 4.957 5.188 5.093 5.868 5.776 5.475 5.715 5.543
   6109   1396132_at   5.196 5.39 4.572 4.713 5.066 3.388 3.877 3.999 3.359 4.024
   6110   1391301_at   4.406 4.969 5.287 3.648 4.971 0 1.718 2.606 0 1.506
   6111   1379387_at   7.178 7.356 6.832 6.876 7.127 5.171 5.158 3.494 5.483 5.135
   6112   1367638_at   8.215 8.183 8.48 8.284 8.142 9.042 8.774 8.933 9.11 8.624
   6113   1394320_at   7.045 7.12 7.255 7.235 7.063 6.811 6.541 6.467 6.734 6.357
   6114   1374100_at   6.44 6.661 6.399 6.461 6.482 7.417 7.353 7.372 6.907 6.955
   6115   1370208_at   9.5 9.418 9.553 9.532 9.456 9.169 8.968 8.948 9.259 8.892
   6116   1389580_at   7.699 7.526 7.386 7.678 7.814 6.915 7.05 7.256 6.961 6.829
   6117   1393279_at   4.637 4.123 4.792 4.688 4.153 6.037 5.745 6.326 5.855 5.236
   6118   1399166_a_at   7.939 7.905 8.024 8.097 8.082 8.898 8.714 8.497 8.47 8.406
   6119   1371251_at   8.18 8.391 8.382 8.297 8.511 9.105 8.874 9.285 8.934 8.757
   6120   1372417_at   8.474 8.463 8.504 8.659 8.661 7.134 7.198 7.852 7.907 7.664
   6121   1383041_x_at   7.607 7.588 7.537 7.596 7.871 8.459 8.79 8.426 8.209 8.137
   6122   1384981_at   7.315 6.967 7.038 7.344 6.547 4.743 5.116 5.062 4.993 3.079
   6123   1382384_at   5.894 6.158 5.934 6.035 5.815 6.457 6.779 6.978 6.528 6.514
   6124   1379668_at   7.851 7.66 7.627 7.795 7.642 8.214 8.066 8.444 8.208 8.075
   6125   1381233_at   7.156 6.912 7.284 7.429 4.249 10.348 10.374 10.581 10.039 9.818
   6126   1391397_at   6.338 6.117 6.602 6.287 6.3 7.028 7.016 7.4 7.006 6.825
   6127   1369347_s_at   6.099 6.233 6.111 6.147 6.334 7.523 7.46 8.287 6.938 7.349
   6128   1381189_at   6.224 5.862 6.64 6.391 6.366 5.321 5.651 5.376 5.545 5.334
   6129   1372743_at   10.834 10.849 10.991 10.886 11.012 10.528 10.415 10.067 10.416 10.484
   6130   1392538_at   6.407 6.555 6.809 6.819 6.761 7.18 7.344 7.352 7.359 7.073
   6131   1376098_a_at   7.549 7.489 7.549 7.508 7.414 6.071 5.651 6.39 4.612 5.817
   6132   1370076_at   9.735 9.781 10.069 9.804 9.418 10.714 10.748 10.231 10.824 10.667
   6133   1377788_at   2.511 3.458 4.049 3.602 3.932 4.911 5.505 5.582 5.355 5.092
   6134   1392784_at   5.318 5.131 5.482 5.341 5.425 5.86 5.719 6.005 5.702 5.965
   6135   1376887_at   7.246 7.314 7.227 7.252 7.611 6.945 6.769 6.686 6.7 6.904
   6136   1369069_at   6.798 7.155 7.069 7.242 6.943 7.859 7.754 7.54 7.545 7.551
   6137   1383894_at   5.828 6.042 5.969 5.823 5.724 6.468 6.386 6.158 6.361 6.528
   6138   1378135_at   2.753 2.952 3.636 3.763 3.023 5.586 4.786 5.736 5.243 4.363
   6139   1395027_at   6.061 6.083 6.505 6.519 5.926 7.391 7.647 7.877 6.945 7.239
   6140   1399048_at   7.398 7.301 7.131 7.344 7.52 6.832 6.391 6.43 6.48 5.94
   6141   1399087_at   7.416 7.369 7.65 7.497 7.525 7.184 7.011 6.909 7.023 6.744
   6142   1393881_at   8.046 7.994 8.107 8.296 8.5 7.226 7.707 7.459 7.213 7.101
   6143   1390915_at   5.492 5.434 4.666 5.126 5.211 6.15 6.068 6.213 6.008 6.488
   6144   1386275_at   5.458 5.981 5.909 5.996 6.104 3.034 2.438 4.595 1.566 2.774
   6145   1374426_at   11.997 11.867 11.972 11.939 12.117 12.21 12.231 12.338 12.298 12.228
   6146   1370032_at   7.89 8.099 8.392 8.308 7.821 8.934 8.879 8.939 9.21 8.688
   6147   1395398_at   5.012 4.444 4.315 4.529 4.1 5.623 5.885 5.939 5.224 5.591
   6148   1384427_at   8.503 8.624 8.601 8.6 8.559 7.809 7.88 8.221 7.672 8.153
   6149   1376498_at   6.681 6.509 6.677 6.931 7.079 7.33 7.626 7.635 7.426 7.39
   6150   1383278_at   7.032 7.101 7.272 7.295 7.216 7.821 7.978 7.844 7.462 7.972
   6151   1395026_at   6.653 6.816 7.216 6.589 5.71 8.382 8.24 7.898 8.575 8.03
   6152   1393197_at   6.568 6.483 6.288 6.222 6.657 7.089 7.261 7.037 7.08 6.856
   6153   1369259_at   6.31 6.346 6.335 6.402 4.536 8.053 8.16 8.191 8.158 8.123
   6154   1378487_at   4.59 5.054 5.009 4.861 4.37 5.803 5.692 5.479 5.529 5.597
   6155   1398327_at   9.822 9.825 10.003 9.965 9.947 10.34 10.361 10.3 10.149 10.156
   6156   1388856_at   7.501 7.496 7.683 7.491 7.371 8.221 8.18 7.955 7.843 8.307
   6157   1388487_at   10.283 10.295 10.35 10.362 10.248 10.752 10.885 11.01 10.598 10.642
   6158   1385753_at   7.517 7.187 7.554 7.387 7.337 8.11 8.154 8.048 7.931 7.726
   6159   1390111_at   9.113 9.182 9.2 9.182 9.212 9.439 9.688 9.439 9.403 9.522
   6160   1392719_at   8.731 8.606 8.61 8.686 8.674 8.809 8.939 8.836 8.861 8.831
   6161   1368503_at   5.646 5.524 5.755 5.793 6.308 3.146 3.234 0.287 0.333 0
   6162   1382306_at   6.683 6.835 6.971 6.773 6.542 5.983 6.218 5.826 6.028 6.339
   6163   1383246_at   6.173 6.403 6.421 6.738 6.472 5.742 5.73 5.32 5.452 5.889
   6164   1368372_at   6.229 6.44 6.454 6.476 6.48 7.396 7.122 7.693 7.305 6.868
   6165   1370519_at   6.541 6.484 6.567 6.733 6.653 5.884 5.793 5.66 6.078 5.245
   6166   1383939_at   6.057 6.471 6.535 6.572 6.002 7.386 7.406 6.915 7.167 7.248
   6167   1378977_at   7.428 7.441 7.347 7.317 7.118 6.663 6.746 6.933 7.006 6.748
   6168   1368210_at   5.044 5.431 4.01 4.702 5.563 3.348 2.491 3.126 3.339 2.976
   6169   1390272_at   6.64 6.753 6.739 6.881 6.927 5.857 6.443 5.76 6.09 5.871
   6170   1384428_at   7.346 7.279 7.698 7.502 7.745 6.636 6.607 6.339 6.993 6.796
   6171   1376468_at   7.641 7.832 7.535 7.65 8.037 7.198 7.235 7.003 6.975 6.813
   6172   1373052_at   9.062 9.052 9.085 9.253 9.204 8.867 8.885 8.658 8.734 8.782
   6173   1393559_at   6.143 6.368 6.146 6.703 6.563 7.606 7.508 7.026 7.851 7.263
   6174   1393035_at   7.444 7.705 7.284 7.456 7.412 6.488 6.868 6.311 6.93 6.338
   6175   1383091_at   7.67 7.801 7.842 7.872 8.062 7.445 7.37 7.373 7.085 7.123
   6176   1385924_at   5.406 5.796 5.416 5.386 5.473 6.564 6.833 6.467 6.162 6.081
   6177   1375011_at   7.365 7.124 7.12 7.352 7.425 7.826 8.097 8.063 7.805 7.667
   6178   1369061_at   8.15 8.287 8.639 8.47 8.254 9.072 9.143 8.775 9.167 8.961
   6179   1383047_at   8.053 8.034 8.329 8.371 8.725 9.05 9.22 9.277 8.949 9.117
   6180   1382434_at   8.499 8.644 9.509 9.265 8.928 10.067 10.61 10.316 10.26 9.992
   6181   1388320_at   11.418 11.308 11.122 11.316 11.287 11.679 11.521 11.693 11.592 11.671
   6182   1390116_at   8.16 8.287 8.591 8.419 8.345 7.837 7.455 7.097 7.689 7.467
   6183   1369963_at   7.173 7.352 7.525 7.871 7.645 6.703 6.434 6.552 6.793 6.863
   6184   1385043_at   7.225 6.969 7.03 7.38 7.174 7.753 7.976 7.971 7.664 7.587
   6185   1370070_at   9.146 8.842 9.344 9.137 9.154 8.453 8.6 8.724 8.471 8.595
   6186   1370300_at   8.221 8.213 8.195 8.418 8.24 8.57 8.61 8.617 8.459 8.516
   6187   1372058_at   9.018 9.064 8.982 9.091 8.981 8.642 8.762 8.864 8.746 8.6
   6188   1389279_at   8.079 7.861 7.728 8.055 8.062 7.108 7.397 7.553 7.033 7.115
   6189   1372262_at   9.259 9.195 9.478 9.224 9.3 9.736 10.085 9.753 9.662 9.82
   6190   1380811_at   6.666 6.621 7.143 6.687 6.892 7.43 7.444 7.551 7.271 7.483
   6191   1393382_at   3.97 3.954 4.087 4.349 3.963 4.866 4.753 5.627 5.048 4.998
   6192   1385487_at   8.115 8.078 8.626 8.56 8.681 7.496 7.768 7.309 7.579 7.492
   6193   1371637_at   9.022 9.104 9.138 9.233 9.356 9.53 9.589 9.505 9.543 9.471
   6194   1372504_at   8.744 8.802 8.685 8.918 8.926 9.273 9.292 9.255 9.312 9.025
   6195   1375998_at   7.388 7.218 7.609 7.85 7.604 6.825 6.911 6.871 6.799 6.529
   6196   1396708_at   4.794 5.021 3.806 5.135 4.56 2.345 2.864 3.146 1.356 2.114
   6197   1389144_at   9.015 9.018 9.075 8.962 8.953 8.49 8.557 8.143 8.671 8.278
   6198   1382353_at   5.394 5.228 5.284 5.26 5.317 4.911 4.839 4.413 4.422 4.726
   6199   1383332_at   8.075 8.121 8.608 8.453 8.204 9.08 9.728 9.544 9.476 8.934
   6200   1371658_at   8.849 8.691 8.493 8.604 8.69 8.052 8.163 8.188 7.819 7.668
   6201   1383485_at   6.843 6.773 7.173 6.696 6.828 6.172 6.147 6.147 6.377 6.44
   6202   1387415_a_at   6.102 5.946 6.152 6.343 6.104 6.724 6.87 6.475 6.597 6.664
   6203   1380635_at   3.945 3.796 5.483 4.752 4.681 1.614 3.159 1.176 1.651 1.316
   6204   1371045_at   4.902 4.501 5.029 4.895 4.457 5.894 5.821 5.848 5.891 5.26
   6205   1369678_a_at   6.455 6.361 6.119 6.773 6.819 7.42 7.32 7.294 7.178 7.323
   6206   1372074_at   8.157 8.162 8.312 8.436 8.261 8.744 8.816 8.754 8.545 8.601
   6207   1378508_at   5.928 6.432 6.227 6.369 6.582 5.616 5.356 5.673 5.668 5.552
   6208   1368318_at   6.508 6.269 6.527 6.703 6.885 7.294 7.126 7.293 7.156 7.215
   6209   1388056_at   5.694 5.396 5.021 5.623 5.885 1.521 2.245 1.683 3.563 3.852
   6210   1383943_at   6.643 6.835 7.034 7.168 6.595 8.619 8.755 7.459 8.54 8.721
   6211   1384283_at   7.014 6.897 7.054 7.142 6.598 7.93 8.118 7.683 7.718 7.484
   6212   1372202_at   7.345 7.475 7.305 7.762 8.085 6.606 6.746 6.739 6.631 6.277
   6213   1372708_at   5.126 5.081 5.056 5.135 5.697 5.908 6.199 5.992 5.869 6.045
   6214   1376630_at   5.422 5.536 5.771 5.871 5.431 6.089 6.313 6.19 6.154 6.167
   6215   1371560_at   8.078 8.001 8.297 8.234 8.326 9.062 8.576 8.868 8.692 8.779
   6216   1383880_at   6.957 7.278 6.839 7.041 7.082 6.673 6.156 6.422 6.237 6.34
   6217   1370724_a_at   6.006 6.346 5.861 6.563 6.481 7.307 7.219 7.098 7.381 6.913
   6218   1370173_at   9.858 9.887 9.693 9.856 9.911 9.216 9.448 9.319 8.904 8.904
   6219   1383620_at   2.325 1.918 2.121 3.35 3.741 4.546 5.135 4.683 5.244 5.216
   6220   1380349_at   6.763 6.716 6.933 7.051 7.047 6.488 6.321 6.158 6.354 6.008
   6221   1387417_a_at   6.771 7.117 6.985 7.003 6.791 7.564 7.475 7.552 7.605 7.231
   6222   1375652_at   9.37 9.367 9.462 9.399 9.651 8.876 9.105 9.055 9.062 9.126
   6223   1375551_at   7.351 7.042 7.152 7.278 7.283 6.841 6.639 6.876 6.522 6.553
   6224   1393516_at   8.855 8.804 8.949 8.703 8.502 10.191 10.176 9.21 10.228 10.091
   6225   1394643_at   5.878 5.454 5.514 5.236 5.478 4.424 5.019 4.268 4.58 4.374
   6226   1399098_at   9.436 9.423 9.397 9.642 9.73 10.024 10.213 9.967 9.964 9.927
   6227   1387114_at   8.615 8.685 8.644 8.979 8.953 8.175 8.258 8.362 8.001 8.018
   6228   1383621_at   7.648 7.964 7.549 7.829 7.834 6.985 7.442 6.977 6.946 6.988
   6229   1374957_at   5.268 5.456 5.327 5.593 5.422 5.767 6.461 6.33 6.099 6.264
   6230   1395465_at   8.085 7.98 8.003 7.813 8.048 7.414 6.901 6.608 7.38 6.926
   6231   1367713_at   9.838 9.851 9.728 9.65 10.054 9.008 9.406 9.069 8.714 8.741
   6232   1383963_at   7.904 7.712 7.87 7.939 7.966 8.395 8.491 8.637 8.138 8.442
   6233   1372441_at   8.887 8.927 9.02 9.219 9.074 9.465 9.507 9.47 9.407 9.297
   6234   1383423_at   7.085 6.788 6.753 7.001 6.929 7.392 7.785 7.849 7.404 7.496
   6235   1391260_at   5.149 5.388 5.396 5.444 5.209 4.295 3.698 2.733 2.365 2.575
   6236   1368436_at   9.417 9.499 9.413 9.751 9.892 8.799 8.95 9.142 8.74 8.764
   6237   1371418_at   10.299 10.385 10.277 10.526 10.505 10.056 10.098 10.065 10.068 9.903
   6238   1368648_at   3.546 3.94 4.394 4.128 3.65 5.007 5.3 5.445 4.735 4.989
   6239   1387920_at   7.528 7.574 7.753 7.748 7.755 8.153 8.21 8.375 8.123 7.954
   6240   1380815_at   6.919 7.002 6.896 7.011 7.077 6.707 6.715 6.406 6.597 6.512
   6241   1371643_at   9.578 9.512 9.729 9.807 9.255 10.173 10.11 10.323 10.117 10.171
   6242   1376042_at   5.75 5.821 5.748 5.787 5.972 6.319 6.372 6.249 6.276 6.016
   6243   1392944_at   5.529 5.411 5.717 5.876 5.531 6.578 6.634 6.689 6.631 5.993
   6244   1367504_at   8.069 8.175 8.081 7.983 7.931 8.538 8.788 8.442 8.591 8.354
   6245   1378088_at   6.704 6.641 6.25 6.375 6.819 7.422 7.327 7.407 7.048 7.255
   6246   1398905_at   9.759 9.936 9.778 9.854 10.029 9.577 9.554 9.497 9.521 9.357
   6247   1372490_at   8.321 8.547 8.542 8.552 8.524 9.394 9.524 9.64 8.955 9.055
   6248   1390788_a_at   7.847 7.85 7.861 7.931 8.092 7.291 7.595 7.371 7.248 7.012
   6249   1376593_at   8.46 8.491 8.439 8.553 8.74 9.355 9.16 9.19 8.815 9.211
   6250   1382972_at   6.883 6.897 7.097 7.166 6.765 6.439 5.925 6.183 6.47 6.139
   6251   1368593_at   6.919 7.17 6.775 6.766 6.998 6.478 6.446 6.543 6.324 6.381
   6252   1377918_at   6.889 6.893 6.21 6.573 7.109 5.367 5.992 5.716 5.487 5.574
   6253   1384778_at   6.812 6.909 7.054 7.02 7.427 4.253 3.883 4.741 4.042 1.485
   6254   1385087_at   5.261 5.445 5.26 5.655 5.138 6.006 6.128 5.857 6.386 6.373
   6255   1367950_at   9.823 9.463 9.919 9.569 9.385 10.548 10.717 10.086 10.658 10.677
   6256   1372436_at   7.552 7.188 7.306 7.411 7.338 6.848 6.936 6.777 6.958 6.563
   6257   1386166_at   7.496 7.474 7.571 7.817 7.513 8.318 8.258 8.616 8.241 7.966
   6258   1372247_at   10.413 10.496 10.319 10.528 10.713 9.99 10.067 10.129 10.021 9.818
   6259   1383571_at   3.312 3.225 3.773 4.233 2.478 5.238 5.281 5.296 5.139 4.886
   6260   1386996_at   11.392 11.42 11.465 11.562 11.641 11.159 11.224 11.246 11.087 11.068
   6261   1376058_at   6.542 6.481 6.77 6.821 6.551 7.734 7.315 7.417 7.193 7.143
   6262   1372814_at   8.894 8.954 8.87 9.064 9.056 9.518 9.525 9.382 9.173 9.486
   6263   1390515_at   8.578 8.441 8.239 8.359 8.458 7.768 7.975 8.07 7.644 7.672
   6264   1398143_at   5.954 5.769 6.16 5.827 6.129 5.432 4.655 4.946 4.357 4.549
   6265   1375806_at   7.122 7.286 6.825 6.799 6.803 5.272 3.403 4.543 5.212 5.342
   6266   1378484_at   5.832 6.079 5.171 5.673 5.874 7.576 6.987 6.745 7.459 6.715
   6267   1376230_at   6.731 6.618 6.456 6.563 6.816 7.699 7.044 7.595 7.574 7.262
   6268   1370223_at   8.298 8.225 8.367 8.537 8.53 7.829 7.837 7.928 7.799 7.419
   6269   1381729_at   3.76 3.259 2.962 4.485 4.07 5.05 5.347 5.536 5.449 5.674
   6270   1371928_at   6.879 7.131 6.994 7.273 7.319 2.436 4.76 5.128 4.496 4.688
   6271   1388410_at   10.046 10.05 9.986 10.004 10.103 10.373 10.394 10.479 10.222 10.243
   6272   1393641_at   8.229 8.467 8.135 8.223 8.561 7.806 7.734 7.892 7.549 7.759
   6273   1377627_at   7.067 6.989 6.904 7.356 7.073 6.53 6.521 6.681 6.306 6.308
   6274   1387773_at   12.241 12.13 12.242 11.986 12.021 12.57 12.575 12.365 12.661 12.664
   6275   1381982_at   8.987 8.916 9.007 9.196 9.083 9.516 9.436 9.293 9.336 9.393
   6276   1377872_at   8.692 8.853 8.757 8.822 8.809 9.086 9.204 9.093 9.13 8.936
   6277   1387013_at   11.796 11.727 11.854 11.549 11.702 12.293 12.491 12.011 12.537 12.457
   6278   1367811_at   8.636 8.658 8.981 8.96 8.767 10.074 10.036 9.204 10.112 9.945
   6279   1389742_at   7.904 7.834 7.548 7.779 7.918 7.251 7.397 6.996 7.259 7.321
   6280   1371708_at   3.452 3.441 3.955 3.686 3.772 4.559 4.198 4.485 4.743 4.948
   6281   1373263_at   7.81 7.883 7.553 7.788 7.9 6.848 7.333 7.274 7.069 6.803
   6282   1368695_at   6.609 6.569 5.552 5.999 5.767 3.885 1.104 3.328 1.521 0.422
   6283   1382699_s_at   8.248 8.241 8.289 8.483 8.356 8.551 8.641 8.773 8.645 8.665
   6284   1387854_at   9.264 9.26 8.903 9.49 10.33 8.035 7.954 8.112 7.804 8.161
   6285   1369768_at   6.038 6.301 6.26 6.021 6.099 7.418 7.433 7.491 6.908 6.651
   6286   1395355_at   7.552 7.742 7.622 7.804 7.615 7.029 7.34 7.034 6.932 7.299
   6287   1384617_at   7.046 6.987 7.151 7.029 7.383 6.577 6.397 5.77 6.221 6.219
   6288   1368763_at   4.785 5.147 4.178 3.394 4.747 1.305 2.995 1.17 0.411 0.918
   6289   1370662_a_at   7.855 7.653 7.531 7.516 7.592 7.31 6.935 7.159 7.081 7.155
   6290   1399120_at   7.626 7.272 7.493 7.459 7.522 6.862 7.118 6.799 7.101 6.783
   6291   1381251_at   8.489 8.457 8.865 8.653 6.627 10.863 10.667 10.984 10.502 10.31
   6292   1373082_at   6.822 6.895 6.809 6.826 7.046 6.323 6.038 5.812 6.337 6.403
   6293   1369718_at   6.583 6.277 6.65 6.541 6.59 6.071 4.942 5.294 5.361 5.299
   6294   1391082_at   4.716 4.74 4.505 4.694 4.511 2.787 3.331 3.761 3.897 3.55
   6295   1384119_at   6.528 6.244 6.465 6.201 6.345 6.684 6.799 6.777 6.762 6.692
   6296   1380226_at   4.544 4.374 4.386 5.066 4.583 5.878 6.07 5.84 5.428 5.3
   6297   1383211_at   4.223 4.65 4.172 3.716 4.324 5.554 5.928 5.311 5.789 4.999
   6298   1385336_at   5.587 5.493 5.728 6.012 6.193 0.632 3.559 2.032 2.014 3.751
   6299   1395410_at   7.785 7.843 7.707 7.96 7.925 8.481 8.7 8.887 8.288 8.353
   6300   1392652_at   3.363 4.256 4.168 3.279 4.153 4.902 5.805 5.595 5.609 5.158
   6301   1390262_a_at   0 0.299 0.138 0.546 0 2.639 3.359 1.856 2.653 4.721
   6302   1379420_at   8.046 7.842 7.679 8.103 8.332 6.816 7.387 7.201 6.789 7.152
   6303   1381470_at   6.487 6.539 6.66 6.726 6.875 7.562 7.12 7.488 7.17 7.213
   6304   1368412_a_at   8.589 8.693 8.576 8.475 8.359 8.095 8.184 8.006 7.741 7.896
   6305   1374733_at   7.331 7.197 7.097 7.264 7.367 6.501 6.661 6.777 6.053 6.357
   6306   1376155_at   7.055 7.008 6.605 6.758 6.728 7.438 7.516 7.583 7.215 7.588
   6307   1385865_at   5.374 5.437 5.511 5.474 5.933 4.301 4.25 2.903 2.862 3.841
   6308   1393695_at   5.548 5.656 5.524 5.741 5.753 6.685 6.902 6.632 6.578 5.997
   6309   1374798_at   8.763 8.81 8.691 8.897 8.975 8.543 8.504 8.297 8.475 8.415
   6310   1381572_at   4.667 4.734 5.194 5.164 5.111 3.203 3.398 4.035 2.991 4.083
   6311   1367635_at   11.724 11.665 11.753 11.77 11.706 11.527 11.523 11.629 11.483 11.459
   6312   1374594_at   7.369 7.456 7.65 7.526 7.59 8.44 8.396 9.12 8.098 8.596
   6313   1378638_a_at   8.525 8.586 8.715 8.768 8.943 8.013 8.205 8.293 7.933 7.823
   6314   1394112_at   4.927 4.906 3.932 5.196 4.53 6.384 7.294 5.813 7.119 6.958
   6315   1399015_at   9.236 9.275 9.35 9.358 9.439 9.009 9.061 8.703 8.921 8.925
   6316   1376163_at   8.349 8.414 8.563 8.538 8.211 9.069 9.344 8.747 9.114 9.186
   6317   1388492_at   9.581 9.557 9.575 9.601 9.588 9.095 9.089 9.276 8.721 8.83
   6318   1382853_at   5.309 4.745 5.148 5.34 5.797 6.472 6.428 6.52 6.412 6.024
   6319   1382615_at   8.548 8.445 8.576 8.849 8.64 8.277 7.991 7.893 8.135 7.915
   6320   1369310_at   7.333 7.676 7.651 7.947 7.905 8.293 8.282 8.523 8.521 8.398
   6321   1398983_at   8.297 8.363 8.424 8.485 8.547 8.801 8.887 8.632 8.776 8.76
   6322   1397450_at   4.442 4.943 4.517 5.607 4.421 6.184 5.986 6.024 6.161 6.262
   6323   1382791_at   4.64 4.893 4.649 4.768 5.258 3.724 3.591 3.916 3.899 2.932
   6324   1388647_at   8.774 8.738 8.824 8.959 9.047 8.214 8.252 8.44 8.27 7.834
   6325   1389265_at   9.394 9.584 9.444 9.363 9.351 9.828 10.057 9.963 9.758 9.721
   6326   1384398_at   5.857 5.966 6.027 5.887 5.693 6.433 6.488 6.434 6.193 6.241
   6327   1387037_at   9.775 9.897 9.732 10.253 10.178 10.652 11.067 10.911 10.534 11.09
   6328   1386959_a_at   7.767 7.761 7.669 7.823 7.75 7.996 7.962 8.252 8.109 8.076
   6329   1397437_at   6.289 6.217 6.62 6.538 6.428 2.878 2.824 4.55 3.878 1.124
   6330   1376975_at   5.917 5.962 6.011 6.16 5.714 6.486 6.554 6.635 6.66 7.081
   6331   1393096_at   7.596 7.832 7.78 8.029 7.821 8.232 8.723 8.484 8.313 8.438
   6332   1379411_at   9.195 9.183 9.313 9.714 7.95 10.965 10.549 10.799 11.414 11.243
   6333   1383947_at   5.611 6.234 5.788 5.448 5.746 7.063 6.835 6.739 6.637 6.418
   6334   1392465_at   10.443 10.444 10.402 10.401 10.514 10.287 10.246 10.216 10.157 10.073
   6335   1395601_at   6.059 6.596 5.866 6.848 6.872 7.638 8.25 7.755 7.899 7.57
   6336   1391131_at   5.241 5.247 5.263 5.2 5.345 6.415 6.721 6.717 6.271 5.685
   6337   1385798_at   7.032 6.909 7.693 7.043 7.012 6.001 6.026 5.614 6.404 5.509
   6338   1390355_at   1.444 0.856 2.736 3.508 2.763 4.646 5.235 5.282 5.139 5.407
   6339   1372465_at   6.857 7.028 6.953 7.027 6.714 7.342 7.323 7.605 7.283 7.388
   6340   1370132_at   4.065 4.743 3.699 4.668 5.064 5.841 5.811 6.25 6.359 5.841
   6341   1386900_at   9.819 9.8 9.996 9.914 9.998 9.636 9.339 9.077 9.325 9.333
   6342   1372449_at   8.118 8.29 8.513 8.4 8.411 9.153 8.801 9.211 8.874 8.83
   6343   1376316_at   5.767 5.293 5.629 5.616 5.964 3.552 2.467 2.969 4.442 4.074
   6344   1393914_at   6.17 5.862 5.804 6.09 6.16 5.417 5.435 5.396 5.553 5.657
   6345   1395045_at   6.427 6.063 6.548 6.025 6.253 7.235 7.19 6.943 6.875 6.833
   6346   1377675_at   8.255 8.403 8.516 8.607 8.331 7.647 7.906 8.086 7.622 7.648
   6347   1389713_at   8.099 8.305 8.239 8.169 8.159 8.617 8.634 8.437 8.41 8.623
   6348   1387485_a_at   8.796 8.747 8.865 8.944 8.922 8.61 8.562 8.506 8.664 8.471
   6349   1371848_at   10.29 10.299 10.177 10.418 10.423 9.92 9.965 10.06 9.846 9.723
   6350   1389687_at   6.488 6.277 6.537 6.575 6.416 7.29 6.842 7.188 6.965 6.901
   6351   1385070_at   7.616 7.685 7.447 7.865 7.909 7.027 7.154 7.025 7.115 7.322
   6352   1368829_at   8.748 8.672 8.692 9.288 9.666 7.853 8.048 7.749 7.716 7.606
   6353   1372015_at   8.886 8.962 9.036 9.056 9.241 8.678 8.674 8.496 8.457 8.67
   6354   1384281_at   6.992 6.868 6.792 7.052 6.979 6.436 6.572 6.196 6.562 6.517
   6355   1388413_at   8.538 8.607 8.592 8.95 8.923 7.784 8.048 8.257 7.653 7.683
   6356   1391574_at   6.799 6.865 6.986 7.069 6.548 7.879 7.739 7.411 7.369 7.774
   6357   1392921_at   5.205 5.157 4.86 3.903 4.072 6.441 6.412 6.686 6.037 6.141
   6358   1384388_at   8.768 8.763 8.542 9.043 8.966 8.018 8.074 8.398 8.173 7.875
   6359   1374278_at   6.117 6.377 6.271 6.958 6.368 7.36 7.608 7.558 7.255 7.136
   6360   1373706_at   7.125 7.059 7.113 7.099 7.32 6.697 6.585 6.612 6.671 6.189
   6361   1382934_at   6.935 6.75 6.397 6.608 6.713 5.936 5.326 5.546 6.136 5.599
   6362   1384017_at   7.25 7.397 7.413 7.443 7.621 6.985 6.824 7.141 6.827 6.778
   6363   1392607_at   4.672 4.383 5.122 4.909 4.486 6.185 6.213 5.917 5.39 5.727
   6364   1378605_at   8.164 8.018 7.947 7.964 7.83 8.732 8.906 8.459 8.625 8.365
   6365   1372258_at   9.2 9.25 9.259 9.303 9.181 9.498 9.725 9.673 9.474 9.479
   6366   1368308_at   8.44 8.574 8.064 8.443 8.284 7.216 7.182 7.086 5.922 7.009
   6367   1373578_at   7.556 7.557 7.8 7.862 7.604 8.23 8.336 8.709 8.239 8.178
   6368   1377337_at   4.098 4.155 3.909 4.233 4.252 4.747 4.982 5.065 4.854 4.467
   6369   1383616_at   9.021 9.181 8.884 9.03 9.355 8.047 8.104 8.645 8.345 8.436
   6370   1377837_at   7.272 7.123 7.169 7.147 7.367 7.755 7.866 7.481 7.738 7.581
   6371   1391212_at   6.562 6.63 6.488 6.839 6.808 7.638 7.771 7.093 7.432 7.319
   6372   1368453_at   6.829 6.973 6.977 7.228 6.996 8.06 7.5 7.639 8.242 7.84
   6373   1387824_at   6.542 6.442 6.144 6.149 6.585 5.823 5.787 5.872 5.616 5.584
   6374   1398439_a_at   5.372 5.314 5.266 5.391 4.812 0.546 2.541 2.901 0.766 3.202
   6375   1374255_at   7.636 7.752 7.771 7.869 7.79 7.306 7.567 7.276 7.407 7.435
   6376   1399083_at   7.883 7.79 7.929 7.898 8.038 8.467 8.383 8.586 8.374 8.135
   6377   1384775_s_at   6.883 6.603 7.071 6.936 5.868 8.414 8.416 8.801 8.123 7.693
   6378   1378652_at   5.562 5.406 5.856 5.537 5.48 6.285 6.081 6.204 5.963 6.048
   6379   1378085_at   6.022 6.221 6.35 6.231 6.137 7.25 7.14 7.818 6.822 7.001
   6380   1387343_at   8.974 8.761 8.888 8.89 9.209 8.473 8.06 7.718 7.739 8.17
   6381   1385838_a_at   8.372 8.385 8.488 8.538 8.594 8.941 9.016 8.856 8.925 8.679
   6382   1373869_at   7.671 7.926 7.887 8.132 8.004 7.508 7.353 7.277 7.301 7.006
   6383   1390259_at   7.117 6.988 6.455 6.789 7.301 5.639 5.824 6.057 6.041 6.156
   6384   1371676_at   8.102 8.103 8.344 8.396 8.356 7.667 7.423 7.894 7.777 7.57
   6385   1383931_at   6.546 6.292 6.641 6.769 6.824 7.342 7.551 7.256 7.14 7.182
   6386   1373013_at   10.328 10.291 10.261 10.36 10.358 10.578 10.595 10.422 10.619 10.525
   6387   1383874_at   5.616 6.248 6.142 6.513 6.461 5.211 5.213 4.596 4.931 5.242
   6388   1388002_at   7.388 7.492 7.11 7.451 7.49 6.957 6.626 6.598 6.902 6.463
   6389   1372523_at   12.079 12.027 12.205 12.003 11.486 12.73 12.609 12.613 12.802 12.876
   6390   1397049_at   4.545 4.152 4.648 4.891 4.013 5.834 5.398 5.97 5.643 5.289
   6391   1389430_at   5.011 5.081 5.477 5.371 5.293 6.413 6.56 6.318 5.748 6.032
   6392   1374735_at   6.541 6.89 7.069 6.337 6.233 3.461 3.013 5.299 3.999 2.348
   6393   1394213_at   6.59 6.78 6.5 6.711 6.705 5.894 6.366 5.99 6.068 5.768
   6394   1374103_at   6.144 6.089 6.299 6.507 6.357 7.002 7.304 7.445 7.098 6.72
   6395   1374758_at   8.371 8.5 8.307 8.616 8.57 9.086 9.217 9.075 8.793 8.901
   6396   1373020_at   9.216 9.154 9.338 9.343 9.38 9.667 9.895 9.9 9.747 9.539
   6397   1371527_at   9.758 9.918 10.413 10.278 9.76 8.902 9.333 8.712 9.001 9.206
   6398   1372860_at   8.115 8.373 8.229 8.508 8.29 8.992 9.082 9.225 8.908 8.647
   6399   1385558_at   4.274 4.657 3.577 4.424 4.209 5.97 5.958 6.098 5.271 5.238
   6400   1396460_at   3.978 4.604 4.452 4.851 4.27 5.714 5.198 5.405 5.637 5.285
   6401   1382244_at   4.271 4.539 5.189 5.468 4.692 6.285 6.403 6.049 6.132 5.939
   6402   1374822_at   6.421 6.644 6.433 6.506 6.578 7.702 7.828 7.929 7.014 7.258
   6403   1373901_at   10.29 10.201 10.333 10.078 10.329 10.612 10.615 10.722 10.617 10.466
   6404   1372624_at   9.361 9.351 9.303 9.531 9.502 9.699 9.897 10.004 9.735 9.771
   6405   1373426_at   8.514 8.454 8.629 8.843 8.637 7.941 8.295 8.145 7.994 7.909
   6406   1374546_at   5.341 5.368 4.827 5.711 5.652 6.398 6.738 7.169 6.261 7.036
   6407   1376240_at   5.911 5.695 6.098 6.504 6.125 6.779 6.898 7.202 6.954 6.841
   6408   1373518_at   8.035 8.612 8.039 8.425 8.341 8.937 9.157 9.309 8.961 8.936
   6409   1371655_at   7.918 7.958 8.233 8.246 8.148 8.519 8.639 8.877 8.746 8.51
   6410   1372196_at   8.161 8.442 8.419 8.622 8.378 9.103 9.012 9.387 8.921 8.892
   6411   1383933_at   7.632 7.67 7.221 7.515 7.756 8.999 9.808 8.592 8.487 9.35
   6412   1376937_at   7.621 7.16 7.379 7.346 7.179 6.789 6.575 6.777 6.917 6.785
   6413   1377718_at   4.202 4.895 5.309 5.344 5.278 2.971 4.087 3.077 3.35 3.055
   6414   1395347_at   7.29 7.262 7.121 7.138 7.415 7.917 8.054 8.156 7.969 7.531
   6415   1377637_at   4.529 5.226 4.789 4.949 5.021 3.907 3.608 2.531 3.243 3.652
   6416   1395409_at   8.081 8.168 8.354 8.289 8.435 7.796 7.362 7.635 7.861 7.515
   6417   1378152_at   6.895 6.461 6.481 6.431 6.436 7.182 6.972 7.222 7.117 7.383
   6418   1373488_at   9.158 9.145 8.985 9.213 9.224 8.334 8.805 8.571 8.119 8.391
   6419   1388317_at   8.18 8.164 8.182 8.324 8.285 8.439 8.476 8.642 8.463 8.492
   6420   1377180_at   5.355 6.075 5.747 5.607 5.435 6.597 6.664 6.845 6.456 6.253
   6421   1383163_at   6.025 5.849 5.218 5.332 5.441 4.336 4.672 4.555 4.526 3.892
   6422   1398870_at   10.808 10.55 10.721 10.758 10.802 11.145 11.171 10.952 11.049 11.059
   6423   1390546_at   6.713 6.601 7.035 7.447 7.333 7.966 8.054 8.194 7.968 7.886
   6424   1379899_at   1.511 0.202 2.95 0.687 2.422 4.763 4.388 4.833 4.242 4.494
   6425   1376184_at   5.714 4.86 5.894 5.292 5.538 6.478 6.665 7.28 6.687 6.627
   6426   1373627_at   6.783 6.982 7.259 6.773 6.944 7.64 7.582 7.372 7.481 7.493
   6427   1389481_at   9.44 9.486 9.408 9.433 9.425 9.778 9.842 9.822 9.833 9.545
   6428   1382252_at   6.545 6.662 6.472 6.446 6.779 5.553 6.093 5.478 4.996 5.538
   6429   1376678_at   6.363 6.16 6.532 6.597 6.463 6.916 7.238 6.859 6.957 6.994
   6430   1378037_at   7.648 7.768 7.778 7.827 7.714 8.171 7.92 8.094 8.258 8.096
   6431   1379321_at   5.224 5.375 5.347 5.098 4.782 5.758 6.31 6.475 6.238 5.9
   6432   1372579_at   8.175 8 8.09 8.346 8.427 8.859 9.111 8.873 8.844 8.591
   6433   1375900_at   7.059 7.7 7.66 8.165 7.417 2.614 5.166 4.657 4.793 5.509
   6434   1375341_at   7.936 7.726 7.851 7.866 7.891 7.284 7.124 6.442 7.038 7.126
   6435   1389689_at   5.821 5.941 5.882 5.992 5.912 6.564 6.732 6.856 6.259 6.372
   6436   1376497_a_at   7.014 7.059 7.462 7.024 6.839 6.341 6.588 6.169 6.023 5.986
   6437   1378021_at   5.872 5.54 5.688 5.507 5.431 6.1 6.506 6.469 6.131 6.66
   6438   1393139_at   4.509 4.935 5.233 4.761 3.31 7.422 6.937 7.187 7.102 5.951
   6439   1385517_at   6.778 6.891 6.924 6.918 6.657 7.658 7.621 7.149 7.454 7.329
   6440   1370026_at   10.615 10.625 11.273 10.984 10.454 11.793 11.706 12.184 11.619 11.713
   6441   1377117_at   6.135 5.702 6.245 6.532 6.937 4.938 5.213 5.152 4.516 4.522
   6442   1381441_at   6.945 7.057 7.279 7.095 6.553 2.365 3.654 4.195 3.396 0
   6443   1374461_at   7.587 7.51 7.561 7.576 7.751 7.802 7.881 8.031 7.898 7.966
   6444   1379781_at   5.609 5.389 5.809 5.677 5.295 6.504 6.223 6.212 6.171 6.057
   6445   1379950_at   4.111 4.147 4.598 3.869 2.556 6.451 5.814 6.361 6.471 5.482
   6446   1368668_at   8.201 8.065 8.212 8.407 8.57 7.619 7.896 7.674 7.683 7.736
   6447   1385797_at   6.458 6.895 6.759 6.379 6.83 4.04 3.548 4.615 5.768 4.467
   6448   1378733_at   6.605 6.494 5.902 6.63 6.324 4.504 5.015 4.962 4.671 3.328
   6449   1388445_at   10.301 10.289 10.333 10.317 10.12 10.772 10.723 10.738 10.489 10.559
   6450   1388680_at   8.694 8.707 8.654 8.617 8.846 9.282 9.439 9.287 9.07 8.989
   6451   1391077_at   4.86 5.187 5.4 6.194 5.07 0.807 2.214 3.521 1.88 0
   6452   1373266_at   6.616 6.655 6.762 7 6.306 7.675 7.436 7.885 7.242 7.528
   6453   1377194_a_at   8.757 8.731 8.677 8.75 9.129 8.351 8.28 8.236 8.42 8.29
   6454   1397917_at   6.44 6.136 6.514 6.493 6.692 5.616 5.462 5.38 5.008 4.471
   6455   1378345_at   6.624 6.598 6.66 6.372 6.565 6.192 6.045 5.641 5.719 5.983
   6456   1379755_at   6.654 6.722 7.191 7.084 7.041 7.801 8.227 8.074 7.625 7.591
   6457   1377077_at   6.203 5.848 6.065 6.1 6.026 5.346 5.511 5.379 5.381 4.812
   6458   1393173_at   7.843 8.004 8.187 7.927 7.64 7.105 7.498 6.765 7 6.957
   6459   1383951_at   8.43 8.417 8.259 8.335 8.398 8.032 8.048 7.609 7.846 7.722
   6460   1398424_at   8.659 8.649 8.795 8.985 8.75 9.332 9.419 9.484 9.132 9.137
   6461   1392044_at   8.236 8.248 8.068 8.554 8.501 8.779 9.155 8.931 8.877 8.96
   6462   1376272_s_at   9.477 9.521 9.048 9.376 9.728 8.699 8.808 8.661 8.902 8.663
   6463   1395604_at   5.041 5.376 5.473 5.707 5.105 6.136 6.358 6.058 6.092 6.62
   6464   1368779_a_at   4.854 4.94 4.193 5.292 1.824 7.881 7.504 8.144 7.805 7.682
   6465   1383074_at   8.377 8.366 8.571 8.517 8.439 7.928 8.143 7.95 7.902 7.588
   6466   1367891_a_at   6.495 6.509 6.748 6.611 6.267 5.63 5.316 4.888 5.96 5.278
   6467   AFFX_Rat_beta-actin_3_at   13.254 13.143 13.162 12.804 12.956 12.61 12.523 12.48 12.671 12.562
   6468   1378156_at   8.376 8.263 8.435 8.8 8.079 9.29 9.026 9.314 9.803 9.409
   6469   1388174_at   5.322 5.33 5.511 5.149 5.006 5.921 5.712 6.115 5.99 5.769
   6470   1395132_at   5.876 5.344 5.34 5.749 5.506 6.377 6.465 6.243 6.168 6.118
   6471   1385462_at   5.848 5.621 5.761 5.822 5.827 6.056 6.096 5.99 6.23 6.155
   6472   1376217_at   5.406 5.107 5.116 4.899 5.035 5.904 5.556 6.022 5.628 5.923
   6473   1370460_at   7.806 7.768 7.798 7.884 7.849 7.245 7.635 7.185 7.158 7.084
   6474   1390351_at   6.391 6.633 6.61 6.614 6.513 5.984 6.227 6.022 6.119 6.298
   6475   1388478_at   8.706 8.802 8.499 8.612 8.843 8.011 8.374 8.14 7.963 8.229
   6476   1380741_at   4.778 5.193 5.808 5.539 5.018 6.292 6.451 6.475 6.297 6.2
   6477   1392847_at   5.735 6.119 6.144 6.249 6.038 5.4 5.213 5.167 5.675 5.261
   6478   1373567_at   1.316 2.731 4.082 1.692 3.094 5.746 5.189 5.512 5.491 5.252
   6479   1382602_at   7.159 7.348 7.248 7.335 7.245 7.725 8.009 7.781 7.63 7.562
   6480   1370334_at   4.564 5.523 5.511 5.215 5.762 6.511 6.663 7.313 6.716 6.526
   6481   1381070_at   3.995 2.907 4.031 3.422 3.583 5.081 5.224 5.083 4.463 4.996
   6482   1390969_at   5.358 5.761 5.648 5.493 5.621 6.928 6.936 6.425 6.21 6.265
   6483   1389478_at   7.588 7.672 7.368 7.924 7.746 8.083 8.279 8.25 8.327 8.197
   6484   1398933_at   9.455 9.482 9.609 9.889 9.826 10.251 10.297 10.297 10.183 10.048
   6485   1383958_at   5.017 5.635 5.847 5.879 5.597 4.052 4.32 4.679 4.804 4.278
   6486   1385564_at   4.947 5.315 5.318 5.208 5.099 4.681 4.306 3.926 4.416 3.89
   6487   1397185_at   8.819 8.587 8.887 8.715 8.128 9.436 9.491 9.274 9.483 9.401
   6488   1377011_at   5.693 5.417 6.035 5.841 5.825 6.538 6.898 6.617 6.244 6.632
   6489   1380773_at   4.586 5.308 5.304 5.314 5.003 6.202 6.139 6.255 6.28 5.687
   6490   1387269_s_at   6.237 5.609 6.738 6.498 6.222 1.986 3.921 3.605 1.945 4.444
   6491   1367828_at   8.713 8.259 8.844 8.695 8.487 9.129 9.195 9.196 9.44 9.374
   6492   1368712_at   7.518 7.449 7.443 7.137 7.324 8.025 7.869 8.012 7.803 7.709
   6493   1385369_at   5.964 5.695 5.548 5.819 5.977 6.595 6.703 7.064 6.548 6.295
   6494   1383069_at   6.572 6.731 6.819 6.853 7.147 7.406 7.555 7.545 7.309 7.332
   6495   1385504_at   6.033 6.321 6.106 6.333 6.173 5.926 5.424 5.329 5.381 5.436
   6496   1374745_at   5.596 5.296 5.207 5.619 5.803 6.391 6.652 6.969 6.196 6.3
   6497   1370230_at   11.067 11.121 11.312 11.256 11.296 11.546 11.595 11.575 11.606 11.416
   6498   1376464_at   5.162 4.999 5.452 5.091 4.826 5.816 6.084 5.955 5.6 5.78
   6499   1368489_at   5.339 4.672 5.843 6.111 5.702 3.871 3.59 4.36 3.482 4.066
   6500   1382643_at   8.111 8.322 7.936 8.061 8.113 7.741 7.676 7.553 7.803 7.586
   6501   1389717_at   8.367 8.38 8.457 8.552 8.365 8.038 8.165 8.18 8.054 8.228
   6502   1398312_s_at   5.076 5.377 5.396 5.079 5.593 6.271 6.077 6.906 6.376 6.055
   6503   1396302_at   5.181 5.356 5.421 5.577 4.991 6.058 6.002 5.817 5.873 5.878
   6504   1391621_at   4.619 4.838 4.813 4.616 4.781 4.418 4.059 3.945 4.323 4.043
   6505   1388301_at   11.484 11.528 11.44 11.322 11.342 11.653 11.677 11.814 11.717 11.656
   6506   1371337_at   10.191 10.307 10.282 10.274 10.468 9.927 10.074 10.042 9.897 9.866
   6507   1384487_at   5.564 4.804 4.556 4.708 4.327 5.866 5.909 6.459 6.422 6.136
   6508   1380127_at   0 1.915 1.604 0.941 2.807 4.71 3.968 3.889 4.168 4.438
   6509   1390481_a_at   5.768 6.128 5.73 5.764 5.767 1.731 2.803 1.379 4.559 1.263
   6510   1371196_at   5.798 6.069 6.086 5.86 5.818 3.631 2.387 3.908 1.891 0.536
   6511   1374085_at   7.512 7.375 7.639 7.588 7.913 8.102 8.26 8.157 8.154 8.042
   6512   1391195_at   6.654 6.496 6.856 6.814 6.716 6.13 6.049 6.319 6.326 5.94
   6513   1375978_at   4.183 4.32 4.977 4.92 4.708 1.561 0.632 1.705 3.536 1.111
   6514   1372680_at   8.815 8.73 8.839 9.004 8.829 8.553 8.639 8.462 8.433 8.513
   6515   1375337_at   8.45 8.487 8.311 8.498 8.507 8.664 8.817 8.675 8.857 8.725
   6516   1397540_at   7.399 7.322 7.468 7.376 7.45 6.502 7.007 6.742 7.066 6.726
   6517   1381474_at   5.386 5.19 5.311 5.713 5.901 2.011 0.731 3.194 3.736 2.746
   6518   1390113_a_at   10.273 10.438 10.166 10.522 10.501 9.756 9.78 9.981 9.491 9.416
   6519   1368129_at   7.337 7.466 7.084 7.31 7.376 6.831 6.654 6.586 6.651 6.135
   6520   1388383_at   8.553 8.586 8.422 8.4 8.204 8.944 9.212 8.986 8.849 8.844
   6521   1374072_at   8.403 8.524 8.567 8.692 8.674 7.998 8.258 8.105 7.866 7.755
   6522   1385668_at   5.793 5.577 5.973 5.469 5.374 7.021 7.113 7.232 6.153 6.83
   6523   1368331_at   9.937 9.892 9.766 9.806 9.92 9.442 9.522 9.03 9.433 9.27
   6524   1377100_at   7.511 7.599 7.613 7.753 7.681 7.187 7.353 6.842 7.103 6.986
   6525   1377776_at   6.055 6.36 6.217 6.477 5.807 7.439 7.118 7.345 7.266 6.689
   6526   1388069_at   6.162 6.138 6.106 5.9 6.087 3.555 3.787 1.351 4.044 3.77
   6527   1398845_at   9.749 9.715 9.683 9.857 9.813 10.269 10.304 10.246 9.943 10.115
   6528   1389220_at   8.171 8.231 8.334 8.299 8.106 7.702 7.821 7.997 7.811 7.592
   6529   1391553_at   5.409 4.665 5.157 5.264 4.935 6.005 5.964 6.057 5.819 5.692
   6530   1385831_at   5.444 5.105 5.681 5.786 5.739 2.852 1.7 3.662 0.367 2.814
   6531   1373576_at   8.674 8.618 8.408 8.633 8.67 8.322 8.327 8.306 8.201 8.317
   6532   1371463_at   9.389 9.481 9.284 9.464 9.493 8.925 9.169 9.122 8.848 8.852
   6533   1385522_at   4.802 5.185 5.044 3.994 4.834 0 0 2.47 0 2.707
   6534   1391158_a_at   8.536 8.303 8.612 8.565 8.664 8.987 9.164 9.159 9.031 8.82
   6535   1395052_at   4.159 5.619 4.644 4.626 5.213 6.104 6.46 6.55 6.261 6.322
   6536   1395725_at   8.281 8.268 8.316 8.282 8.313 8.957 9.237 8.631 8.738 8.829
   6537   1377390_at   6.661 6.592 6.536 6.863 7.422 5.758 5.672 5.952 5.805 6.025
   6538   1398519_at   4.679 5.46 4.243 4.236 4.553 0 0 2.459 0.433 2.609
   6539   1396231_at   5.997 6.111 6.275 5.689 5.15 1.417 1.637 4.652 1.449 1.345
   6540   1382356_at   4.583 4.627 4.762 4.468 4.548 4.985 5.381 5.451 5.853 5.703
   6541   1391388_at   5.545 5.446 5.339 5.401 5.375 4.068 3.778 4.964 4.045 3.596
   6542   1387391_at   7.068 7.69 7.418 7.443 7.295 6.345 5.431 5.407 4.202 4.476
   6543   1379518_at   4 5.618 5.277 4.923 4.285 6.336 6.405 6.711 6.637 6.881
   6544   1368346_at   6.487 6.051 6.167 6.053 6.247 5.228 5.292 5.275 5.392 4.43
   6545   1369468_at   6.057 6.338 6.029 6.34 6.02 7.162 7.024 7.459 6.862 6.646
   6546   1373489_at   6.807 6.565 6.345 6.553 6.587 1.832 1.88 2.932 4.766 4.331
   6547   1389412_at   9.039 8.894 8.949 9.088 8.711 9.707 10.206 10.063 9.575 9.476
   6548   1379878_at   4.565 4.455 4.689 4.458 4.963 3.834 3.823 4.229 3.847 3.647
   6549   1385220_at   6.438 6.724 6.404 6.538 6.442 5.353 5.268 3.86 5.202 5.001
   6550   1385727_at   7.551 7.504 7.817 7.92 8.06 7.284 7.143 7.084 7.089 7.006
   6551   1371534_at   7.356 7.749 7.629 7.542 7.382 6.929 6.839 7.217 6.819 6.729
   6552   1370210_at   1.714 1.536 1.345 1.77 1.506 1.176 1.091 1.007 0.941 1.17
   6553   1388870_at   9.332 9.198 9.335 9.271 9.477 10.199 10.37 10.339 9.995 9.647
   6554   1378540_at   6.385 6.117 6.677 6.148 6.339 5.245 5.247 5.357 4.41 4.247
   6555   1397569_at   5.438 5.452 5.662 5.862 5.385 6.519 6.26 5.943 6.36 6.392
   6556   1374839_at   7.876 7.568 7.285 7.744 7.635 8.334 8.923 8.502 8.443 8.227
   6557   1380387_at   3.449 4.033 4.628 4.344 3.121 5.535 5.617 5.542 5.244 5.733
   6558   1375028_at   7.389 7.37 7.032 7.163 7.106 4.367 3.685 5.31 3.636 1.516
   6559   1397268_at   5.217 5.657 5.488 6.008 5.571 6.519 6.634 6.163 6.835 6.491
   6560   1388467_at   9.013 8.938 9.132 9.211 9.022 9.645 9.622 9.457 9.5 9.298
   6561   1373162_at   7.758 7.786 7.722 8.119 8.11 8.437 8.656 8.761 8.428 8.402
   6562   1371395_at   10.442 10.377 10.482 10.545 10.503 10.255 10.299 10.158 10.159 10.057
   6563   1367475_at   11.372 11.381 11.229 11.232 11.277 11.12 11.134 11.053 11.073 11.099
   6564   1388390_at   10.95 10.965 10.821 10.881 11.014 10.759 10.689 10.75 10.666 10.66
   6565   1375904_at   9.771 9.815 9.733 9.668 9.844 9.327 9.388 8.879 9.21 9.33
   6566   1378198_at   6.185 6.177 6.115 6.067 6.159 4.818 4.925 5.55 5.295 4.354
   6567   1376848_at   7.604 7.353 7.908 7.804 7.596 8.329 8.47 8.192 8.209 8.167
   6568   1377387_a_at   6.247 6.4 6.045 6.478 6.12 5.585 5.763 5.055 5.475 5.228
   6569   1369494_a_at   3.684 4.465 4.391 4.309 4.468 5.572 5.623 6.043 5.216 5.091
   6570   1369443_at   7.606 7.513 7.868 7.52 7.789 7.142 6.489 6.355 7.004 6.497
   6571   1368321_at   8.76 8.676 8.549 8.717 8.217 10.386 10.176 11.985 9.945 10.865
   6572   1395039_at   6.62 6.832 6.515 7.006 6.976 5.636 5.634 6.372 5.882 5.725
   6573   1369382_at   7.082 6.926 7.25 7.313 6.95 6.219 5.851 4.912 6.106 5.626
   6574   1391086_at   7.124 7.21 7.157 7.328 7.411 6.87 6.539 6.712 6.923 6.575
   6575   1376185_at   7.334 7.315 6.941 7.405 7.504 6.733 6.46 6.835 6.382 6.522
   6576   1388405_at   8.166 8.388 7.98 7.949 8.149 7.531 7.749 7.226 7.244 7.242
   6577   1385113_at   7.118 6.691 7.119 7.228 6.948 7.694 7.456 7.583 7.581 7.731
   6578   1377029_at   5.868 6.541 6.356 6.817 6.508 7.387 7.777 7.617 7.209 7.319
   6579   1378040_at   6.849 7.162 6.841 6.932 6.972 6.594 6.157 6.098 6.404 6.449
   6580   1391479_at   4.825 4.685 4.917 5.116 4.216 5.485 6.034 5.932 5.633 5.714
   6581   1376664_at   7.016 7.123 7.413 7.322 7.281 6.7 6.682 6.895 6.786 6.824
   6582   1395445_at   6.893 6.985 7.119 7.152 7.235 6.468 6.27 6.733 6.633 6.355
   6583   1373501_at   9.608 9.632 9.567 9.676 9.49 10.03 9.87 10.004 9.904 9.764
   6584   1371612_at   10.133 10.156 10.082 10.138 10.189 10.34 10.293 10.363 10.225 10.295
   6585   1374616_at   5.629 5.095 5.386 5.033 5.728 4.599 3.626 4.396 4.128 4.17
   6586   1367595_s_at   13.487 13.337 13.187 12.913 13.12 12.641 12.561 12.56 12.713 12.706
   6587   1371688_at   10.294 10.323 10.332 10.419 10.54 10.128 10.119 10.06 9.906 9.981
   6588   1382134_at   7.756 7.648 7.787 7.511 7.942 8.487 8.447 8.106 8.726 8.788
   6589   1374783_at   6.69 6.728 7.313 6.987 6.715 7.444 7.614 7.66 7.575 7.756
   6590   1373481_at   8.735 8.837 9.081 9.023 8.879 8.259 8.478 8.5 8.174 8.517
   6591   1389027_at   7.614 7.525 7.645 7.728 7.91 8.337 8.487 8.449 8.07 8.099
   6592   1374160_at   5.288 5.168 5.488 5.499 5.943 6.36 6.115 6.466 6.644 6.249
   6593   1384346_at   4.612 4.895 5.079 4.555 4.743 6.023 6.478 6.241 5.273 6.102
   6594   1398981_at   8.534 8.708 8.681 8.929 8.625 9.188 9.359 9.114 9.16 9.027
   6595   1382077_at   6.276 5.844 5.935 5.94 6.141 5.601 5.432 5.112 5.371 5.477
   6596   1387033_at   1.852 3.363 3.394 3.485 1.678 6.248 6.725 8.792 5.976 5.5
   6597   1376800_at   7.056 6.955 7.405 6.968 6.776 7.751 7.808 7.749 7.65 7.487
   6598   1393138_at   10.702 10.684 10.615 10.774 10.716 11.424 11.178 11.43 10.914 11.314
   6599   1388475_at   7.311 7.563 7.322 7.477 7.522 8.015 8.046 8.088 7.744 7.774
   6600   1385020_at   7.344 7.47 7.479 7.892 7.96 6.66 7.05 6.94 6.69 6.616
   6601   1394953_at   6.005 5.621 5.919 6.279 6.317 4.891 4.564 3.057 4.058 4.242
   6602   1395329_at   6.317 6.744 6.533 6.924 6.888 5.681 5.882 5.65 5.693 6.207
   6603   1371731_at   2.414 4.405 3.349 3.425 3.704 6.238 5.211 5.97 5.037 5.752
   6604   1387041_at   9.51 9.534 9.59 9.659 9.669 9.369 9.379 9.188 9.2 9.35
   6605   1379578_at   8.528 8.536 8.759 8.928 9.023 9.292 9.907 9.684 9.394 9.597
   6606   1370615_at   4.154 4.323 4.279 4.305 4.845 4.872 5.383 5.643 5.463 5.554
   6607   1378287_at   7.842 7.932 8.005 8.042 7.927 8.502 8.6 8.265 8.288 8.265
   6608   1371465_at   9.236 9.288 9.218 9.42 9.412 9.573 9.715 9.752 9.58 9.57
   6609   1386004_s_at   11.36 11.231 11.218 11.236 11.347 10.981 11.088 10.829 10.999 11.027
   6610   1371374_at   8.938 8.611 8.807 9.018 8.741 9.335 9.318 9.349 9.249 9.136
   6611   1367935_at   9.277 9.229 9.294 9.374 9.424 9.08 9.091 8.856 9.045 8.992
   6612   1383089_at   9.098 9.103 9.356 9.252 9.407 8.674 8.941 8.521 8.632 8.406
   6613   1398277_at   6.431 6.051 6.317 6.533 5.95 7.121 7.094 6.86 6.834 6.889
   6614   1390323_at   5.778 5.919 5.589 5.941 6.194 6.296 6.583 6.559 6.689 6.637
   6615   1378915_x_at   6.277 6.525 6.742 6.359 6.518 3.478 2.848 3.388 0 3.666
   6616   1398719_at   5.665 5.118 5.549 5.889 5.638 2.703 2.128 4.065 4.064 2.081
   6617   1389302_at   7.63 7.814 8.004 7.882 7.899 7.451 7.311 7.016 7.161 6.835
   6618   1390530_at   10.281 10.402 10.409 10.293 10.068 11.377 11.393 10.607 11.415 11.475
   6619   1391317_at   6.685 6.945 6.462 6.658 6.817 2.991 3.838 0.111 0.263 3.601
   6620   1373293_at   7.405 7.42 7.037 7.366 7.445 6.497 6.66 7.005 6.631 6.745
   6621   1370310_at   6.193 6.155 5.387 5.338 5.577 7.679 7.194 6.669 8.177 7.758
   6622   1385759_at   3.472 3.892 3.162 3.367 2.947 1.623 1.811 0 1.655 1.516
   6623   1398062_at   6.178 6.397 6.434 6.375 6.38 5.905 5.262 4.643 5.005 4.973
   6624   1379603_at   6.122 6.489 7.026 6.766 7.239 5.62 5.702 5.28 5.7 5.346
   6625   1399052_at   8.289 8.312 8.316 8.646 8.423 8.879 9.095 9.005 8.706 8.885
   6626   1391813_at   5.727 5.434 5.945 6.064 5.735 6.827 7.034 6.614 6.401 6.454
   6627   1383019_at   7.188 7.072 7.191 7.412 7.255 7.62 8.077 7.748 7.626 7.858
   6628   1388975_at   10.13 10.178 10.452 10.472 10.267 10.762 10.907 10.617 10.797 10.744
   6629   1383922_a_at   6.874 6.696 6.654 6.825 6.723 6.042 6.22 6.047 5.477 6.157
   6630   1380136_at   4.641 4.06 5.169 4.571 4.482 6.22 6.166 5.72 5.371 6.151
   6631   1384285_at   5.835 6.014 5.833 6.419 6.468 5.307 5.236 5.051 5.459 5.275
   6632   1398266_a_at   4.997 5.211 5.748 5.18 5.256 4.403 3.664 4.596 4.024 3.948
   6633   1395363_at   6.964 6.825 6.971 7.222 7.011 6.501 5.959 5.764 6.425 6.118
   6634   1377877_at   5.005 5.317 4.932 5.093 5.649 6.777 6.809 7.621 6.301 6.231
   6635   1373267_at   7.041 7.184 7.236 7.099 7.015 7.777 8.306 7.712 7.784 7.589
   6636   1388315_at   11.701 11.726 11.793 11.662 11.654 12.18 12.073 11.841 12.099 12.165
   6637   1373669_at   5.98 6.004 6.217 6.048 6.134 5.23 4.409 4.119 5.02 5.223
   6638   1386530_at   4.581 5.307 4.233 5.078 5.285 6.182 5.962 6.001 6.435 6.179
   6639   1367813_at   6.09 5.638 5.942 5.9 5.863 6.579 6.562 6.478 6.177 6.479
   6640   1393046_at   7.287 7.207 7.38 7.289 7.587 7.038 6.855 6.742 6.811 6.586
   6641   1398836_s_at   13.255 13.16 13.059 12.838 13.004 12.605 12.388 12.475 12.695 12.646
   6642   1390816_at   5.491 5.336 5.87 5.539 5.802 4.994 4.21 3.462 3.873 3.991
   6643   1390832_at   8.757 8.636 8.259 8.933 9.036 7.93 8.113 7.845 7.668 7.83
   6644   1373194_at   7.977 8.139 8.149 7.841 8.072 8.435 8.647 8.387 8.437 8.37
   6645   1390623_at   5.983 6.205 5.746 5.994 6.212 3.018 3.178 2.214 4.747 1.395
   6646   1373329_at   8.395 8.557 8.159 8.604 8.798 9.286 9.642 10.067 9.232 9.418
   6647   1375015_at   6.877 7.116 7.338 7.317 7.296 6.495 5.911 6.678 6.032 6.027
   6648   1373808_at   8.068 8.172 8.348 8.475 8.369 8.715 8.976 8.825 8.675 9.029
   6649   1381201_at   6.107 5.564 5.722 5.974 6.378 6.989 7.435 7.632 6.809 6.87
   6650   1383790_at   6.422 6.519 6.417 6.212 6.514 6.776 7.125 6.987 7.03 6.737
   6651   1387046_at   6.388 6.472 6.463 6.123 6.346 6.966 7.038 7.402 6.814 6.888
   6652   1399109_at   6.945 6.8 6.997 6.722 6.517 7.388 7.632 7.879 7.36 7.375
   6653   1373119_at   7.174 6.676 6.687 6.895 7.018 7.483 7.632 7.648 7.331 7.457
   6654   1367969_at   10.077 10.009 10.268 10.514 10.38 10.847 10.77 10.833 10.834 10.681
   6655   1382148_at   6.67 6.415 6.556 6.597 6.71 5.985 5.918 5.937 6.332 5.73
   6656   1391009_a_at   4.985 4.173 5.572 5.603 5.385 1.996 3.309 3.053 1.104 0.911
   6657   1388401_at   8.705 8.736 8.546 8.876 8.826 9.245 9.104 9.031 9.115 9.319
   6658   1394710_at   5.886 6.182 6.25 6.156 6.051 5.041 5.046 5.545 5.545 5.543
   6659   1378471_at   3.864 4.266 4.92 3.856 4.078 5.485 5.168 5.202 5.411 5.379
   6660   1394948_at   7.206 7.207 7.19 7.127 7.061 7.997 7.958 8.316 7.693 7.552
   6661   1391220_at   8.139 8.076 7.941 8.212 8.468 7.59 7.695 7.693 7.453 7.678
   6662   1383293_at   5.07 3.777 4.074 4.473 4.643 6.05 6.418 5.498 5.901 5.702
   6663   1388876_at   4.576 4.407 4.899 4.318 4.938 5.741 5.209 5.731 5.654 5.362
   6664   1390820_at   6.542 6.338 6.486 6.615 6.985 6.037 5.989 5.511 5.713 5.65
   6665   1389805_at   5.643 5.334 5.918 5.894 6.135 6.801 6.855 7.419 6.875 6.51
   6666   1389966_at   9.348 9.553 9.557 9.9 10.227 8.885 8.761 8.487 8.434 8.922
   6667   1371635_at   7.131 6.919 7.089 7.411 7.184 7.815 7.666 8.068 7.816 7.568
   6668   1385626_at   4.39 5.657 5.335 5.127 5.067 6.876 6.76 6.835 6.488 5.974
   6669   1389537_at   6.874 6.708 6.68 6.909 6.576 5.767 5.492 5.94 5.715 4.725
   6670   1384621_at   6.055 6.192 5.835 6.161 5.961 6.565 6.68 6.912 6.582 6.399
   6671   1399132_at   9.937 9.965 9.926 10.116 9.976 10.268 10.447 10.35 10.176 10.256
   6672   1393545_at   4.797 4.943 5.109 5.89 5.199 6.463 6.212 6.068 6.618 6.405
   6673   1397363_at   6.672 6.856 6.85 6.866 6.787 7.424 7.828 7.286 7.226 7.356
   6674   1368943_at   8.005 8.104 7.944 8.018 8.595 7.01 6.641 5.659 7.098 6.507
   6675   1393910_at   4.886 5.124 5.058 5.472 5.428 6.103 6.211 5.943 6.417 5.733
   6676   1385320_at   2.511 4.009 3.921 4.268 3.833 5.243 5.577 5.879 5.306 5.562
   6677   1391491_a_at   7.621 7.707 7.895 7.928 7.83 7.444 7.137 6.98 7.389 7.062
   6678   1372221_at   8.141 8.277 8.069 8.379 8.385 8.724 8.9 8.859 8.674 8.552
   6679   1379078_at   3.822 3.794 4.348 4.07 3.141 5.295 5.299 5.093 5.854 4.829
   6680   1389206_at   2.886 3.319 3.675 2.852 1.454 5.176 5.408 5.768 4.916 4.622
   6681   1390202_at   6.481 6.372 6.693 6.753 6.48 7.188 7.062 6.919 7 6.962
   6682   1371310_s_at   10.275 10.407 10.048 10.409 10.744 9.373 9.582 9.725 9.453 9.845
   6683   1390101_at   6.15 6.082 6.296 6.528 6.407 6.825 7.417 7.714 7.31 7.016
   6684   1393387_at   0 0.536 0 2.128 0.465 3.802 3.457 4.409 2.35 3.31
   6685   1369090_at   3.057 2.803 3.511 3.037 4.405 5.13 5.255 4.574 5.227 5.198
   6686   1377926_at   4.163 4.408 5.146 5.408 4.851 6.176 6.197 6.259 6.047 5.87
   6687   1388430_at   8.503 8.288 8.117 7.933 8.234 8.756 8.674 8.789 8.759 8.738
   6688   1376159_at   7.176 6.932 7.069 6.934 7.017 7.349 7.366 7.209 7.369 7.423
   6689   1397411_at   6.617 6.378 6.386 6.77 6.248 3.099 3.955 4.2 2.916 0.614
   6690   1368947_at   9.408 9.577 9.669 9.786 9.753 10.927 10.735 11.075 10.039 10.642
   6691   1389596_at   7.925 8.06 8.189 8.33 8.06 7.608 7.712 7.808 7.685 7.595
   6692   1396280_at   6.478 6.745 6.844 6.845 6.993 5.633 6.166 5.553 5.202 4.847
   6693   1373129_at   8.073 8.029 7.913 8.156 8.128 7.525 7.763 7.756 7.379 7.526
   6694   1387373_at   5.443 5.665 5.695 5.022 5.302 7.003 6.689 5.938 6.867 6.81
   6695   1387164_at   0.66 0.084 1.811 1.227 0 3.268 3.695 3.799 3.517 2.114
   6696   1398233_at   3.748 4.673 4.649 4.719 4.651 5.376 5.478 5.651 5.59 5.785
   6697   1382048_at   9.082 9.222 9.441 9.365 9.363 9.752 10.039 9.709 9.957 9.671
   6698   1372067_at   9.48 9.483 9.454 9.505 9.576 9.128 9.322 9.293 9.086 9.264
   6699   1370286_at   8.948 9.095 9.18 9.547 9.281 9.814 9.728 9.731 9.902 9.766
   6700   1398867_at   8.619 8.654 8.685 8.899 8.88 8.336 8.32 8.393 8.247 8.015
   6701   1398345_at   9.264 9.283 9.125 9.409 9.474 8.796 8.743 8.136 8.69 8.508
   6702   1374764_at   6.456 6.396 6.676 6.988 6.859 5.376 6.156 5.824 5.668 5.507
   6703   1368521_at   11.457 11.398 11.4 11.444 11.488 11.788 11.649 11.596 11.873 11.709
   6704   1374707_at   7.603 7.489 7.472 7.739 7.65 6.919 7.241 7.287 6.97 7.126
   6705   1389463_at   5.933 6.226 5.918 6.273 6.548 6.911 6.899 7.513 7.012 7.027
   6706   1378860_at   7.483 7.555 7.605 7.797 7.655 8.024 8.362 8.473 8.178 7.995
   6707   1381567_at   5.48 5.795 5.501 5.561 5.926 4.747 4.654 4.641 4.099 3.585
   6708   1392726_at   4.675 4.599 4.785 4.848 4.601 5.415 5.268 5.186 5.404 4.948
   6709   1371521_at   9.315 9.283 9.396 9.503 9.638 8.905 8.915 9.139 8.815 8.7
   6710   1382745_at   6.308 6.209 6.289 6.744 6.725 5.743 5.939 5.697 5.739 5.526
   6711   1396467_at   3.678 3.819 3.86 3.596 3.811 4.23 4.951 4.71 4.298 4.892
   6712   1372040_at   6.638 6.644 6.827 7.145 6.614 7.556 7.582 7.217 7.424 7.375
   6713   1378978_a_at   5.258 4.989 4.974 5.535 5.155 5.764 6.148 6.135 5.79 5.789
   6714   1387261_at   6.396 6.58 6.737 6.359 6.621 5.808 5.887 6.028 5.517 6.089
   6715   1397911_at   4.85 5.418 5.382 5.304 4.929 6.16 5.891 5.842 5.846 5.828
   6716   1371058_at   6.226 5.924 7 6.131 5.657 4.151 4.591 5.21 4.666 4.156
   6717   1370299_at   12.796 12.714 12.676 12.524 12.502 13.21 13.031 12.923 13.288 13.287
   6718   1375085_at   6.017 6.138 6.193 6.584 6.431 6.974 6.999 7.234 6.823 6.822
   6719   1391078_at   7.023 7.153 7.263 7.31 7.29 6.579 6.717 6.607 6.761 6.174
   6720   1386755_at   7.229 7.213 7.29 7.615 7.236 7.936 8.001 7.904 7.739 7.674
   6721   1382472_at   5.38 5.441 5.892 6.12 5.59 4.371 4.271 3.462 4.815 4.371
   6722   1384002_at   7.603 7.558 7.568 7.59 7.376 7.279 7.329 7.252 7.147 7.121
   6723   1399102_at   8.087 8.057 8.323 8.333 8.379 8.756 8.599 8.59 8.608 8.662
   6724   1387390_at   6.011 6.299 6.755 6.824 6.429 4.21 4.971 5.229 5.223 5.471
   6725   1383288_at   8.865 9.05 8.916 8.93 8.759 8.353 8.304 8.504 8.569 8.636
   6726   1387351_at   7.854 7.908 7.787 8.402 8.8 7.082 7.019 7.088 7.127 7.076
   6727   1371419_at   11.423 11.546 11.505 11.607 11.468 11.749 11.928 11.716 11.864 12.006
   6728   1392999_at   6.514 6.802 6.609 5.849 6.801 7.619 7.778 7.461 7.34 7.581
   6729   1386797_at   5.557 4.879 5.231 5.344 4.902 3.415 3.154 4.412 3.463 2.566
   6730   1384523_at   6.714 6.466 6.651 6.578 6.66 5.711 5.736 6.214 5.633 5.194
   6731   1372578_at   7.404 7.832 7.628 7.676 7.786 6.598 6.984 7.198 6.391 6.502
   6732   1370356_at   8.136 8.255 7.938 8.389 8.407 7.726 7.721 7.774 7.477 7.496
   6733   1377809_at   8.153 8.066 8.131 8.371 8.4 8.595 8.737 8.684 8.564 8.59
   6734   1374357_at   7.26 7.357 7.317 7.382 7.446 7.038 6.752 7.068 6.795 6.625
   6735   1372481_at   6.695 7.242 6.802 6.998 7.371 7.816 7.636 7.771 7.736 7.727
   6736   1391516_at   4.969 5.458 5.251 4.769 5.362 6.517 6.049 6.601 6.164 5.808
   6737   1375896_at   8.998 9.106 9.106 8.802 8.866 9.531 9.366 9.262 9.364 9.431
   6738   1390385_at   7.257 7.496 7.393 7.643 7.6 7.155 6.965 6.678 6.844 6.881
   6739   1376060_at   7.663 7.791 7.574 7.878 7.755 8.322 8.478 8.085 8.058 8.344
   6740   1387740_at   6.428 5.899 5.929 5.62 5.82 7.535 7.018 6.526 7.391 7.358
   6741   1373133_at   9.847 9.937 10.061 9.967 10.032 9.648 9.711 9.4 9.506 9.678
   6742   1375936_at   5.786 5.394 5.374 5.582 5.774 4.358 4.189 5.185 4.222 4.426
   6743   1383760_at   2.832 4.054 2.521 4.453 3.815 5.648 5.486 5.768 5.706 5.334
   6744   1367913_at   9.4 9.37 9.1 9.667 9.836 8.737 8.552 8.335 8.756 8.799
   6745   1367721_at   11.886 11.825 11.636 11.89 11.88 12.215 12.1 12.254 12.119 12.067
   6746   1391944_at   5.477 5.233 5.05 5.57 5.598 6.004 6.212 6.332 6.501 5.931
   6747   1398770_at   10.979 10.832 10.923 10.898 11.129 10.657 10.665 10.648 10.562 10.442
   6748   1394702_at   4.487 4.881 5.066 4.549 4.807 5.527 5.567 5.43 5.22 5.398
   6749   1378998_at   4.612 5.436 4.991 4.793 5.057 6.614 6.247 6.852 5.902 5.837
   6750   1383469_at   6.757 6.529 7.462 6.095 6.8 7.851 8.286 8.366 7.981 7.873
   6751   1382234_at   6.756 6.651 6.504 6.782 6.559 6.899 7.075 7.013 6.991 6.951
   6752   1384277_at   6.534 6.849 6.624 6.803 6.525 7.239 7.375 7.797 7.156 7.315
   6753   1388852_at   8.916 8.82 8.828 9.151 8.849 9.348 9.633 9.744 9.388 9.318
   6754   1387771_a_at   8.724 8.619 8.621 8.652 8.499 9.219 8.914 9.008 9.172 8.885
   6755   1380028_at   6.835 7.119 7.378 7.316 7.105 6.269 6.264 6.704 6.037 6.471
   6756   1380089_at   5.398 4.769 5.195 5.016 4.351 6.441 6.152 6.261 5.678 6.447
   6757   1388833_at   8.498 8.753 8.791 8.795 8.532 9.167 9.098 9.112 9.048 8.96
   6758   1369212_s_at   7.237 7.105 7.383 7.5 7.407 8.31 8.398 8.191 7.936 7.713
   6759   1387834_at   5.015 5.468 5.258 4.938 5.359 4.401 3.959 4.55 4.099 3.558
   6760   1368843_at   10.208 9.954 10.042 9.973 10.045 9.618 9.809 9.33 9.469 9.475
   6761   1379483_at   8.01 7.664 7.937 7.655 7.352 8.312 8.405 8.324 8.423 8.563
   6762   1393719_at   7.517 7.555 7.495 7.941 7.637 8.076 8.388 8.047 8.205 8.188
   6763   1393309_at   6.483 6.563 6.412 6.683 6.448 7.216 7.419 7.356 7.263 6.759
   6764   1374270_at   5.446 5.768 5.438 5.581 5.686 4.74 4.688 4.796 4.431 5.248
   6765   1385644_at   6.839 6.806 6.734 7.212 6.988 6.45 6.421 6.088 6.265 5.991
   6766   1370848_at   9.06 8.973 9 9.259 8.99 8.35 8.307 8.752 7.993 8.167
   6767   1373002_at   7.411 7.319 7.341 7.663 7.45 7.716 8.091 8.154 8.002 7.897
   6768   1369364_at   2.48 0.986 2.459 0.496 2.359 4.34 3.73 4.303 4.067 4.191
   6769   1389129_at   8.082 8.122 8.41 8.693 8.081 9.332 9.467 8.751 9.555 9.585
   6770   1390364_at   6.328 6.321 6.323 6.686 6.772 5.891 5.61 5.776 5.3 5.141
   6771   1394939_at   5.792 5.762 6.333 5.88 5.45 7.561 6.913 6.707 6.933 6.766
   6772   1380313_at   5.135 5.603 5.804 5.394 4.756 3.474 0.941 0.918 3.406 1.705
   6773   1370534_at   0.642 0.014 0.774 0.66 0.722 1.47 4.318 3.59 4.029 3.516
   6774   1387917_at   9.691 9.866 9.861 9.898 9.861 9.296 9.471 9.54 9.317 9.052
   6775   1388058_at   7.059 7.016 7.192 7.328 7.184 7.646 7.504 7.396 7.526 7.586
   6776   1382629_at   7.677 7.765 7.718 7.51 8.051 6.943 6.962 6.062 6.77 6.851
   6777   1396057_at   4.129 3.944 4.594 4.631 4.57 5.751 5.892 5.193 5.107 5.771
   6778   1389019_at   7.216 7.068 7.057 7.201 7.308 7.643 7.855 8.171 7.545 7.825
   6779   1376882_at   7.609 7.783 7.665 7.967 7.823 8.28 8.34 8.511 8.062 8.252
   6780   1376084_a_at   6.262 6.303 6.337 6.42 6.314 2.111 3.955 3.391 0.748 4.131
   6781   1389372_at   8.777 9.117 8.971 8.978 8.74 8.431 8.52 8.555 8.275 8.488
   6782   1373424_at   6.81 6.557 6.94 7.201 7.043 7.903 7.992 8.173 7.476 7.588
   6783   1372296_at   4.262 4.403 4.489 4.692 4.547 5.133 4.887 5.348 5.033 5.48
   6784   1394136_at   7.456 7.252 7.239 7.165 7.257 6.995 7.059 6.858 6.868 6.895
   6785   1375686_at   8.353 8.268 8.351 8.394 8.588 7.755 8.156 7.684 7.583 7.616
   6786   1373394_at   6.598 6.586 6.535 6.464 6.732 6.357 6.307 6.295 6.322 6.174
   6787   1382400_at   6.602 6.359 6.107 6.498 6.709 4.984 5.388 5.749 5.545 5.775
   6788   1391427_at   6.94 6.844 7.355 7.026 7.218 6.574 6.347 5.76 6.048 6.161
   6789   1390399_at   6.252 6.233 6.535 6.648 6.316 6.944 7.405 6.93 7.461 7.058
   6790   1392040_at   5.229 5.25 5.504 5.539 4.531 0.757 3.004 3.383 2.82 2.718
   6791   1379943_at   4.061 3.885 3.559 4.089 4.184 5.261 5.528 4.55 4.982 4.878
   6792   1367621_at   9.273 9.194 9.495 9.634 9.648 8.819 8.759 9.09 8.728 8.747
   6793   1377469_at   5.738 5.672 5.457 6.148 6.258 6.697 7.057 6.817 6.565 6.773
   6794   1385374_at   5.563 5.423 5.555 5.087 5.274 6.478 6.623 6.524 6.144 5.823
   6795   1382461_at   6.997 7.071 7.38 7.315 7.07 6.642 6.523 6.106 6.331 5.849
   6796   1376265_at   10.097 10.072 9.717 9.874 9.917 10.249 10.425 10.408 10.3 10.443
   6797   1377334_at   5.227 5.047 5.209 5.501 5.888 1.678 2.316 0.757 3.9 3.03
   6798   1389423_at   7.482 7.484 7.433 7.485 7.607 7.331 7.089 7.073 6.935 7.15
   6799   1390502_at   6.58 6.827 5.934 6.815 6.739 5.417 5.424 5.861 5.65 5.414
   6800   1377699_at   3.976 4.517 5.238 4.8 4.668 3.802 2.302 1.884 2.367 2.428
   6801   1388774_at   10.995 10.959 11.282 11.119 11.092 10.732 10.837 10.721 10.777 10.751
   6802   1390197_at   6.852 6.836 6.788 6.985 6.992 6.623 6.455 6.432 6.644 6.329
   6803   1393098_at   7.424 7.759 7.663 7.959 7.968 8.33 8.445 8.584 8.435 8.206
   6804   1387432_at   6.919 7.317 7.233 7.289 6.778 6.145 5.393 5.036 6.317 5.384
   6805   1367496_at   10.531 10.54 10.594 10.638 10.71 10.792 10.943 10.858 10.776 10.842
   6806   1368012_at   7.767 7.64 7.556 8.084 7.973 6.764 7.197 7.309 6.756 6.934
   6807   1389659_at   7.504 7.553 7.931 8 7.578 7.12 7.016 7.043 6.584 6.608
   6808   1382190_at   4.536 4.379 4.122 4.219 4.925 5.418 5.417 5.16 5.371 5.08
   6809   1396449_at   6.526 6.618 6.896 6.778 6.561 6.175 6.142 6.054 6.376 6.242
   6810   1373862_at   10.597 10.629 10.544 10.415 10.622 10.044 10.277 10.16 9.928 10.278
   6811   1376049_at   6.36 7.093 6.538 6.592 6.798 7.294 7.609 7.373 7.354 7.627
   6812   1383952_at   5.176 5.449 5.446 4.81 5.502 3.849 3.142 3.445 4.236 2.356
   6813   1389208_at   4.686 4.114 4.719 4.984 4.948 5.669 5.903 5.576 5.482 5.51
   6814   1393192_at   6.887 6.741 6.969 6.928 6.954 7.741 7.661 7.634 7.43 7.141
   6815   1391921_at   6.89 7.212 7.14 7.136 7.302 6.847 6.545 6.614 6.714 6.546
   6816   1391511_at   4.803 4.249 4.424 4.713 4.709 3.487 3.027 4.037 3.568 3.599
   6817   1397221_s_at   5.151 5.075 5.065 5.043 5.512 4.218 4.121 2.975 3.007 3.86
   6818   1398835_at   13.275 13.176 13.17 12.824 12.998 12.589 12.51 12.391 12.648 12.7
   6819   1382132_at   3.67 0.799 0.978 2.365 0.774 4.782 5.624 4.279 5.181 5.596
   6820   1367863_at   7.698 7.518 7.436 7.765 7.688 7.167 7.172 7.333 6.954 6.995
   6821   1394327_at   6.576 6.55 6.42 6.724 6.522 6.193 6.119 5.765 6.143 5.819
   6822   1376274_at   7.434 7.174 7.221 7.529 7.464 6.945 6.872 6.935 7.064 6.829
   6823   1371428_at   11.294 11.402 11.236 11.184 11.287 10.981 11.115 10.939 10.844 10.99
   6824   1369849_at   5.783 5.477 5.829 5.961 5.773 4.394 3.589 2.913 2.266 4.348
   6825   1383361_at   9.747 9.744 9.664 9.653 9.756 9.365 9.452 9.253 9.301 8.986
   6826   1372836_at   7.734 8.171 8.278 8.408 8.012 9.078 9.127 8.584 8.909 8.964
   6827   1383849_at   6.071 6.056 6.272 6.164 6.389 5.161 5.834 5.212 5.597 5.295
   6828   1399029_at   7.79 7.762 7.701 7.776 7.77 7.657 7.584 7.543 7.521 7.648
   6829   1373159_at   5.485 5.071 4.876 5.301 5.164 6.719 6.905 5.802 6.121 6.392
   6830   1388818_at   5.737 5.881 5.973 6.1 6.299 6.846 7.062 6.557 6.598 6.601
   6831   1368181_at   10.154 10.139 10.149 10.231 10.046 10.665 10.781 10.76 10.305 10.65
   6832   1382433_at   3.099 3.338 3.461 4.436 3.934 5.334 5.32 5.021 4.669 5.133
   6833   1374976_a_at   8.354 8.455 8.632 8.78 8.499 8.003 8.156 7.753 7.837 7.558
   6834   1392492_at   8.137 8.175 7.98 8.372 8.279 8.689 8.999 8.956 8.637 8.582
   6835   1376419_at   6.204 6.331 6.732 6.47 6.538 5.543 5.86 5.763 5.76 5.152
   6836   1371660_at   9.354 9.412 9.235 9.485 9.652 9.018 9.035 9.094 8.869 8.857
   6837   1371709_at   10.225 10.215 10.233 10.167 10.191 9.962 10.048 9.914 9.814 9.721
   6838   1381956_at   3.512 3.991 4.642 4.074 4.899 6.148 5.589 5.32 5.806 5.886
   6839   1372077_at   10.228 10.237 10.343 10.488 10.34 10.126 10.041 9.981 10.047 9.981
   6840   1376698_at   6.548 6.803 6.764 6.901 6.741 7.356 7.318 7.217 7.208 6.987
   6841   1373930_at   6.644 6.494 6.277 6.732 6.794 7.407 7.508 7.012 7.222 7.202
   6842   1398841_at   11.889 11.889 11.863 11.745 11.837 11.6 11.628 11.371 11.602 11.572
   6843   1398778_at   10.56 10.733 10.65 10.491 10.626 10.213 10.21 9.639 10.006 9.935
   6844   1385451_at   5.553 5.197 5.06 5.336 5.092 0 3.119 1.731 3.173 0.516
   6845   1393310_at   8.151 8.145 8.223 7.979 7.783 8.679 8.66 8.359 8.612 8.73
   6846   1373542_at   7.736 7.773 7.814 7.866 7.55 8.339 8.631 8.7 8.201 8.165
   6847   1376668_at   6.668 6.432 5.94 6.573 6.313 4.168 3.768 3.769 4.512 1.609
   6848   1389005_at   6.481 6.588 6.61 6.924 7.006 7.765 7.574 7.564 7.545 7.123
   6849   1369942_at   10.561 10.51 10.623 10.811 10.542 11.079 11.059 10.955 10.973 10.836
   6850   1390409_at   8.08 8.001 8.367 8.305 8.195 7.553 7.821 7.586 7.646 7.251
   6851   1394025_at   5.097 5.8 5.4 5.14 5.05 6.463 5.87 6.239 6.203 6.439
   6852   1384284_at   4.64 4.013 4.104 4.107 4.412 6.013 5.673 6.169 5.278 4.962
   6853   1396185_at   6.678 6.547 6.451 6.751 6.981 6.057 5.603 5.515 6.185 5.561
   6854   1396866_s_at   8.089 8.234 8.093 7.755 8.148 7.596 7.577 7.112 7.452 7.3
   6855   1388084_at   6.161 6.162 6.766 5.886 6.224 4.155 1.978 4.302 2.011 1.111
   6856   1389065_at   7.202 7.273 6.981 7.256 6.958 6.383 6.864 6.404 6.323 6.245
   6857   1382544_at   7.563 7.406 7.95 7.631 7.162 8.671 8.272 8.194 8.796 8.436
   6858   1375632_at   12.028 11.977 12.05 12.018 12.071 11.952 11.858 11.817 11.836 11.771
   6859   1376321_at   7.355 7.439 7.401 7.727 7.696 6.5 6.277 7.155 6.36 6.148
   6860   1382041_at   7.156 7.496 7.764 7.487 7.201 8.285 8.164 8.125 8.092 7.888
   6861   1388704_at   9.476 9.546 9.55 9.595 9.607 9.251 9.4 9.203 9.032 9.161
   6862   1368819_at   11.059 11.081 11.001 11.106 11.132 10.749 10.797 10.82 10.5 10.803
   6863   1379725_at   8.114 8.119 8.211 8.363 8.196 7.927 7.834 7.505 7.772 7.683
   6864   1390278_at   8.169 8.124 7.871 8.175 8.47 7.741 7.598 7.619 7.603 7.53
   6865   1390214_a_at   7.646 7.704 7.61 7.996 7.818 7.226 7.249 7.482 7.141 7.116
   6866   1387412_at   6.994 7.039 7.252 7.081 7.23 6.43 6.045 6.558 6.755 6.382
   6867   1395809_at   5.91 5.785 6.023 5.698 6.34 7.222 6.739 6.647 7.237 6.645
   6868   1371962_at   10.074 9.996 10.232 10.214 10.197 10.457 10.341 10.402 10.506 10.448
   6869   1376401_at   7.799 7.988 7.854 8.076 7.978 8.441 8.364 8.525 8.255 8.197
   6870   1396847_at   5.009 4.806 5.106 4.961 4.659 6.002 5.809 5.543 5.892 5.304
   6871   1384504_at   5.969 5.591 5.307 5.236 5.476 6.208 6.499 6.334 6.115 6.302
   6872   1387483_at   6.189 6.309 5.794 6.158 6.439 4.184 4.498 5.499 5.135 4.679
   6873   1399071_at   8.381 8.302 8.31 8.487 8.448 7.933 8.006 8.169 8.12 7.854
   6874   1388097_at   4.372 5.412 5.05 5.17 2.616 7.765 6.997 7.578 7.583 6.993
   6875   1384897_at   6.296 6.436 5.862 6.179 5.699 5.405 5.164 4.502 4.931 4.57
   6876   1374502_at   7.498 7.585 7.391 7.429 7.247 7.174 6.892 7.097 6.952 7.009
   6877   1389500_at   5.637 6.3 5.887 6.44 6.279 4.566 4.515 5.376 4.223 5.044
   6878   1374695_at   8.611 8.798 8.726 8.323 8.5 7.947 8.121 7.784 8.105 8.122
   6879   1389674_at   5.749 6.024 6.121 6.018 6.137 5.642 5.569 5.597 5.574 5.346
   6880   1391412_at   9.026 8.926 9.052 9.131 9.052 8.797 8.806 8.485 8.633 8.625
   6881   1380336_at   7.016 6.661 6.662 6.707 7.034 4.906 5.206 5.327 4.892 3.213
   6882   1382402_at   5.449 5.337 5.568 5.884 6.124 6.642 6.422 6.448 6.796 6.427
   6883   1372152_at   7.58 7.763 7.861 7.805 7.842 8.163 8.395 8.334 8.195 8.013
   6884   1370866_at   12.569 12.465 12.366 12.246 12.386 12.086 12.107 12.069 12.112 12.162
   6885   1373590_at   9.016 9.063 9.126 9.028 8.946 9.262 9.325 9.644 9.451 9.414
   6886   1375428_at   8.89 8.699 9.391 9.195 9.269 8.403 8.166 8.536 8.174 8.243
   6887   1388177_at   4.995 5.59 5.566 5.797 5.83 6.546 6.714 6.238 6.492 6.354
   6888   1389648_at   7.964 7.987 7.711 8.235 8.2 8.847 8.777 9.015 8.417 8.695
   6889   1375914_at   5.24 5.282 5.314 4.904 5.212 5.681 5.727 6.155 5.667 5.851
   6890   1373452_at   7.754 7.902 7.97 8.065 7.899 7.281 7.523 7.419 7.515 7.026
   6891   1380414_at   6.694 6.919 6.674 6.976 6.891 2.585 0.949 4.262 4.875 1.926
   6892   1371429_at   9.114 9.169 9.11 9.215 9.259 9.687 9.83 9.854 9.521 9.41
   6893   1398585_at   5.534 5.822 5.845 6.026 5.427 6.9 6.243 6.987 6.779 6.475
   6894   1369213_at   5.906 6.359 6.045 6.741 6.111 7.422 7.401 8.081 6.98 7.416
   6895   1367540_at   8.369 8.385 7.913 8.259 8.24 7.431 7.919 7.494 7.472 7.571
   6896   1373215_at   6.805 6.806 6.781 6.961 7.381 6.18 6.105 5.576 5.258 6.009
   6897   1387658_at   6.219 6.243 5.556 6.161 6.31 5.298 4.888 5.048 4.171 4.863
   6898   1395038_at   3.931 4.173 3.667 3.484 4.027 3.26 3.057 2.795 3.044 2.599
   6899   1387945_at   6.537 6.196 6.427 6.247 6.489 4.171 2.606 3.815 4.232 1.131
   6900   1375001_at   4.196 1.718 1.824 3.23 3.076 5.768 5.401 5.732 5.025 5.173
   6901   1382319_at   5.729 5.883 5.487 5.863 5.552 4.959 4.986 5.193 5.33 4.822
   6902   1370114_a_at   7.352 7.566 7.222 7.719 7.551 6.856 6.691 6.912 6.607 6.185
   6903   1399137_at   8.96 8.949 8.718 9.061 9.01 8.586 8.685 8.502 8.532 8.604
   6904   1370691_a_at   4.484 4.233 5.207 4.576 4.767 6.425 6.683 7.001 6.381 5.355
   6905   1391177_at   6.202 6.417 6.273 6.416 6.374 5.929 5.717 5.425 5.872 5.32
   6906   1382566_at   5.498 5.989 6.06 5.74 5.384 5.186 4.294 4.132 4.58 4.151
   6907   1374151_at   7.04 7.067 7.037 6.988 7.311 6.823 6.775 6.763 6.646 6.718
   6908   1373738_at   8.164 8.171 8.366 8.631 8.467 7.819 7.873 7.743 7.732 7.405
   6909   1377884_at   7.076 7.044 6.924 7.095 7.076 7.371 7.808 7.713 7.332 7.662
   6910   1374143_at   6.151 6.398 6.752 6.858 6.662 7.753 7.817 7.804 7.024 7.878
   6911   1388542_at   9.31 9.163 9.108 9.424 9.296 9.708 9.591 9.861 9.57 9.629
   6912   1385929_at   5.724 6.324 5.895 6.17 5.694 6.855 7.035 6.433 7.115 6.969
   6913   1376701_a_at   7.31 7.235 7.538 7.031 7.122 6.384 6.887 6.35 6.464 6.14
   6914   1384174_at   4.698 4.946 4.694 5.614 5.559 2.789 3.372 2.254 1.157 0.345
   6915   1391044_s_at   5.96 5.461 5.462 5.891 5.562 5.119 4.622 4.864 4.295 4.797
   6916   1372227_at   7.942 8.216 8.06 7.995 8.169 8.446 8.521 8.53 8.362 8.311
   6917   1390928_at   6.415 6.659 6.535 6.578 6.699 6.16 6.359 6.15 6.006 5.985
   6918   1394605_at   7.272 7.417 7.53 7.582 7.599 7.054 7.217 7.102 6.961 7.099
   6919   1377819_at   7.01 6.945 7.028 7.112 7.1 6.51 6.47 6.896 6.52 6.449
   6920   1389351_at   9.088 9.026 8.902 9.174 9.046 8.151 8.332 8.711 7.809 8.111
   6921   1391444_at   4.306 5.091 4.488 5.106 4.679 6.208 6.32 6.25 5.451 5.708
   6922   1376866_at   5.783 5.318 5.656 5.998 5.317 4.497 3.717 4.829 4.212 4.606
   6923   1395086_at   5.376 5.348 5.846 5.637 5.484 4.79 4.029 4.485 3.869 4.812
   6924   1373003_at   7.807 7.726 7.538 7.98 7.888 6.96 7.248 7.37 6.715 6.919
   6925   1385887_at   7.383 7.697 7.589 7.756 7.535 8.048 8.384 8.415 7.975 8.099
   6926   1387959_at   6.989 6.748 7.165 7.329 6.865 8.427 8.322 9.089 8.217 7.675
   6927   1371546_at   8.872 8.685 8.467 8.89 8.967 7.936 8.146 8.25 8.01 7.551
   6928   1376125_at   6.205 6.57 6.315 6.556 6.796 7.323 7.256 7.408 6.871 7.32
   6929   1367494_at   10.377 10.415 10.434 10.382 10.406 10.281 10.139 10.202 10.014 10.178
   6930   1383156_at   7.89 7.918 8.046 8.312 8.05 7.361 7.532 7.639 7.418 7.088
   6931   1373195_at   10.227 10.208 10.278 10.426 10.447 9.849 9.905 9.986 9.702 9.499
   6932   1395555_at   7.252 7.426 7.381 7.364 7.336 7.03 6.585 6.871 7.013 6.615
   6933   1370352_at   10.555 10.546 10.603 10.22 10.039 11.386 11.55 10.825 11.391 11.241
   6934   1374597_at   8.071 8.171 8.057 8.109 7.979 8.551 8.691 8.231 8.656 8.6
   6935   1371940_at   8.427 8.47 8.361 8.773 8.511 9.02 9.403 9.333 8.851 9.267
   6936   1387775_at   9.418 9.453 9.408 9.412 9.387 9.196 9.253 9.218 9.031 9.01
   6937   1383269_at   7.819 7.87 7.72 8.021 8.282 7.495 7.458 7.23 7.2 7.244
   6938   1381391_at   4.32 4.133 4.138 4.15 4.737 5.344 5.702 4.806 5.77 5.87
   6939   1383753_at   4.704 4.64 4.903 4.862 4.768 4.99 5.27 5.482 5.284 5.353
   6940   1388905_at   7.615 7.584 7.998 7.79 8.006 8.392 8.546 8.531 8.147 8.44
   6941   1396646_at   4.246 2.074 3.359 3.194 3.721 5.354 5.544 5.063 5.255 5.175
   6942   1390392_at   9.051 9.049 9.008 9.42 9.033 9.723 9.77 9.574 9.44 9.62
   6943   1371716_at   6.667 6.747 6.45 6.914 6.833 7.481 7.478 7.688 7.297 7.085
   6944   1367518_at   8.135 8.019 7.989 8.226 8.191 8.312 8.407 8.363 8.43 8.443
   6945   1383232_at   7.471 7.437 7.394 7.342 7.481 7.052 7.212 7.133 6.932 7.217
   6946   1378098_at   8.328 8.248 8.387 8.438 8.317 8.783 8.814 8.645 8.532 8.605
   6947   1387287_a_at   3.869 4.151 3.508 3.432 3.926 5.213 5.247 4.271 5.361 5.116
   6948   1371894_at   10.977 10.975 11.028 11.064 11.024 11.294 11.337 11.108 11.297 11.4
   6949   1383539_at   4.964 4.729 4.551 5.068 4.844 3.36 3.718 3.826 3.737 4.411
   6950   1391296_at   4.976 4.275 4.984 4.505 4.527 3.349 2.868 3.82 3.827 3.093
   6951   1399104_at   7.641 7.54 7.447 7.548 7.569 7.781 8.041 7.994 7.793 7.813
   6952   1380044_x_at   5.436 5.266 5.152 4.376 5.806 6.559 6.524 6.532 6.72 6.251
   6953   1387057_at   7.469 7.635 7.634 7.972 7.468 8.736 8.918 8.037 8.607 8.5
   6954   1389482_at   9.686 9.772 9.655 9.828 9.889 9.416 9.489 9.423 9.166 9.161
   6955   1374945_at   7.32 7.277 7.063 7.639 7.544 6.905 6.791 6.431 6.656 6.623
   6956   1367992_at   4.725 5.166 4.587 4.501 4.869 5.365 6.07 5.942 5.549 5.583
   6957   1376446_at   7.789 7.747 8.038 7.906 7.833 8.323 8.302 8.849 8.597 8.35
   6958   1372473_at   9.219 9.235 9.106 9.118 9.174 9.374 9.392 9.338 9.275 9.413
   6959   1381296_at   5.47 4.974 5.319 5.6 5.085 5.852 6.687 6.33 6.323 6.076
   6960   1395721_at   7.46 7.464 7.494 7.288 7.151 8.217 8.454 8.095 8.224 7.678
   6961   1394871_at   8.091 8.493 8.231 8.488 7.954 8.869 8.896 8.72 8.91 8.836
   6962   1367761_at   9.028 9.058 8.973 9.052 8.879 9.347 9.197 9.181 9.403 9.375
   6963   1382004_at   5.842 6.283 5.901 5.988 6.003 7.001 6.936 7.123 6.372 6.67
   6964   1376913_at   3.988 2.328 3.552 2.425 0 6.86 6.778 5.822 5.973 6.059
   6965   1371639_at   10.412 10.567 10.516 10.406 10.428 10.766 10.874 10.605 10.741 10.832
   6966   1383669_at   5.005 4.981 4.737 5.052 4.663 5.841 5.266 5.472 5.715 5.505
   6967   1374774_at   6.708 7.061 7.05 7.331 7.195 7.827 7.664 7.947 7.514 7.754
   6968   1376762_at   8.369 8.126 8.234 8.289 8.006 8.944 9.159 8.617 8.678 8.743
   6969   1373084_at   6.297 6.088 6.361 6.156 6.063 6.877 6.823 6.643 6.549 6.521
   6970   1377266_at   6.369 6.344 6.176 6.563 6.437 6.784 7.603 7.604 7.119 7.217
   6971   1375531_at   6.274 6.063 6.129 6.171 6.111 6.767 6.76 6.537 6.594 6.349
   6972   1392852_at   3.788 4.078 4.256 3.622 3.16 4.963 5.421 5.047 4.87 4.648
   6973   1390208_at   7.993 8.103 8.366 8.138 8.191 7.221 7.601 7.859 7.453 7.431
   6974   1367502_at   8.643 8.71 8.819 8.858 8.837 8.308 8.51 8.565 8.431 8.319
   6975   1368800_at   4.88 4.734 4.556 4.755 5.101 2.722 3.24 0.367 2.467 2.06
   6976   1371358_at   9.703 9.635 9.653 9.698 9.921 10.253 10.205 10.375 10.092 9.968
   6977   1398471_at   8.196 8.289 8.203 8.348 8.499 8.013 7.785 7.99 7.993 7.893
   6978   1398894_at   10.458 10.428 10.548 10.578 10.566 10.898 10.872 10.658 10.747 10.802
   6979   1396614_at   5.79 4.5 4.127 4.424 4.351 6.159 6.526 6.27 6.279 6.033
   6980   1378172_at   7.091 7.046 6.701 7.387 7.417 5.962 6.352 6.484 5.856 6.301
   6981   1391476_at   7.081 7.269 7.481 7.297 7.05 7.936 7.9 7.53 7.923 7.78
   6982   1387568_at   4.673 5.025 5.2 4.267 5.169 2.411 3.131 1.144 3.511 1.74
   6983   1367787_at   6.17 6.397 5.911 6.263 6.477 7.048 6.821 7.344 6.838 6.861
   6984   1380640_at   4.08 4.042 4.025 4.615 4.388 5.058 4.834 5.111 4.78 4.887
   6985   1389237_at   6.939 6.712 6.72 6.901 6.963 6.519 6.322 6.194 6.411 5.994
   6986   1374144_at   8.195 8.215 8.256 8.43 8.196 9.061 9.046 9.019 8.583 8.633
   6987   1383161_a_at   9.993 10.021 10.578 10.479 10.136 10.928 10.779 11.139 11.218 10.946
   6988   1369867_at   4.291 4.599 4.715 5.198 4.479 2.128 2.878 0.799 1.82 3.348
   6989   1390442_at   5.417 5.724 5.871 5.818 5.546 5.282 5.121 4.98 5.073 5.243
   6990   1368397_at   9.771 9.916 10.407 9.87 9.253 10.844 11.366 10.649 11.118 11.342
   6991   1374590_at   5.992 6.299 6.434 6.508 6.351 5.395 5.67 5.393 5.888 5.748
   6992   1369305_at   6.707 6.854 7.026 7.041 7.123 6.523 6.317 5.944 6.303 5.872
   6993   1389664_at   6.406 6.117 6.435 6.218 6.655 5.669 5.57 5.851 5.947 5.512
   6994   1369191_at   5.815 5.769 5.676 5.246 5.067 0.595 3.091 3.638 2.558 3.139
   6995   1382610_at   4.975 5.301 5.191 5.169 5.183 4.419 4.358 4.242 4.33 3.43
   6996   1379462_at   6.458 6.175 6.14 6.127 6.313 5.824 5.754 5.319 5.165 5.352
   6997   1378681_at   3.266 4.049 3.445 3.781 2.926 5.216 4.4 5.266 4.61 4.794
   6998   1389313_at   7.362 7.533 7.408 7.552 7.421 7.826 7.91 7.797 7.627 7.718
   6999   1377126_at   7.042 7.21 7.007 7.394 7.326 6.633 6.796 6.253 6.658 6.48
   7000   1367487_at   8.776 8.81 8.734 8.802 8.846 8.556 8.575 8.703 8.657 8.615
   7001   1378386_at   6.19 6.179 6.747 6.493 6.779 0.401 2.778 3.743 4.141 3.475
   7002   1390689_at   5.778 5.496 5.84 5.828 5.153 5.069 4.638 4.902 4.719 4.718
   7003   1386073_at   5.801 5.583 5.235 5.291 5.15 4.083 4.686 4.088 4.639 4.658
   7004   1382288_at   7 7.138 7.074 6.965 7.399 6.469 6.736 6.197 6.61 6.346
   7005   1384107_at   6.524 6.971 6.221 6.486 6.859 5.916 5.942 5.764 5.564 5.873
   7006   1376768_at   4.728 5.275 5.272 5.029 5.443 5.831 6.425 6.284 5.989 5.815
   7007   1377407_at   4.979 4.645 5.436 5.71 4.758 6.585 6.295 6.239 6.259 6.011
   7008   1391626_at   4.643 5.342 4.964 5.482 4.552 0 2.465 0 1.77 3.159
   7009   1399020_at   8.334 8.338 8.373 8.666 8.38 8.172 8.014 8.024 7.972 7.915
   7010   1371634_at   9.446 9.596 9.501 9.4 9.44 9.757 9.805 9.713 9.659 9.644
   7011   1393477_at   3.792 4.312 4.771 4.108 3.424 5.63 5.375 5.351 5.25 5.162
   7012   1379401_a_at   7.688 7.66 7.978 7.995 7.988 7.232 7.479 7.169 7.427 7.031
   7013   1397458_at   6.771 7.065 7.235 7.555 7.197 6.395 5.969 5.755 6.533 6.187
   7014   1368057_at   9.173 8.447 8.683 8.983 9.027 9.599 10.354 10.306 9.992 9.592
   7015   1374855_at   5.379 4.078 5.928 6.144 6.177 8.682 7.96 8.554 7.109 8.047
   7016   1390132_at   7.605 7.517 7.237 7.457 7.391 7.839 8.097 8.033 7.877 8.383
   7017   1369010_at   6.167 5.957 5.785 5.924 5.437 4.885 4.739 5.236 4.148 4.611
   7018   1395642_at   7.78 7.81 8.139 8.081 8.071 7.411 7.161 7.276 7.212 6.582
   7019   1372377_at   8.384 8.424 8.204 8.438 8.553 8.065 7.86 7.886 8.078 7.681
   7020   1381612_at   3.536 2.88 3.661 3.742 3.471 4.279 4.424 4.341 4.233 4.169
   7021   1383129_at   7.915 7.844 7.755 7.86 7.876 8.429 8.537 8.866 8.169 8.357
   7022   1374198_at   7.08 6.866 6.141 6.386 7.074 5.481 5.522 5.764 5.714 5.035
   7023   1375206_at   5.543 5.83 5.174 5.881 5.415 6.425 6.376 6.617 6.486 6.088
   7024   1371919_at   7.936 7.651 7.713 7.734 7.499 8.092 8.41 8.177 8.24 8.069
   7025   1398481_at   8.426 8.191 7.985 8.363 8.235 7.68 7.701 7.77 7.495 7.901
   7026   1389177_at   8.452 8.434 8.622 8.411 8.669 9.299 9.249 8.734 9.229 9.103
   7027   1383607_at   6.177 6.07 6.356 6.391 6.137 7.08 7.286 7.152 6.579 6.788
   7028   1367478_at   8.565 8.749 8.775 8.714 8.435 8.195 8.176 8.416 8.175 8.138
   7029   1369839_at   4.957 4.995 4.787 4.919 4.935 5.713 6.021 5.888 5.788 5.161
   7030   1382581_at   5.725 5.832 5.604 5.945 6.328 5.059 4.512 5.173 5.188 4.885
   7031   1398871_at   12.561 12.448 12.417 12.256 12.531 12.107 12.102 12.201 12.07 11.935
   7032   1390199_at   7.654 7.611 7.68 7.568 7.684 8.118 8.606 8.08 8.161 8.026
   7033   1391414_at   7.174 7.171 7.213 7.343 7.163 6.672 6.469 6.776 6.313 6.908
   7034   1390232_at   6.224 6.105 6.201 6.205 6.845 4.76 5.388 5.721 5.098 4.927
   7035   1383068_at   9.328 9.372 9.257 9.392 9.28 8.597 8.828 9.137 8.562 8.653
   7036   1387121_a_at   7.884 8.038 8.112 8.26 8.019 9.032 9.247 9.791 8.928 8.632
   7037   1380067_at   3.854 3.986 4.07 4.555 3.714 4.893 4.873 4.932 4.967 4.611
   7038   1372787_at   8.912 9.164 8.656 9.079 9.185 8.453 8.57 8.414 8.209 8.283
   7039   1397841_at   2.954 2.257 1.077 1.718 2.192 4.303 3.98 4.323 3.754 3.328
   7040   1383396_at   7.601 7.881 7.686 7.772 7.872 6.961 7.315 6.451 7.058 7.052
   7041   1380824_at   6.507 6.71 6.772 6.806 6.859 6.068 6.373 6.167 6.07 6.429
   7042   1376323_at   6.424 6.412 6.68 6.405 6.528 7.226 7.081 6.756 7.198 6.906
   7043   1385912_at   6.055 6.314 6.973 6.467 5.823 8.037 7.968 7.092 8.034 7.545
   7044   1373241_at   8.54 8.489 8.55 8.693 8.668 8.195 8.326 8.328 8.255 8.017
   7045   1390246_at   6.047 6.316 6.488 6.252 6.069 6.998 7.594 6.707 7.076 7.108
   7046   1381190_at   8.709 8.57 8.512 9.048 9.011 9.473 9.459 9.661 9.258 9.377
   7047   1371632_at   8.736 8.788 8.755 9.1 9.056 8.394 8.497 8.015 8.163 7.975
   7048   1385090_at   7.714 7.294 7.162 7.39 7.558 6.749 7.053 6.735 6.747 6.57
   7049   1372784_at   8.478 8.658 8.528 8.755 8.507 8.381 8.182 8.174 8.197 8.278
   7050   1377263_at   7.513 7.71 7.594 7.704 7.958 7.08 7.299 6.559 6.858 6.69
   7051   1398318_at   8.26 8.135 8.423 8.449 8.047 8.977 9.118 9.761 9.03 8.926
   7052   1388474_at   8.541 8.654 8.453 8.633 8.761 8.303 8.292 8.342 8.145 8.318
   7053   1376776_at   6.553 6.179 6.794 6.732 6.552 7.669 7.235 7.672 7.173 7.188
   7054   1377250_at   7.901 7.811 7.681 7.956 7.626 7.109 7.189 7.501 6.829 7.073
   7055   1368067_at   7.998 8.218 8.334 8.302 8.169 8.605 8.561 8.488 8.506 8.509
   7056   1374027_at   7.785 7.859 7.703 7.259 7.605 8.485 8.284 8.215 8.1 8.336
   7057   1387549_at   6.493 6.152 6.728 6.687 6.284 7.136 7.003 7.272 7.238 6.989
   7058   1368246_at   5.574 5.469 5.648 5.951 5.789 4.941 4.678 5.077 5.194 5.193
   7059   1383700_at   7.796 7.703 7.715 7.732 7.863 7.524 7.576 7.508 7.42 7.614
   7060   1389940_at   10.714 10.675 10.584 10.662 10.792 10.467 10.55 10.436 10.493 10.439
   7061   1388625_at   8.304 7.868 8.489 7.926 8.257 7.505 7.502 7.342 6.898 6.935
   7062   1382204_at   1.465 0.345 0 2.319 0 3.093 3.16 3.696 3.938 3.101
   7063   1390123_at   5.746 6.175 6.723 6.27 6.254 7.271 7.33 7.746 7.325 6.887
   7064   1375042_at   7.796 8.02 7.847 7.978 8.023 7.436 7.57 7.707 7.33 7.555
   7065   1384720_at   3.263 3.982 4.551 3.639 3.851 5.572 5.473 4.769 5.031 5.161
   7066   1375224_at   6.479 6.626 6.889 6.89 7.268 5.572 5.651 6.332 5.893 5.679
   7067   1390577_at   7.808 7.794 7.629 7.786 7.799 7.465 7.492 7.507 7.295 7.186
   7068   1373566_at   8.632 8.424 8.498 8.419 8.347 9.567 9.619 9.534 8.732 9.32
   7069   1392569_at   7.117 7.245 6.965 7.432 7.907 8.097 8.374 8.622 8.143 8.324
   7070   1377090_at   5.567 5.726 5.319 5.45 5.711 6.374 6.407 6.336 6.292 5.823
   7071   1374088_at   0 0.239 2.711 0 0 4.005 3.336 3.406 4.267 3.036
   7072   1375183_at   5.114 5.401 5.383 5.425 4.997 5.806 6.368 6.319 5.87 5.869
   7073   1371173_a_at   6.745 7.496 7.547 7.062 7.404 6.588 6.268 5.735 6.087 6.216
   7074   1375625_at   5.684 5.868 5.778 5.664 5.552 5.009 4.841 4.865 3.911 4.658
   7075   1372434_at   5.191 3.195 4.228 3.365 4.923 6.56 6.503 7.185 5.895 6.477
   7076   1382240_at   7.015 7.073 7.084 7.094 7.194 7.434 7.692 7.371 7.316 7.511
   7077   1377032_at   5.911 6.068 6.062 6.17 5.868 7.07 6.432 7.129 6.52 6.916
   7078   1368258_at   5.805 5.862 6.266 5.872 5.865 5.045 5.065 5.149 5.433 4.51
   7079   1397615_at   4.569 4.433 4.77 4.974 4.708 5.622 6.181 5.552 5.567 5.222
   7080   1372443_at   8.125 7.992 7.998 8.132 8.261 8.733 8.895 8.307 8.863 8.741
   7081   1389536_at   6.657 6.484 6.786 6.995 6.581 7.212 7.26 7.564 7.149 7.283
   7082   1394268_at   6.442 6.346 6.222 5.983 5.807 5.648 5.544 5.505 5.327 5.392
   7083   1375057_at   4.38 3.123 4.739 5.025 4.71 6.386 6.237 6.313 6.143 5.885
   7084   1397859_x_at   5.789 5.665 5.064 5.829 6.916 3.414 3.45 3.976 3.114 1.384
   7085   1383896_at   6.906 6.933 6.869 6.739 6.924 6.028 6.349 5.781 6.55 6.062
   7086   1382388_at   6.596 6.197 6.604 6.638 6.496 7.105 6.795 7.189 7.273 7.223
   7087   1375894_at   8.507 8.678 8.858 8.617 8.811 9.452 9.358 9.007 9.762 9.458
   7088   1372653_at   7.095 7.044 6.675 7.096 7.378 6.183 5.861 5.955 5.946 4.92
   7089   1376553_at   6.704 6.637 6.531 6.676 6.781 6.019 6.107 6.124 6.261 5.572
   7090   1398928_at   9.784 9.753 9.783 10.012 10.056 10.181 10.385 10.509 10.228 10.344
   7091   1373946_at   7.942 7.973 7.981 8.165 8.277 7.598 7.627 7.82 7.592 7.482
   7092   1384484_at   4.484 4.91 4.532 4.788 4.436 5.501 5.519 5.257 5.655 5.018
   7093   1387427_at   6.146 6.403 6.222 6.464 6.018 5.478 5.608 5.109 5.741 4.987
   7094   1393308_at   5.456 5.734 5.85 5.803 5.656 6.466 6.117 6.027 6.306 6.259
   7095   1369265_at   7.124 7.245 7.258 7.203 7.024 7.006 6.767 6.626 6.747 6.718
   7096   1383848_at   4.6 5.116 4.455 4.668 3.527 5.937 5.725 6.026 5.692 6.037
   7097   1378560_at   4.405 3.899 2.68 4.573 2.816 5.592 5.747 5.913 6.715 5.857
   7098   1372858_at   4.431 3.635 4.453 4.578 4.566 5.383 5.854 5.346 5.941 5.177
   7099   1398601_at   7.554 7.498 7.602 7.762 7.535 8.102 8.18 8.489 8.012 7.934
   7100   1388038_at   6.649 7.287 7.3 7.202 7.502 8.013 8.231 7.977 7.867 7.919
   7101   1380770_at   3.116 3.951 3.398 3.352 2.895 4.125 4.405 4.269 4.301 4.521
   7102   1376037_at   6.827 6.663 6.817 6.901 7.01 5.846 5.839 6.569 5.884 5.609
   7103   1389990_at   6.209 6.112 6.246 6.122 6.243 5.72 5.274 5.214 5.817 5.102
   7104   1379761_at   4.831 4.27 3.731 3.704 4.338 5.685 5.291 5.848 5.306 5.17
   7105   1392986_at   7.48 7.552 7.521 7.641 7.761 6.975 7.287 7.188 6.81 7.144
   7106   1371043_a_at   8.294 8.255 8.022 8.448 8.532 8.801 8.99 9.227 8.748 8.942
   7107   1392729_at   4.353 4.894 5.333 4.705 5.407 6.112 6.248 5.979 6.075 5.777
   7108   1371109_at   4.194 3.406 4.172 4.163 3.955 5.153 4.933 4.835 5.317 4.631
   7109   1394209_at   5.649 5.691 5.728 5.504 5.696 5.359 5.178 4.692 4.842 4.866
   7110   1377260_at   4.42 4.876 4.977 5.057 5.054 4.32 4.285 4.066 3.842 3.95
   7111   1386944_a_at   10.843 10.823 11.25 10.862 10.114 11.835 11.89 11.516 12.145 12.035
   7112   1390469_at   4.975 5.259 5.646 5.808 5.883 3.949 3.725 4.253 2.872 4.422
   7113   1375705_at   9.512 9.54 9.402 9.309 9.513 9.264 9.113 9.078 9.139 8.952
   7114   1393207_at   6.397 6.4 6.421 6.098 6.42 5.583 5.783 5.895 5.87 6.046
   7115   1387948_at   6.925 6.711 6.753 6.788 6.824 7.176 7.102 7.345 6.99 7.158
   7116   1389021_at   8.232 8.294 8.242 8.435 8.375 7.896 8.018 8.165 7.926 7.987
   7117   1371545_at   6.786 6.989 6.362 7.128 7.093 7.813 7.546 7.499 7.73 7.751
   7118   1368643_at   7.288 7.189 6.935 7.188 7.138 7.641 7.766 7.566 7.472 7.442
   7119   1373448_at   8.039 7.971 8.25 8.221 7.941 8.454 8.811 8.878 8.565 8.493
   7120   1388585_at   8.49 8.437 8.424 8.299 8.645 7.899 8.254 8.028 8.019 7.98
   7121   1379311_at   7.463 7.539 7.451 7.659 7.71 4.409 4.638 5.288 4.507 1.428
   7122   1368271_a_at   5.288 4.954 4.544 5.133 4.891 6.87 6.823 8.629 6.518 6.61
   7123   1374896_at   5.411 5.947 5.503 5.79 5.412 6.37 6.431 6.606 6.43 6.014
   7124   1373454_at   8.912 8.889 8.756 8.863 8.95 8.459 8.473 8.755 8.499 8.427
   7125   1369955_at   7.752 7.722 7.803 8.103 8.73 7.109 6.946 6.967 7.054 7.052
   7126   1376258_at   5.323 5.073 5.347 5.3 4.984 4.659 3.861 3.527 4.268 3.426
   7127   1371368_at   9.415 9.466 9.416 9.585 9.665 9.059 9.225 9.285 9.005 9.035
   7128   1371415_at   11.35 11.267 11.383 11.295 11.493 11.636 11.777 11.76 11.573 11.576
   7129   1384116_at   6.569 7.278 6.791 7.254 7.167 7.926 7.92 8.341 7.831 7.679
   7130   1374352_at   4.491 4.165 5.945 4.88 5.155 2.111 1.281 3.475 1.214 0.138
   7131   1392803_at   4.063 1.941 4.256 3.903 2.496 5.67 5.722 5.724 5.809 5.852
   7132   1373780_at   8.861 8.877 8.897 8.992 9.307 7.87 8.094 8.566 8.178 7.632
   7133   1388720_at   8.425 8.6 8.215 8.387 8.359 7.887 8.163 7.768 7.669 7.767
   7134   1385601_at   5.904 6.064 6.207 6.38 6.122 5.579 5.502 5.289 5.788 5.598
   7135   1389338_at   8.771 8.729 8.823 8.77 8.944 9.291 9.454 9.06 9.312 9.081
   7136   1373664_at   7.628 7.672 7.615 7.66 7.742 7.381 7.523 7.29 7.486 7.312
   7137   1390722_at   7.998 7.951 8.021 7.984 7.907 7.585 7.462 7.776 7.703 7.386
   7138   1367661_at   11.126 11.045 11.106 10.926 11.127 10.184 10.48 10.79 10.019 10.27
   7139   1393454_at   4.738 4.539 4.474 4.187 5.326 5.592 6.454 6.223 5.608 5.974
   7140   1397864_at   3.303 2.848 2.886 1.111 2.637 0 0 1.202 0 0
   7141   1372521_at   5.417 5.943 5.558 5.621 5.426 6.419 6.471 6.298 6.946 6.136
   7142   1371873_at   9.301 9.26 9.424 9.406 9.198 8.763 9.116 8.855 8.681 8.59
   7143   1398960_at   10.485 10.58 10.529 10.77 10.767 10.089 10.334 10.196 10.303 10.28
   7144   1371422_at   11.263 11.254 11.137 11.154 11.362 10.628 10.719 10.676 10.624 11.078
   7145   1399041_at   7.742 7.508 7.743 8.035 7.409 8.337 8.759 8.388 8.384 8.226
   7146   1377554_at   5.1 4.976 4.807 5.506 5.026 3.493 4.018 4.117 4.39 4.318
   7147   1398592_at   4.093 1.655 4.916 3.996 3.152 6.788 6.657 6.42 6.404 6.138
   7148   1383377_at   7.539 7.613 7.581 7.665 7.649 7.117 7.394 6.961 7.219 6.864
   7149   1399019_at   9.403 9.43 9.465 9.531 9.495 9.649 9.557 9.749 9.693 9.686
   7150   1370314_at   9.689 9.73 9.697 9.81 9.886 9.087 8.923 9.324 8.345 8.792
   7151   1387120_at   10.174 10.165 10.028 10.32 10.418 9.815 9.816 9.909 9.598 9.623
   7152   1367573_at   12.897 12.844 12.743 12.465 12.646 12.195 12.139 12.327 12.388 12.254
   7153   1368941_at   6.616 6.107 6.585 6.452 6.188 5.451 5.024 5.573 5.916 5.224
   7154   1371830_at   9.325 9.606 9.31 9.527 9.719 8.678 9.065 9.046 8.898 8.596
   7155   1380753_at   6.477 6.418 6.593 6.768 6.387 5.953 6.149 5.846 6.116 5.668
   7156   1387548_at   5.395 5.848 5.829 6.071 5.393 0.669 3.593 4.037 2.29 1.791
   7157   1388900_at   8.258 8.305 8.223 8.293 8.308 7.933 7.61 7.913 7.343 7.743
   7158   1393140_at   5.949 6.378 5.955 6.152 6.344 5.252 5.494 5.81 5.451 5.298
   7159   1394039_at   6.898 6.875 5.455 6.553 7.435 5.057 4.553 5.195 4.664 4.462
   7160   1377982_at   5.477 5.935 5.667 6.081 6.232 5.011 4.82 4.464 5.115 4.146
   7161   1382954_at   3.093 3.372 3.681 3.625 2.962 4.338 3.914 4.855 4.496 4.69
   7162   1379662_a_at   8.37 8.21 8.344 8.681 8.646 7.938 8.025 7.729 7.87 7.978
   7163   1392072_at   6.461 6.104 6.013 6.307 5.768 7.235 7.348 7.311 6.644 6.859
   7164   1386399_at   1.339 0.782 2.707 2.118 0.546 3.356 3.834 3.589 4.113 3.589
   7165   1391443_at   7.358 7.462 7.67 7.566 7.474 7.85 7.802 7.742 7.826 7.762
   7166   1393653_at   7.443 7.286 6.992 7.5 7.566 6.39 6.636 6.827 6.097 6.643
   7167   1399006_at   7.752 7.614 7.785 7.94 8.167 6.887 7.191 7.391 7.293 7.222
   7168   1372126_at   9.314 9.483 9.349 9.529 9.575 9.016 9.214 9.155 8.922 9.08
   7169   1393822_at   6.787 6.423 5.975 6.624 6.743 5.815 5.793 5.469 5.239 5.294
   7170   1395813_at   8.954 8.952 9.001 9.035 9.288 8.717 8.502 8.572 8.378 8.165
   7171   1369974_at   7.083 6.654 7.074 7.194 6.639 7.896 8.194 8.062 7.372 7.832
   7172   1383961_a_at   6.906 6.694 6.862 6.916 6.868 6.556 6.433 5.989 6.292 6.327
   7173   1370181_at   6.969 6.982 7.008 7.482 7.325 7.919 8.107 7.72 7.677 7.748
   7174   1370894_at   8.091 8.31 8.399 8.861 8.497 7.246 7.293 8.011 7.179 7.323
   7175   1399155_at   6.975 6.751 6.565 6.829 6.461 7.726 7.605 7.45 7.096 7.362
   7176   1398384_at   6.726 7.06 7.033 7.176 6.843 6.456 6.462 6.452 5.991 6.078
   7177   1374856_at   5.955 5.898 5.985 6.065 6.295 5.253 5.76 5.296 5.393 5.531
   7178   1385676_at   7.742 7.586 7.647 7.779 7.663 7.244 6.958 7.115 7.352 6.709
   7179   1371482_at   10.373 10.354 10.322 10.466 10.513 10.864 11.073 11.015 10.78 10.614
   7180   1398401_at   6.919 6.377 6.728 6.974 7.236 7.582 7.924 8.208 7.61 7.685
   7181   1371698_at   9.17 9.277 9.47 9.9 9.457 8.796 8.841 8.748 8.813 8.51
   7182   1387863_at   10.24 10.183 10.045 10.193 10.215 10.45 10.714 10.598 10.375 10.498
   7183   1388662_at   7.466 7.64 7.638 7.705 7.449 8.503 8.665 7.958 8.068 8.35
   7184   1396628_at   1.406 2.811 2.628 2.202 1.941 3.956 3.714 4.242 3.946 3.144
   7185   1376560_at   7.367 7.323 7.087 7.278 7.414 6.733 7.071 6.731 6.9 6.69
   7186   1395460_at   6.995 6.939 6.743 6.904 6.851 7.616 7.576 7.974 7.764 7.154
   7187   1370665_at   6.973 7.106 7.027 7.229 7.06 6.639 6.097 6.292 6.742 6.432
   7188   1383227_at   7.439 7.537 7.766 7.667 7.83 7.302 7.284 7.196 7.01 7.159
   7189   1374334_at   6.598 6.792 6.14 6.816 6.65 7.43 7.505 7.058 7.377 7.334
   7190   1372572_at   5.708 5.992 5.657 5.736 5.436 6.661 6.044 6.594 6.414 6.403
   7191   1371585_at   10.779 10.795 10.655 10.642 10.495 10.304 10.378 10.315 10.324 10.46
   7192   1390684_at   7.695 7.832 7.71 7.758 7.864 8.127 8.428 8.211 8.014 8.093
   7193   1387214_at   4.864 4.904 4.225 4.996 0.651 8.26 8.545 8.278 8.179 7.91
   7194   1374835_at   7.051 7.277 7.445 7.506 7.55 8.158 7.984 8.39 7.777 8.03
   7195   1385695_at   5.066 5.277 5.417 5.445 5.464 4.897 4.391 3.71 3.888 3.946
   7196   1383832_at   7.518 7.626 8.084 7.615 7.03 9.504 9.425 8.147 9.66 9.389
   7197   1396993_at   5.772 5.637 5.787 6.093 6.166 4.879 4.336 4.047 5.093 3.585
   7198   1374782_at   4.083 4.449 4.671 5.5 4.257 6.2 6.564 6.623 6.268 5.495
   7199   1387020_at   7.271 7.299 7.224 7.4 7.456 6.96 7.072 6.615 6.595 6.67
   7200   1376953_at   7.829 8.169 8.228 8.235 8.042 8.686 8.59 8.456 8.507 8.492
   7201   1390128_at   8.31 8.256 8.28 8.404 8.283 8.66 8.755 8.492 8.603 8.893
   7202   1376747_at   5.452 5.386 5.178 5.22 4.789 5.832 6.188 5.882 5.724 6.037
   7203   1387808_at   10.24 10.256 10.384 10.365 10.133 11.444 11.316 10.527 11.534 11.429
   7204   1396053_at   7.183 7.107 7.284 7.368 7.24 8.01 8.039 7.811 7.545 7.607
   7205   1377691_at   8.713 8.784 8.899 9.274 8.905 8.43 8.13 8.118 8.495 8.13
   7206   1389360_at   5.484 5.594 5.55 6.117 6.108 4.831 4.854 5.076 4.455 5.043
   7207   1372491_at   7.795 7.782 8.007 7.708 7.921 8.349 8.151 8.062 8.277 8.284
   7208   1391885_at   6.073 6.025 5.94 6.315 6.61 7.42 7.192 7.071 6.766 6.852
   7209   1386705_at   4.418 4.044 3.934 1.868 4.534 6.983 6.397 6.291 6.196 6.056
   7210   1374216_at   6.168 6.421 6.266 5.864 6.405 4.951 4.823 5.61 5.469 5.467
   7211   1371121_at   3.16 3.551 4.168 4.151 4.321 5.251 5.112 5.209 5.196 4.738
   7212   1372348_at   6.507 6.293 6.289 6.505 6.427 6.783 7.175 7.272 6.793 6.885
   7213   1371386_at   11.579 11.564 11.624 11.493 11.61 11.291 11.402 11.306 11.339 11.476
   7214   1376124_at   4.372 3.94 4.365 4.861 3.22 5.566 5.486 5.475 5.957 5.677
   7215   1367772_at   9.154 9.219 9.25 9.113 9.332 8.934 8.86 8.768 9.06 8.865
   7216   1376662_at   8.362 8.449 8.535 8.697 8.595 8.166 8.247 7.848 8.064 7.945
   7217   1367851_at   8.469 8.487 8.019 7.803 7.113 9.648 9.849 8.884 10.18 9.978
   7218   1387316_at   8.117 7.957 6.1 7.145 8.337 4.712 4.943 5.848 3.662 4.693
   7219   1393972_at   6.583 7.069 6.304 6.563 6.546 6.031 5.812 5.649 6.04 5.662
   7220   1385556_at   6.017 6.013 6.539 6.621 6.539 7.112 7.478 7.479 7.173 6.928
   7221   1377262_at   7.552 7.704 7.777 7.69 7.723 7.141 6.913 6.913 6.959 7.5
   7222   1384099_at   4.996 4.738 5.09 4.995 5.256 4.456 4.43 3.617 3.93 3.794
   7223   1371297_at   13.091 12.844 12.814 12.776 12.815 12.594 12.504 12.545 12.596 12.42
   7224   1371444_at   9.615 9.693 10.013 9.776 9.572 10.134 10.135 10.245 10.245 10.079
   7225   1373061_at   8.542 8.582 8.485 8.74 8.669 9.025 8.947 8.946 9.172 8.816
   7226   1382239_at   2.639 0.782 3.595 3.497 3.343 5.365 5.805 5.119 5.984 5.57
   7227   1399143_at   10.302 10.326 10.432 10.364 10.734 9.409 10.049 9.909 9.603 9.569
   7228   1376194_at   8.539 8.461 8.22 8.833 8.706 8.989 9.516 9.277 9.154 9.359
   7229   1390311_at   6.974 6.592 6.408 6.93 6.973 7.391 7.664 7.596 7.597 7.236
   7230   1393761_at   2.893 3.163 2.685 3.013 2.68 4.291 4.294 3.718 3.612 3.5
   7231   1395118_at   6.487 6.511 6.059 6.273 6.429 5.585 5.716 5.403 5.579 4.796
   7232   1372470_at   5.99 6.132 6.306 6.171 6.647 7.125 7.022 6.943 6.891 6.683
   7233   1370684_s_at   8.556 8.492 8.572 8.716 8.631 9.029 9.333 9.081 8.826 9.002
   7234   1397641_at   5.261 4.761 5.129 5.202 5.413 4.647 4.432 4.283 4.463 4.646
   7235   1380637_at   5.561 5.809 5.138 5.824 5.354 6.305 6.277 6.156 6.145 6.304
   7236   1385344_at   6.32 6.397 6.638 6.628 6.495 5.975 5.455 5.371 6.018 5.238
   7237   1391139_at   6.005 5.916 5.388 6.354 5.928 5.147 5.131 4.992 5.172 5.115
   7238   1368102_at   10.739 10.747 10.683 10.537 10.8 11.217 11.095 11.206 10.981 10.917
   7239   1370465_at   4.779 4.937 5.551 5.65 4.864 6.345 6.268 6.235 5.884 6.163
   7240   1374109_at   7.686 7.763 7.851 7.856 7.731 8.382 8.164 8.238 8.083 7.975
   7241   1374700_at   8.435 8.077 8.107 8.225 8.425 7.73 7.855 7.706 7.954 7.731
   7242   1389595_at   6.971 7.184 7.327 7.121 7.398 6.705 6.834 6.766 6.438 6.675
   7243   1375915_at   6.983 6.917 6.581 6.967 6.828 7.689 7.798 7.318 7.739 7.237
   7244   1391280_at   6.845 6.737 6.723 7.174 7.06 7.653 8.124 7.594 7.763 7.354
   7245   1390457_at   6.323 6.556 6.39 6.648 6.68 7.393 7.171 7.347 6.991 6.859
   7246   1368886_at   7.484 7.689 7.647 7.713 7.538 7.921 7.88 7.878 7.971 8.157
   7247   1367560_at   12.805 12.543 12.493 12.347 12.453 12.201 12.132 11.978 12.068 12.101
   7248   1386538_at   8.797 8.85 9.338 9.086 9.059 9.593 9.689 9.459 9.99 9.727
   7249   1370351_at   8.596 8.581 8.148 8.563 8.598 7.958 8.062 8.13 7.96 7.888
   7250   1398706_at   6.295 4.938 6.404 5.524 6.368 4.057 4.098 4.605 3.873 3.046
   7251   1374585_at   8.139 8.176 7.998 8.305 8.435 8.678 8.922 8.759 8.586 8.589
   7252   1368967_at   7.177 7.397 7.475 7.49 7.442 6.802 6.706 7.052 6.279 6.561
   7253   1375943_at   5.897 5.717 5.977 6.03 5.761 5.435 5.034 5.542 5.238 5.4
   7254   1380193_at   6.317 6.179 6.165 6.288 6.019 6.665 7.023 7.201 6.674 6.634
   7255   1370509_at   7.985 8.028 8.567 8.321 8.264 8.799 9.376 8.833 9.057 8.907
   7256   1373789_at   5.706 5.757 5.579 6.116 6.017 6.478 6.457 6.315 6.639 6.295
   7257   1388289_s_at   0.807 0.986 1.59 0.575 1.722 3.45 2.032 4.393 3.82 3.43
   7258   1374566_at   5.778 6.461 5.773 5.931 5.626 6.82 6.613 6.584 6.768 6.658
   7259   1396249_at   1.183 3.77 2.573 2.498 1.956 5.331 5.109 4.336 4.797 4.351
   7260   1392791_at   5.852 5.751 5.807 5.823 5.64 3.618 4.061 5.171 3.281 2.995
   7261   1386930_at   9.145 9.09 9.22 9.268 9.372 8.748 8.868 8.817 8.958 8.549
   7262   1395928_at   7.016 6.866 7.248 7.316 7.42 7.995 8.205 7.766 7.945 7.611
   7263   1376451_at   6.516 6.686 7.195 6.963 7.009 6.067 6.353 6.116 6.263 5.907
   7264   1372723_at   8.943 8.933 8.75 9.091 9.096 8.419 8.707 8.605 8.391 8.449
   7265   1389647_at   8.305 8.512 8.313 8.286 8.424 8.808 8.989 8.699 8.611 8.71
   7266   1383437_at   8.247 8.288 8.461 8.846 8.473 9.234 9.323 9.561 9.039 8.968
   7267   1379299_at   6.337 6.324 6.261 6.545 6.165 6.044 5.775 5.904 5.843 5.594
   7268   1398440_at   6.131 6.036 6.344 5.753 5.885 7.019 7.013 6.875 6.521 6.542
   7269   1369982_at   9.775 9.738 9.712 9.765 9.793 10.176 10.219 9.876 10.041 10.238
   7270   1398779_at   9.945 9.906 10.036 10.099 10.02 10.348 10.341 10.182 10.292 10.165
   7271   1380437_at   5.177 4.571 4.515 5.146 4.928 5.6 5.607 5.51 5.619 5.588
   7272   1372954_at   7.041 6.932 6.678 6.808 6.925 7.372 7.459 7.5 7.339 7.098
   7273   1372722_at   8.105 7.998 7.977 8.4 8.323 8.842 8.771 8.887 8.493 8.648
   7274   1393214_at   7.571 7.482 7.544 7.464 7.506 7.253 7.335 7.435 7.267 7.316
   7275   1368250_at   5.797 6.138 4.926 5.944 6.463 1.911 3.449 4.375 3.901 3.18
   7276   1380066_at   4.16 3.869 4.93 3.863 4.791 3.349 3.211 2.888 3.016 2.753
   7277   1378378_at   7.625 7.552 7.559 7.612 7.808 7.024 7.291 7.317 6.968 6.809
   7278   1388471_at   7.383 7.549 7.76 7.732 7.863 8.159 8.425 8.206 8.238 8.012
   7279   1367911_at   9.61 9.668 9.678 9.617 9.69 9.835 10.121 10.13 9.851 9.99
   7280   1377735_at   9.294 9.287 9.18 9.294 9.169 9.05 8.977 8.745 8.841 9.001
   7281   1388386_at   8.691 8.803 8.822 8.93 8.973 8.423 8.673 8.44 8.448 8.343
   7282   1395551_at   6.954 6.893 7.297 7.033 6.847 7.814 8.09 7.341 7.689 7.758
   7283   1387363_at   6.177 6.288 5.908 5.928 4.474 8.377 8.388 6.85 8.479 8.056
   7284   1374448_at   7.649 7.878 8.094 8.19 8.054 8.616 8.643 8.298 8.69 8.713
   7285   1374051_at   7.707 7.957 7.697 7.911 8.06 7.331 7.504 7.547 7.283 7.203
   7286   1368879_a_at   4.313 3.868 3.826 4.045 3.388 4.707 4.574 4.64 5.006 4.835
   7287   1387983_at   4.472 4.116 4.775 4.779 4.624 5.699 5.089 5.631 5.796 5.244
   7288   1383988_at   5.568 5.866 5.442 5.648 5.511 6.438 6.385 6.538 6.133 5.928
   7289   1374003_at   6.618 6.339 6.362 6.624 6.861 5.64 5.903 6.064 6.079 5.95
   7290   1383566_at   4.6 4.614 3.278 4.808 4.804 6.022 6.144 6.594 5.753 5.84
   7291   1389681_at   6.609 6.343 6.615 6.49 6.696 6.271 5.91 6.099 6.156 5.888
   7292   1376799_a_at   5.12 4.907 4.652 5.158 6.052 1.511 1.417 1.551 2.06 4.294
   7293   1371906_at   6.866 7.127 6.848 7.042 6.979 7.392 7.462 7.31 7.261 7.207
   7294   1378537_at   5.079 4.489 4.593 4.681 4.437 2.606 2.513 3.086 3.306 4.074
   7295   1390097_at   5.377 5.92 6.144 6.252 6.047 6.864 6.833 6.903 6.898 6.524
   7296   1384138_at   5.513 5.41 5.19 5.354 5.679 6.807 6.548 7.246 6.413 5.951
   7297   1370040_at   6.651 6.705 6.703 6.657 6.693 6 5.929 6.088 6.412 5.585
   7298   1398808_at   9.039 9.115 9.173 9.28 9.285 8.73 8.823 8.899 8.989 8.812
   7299   1371020_at   5.362 5.275 5.192 5.304 5.495 6.668 6.491 6.589 6.29 5.608
   7300   1393998_at   5.873 5.623 5.675 5.802 5.414 5.309 4.906 4.733 5.188 4.957
   7301   1371227_at   4.24 4.173 3.65 2.705 2.17 1.373 0.864 1.599 1.208 0.774
   7302   1384920_at   4.558 4.251 4.265 3.16 4.358 0.151 3.119 0.454 0.39 1.305
   7303   1392582_at   4.015 3.967 3.783 4.216 4.071 0.516 0.356 0.807 1.183 3.331
   7304   1367506_at   9.468 9.423 9.363 9.656 9.505 9.783 9.799 9.951 9.902 9.691
   7305   1384252_at   6.398 6.307 5.62 6.024 6.198 5.369 5.021 4.767 5.302 4.413
   7306   1371682_at   8.308 8.577 8.208 8.978 8.507 9.205 9.078 9.277 9.252 9.406
   7307   1382409_at   5.437 5.395 5.877 5.77 5.602 4.621 3.872 5.12 4.053 3.993
   7308   1379423_at   7.331 7.383 7.229 7.774 7.682 6.985 6.435 6.799 6.651 6.229
   7309   1397885_at   3.173 4.195 3.89 4.291 4.093 5.272 4.939 4.816 5.039 5
   7310   1396106_at   3.842 4.186 3.78 3.888 3.409 4.365 4.671 5.091 4.574 4.801
   7311   1374621_at   6.066 6.396 5.678 5.869 6.179 5.538 4.972 5.123 5.283 4.821
   7312   1395828_at   4.727 5.064 5.743 5.474 5.242 1.604 3.614 2.83 2.124 3.985
   7313   1380883_at   3.954 4.063 3.686 3.096 4.527 5.056 5.219 5.319 4.849 5.198
   7314   1394778_at   4.68 4.529 4.142 4.975 4.451 6.158 5.832 6.259 5.071 5.779
   7315   1369732_a_at   7.577 7.433 7.575 7.596 7.563 7.759 7.951 7.933 7.779 7.724
   7316   1390341_at   5.912 5.951 5.617 5.76 6.032 6.703 6.511 6.501 6.13 6.436
   7317   1373207_at   8.082 8.305 8.002 8.012 8.262 7.48 7.815 7.747 7.435 7.696
   7318   1370025_at   8.778 8.936 8.916 9.203 9.022 9.404 9.396 9.357 9.315 9.268
   7319   1383813_at   7.86 7.801 8.109 8.068 8.006 8.807 8.591 8.318 8.641 8.325
   7320   1389312_at   8.638 8.739 8.788 8.825 8.833 8.306 8.619 8.251 8.132 8.236
   7321   1369614_at   4.621 5.084 5.274 5.446 5.249 4.173 4.414 4.5 4.372 4.019
   7322   1371717_at   8.503 8.395 8.663 8.703 8.45 8.888 8.929 9.149 8.973 8.828
   7323   1374522_at   9.321 9.275 9.068 9.307 9.318 9.635 9.871 9.722 9.484 9.609
   7324   1375040_at   6.002 6.227 6.084 6.051 6.354 0.774 0.714 3.342 3.591 4.012
   7325   1386910_a_at   9.066 9.196 9.03 9.149 9.248 8.903 8.887 8.634 8.864 8.787
   7326   1370211_at   4.83 5.421 5.583 5.427 4.537 6.152 6.111 6.622 6.391 6.177
   7327   1378248_at   6.385 6.458 6.778 6.654 6.521 7.047 7.304 6.889 6.963 7.14
   7328   1371059_at   6.179 6.116 6.102 5.852 5.722 7.399 6.717 6.627 7.227 6.655
   7329   1384797_at   7.591 7.503 7.678 7.593 7.787 7.174 6.973 6.519 7.081 6.449
   7330   1391906_at   5.198 5.405 5.134 5.185 5.078 6.359 5.5 6.079 5.861 6.057
   7331   1377769_at   9.82 9.816 9.804 9.63 9.832 9.554 9.536 9.365 9.484 9.259
   7332   1370974_at   8.477 8.489 8.315 8.621 8.543 7.757 8 8.281 7.815 7.956
   7333   1374349_at   8.46 8.412 8.234 8.49 8.432 8.822 8.681 9.095 8.717 8.929
   7334   1380730_at   7.459 7.617 7.742 7.589 7.17 6.917 6.883 6.741 7.163 6.834
   7335   1372439_at   10.55 10.547 10.591 10.648 10.793 10.111 10.125 9.579 9.964 10.235
   7336   1389289_at   8.894 8.831 9.165 9.322 9.241 8.613 8.559 8.679 8.497 8.449
   7337   1372171_at   6.198 6.569 6.076 6.365 6.013 7.402 7.134 6.855 6.9 6.778
   7338   1384059_at   5.896 5.797 5.582 5.619 5.555 5.152 5.102 4.748 4.228 4.617
   7339   1392920_at   6.88 7.027 6.696 6.774 6.83 6.136 6.311 6.313 5.981 5.525
   7340   1395480_at   3.086 3.816 4.039 4.232 4.149 0.934 2.417 0.433 2.248 0
   7341   1368380_at   2.007 2.639 1.104 3.581 1.151 4.837 4.139 4.52 4.6 4.551
   7342   1387101_at   10.028 10.046 9.775 9.967 10.216 9.374 9.746 9.368 9.15 9.381
   7343   1388194_at   8.812 8.807 8.761 8.823 8.79 9.229 9.589 9.13 9.108 9.176
   7344   1375379_at   5.477 5.32 4.904 4.517 5.048 3.063 2.709 3.376 3.949 1.664
   7345   1377351_at   7.547 7.906 8.14 7.986 7.624 8.813 9.063 9.136 8.504 8.381
   7346   1382057_at   5.598 5.517 5.913 5.84 5.678 6.893 6.282 6.91 6.207 6.458
   7347   1394490_at   6.199 6.223 6.515 6.299 5.756 4.923 5.066 4.993 5.805 5.104
   7348   1392340_at   3.793 4.219 4.86 4.507 4.154 5.465 5.342 5.23 5.093 5.198
   7349   1397077_at   4.049 3.206 4.001 3.121 3.323 1.163 0.678 1.433 0.971 2.811
   7350   1373157_at   7.625 7.585 7.473 7.742 7.533 8.015 8.138 7.954 7.847 7.833
   7351   1373915_at   5.857 5.797 6.049 6.401 6.552 7.471 7.026 7.873 6.937 7.108
   7352   1379584_at   5.674 5.679 6.052 5.555 5.692 5.19 5.013 4.351 4.434 4.137
   7353   1383589_at   7.243 6.609 6.643 6.971 7.467 5.256 4.51 4.931 4.508 2.392
   7354   1376267_at   6.654 6.802 6.681 6.876 6.686 7.804 8.363 7.206 7.616 7.589
   7355   1376680_at   7.537 7.62 7.333 7.739 7.821 7.169 6.955 7.19 7.215 7.074
   7356   1373502_at   8.185 8.13 8.164 8.236 8.18 8.533 8.645 8.711 8.341 8.428
   7357   1386478_at   8.288 8.144 8.667 8.685 8.428 8.928 9.303 9.431 9.106 8.999
   7358   1390026_at   8.105 8.295 8.186 8.429 8.249 8.945 9.148 8.579 8.641 8.956
   7359   1369058_at   2.692 0.791 1.251 2.305 1.811 3.445 3.312 3.82 3.884 4.491
   7360   1375906_at   7.571 7.581 7.76 7.552 7.765 7.255 7.355 7.1 7.17 6.837
   7361   1385551_at   4.71 5.603 5.758 5.271 5.803 4.206 3.014 4.193 4.146 3.214
   7362   1374123_at   4.267 4.72 4.869 5.097 4.381 6.069 5.551 6.032 5.293 5.731
   7363   1370530_a_at   8.321 8.383 8.496 8.388 8.184 9.12 8.738 9.165 8.889 9.541
   7364   1387704_at   3.697 4.43 4.424 5.111 4.031 6.366 5.881 5.264 6.382 5.749
   7365   1376067_at   8.548 8.468 8.39 8.222 8.443 8.74 8.751 8.677 8.725 8.642
   7366   1387002_at   9.286 9.298 9.454 9.53 9.376 8.949 9.121 9.197 9.071 9.007
   7367   1388454_at   9.085 8.933 8.863 9.306 9.231 9.569 9.716 9.824 9.575 9.44
   7368   1392301_at   8.92 8.997 8.789 8.942 8.563 9.411 9.474 9.575 9.138 9.32
   7369   1368005_at   7.331 7.616 6.959 7.572 7.538 6.511 6.4 6.887 5.91 6.225
   7370   1370261_at   7.869 7.821 7.601 7.964 7.826 6.88 6.885 7.601 6.973 6.833
   7371   1377410_at   5.424 5.882 5.666 5.619 5.13 3.376 4.467 4.8 3.428 3.862
   7372   1374117_at   6.087 6.437 6.477 6.605 6.422 7.236 6.717 7.103 6.97 7.185
   7373   1377874_at   6.206 6.288 6.414 6.551 6.549 7.303 7.653 6.98 7.094 6.869
   7374   1389215_at   7.532 7.661 7.862 7.853 7.64 8.671 8.466 7.967 8.516 8.33
   7375   1380600_at   6.212 6.17 5.84 6.468 6.432 6.952 7.226 7.316 7.172 6.634
   7376   1376625_at   7.061 6.923 6.951 7.097 7.156 6.583 6.833 6.768 6.588 6.503
   7377   1368867_at   6.842 6.752 7.042 7.049 7.299 5.51 6.231 6.187 6.43 5.759
   7378   1384309_at   4.436 4.621 4.703 3.684 4.505 5.482 5.7 5.295 5.262 5.269
   7379   1368133_at   6.492 6.536 6.295 6.59 6.813 7.364 7.603 6.99 7.017 7.322
   7380   1398968_at   8.796 8.768 8.698 8.678 8.633 9.243 9.408 9.043 8.958 9.075
   7381   1389160_at   0.918 0.556 1.491 0 1.433 1.884 2.842 3.118 2.556 2.731
   7382   1372317_at   8.968 8.982 9.174 9.133 9.088 9.616 9.616 9.234 9.493 9.436
   7383   1371741_at   8.992 8.947 8.906 9.13 9.021 8.567 8.716 8.844 8.615 8.716
   7384   1388769_at   7.967 7.994 8.334 8.146 8.249 7.746 7.749 7.65 7.821 7.525
   7385   1398463_at   8.715 8.597 9.025 8.565 8.051 10.307 10.462 9.094 10.497 10.253
   7386   1398710_at   5.259 6.029 5.513 5.741 6.06 6.594 6.578 7.018 6.498 6.482
   7387   1380835_at   5.558 5.736 6.062 5.721 5.541 3.611 4.098 3.124 4.832 4.642
   7388   1382281_at   4.663 3.803 3.562 4.342 4.584 5.589 5.642 5.599 5.077 5.228
   7389   1379519_at   7.182 6.991 6.896 7.397 6.998 7.519 7.64 7.647 8.027 7.94
   7390   1397383_at   6.053 6.595 6.524 6.324 6.248 7.308 7.285 7.439 7.07 6.702
   7391   1389307_at   5.903 5.622 5.912 5.838 5.793 7.026 6.688 7.087 6.498 6.184
   7392   1369064_a_at   3.656 0.911 0 1.251 2.403 5.349 4.369 4.791 5.095 5.053
   7393   1387648_at   6.297 6.428 4.572 5.413 6.877 1.501 3.096 3.426 2.975 3.975
   7394   1376518_at   5.93 6.418 6.877 6.858 6.539 7.593 7.766 7.285 7.47 7.358
   7395   1388199_at   11.808 11.798 11.744 11.802 11.918 11.908 12.019 12.071 12.075 12.076
   7396   1382785_at   1.31 0.098 1.07 1.091 1.379 4.019 2.519 3.662 3.167 2.121
   7397   1372450_at   7.298 7.528 7.67 7.604 7.582 8.065 7.956 8.254 7.856 7.921
   7398   1370887_at   7.304 7.628 8.015 7.681 8.042 7.115 6.89 7.152 6.865 6.851
   7399   1374860_at   7.249 7.145 6.993 7.131 7.216 7.543 7.571 7.647 7.501 7.301
   7400   1384556_at   4.447 3.648 3.797 3.497 4.815 2.911 2.339 1.736 0.956 1.952
   7401   1378055_at   3.381 4.037 4.239 4.209 3.779 4.866 4.721 5.434 4.772 4.975
   7402   1383877_a_at   6.01 6.405 6.115 6.262 6.345 5.481 5.719 5.843 5.765 5.846
   7403   1384888_at   4.405 4.82 4.509 4.966 4.083 6.111 5.492 5.507 5.439 5.378
   7404   1383208_at   8.051 7.886 8.271 8.19 8.013 8.515 8.517 8.352 8.421 8.48
   7405   1371904_at   8.302 8.392 8.295 8.542 8.515 7.989 7.943 8.162 7.711 7.709
   7406   1381049_at   5.554 6.044 6.045 5.894 5.635 6.315 6.612 6.641 6.451 6.296
   7407   1368722_at   4.128 4.356 4.089 4.341 3.658 2.007 0.546 0.963 0.379 3.175
   7408   1383060_at   5.496 5.562 5.974 5.694 5.858 6.422 7.058 6.318 6.328 6.561
   7409   1383630_at   7.104 6.859 6.928 7.017 7.284 2.982 2.768 5.158 5.47 4.655
   7410   1367492_at   9.43 9.389 9.404 9.565 9.643 9.2 9.111 9.25 9.105 8.94
   7411   1372226_at   5.687 5.251 5.721 5.612 5.338 6.293 6.05 6.124 5.895 6.143
   7412   1385322_at   5.076 4.331 5.301 5.42 5.25 6.81 6.543 6.623 6.052 5.897
   7413   1373087_at   9.078 8.962 9.128 9.12 9.039 9.741 9.742 9.388 9.315 9.489
   7414   1370086_at   5.711 5.706 4.313 5.742 5.656 2.854 3.02 4.425 3.534 2.59
   7415   1388713_at   8.109 8.181 8.158 8.31 8.35 8.782 8.703 8.95 8.657 8.432
   7416   1394869_at   5.35 4.986 6.144 5.485 5.943 4.077 4.357 4.235 4.747 3.671
   7417   1367520_at   9.033 9.313 9.247 9.311 9.337 9.777 9.69 9.79 9.587 9.466
   7418   1387035_a_at   10.345 10.334 10.427 10.317 10.269 10.085 10.05 9.883 10.202 9.957
   7419   1376950_at   6.146 6.647 6.474 6.468 6.621 5.945 5.742 6.12 5.755 5.59
   7420   1388715_at   10.218 10.09 10.03 10.344 10.193 9.653 9.672 9.977 9.478 9.524
   7421   1383884_at   7.427 7.308 6.83 7.453 7.169 7.651 8.011 8.09 7.904 7.928
   7422   1375852_at   8.314 8.298 8.339 8.601 8.577 7.808 7.997 8.125 7.693 7.622
   7423   1388759_at   11.656 11.583 11.549 11.584 11.585 11.332 11.405 11.086 11.293 11.37
   7424   1387840_at   9.273 9.352 9.332 9.344 9.178 9.779 9.692 9.495 9.816 9.524
   7425   1375305_at   4.548 4.007 5.287 3.822 5.037 6.516 5.983 6.804 6.101 5.776
   7426   1373918_at   7.203 7.15 7.423 7.21 7.332 7.657 7.58 7.629 7.431 7.658
   7427   1368119_at   6.008 6.296 6.647 6.314 6.217 7.125 7.227 7.849 6.908 7.122
   7428   1378547_at   5.171 5.187 5.099 5.594 5.596 6.015 6.053 6.096 6.327 5.746
   7429   1373948_at   6.538 6.731 6.883 6.753 6.587 7.165 7.183 7.436 6.983 7.12
   7430   1388729_at   8.368 8.5 8.351 8.661 8.739 8.166 8.138 8.08 7.935 7.857
   7431   1388422_at   8.067 8.216 8.416 8.152 7.902 9.228 9.156 8.437 9.312 9.374
   7432   1367561_at   12.418 12.439 12.411 12.174 12.334 12.105 12.06 11.944 12.092 11.851
   7433   1377632_at   2.926 4.223 4.295 4.053 4.619 5.781 5.865 5.31 5.765 5.28
   7434   1380526_at   7.28 7.184 7.385 6.931 6.927 8.329 7.922 7.576 8.387 7.899
   7435   1399064_at   6.318 6.254 6.29 6.5 6.184 7.429 6.737 7.104 6.762 6.953
   7436   1392763_at   8.115 8.137 8.192 8.446 8.159 8.758 8.841 8.933 8.727 8.422
   7437   1388966_at   6.728 6.713 6.693 6.741 6.506 6.419 5.978 5.681 5.526 5.765
   7438   1391363_at   2.698 3.503 4.294 3.917 4.689 2 2.245 1.922 2.227 1.989
   7439   1372160_at   8.012 7.947 8.182 8.292 8.252 8.8 8.988 9.022 8.552 8.499
   7440   1376381_at   5.405 5.572 5.197 5.946 5.786 2.884 4.335 4.358 4.137 4.343
   7441   1374148_at   1.144 3.114 4.063 2.239 3.027 6.349 5.938 5.111 4.806 5.416
   7442   1385624_at   5.851 5.431 5.638 5.896 5.643 6.87 6.605 6.405 6.313 6.112
   7443   1382417_at   5.742 5.688 6.254 5.657 5.678 4.793 5.01 4.326 5.288 4.642
   7444   1379637_at   5.779 6.082 5.507 5.707 5.676 4.843 5.06 4.716 5.111 5.364
   7445   1393612_a_at   6.355 6.2 6.68 6.22 6.016 6.854 7.217 6.834 6.967 6.874
   7446   1390982_at   6.244 6.031 6.307 6.19 6.366 5.989 5.439 5.771 5.558 5.6
   7447   1383301_at   6.927 7.018 6.847 6.799 7.174 6.489 6.53 6.626 6.315 6.622
   7448   1370017_at   7.779 7.916 8.095 8.08 7.874 7.149 7.008 7.167 6.636 5.972
   7449   1387880_at   5.602 5.422 6.066 5.178 5.696 3.661 0.88 3.26 0 0
   7450   1392615_at   6.556 6.489 6.7 6.462 6.231 6.025 5.992 5.976 5.476 5.709
   7451   1389393_at   10.405 10.478 10.433 10.503 10.516 11.094 11.278 10.651 10.91 11.102
   7452   1395654_at   6.336 6.254 6.128 6.455 5.99 5.295 5.535 4.605 5.482 5.478
   7453   1367915_at   7.714 7.751 7.735 8.046 8.222 8.395 8.424 8.514 8.338 8.447
   7454   1396066_at   3.3 3.89 3.355 3.178 2.962 0 1.316 2.307 0.401 1.609
   7455   1388469_at   7.348 7.435 6.929 6.948 7.919 8.145 8.5 8.334 8.139 8.299
   7456   1397855_at   0.651 0 3.728 3.005 0 5.721 5.781 5.129 5.287 5.371
   7457   1379294_at   8.411 8.213 8.459 8.252 8.245 8.917 8.621 8.692 8.6 8.937
   7458   1393507_at   4.467 3.369 4.456 4.374 3.987 5.761 5.734 4.866 5.453 5.425
   7459   1368399_a_at   10.066 9.896 10.224 10.146 10.052 10.532 10.587 10.327 10.863 10.756
   7460   1388817_at   8.146 8.099 8.113 8.216 8.332 8.461 8.613 8.773 8.398 8.675
   7461   1394459_at   5.938 5.99 5.856 5.851 6.055 6.649 6.936 7.147 6.228 6.69
   7462   1396205_at   7.611 7.545 7.112 7.29 7.219 4.033 4.699 0.595 4.062 4.459
   7463   1388354_at   8.983 8.845 8.748 8.797 8.772 8.051 8.397 8.538 7.945 8.297
   7464   1398796_at   10.353 10.354 10.351 10.379 10.45 10.062 10.003 10.243 9.913 9.819
   7465   1389336_at   7.564 7.572 7.794 7.499 7.466 8.089 8.214 8.012 7.97 7.807
   7466   1375209_at   7.684 7.379 7.801 7.748 7.922 7.177 7.111 7.158 7.338 6.905
   7467   1374485_at   7.551 7.538 7.44 7.729 7.71 7.92 8.169 7.833 7.986 8.002
   7468   1383920_at   8.843 8.766 8.52 8.628 8.769 9.184 9.583 8.968 9.447 9.457
   7469   1394409_at   5.978 6.42 5.632 5.75 5.778 4.916 4.975 5.384 4.94 5.211
   7470   1398683_at   6.103 5.967 6.26 6.255 6.227 5.849 5.567 5.592 5.902 5.727
   7471   1398317_at   9.07 9.052 9.101 9.158 9.156 9.585 10.018 9.666 9.543 9.388
   7472   1374167_at   4.209 1.88 1.227 4.093 4.367 6.65 6.402 6.877 6.237 6.353
   7473   1371384_at   11.679 11.621 11.613 11.424 11.572 11.372 11.339 11.214 11.337 11.364
   7474   1374192_at   3.586 4.11 3.967 2.88 3.974 1.202 0.848 1.971 1.744 2.782
   7475   1392451_at   1.144 1.131 2.313 2.242 2.094 0.151 0 0.864 0.176 0
   7476   1381803_at   4.591 5.044 4.954 5.332 5.454 6.159 5.875 6.006 5.805 5.736
   7477   1395704_at   6.721 6.816 6.551 6.548 6.631 6.311 6.391 6.082 6.041 5.922
   7478   1392841_at   5.634 5.483 5.433 5.283 5.003 6.582 5.907 6.009 6.295 6.055
   7479   1389321_at   6.634 6.721 6.523 7.198 6.935 7.492 7.71 7.764 7.594 7.232
   7480   1385761_s_at   6.994 7.186 7.257 7.343 7.478 6.538 6.658 6.993 6.48 6.516
   7481   1381972_at   8.828 9.076 9.33 9.073 9.007 8.497 8.584 8.131 8.595 8.559
   7482   1379904_at   8.2 8.164 7.872 8.324 8.482 9.041 8.964 9.3 8.877 8.608
   7483   1382800_at   4.348 4.03 4.857 3.894 4.24 5.27 5.339 4.848 5.519 5.522
   7484   1374456_at   8.555 8.567 8.596 8.794 8.758 8.244 8.46 8.281 8.068 8.154
   7485   1370916_at   6.682 6.963 6.813 7.444 7.171 6.189 6.286 6.083 6.491 5.918
   7486   1388777_at   10.964 10.762 10.605 10.803 10.921 10.391 10.588 10.33 10.326 10.442
   7487   1375338_at   10.157 10.165 10.382 10.391 10.21 9.971 9.934 9.961 10.064 9.87
   7488   1394195_at   4.353 4.775 4.314 5.067 4.672 5.291 5.704 6.084 5.376 5.62
   7489   1388698_at   7.207 7.629 7.447 7.434 7.792 8.104 8.035 7.869 8.165 8.077
   7490   1370277_at   12.35 12.294 12.342 12.228 12.264 12.528 12.424 12.512 12.701 12.58
   7491   1378243_at   5.97 5.85 5.853 5.851 6.095 6.443 6.591 6.668 6.588 6.102
   7492   1379782_a_at   6.251 6.379 6.309 6.642 7.134 7.552 7.731 7.856 7.243 7.306
   7493   1391183_at   6.031 6.446 6.122 6.553 6.331 6.905 6.997 6.95 6.626 6.905
   7494   1371055_at   6.788 6.936 6.805 6.872 6.775 6.588 6.502 6.075 6.385 6.277
   7495   1393707_at   6.866 6.451 6.799 7.258 7.014 7.593 7.642 7.644 7.51 7.434
   7496   1379810_at   8.705 8.859 8.799 8.962 8.901 8.398 8.588 8.405 8.072 8.418
   7497   1392991_at   6.938 6.997 6.397 6.953 7.143 6.165 6.338 6.182 5.934 6.243
   7498   1376132_at   6.878 6.988 6.816 6.795 7.017 6.699 6.61 6.528 6.456 6.354
   7499   1374350_at   6.888 7.456 7.367 7.654 7.358 7.85 8.267 8.009 8.023 8.128
   7500   1368134_a_at   8.556 8.57 8.653 8.714 8.632 8.212 8.134 8.36 7.919 7.757
   7501   1383604_at   6.577 6.558 6.458 6.884 6.642 7.014 7.063 6.898 7.121 7.009
   7502   1373845_at   8.581 8.591 8.352 8.434 8.506 8.152 8.274 8.284 8.067 8.07
   7503   1397768_at   4.547 5.374 4.503 4.845 4.464 5.998 5.424 5.655 6 5.686
   7504   1381997_at   5.189 5.671 4.839 5.236 4.75 5.951 5.98 6.496 6.177 5.924
   7505   1399088_at   7.544 7.654 7.661 7.806 7.764 6.771 7.258 7.331 6.7 7.046
   7506   1373387_at   9.162 9.12 9.083 9.079 9.248 8.667 8.991 8.568 8.741 8.512
   7507   1398662_at   3.756 4.733 4.355 3.605 4.279 5.354 5.191 5.085 5.114 5.223
   7508   1384878_at   5.801 5.808 6.142 6.05 6.144 5.36 5.643 5.508 5.641 5.454
   7509   1373333_at   2.032 3.383 2.101 3.209 3.205 4.831 4.337 4.631 4.558 3.892
   7510   1379784_at   7.838 8.086 7.937 7.846 7.98 8.376 8.681 8.412 8.16 8.404
   7511   1368065_at   8.508 8.432 8.484 8.717 8.611 8.316 8.067 8.164 8.046 7.832
   7512   1384965_at   6.702 6.654 6.531 6.773 6.386 6.257 5.859 5.635 6.144 5.969
   7513   1374172_at   7.065 6.988 6.849 7.11 7.374 8.055 8.268 7.387 7.977 7.895
   7514   1394093_at   7.278 7.773 7.534 7.932 7.486 7.033 6.98 6.951 6.991 6.592
   7515   1391026_at   6.885 7.143 6.66 6.899 7.177 6.138 5.35 6.356 6.151 5.711
   7516   1380786_at   4.406 4.231 5.056 4.561 4.05 5.811 5.299 5.757 5.301 5.299
   7517   1398634_at   6.766 6.751 7.066 6.935 6.534 7.793 8.303 7.597 7.798 7.271
   7518   1398800_at   10.806 10.777 10.664 10.737 10.73 10.451 10.574 10.614 10.405 10.541
   7519   1383099_at   7.296 7.536 7.356 7.47 7.609 7.217 6.958 6.684 6.823 6.638
   7520   1382062_at   6.825 6.68 6.637 6.731 6.634 6.324 6.079 6.471 6.062 5.897
   7521   1390848_at   6.215 6.425 6.663 6.976 6.636 5.447 6.096 5.721 5.178 5.572
   7522   1394101_at   5.462 4.72 5.454 5.311 5.52 6.162 6.319 6.413 5.802 6.245
   7523   1382403_at   4.817 4.873 4.474 4.424 5.198 5.749 5.441 5.635 6.225 6.384
   7524   1377162_at   6.511 6.444 6.245 6.465 6.415 7.083 7.308 6.761 6.75 6.997
   7525   1372139_at   5.928 5.726 6.254 6.602 6.214 6.787 6.864 7.381 7.117 7.09
   7526   1388699_at   9.552 9.518 9.266 9.677 9.615 9.981 9.808 9.867 9.952 9.918
   7527   1388369_at   10.096 9.912 9.78 10.137 10.29 9.445 9.579 9.669 9.272 9.494
   7528   1369485_at   5.029 4.664 4.897 5.055 4.291 6.151 5.936 5.319 6.608 6.259
   7529   1372187_at   8.37 8.665 8.509 8.68 8.61 7.953 8.002 7.485 8.08 8.129
   7530   1370235_at   11.511 11.476 11.619 11.588 11.681 11.791 11.833 11.83 11.835 11.7
   7531   1367653_a_at   12.043 12.059 12.09 11.96 11.954 12.373 12.263 12.193 12.521 12.516
   7532   1399004_at   9.458 9.591 9.578 9.722 9.757 9.92 10.101 9.976 10.119 9.846
   7533   1383729_at   5.273 5.574 5.246 4.895 5.228 4.243 4.327 4.351 4.706 4.789
   7534   1395173_at   8.697 8.672 8.783 8.72 8.716 8.263 7.591 7.382 8.213 7.478
   7535   1381847_at   6.558 6.78 6.438 6.682 7.097 5.574 6.278 5.693 6.031 5.604
   7536   1392744_at   7.343 7.41 7.505 7.358 7.526 6.948 7.22 6.873 7.03 7.184
   7537   1370276_at   12.107 12.044 11.945 11.678 11.831 12.27 12.291 12.289 12.389 12.328
   7538   1373984_at   8.433 8.271 7.935 8.195 8.002 7.453 7.617 7.574 7.218 6.855
   7539   1372600_at   7.915 7.945 8.12 8.051 7.881 8.393 8.444 8.255 8.361 8.153
   7540   1396145_at   8.269 8.304 8.513 8.433 8.307 7.796 8.037 7.422 7.807 7.942
   7541   1371920_at   8.91 8.888 8.892 8.781 8.768 9.362 9.511 9.449 9.159 9.047
   7542   1381725_at   1.787 3.194 2.993 3.046 3.583 1.632 0.526 0.888 1.485 0.356
   7543   1388447_at   8.749 8.813 9.04 8.884 8.978 8.456 8.6 8.445 8.505 8.137
   7544   1382463_at   8.014 8.414 8.488 8.142 8.026 7.273 7.49 6.63 7.304 7.591
   7545   1368784_at   4.783 4.957 5.831 5.181 5.259 6.197 6.038 6.442 6.799 6.103
   7546   1367907_a_at   8.301 8.251 8.199 8.385 8.328 8.507 8.683 8.757 8.54 8.492
   7547   1386998_at   5.663 5.912 5.6 6.204 6.102 4.758 4.469 3.205 4.455 4.812
   7548   1373539_at   7.945 7.597 7.578 7.786 7.776 8.231 8.382 8.077 8.49 8.094
   7549   1377166_at   7.622 7.87 7.664 8.081 7.935 8.317 8.7 8.634 8.447 8.2
   7550   1393271_at   5.2 4.953 5.108 5.758 4.793 6.375 6.1 7.045 6.838 5.97
   7551   1369691_at   0.546 0 0 0 0 4.168 1.063 3.807 3.065 2.345
   7552   1383624_at   6.804 6.915 7.101 7.234 7.046 7.733 8.17 8.485 7.825 7.507
   7553   1398764_at   11.046 11.11 11.31 11.085 11.183 10.789 10.917 10.477 10.677 10.494
   7554   1383981_at   6.533 6.313 6.657 6.563 6.786 7.474 7.622 7.538 6.976 7.045
   7555   1380760_at   5.973 6.469 5.9 5.969 6.246 5.448 5.316 4.96 5.545 5.536
   7556   1388604_at   5.479 5.973 5.362 5.572 4.128 6.906 6.902 7.084 6.63 6.854
   7557   1369433_at   5.949 5.898 5.851 5.488 5.795 6.451 6.409 6.276 6.099 6.261
   7558   1384404_at   5.799 5.515 5.799 5.789 5.803 5.231 4.495 5.272 5.071 4.9
   7559   1388326_at   10.647 10.634 10.632 10.538 10.64 10.923 11.028 10.747 10.936 10.797
   7560   1378887_at   5.437 5.207 5.111 4.979 5.418 4.341 2.425 2.976 3.745 3.92
   7561   1395838_at   3.133 3.834 4.178 3.268 2.746 0.07 1.496 2.254 1.287 0.251
   7562   1386621_at   5.281 5.207 5.686 5.649 5.707 4.963 4.974 4.373 4.392 4.341
   7563   1375649_at   5.009 5.631 5.708 5.075 5.044 3.529 4.202 4.446 3.702 4.481
   7564   1376726_at   0.506 0.345 0.251 2.151 0 2.124 2.731 2.934 3.722 3.553
   7565   1367997_at   7.825 7.984 7.792 8.355 8.204 8.529 8.739 9.06 8.695 8.614
   7566   1379703_at   8.524 8.372 8.154 8.442 8.422 9.145 8.994 8.953 8.784 8.649
   7567   1368313_a_at   6.552 6.135 6.56 6.696 6.278 7.865 8.101 6.867 8.192 7.578
   7568   1379704_at   6.531 6.681 6.802 6.537 6.909 5.528 6.246 6.035 5.682 6.136
   7569   1398589_at   5.987 5.673 6.139 5.518 5.741 4.517 1.696 1.868 3.746 1.22
   7570   1385222_at   4.191 4.186 4.881 4.648 3.964 2.438 0.722 2.398 2.531 3.108
   7571   1375280_at   8.593 8.553 8.945 8.835 8.997 8.082 8.386 8.396 8.096 8.084
   7572   1373896_at   3.698 2.791 3.943 3.899 3.236 4.831 4.342 5.223 4.822 4.753
   7573   1372179_at   9.336 9.367 9.524 9.739 9.63 10.114 9.788 10.009 9.963 10.018
   7574   1387903_at   8.274 8.54 8.275 8.374 8.209 8.87 8.91 9.068 8.525 8.936
   7575   1387185_at   5.602 5.541 5.426 5.609 5.366 6.258 6.284 6.54 5.786 6.027
   7576   1369057_at   8.431 8.424 8.438 8.62 8.458 8.117 7.742 8.03 8.117 7.594
   7577   1375934_at   9.64 9.588 9.583 9.478 9.625 9.981 10.112 9.862 9.731 10.064
   7578   1390563_at   8.054 7.954 8.182 8.268 8.371 8.599 8.731 8.721 8.448 8.562
   7579   1393422_at   4.022 5.209 5.124 5.648 5.668 6.434 6.953 6.501 6.836 6.673
   7580   1375188_at   6.784 6.87 6.663 7.031 7.165 6.229 5.827 6.294 5.928 6.503
   7581   1371471_at   9.547 9.661 9.46 9.638 9.716 9.392 9.358 9.381 9.236 9.219
   7582   1378692_at   9.045 9.09 8.953 9.338 9.11 9.636 9.676 9.842 9.395 9.472
   7583   1388485_at   5.378 5.341 5.743 5.517 5.539 6.435 6.296 5.765 6.66 6.46
   7584   1391206_at   5.914 6.296 6.205 6.128 6.237 6.541 6.66 6.671 6.471 6.919
   7585   1378441_at   6.263 6.028 6.254 6.24 5.88 7.126 6.825 7.058 6.585 6.542
   7586   1371605_at   10.997 11.02 10.989 11.037 11.062 11.132 11.265 11.362 11.186 11.19
   7587   1384338_at   6.287 5.896 5.464 5.877 5.993 1.063 4.059 1.891 3.81 3.674
   7588   1397663_at   5.771 5.301 5.364 5.789 5.019 3.141 1.379 3.797 0.872 3.366
   7589   1373639_at   8.309 8.302 8.345 8.502 8.595 9.033 9.135 8.908 8.791 8.671
   7590   1373169_at   7.882 8.284 8.03 8.049 7.849 7.502 7.551 7.717 7.426 7.636
   7591   1376774_at   7.642 7.614 7.388 7.61 7.47 8.12 8.39 8.346 7.922 7.855
   7592   1372451_at   7.961 7.89 8.032 7.964 8.037 7.742 7.746 7.798 7.777 7.55
   7593   1374694_at   6.793 7.036 7.011 6.779 7.013 7.41 7.715 7.48 7.153 7.496
   7594   1372590_at   6.573 6.64 5.752 6.426 6.857 5.532 5.469 5.317 5.131 5.671
   7595   1371375_at   11.679 11.637 11.813 11.612 11.825 11.451 11.524 11.517 11.421 11.456
   7596   1389862_at   6.91 6.171 6.553 6.636 6.907 5.61 5.87 6.163 5.552 5.669
   7597   1372706_at   10.697 10.681 10.599 10.555 10.597 10.995 11.038 10.721 11.068 10.957
   7598   1373819_at   9.974 10.128 10.291 10.289 10.249 10.641 10.661 10.414 10.663 10.494
   7599   1378492_at   4.94 4.374 4.309 5.33 5.191 5.872 6.035 6.383 6.067 5.664
   7600   1394815_at   6.547 6.806 6.706 7.02 6.768 7.158 7.434 7.338 7.736 7.253
   7601   1390098_at   7.475 7.753 7.808 7.932 8.05 7.043 7.184 7.456 6.838 7.064
   7602   1397709_at   4.926 4.803 5.08 4.535 4.453 3.623 4.34 3.35 3.058 3.422
   7603   1376010_at   8.675 8.67 8.444 8.503 8.336 8.255 8.11 8.119 8.096 8.237
   7604   1390102_at   8.427 8.277 8.504 8.771 8.55 9.267 9.248 8.747 9.148 9.297
   7605   1380001_at   6.955 6.811 6.928 6.803 6.89 6.579 5.854 6 6.359 5.783
   7606   1397812_at   7.076 7.259 7.16 7.249 7.304 7.569 7.771 7.494 7.501 7.441
   7607   1385297_at   3.795 3.9 4.048 3.857 4.454 0.722 0.911 3.242 0 1.496
   7608   1377865_at   5.241 5.366 5.92 6.087 5.33 6.515 6.779 6.518 6.378 6.355
   7609   1389542_at   5.122 4.822 5.248 5.279 5.6 3.111 1.17 1.709 3.848 0.356
   7610   1369431_at   5.551 5.695 5.777 5.603 5.796 4.986 5.059 4.783 5.319 5.372
   7611   1369441_at   5.616 5.545 5.587 5.536 5.893 5.344 5.075 4.77 5.168 4.975
   7612   1377104_at   0 0.31 0 0.07 1.07 1.057 3.251 2.37 2.342 2.325
   7613   1374332_at   7.537 7.399 7.446 7.447 7.816 7.189 6.965 7.004 7.221 7.051
   7614   1395517_at   6.399 6.46 6.237 6.513 6.181 5.589 5.049 4.635 5.858 5.246
   7615   1376324_at   5.651 5.101 5.304 5.685 5.748 4.123 4.738 4.934 4.648 4.547
   7616   1372155_at   9.865 9.782 9.981 9.885 9.772 9.736 9.53 9.571 9.606 9.549
   7617   1375376_at   3.07 1.551 1.575 1.903 1.618 3.694 3.593 2.967 3.602 3.953
   7618   1387816_at   5.844 5.42 5.821 5.387 5.844 6.879 6.988 6.017 7.218 7.214
   7619   1372898_at   6.503 6.6 6.632 6.717 6.635 6.426 6.031 5.888 5.917 6.22
   7620   1372826_at   7.596 7.158 7.558 7.461 7.203 7.015 6.921 6.894 6.709 6.637
   7621   1372714_at   9.113 8.998 8.91 8.869 9.063 8.576 8.843 8.429 8.448 8.514
   7622   1383442_at   3.535 4.081 2.918 4.213 3.493 5.53 5.449 5.663 4.483 4.93
   7623   1376205_at   7.43 7.67 7.553 7.762 7.761 6.961 6.677 7.415 6.829 6.807
   7624   1389314_at   6.78 6.572 6.497 6.646 6.908 6.198 6.161 5.897 5.853 6.357
   7625   1373922_at   8.121 8.067 8.169 8.267 8.194 9.046 9.157 9.591 8.537 8.837
   7626   1395297_at   6.568 5.951 5.403 6.565 6.472 5.235 4.698 4.554 5.118 4.98
   7627   1376737_at   8.684 8.664 8.681 8.497 8.64 8.283 7.943 8.248 7.621 7.753
   7628   1398880_at   11.453 11.424 11.535 11.522 11.466 11.364 11.256 11.04 11.11 11.14
   7629   1395440_at   6.971 7.013 6.896 7.073 7.154 7.405 7.391 7.166 7.369 7.296
   7630   1368074_at   5.743 6.268 6.391 5.998 6.369 4.992 5.146 5.131 5.513 5.659
   7631   1376156_at   5.198 5.706 5.489 5.747 6.046 6.461 6.2 6.314 6.536 6.401
   7632   1377860_at   6.998 6.889 6.658 7.246 7.173 6.217 6.665 6.143 6.052 6.26
   7633   1384390_at   6.958 7.106 7.06 7.408 7.228 6.747 6.765 6.589 6.29 6.558
   7634   1368166_at   6.987 6.672 6.548 6.864 6.725 7.481 7.133 7.472 7.267 7.118
   7635   1390856_at   4.998 4.709 4.559 4.91 4.871 5.245 5.196 5.216 5.302 5.124
   7636   1371132_a_at   6.611 6.311 6.286 5.971 6.438 6.745 7.446 6.94 7.18 7.198
   7637   1369484_at   5.668 5.344 4.839 5.437 5.361 3.332 4.384 3.349 4.493 4.137
   7638   1387031_at   9.978 9.865 9.821 9.939 10.17 9.559 9.724 9.652 9.485 9.485
   7639   1371985_a_at   9.24 9.127 9.002 9.131 9.27 8.666 8.758 8.854 8.556 8.286
   7640   1372701_at   12.397 12.372 12.261 12.071 12.18 11.64 11.835 11.585 11.544 12.028
   7641   1398567_at   1.628 1.945 2.325 4.142 3.717 5.39 5.186 4.933 5.151 5.972
   7642   1379397_at   5.785 5.996 6.041 6.408 6.558 7.283 7.364 6.824 6.856 6.902
   7643   1391410_at   7.195 7.438 7.527 7.239 7.437 7.911 7.935 7.755 7.631 7.712
   7644   1391878_at   5.024 5.246 6.049 5.938 5.362 2.836 0.595 2.996 0 3.465
   7645   1377998_at   8.348 8.47 8.064 8.209 8.291 7.984 7.85 7.471 7.734 7.655
   7646   1375121_at   5.685 6.084 6.26 6.593 6.382 7.008 6.958 7.034 6.913 6.882
   7647   1399036_at   7.914 8.052 7.94 8.276 7.881 8.442 8.55 8.744 8.429 8.335
   7648   1381936_at   3.42 3.714 2.729 3.03 2.272 4.89 5.027 4.883 3.814 4.69
   7649   1398242_at   8.005 7.533 8.011 8.054 8.05 8.445 8.423 8.51 8.452 8.342
   7650   1376611_at   6.712 7.029 6.522 6.748 6.644 6.216 6.238 5.882 5.665 6.201
   7651   1383439_at   5.279 4.938 5.071 5.163 4.806 7.043 6.849 5.852 5.977 5.968
   7652   1370011_at   5.674 5.405 5.663 6.072 5.942 6.415 6.566 6.455 6.526 6.148
   7653   1378027_at   7.408 7.537 7.745 7.749 7.929 8.34 8.714 8.144 8.229 8.232
   7654   1372410_at   6.368 6.584 6.535 6.875 7.218 5.659 5.745 6.193 5.923 5.639
   7655   1382510_at   4.558 4.441 4.716 4.511 4.452 5.277 5.555 5.093 4.95 4.914
   7656   1382593_at   6.445 6.76 6.672 6.544 6.753 7.24 7.515 6.893 7.635 7.332
   7657   1367800_at   9.425 9.366 9.448 9.515 9.466 9.771 9.836 9.653 9.611 9.948
   7658   1381986_at   6.278 6.01 6.141 6.457 6.044 5.65 5.671 5.8 5.863 5.731
   7659   1397678_at   0.956 0 3.489 0.227 1.526 5.559 4.945 5.018 3.466 4.563
   7660   1383686_at   5.624 5.289 5.95 6.027 6.142 6.905 7.072 6.693 6.996 6.331
   7661   1372304_at   9.208 9.255 9.464 9.497 9.48 9.094 9.008 8.995 8.955 8.721
   7662   1383127_at   9.098 9.412 9.066 9.242 9.684 8.54 8.736 8.227 8.449 8.81
   7663   1373832_at   7.052 6.778 7.045 7.152 6.963 7.326 7.544 7.336 7.487 7.272
   7664   1398451_at   7.428 6.996 7.162 7.626 7.622 6.241 6.836 6.623 6.685 6.267
   7665   1374032_at   6.144 6.282 6.201 6.481 6.307 6.666 6.629 6.675 6.566 6.495
   7666   1396114_at   0 0 0 0.632 0.934 1.31 2.905 3.706 1.96 2.751
   7667   1382118_at   4.924 5.334 4.768 5.18 5.335 6.234 6.335 6.123 5.509 5.867
   7668   1367585_a_at   12.322 12.296 12.29 12.116 12.234 12.435 12.445 12.444 12.508 12.406
   7669   1391228_at   4.09 3.406 3.466 3.654 3.754 5.223 4.824 5.766 5.252 4.215
   7670   1370725_a_at   9.88 9.895 10.335 9.729 8.873 11.182 11.31 10.518 11.417 11.268
   7671   1372870_at   7.136 7.104 6.978 7.031 7.505 6.404 6.498 6.841 6.618 6.431
   7672   1371064_at   7.173 7.273 7.258 7.261 7.117 7.856 7.777 7.916 7.399 7.572
   7673   1391315_at   6.027 6.169 6.84 6.607 6.188 7.409 7.238 7.047 7.345 6.992
   7674   1371665_at   6.398 6.558 6.175 6.414 6.116 7.302 7.077 7.883 6.974 6.989
   7675   1378783_at   4.271 4.255 3.808 4.155 4.153 5.198 4.923 5.822 5.045 4.692
   7676   1382398_at   2.83 2.746 2.342 2.536 3.214 4.513 4.624 4.405 3.225 4.016
   7677   1398330_at   7.7 7.723 7.957 8.167 7.891 7.255 7.345 7.555 7.492 7.27
   7678   1379713_at   3.699 3.664 1.766 3.995 4.084 5.498 5.432 5.721 5.601 5.388
   7679   1374116_at   6.784 6.798 6.958 6.846 6.888 7.162 7.533 7.245 7.074 7.283
   7680   1393620_at   7.058 6.742 7.45 7.457 7.137 6.1 6.522 6.307 6.607 5.993
   7681   1384602_at   6.057 6.122 6.013 6.4 6.139 5.635 5.195 5.317 5.857 5.249
   7682   1374323_at   9.456 9.336 8.966 9.328 9.451 8.802 8.782 8.92 8.752 8.473
   7683   1377713_at   8.179 8.218 7.768 8.275 8.37 7.464 7.731 7.707 7.55 7.577
   7684   1379768_at   7.561 7.668 7.621 7.57 7.517 7.019 6.971 6.643 7.362 7.086
   7685   1392562_at   8.021 7.834 7.771 8.072 8.021 7.724 7.602 7.619 7.62 7.516
   7686   1368596_at   5.799 5.955 6.17 6.221 5.715 8.123 8.097 8.413 6.388 7.801
   7687   1371427_at   11.532 11.522 11.556 11.551 11.618 11.409 11.248 11.242 11.432 11.384
   7688   1382444_at   3.527 3.687 3.603 3.77 4.287 5.107 4.8 5.198 4.408 4.452
   7689   1379148_at   2.618 3.472 3.256 3.602 3.666 4.469 4.698 5.004 3.986 4.543
   7690   1398610_at   6.323 6.422 7.048 6.758 6.895 7.433 7.618 7.422 7.21 7.444
   7691   1379731_at   6.734 6.944 6.728 6.825 6.84 7.014 7.069 7.024 6.967 7.092
   7692   1378002_at   6.201 6.378 5.883 6.3 6.304 6.771 7.338 6.986 6.673 6.818
   7693   1373580_at   5.029 5.318 5.644 5.781 5.375 5.944 6.37 6.802 6.264 6.357
   7694   1392789_at   7.368 7.501 7.758 7.162 7.102 8.533 8.137 7.881 8.381 8.016
   7695   1369439_at   5.223 5.531 5.195 5.188 5.755 6.083 6.175 6.073 6.033 5.759
   7696   1380669_at   1.401 0.88 2.68 2.854 0.595 4.511 4.044 4.338 4.357 3.362
   7697   1370895_at   8.88 8.766 8.584 9.009 9.724 8.067 7.91 7.596 7.925 8.181
   7698   1398899_at   8.387 8.493 8.521 8.824 8.622 8.187 8.016 8.217 8.256 8.114
   7699   1382865_at   5.793 5.696 6 6.015 6.33 5.06 5.605 5.27 4.999 5.23
   7700   1379541_at   6.679 6.942 6.906 7.091 7.253 7.63 7.61 7.698 7.342 7.421
   7701   1372736_at   7.129 7.119 7.029 7.377 7.215 7.83 8.085 7.95 7.534 7.522
   7702   1398618_s_at   7.616 7.855 8.163 8.058 8.208 8.598 8.666 8.79 8.432 8.479
   7703   1383798_at   10.085 10.093 10.433 10.161 9.81 11.111 11.389 10.443 11.379 11.05
   7704   1380969_at   3.116 4.42 3.114 3.256 2.96 5.694 5.236 6.268 4.692 4.692
   7705   1372193_at   7.183 6.936 7.401 6.966 6.854 7.43 7.568 7.674 7.655 7.638
   7706   1398813_at   9.655 9.638 9.63 9.58 9.46 9.331 9.467 9.359 9.265 9.378
   7707   1390940_at   5.412 5.769 6.1 6.077 6.397 5.073 5.025 4.489 5.174 4.395
   7708   1392746_x_at   9.081 8.917 8.989 9.344 9.101 8.51 8.77 8.701 8.292 8.444
   7709   1371627_at   7.138 7.04 7.214 7.287 7.321 7.762 7.526 7.747 7.391 7.663
   7710   1378506_at   7.719 7.659 7.387 7.451 7.513 7.878 8.007 7.941 7.761 7.976
   7711   1367642_at   10.609 10.598 10.604 10.652 10.765 10.952 11.066 10.755 11.008 10.918
   7712   1379485_at   6.915 6.724 7.178 7.133 7.129 6.433 6.242 6.219 6.748 6.218
   7713   1393549_at   1.74 0.043 0.971 0.043 2.57 4.23 3.894 4.037 4.846 2.751
   7714   1385074_at   6.51 6.251 5.983 6.116 6.015 6.94 7.289 8.025 6.917 7.114
   7715   1373730_at   7.756 7.715 7.615 7.699 7.66 7.891 8.106 8.076 7.825 7.958
   7716   1370956_at   11.523 11.43 11.183 11.019 11.451 10.651 10.447 9.905 10.756 10.586
   7717   1378459_at   2.761 3.492 4.157 3.602 3.38 5.087 4.417 4.438 4.862 5.159
   7718   1390710_x_at   8.253 8.458 8.483 8.766 8.339 8.837 9.224 9.57 9.097 9.114
   7719   1370176_at   7.586 7.687 7.846 7.907 7.727 8.254 8.122 8.048 8.005 8.052
   7720   1387679_at   2.192 0 0 2.186 0.888 3.348 3.459 3.22 3.525 3.658
   7721   1397015_at   4.745 5.068 4.668 5.605 5.715 6.237 6.801 5.932 6.636 6.611
   7722   1381825_at   5.856 5.897 6.263 6.118 5.883 6.657 6.524 6.934 6.533 6.36
   7723   1377015_at   6.563 6.596 6.423 6.734 6.889 7.288 7.665 7.964 7.397 7.071
   7724   1379816_at   6.029 6.24 6.497 6.541 6.378 7.02 6.82 6.928 6.785 6.715
   7725   1384323_at   8.829 8.824 9.021 8.954 9.064 8.593 8.637 8.429 8.748 8.479
   7726   1381019_x_at   5.978 6 6.485 6.299 6.507 5.591 5.625 5.171 5.726 5.582
   7727   1368033_at   6.766 6.552 6.404 6.338 6.634 6.151 5.139 4.995 5.715 5.338
   7728   1387370_at   1.556 3.512 2.459 3.356 0 5.795 5.174 4.866 5.609 5.476
   7729   1393427_s_at   6.013 6.089 6.441 6.29 6.872 7.318 7.07 7.456 7.514 6.918
   7730   1370054_at   7.955 7.954 7.769 8.02 7.997 6.562 7.404 7.354 6.964 7.364
   7731   1390647_at   6.849 7.154 6.915 6.847 6.835 6.637 6.66 6.389 6.438 6.547
   7732   1379031_at   7.104 7.413 7.325 7.27 7.537 7.727 7.891 8.092 7.679 7.754
   7733   1373270_at   7.115 7.274 7.318 7.343 7.615 7.936 7.855 7.638 7.966 7.724
   7734   1379385_at   7.062 6.902 7.081 7.275 7.269 6.445 6.509 6.594 6 6.7
   7735   1391437_at   7.787 7.752 7.667 7.809 7.838 7.574 7.623 7.551 7.385 7.429
   7736   1397251_at   5.405 5.05 5.801 5.503 5.511 7.412 6.53 7.298 6.694 6.047
   7737   1380822_at   5.453 5.577 5.552 4.646 5.22 4.216 4.088 2.911 3.989 4.121
   7738   1371689_at   8.781 8.852 8.831 9.105 8.885 9.434 9.319 9.191 9.13 9.235
   7739   1374386_at   7.271 6.852 6.934 6.928 6.769 6.573 6.573 6.372 6.53 6.369
   7740   1381203_at   7.617 7.559 7.448 7.717 7.782 6.92 6.989 7.413 6.776 6.677
   7741   1373880_at   6.258 6.256 6.291 6.318 6.061 6.565 6.455 6.874 6.668 6.611
   7742   1381975_at   6.643 6.399 6.624 6.424 6.449 6.245 5.922 6.149 5.917 6.206
   7743   1387875_at   7.965 8.111 8.26 8.216 8.074 8.513 8.762 8.435 8.405 8.483
   7744   1395418_at   5.978 6.275 6.293 6.359 6.408 6.015 5.654 5.64 5.828 5.614
   7745   1383390_at   3.667 3.043 3.38 3.815 0.949 6.049 6.521 4.934 5.681 5.664
   7746   1370287_a_at   9.9 9.992 9.904 10.295 10.4 9.636 9.623 9.523 9.247 9.419
   7747   1392886_a_at   6.585 6.341 6.464 6.266 6.271 6.033 5.248 5.37 5.489 4.801
   7748   1384647_at   1.007 0 1.351 0.986 0.84 4.463 3.977 1.705 3.027 4.111
   7749   1368241_a_at   8.729 8.701 8.527 8.912 8.969 8.102 8.142 8.518 8.147 8.292
   7750   1384696_at   0.411 2.183 1.669 0 0.367 3.998 2.937 2.88 3.018 3.063
   7751   1374049_at   5.683 5.736 5.09 5.021 4.975 6.547 6.258 6.171 5.943 6.227
   7752   1368523_at   5.486 5.788 4.845 5.115 6.135 4.285 4.657 4.067 3.732 3.96
   7753   1387507_at   4.952 5.111 5.332 4.653 5.174 4.144 4.346 3.445 4.31 4.175
   7754   1376790_at   7.956 8.076 8.228 8.017 7.883 8.376 8.435 8.344 8.303 8.267
   7755   1394472_at   8.19 8.201 8.544 8.323 8.385 9.282 9.436 8.53 9.245 9.285
   7756   1367482_at   9.036 9.135 9.059 9.173 9.285 8.752 8.878 8.98 8.768 8.7
   7757   1397164_at   8.42 8.615 8.893 8.66 8.914 10.607 9.915 10.546 9.454 9.439
   7758   1383057_at   5.275 4.536 4.777 4.582 4.305 5.516 5.482 5.501 5.384 5.565
   7759   1374318_at   9.475 9.612 9.75 9.711 9.733 10.139 10.205 9.883 9.917 10.063
   7760   1376936_at   7.532 7.493 7.589 7.639 7.721 7.103 7.027 7.406 6.841 6.778
   7761   1388328_at   10.052 9.855 10.116 9.889 10.075 9.444 9.524 9.694 9.627 9.165
   7762   1373522_at   6.019 6.108 6.005 6.096 6.04 5.389 5.621 5.623 5.373 4.849
   7763   1378979_x_at   5.074 5.154 5.331 5.579 5.394 5.805 5.932 6.429 5.825 6.315
   7764   1372690_at   4.921 4.756 4.633 4.92 5.211 5.663 5.187 5.646 5.541 5.5
   7765   1390092_at   1.459 2.319 3.824 3.563 1.868 5.721 5.278 4.35 5.333 4.828
   7766   1368901_at   4.98 5.247 5.207 4.868 5.076 5.57 5.769 5.295 5.711 5.787
   7767   1382371_at   6.951 6.746 7.169 7.06 7.109 7.554 8.006 7.305 7.714 7.734
   7768   1368599_at   5.501 5.337 5.183 6.063 5.153 6.391 6.514 6.033 6.575 6.295
   7769   1369680_at   5.747 5.934 5.776 5.912 6 6.502 6.1 6.567 6.564 6.22
   7770   1369119_a_at   5.838 5.655 5.405 6.024 5.991 4.113 3.891 3.772 3.637 1.422
   7771   1375833_at   4.837 4.997 4.717 5.214 4.592 3.227 4.285 3.73 3.936 2.987
   7772   1380675_s_at   4.858 5.327 5.507 5.633 5.856 6.278 6.281 6.731 6.254 6.241
   7773   1375951_at   6.351 6.822 6.778 6.55 6.564 7.186 7.116 6.898 7.106 7.107
   7774   1398265_at   4.144 4.781 4.89 4.271 5.601 6.067 6.026 5.873 5.969 5.98
   7775   1367791_at   5.639 5.756 5.834 6.132 6.367 3.979 4.815 4.782 5.198 5.03
   7776   1374454_at   7.055 7.204 7.212 7.413 7.488 7.874 7.834 7.52 7.734 7.793
   7777   1376107_at   5.297 5.365 5.164 4.983 4.854 5.592 5.823 5.864 5.556 6.094
   7778   1398517_at   5.703 5.757 5.725 5.722 5.586 6.247 6.742 6.328 6.246 5.974
   7779   1388173_at   3.977 5.027 4.529 4.782 5.07 1.945 1.316 1.651 0 3.654
   7780   1380596_at   7.093 7.44 6.955 7.788 7.697 6.144 6.7 6.662 5.902 6.175
   7781   1371593_at   6.852 7.11 7.049 7.14 7.029 7.557 7.297 7.645 7.428 7.297
   7782   1392168_at   5.763 5.708 5.74 5.623 5.696 5.385 4.455 4.957 4.918 4.369
   7783   1374847_at   7.679 7.741 7.742 7.723 7.566 8.175 8.098 7.993 8.112 7.806
   7784   1377093_at   5.795 5.648 5.781 6.02 5.924 6.284 6.473 6.137 6.284 6.674
   7785   1388701_at   7.998 7.956 7.719 8.184 7.857 8.563 8.561 8.949 8.414 8.338
   7786   1385722_at   6.834 6.919 6.636 6.668 6.838 7.599 7.577 8.158 7.182 7.423
   7787   1372002_at   9.454 9.574 9.042 9.483 9.933 8.845 8.781 8.772 8.569 8.885
   7788   1383216_at   6.807 6.865 6.808 6.948 7.197 7.473 7.607 7.446 7.202 7.305
   7789   1380203_at   5.955 5.919 6.03 6.335 6.367 6.872 7.098 7.23 6.703 6.521
   7790   1373879_at   8.03 7.95 8.045 8.066 7.856 7.111 7.557 7.412 7.68 7.611
   7791   1377353_a_at   8.419 8.368 8.206 8.328 8.455 8.72 9.155 8.715 8.916 8.667
   7792   1389558_at   8.403 8.315 8.619 8.264 8.485 7.943 7.792 6.946 7.599 7.426
   7793   1374152_at   7.428 7.186 7.253 7.394 7.245 7.073 7.079 6.994 6.98 6.826
   7794   1382774_at   3.925 5.377 4.932 5.149 5.086 0.485 2.124 3.598 1.664 2.759
   7795   1398934_at   8.754 8.827 8.867 8.991 8.865 8.7 8.662 8.602 8.478 8.562
   7796   1392924_at   5.889 6.21 6.483 6.334 6.076 6.731 7.005 6.719 6.605 6.896
   7797   1376177_at   6.671 6.862 6.812 6.846 6.746 7.137 7.195 7.143 7.148 6.882
   7798   1377208_at   4.371 4.45 4.038 3.678 4.35 4.651 5.19 5.282 5.238 4.943
   7799   1371982_at   9.17 9.169 9.072 9.17 9.172 8.708 8.857 9.013 8.873 8.626
   7800   1377943_at   5.756 5.936 5.876 5.911 5.657 5.367 5.022 4.749 5.488 4.832
   7801   1388545_at   4.119 3.798 3.926 5.113 2.891 5.964 6.02 6.411 6.056 5.161
   7802   1385808_at   4.279 4.122 4.601 4.884 4.241 5.202 5.586 5.259 4.953 5.22
   7803   1380869_at   5.45 5.368 5.608 4.844 5.247 6.336 6.241 6.059 6.037 5.745
   7804   1378259_at   7.211 7.141 7.316 7.292 7.399 7.112 6.604 6.669 6.605 6.841
   7805   1373632_at   7.822 8.108 7.852 7.983 7.804 7.39 7.443 7.31 7.411 6.783
   7806   1379051_at   4.08 3.602 4.602 3.591 4.198 2.459 2.876 1.275 2.478 2.784
   7807   1371559_at   10.042 10.147 10.108 10.065 10.168 9.761 9.962 9.49 9.749 9.647
   7808   1398432_at   7.585 7.449 7.731 7.77 7.476 8.081 8.12 7.998 7.917 7.847
   7809   1372892_at   5.402 5.223 5.844 6.091 4.687 6.709 6.713 7.099 6.599 6.468
   7810   1392025_x_at   6.18 5.837 6.679 6.026 6.712 7.317 6.921 7.233 7.508 7.201
   7811   1376436_at   7.61 7.772 7.959 7.944 8.238 7.126 6.921 7.447 7.239 6.622
   7812   1375526_at   9.284 9.191 9.453 9.36 9.5 9.79 9.991 9.883 9.69 9.584
   7813   1369159_at   5.793 5.733 6.158 6.41 6.376 7.059 7.211 6.877 6.814 6.633
   7814   1384479_at   5.8 5.423 5.515 5.435 5.699 6.099 6.328 5.887 6.566 6.218
   7815   1385335_at   5.511 5.555 5.597 5.567 5.986 6.196 6.083 5.95 6.154 6.281
   7816   1387456_at   5.409 5.675 5.027 5.551 5.222 6.12 6.186 5.826 5.914 5.991
   7817   1387454_at   4.365 5.069 5.18 5.059 5.49 2.214 3.302 2.899 3.046 0.367
   7818   1389017_at   7.144 7.279 6.881 7.56 7.168 6.64 6.783 6.476 6.369 6.646
   7819   1394359_at   6.681 6.678 6.781 6.901 6.839 4.436 4.459 1.655 4.685 1.531
   7820   1395251_at   6.04 6.269 6.381 6.271 6.415 7.257 7.149 7.125 7.171 6.457
   7821   1374159_at   5.553 5.822 5.506 5.317 5.496 6.128 6.178 6.448 6.17 5.821
   7822   1374075_at   8.486 8.509 8.39 8.4 8.387 8.57 8.67 8.831 8.663 8.636
   7823   1372924_at   7.173 7.155 6.723 6.936 7.079 6.686 6.53 6.28 6.336 6.567
   7824   1384136_at   7.121 7.249 7.273 7.183 7.391 7.794 7.619 7.697 7.885 7.414
   7825   1390019_at   12.157 12.088 12.014 11.956 12.082 11.772 11.636 11.709 11.952 11.736
   7826   1389562_at   6.943 7.03 6.675 6.775 6.957 7.558 7.784 7.254 7.269 7.356
   7827   1398617_at   5.776 6.089 5.893 5.998 6.325 5.313 5.322 5.708 5.033 5.336
   7828   1390535_at   7.477 7.531 7.448 7.566 7.125 7.789 8.007 8.061 7.814 8.265
   7829   1375970_at   8.629 8.558 8.572 8.454 8.572 8.457 8.073 8.074 8.067 8.211
   7830   1377041_at   6.818 6.561 6.818 6.806 6.914 6.224 5.637 5.575 5.77 6.439
   7831   1395767_at   7.32 7.186 7.462 7.253 7.202 6.492 6.117 6.271 7.071 6.46
   7832   1373824_at   8.53 8.558 8.378 8.736 8.782 8.154 8.167 8.246 7.767 7.952
   7833   1370531_a_at   6.294 5.897 6.191 6.669 6.169 7.082 6.732 7.618 7.122 7.569
   7834   1398508_at   8.925 9.087 9.142 9.449 9.362 9.792 9.87 10.196 9.626 9.71
   7835   1371768_at   10.158 9.979 10.051 9.972 9.827 9.747 9.559 9.758 9.608 9.719
   7836   1377786_at   3.5 4.274 4.057 4.197 3.855 5.17 5.403 5.02 4.459 4.833
   7837   1392061_at   5.787 5.202 5.245 5.666 5.439 3.302 1.227 3.638 3.694 0.986
   7838   1396221_at   5.671 5.666 5.343 5.824 6.422 4.635 5.263 4.663 4.542 4.554
   7839   1397300_at   8.124 8.183 8.258 8.29 7.974 8.976 9.238 8.493 8.748 8.684
   7840   1398611_at   6.977 6.774 6.755 6.791 6.708 6.446 6.503 6.072 6.056 6.407
   7841   1374054_at   7.747 7.595 7.743 7.816 7.675 8.073 8.324 8.18 7.865 8.114
   7842   1389639_at   7.856 7.985 7.945 8.214 7.866 8.265 8.389 8.55 8.283 8.599
   7843   1383126_at   9.217 9.23 9.009 9.321 9.451 8.661 8.97 8.917 8.687 8.707
   7844   1393376_at   3.335 2.66 3.612 4.076 3.998 5.923 5.386 4.812 4.64 5.036
   7845   1377736_at   5.363 4.972 4.545 5.116 5.069 6.261 5.876 5.859 5.46 6.108
   7846   1395199_at   7.454 7.548 8.032 7.726 7.774 7.097 7.401 7.019 6.891 7.022
   7847   1380301_at   6.707 6.861 6.851 6.624 6.076 5.326 5.896 6.012 5.828 5.596
   7848   1376237_at   6.677 6.477 6.398 6.401 7.099 6.001 5.311 5.66 5.041 5.846
   7849   1373417_at   7.32 7.638 7.434 8.045 7.679 7.004 6.815 6.962 7.171 6.919
   7850   1378309_at   6.482 6.885 6.567 7.069 6.438 7.295 7.529 7.241 7.2 7.515
   7851   1386817_at   5.941 5.925 6.656 6.956 6.213 7.328 7.426 7.274 7.337 7.204
   7852   1398755_at   11.832 11.81 11.841 11.786 11.893 11.978 11.92 12.142 12.047 12.056
   7853   1374666_at   5.945 6.304 6.397 6.231 6.323 7.515 7.205 7.582 6.853 6.667
   7854   1376288_at   6.523 6.707 6.596 6.707 6.834 6.028 6.498 6.079 6.262 6.176
   7855   1395503_at   5.754 6.164 5.606 6 5.718 5.17 5.262 5.106 4.658 5.342
   7856   1378100_at   8.248 8.161 8.145 8.339 8.551 7.667 7.972 7.906 7.731 7.925
   7857   1387015_at   7.981 7.696 7.81 7.824 8.024 8.518 8.752 9.062 8.271 8.372
   7858   1388305_at   5.907 5.944 5.821 6.27 5.647 6.868 6.683 6.369 6.352 6.75
   7859   1386120_at   4.227 4.087 5.015 4.134 3.932 7.287 7.211 5.109 6.434 5.954
   7860   1372801_at   9.144 9.278 9.122 9.204 9.312 8.805 9.112 8.821 8.875 8.876
   7861   1384240_at   9.051 8.879 8.83 8.486 8.756 9.7 9.776 9.044 9.718 9.604
   7862   1374517_at   8.056 8.061 8.197 8.186 8.233 7.965 7.831 7.837 7.571 7.788
   7863   1373171_at   7.092 7.106 7.255 7.307 7.241 6.718 6.169 6.878 6.604 6.239
   7864   1369473_at   7.759 7.851 7.566 7.548 7.503 7.953 8.366 8.018 8.33 8.089
   7865   1379019_at   5.864 6.305 6.238 6.255 6.402 5.497 4.788 5.539 5.11 5.688
   7866   1390121_at   8.692 8.844 9.043 8.939 8.703 9.726 9.686 9.508 9.427 9.068
   7867   1367523_at   9.488 9.466 9.592 9.39 9.694 9.268 9.319 9.055 9.173 9.082
   7868   1383122_at   6.693 6.393 6.799 6.576 6.469 7.235 7.026 7.371 7.165 6.851
   7869   1372484_at   10.598 10.695 10.625 10.663 10.547 11.057 11.049 10.876 10.811 10.799
   7870   1368862_at   8.06 8.164 7.994 8.482 8.325 7.779 7.719 7.754 7.705 7.392
   7871   1370423_at   6.14 6.849 6.479 6.407 6.658 5.035 4.445 2.611 4.805 2.548
   7872   1380303_at   6.368 6.169 6.267 6.338 6.175 4.343 4.733 4.158 4.601 2.104
   7873   1394744_at   5.161 5.547 5.864 5.424 5.152 4.465 4.554 4.376 4.608 3.529
   7874   1372866_at   6.895 6.503 7.004 7.278 6.891 7.593 7.667 7.771 7.661 7.319
   7875   1388837_at   8.347 8.715 8.933 8.78 8.662 8.02 8.104 8.37 7.813 7.841
   7876   1385352_at   4.804 3.087 5.073 4.928 4.182 6.524 6.019 6.873 6.209 5.942
   7877   1397312_at   6.733 6.733 6.707 6.883 6.634 6.984 7.238 6.988 6.951 7.077
   7878   1388217_a_at   9.185 9.14 9.174 9.374 9.513 8.968 8.927 8.957 8.773 8.756
   7879   1389340_at   6.238 5.821 6.148 6.186 6.239 6.396 6.729 6.781 6.526 6.682
   7880   1391812_at   5.492 5.403 5.518 5.432 5.845 6.046 6.119 5.861 6.48 6.278
   7881   1397766_at   10.504 10.569 10.311 10.341 10.741 9.894 9.902 9.343 9.49 10.082
   7882   1386701_at   6.398 6.229 6.222 6.562 5.931 5.52 5.776 5.454 5.8 5.198
   7883   1388893_at   7.725 7.81 7.49 7.571 7.818 7.303 7.468 7.239 7.099 7.192
   7884   1393459_at   7.277 7.444 7.298 7.17 7.233 6.92 7.033 6.437 6.6 6.685
   7885   1377620_at   6.505 6.582 6.784 6.665 6.426 7.466 7.179 6.933 6.946 7.147
   7886   1398252_at   7.45 7.387 7.547 7.405 7.323 8.05 8.213 7.82 8.078 7.609
   7887   1396864_at   4.346 4.047 4.126 4.343 2.296 1.705 1.077 1.722 2.084 2.266
   7888   1377980_at   4.292 3.457 4.098 4.379 4.123 1.651 3.138 2.86 1.807 2.828
   7889   1371837_at   11.415 11.438 11.477 11.343 11.369 11.635 11.597 11.491 11.556 11.658
   7890   1372682_at   5.749 5.946 5.823 6.006 6.241 6.418 6.666 7.207 6.528 6.696
   7891   1374266_at   6.262 6.258 6.064 5.918 6.265 6.894 6.516 6.868 6.456 6.694
   7892   1388742_at   7.542 7.466 8.001 7.995 7.426 7.24 6.921 6.793 6.717 7.04
   7893   1368075_at   9.078 8.974 8.915 9.022 8.991 9.178 9.395 9.272 9.171 9.46
   7894   1383677_at   5.43 5.959 6.029 5.367 5.985 4.925 5.065 5.078 5.161 4.794
   7895   1376368_at   7.792 7.881 7.566 7.841 8.017 7.065 7.569 7.117 6.983 6.731
   7896   1367473_at   10.612 10.522 10.664 10.583 10.479 10.465 10.241 10.269 10.331 10.359
   7897   1375707_at   4.189 5.6 4.079 4.455 6.274 7.02 6.568 7.488 7.315 7.008
   7898   1375449_at   5.543 5.787 6.215 5.618 6.527 4.957 4.903 4.752 5.311 4.703
   7899   1372108_at   9.35 9.364 9.334 9.252 9.23 10.001 9.868 9.473 9.69 9.693
   7900   1393064_at   4.965 5.516 5.768 5.252 5.377 6.263 6.371 5.896 5.97 6.474
   7901   1382807_at   7.804 8.006 7.862 8.035 7.775 8.518 8.544 8.289 8.395 8.079
   7902   1393365_at   6.503 6.295 6.266 6.505 6.517 5.298 4.669 4.995 5.68 5.939
   7903   1394779_at   4.951 4.883 5.12 5.208 4.918 6.49 5.636 6.097 5.538 5.708
   7904   1397172_at   0 0 2.275 0 0 2.281 2.648 2.935 4.125 4.024
   7905   1369772_at   4.896 4.773 4.866 5.342 4.866 3.976 4.651 4.038 3.833 4.201
   7906   1395135_at   6.24 6.295 6.039 6.795 6.513 5.682 5.123 5.855 5.563 5.204
   7907   1382085_at   7.702 7.722 7.899 7.808 7.893 8.197 8.449 8.14 8.224 7.993
   7908   1379062_at   5.99 6.127 6.519 6.347 7.064 4.968 5.52 4.413 5.521 5.131
   7909   1375988_at   6.445 6.449 6.606 6.516 6.583 5.015 5.387 5.459 6.043 6.019
   7910   1398324_at   12.931 12.844 12.711 12.549 12.673 12.414 12.296 12.376 12.393 12.497
   7911   1397349_at   7.032 7.618 7.211 7.217 7.562 7.871 8.14 8.036 7.952 7.732
   7912   1373656_at   5.895 5.922 6.067 6.06 6.335 5.598 5.518 5.785 5.615 5.665
   7913   1389090_at   7.388 7.437 7.452 7.589 7.478 7.326 7.316 7.27 7.17 7.205
   7914   1389449_at   2.903 3.307 3.511 4.42 4.108 5.105 5.358 4.865 5.708 4.761
   7915   1382184_at   6.358 6.202 5.643 6.064 6.332 7.114 6.667 6.769 6.714 6.926
   7916   1380269_at   3.467 4.151 3.282 4.045 3.165 2.007 2.387 2.157 1.144 2.705
   7917   1392546_at   6.143 6.428 6.147 6.725 6.466 5.781 6.023 5.563 5.622 5.43
   7918   1370183_at   7.457 7.603 7.55 7.525 7.606 7.351 7.411 7.43 7.409 7.42
   7919   1393891_at   4.817 4.827 4.996 4.48 5.358 3.497 3.42 3.92 3.507 2.077
   7920   1382722_at   5.811 5.871 6.026 5.913 5.736 5.225 5.546 5.657 5.264 5.437
   7921   1382045_at   8.883 9.04 8.794 8.685 8.841 8.361 8.53 7.983 8.358 8.443
   7922   1375413_at   9.225 9.206 9.362 9.237 9.087 9.621 9.461 9.46 9.516 9.4
   7923   1376574_at   7.526 7.748 7.859 7.858 7.631 7.103 7.317 7.451 7.41 7.2
   7924   1372584_at   6.484 6.372 5.912 6.653 6.926 5.605 5.776 5.552 5.771 5.555
   7925   1387836_at   7.848 7.944 8.008 7.943 8.042 7.649 7.382 7.211 7.51 6.896
   7926   1382197_at   7.043 7.455 7.285 7.528 7.139 8.111 7.894 8.123 8.286 7.582
   7927   1373469_at   8.227 8.264 8.086 8.289 8.365 8.79 8.858 8.845 8.416 8.59
   7928   1383799_at   6.329 6.681 6.653 6.559 6.322 7.193 7.109 6.799 7.227 6.891
   7929   1383772_at   6.227 6.201 6.15 5.947 5.878 5.486 5.702 5.839 5.483 5.616
   7930   1368830_at   5.202 5.106 5.135 5.566 5.519 6.202 5.864 5.669 5.931 5.896
   7931   1373814_at   6.055 6.649 6.502 6.637 6.226 7.263 7.2 7.083 6.769 7.158
   7932   1395170_at   2.915 3.677 3.128 2.918 2.651 5.028 4.441 3.625 4.757 4.285
   7933   1375448_at   6.719 7.192 6.61 7.313 7.508 8.167 8.272 7.845 8.209 7.687
   7934   1376731_at   7.069 7.061 7.079 7.316 7.432 7.738 8.014 7.632 7.688 7.516
   7935   1375679_at   1.373 3.146 0.604 1.614 1.88 4.405 3.794 3.666 3.429 3.7
   7936   1370002_at   8.659 8.67 8.734 8.844 8.563 8.04 8.324 8.468 8.019 7.881
   7937   1377340_at   6.391 6.347 6.83 6.639 6.352 3.482 0.84 4.278 3.53 4.673
   7938   1387794_at   6.295 6.349 6.219 6.532 6.454 3.305 3.786 4.43 2.014 0
   7939   1382866_at   7.081 7.133 7.416 7.256 7.008 7.766 7.709 7.554 7.715 7.411
   7940   1380106_at   7.462 7.447 7.415 7.739 7.496 7.254 7.238 7.163 7.275 7.19
   7941   1392180_at   7.743 7.648 8.119 7.745 7.886 7.319 7.42 7.393 7.539 7.255
   7942   AFFX_Rat_Hexokinase_5_at   6.486 6.697 6.879 7.083 6.721 6.168 5.705 6.163 6.129 5.395
   7943   1377174_at   7.199 7.14 7.13 7.103 6.67 6.456 6.129 5.953 6.453 5.481
   7944   1376708_at   6.746 6.845 6.796 7.137 7.225 5.888 6.452 6.523 6.097 5.863
   7945   1397296_at   4.177 4.182 3.547 4.145 4.68 5.694 5.272 4.707 5.251 5.462
   7946   1370043_at   7.524 7.345 7.425 7.562 7.773 8.18 8.424 8.557 8.162 7.791
   7947   1375760_at   7.519 7.542 7.689 7.802 7.753 8.565 8.631 8.303 8.082 7.981
   7948   1388912_at   8.588 8.515 8.538 8.539 8.597 8.366 8.436 8.128 8.305 8.298
   7949   1393437_at   4.514 3.903 3.383 2.678 4.034 5.029 5.203 5.349 5.128 5.184
   7950   1374316_at   8.349 8.461 8.454 8.7 8.563 8 8.078 8.317 7.817 7.943
   7951   1393367_at   7.64 7.775 7.598 8.005 7.816 7.074 7.175 7.583 7.172 7.098
   7952   1373849_at   8.667 8.752 8.748 8.868 8.892 8.585 8.422 8.46 8.182 8.462
   7953   1367480_at   10.274 10.239 10.126 10.207 10.259 10.066 10.02 10.072 9.973 9.851
   7954   1370392_at   5.234 4.964 5.135 5.557 5.57 5.935 6.04 6.072 5.773 5.773
   7955   1388954_at   6.759 6.744 6.773 7.175 7.24 5.748 6.312 6.517 5.85 5.733
   7956   1372347_at   10.127 10.253 10.344 10.39 10.026 10.607 10.714 10.618 10.458 10.579
   7957   1378291_at   5.231 5.069 4.943 5.034 5.456 6.282 5.862 5.906 5.848 5.474
   7958   1383267_at   6.814 6.785 6.854 6.83 6.895 6.351 6.573 6.715 6.392 6.556
   7959   1385133_at   5.421 5.786 5.423 5.682 5.361 4.879 4.962 4.947 4.122 4.501
   7960   1373004_at   7.477 7.541 7.305 7.514 7.551 6.631 7.108 7.215 6.703 6.916
   7961   1373877_at   11.457 11.371 11.445 11.464 11.501 11.267 11.364 11.111 11.211 11.176
   7962   1374486_at   6.918 7.278 6.994 7.333 6.969 8.07 7.651 7.843 7.45 7.633
   7963   1376214_at   8.106 8.115 8.169 7.657 7.876 8.832 8.934 8.309 8.617 9.089
   7964   1372253_at   7.72 7.923 7.993 8.184 8.325 7.407 7.39 7.259 7.697 7.45
   7965   1391170_at   6.346 6.585 6.837 6.998 7.077 6.226 6.03 6.201 5.845 6.019
   7966   1399107_at   7.759 7.633 7.801 7.703 7.747 8.348 8.438 8.336 7.91 8.045
   7967   1390769_at   7.71 7.392 7.945 7.794 7.554 7.264 7.208 7.219 7.168 7.271
   7968   1382037_at   6.816 6.885 6.795 6.936 6.897 7.388 7.338 7.167 7.083 7.046
   7969   1367972_at   9.819 9.805 9.749 9.994 9.921 9.472 9.723 9.493 9.341 9.342
   7970   1374496_at   6.618 6.635 6.381 6.749 7.395 7.547 7.821 7.626 7.694 7.388
   7971   1387852_at   3.957 2.934 1.305 3.513 4.54 5.767 5.99 7.159 6.15 5.543
   7972   1382141_at   8.32 8.396 8.108 8.202 8.314 8.019 7.988 8.064 7.843 7.975
   7973   1379803_at   6.202 5.803 5.556 5.944 5.622 6.869 6.636 6.847 7.2 6.211
   7974   1376872_at   6.293 6.21 6.192 6.441 6.232 3.664 3.243 3.845 0 3.69
   7975   1396320_at   6.421 6.027 6.382 6.282 6.29 5.964 5.886 5.684 5.989 5.707
   7976   1392865_at   7.085 7.182 7.108 7.597 7.118 8.08 8.558 8.978 7.764 8.104
   7977   1368350_at   5.935 6.031 4.719 5.995 6.171 4.583 3.812 4.668 4.08 3.356
   7978   1398579_at   7.161 7.148 7.344 7.49 7.081 7.776 7.839 7.552 7.605 7.611
   7979   1374260_at   0.176 0.138 0.651 0 0 2.021 1.157 1.585 3.478 2.832
   7980   1377851_at   5.978 5.591 5.946 5.382 5.623 4.977 4.482 5.027 4.513 3.811
   7981   1376983_at   5.682 4.785 5.675 5.34 5.51 4.138 4.434 3.875 3.862 2.642
   7982   1372412_at   7.778 7.837 7.861 8.083 7.942 8.276 8.323 8.403 8.227 8.064
   7983   1377151_at   7.343 7.452 7.769 7.644 7.455 7.174 7.056 7.218 7.199 7.019
   7984   1378426_at   0.111 0.333 0.696 0.986 1.275 2.563 3.532 3.081 2.939 1.305
   7985   1386942_at   7.023 7.109 7.296 7.061 6.923 7.368 7.498 7.514 7.301 7.424
   7986   1383014_at   2.648 2.941 3.847 4.045 3.331 4.656 5.51 4.43 4.71 4.939
   7987   1397255_at   7.128 6.967 6.821 6.89 6.929 6.372 6.033 5.757 6.048 6.676
   7988   1393433_at   4.149 5.078 4.008 5.207 4.702 6.162 5.826 5.574 5.763 5.799
   7989   1376303_a_at   7.747 7.581 7.763 7.845 7.586 8.337 8.035 8.097 8.115 7.921
   7990   1374141_at   7.548 7.613 7.33 7.568 7.697 7.011 7.09 7.272 6.937 6.629
   7991   1370219_at   11.695 11.752 11.82 11.576 11.588 10.891 11.017 10.152 11.113 11.041
   7992   1390555_at   7.44 7.54 7.781 7.651 7.565 7.95 8.077 8.271 7.972 7.842
   7993   1367464_at   9.44 9.38 9.313 9.513 9.565 8.988 9.179 9.155 8.896 8.698
   7994   1395362_at   7.994 8.044 7.93 8.065 7.808 8.354 8.68 8.244 8.358 8.285
   7995   1373963_at   6.54 6.303 6.565 5.717 5.606 7.384 7.434 7.291 7.487 6.754
   7996   1390847_at   6.778 6.811 7.371 7.053 6.811 8.326 7.954 7.401 8.659 7.987
   7997   1391370_at   5.934 6.157 6.307 6.307 5.952 6.585 6.987 6.542 6.661 6.561
   7998   1389981_at   6.922 6.715 6.836 6.928 7.01 6.538 6.604 6.253 6.209 6.562
   7999   1372551_at   7.671 7.952 7.96 7.888 8.038 8.177 8.338 8.499 8.345 8.169
   8000   1368219_at   5.416 5.808 5.302 5.413 6.081 6.535 6.157 6.876 6.589 6.302
   8001   1388561_at   5.218 6.43 5.158 6.365 6.292 7.433 7.297 6.913 7.485 7.41
   8002   1397688_at   2.874 4.123 1.029 4.615 3.349 5.935 6.034 6.365 6.342 5.836
   8003   1379249_at   7.668 7.942 7.527 7.706 7.559 7.365 7.164 6.777 7.161 7.159
   8004   1396742_at   6.507 6.338 6.523 6.092 6.213 5.399 4.719 4.57 5.789 5.476
   8005   1391147_at   3.401 4.09 2.189 3.263 3.557 5.423 5.219 4.25 5.375 4.95
   8006   1373846_at   9.1 9.051 9.059 9.089 9.209 9.343 9.513 9.206 9.36 9.428
   8007   1375130_at   6.833 6.734 7.009 7.247 7.162 6.646 6.507 6.137 6.057 6.128
   8008   1376266_at   6.112 6.528 6.449 6.311 6.281 5.826 6.068 6.015 5.737 5.76
   8009   1383443_at   6.763 6.964 6.933 7.069 6.902 7.582 7.183 7.175 7.532 7.482
   8010   1392551_at   5.581 5.313 5.757 5.615 6.138 6.493 6.826 6.39 6.239 6.338
   8011   1396304_at   4.098 4.65 3.67 3.989 4.2 2.88 1.811 0 0 2.074
   8012   1380598_at   8.196 8.233 8.249 8.243 8.475 9.105 9.029 8.865 8.443 8.834
   8013   1370429_at   7.48 7.535 7.776 8.019 8.093 0.124 4.065 5.038 4.702 4.216
   8014   1390466_at   5.679 6.148 5.967 5.453 5.731 6.708 6.438 6.705 6.546 6.172
   8015   1382919_at   6.277 6.6 6.365 6.31 6.006 5.325 5.288 4.844 5.178 3.733
   8016   1376231_at   6.398 6.166 6.153 6.495 6.762 2.854 4.064 4.414 3.081 0.164
   8017   1397322_at   2.26 1.993 4.119 2.441 3.093 5.236 4.449 5.033 4.139 4.991
   8018   1374489_at   7.795 7.967 7.909 7.97 8.107 7.331 7.348 7.823 7.444 7.311
   8019   1383929_at   7.592 7.767 7.771 7.819 7.867 7.324 7.549 7.082 7.171 7.446
   8020   1369086_a_at   3.333 4.115 4.162 3.919 3.553 4.55 4.702 4.601 4.469 4.577
   8021   1376677_a_at   4.368 3.471 3.628 4.242 4.581 5.169 5.673 5.347 5.563 4.753
   8022   1389395_at   8.053 8.07 7.953 8.187 8.515 7.469 7.745 7.771 7.648 7.539
   8023   1380163_at   7.038 7.249 6.493 6.731 6.841 6.143 6.291 6.115 6.095 6.415
   8024   1386191_a_at   6.545 6.374 6.772 6.883 6.615 5.764 5.113 4.089 5.799 4.89
   8025   1380690_at   5.66 6.207 6.159 6.039 6.131 5.264 5.185 5.371 4.856 4.221
   8026   1368272_at   9.946 9.94 10.1 9.722 9.903 10.445 10.488 10.459 10.369 10.075
   8027   1388578_at   6.719 6.764 6.813 6.683 6.532 6.544 6.336 6.32 6.303 6.125
   8028   1395061_at   7.774 7.856 8.197 8.109 8.337 7.722 7.245 7.494 7.332 7.175
   8029   1397617_at   6.377 6.596 6.385 6.313 6.225 5.951 5.6 5.489 5.922 6.054
   8030   1380172_at   1.124 1.753 3.98 3.539 4.002 5.535 5.92 5.592 5.982 5.435
   8031   1393216_at   8.643 8.961 8.88 9.076 9.109 9.275 9.648 9.651 9.305 9.455
   8032   1377084_at   5.99 6.231 6.326 6.06 6.404 6.836 6.468 7.168 6.808 6.846
   8033   1372792_at   6.337 6.468 6.463 6.416 6.449 6.129 5.952 6.013 5.441 5.675
   8034   1398362_at   8.629 8.589 8.596 8.784 8.846 7.914 8.201 8.278 7.391 7.865
   8035   1384858_a_at   7.619 7.667 7.816 8.129 7.931 7.306 7.094 6.882 7.521 7.041
   8036   1371918_at   10.034 9.988 9.897 10.09 10.179 9.609 9.458 9.85 9.569 9.274
   8037   1390671_at   4.159 4.212 4.172 4.738 4.873 6.343 5.075 6.225 5.614 5.441
   8038   1368898_at   4.45 3.236 3.808 3.622 4.638 5.985 5.877 4.864 5.507 5.195
   8039   1396692_at   6.478 6.627 6.508 6.757 6.931 6.335 6.278 6.081 5.978 5.895
   8040   1379831_at   6.802 7.029 6.959 7.12 7.022 6.549 6.728 6.405 6.556 6.142
   8041   1378215_at   6.396 6.177 6.371 6.391 6.124 6.933 6.868 6.653 6.691 6.512
   8042   1381145_at   6.998 7.123 7.036 7.309 7.403 6.261 6.762 6.388 6.656 6.791
   8043   1393142_at   6.434 6.273 5.865 6.318 6.326 7.198 6.898 7.302 6.774 6.657
   8044   1388632_at   3.548 3.796 4.007 2.814 3.784 5.096 4.978 5.425 5.269 4.148
   8045   1369543_s_at   5.5 5.435 5.07 5.588 5.609 6.15 6.298 6.119 5.697 6.251
   8046   1374664_at   7.542 7.581 7.65 7.771 7.713 7.914 7.968 8.155 8.154 7.832
   8047   1384217_at   7.219 7.166 6.641 7.33 7.259 7.991 8.375 7.795 8.191 7.572
   8048   1381618_at   4.53 4.614 4.324 5.307 4.864 5.922 5.98 6.377 5.325 5.633
   8049   1395376_at   6.188 6.16 6.061 5.843 5.983 5.433 5.324 5.529 5.078 4.53
   8050   1384063_at   5.902 6.341 5.504 6.237 7.504 3.802 4.374 4.663 2.192 3.969
   8051   1378395_at   6.492 6.601 6.742 6.754 6.872 6.933 7.244 7.21 7.494 7.143
   8052   1388468_at   8.882 8.781 9.037 9.015 8.877 8.733 8.566 8.605 8.338 8.427
   8053   1375014_at   5.949 5.917 6.31 6.519 6.108 5.472 4.268 4.083 5.351 4.726
   8054   1392588_at   6.32 6.364 6.476 6.628 6.775 6.956 6.879 7.085 6.999 6.831
   8055   1392099_at   4.935 4.48 4.724 4.591 5.151 3.294 4.077 3.459 3.825 4.252
   8056   1373219_at   5.637 5.937 5.775 5.718 6.228 5.055 5.071 4.39 5.36 4.445
   8057   1390201_at   6.691 6.835 6.518 6.645 6.732 6.342 6.393 6.49 6.269 6.148
   8058   1392468_at   7.706 7.991 7.801 7.757 7.89 8.205 8.315 8.356 7.963 8.337
   8059   1385691_at   5.92 6.125 6.008 6.069 5.951 5.591 5.597 5.333 5.798 5.734
   8060   1370601_a_at   0.536 2.121 0.687 1.379 2.063 3.858 2.768 2.744 3.172 3.134
   8061   1367970_at   6.259 6.145 6.092 6.219 6.517 6.902 6.879 6.88 7.016 6.427
   8062   1368083_at   8.328 8.532 8.483 8.603 8.597 8.328 8.135 8.197 8.291 8.169
   8063   1382901_at   3.806 3.245 3.461 3.454 4.252 0.202 2.411 2.248 1.949 0.978
   8064   1379814_at   5.15 5.46 5.724 6.065 5.862 6.571 7.257 6.412 6.552 6.506
   8065   1372931_at   7.873 8.103 7.784 7.915 8.086 7.412 7.551 7.726 7.218 7.232
   8066   1394765_at   2.621 0.124 1.664 0.345 0 3.541 3.305 2.814 4.048 3.666
   8067   1383230_at   7.075 7.22 7.525 7.491 7.487 6.608 6.042 6.999 6.572 6.534
   8068   1398875_at   9.043 9.168 9.098 9.178 9.339 8.977 8.918 8.95 8.867 8.865
   8069   1382200_at   5.89 6.075 5.62 6.175 6.017 6.569 6.598 6.73 6.761 6.22
   8070   1371433_at   9.34 9.275 9.214 9.477 9.519 9.664 9.647 9.573 9.685 9.766
   8071   1368281_at   7.537 7.662 7.439 7.488 7.507 8.615 8.645 7.654 8.603 8.583
   8072   1385793_at   2.731 0.941 2.114 1.214 1.761 5.157 3.489 3.189 4.059 3.597
   8073   1397336_at   4.957 5.377 5.207 4.773 4.43 4.06 3.48 3.758 4.123 4.295
   8074   1397365_at   5.004 4.541 5.07 5.464 5.071 6.481 5.604 6.404 6.292 5.718
   8075   1378003_at   6.713 6.691 7.049 6.908 6.77 7.679 8.136 7.362 7.58 7.238
   8076   1385758_at   8.617 8.748 8.818 8.924 8.533 8.427 8.188 8.228 8.257 7.844
   8077   1392995_at   5.5 5.499 5.228 4.893 4.923 3.788 4.418 4.716 4.344 4.124
   8078   1382971_at   9.266 9.193 9.367 9.615 9.312 9.762 9.904 10 9.66 9.668
   8079   1374620_at   7.462 7.491 7.25 7.468 7.07 7.057 6.88 6.626 6.757 6.912
   8080   1377961_at   5.365 5.68 5.828 5.834 6.116 6.24 7.005 6.886 6.451 6.517
   8081   1398192_at   1.655 0.816 1.575 3.257 1.257 3.187 3.696 3.727 4.488 4.177
   8082   1390867_at   5.076 5.708 5.616 5.33 5.139 4.453 4.063 4.108 4.866 4.726
   8083   1396150_at   6.207 6.652 5.997 6.34 6.258 5.688 5.94 5.379 5.374 5.641
   8084   1379971_at   4.51 5.223 4.938 4.532 4.664 5.615 5.633 5.289 5.36 5.438
   8085   1380022_at   7.217 7.511 7.637 7.282 7.117 8.659 8.044 7.788 8.012 8.326
   8086   1372240_at   4.559 4.055 4.972 5.148 3.445 5.972 6.138 6.505 6.03 5.51
   8087   1387860_at   9.653 9.621 9.539 9.807 9.833 8.986 9.079 9.532 8.828 9.067
