# Supplementary material for: Activation of counter-regulatory mechanisms in a rat renal acute rejection model
Source: BMC Genomics. 2008 Feb 8;9:71. doi: 10.1186/1471-2164-9-71 (PMC2262896; doi:10.1186/1471-2164-9-71)
Supplement: Additional file 1 — Gene lists from the class comparison between the groups analyzed with BRB ArrayTools. [file 1471-2164-9-71-S1.zip › ControlvsSyngeneic/Annotations.html]

   TABLE {font-size: 9pt}    

       Gene Info     


    Gene Ontology   
    Biocarta Pathways         Kegg Pathways     
    Probe set : 1370155_at    Name:   procollagen, type I, alpha 2    GB acc:   BM388837    UniGene:   Rn.107239    Gene Symbol:   Col1a2    Locus Link:   84352    Chromosome:   4    Cytoband:   Chr:4q13    SOURCE    GENECARD       Gene Ontology:     BioCarta Pathways:     KEGG Pathways:  
    Probe set : 1371369_at    Name:   procollagen, type VI, alpha 2 (predicted)    GB acc:   BI287851    UniGene:   Rn.128903    Gene Symbol:   Col6a2_predicted    Locus Link:   361821    Chromosome:   20    Cytoband:   Chr:20p12    SOURCE    GENECARD       Gene Ontology:     BioCarta Pathways:     KEGG Pathways:  
    Probe set : 1370959_at    Name:   collagen, type III, alpha 1    GB acc:   BI275716    UniGene:   Rn.3247    Gene Symbol:   Col3a1    Locus Link:   84032    Chromosome:   9    Cytoband:   Chr:9q22    SOURCE    GENECARD       Gene Ontology:     BioCarta Pathways:     KEGG Pathways:  
    Probe set : 1367784_a_at    Name:   clusterin    GB acc:   AF314657    UniGene:   Rn.1780    Gene Symbol:   Clu    Locus Link:   24854    Chromosome:   15    Cytoband:   Chr:15p12    SOURCE    GENECARD       Gene Ontology:   cellular component:  extracellular region   cellular component:  extracellular space      BioCarta Pathways:     KEGG Pathways:  
    Probe set : 1372213_at    Name:   similar to hypothetical protein MGC6835    GB acc:   BM390487    UniGene:   Rn.22047    Gene Symbol:   LOC500300    Locus Link:   500300    Chromosome:   4    Cytoband:   Chr:4q42    SOURCE    GENECARD       Gene Ontology:     BioCarta Pathways:     KEGG Pathways:  
    Probe set : 1370167_at    Name:   syndecan 2    GB acc:   BG668421    UniGene:   Rn.11127    Gene Symbol:   Sdc2    Locus Link:   25615    Chromosome:   7    Cytoband:   Chr:7q22    SOURCE    GENECARD       Gene Ontology:   cellular component:  integral to plasma membrane   cellular component:  membrane   cellular component:  integral to membrane   molecular function:  cytoskeletal protein binding      BioCarta Pathways:     KEGG Pathways:  
    Probe set : 1367651_at    Name:   cathepsin D    GB acc:   NM_134334    UniGene:   Rn.11085    Gene Symbol:   Ctsd    Locus Link:   171293    Chromosome:   1    Cytoband:   Chr:1q41    SOURCE    GENECARD       Gene Ontology:   biological process:  proteolysis   biological process:  proteolysis   cellular component:  mitochondrion   cellular component:  lysosome   cellular component:  lysosome      BioCarta Pathways:     KEGG Pathways:  
    Probe set : 1387854_at    Name:   procollagen, type I, alpha 2    GB acc:   BI282748    UniGene:   Rn.107239    Gene Symbol:   Col1a2    Locus Link:   84352    Chromosome:   4    Cytoband:   Chr:4q13    SOURCE    GENECARD       Gene Ontology:     BioCarta Pathways:     KEGG Pathways:  
    Probe set : 1372765_a_at    Name:   similar to Peci protein /// similar to RIKEN cDNA 1810022C23 (predicted)    GB acc:   BM390774    UniGene:   Rn.3194    Gene Symbol:   RGD:1359427 /// RGD1310224_predicted    Locus Link:   291075 /// 291076    Chromosome:   17    Cytoband:   Chr:17p12    SOURCE    GENECARD       Gene Ontology:   biological process:  metabolism   cellular component:  peroxisome      BioCarta Pathways:     KEGG Pathways:  
    Probe set : 1372406_at    Name:   minichromosome maintenance deficient 3 (S. cerevisiae) (predicted) /// similar to DNA replication licensing factor MCM3 (DNA polymerase alpha holoenzyme-associated protein P1) (P1-MCM3)    GB acc:   BM383428    UniGene:   Rn.101621    Gene Symbol:   Mcm3_predicted /// LOC367976    Locus Link:   316273 /// 367976    Chromosome:   9    Cytoband:   Chr:9q13    SOURCE    GENECARD       Gene Ontology:   cellular component:  nucleus   cellular component:  alpha DNA polymerase:primase complex      BioCarta Pathways:     KEGG Pathways:  
    Probe set : 1374649_at    Name:   RAS guanyl releasing protein 2 (calcium and DAG-regulated) (predicted)    GB acc:   BG380629    UniGene:   Rn.38473    Gene Symbol:   Rasgrp2_predicted    Locus Link:   361714    Chromosome:   1    Cytoband:   Chr:1q43    SOURCE    GENECARD       Gene Ontology:   biological process:  regulation of cell growth   biological process:  small GTPase mediated signal transduction   biological process:  Ras protein signal transduction   cellular component:  cytosol   cellular component:  plasma membrane      BioCarta Pathways:     KEGG Pathways:  
    Probe set : 1378477_a_at    Name:   similar to Formin homology 2 domain containing 1    GB acc:   BI281796    UniGene:   Rn.105655    Gene Symbol:   LOC291964    Locus Link:   291964    Chromosome:   19    Cytoband:   Chr:19q11    SOURCE    GENECARD       Gene Ontology:     BioCarta Pathways:     KEGG Pathways:  
    Probe set : 1389555_at    Name:   transcription factor 19    GB acc:   BM387190    UniGene:   Rn.145385    Gene Symbol:   RGD:1302974    Locus Link:   406195    Chromosome:   20    Cytoband:   Chr:20p12    SOURCE    GENECARD       Gene Ontology:   molecular function:  transcription factor activity      BioCarta Pathways:     KEGG Pathways:  
    Probe set : 1388116_at    Name:   collagen, type 1, alpha 1    GB acc:   BI285575    UniGene:   Rn.2953    Gene Symbol:   Col1a1    Locus Link:   29393    Chromosome:   10    Cytoband:   Chr:10q31    SOURCE    GENECARD       Gene Ontology:     BioCarta Pathways:     KEGG Pathways:  
    Probe set : 1372621_at    Name:   similar to 2610027C15Rik protein (predicted)    GB acc:   AI179588    UniGene:   Rn.22919    Gene Symbol:   RGD1308876_predicted    Locus Link:   362597    Chromosome:   5    Cytoband:   Chr:5q36    SOURCE    GENECARD       Gene Ontology:     BioCarta Pathways:     KEGG Pathways:  
    Probe set : 1371349_at    Name:   similar to collagen alpha1 type VI-precursor    GB acc:   AI598402    UniGene:   Rn.107165    Gene Symbol:   LOC294337    Locus Link:   294337    Chromosome:   20    Cytoband:   Chr:20p12    SOURCE    GENECARD       Gene Ontology:     BioCarta Pathways:     KEGG Pathways:  
    Probe set : 1386128_at    Name:   tetranectin (plasminogen binding protein) (predicted)    GB acc:   BF524010    UniGene:   Rn.23478    Gene Symbol:   Tna_predicted    Locus Link:   316099    Chromosome:   8    Cytoband:   Chr:8q32    SOURCE    GENECARD       Gene Ontology:   biological process:  skeletal development   cellular component:  extracellular region   cellular component:  extracellular matrix (sensu Metazoa)   cellular component:  extracellular space   molecular function:  calcium ion binding   molecular function:  sugar binding      BioCarta Pathways:     KEGG Pathways:  
    Probe set : 1368288_at    Name:   group specific component    GB acc:   NM_012564    UniGene:   Rn.1437    Gene Symbol:   Gc    Locus Link:   24384    Chromosome:   14    Cytoband:   Chr:14p22    SOURCE    GENECARD       Gene Ontology:   biological process:  transport   biological process:  vitamin D metabolism   biological process:  vitamin transport   cellular component:  extracellular space      BioCarta Pathways:     KEGG Pathways:  
    Probe set : 1369955_at    Name:   Collagen, type V, alpha 1    GB acc:   NM_134452    UniGene:   Rn.117    Gene Symbol:   Col5a1    Locus Link:   85490    Chromosome:   3    Cytoband:   Chr:3p12    SOURCE    GENECARD       Gene Ontology:   biological process:  phosphate transport   biological process:  cell adhesion      BioCarta Pathways:     KEGG Pathways:  
    Probe set : 1389123_at    Name:   chemokine (C-C motif) ligand 6    GB acc:   BE095824    UniGene:   Rn.7857    Gene Symbol:   RGD:1303200    Locus Link:   287910    Chromosome:   10    Cytoband:   Chr:10q26    SOURCE    GENECARD       Gene Ontology:   biological process:  chemotaxis   biological process:  immune response   biological process:  sensory perception   cellular component:  extracellular region   cellular component:  extracellular space   molecular function:  cytokine activity   molecular function:  chemokine activity      BioCarta Pathways:     KEGG Pathways:  
    Probe set : 1367794_at    Name:   alpha-2-macroglobulin    GB acc:   NM_012488    UniGene:   Rn.780    Gene Symbol:   A2m    Locus Link:   24153    Chromosome:   4    Cytoband:   Chr:4q42    SOURCE    GENECARD       Gene Ontology:   cellular component:  extracellular region   cellular component:  extracellular space   cellular component:  extracellular space      BioCarta Pathways:     KEGG Pathways:  
    Probe set : 1370864_at    Name:   collagen, type 1, alpha 1    GB acc:   Z78279    UniGene:   Rn.2953    Gene Symbol:   Col1a1    Locus Link:   29393    Chromosome:   10    Cytoband:   Chr:10q31    SOURCE    GENECARD       Gene Ontology:     BioCarta Pathways:     KEGG Pathways:  
    Probe set : 1389295_at    Name:   olfactomedin-like 2B (predicted)    GB acc:   AI411761    UniGene:   Rn.13880    Gene Symbol:   Olfml2b_predicted    Locus Link:   304960    Chromosome:   13    Cytoband:   Chr:13q24    SOURCE    GENECARD       Gene Ontology:   cellular component:  membrane   molecular function:  latrotoxin receptor activity      BioCarta Pathways:     KEGG Pathways:  
    Probe set : 1387351_at    Name:   fibrillin 1    GB acc:   NM_031825    UniGene:   Rn.12759    Gene Symbol:   Fbn1    Locus Link:   83727    Chromosome:   3    Cytoband:   Chr:3q36    SOURCE    GENECARD       Gene Ontology:   biological process:  skeletal development   biological process:  development   biological process:  visual perception   cellular component:  microfibril   cellular component:  extracellular matrix (sensu Metazoa)   cellular component:  extracellular space   molecular function:  extracellular matrix structural constituent   molecular function:  calcium ion binding   molecular function:  growth factor binding      BioCarta Pathways:     KEGG Pathways:  
    Probe set : 1386912_at    Name:   procollagen C-proteinase enhancer protein    GB acc:   NM_019237    UniGene:   Rn.2910    Gene Symbol:   Pcolce    Locus Link:   29569    Chromosome:   12    Cytoband:   Chr:12q12    SOURCE    GENECARD       Gene Ontology:   biological process:  proteolysis   biological process:  development   cellular component:  extracellular space   molecular function:  nucleic acid binding   molecular function:  collagen binding      BioCarta Pathways:     KEGG Pathways:  
    Probe set : 1398347_at    Name:   AXL receptor tyrosine kinase (predicted)    GB acc:   AI406520     Gene Symbol:   Axl_predicted    Locus Link:   308444    Chromosome:   1    Cytoband:   Chr:1q21     GENECARD       Gene Ontology:     BioCarta Pathways:     KEGG Pathways:  
    Probe set : 1377367_at    Name:   Kazal-type serine protease inhibitor domain 1 (predicted)    GB acc:   BE099439    UniGene:   Rn.138194    Gene Symbol:   Kazald1_predicted    Locus Link:   293997    Chromosome:   1    Cytoband:   Chr:1q54    SOURCE    GENECARD       Gene Ontology:   biological process:  regulation of cell growth   cellular component:  extracellular region   molecular function:  insulin-like growth factor binding   molecular function:  peptidase activity      BioCarta Pathways:     KEGG Pathways:  
    Probe set : 1370290_at    Name:   tubulin, beta 5    GB acc:   AB011679    UniGene:   Rn.2458    Gene Symbol:   Tubb5    Locus Link:   29214    Chromosome:   20    Cytoband:   Chr:20p12    SOURCE    GENECARD       Gene Ontology:     BioCarta Pathways:     KEGG Pathways:  
    Probe set : 1374742_at    Name:   immunoglobulin superfamily, member 4C (predicted)    GB acc:   AA943983     Gene Symbol:   Igsf4c_predicted    Locus Link:   365216    Chromosome:   1    Cytoband:   Chr:1q21     GENECARD       Gene Ontology:   cellular component:  extracellular space   cellular component:  integral to membrane      BioCarta Pathways:     KEGG Pathways:  
    Probe set : 1384917_at    Name:   Similar to uracil-DNA glycosylase 2    GB acc:   BF547287    UniGene:   Rn.61269     Locus Link:   499528      SOURCE        Gene Ontology:     BioCarta Pathways:     KEGG Pathways:  
    Probe set : 1370347_at    Name:   PDZ and LIM domain 7    GB acc:   AF095585    UniGene:   Rn.7274    Gene Symbol:   Pdlim7    Locus Link:   286908    Chromosome:   17    Cytoband:   Chr:17p14    SOURCE    GENECARD       Gene Ontology:   biological process:  positive regulation of osteoblast differentiation   molecular function:  protein binding   molecular function:  zinc ion binding   molecular function:  metal ion binding      BioCarta Pathways:     KEGG Pathways:  
    Probe set : 1367562_at    Name:   secreted acidic cysteine rich glycoprotein    GB acc:   NM_012656    UniGene:   Rn.98989    Gene Symbol:   Sparc    Locus Link:   24791    Chromosome:   10    Cytoband:   Chr:10q22    SOURCE    GENECARD       Gene Ontology:   biological process:  ossification      BioCarta Pathways:     KEGG Pathways:  
    Probe set : 1388182_at    Name:   DNA primase, p49 subunit    GB acc:   BE108911    UniGene:   Rn.92497    Gene Symbol:   RGD:621380    Locus Link:   246327    Chromosome:   7    Cytoband:   Chr:7q11    SOURCE    GENECARD       Gene Ontology:   biological process:  DNA replication   biological process:  DNA replication, synthesis of RNA primer      BioCarta Pathways:     KEGG Pathways:  
    Probe set : 1367914_at    Name:   epithelial membrane protein 3    GB acc:   NM_030847    UniGene:   Rn.6782    Gene Symbol:   Emp3    Locus Link:   81505    Chromosome:   1    Cytoband:   Chr:1q22    SOURCE    GENECARD       Gene Ontology:     BioCarta Pathways:     KEGG Pathways:  
    Probe set : 1371318_at    Name:   ribosomal protein S16    GB acc:   BG666872    UniGene:   Rn.144646    Gene Symbol:   Rps16    Locus Link:   140655    Chromosome:   1    Cytoband:   Chr:1q21    SOURCE    GENECARD       Gene Ontology:   biological process:  protein biosynthesis   biological process:  ribosome biogenesis   molecular function:  RNA binding   molecular function:  structural constituent of ribosome      BioCarta Pathways:     KEGG Pathways:  
    Probe set : 1383716_at    Name:   Transcribed locus, strongly similar to NP_777569.1 hypothetical protein MGC23909 [Homo sapiens]    GB acc:   BF556301    UniGene:   Rn.137995        SOURCE        Gene Ontology:     BioCarta Pathways:     KEGG Pathways:  
    Probe set : 1384331_at    Name:   Neoplastic progression 3 (predicted)    GB acc:   H31896    UniGene:   Rn.2835     Locus Link:   296271    Chromosome:   3    Cytoband:   Chr:3q41    SOURCE        Gene Ontology:   cellular component:  extrachromosomal circular DNA   molecular function:  DNA binding      BioCarta Pathways:     KEGG Pathways:  
    Probe set : 1371754_at    Name:   solute carrier family 25 (mitochondrial carrier, phosphate carrier), member 25    GB acc:   AI177358    UniGene:   Rn.17644    Gene Symbol:   Slc25a25    Locus Link:   246771    Chromosome:   3    Cytoband:   Chr:3p11    SOURCE    GENECARD       Gene Ontology:   biological process:  transport   molecular function:  binding   molecular function:  calcium ion binding      BioCarta Pathways:     KEGG Pathways:  
    Probe set : 1387859_at    Name:   nitrogen fixation gene 1 (S. cerevisiae)    GB acc:   AI410876    UniGene:   Rn.3151    Gene Symbol:   Nfs1    Locus Link:   84594    Chromosome:   3    Cytoband:   Chr:3q42    SOURCE    GENECARD       Gene Ontology:   biological process:  metabolism   cellular component:  extracellular space   cellular component:  mitochondrion   cellular component:  mitochondrial matrix   molecular function:  transaminase activity   molecular function:  transferase activity   molecular function:  cysteine desulfurase activity      BioCarta Pathways:     KEGG Pathways:  
    Probe set : 1381370_at    Name:   Cysteine and glycine-rich protein 2    GB acc:   AW254502    UniGene:   Rn.94754    Gene Symbol:   Csrp2    Locus Link:   29317    Chromosome:   7    Cytoband:   Chr:7q13    SOURCE    GENECARD       Gene Ontology:   biological process:  development   biological process:  cell differentiation   biological process:  myoblast differentiation   cellular component:  nucleus   cellular component:  nucleus   molecular function:  zinc ion binding   molecular function:  metal ion binding      BioCarta Pathways:     KEGG Pathways:  
    Probe set : 1384211_at    Name:   procollagen, type XI, alpha 1    GB acc:   BM388456    UniGene:   Rn.260    Gene Symbol:   Col11a1    Locus Link:   25654    Chromosome:   2    Cytoband:   Chr:2q41    SOURCE    GENECARD       Gene Ontology:     BioCarta Pathways:     KEGG Pathways:  
    Probe set : 1392899_at    Name:   protein regulator of cytokinesis 1 (predicted)    GB acc:   BI284262    UniGene:   Rn.138438    Gene Symbol:   Prc1_predicted    Locus Link:   308761    Chromosome:   1    Cytoband:   Chr:1q31    SOURCE    GENECARD       Gene Ontology:   biological process:  mitotic spindle elongation   biological process:  cytokinesis   cellular component:  nucleus   cellular component:  spindle   cellular component:  spindle microtubule      BioCarta Pathways:     KEGG Pathways:  
    Probe set : 1379967_at    Name:   zinc finger protein 367 (predicted)    GB acc:   AI501039    UniGene:   Rn.35247    Gene Symbol:   Zfp367_predicted    Locus Link:   306695    Chromosome:   17    Cytoband:   Chr:17p14    SOURCE    GENECARD       Gene Ontology:   biological process:  regulation of transcription from RNA polymerase II promoter   cellular component:  nucleus      BioCarta Pathways:     KEGG Pathways:  
    Probe set : 1367780_at    Name:   pituitary tumor-transforming 1    GB acc:   NM_022391    UniGene:   Rn.271    Gene Symbol:   Pttg1    Locus Link:   64193    Chromosome:   10    Cytoband:   Chr:10q21    SOURCE    GENECARD       Gene Ontology:   biological process:  DNA replication and chromosome cycle   biological process:  regulation of cell growth   biological process:  regulation of cell growth   biological process:  DNA metabolism   biological process:  DNA repair   biological process:  transcription from RNA polymerase II promoter   biological process:  chromosome segregation   biological process:  mitosis   biological process:  spermatogenesis   biological process:  negative regulation of cell proliferation   biological process:  negative regulation of cell proliferation   biological process:  homologous chromosome segregation   biological process:  cellular physiological process   biological process:  response to DNA damage stimulus   biological process:  cell cycle   biological process:  cell division   cellular component:  nucleus   cellular component:  cytoplasm   molecular function:  transcription factor activity   molecular function:  cysteine protease inhibitor activity   molecular function:  protein binding      BioCarta Pathways:     KEGG Pathways:  
    Probe set : 1383578_at    Name:   Transcribed locus    GB acc:   AI071227    UniGene:   Rn.20474        SOURCE        Gene Ontology:     BioCarta Pathways:     KEGG Pathways:  
    Probe set : 1368545_at    Name:   CASP8 and FADD-like apoptosis regulator    GB acc:   NM_057138    UniGene:   Rn.28010    Gene Symbol:   Cflar    Locus Link:   117279    Chromosome:   9    Cytoband:   Chr:9q31    SOURCE    GENECARD       Gene Ontology:   molecular function:  protein binding   molecular function:  caspase activity      BioCarta Pathways:     KEGG Pathways:  
    Probe set : 1388340_at    Name:   Ns5atp9 protein    GB acc:   BF281153    UniGene:   Rn.7233    Gene Symbol:   RGD:1303041    Locus Link:   300795    Chromosome:   8    Cytoband:   Chr:8q24    SOURCE    GENECARD       Gene Ontology:     BioCarta Pathways:     KEGG Pathways:  
    Probe set : 1372125_at    Name:   glutathione peroxidase 7 (predicted)    GB acc:   BF408445    UniGene:   Rn.4130    Gene Symbol:   Gpx7_predicted    Locus Link:   298376    Chromosome:   5    Cytoband:   Chr:5q35    SOURCE    GENECARD       Gene Ontology:   biological process:  response to oxidative stress   cellular component:  extracellular space   molecular function:  peroxidase activity   molecular function:  glutathione peroxidase activity   molecular function:  oxidoreductase activity      BioCarta Pathways:     KEGG Pathways:  
    Probe set : 1373026_at    Name:   similar to RIKEN cDNA 2410030K01 (predicted)    GB acc:   BI303598    UniGene:   Rn.18387    Gene Symbol:   RGD1307631_predicted    Locus Link:   363028    Chromosome:   8    Cytoband:   Chr:8q13    SOURCE    GENECARD       Gene Ontology:     BioCarta Pathways:     KEGG Pathways:  
    Probe set : 1392965_a_at    Name:   SPARC related modular calcium binding 2 (predicted)    GB acc:   AI028877    UniGene:   Rn.6092    Gene Symbol:   Smoc2_predicted    Locus Link:   292401    Chromosome:   1    Cytoband:   Chr:1q12    SOURCE    GENECARD       Gene Ontology:   cellular component:  extracellular matrix (sensu Metazoa)   cellular component:  extracellular space   molecular function:  calcium ion binding      BioCarta Pathways:     KEGG Pathways:  
    Probe set : 1371775_at    Name:   Acyl-Coenzyme A dehydrogenase, short/branched chain    GB acc:   AI172057    UniGene:   Rn.44423    Gene Symbol:   Acadsb    Locus Link:   25618    Chromosome:   1    Cytoband:   Chr:1q41    SOURCE    GENECARD       Gene Ontology:   cellular component:  mitochondrion   cellular component:  mitochondrion      BioCarta Pathways:     KEGG Pathways:  
    Probe set : 1373463_at    Name:   Collagen, type V, alpha 2    GB acc:   AI172281    UniGene:   Rn.2875    Gene Symbol:   Col5a2    Locus Link:   85250    Chromosome:   9    Cytoband:   Chr:9q22    SOURCE    GENECARD       Gene Ontology:   molecular function:  extracellular matrix structural constituent   molecular function:  extracellular matrix structural constituent conferring tensile strength      BioCarta Pathways:     KEGG Pathways:  
    Probe set : 1384361_at    Name:   mal, T-cell differentiation protein 2    GB acc:   BF555523    UniGene:   Rn.103382    Gene Symbol:   Mal2    Locus Link:   362911    Chromosome:   7    Cytoband:   Chr:7q31    SOURCE    GENECARD       Gene Ontology:   biological process:  transcytosis   cellular component:  lipid raft   cellular component:  membrane   molecular function:  protein transporter activity      BioCarta Pathways:     KEGG Pathways:  
    Probe set : 1372006_at    Name:   Transcribed locus    GB acc:   BM391274    UniGene:   Rn.954        SOURCE        Gene Ontology:     BioCarta Pathways:     KEGG Pathways:  
    Probe set : 1367741_at    Name:   homocysteine-inducible, endoplasmic reticulum stress-inducible, ubiquitin-like domain member 1    GB acc:   NM_053523    UniGene:   Rn.4028    Gene Symbol:   Herpud1    Locus Link:   85430    Chromosome:   19    Cytoband:   Chr:19p12    SOURCE    GENECARD       Gene Ontology:   biological process:  protein modification   biological process:  response to stress   biological process:  response to unfolded protein   cellular component:  endoplasmic reticulum   cellular component:  endoplasmic reticulum membrane   cellular component:  integral to membrane      BioCarta Pathways:     KEGG Pathways:  
    Probe set : 1367628_at    Name:   lectin, galactose binding, soluble 1    GB acc:   NM_019904    UniGene:   Rn.57    Gene Symbol:   Lgals1    Locus Link:   56646    Chromosome:   7    Cytoband:   Chr:7q34    SOURCE    GENECARD       Gene Ontology:   biological process:  heterophilic cell adhesion   biological process:  myoblast differentiation   cellular component:  extracellular space   molecular function:  sugar binding      BioCarta Pathways:     KEGG Pathways:  
    Probe set : 1370895_at    Name:   collagen, type V, alpha 2    GB acc:   AI179399    UniGene:   Rn.2875    Gene Symbol:   Col5a2    Locus Link:   85250    Chromosome:   9    Cytoband:   Chr:9q22    SOURCE    GENECARD       Gene Ontology:   molecular function:  extracellular matrix structural constituent   molecular function:  extracellular matrix structural constituent conferring tensile strength      BioCarta Pathways:     KEGG Pathways:  
    Probe set : 1371331_at    Name:   follistatin-like 1    GB acc:   BG665037    UniGene:   Rn.95652    Gene Symbol:   Fstl1    Locus Link:   79210    Chromosome:   11    Cytoband:   Chr:11q21    SOURCE    GENECARD       Gene Ontology:   cellular component:  extracellular space   molecular function:  calcium ion binding   molecular function:  heparin binding      BioCarta Pathways:     KEGG Pathways:  
    Probe set : 1387891_at    Name:   peroxiredoxin 4    GB acc:   BI282076    UniGene:   Rn.17958    Gene Symbol:   Prdx4    Locus Link:   85274      SOURCE    GENECARD       Gene Ontology:   biological process:  I-kappaB phosphorylation   cellular component:  extracellular space   cellular component:  extracellular space   cellular component:  mitochondrion      BioCarta Pathways:     KEGG Pathways:  
    Probe set : 1373658_at    Name:   Rac GTPase-activating protein 1 (predicted)    GB acc:   AI409259    UniGene:   Rn.101301     Locus Link:   315298    Chromosome:   7    Cytoband:   Chr:7q36    SOURCE        Gene Ontology:   biological process:  electron transport   biological process:  transport   biological process:  intracellular signaling cascade   cellular component:  mitochondrion      BioCarta Pathways:     KEGG Pathways:  
    Probe set : 1374938_at    Name:   ZW10 homolog, centromere/kinetochore protein (Drosophila) (predicted)    GB acc:   BI295067    UniGene:   Rn.25242    Gene Symbol:   Zw10_predicted    Locus Link:   363059    Chromosome:   8    Cytoband:   Chr:8q23    SOURCE    GENECARD       Gene Ontology:   molecular function:  protein binding   molecular function:  centromeric DNA binding      BioCarta Pathways:     KEGG Pathways:  
    Probe set : 1385190_at    Name:   Transcribed locus    GB acc:   BG376453    UniGene:   Rn.66394        SOURCE        Gene Ontology:     BioCarta Pathways:     KEGG Pathways:  
    Probe set : 1387878_at    Name:   glutamate dehydrogenase 1    GB acc:   AW916644    UniGene:   Rn.55106    Gene Symbol:   Glud1    Locus Link:   24399    Chromosome:   16    Cytoband:   Chr:16p16    SOURCE    GENECARD       Gene Ontology:   biological process:  amino acid metabolism   biological process:  glutamate catabolism   cellular component:  mitochondrion      BioCarta Pathways:     KEGG Pathways:  
    Probe set : 1371970_at    Name:   similar to expressed sequence AW413625    GB acc:   AA799328    UniGene:   Rn.112856    Gene Symbol:   LOC499322    Locus Link:   499322    Chromosome:   1    Cytoband:   Chr:1q43    SOURCE    GENECARD       Gene Ontology:     BioCarta Pathways:     KEGG Pathways:  
    Probe set : 1388335_at    Name:   transgelin 2 (predicted)    GB acc:   BI285865    UniGene:   Rn.145963    Gene Symbol:   Tagln2_predicted    Locus Link:   304983    Chromosome:   13    Cytoband:   Chr:13q24    SOURCE    GENECARD       Gene Ontology:   biological process:  muscle development      BioCarta Pathways:     KEGG Pathways:  
    Probe set : 1391450_at    Name:   lysyl oxidase-like 2 (predicted)    GB acc:   AI044651    UniGene:   Rn.19018    Gene Symbol:   Loxl2_predicted    Locus Link:   290350    Chromosome:   15    Cytoband:   Chr:15p11    SOURCE    GENECARD       Gene Ontology:   biological process:  protein modification   biological process:  cell adhesion   biological process:  aging   cellular component:  extracellular space   cellular component:  membrane      BioCarta Pathways:     KEGG Pathways:  
    Probe set : 1377907_at    Name:   similar to RIKEN cDNA 6530403A03 (predicted)    GB acc:   BF287924    UniGene:   Rn.39699    Gene Symbol:   RGD1309020_predicted    Locus Link:   291060    Chromosome:   17    Cytoband:   Chr:17p12    SOURCE    GENECARD       Gene Ontology:     BioCarta Pathways:     KEGG Pathways:  
    Probe set : 1368990_at    Name:   cytochrome P450, family 1, subfamily b, polypeptide 1    GB acc:   NM_012940    UniGene:   Rn.10125    Gene Symbol:   Cyp1b1    Locus Link:   25426    Chromosome:   6    Cytoband:   Chr:6q11    SOURCE    GENECARD       Gene Ontology:     BioCarta Pathways:     KEGG Pathways:  
    Probe set : 1368420_at    Name:   ceruloplasmin    GB acc:   NM_012532    UniGene:   Rn.32777    Gene Symbol:   Cp    Locus Link:   24268    Chromosome:   2    Cytoband:   Chr:2q24    SOURCE    GENECARD       Gene Ontology:     BioCarta Pathways:     KEGG Pathways:  
    Probe set : 1367570_at    Name:   transgelin    GB acc:   NM_031549    UniGene:   Rn.34397    Gene Symbol:   Tagln    Locus Link:   25123    Chromosome:   8    Cytoband:   Chr:8q24    SOURCE    GENECARD       Gene Ontology:   biological process:  cytoskeleton organization and biogenesis   biological process:  muscle development   molecular function:  actin binding   molecular function:  protein binding, bridging   molecular function:  actin filament binding      BioCarta Pathways:     KEGG Pathways:  
    Probe set : 1367946_at    Name:   PDZ and LIM domain 1 (elfin)    GB acc:   NM_017365    UniGene:   Rn.11170    Gene Symbol:   Pdlim1    Locus Link:   54133    Chromosome:   1    Cytoband:   Chr:1q53    SOURCE    GENECARD       Gene Ontology:   cellular component:  transcription factor complex   cellular component:  cytoskeleton      BioCarta Pathways:     KEGG Pathways:  
    Probe set : 1374449_at    Name:   cell division cycle associated 3 /// similar to cell division cycle associated 3    GB acc:   BF417638    UniGene:   Rn.3246    Gene Symbol:   RGD:1359093 /// LOC501134    Locus Link:   297594 /// 501134    Chromosome:   4    Cytoband:   Chr:4q42 /// Chr:9q22    SOURCE    GENECARD       Gene Ontology:   biological process:  cell division      BioCarta Pathways:     KEGG Pathways:  
    Probe set : 1370288_a_at    Name:   tropomyosin 1, alpha    GB acc:   AF372216    UniGene:   Rn.87540    Gene Symbol:   Tpm1    Locus Link:   24851    Chromosome:   8    Cytoband:   Chr:8q24    SOURCE    GENECARD       Gene Ontology:   cellular component:  cytoskeleton   cellular component:  muscle thin filament tropomyosin   cellular component:  kinesin complex      BioCarta Pathways:     KEGG Pathways:  
    Probe set : 1374036_at    Name:   minichromosome maintenance deficient 2 mitotin (S. cerevisiae) (predicted)    GB acc:   AI229508    UniGene:   Rn.2715    Gene Symbol:   Mcm2_predicted    Locus Link:   312538    Chromosome:   4    Cytoband:   Chr:4q34    SOURCE    GENECARD       Gene Ontology:   cellular component:  chromatin   cellular component:  nucleus   cellular component:  nuclear origin of replication recognition complex      BioCarta Pathways:     KEGG Pathways:  
    Probe set : 1377832_at    Name:   polo-like kinase 4 (Drosophila) (predicted)    GB acc:   BE109322    UniGene:   Rn.14848    Gene Symbol:   Plk4_predicted    Locus Link:   310344    Chromosome:   2    Cytoband:   Chr:2q26    SOURCE    GENECARD       Gene Ontology:   biological process:  regulation of progression through cell cycle   biological process:  protein amino acid phosphorylation   biological process:  cell cycle      BioCarta Pathways:     KEGG Pathways:  
    Probe set : 1386660_at    Name:   Transcribed locus    GB acc:   BF565021    UniGene:   Rn.146221        SOURCE        Gene Ontology:     BioCarta Pathways:     KEGG Pathways:  
    Probe set : 1373286_at    Name:   CSX-associated LIM    GB acc:   AA875261    UniGene:   Rn.2851    Gene Symbol:   RGD:1359551    Locus Link:   362650    Chromosome:   5    Cytoband:   Chr:5q36    SOURCE    GENECARD       Gene Ontology:   molecular function:  zinc ion binding   molecular function:  metal ion binding      BioCarta Pathways:     KEGG Pathways:  
    Probe set : 1389408_at    Name:   ribonucleotide reductase M2    GB acc:   BG379338    UniGene:   Rn.101236    Gene Symbol:   Rrm2    Locus Link:   362720    Chromosome:   6    Cytoband:   Chr:6q16    SOURCE    GENECARD       Gene Ontology:   biological process:  DNA replication   biological process:  deoxyribonucleoside diphosphate metabolism   biological process:  deoxyribonucleotide metabolism   cellular component:  cytoplasm   cellular component:  ribonucleoside-diphosphate reductase complex      BioCarta Pathways:     KEGG Pathways:  
    Probe set : 1392915_at    Name:   procollagen, type XI, alpha 1    GB acc:   BM389291    UniGene:   Rn.260    Gene Symbol:   Col11a1    Locus Link:   25654    Chromosome:   2    Cytoband:   Chr:2q41    SOURCE    GENECARD       Gene Ontology:     BioCarta Pathways:     KEGG Pathways:  
    Probe set : 1387870_at    Name:   zinc finger protein 36    GB acc:   AB025017    UniGene:   Rn.82737    Gene Symbol:   Zfp36    Locus Link:   79426    Chromosome:   1    Cytoband:   Chr:1q21    SOURCE    GENECARD       Gene Ontology:   cellular component:  nucleus   cellular component:  nucleus   cellular component:  cytoplasm   cellular component:  cytoplasm      BioCarta Pathways:     KEGG Pathways:  
    Probe set : 1377967_at    Name:   Retroviral integration site 2 (predicted)    GB acc:   AI112987    UniGene:   Rn.8884     Locus Link:   292071    Chromosome:   19    Cytoband:   Chr:19q12    SOURCE        Gene Ontology:   biological process:  DNA replication   biological process:  cell cycle   biological process:  regulation of S phase of mitotic cell cycle   cellular component:  nucleus   molecular function:  DNA binding      BioCarta Pathways:     KEGG Pathways:  
    Probe set : 1380088_at    Name:   Transcribed locus    GB acc:   AW533021    UniGene:   Rn.28360        SOURCE        Gene Ontology:     BioCarta Pathways:     KEGG Pathways:  
    Probe set : 1373628_at    Name:   Transcribed locus    GB acc:   AA818342    UniGene:   Rn.1026        SOURCE        Gene Ontology:     BioCarta Pathways:     KEGG Pathways:  
    Probe set : 1388236_x_at    Name:   RT1 class I, CE12    GB acc:   M24026    UniGene:   Rn.116792    Gene Symbol:   RGD:1359103    Locus Link:   309600    Chromosome:   20    Cytoband:   Chr:20p12    SOURCE    GENECARD       Gene Ontology:   biological process:  antigen presentation   cellular component:  MHC class I protein complex   molecular function:  MHC class I receptor activity      BioCarta Pathways:     KEGG Pathways:  
    Probe set : 1388985_at    Name:   hypothetical protein LOC310926 /// LOC501211    GB acc:   AI012869     Gene Symbol:   LOC310926 /// LOC501211    Locus Link:   310926 /// 501211    Chromosome:   2    Cytoband:   Chr:2q44     GENECARD       Gene Ontology:     BioCarta Pathways:     KEGG Pathways:  
    Probe set : 1368171_at    Name:   lysyl oxidase    GB acc:   NM_017061    UniGene:   Rn.11372    Gene Symbol:   Lox    Locus Link:   24914    Chromosome:   18    Cytoband:   Chr:18q11    SOURCE    GENECARD       Gene Ontology:   biological process:  protein modification   cellular component:  extracellular matrix (sensu Metazoa)   cellular component:  extracellular space      BioCarta Pathways:     KEGG Pathways:  
    Probe set : 1377908_at    Name:   CD4 antigen    GB acc:   AI408225    UniGene:   Rn.10748    Gene Symbol:   Cd4    Locus Link:   24932    Chromosome:   4    Cytoband:   Chr:4q42    SOURCE    GENECARD       Gene Ontology:   biological process:  defense response   biological process:  immune response   biological process:  cell adhesion   biological process:  transmembrane receptor protein tyrosine kinase signaling pathway   biological process:  T cell differentiation   biological process:  T cell selection   biological process:  positive regulation of interleukin-2 biosynthesis   biological process:  immune response   biological process:  transmembrane receptor protein tyrosine kinase signaling pathway   biological process:  T cell differentiation   biological process:  T cell selection   biological process:  positive regulation of interleukin-2 biosynthesis      BioCarta Pathways:     KEGG Pathways:  
    Probe set : 1376765_at    Name:   similar to maestro    GB acc:   BF283408    UniGene:   Rn.16360    Gene Symbol:   LOC361348    Locus Link:   361348    Chromosome:   18    Cytoband:   Chr:18q12.2    SOURCE    GENECARD       Gene Ontology:     BioCarta Pathways:     KEGG Pathways:  
    Probe set : 1383962_at    Name:   Cd27 binding protein (Hindu God of destruction)    GB acc:   BI275530    UniGene:   Rn.23843    Gene Symbol:   Siva    Locus Link:   362791    Chromosome:   6    Cytoband:   Chr:6q32    SOURCE    GENECARD       Gene Ontology:     BioCarta Pathways:     KEGG Pathways:  
    Probe set : 1372935_at    Name:   Similar to Coronin, actin binding protein 1C    GB acc:   AI598550    UniGene:   Rn.45957     Locus Link:   363930    Chromosome:   12    Cytoband:   Chr:12q16    SOURCE        Gene Ontology:   biological process:  cell adhesion      BioCarta Pathways:     KEGG Pathways:  
    Probe set : 1370234_at    Name:   fibronectin 1    GB acc:   AA893484    UniGene:   Rn.1604    Gene Symbol:   Fn1    Locus Link:   25661    Chromosome:   9    Cytoband:   Chr:9q33    SOURCE    GENECARD       Gene Ontology:     BioCarta Pathways:     KEGG Pathways:  
    Probe set : 1393436_at    Name:   similar to potential ligand-binding protein    GB acc:   AI549033    UniGene:   Rn.41707    Gene Symbol:   LOC309100    Locus Link:   309100    Chromosome:   1    Cytoband:   Chr:1q41    SOURCE    GENECARD       Gene Ontology:     BioCarta Pathways:     KEGG Pathways:  
    Probe set : 1373894_at     GB acc:   BF410946              Gene Ontology:     BioCarta Pathways:     KEGG Pathways:  
    Probe set : 1375926_at    Name:   Transcribed locus    GB acc:   BG372589    UniGene:   Rn.22507        SOURCE        Gene Ontology:     BioCarta Pathways:     KEGG Pathways:  
    Probe set : 1398380_at    Name:   similar to von Willebrand factor A-domain containing 1 (predicted)    GB acc:   BF413643    UniGene:   Rn.62409    Gene Symbol:   RGD1311476_predicted    Locus Link:   298683    Chromosome:   5    Cytoband:   Chr:5q36    SOURCE    GENECARD       Gene Ontology:   cellular component:  extracellular region   cellular component:  extracellular space      BioCarta Pathways:     KEGG Pathways:  
    Probe set : 1382590_at    Name:   Similar to RIKEN cDNA 2310015N21    GB acc:   BI277914    UniGene:   Rn.126330     Locus Link:   501095      SOURCE        Gene Ontology:     BioCarta Pathways:     KEGG Pathways:  
    Probe set : 1371074_a_at    Name:   mini chromosome maintenance deficient 6 (S. cerevisiae)    GB acc:   U17565    UniGene:   Rn.33226    Gene Symbol:   Mcmd6    Locus Link:   29685    Chromosome:   13    Cytoband:   Chr:13q12    SOURCE    GENECARD       Gene Ontology:   cellular component:  nucleus      BioCarta Pathways:     KEGG Pathways:  
    Probe set : 1367776_at    Name:   cell division cycle 2 homolog A (S. pombe)    GB acc:   NM_019296    UniGene:   Rn.6934    Gene Symbol:   Cdc2a    Locus Link:   54237    Chromosome:   20    Cytoband:   Chr:20p11    SOURCE    GENECARD       Gene Ontology:   cellular component:  nucleus      BioCarta Pathways:     KEGG Pathways:  
    Probe set : 1388763_at    Name:   Transcribed locus, strongly similar to NP_031751.1 calponin 2 [Mus musculus]    GB acc:   AI230762    UniGene:   Rn.43993        SOURCE        Gene Ontology:     BioCarta Pathways:     KEGG Pathways:  
    Probe set : 1388955_at    Name:   Putative eps protein    GB acc:   BE110691    UniGene:   Rn.16016    Gene Symbol:   MGEPS    Locus Link:   361512    Chromosome:   1    Cytoband:   Chr:1q21    SOURCE    GENECARD       Gene Ontology:     BioCarta Pathways:     KEGG Pathways:  
    Probe set : 1371974_at    Name:   phytanoyl-CoA dioxygenase domain containing 1 (predicted)    GB acc:   BI275763    UniGene:   Rn.32623    Gene Symbol:   Phyhd1_predicted    Locus Link:   296621    Chromosome:   3    Cytoband:   Chr:3p12    SOURCE    GENECARD       Gene Ontology:   molecular function:  oxidoreductase activity, acting on single donors with incorporation of molecular oxygen, incorporation of two atoms of oxygen      BioCarta Pathways:     KEGG Pathways:  
    Probe set : 1393240_at    Name:   EGF-containing fibulin-like extracellular matrix protein 2    GB acc:   AI112299    UniGene:   Rn.16151    Gene Symbol:   RGD:1359496    Locus Link:   293677    Chromosome:   1    Cytoband:   Chr:1q43    SOURCE    GENECARD       Gene Ontology:   biological process:  homophilic cell adhesion   biological process:  blood coagulation   cellular component:  membrane   molecular function:  transmembrane receptor activity   molecular function:  structural molecule activity   molecular function:  calcium ion binding      BioCarta Pathways:     KEGG Pathways:  
    Probe set : 1376198_at    Name:   adipocyte-specific adhesion molecule    GB acc:   BI303342    UniGene:   Rn.40141    Gene Symbol:   Asam    Locus Link:   286939    Chromosome:   8    Cytoband:   Chr:8q22    SOURCE    GENECARD       Gene Ontology:   biological process:  cell-cell adhesion   cellular component:  tight junction   cellular component:  integral to membrane      BioCarta Pathways:     KEGG Pathways:  
    Probe set : 1369944_at    Name:   MARCKS-like protein    GB acc:   NM_030862    UniGene:   Rn.2486    Gene Symbol:   Mlp    Locus Link:   81520    Chromosome:   5    Cytoband:   Chr:5q36    SOURCE    GENECARD       Gene Ontology:   biological process:  vesicle-mediated transport   molecular function:  calmodulin binding      BioCarta Pathways:     KEGG Pathways:  
    Probe set : 1390707_at    Name:   regulator of G-protein signalling 10    GB acc:   BI281632    UniGene:   Rn.23957    Gene Symbol:   Rgs10    Locus Link:   54290    Chromosome:   1    Cytoband:   Chr:1q36    SOURCE    GENECARD       Gene Ontology:   molecular function:  signal transducer activity   molecular function:  GTPase activator activity      BioCarta Pathways:     KEGG Pathways:  
    Probe set : 1368961_at    Name:   Matrix metalloproteinase 23    GB acc:   NM_053606    UniGene:   Rn.22562    Gene Symbol:   Mmp23    Locus Link:   94339    Chromosome:   5    Cytoband:   Chr:5q36    SOURCE    GENECARD       Gene Ontology:   biological process:  proteolysis   biological process:  transport   cellular component:  extracellular matrix (sensu Metazoa)   cellular component:  extracellular space   cellular component:  intracellular      BioCarta Pathways:     KEGG Pathways:  
    Probe set : 1368840_at    Name:   LR8 protein    GB acc:   NM_134390    UniGene:   Rn.26876    Gene Symbol:   Lr8    Locus Link:   171411    Chromosome:   4    Cytoband:   Chr:4q24    SOURCE    GENECARD       Gene Ontology:   cellular component:  integral to membrane      BioCarta Pathways:     KEGG Pathways:  
    Probe set : 1386940_at    Name:   tissue inhibitor of metalloproteinase 2    GB acc:   NM_021989    UniGene:   Rn.10161    Gene Symbol:   Timp2    Locus Link:   29543    Chromosome:   10    Cytoband:   Chr:10q32.3    SOURCE    GENECARD       Gene Ontology:   biological process:  spermatogenesis   biological process:  spermatogenesis   cellular component:  extracellular matrix (sensu Metazoa)   cellular component:  basement membrane   cellular component:  extracellular space   molecular function:  enzyme activator activity   molecular function:  metalloendopeptidase inhibitor activity   molecular function:  enzyme inhibitor activity      BioCarta Pathways:     KEGG Pathways:  
    Probe set : 1370346_at    Name:   cyclin B1    GB acc:   X64589    UniGene:   Rn.145413    Gene Symbol:   Ccnb1    Locus Link:   25203    Chromosome:   2    Cytoband:   Chr:2q12    SOURCE    GENECARD       Gene Ontology:   cellular component:  nucleus   molecular function:  protein binding   molecular function:  kinase activity   molecular function:  cyclin-dependent protein kinase regulator activity      BioCarta Pathways:     KEGG Pathways:  
    Probe set : 1379345_at    Name:   procollagen, type XV (predicted)    GB acc:   BM386752    UniGene:   Rn.31832    Gene Symbol:   Col15a1_predicted    Locus Link:   298069    Chromosome:   5    Cytoband:   Chr:5q22    SOURCE    GENECARD       Gene Ontology:   biological process:  phosphate transport   biological process:  cell adhesion      BioCarta Pathways:     KEGG Pathways:  
    Probe set : 1372516_at    Name:   kinesin family member 22 (predicted)    GB acc:   AI317842    UniGene:   Rn.8601    Gene Symbol:   Kif22_predicted    Locus Link:   293502    Chromosome:   1    Cytoband:   Chr:1q36    SOURCE    GENECARD       Gene Ontology:     BioCarta Pathways:     KEGG Pathways:  
    Probe set : 1371861_at    Name:   Transcribed locus    GB acc:   BE117558    UniGene:   Rn.19802        SOURCE        Gene Ontology:     BioCarta Pathways:     KEGG Pathways:  
    Probe set : 1374784_at    Name:   phosphoribosyl transferase domain containing 1 (predicted)    GB acc:   AI407006    UniGene:   Rn.33792    Gene Symbol:   Prtfdc1_predicted    Locus Link:   291355    Chromosome:   17    Cytoband:   Chr:17q12.3    SOURCE    GENECARD       Gene Ontology:   biological process:  purine ribonucleoside salvage   biological process:  nucleoside metabolism   cellular component:  cytoplasm   molecular function:  hypoxanthine phosphoribosyltransferase activity   molecular function:  transferase activity      BioCarta Pathways:     KEGG Pathways:  
    Probe set : 1371391_at    Name:   thioredoxin domain containing 5 (predicted)    GB acc:   BM390196    UniGene:   Rn.128652    Gene Symbol:   Txndc5_predicted    Locus Link:   306869    Chromosome:   17    Cytoband:   Chr:17p12    SOURCE    GENECARD       Gene Ontology:   biological process:  electron transport   cellular component:  endoplasmic reticulum   molecular function:  electron transporter activity   molecular function:  isomerase activity      BioCarta Pathways:     KEGG Pathways:  
    Probe set : 1398788_at    Name:   glucose regulated protein, 58 kDa    GB acc:   NM_017319    UniGene:   Rn.11527    Gene Symbol:   Grp58    Locus Link:   29468    Chromosome:   3    Cytoband:   Chr:3q35    SOURCE    GENECARD       Gene Ontology:   cellular component:  extracellular space   cellular component:  endoplasmic reticulum      BioCarta Pathways:     KEGG Pathways:  
    Probe set : 1384344_at    Name:   FK506 binding protein 7 (predicted)    GB acc:   AI060231    UniGene:   Rn.82775    Gene Symbol:   Fkbp7_predicted    Locus Link:   295672    Chromosome:   3    Cytoband:   Chr:3q23    SOURCE    GENECARD       Gene Ontology:   biological process:  protein folding   cellular component:  extracellular space   cellular component:  endoplasmic reticulum   molecular function:  peptidyl-prolyl cis-trans isomerase activity   molecular function:  calcium ion binding   molecular function:  isomerase activity      BioCarta Pathways:     KEGG Pathways:  
    Probe set : 1376648_at    Name:   v-myc myelocytomatosis viral related oncogene, neuroblastoma derived (avian)    GB acc:   BI275570    UniGene:   Rn.81116    Gene Symbol:   Mycn    Locus Link:   298894    Chromosome:   6    Cytoband:   Chr:6q14    SOURCE    GENECARD       Gene Ontology:   cellular component:  intracellular   cellular component:  nucleus   molecular function:  DNA binding   molecular function:  transcription factor activity   molecular function:  protein binding      BioCarta Pathways:     KEGG Pathways:  
    Probe set : 1371475_at    Name:   ribonuclease, RNase A family 4    GB acc:   BI284831    UniGene:   Rn.1742    Gene Symbol:   Rnase4    Locus Link:   56759    Chromosome:   15    Cytoband:   Chr:15p14    SOURCE    GENECARD       Gene Ontology:   biological process:  mRNA cleavage      BioCarta Pathways:     KEGG Pathways:  
    Probe set : 1371480_at    Name:   LOC499655    GB acc:   AI407985    UniGene:   Rn.128820    Gene Symbol:   LOC499655    Locus Link:   499655    Chromosome:   2    Cytoband:   Chr:2q34    SOURCE    GENECARD       Gene Ontology:     BioCarta Pathways:     KEGG Pathways:  
    Probe set : 1373882_at     GB acc:   AI170324              Gene Ontology:     BioCarta Pathways:     KEGG Pathways:  
    Probe set : 1368409_at    Name:   glutathione S-transferase, theta 2    GB acc:   NM_012796    UniGene:   Rn.87212    Gene Symbol:   Gstt2    Locus Link:   29487    Chromosome:   20    Cytoband:   Chr:20p12    SOURCE    GENECARD       Gene Ontology:   biological process:  glutathione metabolism   biological process:  glutathione metabolism   cellular component:  nucleus      BioCarta Pathways:     KEGG Pathways:  
    Probe set : 1367744_at    Name:   melanoma antigen, family D, 2    GB acc:   NM_080479    UniGene:   Rn.11984    Gene Symbol:   Maged2    Locus Link:   113947    Chromosome:   X    Cytoband:   Chr:Xq21    SOURCE    GENECARD       Gene Ontology:     BioCarta Pathways:     KEGG Pathways:  
    Probe set : 1369788_s_at    Name:   v-jun sarcoma virus 17 oncogene homolog (avian)    GB acc:   NM_021835    UniGene:   Rn.93714    Gene Symbol:   Jun    Locus Link:   24516    Chromosome:   5    Cytoband:   Chr:5q34.1    SOURCE    GENECARD       Gene Ontology:     BioCarta Pathways:     KEGG Pathways:  
    Probe set : 1388792_at    Name:   growth arrest and DNA-damage-inducible 45 gamma (predicted)    GB acc:   AI599423    UniGene:   Rn.16950    Gene Symbol:   Gadd45g_predicted    Locus Link:   291005    Chromosome:   17    Cytoband:   Chr:17p14    SOURCE    GENECARD       Gene Ontology:   cellular component:  nucleus   molecular function:  protein binding      BioCarta Pathways:     KEGG Pathways:  
    Probe set : 1389966_at    Name:   procollagen, type VI, alpha 3 (predicted)    GB acc:   AI176126    UniGene:   Rn.2157    Gene Symbol:   Col6a3_predicted    Locus Link:   367313    Chromosome:   9    Cytoband:   Chr:9q35-q36    SOURCE    GENECARD       Gene Ontology:   biological process:  phosphate transport   biological process:  cell adhesion   biological process:  muscle development      BioCarta Pathways:     KEGG Pathways:  
    Probe set : 1371099_at    Name:   Polymeric immunoglobulin receptor AATTAA-containing 3'UTR mRNA sequence    GB acc:   U07886    UniGene:   Rn.107069        SOURCE        Gene Ontology:     BioCarta Pathways:     KEGG Pathways:  
    Probe set : 1367567_at    Name:   ribosomal protein L6    GB acc:   NM_053971    UniGene:   Rn.2660    Gene Symbol:   Rpl6    Locus Link:   117042    Chromosome:   12    Cytoband:   Chr:12q16    SOURCE    GENECARD       Gene Ontology:   biological process:  regulation of transcription, DNA-dependent   biological process:  protein biosynthesis   biological process:  ribosome biogenesis   molecular function:  DNA binding   molecular function:  RNA binding   molecular function:  structural constituent of ribosome      BioCarta Pathways:     KEGG Pathways:  
    Probe set : 1372870_at    Name:   KDEL (Lys-Asp-Glu-Leu) endoplasmic reticulum protein retention receptor 3 (predicted)    GB acc:   BM391302    UniGene:   Rn.144441    Gene Symbol:   Kdelr3_predicted    Locus Link:   315131    Chromosome:   7    Cytoband:   Chr:7q34    SOURCE    GENECARD       Gene Ontology:   biological process:  intracellular protein transport   biological process:  protein transport   cellular component:  extracellular space   cellular component:  endoplasmic reticulum   cellular component:  integral to membrane   molecular function:  receptor activity   molecular function:  protein transporter activity      BioCarta Pathways:     KEGG Pathways:  
    Probe set : 1374927_at    Name:   Transcribed locus    GB acc:   AI714124    UniGene:   Rn.14945        SOURCE        Gene Ontology:     BioCarta Pathways:     KEGG Pathways:  
    Probe set : 1368924_at    Name:   growth hormone receptor    GB acc:   NM_017094    UniGene:   Rn.2178    Gene Symbol:   Ghr    Locus Link:   25235    Chromosome:   2    Cytoband:   Chr:2q16    SOURCE    GENECARD       Gene Ontology:     BioCarta Pathways:     KEGG Pathways:  
    Probe set : 1370594_at    Name:   immunoglobulin superfamily, member 1    GB acc:   AF322217    UniGene:   Rn.23738    Gene Symbol:   Igsf1    Locus Link:   302822    Chromosome:   X    Cytoband:   Chr:Xq36    SOURCE    GENECARD       Gene Ontology:   molecular function:  coreceptor activity      BioCarta Pathways:     KEGG Pathways:  
    Probe set : 1387062_a_at    Name:   checkpoint kinase 1 homolog (S. pombe)    GB acc:   NM_080400    UniGene:   Rn.33267    Gene Symbol:   Chek1    Locus Link:   140583    Chromosome:   8    Cytoband:   Chr:8q21    SOURCE    GENECARD       Gene Ontology:   cellular component:  condensed nuclear chromosome   cellular component:  nucleus      BioCarta Pathways:     KEGG Pathways:  
    Probe set : 1389395_at    Name:   Transcribed locus, moderately similar to NP_065184.2 selenoprotein N, 1 isoform 1 precursor; muscular dystrophy with rigid spine 1; rigid spine muscular dystrophy 1 [Homo sapiens]    GB acc:   BI275654    UniGene:   Rn.15108        SOURCE        Gene Ontology:     BioCarta Pathways:     KEGG Pathways:  
    Probe set : 1373672_at     GB acc:   BM384419              Gene Ontology:     BioCarta Pathways:     KEGG Pathways:  
    Probe set : 1370200_at    Name:   glutamate dehydrogenase 1    GB acc:   BI284411    UniGene:   Rn.55106    Gene Symbol:   Glud1    Locus Link:   24399    Chromosome:   16    Cytoband:   Chr:16p16    SOURCE    GENECARD       Gene Ontology:   biological process:  amino acid metabolism   biological process:  glutamate catabolism   cellular component:  mitochondrion      BioCarta Pathways:     KEGG Pathways:  
    Probe set : 1367618_a_at    Name:   discs, large homolog 5 (Drosophila) (predicted)    GB acc:   NM_130734    UniGene:   Rn.110214    Gene Symbol:   Dlg5_predicted    Locus Link:   305645    Chromosome:   15    Cytoband:   Chr:15p16    SOURCE    GENECARD       Gene Ontology:   biological process:  regulation of progression through cell cycle   biological process:  intracellular signaling cascade   biological process:  cell-cell adhesion   cellular component:  plasma membrane   cellular component:  cell-cell adherens junction      BioCarta Pathways:     KEGG Pathways:  
    Probe set : 1373128_at    Name:   reticulocalbin 3, EF-hand calcium binding domain    GB acc:   BI278379    UniGene:   Rn.2997    Gene Symbol:   RGD:1359365    Locus Link:   494125    Chromosome:   1    Cytoband:   Chr:1q22    SOURCE    GENECARD       Gene Ontology:   cellular component:  endoplasmic reticulum   molecular function:  calcium ion binding      BioCarta Pathways:     KEGG Pathways:  
    Probe set : 1398853_at    Name:   proteasome (prosome, macropain) subunit, beta type 3    GB acc:   AI169273    UniGene:   Rn.144576    Gene Symbol:   Psmb3    Locus Link:   29676    Chromosome:   10    Cytoband:   Chr:10q31    SOURCE    GENECARD       Gene Ontology:   biological process:  ubiquitin-dependent protein catabolism   biological process:  protein catabolism   cellular component:  cytosol   cellular component:  proteasome core complex (sensu Eukaryota)   cellular component:  protein complex      BioCarta Pathways:     KEGG Pathways:  
    Probe set : 1367581_a_at    Name:   secreted phosphoprotein 1    GB acc:   AB001382    UniGene:   Rn.8871    Gene Symbol:   Spp1    Locus Link:   25353    Chromosome:   14    Cytoband:   Chr:14p22    SOURCE    GENECARD       Gene Ontology:   biological process:  ossification   biological process:  anti-apoptosis   biological process:  inflammatory response   biological process:  cell adhesion   biological process:  cell adhesion   biological process:  cell-matrix adhesion   biological process:  cell-cell signaling   biological process:  negative regulation of bone mineralization   biological process:  immune cell chemotaxis   biological process:  T-helper 1 type immune response   biological process:  positive regulation of T cell proliferation   biological process:  regulation of myeloid cell differentiation   biological process:  induction of positive chemotaxis   biological process:  anti-apoptosis   biological process:  inflammatory response   biological process:  cell-matrix adhesion   biological process:  cell-cell signaling   biological process:  negative regulation of bone mineralization   biological process:  immune cell chemotaxis   biological process:  T-helper 1 type immune response   biological process:  positive regulation of T cell proliferation   biological process:  regulation of myeloid cell differentiation   biological process:  induction of positive chemotaxis      BioCarta Pathways:     KEGG Pathways:  
    Probe set : 1368236_at    Name:   meprin 1 alpha    GB acc:   NM_013143    UniGene:   Rn.16265    Gene Symbol:   Mep1a    Locus Link:   25684    Chromosome:   9    Cytoband:   Chr:9q12    SOURCE    GENECARD       Gene Ontology:   biological process:  proteolysis   biological process:  proteolysis   biological process:  digestion      BioCarta Pathways:     KEGG Pathways:  
    Probe set : 1384280_at    Name:   Nucleolar and spindle associated protein 1 (predicted)    GB acc:   AA964152    UniGene:   Rn.106933     Locus Link:   311336    Chromosome:   3    Cytoband:   Chr:3q35    SOURCE        Gene Ontology:   cellular component:  nucleus   cellular component:  nucleolus   cellular component:  spindle microtubule   molecular function:  microtubule binding      BioCarta Pathways:     KEGG Pathways:  
    Probe set : 1369499_at    Name:   thymidylate synthase    GB acc:   NM_019179    UniGene:   Rn.53975    Gene Symbol:   Tyms    Locus Link:   29261    Chromosome:   9    Cytoband:   Chr:9q38    SOURCE    GENECARD       Gene Ontology:     BioCarta Pathways:     KEGG Pathways:  
    Probe set : 1398117_at    Name:   Similar to RIKEN cDNA 2700067D09 (predicted)    GB acc:   AW533235    UniGene:   Rn.23071    Gene Symbol:   MGC94228    Locus Link:   302937    Chromosome:   10    Cytoband:   Chr:10q12    SOURCE    GENECARD       Gene Ontology:   biological process:  regulation of transcription, DNA-dependent   molecular function:  DNA binding   molecular function:  transcription factor activity      BioCarta Pathways:     KEGG Pathways:  
    Probe set : 1383940_at    Name:   cell division cycle associated 1 (predicted)    GB acc:   BG375704    UniGene:   Rn.41428    Gene Symbol:   Cdca1_predicted    Locus Link:   304951    Chromosome:   13    Cytoband:   Chr:13q24    SOURCE    GENECARD       Gene Ontology:   biological process:  chromosome segregation   biological process:  attachment of spindle microtubules to kinetochore   biological process:  cell division   cellular component:  chromosome, pericentric region   cellular component:  kinetochore      BioCarta Pathways:     KEGG Pathways:  
    Probe set : 1388439_at    Name:   similar to 65kDa FK506-binding protein    GB acc:   BI282959    UniGene:   Rn.62446    Gene Symbol:   LOC360627    Locus Link:   360627    Chromosome:   10    Cytoband:   Chr:10q32.1    SOURCE    GENECARD       Gene Ontology:   biological process:  protein folding   molecular function:  calcium ion binding      BioCarta Pathways:     KEGG Pathways:  
    Probe set : 1387892_at    Name:   tubulin, beta 5    GB acc:   BI285440    UniGene:   Rn.2458    Gene Symbol:   Tubb5    Locus Link:   29214    Chromosome:   20    Cytoband:   Chr:20p12    SOURCE    GENECARD       Gene Ontology:     BioCarta Pathways:     KEGG Pathways:  
    Probe set : 1368207_at    Name:   FXYD domain-containing ion transport regulator 5    GB acc:   NM_021909    UniGene:   Rn.24997    Gene Symbol:   Fxyd5    Locus Link:   60338    Chromosome:   1    Cytoband:   Chr:1q21    SOURCE    GENECARD       Gene Ontology:   biological process:  ion transport   biological process:  transport   cellular component:  extracellular space   cellular component:  membrane   cellular component:  integral to membrane   molecular function:  ion channel activity      BioCarta Pathways:     KEGG Pathways:  
    Probe set : 1373401_at    Name:   Transcribed locus    GB acc:   AI176034    UniGene:   Rn.152256        SOURCE        Gene Ontology:     BioCarta Pathways:     KEGG Pathways:  
    Probe set : 1373838_at    Name:   fucosyltransferase 4    GB acc:   AI237192    UniGene:   Rn.44467    Gene Symbol:   Fut4    Locus Link:   60670    Chromosome:   8    Cytoband:   Chr:8q11    SOURCE    GENECARD       Gene Ontology:   biological process:  protein amino acid glycosylation      BioCarta Pathways:     KEGG Pathways:  
    Probe set : 1387946_at    Name:   lectin, galactoside-binding, soluble, 3 binding protein    GB acc:   AF065438    UniGene:   Rn.3251    Gene Symbol:   Lgals3bp    Locus Link:   245955    Chromosome:   10    Cytoband:   Chr:10q32.3    SOURCE    GENECARD       Gene Ontology:   cellular component:  extracellular space   cellular component:  membrane   molecular function:  scavenger receptor activity   molecular function:  protein binding   molecular function:  isomerase activity      BioCarta Pathways:     KEGG Pathways:  
    Probe set : 1396055_at    Name:   procollagen, type VI, alpha 3 (predicted)    GB acc:   AW530165    UniGene:   Rn.2157    Gene Symbol:   Col6a3_predicted    Locus Link:   367313    Chromosome:   9    Cytoband:   Chr:9q35-q36    SOURCE    GENECARD       Gene Ontology:   biological process:  phosphate transport   biological process:  cell adhesion   biological process:  muscle development      BioCarta Pathways:     KEGG Pathways:  
    Probe set : 1383805_at    Name:   similar to Traf2 and NCK interacting kinase, splice variant 4    GB acc:   BG666454    UniGene:   Rn.23142    Gene Symbol:   LOC294917    Locus Link:   294917    Chromosome:   2    Cytoband:   Chr:2q24    SOURCE    GENECARD       Gene Ontology:     BioCarta Pathways:     KEGG Pathways:  
    Probe set : 1372685_at    Name:   cyclin-dependent kinase inhibitor 3 (predicted)    GB acc:   BE113362    UniGene:   Rn.107220    Gene Symbol:   Cdkn3_predicted    Locus Link:   289993    Chromosome:   15    Cytoband:   Chr:15p14    SOURCE    GENECARD       Gene Ontology:     BioCarta Pathways:     KEGG Pathways:  
    Probe set : 1389538_at    Name:   nuclear factor of kappa light chain gene enhancer in B-cells inhibitor, alpha    GB acc:   AW672589    UniGene:   Rn.12550    Gene Symbol:   Nfkbia    Locus Link:   25493    Chromosome:   6    Cytoband:   Chr:6q23    SOURCE    GENECARD       Gene Ontology:   cellular component:  nucleus   cellular component:  cytoplasm   cellular component:  cytosol   molecular function:  protein binding   molecular function:  transcription factor binding      BioCarta Pathways:     KEGG Pathways:  
    Probe set : 1394129_at    Name:   Activin receptor interacting protein 1    GB acc:   AA850692    UniGene:   Rn.7033    Gene Symbol:   Acvrip1    Locus Link:   113970    Chromosome:   4    Cytoband:   Chr:4q11    SOURCE    GENECARD       Gene Ontology:   biological process:  signal transduction   biological process:  intracellular signaling cascade   biological process:  intracellular signaling cascade   molecular function:  signal transducer activity   molecular function:  protein binding   molecular function:  protein binding      BioCarta Pathways:     KEGG Pathways:  
    Probe set : 1388484_at    Name:   ubiquitin-conjugating enzyme E2C (predicted)    GB acc:   BI296084    UniGene:   Rn.3102    Gene Symbol:   Ube2c_predicted    Locus Link:   296368    Chromosome:   3    Cytoband:   Chr:3q42    SOURCE    GENECARD       Gene Ontology:   molecular function:  ubiquitin conjugating enzyme activity   molecular function:  ubiquitin-protein ligase activity   molecular function:  ligase activity      BioCarta Pathways:     KEGG Pathways:  
    Probe set : 1391387_s_at    Name:   Transcribed locus    GB acc:   AI072956    UniGene:   Rn.20311        SOURCE        Gene Ontology:     BioCarta Pathways:     KEGG Pathways:  
    Probe set : 1382153_at    Name:   C-type (calcium dependent, carbohydrate recognition domain) lectin, superfamily member 6    GB acc:   AI171821    UniGene:   Rn.24032    Gene Symbol:   RGD:1359662    Locus Link:   474143    Chromosome:   4    Cytoband:   Chr:4q42    SOURCE    GENECARD       Gene Ontology:   biological process:  immune response   cellular component:  integral to membrane   molecular function:  sugar binding   molecular function:  receptor activity      BioCarta Pathways:     KEGG Pathways:  
    Probe set : 1388866_at    Name:   Transcribed locus    GB acc:   AA799392    UniGene:   Rn.6181        SOURCE        Gene Ontology:     BioCarta Pathways:     KEGG Pathways:  
    Probe set : 1373785_at    Name:   similar to expressed sequence AI132321    GB acc:   AA848738    UniGene:   Rn.6379    Gene Symbol:   LOC362792    Locus Link:   362792    Chromosome:   6    Cytoband:   Chr:6q32    SOURCE    GENECARD       Gene Ontology:     BioCarta Pathways:     KEGG Pathways:  
    Probe set : 1372565_at    Name:   similar to toll-associated serine protease (predicted)    GB acc:   BI274192    UniGene:   Rn.15228    Gene Symbol:   RGD1308120_predicted    Locus Link:   360959    Chromosome:   14    Cytoband:   Chr:14q21    SOURCE    GENECARD       Gene Ontology:     BioCarta Pathways:     KEGG Pathways:  
    Probe set : 1368822_at    Name:   follistatin-like 1    GB acc:   BI290885    UniGene:   Rn.95652    Gene Symbol:   Fstl1    Locus Link:   79210    Chromosome:   11    Cytoband:   Chr:11q21    SOURCE    GENECARD       Gene Ontology:   cellular component:  extracellular space   molecular function:  calcium ion binding   molecular function:  heparin binding      BioCarta Pathways:     KEGG Pathways:  
    Probe set : 1388939_at    Name:   procollagen, type XV (predicted)    GB acc:   AA800298    UniGene:   Rn.31832    Gene Symbol:   Col15a1_predicted    Locus Link:   298069    Chromosome:   5    Cytoband:   Chr:5q22    SOURCE    GENECARD       Gene Ontology:   biological process:  phosphate transport   biological process:  cell adhesion      BioCarta Pathways:     KEGG Pathways:  
    Probe set : 1376943_at     GB acc:   BM386570              Gene Ontology:     BioCarta Pathways:     KEGG Pathways:  
    Probe set : 1373000_at    Name:   similar to Sushi-repeat containing protein    GB acc:   AA818334    UniGene:   Rn.33831    Gene Symbol:   LOC317181    Locus Link:   317181    Chromosome:   X    Cytoband:   Chr:Xq34    SOURCE    GENECARD       Gene Ontology:     BioCarta Pathways:     KEGG Pathways:  
    Probe set : 1370462_at    Name:   hyaluronan mediated motility receptor    GB acc:   AI171185    UniGene:   Rn.92304    Gene Symbol:   Hmmr    Locus Link:   25460    Chromosome:   10    Cytoband:   Chr:10q12    SOURCE    GENECARD       Gene Ontology:   biological process:  cell motility   biological process:  cytoskeleton organization and biogenesis   biological process:  cell-cell signaling   molecular function:  hyaluronic acid binding   molecular function:  hyaluronic acid binding   molecular function:  receptor activity      BioCarta Pathways:     KEGG Pathways:  
    Probe set : 1369947_at    Name:   cathepsin K    GB acc:   NM_031560    UniGene:   Rn.5598    Gene Symbol:   Ctsk    Locus Link:   29175    Chromosome:   2    Cytoband:   Chr:2q34    SOURCE    GENECARD       Gene Ontology:   biological process:  proteolysis   biological process:  proteolysis   cellular component:  extracellular space   cellular component:  lysosome      BioCarta Pathways:     KEGG Pathways:  
    Probe set : 1377369_at    Name:   cytochrome b reductase 1 (predicted)    GB acc:   BF419070    UniGene:   Rn.13660    Gene Symbol:   Cybrd1_predicted    Locus Link:   295669    Chromosome:   3    Cytoband:   Chr:3q21    SOURCE    GENECARD       Gene Ontology:   biological process:  electron transport   cellular component:  integral to membrane   molecular function:  transferase activity      BioCarta Pathways:     KEGG Pathways:  
    Probe set : 1397704_at    Name:   similar to establishment of cohesion 1 homolog 2    GB acc:   BF407449     Gene Symbol:   LOC498536    Locus Link:   498536    Chromosome:   15    Cytoband:   Chr:15p12     GENECARD       Gene Ontology:     BioCarta Pathways:     KEGG Pathways:  
    Probe set : 1368404_at    Name:   drebrin 1    GB acc:   NM_031024    UniGene:   Rn.11247    Gene Symbol:   Dbn1    Locus Link:   81653    Chromosome:   17    Cytoband:   Chr:17p14    SOURCE    GENECARD       Gene Ontology:   molecular function:  actin binding   molecular function:  profilin binding   molecular function:  profilin binding      BioCarta Pathways:     KEGG Pathways:  
    Probe set : 1386935_at    Name:   nuclear receptor subfamily 4, group A, member 1    GB acc:   NM_024388    UniGene:   Rn.10000    Gene Symbol:   Nr4a1    Locus Link:   79240    Chromosome:   7    Cytoband:   Chr:7q36    SOURCE    GENECARD       Gene Ontology:   cellular component:  nucleus      BioCarta Pathways:     KEGG Pathways:  
    Probe set : 1373914_at    Name:   Stem cell growth factor    GB acc:   BM389075    UniGene:   Rn.30828    Gene Symbol:   Scgf    Locus Link:   29313    Chromosome:   1    Cytoband:   Chr:1q22    SOURCE    GENECARD       Gene Ontology:   biological process:  positive regulation of cell proliferation   cellular component:  extracellular region   cellular component:  extracellular space   molecular function:  nucleic acid binding   molecular function:  sugar binding   molecular function:  growth factor activity      BioCarta Pathways:     KEGG Pathways:  
    Probe set : 1378819_at    Name:   similar to RIKEN cDNA 0610012D14 (predicted)    GB acc:   AW435096    UniGene:   Rn.54118    Gene Symbol:   RGD1310111_predicted    Locus Link:   292875    Chromosome:   1    Cytoband:   Chr:1q22    SOURCE    GENECARD       Gene Ontology:   biological process:  lysine biosynthesis   biological process:  diaminopimelate biosynthesis   biological process:  pyridine nucleotide biosynthesis   molecular function:  oxidoreductase activity      BioCarta Pathways:     KEGG Pathways:  
    Probe set : 1367712_at    Name:   tissue inhibitor of metalloproteinase 1    GB acc:   NM_053819    UniGene:   Rn.25754    Gene Symbol:   Timp1    Locus Link:   116510    Chromosome:   X    Cytoband:   Chr:Xq12    SOURCE    GENECARD       Gene Ontology:   biological process:  C21-steroid hormone biosynthesis   biological process:  erythrocyte maturation   cellular component:  extracellular matrix (sensu Metazoa)   cellular component:  basement membrane   cellular component:  extracellular space   molecular function:  metalloendopeptidase inhibitor activity   molecular function:  metalloendopeptidase inhibitor activity   molecular function:  enzyme inhibitor activity      BioCarta Pathways:     KEGG Pathways:  
    Probe set : 1367655_at    Name:   similar to thymosin, beta 10    GB acc:   NM_021261     Gene Symbol:   LOC502600    Locus Link:   502600    Chromosome:   2    Cytoband:   Chr:2q44     GENECARD       Gene Ontology:     BioCarta Pathways:     KEGG Pathways:  
    Probe set : 1382580_at    Name:   Transcribed locus    GB acc:   AI555526    UniGene:   Rn.38384        SOURCE        Gene Ontology:     BioCarta Pathways:     KEGG Pathways:  
    Probe set : 1388392_at    Name:   Tax1 (human T-cell leukemia virus type I) binding protein 3 (predicted)    GB acc:   BI274159    UniGene:   Rn.145939    Gene Symbol:   Tax1bp3_predicted    Locus Link:   360564    Chromosome:   10    Cytoband:   Chr:10q24    SOURCE    GENECARD       Gene Ontology:   biological process:  intracellular signaling cascade   biological process:  negative regulation of cell proliferation   cellular component:  nucleus   cellular component:  cytoplasm   molecular function:  protein binding   molecular function:  beta-catenin binding   molecular function:  transcription regulator activity      BioCarta Pathways:     KEGG Pathways:  
    Probe set : 1367725_at    Name:   serine/threonine-protein kinase pim-3    GB acc:   NM_022602    UniGene:   Rn.6343    Gene Symbol:   Pim3    Locus Link:   64534    Chromosome:   7    Cytoband:   Chr:7q34    SOURCE    GENECARD       Gene Ontology:     BioCarta Pathways:     KEGG Pathways:  
    Probe set : 1370353_at    Name:   translocase of inner mitochondrial membrane 22 homolog (yeast)    GB acc:   AF223951    UniGene:   Rn.41817    Gene Symbol:   Timm22    Locus Link:   79463    Chromosome:   10    Cytoband:   Chr:10q26    SOURCE    GENECARD       Gene Ontology:   biological process:  intracellular protein transport   biological process:  protein transport   molecular function:  protein transporter activity   molecular function:  protein translocase activity   molecular function:  protein translocase activity      BioCarta Pathways:     KEGG Pathways:  
    Probe set : 1378296_at     GB acc:   AI012651              Gene Ontology:     BioCarta Pathways:     KEGG Pathways:  
    Probe set : 1374726_at    Name:   fibronectin type III domain containing 1 (predicted)    GB acc:   AI411941    UniGene:   Rn.20633    Gene Symbol:   Fndc1_predicted    Locus Link:   308099    Chromosome:   1    Cytoband:   Chr:1q11    SOURCE    GENECARD       Gene Ontology:     BioCarta Pathways:     KEGG Pathways:  
    Probe set : 1399027_at    Name:   ras homolog gene family, member A    GB acc:   AI408053    UniGene:   Rn.107401    Gene Symbol:   RGD:619921    Locus Link:   117273    Chromosome:   16    Cytoband:   Chr:16q12.5    SOURCE    GENECARD       Gene Ontology:   biological process:  cell adhesion   biological process:  cell-matrix adhesion   biological process:  integrin-mediated signaling pathway   biological process:  small GTPase mediated signal transduction   biological process:  Rho protein signal transduction   biological process:  striated muscle development   biological process:  protein transport   biological process:  actin cytoskeleton organization and biogenesis   biological process:  cell differentiation   biological process:  positive regulation of NF-kappaB import into nucleus   biological process:  positive regulation of I-kappaB kinase/NF-kappaB cascade   biological process:  stress fiber formation   biological process:  Rho protein signal transduction   biological process:  actin cytoskeleton organization and biogenesis   biological process:  positive regulation of NF-kappaB import into nucleus   biological process:  positive regulation of I-kappaB kinase/NF-kappaB cascade   cellular component:  cytosol   cellular component:  cytoskeleton   cellular component:  membrane      BioCarta Pathways:     KEGG Pathways:  
    Probe set : 1373204_at    Name:   hypothetical LOC297077 (predicted)    GB acc:   BM388911    UniGene:   Rn.17071    Gene Symbol:   RGD1310725_predicted    Locus Link:   297077    Chromosome:   4    Cytoband:   Chr:4q24    SOURCE    GENECARD       Gene Ontology:   cellular component:  integral to membrane      BioCarta Pathways:     KEGG Pathways:  
    Probe set : 1389858_at    Name:   Thymidine kinase 1    GB acc:   BM389006    UniGene:   Rn.4191    Gene Symbol:   Tk1    Locus Link:   24834    Chromosome:   10    Cytoband:   Chr:10    SOURCE    GENECARD       Gene Ontology:   biological process:  DNA metabolism   biological process:  DNA replication   cellular component:  cytoplasm      BioCarta Pathways:     KEGG Pathways:  
    Probe set : 1368877_at    Name:   zinc finger protein 354A    GB acc:   NM_052798    UniGene:   Rn.11049    Gene Symbol:   Znf354a    Locus Link:   24522    Chromosome:   10    Cytoband:   Chr:10q21    SOURCE    GENECARD       Gene Ontology:   biological process:  regulation of transcription, DNA-dependent   biological process:  development   biological process:  transcription   cellular component:  intracellular   cellular component:  nucleus   cellular component:  transcription factor complex      BioCarta Pathways:     KEGG Pathways:  
    Probe set : 1388879_at     GB acc:   BG669292              Gene Ontology:     BioCarta Pathways:     KEGG Pathways:  
    Probe set : 1369957_at    Name:   regulator of G-protein signaling 5    GB acc:   NM_019341    UniGene:   Rn.1150    Gene Symbol:   Rgs5    Locus Link:   54294    Chromosome:   13    Cytoband:   Chr:13q24    SOURCE    GENECARD       Gene Ontology:   molecular function:  signal transducer activity   molecular function:  GTPase activator activity      BioCarta Pathways:     KEGG Pathways:  
    Probe set : 1367829_at    Name:   enoyl Coenzyme A hydratase, short chain, 1, mitochondrial    GB acc:   NM_078623    UniGene:   Rn.6847    Gene Symbol:   Echs1    Locus Link:   140547    Chromosome:   1    Cytoband:   Chr:1q41    SOURCE    GENECARD       Gene Ontology:   cellular component:  mitochondrion   cellular component:  mitochondrion      BioCarta Pathways:     KEGG Pathways:  
    Probe set : 1393252_at    Name:   Fibulin 1 (predicted)    GB acc:   AA996804    UniGene:   Rn.9375     Locus Link:   315191    Chromosome:   7    Cytoband:   Chr:7q34    SOURCE        Gene Ontology:   biological process:  chitin metabolism   molecular function:  extracellular matrix structural constituent   molecular function:  calcium ion binding   molecular function:  chitin binding      BioCarta Pathways:     KEGG Pathways:  
    Probe set : 1389114_at    Name:   similar to Hypothetical protein MGC59076 (predicted)    GB acc:   AW523874    UniGene:   Rn.16112    Gene Symbol:   RGD1309144_predicted    Locus Link:   297332    Chromosome:   4    Cytoband:   Chr:4q32    SOURCE    GENECARD       Gene Ontology:   biological process:  threonine biosynthesis   biological process:  metabolism   molecular function:  catalytic activity   molecular function:  threonine synthase activity      BioCarta Pathways:     KEGG Pathways:  
    Probe set : 1379235_x_at    Name:   similar to CDC45L    GB acc:   AI179539    UniGene:   Rn.98535    Gene Symbol:   LOC287961    Locus Link:   287961    Chromosome:   11    Cytoband:   Chr:11p12    SOURCE    GENECARD       Gene Ontology:     BioCarta Pathways:     KEGG Pathways:  
    Probe set : 1371071_at    Name:   guanine nucleotide binding protein beta 4 subunit    GB acc:   BI284800    UniGene:   Rn.10844    Gene Symbol:   RGD:1359321    Locus Link:   294962    Chromosome:   2    Cytoband:   Chr:2q25    SOURCE    GENECARD       Gene Ontology:   biological process:  signal transduction   biological process:  G-protein coupled receptor protein signaling pathway   cellular component:  heterotrimeric G-protein complex   molecular function:  signal transducer activity      BioCarta Pathways:     KEGG Pathways:  
    Probe set : 1372186_a_at    Name:   topoisomerase (DNA) 2 alpha    GB acc:   BM385445    UniGene:   Rn.90996    Gene Symbol:   Top2a    Locus Link:   360243    Chromosome:   10    Cytoband:   Chr:10q31    SOURCE    GENECARD       Gene Ontology:     BioCarta Pathways:     KEGG Pathways:  
    Probe set : 1398514_at    Name:   homogentisate 1, 2-dioxygenase (predicted)    GB acc:   AI232328    UniGene:   Rn.145148    Gene Symbol:   Hgd_predicted    Locus Link:   360719    Chromosome:   11    Cytoband:   Chr:11q21    SOURCE    GENECARD       Gene Ontology:   biological process:  L-phenylalanine catabolism   biological process:  tyrosine metabolism   biological process:  tyrosine catabolism      BioCarta Pathways:     KEGG Pathways:  
    Probe set : 1383420_at    Name:   Similar to lactation elevated 1    GB acc:   BM390554    UniGene:   Rn.24314     Locus Link:   502479      SOURCE        Gene Ontology:     BioCarta Pathways:     KEGG Pathways:  
    Probe set : 1383404_at    Name:   Transcribed locus    GB acc:   BI295203    UniGene:   Rn.147831        SOURCE        Gene Ontology:     BioCarta Pathways:     KEGG Pathways:  
    Probe set : 1367732_at    Name:   guanine nucleotide binding protein, beta 1    GB acc:   U34958    UniGene:   Rn.126047    Gene Symbol:   Gnb1    Locus Link:   24400    Chromosome:   5    Cytoband:   Chr:5q36    SOURCE    GENECARD       Gene Ontology:   cellular component:  heterotrimeric G-protein complex   cellular component:  heterotrimeric G-protein complex   cellular component:  membrane   molecular function:  GTPase activity   molecular function:  signal transducer activity      BioCarta Pathways:     KEGG Pathways:  
    Probe set : 1397769_at    Name:   Similar to RIKEN cDNA 6330512M04 gene    GB acc:   BI303199    UniGene:   Rn.14618     Locus Link:   293632    Chromosome:   1    Cytoband:   Chr:1q41    SOURCE        Gene Ontology:     BioCarta Pathways:     KEGG Pathways:  
    Probe set : 1383246_at    Name:   Similar to Hypothetical protein A630084C13 (predicted)    GB acc:   AI146111    UniGene:   Rn.144716     Locus Link:   287827    Chromosome:   10    Cytoband:   Chr:10q32.3    SOURCE        Gene Ontology:     BioCarta Pathways:     KEGG Pathways:  
    Probe set : 1372042_at    Name:   chemokine-like factor super family 3 (predicted)    GB acc:   BI294844    UniGene:   Rn.15332    Gene Symbol:   Cklfsf3_predicted    Locus Link:   291813    Chromosome:   19    Cytoband:   Chr:19p14    SOURCE    GENECARD       Gene Ontology:   biological process:  chemotaxis   cellular component:  membrane   cellular component:  integral to membrane   molecular function:  cytokine activity      BioCarta Pathways:     KEGG Pathways:  
    Probe set : 1398343_at    Name:   DnaJ (Hsp40) homolog, subfamily A, member 4 (predicted)    GB acc:   AI104324    UniGene:   Rn.27927     Locus Link:   300721    Chromosome:   8    Cytoband:   Chr:8q24    SOURCE        Gene Ontology:   biological process:  protein folding   molecular function:  heat shock protein binding   molecular function:  unfolded protein binding      BioCarta Pathways:     KEGG Pathways:  
    Probe set : 1377812_a_at    Name:   transmembrane protein 24 (predicted)    GB acc:   BG378195    UniGene:   Rn.57410    Gene Symbol:   Tmem24_predicted    Locus Link:   300666    Chromosome:   8    Cytoband:   Chr:8q22    SOURCE    GENECARD       Gene Ontology:   cellular component:  integral to membrane      BioCarta Pathways:     KEGG Pathways:  
    Probe set : 1371700_at    Name:   Microfibrillar-associated protein 4 (predicted)    GB acc:   AI177059    UniGene:   Rn.12328     Locus Link:   287382    Chromosome:   10    Cytoband:   Chr:10q23    SOURCE        Gene Ontology:   biological process:  cell adhesion   cellular component:  microfibril   cellular component:  extracellular matrix (sensu Metazoa)   cellular component:  extracellular space   molecular function:  protein binding      BioCarta Pathways:     KEGG Pathways:  
    Probe set : 1383085_at    Name:   Similar to Sh3bgrl protein    GB acc:   BG672648    UniGene:   Rn.58623     Locus Link:   302363    Chromosome:   X    Cytoband:   Chr:Xq31    SOURCE        Gene Ontology:     BioCarta Pathways:     KEGG Pathways:  
    Probe set : 1383323_at    Name:   similar to RIKEN cDNA 1700022L09    GB acc:   AW435234    UniGene:   Rn.117385    Gene Symbol:   LOC499933    Locus Link:   499933    Chromosome:   3    Cytoband:   Chr:3q42    SOURCE    GENECARD       Gene Ontology:     BioCarta Pathways:     KEGG Pathways:  
    Probe set : 1371012_at    Name:   2-hydroxyphytanoyl-Coenzyme A lyase    GB acc:   AJ245707    UniGene:   Rn.21425    Gene Symbol:   Hpcl2    Locus Link:   85255    Chromosome:   16    Cytoband:   Chr:16p16    SOURCE    GENECARD       Gene Ontology:     BioCarta Pathways:     KEGG Pathways:  
    Probe set : 1370209_at    Name:   basic transcription element binding protein 1    GB acc:   BE101336    UniGene:   Rn.19481    Gene Symbol:   Bteb1    Locus Link:   117560    Chromosome:   1    Cytoband:   Chr:1q51    SOURCE    GENECARD       Gene Ontology:   cellular component:  nucleus      BioCarta Pathways:     KEGG Pathways:  
    Probe set : 1367749_at    Name:   lumican    GB acc:   NM_031050    UniGene:   Rn.3087    Gene Symbol:   Lum    Locus Link:   81682    Chromosome:   7    Cytoband:   Chr:7q13    SOURCE    GENECARD       Gene Ontology:   cellular component:  extracellular matrix (sensu Metazoa)   cellular component:  extracellular space      BioCarta Pathways:     KEGG Pathways:  
    Probe set : 1392672_at    Name:   stem cell growth factor    GB acc:   AI576758    UniGene:   Rn.30828    Gene Symbol:   Scgf    Locus Link:   29313    Chromosome:   1    Cytoband:   Chr:1q22    SOURCE    GENECARD       Gene Ontology:   biological process:  positive regulation of cell proliferation   cellular component:  extracellular region   cellular component:  extracellular space   molecular function:  nucleic acid binding   molecular function:  sugar binding   molecular function:  growth factor activity      BioCarta Pathways:     KEGG Pathways:  
    Probe set : 1370127_at    Name:   polymerase (DNA directed), delta 1, catalytic subunit    GB acc:   NM_021662    UniGene:   Rn.88690    Gene Symbol:   Pold1    Locus Link:   59294    Chromosome:   1    Cytoband:   Chr:1q22    SOURCE    GENECARD       Gene Ontology:   cellular component:  nucleus   cellular component:  delta DNA polymerase complex   molecular function:  nucleotide binding   molecular function:  DNA binding   molecular function:  DNA-directed DNA polymerase activity   molecular function:  alpha DNA polymerase activity   molecular function:  beta DNA polymerase activity   molecular function:  delta DNA polymerase activity   molecular function:  epsilon DNA polymerase activity   molecular function:  zeta DNA polymerase activity   molecular function:  gamma DNA-directed DNA polymerase activity   molecular function:  exonuclease activity   molecular function:  3'-5' exonuclease activity   molecular function:  eta DNA polymerase activity   molecular function:  iota DNA polymerase activity   molecular function:  mu DNA polymerase activity   molecular function:  lambda DNA polymerase activity   molecular function:  kappa DNA polymerase activity   molecular function:  nu DNA polymerase activity   molecular function:  theta DNA polymerase activity   molecular function:  transferase activity   molecular function:  hydrolase activity   molecular function:  sigma DNA polymerase activity   molecular function:  protein binding   molecular function:  nuclease activity   molecular function:  zinc ion binding   molecular function:  nucleotidyltransferase activity   molecular function:  metal ion binding      BioCarta Pathways:     KEGG Pathways:  
    Probe set : 1389566_at    Name:   cyclin B2 (predicted)    GB acc:   AW253821    UniGene:   Rn.6743    Gene Symbol:   Ccnb2_predicted    Locus Link:   363088    Chromosome:   8    Cytoband:   Chr:8q24    SOURCE    GENECARD       Gene Ontology:   cellular component:  nucleus   molecular function:  cyclin-dependent protein kinase regulator activity      BioCarta Pathways:     KEGG Pathways:  
    Probe set : 1371573_at    Name:   ribosomal protein L36a (predicted)    GB acc:   AW142090    UniGene:   Rn.144788    Gene Symbol:   Rpl36a_predicted    Locus Link:   292964    Chromosome:   X    Cytoband:   Chr:Xq34    SOURCE    GENECARD       Gene Ontology:   biological process:  protein biosynthesis   biological process:  ribosome biogenesis   cellular component:  intracellular   cellular component:  cytosolic ribosome (sensu Eukaryota)   cellular component:  ribosome   molecular function:  structural constituent of ribosome      BioCarta Pathways:     KEGG Pathways:  
    Probe set : 1371365_at    Name:   similar to Ubiquitin-conjugating enzyme E2S    GB acc:   BI279788    UniGene:   Rn.120398    Gene Symbol:   LOC292588    Locus Link:   292588    Chromosome:   1    Cytoband:   Chr:1q12    SOURCE    GENECARD       Gene Ontology:     BioCarta Pathways:     KEGG Pathways:  
    Probe set : 1389413_at    Name:   Oligodendrocyte-myelin glycoprotein    GB acc:   BI279325    UniGene:   Rn.11850    Gene Symbol:   Omg    Locus Link:   450224      SOURCE    GENECARD       Gene Ontology:   cellular component:  integral to membrane      BioCarta Pathways:     KEGG Pathways:  
    Probe set : 1388650_at    Name:   topoisomerase (DNA) 2 alpha    GB acc:   BM385445    UniGene:   Rn.90996    Gene Symbol:   Top2a    Locus Link:   360243    Chromosome:   10    Cytoband:   Chr:10q31    SOURCE    GENECARD       Gene Ontology:     BioCarta Pathways:     KEGG Pathways:  
    Probe set : 1368773_at    Name:   follicle stimulating hormone primary response gene 1    GB acc:   NM_012955    UniGene:   Rn.10411    Gene Symbol:   Fshprh1    Locus Link:   25448    Chromosome:   X    Cytoband:   Chr:Xq34    SOURCE    GENECARD       Gene Ontology:   biological process:  spermatogenesis   biological process:  female gamete generation   biological process:  sex differentiation      BioCarta Pathways:     KEGG Pathways:  
    Probe set : 1367802_at    Name:   serum/glucocorticoid regulated kinase    GB acc:   NM_019232    UniGene:   Rn.4636    Gene Symbol:   Sgk    Locus Link:   29517    Chromosome:   1    Cytoband:   Chr:1p12    SOURCE    GENECARD       Gene Ontology:   cellular component:  nucleus      BioCarta Pathways:     KEGG Pathways:  
    Probe set : 1372013_at    Name:   interferon induced transmembrane protein 1 (predicted)    GB acc:   BG380285    UniGene:   Rn.22087    Gene Symbol:   Ifitm1_predicted    Locus Link:   293618    Chromosome:   1    Cytoband:   Chr:1q41    SOURCE    GENECARD       Gene Ontology:   cellular component:  plasma membrane   cellular component:  integral to membrane   molecular function:  receptor signaling protein activity      BioCarta Pathways:     KEGG Pathways:  
    Probe set : 1386045_at    Name:   Transcribed locus    GB acc:   AA893205    UniGene:   Rn.143823        SOURCE        Gene Ontology:     BioCarta Pathways:     KEGG Pathways:  
    Probe set : 1367691_at    Name:   protein kinase C, delta binding protein    GB acc:   NM_134449    UniGene:   Rn.12281    Gene Symbol:   Prkcdbp    Locus Link:   85332    Chromosome:   1    Cytoband:   Chr:1q33    SOURCE    GENECARD       Gene Ontology:   biological process:  regulation of cell growth   molecular function:  protein kinase C binding   molecular function:  kinase activity      BioCarta Pathways:     KEGG Pathways:  
    Probe set : 1387531_at    Name:   methionine sulfoxide reductase A    GB acc:   NM_053307    UniGene:   Rn.41704    Gene Symbol:   Msra    Locus Link:   29447    Chromosome:   15    Cytoband:   Chr:15p12    SOURCE    GENECARD       Gene Ontology:     BioCarta Pathways:     KEGG Pathways:  
    Probe set : 1368025_at    Name:   DNA-damage-inducible transcript 4    GB acc:   NM_080906    UniGene:   Rn.9775    Gene Symbol:   Ddit4    Locus Link:   140942    Chromosome:   20    Cytoband:   Chr:20q11    SOURCE    GENECARD       Gene Ontology:     BioCarta Pathways:     KEGG Pathways:  
    Probe set : 1376702_at    Name:   Megalencephalic leukoencephalopathy with subcortical cysts 1 (predicted)    GB acc:   AI228307    UniGene:   Rn.98819     Locus Link:   315215    Chromosome:   7    Cytoband:   Chr:7q34    SOURCE        Gene Ontology:   biological process:  transport   cellular component:  integral to membrane of membrane fraction   cellular component:  integral to membrane   molecular function:  ion channel activity      BioCarta Pathways:     KEGG Pathways:  
    Probe set : 1373897_at     GB acc:   AI103106              Gene Ontology:     BioCarta Pathways:     KEGG Pathways:  
    Probe set : 1376889_at     GB acc:   AI599549              Gene Ontology:     BioCarta Pathways:     KEGG Pathways:  
    Probe set : 1372341_at    Name:   Transcribed locus, strongly similar to XP_484968.2 PREDICTED: similar to RIKEN cDNA C330005L02 [Mus musculus]    GB acc:   AI233213    UniGene:   Rn.17305        SOURCE        Gene Ontology:     BioCarta Pathways:     KEGG Pathways:  
    Probe set : 1367568_a_at    Name:   matrix Gla protein    GB acc:   NM_012862    UniGene:   Rn.2379    Gene Symbol:   Mgp    Locus Link:   25333    Chromosome:   4    Cytoband:   Chr:4q43    SOURCE    GENECARD       Gene Ontology:   cellular component:  extracellular region   cellular component:  extracellular matrix (sensu Metazoa)   cellular component:  extracellular space   molecular function:  extracellular matrix structural constituent   molecular function:  calcium ion binding   molecular function:  structural constituent of bone      BioCarta Pathways:     KEGG Pathways:  
    Probe set : 1374013_at    Name:   C1q and tumor necrosis factor related protein 5 (predicted)    GB acc:   BI278078    UniGene:   Rn.99884    Gene Symbol:   C1qtnf5_predicted    Locus Link:   315598    Chromosome:   8    Cytoband:   Chr:8q22    SOURCE    GENECARD       Gene Ontology:   biological process:  phosphate transport   cellular component:  cytoplasm      BioCarta Pathways:     KEGG Pathways:  
    Probe set : 1391139_at    Name:   Transcribed locus    GB acc:   BF397925    UniGene:   Rn.49618        SOURCE        Gene Ontology:     BioCarta Pathways:     KEGG Pathways:  
    Probe set : 1379425_at    Name:   similar to RIKEN cDNA 3110037I16 (predicted)    GB acc:   AI549208    UniGene:   Rn.35470    Gene Symbol:   RGD1309802_predicted    Locus Link:   313563    Chromosome:   5    Cytoband:   Chr:5q36    SOURCE    GENECARD       Gene Ontology:     BioCarta Pathways:     KEGG Pathways:  
    Probe set : 1370585_a_at    Name:   protein kinase C, beta 1    GB acc:   X04440    UniGene:   Rn.91118    Gene Symbol:   Prkcb1    Locus Link:   25023    Chromosome:   1    Cytoband:   Chr:1q36    SOURCE    GENECARD       Gene Ontology:   cellular component:  nucleus   cellular component:  cytoplasm   cellular component:  plasma membrane   cellular component:  membrane   molecular function:  protein kinase activity   molecular function:  protein serine/threonine kinase activity   molecular function:  protein kinase CK2 activity   molecular function:  cAMP-dependent protein kinase activity   molecular function:  protein kinase C activity   molecular function:  protein kinase C activity   molecular function:  protein-tyrosine kinase activity   molecular function:  calcium channel regulator activity   molecular function:  calcium ion binding   molecular function:  protein binding   molecular function:  ATP binding   molecular function:  transferase activity   molecular function:  diacylglycerol binding   molecular function:  nucleotide binding   molecular function:  zinc ion binding   molecular function:  kinase activity   molecular function:  metal ion binding      BioCarta Pathways:     KEGG Pathways:  
    Probe set : 1367658_at    Name:   SH3/ankyrin domain gene 3 /// hypothetical gene supported by NM_021676    GB acc:   NM_021676    UniGene:   Rn.42876    Gene Symbol:   Shank3 /// LOC497826    Locus Link:   497826 /// 59312    Chromosome:   7    Cytoband:   Chr:7q34    SOURCE    GENECARD       Gene Ontology:   molecular function:  DNA binding   molecular function:  transcription factor activity   molecular function:  protein binding      BioCarta Pathways:     KEGG Pathways:  
    Probe set : 1388784_at    Name:   colony stimulating factor 1 receptor    GB acc:   BI285793    UniGene:   Rn.72599    Gene Symbol:   Csf1r    Locus Link:   307403    Chromosome:   18    Cytoband:   Chr:18q12.1    SOURCE    GENECARD       Gene Ontology:     BioCarta Pathways:     KEGG Pathways:  
    Probe set : 1389873_at    Name:   apoptosis-associated speck-like protein containing a CARD    GB acc:   BI282953    UniGene:   Rn.7817    Gene Symbol:   Pycard    Locus Link:   282817    Chromosome:   1    Cytoband:   Chr:1q36    SOURCE    GENECARD       Gene Ontology:   biological process:  induction of apoptosis   biological process:  regulation of apoptosis   cellular component:  intracellular   molecular function:  protein binding      BioCarta Pathways:     KEGG Pathways:  
    Probe set : 1378633_at    Name:   leupaxin (predicted)    GB acc:   BE106297    UniGene:   Rn.29200    Gene Symbol:   Lpxn_predicted    Locus Link:   293783    Chromosome:   1    Cytoband:   Chr:1q43    SOURCE    GENECARD       Gene Ontology:   biological process:  protein complex assembly   biological process:  cell adhesion   biological process:  signal transduction   cellular component:  cytoplasm   cellular component:  integral to membrane   molecular function:  zinc ion binding   molecular function:  metal ion binding      BioCarta Pathways:     KEGG Pathways:  
    Probe set : 1367563_at    Name:   secreted acidic cysteine rich glycoprotein    GB acc:   D28875    UniGene:   Rn.98989    Gene Symbol:   Sparc    Locus Link:   24791    Chromosome:   10    Cytoband:   Chr:10q22    SOURCE    GENECARD       Gene Ontology:   biological process:  ossification      BioCarta Pathways:     KEGG Pathways:  
    Probe set : 1372158_at    Name:   LRP16 protein    GB acc:   BI295768    UniGene:   Rn.98178    Gene Symbol:   Lrp16    Locus Link:   246233    Chromosome:   1    Cytoband:   Chr:1q43    SOURCE    GENECARD       Gene Ontology:   cellular component:  mitochondrion      BioCarta Pathways:     KEGG Pathways:  
    Probe set : 1377966_at    Name:   Transcribed locus, weakly similar to XP_579465.1 PREDICTED: DNA binding protein N5 [Rattus norvegicus]    GB acc:   BI275560    UniGene:   Rn.22923        SOURCE        Gene Ontology:     BioCarta Pathways:     KEGG Pathways:  
    Probe set : 1393643_at    Name:   reticulocalbin (predicted)    GB acc:   AI112962    UniGene:   Rn.103345    Gene Symbol:   Rcn_predicted    Locus Link:   362182    Chromosome:   3    Cytoband:   Chr:3q32    SOURCE    GENECARD       Gene Ontology:   cellular component:  extracellular space   cellular component:  endoplasmic reticulum   cellular component:  endoplasmic reticulum lumen   molecular function:  calcium ion binding      BioCarta Pathways:     KEGG Pathways:  
    Probe set : 1371340_at    Name:   ribosomal protein, large P2 /// similar to 60S acidic ribosomal protein P2    GB acc:   AW919054    UniGene:   Rn.976    Gene Symbol:   Rplp2 /// LOC498363    Locus Link:   140662 /// 498363    Chromosome:   1    Cytoband:   Chr:1q41 /// Chr:14p11    SOURCE    GENECARD       Gene Ontology:   biological process:  protein biosynthesis   biological process:  translational elongation   molecular function:  RNA binding   molecular function:  structural constituent of ribosome   molecular function:  structural constituent of ribosome      BioCarta Pathways:     KEGG Pathways:  
    Probe set : 1375642_at    Name:   ubiquitin-conjugating enzyme E2B, RAD6 homolog (S. cerevisiae)    GB acc:   AA892795    UniGene:   Rn.20766    Gene Symbol:   Ube2b    Locus Link:   81816    Chromosome:   10    Cytoband:   Chr:10q22    SOURCE    GENECARD       Gene Ontology:   cellular component:  nucleus   cellular component:  membrane      BioCarta Pathways:     KEGG Pathways:  
    Probe set : 1398500_at    Name:   G protein-coupled receptor 116    GB acc:   BI295477    UniGene:   Rn.25073    Gene Symbol:   Gpr116    Locus Link:   245977    Chromosome:   9    Cytoband:   Chr:9q12    SOURCE    GENECARD       Gene Ontology:   biological process:  signal transduction   biological process:  G-protein coupled receptor protein signaling pathway   biological process:  neuropeptide signaling pathway   cellular component:  membrane   cellular component:  integral to membrane      BioCarta Pathways:     KEGG Pathways:  
    Probe set : 1373750_at    Name:   leprecan-like 2 (predicted)    GB acc:   BF406832    UniGene:   Rn.15264    Gene Symbol:   Leprel2_predicted    Locus Link:   297595    Chromosome:   4    Cytoband:   Chr:4q42    SOURCE    GENECARD       Gene Ontology:   biological process:  protein metabolism      BioCarta Pathways:     KEGG Pathways:  
    Probe set : 1368390_at    Name:   v-raf oncogene homolog 1 (murine sarcoma 3611 virus)    GB acc:   NM_022532    UniGene:   Rn.1714    Gene Symbol:   Araf1    Locus Link:   64363    Chromosome:   X    Cytoband:   Chr:Xq11.1    SOURCE    GENECARD       Gene Ontology:   cellular component:  intracellular   cellular component:  insoluble fraction   cellular component:  mitochondrion   cellular component:  cytosol      BioCarta Pathways:     KEGG Pathways:  
    Probe set : 1394419_at    Name:   similar to RIKEN cDNA 6530401L14 gene (predicted)    GB acc:   BE109109    UniGene:   Rn.51673    Gene Symbol:   RGD1309107_predicted    Locus Link:   296060    Chromosome:   3    Cytoband:   Chr:3q34-q35    SOURCE    GENECARD       Gene Ontology:   molecular function:  GTPase activator activity      BioCarta Pathways:     KEGG Pathways:  
    Probe set : 1390680_at    Name:   Transcribed locus    GB acc:   AI059409    UniGene:   Rn.2136        SOURCE        Gene Ontology:     BioCarta Pathways:     KEGG Pathways:  
    Probe set : 1374805_at    Name:   similar to hypothetical protein MGC5528    GB acc:   AW251849    UniGene:   Rn.8559    Gene Symbol:   LOC299933    Locus Link:   299933    Chromosome:   7    Cytoband:   Chr:7q31    SOURCE    GENECARD       Gene Ontology:     BioCarta Pathways:     KEGG Pathways:  
    Probe set : 1370308_at    Name:   RS21-C6 protein    GB acc:   AY029335    UniGene:   Rn.109806    Gene Symbol:   Rs21c6    Locus Link:   192252    Chromosome:   1    Cytoband:   Chr:1q36    SOURCE    GENECARD       Gene Ontology:     BioCarta Pathways:     KEGG Pathways:  
    Probe set : 1384188_at     GB acc:   AW142766              Gene Ontology:     BioCarta Pathways:     KEGG Pathways:  
    Probe set : 1373575_at    Name:   similar to NADH dehydrogenase (ubiquinone) Fe-S protein 2    GB acc:   BE111722     Gene Symbol:   LOC498279    Locus Link:   498279    Chromosome:   13    Cytoband:   Chr:13q24     GENECARD       Gene Ontology:     BioCarta Pathways:     KEGG Pathways:  
    Probe set : 1370813_at    Name:   glutathione S-transferase, mu 5    GB acc:   U86635    UniGene:   Rn.9158    Gene Symbol:   Gstm5    Locus Link:   64352    Chromosome:   2    Cytoband:   Chr:2q34    SOURCE    GENECARD       Gene Ontology:   biological process:  metabolism   molecular function:  glutathione transferase activity   molecular function:  glutathione transferase activity   molecular function:  transferase activity      BioCarta Pathways:     KEGG Pathways:  
    Probe set : 1371487_at    Name:   SH3 domain binding glutamic acid-rich protein-like 3 (predicted)    GB acc:   BG666075    UniGene:   Rn.18564    Gene Symbol:   Sh3bgrl3_predicted    Locus Link:   298544    Chromosome:   5    Cytoband:   Chr:5q36    SOURCE    GENECARD       Gene Ontology:   cellular component:  nucleus      BioCarta Pathways:     KEGG Pathways:  
    Probe set : 1383395_at    Name:   agmatine ureohydrolase (agmatinase) (predicted)    GB acc:   AI028979    UniGene:   Rn.7880    Gene Symbol:   Agmat_predicted    Locus Link:   298607    Chromosome:   5    Cytoband:   Chr:5q36    SOURCE    GENECARD       Gene Ontology:   cellular component:  extracellular space   cellular component:  mitochondrion      BioCarta Pathways:     KEGG Pathways:  
    Probe set : 1373093_at    Name:   similar to Mitogen-inducible gene 6 protein homolog (Mig-6) (Gene 33 polypeptide) (predicted)    GB acc:   AI169756    UniGene:   Rn.100336    Gene Symbol:   RGD1307599_predicted    Locus Link:   313729    Chromosome:   5    Cytoband:   Chr:5q36    SOURCE    GENECARD       Gene Ontology:     BioCarta Pathways:     KEGG Pathways:  
    Probe set : 1371266_at    Name:   afamin    GB acc:   X76456    UniGene:   Rn.126589    Gene Symbol:   Afm    Locus Link:   282708    Chromosome:   14    Cytoband:   Chr:14p22    SOURCE    GENECARD       Gene Ontology:   biological process:  transport   cellular component:  extracellular space   cellular component:  extracellular space   molecular function:  carrier activity      BioCarta Pathways:     KEGG Pathways:  
    Probe set : 1376937_at    Name:   similar to 4631422O05Rik protein    GB acc:   BM389685    UniGene:   Rn.8383    Gene Symbol:   LOC304020    Locus Link:   304020    Chromosome:   11    Cytoband:   Chr:11q12    SOURCE    GENECARD       Gene Ontology:     BioCarta Pathways:     KEGG Pathways:  
    Probe set : 1370539_at    Name:   RAB8B, member RAS oncogene family    GB acc:   U53475    UniGene:   Rn.10995    Gene Symbol:   Rab8b    Locus Link:   266688    Chromosome:   8    Cytoband:   Chr:8q24    SOURCE    GENECARD       Gene Ontology:     BioCarta Pathways:     KEGG Pathways:  
    Probe set : 1379582_a_at    Name:   cyclin A2    GB acc:   AA998516    UniGene:   Rn.13094    Gene Symbol:   Ccna2    Locus Link:   114494    Chromosome:   2    Cytoband:   Chr:2q25    SOURCE    GENECARD       Gene Ontology:   biological process:  regulation of progression through cell cycle   biological process:  cell cycle   biological process:  cell division   cellular component:  nucleus      BioCarta Pathways:     KEGG Pathways:  
    Probe set : 1385625_at    Name:   EGF-containing fibulin-like extracellular matrix protein 2    GB acc:   BF551426    UniGene:   Rn.16151    Gene Symbol:   RGD:1359496    Locus Link:   293677    Chromosome:   1    Cytoband:   Chr:1q43    SOURCE    GENECARD       Gene Ontology:   biological process:  homophilic cell adhesion   biological process:  blood coagulation   cellular component:  membrane   molecular function:  transmembrane receptor activity   molecular function:  structural molecule activity   molecular function:  calcium ion binding      BioCarta Pathways:     KEGG Pathways:  
    Probe set : 1371300_at    Name:   ribosomal protein L3    GB acc:   AA892367    UniGene:   Rn.107726    Gene Symbol:   Rpl3    Locus Link:   300079    Chromosome:   7    Cytoband:   Chr:7q34    SOURCE    GENECARD       Gene Ontology:   biological process:  protein biosynthesis   cellular component:  intracellular   cellular component:  ribosome   cellular component:  ribonucleoprotein complex   molecular function:  structural constituent of ribosome      BioCarta Pathways:     KEGG Pathways:  
    Probe set : 1372410_at    Name:   C1q and tumor necrosis factor related protein 6 (predicted)    GB acc:   AW251360    UniGene:   Rn.17708     Locus Link:   315114    Chromosome:   7    Cytoband:   Chr:7q34    SOURCE        Gene Ontology:   biological process:  phosphate transport   cellular component:  cytoplasm      BioCarta Pathways:     KEGG Pathways:  
    Probe set : 1370328_at    Name:   Dickkopf homolog 3 (Xenopus laevis)    GB acc:   AF245040    UniGene:   Rn.12516    Gene Symbol:   Dkk3    Locus Link:   171548    Chromosome:   1    Cytoband:   Chr:1q33    SOURCE    GENECARD       Gene Ontology:   biological process:  development   biological process:  Wnt receptor signaling pathway   biological process:  negative regulation of Wnt receptor signaling pathway   cellular component:  extracellular region   cellular component:  extracellular space      BioCarta Pathways:     KEGG Pathways:  
    Probe set : 1370642_s_at    Name:   platelet derived growth factor receptor, beta polypeptide /// hypothetical gene supported by NM_031525    GB acc:   BM389426    UniGene:   Rn.98311    Gene Symbol:   Pdgfrb /// LOC497724    Locus Link:   24629 /// 497724    Chromosome:   18    Cytoband:   Chr:18q12.1    SOURCE    GENECARD       Gene Ontology:   cellular component:  extracellular space   cellular component:  membrane   cellular component:  integral to membrane   molecular function:  protein kinase activity   molecular function:  protein kinase activity   molecular function:  protein serine/threonine kinase activity   molecular function:  protein-tyrosine kinase activity   molecular function:  protein-tyrosine kinase activity   molecular function:  transmembrane receptor protein tyrosine kinase activity   molecular function:  signal transducer activity   molecular function:  receptor activity   molecular function:  platelet activating factor receptor activity   molecular function:  platelet-derived growth factor receptor activity   molecular function:  platelet-derived growth factor receptor activity   molecular function:  platelet-derived growth factor receptor activity   molecular function:  vascular endothelial growth factor receptor activity   molecular function:  ATP binding   molecular function:  kinase activity   molecular function:  transferase activity   molecular function:  nucleotide binding      BioCarta Pathways:     KEGG Pathways:  
    Probe set : 1372518_at    Name:   fibulin 1 (predicted)    GB acc:   AI176918    UniGene:   Rn.9375    Gene Symbol:   Fbln1_predicted    Locus Link:   315191    Chromosome:   7    Cytoband:   Chr:7q34    SOURCE    GENECARD       Gene Ontology:   biological process:  chitin metabolism   molecular function:  extracellular matrix structural constituent   molecular function:  calcium ion binding   molecular function:  chitin binding      BioCarta Pathways:     KEGG Pathways:  
    Probe set : 1387357_at    Name:   trimethyllysine hydroxylase, epsilon    GB acc:   NM_133387    UniGene:   Rn.3607    Gene Symbol:   Tmlhe    Locus Link:   170898    Chromosome:   20    Cytoband:   Chr:20p12    SOURCE    GENECARD       Gene Ontology:   biological process:  carnitine biosynthesis   biological process:  carnitine biosynthesis   cellular component:  mitochondrion   cellular component:  mitochondrion      BioCarta Pathways:     KEGG Pathways:  
    Probe set : 1372818_at    Name:   collectin sub-family member 12    GB acc:   BI284441    UniGene:   Rn.31273    Gene Symbol:   Colec12    Locus Link:   361289    Chromosome:   18    Cytoband:   Chr:18p13    SOURCE    GENECARD       Gene Ontology:   biological process:  phosphate transport   cellular component:  cytoplasm      BioCarta Pathways:     KEGG Pathways:  
    Probe set : 1371310_s_at    Name:   serine (or cysteine) proteinase inhibitor, clade H, member 1    GB acc:   BI285495    UniGene:   Rn.98199    Gene Symbol:   Serpinh1    Locus Link:   29345    Chromosome:   1    Cytoband:   Chr:1q32    SOURCE    GENECARD       Gene Ontology:     BioCarta Pathways:     KEGG Pathways:  
    Probe set : 1372475_at    Name:   PTEN induced putative kinase 1 (predicted)    GB acc:   AI410877    UniGene:   Rn.103380    Gene Symbol:   Pink1_predicted    Locus Link:   298575    Chromosome:   5    Cytoband:   Chr:5q36    SOURCE    GENECARD       Gene Ontology:   biological process:  protein amino acid phosphorylation   biological process:  response to stress   biological process:  protein kinase cascade   cellular component:  mitochondrion      BioCarta Pathways:     KEGG Pathways:  
    Probe set : 1379587_at     GB acc:   AI717097              Gene Ontology:     BioCarta Pathways:     KEGG Pathways:  
    Probe set : 1383284_at    Name:   Transcribed locus    GB acc:   BG372598    UniGene:   Rn.147831        SOURCE        Gene Ontology:     BioCarta Pathways:     KEGG Pathways:  
    Probe set : 1367593_at    Name:   selenoprotein W, muscle 1    GB acc:   NM_013027    UniGene:   Rn.37192    Gene Symbol:   Sepw1    Locus Link:   25545    Chromosome:   1    Cytoband:   Chr:1q21    SOURCE    GENECARD       Gene Ontology:   biological process:  cell redox homeostasis   molecular function:  selenium binding   molecular function:  oxidoreductase activity   molecular function:  oxidoreductase activity      BioCarta Pathways:     KEGG Pathways:  
    Probe set : 1374474_at    Name:   Transcribed locus, strongly similar to NP_080091.1 copine family member [Mus musculus]    GB acc:   BE099085    UniGene:   Rn.11746        SOURCE        Gene Ontology:     BioCarta Pathways:     KEGG Pathways:  
    Probe set : 1392489_at    Name:   Transcribed locus    GB acc:   BE108956    UniGene:   Rn.8458        SOURCE        Gene Ontology:     BioCarta Pathways:     KEGG Pathways:  
    Probe set : 1387178_a_at    Name:   cystathionine beta synthase    GB acc:   NM_012522    UniGene:   Rn.87853    Gene Symbol:   Cbs    Locus Link:   24250    Chromosome:   20    Cytoband:   Chr:20p12    SOURCE    GENECARD       Gene Ontology:   cellular component:  cytoplasm      BioCarta Pathways:     KEGG Pathways:  
    Probe set : 1369186_at    Name:   caspase 1    GB acc:   D85899    UniGene:   Rn.37508    Gene Symbol:   Casp1    Locus Link:   25166    Chromosome:   8    Cytoband:   Chr:8q11    SOURCE    GENECARD       Gene Ontology:   cellular component:  extracellular region   cellular component:  intracellular   cellular component:  cytoplasm      BioCarta Pathways:     KEGG Pathways:  
    Probe set : 1367676_at    Name:   high mobility group box 2 /// similar to High mobility group protein 2 (HMG-2) /// similar to High mobility group protein 2 (HMG-2) /// similar to High mobility group protein 2 (HMG-2)    GB acc:   NM_017187    UniGene:   Rn.141159    Gene Symbol:   Hmgb2 /// LOC498072 /// LOC498388 /// LOC498988    Locus Link:   29395 /// 498072 /// 498388 /// 498988    Chromosome:   16    Cytoband:   Chr:16p11 /// Chr:11q12 /// Chr:14q21 /// Chr:1p12    SOURCE    GENECARD       Gene Ontology:   biological process:  DNA replication   biological process:  DNA unwinding during replication   biological process:  DNA repair   biological process:  base-excision repair, DNA ligation   biological process:  DNA packaging   biological process:  establishment and/or maintenance of chromatin architecture   biological process:  nucleosome assembly   biological process:  regulation of transcription, DNA-dependent   biological process:  regulation of transcription from RNA polymerase II promoter   biological process:  nervous system development   biological process:  nervous system development   biological process:  DNA replication   biological process:  DNA unwinding during replication   biological process:  DNA repair   biological process:  base-excision repair, DNA ligation   biological process:  establishment and/or maintenance of chromatin architecture   biological process:  nucleosome assembly   biological process:  regulation of transcription from RNA polymerase II promoter      BioCarta Pathways:     KEGG Pathways:  
    Probe set : 1368418_a_at    Name:   ceruloplasmin    GB acc:   AF202115    UniGene:   Rn.32777    Gene Symbol:   Cp    Locus Link:   24268    Chromosome:   2    Cytoband:   Chr:2q24    SOURCE    GENECARD       Gene Ontology:     BioCarta Pathways:     KEGG Pathways:  
    Probe set : 1376951_at    Name:   MAD2 (mitotic arrest deficient, homolog)-like 1 (yeast) (predicted)    GB acc:   AI101906    UniGene:   Rn.19771     Locus Link:   297176    Chromosome:   4    Cytoband:   Chr:4q31    SOURCE        Gene Ontology:     BioCarta Pathways:     KEGG Pathways:  
    Probe set : 1377299_at    Name:   nuclear autoantigenic sperm protein    GB acc:   AI137618    UniGene:   Rn.48344    Gene Symbol:   RGD:1359609    Locus Link:   298441    Chromosome:   5    Cytoband:   Chr:5q36    SOURCE    GENECARD       Gene Ontology:   molecular function:  binding      BioCarta Pathways:     KEGG Pathways:  
    Probe set : 1388778_at    Name:   Transcribed locus    GB acc:   BF284876    UniGene:   Rn.17629        SOURCE        Gene Ontology:     BioCarta Pathways:     KEGG Pathways:  
    Probe set : 1369677_at    Name:   cannabinoid receptor 1 (brain)    GB acc:   X55812    UniGene:   Rn.89774    Gene Symbol:   Cnr1    Locus Link:   25248    Chromosome:   5    Cytoband:   Chr:5q21    SOURCE    GENECARD       Gene Ontology:   cellular component:  plasma membrane   cellular component:  integral to plasma membrane   cellular component:  integral to membrane      BioCarta Pathways:     KEGG Pathways:  
    Probe set : 1368139_s_at    Name:   alkaline phosphatase, tissue-nonspecific    GB acc:   Y00714    UniGene:   Rn.82764    Gene Symbol:   Alpl    Locus Link:   25586    Chromosome:   5    Cytoband:   Chr:5q36    SOURCE    GENECARD       Gene Ontology:   biological process:  metabolism   cellular component:  extracellular space   cellular component:  membrane   cellular component:  integral to membrane      BioCarta Pathways:     KEGG Pathways:  
    Probe set : 1375972_at    Name:   Transcribed locus    GB acc:   AA851327    UniGene:   Rn.146380        SOURCE        Gene Ontology:     BioCarta Pathways:     KEGG Pathways:  
    Probe set : 1371304_a_at    Name:   similar to myosin, light polypeptide 6, alkali, smooth muscle and non-muscle /// myosin, light polypeptide 6, alkali, smooth muscle and non-muscle (predicted)    GB acc:   BI285502    UniGene:   Rn.105641    Gene Symbol:   LOC304447 /// Myl6_predicted    Locus Link:   304447 /// 362816    Chromosome:   12    Cytoband:   Chr:12q14 /// Chr:13p11    SOURCE    GENECARD       Gene Ontology:   biological process:  cytoskeleton organization and biogenesis   biological process:  muscle development   biological process:  striated muscle development   cellular component:  cytoskeleton   cellular component:  myosin   molecular function:  calcium ion binding      BioCarta Pathways:     KEGG Pathways:  
    Probe set : 1368150_at    Name:   solute carrier family 27 (fatty acid transporter), member 2 /// hypothetical gene supported by NM_031736    GB acc:   NM_031736    UniGene:   Rn.3608    Gene Symbol:   Slc27a2 /// LOC497779    Locus Link:   497779 /// 65192    Chromosome:   3    Cytoband:   Chr:3q36    SOURCE    GENECARD       Gene Ontology:   biological process:  lipid metabolism   biological process:  fatty acid metabolism   biological process:  metabolism      BioCarta Pathways:     KEGG Pathways:  
    Probe set : 1387805_at    Name:   BCL2/adenovirus E1B 19 kDa-interacting protein 3    GB acc:   NM_053420    UniGene:   Rn.2060    Gene Symbol:   Bnip3    Locus Link:   84480    Chromosome:   1    Cytoband:   Chr:1q41    SOURCE    GENECARD       Gene Ontology:   biological process:  apoptosis   biological process:  regulation of apoptosis   biological process:  positive regulation of apoptosis   cellular component:  mitochondrion   cellular component:  integral to membrane   cellular component:  mitochondrial membrane      BioCarta Pathways:     KEGG Pathways:  
    Probe set : 1369415_at    Name:   basic helix-loop-helix domain containing, class B2    GB acc:   NM_053328    UniGene:   Rn.81055    Gene Symbol:   Bhlhb2    Locus Link:   79431    Chromosome:   4    Cytoband:   Chr:4q41    SOURCE    GENECARD       Gene Ontology:   cellular component:  nucleus   cellular component:  nucleus      BioCarta Pathways:     KEGG Pathways:  
    Probe set : 1386274_at    Name:   solute carrier family 17 (sodium phosphate), member 2 (predicted)    GB acc:   AI169925    UniGene:   Rn.28363    Gene Symbol:   Slc17a2_predicted    Locus Link:   306950    Chromosome:   17    Cytoband:   Chr:17p11    SOURCE    GENECARD       Gene Ontology:   biological process:  phosphate metabolism   biological process:  transport   biological process:  sodium ion transport   molecular function:  transporter activity   molecular function:  sodium:phosphate symporter activity   molecular function:  symporter activity      BioCarta Pathways:     KEGG Pathways:  
    Probe set : 1369527_at    Name:   chemokine (C-X3-C) receptor 1    GB acc:   NM_133534    UniGene:   Rn.10482    Gene Symbol:   Cx3cr1    Locus Link:   171056    Chromosome:   8    Cytoband:   Chr:8q32    SOURCE    GENECARD       Gene Ontology:   biological process:  G-protein coupled receptor protein signaling pathway   biological process:  signal transduction   cellular component:  integral to membrane      BioCarta Pathways:     KEGG Pathways:  
    Probe set : 1387687_at    Name:   immunoglobulin superfamily, member 6    GB acc:   NM_133542    UniGene:   Rn.16316    Gene Symbol:   Igsf6    Locus Link:   171064    Chromosome:   1    Cytoband:   Chr:1q36    SOURCE    GENECARD       Gene Ontology:   cellular component:  integral to membrane      BioCarta Pathways:     KEGG Pathways:  
    Probe set : 1377639_at    Name:   adipocyte-specific adhesion molecule    GB acc:   BI275896    UniGene:   Rn.40141    Gene Symbol:   Asam    Locus Link:   286939    Chromosome:   8    Cytoband:   Chr:8q22    SOURCE    GENECARD       Gene Ontology:   biological process:  cell-cell adhesion   cellular component:  tight junction   cellular component:  integral to membrane      BioCarta Pathways:     KEGG Pathways:  
    Probe set : 1374897_at     GB acc:   AI406842              Gene Ontology:     BioCarta Pathways:     KEGG Pathways:  
    Probe set : 1379448_at    Name:   Ttk protein kinase (predicted)    GB acc:   BE110883    UniGene:   Rn.107533    Gene Symbol:   Ttk_predicted    Locus Link:   315852    Chromosome:   8    Cytoband:   Chr:8q31    SOURCE    GENECARD       Gene Ontology:   cellular component:  spindle      BioCarta Pathways:     KEGG Pathways:  
    Probe set : 1381528_at     GB acc:   BF387076              Gene Ontology:     BioCarta Pathways:     KEGG Pathways:  
    Probe set : 1386956_at    Name:   scavenger receptor class B, member 1    GB acc:   NM_031541    UniGene:   Rn.88169    Gene Symbol:   Scarb1    Locus Link:   25073    Chromosome:   12    Cytoband:   Chr:12q15-q16    SOURCE    GENECARD       Gene Ontology:   biological process:  cell adhesion   molecular function:  receptor activity      BioCarta Pathways:     KEGG Pathways:  
    Probe set : 1387263_at    Name:   pyruvate kinase, liver and RBC    GB acc:   NM_012624    UniGene:   Rn.48821    Gene Symbol:   Pklr    Locus Link:   24651    Chromosome:   2    Cytoband:   Chr:2q34    SOURCE    GENECARD       Gene Ontology:   biological process:  glycolysis   biological process:  response to pathogen      BioCarta Pathways:     KEGG Pathways:  
    Probe set : 1380775_at    Name:   M-phase phosphoprotein 1 (predicted)    GB acc:   BE110723    UniGene:   Rn.138477    Gene Symbol:   Mphosph1_predicted    Locus Link:   309523    Chromosome:   1    Cytoband:   Chr:1q52    SOURCE    GENECARD       Gene Ontology:   biological process:  microtubule-based movement   biological process:  cell cycle arrest   biological process:  regulation of mitosis      BioCarta Pathways:     KEGG Pathways:  
    Probe set : 1392648_at    Name:   mannose receptor, C type 1 (predicted)    GB acc:   BI294018    UniGene:   Rn.9197    Gene Symbol:   Mrc1_predicted    Locus Link:   291327    Chromosome:   17    Cytoband:   Chr:17q12.3    SOURCE    GENECARD       Gene Ontology:   biological process:  endocytosis   biological process:  receptor mediated endocytosis   biological process:  pinocytosis      BioCarta Pathways:     KEGG Pathways:  
    Probe set : 1368147_at    Name:   dual specificity phosphatase 1    GB acc:   BE110108    UniGene:   Rn.98260    Gene Symbol:   Dusp1    Locus Link:   114856    Chromosome:   10    Cytoband:   Chr:10q12    SOURCE    GENECARD       Gene Ontology:     BioCarta Pathways:     KEGG Pathways:  
    Probe set : 1372584_at    Name:   similar to DKFZP566K1924 protein (predicted)    GB acc:   BG672517    UniGene:   Rn.8173    Gene Symbol:   RGD1308373_predicted    Locus Link:   364208    Chromosome:   14    Cytoband:   Chr:14q22    SOURCE    GENECARD       Gene Ontology:     BioCarta Pathways:     KEGG Pathways:  
    Probe set : 1388936_at    Name:   cadherin 11    GB acc:   BI296340    UniGene:   Rn.8900    Gene Symbol:   Cdh11    Locus Link:   84407    Chromosome:   19    Cytoband:   Chr:19p14    SOURCE    GENECARD       Gene Ontology:   biological process:  cell-cell adhesion   biological process:  cell-cell adhesion   biological process:  homophilic cell adhesion   cellular component:  membrane   molecular function:  calcium ion binding      BioCarta Pathways:     KEGG Pathways:  
    Probe set : 1398602_at    Name:   MAD2 (mitotic arrest deficient, homolog)-like 1 (yeast) (predicted)    GB acc:   AW143296    UniGene:   Rn.19771     Locus Link:   297176    Chromosome:   4    Cytoband:   Chr:4q31    SOURCE        Gene Ontology:     BioCarta Pathways:     KEGG Pathways:  
    Probe set : 1373693_at    Name:   G protein-coupled receptor, family C, group 5, member C (predicted)    GB acc:   BF414143    UniGene:   Rn.3658    Gene Symbol:   Gprc5c_predicted    Locus Link:   287805    Chromosome:   10    Cytoband:   Chr:10q32.2    SOURCE    GENECARD       Gene Ontology:   biological process:  G-protein coupled receptor protein signaling pathway   biological process:  visual perception   cellular component:  mitochondrion   cellular component:  membrane   cellular component:  integral to membrane      BioCarta Pathways:     KEGG Pathways:  
    Probe set : 1370949_at    Name:   myristoylated alanine rich protein kinase C substrate    GB acc:   M59859    UniGene:   Rn.9560    Gene Symbol:   Marcks    Locus Link:   25603      SOURCE    GENECARD       Gene Ontology:   biological process:  cell motility   cellular component:  plasma membrane   cellular component:  actin cytoskeleton   cellular component:  membrane      BioCarta Pathways:     KEGG Pathways:  
    Probe set : 1389528_s_at    Name:   v-jun sarcoma virus 17 oncogene homolog (avian)    GB acc:   BI288619    UniGene:   Rn.93714    Gene Symbol:   Jun    Locus Link:   24516    Chromosome:   5    Cytoband:   Chr:5q34.1    SOURCE    GENECARD       Gene Ontology:     BioCarta Pathways:     KEGG Pathways:  
    Probe set : 1389548_at    Name:   alcohol dehydrogenase, iron containing, 1 (predicted)    GB acc:   BF288683    UniGene:   Rn.17173    Gene Symbol:   Adhfe1_predicted    Locus Link:   362474    Chromosome:   5    Cytoband:   Chr:5q11    SOURCE    GENECARD       Gene Ontology:   biological process:  metabolism   molecular function:  iron ion binding   molecular function:  oxidoreductase activity      BioCarta Pathways:     KEGG Pathways:  
    Probe set : 1370294_a_at    Name:   cell division cycle 20 homolog (S. cerevisiae)    GB acc:   U05341    UniGene:   Rn.9262    Gene Symbol:   Cdc20    Locus Link:   64515    Chromosome:   5    Cytoband:   Chr:5q36    SOURCE    GENECARD       Gene Ontology:   cellular component:  spindle   molecular function:  protein binding      BioCarta Pathways:     KEGG Pathways:  
    Probe set : 1371389_at    Name:   hypothetical LOC306766    GB acc:   AI170668    UniGene:   Rn.105613    Gene Symbol:   RGD:1359256    Locus Link:   306766    Chromosome:   17    Cytoband:   Chr:17p14    SOURCE    GENECARD       Gene Ontology:     BioCarta Pathways:     KEGG Pathways:  
    Probe set : 1368419_at    Name:   ceruloplasmin    GB acc:   AF202115    UniGene:   Rn.32777    Gene Symbol:   Cp    Locus Link:   24268    Chromosome:   2    Cytoband:   Chr:2q24    SOURCE    GENECARD       Gene Ontology:     BioCarta Pathways:     KEGG Pathways:  
    Probe set : 1383747_at    Name:   Ect2 oncogene (predicted)    GB acc:   AI578135    UniGene:   Rn.27564     Locus Link:   361921    Chromosome:   2    Cytoband:   Chr:2q24    SOURCE        Gene Ontology:   cellular component:  intracellular   cellular component:  nucleus   molecular function:  signal transducer activity   molecular function:  guanyl-nucleotide exchange factor activity      BioCarta Pathways:     KEGG Pathways:  
    Probe set : 1382061_at    Name:   lactate dehydrogenase D (predicted)    GB acc:   AI501131    UniGene:   Rn.137462    Gene Symbol:   Ldhd_predicted    Locus Link:   307858    Chromosome:   19    Cytoband:   Chr:19q12    SOURCE    GENECARD       Gene Ontology:   cellular component:  mitochondrion   cellular component:  mitochondrion      BioCarta Pathways:     KEGG Pathways:  
    Probe set : 1374217_at    Name:   similar to chromosome 16 open reading frame 5 (predicted)    GB acc:   BG375463    UniGene:   Rn.101805    Gene Symbol:   RGD1310686_predicted    Locus Link:   360480    Chromosome:   10    Cytoband:   Chr:10q12    SOURCE    GENECARD       Gene Ontology:   cellular component:  nucleus   cellular component:  integral to membrane      BioCarta Pathways:     KEGG Pathways:  
    Probe set : 1368260_at    Name:   aurora kinase B    GB acc:   NM_053749    UniGene:   Rn.10865    Gene Symbol:   Aurkb    Locus Link:   114592    Chromosome:   10    Cytoband:   Chr:10q24    SOURCE    GENECARD       Gene Ontology:   cellular component:  spindle   cellular component:  spindle   cellular component:  midbody      BioCarta Pathways:     KEGG Pathways:  
    Probe set : 1367634_at    Name:   ribosomal protein L31    GB acc:   NM_022506    UniGene:   Rn.1101    Gene Symbol:   Rpl31    Locus Link:   64298    Chromosome:   9    Cytoband:   Chr:9q21    SOURCE    GENECARD       Gene Ontology:   biological process:  protein biosynthesis   molecular function:  RNA binding   molecular function:  structural constituent of ribosome      BioCarta Pathways:     KEGG Pathways:  
    Probe set : 1378592_at    Name:   Similar to RIKEN cDNA 2310035M22 (predicted)    GB acc:   BF396210    UniGene:   Rn.145316     Locus Link:   365813    Chromosome:   2    Cytoband:   Chr:2q31    SOURCE        Gene Ontology:     BioCarta Pathways:     KEGG Pathways:  
    Probe set : 1368146_at    Name:   dual specificity phosphatase 1    GB acc:   U02553    UniGene:   Rn.98260    Gene Symbol:   Dusp1    Locus Link:   114856    Chromosome:   10    Cytoband:   Chr:10q12    SOURCE    GENECARD       Gene Ontology:     BioCarta Pathways:     KEGG Pathways:  
    Probe set : 1374628_at    Name:   crystallin, zeta (predicted)    GB acc:   AI232098    UniGene:   Rn.6746    Gene Symbol:   Cryz_predicted    Locus Link:   362061    Chromosome:   2    Cytoband:   Chr:2q45    SOURCE    GENECARD       Gene Ontology:   biological process:  sensory organ development   biological process:  visual perception   cellular component:  cytoplasm      BioCarta Pathways:     KEGG Pathways:  
    Probe set : 1381311_at    Name:   EGF-like module containing, mucin-like, hormone receptor-like sequence 1    GB acc:   BE100625    UniGene:   Rn.43404    Gene Symbol:   RGD:1359214    Locus Link:   316137    Chromosome:   9    Cytoband:   Chr:9q11    SOURCE    GENECARD       Gene Ontology:   biological process:  neuropeptide signaling pathway   cellular component:  membrane   molecular function:  G-protein coupled receptor activity   molecular function:  calcium ion binding      BioCarta Pathways:     KEGG Pathways:  
    Probe set : 1383188_at    Name:   Transcribed locus    GB acc:   BM390716    UniGene:   Rn.22635        SOURCE        Gene Ontology:     BioCarta Pathways:     KEGG Pathways:  
    Probe set : 1373490_at    Name:   glia maturation factor, gamma    GB acc:   BG666787    UniGene:   Rn.15333    Gene Symbol:   Gmfg    Locus Link:   113940    Chromosome:   1    Cytoband:   Chr:1q21    SOURCE    GENECARD       Gene Ontology:   biological process:  protein amino acid phosphorylation   cellular component:  intracellular      BioCarta Pathways:     KEGG Pathways:  
    Probe set : 1367574_at    Name:   vimentin    GB acc:   NM_031140    UniGene:   Rn.2710    Gene Symbol:   Vim    Locus Link:   81818    Chromosome:   17    Cytoband:   Chr:17q12.3    SOURCE    GENECARD       Gene Ontology:   biological process:  intermediate filament-based process      BioCarta Pathways:     KEGG Pathways:  
    Probe set : 1369986_at    Name:   hydroxyacyl glutathione hydrolase    GB acc:   NM_033349    UniGene:   Rn.11048    Gene Symbol:   Hagh    Locus Link:   24439    Chromosome:   10    Cytoband:   Chr:10q12    SOURCE    GENECARD       Gene Ontology:   biological process:  spermatogenesis   biological process:  spermatogenesis   molecular function:  hydroxyacylglutathione hydrolase activity   molecular function:  hydrolase activity      BioCarta Pathways:     KEGG Pathways:  
    Probe set : 1385559_at    Name:   Transcribed locus    GB acc:   BF562977    UniGene:   Rn.148168        SOURCE        Gene Ontology:     BioCarta Pathways:     KEGG Pathways:  
    Probe set : 1383786_at    Name:   Transcribed locus    GB acc:   AW915417    UniGene:   Rn.49241        SOURCE        Gene Ontology:     BioCarta Pathways:     KEGG Pathways:  
    Probe set : 1387883_a_at    Name:   thymosin, beta 4    GB acc:   BG668902    UniGene:   Rn.129715    Gene Symbol:   Tmsb4x    Locus Link:   81814    Chromosome:   X    Cytoband:   Chr:Xq31    SOURCE    GENECARD       Gene Ontology:   cellular component:  cytoskeleton   cellular component:  cytoplasm   molecular function:  actin binding      BioCarta Pathways:     KEGG Pathways:  
    Probe set : 1372093_at    Name:   Max interacting protein 1    GB acc:   AI409308    UniGene:   Rn.37510    Gene Symbol:   Mxi1    Locus Link:   25701    Chromosome:   1    Cytoband:   Chr:1q55    SOURCE    GENECARD       Gene Ontology:   cellular component:  nucleus   cellular component:  nucleus      BioCarta Pathways:     KEGG Pathways:  
    Probe set : 1369977_at    Name:   ubiquitin carboxy-terminal hydrolase L1    GB acc:   NM_017237    UniGene:   Rn.107213    Gene Symbol:   Uchl1    Locus Link:   29545    Chromosome:   14    Cytoband:   Chr:14p11    SOURCE    GENECARD       Gene Ontology:   cellular component:  intracellular   cellular component:  cytoplasm      BioCarta Pathways:     KEGG Pathways:  
    Probe set : 1368821_at    Name:   follistatin-like 1    GB acc:   NM_024369    UniGene:   Rn.95652    Gene Symbol:   Fstl1    Locus Link:   79210    Chromosome:   11    Cytoband:   Chr:11q21    SOURCE    GENECARD       Gene Ontology:   cellular component:  extracellular space   molecular function:  calcium ion binding   molecular function:  heparin binding      BioCarta Pathways:     KEGG Pathways:  
    Probe set : 1374101_at    Name:   TBC1 domain family, member 10 (predicted)    GB acc:   BF282437    UniGene:   Rn.105859    Gene Symbol:   Tbc1d10_predicted    Locus Link:   360968    Chromosome:   14    Cytoband:   Chr:14q21    SOURCE    GENECARD       Gene Ontology:   cellular component:  microvillus   molecular function:  PDZ domain binding      BioCarta Pathways:     KEGG Pathways:  
    Probe set : 1384195_at     GB acc:   BM388427              Gene Ontology:     BioCarta Pathways:     KEGG Pathways:  
    Probe set : 1389553_at    Name:   dendritic cell inhibitory receptor 3    GB acc:   BF393825    UniGene:   Rn.137522    Gene Symbol:   RGD:1359528    Locus Link:   362431    Chromosome:   4    Cytoband:   Chr:4q42    SOURCE    GENECARD       Gene Ontology:   molecular function:  receptor activity   molecular function:  sugar binding      BioCarta Pathways:     KEGG Pathways:  
    Probe set : 1387087_at    Name:   CCAAT/enhancer binding protein (C/EBP), beta    GB acc:   NM_024125    UniGene:   Rn.6479    Gene Symbol:   Cebpb    Locus Link:   24253    Chromosome:   3    Cytoband:   Chr:3q42    SOURCE    GENECARD       Gene Ontology:   cellular component:  nucleus   cellular component:  nucleus   cellular component:  cytoplasm      BioCarta Pathways:     KEGG Pathways:  
    Probe set : 1368180_s_at    Name:   glutathione-S-transferase, alpha type2    GB acc:   NM_017013    UniGene:   Rn.144550    Gene Symbol:   Gsta2    Locus Link:   24422    Chromosome:   8    Cytoband:   Chr:8q31    SOURCE    GENECARD       Gene Ontology:   biological process:  metabolism   molecular function:  glutathione transferase activity   molecular function:  glutathione transferase activity   molecular function:  transferase activity      BioCarta Pathways:     KEGG Pathways:  
    Probe set : 1393061_at    Name:   similar to cDNA sequence BC021608    GB acc:   AI030103    UniGene:   Rn.107180    Gene Symbol:   RGD:1359544    Locus Link:   300676    Chromosome:   8    Cytoband:   Chr:8q22    SOURCE    GENECARD       Gene Ontology:   molecular function:  binding      BioCarta Pathways:     KEGG Pathways:  
    Probe set : 1387214_at    Name:   carboxylesterase 1    GB acc:   NM_031565    UniGene:   Rn.144568    Gene Symbol:   RGD:621508    Locus Link:   29225    Chromosome:   19    Cytoband:   Chr:19p11    SOURCE    GENECARD       Gene Ontology:   cellular component:  extracellular space   cellular component:  endoplasmic reticulum      BioCarta Pathways:     KEGG Pathways:  
    Probe set : 1373245_at    Name:   procollagen, type IV, alpha 1 (predicted)    GB acc:   BE111752    UniGene:   Rn.53801    Gene Symbol:   Col4a1_predicted    Locus Link:   290905    Chromosome:   16    Cytoband:   Chr:16q12.5    SOURCE    GENECARD       Gene Ontology:   biological process:  phosphate transport   biological process:  cell adhesion      BioCarta Pathways:     KEGG Pathways:  
    Probe set : 1373504_at    Name:   GLI pathogenesis-related 1 (glioma) (predicted)    GB acc:   BF287967    UniGene:   Rn.65764    Gene Symbol:   Glipr1_predicted    Locus Link:   299783    Chromosome:   7    Cytoband:   Chr:7q21    SOURCE    GENECARD       Gene Ontology:   cellular component:  extracellular region   cellular component:  extracellular space   cellular component:  integral to membrane      BioCarta Pathways:     KEGG Pathways:  
    Probe set : 1391518_at     GB acc:   AI579823              Gene Ontology:     BioCarta Pathways:     KEGG Pathways:  
    Probe set : 1383606_at    Name:   membrane targeting (tandem) C2 domain containing 1    GB acc:   BI302544    UniGene:   Rn.43919    Gene Symbol:   Mtac2d1    Locus Link:   500707    Chromosome:   6    Cytoband:   Chr:6q32    SOURCE    GENECARD       Gene Ontology:   biological process:  transport   cellular component:  synaptic vesicle   cellular component:  membrane   molecular function:  transporter activity      BioCarta Pathways:     KEGG Pathways:  
    Probe set : 1377756_at    Name:   similar to RIKEN cDNA 5830446M03 (predicted)    GB acc:   AI232228    UniGene:   Rn.145210    Gene Symbol:   RGD1307688_predicted    Locus Link:   362394    Chromosome:   4    Cytoband:   Chr:4q34    SOURCE    GENECARD       Gene Ontology:     BioCarta Pathways:     KEGG Pathways:  
    Probe set : 1390116_at    Name:   Polymerase I and transcript release factor (predicted)    GB acc:   AA899937    UniGene:   Rn.13589     Locus Link:   287710    Chromosome:   10    Cytoband:   Chr:10q32.1    SOURCE        Gene Ontology:   cellular component:  nucleus   cellular component:  membrane      BioCarta Pathways:     KEGG Pathways:  
    Probe set : 1379479_at    Name:   kinesin family member 4    GB acc:   AA859926    UniGene:   Rn.22652    Gene Symbol:   Kif4    Locus Link:   84393    Chromosome:   X    Cytoband:   Chr:Xq31    SOURCE    GENECARD       Gene Ontology:   molecular function:  microtubule motor activity   molecular function:  microtubule binding      BioCarta Pathways:     KEGG Pathways:  
    Probe set : 1390801_at    Name:   similar to hypothetical protein MGC34760    GB acc:   AI412174    UniGene:   Rn.23870    Gene Symbol:   RGD:1359349    Locus Link:   361744    Chromosome:   1    Cytoband:   Chr:1q51    SOURCE    GENECARD       Gene Ontology:   cellular component:  integral to membrane      BioCarta Pathways:     KEGG Pathways:  
    Probe set : 1380573_a_at    Name:   similar to C19orf36 protein    GB acc:   AA964213    UniGene:   Rn.11739    Gene Symbol:   LOC362832    Locus Link:   362832    Chromosome:   7    Cytoband:   Chr:7q11    SOURCE    GENECARD       Gene Ontology:     BioCarta Pathways:     KEGG Pathways:  
    Probe set : 1388717_at    Name:   protein O-fucosyltransferase 2 (predicted)    GB acc:   BM384163    UniGene:   Rn.11702    Gene Symbol:   Pofut2_predicted    Locus Link:   309686    Chromosome:   20    Cytoband:   Chr:20p12    SOURCE    GENECARD       Gene Ontology:   biological process:  fucose metabolism      BioCarta Pathways:     KEGG Pathways:  
    Probe set : 1371653_at     GB acc:   BI281955              Gene Ontology:     BioCarta Pathways:     KEGG Pathways:  
    Probe set : 1376599_at    Name:   ATPase family, AAA domain containing 2 (predicted)    GB acc:   AW915567    UniGene:   Rn.6690     Locus Link:   314993    Chromosome:   7    Cytoband:   Chr:7q33    SOURCE        Gene Ontology:   molecular function:  nucleotide binding   molecular function:  ATP binding   molecular function:  nucleoside-triphosphatase activity      BioCarta Pathways:     KEGG Pathways:  
    Probe set : 1382078_at    Name:   Transcribed locus    GB acc:   BF283802    UniGene:   Rn.128184        SOURCE        Gene Ontology:     BioCarta Pathways:     KEGG Pathways:  
    Probe set : 1368661_at    Name:   solute carrier family 13 (sodium-dependent dicarboxylate transporter), member 2    GB acc:   U51153    UniGene:   Rn.10821    Gene Symbol:   Slc13a2    Locus Link:   65202    Chromosome:   10    Cytoband:   Chr:10q25    SOURCE    GENECARD       Gene Ontology:   biological process:  transport   biological process:  sodium ion transport   biological process:  ion transport      BioCarta Pathways:     KEGG Pathways:  
    Probe set : 1367671_at    Name:   proliferating cell nuclear antigen    GB acc:   NM_022381    UniGene:   Rn.223    Gene Symbol:   Pcna    Locus Link:   25737    Chromosome:   3    Cytoband:   Chr:3q36    SOURCE    GENECARD       Gene Ontology:   molecular function:  DNA binding   molecular function:  protein binding   molecular function:  DNA polymerase processivity factor activity      BioCarta Pathways:     KEGG Pathways:  
    Probe set : 1387868_at    Name:   lipopolysaccharide binding protein    GB acc:   BF289368    UniGene:   Rn.48863    Gene Symbol:   Lbp    Locus Link:   29469    Chromosome:   3    Cytoband:   Chr:3q42    SOURCE    GENECARD       Gene Ontology:   cellular component:  extracellular space   cellular component:  integral to membrane   molecular function:  lipopolysaccharide binding   molecular function:  lipid binding      BioCarta Pathways:     KEGG Pathways:  
    Probe set : 1367855_at    Name:   scavenger receptor class B, member 1    GB acc:   AF071495    UniGene:   Rn.88169    Gene Symbol:   Scarb1    Locus Link:   25073    Chromosome:   12    Cytoband:   Chr:12q15-q16    SOURCE    GENECARD       Gene Ontology:   biological process:  cell adhesion   molecular function:  receptor activity      BioCarta Pathways:     KEGG Pathways:  
    Probe set : 1398878_at    Name:   similar to CPSF4 protein    GB acc:   Y17326    UniGene:   Rn.142318    Gene Symbol:   MGC108785    Locus Link:   304277    Chromosome:   12    Cytoband:   Chr:12p11    SOURCE    GENECARD       Gene Ontology:   molecular function:  nucleic acid binding      BioCarta Pathways:     KEGG Pathways:  
    Probe set : 1380577_at    Name:   ATP-binding cassette, sub-family G (WHITE), member 2    GB acc:   AI175616    UniGene:   Rn.13131    Gene Symbol:   Abcg2    Locus Link:   312382    Chromosome:   4    Cytoband:   Chr:4q24    SOURCE    GENECARD       Gene Ontology:   biological process:  transport   cellular component:  membrane   cellular component:  integral to membrane      BioCarta Pathways:     KEGG Pathways:  
    Probe set : 1389355_at    Name:   immediate early response 5    GB acc:   BF285187    UniGene:   Rn.8590    Gene Symbol:   Ier5    Locus Link:   498256    Chromosome:   13    Cytoband:   Chr:13q21    SOURCE    GENECARD       Gene Ontology:     BioCarta Pathways:     KEGG Pathways:  
    Probe set : 1380909_at    Name:   Transcribed locus    GB acc:   BM385894    UniGene:   Rn.81275        SOURCE        Gene Ontology:     BioCarta Pathways:     KEGG Pathways:  
    Probe set : 1371080_at    Name:   kallikrein    GB acc:   AI169562    UniGene:   Rn.21391    Gene Symbol:   RGD:735228    Locus Link:   292861    Chromosome:   1    Cytoband:   Chr:1q22    SOURCE    GENECARD       Gene Ontology:   biological process:  proteolysis   molecular function:  serine-type endopeptidase activity      BioCarta Pathways:     KEGG Pathways:  
    Probe set : 1376746_at    Name:   lactate dehydrogenase D (predicted)    GB acc:   AI145761    UniGene:   Rn.137462    Gene Symbol:   Ldhd_predicted    Locus Link:   307858    Chromosome:   19    Cytoband:   Chr:19q12    SOURCE    GENECARD       Gene Ontology:   cellular component:  mitochondrion   cellular component:  mitochondrion      BioCarta Pathways:     KEGG Pathways:  
    Probe set : 1378174_at     GB acc:   BI274989              Gene Ontology:     BioCarta Pathways:     KEGG Pathways:  
    Probe set : 1374496_at    Name:   similar to RIKEN cDNA 1200009O22; EST AI316813 (predicted)    GB acc:   BG380570    UniGene:   Rn.25045    Gene Symbol:   RGD1310827_predicted    Locus Link:   362364    Chromosome:   4    Cytoband:   Chr:4q24    SOURCE    GENECARD       Gene Ontology:   biological process:  chemotaxis      BioCarta Pathways:     KEGG Pathways:  
    Probe set : 1397341_at    Name:   PDZ binding kinase (predicted)    GB acc:   BG663837    UniGene:   Rn.137333     Locus Link:   290326    Chromosome:   15    Cytoband:   Chr:15p12    SOURCE        Gene Ontology:   biological process:  protein amino acid phosphorylation   biological process:  peptidyl-serine phosphorylation   biological process:  peptidyl-threonine phosphorylation      BioCarta Pathways:     KEGG Pathways:  
    Probe set : 1388298_at    Name:   myosin, light polypeptide 9, regulatory (predicted)    GB acc:   BI279044    UniGene:   Rn.6870    Gene Symbol:   Myl9_predicted    Locus Link:   296313    Chromosome:   3    Cytoband:   Chr:3q42    SOURCE    GENECARD       Gene Ontology:   biological process:  regulation of muscle contraction   biological process:  muscle development   cellular component:  muscle myosin   cellular component:  myosin   molecular function:  calcium ion binding   molecular function:  structural constituent of muscle      BioCarta Pathways:     KEGG Pathways:  
    Probe set : 1390177_at    Name:   Transcribed locus    GB acc:   AI233857    UniGene:   Rn.6397        SOURCE        Gene Ontology:     BioCarta Pathways:     KEGG Pathways:  
    Probe set : 1376871_at     GB acc:   AA891475    UniGene:   Rn.145774    Gene Symbol:   LOC288549 /// LOC363890 /// LOC363894 /// LOC363899 /// LOC498168 /// LOC498169 /// LOC498199 /// LOC501818 /// LOC501821 /// LOC501828    Locus Link:   288549 /// 363890 /// 363894 /// 363899 /// 498168 /// 498169 /// 498199 /// 501818 /// 501821 /// 501828    Chromosome:   12    Cytoband:   Chr:12q12    SOURCE    GENECARD       Gene Ontology:     BioCarta Pathways:     KEGG Pathways:  
    Probe set : 1378997_at    Name:   Eph receptor B6 (predicted)    GB acc:   BM391684    UniGene:   Rn.125930    Gene Symbol:   Ephb6_predicted    Locus Link:   312275    Chromosome:   4    Cytoband:   Chr:4q22    SOURCE    GENECARD       Gene Ontology:   biological process:  protein amino acid phosphorylation   biological process:  transmembrane receptor protein tyrosine kinase signaling pathway   cellular component:  extracellular space   cellular component:  membrane   cellular component:  integral to membrane      BioCarta Pathways:     KEGG Pathways:  
    Probe set : 1388458_at    Name:   replication factor C (activator 1) 4 (predicted)    GB acc:   BI291451    UniGene:   Rn.17046    Gene Symbol:   Rfc4_predicted    Locus Link:   288003    Chromosome:   11    Cytoband:   Chr:11q23    SOURCE    GENECARD       Gene Ontology:   biological process:  DNA replication   biological process:  DNA strand elongation   cellular component:  nucleus   cellular component:  delta-DNA polymerase cofactor complex   cellular component:  DNA replication factor C complex      BioCarta Pathways:     KEGG Pathways:  
    Probe set : 1372478_at    Name:   similar to LNV    GB acc:   AI137605    UniGene:   Rn.12081    Gene Symbol:   LOC501065    Locus Link:   501065    Chromosome:   8    Cytoband:   Chr:8q32    SOURCE    GENECARD       Gene Ontology:     BioCarta Pathways:     KEGG Pathways:  
    Probe set : 1372323_at    Name:   sarcosine dehydrogenase    GB acc:   AI103641    UniGene:   Rn.89832    Gene Symbol:   Sardh    Locus Link:   114123    Chromosome:   3    Cytoband:   Chr:3p12    SOURCE    GENECARD       Gene Ontology:   biological process:  electron transport   biological process:  glycine catabolism   biological process:  choline catabolism      BioCarta Pathways:     KEGG Pathways:  
    Probe set : 1367695_at    Name:   quinoid dihydropteridine reductase    GB acc:   NM_022390    UniGene:   Rn.241    Gene Symbol:   Qdpr    Locus Link:   64192    Chromosome:   14    Cytoband:   Chr:14q21    SOURCE    GENECARD       Gene Ontology:     BioCarta Pathways:     KEGG Pathways:  
    Probe set : 1382069_at    Name:   ATPase, class VI, type 11A (predicted)    GB acc:   AA946031    UniGene:   Rn.98540     Locus Link:   306600    Chromosome:   16    Cytoband:   Chr:16q12.5    SOURCE        Gene Ontology:   biological process:  cation transport   biological process:  metabolism   cellular component:  membrane   cellular component:  integral to membrane      BioCarta Pathways:     KEGG Pathways:  
    Probe set : 1370287_a_at    Name:   tropomyosin 1, alpha    GB acc:   M23764    UniGene:   Rn.87540    Gene Symbol:   Tpm1    Locus Link:   24851    Chromosome:   8    Cytoband:   Chr:8q24    SOURCE    GENECARD       Gene Ontology:   cellular component:  cytoskeleton   cellular component:  muscle thin filament tropomyosin   cellular component:  kinesin complex      BioCarta Pathways:     KEGG Pathways:  
    Probe set : 1387995_a_at    Name:   interferon induced transmembrane protein 3    GB acc:   BI285494    UniGene:   Rn.145191    Gene Symbol:   Ifitm3    Locus Link:   361673    Chromosome:   1    Cytoband:   Chr:1q41    SOURCE    GENECARD       Gene Ontology:     BioCarta Pathways:     KEGG Pathways:  
    Probe set : 1389695_at     GB acc:   AI410976              Gene Ontology:     BioCarta Pathways:     KEGG Pathways:  
    Probe set : 1393793_at    Name:   Transcribed locus    GB acc:   AW524669    UniGene:   Rn.125399        SOURCE        Gene Ontology:     BioCarta Pathways:     KEGG Pathways:  
    Probe set : 1371782_at    Name:   NIPSNAP-related protein    GB acc:   BF283798    UniGene:   Rn.8287    Gene Symbol:   RGD:1359688    Locus Link:   313211    Chromosome:   5    Cytoband:   Chr:5q24    SOURCE    GENECARD       Gene Ontology:     BioCarta Pathways:     KEGG Pathways:  
    Probe set : 1374431_at    Name:   ATPase, H+ transporting, V1 subunit A, isoform 1 (predicted)    GB acc:   BE120644    UniGene:   Rn.1431     Locus Link:   360716    Chromosome:   11    Cytoband:   Chr:11q21    SOURCE        Gene Ontology:     BioCarta Pathways:     KEGG Pathways:  
    Probe set : 1373557_at    Name:   mini chromosome maintenance deficient 4 homolog (S. cerevisiae) /// similar to mcdc21 protein    GB acc:   AI145081    UniGene:   Rn.8341    Gene Symbol:   Mcmd4 /// LOC310429    Locus Link:   29728 /// 310429    Chromosome:   2    Cytoband:   Chr:2q31    SOURCE    GENECARD       Gene Ontology:   cellular component:  nucleus      BioCarta Pathways:     KEGG Pathways:  
    Probe set : 1389857_at    Name:   similar to WW domain binding protein 5    GB acc:   BG378874    UniGene:   Rn.6930    Gene Symbol:   LOC294067    Locus Link:   294067    Chromosome:   X    Cytoband:   Chr:Xq35    SOURCE    GENECARD       Gene Ontology:     BioCarta Pathways:     KEGG Pathways:  
    Probe set : 1372294_at    Name:   PE responsive protein c64    GB acc:   BI278571    UniGene:   Rn.40541     Locus Link:   554172      SOURCE        Gene Ontology:     BioCarta Pathways:     KEGG Pathways:  
    Probe set : 1373506_at    Name:   Guanylate nucleotide binding protein 2    GB acc:   AA944568    UniGene:   Rn.25736    Gene Symbol:   Gbp2    Locus Link:   171164    Chromosome:   2    Cytoband:   Chr:2q44    SOURCE    GENECARD       Gene Ontology:   biological process:  immune response   biological process:  regulation of transcription, DNA-dependent      BioCarta Pathways:     KEGG Pathways:  
    Probe set : 1387793_at    Name:   ERM-binding phosphoprotein    GB acc:   NM_021594    UniGene:   Rn.35142    Gene Symbol:   Slc9a3r1    Locus Link:   59114    Chromosome:   10    Cytoband:   Chr:10q32.2    SOURCE    GENECARD       Gene Ontology:   biological process:  Wnt receptor signaling pathway   biological process:  cAMP-mediated signaling   biological process:  phosphate ion homeostasis   cellular component:  apical plasma membrane   cellular component:  apical part of cell   molecular function:  protein binding      BioCarta Pathways:     KEGG Pathways:  
    Probe set : 1386861_at    Name:   H2A histone family, member Z    GB acc:   NM_022674    UniGene:   Rn.112573    Gene Symbol:   H2afz    Locus Link:   58940    Chromosome:   2    Cytoband:   Chr:2q44    SOURCE    GENECARD       Gene Ontology:   molecular function:  DNA binding   molecular function:  DNA binding      BioCarta Pathways:     KEGG Pathways:  
    Probe set : 1388705_at    Name:   similar to selenoprotein SelM    GB acc:   BI282694    UniGene:   Rn.8166    Gene Symbol:   LOC498398    Locus Link:   498398    Chromosome:   14    Cytoband:   Chr:14q21    SOURCE    GENECARD       Gene Ontology:     BioCarta Pathways:     KEGG Pathways:  
    Probe set : 1383181_at    Name:   DnaJ (Hsp40) homolog, subfamily C, member 9 (predicted)    GB acc:   AI704947    UniGene:   Rn.16186    Gene Symbol:   Dnajc9_predicted    Locus Link:   364240    Chromosome:   15    Cytoband:   Chr:15p16    SOURCE    GENECARD       Gene Ontology:   biological process:  protein folding   molecular function:  heat shock protein binding   molecular function:  unfolded protein binding      BioCarta Pathways:     KEGG Pathways:  
    Probe set : 1375170_at    Name:   S100 calcium binding protein A11 (calizzarin) (predicted)    GB acc:   BG378926    UniGene:   Rn.145400    Gene Symbol:   S100a11_predicted    Locus Link:   445415    Chromosome:   2    Cytoband:   Chr:2q34    SOURCE    GENECARD       Gene Ontology:   biological process:  negative regulation of DNA replication   biological process:  negative regulation of cell proliferation   cellular component:  nucleus   cellular component:  cytoplasm   cellular component:  extracellular space   molecular function:  cytokine activity   molecular function:  calcium ion binding      BioCarta Pathways:     KEGG Pathways:  
    Probe set : 1370345_at    Name:   cyclin B1    GB acc:   L11995    UniGene:   Rn.145413    Gene Symbol:   Ccnb1    Locus Link:   25203    Chromosome:   2    Cytoband:   Chr:2q12    SOURCE    GENECARD       Gene Ontology:   cellular component:  nucleus   molecular function:  protein binding   molecular function:  kinase activity   molecular function:  cyclin-dependent protein kinase regulator activity      BioCarta Pathways:     KEGG Pathways:  
    Probe set : 1387600_at    Name:   gamma-aminobutyric acid (GABA) A receptor, pi    GB acc:   NM_031029    UniGene:   Rn.21401    Gene Symbol:   Gabrp    Locus Link:   81658    Chromosome:   10    Cytoband:   Chr:10q12    SOURCE    GENECARD       Gene Ontology:     BioCarta Pathways:     KEGG Pathways:  
    Probe set : 1385706_at    Name:   Similar to Testis derived transcript    GB acc:   BI292758    UniGene:   Rn.44847     Locus Link:   500040      SOURCE        Gene Ontology:     BioCarta Pathways:     KEGG Pathways:  
    Probe set : 1387372_at    Name:   solute carrier family 6 (neurotransmitter transporter, GABA), member 13    GB acc:   NM_133623    UniGene:   Rn.10527    Gene Symbol:   Slc6a13    Locus Link:   171163    Chromosome:   4    Cytoband:   Chr:4q42    SOURCE    GENECARD       Gene Ontology:     BioCarta Pathways:     KEGG Pathways:  
    Probe set : 1392265_s_at    Name:   Matrix metalloproteinase 23    GB acc:   AI548892    UniGene:   Rn.22562    Gene Symbol:   Mmp23    Locus Link:   94339    Chromosome:   5    Cytoband:   Chr:5q36    SOURCE    GENECARD       Gene Ontology:   biological process:  proteolysis   biological process:  transport   cellular component:  extracellular matrix (sensu Metazoa)   cellular component:  extracellular space   cellular component:  intracellular      BioCarta Pathways:     KEGG Pathways:  
    Probe set : 1389130_at    Name:   E74-like factor 2 (predicted)    GB acc:   AW525131    UniGene:   Rn.107459     Locus Link:   361944    Chromosome:   2    Cytoband:   Chr:2q26    SOURCE        Gene Ontology:   biological process:  regulation of transcription, DNA-dependent   cellular component:  nucleus   molecular function:  DNA binding   molecular function:  transcription factor activity   molecular function:  transcriptional activator activity      BioCarta Pathways:     KEGG Pathways:  
    Probe set : 1389668_at    Name:   similar to AD024 protein (predicted)    GB acc:   AI408269    UniGene:   Rn.16268    Gene Symbol:   RGD1307811_predicted    Locus Link:   295661    Chromosome:   3    Cytoband:   Chr:3q21    SOURCE    GENECARD       Gene Ontology:     BioCarta Pathways:     KEGG Pathways:  
    Probe set : 1385070_at    Name:   Transcribed locus, moderately similar to XP_488956.1 PREDICTED: hypothetical protein LOC66602 [Mus musculus]    GB acc:   BE111420    UniGene:   Rn.4180        SOURCE        Gene Ontology:     BioCarta Pathways:     KEGG Pathways:  
    Probe set : 1376574_at    Name:   Lymphocyte antigen 68    GB acc:   BI282932    UniGene:   Rn.17490    Gene Symbol:   C1qr1    Locus Link:   84398    Chromosome:   3    Cytoband:   Chr:3q41    SOURCE    GENECARD       Gene Ontology:     BioCarta Pathways:     KEGG Pathways:  
    Probe set : 1368270_at    Name:   apolipoprotein B editing complex 1    GB acc:   NM_012907    UniGene:   Rn.10002    Gene Symbol:   Apobec1    Locus Link:   25383    Chromosome:   4    Cytoband:   Chr:4q42    SOURCE    GENECARD       Gene Ontology:   cellular component:  nucleus   cellular component:  nucleus      BioCarta Pathways:     KEGG Pathways:  
    Probe set : 1378070_at    Name:   Transcribed locus    GB acc:   BE099891    UniGene:   Rn.18121        SOURCE        Gene Ontology:     BioCarta Pathways:     KEGG Pathways:  
    Probe set : 1388339_at    Name:   phosphoprotein enriched in astrocytes 15 (predicted)    GB acc:   BE112895    UniGene:   Rn.7282    Gene Symbol:   Pea15_predicted    Locus Link:   364052    Chromosome:   13    Cytoband:   Chr:13q24    SOURCE    GENECARD       Gene Ontology:   cellular component:  membrane fraction   cellular component:  cytosol   cellular component:  microtubule associated complex   molecular function:  protein kinase C binding   molecular function:  sugar porter activity   molecular function:  protein binding      BioCarta Pathways:     KEGG Pathways:  
    Probe set : 1397526_at    Name:   glutaryl-Coenzyme A dehydrogenase (predicted)    GB acc:   AI412068    UniGene:   Rn.99039    Gene Symbol:   Gcdh_predicted    Locus Link:   364975    Chromosome:   19    Cytoband:   Chr:19q11    SOURCE    GENECARD       Gene Ontology:   biological process:  electron transport   biological process:  regulation of transcription, DNA-dependent   cellular component:  mitochondrion      BioCarta Pathways:     KEGG Pathways:  
    Probe set : 1368897_at    Name:   MAD homolog 7 (Drosophila)    GB acc:   AF042499    UniGene:   Rn.29980    Gene Symbol:   Madh7    Locus Link:   81516    Chromosome:   18    Cytoband:   Chr:18q12.3    SOURCE    GENECARD       Gene Ontology:   cellular component:  intracellular   cellular component:  nucleus      BioCarta Pathways:     KEGG Pathways:  
    Probe set : 1371815_at    Name:   microfibrillar-associated protein 2 (predicted)    GB acc:   BM386326    UniGene:   Rn.17012    Gene Symbol:   Mfap2_predicted    Locus Link:   313662    Chromosome:   5    Cytoband:   Chr:5q36    SOURCE    GENECARD       Gene Ontology:   cellular component:  microfibril   cellular component:  extracellular matrix (sensu Metazoa)   cellular component:  extracellular space   molecular function:  extracellular matrix structural constituent      BioCarta Pathways:     KEGG Pathways:  
    Probe set : 1373592_at    Name:   similar to SPI6    GB acc:   AI407094    UniGene:   Rn.95177    Gene Symbol:   MGC94010    Locus Link:   361241    Chromosome:   17    Cytoband:   Chr:17p12    SOURCE    GENECARD       Gene Ontology:   molecular function:  serine-type endopeptidase inhibitor activity      BioCarta Pathways:     KEGG Pathways:  
    Probe set : 1387029_at    Name:   complement component factor H    GB acc:   NM_130409    UniGene:   Rn.101777    Gene Symbol:   Cfh    Locus Link:   155012    Chromosome:   13    Cytoband:   Chr:13q13    SOURCE    GENECARD       Gene Ontology:   molecular function:  protein binding      BioCarta Pathways:     KEGG Pathways:  
    Probe set : 1379498_at    Name:   Transcribed locus, weakly similar to NP_060380.2 hypothetical protein FLJ20628 [Homo sapiens]    GB acc:   AW534136    UniGene:   Rn.48269        SOURCE        Gene Ontology:     BioCarta Pathways:     KEGG Pathways:  
    Probe set : 1372439_at    Name:   procollagen, type IV, alpha 1 (predicted)    GB acc:   AI176393    UniGene:   Rn.53801    Gene Symbol:   Col4a1_predicted    Locus Link:   290905    Chromosome:   16    Cytoband:   Chr:16q12.5    SOURCE    GENECARD       Gene Ontology:   biological process:  phosphate transport   biological process:  cell adhesion      BioCarta Pathways:     KEGG Pathways:  
    Probe set : 1368317_at    Name:   aquaporin 7    GB acc:   NM_019157    UniGene:   Rn.11111    Gene Symbol:   Aqp7    Locus Link:   29171    Chromosome:   5    Cytoband:   Chr:5q22    SOURCE    GENECARD       Gene Ontology:   cellular component:  integral to plasma membrane   cellular component:  membrane   cellular component:  integral to membrane      BioCarta Pathways:     KEGG Pathways:  
    Probe set : 1379440_at    Name:   follistatin-like 3    GB acc:   AW144239    UniGene:   Rn.48835    Gene Symbol:   Fstl3    Locus Link:   114031    Chromosome:   7    Cytoband:   Chr:7q11    SOURCE    GENECARD       Gene Ontology:   biological process:  spermatogenesis   biological process:  negative regulation of BMP signaling pathway   biological process:  secretory pathway   cellular component:  extracellular space   cellular component:  secretory granule   molecular function:  protein binding   molecular function:  activin inhibitor activity   molecular function:  activin binding      BioCarta Pathways:     KEGG Pathways:  
    Probe set : 1371924_at    Name:   olfactomedin-like 3 (predicted)    GB acc:   BI274355    UniGene:   Rn.106761    Gene Symbol:   Olfml3_predicted    Locus Link:   310743    Chromosome:   2    Cytoband:   Chr:2q34    SOURCE    GENECARD       Gene Ontology:   cellular component:  extracellular space      BioCarta Pathways:     KEGG Pathways:  
    Probe set : 1390839_at    Name:   similar to RIKEN cDNA E030024M05 (predicted)    GB acc:   AW915795    UniGene:   Rn.12743    Gene Symbol:   RGD1306818_predicted    Locus Link:   298906    Chromosome:   6    Cytoband:   Chr:6q15    SOURCE    GENECARD       Gene Ontology:     BioCarta Pathways:     KEGG Pathways:  
    Probe set : 1391437_at    Name:   similar to BC002216 protein    GB acc:   AA874995    UniGene:   Rn.3197    Gene Symbol:   RGD:1359656    Locus Link:   313771    Chromosome:   5    Cytoband:   Chr:5q36    SOURCE    GENECARD       Gene Ontology:     BioCarta Pathways:     KEGG Pathways:  
    Probe set : 1369100_at    Name:   angiotensin/vasopressin receptor    GB acc:   NM_134375    UniGene:   Rn.9669    Gene Symbol:   Nalp6    Locus Link:   171390    Chromosome:   1    Cytoband:   Chr:1q41    SOURCE    GENECARD       Gene Ontology:   cellular component:  integral to membrane      BioCarta Pathways:     KEGG Pathways:  
    Probe set : 1398644_at    Name:   Transcribed locus    GB acc:   AI535104    UniGene:   Rn.34740        SOURCE        Gene Ontology:     BioCarta Pathways:     KEGG Pathways:  
    Probe set : 1390384_at    Name:   similar to Histone H2A.x (H2a/x)    GB acc:   AA875257    UniGene:   Rn.2850    Gene Symbol:   LOC500987    Locus Link:   500987    Chromosome:   8    Cytoband:   Chr:8q22    SOURCE    GENECARD       Gene Ontology:     BioCarta Pathways:     KEGG Pathways:  
    Probe set : 1368172_a_at    Name:   lysyl oxidase    GB acc:   BI304009    UniGene:   Rn.11372    Gene Symbol:   Lox    Locus Link:   24914    Chromosome:   18    Cytoband:   Chr:18q11    SOURCE    GENECARD       Gene Ontology:   biological process:  protein modification   cellular component:  extracellular matrix (sensu Metazoa)   cellular component:  extracellular space      BioCarta Pathways:     KEGG Pathways:  
    Probe set : 1367569_at    Name:   laminin receptor 1 (67kD, ribosomal protein SA)    GB acc:   NM_017138    UniGene:   Rn.999    Gene Symbol:   Lamr1    Locus Link:   29236    Chromosome:   8    Cytoband:   Chr:8q32    SOURCE    GENECARD       Gene Ontology:   biological process:  protein biosynthesis      BioCarta Pathways:     KEGG Pathways:  
    Probe set : 1386885_at    Name:   enoyl coenzyme A hydratase 1, peroxisomal    GB acc:   NM_022594    UniGene:   Rn.6148    Gene Symbol:   Ech1    Locus Link:   64526    Chromosome:   1    Cytoband:   Chr:1q21    SOURCE    GENECARD       Gene Ontology:   cellular component:  mitochondrion   cellular component:  peroxisome      BioCarta Pathways:     KEGG Pathways:  
    Probe set : 1373151_at    Name:   similar to RIKEN cDNA 2810489O06    GB acc:   AW252169    UniGene:   Rn.6635    Gene Symbol:   MGC109397    Locus Link:   499615    Chromosome:   2    Cytoband:   Chr:2q26    SOURCE    GENECARD       Gene Ontology:     BioCarta Pathways:     KEGG Pathways:  
    Probe set : 1379741_at    Name:   ATPase, H+ transporting, lysosomal V0 subunit A isoform 4 (predicted)    GB acc:   BG377116    UniGene:   Rn.24723    Gene Symbol:   Atp6v0a4_predicted    Locus Link:   296981    Chromosome:   4    Cytoband:   Chr:4q22    SOURCE    GENECARD       Gene Ontology:   cellular component:  membrane   cellular component:  integral to membrane   cellular component:  hydrogen-translocating V-type ATPase complex   molecular function:  hydrogen-exporting ATPase activity, phosphorylative mechanism   molecular function:  hydrogen ion transporter activity      BioCarta Pathways:     KEGG Pathways:  
    Probe set : 1377702_at    Name:   Retinoblastoma 1    GB acc:   BG380173    UniGene:   Rn.55115    Gene Symbol:   Rb1    Locus Link:   24708    Chromosome:   15    Cytoband:   Chr:15q12    SOURCE    GENECARD       Gene Ontology:     BioCarta Pathways:     KEGG Pathways:  
    Probe set : 1384200_at     GB acc:   BE107328              Gene Ontology:     BioCarta Pathways:     KEGG Pathways:  
    Probe set : 1367705_at    Name:   glutaredoxin 1 (thioltransferase)    GB acc:   AF319950    UniGene:   Rn.1484    Gene Symbol:   Glrx1    Locus Link:   64045    Chromosome:   2    Cytoband:   Chr:2q11    SOURCE    GENECARD       Gene Ontology:   biological process:  electron transport   biological process:  transport   biological process:  cell redox homeostasis      BioCarta Pathways:     KEGG Pathways:  
    Probe set : 1381162_at    Name:   Similar to septin 10 isoform 1    GB acc:   AI136863    UniGene:   Rn.27246     Locus Link:   309891    Chromosome:   20    Cytoband:   Chr:20q13    SOURCE        Gene Ontology:   biological process:  cell cycle   biological process:  cell division   cellular component:  nucleus   molecular function:  nucleotide binding   molecular function:  GTP binding      BioCarta Pathways:     KEGG Pathways:  
    Probe set : 1393848_at    Name:   ribonucleotide reductase M2    GB acc:   AA957260    UniGene:   Rn.101236    Gene Symbol:   Rrm2    Locus Link:   362720    Chromosome:   6    Cytoband:   Chr:6q16    SOURCE    GENECARD       Gene Ontology:   biological process:  DNA replication   biological process:  deoxyribonucleoside diphosphate metabolism   biological process:  deoxyribonucleotide metabolism   cellular component:  cytoplasm   cellular component:  ribonucleoside-diphosphate reductase complex      BioCarta Pathways:     KEGG Pathways:  
    Probe set : 1367578_at    Name:   peroxiredoxin 2    GB acc:   NM_017169    UniGene:   Rn.2511    Gene Symbol:   Prdx2    Locus Link:   29338    Chromosome:   19    Cytoband:   Chr:19q11    SOURCE    GENECARD       Gene Ontology:   biological process:  oxygen and reactive oxygen species metabolism   biological process:  anti-apoptosis   biological process:  response to oxidative stress   cellular component:  cytoplasm      BioCarta Pathways:     KEGG Pathways:  
    Probe set : 1376055_at    Name:   Transcribed locus    GB acc:   AA859768    UniGene:   Rn.123        SOURCE        Gene Ontology:     BioCarta Pathways:     KEGG Pathways:  
    Probe set : 1367672_at    Name:   hydroxysteroid (17-beta) dehydrogenase 4    GB acc:   NM_024392    UniGene:   Rn.2082    Gene Symbol:   Hsd17b4    Locus Link:   79244    Chromosome:   18    Cytoband:   Chr:18q11    SOURCE    GENECARD       Gene Ontology:   cellular component:  mitochondrion   cellular component:  peroxisome      BioCarta Pathways:     KEGG Pathways:  
    Probe set : 1378008_at    Name:   Transcribed locus    GB acc:   BF417386    UniGene:   Rn.105995        SOURCE        Gene Ontology:     BioCarta Pathways:     KEGG Pathways:  
    Probe set : 1388611_at    Name:   transcription elongation factor A (SII), 3 (predicted)    GB acc:   AW252817    UniGene:   Rn.11547    Gene Symbol:   Tcea3_predicted    Locus Link:   298559    Chromosome:   5    Cytoband:   Chr:5q36    SOURCE    GENECARD       Gene Ontology:   cellular component:  nucleus   molecular function:  DNA binding   molecular function:  transcription factor activity   molecular function:  translation elongation factor activity      BioCarta Pathways:     KEGG Pathways:  
    Probe set : 1376105_at    Name:   collagen, type XIV, alpha 1 (undulin) (predicted)    GB acc:   AI599143    UniGene:   Rn.99441    Gene Symbol:   Col14a1_predicted    Locus Link:   314981    Chromosome:   7    Cytoband:   Chr:7q32    SOURCE    GENECARD       Gene Ontology:   biological process:  phosphate transport   biological process:  cell adhesion   biological process:  collagen fibril organization   cellular component:  extracellular matrix (sensu Metazoa)   cellular component:  cytoplasm   molecular function:  extracellular matrix structural constituent   molecular function:  protein binding      BioCarta Pathways:     KEGG Pathways:  
    Probe set : 1390172_at    Name:   dehydrogenase E1 and transketolase domain containing 1 (predicted)    GB acc:   AI409946    UniGene:   Rn.13539    Gene Symbol:   Dhtkd1_predicted    Locus Link:   361272    Chromosome:   17    Cytoband:   Chr:17q12.3    SOURCE    GENECARD       Gene Ontology:   biological process:  metabolism   biological process:  glycolysis   biological process:  electron transport      BioCarta Pathways:     KEGG Pathways:  
    Probe set : 1398294_at    Name:   actinin, alpha 1    GB acc:   NM_031005    UniGene:   Rn.6401    Gene Symbol:   Actn1    Locus Link:   81634    Chromosome:   6    Cytoband:   Chr:6q24    SOURCE    GENECARD       Gene Ontology:   cellular component:  cytoskeleton   cellular component:  actin cytoskeleton      BioCarta Pathways:     KEGG Pathways:  
    Probe set : 1369407_at    Name:   tumor necrosis factor receptor superfamily, member 11b (osteoprotegerin)    GB acc:   NM_012870    UniGene:   Rn.9792    Gene Symbol:   Tnfrsf11b    Locus Link:   25341    Chromosome:   7    Cytoband:   Chr:7q31    SOURCE    GENECARD       Gene Ontology:   cellular component:  extracellular region   cellular component:  extracellular matrix (sensu Metazoa)   cellular component:  extracellular space   molecular function:  receptor activity   molecular function:  cytokine activity   molecular function:  protein binding      BioCarta Pathways:     KEGG Pathways:  
    Probe set : 1389103_at    Name:   similar to RIKEN cDNA 2810037C03 (predicted)    GB acc:   BM391005    UniGene:   Rn.103411    Gene Symbol:   RGD1307397_predicted    Locus Link:   290842    Chromosome:   16    Cytoband:   Chr:16q12.5    SOURCE    GENECARD       Gene Ontology:     BioCarta Pathways:     KEGG Pathways:  
    Probe set : 1397609_at    Name:   TRAF4 associated factor 1    GB acc:   BF287466    UniGene:   Rn.20235    Gene Symbol:   RGD:1303240    Locus Link:   311325    Chromosome:   3    Cytoband:   Chr:3q35    SOURCE    GENECARD       Gene Ontology:     BioCarta Pathways:     KEGG Pathways:  
    Probe set : 1374775_at    Name:   antigen identified by monoclonal antibody Ki-67 (predicted) /// similar to Mki67 protein /// similar to Ki-67    GB acc:   AI714002    UniGene:   Rn.73551    Gene Symbol:   Mki67_predicted /// LOC310382 /// LOC366937    Locus Link:   291234 /// 310382 /// 366937    Chromosome:   1    Cytoband:   Chr:1q41 /// Chr:2q26 /// Chr:7q32    SOURCE    GENECARD       Gene Ontology:   biological process:  regulation of progression through cell cycle   biological process:  meiosis   biological process:  cell proliferation   molecular function:  ATP binding      BioCarta Pathways:     KEGG Pathways:  
    Probe set : 1388628_at    Name:   integral type I protein    GB acc:   BI284430    UniGene:   Rn.98226    Gene Symbol:   RGD:1303327    Locus Link:   300888    Chromosome:   8    Cytoband:   Chr:8q31    SOURCE    GENECARD       Gene Ontology:   biological process:  transport   biological process:  intracellular protein transport   biological process:  protein transport   cellular component:  Golgi stack   cellular component:  membrane   cellular component:  integral to membrane   molecular function:  protein carrier activity      BioCarta Pathways:     KEGG Pathways:  
    Probe set : 1370811_at    Name:   mercaptopyruvate sulfurtransferase    GB acc:   D50564    UniGene:   Rn.32263    Gene Symbol:   Mpst    Locus Link:   192172    Chromosome:   7    Cytoband:   Chr:7q34    SOURCE    GENECARD       Gene Ontology:   biological process:  sulfate transport   biological process:  cyanate catabolism   biological process:  response to toxin   cellular component:  extracellular space   cellular component:  mitochondrion   cellular component:  mitochondrial matrix      BioCarta Pathways:     KEGG Pathways:  
    Probe set : 1386879_at    Name:   lectin, galactose binding, soluble 3    GB acc:   NM_031832    UniGene:   Rn.764    Gene Symbol:   Lgals3    Locus Link:   83781    Chromosome:   15    Cytoband:   Chr:15p14    SOURCE    GENECARD       Gene Ontology:   biological process:  heterophilic cell adhesion   cellular component:  extracellular region   cellular component:  nucleus   cellular component:  cytoplasm   cellular component:  plasma membrane   molecular function:  protein binding   molecular function:  sugar binding   molecular function:  IgE binding      BioCarta Pathways:     KEGG Pathways:  
    Probe set : 1369621_s_at    Name:   FK506 binding protein 1a    GB acc:   NM_053308    UniGene:   Rn.80611    Gene Symbol:   Fkbp1a    Locus Link:   25639    Chromosome:   3    Cytoband:   Chr:3q41    SOURCE    GENECARD       Gene Ontology:   biological process:  protein folding   biological process:  transport   biological process:  positive regulation of I-kappaB kinase/NF-kappaB cascade   cellular component:  microsome   cellular component:  membrane      BioCarta Pathways:     KEGG Pathways:  
    Probe set : 1368838_at    Name:   tropomyosin 4    GB acc:   NM_012678    UniGene:   Rn.108199    Gene Symbol:   Tpm4    Locus Link:   24852    Chromosome:   16    Cytoband:   Chr:16p14    SOURCE    GENECARD       Gene Ontology:   biological process:  muscle contraction   biological process:  muscle contraction   biological process:  muscle development   cellular component:  cytoskeleton   cellular component:  kinesin complex   molecular function:  actin binding      BioCarta Pathways:     KEGG Pathways:  
    Probe set : 1382185_at    Name:   similar to C1q and tumor necrosis factor related protein 2    GB acc:   BG377259    UniGene:   Rn.107489    Gene Symbol:   LOC497886    Locus Link:   497886    Chromosome:   10    Cytoband:   Chr:10q21    SOURCE    GENECARD       Gene Ontology:     BioCarta Pathways:     KEGG Pathways:  
    Probe set : 1389682_at    Name:   similar to RIKEN cDNA 2510006C20 (predicted)    GB acc:   BF284967    UniGene:   Rn.113823    Gene Symbol:   RGD1309120_predicted    Locus Link:   360994    Chromosome:   14    Cytoband:   Chr:14q22    SOURCE    GENECARD       Gene Ontology:     BioCarta Pathways:     KEGG Pathways:  
    Probe set : 1388884_at    Name:   similar to RIKEN cDNA 1810022C23 (predicted)    GB acc:   BM390774    UniGene:   Rn.3194    Gene Symbol:   RGD1310224_predicted    Locus Link:   291076    Chromosome:   17    Cytoband:   Chr:17p12    SOURCE    GENECARD       Gene Ontology:   biological process:  metabolism   cellular component:  peroxisome   molecular function:  catalytic activity   molecular function:  nucleotide binding   molecular function:  RNA binding      BioCarta Pathways:     KEGG Pathways:  
    Probe set : 1370080_at    Name:   heme oxygenase (decycling) 1    GB acc:   NM_012580    UniGene:   Rn.3160    Gene Symbol:   Hmox1    Locus Link:   24451    Chromosome:   19    Cytoband:   Chr:19p11    SOURCE    GENECARD       Gene Ontology:     BioCarta Pathways:     KEGG Pathways:  
    Probe set : 1392837_at    Name:   P34 protein    GB acc:   AA859108    UniGene:   Rn.144663    Gene Symbol:   P34    Locus Link:   171435    Chromosome:   8    Cytoband:   Chr:8q24    SOURCE    GENECARD       Gene Ontology:   biological process:  transport   biological process:  protein transport      BioCarta Pathways:     KEGG Pathways:  
    Probe set : 1373447_at    Name:   similar to HN1-like protein (predicted)    GB acc:   BF281350    UniGene:   Rn.19357    Gene Symbol:   RGD1305117_predicted    Locus Link:   360492    Chromosome:   10    Cytoband:   Chr:10q12    SOURCE    GENECARD       Gene Ontology:   biological process:  regulation of transcription, DNA-dependent   cellular component:  nucleus   molecular function:  DNA binding      BioCarta Pathways:     KEGG Pathways:  
    Probe set : 1372481_at    Name:   Transcribed locus, moderately similar to NP_598415.1 CD34 antigen [Mus musculus]    GB acc:   AI102873    UniGene:   Rn.2721        SOURCE        Gene Ontology:     BioCarta Pathways:     KEGG Pathways:  
    Probe set : 1383879_at    Name:   Transcribed locus    GB acc:   BG377332    UniGene:   Rn.146446        SOURCE        Gene Ontology:     BioCarta Pathways:     KEGG Pathways:  
    Probe set : 1375944_at     GB acc:   AA849497              Gene Ontology:     BioCarta Pathways:     KEGG Pathways:  
    Probe set : 1369968_at    Name:   pleiotrophin    GB acc:   NM_017066    UniGene:   Rn.1653    Gene Symbol:   Ptn    Locus Link:   24924    Chromosome:   4    Cytoband:   Chr:4q22    SOURCE    GENECARD       Gene Ontology:   cellular component:  extracellular matrix (sensu Metazoa)   cellular component:  extracellular space      BioCarta Pathways:     KEGG Pathways:  
    Probe set : 1376218_a_at    Name:   Atpase, class I, type 8B, member 2 (predicted)    GB acc:   BI273855    UniGene:   Rn.20321     Locus Link:   361984    Chromosome:   2    Cytoband:   Chr:2q34    SOURCE        Gene Ontology:   biological process:  cation transport   biological process:  metabolism   cellular component:  membrane   cellular component:  integral to membrane      BioCarta Pathways:     KEGG Pathways:  
    Probe set : 1374198_at    Name:   B7 homlog 3    GB acc:   BF398424    UniGene:   Rn.76129    Gene Symbol:   B7h3    Locus Link:   315716    Chromosome:   8    Cytoband:   Chr:8q24    SOURCE    GENECARD       Gene Ontology:   biological process:  negative regulation of T cell proliferation      BioCarta Pathways:     KEGG Pathways:  
    Probe set : 1371912_at    Name:   NADH dehydrogenase (ubiquinone) Fe-S protein 7 (predicted)    GB acc:   AI231358    UniGene:   Rn.2855    Gene Symbol:   Ndufs7_predicted    Locus Link:   362837    Chromosome:   7    Cytoband:   Chr:7q11    SOURCE    GENECARD       Gene Ontology:   biological process:  mitochondrial electron transport, NADH to ubiquinone   cellular component:  extracellular space   cellular component:  mitochondrion   molecular function:  NADH dehydrogenase activity   molecular function:  NADH dehydrogenase (ubiquinone) activity   molecular function:  oxidoreductase activity      BioCarta Pathways:     KEGG Pathways:  
    Probe set : 1371947_at    Name:   necdin (predicted)    GB acc:   BG671865    UniGene:   Rn.3096    Gene Symbol:   Ndn_predicted    Locus Link:   308690    Chromosome:   1    Cytoband:   Chr:1q22    SOURCE    GENECARD       Gene Ontology:   cellular component:  nucleus   molecular function:  DNA binding   molecular function:  protein binding      BioCarta Pathways:     KEGG Pathways:  
    Probe set : 1379957_at    Name:   similar to schlafen 8    GB acc:   BE107457    UniGene:   Rn.137139    Gene Symbol:   RGD:1359139    Locus Link:   303378    Chromosome:   10    Cytoband:   Chr:10q26    SOURCE    GENECARD       Gene Ontology:     BioCarta Pathways:     KEGG Pathways:  
    Probe set : 1386882_at    Name:   t-complex testis expressed 1    GB acc:   NM_031318    UniGene:   Rn.29901    Gene Symbol:   Tctex1    Locus Link:   83462    Chromosome:   1    Cytoband:   Chr:1q11    SOURCE    GENECARD       Gene Ontology:   biological process:  microtubule-based process   molecular function:  motor activity   molecular function:  microtubule motor activity   molecular function:  protein binding      BioCarta Pathways:     KEGG Pathways:  
    Probe set : 1373054_at    Name:   CDW92 antigen    GB acc:   AA801076    UniGene:   Rn.97686    Gene Symbol:   Cdw92    Locus Link:   85254    Chromosome:   5    Cytoband:   Chr:5q24    SOURCE    GENECARD       Gene Ontology:   biological process:  choline transport   biological process:  transport   cellular component:  integral to membrane   molecular function:  choline transporter activity      BioCarta Pathways:     KEGG Pathways:  
    Probe set : 1373048_at    Name:   ARP10 actin-related protein 10 homolog (S. cerevisiae) (predicted)    GB acc:   AI177051    UniGene:   Rn.36338    Gene Symbol:   Actr10_predicted    Locus Link:   299121    Chromosome:   6    Cytoband:   Chr:6q24    SOURCE    GENECARD       Gene Ontology:   biological process:  microtubule-based movement   cellular component:  cytoskeleton   cellular component:  dynactin complex   cellular component:  actin cytoskeleton   molecular function:  structural molecule activity   molecular function:  structural constituent of cytoskeleton   molecular function:  protein binding      BioCarta Pathways:     KEGG Pathways:  
    Probe set : 1390901_at    Name:   bone specific CMF608    GB acc:   BF543355    UniGene:   Rn.23485    Gene Symbol:   Igsf10    Locus Link:   310448    Chromosome:   2    Cytoband:   Chr:2q31    SOURCE    GENECARD       Gene Ontology:   biological process:  tissue regeneration   biological process:  ossification   biological process:  protein amino acid phosphorylation   molecular function:  vascular endothelial growth factor receptor activity   molecular function:  ATP binding      BioCarta Pathways:     KEGG Pathways:  
    Probe set : 1369989_at    Name:   pyridoxine 5'-phosphate oxidase    GB acc:   NM_022601    UniGene:   Rn.6299    Gene Symbol:   Pnpo    Locus Link:   64533      SOURCE    GENECARD       Gene Ontology:   biological process:  pyridoxine biosynthesis      BioCarta Pathways:     KEGG Pathways:  
    Probe set : 1370941_at    Name:   platelet derived growth factor receptor, alpha polypeptide    GB acc:   AI232379    UniGene:   Rn.55127    Gene Symbol:   Pdgfra    Locus Link:   25267    Chromosome:   14    Cytoband:   Chr:14p11    SOURCE    GENECARD       Gene Ontology:   molecular function:  protein kinase activity   molecular function:  protein serine/threonine kinase activity   molecular function:  protein-tyrosine kinase activity   molecular function:  transmembrane receptor protein tyrosine kinase activity   molecular function:  receptor activity   molecular function:  platelet-derived growth factor alpha-receptor activity   molecular function:  platelet-derived growth factor alpha-receptor activity   molecular function:  vascular endothelial growth factor receptor activity   molecular function:  ATP binding   molecular function:  kinase activity   molecular function:  transferase activity   molecular function:  protein dimerization activity   molecular function:  protein dimerization activity   molecular function:  platelet-derived growth factor binding   molecular function:  nucleotide binding      BioCarta Pathways:     KEGG Pathways:  
    Probe set : 1368224_at    Name:   Serine protease inhibitor    GB acc:   NM_031531    UniGene:   Rn.128    Gene Symbol:   Spin2c    Locus Link:   24795    Chromosome:   6    Cytoband:   Chr:6q32    SOURCE    GENECARD       Gene Ontology:   cellular component:  extracellular space   molecular function:  endopeptidase inhibitor activity   molecular function:  serine-type endopeptidase inhibitor activity      BioCarta Pathways:     KEGG Pathways:  
    Probe set : 1388393_at    Name:   proteolipid protein 2    GB acc:   BM385031    UniGene:   Rn.96974    Gene Symbol:   Plp2    Locus Link:   302562    Chromosome:   X    Cytoband:   Chr:Xq13    SOURCE    GENECARD       Gene Ontology:   cellular component:  integral to membrane   cellular component:  membrane   molecular function:  zinc ion binding   molecular function:  metal ion binding      BioCarta Pathways:     KEGG Pathways:  
    Probe set : 1387952_a_at    Name:   CD44 antigen    GB acc:   AF065147    UniGene:   Rn.1120    Gene Symbol:   Cd44    Locus Link:   25406    Chromosome:   3    Cytoband:   Chr:3q31    SOURCE    GENECARD       Gene Ontology:     BioCarta Pathways:     KEGG Pathways:  
    Probe set : 1393041_at    Name:   SMC2 structural maintenance of chromosomes 2-like 1 (yeast) (predicted)    GB acc:   AW535052    UniGene:   Rn.94452    Gene Symbol:   Smc2l1_predicted    Locus Link:   362519    Chromosome:   5    Cytoband:   Chr:5q23    SOURCE    GENECARD       Gene Ontology:   cellular component:  nucleus   cellular component:  chromosome   cellular component:  membrane      BioCarta Pathways:     KEGG Pathways:  
    Probe set : 1389326_at    Name:   replication factor C (activator 1) 3 (predicted)    GB acc:   AI103093    UniGene:   Rn.3711    Gene Symbol:   Rfc3_predicted    Locus Link:   288414    Chromosome:   12    Cytoband:   Chr:12p12    SOURCE    GENECARD       Gene Ontology:   biological process:  DNA replication   biological process:  DNA strand elongation      BioCarta Pathways:     KEGG Pathways:  
    Probe set : 1368992_a_at    Name:   splicing factor, arginine/serine-rich 5    GB acc:   AI104005    UniGene:   Rn.54448    Gene Symbol:   Sfrs5    Locus Link:   29667    Chromosome:   6    Cytoband:   Chr:6q24    SOURCE    GENECARD       Gene Ontology:   cellular component:  nucleus   cellular component:  spliceosome complex   molecular function:  nucleic acid binding   molecular function:  RNA binding   molecular function:  nucleotide binding      BioCarta Pathways:     KEGG Pathways:  
    Probe set : 1388479_at    Name:   Dihydropyrimidinase-like 3    GB acc:   BI294841    UniGene:   Rn.93365    Gene Symbol:   Dpysl3    Locus Link:   25418    Chromosome:   18    Cytoband:   Chr:18p11    SOURCE    GENECARD       Gene Ontology:   molecular function:  dihydropyrimidinase activity   molecular function:  hydrolase activity      BioCarta Pathways:     KEGG Pathways:  
    Probe set : 1385781_at    Name:   similar to SNF2/RAD54 family protein    GB acc:   BF397986    UniGene:   Rn.72855    Gene Symbol:   LOC317252    Locus Link:   317252    Chromosome:   X    Cytoband:   Chr:Xq31    SOURCE    GENECARD       Gene Ontology:     BioCarta Pathways:     KEGG Pathways:  
    Probe set : 1369950_at    Name:   cyclin-dependent kinase 4    GB acc:   NM_053593    UniGene:   Rn.6115    Gene Symbol:   Cdk4    Locus Link:   94201    Chromosome:   7    Cytoband:   Chr:7q22    SOURCE    GENECARD       Gene Ontology:   cellular component:  cyclin-dependent protein kinase holoenzyme complex   cellular component:  nucleus   cellular component:  transcription factor complex      BioCarta Pathways:     KEGG Pathways:  
    Probe set : 1374902_at    Name:   IQ motif containing GTPase activating protein 3 (predicted)    GB acc:   AI071398    UniGene:   Rn.17539    Gene Symbol:   Iqgap3_predicted    Locus Link:   310621    Chromosome:   2    Cytoband:   Chr:2q34    SOURCE    GENECARD       Gene Ontology:   biological process:  small GTPase mediated signal transduction   molecular function:  Ras GTPase activator activity   molecular function:  calmodulin binding      BioCarta Pathways:     KEGG Pathways:  
    Probe set : 1386933_at    Name:   glycoprotein 2 (zymogen granule membrane)    GB acc:   NM_134418    UniGene:   Rn.11223    Gene Symbol:   Gp2    Locus Link:   171459    Chromosome:   1    Cytoband:   Chr:1q35    SOURCE    GENECARD       Gene Ontology:   cellular component:  extracellular space   cellular component:  membrane fraction   cellular component:  membrane      BioCarta Pathways:     KEGG Pathways:  
    Probe set : 1378802_at    Name:   similar to hypothetical protein MGC13047    GB acc:   AI105366    UniGene:   Rn.102445    Gene Symbol:   RGD:1303232    Locus Link:   309381    Chromosome:   1    Cytoband:   Chr:1q54    SOURCE    GENECARD       Gene Ontology:   biological process:  electron transport   biological process:  carotenoid biosynthesis      BioCarta Pathways:     KEGG Pathways:  
    Probe set : 1375862_at     GB acc:   BM384701              Gene Ontology:     BioCarta Pathways:     KEGG Pathways:  
    Probe set : 1370566_at    Name:   retinol dehydrogenase 2    GB acc:   U33500    UniGene:   Rn.94108    Gene Symbol:   Rdh2    Locus Link:   299511    Chromosome:   7    Cytoband:   Chr:7q22    SOURCE    GENECARD       Gene Ontology:   biological process:  retinol metabolism   biological process:  retinoic acid metabolism   biological process:  metabolism   cellular component:  endoplasmic reticulum   cellular component:  microsome   molecular function:  retinol dehydrogenase activity   molecular function:  retinol dehydrogenase activity   molecular function:  oxidoreductase activity      BioCarta Pathways:     KEGG Pathways:  
    Probe set : 1393059_at    Name:   Transcribed locus    GB acc:   AI137360    UniGene:   Rn.24086        SOURCE        Gene Ontology:     BioCarta Pathways:     KEGG Pathways:  
    Probe set : 1376652_at    Name:   complement component 1, q subcomponent, alpha polypeptide (predicted)    GB acc:   BF418957    UniGene:   Rn.105647    Gene Symbol:   C1qa_predicted    Locus Link:   298566    Chromosome:   5    Cytoband:   Chr:5q36    SOURCE    GENECARD       Gene Ontology:   cellular component:  complement component C1q complex   cellular component:  extracellular space   cellular component:  cytoplasm      BioCarta Pathways:     KEGG Pathways:  
    Probe set : 1377452_at    Name:   tetranectin (plasminogen binding protein) (predicted)    GB acc:   AW251280    UniGene:   Rn.23478    Gene Symbol:   Tna_predicted    Locus Link:   316099    Chromosome:   8    Cytoband:   Chr:8q32    SOURCE    GENECARD       Gene Ontology:   biological process:  skeletal development   cellular component:  extracellular region   cellular component:  extracellular matrix (sensu Metazoa)   cellular component:  extracellular space   molecular function:  calcium ion binding   molecular function:  sugar binding      BioCarta Pathways:     KEGG Pathways:  
    Probe set : 1385458_a_at    Name:   similar to C11orf17 protein (predicted)    GB acc:   AA799992    UniGene:   Rn.106344    Gene Symbol:   RGD1306959_predicted    Locus Link:   361624    Chromosome:   1    Cytoband:   Chr:1q33    SOURCE    GENECARD       Gene Ontology:     BioCarta Pathways:     KEGG Pathways:  
    Probe set : 1379347_at    Name:   Similar to RIKEN cDNA 1700023M03 (predicted)    GB acc:   BM389290    UniGene:   Rn.13471    Gene Symbol:   MGC108928    Locus Link:   314730    Chromosome:   7    Cytoband:   Chr:7q13    SOURCE    GENECARD       Gene Ontology:     BioCarta Pathways:     KEGG Pathways:  
    Probe set : 1370550_at    Name:   limbic system-associated membrane protein    GB acc:   U31554    UniGene:   Rn.10176    Gene Symbol:   Lsamp    Locus Link:   29561    Chromosome:   11    Cytoband:   Chr:11q21    SOURCE    GENECARD       Gene Ontology:   biological process:  cell adhesion   cellular component:  extracellular space   cellular component:  membrane   molecular function:  protein binding      BioCarta Pathways:     KEGG Pathways:  
    Probe set : 1369976_at    Name:   dynein, cytoplasmic, light chain 1    GB acc:   NM_053319    UniGene:   Rn.35769    Gene Symbol:   Dnclc1    Locus Link:   58945    Chromosome:   12    Cytoband:   Chr:12q16    SOURCE    GENECARD       Gene Ontology:     BioCarta Pathways:     KEGG Pathways:  
    Probe set : 1371763_at    Name:   similar to RIKEN cDNA 4931406C07 (predicted)    GB acc:   BI274533    UniGene:   Rn.3085    Gene Symbol:   RGD1309534_predicted    Locus Link:   363016    Chromosome:   8    Cytoband:   Chr:8q12    SOURCE    GENECARD       Gene Ontology:     BioCarta Pathways:     KEGG Pathways:  
    Probe set : 1368304_at    Name:   Flavin containing monooxygenase 3    GB acc:   NM_053433    UniGene:   Rn.28357    Gene Symbol:   Fmo3    Locus Link:   84493    Chromosome:   13    Cytoband:   Chr:13q22    SOURCE    GENECARD       Gene Ontology:   biological process:  electron transport      BioCarta Pathways:     KEGG Pathways:  
    Probe set : 1374289_at    Name:   RNA polymerase 1-1 (predicted)    GB acc:   AA818183    UniGene:   Rn.107990    Gene Symbol:   Rpo1-1_predicted    Locus Link:   301246    Chromosome:   9    Cytoband:   Chr:9q12    SOURCE    GENECARD       Gene Ontology:   biological process:  transcription   biological process:  transcription from RNA polymerase I promoter   cellular component:  nucleus   cellular component:  DNA-directed RNA polymerase I complex      BioCarta Pathways:     KEGG Pathways:  
    Probe set : 1369259_at    Name:   deiodinase, iodothyronine, type I    GB acc:   NM_021653    UniGene:   Rn.87549    Gene Symbol:   Dio1    Locus Link:   25430    Chromosome:   5    Cytoband:   Chr:5q34    SOURCE    GENECARD       Gene Ontology:   cellular component:  extracellular space   cellular component:  integral to membrane   cellular component:  endoplasmic reticulum      BioCarta Pathways:     KEGG Pathways:  
    Probe set : 1388776_at    Name:   scotin    GB acc:   AI169176    UniGene:   Rn.102027    Gene Symbol:   RGD:1359661    Locus Link:   301013    Chromosome:   8    Cytoband:   Chr:8q32    SOURCE    GENECARD       Gene Ontology:     BioCarta Pathways:     KEGG Pathways:  
    Probe set : 1367614_at    Name:   annexin A1    GB acc:   NM_012904    UniGene:   Rn.1792    Gene Symbol:   Anxa1    Locus Link:   25380    Chromosome:   1    Cytoband:   Chr:1q51    SOURCE    GENECARD       Gene Ontology:   cellular component:  sarcolemma      BioCarta Pathways:     KEGG Pathways:  
    Probe set : 1370857_at    Name:   smooth muscle alpha-actin    GB acc:   BI282702    UniGene:   Rn.98160    Gene Symbol:   RGD:621676    Locus Link:   81633      SOURCE    GENECARD       Gene Ontology:   biological process:  muscle contraction   biological process:  muscle development   cellular component:  cytoskeleton   cellular component:  actin filament      BioCarta Pathways:     KEGG Pathways:  
    Probe set : 1373250_at     GB acc:   AI229404              Gene Ontology:     BioCarta Pathways:     KEGG Pathways:  
    Probe set : 1388271_at     GB acc:   BM383531              Gene Ontology:     BioCarta Pathways:     KEGG Pathways:  
    Probe set : 1371712_at    Name:   similar to snRNP core protein SMX5d    GB acc:   AI176503     Gene Symbol:   LOC292030    Locus Link:   292030    Chromosome:   19    Cytoband:   Chr:19q12     GENECARD       Gene Ontology:     BioCarta Pathways:     KEGG Pathways:  
    Probe set : 1388622_at    Name:   nucleolar protein 5A (predicted)    GB acc:   AW535890    UniGene:   Rn.145204    Gene Symbol:   Nol5a_predicted    Locus Link:   362214    Chromosome:   3    Cytoband:   Chr:3q36    SOURCE    GENECARD       Gene Ontology:   biological process:  rRNA processing   biological process:  ribosome biogenesis   cellular component:  nucleus   cellular component:  nucleolus   molecular function:  RNA binding      BioCarta Pathways:     KEGG Pathways:  
    Probe set : 1367693_at    Name:   tyrosine 3-monooxygenase/tryptophan 5-monooxygenase activation protein, eta polypeptide    GB acc:   NM_013052    UniGene:   Rn.3324    Gene Symbol:   Ywhah    Locus Link:   25576    Chromosome:   14    Cytoband:   Chr:14q21    SOURCE    GENECARD       Gene Ontology:   cellular component:  cytoplasm      BioCarta Pathways:     KEGG Pathways:  
    Probe set : 1367584_at    Name:   annexin A2    GB acc:   NM_019905    UniGene:   Rn.90546    Gene Symbol:   Anxa2    Locus Link:   56611    Chromosome:   8    Cytoband:   Chr:8q24    SOURCE    GENECARD       Gene Ontology:     BioCarta Pathways:     KEGG Pathways:  
    Probe set : 1369105_a_at    Name:   protein kinase (cAMP dependent, catalytic) inhibitor beta    GB acc:   AF413572    UniGene:   Rn.9748    Gene Symbol:   Pkib    Locus Link:   24678    Chromosome:   20    Cytoband:   Chr:20q12-q13    SOURCE    GENECARD       Gene Ontology:   biological process:  negative regulation of protein kinase activity   molecular function:  protein kinase inhibitor activity   molecular function:  cAMP-dependent protein kinase inhibitor activity   molecular function:  kinase activity      BioCarta Pathways:     KEGG Pathways:  
    Probe set : 1370215_at    Name:   complement component 1, q subcomponent, beta polypeptide    GB acc:   AW434057    UniGene:   Rn.6702    Gene Symbol:   C1qb    Locus Link:   29687    Chromosome:   5    Cytoband:   Chr:5q36    SOURCE    GENECARD       Gene Ontology:   cellular component:  complement component C1q complex   cellular component:  extracellular space   cellular component:  cytoplasm      BioCarta Pathways:     KEGG Pathways:  
    Probe set : 1370301_at    Name:   matrix metallopeptidase 2    GB acc:   U65656    UniGene:   Rn.6422    Gene Symbol:   Mmp2    Locus Link:   81686    Chromosome:   19    Cytoband:   Chr:19p11    SOURCE    GENECARD       Gene Ontology:   biological process:  proteolysis   biological process:  collagen catabolism   biological process:  peptidoglycan metabolism   cellular component:  extracellular matrix (sensu Metazoa)   cellular component:  extracellular space      BioCarta Pathways:     KEGG Pathways:  
    Probe set : 1383401_at    Name:   similar to Testis derived transcript    GB acc:   BI288769    UniGene:   Rn.44847    Gene Symbol:   LOC500040    Locus Link:   500040    Chromosome:   4    Cytoband:   Chr:4q21    SOURCE    GENECARD       Gene Ontology:     BioCarta Pathways:     KEGG Pathways:  
    Probe set : 1379770_at    Name:   similar to choline phosphotransferase 1; cholinephosphotransferase 1 alpha; cholinephosphotransferase 1    GB acc:   BF282951    UniGene:   Rn.16261    Gene Symbol:   RGD:1359283    Locus Link:   362866    Chromosome:   7    Cytoband:   Chr:7q13    SOURCE    GENECARD       Gene Ontology:   biological process:  phospholipid biosynthesis   molecular function:  transferase activity      BioCarta Pathways:     KEGG Pathways:  
    Probe set : 1368464_at    Name:   macrophage galactose N-acetyl-galactosamine specific lectin 1    GB acc:   NM_022393    UniGene:   Rn.779    Gene Symbol:   Mgl1    Locus Link:   64195    Chromosome:   10    Cytoband:   Chr:10q24    SOURCE    GENECARD       Gene Ontology:   cellular component:  membrane   cellular component:  integral to membrane   molecular function:  sugar binding   molecular function:  sugar binding   molecular function:  calcium ion binding      BioCarta Pathways:     KEGG Pathways:  
    Probe set : 1370228_at    Name:   Transferrin /// signal recognition particle receptor, B subunit (predicted)    GB acc:   AA945178    UniGene:   Rn.91296    Gene Symbol:   Tf /// Srprb_predicted    Locus Link:   24825 /// 300965    Chromosome:   8    Cytoband:   Chr:8q32    SOURCE    GENECARD       Gene Ontology:     BioCarta Pathways:     KEGG Pathways:  
    Probe set : 1392713_a_at     GB acc:   AA892541              Gene Ontology:     BioCarta Pathways:     KEGG Pathways:  
    Probe set : 1372154_at    Name:   similar to 2010321M09Rik protein    GB acc:   AW521019    UniGene:   Rn.9525    Gene Symbol:   RGD:1359616    Locus Link:   300782    Chromosome:   8    Cytoband:   Chr:8q24    SOURCE    GENECARD       Gene Ontology:     BioCarta Pathways:     KEGG Pathways:  
    Probe set : 1373595_at    Name:   similar to RIKEN cDNA 1200015A22    GB acc:   BM385463    UniGene:   Rn.15201    Gene Symbol:   MGC94413    Locus Link:   362401    Chromosome:   4    Cytoband:   Chr:4q34    SOURCE    GENECARD       Gene Ontology:     BioCarta Pathways:     KEGG Pathways:  
    Probe set : 1373172_at    Name:   Transcribed locus    GB acc:   AA944483    UniGene:   Rn.32516        SOURCE        Gene Ontology:     BioCarta Pathways:     KEGG Pathways:  
    Probe set : 1376707_at    Name:   C1q and tumor necrosis factor related protein 4 (predicted)    GB acc:   AW918311    UniGene:   Rn.24378    Gene Symbol:   C1qtnf4_predicted    Locus Link:   311184    Chromosome:   3    Cytoband:   Chr:3q24    SOURCE    GENECARD       Gene Ontology:     BioCarta Pathways:     KEGG Pathways:  
    Probe set : 1398751_at    Name:   similar to ribosomal protein S7    GB acc:   NM_031570     Gene Symbol:   LOC497813    Locus Link:   497813    Chromosome:   6    Cytoband:   Chr:6q16     GENECARD       Gene Ontology:     BioCarta Pathways:     KEGG Pathways:  
    Probe set : 1386857_at    Name:   stathmin 1    GB acc:   NM_017166    UniGene:   Rn.555    Gene Symbol:   Stmn1    Locus Link:   29332    Chromosome:   5    Cytoband:   Chr:5q36    SOURCE    GENECARD       Gene Ontology:   cellular component:  cytoplasm   cellular component:  cytosol   cellular component:  kinesin complex   cellular component:  microtubule   molecular function:  signal transducer activity   molecular function:  tubulin binding   molecular function:  tubulin binding      BioCarta Pathways:     KEGG Pathways:  
    Probe set : 1379059_at    Name:   similar to FLJ23471 protein (predicted)    GB acc:   BG373522    UniGene:   Rn.108004    Gene Symbol:   RGD1307875_predicted    Locus Link:   288515    Chromosome:   12    Cytoband:   Chr:12q11    SOURCE    GENECARD       Gene Ontology:     BioCarta Pathways:     KEGG Pathways:  
    Probe set : 1393280_at    Name:   lymphocyte antigen 86 (predicted)    GB acc:   AA874924    UniGene:   Rn.3176    Gene Symbol:   Ly86_predicted    Locus Link:   291359    Chromosome:   17    Cytoband:   Chr:17p12    SOURCE    GENECARD       Gene Ontology:   cellular component:  extracellular space   cellular component:  plasma membrane   molecular function:  signal transducer activity      BioCarta Pathways:     KEGG Pathways:  
    Probe set : 1387965_at    Name:   kidney injury molecule 1    GB acc:   AF035963    UniGene:   Rn.11154    Gene Symbol:   Havcr1    Locus Link:   286934    Chromosome:   10    Cytoband:   Chr:10q21    SOURCE    GENECARD       Gene Ontology:   biological process:  cell adhesion   cellular component:  integral to membrane      BioCarta Pathways:     KEGG Pathways:  
    Probe set : 1372869_at    Name:   similar to GTP-binding protein NGB    GB acc:   BE109202     Gene Symbol:   LOC498786    Locus Link:   498786    Chromosome:   17    Cytoband:   Chr:17q12.1     GENECARD       Gene Ontology:     BioCarta Pathways:     KEGG Pathways:  
    Probe set : 1387737_at    Name:   methionine adenosyltransferase II, alpha    GB acc:   NM_134351    UniGene:   Rn.41420    Gene Symbol:   Mat2a    Locus Link:   171347    Chromosome:   4    Cytoband:   Chr:4q33    SOURCE    GENECARD       Gene Ontology:   biological process:  S-adenosylmethionine biosynthesis   biological process:  one-carbon compound metabolism      BioCarta Pathways:     KEGG Pathways:  
    Probe set : 1391643_at     GB acc:   BI290758              Gene Ontology:     BioCarta Pathways:     KEGG Pathways:  
    Probe set : 1389306_at    Name:   matrilin 2 (predicted)    GB acc:   BM387419    UniGene:   Rn.100742    Gene Symbol:   Matn2_predicted    Locus Link:   299996    Chromosome:   7    Cytoband:   Chr:7q22    SOURCE    GENECARD       Gene Ontology:   cellular component:  extracellular matrix (sensu Metazoa)   cellular component:  extracellular space   molecular function:  calcium ion binding      BioCarta Pathways:     KEGG Pathways:  
    Probe set : 1374584_at    Name:   similar to Serine/threonine protein kinase 24    GB acc:   BM388112    UniGene:   Rn.105397    Gene Symbol:   LOC361092    Locus Link:   361092    Chromosome:   15    Cytoband:   Chr:15q25    SOURCE    GENECARD       Gene Ontology:     BioCarta Pathways:     KEGG Pathways:  
    Probe set : 1385829_at    Name:   similar to mKIAA0657 protein    GB acc:   BI294746    UniGene:   Rn.43633    Gene Symbol:   LOC363257    Locus Link:   363257    Chromosome:   9    Cytoband:   Chr:9q33    SOURCE    GENECARD       Gene Ontology:     BioCarta Pathways:     KEGG Pathways:  
    Probe set : 1389885_at    Name:   similar to RIKEN cDNA 0610025L06 (predicted)    GB acc:   BI294855    UniGene:   Rn.112761    Gene Symbol:   RGD1309967_predicted    Locus Link:   360646    Chromosome:   10    Cytoband:   Chr:10q32.1    SOURCE    GENECARD       Gene Ontology:   molecular function:  zinc ion binding      BioCarta Pathways:     KEGG Pathways:  
    Probe set : 1388867_at    Name:   similar to transcription factor    GB acc:   AI227852    UniGene:   Rn.13772    Gene Symbol:   MGC112830    Locus Link:   361178    Chromosome:   16    Cytoband:   Chr:16q12.5    SOURCE    GENECARD       Gene Ontology:   biological process:  regulation of progression through cell cycle   biological process:  regulation of transcription, DNA-dependent   cellular component:  transcription factor complex   molecular function:  transcription factor activity      BioCarta Pathways:     KEGG Pathways:  
    Probe set : 1379317_a_at    Name:   programmed cell death protein 11 (predicted)    GB acc:   BI280308    UniGene:   Rn.136636    Gene Symbol:   Pdcd11_predicted    Locus Link:   309458    Chromosome:   1    Cytoband:   Chr:1q54    SOURCE    GENECARD       Gene Ontology:   biological process:  regulation of transcription, DNA-dependent   biological process:  mRNA processing   biological process:  apoptosis   cellular component:  cytosol      BioCarta Pathways:     KEGG Pathways:  
    Probe set : 1383643_at    Name:   similar to UPF0197 protein C11orf10 homolog    GB acc:   BI282584    UniGene:   Rn.129769    Gene Symbol:   LOC499317    Locus Link:   499317    Chromosome:   1    Cytoband:   Chr:1q43    SOURCE    GENECARD       Gene Ontology:     BioCarta Pathways:     KEGG Pathways:  
    Probe set : 1374307_at    Name:   CaM-kinase II inhibitor alpha    GB acc:   AI236027    UniGene:   Rn.12600    Gene Symbol:   LOC287005    Locus Link:   287005    Chromosome:   5    Cytoband:   Chr:5q36    SOURCE    GENECARD       Gene Ontology:   cellular component:  dendrite   cellular component:  cell soma   molecular function:  protein kinase binding   molecular function:  kinase activity      BioCarta Pathways:     KEGG Pathways:  
    Probe set : 1374244_at    Name:   Ab2-060    GB acc:   AI411141    UniGene:   Rn.43844     Locus Link:   501038      SOURCE        Gene Ontology:     BioCarta Pathways:     KEGG Pathways:  
    Probe set : 1374959_at    Name:   NAD(P)H dehydrogenase, quinone 2    GB acc:   AA945624    UniGene:   Rn.7882    Gene Symbol:   RGD:1303320    Locus Link:   291084    Chromosome:   17    Cytoband:   Chr:17p12    SOURCE    GENECARD       Gene Ontology:   biological process:  myeloid cell differentiation   biological process:  electron transport      BioCarta Pathways:     KEGG Pathways:  
    Probe set : 1367597_at    Name:   ribosomal protein S8    GB acc:   NM_031706    UniGene:   Rn.3198    Gene Symbol:   Rps8    Locus Link:   65136    Chromosome:   5    Cytoband:   Chr:5q36    SOURCE    GENECARD       Gene Ontology:   biological process:  protein biosynthesis   biological process:  ribosome biogenesis   molecular function:  RNA binding   molecular function:  structural constituent of ribosome      BioCarta Pathways:     KEGG Pathways:  
    Probe set : 1384581_at    Name:   actin, gamma, cytoplasmic (predicted) /// similar to Actin, cytoplasmic 2 (Gamma-actin) /// similar to put. type 5 nonmuscle actin    GB acc:   BI850151    UniGene:   Rn.101464    Gene Symbol:   Actg_predicted /// LOC295810 /// LOC360883    Locus Link:   287876 /// 295810 /// 360883    Chromosome:   3    Cytoband:   Chr:3q24 /// Chr:10q32.3 /// Chr:13q24    SOURCE    GENECARD       Gene Ontology:   biological process:  cytoskeleton organization and biogenesis   cellular component:  cytoskeleton   cellular component:  actin filament   cellular component:  actin cytoskeleton   molecular function:  motor activity   molecular function:  structural molecule activity   molecular function:  structural constituent of cytoskeleton      BioCarta Pathways:     KEGG Pathways:  
    Probe set : 1388802_at    Name:   similar to EG2RVC    GB acc:   AI579422    UniGene:   Rn.9190    Gene Symbol:   LOC501625    Locus Link:   501625    Chromosome:   X    Cytoband:   Chr:Xq35    SOURCE    GENECARD       Gene Ontology:     BioCarta Pathways:     KEGG Pathways:  
    Probe set : 1368671_at    Name:   sushi-repeat-containing protein    GB acc:   NM_022524    UniGene:   Rn.1489    Gene Symbol:   Srpx    Locus Link:   64316    Chromosome:   X    Cytoband:   Chr:Xq13    SOURCE    GENECARD       Gene Ontology:   biological process:  cell adhesion   cellular component:  extracellular space   cellular component:  membrane   molecular function:  protein binding      BioCarta Pathways:     KEGG Pathways:  
    Probe set : 1372493_at    Name:   similar to RIKEN cDNA 2410005K17 (predicted)    GB acc:   AI102862    UniGene:   Rn.2954    Gene Symbol:   RGD1311232_predicted    Locus Link:   362578    Chromosome:   5    Cytoband:   Chr:5q36    SOURCE    GENECARD       Gene Ontology:     BioCarta Pathways:     KEGG Pathways:  
    Probe set : 1387893_at    Name:   complement component 1, s subcomponent /// similar to complement component 1, s subcomponent    GB acc:   D88250    UniGene:   Rn.145641    Gene Symbol:   C1s /// LOC500313    Locus Link:   192262 /// 500313    Chromosome:   4    Cytoband:   Chr:4q42    SOURCE    GENECARD       Gene Ontology:   cellular component:  extracellular region   cellular component:  extracellular region   cellular component:  extracellular space      BioCarta Pathways:     KEGG Pathways:  
    Probe set : 1392829_at    Name:   Transcribed locus, strongly similar to XP_529072.1 PREDICTED: similar to cleavage stimulation factor subunit 2 [Pan troglodytes]    GB acc:   BI289495    UniGene:   Rn.139019        SOURCE        Gene Ontology:     BioCarta Pathways:     KEGG Pathways:  
    Probe set : 1386938_at    Name:   alanyl (membrane) aminopeptidase    GB acc:   NM_031012    UniGene:   Rn.11132    Gene Symbol:   Anpep    Locus Link:   81641    Chromosome:   1    Cytoband:   Chr:1q31    SOURCE    GENECARD       Gene Ontology:   cellular component:  integral to plasma membrane   cellular component:  integral to membrane      BioCarta Pathways:     KEGG Pathways:  
    Probe set : 1370991_at    Name:   camello-like 3    GB acc:   AF187814    UniGene:   Rn.43582    Gene Symbol:   Cml3    Locus Link:   113892    Chromosome:   4    Cytoband:   Chr:4q34    SOURCE    GENECARD       Gene Ontology:   biological process:  regulation of transcription, DNA-dependent   biological process:  negative regulation of cell adhesion      BioCarta Pathways:     KEGG Pathways:  
    Probe set : 1384695_at    Name:   uroplakin 3A (predicted)    GB acc:   BM387108    UniGene:   Rn.81907    Gene Symbol:   Upk3a_predicted    Locus Link:   315190    Chromosome:   7    Cytoband:   Chr:7q34    SOURCE    GENECARD       Gene Ontology:     BioCarta Pathways:     KEGG Pathways:  
    Probe set : 1383917_at    Name:   ABI gene family, member 3 (predicted)    GB acc:   BE110730    UniGene:   Rn.95169    Gene Symbol:   Abi3_predicted    Locus Link:   303476    Chromosome:   10    Cytoband:   Chr:10q31    SOURCE    GENECARD       Gene Ontology:   biological process:  cell motility   biological process:  regulation of cell migration   cellular component:  cytoplasm   molecular function:  protein binding      BioCarta Pathways:     KEGG Pathways:  
    Probe set : 1388217_a_at    Name:   calumenin    GB acc:   AJ001929    UniGene:   Rn.1841    Gene Symbol:   Calu    Locus Link:   64366    Chromosome:   4    Cytoband:   Chr:4q22    SOURCE    GENECARD       Gene Ontology:   biological process:  muscle development   molecular function:  calcium ion binding   molecular function:  calcium ion binding      BioCarta Pathways:     KEGG Pathways:  
    Probe set : 1382266_at    Name:   similar to G protein-coupled receptor 146    GB acc:   AI500893    UniGene:   Rn.41446    Gene Symbol:   LOC498153    Locus Link:   498153    Chromosome:   12    Cytoband:   Chr:12q11    SOURCE    GENECARD       Gene Ontology:     BioCarta Pathways:     KEGG Pathways:  
    Probe set : 1387769_a_at    Name:   Inhibitor of DNA binding 3, dominant negative helix-loop-helix protein    GB acc:   AF000942    UniGene:   Rn.2760    Gene Symbol:   Id3    Locus Link:   25585    Chromosome:   5    Cytoband:   Chr:5q36    SOURCE    GENECARD       Gene Ontology:   cellular component:  nucleus      BioCarta Pathways:     KEGG Pathways:  
    Probe set : 1376816_at    Name:   Hypothetical gene supported by NM_012514    GB acc:   BF284903    UniGene:   Rn.48840    Gene Symbol:   Brca1    Locus Link:   24227    Chromosome:   10    Cytoband:   Chr:10q32.1    SOURCE    GENECARD       Gene Ontology:   biological process:  DNA replication and chromosome cycle   biological process:  cell cycle checkpoint   biological process:  carbohydrate metabolism   biological process:  DNA repair   biological process:  DNA repair   biological process:  regulation of transcription from RNA polymerase II promoter   biological process:  regulation of transcription from RNA polymerase III promoter   biological process:  DNA damage response, signal transduction by p53 class mediator resulting in transcription of p21 class mediator   biological process:  centrosome cycle   biological process:  DNA damage response, signal transduction resulting in induction of apoptosis   biological process:  dosage compensation, by inactivation of X chromosome   biological process:  protein ubiquitination   biological process:  regulation of cell proliferation   biological process:  regulation of apoptosis   biological process:  positive regulation of DNA repair   biological process:  negative regulation of progression through cell cycle   biological process:  negative regulation of centriole replication   biological process:  response to DNA damage stimulus   biological process:  cell cycle      BioCarta Pathways:     KEGG Pathways:  
    Probe set : 1370948_a_at    Name:   myristoylated alanine rich protein kinase C substrate    GB acc:   M59859    UniGene:   Rn.9560    Gene Symbol:   Marcks    Locus Link:   25603      SOURCE    GENECARD       Gene Ontology:   biological process:  cell motility   cellular component:  plasma membrane   cellular component:  actin cytoskeleton   cellular component:  membrane      BioCarta Pathways:     KEGG Pathways:  
    Probe set : 1397476_at    Name:   Ttk protein kinase (predicted)    GB acc:   BM383386    UniGene:   Rn.107533    Gene Symbol:   Ttk_predicted    Locus Link:   315852    Chromosome:   8    Cytoband:   Chr:8q31    SOURCE    GENECARD       Gene Ontology:   cellular component:  spindle      BioCarta Pathways:     KEGG Pathways:  
    Probe set : 1390692_at    Name:   cytidine 5'-triphosphate synthase (predicted)    GB acc:   BE105721    UniGene:   Rn.46765    Gene Symbol:   Ctps_predicted    Locus Link:   313560    Chromosome:   5    Cytoband:   Chr:5q36    SOURCE    GENECARD       Gene Ontology:   molecular function:  catalytic activity   molecular function:  CTP synthase activity   molecular function:  ligase activity      BioCarta Pathways:     KEGG Pathways:  
    Probe set : 1389732_at    Name:   Transcribed locus, strongly similar to NP_082154.2 hypothetical protein LOC71712 [Mus musculus]    GB acc:   BM386323    UniGene:   Rn.1826        SOURCE        Gene Ontology:     BioCarta Pathways:     KEGG Pathways:  
    Probe set : 1375657_at    Name:   Transcribed locus    GB acc:   BE107438    UniGene:   Rn.144180        SOURCE        Gene Ontology:     BioCarta Pathways:     KEGG Pathways:  
    Probe set : 1384900_at    Name:   MOB1, Mps One Binder kinase activator-like 2B (yeast) (predicted)    GB acc:   AW530806    UniGene:   Rn.9147    Gene Symbol:   Mobkl2b_predicted    Locus Link:   362833    Chromosome:   7    Cytoband:   Chr:7q11    SOURCE    GENECARD       Gene Ontology:     BioCarta Pathways:     KEGG Pathways:  
    Probe set : 1381971_at    Name:   SRY-box containing gene 18 (predicted)    GB acc:   AW528157    UniGene:   Rn.22446    Gene Symbol:   Sox18_predicted    Locus Link:   311723    Chromosome:   3    Cytoband:   Chr:3q43    SOURCE    GENECARD       Gene Ontology:   biological process:  regulation of transcription, DNA-dependent   biological process:  regulation of transcription from RNA polymerase II promoter   cellular component:  nucleus   molecular function:  DNA binding   molecular function:  transcription factor activity   molecular function:  RNA polymerase II transcription factor activity      BioCarta Pathways:     KEGG Pathways:  
    Probe set : 1387382_at    Name:   histamine N-methyltransferase    GB acc:   NM_031044    UniGene:   Rn.13145    Gene Symbol:   Hnmt    Locus Link:   81676    Chromosome:   3    Cytoband:   Chr:3p13    SOURCE    GENECARD       Gene Ontology:   biological process:  regulation of neurotransmitter levels   biological process:  respiratory gaseous exchange      BioCarta Pathways:     KEGG Pathways:  
    Probe set : 1386862_at    Name:   annexin A5    GB acc:   NM_013132    UniGene:   Rn.3318    Gene Symbol:   Anxa5    Locus Link:   25673    Chromosome:   2    Cytoband:   Chr:2q25    SOURCE    GENECARD       Gene Ontology:   biological process:  blood coagulation   biological process:  negative regulation of coagulation   molecular function:  phospholipase inhibitor activity   molecular function:  calcium ion binding   molecular function:  calcium-dependent phospholipid binding      BioCarta Pathways:     KEGG Pathways:  
    Probe set : 1388324_at    Name:   nitrilase 1    GB acc:   BG668463    UniGene:   Rn.64425    Gene Symbol:   Nit1    Locus Link:   289222    Chromosome:   13    Cytoband:   Chr:13q24    SOURCE    GENECARD       Gene Ontology:   biological process:  nitrogen compound metabolism      BioCarta Pathways:     KEGG Pathways:  
    Probe set : 1378507_at    Name:   Transcribed locus    GB acc:   BE108914    UniGene:   Rn.88974        SOURCE        Gene Ontology:     BioCarta Pathways:     KEGG Pathways:  
    Probe set : 1371969_at    Name:   Caldesmon 1    GB acc:   BI291848    UniGene:   Rn.87384    Gene Symbol:   Cald1    Locus Link:   25687    Chromosome:   4    Cytoband:   Chr:4q22    SOURCE    GENECARD       Gene Ontology:   cellular component:  membrane fraction   cellular component:  cytoskeleton   cellular component:  actin cap      BioCarta Pathways:     KEGG Pathways:  
    Probe set : 1370289_x_at    Name:   kallikrein, submaxillary gland S3    GB acc:   M11566    UniGene:   Rn.34527    Gene Symbol:   Klks3    Locus Link:   292868    Chromosome:   1    Cytoband:   Chr:1q22    SOURCE    GENECARD       Gene Ontology:   biological process:  proteolysis   biological process:  positive regulation of vasoconstriction      BioCarta Pathways:     KEGG Pathways:  
    Probe set : 1392732_at    Name:   nucleolar and spindle associated protein 1 (predicted)    GB acc:   AI711233    UniGene:   Rn.106933    Gene Symbol:   Nusap1_predicted    Locus Link:   311336    Chromosome:   3    Cytoband:   Chr:3q35    SOURCE    GENECARD       Gene Ontology:   cellular component:  nucleus   cellular component:  nucleolus   cellular component:  spindle microtubule   molecular function:  microtubule binding      BioCarta Pathways:     KEGG Pathways:  
    Probe set : 1388114_at    Name:   myosin light chain, regulatory B    GB acc:   X05566    UniGene:   Rn.103179    Gene Symbol:   Mrlcb    Locus Link:   50685    Chromosome:   9    Cytoband:   Chr:9q38    SOURCE    GENECARD       Gene Ontology:   biological process:  cytoskeleton organization and biogenesis   biological process:  muscle development   cellular component:  cytoskeleton   cellular component:  apical part of cell   cellular component:  myosin   molecular function:  motor activity   molecular function:  calcium ion binding      BioCarta Pathways:     KEGG Pathways:  
    Probe set : 1380420_at    Name:   Internexin, alpha    GB acc:   AI549585    UniGene:   Rn.88941    Gene Symbol:   Inexa    Locus Link:   24503    Chromosome:   1    Cytoband:   Chr:1q54    SOURCE    GENECARD       Gene Ontology:   molecular function:  structural molecule activity   molecular function:  structural constituent of cytoskeleton      BioCarta Pathways:     KEGG Pathways:  
    Probe set : 1370887_at    Name:   transforming growth factor beta 1 induced transcript 1    GB acc:   BI279862    UniGene:   Rn.103260    Gene Symbol:   Tgfb1i1    Locus Link:   84574    Chromosome:   1    Cytoband:   Chr:1q36    SOURCE    GENECARD       Gene Ontology:     BioCarta Pathways:     KEGG Pathways:  
    Probe set : 1382903_at    Name:   similar to mFLJ00191 protein    GB acc:   BF420492    UniGene:   Rn.136946    Gene Symbol:   LOC310958    Locus Link:   310958    Chromosome:   2    Cytoband:   Chr:2q45    SOURCE    GENECARD       Gene Ontology:     BioCarta Pathways:     KEGG Pathways:  
    Probe set : 1369181_at    Name:   cytochrome b-245, beta polypeptide    GB acc:   NM_023965    UniGene:   Rn.98491    Gene Symbol:   Cybb    Locus Link:   66021    Chromosome:   X    Cytoband:   Chr:Xq13    SOURCE    GENECARD       Gene Ontology:   biological process:  electron transport   cellular component:  intracellular   cellular component:  membrane   cellular component:  integral to membrane      BioCarta Pathways:     KEGG Pathways:  
    Probe set : 1373024_at    Name:   adaptor-related protein complex 3, sigma 1 subunit (predicted)    GB acc:   BI296511    UniGene:   Rn.3070    Gene Symbol:   Ap3s1_predicted    Locus Link:   302290    Chromosome:   18    Cytoband:   Chr:18q11    SOURCE    GENECARD       Gene Ontology:   molecular function:  transporter activity      BioCarta Pathways:     KEGG Pathways:  
    Probe set : 1373099_at    Name:   phosphatidylinositol glycan, class S    GB acc:   BI284221    UniGene:   Rn.59136    Gene Symbol:   RGD:1359436    Locus Link:   303277    Chromosome:   10    Cytoband:   Chr:10q25    SOURCE    GENECARD       Gene Ontology:   cellular component:  endoplasmic reticulum   cellular component:  integral to membrane      BioCarta Pathways:     KEGG Pathways:  
    Probe set : 1370504_a_at    Name:   peripheral myelin protein 22    GB acc:   AW252810    UniGene:   Rn.1476    Gene Symbol:   Pmp22    Locus Link:   24660    Chromosome:   10    Cytoband:   Chr:10q22    SOURCE    GENECARD       Gene Ontology:     BioCarta Pathways:     KEGG Pathways:  
    Probe set : 1372488_at    Name:   similar to RIKEN cDNA 2310057D15 (predicted)    GB acc:   BM386249    UniGene:   Rn.98105    Gene Symbol:   RGD1309016_predicted    Locus Link:   305177    Chromosome:   14    Cytoband:   Chr:14p22    SOURCE    GENECARD       Gene Ontology:   biological process:  metabolism   molecular function:  hydrolase activity   molecular function:  catalytic activity      BioCarta Pathways:     KEGG Pathways:  
    Probe set : 1388751_at    Name:   RNA binding motif protein 24 (predicted)    GB acc:   AW433787     Gene Symbol:   Rbm24_predicted    Locus Link:   291031    Chromosome:   17    Cytoband:   Chr:17p13     GENECARD       Gene Ontology:   biological process:  nuclear mRNA splicing, via spliceosome   molecular function:  nucleic acid binding      BioCarta Pathways:     KEGG Pathways:  
    Probe set : 1386989_at    Name:   endothelial differentiation, sphingolipid G-protein-coupled receptor, 5    GB acc:   NM_017192    UniGene:   Rn.2491    Gene Symbol:   Edg5    Locus Link:   29415    Chromosome:   8    Cytoband:   Chr:8q13    SOURCE    GENECARD       Gene Ontology:   biological process:  G-protein coupled receptor protein signaling pathway   biological process:  behavior   biological process:  signal transduction   cellular component:  integral to plasma membrane   cellular component:  integral to membrane      BioCarta Pathways:     KEGG Pathways:  
    Probe set : 1377869_at    Name:   similar to carbon catabolite repression 4 protein homolog    GB acc:   BI284261    UniGene:   Rn.15040    Gene Symbol:   LOC310395    Locus Link:   310395    Chromosome:   2    Cytoband:   Chr:2q26    SOURCE    GENECARD       Gene Ontology:   biological process:  rhythmic process      BioCarta Pathways:     KEGG Pathways:  
    Probe set : 1372308_at    Name:   CDNA clone IMAGE:7366335    GB acc:   BM386869    UniGene:   Rn.8110        SOURCE        Gene Ontology:     BioCarta Pathways:     KEGG Pathways:  
    Probe set : 1376100_at    Name:   similar to RIKEN cDNA 2310057H16 (predicted)    GB acc:   BI274903    UniGene:   Rn.98430    Gene Symbol:   RGD1305887_predicted    Locus Link:   307351    Chromosome:   18    Cytoband:   Chr:18q12.1    SOURCE    GENECARD       Gene Ontology:   biological process:  microtubule-based process   biological process:  microtubule-based movement   biological process:  protein polymerization   cellular component:  microtubule   cellular component:  cytoplasm   cellular component:  protein complex      BioCarta Pathways:     KEGG Pathways:  
    Probe set : 1368393_at    Name:   lymphocyte antigen 68    GB acc:   NM_053383    UniGene:   Rn.17490    Gene Symbol:   C1qr1    Locus Link:   84398    Chromosome:   3    Cytoband:   Chr:3q41    SOURCE    GENECARD       Gene Ontology:     BioCarta Pathways:     KEGG Pathways:  
    Probe set : 1388010_at    Name:   Pro-melanin-concentrating hormone    GB acc:   AF303035    UniGene:   Rn.9675    Gene Symbol:   Pmch    Locus Link:   24659    Chromosome:   7    Cytoband:   Chr:7q13    SOURCE    GENECARD       Gene Ontology:   cellular component:  extracellular region   cellular component:  extracellular space      BioCarta Pathways:     KEGG Pathways:  
    Probe set : 1372510_at    Name:   Neoplastic progression 3 (predicted)    GB acc:   AI172302    UniGene:   Rn.2835     Locus Link:   296271    Chromosome:   3    Cytoband:   Chr:3q41    SOURCE        Gene Ontology:   cellular component:  extrachromosomal circular DNA   molecular function:  DNA binding      BioCarta Pathways:     KEGG Pathways:  
    Probe set : 1395532_at    Name:   Transcribed locus, strongly similar to NP_035873.1 SH3-domain binding protein 3 [Mus musculus]    GB acc:   AI137456    UniGene:   Rn.6467        SOURCE        Gene Ontology:     BioCarta Pathways:     KEGG Pathways:  
    Probe set : 1372325_at    Name:   elastin microfibril interfacer 1 (predicted)    GB acc:   BI303596    UniGene:   Rn.99346    Gene Symbol:   Emilin1_predicted    Locus Link:   298845    Chromosome:   6    Cytoband:   Chr:6q14    SOURCE    GENECARD       Gene Ontology:   biological process:  phosphate transport   biological process:  cell adhesion   cellular component:  extracellular matrix (sensu Metazoa)   cellular component:  extracellular space   cellular component:  cytoplasm      BioCarta Pathways:     KEGG Pathways:  
    Probe set : 1385573_at    Name:   Homer homolog 3 (Drosophila)    GB acc:   AI070797    UniGene:   Rn.144573    Gene Symbol:   Homer3    Locus Link:   29548    Chromosome:   16    Cytoband:   Chr:16p14    SOURCE    GENECARD       Gene Ontology:   biological process:  protein targeting   biological process:  defense response   biological process:  metabotropic glutamate receptor signaling pathway   cellular component:  outer membrane (sensu Gram-negative Bacteria)   molecular function:  protein binding      BioCarta Pathways:     KEGG Pathways:  
    Probe set : 1387145_at    Name:   gap junction membrane channel protein beta 1    GB acc:   NM_017251    UniGene:   Rn.10444    Gene Symbol:   Gjb1    Locus Link:   29584    Chromosome:   X    Cytoband:   Chr:Xq31    SOURCE    GENECARD       Gene Ontology:   molecular function:  gap-junction forming channel activity   molecular function:  gap-junction forming channel activity   molecular function:  connexon channel activity      BioCarta Pathways:     KEGG Pathways:  
    Probe set : 1367999_at    Name:   aldehyde dehydrogenase 2    GB acc:   NM_032416    UniGene:   Rn.101781    Gene Symbol:   Aldh2    Locus Link:   29539    Chromosome:   12    Cytoband:   Chr:12q16    SOURCE    GENECARD       Gene Ontology:   biological process:  carbohydrate metabolism   biological process:  alcohol metabolism   biological process:  metabolism   cellular component:  mitochondrion   cellular component:  mitochondrion      BioCarta Pathways:     KEGG Pathways:  
    Probe set : 1383291_at     GB acc:   BF282631              Gene Ontology:     BioCarta Pathways:     KEGG Pathways:  
    Probe set : 1395663_at    Name:   similar to BENE protein (predicted)    GB acc:   BM390763    UniGene:   Rn.48232    Gene Symbol:   RGD1310869_predicted    Locus Link:   362211    Chromosome:   3    Cytoband:   Chr:3q36    SOURCE    GENECARD       Gene Ontology:   cellular component:  membrane   cellular component:  integral to membrane      BioCarta Pathways:     KEGG Pathways:  
    Probe set : 1371864_at    Name:   Basic transcription element binding protein 1    GB acc:   AW524563    UniGene:   Rn.19481    Gene Symbol:   Bteb1    Locus Link:   117560    Chromosome:   1    Cytoband:   Chr:1q51    SOURCE    GENECARD       Gene Ontology:   cellular component:  nucleus      BioCarta Pathways:     KEGG Pathways:  
    Probe set : 1369952_at    Name:   poly(A) binding protein, cytoplasmic 1    GB acc:   NM_134353    UniGene:   Rn.127813    Gene Symbol:   Pabpc1    Locus Link:   171350    Chromosome:   7    Cytoband:   Chr:7q22    SOURCE    GENECARD       Gene Ontology:   biological process:  mRNA polyadenylylation   biological process:  mRNA metabolism   cellular component:  cytoplasm      BioCarta Pathways:     KEGG Pathways:  
    Probe set : 1388469_at    Name:   Insulin-like growth factor I mRNA, 3' end of mRNA    GB acc:   AA945615    UniGene:   Rn.25124        SOURCE        Gene Ontology:     BioCarta Pathways:     KEGG Pathways:  
    Probe set : 1390416_at    Name:   solute carrier family 25, member 30    GB acc:   H35736    UniGene:   Rn.19440    Gene Symbol:   RGD:1359702    Locus Link:   361074    Chromosome:   15    Cytoband:   Chr:15q11    SOURCE    GENECARD       Gene Ontology:   biological process:  transport   biological process:  mitochondrial transport   molecular function:  transporter activity   molecular function:  binding      BioCarta Pathways:     KEGG Pathways:  
    Probe set : 1367680_at    Name:   acyl-Coenzyme A oxidase 1, palmitoyl    GB acc:   NM_017340    UniGene:   Rn.31796    Gene Symbol:   Acox1    Locus Link:   50681    Chromosome:   10    Cytoband:   Chr:10q32.3    SOURCE    GENECARD       Gene Ontology:   cellular component:  mitochondrion   cellular component:  peroxisome      BioCarta Pathways:     KEGG Pathways:  
    Probe set : 1382419_at    Name:   SoxLZ/Sox6 leucine zipper binding protein in testis (predicted)    GB acc:   BI276776    UniGene:   Rn.40474    Gene Symbol:   Solt_predicted    Locus Link:   294712    Chromosome:   2    Cytoband:   Chr:2q13    SOURCE    GENECARD       Gene Ontology:     BioCarta Pathways:     KEGG Pathways:  
    Probe set : 1373803_a_at    Name:   growth hormone receptor    GB acc:   AI170771    UniGene:   Rn.2178    Gene Symbol:   Ghr    Locus Link:   25235    Chromosome:   2    Cytoband:   Chr:2q16    SOURCE    GENECARD       Gene Ontology:     BioCarta Pathways:     KEGG Pathways:  
    Probe set : 1377239_at    Name:   amyloid beta (A4) precursor protein-binding, family B, member 1 interacting protein (predicted)    GB acc:   BM386169    UniGene:   Rn.38834    Gene Symbol:   Apbb1ip_predicted    Locus Link:   307171    Chromosome:   17    Cytoband:   Chr:17q12.3    SOURCE    GENECARD       Gene Ontology:   biological process:  signal transduction   biological process:  neuropeptide signaling pathway      BioCarta Pathways:     KEGG Pathways:  
    Probe set : 1371237_a_at    Name:   Metallothionein    GB acc:   AF411318    UniGene:   Rn.16133    Gene Symbol:   Mt1a    Locus Link:   24567    Chromosome:   19    Cytoband:   Chr:19p12    SOURCE    GENECARD       Gene Ontology:   cellular component:  cytoplasm   cellular component:  lysosome   cellular component:  cytosol   cellular component:  nucleus      BioCarta Pathways:     KEGG Pathways:  
    Probe set : 1378140_at    Name:   ADP-ribosylation factor-like 11 (predicted)    GB acc:   BI296317    UniGene:   Rn.23090    Gene Symbol:   Arl11_predicted    Locus Link:   364396    Chromosome:   15    Cytoband:   Chr:15p12    SOURCE    GENECARD       Gene Ontology:   biological process:  small GTPase mediated signal transduction   molecular function:  GTP binding   molecular function:  nucleotide binding      BioCarta Pathways:     KEGG Pathways:  
    Probe set : 1373473_a_at    Name:   nucleosome assembly protein 1-like 1    GB acc:   BM386384    UniGene:   Rn.18480    Gene Symbol:   Nap1l1    Locus Link:   89825    Chromosome:   7    Cytoband:   Chr:7q21    SOURCE    GENECARD       Gene Ontology:   biological process:  DNA replication   biological process:  nucleosome assembly   biological process:  positive regulation of cell proliferation   cellular component:  nucleus   cellular component:  chromatin assembly complex      BioCarta Pathways:     KEGG Pathways:  
    Probe set : 1377804_at    Name:   Transcribed locus    GB acc:   AI030809    UniGene:   Rn.12313        SOURCE        Gene Ontology:     BioCarta Pathways:     KEGG Pathways:  
    Probe set : 1376153_at    Name:   Transcribed locus    GB acc:   BE102621    UniGene:   Rn.22233        SOURCE        Gene Ontology:     BioCarta Pathways:     KEGG Pathways:  
    Probe set : 1372631_at    Name:   Thymidine kinase 1    GB acc:   AI170596    UniGene:   Rn.4191    Gene Symbol:   Tk1    Locus Link:   24834    Chromosome:   10    Cytoband:   Chr:10    SOURCE    GENECARD       Gene Ontology:   biological process:  DNA metabolism   biological process:  DNA replication   cellular component:  cytoplasm      BioCarta Pathways:     KEGG Pathways:  
    Probe set : 1368016_at    Name:   peroxisomal trans-2-enoyl-CoA reductase    GB acc:   NM_133299    UniGene:   Rn.19267    Gene Symbol:   Pecr    Locus Link:   113956    Chromosome:   9    Cytoband:   Chr:9q33    SOURCE    GENECARD       Gene Ontology:   cellular component:  mitochondrion   cellular component:  peroxisome   cellular component:  mitochondrion   molecular function:  oxidoreductase activity   molecular function:  trans-2-enoyl-CoA reductase (NADPH) activity      BioCarta Pathways:     KEGG Pathways:  
    Probe set : 1368908_at    Name:   annexin A4    GB acc:   NM_024155    UniGene:   Rn.19270    Gene Symbol:   Anxa4    Locus Link:   79124    Chromosome:   4    Cytoband:   Chr:4q34    SOURCE    GENECARD       Gene Ontology:   biological process:  kidney development   biological process:  exocytosis   cellular component:  apical plasma membrane   molecular function:  phospholipase inhibitor activity   molecular function:  calcium ion binding   molecular function:  calcium-dependent phospholipid binding      BioCarta Pathways:     KEGG Pathways:  
    Probe set : 1376109_at    Name:   Transcribed locus, weakly similar to NP_062289.1 DNA binding protein with his-thr domain [Mus musculus]    GB acc:   BM387711    UniGene:   Rn.48053        SOURCE        Gene Ontology:     BioCarta Pathways:     KEGG Pathways:  
    Probe set : 1369097_s_at    Name:   guanylate cyclase 1, soluble, beta 3    GB acc:   NM_012769    UniGene:   Rn.87228    Gene Symbol:   Gucy1b3    Locus Link:   25202    Chromosome:   2    Cytoband:   Chr:2q31-q33    SOURCE    GENECARD       Gene Ontology:   cellular component:  cytoplasm   cellular component:  guanylate cyclase complex, soluble      BioCarta Pathways:     KEGG Pathways:  
    Probe set : 1374912_at     GB acc:   BI274467              Gene Ontology:     BioCarta Pathways:     KEGG Pathways:  
    Probe set : 1375695_at    Name:   YTH domain family 2 (predicted)    GB acc:   AW535358    UniGene:   Rn.21737    Gene Symbol:   Ythdf2_predicted    Locus Link:   313053    Chromosome:   5    Cytoband:   Chr:5q36    SOURCE    GENECARD       Gene Ontology:     BioCarta Pathways:     KEGG Pathways:  
    Probe set : 1367721_at    Name:   syndecan 4    GB acc:   NM_012649    UniGene:   Rn.2029    Gene Symbol:   Sdc4    Locus Link:   24771    Chromosome:   3    Cytoband:   Chr:3q42    SOURCE    GENECARD       Gene Ontology:   molecular function:  cytoskeletal protein binding      BioCarta Pathways:     KEGG Pathways:  
    Probe set : 1395958_at    Name:   similar to KIAA0460 protein (predicted)    GB acc:   BF396619    UniGene:   Rn.59347    Gene Symbol:   RGD1308268_predicted    Locus Link:   365869    Chromosome:   2    Cytoband:   Chr:2q34    SOURCE    GENECARD       Gene Ontology:     BioCarta Pathways:     KEGG Pathways:  
    Probe set : 1374704_at    Name:   KDEL (Lys-Asp-Glu-Leu) containing 2    GB acc:   BM384752    UniGene:   Rn.98686    Gene Symbol:   Kdelc2    Locus Link:   315664    Chromosome:   8    Cytoband:   Chr:8q24    SOURCE    GENECARD       Gene Ontology:     BioCarta Pathways:     KEGG Pathways:  
    Probe set : 1388994_at    Name:   Transcribed locus    GB acc:   AW917849    UniGene:   Rn.2740        SOURCE        Gene Ontology:     BioCarta Pathways:     KEGG Pathways:  
    Probe set : 1380393_at    Name:   crystallin, zeta (predicted)    GB acc:   AI409083    UniGene:   Rn.6746    Gene Symbol:   Cryz_predicted    Locus Link:   362061    Chromosome:   2    Cytoband:   Chr:2q45    SOURCE    GENECARD       Gene Ontology:   biological process:  sensory organ development   biological process:  visual perception   cellular component:  cytoplasm      BioCarta Pathways:     KEGG Pathways:  
    Probe set : 1368829_at    Name:   fibrillin 1    GB acc:   BM389019    UniGene:   Rn.12759    Gene Symbol:   Fbn1    Locus Link:   83727    Chromosome:   3    Cytoband:   Chr:3q36    SOURCE    GENECARD       Gene Ontology:   biological process:  skeletal development   biological process:  development   biological process:  visual perception   cellular component:  microfibril   cellular component:  extracellular matrix (sensu Metazoa)   cellular component:  extracellular space   molecular function:  extracellular matrix structural constituent   molecular function:  calcium ion binding   molecular function:  growth factor binding      BioCarta Pathways:     KEGG Pathways:  
    Probe set : 1376248_at    Name:   sulfotransferase family, cytosolic, 2B, member 1 (predicted)    GB acc:   BM390571    UniGene:   Rn.81913    Gene Symbol:   Sult2b1_predicted    Locus Link:   292915    Chromosome:   1    Cytoband:   Chr:1q22    SOURCE    GENECARD       Gene Ontology:   biological process:  sulfate assimilation   biological process:  steroid metabolism   molecular function:  alcohol sulfotransferase activity   molecular function:  sulfotransferase activity   molecular function:  transferase activity      BioCarta Pathways:     KEGG Pathways:  
    Probe set : 1376219_at    Name:   Atpase, class I, type 8B, member 2 (predicted)    GB acc:   BI273855    UniGene:   Rn.20321     Locus Link:   361984    Chromosome:   2    Cytoband:   Chr:2q34    SOURCE        Gene Ontology:   biological process:  cation transport   biological process:  metabolism   cellular component:  membrane   cellular component:  integral to membrane      BioCarta Pathways:     KEGG Pathways:  
    Probe set : 1398828_at    Name:   FK506 binding protein 1a    GB acc:   U09386    UniGene:   Rn.80611    Gene Symbol:   Fkbp1a    Locus Link:   25639    Chromosome:   3    Cytoband:   Chr:3q41    SOURCE    GENECARD       Gene Ontology:   biological process:  protein folding   biological process:  transport   biological process:  positive regulation of I-kappaB kinase/NF-kappaB cascade   cellular component:  microsome   cellular component:  membrane      BioCarta Pathways:     KEGG Pathways:  
    Probe set : 1367899_at    Name:   coagulation factor II (thrombin) receptor    GB acc:   NM_012950    UniGene:   Rn.2609    Gene Symbol:   F2r    Locus Link:   25439    Chromosome:   2    Cytoband:   Chr:2q12    SOURCE    GENECARD       Gene Ontology:   biological process:  regulation of progression through cell cycle   biological process:  apoptosis   biological process:  caspase activation   biological process:  cell motility   biological process:  signal transduction   biological process:  G-protein coupled receptor protein signaling pathway   biological process:  G-protein coupled receptor protein signaling pathway   biological process:  tyrosine phosphorylation of STAT protein   biological process:  STAT protein nuclear translocation   biological process:  blood coagulation   biological process:  blood coagulation   biological process:  response to wounding   biological process:  morphogenesis   biological process:  positive regulation of I-kappaB kinase/NF-kappaB cascade      BioCarta Pathways:     KEGG Pathways:  
    Probe set : 1380822_at    Name:   Transcribed locus    GB acc:   BE097945    UniGene:   Rn.148632        SOURCE        Gene Ontology:     BioCarta Pathways:     KEGG Pathways:  
    Probe set : 1388447_at    Name:   Transcribed locus, strongly similar to NP_084275.3 limb-bud and heart [Mus musculus]    GB acc:   AA800701    UniGene:   Rn.97659        SOURCE        Gene Ontology:     BioCarta Pathways:     KEGG Pathways:  
    Probe set : 1373990_at    Name:   solute carrier family 7 (cationic amino acid transporter, y+ system), member 12 (predicted) /// similar to solute carrier family 7 (cationic amino acid transporter, y+ system), member 12    GB acc:   AI411935    UniGene:   Rn.133380    Gene Symbol:   Slc7a12_predicted /// LOC361914    Locus Link:   294881 /// 361914    Chromosome:   2    Cytoband:   Chr:2q23    SOURCE    GENECARD       Gene Ontology:   biological process:  transport   biological process:  amino acid transport   molecular function:  amino acid-polyamine transporter activity   molecular function:  amino acid transporter activity      BioCarta Pathways:     KEGG Pathways:  
    Probe set : 1384874_at    Name:   Transcribed locus    GB acc:   BF558516    UniGene:   Rn.96188        SOURCE        Gene Ontology:     BioCarta Pathways:     KEGG Pathways:  
    Probe set : 1368293_at    Name:   carboxypeptidase Z    GB acc:   NM_031766    UniGene:   Rn.11056    Gene Symbol:   Cpz    Locus Link:   83575    Chromosome:   14    Cytoband:   Chr:14q21    SOURCE    GENECARD       Gene Ontology:   biological process:  proteolysis   biological process:  development   biological process:  protein processing   cellular component:  extracellular matrix (sensu Metazoa)   cellular component:  integral to membrane      BioCarta Pathways:     KEGG Pathways:  
    Probe set : 1389815_at    Name:   protein phosphatase 1, regulatory (inhibitor) subunit 14B    GB acc:   BF547738    UniGene:   Rn.2773    Gene Symbol:   Ppp1r14b    Locus Link:   259225    Chromosome:   1    Cytoband:   Chr:1q43    SOURCE    GENECARD       Gene Ontology:   molecular function:  protein phosphatase inhibitor activity   molecular function:  protein phosphatase type 1 regulator activity   molecular function:  phosphatase inhibitor activity      BioCarta Pathways:     KEGG Pathways:  
    Probe set : 1383241_at    Name:   complement component 1, r subcomponent (predicted)    GB acc:   BI292425    UniGene:   Rn.70397    Gene Symbol:   C1r_predicted    Locus Link:   312705    Chromosome:   4    Cytoband:   Chr:4q42    SOURCE    GENECARD       Gene Ontology:   biological process:  proteolysis   biological process:  immune response   biological process:  complement activation, classical pathway   cellular component:  extracellular region      BioCarta Pathways:     KEGG Pathways:  
    Probe set : 1384405_at    Name:   Transcribed locus    GB acc:   BI283128    UniGene:   Rn.137455        SOURCE        Gene Ontology:     BioCarta Pathways:     KEGG Pathways:  
    Probe set : 1391201_at    Name:   WD repeat and HMG-box DNA binding protein 1 (predicted)    GB acc:   AI012084    UniGene:   Rn.98955     Locus Link:   305827    Chromosome:   15    Cytoband:   Chr:15p14    SOURCE        Gene Ontology:   biological process:  regulation of transcription, DNA-dependent   cellular component:  nucleus   cellular component:  nucleoplasm   cellular component:  cytoplasm   molecular function:  DNA binding      BioCarta Pathways:     KEGG Pathways:  
    Probe set : 1392946_at    Name:   Transcribed locus    GB acc:   AI029194    UniGene:   Rn.121260        SOURCE        Gene Ontology:     BioCarta Pathways:     KEGG Pathways:  
    Probe set : 1372219_at    Name:   similar to tropomyosin 1, embryonic fibroblast - rat    GB acc:   AA012755    UniGene:   Rn.146072    Gene Symbol:   MGC109519    Locus Link:   500450    Chromosome:   5    Cytoband:   Chr:5q22    SOURCE    GENECARD       Gene Ontology:     BioCarta Pathways:     KEGG Pathways:  
    Probe set : 1373599_at    Name:   BRF2, subunit of RNA polymerase III transcription initiation factor, BRF1-like (predicted)    GB acc:   AI599525    UniGene:   Rn.13322     Locus Link:   306542    Chromosome:   16    Cytoband:   Chr:16q12.4    SOURCE        Gene Ontology:   biological process:  cell cycle   molecular function:  translation initiation factor activity      BioCarta Pathways:     KEGG Pathways:  
    Probe set : 1380631_at     GB acc:   BM384935              Gene Ontology:     BioCarta Pathways:     KEGG Pathways:  
    Probe set : 1368187_at    Name:   glycoprotein (transmembrane) nmb    GB acc:   NM_133298    UniGene:   Rn.13778    Gene Symbol:   Gpnmb    Locus Link:   113955    Chromosome:   4    Cytoband:   Chr:4q24    SOURCE    GENECARD       Gene Ontology:   molecular function:  integrin binding   molecular function:  heparin binding   molecular function:  carbon-nitrogen ligase activity, with glutamine as amido-N-donor      BioCarta Pathways:     KEGG Pathways:  
    Probe set : 1372886_at    Name:   transforming acidic coiled coil 3    GB acc:   AI556917    UniGene:   Rn.58838    Gene Symbol:   RGD:1302948    Locus Link:   360962    Chromosome:   14    Cytoband:   Chr:14q21    SOURCE    GENECARD       Gene Ontology:     BioCarta Pathways:     KEGG Pathways:  
    Probe set : 1375216_at    Name:   poliovirus receptor-related 2 (herpesvirus entry mediator B) (predicted)    GB acc:   AA850909    UniGene:   Rn.7444    Gene Symbol:   Pvrl2_predicted    Locus Link:   308417    Chromosome:   1    Cytoband:   Chr:1q21    SOURCE    GENECARD       Gene Ontology:   cellular component:  plasma membrane   cellular component:  integral to membrane   molecular function:  coreceptor activity   molecular function:  receptor activity      BioCarta Pathways:     KEGG Pathways:  
    Probe set : 1387048_at    Name:   nuclear RNA helicase, DECD variant of DEAD box family    GB acc:   NM_053563    UniGene:   Rn.14550    Gene Symbol:   Ddx39    Locus Link:   89827    Chromosome:   19    Cytoband:   Chr:19q11    SOURCE    GENECARD       Gene Ontology:   biological process:  nuclear mRNA splicing, via spliceosome   biological process:  mRNA processing   cellular component:  nucleus      BioCarta Pathways:     KEGG Pathways:  
    Probe set : 1387895_s_at    Name:   cell division cycle 20 homolog (S. cerevisiae)    GB acc:   U05341    UniGene:   Rn.9262    Gene Symbol:   Cdc20    Locus Link:   64515    Chromosome:   5    Cytoband:   Chr:5q36    SOURCE    GENECARD       Gene Ontology:   cellular component:  spindle   molecular function:  protein binding      BioCarta Pathways:     KEGG Pathways:  
    Probe set : 1380546_at    Name:   similar to hypothetical protein FLJ10986 /// similar to hypothetical protein FLJ10986    GB acc:   AA926048    UniGene:   Rn.8436    Gene Symbol:   LOC298250 /// LOC298251    Locus Link:   298250 /// 298251    Chromosome:   5    Cytoband:   Chr:5q33    SOURCE    GENECARD       Gene Ontology:   biological process:  carbohydrate metabolism      BioCarta Pathways:     KEGG Pathways:  
    Probe set : 1370309_a_at    Name:   heterogeneous nuclear ribonucleoprotein A/B    GB acc:   AJ238854    UniGene:   Rn.1427    Gene Symbol:   Hnrpab    Locus Link:   83498    Chromosome:   10    Cytoband:   Chr:10q22    SOURCE    GENECARD       Gene Ontology:   cellular component:  nucleus   cellular component:  viral nucleocapsid   cellular component:  ribonucleoprotein complex      BioCarta Pathways:     KEGG Pathways:  
    Probe set : 1376613_at    Name:   similar to hypothetical protein    GB acc:   BI279201    UniGene:   Rn.107832    Gene Symbol:   MGC73022    Locus Link:   309196    Chromosome:   1    Cytoband:   Chr:1q43    SOURCE    GENECARD       Gene Ontology:   molecular function:  binding      BioCarta Pathways:     KEGG Pathways:  
    Probe set : 1383324_at    Name:   Similar to PALS2-alpha splice variant    GB acc:   AI703599    UniGene:   Rn.49269     Locus Link:   500121      SOURCE        Gene Ontology:     BioCarta Pathways:     KEGG Pathways:  
    Probe set : 1393891_at    Name:   procollagen, type VIII, alpha 1 (predicted)    GB acc:   BE128699    UniGene:   Rn.53843    Gene Symbol:   Col8a1_predicted    Locus Link:   304021    Chromosome:   11    Cytoband:   Chr:11q12    SOURCE    GENECARD       Gene Ontology:   biological process:  phosphate transport   biological process:  cell adhesion      BioCarta Pathways:     KEGG Pathways:  
    Probe set : 1370493_a_at    Name:   leukocyte immunoglobulin-like receptor, subfamily B (with TM and ITIM domains), member 3 (predicted)    GB acc:   AF169637    UniGene:   Rn.143808    Gene Symbol:   Lilrb3_predicted    Locus Link:   361493    Chromosome:   1    Cytoband:   Chr:1q12    SOURCE    GENECARD       Gene Ontology:   biological process:  immune response   cellular component:  integral to membrane   molecular function:  receptor activity      BioCarta Pathways:     KEGG Pathways:  
    Probe set : 1386925_at    Name:   actin related protein 2/3 complex, subunit 1B    GB acc:   NM_019289    UniGene:   Rn.2090    Gene Symbol:   Arpc1b    Locus Link:   54227    Chromosome:   12    Cytoband:   Chr:12p11    SOURCE    GENECARD       Gene Ontology:   biological process:  cell motility   cellular component:  Arp2/3 protein complex   cellular component:  Arp2/3 protein complex   molecular function:  structural constituent of cytoskeleton      BioCarta Pathways:     KEGG Pathways:  
    Probe set : 1372179_at    Name:   neural visinin-like Ca2+-binding protein type 3    GB acc:   BM389406    UniGene:   Rn.102820    Gene Symbol:   Hpcal1    Locus Link:   50871    Chromosome:   6    Cytoband:   Chr:6q16    SOURCE    GENECARD       Gene Ontology:   molecular function:  calcium ion binding      BioCarta Pathways:     KEGG Pathways:  
    Probe set : 1372024_at    Name:   Transcribed locus    GB acc:   AI104846    UniGene:   Rn.23401        SOURCE        Gene Ontology:     BioCarta Pathways:     KEGG Pathways:  
    Probe set : 1371352_at    Name:   high mobility group protein 17    GB acc:   BM391736    UniGene:   Rn.140742    Gene Symbol:   RGD:620649    Locus Link:   114637    Chromosome:   5    Cytoband:   Chr:5q36    SOURCE    GENECARD       Gene Ontology:   cellular component:  chromosome   cellular component:  chromatin   cellular component:  nucleus   molecular function:  DNA binding      BioCarta Pathways:     KEGG Pathways:  
    Probe set : 1389073_at    Name:   similar to RIKEN cDNA 2700029E10 (predicted)    GB acc:   AA851248    UniGene:   Rn.6988    Gene Symbol:   RGD1306561_predicted    Locus Link:   306014    Chromosome:   15    Cytoband:   Chr:15p11    SOURCE    GENECARD       Gene Ontology:   cellular component:  integral to membrane   molecular function:  receptor activity      BioCarta Pathways:     KEGG Pathways:  
    Probe set : 1390355_at    Name:   ryanodine receptor 1, skeletal muscle    GB acc:   AI575442    UniGene:   Rn.87520    Gene Symbol:   Ryr1    Locus Link:   114207    Chromosome:   1    Cytoband:   Chr:1q21    SOURCE    GENECARD       Gene Ontology:   cellular component:  membrane   cellular component:  integral to membrane      BioCarta Pathways:     KEGG Pathways:  
    Probe set : 1371359_at    Name:   myeloid leukemia factor 2 (predicted)    GB acc:   BG381670    UniGene:   Rn.3678    Gene Symbol:   Mlf2_predicted    Locus Link:   312709    Chromosome:   4    Cytoband:   Chr:4q42    SOURCE    GENECARD       Gene Ontology:   cellular component:  nucleus      BioCarta Pathways:     KEGG Pathways:  
    Probe set : 1377504_at    Name:   Similar to Hypothetical protein MGC28888    GB acc:   BG376478    UniGene:   Rn.131417     Locus Link:   304361    Chromosome:   12    Cytoband:   Chr:12q12    SOURCE        Gene Ontology:   molecular function:  methyltransferase activity   molecular function:  S-adenosylmethionine-dependent methyltransferase activity      BioCarta Pathways:     KEGG Pathways:  
    Probe set : 1388988_at    Name:   similar to RIKEN cDNA 1810013B01    GB acc:   AI175070    UniGene:   Rn.2080    Gene Symbol:   RGD:1359649    Locus Link:   300983    Chromosome:   8    Cytoband:   Chr:8q32    SOURCE    GENECARD       Gene Ontology:   molecular function:  catalytic activity   molecular function:  hydrolase activity      BioCarta Pathways:     KEGG Pathways:  
    Probe set : 1367631_at    Name:   connective tissue growth factor    GB acc:   NM_022266    UniGene:   Rn.17145    Gene Symbol:   Ctgf    Locus Link:   64032    Chromosome:   1    Cytoband:   Chr:1p12    SOURCE    GENECARD       Gene Ontology:   biological process:  cartilage condensation   biological process:  ossification   biological process:  angiogenesis   biological process:  angiogenesis   biological process:  regulation of cell growth   biological process:  DNA metabolism   biological process:  cell motility   biological process:  cell adhesion   biological process:  cell adhesion   biological process:  cell-matrix adhesion   biological process:  integrin-mediated signaling pathway   biological process:  intracellular signaling cascade   biological process:  fibroblast growth factor receptor signaling pathway   biological process:  epidermis development   biological process:  response to wounding   biological process:  cell migration   biological process:  cell differentiation   biological process:  DNA replication      BioCarta Pathways:     KEGG Pathways:  
    Probe set : 1393672_at    Name:   Similar to OTTHUMP00000065631    GB acc:   BE117954    UniGene:   Rn.52931     Locus Link:   289094    Chromosome:   13    Cytoband:   Chr:13q21    SOURCE        Gene Ontology:     BioCarta Pathways:     KEGG Pathways:  
    Probe set : 1389852_at    Name:   similar to proliferation associated nuclear element 1 isoform 1 (predicted)    GB acc:   AI598316    UniGene:   Rn.11654    Gene Symbol:   RGD1307309_predicted    Locus Link:   315164    Chromosome:   7    Cytoband:   Chr:7q34    SOURCE    GENECARD       Gene Ontology:     BioCarta Pathways:     KEGG Pathways:  
    Probe set : 1372264_at    Name:   phosphoenolpyruvate carboxykinase 1    GB acc:   BI277460    UniGene:   Rn.104376    Gene Symbol:   Pck1    Locus Link:   362282    Chromosome:   3    Cytoband:   Chr:3q42    SOURCE    GENECARD       Gene Ontology:     BioCarta Pathways:     KEGG Pathways:  
    Probe set : 1370623_at    Name:   fibrinogen-like 2    GB acc:   AF323608    UniGene:   Rn.64635    Gene Symbol:   Fgl2    Locus Link:   84586    Chromosome:   4    Cytoband:   Chr:4q11    SOURCE    GENECARD       Gene Ontology:   biological process:  parturition   biological process:  cytolysis   cellular component:  extracellular space   molecular function:  peptidase activity      BioCarta Pathways:     KEGG Pathways:  
    Probe set : 1388429_at    Name:   similar to gem (nuclear organelle) associated protein 7    GB acc:   BM384085    UniGene:   Rn.13265    Gene Symbol:   LOC499391    Locus Link:   499391      SOURCE    GENECARD       Gene Ontology:     BioCarta Pathways:     KEGG Pathways:  
    Probe set : 1376914_at    Name:   DEP domain containing 1 (predicted)    GB acc:   AW253690    UniGene:   Rn.27037    Gene Symbol:   Depdc1_predicted    Locus Link:   295538    Chromosome:   2    Cytoband:   Chr:2q45    SOURCE    GENECARD       Gene Ontology:   biological process:  intracellular signaling cascade   molecular function:  GTPase activator activity      BioCarta Pathways:     KEGG Pathways:  
    Probe set : 1382003_at    Name:   solute carrier family 16 (monocarboxylic acid transporters), member 13    GB acc:   AI704067    UniGene:   Rn.23260    Gene Symbol:   RGD:1359138    Locus Link:   287451    Chromosome:   10    Cytoband:   Chr:10q24    SOURCE    GENECARD       Gene Ontology:   biological process:  transport   cellular component:  integral to membrane   molecular function:  transporter activity      BioCarta Pathways:     KEGG Pathways:  
    Probe set : 1388463_at    Name:   putative secreted protein ZSIG11    GB acc:   AW252660    UniGene:   Rn.13429    Gene Symbol:   RGD:1359353    Locus Link:   300988    Chromosome:   8    Cytoband:   Chr:8q32    SOURCE    GENECARD       Gene Ontology:     BioCarta Pathways:     KEGG Pathways:  
    Probe set : 1369013_a_at    Name:   mitochondrial ribosomal protein L17    GB acc:   AI103095    UniGene:   Rn.40354    Gene Symbol:   Mrpl17    Locus Link:   171061    Chromosome:   1    Cytoband:   Chr:1q33    SOURCE    GENECARD       Gene Ontology:   biological process:  mitochondrial genome maintenance   biological process:  protein biosynthesis   molecular function:  structural constituent of ribosome      BioCarta Pathways:     KEGG Pathways:  
    Probe set : 1371322_at    Name:   Laminin, gamma 1    GB acc:   BI275624    UniGene:   Rn.7145    Gene Symbol:   Lamc1    Locus Link:   117036    Chromosome:   13    Cytoband:   Chr:13q21    SOURCE    GENECARD       Gene Ontology:   cellular component:  extracellular matrix (sensu Metazoa)   cellular component:  laminin-1      BioCarta Pathways:     KEGG Pathways:  
    Probe set : 1372103_at    Name:   DnaJ (Hsp40) homolog, subfamily A, member 4 (predicted)    GB acc:   BM386931    UniGene:   Rn.27927     Locus Link:   300721    Chromosome:   8    Cytoband:   Chr:8q24    SOURCE        Gene Ontology:   biological process:  protein folding   molecular function:  heat shock protein binding   molecular function:  unfolded protein binding      BioCarta Pathways:     KEGG Pathways:  
    Probe set : 1398378_at    Name:   glutathione S-transferase kappa 1    GB acc:   AI231779    UniGene:   Rn.109452    Gene Symbol:   Gstk1    Locus Link:   297029    Chromosome:   4    Cytoband:   Chr:4q23    SOURCE    GENECARD       Gene Ontology:   biological process:  protein targeting   biological process:  glutathione metabolism   cellular component:  mitochondrion   cellular component:  mitochondrial matrix   cellular component:  periplasmic space (sensu Gram-negative Bacteria)      BioCarta Pathways:     KEGG Pathways:  
    Probe set : 1371539_at    Name:   nucleolar protein family A, member 2 (predicted)    GB acc:   AW921151    UniGene:   Rn.4216    Gene Symbol:   Nola2_predicted    Locus Link:   287273    Chromosome:   10    Cytoband:   Chr:10q22    SOURCE    GENECARD       Gene Ontology:   biological process:  nuclear mRNA splicing, via spliceosome   biological process:  mRNA processing   biological process:  protein biosynthesis   molecular function:  RNA binding   molecular function:  structural constituent of ribosome      BioCarta Pathways:     KEGG Pathways:  
    Probe set : 1384051_at    Name:   Transcribed locus    GB acc:   BF390066    UniGene:   Rn.26623        SOURCE        Gene Ontology:     BioCarta Pathways:     KEGG Pathways:  
    Probe set : 1392544_at    Name:   rcd1 (required for cell differentiation) homolog 1 (S. pombe) (predicted)    GB acc:   AI044879    UniGene:   Rn.18033    Gene Symbol:   Rqcd1_predicted    Locus Link:   301513    Chromosome:   9    Cytoband:   Chr:9q33    SOURCE    GENECARD       Gene Ontology:   biological process:  sex differentiation   biological process:  cytokine and chemokine mediated signaling pathway   molecular function:  binding      BioCarta Pathways:     KEGG Pathways:  
    Probe set : 1388137_a_at    Name:   zygin-related protein    GB acc:   AA955230     Gene Symbol:   RGD:628711    Locus Link:   286909       GENECARD       Gene Ontology:     BioCarta Pathways:     KEGG Pathways:  
    Probe set : 1391466_at    Name:   Nuclear ubiquitous casein kinase and cyclin-dependent kinase substrate    GB acc:   AI170347    UniGene:   Rn.21324    Gene Symbol:   Nucks    Locus Link:   64709      SOURCE    GENECARD       Gene Ontology:   biological process:  regulation of progression through cell cycle   cellular component:  nucleus   cellular component:  nucleus   cellular component:  cytoplasm   molecular function:  double-stranded DNA binding   molecular function:  single-stranded DNA binding   molecular function:  kinase activity      BioCarta Pathways:     KEGG Pathways:  
    Probe set : 1384960_at    Name:   Transcribed locus    GB acc:   BI281250    UniGene:   Rn.124539        SOURCE        Gene Ontology:     BioCarta Pathways:     KEGG Pathways:  
    Probe set : 1369182_at    Name:   coagulation factor 3    GB acc:   NM_013057    UniGene:   Rn.9980    Gene Symbol:   F3    Locus Link:   25584    Chromosome:   2    Cytoband:   Chr:2q41    SOURCE    GENECARD       Gene Ontology:   biological process:  immune response   biological process:  blood coagulation   molecular function:  hematopoietin/interferon-class (D200-domain) cytokine receptor activity      BioCarta Pathways:     KEGG Pathways:  
    Probe set : 1398775_at    Name:   ribosomal protein S15a    GB acc:   NM_053982    UniGene:   Rn.2890    Gene Symbol:   Rps15a    Locus Link:   117053    Chromosome:   1    Cytoband:   Chr:1q35    SOURCE    GENECARD       Gene Ontology:   biological process:  RNA processing   biological process:  protein biosynthesis      BioCarta Pathways:     KEGG Pathways:  
    Probe set : 1377887_at    Name:   Acyl-Coenzyme A oxidase 3, pristanoyl    GB acc:   AI013044    UniGene:   Rn.10546    Gene Symbol:   Acox3    Locus Link:   83522    Chromosome:   14    Cytoband:   Chr:14q21    SOURCE    GENECARD       Gene Ontology:   cellular component:  peroxisome   cellular component:  peroxisome   cellular component:  peroxisomal matrix      BioCarta Pathways:     KEGG Pathways:  
    Probe set : 1393545_at    Name:   transcription factor CP2-like 2 (predicted)    GB acc:   AI043906    UniGene:   Rn.18589    Gene Symbol:   Tcfcp2l2_predicted    Locus Link:   313994    Chromosome:   6    Cytoband:   Chr:6q16    SOURCE    GENECARD       Gene Ontology:   cellular component:  nucleus      BioCarta Pathways:     KEGG Pathways:  
    Probe set : 1375910_at    Name:   CDC42 effector protein (Rho GTPase binding) 3 (predicted)    GB acc:   AA874943    UniGene:   Rn.4040    Gene Symbol:   Cdc42ep3_predicted    Locus Link:   313838    Chromosome:   6    Cytoband:   Chr:6q11    SOURCE    GENECARD       Gene Ontology:   biological process:  signal transduction   molecular function:  protein binding   molecular function:  cytoskeletal regulatory protein binding      BioCarta Pathways:     KEGG Pathways:  
    Probe set : 1392001_at    Name:   Pannexin 1    GB acc:   AI232305    UniGene:   Rn.45097    Gene Symbol:   Panx1    Locus Link:   315435    Chromosome:   8    Cytoband:   Chr:8q11-q12    SOURCE    GENECARD       Gene Ontology:   biological process:  cell-cell signaling   cellular component:  gap junction   cellular component:  gap junction   cellular component:  integral to membrane   molecular function:  gap-junction forming channel activity   molecular function:  protein heterodimerization activity      BioCarta Pathways:     KEGG Pathways:  
    Probe set : 1389632_at    Name:   Transcribed locus, weakly similar to XP_228131.3 PREDICTED: similar to Rho-related BTB domain-containing protein 1 [Rattus norvegicus]    GB acc:   AA799294    UniGene:   Rn.3782        SOURCE        Gene Ontology:     BioCarta Pathways:     KEGG Pathways:  
    Probe set : 1383253_at    Name:   Solute carrier family 30 (zinc transporter), member 1    GB acc:   AI179795    UniGene:   Rn.10120    Gene Symbol:   Slc30a1    Locus Link:   58976    Chromosome:   13    Cytoband:   Chr:13q27    SOURCE    GENECARD       Gene Ontology:     BioCarta Pathways:     KEGG Pathways:  
    Probe set : 1372233_at    Name:   serologically defined breast cancer antigen 84 (predicted)    GB acc:   AI228649    UniGene:   Rn.22956    Gene Symbol:   Sdbcag84_predicted    Locus Link:   296306    Chromosome:   3    Cytoband:   Chr:3q42    SOURCE    GENECARD       Gene Ontology:   cellular component:  integral to membrane      BioCarta Pathways:     KEGG Pathways:  
    Probe set : 1373605_at    Name:   similar to 106 kDa O-GlcNAc transferase-interacting protein (predicted)    GB acc:   AI176540    UniGene:   Rn.3090    Gene Symbol:   RGD1307844_predicted    Locus Link:   316085    Chromosome:   8    Cytoband:   Chr:8q32    SOURCE    GENECARD       Gene Ontology:     BioCarta Pathways:     KEGG Pathways:  
    Probe set : 1381776_at    Name:   similar to RIKEN cDNA 1810015P03 (predicted)    GB acc:   BI294972    UniGene:   Rn.19837    Gene Symbol:   RGD1305260_predicted    Locus Link:   291991    Chromosome:   19    Cytoband:   Chr:19q12    SOURCE    GENECARD       Gene Ontology:     BioCarta Pathways:     KEGG Pathways:  
    Probe set : 1373156_at    Name:   similar to armadillo repeat protein ALEX2    GB acc:   AI385330    UniGene:   Rn.15754    Gene Symbol:   RGD:1359132    Locus Link:   367903    Chromosome:   X    Cytoband:   Chr:Xq34    SOURCE    GENECARD       Gene Ontology:   molecular function:  binding      BioCarta Pathways:     KEGG Pathways:  
    Probe set : 1386944_a_at    Name:   glucose-6-phosphatase, catalytic    GB acc:   NM_013098    UniGene:   Rn.10992    Gene Symbol:   G6pc    Locus Link:   25634    Chromosome:   10    Cytoband:   Chr:10q32.1    SOURCE    GENECARD       Gene Ontology:     BioCarta Pathways:     KEGG Pathways:  
    Probe set : 1368743_a_at    Name:   dentin sialophosphoprotein    GB acc:   NM_012790    UniGene:   Rn.21392    Gene Symbol:   Dspp    Locus Link:   25254    Chromosome:   14    Cytoband:   Chr:14p22    SOURCE    GENECARD       Gene Ontology:   cellular component:  extracellular matrix (sensu Metazoa)   cellular component:  extracellular space   molecular function:  calcium ion binding      BioCarta Pathways:     KEGG Pathways:  
    Probe set : 1372820_at    Name:   Transcribed locus    GB acc:   BE109102    UniGene:   Rn.3225        SOURCE        Gene Ontology:     BioCarta Pathways:     KEGG Pathways:  
    Probe set : 1379234_a_at    Name:   similar to CDC45L    GB acc:   AI179539    UniGene:   Rn.98535    Gene Symbol:   LOC287961    Locus Link:   287961    Chromosome:   11    Cytoband:   Chr:11p12    SOURCE    GENECARD       Gene Ontology:     BioCarta Pathways:     KEGG Pathways:  
    Probe set : 1383133_at    Name:   Transcribed locus    GB acc:   BI295765    UniGene:   Rn.18130        SOURCE        Gene Ontology:     BioCarta Pathways:     KEGG Pathways:  
    Probe set : 1383371_at    Name:   Transcribed locus    GB acc:   AI712305    UniGene:   Rn.35187        SOURCE        Gene Ontology:     BioCarta Pathways:     KEGG Pathways:  
    Probe set : 1388557_at    Name:   Transcribed locus    GB acc:   BF284922    UniGene:   Rn.37683        SOURCE        Gene Ontology:     BioCarta Pathways:     KEGG Pathways:  
    Probe set : 1367696_at    Name:   interferon induced transmembrane protein 2 (1-8D)    GB acc:   NM_030833    UniGene:   Rn.108174    Gene Symbol:   Ifitm2    Locus Link:   114709    Chromosome:   1    Cytoband:   Chr:1q41    SOURCE    GENECARD       Gene Ontology:   biological process:  response to biotic stimulus   cellular component:  integral to membrane      BioCarta Pathways:     KEGG Pathways:  
    Probe set : 1395572_at    Name:   DnaJ (Hsp40) homolog, subfamily A, member 4 (predicted)    GB acc:   BF565278    UniGene:   Rn.27927    Gene Symbol:   Dnaja4_predicted    Locus Link:   300721    Chromosome:   8    Cytoband:   Chr:8q24    SOURCE    GENECARD       Gene Ontology:   biological process:  protein folding   molecular function:  heat shock protein binding   molecular function:  unfolded protein binding      BioCarta Pathways:     KEGG Pathways:  
    Probe set : 1368779_a_at    Name:   guanylate cyclase 1, soluble, beta 2    GB acc:   NM_012770    UniGene:   Rn.10933    Gene Symbol:   Gucy1b2    Locus Link:   25206    Chromosome:   5    Cytoband:   Chr:5q36    SOURCE    GENECARD       Gene Ontology:   biological process:  cGMP biosynthesis   biological process:  intracellular signaling cascade   biological process:  cyclic nucleotide biosynthesis      BioCarta Pathways:     KEGG Pathways:  
    Probe set : 1388581_at    Name:   hematological and neurological expressed sequence 1    GB acc:   AI317868    UniGene:   Rn.138819    Gene Symbol:   RGD:1359325    Locus Link:   287828    Chromosome:   10    Cytoband:   Chr:10q32.3    SOURCE    GENECARD       Gene Ontology:     BioCarta Pathways:     KEGG Pathways:  
    Probe set : 1383406_at    Name:   Transcribed locus    GB acc:   BE103634    UniGene:   Rn.1961        SOURCE        Gene Ontology:     BioCarta Pathways:     KEGG Pathways:  
    Probe set : 1399059_at    Name:   Enhancer of zeste homolog 1 (Drosophila) (predicted)    GB acc:   BI283868    UniGene:   Rn.12929     Locus Link:   303547    Chromosome:   10    Cytoband:   Chr:10q32.1    SOURCE        Gene Ontology:   biological process:  regulation of transcription, DNA-dependent   biological process:  morphogenesis   cellular component:  nucleus   molecular function:  DNA binding   molecular function:  chromatin binding   molecular function:  protein binding      BioCarta Pathways:     KEGG Pathways:  
    Probe set : 1390292_at    Name:   transmembrane protein 8 (five membrane-spanning domains) (predicted)    GB acc:   AW527467    UniGene:   Rn.21890    Gene Symbol:   Tmem8_predicted    Locus Link:   303004    Chromosome:   10    Cytoband:   Chr:10q12    SOURCE    GENECARD       Gene Ontology:   biological process:  cell adhesion   cellular component:  extracellular space   cellular component:  integral to plasma membrane   cellular component:  integral to membrane   molecular function:  protein binding      BioCarta Pathways:     KEGG Pathways:  
    Probe set : 1377689_at    Name:   kinetochore associated 1 (predicted)    GB acc:   BF564273    UniGene:   Rn.47918    Gene Symbol:   Kntc1_predicted    Locus Link:   304477    Chromosome:   12    Cytoband:   Chr:12q15-q16    SOURCE    GENECARD       Gene Ontology:   cellular component:  spindle pole   cellular component:  nucleus   cellular component:  kinetochore microtubule   molecular function:  DNA binding   molecular function:  protein binding      BioCarta Pathways:     KEGG Pathways:  
    Probe set : 1384063_at    Name:   collagen triple helix repeat containing 1    GB acc:   AA958001    UniGene:   Rn.9468    Gene Symbol:   Cthrc1    Locus Link:   282836    Chromosome:   7    Cytoband:   Chr:7q22    SOURCE    GENECARD       Gene Ontology:   biological process:  phosphate transport   cellular component:  extracellular matrix (sensu Metazoa)   cellular component:  cytoplasm   molecular function:  structural molecule activity      BioCarta Pathways:     KEGG Pathways:  
    Probe set : 1373597_at    Name:   processing of precursor 7, ribonuclease P family, (S. cerevisiae) (predicted)    GB acc:   AI102689    UniGene:   Rn.16344    Gene Symbol:   Pop7_predicted    Locus Link:   288564    Chromosome:   12    Cytoband:   Chr:12q12    SOURCE    GENECARD       Gene Ontology:   biological process:  tRNA processing   cellular component:  nucleus   cellular component:  nucleolar ribonuclease P complex   molecular function:  ribonuclease P activity   molecular function:  hydrolase activity      BioCarta Pathways:     KEGG Pathways:  
    Probe set : 1379972_at    Name:   similar to 1700060H10Rik protein (predicted)    GB acc:   AI070438    UniGene:   Rn.33054    Gene Symbol:   RGD1308699_predicted    Locus Link:   309790    Chromosome:   20    Cytoband:   Chr:20q11    SOURCE    GENECARD       Gene Ontology:   cellular component:  nucleus      BioCarta Pathways:     KEGG Pathways:  
    Probe set : 1374389_at    Name:   guanylate cyclase 1, soluble, beta 3    GB acc:   BF399387    UniGene:   Rn.87228    Gene Symbol:   Gucy1b3    Locus Link:   25202    Chromosome:   2    Cytoband:   Chr:2q31-q33    SOURCE    GENECARD       Gene Ontology:   cellular component:  cytoplasm   cellular component:  guanylate cyclase complex, soluble      BioCarta Pathways:     KEGG Pathways:  
    Probe set : 1387081_at    Name:   reticulocalbin 2    GB acc:   NM_017132    UniGene:   Rn.6133    Gene Symbol:   Rcn2    Locus Link:   29218    Chromosome:   8    Cytoband:   Chr:8q24    SOURCE    GENECARD       Gene Ontology:   biological process:  muscle development   cellular component:  extracellular space   cellular component:  endoplasmic reticulum   molecular function:  calcium ion binding   molecular function:  calcium ion binding   molecular function:  protein binding      BioCarta Pathways:     KEGG Pathways:  
    Probe set : 1368509_at    Name:   Bardet-Biedl syndrome 2 (human)    GB acc:   NM_053618    UniGene:   Rn.15987    Gene Symbol:   Bbs2    Locus Link:   113948    Chromosome:   19    Cytoband:   Chr:19p12    SOURCE    GENECARD       Gene Ontology:   cellular component:  integrin complex      BioCarta Pathways:     KEGG Pathways:  
    Probe set : 1394464_at    Name:   LOC500352 /// LOC500353    GB acc:   AW528921    UniGene:   Rn.47043    Gene Symbol:   LOC500352 /// LOC500353    Locus Link:   500352 /// 500353    Chromosome:   4    Cytoband:   Chr:4q43    SOURCE    GENECARD       Gene Ontology:     BioCarta Pathways:     KEGG Pathways:  
    Probe set : 1389189_at    Name:   actinin, alpha 1    GB acc:   BF555956    UniGene:   Rn.6401    Gene Symbol:   Actn1    Locus Link:   81634    Chromosome:   6    Cytoband:   Chr:6q24    SOURCE    GENECARD       Gene Ontology:   cellular component:  cytoskeleton   cellular component:  actin cytoskeleton      BioCarta Pathways:     KEGG Pathways:  
    Probe set : 1388677_at    Name:   ubiquitin-associated protein 1 (predicted)    GB acc:   BI275991    UniGene:   Rn.98217    Gene Symbol:   Ubap1_predicted    Locus Link:   362502    Chromosome:   5    Cytoband:   Chr:5q22    SOURCE    GENECARD       Gene Ontology:     BioCarta Pathways:     KEGG Pathways:  
    Probe set : 1374817_at    Name:   Transcribed locus    GB acc:   BE103235    UniGene:   Rn.19071        SOURCE        Gene Ontology:     BioCarta Pathways:     KEGG Pathways:  
    Probe set : 1373947_at    Name:   dermatopontin (predicted)    GB acc:   BI278545    UniGene:   Rn.8396    Gene Symbol:   Dpt_predicted    Locus Link:   289178    Chromosome:   13    Cytoband:   Chr:13q23    SOURCE    GENECARD       Gene Ontology:   biological process:  cell adhesion   cellular component:  extracellular matrix (sensu Metazoa)   cellular component:  extracellular space   molecular function:  protein binding      BioCarta Pathways:     KEGG Pathways:  
    Probe set : 1378551_at    Name:   cytochrome P450, family 20, subfamily A, polypeptide 1    GB acc:   BE118606    UniGene:   Rn.101629    Gene Symbol:   Cyp20a1    Locus Link:   316435    Chromosome:   9    Cytoband:   Chr:9q31    SOURCE    GENECARD       Gene Ontology:   biological process:  electron transport   cellular component:  membrane      BioCarta Pathways:     KEGG Pathways:  
    Probe set : 1367881_at    Name:   protein tyrosine phosphatase, non-receptor type substrate 1    GB acc:   NM_013016    UniGene:   Rn.53971    Gene Symbol:   Ptpns1    Locus Link:   25528    Chromosome:   3    Cytoband:   Chr:3q36    SOURCE    GENECARD       Gene Ontology:   cellular component:  extracellular space   cellular component:  integral to plasma membrane   cellular component:  integral to membrane   molecular function:  protein binding   molecular function:  protein phosphorylated amino acid binding      BioCarta Pathways:     KEGG Pathways:  
    Probe set : 1386994_at    Name:   B-cell translocation gene 2, anti-proliferative    GB acc:   NM_017259    UniGene:   Rn.27923    Gene Symbol:   Btg2    Locus Link:   29619      SOURCE    GENECARD       Gene Ontology:   molecular function:  protein binding      BioCarta Pathways:     KEGG Pathways:  
    Probe set : 1372076_at    Name:   hepatitis B virus x interacting protein (predicted)    GB acc:   BI294704    UniGene:   Rn.3763    Gene Symbol:   Hbxip_predicted    Locus Link:   295357    Chromosome:   2    Cytoband:   Chr:2q34    SOURCE    GENECARD       Gene Ontology:   biological process:  response to virus   biological process:  viral life cycle   biological process:  viral genome replication      BioCarta Pathways:     KEGG Pathways:  
    Probe set : 1397584_at    Name:   Transcribed locus    GB acc:   BF289504    UniGene:   Rn.55786        SOURCE        Gene Ontology:     BioCarta Pathways:     KEGG Pathways:  
    Probe set : 1395003_at    Name:   dendritic cell inhibitory receptor 4    GB acc:   BI278559    UniGene:   Rn.47351    Gene Symbol:   RGD:1359109    Locus Link:   362430    Chromosome:   4    Cytoband:   Chr:4q42    SOURCE    GENECARD       Gene Ontology:   molecular function:  receptor activity   molecular function:  sugar binding      BioCarta Pathways:     KEGG Pathways:  
    Probe set : 1367596_at    Name:   ribosomal protein S26    GB acc:   NM_013224    UniGene:   Rn.2388    Gene Symbol:   Rps26    Locus Link:   27139    Chromosome:   7    Cytoband:   Chr:7q11    SOURCE    GENECARD       Gene Ontology:   biological process:  protein biosynthesis   biological process:  tRNA aminoacylation for protein translation   biological process:  aspartyl-tRNA aminoacylation      BioCarta Pathways:     KEGG Pathways:  
    Probe set : 1371017_at    Name:   T-cell receptor gamma chain    GB acc:   Z27087    UniGene:   Rn.10427    Gene Symbol:   Tcrg    Locus Link:   24821    Chromosome:   17    Cytoband:   Chr:17q11    SOURCE    GENECARD       Gene Ontology:   biological process:  immune response   biological process:  cellular defense response   cellular component:  integral to plasma membrane   cellular component:  gamma-delta T cell receptor complex   molecular function:  transmembrane receptor activity      BioCarta Pathways:     KEGG Pathways:  
    Probe set : 1385072_at    Name:   galactose mutarotase (aldose 1-epimerase)    GB acc:   AA997048    UniGene:   Rn.83639    Gene Symbol:   RGD:1359459    Locus Link:   313843    Chromosome:   6    Cytoband:   Chr:6q11    SOURCE    GENECARD       Gene Ontology:   biological process:  carbohydrate metabolism   biological process:  galactose metabolism   molecular function:  aldose 1-epimerase activity   molecular function:  isomerase activity      BioCarta Pathways:     KEGG Pathways:  
    Probe set : 1399015_at    Name:   Transmembrane trafficking protein 21    GB acc:   BI281753    UniGene:   Rn.8509    Gene Symbol:   Tmp21    Locus Link:   84599    Chromosome:   6    Cytoband:   Chr:6q31    SOURCE    GENECARD       Gene Ontology:   molecular function:  protein carrier activity   molecular function:  protein transporter activity      BioCarta Pathways:     KEGG Pathways:  
    Probe set : 1393777_at    Name:   Transcribed locus    GB acc:   AA998104    UniGene:   Rn.92969        SOURCE        Gene Ontology:     BioCarta Pathways:     KEGG Pathways:  
    Probe set : 1372199_at    Name:   PTEN induced putative kinase 1 (predicted)    GB acc:   AI233191    UniGene:   Rn.103380    Gene Symbol:   Pink1_predicted    Locus Link:   298575    Chromosome:   5    Cytoband:   Chr:5q36    SOURCE    GENECARD       Gene Ontology:   biological process:  protein amino acid phosphorylation   biological process:  response to stress   biological process:  protein kinase cascade   cellular component:  mitochondrion      BioCarta Pathways:     KEGG Pathways:  
    Probe set : 1370036_at    Name:   sulfite oxidase    GB acc:   NM_031127    UniGene:   Rn.25720    Gene Symbol:   Suox    Locus Link:   81805    Chromosome:   7    Cytoband:   Chr:7q11    SOURCE    GENECARD       Gene Ontology:   biological process:  electron transport   biological process:  methionine catabolism   cellular component:  mitochondrion      BioCarta Pathways:     KEGG Pathways:  
    Probe set : 1387218_at    Name:   trefoil factor 3    GB acc:   NM_013042    UniGene:   Rn.87477    Gene Symbol:   Tff3    Locus Link:   25563    Chromosome:   20    Cytoband:   Chr:20p12    SOURCE    GENECARD       Gene Ontology:   biological process:  defense response   biological process:  digestion   biological process:  wound healing   biological process:  wound healing   cellular component:  extracellular region   cellular component:  extracellular space   molecular function:  receptor activity      BioCarta Pathways:     KEGG Pathways:  
    Probe set : 1399052_at    Name:   Toll interacting protein (predicted)    GB acc:   AA957069    UniGene:   Rn.137040     Locus Link:   361677    Chromosome:   1    Cytoband:   Chr:1q41    SOURCE        Gene Ontology:   cellular component:  cytoplasm   cellular component:  interleukin-18 receptor complex   cellular component:  interleukin-1 receptor complex   molecular function:  signal transducer activity   molecular function:  Toll binding   molecular function:  protein binding      BioCarta Pathways:     KEGG Pathways:  
    Probe set : 1379340_at    Name:   lamimin, gamma 2    GB acc:   BM385282    UniGene:   Rn.9278    Gene Symbol:   Lamc2    Locus Link:   192362    Chromosome:   13    Cytoband:   Chr:13q21    SOURCE    GENECARD       Gene Ontology:   biological process:  cell adhesion   cellular component:  extracellular matrix (sensu Metazoa)   cellular component:  laminin-2   cellular component:  extracellular matrix      BioCarta Pathways:     KEGG Pathways:  
    Probe set : 1371420_at    Name:   92Aa-Protein    GB acc:   AI412054    UniGene:   Rn.7336    Gene Symbol:   RGD:1359519    Locus Link:   494529      SOURCE    GENECARD       Gene Ontology:     BioCarta Pathways:     KEGG Pathways:  
    Probe set : 1399050_at    Name:   adenylosuccinate synthetase 2, non muscle (predicted)    GB acc:   AI232106    UniGene:   Rn.9047    Gene Symbol:   Adss2_predicted    Locus Link:   289276    Chromosome:   13    Cytoband:   Chr:13q25    SOURCE    GENECARD       Gene Ontology:   biological process:  purine nucleotide biosynthesis   biological process:  AMP biosynthesis      BioCarta Pathways:     KEGG Pathways:  
    Probe set : 1380474_at     GB acc:   AI013470              Gene Ontology:     BioCarta Pathways:     KEGG Pathways:  
    Probe set : 1373025_at    Name:   complement component 1, q subcomponent, gamma polypeptide (predicted)    GB acc:   AI411618    UniGene:   Rn.2393    Gene Symbol:   C1qg_predicted    Locus Link:   362634    Chromosome:   5    Cytoband:   Chr:5q36    SOURCE    GENECARD       Gene Ontology:   cellular component:  extracellular region   cellular component:  extracellular space   cellular component:  cytoplasm      BioCarta Pathways:     KEGG Pathways:  
    Probe set : 1367717_at    Name:   ribosomal protein S27    GB acc:   NM_053597    UniGene:   Rn.144623    Gene Symbol:   Rps27    Locus Link:   94266    Chromosome:   2    Cytoband:   Chr:2q34    SOURCE    GENECARD       Gene Ontology:   biological process:  protein biosynthesis   biological process:  signal transduction   biological process:  cell proliferation      BioCarta Pathways:     KEGG Pathways:  
    Probe set : 1370258_at    Name:   basic leucine zipper and W2 domains 2    GB acc:   BI303268    UniGene:   Rn.13196    Gene Symbol:   Bzw2    Locus Link:   171439    Chromosome:   6    Cytoband:   Chr:6q16    SOURCE    GENECARD       Gene Ontology:   biological process:  regulation of translational initiation   molecular function:  translation initiation factor activity      BioCarta Pathways:     KEGG Pathways:  
    Probe set : 1382918_at    Name:   Transcribed locus    GB acc:   AA956561    UniGene:   Rn.147475        SOURCE        Gene Ontology:     BioCarta Pathways:     KEGG Pathways:  
    Probe set : 1370000_at    Name:   nucleobindin 2    GB acc:   NM_021663    UniGene:   Rn.41602    Gene Symbol:   Nucb2    Locus Link:   59295    Chromosome:   1    Cytoband:   Chr:1q35    SOURCE    GENECARD       Gene Ontology:   biological process:  calcium ion homeostasis   molecular function:  DNA binding   molecular function:  calcium ion binding      BioCarta Pathways:     KEGG Pathways:  
    Probe set : 1372957_at    Name:   Similar to hypothetical protein A730008H23 (predicted)    GB acc:   BE101323    UniGene:   Rn.19499     Locus Link:   316602    Chromosome:   9    Cytoband:   Chr:9q35    SOURCE        Gene Ontology:     BioCarta Pathways:     KEGG Pathways:  
    Probe set : 1373866_at    Name:   similar to hypothetical protein FLJ13448    GB acc:   AI228596    UniGene:   Rn.101706    Gene Symbol:   RGD:1359509    Locus Link:   301416    Chromosome:   9    Cytoband:   Chr:9q31    SOURCE    GENECARD       Gene Ontology:   biological process:  antibiotic biosynthesis   molecular function:  polyketide synthase activity      BioCarta Pathways:     KEGG Pathways:  
    Probe set : 1384259_at    Name:   Rac GTPase-activating protein 1 (predicted)    GB acc:   BF559151    UniGene:   Rn.101301    Gene Symbol:   Racgap1_predicted    Locus Link:   315298    Chromosome:   7    Cytoband:   Chr:7q36    SOURCE    GENECARD       Gene Ontology:   biological process:  electron transport   biological process:  transport   biological process:  intracellular signaling cascade   cellular component:  mitochondrion      BioCarta Pathways:     KEGG Pathways:  
    Probe set : 1388389_at    Name:   septin 2    GB acc:   BF282620    UniGene:   Rn.98570    Gene Symbol:   Sept2    Locus Link:   117515    Chromosome:   9    Cytoband:   Chr:9q36    SOURCE    GENECARD       Gene Ontology:   biological process:  cell cycle   molecular function:  nucleotide binding   molecular function:  GTP binding      BioCarta Pathways:     KEGG Pathways:  
    Probe set : 1391862_at    Name:   Transcribed locus    GB acc:   BG377635    UniGene:   Rn.54356        SOURCE        Gene Ontology:     BioCarta Pathways:     KEGG Pathways:  
    Probe set : 1391417_at    Name:   similar to betaine-homocysteine methyltransferase 2    GB acc:   AA925145    UniGene:   Rn.8494    Gene Symbol:   LOC365972    Locus Link:   365972      SOURCE    GENECARD       Gene Ontology:   molecular function:  homocysteine S-methyltransferase activity      BioCarta Pathways:     KEGG Pathways:  
    Probe set : 1377968_at    Name:   Transcribed locus    GB acc:   AI069912    UniGene:   Rn.13003        SOURCE        Gene Ontology:     BioCarta Pathways:     KEGG Pathways:  
    Probe set : 1375997_at    Name:   Transcribed locus    GB acc:   AA799503    UniGene:   Rn.3768        SOURCE        Gene Ontology:     BioCarta Pathways:     KEGG Pathways:  
    Probe set : 1370377_at    Name:   cytochrome P450, family 2, subfamily d, polypeptide 9 /// cytochrome P450, family 2, subfamily d, polypeptide 10    GB acc:   M25143    UniGene:   Rn.91133    Gene Symbol:   Cyp2d9 /// Cyp2d10    Locus Link:   266684 /// 286963    Chromosome:   7    Cytoband:   Chr:7q34    SOURCE    GENECARD       Gene Ontology:   biological process:  electron transport   cellular component:  endoplasmic reticulum   cellular component:  microsome   cellular component:  membrane      BioCarta Pathways:     KEGG Pathways:  
    Probe set : 1397767_at    Name:   Transcribed locus    GB acc:   BG377356    UniGene:   Rn.41665        SOURCE        Gene Ontology:     BioCarta Pathways:     KEGG Pathways:  
    Probe set : 1382228_at    Name:   similar to Zinc finger protein 431    GB acc:   BE095590    UniGene:   Rn.24101    Gene Symbol:   LOC499597    Locus Link:   499597    Chromosome:   2    Cytoband:   Chr:2q25    SOURCE    GENECARD       Gene Ontology:     BioCarta Pathways:     KEGG Pathways:  
    Probe set : 1379261_at    Name:   family with sequence similarity 33, member A (predicted)    GB acc:   BE111490    UniGene:   Rn.40571    Gene Symbol:   Fam33a_predicted    Locus Link:   287598    Chromosome:   10    Cytoband:   Chr:10q26    SOURCE    GENECARD       Gene Ontology:     BioCarta Pathways:     KEGG Pathways:  
    Probe set : 1388436_at    Name:   small nuclear ribonucleoprotein polypeptide A (predicted)    GB acc:   AI230728    UniGene:   Rn.9347    Gene Symbol:   Snrpa_predicted    Locus Link:   292729    Chromosome:   1    Cytoband:   Chr:1q21    SOURCE    GENECARD       Gene Ontology:   biological process:  nuclear mRNA splicing, via spliceosome   biological process:  mRNA processing   molecular function:  RNA binding   molecular function:  nucleotide binding   molecular function:  nucleic acid binding      BioCarta Pathways:     KEGG Pathways:  
    Probe set : 1387966_at    Name:   asparaginase-like sperm autoantigen    GB acc:   AF329099    UniGene:   Rn.22774    Gene Symbol:   Asrgl1    Locus Link:   246307    Chromosome:   1    Cytoband:   Chr:1q43    SOURCE    GENECARD       Gene Ontology:   biological process:  glycoprotein catabolism   cellular component:  cytoplasm   molecular function:  N4-(beta-N-acetylglucosaminyl)-L-asparaginase activity   molecular function:  asparaginase activity      BioCarta Pathways:     KEGG Pathways:  
    Probe set : 1383227_at    Name:   Transcribed locus    GB acc:   BI288882    UniGene:   Rn.147727        SOURCE        Gene Ontology:     BioCarta Pathways:     KEGG Pathways:  
    Probe set : 1373337_at    Name:   glyoxylate reductase/hydroxypyruvate reductase (predicted)    GB acc:   AI412065    UniGene:   Rn.7815    Gene Symbol:   Grhpr_predicted    Locus Link:   298085    Chromosome:   5    Cytoband:   Chr:5q22    SOURCE    GENECARD       Gene Ontology:   biological process:  L-serine biosynthesis   biological process:  metabolism      BioCarta Pathways:     KEGG Pathways:  
    Probe set : 1381933_at    Name:   Transcribed locus    GB acc:   AW434510    UniGene:   Rn.146519        SOURCE        Gene Ontology:     BioCarta Pathways:     KEGG Pathways:  
    Probe set : 1390659_at    Name:   CD44 antigen    GB acc:   BI302830    UniGene:   Rn.1120    Gene Symbol:   Cd44    Locus Link:   25406    Chromosome:   3    Cytoband:   Chr:3q31    SOURCE    GENECARD       Gene Ontology:     BioCarta Pathways:     KEGG Pathways:  
    Probe set : 1379592_at    Name:   similar to citrin    GB acc:   AI045151    UniGene:   Rn.14686    Gene Symbol:   LOC362322    Locus Link:   362322    Chromosome:   4    Cytoband:   Chr:4q13    SOURCE    GENECARD       Gene Ontology:     BioCarta Pathways:     KEGG Pathways:  
    Probe set : 1367775_at    Name:   alpha-methylacyl-CoA racemase    GB acc:   NM_012816    UniGene:   Rn.2590    Gene Symbol:   Amacr    Locus Link:   25284    Chromosome:   2    Cytoband:   Chr:2q16    SOURCE    GENECARD       Gene Ontology:   cellular component:  mitochondrion   cellular component:  peroxisome      BioCarta Pathways:     KEGG Pathways:  
    Probe set : 1390918_at    Name:   GH regulated TBC protein 1 (predicted)    GB acc:   BE099056    UniGene:   Rn.19589    Gene Symbol:   Grtp1_predicted    Locus Link:   361180    Chromosome:   16    Cytoband:   Chr:16q12.5    SOURCE    GENECARD       Gene Ontology:   cellular component:  extracellular region   molecular function:  hormone activity      BioCarta Pathways:     KEGG Pathways:  
    Probe set : 1387777_at    Name:   integrin linked kinase    GB acc:   NM_133409    UniGene:   Rn.95042    Gene Symbol:   Ilk    Locus Link:   170922    Chromosome:   1    Cytoband:   Chr:1q33    SOURCE    GENECARD       Gene Ontology:   cellular component:  focal adhesion   cellular component:  focal adhesion      BioCarta Pathways:     KEGG Pathways:  
    Probe set : 1370048_at    Name:   endothelial differentiation, lysophosphatidic acid G-protein-coupled receptor, 2    GB acc:   NM_053936    UniGene:   Rn.11200    Gene Symbol:   Edg2    Locus Link:   116744    Chromosome:   5    Cytoband:   Chr:5q24    SOURCE    GENECARD       Gene Ontology:     BioCarta Pathways:     KEGG Pathways:  
    Probe set : 1373223_at    Name:   Transcribed locus    GB acc:   AI101361    UniGene:   Rn.22383        SOURCE        Gene Ontology:     BioCarta Pathways:     KEGG Pathways:  
    Probe set : 1374961_at     GB acc:   BI274511              Gene Ontology:     BioCarta Pathways:     KEGG Pathways:  
    Probe set : 1368134_a_at    Name:   interleukin 4 receptor    GB acc:   NM_133380    UniGene:   Rn.10471    Gene Symbol:   Il4r    Locus Link:   25084    Chromosome:   1    Cytoband:   Chr:1q36    SOURCE    GENECARD       Gene Ontology:     BioCarta Pathways:     KEGG Pathways:  
    Probe set : 1382778_at    Name:   Dual specificity phosphatase 6    GB acc:   AI231350    UniGene:   Rn.4313    Gene Symbol:   Dusp6    Locus Link:   116663    Chromosome:   7    Cytoband:   Chr:7q13    SOURCE    GENECARD       Gene Ontology:   cellular component:  soluble fraction   cellular component:  cytoplasm   cellular component:  cytoplasm   molecular function:  protein phosphatase type 2A activity   molecular function:  protein phosphatase type 1 activity   molecular function:  phosphoprotein phosphatase activity   molecular function:  protein serine/threonine phosphatase activity   molecular function:  calcium-dependent protein serine/threonine phosphatase activity   molecular function:  magnesium-dependent protein serine/threonine phosphatase activity   molecular function:  protein tyrosine phosphatase activity   molecular function:  protein tyrosine/serine/threonine phosphatase activity   molecular function:  protein tyrosine/serine/threonine phosphatase activity   molecular function:  CTD phosphatase activity   molecular function:  protein phosphatase type 2C activity   molecular function:  hydrolase activity   molecular function:  MAP kinase phosphatase activity   molecular function:  myosin phosphatase activity   molecular function:  protein phosphatase type 2B activity      BioCarta Pathways:     KEGG Pathways:  
    Probe set : 1378028_at    Name:   MAD2 (mitotic arrest deficient, homolog)-like 1 (yeast) (predicted)    GB acc:   BM384425    UniGene:   Rn.19771    Gene Symbol:   Mad2l1_predicted    Locus Link:   297176    Chromosome:   4    Cytoband:   Chr:4q31    SOURCE    GENECARD       Gene Ontology:     BioCarta Pathways:     KEGG Pathways:  
    Probe set : 1387665_at    Name:   betaine-homocysteine methyltransferase    GB acc:   U96133    UniGene:   Rn.11406    Gene Symbol:   Bhmt    Locus Link:   81508    Chromosome:   2    Cytoband:   Chr:2q12    SOURCE    GENECARD       Gene Ontology:     BioCarta Pathways:     KEGG Pathways:  
    Probe set : 1375010_at    Name:   CD68 antigen (predicted)    GB acc:   AI177761    UniGene:   Rn.12478    Gene Symbol:   Cd68_predicted    Locus Link:   287435    Chromosome:   10    Cytoband:   Chr:10q24    SOURCE    GENECARD       Gene Ontology:     BioCarta Pathways:     KEGG Pathways:  
    Probe set : 1367838_at    Name:   CTL target antigen    GB acc:   NM_017074    UniGene:   Rn.3881    Gene Symbol:   Cth    Locus Link:   24962    Chromosome:   2    Cytoband:   Chr:2q45    SOURCE    GENECARD       Gene Ontology:   biological process:  amino acid metabolism   biological process:  cysteine biosynthesis   biological process:  amino acid biosynthesis   molecular function:  cystathionine gamma-lyase activity   molecular function:  cystathionine gamma-lyase activity   molecular function:  lyase activity      BioCarta Pathways:     KEGG Pathways:  
    Probe set : 1393666_at    Name:   Transcribed locus    GB acc:   BE101321    UniGene:   Rn.6458        SOURCE        Gene Ontology:     BioCarta Pathways:     KEGG Pathways:  
    Probe set : 1390441_at    Name:   Development and differentiation enhancing (predicted)    GB acc:   AW527409    UniGene:   Rn.63466     Locus Link:   314961    Chromosome:   7    Cytoband:   Chr:7q33    SOURCE        Gene Ontology:   biological process:  regulation of transcription, DNA-dependent   biological process:  regulation of GTPase activity   cellular component:  nucleus   cellular component:  membrane   molecular function:  DNA binding   molecular function:  transcription factor activity   molecular function:  GTPase activator activity      BioCarta Pathways:     KEGG Pathways:  
    Probe set : 1369313_at    Name:   four and a half LIM domains 2    GB acc:   NM_031677    UniGene:   Rn.3849    Gene Symbol:   Fhl2    Locus Link:   63839    Chromosome:   9    Cytoband:   Chr:9q22    SOURCE    GENECARD       Gene Ontology:   biological process:  regulation of transcription from RNA polymerase II promoter   cellular component:  nucleus   molecular function:  protein binding   molecular function:  zinc ion binding   molecular function:  metal ion binding      BioCarta Pathways:     KEGG Pathways:  
    Probe set : 1370242_at    Name:   ribosomal protein S23    GB acc:   AA848284    UniGene:   Rn.144643    Gene Symbol:   Rps23    Locus Link:   124323    Chromosome:   2    Cytoband:   Chr:2q12    SOURCE    GENECARD       Gene Ontology:   biological process:  protein biosynthesis   molecular function:  nucleic acid binding   molecular function:  RNA binding   molecular function:  structural constituent of ribosome      BioCarta Pathways:     KEGG Pathways:  
    Probe set : 1373932_at    Name:   Cytochrome b-245, beta polypeptide    GB acc:   BE098739    UniGene:   Rn.98491    Gene Symbol:   Cybb    Locus Link:   66021    Chromosome:   X    Cytoband:   Chr:Xq13    SOURCE    GENECARD       Gene Ontology:   biological process:  electron transport   cellular component:  intracellular   cellular component:  membrane   cellular component:  integral to membrane      BioCarta Pathways:     KEGG Pathways:  
    Probe set : 1389360_at    Name:   FXYD domain-containing ion transport regulator 3    GB acc:   AI009597    UniGene:   Rn.3896    Gene Symbol:   Fxyd3    Locus Link:   116831    Chromosome:   1    Cytoband:   Chr:1q21    SOURCE    GENECARD       Gene Ontology:   biological process:  ion transport   biological process:  transport   biological process:  ion transport   cellular component:  membrane   cellular component:  membrane   cellular component:  integral to membrane   molecular function:  ion channel activity      BioCarta Pathways:     KEGG Pathways:  
    Probe set : 1383684_at    Name:   ASF1 anti-silencing function 1 homolog B (S. cerevisiae) (predicted)    GB acc:   BI288761    UniGene:   Rn.899    Gene Symbol:   Asf1b_predicted    Locus Link:   304648    Chromosome:   19    Cytoband:   Chr:19q11    SOURCE    GENECARD       Gene Ontology:   biological process:  nucleosome assembly   cellular component:  chromatin   molecular function:  histone binding      BioCarta Pathways:     KEGG Pathways:  
    Probe set : 1374070_at    Name:   glutathione peroxidase 2    GB acc:   AA800587    UniGene:   Rn.3503    Gene Symbol:   Gpx2    Locus Link:   29326    Chromosome:   6    Cytoband:   Chr:6q24    SOURCE    GENECARD       Gene Ontology:   biological process:  response to oxidative stress   cellular component:  cytosol      BioCarta Pathways:     KEGG Pathways:  
    Probe set : 1368794_at    Name:   3-hydroxyanthranilate 3,4-dioxygenase    GB acc:   NM_020076    UniGene:   Rn.48675    Gene Symbol:   Haao    Locus Link:   56823    Chromosome:   6    Cytoband:   Chr:6q12    SOURCE    GENECARD       Gene Ontology:   biological process:  regulation of transcription, DNA-dependent   biological process:  metabolism      BioCarta Pathways:     KEGG Pathways:  
    Probe set : 1376749_at    Name:   osteoglycin (predicted)    GB acc:   AA945955    UniGene:   Rn.103114    Gene Symbol:   Ogn_predicted    Locus Link:   291015    Chromosome:   17    Cytoband:   Chr:17p14    SOURCE    GENECARD       Gene Ontology:   cellular component:  extracellular space   molecular function:  growth factor activity      BioCarta Pathways:     KEGG Pathways:  
    Probe set : 1380809_at     GB acc:   BM388375              Gene Ontology:     BioCarta Pathways:     KEGG Pathways:  
    Probe set : 1367917_at    Name:   cytochrome P450, family 2, subfamily d, polypeptide 26    GB acc:   NM_012730    UniGene:   Rn.91355    Gene Symbol:   Cyp2d26    Locus Link:   25053    Chromosome:   7    Cytoband:   Chr:7q34    SOURCE    GENECARD       Gene Ontology:   biological process:  electron transport   cellular component:  endoplasmic reticulum   cellular component:  microsome   cellular component:  membrane      BioCarta Pathways:     KEGG Pathways:  
    Probe set : 1389787_at    Name:   Transcribed locus    GB acc:   AI549469    UniGene:   Rn.63680        SOURCE        Gene Ontology:     BioCarta Pathways:     KEGG Pathways:  
    Probe set : 1387867_at    Name:   aldehyde dehydrogenase family 9, subfamily A1    GB acc:   BG672075    UniGene:   Rn.98155    Gene Symbol:   Aldh9a1    Locus Link:   64040    Chromosome:   13    Cytoband:   Chr:13q24    SOURCE    GENECARD       Gene Ontology:   cellular component:  cytosol   cellular component:  cytosol      BioCarta Pathways:     KEGG Pathways:  
    Probe set : 1390348_at    Name:   folate receptor 2 (fetal) (predicted)    GB acc:   BI274054    UniGene:   Rn.8801    Gene Symbol:   Folr2_predicted    Locus Link:   293154    Chromosome:   1    Cytoband:   Chr:1q32    SOURCE    GENECARD       Gene Ontology:   biological process:  posttranslational protein targeting to membrane   biological process:  folic acid transport   cellular component:  extracellular space   cellular component:  membrane fraction   cellular component:  membrane   molecular function:  receptor activity   molecular function:  folic acid binding   molecular function:  folic acid transporter activity      BioCarta Pathways:     KEGG Pathways:  
    Probe set : 1369672_at    Name:   arachidonate 5-lipoxygenase activating protein    GB acc:   NM_017260    UniGene:   Rn.18399    Gene Symbol:   Alox5ap    Locus Link:   29624    Chromosome:   12    Cytoband:   Chr:12p11    SOURCE    GENECARD       Gene Ontology:   cellular component:  extracellular space   cellular component:  membrane fraction   cellular component:  integral to membrane   molecular function:  binding   molecular function:  enzyme activator activity   molecular function:  enzyme activator activity      BioCarta Pathways:     KEGG Pathways:  
    Probe set : 1378180_at    Name:   similar to 6030466N05Rik protein    GB acc:   BI284767    UniGene:   Rn.105816    Gene Symbol:   RGD:1359671    Locus Link:   290741    Chromosome:   16    Cytoband:   Chr:16q11    SOURCE    GENECARD       Gene Ontology:   biological process:  nucleotide biosynthesis      BioCarta Pathways:     KEGG Pathways:  
    Probe set : 1391063_at    Name:   kinesin family member 23 (predicted)    GB acc:   BE113443    UniGene:   Rn.63734    Gene Symbol:   Kif23_predicted    Locus Link:   315740    Chromosome:   8    Cytoband:   Chr:8q24    SOURCE    GENECARD       Gene Ontology:   molecular function:  motor activity   molecular function:  microtubule motor activity   molecular function:  ATP binding      BioCarta Pathways:     KEGG Pathways:  
    Probe set : 1379603_at    Name:   similar to CBL E3 ubiquitin protein ligase (Signal transduction protein CBL) (Proto-oncogene c-CBL) (Casitas B-lineage lymphoma proto-oncogene)    GB acc:   BE116890    UniGene:   Rn.35589    Gene Symbol:   LOC500985    Locus Link:   500985    Chromosome:   8    Cytoband:   Chr:8q22    SOURCE    GENECARD       Gene Ontology:     BioCarta Pathways:     KEGG Pathways:  
    Probe set : 1368460_at    Name:   solute carrier family 2, member 5    GB acc:   NM_031741    UniGene:   Rn.10900    Gene Symbol:   Slc2a5    Locus Link:   65197    Chromosome:   5    Cytoband:   Chr:5q36    SOURCE    GENECARD       Gene Ontology:     BioCarta Pathways:     KEGG Pathways:  
    Probe set : 1368915_at    Name:   kynurenine 3-monooxygenase (kynurenine 3-hydroxylase)    GB acc:   NM_021593    UniGene:   Rn.35029    Gene Symbol:   Kmo    Locus Link:   59113    Chromosome:   13    Cytoband:   Chr:13q25    SOURCE    GENECARD       Gene Ontology:   biological process:  electron transport   biological process:  aromatic compound metabolism   biological process:  metabolism   cellular component:  mitochondrion   cellular component:  integral to membrane      BioCarta Pathways:     KEGG Pathways:  
    Probe set : 1382192_at    Name:   extra cellular link domain-containing 1 (predicted)    GB acc:   AI179260    UniGene:   Rn.22111    Gene Symbol:   Xlkd1_predicted    Locus Link:   293186    Chromosome:   1    Cytoband:   Chr:1q33    SOURCE    GENECARD       Gene Ontology:   molecular function:  receptor activity   molecular function:  transmembrane receptor activity   molecular function:  hyaluronic acid binding      BioCarta Pathways:     KEGG Pathways:  
    Probe set : 1371916_at    Name:   CDNA clone MGC:105753 IMAGE:7316479    GB acc:   AI409380    UniGene:   Rn.11960        SOURCE        Gene Ontology:     BioCarta Pathways:     KEGG Pathways:  
    Probe set : 1367913_at    Name:   cytoglobin    GB acc:   NM_130744    UniGene:   Rn.105938    Gene Symbol:   Cygb    Locus Link:   170520    Chromosome:   10    Cytoband:   Chr:10q32.3    SOURCE    GENECARD       Gene Ontology:   cellular component:  cytoplasm   cellular component:  cytoplasm      BioCarta Pathways:     KEGG Pathways:  
    Probe set : 1392407_at    Name:   Similar to RIKEN cDNA 6030405P05 gene    GB acc:   AI012031    UniGene:   Rn.131539     Locus Link:   498282      SOURCE        Gene Ontology:     BioCarta Pathways:     KEGG Pathways:  
    Probe set : 1390861_at    Name:   similar to hypothetical protein FLJ38281    GB acc:   AW524684    UniGene:   Rn.41593    Gene Symbol:   LOC362845    Locus Link:   362845    Chromosome:   7    Cytoband:   Chr:7q11    SOURCE    GENECARD       Gene Ontology:     BioCarta Pathways:     KEGG Pathways:  
    Probe set : 1382496_at     GB acc:   BI288517              Gene Ontology:     BioCarta Pathways:     KEGG Pathways:  
    Probe set : 1371377_at    Name:   ribosomal protein S19    GB acc:   BG668512    UniGene:   Rn.127775    Gene Symbol:   Rps19    Locus Link:   29287    Chromosome:   1    Cytoband:   Chr:1q21    SOURCE    GENECARD       Gene Ontology:   biological process:  protein biosynthesis   biological process:  ribosome biogenesis   biological process:  hemocyte development   molecular function:  RNA binding   molecular function:  structural constituent of ribosome      BioCarta Pathways:     KEGG Pathways:  
    Probe set : 1384824_at    Name:   protocadherin 18 (predicted)    GB acc:   BF408099    UniGene:   Rn.61115    Gene Symbol:   Pcdh18_predicted    Locus Link:   295027    Chromosome:   2    Cytoband:   Chr:2q26    SOURCE    GENECARD       Gene Ontology:   molecular function:  calcium ion binding   molecular function:  protein binding      BioCarta Pathways:     KEGG Pathways:  
    Probe set : 1369956_at    Name:   interferon gamma receptor 1    GB acc:   NM_053783    UniGene:   Rn.19927    Gene Symbol:   Ifngr    Locus Link:   116465    Chromosome:   1    Cytoband:   Chr:1p12    SOURCE    GENECARD       Gene Ontology:   cellular component:  extracellular space   cellular component:  membrane   cellular component:  integral to membrane      BioCarta Pathways:     KEGG Pathways:  
    Probe set : 1368651_at    Name:   pyruvate kinase, liver and RBC    GB acc:   M17685    UniGene:   Rn.48821    Gene Symbol:   Pklr    Locus Link:   24651    Chromosome:   2    Cytoband:   Chr:2q34    SOURCE    GENECARD       Gene Ontology:   biological process:  glycolysis   biological process:  response to pathogen      BioCarta Pathways:     KEGG Pathways:  
    Probe set : 1370243_a_at    Name:   prothymosin alpha    GB acc:   AI178772    UniGene:   Rn.817    Gene Symbol:   Ptma    Locus Link:   29222    Chromosome:   9    Cytoband:   Chr:9q35    SOURCE    GENECARD       Gene Ontology:   cellular component:  nucleus      BioCarta Pathways:     KEGG Pathways:  
    Probe set : 1376128_at    Name:   Transcribed locus    GB acc:   AI103937    UniGene:   Rn.17851        SOURCE        Gene Ontology:     BioCarta Pathways:     KEGG Pathways:  
    Probe set : 1393421_at    Name:   peroxisomal membrane protein 4    GB acc:   BG664011    UniGene:   Rn.7752    Gene Symbol:   Pxmp4    Locus Link:   282634    Chromosome:   3    Cytoband:   Chr:3q41    SOURCE    GENECARD       Gene Ontology:   cellular component:  peroxisome   cellular component:  integral to membrane      BioCarta Pathways:     KEGG Pathways:  
    Probe set : 1388761_at    Name:   histone deacetylase 1 (predicted)    GB acc:   AI180339    UniGene:   Rn.123394    Gene Symbol:   Hdac1_predicted    Locus Link:   297893    Chromosome:   5    Cytoband:   Chr:5q36    SOURCE    GENECARD       Gene Ontology:     BioCarta Pathways:     KEGG Pathways:  
    Probe set : 1373852_at    Name:   similar to SRD5A2L (predicted)    GB acc:   AA859922    UniGene:   Rn.819    Gene Symbol:   RGD1308828_predicted    Locus Link:   305291    Chromosome:   14    Cytoband:   Chr:14p11    SOURCE    GENECARD       Gene Ontology:     BioCarta Pathways:     KEGG Pathways:  
    Probe set : 1370725_a_at    Name:   glucose-6-phosphatase, catalytic    GB acc:   U07993    UniGene:   Rn.10992    Gene Symbol:   G6pc    Locus Link:   25634    Chromosome:   10    Cytoband:   Chr:10q32.1    SOURCE    GENECARD       Gene Ontology:     BioCarta Pathways:     KEGG Pathways:  
    Probe set : 1398860_at    Name:   neural precursor cell expressed, developmentally down-regulated gene 8    GB acc:   AF095740    UniGene:   Rn.2079    Gene Symbol:   Nedd8    Locus Link:   25490    Chromosome:   15    Cytoband:   Chr:15p13    SOURCE    GENECARD       Gene Ontology:   cellular component:  nucleus   molecular function:  ubiquitin conjugating enzyme activity   molecular function:  protein binding      BioCarta Pathways:     KEGG Pathways:  
    Probe set : 1381993_at    Name:   chloride intracellular channel 2 (predicted)    GB acc:   BI293548    UniGene:   Rn.103254    Gene Symbol:   Clic2_predicted    Locus Link:   294141    Chromosome:   20    Cytoband:   Chr:20p12    SOURCE    GENECARD       Gene Ontology:   biological process:  ion transport   biological process:  chloride transport   biological process:  transport   cellular component:  membrane fraction   cellular component:  membrane   molecular function:  voltage-gated chloride channel activity   molecular function:  ion channel activity   molecular function:  voltage-gated ion channel activity      BioCarta Pathways:     KEGG Pathways:  
    Probe set : 1379074_at    Name:   Transcribed locus    GB acc:   BE329032    UniGene:   Rn.15258        SOURCE        Gene Ontology:     BioCarta Pathways:     KEGG Pathways:  
    Probe set : 1384603_at    Name:   ATP-binding cassette, sub-family A (ABC1), member 4 (predicted)    GB acc:   AI602131    UniGene:   Rn.40415    Gene Symbol:   Abca4_predicted    Locus Link:   310836    Chromosome:   2    Cytoband:   Chr:2q41    SOURCE    GENECARD       Gene Ontology:   biological process:  transport   biological process:  visual perception   biological process:  phototransduction, visible light   cellular component:  membrane fraction   cellular component:  integral to plasma membrane   molecular function:  ATP binding   molecular function:  ATPase activity   molecular function:  ATPase activity, coupled to transmembrane movement of substances      BioCarta Pathways:     KEGG Pathways:  
    Probe set : 1383008_at    Name:   SMC4 structural maintenance of chromosomes 4-like 1 (yeast) (predicted)    GB acc:   AW535494    UniGene:   Rn.3162    Gene Symbol:   Smc4l1_predicted    Locus Link:   295107    Chromosome:   2    Cytoband:   Chr:2q31    SOURCE    GENECARD       Gene Ontology:     BioCarta Pathways:     KEGG Pathways:  
    Probe set : 1373219_at    Name:   snail homolog, (Drosophila)    GB acc:   AA946199    UniGene:   Rn.8008    Gene Symbol:   Snai1    Locus Link:   116490    Chromosome:   3    Cytoband:   Chr:3q42    SOURCE    GENECARD       Gene Ontology:   biological process:  epithelial to mesenchymal transition   biological process:  negative regulation of progression through cell cycle   cellular component:  nucleus   molecular function:  nucleic acid binding   molecular function:  zinc ion binding   molecular function:  metal ion binding      BioCarta Pathways:     KEGG Pathways:  
    Probe set : 1372569_at    Name:   four and a half LIM domains 3 (predicted)    GB acc:   AI171676    UniGene:   Rn.100732    Gene Symbol:   Fhl3_predicted    Locus Link:   313582    Chromosome:   5    Cytoband:   Chr:5q36    SOURCE    GENECARD       Gene Ontology:   biological process:  muscle development   biological process:  actin cytoskeleton organization and biogenesis   cellular component:  stress fiber   cellular component:  nucleus   cellular component:  Z disc   molecular function:  actin binding   molecular function:  zinc ion binding      BioCarta Pathways:     KEGG Pathways:  
    Probe set : 1368003_at    Name:   aldehyde dehydrogenase family 1, subfamily A2    GB acc:   NM_053896    UniGene:   Rn.10514    Gene Symbol:   Aldh1a2    Locus Link:   116676    Chromosome:   8    Cytoband:   Chr:8q24    SOURCE    GENECARD       Gene Ontology:     BioCarta Pathways:     KEGG Pathways:  
    Probe set : 1375523_at    Name:   Similar to Myristoylated alanine-rich C-kinase substrate (MARCKS) (ACAMP-81)    GB acc:   BE108178    UniGene:   Rn.9560    Gene Symbol:   Marcks    Locus Link:   25603    Chromosome:   6    Cytoband:   Chr:6    SOURCE    GENECARD       Gene Ontology:   biological process:  cell motility   cellular component:  plasma membrane   cellular component:  actin cytoskeleton   cellular component:  membrane      BioCarta Pathways:     KEGG Pathways:  
    Probe set : 1391538_at    Name:   cell death-inducing DNA fragmentation factor, alpha subunit-like effector B (predicted)    GB acc:   AI137938    UniGene:   Rn.145275    Gene Symbol:   Cideb_predicted    Locus Link:   364388    Chromosome:   15    Cytoband:   Chr:15p13    SOURCE    GENECARD       Gene Ontology:   cellular component:  intracellular   cellular component:  cytosol   molecular function:  protein binding      BioCarta Pathways:     KEGG Pathways:  
    Probe set : 1370892_at    Name:   complement component 4a /// complement component 4, gene 2    GB acc:   BI285347    UniGene:   Rn.81052    Gene Symbol:   C4a /// C4-2    Locus Link:   24233 /// 406161    Chromosome:   20    Cytoband:   Chr:20p12    SOURCE    GENECARD       Gene Ontology:   cellular component:  extracellular region   cellular component:  extracellular region   molecular function:  endopeptidase inhibitor activity      BioCarta Pathways:     KEGG Pathways:  
    Probe set : 1377664_at    Name:   KARP-1 binding protein 1    GB acc:   AI101205    UniGene:   Rn.6424    Gene Symbol:   Kab    Locus Link:   171457    Chromosome:   13    Cytoband:   Chr:13q24    SOURCE    GENECARD       Gene Ontology:     BioCarta Pathways:     KEGG Pathways:  
    Probe set : 1373386_at    Name:   gap junction membrane channel protein beta 2    GB acc:   AI179953    UniGene:   Rn.9230    Gene Symbol:   Gjb2    Locus Link:   394266    Chromosome:   15    Cytoband:   Chr:15p12    SOURCE    GENECARD       Gene Ontology:   biological process:  cell-cell signaling   biological process:  male genitalia morphogenesis   biological process:  cell communication   molecular function:  connexon channel activity   molecular function:  connexon channel activity      BioCarta Pathways:     KEGG Pathways:  
    Probe set : 1373833_at    Name:   similar to RIKEN cDNA 3110040N11 (predicted)    GB acc:   BI288945    UniGene:   Rn.8483    Gene Symbol:   RGD1305713_predicted    Locus Link:   293059    Chromosome:   1    Cytoband:   Chr:1q31    SOURCE    GENECARD       Gene Ontology:     BioCarta Pathways:     KEGG Pathways:  
    Probe set : 1380079_at    Name:   similar to mKIAA2005 protein    GB acc:   AW533923    UniGene:   Rn.118248    Gene Symbol:   LOC500015    Locus Link:   500015    Chromosome:   4    Cytoband:   Chr:4q13    SOURCE    GENECARD       Gene Ontology:     BioCarta Pathways:     KEGG Pathways:  
    Probe set : 1371250_at    Name:   platelet factor 4    GB acc:   AI169104    UniGene:   Rn.6800    Gene Symbol:   Pf4    Locus Link:   360918    Chromosome:   14    Cytoband:   Chr:14p22    SOURCE    GENECARD       Gene Ontology:   cellular component:  extracellular region   cellular component:  extracellular space   molecular function:  cytokine activity   molecular function:  chemokine activity   molecular function:  heparin binding      BioCarta Pathways:     KEGG Pathways:  
    Probe set : 1368742_at    Name:   complement component 5, receptor 1    GB acc:   NM_053619    UniGene:   Rn.10680    Gene Symbol:   C5r1    Locus Link:   113959    Chromosome:   1    Cytoband:   Chr:1q12    SOURCE    GENECARD       Gene Ontology:   cellular component:  cell surface   cellular component:  cell surface   cellular component:  integral to membrane      BioCarta Pathways:     KEGG Pathways:  
    Probe set : 1382569_at    Name:   Transcribed locus    GB acc:   BF386242    UniGene:   Rn.64169        SOURCE        Gene Ontology:     BioCarta Pathways:     KEGG Pathways:  
    Probe set : 1377018_at    Name:   Similar to E430002G05Rik protein (predicted)    GB acc:   BF284124    UniGene:   Rn.33642     Locus Link:   311252    Chromosome:   3    Cytoband:   Chr:3q31    SOURCE        Gene Ontology:     BioCarta Pathways:     KEGG Pathways:  
    Probe set : 1387284_at    Name:   dihydropyrimidinase    GB acc:   NM_031705    UniGene:   Rn.10586    Gene Symbol:   Dpys    Locus Link:   65135    Chromosome:   7    Cytoband:   Chr:7q22    SOURCE    GENECARD       Gene Ontology:   biological process:  nucleobase, nucleoside, nucleotide and nucleic acid metabolism   biological process:  response to toxin   molecular function:  dihydropyrimidinase activity   molecular function:  dihydropyrimidinase activity   molecular function:  hydrolase activity      BioCarta Pathways:     KEGG Pathways:  
    Probe set : 1367683_at    Name:   karyopherin (importin) alpha 2    GB acc:   NM_053483    UniGene:   Rn.2949    Gene Symbol:   Kpna2    Locus Link:   85245    Chromosome:   10    Cytoband:   Chr:10q32.1    SOURCE    GENECARD       Gene Ontology:   cellular component:  nucleus   cellular component:  cytoskeleton      BioCarta Pathways:     KEGG Pathways:  
    Probe set : 1367763_at    Name:   acetyl-coenzyme A acetyltransferase 1    GB acc:   D13921    UniGene:   Rn.4054    Gene Symbol:   Acat1    Locus Link:   25014    Chromosome:   8    Cytoband:   Chr:8q24.1    SOURCE    GENECARD       Gene Ontology:   biological process:  lipoprotein biosynthesis   cellular component:  mitochondrion   cellular component:  mitochondrion      BioCarta Pathways:     KEGG Pathways:  
    Probe set : 1385029_at    Name:   Transcribed locus    GB acc:   BE110921    UniGene:   Rn.124278        SOURCE        Gene Ontology:     BioCarta Pathways:     KEGG Pathways:  
    Probe set : 1374695_at    Name:   Transcribed locus    GB acc:   BF564593    UniGene:   Rn.29900        SOURCE        Gene Ontology:     BioCarta Pathways:     KEGG Pathways:  
    Probe set : 1380794_at    Name:   Aquarius (predicted)    GB acc:   BE106342    UniGene:   Rn.37299     Locus Link:   366163    Chromosome:   3    Cytoband:   Chr:3q35    SOURCE        Gene Ontology:     BioCarta Pathways:     KEGG Pathways:  
    Probe set : 1368143_at    Name:   annexin A7    GB acc:   AI013902    UniGene:   Rn.106184    Gene Symbol:   Anxa7    Locus Link:   155423    Chromosome:   15    Cytoband:   Chr:15p16    SOURCE    GENECARD       Gene Ontology:   biological process:  calcium ion homeostasis   biological process:  cell proliferation   biological process:  negative regulation of coagulation   cellular component:  nuclear membrane   cellular component:  plasma membrane      BioCarta Pathways:     KEGG Pathways:  
    Probe set : 1393163_at    Name:   Transcribed locus    GB acc:   AI112184    UniGene:   Rn.146411        SOURCE        Gene Ontology:     BioCarta Pathways:     KEGG Pathways:  
    Probe set : 1395687_at    Name:   activating transcription factor 7 interacting protein (predicted)    GB acc:   BE113420    UniGene:   Rn.98469    Gene Symbol:   Atf7ip_predicted    Locus Link:   312800    Chromosome:   4    Cytoband:   Chr:4q43    SOURCE    GENECARD       Gene Ontology:   cellular component:  nucleus   molecular function:  protein binding      BioCarta Pathways:     KEGG Pathways:  
    Probe set : 1372613_at    Name:   dehydrogenase/reductase (SDR family) member 6 (predicted)    GB acc:   AI232784    UniGene:   Rn.3651    Gene Symbol:   Dhrs6_predicted    Locus Link:   295458    Chromosome:   2    Cytoband:   Chr:2q43    SOURCE    GENECARD       Gene Ontology:   biological process:  metabolism   molecular function:  oxidoreductase activity      BioCarta Pathways:     KEGG Pathways:  
    Probe set : 1389965_at    Name:   trans-golgi network protein 1    GB acc:   AA799818    UniGene:   Rn.11349    Gene Symbol:   Tgoln2    Locus Link:   192152    Chromosome:   4    Cytoband:   Chr:4q33    SOURCE    GENECARD       Gene Ontology:   biological process:  Golgi to endosome transport   molecular function:  protein binding      BioCarta Pathways:     KEGG Pathways:  
    Probe set : 1382478_at    Name:   BTB (POZ) domain containing 3 (predicted)    GB acc:   AI230481    UniGene:   Rn.12235    Gene Symbol:   Btbd3_predicted    Locus Link:   311462    Chromosome:   3    Cytoband:   Chr:3q36    SOURCE    GENECARD       Gene Ontology:   molecular function:  protein binding      BioCarta Pathways:     KEGG Pathways:  
    Probe set : 1390357_at    Name:   zinc finger protein 574 (predicted)    GB acc:   AW525082    UniGene:   Rn.15466    Gene Symbol:   Znf574_predicted    Locus Link:   308434    Chromosome:   1    Cytoband:   Chr:1q21    SOURCE    GENECARD       Gene Ontology:   cellular component:  nucleus   molecular function:  nucleic acid binding   molecular function:  zinc ion binding   molecular function:  metal ion binding      BioCarta Pathways:     KEGG Pathways:  
    Probe set : 1391106_at    Name:   Transcribed locus    GB acc:   BM385061    UniGene:   Rn.20457        SOURCE        Gene Ontology:     BioCarta Pathways:     KEGG Pathways:  
    Probe set : 1383596_at    Name:   similar to RIKEN cDNA 5830446M03 (predicted)    GB acc:   AA956300    UniGene:   Rn.145210    Gene Symbol:   RGD1307688_predicted    Locus Link:   362394    Chromosome:   4    Cytoband:   Chr:4q34    SOURCE    GENECARD       Gene Ontology:     BioCarta Pathways:     KEGG Pathways:  
    Probe set : 1368153_a_at    Name:   nasal embryonic LHRH factor    GB acc:   AJ293698    UniGene:   Rn.7504    Gene Symbol:   Nelf    Locus Link:   117536    Chromosome:   3    Cytoband:   Chr:3p13    SOURCE    GENECARD       Gene Ontology:     BioCarta Pathways:     KEGG Pathways:  
    Probe set : 1368321_at    Name:   early growth response 1    GB acc:   NM_012551    UniGene:   Rn.9096    Gene Symbol:   Egr1    Locus Link:   24330    Chromosome:   18    Cytoband:   Chr:18q    SOURCE    GENECARD       Gene Ontology:   cellular component:  nucleus      BioCarta Pathways:     KEGG Pathways:  
    Probe set : 1378104_at    Name:   similar to RIKEN cDNA 8430406I07 (predicted)    GB acc:   AI105459    UniGene:   Rn.23965    Gene Symbol:   RGD1307465_predicted    Locus Link:   296200    Chromosome:   3    Cytoband:   Chr:3q41    SOURCE    GENECARD       Gene Ontology:     BioCarta Pathways:     KEGG Pathways:  
    Probe set : 1383640_at    Name:   Phosphatidylinositol glycan, class N (predicted)    GB acc:   BI294569    UniGene:   Rn.131689     Locus Link:   309051    Chromosome:   13    Cytoband:   Chr:13p13    SOURCE        Gene Ontology:   cellular component:  extracellular space   cellular component:  integral to membrane      BioCarta Pathways:     KEGG Pathways:  
    Probe set : 1372347_at    Name:   Transcribed locus    GB acc:   BI289462    UniGene:   Rn.116800        SOURCE        Gene Ontology:     BioCarta Pathways:     KEGG Pathways:  
    Probe set : 1386529_at    Name:   Transcribed locus    GB acc:   BF564309    UniGene:   Rn.146907        SOURCE        Gene Ontology:     BioCarta Pathways:     KEGG Pathways:  
    Probe set : 1371527_at    Name:   epithelial membrane protein 1    GB acc:   BI275741    UniGene:   Rn.19723    Gene Symbol:   Emp1    Locus Link:   25314    Chromosome:   4    Cytoband:   Chr:4q43    SOURCE    GENECARD       Gene Ontology:     BioCarta Pathways:     KEGG Pathways:  
    Probe set : 1368154_at    Name:   guanylate cyclase 1, soluble, alpha 3 /// hypothetical gene supported by NM_017090    GB acc:   NM_017090    UniGene:   Rn.1974    Gene Symbol:   Gucy1a3 /// LOC497757    Locus Link:   25201 /// 497757    Chromosome:   2    Cytoband:   Chr:2q31 /// Chr:2q33    SOURCE    GENECARD       Gene Ontology:   cellular component:  guanylate cyclase complex, soluble   cellular component:  guanylate cyclase complex, soluble      BioCarta Pathways:     KEGG Pathways:  
    Probe set : 1368824_at    Name:   caldesmon 1    GB acc:   BM392106    UniGene:   Rn.87384    Gene Symbol:   Cald1    Locus Link:   25687    Chromosome:   4    Cytoband:   Chr:4q22    SOURCE    GENECARD       Gene Ontology:   cellular component:  membrane fraction   cellular component:  cytoskeleton   cellular component:  actin cap      BioCarta Pathways:     KEGG Pathways:  
    Probe set : 1392509_at    Name:   Transcribed locus    GB acc:   AA925469    UniGene:   Rn.92651        SOURCE        Gene Ontology:     BioCarta Pathways:     KEGG Pathways:  
    Probe set : 1369934_at    Name:   peptidylprolyl isomerase B    GB acc:   NM_022536    UniGene:   Rn.1893    Gene Symbol:   Ppib    Locus Link:   64367    Chromosome:   8    Cytoband:   Chr:8q24    SOURCE    GENECARD       Gene Ontology:   biological process:  protein folding   cellular component:  extracellular space   cellular component:  endoplasmic reticulum   cellular component:  endoplasmic reticulum lumen      BioCarta Pathways:     KEGG Pathways:  
    Probe set : 1388672_at    Name:   Transcribed locus    GB acc:   AI101373    UniGene:   Rn.9024        SOURCE        Gene Ontology:     BioCarta Pathways:     KEGG Pathways:  
    Probe set : 1371131_a_at    Name:   upregulated by 1,25-dihydroxyvitamin D-3    GB acc:   U30789    UniGene:   Rn.2758    Gene Symbol:   Txnip    Locus Link:   117514    Chromosome:   2    Cytoband:   Chr:2q34    SOURCE    GENECARD       Gene Ontology:   biological process:  response to oxidative stress   biological process:  regulation of cell proliferation   cellular component:  cytoplasm   molecular function:  enzyme inhibitor activity   molecular function:  protein binding      BioCarta Pathways:     KEGG Pathways:  
    Probe set : 1388504_at    Name:   similar to HR21spA    GB acc:   AI231313    UniGene:   Rn.3991    Gene Symbol:   MGC116373    Locus Link:   314949    Chromosome:   7    Cytoband:   Chr:7q31    SOURCE    GENECARD       Gene Ontology:   biological process:  chromosome organization and biogenesis (sensu Eukaryota)   cellular component:  nuclear chromosome      BioCarta Pathways:     KEGG Pathways:  
    Probe set : 1388336_at    Name:   Similar to hypothetical protein D2Ertd391e (predicted)    GB acc:   AI411514    UniGene:   Rn.98797     Locus Link:   362164    Chromosome:   3    Cytoband:   Chr:3q24    SOURCE        Gene Ontology:     BioCarta Pathways:     KEGG Pathways:  
    Probe set : 1384707_at    Name:   Transcribed locus    GB acc:   AI600020    UniGene:   Rn.34140        SOURCE        Gene Ontology:     BioCarta Pathways:     KEGG Pathways:  
    Probe set : 1370213_at    Name:   nuclease sensitive element binding protein 1    GB acc:   BI282111    UniGene:   Rn.110976    Gene Symbol:   Nsep1    Locus Link:   29206    Chromosome:   10    Cytoband:   Chr:10q22    SOURCE    GENECARD       Gene Ontology:   cellular component:  nucleus      BioCarta Pathways:     KEGG Pathways:  
    Probe set : 1389651_at    Name:   apelin, AGTRL1 ligand    GB acc:   AI177057    UniGene:   Rn.22847    Gene Symbol:   Apln    Locus Link:   58812    Chromosome:   X    Cytoband:   Chr:Xq35    SOURCE    GENECARD       Gene Ontology:   biological process:  G-protein coupled receptor protein signaling pathway   cellular component:  extracellular space   molecular function:  receptor binding   molecular function:  hormone activity      BioCarta Pathways:     KEGG Pathways:  
    Probe set : 1398540_at    Name:   regulator of G-protein signaling 1    GB acc:   BM386789    UniGene:   Rn.14787    Gene Symbol:   Rgs1    Locus Link:   54289    Chromosome:   13    Cytoband:   Chr:13q21    SOURCE    GENECARD       Gene Ontology:   cellular component:  plasma membrane   molecular function:  signal transducer activity   molecular function:  GTPase activator activity   molecular function:  calmodulin binding      BioCarta Pathways:     KEGG Pathways:  
    Probe set : 1369018_at    Name:   forkhead box M1    GB acc:   NM_031633    UniGene:   Rn.10665    Gene Symbol:   Foxm1    Locus Link:   58921    Chromosome:   4    Cytoband:   Chr:4q42    SOURCE    GENECARD       Gene Ontology:   biological process:  regulation of transcription, DNA-dependent   biological process:  transcription   cellular component:  nucleus   cellular component:  transcription factor complex   molecular function:  DNA binding   molecular function:  transcription factor activity      BioCarta Pathways:     KEGG Pathways:  
    Probe set : 1374172_at    Name:   Procollagen, type VIII, alpha 2 (predicted)    GB acc:   AI010883    UniGene:   Rn.12548     Locus Link:   313592    Chromosome:   5    Cytoband:   Chr:5q36    SOURCE        Gene Ontology:     BioCarta Pathways:     KEGG Pathways:  
    Probe set : 1390855_at    Name:   Prolyl endopeptidase    GB acc:   AI235923    UniGene:   Rn.11058    Gene Symbol:   Prep    Locus Link:   83471    Chromosome:   20    Cytoband:   Chr:20q13    SOURCE    GENECARD       Gene Ontology:   biological process:  proteolysis   biological process:  proteolysis   biological process:  protein metabolism   cellular component:  nucleus   cellular component:  cytoplasm   cellular component:  cytosol      BioCarta Pathways:     KEGG Pathways:  
    Probe set : 1371297_at    Name:   ribosomal protein L7a (predicted)    GB acc:   BI281697    UniGene:   Rn.144857    Gene Symbol:   Rpl7a_predicted    Locus Link:   296596    Chromosome:   3    Cytoband:   Chr:3p12    SOURCE    GENECARD       Gene Ontology:   biological process:  protein biosynthesis   cellular component:  intracellular   cellular component:  ribosome   cellular component:  ribonucleoprotein complex   molecular function:  structural constituent of ribosome      BioCarta Pathways:     KEGG Pathways:  
    Probe set : 1368164_at    Name:   biliverdin reductase A    GB acc:   NM_053850    UniGene:   Rn.9865    Gene Symbol:   Blvra    Locus Link:   116599    Chromosome:   3    Cytoband:   Chr:3q36    SOURCE    GENECARD       Gene Ontology:   biological process:  electron transport   biological process:  metabolism   biological process:  heme catabolism   cellular component:  soluble fraction      BioCarta Pathways:     KEGG Pathways:  
    Probe set : 1389648_at    Name:   Receptor-interacting serine-threonine kinase 4 (predicted)    GB acc:   AI170382    UniGene:   Rn.28403     Locus Link:   304053    Chromosome:   11    Cytoband:   Chr:11q12    SOURCE        Gene Ontology:   biological process:  regulation of transcription, DNA-dependent   biological process:  protein amino acid phosphorylation      BioCarta Pathways:     KEGG Pathways:  
    Probe set : 1371691_at     GB acc:   BI282993              Gene Ontology:     BioCarta Pathways:     KEGG Pathways:  
    Probe set : 1367925_at    Name:   major vault protein    GB acc:   NM_022715    UniGene:   Rn.10028    Gene Symbol:   Mvp    Locus Link:   64681    Chromosome:   1    Cytoband:   Chr:1q36    SOURCE    GENECARD       Gene Ontology:   biological process:  response to drug   cellular component:  nucleus   cellular component:  cytoplasm   cellular component:  ribonucleoprotein complex   molecular function:  calcium ion binding      BioCarta Pathways:     KEGG Pathways:  
    Probe set : 1388455_at    Name:   guanine nucleotide binding protein (G protein), gamma 10    GB acc:   AA799996    UniGene:   Rn.3319    Gene Symbol:   Gng10    Locus Link:   114119    Chromosome:   5    Cytoband:   Chr:5q24    SOURCE    GENECARD       Gene Ontology:   biological process:  G-protein coupled receptor protein signaling pathway   cellular component:  heterotrimeric G-protein complex   cellular component:  heterotrimeric G-protein complex   molecular function:  signal transducer activity      BioCarta Pathways:     KEGG Pathways:  
    Probe set : 1376905_at    Name:   Transcribed locus    GB acc:   BI304147    UniGene:   Rn.3706        SOURCE        Gene Ontology:     BioCarta Pathways:     KEGG Pathways:  
    Probe set : 1372409_at    Name:   MAD2L1 binding protein (predicted)    GB acc:   BI286562    UniGene:   Rn.9596    Gene Symbol:   Mad2l1bp_predicted    Locus Link:   316237    Chromosome:   9    Cytoband:   Chr:9q12    SOURCE    GENECARD       Gene Ontology:   biological process:  mitotic spindle checkpoint   biological process:  regulation of exit from mitosis   cellular component:  nucleus   molecular function:  protein binding      BioCarta Pathways:     KEGG Pathways:  
    Probe set : 1368289_at    Name:   group specific component    GB acc:   AA944965    UniGene:   Rn.1437    Gene Symbol:   Gc    Locus Link:   24384    Chromosome:   14    Cytoband:   Chr:14p22    SOURCE    GENECARD       Gene Ontology:   biological process:  transport   biological process:  vitamin D metabolism   biological process:  vitamin transport   cellular component:  extracellular space      BioCarta Pathways:     KEGG Pathways:  
    Probe set : 1389488_at    Name:   hypothetical protein LOC498544    GB acc:   AA800750    UniGene:   Rn.132048    Gene Symbol:   LOC498544    Locus Link:   498544    Chromosome:   15    Cytoband:   Chr:15q11    SOURCE    GENECARD       Gene Ontology:     BioCarta Pathways:     KEGG Pathways:  
    Probe set : 1375859_a_at    Name:   Similar to zinc finger protein 565    GB acc:   BM386823    UniGene:   Rn.869     Locus Link:   308488    Chromosome:   1    Cytoband:   Chr:1q21    SOURCE        Gene Ontology:     BioCarta Pathways:     KEGG Pathways:  
    Probe set : 1381428_a_at    Name:   Histone 1, H2bh (predicted)    GB acc:   AI177364    UniGene:   Rn.42931    Gene Symbol:   Hfe    Locus Link:   29199    Chromosome:   17    Cytoband:   Chr:17p11    SOURCE    GENECARD       Gene Ontology:   molecular function:  MHC class I receptor activity      BioCarta Pathways:     KEGG Pathways:  
    Probe set : 1368799_at    Name:   baculoviral IAP repeat-containing 5    GB acc:   NM_022274    UniGene:   Rn.54471    Gene Symbol:   Birc5    Locus Link:   64041      SOURCE    GENECARD       Gene Ontology:     BioCarta Pathways:     KEGG Pathways:  
    Probe set : 1370935_at    Name:   CDW92 antigen    GB acc:   AI105205    UniGene:   Rn.97686    Gene Symbol:   Cdw92    Locus Link:   85254    Chromosome:   5    Cytoband:   Chr:5q24    SOURCE    GENECARD       Gene Ontology:   biological process:  choline transport   biological process:  transport   cellular component:  integral to membrane   molecular function:  choline transporter activity      BioCarta Pathways:     KEGG Pathways:  
    Probe set : 1374247_at    Name:   stabilin 1 (predicted)    GB acc:   BI279869    UniGene:   Rn.144136    Gene Symbol:   Stab1_predicted    Locus Link:   290559    Chromosome:   16    Cytoband:   Chr:16p16    SOURCE    GENECARD       Gene Ontology:   biological process:  electron transport   biological process:  inflammatory response   biological process:  cell adhesion      BioCarta Pathways:     KEGG Pathways:  
    Probe set : 1383175_a_at    Name:   similar to C11orf17 protein (predicted)    GB acc:   BF561368    UniGene:   Rn.106344    Gene Symbol:   RGD1306959_predicted    Locus Link:   361624    Chromosome:   1    Cytoband:   Chr:1q33    SOURCE    GENECARD       Gene Ontology:     BioCarta Pathways:     KEGG Pathways:  
    Probe set : 1387605_at    Name:   caspase 12    GB acc:   NM_130422    UniGene:   Rn.81078    Gene Symbol:   Casp12    Locus Link:   156117    Chromosome:   8    Cytoband:   Chr:8q11    SOURCE    GENECARD       Gene Ontology:   cellular component:  intracellular   cellular component:  endoplasmic reticulum   cellular component:  endoplasmic reticulum      BioCarta Pathways:     KEGG Pathways:  
    Probe set : 1387811_at    Name:   angiotensinogen    GB acc:   NM_134432    UniGene:   Rn.6319    Gene Symbol:   Agt    Locus Link:   24179    Chromosome:   19    Cytoband:   Chr:19q12    SOURCE    GENECARD       Gene Ontology:   cellular component:  extracellular region   cellular component:  extracellular space   cellular component:  soluble fraction      BioCarta Pathways:     KEGG Pathways:  
    Probe set : 1373538_at    Name:   ubiquitin specific protease 1 (predicted)    GB acc:   AW525342    UniGene:   Rn.125994    Gene Symbol:   Usp1_predicted    Locus Link:   313387    Chromosome:   5    Cytoband:   Chr:5q33    SOURCE    GENECARD       Gene Ontology:   biological process:  ubiquitin-dependent protein catabolism   biological process:  ubiquitin cycle      BioCarta Pathways:     KEGG Pathways:  
    Probe set : 1370433_at    Name:   CCA2 protein    GB acc:   AB000199    UniGene:   Rn.11380    Gene Symbol:   Hsd3b7    Locus Link:   246211    Chromosome:   1    Cytoband:   Chr:1q36    SOURCE    GENECARD       Gene Ontology:   cellular component:  endoplasmic reticulum   cellular component:  integral to membrane      BioCarta Pathways:     KEGG Pathways:  
    Probe set : 1388902_at    Name:   lysyl oxidase-like 1 (predicted)    GB acc:   AI599031    UniGene:   Rn.770    Gene Symbol:   Loxl1_predicted    Locus Link:   315714    Chromosome:   8    Cytoband:   Chr:8q24    SOURCE    GENECARD       Gene Ontology:   biological process:  protein modification   cellular component:  extracellular region      BioCarta Pathways:     KEGG Pathways:  
    Probe set : 1377048_at    Name:   similar to cDNA sequence BC021917 (predicted)    GB acc:   H31813    UniGene:   Rn.55630    Gene Symbol:   RGD1311026_predicted    Locus Link:   361730    Chromosome:   1    Cytoband:   Chr:1q43    SOURCE    GENECARD       Gene Ontology:   biological process:  glycerol metabolism   molecular function:  glycerone kinase activity      BioCarta Pathways:     KEGG Pathways:  
    Probe set : 1390866_at    Name:   Transcribed locus    GB acc:   AI168981    UniGene:   Rn.146252        SOURCE        Gene Ontology:     BioCarta Pathways:     KEGG Pathways:  
    Probe set : 1367837_at    Name:   proteasome (prosome, macropain) subunit, alpha type 4    GB acc:   NM_017281    UniGene:   Rn.11076    Gene Symbol:   Psma4    Locus Link:   29671    Chromosome:   8    Cytoband:   Chr:8q24    SOURCE    GENECARD       Gene Ontology:   biological process:  ubiquitin-dependent protein catabolism   cellular component:  cytosol   cellular component:  proteasome core complex (sensu Eukaryota)   cellular component:  protein complex      BioCarta Pathways:     KEGG Pathways:  
    Probe set : 1388135_at    Name:   replication protein A2    GB acc:   BM385924    UniGene:   Rn.40389    Gene Symbol:   Rpa2    Locus Link:   59102    Chromosome:   5    Cytoband:   Chr:5q36    SOURCE    GENECARD       Gene Ontology:   biological process:  DNA replication   biological process:  base-excision repair   cellular component:  nucleus   molecular function:  nucleic acid binding   molecular function:  protein N-terminus binding      BioCarta Pathways:     KEGG Pathways:  
    Probe set : 1377350_at    Name:   Transcribed locus    GB acc:   AA859898    UniGene:   Rn.809        SOURCE        Gene Ontology:     BioCarta Pathways:     KEGG Pathways:  
    Probe set : 1373484_at    Name:   kelch-like 13    GB acc:   AI179828    UniGene:   Rn.24229    Gene Symbol:   Klhl13    Locus Link:   313445    Chromosome:   X    Cytoband:   Chr:Xq12    SOURCE    GENECARD       Gene Ontology:     BioCarta Pathways:     KEGG Pathways:  
    Probe set : 1370891_at    Name:   CD48 antigen    GB acc:   X13016    UniGene:   Rn.3705    Gene Symbol:   Cd48    Locus Link:   245962    Chromosome:   13    Cytoband:   Chr:13q24    SOURCE    GENECARD       Gene Ontology:   biological process:  mast cell activation      BioCarta Pathways:     KEGG Pathways:  
    Probe set : 1372234_at    Name:   similar to Lix1 homolog (mouse) like    GB acc:   AA800950    UniGene:   Rn.3632    Gene Symbol:   LOC499677    Locus Link:   499677    Chromosome:   2    Cytoband:   Chr:2q34    SOURCE    GENECARD       Gene Ontology:     BioCarta Pathways:     KEGG Pathways:  
    Probe set : 1372993_at    Name:   Insulin-like growth factor 2 receptor    GB acc:   BI299621    UniGene:   Rn.270    Gene Symbol:   Igf2r    Locus Link:   25151    Chromosome:   1    Cytoband:   Chr:1q11    SOURCE    GENECARD       Gene Ontology:     BioCarta Pathways:     KEGG Pathways:  
    Probe set : 1382459_at     GB acc:   BF287235              Gene Ontology:     BioCarta Pathways:     KEGG Pathways:  
    Probe set : 1387913_at    Name:   cytochrome P450, family 2, subfamily d, polypeptide 22    GB acc:   U48220    UniGene:   Rn.26060    Gene Symbol:   Cyp2d22    Locus Link:   171522    Chromosome:   7    Cytoband:   Chr:7q34    SOURCE    GENECARD       Gene Ontology:   biological process:  electron transport   biological process:  drug metabolism      BioCarta Pathways:     KEGG Pathways:  
    Probe set : 1383321_at    Name:   tyrosylprotein sulfotransferase 1 (predicted)    GB acc:   BG376248    UniGene:   Rn.144728    Gene Symbol:   Tpst1_predicted    Locus Link:   288617    Chromosome:   12    Cytoband:   Chr:12q13    SOURCE    GENECARD       Gene Ontology:   biological process:  peptidyl-tyrosine sulfation   biological process:  inflammatory response   molecular function:  sulfotransferase activity   molecular function:  protein-tyrosine sulfotransferase activity   molecular function:  transferase activity      BioCarta Pathways:     KEGG Pathways:  
    Probe set : 1377573_at    Name:   carbonic anhydrase VB, mitochondrial    GB acc:   AI411132    UniGene:   Rn.22441    Gene Symbol:   Ca5b    Locus Link:   302669    Chromosome:   X    Cytoband:   Chr:Xq21    SOURCE    GENECARD       Gene Ontology:   biological process:  one-carbon compound metabolism   cellular component:  mitochondrion      BioCarta Pathways:     KEGG Pathways:  
    Probe set : 1374007_at    Name:   Transcribed locus    GB acc:   AI178499    UniGene:   Rn.131007        SOURCE        Gene Ontology:     BioCarta Pathways:     KEGG Pathways:  
    Probe set : 1388521_at    Name:   pyrroline-5-carboxylate synthetase (glutamate gamma-semialdehyde synthetase) (predicted)    GB acc:   BG380693    UniGene:   Rn.3148    Gene Symbol:   Pycs_predicted    Locus Link:   361755    Chromosome:   1    Cytoband:   Chr:1q54    SOURCE    GENECARD       Gene Ontology:   biological process:  proline biosynthesis   biological process:  amino acid biosynthesis   cellular component:  cytoplasm   cellular component:  mitochondrion   cellular component:  inner membrane      BioCarta Pathways:     KEGG Pathways:  
    Probe set : 1398787_at    Name:   Ras homolog enriched in brain    GB acc:   NM_013216    UniGene:   Rn.859    Gene Symbol:   Rheb    Locus Link:   26954      SOURCE    GENECARD       Gene Ontology:   cellular component:  spliceosome complex   cellular component:  membrane      BioCarta Pathways:     KEGG Pathways:  
    Probe set : 1390944_at    Name:   Snf7 homologue associated with Alix 3    GB acc:   BM384937    UniGene:   Rn.65511    Gene Symbol:   MGC108776    Locus Link:   361916    Chromosome:   2    Cytoband:   Chr:2q23    SOURCE    GENECARD       Gene Ontology:   biological process:  transport   biological process:  protein transport      BioCarta Pathways:     KEGG Pathways:  
    Probe set : 1377814_at    Name:   Similar to Nedd4-binding brain specific protein BEAN    GB acc:   AA926368    UniGene:   Rn.13040     Locus Link:   361358    Chromosome:   19    Cytoband:   Chr:19p14    SOURCE        Gene Ontology:     BioCarta Pathways:     KEGG Pathways:  
    Probe set : 1373642_at     GB acc:   AI145380              Gene Ontology:     BioCarta Pathways:     KEGG Pathways:  
    Probe set : 1367582_at    Name:   ribosomal protein L29    GB acc:   NM_017150    UniGene:   Rn.3973    Gene Symbol:   Rpl29    Locus Link:   29283    Chromosome:   8    Cytoband:   Chr:8q32    SOURCE    GENECARD       Gene Ontology:     BioCarta Pathways:     KEGG Pathways:  
    Probe set : 1392499_at     GB acc:   H32997              Gene Ontology:     BioCarta Pathways:     KEGG Pathways:  
    Probe set : 1368065_at    Name:   regulator of G-protein signaling 19 interacting protein 1    GB acc:   NM_053341    UniGene:   Rn.30044    Gene Symbol:   Rgs19ip1    Locus Link:   83823    Chromosome:   19    Cytoband:   Chr:19q11    SOURCE    GENECARD       Gene Ontology:   biological process:  protein targeting   biological process:  G-protein coupled receptor protein signaling pathway   cellular component:  membrane fraction   cellular component:  soluble fraction   cellular component:  cytosol   cellular component:  membrane   molecular function:  GTPase activator activity   molecular function:  protein binding      BioCarta Pathways:     KEGG Pathways:  
    Probe set : 1368947_at    Name:   growth arrest and DNA-damage-inducible 45 alpha    GB acc:   NM_024127    UniGene:   Rn.10250    Gene Symbol:   Gadd45a    Locus Link:   25112    Chromosome:   4    Cytoband:   Chr:4q31-q33    SOURCE    GENECARD       Gene Ontology:   cellular component:  nucleus   cellular component:  ribosome   molecular function:  structural constituent of ribosome   molecular function:  protein binding      BioCarta Pathways:     KEGG Pathways:  
    Probe set : 1374456_at    Name:   Transcribed locus, strongly similar to XP_619138.1 PREDICTED: similar to Ras GTPase-activating-like protein IQGAP2 [Mus musculus]    GB acc:   AI179562    UniGene:   Rn.137731        SOURCE        Gene Ontology:     BioCarta Pathways:     KEGG Pathways:  
    Probe set : 1385444_at    Name:   Transcribed locus    GB acc:   AI071994    UniGene:   Rn.147230        SOURCE        Gene Ontology:     BioCarta Pathways:     KEGG Pathways:  
    Probe set : 1393587_a_at     GB acc:   AI578061              Gene Ontology:     BioCarta Pathways:     KEGG Pathways:  
    Probe set : 1367723_a_at    Name:   linker of T-cell receptor pathways    GB acc:   NM_031621    UniGene:   Rn.11228    Gene Symbol:   Lnk    Locus Link:   58838    Chromosome:   12    Cytoband:   Chr:12q16    SOURCE    GENECARD       Gene Ontology:   molecular function:  protein binding      BioCarta Pathways:     KEGG Pathways:  
    Probe set : 1372461_at    Name:   SET translocation (predicted) /// LOC499767    GB acc:   BG381708    UniGene:   Rn.144530    Gene Symbol:   Set_predicted /// LOC499767    Locus Link:   307947 /// 499767    Chromosome:   19    Cytoband:   Chr:19q12 /// Chr:3p12    SOURCE    GENECARD       Gene Ontology:   cellular component:  nucleus   cellular component:  endoplasmic reticulum   cellular component:  perinuclear region      BioCarta Pathways:     KEGG Pathways:  
    Probe set : 1376377_at    Name:   Transcribed locus    GB acc:   BF398599    UniGene:   Rn.9010        SOURCE        Gene Ontology:     BioCarta Pathways:     KEGG Pathways:  
    Probe set : 1377761_at    Name:   glutamine-fructose-6-phosphate transaminase 2    GB acc:   BI296057    UniGene:   Rn.16089    Gene Symbol:   RGD:1303097    Locus Link:   360518    Chromosome:   10    Cytoband:   Chr:10q22    SOURCE    GENECARD       Gene Ontology:   cellular component:  cytoplasm      BioCarta Pathways:     KEGG Pathways:  
    Probe set : 1374404_at    Name:   v-jun sarcoma virus 17 oncogene homolog (avian)    GB acc:   BI288619    UniGene:   Rn.93714    Gene Symbol:   Jun    Locus Link:   24516    Chromosome:   5    Cytoband:   Chr:5q34.1    SOURCE    GENECARD       Gene Ontology:     BioCarta Pathways:     KEGG Pathways:  
    Probe set : 1372905_at     GB acc:   AW433888              Gene Ontology:     BioCarta Pathways:     KEGG Pathways:  
    Probe set : 1367860_a_at    Name:   matrix metalloproteinase 14 (membrane-inserted)    GB acc:   X83537    UniGene:   Rn.10371    Gene Symbol:   Mmp14    Locus Link:   81707    Chromosome:   15    Cytoband:   Chr:15p13    SOURCE    GENECARD       Gene Ontology:   biological process:  proteolysis   biological process:  collagen catabolism   biological process:  peptidoglycan metabolism      BioCarta Pathways:     KEGG Pathways:  
    Probe set : 1382943_at    Name:   protocadherin gamma subfamily A, 10 (predicted)    GB acc:   BE097805    UniGene:   Rn.8628    Gene Symbol:   Pcdhga10_predicted    Locus Link:   364844    Chromosome:   18    Cytoband:   Chr:18p11    SOURCE    GENECARD       Gene Ontology:   biological process:  cell adhesion   biological process:  homophilic cell adhesion   cellular component:  integral to membrane   cellular component:  membrane   molecular function:  calcium ion binding   molecular function:  protein binding      BioCarta Pathways:     KEGG Pathways:  
    Probe set : 1372111_at     GB acc:   BI285449              Gene Ontology:     BioCarta Pathways:     KEGG Pathways:  
    Probe set : 1368862_at    Name:   v-akt murine thymoma viral oncogene homolog 1    GB acc:   NM_033230    UniGene:   Rn.11422    Gene Symbol:   Akt1    Locus Link:   24185    Chromosome:   6    Cytoband:   Chr:6q32    SOURCE    GENECARD       Gene Ontology:   biological process:  regulation of progression through cell cycle   biological process:  protein amino acid phosphorylation   biological process:  nitric oxide biosynthesis   biological process:  apoptosis   biological process:  anti-apoptosis   biological process:  anti-apoptosis   biological process:  anti-apoptosis   biological process:  signal transduction   biological process:  signal transduction   biological process:  G-protein coupled receptor protein signaling pathway   biological process:  germ cell development   biological process:  apoptotic mitochondrial changes   biological process:  response to heat   biological process:  protein ubiquitination   biological process:  protein catabolism   biological process:  positive regulation of cell growth   biological process:  positive regulation of cell growth   biological process:  negative regulation of apoptosis   biological process:  cellular physiological process      BioCarta Pathways:     KEGG Pathways:  
    Probe set : 1386982_at    Name:   Alpha-1,6-mannosyl-glycoprotein 2-beta-N-acetylglucosaminyltransferase    GB acc:   NM_053604    UniGene:   Rn.2342    Gene Symbol:   Mgat2    Locus Link:   94273    Chromosome:   6    Cytoband:   Chr:6q24    SOURCE    GENECARD       Gene Ontology:   biological process:  protein amino acid N-linked glycosylation   biological process:  oligosaccharide biosynthesis   biological process:  methane metabolism      BioCarta Pathways:     KEGG Pathways:  
    Probe set : 1373823_at    Name:   similar to Cyclin-dependent kinases regulatory subunit 2 (CKS-2)    GB acc:   AA944180    UniGene:   Rn.147433    Gene Symbol:   LOC498709    Locus Link:   498709    Chromosome:   17    Cytoband:   Chr:17p14    SOURCE    GENECARD       Gene Ontology:     BioCarta Pathways:     KEGG Pathways:  
    Probe set : 1370956_at    Name:   decorin    GB acc:   BM390253    UniGene:   Rn.106103    Gene Symbol:   Dcn    Locus Link:   29139    Chromosome:   7    Cytoband:   Chr:7q13    SOURCE    GENECARD       Gene Ontology:   biological process:  extracellular matrix organization and biogenesis   biological process:  extracellular matrix organization and biogenesis   cellular component:  extracellular matrix (sensu Metazoa)   cellular component:  extracellular matrix (sensu Metazoa)   cellular component:  extracellular space      BioCarta Pathways:     KEGG Pathways:  
    Probe set : 1368518_at    Name:   CD53 antigen    GB acc:   NM_012523    UniGene:   Rn.31988    Gene Symbol:   Cd53    Locus Link:   24251    Chromosome:   2    Cytoband:   Chr:2q34-q41    SOURCE    GENECARD       Gene Ontology:   biological process:  signal transduction   biological process:  antimicrobial humoral response (sensu Vertebrata)   biological process:  regulation of growth      BioCarta Pathways:     KEGG Pathways:  
    Probe set : 1376161_at    Name:   similar to RIKEN cDNA 1110014L17 (predicted)    GB acc:   AI235294    UniGene:   Rn.21479    Gene Symbol:   RGD1306319_predicted    Locus Link:   305502    Chromosome:   14    Cytoband:   Chr:14q21    SOURCE    GENECARD       Gene Ontology:     BioCarta Pathways:     KEGG Pathways:  
    Probe set : 1369651_at    Name:   thymus cell antigen 1, theta    GB acc:   NM_012673    UniGene:   Rn.108198    Gene Symbol:   Thy1    Locus Link:   24832    Chromosome:   8    Cytoband:   Chr:8q22    SOURCE    GENECARD       Gene Ontology:     BioCarta Pathways:     KEGG Pathways:  
    Probe set : 1376185_at    Name:   kinesin family member C1    GB acc:   AW253880    UniGene:   Rn.38181    Gene Symbol:   RGD:1359118    Locus Link:   294286    Chromosome:   20    Cytoband:   Chr:20p12    SOURCE    GENECARD       Gene Ontology:   biological process:  microtubule-based movement   cellular component:  microtubule   cellular component:  microtubule associated complex      BioCarta Pathways:     KEGG Pathways:  
    Probe set : 1373934_at    Name:   LOC500840    GB acc:   AI178214    UniGene:   Rn.3702    Gene Symbol:   LOC500840    Locus Link:   500840    Chromosome:   7    Cytoband:   Chr:7q22    SOURCE    GENECARD       Gene Ontology:     BioCarta Pathways:     KEGG Pathways:  
    Probe set : 1380995_at    Name:   Transcribed locus    GB acc:   BF412118    UniGene:   Rn.131122        SOURCE        Gene Ontology:     BioCarta Pathways:     KEGG Pathways:  
    Probe set : 1389017_at    Name:   similar to Antxr2 protein    GB acc:   AI013888    UniGene:   Rn.6715    Gene Symbol:   LOC305633    Locus Link:   305633    Chromosome:   14    Cytoband:   Chr:14p22    SOURCE    GENECARD       Gene Ontology:     BioCarta Pathways:     KEGG Pathways:  
    Probe set : 1388744_at    Name:   minichromosome maintenance protein 7    GB acc:   AI599678    UniGene:   Rn.113    Gene Symbol:   RGD:1303018    Locus Link:   288532    Chromosome:   12    Cytoband:   Chr:12q11    SOURCE    GENECARD       Gene Ontology:   biological process:  DNA replication initiation   cellular component:  nucleus      BioCarta Pathways:     KEGG Pathways:  
    Probe set : 1373772_at    Name:   DNA (cytosine-5-)-methyltransferase 1    GB acc:   AI179516    UniGene:   Rn.6955    Gene Symbol:   Dnmt1    Locus Link:   84350    Chromosome:   8    Cytoband:   Chr:8q13    SOURCE    GENECARD       Gene Ontology:   cellular component:  nucleus      BioCarta Pathways:     KEGG Pathways:  
    Probe set : 1394479_at    Name:   Transcribed locus    GB acc:   BI288580    UniGene:   Rn.148355        SOURCE        Gene Ontology:     BioCarta Pathways:     KEGG Pathways:  
    Probe set : 1387567_at    Name:   solute carrier family 21, member 1 /// hypothetical gene supported by NM_017111    GB acc:   NM_017111    UniGene:   Rn.9912    Gene Symbol:   Slc21a1 /// LOC497799    Locus Link:   497799 /// 50572    Chromosome:   4    Cytoband:   Chr:4q44    SOURCE    GENECARD       Gene Ontology:     BioCarta Pathways:     KEGG Pathways:  
    Probe set : 1383362_at    Name:   Transcribed locus    GB acc:   BI275850    UniGene:   Rn.30219        SOURCE        Gene Ontology:     BioCarta Pathways:     KEGG Pathways:  
    Probe set : 1381973_at    Name:   solute carrier family 25, member 30    GB acc:   AW530647    UniGene:   Rn.19440    Gene Symbol:   RGD:1359702    Locus Link:   361074    Chromosome:   15    Cytoband:   Chr:15q11    SOURCE    GENECARD       Gene Ontology:   biological process:  transport   biological process:  mitochondrial transport   molecular function:  transporter activity   molecular function:  binding      BioCarta Pathways:     KEGG Pathways:  
    Probe set : 1369200_at    Name:   5 nucleotidase    GB acc:   NM_021576    UniGene:   Rn.40132    Gene Symbol:   Nt5    Locus Link:   58813    Chromosome:   8    Cytoband:   Chr:8q31    SOURCE    GENECARD       Gene Ontology:   biological process:  DNA metabolism   biological process:  nucleotide catabolism      BioCarta Pathways:     KEGG Pathways:  
    Probe set : 1388246_at    Name:   clusterin    GB acc:   AF314657    UniGene:   Rn.1780    Gene Symbol:   Clu    Locus Link:   24854    Chromosome:   15    Cytoband:   Chr:15p12    SOURCE    GENECARD       Gene Ontology:   cellular component:  extracellular region   cellular component:  extracellular space      BioCarta Pathways:     KEGG Pathways:  
    Probe set : 1371951_at    Name:   four and a half LIM domains 2    GB acc:   AA800031    UniGene:   Rn.3849    Gene Symbol:   Fhl2    Locus Link:   63839    Chromosome:   9    Cytoband:   Chr:9q22    SOURCE    GENECARD       Gene Ontology:   biological process:  regulation of transcription from RNA polymerase II promoter   cellular component:  nucleus   molecular function:  protein binding   molecular function:  zinc ion binding   molecular function:  metal ion binding      BioCarta Pathways:     KEGG Pathways:  
    Probe set : 1370320_at    Name:   MAWD binding protein    GB acc:   AY083160    UniGene:   Rn.2417    Gene Symbol:   Mawbp    Locus Link:   171564    Chromosome:   20    Cytoband:   Chr:20p11    SOURCE    GENECARD       Gene Ontology:   biological process:  biosynthesis   molecular function:  catalytic activity   molecular function:  oxidoreductase activity      BioCarta Pathways:     KEGG Pathways:  
    Probe set : 1370282_at    Name:   cysteine and glycine-rich protein 2    GB acc:   U44948    UniGene:   Rn.94754    Gene Symbol:   RGD:61950    Locus Link:   29317    Chromosome:   7    Cytoband:   Chr:7q13    SOURCE    GENECARD       Gene Ontology:   biological process:  development   biological process:  cell differentiation   biological process:  myoblast differentiation   cellular component:  nucleus   cellular component:  nucleus   molecular function:  zinc ion binding   molecular function:  metal ion binding      BioCarta Pathways:     KEGG Pathways:  
    Probe set : 1384231_at    Name:   Transcribed locus    GB acc:   BE108276    UniGene:   Rn.88359        SOURCE        Gene Ontology:     BioCarta Pathways:     KEGG Pathways:  
    Probe set : 1373667_at    Name:   cysteine conjugate-beta lyase (predicted)    GB acc:   BF400674    UniGene:   Rn.110564    Gene Symbol:   Ccbl1_predicted    Locus Link:   311844    Chromosome:   3    Cytoband:   Chr:3p12    SOURCE    GENECARD       Gene Ontology:   biological process:  amino acid derivative metabolism   biological process:  biosynthesis   cellular component:  cytosol   cellular component:  mitochondrion      BioCarta Pathways:     KEGG Pathways:  
    Probe set : 1383176_at    Name:   similar to C11orf17 protein (predicted)    GB acc:   BF561368    UniGene:   Rn.106344    Gene Symbol:   RGD1306959_predicted    Locus Link:   361624    Chromosome:   1    Cytoband:   Chr:1q33    SOURCE    GENECARD       Gene Ontology:     BioCarta Pathways:     KEGG Pathways:  
    Probe set : 1376615_at    Name:   TEA domain family member 3 (predicted)    GB acc:   BI289650    UniGene:   Rn.254    Gene Symbol:   Tead3_predicted    Locus Link:   294299    Chromosome:   20    Cytoband:   Chr:20p12    SOURCE    GENECARD       Gene Ontology:   cellular component:  nucleus   cellular component:  transcription factor complex   molecular function:  DNA binding   molecular function:  transcription factor activity   molecular function:  RNA polymerase II transcription factor activity      BioCarta Pathways:     KEGG Pathways:  
    Probe set : 1372901_at    Name:   Similar to RIKEN cDNA 8030451K01    GB acc:   BM386850    UniGene:   Rn.11681     Locus Link:   310416    Chromosome:   2    Cytoband:   Chr:2q26    SOURCE        Gene Ontology:     BioCarta Pathways:     KEGG Pathways:  
    Probe set : 1375677_at    Name:   transducer of ERBB2, 2    GB acc:   BI275222    UniGene:   Rn.18247    Gene Symbol:   RGD:1359722    Locus Link:   315159    Chromosome:   7    Cytoband:   Chr:7q34    SOURCE    GENECARD       Gene Ontology:     BioCarta Pathways:     KEGG Pathways:  
    Probe set : 1395030_at    Name:   Basic transcription element binding protein 1    GB acc:   BM390366    UniGene:   Rn.19481    Gene Symbol:   Bteb1    Locus Link:   117560    Chromosome:   1    Cytoband:   Chr:1q51    SOURCE    GENECARD       Gene Ontology:   cellular component:  nucleus      BioCarta Pathways:     KEGG Pathways:  
    Probe set : 1372070_at    Name:   interferon gamma inducible protein 30 (predicted)    GB acc:   BM389261    UniGene:   Rn.3370    Gene Symbol:   Ifi30_predicted    Locus Link:   290644    Chromosome:   16    Cytoband:   Chr:16p14    SOURCE    GENECARD       Gene Ontology:   biological process:  immune response   biological process:  antigen processing, exogenous antigen via MHC class II   cellular component:  extracellular region   cellular component:  extracellular space   cellular component:  lysosome   molecular function:  disulfide oxidoreductase activity   molecular function:  oxidoreductase activity      BioCarta Pathways:     KEGG Pathways:  
    Probe set : 1369010_at    Name:   protein kinase Chk2    GB acc:   NM_053677    UniGene:   Rn.18487    Gene Symbol:   Chek2    Locus Link:   114212    Chromosome:   12    Cytoband:   Chr:12q16    SOURCE    GENECARD       Gene Ontology:   cellular component:  nucleus   cellular component:  membrane      BioCarta Pathways:     KEGG Pathways:  
    Probe set : 1368534_at    Name:   adrenergic receptor, alpha 1d    GB acc:   NM_024483    UniGene:   Rn.11314    Gene Symbol:   Adra1d    Locus Link:   29413    Chromosome:   3    Cytoband:   Chr:3q36    SOURCE    GENECARD       Gene Ontology:   cellular component:  membrane fraction   cellular component:  integral to plasma membrane   cellular component:  integral to membrane      BioCarta Pathways:     KEGG Pathways:  
    Probe set : 1379665_at    Name:   similar to RIKEN cDNA 4632422M10 (predicted)    GB acc:   AI177835    UniGene:   Rn.144258    Gene Symbol:   RGD1310204_predicted    Locus Link:   294711    Chromosome:   2    Cytoband:   Chr:2q13    SOURCE    GENECARD       Gene Ontology:     BioCarta Pathways:     KEGG Pathways:  
    Probe set : 1372339_at    Name:   cell division cycle 26 (predicted)    GB acc:   AA866438    UniGene:   Rn.95113    Gene Symbol:   Cdc26_predicted    Locus Link:   366381    Chromosome:   5    Cytoband:   Chr:5q24    SOURCE    GENECARD       Gene Ontology:   biological process:  cytokinesis   biological process:  cell division   cellular component:  anaphase-promoting complex      BioCarta Pathways:     KEGG Pathways:  
    Probe set : 1373324_at    Name:   dual specificity phosphatase 14 (predicted) /// similar to Dual specificity phosphatase 14    GB acc:   AI236997    UniGene:   Rn.25406    Gene Symbol:   Dusp14_predicted /// LOC499736    Locus Link:   360580 /// 499736    Chromosome:   10    Cytoband:   Chr:10q26    SOURCE    GENECARD       Gene Ontology:   biological process:  protein amino acid dephosphorylation      BioCarta Pathways:     KEGG Pathways:  
    Probe set : 1375060_at    Name:   similar to Cc2-27 (predicted)    GB acc:   BE112912    UniGene:   Rn.8751    Gene Symbol:   RGD1310244_predicted    Locus Link:   366505    Chromosome:   5    Cytoband:   Chr:5q36    SOURCE    GENECARD       Gene Ontology:     BioCarta Pathways:     KEGG Pathways:  
    Probe set : 1367452_at    Name:   SMT3 suppressor of mif two 3 homolog 2 (yeast)    GB acc:   NM_133594    UniGene:   Rn.5958    Gene Symbol:   Sumo2    Locus Link:   171127    Chromosome:   5    Cytoband:   Chr:5q11    SOURCE    GENECARD       Gene Ontology:   biological process:  protein modification   biological process:  ubiquitin cycle      BioCarta Pathways:     KEGG Pathways:  
    Probe set : 1373037_at    Name:   ubiquitin-conjugating enzyme E2L 6 (predicted)    GB acc:   BI279216    UniGene:   Rn.8609    Gene Symbol:   Ube2l6_predicted    Locus Link:   295704    Chromosome:   3    Cytoband:   Chr:3q24    SOURCE    GENECARD       Gene Ontology:   biological process:  protein modification   biological process:  ubiquitin cycle      BioCarta Pathways:     KEGG Pathways:  
    Probe set : 1369652_at    Name:   thymus cell antigen 1, theta    GB acc:   AI145313    UniGene:   Rn.108198    Gene Symbol:   Thy1    Locus Link:   24832    Chromosome:   8    Cytoband:   Chr:8q22    SOURCE    GENECARD       Gene Ontology:     BioCarta Pathways:     KEGG Pathways:  
    Probe set : 1370161_at    Name:   steroid sensitive gene 1    GB acc:   AI235465    UniGene:   Rn.2193    Gene Symbol:   Ssg1    Locus Link:   64387    Chromosome:   11    Cytoband:   Chr:11q21    SOURCE    GENECARD       Gene Ontology:     BioCarta Pathways:     KEGG Pathways:  
    Probe set : 1383486_at    Name:   Transcribed locus    GB acc:   AA926109    UniGene:   Rn.35842        SOURCE        Gene Ontology:     BioCarta Pathways:     KEGG Pathways:  
    Probe set : 1389350_at    Name:   apolipoprotein H (predicted)    GB acc:   AI180413    UniGene:   Rn.1824    Gene Symbol:   Apoh_predicted    Locus Link:   287774    Chromosome:   10    Cytoband:   Chr:10q32.1    SOURCE    GENECARD       Gene Ontology:   biological process:  defense response   cellular component:  extracellular region   cellular component:  extracellular space   molecular function:  lipid transporter activity   molecular function:  heparin binding      BioCarta Pathways:     KEGG Pathways:  
    Probe set : 1389760_at    Name:   ring finger protein 134 (predicted)    GB acc:   AA858786    UniGene:   Rn.16897    Gene Symbol:   Rnf134_predicted    Locus Link:   309457    Chromosome:   1    Cytoband:   Chr:1q54    SOURCE    GENECARD       Gene Ontology:   cellular component:  nucleus   cellular component:  ubiquitin ligase complex      BioCarta Pathways:     KEGG Pathways:  
    Probe set : 1395126_at     GB acc:   AI011393              Gene Ontology:     BioCarta Pathways:     KEGG Pathways:  
    Probe set : 1390931_at    Name:   Transcribed locus    GB acc:   AI704285    UniGene:   Rn.41227        SOURCE        Gene Ontology:     BioCarta Pathways:     KEGG Pathways:  
    Probe set : 1373290_at    Name:   similar to Ezh2 protein    GB acc:   AI012781    UniGene:   Rn.9027    Gene Symbol:   LOC312299    Locus Link:   312299    Chromosome:   4    Cytoband:   Chr:4q24    SOURCE    GENECARD       Gene Ontology:     BioCarta Pathways:     KEGG Pathways:  
    Probe set : 1376099_at    Name:   Collagen, type V, alpha 1    GB acc:   AW254017    UniGene:   Rn.117    Gene Symbol:   Col5a1    Locus Link:   85490    Chromosome:   3    Cytoband:   Chr:3p12    SOURCE    GENECARD       Gene Ontology:   biological process:  phosphate transport   biological process:  cell adhesion      BioCarta Pathways:     KEGG Pathways:  
    Probe set : 1385925_at    Name:   Transcribed locus    GB acc:   AA963975    UniGene:   Rn.23216        SOURCE        Gene Ontology:     BioCarta Pathways:     KEGG Pathways:  
    Probe set : 1370936_at    Name:   dimethylglycine dehydrogenase precursor    GB acc:   AA892345    UniGene:   Rn.3646    Gene Symbol:   Dmgdh    Locus Link:   245961    Chromosome:   2    Cytoband:   Chr:2q12    SOURCE    GENECARD       Gene Ontology:   cellular component:  mitochondrion   cellular component:  mitochondrion   cellular component:  cytoplasm      BioCarta Pathways:     KEGG Pathways:  
    Probe set : 1371824_at    Name:   adenylate kinase 3-like 1    GB acc:   AA891949    UniGene:   Rn.1086    Gene Symbol:   Ak3l1    Locus Link:   29223    Chromosome:   5    Cytoband:   Chr:5q33    SOURCE    GENECARD       Gene Ontology:   biological process:  nucleobase, nucleoside, nucleotide and nucleic acid metabolism   cellular component:  mitochondrion      BioCarta Pathways:     KEGG Pathways:  
    Probe set : 1378144_at    Name:   Transcribed locus    GB acc:   BI296384    UniGene:   Rn.21560        SOURCE        Gene Ontology:     BioCarta Pathways:     KEGG Pathways:  
    Probe set : 1378076_at    Name:   Transcribed locus    GB acc:   AA963273    UniGene:   Rn.11429        SOURCE        Gene Ontology:     BioCarta Pathways:     KEGG Pathways:  
    Probe set : 1371606_at    Name:   Transcribed locus    GB acc:   AA819633    UniGene:   Rn.16646        SOURCE        Gene Ontology:     BioCarta Pathways:     KEGG Pathways:  
    Probe set : 1378244_at    Name:   Transcribed locus    GB acc:   AI072663    UniGene:   Rn.20215        SOURCE        Gene Ontology:     BioCarta Pathways:     KEGG Pathways:  
    Probe set : 1369917_at    Name:   neurotrophin receptor associated death domain    GB acc:   NM_139259    UniGene:   Rn.40242    Gene Symbol:   Nradd    Locus Link:   246143    Chromosome:   8    Cytoband:   Chr:8q32    SOURCE    GENECARD       Gene Ontology:   molecular function:  neurotrophin p75 receptor binding   molecular function:  caspase activator activity   molecular function:  protein binding      BioCarta Pathways:     KEGG Pathways:  
    Probe set : 1369649_at    Name:   calcium channel, voltage-dependent, alpha2/delta subunit 1    GB acc:   AF400662    UniGene:   Rn.11276    Gene Symbol:   Cacna2d1    Locus Link:   25399    Chromosome:   4    Cytoband:   Chr:4q11    SOURCE    GENECARD       Gene Ontology:   biological process:  ion transport   biological process:  transport   biological process:  calcium ion transport      BioCarta Pathways:     KEGG Pathways:  
    Probe set : 1377826_at    Name:   Transcribed locus, weakly similar to NP_036457.1 microtubule-associated protein, RP/EB family, member 1; adenomatous polyposis coli-binding protein EB1 [Homo sapiens]    GB acc:   AI176721    UniGene:   Rn.17666        SOURCE        Gene Ontology:     BioCarta Pathways:     KEGG Pathways:  
    Probe set : 1370154_at    Name:   lysozyme    GB acc:   L12458    UniGene:   Rn.2283    Gene Symbol:   Lyz    Locus Link:   25211    Chromosome:   7    Cytoband:   Chr:7q22    SOURCE    GENECARD       Gene Ontology:   cellular component:  extracellular region   cellular component:  extracellular space   cellular component:  mitochondrion      BioCarta Pathways:     KEGG Pathways:  
    Probe set : 1374565_at    Name:   NIMA (never in mitosis gene a)-related expressed kinase 6    GB acc:   BF282365    UniGene:   Rn.18744    Gene Symbol:   Nek6    Locus Link:   360161    Chromosome:   3    Cytoband:   Chr:3q11    SOURCE    GENECARD       Gene Ontology:   biological process:  protein amino acid phosphorylation      BioCarta Pathways:     KEGG Pathways:  
    Probe set : 1391849_at    Name:   Similar to MBD2 (methyl-CpG-binding protein)-interacting zinc finger protein (predicted)    GB acc:   BF547270    UniGene:   Rn.38367     Locus Link:   300665    Chromosome:   8    Cytoband:   Chr:8q22    SOURCE        Gene Ontology:     BioCarta Pathways:     KEGG Pathways:  
    Probe set : 1392037_at    Name:   Transcribed locus    GB acc:   BM383048    UniGene:   Rn.43297        SOURCE        Gene Ontology:     BioCarta Pathways:     KEGG Pathways:  
    Probe set : 1373345_at    Name:   amphoterin induced gene 2    GB acc:   AW523747    UniGene:   Rn.98123    Gene Symbol:   Amigo2    Locus Link:   300186    Chromosome:   7    Cytoband:   Chr:7q35    SOURCE    GENECARD       Gene Ontology:   cellular component:  extracellular space   cellular component:  integral to membrane   cellular component:  integral to plasma membrane   molecular function:  protein binding   molecular function:  protein binding      BioCarta Pathways:     KEGG Pathways:  
    Probe set : 1371350_at    Name:   similar to S-adenosylmethionine synthetase gamma form (Methionine adenosyltransferase) (AdoMet synthetase) (MAT-II)    GB acc:   AI412159     Gene Symbol:   LOC498211    Locus Link:   498211    Chromosome:   13    Cytoband:   Chr:13p13     GENECARD       Gene Ontology:     BioCarta Pathways:     KEGG Pathways:  
    Probe set : 1384103_at    Name:   Similar to novel protein (HT036)    GB acc:   BE106350    UniGene:   Rn.40997     Locus Link:   500536      SOURCE        Gene Ontology:     BioCarta Pathways:     KEGG Pathways:  
    Probe set : 1397684_at    Name:   dickkopf homolog 4 (Xenopus laevis) (predicted)    GB acc:   BF549625    UniGene:   Rn.44861    Gene Symbol:   Dkk4_predicted    Locus Link:   295445    Chromosome:   2    Cytoband:   Chr:2q43    SOURCE    GENECARD       Gene Ontology:   biological process:  development   biological process:  Wnt receptor signaling pathway   biological process:  negative regulation of Wnt receptor signaling pathway   cellular component:  extracellular region   cellular component:  extracellular space      BioCarta Pathways:     KEGG Pathways:  
    Probe set : 1373242_at    Name:   TATA box binding protein-like 1 (predicted)    GB acc:   AW434020    UniGene:   Rn.25148    Gene Symbol:   Tbpl1_predicted    Locus Link:   292173    Chromosome:   1    Cytoband:   Chr:1p12    SOURCE    GENECARD       Gene Ontology:   cellular component:  nucleus   cellular component:  transcription factor TFIID complex   cellular component:  cytoplasm   molecular function:  DNA binding   molecular function:  RNA polymerase II transcription factor activity      BioCarta Pathways:     KEGG Pathways:  
    Probe set : 1392521_at    Name:   Transcribed locus    GB acc:   AI030119    UniGene:   Rn.18407        SOURCE        Gene Ontology:     BioCarta Pathways:     KEGG Pathways:  
    Probe set : 1398286_at    Name:   cysteine sulfinic acid decarboxylase    GB acc:   M64755    UniGene:   Rn.43232    Gene Symbol:   Csad    Locus Link:   60356    Chromosome:   7    Cytoband:   Chr:7q36    SOURCE    GENECARD       Gene Ontology:     BioCarta Pathways:     KEGG Pathways:  
    Probe set : 1384588_at    Name:   Transcribed locus    GB acc:   BI295602    UniGene:   Rn.79724        SOURCE        Gene Ontology:     BioCarta Pathways:     KEGG Pathways:  
    Probe set : 1381261_at    Name:   Nicotinamide nucleotide transhydrogenase    GB acc:   BE113729    UniGene:   Rn.3128    Gene Symbol:   Nnt    Locus Link:   310378    Chromosome:   2    Cytoband:   Chr:2q16    SOURCE    GENECARD       Gene Ontology:   biological process:  electron transport   biological process:  proton transport   cellular component:  mitochondrion   cellular component:  integral to membrane      BioCarta Pathways:     KEGG Pathways:  
    Probe set : 1369799_at    Name:   4-aminobutyrate aminotransferase    GB acc:   U29701    UniGene:   Rn.10090    Gene Symbol:   Abat    Locus Link:   81632    Chromosome:   10    Cytoband:   Chr:10q12    SOURCE    GENECARD       Gene Ontology:   biological process:  synaptic transmission   biological process:  gamma-aminobutyric acid metabolism   biological process:  neurotransmitter catabolism   cellular component:  mitochondrion   cellular component:  mitochondrial matrix      BioCarta Pathways:     KEGG Pathways:  
    Probe set : 1368921_a_at    Name:   CD44 antigen    GB acc:   NM_012924    UniGene:   Rn.1120    Gene Symbol:   Cd44    Locus Link:   25406    Chromosome:   3    Cytoband:   Chr:3q31    SOURCE    GENECARD       Gene Ontology:     BioCarta Pathways:     KEGG Pathways:  
    Probe set : 1373238_at    Name:   similar to SPT3-associated factor 42 (predicted)    GB acc:   AI234844    UniGene:   Rn.23978    Gene Symbol:   RGD1306774_predicted    Locus Link:   360874    Chromosome:   13    Cytoband:   Chr:13q23    SOURCE    GENECARD       Gene Ontology:     BioCarta Pathways:     KEGG Pathways:  
    Probe set : 1373152_at    Name:   protease, serine, 23    GB acc:   AI177099    UniGene:   Rn.137661    Gene Symbol:   RGD:1359545    Locus Link:   308807    Chromosome:   1    Cytoband:   Chr:1q32    SOURCE    GENECARD       Gene Ontology:   biological process:  nucleosome assembly   biological process:  proteolysis   biological process:  chromosome organization and biogenesis (sensu Eukaryota)   cellular component:  nucleosome   cellular component:  nucleus      BioCarta Pathways:     KEGG Pathways:  
    Probe set : 1374672_at    Name:   cardiac ankyrin repeat kinase    GB acc:   BI293060    UniGene:   Rn.3434    Gene Symbol:   Cark    Locus Link:   295531    Chromosome:   2    Cytoband:   Chr:2q45    SOURCE    GENECARD       Gene Ontology:   biological process:  protein amino acid phosphorylation   cellular component:  nucleus      BioCarta Pathways:     KEGG Pathways:  
    Probe set : 1384580_at    Name:   complement component 6    GB acc:   AI045191    UniGene:   Rn.16145    Gene Symbol:   C6    Locus Link:   24237    Chromosome:   2    Cytoband:   Chr:2q16    SOURCE    GENECARD       Gene Ontology:   cellular component:  membrane attack complex   cellular component:  extracellular space   cellular component:  integral to membrane      BioCarta Pathways:     KEGG Pathways:  
    Probe set : 1368544_a_at    Name:   nucleolar protein 3 (apoptosis repressor with CARD domain)    GB acc:   NM_053516    UniGene:   Rn.86956    Gene Symbol:   Nol3    Locus Link:   85383    Chromosome:   19    Cytoband:   Chr:19q11    SOURCE    GENECARD       Gene Ontology:   cellular component:  nucleus   cellular component:  intracellular   molecular function:  protein binding      BioCarta Pathways:     KEGG Pathways:  
    Probe set : 1370144_at    Name:   G protein-binding protein CRFG /// similar to GTP-binding protein NGB /// similar to GTP-binding protein NGB    GB acc:   NM_053689    UniGene:   Rn.106958    Gene Symbol:   Gtpbp4 /// LOC364763 /// LOC498786    Locus Link:   114300 /// 364763 /// 498786    Chromosome:   17    Cytoband:   Chr:17q12.1    SOURCE    GENECARD       Gene Ontology:   biological process:  ribosome biogenesis   biological process:  small GTPase mediated signal transduction   cellular component:  nucleus   cellular component:  nucleus   molecular function:  GTP binding   molecular function:  GTP binding   molecular function:  nucleotide binding      BioCarta Pathways:     KEGG Pathways:  
    Probe set : 1370253_at    Name:   ribosomal protein L22    GB acc:   AI228150    UniGene:   Rn.21738    Gene Symbol:   Rpl22    Locus Link:   81768    Chromosome:   5    Cytoband:   Chr:5q36    SOURCE    GENECARD       Gene Ontology:   biological process:  protein biosynthesis   biological process:  ribosome biogenesis      BioCarta Pathways:     KEGG Pathways:  
    Probe set : 1372292_at    Name:   Similar to Serine/threonine protein kinase 24    GB acc:   AI009732    UniGene:   Rn.105397     Locus Link:   361092    Chromosome:   15    Cytoband:   Chr:15q25    SOURCE        Gene Ontology:     BioCarta Pathways:     KEGG Pathways:  
    Probe set : 1390141_at    Name:   formyltetrahydrofolate synthetase domain containing 1 (predicted)    GB acc:   BF281848    UniGene:   Rn.18065    Gene Symbol:   Fthfsdc1_predicted    Locus Link:   361472    Chromosome:   1    Cytoband:   Chr:1p11    SOURCE    GENECARD       Gene Ontology:   biological process:  10-formyltetrahydrofolate biosynthesis   biological process:  folic acid and derivative biosynthesis   cellular component:  mitochondrion      BioCarta Pathways:     KEGG Pathways:  
    Probe set : 1385868_at     GB acc:   AA875124              Gene Ontology:     BioCarta Pathways:     KEGG Pathways:  
    Probe set : 1373403_at    Name:   Transcribed locus    GB acc:   AI230625    UniGene:   Rn.146058        SOURCE        Gene Ontology:     BioCarta Pathways:     KEGG Pathways:  
    Probe set : 1390440_at    Name:   similar to mKIAA0267 protein    GB acc:   BG380512    UniGene:   Rn.25009    Gene Symbol:   LOC302863    Locus Link:   302863    Chromosome:   X    Cytoband:   Chr:Xq36    SOURCE    GENECARD       Gene Ontology:     BioCarta Pathways:     KEGG Pathways:  
    Probe set : 1387011_at    Name:   lipocalin 2    GB acc:   NM_130741    UniGene:   Rn.11303    Gene Symbol:   Lcn2    Locus Link:   170496    Chromosome:   3    Cytoband:   Chr:3p11    SOURCE    GENECARD       Gene Ontology:   biological process:  transport   biological process:  apoptosis   cellular component:  extracellular space   cellular component:  soluble fraction   cellular component:  cytoplasm   molecular function:  transporter activity   molecular function:  binding      BioCarta Pathways:     KEGG Pathways:  
    Probe set : 1395184_at     GB acc:   AI411699              Gene Ontology:     BioCarta Pathways:     KEGG Pathways:  
    Probe set : 1390543_at    Name:   Transcribed locus    GB acc:   BF396416    UniGene:   Rn.8770        SOURCE        Gene Ontology:     BioCarta Pathways:     KEGG Pathways:  
    Probe set : 1395781_at    Name:   v-myc myelocytomatosis viral oncogene homolog 1, lung carcinoma derived (avian)    GB acc:   BI300996    UniGene:   Rn.41400    Gene Symbol:   Mycl1    Locus Link:   298506    Chromosome:   5    Cytoband:   Chr:5q36    SOURCE    GENECARD       Gene Ontology:   cellular component:  intracellular   cellular component:  nucleus      BioCarta Pathways:     KEGG Pathways:  
    Probe set : 1395039_at    Name:   Transcribed locus    GB acc:   BI289224    UniGene:   Rn.146198        SOURCE        Gene Ontology:     BioCarta Pathways:     KEGG Pathways:  
    Probe set : 1368627_at    Name:   regucalcin    GB acc:   NM_031546    UniGene:   Rn.10006    Gene Symbol:   Rgn    Locus Link:   25106    Chromosome:   X    Cytoband:   Chr:Xq11-q12    SOURCE    GENECARD       Gene Ontology:   cellular component:  nucleus   cellular component:  cytoplasm   molecular function:  calcium ion binding   molecular function:  enzyme regulator activity      BioCarta Pathways:     KEGG Pathways:  
    Probe set : 1368395_at    Name:   glypican 3    GB acc:   NM_012774    UniGene:   Rn.9717    Gene Symbol:   Gpc3    Locus Link:   25236    Chromosome:   X    Cytoband:   Chr:Xq36    SOURCE    GENECARD       Gene Ontology:   molecular function:  transmembrane receptor activity      BioCarta Pathways:     KEGG Pathways:  
    Probe set : 1390846_at    Name:   procollagen, type XVI, alpha 1 (predicted)    GB acc:   BG380309    UniGene:   Rn.74081    Gene Symbol:   Col16a1_predicted    Locus Link:   366474    Chromosome:   5    Cytoband:   Chr:5q36    SOURCE    GENECARD       Gene Ontology:   biological process:  phosphate transport   biological process:  cell adhesion   biological process:  pregnancy   molecular function:  structural molecule activity   molecular function:  extracellular matrix structural constituent conferring tensile strength      BioCarta Pathways:     KEGG Pathways:  
    Probe set : 1371737_at    Name:   tripartite motif protein 27 (predicted)    GB acc:   BI281720    UniGene:   Rn.8393    Gene Symbol:   Trim27_predicted    Locus Link:   291171    Chromosome:   17    Cytoband:   Chr:17q11    SOURCE    GENECARD       Gene Ontology:     BioCarta Pathways:     KEGG Pathways:  
    Probe set : 1379361_at    Name:   peroxisomal biogenesis factor 11A    GB acc:   BI273703    UniGene:   Rn.14519    Gene Symbol:   Pex11a    Locus Link:   85249    Chromosome:   1    Cytoband:   Chr:1q31    SOURCE    GENECARD       Gene Ontology:   biological process:  peroxisome organization and biogenesis   biological process:  peroxisome membrane biogenesis   biological process:  peroxisome fission      BioCarta Pathways:     KEGG Pathways:  
    Probe set : 1391347_at    Name:   RAB8B, member RAS oncogene family    GB acc:   BF398743    UniGene:   Rn.10995    Gene Symbol:   Rab8b    Locus Link:   266688    Chromosome:   8    Cytoband:   Chr:8q24    SOURCE    GENECARD       Gene Ontology:     BioCarta Pathways:     KEGG Pathways:  
    Probe set : 1374638_at    Name:   peroxisomal biogenesis factor 13 (predicted)    GB acc:   AI012951    UniGene:   Rn.144057    Gene Symbol:   Pex13_predicted    Locus Link:   305581    Chromosome:   14    Cytoband:   Chr:14q22    SOURCE    GENECARD       Gene Ontology:   molecular function:  protein transporter activity      BioCarta Pathways:     KEGG Pathways:  
    Probe set : 1388132_at    Name:   NonO/p54nrb homolog    GB acc:   BE107056    UniGene:   Rn.54645    Gene Symbol:   Sfpq    Locus Link:   252855    Chromosome:   5    Cytoband:   Chr:5q36    SOURCE    GENECARD       Gene Ontology:   molecular function:  nucleic acid binding      BioCarta Pathways:     KEGG Pathways:  
    Probe set : 1388753_at    Name:   sulfatase 2 (predicted)    GB acc:   AA963797    UniGene:   Rn.4228    Gene Symbol:   Sulf2_predicted    Locus Link:   311642    Chromosome:   3    Cytoband:   Chr:3q42    SOURCE    GENECARD       Gene Ontology:     BioCarta Pathways:     KEGG Pathways:  
    Probe set : 1383833_at    Name:   hypothetical protein LOC500991    GB acc:   BI291641    UniGene:   Rn.29872    Gene Symbol:   LOC500991    Locus Link:   500991    Chromosome:   8    Cytoband:   Chr:8q23    SOURCE    GENECARD       Gene Ontology:     BioCarta Pathways:     KEGG Pathways:  
    Probe set : 1387160_at    Name:   potassium voltage-gated channel, Isk-related subfamily, member 3    GB acc:   NM_022235    UniGene:   Rn.44843    Gene Symbol:   Kcne3    Locus Link:   63883    Chromosome:   1    Cytoband:   Chr:1q32    SOURCE    GENECARD       Gene Ontology:   biological process:  transport   biological process:  ion transport   biological process:  potassium ion transport   cellular component:  voltage-gated potassium channel complex   cellular component:  membrane   cellular component:  integral to membrane      BioCarta Pathways:     KEGG Pathways:  
    Probe set : 1392938_s_at    Name:   similar to C11orf17 protein (predicted)    GB acc:   BF561368    UniGene:   Rn.106344    Gene Symbol:   RGD1306959_predicted    Locus Link:   361624    Chromosome:   1    Cytoband:   Chr:1q33    SOURCE    GENECARD       Gene Ontology:     BioCarta Pathways:     KEGG Pathways:  
    Probe set : 1395953_at    Name:   Transcribed locus    GB acc:   AA955517    UniGene:   Rn.8838        SOURCE        Gene Ontology:     BioCarta Pathways:     KEGG Pathways:  
    Probe set : 1374484_at    Name:   similar to RIKEN cDNA 2610033C09 (predicted)    GB acc:   AI230318    UniGene:   Rn.13663    Gene Symbol:   RGD1306421_predicted    Locus Link:   288092    Chromosome:   11    Cytoband:   Chr:11q21    SOURCE    GENECARD       Gene Ontology:     BioCarta Pathways:     KEGG Pathways:  
    Probe set : 1373722_at    Name:   kinesin family member 20A (predicted)    GB acc:   BE111697    UniGene:   Rn.101107    Gene Symbol:   Kif20a_predicted    Locus Link:   361308    Chromosome:   18    Cytoband:   Chr:18p12    SOURCE    GENECARD       Gene Ontology:   cellular component:  Golgi apparatus   cellular component:  microtubule associated complex      BioCarta Pathways:     KEGG Pathways:  
    Probe set : 1372365_at    Name:   Transcribed locus    GB acc:   BM392293    UniGene:   Rn.34193        SOURCE        Gene Ontology:     BioCarta Pathways:     KEGG Pathways:  
    Probe set : 1390492_a_at    Name:   Ubiquitin specific protease 40 (predicted)    GB acc:   BM391750    UniGene:   Rn.9343     Locus Link:   316599    Chromosome:   9    Cytoband:   Chr:9q35    SOURCE        Gene Ontology:   biological process:  ubiquitin-dependent protein catabolism   biological process:  ubiquitin cycle   cellular component:  mitochondrion      BioCarta Pathways:     KEGG Pathways:  
    Probe set : 1367983_at    Name:   flap structure-specific endonuclease 1    GB acc:   NM_053430    UniGene:   Rn.16664    Gene Symbol:   Fen1    Locus Link:   84490    Chromosome:   1    Cytoband:   Chr:1q43    SOURCE    GENECARD       Gene Ontology:   cellular component:  nucleus      BioCarta Pathways:     KEGG Pathways:  
    Probe set : 1383964_at    Name:   Transcribed locus    GB acc:   AI549551    UniGene:   Rn.7482        SOURCE        Gene Ontology:     BioCarta Pathways:     KEGG Pathways:  
    Probe set : 1393550_at    Name:   Transcribed locus    GB acc:   BF564195    UniGene:   Rn.138753        SOURCE        Gene Ontology:     BioCarta Pathways:     KEGG Pathways:  
    Probe set : 1395364_at    Name:   interferon (alpha and beta) receptor 1 (predicted)    GB acc:   BE099982    UniGene:   Rn.105738    Gene Symbol:   Ifnar1_predicted    Locus Link:   288264    Chromosome:   11    Cytoband:   Chr:11q11    SOURCE    GENECARD       Gene Ontology:   biological process:  cell surface receptor linked signal transduction   biological process:  JAK-STAT cascade   biological process:  response to virus      BioCarta Pathways:     KEGG Pathways:  
    Probe set : 1391067_at     GB acc:   BF389648              Gene Ontology:     BioCarta Pathways:     KEGG Pathways:  
    Probe set : 1378793_at    Name:   Forkhead box P4 (predicted)    GB acc:   BE121233    UniGene:   Rn.64329     Locus Link:   363185    Chromosome:   9    Cytoband:   Chr:9q12    SOURCE        Gene Ontology:   biological process:  regulation of transcription, DNA-dependent   cellular component:  nucleus   molecular function:  DNA binding   molecular function:  transcription factor activity   molecular function:  zinc ion binding      BioCarta Pathways:     KEGG Pathways:  
    Probe set : 1388243_at    Name:   gene model 1012, (NCBI)    GB acc:   BG663422    UniGene:   Rn.44656    Gene Symbol:   Gm1012    Locus Link:   117257    Chromosome:   3    Cytoband:   Chr:3q35    SOURCE    GENECARD       Gene Ontology:   biological process:  G-protein coupled receptor protein signaling pathway   biological process:  synaptic transmission   biological process:  signal transduction   cellular component:  integral to plasma membrane   cellular component:  integral to membrane      BioCarta Pathways:     KEGG Pathways:  
    Probe set : 1382138_at    Name:   similar to hypothetical protein FLJ20433    GB acc:   BG380221    UniGene:   Rn.22790    Gene Symbol:   LOC499745    Locus Link:   499745    Chromosome:   3    Cytoband:   Chr:3p13    SOURCE    GENECARD       Gene Ontology:     BioCarta Pathways:     KEGG Pathways:  
    Probe set : 1386868_at    Name:   ribosomal protein S10    GB acc:   NM_031109    UniGene:   Rn.3359    Gene Symbol:   Rps10    Locus Link:   81773    Chromosome:   20    Cytoband:   Chr:20p12    SOURCE    GENECARD       Gene Ontology:   biological process:  regulation of transcription, DNA-dependent   biological process:  protein biosynthesis   cellular component:  nucleus   cellular component:  cytosolic small ribosomal subunit (sensu Eukaryota)   cellular component:  ribonucleoprotein complex   molecular function:  DNA binding   molecular function:  RNA binding   molecular function:  structural constituent of ribosome      BioCarta Pathways:     KEGG Pathways:  
    Probe set : 1395268_at    Name:   WW domain containing E3 ubiquitin protein ligase 1 (predicted)    GB acc:   BM383223    UniGene:   Rn.81239    Gene Symbol:   Wwp1_predicted    Locus Link:   297930    Chromosome:   5    Cytoband:   Chr:5q13    SOURCE    GENECARD       Gene Ontology:   cellular component:  ubiquitin ligase complex   cellular component:  intracellular   molecular function:  ubiquitin-protein ligase activity   molecular function:  protein binding   molecular function:  ligase activity      BioCarta Pathways:     KEGG Pathways:  
    Probe set : 1368576_at    Name:   cartilage homeo protein 1    GB acc:   NM_012921    UniGene:   Rn.9282    Gene Symbol:   Cart1    Locus Link:   25401    Chromosome:   7    Cytoband:   Chr:7q21    SOURCE    GENECARD       Gene Ontology:   cellular component:  intracellular   cellular component:  nucleus   cellular component:  nucleus      BioCarta Pathways:     KEGG Pathways:  
    Probe set : 1382370_at    Name:   cyclin F    GB acc:   BE113938    UniGene:   Rn.15126    Gene Symbol:   Ccnf    Locus Link:   117524    Chromosome:   10    Cytoband:   Chr:10q12    SOURCE    GENECARD       Gene Ontology:   cellular component:  nucleus   molecular function:  cyclin-dependent protein kinase regulator activity      BioCarta Pathways:     KEGG Pathways:  
    Probe set : 1375362_at     GB acc:   BI304125              Gene Ontology:     BioCarta Pathways:     KEGG Pathways:  
    Probe set : 1377390_at    Name:   beta-site APP-cleaving enzyme 2    GB acc:   BG381587    UniGene:   Rn.38581    Gene Symbol:   RGD:1303241    Locus Link:   288227    Chromosome:   11    Cytoband:   Chr:11q12    SOURCE    GENECARD       Gene Ontology:     BioCarta Pathways:     KEGG Pathways:  
    Probe set : 1393682_at    Name:   G-protein-coupled receptor GPR34    GB acc:   BF410504     Gene Symbol:   GPR34    Locus Link:   554353    Chromosome:   X    Cytoband:   Chr:Xq12     GENECARD       Gene Ontology:     BioCarta Pathways:     KEGG Pathways:  
    Probe set : 1390021_at    Name:   histone 1, H2bh (predicted)    GB acc:   BM391206     Gene Symbol:   Hist1h2bh_predicted    Locus Link:   306945    Chromosome:   17    Cytoband:   Chr:17p11     GENECARD       Gene Ontology:     BioCarta Pathways:     KEGG Pathways:  
    Probe set : 1370297_at    Name:   polo-like kinase 1 (Drosophila)    GB acc:   U10188    UniGene:   Rn.11034    Gene Symbol:   Plk1    Locus Link:   25515    Chromosome:   1    Cytoband:   Chr:1q36    SOURCE    GENECARD       Gene Ontology:   cellular component:  nucleus      BioCarta Pathways:     KEGG Pathways:  
    Probe set : 1373150_at    Name:   catechol-O-methyltransferase domain containing 1 (predicted)    GB acc:   AW251225    UniGene:   Rn.7399    Gene Symbol:   Comtd1_predicted    Locus Link:   305685    Chromosome:   15    Cytoband:   Chr:15p16    SOURCE    GENECARD       Gene Ontology:   biological process:  protein prenylation   cellular component:  extracellular space      BioCarta Pathways:     KEGG Pathways:  
    Probe set : 1388143_at    Name:   collagen, type XVIII, alpha 1    GB acc:   BI288582    UniGene:   Rn.12030    Gene Symbol:   Col18a1    Locus Link:   85251    Chromosome:   20    Cytoband:   Chr:20p12    SOURCE    GENECARD       Gene Ontology:     BioCarta Pathways:     KEGG Pathways:  
    Probe set : 1367759_at    Name:   H1 histone family, member 0    GB acc:   NM_012578    UniGene:   Rn.3129    Gene Symbol:   H1f0    Locus Link:   24437    Chromosome:   7    Cytoband:   Chr:7q34    SOURCE    GENECARD       Gene Ontology:   biological process:  nucleosome assembly   biological process:  chromosome organization and biogenesis (sensu Eukaryota)   cellular component:  nucleosome   cellular component:  nucleus   cellular component:  chromosome   molecular function:  DNA binding      BioCarta Pathways:     KEGG Pathways:  
    Probe set : 1382130_at    Name:   similar to Protocadherin 19 precursor    GB acc:   AA997710    UniGene:   Rn.24970    Gene Symbol:   LOC317183    Locus Link:   317183    Chromosome:   X    Cytoband:   Chr:Xq34    SOURCE    GENECARD       Gene Ontology:     BioCarta Pathways:     KEGG Pathways:  
    Probe set : 1392914_at     GB acc:   BI275467              Gene Ontology:     BioCarta Pathways:     KEGG Pathways:  
    Probe set : 1374739_at    Name:   similar to hematopoietic progenitor protein    GB acc:   AI236771    UniGene:   Rn.32426    Gene Symbol:   LOC500727    Locus Link:   500727    Chromosome:   6    Cytoband:   Chr:6q32    SOURCE    GENECARD       Gene Ontology:     BioCarta Pathways:     KEGG Pathways:  
    Probe set : 1388588_at    Name:   mammary tumor virus receptor 2 (predicted)    GB acc:   AI236101    UniGene:   Rn.6896    Gene Symbol:   Mtvr2_predicted    Locus Link:   309170    Chromosome:   1    Cytoband:   Chr:1q43    SOURCE    GENECARD       Gene Ontology:   molecular function:  receptor activity      BioCarta Pathways:     KEGG Pathways:  
    Probe set : 1395427_at    Name:   Transcribed locus    GB acc:   AW918981    UniGene:   Rn.2860        SOURCE        Gene Ontology:     BioCarta Pathways:     KEGG Pathways:  
    Probe set : 1388658_at    Name:   surfeit 2    GB acc:   AA942704    UniGene:   Rn.107863    Gene Symbol:   Surf2    Locus Link:   619345    Chromosome:   3    Cytoband:   Chr:3p12    SOURCE    GENECARD       Gene Ontology:     BioCarta Pathways:     KEGG Pathways:  
    Probe set : 1389061_at    Name:   similar to NOL1R protein; Williams-Beuren syndrome critical region protein 20 (predicted)    GB acc:   AI171164    UniGene:   Rn.128392    Gene Symbol:   RGD1309268_predicted    Locus Link:   288595    Chromosome:   12    Cytoband:   Chr:12q12    SOURCE    GENECARD       Gene Ontology:     BioCarta Pathways:     KEGG Pathways:  
    Probe set : 1367702_at    Name:   acetyl-Coenzyme A dehydrogenase, medium chain    GB acc:   NM_016986    UniGene:   Rn.6302    Gene Symbol:   Acadm    Locus Link:   24158    Chromosome:   2    Cytoband:   Chr:2q45    SOURCE    GENECARD       Gene Ontology:     BioCarta Pathways:     KEGG Pathways:  
    Probe set : 1384591_at    Name:   Similar to hypothetical protein FLJ32884 (predicted)    GB acc:   BG374055    UniGene:   Rn.105776     Locus Link:   310689    Chromosome:   2    Cytoband:   Chr:2q34    SOURCE        Gene Ontology:     BioCarta Pathways:     KEGG Pathways:  
    Probe set : 1368808_at    Name:   CAP, adenylate cyclase-associated protein 1 (yeast)    GB acc:   NM_022383    UniGene:   Rn.112585    Gene Symbol:   Cap1    Locus Link:   64185    Chromosome:   5    Cytoband:   Chr:5q36    SOURCE    GENECARD       Gene Ontology:   cellular component:  cytoplasm   cellular component:  membrane   cellular component:  cortical actin cytoskeleton   molecular function:  adenylate cyclase binding   molecular function:  actin binding      BioCarta Pathways:     KEGG Pathways:  
    Probe set : 1388494_at    Name:   Procollagen, type IV, alpha 2 (predicted)    GB acc:   BI281705    UniGene:   Rn.2237     Locus Link:   306628    Chromosome:   16    Cytoband:   Chr:16q12.5    SOURCE        Gene Ontology:   biological process:  phosphate transport   biological process:  cell adhesion   biological process:  extracellular matrix organization and biogenesis      BioCarta Pathways:     KEGG Pathways:  
    Probe set : 1372132_at    Name:   CNDP dipeptidase 2 (metallopeptidase M20 family) (predicted)    GB acc:   BI279729    UniGene:   Rn.98790    Gene Symbol:   Cndp2_predicted    Locus Link:   291394    Chromosome:   18    Cytoband:   Chr:18q12.3    SOURCE    GENECARD       Gene Ontology:   biological process:  proteolysis   cellular component:  cytosol      BioCarta Pathways:     KEGG Pathways:  
    Probe set : 1370166_at    Name:   syndecan 2    GB acc:   AI169682    UniGene:   Rn.11127    Gene Symbol:   Sdc2    Locus Link:   25615    Chromosome:   7    Cytoband:   Chr:7q22    SOURCE    GENECARD       Gene Ontology:   cellular component:  integral to plasma membrane   cellular component:  membrane   cellular component:  integral to membrane   molecular function:  cytoskeletal protein binding      BioCarta Pathways:     KEGG Pathways:  
    Probe set : 1388306_at    Name:   similar to RIKEN cDNA 1810042K04 (predicted)    GB acc:   BM383758    UniGene:   Rn.2926    Gene Symbol:   RGD1305593_predicted    Locus Link:   288513    Chromosome:   12    Cytoband:   Chr:12q11    SOURCE    GENECARD       Gene Ontology:     BioCarta Pathways:     KEGG Pathways:  
    Probe set : 1389154_at    Name:   lipoic acid synthetase (predicted)    GB acc:   AW524453    UniGene:   Rn.25547    Gene Symbol:   Lias_predicted    Locus Link:   305348    Chromosome:   14    Cytoband:   Chr:14p11    SOURCE    GENECARD       Gene Ontology:   biological process:  lipoic acid biosynthesis   biological process:  lipoate biosynthesis   cellular component:  mitochondrion      BioCarta Pathways:     KEGG Pathways:  
    Probe set : 1373975_at    Name:   similar to thioether S-methyltransferase    GB acc:   AI232716    UniGene:   Rn.19133    Gene Symbol:   LOC368066    Locus Link:   368066    Chromosome:   4    Cytoband:   Chr:4q24    SOURCE    GENECARD       Gene Ontology:     BioCarta Pathways:     KEGG Pathways:  
    Probe set : 1372997_at    Name:   Transcribed locus    GB acc:   AI105243    UniGene:   Rn.137692        SOURCE        Gene Ontology:     BioCarta Pathways:     KEGG Pathways:  
    Probe set : 1375277_at    Name:   similar to hypothetical protein FLJ20433    GB acc:   AA800680    UniGene:   Rn.22790    Gene Symbol:   LOC499745    Locus Link:   499745    Chromosome:   3    Cytoband:   Chr:3p13    SOURCE    GENECARD       Gene Ontology:     BioCarta Pathways:     KEGG Pathways:  
    Probe set : 1378917_at    Name:   Transcription elongation factor B polypeptide 3 binding protein 1 (predicted)    GB acc:   BF406470    UniGene:   Rn.12613     Locus Link:   314630    Chromosome:   7    Cytoband:   Chr:7q11    SOURCE        Gene Ontology:   cellular component:  intracellular   cellular component:  nucleus      BioCarta Pathways:     KEGG Pathways:  
    Probe set : 1371908_at    Name:   NTF2-related export protein 1 (predicted)    GB acc:   AA891920    UniGene:   Rn.8541    Gene Symbol:   Nxt1_predicted    Locus Link:   296219    Chromosome:   3    Cytoband:   Chr:3q41    SOURCE    GENECARD       Gene Ontology:   cellular component:  intracellular   cellular component:  nucleus   cellular component:  nuclear pore   cellular component:  cytoplasm   molecular function:  protein binding   molecular function:  Ran GTPase binding   molecular function:  protein transporter activity      BioCarta Pathways:     KEGG Pathways:  
    Probe set : 1393405_at    Name:   Transcribed locus    GB acc:   AA925444    UniGene:   Rn.8224        SOURCE        Gene Ontology:     BioCarta Pathways:     KEGG Pathways:  
    Probe set : 1373198_at    Name:   similar to RIKEN cDNA 2810451A06 (predicted)    GB acc:   BF283018    UniGene:   Rn.19564    Gene Symbol:   RGD1311098_predicted    Locus Link:   362998    Chromosome:   7    Cytoband:   Chr:7q36    SOURCE    GENECARD       Gene Ontology:   biological process:  protein folding   cellular component:  integral to membrane   molecular function:  unfolded protein binding   molecular function:  heat shock protein binding      BioCarta Pathways:     KEGG Pathways:  
    Probe set : 1382751_at     GB acc:   BF549536              Gene Ontology:     BioCarta Pathways:     KEGG Pathways:  
    Probe set : 1380321_at    Name:   mitochondrial tumor suppressor 1    GB acc:   BI287786    UniGene:   Rn.95344    Gene Symbol:   Mtus1    Locus Link:   306487    Chromosome:   16    Cytoband:   Chr:16q12.1    SOURCE    GENECARD       Gene Ontology:   molecular function:  receptor activity      BioCarta Pathways:     KEGG Pathways:  
    Probe set : 1372903_at    Name:   similar to 3000004C01Rik protein (predicted)    GB acc:   AI410054    UniGene:   Rn.112739    Gene Symbol:   RGD1310360_predicted    Locus Link:   303575    Chromosome:   10    Cytoband:   Chr:10q32.1    SOURCE    GENECARD       Gene Ontology:   biological process:  microtubule-based movement   cellular component:  microtubule associated complex   molecular function:  motor activity   molecular function:  ATP binding   molecular function:  microtubule motor activity      BioCarta Pathways:     KEGG Pathways:  
    Probe set : 1370367_at    Name:   solute carrier family 1 (neuronal/epithelial high affinity glutamate transporter, system Xag), member 1    GB acc:   D63772    UniGene:   Rn.6384    Gene Symbol:   Slc1a1    Locus Link:   25550    Chromosome:   1    Cytoband:   Chr:1q52    SOURCE    GENECARD       Gene Ontology:     BioCarta Pathways:     KEGG Pathways:  
    Probe set : 1378307_at    Name:   Pleckstrin homology domain containing, family H (with MyTH4 domain) member 1 (predicted)    GB acc:   AI412126    UniGene:   Rn.35024     Locus Link:   314262    Chromosome:   6    Cytoband:   Chr:6q24    SOURCE        Gene Ontology:   biological process:  cell adhesion   cellular component:  cytoskeleton      BioCarta Pathways:     KEGG Pathways:  
    Probe set : 1384334_at     GB acc:   AI547761              Gene Ontology:     BioCarta Pathways:     KEGG Pathways:  
    Probe set : 1385143_at     GB acc:   AI548940              Gene Ontology:     BioCarta Pathways:     KEGG Pathways:  
    Probe set : 1380182_at    Name:   similar to KIAA1217    GB acc:   BF291123    UniGene:   Rn.43612    Gene Symbol:   LOC291356    Locus Link:   291356    Chromosome:   17    Cytoband:   Chr:17q12.3    SOURCE    GENECARD       Gene Ontology:     BioCarta Pathways:     KEGG Pathways:  
    Probe set : 1374582_at    Name:   potassium channel tetramerisation domain containing 9 (predicted)    GB acc:   AI175423    UniGene:   Rn.9317    Gene Symbol:   Kctd9_predicted    Locus Link:   364410    Chromosome:   15    Cytoband:   Chr:15p12    SOURCE    GENECARD       Gene Ontology:   biological process:  potassium ion transport   cellular component:  voltage-gated potassium channel complex   cellular component:  membrane   molecular function:  voltage-gated potassium channel activity   molecular function:  protein binding      BioCarta Pathways:     KEGG Pathways:  
    Probe set : 1384378_at    Name:   HCR (a-helix coiled-coil rod homolog)    GB acc:   BI293046    UniGene:   Rn.127116    Gene Symbol:   RGD:1302992    Locus Link:   406196    Chromosome:   20    Cytoband:   Chr:20p12    SOURCE    GENECARD       Gene Ontology:     BioCarta Pathways:     KEGG Pathways:  
    Probe set : 1368522_at    Name:   timeless homolog (Drosophila)    GB acc:   NM_031340    UniGene:   Rn.31755    Gene Symbol:   Timeless    Locus Link:   83508    Chromosome:   7    Cytoband:   Chr:7q11    SOURCE    GENECARD       Gene Ontology:   cellular component:  nucleus   cellular component:  nucleus      BioCarta Pathways:     KEGG Pathways:  
    Probe set : 1376605_at    Name:   solute carrier family 17 (anion/sugar transporter), member 5 (predicted)    GB acc:   AA900983    UniGene:   Rn.74591    Gene Symbol:   Slc17a5_predicted    Locus Link:   363103    Chromosome:   8    Cytoband:   Chr:8q31    SOURCE    GENECARD       Gene Ontology:   biological process:  transport   biological process:  sialic acid transport      BioCarta Pathways:     KEGG Pathways:  
    Probe set : 1367768_at    Name:   latexin    GB acc:   NM_031655    UniGene:   Rn.11404    Gene Symbol:   Lxn    Locus Link:   59073    Chromosome:   2    Cytoband:   Chr:2q31    SOURCE    GENECARD       Gene Ontology:   molecular function:  enzyme inhibitor activity   molecular function:  metalloendopeptidase inhibitor activity      BioCarta Pathways:     KEGG Pathways:  
    Probe set : 1373392_at    Name:   TPA regulated locus (predicted)    GB acc:   AW523924    UniGene:   Rn.2779    Gene Symbol:   Tparl_predicted    Locus Link:   364137    Chromosome:   14    Cytoband:   Chr:14p11    SOURCE    GENECARD       Gene Ontology:   cellular component:  extracellular space   cellular component:  membrane   cellular component:  integral to membrane      BioCarta Pathways:     KEGG Pathways:  
    Probe set : 1374630_at    Name:   chloride intracellular channel 3 (predicted)    GB acc:   AI234249    UniGene:   Rn.25003    Gene Symbol:   Clic3_predicted    Locus Link:   296566    Chromosome:   3    Cytoband:   Chr:3p13    SOURCE    GENECARD       Gene Ontology:   cellular component:  membrane fraction   cellular component:  nucleus   cellular component:  membrane      BioCarta Pathways:     KEGG Pathways:  
    Probe set : 1387109_at    Name:   P450 (cytochrome) oxidoreductase    GB acc:   NM_031576    UniGene:   Rn.11359    Gene Symbol:   Por    Locus Link:   29441    Chromosome:   12    Cytoband:   Chr:12q12    SOURCE    GENECARD       Gene Ontology:   biological process:  electron transport      BioCarta Pathways:     KEGG Pathways:  
    Probe set : 1392906_at    Name:   similar to Ubiquitin-like protein 4 (Ubiquitin-like protein GDX)    GB acc:   AW528860    UniGene:   Rn.1138    Gene Symbol:   LOC293864    Locus Link:   293864    Chromosome:   X    Cytoband:   Chr:Xq37    SOURCE    GENECARD       Gene Ontology:     BioCarta Pathways:     KEGG Pathways:  
    Probe set : 1383202_at    Name:   similar to U6 snRNA-associated Sm-like protein LSm6 (Sm protein F)    GB acc:   AA818406     Gene Symbol:   LOC498934    Locus Link:   498934    Chromosome:   19    Cytoband:   Chr:19q11     GENECARD       Gene Ontology:     BioCarta Pathways:     KEGG Pathways:  
    Probe set : 1376120_at    Name:   similar to pancreatitis-induced protein 49 (predicted)    GB acc:   BG671875    UniGene:   Rn.92162    Gene Symbol:   RGD1308600_predicted    Locus Link:   362090    Chromosome:   3    Cytoband:   Chr:3p13    SOURCE    GENECARD       Gene Ontology:     BioCarta Pathways:     KEGG Pathways:  
    Probe set : 1389354_at    Name:   similar to semaF cytoplasmic domain associated protein 2    GB acc:   BF285490    UniGene:   Rn.20103    Gene Symbol:   LOC362057    Locus Link:   362057    Chromosome:   2    Cytoband:   Chr:2q45    SOURCE    GENECARD       Gene Ontology:   molecular function:  protein binding      BioCarta Pathways:     KEGG Pathways:  
    Probe set : 1378640_at    Name:   ubiquitin-like, containing PHD and RING finger domains, 1    GB acc:   BE098732    UniGene:   Rn.54318    Gene Symbol:   Uhrf1    Locus Link:   316129    Chromosome:   9    Cytoband:   Chr:9q11    SOURCE    GENECARD       Gene Ontology:   cellular component:  nucleus   cellular component:  ubiquitin ligase complex      BioCarta Pathways:     KEGG Pathways:  
    Probe set : 1376726_at    Name:   similar to Carbonic anhydrase-related protein 10 (Carbonic anhydrase-related protein X) (CARP X) (CA-RP X)    GB acc:   AI101117    UniGene:   Rn.12712    Gene Symbol:   LOC360599    Locus Link:   360599    Chromosome:   10    Cytoband:   Chr:10q26    SOURCE    GENECARD       Gene Ontology:     BioCarta Pathways:     KEGG Pathways:  
    Probe set : 1374574_at    Name:   Similar to hypothetical protein MGC38960 (predicted)    GB acc:   AI547611    UniGene:   Rn.23973     Locus Link:   300836    Chromosome:   8    Cytoband:   Chr:8q24    SOURCE        Gene Ontology:     BioCarta Pathways:     KEGG Pathways:  
    Probe set : 1372247_at    Name:   dolichyl-di-phosphooligosaccharide-protein glycotransferase (predicted)    GB acc:   BF282907    UniGene:   Rn.102842    Gene Symbol:   Ddost_predicted    Locus Link:   313648    Chromosome:   5    Cytoband:   Chr:5q36    SOURCE    GENECARD       Gene Ontology:   biological process:  protein amino acid N-linked glycosylation via asparagine   molecular function:  dolichyl-diphosphooligosaccharide-protein glycotransferase activity   molecular function:  transferase activity      BioCarta Pathways:     KEGG Pathways:  
    Probe set : 1395396_at    Name:   similar to CG9135-PA (predicted)    GB acc:   AA848942    UniGene:   Rn.98282    Gene Symbol:   RGD1309986_predicted    Locus Link:   298594    Chromosome:   5    Cytoband:   Chr:5q36    SOURCE    GENECARD       Gene Ontology:     BioCarta Pathways:     KEGG Pathways:  
    Probe set : 1369153_at    Name:   nephrosis 1 homolog, nephrin (human)    GB acc:   NM_022628    UniGene:   Rn.48745    Gene Symbol:   Nphs1    Locus Link:   64563      SOURCE    GENECARD       Gene Ontology:   biological process:  MAPKKK cascade   biological process:  cell adhesion   biological process:  JNK cascade   biological process:  excretion   molecular function:  protein binding      BioCarta Pathways:     KEGG Pathways:  
    Probe set : 1383958_at    Name:   cell division cycle associated 2 (predicted)    GB acc:   BG381524    UniGene:   Rn.49950    Gene Symbol:   Cdca2_predicted    Locus Link:   305984    Chromosome:   15    Cytoband:   Chr:15p12    SOURCE    GENECARD       Gene Ontology:     BioCarta Pathways:     KEGG Pathways:  
    Probe set : 1378482_at    Name:   kidney cell line derived transcript 1 (predicted)    GB acc:   AI411065    UniGene:   Rn.16336    Gene Symbol:   Kdt1_predicted    Locus Link:   362325    Chromosome:   4    Cytoband:   Chr:4q21    SOURCE    GENECARD       Gene Ontology:     BioCarta Pathways:     KEGG Pathways:  
    Probe set : 1377194_a_at    Name:   similar to RIKEN cDNA 2310015N07 (predicted)    GB acc:   BE107471    UniGene:   Rn.145915    Gene Symbol:   RGD1308637_predicted    Locus Link:   308820    Chromosome:   1    Cytoband:   Chr:1q32    SOURCE    GENECARD       Gene Ontology:     BioCarta Pathways:     KEGG Pathways:  
    Probe set : 1376341_at    Name:   Transcribed locus    GB acc:   BM388895    UniGene:   Rn.28603        SOURCE        Gene Ontology:     BioCarta Pathways:     KEGG Pathways:  
    Probe set : 1386904_a_at    Name:   cytochrome b-5    GB acc:   AF007107    UniGene:   Rn.1055    Gene Symbol:   Cyb5    Locus Link:   64001    Chromosome:   18    Cytoband:   Chr:18q12.3    SOURCE    GENECARD       Gene Ontology:   biological process:  electron transport   biological process:  fatty acid metabolism   biological process:  transport      BioCarta Pathways:     KEGG Pathways:  
    Probe set : 1369401_at    Name:   solute carrier family 21, member 13    GB acc:   NM_130736     Gene Symbol:   Slc21a13    Locus Link:   84608    Chromosome:   4    Cytoband:   Chr:4q44     GENECARD       Gene Ontology:   biological process:  transport   biological process:  ion transport   cellular component:  extracellular space   cellular component:  membrane   cellular component:  integral to membrane   molecular function:  transporter activity      BioCarta Pathways:     KEGG Pathways:  
    Probe set : 1375538_at    Name:   Vinculin (predicted)    GB acc:   AI230737    UniGene:   Rn.72459     Locus Link:   305679    Chromosome:   15    Cytoband:   Chr:15p16    SOURCE        Gene Ontology:     BioCarta Pathways:     KEGG Pathways:  
    Probe set : 1375732_at    Name:   Transcribed locus    GB acc:   BE109509    UniGene:   Rn.2378        SOURCE        Gene Ontology:     BioCarta Pathways:     KEGG Pathways:  
    Probe set : 1370421_a_at    Name:   TBP-interacting protein 120B    GB acc:   AB029342    UniGene:   Rn.118129    Gene Symbol:   Tip120B    Locus Link:   192226    Chromosome:   4    Cytoband:   Chr:4q42    SOURCE    GENECARD       Gene Ontology:   biological process:  transcription   biological process:  regulation of transcription, DNA-dependent   biological process:  ubiquitin cycle   cellular component:  intracellular   cellular component:  nucleus   cellular component:  nucleus      BioCarta Pathways:     KEGG Pathways:  
    Probe set : 1382325_at    Name:   glycine C-acetyltransferase (2-amino-3-ketobutyrate-coenzyme A ligase) (predicted)    GB acc:   AW525471    UniGene:   Rn.43940    Gene Symbol:   Gcat_predicted    Locus Link:   366959    Chromosome:   7    Cytoband:   Chr:7q34    SOURCE    GENECARD       Gene Ontology:   cellular component:  mitochondrion      BioCarta Pathways:     KEGG Pathways:  
    Probe set : 1368096_at    Name:   Ras-related GTP-binding protein Rab29    GB acc:   NM_133590    UniGene:   Rn.4323    Gene Symbol:   Rab7l1    Locus Link:   171122      SOURCE    GENECARD       Gene Ontology:   biological process:  intracellular protein transport   biological process:  small GTPase mediated signal transduction   biological process:  protein transport      BioCarta Pathways:     KEGG Pathways:  
    Probe set : 1382865_at    Name:   testis specific, 14    GB acc:   BM390858    UniGene:   Rn.11914    Gene Symbol:   Tsga14    Locus Link:   500069    Chromosome:   4    Cytoband:   Chr:4q22    SOURCE    GENECARD       Gene Ontology:     BioCarta Pathways:     KEGG Pathways:  
    Probe set : 1396739_at     GB acc:   BE120455              Gene Ontology:     BioCarta Pathways:     KEGG Pathways:  
    Probe set : 1393451_at    Name:   similar to RIKEN cDNA 2610510J17 (predicted)    GB acc:   BI295149    UniGene:   Rn.65502    Gene Symbol:   RGD1310953_predicted    Locus Link:   361416    Chromosome:   19    Cytoband:   Chr:19q12    SOURCE    GENECARD       Gene Ontology:     BioCarta Pathways:     KEGG Pathways:  
    Probe set : 1389503_at    Name:   similar to angiopoietin 1 /// similar to uroplakin 3B isoform b    GB acc:   BI285447    UniGene:   Rn.9193    Gene Symbol:   LOC288748 /// LOC367994    Locus Link:   288748 /// 367994    Chromosome:   12    Cytoband:   Chr:12q12    SOURCE    GENECARD       Gene Ontology:     BioCarta Pathways:     KEGG Pathways:  
    Probe set : 1398807_at    Name:   protein phosphatase 1B, magnesium dependent, beta isoform    GB acc:   NM_033096    UniGene:   Rn.4143    Gene Symbol:   Ppm1b    Locus Link:   24667    Chromosome:   6    Cytoband:   Chr:6q12    SOURCE    GENECARD       Gene Ontology:   biological process:  protein amino acid dephosphorylation   cellular component:  protein serine/threonine phosphatase complex      BioCarta Pathways:     KEGG Pathways:  
    Probe set : 1381925_x_at    Name:   AT rich interactive domain 1B (Swi1 like) /// hypothetical gene supported by NM_172157    GB acc:   AI412401    UniGene:   Rn.22598    Gene Symbol:   Arid1b /// LOC497729    Locus Link:   282546 /// 497729    Chromosome:   1    Cytoband:   Chr:1q11    SOURCE    GENECARD       Gene Ontology:   cellular component:  intracellular   cellular component:  SWI/SNF complex   cellular component:  SWI/SNF complex      BioCarta Pathways:     KEGG Pathways:  
    Probe set : 1379889_at    Name:   lamimin, gamma 2    GB acc:   AW527269    UniGene:   Rn.9278    Gene Symbol:   Lamc2    Locus Link:   192362    Chromosome:   13    Cytoband:   Chr:13q21    SOURCE    GENECARD       Gene Ontology:   biological process:  cell adhesion   cellular component:  extracellular matrix (sensu Metazoa)   cellular component:  laminin-2   cellular component:  extracellular matrix      BioCarta Pathways:     KEGG Pathways:  
    Probe set : 1389506_x_at     GB acc:   BI298458              Gene Ontology:     BioCarta Pathways:     KEGG Pathways:  
    Probe set : 1371680_at     GB acc:   BI303631              Gene Ontology:     BioCarta Pathways:     KEGG Pathways:  
    Probe set : 1387872_at    Name:   heterogeneous nuclear ribonucleoprotein A1    GB acc:   BF396270    UniGene:   Rn.25771    Gene Symbol:   Hnrpa1    Locus Link:   29578    Chromosome:   7    Cytoband:   Chr:7q36    SOURCE    GENECARD       Gene Ontology:   cellular component:  nucleus   cellular component:  nucleus   cellular component:  ribonucleoprotein complex      BioCarta Pathways:     KEGG Pathways:  
    Probe set : 1372282_at    Name:   similar to RIKEN cDNA 2310016C16 (predicted)    GB acc:   BF283238    UniGene:   Rn.106264    Gene Symbol:   RGD1307506_predicted    Locus Link:   294744    Chromosome:   2    Cytoband:   Chr:2q14    SOURCE    GENECARD       Gene Ontology:     BioCarta Pathways:     KEGG Pathways:  
    Probe set : 1385086_at    Name:   budding uninhibited by benzimidazoles 1 homolog (S. cerevisiae) (predicted)    GB acc:   BF388785    UniGene:   Rn.56816    Gene Symbol:   Bub1_predicted    Locus Link:   296137    Chromosome:   3    Cytoband:   Chr:3q36    SOURCE    GENECARD       Gene Ontology:     BioCarta Pathways:     KEGG Pathways:  
    Probe set : 1377794_at    Name:   Transcribed locus    GB acc:   BF288862    UniGene:   Rn.41483        SOURCE        Gene Ontology:     BioCarta Pathways:     KEGG Pathways:  
    Probe set : 1367974_at    Name:   annexin A3    GB acc:   NM_012823    UniGene:   Rn.6589    Gene Symbol:   Anxa3    Locus Link:   25291    Chromosome:   14    Cytoband:   Chr:14p22    SOURCE    GENECARD       Gene Ontology:   cellular component:  cytoplasm      BioCarta Pathways:     KEGG Pathways:  
    Probe set : 1388629_at    Name:   inosine 5-monophosphate dehydrogenase 2    GB acc:   BF281278    UniGene:   Rn.8093    Gene Symbol:   Impdh2    Locus Link:   301005    Chromosome:   8    Cytoband:   Chr:8q32    SOURCE    GENECARD       Gene Ontology:   biological process:  GMP biosynthesis   molecular function:  IMP dehydrogenase activity   molecular function:  catalytic activity   molecular function:  IMP dehydrogenase activity      BioCarta Pathways:     KEGG Pathways:  
    Probe set : 1375723_at    Name:   Transcribed locus    GB acc:   AI385171    UniGene:   Rn.21939        SOURCE        Gene Ontology:     BioCarta Pathways:     KEGG Pathways:  
    Probe set : 1367835_at    Name:   proprotein convertase subtilisin/kexin type 1 inhibitor    GB acc:   NM_019279    UniGene:   Rn.25745    Gene Symbol:   Pcsk1n    Locus Link:   246333    Chromosome:   X    Cytoband:   Chr:Xq13    SOURCE    GENECARD       Gene Ontology:   biological process:  peptide hormone processing   biological process:  peptide hormone processing      BioCarta Pathways:     KEGG Pathways:  
    Probe set : 1382447_at     GB acc:   AI170441              Gene Ontology:     BioCarta Pathways:     KEGG Pathways:  
    Probe set : 1382203_at    Name:   growth differentiation factor 1 (predicted)    GB acc:   AI549010    UniGene:   Rn.144974    Gene Symbol:   Gdf1_predicted    Locus Link:   306351    Chromosome:   16    Cytoband:   Chr:16p14    SOURCE    GENECARD       Gene Ontology:   cellular component:  extracellular region   molecular function:  cytokine activity   molecular function:  growth factor activity      BioCarta Pathways:     KEGG Pathways:  
    Probe set : 1389785_at    Name:   similar to RIKEN cDNA 0610006H10 gene (predicted)    GB acc:   AA892933    UniGene:   Rn.22552    Gene Symbol:   RGD1305394_predicted    Locus Link:   293653    Chromosome:   1    Cytoband:   Chr:1q42    SOURCE    GENECARD       Gene Ontology:   biological process:  metabolism   molecular function:  hydrolase activity   molecular function:  hydrolase activity, acting on ester bonds      BioCarta Pathways:     KEGG Pathways:  
    Probe set : 1392128_at    Name:   Transcribed locus    GB acc:   BE104541    UniGene:   Rn.50647        SOURCE        Gene Ontology:     BioCarta Pathways:     KEGG Pathways:  
    Probe set : 1399091_at    Name:   F-actin capping protein beta subunit    GB acc:   AI232101    UniGene:   Rn.105999    Gene Symbol:   RGD:1359099    Locus Link:   298584    Chromosome:   5    Cytoband:   Chr:5q36    SOURCE    GENECARD       Gene Ontology:   biological process:  actin cytoskeleton organization and biogenesis   cellular component:  F-actin capping protein complex   molecular function:  actin binding      BioCarta Pathways:     KEGG Pathways:  
    Probe set : 1384638_at    Name:   Similar to RIKEN cDNA 1700013H19 (predicted)    GB acc:   AI030545    UniGene:   Rn.145286     Locus Link:   364976    Chromosome:   19    Cytoband:   Chr:19q11    SOURCE        Gene Ontology:     BioCarta Pathways:     KEGG Pathways:  
    Probe set : 1389440_at    Name:   Transcribed locus    GB acc:   AI178476    UniGene:   Rn.16546        SOURCE        Gene Ontology:     BioCarta Pathways:     KEGG Pathways:  
    Probe set : 1378731_at    Name:   similar to CDNA sequence BC006909    GB acc:   BF404871    UniGene:   Rn.7518    Gene Symbol:   LOC499267    Locus Link:   499267    Chromosome:   1    Cytoband:   Chr:1q36    SOURCE    GENECARD       Gene Ontology:     BioCarta Pathways:     KEGG Pathways:  
    Probe set : 1379861_at    Name:   similar to RIKEN cDNA 6720485C15    GB acc:   BE107793    UniGene:   Rn.102152    Gene Symbol:   RGD:1359211    Locus Link:   360639    Chromosome:   10    Cytoband:   Chr:10q32.1    SOURCE    GENECARD       Gene Ontology:   biological process:  nucleosome assembly   biological process:  chromosome organization and biogenesis (sensu Eukaryota)   cellular component:  nucleosome   cellular component:  nucleus   molecular function:  DNA binding   molecular function:  ATP binding      BioCarta Pathways:     KEGG Pathways:  
    Probe set : 1374855_at    Name:   period homolog 1 (Drosophila)    GB acc:   BI279017    UniGene:   Rn.34433    Gene Symbol:   Per1    Locus Link:   287422    Chromosome:   10    Cytoband:   Chr:10q24    SOURCE    GENECARD       Gene Ontology:   cellular component:  nucleus   molecular function:  protein binding   molecular function:  signal transducer activity      BioCarta Pathways:     KEGG Pathways:  
    Probe set : 1388851_at    Name:   heat shock protein, A (predicted)    GB acc:   BI282281    UniGene:   Rn.7535    Gene Symbol:   Hspa9a_predicted    Locus Link:   291671    Chromosome:   18    Cytoband:   Chr:18p12    SOURCE    GENECARD       Gene Ontology:   cellular component:  cytoplasm   cellular component:  mitochondrion   molecular function:  protein binding   molecular function:  ATP binding   molecular function:  unfolded protein binding      BioCarta Pathways:     KEGG Pathways:  
    Probe set : 1389674_at    Name:   zinc finger protein 282 (predicted)    GB acc:   BI289654    UniGene:   Rn.9537    Gene Symbol:   Zfp282_predicted    Locus Link:   297065    Chromosome:   4    Cytoband:   Chr:4q24    SOURCE    GENECARD       Gene Ontology:   biological process:  regulation of transcription, DNA-dependent   biological process:  microtubule-based movement   cellular component:  intracellular   cellular component:  nucleus   cellular component:  microtubule      BioCarta Pathways:     KEGG Pathways:  
    Probe set : 1379260_at    Name:   Transcribed locus    GB acc:   BE115626    UniGene:   Rn.8167        SOURCE        Gene Ontology:     BioCarta Pathways:     KEGG Pathways:  
    Probe set : 1392900_at    Name:   Similar to mKIAA1631 protein    GB acc:   AW888327    UniGene:   Rn.13838     Locus Link:   310756    Chromosome:   2    Cytoband:   Chr:2q34    SOURCE        Gene Ontology:     BioCarta Pathways:     KEGG Pathways:  
    Probe set : 1369728_at    Name:   histone 1, H4m (predicted)    GB acc:   NM_022686     Gene Symbol:   Hist1h4m_predicted    Locus Link:   291152    Chromosome:   17    Cytoband:   Chr:17q11     GENECARD       Gene Ontology:     BioCarta Pathways:     KEGG Pathways:  
    Probe set : 1383605_at    Name:   similar to alpha-fetoprotein    GB acc:   BE119842    UniGene:   Rn.30321    Gene Symbol:   LOC360919    Locus Link:   360919    Chromosome:   14    Cytoband:   Chr:14p22    SOURCE    GENECARD       Gene Ontology:   biological process:  transport   cellular component:  extracellular space   molecular function:  carrier activity      BioCarta Pathways:     KEGG Pathways:  
    Probe set : 1381986_at    Name:   Neuron navigator 1 (predicted)    GB acc:   BE112452    UniGene:   Rn.45998     Locus Link:   304818    Chromosome:   13    Cytoband:   Chr:13q13    SOURCE        Gene Ontology:   biological process:  DNA methylation      BioCarta Pathways:     KEGG Pathways:  
    Probe set : 1374799_at    Name:   similar to mKIAA0159 protein    GB acc:   AI407418    UniGene:   Rn.7870    Gene Symbol:   LOC362438    Locus Link:   362438    Chromosome:   4    Cytoband:   Chr:4q42    SOURCE    GENECARD       Gene Ontology:     BioCarta Pathways:     KEGG Pathways:  
    Probe set : 1370057_at    Name:   cysteine and glycine-rich protein 1    GB acc:   NM_017148    UniGene:   Rn.108075    Gene Symbol:   Csrp1    Locus Link:   29276    Chromosome:   13    Cytoband:   Chr:13q13    SOURCE    GENECARD       Gene Ontology:   biological process:  nervous system development   biological process:  actin cytoskeleton organization and biogenesis   cellular component:  nucleus   cellular component:  actin cytoskeleton      BioCarta Pathways:     KEGG Pathways:  
    Probe set : 1377064_at    Name:   dual specificity phosphatase 6    GB acc:   AI602811    UniGene:   Rn.4313    Gene Symbol:   Dusp6    Locus Link:   116663    Chromosome:   7    Cytoband:   Chr:7q13    SOURCE    GENECARD       Gene Ontology:   cellular component:  soluble fraction   cellular component:  cytoplasm   cellular component:  cytoplasm   molecular function:  protein phosphatase type 2A activity   molecular function:  protein phosphatase type 1 activity   molecular function:  phosphoprotein phosphatase activity   molecular function:  protein serine/threonine phosphatase activity   molecular function:  calcium-dependent protein serine/threonine phosphatase activity   molecular function:  magnesium-dependent protein serine/threonine phosphatase activity   molecular function:  protein tyrosine phosphatase activity   molecular function:  protein tyrosine/serine/threonine phosphatase activity   molecular function:  protein tyrosine/serine/threonine phosphatase activity   molecular function:  CTD phosphatase activity   molecular function:  protein phosphatase type 2C activity   molecular function:  hydrolase activity   molecular function:  MAP kinase phosphatase activity   molecular function:  myosin phosphatase activity   molecular function:  protein phosphatase type 2B activity      BioCarta Pathways:     KEGG Pathways:  
    Probe set : 1382128_at    Name:   Transcribed locus    GB acc:   BI289196    UniGene:   Rn.144313        SOURCE        Gene Ontology:     BioCarta Pathways:     KEGG Pathways:  
    Probe set : 1371518_at    Name:   Nidogen (entactin)    GB acc:   AI235948    UniGene:   Rn.105658    Gene Symbol:   Nid    Locus Link:   25494    Chromosome:   17    Cytoband:   Chr:17q12.1    SOURCE    GENECARD       Gene Ontology:   biological process:  cell adhesion   biological process:  cell-matrix adhesion   molecular function:  extracellular matrix structural constituent   molecular function:  calcium ion binding   molecular function:  protein binding      BioCarta Pathways:     KEGG Pathways:  
    Probe set : 1375767_at    Name:   similar to common-site lymphoma leukemia guanine nucleotide exchange factor like (5N754) (predicted)    GB acc:   BI287681    UniGene:   Rn.19912    Gene Symbol:   RGD1308089_predicted    Locus Link:   314249    Chromosome:   6    Cytoband:   Chr:6q24    SOURCE    GENECARD       Gene Ontology:     BioCarta Pathways:     KEGG Pathways:  
    Probe set : 1374537_at    Name:   Carbohydrate (chondroitin) synthase 1 (predicted)    GB acc:   AI406533    UniGene:   Rn.13788     Locus Link:   292999    Chromosome:   1    Cytoband:   Chr:1q22    SOURCE        Gene Ontology:   biological process:  chondroitin sulfate biosynthesis   molecular function:  glucuronosyl-N-acetylgalactosaminyl-proteoglycan 4-beta-N-acetylgalactosaminyltransferase activity      BioCarta Pathways:     KEGG Pathways:  
    Probe set : 1377151_at    Name:   Transcribed locus    GB acc:   AI102833    UniGene:   Rn.22330        SOURCE        Gene Ontology:     BioCarta Pathways:     KEGG Pathways:  
    Probe set : 1398482_at    Name:   B-cell leukemia/lymphoma 3 (predicted)    GB acc:   AI411774    UniGene:   Rn.68084    Gene Symbol:   Bcl3_predicted    Locus Link:   292700    Chromosome:   1    Cytoband:   Chr:1q21    SOURCE    GENECARD       Gene Ontology:   cellular component:  nucleus   molecular function:  DNA binding   molecular function:  transcription factor activity      BioCarta Pathways:     KEGG Pathways:  
    Probe set : 1382115_at    Name:   similar to RIKEN cDNA 4930521E07 (predicted)    GB acc:   BE099174    UniGene:   Rn.22713    Gene Symbol:   RGD1305166_predicted    Locus Link:   309486    Chromosome:   1    Cytoband:   Chr:1q54    SOURCE    GENECARD       Gene Ontology:     BioCarta Pathways:     KEGG Pathways:  
    Probe set : 1372038_at    Name:   MAP kinase-interacting serine/threonine kinase 2 (predicted)    GB acc:   BM383395    UniGene:   Rn.6769    Gene Symbol:   Mknk2_predicted    Locus Link:   299618    Chromosome:   7    Cytoband:   Chr:7q11    SOURCE    GENECARD       Gene Ontology:     BioCarta Pathways:     KEGG Pathways:  
    Probe set : 1397458_at    Name:   phosphomannomutase 2 (predicted)    GB acc:   AW533978    UniGene:   Rn.58278    Gene Symbol:   Pmm2_predicted    Locus Link:   302915    Chromosome:   10    Cytoband:   Chr:10q12    SOURCE    GENECARD       Gene Ontology:   biological process:  protein amino acid N-linked glycosylation   biological process:  metabolism   biological process:  GDP-mannose biosynthesis   cellular component:  cytoplasm   molecular function:  phosphomannomutase activity   molecular function:  isomerase activity      BioCarta Pathways:     KEGG Pathways:  
    Probe set : 1374272_at    Name:   Transcribed locus    GB acc:   BI280268    UniGene:   Rn.138406        SOURCE        Gene Ontology:     BioCarta Pathways:     KEGG Pathways:  
    Probe set : 1384483_at    Name:   Similar to RIKEN cDNA E030034P13 (predicted)    GB acc:   BI274216    UniGene:   Rn.49088     Locus Link:   360488    Chromosome:   10    Cytoband:   Chr:10q12    SOURCE        Gene Ontology:     BioCarta Pathways:     KEGG Pathways:  
    Probe set : 1382277_at     GB acc:   AA894336              Gene Ontology:     BioCarta Pathways:     KEGG Pathways:  
    Probe set : 1379866_at    Name:   Septin 6 (predicted)    GB acc:   AW532098    UniGene:   Rn.27798     Locus Link:   305227    Chromosome:   14    Cytoband:   Chr:14p22    SOURCE        Gene Ontology:   biological process:  cytokinesis   biological process:  cell cycle   molecular function:  protein binding   molecular function:  GTP binding      BioCarta Pathways:     KEGG Pathways:  
    Probe set : 1388927_at    Name:   RAB, member of RAS oncogene family-like 4 (predicted)    GB acc:   BE109617    UniGene:   Rn.9036    Gene Symbol:   Rabl4_predicted    Locus Link:   300062    Chromosome:   7    Cytoband:   Chr:7q34    SOURCE    GENECARD       Gene Ontology:   biological process:  small GTPase mediated signal transduction   biological process:  protein transport   molecular function:  GTP binding      BioCarta Pathways:     KEGG Pathways:  
    Probe set : 1368234_at    Name:   prolyl endopeptidase    GB acc:   NM_031324    UniGene:   Rn.11058    Gene Symbol:   Prep    Locus Link:   83471    Chromosome:   20    Cytoband:   Chr:20q13    SOURCE    GENECARD       Gene Ontology:   biological process:  proteolysis   biological process:  proteolysis   biological process:  protein metabolism   cellular component:  nucleus   cellular component:  cytoplasm   cellular component:  cytosol      BioCarta Pathways:     KEGG Pathways:  
    Probe set : 1395973_at    Name:   Similar to RIKEN cDNA 5730596K20 (predicted)    GB acc:   AI236099    UniGene:   Rn.140524     Locus Link:   309187    Chromosome:   1    Cytoband:   Chr:1q43    SOURCE        Gene Ontology:     BioCarta Pathways:     KEGG Pathways:  
    Probe set : 1385926_at    Name:   Transcribed locus    GB acc:   BF550565    UniGene:   Rn.23216        SOURCE        Gene Ontology:     BioCarta Pathways:     KEGG Pathways:  
    Probe set : 1388930_at    Name:   similar to RIKEN cDNA 2310075C12 (predicted)    GB acc:   AI171651    UniGene:   Rn.12767    Gene Symbol:   RGD1305625_predicted    Locus Link:   363013    Chromosome:   8    Cytoband:   Chr:8q11    SOURCE    GENECARD       Gene Ontology:   cellular component:  extracellular space   cellular component:  integral to membrane   molecular function:  receptor activity      BioCarta Pathways:     KEGG Pathways:  
    Probe set : 1383372_at    Name:   platelet-activating factor receptor    GB acc:   BF407234    UniGene:   Rn.10137    Gene Symbol:   Ptafr    Locus Link:   58949    Chromosome:   5    Cytoband:   Chr:5q36    SOURCE    GENECARD       Gene Ontology:   cellular component:  integral to plasma membrane   cellular component:  integral to membrane      BioCarta Pathways:     KEGG Pathways:  
    Probe set : 1392127_at    Name:   Transcribed locus    GB acc:   BF400081    UniGene:   Rn.47529        SOURCE        Gene Ontology:     BioCarta Pathways:     KEGG Pathways:  
    Probe set : 1368894_at    Name:   CAP, adenylate cyclase-associated protein, 2 (yeast)    GB acc:   AW919109    UniGene:   Rn.10229    Gene Symbol:   Cap2    Locus Link:   116653      SOURCE    GENECARD       Gene Ontology:   cellular component:  membrane      BioCarta Pathways:     KEGG Pathways:  
    Probe set : 1367761_at    Name:   nudE nuclear distribution gene E homolog like 1 (A. nidulans)    GB acc:   NM_133320    UniGene:   Rn.2947    Gene Symbol:   Ndel1    Locus Link:   170845    Chromosome:   10    Cytoband:   Chr:10q24    SOURCE    GENECARD       Gene Ontology:   biological process:  retrograde axon cargo transport   cellular component:  centrosome   cellular component:  microtubule associated complex   molecular function:  protein binding   molecular function:  protein binding   molecular function:  microtubule binding      BioCarta Pathways:     KEGG Pathways:  
    Probe set : 1390046_at    Name:   a disintegrin and metalloprotease domain 8 (predicted)    GB acc:   BI288110    UniGene:   Rn.138249    Gene Symbol:   Adam8_predicted    Locus Link:   309098    Chromosome:   1    Cytoband:   Chr:1q41    SOURCE    GENECARD       Gene Ontology:     BioCarta Pathways:     KEGG Pathways:  
    Probe set : 1390411_at    Name:   claudin 19 (predicted)    GB acc:   BG378563    UniGene:   Rn.106342    Gene Symbol:   Cldn19_predicted    Locus Link:   298487    Chromosome:   5    Cytoband:   Chr:5q36    SOURCE    GENECARD       Gene Ontology:   cellular component:  tight junction   cellular component:  membrane   cellular component:  integral to membrane   molecular function:  structural molecule activity      BioCarta Pathways:     KEGG Pathways:  
    Probe set : 1377004_at    Name:   Transcribed locus    GB acc:   AA800184    UniGene:   Rn.6294        SOURCE        Gene Ontology:     BioCarta Pathways:     KEGG Pathways:  
    Probe set : 1374903_at    Name:   glucosaminyl (N-acetyl) transferase 2, I-branching enzyme /// hypothetical gene supported by NM_001001511    GB acc:   AI234819    UniGene:   Rn.8807    Gene Symbol:   Gcnt2 /// LOC497712    Locus Link:   306860 /// 497712    Chromosome:   17    Cytoband:   Chr:17p12    SOURCE    GENECARD       Gene Ontology:   cellular component:  membrane   molecular function:  acetylglucosaminyltransferase activity   molecular function:  transferase activity      BioCarta Pathways:     KEGG Pathways:  
    Probe set : 1371344_at    Name:   ribosomal protein L27a (predicted)    GB acc:   AI177054    UniGene:   Rn.144797    Gene Symbol:   Rpl27a_predicted    Locus Link:   293418    Chromosome:   1    Cytoband:   Chr:1q33    SOURCE    GENECARD       Gene Ontology:   biological process:  protein biosynthesis   cellular component:  intracellular   cellular component:  ribosome   cellular component:  cytosolic large ribosomal subunit (sensu Eukaryota)   molecular function:  RNA binding   molecular function:  structural constituent of ribosome      BioCarta Pathways:     KEGG Pathways:  
    Probe set : 1395662_at    Name:   Transcribed locus    GB acc:   AW531934    UniGene:   Rn.153738        SOURCE        Gene Ontology:     BioCarta Pathways:     KEGG Pathways:  
    Probe set : 1371541_at    Name:   Myosin, light polypeptide kinase (predicted)    GB acc:   AI177055    UniGene:   Rn.131171     Locus Link:   288057    Chromosome:   11    Cytoband:   Chr:11q22    SOURCE        Gene Ontology:   biological process:  protein amino acid phosphorylation      BioCarta Pathways:     KEGG Pathways:  
    Probe set : 1390420_at    Name:   carboxypeptidase X 1 (M14 family) (predicted)    GB acc:   AI577849    UniGene:   Rn.19382    Gene Symbol:   Cpxm1_predicted    Locus Link:   296156    Chromosome:   3    Cytoband:   Chr:3q36    SOURCE    GENECARD       Gene Ontology:   biological process:  proteolysis   biological process:  transport   biological process:  cell adhesion   cellular component:  extracellular space   cellular component:  mitochondrial inner membrane      BioCarta Pathways:     KEGG Pathways:  
    Probe set : 1380768_at    Name:   Transcribed locus    GB acc:   BM389322    UniGene:   Rn.121802        SOURCE        Gene Ontology:     BioCarta Pathways:     KEGG Pathways:  
    Probe set : 1391744_at    Name:   Transcribed locus    GB acc:   AI548028    UniGene:   Rn.148269        SOURCE        Gene Ontology:     BioCarta Pathways:     KEGG Pathways:  
    Probe set : 1389447_at    Name:   similar to prolyl-4-hydroxylase-alpha NE2 CG9720-PA (predicted)    GB acc:   AA955470    UniGene:   Rn.101391    Gene Symbol:   RGD1311848_predicted    Locus Link:   301008    Chromosome:   8    Cytoband:   Chr:8q32    SOURCE    GENECARD       Gene Ontology:     BioCarta Pathways:     KEGG Pathways:  
    Probe set : 1382924_at    Name:   pantothenate kinase 1 (predicted)    GB acc:   AA850195    UniGene:   Rn.6581    Gene Symbol:   Pank1_predicted    Locus Link:   294088    Chromosome:   1    Cytoband:   Chr:1q52    SOURCE    GENECARD       Gene Ontology:   biological process:  coenzyme A biosynthesis      BioCarta Pathways:     KEGG Pathways:  
    Probe set : 1371914_at    Name:   SWI/SNF related, matrix associated, actin dependent regulator of chromatin, subfamily b, member 1 (predicted)    GB acc:   AI406553    UniGene:   Rn.139601    Gene Symbol:   Smarcb1_predicted    Locus Link:   361825    Chromosome:   20    Cytoband:   Chr:20p12    SOURCE    GENECARD       Gene Ontology:   cellular component:  nuclear chromosome   cellular component:  nucleus   cellular component:  nucleoplasm   molecular function:  protein binding      BioCarta Pathways:     KEGG Pathways:  
    Probe set : 1367890_at    Name:   caspase 2    GB acc:   NM_022522    UniGene:   Rn.1438    Gene Symbol:   Casp2    Locus Link:   64314    Chromosome:   4    Cytoband:   Chr:4q23    SOURCE    GENECARD       Gene Ontology:   cellular component:  intracellular   cellular component:  nucleus      BioCarta Pathways:     KEGG Pathways:  
    Probe set : 1389469_at    Name:   chromodomain helicase DNA binding protein 1-like (predicted)    GB acc:   AA858645    UniGene:   Rn.1478    Gene Symbol:   Chd1l_predicted    Locus Link:   310707    Chromosome:   2    Cytoband:   Chr:2q34    SOURCE    GENECARD       Gene Ontology:   cellular component:  nucleus      BioCarta Pathways:     KEGG Pathways:  
    Probe set : 1374682_at    Name:   Transcribed locus, weakly similar to NP_035569.1 SRY-box 13 [Mus musculus]    GB acc:   BM383170    UniGene:   Rn.30532        SOURCE        Gene Ontology:     BioCarta Pathways:     KEGG Pathways:  
    Probe set : 1370054_at    Name:   cyclin-dependent kinase inhibitor 2C (p18, inhibits CDK4)    GB acc:   NM_131902    UniGene:   Rn.63865    Gene Symbol:   Cdkn2c    Locus Link:   54238      SOURCE    GENECARD       Gene Ontology:   biological process:  regulation of transcription, DNA-dependent   biological process:  cell cycle      BioCarta Pathways:     KEGG Pathways:  
    Probe set : 1398321_a_at    Name:   procollagen, type XII, alpha 1    GB acc:   U57361    UniGene:   Rn.11218    Gene Symbol:   Col12a1    Locus Link:   25683    Chromosome:   8    Cytoband:   Chr:8q31    SOURCE    GENECARD       Gene Ontology:     BioCarta Pathways:     KEGG Pathways:  
    Probe set : 1371959_at    Name:   histone 2, H2aa (predicted) /// similar to Hist2h2aa1 protein    GB acc:   AI235236    UniGene:   Rn.8353    Gene Symbol:   Hist2h2aa_predicted /// LOC499676    Locus Link:   365877 /// 499676    Chromosome:   2    Cytoband:   Chr:2q34    SOURCE    GENECARD       Gene Ontology:     BioCarta Pathways:     KEGG Pathways:  
    Probe set : 1379482_at    Name:   transmembrane 6 superfamily member 1 (predicted)    GB acc:   BI283829    UniGene:   Rn.130366    Gene Symbol:   Tm6sf1_predicted    Locus Link:   361600    Chromosome:   1    Cytoband:   Chr:1q31    SOURCE    GENECARD       Gene Ontology:   cellular component:  integral to membrane      BioCarta Pathways:     KEGG Pathways:  
    Probe set : 1373881_at    Name:   Rho, GDP dissociation inhibitor (GDI) beta (predicted)    GB acc:   BF285771    UniGene:   Rn.15842    Gene Symbol:   Arhgdib_predicted    Locus Link:   362456    Chromosome:   4    Cytoband:   Chr:4q43    SOURCE    GENECARD       Gene Ontology:   cellular component:  cytoplasm   cellular component:  cytoplasmic membrane-bound vesicle   molecular function:  Rho GDP-dissociation inhibitor activity   molecular function:  GTPase activator activity      BioCarta Pathways:     KEGG Pathways:  
    Probe set : 1389540_at    Name:   TG interacting factor (predicted)    GB acc:   AI236141    UniGene:   Rn.144418    Gene Symbol:   Tfig    Locus Link:   316742    Chromosome:   9    Cytoband:   Chr:9q38    SOURCE    GENECARD       Gene Ontology:   cellular component:  nucleus   cellular component:  transcription factor complex   molecular function:  DNA binding   molecular function:  transcription factor activity   molecular function:  transcription corepressor activity      BioCarta Pathways:     KEGG Pathways:  
    Probe set : 1369113_at    Name:   gremlin 1 homolog, cysteine knot superfamily (Xenopus laevis)    GB acc:   NM_019282    UniGene:   Rn.42929    Gene Symbol:   Grem1    Locus Link:   50566    Chromosome:   3    Cytoband:   Chr:3q34    SOURCE    GENECARD       Gene Ontology:   cellular component:  extracellular space   molecular function:  cytokine activity      BioCarta Pathways:     KEGG Pathways:  
    Probe set : 1368467_at    Name:   cytochrome P450, family 4, subfamily F, polypeptide 2    GB acc:   NM_019623    UniGene:   Rn.5722    Gene Symbol:   Cyp4f2    Locus Link:   56266    Chromosome:   7    Cytoband:   Chr:7q11    SOURCE    GENECARD       Gene Ontology:   biological process:  electron transport      BioCarta Pathways:     KEGG Pathways:  
    Probe set : 1382173_at    Name:   Transcribed locus    GB acc:   BF283569    UniGene:   Rn.51063        SOURCE        Gene Ontology:     BioCarta Pathways:     KEGG Pathways:  
    Probe set : 1372536_at    Name:   chaperone, ABC1 activity of bc1 complex like (S. pombe) (predicted)    GB acc:   AI105042     Gene Symbol:   Cabc1_predicted    Locus Link:   360887    Chromosome:   13    Cytoband:   Chr:13q26     GENECARD       Gene Ontology:   biological process:  protein folding   cellular component:  mitochondrion   molecular function:  unfolded protein binding      BioCarta Pathways:     KEGG Pathways:  
    Probe set : 1368593_at    Name:   CD1d1 antigen    GB acc:   NM_017079    UniGene:   Rn.11120    Gene Symbol:   Cd1d1    Locus Link:   25109    Chromosome:   2    Cytoband:   Chr:2q34    SOURCE    GENECARD       Gene Ontology:   cellular component:  integral to plasma membrane   cellular component:  integral to plasma membrane   cellular component:  integral to membrane      BioCarta Pathways:     KEGG Pathways:  
    Probe set : 1391979_at    Name:   Transcribed locus    GB acc:   AA851345    UniGene:   Rn.6731        SOURCE        Gene Ontology:     BioCarta Pathways:     KEGG Pathways:  
    Probe set : 1368463_at    Name:   vascular endothelial growth factor C    GB acc:   NM_053653    UniGene:   Rn.6913    Gene Symbol:   Vegfc    Locus Link:   114111    Chromosome:   16    Cytoband:   Chr:16p11    SOURCE    GENECARD       Gene Ontology:   cellular component:  extracellular space   cellular component:  membrane   molecular function:  growth factor activity   molecular function:  growth factor activity   molecular function:  vascular endothelial growth factor receptor 3 binding      BioCarta Pathways:     KEGG Pathways:  
    Probe set : 1371729_at    Name:   Yippee-like 5 (Drosophila) (predicted)    GB acc:   AI169150    UniGene:   Rn.1136     Locus Link:   298792    Chromosome:   6    Cytoband:   Chr:6q13    SOURCE        Gene Ontology:     BioCarta Pathways:     KEGG Pathways:  
    Probe set : 1387375_at    Name:   ketohexokinase    GB acc:   NM_031855    UniGene:   Rn.33324    Gene Symbol:   Khk    Locus Link:   25659      SOURCE    GENECARD       Gene Ontology:   biological process:  carbohydrate metabolism   biological process:  carbohydrate catabolism   biological process:  carbohydrate catabolism      BioCarta Pathways:     KEGG Pathways:  
    Probe set : 1373826_at     GB acc:   AI170754              Gene Ontology:     BioCarta Pathways:     KEGG Pathways:  
    Probe set : 1376684_at    Name:   discs, large homolog 7 (Drosophila) (predicted)    GB acc:   AA874827    UniGene:   Rn.3137    Gene Symbol:   Dlg7_predicted    Locus Link:   289997    Chromosome:   15    Cytoband:   Chr:15p14    SOURCE    GENECARD       Gene Ontology:   biological process:  cell-cell signaling   molecular function:  kinase activity      BioCarta Pathways:     KEGG Pathways:  
    Probe set : 1388314_at    Name:   high mobility group nucleosomal binding domain 1 (predicted)    GB acc:   BF281178    UniGene:   Rn.4120    Gene Symbol:   Hmgn1_predicted    Locus Link:   360704    Chromosome:   11    Cytoband:   Chr:11q11    SOURCE    GENECARD       Gene Ontology:   biological process:  DNA packaging   cellular component:  chromatin   cellular component:  nucleus      BioCarta Pathways:     KEGG Pathways:  
    Probe set : 1390228_at    Name:   Transcribed locus    GB acc:   BM384446    UniGene:   Rn.31695        SOURCE        Gene Ontology:     BioCarta Pathways:     KEGG Pathways:  
    Probe set : 1379885_at    Name:   flavin containing monooxygenase 4    GB acc:   BF282998    UniGene:   Rn.6404    Gene Symbol:   Fmo4    Locus Link:   246247    Chromosome:   13    Cytoband:   Chr:13q22    SOURCE    GENECARD       Gene Ontology:   biological process:  electron transport   biological process:  xenobiotic metabolism      BioCarta Pathways:     KEGG Pathways:  
    Probe set : 1388076_at    Name:   PAI-1 mRNA-binding protein    GB acc:   AF388527    UniGene:   Rn.7370    Gene Symbol:   Pairbp1    Locus Link:   246303    Chromosome:   4    Cytoband:   Chr:4q31    SOURCE    GENECARD       Gene Ontology:   cellular component:  nucleus   molecular function:  RNA binding   molecular function:  mRNA 3'-UTR binding      BioCarta Pathways:     KEGG Pathways:  
    Probe set : 1376038_at    Name:   testis expressed gene 2 (predicted)    GB acc:   AI411054    UniGene:   Rn.94152    Gene Symbol:   Tex2_predicted    Locus Link:   303611    Chromosome:   10    Cytoband:   Chr:10q32.1    SOURCE    GENECARD       Gene Ontology:     BioCarta Pathways:     KEGG Pathways:  
    Probe set : 1370174_at    Name:   myeloid differentiation primary response gene 116    GB acc:   BI284349    UniGene:   Rn.2232    Gene Symbol:   Myd116    Locus Link:   171071    Chromosome:   1    Cytoband:   Chr:1q22    SOURCE    GENECARD       Gene Ontology:   biological process:  cell differentiation      BioCarta Pathways:     KEGG Pathways:  
    Probe set : 1383703_at    Name:   FUS interacting protein (serine-arginine rich) 1 (predicted)    GB acc:   BE109151    UniGene:   Rn.105889     Locus Link:   362630    Chromosome:   5    Cytoband:   Chr:5q36    SOURCE        Gene Ontology:   cellular component:  nucleus   cellular component:  nucleoplasm   cellular component:  cytoplasm      BioCarta Pathways:     KEGG Pathways:  
    Probe set : 1376723_a_at     GB acc:   AW142765              Gene Ontology:     BioCarta Pathways:     KEGG Pathways:  
    Probe set : 1388840_at    Name:   similar to common-site lymphoma/leukemia GEF (predicted)    GB acc:   BF397719    UniGene:   Rn.140555    Gene Symbol:   RGD1310112_predicted    Locus Link:   292750    Chromosome:   1    Cytoband:   Chr:1q21    SOURCE    GENECARD       Gene Ontology:     BioCarta Pathways:     KEGG Pathways:  
    Probe set : 1370574_a_at    Name:   protein tyrosine phosphatase, non-receptor type substrate 1    GB acc:   D38468    UniGene:   Rn.53971    Gene Symbol:   Ptpns1    Locus Link:   25528    Chromosome:   3    Cytoband:   Chr:3q36    SOURCE    GENECARD       Gene Ontology:   cellular component:  extracellular space   cellular component:  integral to plasma membrane   cellular component:  integral to membrane   molecular function:  protein binding   molecular function:  protein phosphorylated amino acid binding      BioCarta Pathways:     KEGG Pathways:  
    Probe set : 1398174_at    Name:   methyl CpG binding protein 2    GB acc:   BE114216    UniGene:   Rn.9680    Gene Symbol:   Mecp2    Locus Link:   29386    Chromosome:   X    Cytoband:   Chr:Xq37    SOURCE    GENECARD       Gene Ontology:   cellular component:  heterochromatin   cellular component:  nucleus   molecular function:  DNA binding   molecular function:  methyl-CpG binding      BioCarta Pathways:     KEGG Pathways:  
    Probe set : 1371747_at    Name:   similar to RIKEN cDNA 2700038C09 (predicted)    GB acc:   AI406304    UniGene:   Rn.1389    Gene Symbol:   RGD1310660_predicted    Locus Link:   296470    Chromosome:   3    Cytoband:   Chr:3q43    SOURCE    GENECARD       Gene Ontology:     BioCarta Pathways:     KEGG Pathways:  
    Probe set : 1388665_at    Name:   similar to RIKEN cDNA 1110020A23 (predicted)    GB acc:   BM390489    UniGene:   Rn.7220    Gene Symbol:   RGD1308134_predicted    Locus Link:   287452    Chromosome:   10    Cytoband:   Chr:10q24    SOURCE    GENECARD       Gene Ontology:     BioCarta Pathways:     KEGG Pathways:  
    Probe set : 1370163_at    Name:   ornithine decarboxylase 1    GB acc:   BF281299    UniGene:   Rn.874    Gene Symbol:   Odc1    Locus Link:   24609    Chromosome:   6    Cytoband:   Chr:6q16    SOURCE    GENECARD       Gene Ontology:   biological process:  polyamine biosynthesis      BioCarta Pathways:     KEGG Pathways:  
    Probe set : 1379250_at    Name:   galactose mutarotase (aldose 1-epimerase)    GB acc:   AI012955    UniGene:   Rn.83639    Gene Symbol:   RGD:1359459    Locus Link:   313843    Chromosome:   6    Cytoband:   Chr:6q11    SOURCE    GENECARD       Gene Ontology:   biological process:  carbohydrate metabolism   biological process:  galactose metabolism   molecular function:  aldose 1-epimerase activity   molecular function:  isomerase activity      BioCarta Pathways:     KEGG Pathways:  
    Probe set : 1389234_at    Name:   von Willebrand factor    GB acc:   BI298314    UniGene:   Rn.35561    Gene Symbol:   RGD:621759    Locus Link:   116669    Chromosome:   4    Cytoband:   Chr:4q42    SOURCE    GENECARD       Gene Ontology:   cellular component:  extracellular matrix (sensu Metazoa)   molecular function:  protein binding      BioCarta Pathways:     KEGG Pathways:  
    Probe set : 1367600_at    Name:   desmin    GB acc:   NM_022531    UniGene:   Rn.39196    Gene Symbol:   Des    Locus Link:   64362    Chromosome:   9    Cytoband:   Chr:9q33    SOURCE    GENECARD       Gene Ontology:   biological process:  muscle development   biological process:  muscle cell differentiation   cellular component:  kinesin complex   cellular component:  intermediate filament   cellular component:  intermediate filament   molecular function:  structural molecule activity      BioCarta Pathways:     KEGG Pathways:  
    Probe set : 1377772_at    Name:   Transmembrane protein with EGF-like and two follistatin-like domains 1    GB acc:   BI295864    UniGene:   Rn.44829    Gene Symbol:   Tmeff1    Locus Link:   63845    Chromosome:   5    Cytoband:   Chr:5q22    SOURCE    GENECARD       Gene Ontology:   cellular component:  integral to membrane   molecular function:  serine-type endopeptidase inhibitor activity   molecular function:  growth factor activity      BioCarta Pathways:     KEGG Pathways:  
    Probe set : 1367604_at    Name:   cysteine-rich protein 2    GB acc:   NM_022501    UniGene:   Rn.4267    Gene Symbol:   RGD:1302959    Locus Link:   338401    Chromosome:   6    Cytoband:   Chr:6q32    SOURCE    GENECARD       Gene Ontology:   biological process:  positive regulation of cell proliferation   biological process:  hemopoiesis   molecular function:  zinc ion binding   molecular function:  PDZ domain binding   molecular function:  metal ion binding      BioCarta Pathways:     KEGG Pathways:  
    Probe set : 1385909_at    Name:   Transcribed locus    GB acc:   BE118035    UniGene:   Rn.51363        SOURCE        Gene Ontology:     BioCarta Pathways:     KEGG Pathways:  
    Probe set : 1390137_at    Name:   TRAF4 associated factor 1    GB acc:   BM388478    UniGene:   Rn.20235    Gene Symbol:   RGD:1303240    Locus Link:   311325    Chromosome:   3    Cytoband:   Chr:3q35    SOURCE    GENECARD       Gene Ontology:     BioCarta Pathways:     KEGG Pathways:  
    Probe set : 1390812_a_at    Name:   similar to RAS-like, estrogen-regulated, growth-inhibitor    GB acc:   AI170076    UniGene:   Rn.19093    Gene Symbol:   LOC502916    Locus Link:   502916    Chromosome:   4    Cytoband:   Chr:4q43-q44    SOURCE    GENECARD       Gene Ontology:     BioCarta Pathways:     KEGG Pathways:  
    Probe set : 1388119_at    Name:   hypothetical gene supported by Y16641 /// heterogeneous nuclear ribonucleoprotein A3 /// similar to Hnrpa3 protein /// similar to Hnrpa3 protein    GB acc:   BM392140    UniGene:   Rn.99947    Gene Symbol:   LOC292273 /// Hnrpa3 /// LOC363971 /// LOC499592    Locus Link:   292273 /// 362152 /// 363971 /// 499592    Chromosome:   2    Cytoband:   Chr:2q45 /// Chr:3q23 /// Chr:13q11 /// Chr:2q25    SOURCE    GENECARD       Gene Ontology:   biological process:  mRNA transport      BioCarta Pathways:     KEGG Pathways:  
    Probe set : 1367594_at    Name:   biglycan    GB acc:   NM_017087    UniGene:   Rn.783    Gene Symbol:   Bgn    Locus Link:   25181    Chromosome:   X    Cytoband:   Chr:Xq37.2    SOURCE    GENECARD       Gene Ontology:   cellular component:  extracellular matrix (sensu Metazoa)   cellular component:  extracellular space   molecular function:  extracellular matrix structural constituent      BioCarta Pathways:     KEGG Pathways:  
    Probe set : 1380692_at    Name:   Transcribed locus    GB acc:   BM387127    UniGene:   Rn.148725        SOURCE        Gene Ontology:     BioCarta Pathways:     KEGG Pathways:  
    Probe set : 1389392_at    Name:   Transcribed locus    GB acc:   BF399468    UniGene:   Rn.7226        SOURCE        Gene Ontology:     BioCarta Pathways:     KEGG Pathways:  
    Probe set : 1370461_at    Name:   hyaluronan mediated motility receptor    GB acc:   AF336825    UniGene:   Rn.92304    Gene Symbol:   Hmmr    Locus Link:   25460    Chromosome:   10    Cytoband:   Chr:10q12    SOURCE    GENECARD       Gene Ontology:   biological process:  cell motility   biological process:  cytoskeleton organization and biogenesis   biological process:  cell-cell signaling   molecular function:  hyaluronic acid binding   molecular function:  hyaluronic acid binding   molecular function:  receptor activity      BioCarta Pathways:     KEGG Pathways:  
    Probe set : 1380721_at    Name:   Transcribed locus    GB acc:   BE103115    UniGene:   Rn.149616        SOURCE        Gene Ontology:     BioCarta Pathways:     KEGG Pathways:  
    Probe set : 1384628_at    Name:   similar to RIKEN cDNA 0610009A07 (predicted)    GB acc:   AI549292    UniGene:   Rn.34836    Gene Symbol:   RGD1309288_predicted    Locus Link:   308129    Chromosome:   1    Cytoband:   Chr:1p11    SOURCE    GENECARD       Gene Ontology:   biological process:  electron transport   molecular function:  oxidoreductase activity      BioCarta Pathways:     KEGG Pathways:  
    Probe set : 1371498_at    Name:   similar to Jtv1-pending protein    GB acc:   AI412685    UniGene:   Rn.3333    Gene Symbol:   LOC288480    Locus Link:   288480    Chromosome:   12    Cytoband:   Chr:12p11    SOURCE    GENECARD       Gene Ontology:     BioCarta Pathways:     KEGG Pathways:  
    Probe set : 1376811_a_at    Name:   cleavage and polyadenylation specific factor 6, 68kDa (predicted)    GB acc:   BE108383    UniGene:   Rn.32433    Gene Symbol:   Cpsf6_predicted    Locus Link:   299811    Chromosome:   7    Cytoband:   Chr:7q22    SOURCE    GENECARD       Gene Ontology:   biological process:  nuclear mRNA splicing, via spliceosome   cellular component:  nucleus   molecular function:  nucleic acid binding   molecular function:  RNA binding      BioCarta Pathways:     KEGG Pathways:  
    Probe set : 1390386_at    Name:   caspase 3    GB acc:   BM387008    UniGene:   Rn.10562    Gene Symbol:   Casp3    Locus Link:   25402    Chromosome:   16    Cytoband:   Chr:16q11    SOURCE    GENECARD       Gene Ontology:   biological process:  B cell homeostasis   biological process:  proteolysis   biological process:  apoptosis   biological process:  induction of apoptosis   biological process:  induction of apoptosis   biological process:  intracellular signaling cascade   biological process:  intracellular signaling cascade   biological process:  induction of apoptosis via death domain receptors   biological process:  induction of apoptosis by oxidative stress   biological process:  response to wounding   biological process:  neuron differentiation   biological process:  keratinocyte differentiation   biological process:  negative regulation of B cell proliferation   biological process:  T cell homeostasis   biological process:  cell fate commitment   biological process:  negative regulation of cyclin dependent protein kinase activity   biological process:  negative regulation of progression through cell cycle   biological process:  negative regulation of activated T cell proliferation   cellular component:  cytoplasm   cellular component:  cytosol      BioCarta Pathways:     KEGG Pathways:  
    Probe set : 1369960_at    Name:   FXYD domain-containing ion transport regulator 1    GB acc:   NM_031648    UniGene:   Rn.3828    Gene Symbol:   Fxyd1    Locus Link:   58971    Chromosome:   1    Cytoband:   Chr:1q21    SOURCE    GENECARD       Gene Ontology:   molecular function:  ion channel activity   molecular function:  ion channel activity   molecular function:  chloride channel activity      BioCarta Pathways:     KEGG Pathways:  
    Probe set : 1390036_at    Name:   solute carrier family 16 (monocarboxylic acid transporters), member 6    GB acc:   BI289867    UniGene:   Rn.54795    Gene Symbol:   Slc16a6    Locus Link:   303772    Chromosome:   10    Cytoband:   Chr:10q32.1    SOURCE    GENECARD       Gene Ontology:   biological process:  monocarboxylic acid transport   biological process:  transport   cellular component:  integral to membrane   molecular function:  monocarboxylic acid transporter activity   molecular function:  transporter activity      BioCarta Pathways:     KEGG Pathways:  
    Probe set : 1381233_at    Name:   Transcribed locus    GB acc:   AW528010    UniGene:   Rn.21708        SOURCE        Gene Ontology:     BioCarta Pathways:     KEGG Pathways:  
    Probe set : 1372773_at    Name:   neural proliferation, differentiation and control, 1    GB acc:   BM389728    UniGene:   Rn.5802    Gene Symbol:   RGD:1303156    Locus Link:   296562    Chromosome:   3    Cytoband:   Chr:3p13    SOURCE    GENECARD       Gene Ontology:   biological process:  regulation of cell proliferation   biological process:  retinoic acid metabolism   cellular component:  integral to membrane      BioCarta Pathways:     KEGG Pathways:  
    Probe set : 1384232_at    Name:   Transcribed locus    GB acc:   AI709768    UniGene:   Rn.12808        SOURCE        Gene Ontology:     BioCarta Pathways:     KEGG Pathways:  
    Probe set : 1384488_at    Name:   similar to cyclin-dependent kinase inhibitor 2D    GB acc:   BI290067    UniGene:   Rn.122002    Gene Symbol:   Cdkn2d    Locus Link:   494444      SOURCE    GENECARD       Gene Ontology:   molecular function:  kinase activity      BioCarta Pathways:     KEGG Pathways:  
    Probe set : 1367750_at    Name:   phosphoribosyl pyrophosphate synthetase-associated protein 1    GB acc:   NM_022545    UniGene:   Rn.2290    Gene Symbol:   Prpsap1    Locus Link:   64390    Chromosome:   10    Cytoband:   Chr:10q32.3    SOURCE    GENECARD       Gene Ontology:   biological process:  nucleoside metabolism   biological process:  nucleotide biosynthesis   biological process:  nucleotide biosynthesis   cellular component:  extracellular space      BioCarta Pathways:     KEGG Pathways:  
    Probe set : 1389568_at    Name:   family with sequence similarity 26, member B (predicted)    GB acc:   BM391099    UniGene:   Rn.25356    Gene Symbol:   Fam26b_predicted    Locus Link:   294019    Chromosome:   1    Cytoband:   Chr:1q54    SOURCE    GENECARD       Gene Ontology:   cellular component:  integral to membrane      BioCarta Pathways:     KEGG Pathways:  
    Probe set : 1374359_at    Name:   Similar to RIKEN cDNA 2810013E07 (predicted)    GB acc:   BM383329    UniGene:   Rn.98992     Locus Link:   297823    Chromosome:   5    Cytoband:   Chr:5q13    SOURCE        Gene Ontology:     BioCarta Pathways:     KEGG Pathways:  
    Probe set : 1370055_at    Name:   RAB3D, member RAS oncogene family    GB acc:   M83681    UniGene:   Rn.9822    Gene Symbol:   Rab3d    Locus Link:   140665    Chromosome:   8    Cytoband:   Chr:8q13    SOURCE    GENECARD       Gene Ontology:   cellular component:  intracellular   cellular component:  zymogen granule      BioCarta Pathways:     KEGG Pathways:  
    Probe set : 1392219_at     GB acc:   AI145523              Gene Ontology:     BioCarta Pathways:     KEGG Pathways:  
    Probe set : 1396752_at    Name:   Tumor necrosis factor receptor superfamily, member 21 (predicted)    GB acc:   BF405147    UniGene:   Rn.98293     Locus Link:   316256    Chromosome:   9    Cytoband:   Chr:9q12    SOURCE        Gene Ontology:   biological process:  apoptosis   biological process:  signal transduction   cellular component:  integral to membrane   molecular function:  receptor activity   molecular function:  protein binding      BioCarta Pathways:     KEGG Pathways:  
    Probe set : 1395274_at    Name:   dystonin (predicted)    GB acc:   AI235468    UniGene:   Rn.79807    Gene Symbol:   Dst_predicted    Locus Link:   316313    Chromosome:   9    Cytoband:   Chr:9q21    SOURCE    GENECARD       Gene Ontology:   biological process:  cell cycle arrest   biological process:  cell adhesion   biological process:  retrograde axon cargo transport   cellular component:  cytoskeleton   cellular component:  cytoplasmic membrane-bound vesicle   cellular component:  hemidesmosome      BioCarta Pathways:     KEGG Pathways:  
    Probe set : 1390415_at    Name:   Thyroid hormone receptor interactor 13 (predicted)    GB acc:   BM392319    UniGene:   Rn.11751     Locus Link:   292206    Chromosome:   1    Cytoband:   Chr:1p11    SOURCE        Gene Ontology:   biological process:  transcription from RNA polymerase II promoter   cellular component:  nucleus      BioCarta Pathways:     KEGG Pathways:  
    Probe set : 1367630_at    Name:   ribosomal protein S11    GB acc:   NM_031110    UniGene:   Rn.2177    Gene Symbol:   Rps11    Locus Link:   81774    Chromosome:   1    Cytoband:   Chr:1q22    SOURCE    GENECARD       Gene Ontology:   biological process:  protein biosynthesis   cellular component:  intracellular   cellular component:  ribosome   cellular component:  ribonucleoprotein complex      BioCarta Pathways:     KEGG Pathways:  
    Probe set : 1367798_at    Name:   S-adenosylhomocysteine hydrolase    GB acc:   NM_017201    UniGene:   Rn.5878    Gene Symbol:   Ahcy    Locus Link:   29443    Chromosome:   3    Cytoband:   Chr:3q42    SOURCE    GENECARD       Gene Ontology:   cellular component:  cytoplasm   molecular function:  adenosylhomocysteinase activity   molecular function:  adenosylhomocysteinase activity   molecular function:  hydrolase activity      BioCarta Pathways:     KEGG Pathways:  
    Probe set : 1375452_at    Name:   meningioma expressed antigen 6 (coiled-coil proline-rich) (predicted)    GB acc:   AI177180    UniGene:   Rn.58607    Gene Symbol:   Mgea6_predicted    Locus Link:   299078    Chromosome:   6    Cytoband:   Chr:6q23    SOURCE    GENECARD       Gene Ontology:   biological process:  RNA processing   cellular component:  integral to membrane      BioCarta Pathways:     KEGG Pathways:  
    Probe set : 1380596_at    Name:   Transcribed locus    GB acc:   BE098869    UniGene:   Rn.39043        SOURCE        Gene Ontology:     BioCarta Pathways:     KEGG Pathways:  
    Probe set : 1382127_at     GB acc:   AI716708              Gene Ontology:     BioCarta Pathways:     KEGG Pathways:  
    Probe set : 1397620_at    Name:   putative scaffolding protein POSH    GB acc:   BG372003    UniGene:   Rn.74468    Gene Symbol:   Sh3md2    Locus Link:   306417    Chromosome:   16    Cytoband:   Chr:16p12    SOURCE    GENECARD       Gene Ontology:   biological process:  induction of apoptosis   biological process:  protein ubiquitination   cellular component:  ubiquitin ligase complex   molecular function:  protein binding   molecular function:  ubiquitin-protein ligase activity   molecular function:  zinc ion binding      BioCarta Pathways:     KEGG Pathways:  
    Probe set : 1393148_at    Name:   transmembrane 7 superfamily member 1 (predicted)    GB acc:   BF542593    UniGene:   Rn.60453    Gene Symbol:   Tm7sf1_predicted    Locus Link:   289287    Chromosome:   17    Cytoband:   Chr:17q12.1    SOURCE    GENECARD       Gene Ontology:   cellular component:  membrane fraction   cellular component:  integral to plasma membrane   cellular component:  integral to membrane      BioCarta Pathways:     KEGG Pathways:  
    Probe set : 1388592_at    Name:   Transcribed locus    GB acc:   BI298958    UniGene:   Rn.49412        SOURCE        Gene Ontology:     BioCarta Pathways:     KEGG Pathways:  
    Probe set : 1398773_at    Name:   src associated in mitosis, 68 kDa    GB acc:   NM_130405    UniGene:   Rn.108177    Gene Symbol:   Khdrbs1    Locus Link:   117268    Chromosome:   5    Cytoband:   Chr:5q36    SOURCE    GENECARD       Gene Ontology:   biological process:  G1/S transition of mitotic cell cycle   biological process:  mRNA processing   biological process:  transport   biological process:  cell cycle arrest   biological process:  signal transduction   biological process:  Ras protein signal transduction   biological process:  cell proliferation   biological process:  negative regulation of transcription   biological process:  regulation of RNA export from nucleus   biological process:  G2/M transition of mitotic cell cycle   biological process:  negative regulation of transcription   biological process:  regulation of RNA export from nucleus   biological process:  cell surface receptor linked signal transduction   biological process:  transcription   biological process:  regulation of transcription, DNA-dependent   biological process:  cell cycle      BioCarta Pathways:     KEGG Pathways:  
    Probe set : 1375525_at    Name:   Microtubule-associated protein, RP/EB family, member 1    GB acc:   BI294783    UniGene:   Rn.7652    Gene Symbol:   Mapre1    Locus Link:   114764    Chromosome:   3    Cytoband:   Chr:3q41    SOURCE    GENECARD       Gene Ontology:   biological process:  cell cycle   biological process:  mitosis   biological process:  cell division   cellular component:  microtubule   molecular function:  microtubule binding      BioCarta Pathways:     KEGG Pathways:  
    Probe set : 1376755_at    Name:   retinoic acid receptor, beta    GB acc:   BF419646    UniGene:   Rn.38896    Gene Symbol:   Rarb    Locus Link:   24706      SOURCE    GENECARD       Gene Ontology:   cellular component:  nucleus      BioCarta Pathways:     KEGG Pathways:  
    Probe set : 1379195_s_at    Name:   ligase I, DNA, ATP-dependent /// ligase I, DNA, ATP-dependent (predicted)    GB acc:   AI556346    UniGene:   Rn.20467    Gene Symbol:   Lig1 /// Lig1_predicted    Locus Link:   360304 /// 81513    Chromosome:   1    Cytoband:   Chr:1q12 /// Chr:1q21    SOURCE    GENECARD       Gene Ontology:   cellular component:  nucleus      BioCarta Pathways:     KEGG Pathways:  
    Probe set : 1390650_at    Name:   pericentrin 1 (predicted)    GB acc:   BI290593    UniGene:   Rn.67936    Gene Symbol:   Pcnt1_predicted    Locus Link:   287830    Chromosome:   10    Cytoband:   Chr:10q32.3    SOURCE    GENECARD       Gene Ontology:   cellular component:  integral to membrane   cellular component:  outer membrane   molecular function:  porin activity      BioCarta Pathways:     KEGG Pathways:  
    Probe set : 1369434_at    Name:   deoxynucleotidyltransferase, terminal, interacting protein 1    GB acc:   NM_134400    UniGene:   Rn.82702    Gene Symbol:   Dnttip1    Locus Link:   171437    Chromosome:   3    Cytoband:   Chr:3q42    SOURCE    GENECARD       Gene Ontology:   biological process:  regulation of transcription, DNA-dependent   biological process:  transcription   cellular component:  nucleus      BioCarta Pathways:     KEGG Pathways:  
    Probe set : 1387108_at    Name:   casein kinase 2, beta subunit    GB acc:   BF281311    UniGene:   Rn.11095    Gene Symbol:   Csnk2b    Locus Link:   81650    Chromosome:   20    Cytoband:   Chr:20p12    SOURCE    GENECARD       Gene Ontology:   cellular component:  protein kinase CK2 complex      BioCarta Pathways:     KEGG Pathways:  
    Probe set : 1390163_at     GB acc:   BF282174              Gene Ontology:     BioCarta Pathways:     KEGG Pathways:  
    Probe set : 1383315_at    Name:   hepatic protein EIIH    GB acc:   AW434208    UniGene:   Rn.145009    Gene Symbol:   RGD:1359311    Locus Link:   308843    Chromosome:   1    Cytoband:   Chr:1q32    SOURCE    GENECARD       Gene Ontology:     BioCarta Pathways:     KEGG Pathways:  
    Probe set : 1389349_s_at    Name:   similar to interleukin 17 receptor E isoform 1    GB acc:   BM383766    UniGene:   Rn.129068    Gene Symbol:   Il17re    Locus Link:   362417    Chromosome:   4    Cytoband:   Chr:4q42    SOURCE    GENECARD       Gene Ontology:   molecular function:  receptor activity      BioCarta Pathways:     KEGG Pathways:  
    Probe set : 1385690_at    Name:   similar to Mut protein /// similar to Mut protein    GB acc:   BI293631    UniGene:   Rn.54738    Gene Symbol:   LOC301276 /// LOC363201    Locus Link:   301276 /// 363201    Chromosome:   9    Cytoband:   Chr:9q12    SOURCE    GENECARD       Gene Ontology:     BioCarta Pathways:     KEGG Pathways:  
    Probe set : 1376709_at    Name:   solute carrier family 39 (metal ion transporter), member 8 (predicted)    GB acc:   BM388442    UniGene:   Rn.28321    Gene Symbol:   Slc39a8_predicted    Locus Link:   295455    Chromosome:   2    Cytoband:   Chr:2q43    SOURCE    GENECARD       Gene Ontology:   biological process:  transport   biological process:  metal ion transport   cellular component:  extracellular space   cellular component:  membrane   cellular component:  integral to membrane   molecular function:  metal ion transporter activity      BioCarta Pathways:     KEGG Pathways:  
    Probe set : 1398882_at    Name:   ribosomal protein S5    GB acc:   BI282255    UniGene:   Rn.2100    Gene Symbol:   Rps5    Locus Link:   25538    Chromosome:   1    Cytoband:   Chr:1q21    SOURCE    GENECARD       Gene Ontology:   biological process:  protein biosynthesis   biological process:  ribosome biogenesis   molecular function:  RNA binding   molecular function:  structural constituent of ribosome      BioCarta Pathways:     KEGG Pathways:  
    Probe set : 1387849_at    Name:   epsin 2    GB acc:   NM_021852    UniGene:   Rn.44273    Gene Symbol:   Epn2    Locus Link:   60443    Chromosome:   10    Cytoband:   Chr:10q23    SOURCE    GENECARD       Gene Ontology:   biological process:  endocytosis   biological process:  regulation of endocytosis   biological process:  regulation of endocytosis   cellular component:  clathrin coat of endocytic vesicle   cellular component:  clathrin coat of endocytic vesicle   molecular function:  lipid binding      BioCarta Pathways:     KEGG Pathways:  
    Probe set : 1388827_at    Name:   H2A histone family, member V (predicted) /// similar to H2A histone family, member V isoform 1    GB acc:   AW523419    UniGene:   Rn.100938    Gene Symbol:   H2afv_predicted /// LOC498894    Locus Link:   289784 /// 498894    Chromosome:   14    Cytoband:   Chr:14q21 /// Chr:18q13    SOURCE    GENECARD       Gene Ontology:   biological process:  nucleosome assembly   biological process:  chromosome organization and biogenesis (sensu Eukaryota)   cellular component:  nucleosome   cellular component:  nucleus   molecular function:  DNA binding      BioCarta Pathways:     KEGG Pathways:  
    Probe set : 1370993_at    Name:   laminin, gamma 1    GB acc:   AA997129    UniGene:   Rn.7145    Gene Symbol:   Lamc1    Locus Link:   117036    Chromosome:   13    Cytoband:   Chr:13q21    SOURCE    GENECARD       Gene Ontology:   cellular component:  extracellular matrix (sensu Metazoa)   cellular component:  laminin-1      BioCarta Pathways:     KEGG Pathways:  
    Probe set : 1385574_at    Name:   similar to cDNA sequence AF397014 (predicted)    GB acc:   AI501346    UniGene:   Rn.105406    Gene Symbol:   RGD1308114_predicted    Locus Link:   361253    Chromosome:   17    Cytoband:   Chr:17q12.1    SOURCE    GENECARD       Gene Ontology:   biological process:  metabolism   molecular function:  transferase activity      BioCarta Pathways:     KEGG Pathways:  
    Probe set : 1377662_at    Name:   pirin    GB acc:   AI176041    UniGene:   Rn.103685    Gene Symbol:   RGD:1359212    Locus Link:   363465    Chromosome:   X    Cytoband:   Chr:Xq21    SOURCE    GENECARD       Gene Ontology:     BioCarta Pathways:     KEGG Pathways:  
    Probe set : 1382243_at    Name:   Transcribed locus    GB acc:   BE120628    UniGene:   Rn.21747        SOURCE        Gene Ontology:     BioCarta Pathways:     KEGG Pathways:  
    Probe set : 1395036_at    Name:   similar to Ac2-190 (predicted)    GB acc:   BF386788    UniGene:   Rn.129949    Gene Symbol:   RGD1306455_predicted    Locus Link:   312952    Chromosome:   5    Cytoband:   Chr:5q12    SOURCE    GENECARD       Gene Ontology:   molecular function:  hydrolase activity      BioCarta Pathways:     KEGG Pathways:  
    Probe set : 1386247_at    Name:   sorting nexin 25 (predicted)    GB acc:   AA859100    UniGene:   Rn.16962    Gene Symbol:   Snx25_predicted    Locus Link:   306471    Chromosome:   16    Cytoband:   Chr:16q11    SOURCE    GENECARD       Gene Ontology:   biological process:  transport   biological process:  intracellular signaling cascade   biological process:  protein transport   molecular function:  signal transducer activity      BioCarta Pathways:     KEGG Pathways:  
    Probe set : 1368092_at    Name:   fumarylacetoacetate hydrolase    GB acc:   NM_017181    UniGene:   Rn.9195    Gene Symbol:   Fah    Locus Link:   29383    Chromosome:   1    Cytoband:   Chr:1q31    SOURCE    GENECARD       Gene Ontology:   cellular component:  nucleus   cellular component:  nucleus      BioCarta Pathways:     KEGG Pathways:  
    Probe set : 1373394_at     GB acc:   BM391986              Gene Ontology:     BioCarta Pathways:     KEGG Pathways:  
    Probe set : 1388440_at    Name:   presenilin stabilization factor-like    GB acc:   AI408482    UniGene:   Rn.103390    Gene Symbol:   Aph1b    Locus Link:   300802    Chromosome:   8    Cytoband:   Chr:8q24    SOURCE    GENECARD       Gene Ontology:   biological process:  protein processing   biological process:  positive regulation of enzyme activity   cellular component:  integral to membrane   molecular function:  protein binding      BioCarta Pathways:     KEGG Pathways:  
    Probe set : 1389622_at    Name:   similar to citrin    GB acc:   AI232643    UniGene:   Rn.14686    Gene Symbol:   LOC362322    Locus Link:   362322    Chromosome:   4    Cytoband:   Chr:4q13    SOURCE    GENECARD       Gene Ontology:     BioCarta Pathways:     KEGG Pathways:  
    Probe set : 1391302_at     GB acc:   BE098463              Gene Ontology:     BioCarta Pathways:     KEGG Pathways:  
    Probe set : 1391582_at    Name:   Transcribed locus    GB acc:   AI180053    UniGene:   Rn.31458        SOURCE        Gene Ontology:     BioCarta Pathways:     KEGG Pathways:  
    Probe set : 1388111_at    Name:   elastin    GB acc:   J04035    UniGene:   Rn.54384    Gene Symbol:   Eln    Locus Link:   25043    Chromosome:   12    Cytoband:   Chr:12q12    SOURCE    GENECARD       Gene Ontology:     BioCarta Pathways:     KEGG Pathways:  
    Probe set : 1387328_at    Name:   Cytochrome P450, subfamily IIC (mephenytoin 4-hydroxylase)    GB acc:   NM_019184    UniGene:   Rn.10870    Gene Symbol:   Cyp2c    Locus Link:   29277    Chromosome:   1    Cytoband:   Chr:1q53    SOURCE    GENECARD       Gene Ontology:   biological process:  electron transport   biological process:  xenobiotic metabolism   cellular component:  endoplasmic reticulum   cellular component:  microsome   cellular component:  membrane      BioCarta Pathways:     KEGG Pathways:  
    Probe set : 1367728_at    Name:   translin    GB acc:   NM_021762    UniGene:   Rn.38875    Gene Symbol:   Tsn    Locus Link:   60381    Chromosome:   13    Cytoband:   Chr:13q11    SOURCE    GENECARD       Gene Ontology:   molecular function:  mRNA binding      BioCarta Pathways:     KEGG Pathways:  
    Probe set : 1367751_at    Name:   dolichol-phosphate mannosyltransferase 2    GB acc:   NM_019252    UniGene:   Rn.2122    Gene Symbol:   Dpm2    Locus Link:   29640      SOURCE    GENECARD       Gene Ontology:   biological process:  protein amino acid glycosylation   biological process:  macromolecule biosynthesis   cellular component:  endoplasmic reticulum   cellular component:  integral to membrane   cellular component:  integral to endoplasmic reticulum membrane   molecular function:  transferase activity, transferring glycosyl groups      BioCarta Pathways:     KEGG Pathways:  
    Probe set : 1376902_x_at    Name:   Similar to Hypothetical protein 6330514E13    GB acc:   AA964675    UniGene:   Rn.93288     Locus Link:   315117    Chromosome:   7    Cytoband:   Chr:7q34    SOURCE        Gene Ontology:     BioCarta Pathways:     KEGG Pathways:  
    Probe set : 1370871_at    Name:   hypothetical gene supported by Y16641; Y16641    GB acc:   BM384165     Gene Symbol:   LOC288146    Locus Link:   288146    Chromosome:   1    Cytoband:   Chr:1p11     GENECARD       Gene Ontology:     BioCarta Pathways:     KEGG Pathways:  
    Probe set : 1393281_at    Name:   caveolin    GB acc:   AW915173    UniGene:   Rn.22518    Gene Symbol:   Cav    Locus Link:   25404    Chromosome:   4    Cytoband:   Chr:4q21    SOURCE    GENECARD       Gene Ontology:   biological process:  inactivation of MAPK activity   biological process:  negative regulation of endothelial cell proliferation   biological process:  triacylglycerol metabolism   biological process:  endocytosis   biological process:  negative regulation of cell proliferation   biological process:  negative regulation of signal transduction   biological process:  regulation of fatty acid metabolism   biological process:  sequestering of lipid   biological process:  negative regulation of nitric oxide biosynthesis   biological process:  positive regulation of vasoconstriction   biological process:  negative regulation of vasodilation   biological process:  protein oligomerization   biological process:  protein homooligomerization   molecular function:  structural molecule activity   molecular function:  protein binding      BioCarta Pathways:     KEGG Pathways:  
    Probe set : 1374225_at    Name:   CDNA clone IMAGE:7389359    GB acc:   BE112921    UniGene:   Rn.27063        SOURCE        Gene Ontology:     BioCarta Pathways:     KEGG Pathways:  
    Probe set : 1394643_at    Name:   Transcribed locus    GB acc:   AA819409    UniGene:   Rn.34150        SOURCE        Gene Ontology:     BioCarta Pathways:     KEGG Pathways:  
    Probe set : 1374558_at    Name:   Similar to B7-like protein GL50-B    GB acc:   AI010316    UniGene:   Rn.17428     Locus Link:   499415      SOURCE        Gene Ontology:     BioCarta Pathways:     KEGG Pathways:  
    Probe set : 1392995_at    Name:   Transcribed locus    GB acc:   BG669396    UniGene:   Rn.16013        SOURCE        Gene Ontology:     BioCarta Pathways:     KEGG Pathways:  
    Probe set : 1369958_at    Name:   rhoB gene    GB acc:   NM_022542    UniGene:   Rn.2042    Gene Symbol:   Rhob    Locus Link:   64373    Chromosome:   6    Cytoband:   Chr:6q14    SOURCE    GENECARD       Gene Ontology:   biological process:  small GTPase mediated signal transduction   biological process:  Rho protein signal transduction   biological process:  programmed cell death, transformed cells   biological process:  cell adhesion   biological process:  endosome to lysosome transport   biological process:  positive regulation of angiogenesis   biological process:  negative regulation of progression through cell cycle   biological process:  angiogenesis   biological process:  transport   biological process:  apoptosis   biological process:  cell cycle   biological process:  cell adhesion   biological process:  development   biological process:  protein transport   biological process:  cell differentiation   biological process:  negative regulation of progression through cell cycle   molecular function:  GTP binding   molecular function:  GTP binding   molecular function:  nucleotide binding   molecular function:  protein binding      BioCarta Pathways:     KEGG Pathways:  
    Probe set : 1374603_at    Name:   SET domain and mariner transposase fusion gene    GB acc:   AA943793    UniGene:   Rn.7640    Gene Symbol:   Setmar    Locus Link:   500281    Chromosome:   4    Cytoband:   Chr:4q41    SOURCE    GENECARD       Gene Ontology:   biological process:  chromatin modification   cellular component:  nucleus   molecular function:  zinc ion binding   molecular function:  histone-lysine N-methyltransferase activity      BioCarta Pathways:     KEGG Pathways:  
    Probe set : 1372155_at    Name:   tripartite motif protein 28    GB acc:   BI283697    UniGene:   Rn.49293    Gene Symbol:   Trim28    Locus Link:   116698    Chromosome:   1    Cytoband:   Chr:1q21    SOURCE    GENECARD       Gene Ontology:     BioCarta Pathways:     KEGG Pathways:  
    Probe set : 1390783_at    Name:   ATP-binding cassette, sub-family A (ABC1), member 8a (predicted)    GB acc:   BG671264    UniGene:   Rn.22789    Gene Symbol:   Abca8a_predicted    Locus Link:   303638    Chromosome:   10    Cytoband:   Chr:10q32.1    SOURCE    GENECARD       Gene Ontology:   biological process:  transport   cellular component:  membrane   cellular component:  integral to membrane   molecular function:  nucleotide binding   molecular function:  ATP binding   molecular function:  ATPase activity, coupled to transmembrane movement of substances      BioCarta Pathways:     KEGG Pathways:  
    Probe set : 1384897_at    Name:   Transcribed locus    GB acc:   AA858696    UniGene:   Rn.16878        SOURCE        Gene Ontology:     BioCarta Pathways:     KEGG Pathways:  
    Probe set : 1376018_at    Name:   similar to Lmnb2 protein    GB acc:   BF398513    UniGene:   Rn.12408    Gene Symbol:   LOC299625    Locus Link:   299625    Chromosome:   7    Cytoband:   Chr:7q11    SOURCE    GENECARD       Gene Ontology:     BioCarta Pathways:     KEGG Pathways:  
    Probe set : 1391514_at    Name:   Transcribed locus    GB acc:   AI575440    UniGene:   Rn.147659        SOURCE        Gene Ontology:     BioCarta Pathways:     KEGG Pathways:  
    Probe set : 1372549_at    Name:   similar to RIKEN cDNA 2600017H02    GB acc:   AI409460    UniGene:   Rn.11718    Gene Symbol:   LOC497936    Locus Link:   497936    Chromosome:   10    Cytoband:   Chr:10q24    SOURCE    GENECARD       Gene Ontology:     BioCarta Pathways:     KEGG Pathways:  
    Probe set : 1377172_at    Name:   similar to Pins    GB acc:   BE117002    UniGene:   Rn.144235    Gene Symbol:   LOC362021    Locus Link:   362021    Chromosome:   2    Cytoband:   Chr:2q34    SOURCE    GENECARD       Gene Ontology:     BioCarta Pathways:     KEGG Pathways:  
    Probe set : 1370153_at    Name:   growth differentiation factor 15    GB acc:   NM_019216    UniGene:   Rn.44228    Gene Symbol:   Gdf15    Locus Link:   29455    Chromosome:   16    Cytoband:   Chr:16p14    SOURCE    GENECARD       Gene Ontology:   biological process:  transforming growth factor beta receptor signaling pathway   cellular component:  extracellular space   molecular function:  cytokine activity   molecular function:  growth factor activity   molecular function:  growth factor activity      BioCarta Pathways:     KEGG Pathways:  
    Probe set : 1379362_at    Name:   Transcribed locus    GB acc:   BE111121    UniGene:   Rn.13585        SOURCE        Gene Ontology:     BioCarta Pathways:     KEGG Pathways:  
    Probe set : 1369304_at    Name:   6-pyruvoyl-tetrahydropterin synthase    GB acc:   NM_017220    UniGene:   Rn.87164    Gene Symbol:   Pts    Locus Link:   29498    Chromosome:   8    Cytoband:   Chr:8q23    SOURCE    GENECARD       Gene Ontology:     BioCarta Pathways:     KEGG Pathways:  
    Probe set : 1381396_s_at    Name:   Kruppel-like factor 15    GB acc:   AA998067    UniGene:   Rn.22556    Gene Symbol:   Klf15    Locus Link:   85497    Chromosome:   4    Cytoband:   Chr:4q34    SOURCE    GENECARD       Gene Ontology:   biological process:  regulation of transcription, DNA-dependent   cellular component:  nucleus      BioCarta Pathways:     KEGG Pathways:  
    Probe set : 1367588_a_at    Name:   ribosomal protein L13A    GB acc:   NM_022179    UniGene:   Rn.92211    Gene Symbol:   Rpl13a    Locus Link:   317646    Chromosome:   1    Cytoband:   Chr:1q22    SOURCE    GENECARD       Gene Ontology:   biological process:  protein biosynthesis   molecular function:  structural constituent of ribosome   molecular function:  structural constituent of ribosome      BioCarta Pathways:     KEGG Pathways:  
    Probe set : 1384234_at    Name:   paired related homeobox 1    GB acc:   BE126475    UniGene:   Rn.21468    Gene Symbol:   Prrx1    Locus Link:   266813    Chromosome:   13    Cytoband:   Chr:13q22    SOURCE    GENECARD       Gene Ontology:   cellular component:  nucleus   molecular function:  DNA binding   molecular function:  transcription factor activity   molecular function:  transcriptional repressor activity      BioCarta Pathways:     KEGG Pathways:  
    Probe set : 1387900_at    Name:   CDP-diacylglycerol--inositol 3-phosphatidyltransferase (phosphatidylinositol synthase)    GB acc:   D82928    UniGene:   Rn.10598    Gene Symbol:   Cdipt    Locus Link:   192260    Chromosome:   1    Cytoband:   Chr:1q36    SOURCE    GENECARD       Gene Ontology:   cellular component:  integral to membrane   cellular component:  endoplasmic reticulum      BioCarta Pathways:     KEGG Pathways:  
    Probe set : 1376427_a_at    Name:   glycine dehydrogenase (decarboxylating; glycine decarboxylase, glycine cleavage system protein P) (predicted)    GB acc:   AI029729    UniGene:   Rn.17101    Gene Symbol:   Gldc_predicted    Locus Link:   309312    Chromosome:   1    Cytoband:   Chr:1q52    SOURCE    GENECARD       Gene Ontology:   biological process:  glycine catabolism   cellular component:  mitochondrion   cellular component:  glycine dehydrogenase complex (decarboxylating)   molecular function:  glycine dehydrogenase (decarboxylating) activity   molecular function:  electron transporter activity   molecular function:  oxidoreductase activity      BioCarta Pathways:     KEGG Pathways:  
    Probe set : 1383969_at    Name:   similar to CDNA sequence BC043301    GB acc:   BI294901    UniGene:   Rn.47393    Gene Symbol:   LOC499143    Locus Link:   499143    Chromosome:   1    Cytoband:   Chr:1q22    SOURCE    GENECARD       Gene Ontology:     BioCarta Pathways:     KEGG Pathways:  
    Probe set : 1373082_at    Name:   Protein kinase inhibitor, alpha    GB acc:   AA893743    UniGene:   Rn.94808    Gene Symbol:   Pkia    Locus Link:   114906    Chromosome:   2    Cytoband:   Chr:2q23    SOURCE    GENECARD       Gene Ontology:   biological process:  negative regulation of protein kinase activity   biological process:  negative regulation of protein kinase activity      BioCarta Pathways:     KEGG Pathways:  
    Probe set : 1371308_at    Name:   ribosomal protein S4, X-linked    GB acc:   AA799501    UniGene:   Rn.144572    Gene Symbol:   Rps4x    Locus Link:   29426    Chromosome:   X    Cytoband:   Chr:Xq31    SOURCE    GENECARD       Gene Ontology:   molecular function:  RNA binding   molecular function:  structural constituent of ribosome      BioCarta Pathways:     KEGG Pathways:  
    Probe set : 1393203_at    Name:   similar to RIKEN cDNA 2010309E21 (predicted)    GB acc:   BG665886    UniGene:   Rn.2397    Gene Symbol:   RGD1304825_predicted    Locus Link:   297415    Chromosome:   4    Cytoband:   Chr:4q34    SOURCE    GENECARD       Gene Ontology:     BioCarta Pathways:     KEGG Pathways:  
    Probe set : 1373309_at    Name:   Similar to RIKEN cDNA 1810054O13 (predicted)    GB acc:   BI281129    UniGene:   Rn.23056     Locus Link:   308602    Chromosome:   1    Cytoband:   Chr:1q22    SOURCE        Gene Ontology:     BioCarta Pathways:     KEGG Pathways:  
    Probe set : 1390133_at    Name:   BCL2/adenovirus E1B 19kDa-interacting protein 1, NIP2 (predicted)    GB acc:   AI178277    UniGene:   Rn.13297    Gene Symbol:   Bnip2_predicted    Locus Link:   300811    Chromosome:   8    Cytoband:   Chr:8q24    SOURCE    GENECARD       Gene Ontology:   biological process:  apoptosis   biological process:  anti-apoptosis   cellular component:  cytoplasm   molecular function:  GTPase activator activity      BioCarta Pathways:     KEGG Pathways:  
    Probe set : 1374540_at    Name:   cell division cycle associated 7 (predicted)    GB acc:   AA859235    UniGene:   Rn.16408    Gene Symbol:   Cdca7_predicted    Locus Link:   311742    Chromosome:   3    Cytoband:   Chr:3q22    SOURCE    GENECARD       Gene Ontology:   biological process:  cytokinesis   biological process:  regulation of cell proliferation   biological process:  cell division   cellular component:  nucleus      BioCarta Pathways:     KEGG Pathways:  
    Probe set : 1371305_at    Name:   Ribosomal protein L8    GB acc:   AI409193    UniGene:   Rn.127774    Gene Symbol:   Rpl8    Locus Link:   26962    Chromosome:   7    Cytoband:   Chr:7q34    SOURCE    GENECARD       Gene Ontology:   biological process:  protein biosynthesis   cellular component:  intracellular   cellular component:  ribosome   cellular component:  cytosolic large ribosomal subunit (sensu Eukaryota)      BioCarta Pathways:     KEGG Pathways:  
    Probe set : 1379294_at    Name:   T-cell receptor rearranged alpha-chain V-region mRNA (V-alpha-3-V-J-C) partial cds, hybridoma KL    GB acc:   AA957424    UniGene:   Rn.13051        SOURCE        Gene Ontology:     BioCarta Pathways:     KEGG Pathways:  
    Probe set : 1372749_at    Name:   Transcribed locus    GB acc:   AI009786    UniGene:   Rn.95229        SOURCE        Gene Ontology:     BioCarta Pathways:     KEGG Pathways:  
    Probe set : 1369964_at    Name:   coronin, actin binding protein 1A    GB acc:   NM_130411    UniGene:   Rn.6990    Gene Symbol:   Coro1a    Locus Link:   155151    Chromosome:   1    Cytoband:   Chr:1q36    SOURCE    GENECARD       Gene Ontology:   biological process:  transport   biological process:  cell motility   biological process:  mitosis   biological process:  T cell differentiation   molecular function:  actin binding   molecular function:  actin binding   molecular function:  structural molecule activity      BioCarta Pathways:     KEGG Pathways:  
    Probe set : 1389006_at    Name:   Macrophage expressed gene 1    GB acc:   AI170394    UniGene:   Rn.4301    Gene Symbol:   Mpeg1    Locus Link:   64552    Chromosome:   1    Cytoband:   Chr:1q43    SOURCE    GENECARD       Gene Ontology:     BioCarta Pathways:     KEGG Pathways:  
    Probe set : 1390802_at    Name:   similar to RIKEN cDNA 5033414D02 (predicted)    GB acc:   AI170769    UniGene:   Rn.106153    Gene Symbol:   RGD1306839_predicted    Locus Link:   293888    Chromosome:   1    Cytoband:   Chr:1q52    SOURCE    GENECARD       Gene Ontology:     BioCarta Pathways:     KEGG Pathways:  
    Probe set : 1388451_at    Name:   Transcribed locus    GB acc:   AA817802    UniGene:   Rn.1110        SOURCE        Gene Ontology:     BioCarta Pathways:     KEGG Pathways:  
    Probe set : 1390450_a_at    Name:   osteoglycin (predicted)    GB acc:   AA945955    UniGene:   Rn.103114    Gene Symbol:   Ogn_predicted    Locus Link:   291015    Chromosome:   17    Cytoband:   Chr:17p14    SOURCE    GENECARD       Gene Ontology:   cellular component:  extracellular space   molecular function:  growth factor activity      BioCarta Pathways:     KEGG Pathways:  
    Probe set : 1373110_at    Name:   Transcribed locus    GB acc:   AI407487    UniGene:   Rn.1452        SOURCE        Gene Ontology:     BioCarta Pathways:     KEGG Pathways:  
    Probe set : 1368245_at    Name:   ureidopropionase, beta    GB acc:   NM_053845    UniGene:   Rn.11110    Gene Symbol:   Upb1    Locus Link:   116593    Chromosome:   20    Cytoband:   Chr:20p12    SOURCE    GENECARD       Gene Ontology:   biological process:  nitrogen compound metabolism   biological process:  beta-alanine biosynthesis   biological process:  beta-alanine biosynthesis      BioCarta Pathways:     KEGG Pathways:  
    Probe set : 1380115_at     GB acc:   AA850751              Gene Ontology:     BioCarta Pathways:     KEGG Pathways:  
    Probe set : 1373114_at    Name:   Transcribed locus    GB acc:   AI408442    UniGene:   Rn.18597        SOURCE        Gene Ontology:     BioCarta Pathways:     KEGG Pathways:  
    Probe set : 1391823_at    Name:   Ras association (RalGDS/AF-6) domain family 3 (predicted)    GB acc:   AA956842    UniGene:   Rn.137557     Locus Link:   362886    Chromosome:   7    Cytoband:   Chr:7q22    SOURCE        Gene Ontology:   biological process:  signal transduction   biological process:  neuropeptide signaling pathway   molecular function:  protein binding      BioCarta Pathways:     KEGG Pathways:  
    Probe set : 1376831_at    Name:   similar to hypothetical protein FLJ10156 (predicted)    GB acc:   AI712694    UniGene:   Rn.35007    Gene Symbol:   RGD1308747_predicted    Locus Link:   360559    Chromosome:   10    Cytoband:   Chr:10q24    SOURCE    GENECARD       Gene Ontology:     BioCarta Pathways:     KEGG Pathways:  
    Probe set : 1384187_at    Name:   similar to Adapter-related protein complex 1 sigma 1B subunit (Sigma-adaptin 1B) (Adaptor protein complex AP-1 sigma-1B subunit) (Golgi adaptor HA1/AP1 adaptin sigma-1B subunit) (Clathrin assembly protein complex 1 sigma-1B small chain) (Sigma 1B s...    GB acc:   BF551686    UniGene:   Rn.17309    Gene Symbol:   LOC302671    Locus Link:   302671    Chromosome:   X    Cytoband:   Chr:Xq21    SOURCE    GENECARD       Gene Ontology:     BioCarta Pathways:     KEGG Pathways:  
    Probe set : 1377189_at    Name:   similar to BC282485_1 (predicted)    GB acc:   BI295093    UniGene:   Rn.34772    Gene Symbol:   RGD1307390_predicted    Locus Link:   292680    Chromosome:   1    Cytoband:   Chr:1q21    SOURCE    GENECARD       Gene Ontology:     BioCarta Pathways:     KEGG Pathways:  
    Probe set : 1373382_at    Name:   similar to RER1 homolog (predicted)    GB acc:   AI408771    UniGene:   Rn.129142    Gene Symbol:   RGD1306324_predicted    Locus Link:   298675    Chromosome:   5    Cytoband:   Chr:5q36    SOURCE    GENECARD       Gene Ontology:   biological process:  retrograde transport, Golgi to ER   cellular component:  Golgi apparatus   cellular component:  integral to membrane   cellular component:  integral to Golgi membrane      BioCarta Pathways:     KEGG Pathways:  
    Probe set : 1390126_at     GB acc:   BE112913              Gene Ontology:     BioCarta Pathways:     KEGG Pathways:  
    Probe set : 1372667_at    Name:   similar to RIKEN cDNA 1110059E24    GB acc:   AI170752    UniGene:   Rn.105582    Gene Symbol:   RGD:1359158    Locus Link:   361740    Chromosome:   1    Cytoband:   Chr:1q51    SOURCE    GENECARD       Gene Ontology:     BioCarta Pathways:     KEGG Pathways:  
    Probe set : 1370407_at    Name:   chloride ion pump-associated 55 kDa protein    GB acc:   AF332142    UniGene:   Rn.34505    Gene Symbol:   Pcyox1    Locus Link:   246302    Chromosome:   4    Cytoband:   Chr:4q34    SOURCE    GENECARD       Gene Ontology:   biological process:  chloride transport   biological process:  prenylcysteine catabolism   cellular component:  extracellular space   cellular component:  plasma membrane   cellular component:  lysosome   molecular function:  prenylcysteine oxidase activity   molecular function:  chloride-transporting ATPase activity   molecular function:  oxidoreductase activity      BioCarta Pathways:     KEGG Pathways:  
    Probe set : 1370561_at    Name:   iGb3 synthase    GB acc:   AF248543    UniGene:   Rn.37737    Gene Symbol:   RGD:727913    Locus Link:   171553    Chromosome:   5    Cytoband:   Chr:5q36    SOURCE    GENECARD       Gene Ontology:   biological process:  glycosphingolipid biosynthesis   molecular function:  transferase activity, transferring glycosyl groups      BioCarta Pathways:     KEGG Pathways:  
    Probe set : 1371373_at    Name:   similar to RIKEN cDNA 2500002L14; EST C77350    GB acc:   AI010460    UniGene:   Rn.94883    Gene Symbol:   RGD:1359698    Locus Link:   297337    Chromosome:   4    Cytoband:   Chr:4q33    SOURCE    GENECARD       Gene Ontology:   biological process:  protein ubiquitination   cellular component:  ubiquitin ligase complex   molecular function:  ubiquitin-protein ligase activity   molecular function:  zinc ion binding   molecular function:  metal ion binding      BioCarta Pathways:     KEGG Pathways:  
    Probe set : 1383057_at    Name:   ASF1 anti-silencing function 1 homolog A (S. cerevisiae) (predicted)    GB acc:   BF558602    UniGene:   Rn.7111    Gene Symbol:   Asf1a_predicted    Locus Link:   294408    Chromosome:   20    Cytoband:   Chr:20q11    SOURCE    GENECARD       Gene Ontology:   biological process:  DNA repair   biological process:  nucleosome assembly   biological process:  loss of chromatin silencing   cellular component:  nucleus   cellular component:  chromatin remodeling complex   molecular function:  chromatin binding   molecular function:  histone binding      BioCarta Pathways:     KEGG Pathways:  
    Probe set : 1371891_at    Name:   similar to 1700123O20Rik protein (predicted)    GB acc:   BG371476    UniGene:   Rn.2583    Gene Symbol:   RGD1308430_predicted    Locus Link:   361038    Chromosome:   15    Cytoband:   Chr:15p13    SOURCE    GENECARD       Gene Ontology:     BioCarta Pathways:     KEGG Pathways:  
    Probe set : 1384762_at    Name:   Transcribed locus    GB acc:   BE103873    UniGene:   Rn.147915        SOURCE        Gene Ontology:     BioCarta Pathways:     KEGG Pathways:  
    Probe set : 1386881_at    Name:   insulin-like growth factor binding protein 3    GB acc:   NM_012588    UniGene:   Rn.26369    Gene Symbol:   Igfbp3    Locus Link:   24484    Chromosome:   14    Cytoband:   Chr:14q21    SOURCE    GENECARD       Gene Ontology:   cellular component:  extracellular region   cellular component:  extracellular space   cellular component:  extracellular region      BioCarta Pathways:     KEGG Pathways:  
    Probe set : 1391224_at    Name:   similar to 4921517L17Rik protein (predicted)    GB acc:   AI169436    UniGene:   Rn.17118    Gene Symbol:   RGD1311678_predicted    Locus Link:   296311    Chromosome:   3    Cytoband:   Chr:3q42    SOURCE    GENECARD       Gene Ontology:   biological process:  protein biosynthesis   cellular component:  intracellular   cellular component:  ribosome   molecular function:  nucleotide binding   molecular function:  structural constituent of ribosome      BioCarta Pathways:     KEGG Pathways:  
    Probe set : 1367892_at    Name:   pyruvate dehydrogenase kinase, isoenzyme 2    GB acc:   NM_030872    UniGene:   Rn.88597    Gene Symbol:   Pdk2    Locus Link:   81530    Chromosome:   10    Cytoband:   Chr:10q31    SOURCE    GENECARD       Gene Ontology:   cellular component:  mitochondrion      BioCarta Pathways:     KEGG Pathways:  
    Probe set : 1372672_at    Name:   quinolinate phosphoribosyltransferase (predicted)    GB acc:   AI407095    UniGene:   Rn.9220    Gene Symbol:   Qprt_predicted    Locus Link:   293504    Chromosome:   1    Cytoband:   Chr:1q36    SOURCE    GENECARD       Gene Ontology:   cellular component:  extracellular space      BioCarta Pathways:     KEGG Pathways:  
    Probe set : 1387276_at    Name:   activity and neurotransmitter-induced early gene protein 4 (ania-4)    GB acc:   NM_021584    UniGene:   Rn.80575    Gene Symbol:   RGD:621187    Locus Link:   360341    Chromosome:   2    Cytoband:   Chr:2q26    SOURCE    GENECARD       Gene Ontology:     BioCarta Pathways:     KEGG Pathways:  
    Probe set : 1371732_at    Name:   dermatopontin (predicted)    GB acc:   BI285485    UniGene:   Rn.8396    Gene Symbol:   Dpt_predicted    Locus Link:   289178    Chromosome:   13    Cytoband:   Chr:13q23    SOURCE    GENECARD       Gene Ontology:   biological process:  cell adhesion   cellular component:  extracellular matrix (sensu Metazoa)   cellular component:  extracellular space   molecular function:  protein binding      BioCarta Pathways:     KEGG Pathways:  
    Probe set : 1376355_at    Name:   similar to Protein CXorf17 homolog    GB acc:   BF399639    UniGene:   Rn.41046    Gene Symbol:   LOC317423    Locus Link:   317423    Chromosome:   X    Cytoband:   Chr:Xq21    SOURCE    GENECARD       Gene Ontology:     BioCarta Pathways:     KEGG Pathways:  
    Probe set : 1390883_at    Name:   Similar to A-kinase anchor protein 11 (Protein kinase A anchoring protein 11) (PRKA11) (A kinase anchor protein 220 kDa) (AKAP 220) (hAKAP220)    GB acc:   AI501759    UniGene:   Rn.10557    Gene Symbol:   Akap11    Locus Link:   25228    Chromosome:   2    Cytoband:   Chr:2    SOURCE    GENECARD       Gene Ontology:   biological process:  protein kinase cascade   cellular component:  peroxisome   cellular component:  peroxisome      BioCarta Pathways:     KEGG Pathways:  
    Probe set : 1374435_at    Name:   Transcribed locus, strongly similar to NP_954666.1 suppressor of zeste 12 homolog [Mus musculus]    GB acc:   AA996970    UniGene:   Rn.13041        SOURCE        Gene Ontology:     BioCarta Pathways:     KEGG Pathways:  
    Probe set : 1371390_at    Name:   tubulin, beta, 2    GB acc:   AA892044    UniGene:   Rn.110562    Gene Symbol:   Tubb2    Locus Link:   296554    Chromosome:   3    Cytoband:   Chr:3p13    SOURCE    GENECARD       Gene Ontology:   biological process:  microtubule-based movement   biological process:  protein polymerization   cellular component:  cytoplasm   cellular component:  microtubule   cellular component:  protein complex      BioCarta Pathways:     KEGG Pathways:  
    Probe set : 1384988_at    Name:   F-box only protein 5 (predicted)    GB acc:   BM385403    UniGene:   Rn.81585    Gene Symbol:   Fbxo5_predicted    Locus Link:   292263    Chromosome:   1    Cytoband:   Chr:1p11    SOURCE    GENECARD       Gene Ontology:   biological process:  proteolysis   biological process:  ubiquitin cycle   molecular function:  ubiquitin conjugating enzyme activity   molecular function:  ubiquitin-protein ligase activity      BioCarta Pathways:     KEGG Pathways:  
    Probe set : 1375119_at    Name:   neural precursor cell expressed, developmentally down-regulated gene 4A    GB acc:   BI284798    UniGene:   Rn.99540    Gene Symbol:   Nedd4a    Locus Link:   25489    Chromosome:   8    Cytoband:   Chr:8q24    SOURCE    GENECARD       Gene Ontology:   biological process:  protein modification   biological process:  ubiquitin cycle   cellular component:  ubiquitin ligase complex   cellular component:  intracellular   cellular component:  cytosol      BioCarta Pathways:     KEGG Pathways:  
    Probe set : 1379984_at    Name:   Transcribed locus    GB acc:   AI411147    UniGene:   Rn.146220        SOURCE        Gene Ontology:     BioCarta Pathways:     KEGG Pathways:  
    Probe set : 1385211_at     GB acc:   BE116091              Gene Ontology:     BioCarta Pathways:     KEGG Pathways:  
    Probe set : 1375306_at    Name:   similar to toll-associated serine protease (predicted)    GB acc:   BI293698    UniGene:   Rn.15228    Gene Symbol:   RGD1308120_predicted    Locus Link:   360959    Chromosome:   14    Cytoband:   Chr:14q21    SOURCE    GENECARD       Gene Ontology:     BioCarta Pathways:     KEGG Pathways:  
    Probe set : 1388453_at    Name:   myeloid-associated differentiation marker    GB acc:   BI296048    UniGene:   Rn.106161    Gene Symbol:   Myadm    Locus Link:   369016    Chromosome:   1    Cytoband:   Chr:1q12    SOURCE    GENECARD       Gene Ontology:   biological process:  myeloid cell differentiation   cellular component:  membrane      BioCarta Pathways:     KEGG Pathways:  
    Probe set : 1389709_at    Name:   similar to Zinc finger protein 133    GB acc:   AI146063    UniGene:   Rn.117293    Gene Symbol:   LOC499900    Locus Link:   499900    Chromosome:   3    Cytoband:   Chr:3q41    SOURCE    GENECARD       Gene Ontology:   biological process:  transcription   biological process:  regulation of transcription, DNA-dependent   cellular component:  intracellular   cellular component:  nucleus   molecular function:  nucleic acid binding   molecular function:  zinc ion binding   molecular function:  metal ion binding      BioCarta Pathways:     KEGG Pathways:  
    Probe set : 1389582_at    Name:   Transcribed locus    GB acc:   AI407405    UniGene:   Rn.125129        SOURCE        Gene Ontology:     BioCarta Pathways:     KEGG Pathways:  
    Probe set : 1398348_at    Name:   Transcribed locus    GB acc:   AA945604    UniGene:   Rn.111929        SOURCE        Gene Ontology:     BioCarta Pathways:     KEGG Pathways:  
    Probe set : 1377244_at    Name:   Zinc finger protein 95 (predicted)    GB acc:   BM390979    UniGene:   Rn.38399     Locus Link:   304275    Chromosome:   12    Cytoband:   Chr:12p11    SOURCE        Gene Ontology:   biological process:  regulation of transcription, DNA-dependent   cellular component:  intracellular   cellular component:  nucleus      BioCarta Pathways:     KEGG Pathways:  
    Probe set : 1371441_at    Name:   phosphoprotein enriched in astrocytes 15 (predicted)    GB acc:   BG662875    UniGene:   Rn.7282    Gene Symbol:   Pea15_predicted    Locus Link:   364052    Chromosome:   13    Cytoband:   Chr:13q24    SOURCE    GENECARD       Gene Ontology:   cellular component:  membrane fraction   cellular component:  cytosol   cellular component:  microtubule associated complex   molecular function:  protein kinase C binding   molecular function:  sugar porter activity   molecular function:  protein binding      BioCarta Pathways:     KEGG Pathways:  
    Probe set : 1385439_x_at    Name:   Transcribed locus, strongly similar to XP_220978.3 PREDICTED: similar to Probable ATP-dependent helicase LGP2 (Protein D11Lgp2) [Rattus norvegicus]    GB acc:   BF417541    UniGene:   Rn.105654        SOURCE        Gene Ontology:     BioCarta Pathways:     KEGG Pathways:  
    Probe set : 1385226_at     GB acc:   AI137059              Gene Ontology:     BioCarta Pathways:     KEGG Pathways:  
    Probe set : 1368527_at    Name:   prostaglandin-endoperoxide synthase 2    GB acc:   U03389    UniGene:   Rn.44369    Gene Symbol:   Ptgs2    Locus Link:   29527    Chromosome:   13    Cytoband:   Chr:13q21    SOURCE    GENECARD       Gene Ontology:   biological process:  regulation of progression through cell cycle   biological process:  regulation of progression through cell cycle   biological process:  prostaglandin biosynthesis   biological process:  prostaglandin biosynthesis   biological process:  cell motility   biological process:  response to oxidative stress   biological process:  physiological process   biological process:  physiological process   biological process:  regulation of blood pressure   biological process:  positive regulation of cell proliferation   biological process:  positive regulation of cell proliferation   biological process:  negative regulation of cell proliferation   biological process:  negative regulation of cell proliferation   biological process:  cyclooxygenase pathway   biological process:  keratinocyte differentiation   biological process:  regulation of inflammatory response   biological process:  regulation of blood pressure   biological process:  keratinocyte differentiation   biological process:  fatty acid biosynthesis   biological process:  lipid biosynthesis      BioCarta Pathways:     KEGG Pathways:  
    Probe set : 1370693_a_at    Name:   cyclic nucleotide phosphodiesterase 1    GB acc:   M18630    UniGene:   Rn.31762    Gene Symbol:   Cnp1    Locus Link:   25275    Chromosome:   10    Cytoband:   Chr:10q32.1    SOURCE    GENECARD       Gene Ontology:   biological process:  synaptic transmission   biological process:  cyclic nucleotide catabolism   cellular component:  membrane   molecular function:  2',3'-cyclic-nucleotide 3'-phosphodiesterase activity   molecular function:  hydrolase activity      BioCarta Pathways:     KEGG Pathways:  
    Probe set : 1372168_s_at    Name:   insulin-like growth factor binding protein 6    GB acc:   BG673588    UniGene:   Rn.6431    Gene Symbol:   Igfbp6    Locus Link:   25641    Chromosome:   7    Cytoband:   Chr:7q36    SOURCE    GENECARD       Gene Ontology:   biological process:  regulation of cell growth   biological process:  signal transduction   biological process:  negative regulation of cell proliferation   cellular component:  extracellular region   cellular component:  extracellular space   molecular function:  insulin-like growth factor binding   molecular function:  growth factor binding      BioCarta Pathways:     KEGG Pathways:  
    Probe set : 1375120_at    Name:   inhibitor of DNA binding 4    GB acc:   AI412150    UniGene:   Rn.145407    Gene Symbol:   Idb4    Locus Link:   291023    Chromosome:   17    Cytoband:   Chr:17p14    SOURCE    GENECARD       Gene Ontology:   biological process:  regulation of transcription from RNA polymerase II promoter   cellular component:  nucleus   molecular function:  transcription corepressor activity      BioCarta Pathways:     KEGG Pathways:  
    Probe set : 1383926_at    Name:   budding uninhibited by benzimidazoles 1 homolog, beta (S. cerevisiae)    GB acc:   BF557145    UniGene:   Rn.49845    Gene Symbol:   Bub1b    Locus Link:   171576    Chromosome:   3    Cytoband:   Chr:3q35    SOURCE    GENECARD       Gene Ontology:   molecular function:  kinase activity      BioCarta Pathways:     KEGG Pathways:  
    Probe set : 1373241_at     GB acc:   AW252569              Gene Ontology:     BioCarta Pathways:     KEGG Pathways:  
    Probe set : 1392479_at    Name:   FK506-binding protein 1a    GB acc:   AA925694    UniGene:   Rn.80611    Gene Symbol:   Fkbp1a    Locus Link:   25639    Chromosome:   3    Cytoband:   Chr:3q41    SOURCE    GENECARD       Gene Ontology:   biological process:  protein folding   biological process:  transport   biological process:  positive regulation of I-kappaB kinase/NF-kappaB cascade   cellular component:  microsome   cellular component:  membrane      BioCarta Pathways:     KEGG Pathways:  
    Probe set : 1390408_at    Name:   similar to Hccs protein    GB acc:   BF405032    UniGene:   Rn.64326    Gene Symbol:   LOC317444    Locus Link:   317444    Chromosome:   X    Cytoband:   Chr:Xq21    SOURCE    GENECARD       Gene Ontology:     BioCarta Pathways:     KEGG Pathways:  
    Probe set : 1376013_at    Name:   similar to mKIAA0227 protein    GB acc:   BF282700    UniGene:   Rn.8149    Gene Symbol:   LOC500689    Locus Link:   500689    Chromosome:   6    Cytoband:   Chr:6q24    SOURCE    GENECARD       Gene Ontology:     BioCarta Pathways:     KEGG Pathways:  
    Probe set : 1387566_at    Name:   phospholipase A2, group IVA (cytosolic, calcium-dependent)    GB acc:   NM_133551    UniGene:   Rn.10162    Gene Symbol:   Pla2g4a    Locus Link:   24653    Chromosome:   13    Cytoband:   Chr:13q21    SOURCE    GENECARD       Gene Ontology:   cellular component:  cytosol   cellular component:  integral to membrane      BioCarta Pathways:     KEGG Pathways:  
    Probe set : 1368596_at    Name:   SNF1-like kinase    GB acc:   NM_021693    UniGene:   Rn.42905    Gene Symbol:   Snf1lk    Locus Link:   59329    Chromosome:   20    Cytoband:   Chr:20p12    SOURCE    GENECARD       Gene Ontology:   biological process:  negative regulation of transcription from RNA polymerase II promoter   biological process:  protein amino acid phosphorylation   biological process:  protein amino acid phosphorylation   biological process:  cell cycle   biological process:  protein kinase cascade   biological process:  regulation of progression through mitotic cell cycle   biological process:  regulation of cell differentiation   biological process:  protein amino acid phosphorylation   biological process:  protein kinase cascade   biological process:  regulation of progression through mitotic cell cycle   biological process:  regulation of cell differentiation   biological process:  development   biological process:  cell differentiation   cellular component:  nucleus   cellular component:  cytoplasm   cellular component:  cytosol   cellular component:  cytoplasm   molecular function:  magnesium ion binding   molecular function:  protein kinase activity   molecular function:  protein serine/threonine kinase activity   molecular function:  protein serine/threonine kinase activity   molecular function:  protein-tyrosine kinase activity   molecular function:  ATP binding   molecular function:  kinase activity   molecular function:  transcriptional repressor activity   molecular function:  transferase activity   molecular function:  magnesium ion binding   molecular function:  protein serine/threonine kinase activity   molecular function:  ATP binding   molecular function:  nucleotide binding   molecular function:  metal ion binding      BioCarta Pathways:     KEGG Pathways:  
    Probe set : 1370909_at    Name:   nucleoporin 62    GB acc:   BI286040    UniGene:   Rn.54450    Gene Symbol:   Nup62    Locus Link:   65274    Chromosome:   1    Cytoband:   Chr:1q22    SOURCE    GENECARD       Gene Ontology:   biological process:  transport   cellular component:  nucleus   cellular component:  nuclear pore   cellular component:  kinesin complex   molecular function:  structural constituent of nuclear pore   molecular function:  protein binding      BioCarta Pathways:     KEGG Pathways:  
    Probe set : 1367820_at    Name:   barrier to autointegration factor 1    GB acc:   NM_053631    UniGene:   Rn.19921    Gene Symbol:   Banf1    Locus Link:   114087    Chromosome:   1    Cytoband:   Chr:1q43    SOURCE    GENECARD       Gene Ontology:   cellular component:  nucleus   cellular component:  chromosome   molecular function:  DNA binding      BioCarta Pathways:     KEGG Pathways:  
    Probe set : 1375921_at    Name:   Reticulocalbin (predicted)    GB acc:   BF281577    UniGene:   Rn.103345     Locus Link:   362182    Chromosome:   3    Cytoband:   Chr:3q32    SOURCE        Gene Ontology:   cellular component:  extracellular space   cellular component:  endoplasmic reticulum   cellular component:  endoplasmic reticulum lumen   molecular function:  calcium ion binding      BioCarta Pathways:     KEGG Pathways:  
    Probe set : 1387703_a_at    Name:   ubiquitin specific protease 2    GB acc:   AF106659    UniGene:   Rn.144632    Gene Symbol:   Usp2    Locus Link:   115771    Chromosome:   8    Cytoband:   Chr:8q22    SOURCE    GENECARD       Gene Ontology:   biological process:  ubiquitin-dependent protein catabolism   biological process:  ubiquitin cycle   cellular component:  centrosome      BioCarta Pathways:     KEGG Pathways:  
    Probe set : 1373807_at    Name:   Vascular endothelial growth factor A    GB acc:   AI175732    UniGene:   Rn.1923    Gene Symbol:   Vegf    Locus Link:   83785    Chromosome:   9    Cytoband:   Chr:9q12    SOURCE    GENECARD       Gene Ontology:   biological process:  regulation of progression through cell cycle   biological process:  angiogenesis   biological process:  angiogenesis   biological process:  vasculogenesis   biological process:  anti-apoptosis   biological process:  response to stress   biological process:  signal transduction   biological process:  nervous system development   biological process:  nervous system development   biological process:  mesoderm development   biological process:  cell proliferation   biological process:  positive regulation of cell proliferation   biological process:  positive regulation of cell proliferation   biological process:  positive regulation of signal transduction   biological process:  cell migration   biological process:  positive regulation of vascular endothelial growth factor receptor signaling pathway   biological process:  negative regulation of apoptosis   biological process:  induction of positive chemotaxis   biological process:  development   biological process:  cell differentiation   biological process:  nervous system development      BioCarta Pathways:     KEGG Pathways:  
    Probe set : 1370927_at    Name:   procollagen, type XII, alpha 1    GB acc:   BE108345    UniGene:   Rn.11218    Gene Symbol:   Col12a1    Locus Link:   25683    Chromosome:   8    Cytoband:   Chr:8q31    SOURCE    GENECARD       Gene Ontology:     BioCarta Pathways:     KEGG Pathways:  
    Probe set : 1368173_at    Name:   nucleolar protein 5    GB acc:   NM_021754    UniGene:   Rn.43930    Gene Symbol:   Nol5    Locus Link:   60373    Chromosome:   9    Cytoband:   Chr:9q31    SOURCE    GENECARD       Gene Ontology:     BioCarta Pathways:     KEGG Pathways:  
    Probe set : 1374379_at    Name:   Dual specificity phosphatase 23 (predicted)    GB acc:   BF396810    UniGene:   Rn.45931     Locus Link:   360881    Chromosome:   13    Cytoband:   Chr:13q24    SOURCE        Gene Ontology:   biological process:  protein amino acid dephosphorylation   cellular component:  nucleus      BioCarta Pathways:     KEGG Pathways:  
    Probe set : 1368381_at    Name:   cartilage acidic protein 1    GB acc:   NM_134401    UniGene:   Rn.107122    Gene Symbol:   Crtac1    Locus Link:   171438    Chromosome:   1    Cytoband:   Chr:1q54    SOURCE    GENECARD       Gene Ontology:   biological process:  cell adhesion   biological process:  cell-matrix adhesion   cellular component:  extracellular space   cellular component:  integrin complex   molecular function:  calcium ion binding      BioCarta Pathways:     KEGG Pathways:  
    Probe set : 1387182_at    Name:   G protein-coupled receptor 37    GB acc:   NM_057201    UniGene:   Rn.28035    Gene Symbol:   Gpr37    Locus Link:   117549    Chromosome:   4    Cytoband:   Chr:4q22    SOURCE    GENECARD       Gene Ontology:   biological process:  electron transport   biological process:  G-protein coupled receptor protein signaling pathway   biological process:  signal transduction   cellular component:  extracellular space   cellular component:  integral to plasma membrane   cellular component:  integral to membrane      BioCarta Pathways:     KEGG Pathways:  
    Probe set : 1371581_at    Name:   Vascular endothelial zinc finger 1 (predicted)    GB acc:   BM388899    UniGene:   Rn.84878     Locus Link:   287615    Chromosome:   10    Cytoband:   Chr:10q26    SOURCE        Gene Ontology:   biological process:  regulation of transcription from RNA polymerase II promoter   biological process:  cellular defense response   cellular component:  nucleus      BioCarta Pathways:     KEGG Pathways:  
    Probe set : 1371786_at    Name:   tripartite motif-containing 35    GB acc:   BE111791    UniGene:   Rn.105225    Gene Symbol:   Trim35    Locus Link:   498538    Chromosome:   15    Cytoband:   Chr:15p12    SOURCE    GENECARD       Gene Ontology:   biological process:  protein ubiquitination   cellular component:  ubiquitin ligase complex   cellular component:  intracellular   molecular function:  ubiquitin-protein ligase activity   molecular function:  zinc ion binding   molecular function:  metal ion binding      BioCarta Pathways:     KEGG Pathways:  
    Probe set : 1374626_at    Name:   similar to leucine-rich alpha-2-glycoprotein    GB acc:   BG371585    UniGene:   Rn.17847    Gene Symbol:   RGD:1359464    Locus Link:   367455      SOURCE    GENECARD       Gene Ontology:     BioCarta Pathways:     KEGG Pathways:  
    Probe set : 1371794_at    Name:   Transcribed locus, strongly similar to NP_035873.1 SH3-domain binding protein 3 [Mus musculus]    GB acc:   BM391449    UniGene:   Rn.6467        SOURCE        Gene Ontology:     BioCarta Pathways:     KEGG Pathways:  
    Probe set : 1390609_at    Name:   Transcribed locus    GB acc:   BI296683    UniGene:   Rn.27672        SOURCE        Gene Ontology:     BioCarta Pathways:     KEGG Pathways:  
    Probe set : 1370699_a_at    Name:   epidermal growth factor receptor /// peptidase D    GB acc:   AF187818    UniGene:   Rn.37227    Gene Symbol:   Egfr /// Pepd    Locus Link:   24329 /// 292808    Chromosome:   14    Cytoband:   Chr:14q22 /// Chr:1q21    SOURCE    GENECARD       Gene Ontology:   biological process:  regulation of progression through cell cycle   biological process:  cellular morphogenesis   biological process:  electron transport   biological process:  protein biosynthesis   biological process:  protein amino acid phosphorylation   biological process:  signal transduction   biological process:  signal transduction   biological process:  transmembrane receptor protein tyrosine kinase signaling pathway   biological process:  epidermal growth factor receptor signaling pathway   biological process:  epidermal growth factor receptor signaling pathway   biological process:  protein kinase cascade   biological process:  cell proliferation   biological process:  negative regulation of progression through cell cycle   biological process:  regulation of peptidyl-tyrosine phosphorylation   biological process:  cellular physiological process   biological process:  proteolysis   biological process:  collagen catabolism   molecular function:  protein kinase activity   molecular function:  protein serine/threonine kinase activity   molecular function:  protein-tyrosine kinase activity   molecular function:  transmembrane receptor protein tyrosine kinase activity   molecular function:  signal transducer activity   molecular function:  receptor activity   molecular function:  epidermal growth factor receptor activity   molecular function:  epidermal growth factor receptor activity   molecular function:  epidermal growth factor receptor activity   molecular function:  electron transporter activity   molecular function:  protein binding   molecular function:  ATP binding   molecular function:  kinase activity   molecular function:  transferase activity   molecular function:  X-Pro dipeptidase activity   molecular function:  peptidase activity   molecular function:  metalloexopeptidase activity   molecular function:  metallopeptidase activity   molecular function:  hydrolase activity   molecular function:  dipeptidase activity   molecular function:  manganese ion binding   molecular function:  iron ion binding      BioCarta Pathways:     KEGG Pathways:  
    Probe set : 1388118_at    Name:   3-hydroxyisobutyrate dehydrogenase    GB acc:   AI176586    UniGene:   Rn.73    Gene Symbol:   Hibadh    Locus Link:   63938    Chromosome:   4    Cytoband:   Chr:4q24    SOURCE    GENECARD       Gene Ontology:   biological process:  pentose-phosphate shunt   biological process:  valine metabolism   cellular component:  mitochondrion      BioCarta Pathways:     KEGG Pathways:  
    Probe set : 1372642_at    Name:   Transcribed locus    GB acc:   BE113397    UniGene:   Rn.13357        SOURCE        Gene Ontology:     BioCarta Pathways:     KEGG Pathways:  
    Probe set : 1369161_at    Name:   ATP-binding cassette, sub-family B (MDR/TAP), member 4    GB acc:   NM_012690    UniGene:   Rn.82691    Gene Symbol:   Abcb4    Locus Link:   24891    Chromosome:   4    Cytoband:   Chr:4q11-q12    SOURCE    GENECARD       Gene Ontology:     BioCarta Pathways:     KEGG Pathways:  
    Probe set : 1384073_at    Name:   alcohol dehydrogenase, iron containing, 1 (predicted)    GB acc:   BF543577    UniGene:   Rn.17173    Gene Symbol:   Adhfe1_predicted    Locus Link:   362474    Chromosome:   5    Cytoband:   Chr:5q11    SOURCE    GENECARD       Gene Ontology:   biological process:  metabolism   molecular function:  iron ion binding   molecular function:  oxidoreductase activity      BioCarta Pathways:     KEGG Pathways:  
    Probe set : 1376625_at     GB acc:   BI296015              Gene Ontology:     BioCarta Pathways:     KEGG Pathways:  
    Probe set : 1384276_at    Name:   Transcribed locus    GB acc:   BI283340    UniGene:   Rn.153904        SOURCE        Gene Ontology:     BioCarta Pathways:     KEGG Pathways:  
    Probe set : 1371740_at    Name:   similar to RIKEN cDNA 5730470L24 (predicted)    GB acc:   AI178732    UniGene:   Rn.9125    Gene Symbol:   RGD1311316_predicted    Locus Link:   362007    Chromosome:   2    Cytoband:   Chr:2q34    SOURCE    GENECARD       Gene Ontology:     BioCarta Pathways:     KEGG Pathways:  
    Probe set : 1398597_at    Name:   Transcribed locus    GB acc:   AI044699    UniGene:   Rn.30788        SOURCE        Gene Ontology:     BioCarta Pathways:     KEGG Pathways:  
    Probe set : 1390907_at    Name:   Transcribed locus    GB acc:   AI145039    UniGene:   Rn.147757        SOURCE        Gene Ontology:     BioCarta Pathways:     KEGG Pathways:  
    Probe set : 1368811_at    Name:   lamin B1    GB acc:   NM_053905    UniGene:   Rn.11362    Gene Symbol:   Lmnb1    Locus Link:   116685    Chromosome:   18    Cytoband:   Chr:18p12    SOURCE    GENECARD       Gene Ontology:   molecular function:  structural molecule activity      BioCarta Pathways:     KEGG Pathways:  
    Probe set : 1367573_at    Name:   ribosomal protein S6    GB acc:   NM_017160    UniGene:   Rn.34341    Gene Symbol:   Rps6    Locus Link:   29304      SOURCE    GENECARD       Gene Ontology:   biological process:  protein biosynthesis   biological process:  ribosome biogenesis   molecular function:  RNA binding   molecular function:  structural constituent of ribosome   molecular function:  structural constituent of ribosome      BioCarta Pathways:     KEGG Pathways:  
    Probe set : 1372202_at    Name:   similar to expressed sequence AI597479 (predicted)    GB acc:   BF284058    UniGene:   Rn.12996    Gene Symbol:   RGD1310553_predicted    Locus Link:   301374    Chromosome:   9    Cytoband:   Chr:9q22    SOURCE    GENECARD       Gene Ontology:     BioCarta Pathways:     KEGG Pathways:  
    Probe set : 1367590_at    Name:   RAN, member RAS oncogene family    GB acc:   NM_053439    UniGene:   Rn.107698    Gene Symbol:   Ran    Locus Link:   84509    Chromosome:   12    Cytoband:   Chr:12q14    SOURCE    GENECARD       Gene Ontology:   biological process:  regulation of progression through cell cycle   biological process:  microtubule cytoskeleton organization and biogenesis   biological process:  DNA metabolism   biological process:  RNA export from nucleus   biological process:  protein import into nucleus   biological process:  protein export from nucleus   biological process:  protein export from nucleus   biological process:  intracellular protein transport   biological process:  nucleocytoplasmic transport   biological process:  mitotic spindle organization and biogenesis   biological process:  mitosis   biological process:  signal transduction   biological process:  small GTPase mediated signal transduction   biological process:  spermatid development   biological process:  protein transport   biological process:  transport   cellular component:  nucleus   cellular component:  nuclear pore      BioCarta Pathways:     KEGG Pathways:  
    Probe set : 1370770_s_at    Name:   kit ligand    GB acc:   AF071205    UniGene:   Rn.44216    Gene Symbol:   Kitl    Locus Link:   60427    Chromosome:   7    Cytoband:   Chr:7q13    SOURCE    GENECARD       Gene Ontology:   molecular function:  stem cell factor receptor binding   molecular function:  protein binding   molecular function:  growth factor activity      BioCarta Pathways:     KEGG Pathways:  
    Probe set : 1375760_at    Name:   Transducer of ERBB2, 2    GB acc:   AI070270    UniGene:   Rn.18247    Gene Symbol:   Tob2    Locus Link:   315159    Chromosome:   7    Cytoband:   Chr:7q34    SOURCE    GENECARD       Gene Ontology:     BioCarta Pathways:     KEGG Pathways:  
    Probe set : 1398830_at    Name:   ribosomal protein L28    GB acc:   NM_022697    UniGene:   Rn.36614    Gene Symbol:   Rpl28    Locus Link:   64638    Chromosome:   1    Cytoband:   Chr:1q12    SOURCE    GENECARD       Gene Ontology:   biological process:  protein biosynthesis   biological process:  ribosome biogenesis   biological process:  protein biosynthesis      BioCarta Pathways:     KEGG Pathways:  
    Probe set : 1382898_at    Name:   Transcribed locus    GB acc:   BG376849    UniGene:   Rn.66436        SOURCE        Gene Ontology:     BioCarta Pathways:     KEGG Pathways:  
    Probe set : 1387690_at    Name:   caspase 3    GB acc:   U84410    UniGene:   Rn.10562    Gene Symbol:   Casp3    Locus Link:   25402    Chromosome:   16    Cytoband:   Chr:16q11    SOURCE    GENECARD       Gene Ontology:   biological process:  B cell homeostasis   biological process:  proteolysis   biological process:  apoptosis   biological process:  induction of apoptosis   biological process:  induction of apoptosis   biological process:  intracellular signaling cascade   biological process:  intracellular signaling cascade   biological process:  induction of apoptosis via death domain receptors   biological process:  induction of apoptosis by oxidative stress   biological process:  response to wounding   biological process:  neuron differentiation   biological process:  keratinocyte differentiation   biological process:  negative regulation of B cell proliferation   biological process:  T cell homeostasis   biological process:  cell fate commitment   biological process:  negative regulation of cyclin dependent protein kinase activity   biological process:  negative regulation of progression through cell cycle   biological process:  negative regulation of activated T cell proliferation   cellular component:  cytoplasm   cellular component:  cytosol      BioCarta Pathways:     KEGG Pathways:  
    Probe set : 1370809_at    Name:   tubulin, gamma 1    GB acc:   AB015946    UniGene:   Rn.138701    Gene Symbol:   Tubg1    Locus Link:   252921    Chromosome:   10    Cytoband:   Chr:10q32.1    SOURCE    GENECARD       Gene Ontology:   cellular component:  pericentriolar material   cellular component:  condensed nuclear chromosome   cellular component:  gamma-tubulin complex   cellular component:  gamma-tubulin complex   cellular component:  centrosome   cellular component:  microtubule organizing center   cellular component:  spindle pole body   cellular component:  polar microtubule   cellular component:  microtubule   cellular component:  spindle microtubule   cellular component:  cytoplasmic microtubule   cellular component:  tubulin   cellular component:  gamma-tubulin complex   cellular component:  cytoplasm   cellular component:  centrosome   cellular component:  spindle pole body   cellular component:  polar microtubule   cellular component:  protein complex      BioCarta Pathways:     KEGG Pathways:  
    Probe set : 1387939_at    Name:   proline rich 2    GB acc:   BF409210    UniGene:   Rn.10967    Gene Symbol:   Pnrc1    Locus Link:   286988    Chromosome:   5    Cytoband:   Chr:5q21    SOURCE    GENECARD       Gene Ontology:   biological process:  transcription   biological process:  regulation of transcription, DNA-dependent   cellular component:  nucleus      BioCarta Pathways:     KEGG Pathways:  
    Probe set : 1373831_at    Name:   hypothetical protein LOC619574    GB acc:   AW920524     Gene Symbol:   LOC619574    Locus Link:   619574    Chromosome:   10    Cytoband:   Chr:10q32     GENECARD       Gene Ontology:     BioCarta Pathways:     KEGG Pathways:  
    Probe set : 1387877_at    Name:   formiminotransferase cyclodeaminase    GB acc:   BE096729    UniGene:   Rn.20140    Gene Symbol:   Ftcd    Locus Link:   89833    Chromosome:   20    Cytoband:   Chr:20p12    SOURCE    GENECARD       Gene Ontology:   cellular component:  cytoplasm   cellular component:  Golgi apparatus   cellular component:  Golgi apparatus   cellular component:  Golgi stack      BioCarta Pathways:     KEGG Pathways:  
    Probe set : 1372112_at    Name:   similar to 9230105E10Rik protein (predicted)    GB acc:   AA945761    UniGene:   Rn.12117    Gene Symbol:   RGD1304579_predicted    Locus Link:   308906    Chromosome:   1    Cytoband:   Chr:1q32    SOURCE    GENECARD       Gene Ontology:   biological process:  regulation of transcription, DNA-dependent   biological process:  protein ubiquitination   cellular component:  nucleus   cellular component:  ubiquitin ligase complex   cellular component:  intracellular   molecular function:  ubiquitin-protein ligase activity   molecular function:  zinc ion binding   molecular function:  metal ion binding      BioCarta Pathways:     KEGG Pathways:  
    Probe set : 1377748_at    Name:   Transcribed locus    GB acc:   BF410460    UniGene:   Rn.149063        SOURCE        Gene Ontology:     BioCarta Pathways:     KEGG Pathways:  
    Probe set : 1374848_at    Name:   Similar to RIKEN cDNA 2410016F19 (predicted)    GB acc:   AI599867    UniGene:   Rn.9497     Locus Link:   361858    Chromosome:   20    Cytoband:   Chr:20q12    SOURCE        Gene Ontology:     BioCarta Pathways:     KEGG Pathways:  
    Probe set : 1376946_at    Name:   Transcribed locus    GB acc:   BG380358    UniGene:   Rn.23622        SOURCE        Gene Ontology:     BioCarta Pathways:     KEGG Pathways:  
    Probe set : 1371468_at    Name:   Transcribed locus, strongly similar to NP_001003955.1 RAB11 family interacting protein 5 (class I) isoform 1 [Mus musculus]    GB acc:   BI296378    UniGene:   Rn.3496        SOURCE        Gene Ontology:     BioCarta Pathways:     KEGG Pathways:  
    Probe set : 1387202_at    Name:   intercellular adhesion molecule 1    GB acc:   NM_012967    UniGene:   Rn.12    Gene Symbol:   Icam1    Locus Link:   25464    Chromosome:   8    Cytoband:   Chr:8q13    SOURCE    GENECARD       Gene Ontology:   molecular function:  transmembrane receptor activity   molecular function:  protein binding   molecular function:  protein binding      BioCarta Pathways:     KEGG Pathways:  
    Probe set : 1374333_at    Name:   similar to RIKEN cDNA 1110007C09 (predicted)    GB acc:   AI227700    UniGene:   Rn.3059    Gene Symbol:   RGD1306058_predicted    Locus Link:   361224    Chromosome:   17    Cytoband:   Chr:17p14    SOURCE    GENECARD       Gene Ontology:     BioCarta Pathways:     KEGG Pathways:  
    Probe set : 1371977_at    Name:   actin related protein 2/3 complex, subunit 3 (predicted)    GB acc:   BG381477    UniGene:   Rn.4233    Gene Symbol:   Arpc3_predicted    Locus Link:   288669    Chromosome:   12    Cytoband:   Chr:12q16    SOURCE    GENECARD       Gene Ontology:   biological process:  cell motility   biological process:  regulation of actin filament polymerization   cellular component:  Arp2/3 protein complex   cellular component:  actin cytoskeleton   cellular component:  lamellipodium   molecular function:  structural constituent of cytoskeleton      BioCarta Pathways:     KEGG Pathways:  
    Probe set : 1374388_at    Name:   EF hand domain containing 2 (predicted)    GB acc:   AI408294    UniGene:   Rn.92713    Gene Symbol:   Efhd2_predicted    Locus Link:   298609    Chromosome:   5    Cytoband:   Chr:5q36    SOURCE    GENECARD       Gene Ontology:   biological process:  muscle development   molecular function:  calcium ion binding      BioCarta Pathways:     KEGG Pathways:  
    Probe set : 1389001_at    Name:   Transcribed locus, strongly similar to XP_422815.1 PREDICTED: similar to Importin alpha-4 subunit (Karyopherin alpha-4 subunit) (Qip1 protein) [Gallus gallus]    GB acc:   AW433944    UniGene:   Rn.8372        SOURCE        Gene Ontology:     BioCarta Pathways:     KEGG Pathways:  
    Probe set : 1385354_at    Name:   similar to integrin alpha 8    GB acc:   BE120766     Gene Symbol:   LOC364786    Locus Link:   364786    Chromosome:   17    Cytoband:   Chr:17q12.3     GENECARD       Gene Ontology:     BioCarta Pathways:     KEGG Pathways:  
    Probe set : 1385791_at    Name:   similar to villin-like protein (predicted)    GB acc:   BE112294    UniGene:   Rn.52066    Gene Symbol:   RGD1304843_predicted    Locus Link:   301057    Chromosome:   8    Cytoband:   Chr:8q32    SOURCE    GENECARD       Gene Ontology:     BioCarta Pathways:     KEGG Pathways:  
    Probe set : 1393277_at     GB acc:   AI579547              Gene Ontology:     BioCarta Pathways:     KEGG Pathways:  
    Probe set : 1390250_x_at    Name:   Atpase, class I, type 8B, member 2 (predicted)    GB acc:   BI273855    UniGene:   Rn.20321     Locus Link:   361984    Chromosome:   2    Cytoband:   Chr:2q34    SOURCE        Gene Ontology:   biological process:  cation transport   biological process:  metabolism   cellular component:  membrane   cellular component:  integral to membrane      BioCarta Pathways:     KEGG Pathways:  
    Probe set : 1387659_at    Name:   guanine deaminase    GB acc:   AF245172    UniGene:   Rn.24783    Gene Symbol:   Gda    Locus Link:   83585    Chromosome:   1    Cytoband:   Chr:1q51    SOURCE    GENECARD       Gene Ontology:   cellular component:  intracellular      BioCarta Pathways:     KEGG Pathways:  
    Probe set : 1395056_at    Name:   Similar to leucine-rich repeats and IQ motif containing 1    GB acc:   BM389046    UniGene:   Rn.82216     Locus Link:   299759    Chromosome:   7    Cytoband:   Chr:7q21    SOURCE        Gene Ontology:     BioCarta Pathways:     KEGG Pathways:  
    Probe set : 1371945_at    Name:   ubiquitin-conjugating enzyme E2L 3 (predicted)    GB acc:   AI407788    UniGene:   Rn.131117    Gene Symbol:   Ube2l3_predicted    Locus Link:   363836    Chromosome:   11    Cytoband:   Chr:11q23    SOURCE    GENECARD       Gene Ontology:   biological process:  ubiquitin-dependent protein catabolism   biological process:  ubiquitin cycle   cellular component:  ubiquitin ligase complex      BioCarta Pathways:     KEGG Pathways:  
    Probe set : 1396066_at    Name:   similar to Laminin alpha-4 chain precursor    GB acc:   AI170423    UniGene:   Rn.32891    Gene Symbol:   LOC309816    Locus Link:   309816    Chromosome:   20    Cytoband:   Chr:20q12    SOURCE    GENECARD       Gene Ontology:     BioCarta Pathways:     KEGG Pathways:  
    Probe set : 1388688_at    Name:   Transcribed locus    GB acc:   BI296599    UniGene:   Rn.115416        SOURCE        Gene Ontology:     BioCarta Pathways:     KEGG Pathways:  
    Probe set : 1377646_at    Name:   Transcribed locus    GB acc:   AI177360    UniGene:   Rn.3093        SOURCE        Gene Ontology:     BioCarta Pathways:     KEGG Pathways:  
    Probe set : 1393183_at    Name:   similar to StAR-related protein 1-4E    GB acc:   H33503    UniGene:   Rn.62294    Gene Symbol:   LOC502348    Locus Link:   502348    Chromosome:   1    Cytoband:   Chr:1q31    SOURCE    GENECARD       Gene Ontology:     BioCarta Pathways:     KEGG Pathways:  
    Probe set : 1387908_at    Name:   RAS, dexamethasone-induced 1    GB acc:   AF239157    UniGene:   Rn.54720    Gene Symbol:   Rasd1    Locus Link:   64455    Chromosome:   10    Cytoband:   Chr:10q22    SOURCE    GENECARD       Gene Ontology:   cellular component:  soluble fraction   cellular component:  cytoplasm      BioCarta Pathways:     KEGG Pathways:  
    Probe set : 1392280_at    Name:   toll-like receptor 2    GB acc:   AW526982    UniGene:   Rn.46387    Gene Symbol:   Tlr2    Locus Link:   310553    Chromosome:   2    Cytoband:   Chr:2q34    SOURCE    GENECARD       Gene Ontology:   biological process:  immune response   cellular component:  membrane   molecular function:  receptor activity   molecular function:  receptor activity   molecular function:  transmembrane receptor activity      BioCarta Pathways:     KEGG Pathways:  
    Probe set : 1375851_at    Name:   kelch-like 8 (Drosophila) (predicted)    GB acc:   AW916023    UniGene:   Rn.6788    Gene Symbol:   Klhl8_predicted    Locus Link:   289457    Chromosome:   14    Cytoband:   Chr:14p22    SOURCE    GENECARD       Gene Ontology:   molecular function:  protein binding      BioCarta Pathways:     KEGG Pathways:  
    Probe set : 1389644_at    Name:   similar to CG16896-PA    GB acc:   BI289857    UniGene:   Rn.39013    Gene Symbol:   LOC299949    Locus Link:   299949    Chromosome:   7    Cytoband:   Chr:7q33    SOURCE    GENECARD       Gene Ontology:     BioCarta Pathways:     KEGG Pathways:  
    Probe set : 1369793_a_at    Name:   melanoma cell adhesion molecule    GB acc:   AB035507    UniGene:   Rn.2694    Gene Symbol:   Mcam    Locus Link:   78967    Chromosome:   8    Cytoband:   Chr:8q22    SOURCE    GENECARD       Gene Ontology:   biological process:  cell adhesion   biological process:  heterophilic cell adhesion   biological process:  morphogenesis   molecular function:  protein binding      BioCarta Pathways:     KEGG Pathways:  
    Probe set : 1376597_at    Name:   zinc finger, CCHC domain containing 10 (predicted)    GB acc:   AI100951    UniGene:   Rn.53927    Gene Symbol:   Zcchc10_predicted    Locus Link:   360524    Chromosome:   10    Cytoband:   Chr:10q22    SOURCE    GENECARD       Gene Ontology:   molecular function:  nucleic acid binding   molecular function:  zinc ion binding   molecular function:  metal ion binding      BioCarta Pathways:     KEGG Pathways:  
    Probe set : 1383586_at     GB acc:   BI295793              Gene Ontology:     BioCarta Pathways:     KEGG Pathways:  
    Probe set : 1384371_at    Name:   TEA domain family member 3 (predicted)    GB acc:   AW921429    UniGene:   Rn.254    Gene Symbol:   Tead3_predicted    Locus Link:   294299    Chromosome:   20    Cytoband:   Chr:20p12    SOURCE    GENECARD       Gene Ontology:   cellular component:  nucleus   cellular component:  transcription factor complex   molecular function:  DNA binding   molecular function:  transcription factor activity   molecular function:  RNA polymerase II transcription factor activity      BioCarta Pathways:     KEGG Pathways:  
    Probe set : 1369630_at    Name:   adenosine kinase    GB acc:   BF283118    UniGene:   Rn.64165    Gene Symbol:   Adk    Locus Link:   25368    Chromosome:   15    Cytoband:   Chr:15p16    SOURCE    GENECARD       Gene Ontology:   biological process:  purine ribonucleoside salvage   biological process:  purine ribonucleoside salvage   biological process:  ribonucleoside monophosphate biosynthesis   cellular component:  nucleus      BioCarta Pathways:     KEGG Pathways:  
    Probe set : 1393027_at    Name:   similar to RIKEN cDNA 1700052N19 (predicted)    GB acc:   AI058848    UniGene:   Rn.50425    Gene Symbol:   RGD1305235_predicted    Locus Link:   292267    Chromosome:   1    Cytoband:   Chr:1p11    SOURCE    GENECARD       Gene Ontology:     BioCarta Pathways:     KEGG Pathways:  
    Probe set : 1395697_at    Name:   similar to Ezh2 protein    GB acc:   BF561171    UniGene:   Rn.9027    Gene Symbol:   LOC312299    Locus Link:   312299    Chromosome:   4    Cytoband:   Chr:4q24    SOURCE    GENECARD       Gene Ontology:     BioCarta Pathways:     KEGG Pathways:  
    Probe set : 1371330_at    Name:   ribosomal protein L11 (predicted)    GB acc:   BI284252    UniGene:   Rn.14687    Gene Symbol:   Rpl11_predicted    Locus Link:   362631    Chromosome:   5    Cytoband:   Chr:5q36    SOURCE    GENECARD       Gene Ontology:   biological process:  protein biosynthesis   molecular function:  RNA binding   molecular function:  structural constituent of ribosome   molecular function:  rRNA binding      BioCarta Pathways:     KEGG Pathways:  
    Probe set : 1373057_at    Name:   ProSAPiP1 protein    GB acc:   AI229142    UniGene:   Rn.7646    Gene Symbol:   ProSAPiP1    Locus Link:   280670    Chromosome:   3    Cytoband:   Chr:3q36    SOURCE    GENECARD       Gene Ontology:   cellular component:  cytoplasm   cellular component:  membrane      BioCarta Pathways:     KEGG Pathways:  
    Probe set : 1385126_at    Name:   Transcribed locus    GB acc:   AI060037    UniGene:   Rn.147472        SOURCE        Gene Ontology:     BioCarta Pathways:     KEGG Pathways:  
    Probe set : 1398628_at     GB acc:   AI059960              Gene Ontology:     BioCarta Pathways:     KEGG Pathways:  
    Probe set : 1389047_at    Name:   Zinc finger protein 451 (predicted)    GB acc:   AI009167    UniGene:   Rn.6909     Locus Link:   316312    Chromosome:   9    Cytoband:   Chr:9q21    SOURCE        Gene Ontology:   biological process:  regulation of transcription, DNA-dependent   biological process:  phosphoenolpyruvate-dependent sugar phosphotransferase system   cellular component:  nucleus      BioCarta Pathways:     KEGG Pathways:  
    Probe set : 1389176_at    Name:   Inositol polyphosphate-5-phosphatase F (predicted)    GB acc:   BF282674    UniGene:   Rn.60475     Locus Link:   309008    Chromosome:   1    Cytoband:   Chr:1q36-q37    SOURCE        Gene Ontology:     BioCarta Pathways:     KEGG Pathways:  
    Probe set : 1374578_at    Name:   Transcribed locus    GB acc:   AI409501    UniGene:   Rn.17879        SOURCE        Gene Ontology:     BioCarta Pathways:     KEGG Pathways:  
    Probe set : 1391925_at    Name:   chemokine (C-C motif) ligand 19 (predicted)    GB acc:   AA996885    UniGene:   Rn.12445    Gene Symbol:   Ccl19_predicted    Locus Link:   362506    Chromosome:   5    Cytoband:   Chr:5q22    SOURCE    GENECARD       Gene Ontology:   cellular component:  extracellular region   cellular component:  extracellular space   molecular function:  cytokine activity   molecular function:  chemokine activity      BioCarta Pathways:     KEGG Pathways:  
    Probe set : 1375987_at    Name:   ceramide kinase (predicted)    GB acc:   AW525194    UniGene:   Rn.99537    Gene Symbol:   Cerk_predicted    Locus Link:   300129    Chromosome:   7    Cytoband:   Chr:7q34    SOURCE    GENECARD       Gene Ontology:   biological process:  ceramide metabolism   biological process:  protein kinase C activation   cellular component:  integral to membrane of membrane fraction   cellular component:  membrane fraction      BioCarta Pathways:     KEGG Pathways:  
    Probe set : 1383601_at    Name:   DUF729 domain containing 1 (predicted)    GB acc:   BI295614    UniGene:   Rn.130657     Locus Link:   308720    Chromosome:   1    Cytoband:   Chr:1p12    SOURCE        Gene Ontology:     BioCarta Pathways:     KEGG Pathways:  
    Probe set : 1371314_at    Name:   neural precursor cell expressed, developmentally down-regulated gene 4A    GB acc:   BM389736    UniGene:   Rn.99540    Gene Symbol:   Nedd4a    Locus Link:   25489    Chromosome:   8    Cytoband:   Chr:8q24    SOURCE    GENECARD       Gene Ontology:   biological process:  protein modification   biological process:  ubiquitin cycle   cellular component:  ubiquitin ligase complex   cellular component:  intracellular   cellular component:  cytosol      BioCarta Pathways:     KEGG Pathways:  
    Probe set : 1371611_at    Name:   exostoses (multiple) 2 (predicted)    GB acc:   BM387363    UniGene:   Rn.2878    Gene Symbol:   Ext2_predicted    Locus Link:   311215    Chromosome:   3    Cytoband:   Chr:3q24    SOURCE    GENECARD       Gene Ontology:     BioCarta Pathways:     KEGG Pathways:  
    Probe set : 1391953_at    Name:   Msh homeo box homolog 2 (Drosophila)    GB acc:   BI284209    UniGene:   Rn.10414    Gene Symbol:   Msx2    Locus Link:   25483    Chromosome:   17    Cytoband:   Chr:17p14    SOURCE    GENECARD       Gene Ontology:   biological process:  skeletal development   biological process:  regulation of transcription, DNA-dependent   biological process:  development   cellular component:  nucleus   cellular component:  transcription factor complex   molecular function:  DNA binding   molecular function:  transcription factor activity   molecular function:  protein binding      BioCarta Pathways:     KEGG Pathways:  
    Probe set : 1370311_at    Name:   eukaryotic translation initiation factor 2B, subunit 1 alpha    GB acc:   U05821    UniGene:   Rn.9181    Gene Symbol:   Eif2b1    Locus Link:   64514    Chromosome:   12    Cytoband:   Chr:12q15    SOURCE    GENECARD       Gene Ontology:   biological process:  protein biosynthesis   biological process:  translational initiation   biological process:  cellular biosynthesis   cellular component:  eukaryotic translation initiation factor 2B complex   molecular function:  translation initiation factor activity   molecular function:  guanyl-nucleotide exchange factor activity   molecular function:  GTP binding      BioCarta Pathways:     KEGG Pathways:  
    Probe set : 1373839_at    Name:   Transcribed locus    GB acc:   BG372386    UniGene:   Rn.7944        SOURCE        Gene Ontology:     BioCarta Pathways:     KEGG Pathways:  
    Probe set : 1368296_at    Name:   solute carrier organic anion transporter family, member 2b1    GB acc:   AF169410    UniGene:   Rn.44538    Gene Symbol:   Slco2b1    Locus Link:   140860    Chromosome:   1    Cytoband:   Chr:1q32    SOURCE    GENECARD       Gene Ontology:   biological process:  transport   biological process:  ion transport   cellular component:  membrane   cellular component:  integral to membrane   molecular function:  transporter activity      BioCarta Pathways:     KEGG Pathways:  
    Probe set : 1390366_at    Name:   similar to PC-LKC gene product    GB acc:   BI291884    UniGene:   Rn.66752    Gene Symbol:   LOC291002    Locus Link:   291002    Chromosome:   17    Cytoband:   Chr:17p14    SOURCE    GENECARD       Gene Ontology:     BioCarta Pathways:     KEGG Pathways:  
    Probe set : 1393046_at    Name:   similar to Protein C20orf20 (predicted)    GB acc:   AI709474    UniGene:   Rn.28993    Gene Symbol:   RGD1308612_predicted    Locus Link:   311713    Chromosome:   3    Cytoband:   Chr:3q43    SOURCE    GENECARD       Gene Ontology:     BioCarta Pathways:     KEGG Pathways:  
    Probe set : 1388883_at    Name:   polymerase (DNA-directed), delta 4 (predicted)    GB acc:   BF281976    UniGene:   Rn.104646    Gene Symbol:   Pold4_predicted    Locus Link:   361698    Chromosome:   1    Cytoband:   Chr:1q42    SOURCE    GENECARD       Gene Ontology:   biological process:  DNA replication   cellular component:  nucleus   molecular function:  DNA-directed DNA polymerase activity   molecular function:  delta DNA polymerase activity      BioCarta Pathways:     KEGG Pathways:  
    Probe set : 1388071_x_at    Name:   RT1 class Ib, locus Aw2    GB acc:   M24024    UniGene:   Rn.116787    Gene Symbol:   RT1-Aw2    Locus Link:   24737    Chromosome:   20    Cytoband:   Chr:20p12    SOURCE    GENECARD       Gene Ontology:   cellular component:  integral to membrane   cellular component:  MHC class I protein complex   molecular function:  MHC class I receptor activity      BioCarta Pathways:     KEGG Pathways:  
    Probe set : 1373417_at    Name:   phospholipid scramblase 3 (predicted)    GB acc:   BI285973    UniGene:   Rn.22784    Gene Symbol:   Plscr3_predicted    Locus Link:   360549    Chromosome:   10    Cytoband:   Chr:10q24    SOURCE    GENECARD       Gene Ontology:   biological process:  phospholipid scrambling   cellular component:  plasma membrane   cellular component:  integral to membrane   molecular function:  calcium ion binding   molecular function:  phospholipid scramblase activity      BioCarta Pathways:     KEGG Pathways:  
    Probe set : 1385250_at    Name:   chromodomain helicase DNA binding protein 6 (predicted)    GB acc:   BF415132    UniGene:   Rn.43055    Gene Symbol:   Chd6_predicted    Locus Link:   311607    Chromosome:   3    Cytoband:   Chr:3q42    SOURCE    GENECARD       Gene Ontology:   biological process:  chromatin assembly or disassembly   cellular component:  chromatin   cellular component:  nucleus   molecular function:  chromatin binding   molecular function:  ATP binding      BioCarta Pathways:     KEGG Pathways:  
    Probe set : 1367978_at    Name:   adenylate cyclase 2    GB acc:   NM_031007    UniGene:   Rn.10731    Gene Symbol:   Adcy2    Locus Link:   81636    Chromosome:   17    Cytoband:   Chr:17p14    SOURCE    GENECARD       Gene Ontology:     BioCarta Pathways:     KEGG Pathways:  
    Probe set : 1374794_at    Name:   kinesin-like 7    GB acc:   BF396282    UniGene:   Rn.45205    Gene Symbol:   Knsl7    Locus Link:   353302    Chromosome:   8    Cytoband:   Chr:8q32    SOURCE    GENECARD       Gene Ontology:   biological process:  microtubule-based movement   cellular component:  microtubule associated complex      BioCarta Pathways:     KEGG Pathways:  
    Probe set : 1384440_at    Name:   Similar to LRRGT00021    GB acc:   BM385619    UniGene:   Rn.145523     Locus Link:   498993      SOURCE        Gene Ontology:     BioCarta Pathways:     KEGG Pathways:  
    Probe set : 1383232_at    Name:   Transcribed locus    GB acc:   AI716944    UniGene:   Rn.17886        SOURCE        Gene Ontology:     BioCarta Pathways:     KEGG Pathways:  
    Probe set : 1371244_at    Name:   adenine phosphoribosyl transferase (predicted)    GB acc:   AI412934    UniGene:   Rn.2498    Gene Symbol:   Aprt_predicted    Locus Link:   292072    Chromosome:   19    Cytoband:   Chr:19q12    SOURCE    GENECARD       Gene Ontology:   biological process:  purine ribonucleoside salvage   biological process:  adenine salvage   biological process:  nucleoside metabolism      BioCarta Pathways:     KEGG Pathways:  
    Probe set : 1388729_at    Name:   Harvey rat sarcoma oncogene, subgroup R (predicted)    GB acc:   BF282474    UniGene:   Rn.71224    Gene Symbol:   Rras_predicted    Locus Link:   361568    Chromosome:   1    Cytoband:   Chr:1q22    SOURCE    GENECARD       Gene Ontology:   cellular component:  intracellular   molecular function:  GTPase activity   molecular function:  GTP binding      BioCarta Pathways:     KEGG Pathways:  
    Probe set : 1383757_at    Name:   Transcribed locus    GB acc:   AI044802    UniGene:   Rn.20709        SOURCE        Gene Ontology:     BioCarta Pathways:     KEGG Pathways:  
    Probe set : 1369625_at    Name:   aquaporin 1    GB acc:   AA891661    UniGene:   Rn.1618    Gene Symbol:   Aqp1    Locus Link:   25240    Chromosome:   4    Cytoband:   Chr:4q24    SOURCE    GENECARD       Gene Ontology:   biological process:  transport   biological process:  water transport   biological process:  excretion      BioCarta Pathways:     KEGG Pathways:  
    Probe set : 1375847_at    Name:   Transcribed locus    GB acc:   BF282594    UniGene:   Rn.16063        SOURCE        Gene Ontology:     BioCarta Pathways:     KEGG Pathways:  
    Probe set : 1370358_at    Name:   two pore channel 1    GB acc:   AB018253    UniGene:   Rn.24484    Gene Symbol:   Tpcn1    Locus Link:   246215    Chromosome:   12    Cytoband:   Chr:12q16    SOURCE    GENECARD       Gene Ontology:   biological process:  transport   biological process:  ion transport   biological process:  cation transport   cellular component:  integral to membrane   cellular component:  membrane      BioCarta Pathways:     KEGG Pathways:  
    Probe set : 1385670_at    Name:   serine dehydratase-like (predicted)    GB acc:   BF521655    UniGene:   Rn.54338    Gene Symbol:   Sdsl_predicted    Locus Link:   360816    Chromosome:   12    Cytoband:   Chr:12q16    SOURCE    GENECARD       Gene Ontology:   biological process:  amino acid metabolism   biological process:  metabolism   molecular function:  catalytic activity   molecular function:  lyase activity      BioCarta Pathways:     KEGG Pathways:  
    Probe set : 1398553_at    Name:   similar to CGI-100-like protein    GB acc:   AI137113    UniGene:   Rn.85410    Gene Symbol:   RGD:1359437    Locus Link:   289883    Chromosome:   14    Cytoband:   Chr:14p22    SOURCE    GENECARD       Gene Ontology:   biological process:  intracellular protein transport   cellular component:  endoplasmic reticulum   cellular component:  membrane   cellular component:  integral to membrane   molecular function:  protein carrier activity      BioCarta Pathways:     KEGG Pathways:  
    Probe set : 1393684_at    Name:   TBC1D12: TBC1 domain family, member 12 (predicted)    GB acc:   BF389489    UniGene:   Rn.23113    Gene Symbol:   Tbc1d12_predicted    Locus Link:   294071    Chromosome:   1    Cytoband:   Chr:1q53    SOURCE    GENECARD       Gene Ontology:     BioCarta Pathways:     KEGG Pathways:  
    Probe set : 1375895_at    Name:   Serine racemase    GB acc:   BI275908    UniGene:   Rn.101331    Gene Symbol:   Srr    Locus Link:   303306    Chromosome:   10    Cytoband:   Chr:10q24    SOURCE    GENECARD       Gene Ontology:   biological process:  amino acid metabolism   biological process:  metabolism   molecular function:  serine racemase activity   molecular function:  catalytic activity      BioCarta Pathways:     KEGG Pathways:  
    Probe set : 1375067_at    Name:   Transcribed locus    GB acc:   AI555354    UniGene:   Rn.20804        SOURCE        Gene Ontology:     BioCarta Pathways:     KEGG Pathways:  
    Probe set : 1373350_at    Name:   PC4 and SFRS1 interacting protein 1    GB acc:   H31668    UniGene:   Rn.93075    Gene Symbol:   Psip1    Locus Link:   313323    Chromosome:   5    Cytoband:   Chr:5q31    SOURCE    GENECARD       Gene Ontology:   biological process:  transcription   biological process:  regulation of transcription, DNA-dependent   cellular component:  nucleus   molecular function:  DNA binding      BioCarta Pathways:     KEGG Pathways:  
    Probe set : 1381469_a_at    Name:   PERQ amino acid rich, with GYF domain 1 (predicted)    GB acc:   AW526967    UniGene:   Rn.100422    Gene Symbol:   Perq1_predicted    Locus Link:   304378    Chromosome:   12    Cytoband:   Chr:12q12    SOURCE    GENECARD       Gene Ontology:   biological process:  insulin-like growth factor receptor signaling pathway   molecular function:  protein binding      BioCarta Pathways:     KEGG Pathways:  
    Probe set : 1388945_at    Name:   similar to 1300014I06Rik protein (predicted)    GB acc:   BM385779    UniGene:   Rn.6535    Gene Symbol:   RGD1311307_predicted    Locus Link:   361238    Chromosome:   17    Cytoband:   Chr:17p12    SOURCE    GENECARD       Gene Ontology:   biological process:  intracellular signaling cascade      BioCarta Pathways:     KEGG Pathways:  
    Probe set : 1373556_at    Name:   similar to CG14903-PA    GB acc:   AI237593    UniGene:   Rn.8319    Gene Symbol:   LOC298861    Locus Link:   298861    Chromosome:   6    Cytoband:   Chr:6q14    SOURCE    GENECARD       Gene Ontology:     BioCarta Pathways:     KEGG Pathways:  
    Probe set : 1369323_at    Name:   leptin receptor overlapping transcript    GB acc:   NM_020099    UniGene:   Rn.144583    Gene Symbol:   Leprot    Locus Link:   56766    Chromosome:   5    Cytoband:   Chr:5q33    SOURCE    GENECARD       Gene Ontology:   cellular component:  integral to membrane   molecular function:  receptor activity      BioCarta Pathways:     KEGG Pathways:  
    Probe set : 1378423_at    Name:   ataxia, cerebellar, Cayman type (caytaxin) (predicted)    GB acc:   AI639060    UniGene:   Rn.16235    Gene Symbol:   Atcay_predicted    Locus Link:   362826    Chromosome:   7    Cytoband:   Chr:7q11    SOURCE    GENECARD       Gene Ontology:   biological process:  transport      BioCarta Pathways:     KEGG Pathways:  
    Probe set : 1370806_at    Name:   all-trans-13,14-dihydroretinol saturase    GB acc:   AF465614    UniGene:   Rn.55275    Gene Symbol:   Retsat    Locus Link:   246298    Chromosome:   4    Cytoband:   Chr:4q33    SOURCE    GENECARD       Gene Ontology:   biological process:  electron transport   molecular function:  oxidoreductase activity      BioCarta Pathways:     KEGG Pathways:  
    Probe set : 1368265_at    Name:   cytochrome P450 monooxygenase CYP2T1    GB acc:   NM_134369    UniGene:   Rn.144661    Gene Symbol:   RGD:620086    Locus Link:   171380    Chromosome:   1    Cytoband:   Chr:1q21    SOURCE    GENECARD       Gene Ontology:   biological process:  electron transport   cellular component:  membrane      BioCarta Pathways:     KEGG Pathways:  
    Probe set : 1369533_a_at    Name:   5-hydroxytryptamine (serotonin) receptor 4    GB acc:   U20906    UniGene:   Rn.10094    Gene Symbol:   Htr4    Locus Link:   25324    Chromosome:   18    Cytoband:   Chr:18q12.1    SOURCE    GENECARD       Gene Ontology:   cellular component:  integral to plasma membrane   cellular component:  integral to membrane      BioCarta Pathways:     KEGG Pathways:  
    Probe set : 1368160_at    Name:   insulin-like growth factor binding protein 1    GB acc:   NM_013144    UniGene:   Rn.34026    Gene Symbol:   Igfbp1    Locus Link:   25685    Chromosome:   14    Cytoband:   Chr:14q21    SOURCE    GENECARD       Gene Ontology:   biological process:  regulation of cell growth   biological process:  signal transduction   cellular component:  extracellular region   cellular component:  extracellular space   molecular function:  insulin-like growth factor binding   molecular function:  growth factor binding      BioCarta Pathways:     KEGG Pathways:  
    Probe set : 1389971_at    Name:   sarcoglycan, epsilon    GB acc:   AI179438    UniGene:   Rn.3825    Gene Symbol:   RGD:1303201    Locus Link:   432360      SOURCE    GENECARD       Gene Ontology:   cellular component:  sarcoglycan complex   molecular function:  calcium ion binding   molecular function:  protein binding      BioCarta Pathways:     KEGG Pathways:  
    Probe set : 1369483_at    Name:   CD4 antigen    GB acc:   NM_012705    UniGene:   Rn.10748    Gene Symbol:   Cd4    Locus Link:   24932    Chromosome:   4    Cytoband:   Chr:4q42    SOURCE    GENECARD       Gene Ontology:   biological process:  defense response   biological process:  immune response   biological process:  cell adhesion   biological process:  transmembrane receptor protein tyrosine kinase signaling pathway   biological process:  T cell differentiation   biological process:  T cell selection   biological process:  positive regulation of interleukin-2 biosynthesis   biological process:  immune response   biological process:  transmembrane receptor protein tyrosine kinase signaling pathway   biological process:  T cell differentiation   biological process:  T cell selection   biological process:  positive regulation of interleukin-2 biosynthesis      BioCarta Pathways:     KEGG Pathways:  
    Probe set : 1367849_at    Name:   syndecan 1    GB acc:   NM_013026    UniGene:   Rn.11176    Gene Symbol:   Sdc1    Locus Link:   25216    Chromosome:   6    Cytoband:   Chr:6q14    SOURCE    GENECARD       Gene Ontology:   molecular function:  cytoskeletal protein binding      BioCarta Pathways:     KEGG Pathways:  
    Probe set : 1379344_at    Name:   Cytochrome b-245, beta polypeptide    GB acc:   AI176057    UniGene:   Rn.98491    Gene Symbol:   Cybb    Locus Link:   66021    Chromosome:   X    Cytoband:   Chr:Xq13    SOURCE    GENECARD       Gene Ontology:   biological process:  electron transport   cellular component:  intracellular   cellular component:  membrane   cellular component:  integral to membrane      BioCarta Pathways:     KEGG Pathways:  
    Probe set : 1391411_at    Name:   Formin-like 2 (predicted)    GB acc:   AI179941    UniGene:   Rn.7075     Locus Link:   311033    Chromosome:   3    Cytoband:   Chr:3q12    SOURCE        Gene Ontology:   cellular component:  nucleus      BioCarta Pathways:     KEGG Pathways:  
    Probe set : 1374352_at    Name:   Actin alpha cardiac 1    GB acc:   AI711147    UniGene:   Rn.3114    Gene Symbol:   Actc1    Locus Link:   29275      SOURCE    GENECARD       Gene Ontology:   cellular component:  cytoskeleton   cellular component:  actin filament   cellular component:  actin cytoskeleton      BioCarta Pathways:     KEGG Pathways:  
    Probe set : 1371833_at    Name:   brain protein I3 (predicted)    GB acc:   AI230753    UniGene:   Rn.7700    Gene Symbol:   Bri3_predicted    Locus Link:   304284    Chromosome:   12    Cytoband:   Chr:12p11    SOURCE    GENECARD       Gene Ontology:   cellular component:  integral to membrane      BioCarta Pathways:     KEGG Pathways:  
    Probe set : 1384724_at     GB acc:   AA850766              Gene Ontology:     BioCarta Pathways:     KEGG Pathways:  
    Probe set : 1368366_at    Name:   Camello-like 2    GB acc:   NM_021668    UniGene:   Rn.20565    Gene Symbol:   Cml2    Locus Link:   59300    Chromosome:   4    Cytoband:   Chr:4q34    SOURCE    GENECARD       Gene Ontology:   molecular function:  N-acetyltransferase activity   molecular function:  N-acetyltransferase activity   molecular function:  transferase activity      BioCarta Pathways:     KEGG Pathways:  
    Probe set : 1389220_at     GB acc:   BE112918              Gene Ontology:     BioCarta Pathways:     KEGG Pathways:  
    Probe set : 1386890_at    Name:   S100 calcium binding protein A10 (calpactin)    GB acc:   NM_031114    UniGene:   Rn.4083    Gene Symbol:   S100a10    Locus Link:   81778      SOURCE    GENECARD       Gene Ontology:   biological process:  regulation of cell growth   biological process:  regulation of cell differentiation   molecular function:  calcium ion binding      BioCarta Pathways:     KEGG Pathways:  
    Probe set : 1376381_at    Name:   XK-related protein 5    GB acc:   AW252094    UniGene:   Rn.33372    Gene Symbol:   RGD:1359335    Locus Link:   497083    Chromosome:   16    Cytoband:   Chr:16q12.5    SOURCE    GENECARD       Gene Ontology:     BioCarta Pathways:     KEGG Pathways:  
    Probe set : 1372809_at    Name:   hypothetical gene supported by AF152002    GB acc:   AI716200    UniGene:   Rn.103148    Gene Symbol:   LOC290595    Locus Link:   290595    Chromosome:   16    Cytoband:   Chr:16p14    SOURCE    GENECARD       Gene Ontology:     BioCarta Pathways:     KEGG Pathways:  
    Probe set : 1377752_at    Name:   epithelial membrane protein 2    GB acc:   BE112998    UniGene:   Rn.21730    Gene Symbol:   RGD:1359629    Locus Link:   360468    Chromosome:   10    Cytoband:   Chr:10q11    SOURCE    GENECARD       Gene Ontology:   cellular component:  membrane   cellular component:  integral to membrane      BioCarta Pathways:     KEGG Pathways:  
    Probe set : 1374038_at    Name:   Transcribed locus    GB acc:   AA799964    UniGene:   Rn.6261        SOURCE        Gene Ontology:     BioCarta Pathways:     KEGG Pathways:  
    Probe set : 1387897_at    Name:   cyclic nucleotide phosphodiesterase 1    GB acc:   L16532    UniGene:   Rn.31762    Gene Symbol:   Cnp1    Locus Link:   25275    Chromosome:   10    Cytoband:   Chr:10q32.1    SOURCE    GENECARD       Gene Ontology:   biological process:  synaptic transmission   biological process:  cyclic nucleotide catabolism   cellular component:  membrane   molecular function:  2',3'-cyclic-nucleotide 3'-phosphodiesterase activity   molecular function:  hydrolase activity      BioCarta Pathways:     KEGG Pathways:  
    Probe set : 1371341_at    Name:   small nuclear ribonucleoprotein D2 (predicted)    GB acc:   BG372885    UniGene:   Rn.110465    Gene Symbol:   Snrpd2_predicted    Locus Link:   292688    Chromosome:   1    Cytoband:   Chr:1q21    SOURCE    GENECARD       Gene Ontology:   biological process:  spliceosome assembly   biological process:  nuclear mRNA splicing, via spliceosome   biological process:  mRNA processing      BioCarta Pathways:     KEGG Pathways:  
    Probe set : 1382878_at    Name:   secreted frizzled-related protein 1    GB acc:   AI716896    UniGene:   Rn.34957    Gene Symbol:   Sfrp1    Locus Link:   84402    Chromosome:   16    Cytoband:   Chr:16q12.5    SOURCE    GENECARD       Gene Ontology:   cellular component:  extracellular space   cellular component:  membrane   molecular function:  transmembrane receptor activity      BioCarta Pathways:     KEGG Pathways:  
    Probe set : 1376344_at    Name:   Cytochrome b reductase 1 (predicted)    GB acc:   AI010267    UniGene:   Rn.13660     Locus Link:   295669    Chromosome:   3    Cytoband:   Chr:3q21    SOURCE        Gene Ontology:   biological process:  electron transport   cellular component:  integral to membrane   molecular function:  transferase activity      BioCarta Pathways:     KEGG Pathways:  
    Probe set : 1371462_at    Name:   insulin-like growth factor binding protein 4    GB acc:   BE108969    UniGene:   Rn.112691    Gene Symbol:   Igfbp4    Locus Link:   360622    Chromosome:   10    Cytoband:   Chr:10q31    SOURCE    GENECARD       Gene Ontology:   cellular component:  extracellular region   cellular component:  extracellular space   molecular function:  insulin-like growth factor binding   molecular function:  growth factor binding      BioCarta Pathways:     KEGG Pathways:  
    Probe set : 1383343_at    Name:   Putative pheromone receptor V2R4    GB acc:   BI300511    UniGene:   Rn.137680    Gene Symbol:   LOC292498    Locus Link:   292498    Chromosome:   1    Cytoband:   Chr:1q12    SOURCE    GENECARD       Gene Ontology:   biological process:  metabotropic glutamate receptor signaling pathway   cellular component:  membrane   molecular function:  receptor activity   molecular function:  metabotropic glutamate, GABA-B-like receptor activity      BioCarta Pathways:     KEGG Pathways:  
    Probe set : 1388309_at    Name:   high mobility group AT-hook 1    GB acc:   BG378885    UniGene:   Rn.83614    Gene Symbol:   Hmga1    Locus Link:   117062    Chromosome:   20    Cytoband:   Chr:20p12    SOURCE    GENECARD       Gene Ontology:   biological process:  regulation of transcription, DNA-dependent   biological process:  chromosome organization and biogenesis (sensu Eukaryota)   cellular component:  chromatin   cellular component:  nucleus   molecular function:  DNA binding      BioCarta Pathways:     KEGG Pathways:  
    Probe set : 1382732_at    Name:   extra cellular link domain-containing 1 (predicted)    GB acc:   BE116084    UniGene:   Rn.22111    Gene Symbol:   Xlkd1_predicted    Locus Link:   293186    Chromosome:   1    Cytoband:   Chr:1q33    SOURCE    GENECARD       Gene Ontology:   molecular function:  receptor activity   molecular function:  transmembrane receptor activity   molecular function:  hyaluronic acid binding      BioCarta Pathways:     KEGG Pathways:  
    Probe set : 1382651_at    Name:   Transcribed locus    GB acc:   AA998677    UniGene:   Rn.34130        SOURCE        Gene Ontology:     BioCarta Pathways:     KEGG Pathways:  
    Probe set : 1395052_at    Name:   Transcribed locus    GB acc:   BE118917    UniGene:   Rn.53085        SOURCE        Gene Ontology:     BioCarta Pathways:     KEGG Pathways:  
    Probe set : 1371302_at    Name:   Similar to RIKEN cDNA A130038L21    GB acc:   AI406651    UniGene:   Rn.109370     Locus Link:   311358    Chromosome:   3    Cytoband:   Chr:3q35    SOURCE        Gene Ontology:     BioCarta Pathways:     KEGG Pathways:  
    Probe set : 1393149_at     GB acc:   BG663483    UniGene:   Rn.22308    Gene Symbol:   Multiple Gene Symbols    Locus Link:   116741 /// 116742 /// 116778 /// 116779 /// 116780 /// 116781 /// 393085 /// 393086 /// 393088 /// 393089 /// 393091 /// 393092 /// 394223    Chromosome:   18    Cytoband:   Chr:18p11    SOURCE    GENECARD       Gene Ontology:     BioCarta Pathways:     KEGG Pathways:  
    Probe set : 1388762_at     GB acc:   BF284125              Gene Ontology:     BioCarta Pathways:     KEGG Pathways:  
    Probe set : 1394438_at    Name:   ARP1 actin-related protein 1 homolog A (yeast) (predicted)    GB acc:   BI294346    UniGene:   Rn.32312    Gene Symbol:   Actr1a_predicted    Locus Link:   294010    Chromosome:   1    Cytoband:   Chr:1q54    SOURCE    GENECARD       Gene Ontology:   biological process:  vesicle-mediated transport   molecular function:  motor activity   molecular function:  structural molecule activity   molecular function:  structural constituent of cytoskeleton      BioCarta Pathways:     KEGG Pathways:  
    Probe set : 1385529_at    Name:   family with sequence similarity 3, member B (predicted)    GB acc:   BF411402    UniGene:   Rn.19690    Gene Symbol:   Fam3b_predicted    Locus Link:   304050    Chromosome:   11    Cytoband:   Chr:11q12    SOURCE    GENECARD       Gene Ontology:   biological process:  insulin secretion   cellular component:  extracellular region   cellular component:  extracellular space   cellular component:  nuclear membrane lumen   molecular function:  cytokine activity      BioCarta Pathways:     KEGG Pathways:  
    Probe set : 1392731_at    Name:   hypothetical gene supported by NM_130426    GB acc:   BM390522     Gene Symbol:   LOC497808    Locus Link:   497808    Chromosome:   5    Cytoband:   Chr:5q36     GENECARD       Gene Ontology:     BioCarta Pathways:     KEGG Pathways:  
    Probe set : 1383073_at    Name:   ubiquitin specific protease 14 (predicted)    GB acc:   BG666028    UniGene:   Rn.11790    Gene Symbol:   Usp14_predicted    Locus Link:   291796    Chromosome:   18    Cytoband:   Chr:18p13    SOURCE    GENECARD       Gene Ontology:   cellular component:  soluble fraction   cellular component:  synaptosome      BioCarta Pathways:     KEGG Pathways:  
    Probe set : 1378078_at    Name:   Neuronal PAS domain protein 1 (predicted)    GB acc:   BE095857    UniGene:   Rn.140563     Locus Link:   308387    Chromosome:   1    Cytoband:   Chr:1q21    SOURCE        Gene Ontology:   biological process:  regulation of transcription, DNA-dependent   biological process:  signal transduction   biological process:  central nervous system development   cellular component:  nucleus   molecular function:  DNA binding   molecular function:  transcription factor activity   molecular function:  signal transducer activity      BioCarta Pathways:     KEGG Pathways:  
    Probe set : 1373454_at    Name:   similar to RIKEN cDNA 0610037P05 (predicted)    GB acc:   AI230689    UniGene:   Rn.3055    Gene Symbol:   RGD1305823_predicted    Locus Link:   360461    Chromosome:   10    Cytoband:   Chr:10q11    SOURCE    GENECARD       Gene Ontology:     BioCarta Pathways:     KEGG Pathways:  
    Probe set : 1373780_at    Name:   tetraspan 1    GB acc:   BE349699    UniGene:   Rn.33830    Gene Symbol:   RGD:1303308    Locus Link:   298436    Chromosome:   5    Cytoband:   Chr:5q36    SOURCE    GENECARD       Gene Ontology:   cellular component:  membrane   cellular component:  integral to membrane      BioCarta Pathways:     KEGG Pathways:  
    Probe set : 1389617_at     GB acc:   BM392135              Gene Ontology:     BioCarta Pathways:     KEGG Pathways:  
    Probe set : 1369686_at    Name:   double cortin and calcium/calmodulin-dependent protein kinase-like 1    GB acc:   U78857     Gene Symbol:   Dcamkl1    Locus Link:   83825    Chromosome:   2    Cytoband:   Chr:2q26     GENECARD       Gene Ontology:   cellular component:  integral to plasma membrane      BioCarta Pathways:     KEGG Pathways:  
    Probe set : 1394483_at    Name:   A disintegrin-like and metalloprotease (reprolysin type) with thrombospondin type 1 motif, 5 (aggrecanase-2)    GB acc:   AW535310    UniGene:   Rn.107051    Gene Symbol:   Adamts5    Locus Link:   304135    Chromosome:   11    Cytoband:   Chr:11q11    SOURCE    GENECARD       Gene Ontology:   biological process:  proteolysis   biological process:  proteolysis   cellular component:  extracellular matrix   molecular function:  metalloendopeptidase activity   molecular function:  metalloendopeptidase activity      BioCarta Pathways:     KEGG Pathways:  
    Probe set : 1377359_at    Name:   similar to protein P3    GB acc:   BM384440    UniGene:   Rn.140462    Gene Symbol:   LOC501665    Locus Link:   501665    Chromosome:   X    Cytoband:   Chr:Xq37    SOURCE    GENECARD       Gene Ontology:   biological process:  sodium ion transport   biological process:  organic anion transport   cellular component:  membrane   cellular component:  integral to membrane   molecular function:  bile acid:sodium symporter activity      BioCarta Pathways:     KEGG Pathways:  
    Probe set : 1396962_at    Name:   Transcribed locus    GB acc:   BI296196    UniGene:   Rn.146747        SOURCE        Gene Ontology:     BioCarta Pathways:     KEGG Pathways:  
    Probe set : 1380243_at    Name:   similar to CG14803-PA (predicted)    GB acc:   BE098614    UniGene:   Rn.139999    Gene Symbol:   RGD1304693_predicted    Locus Link:   313108    Chromosome:   5    Cytoband:   Chr:5q21    SOURCE    GENECARD       Gene Ontology:     BioCarta Pathways:     KEGG Pathways:  
    Probe set : 1367859_at    Name:   transforming growth factor, beta 3    GB acc:   NM_013174    UniGene:   Rn.7018    Gene Symbol:   Tgfb3    Locus Link:   25717    Chromosome:   6    Cytoband:   Chr:6q31    SOURCE    GENECARD       Gene Ontology:   biological process:  regulation of progression through cell cycle   biological process:  embryonic development (sensu Mammalia)   biological process:  signal transduction   biological process:  cell-cell signaling   biological process:  cell proliferation   biological process:  negative regulation of cell proliferation   biological process:  embryonic development   biological process:  organ morphogenesis   biological process:  cell growth   biological process:  lung development   biological process:  lung development   biological process:  regulation of epithelial cell differentiation   biological process:  regulation of epithelial cell differentiation   biological process:  growth   biological process:  regulation of endothelial cell differentiation   biological process:  regulation of endothelial cell differentiation   biological process:  regulation of epithelial cell proliferation   biological process:  regulation of epithelial cell proliferation   cellular component:  extracellular space      BioCarta Pathways:     KEGG Pathways:  
    Probe set : 1387871_at    Name:   cofilin 1    GB acc:   BM389673    UniGene:   Rn.11675    Gene Symbol:   Cfl1    Locus Link:   29271    Chromosome:   1    Cytoband:   Chr:1q43    SOURCE    GENECARD       Gene Ontology:   biological process:  protein import into nucleus   biological process:  Rho protein signal transduction   biological process:  actin cytoskeleton organization and biogenesis   molecular function:  actin binding   molecular function:  actin binding      BioCarta Pathways:     KEGG Pathways:  
    Probe set : 1390993_at    Name:   MAWD binding protein    GB acc:   AW532101    UniGene:   Rn.2417    Gene Symbol:   Mawbp    Locus Link:   171564    Chromosome:   20    Cytoband:   Chr:20p11    SOURCE    GENECARD       Gene Ontology:   biological process:  biosynthesis   molecular function:  catalytic activity   molecular function:  oxidoreductase activity      BioCarta Pathways:     KEGG Pathways:  
    Probe set : 1384183_at     GB acc:   AA996869              Gene Ontology:     BioCarta Pathways:     KEGG Pathways:  
    Probe set : 1395166_at    Name:   putative eps protein    GB acc:   AW252627    UniGene:   Rn.16016    Gene Symbol:   MGEPS    Locus Link:   361512    Chromosome:   1    Cytoband:   Chr:1q21    SOURCE    GENECARD       Gene Ontology:     BioCarta Pathways:     KEGG Pathways:  
    Probe set : 1368658_at    Name:   ciliary neurotrophic factor    GB acc:   NM_013166    UniGene:   Rn.6067    Gene Symbol:   Cntf    Locus Link:   25707    Chromosome:   1    Cytoband:   Chr:1q43    SOURCE    GENECARD       Gene Ontology:   molecular function:  ciliary neurotrophic factor receptor binding   molecular function:  growth factor activity   molecular function:  growth factor activity      BioCarta Pathways:     KEGG Pathways:  
    Probe set : 1388782_at    Name:   transcription factor 21    GB acc:   BE113336    UniGene:   Rn.1671    Gene Symbol:   Tcf21    Locus Link:   252856    Chromosome:   1    Cytoband:   Chr:1p12    SOURCE    GENECARD       Gene Ontology:   biological process:  regulation of transcription   cellular component:  nucleus   molecular function:  transcription regulator activity      BioCarta Pathways:     KEGG Pathways:  
    Probe set : 1398592_at     GB acc:   AI045966              Gene Ontology:     BioCarta Pathways:     KEGG Pathways:  
    Probe set : 1376688_a_at    Name:   Transcribed locus, moderately similar to NP_705730.1 paired immunoglobin-like type 2 receptor alpha [Mus musculus]    GB acc:   BF288115    UniGene:   Rn.18754        SOURCE        Gene Ontology:     BioCarta Pathways:     KEGG Pathways:  
    Probe set : 1392582_at    Name:   similar to NAG14 protein (predicted)    GB acc:   AA819053    UniGene:   Rn.24122    Gene Symbol:   RGD1311013_predicted    Locus Link:   311236    Chromosome:   3    Cytoband:   Chr:3q31    SOURCE    GENECARD       Gene Ontology:     BioCarta Pathways:     KEGG Pathways:  
    Probe set : 1371584_at    Name:   transient receptor potential cation channel, subfamily C, member 4 associated protein (predicted)    GB acc:   BM390843    UniGene:   Rn.100109    Gene Symbol:   Trpc4ap_predicted    Locus Link:   362247    Chromosome:   3    Cytoband:   Chr:3q42    SOURCE    GENECARD       Gene Ontology:   cellular component:  endoplasmic reticulum lumen   cellular component:  cytosol      BioCarta Pathways:     KEGG Pathways:  
    Probe set : 1398768_at    Name:   retinoblastoma binding protein 7    GB acc:   NM_031816    UniGene:   Rn.3600    Gene Symbol:   Rbbp7    Locus Link:   83712    Chromosome:   X    Cytoband:   Chr:Xq21    SOURCE    GENECARD       Gene Ontology:   cellular component:  nucleus   cellular component:  NuRD complex   molecular function:  protein binding   molecular function:  transcriptional repressor activity      BioCarta Pathways:     KEGG Pathways:  
    Probe set : 1391390_at     GB acc:   AA894192              Gene Ontology:     BioCarta Pathways:     KEGG Pathways:  
    Probe set : 1369614_at    Name:   RAP2B, member of RAS oncogene family    GB acc:   NM_133410    UniGene:   Rn.81035    Gene Symbol:   Rap2b    Locus Link:   170923    Chromosome:   2    Cytoband:   Chr:2q31    SOURCE    GENECARD       Gene Ontology:   biological process:  signal transduction   biological process:  small GTPase mediated signal transduction   biological process:  protein transport   molecular function:  GTP binding   molecular function:  nucleotide binding      BioCarta Pathways:     KEGG Pathways:  
    Probe set : 1370363_at    Name:   carboxylesterase 3    GB acc:   L46791    UniGene:   Rn.34885    Gene Symbol:   Ces3    Locus Link:   113902    Chromosome:   19    Cytoband:   Chr:19p11    SOURCE    GENECARD       Gene Ontology:   cellular component:  extracellular space   cellular component:  endoplasmic reticulum   cellular component:  endoplasmic reticulum lumen      BioCarta Pathways:     KEGG Pathways:  
    Probe set : 1399032_at    Name:   excision repair cross-complementing rodent repair deficiency, complementation group 1 (predicted)    GB acc:   AA892791    UniGene:   Rn.7320    Gene Symbol:   Ercc1_predicted    Locus Link:   292673    Chromosome:   1    Cytoband:   Chr:1q21    SOURCE    GENECARD       Gene Ontology:   biological process:  DNA repair   biological process:  nucleotide-excision repair   biological process:  morphogenesis   cellular component:  nucleotide-excision repair complex   cellular component:  intracellular   cellular component:  nucleus      BioCarta Pathways:     KEGG Pathways:  
    Probe set : 1382589_at    Name:   similar to hypothetical protein FLJ11795 (predicted)    GB acc:   AI072262    UniGene:   Rn.21785    Gene Symbol:   RGD1307735_predicted    Locus Link:   361898    Chromosome:   2    Cytoband:   Chr:2q14    SOURCE    GENECARD       Gene Ontology:     BioCarta Pathways:     KEGG Pathways:  
    Probe set : 1370379_at    Name:   protease, serine, 8 (prostasin)    GB acc:   AF202076    UniGene:   Rn.107364    Gene Symbol:   Prss8    Locus Link:   192107    Chromosome:   1    Cytoband:   Chr:1q36    SOURCE    GENECARD       Gene Ontology:     BioCarta Pathways:     KEGG Pathways:  
    Probe set : 1389836_a_at    Name:   Synapsin III    GB acc:   AI599265    UniGene:   Rn.98839    Gene Symbol:   Syn3    Locus Link:   29130    Chromosome:   7    Cytoband:   Chr:7q13    SOURCE    GENECARD       Gene Ontology:   biological process:  neurotransmitter secretion   biological process:  neurotransmitter secretion      BioCarta Pathways:     KEGG Pathways:  
    Probe set : 1379019_at    Name:   properdin factor, complement    GB acc:   AI639534    UniGene:   Rn.18940    Gene Symbol:   Pfc    Locus Link:   299314    Chromosome:   X    Cytoband:   Chr:Xq12    SOURCE    GENECARD       Gene Ontology:     BioCarta Pathways:     KEGG Pathways:  
    Probe set : 1389096_at     GB acc:   BI274243              Gene Ontology:     BioCarta Pathways:     KEGG Pathways:  
    Probe set : 1373377_at    Name:   sodium channel modifier 1 (predicted)    GB acc:   BG376162    UniGene:   Rn.40268    Gene Symbol:   Scnm1_predicted    Locus Link:   310662    Chromosome:   2    Cytoband:   Chr:2q34    SOURCE    GENECARD       Gene Ontology:     BioCarta Pathways:     KEGG Pathways:  
    Probe set : 1391453_at    Name:   Transcribed locus, strongly similar to XP_576700.1 PREDICTED: similar to RIKEN cDNA 2900016D05 [Rattus norvegicus]    GB acc:   BF390736    UniGene:   Rn.14957        SOURCE        Gene Ontology:     BioCarta Pathways:     KEGG Pathways:  
    Probe set : 1370432_at    Name:   POU domain, class 3, transcription factor 1    GB acc:   M72711    UniGene:   Rn.82720    Gene Symbol:   Pou3f1    Locus Link:   192110    Chromosome:   5    Cytoband:   Chr:5q36    SOURCE    GENECARD       Gene Ontology:   cellular component:  nucleus   cellular component:  nucleus   cellular component:  transcription factor complex   molecular function:  DNA binding   molecular function:  transcription factor activity   molecular function:  transcription factor activity      BioCarta Pathways:     KEGG Pathways:  
    Probe set : 1367834_at    Name:   spermidine synthase    GB acc:   NM_053464    UniGene:   Rn.144616    Gene Symbol:   Srm    Locus Link:   84596    Chromosome:   5    Cytoband:   Chr:5q36    SOURCE    GENECARD       Gene Ontology:   biological process:  spermidine biosynthesis      BioCarta Pathways:     KEGG Pathways:  
    Probe set : 1372492_at    Name:   claudin 10 (predicted)    GB acc:   AI412937    UniGene:   Rn.99994    Gene Symbol:   Cldn10_predicted    Locus Link:   290485    Chromosome:   15    Cytoband:   Chr:15q24    SOURCE    GENECARD       Gene Ontology:   biological process:  cell adhesion   molecular function:  structural molecule activity      BioCarta Pathways:     KEGG Pathways:  
    Probe set : 1390982_at    Name:   chromatin assembly factor 1, subunit B (p60) (predicted)    GB acc:   BM389435    UniGene:   Rn.19317    Gene Symbol:   Chaf1b_predicted    Locus Link:   288242    Chromosome:   11    Cytoband:   Chr:11q11    SOURCE    GENECARD       Gene Ontology:     BioCarta Pathways:     KEGG Pathways:  
    Probe set : 1384298_at    Name:   myosin IF (predicted)    GB acc:   AI764088    UniGene:   Rn.28908    Gene Symbol:   Myo1f_predicted    Locus Link:   314654    Chromosome:   7    Cytoband:   Chr:7q12    SOURCE    GENECARD       Gene Ontology:   cellular component:  myosin   cellular component:  unconventional myosin      BioCarta Pathways:     KEGG Pathways:  
    Probe set : 1367793_at    Name:   D-dopachrome tautomerase    GB acc:   NM_024131    UniGene:   Rn.3464    Gene Symbol:   Ddt    Locus Link:   29318    Chromosome:   20    Cytoband:   Chr:20p12    SOURCE    GENECARD       Gene Ontology:   biological process:  melanin biosynthesis from tyrosine   biological process:  inflammatory response   molecular function:  dopachrome isomerase activity   molecular function:  cytokine activity   molecular function:  isomerase activity      BioCarta Pathways:     KEGG Pathways:  
    Probe set : 1368322_at    Name:   superoxide dismutase 3, extracellular    GB acc:   NM_012880    UniGene:   Rn.10358    Gene Symbol:   Sod3    Locus Link:   25352    Chromosome:   14    Cytoband:   Chr:14q11    SOURCE    GENECARD       Gene Ontology:     BioCarta Pathways:     KEGG Pathways:  
    Probe set : 1377192_a_at    Name:   caseinolytic protease X (E.coli) (predicted)    GB acc:   BM384629    UniGene:   Rn.66864    Gene Symbol:   Clpx_predicted    Locus Link:   300786    Chromosome:   8    Cytoband:   Chr:8q24    SOURCE    GENECARD       Gene Ontology:   biological process:  protein folding   biological process:  protein transport   cellular component:  mitochondrion      BioCarta Pathways:     KEGG Pathways:  
    Probe set : 1396279_at    Name:   ATPase, H+ transporting, lysosomal accessory protein 2    GB acc:   BG663051    UniGene:   Rn.12944    Gene Symbol:   Atp6ap2    Locus Link:   302526    Chromosome:   X    Cytoband:   Chr:Xq13    SOURCE    GENECARD       Gene Ontology:   cellular component:  integral to membrane   molecular function:  receptor activity      BioCarta Pathways:     KEGG Pathways:  
    Probe set : 1386805_at    Name:   Similar to OTTHUMP00000028696    GB acc:   BF554446    UniGene:   Rn.137233     Locus Link:   304558    Chromosome:   12    Cytoband:   Chr:12q16    SOURCE        Gene Ontology:     BioCarta Pathways:     KEGG Pathways:  
    Probe set : 1368558_s_at    Name:   allograft inflammatory factor 1    GB acc:   NM_017196    UniGene:   Rn.32080    Gene Symbol:   Aif1    Locus Link:   29427    Chromosome:   20    Cytoband:   Chr:20p12    SOURCE    GENECARD       Gene Ontology:   molecular function:  calcium ion binding   molecular function:  protein binding      BioCarta Pathways:     KEGG Pathways:  
    Probe set : 1393531_at    Name:   Transcribed locus    GB acc:   AA964815    UniGene:   Rn.23234        SOURCE        Gene Ontology:     BioCarta Pathways:     KEGG Pathways:  
    Probe set : 1393348_at    Name:   Transcribed locus    GB acc:   AI007775    UniGene:   Rn.23340        SOURCE        Gene Ontology:     BioCarta Pathways:     KEGG Pathways:  
    Probe set : 1368946_at    Name:   ADP-ribosylation factor 2    GB acc:   NM_024150    UniGene:   Rn.62175    Gene Symbol:   Arf2    Locus Link:   79119    Chromosome:   10    Cytoband:   Chr:10q32.1    SOURCE    GENECARD       Gene Ontology:   cellular component:  Golgi apparatus   cellular component:  Golgi apparatus   cellular component:  Golgi stack      BioCarta Pathways:     KEGG Pathways:  
    Probe set : 1379688_at    Name:   Transcribed locus    GB acc:   BM389813    UniGene:   Rn.147380        SOURCE        Gene Ontology:     BioCarta Pathways:     KEGG Pathways:  
    Probe set : 1368870_at    Name:   Inhibitor of DNA binding 2, dominant negative helix-loop-helix protein    GB acc:   NM_013060    UniGene:   Rn.3272    Gene Symbol:   Id2    Locus Link:   25587    Chromosome:   6    Cytoband:   Chr:6q16    SOURCE    GENECARD       Gene Ontology:   cellular component:  nucleus   cellular component:  nucleus   cellular component:  cytoplasm   molecular function:  protein binding   molecular function:  protein binding   molecular function:  transcription regulator activity      BioCarta Pathways:     KEGG Pathways:  
    Probe set : 1389209_at    Name:   similar to hypothetical protein BC002942 (predicted)    GB acc:   BG666095    UniGene:   Rn.38271    Gene Symbol:   RGD1306274_predicted    Locus Link:   315218    Chromosome:   7    Cytoband:   Chr:7q34    SOURCE    GENECARD       Gene Ontology:     BioCarta Pathways:     KEGG Pathways:  
    Probe set : 1395167_at    Name:   LOC362424 (predicted)    GB acc:   BG672109    UniGene:   Rn.60747    Gene Symbol:   RGD1308850_predicted    Locus Link:   362424    Chromosome:   4    Cytoband:   Chr:4q42    SOURCE    GENECARD       Gene Ontology:     BioCarta Pathways:     KEGG Pathways:  
    Probe set : 1393461_at    Name:   Similar to zinc finger protein 75    GB acc:   BM392157    UniGene:   Rn.96156     Locus Link:   500950      SOURCE        Gene Ontology:     BioCarta Pathways:     KEGG Pathways:  
    Probe set : 1385001_at    Name:   gasdermin domain containing 1 (predicted)    GB acc:   AI179373    UniGene:   Rn.105659    Gene Symbol:   Gsdmdc1_predicted    Locus Link:   315084    Chromosome:   7    Cytoband:   Chr:7q34    SOURCE    GENECARD       Gene Ontology:     BioCarta Pathways:     KEGG Pathways:  
    Probe set : 1373791_at    Name:   similar to RIKEN cDNA 2310011J03    GB acc:   BI289399    UniGene:   Rn.39052    Gene Symbol:   RGD:1359127    Locus Link:   299612    Chromosome:   7    Cytoband:   Chr:7q11    SOURCE    GENECARD       Gene Ontology:     BioCarta Pathways:     KEGG Pathways:  
    Probe set : 1374148_at    Name:   LOC498009    GB acc:   BI296414    UniGene:   Rn.7290    Gene Symbol:   LOC498009    Locus Link:   498009    Chromosome:   10    Cytoband:   Chr:10q32.1    SOURCE    GENECARD       Gene Ontology:     BioCarta Pathways:     KEGG Pathways:  
    Probe set : 1385089_at    Name:   Transcribed locus    GB acc:   BM386731    UniGene:   Rn.152707        SOURCE        Gene Ontology:     BioCarta Pathways:     KEGG Pathways:  
    Probe set : 1375066_at    Name:   similar to RIKEN cDNA 6330512M04 gene    GB acc:   H33003    UniGene:   Rn.14618    Gene Symbol:   LOC293632    Locus Link:   293632    Chromosome:   1    Cytoband:   Chr:1q41    SOURCE    GENECARD       Gene Ontology:     BioCarta Pathways:     KEGG Pathways:  
    Probe set : 1387974_a_at    Name:   kidney specific organic anion transporter    GB acc:   AF445995    UniGene:   Rn.127795    Gene Symbol:   RGD:621387    Locus Link:   80899    Chromosome:   4    Cytoband:   Chr:4q44    SOURCE    GENECARD       Gene Ontology:   cellular component:  membrane   cellular component:  integral to membrane      BioCarta Pathways:     KEGG Pathways:  
    Probe set : 1398398_at    Name:   similar to Homeobox protein A10    GB acc:   BI295741    UniGene:   Rn.852    Gene Symbol:   LOC368057    Locus Link:   368057    Chromosome:   4    Cytoband:   Chr:4q24    SOURCE    GENECARD       Gene Ontology:     BioCarta Pathways:     KEGG Pathways:  
    Probe set : 1389980_at    Name:   similar to Protein HSPC163    GB acc:   AI412591    UniGene:   Rn.24859    Gene Symbol:   LOC289324    Locus Link:   289324    Chromosome:   13    Cytoband:   Chr:13q26    SOURCE    GENECARD       Gene Ontology:     BioCarta Pathways:     KEGG Pathways:  
    Probe set : 1372087_at    Name:   hypertrophic agonist responsive protein B64    GB acc:   BG666916    UniGene:   Rn.8209    Gene Symbol:   Harpb64    Locus Link:   298917    Chromosome:   6    Cytoband:   Chr:6q16    SOURCE    GENECARD       Gene Ontology:   biological process:  lipid metabolism   molecular function:  hydrolase activity, acting on ester bonds      BioCarta Pathways:     KEGG Pathways:  
    Probe set : 1373777_at    Name:   Transcribed locus    GB acc:   BF391820    UniGene:   Rn.1141        SOURCE        Gene Ontology:     BioCarta Pathways:     KEGG Pathways:  
    Probe set : 1377911_at    Name:   Transcribed locus    GB acc:   AI029434    UniGene:   Rn.18226        SOURCE        Gene Ontology:     BioCarta Pathways:     KEGG Pathways:  
    Probe set : 1373830_at    Name:   hypothetical protein LOC619574    GB acc:   AA893539     Gene Symbol:   LOC619574    Locus Link:   619574    Chromosome:   10    Cytoband:   Chr:10q32     GENECARD       Gene Ontology:     BioCarta Pathways:     KEGG Pathways:  
    Probe set : 1398612_at    Name:   aldo-keto reductase family 1, member C12 (predicted)    GB acc:   BM383010     Gene Symbol:   Akr1c12_predicted    Locus Link:   361266    Chromosome:   17    Cytoband:   Chr:17q12.3     GENECARD       Gene Ontology:   biological process:  xenobiotic metabolism   molecular function:  aldo-keto reductase activity   molecular function:  oxidoreductase activity      BioCarta Pathways:     KEGG Pathways:  
    Probe set : 1368006_at    Name:   lysosomal-associated protein transmembrane 5    GB acc:   NM_053538    UniGene:   Rn.24799    Gene Symbol:   Laptm5    Locus Link:   89783    Chromosome:   5    Cytoband:   Chr:5q36    SOURCE    GENECARD       Gene Ontology:   cellular component:  lysosome   cellular component:  lysosome   cellular component:  integral to membrane      BioCarta Pathways:     KEGG Pathways:  
    Probe set : 1380610_at    Name:   Transcribed locus    GB acc:   AI406521    UniGene:   Rn.148591        SOURCE        Gene Ontology:     BioCarta Pathways:     KEGG Pathways:  
    Probe set : 1391856_at    Name:   similar to semaphorin sem2 (predicted)    GB acc:   BE108859    UniGene:   Rn.32183    Gene Symbol:   RGD1306079_predicted    Locus Link:   290562    Chromosome:   16    Cytoband:   Chr:16p16    SOURCE    GENECARD       Gene Ontology:     BioCarta Pathways:     KEGG Pathways:  
    Probe set : 1381940_at    Name:   Transcribed locus    GB acc:   AW527798    UniGene:   Rn.28619        SOURCE        Gene Ontology:     BioCarta Pathways:     KEGG Pathways:  
    Probe set : 1371337_at    Name:   cytochrome c oxidase subunit VIIa polypeptide 2 like (predicted)    GB acc:   BG378939    UniGene:   Rn.3907    Gene Symbol:   Cox7a2l_predicted    Locus Link:   298762    Chromosome:   6    Cytoband:   Chr:6q12    SOURCE    GENECARD       Gene Ontology:   biological process:  electron transport   cellular component:  mitochondrion   molecular function:  cytochrome-c oxidase activity   molecular function:  electron transporter activity      BioCarta Pathways:     KEGG Pathways:  
    Probe set : 1373566_at    Name:   DD6A4-12(7)-1 mRNA, partial sequence    GB acc:   AI103026    UniGene:   Rn.146347        SOURCE        Gene Ontology:     BioCarta Pathways:     KEGG Pathways:  
    Probe set : 1367957_at    Name:   regulator of G-protein signalling 3    GB acc:   NM_019340    UniGene:   Rn.53900    Gene Symbol:   Rgs3    Locus Link:   54293    Chromosome:   5    Cytoband:   Chr:5q24    SOURCE    GENECARD       Gene Ontology:   molecular function:  signal transducer activity   molecular function:  GTPase activator activity   molecular function:  protein binding      BioCarta Pathways:     KEGG Pathways:  
    Probe set : 1380072_at    Name:   similar to 2310044H10Rik protein    GB acc:   AI233871    UniGene:   Rn.1470    Gene Symbol:   RGD:1303214    Locus Link:   292878    Chromosome:   1    Cytoband:   Chr:1q22    SOURCE    GENECARD       Gene Ontology:     BioCarta Pathways:     KEGG Pathways:  
    Probe set : 1388512_at    Name:   phosphodiesterase 6D, cGMP-specific, rod, delta (predicted)    GB acc:   BM391512    UniGene:   Rn.44816    Gene Symbol:   Pde6d_predicted    Locus Link:   363272    Chromosome:   9    Cytoband:   Chr:9q35    SOURCE    GENECARD       Gene Ontology:   biological process:  visual perception   biological process:  phototransduction      BioCarta Pathways:     KEGG Pathways:  
    Probe set : 1373262_at    Name:   Similar to 2310014H01Rik protein (predicted)    GB acc:   AI102745    UniGene:   Rn.16324     Locus Link:   361790    Chromosome:   20    Cytoband:   Chr:20p12    SOURCE        Gene Ontology:     BioCarta Pathways:     KEGG Pathways:  
    Probe set : 1373986_at    Name:   Transcribed locus    GB acc:   AI410107    UniGene:   Rn.20036        SOURCE        Gene Ontology:     BioCarta Pathways:     KEGG Pathways:  
    Probe set : 1369073_at    Name:   nuclear receptor subfamily 1, group H, member 4    GB acc:   NM_021745    UniGene:   Rn.42943    Gene Symbol:   Nr1h4    Locus Link:   60351    Chromosome:   7    Cytoband:   Chr:7q13    SOURCE    GENECARD       Gene Ontology:   biological process:  regulation of transcription, DNA-dependent   biological process:  bile acid metabolism   biological process:  transcription   cellular component:  nucleus      BioCarta Pathways:     KEGG Pathways:  
    Probe set : 1373876_at     GB acc:   BI295945              Gene Ontology:     BioCarta Pathways:     KEGG Pathways:  
    Probe set : 1373368_at    Name:   Transcribed locus    GB acc:   BI279680    UniGene:   Rn.136571        SOURCE        Gene Ontology:     BioCarta Pathways:     KEGG Pathways:  
    Probe set : 1381434_s_at    Name:   similar to nidogen 2    GB acc:   AW253721    UniGene:   Rn.145062    Gene Symbol:   LOC311772    Locus Link:   311772    Chromosome:   3    Cytoband:   Chr:3p13    SOURCE    GENECARD       Gene Ontology:     BioCarta Pathways:     KEGG Pathways:  
    Probe set : 1377060_at    Name:   methylcrotonoyl-Coenzyme A carboxylase 2 (beta) (predicted)    GB acc:   AI410674    UniGene:   Rn.33635    Gene Symbol:   Mccc2_predicted    Locus Link:   361884    Chromosome:   2    Cytoband:   Chr:2q12    SOURCE    GENECARD       Gene Ontology:   cellular component:  mitochondrion   molecular function:  ligase activity      BioCarta Pathways:     KEGG Pathways:  
    Probe set : 1396035_at    Name:   similar to RPE-spondin    GB acc:   BF407272    UniGene:   Rn.144410    Gene Symbol:   LOC297757    Locus Link:   297757    Chromosome:   5    Cytoband:   Chr:5q11    SOURCE    GENECARD       Gene Ontology:     BioCarta Pathways:     KEGG Pathways:  
    Probe set : 1380699_at    Name:   Zinc finger homeobox 1b (predicted)    GB acc:   BE110761    UniGene:   Rn.59710     Locus Link:   311071    Chromosome:   3    Cytoband:   Chr:3q12    SOURCE        Gene Ontology:   cellular component:  nucleus      BioCarta Pathways:     KEGG Pathways:  
    Probe set : 1387242_at    Name:   Protein kinase, interferon-inducible double stranded RNA dependent    GB acc:   NM_019335    UniGene:   Rn.10022    Gene Symbol:   Prkr    Locus Link:   54287    Chromosome:   6    Cytoband:   Chr:6q11    SOURCE    GENECARD       Gene Ontology:   biological process:  protein amino acid phosphorylation   cellular component:  intracellular      BioCarta Pathways:     KEGG Pathways:  
    Probe set : 1369064_a_at    Name:   sodium channel, voltage-gated, type 8, alpha polypeptide    GB acc:   NM_019266    UniGene:   Rn.91216    Gene Symbol:   Scn8a    Locus Link:   29710    Chromosome:   7    Cytoband:   Chr:7q36    SOURCE    GENECARD       Gene Ontology:   cellular component:  voltage-gated sodium channel complex   cellular component:  membrane   cellular component:  integral to membrane      BioCarta Pathways:     KEGG Pathways:  
    Probe set : 1390066_at    Name:   nuclear receptor subfamily 2, group F, member 6    GB acc:   AI102726    UniGene:   Rn.25840    Gene Symbol:   Nr2f6    Locus Link:   245980    Chromosome:   16    Cytoband:   Chr:16p14    SOURCE    GENECARD       Gene Ontology:   cellular component:  intracellular   cellular component:  nucleus      BioCarta Pathways:     KEGG Pathways:  
    Probe set : 1393048_at    Name:   Adrenergic receptor, alpha 2a    GB acc:   BF561277    UniGene:   Rn.127118    Gene Symbol:   Adra2a    Locus Link:   25083    Chromosome:   1    Cytoband:   Chr:1    SOURCE    GENECARD       Gene Ontology:     BioCarta Pathways:     KEGG Pathways:  
    Probe set : 1371923_at    Name:   similar to hypothetical protein FLJ20481 (predicted)    GB acc:   BG380281    UniGene:   Rn.16547    Gene Symbol:   RGD1311599_predicted    Locus Link:   361467    Chromosome:   1    Cytoband:   Chr:1p11    SOURCE    GENECARD       Gene Ontology:     BioCarta Pathways:     KEGG Pathways:  
    Probe set : 1373460_at    Name:   similar to embryonic seven-span transmembrane protein-like protein    GB acc:   AI178790    UniGene:   Rn.145714    Gene Symbol:   LOC501103    Locus Link:   501103    Chromosome:   9    Cytoband:   Chr:9q12    SOURCE    GENECARD       Gene Ontology:     BioCarta Pathways:     KEGG Pathways:  
    Probe set : 1388989_at    Name:   similar to RIKEN cDNA 3110001N18 (predicted)    GB acc:   BI302675    UniGene:   Rn.112006    Gene Symbol:   RGD1309517_predicted    Locus Link:   361923    Chromosome:   2    Cytoband:   Chr:2q24    SOURCE    GENECARD       Gene Ontology:     BioCarta Pathways:     KEGG Pathways:  
    Probe set : 1383531_at     GB acc:   AA901061              Gene Ontology:     BioCarta Pathways:     KEGG Pathways:  
    Probe set : 1382263_at    Name:   similar to DNA segment, Chr 3, ERATO Doi 250, expressed (predicted)    GB acc:   AI511280    UniGene:   Rn.139568    Gene Symbol:   RGD1308397_predicted    Locus Link:   308017    Chromosome:   2    Cytoband:   Chr:2q44    SOURCE    GENECARD       Gene Ontology:     BioCarta Pathways:     KEGG Pathways:  
    Probe set : 1374685_at    Name:   Similar to ALEX3 protein    GB acc:   BE117446    UniGene:   Rn.44741     Locus Link:   367902    Chromosome:   X    Cytoband:   Chr:Xq34    SOURCE        Gene Ontology:   molecular function:  binding      BioCarta Pathways:     KEGG Pathways:  
    Probe set : 1378248_at    Name:   Similar to amyloid beta (A4) precursor protein-binding, family B, member 2    GB acc:   BE110453    UniGene:   Rn.24283     Locus Link:   305338    Chromosome:   14    Cytoband:   Chr:14p11    SOURCE        Gene Ontology:     BioCarta Pathways:     KEGG Pathways:  
    Probe set : 1398889_at    Name:   glutamate receptor, ionotropic, N-methyl D-aspartate-like 1A    GB acc:   BE113383    UniGene:   Rn.144666    Gene Symbol:   Grinl1a    Locus Link:   192147    Chromosome:   8    Cytoband:   Chr:8q24    SOURCE    GENECARD       Gene Ontology:   molecular function:  receptor activity      BioCarta Pathways:     KEGG Pathways:  
    Probe set : 1377660_at    Name:   similar to transthyretin (4L369) (predicted)    GB acc:   BM387540    UniGene:   Rn.15414    Gene Symbol:   RGD1309350_predicted    Locus Link:   293613    Chromosome:   1    Cytoband:   Chr:1q41    SOURCE    GENECARD       Gene Ontology:     BioCarta Pathways:     KEGG Pathways:  
    Probe set : 1387732_at    Name:   mitochondrial transcription termination factor 1    GB acc:   NM_053499    UniGene:   Rn.64629    Gene Symbol:   Mterf    Locus Link:   85261    Chromosome:   4    Cytoband:   Chr:4q13    SOURCE    GENECARD       Gene Ontology:   cellular component:  mitochondrion   molecular function:  double-stranded DNA binding   molecular function:  transcription termination factor activity   molecular function:  DNA binding      BioCarta Pathways:     KEGG Pathways:  
    Probe set : 1372377_at    Name:   DEAD (Asp-Glu-Ala-Asp) box polypeptide 41 (predicted)    GB acc:   BM389310    UniGene:   Rn.114971    Gene Symbol:   Ddx41_predicted    Locus Link:   314336    Chromosome:   17    Cytoband:   Chr:17p14    SOURCE    GENECARD       Gene Ontology:   cellular component:  nucleus      BioCarta Pathways:     KEGG Pathways:  
    Probe set : 1374033_at    Name:   proteasome (prosome, macropain) subunit, beta type 10 (predicted)    GB acc:   BG373505    UniGene:   Rn.24968    Gene Symbol:   Psmb10_predicted    Locus Link:   291983    Chromosome:   19    Cytoband:   Chr:19q12    SOURCE    GENECARD       Gene Ontology:   biological process:  proteolysis   biological process:  ubiquitin-dependent protein catabolism   biological process:  humoral immune response   cellular component:  cytosol   cellular component:  proteasome core complex (sensu Eukaryota)   cellular component:  protein complex      BioCarta Pathways:     KEGG Pathways:  
    Probe set : 1367920_at    Name:   endothelial differentiation, sphingolipid G-protein-coupled receptor, 5    GB acc:   AB016931    UniGene:   Rn.2491    Gene Symbol:   Edg5    Locus Link:   29415    Chromosome:   8    Cytoband:   Chr:8q13    SOURCE    GENECARD       Gene Ontology:   biological process:  G-protein coupled receptor protein signaling pathway   biological process:  behavior   biological process:  signal transduction   cellular component:  integral to plasma membrane   cellular component:  integral to membrane      BioCarta Pathways:     KEGG Pathways:  
    Probe set : 1369910_at    Name:   G protein-coupled receptor 10    GB acc:   NM_139193    UniGene:   Rn.138127    Gene Symbol:   Gpr10    Locus Link:   246075    Chromosome:   1    Cytoband:   Chr:1q55    SOURCE    GENECARD       Gene Ontology:   biological process:  G-protein coupled receptor protein signaling pathway   biological process:  pregnancy   cellular component:  integral to plasma membrane   cellular component:  integral to membrane   cellular component:  integral to membrane      BioCarta Pathways:     KEGG Pathways:  
    Probe set : 1376887_at    Name:   Similar to zinc finger protein 445    GB acc:   AI555799    UniGene:   Rn.100996     Locus Link:   301076    Chromosome:   8    Cytoband:   Chr:8q32    SOURCE        Gene Ontology:     BioCarta Pathways:     KEGG Pathways:  
    Probe set : 1374201_at     GB acc:   AI712727              Gene Ontology:     BioCarta Pathways:     KEGG Pathways:  
    Probe set : 1386996_at    Name:   myosin light chain, regulatory B    GB acc:   D14688    UniGene:   Rn.144581    Gene Symbol:   Mrlcb    Locus Link:   50685    Chromosome:   9    Cytoband:   Chr:9q38    SOURCE    GENECARD       Gene Ontology:   biological process:  cytoskeleton organization and biogenesis   biological process:  muscle development   cellular component:  cytoskeleton   cellular component:  apical part of cell   cellular component:  myosin   molecular function:  motor activity   molecular function:  calcium ion binding      BioCarta Pathways:     KEGG Pathways:  
    Probe set : 1368045_at    Name:   solute carrier family 31 (copper transporters), member 1    GB acc:   NM_133600    UniGene:   Rn.2789    Gene Symbol:   RGD:620059    Locus Link:   171135    Chromosome:   5    Cytoband:   Chr:5q24    SOURCE    GENECARD       Gene Ontology:   biological process:  transport   biological process:  copper ion transport   biological process:  ion transport   cellular component:  integral to plasma membrane   cellular component:  integral to membrane   molecular function:  copper ion transporter activity   molecular function:  carrier activity   molecular function:  copper ion binding      BioCarta Pathways:     KEGG Pathways:  
    Probe set : 1389158_at    Name:   Transcribed locus    GB acc:   BE329196    UniGene:   Rn.22482        SOURCE        Gene Ontology:     BioCarta Pathways:     KEGG Pathways:  
    Probe set : 1368432_a_at    Name:   v-ros UR2 sarcoma virus oncogene homolog 1 (avian)    GB acc:   NM_012874    UniGene:   Rn.87436    Gene Symbol:   Ros1    Locus Link:   25346    Chromosome:   20    Cytoband:   Chr:20q11    SOURCE    GENECARD       Gene Ontology:     BioCarta Pathways:     KEGG Pathways:  
    Probe set : 1383729_at    Name:   ankyrin repeat and SOCS box-containing 16 (predicted)    GB acc:   BE114522    UniGene:   Rn.46012    Gene Symbol:   Asb16_predicted    Locus Link:   303566    Chromosome:   10    Cytoband:   Chr:10q32.1    SOURCE    GENECARD       Gene Ontology:   biological process:  intracellular signaling cascade      BioCarta Pathways:     KEGG Pathways:  
    Probe set : 1383883_at    Name:   Similar to Hypothetical protein KIAA0469 (predicted)    GB acc:   BG380058    UniGene:   Rn.21220     Locus Link:   316624    Chromosome:   9    Cytoband:   Chr:9q36    SOURCE        Gene Ontology:     BioCarta Pathways:     KEGG Pathways:  
    Probe set : 1382043_at    Name:   unc-93 homolog B (C. elegans) (predicted)    GB acc:   AI234533    UniGene:   Rn.23633    Gene Symbol:   Unc93b_predicted    Locus Link:   361689    Chromosome:   1    Cytoband:   Chr:1q42    SOURCE    GENECARD       Gene Ontology:     BioCarta Pathways:     KEGG Pathways:  
    Probe set : 1398616_at    Name:   similar to Adapter-related protein complex 1 sigma 1B subunit (Sigma-adaptin 1B) (Adaptor protein complex AP-1 sigma-1B subunit) (Golgi adaptor HA1/AP1 adaptin sigma-1B subunit) (Clathrin assembly protein complex 1 sigma-1B small chain) (Sigma 1B s...    GB acc:   AI045228    UniGene:   Rn.17309    Gene Symbol:   LOC302671    Locus Link:   302671    Chromosome:   X    Cytoband:   Chr:Xq21    SOURCE    GENECARD       Gene Ontology:     BioCarta Pathways:     KEGG Pathways:  
    Probe set : 1370427_at    Name:   platelet derived growth factor, alpha    GB acc:   L06238    UniGene:   Rn.10999    Gene Symbol:   Pdgfa    Locus Link:   25266    Chromosome:   12    Cytoband:   Chr:12q11    SOURCE    GENECARD       Gene Ontology:   cellular component:  extracellular space   cellular component:  membrane   molecular function:  platelet-derived growth factor receptor binding   molecular function:  growth factor activity   molecular function:  growth factor activity      BioCarta Pathways:     KEGG Pathways:  
    Probe set : 1373304_at    Name:   alpha-N-acetylglucosaminidase    GB acc:   BM392002    UniGene:   Rn.36938    Gene Symbol:   Naglu    Locus Link:   287711    Chromosome:   10    Cytoband:   Chr:10q32.1    SOURCE    GENECARD       Gene Ontology:   biological process:  biosynthesis   molecular function:  ATP binding   molecular function:  nucleotidyltransferase activity      BioCarta Pathways:     KEGG Pathways:  
    Probe set : 1372513_at    Name:   ras-related C3 botulinum toxin substrate 1    GB acc:   AI103616    UniGene:   Rn.29157    Gene Symbol:   Rac1    Locus Link:   363875    Chromosome:   12    Cytoband:   Chr:12p11    SOURCE    GENECARD       Gene Ontology:   molecular function:  GTPase activity   molecular function:  GTP binding   molecular function:  nucleotide binding   molecular function:  GTP binding      BioCarta Pathways:     KEGG Pathways:  
    Probe set : 1372016_at    Name:   growth arrest and DNA-damage-inducible 45 beta (predicted)    GB acc:   BI287978    UniGene:   Rn.35886    Gene Symbol:   Gadd45b_predicted    Locus Link:   299626    Chromosome:   7    Cytoband:   Chr:7q11    SOURCE    GENECARD       Gene Ontology:   cellular component:  nucleus   molecular function:  protein binding      BioCarta Pathways:     KEGG Pathways:  
    Probe set : 1373656_at    Name:   Transcribed locus    GB acc:   AI713313    UniGene:   Rn.9612        SOURCE        Gene Ontology:     BioCarta Pathways:     KEGG Pathways:  
    Probe set : 1371549_at    Name:   WD repeat domain 23 (predicted)    GB acc:   AI228630    UniGene:   Rn.16542    Gene Symbol:   Wdr23_predicted    Locus Link:   305895    Chromosome:   15    Cytoband:   Chr:15p13    SOURCE    GENECARD       Gene Ontology:     BioCarta Pathways:     KEGG Pathways:  
    Probe set : 1368337_at    Name:   glycosylation dependent cell adhesion molecule 1    GB acc:   NM_012794    UniGene:   Rn.10939    Gene Symbol:   Glycam1    Locus Link:   25258    Chromosome:   7    Cytoband:   Chr:7q36    SOURCE    GENECARD       Gene Ontology:   biological process:  cell adhesion   biological process:  cell adhesion   biological process:  cell adhesion   cellular component:  extracellular space   cellular component:  plasma membrane   molecular function:  protein binding   molecular function:  protein binding      BioCarta Pathways:     KEGG Pathways:  
    Probe set : 1372924_at    Name:   Transcribed locus    GB acc:   AA891479    UniGene:   Rn.1743        SOURCE        Gene Ontology:     BioCarta Pathways:     KEGG Pathways:  
    Probe set : 1389598_at    Name:   ceroid-lipofuscinosis, neuronal 6 (predicted)    GB acc:   BI280367    UniGene:   Rn.14593    Gene Symbol:   Cln6_predicted    Locus Link:   315746    Chromosome:   8    Cytoband:   Chr:8q24    SOURCE    GENECARD       Gene Ontology:   biological process:  lysosome organization and biogenesis   biological process:  neuron differentiation   cellular component:  integral to membrane   cellular component:  integral to membrane      BioCarta Pathways:     KEGG Pathways:  
    Probe set : 1378040_at     GB acc:   BE111867              Gene Ontology:     BioCarta Pathways:     KEGG Pathways:  
    Probe set : 1373736_at    Name:   Transcribed locus, weakly similar to NP_082606.1 hypothetical protein LOC72704 [Mus musculus]    GB acc:   AI178446    UniGene:   Rn.13717        SOURCE        Gene Ontology:     BioCarta Pathways:     KEGG Pathways:  
    Probe set : 1378418_at    Name:   similar to MGC37193 protein (predicted)    GB acc:   BI285027    UniGene:   Rn.139264    Gene Symbol:   RGD1311681_predicted    Locus Link:   364674    Chromosome:   17    Cytoband:   Chr:17p14    SOURCE    GENECARD       Gene Ontology:     BioCarta Pathways:     KEGG Pathways:  
    Probe set : 1368223_at    Name:   a disintegrin-like and metalloprotease (reprolysin type) with thrombospondin type 1 motif, 1    GB acc:   NM_024400    UniGene:   Rn.7897    Gene Symbol:   Adamts1    Locus Link:   79252      SOURCE    GENECARD       Gene Ontology:   biological process:  proteolysis   biological process:  integrin-mediated signaling pathway   biological process:  negative regulation of cell proliferation   cellular component:  extracellular matrix (sensu Metazoa)   cellular component:  plasma membrane   cellular component:  extracellular matrix      BioCarta Pathways:     KEGG Pathways:  
    Probe set : 1374167_at    Name:   similar to autoantigen    GB acc:   AW532828    UniGene:   Rn.145173    Gene Symbol:   LOC361399    Locus Link:   361399    Chromosome:   19    Cytoband:   Chr:19q12    SOURCE    GENECARD       Gene Ontology:     BioCarta Pathways:     KEGG Pathways:  
    Probe set : 1394499_at    Name:   Taube nuss (predicted)    GB acc:   AI501768    UniGene:   Rn.35557     Locus Link:   316216    Chromosome:   9    Cytoband:   Chr:9q12    SOURCE        Gene Ontology:   biological process:  regulation of fat cell differentiation   biological process:  positive regulation of transcription, DNA-dependent   cellular component:  nucleus   cellular component:  transcription factor TFIID complex   molecular function:  protein binding   molecular function:  transcriptional activator activity      BioCarta Pathways:     KEGG Pathways:  
    Probe set : 1392741_at    Name:   Protein phosphatase 2 (formerly 2A), regulatory subunit B (PR 52), alpha isoform    GB acc:   AI145364    UniGene:   Rn.81155    Gene Symbol:   Ppp2r2a    Locus Link:   117104    Chromosome:   15    Cytoband:   Chr:15p12    SOURCE    GENECARD       Gene Ontology:   biological process:  protein amino acid dephosphorylation   biological process:  signal transduction   cellular component:  protein phosphatase type 2A complex   cellular component:  protein phosphatase type 2A complex      BioCarta Pathways:     KEGG Pathways:  
    Probe set : 1371332_at    Name:   Histone 1, H4a (predicted)    GB acc:   AA848821    UniGene:   Rn.3987     Locus Link:   364716    Chromosome:   17    Cytoband:   Chr:17p11    SOURCE        Gene Ontology:     BioCarta Pathways:     KEGG Pathways:  
    Probe set : 1373277_at    Name:   similar to BBP-like protein 2    GB acc:   BG373457    UniGene:   Rn.15627    Gene Symbol:   LOC292995    Locus Link:   292995    Chromosome:   1    Cytoband:   Chr:1q22    SOURCE    GENECARD       Gene Ontology:     BioCarta Pathways:     KEGG Pathways:  
    Probe set : 1372167_at     GB acc:   AW525656              Gene Ontology:     BioCarta Pathways:     KEGG Pathways:  
    Probe set : 1383766_at    Name:   Transcribed locus, strongly similar to NP_036020.1 sarcoglycan, beta (dystrophin-associated glycoprotein) [Mus musculus]    GB acc:   BG663338    UniGene:   Rn.98258        SOURCE        Gene Ontology:     BioCarta Pathways:     KEGG Pathways:  
    Probe set : 1379483_at    Name:   Basic helix-loop-helix domain containing, class B2    GB acc:   AI548256    UniGene:   Rn.81055    Gene Symbol:   Bhlhb2    Locus Link:   79431    Chromosome:   4    Cytoband:   Chr:4q41    SOURCE    GENECARD       Gene Ontology:   cellular component:  nucleus   cellular component:  nucleus      BioCarta Pathways:     KEGG Pathways:  
    Probe set : 1386820_at    Name:   Transcribed locus    GB acc:   AA851099    UniGene:   Rn.146087        SOURCE        Gene Ontology:     BioCarta Pathways:     KEGG Pathways:  
    Probe set : 1372920_at     GB acc:   AI411345              Gene Ontology:     BioCarta Pathways:     KEGG Pathways:  
    Probe set : 1397644_at     GB acc:   BI278369              Gene Ontology:     BioCarta Pathways:     KEGG Pathways:  
    Probe set : 1398315_at    Name:   ribosomal protein L15    GB acc:   AA800007    UniGene:   Rn.27505    Gene Symbol:   Rpl15    Locus Link:   245981    Chromosome:   15    Cytoband:   Chr:15p16    SOURCE    GENECARD       Gene Ontology:   biological process:  protein biosynthesis   cellular component:  intracellular   cellular component:  ribosome   cellular component:  ribonucleoprotein complex   molecular function:  RNA binding   molecular function:  structural constituent of ribosome   molecular function:  structural constituent of ribosome      BioCarta Pathways:     KEGG Pathways:  
    Probe set : 1384250_a_at    Name:   Transcribed locus    GB acc:   AA818098    UniGene:   Rn.23945        SOURCE        Gene Ontology:     BioCarta Pathways:     KEGG Pathways:  
    Probe set : 1370825_a_at    Name:   cell division cycle 42 homolog (S. cerevisiae)    GB acc:   AA925473    UniGene:   Rn.60067    Gene Symbol:   Cdc42    Locus Link:   64465    Chromosome:   5    Cytoband:   Chr:5q36    SOURCE    GENECARD       Gene Ontology:   biological process:  regulation of progression through cell cycle   biological process:  cytokinesis   biological process:  regulation of cell growth   biological process:  actin filament organization   biological process:  establishment and/or maintenance of cell polarity   biological process:  small GTPase mediated signal transduction   biological process:  Rho protein signal transduction   biological process:  protein transport   biological process:  establishment of cell polarity   biological process:  macrophage differentiation   biological process:  filopodium formation   biological process:  cell division   biological process:  response to glucose stimulus   biological process:  regulation of exocytosis   biological process:  establishment and/or maintenance of cell polarity   biological process:  endosome transport   biological process:  cell-cell adhesion   cellular component:  cytoplasm   cellular component:  filopodium   cellular component:  plasma membrane   cellular component:  secretory granule      BioCarta Pathways:     KEGG Pathways:  
    Probe set : 1385359_at    Name:   Similar to RIKEN cDNA 4931406C07 (predicted)    GB acc:   BF406890    UniGene:   Rn.3085     Locus Link:   363016    Chromosome:   8    Cytoband:   Chr:8q12    SOURCE        Gene Ontology:     BioCarta Pathways:     KEGG Pathways:  
    Probe set : 1374571_at    Name:   similar to hypothetical protein FLJ20522 (predicted)    GB acc:   AI172192    UniGene:   Rn.34037    Gene Symbol:   RGD1307289_predicted    Locus Link:   288041    Chromosome:   11    Cytoband:   Chr:11q22    SOURCE    GENECARD       Gene Ontology:   molecular function:  transferase activity   molecular function:  transferase activity, transferring glycosyl groups      BioCarta Pathways:     KEGG Pathways:  
    Probe set : 1378457_at    Name:   Transcribed locus    GB acc:   AI179450    UniGene:   Rn.34019        SOURCE        Gene Ontology:     BioCarta Pathways:     KEGG Pathways:  
    Probe set : 1389337_at    Name:   Transcribed locus    GB acc:   AA946032    UniGene:   Rn.7972        SOURCE        Gene Ontology:     BioCarta Pathways:     KEGG Pathways:  
    Probe set : 1382086_at    Name:   Transcribed locus    GB acc:   AI235883    UniGene:   Rn.19680        SOURCE        Gene Ontology:     BioCarta Pathways:     KEGG Pathways:  
    Probe set : 1396219_at     GB acc:   BF542315              Gene Ontology:     BioCarta Pathways:     KEGG Pathways:  
    Probe set : 1369286_at    Name:   protein C    GB acc:   NM_012803    UniGene:   Rn.91064    Gene Symbol:   Proc    Locus Link:   25268    Chromosome:   18    Cytoband:   Chr:18p12    SOURCE    GENECARD       Gene Ontology:   cellular component:  extracellular region   cellular component:  extracellular space      BioCarta Pathways:     KEGG Pathways:  
    Probe set : 1386932_at    Name:   glutaminase    GB acc:   AI577681    UniGene:   Rn.5762    Gene Symbol:   Gls    Locus Link:   24398    Chromosome:   9    Cytoband:   Chr:9q31-q32    SOURCE    GENECARD       Gene Ontology:   biological process:  glutamine metabolism   biological process:  glutamine catabolism   biological process:  glutamine catabolism   cellular component:  mitochondrion   cellular component:  mitochondrion   cellular component:  mitochondrial inner membrane   molecular function:  glutaminase activity   molecular function:  glutaminase activity   molecular function:  hydrolase activity      BioCarta Pathways:     KEGG Pathways:  
    Probe set : 1382592_at     GB acc:   AI059054              Gene Ontology:     BioCarta Pathways:     KEGG Pathways:  
    Probe set : 1388789_at    Name:   similar to hypothetical protein MGC17943    GB acc:   AI410352    UniGene:   Rn.19351    Gene Symbol:   LOC362857    Locus Link:   362857    Chromosome:   7    Cytoband:   Chr:7q13    SOURCE    GENECARD       Gene Ontology:     BioCarta Pathways:     KEGG Pathways:  
    Probe set : 1397328_at    Name:   Transcribed locus    GB acc:   AI555961    UniGene:   Rn.39181        SOURCE        Gene Ontology:     BioCarta Pathways:     KEGG Pathways:  
    Probe set : 1372875_at    Name:   Transcribed locus    GB acc:   AI408520    UniGene:   Rn.8905        SOURCE        Gene Ontology:     BioCarta Pathways:     KEGG Pathways:  
    Probe set : 1376644_at    Name:   similar to lung cancer metastasis-related protein 1 (predicted)    GB acc:   BG380933    UniGene:   Rn.19551    Gene Symbol:   RGD1311926_predicted    Locus Link:   311165    Chromosome:   3    Cytoband:   Chr:3q24    SOURCE    GENECARD       Gene Ontology:     BioCarta Pathways:     KEGG Pathways:  
    Probe set : 1385242_at    Name:   SPARC related modular calcium binding 2 (predicted)    GB acc:   AA900354    UniGene:   Rn.6092    Gene Symbol:   Smoc2_predicted    Locus Link:   292401    Chromosome:   1    Cytoband:   Chr:1q12    SOURCE    GENECARD       Gene Ontology:   cellular component:  extracellular matrix (sensu Metazoa)   cellular component:  extracellular space   molecular function:  calcium ion binding      BioCarta Pathways:     KEGG Pathways:  
    Probe set : 1398387_at    Name:   Unknown (protein for MGC:72614)    GB acc:   AI009530    UniGene:   Rn.20514    Gene Symbol:   RGD:735075    Locus Link:   310540    Chromosome:   2    Cytoband:   Chr:2q33    SOURCE    GENECARD       Gene Ontology:   cellular component:  integral to membrane      BioCarta Pathways:     KEGG Pathways:  
    Probe set : 1395157_at    Name:   neural precursor cell expressed, developmentally down-regulated gene 4A    GB acc:   BI286685    UniGene:   Rn.99540    Gene Symbol:   Nedd4a    Locus Link:   25489    Chromosome:   8    Cytoband:   Chr:8q24    SOURCE    GENECARD       Gene Ontology:   biological process:  protein modification   biological process:  ubiquitin cycle   cellular component:  ubiquitin ligase complex   cellular component:  intracellular   cellular component:  cytosol      BioCarta Pathways:     KEGG Pathways:  
    Probe set : 1371640_at    Name:   hypothetical protein LOC500855    GB acc:   BF289183    UniGene:   Rn.40403    Gene Symbol:   LOC500855    Locus Link:   500855    Chromosome:   7    Cytoband:   Chr:7q22    SOURCE    GENECARD       Gene Ontology:     BioCarta Pathways:     KEGG Pathways:  
    Probe set : 1375043_at    Name:   FBJ murine osteosarcoma viral oncogene homolog    GB acc:   BF415939    UniGene:   Rn.103750    Gene Symbol:   Fos    Locus Link:   314322    Chromosome:   6    Cytoband:   Chr:6q31    SOURCE    GENECARD       Gene Ontology:     BioCarta Pathways:     KEGG Pathways:  
    Probe set : 1372931_at    Name:   Similar to DXImx39e protein    GB acc:   AI178741    UniGene:   Rn.6226     Locus Link:   367743    Chromosome:   X    Cytoband:   Chr:Xq13    SOURCE        Gene Ontology:     BioCarta Pathways:     KEGG Pathways:  
    Probe set : 1394363_at    Name:   RIO kinase 3 (yeast) (predicted)    GB acc:   H34775    UniGene:   Rn.4181    Gene Symbol:   Riok3_predicted    Locus Link:   361293    Chromosome:   18    Cytoband:   Chr:18p13    SOURCE    GENECARD       Gene Ontology:   biological process:  protein amino acid phosphorylation   biological process:  chromosome segregation      BioCarta Pathways:     KEGG Pathways:  
    Probe set : 1372333_at    Name:   small nuclear ribonucleoprotein E (predicted) /// similar to small nuclear ribonucleoprotein E    GB acc:   BF555033    UniGene:   Rn.145709    Gene Symbol:   Snrpe_predicted /// LOC501075    Locus Link:   360844 /// 501075    Chromosome:   X    Cytoband:   Chr:Xq36 /// Chr:8q32    SOURCE    GENECARD       Gene Ontology:   biological process:  nuclear mRNA splicing, via spliceosome   biological process:  mRNA processing   molecular function:  RNA binding      BioCarta Pathways:     KEGG Pathways:  
    Probe set : 1389010_at    Name:   leukotriene A4 hydrolase (predicted)    GB acc:   AW918009    UniGene:   Rn.104990    Gene Symbol:   Lta4h_predicted    Locus Link:   299732    Chromosome:   7    Cytoband:   Chr:7q13    SOURCE    GENECARD       Gene Ontology:     BioCarta Pathways:     KEGG Pathways:  
    Probe set : 1388344_at    Name:   similar to RIKEN cDNA 1110005A23 (predicted)    GB acc:   BI283112    UniGene:   Rn.1506    Gene Symbol:   RGD1305692_predicted    Locus Link:   362819    Chromosome:   7    Cytoband:   Chr:7q11    SOURCE    GENECARD       Gene Ontology:   cellular component:  nucleus   molecular function:  DNA binding      BioCarta Pathways:     KEGG Pathways:  
    Probe set : 1368851_at    Name:   v-ets erythroblastosis virus E26 oncogene homolog 1 (avian)    GB acc:   NM_012555    UniGene:   Rn.88756    Gene Symbol:   Ets1    Locus Link:   24356    Chromosome:   8    Cytoband:   Chr:8q21    SOURCE    GENECARD       Gene Ontology:   cellular component:  nucleus      BioCarta Pathways:     KEGG Pathways:  
    Probe set : 1386934_at    Name:   choline transporter    GB acc:   NM_017348    UniGene:   Rn.10336    Gene Symbol:   Slc6a8    Locus Link:   50690    Chromosome:   X    Cytoband:   Chr:Xq37    SOURCE    GENECARD       Gene Ontology:   cellular component:  integral to plasma membrane   cellular component:  membrane   cellular component:  integral to membrane      BioCarta Pathways:     KEGG Pathways:  
    Probe set : 1388874_at    Name:   Transcribed locus    GB acc:   BE113032    UniGene:   Rn.12182        SOURCE        Gene Ontology:     BioCarta Pathways:     KEGG Pathways:  
    Probe set : 1378171_at    Name:   Neuropilin 2    GB acc:   BF409060    UniGene:   Rn.10816    Gene Symbol:   Nrp2    Locus Link:   81527    Chromosome:   9    Cytoband:   Chr:9q31    SOURCE    GENECARD       Gene Ontology:   cellular component:  membrane fraction   cellular component:  membrane   cellular component:  integral to membrane      BioCarta Pathways:     KEGG Pathways:  
    Probe set : 1379301_at    Name:   Transcribed locus    GB acc:   BM384812    UniGene:   Rn.28015        SOURCE        Gene Ontology:     BioCarta Pathways:     KEGG Pathways:  
    Probe set : 1370999_at    Name:   sperm associated antigen 5    GB acc:   AF111111    UniGene:   Rn.35359    Gene Symbol:   Spag5    Locus Link:   252918    Chromosome:   10    Cytoband:   Chr:10q25    SOURCE    GENECARD       Gene Ontology:   biological process:  cytokinesis   biological process:  proteolysis   biological process:  cell cycle   biological process:  mitosis   cellular component:  kinetochore   cellular component:  spindle   cellular component:  spindle microtubule   cellular component:  membrane   molecular function:  microtubule binding   molecular function:  peptidase activity   molecular function:  metallopeptidase activity      BioCarta Pathways:     KEGG Pathways:  
    Probe set : 1368896_at    Name:   MAD homolog 7 (Drosophila)    GB acc:   NM_030858    UniGene:   Rn.29980    Gene Symbol:   Madh7    Locus Link:   81516    Chromosome:   18    Cytoband:   Chr:18q12.3    SOURCE    GENECARD       Gene Ontology:   cellular component:  intracellular   cellular component:  nucleus      BioCarta Pathways:     KEGG Pathways:  
    Probe set : 1371818_at    Name:   Exportin, tRNA (nuclear export receptor for tRNAs) (predicted)    GB acc:   AW251204    UniGene:   Rn.2238     Locus Link:   314879    Chromosome:   7    Cytoband:   Chr:7q22    SOURCE        Gene Ontology:   biological process:  transport   cellular component:  nucleus   molecular function:  tRNA binding   molecular function:  binding      BioCarta Pathways:     KEGG Pathways:  
    Probe set : 1398869_at    Name:   proteasome (prosome, macropain) 26S subunit, ATPase, 4    GB acc:   BG378826    UniGene:   Rn.11341    Gene Symbol:   Psmc4    Locus Link:   117262    Chromosome:   1    Cytoband:   Chr:1q21    SOURCE    GENECARD       Gene Ontology:   biological process:  proteolysis   biological process:  protein catabolism      BioCarta Pathways:     KEGG Pathways:  
    Probe set : 1375030_at    Name:   Transcribed locus    GB acc:   AI639285    UniGene:   Rn.28077        SOURCE        Gene Ontology:     BioCarta Pathways:     KEGG Pathways:  
    Probe set : 1378487_at    Name:   E1A binding protein p300    GB acc:   BF403191    UniGene:   Rn.12447    Gene Symbol:   Ep300    Locus Link:   170915    Chromosome:   7    Cytoband:   Chr:7q34    SOURCE    GENECARD       Gene Ontology:   biological process:  regulation of transcription, DNA-dependent   cellular component:  nucleus   molecular function:  transcription cofactor activity      BioCarta Pathways:     KEGG Pathways:  
    Probe set : 1376050_at    Name:   TAF5 RNA polymerase II, TATA box binding protein (TBP)-associated factor (predicted)    GB acc:   AI177663    UniGene:   Rn.144813    Gene Symbol:   Taf5_predicted    Locus Link:   294018    Chromosome:   1    Cytoband:   Chr:1q54    SOURCE    GENECARD       Gene Ontology:   biological process:  regulation of transcription, DNA-dependent   biological process:  regulation of transcription   cellular component:  nucleus   cellular component:  transcription factor TFIID complex   molecular function:  transcription factor activity   molecular function:  transcription regulator activity      BioCarta Pathways:     KEGG Pathways:  
    Probe set : 1382443_at    Name:   similar to Pabpc4_predicted protein /// poly A binding protein, cytoplasmic 4 (predicted)    GB acc:   BG376950    UniGene:   Rn.2995    Gene Symbol:   LOC288398 /// Pabpc4_predicted    Locus Link:   288398 /// 298510    Chromosome:   12    Cytoband:   Chr:12p12 /// Chr:5q36    SOURCE    GENECARD       Gene Ontology:   cellular component:  cytoplasm      BioCarta Pathways:     KEGG Pathways:  
    Probe set : 1386138_at    Name:   Similar to RAS-like, estrogen-regulated, growth-inhibitor    GB acc:   AI172174    UniGene:   Rn.19093     Locus Link:   502916      SOURCE        Gene Ontology:     BioCarta Pathways:     KEGG Pathways:  
    Probe set : 1367982_at    Name:   aminolevulinic acid synthase 1    GB acc:   NM_024484    UniGene:   Rn.97126    Gene Symbol:   Alas1    Locus Link:   65155      SOURCE    GENECARD       Gene Ontology:   biological process:  heme biosynthesis   biological process:  metabolism   biological process:  biosynthesis   cellular component:  mitochondrion      BioCarta Pathways:     KEGG Pathways:  
    Probe set : 1387542_at    Name:   solute carrier family 9, member 3    GB acc:   NM_012654    UniGene:   Rn.9706    Gene Symbol:   Slc9a3    Locus Link:   24784    Chromosome:   1    Cytoband:   Chr:1p11    SOURCE    GENECARD       Gene Ontology:     BioCarta Pathways:     KEGG Pathways:  
    Probe set : 1369007_at    Name:   nuclear receptor subfamily 4, group A, member 2    GB acc:   L08595    UniGene:   Rn.88129    Gene Symbol:   Nr4a2    Locus Link:   54278    Chromosome:   3    Cytoband:   Chr:3q12    SOURCE    GENECARD       Gene Ontology:   cellular component:  nucleus   cellular component:  nucleus   cellular component:  cytoplasm      BioCarta Pathways:     KEGG Pathways:  
    Probe set : 1386897_at    Name:   heterogeneous nuclear ribonucleoproteins methyltransferase-like 2 (S. cerevisiae)    GB acc:   NM_024363    UniGene:   Rn.5870    Gene Symbol:   Hrmt1l2    Locus Link:   60421    Chromosome:   1    Cytoband:   Chr:1q22    SOURCE    GENECARD       Gene Ontology:   molecular function:  methyltransferase activity   molecular function:  N-methyltransferase activity   molecular function:  protein methyltransferase activity   molecular function:  protein methyltransferase activity   molecular function:  histone-arginine N-methyltransferase activity   molecular function:  S-adenosylmethionine-dependent methyltransferase activity   molecular function:  [cytochrome c]-arginine N-methyltransferase activity   molecular function:  transferase activity   molecular function:  protein-arginine omega-N monomethyltransferase activity   molecular function:  protein-arginine omega-N asymmetric methyltransferase activity   molecular function:  protein binding   molecular function:  histone-arginine N-methyltransferase activity   molecular function:  [cytochrome c]-arginine N-methyltransferase activity   molecular function:  protein-arginine omega-N monomethyltransferase activity   molecular function:  protein-arginine omega-N asymmetric methyltransferase activity      BioCarta Pathways:     KEGG Pathways:  
    Probe set : 1371792_at    Name:   Transcribed locus    GB acc:   AI102045    UniGene:   Rn.147985        SOURCE        Gene Ontology:     BioCarta Pathways:     KEGG Pathways:  
    Probe set : 1390396_at    Name:   Similar to eukaryotic translation initiation factor 4E member 3    GB acc:   AI101338    UniGene:   Rn.22427     Locus Link:   297481    Chromosome:   4    Cytoband:   Chr:4q34    SOURCE        Gene Ontology:     BioCarta Pathways:     KEGG Pathways:  
    Probe set : 1392698_a_at    Name:   Transcribed locus    GB acc:   AA899844    UniGene:   Rn.12334        SOURCE        Gene Ontology:     BioCarta Pathways:     KEGG Pathways:  
    Probe set : 1379542_at    Name:   similar to Small nuclear ribonucleoprotein F (snRNP-F) (Sm protein F) (Sm-F) (SmF) /// small nuclear ribonucleoprotein polypeptide F (predicted)    GB acc:   BF522334    UniGene:   Rn.145343    Gene Symbol:   LOC296377 /// Snrpf_predicted    Locus Link:   296377 /// 366871    Chromosome:   3    Cytoband:   Chr:3q42 /// Chr:7q13    SOURCE    GENECARD       Gene Ontology:   biological process:  mRNA processing   cellular component:  nucleus   cellular component:  small nucleolar ribonucleoprotein complex      BioCarta Pathways:     KEGG Pathways:  
    Probe set : 1367701_at    Name:   receptor (calcitonin) activity modifying protein 2    GB acc:   NM_031646    UniGene:   Rn.107168    Gene Symbol:   Ramp2    Locus Link:   58966    Chromosome:   10    Cytoband:   Chr:10q32.1    SOURCE    GENECARD       Gene Ontology:     BioCarta Pathways:     KEGG Pathways:  
    Probe set : 1370393_at    Name:   coiled-coil domain containing 5    GB acc:   BF418951    UniGene:   Rn.29736    Gene Symbol:   Ccdc5    Locus Link:   192228    Chromosome:   18    Cytoband:   Chr:18q12.3    SOURCE    GENECARD       Gene Ontology:   biological process:  cell cycle   biological process:  mitosis   biological process:  cell division   cellular component:  microtubule      BioCarta Pathways:     KEGG Pathways:  
    Probe set : 1371516_at    Name:   exportin 7 (predicted)    GB acc:   BM391834    UniGene:   Rn.32535    Gene Symbol:   Xpo7_predicted    Locus Link:   361070    Chromosome:   15    Cytoband:   Chr:15p11    SOURCE    GENECARD       Gene Ontology:   cellular component:  nucleus   cellular component:  nuclear pore   cellular component:  cytoplasm      BioCarta Pathways:     KEGG Pathways:  
    Probe set : 1372156_at    Name:   similar to RIKEN cDNA 1810014L12 (predicted)    GB acc:   BF414018    UniGene:   Rn.20057    Gene Symbol:   RGD1307423_predicted    Locus Link:   303330    Chromosome:   10    Cytoband:   Chr:10q25    SOURCE    GENECARD       Gene Ontology:   cellular component:  extracellular space   cellular component:  integral to membrane      BioCarta Pathways:     KEGG Pathways:  
    Probe set : 1392699_at    Name:   Transcribed locus    GB acc:   BF411888    UniGene:   Rn.17764        SOURCE        Gene Ontology:     BioCarta Pathways:     KEGG Pathways:  
    Probe set : 1373072_at    Name:   Transcribed locus    GB acc:   AI170552    UniGene:   Rn.1897        SOURCE        Gene Ontology:     BioCarta Pathways:     KEGG Pathways:  
    Probe set : 1383266_at    Name:   secreted frizzled-related protein 1    GB acc:   AW144660    UniGene:   Rn.34957    Gene Symbol:   Sfrp1    Locus Link:   84402    Chromosome:   16    Cytoband:   Chr:16q12.5    SOURCE    GENECARD       Gene Ontology:   cellular component:  extracellular space   cellular component:  membrane   molecular function:  transmembrane receptor activity      BioCarta Pathways:     KEGG Pathways:  
    Probe set : 1373432_at    Name:   myristoylated alanine rich protein kinase C substrate    GB acc:   BE111604    UniGene:   Rn.9560    Gene Symbol:   Marcks    Locus Link:   25603      SOURCE    GENECARD       Gene Ontology:   biological process:  cell motility   cellular component:  plasma membrane   cellular component:  actin cytoskeleton   cellular component:  membrane      BioCarta Pathways:     KEGG Pathways:  
    Probe set : 1379694_at    Name:   Enhancer of polycomb homolog 2 (Drosophila) (predicted)    GB acc:   AI171596    UniGene:   Rn.23462     Locus Link:   362132    Chromosome:   3    Cytoband:   Chr:3q12    SOURCE        Gene Ontology:     BioCarta Pathways:     KEGG Pathways:  
    Probe set : 1375855_at    Name:   ependymin related protein 2    GB acc:   AI171799    UniGene:   Rn.2881    Gene Symbol:   RGD:1359161    Locus Link:   291180    Chromosome:   17    Cytoband:   Chr:17q11    SOURCE    GENECARD       Gene Ontology:   biological process:  cell-matrix adhesion   cellular component:  extracellular region   molecular function:  calcium ion binding      BioCarta Pathways:     KEGG Pathways:  
    Probe set : 1384044_at     GB acc:   AA964882              Gene Ontology:     BioCarta Pathways:     KEGG Pathways:  
    Probe set : 1397737_at    Name:   Transcribed locus    GB acc:   BF391084    UniGene:   Rn.61385        SOURCE        Gene Ontology:     BioCarta Pathways:     KEGG Pathways:  
    Probe set : 1372661_at    Name:   transducin (beta)-like 3 (predicted)    GB acc:   AI406314    UniGene:   Rn.32013    Gene Symbol:   Tbl3_predicted    Locus Link:   287120    Chromosome:   10    Cytoband:   Chr:10q12    SOURCE    GENECARD       Gene Ontology:   biological process:  G-protein signaling, coupled to cGMP nucleotide second messenger   molecular function:  receptor signaling protein activity      BioCarta Pathways:     KEGG Pathways:  
    Probe set : 1368339_at    Name:   calbindin 3, (vitamin D-dependent calcium binding protein)    GB acc:   NM_012521    UniGene:   Rn.9974    Gene Symbol:   Calb3    Locus Link:   24249    Chromosome:   X    Cytoband:   Chr:Xq21    SOURCE    GENECARD       Gene Ontology:   cellular component:  basolateral plasma membrane   cellular component:  apical plasma membrane   molecular function:  calcium ion binding   molecular function:  calcium ion binding   molecular function:  vitamin D binding      BioCarta Pathways:     KEGG Pathways:  
    Probe set : 1382493_at    Name:   Transcribed locus, strongly similar to XP_223060.3 PREDICTED: similar to LEK1 [Rattus norvegicus]    GB acc:   BG377427    UniGene:   Rn.41236        SOURCE        Gene Ontology:     BioCarta Pathways:     KEGG Pathways:  
    Probe set : 1386002_at    Name:   Transcribed locus, weakly similar to XP_128440.2 PREDICTED: RIKEN cDNA 4930432O21 [Mus musculus]    GB acc:   AI236841    UniGene:   Rn.25394        SOURCE        Gene Ontology:     BioCarta Pathways:     KEGG Pathways:  
    Probe set : 1371544_at    Name:   similar to Enhancer of rudimentary homolog    GB acc:   BE109666    UniGene:   Rn.7881    Gene Symbol:   LOC503047    Locus Link:   503047    Chromosome:   6    Cytoband:   Chr:6q24    SOURCE    GENECARD       Gene Ontology:     BioCarta Pathways:     KEGG Pathways:  
    Probe set : 1392861_at    Name:   Transcribed locus    GB acc:   BG373422    UniGene:   Rn.8902        SOURCE        Gene Ontology:     BioCarta Pathways:     KEGG Pathways:  
    Probe set : 1398255_at    Name:   solute carrier family 15 (H+/peptide transporter), member 2    GB acc:   NM_031672    UniGene:   Rn.89268    Gene Symbol:   Slc15a2    Locus Link:   60577    Chromosome:   11    Cytoband:   Chr:11q22    SOURCE    GENECARD       Gene Ontology:   biological process:  transport   biological process:  oligopeptide transport   biological process:  protein transport      BioCarta Pathways:     KEGG Pathways:  
    Probe set : 1382252_at    Name:   Transcribed locus    GB acc:   BE099808    UniGene:   Rn.9295        SOURCE        Gene Ontology:     BioCarta Pathways:     KEGG Pathways:  
    Probe set : 1397673_at    Name:   CUG triplet repeat,RNA-binding protein 2    GB acc:   AW523660    UniGene:   Rn.30004    Gene Symbol:   Cugbp2    Locus Link:   29428    Chromosome:   17    Cytoband:   Chr:17q12.3    SOURCE    GENECARD       Gene Ontology:   cellular component:  nucleus   molecular function:  RNA binding   molecular function:  RNA binding   molecular function:  nucleotide binding   molecular function:  nucleic acid binding      BioCarta Pathways:     KEGG Pathways:  
    Probe set : 1375560_at    Name:   similar to RIKEN cDNA 0610010D20 (predicted)    GB acc:   AA945579    UniGene:   Rn.13905    Gene Symbol:   RGD1310475_predicted    Locus Link:   293949    Chromosome:   1    Cytoband:   Chr:1q54    SOURCE    GENECARD       Gene Ontology:     BioCarta Pathways:     KEGG Pathways:  
    Probe set : 1389163_at    Name:   tripartite motif protein 32 (predicted)    GB acc:   BG372673    UniGene:   Rn.97414    Gene Symbol:   Trim32_predicted    Locus Link:   313264    Chromosome:   5    Cytoband:   Chr:5q24    SOURCE    GENECARD       Gene Ontology:   biological process:  ubiquitin cycle   biological process:  protein ubiquitination   cellular component:  ubiquitin ligase complex   cellular component:  intracellular   cellular component:  nucleus      BioCarta Pathways:     KEGG Pathways:  
    Probe set : 1391649_a_at    Name:   Transcribed locus    GB acc:   AW915522    UniGene:   Rn.128940        SOURCE        Gene Ontology:     BioCarta Pathways:     KEGG Pathways:  
    Probe set : 1368358_a_at    Name:   protein tyrosine phosphatase, receptor type, R    GB acc:   NM_053594    UniGene:   Rn.6277    Gene Symbol:   Ptprr    Locus Link:   94202    Chromosome:   7    Cytoband:   Chr:7q22    SOURCE    GENECARD       Gene Ontology:     BioCarta Pathways:     KEGG Pathways:  
    Probe set : 1373185_at    Name:   signal sequence receptor, beta (predicted)    GB acc:   BE329347    UniGene:   Rn.17458    Gene Symbol:   Ssr2_predicted    Locus Link:   295235    Chromosome:   2    Cytoband:   Chr:2q34    SOURCE    GENECARD       Gene Ontology:   biological process:  cotranslational protein targeting to membrane   cellular component:  extracellular space   cellular component:  endoplasmic reticulum   cellular component:  integral to membrane   molecular function:  receptor activity   molecular function:  signal sequence binding      BioCarta Pathways:     KEGG Pathways:  
    Probe set : 1373911_at    Name:   periostin, osteoblast specific factor (predicted)    GB acc:   BM389026    UniGene:   Rn.30516    Gene Symbol:   Postn_predicted    Locus Link:   361945    Chromosome:   2    Cytoband:   Chr:2q26    SOURCE    GENECARD       Gene Ontology:   biological process:  skeletal development   biological process:  cell adhesion   cellular component:  extracellular matrix (sensu Metazoa)   cellular component:  extracellular space   molecular function:  protein binding   molecular function:  heparin binding      BioCarta Pathways:     KEGG Pathways:  
    Probe set : 1369941_at    Name:   death-associated protein    GB acc:   NM_022526    UniGene:   Rn.1531    Gene Symbol:   Dap    Locus Link:   64322    Chromosome:   2    Cytoband:   Chr:2q22    SOURCE    GENECARD       Gene Ontology:   biological process:  apoptosis   biological process:  induction of apoptosis by extracellular signals   biological process:  programmed cell death      BioCarta Pathways:     KEGG Pathways:  
    Probe set : 1383137_at    Name:   SRY-box containing gene 4 (predicted)    GB acc:   AA956294    UniGene:   Rn.15099     Locus Link:   364712    Chromosome:   17    Cytoband:   Chr:17p12    SOURCE        Gene Ontology:   biological process:  regulation of transcription, DNA-dependent   cellular component:  nucleus   cellular component:  transcription factor complex   molecular function:  DNA binding   molecular function:  transcription factor activity      BioCarta Pathways:     KEGG Pathways:  
    Probe set : 1376785_at    Name:   Synaptonemal complex protein 3    GB acc:   AI104799    UniGene:   Rn.34889    Gene Symbol:   Sycp3    Locus Link:   25561    Chromosome:   7    Cytoband:   Chr:7q13    SOURCE    GENECARD       Gene Ontology:   biological process:  mitosis   biological process:  meiosis   molecular function:  DNA binding   molecular function:  protein binding   molecular function:  protein binding      BioCarta Pathways:     KEGG Pathways:  
    Probe set : 1393138_at    Name:   Jun D proto-oncogene    GB acc:   BE329377    UniGene:   Rn.46225    Gene Symbol:   Jund    Locus Link:   24518    Chromosome:   16    Cytoband:   Chr:16p14    SOURCE    GENECARD       Gene Ontology:   cellular component:  chromatin   cellular component:  intracellular   cellular component:  nucleus      BioCarta Pathways:     KEGG Pathways:  
    Probe set : 1376334_at    Name:   Similar to hypothetical protein FLJ20276 (predicted)    GB acc:   BF394214    UniGene:   Rn.58227     Locus Link:   313331    Chromosome:   5    Cytoband:   Chr:5q31    SOURCE        Gene Ontology:     BioCarta Pathways:     KEGG Pathways:  
    Probe set : 1397450_at    Name:   Transcribed locus    GB acc:   BF388728    UniGene:   Rn.148194        SOURCE        Gene Ontology:     BioCarta Pathways:     KEGG Pathways:  
    Probe set : 1371696_at    Name:   G protein-coupled receptor 56    GB acc:   AI412938    UniGene:   Rn.1677    Gene Symbol:   Gpr56    Locus Link:   260326    Chromosome:   19    Cytoband:   Chr:19p13    SOURCE    GENECARD       Gene Ontology:     BioCarta Pathways:     KEGG Pathways:  
    Probe set : 1398359_at    Name:   similar to RIKEN cDNA 2500002L14; EST C77350    GB acc:   AI008549    UniGene:   Rn.94883    Gene Symbol:   RGD:1359698    Locus Link:   297337    Chromosome:   4    Cytoband:   Chr:4q33    SOURCE    GENECARD       Gene Ontology:   biological process:  protein ubiquitination   cellular component:  ubiquitin ligase complex   molecular function:  ubiquitin-protein ligase activity   molecular function:  zinc ion binding   molecular function:  metal ion binding      BioCarta Pathways:     KEGG Pathways:  
    Probe set : 1390827_at    Name:   MAD homolog 3 (Drosophila)    GB acc:   AA997679    UniGene:   Rn.10636    Gene Symbol:   Madh3    Locus Link:   25631    Chromosome:   8    Cytoband:   Chr:8q24    SOURCE    GENECARD       Gene Ontology:   biological process:  negative regulation of transcription from RNA polymerase II promoter   biological process:  mesoderm formation   biological process:  regulation of transcription, DNA-dependent   biological process:  transcription from RNA polymerase II promoter   biological process:  transforming growth factor beta receptor signaling pathway   biological process:  common-partner SMAD protein phosphorylation   biological process:  SMAD protein heteromerization   biological process:  organ morphogenesis   biological process:  regulation of striated muscle development   biological process:  regulation of transforming growth factor beta receptor signaling pathway   biological process:  positive regulation of transcription   biological process:  positive regulation of transcription from RNA polymerase II promoter   biological process:  paraxial mesoderm morphogenesis   biological process:  regulation of epithelial cell proliferation   biological process:  transcription   cellular component:  intracellular   cellular component:  nucleus   cellular component:  plasma membrane      BioCarta Pathways:     KEGG Pathways:  
    Probe set : 1373516_at    Name:   Similar to RIKEN cDNA 2600005C20 (predicted)    GB acc:   BE099830    UniGene:   Rn.54275     Locus Link:   309673    Chromosome:   20    Cytoband:   Chr:20p12    SOURCE        Gene Ontology:     BioCarta Pathways:     KEGG Pathways:  
    Probe set : 1385716_at    Name:   Transcribed locus    GB acc:   AW522166    UniGene:   Rn.45316        SOURCE        Gene Ontology:     BioCarta Pathways:     KEGG Pathways:  
    Probe set : 1379683_at    Name:   Similar to hypothetical protein MGC17839    GB acc:   BG378155    UniGene:   Rn.24072     Locus Link:   315593    Chromosome:   8    Cytoband:   Chr:8q22    SOURCE        Gene Ontology:     BioCarta Pathways:     KEGG Pathways:  
    Probe set : 1372550_at     GB acc:   AI169278              Gene Ontology:     BioCarta Pathways:     KEGG Pathways:  
    Probe set : 1372426_at    Name:   thrombospondin repeat protein 1    GB acc:   AI411997    UniGene:   Rn.62115    Gene Symbol:   Tsrc1    Locus Link:   310670    Chromosome:   2    Cytoband:   Chr:2q34    SOURCE    GENECARD       Gene Ontology:   cellular component:  extracellular matrix   molecular function:  metalloendopeptidase activity   molecular function:  peptidase activity      BioCarta Pathways:     KEGG Pathways:  
    Probe set : 1374691_at    Name:   sulfotransferase family 5A, member 1 (predicted)    GB acc:   BF283670    UniGene:   Rn.107747    Gene Symbol:   Sult5a1_predicted    Locus Link:   292077    Chromosome:   19    Cytoband:   Chr:19q12    SOURCE    GENECARD       Gene Ontology:   molecular function:  transferase activity      BioCarta Pathways:     KEGG Pathways:  
    Probe set : 1398246_s_at    Name:   Fc receptor, IgG, low affinity III /// Fc gamma receptor II beta    GB acc:   NM_053843    UniGene:   Rn.6050    Gene Symbol:   Fcgr3 /// LOC498276    Locus Link:   116591 /// 498276    Chromosome:   13    Cytoband:   Chr:13q24    SOURCE    GENECARD       Gene Ontology:   biological process:  antibody-dependent cellular cytotoxicity   biological process:  positive regulation of type IIa hypersensitivity   biological process:  positive regulation of type III hypersensitivity   biological process:  positive regulation of type I hypersensitivity   biological process:  serotonin secretion   biological process:  phagocytosis, recognition   biological process:  phagocytosis, engulfment   biological process:  immune response   biological process:  cell surface receptor linked signal transduction   biological process:  neutrophil chemotaxis   biological process:  cell-mediated immune response   biological process:  cell-mediated immune response   biological process:  positive regulation of tumor necrosis factor-alpha biosynthesis   biological process:  antigen presentation, exogenous antigen via MHC class I   biological process:  mast cell activation   biological process:  positive regulation of phagocytosis   biological process:  regulation of immune response   cellular component:  extracellular space   cellular component:  plasma membrane   cellular component:  integral to membrane      BioCarta Pathways:     KEGG Pathways:  
    Probe set : 1386995_at    Name:   B-cell translocation gene 2, anti-proliferative    GB acc:   BI288701    UniGene:   Rn.27923    Gene Symbol:   Btg2    Locus Link:   29619      SOURCE    GENECARD       Gene Ontology:   molecular function:  protein binding      BioCarta Pathways:     KEGG Pathways:  
    Probe set : 1396018_at    Name:   Cullin 5    GB acc:   BF416830    UniGene:   Rn.48797    Gene Symbol:   Cul5    Locus Link:   64624    Chromosome:   8    Cytoband:   Chr:8q24    SOURCE    GENECARD       Gene Ontology:     BioCarta Pathways:     KEGG Pathways:  
    Probe set : 1383623_at    Name:   thymocyte protein thy28    GB acc:   BM383909    UniGene:   Rn.87291    Gene Symbol:   RGD:1359525    Locus Link:   300470    Chromosome:   8    Cytoband:   Chr:8q13    SOURCE    GENECARD       Gene Ontology:     BioCarta Pathways:     KEGG Pathways:  
    Probe set : 1369250_at    Name:   solute carrier family 28 (sodium-coupled nucleoside transporter), member 1    GB acc:   NM_053863    UniGene:   Rn.10517    Gene Symbol:   Slc28a1    Locus Link:   116642    Chromosome:   1    Cytoband:   Chr:1q31    SOURCE    GENECARD       Gene Ontology:     BioCarta Pathways:     KEGG Pathways:  
    Probe set : 1378247_at    Name:   ELL associated factor 2    GB acc:   BI285131    UniGene:   Rn.20681    Gene Symbol:   Eaf2    Locus Link:   266787    Chromosome:   11    Cytoband:   Chr:11q22    SOURCE    GENECARD       Gene Ontology:   cellular component:  nucleus   cellular component:  nucleoplasm   molecular function:  protein binding   molecular function:  transcriptional activator activity      BioCarta Pathways:     KEGG Pathways:  
    Probe set : 1393983_at    Name:   exportin, tRNA (nuclear export receptor for tRNAs) (predicted)    GB acc:   AA998204    UniGene:   Rn.2238    Gene Symbol:   Xpot_predicted    Locus Link:   314879    Chromosome:   7    Cytoband:   Chr:7q22    SOURCE    GENECARD       Gene Ontology:   biological process:  transport   cellular component:  nucleus   molecular function:  tRNA binding   molecular function:  binding      BioCarta Pathways:     KEGG Pathways:  
    Probe set : 1373931_at    Name:   similar to RIKEN cDNA C530028O21 gene (predicted)    GB acc:   C07069    UniGene:   Rn.13411    Gene Symbol:   RGD1304952_predicted    Locus Link:   312711    Chromosome:   4    Cytoband:   Chr:4q42    SOURCE    GENECARD       Gene Ontology:     BioCarta Pathways:     KEGG Pathways:  
    Probe set : 1378365_at    Name:   Transcribed locus    GB acc:   AW529483    UniGene:   Rn.138007        SOURCE        Gene Ontology:     BioCarta Pathways:     KEGG Pathways:  
    Probe set : 1377635_at    Name:   flavin containing monooxygenase 2    GB acc:   BM389350    UniGene:   Rn.3928    Gene Symbol:   Fmo2    Locus Link:   246245    Chromosome:   13    Cytoband:   Chr:13q22    SOURCE    GENECARD       Gene Ontology:   biological process:  electron transport   biological process:  oxygen and reactive oxygen species metabolism      BioCarta Pathways:     KEGG Pathways:  
    Probe set : 1382402_at    Name:   Unc-51 like kinase 1 (C. elegans)    GB acc:   AI230479    UniGene:   Rn.24509    Gene Symbol:   Ulk1    Locus Link:   360827    Chromosome:   12    Cytoband:   Chr:12q16    SOURCE    GENECARD       Gene Ontology:     BioCarta Pathways:     KEGG Pathways:  
    Probe set : 1394014_at    Name:   PHD finger protein 12 (predicted)    GB acc:   AA924840    UniGene:   Rn.101513    Gene Symbol:   Phf12_predicted    Locus Link:   303274    Chromosome:   10    Cytoband:   Chr:10q25    SOURCE    GENECARD       Gene Ontology:   biological process:  transcription   biological process:  regulation of transcription, DNA-dependent   cellular component:  nucleus   molecular function:  DNA binding      BioCarta Pathways:     KEGG Pathways:  
    Probe set : 1397892_at    Name:   eukaryotic translation initiation factor 1A (predicted)    GB acc:   BI301150    UniGene:   Rn.3361    Gene Symbol:   Eif1a_predicted    Locus Link:   317163    Chromosome:   18    Cytoband:   Chr:18q11    SOURCE    GENECARD       Gene Ontology:   biological process:  protein biosynthesis   biological process:  translational initiation   cellular component:  eukaryotic translation initiation factor 4F complex   molecular function:  translation factor activity, nucleic acid binding   molecular function:  RNA binding   molecular function:  translation initiation factor activity      BioCarta Pathways:     KEGG Pathways:  
    Probe set : 1376016_at    Name:   similar to RIKEN cDNA 1110007L15 (predicted)    GB acc:   BF285425    UniGene:   Rn.102176    Gene Symbol:   RGD1311660_predicted    Locus Link:   288518    Chromosome:   12    Cytoband:   Chr:12q11    SOURCE    GENECARD       Gene Ontology:     BioCarta Pathways:     KEGG Pathways:  
    Probe set : 1388103_at    Name:   protein distantly related to to the gamma subunit family    GB acc:   AF361355    UniGene:   Rn.92180    Gene Symbol:   Pr1    Locus Link:   245953      SOURCE    GENECARD       Gene Ontology:   biological process:  ion transport   cellular component:  voltage-gated calcium channel complex   cellular component:  integral to membrane   molecular function:  ion channel activity   molecular function:  voltage-gated ion channel activity   molecular function:  calcium channel activity      BioCarta Pathways:     KEGG Pathways:  
    Probe set : 1391505_x_at     GB acc:   BI275261              Gene Ontology:     BioCarta Pathways:     KEGG Pathways:  
    Probe set : 1379254_at    Name:   similar to RIKEN cDNA 1300007B12; clone MNCb-2755 (predicted)    GB acc:   AA945882     Gene Symbol:   RGD1309892_predicted    Locus Link:   289034    Chromosome:   13    Cytoband:   Chr:13q13     GENECARD       Gene Ontology:   cellular component:  integral to membrane      BioCarta Pathways:     KEGG Pathways:  
    Probe set : 1390820_at    Name:   zinc finger, CCHC domain containing 11 (predicted)    GB acc:   BE108198    UniGene:   Rn.15558    Gene Symbol:   Zcchc11_predicted    Locus Link:   313481    Chromosome:   5    Cytoband:   Chr:5q35    SOURCE    GENECARD       Gene Ontology:   cellular component:  nucleus   molecular function:  nucleic acid binding   molecular function:  zinc ion binding   molecular function:  nucleotidyltransferase activity      BioCarta Pathways:     KEGG Pathways:  
    Probe set : 1370810_at    Name:   cyclin D2    GB acc:   L09752    UniGene:   Rn.96083    Gene Symbol:   Ccnd2    Locus Link:   64033    Chromosome:   4    Cytoband:   Chr:4q42    SOURCE    GENECARD       Gene Ontology:   cellular component:  nucleus   molecular function:  protein binding   molecular function:  cyclin-dependent protein kinase regulator activity      BioCarta Pathways:     KEGG Pathways:  
    Probe set : 1384617_at    Name:   Unknown (protein for MGC:72614)    GB acc:   AI385260    UniGene:   Rn.20514    Gene Symbol:   RGD:735075    Locus Link:   310540    Chromosome:   2    Cytoband:   Chr:2q33    SOURCE    GENECARD       Gene Ontology:   cellular component:  integral to membrane      BioCarta Pathways:     KEGG Pathways:  
    Probe set : 1373942_at    Name:   Transcribed locus    GB acc:   BF396312    UniGene:   Rn.2860        SOURCE        Gene Ontology:     BioCarta Pathways:     KEGG Pathways:  
    Probe set : 1384714_x_at    Name:   similar to 60S ribosomal protein L8 /// similar to mitochondrial tryptophanyl tRNA synthetase 2    GB acc:   BI850152    UniGene:   Rn.32144    Gene Symbol:   LOC499374 /// LOC499679    Locus Link:   499374 /// 499679    Chromosome:   1    Cytoband:   Chr:1q55 /// Chr:2q34    SOURCE    GENECARD       Gene Ontology:     BioCarta Pathways:     KEGG Pathways:  
    Probe set : 1371694_at     GB acc:   AI317805              Gene Ontology:     BioCarta Pathways:     KEGG Pathways:  
    Probe set : 1374216_at    Name:   Transcribed locus    GB acc:   BI280277    UniGene:   Rn.24598        SOURCE        Gene Ontology:     BioCarta Pathways:     KEGG Pathways:  
    Probe set : 1390510_at    Name:   membrane-spanning 4-domains, subfamily A, member 6B    GB acc:   BI294706    UniGene:   Rn.17885    Gene Symbol:   RGD:1359381    Locus Link:   293749    Chromosome:   1    Cytoband:   Chr:1q43    SOURCE    GENECARD       Gene Ontology:   biological process:  signal transduction   cellular component:  integral to membrane   molecular function:  receptor activity      BioCarta Pathways:     KEGG Pathways:  
    Probe set : 1370816_at    Name:   nuclear receptor subfamily 1, group D, member 1    GB acc:   M25804    UniGene:   Rn.29848    Gene Symbol:   Nr1d1    Locus Link:   252917    Chromosome:   10    Cytoband:   Chr:10q31    SOURCE    GENECARD       Gene Ontology:   biological process:  transcription   biological process:  regulation of transcription, DNA-dependent   biological process:  circadian rhythm   cellular component:  nucleus      BioCarta Pathways:     KEGG Pathways:  
    Probe set : 1368605_at    Name:   adaptor protein with pleckstrin homology and src homology 2 domains    GB acc:   NM_053669    UniGene:   Rn.30041    Gene Symbol:   Aps    Locus Link:   114203    Chromosome:   12    Cytoband:   Chr:12q12    SOURCE    GENECARD       Gene Ontology:   biological process:  B-1 B cell homeostasis   biological process:  intracellular signaling cascade   biological process:  nervous system development   biological process:  insulin receptor signaling pathway   biological process:  cytokine and chemokine mediated signaling pathway   biological process:  regulation of metabolism   biological process:  antimicrobial humoral response (sensu Vertebrata)   biological process:  actin cytoskeleton organization and biogenesis   biological process:  glucose homeostasis   biological process:  negative regulation of glucose import   biological process:  regulation of Ras protein signal transduction   biological process:  regulation of immune response   biological process:  antigen receptor-mediated signaling pathway   cellular component:  cytoplasm   cellular component:  actin filament   cellular component:  plasma membrane   cellular component:  membrane      BioCarta Pathways:     KEGG Pathways:  
    Probe set : 1393221_at    Name:   similar to 20-alpha-hydroxysteroid dehydrogenase    GB acc:   AA866264    UniGene:   Rn.14713    Gene Symbol:   LOC498789    Locus Link:   498789    Chromosome:   17    Cytoband:   Chr:17q12.3    SOURCE    GENECARD       Gene Ontology:     BioCarta Pathways:     KEGG Pathways:  
    Probe set : 1382783_at    Name:   Bloom syndrome (predicted)    GB acc:   BF408187    UniGene:   Rn.61121    Gene Symbol:   Blm_predicted    Locus Link:   308755    Chromosome:   1    Cytoband:   Chr:1q31    SOURCE    GENECARD       Gene Ontology:   cellular component:  intracellular   cellular component:  nucleus      BioCarta Pathways:     KEGG Pathways:  
    Probe set : 1372590_at    Name:   Transcribed locus    GB acc:   BM386449    UniGene:   Rn.53880        SOURCE        Gene Ontology:     BioCarta Pathways:     KEGG Pathways:  
    Probe set : 1368428_at    Name:   X-prolyl aminopeptidase (aminopeptidase P) 2, membrane-bound    GB acc:   NM_057155    UniGene:   Rn.56142    Gene Symbol:   Xpnpep2    Locus Link:   117522    Chromosome:   X    Cytoband:   Chr:Xq35    SOURCE    GENECARD       Gene Ontology:   biological process:  proteolysis   cellular component:  membrane      BioCarta Pathways:     KEGG Pathways:  
    Probe set : 1368597_at    Name:   SNF1-like kinase    GB acc:   AB020480    UniGene:   Rn.42905    Gene Symbol:   Snf1lk    Locus Link:   59329    Chromosome:   20    Cytoband:   Chr:20p12    SOURCE    GENECARD       Gene Ontology:   biological process:  negative regulation of transcription from RNA polymerase II promoter   biological process:  protein amino acid phosphorylation   biological process:  protein amino acid phosphorylation   biological process:  cell cycle   biological process:  protein kinase cascade   biological process:  regulation of progression through mitotic cell cycle   biological process:  regulation of cell differentiation   biological process:  protein amino acid phosphorylation   biological process:  protein kinase cascade   biological process:  regulation of progression through mitotic cell cycle   biological process:  regulation of cell differentiation   biological process:  development   biological process:  cell differentiation   cellular component:  nucleus   cellular component:  cytoplasm   cellular component:  cytosol   cellular component:  cytoplasm   molecular function:  magnesium ion binding   molecular function:  protein kinase activity   molecular function:  protein serine/threonine kinase activity   molecular function:  protein serine/threonine kinase activity   molecular function:  protein-tyrosine kinase activity   molecular function:  ATP binding   molecular function:  kinase activity   molecular function:  transcriptional repressor activity   molecular function:  transferase activity   molecular function:  magnesium ion binding   molecular function:  protein serine/threonine kinase activity   molecular function:  ATP binding   molecular function:  nucleotide binding   molecular function:  metal ion binding      BioCarta Pathways:     KEGG Pathways:  
    Probe set : 1391240_at     GB acc:   AI013954              Gene Ontology:     BioCarta Pathways:     KEGG Pathways:  
    Probe set : 1370312_at    Name:   spondin 1    GB acc:   M88469    UniGene:   Rn.7546    Gene Symbol:   Spon1    Locus Link:   64456    Chromosome:   1    Cytoband:   Chr:1q34    SOURCE    GENECARD       Gene Ontology:   biological process:  cell adhesion   biological process:  development   cellular component:  extracellular matrix (sensu Metazoa)   cellular component:  extracellular space   molecular function:  protein binding      BioCarta Pathways:     KEGG Pathways:  
    Probe set : 1385793_at    Name:   Similar to RIKEN cDNA 2310067G05    GB acc:   AI045120    UniGene:   Rn.41     Locus Link:   301113    Chromosome:   9    Cytoband:   Chr:9q11    SOURCE        Gene Ontology:     BioCarta Pathways:     KEGG Pathways:  
    Probe set : 1376852_at    Name:   methylcrotonoyl-Coenzyme A carboxylase 1 (alpha) (predicted)    GB acc:   BI274460    UniGene:   Rn.15587    Gene Symbol:   Mccc1_predicted    Locus Link:   294972    Chromosome:   2    Cytoband:   Chr:2q25    SOURCE    GENECARD       Gene Ontology:   biological process:  leucine catabolism   biological process:  biotin metabolism   biological process:  metabolism   cellular component:  mitochondrion   cellular component:  mitochondrial matrix      BioCarta Pathways:     KEGG Pathways:  
    Probe set : 1374169_at    Name:   similar to chromosome 16 open reading frame 5 (predicted)    GB acc:   AI236128    UniGene:   Rn.101805    Gene Symbol:   RGD1310686_predicted    Locus Link:   360480    Chromosome:   10    Cytoband:   Chr:10q12    SOURCE    GENECARD       Gene Ontology:   cellular component:  nucleus   cellular component:  integral to membrane      BioCarta Pathways:     KEGG Pathways:  
    Probe set : 1398434_at    Name:   Death associated transcription factor 1 (predicted)    GB acc:   BE107465    UniGene:   Rn.8782     Locus Link:   362286    Chromosome:   3    Cytoband:   Chr:3q43    SOURCE        Gene Ontology:   biological process:  transcription   biological process:  regulation of transcription, DNA-dependent   biological process:  apoptosis   cellular component:  nucleus   molecular function:  DNA binding      BioCarta Pathways:     KEGG Pathways:  
    Probe set : 1373955_at    Name:   karyopherin (importin) beta 3 (predicted)    GB acc:   BI280339    UniGene:   Rn.79893    Gene Symbol:   Kpnb3_predicted    Locus Link:   306182    Chromosome:   15    Cytoband:   Chr:15q24    SOURCE    GENECARD       Gene Ontology:   cellular component:  nucleus   cellular component:  nuclear pore   cellular component:  cytoplasm      BioCarta Pathways:     KEGG Pathways:  
    Probe set : 1372065_at    Name:   ADP-ribosyltransferase 3 (predicted)    GB acc:   AI104393    UniGene:   Rn.4077    Gene Symbol:   Art3_predicted    Locus Link:   305235    Chromosome:   14    Cytoband:   Chr:14p22    SOURCE    GENECARD       Gene Ontology:   biological process:  protein amino acid ADP-ribosylation   cellular component:  extracellular space   cellular component:  integral to plasma membrane      BioCarta Pathways:     KEGG Pathways:  
    Probe set : 1375631_at    Name:   pleckstrin homology domain containing, family B (evectins) member 2 (predicted)    GB acc:   AI177176    UniGene:   Rn.1410    Gene Symbol:   Plekhb2_predicted    Locus Link:   301337    Chromosome:   9    Cytoband:   Chr:9q21    SOURCE    GENECARD       Gene Ontology:   cellular component:  membrane      BioCarta Pathways:     KEGG Pathways:  
    Probe set : 1383850_at    Name:   Similar to RIKEN cDNA 1600014C10    GB acc:   BF548042    UniGene:   Rn.2361     Locus Link:   502324      SOURCE        Gene Ontology:     BioCarta Pathways:     KEGG Pathways:  
    Probe set : 1389039_at    Name:   Transcribed locus    GB acc:   BM386520    UniGene:   Rn.7736        SOURCE        Gene Ontology:     BioCarta Pathways:     KEGG Pathways:  
    Probe set : 1388400_at    Name:   cell division cycle 34 homolog (S. cerevisiae) (predicted)    GB acc:   BG375350    UniGene:   Rn.2427    Gene Symbol:   Cdc34_predicted    Locus Link:   299602    Chromosome:   7    Cytoband:   Chr:7q11    SOURCE    GENECARD       Gene Ontology:   cellular component:  nucleus      BioCarta Pathways:     KEGG Pathways:  
    Probe set : 1371336_at    Name:   hematological and neurological expressed sequence 1    GB acc:   AA942716    UniGene:   Rn.138819    Gene Symbol:   RGD:1359325    Locus Link:   287828    Chromosome:   10    Cytoband:   Chr:10q32.3    SOURCE    GENECARD       Gene Ontology:     BioCarta Pathways:     KEGG Pathways:  
    Probe set : 1391014_at    Name:   zinc finger, MYND domain containing 19    GB acc:   AW530427    UniGene:   Rn.40409    Gene Symbol:   Zmynd19    Locus Link:   311791    Chromosome:   3    Cytoband:   Chr:3p13    SOURCE    GENECARD       Gene Ontology:   cellular component:  cytoplasm   cellular component:  internal side of plasma membrane   molecular function:  protein binding   molecular function:  zinc ion binding   molecular function:  metal ion binding      BioCarta Pathways:     KEGG Pathways:  
    Probe set : 1368205_at    Name:   complement factor I    GB acc:   NM_024157    UniGene:   Rn.7424    Gene Symbol:   Cfi    Locus Link:   79126    Chromosome:   2    Cytoband:   Chr:2q43    SOURCE    GENECARD       Gene Ontology:   cellular component:  extracellular region   cellular component:  extracellular space   cellular component:  membrane      BioCarta Pathways:     KEGG Pathways:  
    Probe set : 1373530_at    Name:   cyclin E    GB acc:   AW913890    UniGene:   Rn.15455    Gene Symbol:   Ccne1    Locus Link:   25729    Chromosome:   1    Cytoband:   Chr:1q22    SOURCE    GENECARD       Gene Ontology:   cellular component:  cyclin-dependent protein kinase holoenzyme complex   cellular component:  nucleus   molecular function:  protein binding   molecular function:  kinase activity   molecular function:  cyclin-dependent protein kinase regulator activity      BioCarta Pathways:     KEGG Pathways:  
    Probe set : 1370237_at    Name:   L-3-hydroxyacyl-Coenzyme A dehydrogenase, short chain    GB acc:   AA799574    UniGene:   Rn.92789    Gene Symbol:   Hadhsc    Locus Link:   113965    Chromosome:   2    Cytoband:   Chr:2q43    SOURCE    GENECARD       Gene Ontology:   biological process:  fatty acid metabolism   biological process:  lipid metabolism   cellular component:  mitochondrion      BioCarta Pathways:     KEGG Pathways:  
    Probe set : 1369120_a_at    Name:   luteinizing hormone beta    GB acc:   NM_012858    UniGene:   Rn.10108    Gene Symbol:   Lhb    Locus Link:   25329    Chromosome:   1    Cytoband:   Chr:1q22    SOURCE    GENECARD       Gene Ontology:   cellular component:  extracellular region   cellular component:  extracellular space   cellular component:  soluble fraction   molecular function:  hormone activity      BioCarta Pathways:     KEGG Pathways:  
    Probe set : 1387565_at    Name:   transient receptor potential cation channel, subfamily V, member 6    GB acc:   NM_053686    UniGene:   Rn.22483    Gene Symbol:   Trpv6    Locus Link:   114246    Chromosome:   4    Cytoband:   Chr:4q22    SOURCE    GENECARD       Gene Ontology:   cellular component:  integral to plasma membrane   cellular component:  membrane   cellular component:  integral to membrane      BioCarta Pathways:     KEGG Pathways:  
    Probe set : 1376324_at    Name:   Similar to zinc finger protein 21 isoform 2    GB acc:   BF406329    UniGene:   Rn.21511     Locus Link:   501442      SOURCE        Gene Ontology:     BioCarta Pathways:     KEGG Pathways:  
    Probe set : 1369935_at    Name:   cyclin D3    GB acc:   NM_012766    UniGene:   Rn.3483    Gene Symbol:   Ccnd3    Locus Link:   25193      SOURCE    GENECARD       Gene Ontology:   cellular component:  nucleus      BioCarta Pathways:     KEGG Pathways:  
    Probe set : 1392548_at    Name:   similar to mKIAA1604 protein    GB acc:   BF394282     Gene Symbol:   LOC362153    Locus Link:   362153    Chromosome:   3    Cytoband:   Chr:3q24     GENECARD       Gene Ontology:     BioCarta Pathways:     KEGG Pathways:  
    Probe set : 1389008_at    Name:   Sprouty-related, EVH1 domain containing 2 (predicted)    GB acc:   AI409218    UniGene:   Rn.7582     Locus Link:   305539    Chromosome:   14    Cytoband:   Chr:14q22    SOURCE        Gene Ontology:   biological process:  inactivation of MAPK activity   biological process:  development   biological process:  regulation of signal transduction   cellular component:  plasma membrane   cellular component:  membrane   molecular function:  stem cell factor receptor binding   molecular function:  protein binding      BioCarta Pathways:     KEGG Pathways:  
    Probe set : 1392451_at    Name:   similar to hypothetical protein FLJ37118    GB acc:   BI293843    UniGene:   Rn.108028    Gene Symbol:   LOC362659    Locus Link:   362659    Chromosome:   5    Cytoband:   Chr:5q36    SOURCE    GENECARD       Gene Ontology:     BioCarta Pathways:     KEGG Pathways:  
    Probe set : 1394275_at     GB acc:   BF559096              Gene Ontology:     BioCarta Pathways:     KEGG Pathways:  
    Probe set : 1390709_at    Name:   Transcribed locus    GB acc:   AI102364    UniGene:   Rn.21406        SOURCE        Gene Ontology:     BioCarta Pathways:     KEGG Pathways:  
    Probe set : 1398265_at    Name:   ATP-binding cassette, sub-family C (CFTR/MRP), member 9    GB acc:   NM_013040    UniGene:   Rn.54961    Gene Symbol:   Abcc9    Locus Link:   25560    Chromosome:   4    Cytoband:   Chr:4q44    SOURCE    GENECARD       Gene Ontology:     BioCarta Pathways:     KEGG Pathways:  
    Probe set : 1391652_at    Name:   glycine-N-acyltransferase (predicted)    GB acc:   AI411140    UniGene:   Rn.21872    Gene Symbol:   Glyat_predicted    Locus Link:   293779    Chromosome:   1    Cytoband:   Chr:1q43    SOURCE    GENECARD       Gene Ontology:   biological process:  acyl-CoA metabolism   biological process:  transport   biological process:  response to toxin   cellular component:  mitochondrion   cellular component:  membrane      BioCarta Pathways:     KEGG Pathways:  
    Probe set : 1373446_at    Name:   PHD finger protein 13 (predicted)    GB acc:   AI102512    UniGene:   Rn.13782    Gene Symbol:   Phf13_predicted    Locus Link:   313742    Chromosome:   5    Cytoband:   Chr:5q36    SOURCE    GENECARD       Gene Ontology:   biological process:  regulation of transcription, DNA-dependent   molecular function:  DNA binding      BioCarta Pathways:     KEGG Pathways:  
    Probe set : 1389421_at    Name:   polymerase (DNA directed), epsilon    GB acc:   BE101140    UniGene:   Rn.40977    Gene Symbol:   Pole    Locus Link:   304573    Chromosome:   12    Cytoband:   Chr:12q16    SOURCE    GENECARD       Gene Ontology:   biological process:  DNA replication   biological process:  DNA repair   cellular component:  nucleus      BioCarta Pathways:     KEGG Pathways:  
    Probe set : 1383067_a_at    Name:   deoxythymidylate kinase (predicted)    GB acc:   AW141143    UniGene:   Rn.95    Gene Symbol:   Dtymk_predicted    Locus Link:   301622    Chromosome:   9    Cytoband:   Chr:9q36    SOURCE    GENECARD       Gene Ontology:     BioCarta Pathways:     KEGG Pathways:  
    Probe set : 1392158_at     GB acc:   BF402756              Gene Ontology:     BioCarta Pathways:     KEGG Pathways:  
    Probe set : 1370355_at    Name:   stearoyl-Coenzyme A desaturase 1    GB acc:   J02585    UniGene:   Rn.1023    Gene Symbol:   Scd1    Locus Link:   246074    Chromosome:   1    Cytoband:   Chr:1q54    SOURCE    GENECARD       Gene Ontology:   biological process:  fatty acid biosynthesis   biological process:  lipid biosynthesis   cellular component:  endoplasmic reticulum   cellular component:  membrane   cellular component:  integral to membrane      BioCarta Pathways:     KEGG Pathways:  
    Probe set : 1389127_at     GB acc:   BF552908              Gene Ontology:     BioCarta Pathways:     KEGG Pathways:  
    Probe set : 1391222_at    Name:   similar to Nedd4 binding protein 1 (predicted)    GB acc:   AA965076    UniGene:   Rn.137980    Gene Symbol:   RGD1305179_predicted    Locus Link:   291921    Chromosome:   19    Cytoband:   Chr:19p11    SOURCE    GENECARD       Gene Ontology:     BioCarta Pathways:     KEGG Pathways:  
    Probe set : 1373730_at    Name:   Similar to hypothetical protein MGC20460 (predicted)    GB acc:   BI282077    UniGene:   Rn.23554     Locus Link:   362297    Chromosome:   4    Cytoband:   Chr:4q11    SOURCE        Gene Ontology:     BioCarta Pathways:     KEGG Pathways:  
    Probe set : 1379107_at    Name:   Solute carrier family 4, member 4    GB acc:   BF416861    UniGene:   Rn.11114    Gene Symbol:   Slc4a4    Locus Link:   84484    Chromosome:   14    Cytoband:   Chr:14p22    SOURCE    GENECARD       Gene Ontology:     BioCarta Pathways:     KEGG Pathways:  
    Probe set : 1384935_at    Name:   Cleavage stimulation factor, 3' pre-RNA, subunit 3, 77kDa (predicted)    GB acc:   AI070621    UniGene:   Rn.46377     Locus Link:   362178    Chromosome:   3    Cytoband:   Chr:3q32    SOURCE        Gene Ontology:   biological process:  mRNA polyadenylylation   biological process:  mRNA cleavage   cellular component:  nucleus   molecular function:  RNA binding   molecular function:  binding      BioCarta Pathways:     KEGG Pathways:  
    Probe set : 1387888_at    Name:   ribosomal protein S9    GB acc:   AA799768    UniGene:   Rn.109735    Gene Symbol:   Rps9    Locus Link:   81772    Chromosome:   1    Cytoband:   Chr:1q12    SOURCE    GENECARD       Gene Ontology:   biological process:  protein biosynthesis   molecular function:  RNA binding   molecular function:  structural constituent of ribosome      BioCarta Pathways:     KEGG Pathways:  
    Probe set : 1387033_at    Name:   uncoupling protein 1    GB acc:   NM_012682    UniGene:   Rn.10281    Gene Symbol:   Ucp1    Locus Link:   24860    Chromosome:   19    Cytoband:   Chr:19p11-q11    SOURCE    GENECARD       Gene Ontology:     BioCarta Pathways:     KEGG Pathways:  
    Probe set : 1391452_at    Name:   Similar to RIKEN cDNA A230102I05 (predicted)    GB acc:   AI044680    UniGene:   Rn.15995     Locus Link:   292548    Chromosome:   1    Cytoband:   Chr:1q12    SOURCE        Gene Ontology:     BioCarta Pathways:     KEGG Pathways:  
    Probe set : 1376667_at    Name:   cytochrome P450, family 26, subfamily b, polypeptide 1    GB acc:   BE105541    UniGene:   Rn.19898    Gene Symbol:   Cyp26b1    Locus Link:   312495    Chromosome:   4    Cytoband:   Chr:4q34    SOURCE    GENECARD       Gene Ontology:   cellular component:  endoplasmic reticulum   cellular component:  microsome   cellular component:  membrane      BioCarta Pathways:     KEGG Pathways:  
    Probe set : 1368652_at    Name:   caspase 9    GB acc:   AF262319    UniGene:   Rn.32199    Gene Symbol:   Casp9    Locus Link:   58918    Chromosome:   5    Cytoband:   Chr:5q36    SOURCE    GENECARD       Gene Ontology:     BioCarta Pathways:     KEGG Pathways:  
    Probe set : 1398827_at    Name:   CD 81 antigen    GB acc:   NM_013087    UniGene:   Rn.1975    Gene Symbol:   Cd81    Locus Link:   25621    Chromosome:   1    Cytoband:   Chr:1q41    SOURCE    GENECARD       Gene Ontology:   biological process:  activation of MAPK activity   biological process:  phosphatidylinositol biosynthesis   biological process:  protein localization   biological process:  positive regulation of cell proliferation   biological process:  response to wounding   biological process:  response to wounding   biological process:  phosphoinositide metabolism   biological process:  positive regulation of B cell proliferation   biological process:  regulation of growth   biological process:  regulation of growth   biological process:  regulation of cell proliferation   biological process:  positive regulation of 1-phosphatidylinositol 4-kinase activity   biological process:  viral entry into host cell   biological process:  virion attachment, binding of host cell surface receptor   biological process:  positive regulation of peptidyl-tyrosine phosphorylation   biological process:  activation of MAPK activity   biological process:  phosphatidylinositol biosynthesis   biological process:  protein localization   biological process:  positive regulation of cell proliferation   biological process:  phosphoinositide metabolism   biological process:  positive regulation of B cell proliferation   biological process:  positive regulation of 1-phosphatidylinositol 4-kinase activity   biological process:  viral entry into host cell   biological process:  virion attachment, binding of host cell surface receptor   biological process:  positive regulation of peptidyl-tyrosine phosphorylation   molecular function:  protein binding   molecular function:  protein binding   molecular function:  protein binding      BioCarta Pathways:     KEGG Pathways:  
    Probe set : 1379339_at    Name:   similar to tumor-related protein (predicted)    GB acc:   BM390448    UniGene:   Rn.1166    Gene Symbol:   RGD1307632_predicted    Locus Link:   364879    Chromosome:   18    Cytoband:   Chr:18q12.1    SOURCE    GENECARD       Gene Ontology:   biological process:  metabolism   molecular function:  catalytic activity      BioCarta Pathways:     KEGG Pathways:  
    Probe set : 1369099_at    Name:   solute carrier family 30 (zinc transporter), member 1    GB acc:   NM_022853    UniGene:   Rn.10120    Gene Symbol:   Slc30a1    Locus Link:   58976    Chromosome:   13    Cytoband:   Chr:13q27    SOURCE    GENECARD       Gene Ontology:     BioCarta Pathways:     KEGG Pathways:  
    Probe set : 1398169_at    Name:   Transcribed locus    GB acc:   BF405940    UniGene:   Rn.131131        SOURCE        Gene Ontology:     BioCarta Pathways:     KEGG Pathways:  
    Probe set : 1384770_at    Name:   Transcribed locus    GB acc:   AI112057    UniGene:   Rn.147581        SOURCE        Gene Ontology:     BioCarta Pathways:     KEGG Pathways:  
    Probe set : 1369562_at    Name:   neural visinin-like Ca2+-binding protein type 3    GB acc:   NM_017356    UniGene:   Rn.102820    Gene Symbol:   Hpcal1    Locus Link:   50871    Chromosome:   6    Cytoband:   Chr:6q16    SOURCE    GENECARD       Gene Ontology:   molecular function:  calcium ion binding      BioCarta Pathways:     KEGG Pathways:  
    Probe set : 1373182_at     GB acc:   BF281899              Gene Ontology:     BioCarta Pathways:     KEGG Pathways:  
    Probe set : 1374212_at    Name:   Transcribed locus    GB acc:   AI012753    UniGene:   Rn.14344        SOURCE        Gene Ontology:     BioCarta Pathways:     KEGG Pathways:  
    Probe set : 1372790_at    Name:   Malate dehydrogenase 1, NAD (soluble)    GB acc:   BG671530    UniGene:   Rn.13492    Gene Symbol:   Mdh1    Locus Link:   24551    Chromosome:   14    Cytoband:   Chr:14q22    SOURCE    GENECARD       Gene Ontology:   cellular component:  cytosol      BioCarta Pathways:     KEGG Pathways:  
    Probe set : 1390481_a_at    Name:   similar to RIKEN cDNA 2700084L22 (predicted)    GB acc:   AW531714    UniGene:   Rn.18835    Gene Symbol:   RGD1310816_predicted    Locus Link:   360847    Chromosome:   13    Cytoband:   Chr:13q13    SOURCE    GENECARD       Gene Ontology:     BioCarta Pathways:     KEGG Pathways:  
    Probe set : 1384215_at     GB acc:   AI144991              Gene Ontology:     BioCarta Pathways:     KEGG Pathways:  
    Probe set : 1370130_at    Name:   ras homolog gene family, member A    GB acc:   NM_057132    UniGene:   Rn.107401    Gene Symbol:   RGD:619921    Locus Link:   117273    Chromosome:   16    Cytoband:   Chr:16q12.5    SOURCE    GENECARD       Gene Ontology:   biological process:  cell adhesion   biological process:  cell-matrix adhesion   biological process:  integrin-mediated signaling pathway   biological process:  small GTPase mediated signal transduction   biological process:  Rho protein signal transduction   biological process:  striated muscle development   biological process:  protein transport   biological process:  actin cytoskeleton organization and biogenesis   biological process:  cell differentiation   biological process:  positive regulation of NF-kappaB import into nucleus   biological process:  positive regulation of I-kappaB kinase/NF-kappaB cascade   biological process:  stress fiber formation   biological process:  Rho protein signal transduction   biological process:  actin cytoskeleton organization and biogenesis   biological process:  positive regulation of NF-kappaB import into nucleus   biological process:  positive regulation of I-kappaB kinase/NF-kappaB cascade   cellular component:  cytosol   cellular component:  cytoskeleton   cellular component:  membrane      BioCarta Pathways:     KEGG Pathways:  
    Probe set : 1371602_at    Name:   Transcribed locus    GB acc:   BI274110    UniGene:   Rn.16430        SOURCE        Gene Ontology:     BioCarta Pathways:     KEGG Pathways:  
    Probe set : 1390968_at    Name:   Similar to hypothetical protein A430031N04    GB acc:   AI706441    UniGene:   Rn.11815     Locus Link:   498100      SOURCE        Gene Ontology:     BioCarta Pathways:     KEGG Pathways:  
    Probe set : 1377310_at    Name:   Transcribed locus, weakly similar to XP_575554.1 PREDICTED: similar to RNA binding motif and ELMO domain 1 [Rattus norvegicus]    GB acc:   BG374304    UniGene:   Rn.22341        SOURCE        Gene Ontology:     BioCarta Pathways:     KEGG Pathways:  
    Probe set : 1390535_at    Name:   CTD (carboxy-terminal domain, RNA polymerase II, polypeptide A) phosphatase, subunit 1 (predicted)    GB acc:   BE098745    UniGene:   Rn.137952     Locus Link:   291414    Chromosome:   18    Cytoband:   Chr:18q12.3    SOURCE        Gene Ontology:   cellular component:  intracellular   cellular component:  nucleus   molecular function:  phosphoprotein phosphatase activity   molecular function:  protein binding   molecular function:  hydrolase activity      BioCarta Pathways:     KEGG Pathways:  
    Probe set : 1368253_at    Name:   guanidinoacetate methyltransferase    GB acc:   NM_012793    UniGene:   Rn.33890    Gene Symbol:   Gamt    Locus Link:   25257    Chromosome:   7    Cytoband:   Chr:7q11    SOURCE    GENECARD       Gene Ontology:     BioCarta Pathways:     KEGG Pathways:  
    Probe set : 1380445_at    Name:   Transcribed locus, strongly similar to XP_515868.1 PREDICTED: sodium channel, voltage-gated, type III, alpha [Pan troglodytes]    GB acc:   BI294409    UniGene:   Rn.37544        SOURCE        Gene Ontology:     BioCarta Pathways:     KEGG Pathways:  
    Probe set : 1385382_at    Name:   Transcribed locus    GB acc:   BI294158    UniGene:   Rn.113277        SOURCE        Gene Ontology:     BioCarta Pathways:     KEGG Pathways:  
    Probe set : 1377836_at    Name:   Similar to suppressor of hairy wing homolog 4 isoform 1    GB acc:   AI638997    UniGene:   Rn.32520     Locus Link:   315798    Chromosome:   8    Cytoband:   Chr:8q24    SOURCE        Gene Ontology:     BioCarta Pathways:     KEGG Pathways:  
    Probe set : 1397154_at    Name:   Myeloid/lymphoid or mixed-lineage leukemia (trithorax homolog, Drosophila); translocated to, 2 (predicted)    GB acc:   BF403248    UniGene:   Rn.16122     Locus Link:   305152    Chromosome:   14    Cytoband:   Chr:14p22    SOURCE        Gene Ontology:   cellular component:  nucleus   molecular function:  transcription factor activity      BioCarta Pathways:     KEGG Pathways:  
    Probe set : 1381019_x_at    Name:   development and differentiation enhancing (predicted)    GB acc:   BG378709    UniGene:   Rn.63466    Gene Symbol:   Ddef1_predicted    Locus Link:   314961    Chromosome:   7    Cytoband:   Chr:7q33    SOURCE    GENECARD       Gene Ontology:   biological process:  regulation of transcription, DNA-dependent   biological process:  regulation of GTPase activity   cellular component:  nucleus   cellular component:  membrane   molecular function:  DNA binding   molecular function:  transcription factor activity   molecular function:  GTPase activator activity      BioCarta Pathways:     KEGG Pathways:  
    Probe set : 1374270_at    Name:   Testis specific, 14    GB acc:   AA964789    UniGene:   Rn.11914     Locus Link:   500069      SOURCE        Gene Ontology:     BioCarta Pathways:     KEGG Pathways:  
    Probe set : 1378199_at    Name:   Transcribed locus    GB acc:   AI408485    UniGene:   Rn.35615        SOURCE        Gene Ontology:     BioCarta Pathways:     KEGG Pathways:  
    Probe set : 1386858_at    Name:   ribosomal protein L13    GB acc:   NM_031101    UniGene:   Rn.908    Gene Symbol:   Rpl13    Locus Link:   81765    Chromosome:   19    Cytoband:   Chr:19q12    SOURCE    GENECARD       Gene Ontology:   biological process:  protein biosynthesis   biological process:  ribosome biogenesis   biological process:  protein biosynthesis      BioCarta Pathways:     KEGG Pathways:  
    Probe set : 1397168_at    Name:   Similar to CD2-associated protein (predicted)    GB acc:   AI180093    UniGene:   Rn.42478     Locus Link:   362598    Chromosome:   5    Cytoband:   Chr:5q36    SOURCE        Gene Ontology:     BioCarta Pathways:     KEGG Pathways:  
    Probe set : 1386860_at    Name:   milk fat globule-EGF factor 8 protein    GB acc:   NM_012811    UniGene:   Rn.3742    Gene Symbol:   Mfge8    Locus Link:   25277    Chromosome:   1    Cytoband:   Chr:1q31    SOURCE    GENECARD       Gene Ontology:     BioCarta Pathways:     KEGG Pathways:  
    Probe set : 1367967_at    Name:   leprecan 1    GB acc:   NM_053667    UniGene:   Rn.13741    Gene Symbol:   Lepre1    Locus Link:   114200    Chromosome:   5    Cytoband:   Chr:5q36    SOURCE    GENECARD       Gene Ontology:   biological process:  cell growth   biological process:  protein metabolism   cellular component:  nucleus   cellular component:  cytoplasm   cellular component:  plasma membrane   cellular component:  endoplasmic reticulum      BioCarta Pathways:     KEGG Pathways:  
    Probe set : 1392922_at    Name:   Transcribed locus    GB acc:   BE116619    UniGene:   Rn.23625        SOURCE        Gene Ontology:     BioCarta Pathways:     KEGG Pathways:  
    Probe set : 1389804_at    Name:   Transcribed locus    GB acc:   BF392605    UniGene:   Rn.16770        SOURCE        Gene Ontology:     BioCarta Pathways:     KEGG Pathways:  
    Probe set : 1370156_at    Name:   prion protein    GB acc:   BI278802    UniGene:   Rn.3936    Gene Symbol:   Prnp    Locus Link:   24686    Chromosome:   3    Cytoband:   Chr:3q36    SOURCE    GENECARD       Gene Ontology:   molecular function:  copper ion binding   molecular function:  copper ion binding      BioCarta Pathways:     KEGG Pathways:  
    Probe set : 1398885_at    Name:   ribosomal protein L23    GB acc:   AA925327    UniGene:   Rn.103172    Gene Symbol:   Rpl23    Locus Link:   29282    Chromosome:   10    Cytoband:   Chr:10q31    SOURCE    GENECARD       Gene Ontology:   biological process:  protein biosynthesis   biological process:  ribosomal protein import into nucleus   molecular function:  structural constituent of ribosome   molecular function:  structural constituent of ribosome      BioCarta Pathways:     KEGG Pathways:  
    Probe set : 1397527_at    Name:   tripartite motif protein 47 (predicted)    GB acc:   AI578294    UniGene:   Rn.91719    Gene Symbol:   Trim47_predicted    Locus Link:   287824    Chromosome:   10    Cytoband:   Chr:10q32.3    SOURCE    GENECARD       Gene Ontology:   biological process:  protein ubiquitination   cellular component:  ubiquitin ligase complex   cellular component:  intracellular   cellular component:  nucleus   molecular function:  ubiquitin-protein ligase activity   molecular function:  zinc ion binding      BioCarta Pathways:     KEGG Pathways:  
    Probe set : 1368115_at    Name:   claudin 3    GB acc:   NM_031700    UniGene:   Rn.4513    Gene Symbol:   Cldn3    Locus Link:   65130      SOURCE    GENECARD       Gene Ontology:   biological process:  calcium-independent cell-cell adhesion      BioCarta Pathways:     KEGG Pathways:  
    Probe set : 1373164_at    Name:   Serine/threonine kinase 17b (apoptosis-inducing)    GB acc:   AI012590    UniGene:   Rn.1300    Gene Symbol:   Stk17b    Locus Link:   170904    Chromosome:   9    Cytoband:   Chr:9q31    SOURCE    GENECARD       Gene Ontology:     BioCarta Pathways:     KEGG Pathways:  
    Probe set : 1398852_at    Name:   ribosomal protein S21    GB acc:   BF410811    UniGene:   Rn.4153    Gene Symbol:   Rps21    Locus Link:   81775    Chromosome:   3    Cytoband:   Chr:3q43    SOURCE    GENECARD       Gene Ontology:   biological process:  protein biosynthesis   cellular component:  intracellular   cellular component:  ribosome   cellular component:  ribonucleoprotein complex   molecular function:  structural constituent of ribosome      BioCarta Pathways:     KEGG Pathways:  
    Probe set : 1383784_at    Name:   Transcribed locus    GB acc:   BI289438    UniGene:   Rn.147361        SOURCE        Gene Ontology:     BioCarta Pathways:     KEGG Pathways:  
    Probe set : 1398761_at    Name:   ribosomal protein L5    GB acc:   NM_031099    UniGene:   Rn.92980    Gene Symbol:   Rpl5    Locus Link:   81763    Chromosome:   14    Cytoband:   Chr:14p22    SOURCE    GENECARD       Gene Ontology:   biological process:  protein biosynthesis   biological process:  ribosome biogenesis      BioCarta Pathways:     KEGG Pathways:  
    Probe set : 1383744_at    Name:   Transcribed locus    GB acc:   AI146250    UniGene:   Rn.106882        SOURCE        Gene Ontology:     BioCarta Pathways:     KEGG Pathways:  
    Probe set : 1394605_at    Name:   similar to CDNA sequence BC017647    GB acc:   AI409909    UniGene:   Rn.19152    Gene Symbol:   LOC497960    Locus Link:   497960    Chromosome:   10    Cytoband:   Chr:10q25    SOURCE    GENECARD       Gene Ontology:     BioCarta Pathways:     KEGG Pathways:  
    Probe set : 1382215_at    Name:   Similar to Transcription initiation factor TFIID 105 kDa subunit (TAFII-105) (TAFII105)    GB acc:   AA875331    UniGene:   Rn.2882     Locus Link:   291773    Chromosome:   18    Cytoband:   Chr:18p13    SOURCE        Gene Ontology:     BioCarta Pathways:     KEGG Pathways:  
    Probe set : 1382647_at    Name:   Transcribed locus    GB acc:   BF290662    UniGene:   Rn.17474        SOURCE        Gene Ontology:     BioCarta Pathways:     KEGG Pathways:  
    Probe set : 1374730_at    Name:   Tyro protein tyrosine kinase binding protein    GB acc:   AI102519    UniGene:   Rn.11534    Gene Symbol:   Tyrobp    Locus Link:   361537    Chromosome:   1    Cytoband:   Chr:1q21    SOURCE    GENECARD       Gene Ontology:   cellular component:  integral to membrane      BioCarta Pathways:     KEGG Pathways:  
    Probe set : 1371776_at    Name:   Phosphatidylinositol 3-kinase, regulatory subunit, polypeptide 1    GB acc:   AA819268    UniGene:   Rn.10599    Gene Symbol:   Pik3r1    Locus Link:   25513    Chromosome:   2    Cytoband:   Chr:2q12    SOURCE    GENECARD       Gene Ontology:   cellular component:  phosphoinositide 3-kinase complex      BioCarta Pathways:     KEGG Pathways:  
    Probe set : 1368342_at    Name:   adenosine monophosphate deaminase 3    GB acc:   NM_031544    UniGene:   Rn.11106    Gene Symbol:   Ampd3    Locus Link:   25095    Chromosome:   1    Cytoband:   Chr:1q35-q36    SOURCE    GENECARD       Gene Ontology:     BioCarta Pathways:     KEGG Pathways:  
    Probe set : 1373664_at    Name:   phosphatidylinositol glycan, class C (predicted)    GB acc:   AI228670    UniGene:   Rn.3171    Gene Symbol:   Pigc_predicted    Locus Link:   364032    Chromosome:   13    Cytoband:   Chr:13q22    SOURCE    GENECARD       Gene Ontology:   biological process:  GPI anchor biosynthesis   cellular component:  endoplasmic reticulum   cellular component:  endoplasmic reticulum membrane   cellular component:  integral to membrane      BioCarta Pathways:     KEGG Pathways:  
    Probe set : 1373590_at    Name:   stomatin (predicted)    GB acc:   BI295949    UniGene:   Rn.16958    Gene Symbol:   Stom_predicted    Locus Link:   296655    Chromosome:   3    Cytoband:   Chr:3p11    SOURCE    GENECARD       Gene Ontology:     BioCarta Pathways:     KEGG Pathways:  
    Probe set : 1376673_at    Name:   Transcription factor 8 (represses interleukin 2 expression)    GB acc:   BM391853    UniGene:   Rn.10600    Gene Symbol:   Tcf8    Locus Link:   25705    Chromosome:   17    Cytoband:   Chr:17q12.1    SOURCE    GENECARD       Gene Ontology:   cellular component:  nucleus      BioCarta Pathways:     KEGG Pathways:  
    Probe set : 1376005_at    Name:   Kinesin family member 1B    GB acc:   BE109334    UniGene:   Rn.6526    Gene Symbol:   Kif1b    Locus Link:   117548    Chromosome:   5    Cytoband:   Chr:5q36    SOURCE    GENECARD       Gene Ontology:   biological process:  microtubule-based process   biological process:  microtubule-based movement   biological process:  nerve-nerve synaptic transmission   biological process:  neuromuscular synaptic transmission   biological process:  anterograde axon cargo transport   biological process:  embryonic development   biological process:  microtubule-based movement   biological process:  nerve-nerve synaptic transmission   biological process:  neuromuscular synaptic transmission   biological process:  anterograde axon cargo transport   biological process:  embryonic development   biological process:  cytoskeleton-dependent intracellular transport      BioCarta Pathways:     KEGG Pathways:  
    Probe set : 1380129_at    Name:   Transcribed locus    GB acc:   AA818937    UniGene:   Rn.148708        SOURCE        Gene Ontology:     BioCarta Pathways:     KEGG Pathways:  
    Probe set : 1367791_at    Name:   receptor (calcitonin) activity modifying protein 1    GB acc:   NM_031645    UniGene:   Rn.12265    Gene Symbol:   Ramp1    Locus Link:   58965    Chromosome:   9    Cytoband:   Chr:9q36    SOURCE    GENECARD       Gene Ontology:     BioCarta Pathways:     KEGG Pathways:  
    Probe set : 1375482_at    Name:   similar to plexin 1    GB acc:   BE113029    UniGene:   Rn.91736    Gene Symbol:   LOC362398    Locus Link:   362398    Chromosome:   4    Cytoband:   Chr:4q34    SOURCE    GENECARD       Gene Ontology:     BioCarta Pathways:     KEGG Pathways:  
    Probe set : 1392638_at    Name:   Similar to myeloid leukemia factor 1 interacting protein    GB acc:   AI599308    UniGene:   Rn.128609     Locus Link:   306464    Chromosome:   16    Cytoband:   Chr:16q11    SOURCE        Gene Ontology:     BioCarta Pathways:     KEGG Pathways:  
    Probe set : 1386917_at    Name:   Pyruvate carboxylase    GB acc:   NM_012744    UniGene:   Rn.11094    Gene Symbol:   Pc    Locus Link:   25104    Chromosome:   1    Cytoband:   Chr:1q42    SOURCE    GENECARD       Gene Ontology:   biological process:  gluconeogenesis   biological process:  metabolism   biological process:  lipid biosynthesis   cellular component:  cytoplasm   cellular component:  mitochondrion      BioCarta Pathways:     KEGG Pathways:  
    Probe set : 1370030_at    Name:   glutamate cysteine ligase, modifier subunit    GB acc:   NM_017305    UniGene:   Rn.2460    Gene Symbol:   Gclm    Locus Link:   29739    Chromosome:   2    Cytoband:   Chr:2q41    SOURCE    GENECARD       Gene Ontology:   biological process:  cysteine metabolism   biological process:  glutathione biosynthesis      BioCarta Pathways:     KEGG Pathways:  
    Probe set : 1393129_at     GB acc:   AI711403              Gene Ontology:     BioCarta Pathways:     KEGG Pathways:  
    Probe set : 1374143_at    Name:   Transcribed locus    GB acc:   AI602491    UniGene:   Rn.18976        SOURCE        Gene Ontology:     BioCarta Pathways:     KEGG Pathways:  
    Probe set : 1380431_at    Name:   similar to KIAA0564 protein (predicted)    GB acc:   AA893000     Gene Symbol:   RGD1308772_predicted    Locus Link:   290381    Chromosome:   15    Cytoband:   Chr:15q11-q12     GENECARD       Gene Ontology:     BioCarta Pathways:     KEGG Pathways:  
    Probe set : 1370376_a_at    Name:   cold shock domain protein A    GB acc:   AA866458    UniGene:   Rn.3306    Gene Symbol:   Csda    Locus Link:   83807    Chromosome:   4    Cytoband:   Chr:4q42    SOURCE    GENECARD       Gene Ontology:   cellular component:  nucleus   cellular component:  nucleus   cellular component:  cytoplasm      BioCarta Pathways:     KEGG Pathways:  
    Probe set : 1367706_at    Name:   voltage-dependent anion channel 1    GB acc:   NM_031353    UniGene:   Rn.54594    Gene Symbol:   Vdac1    Locus Link:   83529    Chromosome:   10    Cytoband:   Chr:10q22    SOURCE    GENECARD       Gene Ontology:     BioCarta Pathways:     KEGG Pathways:  
    Probe set : 1368008_at    Name:   prominin 1    GB acc:   NM_021751    UniGene:   Rn.144589    Gene Symbol:   Prom1    Locus Link:   60357    Chromosome:   14    Cytoband:   Chr:14q21    SOURCE    GENECARD       Gene Ontology:   biological process:  phototransduction   biological process:  glucose homeostasis      BioCarta Pathways:     KEGG Pathways:  
    Probe set : 1376687_at    Name:   ubiquitin specific protease 1 (predicted)    GB acc:   AI010241    UniGene:   Rn.125994    Gene Symbol:   Usp1_predicted    Locus Link:   313387    Chromosome:   5    Cytoband:   Chr:5q33    SOURCE    GENECARD       Gene Ontology:   biological process:  ubiquitin-dependent protein catabolism   biological process:  ubiquitin cycle      BioCarta Pathways:     KEGG Pathways:  
    Probe set : 1371721_at    Name:   Transcribed locus, strongly similar to NP_080015.3 hypothetical protein LOC66743 [Mus musculus]    GB acc:   BI281103    UniGene:   Rn.4241        SOURCE        Gene Ontology:     BioCarta Pathways:     KEGG Pathways:  
    Probe set : 1392969_at    Name:   Transcribed locus    GB acc:   BI282309    UniGene:   Rn.12238        SOURCE        Gene Ontology:     BioCarta Pathways:     KEGG Pathways:  
    Probe set : 1398871_at    Name:   ribosomal protein L17    GB acc:   BG671311    UniGene:   Rn.34429    Gene Symbol:   RGD:1303019    Locus Link:   291434    Chromosome:   18    Cytoband:   Chr:18q12.3    SOURCE    GENECARD       Gene Ontology:   biological process:  protein biosynthesis   molecular function:  structural constituent of ribosome      BioCarta Pathways:     KEGG Pathways:  
    Probe set : 1380009_at    Name:   vaccinia related kinase 1 (predicted)    GB acc:   BM392230    UniGene:   Rn.92083    Gene Symbol:   Vrk1_predicted    Locus Link:   362779    Chromosome:   6    Cytoband:   Chr:6q32    SOURCE    GENECARD       Gene Ontology:   biological process:  protein amino acid phosphorylation      BioCarta Pathways:     KEGG Pathways:  
    Probe set : 1383960_at    Name:   peroxisome biogenesis factor 16 (predicted)    GB acc:   BG376773    UniGene:   Rn.98695    Gene Symbol:   Pex16_predicted    Locus Link:   311203    Chromosome:   3    Cytoband:   Chr:3q24    SOURCE    GENECARD       Gene Ontology:   biological process:  peroxisome organization and biogenesis   cellular component:  peroxisome   cellular component:  integral to peroxisomal membrane      BioCarta Pathways:     KEGG Pathways:  
    Probe set : 1387001_at    Name:   v-ral simian leukemia viral oncogene homolog B    GB acc:   NM_053821    UniGene:   Rn.4586    Gene Symbol:   Ralb    Locus Link:   116546    Chromosome:   13    Cytoband:   Chr:13q11    SOURCE    GENECARD       Gene Ontology:   biological process:  small GTPase mediated signal transduction   molecular function:  GTP binding   molecular function:  GTP binding   molecular function:  nucleotide binding      BioCarta Pathways:     KEGG Pathways:  
    Probe set : 1379327_at    Name:   SWI/SNF related, matrix associated, actin dependent regulator of chromatin, subfamily a, member 5 (predicted)    GB acc:   BI292524    UniGene:   Rn.128772    Gene Symbol:   Smarca5_predicted    Locus Link:   307766    Chromosome:   19    Cytoband:   Chr:19q11    SOURCE    GENECARD       Gene Ontology:   biological process:  chromatin remodeling   biological process:  regulation of transcription from RNA polymerase II promoter   biological process:  chromatin modification   cellular component:  nucleoplasm      BioCarta Pathways:     KEGG Pathways:  
    Probe set : 1367579_a_at    Name:   tubulin, alpha 1 /// tubulin, alpha 6 (predicted) /// similar to Tubulin alpha-2 chain (Alpha-tubulin 2)    GB acc:   BI285434    UniGene:   Rn.99661    Gene Symbol:   Tuba1 /// Tuba6_predicted /// LOC500929    Locus Link:   300218 /// 500929 /// 64158    Chromosome:   7    Cytoband:   Chr:7q36    SOURCE    GENECARD       Gene Ontology:     BioCarta Pathways:     KEGG Pathways:  
    Probe set : 1389518_at    Name:   F-box only protein 10 (predicted)    GB acc:   AI009763    UniGene:   Rn.7385    Gene Symbol:   Fbxo10_predicted    Locus Link:   362511    Chromosome:   5    Cytoband:   Chr:5q22    SOURCE    GENECARD       Gene Ontology:   biological process:  ubiquitin cycle   biological process:  protein ubiquitination   cellular component:  ubiquitin ligase complex   cellular component:  ruffle   cellular component:  cytoplasm   molecular function:  ubiquitin-protein ligase activity   molecular function:  protein binding      BioCarta Pathways:     KEGG Pathways:  
    Probe set : 1367687_a_at    Name:   peptidylglycine alpha-amidating monooxygenase    GB acc:   M25719    UniGene:   Rn.1121    Gene Symbol:   Pam    Locus Link:   25508    Chromosome:   9    Cytoband:   Chr:9q36    SOURCE    GENECARD       Gene Ontology:   biological process:  peptide amidation   biological process:  protein modification   biological process:  peptide metabolism      BioCarta Pathways:     KEGG Pathways:  
    Probe set : 1393158_at    Name:   similar to 2610528M18Rik protein    GB acc:   BI298434    UniGene:   Rn.145876    Gene Symbol:   RGD:1359451    Locus Link:   363198    Chromosome:   9    Cytoband:   Chr:9q12    SOURCE    GENECARD       Gene Ontology:     BioCarta Pathways:     KEGG Pathways:  
    Probe set : 1369720_at    Name:   myosin Ib    GB acc:   NM_053986    UniGene:   Rn.36339    Gene Symbol:   Myo1b    Locus Link:   117057    Chromosome:   9    Cytoband:   Chr:9q22    SOURCE    GENECARD       Gene Ontology:   biological process:  cytoskeleton organization and biogenesis   biological process:  nervous system development      BioCarta Pathways:     KEGG Pathways:  
    Probe set : 1395440_at    Name:   similar to RIKEN cDNA 5133401N09    GB acc:   AW915843    UniGene:   Rn.114944    Gene Symbol:   LOC498695    Locus Link:   498695    Chromosome:   17    Cytoband:   Chr:17p14    SOURCE    GENECARD       Gene Ontology:     BioCarta Pathways:     KEGG Pathways:  
    Probe set : 1382010_at     GB acc:   AI711537              Gene Ontology:     BioCarta Pathways:     KEGG Pathways:  
    Probe set : 1374524_at    Name:   selenocysteine lyase    GB acc:   BM384384    UniGene:   Rn.23954    Gene Symbol:   RGD:1359514    Locus Link:   363285    Chromosome:   9    Cytoband:   Chr:9q36    SOURCE    GENECARD       Gene Ontology:   biological process:  metabolism   molecular function:  transaminase activity   molecular function:  lyase activity      BioCarta Pathways:     KEGG Pathways:  
    Probe set : 1382050_at    Name:   Transcribed locus    GB acc:   AI410262    UniGene:   Rn.7315        SOURCE        Gene Ontology:     BioCarta Pathways:     KEGG Pathways:  
    Probe set : 1387158_at    Name:   meprin 1 beta    GB acc:   NM_013183    UniGene:   Rn.3520    Gene Symbol:   Mep1b    Locus Link:   25727      SOURCE    GENECARD       Gene Ontology:   biological process:  proteolysis   biological process:  proteolysis   biological process:  digestion      BioCarta Pathways:     KEGG Pathways:  
    Probe set : 1392082_a_at    Name:   CD7 antigen (predicted)    GB acc:   BI290548    UniGene:   Rn.8175    Gene Symbol:   Cd7_predicted    Locus Link:   303747    Chromosome:   10    Cytoband:   Chr:10q32.3    SOURCE    GENECARD       Gene Ontology:   molecular function:  receptor activity      BioCarta Pathways:     KEGG Pathways:  
    Probe set : 1371382_at    Name:   similar to Filamin A (Alpha-filamin) (Filamin 1) (Endothelial actin-binding protein) (Actin-binding protein 280) (ABP-280) (Nonmuscle filamin)    GB acc:   BI283060    UniGene:   Rn.4213    Gene Symbol:   LOC293860    Locus Link:   293860    Chromosome:   X    Cytoband:   Chr:Xq37    SOURCE    GENECARD       Gene Ontology:     BioCarta Pathways:     KEGG Pathways:  
    Probe set : 1395409_at    Name:   protein phosphatase 2A, regulatory subunit B' (PR 53) (predicted)    GB acc:   BF522283    UniGene:   Rn.24600    Gene Symbol:   Ppp2r4_predicted    Locus Link:   362102    Chromosome:   3    Cytoband:   Chr:3p12    SOURCE    GENECARD       Gene Ontology:   biological process:  protein amino acid dephosphorylation   cellular component:  soluble fraction      BioCarta Pathways:     KEGG Pathways:  
    Probe set : 1393584_at     GB acc:   AI045201              Gene Ontology:     BioCarta Pathways:     KEGG Pathways:  
    Probe set : 1387144_at    Name:   integrin alpha 1    GB acc:   NM_030994    UniGene:   Rn.91044    Gene Symbol:   Itga1    Locus Link:   25118    Chromosome:   2    Cytoband:   Chr:2q16-q21    SOURCE    GENECARD       Gene Ontology:     BioCarta Pathways:     KEGG Pathways:  
    Probe set : 1393038_at    Name:   Fc receptor, IgG, high affinity I (predicted)    GB acc:   BM391631    UniGene:   Rn.16643    Gene Symbol:   Fcgr1_predicted    Locus Link:   295279    Chromosome:   2    Cytoband:   Chr:2q34    SOURCE    GENECARD       Gene Ontology:   biological process:  antibody-dependent cellular cytotoxicity   biological process:  positive regulation of type IIa hypersensitivity   biological process:  positive regulation of type III hypersensitivity   biological process:  receptor mediated endocytosis   biological process:  phagocytosis, recognition   biological process:  phagocytosis, engulfment   biological process:  immune response   biological process:  signal transduction   biological process:  cell surface receptor linked signal transduction   biological process:  antigen presentation, exogenous antigen   biological process:  antigen presentation, exogenous antigen via MHC class I   biological process:  defense response to pathogenic bacteria   biological process:  positive regulation of phagocytosis   biological process:  regulation of immune response      BioCarta Pathways:     KEGG Pathways:  
    Probe set : 1396820_at    Name:   histone deacetylase 1 (predicted)    GB acc:   AW530195    UniGene:   Rn.123394    Gene Symbol:   Hdac1_predicted    Locus Link:   297893    Chromosome:   5    Cytoband:   Chr:5q36    SOURCE    GENECARD       Gene Ontology:     BioCarta Pathways:     KEGG Pathways:  
    Probe set : 1391539_at    Name:   Transcribed locus    GB acc:   BI291849    UniGene:   Rn.49247        SOURCE        Gene Ontology:     BioCarta Pathways:     KEGG Pathways:  
    Probe set : 1370649_at    Name:   bradykinin receptor b2    GB acc:   M59967    UniGene:   Rn.9845    Gene Symbol:   Bdkrb2    Locus Link:   25245    Chromosome:   6    Cytoband:   Chr:6q32    SOURCE    GENECARD       Gene Ontology:   cellular component:  integral to plasma membrane   cellular component:  integral to membrane      BioCarta Pathways:     KEGG Pathways:  
    Probe set : 1370682_at    Name:   paired-Ig-like receptor A2 (predicted) /// similar to PIRA5    GB acc:   AF169636    UniGene:   Rn.145293    Gene Symbol:   Pira2_predicted /// LOC365169    Locus Link:   361492 /// 365169    Chromosome:   1    Cytoband:   Chr:1q12    SOURCE    GENECARD       Gene Ontology:     BioCarta Pathways:     KEGG Pathways:  
    Probe set : 1369632_a_at    Name:   ATP-binding cassette, sub-family C (CFTR/MRP), member 8    GB acc:   AB052294    UniGene:   Rn.11187    Gene Symbol:   Abcc8    Locus Link:   25559    Chromosome:   1    Cytoband:   Chr:1q22    SOURCE    GENECARD       Gene Ontology:   biological process:  carbohydrate metabolism   biological process:  transport   biological process:  potassium ion transport      BioCarta Pathways:     KEGG Pathways:  
    Probe set : 1395856_at    Name:   Hypothetical LOC308765 (predicted)    GB acc:   BF289112    UniGene:   Rn.3618     Locus Link:   308765    Chromosome:   1    Cytoband:   Chr:1q31    SOURCE        Gene Ontology:     BioCarta Pathways:     KEGG Pathways:  
    Probe set : 1381942_at    Name:   Transcribed locus    GB acc:   AA963505    UniGene:   Rn.11570        SOURCE        Gene Ontology:     BioCarta Pathways:     KEGG Pathways:  
    Probe set : 1387343_at    Name:   CCAAT/enhancer binding protein (C/EBP), delta    GB acc:   NM_013154    UniGene:   Rn.6975    Gene Symbol:   Cebpd    Locus Link:   25695      SOURCE    GENECARD       Gene Ontology:   cellular component:  nucleus   cellular component:  nucleus   cellular component:  Golgi stack      BioCarta Pathways:     KEGG Pathways:  
    Probe set : 1391068_at    Name:   E030032D13Rik gene    GB acc:   AI136523     Gene Symbol:   RGD:1303180    Locus Link:   415052    Chromosome:   20    Cytoband:   Chr:20p12     GENECARD       Gene Ontology:     BioCarta Pathways:     KEGG Pathways:  
    Probe set : 1386280_at    Name:   similar to RIKEN cDNA 0610006F02 (predicted)    GB acc:   AA892888     Gene Symbol:   RGD1305205_predicted    Locus Link:   366792    Chromosome:   7    Cytoband:   Chr:7q11     GENECARD       Gene Ontology:   molecular function:  methyltransferase activity   molecular function:  S-adenosylmethionine-dependent methyltransferase activity   molecular function:  transferase activity      BioCarta Pathways:     KEGG Pathways:  
    Probe set : 1389509_at    Name:   Similar to hypothetical protein 2610304F09 (predicted)    GB acc:   BE097016    UniGene:   Rn.46066     Locus Link:   307527    Chromosome:   18    Cytoband:   Chr:18p12    SOURCE        Gene Ontology:     BioCarta Pathways:     KEGG Pathways:  
    Probe set : 1387110_at    Name:   N-arginine dibasic convertase 1    GB acc:   NM_012993    UniGene:   Rn.16763    Gene Symbol:   Nrd1    Locus Link:   25499    Chromosome:   5    Cytoband:   Chr:5q35    SOURCE    GENECARD       Gene Ontology:   biological process:  proteolysis   biological process:  neuromuscular junction development      BioCarta Pathways:     KEGG Pathways:  
    Probe set : 1399030_at    Name:   similar to DNA segment, Chr X, Immunex 38, expressed    GB acc:   AA800682    UniGene:   Rn.25900    Gene Symbol:   RGD:1359718    Locus Link:   302559    Chromosome:   X    Cytoband:   Chr:Xq13    SOURCE    GENECARD       Gene Ontology:     BioCarta Pathways:     KEGG Pathways:  
    Probe set : 1386960_at    Name:   solute carrier family 37 (glycerol-6-phosphate transporter), member 4    GB acc:   NM_031589    UniGene:   Rn.1592    Gene Symbol:   Slc37a4    Locus Link:   29573    Chromosome:   8    Cytoband:   Chr:8q22    SOURCE    GENECARD       Gene Ontology:   molecular function:  transporter activity      BioCarta Pathways:     KEGG Pathways:  
    Probe set : 1389131_at    Name:   similar to C330027I04Rik protein    GB acc:   BF412799    UniGene:   Rn.24114    Gene Symbol:   RGD:1359229    Locus Link:   360629    Chromosome:   10    Cytoband:   Chr:10q32.1    SOURCE    GENECARD       Gene Ontology:     BioCarta Pathways:     KEGG Pathways:  
    Probe set : 1370814_at    Name:   dehydrogenase/reductase (SDR family) member 4    GB acc:   AB062758    UniGene:   Rn.12783    Gene Symbol:   Dhrs4    Locus Link:   266686    Chromosome:   15    Cytoband:   Chr:15p13    SOURCE    GENECARD       Gene Ontology:   biological process:  metabolism      BioCarta Pathways:     KEGG Pathways:  
    Probe set : 1385047_x_at     GB acc:   AI012782    UniGene:   Rn.144940    Gene Symbol:   LOC304349 /// LOC360782 /// LOC498168 /// LOC501815 /// LOC501818    Locus Link:   304349 /// 360782 /// 498168 /// 501815 /// 501818    Chromosome:   12    Cytoband:   Chr:12q11 /// Chr:12q12    SOURCE    GENECARD       Gene Ontology:     BioCarta Pathways:     KEGG Pathways:  
    Probe set : 1388877_at    Name:   mitochondrial ribosomal protein S5 (predicted)    GB acc:   BM386940    UniGene:   Rn.9035    Gene Symbol:   Mrps5_predicted    Locus Link:   296134    Chromosome:   3    Cytoband:   Chr:3q36    SOURCE    GENECARD       Gene Ontology:   biological process:  protein biosynthesis   molecular function:  structural constituent of ribosome      BioCarta Pathways:     KEGG Pathways:  
    Probe set : 1372306_at    Name:   ethylmalonic encephalopathy 1 (predicted)    GB acc:   BM384018    UniGene:   Rn.14691    Gene Symbol:   Ethe1_predicted    Locus Link:   292710    Chromosome:   1    Cytoband:   Chr:1q21    SOURCE    GENECARD       Gene Ontology:   cellular component:  mitochondrion      BioCarta Pathways:     KEGG Pathways:  
    Probe set : 1383182_at    Name:   serine/arginine-rich protein specific kinase 1 (predicted)    GB acc:   AW144184    UniGene:   Rn.2688    Gene Symbol:   Srpk1_predicted    Locus Link:   361811    Chromosome:   20    Cytoband:   Chr:20p12    SOURCE    GENECARD       Gene Ontology:   biological process:  regulation of progression through cell cycle   biological process:  protein amino acid phosphorylation   biological process:  RNA splicing   cellular component:  nucleus      BioCarta Pathways:     KEGG Pathways:  
    Probe set : 1372460_at    Name:   LOC499767    GB acc:   AI172405    UniGene:   Rn.144530    Gene Symbol:   LOC499767    Locus Link:   499767    Chromosome:   3    Cytoband:   Chr:3p12    SOURCE    GENECARD       Gene Ontology:     BioCarta Pathways:     KEGG Pathways:  
    Probe set : 1394097_at    Name:   Transcribed locus    GB acc:   AA849706    UniGene:   Rn.20530        SOURCE        Gene Ontology:     BioCarta Pathways:     KEGG Pathways:  
    Probe set : 1388814_at    Name:   DEAD (Asp-Glu-Ala-Asp) box polypeptide 19    GB acc:   AI408110    UniGene:   Rn.96192    Gene Symbol:   Ddx19    Locus Link:   292022    Chromosome:   19    Cytoband:   Chr:19q12    SOURCE    GENECARD       Gene Ontology:   biological process:  induction of apoptosis   biological process:  response to zinc ion   cellular component:  nucleus      BioCarta Pathways:     KEGG Pathways:  
    Probe set : 1396205_at    Name:   Transcribed locus, weakly similar to XP_282988.2 PREDICTED: similar to novel protein [Mus musculus]    GB acc:   BI303419    UniGene:   Rn.42381        SOURCE        Gene Ontology:     BioCarta Pathways:     KEGG Pathways:  
    Probe set : 1391573_at    Name:   Tumor necrosis factor receptor superfamily, member 21 (predicted)    GB acc:   AA891842    UniGene:   Rn.98293     Locus Link:   316256    Chromosome:   9    Cytoband:   Chr:9q12    SOURCE        Gene Ontology:   biological process:  apoptosis   biological process:  signal transduction   cellular component:  integral to membrane   molecular function:  receptor activity   molecular function:  protein binding      BioCarta Pathways:     KEGG Pathways:  
    Probe set : 1376279_at    Name:   processing of precursor 1, ribonuclease P/MRP family, (S. cerevisiae) (predicted)    GB acc:   BM389467    UniGene:   Rn.22746    Gene Symbol:   Pop1_predicted    Locus Link:   315045    Chromosome:   7    Cytoband:   Chr:7q22    SOURCE    GENECARD       Gene Ontology:   biological process:  tRNA 5'-leader removal   biological process:  tRNA catabolism   cellular component:  ribonuclease MRP complex   cellular component:  nucleus   cellular component:  nucleolar ribonuclease P complex   molecular function:  ribonuclease MRP activity   molecular function:  ribonuclease P activity   molecular function:  hydrolase activity      BioCarta Pathways:     KEGG Pathways:  
    Probe set : 1391162_at    Name:   Transcribed locus    GB acc:   AW522589    UniGene:   Rn.45430        SOURCE        Gene Ontology:     BioCarta Pathways:     KEGG Pathways:  
    Probe set : 1383322_at    Name:   RAS-like family 11 member B    GB acc:   BG375198    UniGene:   Rn.36357    Gene Symbol:   RGD:1303099    Locus Link:   305302    Chromosome:   14    Cytoband:   Chr:14p11    SOURCE    GENECARD       Gene Ontology:   biological process:  negative regulation of cell growth   biological process:  small GTPase mediated signal transduction   molecular function:  nucleotide binding   molecular function:  GTP binding      BioCarta Pathways:     KEGG Pathways:  
    Probe set : 1376909_at    Name:   similar to RRP22 (predicted)    GB acc:   AI412866    UniGene:   Rn.50230    Gene Symbol:   RGD1306100_predicted    Locus Link:   364190    Chromosome:   14    Cytoband:   Chr:14q21    SOURCE    GENECARD       Gene Ontology:     BioCarta Pathways:     KEGG Pathways:  
    Probe set : 1367795_at    Name:   interferon-related developmental regulator 1    GB acc:   NM_019242    UniGene:   Rn.3723    Gene Symbol:   Ifrd1    Locus Link:   29596    Chromosome:   6    Cytoband:   Chr:6q16    SOURCE    GENECARD       Gene Ontology:   cellular component:  nucleus   molecular function:  binding      BioCarta Pathways:     KEGG Pathways:  
    Probe set : 1373040_at    Name:   eukaryotic translation initiation factor 3, subunit 5 (epsilon) (predicted)    GB acc:   BI281752    UniGene:   Rn.41724    Gene Symbol:   Eif3s5_predicted    Locus Link:   293427    Chromosome:   1    Cytoband:   Chr:1q33    SOURCE    GENECARD       Gene Ontology:   biological process:  protein biosynthesis   biological process:  regulation of translational initiation   cellular component:  eukaryotic translation initiation factor 3 complex   molecular function:  translation initiation factor activity      BioCarta Pathways:     KEGG Pathways:  
    Probe set : 1391026_at    Name:   Immediate early response 5-like    GB acc:   BI296897    UniGene:   Rn.9531     Locus Link:   499772      SOURCE        Gene Ontology:     BioCarta Pathways:     KEGG Pathways:  
    Probe set : 1389000_at    Name:   Transcribed locus    GB acc:   AA964998    UniGene:   Rn.124985        SOURCE        Gene Ontology:     BioCarta Pathways:     KEGG Pathways:  
    Probe set : 1369081_at    Name:   neuraminidase 1    GB acc:   NM_031522    UniGene:   Rn.128560    Gene Symbol:   Neu1    Locus Link:   24591    Chromosome:   20    Cytoband:   Chr:20p12    SOURCE    GENECARD       Gene Ontology:   biological process:  carbohydrate metabolism   cellular component:  lysosome      BioCarta Pathways:     KEGG Pathways:  
    Probe set : 1387079_at    Name:   guanylate cyclase 1, soluble, alpha 3 /// hypothetical gene supported by NM_017090    GB acc:   M57405    UniGene:   Rn.1974    Gene Symbol:   Gucy1a3 /// LOC497757    Locus Link:   25201 /// 497757    Chromosome:   2    Cytoband:   Chr:2q31 /// Chr:2q33    SOURCE    GENECARD       Gene Ontology:   cellular component:  guanylate cyclase complex, soluble   cellular component:  guanylate cyclase complex, soluble      BioCarta Pathways:     KEGG Pathways:  
    Probe set : 1390728_at     GB acc:   AI501517              Gene Ontology:     BioCarta Pathways:     KEGG Pathways:  
    Probe set : 1388766_at    Name:   metaxin 2 (predicted)    GB acc:   AI409049    UniGene:   Rn.24607    Gene Symbol:   Mtx2_predicted    Locus Link:   288150    Chromosome:   3    Cytoband:   Chr:3q23    SOURCE    GENECARD       Gene Ontology:   biological process:  transport   biological process:  mitochondrial transport   biological process:  protein transport   cellular component:  mitochondrion   cellular component:  mitochondrial outer membrane   cellular component:  outer membrane      BioCarta Pathways:     KEGG Pathways:  
    Probe set : 1368250_at    Name:   tektin 1    GB acc:   NM_053508    UniGene:   Rn.46442    Gene Symbol:   Tekt1    Locus Link:   85270    Chromosome:   10    Cytoband:   Chr:10q24    SOURCE    GENECARD       Gene Ontology:   biological process:  microtubule cytoskeleton organization and biogenesis   biological process:  microtubule-based process   cellular component:  kinesin complex   cellular component:  microtubule   molecular function:  structural molecule activity      BioCarta Pathways:     KEGG Pathways:  
    Probe set : 1390987_at    Name:   Transcribed locus    GB acc:   AI406858    UniGene:   Rn.41998        SOURCE        Gene Ontology:     BioCarta Pathways:     KEGG Pathways:  
    Probe set : 1383472_at    Name:   aldehyde dehydrogenase 1 family, member B1 (predicted)    GB acc:   AA997683    UniGene:   Rn.12547    Gene Symbol:   Aldh1b1_predicted    Locus Link:   298079    Chromosome:   5    Cytoband:   Chr:5q22    SOURCE    GENECARD       Gene Ontology:   biological process:  carbohydrate metabolism   biological process:  metabolism   cellular component:  extracellular space   cellular component:  mitochondrion   molecular function:  aldehyde dehydrogenase activity   molecular function:  aldehyde dehydrogenase (NAD) activity   molecular function:  oxidoreductase activity      BioCarta Pathways:     KEGG Pathways:  
    Probe set : 1382274_at    Name:   retinoic acid receptor responder (tazarotene induced) 1 (predicted)    GB acc:   AA819288    UniGene:   Rn.16737    Gene Symbol:   Rarres1_predicted    Locus Link:   310486    Chromosome:   2    Cytoband:   Chr:2q31    SOURCE    GENECARD       Gene Ontology:   biological process:  negative regulation of cell proliferation   cellular component:  integral to membrane      BioCarta Pathways:     KEGG Pathways:  
    Probe set : 1381376_at    Name:   Similar to [Mouse primary response gene B94 mRNA, 3end.], gene product    GB acc:   AI103284    UniGene:   Rn.34387     Locus Link:   299339    Chromosome:   6    Cytoband:   Chr:6q32    SOURCE        Gene Ontology:     BioCarta Pathways:     KEGG Pathways:  
    Probe set : 1393982_at    Name:   polymerase (DNA directed), epsilon 2 (p59 subunit) (predicted)    GB acc:   AI070935    UniGene:   Rn.34652    Gene Symbol:   Pole2_predicted    Locus Link:   299112    Chromosome:   6    Cytoband:   Chr:6q24    SOURCE    GENECARD       Gene Ontology:   biological process:  DNA replication   biological process:  DNA repair   cellular component:  nucleus      BioCarta Pathways:     KEGG Pathways:  
    Probe set : 1376697_at    Name:   carbohydrate sulfotransferase 12 (predicted)    GB acc:   BI275885    UniGene:   Rn.78863    Gene Symbol:   Chst12_predicted    Locus Link:   304322    Chromosome:   12    Cytoband:   Chr:12q11    SOURCE    GENECARD       Gene Ontology:   biological process:  dermatan sulfate biosynthesis   cellular component:  integral to membrane   cellular component:  integral to Golgi membrane      BioCarta Pathways:     KEGG Pathways:  
    Probe set : 1397205_at    Name:   dehydrogenase/reductase (SDR family) member 7 (predicted) /// similar to Down-regulated in nephrectomized rat kidney #3    GB acc:   BI288203    UniGene:   Rn.119024    Gene Symbol:   Dhrs7_predicted /// LOC500672    Locus Link:   299131 /// 500672    Chromosome:   6    Cytoband:   Chr:6q24    SOURCE    GENECARD       Gene Ontology:   biological process:  metabolism   molecular function:  oxidoreductase activity      BioCarta Pathways:     KEGG Pathways:  
    Probe set : 1372615_at    Name:   amine oxidase, copper containing 3    GB acc:   AI070137    UniGene:   Rn.56603    Gene Symbol:   Aoc3    Locus Link:   29473    Chromosome:   10    Cytoband:   Chr:10q32.1    SOURCE    GENECARD       Gene Ontology:   biological process:  cell adhesion      BioCarta Pathways:     KEGG Pathways:  
    Probe set : 1378342_at    Name:   LOC498937    GB acc:   BF284819    UniGene:   Rn.145516    Gene Symbol:   LOC498937    Locus Link:   498937    Chromosome:   19    Cytoband:   Chr:19q11    SOURCE    GENECARD       Gene Ontology:     BioCarta Pathways:     KEGG Pathways:  
    Probe set : 1377821_at    Name:   V-erb-b2 erythroblastic leukemia viral oncogene homolog 3 (avian)    GB acc:   BI284288    UniGene:   Rn.10228    Gene Symbol:   Erbb3    Locus Link:   29496    Chromosome:   7    Cytoband:   Chr:7q11    SOURCE    GENECARD       Gene Ontology:     BioCarta Pathways:     KEGG Pathways:  
    Probe set : 1386946_at    Name:   carnitine palmitoyltransferase 1, liver    GB acc:   NM_031559    UniGene:   Rn.2856    Gene Symbol:   Cpt1a    Locus Link:   25757    Chromosome:   1    Cytoband:   Chr:1q43    SOURCE    GENECARD       Gene Ontology:     BioCarta Pathways:     KEGG Pathways:  
    Probe set : 1383434_at    Name:   Transcribed locus    GB acc:   AA997640    UniGene:   Rn.12530        SOURCE        Gene Ontology:     BioCarta Pathways:     KEGG Pathways:  
    Probe set : 1384174_at    Name:   Transcribed locus    GB acc:   AI028874    UniGene:   Rn.147284        SOURCE        Gene Ontology:     BioCarta Pathways:     KEGG Pathways:  
    Probe set : 1378225_at    Name:   Matrix metalloproteinase 14 (membrane-inserted)    GB acc:   AI176440    UniGene:   Rn.10371    Gene Symbol:   Mmp14    Locus Link:   81707    Chromosome:   15    Cytoband:   Chr:15p13    SOURCE    GENECARD       Gene Ontology:   biological process:  proteolysis   biological process:  collagen catabolism   biological process:  peptidoglycan metabolism      BioCarta Pathways:     KEGG Pathways:  
    Probe set : 1382571_at    Name:   Transcribed locus    GB acc:   AA925335    UniGene:   Rn.54842        SOURCE        Gene Ontology:     BioCarta Pathways:     KEGG Pathways:  
    Probe set : 1372949_at    Name:   Septin 6 (predicted)    GB acc:   BG663067    UniGene:   Rn.27798     Locus Link:   305227    Chromosome:   14    Cytoband:   Chr:14p22    SOURCE        Gene Ontology:   biological process:  cytokinesis   biological process:  cell cycle   molecular function:  protein binding   molecular function:  GTP binding      BioCarta Pathways:     KEGG Pathways:  
    Probe set : 1371125_at    Name:   kinesin heavy chain member 2    GB acc:   BF564995    UniGene:   Rn.17829    Gene Symbol:   Kif2    Locus Link:   84391    Chromosome:   2    Cytoband:   Chr:2q14    SOURCE    GENECARD       Gene Ontology:   biological process:  microtubule-based process   biological process:  microtubule-based movement   cellular component:  cytoskeleton   cellular component:  kinesin complex   cellular component:  microtubule associated complex   molecular function:  motor activity   molecular function:  microtubule motor activity   molecular function:  ATP binding      BioCarta Pathways:     KEGG Pathways:  
    Probe set : 1374176_at    Name:   similar to DNA segment, Chr 4, Brigham & Womens Genetics 0951 expressed (predicted)    GB acc:   AI408727    UniGene:   Rn.36366    Gene Symbol:   RGD1308059_predicted    Locus Link:   362535    Chromosome:   5    Cytoband:   Chr:5q31    SOURCE    GENECARD       Gene Ontology:     BioCarta Pathways:     KEGG Pathways:  
    Probe set : 1375612_at    Name:   Transcribed locus    GB acc:   AA965147    UniGene:   Rn.151553        SOURCE        Gene Ontology:     BioCarta Pathways:     KEGG Pathways:  
    Probe set : 1387625_at    Name:   insulin-like growth factor binding protein 6    GB acc:   NM_013104    UniGene:   Rn.6431    Gene Symbol:   Igfbp6    Locus Link:   25641    Chromosome:   7    Cytoband:   Chr:7q36    SOURCE    GENECARD       Gene Ontology:   biological process:  regulation of cell growth   biological process:  signal transduction   biological process:  negative regulation of cell proliferation   cellular component:  extracellular region   cellular component:  extracellular space   molecular function:  insulin-like growth factor binding   molecular function:  growth factor binding      BioCarta Pathways:     KEGG Pathways:  
    Probe set : 1377223_at    Name:   Transcribed locus    GB acc:   AI599621    UniGene:   Rn.147389        SOURCE        Gene Ontology:     BioCarta Pathways:     KEGG Pathways:  
    Probe set : 1370329_at    Name:   cytochrome P450, family 2, subfamily d, polypeptide 22    GB acc:   U48219    UniGene:   Rn.26060    Gene Symbol:   Cyp2d22    Locus Link:   171522    Chromosome:   7    Cytoband:   Chr:7q34    SOURCE    GENECARD       Gene Ontology:   biological process:  electron transport   biological process:  drug metabolism      BioCarta Pathways:     KEGG Pathways:  
    Probe set : 1381054_at    Name:   similar to KIAA0980 protein (predicted)    GB acc:   BI275310    UniGene:   Rn.92676    Gene Symbol:   RGD1306152_predicted    Locus Link:   311529    Chromosome:   3    Cytoband:   Chr:3q41    SOURCE    GENECARD       Gene Ontology:     BioCarta Pathways:     KEGG Pathways:  
    Probe set : 1373617_at    Name:   epithelial membrane protein 2    GB acc:   AA818807    UniGene:   Rn.21730    Gene Symbol:   RGD:1359629    Locus Link:   360468    Chromosome:   10    Cytoband:   Chr:10q11    SOURCE    GENECARD       Gene Ontology:   cellular component:  membrane   cellular component:  integral to membrane      BioCarta Pathways:     KEGG Pathways:  
    Probe set : 1367487_at    Name:   similar to UDP-Gal:betaGlcNAc beta 1,4-galactosyltransferase, polypeptide 3    GB acc:   BG378610    UniGene:   Rn.91751    Gene Symbol:   RGD:1359221    Locus Link:   494342    Chromosome:   13    Cytoband:   Chr:13q24    SOURCE    GENECARD       Gene Ontology:   biological process:  carbohydrate metabolism   cellular component:  Golgi stack   cellular component:  integral to membrane      BioCarta Pathways:     KEGG Pathways:  
    Probe set : 1382148_at    Name:   Transcribed locus, moderately similar to XP_576460.1 PREDICTED: similar to hypothetical protein PB402898.00.0 [Rattus norvegicus]    GB acc:   AA923980    UniGene:   Rn.24639        SOURCE        Gene Ontology:     BioCarta Pathways:     KEGG Pathways:  
    Probe set : 1390925_a_at    Name:   Transcribed locus    GB acc:   AI043758    UniGene:   Rn.23173        SOURCE        Gene Ontology:     BioCarta Pathways:     KEGG Pathways:  
    Probe set : 1372643_at    Name:   similar to protein 4.1G    GB acc:   BE110569    UniGene:   Rn.1362    Gene Symbol:   LOC309557    Locus Link:   309557    Chromosome:   1    Cytoband:   Chr:1p12    SOURCE    GENECARD       Gene Ontology:     BioCarta Pathways:     KEGG Pathways:  
    Probe set : 1372574_at    Name:   TEA domain family member 2    GB acc:   AW142018    UniGene:   Rn.130442    Gene Symbol:   Tead2    Locus Link:   308582    Chromosome:   1    Cytoband:   Chr:1q22    SOURCE    GENECARD       Gene Ontology:   biological process:  regulation of transcription, DNA-dependent   cellular component:  nucleus   molecular function:  DNA binding   molecular function:  transcription factor activity      BioCarta Pathways:     KEGG Pathways:  
    Probe set : 1379725_at    Name:   Transcribed locus    GB acc:   AW920459    UniGene:   Rn.28951        SOURCE        Gene Ontology:     BioCarta Pathways:     KEGG Pathways:  
    Probe set : 1390397_at    Name:   similar to chromosome 20 open reading frame 39 (predicted)    GB acc:   BF413152    UniGene:   Rn.44092    Gene Symbol:   RGD1310753_predicted    Locus Link:   362235    Chromosome:   3    Cytoband:   Chr:3q41    SOURCE    GENECARD       Gene Ontology:   cellular component:  integral to membrane      BioCarta Pathways:     KEGG Pathways:  
    Probe set : 1398829_at    Name:   FK506 binding protein 1a    GB acc:   AI598372    UniGene:   Rn.80611    Gene Symbol:   Fkbp1a    Locus Link:   25639    Chromosome:   3    Cytoband:   Chr:3q41    SOURCE    GENECARD       Gene Ontology:   biological process:  protein folding   biological process:  transport   biological process:  positive regulation of I-kappaB kinase/NF-kappaB cascade   cellular component:  microsome   cellular component:  membrane      BioCarta Pathways:     KEGG Pathways:  
    Probe set : 1391356_at    Name:   Similar to hypothetical protein FLJ10006 (predicted)    GB acc:   BI282688    UniGene:   Rn.26585     Locus Link:   291705    Chromosome:   18    Cytoband:   Chr:18p12    SOURCE        Gene Ontology:   biological process:  electron transport   biological process:  regulation of transcription, DNA-dependent   cellular component:  nucleus   molecular function:  acyl-CoA dehydrogenase activity      BioCarta Pathways:     KEGG Pathways:  
    Probe set : 1383946_at    Name:   claudin 1    GB acc:   AI137640    UniGene:   Rn.24293    Gene Symbol:   Cldn1    Locus Link:   65129    Chromosome:   11    Cytoband:   Chr:11q22    SOURCE    GENECARD       Gene Ontology:     BioCarta Pathways:     KEGG Pathways:  
    Probe set : 1367617_at    Name:   aldolase A    GB acc:   NM_012495    UniGene:   Rn.1774    Gene Symbol:   Aldoa    Locus Link:   24189    Chromosome:   1    Cytoband:   Chr:1q36    SOURCE    GENECARD       Gene Ontology:   biological process:  fructose metabolism   biological process:  glycolysis   biological process:  striated muscle contraction   molecular function:  fructose-bisphosphate aldolase activity   molecular function:  lyase activity   molecular function:  aldehyde-lyase activity      BioCarta Pathways:     KEGG Pathways:  
    Probe set : 1372799_at    Name:   deoxyguanosine kinase (predicted)    GB acc:   BF285179    UniGene:   Rn.6770    Gene Symbol:   Dguok_predicted    Locus Link:   297389    Chromosome:   4    Cytoband:   Chr:4q34    SOURCE    GENECARD       Gene Ontology:   cellular component:  cytoplasm   cellular component:  mitochondrion      BioCarta Pathways:     KEGG Pathways:  
    Probe set : 1369292_at    Name:   hydroxysteroid (17-beta) dehydrogenase 1    GB acc:   NM_012851    UniGene:   Rn.10594    Gene Symbol:   Hsd17b1    Locus Link:   25322    Chromosome:   10    Cytoband:   Chr:10q32.1    SOURCE    GENECARD       Gene Ontology:   cellular component:  cytoplasm   molecular function:  estradiol 17-beta-dehydrogenase activity   molecular function:  estradiol 17-beta-dehydrogenase activity   molecular function:  oxidoreductase activity      BioCarta Pathways:     KEGG Pathways:  
    Probe set : 1369262_at    Name:   caspase 8    GB acc:   NM_022277    UniGene:   Rn.54474    Gene Symbol:   Casp8    Locus Link:   64044    Chromosome:   9    Cytoband:   Chr:9q31    SOURCE    GENECARD       Gene Ontology:     BioCarta Pathways:     KEGG Pathways:  
    Probe set : 1368059_at    Name:   crystallin, mu    GB acc:   NM_053955    UniGene:   Rn.24561    Gene Symbol:   Crym    Locus Link:   117024      SOURCE    GENECARD       Gene Ontology:   biological process:  visual perception      BioCarta Pathways:     KEGG Pathways:  
    Probe set : 1376689_at    Name:   similar to RIKEN cDNA 1700023M03 (predicted)    GB acc:   BF282636    UniGene:   Rn.13471    Gene Symbol:   RGD1305457_predicted    Locus Link:   314730    Chromosome:   7    Cytoband:   Chr:7q13    SOURCE    GENECARD       Gene Ontology:     BioCarta Pathways:     KEGG Pathways:  
    Probe set : 1375346_at    Name:   similar to hypothetical protein FLJ20010    GB acc:   BI290002    UniGene:   Rn.12131    Gene Symbol:   LOC500993    Locus Link:   500993    Chromosome:   8    Cytoband:   Chr:8q23    SOURCE    GENECARD       Gene Ontology:     BioCarta Pathways:     KEGG Pathways:  
    Probe set : 1385395_at     GB acc:   AI549268              Gene Ontology:     BioCarta Pathways:     KEGG Pathways:  
    Probe set : 1378663_at    Name:   Transcribed locus    GB acc:   BF401737    UniGene:   Rn.146953        SOURCE        Gene Ontology:     BioCarta Pathways:     KEGG Pathways:  
    Probe set : 1369260_a_at    Name:   membrane protein, palmitoylated 4 (MAGUK p55 subfamily member 4)    GB acc:   NM_021265    UniGene:   Rn.98599    Gene Symbol:   Mpp4    Locus Link:   58808    Chromosome:   9    Cytoband:   Chr:9q31    SOURCE    GENECARD       Gene Ontology:   biological process:  cell adhesion   molecular function:  guanylate kinase activity   molecular function:  protein binding   molecular function:  protein binding      BioCarta Pathways:     KEGG Pathways:  
    Probe set : 1373674_at    Name:   microfibrillar associated protein 5 (predicted)    GB acc:   BI283094    UniGene:   Rn.25217    Gene Symbol:   Mfap5_predicted    Locus Link:   362429    Chromosome:   4    Cytoband:   Chr:4q42    SOURCE    GENECARD       Gene Ontology:   cellular component:  microfibril   cellular component:  extracellular matrix (sensu Metazoa)   cellular component:  extracellular space   molecular function:  extracellular matrix structural constituent   molecular function:  extracellular matrix constituent conferring elasticity      BioCarta Pathways:     KEGG Pathways:  
    Probe set : 1379804_at    Name:   PDZ protein Mrt1    GB acc:   AA900057    UniGene:   Rn.7581    Gene Symbol:   Snx27    Locus Link:   260323    Chromosome:   2    Cytoband:   Chr:2q34    SOURCE    GENECARD       Gene Ontology:   molecular function:  protein binding      BioCarta Pathways:     KEGG Pathways:  
    Probe set : 1392242_at    Name:   PHD finger protein 13 (predicted)    GB acc:   BF408410    UniGene:   Rn.13782    Gene Symbol:   Phf13_predicted    Locus Link:   313742    Chromosome:   5    Cytoband:   Chr:5q36    SOURCE    GENECARD       Gene Ontology:   biological process:  regulation of transcription, DNA-dependent   molecular function:  DNA binding      BioCarta Pathways:     KEGG Pathways:  
    Probe set : 1385157_at    Name:   chromobox homolog 1 (Drosophila HP1 beta) (predicted)    GB acc:   BF389675    UniGene:   Rn.32816    Gene Symbol:   Cbx1_predicted    Locus Link:   360609    Chromosome:   10    Cytoband:   Chr:10q31    SOURCE    GENECARD       Gene Ontology:   biological process:  chromatin assembly or disassembly   biological process:  regulation of transcription, DNA-dependent   biological process:  chromatin modification   molecular function:  chromatin binding   molecular function:  protein binding      BioCarta Pathways:     KEGG Pathways:  
    Probe set : 1380018_at    Name:   Transcribed locus    GB acc:   BF397708    UniGene:   Rn.146177        SOURCE        Gene Ontology:     BioCarta Pathways:     KEGG Pathways:  
    Probe set : 1376795_at    Name:   phosphoinositide-3-kinase adaptor protein 1 (predicted)    GB acc:   AW916092    UniGene:   Rn.15411    Gene Symbol:   Pik3ap1_predicted    Locus Link:   294048    Chromosome:   1    Cytoband:   Chr:1q54    SOURCE    GENECARD       Gene Ontology:   cellular component:  nucleus   cellular component:  cytoplasm   cellular component:  plasma membrane   molecular function:  transmembrane receptor protein tyrosine kinase signaling protein activity   molecular function:  protein binding   molecular function:  kinase activity      BioCarta Pathways:     KEGG Pathways:  
    Probe set : 1387303_at    Name:   solute carrier family 22 (organic cation transporter), member 2    GB acc:   NM_031584    UniGene:   Rn.10555    Gene Symbol:   Slc22a2    Locus Link:   29503    Chromosome:   1    Cytoband:   Chr:1q11    SOURCE    GENECARD       Gene Ontology:     BioCarta Pathways:     KEGG Pathways:  
    Probe set : 1371761_at    Name:   ribosomal protein L34 (predicted)    GB acc:   AI013910     Gene Symbol:   Rpl34_predicted    Locus Link:   362041    Chromosome:   2    Cytoband:   Chr:2q43     GENECARD       Gene Ontology:   biological process:  protein biosynthesis   cellular component:  intracellular   cellular component:  ribosome   cellular component:  cytosolic large ribosomal subunit (sensu Eukaryota)   molecular function:  RNA binding   molecular function:  structural constituent of ribosome      BioCarta Pathways:     KEGG Pathways:  
    Probe set : 1382739_at    Name:   G protein-coupled receptor 19    GB acc:   AI045718    UniGene:   Rn.99370    Gene Symbol:   Gpr19    Locus Link:   312787    Chromosome:   4    Cytoband:   Chr:4q43    SOURCE    GENECARD       Gene Ontology:   cellular component:  integral to plasma membrane   cellular component:  integral to plasma membrane   cellular component:  integral to membrane      BioCarta Pathways:     KEGG Pathways:  
    Probe set : 1388794_at    Name:   RNA binding motif protein, X chromosome retrogene (predicted)    GB acc:   BE107489    UniGene:   Rn.19966    Gene Symbol:   Rbmxrt_predicted    Locus Link:   307779    Chromosome:   19    Cytoband:   Chr:19q11    SOURCE    GENECARD       Gene Ontology:   biological process:  nuclear mRNA splicing, via spliceosome   biological process:  RNA processing   biological process:  mRNA processing   cellular component:  nucleus   cellular component:  ribonucleoprotein complex   molecular function:  RNA binding      BioCarta Pathways:     KEGG Pathways:  
    Probe set : 1376894_at    Name:   pallidin (predicted)    GB acc:   AA849956    UniGene:   Rn.139756    Gene Symbol:   Pldn_predicted    Locus Link:   317630    Chromosome:   3    Cytoband:   Chr:3q35    SOURCE    GENECARD       Gene Ontology:   cellular component:  endosome   cellular component:  membrane   molecular function:  protein binding   molecular function:  syntaxin-13 binding      BioCarta Pathways:     KEGG Pathways:  
    Probe set : 1370894_at    Name:   claudin 7    GB acc:   AJ011811    UniGene:   Rn.32259    Gene Symbol:   Cldn7    Locus Link:   65132    Chromosome:   10    Cytoband:   Chr:10q24    SOURCE    GENECARD       Gene Ontology:   biological process:  calcium-independent cell-cell adhesion   molecular function:  structural molecule activity   molecular function:  protein binding   molecular function:  protein self binding      BioCarta Pathways:     KEGG Pathways:  
    Probe set : 1372670_at    Name:   similar to hypothetical protein FLJ21827 (predicted)    GB acc:   BI281686    UniGene:   Rn.13688    Gene Symbol:   RGD1307682_predicted    Locus Link:   300675    Chromosome:   8    Cytoband:   Chr:8q22    SOURCE    GENECARD       Gene Ontology:     BioCarta Pathways:     KEGG Pathways:  
    Probe set : 1380217_at     GB acc:   BI288924              Gene Ontology:     BioCarta Pathways:     KEGG Pathways:  
    Probe set : 1394585_at    Name:   Similar to RIKEN cDNA G430041M01    GB acc:   BE116089    UniGene:   Rn.7020     Locus Link:   500116      SOURCE        Gene Ontology:     BioCarta Pathways:     KEGG Pathways:  
    Probe set : 1370247_a_at    Name:   peripheral myelin protein 22    GB acc:   AA943163    UniGene:   Rn.1476    Gene Symbol:   Pmp22    Locus Link:   24660    Chromosome:   10    Cytoband:   Chr:10q22    SOURCE    GENECARD       Gene Ontology:     BioCarta Pathways:     KEGG Pathways:  
    Probe set : 1395012_at    Name:   hect domain and RLD 4 (predicted)    GB acc:   BE106727    UniGene:   Rn.18143    Gene Symbol:   Herc4_predicted    Locus Link:   309758    Chromosome:   20    Cytoband:   Chr:20p11    SOURCE    GENECARD       Gene Ontology:   biological process:  ubiquitin cycle   biological process:  protein modification   cellular component:  intracellular   molecular function:  ubiquitin-protein ligase activity      BioCarta Pathways:     KEGG Pathways:  
    Probe set : 1387770_at    Name:   putative ISG12(a) protein    GB acc:   NM_130743     Gene Symbol:   RGD:1303168    Locus Link:   360415    Chromosome:   6    Cytoband:   Chr:6q32     GENECARD       Gene Ontology:     BioCarta Pathways:     KEGG Pathways:  
    Probe set : 1391077_at    Name:   Claspin homolog (Xenopus laevis) (predicted)    GB acc:   AA851257    UniGene:   Rn.6720     Locus Link:   298534    Chromosome:   5    Cytoband:   Chr:5q36    SOURCE        Gene Ontology:   biological process:  DNA replication checkpoint   cellular component:  nucleus   molecular function:  DNA secondary structure binding   molecular function:  protein binding      BioCarta Pathways:     KEGG Pathways:  
    Probe set : 1392048_at    Name:   Transcribed locus, strongly similar to XP_226953.3 PREDICTED: similar to glucocorticoid induced gene 1 [Rattus norvegicus]    GB acc:   BI288401    UniGene:   Rn.57374        SOURCE        Gene Ontology:     BioCarta Pathways:     KEGG Pathways:  
    Probe set : 1379374_at    Name:   plasticity related gene 1    GB acc:   AW526088    UniGene:   Rn.58355    Gene Symbol:   RGD:727806    Locus Link:   295401    Chromosome:   2    Cytoband:   Chr:2q41    SOURCE    GENECARD       Gene Ontology:   biological process:  axonogenesis   biological process:  phospholipid dephosphorylation   cellular component:  integral to plasma membrane   cellular component:  external side of plasma membrane   molecular function:  lipid phosphatase activity      BioCarta Pathways:     KEGG Pathways:  
    Probe set : 1372254_at    Name:   serine (or cysteine) peptidase inhibitor, clade G, member 1    GB acc:   AW915763    UniGene:   Rn.100285    Gene Symbol:   Serping1    Locus Link:   295703    Chromosome:   3    Cytoband:   Chr:3q24    SOURCE    GENECARD       Gene Ontology:   biological process:  negative regulation of complement activation, classical pathway   molecular function:  serine-type endopeptidase inhibitor activity   molecular function:  serine-type endopeptidase inhibitor activity      BioCarta Pathways:     KEGG Pathways:  
    Probe set : 1395084_at     GB acc:   AI179556              Gene Ontology:     BioCarta Pathways:     KEGG Pathways:  
    Probe set : 1375974_at    Name:   Similar to RNA-binding protein isoform G3BP-2a (predicted)    GB acc:   AI406315    UniGene:   Rn.22084     Locus Link:   305240    Chromosome:   14    Cytoband:   Chr:14p22    SOURCE        Gene Ontology:   cellular component:  intracellular   cellular component:  nucleus      BioCarta Pathways:     KEGG Pathways:  
    Probe set : 1375267_at    Name:   peptidylprolyl isomerase C    GB acc:   BI291292    UniGene:   Rn.144176    Gene Symbol:   RGD:1303221    Locus Link:   291463    Chromosome:   18    Cytoband:   Chr:18q11    SOURCE    GENECARD       Gene Ontology:   biological process:  protein folding   molecular function:  peptidyl-prolyl cis-trans isomerase activity   molecular function:  isomerase activity      BioCarta Pathways:     KEGG Pathways:  
    Probe set : 1368917_at    Name:   nudix (nucleoside diphosphate linked moiety X)-type motif 1    GB acc:   NM_057120    UniGene:   Rn.10669    Gene Symbol:   Nudt1    Locus Link:   117260    Chromosome:   12    Cytoband:   Chr:12q11    SOURCE    GENECARD       Gene Ontology:   biological process:  dGTP catabolism   biological process:  DNA repair   biological process:  response to oxidative stress   cellular component:  extracellular space      BioCarta Pathways:     KEGG Pathways:  
    Probe set : 1389477_at    Name:   Transcribed locus    GB acc:   BI278855    UniGene:   Rn.35620        SOURCE        Gene Ontology:     BioCarta Pathways:     KEGG Pathways:  
    Probe set : 1391148_at    Name:   Transcribed locus    GB acc:   AA957886    UniGene:   Rn.9395        SOURCE        Gene Ontology:     BioCarta Pathways:     KEGG Pathways:  
    Probe set : 1368178_at    Name:   PDZ domain containing 1    GB acc:   NM_031712    UniGene:   Rn.19842    Gene Symbol:   Pdzk1    Locus Link:   65144    Chromosome:   2    Cytoband:   Chr:2q34    SOURCE    GENECARD       Gene Ontology:   molecular function:  scavenger receptor binding   molecular function:  transporter activity   molecular function:  protein binding      BioCarta Pathways:     KEGG Pathways:  
    Probe set : 1371893_at    Name:   Transcribed locus    GB acc:   BE111640    UniGene:   Rn.7805        SOURCE        Gene Ontology:     BioCarta Pathways:     KEGG Pathways:  
    Probe set : 1376106_at    Name:   similar to hypothetical protein MGC33926    GB acc:   AI010157    UniGene:   Rn.17530    Gene Symbol:   RGD:1303057    Locus Link:   362691    Chromosome:   6    Cytoband:   Chr:6q11-q12    SOURCE    GENECARD       Gene Ontology:   cellular component:  tight junction   molecular function:  structural molecule activity      BioCarta Pathways:     KEGG Pathways:  
    Probe set : 1392334_at    Name:   RT1 class II, locus Ba    GB acc:   AW528743    UniGene:   Rn.25717    Gene Symbol:   RT1-Ba    Locus Link:   309621    Chromosome:   20    Cytoband:   Chr:20p12    SOURCE    GENECARD       Gene Ontology:   cellular component:  MHC class II protein complex   cellular component:  membrane   molecular function:  MHC class II receptor activity      BioCarta Pathways:     KEGG Pathways:  
    Probe set : 1389425_at    Name:   similar to 5(3)-deoxyribonucleotidase    GB acc:   AI408674    UniGene:   Rn.12084    Gene Symbol:   LOC501746    Locus Link:   501746    Chromosome:   10    Cytoband:   Chr:10q32.3    SOURCE    GENECARD       Gene Ontology:     BioCarta Pathways:     KEGG Pathways:  
    Probe set : 1369301_at    Name:   angiotensin receptor-like 1    GB acc:   NM_031349    UniGene:   Rn.7965    Gene Symbol:   Agtrl1    Locus Link:   83518    Chromosome:   3    Cytoband:   Chr:3q24    SOURCE    GENECARD       Gene Ontology:   biological process:  G-protein coupled receptor protein signaling pathway   biological process:  signal transduction   cellular component:  integral to plasma membrane   cellular component:  integral to membrane      BioCarta Pathways:     KEGG Pathways:  
    Probe set : 1388846_at     GB acc:   AI227978              Gene Ontology:     BioCarta Pathways:     KEGG Pathways:  
    Probe set : 1388625_at    Name:   similar to RIKEN cDNA 4921521J11 (predicted)    GB acc:   BM390149    UniGene:   Rn.2385    Gene Symbol:   RGD1304711_predicted    Locus Link:   290679    Chromosome:   16    Cytoband:   Chr:16p14    SOURCE    GENECARD       Gene Ontology:     BioCarta Pathways:     KEGG Pathways:  
    Probe set : 1387130_at    Name:   solute carrier family 39 (iron-regulated transporter), member 1    GB acc:   NM_133315    UniGene:   Rn.15324    Gene Symbol:   Slc40a1    Locus Link:   170840    Chromosome:   9    Cytoband:   Chr:9q22    SOURCE    GENECARD       Gene Ontology:   biological process:  transport   biological process:  iron ion transport   cellular component:  mitochondrial inner membrane   cellular component:  synaptic vesicle   cellular component:  integral to membrane   molecular function:  iron ion transporter activity   molecular function:  binding   molecular function:  iron ion binding      BioCarta Pathways:     KEGG Pathways:  
    Probe set : 1375985_at    Name:   RAB2B, member RAS oncogene family (predicted)    GB acc:   BF282984    UniGene:   Rn.85120    Gene Symbol:   Rab2b_predicted    Locus Link:   305853    Chromosome:   15    Cytoband:   Chr:15p14    SOURCE    GENECARD       Gene Ontology:   biological process:  transport   biological process:  small GTPase mediated signal transduction   biological process:  protein transport   cellular component:  endoplasmic reticulum   cellular component:  Golgi apparatus   molecular function:  GTP binding      BioCarta Pathways:     KEGG Pathways:  
    Probe set : 1373334_at    Name:   Similar to RIKEN cDNA 0610039G24 gene (predicted)    GB acc:   AI406609    UniGene:   Rn.137870     Locus Link:   305458    Chromosome:   14    Cytoband:   Chr:14q21    SOURCE        Gene Ontology:     BioCarta Pathways:     KEGG Pathways:  
    Probe set : 1380148_at    Name:   Transcribed locus    GB acc:   BE118688    UniGene:   Rn.146647        SOURCE        Gene Ontology:     BioCarta Pathways:     KEGG Pathways:  
    Probe set : 1390026_at    Name:   Bcl2-associated athanogene 3 (predicted)    GB acc:   AI231792    UniGene:   Rn.46304    Gene Symbol:   Bag3_predicted    Locus Link:   293524    Chromosome:   1    Cytoband:   Chr:1q36    SOURCE    GENECARD       Gene Ontology:   cellular component:  cytosol   molecular function:  protein binding   molecular function:  Hsp70/Hsc70 protein regulator activity   molecular function:  unfolded protein binding      BioCarta Pathways:     KEGG Pathways:  
    Probe set : 1382009_at    Name:   Transcribed locus    GB acc:   AA955721    UniGene:   Rn.146623        SOURCE        Gene Ontology:     BioCarta Pathways:     KEGG Pathways:  
    Probe set : 1389214_at    Name:   similar to Laminin alpha-4 chain precursor    GB acc:   BF406693    UniGene:   Rn.32891    Gene Symbol:   LOC309816    Locus Link:   309816    Chromosome:   20    Cytoband:   Chr:20q12    SOURCE    GENECARD       Gene Ontology:     BioCarta Pathways:     KEGG Pathways:  
    Probe set : 1383376_at    Name:   similar to Williams-Beuren syndrome critical region protein 18    GB acc:   AI717529    UniGene:   Rn.29127    Gene Symbol:   LOC368190    Locus Link:   368190    Chromosome:   12    Cytoband:   Chr:12q12    SOURCE    GENECARD       Gene Ontology:     BioCarta Pathways:     KEGG Pathways:  
    Probe set : 1392525_at    Name:   Transcribed locus    GB acc:   AA859437    UniGene:   Rn.147296        SOURCE        Gene Ontology:     BioCarta Pathways:     KEGG Pathways:  
    Probe set : 1373920_at    Name:   Similar to 106 kDa O-GlcNAc transferase-interacting protein (predicted)    GB acc:   AI175534    UniGene:   Rn.3090     Locus Link:   316085    Chromosome:   8    Cytoband:   Chr:8q32    SOURCE        Gene Ontology:     BioCarta Pathways:     KEGG Pathways:  
    Probe set : 1370960_at    Name:   insulin-like growth factor binding protein 5    GB acc:   BE104060    UniGene:   Rn.1593    Gene Symbol:   Igfbp5    Locus Link:   25285    Chromosome:   9    Cytoband:   Chr:9q33    SOURCE    GENECARD       Gene Ontology:   biological process:  regulation of cell growth   biological process:  signal transduction   cellular component:  extracellular region   cellular component:  extracellular space   cellular component:  insulin-like growth factor binding protein complex   molecular function:  insulin-like growth factor binding   molecular function:  growth factor binding      BioCarta Pathways:     KEGG Pathways:  
    Probe set : 1389378_at    Name:   CDC42 effector protein (Rho GTPase binding) 5 (predicted)    GB acc:   AI599324    UniGene:   Rn.145184    Gene Symbol:   Cdc42ep5_predicted    Locus Link:   361505    Chromosome:   1    Cytoband:   Chr:1q12    SOURCE    GENECARD       Gene Ontology:   biological process:  JNK cascade   biological process:  Rho protein signal transduction   cellular component:  cytoplasm   cellular component:  plasma membrane   molecular function:  protein binding      BioCarta Pathways:     KEGG Pathways:  
    Probe set : 1372404_at    Name:   RAS-related C3 botulinum substrate 2 (predicted)    GB acc:   AI010476    UniGene:   Rn.2863    Gene Symbol:   Rac2_predicted    Locus Link:   366957    Chromosome:   7    Cytoband:   Chr:7q34    SOURCE    GENECARD       Gene Ontology:   cellular component:  membrane fraction   cellular component:  nuclear membrane   cellular component:  cytoplasm   molecular function:  GTPase activity   molecular function:  GTP binding      BioCarta Pathways:     KEGG Pathways:  
    Probe set : 1373977_at    Name:   Kinesin family member 5C (predicted)    GB acc:   BE108253    UniGene:   Rn.50843     Locus Link:   311024    Chromosome:   3    Cytoband:   Chr:3q12    SOURCE        Gene Ontology:   cellular component:  kinesin complex   cellular component:  microtubule associated complex      BioCarta Pathways:     KEGG Pathways:  
    Probe set : 1383153_at    Name:   Transcribed locus    GB acc:   AI555284    UniGene:   Rn.23647        SOURCE        Gene Ontology:     BioCarta Pathways:     KEGG Pathways:  
    Probe set : 1371984_at    Name:   Similar to mKIAA0613 protein    GB acc:   AI598442    UniGene:   Rn.17340     Locus Link:   498587      SOURCE        Gene Ontology:   molecular function:  protein binding      BioCarta Pathways:     KEGG Pathways:  
    Probe set : 1375438_at    Name:   golgi SNAP receptor complex member 2    GB acc:   AA964687    UniGene:   Rn.13518    Gene Symbol:   Gosr2    Locus Link:   64154    Chromosome:   10    Cytoband:   Chr:10q32.1    SOURCE    GENECARD       Gene Ontology:     BioCarta Pathways:     KEGG Pathways:  
    Probe set : 1386916_at    Name:   aconitase 1    GB acc:   NM_017321    UniGene:   Rn.35934    Gene Symbol:   Aco1    Locus Link:   50655    Chromosome:   5    Cytoband:   Chr:5q22    SOURCE    GENECARD       Gene Ontology:   cellular component:  cytoplasm   cellular component:  cytoplasm      BioCarta Pathways:     KEGG Pathways:  
    Probe set : 1374910_at     GB acc:   BE103601              Gene Ontology:     BioCarta Pathways:     KEGG Pathways:  
    Probe set : 1379590_at    Name:   similar to U2 small nuclear ribonucleoprotein auxiliary factor 35 kDa subunit related-protein 1 (U2(RNU2) small nuclear RNA auxillary factor 1-like 1) (SP2)    GB acc:   AW527413    UniGene:   Rn.46376    Gene Symbol:   LOC498425    Locus Link:   498425    Chromosome:   14    Cytoband:   Chr:14q22    SOURCE    GENECARD       Gene Ontology:   biological process:  cell cycle   cellular component:  nucleus   cellular component:  spliceosome complex      BioCarta Pathways:     KEGG Pathways:  
    Probe set : 1390264_at    Name:   similar to RIKEN cDNA 1200014J11 (predicted)    GB acc:   BF413854    UniGene:   Rn.13279    Gene Symbol:   RGD1308139_predicted    Locus Link:   360563    Chromosome:   10    Cytoband:   Chr:10q24    SOURCE    GENECARD       Gene Ontology:     BioCarta Pathways:     KEGG Pathways:  
    Probe set : 1375224_at    Name:   pleckstrin homology-like domain, family A, member 3 (predicted)    GB acc:   AW520812    UniGene:   Rn.17905    Gene Symbol:   Phlda3_predicted    Locus Link:   363989    Chromosome:   13    Cytoband:   Chr:13q13    SOURCE    GENECARD       Gene Ontology:   biological process:  morphogenesis      BioCarta Pathways:     KEGG Pathways:  
    Probe set : 1389254_at    Name:   Similar to hypothetical protein FLJ21924 (predicted)    GB acc:   AA800519    UniGene:   Rn.3883     Locus Link:   311266    Chromosome:   3    Cytoband:   Chr:3q32    SOURCE        Gene Ontology:     BioCarta Pathways:     KEGG Pathways:  
    Probe set : 1370007_at    Name:   protein disulfide isomerase related protein (calcium-binding protein, intestinal-related)    GB acc:   NM_053849    UniGene:   Rn.39305    Gene Symbol:   Erp70    Locus Link:   116598    Chromosome:   4    Cytoband:   Chr:4q24    SOURCE    GENECARD       Gene Ontology:   biological process:  electron transport   biological process:  protein secretion   cellular component:  extracellular space   cellular component:  endoplasmic reticulum   cellular component:  endoplasmic reticulum lumen      BioCarta Pathways:     KEGG Pathways:  
    Probe set : 1383290_at    Name:   hepatocyte growth factor activator inhibitor 1    GB acc:   BI280343    UniGene:   Rn.44189    Gene Symbol:   RGD:1303138    Locus Link:   311331    Chromosome:   3    Cytoband:   Chr:3q35    SOURCE    GENECARD       Gene Ontology:   cellular component:  integral to membrane   molecular function:  serine-type endopeptidase inhibitor activity   molecular function:  serine-type endopeptidase inhibitor activity      BioCarta Pathways:     KEGG Pathways:  
    Probe set : 1396109_at    Name:   Transcribed locus    GB acc:   R47022    UniGene:   Rn.41192        SOURCE        Gene Ontology:     BioCarta Pathways:     KEGG Pathways:  
    Probe set : 1373287_at    Name:   similar to bHLH factor Math6    GB acc:   BE113298    UniGene:   Rn.18505    Gene Symbol:   LOC500200    Locus Link:   500200    Chromosome:   4    Cytoband:   Chr:4q33    SOURCE    GENECARD       Gene Ontology:     BioCarta Pathways:     KEGG Pathways:  
    Probe set : 1397864_at    Name:   Transcribed locus    GB acc:   BF412601    UniGene:   Rn.146428        SOURCE        Gene Ontology:     BioCarta Pathways:     KEGG Pathways:  
    Probe set : 1378016_at    Name:   Echinoderm microtubule associated protein like 4 (predicted)    GB acc:   BE104462    UniGene:   Rn.22926     Locus Link:   313861    Chromosome:   6    Cytoband:   Chr:6q12    SOURCE        Gene Ontology:     BioCarta Pathways:     KEGG Pathways:  
    Probe set : 1374293_at     GB acc:   AI137495              Gene Ontology:     BioCarta Pathways:     KEGG Pathways:  
    Probe set : 1372774_at    Name:   Coenzyme Q6 homolog (yeast) (predicted)    GB acc:   AI170570    UniGene:   Rn.9283     Locus Link:   299195    Chromosome:   6    Cytoband:   Chr:6q31    SOURCE        Gene Ontology:   cellular component:  extracellular space      BioCarta Pathways:     KEGG Pathways:  
    Probe set : 1395757_at    Name:   Transcribed locus    GB acc:   AW529878    UniGene:   Rn.61134        SOURCE        Gene Ontology:     BioCarta Pathways:     KEGG Pathways:  
    Probe set : 1374802_at    Name:   similar to chromosome 14 open reading frame 94 (predicted)    GB acc:   AI010721    UniGene:   Rn.17621    Gene Symbol:   RGD1305288_predicted    Locus Link:   305882    Chromosome:   15    Cytoband:   Chr:15p13    SOURCE    GENECARD       Gene Ontology:     BioCarta Pathways:     KEGG Pathways:  
    Probe set : 1390075_at    Name:   olfactomedin-like 2B (predicted)    GB acc:   AA955964    UniGene:   Rn.13880    Gene Symbol:   Olfml2b_predicted    Locus Link:   304960    Chromosome:   13    Cytoband:   Chr:13q24    SOURCE    GENECARD       Gene Ontology:   cellular component:  membrane   molecular function:  latrotoxin receptor activity      BioCarta Pathways:     KEGG Pathways:  
    Probe set : 1393613_at    Name:   Transcribed locus    GB acc:   BE117871    UniGene:   Rn.52915        SOURCE        Gene Ontology:     BioCarta Pathways:     KEGG Pathways:  
    Probe set : 1387795_at    Name:   polymerase (DNA directed), alpha 2    GB acc:   NM_053480    UniGene:   Rn.91557    Gene Symbol:   Pola2    Locus Link:   85242    Chromosome:   1    Cytoband:   Chr:1q43    SOURCE    GENECARD       Gene Ontology:   biological process:  DNA replication   biological process:  DNA replication   cellular component:  nucleus   cellular component:  alpha DNA polymerase:primase complex   cellular component:  alpha DNA polymerase:primase complex      BioCarta Pathways:     KEGG Pathways:  
    Probe set : 1367961_at    Name:   nerve growth factor, gamma    GB acc:   NM_031523    UniGene:   Rn.11331    Gene Symbol:   Ngfg    Locus Link:   24594    Chromosome:   1    Cytoband:   Chr:1q22    SOURCE    GENECARD       Gene Ontology:   biological process:  proteolysis      BioCarta Pathways:     KEGG Pathways:  
    Probe set : 1385291_a_at    Name:   Synaptonemal complex protein SC65    GB acc:   BI303187    UniGene:   Rn.90100    Gene Symbol:   Sc65    Locus Link:   59101      SOURCE    GENECARD       Gene Ontology:   biological process:  synaptonemal complex formation   cellular component:  synaptonemal complex   cellular component:  nucleus   cellular component:  nucleolus   molecular function:  binding      BioCarta Pathways:     KEGG Pathways:  
    Probe set : 1395828_at    Name:   kinesin family member 23 (predicted)    GB acc:   BF407793    UniGene:   Rn.63734    Gene Symbol:   Kif23_predicted    Locus Link:   315740    Chromosome:   8    Cytoband:   Chr:8q24    SOURCE    GENECARD       Gene Ontology:   molecular function:  motor activity   molecular function:  microtubule motor activity   molecular function:  ATP binding      BioCarta Pathways:     KEGG Pathways:  
    Probe set : 1385814_at    Name:   mitogen activated protein kinase kinase kinase 3 (predicted)    GB acc:   H32486    UniGene:   Rn.72680    Gene Symbol:   Map3k3_predicted    Locus Link:   303604    Chromosome:   10    Cytoband:   Chr:10q32.1    SOURCE    GENECARD       Gene Ontology:   biological process:  MAPKKK cascade   biological process:  protein amino acid phosphorylation   biological process:  positive regulation of I-kappaB kinase/NF-kappaB cascade      BioCarta Pathways:     KEGG Pathways:  
    Probe set : 1384192_at    Name:   carbohydrate (keratan sulfate Gal-6) sulfotransferase 1 (predicted)    GB acc:   AA819913    UniGene:   Rn.144848    Gene Symbol:   Chst1_predicted    Locus Link:   295934    Chromosome:   3    Cytoband:   Chr:3q24    SOURCE    GENECARD       Gene Ontology:   biological process:  polysaccharide metabolism   biological process:  carbohydrate metabolism   biological process:  inflammatory response   cellular component:  extracellular space   cellular component:  integral to membrane   cellular component:  Golgi stack   molecular function:  sulfotransferase activity   molecular function:  transferase activity   molecular function:  keratan sulfotransferase activity      BioCarta Pathways:     KEGG Pathways:  
    Probe set : 1397392_at     GB acc:   AW535120              Gene Ontology:     BioCarta Pathways:     KEGG Pathways:  
    Probe set : 1384773_at    Name:   similar to dendritic cell-derived ubiquitin-like protein (predicted)    GB acc:   AI044323    UniGene:   Rn.95607    Gene Symbol:   RGD1305506_predicted    Locus Link:   287178    Chromosome:   10    Cytoband:   Chr:10q12    SOURCE    GENECARD       Gene Ontology:     BioCarta Pathways:     KEGG Pathways:  
    Probe set : 1371865_at    Name:   similar to RIKEN cDNA 0610039A15 (predicted)    GB acc:   BI283821    UniGene:   Rn.2282    Gene Symbol:   RGD1311566_predicted    Locus Link:   312640    Chromosome:   4    Cytoband:   Chr:4q42    SOURCE    GENECARD       Gene Ontology:   cellular component:  integral to membrane      BioCarta Pathways:     KEGG Pathways:  
    Probe set : 1370826_at    Name:   nucleosome assembly protein 1-like 1    GB acc:   AF062594    UniGene:   Rn.18480    Gene Symbol:   Nap1l1    Locus Link:   89825    Chromosome:   7    Cytoband:   Chr:7q21    SOURCE    GENECARD       Gene Ontology:   biological process:  DNA replication   biological process:  nucleosome assembly   biological process:  positive regulation of cell proliferation   cellular component:  nucleus   cellular component:  chromatin assembly complex      BioCarta Pathways:     KEGG Pathways:  
    Probe set : 1376056_at    Name:   similar to poly (ADP-ribose) polymerase family, member 10    GB acc:   BF291214    UniGene:   Rn.137552    Gene Symbol:   LOC300043    Locus Link:   300043    Chromosome:   7    Cytoband:   Chr:7q34    SOURCE    GENECARD       Gene Ontology:     BioCarta Pathways:     KEGG Pathways:  
    Probe set : 1383548_at    Name:   Similar to hypothetical protein FLJ20189 (predicted)    GB acc:   BI290887    UniGene:   Rn.15268     Locus Link:   315434    Chromosome:   8    Cytoband:   Chr:8q11    SOURCE        Gene Ontology:     BioCarta Pathways:     KEGG Pathways:  
    Probe set : 1387005_at    Name:   cathepsin S    GB acc:   NM_017320    UniGene:   Rn.11347    Gene Symbol:   Ctss    Locus Link:   50654    Chromosome:   2    Cytoband:   Chr:2q34    SOURCE    GENECARD       Gene Ontology:   biological process:  proteolysis   cellular component:  extracellular space   cellular component:  lysosome   cellular component:  membrane      BioCarta Pathways:     KEGG Pathways:  
    Probe set : 1383126_at    Name:   V-akt murine thymoma viral oncogene homolog 1    GB acc:   AA998057    UniGene:   Rn.11422    Gene Symbol:   Akt1    Locus Link:   24185    Chromosome:   6    Cytoband:   Chr:6q32    SOURCE    GENECARD       Gene Ontology:   biological process:  regulation of progression through cell cycle   biological process:  protein amino acid phosphorylation   biological process:  nitric oxide biosynthesis   biological process:  apoptosis   biological process:  anti-apoptosis   biological process:  anti-apoptosis   biological process:  anti-apoptosis   biological process:  signal transduction   biological process:  signal transduction   biological process:  G-protein coupled receptor protein signaling pathway   biological process:  germ cell development   biological process:  apoptotic mitochondrial changes   biological process:  response to heat   biological process:  protein ubiquitination   biological process:  protein catabolism   biological process:  positive regulation of cell growth   biological process:  positive regulation of cell growth   biological process:  negative regulation of apoptosis   biological process:  cellular physiological process      BioCarta Pathways:     KEGG Pathways:  
    Probe set : 1374306_at    Name:   Transcribed locus    GB acc:   BF394933    UniGene:   Rn.25422        SOURCE        Gene Ontology:     BioCarta Pathways:     KEGG Pathways:  
    Probe set : 1383590_at    Name:   Similar to chondroitin beta1,4 N-acetylgalactosaminyltransferase (predicted)    GB acc:   AA963863    UniGene:   Rn.101848     Locus Link:   306375    Chromosome:   16    Cytoband:   Chr:16p14    SOURCE        Gene Ontology:     BioCarta Pathways:     KEGG Pathways:  
    Probe set : 1374545_at    Name:   ring finger and KH domain containing 2 (predicted)    GB acc:   AA945202    UniGene:   Rn.8138    Gene Symbol:   Rkhd2_predicted    Locus Link:   307271    Chromosome:   18    Cytoband:   Chr:18q12.2    SOURCE    GENECARD       Gene Ontology:   biological process:  protein ubiquitination   cellular component:  ubiquitin ligase complex   molecular function:  ubiquitin-protein ligase activity   molecular function:  zinc ion binding      BioCarta Pathways:     KEGG Pathways:  
    Probe set : 1381267_at    Name:   Transcribed locus    GB acc:   BI292529    UniGene:   Rn.28554        SOURCE        Gene Ontology:     BioCarta Pathways:     KEGG Pathways:  
    Probe set : 1389402_at    Name:   Transcribed locus    GB acc:   AI178746    UniGene:   Rn.8793        SOURCE        Gene Ontology:     BioCarta Pathways:     KEGG Pathways:  
    Probe set : 1372440_at    Name:   serine (or cysteine) proteinase inhibitor, clade E, member 2    GB acc:   BI275818    UniGene:   Rn.2271    Gene Symbol:   Serpine2    Locus Link:   29366    Chromosome:   9    Cytoband:   Chr:9q34    SOURCE    GENECARD       Gene Ontology:   cellular component:  extracellular region   cellular component:  extracellular space      BioCarta Pathways:     KEGG Pathways:  
    Probe set : 1375176_at    Name:   similar to Sperm 1 POU-domain transcription factor (SPRM-1) (predicted)    GB acc:   BF409908    UniGene:   Rn.109178    Gene Symbol:   RGD1305526_predicted    Locus Link:   294606    Chromosome:   2    Cytoband:   Chr:2q11    SOURCE    GENECARD       Gene Ontology:   cellular component:  nucleus   molecular function:  DNA binding   molecular function:  transcription factor activity      BioCarta Pathways:     KEGG Pathways:  
    Probe set : 1391825_at    Name:   Similar to part of a novel protein    GB acc:   BE120422    UniGene:   Rn.35202     Locus Link:   497910      SOURCE        Gene Ontology:     BioCarta Pathways:     KEGG Pathways:  
    Probe set : 1391733_at    Name:   Tetratricopeptide repeat domain 4 (predicted)    GB acc:   BE116541    UniGene:   Rn.2919     Locus Link:   362556    Chromosome:   5    Cytoband:   Chr:5q34    SOURCE        Gene Ontology:   molecular function:  binding      BioCarta Pathways:     KEGG Pathways:  
    Probe set : 1368501_s_at    Name:   mast cell protease 8 /// mast cell protease 10 /// mast cell protease 9 /// mast cell protease 8-like 2    GB acc:   NM_019323    UniGene:   Rn.10651    Gene Symbol:   Mcpt8 /// Mcpt10 /// Mcpt9 /// RGD:1302938    Locus Link:   29269 /// 408240 /// 54269 /// 54272    Chromosome:   15    Cytoband:   Chr:15p12 /// Chr:15p13    SOURCE    GENECARD       Gene Ontology:   biological process:  proteolysis   cellular component:  extracellular space      BioCarta Pathways:     KEGG Pathways:  
    Probe set : 1367866_at    Name:   fibulin 5    GB acc:   NM_019153    UniGene:   Rn.1699    Gene Symbol:   Fbln5    Locus Link:   29158    Chromosome:   6    Cytoband:   Chr:6q32    SOURCE    GENECARD       Gene Ontology:     BioCarta Pathways:     KEGG Pathways:  
    Probe set : 1368790_at    Name:   serine (or cysteine) proteinase inhibitor, clade A (alpha-1 antiproteinase, antitrypsin), member 10    GB acc:   NM_133617    UniGene:   Rn.10502    Gene Symbol:   Serpina10    Locus Link:   171154    Chromosome:   6    Cytoband:   Chr:6q32    SOURCE    GENECARD       Gene Ontology:   biological process:  transport   cellular component:  extracellular space   cellular component:  nucleus      BioCarta Pathways:     KEGG Pathways:  
    Probe set : 1383355_at    Name:   ATP-binding cassette, sub-family A (ABC1), member 1 /// hypothetical gene supported by NM_178095    GB acc:   AW918387    UniGene:   Rn.3724    Gene Symbol:   Abca1 /// LOC497803    Locus Link:   313210 /// 497803    Chromosome:   5    Cytoband:   Chr:5q24    SOURCE    GENECARD       Gene Ontology:     BioCarta Pathways:     KEGG Pathways:  
    Probe set : 1372004_at    Name:   heme binding protein 1 (predicted)    GB acc:   AI102065    UniGene:   Rn.6886    Gene Symbol:   Hebp1_predicted    Locus Link:   362454    Chromosome:   4    Cytoband:   Chr:4q43    SOURCE    GENECARD       Gene Ontology:   biological process:  circadian rhythm   biological process:  heme metabolism   cellular component:  cytoplasm   cellular component:  mitochondrion   cellular component:  cytosol   molecular function:  binding   molecular function:  heme binding      BioCarta Pathways:     KEGG Pathways:  
    Probe set : 1393352_at    Name:   similar to hypothetical protein MGC38960 (predicted)    GB acc:   BI293882    UniGene:   Rn.23973    Gene Symbol:   RGD1310552_predicted    Locus Link:   300836    Chromosome:   8    Cytoband:   Chr:8q24    SOURCE    GENECARD       Gene Ontology:     BioCarta Pathways:     KEGG Pathways:  
    Probe set : 1397772_at    Name:   Similar to putative membrane steroid receptor (predicted)    GB acc:   BF390195    UniGene:   Rn.17067     Locus Link:   316275    Chromosome:   9    Cytoband:   Chr:9q13    SOURCE        Gene Ontology:   biological process:  G-protein coupled receptor protein signaling pathway   cellular component:  integral to membrane   molecular function:  receptor activity   molecular function:  G-protein coupled receptor activity   molecular function:  steroid binding      BioCarta Pathways:     KEGG Pathways:  
    Probe set : 1382442_at    Name:   Similar to NF-kappa-B-repressing factor (NFKB-repressing factor) (Transcription factor NRF)    GB acc:   BF288000    UniGene:   Rn.48945     Locus Link:   298316    Chromosome:   X    Cytoband:   Chr:Xq12    SOURCE        Gene Ontology:     BioCarta Pathways:     KEGG Pathways:  
    Probe set : 1374830_at    Name:   similar to Coiled-coil domain containing protein 3 precursor    GB acc:   AA946242    UniGene:   Rn.30331    Gene Symbol:   LOC498795    Locus Link:   498795    Chromosome:   17    Cytoband:   Chr:17q12.3    SOURCE    GENECARD       Gene Ontology:     BioCarta Pathways:     KEGG Pathways:  
    Probe set : 1397628_at    Name:   similar to RIKEN cDNA 2900092E17 (predicted)    GB acc:   BM390965    UniGene:   Rn.38914    Gene Symbol:   RGD1305592_predicted    Locus Link:   293500    Chromosome:   1    Cytoband:   Chr:1q36    SOURCE    GENECARD       Gene Ontology:     BioCarta Pathways:     KEGG Pathways:  
    Probe set : 1368340_at    Name:   inositol polyphosphate multikinase    GB acc:   NM_134417    UniGene:   Rn.15187    Gene Symbol:   Impk    Locus Link:   171458    Chromosome:   20    Cytoband:   Chr:20p11    SOURCE    GENECARD       Gene Ontology:   biological process:  phosphoinositide phosphorylation   cellular component:  nucleus      BioCarta Pathways:     KEGG Pathways:  
    Probe set : 1367850_at    Name:   Fc gamma receptor II beta    GB acc:   NM_053843    UniGene:   Rn.131543    Gene Symbol:   LOC498276    Locus Link:   498276    Chromosome:   13    Cytoband:   Chr:13q24    SOURCE    GENECARD       Gene Ontology:     BioCarta Pathways:     KEGG Pathways:  
    Probe set : 1372770_at     GB acc:   BF281357              Gene Ontology:     BioCarta Pathways:     KEGG Pathways:  
    Probe set : 1372912_at    Name:   Mitogen-activated protein kinase kinase kinase 7 interacting protein 1 (predicted)    GB acc:   AA851280    UniGene:   Rn.17231     Locus Link:   315139    Chromosome:   7    Cytoband:   Chr:7q34    SOURCE        Gene Ontology:   biological process:  activation of MAPKKK activity      BioCarta Pathways:     KEGG Pathways:  
    Probe set : 1382308_at    Name:   Transcribed locus    GB acc:   AI717668    UniGene:   Rn.5854        SOURCE        Gene Ontology:     BioCarta Pathways:     KEGG Pathways:  
    Probe set : 1377153_a_at    Name:   kelch-like 6 (Drosophila) (predicted)    GB acc:   AI178384    UniGene:   Rn.137232    Gene Symbol:   Klhl6_predicted    Locus Link:   287974    Chromosome:   11    Cytoband:   Chr:11q23    SOURCE    GENECARD       Gene Ontology:   molecular function:  protein binding      BioCarta Pathways:     KEGG Pathways:  
    Probe set : 1395342_at    Name:   cytoskeleton-associated protein 4 (predicted)    GB acc:   AI598942    UniGene:   Rn.99508    Gene Symbol:   Ckap4_predicted    Locus Link:   362859    Chromosome:   7    Cytoband:   Chr:7q13    SOURCE    GENECARD       Gene Ontology:   molecular function:  protein binding      BioCarta Pathways:     KEGG Pathways:  
    Probe set : 1385718_at    Name:   similar to RIKEN cDNA 2510048L02 (predicted)    GB acc:   AW530248    UniGene:   Rn.47411    Gene Symbol:   RGD1308031_predicted    Locus Link:   300442    Chromosome:   8    Cytoband:   Chr:8q13    SOURCE    GENECARD       Gene Ontology:   biological process:  rRNA processing   biological process:  RNA processing   cellular component:  exosome (RNase complex)   cellular component:  nucleus      BioCarta Pathways:     KEGG Pathways:  
    Probe set : 1374368_at    Name:   CDNA clone IMAGE:7389555    GB acc:   BI291066    UniGene:   Rn.25222        SOURCE        Gene Ontology:     BioCarta Pathways:     KEGG Pathways:  
    Probe set : 1387788_at    Name:   Jun-B oncogene    GB acc:   NM_021836    UniGene:   Rn.15806    Gene Symbol:   Junb    Locus Link:   24517    Chromosome:   19    Cytoband:   Chr:19q11    SOURCE    GENECARD       Gene Ontology:   cellular component:  chromatin   cellular component:  intracellular   cellular component:  nucleus      BioCarta Pathways:     KEGG Pathways:  
    Probe set : 1376835_at    Name:   Solute carrier family 35, member B2    GB acc:   BI293600    UniGene:   Rn.129334    Gene Symbol:   Slc35b2    Locus Link:   316241    Chromosome:   9    Cytoband:   Chr:9q12    SOURCE    GENECARD       Gene Ontology:   biological process:  nucleotide-sugar transport   molecular function:  nucleotide-sugar transporter activity      BioCarta Pathways:     KEGG Pathways:  
    Probe set : 1380583_s_at    Name:   colony stimulating factor 1 (macrophage)    GB acc:   AW535553    UniGene:   Rn.83632    Gene Symbol:   Csf1    Locus Link:   78965    Chromosome:   2    Cytoband:   Chr:2q34    SOURCE    GENECARD       Gene Ontology:   biological process:  ossification   biological process:  ossification   biological process:  development   biological process:  development   biological process:  cell proliferation   biological process:  positive regulation of cell proliferation   biological process:  hemopoiesis   biological process:  cell differentiation   biological process:  macrophage differentiation   biological process:  macrophage differentiation   biological process:  regulation of ossification   biological process:  osteoclast differentiation   biological process:  osteoclast differentiation   biological process:  osteoclast differentiation   biological process:  positive regulation of body size   biological process:  odontogenesis   biological process:  odontogenesis   biological process:  positive regulation of odontogenesis (sensu Vertebrata)   biological process:  regulation of ossification   biological process:  positive regulation of body size   biological process:  positive regulation of odontogenesis (sensu Vertebrata)   biological process:  positive regulation of osteoclast differentiation   molecular function:  cytokine activity   molecular function:  macrophage colony stimulating factor receptor binding   molecular function:  growth factor activity      BioCarta Pathways:     KEGG Pathways:  
    Probe set : 1376955_at    Name:   procollagen, type IV, alpha 4 (predicted)    GB acc:   AI412936    UniGene:   Rn.22075    Gene Symbol:   Col4a4_predicted    Locus Link:   301562    Chromosome:   9    Cytoband:   Chr:9q34    SOURCE    GENECARD       Gene Ontology:   biological process:  phosphate transport   biological process:  cell adhesion   biological process:  long-term strengthening of neuromuscular junction      BioCarta Pathways:     KEGG Pathways:  
    Probe set : 1398599_at    Name:   similar to integral membrane protein 1    GB acc:   BG664929    UniGene:   Rn.120361    Gene Symbol:   LOC500972    Locus Link:   500972    Chromosome:   8    Cytoband:   Chr:8q21    SOURCE    GENECARD       Gene Ontology:     BioCarta Pathways:     KEGG Pathways:  
    Probe set : 1385292_at    Name:   Synaptonemal complex protein SC65    GB acc:   BI303187    UniGene:   Rn.90100    Gene Symbol:   Sc65    Locus Link:   59101      SOURCE    GENECARD       Gene Ontology:   biological process:  synaptonemal complex formation   cellular component:  synaptonemal complex   cellular component:  nucleus   cellular component:  nucleolus   molecular function:  binding      BioCarta Pathways:     KEGG Pathways:  
    Probe set : 1387227_at    Name:   Wiskott-Aldrich syndrome protein interacting protein    GB acc:   NM_057192    UniGene:   Rn.7914    Gene Symbol:   Waspip    Locus Link:   117538    Chromosome:   3    Cytoband:   Chr:3q23    SOURCE    GENECARD       Gene Ontology:   cellular component:  actin filament   cellular component:  actin cytoskeleton   molecular function:  actin binding   molecular function:  profilin binding      BioCarta Pathways:     KEGG Pathways:  
    Probe set : 1379740_at    Name:   Similar to chromosome 18 open reading frame 54    GB acc:   AW521797    UniGene:   Rn.31250     Locus Link:   361346    Chromosome:   18    Cytoband:   Chr:18q12.1    SOURCE        Gene Ontology:     BioCarta Pathways:     KEGG Pathways:  
    Probe set : 1386096_at    Name:   similar to Metal-response element-binding transcription factor 2 (Metal-response element DNA-binding protein M96) (predicted)    GB acc:   BG666758    UniGene:   Rn.20300    Gene Symbol:   RGD1304727_predicted    Locus Link:   360905    Chromosome:   14    Cytoband:   Chr:14p22    SOURCE    GENECARD       Gene Ontology:   biological process:  regulation of transcription, DNA-dependent   cellular component:  nucleus   cellular component:  transcription factor complex      BioCarta Pathways:     KEGG Pathways:  
    Probe set : 1372430_at    Name:   similar to RIKEN cDNA 1700037H04 (predicted)    GB acc:   H31887    UniGene:   Rn.144317    Gene Symbol:   RGD1311739_predicted    Locus Link:   311428    Chromosome:   3    Cytoband:   Chr:3q36    SOURCE    GENECARD       Gene Ontology:   biological process:  protein biosynthesis   biological process:  aspartyl-tRNA aminoacylation   cellular component:  cytoplasm   molecular function:  aspartate-tRNA ligase activity   molecular function:  ATP binding   molecular function:  ligase activity      BioCarta Pathways:     KEGG Pathways:  
    Probe set : 1370131_at    Name:   caveolin    GB acc:   NM_031556    UniGene:   Rn.22518    Gene Symbol:   Cav    Locus Link:   25404    Chromosome:   4    Cytoband:   Chr:4q21    SOURCE    GENECARD       Gene Ontology:   biological process:  inactivation of MAPK activity   biological process:  negative regulation of endothelial cell proliferation   biological process:  triacylglycerol metabolism   biological process:  endocytosis   biological process:  negative regulation of cell proliferation   biological process:  negative regulation of signal transduction   biological process:  regulation of fatty acid metabolism   biological process:  sequestering of lipid   biological process:  negative regulation of nitric oxide biosynthesis   biological process:  positive regulation of vasoconstriction   biological process:  negative regulation of vasodilation   biological process:  protein oligomerization   biological process:  protein homooligomerization   molecular function:  structural molecule activity   molecular function:  protein binding      BioCarta Pathways:     KEGG Pathways:  
    Probe set : 1391676_at    Name:   Transcribed locus    GB acc:   AI511097    UniGene:   Rn.148006        SOURCE        Gene Ontology:     BioCarta Pathways:     KEGG Pathways:  
    Probe set : 1382431_at    Name:   Hypothetical gene supported by NM_178095    GB acc:   AI103530    UniGene:   Rn.3724    Gene Symbol:   Abca1    Locus Link:   313210    Chromosome:   5    Cytoband:   Chr:5q24    SOURCE    GENECARD       Gene Ontology:     BioCarta Pathways:     KEGG Pathways:  
    Probe set : 1377949_s_at     GB acc:   AA899043              Gene Ontology:     BioCarta Pathways:     KEGG Pathways:  
    Probe set : 1371189_x_at    Name:   laminin receptor 1 (67kD, ribosomal protein SA) /// similar to 40S ribosomal protein SA (p40) (34/67 kDa laminin receptor) /// similar to Ribosomal protein SA    GB acc:   BG153272    UniGene:   Rn.999    Gene Symbol:   Lamr1 /// LOC367030 /// LOC367303    Locus Link:   29236 /// 367030 /// 367303    Chromosome:   8    Cytoband:   Chr:8q32 /// Chr:8q13 /// Chr:9q34    SOURCE    GENECARD       Gene Ontology:   biological process:  protein biosynthesis      BioCarta Pathways:     KEGG Pathways:  
    Probe set : 1392863_at    Name:   Similar to fibronectin leucine rich transmembrane protein 3    GB acc:   AA817953    UniGene:   Rn.95324     Locus Link:   366205    Chromosome:   3    Cytoband:   Chr:3q41    SOURCE        Gene Ontology:     BioCarta Pathways:     KEGG Pathways:  
    Probe set : 1393972_at    Name:   Transcribed locus    GB acc:   BE096871    UniGene:   Rn.146677        SOURCE        Gene Ontology:     BioCarta Pathways:     KEGG Pathways:  
    Probe set : 1371862_at    Name:   ribonucleotide reductase M1    GB acc:   AI412015    UniGene:   Rn.145914    Gene Symbol:   Rrm1    Locus Link:   365320    Chromosome:   1    Cytoband:   Chr:1q32    SOURCE    GENECARD       Gene Ontology:   biological process:  DNA replication   cellular component:  ribonucleoside-diphosphate reductase complex      BioCarta Pathways:     KEGG Pathways:  
    Probe set : 1371431_at    Name:   Transcribed locus, strongly similar to XP_232343.3 PREDICTED: similar to Pex5 protein [Rattus norvegicus]    GB acc:   BI296577    UniGene:   Rn.14316        SOURCE        Gene Ontology:     BioCarta Pathways:     KEGG Pathways:  
    Probe set : 1370705_at    Name:   zinc finger protein HIT-4    GB acc:   AF277900    UniGene:   Rn.64641    Gene Symbol:   RGD:628674    Locus Link:   266774    Chromosome:   10    Cytoband:   Chr:10q12    SOURCE    GENECARD       Gene Ontology:   biological process:  regulation of transcription, DNA-dependent   cellular component:  nucleus   cellular component:  intracellular   molecular function:  nucleic acid binding   molecular function:  zinc ion binding   molecular function:  metal ion binding      BioCarta Pathways:     KEGG Pathways:  
    Probe set : 1397282_at    Name:   Transcribed locus    GB acc:   AW531746    UniGene:   Rn.148424        SOURCE        Gene Ontology:     BioCarta Pathways:     KEGG Pathways:  
    Probe set : 1371313_at    Name:   similar to 60S ribosomal protein L23a    GB acc:   AI172199     Gene Symbol:   LOC501709    Locus Link:   501709    Chromosome:   10    Cytoband:   Chr:10q25     GENECARD       Gene Ontology:     BioCarta Pathways:     KEGG Pathways:  
    Probe set : 1372722_at    Name:   Transcribed locus    GB acc:   AI175031    UniGene:   Rn.13387        SOURCE        Gene Ontology:     BioCarta Pathways:     KEGG Pathways:  
    Probe set : 1374871_at    Name:   asparaginase-like sperm autoantigen    GB acc:   AI233343    UniGene:   Rn.22774    Gene Symbol:   Asrgl1    Locus Link:   246307    Chromosome:   1    Cytoband:   Chr:1q43    SOURCE    GENECARD       Gene Ontology:   biological process:  glycoprotein catabolism   cellular component:  cytoplasm   molecular function:  N4-(beta-N-acetylglucosaminyl)-L-asparaginase activity   molecular function:  asparaginase activity      BioCarta Pathways:     KEGG Pathways:  
    Probe set : 1386252_at    Name:   Transcribed locus    GB acc:   BE349825    UniGene:   Rn.54041        SOURCE        Gene Ontology:     BioCarta Pathways:     KEGG Pathways:  
    Probe set : 1370400_at    Name:   RT1 class Ib gene, H2-TL-like, grc region (N1) /// RT1 class Ib gene, H2-TL-like, grc region (N3) /// RT1 class Ib gene, H2-TL-like, grc region(N2)    GB acc:   L23128    UniGene:   Rn.107070    Gene Symbol:   RT1-N1 /// RT1-N3 /// RT1-N2    Locus Link:   24748 /// 24750 /// 360323    Chromosome:   20    Cytoband:   Chr:20p12    SOURCE    GENECARD       Gene Ontology:   biological process:  antigen presentation   cellular component:  MHC class I protein complex   cellular component:  MHC class I protein complex   molecular function:  MHC class I receptor activity      BioCarta Pathways:     KEGG Pathways:  
    Probe set : 1393732_at    Name:   Transcribed locus    GB acc:   AI071024    UniGene:   Rn.149688        SOURCE        Gene Ontology:     BioCarta Pathways:     KEGG Pathways:  
    Probe set : 1392847_at    Name:   Casitas B-lineage lymphoma-like 1 (predicted)    GB acc:   BE118227    UniGene:   Rn.52970    Gene Symbol:   Cbll1_predicted    Locus Link:   314028    Chromosome:   6    Cytoband:   Chr:6q16    SOURCE    GENECARD       Gene Ontology:   cellular component:  ubiquitin ligase complex   molecular function:  ubiquitin-protein ligase activity   molecular function:  protein binding   molecular function:  zinc ion binding      BioCarta Pathways:     KEGG Pathways:  
    Probe set : 1382601_at    Name:   Similar to scavenger receptor type A SR-A    GB acc:   AI237698    UniGene:   Rn.25444     Locus Link:   498638      SOURCE        Gene Ontology:     BioCarta Pathways:     KEGG Pathways:  
    Probe set : 1382158_at    Name:   Transcribed locus    GB acc:   BF557889    UniGene:   Rn.12986        SOURCE        Gene Ontology:     BioCarta Pathways:     KEGG Pathways:  
    Probe set : 1391858_at    Name:   splicing factor, arginine/serine-rich 8 (suppressor-of-white-apricot homolog, Drosophila) (predicted)    GB acc:   BM385930    UniGene:   Rn.12338    Gene Symbol:   Sfrs8_predicted    Locus Link:   304431    Chromosome:   12    Cytoband:   Chr:12q13    SOURCE    GENECARD       Gene Ontology:   cellular component:  nucleus   cellular component:  spliceosome complex   molecular function:  RNA binding      BioCarta Pathways:     KEGG Pathways:  
    Probe set : 1398472_at    Name:   progressive external ophthalmoplegia 1 (predicted)    GB acc:   AA925995    UniGene:   Rn.105678    Gene Symbol:   Peo1_predicted    Locus Link:   309441    Chromosome:   1    Cytoband:   Chr:1q54    SOURCE    GENECARD       Gene Ontology:   biological process:  DNA unwinding during replication   cellular component:  mitochondrion   molecular function:  DNA helicase activity   molecular function:  helicase activity      BioCarta Pathways:     KEGG Pathways:  
    Probe set : 1384383_at    Name:   similar to Putative lysophosphatidic acid acyltransferase (predicted)    GB acc:   BF557230    UniGene:   Rn.6835    Gene Symbol:   RGD1310520_predicted    Locus Link:   290843    Chromosome:   16    Cytoband:   Chr:16q12.5    SOURCE    GENECARD       Gene Ontology:   biological process:  metabolism   biological process:  phospholipid biosynthesis   cellular component:  integral to membrane   molecular function:  acyltransferase activity   molecular function:  transferase activity      BioCarta Pathways:     KEGG Pathways:  
    Probe set : 1376451_at    Name:   similar to krev interaction trapped-1A    GB acc:   BG377849    UniGene:   Rn.118242    Gene Symbol:   LOC362317    Locus Link:   362317    Chromosome:   4    Cytoband:   Chr:4q13    SOURCE    GENECARD       Gene Ontology:     BioCarta Pathways:     KEGG Pathways:  
    Probe set : 1389197_at    Name:   similar to RIKEN cDNA 9630046K23 (predicted)    GB acc:   BI290535    UniGene:   Rn.7051    Gene Symbol:   RGD1306248_predicted    Locus Link:   288091    Chromosome:   11    Cytoband:   Chr:11q21    SOURCE    GENECARD       Gene Ontology:   cellular component:  extracellular space      BioCarta Pathways:     KEGG Pathways:  
    Probe set : 1374134_at    Name:   PCTAIRE-motif protein kinase 2    GB acc:   BM384057    UniGene:   Rn.94638     Locus Link:   314743    Chromosome:   7    Cytoband:   Chr:7q13    SOURCE        Gene Ontology:     BioCarta Pathways:     KEGG Pathways:  
    Probe set : 1379431_at    Name:   Transcribed locus    GB acc:   AW918368    UniGene:   Rn.8749        SOURCE        Gene Ontology:     BioCarta Pathways:     KEGG Pathways:  
    Probe set : 1390807_at    Name:   carbonic anhydrase VB, mitochondrial    GB acc:   BG375352    UniGene:   Rn.22441    Gene Symbol:   Ca5b    Locus Link:   302669    Chromosome:   X    Cytoband:   Chr:Xq21    SOURCE    GENECARD       Gene Ontology:   biological process:  one-carbon compound metabolism   cellular component:  mitochondrion      BioCarta Pathways:     KEGG Pathways:  
    Probe set : 1368575_at    Name:   solute carrier family 6 (neurotransmitter transporter), member 18    GB acc:   NM_017163    UniGene:   Rn.9998    Gene Symbol:   Slc6a18    Locus Link:   29323    Chromosome:   1    Cytoband:   Chr:1p11    SOURCE    GENECARD       Gene Ontology:     BioCarta Pathways:     KEGG Pathways:  
    Probe set : 1387131_at    Name:   serine (or cysteine) peptidase inhibitor, clade I, member 1    GB acc:   AF193015    UniGene:   Rn.52632    Gene Symbol:   Serpini1    Locus Link:   116459    Chromosome:   2    Cytoband:   Chr:2q32    SOURCE    GENECARD       Gene Ontology:   biological process:  central nervous system development   biological process:  peripheral nervous system development   cellular component:  extracellular space   molecular function:  serine-type endopeptidase inhibitor activity   molecular function:  endopeptidase inhibitor activity      BioCarta Pathways:     KEGG Pathways:  
    Probe set : 1391537_at    Name:   similar to SERTA domain containing 4    GB acc:   AW251654    UniGene:   Rn.101908    Gene Symbol:   LOC360899    Locus Link:   360899    Chromosome:   13    Cytoband:   Chr:13q27    SOURCE    GENECARD       Gene Ontology:     BioCarta Pathways:     KEGG Pathways:  
    Probe set : 1372052_at    Name:   Budding uninhibited by benzimidazoles 3 homolog (S. cerevisiae) (predicted)    GB acc:   BI275918    UniGene:   Rn.6897     Locus Link:   361662    Chromosome:   1    Cytoband:   Chr:1q41    SOURCE        Gene Ontology:   biological process:  mitosis   biological process:  mitotic spindle checkpoint   biological process:  cell proliferation   cellular component:  kinetochore   cellular component:  nucleus      BioCarta Pathways:     KEGG Pathways:  
    Probe set : 1389366_at    Name:   similar to Zinc finger protein 553    GB acc:   BI293689    UniGene:   Rn.12466    Gene Symbol:   LOC308991    Locus Link:   308991    Chromosome:   1    Cytoband:   Chr:1q36    SOURCE    GENECARD       Gene Ontology:     BioCarta Pathways:     KEGG Pathways:  
    Probe set : 1367771_at    Name:   delta sleep inducing peptide, immunoreactor    GB acc:   NM_031345    UniGene:   Rn.21970    Gene Symbol:   Dsipi    Locus Link:   83514    Chromosome:   X    Cytoband:   Chr:Xq35    SOURCE    GENECARD       Gene Ontology:   cellular component:  nucleus   molecular function:  transcription factor activity      BioCarta Pathways:     KEGG Pathways:  
    Probe set : 1387884_at    Name:   proteasome (prosome, macropain) subunit, alpha type 5    GB acc:   AI011191    UniGene:   Rn.1276    Gene Symbol:   Psma5    Locus Link:   29672    Chromosome:   2    Cytoband:   Chr:2q34    SOURCE    GENECARD       Gene Ontology:   biological process:  ubiquitin-dependent protein catabolism   cellular component:  cytosol   cellular component:  proteasome core complex (sensu Eukaryota)   cellular component:  protein complex      BioCarta Pathways:     KEGG Pathways:  
    Probe set : 1367663_at    Name:   protease (prosome, macropain) 28 subunit, alpha    GB acc:   NM_017264    UniGene:   Rn.144575    Gene Symbol:   Psme1    Locus Link:   29630    Chromosome:   15    Cytoband:   Chr:15p13    SOURCE    GENECARD       Gene Ontology:   biological process:  immune response   cellular component:  proteasome complex (sensu Eukaryota)   cellular component:  cytosol   cellular component:  proteasome activator complex   molecular function:  proteasome activator activity   molecular function:  proteasome activator activity   molecular function:  peptidase activity      BioCarta Pathways:     KEGG Pathways:  
    Probe set : 1368379_at    Name:   CD36 antigen (collagen type I receptor, thrombospondin receptor)-like 2    GB acc:   NM_054001    UniGene:   Rn.3957    Gene Symbol:   Scarb2    Locus Link:   117106    Chromosome:   14    Cytoband:   Chr:14p22    SOURCE    GENECARD       Gene Ontology:   biological process:  cell adhesion   biological process:  cell adhesion   molecular function:  receptor activity      BioCarta Pathways:     KEGG Pathways:  
    Probe set : 1388244_s_at    Name:   laminin receptor 1 (67kD, ribosomal protein SA)    GB acc:   BG153272    UniGene:   Rn.999    Gene Symbol:   Lamr1    Locus Link:   29236    Chromosome:   8    Cytoband:   Chr:8q32    SOURCE    GENECARD       Gene Ontology:   biological process:  protein biosynthesis      BioCarta Pathways:     KEGG Pathways:  
    Probe set : 1381481_at    Name:   Similar to regulator of sex-limitation candidate 16    GB acc:   AI028912    UniGene:   Rn.33755     Locus Link:   498682      SOURCE        Gene Ontology:     BioCarta Pathways:     KEGG Pathways:  
    Probe set : 1374207_at    Name:   angiopoietin 2    GB acc:   BI275292    UniGene:   Rn.138360    Gene Symbol:   Agpt2    Locus Link:   89805    Chromosome:   16    Cytoband:   Chr:16q12.5    SOURCE    GENECARD       Gene Ontology:   cellular component:  extracellular space   molecular function:  receptor binding   molecular function:  vascular endothelial growth factor receptor binding      BioCarta Pathways:     KEGG Pathways:  
    Probe set : 1372015_at    Name:   transforming, acidic coiled-coil containing protein 1a    GB acc:   AI008689    UniGene:   Rn.73645    Gene Symbol:   RGD:1303120    Locus Link:   306562    Chromosome:   16    Cytoband:   Chr:16q12.4    SOURCE    GENECARD       Gene Ontology:   biological process:  M phase of mitotic cell cycle      BioCarta Pathways:     KEGG Pathways:  
    Probe set : 1388527_at    Name:   Transcribed locus    GB acc:   BI283685    UniGene:   Rn.41192        SOURCE        Gene Ontology:     BioCarta Pathways:     KEGG Pathways:  
    Probe set : 1389664_at    Name:   similar to hypothetical protein (predicted)    GB acc:   BM387379    UniGene:   Rn.49608    Gene Symbol:   RGD1305937_predicted    Locus Link:   315988    Chromosome:   8    Cytoband:   Chr:8q32    SOURCE    GENECARD       Gene Ontology:     BioCarta Pathways:     KEGG Pathways:  
    Probe set : 1370020_at    Name:   solute carrier family 25 (mitochondrial carrier; dicarboxylate transporter), member 10    GB acc:   NM_133418    UniGene:   Rn.3631    Gene Symbol:   RGD:621430    Locus Link:   170943    Chromosome:   10    Cytoband:   Chr:10q32.3    SOURCE    GENECARD       Gene Ontology:   biological process:  transport   biological process:  mitochondrial transport   molecular function:  binding   molecular function:  electrochemical potential-driven transporter activity      BioCarta Pathways:     KEGG Pathways:  
    Probe set : 1389744_at    Name:   LOC499898    GB acc:   AW527194    UniGene:   Rn.43161    Gene Symbol:   LOC499898    Locus Link:   499898    Chromosome:   3    Cytoband:   Chr:3q41    SOURCE    GENECARD       Gene Ontology:     BioCarta Pathways:     KEGG Pathways:  
    Probe set : 1387434_at    Name:   solute carrier family 22 (organic cation transporter), member 4    GB acc:   NM_022270    UniGene:   Rn.54434    Gene Symbol:   Slc22a4    Locus Link:   64037      SOURCE    GENECARD       Gene Ontology:     BioCarta Pathways:     KEGG Pathways:  
    Probe set : 1392927_at    Name:   progestin and adipoQ receptor family member IV (predicted)    GB acc:   BG381724    UniGene:   Rn.11767    Gene Symbol:   Paqr4_predicted    Locus Link:   302967    Chromosome:   10    Cytoband:   Chr:10q12    SOURCE    GENECARD       Gene Ontology:   cellular component:  integral to membrane   molecular function:  receptor activity      BioCarta Pathways:     KEGG Pathways:  
    Probe set : 1392806_at    Name:   Wolf-Hirschhorn syndrome candidate 1-like 1 (predicted)    GB acc:   AI234807    UniGene:   Rn.19432     Locus Link:   290831    Chromosome:   16    Cytoband:   Chr:16q12.4    SOURCE        Gene Ontology:   cellular component:  ubiquitin ligase complex   cellular component:  nucleus      BioCarta Pathways:     KEGG Pathways:  
    Probe set : 1398361_at    Name:   similar to Rho-GTPase-activating protein PS-GAP-a    GB acc:   BI295127    UniGene:   Rn.40465    Gene Symbol:   LOC498938    Locus Link:   498938    Chromosome:   19    Cytoband:   Chr:19q11    SOURCE    GENECARD       Gene Ontology:     BioCarta Pathways:     KEGG Pathways:  
    Probe set : 1368736_at    Name:   testis specific X-linked gene    GB acc:   NM_019203    UniGene:   Rn.87406    Gene Symbol:   Tsx    Locus Link:   29391    Chromosome:   X    Cytoband:   Chr:Xq31    SOURCE    GENECARD       Gene Ontology:   biological process:  dosage compensation, by inactivation of X chromosome      BioCarta Pathways:     KEGG Pathways:  
    Probe set : 1368943_at    Name:   ribonuclease, RNase A family 4    GB acc:   NM_020082    UniGene:   Rn.1742    Gene Symbol:   Rnase4    Locus Link:   56759    Chromosome:   15    Cytoband:   Chr:15p14    SOURCE    GENECARD       Gene Ontology:   biological process:  mRNA cleavage      BioCarta Pathways:     KEGG Pathways:  
    Probe set : 1385116_at     GB acc:   BF386807              Gene Ontology:     BioCarta Pathways:     KEGG Pathways:  
    Probe set : 1372161_at    Name:   Transcribed locus    GB acc:   BE112719    UniGene:   Rn.3167        SOURCE        Gene Ontology:     BioCarta Pathways:     KEGG Pathways:  
    Probe set : 1384282_at    Name:   zinc finger proliferation 1 (predicted)    GB acc:   BG379149    UniGene:   Rn.18600    Gene Symbol:   Zipro1_predicted    Locus Link:   304342    Chromosome:   12    Cytoband:   Chr:12q11    SOURCE    GENECARD       Gene Ontology:   biological process:  regulation of transcription, DNA-dependent   biological process:  development   biological process:  spermatogenesis   cellular component:  nucleus      BioCarta Pathways:     KEGG Pathways:  
    Probe set : 1371253_at    Name:   electron transferring flavoprotein, alpha polypeptide    GB acc:   AA942749    UniGene:   Rn.32496    Gene Symbol:   Etfa    Locus Link:   300726    Chromosome:   8    Cytoband:   Chr:8q24    SOURCE    GENECARD       Gene Ontology:   biological process:  electron transport   cellular component:  mitochondrion   cellular component:  electron transfer flavoprotein complex (sensu Eukaryota)   molecular function:  electron transporter activity   molecular function:  electron carrier activity      BioCarta Pathways:     KEGG Pathways:  
    Probe set : 1393564_at    Name:   similar to Ubiquitin ligase protein DZIP3 (DAZ-interacting protein 3 homolog)    GB acc:   BF402371    UniGene:   Rn.6213    Gene Symbol:   LOC303963    Locus Link:   303963    Chromosome:   11    Cytoband:   Chr:11q21    SOURCE    GENECARD       Gene Ontology:     BioCarta Pathways:     KEGG Pathways:  
    Probe set : 1370547_at    Name:   pregnancy-zone protein    GB acc:   M84000    UniGene:   Rn.11295    Gene Symbol:   Pzp    Locus Link:   252922    Chromosome:   4    Cytoband:   Chr:4q42    SOURCE    GENECARD       Gene Ontology:   biological process:  pregnancy   cellular component:  extracellular region   cellular component:  extracellular space      BioCarta Pathways:     KEGG Pathways:  
    Probe set : 1390947_at    Name:   Similar to RIKEN cDNA D930036F22 gene    GB acc:   AA891255    UniGene:   Rn.7269     Locus Link:   362737    Chromosome:   6    Cytoband:   Chr:6q22    SOURCE        Gene Ontology:     BioCarta Pathways:     KEGG Pathways:  
    Probe set : 1373230_at    Name:   similar to cDNA sequence BC036718 (predicted)    GB acc:   AI145851    UniGene:   Rn.95207    Gene Symbol:   RGD1311802_predicted    Locus Link:   361068    Chromosome:   15    Cytoband:   Chr:15p11    SOURCE    GENECARD       Gene Ontology:     BioCarta Pathways:     KEGG Pathways:  
    Probe set : 1373214_at    Name:   KDEL (Lys-Asp-Glu-Leu) containing 1 (predicted)    GB acc:   AA945238    UniGene:   Rn.7848    Gene Symbol:   Kdelc1_predicted    Locus Link:   316370    Chromosome:   9    Cytoband:   Chr:9q22    SOURCE    GENECARD       Gene Ontology:     BioCarta Pathways:     KEGG Pathways:  
    Probe set : 1368511_at    Name:   Basic helix-loop-helix domain containing, class B3    GB acc:   NM_133303    UniGene:   Rn.10784    Gene Symbol:   Bhlhb3    Locus Link:   117095    Chromosome:   4    Cytoband:   Chr:4q43    SOURCE    GENECARD       Gene Ontology:   biological process:  negative regulation of transcription from RNA polymerase II promoter   biological process:  regulation of transcription, DNA-dependent   biological process:  nervous system development   biological process:  nervous system development   biological process:  cell proliferation   biological process:  entrainment of circadian clock   biological process:  entrainment of circadian clock   biological process:  organ morphogenesis   biological process:  cell differentiation   biological process:  regulation of neuronal synaptic plasticity   biological process:  regulation of neuronal synaptic plasticity   biological process:  regulation of neuronal synaptic plasticity   biological process:  transcription   biological process:  regulation of transcription   cellular component:  nucleus   cellular component:  transcriptional repressor complex      BioCarta Pathways:     KEGG Pathways:  
    Probe set : 1370333_a_at    Name:   insulin-like growth factor 1    GB acc:   M15481    UniGene:   Rn.6282    Gene Symbol:   Igf1    Locus Link:   24482    Chromosome:   7    Cytoband:   Chr:7q12-q13    SOURCE    GENECARD       Gene Ontology:   biological process:  skeletal development   biological process:  DNA replication   biological process:  anti-apoptosis   biological process:  cell motility   biological process:  signal transduction   biological process:  Ras protein signal transduction   biological process:  nervous system development   biological process:  muscle development   biological process:  physiological process   biological process:  sensory perception of sound   biological process:  positive regulation of cell proliferation   biological process:  glycolate metabolism   biological process:  organ morphogenesis   biological process:  glial cell differentiation   biological process:  insulin-like growth factor receptor signaling pathway   cellular component:  extracellular region      BioCarta Pathways:     KEGG Pathways:  
    Probe set : 1367977_at    Name:   synuclein, alpha    GB acc:   NM_019169    UniGene:   Rn.1827    Gene Symbol:   Snca    Locus Link:   29219    Chromosome:   4    Cytoband:   Chr:4q24    SOURCE    GENECARD       Gene Ontology:   biological process:  anti-apoptosis   biological process:  central nervous system development   biological process:  synaptic vesicle transport   cellular component:  cytoplasm   cellular component:  cytoplasm   molecular function:  protein N-terminus binding      BioCarta Pathways:     KEGG Pathways:  
    Probe set : 1396942_at    Name:   hypothetical LOC301267    GB acc:   AI408959    UniGene:   Rn.91400    Gene Symbol:   LOC301267    Locus Link:   301267    Chromosome:   9    Cytoband:   Chr:9q12    SOURCE    GENECARD       Gene Ontology:     BioCarta Pathways:     KEGG Pathways:  
    Probe set : 1384548_at    Name:   ribosomal protein L32    GB acc:   BM388159    UniGene:   Rn.110966    Gene Symbol:   Rpl32    Locus Link:   28298    Chromosome:   6    Cytoband:   Chr:6q33    SOURCE    GENECARD       Gene Ontology:   biological process:  protein biosynthesis   molecular function:  RNA binding   molecular function:  structural constituent of ribosome      BioCarta Pathways:     KEGG Pathways:  
    Probe set : 1397554_at    Name:   Transcribed locus    GB acc:   BE108776    UniGene:   Rn.46626        SOURCE        Gene Ontology:     BioCarta Pathways:     KEGG Pathways:  
    Probe set : 1374396_at    Name:   ATPase, H+ transporting, V1 subunit C, isoform 1 (predicted)    GB acc:   BI293306    UniGene:   Rn.32254    Gene Symbol:   Atp6v1c1_predicted    Locus Link:   299971    Chromosome:   7    Cytoband:   Chr:7q22    SOURCE    GENECARD       Gene Ontology:     BioCarta Pathways:     KEGG Pathways:  
    Probe set : 1383581_at     GB acc:   BI290662              Gene Ontology:     BioCarta Pathways:     KEGG Pathways:  
    Probe set : 1385994_at    Name:   sporulation protein, meiosis-specific, SPO11 homolog (S. cerevisiae) (predicted)    GB acc:   AI232279    UniGene:   Rn.31584    Gene Symbol:   Spo11_predicted    Locus Link:   366261    Chromosome:   3    Cytoband:   Chr:3q42    SOURCE    GENECARD       Gene Ontology:   cellular component:  nucleus      BioCarta Pathways:     KEGG Pathways:  
    Probe set : 1382083_at    Name:   coagulation factor C homolog (Limulus polyphemus) (predicted)    GB acc:   BF287593    UniGene:   Rn.8204    Gene Symbol:   Coch_predicted    Locus Link:   362735    Chromosome:   6    Cytoband:   Chr:6q22    SOURCE    GENECARD       Gene Ontology:   biological process:  blood coagulation   biological process:  sensory perception of sound   cellular component:  extracellular space      BioCarta Pathways:     KEGG Pathways:  
    Probe set : 1368927_at    Name:   membrane bound C2 domain containing protein    GB acc:   NM_017249    UniGene:   Rn.11636    Gene Symbol:   Mbc2    Locus Link:   29579    Chromosome:   7    Cytoband:   Chr:7q11    SOURCE    GENECARD       Gene Ontology:   cellular component:  integral to membrane      BioCarta Pathways:     KEGG Pathways:  
    Probe set : 1377286_at    Name:   GTPase, IMAP family member 4    GB acc:   BM389208    UniGene:   Rn.43169    Gene Symbol:   Gimap4    Locus Link:   286938    Chromosome:   4    Cytoband:   Chr:4q24    SOURCE    GENECARD       Gene Ontology:   molecular function:  GTP binding   molecular function:  nucleotide binding      BioCarta Pathways:     KEGG Pathways:  
    Probe set : 1383855_at     GB acc:   AI136525              Gene Ontology:     BioCarta Pathways:     KEGG Pathways:  
    Probe set : 1373089_at    Name:   cadherin 3, type 1, P-cadherin (placental)    GB acc:   AI010270    UniGene:   Rn.105829    Gene Symbol:   Cdh3    Locus Link:   116777    Chromosome:   19    Cytoband:   Chr:19q12    SOURCE    GENECARD       Gene Ontology:   biological process:  cell-cell adhesion   biological process:  homophilic cell adhesion   cellular component:  membrane   molecular function:  calcium ion binding      BioCarta Pathways:     KEGG Pathways:  
    Probe set : 1383248_at    Name:   flavin containing monooxygenase 5    GB acc:   AI454611    UniGene:   Rn.7038    Gene Symbol:   Fmo5    Locus Link:   246248    Chromosome:   2    Cytoband:   Chr:2q34    SOURCE    GENECARD       Gene Ontology:   biological process:  electron transport      BioCarta Pathways:     KEGG Pathways:  
    Probe set : 1373302_at    Name:   LOC500488    GB acc:   BM387041    UniGene:   Rn.6387    Gene Symbol:   LOC500488    Locus Link:   500488    Chromosome:   5    Cytoband:   Chr:5q32    SOURCE    GENECARD       Gene Ontology:     BioCarta Pathways:     KEGG Pathways:  
    Probe set : 1382611_at    Name:   Transcribed locus    GB acc:   AA945938    UniGene:   Rn.24100        SOURCE        Gene Ontology:     BioCarta Pathways:     KEGG Pathways:  
    Probe set : 1379799_at    Name:   Transcribed locus    GB acc:   BF415056    UniGene:   Rn.47821        SOURCE        Gene Ontology:     BioCarta Pathways:     KEGG Pathways:  
    Probe set : 1374683_at    Name:   gamma sarcoglycan    GB acc:   AA850867    UniGene:   Rn.7061    Gene Symbol:   RGD:1359577    Locus Link:   305941    Chromosome:   15    Cytoband:   Chr:15p12    SOURCE    GENECARD       Gene Ontology:   biological process:  cytoskeleton organization and biogenesis   cellular component:  sarcoglycan complex   cellular component:  integral to membrane      BioCarta Pathways:     KEGG Pathways:  
    Probe set : 1372029_at    Name:   similar to mannose-6-phosphate receptor binding protein 1    GB acc:   BI281957    UniGene:   Rn.9506    Gene Symbol:   LOC316130    Locus Link:   316130    Chromosome:   9    Cytoband:   Chr:9q11    SOURCE    GENECARD       Gene Ontology:     BioCarta Pathways:     KEGG Pathways:  
    Probe set : 1392235_at    Name:   GLI-Kruppel family member GLI3    GB acc:   BE105570    UniGene:   Rn.12004    Gene Symbol:   Gli3    Locus Link:   140588    Chromosome:   17    Cytoband:   Chr:17q12.1    SOURCE    GENECARD       Gene Ontology:   biological process:  smoothened signaling pathway   biological process:  development   molecular function:  transcriptional repressor activity      BioCarta Pathways:     KEGG Pathways:  
    Probe set : 1389299_at    Name:   Phosphate cytidylyltransferase 1, choline, alpha isoform    GB acc:   BG673255    UniGene:   Rn.81192    Gene Symbol:   Pcyt1a    Locus Link:   140544    Chromosome:   11    Cytoband:   Chr:11q22    SOURCE    GENECARD       Gene Ontology:     BioCarta Pathways:     KEGG Pathways:  
    Probe set : 1376913_at     GB acc:   AA925375              Gene Ontology:     BioCarta Pathways:     KEGG Pathways:  
    Probe set : 1387064_at    Name:   peroxisomal membrane protein 3    GB acc:   NM_017234    UniGene:   Rn.4065    Gene Symbol:   Pxmp3    Locus Link:   29534    Chromosome:   2    Cytoband:   Chr:2q24    SOURCE    GENECARD       Gene Ontology:   molecular function:  ubiquitin-protein ligase activity   molecular function:  zinc ion binding   molecular function:  metal ion binding      BioCarta Pathways:     KEGG Pathways:  
    Probe set : 1382294_at    Name:   Activated leukocyte cell adhesion molecule    GB acc:   AI576111    UniGene:   Rn.5789    Gene Symbol:   Alcam    Locus Link:   79559    Chromosome:   11    Cytoband:   Chr:11q21    SOURCE    GENECARD       Gene Ontology:   biological process:  cell adhesion   biological process:  signal transduction   biological process:  antimicrobial humoral response (sensu Vertebrata)   molecular function:  guanyl-nucleotide exchange factor adaptor activity   molecular function:  receptor binding   molecular function:  protein binding      BioCarta Pathways:     KEGG Pathways:  
    Probe set : 1395146_at    Name:   adiponectin receptor 2 (predicted)    GB acc:   BI290608    UniGene:   Rn.101984    Gene Symbol:   Adipor2_predicted    Locus Link:   312670    Chromosome:   4    Cytoband:   Chr:4q42    SOURCE    GENECARD       Gene Ontology:   cellular component:  integral to membrane      BioCarta Pathways:     KEGG Pathways:  
    Probe set : 1371395_at    Name:   chromobox homolog 3 (HP1 gamma homolog, Drosophila)    GB acc:   AA851329     Gene Symbol:   Cbx3    Locus Link:   297093    Chromosome:   4    Cytoband:   Chr:4q24     GENECARD       Gene Ontology:     BioCarta Pathways:     KEGG Pathways:  
    Probe set : 1399148_s_at    Name:   similar to hypothetical protein MGC38524 (predicted)    GB acc:   AI013567    UniGene:   Rn.105830    Gene Symbol:   RGD1309002_predicted    Locus Link:   290637    Chromosome:   16    Cytoband:   Chr:16p14    SOURCE    GENECARD       Gene Ontology:     BioCarta Pathways:     KEGG Pathways:  
    Probe set : 1388134_at    Name:   eukaryotic translation elongation factor 1 delta (guanine nucleotide exchange protein)    GB acc:   BG380216    UniGene:   Rn.146086    Gene Symbol:   Eef1d    Locus Link:   300033    Chromosome:   7    Cytoband:   Chr:7q34    SOURCE    GENECARD       Gene Ontology:   biological process:  protein biosynthesis   biological process:  translational elongation   cellular component:  eukaryotic translation elongation factor 1 complex   molecular function:  translation elongation factor activity      BioCarta Pathways:     KEGG Pathways:  
    Probe set : 1373053_at     GB acc:   AI169620              Gene Ontology:     BioCarta Pathways:     KEGG Pathways:  
    Probe set : 1374763_at    Name:   cerebral endothelial cell adhesion molecule 1 (predicted)    GB acc:   BI274117    UniGene:   Rn.93530    Gene Symbol:   Ceecam1_predicted    Locus Link:   296616    Chromosome:   3    Cytoband:   Chr:3p12    SOURCE    GENECARD       Gene Ontology:   cellular component:  membrane fraction   cellular component:  plasma membrane   cellular component:  integral to membrane      BioCarta Pathways:     KEGG Pathways:  
    Probe set : 1373321_at    Name:   Transcribed locus, strongly similar to XP_574622.1 PREDICTED: similar to UPF0197 protein C11orf10 homolog [Rattus norvegicus]    GB acc:   BE111682    UniGene:   Rn.96        SOURCE        Gene Ontology:     BioCarta Pathways:     KEGG Pathways:  
    Probe set : 1376215_at    Name:   similar to hypothetical protein 9630025C22    GB acc:   AW528861     Gene Symbol:   LOC499558    Locus Link:   499558    Chromosome:   2    Cytoband:   Chr:2q22     GENECARD       Gene Ontology:     BioCarta Pathways:     KEGG Pathways:  
    Probe set : 1390437_at    Name:   Transcribed locus    GB acc:   BE116385    UniGene:   Rn.14619        SOURCE        Gene Ontology:     BioCarta Pathways:     KEGG Pathways:  
    Probe set : 1374438_at     GB acc:   AI103962              Gene Ontology:     BioCarta Pathways:     KEGG Pathways:  
    Probe set : 1375684_at    Name:   neuraminidase 1    GB acc:   BE111846    UniGene:   Rn.128560    Gene Symbol:   Neu1    Locus Link:   24591    Chromosome:   20    Cytoband:   Chr:20p12    SOURCE    GENECARD       Gene Ontology:   biological process:  carbohydrate metabolism   cellular component:  lysosome      BioCarta Pathways:     KEGG Pathways:  
    Probe set : 1382993_at    Name:   Bcl-2 binding component 3    GB acc:   AI236152    UniGene:   Rn.25176    Gene Symbol:   Bbc3    Locus Link:   317673      SOURCE    GENECARD       Gene Ontology:   biological process:  apoptosis   biological process:  induction of apoptosis   cellular component:  mitochondrion      BioCarta Pathways:     KEGG Pathways:  
    Probe set : 1369208_at    Name:   interleukin 7    GB acc:   AF367210    UniGene:   Rn.10793    Gene Symbol:   Il7    Locus Link:   25647    Chromosome:   2    Cytoband:   Chr:2q23    SOURCE    GENECARD       Gene Ontology:   cellular component:  extracellular region   cellular component:  extracellular space      BioCarta Pathways:     KEGG Pathways:  
    Probe set : 1368679_a_at    Name:   Yamaguchi sarcoma viral (v-yes-1) oncogene homolog    GB acc:   L14782    UniGene:   Rn.4338    Gene Symbol:   Lyn    Locus Link:   81515    Chromosome:   5    Cytoband:   Chr:5q12    SOURCE    GENECARD       Gene Ontology:   cellular component:  intracellular      BioCarta Pathways:     KEGG Pathways:  
    Probe set : 1373864_at    Name:   mitogen-activated protein kinase kinase kinase kinase 4 (predicted)    GB acc:   BM388810    UniGene:   Rn.101820    Gene Symbol:   Map4k4_predicted    Locus Link:   301363    Chromosome:   9    Cytoband:   Chr:9q21    SOURCE    GENECARD       Gene Ontology:   biological process:  protein amino acid phosphorylation   biological process:  response to stress   biological process:  protein kinase cascade      BioCarta Pathways:     KEGG Pathways:  
    Probe set : 1368329_at    Name:   solute carrier family 22 (organic anion transporter), member 6    GB acc:   NM_017224    UniGene:   Rn.87849    Gene Symbol:   Slc22a6    Locus Link:   29509    Chromosome:   1    Cytoband:   Chr:1q43    SOURCE    GENECARD       Gene Ontology:     BioCarta Pathways:     KEGG Pathways:  
    Probe set : 1372007_at    Name:   similar to AOF2 protein    GB acc:   BE099064    UniGene:   Rn.6607    Gene Symbol:   LOC500569    Locus Link:   500569    Chromosome:   5    Cytoband:   Chr:5q36    SOURCE    GENECARD       Gene Ontology:     BioCarta Pathways:     KEGG Pathways:  
    Probe set : 1392952_at    Name:   hypothetical protein LOC619561    GB acc:   BI277131     Gene Symbol:   LOC619561    Locus Link:   619561    Chromosome:   10    Cytoband:   Chr:10q26     GENECARD       Gene Ontology:     BioCarta Pathways:     KEGG Pathways:  
    Probe set : 1368162_at    Name:   cystatin E/M    GB acc:   NM_133566    UniGene:   Rn.9609    Gene Symbol:   Cst6    Locus Link:   171096    Chromosome:   1    Cytoband:   Chr:1q43    SOURCE    GENECARD       Gene Ontology:   biological process:  epidermis development   cellular component:  cornified envelope   cellular component:  extracellular space   molecular function:  endopeptidase inhibitor activity   molecular function:  cysteine protease inhibitor activity      BioCarta Pathways:     KEGG Pathways:  
    Probe set : 1370385_at    Name:   phospholipase A2, group VI    GB acc:   U51898    UniGene:   Rn.44692    Gene Symbol:   Pla2g6    Locus Link:   360426    Chromosome:   7    Cytoband:   Chr:7q34    SOURCE    GENECARD       Gene Ontology:   cellular component:  cytoplasm   cellular component:  membrane      BioCarta Pathways:     KEGG Pathways:  
    Probe set : 1389596_at     GB acc:   BM389642              Gene Ontology:     BioCarta Pathways:     KEGG Pathways:  
    Probe set : 1382334_at    Name:   Transcribed locus    GB acc:   AI170308    UniGene:   Rn.6650        SOURCE        Gene Ontology:     BioCarta Pathways:     KEGG Pathways:  
    Probe set : 1374296_at    Name:   Leucine-rich repeat-containing 8 (predicted)    GB acc:   BM387574    UniGene:   Rn.33512     Locus Link:   311846    Chromosome:   3    Cytoband:   Chr:3p12    SOURCE        Gene Ontology:   cellular component:  integral to membrane      BioCarta Pathways:     KEGG Pathways:  
    Probe set : 1372668_at    Name:   LIM domains containing 1 (predicted)    GB acc:   BI289676    UniGene:   Rn.3966     Locus Link:   316101    Chromosome:   8    Cytoband:   Chr:8q32    SOURCE        Gene Ontology:   biological process:  regulation of transcription, DNA-dependent   biological process:  signal transduction   biological process:  development   molecular function:  protein binding   molecular function:  zinc ion binding      BioCarta Pathways:     KEGG Pathways:  
    Probe set : 1380277_at    Name:   RAD51 associated protein 1 (predicted)    GB acc:   AI639142    UniGene:   Rn.42022     Locus Link:   312721    Chromosome:   4    Cytoband:   Chr:4q42    SOURCE        Gene Ontology:   biological process:  DNA recombination   cellular component:  nucleus      BioCarta Pathways:     KEGG Pathways:  
    Probe set : 1381014_at    Name:   interferon-induced protein 44 (predicted)    GB acc:   BI274623    UniGene:   Rn.43876    Gene Symbol:   Ifi44_predicted    Locus Link:   310969    Chromosome:   2    Cytoband:   Chr:2q45    SOURCE    GENECARD       Gene Ontology:   biological process:  response to virus   cellular component:  cytoplasm      BioCarta Pathways:     KEGG Pathways:  
    Probe set : 1381771_at    Name:   Transcribed locus    GB acc:   AI178422    UniGene:   Rn.20519        SOURCE        Gene Ontology:     BioCarta Pathways:     KEGG Pathways:  
    Probe set : 1370060_at    Name:   solute carrier family 25 (mitochondrial carrier; oxoglutarate carrier), member 11    GB acc:   NM_022398    UniGene:   Rn.853    Gene Symbol:   Slc25a11    Locus Link:   64201    Chromosome:   10    Cytoband:   Chr:10q24    SOURCE    GENECARD       Gene Ontology:   biological process:  transport      BioCarta Pathways:     KEGG Pathways:  
    Probe set : 1374015_at    Name:   Transcribed locus    GB acc:   BF281697    UniGene:   Rn.7770        SOURCE        Gene Ontology:     BioCarta Pathways:     KEGG Pathways:  
    Probe set : 1370210_at    Name:   Ras-induced senescence 1    GB acc:   BF414025    UniGene:   Rn.4294    Gene Symbol:   Ris1    Locus Link:   117582    Chromosome:   8    Cytoband:   Chr:8q32    SOURCE    GENECARD       Gene Ontology:   molecular function:  receptor activity   molecular function:  peptide binding      BioCarta Pathways:     KEGG Pathways:  
    Probe set : 1387624_at    Name:   upstream transcription factor 1    GB acc:   NM_031777    UniGene:   Rn.10845    Gene Symbol:   Usf1    Locus Link:   83586    Chromosome:   13    Cytoband:   Chr:13q24    SOURCE    GENECARD       Gene Ontology:   cellular component:  nucleus      BioCarta Pathways:     KEGG Pathways:  
    Probe set : 1393247_at    Name:   Similar to zinc finger protein 560    GB acc:   BI298190    UniGene:   Rn.12638     Locus Link:   367033    Chromosome:   8    Cytoband:   Chr:8q13    SOURCE        Gene Ontology:     BioCarta Pathways:     KEGG Pathways:  
    Probe set : 1390128_at     GB acc:   BF557618              Gene Ontology:     BioCarta Pathways:     KEGG Pathways:  
    Probe set : 1380537_at    Name:   hematopoietic protein 1 (predicted)    GB acc:   BI293796    UniGene:   Rn.19580    Gene Symbol:   Hem1_predicted    Locus Link:   315348    Chromosome:   7    Cytoband:   Chr:7q36    SOURCE    GENECARD       Gene Ontology:   cellular component:  membrane fraction   cellular component:  integral to plasma membrane      BioCarta Pathways:     KEGG Pathways:  
    Probe set : 1381833_at    Name:   cell division cycle associated 2 (predicted)    GB acc:   AW532628    UniGene:   Rn.49950    Gene Symbol:   Cdca2_predicted    Locus Link:   305984    Chromosome:   15    Cytoband:   Chr:15p12    SOURCE    GENECARD       Gene Ontology:     BioCarta Pathways:     KEGG Pathways:  
    Probe set : 1384544_at    Name:   paraoxonase 3    GB acc:   BM385409    UniGene:   Rn.16469    Gene Symbol:   RGD:1302965    Locus Link:   312086    Chromosome:   4    Cytoband:   Chr:4q13    SOURCE    GENECARD       Gene Ontology:   cellular component:  microsome   cellular component:  extracellular region   molecular function:  aryldialkylphosphatase activity   molecular function:  arylesterase activity   molecular function:  hydrolase activity      BioCarta Pathways:     KEGG Pathways:  
    Probe set : 1388774_at     GB acc:   BM389032              Gene Ontology:     BioCarta Pathways:     KEGG Pathways:  
    Probe set : 1397372_at    Name:   similar to Mob4B protein /// MOB1, Mps One Binder kinase activator-like 1B (yeast) (predicted)    GB acc:   BI289004    UniGene:   Rn.129443    Gene Symbol:   LOC308302 /// Mobk1b_predicted    Locus Link:   308302 /// 362389    Chromosome:   1    Cytoband:   Chr:1q12 /// Chr:4q34    SOURCE    GENECARD       Gene Ontology:     BioCarta Pathways:     KEGG Pathways:  
    Probe set : 1391170_at    Name:   similar to mKIAA1757 protein (predicted)    GB acc:   BE115521    UniGene:   Rn.49964    Gene Symbol:   RGD1310433_predicted    Locus Link:   297514    Chromosome:   4    Cytoband:   Chr:4q42    SOURCE    GENECARD       Gene Ontology:     BioCarta Pathways:     KEGG Pathways:  
    Probe set : 1392142_at    Name:   Transcribed locus    GB acc:   AI764196    UniGene:   Rn.148234        SOURCE        Gene Ontology:     BioCarta Pathways:     KEGG Pathways:  
    Probe set : 1370249_at    Name:   benzodiazepin receptor    GB acc:   AI008680    UniGene:   Rn.1820    Gene Symbol:   Bzrp    Locus Link:   24230    Chromosome:   7    Cytoband:   Chr:7q34    SOURCE    GENECARD       Gene Ontology:   biological process:  protein targeting to mitochondrion   biological process:  ion transport   biological process:  signal transduction   molecular function:  receptor activity   molecular function:  benzodiazepine receptor activity   molecular function:  benzodiazepine receptor activity      BioCarta Pathways:     KEGG Pathways:  
    Probe set : 1384699_at    Name:   Transcribed locus    GB acc:   BM384930    UniGene:   Rn.81485        SOURCE        Gene Ontology:     BioCarta Pathways:     KEGG Pathways:  
    Probe set : 1369173_at    Name:   complement component 3a receptor 1    GB acc:   NM_032060    UniGene:   Rn.9772    Gene Symbol:   C3ar1    Locus Link:   84007    Chromosome:   4    Cytoband:   Chr:4q42    SOURCE    GENECARD       Gene Ontology:   cellular component:  integral to membrane   cellular component:  integral to membrane      BioCarta Pathways:     KEGG Pathways:  
    Probe set : 1387234_at    Name:   alpha-2-glycoprotein 1, zinc    GB acc:   NM_012826    UniGene:   Rn.5007    Gene Symbol:   Azgp1    Locus Link:   25294    Chromosome:   12    Cytoband:   Chr:12q11    SOURCE    GENECARD       Gene Ontology:     BioCarta Pathways:     KEGG Pathways:  
    Probe set : 1389580_at    Name:   LOC499580    GB acc:   BM384827    UniGene:   Rn.1039    Gene Symbol:   LOC499580    Locus Link:   499580    Chromosome:   2    Cytoband:   Chr:2q24    SOURCE    GENECARD       Gene Ontology:     BioCarta Pathways:     KEGG Pathways:  
    Probe set : 1382587_at    Name:   Family with sequence similarity 33, member A (predicted)    GB acc:   AA901257    UniGene:   Rn.40571    Gene Symbol:   MGC109093    Locus Link:   287598    Chromosome:   10    Cytoband:   Chr:10q26    SOURCE    GENECARD       Gene Ontology:     BioCarta Pathways:     KEGG Pathways:  
    Probe set : 1374538_at    Name:   O-linked mannose beta1,2-N-acetylglucosaminyltransferase    GB acc:   AW915001    UniGene:   Rn.13079    Gene Symbol:   RGD:1359396    Locus Link:   362567    Chromosome:   5    Cytoband:   Chr:5q36    SOURCE    GENECARD       Gene Ontology:   biological process:  protein amino acid N-linked glycosylation   cellular component:  Golgi membrane   cellular component:  Golgi stack   cellular component:  integral to membrane      BioCarta Pathways:     KEGG Pathways:  
    Probe set : 1371032_at    Name:   nidogen (entactin)    GB acc:   BG372942    UniGene:   Rn.105658    Gene Symbol:   Nid    Locus Link:   25494    Chromosome:   17    Cytoband:   Chr:17q12.1    SOURCE    GENECARD       Gene Ontology:   biological process:  cell adhesion   biological process:  cell-matrix adhesion   molecular function:  extracellular matrix structural constituent   molecular function:  calcium ion binding   molecular function:  protein binding      BioCarta Pathways:     KEGG Pathways:  
    Probe set : 1370818_at    Name:   2-4-dienoyl-Coenzyme A reductase 2, peroxisomal    GB acc:   AF044574    UniGene:   Rn.144598    Gene Symbol:   Decr2    Locus Link:   64461    Chromosome:   10    Cytoband:   Chr:10q12    SOURCE    GENECARD       Gene Ontology:   biological process:  peroxisome organization and biogenesis   biological process:  metabolism   cellular component:  peroxisome   molecular function:  2,4-dienoyl-CoA reductase (NADPH) activity   molecular function:  oxidoreductase activity   molecular function:  2,4-dienoyl-CoA reductase (NADPH) activity      BioCarta Pathways:     KEGG Pathways:  
    Probe set : 1374635_at    Name:   A disintegrin and metalloprotease domain 23 (predicted)    GB acc:   AW527151    UniGene:   Rn.24184     Locus Link:   301460    Chromosome:   9    Cytoband:   Chr:9q32    SOURCE        Gene Ontology:   biological process:  proteolysis   biological process:  cell adhesion   biological process:  central nervous system development   cellular component:  plasma membrane   cellular component:  integral to plasma membrane   cellular component:  integral to membrane      BioCarta Pathways:     KEGG Pathways:  
    Probe set : 1387765_at    Name:   mannose binding lectin 1, protein A    GB acc:   NM_012599    UniGene:   Rn.10273    Gene Symbol:   Mbl1    Locus Link:   24548    Chromosome:   16    Cytoband:   Chr:16p14    SOURCE    GENECARD       Gene Ontology:     BioCarta Pathways:     KEGG Pathways:  
    Probe set : 1394826_at    Name:   Similar to Fanconi anemia, complementation group F    GB acc:   BF417759    UniGene:   Rn.8304     Locus Link:   499155      SOURCE        Gene Ontology:     BioCarta Pathways:     KEGG Pathways:  
    Probe set : 1388623_at    Name:   EH-domain containing 1 (predicted)    GB acc:   BI277003    UniGene:   Rn.8141     Locus Link:   293692    Chromosome:   1    Cytoband:   Chr:1q43    SOURCE        Gene Ontology:   biological process:  endosome transport   cellular component:  early endosome   cellular component:  endocytic vesicle      BioCarta Pathways:     KEGG Pathways:  
    Probe set : 1387882_at    Name:   basic transcription element binding protein 1    GB acc:   AI071565    UniGene:   Rn.19481    Gene Symbol:   Bteb1    Locus Link:   117560    Chromosome:   1    Cytoband:   Chr:1q51    SOURCE    GENECARD       Gene Ontology:   cellular component:  nucleus      BioCarta Pathways:     KEGG Pathways:  
    Probe set : 1387678_at    Name:   neuroblastoma RAS viral (v-ras) oncogene homolog    GB acc:   NM_080766    UniGene:   Rn.11271    Gene Symbol:   Nras    Locus Link:   24605    Chromosome:   2    Cytoband:   Chr:2q34    SOURCE    GENECARD       Gene Ontology:   cellular component:  membrane fraction   cellular component:  Golgi stack   cellular component:  membrane      BioCarta Pathways:     KEGG Pathways:  
    Probe set : 1372969_at    Name:   F-box and leucine-rich repeat protein 3a (predicted)    GB acc:   BI289651    UniGene:   Rn.36356    Gene Symbol:   Fbxl3a_predicted    Locus Link:   306129    Chromosome:   15    Cytoband:   Chr:15q22    SOURCE    GENECARD       Gene Ontology:     BioCarta Pathways:     KEGG Pathways:  
    Probe set : 1384310_at    Name:   Transcribed locus    GB acc:   BM383081    UniGene:   Rn.23945        SOURCE        Gene Ontology:     BioCarta Pathways:     KEGG Pathways:  
    Probe set : 1379304_at    Name:   similar to PRKR interacting protein 1 (IL11 inducible)    GB acc:   BM388446    UniGene:   Rn.44767    Gene Symbol:   LOC498171    Locus Link:   498171    Chromosome:   12    Cytoband:   Chr:12q12    SOURCE    GENECARD       Gene Ontology:     BioCarta Pathways:     KEGG Pathways:  
    Probe set : 1382387_at    Name:   Transmembrane protein 16A (predicted)    GB acc:   BF288508    UniGene:   Rn.141722     Locus Link:   309135    Chromosome:   1    Cytoband:   Chr:1q42    SOURCE        Gene Ontology:   cellular component:  integral to membrane      BioCarta Pathways:     KEGG Pathways:  
    Probe set : 1378198_at    Name:   Transcribed locus    GB acc:   BG372528    UniGene:   Rn.147966        SOURCE        Gene Ontology:     BioCarta Pathways:     KEGG Pathways:  
    Probe set : 1388460_at    Name:   capping protein (actin filament), gelsolin-like (predicted)    GB acc:   BM384693    UniGene:   Rn.8945    Gene Symbol:   Capg_predicted    Locus Link:   297339    Chromosome:   4    Cytoband:   Chr:4q33    SOURCE    GENECARD       Gene Ontology:   cellular component:  nucleus   cellular component:  F-actin capping protein complex   molecular function:  actin binding      BioCarta Pathways:     KEGG Pathways:  
    Probe set : 1372299_at    Name:   cyclin-dependent kinase inhibitor 1C (P57)    GB acc:   AI013919    UniGene:   Rn.92509    Gene Symbol:   Cdkn1c    Locus Link:   246060    Chromosome:   1    Cytoband:   Chr:1q42    SOURCE    GENECARD       Gene Ontology:   cellular component:  nucleus      BioCarta Pathways:     KEGG Pathways:  
    Probe set : 1380045_at    Name:   pyruvate dehydrogenase phosphatase isoenzyme 2    GB acc:   AI411446    UniGene:   Rn.30021    Gene Symbol:   Pdp2    Locus Link:   246311    Chromosome:   19    Cytoband:   Chr:19p14    SOURCE    GENECARD       Gene Ontology:   biological process:  protein amino acid dephosphorylation   biological process:  carbohydrate utilization   biological process:  carbohydrate utilization      BioCarta Pathways:     KEGG Pathways:  
    Probe set : 1369169_at    Name:   solute carrier family 23 (nucleobase transporters), member 1    GB acc:   NM_017315    UniGene:   Rn.144579    Gene Symbol:   Slc23a1    Locus Link:   50621    Chromosome:   18    Cytoband:   Chr:18p11    SOURCE    GENECARD       Gene Ontology:   biological process:  transport   biological process:  sodium ion transport   biological process:  ion transport      BioCarta Pathways:     KEGG Pathways:  
    Probe set : 1388636_at    Name:   similar to RIKEN cDNA 5730408C10 (predicted)    GB acc:   BI280272    UniGene:   Rn.26488    Gene Symbol:   RGD1305972_predicted    Locus Link:   360554    Chromosome:   10    Cytoband:   Chr:10q24    SOURCE    GENECARD       Gene Ontology:   biological process:  proteolysis   biological process:  protein ubiquitination   cellular component:  ubiquitin ligase complex   molecular function:  ubiquitin-protein ligase activity   molecular function:  peptidase activity   molecular function:  zinc ion binding      BioCarta Pathways:     KEGG Pathways:  
    Probe set : 1374081_at    Name:   Similar to H63 breast cancer expressed gene isoform a; gene associated with HER-2/neu overexpression (predicted)    GB acc:   AI011704    UniGene:   Rn.55346     Locus Link:   362204    Chromosome:   3    Cytoband:   Chr:3q35    SOURCE        Gene Ontology:     BioCarta Pathways:     KEGG Pathways:  
    Probe set : 1392612_at    Name:   Prolactin receptor    GB acc:   AW142962    UniGene:   Rn.9757    Gene Symbol:   Prlr    Locus Link:   24684    Chromosome:   2    Cytoband:   Chr:2q16    SOURCE    GENECARD       Gene Ontology:   biological process:  steroid biosynthesis   biological process:  anti-apoptosis   biological process:  cell surface receptor linked signal transduction   biological process:  transmembrane receptor protein tyrosine kinase activation (dimerization)   biological process:  embryo implantation   biological process:  lactation   biological process:  regulation of cell adhesion   biological process:  regulation of epithelial cell differentiation   biological process:  T cell activation   biological process:  tyrosine phosphorylation of JAK2 protein   biological process:  steroid biosynthesis   biological process:  anti-apoptosis   biological process:  cell surface receptor linked signal transduction   biological process:  transmembrane receptor protein tyrosine kinase activation (dimerization)   biological process:  lactation   biological process:  T cell activation   biological process:  tyrosine phosphorylation of JAK2 protein      BioCarta Pathways:     KEGG Pathways:  
    Probe set : 1372399_at    Name:   similar to cofactor of BRCA1; negative elongation factor protein B (predicted)    GB acc:   BM392127    UniGene:   Rn.8119    Gene Symbol:   RGD1307832_predicted    Locus Link:   311796    Chromosome:   3    Cytoband:   Chr:3p13    SOURCE    GENECARD       Gene Ontology:     BioCarta Pathways:     KEGG Pathways:  
    Probe set : 1388726_a_at     GB acc:   AA800242              Gene Ontology:     BioCarta Pathways:     KEGG Pathways:  
    Probe set : 1374849_at    Name:   a disintegrin-like and metalloprotease (reprolysin type) with thrombospondin type 1 motif, 7 (predicted)    GB acc:   BF285745    UniGene:   Rn.23850    Gene Symbol:   Adamts7_predicted    Locus Link:   315879    Chromosome:   8    Cytoband:   Chr:8q31    SOURCE    GENECARD       Gene Ontology:   biological process:  proteolysis   biological process:  integrin-mediated signaling pathway      BioCarta Pathways:     KEGG Pathways:  
    Probe set : 1393165_at    Name:   similar to RIKEN cDNA 2310028N02    GB acc:   BG377684    UniGene:   Rn.18267    Gene Symbol:   RGD:1359339    Locus Link:   305070    Chromosome:   13    Cytoband:   Chr:13q27    SOURCE    GENECARD       Gene Ontology:     BioCarta Pathways:     KEGG Pathways:  
    Probe set : 1382746_s_at    Name:   Similar to lymphocyte alpha-kinase (predicted)    GB acc:   BE108581    UniGene:   Rn.17057     Locus Link:   310879    Chromosome:   2    Cytoband:   Chr:2q42    SOURCE        Gene Ontology:     BioCarta Pathways:     KEGG Pathways:  
    Probe set : 1388949_at    Name:   Transcribed locus    GB acc:   BI297693    UniGene:   Rn.57310        SOURCE        Gene Ontology:     BioCarta Pathways:     KEGG Pathways:  
    Probe set : 1387928_at    Name:   Rap2 interacting protein    GB acc:   AF288611    UniGene:   Rn.102473    Gene Symbol:   Rap2ip    Locus Link:   303569    Chromosome:   10    Cytoband:   Chr:10q32.1    SOURCE    GENECARD       Gene Ontology:   cellular component:  cytosol   cellular component:  internal side of plasma membrane   molecular function:  protein binding   molecular function:  guanylate cyclase activator activity      BioCarta Pathways:     KEGG Pathways:  
    Probe set : 1368474_at    Name:   vascular cell adhesion molecule 1    GB acc:   NM_012889    UniGene:   Rn.11267    Gene Symbol:   Vcam1    Locus Link:   25361    Chromosome:   2    Cytoband:   Chr:2q41    SOURCE    GENECARD       Gene Ontology:   biological process:  cell adhesion   biological process:  cell adhesion   biological process:  cell adhesion   biological process:  cell-cell adhesion   molecular function:  integrin binding   molecular function:  integrin binding   molecular function:  protein binding      BioCarta Pathways:     KEGG Pathways:  
    Probe set : 1369991_at    Name:   signal peptidase complex 18kD    GB acc:   NM_031723    UniGene:   Rn.13070    Gene Symbol:   Spc18    Locus Link:   65166    Chromosome:   1    Cytoband:   Chr:1q31    SOURCE    GENECARD       Gene Ontology:   biological process:  signal peptide processing   biological process:  proteolysis      BioCarta Pathways:     KEGG Pathways:  
    Probe set : 1394020_at    Name:   Transcribed locus    GB acc:   AI412164    UniGene:   Rn.44086        SOURCE        Gene Ontology:     BioCarta Pathways:     KEGG Pathways:  
    Probe set : 1367825_at    Name:   ral guanine nucleotide dissociation stimulator    GB acc:   NM_019250    UniGene:   Rn.40174    Gene Symbol:   Ralgds    Locus Link:   29622    Chromosome:   3    Cytoband:   Chr:3p12    SOURCE    GENECARD       Gene Ontology:     BioCarta Pathways:     KEGG Pathways:  
    Probe set : 1369518_at    Name:   phosphatidylinositol 3 kinase, regulatory subunit, polypeptide 3    GB acc:   NM_022213    UniGene:   Rn.44448    Gene Symbol:   Pik3r3    Locus Link:   60664    Chromosome:   5    Cytoband:   Chr:5q36    SOURCE    GENECARD       Gene Ontology:   biological process:  intracellular signaling cascade   cellular component:  phosphoinositide 3-kinase complex   molecular function:  phosphatidylinositol 3-kinase regulator activity   molecular function:  phosphatidylinositol 3-kinase activity      BioCarta Pathways:     KEGG Pathways:  
    Probe set : 1380365_at    Name:   Transcribed locus    GB acc:   BF403932    UniGene:   Rn.147925        SOURCE        Gene Ontology:     BioCarta Pathways:     KEGG Pathways:  
    Probe set : 1375319_at    Name:   Transmembrane protein 16D (eight membrane-spanning domains) (predicted)    GB acc:   BF406138    UniGene:   Rn.23908     Locus Link:   299714    Chromosome:   7    Cytoband:   Chr:7q13    SOURCE        Gene Ontology:     BioCarta Pathways:     KEGG Pathways:  
    Probe set : 1383020_at     GB acc:   AA899161              Gene Ontology:     BioCarta Pathways:     KEGG Pathways:  
    Probe set : 1394940_at    Name:   similar to hypothetical protein FLJ20037 (predicted)    GB acc:   BI294811    UniGene:   Rn.788    Gene Symbol:   RGD1311381_predicted    Locus Link:   300870    Chromosome:   8    Cytoband:   Chr:8q31    SOURCE    GENECARD       Gene Ontology:     BioCarta Pathways:     KEGG Pathways:  
    Probe set : 1374200_at    Name:   solute carrier family 29 (nucleoside transporters), member 3    GB acc:   BM392280    UniGene:   Rn.15870    Gene Symbol:   Slc29a3    Locus Link:   353307    Chromosome:   20    Cytoband:   Chr:20q11    SOURCE    GENECARD       Gene Ontology:   biological process:  nucleoside transport   biological process:  transport   cellular component:  membrane   cellular component:  integral to membrane   molecular function:  nucleoside transporter activity   molecular function:  nucleoside transporter activity      BioCarta Pathways:     KEGG Pathways:  
    Probe set : 1391507_at    Name:   similar to zinc finger protein EZI    GB acc:   BF417765    UniGene:   Rn.27249    Gene Symbol:   LOC500110    Locus Link:   500110    Chromosome:   4    Cytoband:   Chr:4q24    SOURCE    GENECARD       Gene Ontology:   cellular component:  nucleus   molecular function:  nucleic acid binding   molecular function:  zinc ion binding   molecular function:  metal ion binding      BioCarta Pathways:     KEGG Pathways:  
    Probe set : 1376808_at    Name:   Transcribed locus    GB acc:   BG672572    UniGene:   Rn.21871        SOURCE        Gene Ontology:     BioCarta Pathways:     KEGG Pathways:  
    Probe set : 1372748_at    Name:   similar to KIAA1055 protein (predicted)    GB acc:   BE113335     Gene Symbol:   RGD1307436_predicted    Locus Link:   315880    Chromosome:   8    Cytoband:   Chr:8q31     GENECARD       Gene Ontology:     BioCarta Pathways:     KEGG Pathways:  
    Probe set : 1393653_at    Name:   similar to putative protein product of HMFN2073    GB acc:   BM384831    UniGene:   Rn.127094    Gene Symbol:   LOC501174    Locus Link:   501174    Chromosome:   9    Cytoband:   Chr:9q34    SOURCE    GENECARD       Gene Ontology:     BioCarta Pathways:     KEGG Pathways:  
    Probe set : 1370134_at    Name:   acetyl-CoA transporter    GB acc:   NM_022252    UniGene:   Rn.144592    Gene Symbol:   Slc33a1    Locus Link:   64018    Chromosome:   2    Cytoband:   Chr:2q31    SOURCE    GENECARD       Gene Ontology:   biological process:  transport   biological process:  central nervous system development   molecular function:  acetyl-CoA transporter activity   molecular function:  acetyl-CoA transporter activity      BioCarta Pathways:     KEGG Pathways:  
    Probe set : 1393249_at    Name:   Friend leukemia integration 1 (predicted)    GB acc:   BF407924    UniGene:   Rn.65827     Locus Link:   315532    Chromosome:   8    Cytoband:   Chr:8q21    SOURCE        Gene Ontology:   cellular component:  nucleus   molecular function:  DNA binding   molecular function:  transcription factor activity   molecular function:  transcriptional activator activity      BioCarta Pathways:     KEGG Pathways:  
    Probe set : 1393799_at    Name:   Unc-5 homolog B (C. elegans)    GB acc:   BI303989    UniGene:   Rn.34617    Gene Symbol:   Unc5b    Locus Link:   60630    Chromosome:   20    Cytoband:   Chr:20q11    SOURCE    GENECARD       Gene Ontology:   biological process:  apoptosis   biological process:  signal transduction   biological process:  development   biological process:  axon guidance   cellular component:  integral to membrane   molecular function:  receptor activity   molecular function:  netrin receptor activity   molecular function:  protein binding      BioCarta Pathways:     KEGG Pathways:  
    Probe set : 1373363_at    Name:   microtubule-associated protein 1b    GB acc:   BI281702    UniGene:   Rn.98152    Gene Symbol:   Map1b    Locus Link:   29456    Chromosome:   2    Cytoband:   Chr:2q12    SOURCE    GENECARD       Gene Ontology:   cellular component:  microtubule associated complex   cellular component:  microtubule associated complex   cellular component:  microtubule      BioCarta Pathways:     KEGG Pathways:  
    Probe set : 1371922_at    Name:   Transcribed locus    GB acc:   AI169140    UniGene:   Rn.18638        SOURCE        Gene Ontology:     BioCarta Pathways:     KEGG Pathways:  
    Probe set : 1375432_at    Name:   DD6A4-12(7)-2 mRNA, partial sequence    GB acc:   AI171599    UniGene:   Rn.22355        SOURCE        Gene Ontology:     BioCarta Pathways:     KEGG Pathways:  
    Probe set : 1396646_at    Name:   similar to RIKEN cDNA 4930431B11 gene (predicted)    GB acc:   BF400757    UniGene:   Rn.135789    Gene Symbol:   RGD1308555_predicted    Locus Link:   300866    Chromosome:   8    Cytoband:   Chr:8q31    SOURCE    GENECARD       Gene Ontology:     BioCarta Pathways:     KEGG Pathways:  
    Probe set : 1391702_at    Name:   Similar to hypothetical protein FLJ20626 (predicted)    GB acc:   BM389392    UniGene:   Rn.66269     Locus Link:   308365    Chromosome:   1    Cytoband:   Chr:1q21    SOURCE        Gene Ontology:     BioCarta Pathways:     KEGG Pathways:  
    Probe set : 1370422_at    Name:   receptor-interacting serine-threonine kinase 3    GB acc:   AF036537    UniGene:   Rn.7110    Gene Symbol:   Ripk3    Locus Link:   246240    Chromosome:   15    Cytoband:   Chr:15p13    SOURCE    GENECARD       Gene Ontology:   cellular component:  intracellular      BioCarta Pathways:     KEGG Pathways:  
    Probe set : 1378321_at    Name:   Ras association (RalGDS/AF-6) domain family 4 (predicted)    GB acc:   BG375029    UniGene:   Rn.11482    Gene Symbol:   Rassf4_predicted    Locus Link:   362423    Chromosome:   4    Cytoband:   Chr:4q42    SOURCE    GENECARD       Gene Ontology:   biological process:  signal transduction   biological process:  neuropeptide signaling pathway   molecular function:  protein binding   molecular function:  oxidoreductase activity      BioCarta Pathways:     KEGG Pathways:  
    Probe set : 1383171_at    Name:   transcription factor B2, mitochondrial (predicted)    GB acc:   BG373674    UniGene:   Rn.96104    Gene Symbol:   Tfb2m_predicted    Locus Link:   289307    Chromosome:   13    Cytoband:   Chr:13q26    SOURCE    GENECARD       Gene Ontology:   biological process:  rRNA modification   cellular component:  extracellular space   molecular function:  rRNA (adenine-N6,N6-)-dimethyltransferase activity   molecular function:  rRNA methyltransferase activity      BioCarta Pathways:     KEGG Pathways:  
    Probe set : 1369682_at    Name:   transcription factor 2    GB acc:   NM_013103    UniGene:   Rn.11342    Gene Symbol:   Tcf2    Locus Link:   25640    Chromosome:   10    Cytoband:   Chr:10q26    SOURCE    GENECARD       Gene Ontology:   cellular component:  nucleus   cellular component:  nucleus   cellular component:  transcription factor complex      BioCarta Pathways:     KEGG Pathways:  
    Probe set : 1379423_at    Name:   Transcribed locus, strongly similar to NP_033131.2 Ras suppressor protein 1 [Mus musculus]    GB acc:   AA963364    UniGene:   Rn.11453        SOURCE        Gene Ontology:     BioCarta Pathways:     KEGG Pathways:  
    Probe set : 1390951_at    Name:   Transcribed locus    GB acc:   BE107735    UniGene:   Rn.9566        SOURCE        Gene Ontology:     BioCarta Pathways:     KEGG Pathways:  
    Probe set : 1372990_at    Name:   cAMP responsive element binding protein 3 (predicted)    GB acc:   AI179264    UniGene:   Rn.144871    Gene Symbol:   Creb3_predicted    Locus Link:   298400    Chromosome:   5    Cytoband:   Chr:5q22    SOURCE    GENECARD       Gene Ontology:   cellular component:  nucleus   cellular component:  endoplasmic reticulum   cellular component:  integral to membrane   molecular function:  DNA binding   molecular function:  translation activator activity      BioCarta Pathways:     KEGG Pathways:  
    Probe set : 1373577_at    Name:   Neuropilin 1    GB acc:   BE116566    UniGene:   Rn.10815    Gene Symbol:   Nrp    Locus Link:   246331    Chromosome:   19    Cytoband:   Chr:19q12    SOURCE    GENECARD       Gene Ontology:   biological process:  angiogenesis   biological process:  cell adhesion   biological process:  signal transduction   biological process:  cell-cell signaling   biological process:  nervous system development   biological process:  nervous system development   biological process:  axon guidance   biological process:  heart development   biological process:  positive regulation of cell proliferation   biological process:  dendrite morphogenesis   biological process:  cell migration   biological process:  negative regulation of axon extension   biological process:  vascular endothelial growth factor receptor signaling pathway   biological process:  development   biological process:  cell differentiation      BioCarta Pathways:     KEGG Pathways:  
    Probe set : 1398789_at    Name:   ribosomal protein L37    GB acc:   NM_031106    UniGene:   Rn.65053    Gene Symbol:   Rpl37    Locus Link:   81770    Chromosome:   2    Cytoband:   Chr:2q16    SOURCE    GENECARD       Gene Ontology:   biological process:  protein biosynthesis      BioCarta Pathways:     KEGG Pathways:  
    Probe set : 1377945_at    Name:   similar to RIKEN cDNA 2310005B10    GB acc:   AI111512    UniGene:   Rn.26598    Gene Symbol:   MGC95311    Locus Link:   308490    Chromosome:   13    Cytoband:   Chr:13q11    SOURCE    GENECARD       Gene Ontology:     BioCarta Pathways:     KEGG Pathways:  
    Probe set : 1370897_at    Name:   Branched chain ketoacid dehydrogenase E1, alpha polypeptide    GB acc:   J02827    UniGene:   Rn.49145    Gene Symbol:   Bckdha    Locus Link:   25244    Chromosome:   1    Cytoband:   Chr:1q21    SOURCE    GENECARD       Gene Ontology:   biological process:  metabolism   biological process:  branched chain family amino acid catabolism   biological process:  branched chain family amino acid catabolism      BioCarta Pathways:     KEGG Pathways:  
    Probe set : 1375857_at    Name:   similar to Myoferlin (Fer-1 like protein 3)    GB acc:   AW917760    UniGene:   Rn.73651    Gene Symbol:   LOC309499    Locus Link:   309499    Chromosome:   1    Cytoband:   Chr:1q53    SOURCE    GENECARD       Gene Ontology:     BioCarta Pathways:     KEGG Pathways:  
    Probe set : 1380117_at    Name:   Transcribed locus    GB acc:   BF397842    UniGene:   Rn.148018        SOURCE        Gene Ontology:     BioCarta Pathways:     KEGG Pathways:  
    Probe set : 1392040_at    Name:   similar to 2810453L12Rik protein (predicted)    GB acc:   BE107904    UniGene:   Rn.51026    Gene Symbol:   RGD1306255_predicted    Locus Link:   310807    Chromosome:   2    Cytoband:   Chr:2q41    SOURCE    GENECARD       Gene Ontology:     BioCarta Pathways:     KEGG Pathways:  
    Probe set : 1379560_at    Name:   nuclear receptor binding SET domain protein 1 (predicted)    GB acc:   BE099674    UniGene:   Rn.96085    Gene Symbol:   Nsd1_predicted    Locus Link:   306764    Chromosome:   17    Cytoband:   Chr:17p14    SOURCE    GENECARD       Gene Ontology:   biological process:  regulation of transcription, DNA-dependent   biological process:  protein ubiquitination   cellular component:  ubiquitin ligase complex   cellular component:  nucleus   molecular function:  DNA binding   molecular function:  ubiquitin-protein ligase activity   molecular function:  zinc ion binding      BioCarta Pathways:     KEGG Pathways:  
    Probe set : 1388480_at    Name:   similar to Glycolipid transfer protein    GB acc:   AI412863    UniGene:   Rn.4111    Gene Symbol:   LOC288707    Locus Link:   288707    Chromosome:   12    Cytoband:   Chr:12q16    SOURCE    GENECARD       Gene Ontology:     BioCarta Pathways:     KEGG Pathways:  
    Probe set : 1368598_at    Name:   somatostatin receptor 3 /// hypothetical gene supported by NM_133522    GB acc:   NM_133522    UniGene:   Rn.10458    Gene Symbol:   Sstr3 /// SSTR    Locus Link:   171044 /// 497823    Chromosome:   7    Cytoband:   Chr:7q34    SOURCE    GENECARD       Gene Ontology:   biological process:  G-protein coupled receptor protein signaling pathway   biological process:  signal transduction   cellular component:  integral to membrane      BioCarta Pathways:     KEGG Pathways:  
    Probe set : 1376812_at    Name:   Transcribed locus    GB acc:   BF419731    UniGene:   Rn.32457        SOURCE        Gene Ontology:     BioCarta Pathways:     KEGG Pathways:  
    Probe set : 1376317_at     GB acc:   BI282917              Gene Ontology:     BioCarta Pathways:     KEGG Pathways:  
    Probe set : 1370859_at    Name:   thioredoxin domain containing 7    GB acc:   BI284965    UniGene:   Rn.2685    Gene Symbol:   Txndc7    Locus Link:   286906      SOURCE    GENECARD       Gene Ontology:   biological process:  electron transport   biological process:  protein folding   cellular component:  extracellular space   cellular component:  endoplasmic reticulum      BioCarta Pathways:     KEGG Pathways:  
    Probe set : 1385557_at    Name:   Transcribed locus    GB acc:   AA925349    UniGene:   Rn.128108        SOURCE        Gene Ontology:     BioCarta Pathways:     KEGG Pathways:  
    Probe set : 1383654_a_at    Name:   similar to fructosamine-3-kinase    GB acc:   BE112523    UniGene:   Rn.14724    Gene Symbol:   Fnsk    Locus Link:   498034    Chromosome:   10    Cytoband:   Chr:10q32.3    SOURCE    GENECARD       Gene Ontology:     BioCarta Pathways:     KEGG Pathways:  
    Probe set : 1392449_at    Name:   RAD18 homolog (S. cerevisiae) (predicted)    GB acc:   BI282152    UniGene:   Rn.139798    Gene Symbol:   Rad18_predicted    Locus Link:   362412    Chromosome:   4    Cytoband:   Chr:4q42    SOURCE    GENECARD       Gene Ontology:   biological process:  DNA repair   biological process:  response to DNA damage stimulus   biological process:  protein ubiquitination   cellular component:  ubiquitin ligase complex   cellular component:  nucleus      BioCarta Pathways:     KEGG Pathways:  
    Probe set : 1377201_at    Name:   Similar to 1700054N08Rik protein    GB acc:   BE109156    UniGene:   Rn.46749     Locus Link:   307926    Chromosome:   19    Cytoband:   Chr:19q12    SOURCE        Gene Ontology:     BioCarta Pathways:     KEGG Pathways:  
    Probe set : 1388312_at    Name:   Transcribed locus    GB acc:   BI274487    UniGene:   Rn.146338        SOURCE        Gene Ontology:     BioCarta Pathways:     KEGG Pathways:  
    Probe set : 1377982_at    Name:   deltex 4 homolog (Drosophila) (predicted)    GB acc:   AW915930    UniGene:   Rn.23314    Gene Symbol:   Dtx4_predicted    Locus Link:   293774    Chromosome:   1    Cytoband:   Chr:1q43    SOURCE    GENECARD       Gene Ontology:   biological process:  protein ubiquitination   cellular component:  ubiquitin ligase complex   molecular function:  ubiquitin-protein ligase activity   molecular function:  zinc ion binding   molecular function:  metal ion binding      BioCarta Pathways:     KEGG Pathways:  
    Probe set : 1373351_at    Name:   Transcribed locus    GB acc:   BI286775    UniGene:   Rn.26749        SOURCE        Gene Ontology:     BioCarta Pathways:     KEGG Pathways:  
    Probe set : 1394031_at    Name:   similar to Protein C20orf129 homolog    GB acc:   AA800742    UniGene:   Rn.55931    Gene Symbol:   LOC311598    Locus Link:   311598    Chromosome:   3    Cytoband:   Chr:3q42    SOURCE    GENECARD       Gene Ontology:     BioCarta Pathways:     KEGG Pathways:  
    Probe set : 1367739_at    Name:   Cytochrom c oxidase subunit VIII-H (heart/muscle)    GB acc:   NM_012786    UniGene:   Rn.10325    Gene Symbol:   Cox8h    Locus Link:   25250    Chromosome:   1    Cytoband:   Chr:1q41    SOURCE    GENECARD       Gene Ontology:   molecular function:  cytochrome-c oxidase activity   molecular function:  cytochrome-c oxidase activity   molecular function:  oxidoreductase activity      BioCarta Pathways:     KEGG Pathways:  
    Probe set : 1386162_at    Name:   TXK tyrosine kinase (predicted)    GB acc:   AI009944    UniGene:   Rn.17505    Gene Symbol:   Txk_predicted    Locus Link:   305311    Chromosome:   14    Cytoband:   Chr:14p11    SOURCE    GENECARD       Gene Ontology:   biological process:  protein amino acid phosphorylation   biological process:  intracellular signaling cascade   cellular component:  cytoplasm      BioCarta Pathways:     KEGG Pathways:  
    Probe set : 1374174_at    Name:   Transcribed locus    GB acc:   AI555195    UniGene:   Rn.114503        SOURCE        Gene Ontology:     BioCarta Pathways:     KEGG Pathways:  
    Probe set : 1383465_at    Name:   F-box and leucine-rich repeat protein 14 (predicted)    GB acc:   AI058747    UniGene:   Rn.18972    Gene Symbol:   Fbxl14_predicted    Locus Link:   312675    Chromosome:   4    Cytoband:   Chr:4q42    SOURCE    GENECARD       Gene Ontology:   biological process:  ubiquitin cycle      BioCarta Pathways:     KEGG Pathways:  
    Probe set : 1380374_at    Name:   Transcribed locus    GB acc:   BI282293    UniGene:   Rn.146589        SOURCE        Gene Ontology:     BioCarta Pathways:     KEGG Pathways:  
    Probe set : 1387918_at    Name:   tropomyosin 3, gamma    GB acc:   BI295970    UniGene:   Rn.37575    Gene Symbol:   Tpm3    Locus Link:   117557    Chromosome:   2    Cytoband:   Chr:2q34    SOURCE    GENECARD       Gene Ontology:   biological process:  regulation of muscle contraction   biological process:  muscle development   cellular component:  cytoskeleton   cellular component:  muscle thin filament tropomyosin   molecular function:  actin binding      BioCarta Pathways:     KEGG Pathways:  
    Probe set : 1380219_at     GB acc:   BE117902              Gene Ontology:     BioCarta Pathways:     KEGG Pathways:  
    Probe set : 1393683_at    Name:   similar to KIAA0368 (predicted)    GB acc:   BF415479    UniGene:   Rn.22555    Gene Symbol:   RGD1306148_predicted    Locus Link:   313196    Chromosome:   5    Cytoband:   Chr:5q24    SOURCE    GENECARD       Gene Ontology:     BioCarta Pathways:     KEGG Pathways:  
    Probe set : 1383559_at    Name:   DEAD (Asp-Glu-Ala-Asp) box polypeptide 19    GB acc:   AI144819    UniGene:   Rn.96192    Gene Symbol:   Ddx19    Locus Link:   292022    Chromosome:   19    Cytoband:   Chr:19q12    SOURCE    GENECARD       Gene Ontology:   biological process:  induction of apoptosis   biological process:  response to zinc ion   cellular component:  nucleus      BioCarta Pathways:     KEGG Pathways:  
    Probe set : 1376814_at    Name:   Transcribed locus    GB acc:   BE107949    UniGene:   Rn.50879        SOURCE        Gene Ontology:     BioCarta Pathways:     KEGG Pathways:  
    Probe set : 1369493_at    Name:   prolactin receptor    GB acc:   NM_012630    UniGene:   Rn.9757    Gene Symbol:   Prlr    Locus Link:   24684    Chromosome:   2    Cytoband:   Chr:2q16    SOURCE    GENECARD       Gene Ontology:   biological process:  steroid biosynthesis   biological process:  anti-apoptosis   biological process:  cell surface receptor linked signal transduction   biological process:  transmembrane receptor protein tyrosine kinase activation (dimerization)   biological process:  embryo implantation   biological process:  lactation   biological process:  regulation of cell adhesion   biological process:  regulation of epithelial cell differentiation   biological process:  T cell activation   biological process:  tyrosine phosphorylation of JAK2 protein   biological process:  steroid biosynthesis   biological process:  anti-apoptosis   biological process:  cell surface receptor linked signal transduction   biological process:  transmembrane receptor protein tyrosine kinase activation (dimerization)   biological process:  lactation   biological process:  T cell activation   biological process:  tyrosine phosphorylation of JAK2 protein      BioCarta Pathways:     KEGG Pathways:  
    Probe set : 1392794_at    Name:   Transcribed locus    GB acc:   AA893579    UniGene:   Rn.148291        SOURCE        Gene Ontology:     BioCarta Pathways:     KEGG Pathways:  
    Probe set : 1374646_at    Name:   Casein kinase 1, alpha 1    GB acc:   BM387173    UniGene:   Rn.23810    Gene Symbol:   Csnk1a1    Locus Link:   113927    Chromosome:   18    Cytoband:   Chr:18q12.1    SOURCE    GENECARD       Gene Ontology:   biological process:  protein amino acid phosphorylation   biological process:  Wnt receptor signaling pathway      BioCarta Pathways:     KEGG Pathways:  
    Probe set : 1385883_at    Name:   Transcribed locus    GB acc:   AI144842    UniGene:   Rn.27701        SOURCE        Gene Ontology:     BioCarta Pathways:     KEGG Pathways:  
    Probe set : 1372739_at    Name:   sarcoma amplified sequence (predicted)    GB acc:   AA818865    UniGene:   Rn.129220    Gene Symbol:   Sas_predicted    Locus Link:   362890    Chromosome:   7    Cytoband:   Chr:7q22    SOURCE    GENECARD       Gene Ontology:   biological process:  positive regulation of cell proliferation   cellular component:  membrane fraction   cellular component:  integral to plasma membrane   cellular component:  integral to membrane      BioCarta Pathways:     KEGG Pathways:  
    Probe set : 1373818_at    Name:   Potassium channel tetramerisation domain containing 12 (predicted)    GB acc:   AI408351    UniGene:   Rn.13832     Locus Link:   364458    Chromosome:   15    Cytoband:   Chr:15q22    SOURCE        Gene Ontology:   biological process:  potassium ion transport   cellular component:  voltage-gated potassium channel complex   cellular component:  membrane   molecular function:  voltage-gated potassium channel activity      BioCarta Pathways:     KEGG Pathways:  
    Probe set : 1393144_at    Name:   N-myc (and STAT) interactor (predicted)    GB acc:   BM388202    UniGene:   Rn.18496     Locus Link:   311021    Chromosome:   3    Cytoband:   Chr:3q12    SOURCE        Gene Ontology:   biological process:  transcription from RNA polymerase II promoter   biological process:  inflammatory response   biological process:  JAK-STAT cascade   cellular component:  cytoplasm   molecular function:  transcription cofactor activity      BioCarta Pathways:     KEGG Pathways:  
    Probe set : 1388166_at    Name:   similar to Igh-6 protein /// similar to Ig H-chain V-region precursor /// similar to single chain Fv antibody fragment scFv 7-10A /// similar to Ig heavy chain V region MC101 precursor    GB acc:   AI411693    UniGene:   Rn.10956    Gene Symbol:   RGD:1359202 /// LOC299458 /// LOC314509 /// LOC366747    Locus Link:   299357 /// 299458 /// 314509 /// 366747    Chromosome:   6    Cytoband:   Chr:6q32 /// Chr:6q33    SOURCE    GENECARD       Gene Ontology:   molecular function:  antigen binding      BioCarta Pathways:     KEGG Pathways:  
    Probe set : 1398883_at    Name:   heterogeneous nuclear ribonucleoprotein A2/B1 (predicted)    GB acc:   BI296284    UniGene:   Rn.129934    Gene Symbol:   Hnrpa2b1_predicted    Locus Link:   362361    Chromosome:   4    Cytoband:   Chr:4q24    SOURCE    GENECARD       Gene Ontology:   biological process:  nuclear mRNA splicing, via spliceosome   biological process:  RNA processing   biological process:  mRNA processing   cellular component:  nucleus   cellular component:  ribonucleoprotein complex   cellular component:  heterogeneous nuclear ribonucleoprotein complex   molecular function:  nucleic acid binding   molecular function:  RNA binding      BioCarta Pathways:     KEGG Pathways:  
    Probe set : 1394922_at     GB acc:   AI716205              Gene Ontology:     BioCarta Pathways:     KEGG Pathways:  
    Probe set : 1368643_at    Name:   spermatogenesis associated 6    GB acc:   NM_134392    UniGene:   Rn.8467    Gene Symbol:   Spata6    Locus Link:   171413    Chromosome:   5    Cytoband:   Chr:5q35    SOURCE    GENECARD       Gene Ontology:   molecular function:  motor activity      BioCarta Pathways:     KEGG Pathways:  
    Probe set : 1378914_a_at    Name:   hypothetical protein LOC308990    GB acc:   AI059468    UniGene:   Rn.98391    Gene Symbol:   LOC308990    Locus Link:   308990    Chromosome:   1    Cytoband:   Chr:1q36    SOURCE    GENECARD       Gene Ontology:     BioCarta Pathways:     KEGG Pathways:  
    Probe set : 1392921_at    Name:   Transcribed locus    GB acc:   BI294990    UniGene:   Rn.12985        SOURCE        Gene Ontology:     BioCarta Pathways:     KEGG Pathways:  
    Probe set : 1388397_at    Name:   EBNA1 binding protein 2 (predicted)    GB acc:   BI281160    UniGene:   Rn.9598    Gene Symbol:   Ebna1bp2_predicted    Locus Link:   114021    Chromosome:   5    Cytoband:   Chr:5q36    SOURCE    GENECARD       Gene Ontology:   biological process:  rRNA processing   biological process:  ribosome biogenesis   biological process:  chromosome segregation   cellular component:  membrane fraction   cellular component:  nucleus   cellular component:  nucleolus   molecular function:  protein binding      BioCarta Pathways:     KEGG Pathways:  
    Probe set : 1387395_at    Name:   adenosine A2B receptor    GB acc:   NM_017161    UniGene:   Rn.89544    Gene Symbol:   Adora2b    Locus Link:   29316    Chromosome:   10    Cytoband:   Chr:10q23    SOURCE    GENECARD       Gene Ontology:   cellular component:  extracellular space   cellular component:  integral to plasma membrane   cellular component:  integral to membrane      BioCarta Pathways:     KEGG Pathways:  
    Probe set : 1371500_at    Name:   latent transforming growth factor beta binding protein 4 (predicted)    GB acc:   BG375362    UniGene:   Rn.7961    Gene Symbol:   Ltbp4_predicted    Locus Link:   292734    Chromosome:   1    Cytoband:   Chr:1q21    SOURCE    GENECARD       Gene Ontology:   cellular component:  extracellular matrix (sensu Metazoa)      BioCarta Pathways:     KEGG Pathways:  
    Probe set : 1370068_at    Name:   phospholipase A2, group V    GB acc:   NM_017174    UniGene:   Rn.20244    Gene Symbol:   Pla2g5    Locus Link:   29354    Chromosome:   5    Cytoband:   Chr:5q36    SOURCE    GENECARD       Gene Ontology:   biological process:  phospholipid metabolism   biological process:  lipid catabolism   cellular component:  extracellular region   cellular component:  extracellular space      BioCarta Pathways:     KEGG Pathways:  
    Probe set : 1372459_at    Name:   vasodilator-stimulated phosphoprotein (predicted)    GB acc:   AW520792    UniGene:   Rn.98750    Gene Symbol:   Vasp_predicted    Locus Link:   361517    Chromosome:   1    Cytoband:   Chr:1q21    SOURCE    GENECARD       Gene Ontology:   molecular function:  actin binding   molecular function:  protein binding      BioCarta Pathways:     KEGG Pathways:
