# Supplementary material for: Activation of counter-regulatory mechanisms in a rat renal acute rejection model
Source: BMC Genomics. 2008 Feb 8;9:71. doi: 10.1186/1471-2164-9-71 (PMC2262896; doi:10.1186/1471-2164-9-71)
Supplement: Additional file 1 — Gene lists from the class comparison between the groups analyzed with BRB ArrayTools. [file 1471-2164-9-71-S1.zip › ControlvsSyngeneic/ControlvsSyngeneic.html]

 

 
 
 Class Comparison Results 
 
P {font-size: 9pt}
TABLE {font-size: 9pt}
 
 
 
 
function popUp(text) {
   var prop =
   "location=no,scrollbars=yes,menubars=no,toolbars=no,resizable=yes";
   popup = window.open("","mywin",prop);
   popup.document.open();
   popup.document.write(text);
   popup.document.close();
   popup.focus();
   }
    
 HELP   
   Description of the problem:   
 
Number of classes: 2
 
Number of genes that passed filtering criteria: 31099
 
Type of univariate test used: Two-sample T-test (with random variance model)
 
 
Column of the Experiment Descriptors sheet that defines class variable : Control vs syngeneic
 
 
WARNING: Distribution assumptions underlying the univariate test random variance model are not satisfied. Random variance model was not used
 
 
Exact Multivariate Permutations test was computed based on 126 available permutations
 
 
 
Nominal significance level of each univariate test: 0.001
 
 
Confidence level of false discovery rate assessment: 90 %
 
Maximum allowed number of false-positive genes: 10
 
Maximum allowed proportion of false-positive genes: 0.1
 
 
   Summary of Results:   
 
 
Number of genes significant at 0.001 level of the univariate test: 1855
 
 
Probability of getting at least 1855 genes significant by chance (at the 0.001 level) if there are no real differences between the classes: 0.00794
 
 
   Genes which discriminate among classes:   
  Table 1  - Sorted by p-value of the univariate test.
 
The first 1855 genes are significant at the nominal 0.001 level of the univariate test
 
With probability of 90 % the first 739 genes contain no more than 10 false discoveries.
 
 
With probability of 90% the first 2328 genes contain no more than 10% of false discoveries. 
Further extension of the list was halted because the list would contain more than 100 false discoveries
 
 
  
    Parametric  p-value   Geom mean of  intensities  in class  1 :  control  Geom mean of  intensities  in class  2 :  syngeneic  Fold difference of geom means   Probe set Description Anno- tations Gene symbol
   1 p  584.3 2443.6 0.239  1370155_at  procollagen, type I, alpha 2  Info   Col1a2 
   2 p  150.9 392.4 0.385  1371369_at  procollagen, type VI, alpha 2 (predicted)  Info   Col6a2_predicted 
   3 p  755.7 3285 0.23  1370959_at  collagen, type III, alpha 1  Info   Col3a1 
   4 p  871.1 6839.6 0.127  1367784_a_at  clusterin  Info   Clu 
   5 p  634.5 96 6.609  1372213_at  similar to hypothetical protein MGC6835  Info   LOC500300 
   6 p  1730.7 1212.2 1.428  1370167_at  syndecan 2  Info   Sdc2 
   7 1e-07 2367.2 4144.9 0.571  1367651_at  cathepsin D  Info   Ctsd 
   8 1e-07 258.3 1246.8 0.207  1387854_at  procollagen, type I, alpha 2  Info   Col1a2 
   9 1e-07 2298.2 1386.4 1.658  1372765_a_at  similar to Peci protein /// similar to RIKEN cDNA 1810022C23 (predicted)  Info   RGD:1359427 /// RGD1310224_predicted 
   10 1e-07 24.7 129.3 0.191  1372406_at  minichromosome maintenance deficient 3 (S. cerevisiae) (predicted) /// similar to DNA replication licensing factor MCM3 (DNA polymerase alpha holoenzyme-associated protein P1) (P1-MCM3)  Info   Mcm3_predicted /// LOC367976 
   11 1e-07 74.7 134.4 0.556  1374649_at  RAS guanyl releasing protein 2 (calcium and DAG-regulated) (predicted)  Info   Rasgrp2_predicted 
   12 1e-07 58.6 98.7 0.594  1378477_a_at  similar to Formin homology 2 domain containing 1  Info   LOC291964 
   13 1e-07 121.7 368.7 0.33  1389555_at  transcription factor 19  Info   RGD:1302974 
   14 1e-07 253.3 1597.8 0.159  1388116_at  collagen, type 1, alpha 1  Info   Col1a1 
   15 1e-07 274.8 578.5 0.475  1372621_at  similar to 2610027C15Rik protein (predicted)  Info   RGD1308876_predicted 
   16 1e-07 229.5 755.4 0.304  1371349_at  similar to collagen alpha1 type VI-precursor  Info   LOC294337 
   17 1e-07 34 307.5 0.111  1386128_at  tetranectin (plasminogen binding protein) (predicted)  Info   Tna_predicted 
   18 1e-07 1624 469.2 3.461  1368288_at  group specific component  Info   Gc 
   19 2e-07 130.3 488.4 0.267  1369955_at  Collagen, type V, alpha 1  Info   Col5a1 
   20 2e-07 114.4 510.8 0.224  1389123_at  chemokine (C-C motif) ligand 6  Info   RGD:1303200 
   21 2e-07 18.4 162.4 0.113  1367794_at  alpha-2-macroglobulin  Info   A2m 
   22 2e-07 109.4 660.9 0.166  1370864_at  collagen, type 1, alpha 1  Info   Col1a1 
   23 2e-07 96 247.7 0.388  1389295_at  olfactomedin-like 2B (predicted)  Info   Olfml2b_predicted 
   24 2e-07 135.1 495.6 0.273  1387351_at  fibrillin 1  Info   Fbn1 
   25 3e-07 254.8 802.2 0.318  1386912_at  procollagen C-proteinase enhancer protein  Info   Pcolce 
   26 3e-07 200.2 480.9 0.416  1398347_at  AXL receptor tyrosine kinase (predicted)  Info   Axl_predicted 
   27 3e-07 19.4 60.8 0.319  1377367_at  Kazal-type serine protease inhibitor domain 1 (predicted)  Info   Kazald1_predicted 
   28 3e-07 265.9 937 0.284  1370290_at  tubulin, beta 5  Info   Tubb5 
   29 3e-07 87.4 179.1 0.488  1374742_at  immunoglobulin superfamily, member 4C (predicted)  Info   Igsf4c_predicted 
   30 3e-07 45 1.2 37.5  1384917_at  Similar to uracil-DNA glycosylase 2  Info    
   31 3e-07 99.4 186.4 0.533  1370347_at  PDZ and LIM domain 7  Info   Pdlim7 
   32 4e-07 1342.7 2406.3 0.558  1367562_at  secreted acidic cysteine rich glycoprotein  Info   Sparc 
   33 4e-07 137.7 332.3 0.414  1388182_at  DNA primase, p49 subunit  Info   RGD:621380 
   34 4e-07 109.1 352.5 0.31  1367914_at  epithelial membrane protein 3  Info   Emp3 
   35 4e-07 4094.3 5595.3 0.732  1371318_at  ribosomal protein S16  Info   Rps16 
   36 4e-07 62.4 97.2 0.642  1383716_at  Transcribed locus, strongly similar to NP_777569.1 hypothetical protein MGC23909 [Homo sapiens]  Info    
   37 5e-07 51.6 131.4 0.393  1384331_at  Neoplastic progression 3 (predicted)  Info    
   38 5e-07 1092.5 71.3 15.323  1371754_at  solute carrier family 25 (mitochondrial carrier, phosphate carrier), member 25  Info   Slc25a25 
   39 5e-07 2103.1 1454.7 1.446  1387859_at  nitrogen fixation gene 1 (S. cerevisiae)  Info   Nfs1 
   40 6e-07 8.3 1 8.3  1381370_at  Cysteine and glycine-rich protein 2  Info   Csrp2 
   41 6e-07 21.5 82.4 0.261  1384211_at  procollagen, type XI, alpha 1  Info   Col11a1 
   42 6e-07 48.2 349 0.138  1392899_at  protein regulator of cytokinesis 1 (predicted)  Info   Prc1_predicted 
   43 6e-07 41.2 155.5 0.265  1379967_at  zinc finger protein 367 (predicted)  Info   Zfp367_predicted 
   44 6e-07 58.8 419.4 0.14  1367780_at  pituitary tumor-transforming 1  Info   Pttg1 
   45 6e-07 25.8 119.2 0.216  1383578_at  Transcribed locus  Info    
   46 6e-07 278.9 132.3 2.108  1368545_at  CASP8 and FADD-like apoptosis regulator  Info   Cflar 
   47 6e-07 33.2 294.6 0.113  1388340_at  Ns5atp9 protein  Info   RGD:1303041 
   48 7e-07 81.9 234.1 0.35  1372125_at  glutathione peroxidase 7 (predicted)  Info   Gpx7_predicted 
   49 7e-07 34.4 172.6 0.199  1373026_at  similar to RIKEN cDNA 2410030K01 (predicted)  Info   RGD1307631_predicted 
   50 7e-07 313.1 898.1 0.349  1392965_a_at  SPARC related modular calcium binding 2 (predicted)  Info   Smoc2_predicted 
   51 7e-07 2393.5 1576.2 1.519  1371775_at  Acyl-Coenzyme A dehydrogenase, short/branched chain  Info   Acadsb 
   52 7e-07 164.1 600.4 0.273  1373463_at  Collagen, type V, alpha 2  Info   Col5a2 
   53 7e-07 97.3 159.9 0.609  1384361_at  mal, T-cell differentiation protein 2  Info   Mal2 
   54 7e-07 177.1 760.3 0.233  1372006_at  Transcribed locus  Info    
   55 7e-07 1715.6 719 2.386  1367741_at  homocysteine-inducible, endoplasmic reticulum stress-inducible, ubiquitin-like domain member 1  Info   Herpud1 
   56 8e-07 555.3 1899.9 0.292  1367628_at  lectin, galactose binding, soluble 1  Info   Lgals1 
   57 8e-07 244.8 905 0.27  1370895_at  collagen, type V, alpha 2  Info   Col5a2 
   58 8e-07 511.4 1017.2 0.503  1371331_at  follistatin-like 1  Info   Fstl1 
   59 8e-07 363.1 675.4 0.538  1387891_at  peroxiredoxin 4  Info   Prdx4 
   60 9e-07 42.5 186 0.228  1373658_at  Rac GTPase-activating protein 1 (predicted)  Info    
   61 9e-07 116.3 185.9 0.626  1374938_at  ZW10 homolog, centromere/kinetochore protein (Drosophila) (predicted)  Info   Zw10_predicted 
   62 9e-07 84.7 217 0.39  1385190_at  Transcribed locus  Info    
   63 9e-07 3390.1 2569.6 1.319  1387878_at  glutamate dehydrogenase 1  Info   Glud1 
   64 9e-07 14.4 110.3 0.131  1371970_at  similar to expressed sequence AW413625  Info   LOC499322 
   65 1e-06 1232.6 2120.8 0.581  1388335_at  transgelin 2 (predicted)  Info   Tagln2_predicted 
   66 1e-06 44.2 199.2 0.222  1391450_at  lysyl oxidase-like 2 (predicted)  Info   Loxl2_predicted 
   67 1.1e-06 9.3 25.4 0.366  1377907_at  similar to RIKEN cDNA 6530403A03 (predicted)  Info   RGD1309020_predicted 
   68 1.1e-06 43 184.2 0.233  1368990_at  cytochrome P450, family 1, subfamily b, polypeptide 1  Info   Cyp1b1 
   69 1.1e-06 148.8 462.7 0.322  1368420_at  ceruloplasmin  Info   Cp 
   70 1.1e-06 395.4 1134.3 0.349  1367570_at  transgelin  Info   Tagln 
   71 1.1e-06 160.1 299 0.535  1367946_at  PDZ and LIM domain 1 (elfin)  Info   Pdlim1 
   72 1.1e-06 42.9 300.1 0.143  1374449_at  cell division cycle associated 3 /// similar to cell division cycle associated 3  Info   RGD:1359093 /// LOC501134 
   73 1.2e-06 272.9 635 0.43  1370288_a_at  tropomyosin 1, alpha  Info   Tpm1 
   74 1.2e-06 96.3 278.3 0.346  1374036_at  minichromosome maintenance deficient 2 mitotin (S. cerevisiae) (predicted)  Info   Mcm2_predicted 
   75 1.2e-06 9.7 93.6 0.104  1377832_at  polo-like kinase 4 (Drosophila) (predicted)  Info   Plk4_predicted 
   76 1.3e-06 13.9 36.5 0.381  1386660_at  Transcribed locus  Info    
   77 1.3e-06 52.4 194 0.27  1373286_at  CSX-associated LIM  Info   RGD:1359551 
   78 1.3e-06 92.7 635.3 0.146  1389408_at  ribonucleotide reductase M2  Info   Rrm2 
   79 1.3e-06 57.6 183.9 0.313  1392915_at  procollagen, type XI, alpha 1  Info   Col11a1 
   80 1.4e-06 2400.2 333.5 7.197  1387870_at  zinc finger protein 36  Info   Zfp36 
   81 1.4e-06 17.1 98.8 0.173  1377967_at  Retroviral integration site 2 (predicted)  Info    
   82 1.4e-06 31.7 97.6 0.325  1380088_at  Transcribed locus  Info    
   83 1.5e-06 13 85.4 0.152  1373628_at  Transcribed locus  Info    
   84 1.5e-06 259.3 419.2 0.619  1388236_x_at  RT1 class I, CE12  Info   RGD:1359103 
   85 1.5e-06 413.1 1474.1 0.28  1388985_at  hypothetical protein LOC310926 /// LOC501211  Info   LOC310926 /// LOC501211 
   86 1.5e-06 69.7 288.7 0.241  1368171_at  lysyl oxidase  Info   Lox 
   87 1.5e-06 62.8 114.8 0.547  1377908_at  CD4 antigen  Info   Cd4 
   88 1.6e-06 2187.8 1298.4 1.685  1376765_at  similar to maestro  Info   LOC361348 
   89 1.6e-06 82.1 196.9 0.417  1383962_at  Cd27 binding protein (Hindu God of destruction)  Info   Siva 
   90 1.6e-06 32.2 109.9 0.293  1372935_at  Similar to Coronin, actin binding protein 1C  Info    
   91 1.6e-06 658.2 1847.8 0.356  1370234_at  fibronectin 1  Info   Fn1 
   92 1.7e-06 26.3 3 8.767  1393436_at  similar to potential ligand-binding protein  Info   LOC309100 
   93 1.7e-06 156.4 241.6 0.647  1373894_at    Info    
   94 1.7e-06 893.4 430.8 2.074  1375926_at  Transcribed locus  Info    
   95 1.7e-06 100.2 197.5 0.507  1398380_at  similar to von Willebrand factor A-domain containing 1 (predicted)  Info   RGD1311476_predicted 
   96 1.8e-06 52 121.1 0.429  1382590_at  Similar to RIKEN cDNA 2310015N21  Info    
   97 1.8e-06 47 227.6 0.207  1371074_a_at  mini chromosome maintenance deficient 6 (S. cerevisiae)  Info   Mcmd6 
   98 1.9e-06 50.2 355.9 0.141  1367776_at  cell division cycle 2 homolog A (S. pombe)  Info   Cdc2a 
   99 2e-06 465.4 897.1 0.519  1388763_at  Transcribed locus, strongly similar to NP_031751.1 calponin 2 [Mus musculus]  Info    
   100 2e-06 197 365.7 0.539  1388955_at  Putative eps protein  Info   MGEPS 
   101 2e-06 2084.1 1514.3 1.376  1371974_at  phytanoyl-CoA dioxygenase domain containing 1 (predicted)  Info   Phyhd1_predicted 
   102 2.1e-06 103.3 307.3 0.336  1393240_at  EGF-containing fibulin-like extracellular matrix protein 2  Info   RGD:1359496 
   103 2.1e-06 66.7 148.5 0.449  1376198_at  adipocyte-specific adhesion molecule  Info   Asam 
   104 2.2e-06 271.4 683 0.397  1369944_at  MARCKS-like protein  Info   Mlp 
   105 2.3e-06 90.5 214.9 0.421  1390707_at  regulator of G-protein signalling 10  Info   Rgs10 
   106 2.3e-06 65 152.7 0.426  1368961_at  Matrix metalloproteinase 23  Info   Mmp23 
   107 2.4e-06 1097.7 2375.6 0.462  1368840_at  LR8 protein  Info   Lr8 
   108 2.4e-06 507.2 1011 0.502  1386940_at  tissue inhibitor of metalloproteinase 2  Info   Timp2 
   109 2.4e-06 32 218.6 0.146  1370346_at  cyclin B1  Info   Ccnb1 
   110 2.7e-06 46.5 192.5 0.242  1379345_at  procollagen, type XV (predicted)  Info   Col15a1_predicted 
   111 2.7e-06 18.7 130.6 0.143  1372516_at  kinesin family member 22 (predicted)  Info   Kif22_predicted 
   112 2.8e-06 737.1 1355.5 0.544  1371861_at  Transcribed locus  Info    
   113 2.8e-06 55.2 168.9 0.327  1374784_at  phosphoribosyl transferase domain containing 1 (predicted)  Info   Prtfdc1_predicted 
   114 2.9e-06 392.1 633.5 0.619  1371391_at  thioredoxin domain containing 5 (predicted)  Info   Txndc5_predicted 
   115 2.9e-06 1952 2711.2 0.72  1398788_at  glucose regulated protein, 58 kDa  Info   Grp58 
   116 2.9e-06 36 68.3 0.527  1384344_at  FK506 binding protein 7 (predicted)  Info   Fkbp7_predicted 
   117 2.9e-06 12.3 40.7 0.302  1376648_at  v-myc myelocytomatosis viral related oncogene, neuroblastoma derived (avian)  Info   Mycn 
   118 3e-06 201.3 437.1 0.461  1371475_at  ribonuclease, RNase A family 4  Info   Rnase4 
   119 3e-06 88.7 252.1 0.352  1371480_at  LOC499655  Info   LOC499655 
   120 3e-06 158.9 289.3 0.549  1373882_at    Info    
   121 3.1e-06 339.6 214.4 1.584  1368409_at  glutathione S-transferase, theta 2  Info   Gstt2 
   122 3.1e-06 148.8 310.5 0.479  1367744_at  melanoma antigen, family D, 2  Info   Maged2 
   123 3.1e-06 1684.4 357.1 4.717  1369788_s_at  v-jun sarcoma virus 17 oncogene homolog (avian)  Info   Jun 
   124 3.3e-06 755 146.9 5.14  1388792_at  growth arrest and DNA-damage-inducible 45 gamma (predicted)  Info   Gadd45g_predicted 
   125 3.3e-06 415.2 1248.2 0.333  1389966_at  procollagen, type VI, alpha 3 (predicted)  Info   Col6a3_predicted 
   126 3.4e-06 1313.8 744.5 1.765  1371099_at  Polymeric immunoglobulin receptor AATTAA-containing 3'UTR mRNA sequence  Info    
   127 3.4e-06 3351.8 4245.9 0.789  1367567_at  ribosomal protein L6  Info   Rpl6 
   128 3.5e-06 94.2 221.4 0.425  1372870_at  KDEL (Lys-Asp-Glu-Leu) endoplasmic reticulum protein retention receptor 3 (predicted)  Info   Kdelr3_predicted 
   129 3.5e-06 45.4 106.9 0.425  1374927_at  Transcribed locus  Info    
   130 3.5e-06 339.5 171.4 1.981  1368924_at  growth hormone receptor  Info   Ghr 
   131 3.6e-06 2.4 21.1 0.114  1370594_at  immunoglobulin superfamily, member 1  Info   Igsf1 
   132 3.7e-06 21.4 92 0.233  1387062_a_at  checkpoint kinase 1 homolog (S. pombe)  Info   Chek1 
   133 3.7e-06 198.7 384.7 0.517  1389395_at  Transcribed locus, moderately similar to NP_065184.2 selenoprotein N, 1 isoform 1 precursor; muscular dystrophy with rigid spine 1; rigid spine muscular dystrophy 1 [Homo sapiens]  Info    
   134 3.7e-06 231.5 395.4 0.585  1373672_at    Info    
   135 3.8e-06 2507.2 1958.4 1.28  1370200_at  glutamate dehydrogenase 1  Info   Glud1 
   136 3.8e-06 3121 3988.4 0.783  1367618_a_at  discs, large homolog 5 (Drosophila) (predicted)  Info   Dlg5_predicted 
   137 3.8e-06 138.8 305 0.455  1373128_at  reticulocalbin 3, EF-hand calcium binding domain  Info   RGD:1359365 
   138 3.8e-06 1425 1708.4 0.834  1398853_at  proteasome (prosome, macropain) subunit, beta type 3  Info   Psmb3 
   139 3.8e-06 2964.7 8728.3 0.34  1367581_a_at  secreted phosphoprotein 1  Info   Spp1 
   140 3.9e-06 3590 2843.8 1.262  1368236_at  meprin 1 alpha  Info   Mep1a 
   141 4e-06 43.7 205.7 0.212  1384280_at  Nucleolar and spindle associated protein 1 (predicted)  Info    
   142 4.2e-06 32.4 94.3 0.344  1369499_at  thymidylate synthase  Info   Tyms 
   143 4.2e-06 8.8 1.1 8  1398117_at  Similar to RIKEN cDNA 2700067D09 (predicted)  Info   MGC94228 
   144 4.3e-06 24.5 128.1 0.191  1383940_at  cell division cycle associated 1 (predicted)  Info   Cdca1_predicted 
   145 4.3e-06 40.7 120.7 0.337  1388439_at  similar to 65kDa FK506-binding protein  Info   LOC360627 
   146 4.3e-06 460.3 1363.9 0.337  1387892_at  tubulin, beta 5  Info   Tubb5 
   147 4.6e-06 133.4 287.7 0.464  1368207_at  FXYD domain-containing ion transport regulator 5  Info   Fxyd5 
   148 4.6e-06 50.1 187 0.268  1373401_at  Transcribed locus  Info    
   149 4.6e-06 42.1 86.1 0.489  1373838_at  fucosyltransferase 4  Info   Fut4 
   150 4.6e-06 372.2 856.7 0.434  1387946_at  lectin, galactoside-binding, soluble, 3 binding protein  Info   Lgals3bp 
   151 4.8e-06 51.6 175.3 0.294  1396055_at  procollagen, type VI, alpha 3 (predicted)  Info   Col6a3_predicted 
   152 4.9e-06 26.2 49.7 0.527  1383805_at  similar to Traf2 and NCK interacting kinase, splice variant 4  Info   LOC294917 
   153 4.9e-06 37.3 351.1 0.106  1372685_at  cyclin-dependent kinase inhibitor 3 (predicted)  Info   Cdkn3_predicted 
   154 4.9e-06 967.2 329.4 2.936  1389538_at  nuclear factor of kappa light chain gene enhancer in B-cells inhibitor, alpha  Info   Nfkbia 
   155 5e-06 33.6 20.4 1.647  1394129_at  Activin receptor interacting protein 1  Info   Acvrip1 
   156 5.1e-06 50.8 285.9 0.178  1388484_at  ubiquitin-conjugating enzyme E2C (predicted)  Info   Ube2c_predicted 
   157 5.1e-06 58 137.8 0.421  1391387_s_at  Transcribed locus  Info    
   158 5.1e-06 21.8 92.5 0.236  1382153_at  C-type (calcium dependent, carbohydrate recognition domain) lectin, superfamily member 6  Info   RGD:1359662 
   159 5.1e-06 47.1 75.3 0.625  1388866_at  Transcribed locus  Info    
   160 5.2e-06 97.3 140.1 0.695  1373785_at  similar to expressed sequence AI132321  Info   LOC362792 
   161 5.3e-06 61.2 160.3 0.382  1372565_at  similar to toll-associated serine protease (predicted)  Info   RGD1308120_predicted 
   162 5.4e-06 517.6 968.6 0.534  1368822_at  follistatin-like 1  Info   Fstl1 
   163 5.4e-06 130.1 401.7 0.324  1388939_at  procollagen, type XV (predicted)  Info   Col15a1_predicted 
   164 5.5e-06 23.4 71.8 0.326  1376943_at    Info    
   165 5.5e-06 45.1 100.8 0.447  1373000_at  similar to Sushi-repeat containing protein  Info   LOC317181 
   166 5.5e-06 28.4 130.6 0.217  1370462_at  hyaluronan mediated motility receptor  Info   Hmmr 
   167 5.6e-06 70.3 180.9 0.389  1369947_at  cathepsin K  Info   Ctsk 
   168 5.7e-06 33.9 81.3 0.417  1377369_at  cytochrome b reductase 1 (predicted)  Info   Cybrd1_predicted 
   169 5.8e-06 12.9 80.1 0.161  1397704_at  similar to establishment of cohesion 1 homolog 2  Info   LOC498536 
   170 5.8e-06 77.9 202.6 0.385  1368404_at  drebrin 1  Info   Dbn1 
   171 5.9e-06 916.4 90 10.182  1386935_at  nuclear receptor subfamily 4, group A, member 1  Info   Nr4a1 
   172 5.9e-06 36.9 114.1 0.323  1373914_at  Stem cell growth factor  Info   Scgf 
   173 5.9e-06 614 340.3 1.804  1378819_at  similar to RIKEN cDNA 0610012D14 (predicted)  Info   RGD1310111_predicted 
   174 6.3e-06 78.1 528.4 0.148  1367712_at  tissue inhibitor of metalloproteinase 1  Info   Timp1 
   175 6.3e-06 264.9 1068.5 0.248  1367655_at  similar to thymosin, beta 10  Info   LOC502600 
   176 6.5e-06 35.3 86.2 0.41  1382580_at  Transcribed locus  Info    
   177 6.5e-06 529 1031.1 0.513  1388392_at  Tax1 (human T-cell leukemia virus type I) binding protein 3 (predicted)  Info   Tax1bp3_predicted 
   178 6.7e-06 1188.6 272.9 4.355  1367725_at  serine/threonine-protein kinase pim-3  Info   Pim3 
   179 6.7e-06 214.4 256.5 0.836  1370353_at  translocase of inner mitochondrial membrane 22 homolog (yeast)  Info   Timm22 
   180 6.7e-06 67.5 170.8 0.395  1378296_at    Info    
   181 6.7e-06 28.6 135.3 0.211  1374726_at  fibronectin type III domain containing 1 (predicted)  Info   Fndc1_predicted 
   182 6.8e-06 1581.4 2055.2 0.769  1399027_at  ras homolog gene family, member A  Info   RGD:619921 
   183 6.8e-06 593.9 1101 0.539  1373204_at  hypothetical LOC297077 (predicted)  Info   RGD1310725_predicted 
   184 7.2e-06 109.9 302.3 0.364  1389858_at  Thymidine kinase 1  Info   Tk1 
   185 7.3e-06 1214.9 503.4 2.413  1368877_at  zinc finger protein 354A  Info   Znf354a 
   186 7.3e-06 40.7 105.1 0.387  1388879_at    Info    
   187 7.4e-06 82.7 143.9 0.575  1369957_at  regulator of G-protein signaling 5  Info   Rgs5 
   188 7.6e-06 1854.1 1309.5 1.416  1367829_at  enoyl Coenzyme A hydratase, short chain, 1, mitochondrial  Info   Echs1 
   189 7.6e-06 83.6 270.8 0.309  1393252_at  Fibulin 1 (predicted)  Info    
   190 7.8e-06 1617.4 1047.5 1.544  1389114_at  similar to Hypothetical protein MGC59076 (predicted)  Info   RGD1309144_predicted 
   191 8e-06 35.6 113.8 0.313  1379235_x_at  similar to CDC45L  Info   LOC287961 
   192 8.1e-06 46.5 112.3 0.414  1371071_at  guanine nucleotide binding protein beta 4 subunit  Info   RGD:1359321 
   193 8.1e-06 14.2 102.9 0.138  1372186_a_at  topoisomerase (DNA) 2 alpha  Info   Top2a 
   194 8.1e-06 2876.3 2284.5 1.259  1398514_at  homogentisate 1, 2-dioxygenase (predicted)  Info   Hgd_predicted 
   195 8.2e-06 457.1 260.8 1.753  1383420_at  Similar to lactation elevated 1  Info    
   196 8.3e-06 77 170 0.453  1383404_at  Transcribed locus  Info    
   197 8.4e-06 313.9 510.4 0.615  1367732_at  guanine nucleotide binding protein, beta 1  Info   Gnb1 
   198 8.5e-06 9.5 95.2 0.1  1397769_at  Similar to RIKEN cDNA 6330512M04 gene  Info    
   199 8.5e-06 49.4 114 0.433  1383246_at  Similar to Hypothetical protein A630084C13 (predicted)  Info    
   200 8.6e-06 81.1 239.5 0.339  1372042_at  chemokine-like factor super family 3 (predicted)  Info   Cklfsf3_predicted 
   201 8.7e-06 663.7 391.2 1.697  1398343_at  DnaJ (Hsp40) homolog, subfamily A, member 4 (predicted)  Info    
   202 8.8e-06 498.7 345.1 1.445  1377812_a_at  transmembrane protein 24 (predicted)  Info   Tmem24_predicted 
   203 8.8e-06 158.9 716.6 0.222  1371700_at  Microfibrillar-associated protein 4 (predicted)  Info    
   204 8.9e-06 323.1 581.7 0.555  1383085_at  Similar to Sh3bgrl protein  Info    
   205 8.9e-06 13 54.4 0.239  1383323_at  similar to RIKEN cDNA 1700022L09  Info   LOC499933 
   206 9e-06 783.6 374.9 2.09  1371012_at  2-hydroxyphytanoyl-Coenzyme A lyase  Info   Hpcl2 
   207 9e-06 2372.8 1205.2 1.969  1370209_at  basic transcription element binding protein 1  Info   Bteb1 
   208 9.1e-06 47.4 305.8 0.155  1367749_at  lumican  Info   Lum 
   209 9.1e-06 73.7 200.5 0.368  1392672_at  stem cell growth factor  Info   Scgf 
   210 9.2e-06 38.7 84.1 0.46  1370127_at  polymerase (DNA directed), delta 1, catalytic subunit  Info   Pold1 
   211 9.3e-06 46.4 305.1 0.152  1389566_at  cyclin B2 (predicted)  Info   Ccnb2_predicted 
   212 9.3e-06 1535.8 2174.6 0.706  1371573_at  ribosomal protein L36a (predicted)  Info   Rpl36a_predicted 
   213 9.3e-06 593.3 963.9 0.616  1371365_at  similar to Ubiquitin-conjugating enzyme E2S  Info   LOC292588 
   214 9.4e-06 25 112.4 0.222  1389413_at  Oligodendrocyte-myelin glycoprotein  Info   Omg 
   215 9.4e-06 44.5 195.2 0.228  1388650_at  topoisomerase (DNA) 2 alpha  Info   Top2a 
   216 9.5e-06 13.4 62.4 0.215  1368773_at  follicle stimulating hormone primary response gene 1  Info   Fshprh1 
   217 9.5e-06 4761.9 2077.8 2.292  1367802_at  serum/glucocorticoid regulated kinase  Info   Sgk 
   218 9.6e-06 568.6 1164.1 0.488  1372013_at  interferon induced transmembrane protein 1 (predicted)  Info   Ifitm1_predicted 
   219 9.7e-06 84.2 40.3 2.089  1386045_at  Transcribed locus  Info    
   220 9.9e-06 352.5 667.9 0.528  1367691_at  protein kinase C, delta binding protein  Info   Prkcdbp 
   221 1e-05 945.5 644.9 1.466  1387531_at  methionine sulfoxide reductase A  Info   Msra 
   222 1.01e-05 699.1 196.6 3.556  1368025_at  DNA-damage-inducible transcript 4  Info   Ddit4 
   223 1.01e-05 69.6 18.9 3.683  1376702_at  Megalencephalic leukoencephalopathy with subcortical cysts 1 (predicted)  Info    
   224 1.02e-05 70.7 255.3 0.277  1373897_at    Info    
   225 1.02e-05 37.3 71.6 0.521  1376889_at    Info    
   226 1.02e-05 995.1 653.9 1.522  1372341_at  Transcribed locus, strongly similar to XP_484968.2 PREDICTED: similar to RIKEN cDNA C330005L02 [Mus musculus]  Info    
   227 1.05e-05 3330.2 5632.5 0.591  1367568_a_at  matrix Gla protein  Info   Mgp 
   228 1.1e-05 98.7 236.6 0.417  1374013_at  C1q and tumor necrosis factor related protein 5 (predicted)  Info   C1qtnf5_predicted 
   229 1.12e-05 34.6 63.5 0.545  1391139_at  Transcribed locus  Info    
   230 1.12e-05 23.3 56.3 0.414  1379425_at  similar to RIKEN cDNA 3110037I16 (predicted)  Info   RGD1309802_predicted 
   231 1.13e-05 50.2 111.7 0.449  1370585_a_at  protein kinase C, beta 1  Info   Prkcb1 
   232 1.16e-05 182.6 124.8 1.463  1367658_at  SH3/ankyrin domain gene 3 /// hypothetical gene supported by NM_021676  Info   Shank3 /// LOC497826 
   233 1.17e-05 204.9 373.6 0.548  1388784_at  colony stimulating factor 1 receptor  Info   Csf1r 
   234 1.18e-05 67.2 137.5 0.489  1389873_at  apoptosis-associated speck-like protein containing a CARD  Info   Pycard 
   235 1.18e-05 79.9 139.9 0.571  1378633_at  leupaxin (predicted)  Info   Lpxn_predicted 
   236 1.18e-05 1645.6 3464.8 0.475  1367563_at  secreted acidic cysteine rich glycoprotein  Info   Sparc 
   237 1.2e-05 756.8 442.7 1.71  1372158_at  LRP16 protein  Info   Lrp16 
   238 1.2e-05 1486.5 715.9 2.076  1377966_at  Transcribed locus, weakly similar to XP_579465.1 PREDICTED: DNA binding protein N5 [Rattus norvegicus]  Info    
   239 1.21e-05 146.1 313 0.467  1393643_at  reticulocalbin (predicted)  Info   Rcn_predicted 
   240 1.21e-05 3659 4305.6 0.85  1371340_at  ribosomal protein, large P2 /// similar to 60S acidic ribosomal protein P2  Info   Rplp2 /// LOC498363 
   241 1.22e-05 1225.2 960.2 1.276  1375642_at  ubiquitin-conjugating enzyme E2B, RAD6 homolog (S. cerevisiae)  Info   Ube2b 
   242 1.22e-05 370.4 274.8 1.348  1398500_at  G protein-coupled receptor 116  Info   Gpr116 
   243 1.22e-05 55.1 101.2 0.544  1373750_at  leprecan-like 2 (predicted)  Info   Leprel2_predicted 
   244 1.24e-05 395.3 312 1.267  1368390_at  v-raf oncogene homolog 1 (murine sarcoma 3611 virus)  Info   Araf1 
   245 1.24e-05 32.2 199 0.162  1394419_at  similar to RIKEN cDNA 6530401L14 gene (predicted)  Info   RGD1309107_predicted 
   246 1.24e-05 11.8 23.3 0.506  1390680_at  Transcribed locus  Info    
   247 1.25e-05 16 70.2 0.228  1374805_at  similar to hypothetical protein MGC5528  Info   LOC299933 
   248 1.27e-05 127.5 310.8 0.41  1370308_at  RS21-C6 protein  Info   Rs21c6 
   249 1.28e-05 357 207.7 1.719  1384188_at    Info    
   250 1.28e-05 760.1 1404.9 0.541  1373575_at  similar to NADH dehydrogenase (ubiquinone) Fe-S protein 2  Info   LOC498279 
   251 1.28e-05 68.3 137.2 0.498  1370813_at  glutathione S-transferase, mu 5  Info   Gstm5 
   252 1.29e-05 526.7 852 0.618  1371487_at  SH3 domain binding glutamic acid-rich protein-like 3 (predicted)  Info   Sh3bgrl3_predicted 
   253 1.3e-05 1773.5 1079.8 1.642  1383395_at  agmatine ureohydrolase (agmatinase) (predicted)  Info   Agmat_predicted 
   254 1.32e-05 3308.3 941.1 3.515  1373093_at  similar to Mitogen-inducible gene 6 protein homolog (Mig-6) (Gene 33 polypeptide) (predicted)  Info   RGD1307599_predicted 
   255 1.32e-05 852.8 448.2 1.903  1371266_at  afamin  Info   Afm 
   256 1.32e-05 109 180.7 0.603  1376937_at  similar to 4631422O05Rik protein  Info   LOC304020 
   257 1.33e-05 57.3 112 0.512  1370539_at  RAB8B, member RAS oncogene family  Info   Rab8b 
   258 1.33e-05 11.4 112 0.102  1379582_a_at  cyclin A2  Info   Ccna2 
   259 1.33e-05 96.4 270.9 0.356  1385625_at  EGF-containing fibulin-like extracellular matrix protein 2  Info   RGD:1359496 
   260 1.34e-05 4406.5 5485.7 0.803  1371300_at  ribosomal protein L3  Info   Rpl3 
   261 1.36e-05 57 132.4 0.431  1372410_at  C1q and tumor necrosis factor related protein 6 (predicted)  Info    
   262 1.37e-05 62.6 178.5 0.351  1370328_at  Dickkopf homolog 3 (Xenopus laevis)  Info   Dkk3 
   263 1.38e-05 119.5 222 0.538  1370642_s_at  platelet derived growth factor receptor, beta polypeptide /// hypothetical gene supported by NM_031525  Info   Pdgfrb /// LOC497724 
   264 1.4e-05 31.6 121.5 0.26  1372518_at  fibulin 1 (predicted)  Info   Fbln1_predicted 
   265 1.42e-05 1406.4 840 1.674  1387357_at  trimethyllysine hydroxylase, epsilon  Info   Tmlhe 
   266 1.43e-05 227.3 473.3 0.48  1372818_at  collectin sub-family member 12  Info   Colec12 
   267 1.43e-05 773.6 1527.3 0.507  1371310_s_at  serine (or cysteine) proteinase inhibitor, clade H, member 1  Info   Serpinh1 
   268 1.44e-05 1451.2 874.7 1.659  1372475_at  PTEN induced putative kinase 1 (predicted)  Info   Pink1_predicted 
   269 1.44e-05 193.6 127.5 1.518  1379587_at    Info    
   270 1.47e-05 60.4 147.6 0.409  1383284_at  Transcribed locus  Info    
   271 1.49e-05 165.4 297 0.557  1367593_at  selenoprotein W, muscle 1  Info   Sepw1 
   272 1.5e-05 145 243.9 0.595  1374474_at  Transcribed locus, strongly similar to NP_080091.1 copine family member [Mus musculus]  Info    
   273 1.51e-05 30.1 85.7 0.351  1392489_at  Transcribed locus  Info    
   274 1.51e-05 968.6 682 1.42  1387178_a_at  cystathionine beta synthase  Info   Cbs 
   275 1.52e-05 73.5 156.4 0.47  1369186_at  caspase 1  Info   Casp1 
   276 1.53e-05 192.9 582.2 0.331  1367676_at  high mobility group box 2 /// similar to High mobility group protein 2 (HMG-2) /// similar to High mobility group protein 2 (HMG-2) /// similar to High mobility group protein 2 (HMG-2)  Info   Hmgb2 /// LOC498072 /// LOC498388 /// LOC498988 
   277 1.54e-05 357.4 893.8 0.4  1368418_a_at  ceruloplasmin  Info   Cp 
   278 1.54e-05 21 60.6 0.347  1376951_at  MAD2 (mitotic arrest deficient, homolog)-like 1 (yeast) (predicted)  Info    
   279 1.55e-05 83.8 166.3 0.504  1377299_at  nuclear autoantigenic sperm protein  Info   RGD:1359609 
   280 1.56e-05 1160.4 568.9 2.04  1388778_at  Transcribed locus  Info    
   281 1.57e-05 61.2 30.9 1.981  1369677_at  cannabinoid receptor 1 (brain)  Info   Cnr1 
   282 1.58e-05 737.7 302.7 2.437  1368139_s_at  alkaline phosphatase, tissue-nonspecific  Info   Alpl 
   283 1.59e-05 26.5 74 0.358  1375972_at  Transcribed locus  Info    
   284 1.59e-05 4266.8 4685.2 0.911  1371304_a_at  similar to myosin, light polypeptide 6, alkali, smooth muscle and non-muscle /// myosin, light polypeptide 6, alkali, smooth muscle and non-muscle (predicted)  Info   LOC304447 /// Myl6_predicted 
   285 1.59e-05 4456.9 3452.4 1.291  1368150_at  solute carrier family 27 (fatty acid transporter), member 2 /// hypothetical gene supported by NM_031736  Info   Slc27a2 /// LOC497779 
   286 1.6e-05 1810 1323 1.368  1387805_at  BCL2/adenovirus E1B 19 kDa-interacting protein 3  Info   Bnip3 
   287 1.61e-05 413.2 158.7 2.604  1369415_at  basic helix-loop-helix domain containing, class B2  Info   Bhlhb2 
   288 1.67e-05 177.2 109.4 1.62  1386274_at  solute carrier family 17 (sodium phosphate), member 2 (predicted)  Info   Slc17a2_predicted 
   289 1.67e-05 26.9 71.7 0.375  1369527_at  chemokine (C-X3-C) receptor 1  Info   Cx3cr1 
   290 1.67e-05 21.7 65.5 0.331  1387687_at  immunoglobulin superfamily, member 6  Info   Igsf6 
   291 1.69e-05 38.1 88.8 0.429  1377639_at  adipocyte-specific adhesion molecule  Info   Asam 
   292 1.7e-05 290.1 406.2 0.714  1374897_at    Info    
   293 1.71e-05 8.3 78.3 0.106  1379448_at  Ttk protein kinase (predicted)  Info   Ttk_predicted 
   294 1.73e-05 106.1 156 0.68  1381528_at    Info    
   295 1.75e-05 38.7 72.6 0.533  1386956_at  scavenger receptor class B, member 1  Info   Scarb1 
   296 1.75e-05 193.6 76.7 2.524  1387263_at  pyruvate kinase, liver and RBC  Info   Pklr 
   297 1.76e-05 35.9 86.1 0.417  1380775_at  M-phase phosphoprotein 1 (predicted)  Info   Mphosph1_predicted 
   298 1.77e-05 23.7 150.4 0.158  1392648_at  mannose receptor, C type 1 (predicted)  Info   Mrc1_predicted 
   299 1.79e-05 463.6 76.5 6.06  1368147_at  dual specificity phosphatase 1  Info   Dusp1 
   300 1.8e-05 50.3 108.6 0.463  1372584_at  similar to DKFZP566K1924 protein (predicted)  Info   RGD1308373_predicted 
   301 1.81e-05 245 758.4 0.323  1388936_at  cadherin 11  Info   Cdh11 
   302 1.81e-05 31.2 120.1 0.26  1398602_at  MAD2 (mitotic arrest deficient, homolog)-like 1 (yeast) (predicted)  Info    
   303 1.82e-05 972.1 743.9 1.307  1373693_at  G protein-coupled receptor, family C, group 5, member C (predicted)  Info   Gprc5c_predicted 
   304 1.84e-05 84.5 226.5 0.373  1370949_at  myristoylated alanine rich protein kinase C substrate  Info   Marcks 
   305 1.85e-05 1780.4 588 3.028  1389528_s_at  v-jun sarcoma virus 17 oncogene homolog (avian)  Info   Jun 
   306 1.86e-05 1328.9 796.4 1.669  1389548_at  alcohol dehydrogenase, iron containing, 1 (predicted)  Info   Adhfe1_predicted 
   307 1.87e-05 57 190.1 0.3  1370294_a_at  cell division cycle 20 homolog (S. cerevisiae)  Info   Cdc20 
   308 1.87e-05 4205.3 2792.9 1.506  1371389_at  hypothetical LOC306766  Info   RGD:1359256 
   309 1.87e-05 401.5 887.1 0.453  1368419_at  ceruloplasmin  Info   Cp 
   310 1.9e-05 43.9 368.8 0.119  1383747_at  Ect2 oncogene (predicted)  Info    
   311 1.91e-05 810.5 458.6 1.767  1382061_at  lactate dehydrogenase D (predicted)  Info   Ldhd_predicted 
   312 1.96e-05 467.9 295.3 1.584  1374217_at  similar to chromosome 16 open reading frame 5 (predicted)  Info   RGD1310686_predicted 
   313 1.96e-05 20.9 119.8 0.174  1368260_at  aurora kinase B  Info   Aurkb 
   314 1.98e-05 4826.3 5745.5 0.84  1367634_at  ribosomal protein L31  Info   Rpl31 
   315 1.99e-05 64.6 201.9 0.32  1378592_at  Similar to RIKEN cDNA 2310035M22 (predicted)  Info    
   316 2e-05 2158.2 452 4.775  1368146_at  dual specificity phosphatase 1  Info   Dusp1 
   317 2e-05 2953.4 2057 1.436  1374628_at  crystallin, zeta (predicted)  Info   Cryz_predicted 
   318 2e-05 71.4 187 0.382  1381311_at  EGF-like module containing, mucin-like, hormone receptor-like sequence 1  Info   RGD:1359214 
   319 2.02e-05 186.4 117.1 1.592  1383188_at  Transcribed locus  Info    
   320 2.04e-05 186.6 311 0.6  1373490_at  glia maturation factor, gamma  Info   Gmfg 
   321 2.04e-05 635.5 1875.9 0.339  1367574_at  vimentin  Info   Vim 
   322 2.04e-05 2040.8 1414.9 1.442  1369986_at  hydroxyacyl glutathione hydrolase  Info   Hagh 
   323 2.05e-05 20.8 50.5 0.412  1385559_at  Transcribed locus  Info    
   324 2.06e-05 184.6 340.3 0.542  1383786_at  Transcribed locus  Info    
   325 2.08e-05 5495 7869.4 0.698  1387883_a_at  thymosin, beta 4  Info   Tmsb4x 
   326 2.09e-05 1291.7 942 1.371  1372093_at  Max interacting protein 1  Info   Mxi1 
   327 2.1e-05 33.5 92.8 0.361  1369977_at  ubiquitin carboxy-terminal hydrolase L1  Info   Uchl1 
   328 2.12e-05 227 531.3 0.427  1368821_at  follistatin-like 1  Info   Fstl1 
   329 2.14e-05 521.8 412 1.267  1374101_at  TBC1 domain family, member 10 (predicted)  Info   Tbc1d10_predicted 
   330 2.16e-05 34.3 87.4 0.392  1384195_at    Info    
   331 2.17e-05 120 321.9 0.373  1389553_at  dendritic cell inhibitory receptor 3  Info   RGD:1359528 
   332 2.19e-05 899.2 237.6 3.785  1387087_at  CCAAT/enhancer binding protein (C/EBP), beta  Info   Cebpb 
   333 2.2e-05 5783 4738.7 1.22  1368180_s_at  glutathione-S-transferase, alpha type2  Info   Gsta2 
   334 2.21e-05 1113.4 708.4 1.572  1393061_at  similar to cDNA sequence BC021608  Info   RGD:1359544 
   335 2.24e-05 301.2 76.7 3.927  1387214_at  carboxylesterase 1  Info   RGD:621508 
   336 2.25e-05 1210.6 2602.3 0.465  1373245_at  procollagen, type IV, alpha 1 (predicted)  Info   Col4a1_predicted 
   337 2.26e-05 97.8 203.4 0.481  1373504_at  GLI pathogenesis-related 1 (glioma) (predicted)  Info   Glipr1_predicted 
   338 2.27e-05 19.6 102.9 0.19  1391518_at    Info    
   339 2.27e-05 84.7 139 0.609  1383606_at  membrane targeting (tandem) C2 domain containing 1  Info   Mtac2d1 
   340 2.27e-05 145.5 212.6 0.684  1377756_at  similar to RIKEN cDNA 5830446M03 (predicted)  Info   RGD1307688_predicted 
   341 2.28e-05 182.2 408.8 0.446  1390116_at  Polymerase I and transcript release factor (predicted)  Info    
   342 2.3e-05 14.6 67.3 0.217  1379479_at  kinesin family member 4  Info   Kif4 
   343 2.3e-05 1188.9 394.5 3.014  1390801_at  similar to hypothetical protein MGC34760  Info   RGD:1359349 
   344 2.33e-05 5.6 16.6 0.337  1380573_a_at  similar to C19orf36 protein  Info   LOC362832 
   345 2.37e-05 132.1 204.7 0.645  1388717_at  protein O-fucosyltransferase 2 (predicted)  Info   Pofut2_predicted 
   346 2.39e-05 1244 2102.6 0.592  1371653_at    Info    
   347 2.44e-05 62.3 164.2 0.379  1376599_at  ATPase family, AAA domain containing 2 (predicted)  Info    
   348 2.45e-05 30.4 81.7 0.372  1382078_at  Transcribed locus  Info    
   349 2.45e-05 895.4 444.4 2.015  1368661_at  solute carrier family 13 (sodium-dependent dicarboxylate transporter), member 2  Info   Slc13a2 
   350 2.45e-05 1088.4 1857.9 0.586  1367671_at  proliferating cell nuclear antigen  Info   Pcna 
   351 2.51e-05 47.2 111.3 0.424  1387868_at  lipopolysaccharide binding protein  Info   Lbp 
   352 2.52e-05 45.6 88.9 0.513  1367855_at  scavenger receptor class B, member 1  Info   Scarb1 
   353 2.54e-05 538.4 380.4 1.415  1398878_at  similar to CPSF4 protein  Info   MGC108785 
   354 2.55e-05 1419.9 1014.7 1.399  1380577_at  ATP-binding cassette, sub-family G (WHITE), member 2  Info   Abcg2 
   355 2.57e-05 120.2 236.7 0.508  1389355_at  immediate early response 5  Info   Ier5 
   356 2.57e-05 124.8 317.6 0.393  1380909_at  Transcribed locus  Info    
   357 2.57e-05 829 139.3 5.951  1371080_at  kallikrein  Info   RGD:735228 
   358 2.6e-05 638 269 2.372  1376746_at  lactate dehydrogenase D (predicted)  Info   Ldhd_predicted 
   359 2.6e-05 2.3 9.4 0.245  1378174_at    Info    
   360 2.63e-05 196.1 415.5 0.472  1374496_at  similar to RIKEN cDNA 1200009O22; EST AI316813 (predicted)  Info   RGD1310827_predicted 
   361 2.64e-05 17 135.9 0.125  1397341_at  PDZ binding kinase (predicted)  Info    
   362 2.65e-05 390.1 740.2 0.527  1388298_at  myosin, light polypeptide 9, regulatory (predicted)  Info   Myl9_predicted 
   363 2.65e-05 140 236.8 0.591  1390177_at  Transcribed locus  Info    
   364 2.68e-05 28.9 72.5 0.399  1376871_at    Info   LOC288549 /// LOC363890 /// LOC363894 /// LOC363899 /// LOC498168 /// LOC498169 /// LOC498199 /// LOC501818 /// LOC501821 /// LOC501828 
   365 2.69e-05 4.2 33 0.127  1378997_at  Eph receptor B6 (predicted)  Info   Ephb6_predicted 
   366 2.73e-05 124.4 205 0.607  1388458_at  replication factor C (activator 1) 4 (predicted)  Info   Rfc4_predicted 
   367 2.75e-05 177.1 329.6 0.537  1372478_at  similar to LNV  Info   LOC501065 
   368 2.76e-05 640.4 454.1 1.41  1372323_at  sarcosine dehydrogenase  Info   Sardh 
   369 2.76e-05 3258.4 2500.6 1.303  1367695_at  quinoid dihydropteridine reductase  Info   Qdpr 
   370 2.8e-05 383.8 213.9 1.794  1382069_at  ATPase, class VI, type 11A (predicted)  Info    
   371 2.8e-05 718.9 1526.4 0.471  1370287_a_at  tropomyosin 1, alpha  Info   Tpm1 
   372 2.81e-05 1119.1 1773.2 0.631  1387995_a_at  interferon induced transmembrane protein 3  Info   Ifitm3 
   373 2.82e-05 792.4 569.3 1.392  1389695_at    Info    
   374 2.83e-05 1041.4 552 1.887  1393793_at  Transcribed locus  Info    
   375 2.83e-05 64 172.1 0.372  1371782_at  NIPSNAP-related protein  Info   RGD:1359688 
   376 2.84e-05 2439.9 2104.5 1.159  1374431_at  ATPase, H+ transporting, V1 subunit A, isoform 1 (predicted)  Info    
   377 2.86e-05 43.5 126.9 0.343  1373557_at  mini chromosome maintenance deficient 4 homolog (S. cerevisiae) /// similar to mcdc21 protein  Info   Mcmd4 /// LOC310429 
   378 2.88e-05 1025.2 1268.4 0.808  1389857_at  similar to WW domain binding protein 5  Info   LOC294067 
   379 2.88e-05 190.1 501.9 0.379  1372294_at  PE responsive protein c64  Info    
   380 2.92e-05 392.6 256.1 1.533  1373506_at  Guanylate nucleotide binding protein 2  Info   Gbp2 
   381 2.92e-05 3341.4 2921.8 1.144  1387793_at  ERM-binding phosphoprotein  Info   Slc9a3r1 
   382 2.94e-05 2114.5 3385.7 0.625  1386861_at  H2A histone family, member Z  Info   H2afz 
   383 2.95e-05 381.7 583.2 0.654  1388705_at  similar to selenoprotein SelM  Info   LOC498398 
   384 2.97e-05 258 440.3 0.586  1383181_at  DnaJ (Hsp40) homolog, subfamily C, member 9 (predicted)  Info   Dnajc9_predicted 
   385 2.98e-05 1895.8 3628 0.523  1375170_at  S100 calcium binding protein A11 (calizzarin) (predicted)  Info   S100a11_predicted 
   386 3e-05 22.3 162.9 0.137  1370345_at  cyclin B1  Info   Ccnb1 
   387 3e-05 26.8 92 0.291  1387600_at  gamma-aminobutyric acid (GABA) A receptor, pi  Info   Gabrp 
   388 3.04e-05 25.5 53.1 0.48  1385706_at  Similar to Testis derived transcript  Info    
   389 3.06e-05 514.8 269.6 1.909  1387372_at  solute carrier family 6 (neurotransmitter transporter, GABA), member 13  Info   Slc6a13 
   390 3.07e-05 99 193.9 0.511  1392265_s_at  Matrix metalloproteinase 23  Info   Mmp23 
   391 3.1e-05 148.6 233.9 0.635  1389130_at  E74-like factor 2 (predicted)  Info    
   392 3.12e-05 30.1 160.9 0.187  1389668_at  similar to AD024 protein (predicted)  Info   RGD1307811_predicted 
   393 3.15e-05 139.9 206.6 0.677  1385070_at  Transcribed locus, moderately similar to XP_488956.1 PREDICTED: hypothetical protein LOC66602 [Mus musculus]  Info    
   394 3.15e-05 157.2 258.4 0.608  1376574_at  Lymphocyte antigen 68  Info   C1qr1 
   395 3.16e-05 37.5 101 0.371  1368270_at  apolipoprotein B editing complex 1  Info   Apobec1 
   396 3.16e-05 98.4 142 0.693  1378070_at  Transcribed locus  Info    
   397 3.17e-05 214.4 367 0.584  1388339_at  phosphoprotein enriched in astrocytes 15 (predicted)  Info   Pea15_predicted 
   398 3.19e-05 1158.6 795 1.457  1397526_at  glutaryl-Coenzyme A dehydrogenase (predicted)  Info   Gcdh_predicted 
   399 3.2e-05 105.2 52.8 1.992  1368897_at  MAD homolog 7 (Drosophila)  Info   Madh7 
   400 3.21e-05 21.7 77.8 0.279  1371815_at  microfibrillar-associated protein 2 (predicted)  Info   Mfap2_predicted 
   401 3.22e-05 200.3 346.5 0.578  1373592_at  similar to SPI6  Info   MGC94010 
   402 3.24e-05 475 943.4 0.503  1387029_at  complement component factor H  Info   Cfh 
   403 3.24e-05 93.6 57 1.642  1379498_at  Transcribed locus, weakly similar to NP_060380.2 hypothetical protein FLJ20628 [Homo sapiens]  Info    
   404 3.24e-05 1026.2 2296 0.447  1372439_at  procollagen, type IV, alpha 1 (predicted)  Info   Col4a1_predicted 
   405 3.28e-05 1930 1619.9 1.191  1368317_at  aquaporin 7  Info   Aqp7 
   406 3.3e-05 42.6 88.4 0.482  1379440_at  follistatin-like 3  Info   Fstl3 
   407 3.34e-05 314.8 891.1 0.353  1371924_at  olfactomedin-like 3 (predicted)  Info   Olfml3_predicted 
   408 3.37e-05 109.1 379.5 0.287  1390839_at  similar to RIKEN cDNA E030024M05 (predicted)  Info   RGD1306818_predicted 
   409 3.37e-05 182.5 243.7 0.749  1391437_at  similar to BC002216 protein  Info   RGD:1359656 
   410 3.37e-05 461.4 253.4 1.821  1369100_at  angiotensin/vasopressin receptor  Info   Nalp6 
   411 3.37e-05 1.9 14.4 0.132  1398644_at  Transcribed locus  Info    
   412 3.38e-05 94.8 190 0.499  1390384_at  similar to Histone H2A.x (H2a/x)  Info   LOC500987 
   413 3.4e-05 45.9 167.8 0.274  1368172_a_at  lysyl oxidase  Info   Lox 
   414 3.43e-05 3696.4 4551.2 0.812  1367569_at  laminin receptor 1 (67kD, ribosomal protein SA)  Info   Lamr1 
   415 3.46e-05 2323.4 1746.7 1.33  1386885_at  enoyl coenzyme A hydratase 1, peroxisomal  Info   Ech1 
   416 3.46e-05 253.7 397.7 0.638  1373151_at  similar to RIKEN cDNA 2810489O06  Info   MGC109397 
   417 3.48e-05 2314.8 1833.4 1.263  1379741_at  ATPase, H+ transporting, lysosomal V0 subunit A isoform 4 (predicted)  Info   Atp6v0a4_predicted 
   418 3.5e-05 86.3 182.8 0.472  1377702_at  Retinoblastoma 1  Info   Rb1 
   419 3.51e-05 97.2 167.7 0.58  1384200_at    Info    
   420 3.53e-05 121.3 257.9 0.47  1367705_at  glutaredoxin 1 (thioltransferase)  Info   Glrx1 
   421 3.55e-05 192.8 369.6 0.522  1381162_at  Similar to septin 10 isoform 1  Info    
   422 3.55e-05 20.7 121.8 0.17  1393848_at  ribonucleotide reductase M2  Info   Rrm2 
   423 3.59e-05 2297 2928.9 0.784  1367578_at  peroxiredoxin 2  Info   Prdx2 
   424 3.59e-05 46.3 156.7 0.295  1376055_at  Transcribed locus  Info    
   425 3.59e-05 2855.8 2280.2 1.252  1367672_at  hydroxysteroid (17-beta) dehydrogenase 4  Info   Hsd17b4 
   426 3.64e-05 80.4 120.5 0.667  1378008_at  Transcribed locus  Info    
   427 3.67e-05 433.9 245.4 1.768  1388611_at  transcription elongation factor A (SII), 3 (predicted)  Info   Tcea3_predicted 
   428 3.69e-05 42.4 170.9 0.248  1376105_at  collagen, type XIV, alpha 1 (undulin) (predicted)  Info   Col14a1_predicted 
   429 3.7e-05 1373.7 919.1 1.495  1390172_at  dehydrogenase E1 and transketolase domain containing 1 (predicted)  Info   Dhtkd1_predicted 
   430 3.72e-05 165.2 377.2 0.438  1398294_at  actinin, alpha 1  Info   Actn1 
   431 3.75e-05 286.9 84 3.415  1369407_at  tumor necrosis factor receptor superfamily, member 11b (osteoprotegerin)  Info   Tnfrsf11b 
   432 3.77e-05 47.8 100.8 0.474  1389103_at  similar to RIKEN cDNA 2810037C03 (predicted)  Info   RGD1307397_predicted 
   433 3.77e-05 2.2 48.5 0.045  1397609_at  TRAF4 associated factor 1  Info   RGD:1303240 
   434 3.82e-05 31.7 216.7 0.146  1374775_at  antigen identified by monoclonal antibody Ki-67 (predicted) /// similar to Mki67 protein /// similar to Ki-67  Info   Mki67_predicted /// LOC310382 /// LOC366937 
   435 3.84e-05 123.1 283.1 0.435  1388628_at  integral type I protein  Info   RGD:1303327 
   436 3.84e-05 1520 1057 1.438  1370811_at  mercaptopyruvate sulfurtransferase  Info   Mpst 
   437 3.86e-05 598.3 1251.2 0.478  1386879_at  lectin, galactose binding, soluble 3  Info   Lgals3 
   438 3.89e-05 483.3 745.8 0.648  1369621_s_at  FK506 binding protein 1a  Info   Fkbp1a 
   439 3.89e-05 418.4 849.6 0.492  1368838_at  tropomyosin 4  Info   Tpm4 
   440 3.9e-05 64.2 181.6 0.354  1382185_at  similar to C1q and tumor necrosis factor related protein 2  Info   LOC497886 
   441 3.92e-05 96.3 157 0.613  1389682_at  similar to RIKEN cDNA 2510006C20 (predicted)  Info   RGD1309120_predicted 
   442 3.96e-05 3099.1 2140.4 1.448  1388884_at  similar to RIKEN cDNA 1810022C23 (predicted)  Info   RGD1310224_predicted 
   443 3.99e-05 53.7 151.8 0.354  1370080_at  heme oxygenase (decycling) 1  Info   Hmox1 
   444 4.02e-05 127.7 229.4 0.557  1392837_at  P34 protein  Info   P34 
   445 4.03e-05 235.4 374.9 0.628  1373447_at  similar to HN1-like protein (predicted)  Info   RGD1305117_predicted 
   446 4.06e-05 213.4 291.4 0.732  1372481_at  Transcribed locus, moderately similar to NP_598415.1 CD34 antigen [Mus musculus]  Info    
   447 4.07e-05 818 560.3 1.46  1383879_at  Transcribed locus  Info    
   448 4.1e-05 4007.9 2977.1 1.346  1375944_at    Info    
   449 4.1e-05 265.9 496.3 0.536  1369968_at  pleiotrophin  Info   Ptn 
   450 4.11e-05 16.6 36.1 0.46  1376218_a_at  Atpase, class I, type 8B, member 2 (predicted)  Info    
   451 4.11e-05 45.4 110.1 0.412  1374198_at  B7 homlog 3  Info   B7h3 
   452 4.13e-05 2956.1 2581.9 1.145  1371912_at  NADH dehydrogenase (ubiquinone) Fe-S protein 7 (predicted)  Info   Ndufs7_predicted 
   453 4.15e-05 28.9 60.1 0.481  1371947_at  necdin (predicted)  Info   Ndn_predicted 
   454 4.17e-05 38 80.5 0.472  1379957_at  similar to schlafen 8  Info   RGD:1359139 
   455 4.17e-05 291.2 507.6 0.574  1386882_at  t-complex testis expressed 1  Info   Tctex1 
   456 4.25e-05 276.1 432.4 0.639  1373054_at  CDW92 antigen  Info   Cdw92 
   457 4.26e-05 600 695.4 0.863  1373048_at  ARP10 actin-related protein 10 homolog (S. cerevisiae) (predicted)  Info   Actr10_predicted 
   458 4.27e-05 105.4 272.6 0.387  1390901_at  bone specific CMF608  Info   Igsf10 
   459 4.28e-05 682.8 450.4 1.516  1369989_at  pyridoxine 5'-phosphate oxidase  Info   Pnpo 
   460 4.28e-05 594 985.6 0.603  1370941_at  platelet derived growth factor receptor, alpha polypeptide  Info   Pdgfra 
   461 4.28e-05 20 103.3 0.194  1368224_at  Serine protease inhibitor  Info   Spin2c 
   462 4.31e-05 422.7 920.7 0.459  1388393_at  proteolipid protein 2  Info   Plp2 
   463 4.33e-05 48.4 191.8 0.252  1387952_a_at  CD44 antigen  Info   Cd44 
   464 4.35e-05 48.9 153.9 0.318  1393041_at  SMC2 structural maintenance of chromosomes 2-like 1 (yeast) (predicted)  Info   Smc2l1_predicted 
   465 4.37e-05 62.4 122.8 0.508  1389326_at  replication factor C (activator 1) 3 (predicted)  Info   Rfc3_predicted 
   466 4.38e-05 1668.8 1030.5 1.619  1368992_a_at  splicing factor, arginine/serine-rich 5  Info   Sfrs5 
   467 4.38e-05 68.1 164.3 0.414  1388479_at  Dihydropyrimidinase-like 3  Info   Dpysl3 
   468 4.39e-05 9.4 29.8 0.315  1385781_at  similar to SNF2/RAD54 family protein  Info   LOC317252 
   469 4.4e-05 474.2 792.9 0.598  1369950_at  cyclin-dependent kinase 4  Info   Cdk4 
   470 4.43e-05 6.6 60.1 0.11  1374902_at  IQ motif containing GTPase activating protein 3 (predicted)  Info   Iqgap3_predicted 
   471 4.44e-05 928.2 293 3.168  1386933_at  glycoprotein 2 (zymogen granule membrane)  Info   Gp2 
   472 4.45e-05 1685.4 1047.7 1.609  1378802_at  similar to hypothetical protein MGC13047  Info   RGD:1303232 
   473 4.46e-05 55.4 157.6 0.352  1375862_at    Info    
   474 4.47e-05 595.6 310.9 1.916  1370566_at  retinol dehydrogenase 2  Info   Rdh2 
   475 4.47e-05 38.6 67.3 0.574  1393059_at  Transcribed locus  Info    
   476 4.48e-05 270 694.7 0.389  1376652_at  complement component 1, q subcomponent, alpha polypeptide (predicted)  Info   C1qa_predicted 
   477 4.49e-05 29.8 136.3 0.219  1377452_at  tetranectin (plasminogen binding protein) (predicted)  Info   Tna_predicted 
   478 4.49e-05 119.5 191.9 0.623  1385458_a_at  similar to C11orf17 protein (predicted)  Info   RGD1306959_predicted 
   479 4.51e-05 92.3 145.3 0.635  1379347_at  Similar to RIKEN cDNA 1700023M03 (predicted)  Info   MGC108928 
   480 4.51e-05 5.5 43.3 0.127  1370550_at  limbic system-associated membrane protein  Info   Lsamp 
   481 4.52e-05 1242.6 2129.8 0.583  1369976_at  dynein, cytoplasmic, light chain 1  Info   Dnclc1 
   482 4.55e-05 3638.7 2440 1.491  1371763_at  similar to RIKEN cDNA 4931406C07 (predicted)  Info   RGD1309534_predicted 
   483 4.57e-05 3629.6 2302.7 1.576  1368304_at  Flavin containing monooxygenase 3  Info   Fmo3 
   484 4.65e-05 284.3 362.7 0.784  1374289_at  RNA polymerase 1-1 (predicted)  Info   Rpo1-1_predicted 
   485 4.66e-05 281.5 160.5 1.754  1369259_at  deiodinase, iodothyronine, type I  Info   Dio1 
   486 4.69e-05 710 1091.1 0.651  1388776_at  scotin  Info   RGD:1359661 
   487 4.69e-05 1022.4 1962.3 0.521  1367614_at  annexin A1  Info   Anxa1 
   488 4.72e-05 277.5 619.9 0.448  1370857_at  smooth muscle alpha-actin  Info   RGD:621676 
   489 4.74e-05 31 131.2 0.236  1373250_at    Info    
   490 4.76e-05 6750.2 4167.3 1.62  1388271_at    Info    
   491 4.78e-05 192.4 276.5 0.696  1371712_at  similar to snRNP core protein SMX5d  Info   LOC292030 
   492 4.8e-05 251.9 372.9 0.676  1388622_at  nucleolar protein 5A (predicted)  Info   Nol5a_predicted 
   493 4.8e-05 748.9 1215.5 0.616  1367693_at  tyrosine 3-monooxygenase/tryptophan 5-monooxygenase activation protein, eta polypeptide  Info   Ywhah 
   494 4.83e-05 978 2176.4 0.449  1367584_at  annexin A2  Info   Anxa2 
   495 4.84e-05 4.5 22.5 0.2  1369105_a_at  protein kinase (cAMP dependent, catalytic) inhibitor beta  Info   Pkib 
   496 4.86e-05 138.4 363.8 0.38  1370215_at  complement component 1, q subcomponent, beta polypeptide  Info   C1qb 
   497 4.86e-05 157.7 409.6 0.385  1370301_at  matrix metallopeptidase 2  Info   Mmp2 
   498 4.88e-05 306.7 565.9 0.542  1383401_at  similar to Testis derived transcript  Info   LOC500040 
   499 4.89e-05 1949 1405.1 1.387  1379770_at  similar to choline phosphotransferase 1; cholinephosphotransferase 1 alpha; cholinephosphotransferase 1  Info   RGD:1359283 
   500 4.9e-05 33.8 128.3 0.263  1368464_at  macrophage galactose N-acetyl-galactosamine specific lectin 1  Info   Mgl1 
   501 4.91e-05 224.4 441.1 0.509  1370228_at  Transferrin /// signal recognition particle receptor, B subunit (predicted)  Info   Tf /// Srprb_predicted 
   502 4.98e-05 545.6 310.3 1.758  1392713_a_at    Info    
   503 4.99e-05 298.8 390.6 0.765  1372154_at  similar to 2010321M09Rik protein  Info   RGD:1359616 
   504 5e-05 341.1 469.9 0.726  1373595_at  similar to RIKEN cDNA 1200015A22  Info   MGC94413 
   505 5.01e-05 41.9 71.9 0.583  1373172_at  Transcribed locus  Info    
   506 5.01e-05 74.9 51.1 1.466  1376707_at  C1q and tumor necrosis factor related protein 4 (predicted)  Info   C1qtnf4_predicted 
   507 5.03e-05 2901.7 3960.6 0.733  1398751_at  similar to ribosomal protein S7  Info   LOC497813 
   508 5.05e-05 1396.9 2346.3 0.595  1386857_at  stathmin 1  Info   Stmn1 
   509 5.07e-05 23.4 48.6 0.481  1379059_at  similar to FLJ23471 protein (predicted)  Info   RGD1307875_predicted 
   510 5.11e-05 106.2 216.5 0.491  1393280_at  lymphocyte antigen 86 (predicted)  Info   Ly86_predicted 
   511 5.12e-05 14.7 1418.9 0.01  1387965_at  kidney injury molecule 1  Info   Havcr1 
   512 5.13e-05 2892.2 1039 2.784  1372869_at  similar to GTP-binding protein NGB  Info   LOC498786 
   513 5.14e-05 1496 779.8 1.918  1387737_at  methionine adenosyltransferase II, alpha  Info   Mat2a 
   514 5.14e-05 292.2 179.9 1.624  1391643_at    Info    
   515 5.15e-05 95.2 246 0.387  1389306_at  matrilin 2 (predicted)  Info   Matn2_predicted 
   516 5.16e-05 965.4 688.2 1.403  1374584_at  similar to Serine/threonine protein kinase 24  Info   LOC361092 
   517 5.21e-05 112.3 66.9 1.679  1385829_at  similar to mKIAA0657 protein  Info   LOC363257 
   518 5.21e-05 112.1 253.6 0.442  1389885_at  similar to RIKEN cDNA 0610025L06 (predicted)  Info   RGD1309967_predicted 
   519 5.23e-05 305.8 543.3 0.563  1388867_at  similar to transcription factor  Info   MGC112830 
   520 5.24e-05 109.6 168.9 0.649  1379317_a_at  programmed cell death protein 11 (predicted)  Info   Pdcd11_predicted 
   521 5.28e-05 673 878 0.767  1383643_at  similar to UPF0197 protein C11orf10 homolog  Info   LOC499317 
   522 5.28e-05 982 610.8 1.608  1374307_at  CaM-kinase II inhibitor alpha  Info   LOC287005 
   523 5.31e-05 721.3 412.6 1.748  1374244_at  Ab2-060  Info    
   524 5.38e-05 1083.3 747.8 1.449  1374959_at  NAD(P)H dehydrogenase, quinone 2  Info   RGD:1303320 
   525 5.43e-05 3855.8 4789.2 0.805  1367597_at  ribosomal protein S8  Info   Rps8 
   526 5.47e-05 72.6 114.7 0.633  1384581_at  actin, gamma, cytoplasmic (predicted) /// similar to Actin, cytoplasmic 2 (Gamma-actin) /// similar to put. type 5 nonmuscle actin  Info   Actg_predicted /// LOC295810 /// LOC360883 
   527 5.47e-05 65.8 110 0.598  1388802_at  similar to EG2RVC  Info   LOC501625 
   528 5.51e-05 67.3 141.6 0.475  1368671_at  sushi-repeat-containing protein  Info   Srpx 
   529 5.54e-05 95.4 160.2 0.596  1372493_at  similar to RIKEN cDNA 2410005K17 (predicted)  Info   RGD1311232_predicted 
   530 5.54e-05 526 920.8 0.571  1387893_at  complement component 1, s subcomponent /// similar to complement component 1, s subcomponent  Info   C1s /// LOC500313 
   531 5.61e-05 90.5 125.6 0.721  1392829_at  Transcribed locus, strongly similar to XP_529072.1 PREDICTED: similar to cleavage stimulation factor subunit 2 [Pan troglodytes]  Info    
   532 5.62e-05 3409.7 2473.1 1.379  1386938_at  alanyl (membrane) aminopeptidase  Info   Anpep 
   533 5.63e-05 1231.3 685.1 1.797  1370991_at  camello-like 3  Info   Cml3 
   534 5.64e-05 47.2 127 0.372  1384695_at  uroplakin 3A (predicted)  Info   Upk3a_predicted 
   535 5.66e-05 69.1 107.5 0.643  1383917_at  ABI gene family, member 3 (predicted)  Info   Abi3_predicted 
   536 5.66e-05 469.9 705.4 0.666  1388217_a_at  calumenin  Info   Calu 
   537 5.67e-05 80.7 46.3 1.743  1382266_at  similar to G protein-coupled receptor 146  Info   LOC498153 
   538 5.7e-05 568.5 1158.9 0.491  1387769_a_at  Inhibitor of DNA binding 3, dominant negative helix-loop-helix protein  Info   Id3 
   539 5.72e-05 9.8 35.7 0.275  1376816_at  Hypothetical gene supported by NM_012514  Info   Brca1 
   540 5.77e-05 65.1 185.8 0.35  1370948_a_at  myristoylated alanine rich protein kinase C substrate  Info   Marcks 
   541 5.82e-05 3.3 33.7 0.098  1397476_at  Ttk protein kinase (predicted)  Info   Ttk_predicted 
   542 5.85e-05 55.6 105.6 0.527  1390692_at  cytidine 5'-triphosphate synthase (predicted)  Info   Ctps_predicted 
   543 5.86e-05 283.5 440.6 0.643  1389732_at  Transcribed locus, strongly similar to NP_082154.2 hypothetical protein LOC71712 [Mus musculus]  Info    
   544 5.89e-05 474 307.6 1.541  1375657_at  Transcribed locus  Info    
   545 5.9e-05 14.7 44.2 0.333  1384900_at  MOB1, Mps One Binder kinase activator-like 2B (yeast) (predicted)  Info   Mobkl2b_predicted 
   546 5.97e-05 83.1 229.5 0.362  1381971_at  SRY-box containing gene 18 (predicted)  Info   Sox18_predicted 
   547 5.99e-05 2280.4 1181.6 1.93  1387382_at  histamine N-methyltransferase  Info   Hnmt 
   548 6.02e-05 1469.7 2541 0.578  1386862_at  annexin A5  Info   Anxa5 
   549 6.07e-05 2785.6 2200.4 1.266  1388324_at  nitrilase 1  Info   Nit1 
   550 6.1e-05 13.3 54.5 0.244  1378507_at  Transcribed locus  Info    
   551 6.1e-05 627.3 1210.3 0.518  1371969_at  Caldesmon 1  Info   Cald1 
   552 6.1e-05 377.9 64 5.905  1370289_x_at  kallikrein, submaxillary gland S3  Info   Klks3 
   553 6.11e-05 22.5 203.7 0.11  1392732_at  nucleolar and spindle associated protein 1 (predicted)  Info   Nusap1_predicted 
   554 6.15e-05 1578.4 2442.6 0.646  1388114_at  myosin light chain, regulatory B  Info   Mrlcb 
   555 6.15e-05 17.6 3.7 4.757  1380420_at  Internexin, alpha  Info   Inexa 
   556 6.21e-05 125.8 254.7 0.494  1370887_at  transforming growth factor beta 1 induced transcript 1  Info   Tgfb1i1 
   557 6.23e-05 112 168.1 0.666  1382903_at  similar to mFLJ00191 protein  Info   LOC310958 
   558 6.27e-05 97.4 208.6 0.467  1369181_at  cytochrome b-245, beta polypeptide  Info   Cybb 
   559 6.29e-05 270 421 0.641  1373024_at  adaptor-related protein complex 3, sigma 1 subunit (predicted)  Info   Ap3s1_predicted 
   560 6.31e-05 296.2 383.6 0.772  1373099_at  phosphatidylinositol glycan, class S  Info   RGD:1359436 
   561 6.39e-05 100.8 173.9 0.58  1370504_a_at  peripheral myelin protein 22  Info   Pmp22 
   562 6.4e-05 188.4 275 0.685  1372488_at  similar to RIKEN cDNA 2310057D15 (predicted)  Info   RGD1309016_predicted 
   563 6.41e-05 1041.5 795 1.31  1388751_at  RNA binding motif protein 24 (predicted)  Info   Rbm24_predicted 
   564 6.43e-05 45 104.7 0.43  1386989_at  endothelial differentiation, sphingolipid G-protein-coupled receptor, 5  Info   Edg5 
   565 6.45e-05 318.5 81.6 3.903  1377869_at  similar to carbon catabolite repression 4 protein homolog  Info   LOC310395 
   566 6.46e-05 78.1 110.2 0.709  1372308_at  CDNA clone IMAGE:7366335  Info    
   567 6.5e-05 25.3 177.4 0.143  1376100_at  similar to RIKEN cDNA 2310057H16 (predicted)  Info   RGD1305887_predicted 
   568 6.56e-05 154.1 256.7 0.6  1368393_at  lymphocyte antigen 68  Info   C1qr1 
   569 6.57e-05 15 41.3 0.363  1388010_at  Pro-melanin-concentrating hormone  Info   Pmch 
   570 6.57e-05 108.1 219.5 0.492  1372510_at  Neoplastic progression 3 (predicted)  Info    
   571 6.61e-05 738.9 502.1 1.472  1395532_at  Transcribed locus, strongly similar to NP_035873.1 SH3-domain binding protein 3 [Mus musculus]  Info    
   572 6.71e-05 207.6 421.7 0.492  1372325_at  elastin microfibril interfacer 1 (predicted)  Info   Emilin1_predicted 
   573 6.71e-05 73.9 112 0.66  1385573_at  Homer homolog 3 (Drosophila)  Info   Homer3 
   574 6.71e-05 295.6 185.5 1.594  1387145_at  gap junction membrane channel protein beta 1  Info   Gjb1 
   575 6.73e-05 2823.8 2103.5 1.342  1367999_at  aldehyde dehydrogenase 2  Info   Aldh2 
   576 6.75e-05 883.9 2361.9 0.374  1383291_at    Info    
   577 6.76e-05 41.5 71.6 0.58  1395663_at  similar to BENE protein (predicted)  Info   RGD1310869_predicted 
   578 6.84e-05 2387.5 1228 1.944  1371864_at  Basic transcription element binding protein 1  Info   Bteb1 
   579 6.85e-05 2543.4 3642.9 0.698  1369952_at  poly(A) binding protein, cytoplasmic 1  Info   Pabpc1 
   580 7e-05 311.6 177.7 1.754  1388469_at  Insulin-like growth factor I mRNA, 3' end of mRNA  Info    
   581 7.01e-05 1850 1105.2 1.674  1390416_at  solute carrier family 25, member 30  Info   RGD:1359702 
   582 7.03e-05 3550 2337.6 1.519  1367680_at  acyl-Coenzyme A oxidase 1, palmitoyl  Info   Acox1 
   583 7.04e-05 24.8 92.7 0.268  1382419_at  SoxLZ/Sox6 leucine zipper binding protein in testis (predicted)  Info   Solt_predicted 
   584 7.04e-05 507.8 297.9 1.705  1373803_a_at  growth hormone receptor  Info   Ghr 
   585 7.05e-05 59.1 128.7 0.459  1377239_at  amyloid beta (A4) precursor protein-binding, family B, member 1 interacting protein (predicted)  Info   Apbb1ip_predicted 
   586 7.09e-05 6811.4 4194.9 1.624  1371237_a_at  Metallothionein  Info   Mt1a 
   587 7.1e-05 17.9 63.4 0.282  1378140_at  ADP-ribosylation factor-like 11 (predicted)  Info   Arl11_predicted 
   588 7.1e-05 162.7 265.9 0.612  1373473_a_at  nucleosome assembly protein 1-like 1  Info   Nap1l1 
   589 7.12e-05 7.3 13.2 0.553  1377804_at  Transcribed locus  Info    
   590 7.13e-05 134.8 264.3 0.51  1376153_at  Transcribed locus  Info    
   591 7.14e-05 83.8 218.4 0.384  1372631_at  Thymidine kinase 1  Info   Tk1 
   592 7.16e-05 4044.6 3060.5 1.322  1368016_at  peroxisomal trans-2-enoyl-CoA reductase  Info   Pecr 
   593 7.18e-05 450.7 705.3 0.639  1368908_at  annexin A4  Info   Anxa4 
   594 7.22e-05 78.5 209.7 0.374  1376109_at  Transcribed locus, weakly similar to NP_062289.1 DNA binding protein with his-thr domain [Mus musculus]  Info    
   595 7.22e-05 50 123.4 0.405  1369097_s_at  guanylate cyclase 1, soluble, beta 3  Info   Gucy1b3 
   596 7.27e-05 10.1 63.2 0.16  1374912_at    Info    
   597 7.33e-05 180.3 277.7 0.649  1375695_at  YTH domain family 2 (predicted)  Info   Ythdf2_predicted 
   598 7.34e-05 4547.7 3469.2 1.311  1367721_at  syndecan 4  Info   Sdc4 
   599 7.35e-05 122.9 212.9 0.577  1395958_at  similar to KIAA0460 protein (predicted)  Info   RGD1308268_predicted 
   600 7.4e-05 95.7 169.4 0.565  1374704_at  KDEL (Lys-Asp-Glu-Leu) containing 2  Info   Kdelc2 
   601 7.45e-05 78.5 170.6 0.46  1388994_at  Transcribed locus  Info    
   602 7.46e-05 2464 1708 1.443  1380393_at  crystallin, zeta (predicted)  Info   Cryz_predicted 
   603 7.47e-05 222 723 0.307  1368829_at  fibrillin 1  Info   Fbn1 
   604 7.53e-05 194.9 124.4 1.567  1376248_at  sulfotransferase family, cytosolic, 2B, member 1 (predicted)  Info   Sult2b1_predicted 
   605 7.54e-05 92 135 0.681  1376219_at  Atpase, class I, type 8B, member 2 (predicted)  Info    
   606 7.61e-05 959.5 1294.4 0.741  1398828_at  FK506 binding protein 1a  Info   Fkbp1a 
   607 7.68e-05 853.5 1332.2 0.641  1367899_at  coagulation factor II (thrombin) receptor  Info   F2r 
   608 7.71e-05 14.6 54.3 0.269  1380822_at  Transcribed locus  Info    
   609 7.71e-05 344.5 616.9 0.558  1388447_at  Transcribed locus, strongly similar to NP_084275.3 limb-bud and heart [Mus musculus]  Info    
   610 7.73e-05 1890.3 390.9 4.836  1373990_at  solute carrier family 7 (cationic amino acid transporter, y+ system), member 12 (predicted) /// similar to solute carrier family 7 (cationic amino acid transporter, y+ system), member 12  Info   Slc7a12_predicted /// LOC361914 
   611 7.74e-05 261.6 136.8 1.912  1384874_at  Transcribed locus  Info    
   612 7.75e-05 25.5 121.5 0.21  1368293_at  carboxypeptidase Z  Info   Cpz 
   613 7.75e-05 639.5 1077 0.594  1389815_at  protein phosphatase 1, regulatory (inhibitor) subunit 14B  Info   Ppp1r14b 
   614 7.76e-05 551.9 895.4 0.616  1383241_at  complement component 1, r subcomponent (predicted)  Info   C1r_predicted 
   615 7.78e-05 169.7 235.5 0.721  1384405_at  Transcribed locus  Info    
   616 7.79e-05 2.7 22 0.123  1391201_at  WD repeat and HMG-box DNA binding protein 1 (predicted)  Info    
   617 7.79e-05 69.5 117.5 0.591  1392946_at  Transcribed locus  Info    
   618 7.82e-05 205.9 490.1 0.42  1372219_at  similar to tropomyosin 1, embryonic fibroblast - rat  Info   MGC109519 
   619 7.82e-05 95.4 203.7 0.468  1373599_at  BRF2, subunit of RNA polymerase III transcription initiation factor, BRF1-like (predicted)  Info    
   620 7.88e-05 5.5 14.4 0.382  1380631_at    Info    
   621 7.89e-05 83.3 557.1 0.15  1368187_at  glycoprotein (transmembrane) nmb  Info   Gpnmb 
   622 7.91e-05 18.5 96 0.193  1372886_at  transforming acidic coiled coil 3  Info   RGD:1302948 
   623 7.91e-05 96.1 198.8 0.483  1375216_at  poliovirus receptor-related 2 (herpesvirus entry mediator B) (predicted)  Info   Pvrl2_predicted 
   624 7.92e-05 126.8 263.9 0.48  1387048_at  nuclear RNA helicase, DECD variant of DEAD box family  Info   Ddx39 
   625 7.92e-05 34.6 231.7 0.149  1387895_s_at  cell division cycle 20 homolog (S. cerevisiae)  Info   Cdc20 
   626 7.92e-05 775.1 530.2 1.462  1380546_at  similar to hypothetical protein FLJ10986 /// similar to hypothetical protein FLJ10986  Info   LOC298250 /// LOC298251 
   627 7.96e-05 1401.5 1913.3 0.733  1370309_a_at  heterogeneous nuclear ribonucleoprotein A/B  Info   Hnrpab 
   628 8.02e-05 186.9 264.6 0.706  1376613_at  similar to hypothetical protein  Info   MGC73022 
   629 8.03e-05 553.2 374 1.479  1383324_at  Similar to PALS2-alpha splice variant  Info    
   630 8.05e-05 9.7 56.5 0.172  1393891_at  procollagen, type VIII, alpha 1 (predicted)  Info   Col8a1_predicted 
   631 8.07e-05 24.5 65.4 0.375  1370493_a_at  leukocyte immunoglobulin-like receptor, subfamily B (with TM and ITIM domains), member 3 (predicted)  Info   Lilrb3_predicted 
   632 8.08e-05 720.9 1151.7 0.626  1386925_at  actin related protein 2/3 complex, subunit 1B  Info   Arpc1b 
   633 8.2e-05 1008.8 638.5 1.58  1372179_at  neural visinin-like Ca2+-binding protein type 3  Info   Hpcal1 
   634 8.22e-05 143.5 103.6 1.385  1372024_at  Transcribed locus  Info    
   635 8.23e-05 1700 2629.3 0.647  1371352_at  high mobility group protein 17  Info   RGD:620649 
   636 8.26e-05 128.9 216.2 0.596  1389073_at  similar to RIKEN cDNA 2700029E10 (predicted)  Info   RGD1306561_predicted 
   637 8.3e-05 35.3 12.8 2.758  1390355_at  ryanodine receptor 1, skeletal muscle  Info   Ryr1 
   638 8.31e-05 1197 939.5 1.274  1371359_at  myeloid leukemia factor 2 (predicted)  Info   Mlf2_predicted 
   639 8.31e-05 34.5 75.3 0.458  1377504_at  Similar to Hypothetical protein MGC28888  Info    
   640 8.33e-05 3293.7 2718.7 1.211  1388988_at  similar to RIKEN cDNA 1810013B01  Info   RGD:1359649 
   641 8.42e-05 2682.6 848 3.163  1367631_at  connective tissue growth factor  Info   Ctgf 
   642 8.47e-05 15.9 44.1 0.361  1393672_at  Similar to OTTHUMP00000065631  Info    
   643 8.48e-05 16.3 61.6 0.265  1389852_at  similar to proliferation associated nuclear element 1 isoform 1 (predicted)  Info   RGD1307309_predicted 
   644 8.51e-05 5413 3135.8 1.726  1372264_at  phosphoenolpyruvate carboxykinase 1  Info   Pck1 
   645 8.56e-05 52.7 89.7 0.588  1370623_at  fibrinogen-like 2  Info   Fgl2 
   646 8.57e-05 119.2 186.7 0.638  1388429_at  similar to gem (nuclear organelle) associated protein 7  Info   LOC499391 
   647 8.6e-05 8 56.4 0.142  1376914_at  DEP domain containing 1 (predicted)  Info   Depdc1_predicted 
   648 8.6e-05 499.2 363.3 1.374  1382003_at  solute carrier family 16 (monocarboxylic acid transporters), member 13  Info   RGD:1359138 
   649 8.61e-05 1707.4 1333.4 1.28  1388463_at  putative secreted protein ZSIG11  Info   RGD:1359353 
   650 8.62e-05 1837.9 2196.5 0.837  1369013_a_at  mitochondrial ribosomal protein L17  Info   Mrpl17 
   651 8.64e-05 385.6 542.8 0.71  1371322_at  Laminin, gamma 1  Info   Lamc1 
   652 8.75e-05 313.3 186.6 1.679  1372103_at  DnaJ (Hsp40) homolog, subfamily A, member 4 (predicted)  Info    
   653 8.83e-05 1054.1 658.2 1.601  1398378_at  glutathione S-transferase kappa 1  Info   Gstk1 
   654 8.88e-05 340.7 523.1 0.651  1371539_at  nucleolar protein family A, member 2 (predicted)  Info   Nola2_predicted 
   655 8.88e-05 38 77.4 0.491  1384051_at  Transcribed locus  Info    
   656 9.01e-05 123.6 183.9 0.672  1392544_at  rcd1 (required for cell differentiation) homolog 1 (S. pombe) (predicted)  Info   Rqcd1_predicted 
   657 9.05e-05 364.5 459.3 0.794  1388137_a_at  zygin-related protein  Info   RGD:628711 
   658 9.17e-05 65.4 116.2 0.563  1391466_at  Nuclear ubiquitous casein kinase and cyclin-dependent kinase substrate  Info   Nucks 
   659 9.17e-05 81.9 29.6 2.767  1384960_at  Transcribed locus  Info    
   660 9.2e-05 1085.6 440.2 2.466  1369182_at  coagulation factor 3  Info   F3 
   661 9.23e-05 3385.7 4527.5 0.748  1398775_at  ribosomal protein S15a  Info   Rps15a 
   662 9.26e-05 323.7 235.8 1.373  1377887_at  Acyl-Coenzyme A oxidase 3, pristanoyl  Info   Acox3 
   663 9.3e-05 81.7 43.8 1.865  1393545_at  transcription factor CP2-like 2 (predicted)  Info   Tcfcp2l2_predicted 
   664 9.31e-05 89.9 139.5 0.644  1375910_at  CDC42 effector protein (Rho GTPase binding) 3 (predicted)  Info   Cdc42ep3_predicted 
   665 9.32e-05 141.3 61.3 2.305  1392001_at  Pannexin 1  Info   Panx1 
   666 9.39e-05 713.4 447.6 1.594  1389632_at  Transcribed locus, weakly similar to XP_228131.3 PREDICTED: similar to Rho-related BTB domain-containing protein 1 [Rattus norvegicus]  Info    
   667 9.41e-05 1060.5 668.1 1.587  1383253_at  Solute carrier family 30 (zinc transporter), member 1  Info   Slc30a1 
   668 9.46e-05 663.6 784.2 0.846  1372233_at  serologically defined breast cancer antigen 84 (predicted)  Info   Sdbcag84_predicted 
   669 9.46e-05 598.9 317.2 1.888  1373605_at  similar to 106 kDa O-GlcNAc transferase-interacting protein (predicted)  Info   RGD1307844_predicted 
   670 9.57e-05 301.6 586.4 0.514  1381776_at  similar to RIKEN cDNA 1810015P03 (predicted)  Info   RGD1305260_predicted 
   671 9.69e-05 46.4 111.8 0.415  1373156_at  similar to armadillo repeat protein ALEX2  Info   RGD:1359132 
   672 9.83e-05 3780.2 2092.7 1.806  1386944_a_at  glucose-6-phosphatase, catalytic  Info   G6pc 
   673 9.85e-05 6.9 17.8 0.388  1368743_a_at  dentin sialophosphoprotein  Info   Dspp 
   674 9.91e-05 125.3 230 0.545  1372820_at  Transcribed locus  Info    
   675 9.92e-05 52.4 112.8 0.465  1379234_a_at  similar to CDC45L  Info   LOC287961 
   676 9.96e-05 123.9 191.2 0.648  1383133_at  Transcribed locus  Info    
   677 0.0001003 98 142.6 0.687  1383371_at  Transcribed locus  Info    
   678 0.0001005 1383.1 2932.7 0.472  1388557_at  Transcribed locus  Info    
   679 0.0001022 2262.2 2726.8 0.83  1367696_at  interferon induced transmembrane protein 2 (1-8D)  Info   Ifitm2 
   680 0.0001024 153.2 78.3 1.957  1395572_at  DnaJ (Hsp40) homolog, subfamily A, member 4 (predicted)  Info   Dnaja4_predicted 
   681 0.000103 223.4 55.2 4.047  1368779_a_at  guanylate cyclase 1, soluble, beta 2  Info   Gucy1b2 
   682 0.0001034 319 531.7 0.6  1388581_at  hematological and neurological expressed sequence 1  Info   RGD:1359325 
   683 0.0001036 26.4 39.6 0.667  1383406_at  Transcribed locus  Info    
   684 0.0001041 584.2 407 1.435  1399059_at  Enhancer of zeste homolog 1 (Drosophila) (predicted)  Info    
   685 0.0001042 752.6 548.1 1.373  1390292_at  transmembrane protein 8 (five membrane-spanning domains) (predicted)  Info   Tmem8_predicted 
   686 0.0001047 18.4 53.8 0.342  1377689_at  kinetochore associated 1 (predicted)  Info   Kntc1_predicted 
   687 0.0001047 13.9 141.4 0.098  1384063_at  collagen triple helix repeat containing 1  Info   Cthrc1 
   688 0.000105 234.1 306.9 0.763  1373597_at  processing of precursor 7, ribonuclease P family, (S. cerevisiae) (predicted)  Info   Pop7_predicted 
   689 0.0001052 17.9 35.5 0.504  1379972_at  similar to 1700060H10Rik protein (predicted)  Info   RGD1308699_predicted 
   690 0.0001054 94.4 219.2 0.431  1374389_at  guanylate cyclase 1, soluble, beta 3  Info   Gucy1b3 
   691 0.0001057 249.2 374.1 0.666  1387081_at  reticulocalbin 2  Info   Rcn2 
   692 0.0001058 543.9 381.8 1.425  1368509_at  Bardet-Biedl syndrome 2 (human)  Info   Bbs2 
   693 0.0001062 34.3 56.5 0.607  1394464_at  LOC500352 /// LOC500353  Info   LOC500352 /// LOC500353 
   694 0.0001065 368.7 771.1 0.478  1389189_at  actinin, alpha 1  Info   Actn1 
   695 0.0001065 415.8 327.6 1.269  1388677_at  ubiquitin-associated protein 1 (predicted)  Info   Ubap1_predicted 
   696 0.0001065 46.2 71.7 0.644  1374817_at  Transcribed locus  Info    
   697 0.0001068 687.5 1354.6 0.508  1373947_at  dermatopontin (predicted)  Info   Dpt_predicted 
   698 0.0001073 331.6 439.4 0.755  1378551_at  cytochrome P450, family 20, subfamily A, polypeptide 1  Info   Cyp20a1 
   699 0.0001076 164.4 263.6 0.624  1367881_at  protein tyrosine phosphatase, non-receptor type substrate 1  Info   Ptpns1 
   700 0.0001084 914.8 191.5 4.777  1386994_at  B-cell translocation gene 2, anti-proliferative  Info   Btg2 
   701 0.0001091 2782.2 2183.5 1.274  1372076_at  hepatitis B virus x interacting protein (predicted)  Info   Hbxip_predicted 
   702 0.0001094 475.5 345.7 1.375  1397584_at  Transcribed locus  Info    
   703 0.0001099 60.9 123.4 0.494  1395003_at  dendritic cell inhibitory receptor 4  Info   RGD:1359109 
   704 0.0001103 3637.1 4591.2 0.792  1367596_at  ribosomal protein S26  Info   Rps26 
   705 0.0001111 20.3 35 0.58  1371017_at  T-cell receptor gamma chain  Info   Tcrg 
   706 0.0001119 1894.7 1305.9 1.451  1385072_at  galactose mutarotase (aldose 1-epimerase)  Info   RGD:1359459 
   707 0.0001123 485.6 664 0.731  1399015_at  Transmembrane trafficking protein 21  Info   Tmp21 
   708 0.0001123 759.9 535.6 1.419  1393777_at  Transcribed locus  Info    
   709 0.0001124 534.5 354.5 1.508  1372199_at  PTEN induced putative kinase 1 (predicted)  Info   Pink1_predicted 
   710 0.0001125 706.3 459.6 1.537  1370036_at  sulfite oxidase  Info   Suox 
   711 0.0001126 7248.5 3956.9 1.832  1387218_at  trefoil factor 3  Info   Tff3 
   712 0.0001129 482.4 337.8 1.428  1399052_at  Toll interacting protein (predicted)  Info    
   713 0.000113 32.1 111.5 0.288  1379340_at  lamimin, gamma 2  Info   Lamc2 
   714 0.0001132 600.5 346.9 1.731  1371420_at  92Aa-Protein  Info   RGD:1359519 
   715 0.0001132 1451.2 1768.6 0.821  1399050_at  adenylosuccinate synthetase 2, non muscle (predicted)  Info   Adss2_predicted 
   716 0.0001137 44.2 119.5 0.37  1380474_at    Info    
   717 0.0001139 270.6 523.5 0.517  1373025_at  complement component 1, q subcomponent, gamma polypeptide (predicted)  Info   C1qg_predicted 
   718 0.0001147 4490.8 5067.1 0.886  1367717_at  ribosomal protein S27  Info   Rps27 
   719 0.0001148 100.7 181.7 0.554  1370258_at  basic leucine zipper and W2 domains 2  Info   Bzw2 
   720 0.0001153 114.7 62.3 1.841  1382918_at  Transcribed locus  Info    
   721 0.0001162 132.9 211.7 0.628  1370000_at  nucleobindin 2  Info   Nucb2 
   722 0.0001165 6.7 53.5 0.125  1372957_at  Similar to hypothetical protein A730008H23 (predicted)  Info    
   723 0.0001167 742 498.1 1.49  1373866_at  similar to hypothetical protein FLJ13448  Info   RGD:1359509 
   724 0.000117 17.3 59.9 0.289  1384259_at  Rac GTPase-activating protein 1 (predicted)  Info   Racgap1_predicted 
   725 0.0001171 754.7 1125 0.671  1388389_at  septin 2  Info   Sept2 
   726 0.0001177 96.2 170.7 0.564  1391862_at  Transcribed locus  Info    
   727 0.0001184 4548.6 3312.6 1.373  1391417_at  similar to betaine-homocysteine methyltransferase 2  Info   LOC365972 
   728 0.0001185 53.5 103.4 0.517  1377968_at  Transcribed locus  Info    
   729 0.0001188 613.3 407.6 1.505  1375997_at  Transcribed locus  Info    
   730 0.0001198 1817.4 866 2.099  1370377_at  cytochrome P450, family 2, subfamily d, polypeptide 9 /// cytochrome P450, family 2, subfamily d, polypeptide 10  Info   Cyp2d9 /// Cyp2d10 
   731 0.0001201 31.1 64.2 0.484  1397767_at  Transcribed locus  Info    
   732 0.0001202 98.1 144.4 0.679  1382228_at  similar to Zinc finger protein 431  Info   LOC499597 
   733 0.000121 121.8 189.7 0.642  1379261_at  family with sequence similarity 33, member A (predicted)  Info   Fam33a_predicted 
   734 0.0001213 74.5 143.1 0.521  1388436_at  small nuclear ribonucleoprotein polypeptide A (predicted)  Info   Snrpa_predicted 
   735 0.0001216 1148 686.8 1.672  1387966_at  asparaginase-like sperm autoantigen  Info   Asrgl1 
   736 0.0001218 146 202.4 0.721  1383227_at  Transcribed locus  Info    
   737 0.0001219 4095.3 3368.3 1.216  1373337_at  glyoxylate reductase/hydroxypyruvate reductase (predicted)  Info   Grhpr_predicted 
   738 0.0001219 115.1 186.4 0.617  1381933_at  Transcribed locus  Info    
   739 0.0001227 75.4 261 0.289  1390659_at  CD44 antigen  Info   Cd44 
   740 0.0001232 843.5 554.9 1.52  1379592_at  similar to citrin  Info   LOC362322 
   741 0.0001235 3132.2 2338 1.34  1367775_at  alpha-methylacyl-CoA racemase  Info   Amacr 
   742 0.0001239 904.4 623.4 1.451  1390918_at  GH regulated TBC protein 1 (predicted)  Info   Grtp1_predicted 
   743 0.0001239 968.8 1232.6 0.786  1387777_at  integrin linked kinase  Info   Ilk 
   744 0.0001241 74.5 151.8 0.491  1370048_at  endothelial differentiation, lysophosphatidic acid G-protein-coupled receptor, 2  Info   Edg2 
   745 0.0001241 34.6 56.8 0.609  1373223_at  Transcribed locus  Info    
   746 0.0001242 77.2 146.7 0.526  1374961_at    Info    
   747 0.0001242 269.9 127.2 2.122  1368134_a_at  interleukin 4 receptor  Info   Il4r 
   748 0.0001246 138.4 91.6 1.511  1382778_at  Dual specificity phosphatase 6  Info   Dusp6 
   749 0.000125 19.9 52.8 0.377  1378028_at  MAD2 (mitotic arrest deficient, homolog)-like 1 (yeast) (predicted)  Info   Mad2l1_predicted 
   750 0.0001255 214.3 137.4 1.56  1387665_at  betaine-homocysteine methyltransferase  Info   Bhmt 
   751 0.0001261 94.9 241.1 0.394  1375010_at  CD68 antigen (predicted)  Info   Cd68_predicted 
   752 0.0001263 1737.4 1210.5 1.435  1367838_at  CTL target antigen  Info   Cth 
   753 0.0001263 235 141.7 1.658  1393666_at  Transcribed locus  Info    
   754 0.0001264 52.2 88.4 0.59  1390441_at  Development and differentiation enhancing (predicted)  Info    
   755 0.0001264 217.1 417.9 0.52  1369313_at  four and a half LIM domains 2  Info   Fhl2 
   756 0.0001266 5094.8 6189.3 0.823  1370242_at  ribosomal protein S23  Info   Rps23 
   757 0.0001267 107.1 188.1 0.569  1373932_at  Cytochrome b-245, beta polypeptide  Info   Cybb 
   758 0.0001269 28.9 60 0.482  1389360_at  FXYD domain-containing ion transport regulator 3  Info   Fxyd3 
   759 0.0001276 9.4 36.8 0.255  1383684_at  ASF1 anti-silencing function 1 homolog B (S. cerevisiae) (predicted)  Info   Asf1b_predicted 
   760 0.0001277 255.8 718 0.356  1374070_at  glutathione peroxidase 2  Info   Gpx2 
   761 0.000128 1813.2 855.1 2.12  1368794_at  3-hydroxyanthranilate 3,4-dioxygenase  Info   Haao 
   762 0.0001281 456.6 950.4 0.48  1376749_at  osteoglycin (predicted)  Info   Ogn_predicted 
   763 0.0001286 1.7 12.4 0.137  1380809_at    Info    
   764 0.0001288 2934.9 2004.8 1.464  1367917_at  cytochrome P450, family 2, subfamily d, polypeptide 26  Info   Cyp2d26 
   765 0.0001289 146.6 287 0.511  1389787_at  Transcribed locus  Info    
   766 0.0001294 2160.1 1686.9 1.281  1387867_at  aldehyde dehydrogenase family 9, subfamily A1  Info   Aldh9a1 
   767 0.0001294 53.9 149.4 0.361  1390348_at  folate receptor 2 (fetal) (predicted)  Info   Folr2_predicted 
   768 0.0001303 52 112.8 0.461  1369672_at  arachidonate 5-lipoxygenase activating protein  Info   Alox5ap 
   769 0.0001304 45.1 99 0.456  1378180_at  similar to 6030466N05Rik protein  Info   RGD:1359671 
   770 0.0001309 28 106.3 0.263  1391063_at  kinesin family member 23 (predicted)  Info   Kif23_predicted 
   771 0.0001313 46.2 79.1 0.584  1379603_at  similar to CBL E3 ubiquitin protein ligase (Signal transduction protein CBL) (Proto-oncogene c-CBL) (Casitas B-lineage lymphoma proto-oncogene)  Info   LOC500985 
   772 0.0001324 877.9 621.9 1.412  1368460_at  solute carrier family 2, member 5  Info   Slc2a5 
   773 0.0001327 1419.7 944.9 1.502  1368915_at  kynurenine 3-monooxygenase (kynurenine 3-hydroxylase)  Info   Kmo 
   774 0.0001331 183.7 295.5 0.622  1382192_at  extra cellular link domain-containing 1 (predicted)  Info   Xlkd1_predicted 
   775 0.0001333 3913.3 3106.8 1.26  1371916_at  CDNA clone MGC:105753 IMAGE:7316479  Info    
   776 0.0001338 397.8 728.7 0.546  1367913_at  cytoglobin  Info   Cygb 
   777 0.0001339 35.5 91.6 0.388  1392407_at  Similar to RIKEN cDNA 6030405P05 gene  Info    
   778 0.0001341 30.6 47.1 0.65  1390861_at  similar to hypothetical protein FLJ38281  Info   LOC362845 
   779 0.0001342 514 291.6 1.763  1382496_at    Info    
   780 0.0001346 3585.9 5041.8 0.711  1371377_at  ribosomal protein S19  Info   Rps19 
   781 0.0001355 61.3 151.1 0.406  1384824_at  protocadherin 18 (predicted)  Info   Pcdh18_predicted 
   782 0.0001355 541.6 815.8 0.664  1369956_at  interferon gamma receptor 1  Info   Ifngr 
   783 0.0001355 470.9 216.3 2.177  1368651_at  pyruvate kinase, liver and RBC  Info   Pklr 
   784 0.0001356 2832.4 3568.9 0.794  1370243_a_at  prothymosin alpha  Info   Ptma 
   785 0.0001363 557.4 383 1.455  1376128_at  Transcribed locus  Info    
   786 0.0001369 389.3 242.5 1.605  1393421_at  peroxisomal membrane protein 4  Info   Pxmp4 
   787 0.0001371 228.5 330.3 0.692  1388761_at  histone deacetylase 1 (predicted)  Info   Hdac1_predicted 
   788 0.0001376 112.9 178.4 0.633  1373852_at  similar to SRD5A2L (predicted)  Info   RGD1308828_predicted 
   789 0.0001377 2254.7 1006.8 2.239  1370725_a_at  glucose-6-phosphatase, catalytic  Info   G6pc 
   790 0.0001379 2291 2632.9 0.87  1398860_at  neural precursor cell expressed, developmentally down-regulated gene 8  Info   Nedd8 
   791 0.0001379 346 503.7 0.687  1381993_at  chloride intracellular channel 2 (predicted)  Info   Clic2_predicted 
   792 0.0001382 47.8 78.9 0.606  1379074_at  Transcribed locus  Info    
   793 0.0001383 244.5 132.1 1.851  1384603_at  ATP-binding cassette, sub-family A (ABC1), member 4 (predicted)  Info   Abca4_predicted 
   794 0.000139 26.6 95.6 0.278  1383008_at  SMC4 structural maintenance of chromosomes 4-like 1 (yeast) (predicted)  Info   Smc4l1_predicted 
   795 0.0001396 29.1 76.7 0.379  1373219_at  snail homolog, (Drosophila)  Info   Snai1 
   796 0.0001396 113.2 210.3 0.538  1372569_at  four and a half LIM domains 3 (predicted)  Info   Fhl3_predicted 
   797 0.0001398 109.5 183.6 0.596  1368003_at  aldehyde dehydrogenase family 1, subfamily A2  Info   Aldh1a2 
   798 0.0001403 275.7 582.2 0.474  1375523_at  Similar to Myristoylated alanine-rich C-kinase substrate (MARCKS) (ACAMP-81)  Info   Marcks 
   799 0.0001404 395.9 305.2 1.297  1391538_at  cell death-inducing DNA fragmentation factor, alpha subunit-like effector B (predicted)  Info   Cideb_predicted 
   800 0.0001409 525.8 1673.3 0.314  1370892_at  complement component 4a /// complement component 4, gene 2  Info   C4a /// C4-2 
   801 0.0001413 49.3 95.7 0.515  1377664_at  KARP-1 binding protein 1  Info   Kab 
   802 0.0001422 1607.8 1035.3 1.553  1373386_at  gap junction membrane channel protein beta 2  Info   Gjb2 
   803 0.0001425 72.1 102 0.707  1373833_at  similar to RIKEN cDNA 3110040N11 (predicted)  Info   RGD1305713_predicted 
   804 0.0001429 32.5 74.8 0.434  1380079_at  similar to mKIAA2005 protein  Info   LOC500015 
   805 0.0001431 68.2 154.7 0.441  1371250_at  platelet factor 4  Info   Pf4 
   806 0.0001446 70.4 142.9 0.493  1368742_at  complement component 5, receptor 1  Info   C5r1 
   807 0.0001447 196.8 109.9 1.791  1382569_at  Transcribed locus  Info    
   808 0.0001457 399.4 268 1.49  1377018_at  Similar to E430002G05Rik protein (predicted)  Info    
   809 0.0001459 653.8 450.5 1.451  1387284_at  dihydropyrimidinase  Info   Dpys 
   810 0.000146 420.3 731.2 0.575  1367683_at  karyopherin (importin) alpha 2  Info   Kpna2 
   811 0.0001465 2895.5 2386.4 1.213  1367763_at  acetyl-coenzyme A acetyltransferase 1  Info   Acat1 
   812 0.0001467 37.2 103.7 0.359  1385029_at  Transcribed locus  Info    
   813 0.0001467 258.8 381.2 0.679  1374695_at  Transcribed locus  Info    
   814 0.000147 89.8 132 0.68  1380794_at  Aquarius (predicted)  Info    
   815 0.0001471 396 634 0.625  1368143_at  annexin A7  Info   Anxa7 
   816 0.0001481 85.4 47.2 1.809  1393163_at  Transcribed locus  Info    
   817 0.0001483 33.2 48.1 0.69  1395687_at  activating transcription factor 7 interacting protein (predicted)  Info   Atf7ip_predicted 
   818 0.0001485 1914.8 1370.5 1.397  1372613_at  dehydrogenase/reductase (SDR family) member 6 (predicted)  Info   Dhrs6_predicted 
   819 0.0001486 2018.1 1642.9 1.228  1389965_at  trans-golgi network protein 1  Info   Tgoln2 
   820 0.0001486 18.1 32.4 0.559  1382478_at  BTB (POZ) domain containing 3 (predicted)  Info   Btbd3_predicted 
   821 0.00015 85.4 138.2 0.618  1390357_at  zinc finger protein 574 (predicted)  Info   Znf574_predicted 
   822 0.0001504 143.2 342.4 0.418  1391106_at  Transcribed locus  Info    
   823 0.0001522 58.8 112.2 0.524  1383596_at  similar to RIKEN cDNA 5830446M03 (predicted)  Info   RGD1307688_predicted 
   824 0.0001525 65.9 102.6 0.642  1368153_a_at  nasal embryonic LHRH factor  Info   Nelf 
   825 0.0001532 1630.8 174.3 9.356  1368321_at  early growth response 1  Info   Egr1 
   826 0.0001533 95.7 135.4 0.707  1378104_at  similar to RIKEN cDNA 8430406I07 (predicted)  Info   RGD1307465_predicted 
   827 0.0001534 366.2 224.6 1.63  1383640_at  Phosphatidylinositol glycan, class N (predicted)  Info    
   828 0.0001536 1546.9 1115.4 1.387  1372347_at  Transcribed locus  Info    
   829 0.000154 92.4 143 0.646  1386529_at  Transcribed locus  Info    
   830 0.0001554 523 1039 0.503  1371527_at  epithelial membrane protein 1  Info   Emp1 
   831 0.0001555 286.2 606 0.472  1368154_at  guanylate cyclase 1, soluble, alpha 3 /// hypothetical gene supported by NM_017090  Info   Gucy1a3 /// LOC497757 
   832 0.0001556 190 426.2 0.446  1368824_at  caldesmon 1  Info   Cald1 
   833 0.0001558 72.5 121.1 0.599  1392509_at  Transcribed locus  Info    
   834 0.000156 2193.1 2794.3 0.785  1369934_at  peptidylprolyl isomerase B  Info   Ppib 
   835 0.000156 113.1 205.6 0.55  1388672_at  Transcribed locus  Info    
   836 0.0001562 2934.4 1678.3 1.748  1371131_a_at  upregulated by 1,25-dihydroxyvitamin D-3  Info   Txnip 
   837 0.0001566 1085.3 1346.4 0.806  1388504_at  similar to HR21spA  Info   MGC116373 
   838 0.0001591 337.9 240.6 1.404  1388336_at  Similar to hypothetical protein D2Ertd391e (predicted)  Info    
   839 0.0001598 16.2 50.9 0.318  1384707_at  Transcribed locus  Info    
   840 0.0001599 3050.9 3714.1 0.821  1370213_at  nuclease sensitive element binding protein 1  Info   Nsep1 
   841 0.0001605 45.2 97.5 0.464  1389651_at  apelin, AGTRL1 ligand  Info   Apln 
   842 0.0001606 202.5 7.9 25.633  1398540_at  regulator of G-protein signaling 1  Info   Rgs1 
   843 0.0001607 20.8 60.5 0.344  1369018_at  forkhead box M1  Info   Foxm1 
   844 0.0001607 241.6 531.6 0.454  1374172_at  Procollagen, type VIII, alpha 2 (predicted)  Info    
   845 0.0001609 291.3 142.7 2.041  1390855_at  Prolyl endopeptidase  Info   Prep 
   846 0.0001612 5921.8 7069.4 0.838  1371297_at  ribosomal protein L7a (predicted)  Info   Rpl7a_predicted 
   847 0.0001612 851.6 453.9 1.876  1368164_at  biliverdin reductase A  Info   Blvra 
   848 0.0001616 430.7 239.2 1.801  1389648_at  Receptor-interacting serine-threonine kinase 4 (predicted)  Info    
   849 0.000162 628 1724.3 0.364  1371691_at    Info    
   850 0.0001622 381.3 529.7 0.72  1367925_at  major vault protein  Info   Mvp 
   851 0.0001622 342.2 604.2 0.566  1388455_at  guanine nucleotide binding protein (G protein), gamma 10  Info   Gng10 
   852 0.0001633 5.1 35.9 0.142  1376905_at  Transcribed locus  Info    
   853 0.0001636 97.2 148 0.657  1372409_at  MAD2L1 binding protein (predicted)  Info   Mad2l1bp_predicted 
   854 0.0001646 82.2 28.1 2.925  1368289_at  group specific component  Info   Gc 
   855 0.0001648 2376.8 2007.7 1.184  1389488_at  hypothetical protein LOC498544  Info   LOC498544 
   856 0.000165 44.8 67.6 0.663  1375859_a_at  Similar to zinc finger protein 565  Info    
   857 0.0001656 2487 1416.8 1.755  1381428_a_at  Histone 1, H2bh (predicted)  Info   Hfe 
   858 0.0001658 35.1 116.4 0.302  1368799_at  baculoviral IAP repeat-containing 5  Info   Birc5 
   859 0.0001658 263 385.4 0.682  1370935_at  CDW92 antigen  Info   Cdw92 
   860 0.0001668 34.4 81.3 0.423  1374247_at  stabilin 1 (predicted)  Info   Stab1_predicted 
   861 0.000167 109.8 167.9 0.654  1383175_a_at  similar to C11orf17 protein (predicted)  Info   RGD1306959_predicted 
   862 0.0001672 27.4 66.8 0.41  1387605_at  caspase 12  Info   Casp12 
   863 0.0001678 250.2 97.7 2.561  1387811_at  angiotensinogen  Info   Agt 
   864 0.0001678 107.3 187.1 0.573  1373538_at  ubiquitin specific protease 1 (predicted)  Info   Usp1_predicted 
   865 0.000168 122.9 229.9 0.535  1370433_at  CCA2 protein  Info   Hsd3b7 
   866 0.0001688 378.8 819 0.463  1388902_at  lysyl oxidase-like 1 (predicted)  Info   Loxl1_predicted 
   867 0.0001698 1826.5 1166.9 1.565  1377048_at  similar to cDNA sequence BC021917 (predicted)  Info   RGD1311026_predicted 
   868 0.0001699 345.3 736.3 0.469  1390866_at  Transcribed locus  Info    
   869 0.0001703 486.1 624.5 0.778  1367837_at  proteasome (prosome, macropain) subunit, alpha type 4  Info   Psma4 
   870 0.0001705 72.9 150.9 0.483  1388135_at  replication protein A2  Info   Rpa2 
   871 0.0001709 26.5 52.1 0.509  1377350_at  Transcribed locus  Info    
   872 0.0001712 110.1 199.8 0.551  1373484_at  kelch-like 13  Info   Klhl13 
   873 0.0001713 388.2 696.4 0.557  1370891_at  CD48 antigen  Info   Cd48 
   874 0.000172 119.1 186 0.64  1372234_at  similar to Lix1 homolog (mouse) like  Info   LOC499677 
   875 0.0001736 28.9 50.5 0.572  1372993_at  Insulin-like growth factor 2 receptor  Info   Igf2r 
   876 0.0001736 2.2 35.4 0.062  1382459_at    Info    
   877 0.0001738 101.3 57.5 1.762  1387913_at  cytochrome P450, family 2, subfamily d, polypeptide 22  Info   Cyp2d22 
   878 0.0001741 101.9 180.8 0.564  1383321_at  tyrosylprotein sulfotransferase 1 (predicted)  Info   Tpst1_predicted 
   879 0.0001743 910.7 638.6 1.426  1377573_at  carbonic anhydrase VB, mitochondrial  Info   Ca5b 
   880 0.0001743 438.4 505.4 0.867  1374007_at  Transcribed locus  Info    
   881 0.0001766 93.7 140.2 0.668  1388521_at  pyrroline-5-carboxylate synthetase (glutamate gamma-semialdehyde synthetase) (predicted)  Info   Pycs_predicted 
   882 0.0001768 1971.1 2289.7 0.861  1398787_at  Ras homolog enriched in brain  Info   Rheb 
   883 0.0001776 242 193.3 1.252  1390944_at  Snf7 homologue associated with Alix 3  Info   MGC108776 
   884 0.000178 21.4 46.1 0.464  1377814_at  Similar to Nedd4-binding brain specific protein BEAN  Info    
   885 0.0001789 62.2 101.6 0.612  1373642_at    Info    
   886 0.0001806 3241.3 4299.6 0.754  1367582_at  ribosomal protein L29  Info   Rpl29 
   887 0.0001807 111.7 144 0.776  1392499_at    Info    
   888 0.000181 271.6 414.9 0.655  1368065_at  regulator of G-protein signaling 19 interacting protein 1  Info   Rgs19ip1 
   889 0.0001812 1644.6 693 2.373  1368947_at  growth arrest and DNA-damage-inducible 45 alpha  Info   Gadd45a 
   890 0.0001815 302.6 422.5 0.716  1374456_at  Transcribed locus, strongly similar to XP_619138.1 PREDICTED: similar to Ras GTPase-activating-like protein IQGAP2 [Mus musculus]  Info    
   891 0.0001817 357.1 557.4 0.641  1385444_at  Transcribed locus  Info    
   892 0.0001819 731.9 547.2 1.338  1393587_a_at    Info    
   893 0.0001824 190.8 267.4 0.714  1367723_a_at  linker of T-cell receptor pathways  Info   Lnk 
   894 0.0001824 562.4 782.7 0.719  1372461_at  SET translocation (predicted) /// LOC499767  Info   Set_predicted /// LOC499767 
   895 0.0001831 12 37.1 0.323  1376377_at  Transcribed locus  Info    
   896 0.0001832 50.3 82.5 0.61  1377761_at  glutamine-fructose-6-phosphate transaminase 2  Info   RGD:1303097 
   897 0.0001833 634.8 147.2 4.312  1374404_at  v-jun sarcoma virus 17 oncogene homolog (avian)  Info   Jun 
   898 0.0001833 510.8 735.2 0.695  1372905_at    Info    
   899 0.0001837 265.1 551.5 0.481  1367860_a_at  matrix metalloproteinase 14 (membrane-inserted)  Info   Mmp14 
   900 0.0001839 44.1 81.5 0.541  1382943_at  protocadherin gamma subfamily A, 10 (predicted)  Info   Pcdhga10_predicted 
   901 0.0001844 355.5 517.8 0.687  1372111_at    Info    
   902 0.0001854 203.6 292.3 0.697  1368862_at  v-akt murine thymoma viral oncogene homolog 1  Info   Akt1 
   903 0.0001856 207.5 330.6 0.628  1386982_at  Alpha-1,6-mannosyl-glycoprotein 2-beta-N-acetylglucosaminyltransferase  Info   Mgat2 
   904 0.0001861 155 487.2 0.318  1373823_at  similar to Cyclin-dependent kinases regulatory subunit 2 (CKS-2)  Info   LOC498709 
   905 0.0001862 1417.5 2960.1 0.479  1370956_at  decorin  Info   Dcn 
   906 0.0001868 172.7 338.9 0.51  1368518_at  CD53 antigen  Info   Cd53 
   907 0.0001872 502.7 415.5 1.21  1376161_at  similar to RIKEN cDNA 1110014L17 (predicted)  Info   RGD1306319_predicted 
   908 0.0001873 90 210.8 0.427  1369651_at  thymus cell antigen 1, theta  Info   Thy1 
   909 0.0001879 96.1 151.9 0.633  1376185_at  kinesin family member C1  Info   RGD:1359118 
   910 0.0001881 287.2 206.5 1.391  1373934_at  LOC500840  Info   LOC500840 
   911 0.0001881 1.9 10.6 0.179  1380995_at  Transcribed locus  Info    
   912 0.0001883 95.8 163 0.588  1389017_at  similar to Antxr2 protein  Info   LOC305633 
   913 0.000189 222.7 420.1 0.53  1388744_at  minichromosome maintenance protein 7  Info   RGD:1303018 
   914 0.0001893 94.5 169.6 0.557  1373772_at  DNA (cytosine-5-)-methyltransferase 1  Info   Dnmt1 
   915 0.0001897 639.2 409.4 1.561  1394479_at  Transcribed locus  Info    
   916 0.0001898 1544.8 468.8 3.295  1387567_at  solute carrier family 21, member 1 /// hypothetical gene supported by NM_017111  Info   Slc21a1 /// LOC497799 
   917 0.00019 91.8 143.6 0.639  1383362_at  Transcribed locus  Info    
   918 0.00019 762.8 438.2 1.741  1381973_at  solute carrier family 25, member 30  Info   RGD:1359702 
   919 0.0001901 759 547.3 1.387  1369200_at  5 nucleotidase  Info   Nt5 
   920 0.0001902 1.7 22 0.077  1388246_at  clusterin  Info   Clu 
   921 0.0001902 375.1 713 0.526  1371951_at  four and a half LIM domains 2  Info   Fhl2 
   922 0.0001904 2278.6 1670.6 1.364  1370320_at  MAWD binding protein  Info   Mawbp 
   923 0.0001912 1272.7 953.3 1.335  1370282_at  cysteine and glycine-rich protein 2  Info   RGD:61950 
   924 0.0001915 9.5 48.7 0.195  1384231_at  Transcribed locus  Info    
   925 0.0001918 2291.2 1621.7 1.413  1373667_at  cysteine conjugate-beta lyase (predicted)  Info   Ccbl1_predicted 
   926 0.0001934 57.8 104.4 0.554  1383176_at  similar to C11orf17 protein (predicted)  Info   RGD1306959_predicted 
   927 0.0001937 60.5 94.7 0.639  1376615_at  TEA domain family member 3 (predicted)  Info   Tead3_predicted 
   928 0.0001942 171.1 234.5 0.73  1372901_at  Similar to RIKEN cDNA 8030451K01  Info    
   929 0.0001942 379.3 206.3 1.839  1375677_at  transducer of ERBB2, 2  Info   RGD:1359722 
   930 0.0001944 88.8 3.6 24.667  1395030_at  Basic transcription element binding protein 1  Info   Bteb1 
   931 0.0001947 1203.3 1543.6 0.78  1372070_at  interferon gamma inducible protein 30 (predicted)  Info   Ifi30_predicted 
   932 0.0001948 26.4 64.3 0.411  1369010_at  protein kinase Chk2  Info   Chek2 
   933 0.0001955 7.8 75.3 0.104  1368534_at  adrenergic receptor, alpha 1d  Info   Adra1d 
   934 0.0001956 43.5 81 0.537  1379665_at  similar to RIKEN cDNA 4632422M10 (predicted)  Info   RGD1310204_predicted 
   935 0.0001962 162.3 251.1 0.646  1372339_at  cell division cycle 26 (predicted)  Info   Cdc26_predicted 
   936 0.0001965 104.8 166.6 0.629  1373324_at  dual specificity phosphatase 14 (predicted) /// similar to Dual specificity phosphatase 14  Info   Dusp14_predicted /// LOC499736 
   937 0.0001972 41.5 85.9 0.483  1375060_at  similar to Cc2-27 (predicted)  Info   RGD1310244_predicted 
   938 0.0001987 1625.8 1955.4 0.831  1367452_at  SMT3 suppressor of mif two 3 homolog 2 (yeast)  Info   Sumo2 
   939 0.0001988 68.3 128.8 0.53  1373037_at  ubiquitin-conjugating enzyme E2L 6 (predicted)  Info   Ube2l6_predicted 
   940 0.0001992 128.9 218 0.591  1369652_at  thymus cell antigen 1, theta  Info   Thy1 
   941 0.0001993 516.9 1368.9 0.378  1370161_at  steroid sensitive gene 1  Info   Ssg1 
   942 0.0001993 2.7 42 0.064  1383486_at  Transcribed locus  Info    
   943 0.0001995 121 61 1.984  1389350_at  apolipoprotein H (predicted)  Info   Apoh_predicted 
   944 0.0001997 77.7 128.4 0.605  1389760_at  ring finger protein 134 (predicted)  Info   Rnf134_predicted 
   945 0.0001999 16.6 49.7 0.334  1395126_at    Info    
   946 0.0002002 26.5 65.7 0.403  1390931_at  Transcribed locus  Info    
   947 0.0002004 35.1 85.5 0.411  1373290_at  similar to Ezh2 protein  Info   LOC312299 
   948 0.0002004 103.4 210.1 0.492  1376099_at  Collagen, type V, alpha 1  Info   Col5a1 
   949 0.000201 49.9 93.2 0.535  1385925_at  Transcribed locus  Info    
   950 0.0002012 788.3 462 1.706  1370936_at  dimethylglycine dehydrogenase precursor  Info   Dmgdh 
   951 0.0002014 2566.2 1646.5 1.559  1371824_at  adenylate kinase 3-like 1  Info   Ak3l1 
   952 0.0002024 60.3 116.2 0.519  1378144_at  Transcribed locus  Info    
   953 0.0002024 6 45.8 0.131  1378076_at  Transcribed locus  Info    
   954 0.000203 249.2 175.3 1.422  1371606_at  Transcribed locus  Info    
   955 0.0002037 4.3 1.2 3.583  1378244_at  Transcribed locus  Info    
   956 0.0002049 27.4 79.3 0.346  1369917_at  neurotrophin receptor associated death domain  Info   Nradd 
   957 0.0002052 20.6 57.9 0.356  1369649_at  calcium channel, voltage-dependent, alpha2/delta subunit 1  Info   Cacna2d1 
   958 0.0002058 76.9 127.2 0.605  1377826_at  Transcribed locus, weakly similar to NP_036457.1 microtubule-associated protein, RP/EB family, member 1; adenomatous polyposis coli-binding protein EB1 [Homo sapiens]  Info    
   959 0.0002058 2871 4659.1 0.616  1370154_at  lysozyme  Info   Lyz 
   960 0.000206 89.1 174.6 0.51  1374565_at  NIMA (never in mitosis gene a)-related expressed kinase 6  Info   Nek6 
   961 0.0002072 43.4 66.4 0.654  1391849_at  Similar to MBD2 (methyl-CpG-binding protein)-interacting zinc finger protein (predicted)  Info    
   962 0.0002074 19.5 37.7 0.517  1392037_at  Transcribed locus  Info    
   963 0.0002077 71 91.9 0.773  1373345_at  amphoterin induced gene 2  Info   Amigo2 
   964 0.0002091 3058.6 2138.8 1.43  1371350_at  similar to S-adenosylmethionine synthetase gamma form (Methionine adenosyltransferase) (AdoMet synthetase) (MAT-II)  Info   LOC498211 
   965 0.0002098 356.3 228.2 1.561  1384103_at  Similar to novel protein (HT036)  Info    
   966 0.00021 54.9 92.3 0.595  1397684_at  dickkopf homolog 4 (Xenopus laevis) (predicted)  Info   Dkk4_predicted 
   967 0.0002101 217.6 301.4 0.722  1373242_at  TATA box binding protein-like 1 (predicted)  Info   Tbpl1_predicted 
   968 0.0002102 68.6 29.4 2.333  1392521_at  Transcribed locus  Info    
   969 0.0002104 981.3 433.8 2.262  1398286_at  cysteine sulfinic acid decarboxylase  Info   Csad 
   970 0.0002121 110.2 170.8 0.645  1384588_at  Transcribed locus  Info    
   971 0.0002126 12.9 26.2 0.492  1381261_at  Nicotinamide nucleotide transhydrogenase  Info   Nnt 
   972 0.0002127 503.8 375.9 1.34  1369799_at  4-aminobutyrate aminotransferase  Info   Abat 
   973 0.0002128 58.5 167.2 0.35  1368921_a_at  CD44 antigen  Info   Cd44 
   974 0.0002129 102.8 163.1 0.63  1373238_at  similar to SPT3-associated factor 42 (predicted)  Info   RGD1306774_predicted 
   975 0.000214 1225.9 1848.3 0.663  1373152_at  protease, serine, 23  Info   RGD:1359545 
   976 0.0002145 25.3 10 2.53  1374672_at  cardiac ankyrin repeat kinase  Info   Cark 
   977 0.0002147 11.8 24.4 0.484  1384580_at  complement component 6  Info   C6 
   978 0.000216 17.5 60.2 0.291  1368544_a_at  nucleolar protein 3 (apoptosis repressor with CARD domain)  Info   Nol3 
   979 0.0002161 2697.6 800.2 3.371  1370144_at  G protein-binding protein CRFG /// similar to GTP-binding protein NGB /// similar to GTP-binding protein NGB  Info   Gtpbp4 /// LOC364763 /// LOC498786 
   980 0.0002162 3083.2 3899.2 0.791  1370253_at  ribosomal protein L22  Info   Rpl22 
   981 0.0002164 467.5 351 1.332  1372292_at  Similar to Serine/threonine protein kinase 24  Info    
   982 0.0002178 7.2 22.5 0.32  1390141_at  formyltetrahydrofolate synthetase domain containing 1 (predicted)  Info   Fthfsdc1_predicted 
   983 0.0002181 17.9 40.3 0.444  1385868_at    Info    
   984 0.0002184 584.5 328.3 1.78  1373403_at  Transcribed locus  Info    
   985 0.0002188 321.2 220.4 1.457  1390440_at  similar to mKIAA0267 protein  Info   LOC302863 
   986 0.0002192 108.5 847.8 0.128  1387011_at  lipocalin 2  Info   Lcn2 
   987 0.0002199 36.6 91.3 0.401  1395184_at    Info    
   988 0.0002203 785.1 535.1 1.467  1390543_at  Transcribed locus  Info    
   989 0.0002204 36.1 62.2 0.58  1395781_at  v-myc myelocytomatosis viral oncogene homolog 1, lung carcinoma derived (avian)  Info   Mycl1 
   990 0.0002207 57.7 117.4 0.491  1395039_at  Transcribed locus  Info    
   991 0.0002209 2919.7 1302.3 2.242  1368627_at  regucalcin  Info   Rgn 
   992 0.0002215 197.2 291 0.678  1368395_at  glypican 3  Info   Gpc3 
   993 0.0002216 86.2 146.3 0.589  1390846_at  procollagen, type XVI, alpha 1 (predicted)  Info   Col16a1_predicted 
   994 0.0002216 264.5 351 0.754  1371737_at  tripartite motif protein 27 (predicted)  Info   Trim27_predicted 
   995 0.0002217 466.6 251.8 1.853  1379361_at  peroxisomal biogenesis factor 11A  Info   Pex11a 
   996 0.0002221 105.3 215.2 0.489  1391347_at  RAB8B, member RAS oncogene family  Info   Rab8b 
   997 0.0002222 876.6 698.9 1.254  1374638_at  peroxisomal biogenesis factor 13 (predicted)  Info   Pex13_predicted 
   998 0.0002234 219.9 323 0.681  1388132_at  NonO/p54nrb homolog  Info   Sfpq 
   999 0.0002234 364.3 524.7 0.694  1388753_at  sulfatase 2 (predicted)  Info   Sulf2_predicted 
   1000 0.0002235 127.1 210.3 0.604  1383833_at  hypothetical protein LOC500991  Info   LOC500991 
   1001 0.000224 8.2 38.9 0.211  1387160_at  potassium voltage-gated channel, Isk-related subfamily, member 3  Info   Kcne3 
   1002 0.000225 159.3 251.1 0.634  1392938_s_at  similar to C11orf17 protein (predicted)  Info   RGD1306959_predicted 
   1003 0.0002255 41.9 60.6 0.691  1395953_at  Transcribed locus  Info    
   1004 0.0002258 98.5 119 0.828  1374484_at  similar to RIKEN cDNA 2610033C09 (predicted)  Info   RGD1306421_predicted 
   1005 0.0002266 23.8 186.9 0.127  1373722_at  kinesin family member 20A (predicted)  Info   Kif20a_predicted 
   1006 0.0002271 1213 739.5 1.64  1372365_at  Transcribed locus  Info    
   1007 0.0002278 216 271.7 0.795  1390492_a_at  Ubiquitin specific protease 40 (predicted)  Info    
   1008 0.000228 46.7 108.7 0.43  1367983_at  flap structure-specific endonuclease 1  Info   Fen1 
   1009 0.0002282 105.6 139.7 0.756  1383964_at  Transcribed locus  Info    
   1010 0.0002283 64 43.7 1.465  1393550_at  Transcribed locus  Info    
   1011 0.0002287 106.1 160.4 0.661  1395364_at  interferon (alpha and beta) receptor 1 (predicted)  Info   Ifnar1_predicted 
   1012 0.0002304 48.6 118.8 0.409  1391067_at    Info    
   1013 0.0002314 22.7 41 0.554  1378793_at  Forkhead box P4 (predicted)  Info    
   1014 0.0002318 71.3 142.5 0.5  1388243_at  gene model 1012, (NCBI)  Info   Gm1012 
   1015 0.0002319 18.4 52 0.354  1382138_at  similar to hypothetical protein FLJ20433  Info   LOC499745 
   1016 0.0002326 2691.9 3185 0.845  1386868_at  ribosomal protein S10  Info   Rps10 
   1017 0.0002332 512.6 351.2 1.46  1395268_at  WW domain containing E3 ubiquitin protein ligase 1 (predicted)  Info   Wwp1_predicted 
   1018 0.0002333 56.1 124.8 0.45  1368576_at  cartilage homeo protein 1  Info   Cart1 
   1019 0.0002337 24.5 78.3 0.313  1382370_at  cyclin F  Info   Ccnf 
   1020 0.0002341 139.7 240.4 0.581  1375362_at    Info    
   1021 0.0002343 57.4 85.7 0.67  1377390_at  beta-site APP-cleaving enzyme 2  Info   RGD:1303241 
   1022 0.0002349 11.1 44.1 0.252  1393682_at  G-protein-coupled receptor GPR34  Info   GPR34 
   1023 0.0002353 1436.5 1209 1.188  1390021_at  histone 1, H2bh (predicted)  Info   Hist1h2bh_predicted 
   1024 0.0002358 12.6 119.8 0.105  1370297_at  polo-like kinase 1 (Drosophila)  Info   Plk1 
   1025 0.000236 368.6 579.4 0.636  1373150_at  catechol-O-methyltransferase domain containing 1 (predicted)  Info   Comtd1_predicted 
   1026 0.000236 454.8 726.7 0.626  1388143_at  collagen, type XVIII, alpha 1  Info   Col18a1 
   1027 0.0002364 1465 791.1 1.852  1367759_at  H1 histone family, member 0  Info   H1f0 
   1028 0.0002366 40.4 67 0.603  1382130_at  similar to Protocadherin 19 precursor  Info   LOC317183 
   1029 0.000237 276.8 192.4 1.439  1392914_at    Info    
   1030 0.0002372 90 202.2 0.445  1374739_at  similar to hematopoietic progenitor protein  Info   LOC500727 
   1031 0.0002374 273.3 366.5 0.746  1388588_at  mammary tumor virus receptor 2 (predicted)  Info   Mtvr2_predicted 
   1032 0.0002385 136.8 259.2 0.528  1395427_at  Transcribed locus  Info    
   1033 0.0002386 363.4 285.9 1.271  1388658_at  surfeit 2  Info   Surf2 
   1034 0.00024 130.5 159.6 0.818  1389061_at  similar to NOL1R protein; Williams-Beuren syndrome critical region protein 20 (predicted)  Info   RGD1309268_predicted 
   1035 0.0002412 4494.7 3139.1 1.432  1367702_at  acetyl-Coenzyme A dehydrogenase, medium chain  Info   Acadm 
   1036 0.0002414 66.9 101.4 0.66  1384591_at  Similar to hypothetical protein FLJ32884 (predicted)  Info    
   1037 0.0002423 625.2 902.1 0.693  1368808_at  CAP, adenylate cyclase-associated protein 1 (yeast)  Info   Cap1 
   1038 0.0002427 578.3 1065.2 0.543  1388494_at  Procollagen, type IV, alpha 2 (predicted)  Info    
   1039 0.0002429 3314.1 2708.6 1.224  1372132_at  CNDP dipeptidase 2 (metallopeptidase M20 family) (predicted)  Info   Cndp2_predicted 
   1040 0.000243 1928.9 1492.8 1.292  1370166_at  syndecan 2  Info   Sdc2 
   1041 0.000243 151 213.1 0.709  1388306_at  similar to RIKEN cDNA 1810042K04 (predicted)  Info   RGD1305593_predicted 
   1042 0.0002432 592.4 515 1.15  1389154_at  lipoic acid synthetase (predicted)  Info   Lias_predicted 
   1043 0.0002435 1638.4 871.5 1.88  1373975_at  similar to thioether S-methyltransferase  Info   LOC368066 
   1044 0.0002442 890.6 588.4 1.514  1372997_at  Transcribed locus  Info    
   1045 0.0002444 38.1 76.9 0.495  1375277_at  similar to hypothetical protein FLJ20433  Info   LOC499745 
   1046 0.0002452 4.8 19.4 0.247  1378917_at  Transcription elongation factor B polypeptide 3 binding protein 1 (predicted)  Info    
   1047 0.0002454 147.4 238.4 0.618  1371908_at  NTF2-related export protein 1 (predicted)  Info   Nxt1_predicted 
   1048 0.0002463 18.9 35.9 0.526  1393405_at  Transcribed locus  Info    
   1049 0.0002468 529 394.5 1.341  1373198_at  similar to RIKEN cDNA 2810451A06 (predicted)  Info   RGD1311098_predicted 
   1050 0.000247 20.2 60.6 0.333  1382751_at    Info    
   1051 0.0002471 215.6 161.7 1.333  1380321_at  mitochondrial tumor suppressor 1  Info   Mtus1 
   1052 0.000248 5.5 50.9 0.108  1372903_at  similar to 3000004C01Rik protein (predicted)  Info   RGD1310360_predicted 
   1053 0.0002484 1531.1 1131.9 1.353  1370367_at  solute carrier family 1 (neuronal/epithelial high affinity glutamate transporter, system Xag), member 1  Info   Slc1a1 
   1054 0.0002489 500.8 288.5 1.736  1378307_at  Pleckstrin homology domain containing, family H (with MyTH4 domain) member 1 (predicted)  Info    
   1055 0.0002505 9.6 54.4 0.176  1384334_at    Info    
   1056 0.0002508 52.8 95.1 0.555  1385143_at    Info    
   1057 0.000251 226.5 327.5 0.692  1380182_at  similar to KIAA1217  Info   LOC291356 
   1058 0.0002512 350.2 298.9 1.172  1374582_at  potassium channel tetramerisation domain containing 9 (predicted)  Info   Kctd9_predicted 
   1059 0.0002515 56.6 86.7 0.653  1384378_at  HCR (a-helix coiled-coil rod homolog)  Info   RGD:1302992 
   1060 0.0002519 20.1 61.2 0.328  1368522_at  timeless homolog (Drosophila)  Info   Timeless 
   1061 0.0002529 737.2 476.8 1.546  1376605_at  solute carrier family 17 (anion/sugar transporter), member 5 (predicted)  Info   Slc17a5_predicted 
   1062 0.0002538 90 193.5 0.465  1367768_at  latexin  Info   Lxn 
   1063 0.000254 231.8 306.6 0.756  1373392_at  TPA regulated locus (predicted)  Info   Tparl_predicted 
   1064 0.0002542 233 136.1 1.712  1374630_at  chloride intracellular channel 3 (predicted)  Info   Clic3_predicted 
   1065 0.0002542 540.7 409.6 1.32  1387109_at  P450 (cytochrome) oxidoreductase  Info   Por 
   1066 0.0002553 336.1 424.8 0.791  1392906_at  similar to Ubiquitin-like protein 4 (Ubiquitin-like protein GDX)  Info   LOC293864 
   1067 0.0002554 410.7 560.3 0.733  1383202_at  similar to U6 snRNA-associated Sm-like protein LSm6 (Sm protein F)  Info   LOC498934 
   1068 0.0002555 33.3 56 0.595  1376120_at  similar to pancreatitis-induced protein 49 (predicted)  Info   RGD1308600_predicted 
   1069 0.0002558 797.9 696.2 1.146  1389354_at  similar to semaF cytoplasmic domain associated protein 2  Info   LOC362057 
   1070 0.0002564 12 40.7 0.295  1378640_at  ubiquitin-like, containing PHD and RING finger domains, 1  Info   Uhrf1 
   1071 0.0002572 8.1 1.9 4.263  1376726_at  similar to Carbonic anhydrase-related protein 10 (Carbonic anhydrase-related protein X) (CARP X) (CA-RP X)  Info   LOC360599 
   1072 0.0002575 169.7 113.9 1.49  1374574_at  Similar to hypothetical protein MGC38960 (predicted)  Info    
   1073 0.0002576 1027.5 1316.2 0.781  1372247_at  dolichyl-di-phosphooligosaccharide-protein glycotransferase (predicted)  Info   Ddost_predicted 
   1074 0.0002588 63.9 94.7 0.675  1395396_at  similar to CG9135-PA (predicted)  Info   RGD1309986_predicted 
   1075 0.0002588 122.6 72.5 1.691  1369153_at  nephrosis 1 homolog, nephrin (human)  Info   Nphs1 
   1076 0.0002591 21.5 45.7 0.47  1383958_at  cell division cycle associated 2 (predicted)  Info   Cdca2_predicted 
   1077 0.0002593 82.7 124.4 0.665  1378482_at  kidney cell line derived transcript 1 (predicted)  Info   Kdt1_predicted 
   1078 0.0002598 318.5 393.1 0.81  1377194_a_at  similar to RIKEN cDNA 2310015N07 (predicted)  Info   RGD1308637_predicted 
   1079 0.0002603 72.2 42.8 1.687  1376341_at  Transcribed locus  Info    
   1080 0.0002604 3113.5 2445.2 1.273  1386904_a_at  cytochrome b-5  Info   Cyb5 
   1081 0.0002607 2161.6 1169.9 1.848  1369401_at  solute carrier family 21, member 13  Info   Slc21a13 
   1082 0.000261 130.6 233.5 0.559  1375538_at  Vinculin (predicted)  Info    
   1083 0.0002616 69.1 88.6 0.78  1375732_at  Transcribed locus  Info    
   1084 0.0002621 32.4 59.4 0.545  1370421_a_at  TBP-interacting protein 120B  Info   Tip120B 
   1085 0.0002621 463.4 319.5 1.45  1382325_at  glycine C-acetyltransferase (2-amino-3-ketobutyrate-coenzyme A ligase) (predicted)  Info   Gcat_predicted 
   1086 0.0002622 2480.9 1353.4 1.833  1368096_at  Ras-related GTP-binding protein Rab29  Info   Rab7l1 
   1087 0.0002625 37.6 64.8 0.58  1382865_at  testis specific, 14  Info   Tsga14 
   1088 0.0002629 10.8 3.2 3.375  1396739_at    Info    
   1089 0.0002635 76.8 155.3 0.495  1393451_at  similar to RIKEN cDNA 2610510J17 (predicted)  Info   RGD1310953_predicted 
   1090 0.0002646 51.6 100.7 0.512  1389503_at  similar to angiopoietin 1 /// similar to uroplakin 3B isoform b  Info   LOC288748 /// LOC367994 
   1091 0.0002648 1269.9 915.6 1.387  1398807_at  protein phosphatase 1B, magnesium dependent, beta isoform  Info   Ppm1b 
   1092 0.0002648 494.3 659.6 0.749  1381925_x_at  AT rich interactive domain 1B (Swi1 like) /// hypothetical gene supported by NM_172157  Info   Arid1b /// LOC497729 
   1093 0.0002665 9.6 35.3 0.272  1379889_at  lamimin, gamma 2  Info   Lamc2 
   1094 0.0002666 12.8 30.2 0.424  1389506_x_at    Info    
   1095 0.000267 2199.8 1516.1 1.451  1371680_at    Info    
   1096 0.0002674 303.7 517.7 0.587  1387872_at  heterogeneous nuclear ribonucleoprotein A1  Info   Hnrpa1 
   1097 0.000268 285.1 484.1 0.589  1372282_at  similar to RIKEN cDNA 2310016C16 (predicted)  Info   RGD1307506_predicted 
   1098 0.000268 8.8 73.5 0.12  1385086_at  budding uninhibited by benzimidazoles 1 homolog (S. cerevisiae) (predicted)  Info   Bub1_predicted 
   1099 0.0002686 245.2 331.2 0.74  1377794_at  Transcribed locus  Info    
   1100 0.0002694 226.1 419.6 0.539  1367974_at  annexin A3  Info   Anxa3 
   1101 0.00027 355.7 520.2 0.684  1388629_at  inosine 5-monophosphate dehydrogenase 2  Info   Impdh2 
   1102 0.0002705 39.9 61.1 0.653  1375723_at  Transcribed locus  Info    
   1103 0.0002709 292.9 178.4 1.642  1367835_at  proprotein convertase subtilisin/kexin type 1 inhibitor  Info   Pcsk1n 
   1104 0.000271 375.7 228.6 1.643  1382447_at    Info    
   1105 0.0002715 227.5 265.7 0.856  1382203_at  growth differentiation factor 1 (predicted)  Info   Gdf1_predicted 
   1106 0.0002724 4244.5 2606.9 1.628  1389785_at  similar to RIKEN cDNA 0610006H10 gene (predicted)  Info   RGD1305394_predicted 
   1107 0.0002725 13.8 37.2 0.371  1392128_at  Transcribed locus  Info    
   1108 0.0002732 1596.2 1973.8 0.809  1399091_at  F-actin capping protein beta subunit  Info   RGD:1359099 
   1109 0.0002734 1459 968.8 1.506  1384638_at  Similar to RIKEN cDNA 1700013H19 (predicted)  Info    
   1110 0.0002739 17.3 72.9 0.237  1389440_at  Transcribed locus  Info    
   1111 0.0002741 31.5 46.5 0.677  1378731_at  similar to CDNA sequence BC006909  Info   LOC499267 
   1112 0.000275 58 91.6 0.633  1379861_at  similar to RIKEN cDNA 6720485C15  Info   RGD:1359211 
   1113 0.000275 268.8 56.4 4.766  1374855_at  period homolog 1 (Drosophila)  Info   Per1 
   1114 0.0002751 2051.7 1741.8 1.178  1388851_at  heat shock protein, A (predicted)  Info   Hspa9a_predicted 
   1115 0.0002756 46.7 70.4 0.663  1389674_at  zinc finger protein 282 (predicted)  Info   Zfp282_predicted 
   1116 0.0002762 311.2 203.2 1.531  1379260_at  Transcribed locus  Info    
   1117 0.0002765 519.5 761.3 0.682  1392900_at  Similar to mKIAA1631 protein  Info    
   1118 0.0002766 131.9 49.5 2.665  1369728_at  histone 1, H4m (predicted)  Info   Hist1h4m_predicted 
   1119 0.0002767 861.5 461.6 1.866  1383605_at  similar to alpha-fetoprotein  Info   LOC360919 
   1120 0.0002768 53.6 78.4 0.684  1381986_at  Neuron navigator 1 (predicted)  Info    
   1121 0.0002777 30.8 71.2 0.433  1374799_at  similar to mKIAA0159 protein  Info   LOC362438 
   1122 0.0002794 619.5 951.8 0.651  1370057_at  cysteine and glycine-rich protein 1  Info   Csrp1 
   1123 0.0002826 477.7 318.1 1.502  1377064_at  dual specificity phosphatase 6  Info   Dusp6 
   1124 0.0002831 171.2 113.9 1.503  1382128_at  Transcribed locus  Info    
   1125 0.0002839 1270.4 2066.9 0.615  1371518_at  Nidogen (entactin)  Info   Nid 
   1126 0.0002856 427.5 304.4 1.404  1375767_at  similar to common-site lymphoma leukemia guanine nucleotide exchange factor like (5N754) (predicted)  Info   RGD1308089_predicted 
   1127 0.0002857 129.9 222.7 0.583  1374537_at  Carbohydrate (chondroitin) synthase 1 (predicted)  Info    
   1128 0.0002857 140.4 191.4 0.734  1377151_at  Transcribed locus  Info    
   1129 0.0002866 31.6 76.9 0.411  1398482_at  B-cell leukemia/lymphoma 3 (predicted)  Info   Bcl3_predicted 
   1130 0.0002881 178.7 143.8 1.243  1382115_at  similar to RIKEN cDNA 4930521E07 (predicted)  Info   RGD1305166_predicted 
   1131 0.0002883 676.4 460.1 1.47  1372038_at  MAP kinase-interacting serine/threonine kinase 2 (predicted)  Info   Mknk2_predicted 
   1132 0.0002885 71.9 138 0.521  1397458_at  phosphomannomutase 2 (predicted)  Info   Pmm2_predicted 
   1133 0.0002885 345.8 256.2 1.35  1374272_at  Transcribed locus  Info    
   1134 0.0002888 44.6 77.3 0.577  1384483_at  Similar to RIKEN cDNA E030034P13 (predicted)  Info    
   1135 0.000289 75.4 138.4 0.545  1382277_at    Info    
   1136 0.0002902 38 84 0.452  1379866_at  Septin 6 (predicted)  Info    
   1137 0.0002902 129.3 176 0.735  1388927_at  RAB, member of RAS oncogene family-like 4 (predicted)  Info   Rabl4_predicted 
   1138 0.0002909 1301.7 618.6 2.104  1368234_at  prolyl endopeptidase  Info   Prep 
   1139 0.000291 215.8 319.4 0.676  1395973_at  Similar to RIKEN cDNA 5730596K20 (predicted)  Info    
   1140 0.0002916 68.8 123.3 0.558  1385926_at  Transcribed locus  Info    
   1141 0.0002917 1135.1 1499.1 0.757  1388930_at  similar to RIKEN cDNA 2310075C12 (predicted)  Info   RGD1305625_predicted 
   1142 0.0002924 134.6 177.9 0.757  1383372_at  platelet-activating factor receptor  Info   Ptafr 
   1143 0.0002926 1.3 6.8 0.191  1392127_at  Transcribed locus  Info    
   1144 0.0002929 57.2 23.6 2.424  1368894_at  CAP, adenylate cyclase-associated protein, 2 (yeast)  Info   Cap2 
   1145 0.0002938 630.7 471.6 1.337  1367761_at  nudE nuclear distribution gene E homolog like 1 (A. nidulans)  Info   Ndel1 
   1146 0.0002946 138 194.9 0.708  1390046_at  a disintegrin and metalloprotease domain 8 (predicted)  Info   Adam8_predicted 
   1147 0.0002955 210 426.8 0.492  1390411_at  claudin 19 (predicted)  Info   Cldn19_predicted 
   1148 0.0002964 206.5 133 1.553  1377004_at  Transcribed locus  Info    
   1149 0.0002966 785.6 597 1.316  1374903_at  glucosaminyl (N-acetyl) transferase 2, I-branching enzyme /// hypothetical gene supported by NM_001001511  Info   Gcnt2 /// LOC497712 
   1150 0.0002967 4338.1 5202.3 0.834  1371344_at  ribosomal protein L27a (predicted)  Info   Rpl27a_predicted 
   1151 0.0002969 42.5 87.4 0.486  1395662_at  Transcribed locus  Info    
   1152 0.0002983 870 1236.6 0.704  1371541_at  Myosin, light polypeptide kinase (predicted)  Info    
   1153 0.0002984 40.1 84.2 0.476  1390420_at  carboxypeptidase X 1 (M14 family) (predicted)  Info   Cpxm1_predicted 
   1154 0.0002997 40.8 83.1 0.491  1380768_at  Transcribed locus  Info    
   1155 0.0002997 61.1 88.4 0.691  1391744_at  Transcribed locus  Info    
   1156 0.0003014 25.1 46.3 0.542  1389447_at  similar to prolyl-4-hydroxylase-alpha NE2 CG9720-PA (predicted)  Info   RGD1311848_predicted 
   1157 0.0003021 1087.4 728.3 1.493  1382924_at  pantothenate kinase 1 (predicted)  Info   Pank1_predicted 
   1158 0.0003031 316.3 432.6 0.731  1371914_at  SWI/SNF related, matrix associated, actin dependent regulator of chromatin, subfamily b, member 1 (predicted)  Info   Smarcb1_predicted 
   1159 0.0003046 186.5 302.9 0.616  1367890_at  caspase 2  Info   Casp2 
   1160 0.0003052 1163.8 708.2 1.643  1389469_at  chromodomain helicase DNA binding protein 1-like (predicted)  Info   Chd1l_predicted 
   1161 0.0003053 306.2 196.5 1.558  1374682_at  Transcribed locus, weakly similar to NP_035569.1 SRY-box 13 [Mus musculus]  Info    
   1162 0.0003061 140 301.5 0.464  1370054_at  cyclin-dependent kinase inhibitor 2C (p18, inhibits CDK4)  Info   Cdkn2c 
   1163 0.0003062 34.8 82.9 0.42  1398321_a_at  procollagen, type XII, alpha 1  Info   Col12a1 
   1164 0.0003068 595.4 336.6 1.769  1371959_at  histone 2, H2aa (predicted) /// similar to Hist2h2aa1 protein  Info   Hist2h2aa_predicted /// LOC499676 
   1165 0.0003072 40.1 68.5 0.585  1379482_at  transmembrane 6 superfamily member 1 (predicted)  Info   Tm6sf1_predicted 
   1166 0.0003075 613 850.8 0.72  1373881_at  Rho, GDP dissociation inhibitor (GDI) beta (predicted)  Info   Arhgdib_predicted 
   1167 0.0003089 1408.4 906.9 1.553  1389540_at  TG interacting factor (predicted)  Info   Tfig 
   1168 0.0003091 308.4 657.1 0.469  1369113_at  gremlin 1 homolog, cysteine knot superfamily (Xenopus laevis)  Info   Grem1 
   1169 0.0003103 3106.8 2297.5 1.352  1368467_at  cytochrome P450, family 4, subfamily F, polypeptide 2  Info   Cyp4f2 
   1170 0.0003104 64.9 27.8 2.335  1382173_at  Transcribed locus  Info    
   1171 0.0003108 495.8 312.5 1.587  1372536_at  chaperone, ABC1 activity of bc1 complex like (S. pombe) (predicted)  Info   Cabc1_predicted 
   1172 0.0003122 86.5 119.9 0.721  1368593_at  CD1d1 antigen  Info   Cd1d1 
   1173 0.0003127 51.7 99.3 0.521  1391979_at  Transcribed locus  Info    
   1174 0.0003141 146.8 218.1 0.673  1368463_at  vascular endothelial growth factor C  Info   Vegfc 
   1175 0.0003144 935.5 686.6 1.363  1371729_at  Yippee-like 5 (Drosophila) (predicted)  Info    
   1176 0.0003144 2319 1428.8 1.623  1387375_at  ketohexokinase  Info   Khk 
   1177 0.0003155 998.1 732.2 1.363  1373826_at    Info    
   1178 0.0003158 13.1 83.1 0.158  1376684_at  discs, large homolog 7 (Drosophila) (predicted)  Info   Dlg7_predicted 
   1179 0.000316 2658.4 3452.4 0.77  1388314_at  high mobility group nucleosomal binding domain 1 (predicted)  Info   Hmgn1_predicted 
   1180 0.000316 464.4 315.7 1.471  1390228_at  Transcribed locus  Info    
   1181 0.0003164 1515.2 1154 1.313  1379885_at  flavin containing monooxygenase 4  Info   Fmo4 
   1182 0.0003174 1877.9 2257.1 0.832  1388076_at  PAI-1 mRNA-binding protein  Info   Pairbp1 
   1183 0.0003182 587.1 459.1 1.279  1376038_at  testis expressed gene 2 (predicted)  Info   Tex2_predicted 
   1184 0.0003184 339.3 99.9 3.396  1370174_at  myeloid differentiation primary response gene 116  Info   Myd116 
   1185 0.0003185 133.1 194.3 0.685  1383703_at  FUS interacting protein (serine-arginine rich) 1 (predicted)  Info    
   1186 0.0003193 947.9 754.6 1.256  1376723_a_at    Info    
   1187 0.0003198 44.1 81.2 0.543  1388840_at  similar to common-site lymphoma/leukemia GEF (predicted)  Info   RGD1310112_predicted 
   1188 0.0003204 57.5 99.6 0.577  1370574_a_at  protein tyrosine phosphatase, non-receptor type substrate 1  Info   Ptpns1 
   1189 0.0003207 2.3 11.8 0.195  1398174_at  methyl CpG binding protein 2  Info   Mecp2 
   1190 0.0003208 768.4 598.5 1.284  1371747_at  similar to RIKEN cDNA 2700038C09 (predicted)  Info   RGD1310660_predicted 
   1191 0.0003212 232.1 160.6 1.445  1388665_at  similar to RIKEN cDNA 1110020A23 (predicted)  Info   RGD1308134_predicted 
   1192 0.0003216 2872.6 1572.4 1.827  1370163_at  ornithine decarboxylase 1  Info   Odc1 
   1193 0.000322 1342.5 932.8 1.439  1379250_at  galactose mutarotase (aldose 1-epimerase)  Info   RGD:1359459 
   1194 0.0003222 393.8 234.7 1.678  1389234_at  von Willebrand factor  Info   RGD:621759 
   1195 0.0003239 77.3 110.8 0.698  1367600_at  desmin  Info   Des 
   1196 0.000324 19.5 49.8 0.392  1377772_at  Transmembrane protein with EGF-like and two follistatin-like domains 1  Info   Tmeff1 
   1197 0.0003241 753.6 1065.3 0.707  1367604_at  cysteine-rich protein 2  Info   RGD:1302959 
   1198 0.0003243 25.6 43.3 0.591  1385909_at  Transcribed locus  Info    
   1199 0.0003249 6.7 118.6 0.056  1390137_at  TRAF4 associated factor 1  Info   RGD:1303240 
   1200 0.0003258 102.1 172.6 0.592  1390812_a_at  similar to RAS-like, estrogen-regulated, growth-inhibitor  Info   LOC502916 
   1201 0.0003267 1387.7 1791.3 0.775  1388119_at  hypothetical gene supported by Y16641 /// heterogeneous nuclear ribonucleoprotein A3 /// similar to Hnrpa3 protein /// similar to Hnrpa3 protein  Info   LOC292273 /// Hnrpa3 /// LOC363971 /// LOC499592 
   1202 0.0003268 419.4 847.9 0.495  1367594_at  biglycan  Info   Bgn 
   1203 0.0003293 60.9 144.6 0.421  1380692_at  Transcribed locus  Info    
   1204 0.0003295 588 276.6 2.126  1389392_at  Transcribed locus  Info    
   1205 0.0003302 22.4 75.9 0.295  1370461_at  hyaluronan mediated motility receptor  Info   Hmmr 
   1206 0.0003308 19.4 44.9 0.432  1380721_at  Transcribed locus  Info    
   1207 0.0003312 1106.1 800 1.383  1384628_at  similar to RIKEN cDNA 0610009A07 (predicted)  Info   RGD1309288_predicted 
   1208 0.0003339 455.5 596.4 0.764  1371498_at  similar to Jtv1-pending protein  Info   LOC288480 
   1209 0.0003343 64.3 115.6 0.556  1376811_a_at  cleavage and polyadenylation specific factor 6, 68kDa (predicted)  Info   Cpsf6_predicted 
   1210 0.0003346 93.4 202.6 0.461  1390386_at  caspase 3  Info   Casp3 
   1211 0.0003348 93 123.7 0.752  1369960_at  FXYD domain-containing ion transport regulator 1  Info   Fxyd1 
   1212 0.0003353 331.3 198.2 1.672  1390036_at  solute carrier family 16 (monocarboxylic acid transporters), member 6  Info   Slc16a6 
   1213 0.0003354 1202.7 296.4 4.058  1381233_at  Transcribed locus  Info    
   1214 0.0003364 452.5 554.4 0.816  1372773_at  neural proliferation, differentiation and control, 1  Info   RGD:1303156 
   1215 0.0003378 43.8 92.2 0.475  1384232_at  Transcribed locus  Info    
   1216 0.0003388 83.6 204 0.41  1384488_at  similar to cyclin-dependent kinase inhibitor 2D  Info   Cdkn2d 
   1217 0.0003398 544.6 447.5 1.217  1367750_at  phosphoribosyl pyrophosphate synthetase-associated protein 1  Info   Prpsap1 
   1218 0.0003424 50.4 98.3 0.513  1389568_at  family with sequence similarity 26, member B (predicted)  Info   Fam26b_predicted 
   1219 0.0003465 35 107.8 0.325  1374359_at  Similar to RIKEN cDNA 2810013E07 (predicted)  Info    
   1220 0.0003471 96 169.4 0.567  1370055_at  RAB3D, member RAS oncogene family  Info   Rab3d 
   1221 0.0003473 16.9 27.9 0.606  1392219_at    Info    
   1222 0.0003485 54.1 19.1 2.832  1396752_at  Tumor necrosis factor receptor superfamily, member 21 (predicted)  Info    
   1223 0.0003485 231.7 165.3 1.402  1395274_at  dystonin (predicted)  Info   Dst_predicted 
   1224 0.0003488 18.9 55.6 0.34  1390415_at  Thyroid hormone receptor interactor 13 (predicted)  Info    
   1225 0.0003506 3879.7 4553.9 0.852  1367630_at  ribosomal protein S11  Info   Rps11 
   1226 0.0003514 1736.2 1207 1.438  1367798_at  S-adenosylhomocysteine hydrolase  Info   Ahcy 
   1227 0.0003516 1012.1 770.6 1.313  1375452_at  meningioma expressed antigen 6 (coiled-coil proline-rich) (predicted)  Info   Mgea6_predicted 
   1228 0.0003519 79.7 178 0.448  1380596_at  Transcribed locus  Info    
   1229 0.0003528 1270.8 996.1 1.276  1382127_at    Info    
   1230 0.0003544 184.5 80.3 2.298  1397620_at  putative scaffolding protein POSH  Info   Sh3md2 
   1231 0.0003547 764.4 641.3 1.192  1393148_at  transmembrane 7 superfamily member 1 (predicted)  Info   Tm7sf1_predicted 
   1232 0.0003551 169.5 370.3 0.458  1388592_at  Transcribed locus  Info    
   1233 0.0003551 808.1 1049.7 0.77  1398773_at  src associated in mitosis, 68 kDa  Info   Khdrbs1 
   1234 0.0003566 439.3 522.7 0.84  1375525_at  Microtubule-associated protein, RP/EB family, member 1  Info   Mapre1 
   1235 0.000357 242.6 328.2 0.739  1376755_at  retinoic acid receptor, beta  Info   Rarb 
   1236 0.0003576 38.8 71.4 0.543  1379195_s_at  ligase I, DNA, ATP-dependent /// ligase I, DNA, ATP-dependent (predicted)  Info   Lig1 /// Lig1_predicted 
   1237 0.0003576 63.2 106.8 0.592  1390650_at  pericentrin 1 (predicted)  Info   Pcnt1_predicted 
   1238 0.0003578 4.2 10.9 0.385  1369434_at  deoxynucleotidyltransferase, terminal, interacting protein 1  Info   Dnttip1 
   1239 0.0003591 1225.6 1386.8 0.884  1387108_at  casein kinase 2, beta subunit  Info   Csnk2b 
   1240 0.0003594 61.5 88.6 0.694  1390163_at    Info    
   1241 0.0003605 312.5 116.3 2.687  1383315_at  hepatic protein EIIH  Info   RGD:1359311 
   1242 0.0003606 29.8 55.6 0.536  1389349_s_at  similar to interleukin 17 receptor E isoform 1  Info   Il17re 
   1243 0.000361 525.9 375.2 1.402  1385690_at  similar to Mut protein /// similar to Mut protein  Info   LOC301276 /// LOC363201 
   1244 0.0003622 771.2 390.3 1.976  1376709_at  solute carrier family 39 (metal ion transporter), member 8 (predicted)  Info   Slc39a8_predicted 
   1245 0.0003625 3566.6 4439.1 0.803  1398882_at  ribosomal protein S5  Info   Rps5 
   1246 0.0003656 27.2 40.4 0.673  1387849_at  epsin 2  Info   Epn2 
   1247 0.0003669 740.5 947.7 0.781  1388827_at  H2A histone family, member V (predicted) /// similar to H2A histone family, member V isoform 1  Info   H2afv_predicted /// LOC498894 
   1248 0.0003669 136.1 202.9 0.671  1370993_at  laminin, gamma 1  Info   Lamc1 
   1249 0.0003687 506.6 339.1 1.494  1385574_at  similar to cDNA sequence AF397014 (predicted)  Info   RGD1308114_predicted 
   1250 0.0003689 51.6 78.4 0.658  1377662_at  pirin  Info   RGD:1359212 
   1251 0.000369 2.3 14.5 0.159  1382243_at  Transcribed locus  Info    
   1252 0.0003698 47.9 82.3 0.582  1395036_at  similar to Ac2-190 (predicted)  Info   RGD1306455_predicted 
   1253 0.0003703 304 207.8 1.463  1386247_at  sorting nexin 25 (predicted)  Info   Snx25_predicted 
   1254 0.0003729 2559.4 1975 1.296  1368092_at  fumarylacetoacetate hydrolase  Info   Fah 
   1255 0.0003744 78.3 99.6 0.786  1373394_at    Info    
   1256 0.0003749 533.2 684.4 0.779  1388440_at  presenilin stabilization factor-like  Info   Aph1b 
   1257 0.0003751 1625.1 1143.5 1.421  1389622_at  similar to citrin  Info   LOC362322 
   1258 0.0003756 56.3 130.4 0.432  1391302_at    Info    
   1259 0.0003758 20.2 34.6 0.584  1391582_at  Transcribed locus  Info    
   1260 0.0003769 55.3 125.6 0.44  1388111_at  elastin  Info   Eln 
   1261 0.0003771 1726.1 223.6 7.72  1387328_at  Cytochrome P450, subfamily IIC (mephenytoin 4-hydroxylase)  Info   Cyp2c 
   1262 0.0003772 122.7 182.2 0.673  1367728_at  translin  Info   Tsn 
   1263 0.000378 168.9 250.4 0.675  1367751_at  dolichol-phosphate mannosyltransferase 2  Info   Dpm2 
   1264 0.0003795 3 12.4 0.242  1376902_x_at  Similar to Hypothetical protein 6330514E13  Info    
   1265 0.0003817 1468.7 2062.6 0.712  1370871_at  hypothetical gene supported by Y16641; Y16641  Info   LOC288146 
   1266 0.0003818 185.8 282.3 0.658  1393281_at  caveolin  Info   Cav 
   1267 0.0003828 37.1 82.1 0.452  1374225_at  CDNA clone IMAGE:7389359  Info    
   1268 0.0003836 23.2 44.9 0.517  1394643_at  Transcribed locus  Info    
   1269 0.0003843 501.3 333.9 1.501  1374558_at  Similar to B7-like protein GL50-B  Info    
   1270 0.0003852 19.4 40.2 0.483  1392995_at  Transcribed locus  Info    
   1271 0.0003858 1844.4 1169.2 1.577  1369958_at  rhoB gene  Info   Rhob 
   1272 0.0003861 66.9 110.1 0.608  1374603_at  SET domain and mariner transposase fusion gene  Info   Setmar 
   1273 0.0003865 775.3 1027.8 0.754  1372155_at  tripartite motif protein 28  Info   Trim28 
   1274 0.0003867 246.6 446.1 0.553  1390783_at  ATP-binding cassette, sub-family A (ABC1), member 8a (predicted)  Info   Abca8a_predicted 
   1275 0.0003868 30.2 65 0.465  1384897_at  Transcribed locus  Info    
   1276 0.0003874 31.8 75.1 0.423  1376018_at  similar to Lmnb2 protein  Info   LOC299625 
   1277 0.0003877 4.9 22.5 0.218  1391514_at  Transcribed locus  Info    
   1278 0.0003888 136.8 209.3 0.654  1372549_at  similar to RIKEN cDNA 2600017H02  Info   LOC497936 
   1279 0.0003899 68 90.9 0.748  1377172_at  similar to Pins  Info   LOC362021 
   1280 0.00039 370.8 130.1 2.85  1370153_at  growth differentiation factor 15  Info   Gdf15 
   1281 0.0003905 122 72.1 1.692  1379362_at  Transcribed locus  Info    
   1282 0.0003932 368.1 241.4 1.525  1369304_at  6-pyruvoyl-tetrahydropterin synthase  Info   Pts 
   1283 0.0003935 446.5 311.5 1.433  1381396_s_at  Kruppel-like factor 15  Info   Klf15 
   1284 0.0003941 2838.5 3580.2 0.793  1367588_a_at  ribosomal protein L13A  Info   Rpl13a 
   1285 0.0003942 3.3 21.7 0.152  1384234_at  paired related homeobox 1  Info   Prrx1 
   1286 0.0003946 764.5 925.3 0.826  1387900_at  CDP-diacylglycerol--inositol 3-phosphatidyltransferase (phosphatidylinositol synthase)  Info   Cdipt 
   1287 0.0003947 330.9 181 1.828  1376427_a_at  glycine dehydrogenase (decarboxylating; glycine decarboxylase, glycine cleavage system protein P) (predicted)  Info   Gldc_predicted 
   1288 0.0003957 28.7 44.2 0.649  1383969_at  similar to CDNA sequence BC043301  Info   LOC499143 
   1289 0.000396 72.6 154.6 0.47  1373082_at  Protein kinase inhibitor, alpha  Info   Pkia 
   1290 0.0003963 4429.5 5400.2 0.82  1371308_at  ribosomal protein S4, X-linked  Info   Rps4x 
   1291 0.0003963 92.9 129.7 0.716  1393203_at  similar to RIKEN cDNA 2010309E21 (predicted)  Info   RGD1304825_predicted 
   1292 0.0003972 410 178.6 2.296  1373309_at  Similar to RIKEN cDNA 1810054O13 (predicted)  Info    
   1293 0.0003974 166.8 229.4 0.727  1390133_at  BCL2/adenovirus E1B 19kDa-interacting protein 1, NIP2 (predicted)  Info   Bnip2_predicted 
   1294 0.0003984 5.1 58 0.088  1374540_at  cell division cycle associated 7 (predicted)  Info   Cdca7_predicted 
   1295 0.0003987 3259.5 4063.3 0.802  1371305_at  Ribosomal protein L8  Info   Rpl8 
   1296 0.0003995 431.6 309.6 1.394  1379294_at  T-cell receptor rearranged alpha-chain V-region mRNA (V-alpha-3-V-J-C) partial cds, hybridoma KL  Info    
   1297 0.0003999 246.9 302.5 0.816  1372749_at  Transcribed locus  Info    
   1298 0.0004023 225 395.5 0.569  1369964_at  coronin, actin binding protein 1A  Info   Coro1a 
   1299 0.000403 265.8 494.7 0.537  1389006_at  Macrophage expressed gene 1  Info   Mpeg1 
   1300 0.0004039 185.5 244.3 0.759  1390802_at  similar to RIKEN cDNA 5033414D02 (predicted)  Info   RGD1306839_predicted 
   1301 0.0004042 13.6 39.2 0.347  1388451_at  Transcribed locus  Info    
   1302 0.0004048 89.8 264.6 0.339  1390450_a_at  osteoglycin (predicted)  Info   Ogn_predicted 
   1303 0.0004065 623.4 387.6 1.608  1373110_at  Transcribed locus  Info    
   1304 0.0004088 2200 1256.8 1.75  1368245_at  ureidopropionase, beta  Info   Upb1 
   1305 0.000409 121.7 44.5 2.735  1380115_at    Info    
   1306 0.0004093 126.2 221.5 0.57  1373114_at  Transcribed locus  Info    
   1307 0.0004093 1.1 2.4 0.458  1391823_at  Ras association (RalGDS/AF-6) domain family 3 (predicted)  Info    
   1308 0.0004106 5.4 49.8 0.108  1376831_at  similar to hypothetical protein FLJ10156 (predicted)  Info   RGD1308747_predicted 
   1309 0.000413 59.8 127.4 0.469  1384187_at  similar to Adapter-related protein complex 1 sigma 1B subunit (Sigma-adaptin 1B) (Adaptor protein complex AP-1 sigma-1B subunit) (Golgi adaptor HA1/AP1 adaptin sigma-1B subunit) (Clathrin assembly protein complex 1 sigma-1B small chain) (Sigma 1B s...  Info   LOC302671 
   1310 0.0004138 69.3 117 0.592  1377189_at  similar to BC282485_1 (predicted)  Info   RGD1307390_predicted 
   1311 0.0004147 970.8 1163 0.835  1373382_at  similar to RER1 homolog (predicted)  Info   RGD1306324_predicted 
   1312 0.0004159 403.6 325.5 1.24  1390126_at    Info    
   1313 0.0004172 246.6 313.9 0.786  1372667_at  similar to RIKEN cDNA 1110059E24  Info   RGD:1359158 
   1314 0.0004173 158.9 246.4 0.645  1370407_at  chloride ion pump-associated 55 kDa protein  Info   Pcyox1 
   1315 0.0004173 49.8 132.1 0.377  1370561_at  iGb3 synthase  Info   RGD:727913 
   1316 0.0004177 956.3 762.1 1.255  1371373_at  similar to RIKEN cDNA 2500002L14; EST C77350  Info   RGD:1359698 
   1317 0.0004178 44.9 27.5 1.633  1383057_at  ASF1 anti-silencing function 1 homolog A (S. cerevisiae) (predicted)  Info   Asf1a_predicted 
   1318 0.0004189 325.7 402.1 0.81  1371891_at  similar to 1700123O20Rik protein (predicted)  Info   RGD1308430_predicted 
   1319 0.0004214 7.1 42.8 0.166  1384762_at  Transcribed locus  Info    
   1320 0.0004217 802.2 372.5 2.154  1386881_at  insulin-like growth factor binding protein 3  Info   Igfbp3 
   1321 0.0004243 128.8 93.3 1.38  1391224_at  similar to 4921517L17Rik protein (predicted)  Info   RGD1311678_predicted 
   1322 0.000425 2137.5 1613.9 1.324  1367892_at  pyruvate dehydrogenase kinase, isoenzyme 2  Info   Pdk2 
   1323 0.0004277 1441.7 959.9 1.502  1372672_at  quinolinate phosphoribosyltransferase (predicted)  Info   Qprt_predicted 
   1324 0.0004314 26.3 83.1 0.316  1387276_at  activity and neurotransmitter-induced early gene protein 4 (ania-4)  Info   RGD:621187 
   1325 0.0004319 1025.1 1477.6 0.694  1371732_at  dermatopontin (predicted)  Info   Dpt_predicted 
   1326 0.0004323 10 18.1 0.552  1376355_at  similar to Protein CXorf17 homolog  Info   LOC317423 
   1327 0.0004332 227.7 154.9 1.47  1390883_at  Similar to A-kinase anchor protein 11 (Protein kinase A anchoring protein 11) (PRKA11) (A kinase anchor protein 220 kDa) (AKAP 220) (hAKAP220)  Info   Akap11 
   1328 0.0004343 122.4 219.1 0.559  1374435_at  Transcribed locus, strongly similar to NP_954666.1 suppressor of zeste 12 homolog [Mus musculus]  Info    
   1329 0.0004377 1531.4 2205.4 0.694  1371390_at  tubulin, beta, 2  Info   Tubb2 
   1330 0.0004379 20.1 43.3 0.464  1384988_at  F-box only protein 5 (predicted)  Info   Fbxo5_predicted 
   1331 0.0004385 1211.6 1761.8 0.688  1375119_at  neural precursor cell expressed, developmentally down-regulated gene 4A  Info   Nedd4a 
   1332 0.00044 26 40.4 0.644  1379984_at  Transcribed locus  Info    
   1333 0.0004409 92.7 128 0.724  1385211_at    Info    
   1334 0.0004409 11.3 51.7 0.219  1375306_at  similar to toll-associated serine protease (predicted)  Info   RGD1308120_predicted 
   1335 0.0004415 296.7 440.8 0.673  1388453_at  myeloid-associated differentiation marker  Info   Myadm 
   1336 0.0004428 52 86.7 0.6  1389709_at  similar to Zinc finger protein 133  Info   LOC499900 
   1337 0.0004442 131.8 193.3 0.682  1389582_at  Transcribed locus  Info    
   1338 0.0004472 3257.8 2612.1 1.247  1398348_at  Transcribed locus  Info    
   1339 0.0004476 104.1 127.5 0.816  1377244_at  Zinc finger protein 95 (predicted)  Info    
   1340 0.0004483 248.7 362.1 0.687  1371441_at  phosphoprotein enriched in astrocytes 15 (predicted)  Info   Pea15_predicted 
   1341 0.0004488 25 37.6 0.665  1385439_x_at  Transcribed locus, strongly similar to XP_220978.3 PREDICTED: similar to Probable ATP-dependent helicase LGP2 (Protein D11Lgp2) [Rattus norvegicus]  Info    
   1342 0.00045 32.3 62.1 0.52  1385226_at    Info    
   1343 0.0004504 98.3 39.7 2.476  1368527_at  prostaglandin-endoperoxide synthase 2  Info   Ptgs2 
   1344 0.0004504 35.8 64 0.559  1370693_a_at  cyclic nucleotide phosphodiesterase 1  Info   Cnp1 
   1345 0.0004506 60.2 121.9 0.494  1372168_s_at  insulin-like growth factor binding protein 6  Info   Igfbp6 
   1346 0.0004508 366.4 218.8 1.675  1375120_at  inhibitor of DNA binding 4  Info   Idb4 
   1347 0.0004509 4 54.4 0.074  1383926_at  budding uninhibited by benzimidazoles 1 homolog, beta (S. cerevisiae)  Info   Bub1b 
   1348 0.0004516 299.1 393.2 0.761  1373241_at    Info    
   1349 0.0004521 87.3 120.9 0.722  1392479_at  FK506-binding protein 1a  Info   Fkbp1a 
   1350 0.000453 275.8 363.2 0.759  1390408_at  similar to Hccs protein  Info   LOC317444 
   1351 0.0004531 56.8 89.1 0.637  1376013_at  similar to mKIAA0227 protein  Info   LOC500689 
   1352 0.0004548 192.9 289 0.667  1387566_at  phospholipase A2, group IVA (cytosolic, calcium-dependent)  Info   Pla2g4a 
   1353 0.0004558 217.4 47.5 4.577  1368596_at  SNF1-like kinase  Info   Snf1lk 
   1354 0.0004561 155.6 228.3 0.682  1370909_at  nucleoporin 62  Info   Nup62 
   1355 0.0004567 1037.1 1291 0.803  1367820_at  barrier to autointegration factor 1  Info   Banf1 
   1356 0.0004569 21.9 42.7 0.513  1375921_at  Reticulocalbin (predicted)  Info    
   1357 0.0004577 378.6 186 2.035  1387703_a_at  ubiquitin specific protease 2  Info   Usp2 
   1358 0.000458 895.5 570.6 1.569  1373807_at  Vascular endothelial growth factor A  Info   Vegf 
   1359 0.0004587 341.4 799 0.427  1370927_at  procollagen, type XII, alpha 1  Info   Col12a1 
   1360 0.0004591 254.2 393.4 0.646  1368173_at  nucleolar protein 5  Info   Nol5 
   1361 0.0004593 97.8 65 1.505  1374379_at  Dual specificity phosphatase 23 (predicted)  Info    
   1362 0.0004607 14.4 35.6 0.404  1368381_at  cartilage acidic protein 1  Info   Crtac1 
   1363 0.000462 30.1 54.8 0.549  1387182_at  G protein-coupled receptor 37  Info   Gpr37 
   1364 0.000462 744.2 989.4 0.752  1371581_at  Vascular endothelial zinc finger 1 (predicted)  Info    
   1365 0.0004628 1519.2 969.8 1.567  1371786_at  tripartite motif-containing 35  Info   Trim35 
   1366 0.000463 69.8 105.4 0.662  1374626_at  similar to leucine-rich alpha-2-glycoprotein  Info   RGD:1359464 
   1367 0.0004635 645 429.5 1.502  1371794_at  Transcribed locus, strongly similar to NP_035873.1 SH3-domain binding protein 3 [Mus musculus]  Info    
   1368 0.0004644 8.4 16.3 0.515  1390609_at  Transcribed locus  Info    
   1369 0.0004664 2841 2351.7 1.208  1370699_a_at  epidermal growth factor receptor /// peptidase D  Info   Egfr /// Pepd 
   1370 0.0004665 4363.9 3643.9 1.198  1388118_at  3-hydroxyisobutyrate dehydrogenase  Info   Hibadh 
   1371 0.0004667 449.2 314.2 1.43  1372642_at  Transcribed locus  Info    
   1372 0.0004673 37.2 59.1 0.629  1369161_at  ATP-binding cassette, sub-family B (MDR/TAP), member 4  Info   Abcb4 
   1373 0.0004697 819.2 442.6 1.851  1384073_at  alcohol dehydrogenase, iron containing, 1 (predicted)  Info   Adhfe1_predicted 
   1374 0.000472 100.8 137.8 0.731  1376625_at    Info    
   1375 0.0004726 8.7 23.2 0.375  1384276_at  Transcribed locus  Info    
   1376 0.0004738 153.7 193 0.796  1371740_at  similar to RIKEN cDNA 5730470L24 (predicted)  Info   RGD1311316_predicted 
   1377 0.0004751 82.7 118.4 0.698  1398597_at  Transcribed locus  Info    
   1378 0.0004765 34.5 63.3 0.545  1390907_at  Transcribed locus  Info    
   1379 0.0004769 25.9 73.6 0.352  1368811_at  lamin B1  Info   Lmnb1 
   1380 0.0004789 4907 6056.8 0.81  1367573_at  ribosomal protein S6  Info   Rps6 
   1381 0.0004828 97 166 0.584  1372202_at  similar to expressed sequence AI597479 (predicted)  Info   RGD1310553_predicted 
   1382 0.0004839 2182.5 2898.3 0.753  1367590_at  RAN, member RAS oncogene family  Info   Ran 
   1383 0.0004846 30.5 55.7 0.548  1370770_s_at  kit ligand  Info   Kitl 
   1384 0.0004847 317.9 176.1 1.805  1375760_at  Transducer of ERBB2, 2  Info   Tob2 
   1385 0.0004858 4173.6 5094.3 0.819  1398830_at  ribosomal protein L28  Info   Rpl28 
   1386 0.0004868 56.5 37.6 1.503  1382898_at  Transcribed locus  Info    
   1387 0.0004885 46.7 105.8 0.441  1387690_at  caspase 3  Info   Casp3 
   1388 0.0004885 236.6 332.9 0.711  1370809_at  tubulin, gamma 1  Info   Tubg1 
   1389 0.0004913 1.4 4.5 0.311  1387939_at  proline rich 2  Info   Pnrc1 
   1390 0.000492 118.1 199.4 0.592  1373831_at  hypothetical protein LOC619574  Info   LOC619574 
   1391 0.0004928 170.4 86.2 1.977  1387877_at  formiminotransferase cyclodeaminase  Info   Ftcd 
   1392 0.0004934 221.4 346.7 0.639  1372112_at  similar to 9230105E10Rik protein (predicted)  Info   RGD1304579_predicted 
   1393 0.0004939 90.1 125.3 0.719  1377748_at  Transcribed locus  Info    
   1394 0.0004945 153.9 182 0.846  1374848_at  Similar to RIKEN cDNA 2410016F19 (predicted)  Info    
   1395 0.0004945 22.3 41.8 0.533  1376946_at  Transcribed locus  Info    
   1396 0.0004952 693.4 461 1.504  1371468_at  Transcribed locus, strongly similar to NP_001003955.1 RAB11 family interacting protein 5 (class I) isoform 1 [Mus musculus]  Info    
   1397 0.0004953 206.4 337.6 0.611  1387202_at  intercellular adhesion molecule 1  Info   Icam1 
   1398 0.0004958 368.7 525.5 0.702  1374333_at  similar to RIKEN cDNA 1110007C09 (predicted)  Info   RGD1306058_predicted 
   1399 0.0004964 1315.9 1686.9 0.78  1371977_at  actin related protein 2/3 complex, subunit 3 (predicted)  Info   Arpc3_predicted 
   1400 0.0004976 272.3 387.7 0.702  1374388_at  EF hand domain containing 2 (predicted)  Info   Efhd2_predicted 
   1401 0.0004995 375.6 568.8 0.66  1389001_at  Transcribed locus, strongly similar to XP_422815.1 PREDICTED: similar to Importin alpha-4 subunit (Karyopherin alpha-4 subunit) (Qip1 protein) [Gallus gallus]  Info    
   1402 0.0005006 30.3 52.5 0.577  1385354_at  similar to integrin alpha 8  Info   LOC364786 
   1403 0.0005018 466.2 278.7 1.673  1385791_at  similar to villin-like protein (predicted)  Info   RGD1304843_predicted 
   1404 0.000502 42.5 59.3 0.717  1393277_at    Info    
   1405 0.0005024 54.5 86.3 0.632  1390250_x_at  Atpase, class I, type 8B, member 2 (predicted)  Info    
   1406 0.0005037 182.8 343.6 0.532  1387659_at  guanine deaminase  Info   Gda 
   1407 0.000506 24.8 55.5 0.447  1395056_at  Similar to leucine-rich repeats and IQ motif containing 1  Info    
   1408 0.0005068 690.1 805.3 0.857  1371945_at  ubiquitin-conjugating enzyme E2L 3 (predicted)  Info   Ube2l3_predicted 
   1409 0.0005075 2.2 12.3 0.179  1396066_at  similar to Laminin alpha-4 chain precursor  Info   LOC309816 
   1410 0.0005085 478.7 351 1.364  1388688_at  Transcribed locus  Info    
   1411 0.0005093 3035.6 2355.6 1.289  1377646_at  Transcribed locus  Info    
   1412 0.0005098 131.6 210.7 0.625  1393183_at  similar to StAR-related protein 1-4E  Info   LOC502348 
   1413 0.0005108 275 57.2 4.808  1387908_at  RAS, dexamethasone-induced 1  Info   Rasd1 
   1414 0.0005113 33.2 74.8 0.444  1392280_at  toll-like receptor 2  Info   Tlr2 
   1415 0.0005116 114.4 76.7 1.492  1375851_at  kelch-like 8 (Drosophila) (predicted)  Info   Klhl8_predicted 
   1416 0.0005122 68.2 108.1 0.631  1389644_at  similar to CG16896-PA  Info   LOC299949 
   1417 0.000513 91 142.9 0.637  1369793_a_at  melanoma cell adhesion molecule  Info   Mcam 
   1418 0.0005139 90.7 135.4 0.67  1376597_at  zinc finger, CCHC domain containing 10 (predicted)  Info   Zcchc10_predicted 
   1419 0.0005139 400.1 463.2 0.864  1383586_at    Info    
   1420 0.000514 31.8 47.8 0.665  1384371_at  TEA domain family member 3 (predicted)  Info   Tead3_predicted 
   1421 0.0005142 3002.6 2583.9 1.162  1369630_at  adenosine kinase  Info   Adk 
   1422 0.0005159 163.8 212.7 0.77  1393027_at  similar to RIKEN cDNA 1700052N19 (predicted)  Info   RGD1305235_predicted 
   1423 0.0005162 27.4 77.3 0.354  1395697_at  similar to Ezh2 protein  Info   LOC312299 
   1424 0.0005166 4094.5 4825.7 0.848  1371330_at  ribosomal protein L11 (predicted)  Info   Rpl11_predicted 
   1425 0.0005168 704.1 481 1.464  1373057_at  ProSAPiP1 protein  Info   ProSAPiP1 
   1426 0.0005179 60.5 40.5 1.494  1385126_at  Transcribed locus  Info    
   1427 0.0005183 1277.6 815.2 1.567  1398628_at    Info    
   1428 0.0005187 188.6 242.6 0.777  1389047_at  Zinc finger protein 451 (predicted)  Info    
   1429 0.00052 433.6 311.7 1.391  1389176_at  Inositol polyphosphate-5-phosphatase F (predicted)  Info    
   1430 0.0005202 114.9 177.5 0.647  1374578_at  Transcribed locus  Info    
   1431 0.0005219 70.3 174.4 0.403  1391925_at  chemokine (C-C motif) ligand 19 (predicted)  Info   Ccl19_predicted 
   1432 0.0005226 86.9 136.4 0.637  1375987_at  ceramide kinase (predicted)  Info   Cerk_predicted 
   1433 0.000523 8.5 52.2 0.163  1383601_at  DUF729 domain containing 1 (predicted)  Info    
   1434 0.0005249 1429.6 2198.7 0.65  1371314_at  neural precursor cell expressed, developmentally down-regulated gene 4A  Info   Nedd4a 
   1435 0.0005263 340.9 429.2 0.794  1371611_at  exostoses (multiple) 2 (predicted)  Info   Ext2_predicted 
   1436 0.0005265 11.8 4 2.95  1391953_at  Msh homeo box homolog 2 (Drosophila)  Info   Msx2 
   1437 0.0005274 273.3 304.7 0.897  1370311_at  eukaryotic translation initiation factor 2B, subunit 1 alpha  Info   Eif2b1 
   1438 0.0005289 20.6 49.8 0.414  1373839_at  Transcribed locus  Info    
   1439 0.0005296 47.3 84.2 0.562  1368296_at  solute carrier organic anion transporter family, member 2b1  Info   Slco2b1 
   1440 0.000531 396.2 296.3 1.337  1390366_at  similar to PC-LKC gene product  Info   LOC291002 
   1441 0.0005313 111.9 157.6 0.71  1393046_at  similar to Protein C20orf20 (predicted)  Info   RGD1308612_predicted 
   1442 0.0005314 661.6 500.4 1.322  1388883_at  polymerase (DNA-directed), delta 4 (predicted)  Info   Pold4_predicted 
   1443 0.0005328 3282.5 4323.2 0.759  1388071_x_at  RT1 class Ib, locus Aw2  Info   RT1-Aw2 
   1444 0.0005336 125.7 174.7 0.72  1373417_at  phospholipid scramblase 3 (predicted)  Info   Plscr3_predicted 
   1445 0.0005357 47.8 8.7 5.494  1385250_at  chromodomain helicase DNA binding protein 6 (predicted)  Info   Chd6_predicted 
   1446 0.000537 21.6 51.9 0.416  1367978_at  adenylate cyclase 2  Info   Adcy2 
   1447 0.0005385 17.4 57.6 0.302  1374794_at  kinesin-like 7  Info   Knsl7 
   1448 0.0005393 53.6 102.5 0.523  1384440_at  Similar to LRRGT00021  Info    
   1449 0.0005402 138.1 177 0.78  1383232_at  Transcribed locus  Info    
   1450 0.000541 1501.5 1921.3 0.782  1371244_at  adenine phosphoribosyl transferase (predicted)  Info   Aprt_predicted 
   1451 0.0005417 262.3 344.1 0.762  1388729_at  Harvey rat sarcoma oncogene, subgroup R (predicted)  Info   Rras_predicted 
   1452 0.0005442 222.6 132.1 1.685  1383757_at  Transcribed locus  Info    
   1453 0.0005453 4948.8 3938.9 1.256  1369625_at  aquaporin 1  Info   Aqp1 
   1454 0.0005478 135 195.6 0.69  1375847_at  Transcribed locus  Info    
   1455 0.0005501 1040.2 890.1 1.169  1370358_at  two pore channel 1  Info   Tpcn1 
   1456 0.0005513 119.9 60.7 1.975  1385670_at  serine dehydratase-like (predicted)  Info   Sdsl_predicted 
   1457 0.0005514 40.5 59.9 0.676  1398553_at  similar to CGI-100-like protein  Info   RGD:1359437 
   1458 0.0005514 15.3 50.9 0.301  1393684_at  TBC1D12: TBC1 domain family, member 12 (predicted)  Info   Tbc1d12_predicted 
   1459 0.0005515 162.9 222.7 0.731  1375895_at  Serine racemase  Info   Srr 
   1460 0.0005522 62.3 110.5 0.564  1375067_at  Transcribed locus  Info    
   1461 0.0005528 19.7 38.4 0.513  1373350_at  PC4 and SFRS1 interacting protein 1  Info   Psip1 
   1462 0.0005531 82.1 54.9 1.495  1381469_a_at  PERQ amino acid rich, with GYF domain 1 (predicted)  Info   Perq1_predicted 
   1463 0.0005533 264.7 169.5 1.562  1388945_at  similar to 1300014I06Rik protein (predicted)  Info   RGD1311307_predicted 
   1464 0.0005547 104.4 135.8 0.769  1373556_at  similar to CG14903-PA  Info   LOC298861 
   1465 0.0005562 162 231.9 0.699  1369323_at  leptin receptor overlapping transcript  Info   Leprot 
   1466 0.0005566 11 19.7 0.558  1378423_at  ataxia, cerebellar, Cayman type (caytaxin) (predicted)  Info   Atcay_predicted 
   1467 0.0005609 3315.8 2628.3 1.262  1370806_at  all-trans-13,14-dihydroretinol saturase  Info   Retsat 
   1468 0.0005609 159 110.9 1.434  1368265_at  cytochrome P450 monooxygenase CYP2T1  Info   RGD:620086 
   1469 0.0005614 6.4 29.6 0.216  1369533_a_at  5-hydroxytryptamine (serotonin) receptor 4  Info   Htr4 
   1470 0.0005627 1214.3 579 2.097  1368160_at  insulin-like growth factor binding protein 1  Info   Igfbp1 
   1471 0.0005659 153 217.6 0.703  1389971_at  sarcoglycan, epsilon  Info   RGD:1303201 
   1472 0.0005667 56.7 110 0.515  1369483_at  CD4 antigen  Info   Cd4 
   1473 0.000567 116.1 178.2 0.652  1367849_at  syndecan 1  Info   Sdc1 
   1474 0.0005672 389.6 751.3 0.519  1379344_at  Cytochrome b-245, beta polypeptide  Info   Cybb 
   1475 0.0005693 475.1 673.3 0.706  1391411_at  Formin-like 2 (predicted)  Info    
   1476 0.0005696 3.1 30.5 0.102  1374352_at  Actin alpha cardiac 1  Info   Actc1 
   1477 0.0005718 1921.3 1450 1.325  1371833_at  brain protein I3 (predicted)  Info   Bri3_predicted 
   1478 0.0005726 48.6 81.6 0.596  1384724_at    Info    
   1479 0.0005733 2025.7 1154.3 1.755  1368366_at  Camello-like 2  Info   Cml2 
   1480 0.0005746 220.5 311.3 0.708  1389220_at    Info    
   1481 0.0005748 874.2 1599.1 0.547  1386890_at  S100 calcium binding protein A10 (calpactin)  Info   S100a10 
   1482 0.0005766 16.1 48.7 0.331  1376381_at  XK-related protein 5  Info   RGD:1359335 
   1483 0.0005771 7.8 15.8 0.494  1372809_at  hypothetical gene supported by AF152002  Info   LOC290595 
   1484 0.0005772 22.7 43.5 0.522  1377752_at  epithelial membrane protein 2  Info   RGD:1359629 
   1485 0.0005776 503.7 343.2 1.468  1374038_at  Transcribed locus  Info    
   1486 0.0005805 98 181.9 0.539  1387897_at  cyclic nucleotide phosphodiesterase 1  Info   Cnp1 
   1487 0.0005808 1064.1 1634.2 0.651  1371341_at  small nuclear ribonucleoprotein D2 (predicted)  Info   Snrpd2_predicted 
   1488 0.0005811 96.9 185.9 0.521  1382878_at  secreted frizzled-related protein 1  Info   Sfrp1 
   1489 0.000582 37 70.1 0.528  1376344_at  Cytochrome b reductase 1 (predicted)  Info    
   1490 0.0005829 211 135.8 1.554  1371462_at  insulin-like growth factor binding protein 4  Info   Igfbp4 
   1491 0.0005834 44.7 72.4 0.617  1383343_at  Putative pheromone receptor V2R4  Info   LOC292498 
   1492 0.0005834 108.6 155.7 0.697  1388309_at  high mobility group AT-hook 1  Info   Hmga1 
   1493 0.0005854 96 159.8 0.601  1382732_at  extra cellular link domain-containing 1 (predicted)  Info   Xlkd1_predicted 
   1494 0.0005862 13.7 23.3 0.588  1382651_at  Transcribed locus  Info    
   1495 0.0005864 81 55.1 1.47  1395052_at  Transcribed locus  Info    
   1496 0.0005871 3569.7 4076.5 0.876  1371302_at  Similar to RIKEN cDNA A130038L21  Info    
   1497 0.0005876 20.5 43.2 0.475  1393149_at    Info   Multiple Gene Symbols 
   1498 0.0005889 354.4 515.7 0.687  1388762_at    Info    
   1499 0.0005904 251.6 311.3 0.808  1394438_at  ARP1 actin-related protein 1 homolog A (yeast) (predicted)  Info   Actr1a_predicted 
   1500 0.0005914 6.1 19.2 0.318  1385529_at  family with sequence similarity 3, member B (predicted)  Info   Fam3b_predicted 
   1501 0.0005928 2.5 13.4 0.187  1392731_at  hypothetical gene supported by NM_130426  Info   LOC497808 
   1502 0.0005929 799.8 1014.7 0.788  1383073_at  ubiquitin specific protease 14 (predicted)  Info   Usp14_predicted 
   1503 0.0005931 33.5 48 0.698  1378078_at  Neuronal PAS domain protein 1 (predicted)  Info    
   1504 0.0005939 367.8 494.1 0.744  1373454_at  similar to RIKEN cDNA 0610037P05 (predicted)  Info   RGD1305823_predicted 
   1505 0.000595 268.4 541.8 0.495  1373780_at  tetraspan 1  Info   RGD:1303308 
   1506 0.0005964 254.4 398 0.639  1389617_at    Info    
   1507 0.0005968 21.5 43.2 0.498  1369686_at  double cortin and calcium/calmodulin-dependent protein kinase-like 1  Info   Dcamkl1 
   1508 0.000598 95.8 163 0.588  1394483_at  A disintegrin-like and metalloprotease (reprolysin type) with thrombospondin type 1 motif, 5 (aggrecanase-2)  Info   Adamts5 
   1509 0.0005997 117.8 138.4 0.851  1377359_at  similar to protein P3  Info   LOC501665 
   1510 6e-04 2.7 9.2 0.293  1396962_at  Transcribed locus  Info    
   1511 0.0006012 9.3 30.3 0.307  1380243_at  similar to CG14803-PA (predicted)  Info   RGD1304693_predicted 
   1512 0.0006025 42.5 72.1 0.589  1367859_at  transforming growth factor, beta 3  Info   Tgfb3 
   1513 0.0006048 112.6 166.6 0.676  1387871_at  cofilin 1  Info   Cfl1 
   1514 0.0006055 467.8 314.9 1.486  1390993_at  MAWD binding protein  Info   Mawbp 
   1515 0.0006058 34.6 24.1 1.436  1384183_at    Info    
   1516 0.0006068 90.5 170.4 0.531  1395166_at  putative eps protein  Info   MGEPS 
   1517 0.0006071 97.9 217.9 0.449  1368658_at  ciliary neurotrophic factor  Info   Cntf 
   1518 0.0006074 285.6 656.3 0.435  1388782_at  transcription factor 21  Info   Tcf21 
   1519 0.0006079 89.3 46.8 1.908  1398592_at    Info    
   1520 0.000608 27.1 68 0.399  1376688_a_at  Transcribed locus, moderately similar to NP_705730.1 paired immunoglobin-like type 2 receptor alpha [Mus musculus]  Info    
   1521 0.0006096 2.4 19.6 0.122  1392582_at  similar to NAG14 protein (predicted)  Info   RGD1311013_predicted 
   1522 0.000611 534.1 430.8 1.24  1371584_at  transient receptor potential cation channel, subfamily C, member 4 associated protein (predicted)  Info   Trpc4ap_predicted 
   1523 0.0006124 1468.1 1968.2 0.746  1398768_at  retinoblastoma binding protein 7  Info   Rbbp7 
   1524 0.0006131 650.1 465.4 1.397  1391390_at    Info    
   1525 0.0006136 19.6 28.8 0.681  1369614_at  RAP2B, member of RAS oncogene family  Info   Rap2b 
   1526 0.000614 305.7 201.1 1.52  1370363_at  carboxylesterase 3  Info   Ces3 
   1527 0.0006146 229.3 297.9 0.77  1399032_at  excision repair cross-complementing rodent repair deficiency, complementation group 1 (predicted)  Info   Ercc1_predicted 
   1528 0.000615 3.3 14.6 0.226  1382589_at  similar to hypothetical protein FLJ11795 (predicted)  Info   RGD1307735_predicted 
   1529 0.0006155 1474.5 1064.9 1.385  1370379_at  protease, serine, 8 (prostasin)  Info   Prss8 
   1530 0.0006157 3306 2822.7 1.171  1389836_a_at  Synapsin III  Info   Syn3 
   1531 0.0006172 40.1 79 0.508  1379019_at  properdin factor, complement  Info   Pfc 
   1532 0.0006173 36.8 59.1 0.623  1389096_at    Info    
   1533 0.0006188 383.7 237.8 1.614  1373377_at  sodium channel modifier 1 (predicted)  Info   Scnm1_predicted 
   1534 0.000619 25.4 43.6 0.583  1391453_at  Transcribed locus, strongly similar to XP_576700.1 PREDICTED: similar to RIKEN cDNA 2900016D05 [Rattus norvegicus]  Info    
   1535 0.0006192 150.6 36.2 4.16  1370432_at  POU domain, class 3, transcription factor 1  Info   Pou3f1 
   1536 0.0006201 181.5 256.1 0.709  1367834_at  spermidine synthase  Info   Srm 
   1537 0.0006214 2787.6 2189.3 1.273  1372492_at  claudin 10 (predicted)  Info   Cldn10_predicted 
   1538 0.0006236 51 82.2 0.62  1390982_at  chromatin assembly factor 1, subunit B (p60) (predicted)  Info   Chaf1b_predicted 
   1539 0.0006239 26.3 65.4 0.402  1384298_at  myosin IF (predicted)  Info   Myo1f_predicted 
   1540 0.0006243 3313.8 2663.5 1.244  1367793_at  D-dopachrome tautomerase  Info   Ddt 
   1541 0.0006244 1062.8 2041.1 0.521  1368322_at  superoxide dismutase 3, extracellular  Info   Sod3 
   1542 0.0006247 1197 925.6 1.293  1377192_a_at  caseinolytic protease X (E.coli) (predicted)  Info   Clpx_predicted 
   1543 0.0006268 760.2 636.9 1.194  1396279_at  ATPase, H+ transporting, lysosomal accessory protein 2  Info   Atp6ap2 
   1544 0.0006281 20.2 38.3 0.527  1386805_at  Similar to OTTHUMP00000028696  Info    
   1545 0.0006284 122.8 229.8 0.534  1368558_s_at  allograft inflammatory factor 1  Info   Aif1 
   1546 0.00063 332.3 225.7 1.472  1393531_at  Transcribed locus  Info    
   1547 0.0006307 29.7 10.5 2.829  1393348_at  Transcribed locus  Info    
   1548 0.0006324 98 145.2 0.675  1368946_at  ADP-ribosylation factor 2  Info   Arf2 
   1549 0.0006334 37.1 70.9 0.523  1379688_at  Transcribed locus  Info    
   1550 0.0006339 1258.8 842.8 1.494  1368870_at  Inhibitor of DNA binding 2, dominant negative helix-loop-helix protein  Info   Id2 
   1551 0.0006339 198.2 277.1 0.715  1389209_at  similar to hypothetical protein BC002942 (predicted)  Info   RGD1306274_predicted 
   1552 0.0006358 540 311.2 1.735  1395167_at  LOC362424 (predicted)  Info   RGD1308850_predicted 
   1553 0.0006371 88.2 128.8 0.685  1393461_at  Similar to zinc finger protein 75  Info    
   1554 0.0006376 42.1 83.3 0.505  1385001_at  gasdermin domain containing 1 (predicted)  Info   Gsdmdc1_predicted 
   1555 0.0006379 247.8 309.5 0.801  1373791_at  similar to RIKEN cDNA 2310011J03  Info   RGD:1359127 
   1556 0.000638 46 9 5.111  1374148_at  LOC498009  Info   LOC498009 
   1557 0.0006381 56.4 15.7 3.592  1385089_at  Transcribed locus  Info    
   1558 0.00064 34.2 71.8 0.476  1375066_at  similar to RIKEN cDNA 6330512M04 gene  Info   LOC293632 
   1559 0.000643 1930.2 1434.3 1.346  1387974_a_at  kidney specific organic anion transporter  Info   RGD:621387 
   1560 0.000647 341.3 449.3 0.76  1398398_at  similar to Homeobox protein A10  Info   LOC368057 
   1561 0.0006477 220.7 350.3 0.63  1389980_at  similar to Protein HSPC163  Info   LOC289324 
   1562 0.0006489 2637.7 1955.6 1.349  1372087_at  hypertrophic agonist responsive protein B64  Info   Harpb64 
   1563 0.000651 20.4 50.8 0.402  1373777_at  Transcribed locus  Info    
   1564 0.0006513 1027.4 686.2 1.497  1377911_at  Transcribed locus  Info    
   1565 0.0006519 115.5 190.2 0.607  1373830_at  hypothetical protein LOC619574  Info   LOC619574 
   1566 0.0006525 2819.7 1289 2.188  1398612_at  aldo-keto reductase family 1, member C12 (predicted)  Info   Akr1c12_predicted 
   1567 0.0006531 115.2 223.5 0.515  1368006_at  lysosomal-associated protein transmembrane 5  Info   Laptm5 
   1568 0.0006537 312.3 167 1.87  1380610_at  Transcribed locus  Info    
   1569 0.0006542 1288.5 955.1 1.349  1391856_at  similar to semaphorin sem2 (predicted)  Info   RGD1306079_predicted 
   1570 0.0006547 149.1 95.6 1.56  1381940_at  Transcribed locus  Info    
   1571 0.0006551 996.8 1219.9 0.817  1371337_at  cytochrome c oxidase subunit VIIa polypeptide 2 like (predicted)  Info   Cox7a2l_predicted 
   1572 0.0006553 654.5 328.9 1.99  1373566_at  DD6A4-12(7)-1 mRNA, partial sequence  Info    
   1573 0.0006555 138.7 223 0.622  1367957_at  regulator of G-protein signalling 3  Info   Rgs3 
   1574 0.0006566 116.8 153 0.763  1380072_at  similar to 2310044H10Rik protein  Info   RGD:1303214 
   1575 0.000657 258.5 310.5 0.833  1388512_at  phosphodiesterase 6D, cGMP-specific, rod, delta (predicted)  Info   Pde6d_predicted 
   1576 0.0006574 61.7 113 0.546  1373262_at  Similar to 2310014H01Rik protein (predicted)  Info    
   1577 0.0006575 1470.6 1072.9 1.371  1373986_at  Transcribed locus  Info    
   1578 0.0006581 610.6 411.8 1.483  1369073_at  nuclear receptor subfamily 1, group H, member 4  Info   Nr1h4 
   1579 0.0006582 332.9 456 0.73  1373876_at    Info    
   1580 0.0006616 76.3 133 0.574  1373368_at  Transcribed locus  Info    
   1581 0.0006619 38.5 90 0.428  1381434_s_at  similar to nidogen 2  Info   LOC311772 
   1582 0.0006625 1552.5 1246.2 1.246  1377060_at  methylcrotonoyl-Coenzyme A carboxylase 2 (beta) (predicted)  Info   Mccc2_predicted 
   1583 0.0006627 464.5 268.3 1.731  1396035_at  similar to RPE-spondin  Info   LOC297757 
   1584 0.0006638 83 178.7 0.464  1380699_at  Zinc finger homeobox 1b (predicted)  Info    
   1585 0.0006647 69 127.9 0.539  1387242_at  Protein kinase, interferon-inducible double stranded RNA dependent  Info   Prkr 
   1586 0.0006689 30.5 13.5 2.259  1369064_a_at  sodium channel, voltage-gated, type 8, alpha polypeptide  Info   Scn8a 
   1587 0.0006695 3.9 21 0.186  1390066_at  nuclear receptor subfamily 2, group F, member 6  Info   Nr2f6 
   1588 0.0006699 41 111.3 0.368  1393048_at  Adrenergic receptor, alpha 2a  Info   Adra2a 
   1589 0.0006704 43.4 90.8 0.478  1371923_at  similar to hypothetical protein FLJ20481 (predicted)  Info   RGD1311599_predicted 
   1590 0.0006724 166.6 238.1 0.7  1373460_at  similar to embryonic seven-span transmembrane protein-like protein  Info   LOC501103 
   1591 0.0006735 1850.9 2283.7 0.81  1388989_at  similar to RIKEN cDNA 3110001N18 (predicted)  Info   RGD1309517_predicted 
   1592 0.0006737 247.3 159.9 1.547  1383531_at    Info    
   1593 0.0006741 56.2 75.1 0.748  1382263_at  similar to DNA segment, Chr 3, ERATO Doi 250, expressed (predicted)  Info   RGD1308397_predicted 
   1594 0.0006761 61.2 86.6 0.707  1374685_at  Similar to ALEX3 protein  Info    
   1595 0.0006765 134.2 98.5 1.362  1378248_at  Similar to amyloid beta (A4) precursor protein-binding, family B, member 2  Info    
   1596 0.000677 1431.3 1735 0.825  1398889_at  glutamate receptor, ionotropic, N-methyl D-aspartate-like 1A  Info   Grinl1a 
   1597 0.0006771 174.4 29.1 5.993  1377660_at  similar to transthyretin (4L369) (predicted)  Info   RGD1309350_predicted 
   1598 0.000678 238.8 329.7 0.724  1387732_at  mitochondrial transcription termination factor 1  Info   Mterf 
   1599 0.0006789 241.1 333.3 0.723  1372377_at  DEAD (Asp-Glu-Ala-Asp) box polypeptide 41 (predicted)  Info   Ddx41_predicted 
   1600 0.0006797 135.4 318.8 0.425  1374033_at  proteasome (prosome, macropain) subunit, beta type 10 (predicted)  Info   Psmb10_predicted 
   1601 0.0006798 15.7 42.4 0.37  1367920_at  endothelial differentiation, sphingolipid G-protein-coupled receptor, 5  Info   Edg5 
   1602 0.0006809 2.2 13.6 0.162  1369910_at  G protein-coupled receptor 10  Info   Gpr10 
   1603 0.0006831 111.5 171.4 0.651  1376887_at  Similar to zinc finger protein 445  Info    
   1604 0.0006834 46.5 85.1 0.546  1374201_at    Info    
   1605 0.0006835 2283.4 2810 0.813  1386996_at  myosin light chain, regulatory B  Info   Mrlcb 
   1606 0.0006842 951.5 737.5 1.29  1368045_at  solute carrier family 31 (copper transporters), member 1  Info   RGD:620059 
   1607 0.0006857 56.9 99.4 0.572  1389158_at  Transcribed locus  Info    
   1608 0.0006862 158.6 84.4 1.879  1368432_a_at  v-ros UR2 sarcoma virus oncogene homolog 1 (avian)  Info   Ros1 
   1609 0.0006872 22.4 36.2 0.619  1383729_at  ankyrin repeat and SOCS box-containing 16 (predicted)  Info   Asb16_predicted 
   1610 0.0006895 28.9 53.9 0.536  1383883_at  Similar to Hypothetical protein KIAA0469 (predicted)  Info    
   1611 0.0006896 220.3 396.9 0.555  1382043_at  unc-93 homolog B (C. elegans) (predicted)  Info   Unc93b_predicted 
   1612 0.0006908 80.7 143.7 0.562  1398616_at  similar to Adapter-related protein complex 1 sigma 1B subunit (Sigma-adaptin 1B) (Adaptor protein complex AP-1 sigma-1B subunit) (Golgi adaptor HA1/AP1 adaptin sigma-1B subunit) (Clathrin assembly protein complex 1 sigma-1B small chain) (Sigma 1B s...  Info   LOC302671 
   1613 0.0006926 278.8 169.9 1.641  1370427_at  platelet derived growth factor, alpha  Info   Pdgfa 
   1614 0.0006956 335.6 225.8 1.486  1373304_at  alpha-N-acetylglucosaminidase  Info   Naglu 
   1615 0.0006982 992.7 1271.2 0.781  1372513_at  ras-related C3 botulinum toxin substrate 1  Info   Rac1 
   1616 0.0007039 472.2 80.4 5.873  1372016_at  growth arrest and DNA-damage-inducible 45 beta (predicted)  Info   Gadd45b_predicted 
   1617 0.0007043 49.7 72.8 0.683  1373656_at  Transcribed locus  Info    
   1618 0.0007057 1049.5 794.7 1.321  1371549_at  WD repeat domain 23 (predicted)  Info   Wdr23_predicted 
   1619 0.000706 32.8 54.1 0.606  1368337_at  glycosylation dependent cell adhesion molecule 1  Info   Glycam1 
   1620 0.0007078 89.3 132.3 0.675  1372924_at  Transcribed locus  Info    
   1621 0.0007081 256 341 0.751  1389598_at  ceroid-lipofuscinosis, neuronal 6 (predicted)  Info   Cln6_predicted 
   1622 0.0007083 81 125.3 0.646  1378040_at    Info    
   1623 0.00071 178.5 102.7 1.738  1373736_at  Transcribed locus, weakly similar to NP_082606.1 hypothetical protein LOC72704 [Mus musculus]  Info    
   1624 0.0007119 13.8 40.9 0.337  1378418_at  similar to MGC37193 protein (predicted)  Info   RGD1311681_predicted 
   1625 0.0007135 138.8 466.7 0.297  1368223_at  a disintegrin-like and metalloprotease (reprolysin type) with thrombospondin type 1 motif, 1  Info   Adamts1 
   1626 0.0007143 90.7 38.8 2.338  1374167_at  similar to autoantigen  Info   LOC361399 
   1627 0.0007148 48.7 71.5 0.681  1394499_at  Taube nuss (predicted)  Info    
   1628 0.0007153 17.4 3.7 4.703  1392741_at  Protein phosphatase 2 (formerly 2A), regulatory subunit B (PR 52), alpha isoform  Info   Ppp2r2a 
   1629 0.0007156 2710.9 1677.3 1.616  1371332_at  Histone 1, H4a (predicted)  Info    
   1630 0.0007158 699.7 553.8 1.263  1373277_at  similar to BBP-like protein 2  Info   LOC292995 
   1631 0.0007163 634.6 1012 0.627  1372167_at    Info    
   1632 0.0007175 44.3 61.8 0.717  1383766_at  Transcribed locus, strongly similar to NP_036020.1 sarcoglycan, beta (dystrophin-associated glycoprotein) [Mus musculus]  Info    
   1633 0.0007183 339.1 229.7 1.476  1379483_at  Basic helix-loop-helix domain containing, class B2  Info   Bhlhb2 
   1634 0.000719 173.5 130.8 1.326  1386820_at  Transcribed locus  Info    
   1635 0.00072 373 190.5 1.958  1372920_at    Info    
   1636 0.0007204 82.2 134.6 0.611  1397644_at    Info    
   1637 0.0007209 2898.8 4078.9 0.711  1398315_at  ribosomal protein L15  Info   Rpl15 
   1638 0.0007233 58.4 108.5 0.538  1384250_a_at  Transcribed locus  Info    
   1639 0.0007264 1804.5 2427 0.744  1370825_a_at  cell division cycle 42 homolog (S. cerevisiae)  Info   Cdc42 
   1640 0.000727 16.8 36.1 0.465  1385359_at  Similar to RIKEN cDNA 4931406C07 (predicted)  Info    
   1641 0.0007276 534.7 660.7 0.809  1374571_at  similar to hypothetical protein FLJ20522 (predicted)  Info   RGD1307289_predicted 
   1642 0.0007281 387.2 252.3 1.535  1378457_at  Transcribed locus  Info    
   1643 0.0007289 160.7 237.3 0.677  1389337_at  Transcribed locus  Info    
   1644 0.0007323 31.5 81 0.389  1382086_at  Transcribed locus  Info    
   1645 0.0007324 18.4 57.6 0.319  1396219_at    Info    
   1646 0.0007333 468.4 285.3 1.642  1369286_at  protein C  Info   Proc 
   1647 0.0007337 30.9 55.2 0.56  1386932_at  glutaminase  Info   Gls 
   1648 0.0007337 37.4 65.6 0.57  1382592_at    Info    
   1649 0.0007343 301.6 398.3 0.757  1388789_at  similar to hypothetical protein MGC17943  Info   LOC362857 
   1650 0.0007351 60.4 106.7 0.566  1397328_at  Transcribed locus  Info    
   1651 0.0007375 154.3 208.5 0.74  1372875_at  Transcribed locus  Info    
   1652 0.0007379 83.5 135.2 0.618  1376644_at  similar to lung cancer metastasis-related protein 1 (predicted)  Info   RGD1311926_predicted 
   1653 0.0007383 8.4 57.6 0.146  1385242_at  SPARC related modular calcium binding 2 (predicted)  Info   Smoc2_predicted 
   1654 0.0007403 207 339.5 0.61  1398387_at  Unknown (protein for MGC:72614)  Info   RGD:735075 
   1655 0.0007417 186.3 375.5 0.496  1395157_at  neural precursor cell expressed, developmentally down-regulated gene 4A  Info   Nedd4a 
   1656 0.000742 1422.4 1257.9 1.131  1371640_at  hypothetical protein LOC500855  Info   LOC500855 
   1657 0.0007424 460.4 42.3 10.884  1375043_at  FBJ murine osteosarcoma viral oncogene homolog  Info   Fos 
   1658 0.0007429 172.2 249.3 0.691  1372931_at  Similar to DXImx39e protein  Info    
   1659 0.0007441 227.3 170.1 1.336  1394363_at  RIO kinase 3 (yeast) (predicted)  Info   Riok3_predicted 
   1660 0.0007442 1217.8 1550.4 0.785  1372333_at  small nuclear ribonucleoprotein E (predicted) /// similar to small nuclear ribonucleoprotein E  Info   Snrpe_predicted /// LOC501075 
   1661 0.0007445 648.3 885.9 0.732  1389010_at  leukotriene A4 hydrolase (predicted)  Info   Lta4h_predicted 
   1662 0.0007449 562.4 476.7 1.18  1388344_at  similar to RIKEN cDNA 1110005A23 (predicted)  Info   RGD1305692_predicted 
   1663 0.0007459 297.8 422.2 0.705  1368851_at  v-ets erythroblastosis virus E26 oncogene homolog 1 (avian)  Info   Ets1 
   1664 0.0007467 2423.3 1962.6 1.235  1386934_at  choline transporter  Info   Slc6a8 
   1665 0.0007472 1410.5 1162.8 1.213  1388874_at  Transcribed locus  Info    
   1666 0.0007473 37.9 74 0.512  1378171_at  Neuropilin 2  Info   Nrp2 
   1667 0.0007485 69.7 41.7 1.671  1379301_at  Transcribed locus  Info    
   1668 0.0007492 5.3 47.1 0.113  1370999_at  sperm associated antigen 5  Info   Spag5 
   1669 0.0007553 261.3 140.2 1.864  1368896_at  MAD homolog 7 (Drosophila)  Info   Madh7 
   1670 0.0007568 1174.6 906.9 1.295  1371818_at  Exportin, tRNA (nuclear export receptor for tRNAs) (predicted)  Info    
   1671 0.0007595 896.9 1056.8 0.849  1398869_at  proteasome (prosome, macropain) 26S subunit, ATPase, 4  Info   Psmc4 
   1672 0.000761 390.8 214 1.826  1375030_at  Transcribed locus  Info    
   1673 0.0007618 49.2 31.1 1.582  1378487_at  E1A binding protein p300  Info   Ep300 
   1674 0.0007624 35.3 78.6 0.449  1376050_at  TAF5 RNA polymerase II, TATA box binding protein (TBP)-associated factor (predicted)  Info   Taf5_predicted 
   1675 0.000763 203.9 300.8 0.678  1382443_at  similar to Pabpc4_predicted protein /// poly A binding protein, cytoplasmic 4 (predicted)  Info   LOC288398 /// Pabpc4_predicted 
   1676 0.0007642 30.9 47 0.657  1386138_at  Similar to RAS-like, estrogen-regulated, growth-inhibitor  Info    
   1677 0.0007654 1018.3 696.2 1.463  1367982_at  aminolevulinic acid synthase 1  Info   Alas1 
   1678 0.0007693 939.8 648.9 1.448  1387542_at  solute carrier family 9, member 3  Info   Slc9a3 
   1679 0.0007697 70.7 29.2 2.421  1369007_at  nuclear receptor subfamily 4, group A, member 2  Info   Nr4a2 
   1680 0.0007703 520.1 729.9 0.713  1386897_at  heterogeneous nuclear ribonucleoproteins methyltransferase-like 2 (S. cerevisiae)  Info   Hrmt1l2 
   1681 0.0007706 1004.3 1311.8 0.766  1371792_at  Transcribed locus  Info    
   1682 0.0007706 54.6 83.5 0.654  1390396_at  Similar to eukaryotic translation initiation factor 4E member 3  Info    
   1683 0.0007718 79.4 131.2 0.605  1392698_a_at  Transcribed locus  Info    
   1684 0.0007718 382.3 569.3 0.672  1379542_at  similar to Small nuclear ribonucleoprotein F (snRNP-F) (Sm protein F) (Sm-F) (SmF) /// small nuclear ribonucleoprotein polypeptide F (predicted)  Info   LOC296377 /// Snrpf_predicted 
   1685 0.000772 230.4 324 0.711  1367701_at  receptor (calcitonin) activity modifying protein 2  Info   Ramp2 
   1686 0.0007721 44 80.1 0.549  1370393_at  coiled-coil domain containing 5  Info   Ccdc5 
   1687 0.0007727 410.3 311.3 1.318  1371516_at  exportin 7 (predicted)  Info   Xpo7_predicted 
   1688 0.0007752 281.7 405.9 0.694  1372156_at  similar to RIKEN cDNA 1810014L12 (predicted)  Info   RGD1307423_predicted 
   1689 0.0007761 37.4 66 0.567  1392699_at  Transcribed locus  Info    
   1690 0.0007763 434.3 665.9 0.652  1373072_at  Transcribed locus  Info    
   1691 0.0007784 59.4 109 0.545  1383266_at  secreted frizzled-related protein 1  Info   Sfrp1 
   1692 0.0007786 336 626.9 0.536  1373432_at  myristoylated alanine rich protein kinase C substrate  Info   Marcks 
   1693 0.0007787 61 40 1.525  1379694_at  Enhancer of polycomb homolog 2 (Drosophila) (predicted)  Info    
   1694 0.0007814 15.5 29.7 0.522  1375855_at  ependymin related protein 2  Info   RGD:1359161 
   1695 0.0007839 53.7 93 0.577  1384044_at    Info    
   1696 0.0007844 16 26.8 0.597  1397737_at  Transcribed locus  Info    
   1697 0.0007855 115.5 146.2 0.79  1372661_at  transducin (beta)-like 3 (predicted)  Info   Tbl3_predicted 
   1698 0.0007871 96 53.1 1.808  1368339_at  calbindin 3, (vitamin D-dependent calcium binding protein)  Info   Calb3 
   1699 0.0007892 21.8 106.8 0.204  1382493_at  Transcribed locus, strongly similar to XP_223060.3 PREDICTED: similar to LEK1 [Rattus norvegicus]  Info    
   1700 0.0007909 46.9 63 0.744  1386002_at  Transcribed locus, weakly similar to XP_128440.2 PREDICTED: RIKEN cDNA 4930432O21 [Mus musculus]  Info    
   1701 0.0007916 947.1 1346.1 0.704  1371544_at  similar to Enhancer of rudimentary homolog  Info   LOC503047 
   1702 0.0007925 18.6 33.3 0.559  1392861_at  Transcribed locus  Info    
   1703 0.0007927 1199.4 775.1 1.547  1398255_at  solute carrier family 15 (H+/peptide transporter), member 2  Info   Slc15a2 
   1704 0.000794 46.3 92.8 0.499  1382252_at  Transcribed locus  Info    
   1705 0.0007945 9.9 17.5 0.566  1397673_at  CUG triplet repeat,RNA-binding protein 2  Info   Cugbp2 
   1706 0.000797 1007.8 760.1 1.326  1375560_at  similar to RIKEN cDNA 0610010D20 (predicted)  Info   RGD1310475_predicted 
   1707 0.0007978 285.2 381.5 0.748  1389163_at  tripartite motif protein 32 (predicted)  Info   Trim32_predicted 
   1708 0.0007979 62.8 111.3 0.564  1391649_a_at  Transcribed locus  Info    
   1709 0.0007981 279.3 173.5 1.61  1368358_a_at  protein tyrosine phosphatase, receptor type, R  Info   Ptprr 
   1710 8e-04 581.6 772.5 0.753  1373185_at  signal sequence receptor, beta (predicted)  Info   Ssr2_predicted 
   1711 0.0008005 11.4 70.3 0.162  1373911_at  periostin, osteoblast specific factor (predicted)  Info   Postn_predicted 
   1712 0.0008018 891.5 1196.2 0.745  1369941_at  death-associated protein  Info   Dap 
   1713 0.000803 432.1 710.9 0.608  1383137_at  SRY-box containing gene 4 (predicted)  Info    
   1714 0.0008096 799.8 579.2 1.381  1376785_at  Synaptonemal complex protein 3  Info   Sycp3 
   1715 0.0008114 2438.8 1709 1.427  1393138_at  Jun D proto-oncogene  Info   Jund 
   1716 0.0008128 32.5 45.3 0.717  1376334_at  Similar to hypothetical protein FLJ20276 (predicted)  Info    
   1717 0.000814 69.7 45.3 1.539  1397450_at  Transcribed locus  Info    
   1718 0.0008141 1798.6 1519.9 1.183  1371696_at  G protein-coupled receptor 56  Info   Gpr56 
   1719 0.0008149 807.2 657 1.229  1398359_at  similar to RIKEN cDNA 2500002L14; EST C77350  Info   RGD:1359698 
   1720 0.0008161 197.8 257.1 0.769  1390827_at  MAD homolog 3 (Drosophila)  Info   Madh3 
   1721 0.0008165 135.3 192.5 0.703  1373516_at  Similar to RIKEN cDNA 2600005C20 (predicted)  Info    
   1722 0.0008168 446.7 319.4 1.399  1385716_at  Transcribed locus  Info    
   1723 0.0008192 72.1 116.8 0.617  1379683_at  Similar to hypothetical protein MGC17839  Info    
   1724 0.0008193 201.3 305.7 0.658  1372550_at    Info    
   1725 0.0008195 124.4 177.8 0.7  1372426_at  thrombospondin repeat protein 1  Info   Tsrc1 
   1726 0.000824 81.5 125 0.652  1374691_at  sulfotransferase family 5A, member 1 (predicted)  Info   Sult5a1_predicted 
   1727 0.000825 185 501 0.369  1398246_s_at  Fc receptor, IgG, low affinity III /// Fc gamma receptor II beta  Info   Fcgr3 /// LOC498276 
   1728 0.0008302 912.8 215.9 4.228  1386995_at  B-cell translocation gene 2, anti-proliferative  Info   Btg2 
   1729 0.0008305 40.1 57.8 0.694  1396018_at  Cullin 5  Info   Cul5 
   1730 0.0008305 137.2 202.8 0.677  1383623_at  thymocyte protein thy28  Info   RGD:1359525 
   1731 0.0008309 101.6 55.9 1.818  1369250_at  solute carrier family 28 (sodium-coupled nucleoside transporter), member 1  Info   Slc28a1 
   1732 0.0008332 91.4 48.4 1.888  1378247_at  ELL associated factor 2  Info   Eaf2 
   1733 0.0008335 452.8 343.3 1.319  1393983_at  exportin, tRNA (nuclear export receptor for tRNAs) (predicted)  Info   Xpot_predicted 
   1734 0.0008357 27.9 42.3 0.66  1373931_at  similar to RIKEN cDNA C530028O21 gene (predicted)  Info   RGD1304952_predicted 
   1735 0.0008362 71.6 41.9 1.709  1378365_at  Transcribed locus  Info    
   1736 0.0008365 341.3 209.5 1.629  1377635_at  flavin containing monooxygenase 2  Info   Fmo2 
   1737 0.000837 93.5 63.6 1.47  1382402_at  Unc-51 like kinase 1 (C. elegans)  Info   Ulk1 
   1738 0.0008382 71.1 102.9 0.691  1394014_at  PHD finger protein 12 (predicted)  Info   Phf12_predicted 
   1739 0.0008383 176.4 245.7 0.718  1397892_at  eukaryotic translation initiation factor 1A (predicted)  Info   Eif1a_predicted 
   1740 0.0008392 209.7 325.5 0.644  1376016_at  similar to RIKEN cDNA 1110007L15 (predicted)  Info   RGD1311660_predicted 
   1741 0.0008411 1240 776.4 1.597  1388103_at  protein distantly related to to the gamma subunit family  Info   Pr1 
   1742 0.0008421 34.7 66 0.526  1391505_x_at    Info    
   1743 0.0008437 1223.4 984.5 1.243  1379254_at  similar to RIKEN cDNA 1300007B12; clone MNCb-2755 (predicted)  Info   RGD1309892_predicted 
   1744 0.0008439 55 88 0.625  1390820_at  zinc finger, CCHC domain containing 11 (predicted)  Info   Zcchc11_predicted 
   1745 0.000846 27.7 50.7 0.546  1370810_at  cyclin D2  Info   Ccnd2 
   1746 0.0008463 75.4 126.3 0.597  1384617_at  Unknown (protein for MGC:72614)  Info   RGD:735075 
   1747 0.0008477 166.8 258.1 0.646  1373942_at  Transcribed locus  Info    
   1748 0.0008477 366.6 479.6 0.764  1384714_x_at  similar to 60S ribosomal protein L8 /// similar to mitochondrial tryptophanyl tRNA synthetase 2  Info   LOC499374 /// LOC499679 
   1749 0.0008486 518.6 706.2 0.734  1371694_at    Info    
   1750 0.0008496 38.4 86.7 0.443  1374216_at  Transcribed locus  Info    
   1751 0.0008498 62.8 174.7 0.359  1390510_at  membrane-spanning 4-domains, subfamily A, member 6B  Info   RGD:1359381 
   1752 0.0008522 224.9 83.8 2.684  1370816_at  nuclear receptor subfamily 1, group D, member 1  Info   Nr1d1 
   1753 0.0008561 23.4 45.5 0.514  1368605_at  adaptor protein with pleckstrin homology and src homology 2 domains  Info   Aps 
   1754 0.0008574 3221.2 2206.6 1.46  1393221_at  similar to 20-alpha-hydroxysteroid dehydrogenase  Info   LOC498789 
   1755 0.0008582 36.8 57.1 0.644  1382783_at  Bloom syndrome (predicted)  Info   Blm_predicted 
   1756 0.000861 42.9 64.8 0.662  1372590_at  Transcribed locus  Info    
   1757 0.0008623 1163.5 874.9 1.33  1368428_at  X-prolyl aminopeptidase (aminopeptidase P) 2, membrane-bound  Info   Xpnpep2 
   1758 0.0008624 182.5 61.9 2.948  1368597_at  SNF1-like kinase  Info   Snf1lk 
   1759 0.0008644 75.9 45.4 1.672  1391240_at    Info    
   1760 0.0008653 780.7 1190.5 0.656  1370312_at  spondin 1  Info   Spon1 
   1761 0.0008654 14.9 3.5 4.257  1385793_at  Similar to RIKEN cDNA 2310067G05  Info    
   1762 0.0008678 1242.2 849.7 1.462  1376852_at  methylcrotonoyl-Coenzyme A carboxylase 1 (alpha) (predicted)  Info   Mccc1_predicted 
   1763 0.0008678 490.8 343.8 1.428  1374169_at  similar to chromosome 16 open reading frame 5 (predicted)  Info   RGD1310686_predicted 
   1764 0.000868 56.1 79.9 0.702  1398434_at  Death associated transcription factor 1 (predicted)  Info    
   1765 0.0008705 300.1 410.7 0.731  1373955_at  karyopherin (importin) beta 3 (predicted)  Info   Kpnb3_predicted 
   1766 0.0008707 59.6 76.6 0.778  1372065_at  ADP-ribosyltransferase 3 (predicted)  Info   Art3_predicted 
   1767 0.0008717 2540 1833.7 1.385  1375631_at  pleckstrin homology domain containing, family B (evectins) member 2 (predicted)  Info   Plekhb2_predicted 
   1768 0.000873 349.4 240.2 1.455  1383850_at  Similar to RIKEN cDNA 1600014C10  Info    
   1769 0.0008737 88.7 148.1 0.599  1389039_at  Transcribed locus  Info    
   1770 0.0008749 309.1 397.5 0.778  1388400_at  cell division cycle 34 homolog (S. cerevisiae) (predicted)  Info   Cdc34_predicted 
   1771 0.0008757 388.9 644.2 0.604  1371336_at  hematological and neurological expressed sequence 1  Info   RGD:1359325 
   1772 0.0008763 59 77.6 0.76  1391014_at  zinc finger, MYND domain containing 19  Info   Zmynd19 
   1773 0.0008786 52.5 162.3 0.323  1368205_at  complement factor I  Info   Cfi 
   1774 0.0008817 6.3 42.7 0.148  1373530_at  cyclin E  Info   Ccne1 
   1775 0.0008823 2962 2316.1 1.279  1370237_at  L-3-hydroxyacyl-Coenzyme A dehydrogenase, short chain  Info   Hadhsc 
   1776 0.0008833 47 60.3 0.779  1369120_a_at  luteinizing hormone beta  Info   Lhb 
   1777 0.0008835 34.2 63.4 0.539  1387565_at  transient receptor potential cation channel, subfamily V, member 6  Info   Trpv6 
   1778 0.0008854 24.2 42.9 0.564  1376324_at  Similar to zinc finger protein 21 isoform 2  Info    
   1779 0.0008857 303 384.4 0.788  1369935_at  cyclin D3  Info   Ccnd3 
   1780 0.0008869 35.7 56.1 0.636  1392548_at  similar to mKIAA1604 protein  Info   LOC362153 
   1781 0.0008896 557.6 450.7 1.237  1389008_at  Sprouty-related, EVH1 domain containing 2 (predicted)  Info    
   1782 0.0008911 1.2 2.9 0.414  1392451_at  similar to hypothetical protein FLJ37118  Info   LOC362659 
   1783 0.000894 110.6 166.6 0.664  1394275_at    Info    
   1784 0.0008943 220.9 284.1 0.778  1390709_at  Transcribed locus  Info    
   1785 0.0008945 63.3 102.3 0.619  1398265_at  ATP-binding cassette, sub-family C (CFTR/MRP), member 9  Info   Abcc9 
   1786 0.0008946 2425 1868.1 1.298  1391652_at  glycine-N-acyltransferase (predicted)  Info   Glyat_predicted 
   1787 0.0008961 62.2 97.5 0.638  1373446_at  PHD finger protein 13 (predicted)  Info   Phf13_predicted 
   1788 0.0008965 19.4 47.7 0.407  1389421_at  polymerase (DNA directed), epsilon  Info   Pole 
   1789 0.0008976 255.4 351.2 0.727  1383067_a_at  deoxythymidylate kinase (predicted)  Info   Dtymk_predicted 
   1790 0.0008976 16.2 27.7 0.585  1392158_at    Info    
   1791 0.0008989 111.5 21.3 5.235  1370355_at  stearoyl-Coenzyme A desaturase 1  Info   Scd1 
   1792 0.0008993 247.8 313.3 0.791  1389127_at    Info    
   1793 0.0008996 28.8 59.2 0.486  1391222_at  similar to Nedd4 binding protein 1 (predicted)  Info   RGD1305179_predicted 
   1794 0.0009011 250.9 197.3 1.272  1373730_at  Similar to hypothetical protein MGC20460 (predicted)  Info    
   1795 0.0009033 62.4 20.7 3.014  1379107_at  Solute carrier family 4, member 4  Info   Slc4a4 
   1796 0.0009042 144 209.8 0.686  1384935_at  Cleavage stimulation factor, 3' pre-RNA, subunit 3, 77kDa (predicted)  Info    
   1797 0.0009071 3211.3 3786.9 0.848  1387888_at  ribosomal protein S9  Info   Rps9 
   1798 0.0009078 100.3 7.4 13.554  1387033_at  uncoupling protein 1  Info   Ucp1 
   1799 0.0009086 19.9 32.9 0.605  1391452_at  Similar to RIKEN cDNA A230102I05 (predicted)  Info    
   1800 0.0009092 26.2 6 4.367  1376667_at  cytochrome P450, family 26, subfamily b, polypeptide 1  Info   Cyp26b1 
   1801 0.0009097 131.9 157.6 0.837  1368652_at  caspase 9  Info   Casp9 
   1802 0.0009106 2838.2 3440.4 0.825  1398827_at  CD 81 antigen  Info   Cd81 
   1803 0.0009165 461.8 360.2 1.282  1379339_at  similar to tumor-related protein (predicted)  Info   RGD1307632_predicted 
   1804 0.0009168 175.3 111.7 1.569  1369099_at  solute carrier family 30 (zinc transporter), member 1  Info   Slc30a1 
   1805 0.0009174 17.5 3.5 5  1398169_at  Transcribed locus  Info    
   1806 0.0009178 172.7 92.6 1.865  1384770_at  Transcribed locus  Info    
   1807 0.0009181 632.2 409 1.546  1369562_at  neural visinin-like Ca2+-binding protein type 3  Info   Hpcal1 
   1808 0.0009224 335.6 258.9 1.296  1373182_at    Info    
   1809 0.0009234 65.3 95 0.687  1374212_at  Transcribed locus  Info    
   1810 0.0009242 1493.4 1103.4 1.353  1372790_at  Malate dehydrogenase 1, NAD (soluble)  Info   Mdh1 
   1811 0.0009248 5.1 51.9 0.098  1390481_a_at  similar to RIKEN cDNA 2700084L22 (predicted)  Info   RGD1310816_predicted 
   1812 0.0009261 176.5 91.2 1.935  1384215_at    Info    
   1813 0.0009262 875.3 1045.8 0.837  1370130_at  ras homolog gene family, member A  Info   RGD:619921 
   1814 0.0009278 395.3 567.2 0.697  1371602_at  Transcribed locus  Info    
   1815 0.0009292 53.5 70.6 0.758  1390968_at  Similar to hypothetical protein A430031N04  Info    
   1816 0.0009297 137.1 188.8 0.726  1377310_at  Transcribed locus, weakly similar to XP_575554.1 PREDICTED: similar to RNA binding motif and ELMO domain 1 [Rattus norvegicus]  Info    
   1817 0.0009298 253.7 173.6 1.461  1390535_at  CTD (carboxy-terminal domain, RNA polymerase II, polypeptide A) phosphatase, subunit 1 (predicted)  Info    
   1818 0.0009319 1046.8 740.6 1.413  1368253_at  guanidinoacetate methyltransferase  Info   Gamt 
   1819 0.0009335 74.3 105.3 0.706  1380445_at  Transcribed locus, strongly similar to XP_515868.1 PREDICTED: sodium channel, voltage-gated, type III, alpha [Pan troglodytes]  Info    
   1820 0.0009363 16.8 31 0.542  1385382_at  Transcribed locus  Info    
   1821 0.0009374 59.9 81.5 0.735  1377836_at  Similar to suppressor of hairy wing homolog 4 isoform 1  Info    
   1822 0.000938 22 9.8 2.245  1397154_at  Myeloid/lymphoid or mixed-lineage leukemia (trithorax homolog, Drosophila); translocated to, 2 (predicted)  Info    
   1823 0.0009405 46.5 73.3 0.634  1381019_x_at  development and differentiation enhancing (predicted)  Info   Ddef1_predicted 
   1824 0.0009408 27.5 50.5 0.545  1374270_at  Testis specific, 14  Info    
   1825 0.0009419 8.7 52.2 0.167  1378199_at  Transcribed locus  Info    
   1826 0.0009431 2781.7 3601.2 0.772  1386858_at  ribosomal protein L13  Info   Rpl13 
   1827 0.0009488 79.8 133.5 0.598  1397168_at  Similar to CD2-associated protein (predicted)  Info    
   1828 0.0009489 346.7 488.3 0.71  1386860_at  milk fat globule-EGF factor 8 protein  Info   Mfge8 
   1829 0.0009489 110 156.8 0.702  1367967_at  leprecan 1  Info   Lepre1 
   1830 0.000952 107 186 0.575  1392922_at  Transcribed locus  Info    
   1831 0.000953 50 83.8 0.597  1389804_at  Transcribed locus  Info    
   1832 0.0009544 366.6 483.9 0.758  1370156_at  prion protein  Info   Prnp 
   1833 0.0009568 4048.7 4674.4 0.866  1398885_at  ribosomal protein L23  Info   Rpl23 
   1834 0.0009585 32 51.1 0.626  1397527_at  tripartite motif protein 47 (predicted)  Info   Trim47_predicted 
   1835 0.0009598 339.9 180.3 1.885  1368115_at  claudin 3  Info   Cldn3 
   1836 0.0009608 179.7 297.4 0.604  1373164_at  Serine/threonine kinase 17b (apoptosis-inducing)  Info   Stk17b 
   1837 0.0009609 3560.4 4437.4 0.802  1398852_at  ribosomal protein S21  Info   Rps21 
   1838 0.0009613 18.6 39.6 0.47  1383784_at  Transcribed locus  Info    
   1839 0.0009625 4323.5 5052.6 0.856  1398761_at  ribosomal protein L5  Info   Rpl5 
   1840 0.0009677 336.4 180.9 1.86  1383744_at  Transcribed locus  Info    
   1841 0.0009683 135.9 166 0.819  1394605_at  similar to CDNA sequence BC017647  Info   LOC497960 
   1842 0.0009704 242.8 181.6 1.337  1382215_at  Similar to Transcription initiation factor TFIID 105 kDa subunit (TAFII-105) (TAFII105)  Info    
   1843 0.0009762 10.5 20.3 0.517  1382647_at  Transcribed locus  Info    
   1844 0.0009763 108.8 195.2 0.557  1374730_at  Tyro protein tyrosine kinase binding protein  Info   Tyrobp 
   1845 0.0009795 184.3 277.5 0.664  1371776_at  Phosphatidylinositol 3-kinase, regulatory subunit, polypeptide 1  Info   Pik3r1 
   1846 0.0009845 286.9 385.4 0.744  1368342_at  adenosine monophosphate deaminase 3  Info   Ampd3 
   1847 0.0009846 168.7 206.6 0.817  1373664_at  phosphatidylinositol glycan, class C (predicted)  Info   Pigc_predicted 
   1848 0.0009854 684.6 479.5 1.428  1373590_at  stomatin (predicted)  Info   Stom_predicted 
   1849 0.0009871 264.7 373.4 0.709  1376673_at  Transcription factor 8 (represses interleukin 2 expression)  Info   Tcf8 
   1850 0.0009875 63.8 98.7 0.646  1376005_at  Kinesin family member 1B  Info   Kif1b 
   1851 0.0009913 49.1 80.1 0.613  1380129_at  Transcribed locus  Info    
   1852 0.0009924 27.1 64.4 0.421  1367791_at  receptor (calcitonin) activity modifying protein 1  Info   Ramp1 
   1853 0.0009937 3.4 16.3 0.209  1375482_at  similar to plexin 1  Info   LOC362398 
   1854 0.0009953 32.7 61.4 0.533  1392638_at  Similar to myeloid leukemia factor 1 interacting protein  Info    
   1855 0.0009987 1050.5 722.9 1.453  1386917_at  Pyruvate carboxylase  Info   Pc 
   1856 0.001 3236.6 2673.1 1.211  1370030_at  glutamate cysteine ligase, modifier subunit  Info   Gclm 
   1857 0.0010015 2.5 17.4 0.144  1393129_at    Info    
   1858 0.0010017 201.6 107.2 1.881  1374143_at  Transcribed locus  Info    
   1859 0.0010037 244.7 169.2 1.446  1380431_at  similar to KIAA0564 protein (predicted)  Info   RGD1308772_predicted 
   1860 0.0010041 221.6 409.7 0.541  1370376_a_at  cold shock domain protein A  Info   Csda 
   1861 0.0010056 4952.4 4390.5 1.128  1367706_at  voltage-dependent anion channel 1  Info   Vdac1 
   1862 0.0010069 1461.4 1189.3 1.229  1368008_at  prominin 1  Info   Prom1 
   1863 0.0010088 219.3 313.1 0.7  1376687_at  ubiquitin specific protease 1 (predicted)  Info   Usp1_predicted 
   1864 0.00101 268.3 231.8 1.157  1371721_at  Transcribed locus, strongly similar to NP_080015.3 hypothetical protein LOC66743 [Mus musculus]  Info    
   1865 0.0010113 3208.4 2515.7 1.275  1392969_at  Transcribed locus  Info    
   1866 0.0010118 4338.5 5239.6 0.828  1398871_at  ribosomal protein L17  Info   RGD:1303019 
   1867 0.0010125 88.4 119.5 0.74  1380009_at  vaccinia related kinase 1 (predicted)  Info   Vrk1_predicted 
   1868 0.001013 327.1 239.1 1.368  1383960_at  peroxisome biogenesis factor 16 (predicted)  Info   Pex16_predicted 
   1869 0.0010138 414.8 549.8 0.754  1387001_at  v-ral simian leukemia viral oncogene homolog B  Info   Ralb 
   1870 0.0010173 185.3 248.1 0.747  1379327_at  SWI/SNF related, matrix associated, actin dependent regulator of chromatin, subfamily a, member 5 (predicted)  Info   Smarca5_predicted 
   1871 0.0010177 4056.6 5194.1 0.781  1367579_a_at  tubulin, alpha 1 /// tubulin, alpha 6 (predicted) /// similar to Tubulin alpha-2 chain (Alpha-tubulin 2)  Info   Tuba1 /// Tuba6_predicted /// LOC500929 
   1872 0.0010187 398.7 538.4 0.741  1389518_at  F-box only protein 10 (predicted)  Info   Fbxo10_predicted 
   1873 0.0010194 1223 1044.9 1.17  1367687_a_at  peptidylglycine alpha-amidating monooxygenase  Info   Pam 
   1874 0.0010194 17 49.3 0.345  1393158_at  similar to 2610528M18Rik protein  Info   RGD:1359451 
   1875 0.0010232 103.9 165.1 0.629  1369720_at  myosin Ib  Info   Myo1b 
   1876 0.0010234 160.4 132.4 1.211  1395440_at  similar to RIKEN cDNA 5133401N09  Info   LOC498695 
   1877 0.0010262 148.7 214.4 0.694  1382010_at    Info    
   1878 0.0010294 1909.3 1382.7 1.381  1374524_at  selenocysteine lyase  Info   RGD:1359514 
   1879 0.0010304 1630.2 1057.1 1.542  1382050_at  Transcribed locus  Info    
   1880 0.0010329 1397.6 786.7 1.777  1387158_at  meprin 1 beta  Info   Mep1b 
   1881 0.001035 39.1 59.7 0.655  1392082_a_at  CD7 antigen (predicted)  Info   Cd7_predicted 
   1882 0.0010357 478.2 853.3 0.56  1371382_at  similar to Filamin A (Alpha-filamin) (Filamin 1) (Endothelial actin-binding protein) (Actin-binding protein 280) (ABP-280) (Nonmuscle filamin)  Info   LOC293860 
   1883 0.0010397 198.6 284.2 0.699  1395409_at  protein phosphatase 2A, regulatory subunit B' (PR 53) (predicted)  Info   Ppp2r4_predicted 
   1884 0.0010403 732.8 422 1.736  1393584_at    Info    
   1885 0.0010404 63.5 136.2 0.466  1387144_at  integrin alpha 1  Info   Itga1 
   1886 0.0010416 93.2 165.5 0.563  1393038_at  Fc receptor, IgG, high affinity I (predicted)  Info   Fcgr1_predicted 
   1887 0.0010422 270.4 411.1 0.658  1396820_at  histone deacetylase 1 (predicted)  Info   Hdac1_predicted 
   1888 0.0010431 854 519.4 1.644  1391539_at  Transcribed locus  Info    
   1889 0.0010436 42 25.6 1.641  1370649_at  bradykinin receptor b2  Info   Bdkrb2 
   1890 0.0010444 37.4 85.8 0.436  1370682_at  paired-Ig-like receptor A2 (predicted) /// similar to PIRA5  Info   Pira2_predicted /// LOC365169 
   1891 0.0010453 49.5 28.9 1.713  1369632_a_at  ATP-binding cassette, sub-family C (CFTR/MRP), member 8  Info   Abcc8 
   1892 0.0010456 91.1 120.8 0.754  1395856_at  Hypothetical LOC308765 (predicted)  Info    
   1893 0.0010476 58.5 112.8 0.519  1381942_at  Transcribed locus  Info    
   1894 0.0010562 261.8 108.3 2.417  1387343_at  CCAAT/enhancer binding protein (C/EBP), delta  Info   Cebpd 
   1895 0.0010562 31.2 60.7 0.514  1391068_at  E030032D13Rik gene  Info   RGD:1303180 
   1896 0.0010575 1036.2 699.6 1.481  1386280_at  similar to RIKEN cDNA 0610006F02 (predicted)  Info   RGD1305205_predicted 
   1897 0.0010577 309.9 346.9 0.893  1389509_at  Similar to hypothetical protein 2610304F09 (predicted)  Info    
   1898 0.0010625 381.5 291.6 1.308  1387110_at  N-arginine dibasic convertase 1  Info   Nrd1 
   1899 0.0010635 434.1 361.7 1.2  1399030_at  similar to DNA segment, Chr X, Immunex 38, expressed  Info   RGD:1359718 
   1900 0.0010641 1896.1 1265 1.499  1386960_at  solute carrier family 37 (glycerol-6-phosphate transporter), member 4  Info   Slc37a4 
   1901 0.0010647 193.3 266.9 0.724  1389131_at  similar to C330027I04Rik protein  Info   RGD:1359229 
   1902 0.0010673 1273.2 1060 1.201  1370814_at  dehydrogenase/reductase (SDR family) member 4  Info   Dhrs4 
   1903 0.0010682 32.1 62.7 0.512  1385047_x_at    Info   LOC304349 /// LOC360782 /// LOC498168 /// LOC501815 /// LOC501818 
   1904 0.0010686 278.8 403.9 0.69  1388877_at  mitochondrial ribosomal protein S5 (predicted)  Info   Mrps5_predicted 
   1905 0.0010706 2626.5 1790.4 1.467  1372306_at  ethylmalonic encephalopathy 1 (predicted)  Info   Ethe1_predicted 
   1906 0.0010714 86.3 113.3 0.762  1383182_at  serine/arginine-rich protein specific kinase 1 (predicted)  Info   Srpk1_predicted 
   1907 0.0010751 631.5 780.7 0.809  1372460_at  LOC499767  Info   LOC499767 
   1908 0.0010758 35 59.7 0.586  1394097_at  Transcribed locus  Info    
   1909 0.0010762 2002.7 1285.1 1.558  1388814_at  DEAD (Asp-Glu-Ala-Asp) box polypeptide 19  Info   Ddx19 
   1910 0.0010773 11.9 169.5 0.07  1396205_at  Transcribed locus, weakly similar to XP_282988.2 PREDICTED: similar to novel protein [Mus musculus]  Info    
   1911 0.0010804 542.5 320.5 1.693  1391573_at  Tumor necrosis factor receptor superfamily, member 21 (predicted)  Info    
   1912 0.0010808 110.8 69 1.606  1376279_at  processing of precursor 1, ribonuclease P/MRP family, (S. cerevisiae) (predicted)  Info   Pop1_predicted 
   1913 0.0010843 84.4 147.1 0.574  1391162_at  Transcribed locus  Info    
   1914 0.0010914 63.4 109.1 0.581  1383322_at  RAS-like family 11 member B  Info   RGD:1303099 
   1915 0.0010932 42.6 74.6 0.571  1376909_at  similar to RRP22 (predicted)  Info   RGD1306100_predicted 
   1916 0.0010937 475 253.6 1.873  1367795_at  interferon-related developmental regulator 1  Info   Ifrd1 
   1917 0.0010949 1353.6 1799.8 0.752  1373040_at  eukaryotic translation initiation factor 3, subunit 5 (epsilon) (predicted)  Info   Eif3s5_predicted 
   1918 0.0010988 61.4 124.3 0.494  1391026_at  Immediate early response 5-like  Info    
   1919 0.0010993 227.4 274.3 0.829  1389000_at  Transcribed locus  Info    
   1920 0.0010996 753.6 543.9 1.386  1369081_at  neuraminidase 1  Info   Neu1 
   1921 0.0010996 47.3 114.7 0.412  1387079_at  guanylate cyclase 1, soluble, alpha 3 /// hypothetical gene supported by NM_017090  Info   Gucy1a3 /// LOC497757 
   1922 0.0011002 99.3 158.5 0.626  1390728_at    Info    
   1923 0.0011043 497.9 662.7 0.751  1388766_at  metaxin 2 (predicted)  Info   Mtx2_predicted 
   1924 0.0011046 10.3 45.1 0.228  1368250_at  tektin 1  Info   Tekt1 
   1925 0.0011063 152.1 113.8 1.337  1390987_at  Transcribed locus  Info    
   1926 0.0011067 127 68.8 1.846  1383472_at  aldehyde dehydrogenase 1 family, member B1 (predicted)  Info   Aldh1b1_predicted 
   1927 0.0011077 476.2 329.6 1.445  1382274_at  retinoic acid receptor responder (tazarotene induced) 1 (predicted)  Info   Rarres1_predicted 
   1928 0.001108 54.6 76.7 0.712  1381376_at  Similar to [Mouse primary response gene B94 mRNA, 3end.], gene product  Info    
   1929 0.0011104 13.5 68.4 0.197  1393982_at  polymerase (DNA directed), epsilon 2 (p59 subunit) (predicted)  Info   Pole2_predicted 
   1930 0.0011114 40.9 77.7 0.526  1376697_at  carbohydrate sulfotransferase 12 (predicted)  Info   Chst12_predicted 
   1931 0.0011127 4031.7 1963.9 2.053  1397205_at  dehydrogenase/reductase (SDR family) member 7 (predicted) /// similar to Down-regulated in nephrectomized rat kidney #3  Info   Dhrs7_predicted /// LOC500672 
   1932 0.0011165 84 146.1 0.575  1372615_at  amine oxidase, copper containing 3  Info   Aoc3 
   1933 0.0011168 31.2 54.4 0.574  1378342_at  LOC498937  Info   LOC498937 
   1934 0.0011173 695.7 489.3 1.422  1377821_at  V-erb-b2 erythroblastic leukemia viral oncogene homolog 3 (avian)  Info   Erbb3 
   1935 0.001118 1753.1 1462 1.199  1386946_at  carnitine palmitoyltransferase 1, liver  Info   Cpt1a 
   1936 0.0011183 1.8 18.2 0.099  1383434_at  Transcribed locus  Info    
   1937 0.0011186 4 27.6 0.145  1384174_at  Transcribed locus  Info    
   1938 0.0011222 49.2 94 0.523  1378225_at  Matrix metalloproteinase 14 (membrane-inserted)  Info   Mmp14 
   1939 0.0011251 408.8 301.8 1.355  1382571_at  Transcribed locus  Info    
   1940 0.0011289 399.7 534.3 0.748  1372949_at  Septin 6 (predicted)  Info    
   1941 0.0011301 114.4 156.3 0.732  1371125_at  kinesin heavy chain member 2  Info   Kif2 
   1942 0.0011303 181.8 311 0.585  1374176_at  similar to DNA segment, Chr 4, Brigham & Womens Genetics 0951 expressed (predicted)  Info   RGD1308059_predicted 
   1943 0.0011324 64.6 109.1 0.592  1375612_at  Transcribed locus  Info    
   1944 0.0011332 29 88 0.33  1387625_at  insulin-like growth factor binding protein 6  Info   Igfbp6 
   1945 0.001134 114.5 84.3 1.358  1377223_at  Transcribed locus  Info    
   1946 0.0011361 296.6 169.6 1.749  1370329_at  cytochrome P450, family 2, subfamily d, polypeptide 22  Info   Cyp2d22 
   1947 0.0011367 19.9 31.9 0.624  1381054_at  similar to KIAA0980 protein (predicted)  Info   RGD1306152_predicted 
   1948 0.0011378 99.7 155.2 0.642  1373617_at  epithelial membrane protein 2  Info   RGD:1359629 
   1949 0.0011385 393.8 471.9 0.834  1367487_at  similar to UDP-Gal:betaGlcNAc beta 1,4-galactosyltransferase, polypeptide 3  Info   RGD:1359221 
   1950 0.0011386 63.1 90.9 0.694  1382148_at  Transcribed locus, moderately similar to XP_576460.1 PREDICTED: similar to hypothetical protein PB402898.00.0 [Rattus norvegicus]  Info    
   1951 0.0011386 41.6 67.8 0.614  1390925_a_at  Transcribed locus  Info    
   1952 0.0011403 182.5 256.6 0.711  1372643_at  similar to protein 4.1G  Info   LOC309557 
   1953 0.0011403 36.5 102.4 0.356  1372574_at  TEA domain family member 2  Info   Tead2 
   1954 0.0011422 214.4 291.2 0.736  1379725_at  Transcribed locus  Info    
   1955 0.0011429 12.6 30.7 0.41  1390397_at  similar to chromosome 20 open reading frame 39 (predicted)  Info   RGD1310753_predicted 
   1956 0.0011445 1021.5 1267.4 0.806  1398829_at  FK506 binding protein 1a  Info   Fkbp1a 
   1957 0.0011451 193.2 271.3 0.712  1391356_at  Similar to hypothetical protein FLJ10006 (predicted)  Info    
   1958 0.0011469 17.3 41.3 0.419  1383946_at  claudin 1  Info   Cldn1 
   1959 0.0011481 5513.8 6330.2 0.871  1367617_at  aldolase A  Info   Aldoa 
   1960 0.00115 270.6 221.1 1.224  1372799_at  deoxyguanosine kinase (predicted)  Info   Dguok_predicted 
   1961 0.0011505 99.1 56.3 1.76  1369292_at  hydroxysteroid (17-beta) dehydrogenase 1  Info   Hsd17b1 
   1962 0.0011527 64.9 114.8 0.565  1369262_at  caspase 8  Info   Casp8 
   1963 0.0011528 2759.4 2130.2 1.295  1368059_at  crystallin, mu  Info   Crym 
   1964 0.0011542 44.4 87.9 0.505  1376689_at  similar to RIKEN cDNA 1700023M03 (predicted)  Info   RGD1305457_predicted 
   1965 0.0011571 642.6 493.7 1.302  1375346_at  similar to hypothetical protein FLJ20010  Info   LOC500993 
   1966 0.0011587 64.4 86.1 0.748  1385395_at    Info    
   1967 0.0011588 11 2.1 5.238  1378663_at  Transcribed locus  Info    
   1968 0.0011599 13.6 21.3 0.638  1369260_a_at  membrane protein, palmitoylated 4 (MAGUK p55 subfamily member 4)  Info   Mpp4 
   1969 0.0011599 18.4 62.4 0.295  1373674_at  microfibrillar associated protein 5 (predicted)  Info   Mfap5_predicted 
   1970 0.0011665 66.3 87.1 0.761  1379804_at  PDZ protein Mrt1  Info   Snx27 
   1971 0.0011667 22.9 34 0.674  1392242_at  PHD finger protein 13 (predicted)  Info   Phf13_predicted 
   1972 0.0011682 70.8 131.3 0.539  1385157_at  chromobox homolog 1 (Drosophila HP1 beta) (predicted)  Info   Cbx1_predicted 
   1973 0.0011703 2.7 10.5 0.257  1380018_at  Transcribed locus  Info    
   1974 0.0011706 121.7 178.7 0.681  1376795_at  phosphoinositide-3-kinase adaptor protein 1 (predicted)  Info   Pik3ap1_predicted 
   1975 0.0011743 3631 1802.7 2.014  1387303_at  solute carrier family 22 (organic cation transporter), member 2  Info   Slc22a2 
   1976 0.0011756 3785 4319 0.876  1371761_at  ribosomal protein L34 (predicted)  Info   Rpl34_predicted 
   1977 0.0011768 11.9 30.1 0.395  1382739_at  G protein-coupled receptor 19  Info   Gpr19 
   1978 0.0011772 283.8 381 0.745  1388794_at  RNA binding motif protein, X chromosome retrogene (predicted)  Info   Rbmxrt_predicted 
   1979 0.0011777 254.2 216.7 1.173  1376894_at  pallidin (predicted)  Info   Pldn_predicted 
   1980 0.0011787 170.1 313 0.543  1370894_at  claudin 7  Info   Cldn7 
   1981 0.0011828 368.2 284 1.296  1372670_at  similar to hypothetical protein FLJ21827 (predicted)  Info   RGD1307682_predicted 
   1982 0.0011865 1.3 8.4 0.155  1380217_at    Info    
   1983 0.0011938 15.2 45.2 0.336  1394585_at  Similar to RIKEN cDNA G430041M01  Info    
   1984 0.0011941 228.1 414.6 0.55  1370247_a_at  peripheral myelin protein 22  Info   Pmp22 
   1985 0.0011963 16.1 7.1 2.268  1395012_at  hect domain and RLD 4 (predicted)  Info   Herc4_predicted 
   1986 0.0011976 1007 1743.4 0.578  1387770_at  putative ISG12(a) protein  Info   RGD:1303168 
   1987 0.001199 3.2 27.7 0.116  1391077_at  Claspin homolog (Xenopus laevis) (predicted)  Info    
   1988 0.0011996 13.3 24.9 0.534  1392048_at  Transcribed locus, strongly similar to XP_226953.3 PREDICTED: similar to glucocorticoid induced gene 1 [Rattus norvegicus]  Info    
   1989 0.0012002 14.1 26.1 0.54  1379374_at  plasticity related gene 1  Info   RGD:727806 
   1990 0.0012049 1669.8 2195.4 0.761  1372254_at  serine (or cysteine) peptidase inhibitor, clade G, member 1  Info   Serping1 
   1991 0.0012066 2.6 15.1 0.172  1395084_at    Info    
   1992 0.001208 738.6 879 0.84  1375974_at  Similar to RNA-binding protein isoform G3BP-2a (predicted)  Info    
   1993 0.0012086 2011.8 1296 1.552  1375267_at  peptidylprolyl isomerase C  Info   RGD:1303221 
   1994 0.0012122 53.1 80.5 0.66  1368917_at  nudix (nucleoside diphosphate linked moiety X)-type motif 1  Info   Nudt1 
   1995 0.001213 58.3 85.1 0.685  1389477_at  Transcribed locus  Info    
   1996 0.0012144 123.4 83.6 1.476  1391148_at  Transcribed locus  Info    
   1997 0.0012145 2893.6 2455.8 1.178  1368178_at  PDZ domain containing 1  Info   Pdzk1 
   1998 0.0012164 745.2 587.1 1.269  1371893_at  Transcribed locus  Info    
   1999 0.0012182 434.7 605.2 0.718  1376106_at  similar to hypothetical protein MGC33926  Info   RGD:1303057 
   2000 0.0012237 1220.3 1666.3 0.732  1392334_at  RT1 class II, locus Ba  Info   RT1-Ba 
   2001 0.0012248 61.2 99.1 0.618  1389425_at  similar to 5(3)-deoxyribonucleotidase  Info   LOC501746 
   2002 0.001226 9.7 28.6 0.339  1369301_at  angiotensin receptor-like 1  Info   Agtrl1 
   2003 0.0012282 70.3 119.8 0.587  1388846_at    Info    
   2004 0.0012299 150.8 246.2 0.613  1388625_at  similar to RIKEN cDNA 4921521J11 (predicted)  Info   RGD1304711_predicted 
   2005 0.0012309 384.4 555.8 0.692  1387130_at  solute carrier family 39 (iron-regulated transporter), member 1  Info   Slc40a1 
   2006 0.0012314 311.9 216.2 1.443  1375985_at  RAB2B, member RAS oncogene family (predicted)  Info   Rab2b_predicted 
   2007 0.001236 163.2 208.6 0.782  1373334_at  Similar to RIKEN cDNA 0610039G24 gene (predicted)  Info    
   2008 0.0012364 85.5 151.7 0.564  1380148_at  Transcribed locus  Info    
   2009 0.001237 462.7 294.7 1.57  1390026_at  Bcl2-associated athanogene 3 (predicted)  Info   Bag3_predicted 
   2010 0.0012373 104.2 179.3 0.581  1382009_at  Transcribed locus  Info    
   2011 0.0012383 56.1 100.8 0.557  1389214_at  similar to Laminin alpha-4 chain precursor  Info   LOC309816 
   2012 0.0012386 412 335.8 1.227  1383376_at  similar to Williams-Beuren syndrome critical region protein 18  Info   LOC368190 
   2013 0.0012414 26.2 39.5 0.663  1392525_at  Transcribed locus  Info    
   2014 0.0012417 70.7 33.6 2.104  1373920_at  Similar to 106 kDa O-GlcNAc transferase-interacting protein (predicted)  Info    
   2015 0.0012445 3389.2 2514.9 1.348  1370960_at  insulin-like growth factor binding protein 5  Info   Igfbp5 
   2016 0.0012481 90.7 154.9 0.586  1389378_at  CDC42 effector protein (Rho GTPase binding) 5 (predicted)  Info   Cdc42ep5_predicted 
   2017 0.0012519 221.4 356.9 0.62  1372404_at  RAS-related C3 botulinum substrate 2 (predicted)  Info   Rac2_predicted 
   2018 0.0012544 135 62.2 2.17  1373977_at  Kinesin family member 5C (predicted)  Info    
   2019 0.0012545 70.2 43.2 1.625  1383153_at  Transcribed locus  Info    
   2020 0.0012551 24.4 51.2 0.477  1371984_at  Similar to mKIAA0613 protein  Info    
   2021 0.0012557 1047.3 711.1 1.473  1375438_at  golgi SNAP receptor complex member 2  Info   Gosr2 
   2022 0.0012592 1972.5 1437.3 1.372  1386916_at  aconitase 1  Info   Aco1 
   2023 0.0012601 275.1 205 1.342  1374910_at    Info    
   2024 0.0012635 84.5 53.3 1.585  1379590_at  similar to U2 small nuclear ribonucleoprotein auxiliary factor 35 kDa subunit related-protein 1 (U2(RNU2) small nuclear RNA auxillary factor 1-like 1) (SP2)  Info   LOC498425 
   2025 0.0012636 153.8 195.6 0.786  1390264_at  similar to RIKEN cDNA 1200014J11 (predicted)  Info   RGD1308139_predicted 
   2026 0.0012636 56.7 115.9 0.489  1375224_at  pleckstrin homology-like domain, family A, member 3 (predicted)  Info   Phlda3_predicted 
   2027 0.0012652 125.2 198.1 0.632  1389254_at  Similar to hypothetical protein FLJ21924 (predicted)  Info    
   2028 0.0012659 365 518.2 0.704  1370007_at  protein disulfide isomerase related protein (calcium-binding protein, intestinal-related)  Info   Erp70 
   2029 0.0012672 286.3 366.2 0.782  1383290_at  hepatocyte growth factor activator inhibitor 1  Info   RGD:1303138 
   2030 0.0012683 21.6 38.3 0.564  1396109_at  Transcribed locus  Info    
   2031 0.0012685 26.2 50.1 0.523  1373287_at  similar to bHLH factor Math6  Info   LOC500200 
   2032 0.0012698 1.2 6.3 0.19  1397864_at  Transcribed locus  Info    
   2033 0.001273 79.5 109.8 0.724  1378016_at  Echinoderm microtubule associated protein like 4 (predicted)  Info    
   2034 0.0012734 669.6 534.4 1.253  1374293_at    Info    
   2035 0.0012741 1869.2 1190.7 1.57  1372774_at  Coenzyme Q6 homolog (yeast) (predicted)  Info    
   2036 0.0012747 1.8 7.3 0.247  1395757_at  Transcribed locus  Info    
   2037 0.0012756 161 330.3 0.487  1374802_at  similar to chromosome 14 open reading frame 94 (predicted)  Info   RGD1305288_predicted 
   2038 0.0012767 14.8 39.2 0.378  1390075_at  olfactomedin-like 2B (predicted)  Info   Olfml2b_predicted 
   2039 0.0012784 56.4 86.5 0.652  1393613_at  Transcribed locus  Info    
   2040 0.0012801 97.1 185.5 0.523  1387795_at  polymerase (DNA directed), alpha 2  Info   Pola2 
   2041 0.0012803 3552 2263.7 1.569  1367961_at  nerve growth factor, gamma  Info   Ngfg 
   2042 0.0012806 19.2 37 0.519  1385291_a_at  Synaptonemal complex protein SC65  Info   Sc65 
   2043 0.0012848 7.1 36 0.197  1395828_at  kinesin family member 23 (predicted)  Info   Kif23_predicted 
   2044 0.0012854 34.6 52.4 0.66  1385814_at  mitogen activated protein kinase kinase kinase 3 (predicted)  Info   Map3k3_predicted 
   2045 0.0012888 20 33.9 0.59  1384192_at  carbohydrate (keratan sulfate Gal-6) sulfotransferase 1 (predicted)  Info   Chst1_predicted 
   2046 0.0012891 39.7 14.9 2.664  1397392_at    Info    
   2047 0.0012905 33.5 60.7 0.552  1384773_at  similar to dendritic cell-derived ubiquitin-like protein (predicted)  Info   RGD1305506_predicted 
   2048 0.001294 596.3 745 0.8  1371865_at  similar to RIKEN cDNA 0610039A15 (predicted)  Info   RGD1311566_predicted 
   2049 0.001296 647.5 906.8 0.714  1370826_at  nucleosome assembly protein 1-like 1  Info   Nap1l1 
   2050 0.0012993 103.3 168.9 0.612  1376056_at  similar to poly (ADP-ribose) polymerase family, member 10  Info   LOC300043 
   2051 0.0013 94.9 129.7 0.732  1383548_at  Similar to hypothetical protein FLJ20189 (predicted)  Info    
   2052 0.0013053 405.4 650.8 0.623  1387005_at  cathepsin S  Info   Ctss 
   2053 0.001307 442.1 563.2 0.785  1383126_at  V-akt murine thymoma viral oncogene homolog 1  Info   Akt1 
   2054 0.001308 59.3 81.8 0.725  1374306_at  Transcribed locus  Info    
   2055 0.0013116 53.6 82.2 0.652  1383590_at  Similar to chondroitin beta1,4 N-acetylgalactosaminyltransferase (predicted)  Info    
   2056 0.0013124 246.8 390.9 0.631  1374545_at  ring finger and KH domain containing 2 (predicted)  Info   Rkhd2_predicted 
   2057 0.0013143 20.7 31 0.668  1381267_at  Transcribed locus  Info    
   2058 0.0013167 226.7 71.7 3.162  1389402_at  Transcribed locus  Info    
   2059 0.0013172 197.7 375 0.527  1372440_at  serine (or cysteine) proteinase inhibitor, clade E, member 2  Info   Serpine2 
   2060 0.0013184 32 44.9 0.713  1375176_at  similar to Sperm 1 POU-domain transcription factor (SPRM-1) (predicted)  Info   RGD1305526_predicted 
   2061 0.0013189 15.9 41.2 0.386  1391825_at  Similar to part of a novel protein  Info    
   2062 0.0013207 70.5 94.1 0.749  1391733_at  Tetratricopeptide repeat domain 4 (predicted)  Info    
   2063 0.0013236 2.5 14.8 0.169  1368501_s_at  mast cell protease 8 /// mast cell protease 10 /// mast cell protease 9 /// mast cell protease 8-like 2  Info   Mcpt8 /// Mcpt10 /// Mcpt9 /// RGD:1302938 
   2064 0.0013258 692.6 1222.9 0.566  1367866_at  fibulin 5  Info   Fbln5 
   2065 0.0013275 104.2 258.5 0.403  1368790_at  serine (or cysteine) proteinase inhibitor, clade A (alpha-1 antiproteinase, antitrypsin), member 10  Info   Serpina10 
   2066 0.0013284 69.8 111.7 0.625  1383355_at  ATP-binding cassette, sub-family A (ABC1), member 1 /// hypothetical gene supported by NM_178095  Info   Abca1 /// LOC497803 
   2067 0.0013307 80.6 135.5 0.595  1372004_at  heme binding protein 1 (predicted)  Info   Hebp1_predicted 
   2068 0.0013321 226 153.5 1.472  1393352_at  similar to hypothetical protein MGC38960 (predicted)  Info   RGD1310552_predicted 
   2069 0.0013331 34.2 62.1 0.551  1397772_at  Similar to putative membrane steroid receptor (predicted)  Info    
   2070 0.0013346 28.6 53.7 0.533  1382442_at  Similar to NF-kappa-B-repressing factor (NFKB-repressing factor) (Transcription factor NRF)  Info    
   2071 0.001336 82.8 136.6 0.606  1374830_at  similar to Coiled-coil domain containing protein 3 precursor  Info   LOC498795 
   2072 0.001338 122.4 162 0.756  1397628_at  similar to RIKEN cDNA 2900092E17 (predicted)  Info   RGD1305592_predicted 
   2073 0.0013402 1200.4 853 1.407  1368340_at  inositol polyphosphate multikinase  Info   Impk 
   2074 0.001343 116.4 315 0.37  1367850_at  Fc gamma receptor II beta  Info   LOC498276 
   2075 0.0013452 640.3 884.2 0.724  1372770_at    Info    
   2076 0.0013483 123.2 209.4 0.588  1372912_at  Mitogen-activated protein kinase kinase kinase 7 interacting protein 1 (predicted)  Info    
   2077 0.0013487 222.5 334.9 0.664  1382308_at  Transcribed locus  Info    
   2078 0.0013498 35.7 70.8 0.504  1377153_a_at  kelch-like 6 (Drosophila) (predicted)  Info   Klhl6_predicted 
   2079 0.0013503 46.4 87 0.533  1395342_at  cytoskeleton-associated protein 4 (predicted)  Info   Ckap4_predicted 
   2080 0.0013507 39.9 63 0.633  1385718_at  similar to RIKEN cDNA 2510048L02 (predicted)  Info   RGD1308031_predicted 
   2081 0.0013513 190.1 240.7 0.79  1374368_at  CDNA clone IMAGE:7389555  Info    
   2082 0.0013573 529.9 154.8 3.423  1387788_at  Jun-B oncogene  Info   Junb 
   2083 0.0013575 93.5 146.7 0.637  1376835_at  Solute carrier family 35, member B2  Info   Slc35b2 
   2084 0.0013583 84.2 124.6 0.676  1380583_s_at  colony stimulating factor 1 (macrophage)  Info   Csf1 
   2085 0.0013599 138.3 82.2 1.682  1376955_at  procollagen, type IV, alpha 4 (predicted)  Info   Col4a4_predicted 
   2086 0.0013611 120 167.6 0.716  1398599_at  similar to integral membrane protein 1  Info   LOC500972 
   2087 0.0013614 18.2 44.5 0.409  1385292_at  Synaptonemal complex protein SC65  Info   Sc65 
   2088 0.0013619 287.9 369.8 0.779  1387227_at  Wiskott-Aldrich syndrome protein interacting protein  Info   Waspip 
   2089 0.0013635 16.6 40.4 0.411  1379740_at  Similar to chromosome 18 open reading frame 54  Info    
   2090 0.001364 78.4 135.7 0.578  1386096_at  similar to Metal-response element-binding transcription factor 2 (Metal-response element DNA-binding protein M96) (predicted)  Info   RGD1304727_predicted 
   2091 0.0013643 129.5 205.5 0.63  1372430_at  similar to RIKEN cDNA 1700037H04 (predicted)  Info   RGD1311739_predicted 
   2092 0.0013661 77.6 128.8 0.602  1370131_at  caveolin  Info   Cav 
   2093 0.0013671 17.7 29.5 0.6  1391676_at  Transcribed locus  Info    
   2094 0.0013729 135.4 236 0.574  1382431_at  Hypothetical gene supported by NM_178095  Info   Abca1 
   2095 0.0013743 186.5 144.2 1.293  1377949_s_at    Info    
   2096 0.0013745 684.5 832.7 0.822  1371189_x_at  laminin receptor 1 (67kD, ribosomal protein SA) /// similar to 40S ribosomal protein SA (p40) (34/67 kDa laminin receptor) /// similar to Ribosomal protein SA  Info   Lamr1 /// LOC367030 /// LOC367303 
   2097 0.0013748 66.9 116.6 0.574  1392863_at  Similar to fibronectin leucine rich transmembrane protein 3  Info    
   2098 0.0013751 57.2 81.1 0.705  1393972_at  Transcribed locus  Info    
   2099 0.0013764 186.9 323.3 0.578  1371862_at  ribonucleotide reductase M1  Info   Rrm1 
   2100 0.0013778 480.3 386.6 1.242  1371431_at  Transcribed locus, strongly similar to XP_232343.3 PREDICTED: similar to Pex5 protein [Rattus norvegicus]  Info    
   2101 0.0013819 39.3 49.4 0.796  1370705_at  zinc finger protein HIT-4  Info   RGD:628674 
   2102 0.0013835 24.6 36.8 0.668  1397282_at  Transcribed locus  Info    
   2103 0.0013865 2711.5 3301.2 0.821  1371313_at  similar to 60S ribosomal protein L23a  Info   LOC501709 
   2104 0.0013866 424.1 303 1.4  1372722_at  Transcribed locus  Info    
   2105 0.0013877 1532.2 1006.3 1.523  1374871_at  asparaginase-like sperm autoantigen  Info   Asrgl1 
   2106 0.0013881 51.7 90.2 0.573  1386252_at  Transcribed locus  Info    
   2107 0.0013925 37.8 72.2 0.524  1370400_at  RT1 class Ib gene, H2-TL-like, grc region (N1) /// RT1 class Ib gene, H2-TL-like, grc region (N3) /// RT1 class Ib gene, H2-TL-like, grc region(N2)  Info   RT1-N1 /// RT1-N3 /// RT1-N2 
   2108 0.0013928 357.6 148 2.416  1393732_at  Transcribed locus  Info    
   2109 0.0013932 40.6 56.4 0.72  1392847_at  Casitas B-lineage lymphoma-like 1 (predicted)  Info   Cbll1_predicted 
   2110 0.0013939 14.1 55.2 0.255  1382601_at  Similar to scavenger receptor type A SR-A  Info    
   2111 0.0013954 45.6 60.6 0.752  1382158_at  Transcribed locus  Info    
   2112 0.0013984 95 67.2 1.414  1391858_at  splicing factor, arginine/serine-rich 8 (suppressor-of-white-apricot homolog, Drosophila) (predicted)  Info   Sfrs8_predicted 
   2113 0.0014033 76.6 116.6 0.657  1398472_at  progressive external ophthalmoplegia 1 (predicted)  Info   Peo1_predicted 
   2114 0.0014075 107.3 142.7 0.752  1384383_at  similar to Putative lysophosphatidic acid acyltransferase (predicted)  Info   RGD1310520_predicted 
   2115 0.0014116 70.6 118.8 0.594  1376451_at  similar to krev interaction trapped-1A  Info   LOC362317 
   2116 0.0014117 179.9 239.3 0.752  1389197_at  similar to RIKEN cDNA 9630046K23 (predicted)  Info   RGD1306248_predicted 
   2117 0.0014132 96.6 138.1 0.699  1374134_at  PCTAIRE-motif protein kinase 2  Info    
   2118 0.0014146 174.6 123.8 1.41  1379431_at  Transcribed locus  Info    
   2119 0.001415 1417.9 1132.8 1.252  1390807_at  carbonic anhydrase VB, mitochondrial  Info   Ca5b 
   2120 0.0014157 1972.8 1451.2 1.359  1368575_at  solute carrier family 6 (neurotransmitter transporter), member 18  Info   Slc6a18 
   2121 0.0014185 23.9 56 0.427  1387131_at  serine (or cysteine) peptidase inhibitor, clade I, member 1  Info   Serpini1 
   2122 0.0014208 36.6 76.4 0.479  1391537_at  similar to SERTA domain containing 4  Info   LOC360899 
   2123 0.0014229 734.3 860.9 0.853  1372052_at  Budding uninhibited by benzimidazoles 3 homolog (S. cerevisiae) (predicted)  Info    
   2124 0.0014234 68.3 110 0.621  1389366_at  similar to Zinc finger protein 553  Info   LOC308991 
   2125 0.001427 850.9 587.7 1.448  1367771_at  delta sleep inducing peptide, immunoreactor  Info   Dsipi 
   2126 0.0014289 858.7 1055.2 0.814  1387884_at  proteasome (prosome, macropain) subunit, alpha type 5  Info   Psma5 
   2127 0.0014292 1976.5 2558 0.773  1367663_at  protease (prosome, macropain) 28 subunit, alpha  Info   Psme1 
   2128 0.0014321 81.6 120.5 0.677  1368379_at  CD36 antigen (collagen type I receptor, thrombospondin receptor)-like 2  Info   Scarb2 
   2129 0.0014325 2567 3022.4 0.849  1388244_s_at  laminin receptor 1 (67kD, ribosomal protein SA)  Info   Lamr1 
   2130 0.0014347 54.6 90.4 0.604  1381481_at  Similar to regulator of sex-limitation candidate 16  Info    
   2131 0.0014362 114.8 139.3 0.824  1374207_at  angiopoietin 2  Info   Agpt2 
   2132 0.001438 386.7 508.9 0.76  1372015_at  transforming, acidic coiled-coil containing protein 1a  Info   RGD:1303120 
   2133 0.0014412 79.6 145.2 0.548  1388527_at  Transcribed locus  Info    
   2134 0.0014421 52.3 77.9 0.671  1389664_at  similar to hypothetical protein (predicted)  Info   RGD1305937_predicted 
   2135 0.0014434 1908.6 1373.4 1.39  1370020_at  solute carrier family 25 (mitochondrial carrier; dicarboxylate transporter), member 10  Info   RGD:621430 
   2136 0.0014434 440 337.8 1.303  1389744_at  LOC499898  Info   LOC499898 
   2137 0.0014482 205.8 157.9 1.303  1387434_at  solute carrier family 22 (organic cation transporter), member 4  Info   Slc22a4 
   2138 0.0014499 25.7 67.8 0.379  1392927_at  progestin and adipoQ receptor family member IV (predicted)  Info   Paqr4_predicted 
   2139 0.0014509 124.6 91.1 1.368  1392806_at  Wolf-Hirschhorn syndrome candidate 1-like 1 (predicted)  Info    
   2140 0.001453 212.4 165.7 1.282  1398361_at  similar to Rho-GTPase-activating protein PS-GAP-a  Info   LOC498938 
   2141 0.0014531 227.9 171.1 1.332  1368736_at  testis specific X-linked gene  Info   Tsx 
   2142 0.0014556 95.9 239.2 0.401  1368943_at  ribonuclease, RNase A family 4  Info   Rnase4 
   2143 0.0014582 6.8 14.4 0.472  1385116_at    Info    
   2144 0.0014583 19.6 48 0.408  1372161_at  Transcribed locus  Info    
   2145 0.0014603 61.3 92.4 0.663  1384282_at  zinc finger proliferation 1 (predicted)  Info   Zipro1_predicted 
   2146 0.0014606 1965.3 1698.1 1.157  1371253_at  electron transferring flavoprotein, alpha polypeptide  Info   Etfa 
   2147 0.0014607 18.8 34.2 0.55  1393564_at  similar to Ubiquitin ligase protein DZIP3 (DAZ-interacting protein 3 homolog)  Info   LOC303963 
   2148 0.0014615 1371.2 979.7 1.4  1370547_at  pregnancy-zone protein  Info   Pzp 
   2149 0.0014634 117.8 157.2 0.749  1390947_at  Similar to RIKEN cDNA D930036F22 gene  Info    
   2150 0.001465 257.3 193.2 1.332  1373230_at  similar to cDNA sequence BC036718 (predicted)  Info   RGD1311802_predicted 
   2151 0.0014681 75.7 111.5 0.679  1373214_at  KDEL (Lys-Asp-Glu-Leu) containing 1 (predicted)  Info   Kdelc1_predicted 
   2152 0.0014702 77.2 49.7 1.553  1368511_at  Basic helix-loop-helix domain containing, class B3  Info   Bhlhb3 
   2153 0.0014706 628.7 427.7 1.47  1370333_a_at  insulin-like growth factor 1  Info   Igf1 
   2154 0.0014714 266.9 98.4 2.712  1367977_at  synuclein, alpha  Info   Snca 
   2155 0.0014729 152.9 67.5 2.265  1396942_at  hypothetical LOC301267  Info   LOC301267 
   2156 0.0014745 5061.9 6118.1 0.827  1384548_at  ribosomal protein L32  Info   Rpl32 
   2157 0.0014759 19.7 37.8 0.521  1397554_at  Transcribed locus  Info    
   2158 0.0014763 981.3 748.6 1.311  1374396_at  ATPase, H+ transporting, V1 subunit C, isoform 1 (predicted)  Info   Atp6v1c1_predicted 
   2159 0.0014791 12.8 55.1 0.232  1383581_at    Info    
   2160 0.0014793 109.3 74.9 1.459  1385994_at  sporulation protein, meiosis-specific, SPO11 homolog (S. cerevisiae) (predicted)  Info   Spo11_predicted 
   2161 0.0014806 53.9 25.8 2.089  1382083_at  coagulation factor C homolog (Limulus polyphemus) (predicted)  Info   Coch_predicted 
   2162 0.0014848 74.5 120.5 0.618  1368927_at  membrane bound C2 domain containing protein  Info   Mbc2 
   2163 0.0014864 94.4 128.2 0.736  1377286_at  GTPase, IMAP family member 4  Info   Gimap4 
   2164 0.001487 1719.8 2205.4 0.78  1383855_at    Info    
   2165 0.001487 71.8 102.8 0.698  1373089_at  cadherin 3, type 1, P-cadherin (placental)  Info   Cdh3 
   2166 0.0014889 592.1 442.1 1.339  1383248_at  flavin containing monooxygenase 5  Info   Fmo5 
   2167 0.0014896 417.3 608.9 0.685  1373302_at  LOC500488  Info   LOC500488 
   2168 0.0014901 454 307.5 1.476  1382611_at  Transcribed locus  Info    
   2169 0.0014903 20.4 41.7 0.489  1379799_at  Transcribed locus  Info    
   2170 0.0014913 4 20.8 0.192  1374683_at  gamma sarcoglycan  Info   RGD:1359577 
   2171 0.0014918 471.8 680.9 0.693  1372029_at  similar to mannose-6-phosphate receptor binding protein 1  Info   LOC316130 
   2172 0.0014938 1.1 1.9 0.579  1392235_at  GLI-Kruppel family member GLI3  Info   Gli3 
   2173 0.0014965 256.3 186.2 1.376  1389299_at  Phosphate cytidylyltransferase 1, choline, alpha isoform  Info   Pcyt1a 
   2174 0.0014965 78.7 29.9 2.632  1376913_at    Info    
   2175 0.0014976 154.2 206.1 0.748  1387064_at  peroxisomal membrane protein 3  Info   Pxmp3 
   2176 0.0014979 63.4 88.7 0.715  1382294_at  Activated leukocyte cell adhesion molecule  Info   Alcam 
   2177 0.0015 164.9 212.1 0.777  1395146_at  adiponectin receptor 2 (predicted)  Info   Adipor2_predicted 
   2178 0.0015002 1164.6 1426.4 0.816  1371395_at  chromobox homolog 3 (HP1 gamma homolog, Drosophila)  Info   Cbx3 
   2179 0.0015026 77.3 116.8 0.662  1399148_s_at  similar to hypothetical protein MGC38524 (predicted)  Info   RGD1309002_predicted 
   2180 0.0015031 1044.1 1246.3 0.838  1388134_at  eukaryotic translation elongation factor 1 delta (guanine nucleotide exchange protein)  Info   Eef1d 
   2181 0.0015069 359.3 250 1.437  1373053_at    Info    
   2182 0.0015086 22 47.5 0.463  1374763_at  cerebral endothelial cell adhesion molecule 1 (predicted)  Info   Ceecam1_predicted 
   2183 0.0015093 280.2 173.4 1.616  1373321_at  Transcribed locus, strongly similar to XP_574622.1 PREDICTED: similar to UPF0197 protein C11orf10 homolog [Rattus norvegicus]  Info    
   2184 0.0015107 13.4 33.2 0.404  1376215_at  similar to hypothetical protein 9630025C22  Info   LOC499558 
   2185 0.0015128 242.5 177.7 1.365  1390437_at  Transcribed locus  Info    
   2186 0.0015137 233.3 316.4 0.737  1374438_at    Info    
   2187 0.0015143 203.1 149.9 1.355  1375684_at  neuraminidase 1  Info   Neu1 
   2188 0.0015146 74.6 133.9 0.557  1382993_at  Bcl-2 binding component 3  Info   Bbc3 
   2189 0.0015167 23.1 46.7 0.495  1369208_at  interleukin 7  Info   Il7 
   2190 0.0015175 97.1 153.6 0.632  1368679_a_at  Yamaguchi sarcoma viral (v-yes-1) oncogene homolog  Info   Lyn 
   2191 0.0015208 233.8 365.4 0.64  1373864_at  mitogen-activated protein kinase kinase kinase kinase 4 (predicted)  Info   Map4k4_predicted 
   2192 0.0015226 2621.1 1781.8 1.471  1368329_at  solute carrier family 22 (organic anion transporter), member 6  Info   Slc22a6 
   2193 0.0015252 146.1 198.6 0.736  1372007_at  similar to AOF2 protein  Info   LOC500569 
   2194 0.0015254 434.6 283.2 1.535  1392952_at  hypothetical protein LOC619561  Info   LOC619561 
   2195 0.0015272 10.3 25.3 0.407  1368162_at  cystatin E/M  Info   Cst6 
   2196 0.0015292 306.3 188.3 1.627  1370385_at  phospholipase A2, group VI  Info   Pla2g6 
   2197 0.00153 205.3 239.1 0.859  1389596_at    Info    
   2198 0.0015305 82.2 122.1 0.673  1382334_at  Transcribed locus  Info    
   2199 0.0015326 633.8 749 0.846  1374296_at  Leucine-rich repeat-containing 8 (predicted)  Info    
   2200 0.0015331 146.5 194.2 0.754  1372668_at  LIM domains containing 1 (predicted)  Info    
   2201 0.0015334 8.3 23.9 0.347  1380277_at  RAD51 associated protein 1 (predicted)  Info    
   2202 0.0015348 43.6 77.8 0.56  1381014_at  interferon-induced protein 44 (predicted)  Info   Ifi44_predicted 
   2203 0.0015354 2.9 15.8 0.184  1381771_at  Transcribed locus  Info    
   2204 0.0015362 1968.5 1726.7 1.14  1370060_at  solute carrier family 25 (mitochondrial carrier; oxoglutarate carrier), member 11  Info   Slc25a11 
   2205 0.0015364 15.6 51 0.306  1374015_at  Transcribed locus  Info    
   2206 0.0015366 2.1 3.1 0.677  1370210_at  Ras-induced senescence 1  Info   Ris1 
   2207 0.0015366 150.5 201 0.749  1387624_at  upstream transcription factor 1  Info   Usf1 
   2208 0.001538 27.5 56.1 0.49  1393247_at  Similar to zinc finger protein 560  Info    
   2209 0.0015408 410.4 320.5 1.28  1390128_at    Info    
   2210 0.001543 15.9 33.6 0.473  1380537_at  hematopoietic protein 1 (predicted)  Info   Hem1_predicted 
   2211 0.0015459 3.8 35.2 0.108  1381833_at  cell division cycle associated 2 (predicted)  Info   Cdca2_predicted 
   2212 0.0015464 127.5 233.3 0.547  1384544_at  paraoxonase 3  Info   RGD:1302965 
   2213 0.0015487 1738.2 1480.5 1.174  1388774_at    Info    
   2214 0.0015539 45.3 65.9 0.687  1397372_at  similar to Mob4B protein /// MOB1, Mps One Binder kinase activator-like 1B (yeast) (predicted)  Info   LOC308302 /// Mobk1b_predicted 
   2215 0.0015551 66.9 104.7 0.639  1391170_at  similar to mKIAA1757 protein (predicted)  Info   RGD1310433_predicted 
   2216 0.0015558 13.7 24.5 0.559  1392142_at  Transcribed locus  Info    
   2217 0.0015579 557.4 973 0.573  1370249_at  benzodiazepin receptor  Info   Bzrp 
   2218 0.001559 49.9 80.2 0.622  1384699_at  Transcribed locus  Info    
   2219 0.0015631 13.1 32.7 0.401  1369173_at  complement component 3a receptor 1  Info   C3ar1 
   2220 0.0015641 183.5 103.5 1.773  1387234_at  alpha-2-glycoprotein 1, zinc  Info   Azgp1 
   2221 0.0015645 128.2 176.3 0.727  1389580_at  LOC499580  Info   LOC499580 
   2222 0.0015661 18.1 33.6 0.539  1382587_at  Family with sequence similarity 33, member A (predicted)  Info   MGC109093 
   2223 0.0015676 234.8 318.6 0.737  1374538_at  O-linked mannose beta1,2-N-acetylglucosaminyltransferase  Info   RGD:1359396 
   2224 0.00157 136.3 238.8 0.571  1371032_at  nidogen (entactin)  Info   Nid 
   2225 0.0015712 2371.5 1678.8 1.413  1370818_at  2-4-dienoyl-Coenzyme A reductase 2, peroxisomal  Info   Decr2 
   2226 0.001572 22.8 55.8 0.409  1374635_at  A disintegrin and metalloprotease domain 23 (predicted)  Info    
   2227 0.0015721 219.7 151.9 1.446  1387765_at  mannose binding lectin 1, protein A  Info   Mbl1 
   2228 0.0015747 15.4 31.3 0.492  1394826_at  Similar to Fanconi anemia, complementation group F  Info    
   2229 0.0015748 427.5 308.6 1.385  1388623_at  EH-domain containing 1 (predicted)  Info    
   2230 0.0015792 346.8 176.6 1.964  1387882_at  basic transcription element binding protein 1  Info   Bteb1 
   2231 0.0015831 86.5 113.3 0.763  1387678_at  neuroblastoma RAS viral (v-ras) oncogene homolog  Info   Nras 
   2232 0.0015842 199.2 155.1 1.284  1372969_at  F-box and leucine-rich repeat protein 3a (predicted)  Info   Fbxl3a_predicted 
   2233 0.0015849 83.7 140.3 0.597  1384310_at  Transcribed locus  Info    
   2234 0.0015853 52.9 85.7 0.617  1379304_at  similar to PRKR interacting protein 1 (IL11 inducible)  Info   LOC498171 
   2235 0.0015885 31.7 60.9 0.521  1382387_at  Transmembrane protein 16A (predicted)  Info    
   2236 0.0015898 31.7 64.5 0.491  1378198_at  Transcribed locus  Info    
   2237 0.0015902 258.7 418.8 0.618  1388460_at  capping protein (actin filament), gelsolin-like (predicted)  Info   Capg_predicted 
   2238 0.0015918 493 820.3 0.601  1372299_at  cyclin-dependent kinase inhibitor 1C (P57)  Info   Cdkn1c 
   2239 0.0015944 281.2 386.7 0.727  1380045_at  pyruvate dehydrogenase phosphatase isoenzyme 2  Info   Pdp2 
   2240 0.0015965 1037 742.5 1.397  1369169_at  solute carrier family 23 (nucleobase transporters), member 1  Info   Slc23a1 
   2241 0.0016003 449.7 328.1 1.371  1388636_at  similar to RIKEN cDNA 5730408C10 (predicted)  Info   RGD1305972_predicted 
   2242 0.0016017 79.8 133.4 0.598  1374081_at  Similar to H63 breast cancer expressed gene isoform a; gene associated with HER-2/neu overexpression (predicted)  Info    
   2243 0.0016035 466.1 332 1.404  1392612_at  Prolactin receptor  Info   Prlr 
   2244 0.0016037 260.6 329.6 0.791  1372399_at  similar to cofactor of BRCA1; negative elongation factor protein B (predicted)  Info   RGD1307832_predicted 
   2245 0.0016048 852 1319.6 0.646  1388726_a_at    Info    
   2246 0.0016061 59.3 115.6 0.513  1374849_at  a disintegrin-like and metalloprotease (reprolysin type) with thrombospondin type 1 motif, 7 (predicted)  Info   Adamts7_predicted 
   2247 0.001609 265.7 202.8 1.31  1393165_at  similar to RIKEN cDNA 2310028N02  Info   RGD:1359339 
   2248 0.0016138 30.9 51.3 0.602  1382746_s_at  Similar to lymphocyte alpha-kinase (predicted)  Info    
   2249 0.0016146 12.9 28.5 0.453  1388949_at  Transcribed locus  Info    
   2250 0.0016149 44.8 73.2 0.612  1387928_at  Rap2 interacting protein  Info   Rap2ip 
   2251 0.0016156 275.1 426.5 0.645  1368474_at  vascular cell adhesion molecule 1  Info   Vcam1 
   2252 0.0016161 580.9 824 0.705  1369991_at  signal peptidase complex 18kD  Info   Spc18 
   2253 0.0016221 1061.1 684.6 1.55  1394020_at  Transcribed locus  Info    
   2254 0.0016252 46.5 86.5 0.538  1367825_at  ral guanine nucleotide dissociation stimulator  Info   Ralgds 
   2255 0.0016258 75.2 133 0.565  1369518_at  phosphatidylinositol 3 kinase, regulatory subunit, polypeptide 3  Info   Pik3r3 
   2256 0.0016295 82.5 40.7 2.027  1380365_at  Transcribed locus  Info    
   2257 0.0016327 1.3 2.9 0.448  1375319_at  Transmembrane protein 16D (eight membrane-spanning domains) (predicted)  Info    
   2258 0.0016335 22.3 46.9 0.475  1383020_at    Info    
   2259 0.0016372 63.4 98.8 0.642  1394940_at  similar to hypothetical protein FLJ20037 (predicted)  Info   RGD1311381_predicted 
   2260 0.0016409 800.7 668.7 1.197  1374200_at  solute carrier family 29 (nucleoside transporters), member 3  Info   Slc29a3 
   2261 0.0016411 112.2 161.7 0.694  1391507_at  similar to zinc finger protein EZI  Info   LOC500110 
   2262 0.0016433 536.6 265.4 2.022  1376808_at  Transcribed locus  Info    
   2263 0.0016436 168.5 269.2 0.626  1372748_at  similar to KIAA1055 protein (predicted)  Info   RGD1307436_predicted 
   2264 0.0016462 91.7 173.3 0.529  1393653_at  similar to putative protein product of HMFN2073  Info   LOC501174 
   2265 0.0016516 439.7 356.5 1.233  1370134_at  acetyl-CoA transporter  Info   Slc33a1 
   2266 0.0016534 116.8 164.6 0.71  1393249_at  Friend leukemia integration 1 (predicted)  Info    
   2267 0.0016539 70 112.1 0.624  1393799_at  Unc-5 homolog B (C. elegans)  Info   Unc5b 
   2268 0.0016556 82.2 155.8 0.528  1373363_at  microtubule-associated protein 1b  Info   Map1b 
   2269 0.0016571 3802.8 3438 1.106  1371922_at  Transcribed locus  Info    
   2270 0.0016579 163.2 181 0.902  1375432_at  DD6A4-12(7)-2 mRNA, partial sequence  Info    
   2271 0.001658 38.8 6.3 6.159  1396646_at  similar to RIKEN cDNA 4930431B11 gene (predicted)  Info   RGD1308555_predicted 
   2272 0.0016585 71.3 53.2 1.34  1391702_at  Similar to hypothetical protein FLJ20626 (predicted)  Info    
   2273 0.0016614 50.5 113.8 0.444  1370422_at  receptor-interacting serine-threonine kinase 3  Info   Ripk3 
   2274 0.0016619 26.7 50.2 0.532  1378321_at  Ras association (RalGDS/AF-6) domain family 4 (predicted)  Info   Rassf4_predicted 
   2275 0.0016638 400.3 344 1.164  1383171_at  transcription factor B2, mitochondrial (predicted)  Info   Tfb2m_predicted 
   2276 0.0016695 1077.8 662.1 1.628  1369682_at  transcription factor 2  Info   Tcf2 
   2277 0.0016726 98.3 155.6 0.632  1379423_at  Transcribed locus, strongly similar to NP_033131.2 Ras suppressor protein 1 [Mus musculus]  Info    
   2278 0.001674 54.9 16.8 3.268  1390951_at  Transcribed locus  Info    
   2279 0.0016756 181.6 245.3 0.74  1372990_at  cAMP responsive element binding protein 3 (predicted)  Info   Creb3_predicted 
   2280 0.0016766 585.4 739.4 0.792  1373577_at  Neuropilin 1  Info   Nrp 
   2281 0.0016767 3609.2 4308.8 0.838  1398789_at  ribosomal protein L37  Info   Rpl37 
   2282 0.0016768 149.5 188.7 0.792  1377945_at  similar to RIKEN cDNA 2310005B10  Info   MGC95311 
   2283 0.0016782 786.6 601.4 1.308  1370897_at  Branched chain ketoacid dehydrogenase E1, alpha polypeptide  Info   Bckdha 
   2284 0.0016786 264.6 402 0.658  1375857_at  similar to Myoferlin (Fer-1 like protein 3)  Info   LOC309499 
   2285 0.0016807 210.4 127.8 1.646  1380117_at  Transcribed locus  Info    
   2286 0.0016848 5.8 28.3 0.205  1392040_at  similar to 2810453L12Rik protein (predicted)  Info   RGD1306255_predicted 
   2287 0.0016866 75.6 89.5 0.845  1379560_at  nuclear receptor binding SET domain protein 1 (predicted)  Info   Nsd1_predicted 
   2288 0.0016875 307.2 447.6 0.686  1388480_at  similar to Glycolipid transfer protein  Info   LOC288707 
   2289 0.0016885 24.4 39.5 0.618  1368598_at  somatostatin receptor 3 /// hypothetical gene supported by NM_133522  Info   Sstr3 /// SSTR 
   2290 0.0016896 60.2 85.6 0.703  1376812_at  Transcribed locus  Info    
   2291 0.0016907 36.8 65.8 0.559  1376317_at    Info    
   2292 0.0016936 1187 1581.6 0.751  1370859_at  thioredoxin domain containing 7  Info   Txndc7 
   2293 0.0016957 29.5 45.6 0.647  1385557_at  Transcribed locus  Info    
   2294 0.001696 390.7 266.8 1.464  1383654_a_at  similar to fructosamine-3-kinase  Info   Fnsk 
   2295 0.0016974 10.3 36.2 0.285  1392449_at  RAD18 homolog (S. cerevisiae) (predicted)  Info   Rad18_predicted 
   2296 0.0016979 6 48 0.125  1377201_at  Similar to 1700054N08Rik protein  Info    
   2297 0.0017024 366.1 550.9 0.665  1388312_at  Transcribed locus  Info    
   2298 0.0017025 26.2 46.9 0.559  1377982_at  deltex 4 homolog (Drosophila) (predicted)  Info   Dtx4_predicted 
   2299 0.0017042 91.8 68 1.35  1373351_at  Transcribed locus  Info    
   2300 0.0017047 5.6 42.5 0.132  1394031_at  similar to Protein C20orf129 homolog  Info   LOC311598 
   2301 0.0017063 43.3 3.6 12.028  1367739_at  Cytochrom c oxidase subunit VIII-H (heart/muscle)  Info   Cox8h 
   2302 0.0017096 25.3 40.6 0.623  1386162_at  TXK tyrosine kinase (predicted)  Info   Txk_predicted 
   2303 0.0017098 205.3 162.7 1.262  1374174_at  Transcribed locus  Info    
   2304 0.001712 114.3 134.4 0.85  1383465_at  F-box and leucine-rich repeat protein 14 (predicted)  Info   Fbxl14_predicted 
   2305 0.0017124 9.1 14.1 0.645  1380374_at  Transcribed locus  Info    
   2306 0.0017147 497.5 761.9 0.653  1387918_at  tropomyosin 3, gamma  Info   Tpm3 
   2307 0.0017155 87.9 57.3 1.534  1380219_at    Info    
   2308 0.0017189 47.9 73.3 0.653  1393683_at  similar to KIAA0368 (predicted)  Info   RGD1306148_predicted 
   2309 0.0017209 999 563.4 1.773  1383559_at  DEAD (Asp-Glu-Ala-Asp) box polypeptide 19  Info   Ddx19 
   2310 0.0017213 40.5 69.4 0.584  1376814_at  Transcribed locus  Info    
   2311 0.0017229 513.4 328.1 1.565  1369493_at  prolactin receptor  Info   Prlr 
   2312 0.001724 19.8 43 0.46  1392794_at  Transcribed locus  Info    
   2313 0.0017282 898.5 1085.4 0.828  1374646_at  Casein kinase 1, alpha 1  Info   Csnk1a1 
   2314 0.001731 140.5 195.6 0.718  1385883_at  Transcribed locus  Info    
   2315 0.0017318 487.6 584.5 0.834  1372739_at  sarcoma amplified sequence (predicted)  Info   Sas_predicted 
   2316 0.001732 297.1 422.6 0.703  1373818_at  Potassium channel tetramerisation domain containing 12 (predicted)  Info    
   2317 0.0017335 88.4 153.1 0.577  1393144_at  N-myc (and STAT) interactor (predicted)  Info    
   2318 0.0017381 88.2 166.1 0.531  1388166_at  similar to Igh-6 protein /// similar to Ig H-chain V-region precursor /// similar to single chain Fv antibody fragment scFv 7-10A /// similar to Ig heavy chain V region MC101 precursor  Info   RGD:1359202 /// LOC299458 /// LOC314509 /// LOC366747 
   2319 0.0017418 939.8 1285 0.731  1398883_at  heterogeneous nuclear ribonucleoprotein A2/B1 (predicted)  Info   Hnrpa2b1_predicted 
   2320 0.0017424 3.4 16.7 0.204  1394922_at    Info    
   2321 0.0017444 191 252.9 0.755  1368643_at  spermatogenesis associated 6  Info   Spata6 
   2322 0.0017458 24.2 42.4 0.571  1378914_a_at  hypothetical protein LOC308990  Info   LOC308990 
   2323 0.0017461 81.2 28.5 2.849  1392921_at  Transcribed locus  Info    
   2324 0.0017488 237.7 296.2 0.802  1388397_at  EBNA1 binding protein 2 (predicted)  Info   Ebna1bp2_predicted 
   2325 0.001754 43.8 66.7 0.657  1387395_at  adenosine A2B receptor  Info   Adora2b 
   2326 0.0017545 492.9 826.3 0.597  1371500_at  latent transforming growth factor beta binding protein 4 (predicted)  Info   Ltbp4_predicted 
   2327 0.0017562 15.2 19.5 0.779  1370068_at  phospholipase A2, group V  Info   Pla2g5 
   2328 0.0017597 373.6 582.2 0.642  1372459_at  vasodilator-stimulated phosphoprotein (predicted)  Info   Vasp_predicted 
 
  
 
    
'Observed v. Expected' table of GO classes and parent classes, in list of 2328 genes shown above:
    
Only GO classes and parent classes with at least 5 observations in the selected subset and with an 'Observed vs. Expected' ratio of at least 2 are shown.  
 Cellular Component 
 
  
  GO id GO classification Observed in selected subset Expected in selected subset Observed/ Expected
  0001527 microfibril  5 0.46 10.9
  0043205 fibril  5 0.55 9.08
  0005830 cytosolic ribosome (sensu Eukaryota)  5 0.83 6.06
  0005876 spindle microtubule  6 1.01 5.95
  0005788 endoplasmic reticulum lumen  6 1.19 5.03
  0005605 basal lamina  6 1.38 4.36
  0043233 organelle lumen  7 1.93 3.63
  0005884 actin filament  5 1.56 3.21
  0005604 basement membrane  8 2.57 3.11
  0005819 spindle  13 5.5 2.36
  0042579 microbody  12 5.5 2.18
  0005777 peroxisome  12 5.5 2.18
  0031012 extracellular matrix  44 20.73 2.12
  0000228 nuclear chromosome  7 3.3 2.12
  0005578 extracellular matrix (sensu Metazoa)  42 20.28 2.07
  0045177 apical part of cell  5 2.48 2.02
 
  
 Molecular Function 
 
  
  GO id GO classification Observed in selected subset Expected in selected subset Observed/ Expected
  0016538 cyclin-dependent protein kinase regulator activity  6 1.52 3.93
  0005201 extracellular matrix structural constituent  14 3.59 3.9
  0005520 insulin-like growth factor binding  6 1.97 3.04
  0019838 growth factor binding  9 3.23 2.79
  0001871 pattern binding  14 5.38 2.6
  0030247 polysaccharide binding  13 5.29 2.46
  0008757 S-adenosylmethionine-dependent methyltransferase activity  6 2.6 2.31
  0005539 glycosaminoglycan binding  11 4.93 2.23
  0008017 microtubule binding  6 2.96 2.03
 
  
 Biological Process 
 
  
  GO id GO classification Observed in selected subset Expected in selected subset Observed/ Expected
  0050670 regulation of lymphocyte proliferation  5 1.4 3.57
  0051240 positive regulation of organismal physiological process  9 2.6 3.46
  0007519 myogenesis  9 2.6 3.46
  0048747 muscle fiber development  5 1.5 3.33
  0048741 skeletal muscle fiber development  5 1.5 3.33
  0048637 skeletal muscle development  5 1.5 3.33
  0009063 amino acid catabolism  9 2.7 3.33
  0000096 sulfur amino acid metabolism  5 1.5 3.33
  0051241 negative regulation of organismal physiological process  6 1.9 3.16
  0006817 phosphate transport  18 6 3
  0009069 serine family amino acid metabolism  5 1.7 2.94
  0044270 nitrogen compound catabolism  10 3.7 2.7
  0000087 M phase of mitotic cell cycle  12 4.5 2.67
  0006260 DNA replication  22 8.3 2.65
  0046651 lymphocyte proliferation  5 1.9 2.63
  0050778 positive regulation of immune response  6 2.3 2.61
  0009967 positive regulation of signal transduction  9 3.5 2.57
  0009310 amine catabolism  9 3.5 2.57
  0007067 mitosis  11 4.3 2.56
  0043123 positive regulation of I-kappaB kinase/NF-kappaB cascade  8 3.2 2.5
  0042110 T cell activation  9 3.6 2.5
  0007229 integrin-mediated signaling pathway  5 2 2.5
  0050776 regulation of immune response  11 4.5 2.45
  0008284 positive regulation of cell proliferation  13 5.3 2.45
  0015698 inorganic anion transport  21 9 2.33
  0006261 DNA-dependent DNA replication  7 3 2.33
  0000278 mitotic cell cycle  16 7 2.29
  0051301 cell division  14 6.3 2.22
  0000279 M phase  15 6.8 2.21
  0007266 Rho protein signal transduction  6 2.8 2.14
  0008285 negative regulation of cell proliferation  14 6.6 2.12
  0040008 regulation of growth  18 8.6 2.09
  0016049 cell growth  16 7.7 2.08
  0009968 negative regulation of signal transduction  5 2.4 2.08
  0008361 regulation of cell size  16 7.7 2.08
  0030099 myeloid cell differentiation  6 2.9 2.07
  0007517 muscle development  25 12.2 2.05
  0007160 cell-matrix adhesion  8 3.9 2.05
  0042127 regulation of cell proliferation  31 15.2 2.04
  0006820 anion transport  22 10.8 2.04
  0043122 regulation of I-kappaB kinase/NF-kappaB cascade  8 4 2
 
  
  
 
  
 
  
 
 
 
 
   Date and time of the analysis:   
Name of the project file: Project.xls
 
Time of the analysis: Fri Apr 07 13:39:04 2006
 
BRB-ArrayTools Version: 3.3
 
 
   Filtering parameters:   
   Spot Filters: OFF
    
    
Normalization: OFF
   
   Gene Filters: OFF
   
   Gene Subsets: OFF
   
      View expression of significant genes.
