# Supplementary material for: Activation of counter-regulatory mechanisms in a rat renal acute rejection model
Source: BMC Genomics. 2008 Feb 8;9:71. doi: 10.1186/1471-2164-9-71 (PMC2262896; doi:10.1186/1471-2164-9-71)
Supplement: Additional file 1 — Gene lists from the class comparison between the groups analyzed with BRB ArrayTools. [file 1471-2164-9-71-S1.zip › ControlvsSyngeneic/ExpressionTable.html]

  
 
 
    Table.  Normalized Log-Transformed Gene Expressions for Significant Genes  
  
    Experiment  Control1 Control2 Control3 Control4 Control5 Sygeneic1 Sygeneic2 Sygeneic3 Sygeneic4 Sygeneic5
    Class  control control control control control syngeneic syngeneic syngeneic syngeneic syngeneic
   1   1370155_at   9.201 9.123 9.249 9.224 9.156 11.367 11.315 11.193 11.298 11.1
   2   1371369_at   7.183 7.216 7.158 7.285 7.345 8.705 8.599 8.553 8.703 8.521
   3   1370959_at   9.528 9.425 9.627 9.512 9.716 11.841 11.763 11.526 11.761 11.517
   4   1367784_a_at   9.873 9.697 9.871 9.8 9.592 12.936 12.974 12.789 12.616 12.384
   5   1372213_at   9.449 9.297 9.425 9.107 9.27 6.955 6.672 6.642 6.427 6.227
   6   1370167_at   10.8 10.732 10.735 10.698 10.821 10.224 10.275 10.238 10.21 10.269
   7   1367651_at   11.223 11.273 11.169 11.253 11.128 12.052 11.901 11.998 12.059 12.076
   8   1387854_at   8.035 7.954 8.112 7.804 8.161 10.462 10.503 10.253 10.277 9.925
   9   1372765_a_at   11.061 11.262 11.117 11.219 11.173 10.426 10.42 10.501 10.413 10.425
   10   1372406_at   4.385 4.689 4.709 4.703 4.637 7.428 6.991 7.008 6.733 6.912
   11   1374649_at   6.236 6.295 6.184 6.187 6.215 7.198 6.974 7.028 7.14 7.01
   12   1378477_a_at   5.791 5.972 5.878 5.866 5.856 6.583 6.718 6.574 6.676 6.574
   13   1389555_at   6.959 6.958 7.161 6.904 6.651 8.588 8.431 8.541 8.452 8.62
   14   1388116_at   7.823 7.924 8.507 7.798 7.871 10.857 10.595 10.422 10.712 10.622
   15   1372621_at   8.022 8.236 8.157 8.178 7.919 9.224 9.182 9.117 9.21 9.148
   16   1371349_at   7.936 7.78 7.793 7.678 8.023 9.787 9.626 9.458 9.597 9.336
   17   1386128_at   5.318 5.307 4.914 4.684 5.222 8.537 7.969 8.528 8.36 7.929
   18   1368288_at   10.502 10.714 10.81 10.614 10.687 8.595 8.718 9.001 8.989 9.068
   19   1369955_at   7.109 6.946 6.967 7.054 7.052 9.154 8.668 8.935 9.201 8.701
   20   1389123_at   6.89 6.925 6.946 6.938 6.489 8.887 9.013 9.135 9.249 8.698
   21   1367794_at   4.373 3.866 4.485 3.865 4.401 7.581 7.659 7.368 7.171 6.937
   22   1370864_at   6.783 6.522 7.023 6.513 7.022 9.605 9.563 9.288 9.406 8.979
   23   1389295_at   6.462 6.714 6.609 6.605 6.537 8.053 7.866 7.963 8.154 7.726
   24   1387351_at   7.082 7.019 7.088 7.127 7.076 9.176 8.952 9.207 8.865 8.565
   25   1386912_at   8.106 7.956 8.223 7.736 7.946 9.855 9.629 9.605 9.698 9.452
   26   1398347_at   7.726 7.634 7.766 7.594 7.504 9.05 9.038 8.954 8.78 8.725
   27   1377367_at   4.067 4.428 4.235 4.252 4.424 6.171 5.661 5.969 5.882 5.949
   28   1370290_at   8.085 8.126 8.227 8.033 7.801 10.098 10.071 9.836 9.618 9.737
   29   1374742_at   6.417 6.456 6.523 6.432 6.417 7.476 7.418 7.537 7.691 7.302
   30   1384917_at   5.702 5.876 6.166 4.314 5.402 0 0.556 0 0 0.526
   31   1370347_at   6.608 6.67 6.793 6.549 6.557 7.534 7.688 7.483 7.541 7.466
   32   1367562_at   10.382 10.346 10.298 10.433 10.496 11.39 11.168 11.249 11.141 11.215
   33   1388182_at   7.216 7.171 7.236 6.938 6.969 8.397 8.529 8.438 8.205 8.312
   34   1367914_at   6.464 6.667 6.912 6.86 6.946 8.647 8.584 8.43 8.33 8.316
   35   1371318_at   11.992 12.054 11.999 11.989 11.963 12.463 12.543 12.423 12.391 12.429
   36   1383716_at   5.874 6.006 6 6.049 5.893 6.539 6.691 6.612 6.574 6.598
   37   1384331_at   5.685 5.772 5.754 5.802 5.431 7.057 7.097 6.834 6.987 7.216
   38   1371754_at   10.412 10.234 10.551 9.22 10.051 6.407 6.346 6.371 5.946 5.713
   39   1387859_at   11.048 11.126 10.962 11.012 11.045 10.456 10.502 10.581 10.448 10.546
   40   1381370_at   3.109 2.602 2.897 3.829 2.854 0 0.098 0.124 0 0
   41   1384211_at   4.467 4.389 4.382 4.592 4.303 6.386 5.944 6.693 6.53 6.271
   42   1392899_at   5.22 5.548 5.856 5.788 5.537 9.078 8.159 8.428 8.179 8.392
   43   1379967_at   5.027 5.598 5.267 5.249 5.682 7.296 7.323 7.461 7.096 7.228
   44   1367780_at   5.923 6.109 5.796 5.768 5.794 9.405 8.763 8.635 8.33 8.428
   45   1383578_at   4.355 4.749 4.785 4.527 5.032 7.161 6.781 6.953 6.56 7.033
   46   1368545_at   8.363 8.123 7.976 8.029 8.127 7.074 6.986 7.177 7.04 6.962
   47   1388340_at   4.829 4.986 5.33 4.579 5.537 8.652 8.229 8.349 7.86 7.923
   48   1372125_at   6.585 6.314 6.19 6.282 6.407 8.02 7.974 7.824 7.982 7.556
   49   1373026_at   5.254 5.127 4.985 4.831 5.319 7.912 7.2 7.601 7.21 7.233
   50   1392965_a_at   8.316 8.171 8.279 8.32 8.365 9.918 9.575 10.152 9.784 9.625
   51   1371775_at   11.221 11.213 11.224 11.274 11.192 10.661 10.506 10.541 10.704 10.699
   52   1373463_at   7.488 7.48 7.205 7.056 7.563 9.452 9.107 9.336 9.327 8.927
   53   1384361_at   6.59 6.758 6.512 6.568 6.595 7.243 7.247 7.387 7.376 7.353
   54   1372006_at   7.383 7.547 7.122 7.54 7.749 9.769 9.414 9.603 9.832 9.233
   55   1367741_at   10.886 10.658 10.79 10.475 10.914 9.588 9.346 9.451 9.528 9.536
   56   1367628_at   9.21 9.025 9.177 9.096 9.079 11.028 11.248 10.843 10.849 10.491
   57   1370895_at   8.067 7.91 7.596 7.925 8.181 10.007 9.943 9.841 9.863 9.455
   58   1371331_at   9.058 8.983 8.879 9.036 9.034 10.136 9.787 10.025 10.106 9.898
   59   1387891_at   8.51 8.568 8.516 8.555 8.374 9.571 9.412 9.439 9.344 9.232
   60   1373658_at   5.23 5.362 5.556 5.264 5.641 8.058 7.35 7.375 7.356 7.558
   61   1374938_at   6.91 6.851 6.799 6.988 6.759 7.573 7.461 7.555 7.481 7.622
   62   1385190_at   6.305 6.387 6.689 6.239 6.404 7.705 7.638 7.81 7.997 7.659
   63   1387878_at   11.674 11.802 11.722 11.706 11.731 11.27 11.364 11.377 11.34 11.285
   64   1371970_at   3.635 4.132 3.47 4.043 3.963 6.97 7.177 7.055 6.479 6.246
   65   1388335_at   10.2 10.297 10.296 10.368 10.176 11.217 11.018 11.064 10.924 11.028
   66   1391450_at   5.555 5.402 5.22 5.428 5.727 7.754 7.465 8.072 7.669 7.231
   67   1377907_at   3.063 3.355 3.146 3.445 3.103 4.745 4.424 4.911 4.597 4.666
   68   1368990_at   5.224 5.455 5.635 5.47 5.354 7.948 7.561 7.659 7.378 7.08
   69   1368420_at   7.437 7.11 7.09 7.13 7.321 9.222 8.766 8.935 8.694 8.652
   70   1367570_at   9.016 8.554 8.618 8.46 8.488 10.089 10.243 10.243 9.951 10.211
   71   1367946_at   7.349 7.318 7.404 7.242 7.299 8.437 8.159 8.295 8.142 8.087
   72   1374449_at   4.905 5.721 5.629 5.092 5.763 8.629 8.105 8.344 7.909 8.158
   73   1370288_a_at   8.354 7.957 8.074 8.118 7.959 9.466 9.428 9.252 9.263 9.145
   74   1374036_at   6.479 6.411 6.924 6.701 6.435 8.118 8.235 8.247 7.878 8.123
   75   1377832_at   2.508 3.131 3.609 3.508 3.618 6.852 6.582 6.803 6.417 6.087
   76   1386660_at   3.553 3.933 3.837 3.873 3.779 5.493 4.989 5.251 5.081 5.144
   77   1373286_at   5.721 5.566 5.893 5.513 5.87 7.936 7.692 7.658 7.543 7.169
   78   1389408_at   6.167 6.682 7.033 6.282 6.504 9.727 9.53 9.373 8.921 9.005
   79   1392915_at   6.161 5.791 5.935 5.611 5.739 7.624 7.269 7.786 7.562 7.374
   80   1387870_at   11.108 10.987 11.558 10.967 11.525 8.972 8.32 8.429 8.359 7.827
   81   1377967_at   4.082 4.059 4.644 3.878 3.835 6.668 6.974 6.707 6.13 6.652
   82   1380088_at   4.981 5.076 4.888 4.761 5.224 6.742 6.304 6.644 6.88 6.476
   83   1373628_at   3.624 4.308 3.857 3.633 3.06 6.494 6.533 6.284 6.188 6.581
   84   1388236_x_at   8.011 8.023 8.106 7.95 8.002 8.784 8.593 8.597 8.754 8.829
   85   1388985_at   8.527 8.777 8.554 8.681 8.912 10.46 10.102 10.796 10.486 10.785
   86   1368171_at   6.414 5.965 5.959 6.111 6.169 8.323 8.567 8.167 8.099 7.713
   87   1377908_at   5.873 6.036 5.909 5.915 6.133 6.856 6.653 6.938 6.897 6.869
   88   1376765_at   11.135 11.12 11.077 11.076 11.067 10.144 10.285 10.403 10.479 10.402
   89   1383962_at   6.519 6.45 6.532 6.268 6.025 7.708 7.64 7.595 7.649 7.513
   90   1372935_at   4.966 4.887 4.866 5.252 5.069 7.226 6.689 6.655 6.831 6.497
   91   1370234_at   9.305 9.422 9.291 9.261 9.534 11.193 10.792 10.927 10.823 10.523
   92   1393436_at   4.525 4.778 5.234 4.871 4.164 2.173 1.227 1.744 1.233 1.642
   93   1373894_at   7.28 7.269 7.281 7.384 7.231 8.024 7.841 7.839 8.025 7.855
   94   1375926_at   9.858 9.578 9.819 9.769 9.991 8.907 8.63 8.669 8.71 8.838
   95   1398380_at   6.61 6.809 6.487 6.553 6.777 7.69 7.773 7.617 7.523 7.524
   96   1382590_at   5.734 5.769 5.384 5.787 5.826 6.831 6.943 6.762 7.099 6.964
   97   1371074_a_at   5.21 5.735 5.943 5.78 5.107 8.052 7.73 7.861 7.573 7.937
   98   1367776_at   5.058 5.774 5.664 5.786 5.962 9.064 8.551 8.443 8.017 8.301
   99   1388763_at   8.927 8.785 8.906 8.806 8.888 9.985 9.869 9.918 9.647 9.626
   100   1388955_at   7.733 7.542 7.788 7.504 7.542 8.577 8.477 8.627 8.533 8.359
   101   1371974_at   11.028 11.036 10.96 11.009 11.094 10.45 10.567 10.569 10.598 10.638
   102   1393240_at   6.651 6.784 6.72 6.66 6.641 8.483 8.441 8.442 8.133 7.818
   103   1376198_at   6.051 6.139 6.045 6.158 5.903 7.286 7.387 7.239 7.268 6.89
   104   1369944_at   8.298 8.056 8.05 8.09 7.928 9.746 9.302 9.488 9.325 9.218
   105   1390707_at   6.481 6.386 6.639 6.414 6.577 7.763 7.91 7.755 7.91 7.4
   106   1368961_at   6.065 6.069 6.252 6.064 5.658 7.318 7.285 7.31 7.133 7.225
   107   1368840_at   10.197 9.99 9.976 10.297 10.041 11.391 11.256 11.214 11.248 10.961
   108   1386940_at   9.063 9.01 9.064 8.998 8.797 10.022 10.052 10.041 10.082 9.71
   109   1370346_at   4.423 5.246 5.059 4.994 5.285 8.433 7.643 7.779 7.391 7.614
   110   1379345_at   5.693 5.06 5.631 5.538 5.766 7.96 7.496 7.311 7.793 7.384
   111   1372516_at   4.098 4.553 4.361 3.557 4.573 7.585 7.076 6.912 6.721 6.849
   112   1371861_at   9.562 9.516 9.586 9.568 9.396 10.485 10.321 10.626 10.348 10.243
   113   1374784_at   5.84 5.828 5.459 5.812 5.998 7.521 7.357 7.67 7.429 7.024
   114   1371391_at   8.645 8.672 8.629 8.58 8.55 9.449 9.386 9.348 9.171 9.182
   115   1398788_at   10.945 10.955 11.024 10.918 10.812 11.438 11.431 11.453 11.362 11.341
   116   1384344_at   5.243 5.028 5.319 5.24 5.02 6.076 6.24 6.044 6.168 5.939
   117   1376648_at   3.432 3.52 3.862 3.874 3.414 5.408 5.059 5.12 5.564 5.578
   118   1371475_at   7.763 7.691 7.544 7.621 7.645 8.897 8.652 8.986 8.84 8.485
   119   1371480_at   6.349 6.433 6.449 6.692 6.429 8.333 7.892 8.169 7.732 7.763
   120   1373882_at   7.386 7.318 7.26 7.382 7.215 8.263 7.989 8.178 8.374 8.079
   121   1368409_at   8.481 8.418 8.356 8.455 8.33 7.749 7.548 7.804 7.819 7.8
   122   1367744_at   7.424 7.274 7.278 7.027 7.083 8.316 8.313 8.361 8.354 8.047
   123   1369788_s_at   10.797 10.584 11.083 10.094 11.032 8.637 8.631 8.55 8.288 8.295
   124   1388792_at   9.56 9.323 9.953 9.123 9.842 7.519 7.416 7.322 6.821 6.915
   125   1389966_at   8.885 8.761 8.487 8.434 8.922 10.479 10.164 10.459 10.361 9.965
   126   1371099_at   10.486 10.45 10.397 10.248 10.216 9.569 9.513 9.647 9.368 9.603
   127   1367567_at   11.749 11.739 11.75 11.68 11.635 12.079 12.114 12.007 12.037 12.021
   128   1372870_at   6.404 6.498 6.841 6.618 6.431 7.905 7.993 7.787 7.701 7.565
   129   1374927_at   5.629 5.548 5.37 5.583 5.391 6.927 6.894 6.56 6.456 6.864
   130   1368924_at   8.563 8.536 8.132 8.376 8.429 7.423 7.464 7.555 7.319 7.346
   131   1370594_at   1.293 1.491 1.832 1.328 0.367 4.632 4.787 4.354 4.061 4.159
   132   1387062_a_at   4.081 4.502 4.997 4.491 4.019 6.306 6.525 6.725 6.551 6.51
   133   1389395_at   7.469 7.745 7.771 7.648 7.539 8.565 8.463 8.69 8.769 8.45
   134   1373672_at   7.958 7.912 7.688 7.874 7.842 8.823 8.571 8.615 8.61 8.518
   135   1370200_at   11.24 11.337 11.261 11.334 11.287 10.893 10.894 10.996 10.999 10.895
   136   1367618_a_at   11.594 11.598 11.636 11.658 11.553 12.05 11.989 11.911 11.941 11.917
   137   1373128_at   7.212 7.12 7.121 7.007 7.124 8.46 7.935 8.413 8.317 8.139
   138   1398853_at   10.445 10.51 10.461 10.535 10.432 10.767 10.76 10.748 10.718 10.699
   139   1367581_a_at   11.615 11.464 11.799 11.449 11.342 13.268 13.434 13.053 12.884 12.819
   140   1368236_at   11.78 11.796 11.771 11.795 11.907 11.407 11.488 11.471 11.501 11.501
   141   1384280_at   4.91 5.314 5.666 5.666 5.689 8.197 7.693 7.513 7.479 7.539
   142   1369499_at   4.873 5.062 5.05 5.343 4.771 6.756 6.503 6.833 6.361 6.345
   143   1398117_at   3.697 3.497 2.336 3.479 2.648 0 0 0 0.043 0.367
   144   1383940_at   4.204 4.834 4.082 4.902 5.059 7.274 7.057 7.078 6.786 6.812
   145   1388439_at   5.572 5.436 5.174 5.149 5.399 7.132 7.108 7.016 6.831 6.492
   146   1387892_at   8.783 8.974 8.999 8.704 8.772 10.865 10.48 10.36 10.078 10.284
   147   1368207_at   7.096 7.09 7.257 7.148 6.71 8.276 8.233 8.15 8.158 8.025
   148   1373401_at   5.783 5.719 5.65 5.715 5.368 7.822 7.748 7.629 6.93 7.606
   149   1373838_at   5.227 5.24 5.374 5.655 5.489 6.518 6.35 6.564 6.42 6.291
   150   1387946_at   8.621 8.445 8.522 8.525 8.587 10.158 9.651 9.557 9.715 9.632
   151   1396055_at   6.006 5.964 5.226 5.559 5.69 7.694 7.355 7.405 7.57 7.245
   152   1383805_at   4.587 4.873 4.695 4.561 4.838 5.543 5.508 5.657 5.632 5.839
   153   1372685_at   4.588 5.657 5.114 5.02 5.733 9.214 8.63 8.191 8.123 8.122
   154   1389538_at   10.183 10.033 10.052 9.551 9.77 8.619 8.133 8.517 8.291 8.257
   155   1394129_at   5.118 5.216 5.114 4.896 5.01 4.476 4.38 4.334 4.333 4.235
   156   1388484_at   5.523 5.202 5.968 5.937 5.698 8.877 8.105 7.985 7.831 7.997
   157   1391387_s_at   5.696 5.969 5.808 5.728 6.093 7.068 7.064 7.355 7.224 6.824
   158   1382153_at   4.54 4.589 4.437 4.63 4.031 6.864 6.321 6.752 6.724 5.998
   159   1388866_at   5.634 5.625 5.6 5.4 5.529 6.349 6.104 6.333 6.196 6.196
   160   1373785_at   6.661 6.593 6.561 6.627 6.579 7.253 7.054 7.177 7.17 6.997
   161   1372565_at   6.004 5.939 6.029 5.676 6.025 7.451 7.178 7.307 7.671 7.016
   162   1368822_at   9.026 8.993 9.024 9.061 8.973 10.092 9.645 10.096 9.855 9.911
   163   1388939_at   6.793 7.124 6.91 6.914 7.377 8.95 8.626 8.585 8.808 8.28
   164   1376943_at   4.433 4.785 4.731 4.674 4.114 6.344 6.335 6.246 5.973 5.93
   165   1373000_at   5.624 5.577 5.424 5.637 5.206 6.528 6.438 6.816 6.774 6.722
   166   1370462_at   4.634 4.902 4.532 5.043 5.039 7.551 7.139 7.126 6.463 6.866
   167   1369947_at   6.123 5.939 6.274 6.311 6.03 7.549 7.34 7.793 7.632 7.181
   168   1377369_at   5.333 4.782 5.209 5.201 4.9 6.243 6.444 6.22 6.522 6.297
   169   1397704_at   3.917 4.201 2.978 3.64 3.73 6.726 6.486 6.43 5.984 5.991
   170   1368404_at   6.218 6.509 6.32 6.087 6.288 7.997 7.657 7.574 7.767 7.319
   171   1386935_at   10.257 9.766 10.668 8.845 9.663 6.448 6.641 6.323 6.31 6.734
   172   1373914_at   5.212 5.395 5.347 4.942 5.14 7.101 6.986 6.946 6.796 6.341
   173   1378819_at   9.263 9.166 9.433 9.373 9.075 8.276 8.381 8.365 8.476 8.555
   174   1367712_at   6.498 5.984 6.847 6.262 5.849 9.334 9.58 9.078 8.728 8.507
   175   1367655_at   7.996 8.032 8.312 8.06 7.847 10.425 10.382 10.179 9.483 9.838
   176   1382580_at   5.156 5.297 5.136 5.009 5.119 6.587 6.039 6.67 6.548 6.303
   177   1388392_at   8.998 9.023 8.98 9.073 9.16 10.072 10.253 10.102 9.838 9.784
   178   1367725_at   10.517 10.042 10.71 9.641 10.165 8.176 7.755 8.156 8.141 8.233
   179   1370353_at   7.736 7.775 7.784 7.735 7.692 7.966 8.006 8.051 7.955 8.036
   180   1378296_at   6.186 5.843 6.188 5.903 6.263 7.7 7.46 7.528 7.208 7.183
   181   1374726_at   5.07 4.751 5.118 4.167 5.077 7.534 7.132 6.864 6.903 6.969
   182   1399027_at   10.61 10.669 10.606 10.683 10.567 11.068 11.04 10.992 11.029 10.896
   183   1373204_at   9.219 9.056 9.318 9.333 9.144 10.244 10.188 10.156 10.085 9.85
   184   1389858_at   6.812 7.055 6.724 6.715 6.594 8.633 8.305 8.235 7.896 8.129
   185   1368877_at   10.548 10.44 9.884 10.166 10.195 8.806 8.963 9.061 9.045 9.003
   186   1388879_at   5.486 5.303 5.112 5.216 5.626 6.769 6.813 6.973 6.632 6.394
   187   1369957_at   6.359 6.161 6.453 6.516 6.36 7.137 7.36 7.167 7.122 7.061
   188   1367829_at   10.894 10.907 10.788 10.871 10.822 10.403 10.231 10.267 10.431 10.443
   189   1393252_at   6.726 6.478 5.883 6.24 6.599 8.233 8.103 7.993 8.237 7.838
   190   1389114_at   10.633 10.717 10.609 10.7 10.638 9.816 10.015 10.087 10.15 10.097
   191   1379235_x_at   5.184 5.592 5.098 4.811 5.086 7.048 6.451 7.03 6.768 6.859
   192   1371071_at   5.686 5.66 5.593 5.187 5.57 6.992 6.701 6.916 6.931 6.518
   193   1372186_a_at   3.529 4.525 4.268 3.297 3.498 7.08 6.839 6.619 6.164 6.724
   194   1398514_at   11.445 11.534 11.485 11.54 11.446 11.08 11.131 11.156 11.233 11.188
   195   1383420_at   8.889 8.977 8.789 8.839 8.686 7.868 8.213 8.13 8.002 7.919
   196   1383404_at   6.243 6.368 6.376 6.12 6.229 7.631 7.182 7.667 7.365 7.2
   197   1367732_at   8.333 8.34 8.288 8.213 8.296 9.108 9.11 9.069 8.921 8.769
   198   1397769_at   3.96 2.711 2.465 4.056 3.07 6.646 6.768 6.345 6.452 6.654
   199   1383246_at   5.742 5.73 5.32 5.452 5.889 6.705 6.809 6.847 7.057 6.749
   200   1372042_at   6.318 6.1 6.534 6.254 6.506 8.264 7.701 8.172 7.804 7.579
   201   1398343_at   9.409 9.439 9.415 9.203 9.405 8.632 8.369 8.655 8.667 8.736
   202   1377812_a_at   8.989 9.064 8.823 8.88 9.056 8.457 8.421 8.367 8.505 8.405
   203   1371700_at   7.259 7.348 6.828 7.565 7.56 9.67 9.051 9.246 10.04 9.419
   204   1383085_at   8.387 8.447 8.161 8.457 8.226 9.11 9.366 9.266 9.161 9.017
   205   1383323_at   3.284 3.973 4.184 3.561 3.52 5.803 5.575 5.875 5.413 6.157
   206   1371012_at   9.588 9.751 9.809 9.656 9.266 8.51 8.42 8.719 8.586 8.517
   207   1370209_at   11.355 11.226 11.196 10.909 11.376 10.24 10.115 10.379 10.317 10.125
   208   1367749_at   5.62 5.246 5.958 5.347 5.657 8.498 8.968 7.937 8.322 7.557
   209   1392672_at   6.483 5.845 6.223 6.344 6.124 7.966 7.536 7.605 7.744 7.387
   210   1370127_at   5.503 5.267 5.466 5.058 5.078 6.595 6.347 6.317 6.232 6.478
   211   1389566_at   4.982 5.915 5.685 5.072 6.032 8.922 8.036 8.184 8.011 8.113
   212   1371573_at   10.629 10.617 10.687 10.58 10.411 11.113 11.093 11.143 11.054 11.03
   213   1371365_at   9.18 9.249 9.341 9.163 9.13 10.141 9.863 9.913 9.852 9.795
   214   1389413_at   3.84 4.771 5.096 4.846 4.678 6.85 6.941 6.887 6.717 6.665
   215   1388650_at   5.053 5.535 5.531 5.383 5.869 8.23 7.426 7.692 7.463 7.234
   216   1368773_at   3.413 3.521 3.839 4.415 3.529 6.351 6.137 5.888 5.865 5.572
   217   1367802_at   12.492 12.173 11.811 12.275 12.336 11.039 10.924 11.172 10.997 10.972
   218   1372013_at   9.191 9.007 9.331 9.16 9.066 10.282 9.871 10.409 10.203 10.16
   219   1386045_at   6.486 6.243 6.657 6.459 6.136 5.361 5.377 5.503 5.247 5.179
   220   1367691_at   8.54 8.591 8.355 8.339 8.481 9.446 9.553 9.458 9.377 9.084
   221   1387531_at   9.94 9.903 9.885 9.89 9.806 9.206 9.37 9.283 9.292 9.514
   222   1368025_at   9.524 9.751 9.839 8.832 9.3 7.429 7.645 7.669 7.717 7.637
   223   1376702_at   6.084 6.08 6.492 6.079 5.873 3.616 4.209 4.489 4.365 4.51
   224   1373897_at   5.874 6.021 6.793 6.031 5.995 8.269 8.02 8.117 7.785 7.789
   225   1376889_at   5.07 5.305 4.972 5.251 5.513 6.173 6.112 6.125 6.221 6.18
   226   1372341_at   10.143 10.044 9.878 9.833 9.895 9.336 9.369 9.293 9.343 9.424
   227   1367568_a_at   11.743 11.594 11.628 11.667 11.874 12.636 12.565 12.372 12.32 12.406
   228   1374013_at   6.942 6.544 6.375 6.676 6.586 7.938 7.693 8.134 8.008 7.659
   229   1391139_at   5.147 5.131 4.992 5.172 5.115 6.02 6.167 6.17 5.842 5.746
   230   1379425_at   4.408 4.893 4.55 4.564 4.297 5.72 5.641 6.133 5.844 5.737
   231   1370585_a_at   5.75 5.519 5.841 5.496 5.639 6.997 6.623 7.032 6.841 6.526
   232   1367658_at   7.477 7.428 7.628 7.523 7.505 7.006 7.124 6.875 6.938 6.878
   233   1388784_at   7.758 7.761 7.65 7.615 7.61 8.671 8.473 8.481 8.792 8.31
   234   1389873_at   5.914 6.124 6.156 6.182 5.971 7.374 7.143 7.013 7.177 6.807
   235   1378633_at   6.463 6.226 6.456 6.252 6.201 7.292 7.184 7.188 7.033 6.945
   236   1367563_at   10.666 10.756 10.386 11.009 10.605 11.805 11.761 11.575 11.844 11.808
   237   1372158_at   9.756 9.616 9.64 9.437 9.371 8.697 8.688 8.845 8.861 8.86
   238   1377966_at   10.516 10.733 10.609 10.515 10.315 9.217 9.379 9.509 9.718 9.595
   239   1393643_at   7.209 7.167 7.404 7.105 7.069 8.526 8.414 8.387 8.143 7.981
   240   1371340_at   11.853 11.869 11.82 11.844 11.8 12.1 12.112 12 12.046 12.101
   241   1375642_at   10.174 10.225 10.308 10.224 10.362 9.934 9.899 9.853 9.912 9.938
   242   1398500_at   8.578 8.555 8.44 8.468 8.623 8.031 8.099 8.207 8.067 8.106
   243   1373750_at   5.801 5.717 6.081 5.75 5.567 6.735 6.589 6.589 6.624 6.768
   244   1368390_at   8.707 8.523 8.62 8.678 8.608 8.309 8.27 8.316 8.227 8.305
   245   1394419_at   5.11 5.248 5.475 4.247 4.958 8.189 7.618 7.801 7.078 7.497
   246   1390680_at   3.466 3.732 3.373 3.684 3.545 4.5 4.606 4.816 4.457 4.347
   247   1374805_at   4.06 4.172 4.543 3.448 3.8 6.388 5.831 6.442 5.848 6.157
   248   1370308_at   6.92 6.991 7.386 6.889 6.786 8.608 8.296 8.239 8.14 8.117
   249   1384188_at   8.384 8.543 8.566 8.397 8.508 7.667 7.66 7.936 7.747 7.483
   250   1373575_at   9.53 9.523 9.782 9.49 9.526 10.738 10.364 10.405 10.484 10.291
   251   1370813_at   6.083 6.096 6.183 6.253 5.853 7.249 7.221 7.172 7.061 6.798
   252   1371487_at   8.996 9.023 9.275 8.947 8.963 9.796 9.773 9.812 9.713 9.58
   253   1383395_at   10.76 10.798 10.923 10.714 10.766 9.987 9.856 10.201 10.187 10.152
   254   1373093_at   11.777 11.684 11.772 11.403 11.822 10.045 10.131 10.245 9.697 9.273
   255   1371266_at   9.787 9.653 9.942 9.548 9.751 8.796 8.573 8.749 8.975 8.948
   256   1376937_at   6.789 6.575 6.777 6.917 6.785 7.514 7.378 7.666 7.548 7.38
   257   1370539_at   5.806 5.98 5.839 5.958 5.614 7.091 6.748 6.851 6.633 6.713
   258   1379582_a_at   3.181 4.19 2.856 3.531 3.828 7.556 6.967 7.075 6.212 6.229
   259   1385625_at   6.543 6.729 6.314 6.888 6.48 8.409 8.181 8.18 7.958 7.68
   260   1371300_at   12.112 12.142 12.119 12.107 12.047 12.491 12.47 12.445 12.348 12.354
   261   1372410_at   5.659 5.745 6.193 5.923 5.639 6.978 6.805 7.259 7.037 7.165
   262   1370328_at   6.114 5.918 5.577 6.136 6.091 7.721 7.542 7.608 7.518 7.01
   263   1370642_s_at   6.984 6.982 6.818 7.106 6.613 7.873 7.74 7.794 7.898 7.665
   264   1372518_at   4.807 4.949 5.27 4.803 5.074 7.358 6.966 7.022 7.056 6.221
   265   1387357_at   10.505 10.479 10.586 10.356 10.363 9.661 9.512 9.677 9.851 9.871
   266   1372818_at   7.877 7.764 7.525 7.87 8.107 8.997 8.909 8.936 8.949 8.642
   267   1371310_s_at   9.373 9.582 9.725 9.453 9.845 10.722 10.722 10.442 10.491 10.508
   268   1372475_at   10.561 10.49 10.476 10.534 10.453 9.654 9.563 9.767 9.962 9.919
   269   1379587_at   7.661 7.598 7.544 7.593 7.59 6.912 6.826 6.971 7.087 7.175
   270   1383284_at   6.096 5.867 6.026 5.788 5.801 7.431 6.777 7.411 7.33 7.077
   271   1367593_at   7.593 7.277 7.369 7.318 7.291 8.41 8.328 8.174 8.151 8.01
   272   1374474_at   7.129 7.138 7.386 7.201 7.045 8.076 7.904 8 7.938 7.733
   273   1392489_at   5.102 4.807 4.809 5.277 4.553 6.504 6.476 6.369 6.69 6.064
   274   1387178_a_at   9.915 10.011 9.964 9.879 9.83 9.452 9.246 9.426 9.433 9.512
   275   1369186_at   6.035 6.254 6.283 6.386 6.043 7.566 7.165 7.47 7.155 7.091
   276   1367676_at   7.527 7.758 7.641 7.434 7.6 9.701 9.285 9.278 8.86 8.802
   277   1368418_a_at   8.564 8.479 8.106 8.611 8.648 10.043 9.605 9.985 9.871 9.515
   278   1376951_at   4.524 4.331 4.801 3.994 4.317 6.159 5.957 6.087 5.777 5.623
   279   1377299_at   6.54 6.383 6.524 6.319 6.178 7.607 7.311 7.506 7.125 7.339
   280   1388778_at   10.062 10.233 10.446 10.015 10.146 9.07 8.903 9.38 9.143 9.264
   281   1369677_at   5.887 5.935 6.144 5.694 6.012 5.209 4.788 4.795 4.955 4.996
   282   1368139_s_at   9.554 9.477 9.593 9.627 9.383 8.1 7.852 8.191 8.475 8.592
   283   1375972_at   4.441 4.811 4.996 4.679 4.711 6.202 6.71 6.134 5.922 6.079
   284   1371304_a_at   12.038 12.065 12.062 12.059 12.071 12.2 12.203 12.158 12.172 12.236
   285   1368150_at   12.164 12.049 12.064 12.159 12.172 11.657 11.737 11.746 11.837 11.79
   286   1387805_at   10.838 10.862 10.778 10.742 10.889 10.404 10.422 10.469 10.239 10.315
   287   1369415_at   9.016 8.7 8.956 8.316 8.465 7.243 7.486 7.282 7.42 7.119
   288   1386274_at   7.475 7.489 7.48 7.376 7.529 6.842 6.603 6.956 6.605 6.864
   289   1369527_at   4.799 4.472 4.779 5.209 4.476 6.447 6.147 6.155 6.059 6.01
   290   1387687_at   4.161 4.455 4.751 4.38 4.455 6.3 6.464 5.797 5.718 5.893
   291   1377639_at   5.111 5.541 5.034 5.084 5.48 6.568 6.211 6.641 6.58 6.364
   292   1374897_at   8.135 8.258 8.254 8.16 8.095 8.779 8.69 8.707 8.627 8.528
   293   1379448_at   2.483 3.515 2.934 2.444 3.865 6.806 6.685 6.279 6.1 5.583
   294   1381528_at   6.914 6.623 6.761 6.708 6.643 7.297 7.26 7.389 7.288 7.191
   295   1386956_at   5.53 5.331 5.108 5.249 5.156 6.253 6.323 6.25 6.14 5.942
   296   1387263_at   7.745 7.624 7.782 7.595 7.239 6.085 6.187 6.075 6.275 6.683
   297   1380775_at   5.079 5.323 5.161 5.066 5.201 6.78 6.674 6.36 6.076 6.252
   298   1392648_at   4.732 4.218 5.151 4.388 4.346 7.486 7.507 7.485 7.417 6.271
   299   1368147_at   9.256 8.656 9.236 8.244 8.892 6.812 6.757 5.887 5.889 5.94
   300   1372584_at   5.605 5.776 5.552 5.771 5.555 6.966 6.366 6.856 6.951 6.673
   301   1388936_at   8.15 8.15 7.437 7.896 8.048 9.789 9.334 9.791 9.707 9.214
   302   1398602_at   4.499 5.113 4.612 5.352 5.252 7.044 7.158 7.116 6.494 6.725
   303   1373693_at   9.852 9.95 10.047 9.867 9.91 9.456 9.598 9.558 9.515 9.569
   304   1370949_at   6.255 6.297 6.537 6.502 6.415 7.596 7.393 8.026 8.218 7.884
   305   1389528_s_at   10.806 10.785 11.177 10.34 10.881 9.454 9.341 9.33 9.058 8.814
   306   1389548_at   10.437 10.45 10.129 10.467 10.397 9.658 9.436 9.727 9.643 9.723
   307   1370294_a_at   5.734 6.277 5.775 5.91 5.472 8.087 7.422 7.458 7.262 7.623
   308   1371389_at   12.047 11.869 12.027 12.174 12.074 11.405 11.306 11.463 11.559 11.504
   309   1368419_at   8.643 8.706 8.383 8.69 8.823 9.815 9.558 10.039 10.001 9.552
   310   1383747_at   4.492 5.921 5.572 5.421 5.873 9.029 8.76 8.869 8.041 7.935
   311   1382061_at   9.618 9.826 9.655 9.712 9.503 8.659 8.694 8.844 8.965 9.044
   312   1374217_at   8.857 8.894 8.961 8.905 8.733 8.135 8.055 8.197 8.206 8.437
   313   1368260_at   4.421 4.596 4.583 3.644 4.669 7.59 7.091 6.894 6.444 6.501
   314   1367634_at   12.285 12.231 12.276 12.226 12.165 12.524 12.536 12.452 12.446 12.483
   315   1378592_at   5.978 6.296 5.709 6.47 5.616 7.859 7.621 7.834 7.425 7.549
   316   1368146_at   11.199 10.994 11.35 10.473 11.362 9.477 8.712 8.989 8.387 8.536
   317   1374628_at   11.556 11.529 11.522 11.607 11.427 10.878 10.971 11.041 10.96 11.181
   318   1381311_at   6.434 6.092 6.178 6.068 6.016 7.875 7.399 7.587 7.77 7.104
   319   1383188_at   7.743 7.595 7.379 7.567 7.426 6.797 6.811 6.845 7.01 6.897
   320   1373490_at   7.508 7.516 7.607 7.593 7.494 8.481 8.215 8.434 8.231 8.044
   321   1367574_at   9.396 9.058 9.56 9.128 9.416 11.151 11.199 10.853 10.789 10.374
   322   1369986_at   10.994 11.066 11.031 10.978 10.905 10.346 10.331 10.569 10.566 10.52
   323   1385559_at   4.446 4.663 4.287 4.365 4.119 5.912 5.725 5.424 5.36 5.864
   324   1383786_at   7.461 7.614 7.488 7.601 7.477 8.542 8.632 8.494 8.13 8.256
   325   1387883_a_at   12.46 12.37 12.349 12.533 12.407 13.026 13.065 12.815 12.853 12.951
   326   1372093_at   10.349 10.236 10.497 10.264 10.329 9.844 9.805 9.896 9.938 9.915
   327   1369977_at   5.319 5.03 5.473 4.774 4.728 6.516 6.783 6.585 6.493 6.3
   328   1368821_at   8.119 7.783 7.423 8.046 7.762 9.242 8.858 9.107 8.979 9.08
   329   1374101_at   8.911 8.984 9.11 9.088 9.045 8.701 8.668 8.644 8.722 8.697
   330   1384195_at   4.96 5.056 5.129 5.307 5.059 6.678 6.263 6.652 6.678 5.979
   331   1389553_at   6.827 6.929 7.17 6.855 6.756 8.768 8.305 8.498 8.17 7.912
   332   1387087_at   10.137 9.909 10.116 9.069 9.833 8.105 8.001 7.943 7.885 7.527
   333   1368180_s_at   12.46 12.501 12.441 12.564 12.523 12.135 12.217 12.224 12.191 12.284
   334   1393061_at   10.061 10.098 10.205 10.18 10.061 9.298 9.321 9.523 9.644 9.556
   335   1387214_at   8.26 8.545 8.278 8.179 7.91 5.8 5.887 6.813 6.17 6.634
   336   1373245_at   10.278 10.531 9.897 10.199 10.303 11.516 11.173 11.428 11.444 11.166
   337   1373504_at   6.724 6.605 6.681 6.616 6.432 7.989 7.857 7.583 7.514 7.4
   338   1391518_at   3.589 4.406 4.41 4.918 4.126 7.254 6.731 6.678 6.233 6.526
   339   1383606_at   6.59 6.295 6.391 6.41 6.339 7.23 7.146 7.183 7.169 6.869
   340   1377756_at   7.223 7.23 7.243 7.103 7.126 7.645 7.757 7.933 7.699 7.626
   341   1390116_at   7.837 7.455 7.097 7.689 7.467 8.675 8.819 8.533 8.678 8.673
   342   1379479_at   3.438 4.233 4.486 3.399 3.8 6.462 6.092 5.897 5.697 6.215
   343   1390801_at   10.362 10.435 10.265 9.605 10.41 8.603 8.836 8.783 8.607 8.291
   344   1380573_a_at   2.648 2.227 2.698 2.275 2.529 4.148 3.729 3.953 4.603 3.842
   345   1388717_at   7.165 6.975 7.101 6.99 6.995 7.836 7.472 7.761 7.624 7.694
   346   1371653_at   10.338 10.3 10.435 10.062 10.269 11.204 11.132 11.053 10.869 10.932
   347   1376599_at   5.728 5.828 6.069 5.753 6.424 7.497 7.547 7.458 7.033 7.26
   348   1382078_at   4.77 4.617 4.943 5.118 5.183 6.438 6.682 6.016 6.52 6.104
   349   1368661_at   9.863 9.987 9.956 9.774 9.452 8.602 8.682 8.922 8.931 8.843
   350   1367671_at   10.035 10.131 10.295 9.931 10.047 11.012 10.8 10.952 10.64 10.894
   351   1387868_at   5.811 5.67 5.182 5.612 5.535 6.879 6.98 6.976 6.679 6.476
   352   1367855_at   5.456 5.461 5.736 5.469 5.431 6.806 6.544 6.312 6.446 6.266
   353   1398878_at   9.061 9.043 9.17 9.075 9.014 8.495 8.554 8.76 8.586 8.463
   354   1380577_at   10.446 10.521 10.326 10.574 10.491 9.992 10.044 9.997 10.056 9.845
   355   1389355_at   6.597 7.225 7.004 6.827 6.897 7.999 7.962 7.909 7.826 7.738
   356   1380909_at   7.122 7.087 7.094 6.68 6.836 8.492 8.493 8.508 8.223 7.838
   357   1371080_at   9.712 9.969 9.76 9.666 9.369 6.802 6.458 7.348 6.903 8.101
   358   1376746_at   9.224 9.378 9.587 9.364 9.034 7.801 7.812 8.158 8.349 8.239
   359   1378174_at   1.47 1.521 1.395 0.444 1.287 3.374 3.335 3.211 3.442 2.763
   360   1374496_at   7.547 7.821 7.626 7.694 7.388 8.853 8.369 8.684 8.972 8.616
   361   1397341_at   3.005 4.227 4.793 4.018 4.394 7.628 7.309 7.064 6.572 6.86
   362   1388298_at   8.821 8.334 8.683 8.495 8.704 9.626 9.523 9.721 9.365 9.423
   363   1390177_at   7.012 7.187 7.15 7.209 7.088 8.026 7.978 8.034 7.781 7.621
   364   1376871_at   5.303 4.851 4.648 4.765 4.686 6.142 6.26 6.515 5.91 6.076
   365   1378997_at   2.108 1.526 2.694 2.667 1.322 5.364 5.503 4.929 5.08 4.339
   366   1388458_at   6.999 7.052 6.863 6.868 7.012 7.683 7.785 7.86 7.42 7.65
   367   1372478_at   7.615 7.357 7.738 7.312 7.321 8.543 8.319 8.335 8.432 8.195
   368   1372323_at   9.333 9.417 9.441 9.283 9.14 8.782 8.808 8.849 8.903 8.793
   369   1367695_at   11.737 11.739 11.543 11.689 11.641 11.256 11.206 11.331 11.296 11.351
   370   1382069_at   8.558 8.688 8.546 8.549 8.58 8.027 7.55 7.768 7.846 7.513
   371   1370287_a_at   9.636 9.623 9.523 9.247 9.419 10.701 10.918 10.433 10.324 10.504
   372   1387995_a_at   10.268 10.084 9.986 10.17 10.132 10.984 10.623 10.756 10.723 10.876
   373   1389695_at   9.706 9.686 9.57 9.519 9.67 9.159 9.032 9.086 9.262 9.227
   374   1393793_at   10.052 10.126 10.057 9.732 10.155 9.218 9.007 9.356 9.011 8.951
   375   1371782_at   5.743 5.699 6.308 6.106 6.139 7.625 7.483 7.724 7.141 7.161
   376   1374431_at   11.233 11.208 11.266 11.274 11.281 11.104 10.986 11.051 11.053 11.002
   377   1373557_at   4.871 5.823 5.593 5.504 5.427 7.104 7.107 7.002 6.633 7.091
   378   1389857_at   10.016 10.014 10.097 9.912 9.969 10.256 10.308 10.352 10.275 10.353
   379   1372294_at   7.44 7.588 7.454 7.781 7.591 9.178 8.736 9.241 9.219 8.483
   380   1373506_at   8.677 8.626 8.791 8.457 8.533 7.948 8.069 8.135 7.888 7.962
   381   1387793_at   11.738 11.732 11.727 11.705 11.629 11.501 11.492 11.491 11.539 11.54
   382   1386861_at   11.07 11.131 11.068 11.036 10.926 11.95 11.793 11.722 11.512 11.65
   383   1388705_at   8.652 8.573 8.674 8.523 8.46 9.144 9.272 9.364 9.149 9.01
   384   1383181_at   7.857 8.005 8.278 7.992 7.924 8.914 8.691 8.913 8.64 8.753
   385   1375170_at   10.816 10.741 11.273 10.785 10.827 11.893 11.936 11.885 11.642 11.768
   386   1370345_at   3.555 4.656 5.033 4.675 4.471 8.117 7.484 7.419 6.854 6.866
   387   1387600_at   5.169 4.882 4.868 4.769 4.022 6.71 6.695 6.454 6.531 6.226
   388   1385706_at   4.536 4.426 4.925 4.886 4.586 5.84 5.596 5.972 5.689 5.555
   389   1387372_at   9.289 9.081 8.665 9.006 8.998 7.925 8.066 8.122 8.048 8.213
   390   1392265_s_at   6.826 6.61 6.608 6.778 6.328 7.616 7.655 7.653 7.76 7.313
   391   1389130_at   7.005 7.111 7.394 7.344 7.223 7.852 7.784 7.951 7.922 7.84
   392   1389668_at   4.476 5.235 5.274 4.418 5.166 7.854 7.374 7.543 6.552 7.327
   393   1385070_at   7.027 7.154 7.025 7.115 7.322 7.734 7.765 7.738 7.669 7.547
   394   1376574_at   7.103 7.317 7.451 7.41 7.2 7.958 7.886 8.05 8.213 7.961
   395   1368270_at   5.066 5.3 5.152 5.299 5.318 6.977 6.911 6.659 6.692 6.051
   396   1378070_at   6.543 6.713 6.525 6.717 6.601 7.27 7.11 6.987 7.211 7.17
   397   1388339_at   7.925 7.594 7.63 7.919 7.653 8.7 8.606 8.416 8.42 8.456
   398   1397526_at   10.279 10.137 10.019 10.332 10.124 9.517 9.637 9.682 9.705 9.633
   399   1368897_at   7.102 6.795 6.612 6.495 6.583 5.779 5.61 5.701 5.632 5.887
   400   1371815_at   4.583 4.655 4.323 4.608 4.014 6.374 5.666 6.504 6.752 6.111
   401   1373592_at   7.725 7.627 7.534 7.805 7.538 8.746 8.307 8.382 8.371 8.376
   402   1387029_at   8.962 8.899 8.564 8.989 9.045 9.934 9.849 10.024 10.024 9.578
   403   1379498_at   6.488 6.693 6.605 6.538 6.421 5.84 5.663 5.759 6.095 5.808
   404   1372439_at   10.111 10.125 9.579 9.964 10.235 11.371 10.99 11.145 11.322 10.996
   405   1368317_at   10.867 10.908 10.949 10.986 10.863 10.712 10.63 10.699 10.615 10.651
   406   1379440_at   5.618 5.294 5.721 5.132 5.307 6.603 6.539 6.334 6.561 6.296
   407   1371924_at   8.557 8.224 7.754 8.567 8.388 9.972 9.66 9.878 10.011 9.476
   408   1390839_at   6.701 6.68 7.073 6.362 7.031 8.725 8.813 8.954 8.347 8.003
   409   1391437_at   7.574 7.623 7.551 7.385 7.429 7.925 7.985 7.966 7.908 7.86
   410   1369100_at   8.909 9.015 9.03 8.708 8.588 8.046 7.803 7.937 7.992 8.148
   411   1398644_at   1.88 0.39 1.39 0.496 0.604 4.097 3.566 4.43 3.338 3.807
   412   1390384_at   6.673 6.447 6.577 6.683 6.453 7.926 7.497 7.703 7.413 7.311
   413   1368172_a_at   5.573 5.824 5.131 5.49 5.589 7.715 7.559 7.695 7.327 6.658
   414   1367569_at   11.856 11.802 11.941 11.854 11.806 12.227 12.196 12.082 12.12 12.135
   415   1386885_at   11.247 11.127 11.21 11.221 11.105 10.693 10.671 10.848 10.881 10.758
   416   1373151_at   8.072 7.967 8.022 7.974 7.9 8.724 8.422 8.795 8.735 8.501
   417   1379741_at   11.14 11.202 11.206 11.227 11.109 10.775 10.84 10.96 10.854 10.772
   418   1377702_at   6.329 6.296 6.207 6.794 6.533 7.498 7.821 7.401 7.409 7.442
   419   1384200_at   6.819 6.509 6.511 6.414 6.757 7.351 7.551 7.418 7.413 7.216
   420   1367705_at   7.003 6.607 6.994 7.049 6.958 8.31 8.101 8.046 7.921 7.674
   421   1381162_at   7.616 7.629 7.519 7.452 7.74 8.778 8.635 8.66 8.336 8.24
   422   1393848_at   4.133 4.891 4.012 4.406 4.412 7.746 7.331 6.787 6.524 6.252
   423   1367578_at   11.118 11.178 11.276 11.147 11.108 11.542 11.499 11.6 11.524 11.416
   424   1376055_at   5.099 5.309 6.207 5.373 5.671 7.306 7.286 7.573 6.978 7.315
   425   1367672_at   11.465 11.528 11.37 11.532 11.504 11.126 11.098 11.116 11.213 11.221
   426   1378008_at   6.347 6.364 6.34 6.213 6.382 6.956 6.907 6.951 7.07 6.678
   427   1388611_at   8.811 8.582 9.013 8.694 8.704 7.81 7.741 7.994 8.08 8.07
   428   1376105_at   5.668 5.533 5.719 4.548 5.568 7.644 7.642 7.243 7.484 7.07
   429   1390172_at   10.384 10.643 10.261 10.324 10.508 9.771 9.864 9.878 9.856 9.851
   430   1398294_at   7.551 7.317 7.31 7.349 7.312 8.863 8.668 8.792 8.149 8.324
   431   1369407_at   8.176 8.242 8.72 8.038 7.647 6.566 6.171 6.007 6.504 6.712
   432   1389103_at   5.271 5.584 5.714 5.675 5.657 6.795 6.772 6.889 6.339 6.481
   433   1397609_at   2.524 0.872 2.202 0 0 5.981 5.563 5.443 5.26 5.744
   434   1374775_at   3.925 5.586 5.616 5.029 4.787 8.116 7.724 8.036 7.405 7.517
   435   1388628_at   7.018 7.066 7.314 6.588 6.734 8.285 8.047 8.225 8.263 7.906
   436   1370811_at   10.417 10.714 10.522 10.7 10.498 10.001 9.996 10.091 10.127 10.014
   437   1386879_at   9.264 9.085 9.552 9.154 9.069 10.448 10.576 10.114 10.226 10.082
   438   1369621_s_at   8.939 8.839 8.828 9.034 8.944 9.692 9.671 9.552 9.468 9.331
   439   1368838_at   9.074 8.55 8.633 8.725 8.562 9.869 9.967 9.645 9.53 9.643
   440   1382185_at   5.674 6.179 6.297 5.646 6.222 7.672 7.121 7.705 7.695 7.331
   441   1389682_at   6.803 6.666 6.529 6.547 6.401 7.35 7.302 7.411 7.317 7.092
   442   1388884_at   11.521 11.659 11.613 11.686 11.509 10.865 11.023 11.118 11.157 11.156
   443   1370080_at   5.605 5.638 5.996 5.754 5.744 7.691 7.063 6.69 7.45 7.336
   444   1392837_at   6.762 6.979 7.051 7.178 7.012 7.99 7.622 8.053 7.739 7.804
   445   1373447_at   7.863 8.079 7.809 7.76 7.883 8.614 8.6 8.709 8.484 8.345
   446   1372481_at   7.816 7.636 7.771 7.736 7.727 8.138 8.089 8.193 8.363 8.151
   447   1383879_at   9.597 9.768 9.752 9.524 9.738 8.971 9.209 9.085 9.228 9.157
   448   1375944_at   11.963 11.965 12.073 11.844 11.999 11.511 11.417 11.61 11.531 11.63
   449   1369968_at   8.185 8.141 7.762 8.241 7.946 9.015 9.061 9.078 8.911 8.709
   450   1376218_a_at   4.271 3.984 3.975 3.773 4.275 5.246 4.792 5.358 5.291 5.185
   451   1374198_at   5.481 5.522 5.764 5.714 5.035 6.826 6.98 6.943 6.687 6.476
   452   1371912_at   11.482 11.523 11.558 11.553 11.532 11.331 11.322 11.271 11.392 11.356
   453   1371947_at   4.792 4.728 5.185 4.88 4.672 6.105 5.864 6.084 5.919 5.575
   454   1379957_at   5.497 5.041 5.284 5.217 5.209 6.678 6.446 6.336 6.174 6.024
   455   1386882_at   8.145 8.407 8.21 7.905 8.262 8.998 9.094 9.114 8.918 8.814
   456   1373054_at   7.988 8.233 8.182 8.091 8.05 8.786 8.694 8.938 8.826 8.537
   457   1373048_at   9.257 9.202 9.228 9.236 9.221 9.412 9.541 9.425 9.413 9.416
   458   1390901_at   6.6 6.928 6.557 6.815 6.698 8.28 7.987 8.381 8.282 7.523
   459   1369989_at   9.426 9.543 9.403 9.309 9.397 8.722 8.607 8.894 8.902 8.95
   460   1370941_at   9.123 9.258 9.343 9.219 9.129 10.099 9.804 10.103 10.015 9.704
   461   1368224_at   4.111 4.438 5.092 4.041 3.927 7.091 7.233 6.633 6.314 6.182
   462   1388393_at   8.728 8.604 9.171 8.561 8.553 9.967 10.055 9.867 9.633 9.711
   463   1387952_a_at   5.402 5.371 6.229 5.535 5.445 7.881 8.089 7.639 7.191 7.12
   464   1393041_at   5.46 6.266 5.418 5.389 5.53 7.438 7.437 7.518 7.024 6.91
   465   1389326_at   5.818 6.076 6.273 5.873 5.779 7.068 6.897 7.159 6.706 6.87
   466   1368992_a_at   10.753 10.843 10.856 10.502 10.569 9.97 9.966 10.19 9.904 10.015
   467   1388479_at   6.266 6.213 6.229 5.928 5.814 7.752 7.208 7.485 7.374 6.98
   468   1385781_at   3.362 3.128 3.772 2.87 3.004 5.172 5.053 4.725 4.445 5.087
   469   1369950_at   8.901 8.978 8.906 8.985 8.677 9.768 9.597 9.748 9.682 9.36
   470   1374902_at   3.5 1.664 2.513 3.438 2.467 6.666 5.848 5.893 5.41 5.735
   471   1386933_at   9.973 9.728 10.122 9.959 9.509 7.804 7.846 8.308 8.231 8.785
   472   1378802_at   10.842 10.751 10.696 10.776 10.528 9.909 9.858 10.11 10.23 10.058
   473   1375862_at   5.973 5.655 5.61 5.884 5.832 7.848 7.256 7.023 7.517 6.858
   474   1370566_at   9.189 9.108 9.028 9.573 9.193 8.103 8.163 8.485 8.253 8.397
   475   1393059_at   5.322 5.198 5.46 5.275 5.093 6.042 6.238 5.81 6.239 6.029
   476   1376652_at   8.012 8.045 8.383 7.861 8.082 9.874 9.585 9.324 9.445 8.974
   477   1377452_at   4.151 5.52 5.252 4.558 5.007 7.348 6.732 7.059 7.395 6.922
   478   1385458_a_at   7.112 6.805 7.051 6.808 6.727 7.597 7.617 7.711 7.486 7.512
   479   1379347_at   6.425 6.571 6.557 6.544 6.548 7.323 7.229 7.357 7.05 6.957
   480   1370550_at   2.195 3.732 2.236 2.134 1.96 5.365 5.269 5.469 6.116 4.966
   481   1369976_at   10.142 10.175 10.5 10.195 10.384 11.139 11.196 11.166 10.917 10.865
   482   1371763_at   11.774 11.936 11.881 11.813 11.742 11.11 11.092 11.314 11.358 11.39
   483   1368304_at   11.817 11.697 11.819 11.84 11.955 11.07 10.94 11.228 11.284 11.324
   484   1374289_at   8.114 8.124 8.196 8.061 8.262 8.454 8.495 8.503 8.604 8.457
   485   1369259_at   8.053 8.16 8.191 8.158 8.123 7.125 7.089 7.585 7.321 7.511
   486   1388776_at   9.583 9.519 9.586 9.375 9.296 10.205 10.03 10.171 10.133 9.919
   487   1367614_at   9.947 10.175 10.166 9.867 9.833 11.083 11.083 11.077 10.835 10.616
   488   1370857_at   8.232 7.869 8.511 7.93 8.041 9.336 9.341 9.233 8.965 9.504
   489   1373250_at   5.084 4.565 5.494 5.291 4.348 7.527 6.936 7.156 6.927 6.632
   490   1388271_at   12.743 12.48 12.67 12.697 13.012 11.987 12.065 12.046 11.965 12.061
   491   1371712_at   7.687 7.463 7.731 7.489 7.573 8.172 8.168 7.961 8.157 8.097
   492   1388622_at   8.023 8.156 7.827 7.96 7.919 8.464 8.723 8.517 8.5 8.509
   493   1367693_at   9.468 9.596 9.73 9.421 9.527 10.298 10.285 10.246 9.991 10.416
   494   1367584_at   9.81 9.964 10.418 9.693 9.784 11.233 11.264 11.035 11.009 10.897
   495   1369105_a_at   1.736 2.932 1.744 2.23 2.154 4.773 4.316 5.003 3.842 4.519
   496   1370215_at   7.332 6.843 7.125 6.939 7.323 8.919 8.723 8.462 8.361 8.07
   497   1370301_at   7.333 7.044 7.172 7.341 7.616 8.863 8.341 8.641 9.132 8.413
   498   1383401_at   8.202 8.273 8.461 8.004 8.363 9.214 9.342 9.245 9.031 8.89
   499   1379770_at   11.017 10.956 10.801 11 10.869 10.353 10.428 10.465 10.616 10.421
   500   1368464_at   5.002 5.08 5.68 4.981 4.657 7.584 7.04 6.817 7.079 6.494
   501   1370228_at   7.907 7.797 7.75 7.619 7.979 8.804 8.76 8.906 9.048 8.406
   502   1392713_a_at   8.962 9.448 9.057 9.068 8.924 8.2 8.388 8.388 8.217 8.194
   503   1372154_at   8.257 8.357 8.181 8.182 8.137 8.634 8.704 8.603 8.588 8.518
   504   1373595_at   8.393 8.465 8.517 8.392 8.303 8.893 8.866 9.009 8.894 8.72
   505   1373172_at   5.315 5.751 5.368 5.311 5.205 6.061 6.181 6.255 6.183 6.159
   506   1376707_at   6.135 6.281 6.36 6.104 6.255 5.632 5.827 5.515 5.735 5.66
   507   1398751_at   11.59 11.473 11.476 11.504 11.47 11.966 12.069 11.909 11.774 12.039
   508   1386857_at   10.407 10.542 10.29 10.514 10.487 11.434 11.241 11.288 10.966 11.052
   509   1379059_at   4.587 4.685 4.818 4.176 4.487 5.786 5.583 5.712 5.322 5.605
   510   1393280_at   6.545 6.598 6.949 6.798 6.767 8.105 7.851 7.538 7.784 7.512
   511   1387965_at   3.769 1.536 5.761 4.854 3.448 10.97 11.468 11.001 9.932 8.981
   512   1372869_at   11.692 11.38 11.669 11.401 11.348 9.972 9.552 9.861 10.112 10.608
   513   1387737_at   10.674 10.157 10.575 10.59 10.738 9.799 9.491 9.488 9.538 9.719
   514   1391643_at   8.244 8.187 8.258 8.207 8.058 7.469 7.233 7.468 7.54 7.746
   515   1389306_at   6.499 6.523 6.105 6.599 7.136 7.99 7.76 8.03 8.08 7.853
   516   1374584_at   10.065 9.964 9.912 9.789 9.846 9.29 9.44 9.481 9.525 9.398
   517   1385829_at   6.869 6.874 6.881 6.722 6.71 6.108 5.725 6.184 6.207 6.097
   518   1389885_at   6.667 6.737 7.189 7.016 6.433 8.22 7.841 8.065 7.954 7.851
   519   1388867_at   8.332 8.514 8.088 8.166 8.181 9.202 9.172 9.199 9.046 8.81
   520   1379317_a_at   6.587 6.839 6.853 6.767 6.831 7.335 7.368 7.523 7.558 7.216
   521   1383643_at   9.36 9.4 9.504 9.428 9.28 9.831 9.787 9.842 9.771 9.66
   522   1374307_at   9.925 10.088 9.911 9.873 9.901 9.001 9.205 9.218 9.432 9.416
   523   1374244_at   9.496 9.633 9.412 9.432 9.499 8.461 8.472 8.818 8.944 8.747
   524   1374959_at   10.075 10.256 9.969 9.968 10.138 9.384 9.596 9.614 9.594 9.544
   525   1367597_at   11.95 11.93 11.954 11.903 11.827 12.349 12.236 12.177 12.195 12.171
   526   1384581_at   6.272 6.067 6.189 6.223 6.156 7.073 6.743 6.974 6.657 6.762
   527   1388802_at   6.202 6.168 6.097 5.946 5.791 6.868 6.942 6.789 6.646 6.663
   528   1368671_at   6.244 5.934 5.944 6.313 5.932 7.252 7.42 7.042 7.234 6.778
   529   1372493_at   6.586 6.615 6.475 6.835 6.369 7.314 7.236 7.5 7.392 7.178
   530   1387893_at   9.266 9.095 8.902 9.003 8.929 10.118 9.816 9.847 9.843 9.609
   531   1392829_at   6.497 6.423 6.559 6.612 6.406 7.02 6.826 6.953 6.954 7.114
   532   1386938_at   11.599 11.845 11.85 11.667 11.716 11.179 11.2 11.321 11.331 11.329
   533   1370991_at   10.272 10.301 9.962 10.323 10.471 9.138 9.502 9.461 9.503 9.497
   534   1384695_at   5.293 5.762 6.104 5.241 5.405 7.262 6.763 6.877 6.947 7.094
   535   1383917_at   5.984 6.097 6.219 6.203 6.056 6.95 6.596 6.868 6.702 6.622
   536   1388217_a_at   8.968 8.927 8.957 8.773 8.756 9.619 9.477 9.54 9.41 9.264
   537   1382266_at   6.503 6.007 6.404 6.362 6.398 5.658 5.661 5.353 5.455 5.539
   538   1387769_a_at   8.818 9.215 9.196 9.346 9.179 10.509 10.138 10.218 10.141 9.886
   539   1376816_at   3.913 3.652 2.738 2.818 3.346 5.286 5.377 5.029 4.958 5.138
   540   1370948_a_at   6.044 6.364 5.476 6.331 5.906 7.841 7.425 7.537 7.693 7.191
   541   1397476_at   1.585 2.954 0.895 0.856 2.39 5.569 4.968 4.924 4.798 5.107
   542   1390692_at   5.689 5.802 5.973 5.856 5.669 6.974 6.529 6.86 6.414 6.835
   543   1389732_at   8.056 8.294 8.003 8.177 8.207 9.024 8.759 8.782 8.659 8.693
   544   1375657_at   8.84 9.039 8.85 8.835 8.88 8.278 7.996 8.396 8.274 8.38
   545   1384900_at   3.515 4.14 3.784 4.312 3.628 5.854 5.458 5.01 5.368 5.647
   546   1381971_at   5.862 6.2 6.619 6.906 6.3 7.683 7.801 7.733 8.04 7.955
   547   1387382_at   11.235 11.353 11.151 11.065 10.971 10.015 9.952 10.27 10.256 10.54
   548   1386862_at   10.467 10.53 10.881 10.345 10.384 11.327 11.294 11.454 11.238 11.243
   549   1388324_at   11.409 11.436 11.416 11.511 11.448 10.945 11.111 11.16 11.141 11.161
   550   1378507_at   3.048 3.543 4.439 3.539 4.101 5.947 5.963 5.78 5.798 5.354
   551   1371969_at   9.493 9.492 8.988 9.238 9.254 10.373 10.367 10.358 10.136 9.971
   552   1370289_x_at   8.985 8.999 8.527 8.797 7.501 5.699 6.622 5.82 5.63 6.229
   553   1392732_at   4.326 5.15 5.18 3.058 4.754 8.204 7.454 7.72 7.452 7.521
   554   1388114_at   10.623 10.678 10.556 10.51 10.753 11.338 11.439 11.314 11.077 11.102
   555   1380420_at   3.964 4.544 4.719 3.246 4.211 2.22 1.74 2.084 1.39 1.952
   556   1370887_at   7.115 6.89 7.152 6.865 6.851 8.35 7.763 8.189 7.85 7.813
   557   1382903_at   6.836 6.88 6.631 6.968 6.722 7.415 7.486 7.49 7.352 7.226
   558   1369181_at   6.454 6.616 6.831 6.841 6.288 7.977 7.892 7.566 7.604 7.484
   559   1373024_at   8.061 8.105 8.145 8.102 7.971 8.871 8.725 8.86 8.699 8.434
   560   1373099_at   8.175 8.283 8.218 8.288 8.088 8.685 8.607 8.52 8.597 8.509
   561   1370504_a_at   6.84 6.407 6.499 6.741 6.79 7.532 7.457 7.591 7.257 7.373
   562   1372488_at   7.418 7.651 7.631 7.515 7.576 8.21 8.126 8.234 8.024 7.923
   563   1388751_at   10.021 9.977 10.024 9.974 10.125 9.657 9.673 9.76 9.581 9.503
   564   1386989_at   5.53 5.249 5.488 5.435 5.755 6.908 6.375 6.62 7.14 6.509
   565   1377869_at   8.738 8.116 8.903 7.577 8.241 6.434 6.079 6.5 6.12 6.623
   566   1372308_at   6.24 6.389 6.321 6.232 6.257 6.9 6.57 6.823 6.866 6.761
   567   1376100_at   4.135 4.558 5.241 4.148 5.216 8.285 7.932 7.333 6.798 7.005
   568   1368393_at   7.257 7.349 7.037 7.361 7.332 8.145 7.916 8.23 7.862 7.868
   569   1388010_at   3.68 4.009 3.897 4.198 3.748 5.777 5.181 5.692 4.851 5.341
   570   1372510_at   6.707 6.943 7.13 6.39 6.611 7.699 7.789 7.668 7.856 7.878
   571   1395532_at   9.564 9.731 9.562 9.475 9.314 8.994 8.874 9.029 9.023 8.939
   572   1372325_at   7.974 7.264 7.73 7.902 7.617 8.798 8.771 8.728 8.787 8.516
   573   1385573_at   6.189 6.28 6.013 6.326 6.233 6.657 6.727 6.883 6.988 6.782
   574   1387145_at   8.249 8.073 8.078 8.339 8.3 7.383 7.554 7.742 7.379 7.616
   575   1367999_at   11.348 11.456 11.536 11.458 11.519 10.923 10.966 11.07 11.188 11.047
   576   1383291_at   10.001 9.92 9.103 10.028 9.887 11.317 11.321 11.279 11.187 10.925
   577   1395663_at   5.538 5.218 5.581 5.178 5.358 6.141 6.386 6.137 6.176 5.975
   578   1371864_at   11.349 11.266 10.879 11.075 11.537 10.253 10.332 10.348 10.34 10.037
   579   1369952_at   11.36 11.286 11.351 11.272 11.293 11.86 11.959 11.923 11.833 11.58
   580   1388469_at   8.145 8.5 8.334 8.139 8.299 7.48 7.462 7.363 7.781 7.28
   581   1390416_at   10.839 10.677 10.919 10.958 10.873 10.081 9.786 10.24 10.188 10.256
   582   1367680_at   11.736 11.794 11.711 11.899 11.828 11.038 11.056 11.13 11.335 11.394
   583   1382419_at   4.075 4.793 4.955 4.098 5.254 6.593 6.597 6.82 6.286 6.375
   584   1373803_a_at   9.292 9.052 8.748 8.968 8.88 8.099 8.272 8.249 8.351 8.122
   585   1377239_at   6.106 5.791 6.059 5.64 5.831 7.363 7.064 6.871 7.108 6.633
   586   1371237_a_at   12.813 12.585 12.597 12.71 12.964 11.876 11.928 12.026 12.157 12.185
   587   1378140_at   3.756 4.55 3.988 3.736 4.79 6.179 6.042 6.185 5.965 5.556
   588   1373473_a_at   7.295 7.29 7.349 7.19 7.606 8.183 8.059 8.19 7.846 7.995
   589   1377804_at   2.907 2.597 2.685 2.978 3.219 3.66 3.811 3.756 3.713 3.686
   590   1376153_at   6.941 7.164 7.265 7.093 6.909 8.144 7.996 8.16 8.289 7.641
   591   1372631_at   6.045 6.581 6.832 6.201 6.286 8.176 7.671 7.854 7.453 7.698
   592   1368016_at   11.914 11.917 12.047 12.03 12.001 11.611 11.654 11.56 11.663 11.411
   593   1368908_at   8.948 8.965 8.897 8.72 8.55 9.469 9.533 9.517 9.457 9.336
   594   1376109_at   6.344 6.107 6.377 6.469 6.175 8.129 7.834 8.004 7.386 7.208
   595   1369097_s_at   5.903 6.105 5.319 5.488 5.405 7.097 6.881 7.177 6.875 6.704
   596   1374912_at   4.272 3.727 3.206 2.759 2.74 6.346 5.549 6.42 6.117 5.481
   597   1375695_at   7.618 7.394 7.557 7.4 7.503 7.923 8.048 8.355 8.093 8.167
   598   1367721_at   12.215 12.1 12.254 12.119 12.067 11.764 11.676 11.696 11.769 11.896
   599   1395958_at   6.899 7.031 6.821 7.239 6.715 7.749 7.608 7.866 7.845 7.602
   600   1374704_at   6.353 6.435 6.831 6.62 6.663 7.498 7.427 7.581 7.352 7.162
   601   1388994_at   6.07 6.334 6.265 6.806 6.002 7.439 7.48 7.547 7.24 7.368
   602   1380393_at   11.222 11.237 11.375 11.216 11.284 10.534 10.651 10.902 10.8 10.803
   603   1368829_at   7.853 8.048 7.749 7.716 7.606 9.914 9.23 10.007 9.502 8.837
   604   1376248_at   7.807 7.629 7.555 7.553 7.489 7.1 6.703 7.008 6.96 7.027
   605   1376219_at   6.657 6.327 6.717 6.495 6.422 7.008 7.089 7.086 7.093 7.11
   606   1398828_at   9.949 9.797 9.931 9.992 9.862 10.492 10.296 10.399 10.26 10.243
   607   1367899_at   9.708 9.759 10.009 9.519 9.691 10.429 10.388 10.482 10.31 10.289
   608   1380822_at   4.216 4.088 2.911 3.989 4.121 5.664 5.694 5.935 5.999 5.526
   609   1388447_at   8.456 8.6 8.445 8.505 8.137 9.395 8.958 9.379 9.234 9.378
   610   1373990_at   10.886 10.873 11.073 10.687 10.903 8.367 7.909 8.31 8.785 9.682
   611   1384874_at   8.294 8.007 8.001 8.035 7.818 6.932 6.781 7.221 7.289 7.257
   612   1368293_at   3.923 4.792 4.223 5.336 5.101 7.386 6.553 7.125 6.884 6.674
   613   1389815_at   9.374 9.405 9.495 9.311 9.018 10.097 10.219 10.032 10.156 9.861
   614   1383241_at   8.933 9.165 8.989 9.271 9.184 10.054 9.852 9.67 9.683 9.773
   615   1384405_at   7.415 7.413 7.423 7.344 7.439 7.813 7.756 8.005 8.053 7.773
   616   1391201_at   1.428 2.905 1.144 0.506 1.111 4.677 4.482 4.336 4.161 4.636
   617   1392946_at   6.007 6.178 6.197 6.109 6.106 7.219 6.762 6.962 6.701 6.736
   618   1372219_at   8.077 7.451 8.046 7.358 7.496 8.997 8.983 9.133 8.734 8.838
   619   1373599_at   6.586 6.568 6.705 6.739 6.282 7.896 7.247 7.94 7.684 7.584
   620   1380631_at   2.263 2.198 2.406 2.43 2.996 3.869 3.633 4.129 4.102 3.5
   621   1368187_at   5.633 6.401 7.457 6.101 6.314 9.54 9.337 9.349 9.093 8.289
   622   1372886_at   3.213 4.334 4.17 5 4.313 7.134 6.637 6.465 6.279 6.41
   623   1375216_at   6.101 6.826 6.755 6.742 6.509 7.715 7.758 7.648 7.46 7.595
   624   1387048_at   6.697 7.22 6.934 7.018 7.061 8.488 7.868 7.951 8.039 7.874
   625   1387895_s_at   4.953 5.469 4.256 5.871 5.023 7.97 8.035 8.456 7.911 6.908
   626   1380546_at   9.584 9.769 9.594 9.552 9.491 9.065 8.911 9.258 9.038 8.979
   627   1370309_a_at   10.565 10.556 10.393 10.393 10.357 10.899 10.881 10.986 10.984 10.759
   628   1376613_at   7.434 7.516 7.54 7.667 7.573 8.222 7.969 8.144 7.951 7.953
   629   1383324_at   9.015 9.167 9.252 9.079 9.045 8.557 8.308 8.642 8.667 8.559
   630   1393891_at   3.497 3.42 3.92 3.507 2.077 6.261 5.749 5.979 5.715 5.4
   631   1370493_a_at   4.77 4.566 4.786 4.523 4.432 6.565 6.232 5.821 6.029 5.505
   632   1386925_at   9.494 9.541 9.684 9.49 9.259 10.35 10.228 10.199 9.994 10.078
   633   1372179_at   10.114 9.788 10.009 9.963 10.018 9.153 9.141 9.367 9.449 9.482
   634   1372024_at   7.232 7.036 7.226 7.08 7.247 6.79 6.644 6.824 6.596 6.62
   635   1371352_at   10.76 10.794 10.6 10.795 10.708 11.597 11.446 11.376 11.207 11.176
   636   1389073_at   7.065 6.93 7.194 7.05 6.81 7.833 7.702 7.949 7.484 7.812
   637   1390355_at   4.646 5.235 5.282 5.139 5.407 3.525 3.216 4.119 3.762 3.791
   638   1371359_at   10.243 10.199 10.221 10.305 10.158 9.957 9.73 9.842 9.917 9.933
   639   1377504_at   5.372 4.953 5.501 4.967 4.745 6.32 6.033 6.162 6.283 6.376
   640   1388988_at   11.671 11.654 11.644 11.752 11.707 11.374 11.33 11.37 11.471 11.498
   641   1367631_at   11.972 11.652 11.222 10.688 11.412 9.951 9.814 9.689 9.511 9.675
   642   1393672_at   4.059 4.06 3.391 3.959 4.465 5.653 5.476 5.735 5.241 5.207
   643   1389852_at   3.724 3.963 3.557 4.773 4.114 6.154 5.572 6.074 5.572 6.352
   644   1372264_at   12.436 12.39 12.105 12.381 12.698 11.503 11.569 11.747 11.518 11.736
   645   1370623_at   5.767 5.87 5.797 5.449 5.711 6.735 6.516 6.257 6.497 6.431
   646   1388429_at   6.836 7.035 6.917 6.917 6.779 7.663 7.668 7.603 7.541 7.247
   647   1376914_at   1.832 3.459 3.186 3.493 3.074 6.577 5.835 5.967 5.583 5.132
   648   1382003_at   9.053 9.075 8.886 8.94 8.865 8.414 8.41 8.477 8.585 8.64
   649   1388463_at   10.704 10.745 10.655 10.854 10.731 10.259 10.341 10.439 10.459 10.406
   650   1369013_a_at   10.786 10.833 10.856 10.842 10.901 11.175 11.165 11.092 11.029 11.043
   651   1371322_at   8.452 8.676 8.554 8.574 8.699 9.224 9.042 9.146 9.091 8.919
   652   1372103_at   8.374 8.369 8.26 8.028 8.426 7.323 7.52 7.791 7.552 7.532
   653   1398378_at   10.005 10.038 10.244 9.925 9.996 9.156 9.232 9.451 9.585 9.387
   654   1371539_at   8.265 8.526 8.404 8.491 8.375 9.193 9.215 8.962 8.877 8.907
   655   1384051_at   4.84 5.502 5.546 5.06 5.293 6.321 6.117 6.384 6.328 6.224
   656   1392544_at   7.061 6.984 7.082 6.942 6.681 7.593 7.532 7.579 7.405 7.506
   657   1388137_a_at   8.504 8.492 8.557 8.579 8.417 8.876 8.775 8.97 8.821 8.776
   658   1391466_at   6.236 6.163 6.064 5.86 5.83 6.9 6.561 7.025 6.985 6.829
   659   1384960_at   6.359 6.051 6.582 6.515 6.271 5.025 4.682 5.35 5.072 4.299
   660   1369182_at   10.298 10.417 9.691 9.84 10.176 8.936 8.947 8.908 8.792 8.327
   661   1398775_at   11.791 11.785 11.643 11.696 11.711 12.162 12.331 12.055 12.123 12.051
   662   1377887_at   8.292 8.298 8.421 8.454 8.229 7.824 7.739 8.007 7.883 7.954
   663   1393545_at   6.463 6.212 6.068 6.618 6.405 5.457 5.255 5.732 5.358 5.469
   664   1375910_at   6.513 6.484 6.4 6.443 6.609 7.25 6.976 7.268 7.243 6.883
   665   1392001_at   7.403 7.401 7.18 7.091 6.639 5.765 5.736 5.873 6.177 6.134
   666   1389632_at   9.453 9.443 9.733 9.478 9.285 8.912 8.757 8.933 8.824 8.604
   667   1383253_at   10.216 9.987 10.244 9.973 9.832 9.319 9.219 9.44 9.474 9.467
   668   1372233_at   9.342 9.355 9.484 9.359 9.331 9.629 9.627 9.669 9.589 9.561
   669   1373605_at   9.404 9.309 9.384 8.812 9.221 8.159 8.348 8.168 8.524 8.347
   670   1381776_at   8.034 8.017 8.504 8.347 8.28 9.211 9.25 9.509 9.072 8.936
   671   1373156_at   5.555 5.532 5.249 5.311 6.03 7.06 6.612 6.994 6.885 6.475
   672   1386944_a_at   11.835 11.89 11.516 12.145 12.035 11.082 10.845 11.027 11.171 11.031
   673   1368743_a_at   2.658 3.053 2.299 3.053 2.826 3.734 4.214 4.547 4.057 4.235
   674   1372820_at   7.077 7.164 6.619 7.085 6.903 8.004 7.787 7.949 7.903 7.583
   675   1379234_a_at   5.749 5.474 5.795 6.086 5.455 7.013 6.583 7.101 6.631 6.76
   676   1383133_at   6.923 7.108 6.711 6.996 7.028 7.614 7.637 7.729 7.513 7.401
   677   1383371_at   6.779 6.377 6.611 6.662 6.643 7.03 7.239 7.212 7.108 7.187
   678   1388557_at   10.544 10.663 9.895 10.554 10.513 11.657 11.608 11.586 11.448 11.291
   679   1367696_at   11.199 11.179 11.135 11.169 11.034 11.421 11.47 11.323 11.421 11.43
   680   1395572_at   7.475 7.198 7.004 7.37 7.252 6.22 5.961 6.266 6.365 6.644
   681   1368779_a_at   7.881 7.504 8.144 7.805 7.682 5.969 4.744 5.992 6.182 6.053
   682   1388581_at   8.239 8.398 8.579 8.261 8.111 9.227 9.043 9.126 9.054 8.822
   683   1383406_at   4.629 4.75 4.848 4.785 4.609 5.128 5.375 5.503 5.349 5.178
   684   1399059_at   9.143 9.158 9.45 9.106 9.094 8.637 8.579 8.78 8.666 8.684
   685   1390292_at   9.593 9.59 9.416 9.629 9.551 9.136 8.928 9.163 9.23 9.035
   686   1377689_at   3.778 4.296 4.564 4.193 4.196 6.145 6.12 5.586 5.714 5.19
   687   1384063_at   3.802 4.374 4.663 2.192 3.969 7.454 7.574 7.045 7.196 6.447
   688   1373597_at   7.815 7.834 7.989 7.913 7.806 8.263 8.358 8.303 8.103 8.283
   689   1379972_at   3.73 4.121 4.359 4.383 4.206 5.229 4.964 5.012 5.382 5.154
   690   1374389_at   6.628 6.79 5.953 6.794 6.636 7.791 7.813 7.76 7.986 7.53
   691   1387081_at   8.011 8.222 7.749 7.867 7.956 8.602 8.464 8.547 8.524 8.6
   692   1368509_at   9.039 9.17 9.182 9.064 8.98 8.427 8.438 8.658 8.632 8.729
   693   1394464_at   5.187 5.098 5.025 5.112 5.087 5.931 5.653 6.13 5.803 5.582
   694   1389189_at   8.602 8.34 8.62 8.422 8.648 9.73 9.849 9.834 9.393 9.148
   695   1388677_at   8.735 8.769 8.626 8.764 8.604 8.283 8.452 8.312 8.42 8.31
   696   1374817_at   5.232 5.512 5.574 5.76 5.576 6.107 6.146 6.255 6.189 6.119
   697   1373947_at   9.549 9.426 8.939 9.702 9.51 10.542 10.312 10.353 10.503 10.309
   698   1378551_at   8.518 8.424 8.317 8.331 8.278 8.757 8.835 8.897 8.708 8.7
   699   1367881_at   7.379 7.559 7.281 7.513 7.071 8.17 8.059 8.063 7.996 7.922
   700   1386994_at   9.9 9.614 10.871 8.931 9.871 7.811 7.675 7.565 7.44 7.413
   701   1372076_at   11.359 11.437 11.415 11.565 11.434 10.968 11.122 11.176 11.137 11.059
   702   1397584_at   8.898 8.907 8.992 8.766 8.903 8.351 8.318 8.368 8.551 8.578
   703   1395003_at   5.73 5.783 5.934 6.244 5.954 7.306 6.87 6.889 7.052 6.616
   704   1367596_at   11.93 11.834 11.693 11.853 11.832 12.223 12.243 12.113 12.141 12.103
   705   1371017_at   4.218 4.392 4.18 4.586 4.325 5.339 5.272 5.075 5.11 4.845
   706   1385072_at   10.924 11.007 10.765 10.842 10.901 10.348 10.119 10.419 10.514 10.354
   707   1399015_at   9.009 9.061 8.703 8.921 8.925 9.403 9.375 9.395 9.405 9.297
   708   1393777_at   9.565 9.737 9.392 9.542 9.611 9.022 8.983 9.243 9.051 9.026
   709   1372199_at   9.089 9.16 9.087 9.001 8.973 8.423 8.188 8.568 8.624 8.545
   710   1370036_at   9.548 9.649 9.42 9.366 9.337 8.612 8.808 8.973 8.968 8.86
   711   1387218_at   12.743 12.748 12.93 12.813 12.883 11.808 11.585 12.05 12.017 12.291
   712   1399052_at   8.879 9.095 9.005 8.706 8.885 8.391 8.362 8.516 8.412 8.32
   713   1379340_at   4.963 4.819 5.519 4.435 5.291 6.878 7.35 6.928 6.401 6.447
   714   1371420_at   9.291 9.23 9.382 9.063 9.184 8.452 8.083 8.539 8.428 8.69
   715   1399050_at   10.506 10.483 10.555 10.509 10.462 10.822 10.904 10.801 10.688 10.727
   716   1380474_at   5.438 5.197 5.038 5.609 6.052 7.067 7.114 6.947 6.853 6.523
   717   1373025_at   8.18 8.038 8.215 7.874 8.094 9.433 9.096 8.893 9.042 8.695
   718   1367717_at   12.191 12.131 12.159 12.117 12.066 12.302 12.294 12.36 12.283 12.297
   719   1370258_at   6.681 6.726 6.893 6.6 6.373 7.654 7.583 7.635 7.475 7.179
   720   1382918_at   6.809 6.88 6.834 6.908 6.777 5.526 5.858 6.146 6.213 6.062
   721   1370000_at   7.202 7.119 7.114 6.82 7.015 7.863 7.74 7.863 7.685 7.48
   722   1372957_at   2.365 1.824 2.982 2.365 4.147 6.193 5.705 5.941 5.179 5.696
   723   1373866_at   9.711 9.593 9.562 9.235 9.575 8.876 8.971 8.976 8.974 9.004
   724   1384259_at   4.482 3.542 3.709 4.555 4.273 6.469 5.945 5.838 5.574 5.693
   725   1388389_at   9.603 9.621 9.597 9.471 9.507 10.326 10.118 10.262 10.09 9.882
   726   1391862_at   6.28 6.835 6.386 6.718 6.724 7.458 7.229 7.522 7.407 7.462
   727   1391417_at   12.199 12.164 11.996 12.19 12.205 11.486 11.725 11.734 11.745 11.78
   728   1377968_at   5.595 5.546 6.059 5.743 5.766 6.843 6.831 6.9 6.417 6.466
   729   1375997_at   9.324 9.192 9.048 9.36 9.379 8.641 8.505 8.631 8.853 8.725
   730   1370377_at   10.799 10.726 11.044 10.793 10.777 9.463 9.371 9.984 9.885 10.089
   731   1397767_at   5.032 5.261 4.773 5.086 4.647 5.992 6.024 6.307 6.04 5.662
   732   1382228_at   6.625 6.749 6.568 6.715 6.423 7.306 7.043 7.23 7.252 7.037
   733   1379261_at   6.92 6.972 6.97 6.921 6.859 7.667 7.494 7.773 7.255 7.65
   734   1388436_at   5.933 6.316 6.219 6.568 6.063 7.302 7.292 7.282 6.993 6.933
   735   1387966_at   10.165 10.27 10.375 10.046 9.97 9.354 9.162 9.624 9.484 9.494
   736   1383227_at   7.302 7.284 7.196 7.01 7.159 7.632 7.592 7.823 7.585 7.674
   737   1373337_at   11.993 12.045 11.911 12.057 11.992 11.647 11.751 11.66 11.82 11.711
   738   1381933_at   6.94 6.866 6.806 6.962 6.661 7.519 7.8 7.659 7.339 7.395
   739   1390659_at   6.252 6.247 6.581 6.178 5.928 8.246 8.53 8.372 7.758 7.233
   740   1379592_at   9.788 9.823 9.461 9.913 9.616 9.15 9.033 9.192 9.037 9.168
   741   1367775_at   11.588 11.696 11.578 11.721 11.482 11.111 11.09 11.201 11.329 11.224
   742   1390918_at   9.93 9.805 9.585 9.938 9.847 9.193 9.195 9.412 9.358 9.262
   743   1387777_at   9.937 9.927 9.93 9.852 9.953 10.292 10.427 10.266 10.207 10.146
   744   1370048_at   6.455 6.381 6.193 6.212 5.853 7.159 7.001 7.592 7.377 7.1
   745   1373223_at   5.185 4.961 5.267 5.144 5.006 6.098 5.839 5.788 5.554 5.861
   746   1374961_at   5.8 6.269 6.552 6.371 6.359 7.26 7.349 7.133 7.122 7.121
   747   1368134_a_at   8.212 8.134 8.36 7.919 7.757 7.151 6.581 6.923 7.061 7.24
   748   1382778_at   6.92 6.963 7.203 7.265 7.212 6.468 6.403 6.698 6.508 6.512
   749   1378028_at   4.272 4.369 4.899 3.926 4.114 5.949 6.045 5.645 5.596 5.379
   750   1387665_at   7.654 7.902 7.635 7.865 7.66 7.048 6.968 7.371 6.992 7.135
   751   1375010_at   6.516 6.386 7.075 6.484 6.383 8.463 7.802 7.867 7.85 7.586
   752   1367838_at   10.781 10.808 10.854 10.622 10.748 10.206 10.043 10.256 10.253 10.449
   753   1393666_at   7.77 8.102 7.995 7.668 7.845 7.102 7.03 7.414 7.169 7.02
   754   1390441_at   5.501 5.92 5.565 5.963 5.578 6.444 6.628 6.496 6.304 6.461
   755   1369313_at   8.006 7.929 7.367 7.782 7.726 8.878 8.725 8.712 8.813 8.406
   756   1370242_at   12.363 12.32 12.216 12.41 12.265 12.68 12.594 12.579 12.561 12.564
   757   1373932_at   6.702 6.471 6.797 6.913 6.827 7.811 7.561 7.537 7.62 7.248
   758   1389360_at   4.831 4.854 5.076 4.455 5.043 6.096 5.662 5.648 5.981 6.146
   759   1383684_at   2.328 3.184 3.86 3.662 3.126 5.463 5.417 5.194 4.942 4.986
   760   1374070_at   7.355 8.431 8.053 8.145 8.01 9.566 9.855 9.492 9.45 9.076
   761   1368794_at   10.708 11.005 11.008 10.892 10.508 9.584 9.386 9.684 10.012 10.034
   762   1376749_at   9.024 8.916 8.312 9.001 8.92 9.925 9.832 10.03 10.053 9.622
   763   1380809_at   0.632 0 0.687 2.05 0.251 3.905 3.018 3.909 4.183 3.144
   764   1367917_at   11.565 11.432 11.528 11.599 11.471 10.679 10.999 11.051 11.025 11.092
   765   1389787_at   7.323 7.174 7.555 6.897 7.028 8.218 7.945 8.123 8.443 8.096
   766   1387867_at   11.046 11.09 11.159 11.079 11.011 10.609 10.635 10.72 10.779 10.858
   767   1390348_at   5.606 5.69 6.396 5.748 5.327 7.61 7.302 7.25 7.077 6.877
   768   1369672_at   6.126 5.56 5.245 5.753 5.816 6.736 7.016 6.966 6.647 6.722
   769   1378180_at   5.439 5.639 5.18 5.558 5.66 6.733 6.818 6.991 6.295 6.307
   770   1391063_at   5.588 4.021 4.866 4.747 4.824 7.211 6.77 6.649 6.578 6.451
   771   1379603_at   5.62 5.702 5.28 5.7 5.346 6.328 6.274 6.553 6.228 6.147
   772   1368460_at   9.822 9.776 9.773 9.826 9.693 9.294 9.06 9.491 9.301 9.257
   773   1368915_at   10.4 10.529 10.471 10.545 10.412 9.748 9.794 9.727 10.127 10.024
   774   1382192_at   7.539 7.614 7.307 7.509 7.639 8.136 8.193 8.41 8.347 7.949
   775   1371916_at   11.925 11.925 11.801 12.037 11.983 11.553 11.526 11.594 11.669 11.663
   776   1367913_at   8.737 8.552 8.335 8.756 8.799 9.57 9.234 9.718 9.677 9.346
   777   1392407_at   4.826 5.085 5.777 5.127 4.928 6.76 6.704 6.611 6.188 6.319
   778   1390861_at   4.809 4.786 4.88 5.269 4.943 5.529 5.629 5.594 5.532 5.499
   779   1382496_at   9.162 8.71 8.882 9.23 9.044 8.028 8.085 8.106 8.413 8.307
   780   1371377_at   11.819 11.862 11.799 11.714 11.847 12.379 12.508 12.275 12.221 12.115
   781   1384824_at   6.168 5.954 5.462 5.912 6.186 7.671 7.142 7.351 7.216 6.816
   782   1369956_at   9.154 8.989 8.93 9.185 9.147 9.778 9.737 9.739 9.712 9.394
   783   1368651_at   8.849 9.006 9.152 8.808 8.582 7.627 7.386 7.655 7.978 8.137
   784   1370243_a_at   11.477 11.526 11.384 11.453 11.499 11.882 11.906 11.8 11.678 11.741
   785   1376128_at   9.066 9.345 9.05 9.088 9.064 8.523 8.518 8.45 8.769 8.645
   786   1393421_at   8.543 8.733 8.525 8.707 8.517 7.888 7.592 8.009 8.084 8.035
   787   1388761_at   7.834 7.988 7.83 7.744 7.784 8.592 8.35 8.409 8.289 8.197
   788   1373852_at   6.593 6.894 7.06 6.869 6.676 7.501 7.396 7.622 7.339 7.538
   789   1370725_a_at   11.182 11.31 10.518 11.417 11.268 9.964 10.1 9.844 10.128 9.842
   790   1398860_at   11.177 11.179 11.221 11.16 11.072 11.346 11.383 11.306 11.389 11.389
   791   1381993_at   8.403 8.555 8.319 8.399 8.495 8.96 8.841 9.166 9.096 8.82
   792   1379074_at   5.334 5.516 5.841 5.807 5.4 6.252 6.293 6.331 6.367 6.268
   793   1384603_at   8.023 7.903 8.215 7.728 7.8 6.768 6.911 7.223 7.023 7.303
   794   1383008_at   5.232 4.952 4.401 5.107 3.972 6.745 6.275 6.91 6.707 6.256
   795   1373219_at   5.055 5.071 4.39 5.36 4.445 6.039 6.269 6.216 6.527 6.255
   796   1372569_at   6.865 6.689 7.054 6.929 6.576 8.074 7.695 7.664 7.698 7.452
   797   1368003_at   6.85 6.766 7.064 6.767 6.426 7.483 7.436 7.565 7.467 7.65
   798   1375523_at   8.08 8.277 7.842 8.337 7.998 9.38 8.986 9.349 9.441 8.771
   799   1391538_at   8.544 8.772 8.691 8.535 8.603 8.186 8.341 8.317 8.192 8.232
   800   1370892_at   8.859 8.887 9.357 8.966 9.123 10.961 11.079 11.111 10.476 9.916
   801   1377664_at   5.796 5.724 5.713 5.451 5.44 6.774 6.686 6.63 6.701 6.11
   802   1373386_at   10.718 10.831 10.694 10.572 10.44 9.965 9.793 10.156 10.031 10.135
   803   1373833_at   5.993 6.307 6.113 6.29 6.153 6.638 6.756 6.652 6.783 6.529
   804   1380079_at   5.142 5.032 4.825 5.535 4.58 6.385 6.431 6.068 6.064 6.181
   805   1371250_at   6.044 5.964 5.893 6.319 6.235 6.872 7.649 7.5 7.403 6.942
   806   1368742_at   6.076 5.78 6.683 6.129 6.019 7.19 7.239 7.092 7.112 7.16
   807   1382569_at   7.662 7.592 7.513 7.801 7.534 6.65 6.404 6.959 6.873 7.016
   808   1377018_at   8.51 8.8 8.814 8.522 8.561 8.036 8.081 8.256 7.973 7.983
   809   1387284_at   9.385 9.491 9.343 9.279 9.265 8.644 8.663 8.895 8.89 8.986
   810   1367683_at   8.626 8.949 8.451 8.736 8.814 9.785 9.549 9.555 9.312 9.37
   811   1367763_at   11.549 11.558 11.399 11.54 11.452 11.163 11.264 11.296 11.164 11.216
   812   1385029_at   5.184 5.351 5.271 5.121 5.159 7.156 6.948 6.978 6.397 6
   813   1374695_at   7.947 8.121 7.784 8.105 8.122 8.683 8.678 8.555 8.537 8.419
   814   1380794_at   6.436 6.672 6.414 6.512 6.406 6.934 7.18 7.228 6.915 6.965
   815   1368143_at   8.5 8.609 8.874 8.528 8.635 9.29 9.53 9.392 9.26 9.07
   816   1393163_at   6.345 6.407 6.627 6.502 6.197 5.614 5.582 5.174 5.626 5.808
   817   1395687_at   5.069 4.968 4.982 5.29 4.957 5.508 5.494 5.698 5.713 5.519
   818   1372613_at   10.949 10.923 10.694 11.05 10.9 10.388 10.461 10.44 10.533 10.281
   819   1389965_at   10.931 11.08 10.948 10.897 11.037 10.646 10.607 10.744 10.747 10.665
   820   1382478_at   4.265 3.903 4.46 4.186 4.082 4.981 4.928 5.194 5.206 4.77
   821   1390357_at   6.524 6.47 6.466 6.579 6.038 7.066 7.032 7.243 7.135 7.076
   822   1391106_at   7.083 7.25 7.33 7.012 7.133 8.628 8.764 8.632 8.296 7.777
   823   1383596_at   5.774 5.961 6.187 6.013 5.456 6.882 6.927 6.734 6.602 6.901
   824   1368153_a_at   6.23 6.049 6.02 6.045 5.862 6.808 6.59 6.749 6.429 6.828
   825   1368321_at   10.386 10.176 11.985 9.945 10.865 8.426 7.559 7.717 6.91 6.615
   826   1378104_at   6.555 6.611 6.548 6.647 6.538 7.185 6.839 7.246 7.011 7.124
   827   1383640_at   8.314 8.673 8.67 8.453 8.471 7.737 7.769 8.061 7.9 7.59
   828   1372347_at   10.607 10.714 10.618 10.458 10.579 10.254 9.978 10.237 10.143 10.005
   829   1386529_at   6.452 6.461 6.396 6.833 6.511 7.027 7.157 7.196 7.335 7.083
   830   1371527_at   8.902 9.333 8.712 9.001 9.206 10.266 9.952 10.129 10.081 9.677
   831   1368154_at   8.144 8.337 8.065 8.389 7.868 9.339 8.919 9.478 9.538 8.942
   832   1368824_at   7.582 7.491 7.719 7.993 7.062 8.812 8.46 8.728 8.98 8.697
   833   1392509_at   6.087 5.96 6.428 6.13 6.298 6.898 7.166 6.88 6.949 6.705
   834   1369934_at   11.099 11.121 11.249 11.094 10.93 11.444 11.471 11.401 11.455 11.471
   835   1388672_at   6.707 7.022 6.697 6.826 6.854 7.79 7.708 7.858 7.826 7.235
   836   1371131_a_at   11.601 11.282 11.748 11.418 11.545 10.749 10.634 10.974 10.785 10.422
   837   1388504_at   9.964 10.124 10.102 10.117 10.112 10.438 10.354 10.505 10.378 10.299
   838   1388336_at   8.336 8.417 8.585 8.354 8.312 7.92 7.738 7.991 8.052 7.852
   839   1384707_at   4.065 3.463 4.618 3.851 4.072 6.144 5.303 5.815 5.792 5.294
   840   1370213_at   11.598 11.569 11.69 11.576 11.442 11.857 11.874 11.809 11.85 11.904
   841   1389651_at   5.101 5.553 5.849 5.35 5.641 6.982 6.376 6.676 6.56 6.441
   842   1398540_at   8.181 7.914 7.282 6.939 7.993 3.921 3.118 3.5 3.948 0.39
   843   1369018_at   3.574 4.788 4.406 4.438 4.696 6.117 5.978 5.605 5.885 6.011
   844   1374172_at   8.055 8.268 7.387 7.977 7.895 8.949 9.148 9.172 9.246 8.757
   845   1390855_at   8.088 8.281 8.425 8.088 8.048 7.038 6.724 7.322 7.163 7.539
   846   1371297_at   12.594 12.504 12.545 12.596 12.42 12.787 12.815 12.724 12.771 12.841
   847   1368164_at   9.65 9.704 10.076 9.728 9.513 8.742 8.515 8.825 8.935 9.115
   848   1389648_at   8.847 8.777 9.015 8.417 8.695 8.064 8.026 7.985 7.805 7.63
   849   1371691_at   9.392 9.105 9.181 9.265 9.53 10.582 11.496 10.882 10.39 10.409
   850   1367925_at   8.568 8.624 8.617 8.674 8.39 9.205 9.008 9.091 9.052 8.889
   851   1388455_at   8.366 8.563 8.575 8.427 8.162 9.451 9.114 9.502 9.074 9.055
   852   1376905_at   3.407 1.58 2.805 2.147 1.86 5.743 4.188 5.244 5.451 5.202
   853   1372409_at   6.513 6.43 6.616 6.759 6.696 7.286 7.018 7.295 7.377 7.073
   854   1368289_at   6.163 6.362 6.827 6.352 6.102 4.59 4.176 5.196 4.888 5.214
   855   1389488_at   11.165 11.218 11.256 11.143 11.293 10.929 10.96 11.05 10.999 10.92
   856   1375859_a_at   5.39 5.627 5.623 5.358 5.431 5.973 6.002 6.346 6.042 6.028
   857   1381428_a_at   11.233 11.114 11.427 11.098 11.529 10.539 10.22 10.445 10.386 10.752
   858   1368799_at   5.512 5.166 5.728 4.506 4.765 7.362 6.722 6.757 6.633 6.84
   859   1370935_at   8.17 8.038 8.016 8.023 7.948 8.664 8.648 8.681 8.666 8.291
   860   1374247_at   4.64 5.184 5.281 5.047 5.362 6.546 6.187 6.648 6.464 5.884
   861   1383175_a_at   6.885 6.954 6.81 6.681 6.563 7.481 7.54 7.438 7.233 7.266
   862   1387605_at   4.631 4.699 4.864 4.798 4.897 6.353 6.458 5.978 6.132 5.39
   863   1387811_at   7.879 7.772 8.319 7.716 8.15 6.789 5.99 6.904 6.86 6.51
   864   1373538_at   6.768 6.842 6.706 6.85 6.561 7.475 7.252 7.927 7.513 7.572
   865   1370433_at   6.96 6.832 7.054 7.184 6.676 7.927 7.906 8.072 7.872 7.449
   866   1388902_at   8.765 8.391 8.166 8.693 8.811 9.75 9.757 10.005 9.558 9.319
   867   1377048_at   10.717 10.901 10.929 10.771 10.856 10.087 9.961 10.098 10.366 10.431
   868   1390866_at   8.07 8.696 8.568 8.372 8.452 9.666 9.714 9.768 9.385 9.088
   869   1367837_at   9.057 8.888 8.946 8.942 8.791 9.339 9.163 9.35 9.274 9.307
   870   1388135_at   6.144 6.173 5.934 6.543 6.145 7.179 7.301 7.551 6.799 7.359
   871   1377350_at   4.374 4.727 4.713 4.699 5.114 5.635 5.542 5.774 5.541 6.023
   872   1373484_at   6.897 6.909 6.599 6.663 6.85 7.753 7.886 7.794 7.255 7.524
   873   1370891_at   8.66 8.789 8.189 8.731 8.633 9.426 9.356 9.643 9.553 9.241
   874   1372234_at   6.736 6.896 7.088 6.817 6.941 7.582 7.243 7.702 7.595 7.576
   875   1372993_at   4.81 5.207 4.724 4.851 4.68 5.641 5.803 5.556 5.429 5.858
   876   1382459_at   2.278 0 0 2.696 0.696 5.483 5.402 5.498 4.37 4.979
   877   1387913_at   6.826 6.707 6.891 6.638 6.25 5.792 5.684 5.914 5.843 5.997
   878   1383321_at   6.737 6.443 6.71 6.589 6.879 7.672 7.275 7.347 7.805 7.391
   879   1377573_at   9.92 9.96 9.688 9.844 9.743 9.342 9.437 9.446 9.161 9.207
   880   1374007_at   8.701 8.821 8.826 8.752 8.781 8.91 8.987 9.041 8.981 8.988
   881   1388521_at   6.704 6.687 6.47 6.467 6.423 7.204 7.25 7.183 6.881 7.139
   882   1398787_at   10.982 10.887 10.992 10.988 10.874 11.205 11.126 11.196 11.103 11.175
   883   1390944_at   7.861 8.03 7.982 7.96 7.761 7.6 7.55 7.602 7.598 7.622
   884   1377814_at   3.787 4.598 4.571 4.66 4.467 5.474 5.369 5.557 5.702 5.535
   885   1373642_at   6.126 5.917 6.251 5.827 5.676 6.756 6.712 6.621 6.674 6.572
   886   1367582_at   11.723 11.648 11.631 11.751 11.559 12.101 12.233 12.03 12.074 11.911
   887   1392499_at   6.79 6.872 6.817 6.708 6.827 7.274 7.065 7.303 7.11 7.098
   888   1368065_at   8.316 8.067 8.164 8.046 7.832 8.744 8.649 8.825 8.733 8.532
   889   1368947_at   10.927 10.735 11.075 10.039 10.642 9.371 9.269 9.653 9.536 9.355
   890   1374456_at   8.244 8.46 8.281 8.068 8.154 8.729 8.712 8.843 8.671 8.658
   891   1385444_at   8.48 8.569 8.378 8.545 8.431 9.021 9.125 9.401 9.213 8.854
   892   1393587_a_at   9.532 9.677 9.565 9.416 9.387 9.107 9.094 9.181 9.137 8.961
   893   1367723_a_at   7.69 7.617 7.509 7.558 7.506 8.136 8.06 8.16 8.149 7.809
   894   1372461_at   9.2 9.097 9.337 9.019 9.023 9.661 9.581 9.743 9.574 9.502
   895   1376377_at   4.104 3.053 3.453 3.632 3.671 5.773 5.381 5.231 4.676 5.011
   896   1377761_at   5.669 5.484 5.424 5.93 5.758 6.566 6.357 6.275 6.405 6.225
   897   1374404_at   9.536 9.111 9.782 8.817 9.306 7.858 7.809 6.877 7.037 6.427
   898   1372905_at   9.041 9.132 8.923 8.811 9.078 9.585 9.676 9.563 9.383 9.403
   899   1367860_a_at   7.948 7.906 8.291 8.296 7.81 9.38 8.757 9.007 9.414 8.979
   900   1382943_at   5.583 5.793 5.318 5.19 5.438 6.452 6.339 6.025 6.41 6.518
   901   1372111_at   8.425 8.558 8.741 8.268 8.377 8.992 8.998 9.026 9.081 8.984
   902   1368862_at   7.779 7.719 7.754 7.705 7.392 8.254 8.288 8.085 8.197 8.132
   903   1386982_at   7.67 7.85 7.61 7.663 7.693 8.404 8.475 8.607 8.318 8.04
   904   1373823_at   7.592 7.536 6.922 6.688 7.644 9.505 8.889 8.983 8.602 8.663
   905   1370956_at   10.651 10.447 9.905 10.756 10.586 11.637 11.623 11.531 11.585 11.281
   906   1368518_at   7.382 7.178 7.706 7.538 7.358 8.76 8.613 8.238 8.283 8.129
   907   1376161_at   9.003 9.008 8.87 9.016 8.971 8.65 8.703 8.814 8.626 8.701
   908   1369651_at   6.778 6.335 5.911 6.842 6.595 8.044 7.685 7.648 7.609 7.613
   909   1376185_at   6.733 6.46 6.835 6.382 6.522 7.359 7.313 7.088 7.334 7.139
   910   1373934_at   8.09 8.237 8.313 8.051 8.139 7.642 7.53 7.867 7.681 7.731
   911   1380995_at   1.245 0.705 1.029 0.575 0.888 2.014 4.174 3.721 3.398 3.694
   912   1389017_at   6.64 6.783 6.476 6.369 6.646 7.326 7.239 7.532 7.575 7.072
   913   1388744_at   8.053 7.646 8.094 7.549 7.652 8.815 8.578 8.732 8.49 8.958
   914   1373772_at   6.519 6.668 6.857 6.3 6.468 7.49 7.518 7.553 7.061 7.409
   915   1394479_at   9.301 9.422 9.359 9.39 9.129 8.496 8.455 8.761 8.808 8.867
   916   1387567_at   10.546 10.668 10.742 10.503 10.508 8.692 8.025 8.821 9.309 9.517
   917   1383362_at   6.496 6.639 6.437 6.533 6.498 7.384 7.395 6.969 7.091 6.99
   918   1381973_at   9.743 9.385 9.59 9.721 9.437 8.769 8.506 8.613 8.951 9.038
   919   1369200_at   9.604 9.691 9.604 9.478 9.463 9.032 9.128 9.182 9.235 8.905
   920   1388246_at   0 2.573 1.07 0.084 0.263 5.108 4.565 3.428 4.905 4.305
   921   1371951_at   8.807 8.746 8.317 8.589 8.298 9.631 9.199 9.613 9.651 9.294
   922   1370320_at   11.205 11.233 11.054 11.184 11.093 10.552 10.66 10.636 10.887 10.796
   923   1370282_at   10.443 10.313 10.337 10.202 10.274 9.717 9.89 10.018 9.961 9.898
   924   1384231_at   3.206 2.965 4.029 3.05 2.962 6.206 5.962 5.894 4.484 5.482
   925   1373667_at   11.034 11.157 11.095 11.286 11.238 10.599 10.543 10.885 10.708 10.581
   926   1383176_at   6.21 5.751 6.079 5.577 5.652 6.637 6.684 6.594 6.775 6.84
   927   1376615_at   6.183 5.917 5.709 5.931 5.856 6.489 6.578 6.601 6.771 6.386
   928   1372901_at   7.363 7.48 7.411 7.558 7.281 7.884 8.006 7.964 7.754 7.759
   929   1375677_at   8.832 8.76 8.662 8.356 8.225 7.852 7.51 7.824 7.569 7.689
   930   1395030_at   6.886 6.434 6.891 5.972 6.177 1.575 1.501 0.556 4.515 1.111
   931   1372070_at   10.238 10.229 10.254 10.279 10.164 10.711 10.643 10.578 10.626 10.402
   932   1369010_at   4.885 4.739 5.236 4.148 4.611 6.163 5.964 6.056 5.688 6.159
   933   1368534_at   3.119 3.392 2.674 3.57 2.036 6.904 6.994 6.576 6.029 4.674
   934   1379665_at   5.691 5.733 5.209 5.257 5.328 6.542 6.48 6.35 6.083 6.243
   935   1372339_at   7.266 7.563 7.181 7.434 7.269 8.202 7.971 7.992 7.925 7.772
   936   1373324_at   6.856 6.833 6.797 6.462 6.611 7.565 7.293 7.501 7.174 7.369
   937   1375060_at   5.24 5.429 5.855 4.955 5.404 6.532 6.182 6.468 6.366 6.574
   938   1367452_at   10.694 10.75 10.618 10.692 10.58 11.012 10.925 10.981 10.868 10.879
   939   1373037_at   6.117 5.695 6.439 6.186 6.03 7.296 6.962 6.984 6.869 6.931
   940   1369652_at   6.901 6.9 7.356 6.941 6.956 7.835 7.91 7.778 7.855 7.464
   941   1370161_at   9.044 9.195 9.335 8.812 8.683 11.017 10.342 10.507 10.345 9.883
   942   1383486_at   0 1.17 3.249 2.362 0.475 5.751 5.597 5.273 5.185 5.161
   943   1389350_at   6.858 6.98 7.207 6.922 6.629 5.972 5.529 5.802 6.158 6.187
   944   1389760_at   6.398 6.227 5.981 6.302 6.489 7.07 7.1 7.175 6.897 6.783
   945   1395126_at   3.96 3.871 3.516 4.616 4.286 5.378 6.016 5.733 5.882 5.161
   946   1390931_at   4.266 5.133 4.435 5.118 4.699 5.951 6.192 6.269 6.088 5.69
   947   1373290_at   5.351 5.15 5.031 5.535 4.603 6.821 6.435 6.472 6.097 6.261
   948   1376099_at   6.868 6.689 6.768 6.4 6.734 7.822 7.201 8.033 7.771 7.748
   949   1385925_at   5.928 5.498 5.315 5.708 5.748 6.855 6.528 6.556 6.485 6.285
   950   1370936_at   9.711 9.695 9.58 9.651 9.476 8.445 8.925 9.113 8.826 8.951
   951   1371824_at   11.181 11.542 11.419 11.215 11.27 10.592 10.509 10.943 10.691 10.691
   952   1378144_at   6.155 5.922 5.896 5.592 6.008 7.219 6.855 6.864 6.871 6.496
   953   1378076_at   2.932 1.934 1.144 3.484 3.426 5.392 5.594 5.561 5.499 5.539
   954   1371606_at   7.873 8.122 8.049 7.929 7.832 7.474 7.276 7.593 7.378 7.547
   955   1378244_at   2.403 1.727 2.42 1.465 2.536 0 0.227 0 1.007 0.084
   956   1369917_at   5.027 4.659 5.223 4.97 4.003 6.262 6.332 6.704 6.128 6.119
   957   1369649_at   5.018 4.283 4.481 4.101 3.948 6.025 6.289 5.543 5.834 5.588
   958   1377826_at   6.199 6.032 6.469 6.413 6.214 7.239 7.051 6.971 6.737 6.959
   959   1370154_at   11.466 11.29 11.623 11.57 11.488 12.435 12.366 11.94 12.075 12.113
   960   1374565_at   6.546 6.423 6.77 6.358 6.286 7.593 7.77 7.545 7.274 7.06
   961   1391849_at   5.202 5.329 5.656 5.537 5.469 6.134 6.049 5.928 6.212 5.947
   962   1392037_at   4.109 4.535 4.614 4.252 3.914 5.087 5.254 5.48 5.108 5.244
   963   1373345_at   6.204 6.109 6.264 6.011 6.159 6.522 6.519 6.636 6.546 6.391
   964   1371350_at   11.542 11.653 11.596 11.357 11.746 11.151 10.926 11.135 10.968 11.133
   965   1384103_at   8.555 8.506 8.337 8.552 8.433 7.676 7.703 7.683 8.103 8.008
   966   1397684_at   6.078 5.897 5.58 5.828 5.515 6.601 6.384 6.637 6.609 6.408
   967   1373242_at   7.864 7.734 7.871 7.708 7.649 8.155 8.259 8.456 8.16 8.146
   968   1392521_at   5.999 6.062 6.089 6 6.355 4.381 4.505 5.158 5.206 5.145
   969   1398286_at   9.936 9.999 10.291 9.691 9.775 8.59 8.311 9.115 8.708 9.082
   970   1384588_at   6.837 6.944 6.884 6.536 6.719 7.414 7.183 7.506 7.392 7.587
   971   1381261_at   3.72 3.16 4.146 3.645 3.751 4.743 4.685 4.825 4.707 4.611
   972   1369799_at   9.028 9.121 8.761 8.993 8.981 8.584 8.448 8.549 8.564 8.626
   973   1368921_a_at   5.893 5.535 6.194 5.909 5.826 7.684 7.802 7.702 6.94 6.799
   974   1373238_at   6.648 6.592 6.668 7.015 6.492 7.312 7.452 7.504 7.266 7.213
   975   1373152_at   10.075 10.22 10.523 10.147 10.333 10.727 10.906 11.008 10.846 10.772
   976   1374672_at   4.463 4.888 4.274 5.05 4.628 3.37 2.842 3.826 3.322 3.228
   977   1384580_at   3.534 3.358 3.214 4.008 3.72 4.892 4.542 4.535 4.679 4.386
   978   1368544_a_at   4.624 4.2 4.333 3.22 4.254 6.162 5.702 5.919 6.296 5.482
   979   1370144_at   11.526 11.266 11.562 11.415 11.218 9.678 8.89 9.301 9.888 10.465
   980   1370253_at   11.549 11.554 11.773 11.591 11.483 11.939 11.953 11.945 11.961 11.847
   981   1372292_at   8.941 9.015 8.738 8.942 8.708 8.456 8.413 8.445 8.427 8.536
   982   1390141_at   2.524 2.818 3.131 2.467 3.316 4.632 4.954 4.645 4.477 3.766
   983   1385868_at   4.249 3.903 4.482 4.237 3.946 5.626 5.153 5.755 5.119 5.011
   984   1373403_at   9.267 9.193 9.266 9.069 9.16 8.487 8.54 8.595 8.262 7.911
   985   1390440_at   8.244 8.414 8.426 8.101 8.451 7.75 7.755 7.971 7.793 7.65
   986   1387011_at   6.77 6.342 7.833 6.602 6.26 10.459 10.382 9.992 9.327 8.478
   987   1395184_at   4.986 5.026 5.785 5.149 5.013 6.767 6.332 6.926 6.378 6.162
   988   1390543_at   9.673 9.875 9.584 9.442 9.509 8.891 9.08 9.127 9.102 9.119
   989   1395781_at   5.53 5.134 5.252 5.045 4.905 5.936 6.044 5.789 5.871 6.155
   990   1395039_at   5.636 5.634 6.372 5.882 5.725 6.734 7.056 7.045 6.903 6.636
   991   1368627_at   11.317 11.54 11.7 11.483 11.517 10.018 9.872 10.436 10.693 10.716
   992   1368395_at   7.477 7.889 7.694 7.414 7.642 8.219 8.204 8.259 8.115 8.126
   993   1390846_at   6.547 6.349 6.364 6.303 6.583 7.598 7.042 7.067 7.043 7.216
   994   1371737_at   7.941 8.085 8.032 8.05 8.127 8.459 8.498 8.641 8.354 8.325
   995   1379361_at   9.313 8.841 8.651 8.832 8.695 7.803 7.916 8.242 7.884 8.037
   996   1391347_at   6.764 6.419 6.901 7.122 6.389 7.713 7.522 7.902 7.648 7.962
   997   1374638_at   9.778 9.93 9.801 9.704 9.666 9.463 9.386 9.52 9.469 9.408
   998   1388132_at   7.534 7.809 7.868 7.783 7.91 8.371 8.432 8.469 8.16 8.245
   999   1388753_at   8.525 8.515 8.747 8.369 8.389 9.097 8.845 9.045 9.098 9.092
   1000   1383833_at   6.839 7.213 7.205 6.828 6.861 7.644 7.544 7.971 7.74 7.683
   1001   1387160_at   2.373 3.401 3.923 3.048 2.472 5.43 5.661 5.044 4.609 5.658
   1002   1392938_s_at   7.232 7.498 7.534 7.149 7.166 8.154 7.869 8.072 7.824 7.941
   1003   1395953_at   5.557 5.15 5.472 5.409 5.364 5.766 5.99 6.043 5.865 5.947
   1004   1374484_at   6.681 6.55 6.526 6.619 6.738 6.89 6.917 6.93 6.904 6.833
   1005   1373722_at   4.559 4.407 3.123 4.978 5.811 8.077 7.705 7.312 7.076 7.559
   1006   1372365_at   10.477 10.344 9.949 10.146 10.305 9.554 9.408 9.741 9.584 9.365
   1007   1390492_a_at   7.828 7.86 7.759 7.615 7.713 8.119 8.002 8.167 8.041 8.101
   1008   1367983_at   5.307 5.834 5.852 5.586 5.151 6.683 6.856 6.877 6.305 7.102
   1009   1383964_at   6.715 6.779 6.67 6.737 6.707 7.246 6.988 7.297 7.069 7.03
   1010   1393550_at   6.119 6.105 5.794 5.843 6.14 5.583 5.507 5.33 5.387 5.449
   1011   1395364_at   6.687 6.438 6.724 6.897 6.9 7.395 7.347 7.425 7.245 7.214
   1012   1391067_at   6.018 5.064 5.598 5.791 5.55 6.856 6.423 7.19 7.013 6.982
   1013   1378793_at   4.489 4.702 4.067 4.695 4.571 5.29 5.368 5.225 5.288 5.618
   1014   1388243_at   6.242 6.109 6.203 6.087 6.137 7.324 6.711 7.423 7.467 6.853
   1015   1382138_at   3.702 3.864 4.923 4.121 4.391 5.897 5.575 5.938 5.708 5.389
   1016   1386868_at   11.494 11.43 11.373 11.35 11.325 11.686 11.627 11.59 11.697 11.585
   1017   1395268_at   9.041 9.108 9.125 8.733 9.001 8.432 8.278 8.558 8.538 8.476
   1018   1368576_at   5.518 5.463 5.847 6.356 5.864 6.895 6.706 6.899 7.082 7.234
   1019   1382370_at   5.009 4.702 5.139 4.248 3.978 6.573 6.373 6.468 5.724 6.317
   1020   1375362_at   7.071 7.197 7.06 7.29 7.013 7.96 7.817 8.259 7.952 7.559
   1021   1377390_at   5.758 5.672 5.952 5.805 6.025 6.461 6.322 6.662 6.315 6.347
   1022   1393682_at   3.816 4.156 3.009 3.69 2.694 5.241 5.309 5.76 5.935 5.062
   1023   1390021_at   10.594 10.378 10.503 10.463 10.504 10.216 10.214 10.2 10.291 10.276
   1024   1370297_at   4.461 4.328 2.488 4.569 2.438 7.521 6.845 6.923 6.581 6.651
   1025   1373150_at   8.521 8.78 8.636 8.527 8.166 9.157 9.159 9.255 9.188 9.133
   1026   1388143_at   8.527 8.905 9.086 8.751 8.877 9.605 9.379 9.574 9.604 9.364
   1027   1367759_at   10.706 10.552 10.889 10.089 10.347 9.66 9.571 9.685 9.659 9.564
   1028   1382130_at   5.158 5.589 5.166 5.289 5.485 6.073 6.014 6.284 6.143 5.814
   1029   1392914_at   8.139 8.205 8.299 7.997 7.925 7.607 7.398 7.639 7.627 7.67
   1030   1374739_at   6.628 6.299 6.862 6.628 6.044 7.946 7.254 7.821 7.582 7.696
   1031   1388588_at   8.093 8.058 8.212 8.161 7.946 8.668 8.477 8.582 8.486 8.376
   1032   1395427_at   7.35 7.226 7.145 7.121 6.638 8.211 8.043 8.173 7.886 7.776
   1033   1388658_at   8.619 8.377 8.495 8.535 8.502 8.169 8.168 8.293 8.105 8.062
   1034   1389061_at   7.027 6.95 7.069 7.106 6.986 7.396 7.345 7.262 7.204 7.385
   1035   1367702_at   12.068 12.118 12.217 12.176 12.091 11.514 11.396 11.61 11.713 11.848
   1036   1384591_at   6.072 6.004 5.814 6.134 6.293 6.624 6.489 6.763 6.798 6.647
   1037   1368808_at   9.463 9.263 9.164 9.393 9.158 10.025 9.852 9.78 9.698 9.73
   1038   1388494_at   9.014 9.212 9.306 8.992 9.353 10.263 9.626 10.199 10.229 9.968
   1039   1372132_at   11.721 11.714 11.754 11.695 11.588 11.387 11.319 11.333 11.475 11.503
   1040   1370166_at   11.034 10.994 10.756 10.826 10.958 10.566 10.576 10.613 10.467 10.498
   1041   1388306_at   7.374 7.277 7.193 7.321 7.027 7.694 7.679 7.751 7.628 7.925
   1042   1389154_at   9.164 9.255 9.232 9.192 9.21 8.958 8.97 9.056 9.095 8.963
   1043   1373975_at   10.817 10.552 10.807 10.639 10.576 9.759 9.458 9.521 9.9 10.199
   1044   1372997_at   10.029 9.66 9.818 9.605 9.881 9.274 9.208 9.341 9.179 9
   1045   1375277_at   5.185 4.882 5.232 5.462 5.497 6.556 6.038 6.211 5.995 6.524
   1046   1378917_at   1.47 1.761 3.163 2.551 2.31 4.464 4.057 4.206 4.672 3.991
   1047   1371908_at   7.265 7.31 7.216 7.308 6.918 7.628 8.006 8.007 8.067 7.779
   1048   1393405_at   4.109 4.42 4.276 4.534 3.849 5.476 4.942 5.185 5.089 5.145
   1049   1373198_at   9.092 9.209 8.921 9.078 8.936 8.552 8.596 8.689 8.75 8.532
   1050   1382751_at   3.851 4.482 4.985 4.324 4.022 5.668 5.512 6.163 5.872 6.398
   1051   1380321_at   7.928 7.793 7.605 7.642 7.793 7.291 7.435 7.249 7.344 7.366
   1052   1372903_at   3.149 0.872 2.325 2.37 3.586 6.03 5.025 6.168 5.263 5.863
   1053   1370367_at   10.48 10.628 10.733 10.507 10.554 10.008 10.11 10.247 10.287 10.07
   1054   1378307_at   8.975 9.003 9.31 8.88 8.672 7.939 8.154 8.364 8.298 8.109
   1055   1384334_at   3.044 3.016 4.132 2.727 3.36 6.136 6.481 6.242 4.983 4.992
   1056   1385143_at   5.619 5.848 5.837 5.589 5.715 6.908 6.664 6.72 6.253 6.31
   1057   1380182_at   7.656 7.97 7.753 7.87 7.868 8.306 8.384 8.558 8.378 8.15
   1058   1374582_at   8.499 8.538 8.363 8.408 8.452 8.244 8.178 8.266 8.254 8.176
   1059   1384378_at   5.969 5.707 5.941 5.571 5.926 6.381 6.457 6.276 6.43 6.644
   1060   1368522_at   3.688 4.479 4.051 4.697 4.748 5.989 5.876 6.136 5.371 6.309
   1061   1376605_at   9.517 9.688 9.727 9.363 9.335 8.921 8.747 8.852 9.114 8.852
   1062   1367768_at   6.287 6.581 6.755 6.619 6.215 7.792 7.797 7.89 7.342 7.161
   1063   1373392_at   7.949 7.901 7.981 7.756 7.696 8.31 8.237 8.339 8.267 8.147
   1064   1374630_at   7.953 7.821 7.998 7.903 7.646 7.073 6.731 7.05 7.191 7.396
   1065   1387109_at   9.256 9.139 9.025 8.948 9.026 8.567 8.75 8.758 8.644 8.672
   1066   1392906_at   8.341 8.464 8.472 8.429 8.257 8.812 8.747 8.601 8.722 8.771
   1067   1383202_at   8.718 8.703 8.773 8.773 8.443 9.2 9.047 9.24 9.082 9.081
   1068   1376120_at   4.939 5.259 5.132 4.953 5 6.091 5.757 5.65 5.539 6.006
   1069   1389354_at   9.693 9.645 9.529 9.646 9.687 9.414 9.476 9.444 9.46 9.423
   1070   1378640_at   3.664 4.003 3.949 2.866 3.421 5.794 5.471 5.533 4.631 5.312
   1071   1376726_at   2.124 2.731 2.934 3.722 3.553 0.632 0.687 0.66 1.501 1.144
   1072   1374574_at   7.41 7.534 7.547 7.136 7.406 6.687 6.743 6.876 6.839 7.011
   1073   1372247_at   9.99 10.067 10.129 10.021 9.818 10.375 10.288 10.378 10.433 10.337
   1074   1395396_at   6.05 6.101 5.946 5.956 5.932 6.803 6.739 6.382 6.432 6.474
   1075   1369153_at   6.768 7.219 7.138 6.768 6.795 6.102 6.082 6.171 6.084 6.457
   1076   1383958_at   4.052 4.32 4.679 4.804 4.278 5.846 5.633 5.507 5.348 5.229
   1077   1378482_at   6.533 6.383 6.268 6.434 6.231 7.178 6.942 7.077 6.852 6.744
   1078   1377194_a_at   8.351 8.28 8.236 8.42 8.29 8.736 8.596 8.663 8.576 8.522
   1079   1376341_at   6.323 6.141 6.206 5.947 6.251 5.598 5.082 5.637 5.488 5.293
   1080   1386904_a_at   11.639 11.72 11.483 11.624 11.554 11.142 11.2 11.256 11.358 11.322
   1081   1369401_at   11.131 11.027 11.268 10.929 11.035 10.027 9.882 10.259 10.14 10.653
   1082   1375538_at   7.238 7.009 6.733 7.178 6.988 7.747 8.081 8.113 7.822 7.571
   1083   1375732_at   5.989 6.265 6.114 6.058 6.129 6.407 6.531 6.468 6.377 6.564
   1084   1370421_a_at   5.356 5.07 5.184 4.567 4.909 5.976 5.862 5.889 5.985 5.744
   1085   1382325_at   8.795 8.917 9 8.852 8.718 8.406 8.093 8.254 8.328 8.519
   1086   1368096_at   11.218 11.247 11.586 11.2 11.131 10.303 10.025 10.68 10.607 10.396
   1087   1382865_at   5.06 5.605 5.27 4.999 5.23 5.895 5.859 6.245 6 6.095
   1088   1396739_at   3.857 2.858 3.483 3.413 3.557 1.696 1.084 2.131 1.281 2.263
   1089   1393451_at   6.071 6.363 6.132 6.514 6.233 7.557 7.462 7.398 6.746 7.229
   1090   1389503_at   5.46 5.556 6.126 5.834 5.469 6.75 6.793 6.738 6.307 6.683
   1091   1398807_at   10.276 10.43 10.454 10.191 10.201 9.726 9.751 10.016 9.884 9.816
   1092   1381925_x_at   9.005 9.1 8.791 8.885 8.963 9.316 9.331 9.512 9.399 9.269
   1093   1379889_at   3.087 3.946 2.599 2.987 3.694 5.498 5.53 5.157 4.548 4.983
   1094   1389506_x_at   3.612 3.541 3.167 4.029 4.027 5.016 4.565 4.939 4.786 5.271
   1095   1371680_at   11.105 11.183 11.182 10.982 11.064 10.452 10.371 10.824 10.635 10.549
   1096   1387872_at   8.313 8.215 8.468 8.137 8.099 9.353 9.137 8.991 8.786 8.813
   1097   1372282_at   8.221 7.814 8.439 8.199 8.104 9.144 8.84 8.872 9.005 8.735
   1098   1385086_at   3.428 4.279 3.208 3.274 1.506 6.686 6.43 6.422 5.54 5.922
   1099   1377794_at   7.959 8.08 7.959 7.956 7.734 8.291 8.474 8.459 8.266 8.367
   1100   1367974_at   7.945 7.936 7.767 7.706 7.75 9.123 8.83 8.756 8.535 8.32
   1101   1388629_at   8.606 8.479 8.644 8.34 8.303 9.174 9.019 9.125 8.889 8.907
   1102   1375723_at   5.314 5.357 5.545 5.302 5.082 5.712 6.08 5.92 5.892 6.061
   1103   1367835_at   8.424 8.074 7.992 8.343 8.139 7.497 7.245 7.405 7.754 7.494
   1104   1382447_at   8.551 8.748 8.67 8.542 8.255 7.672 7.649 8.013 7.825 8.026
   1105   1382203_at   7.809 7.842 7.788 7.912 7.796 7.968 8.022 8.099 8.13 8.049
   1106   1389785_at   12.118 12.03 12.096 12.095 11.917 11.275 10.983 11.365 11.505 11.613
   1107   1392128_at   3.703 3.094 4.351 3.689 4.082 5.425 5.216 4.903 5.408 5.14
   1108   1399091_at   10.544 10.72 10.69 10.655 10.593 11.048 10.826 10.981 10.978 10.901
   1109   1384638_at   10.526 10.458 10.592 10.497 10.48 9.652 9.756 10.073 9.982 10.138
   1110   1389440_at   4.418 3.198 4.951 3.956 4.027 6.064 6.762 6.119 5.684 6.305
   1111   1378731_at   4.909 4.849 4.945 4.901 5.276 5.51 5.556 5.7 5.553 5.383
   1112   1379861_at   5.835 5.901 5.857 6.135 5.562 6.349 6.554 6.429 6.603 6.652
   1113   1374855_at   8.682 7.96 8.554 7.109 8.047 5.843 5.049 6.558 5.862 5.776
   1114   1388851_at   11.035 11.016 10.972 11.078 10.912 10.712 10.741 10.746 10.769 10.864
   1115   1389674_at   5.642 5.569 5.597 5.574 5.346 6.122 5.894 6.173 6.403 6.099
   1116   1379260_at   8.343 8.313 8.394 8.111 8.248 7.669 7.482 7.929 7.78 7.475
   1117   1392900_at   9.046 9.237 9.152 8.797 8.874 9.608 9.589 9.645 9.58 9.439
   1118   1369728_at   6.912 7.017 7.263 6.873 7.153 5.148 6.204 5.098 5.916 5.789
   1119   1383605_at   9.661 9.69 10.084 9.502 9.816 8.787 8.447 8.982 9.013 9.023
   1120   1381986_at   5.65 5.671 5.8 5.863 5.731 6.29 6.024 6.521 6.271 6.36
   1121   1374799_at   4.758 5.239 5.11 4.828 4.791 6.491 6.106 6.597 5.854 5.721
   1122   1370057_at   9.271 9.281 9.512 8.984 9.327 10.024 9.731 10.001 9.894 9.822
   1123   1377064_at   8.706 8.816 9.21 8.771 8.996 8.33 8.311 8.41 8.277 8.239
   1124   1382128_at   7.473 7.538 7.482 7.175 7.429 6.831 6.798 6.988 6.959 6.582
   1125   1371518_at   10.268 10.564 10.135 10.047 10.541 10.952 10.967 11.186 11.039 10.924
   1126   1375767_at   8.717 8.822 8.962 8.679 8.519 8.315 8.145 8.301 8.271 8.217
   1127   1374537_at   7.082 7.217 6.899 6.922 6.988 7.985 7.924 7.959 7.749 7.378
   1128   1377151_at   7.174 7.056 7.218 7.199 7.019 7.656 7.722 7.653 7.404 7.468
   1129   1398482_at   4.674 5.125 5.123 5.542 4.435 6.465 6.408 6.307 6.072 6.072
   1130   1382115_at   7.641 7.39 7.496 7.411 7.468 7.201 7.116 7.213 7.093 7.216
   1131   1372038_at   9.491 9.358 9.528 9.34 9.293 8.957 8.572 8.8 9.032 8.868
   1132   1397458_at   6.395 5.969 5.755 6.533 6.187 7.067 7.336 6.949 7.107 7.082
   1133   1374272_at   8.336 8.628 8.461 8.357 8.387 7.954 7.859 8.144 8.011 8.039
   1134   1384483_at   5.272 5.632 5.35 5.869 5.266 6.312 6.067 6.302 6.292 6.39
   1135   1382277_at   6.123 6.285 6.269 6.156 6.345 7.492 7.079 7.285 7.042 6.667
   1136   1379866_at   5.619 4.927 4.885 5.571 5.243 6.435 6.135 6.759 6.396 6.239
   1137   1388927_at   7.105 6.851 7.102 7.119 6.898 7.399 7.621 7.417 7.486 7.374
   1138   1368234_at   10.302 10.307 10.48 10.32 10.323 9.095 8.808 9.169 9.471 9.821
   1139   1395973_at   7.582 7.671 7.878 7.786 7.849 8.352 8.158 8.541 8.395 8.15
   1140   1385926_at   6.254 5.916 6.256 6.123 5.972 7.328 6.962 7.049 6.696 6.693
   1141   1388930_at   10.237 10.232 10.1 10.125 10.05 10.541 10.624 10.683 10.54 10.361
   1142   1383372_at   7.151 7.133 7.089 7.094 6.896 7.596 7.394 7.508 7.541 7.337
   1143   1392127_at   1.151 0.872 0 0 0 2.339 3.636 2.074 2.511 3.278
   1144   1368894_at   5.95 5.657 6.274 6.083 5.224 4.364 4.458 4.615 4.43 4.933
   1145   1367761_at   9.347 9.197 9.181 9.403 9.375 8.841 8.986 9.004 8.847 8.73
   1146   1390046_at   7.218 7.172 7.101 6.911 7.143 7.712 7.369 7.667 7.596 7.689
   1147   1390411_at   7.765 7.342 8.266 7.726 7.471 8.734 8.556 8.805 8.703 8.89
   1148   1377004_at   7.554 8.052 7.658 7.616 7.571 6.989 7.137 7.207 6.963 6.98
   1149   1374903_at   9.567 9.683 9.642 9.467 9.729 9.069 9.285 9.34 9.224 9.19
   1150   1371344_at   12.082 12.05 12.122 12.116 12.044 12.454 12.431 12.283 12.279 12.278
   1151   1395662_at   5.59 5.531 5.338 5.59 5.001 6.541 6.77 6.538 5.989 6.407
   1152   1371541_at   9.706 9.755 9.881 9.75 9.733 10.358 9.967 10.388 10.358 10.289
   1153   1390420_at   5.062 5.136 5.303 5.633 5.491 6.599 5.89 6.35 6.44 6.703
   1154   1380768_at   5.371 5.144 5.081 5.514 5.642 6.532 6.467 6.634 6.37 5.88
   1155   1391744_at   6.072 5.675 5.952 5.895 6.071 6.5 6.519 6.602 6.359 6.35
   1156   1389447_at   4.855 4.719 4.518 4.875 4.292 5.427 5.512 5.904 5.447 5.38
   1157   1382924_at   10.048 10.35 9.941 9.992 10.102 9.541 9.267 9.634 9.582 9.517
   1158   1371914_at   8.443 8.312 8.255 8.429 8.087 8.714 8.755 8.669 8.759 8.887
   1159   1367890_at   7.467 7.305 7.642 7.78 7.521 8.496 8.264 8.282 8.192 7.979
   1160   1389469_at   10.247 10.123 10.501 10.032 10.02 9.29 9.336 9.728 9.541 9.445
   1161   1374682_at   8.133 8.277 8.493 8.087 8.301 7.35 7.679 7.703 7.803 7.554
   1162   1370054_at   6.562 7.404 7.354 6.964 7.364 8.45 8.022 8.416 8.15 8.142
   1163   1398321_a_at   5.479 5.142 4.362 5.335 5.281 6.387 6.318 6.238 6.312 6.613
   1164   1371959_at   9.295 9.157 9.377 9.085 9.176 8.121 8.074 8.62 8.483 8.677
   1165   1379482_at   5.515 5.61 5.174 4.972 5.362 6.027 6.179 6.231 6.126 5.927
   1166   1373881_at   9.224 9.145 9.495 9.163 9.271 9.854 9.7 9.771 9.765 9.574
   1167   1389540_at   10.509 10.618 10.447 10.264 10.461 9.939 9.505 9.763 9.938 9.978
   1168   1369113_at   8.375 8.524 8.636 7.795 8.013 9.11 9.359 9.528 9.572 9.232
   1169   1368467_at   11.577 11.692 11.607 11.526 11.604 11.043 11.17 11.048 11.412 11.157
   1170   1382173_at   6.146 6.186 6.209 5.287 6.27 4.836 4.841 4.948 4.888 4.467
   1171   1372536_at   9.016 9.103 9.091 8.931 8.627 8.385 8.283 8.137 8.487 8.147
   1172   1368593_at   6.478 6.446 6.543 6.324 6.381 6.852 6.796 7.17 6.894 6.814
   1173   1391979_at   5.769 5.75 5.785 5.644 5.513 6.999 6.799 6.546 6.704 6.122
   1174   1368463_at   7.197 7.459 7.043 7.291 6.999 7.867 7.651 7.741 7.876 7.709
   1175   1371729_at   9.993 9.948 9.88 9.852 9.675 9.289 9.401 9.571 9.497 9.36
   1176   1387375_at   11.069 11.3 11.292 11.172 11.062 10.343 10.214 10.449 10.577 10.82
   1177   1373826_at   10.112 9.765 9.955 10.13 9.852 9.545 9.522 9.479 9.574 9.461
   1178   1376684_at   2.376 4.369 4.606 3.101 4.123 6.833 6.088 6.21 6.494 6.257
   1179   1388314_at   11.4 11.46 11.178 11.378 11.465 11.87 11.716 11.794 11.685 11.702
   1180   1390228_at   9.013 8.857 8.898 8.747 8.781 8.203 8.088 8.327 8.567 8.328
   1181   1379885_at   10.467 10.513 10.666 10.638 10.544 10.061 10.035 10.247 10.308 10.211
   1182   1388076_at   10.893 10.927 10.895 10.901 10.758 11.077 11.075 11.235 11.197 11.117
   1183   1376038_at   9.258 9.292 9.094 9.152 9.192 8.881 8.694 8.919 8.944 8.776
   1184   1370174_at   8.543 8.342 9.124 7.591 8.432 6.943 6.89 6.708 6.631 6.042
   1185   1383703_at   6.908 7.115 6.992 7.131 7.137 7.374 7.582 7.751 7.797 7.505
   1186   1376723_a_at   9.982 9.834 10.002 9.877 9.748 9.514 9.558 9.572 9.497 9.656
   1187   1388840_at   5.975 5.279 5.322 5.452 5.29 6.517 6.131 6.371 6.41 6.291
   1188   1370574_a_at   6.177 5.694 5.77 6.081 5.51 6.674 6.658 6.516 6.567 6.776
   1189   1398174_at   1.811 1.351 0.766 1.727 0.367 3.997 3.222 4.111 2.644 3.824
   1190   1371747_at   9.521 9.546 9.777 9.507 9.577 9.242 9.112 9.283 9.3 9.189
   1191   1388665_at   7.937 7.72 8.065 7.879 7.693 7.442 7.425 7.217 7.175 7.379
   1192   1370163_at   11.582 11.364 11.768 11.247 11.48 10.663 10.369 10.399 10.665 10.998
   1193   1379250_at   10.283 10.421 10.472 10.482 10.296 9.825 9.607 9.865 9.973 10.058
   1194   1389234_at   8.627 8.601 8.515 8.589 8.774 7.704 7.949 8.297 7.655 7.767
   1195   1367600_at   6.535 6.178 6.334 6.264 6.054 6.843 6.697 6.819 6.738 6.858
   1196   1377772_at   4.601 4.545 4.767 3.598 3.93 5.717 5.695 5.583 5.606 5.59
   1197   1367604_at   9.675 9.534 9.693 9.407 9.479 10.18 9.843 10.066 10.179 10.017
   1198   1385909_at   4.527 4.598 4.529 4.658 5.083 5.357 5.564 5.506 5.192 5.559
   1199   1390137_at   1.239 4.2 4.124 2.996 1.151 7.59 6.718 6.87 6.512 6.761
   1200   1390812_a_at   6.872 6.829 6.259 6.787 6.623 7.431 7.225 7.58 7.498 7.422
   1201   1388119_at   10.498 10.553 10.45 10.362 10.329 10.887 10.811 10.925 10.699 10.713
   1202   1367594_at   8.493 8.844 9.149 8.549 8.525 9.807 9.392 10.016 9.877 9.547
   1203   1380692_at   5.557 6.565 5.935 5.77 5.816 7.257 7.324 7.5 6.886 6.912
   1204   1389392_at   9.538 9.426 8.674 9.203 9.158 7.941 7.819 8.409 8.173 8.217
   1205   1370461_at   3.77 4.32 4.288 5.231 4.817 6.752 6.224 5.831 6.04 6.384
   1206   1380721_at   3.865 4.235 4.455 4.837 3.998 5.826 5.565 5.227 5.302 5.52
   1207   1384628_at   10.111 10.168 10.034 10.114 10.13 9.439 9.486 9.761 9.796 9.738
   1208   1371498_at   8.851 9.002 8.808 8.786 8.709 9.318 9.297 9.251 9.124 9.11
   1209   1376811_a_at   5.964 6.079 5.784 5.963 6.241 6.896 6.917 7.026 7.042 6.386
   1210   1390386_at   6.552 6.265 6.89 6.854 6.164 7.89 7.758 7.749 7.692 7.223
   1211   1369960_at   6.576 6.607 6.499 6.555 6.462 7.191 6.968 6.879 6.849 6.865
   1212   1390036_at   8.462 8.774 8.044 8.296 8.285 7.558 7.673 7.612 7.722 7.59
   1213   1381233_at   10.348 10.374 10.581 10.039 9.818 8.21 7.179 8.13 8.426 9.112
   1214   1372773_at   8.76 8.91 8.835 8.832 8.772 9.177 9.083 9.145 9.2 8.97
   1215   1384232_at   5.11 5.799 5.377 5.416 5.564 6.768 6.254 6.816 6.665 6.132
   1216   1384488_at   6.527 6.399 6.91 6.378 5.714 7.936 7.399 7.508 7.778 7.743
   1217   1367750_at   9.107 9.169 9.133 8.988 9.048 8.875 8.692 8.806 8.882 8.773
   1218   1389568_at   5.367 5.779 5.374 6.157 5.605 6.87 6.632 6.509 6.496 6.59
   1219   1374359_at   4.993 5.396 4.522 5.633 5.104 7.444 6.857 6.554 6.263 6.644
   1220   1370055_at   6.586 6.825 6.842 6.295 6.375 7.493 7.421 7.406 7.592 7.111
   1221   1392219_at   3.991 4.163 4.292 4.149 3.806 5.102 4.759 4.888 4.657 4.61
   1222   1396752_at   5.904 5.584 6.177 5.533 5.584 4.128 4.891 3.818 4.645 3.801
   1223   1395274_at   7.831 8.081 7.756 7.898 7.714 7.472 7.248 7.499 7.274 7.354
   1224   1390415_at   3.903 4.49 3.674 4.136 5.003 6.169 5.754 5.746 5.431 5.88
   1225   1367630_at   11.894 11.875 12.008 11.918 11.914 12.196 12.253 12.126 12.078 12.112
   1226   1367798_at   10.792 10.828 10.943 10.671 10.575 10.275 10.014 10.238 10.27 10.389
   1227   1375452_at   10.041 10.026 10.005 9.937 9.907 9.636 9.432 9.53 9.797 9.555
   1228   1380596_at   6.144 6.7 6.662 5.902 6.175 7.519 7.639 7.79 7.126 7.307
   1229   1382127_at   10.372 10.422 10.186 10.194 10.385 9.923 10.032 9.959 10.022 9.864
   1230   1397620_at   8.041 7.679 7.629 6.888 7.399 6.463 6.196 6.255 6.53 6.196
   1231   1393148_at   9.479 9.68 9.6 9.488 9.643 9.34 9.309 9.371 9.297 9.307
   1232   1388592_at   7.086 7.33 7.993 7.086 7.53 8.599 8.5 8.816 8.473 8.274
   1233   1398773_at   9.641 9.77 9.704 9.479 9.698 10.12 10.028 10.118 10.014 9.9
   1234   1375525_at   8.846 8.781 8.834 8.733 8.701 9.114 9.025 9.086 8.977 8.946
   1235   1376755_at   7.993 7.977 7.94 7.73 7.971 8.444 8.191 8.442 8.454 8.26
   1236   1379195_s_at   5.612 4.927 5.461 5.326 5.058 5.983 6.2 6.443 6.073 6.089
   1237   1390650_at   5.751 6.418 6.05 5.826 5.861 6.796 6.755 6.86 6.589 6.697
   1238   1369434_at   2.536 1.891 1.93 2.275 1.774 3.379 2.897 4.047 3.425 3.482
   1239   1387108_at   10.283 10.256 10.305 10.239 10.212 10.475 10.471 10.451 10.453 10.338
   1240   1390163_at   5.799 5.938 5.919 6.254 5.808 6.539 6.49 6.536 6.375 6.407
   1241   1383315_at   8.573 8.704 7.841 8.081 8.24 6.936 6.25 6.741 7.356 7.027
   1242   1389349_s_at   4.963 4.939 4.984 4.494 5.101 5.91 5.41 5.893 6.058 5.711
   1243   1385690_at   9.216 9.248 8.834 8.969 8.927 8.535 8.577 8.543 8.526 8.576
   1244   1376709_at   9.416 9.649 9.953 9.482 9.454 8.365 8.267 8.597 8.857 8.956
   1245   1398882_at   11.879 11.8 11.72 11.864 11.738 12.188 12.245 12.072 12.052 12.022
   1246   1387849_at   4.838 4.583 4.672 5.044 4.694 5.47 5.364 5.314 5.148 5.382
   1247   1388827_at   9.629 9.507 9.606 9.58 9.34 9.921 9.929 9.95 9.856 9.785
   1248   1370993_at   7.129 7.223 6.754 7.209 7.128 7.673 7.682 7.777 7.699 7.492
   1249   1385574_at   8.828 8.897 9.088 9.088 9.022 8.112 8.331 8.493 8.54 8.551
   1250   1377662_at   5.61 5.993 5.599 5.746 5.502 6.456 6.257 6.16 6.197 6.398
   1251   1382243_at   0.465 2.101 2.266 0.111 0.956 3.667 4.057 3.982 4.196 3.396
   1252   1395036_at   5.766 5.74 5.504 5.731 5.174 6.625 6.293 6.328 6.355 6.211
   1253   1386247_at   8.413 8.192 8.222 8.024 8.388 7.59 7.664 7.558 7.85 7.832
   1254   1368092_at   11.283 11.412 11.317 11.393 11.203 10.803 10.88 10.937 11.027 11.09
   1255   1373394_at   6.357 6.307 6.295 6.322 6.174 6.681 6.71 6.726 6.625 6.449
   1256   1388440_at   9.035 9.026 9.196 9.053 8.983 9.455 9.34 9.589 9.401 9.308
   1257   1389622_at   10.668 10.892 10.408 10.732 10.631 10.119 10.092 10.294 10.171 10.119
   1258   1391302_at   5.9 5.87 6.317 5.809 5.18 7.249 6.718 7.076 6.906 7.184
   1259   1391582_at   4.279 4.41 4.695 3.955 4.348 5.165 5.025 5.31 4.996 5.064
   1260   1388111_at   6.156 5.433 6.029 5.862 5.463 7.063 6.467 6.953 7.303 7.081
   1261   1387328_at   10.615 10.72 11.111 10.883 10.438 7.8 6.249 7.305 8.79 8.879
   1262   1367728_at   7.057 6.77 6.873 7.1 6.894 7.419 7.403 7.771 7.587 7.366
   1263   1367751_at   7.454 7.595 7.193 7.337 7.419 7.87 8.069 8.047 8.116 7.739
   1264   1376902_x_at   1.993 1.736 1.566 1.287 1.299 4.236 3.664 2.531 4.321 3.406
   1265   1370871_at   10.469 10.673 10.501 10.583 10.375 11.213 11.083 10.891 10.842 11.022
   1266   1393281_at   7.647 7.45 7.762 7.602 7.226 8.178 8.1 8.141 8.281 8.004
   1267   1374225_at   5.583 5.493 4.704 5.042 5.237 6.699 6.142 6.566 6.155 6.23
   1268   1394643_at   4.424 5.019 4.268 4.58 4.374 5.564 5.81 5.231 5.409 5.422
   1269   1374558_at   8.953 9.144 8.908 8.98 8.861 8.34 8.127 8.546 8.29 8.611
   1270   1392995_at   3.788 4.418 4.716 4.344 4.124 5.466 5.542 5.411 5.094 5.128
   1271   1369958_at   11.172 10.924 10.931 10.535 10.683 10.146 10.212 10.272 10.148 10.179
   1272   1374603_at   6.035 5.923 6.396 6.115 5.848 6.599 6.891 6.915 6.927 6.584
   1273   1372155_at   9.736 9.53 9.571 9.606 9.549 10.135 9.786 10.039 10.069 9.999
   1274   1390783_at   8.257 7.998 7.473 8.141 7.86 8.912 8.724 8.864 8.882 8.625
   1275   1384897_at   5.405 5.164 4.502 4.931 4.57 6.07 6.145 5.814 6.226 5.86
   1276   1376018_at   4.543 4.674 5.213 5.469 5.062 6.394 6.049 6.603 5.887 6.224
   1277   1391514_at   1.989 2.046 2.696 2.16 2.625 3.329 5.046 4.348 4.423 5.32
   1278   1372549_at   7.114 6.912 7.137 7.23 7.086 7.67 7.403 7.961 7.801 7.713
   1279   1377172_at   6.097 6.207 6.05 6.164 5.924 6.672 6.415 6.585 6.414 6.446
   1280   1370153_at   8.43 7.968 9.265 8.47 8.54 7.523 6.858 7.155 6.977 6.602
   1281   1379362_at   7.03 7.02 7.239 6.498 6.869 6.186 6.138 6.31 6.051 6.176
   1282   1369304_at   8.282 8.392 8.701 8.644 8.601 8.095 7.726 7.913 8.027 7.817
   1283   1381396_s_at   8.994 8.711 8.65 8.752 8.904 8.099 8.269 8.371 8.215 8.46
   1284   1367588_a_at   11.464 11.559 11.539 11.433 11.359 11.85 11.954 11.759 11.77 11.695
   1285   1384234_at   1.93 1.683 0.287 2.17 2.63 4.124 3.632 4.801 4.826 4.799
   1286   1387900_at   9.581 9.606 9.694 9.507 9.503 9.801 9.825 9.975 9.851 9.816
   1287   1376427_a_at   8.398 8.584 8.129 8.669 8.07 7.205 7.588 7.405 7.567 7.734
   1288   1383969_at   4.614 5.005 4.862 5.023 4.699 5.693 5.299 5.535 5.457 5.346
   1289   1373082_at   6.323 6.038 5.812 6.337 6.403 7.347 7.628 7.454 7.188 6.744
   1290   1371308_at   12.12 12.106 12.054 12.146 12.138 12.468 12.535 12.324 12.281 12.387
   1291   1393203_at   6.613 6.36 6.594 6.586 6.538 7.162 7.084 7.097 6.98 6.771
   1292   1373309_at   8.736 8.968 8.059 8.827 8.807 7.517 6.998 7.473 7.66 7.754
   1293   1390133_at   7.334 7.449 7.473 7.228 7.428 7.994 7.762 7.974 7.83 7.65
   1294   1374540_at   2.281 1.118 3.855 1.799 2.66 6.589 6.454 6.154 4.406 5.68
   1295   1371305_at   11.786 11.572 11.565 11.799 11.63 12.001 12.013 11.946 11.942 12.041
   1296   1379294_at   8.917 8.621 8.692 8.6 8.937 8.21 8.273 8.393 8.173 8.322
   1297   1372749_at   7.905 7.914 8.024 7.915 7.98 8.266 8.117 8.354 8.308 8.16
   1298   1369964_at   7.81 7.721 8.047 7.926 7.563 9.049 8.647 8.561 8.478 8.403
   1299   1389006_at   8.113 8.058 8.247 8.172 7.68 9.397 8.852 8.975 8.72 8.808
   1300   1390802_at   7.538 7.54 7.69 7.518 7.39 7.928 7.98 8.09 7.862 7.804
   1301   1388451_at   3.595 3.032 3.816 4.066 4.31 5.19 5.285 5.581 5.603 4.802
   1302   1390450_a_at   6.895 6.73 5.624 6.764 6.428 7.985 7.919 8.305 8.407 7.624
   1303   1373110_at   9.338 9.385 9.125 9.192 9.38 8.444 8.281 8.886 8.69 8.689
   1304   1368245_at   11.103 11.138 11.354 11.094 10.827 10.073 10.036 10.335 10.389 10.644
   1305   1380115_at   7.065 6.929 6.338 7.214 7.089 5.004 5.064 5.752 6.033 5.522
   1306   1373114_at   6.912 7.138 6.967 7.112 6.766 8.005 7.43 7.932 8.023 7.566
   1307   1391823_at   0.485 0 0 0.227 0.029 0.911 1.057 1.475 1.057 1.82
   1308   1376831_at   2.922 3.61 0.782 3.278 1.632 6.098 5.364 5.339 5.698 5.693
   1309   1384187_at   5.805 6.17 6.167 5.552 5.811 7.484 7.06 6.988 6.846 6.588
   1310   1377189_at   6.22 6.024 6.504 5.973 5.853 6.873 6.811 6.744 6.811 7.111
   1311   1373382_at   9.885 10.041 9.915 9.938 9.835 10.191 10.155 10.287 10.177 10.108
   1312   1390126_at   8.748 8.702 8.457 8.7 8.676 8.379 8.388 8.321 8.335 8.308
   1313   1372667_at   7.923 8.11 7.837 7.994 7.867 8.31 8.371 8.362 8.238 8.19
   1314   1370407_at   7.263 7.609 7.225 7.263 7.199 7.943 7.812 8.196 8.026 7.746
   1315   1370561_at   5.735 5.243 5.222 6.348 5.643 7.306 7.121 6.956 7.265 6.581
   1316   1371373_at   9.905 9.9 9.815 9.892 9.993 9.548 9.48 9.753 9.594 9.494
   1317   1383057_at   5.516 5.482 5.501 5.384 5.565 4.442 5.13 4.892 4.835 4.601
   1318   1371891_at   8.437 8.402 8.292 8.254 8.351 8.753 8.676 8.695 8.62 8.513
   1319   1384762_at   1.614 3.498 3.944 2.202 2.884 5.868 5.519 5.477 4.917 5.319
   1320   1386881_at   10.019 9.876 9.008 9.683 9.654 8.477 8.359 8.457 8.843 8.569
   1321   1391224_at   7.036 7.089 6.853 7.036 7.031 6.329 6.499 6.537 6.762 6.591
   1322   1367892_at   11.049 11.067 10.995 11.136 11.062 10.573 10.445 10.749 10.823 10.691
   1323   1372672_at   10.408 10.617 10.594 10.566 10.284 9.735 9.733 10.025 9.904 10.137
   1324   1387276_at   5.202 4.527 3.886 4.526 5.445 6.619 6.198 6.514 6.348 6.204
   1325   1371732_at   10.086 10.057 9.768 10.029 10.069 10.605 10.275 10.627 10.658 10.481
   1326   1376355_at   3.167 3.082 3.415 3.537 3.421 4.566 3.903 4.079 3.999 4.343
   1327   1390883_at   7.827 7.874 7.936 7.607 7.912 7.427 7.033 7.454 7.244 7.219
   1328   1374435_at   7.077 7.144 6.561 6.705 7.191 7.825 7.709 8.029 7.718 7.596
   1329   1371390_at   10.679 10.767 10.529 10.504 10.425 11.311 11.081 10.929 11.013 11.199
   1330   1384988_at   4.76 4.529 4.569 3.87 3.92 5.643 5.505 5.372 5.412 5.254
   1331   1375119_at   10.355 10.173 9.986 10.323 10.376 10.877 10.788 10.931 10.725 10.593
   1332   1379984_at   4.5 4.61 4.901 4.636 4.849 5.506 5.344 5.367 5.436 5.031
   1333   1385211_at   6.511 6.664 6.475 6.417 6.602 7.026 7.087 7.185 6.795 6.906
   1334   1375306_at   3.716 4.174 3.536 3.542 2.536 5.986 6.202 6.15 5.336 4.79
   1335   1388453_at   8.033 8.203 8.439 8.011 8.379 8.954 8.786 8.783 8.741 8.657
   1336   1389709_at   5.578 5.735 5.483 5.686 6.021 6.265 6.644 6.631 6.203 6.444
   1337   1389582_at   6.999 6.988 6.94 6.967 7.318 7.627 7.605 7.722 7.34 7.679
   1338   1398348_at   11.578 11.726 11.654 11.665 11.725 11.225 11.24 11.421 11.433 11.437
   1339   1377244_at   6.584 6.789 6.655 6.684 6.8 6.947 7.001 6.915 7.092 7.014
   1340   1371441_at   7.771 7.97 8.146 8.146 7.759 8.48 8.544 8.617 8.364 8.495
   1341   1385439_x_at   4.717 4.618 4.635 4.527 4.719 5.547 4.98 5.108 5.332 5.205
   1342   1385226_at   5.111 4.854 5.029 4.733 5.336 5.635 6.07 6.349 5.72 6.013
   1343   1368527_at   6.611 6.603 6.934 6.103 6.842 5.702 5.07 5.637 5.396 4.757
   1344   1370693_a_at   5.362 5.109 5.482 5.052 4.812 6.169 6.161 5.888 6.068 5.718
   1345   1372168_s_at   5.694 5.601 6.028 5.8 6.432 7.298 6.779 6.898 6.932 6.739
   1346   1375120_at   8.65 8.398 8.641 8.631 8.265 7.611 7.469 8.055 7.85 7.882
   1347   1383926_at   1.485 1.757 4.525 1.007 1.299 6.283 5.814 5.512 5.816 5.407
   1348   1373241_at   8.195 8.326 8.328 8.255 8.017 8.573 8.619 8.768 8.579 8.556
   1349   1392479_at   6.551 6.366 6.459 6.562 6.302 6.707 7.074 6.842 7.005 6.962
   1350   1390408_at   7.967 8.168 8.164 8.157 8.081 8.366 8.57 8.584 8.365 8.638
   1351   1376013_at   5.681 6.089 5.724 5.888 5.76 6.515 6.732 6.549 6.368 6.22
   1352   1387566_at   7.538 7.705 7.653 7.664 7.396 8.341 7.904 8.362 8.193 8.075
   1353   1368596_at   8.123 8.097 8.413 6.388 7.801 5.857 5.55 5.707 5.031 5.706
   1354   1370909_at   7.063 7.338 7.508 7.274 7.224 8.06 7.664 7.822 7.851 7.779
   1355   1367820_at   10.142 10.103 9.95 9.921 9.976 10.436 10.277 10.355 10.361 10.242
   1356   1375921_at   4.01 4.413 4.394 4.768 4.672 5.315 5.755 5.352 5.531 5.128
   1357   1387703_a_at   8.865 8.556 8.83 7.917 8.654 7.487 7.349 7.599 7.607 7.654
   1358   1373807_at   9.914 9.772 10.14 9.444 9.762 9.184 9.184 9.155 9.113 9.146
   1359   1370927_at   8.602 8.553 7.617 8.509 8.795 9.541 9.734 9.845 9.604 9.487
   1360   1368173_at   8.011 8.019 8.242 7.919 7.759 8.562 8.847 8.75 8.526 8.416
   1361   1374379_at   6.346 6.881 6.658 6.657 6.516 5.986 5.942 6.083 6.201 5.904
   1362   1368381_at   4.316 4.285 3.562 3.816 3.268 5.324 5.292 5.241 5.169 4.746
   1363   1387182_at   4.773 5.181 4.727 4.875 5.001 5.59 5.589 6.092 6.087 5.527
   1364   1371581_at   9.487 9.688 9.668 9.523 9.332 9.967 9.996 10.031 9.896 9.862
   1365   1371786_at   10.808 10.748 10.342 10.482 10.465 9.903 9.708 10.083 10.07 9.843
   1366   1374626_at   6.08 5.983 5.896 6.256 6.408 6.693 6.577 6.875 6.759 6.693
   1367   1371794_at   9.231 9.647 9.391 9.155 9.242 8.723 8.592 8.933 8.716 8.769
   1368   1390609_at   2.87 3.099 3.109 3.05 3.245 3.626 4.574 3.977 4.053 3.883
   1369   1370699_a_at   11.447 11.525 11.372 11.552 11.465 11.113 11.193 11.14 11.233 11.318
   1370   1388118_at   12.141 12.111 11.946 12.064 12.195 11.814 11.78 11.854 11.824 11.884
   1371   1372642_at   9.073 8.826 8.639 8.789 8.729 8.279 8.348 8.477 8.187 8.187
   1372   1369161_at   5.167 5.119 5.476 5.257 5.057 5.589 5.82 6.161 5.921 5.936
   1373   1384073_at   9.905 9.305 9.497 9.956 9.727 8.974 8.536 8.782 8.622 9.036
   1374   1376625_at   6.583 6.833 6.768 6.588 6.503 7.197 7.217 6.964 7.138 7.016
   1375   1384276_at   3.379 3.089 3.469 2.428 3.216 5.098 4.463 4.697 4.123 4.293
   1376   1371740_at   7.213 7.185 7.4 7.179 7.342 7.683 7.681 7.545 7.528 7.526
   1377   1398597_at   6.562 6.501 6.176 6.376 6.233 6.988 6.84 6.885 7.01 6.716
   1378   1390907_at   4.981 5.237 5.154 5.191 4.987 6.154 5.48 6.303 6.124 5.858
   1379   1368811_at   3.956 4.96 4.533 4.794 5.231 6.644 6.269 6.348 6.006 5.736
   1380   1367573_at   12.195 12.139 12.327 12.388 12.254 12.637 12.633 12.491 12.526 12.535
   1381   1372202_at   6.606 6.746 6.739 6.631 6.277 7.701 7.408 7.471 7.078 7.215
   1382   1367590_at   11.13 11.17 11.07 11.079 11.01 11.675 11.634 11.478 11.333 11.386
   1383   1370770_s_at   5.322 4.615 4.789 5.05 4.883 5.93 5.44 5.959 5.765 5.901
   1384   1375760_at   8.565 8.631 8.303 8.082 7.981 7.203 7.381 7.469 7.639 7.611
   1385   1398830_at   12.08 11.928 12.163 12.03 11.934 12.278 12.401 12.274 12.341 12.28
   1386   1382898_at   5.881 5.929 6.078 5.675 5.539 5.104 5.34 5.255 5.183 5.288
   1387   1387690_at   5.525 5.612 5.475 5.773 5.339 7.198 7.195 6.497 6.267 6.469
   1388   1370809_at   7.896 8.068 7.985 7.642 7.84 8.43 8.486 8.322 8.214 8.441
   1389   1387939_at   0 1.281 0.856 0.176 0 2.692 1.88 2.189 2.205 1.824
   1390   1373831_at   6.694 6.626 6.798 7.176 7.125 7.885 7.72 7.568 7.546 7.477
   1391   1387877_at   7.196 7.565 7.814 7.221 7.27 6.291 6.059 6.381 6.632 6.781
   1392   1372112_at   7.839 7.786 8.023 7.721 7.584 8.77 8.344 8.441 8.391 8.241
   1393   1377748_at   6.232 6.5 6.541 6.694 6.503 6.941 7.092 7.003 6.958 6.85
   1394   1374848_at   7.297 7.291 7.222 7.327 7.191 7.505 7.554 7.569 7.536 7.375
   1395   1376946_at   4.608 4.633 4.243 4.779 4.119 5.569 5.013 5.374 5.569 5.407
   1396   1371468_at   9.476 9.596 9.401 9.092 9.623 8.91 8.74 8.926 8.922 8.745
   1397   1387202_at   7.822 7.675 7.802 7.69 7.457 8.63 8.622 8.425 8.245 8.073
   1398   1374333_at   8.524 8.546 8.768 8.445 8.348 9.133 9.025 9.204 8.901 8.925
   1399   1371977_at   10.301 10.464 10.423 10.35 10.271 10.772 10.788 10.845 10.629 10.566
   1400   1374388_at   8 8.18 8.143 8.109 8.014 8.931 8.534 8.501 8.528 8.499
   1401   1389001_at   8.499 8.534 8.327 8.709 8.696 9.184 9.112 9.415 9.133 8.915
   1402   1385354_at   4.662 4.926 4.848 5.301 4.866 5.504 5.779 5.803 5.498 5.992
   1403   1385791_at   9.039 8.861 8.912 8.809 8.703 7.902 7.795 8.169 8.335 8.413
   1404   1393277_at   5.409 5.412 5.449 5.309 5.467 5.765 5.939 6.131 5.958 5.662
   1405   1390250_x_at   5.516 5.787 5.866 6.075 5.591 6.459 6.295 6.317 6.433 6.649
   1406   1387659_at   7.493 7.84 7.413 7.6 7.225 8.587 8.409 8.774 8.327 8.025
   1407   1395056_at   4.705 4.297 4.234 5.144 4.769 5.504 5.593 5.859 5.799 6.213
   1408   1371945_at   9.438 9.472 9.389 9.512 9.342 9.701 9.68 9.695 9.628 9.563
   1409   1396066_at   0 1.316 2.307 0.401 1.609 3.72 4.133 3.603 3.198 3.428
   1410   1388688_at   8.92 8.905 8.792 8.821 9.077 8.464 8.281 8.651 8.509 8.372
   1411   1377646_at   11.611 11.724 11.547 11.434 11.522 11.211 11.264 11.162 11.314 11.058
   1412   1393183_at   7.216 7.276 7.034 6.881 6.792 7.856 7.871 7.448 7.66 7.76
   1413   1387908_at   8.48 8.292 8.144 7.622 7.978 6.841 6.204 6.1 5.407 4.632
   1414   1392280_at   4.649 4.915 5.682 4.997 5.025 6.659 6.001 6.308 6.026 6.131
   1415   1375851_at   6.996 6.708 6.859 7.023 6.607 6.113 6.128 6.248 6.404 6.411
   1416   1389644_at   6.113 6.343 5.912 6.173 5.916 7.004 6.784 6.821 6.474 6.7
   1417   1369793_a_at   6.753 6.527 6.139 6.629 6.495 7.36 7.165 7.043 7.1 7.127
   1418   1376597_at   6.433 6.635 6.392 6.438 6.621 7.256 7.107 7.257 7.008 6.777
   1419   1383586_at   8.692 8.642 8.528 8.728 8.631 8.902 8.806 8.879 8.836 8.855
   1420   1384371_at   5.222 5.018 4.876 4.833 4.998 5.517 5.627 5.864 5.395 5.488
   1421   1369630_at   11.557 11.509 11.477 11.574 11.642 11.356 11.336 11.344 11.401 11.24
   1422   1393027_at   7.317 7.545 7.321 7.38 7.217 7.837 7.787 7.744 7.602 7.695
   1423   1395697_at   5.245 4.971 3.807 5.108 4.756 6.507 6.359 6.279 6.181 6.039
   1424   1371330_at   12.037 12.045 11.99 12.003 11.922 12.312 12.325 12.146 12.169 12.23
   1425   1373057_at   9.727 9.603 9.429 9.235 9.304 8.883 8.879 8.993 8.805 8.99
   1426   1385126_at   5.99 6.13 5.891 5.705 5.873 5.297 5.527 5.508 5.166 5.198
   1427   1398628_at   10.311 10.566 9.949 10.355 10.416 9.608 9.641 9.514 9.795 9.796
   1428   1389047_at   7.602 7.652 7.434 7.532 7.576 7.935 8.075 7.928 7.739 7.937
   1429   1389176_at   8.923 8.736 8.655 8.58 8.907 8.269 8.189 8.349 8.445 8.168
   1430   1374578_at   6.945 6.725 6.677 7.04 6.836 7.718 7.49 7.546 7.164 7.442
   1431   1391925_at   6.771 5.795 6.501 5.754 5.854 7.851 7.263 7.462 7.316 7.339
   1432   1375987_at   6.568 6.362 6.663 6.436 6.181 7.274 6.961 7.291 6.895 7.038
   1433   1383601_at   1.438 3.692 3.542 3.843 2.95 6.091 5.646 6.04 5.273 5.476
   1434   1371314_at   10.476 10.702 10.36 10.459 10.41 11.148 11.389 11.151 11.013 10.811
   1435   1371611_at   8.368 8.522 8.384 8.468 8.325 8.746 8.839 8.841 8.72 8.581
   1436   1391953_at   3.23 3.329 3.564 3.996 3.676 2.444 1.227 2.595 1.945 1.736
   1437   1370311_at   8.094 8.123 8.052 8.134 8.069 8.283 8.193 8.261 8.316 8.203
   1438   1373839_at   3.618 4.515 4.507 4.35 4.845 5.694 5.329 5.887 5.806 5.481
   1439   1368296_at   5.638 5.407 5.459 5.598 5.718 6.583 6.285 6.15 6.837 6.119
   1440   1390366_at   8.608 8.793 8.577 8.683 8.489 8.088 8.172 8.406 8.249 8.14
   1441   1393046_at   7.038 6.855 6.742 6.811 6.586 7.372 7.46 7.247 7.204 7.22
   1442   1388883_at   9.405 9.312 9.305 9.461 9.366 8.876 8.901 8.816 9.172 9.069
   1443   1388071_x_at   11.691 11.672 11.683 11.783 11.574 12.281 12.148 11.913 12.008 12.04
   1444   1373417_at   7.004 6.815 6.962 7.171 6.919 7.618 7.379 7.27 7.422 7.554
   1445   1385250_at   5.498 5.135 5.034 6.178 6.047 2.975 2.935 3.536 4.234 1.963
   1446   1367978_at   4.171 4.92 3.825 4.699 4.555 5.787 5.907 5.947 5.482 5.364
   1447   1374794_at   4.733 3.297 4.285 4.326 3.943 6.257 5.919 6.278 5.5 5.289
   1448   1384440_at   5.835 5.631 5.358 6.106 5.788 7.043 6.427 6.85 6.539 6.538
   1449   1383232_at   7.052 7.212 7.133 6.932 7.217 7.389 7.43 7.551 7.556 7.41
   1450   1371244_at   10.562 10.626 10.651 10.556 10.366 10.959 10.981 10.974 10.844 10.782
   1451   1388729_at   8.166 8.138 8.08 7.935 7.857 8.5 8.283 8.439 8.47 8.44
   1452   1383757_at   7.687 7.937 8.144 7.509 7.715 6.913 6.801 7.208 7.138 7.167
   1453   1369625_at   12.275 12.186 12.201 12.327 12.376 11.91 11.849 11.887 11.953 12.119
   1454   1375847_at   6.906 7.154 7.244 7.213 6.865 7.506 7.465 7.638 7.701 7.75
   1455   1370358_at   10.036 10.064 10.014 9.947 10.052 9.866 9.78 9.822 9.851 9.671
   1456   1385670_at   6.605 6.913 7.379 6.972 6.66 6.002 5.652 5.765 5.897 6.303
   1457   1398553_at   5.309 5.543 5 5.307 5.534 5.872 5.821 5.954 5.941 5.934
   1458   1393684_at   4.121 4.409 4.529 3.266 3.365 5.797 5.724 6.116 5.635 5.075
   1459   1375895_at   7.372 7.46 7.41 7.265 7.232 7.999 7.757 7.915 7.705 7.621
   1460   1375067_at   6.03 6.181 5.788 5.877 5.925 6.734 6.873 7.209 6.744 6.379
   1461   1373350_at   4.521 4.351 3.94 4.142 4.557 5.39 5.289 5.61 5.186 4.831
   1462   1381469_a_at   6.477 6.341 6.355 6.278 6.345 5.666 5.667 6.076 5.948 5.543
   1463   1388945_at   8.176 8.069 8.301 7.708 7.985 7.403 7.603 7.444 7.315 7.26
   1464   1373556_at   6.781 6.808 6.703 6.733 6.507 7.137 7.22 7.06 6.964 7.048
   1465   1369323_at   7.506 7.33 7.201 7.486 7.175 8.047 7.765 7.964 7.795 7.715
   1466   1378423_at   3.497 3.775 3.256 3.707 3.07 4.461 4.22 4.092 4.477 4.263
   1467   1370806_at   11.653 11.641 11.711 11.752 11.72 11.202 11.245 11.491 11.429 11.432
   1468   1368265_at   7.423 7.144 7.347 7.339 7.314 6.778 6.958 6.961 6.757 6.513
   1469   1369533_a_at   1.761 3.254 2.651 3.099 2.664 4.133 5.429 4.32 4.872 5.696
   1470   1368160_at   10.616 10.25 10.444 9.639 10.281 9.317 9.486 9.149 8.941 8.995
   1471   1389971_at   7.361 7.298 7.159 7.297 7.174 8.005 7.711 7.81 7.492 7.809
   1472   1369483_at   6.255 5.576 5.598 6.189 5.502 6.782 6.637 6.849 6.963 6.676
   1473   1367849_at   6.866 6.939 7.114 6.665 6.714 7.487 7.445 7.627 7.625 7.202
   1474   1379344_at   8.685 8.609 8.737 8.383 8.614 10.068 9.509 9.631 9.496 9.062
   1475   1391411_at   8.954 9.058 8.759 8.734 8.956 9.508 9.366 9.488 9.466 9.147
   1476   1374352_at   2.111 1.281 3.475 1.214 0.138 4.988 4.871 5.353 5.305 4.131
   1477   1371833_at   11.005 10.835 10.916 10.933 10.851 10.462 10.263 10.536 10.634 10.614
   1478   1384724_at   5.833 5.781 5.612 5.435 5.356 6.261 6.255 6.651 6.497 6.093
   1479   1368366_at   10.69 10.926 11.092 11.06 11.153 9.96 9.84 10.187 10.417 10.461
   1480   1389220_at   7.702 7.821 7.997 7.811 7.592 8.26 8.425 8.353 8.306 8.067
   1481   1386890_at   9.72 9.826 10.092 9.57 9.651 10.742 10.968 10.815 10.438 10.253
   1482   1376381_at   2.884 4.335 4.358 4.137 4.343 5.612 5.716 5.548 5.718 5.429
   1483   1372809_at   3.242 2.93 3.113 3.044 2.503 3.692 4.39 4.082 3.674 4.076
   1484   1377752_at   4.302 4.787 4.614 4.134 4.689 5.796 5.241 5.243 5.651 5.275
   1485   1374038_at   8.933 9.107 8.997 8.815 9.03 8.364 8.333 8.752 8.429 8.237
   1486   1387897_at   6.186 6.432 6.862 6.905 6.685 7.786 7.296 7.605 7.511 7.336
   1487   1371341_at   10.012 10.189 10.212 10.002 9.862 10.665 10.957 10.627 10.737 10.385
   1488   1382878_at   7.029 6.474 6.285 6.513 6.688 7.884 7.749 7.404 7.285 7.371
   1489   1376344_at   5.244 4.932 4.895 5.611 5.369 6.36 6.037 5.978 6.384 5.903
   1490   1371462_at   7.879 7.811 7.614 7.748 7.555 7.34 6.932 6.899 6.943 7.314
   1491   1383343_at   5.514 5.551 5.367 5.504 5.471 6.373 6.448 6.048 6.253 5.767
   1492   1388309_at   6.811 6.794 6.956 6.715 6.54 7.342 7.251 7.414 7.363 7.041
   1493   1382732_at   6.806 6.722 6.243 6.682 6.472 7.332 7.148 7.63 7.338 7.155
   1494   1382651_at   3.645 4.037 3.912 3.394 3.88 4.582 4.803 4.542 4.491 4.296
   1495   1395052_at   6.104 6.46 6.55 6.261 6.322 5.842 5.73 5.88 5.912 5.553
   1496   1371302_at   11.783 11.821 11.722 11.823 11.86 12.032 12.076 11.948 11.959 11.95
   1497   1393149_at   4.666 4.614 3.905 3.957 4.652 5.593 5.317 5.692 5.293 5.264
   1498   1388762_at   8.661 8.501 8.565 8.263 8.357 9.244 8.981 9.018 8.992 8.817
   1499   1394438_at   8.076 7.963 7.96 8.063 7.813 8.313 8.293 8.247 8.19 8.368
   1500   1385529_at   3.142 1.727 2.975 2.996 2.189 4.41 3.782 4.459 4.376 4.274
   1501   1392731_at   2.163 0.411 0.766 2.011 1.333 4.642 3.54 3.516 4.03 2.991
   1502   1383073_at   9.556 9.685 9.782 9.545 9.65 10.042 9.877 10.115 10.005 9.895
   1503   1378078_at   4.836 4.957 5.288 5.197 5.045 5.448 5.527 5.553 5.708 5.687
   1504   1373454_at   8.459 8.473 8.755 8.499 8.427 9.086 8.957 9.018 8.888 8.795
   1505   1373780_at   7.87 8.094 8.566 8.178 7.632 9.171 8.848 9.315 8.841 9.232
   1506   1389617_at   8.048 8.228 7.787 7.925 7.968 8.734 8.56 8.809 8.774 8.306
   1507   1369686_at   4.209 4.41 4.402 4.602 4.519 5.994 5.224 5.666 5.195 5.088
   1508   1394483_at   6.499 6.592 6.504 6.616 6.7 7.347 7.38 7.635 7.532 6.85
   1509   1377359_at   6.911 6.838 6.94 6.949 6.762 7.096 7.035 7.113 7.144 7.175
   1510   1396962_at   1.373 0.856 2.367 1.07 1.536 2.814 3.642 3.5 2.683 3.402
   1511   1380243_at   3.746 3.838 2.618 2.976 2.943 5.041 4.472 5.561 4.537 5.005
   1512   1367859_at   5.749 5.471 5.3 5.379 5.152 6.262 5.827 6.104 6.278 6.393
   1513   1387871_at   6.861 6.962 6.871 6.71 6.67 7.67 7.408 7.433 7.193 7.198
   1514   1390993_at   8.94 9.021 8.699 8.914 8.774 8.21 8.099 8.377 8.598 8.21
   1515   1384183_at   5.263 5.027 5.273 4.989 5.003 4.689 4.813 4.465 4.444 4.54
   1516   1395166_at   6.74 6.667 6.196 6.74 6.157 7.678 7.329 7.545 7.434 7.078
   1517   1368658_at   6.622 6.646 6.753 6.439 6.609 7.875 8.172 8.151 7.562 7.075
   1518   1388782_at   8.005 8.205 7.8 8.723 8.057 9.441 8.816 9.471 9.769 9.293
   1519   1398592_at   6.788 6.657 6.42 6.404 6.138 5.465 5.517 6.046 5.41 5.309
   1520   1376688_a_at   5.246 4.532 4.692 4.543 4.78 6.509 6.181 6.378 6.046 5.328
   1521   1392582_at   0.516 0.356 0.807 1.183 3.331 4.615 4.026 4.003 4.153 4.684
   1522   1371584_at   9.149 9.198 8.968 8.983 9.006 8.819 8.667 8.83 8.739 8.7
   1523   1398768_at   10.478 10.644 10.526 10.448 10.502 10.993 11.083 11.082 10.746 10.81
   1524   1391390_at   9.529 9.255 9.29 9.234 9.414 8.687 8.794 9.1 8.826 8.906
   1525   1369614_at   4.173 4.414 4.5 4.372 4.019 4.8 4.706 4.907 5.003 4.814
   1526   1370363_at   8.452 8.422 7.942 8.315 8.149 7.808 7.666 7.5 7.74 7.544
   1527   1399032_at   7.9 7.747 8.05 7.823 7.685 8.167 8.307 8.262 8.178 8.179
   1528   1382589_at   1.86 0.956 1.07 2.934 1.864 3.572 4.018 3.54 3.772 4.435
   1529   1370379_at   10.676 10.582 10.302 10.578 10.491 9.893 10 10.013 10.224 10.152
   1530   1389836_a_at   11.631 11.735 11.653 11.713 11.722 11.393 11.502 11.379 11.463 11.577
   1531   1379019_at   5.497 4.788 5.539 5.11 5.688 6.446 6.089 6.256 6.24 6.487
   1532   1389096_at   5.443 4.888 5.222 5.108 5.341 6.159 5.798 5.736 5.752 5.975
   1533   1373377_at   8.701 8.815 8.516 8.465 8.424 7.599 7.706 7.997 8.028 8.139
   1534   1391453_at   4.392 4.928 4.529 4.482 5.012 5.475 5.333 5.263 5.514 5.653
   1535   1370432_at   7.629 7.475 7.78 6.062 7.226 4.43 5.599 5.627 5.243 4.994
   1536   1367834_at   7.724 7.675 7.511 7.25 7.361 7.979 8.021 7.957 8.037 8.009
   1537   1372492_at   11.333 11.403 11.496 11.507 11.485 11.139 10.897 11.084 11.136 11.225
   1538   1390982_at   5.989 5.439 5.771 5.558 5.6 6.125 6.565 6.507 6.217 6.395
   1539   1384298_at   5.102 4.987 4.907 4.589 4.003 6.446 5.91 6.019 5.607 6.176
   1540   1367793_at   11.641 11.664 11.759 11.746 11.662 11.308 11.221 11.381 11.501 11.484
   1541   1368322_at   10.01 10.26 9.795 10.102 10.101 10.995 11.325 11.353 10.729 10.574
   1542   1377192_a_at   10.273 10.383 10.123 10.195 10.152 9.742 9.728 9.914 9.971 9.917
   1543   1396279_at   9.651 9.628 9.533 9.623 9.417 9.372 9.319 9.334 9.272 9.277
   1544   1386805_at   4.013 4.462 4.569 3.979 4.644 5.12 5.125 5.072 5.42 5.553
   1545   1368558_s_at   7.147 7.048 7.083 6.798 6.624 8.354 7.704 7.569 7.823 7.772
   1546   1393531_at   8.491 8.411 8.511 8.385 8.085 7.607 7.711 7.872 7.966 7.934
   1547   1393348_at   5.032 5.323 4.664 5.014 4.429 3.373 3.023 3.981 2.778 3.818
   1548   1368946_at   6.698 6.685 6.346 6.77 6.577 7.098 6.953 7.346 7.178 7.333
   1549   1379688_at   5.019 5.24 5.772 5.02 5.025 6.435 6.125 6.16 6.158 5.856
   1550   1368870_at   10.521 10.45 10.326 10.091 10.102 9.69 9.904 9.808 9.582 9.611
   1551   1389209_at   7.473 7.78 7.851 7.46 7.591 8.165 8.109 8.235 7.997 8.066
   1552   1395167_at   8.999 9.049 9.426 8.78 9.13 8.046 8.098 8.579 8.458 8.226
   1553   1393461_at   6.466 6.469 6.284 6.332 6.759 6.818 6.941 7.144 7.084 7.057
   1554   1385001_at   5.5 5.452 5.408 5.553 5.067 6.99 6.417 6.15 6.16 6.181
   1555   1373791_at   7.866 8.001 8.044 7.809 8.045 8.239 8.17 8.372 8.276 8.311
   1556   1374148_at   6.349 5.938 5.111 4.806 5.416 3.619 3.919 3.243 3.079 1.952
   1557   1385089_at   6.194 5.812 5.072 6.411 5.599 3.68 4.59 4.551 3.413 3.65
   1558   1375066_at   5.326 4.969 5.681 4.626 4.879 6.122 6.28 6.37 5.965 6.088
   1559   1387974_a_at   10.737 10.969 10.929 11.027 10.911 10.477 10.259 10.494 10.615 10.586
   1560   1398398_at   8.361 8.597 8.544 8.33 8.244 8.788 8.727 8.879 8.782 8.883
   1561   1389980_at   7.735 7.965 7.638 7.929 7.665 8.619 8.682 8.498 8.361 8.103
   1562   1372087_at   11.287 11.378 11.183 11.555 11.421 10.897 10.808 10.904 10.948 11.11
   1563   1373777_at   4.296 4.351 4.85 3.698 4.547 5.992 6.043 5.638 5.397 5.268
   1564   1377911_at   9.972 10.289 9.836 9.844 10.082 9.415 9.494 9.599 9.416 9.188
   1565   1373830_at   6.776 6.61 7.072 7.05 6.753 7.916 7.61 7.543 7.46 7.326
   1566   1398612_at   11.357 11.439 11.699 11.367 11.445 10.129 9.76 10.2 10.741 10.829
   1567   1368006_at   6.712 6.889 7.1 6.7 6.838 8.164 7.848 7.915 7.899 7.195
   1568   1380610_at   8.143 8.244 8.814 8.067 8.166 7.189 7.132 7.533 7.412 7.654
   1569   1391856_at   10.41 10.418 10.41 10.23 10.189 9.804 9.717 9.931 9.977 10.069
   1570   1381940_at   7.363 7.534 7.142 6.971 7.091 6.509 6.484 6.554 6.827 6.523
   1571   1371337_at   9.927 10.074 10.042 9.897 9.866 10.29 10.212 10.372 10.21 10.178
   1572   1373566_at   9.567 9.619 9.534 8.732 9.32 8.461 8.17 8.579 8.443 8.154
   1573   1367957_at   6.962 7.189 7.497 7.183 6.75 7.87 7.802 7.832 7.756 7.744
   1574   1380072_at   6.855 6.901 7.059 6.84 6.686 7.334 7.224 7.26 7.122 7.345
   1575   1388512_at   8.048 8.079 7.906 7.927 8.109 8.22 8.344 8.209 8.314 8.304
   1576   1373262_at   5.905 5.747 6.183 6.231 5.67 7.005 6.731 7.018 6.942 6.402
   1577   1373986_at   10.673 10.519 10.31 10.488 10.62 10.009 10.123 10.241 10.064 9.9
   1578   1369073_at   9.335 9.347 8.985 9.291 9.313 8.697 8.838 8.808 8.704 8.383
   1579   1373876_at   8.362 8.378 8.584 8.293 8.279 8.874 8.73 8.981 8.938 8.641
   1580   1373368_at   6.137 6.616 6.32 6.087 6.103 6.784 7.4 7.152 7.095 6.848
   1581   1381434_s_at   5.648 5.068 5.515 4.848 5.255 6.412 7.104 6.158 6.167 6.619
   1582   1377060_at   10.588 10.708 10.723 10.467 10.516 10.303 10.17 10.291 10.307 10.346
   1583   1396035_at   8.901 8.791 9.25 8.723 8.633 7.783 8.049 8.111 7.989 8.407
   1584   1380699_at   6.96 6.284 5.983 6.461 6.186 7.466 7.556 7.758 7.595 7.03
   1585   1387242_at   6.085 5.834 6.009 6.691 5.926 6.987 6.781 7.027 7.202 6.995
   1586   1369064_a_at   5.349 4.369 4.791 5.095 5.053 4.062 3.236 3.931 3.862 3.662
   1587   1390066_at   1.824 1.628 3.197 2.195 0.978 4.933 4.087 3.542 4.463 4.94
   1588   1393048_at   5.981 5.36 5.223 5.723 4.505 6.849 6.524 6.941 7.023 6.654
   1589   1371923_at   5.068 5.27 5.684 5.569 5.6 6.884 6.815 6.424 6.405 5.992
   1590   1373460_at   7.429 7.364 7.404 7.488 7.214 7.827 7.908 8.215 7.786 7.742
   1591   1388989_at   10.81 10.968 10.875 10.876 10.741 11.182 11.183 11.224 11.204 10.992
   1592   1383531_at   8.139 7.966 7.844 7.975 7.826 7.489 7.315 7.292 7.55 6.96
   1593   1382263_at   5.694 5.93 6.013 5.627 5.8 6.258 6.329 6.229 6.178 6.156
   1594   1374685_at   6.176 5.839 5.822 5.89 5.953 6.445 6.648 6.457 6.41 6.222
   1595   1378248_at   7.047 7.304 6.889 6.963 7.14 6.69 6.652 6.635 6.46 6.674
   1596   1398889_at   10.427 10.627 10.491 10.507 10.364 10.805 10.719 10.752 10.838 10.689
   1597   1377660_at   7.062 7.709 8.016 7.328 7.117 5.397 3.195 4.735 5.255 5.727
   1598   1387732_at   7.67 7.927 7.955 8.093 7.853 8.363 8.398 8.491 8.401 8.173
   1599   1372377_at   8.065 7.86 7.886 8.078 7.681 8.343 8.531 8.433 8.253 8.344
   1600   1374033_at   7.168 6.979 7.482 7.109 6.665 8.921 8.555 8.223 7.912 7.974
   1601   1367920_at   3.876 3.877 3.676 4.619 3.826 6.004 5.036 5.535 5.618 4.836
   1602   1369910_at   0 2.566 0.623 1.731 0.696 3.696 3.228 3.581 3.945 4.354
   1603   1376887_at   6.945 6.769 6.686 6.7 6.904 7.349 7.471 7.546 7.674 7.067
   1604   1374201_at   5.417 5.653 5.921 5.692 5.019 6.446 6.37 6.613 6.326 6.303
   1605   1386996_at   11.159 11.224 11.246 11.087 11.068 11.421 11.458 11.615 11.433 11.355
   1606   1368045_at   9.896 10.007 9.65 9.988 9.93 9.553 9.607 9.506 9.5 9.467
   1607   1389158_at   6.079 5.748 5.816 6.152 5.355 6.643 6.483 6.729 6.763 6.555
   1608   1368432_a_at   7.405 7.439 6.968 7.164 7.572 5.941 6.408 6.761 6.47 6.418
   1609   1383729_at   4.243 4.327 4.351 4.706 4.789 5.407 5.172 5.072 5.012 5.217
   1610   1383883_at   5.044 4.854 5.033 4.408 4.926 5.39 5.767 6.031 5.585 5.991
   1611   1382043_at   7.82 8.018 7.72 7.711 7.648 9.027 8.854 8.217 8.642 8.423
   1612   1398616_at   6.175 6.201 6.674 6.151 6.469 7.6 7.012 7.162 7.144 6.915
   1613   1370427_at   8.216 8.097 8.448 8.049 7.806 7.541 7.086 7.473 7.433 7.511
   1614   1373304_at   8.41 8.405 8.583 8.353 8.201 7.7 7.632 8.039 8.024 7.699
   1615   1372513_at   10.093 9.835 9.901 10.077 9.871 10.439 10.359 10.276 10.213 10.274
   1616   1372016_at   9.517 8.987 9.859 7.231 8.822 6.437 6.524 6.755 5.964 5.965
   1617   1373656_at   5.598 5.518 5.785 5.615 5.665 6.479 6.07 6.113 6.313 5.955
   1618   1371549_at   10.132 10.113 10.098 9.91 9.925 9.672 9.406 9.687 9.716 9.691
   1619   1368337_at   5.046 5.298 4.998 4.585 5.26 5.908 5.741 5.699 5.639 5.806
   1620   1372924_at   6.686 6.53 6.28 6.336 6.567 7.231 6.886 7.122 7.152 6.851
   1621   1389598_at   8.033 8.108 8.189 7.843 7.826 8.408 8.395 8.344 8.523 8.397
   1622   1378040_at   6.594 6.157 6.098 6.404 6.449 7.049 6.84 7.219 6.896 6.843
   1623   1373736_at   7.593 7.625 7.718 7.313 7.15 6.389 6.669 6.915 6.918 6.524
   1624   1378418_at   3.331 4.155 3.984 4.411 3.044 5.919 5.131 5.313 5.238 5.172
   1625   1368223_at   7.205 6.934 7.212 7.191 7.043 9.285 9.622 9.193 8.365 7.866
   1626   1374167_at   6.65 6.402 6.877 6.237 6.353 4.92 4.851 5.101 5.77 5.743
   1627   1394499_at   5.713 5.72 5.818 5.359 5.412 6.156 6.036 6.319 6.076 6.209
   1628   1392741_at   4.468 4.011 4.07 3.536 4.515 0.941 1.05 2.924 2.348 2.114
   1629   1371332_at   11.471 11.519 11.454 10.984 11.595 10.941 10.608 10.78 10.716 10.515
   1630   1373277_at   9.464 9.554 9.486 9.336 9.413 9.025 8.989 9.282 9.111 9.159
   1631   1372167_at   9.414 9.456 9.119 9.323 9.235 10.033 10.262 9.975 10.062 9.583
   1632   1383766_at   5.395 5.29 5.487 5.648 5.526 6.149 5.874 6.037 5.937 5.756
   1633   1379483_at   8.312 8.405 8.324 8.423 8.563 8.027 7.824 7.938 7.945 7.482
   1634   1386820_at   7.507 7.402 7.499 7.561 7.226 6.939 7.001 6.975 7.219 7.021
   1635   1372920_at   8.808 8.511 8.835 8.463 8.1 7.657 7.504 7.787 7.125 7.796
   1636   1397644_at   6.347 6.184 6.574 6.387 6.311 7.32 7.201 7.209 6.981 6.653
   1637   1398315_at   11.506 11.499 11.498 11.534 11.47 12.071 12.282 11.999 11.888 11.729
   1638   1384250_a_at   5.857 6.083 6.125 5.677 5.603 6.692 6.502 7.257 6.76 6.594
   1639   1370825_a_at   10.874 10.893 10.752 10.846 10.723 11.224 11.449 11.335 11.207 11.009
   1640   1385359_at   3.517 4.319 3.839 4.539 4.146 5.401 5.206 4.887 4.986 5.391
   1641   1374571_at   9.058 9.141 9.083 9.039 8.993 9.408 9.443 9.471 9.337 9.18
   1642   1378457_at   8.52 8.669 8.724 8.696 8.375 7.972 7.684 8.258 8.09 7.892
   1643   1389337_at   7.393 7.205 7.623 7.263 7.158 8.081 7.944 7.804 7.926 7.698
   1644   1382086_at   4.836 5.403 4.802 4.544 5.296 6.602 6.503 6.472 6.574 5.543
   1645   1396219_at   3.263 4.916 4.599 3.753 4.458 6.027 5.839 5.642 5.707 6.029
   1646   1369286_at   8.783 9.152 8.893 8.742 8.788 7.956 7.902 8.528 8.135 8.26
   1647   1386932_at   5.166 5.116 5.131 4.989 4.342 5.82 5.673 5.752 5.822 5.871
   1648   1382592_at   5.243 5.433 5.496 4.999 4.946 6.34 6.241 5.9 5.848 5.847
   1649   1388789_at   8.254 8.395 8.254 8.163 8.117 8.737 8.49 8.736 8.727 8.497
   1650   1397328_at   5.946 5.96 5.697 6.305 5.676 6.842 6.328 6.897 6.757 6.865
   1651   1372875_at   7.277 7.363 7.29 7.264 7.153 7.862 7.777 7.831 7.507 7.541
   1652   1376644_at   6.152 6.558 6.22 6.656 6.33 6.895 6.952 7.367 7.201 6.978
   1653   1385242_at   3.823 2.595 2.441 1.828 4.694 5.936 5.622 5.793 6.091 5.795
   1654   1398387_at   7.879 7.942 7.347 7.708 7.59 8.498 8.358 8.504 8.57 8.105
   1655   1395157_at   7.866 7.424 6.973 8.005 7.439 8.574 8.61 8.608 8.642 8.329
   1656   1371640_at   10.535 10.426 10.493 10.525 10.391 10.284 10.328 10.231 10.325 10.316
   1657   1375043_at   8.39 8.272 10.247 8.211 9.114 6.694 6.213 5.739 3.951 4.415
   1658   1372931_at   7.412 7.551 7.726 7.218 7.232 7.94 8.057 7.945 7.878 7.99
   1659   1394363_at   7.918 7.971 7.678 7.81 7.767 7.341 7.387 7.556 7.529 7.238
   1660   1372333_at   10.341 10.271 10.274 10.25 10.114 10.569 10.748 10.665 10.59 10.421
   1661   1389010_at   9.36 9.493 9.313 9.316 9.221 9.868 9.961 9.856 9.73 9.539
   1662   1388344_at   9.061 9.143 9.097 9.18 9.196 8.862 8.917 9.034 8.838 8.833
   1663   1368851_at   8.309 8.339 8.007 8.162 8.273 8.848 8.789 8.85 8.661 8.462
   1664   1386934_at   11.344 11.342 11.11 11.19 11.228 10.874 10.876 11.072 10.93 10.941
   1665   1388874_at   10.577 10.492 10.273 10.474 10.495 10.183 10.215 10.215 10.173 10.131
   1666   1378171_at   5.384 4.968 5.403 5.615 4.848 6.482 6.342 5.897 6.333 5.99
   1667   1379301_at   6.062 6.072 6.123 6.222 6.133 5.473 4.891 5.713 5.334 5.505
   1668   1370999_at   1.48 0.816 3.219 3.086 3.436 6.23 5.956 5.802 4.81 4.986
   1669   1368896_at   8.017 8.269 8.295 7.683 7.883 7.012 6.827 7.537 6.998 7.282
   1670   1371818_at   10.127 10.399 10.19 10.089 10.185 9.71 9.747 9.974 9.862 9.832
   1671   1398869_at   9.809 9.807 9.92 9.846 9.661 10.05 10.071 10.017 10.086 10.004
   1672   1375030_at   8.534 8.91 8.958 8.367 8.282 7.693 7.425 7.947 7.863 7.781
   1673   1378487_at   5.803 5.692 5.479 5.529 5.597 5.033 4.958 4.53 5.144 5.12
   1674   1376050_at   5.074 5.105 5.245 5.769 4.525 6.31 6.632 6.186 6.258 6.093
   1675   1382443_at   7.83 7.646 7.463 7.785 7.634 8.22 8.446 8.33 8.232 7.935
   1676   1386138_at   4.87 4.991 5.102 4.844 4.943 5.431 5.34 5.859 5.756 5.389
   1677   1367982_at   9.908 9.953 10.347 9.963 9.789 9.427 9.604 9.323 9.434 9.428
   1678   1387542_at   9.982 9.981 9.729 9.775 9.913 9.31 9.534 9.379 9.458 9.028
   1679   1369007_at   6.501 6.249 6.649 5.414 5.907 5.029 4.508 5.04 4.952 4.816
   1680   1386897_at   8.954 9.106 9.181 9.003 8.869 9.62 9.687 9.576 9.389 9.286
   1681   1371792_at   9.8 9.956 10.114 9.998 9.993 10.49 10.332 10.471 10.27 10.224
   1682   1390396_at   6.007 5.791 5.611 5.574 5.868 6.406 6.413 6.647 6.326 6.123
   1683   1392698_a_at   6.034 6.265 6.097 6.68 6.476 7.059 6.952 7.181 6.819 7.168
   1684   1379542_at   8.653 8.538 8.884 8.448 8.371 9.199 9.283 9.264 9.062 8.956
   1685   1367701_at   7.749 7.886 7.799 7.996 7.81 8.328 8.143 8.514 8.543 8.172
   1686   1370393_at   5.395 5.458 5.302 5.968 5.174 6.407 6.407 6.583 6.11 6.111
   1687   1371516_at   8.708 8.839 8.743 8.44 8.672 8.283 8.186 8.355 8.373 8.213
   1688   1372156_at   8.101 8.234 8.351 8.105 7.9 8.762 8.692 8.675 8.412 8.783
   1689   1392699_at   5.199 5.335 4.786 5.583 5.23 6.206 5.935 6.295 5.956 5.834
   1690   1373072_at   8.705 8.868 8.598 8.724 8.917 9.67 9.394 9.51 9.24 9.082
   1691   1383266_at   6.249 5.834 5.875 5.656 5.844 6.737 6.891 7.137 6.776 6.302
   1692   1373432_at   8.371 8.795 8.146 8.344 8.305 9.57 8.848 9.434 9.478 9.131
   1693   1379694_at   6.088 5.887 6.033 5.743 5.901 5.384 5.368 5.445 4.931 5.474
   1694   1375855_at   3.401 4.365 3.86 4.101 4.046 5.126 4.926 4.688 4.985 4.743
   1695   1384044_at   5.981 5.665 5.537 6.089 5.463 6.454 6.657 6.552 6.772 6.259
   1696   1397737_at   4.222 4.093 3.632 4.299 3.756 4.819 4.932 4.641 4.695 4.646
   1697   1372661_at   6.723 6.897 7.007 6.898 6.731 7.321 7.134 7.192 7.123 7.187
   1698   1368339_at   6.653 6.589 6.608 6.556 6.515 5.144 5.875 5.642 6.04 5.957
   1699   1382493_at   3.432 5.124 3.628 4.833 5.225 7.51 6.421 6.882 6.548 6.334
   1700   1386002_at   5.676 5.371 5.653 5.525 5.538 6.024 5.94 6.095 6.07 5.761
   1701   1371544_at   9.923 10.017 9.765 9.993 9.739 10.548 10.451 10.555 10.228 10.191
   1702   1392861_at   3.929 4.636 4.016 4.357 4.131 5.044 5.421 4.932 5.052 4.84
   1703   1398255_at   10.175 10.276 10.545 9.929 10.216 9.566 9.402 9.802 9.694 9.527
   1704   1382252_at   5.553 6.093 5.478 4.996 5.538 6.573 6.55 6.494 6.788 6.279
   1705   1397673_at   3.743 2.95 3.333 3.191 3.296 3.857 4.206 3.977 4.277 4.349
   1706   1375560_at   10.119 9.973 9.869 10.153 9.771 9.495 9.593 9.658 9.576 9.528
   1707   1389163_at   8.192 8.243 7.903 8.221 8.221 8.608 8.538 8.71 8.607 8.414
   1708   1391649_a_at   6.073 5.997 6.264 5.682 5.849 7.102 6.647 7.019 6.8 6.424
   1709   1368358_a_at   8.23 8.248 8.273 7.771 8.108 7.168 7.308 7.476 7.706 7.534
   1710   1373185_at   9.113 9.263 9.307 9.153 9.083 9.759 9.668 9.652 9.49 9.398
   1711   1373911_at   3.046 2.941 4.461 2.685 4.426 6.406 7.06 5.659 6.354 5.198
   1712   1369941_at   9.749 9.9 9.731 9.867 9.753 10.214 10.358 10.356 10.239 9.955
   1713   1383137_at   8.696 8.97 9.084 8.647 8.379 9.589 9.382 9.577 9.526 9.294
   1714   1376785_at   9.711 9.849 9.629 9.494 9.534 9.077 9.014 9.332 9.308 9.159
   1715   1393138_at   11.424 11.178 11.43 10.914 11.314 10.755 10.726 10.756 10.791 10.668
   1716   1376334_at   4.974 5.026 4.853 5.097 5.16 5.57 5.643 5.574 5.51 5.212
   1717   1397450_at   6.184 5.986 6.024 6.161 6.262 5.208 5.561 5.394 5.481 5.863
   1718   1371696_at   10.888 10.931 10.765 10.728 10.751 10.531 10.554 10.627 10.621 10.516
   1719   1398359_at   9.752 9.566 9.505 9.71 9.751 9.367 9.445 9.317 9.374 9.297
   1720   1390827_at   7.559 7.674 7.586 7.767 7.554 8.033 7.79 8.158 8.009 8.04
   1721   1373516_at   6.877 7.01 7.3 6.939 7.276 7.65 7.501 7.716 7.585 7.49
   1722   1385716_at   8.866 8.933 8.972 8.675 8.569 8.376 8.147 8.267 8.437 8.37
   1723   1379683_at   6.373 6.309 6.287 6.102 5.785 6.922 6.782 7.152 6.678 6.804
   1724   1372550_at   7.728 7.714 7.483 7.696 7.647 8.471 8.117 8.53 8.196 7.966
   1725   1372426_at   7.111 6.839 6.98 6.81 7.053 7.535 7.409 7.609 7.624 7.194
   1726   1374691_at   6.542 6.507 5.935 6.387 6.371 7.117 6.881 7.024 6.958 6.85
   1727   1398246_s_at   7.414 7.533 8.278 7.246 7.188 9.56 9.206 8.852 8.822 8.404
   1728   1386995_at   10.154 9.616 11.052 8.813 9.535 8.008 8.114 7.839 7.391 7.422
   1729   1396018_at   5.261 5.351 5.093 5.361 5.555 5.744 6.072 5.926 5.681 5.842
   1730   1383623_at   7.203 7.321 6.896 7.084 6.997 7.759 7.821 7.714 7.655 7.369
   1731   1369250_at   7.045 6.784 6.292 6.686 6.53 5.926 5.557 5.592 5.811 6.142
   1732   1378247_at   6.616 6.543 6.846 6.523 6.047 5.842 5.154 5.652 5.754 5.587
   1733   1393983_at   8.909 8.961 8.617 8.783 8.844 8.339 8.337 8.605 8.416 8.419
   1734   1373931_at   4.903 5.063 4.734 4.88 4.439 5.395 5.363 5.259 5.548 5.447
   1735   1378365_at   6.207 6.556 6.117 5.983 5.945 5.454 5.31 5.563 5.583 5.03
   1736   1377635_at   8.682 8.535 7.993 8.406 8.458 7.76 7.576 7.546 7.945 7.726
   1737   1382402_at   6.642 6.422 6.448 6.796 6.427 6.2 6.09 5.759 5.887 6.021
   1738   1394014_at   6.299 6.131 6.16 6.362 5.808 6.751 6.572 6.733 6.622 6.746
   1739   1397892_at   7.434 7.455 7.372 7.468 7.583 8.175 7.98 8.013 7.656 7.879
   1740   1376016_at   7.671 7.867 7.318 7.915 7.79 8.117 8.431 8.377 8.45 8.357
   1741   1388103_at   10.503 10.185 10.001 10.42 10.272 9.306 9.548 9.581 9.668 9.899
   1742   1391505_x_at   5.115 5.564 5.005 5.152 4.739 5.853 5.726 6.364 6.022 6.262
   1743   1379254_at   10.251 10.242 10.42 10.085 10.286 9.975 10.027 9.952 9.886 9.876
   1744   1390820_at   6.037 5.989 5.511 5.713 5.65 6.365 6.403 6.695 6.602 6.231
   1745   1370810_at   4.786 4.467 5.278 4.801 4.62 5.801 5.296 5.854 5.621 5.751
   1746   1384617_at   6.577 6.397 5.77 6.221 6.219 7.095 6.893 7.112 6.94 6.866
   1747   1373942_at   7.249 7.495 7.343 7.489 7.332 8.276 7.657 8.162 7.858 8.105
   1748   1384714_x_at   8.549 8.585 8.334 8.734 8.388 8.838 8.941 8.88 8.912 8.957
   1749   1371694_at   9.042 9.177 8.948 8.909 9.015 9.73 9.391 9.482 9.415 9.301
   1750   1374216_at   4.951 4.823 5.61 5.469 5.467 6.93 6.532 6.168 5.996 6.563
   1751   1390510_at   6.019 5.608 6.73 5.808 5.696 8.057 7.471 7.501 7.431 6.783
   1752   1370816_at   7.914 7.772 7.495 7.356 8.53 6.394 5.675 6.657 6.638 6.581
   1753   1368605_at   4.513 4.642 5.018 4.219 4.356 5.759 5.049 5.625 5.651 5.453
   1754   1393221_at   11.576 11.685 11.566 11.676 11.764 10.842 11.117 11.428 10.975 11.176
   1755   1382783_at   5.246 5.122 5.352 5.312 4.97 5.777 5.59 6.211 5.812 5.792
   1756   1372590_at   5.532 5.469 5.317 5.131 5.671 6.042 6.148 5.845 6.181 5.878
   1757   1368428_at   10.19 10.25 10.116 10.277 10.088 9.662 9.64 10.007 9.865 9.691
   1758   1368597_at   8.003 7.695 7.997 6.632 7.231 5.482 5.881 5.831 6.285 6.284
   1759   1391240_at   6.341 6.574 6.269 6.099 5.945 5.687 5.294 5.765 5.463 5.314
   1760   1370312_at   9.609 9.687 9.697 9.544 9.506 10.307 9.907 10.429 10.447 9.997
   1761   1385793_at   5.157 3.489 3.189 4.059 3.597 2.546 1.618 1.428 1.47 2.021
   1762   1376852_at   10.225 10.199 10.607 10.197 10.165 9.627 9.636 9.672 9.732 9.987
   1763   1374169_at   8.933 8.871 9.268 8.729 8.895 8.321 8.364 8.584 8.433 8.425
   1764   1398434_at   5.655 6.074 5.851 5.666 5.803 6.491 6.424 6.322 6.183 6.177
   1765   1373955_at   8.149 8.362 8.371 8.162 8.102 8.646 8.896 8.703 8.687 8.477
   1766   1372065_at   5.862 6.023 6.013 5.766 5.816 6.157 6.296 6.136 6.357 6.35
   1767   1375631_at   11.341 11.257 11.088 11.389 11.479 10.961 10.63 10.914 10.765 10.933
   1768   1383850_at   8.396 8.534 8.447 8.305 8.562 7.688 7.927 8.162 8.057 7.705
   1769   1389039_at   6.247 6.731 6.457 6.376 6.543 7.586 7.023 7.338 7.191 6.914
   1770   1388400_at   8.31 8.205 8.409 8.23 8.206 8.612 8.541 8.549 8.611 8.861
   1771   1371336_at   8.541 8.493 8.78 8.636 8.566 9.634 9.048 9.631 9.311 9.033
   1772   1391014_at   5.969 5.754 5.889 6.029 5.772 6.353 6.151 6.427 6.301 6.154
   1773   1368205_at   6.18 5.657 5.506 5.621 5.602 7.466 7.83 7.923 7.206 6.288
   1774   1373530_at   1.59 2.969 4.5 2.558 1.705 5.328 5.096 5.626 5.488 5.536
   1775   1370237_at   11.534 11.514 11.482 11.617 11.514 11.128 10.953 11.326 11.272 11.208
   1776   1369120_a_at   5.393 5.483 5.589 5.693 5.619 5.916 5.909 5.896 5.779 6.064
   1777   1387565_at   5.179 4.921 4.858 5.198 5.328 6.429 5.608 6.187 5.969 5.74
   1778   1376324_at   4.123 4.738 4.934 4.648 4.547 5.476 5.446 5.694 5.318 5.172
   1779   1369935_at   8.267 8.157 8.39 8.244 8.157 8.785 8.509 8.57 8.553 8.514
   1780   1392548_at   4.75 5.196 5.18 5.482 5.176 5.692 5.771 5.993 5.812 5.783
   1781   1389008_at   9.093 9.183 9.129 9.11 9.101 8.792 8.794 8.983 8.877 8.633
   1782   1392451_at   0.151 0 0.864 0.176 0 0.911 2.05 1.888 1.373 1.566
   1783   1394275_at   6.904 7.125 6.529 6.559 6.832 7.346 7.318 7.482 7.35 7.406
   1784   1390709_at   7.742 7.754 7.726 7.816 7.9 8.165 8.316 8.231 8.094 7.945
   1785   1398265_at   6.067 6.026 5.873 5.969 5.98 6.485 6.752 7.144 6.599 6.4
   1786   1391652_at   11.19 11.297 11.068 11.252 11.411 10.721 10.813 10.929 10.885 10.988
   1787   1373446_at   5.938 5.796 5.954 6.024 6.08 6.471 6.305 6.51 6.797 6.951
   1788   1389421_at   3.608 4.587 4.892 4.424 3.894 5.434 5.672 5.549 5.336 5.885
   1789   1383067_a_at   7.914 8.2 8.124 7.905 7.841 8.572 8.477 8.56 8.265 8.406
   1790   1392158_at   4.353 3.764 3.718 4.312 3.957 4.834 4.791 4.905 4.529 4.908
   1791   1370355_at   6.259 6.766 8.254 6.822 5.901 4.409 3.644 4.209 4.824 4.979
   1792   1389127_at   7.939 8.152 7.869 7.828 7.977 8.401 8.244 8.336 8.27 8.206
   1793   1391222_at   5.162 4.483 4.649 5.265 4.691 6.063 5.82 6.298 5.552 5.705
   1794   1373730_at   7.891 8.106 8.076 7.825 7.958 7.685 7.498 7.702 7.684 7.551
   1795   1379107_at   6.948 5.641 6.185 5.246 5.794 4.223 4.128 4.746 4.233 4.524
   1796   1384935_at   7.024 7.274 7.142 7.133 7.277 7.523 7.913 7.92 7.739 7.47
   1797   1387888_at   11.642 11.61 11.733 11.737 11.523 11.955 11.921 11.877 11.857 11.824
   1798   1387033_at   6.248 6.725 8.792 5.976 5.5 3.318 1.057 3.069 3.631 3.32
   1799   1391452_at   3.865 4.245 4.635 4.408 4.42 5.232 5.072 4.926 5.072 4.899
   1800   1376667_at   5.161 4.169 4.278 5.656 4.297 2.698 1.989 1.844 3.039 3.35
   1801   1368652_at   7.094 7.079 7.159 6.976 6.911 7.341 7.355 7.305 7.279 7.223
   1802   1398827_at   11.56 11.438 11.308 11.522 11.527 11.816 11.738 11.686 11.684 11.818
   1803   1379339_at   9.056 8.91 8.727 8.854 8.709 8.467 8.434 8.595 8.451 8.517
   1804   1369099_at   7.839 7.471 7.398 7.397 7.165 6.637 7.039 6.775 6.761 6.805
   1805   1398169_at   4.545 3.999 4.436 4.605 3.06 1.599 1.263 2.039 3.069 1.151
   1806   1384770_at   7.423 7.311 7.836 7.199 7.391 6.392 6.098 6.598 6.646 6.932
   1807   1369562_at   9.468 9.192 9.168 9.344 9.349 8.416 8.412 8.785 8.934 8.833
   1808   1373182_at   8.204 8.423 8.483 8.365 8.477 7.967 8.073 8.105 7.832 8.102
   1809   1374212_at   6.248 6.141 6.062 5.676 6.016 6.579 6.575 6.643 6.645 6.405
   1810   1372790_at   10.578 10.589 10.726 10.502 10.326 10.082 9.951 10.11 10.101 10.295
   1811   1390481_a_at   1.731 2.803 1.379 4.559 1.263 5.942 5.869 5.996 4.796 5.887
   1812   1384215_at   7.116 7.718 7.485 7.635 7.365 6.506 5.983 6.453 6.716 6.896
   1813   1370130_at   9.874 9.807 9.67 9.827 9.69 10.127 9.992 9.985 10.077 9.971
   1814   1371602_at   8.517 8.823 8.564 8.475 8.755 9.298 8.897 9.301 9.169 9.074
   1815   1390968_at   5.522 5.7 5.853 5.879 5.752 6.24 6.251 6.068 6.02 6.128
   1816   1377310_at   7.22 7.184 7.133 6.962 6.994 7.654 7.551 7.716 7.6 7.283
   1817   1390535_at   7.789 8.007 8.061 7.814 8.265 7.592 7.269 7.556 7.451 7.332
   1818   1368253_at   9.982 9.999 10.195 10.005 9.977 9.417 9.24 9.661 9.711 9.635
   1819   1380445_at   6.107 6.477 6.337 6.153 6.004 6.841 6.842 6.635 6.625 6.647
   1820   1385382_at   4.132 4.434 3.595 4.024 4.187 4.86 5.131 4.878 5.251 4.659
   1821   1377836_at   5.819 5.884 5.8 6.125 5.89 6.472 6.424 6.403 6.346 6.101
   1822   1397154_at   4.77 4.426 4.271 4.726 4.116 3.999 3.291 3.278 2.915 3.016
   1823   1381019_x_at   5.591 5.625 5.171 5.726 5.582 6.341 6.415 6.111 6.184 5.925
   1824   1374270_at   4.74 4.688 4.796 4.431 5.248 5.559 6.06 5.41 5.573 5.683
   1825   1378199_at   2.333 4.366 2.922 4.119 1.899 6.187 5.768 5.73 5.295 5.545
   1826   1386858_at   11.456 11.465 11.568 11.416 11.304 11.921 11.968 11.71 11.816 11.657
   1827   1397168_at   6.392 6.425 6.611 6.19 5.979 6.989 7.106 7.416 6.853 6.94
   1828   1386860_at   8.382 8.313 8.474 8.646 8.372 9.071 8.643 9.06 8.914 8.97
   1829   1367967_at   6.9 6.552 6.9 6.764 6.792 7.495 7.436 7.288 7.089 7.158
   1830   1392922_at   6.468 6.464 7.021 7.137 6.62 7.739 7.444 7.547 7.619 7.348
   1831   1389804_at   5.771 5.559 5.553 5.327 6.006 6.425 6.504 6.626 6.089 6.303
   1832   1370156_at   8.595 8.574 8.623 8.355 8.444 9.009 8.862 9.092 8.884 8.747
   1833   1398885_at   12.009 11.962 12.093 11.956 11.896 12.245 12.24 12.191 12.159 12.117
   1834   1397527_at   4.941 4.833 5.392 4.985 4.845 5.815 5.814 5.53 5.413 5.811
   1835   1368115_at   8.653 8.332 8.812 7.93 8.317 7.7 7.205 7.377 7.706 7.482
   1836   1373164_at   7.509 7.471 7.603 7.244 7.621 8.47 8.311 8.469 7.95 7.882
   1837   1398852_at   11.78 11.899 11.756 11.828 11.725 12.194 12.239 12.167 11.948 12.029
   1838   1383784_at   3.995 4.177 4.448 4.379 4.105 4.999 5.509 5.969 5.178 4.88
   1839   1398761_at   12.067 12.095 12.152 12 12.076 12.246 12.434 12.328 12.229 12.277
   1840   1383744_at   8.742 8.531 8.632 7.905 8.162 7.352 7.427 7.781 7.353 7.582
   1841   1394605_at   7.054 7.217 7.102 6.961 7.099 7.329 7.364 7.496 7.42 7.267
   1842   1382215_at   7.956 8.071 7.922 7.822 7.847 7.619 7.345 7.613 7.624 7.323
   1843   1382647_at   3.126 3.262 3.237 3.428 3.88 4.737 4.022 4.572 4.266 4.122
   1844   1374730_at   6.966 6.511 7.001 6.749 6.599 7.958 7.765 7.559 7.623 7.141
   1845   1371776_at   7.581 7.398 7.659 7.581 7.411 8.181 8.392 8.226 8.002 7.78
   1846   1368342_at   8.162 8.196 8.39 7.955 8.119 8.588 8.663 8.64 8.65 8.41
   1847   1373664_at   7.381 7.523 7.29 7.486 7.312 7.66 7.655 7.799 7.74 7.601
   1848   1373590_at   9.262 9.325 9.644 9.451 9.414 8.91 8.725 9.188 8.895 8.809
   1849   1376673_at   8.151 8.271 7.793 7.911 8.114 8.625 8.429 8.586 8.65 8.434
   1850   1376005_at   5.915 5.652 6.032 6.14 6.237 6.795 6.613 6.782 6.429 6.506
   1851   1380129_at   5.729 5.35 5.553 5.924 5.534 6.72 6.243 6.198 6.181 6.279
   1852   1367791_at   3.979 4.815 4.782 5.198 5.03 6.126 6.047 6.038 6.308 5.521
   1853   1375482_at   1.915 0.895 1.59 2.82 1.531 2.766 4.38 4.594 4.163 4.238
   1854   1392638_at   5.397 4.87 4.902 4.893 5.087 6.131 6.028 6.339 5.503 5.699
   1855   1386917_at   9.922 10.192 9.784 10.039 10.248 9.482 9.461 9.731 9.478 9.336
   1856   1370030_at   11.677 11.683 11.581 11.588 11.771 11.406 11.267 11.369 11.354 11.526
   1857   1393129_at   0 1.299 0.903 1.526 2.86 3.493 5.054 4.36 3.411 4.277
   1858   1374143_at   7.753 7.817 7.804 7.024 7.878 6.89 6.842 6.899 6.626 6.463
   1859   1380431_at   7.727 8.123 7.976 7.819 8.028 7.145 7.366 7.599 7.52 7.38
   1860   1370376_a_at   7.573 7.665 8.181 7.619 7.92 8.683 9.087 8.77 8.592 8.26
   1861   1367706_at   12.241 12.234 12.226 12.322 12.347 12.14 12.098 12.049 12.048 12.166
   1862   1368008_at   10.396 10.467 10.672 10.512 10.519 10.142 10.14 10.343 10.25 10.205
   1863   1376687_at   7.636 8.026 7.879 7.587 7.756 8.419 8.274 8.445 8.205 8.112
   1864   1371721_at   8.053 8.053 8.129 8.156 7.947 7.849 7.937 7.818 7.832 7.848
   1865   1392969_at   11.595 11.645 11.573 11.667 11.758 11.118 11.188 11.334 11.43 11.414
   1866   1398871_at   12.107 12.102 12.201 12.07 11.935 12.351 12.476 12.32 12.351 12.278
   1867   1380009_at   6.525 6.506 6.614 6.355 6.331 7.024 6.659 7.023 6.861 6.937
   1868   1383960_at   8.367 8.426 8.297 8.462 8.216 7.678 7.777 7.95 7.98 8.122
   1869   1387001_at   8.843 8.786 8.702 8.4 8.751 9.106 9.058 9.087 9.187 9.076
   1870   1379327_at   7.718 7.591 7.574 7.413 7.374 7.775 7.902 7.978 8.103 8.016
   1871   1367579_a_at   11.962 11.97 12.041 11.986 11.972 12.542 12.39 12.141 12.24 12.4
   1872   1389518_at   8.702 8.721 8.769 8.547 8.457 9.165 9.049 9.259 8.986 8.904
   1873   1367687_a_at   10.187 10.307 10.222 10.254 10.311 10.005 10.022 10.176 9.986 9.957
   1874   1393158_at   3.667 3.374 5.056 3.925 4.434 5.707 5.5 5.478 5.595 5.839
   1875   1369720_at   6.944 6.765 6.239 6.685 6.866 7.493 7.416 7.262 7.426 7.239
   1876   1395440_at   7.405 7.391 7.166 7.369 7.296 6.961 7.156 7.019 7.024 7.082
   1877   1382010_at   7.399 7.27 7.15 7.262 6.999 7.896 7.826 7.901 7.577 7.522
   1878   1374524_at   10.871 10.973 10.694 10.99 10.966 10.431 10.151 10.479 10.538 10.568
   1879   1382050_at   10.712 10.894 10.338 10.552 10.859 9.964 10.079 10.259 10.092 9.837
   1880   1387158_at   10.369 10.456 10.694 10.388 10.337 9.163 9.371 9.718 9.909 9.937
   1881   1392082_a_at   5.521 5.119 5.136 5.409 5.255 5.955 5.678 5.728 6.204 5.933
   1882   1371382_at   8.777 8.825 9.368 8.63 8.908 9.891 9.873 9.951 9.367 9.603
   1883   1395409_at   7.796 7.362 7.635 7.861 7.515 8.213 8.055 8.258 8.213 8.014
   1884   1393584_at   9.784 9.454 9.386 9.187 9.776 8.615 8.532 9.12 8.783 8.556
   1885   1387144_at   5.674 5.745 5.766 6.613 6.141 6.865 7.143 7.475 7.231 6.736
   1886   1393038_at   6.54 6.287 6.932 6.402 6.553 7.814 7.182 7.223 7.478 7.159
   1887   1396820_at   8.277 8.044 7.896 8.363 7.814 8.86 8.774 8.591 8.633 8.558
   1888   1391539_at   9.708 10.025 9.958 9.388 9.61 8.778 9.087 9.279 9.038 8.922
   1889   1370649_at   5.449 5.202 5.5 5.405 5.401 4.937 4.478 4.584 4.341 5.037
   1890   1370682_at   5.638 4.985 5.795 4.997 4.703 6.678 6.491 6.582 6.355 6.011
   1891   1369632_a_at   5.522 5.669 6.127 5.573 5.253 5.071 4.892 4.727 4.776 4.809
   1892   1395856_at   6.392 6.737 6.552 6.571 6.294 6.843 6.929 6.97 6.976 6.864
   1893   1381942_at   6.377 5.811 6.049 5.359 5.753 6.507 6.939 6.89 6.762 6.991
   1894   1387343_at   8.473 8.06 7.718 7.739 8.17 7.382 7.067 6.737 6.33 6.276
   1895   1391068_at   4.79 4.895 5.399 4.705 5.017 5.924 5.977 5.534 6.438 5.75
   1896   1386280_at   10.026 9.943 10.072 10.046 9.998 9.416 9.091 9.556 9.415 9.774
   1897   1389509_at   8.293 8.304 8.307 8.282 8.193 8.479 8.466 8.481 8.414 8.353
   1898   1387110_at   8.781 8.628 8.531 8.513 8.425 8.234 8.125 8.357 8.13 8.092
   1899   1399030_at   8.769 8.82 8.746 8.74 8.733 8.412 8.637 8.495 8.581 8.367
   1900   1386960_at   11.085 10.946 10.506 10.999 10.907 10.157 10.186 10.446 10.437 10.299
   1901   1389131_at   7.748 7.766 7.561 7.46 7.437 7.973 8.137 8.219 8.102 7.869
   1902   1370814_at   10.274 10.356 10.413 10.315 10.213 9.996 9.931 10.083 10.071 10.169
   1903   1385047_x_at   4.629 4.698 4.96 5.474 5.251 6.314 6.109 5.69 5.854 5.889
   1904   1388877_at   8.091 8.33 8.058 7.908 8.228 8.824 8.38 8.797 8.638 8.65
   1905   1372306_at   11.441 11.308 11.185 11.306 11.555 10.663 10.524 10.914 10.926 11.004
   1906   1383182_at   6.459 6.692 6.247 6.403 6.358 6.888 6.777 6.757 6.811 6.889
   1907   1372460_at   9.236 9.45 9.227 9.358 9.243 9.675 9.7 9.625 9.456 9.587
   1908   1394097_at   5.031 4.744 5.441 5.122 5.298 5.666 5.97 6.157 6.031 5.669
   1909   1388814_at   11.014 11.031 10.528 11.141 11.124 10.213 10.248 10.448 10.504 10.225
   1910   1396205_at   4.033 4.699 0.595 4.062 4.459 7.954 7.537 7.115 7.163 7.257
   1911   1391573_at   9.308 9.146 8.902 8.703 9.359 8.213 8.177 8.634 8.412 8.183
   1912   1376279_at   6.75 6.89 7.036 6.499 6.783 6.083 6.387 6.115 5.744 6.208
   1913   1391162_at   6.199 6.293 6.811 6.539 6.153 7.229 7.28 7.534 6.95 7.01
   1914   1383322_at   5.949 5.606 6.332 6.004 6.045 7.046 6.444 6.722 6.956 6.683
   1915   1376909_at   5.594 5.38 5.355 5.807 4.93 6.1 6.059 6.228 6.252 6.471
   1916   1367795_at   9.125 8.747 9.138 8.639 8.811 8.339 8.195 8.129 7.739 7.53
   1917   1373040_at   10.463 10.48 10.445 10.361 10.265 10.875 10.9 10.887 10.882 10.525
   1918   1391026_at   6.138 5.35 6.356 6.151 5.711 7.305 6.764 6.811 6.989 6.923
   1919   1389000_at   7.903 7.85 7.862 7.831 7.7 8.092 8.158 8.095 8.201 7.952
   1920   1369081_at   9.629 9.456 9.553 9.764 9.386 8.822 9.132 9.119 9.205 9.157
   1921   1387079_at   5.715 5.325 5.162 6.294 5.322 7.012 6.417 7.061 7.2 6.517
   1922   1390728_at   6.583 6.829 6.583 6.908 6.266 7.416 7.432 7.443 7.171 7.081
   1923   1388766_at   8.995 9.158 9.007 8.774 8.865 9.259 9.33 9.56 9.382 9.329
   1924   1368250_at   1.911 3.449 4.375 3.901 3.18 5.548 5.879 5.417 5.324 5.299
   1925   1390987_at   7.33 7.215 7.428 7.194 7.078 6.836 6.743 6.66 6.994 6.917
   1926   1383472_at   7.02 6.622 7.517 6.876 6.906 5.795 6.011 6.377 6.252 6.087
   1927   1382274_at   8.847 8.768 8.984 8.984 8.894 8.442 8.075 8.215 8.455 8.637
   1928   1381376_at   5.951 5.51 5.619 5.84 5.939 6.421 6.242 6.165 6.207 6.273
   1929   1393982_at   4.521 4.231 4.398 2.214 3.405 6.699 6.167 6.013 5.491 6.115
   1930   1376697_at   5.133 5.491 5.68 5.008 5.462 6.408 6.292 6.122 6.708 5.871
   1931   1397205_at   11.903 11.81 12.199 11.844 12.13 10.557 10.487 10.944 11.186 11.523
   1932   1372615_at   6.12 6.583 6.609 6.498 6.151 6.808 7.218 7.536 7.314 7.081
   1933   1378342_at   5.203 4.901 4.866 4.663 5.175 5.638 6.083 5.771 5.969 5.363
   1934   1377821_at   9.417 9.739 9.512 9.367 9.177 8.943 8.974 8.992 9.005 8.759
   1935   1386946_at   10.726 10.8 10.883 10.78 10.688 10.458 10.436 10.668 10.515 10.492
   1936   1383434_at   0.888 0.084 0.926 1.828 0.454 4.367 5.557 4.893 1.945 4.156
   1937   1384174_at   2.789 3.372 2.254 1.157 0.345 5.289 4.864 4.727 4.496 4.561
   1938   1378225_at   5.354 5.343 6.118 5.858 5.423 6.637 6.537 6.804 6.625 6.169
   1939   1382571_at   8.758 8.872 8.546 8.527 8.672 8.169 8.055 8.38 8.23 8.355
   1940   1372949_at   8.775 8.734 8.526 8.567 8.612 9.276 9.033 9.151 8.971 8.876
   1941   1371125_at   6.971 7.051 6.717 6.845 6.604 7.221 7.266 7.252 7.448 7.252
   1942   1374176_at   7.598 7.76 7.317 7.546 7.31 8.484 8.256 8.44 8.442 7.782
   1943   1375612_at   5.817 6.037 5.91 6.042 6.257 6.821 7.042 6.99 6.706 6.29
   1944   1387625_at   4.787 5.027 5.619 3.868 4.978 6.877 6.675 6.225 6.533 5.99
   1945   1377223_at   7.016 6.947 6.766 6.789 6.675 6.176 6.355 6.441 6.556 6.459
   1946   1370329_at   8.213 8.07 8.563 8.246 7.968 7.378 6.927 7.545 7.513 7.668
   1947   1381054_at   3.959 4.329 4.776 4.303 4.219 4.951 5.022 4.936 4.937 5.14
   1948   1373617_at   6.522 6.954 6.653 6.709 6.356 7.588 7.223 7.293 7.148 7.135
   1949   1367487_at   8.556 8.575 8.703 8.657 8.615 9.038 8.887 8.845 8.757 8.885
   1950   1382148_at   5.985 5.918 5.937 6.332 5.73 6.533 6.571 6.599 6.465 6.365
   1951   1390925_a_at   5.078 5.093 5.676 5.44 5.61 6.252 5.959 5.899 6.132 6.17
   1952   1372643_at   7.591 7.531 7.652 7.436 7.349 8.299 7.882 7.967 8.052 7.818
   1953   1372574_at   5.454 5.632 5.018 4.143 5.697 6.601 6.558 6.993 6.773 6.466
   1954   1379725_at   7.927 7.834 7.505 7.772 7.683 8.303 8.186 8.001 8.276 8.162
   1955   1390397_at   4.288 3.401 3.29 3.811 3.467 5.408 5.089 5.183 4.384 4.632
   1956   1398829_at   9.936 9.977 10.111 9.974 9.985 10.46 10.404 10.246 10.151 10.277
   1957   1391356_at   7.536 7.609 7.487 7.623 7.716 8.217 7.883 8.159 8.306 7.853
   1958   1383946_at   3.746 4.247 3.888 4.794 3.888 5.693 5.206 5.815 5.224 4.896
   1959   1367617_at   12.401 12.398 12.568 12.422 12.355 12.591 12.676 12.585 12.643 12.646
   1960   1372799_at   8.054 8.08 7.97 8.084 8.213 7.698 7.845 7.701 7.93 7.767
   1961   1369292_at   6.776 6.835 6.549 6.61 6.384 5.609 5.485 5.655 6.128 6.193
   1962   1369262_at   5.581 6.003 6.132 6.373 6.007 7.047 6.973 6.838 6.919 6.438
   1963   1368059_at   11.368 11.538 11.461 11.414 11.37 10.991 10.834 11.07 11.158 11.231
   1964   1376689_at   5.72 5.757 5.609 5.038 5.232 6.586 6.487 6.884 6.285 6.049
   1965   1375346_at   9.349 9.298 9.525 9.338 9.128 8.854 8.899 8.997 8.893 9.096
   1966   1385395_at   6.083 6.026 5.807 6.116 6.015 6.553 6.394 6.226 6.376 6.59
   1967   1378663_at   3.788 3.058 3.234 3.546 3.678 2.893 0.31 0.88 0.604 0.516
   1968   1369260_a_at   3.957 3.645 3.803 3.909 3.493 4.427 4.041 4.418 4.624 4.553
   1969   1373674_at   5.012 4.091 4.259 3.455 4.172 6.369 6.768 5.595 5.391 5.698
   1970   1379804_at   6.059 5.889 6.174 6.195 5.943 6.427 6.454 6.624 6.302 6.417
   1971   1392242_at   4.482 4.433 4.541 4.361 4.763 4.923 4.823 5.137 5.318 5.246
   1972   1385157_at   5.909 6.028 6.205 6.735 5.846 6.981 6.741 7.061 7.163 7.236
   1973   1380018_at   2.66 1.459 1.345 0.74 1.063 3.111 3.741 3.8 2.653 3.663
   1974   1376795_at   6.716 7.207 6.787 6.81 7.116 7.684 7.474 7.427 7.475 7.348
   1975   1387303_at   11.844 11.801 11.948 11.708 11.83 10.549 10.296 10.726 11.052 11.457
   1976   1371761_at   11.939 11.867 11.905 11.872 11.847 12.113 12.184 11.994 12.087 12.005
   1977   1382739_at   4.184 3.418 3.846 3.313 3.082 5.181 5.289 4.444 4.457 5.182
   1978   1388794_at   8.255 8.323 8.102 8.023 8.041 8.719 8.694 8.599 8.436 8.42
   1979   1376894_at   8.02 8.142 7.932 7.915 7.939 7.742 7.793 7.803 7.769 7.689
   1980   1370894_at   7.246 7.293 8.011 7.179 7.323 8.545 8.396 8.33 7.99 8.188
   1981   1372670_at   8.589 8.542 8.703 8.479 8.31 8.133 8.013 8.146 8.246 8.211
   1982   1380217_at   0.816 0 0.604 0.176 0.263 3.581 4.534 2.551 1.373 3.306
   1983   1394585_at   4.269 2.725 4.369 4.317 3.929 5.361 5.739 5.646 5.513 5.241
   1984   1370247_a_at   8.08 7.559 7.337 7.953 8.237 8.893 8.732 8.628 8.634 8.591
   1985   1395012_at   4.07 3.67 4.173 3.988 4.145 3.057 2.063 3.198 3.233 2.553
   1986   1387770_at   9.924 9.899 9.937 10.048 10.072 11.252 10.483 10.37 10.813 10.92
   1987   1391077_at   0.807 2.214 3.521 1.88 0 4.258 4.687 5.45 4.673 4.888
   1988   1392048_at   3.916 3.974 4.019 3.245 3.503 4.727 4.862 4.24 4.671 4.684
   1989   1379374_at   3.718 3.663 3.989 3.867 3.846 4.736 5.054 5.001 4.09 4.644
   1990   1372254_at   10.704 10.742 10.824 10.661 10.596 11.306 10.986 11.183 11.122 10.904
   1991   1395084_at   1.48 2.214 1.084 2.144 0 3.772 2.945 4.109 3.803 4.943
   1992   1375974_at   9.466 9.654 9.433 9.579 9.512 9.849 9.658 9.788 9.819 9.785
   1993   1375267_at   10.952 11.089 11.285 10.864 10.681 10.224 10.123 10.565 10.319 10.468
   1994   1368917_at   5.688 5.977 5.694 5.923 5.375 6.561 6.329 6.295 6.207 6.263
   1995   1389477_at   6.021 5.845 5.769 6.005 5.684 6.616 6.633 6.357 6.25 6.201
   1996   1391148_at   6.84 7.018 6.978 6.858 7.041 6.121 6.267 6.763 6.424 6.355
   1997   1368178_at   11.471 11.655 11.367 11.53 11.47 11.258 11.27 11.302 11.235 11.245
   1998   1371893_at   9.598 9.493 9.392 9.574 9.649 9.351 9.154 9.297 9.117 9.067
   1999   1376106_at   8.887 8.735 8.691 8.666 8.841 9.159 9.029 9.489 9.407 9.123
   2000   1392334_at   10.38 10.051 10.315 10.257 10.262 10.893 10.797 10.494 10.757 10.571
   2001   1389425_at   5.631 5.851 6.15 6.117 5.927 6.711 6.731 6.511 6.915 6.289
   2002   1369301_at   2.098 3.633 3.424 3.81 3.444 4.979 4.51 4.966 4.734 4.99
   2003   1388846_at   6.379 6.202 6.309 5.991 5.797 7.136 6.955 6.975 6.997 6.46
   2004   1388625_at   7.505 7.502 7.342 6.898 6.935 8.011 7.871 8.068 8.005 7.763
   2005   1387130_at   8.647 8.65 8.23 8.732 8.673 9.111 9.222 9.273 9.055 8.93
   2006   1375985_at   8.239 8.362 8.44 8.113 8.271 7.861 7.452 7.73 8.017 7.722
   2007   1373334_at   7.465 7.339 7.272 7.33 7.346 7.795 7.683 7.528 7.9 7.616
   2008   1380148_at   6.216 6.256 6.544 6.783 6.287 7.407 7.217 7.459 7.395 6.746
   2009   1390026_at   8.945 9.148 8.579 8.641 8.956 8.098 7.989 8.261 8.469 8.198
   2010   1382009_at   6.363 6.58 6.668 7.039 6.869 7.343 7.381 7.64 7.837 7.23
   2011   1389214_at   5.877 5.718 6.037 5.798 5.616 7.129 6.328 6.936 6.481 6.407
   2012   1383376_at   8.808 8.63 8.706 8.629 8.658 8.224 8.327 8.477 8.487 8.441
   2013   1392525_at   4.823 4.426 4.973 4.831 4.496 5.22 5.504 5.301 5.146 5.348
   2014   1373920_at   6.098 6.254 6.383 6.033 5.955 5.248 4.844 5.668 4.432 5.155
   2015   1370960_at   11.807 11.842 11.501 11.799 11.686 11.301 11.059 11.316 11.399 11.405
   2016   1389378_at   6.597 6.34 6.966 6.458 6.153 7.328 7.255 7.497 7.298 6.998
   2017   1372404_at   7.814 7.688 8.168 7.767 7.516 8.83 8.333 8.505 8.367 8.361
   2018   1373977_at   6.968 7.365 7.266 6.989 6.796 5.843 5.22 6.083 6.377 6.265
   2019   1383153_at   6.283 6.181 6.229 6.096 5.876 5.265 5.031 5.699 5.55 5.626
   2020   1371984_at   4.098 5.132 4.109 4.811 4.89 5.833 5.66 5.64 5.763 5.5
   2021   1375438_at   10.127 10.068 9.754 10.223 9.989 9.329 9.243 9.5 9.678 9.621
   2022   1386916_at   10.889 11.058 11.022 10.932 10.828 10.266 10.337 10.506 10.711 10.626
   2023   1374910_at   8.086 8.298 8.108 7.992 8.034 7.714 7.443 7.732 7.641 7.868
   2024   1379590_at   6.525 6.399 6.704 6.045 6.335 6.029 5.741 5.726 5.519 5.659
   2025   1390264_at   7.22 7.4 7.274 7.229 7.2 7.703 7.44 7.756 7.668 7.491
   2026   1375224_at   5.572 5.651 6.332 5.893 5.679 7.303 6.46 7.169 6.632 6.718
   2027   1389254_at   6.768 6.972 6.929 7.071 7.098 7.868 7.426 7.986 7.481 7.391
   2028   1370007_at   8.4 8.468 8.678 8.274 8.738 9.108 9.197 8.959 8.888 8.934
   2029   1383290_at   8.211 8.071 8.303 7.996 8.226 8.521 8.505 8.68 8.491 8.385
   2030   1396109_at   4.44 4.341 4.308 4.454 4.608 5.243 5.266 4.686 5.674 5.434
   2031   1373287_at   4.198 4.885 5.027 4.387 5.054 5.801 5.394 5.577 5.624 5.844
   2032   1397864_at   0 0 1.202 0 0 3.332 2.454 3.841 2.333 1.316
   2033   1378016_at   6.254 6.209 6.487 6.465 6.149 6.785 6.594 6.96 6.886 6.669
   2034   1374293_at   9.217 9.431 9.576 9.41 9.301 9.082 8.971 9.114 9.116 9.026
   2035   1372774_at   10.742 11.139 10.941 10.665 10.854 10.073 9.881 10.297 10.458 10.38
   2036   1395757_at   1.293 0.74 0 0.678 1.48 2.805 2.822 3.667 1.727 3.313
   2037   1374802_at   7.4 7.633 7.817 7.071 6.734 8.612 8.384 8.5 8.206 8.135
   2038   1390075_at   3.503 4.372 3.365 4.593 3.625 5.715 5.141 5.585 4.945 5.082
   2039   1393613_at   5.862 6.032 5.637 5.781 5.771 6.368 6.536 6.728 6.48 6.063
   2040   1387795_at   6.596 6.588 7.122 6.143 6.561 7.445 7.318 7.732 7.872 7.308
   2041   1367961_at   11.826 11.853 11.759 11.918 11.617 10.802 10.984 11.303 11.116 11.517
   2042   1385291_a_at   4.544 4.499 4.535 3.788 3.95 5.415 5.318 5.171 5.323 4.811
   2043   1395828_at   1.604 3.614 2.83 2.124 3.985 5.77 4.597 5.174 5.26 5.043
   2044   1385814_at   5.261 5.012 4.831 5.411 5.043 5.668 5.629 5.965 5.748 5.547
   2045   1384192_at   4.322 4.46 3.888 4.773 4.163 4.959 5.185 5.207 4.956 5.102
   2046   1397392_at   5.135 5.586 5.4 4.995 5.434 3.615 3.197 3.944 4.849 3.873
   2047   1384773_at   5.489 4.753 5.353 4.611 5.132 5.902 6.017 5.728 5.998 5.971
   2048   1371865_at   9.297 9.231 9.117 9.342 9.113 9.595 9.697 9.515 9.465 9.433
   2049   1370826_at   9.302 9.427 9.352 9.255 9.357 10.132 9.861 9.858 9.73 9.543
   2050   1376056_at   6.681 6.503 7.078 6.498 6.693 7.768 7.185 7.452 7.329 7.264
   2051   1383548_at   6.53 6.76 6.632 6.627 6.291 7.079 7.071 7.143 6.871 6.93
   2052   1387005_at   8.664 8.44 8.856 8.458 8.897 9.695 9.207 9.319 9.424 9.085
   2053   1383126_at   8.661 8.97 8.917 8.687 8.707 9.142 9.169 9.229 9.121 9.027
   2054   1374306_at   5.604 5.944 6.083 5.939 5.883 6.474 6.466 6.332 6.178 6.32
   2055   1383590_at   5.75 5.805 5.556 5.904 5.707 6.315 6.745 6.317 6.392 6.033
   2056   1374545_at   7.879 7.94 7.749 8.261 7.906 8.559 8.87 8.844 8.466 8.313
   2057   1381267_at   4.29 4.53 4.602 4.418 4.001 4.985 4.88 4.787 5.143 4.976
   2058   1389402_at   7.881 7.627 8.793 6.874 7.949 6.319 6.238 6.592 5.985 5.686
   2059   1372440_at   7.565 7.673 7.401 7.756 7.743 8.842 7.947 8.899 8.726 8.34
   2060   1375176_at   5.183 4.916 4.835 5.109 4.959 5.361 5.315 5.757 5.453 5.561
   2061   1391825_at   3.998 4.222 4.712 3.912 3.121 5.674 5.128 5.132 5.266 5.622
   2062   1391733_at   6.041 5.939 6.07 6.249 6.398 6.595 6.596 6.54 6.453 6.595
   2063   1368501_s_at   0.872 1.642 1.491 1.864 0.604 4.548 4.318 4.844 3.547 2.16
   2064   1367866_at   9.576 9.425 8.852 9.679 9.649 10.415 9.973 10.312 10.335 10.245
   2065   1368790_at   6.915 6.749 6.881 6.501 6.468 8.425 8.589 8.253 7.421 7.382
   2066   1383355_at   6.186 6.031 6.345 6.152 5.907 6.927 6.891 7.079 6.745 6.373
   2067   1372004_at   6.543 6.082 6.507 6.301 6.231 7.374 7.382 6.941 7 6.715
   2068   1393352_at   7.63 7.861 7.956 7.724 7.928 6.997 7.591 7.183 7.326 7.214
   2069   1397772_at   5.135 5.285 5.402 4.68 4.984 6.345 6.139 5.811 5.636 5.847
   2070   1382442_at   4.733 4.886 5.196 4.785 4.599 6.024 6.193 5.678 5.508 5.332
   2071   1374830_at   6.636 6.395 6.267 6.484 6.072 7.286 6.683 6.991 7.26 7.248
   2072   1397628_at   6.941 6.744 6.965 7.027 6.999 7.559 7.143 7.301 7.31 7.386
   2073   1368340_at   10.166 10.197 10.431 10.217 10.135 9.713 9.723 10.052 9.686 9.508
   2074   1367850_at   6.704 6.799 7.669 6.546 6.598 9.043 8.375 8.294 8.04 7.744
   2075   1372770_at   9.425 9.502 9.225 9.416 9.045 9.904 9.86 9.838 9.65 9.689
   2076   1372912_at   6.763 6.763 7.067 7.457 6.674 7.71 7.453 7.773 7.78 7.835
   2077   1382308_at   7.677 7.988 7.936 7.856 7.529 8.363 8.139 8.67 8.468 8.299
   2078   1377153_a_at   5.139 5.095 4.748 5.719 5.082 6.588 6.234 5.807 6.139 5.958
   2079   1395342_at   5.718 5.864 4.846 5.637 5.616 6.198 6.446 6.516 6.532 6.52
   2080   1385718_at   5.119 5.496 5.395 5.403 5.172 5.635 6.083 5.986 6.334 5.849
   2081   1374368_at   7.49 7.546 7.687 7.493 7.637 7.936 7.845 8.102 7.745 7.928
   2082   1387788_at   8.657 8.668 10.439 8.553 8.931 7.665 7.257 7.224 7.252 6.974
   2083   1376835_at   6.344 6.634 6.964 6.499 6.292 7.391 7.082 7.152 7.071 7.288
   2084   1380583_s_at   6.397 6.07 6.706 6.573 6.23 6.932 6.875 6.999 6.955 7.045
   2085   1376955_at   7.283 7.238 6.778 7.152 7.108 6.225 5.978 6.62 6.672 6.31
   2086   1398599_at   6.883 6.874 7.022 6.857 6.897 7.343 7.75 7.223 7.399 7.231
   2087   1385292_at   3.687 4.821 3.719 4.367 4.34 5.928 5.702 5.256 5.011 5.487
   2088   1387227_at   8.341 8.235 8.019 8.139 8.112 8.725 8.517 8.517 8.462 8.431
   2089   1379740_at   3.975 4.554 3.424 4.343 3.961 5.689 5.656 5.523 4.723 5.099
   2090   1386096_at   6.577 6.328 6.404 6.473 5.68 7.123 7.123 7.101 7.171 6.904
   2091   1372430_at   6.77 7.118 7.18 6.785 7.234 7.826 7.489 7.985 7.502 7.614
   2092   1370131_at   6.453 6.095 6.174 6.433 6.233 7.25 7.221 6.499 7.045 7.032
   2093   1391676_at   4.07 4.574 4.304 4.118 3.645 4.935 4.927 4.806 4.83 4.926
   2094   1382431_at   7.067 6.965 6.824 7.319 7.232 7.877 7.895 8.167 8.109 7.365
   2095   1377949_s_at   7.622 7.499 7.533 7.557 7.506 7.249 7.051 7.072 7.059 7.43
   2096   1371189_x_at   9.508 9.474 9.382 9.486 9.246 9.795 9.758 9.605 9.691 9.66
   2097   1392863_at   6.367 5.744 5.635 6.264 6.309 6.703 6.921 6.814 7.08 6.809
   2098   1393972_at   6.031 5.812 5.649 6.04 5.662 6.458 6.432 6.418 6.141 6.257
   2099   1371862_at   7.221 7.499 7.806 7.235 7.967 8.525 8.4 8.407 8.171 8.181
   2100   1371431_at   8.97 8.952 8.911 8.916 8.79 8.451 8.495 8.689 8.756 8.582
   2101   1370705_at   5.434 5.324 5.099 5.249 5.371 5.581 5.639 5.6 5.771 5.548
   2102   1397282_at   4.663 4.327 4.805 4.734 4.574 4.897 5.129 5.281 5.417 5.291
   2103   1371313_at   11.463 11.542 11.339 11.36 11.32 11.696 11.828 11.597 11.713 11.61
   2104   1372722_at   8.842 8.771 8.887 8.493 8.648 8.24 8.392 8.377 8.215 7.991
   2105   1374871_at   10.492 10.518 10.854 10.508 10.535 10.03 9.552 10.072 10.123 10.097
   2106   1386252_at   5.273 5.987 5.884 5.688 5.622 6.248 6.405 6.726 6.808 6.291
   2107   1370400_at   5.09 5.053 4.898 5.834 5.331 6.453 6.281 5.878 5.978 6.279
   2108   1393732_at   7.923 9.043 8.085 8.331 9.029 7.06 7.331 7.643 6.961 7.051
   2109   1392847_at   5.4 5.213 5.167 5.675 5.261 5.775 5.903 5.888 5.83 5.694
   2110   1382601_at   4.229 3.775 4.452 2.454 4.165 6.444 5.878 5.93 5.309 5.378
   2111   1382158_at   5.595 5.507 5.737 5.455 5.26 5.95 5.801 6.015 5.916 5.93
   2112   1391858_at   6.763 6.519 6.291 6.452 6.825 6.088 6.004 5.981 6.148 6.13
   2113   1398472_at   6.558 6.385 5.916 6.112 6.329 7.088 6.791 6.798 6.887 6.76
   2114   1384383_at   6.859 6.809 6.792 6.629 6.641 7.417 7.17 6.993 7.152 7.054
   2115   1376451_at   6.067 6.353 6.116 6.263 5.907 6.756 6.916 7.407 6.618 6.763
   2116   1389197_at   7.427 7.584 7.61 7.485 7.35 8.103 7.949 7.971 7.691 7.8
   2117   1374134_at   6.412 6.678 6.776 6.446 6.661 7.117 6.823 7.193 7.321 7.093
   2118   1379431_at   7.578 7.659 7.229 7.319 7.455 6.794 6.8 6.972 7.103 7.091
   2119   1390807_at   10.479 10.541 10.418 10.389 10.52 10.09 10.18 10.365 10.088 10.005
   2120   1368575_at   10.929 10.965 11.191 10.845 10.802 10.552 10.252 10.602 10.573 10.536
   2121   1387131_at   3.734 5.009 4.755 4.741 4.643 6.029 5.379 6.156 5.807 5.665
   2122   1391537_at   4.578 4.952 5.4 5.221 5.817 6.422 6.022 6.44 6.19 6.203
   2123   1372052_at   9.54 9.518 9.58 9.548 9.416 9.752 9.829 9.809 9.604 9.756
   2124   1389366_at   6.073 5.864 6.216 6.167 6.149 6.712 7.076 6.651 7.074 6.397
   2125   1367771_at   10.028 9.768 9.464 9.505 9.898 9.178 9.251 9.263 9.153 9.151
   2126   1387884_at   9.635 9.82 9.834 9.796 9.645 10.107 10.046 10.171 9.958 9.934
   2127   1367663_at   10.98 10.959 11.008 10.893 10.904 11.529 11.448 11.251 11.268 11.108
   2128   1368379_at   6.531 6.604 6.416 6.141 6.061 7.074 6.955 6.854 6.909 6.772
   2129   1388244_s_at   11.331 11.353 11.426 11.33 11.189 11.653 11.462 11.563 11.54 11.589
   2130   1381481_at   5.894 5.984 5.34 5.849 5.783 6.791 6.665 6.277 6.488 6.267
   2131   1374207_at   6.963 6.865 6.897 6.766 6.721 7.208 7.051 7.012 7.139 7.198
   2132   1372015_at   8.678 8.674 8.496 8.457 8.67 9.058 8.762 9.106 9.115 8.915
   2133   1388527_at   6.51 6.542 6.395 5.904 6.218 7.461 7.18 7.141 7.443 6.686
   2134   1389664_at   5.669 5.57 5.851 5.947 5.512 5.985 6.208 6.311 6.467 6.443
   2135   1370020_at   10.865 10.759 11.06 10.993 10.814 10.222 10.234 10.519 10.632 10.511
   2136   1389744_at   8.715 8.99 8.84 8.686 8.675 8.232 8.333 8.503 8.415 8.515
   2137   1387434_at   7.744 7.777 7.736 7.519 7.65 7.074 7.286 7.47 7.377 7.31
   2138   1392927_at   4.783 4.598 4.895 3.807 5.321 6.238 5.511 6.474 6.146 6.047
   2139   1392806_at   7.136 7.06 6.831 7.012 6.765 6.453 6.635 6.666 6.479 6.313
   2140   1398361_at   7.862 7.878 7.585 7.713 7.614 7.208 7.408 7.441 7.453 7.35
   2141   1368736_at   7.883 7.998 7.595 7.871 7.813 7.524 7.267 7.325 7.56 7.42
   2142   1368943_at   7.01 6.641 5.659 7.098 6.507 8.095 8.031 7.855 8.032 7.497
   2143   1385116_at   2.905 2.95 2.534 2.703 2.751 4.543 4.029 3.243 3.718 3.697
   2144   1372161_at   3.639 4.207 4.8 4.203 4.618 5.322 5.271 5.955 6.107 5.268
   2145   1384282_at   5.66 5.82 5.982 6.01 6.215 6.471 6.667 6.617 6.667 6.231
   2146   1371253_at   10.999 10.955 10.889 11.01 10.849 10.738 10.79 10.776 10.733 10.611
   2147   1393564_at   4.175 4.255 3.901 4.725 4.126 5.128 4.699 5.459 5.107 5.088
   2148   1370547_at   10.306 10.519 10.334 10.408 10.538 9.598 9.985 10.126 10.047 9.925
   2149   1390947_at   6.991 7.039 6.834 6.746 6.793 7.381 7.192 7.485 7.31 7.112
   2150   1373230_at   8.018 8.083 8.186 7.975 7.775 7.587 7.397 7.601 7.676 7.71
   2151   1373214_at   6.277 6.191 6.401 6.149 6.197 6.942 7.111 6.78 6.707 6.466
   2152   1368511_at   6.045 6.191 6.278 6.207 6.634 5.716 5.935 5.621 5.487 5.412
   2153   1370333_a_at   9.191 9.406 9.505 9.303 9.075 8.655 8.733 8.769 9.046 8.499
   2154   1367977_at   7.581 7.848 8.826 8.075 7.971 6.48 5.896 6.557 7.066 7.102
   2155   1396942_at   7.363 7.127 8.021 7.029 6.74 6.026 5.666 6.029 6.371 6.296
   2156   1384548_at   12.367 12.354 12.351 12.237 12.219 12.542 12.718 12.612 12.599 12.423
   2157   1397554_at   4.255 4.324 3.86 4.756 4.315 5.71 4.868 5.139 5.164 5.322
   2158   1374396_at   10.107 10.058 9.703 9.826 9.999 9.459 9.66 9.546 9.56 9.515
   2159   1383581_at   3.542 3.349 4.663 4.506 2.356 6.25 5.809 5.752 5.352 5.752
   2160   1385994_at   6.887 6.888 6.927 6.693 6.465 6.016 6.377 6.197 6.417 6.123
   2161   1382083_at   6.065 5.594 6.297 5.617 5.185 4.837 4.346 4.731 4.985 4.536
   2162   1368927_at   6.121 6.238 6.647 5.762 6.325 6.976 6.918 6.798 6.956 6.92
   2163   1377286_at   6.635 6.748 6.412 6.458 6.548 7.214 6.865 6.943 7.126 6.865
   2164   1383855_at   10.704 10.704 10.95 10.828 10.553 11.125 10.965 11.153 11.159 11.132
   2165   1373089_at   6.017 5.927 6.255 6.438 6.194 6.759 6.799 6.532 6.531 6.795
   2166   1383248_at   9.277 9.109 9.374 9.198 9.09 8.651 8.677 8.851 9.04 8.721
   2167   1373302_at   8.549 8.608 8.826 8.704 8.837 9.285 9.21 9.611 9.114 9.029
   2168   1382611_at   9.117 8.71 8.855 8.742 8.708 8.168 8.258 8.309 8.567 8.02
   2169   1379799_at   3.912 5.027 4.002 4.217 4.598 5.578 5.272 5.452 5.179 5.426
   2170   1374683_at   2.214 1.506 1.941 1.895 2.373 2.446 4.957 4.887 4.817 4.786
   2171   1372029_at   8.843 9.012 9.234 8.611 8.711 9.358 9.447 9.439 9.432 9.38
   2172   1392235_at   0 0 0 0 0.516 0.705 1.281 0.575 1.281 0.888
   2173   1389299_at   8.039 8.155 7.872 7.728 8.215 7.663 7.502 7.58 7.439 7.519
   2174   1376913_at   6.86 6.778 5.822 5.973 6.059 4.621 4.946 5.153 4.316 5.473
   2175   1387064_at   7.253 7.439 7.159 7.23 7.26 7.851 7.618 7.82 7.713 7.433
   2176   1382294_at   6.022 5.952 5.978 6.084 5.893 6.639 6.763 6.389 6.27 6.297
   2177   1395146_at   7.51 7.375 7.211 7.328 7.402 7.801 7.561 7.761 7.891 7.628
   2178   1371395_at   10.255 10.299 10.158 10.159 10.057 10.583 10.58 10.462 10.357 10.409
   2179   1399148_s_at   6.347 6.486 6.251 6.217 6.064 7.051 7.131 6.749 6.862 6.547
   2180   1388134_at   10.119 10.05 10.099 10.017 9.855 10.332 10.303 10.309 10.297 10.178
   2181   1373053_at   8.283 8.446 8.671 8.539 8.507 7.824 7.764 8.204 8.165 7.873
   2182   1374763_at   4.624 4.309 5.056 4.335 3.982 5.729 6.054 5.211 5.316 5.533
   2183   1373321_at   8.187 8.552 7.791 8.274 7.847 7.509 7.305 7.453 7.531 7.392
   2184   1376215_at   2.866 4.285 3.91 3.67 3.972 5.353 4.921 5.248 5.181 4.563
   2185   1390437_at   7.821 8.073 7.738 7.836 8.141 7.534 7.492 7.517 7.261 7.561
   2186   1374438_at   7.765 7.882 7.91 7.939 7.835 8.38 8.128 8.617 8.197 8.208
   2187   1375684_at   7.665 7.859 7.61 7.744 7.454 7.211 7.011 7.264 7.246 7.408
   2188   1382993_at   5.923 6.21 6.498 6.495 5.98 7.166 7.488 6.96 7.022 6.69
   2189   1369208_at   4.085 4.745 5.026 4.306 4.489 5.948 5.701 5.568 5.391 5.124
   2190   1368679_a_at   7.051 6.565 6.429 6.431 6.532 7.575 7.165 7.218 7.221 7.135
   2191   1373864_at   7.854 7.957 8.239 7.639 7.656 8.68 8.46 8.711 8.44 8.276
   2192   1368329_at   11.33 11.493 11.165 11.389 11.404 10.496 10.678 10.887 11.125 10.81
   2193   1372007_at   7.091 7.034 7.336 7.165 7.327 7.549 7.782 7.832 7.49 7.518
   2194   1392952_at   8.879 8.806 8.475 8.967 8.69 8.121 7.939 7.924 8.417 8.328
   2195   1368162_at   2.446 3.479 3.696 3.601 3.586 4.943 4.236 4.878 4.892 4.357
   2196   1370385_at   8.341 8.301 8.502 8.118 8.03 7.358 7.177 7.674 7.758 7.815
   2197   1389596_at   7.608 7.712 7.808 7.685 7.595 7.946 7.804 7.9 7.95 7.907
   2198   1382334_at   6.387 6.136 6.569 6.386 6.325 7.055 7.021 6.937 7.099 6.548
   2199   1374296_at   9.32 9.364 9.334 9.223 9.298 9.626 9.668 9.55 9.477 9.423
   2200   1372668_at   7.12 7.274 7.218 7.131 7.229 7.861 7.581 7.587 7.629 7.35
   2201   1380277_at   2.842 3.597 3.66 2.328 2.874 4.303 4.664 5.188 3.999 4.733
   2202   1381014_at   5.525 5.328 5.718 5.212 5.452 6.686 5.773 6.125 6.419 6.402
   2203   1381771_at   1.709 2.483 2.609 0.88 0 4.315 4.369 3.751 3.935 3.524
   2204   1370060_at   10.919 10.931 10.947 11.02 10.898 10.795 10.65 10.725 10.744 10.854
   2205   1374015_at   4.229 4.336 4.233 3.467 3.555 5.874 6.137 6.04 5.863 4.442
   2206   1370210_at   1.176 1.091 1.007 0.941 1.17 1.844 1.669 1.58 1.731 1.233
   2207   1387624_at   7.274 7.317 7.161 7.245 7.172 7.865 7.774 7.704 7.438 7.475
   2208   1393247_at   4.888 4.885 4.928 4.559 4.643 5.98 5.624 6.217 6.139 5.096
   2209   1390128_at   8.66 8.755 8.492 8.603 8.893 8.392 8.41 8.283 8.302 8.233
   2210   1380537_at   3.192 4.404 4.271 4.021 4.073 4.961 4.792 5.115 5.306 5.177
   2211   1381833_at   1.978 1.454 4.329 1.328 0.516 5.518 4.633 5.624 5.348 4.555
   2212   1384544_at   6.699 7.078 6.91 7.119 7.163 7.839 8.34 8.122 7.576 7.454
   2213   1388774_at   10.732 10.837 10.721 10.777 10.751 10.656 10.508 10.61 10.411 10.475
   2214   1397372_at   5.519 5.413 5.634 5.536 5.404 5.674 6.161 6.246 6.2 5.929
   2215   1391170_at   6.226 6.03 6.201 5.845 6.019 6.558 6.836 7.12 6.478 6.56
   2216   1392142_at   3.847 3.318 3.911 4.238 3.57 4.655 4.307 4.566 4.782 4.766
   2217   1370249_at   9.058 9.255 9.596 8.91 8.794 10.214 10.04 9.924 9.807 9.647
   2218   1384699_at   5.567 5.716 5.69 5.618 5.608 6.402 6.491 6.306 6.635 5.794
   2219   1369173_at   3.126 3.41 4.14 4.458 3.448 4.769 5.306 4.857 4.845 5.37
   2220   1387234_at   7.59 7.217 7.795 7.401 7.597 6.663 6.263 6.515 6.948 7.076
   2221   1389580_at   6.915 7.05 7.256 6.961 6.829 7.557 7.305 7.628 7.311 7.509
   2222   1382587_at   4.383 4.168 4.383 3.833 4.137 4.676 4.725 5.27 5.177 5.507
   2223   1374538_at   7.658 7.972 8.141 7.878 7.726 8.184 8.383 8.388 8.331 8.292
   2224   1371032_at   7.351 6.992 7.041 7.227 6.842 8.223 7.366 7.899 8.115 7.894
   2225   1370818_at   11.281 11.235 10.85 11.413 11.278 10.546 10.673 10.778 10.813 10.756
   2226   1374635_at   4.552 4.431 4.161 4.544 4.856 6.136 5.879 5.761 6.354 4.881
   2227   1387765_at   7.829 7.825 7.992 7.675 7.575 7.181 7.033 7.386 7.122 7.513
   2228   1394826_at   3.2 3.972 4.252 4.261 4.033 5.004 4.971 4.922 4.657 5.278
   2229   1388623_at   8.77 9.026 8.58 8.792 8.532 8.302 8.158 8.277 8.43 8.181
   2230   1387882_at   8.803 8.065 7.909 8.667 8.745 7.517 7.695 7.28 7.604 7.226
   2231   1387678_at   6.489 6.527 6.515 6.342 6.303 6.999 6.734 6.86 6.919 6.61
   2232   1372969_at   7.709 7.682 7.758 7.576 7.467 7.403 7.069 7.348 7.28 7.287
   2233   1384310_at   5.914 6.481 6.647 6.447 6.449 7.075 7.11 7.506 6.908 7.062
   2234   1379304_at   5.948 5.58 5.789 5.897 5.412 6.831 6.182 6.349 6.315 6.431
   2235   1382387_at   4.858 5.493 5.263 4.633 4.69 6.302 5.68 5.937 5.99 5.728
   2236   1378198_at   4.818 4.925 5.55 5.295 4.354 5.904 5.827 5.991 6.073 6.259
   2237   1388460_at   7.943 8.028 8.479 7.619 8.006 8.917 8.64 8.724 8.687 8.584
   2238   1372299_at   8.949 8.886 9.161 8.78 8.952 9.522 9.386 10.116 9.928 9.448
   2239   1380045_at   7.976 8.065 8.023 8.359 8.254 8.523 8.437 8.8 8.69 8.525
   2240   1369169_at   10.012 10.15 10.16 9.897 9.872 9.58 9.211 9.582 9.625 9.683
   2241   1388636_at   8.808 8.843 9.1 8.557 8.757 8.415 8.276 8.273 8.497 8.328
   2242   1374081_at   6.132 6.638 6.525 6.28 6.016 7.036 7.054 7.388 7.109 6.712
   2243   1392612_at   8.707 8.934 8.97 8.648 9.063 8.35 8.462 8.459 8.484 8.12
   2244   1372399_at   7.921 8.059 8.069 8.234 7.845 8.463 8.316 8.357 8.38 8.308
   2245   1388726_a_at   9.707 9.835 10.093 9.625 9.413 10.34 10.538 10.503 10.327 10.121
   2246   1374849_at   5.646 5.917 5.744 6.495 5.647 6.981 6.743 6.991 6.397 7.152
   2247   1393165_at   8.079 8.339 7.875 8.067 7.908 7.698 7.699 7.651 7.641 7.63
   2248   1382746_s_at   4.481 5.135 4.962 5.22 4.956 5.692 5.439 5.886 5.867 5.518
   2249   1388949_at   4.107 3.85 3.575 3.053 3.866 4.414 4.861 5.159 5.228 4.499
   2250   1387928_at   5.238 5.15 5.885 5.642 5.514 6.157 6.381 6.09 6.336 6.006
   2251   1368474_at   8.278 8.397 7.851 7.897 8.097 9.049 8.574 8.752 8.712 8.595
   2252   1369991_at   9.188 9.197 8.964 9.377 9.184 9.839 9.713 9.894 9.549 9.438
   2253   1394020_at   9.938 10.137 10.325 9.775 10.082 9.303 9.112 9.432 9.579 9.669
   2254   1367825_at   5.509 5.268 5.934 5.183 5.806 6.873 6.462 6.162 6.444 6.23
   2255   1369518_at   6.488 5.973 6.207 6.726 5.767 7.035 6.937 7.139 7.143 7.02
   2256   1380365_at   6.433 6.528 6.603 5.796 6.473 5.287 4.754 5.622 5.66 5.419
   2257   1375319_at   0.848 0.263 0.546 0 0 1.131 1.986 2.074 1.233 1.281
   2258   1383020_at   5.002 4.638 4.702 4.226 3.825 5.688 5.419 5.623 5.216 5.818
   2259   1394940_at   5.895 5.993 6.214 5.957 5.869 6.998 6.535 6.647 6.715 6.239
   2260   1374200_at   9.638 9.726 9.54 9.735 9.586 9.444 9.251 9.479 9.339 9.413
   2261   1391507_at   6.749 6.649 7.212 6.806 6.637 7.412 7.199 7.423 7.362 7.288
   2262   1376808_at   9.015 9.15 9.572 8.842 8.758 7.931 7.462 8.343 8.207 8.318
   2263   1372748_at   7.512 7.549 7.303 7.331 7.289 8.324 7.657 8.295 8.236 7.851
   2264   1393653_at   6.39 6.636 6.827 6.097 6.643 7.804 7.376 7.709 7.354 6.943
   2265   1370134_at   8.911 8.723 8.82 8.791 8.656 8.526 8.492 8.626 8.382 8.362
   2266   1393249_at   6.936 6.935 6.662 6.957 6.848 7.627 7.392 7.231 7.463 7.101
   2267   1393799_at   6.376 6.426 6.119 6.043 5.686 7.022 6.684 6.835 6.699 6.805
   2268   1373363_at   6.127 6.282 6.554 5.92 6.922 7.427 7.306 7.464 7.293 6.926
   2269   1371922_at   11.849 11.973 11.807 11.938 11.896 11.734 11.727 11.777 11.763 11.735
   2270   1375432_at   7.4 7.372 7.394 7.284 7.304 7.525 7.537 7.5 7.417 7.519
   2271   1396646_at   5.354 5.544 5.063 5.255 5.175 3.736 1.124 4.093 2.462 1.856
   2272   1391702_at   6.154 6.037 6.169 6.211 6.212 5.839 5.862 5.602 5.899 5.462
   2273   1370422_at   5.696 5.508 6.061 5.639 5.381 7.565 7.059 6.784 6.373 6.371
   2274   1378321_at   4.348 4.659 5.13 5.178 4.381 5.886 5.387 5.609 5.617 5.756
   2275   1383171_at   8.69 8.681 8.583 8.694 8.578 8.323 8.354 8.516 8.507 8.43
   2276   1369682_at   10.136 10.051 10.479 9.957 9.746 9.225 9.086 9.501 9.571 9.472
   2277   1379423_at   6.985 6.435 6.799 6.651 6.229 7.152 7.29 7.184 7.433 7.347
   2278   1390951_at   5.985 6.056 6.111 5.3 5.439 3.479 3.297 4.222 5.146 4.198
   2279   1372990_at   7.358 7.494 7.566 7.552 7.552 8.042 7.926 8.175 7.884 7.664
   2280   1373577_at   9.174 9.397 9.126 9.161 9.108 9.469 9.584 9.632 9.604 9.362
   2281   1398789_at   11.929 11.805 11.769 11.812 11.773 12.089 12.23 11.973 12.093 11.98
   2282   1377945_at   7.259 7.322 7.26 7.016 7.264 7.542 7.636 7.683 7.397 7.544
   2283   1370897_at   9.576 9.683 9.777 9.598 9.464 9.097 9.089 9.332 9.42 9.223
   2284   1375857_at   8.049 8.266 8.099 7.976 7.849 8.746 8.831 8.9 8.431 8.347
   2285   1380117_at   7.765 7.587 7.814 7.405 8.013 7.069 6.55 7.042 7.118 7.211
   2286   1392040_at   0.757 3.004 3.383 2.82 2.718 4.336 4.86 5.455 4.755 4.703
   2287   1379560_at   6.223 6.116 6.302 6.299 6.265 6.445 6.481 6.473 6.392 6.63
   2288   1388480_at   8.219 8.221 8.596 8.272 8.008 8.846 8.563 8.992 8.808 8.821
   2289   1368598_at   4.703 4.402 4.856 4.804 4.28 5.395 5.137 5.378 5.58 5.031
   2290   1376812_at   5.702 5.892 5.957 6.126 5.885 6.397 6.62 6.406 6.552 6.122
   2291   1376317_at   5.216 5.484 5.264 5.477 4.574 6.107 5.864 6.255 5.908 6.063
   2292   1370859_at   10.113 10.374 10.325 9.992 10.261 10.789 10.694 10.642 10.52 10.491
   2293   1385557_at   4.794 4.758 4.969 4.775 5.109 5.827 5.149 5.681 5.511 5.384
   2294   1383654_a_at   8.818 8.772 8.345 8.752 8.362 7.905 8.166 8.139 8.141 7.947
   2295   1392449_at   3.889 3.883 3.167 1.967 3.924 5.288 5.155 5.401 4.784 5.268
   2296   1377201_at   1.915 1.316 1.47 4.268 3.981 5.883 5.491 5.78 5.184 5.584
   2297   1388312_at   8.68 8.504 8.395 8.518 8.483 9.252 8.738 9.255 9.367 8.917
   2298   1377982_at   5.011 4.82 4.464 5.115 4.146 5.631 5.506 5.555 5.482 5.58
   2299   1373351_at   6.394 6.748 6.45 6.457 6.555 6.275 5.943 6.223 5.938 6.063
   2300   1394031_at   0.614 1.39 3.558 3.297 3.557 5.882 5.424 5.418 5.241 5.086
   2301   1367739_at   5.227 5.096 7.332 5.247 4.274 1.59 3.63 0.189 2.58 1.339
   2302   1386162_at   4.379 4.649 4.672 4.661 4.945 5.211 5.42 5.401 5.692 4.989
   2303   1374174_at   7.627 7.724 7.816 7.752 7.49 7.292 7.202 7.426 7.36 7.451
   2304   1383465_at   6.779 6.788 6.827 6.988 6.805 7.183 7.003 7.053 7.101 7.015
   2305   1380374_at   3.172 2.826 3.24 3.336 3.32 3.733 3.471 4.033 3.996 3.841
   2306   1387918_at   9.004 9.056 8.709 9.225 8.8 9.894 9.667 9.536 9.364 9.407
   2307   1380219_at   6.496 6.534 6.683 6.168 6.404 5.906 5.659 6.086 6.008 5.549
   2308   1393683_at   5.893 5.65 5.37 5.727 5.268 6.147 6.326 6.321 5.967 6.215
   2309   1383559_at   10.196 10.015 9.323 10.312 9.975 9.169 9.105 9.038 9.323 9.056
   2310   1376814_at   5.171 5.323 5.401 5.782 5.027 5.969 6.404 6.083 5.811 6.317
   2311   1369493_at   8.725 9.089 9.316 8.858 9.031 8.247 8.044 8.593 8.495 8.41
   2312   1392794_at   4.547 4.353 3.982 4.759 3.883 6.002 5.615 4.978 5.355 5.183
   2313   1374646_at   9.868 9.883 9.741 9.807 9.759 10.123 10.074 10.233 10.078 9.911
   2314   1385883_at   6.842 7.199 7.141 7.29 7.199 7.656 7.758 7.694 7.595 7.355
   2315   1372739_at   8.864 8.992 8.972 8.912 8.907 9.152 9.283 9.337 9.127 9.056
   2316   1373818_at   8.319 8.148 8.184 8.178 8.245 9.047 8.703 8.407 8.826 8.632
   2317   1393144_at   6.5 6.586 6.918 5.947 6.379 7.396 7.137 7.457 7.161 7.144
   2318   1388166_at   6.34 6.15 6.354 6.927 6.54 7.555 7.58 7.148 7.687 6.909
   2319   1398883_at   9.829 10.108 9.945 9.767 9.733 10.42 10.475 10.405 10.091 10.247
   2320   1394922_at   1.444 1.642 1.063 3.086 1.632 4.794 4.383 4.093 4.328 2.703
   2321   1368643_at   7.641 7.766 7.566 7.472 7.442 7.987 8.092 8.073 8.029 7.731
   2322   1378914_a_at   4.612 4.621 4.906 4.795 4.048 5.348 5.748 5.471 5.248 5.223
   2323   1392921_at   6.441 6.412 6.686 6.037 6.141 5.24 4.866 3.638 5.179 5.246
   2324   1388397_at   7.826 8.032 7.929 7.762 7.916 8.295 8.334 8.242 8.105 8.077
   2325   1387395_at   5.415 5.329 5.906 5.423 5.2 6.157 6.163 6.112 5.893 5.972
   2326   1371500_at   9.214 9.134 8.625 8.889 8.865 9.92 9.256 9.855 9.844 9.577
   2327   1370068_at   3.927 3.771 3.941 4.037 3.952 4.355 4.31 4.038 4.42 4.304
   2328   1372459_at   8.693 8.429 8.97 8.309 8.326 9.353 9.074 9.267 9.19 9.042
