# Supplementary material for: Activation of counter-regulatory mechanisms in a rat renal acute rejection model
Source: BMC Genomics. 2008 Feb 8;9:71. doi: 10.1186/1471-2164-9-71 (PMC2262896; doi:10.1186/1471-2164-9-71)
Supplement: Additional file 1 — Gene lists from the class comparison between the groups analyzed with BRB ArrayTools. [file 1471-2164-9-71-S1.zip › syngeneicvsallogeneic/ExpressionTable.html]

  
 
 
    Table.  Normalized Log-Transformed Gene Expressions for Significant Genes  
  
    Experiment  Allogeneic1 Allogeneic2 Allogeneic3 Allogeneic4 Allogeneic5 Sygeneic1 Sygeneic2 Sygeneic3 Sygeneic4 Sygeneic5
    Class  allogeneic allogeneic allogeneic allogeneic allogeneic syngeneic syngeneic syngeneic syngeneic syngeneic
   1   1391737_at   9.063 9.063 8.896 8.844 9.127 6.169 5.935 5.854 6.03 5.951
   2   1388600_at   8.909 8.885 9.065 9.044 8.968 6.919 6.683 6.838 6.908 6.808
   3   1376795_at   9.71 9.753 9.827 9.812 9.78 7.684 7.474 7.427 7.475 7.348
   4   1384191_at   8.021 8.07 8.106 8.041 7.961 4.982 4.699 4.429 4.692 4.614
   5   1392785_at   9.069 9.099 9.13 8.923 9.211 6.851 6.663 6.837 6.847 6.654
   6   1377759_at   9.519 9.52 9.57 9.629 9.521 7.891 7.717 7.686 7.61 7.637
   7   1385439_x_at   8.782 8.757 8.664 8.888 8.878 5.547 4.98 5.108 5.332 5.205
   8   1373949_at   8.613 8.655 8.733 8.635 8.806 7.028 6.808 6.916 6.994 6.866
   9   1371577_at   10.599 10.578 10.602 10.642 10.581 11.111 11.111 11.142 11.127 11.076
   10   1373672_at   10.557 10.528 10.7 10.621 10.518 8.823 8.571 8.615 8.61 8.518
   11   1379766_at   9.195 9.159 9.566 9.439 9.293 5.947 5.631 5.582 5.737 5.397
   12   1368064_a_at   8.209 8.26 8.259 8.35 8.086 9.822 10.001 9.892 9.836 9.876
   13   1378428_at   10.081 10.075 9.891 9.956 9.996 8.305 8.286 8.126 8.221 8.057
   14   1373115_at   8.042 8.022 8.041 8.1 8.029 7.15 7.086 7.104 7.087 6.968
   15   1391593_at   7.606 7.894 7.846 7.858 7.837 4.76 4.384 4.467 4.326 4.808
   16   1368010_at   9.594 9.619 9.715 9.734 9.836 7.477 7.099 7.224 7.117 7.068
   17   1369387_at   8.035 8.143 7.967 8.088 8.023 5.426 5.262 5.728 5.265 5.473
   18   1390050_at   7.361 7.403 7.28 7.235 7.387 8.478 8.58 8.579 8.522 8.455
   19   1368742_at   9.154 9.14 9.125 9.263 9.484 7.19 7.239 7.092 7.112 7.16
   20   1383574_at   9.171 9.234 9.297 9.243 9.203 8.139 8.058 8.242 8.168 8.094
   21   1371847_at   9.368 9.384 9.464 9.534 9.453 10.506 10.539 10.488 10.58 10.444
   22   1368311_at   7.899 7.843 7.946 7.876 8.005 6.901 6.763 6.787 6.751 6.819
   23   1388740_at   9.047 9.092 9.364 9.212 9.122 6.338 5.924 5.904 5.843 6.189
   24   1373881_at   11.135 11.204 11.275 11.179 11.225 9.854 9.7 9.771 9.765 9.574
   25   1378321_at   8.287 8.183 8.115 8.118 8.121 5.886 5.387 5.609 5.617 5.756
   26   1379604_at   8.916 8.953 8.835 8.966 9.085 6.806 6.925 6.989 6.89 6.618
   27   1388574_at   11.627 11.577 11.42 11.37 11.528 9.262 8.823 8.869 8.991 8.898
   28   1375041_at   8.362 8.471 8.26 8.556 8.593 5.421 5.533 5.394 4.99 5.195
   29   1373961_at   8.752 8.814 8.663 8.784 8.897 7.642 7.509 7.54 7.531 7.563
   30   1369931_at   11.599 11.6 11.604 11.637 11.655 10.618 10.441 10.509 10.395 10.544
   31   1372236_at   8.993 8.879 8.857 9.113 9.028 6.905 6.739 6.953 7.055 6.756
   32   1372093_at   8.981 8.962 8.952 9.059 8.925 9.844 9.805 9.896 9.938 9.915
   33   1392544_at   8.576 8.685 8.716 8.649 8.709 7.593 7.532 7.579 7.405 7.506
   34   1390042_at   10.811 10.807 10.994 11.015 10.779 9.222 9.041 9.204 9.251 9.129
   35   1370913_at   10.679 10.672 10.564 10.81 10.637 7.856 7.605 7.239 7.309 7.565
   36   1368679_a_at   9.482 9.471 9.563 9.423 9.453 7.575 7.165 7.218 7.221 7.135
   37   1371913_at   11.162 11.159 11.388 11.315 11.13 9.375 9.117 9.225 9.185 9.031
   38   1373754_at   7.712 7.561 7.571 7.702 7.609 8.641 8.631 8.712 8.616 8.725
   39   1388460_at   10.515 10.482 10.406 10.355 10.534 8.917 8.64 8.724 8.687 8.584
   40   1367673_at   8.919 8.871 8.92 8.848 8.831 9.855 9.729 9.807 9.916 9.812
   41   1383372_at   8.995 9.083 9.074 9.152 8.947 7.596 7.394 7.508 7.541 7.337
   42   1380377_at   7.699 7.541 7.684 7.572 7.738 5.502 5.008 5.336 5.181 5.155
   43   1388492_at   9.581 9.557 9.575 9.601 9.588 8.839 8.678 8.726 8.733 8.65
   44   1390391_at   8.891 8.901 8.99 9.236 9.115 6.82 6.478 6.644 6.518 6.741
   45   1370882_at   11.771 11.72 11.756 11.61 11.574 9.6 9.209 9.173 9.476 9.301
   46   1380621_at   7.968 7.893 7.984 8.094 8.081 5.782 5.513 5.291 5.348 5.303
   47   1373932_at   10.242 10.262 10.252 10.168 10.005 7.811 7.561 7.537 7.62 7.248
   48   1369557_at   8.198 8.226 8.33 8.079 8.165 6.746 6.544 6.586 6.772 6.662
   49   1384483_at   8.136 8.118 8.203 8.349 8.394 6.312 6.067 6.302 6.292 6.39
   50   1380123_at   7.16 7.118 7.097 7.062 7.116 8.106 8.096 8.004 8.23 8.137
   51   1373037_at   9.541 9.61 9.469 9.358 9.764 7.296 6.962 6.984 6.869 6.931
   52   1376197_at   7.91 8.054 7.811 7.759 7.571 5.388 5.16 4.973 5.196 5.247
   53   1368274_at   10.293 10.242 10.404 10.419 10.376 8.857 8.506 8.662 8.649 8.73
   54   1381376_at   8.237 8.38 8.097 8.119 8.018 6.421 6.242 6.165 6.207 6.273
   55   1369186_at   9.793 9.82 9.867 9.807 10.012 7.566 7.165 7.47 7.155 7.091
   56   1390798_at   9.938 9.991 10.148 9.965 9.946 7.21 6.747 6.762 6.695 6.433
   57   1393400_at   9.205 9.329 9.551 9.132 8.856 5.563 5.006 4.832 5.019 5.114
   58   1379425_at   8.255 8.187 8.087 8.257 8.386 5.72 5.641 6.133 5.844 5.737
   59   1372478_at   9.888 9.947 10 9.87 9.869 8.543 8.319 8.335 8.432 8.195
   60   1378257_at   10.088 10.133 10.109 10.151 10.19 7.959 7.349 7.589 7.632 7.46
   61   1379302_at   8.29 8.292 8.375 8.234 8.293 7.273 7.252 7.242 7.256 7.065
   62   1369669_at   8.69 8.771 8.462 8.705 8.713 7.025 7.002 6.852 6.925 6.755
   63   1376029_at   12.023 11.967 11.916 11.907 12.067 10.017 9.743 9.685 9.631 9.471
   64   1368732_at   9.967 9.932 10.147 10.302 9.974 7.28 6.999 6.833 6.604 6.863
   65   1387087_at   10.295 10.248 10.229 10.278 10.331 8.105 8.001 7.943 7.885 7.527
   66   1398256_at   9.234 9.184 9.18 9.089 9.102 6.354 5.702 5.962 5.726 5.594
   67   1376976_at   10.064 10.058 10.118 10.157 10.095 8.151 7.886 8.218 8.079 7.758
   68   1391925_at   11.111 10.959 10.711 10.578 10.755 7.851 7.263 7.462 7.316 7.339
   69   1370281_at   11.131 11.127 11.331 11.434 11.349 9.304 8.918 8.981 9.118 9.161
   70   1383658_at   9.352 9.264 9.367 9.526 9.563 7.437 7.224 7.257 7.295 6.986
   71   1372468_at   8.856 8.806 8.932 8.95 8.848 7.391 7.007 7.119 7.021 7.096
   72   1373575_at   12.328 12.314 12.312 12.261 12.39 10.738 10.364 10.405 10.484 10.291
   73   1387198_at   7.962 8.239 8.167 8.3 8.085 6.496 6.361 6.195 6.319 6.265
   74   1372182_at   10.351 10.382 10.344 10.529 10.502 9.189 8.984 9.099 8.972 9.131
   75   1394678_at   7.575 7.802 7.599 7.766 7.739 5.909 5.854 5.655 5.996 5.676
   76   1393108_at   12.251 12.242 12.214 12.137 12.197 10.22 9.713 9.702 9.802 9.682
   77   1375560_at   8.78 8.769 8.843 8.834 8.75 9.495 9.593 9.658 9.576 9.528
   78   1372404_at   10.712 10.939 10.988 10.953 10.895 8.83 8.333 8.505 8.367 8.361
   79   1385213_at   7.63 7.598 7.785 7.644 7.881 5.231 5.073 4.975 4.642 5.165
   80   1372135_at   8.696 8.554 8.843 8.812 8.767 7 6.715 6.896 6.661 6.686
   81   1379741_at   9.604 9.634 9.767 9.665 9.527 10.775 10.84 10.96 10.854 10.772
   82   1377826_at   9.056 9.3 9.204 9.316 9.369 7.239 7.051 6.971 6.737 6.959
   83   1380063_at   9.588 9.583 9.701 9.677 9.83 7.666 7.212 7.265 7.196 7.166
   84   1393144_at   9.373 9.431 9.244 9.555 9.607 7.396 7.137 7.457 7.161 7.144
   85   1369973_at   12.17 12.144 12.099 11.979 12.16 10.46 10.251 10.266 10.297 10.006
   86   1373514_at   10.458 10.426 10.216 10.299 10.377 7.504 6.912 7.095 7.194 6.694
   87   1380129_at   9.109 8.959 8.792 9.097 8.943 6.72 6.243 6.198 6.181 6.279
   88   1395810_at   6.731 6.731 6.828 6.843 6.439 8.567 8.761 8.774 8.644 8.572
   89   1398473_at   9.105 9.148 9.16 9.079 9.154 8.353 8.409 8.382 8.386 8.239
   90   1377125_at   6.201 6.091 6.177 6.006 6.029 7.486 7.621 7.625 7.5 7.362
   91   1370096_at   9.066 9.078 9.882 9.622 9.046 5.322 4.891 4.915 4.991 4.973
   92   1369181_at   10.25 10.24 10.493 10.181 10.17 7.977 7.892 7.566 7.604 7.484
   93   1387227_at   9.779 9.814 9.921 9.873 9.798 8.725 8.517 8.517 8.462 8.431
   94   1398786_at   11.516 11.456 11.613 11.51 11.594 10.612 10.681 10.508 10.532 10.557
   95   1389006_at   11.864 11.737 11.791 11.753 11.64 9.397 8.852 8.975 8.72 8.808
   96   1368281_at   7.537 7.662 7.439 7.488 7.507 8.956 8.914 9.201 9.018 8.915
   97   1379748_at   10.256 10.289 10.199 10.232 10.409 8.008 7.514 7.585 7.691 7.331
   98   1370516_at   9.47 9.37 9.421 9.633 9.727 7.168 6.57 6.801 6.814 6.674
   99   1388565_at   9.936 9.919 9.96 9.912 9.857 9.233 9.163 9.198 9.167 9.06
   100   1386925_at   11.593 11.601 11.582 11.66 11.691 10.35 10.228 10.199 9.994 10.078
   101   1368144_at   8.496 8.448 8.532 8.514 8.703 6.591 6.189 6.35 6.04 6.281
   102   1379561_at   10.098 10.109 9.923 10.073 10.104 9.116 8.977 9.086 8.974 8.984
   103   1370987_at   9.112 9.003 9.183 9.342 8.969 7.136 6.962 6.845 7.215 7.079
   104   1382680_at   8.293 8.367 8.355 8.478 8.658 5.63 5.585 6.064 5.834 5.478
   105   1370119_at   9.794 9.643 9.839 9.861 9.872 6.515 6.102 5.983 5.896 5.484
   106   1370904_at   10.662 10.621 10.806 10.616 10.435 8.482 8.152 7.923 8.04 8.215
   107   1373490_at   10.17 10.149 10.305 10.339 10.2 8.481 8.215 8.434 8.231 8.044
   108   1375638_at   7.012 7.236 6.88 6.982 7.06 8.497 8.376 8.534 8.538 8.471
   109   1369113_at   6.616 6.93 6.851 6.878 6.533 9.11 9.359 9.528 9.572 9.232
   110   1369412_a_at   9.044 9.011 8.926 8.946 8.923 9.598 9.556 9.649 9.613 9.607
   111   1393256_at   7.057 6.96 7.05 7.087 6.981 7.816 7.828 7.854 7.916 7.748
   112   1370202_at   11.883 11.797 12.047 11.991 11.99 9.754 9.108 9.328 9.337 9.243
   113   1387277_at   7.082 7.035 7.074 7.089 7.06 5.228 4.75 5.053 4.711 4.958
   114   1389488_at   10.213 10.212 10.143 10.293 10.173 10.929 10.96 11.05 10.999 10.92
   115   1370056_at   12.361 12.145 12.042 11.958 12.032 10.197 9.836 9.917 9.825 9.828
   116   1379688_at   8.228 8.2 8.283 8.335 8.176 6.435 6.125 6.16 6.158 5.856
   117   1391817_at   8.51 8.409 8.618 8.409 8.467 7.209 7.241 7.331 7.354 7.13
   118   1370623_at   8.37 8.249 8.424 8.339 8.482 6.735 6.516 6.257 6.497 6.431
   119   1381968_at   10.417 10.401 10.523 10.45 10.502 9.398 9.137 9.372 9.259 9.349
   120   1370064_at   7.927 7.917 8.185 8.055 8.004 6.459 6.151 6.236 6.11 6.084
   121   1374006_at   10.12 10.31 10.479 10.351 10.021 8.098 8.081 8.076 8.055 7.761
   122   1386943_at   8.917 8.864 8.843 8.967 8.91 9.993 10.067 10.283 10.162 10.036
   123   1388773_at   9.356 9.393 9.477 9.456 9.174 7.548 7.099 7.188 7.148 7.345
   124   1367600_at   7.837 7.887 7.804 7.971 8.025 6.843 6.697 6.819 6.738 6.858
   125   1368693_at   9.759 9.741 9.851 9.988 9.795 7.052 6.341 6.357 6.271 6.245
   126   1373592_at   10.45 10.399 10.523 10.453 10.235 8.746 8.307 8.382 8.371 8.376
   127   1371017_at   7.251 7.08 7.217 7.101 7.059 5.339 5.272 5.075 5.11 4.845
   128   1369110_x_at   10.791 10.617 10.96 10.817 10.765 7.291 7.254 7.375 6.692 7.653
   129   1392407_at   9.06 9.154 9.287 9.393 9.355 6.76 6.704 6.611 6.188 6.319
   130   1392590_at   10.032 10.08 10.206 10.274 10.126 11.135 11.148 11.26 11.165 11.192
   131   1369665_a_at   9.369 9.327 9.333 9.267 9.377 7.09 6.46 6.667 6.659 6.362
   132   1368930_at   7.641 7.647 7.543 7.586 7.346 4.43 3.844 4.295 3.958 3.553
   133   1370440_at   8.712 8.614 8.693 8.878 8.789 7.33 7.08 7.325 7.088 7.108
   134   1369716_s_at   8.823 8.839 8.605 8.776 8.848 7.327 6.977 7.137 6.971 7.121
   135   1369964_at   11.413 11.429 11.619 11.329 11.21 9.049 8.647 8.561 8.478 8.403
   136   1382275_at   8.025 8.028 8.253 8.272 8.265 6.441 6.181 6.49 6.438 6.222
   137   1389732_at   10.709 10.682 10.479 10.627 10.866 9.024 8.759 8.782 8.659 8.693
   138   1384580_at   7.158 7.083 7.169 6.928 7.46 4.892 4.542 4.535 4.679 4.386
   139   1370415_at   8.305 8.345 8.506 8.577 8.303 6.947 6.877 6.798 6.725 6.657
   140   1378418_at   8.468 8.443 8.334 8.416 8.492 5.919 5.131 5.313 5.238 5.172
   141   1368518_at   11.258 11.263 11.403 11.251 11.013 8.76 8.613 8.238 8.283 8.129
   142   1398348_at   10.291 10.21 10.187 10.288 10.204 11.225 11.24 11.421 11.433 11.437
   143   1389885_at   9.81 9.824 9.955 9.78 9.641 8.22 7.841 8.065 7.954 7.851
   144   1368227_at   8.692 8.563 8.86 8.735 8.685 6.783 6.589 6.46 6.43 6.207
   145   1369294_at   9.007 8.924 9.218 9.189 9.098 6.728 6.291 6.258 6.227 5.968
   146   1371111_at   7.798 7.643 7.829 7.654 7.905 6.239 6.308 6.142 6.289 6.448
   147   1389470_at   9.249 9.184 9.07 9.361 9.257 5.934 5.202 5.105 4.952 5.609
   148   1387040_at   9.546 9.668 9.696 9.629 9.583 10.468 10.53 10.687 10.548 10.594
   149   1375423_at   7.339 7.371 7.409 7.434 7.369 8.111 8.183 8.228 8.329 8.176
   150   1379357_at   9.152 9.045 8.904 8.683 8.953 5.855 5.811 5.906 5.236 5.352
   151   1372691_at   10.434 10.48 10.452 10.443 10.561 8.044 7.54 7.581 7.475 7.179
   152   1386529_at   5.592 5.565 5.748 5.799 5.764 7.027 7.157 7.196 7.335 7.083
   153   1385925_at   8.842 8.653 8.554 8.747 8.628 6.855 6.528 6.556 6.485 6.285
   154   1372459_at   10.465 10.482 10.531 10.511 10.393 9.353 9.074 9.267 9.19 9.042
   155   1387074_at   9.571 9.484 9.423 9.403 9.747 7.572 7.265 7.492 7.089 7.294
   156   1367765_at   9.623 9.716 9.801 9.682 9.691 10.98 10.898 10.874 10.852 10.684
   157   1389873_at   9.115 9.126 9.097 8.993 9.16 7.374 7.143 7.013 7.177 6.807
   158   1369204_at   10.186 10.279 10.294 10.253 10.206 7.309 6.786 6.562 6.435 6.32
   159   1371440_at   12.683 12.587 12.532 12.4 12.549 11.191 10.892 10.832 10.854 10.945
   160   1387897_at   9.555 9.401 9.605 9.568 9.7 7.786 7.296 7.605 7.511 7.336
   161   1367816_at   10.062 10.084 10.071 9.939 10.013 8.06 7.862 7.947 7.599 7.484
   162   1369100_at   6.719 6.8 6.746 6.79 6.817 8.046 7.803 7.937 7.992 8.148
   163   1395124_at   10.427 10.387 10.267 10.369 10.444 8.094 7.417 7.383 7.485 7.382
   164   1393297_at   6.398 6.771 6.76 6.762 6.591 4.829 4.576 4.787 4.49 4.542
   165   1387687_at   8.967 9.008 9.125 9.029 9.055 6.3 6.464 5.797 5.718 5.893
   166   1372646_at   8.327 8.173 8.04 8.103 8.144 9.696 9.633 9.748 10.012 9.75
   167   1385572_at   7.9 8.016 7.904 7.931 8.068 5.385 5.619 5.501 5.355 4.877
   168   1382535_at   8.105 8.117 8.1 7.947 8.052 6.508 6.257 5.993 6.183 6.336
   169   1391214_at   8.7 8.653 8.646 8.488 8.291 5.547 4.728 4.591 4.783 4.865
   170   1389538_at   10.197 10.153 10.114 10.151 10.184 8.619 8.133 8.517 8.291 8.257
   171   1392280_at   8.935 8.911 9.002 8.738 8.736 6.659 6.001 6.308 6.026 6.131
   172   1371210_s_at   9.887 9.932 9.984 9.916 9.922 7.447 6.785 6.653 6.571 6.79
   173   1374157_at   9.033 9.024 9.063 9.108 8.971 7.562 7.335 7.359 7.58 7.148
   174   1370428_x_at   11.984 11.928 11.852 11.932 12 9.441 8.604 8.653 8.676 8.768
   175   1369427_at   9.316 9.249 9.435 9.191 9.321 6.137 5.359 5.172 5.712 5.141
   176   1374551_at   10.357 10.312 10.297 10.317 10.347 8.625 8.151 8.061 8.364 8.115
   177   1383320_at   9.757 9.871 10.126 9.952 9.55 7.41 6.884 7.01 6.882 6.807
   178   1370172_at   12.005 11.891 11.94 11.852 12.023 10.529 10.132 10.474 10.357 10.481
   179   1383711_at   8.641 8.644 8.774 8.467 8.663 7.53 7.503 7.449 7.603 7.575
   180   1389014_at   11.464 11.415 11.372 11.175 11.366 8.939 8.3 8.482 8.193 8.173
   181   1373240_at   8.34 8.27 8.264 8.261 8.287 9.332 9.27 9.512 9.56 9.508
   182   1389344_at   8.318 8.357 8.336 8.342 8.389 7.839 7.732 7.808 7.693 7.722
   183   1371662_at   11.089 11.07 11.161 11.061 11.354 9.763 9.631 9.539 9.61 9.411
   184   1374578_at   9.246 9.236 9.354 9.342 9.341 7.718 7.49 7.546 7.164 7.442
   185   1393317_at   8.821 8.858 8.873 8.822 8.89 8.338 8.312 8.234 8.265 8.19
   186   1376723_a_at   8.718 8.632 8.803 8.796 8.759 9.514 9.558 9.572 9.497 9.656
   187   1367710_at   12.28 12.237 12.149 12.104 12.269 10.301 9.959 9.908 9.735 9.651
   188   1383426_at   8.147 8.065 8.283 8.197 8.182 6.058 5.336 5.776 5.669 5.49
   189   1389092_at   10.981 10.959 11.195 11.037 10.847 8.14 7.24 7.263 7.524 7.272
   190   1368723_at   8.723 8.618 8.851 8.87 8.743 5.8 4.972 5.316 4.764 5.062
   191   1386871_at   11.271 11.257 11.415 11.313 11.276 11.932 11.96 11.885 11.964 11.917
   192   1382442_at   8.891 8.898 8.909 9.081 8.901 6.024 6.193 5.678 5.508 5.332
   193   1392863_at   5.329 5.378 5.536 5.492 5.421 6.703 6.921 6.814 7.08 6.809
   194   1375211_at   12.15 12.111 12.112 11.975 12.016 11.009 11.026 10.936 10.865 10.755
   195   1389696_at   8.918 8.915 8.898 9.025 9.291 5.614 5.336 5.218 5.106 4.494
   196   1376977_at   8.335 8.481 8.234 8.284 8.213 9.306 9.237 9.262 9.319 9.319
   197   1382026_at   8.958 9.096 9.317 9.234 9.005 6.897 6.186 6.374 6.495 6.57
   198   1387005_at   11.495 11.49 11.465 11.474 11.7 9.695 9.207 9.319 9.424 9.085
   199   1392881_at   8.789 8.887 8.681 8.732 8.984 6.64 6.136 5.877 6.204 6.257
   200   1373785_at   8.679 8.677 8.755 8.492 8.434 7.253 7.054 7.177 7.17 6.997
   201   1371629_at   9.136 8.968 9.196 9.247 9.103 10.204 10.112 10.296 10.237 10.196
   202   1371070_at   8.645 8.783 8.69 9.026 9.147 5.989 5.105 5.345 5.336 5.288
   203   1390126_at   6.735 6.983 6.643 6.881 6.525 8.379 8.388 8.321 8.335 8.308
   204   1370928_at   11.948 11.906 11.86 11.899 11.833 11.032 11.014 11.025 10.894 10.807
   205   1373642_at   5.778 5.835 5.686 5.623 5.736 6.756 6.712 6.621 6.674 6.572
   206   1376835_at   8.972 8.851 8.64 8.977 8.884 7.391 7.082 7.152 7.071 7.288
   207   1369559_a_at   10.452 10.482 10.558 10.487 10.57 9.418 9.112 9.356 9.427 9.281
   208   1368377_at   7.437 7.55 7.453 7.422 7.246 4.679 4.608 4.855 4.804 4.076
   209   1377830_a_at   7.529 7.73 7.725 7.654 7.628 8.471 8.5 8.623 8.493 8.514
   210   1393957_at   7.065 6.99 7.151 7.114 7.399 4.496 4.055 4.318 4.576 3.858
   211   1387205_at   10.052 10.035 9.854 10.073 10.295 7.855 7.335 7.343 7.291 7.138
   212   1382346_at   8.239 8.14 8.194 8.325 8.377 6.476 6.283 6.175 6.224 5.867
   213   1394577_at   8.133 8.132 8.218 8.209 8.227 9.216 9.077 9.369 9.262 9.145
   214   1389210_at   11.317 11.478 11.54 11.503 11.391 9.409 8.89 8.691 8.864 8.763
   215   1379482_at   7.234 7.296 7.425 7.3 7.263 6.027 6.179 6.231 6.126 5.927
   216   1374388_at   10.28 10.39 10.513 10.574 10.473 8.931 8.534 8.501 8.528 8.499
   217   1371931_at   8.438 8.409 8.57 8.63 8.583 9.482 9.399 9.417 9.405 9.37
   218   1370885_at   11.611 11.579 11.299 11.331 11.599 8.562 7.83 7.711 7.713 7.539
   219   1372302_at   9.309 9.253 9.362 9.269 9.363 8.275 8.124 8.228 7.969 8.016
   220   1367716_at   8.672 8.614 8.816 8.684 8.696 9.507 9.412 9.425 9.408 9.507
   221   1375522_at   9.624 9.55 9.542 9.655 9.606 8.989 8.899 8.813 8.839 8.835
   222   1394724_at   8.66 8.653 8.658 8.56 8.777 7.867 7.764 7.73 7.71 7.659
   223   1398616_at   9.744 9.661 9.47 9.522 9.774 7.6 7.012 7.162 7.144 6.915
   224   1369663_at   8.758 8.726 8.652 8.807 8.709 9.811 10.071 10.096 10.07 9.848
   225   1370400_at   8.55 8.461 8.672 8.376 8.772 6.453 6.281 5.878 5.978 6.279
   226   1372587_at   8.997 9.069 8.998 8.904 8.893 9.969 9.955 10.2 10.138 10.029
   227   1389159_at   7.019 7.038 6.973 7.204 7.259 8.331 8.186 8.356 8.281 8.357
   228   1377086_at   6.209 6.189 6.284 6.511 6.024 8.414 8.469 8.118 8.354 8.104
   229   1376327_at   9.371 9.389 9.758 9.557 9.643 7.515 7.307 7.726 7.679 7.64
   230   1389071_at   7.858 7.819 7.894 8.019 8.067 6.573 6.545 6.353 6.395 6.213
   231   1367856_at   10.139 10.165 10.125 10.244 10.039 9.371 9.291 9.39 9.343 9.444
   232   1376943_at   8.123 8.203 8.508 8.474 8.367 6.344 6.335 6.246 5.973 5.93
   233   1368558_s_at   10.397 10.444 10.615 10.504 10.69 8.354 7.704 7.569 7.823 7.772
   234   1368940_at   6.886 7.164 7.211 7.133 7.179 5.485 5.336 5.307 5.602 5.635
   235   1388109_at   7.77 7.601 7.709 7.697 7.665 8.35 8.431 8.483 8.411 8.335
   236   1397167_at   8.086 8.198 8.173 8.239 8.28 5.551 4.493 4.785 4.981 4.959
   237   1381771_at   7.107 7.535 7.476 7.192 7.463 4.315 4.369 3.751 3.935 3.524
   238   1373900_at   9.035 9.1 9.06 9.221 9.004 10.084 10.1 10.313 10.243 10.291
   239   1370693_a_at   7.902 8.069 7.849 7.888 8.186 6.169 6.161 5.888 6.068 5.718
   240   1373025_at   11.406 11.352 11.438 11.274 11.274 9.433 9.096 8.893 9.042 8.695
   241   1387884_at   11.089 11.121 11.184 11.247 11.339 10.107 10.046 10.171 9.958 9.934
   242   1367805_at   7.271 7.236 7.147 7.564 6.792 9.769 10.024 10.201 9.963 9.748
   243   1388164_at   10.594 10.583 10.618 10.659 10.69 8.126 7.205 7.313 7.514 7.222
   244   1372070_at   11.636 11.632 11.622 11.545 11.679 10.711 10.643 10.578 10.626 10.402
   245   1368808_at   10.916 10.887 10.935 10.996 10.986 10.025 9.852 9.78 9.698 9.73
   246   1372757_at   11.878 11.832 11.889 11.665 11.783 9.701 9.101 9.025 8.956 8.965
   247   1393038_at   9.977 9.823 9.761 9.678 9.89 7.814 7.182 7.223 7.478 7.159
   248   1374429_at   8.218 8.228 7.947 8.072 7.851 5.203 5.253 5.775 5.55 5.068
   249   1367909_at   9.754 9.682 9.891 10.009 9.721 11.073 11.203 11.211 11.346 11.329
   250   1373066_at   8.587 8.707 8.359 8.385 8.548 6.425 5.898 6.029 6.345 5.863
   251   1375137_at   10.939 10.881 11.027 10.882 11.005 10.016 10.048 9.998 9.814 9.863
   252   1368455_at   9.076 9.189 9.663 9.51 8.838 5.965 5.516 5.327 5.254 5.094
   253   1371122_at   7.517 7.613 7.582 7.562 7.522 6.505 6.715 6.448 6.388 6.489
   254   1382014_at   7.432 7.528 7.589 7.519 7.312 8.483 8.389 8.366 8.406 8.425
   255   1377034_at   10.677 10.799 10.81 10.815 10.91 9.934 9.799 9.93 9.869 9.742
   256   1389250_at   8.234 8.306 8.417 8.397 8.553 6.422 6.429 5.876 6.37 6.301
   257   1379760_at   8.309 8.131 8.454 8.306 8.528 6.43 6.34 6.157 5.938 5.945
   258   1367922_at   9.606 9.731 9.513 9.735 9.828 8.422 8.312 8.311 8.374 8.109
   259   1385557_at   7.672 7.723 7.735 7.571 7.709 5.827 5.149 5.681 5.511 5.384
   260   1379281_at   9.67 9.445 9.868 9.591 9.37 11.301 11.251 11.234 11.133 11.265
   261   1388236_x_at   10.039 9.891 10.136 10.198 10.221 8.784 8.593 8.597 8.754 8.829
   262   1391409_at   10.019 9.916 10.046 10.11 10.036 10.949 10.926 11.046 11.016 10.809
   263   1386293_at   5.975 5.956 5.786 5.91 5.859 6.923 6.801 7.07 6.982 6.862
   264   1383662_at   9.08 9.283 9.1 9.262 9.44 7.257 6.8 7.038 7.206 6.72
   265   1370924_at   10.165 10.177 10.728 10.418 9.855 6.596 5.863 5.509 5.768 5.47
   266   1390909_at   9.201 8.992 8.9 9.036 9.146 6.488 6.272 5.832 5.573 5.898
   267   1374544_at   8.477 8.447 8.571 8.571 8.591 6.792 6.498 6.816 6.782 6.298
   268   1376623_at   6.786 6.432 6.45 6.644 6.464 8.573 8.157 8.467 8.628 8.431
   269   1368008_at   9.089 9.142 9.112 9.233 9.331 10.142 10.14 10.343 10.25 10.205
   270   1392900_at   10.385 10.571 10.45 10.573 10.582 9.608 9.589 9.645 9.58 9.439
   271   1370449_at   9.105 9.211 9.145 9.316 9.286 7.345 6.861 7.187 7.268 6.775
   272   1369954_at   11.109 11.11 11.141 11.161 10.892 12.109 12.309 12.112 12.204 12.132
   273   1370383_s_at   11.797 11.769 11.876 11.744 11.698 9.901 9.449 9.1 9.478 9.471
   274   1373670_at   9.131 8.956 9.082 9.153 9.289 7.292 6.796 6.772 7.106 6.714
   275   1387982_at   8.247 8.122 8.071 7.83 8.171 6.266 6.016 5.691 6.108 5.942
   276   1375231_a_at   8.259 8.366 8.219 8.372 8.272 9.427 9.288 9.553 9.583 9.62
   277   1367689_a_at   7.269 7.033 6.786 6.933 7.019 8.599 8.668 8.702 8.556 8.447
   278   1372930_at   9.901 9.95 9.701 9.917 9.946 8.306 7.739 7.758 7.902 7.867
   279   1367705_at   9.885 10.107 10.207 10.051 10.058 8.31 8.101 8.046 7.921 7.674
   280   1369940_at   11.19 11.155 11.147 11.183 11.214 10.722 10.768 10.784 10.66 10.696
   281   1391167_at   8.019 7.964 8.116 7.998 8.156 7.205 7.125 7.294 7.144 7.25
   282   1386934_at   10.019 10.045 9.977 10.062 9.835 10.874 10.876 11.072 10.93 10.941
   283   1379717_at   10.332 10.34 10.228 10.228 10.541 8.236 7.654 7.89 7.711 7.43
   284   1380077_at   8.531 8.56 8.516 8.514 8.425 6.994 6.578 6.783 6.43 6.847
   285   1370113_at   9.434 9.343 8.969 9.493 9.627 6.739 6.092 6.175 6.182 5.867
   286   1381279_at   8.799 8.674 8.333 8.46 8.994 6.331 5.878 6.196 6.132 5.886
   287   1371968_at   11.197 11.184 11.291 11.229 11.349 10.126 9.878 10.169 10 9.844
   288   1387893_at   11.583 11.586 11.433 11.331 11.497 10.118 9.816 9.847 9.843 9.609
   289   1370173_at   9.858 9.887 9.693 9.856 9.911 8.818 8.723 8.989 8.796 8.809
   290   1387202_at   10.616 10.669 10.36 10.759 10.626 8.63 8.622 8.425 8.245 8.073
   291   1372852_at   8.657 8.737 8.839 8.86 8.455 5.535 5.269 4.48 5.406 4.868
   292   1367952_at   10.012 10.009 10.18 10.447 10.1 11.729 11.821 12.013 11.759 11.754
   293   1380521_at   9.577 9.564 9.81 9.972 9.915 7.589 7.073 7.038 7.229 6.846
   294   1384413_at   8.781 8.77 8.785 8.684 8.678 7.526 7.389 7.447 7.458 7.099
   295   1382777_at   7.108 7.123 7.117 7.19 7.073 6.229 6.199 6.253 6.064 5.976
   296   1370972_x_at   10.347 10.271 10.424 10.233 10.339 8.589 7.997 8.111 8.086 7.869
   297   1368568_at   10.483 10.473 10.779 10.649 10.759 11.995 11.853 11.925 11.968 12.106
   298   1393460_at   8.782 8.946 8.912 8.934 9.117 7.082 6.588 6.657 6.794 6.367
   299   1370930_at   7.333 7.177 7.157 7.422 7.319 8.476 8.422 8.491 8.725 8.535
   300   1372896_at   7.741 7.646 7.606 7.601 7.566 6.146 6.228 6.271 6.429 5.959
   301   1373197_at   9.592 9.376 9.161 9.384 9.673 7.685 7.439 7.246 7.503 7.405
   302   1371557_at   9.878 9.836 9.784 9.926 9.943 10.649 10.516 10.601 10.544 10.677
   303   1376077_at   7.408 7.218 7.461 7.562 7.505 6.073 5.702 5.935 5.918 5.73
   304   1384187_at   9.515 9.513 9.508 9.532 9.606 7.484 7.06 6.988 6.846 6.588
   305   1393280_at   9.975 10.051 10.37 9.947 9.979 8.105 7.851 7.538 7.784 7.512
   306   1372112_at   10.055 10.134 10.185 10.152 10.341 8.77 8.344 8.441 8.391 8.241
   307   1373775_at   9.951 9.974 9.828 10.018 10.185 8.422 7.853 8.142 8.196 8.119
   308   1371869_at   11.711 11.606 11.634 11.661 11.828 10.809 10.691 10.864 10.701 10.662
   309   1392508_at   6.618 6.664 6.683 6.669 6.71 6.008 6.049 5.972 5.834 6.018
   310   1390421_at   10.29 10.219 10.162 10.305 10.125 10.948 11.102 11.055 11.161 11.071
   311   1379295_at   8.689 8.847 8.905 9.039 8.835 6.894 6.809 7.255 7.219 6.899
   312   1392960_at   8.959 8.925 8.873 8.932 9.067 7.634 7.225 7.46 7.266 7.151
   313   1382274_at   10.463 10.44 10.637 10.507 10.196 8.442 8.075 8.215 8.455 8.637
   314   1386885_at   9.844 9.918 9.961 9.814 9.779 10.693 10.671 10.848 10.881 10.758
   315   1390396_at   7.929 8.138 8.072 8.168 8.272 6.406 6.413 6.647 6.326 6.123
   316   1387100_at   8.906 8.959 8.986 9.251 9.18 10.709 10.419 10.787 10.767 10.645
   317   1372755_at   8.929 8.885 8.967 8.83 8.991 9.5 9.568 9.57 9.66 9.589
   318   1375010_at   10.436 10.379 10.589 10.556 10.579 8.463 7.802 7.867 7.85 7.586
   319   1368474_at   11.177 11.057 10.777 10.64 10.957 9.049 8.574 8.752 8.712 8.595
   320   1383585_s_at   9.174 9.273 9.483 9.269 9.364 6.582 6.389 6.772 5.938 5.947
   321   1371016_at   10.156 10.229 10.619 10.425 9.977 7.649 7.036 6.812 7.344 7.262
   322   1377239_at   9.167 9.188 9.169 9.112 9.352 7.363 7.064 6.871 7.108 6.633
   323   1369214_a_at   9.223 9.14 9.16 9.097 9.019 6.148 4.951 5.191 5.372 4.948
   324   1369937_at   12.469 12.349 12.362 12.303 12.47 11.591 11.503 11.503 11.345 11.532
   325   1377669_at   8.018 8.068 8.285 8.093 8.024 6.46 5.827 6.262 6.156 6.151
   326   1367936_at   8.367 8.455 8.631 8.687 8.602 7.08 6.949 7.154 7.071 6.758
   327   1387022_at   10.654 10.751 10.91 10.802 10.694 11.834 11.753 11.812 12.019 11.945
   328   1395313_s_at   7.796 7.807 7.962 7.987 7.609 5.938 5.73 6.053 5.468 5.785
   329   1398788_at   11.909 11.898 11.825 11.863 11.922 11.438 11.431 11.453 11.362 11.341
   330   1390579_at   7.287 7.257 7.172 7.492 7.45 5.747 5.732 6.063 5.688 5.729
   331   1388629_at   10.121 10.225 10.025 10.11 10.158 9.174 9.019 9.125 8.889 8.907
   332   1394340_at   8.106 8.134 8.063 8.3 8.116 7.158 7.007 7.146 6.979 6.886
   333   1387221_at   8.041 8.107 8.104 8.271 8.534 5.441 4.87 4.528 5.354 4.946
   334   1372577_at   10.489 10.461 10.48 10.663 10.667 9.684 9.474 9.608 9.536 9.556
   335   1377145_at   7.878 7.625 8.048 7.949 7.852 9.779 9.717 9.717 9.346 9.581
   336   1380583_s_at   8.137 7.998 7.86 7.976 7.851 6.932 6.875 6.999 6.955 7.045
   337   1371213_at   7.876 7.763 7.722 7.93 8.044 6.05 5.481 5.662 5.432 5.38
   338   1368461_at   10.021 10.03 10.275 10.152 9.834 11.427 11.632 11.505 11.721 11.655
   339   1372562_at   8.576 8.666 8.552 8.736 8.565 9.332 9.345 9.439 9.479 9.474
   340   1371209_at   11.64 11.663 11.616 11.416 11.552 9.025 8.195 7.945 8.14 8.072
   341   1371328_at   10.126 10.21 10.246 10.277 10.24 10.751 10.782 10.718 10.718 10.686
   342   1373818_at   10.591 10.642 10.544 10.469 10.546 9.047 8.703 8.407 8.826 8.632
   343   1380537_at   7.601 7.676 7.061 7.617 7.669 4.961 4.792 5.115 5.306 5.177
   344   1373889_at   9.707 9.65 10.185 9.986 9.718 7.206 6.949 6.831 6.463 6.379
   345   1388976_at   8.75 8.657 8.813 8.793 8.871 9.63 9.826 9.755 9.893 9.693
   346   1391979_at   9.101 9.438 9.488 9.498 9.505 6.999 6.799 6.546 6.704 6.122
   347   1367662_at   10.747 10.698 10.689 10.674 10.741 11.304 11.355 11.494 11.469 11.507
   348   1370248_at   8.416 8.363 8.537 8.491 8.572 9.649 9.564 9.668 9.839 9.915
   349   1390510_at   10.802 10.909 10.755 10.809 10.885 8.057 7.471 7.501 7.431 6.783
   350   1397214_at   8.021 7.888 7.97 7.924 7.651 5.706 5.02 5.042 4.891 4.846
   351   1388988_at   10.15 10.205 10.441 10.38 10.149 11.374 11.33 11.37 11.471 11.498
   352   1395003_at   8.821 8.892 8.901 8.943 9.034 7.306 6.87 6.889 7.052 6.616
   353   1369025_at   8.115 8.092 8.392 8.143 7.806 5.27 4.415 4.151 4.713 4.428
   354   1369660_at   10.97 10.934 11.039 10.976 10.835 12.087 12.082 12.113 12.224 12.435
   355   1371537_at   9.81 9.722 9.702 9.735 9.803 8.121 7.816 7.529 7.611 7.435
   356   1372865_at   10.375 10.431 10.413 10.326 10.528 9.324 9.045 9.326 9.1 9.014
   357   1378927_at   9.813 9.842 9.984 10.072 10.138 7.002 6.068 6.195 6.232 5.665
   358   1370215_at   11.029 11.005 11.02 10.866 10.975 8.919 8.723 8.462 8.361 8.07
   359   1368191_a_at   9.446 9.473 9.46 9.452 9.236 10.529 10.434 10.43 10.692 10.455
   360   1380445_at   7.803 8.008 8.013 8.02 8.178 6.841 6.842 6.635 6.625 6.647
   361   1374539_at   8.285 8.257 8.164 8.188 8.372 6.588 6.423 6.945 6.732 6.525
   362   1384874_at   4.877 4.867 5.11 5.12 5.24 6.932 6.781 7.221 7.289 7.257
   363   1370072_at   9.336 9.325 9.364 9.518 9.08 10.714 10.91 11.01 10.843 10.694
   364   1376379_a_at   8.521 8.496 8.515 8.409 8.684 7.203 6.958 7.136 6.764 6.851
   365   1368207_at   9.296 9.389 9.654 9.612 9.583 8.276 8.233 8.15 8.158 8.025
   366   1392722_at   7.962 8.003 7.673 7.889 7.815 5.576 5.015 4.97 4.855 4.558
   367   1374468_at   9.983 9.964 9.741 9.871 9.948 8.635 8.538 8.414 8.424 8.169
   368   1369549_at   9.046 8.927 9.267 8.947 8.907 6.804 6.337 5.942 6.051 6.43
   369   1388403_at   11.426 11.43 11.406 11.399 11.37 11.878 11.906 11.939 11.905 12.053
   370   1382108_at   9.554 9.56 9.814 9.976 9.989 8.133 7.861 8.071 8.098 7.979
   371   1388202_at   9.74 9.815 10.181 10.036 10.086 7.655 6.762 6.962 6.936 6.84
   372   1370603_a_at   9.098 9.085 9.263 9.179 8.961 6.336 5.491 5.036 5.548 5.401
   373   1390687_at   10.472 10.485 10.556 10.581 10.596 7.631 6.411 6.647 6.72 6.335
   374   1393044_at   8.543 8.473 8.306 8.464 8.617 6.204 5.494 5.182 5.583 5.397
   375   1370483_at   7.199 7.377 7.714 7.644 7.302 4.442 4.384 4.241 3.449 4.008
   376   1380425_at   8.284 8.163 8.048 8.292 8.408 6.463 5.803 6.083 6.309 6.042
   377   1388534_at   9.213 9.312 9.469 9.353 9.206 10.186 10.182 10.344 10.22 10.239
   378   1387394_at   8.652 8.672 8.872 8.881 8.624 6.253 5.526 5.525 5.253 5.193
   379   1367861_at   8.478 8.485 8.556 8.7 8.528 7.868 7.796 7.87 7.848 7.784
   380   1371239_s_at   10.9 10.846 10.94 10.939 11.053 9.898 9.734 9.803 9.709 9.498
   381   1386884_at   10.176 10.165 10.06 10.095 10.156 9.216 9.369 9.185 9.424 9.228
   382   1386637_at   7.744 7.775 8.175 7.853 8.019 6.125 5.93 5.706 5.543 5.689
   383   1392547_at   10.261 10.288 10.312 10.231 10.254 6.834 6.133 5.67 5.499 5.334
   384   1368655_at   11.115 11.12 11.345 11.119 11.063 9.343 9.405 9.151 9.677 9.118
   385   1374846_at   9.538 9.541 9.478 9.596 9.488 10.027 9.964 10.019 10.032 9.934
   386   1389903_at   10.597 10.593 10.655 10.68 10.531 11.155 11.194 11.137 11.076 11.121
   387   1394940_at   8.841 8.73 8.943 8.719 8.657 6.998 6.535 6.647 6.715 6.239
   388   1369943_at   11.917 11.883 11.715 11.591 11.757 10.731 10.523 10.618 10.55 10.47
   389   1389571_at   9.811 9.841 9.769 10.078 10.111 8.031 7.286 7.373 7.505 7.496
   390   1379316_at   8.197 8.232 8.08 8.344 8.407 6.724 6.736 6.776 6.566 7.016
   391   1373523_at   12.336 12.163 12.271 11.92 12.073 9.515 8.872 8.517 8.516 8.491
   392   1369815_at   7.484 7.611 8 7.895 7.54 5.406 5.07 5.072 4.739 5.389
   393   1390835_at   10.19 10.113 10.182 10.201 9.908 10.999 11.049 11.036 11.098 11.021
   394   1372034_at   10.772 10.786 10.678 10.81 10.978 8.594 7.7 7.701 7.794 7.717
   395   1387678_at   8.14 8.124 8.026 8.233 8.011 6.999 6.734 6.86 6.919 6.61
   396   1382533_at   8.504 8.574 8.727 8.746 8.699 7.576 7.613 7.378 7.358 7.349
   397   1369766_at   5.911 6.182 6.679 6.222 6.086 1.195 2.147 0.604 0.934 1.736
   398   1369103_at   7.914 7.841 8.285 8.262 8.075 6.322 6.124 6.267 6.077 5.854
   399   1374574_at   5.71 5.828 5.716 5.713 5.562 6.687 6.743 6.876 6.839 7.011
   400   1370340_x_at   10.965 10.961 10.87 10.973 11.107 9.904 9.804 9.713 9.545 9.557
   401   1371146_at   8.608 8.509 8.56 8.443 8.576 6.558 6.638 6.074 5.996 6.031
   402   1395184_at   9.104 9.151 8.954 8.799 9.057 6.767 6.332 6.926 6.378 6.162
   403   1370445_at   9.581 9.571 9.728 9.816 9.649 7.512 7.522 7.182 6.731 7.116
   404   1379275_at   8.583 8.304 8.312 8.47 8.441 6.372 5.977 5.98 5.637 5.527
   405   1387034_at   11.127 11.141 11.323 11.141 10.987 12.225 12.351 12.114 12.153 12.228
   406   1381875_at   8.352 8.408 8.254 8.599 8.743 6.505 6.323 6.242 6.074 5.851
   407   1374033_at   11.398 11.347 11.392 11.337 11.575 8.921 8.555 8.223 7.912 7.974
   408   1385641_at   9.182 9.258 9.138 9.287 9.224 7.257 6.693 6.799 6.381 6.463
   409   1372953_at   6.994 6.918 6.842 6.986 7.054 7.858 7.657 7.83 7.868 7.844
   410   1396268_at   8.496 8.672 8.584 8.491 8.682 7.06 6.527 6.699 6.869 7.037
   411   1397866_at   7.414 7.474 7.853 7.949 7.581 5.955 5.639 5.67 5.665 5.751
   412   1392171_at   9.303 9.155 9.415 9.342 9.061 5.848 5.063 5.498 4.438 4.777
   413   1368765_at   7.086 7.394 7.473 7.395 7.32 8.738 8.556 8.75 8.728 8.92
   414   1372328_at   8.303 8.386 8.447 8.597 8.349 9.349 9.501 9.538 9.611 9.446
   415   1373524_at   7.713 7.773 7.678 7.696 7.514 8.608 8.529 8.757 8.763 8.68
   416   1370585_a_at   8.505 8.482 8.706 8.588 8.467 6.997 6.623 7.032 6.841 6.526
   417   1374172_at   7.065 6.988 6.849 7.11 7.374 8.949 9.148 9.172 9.246 8.757
   418   1371445_at   10.457 10.446 10.455 10.467 10.539 9.865 9.751 9.725 9.629 9.626
   419   1368027_at   8.385 8.443 8.467 8.08 8.136 6.603 6.874 6.519 6.547 6.49
   420   1371123_x_at   11.731 11.632 11.623 11.639 11.792 9.213 8.234 8.074 8.488 8.603
   421   1377023_at   8.303 8.342 8.614 8.592 8.091 6.007 5.224 5.809 5.36 5.386
   422   1389622_at   9.282 9.229 9.345 9.415 9.184 10.119 10.092 10.294 10.171 10.119
   423   1384298_at   8.566 8.498 8.909 8.803 8.463 6.446 5.91 6.019 5.607 6.176
   424   1375220_at   10.103 10.126 10.253 10.105 10.138 10.731 10.642 10.63 10.657 10.685
   425   1376130_a_at   8.389 8.295 8.394 8.432 8.325 7.221 7.299 7.284 7.192 7.544
   426   1368565_at   8.759 8.784 8.904 8.698 8.549 6.221 5.595 5.164 5.173 5.353
   427   1398296_at   10.367 10.373 10.401 10.337 10.161 11.104 11.111 11.136 11.267 11.148
   428   1372111_at   7.683 7.726 8.095 7.825 7.737 8.992 8.998 9.026 9.081 8.984
   429   1386773_at   8.033 8.21 8.32 8.28 8.195 9.182 9.102 9.261 9.336 9.172
   430   1388203_x_at   10.102 10.08 10.302 10.257 10.266 8.121 7.407 7.217 7.741 7.409
   431   1376275_at   7.877 7.958 7.749 8.04 7.845 6.788 6.653 6.689 6.91 6.6
   432   1383424_at   9.411 9.512 9.122 9.646 9.649 7.189 6.308 6.475 6.718 6.637
   433   1373683_at   7.921 7.854 8.162 8.073 7.972 6.529 5.95 6.183 6.327 6.106
   434   1376880_at   8.983 9.08 9.059 9.058 9.213 8.291 8.024 8.089 8.146 8.041
   435   1384180_at   10.024 10.078 9.912 10.004 10.264 7.819 6.829 6.925 7.156 7.059
   436   1371576_at   10.113 10.104 10.188 10.078 9.974 10.666 10.677 10.703 10.709 10.633
   437   1392597_at   10.695 10.62 10.726 10.68 10.579 11.56 11.389 11.601 11.665 11.677
   438   1390285_at   6.364 6.412 6.413 6.659 6.26 8.393 7.909 8.391 8.462 8.364
   439   1370822_at   12.449 12.377 12.421 12.228 12.222 10.522 10.229 9.713 9.984 10.065
   440   1388526_at   8.139 7.966 8.119 8.157 8.269 9.157 9.238 9.358 9.479 9.421
   441   1367943_at   8.679 8.703 8.679 8.91 8.708 7.909 7.833 7.657 7.779 7.764
   442   1388300_at   11.517 11.466 11.692 11.383 11.506 12.35 12.37 12.323 12.356 12.286
   443   1376144_at   10.886 10.911 10.788 10.938 11.113 8.692 8.099 7.76 7.8 7.782
   444   1390022_at   11.833 11.754 11.782 11.68 11.781 10.916 10.805 10.836 10.758 10.568
   445   1387048_at   9.941 9.904 9.864 9.806 9.944 8.488 7.868 7.951 8.039 7.874
   446   1374718_at   10.825 10.879 10.734 10.697 10.83 8.26 7.529 7.352 7.369 7.004
   447   1395411_at   8.636 8.641 8.839 8.859 8.646 9.616 9.67 9.575 9.734 9.597
   448   1367837_at   10.177 10.063 10.139 10.282 10.325 9.339 9.163 9.35 9.274 9.307
   449   1383709_at   7.161 7.294 7.214 7.519 7.329 5.877 5.619 5.386 5.374 5.441
   450   1379987_at   7.104 7.133 7.189 7.136 7.027 6.27 6.173 6.321 6.438 6.36
   451   1367738_at   8.326 8.421 8.414 8.3 8.345 7.232 7.284 6.912 6.93 7.158
   452   1370443_at   8 7.753 8.02 8.046 8.171 6.705 6.784 6.818 6.811 6.921
   453   1388213_a_at   11.222 11.231 11.104 11.163 11.335 8.709 7.659 7.538 7.955 7.768
   454   1384946_at   9.355 9.329 9.245 9.208 9.409 7.162 6.523 6.484 6.498 6.081
   455   1389387_at   10.024 9.975 10.088 10.136 10.192 8.969 9.109 8.953 8.86 8.724
   456   1383189_at   10.722 10.86 10.417 10.645 10.798 8.213 8.008 7.555 7.483 7.262
   457   1373057_at   7.894 7.696 7.765 8.002 7.686 8.883 8.879 8.993 8.805 8.99
   458   1378193_at   10.36 10.352 10.621 10.543 10.569 7.291 6.272 6.242 6.092 5.708
   459   1389107_at   9.822 9.755 10.062 9.936 9.721 10.918 10.891 11.058 10.957 11.063
   460   1371604_at   8.388 8.277 8.508 8.451 8.419 9.13 9.308 9.153 9.274 9.19
   461   1368835_at   12.448 12.431 12.464 12.23 12.365 10.278 9.479 9.4 9.497 9.304
   462   1367825_at   8.59 8.576 8.375 8.721 8.713 6.873 6.462 6.162 6.444 6.23
   463   1374705_at   8.974 9.119 9.087 9.019 9.093 10.224 10.292 10.295 10.321 9.945
   464   1376056_at   9.004 8.996 9.024 9.053 9.16 7.768 7.185 7.452 7.329 7.264
   465   1378137_at   9.203 9.169 9.154 9.308 9.27 8.365 8.372 8.099 8.251 8.147
   466   1397904_at   7.375 7.346 7.501 7.265 7.413 6.397 6.386 6.14 6.099 6.262
   467   1367589_at   11.258 11.238 11.241 11.25 11.155 11.841 11.886 11.815 11.948 12.035
   468   1374670_at   8.803 8.729 8.439 8.683 8.889 7.328 7.207 7.339 7.033 7.095
   469   1388351_at   11.737 11.676 11.666 11.66 11.756 11.155 10.966 11.083 10.975 10.966
   470   1388570_at   9.519 9.454 9.574 9.661 9.624 10.382 10.254 10.434 10.479 10.365
   471   1372741_at   9.32 9.298 9.433 9.475 9.437 10.088 10.22 10.34 10.203 10.226
   472   1368282_at   6.576 6.529 6.369 6.275 6.09 7.698 7.809 7.822 7.793 7.748
   473   1371256_at   9.179 9.377 9.266 9.153 9.419 7.85 7.27 7.428 7.601 7.364
   474   1372613_at   9.348 9.315 9.469 9.179 9.136 10.388 10.461 10.44 10.533 10.281
   475   1383241_at   11.857 11.743 11.411 11.378 11.652 10.054 9.852 9.67 9.683 9.773
   476   1390507_at   6.929 7.109 6.982 7.355 7.238 5.167 4.684 4.986 4.618 5.185
   477   1389365_at   9.174 9.099 9.073 9.285 9.463 7.933 7.698 7.868 7.634 7.884
   478   1383684_at   6.85 6.905 6.908 6.833 6.829 5.463 5.417 5.194 4.942 4.986
   479   1374469_at   8.784 8.788 8.588 8.991 9.13 5.968 4.776 5.508 5.184 4.93
   480   1389355_at   9.27 9.166 8.881 9.121 9.099 7.999 7.962 7.909 7.826 7.738
   481   1383748_at   8.241 8.243 8.242 8.262 8.267 7.615 7.452 7.447 7.295 7.492
   482   1377110_at   7.277 7.305 7.527 7.096 7.229 4.236 3.861 2.852 3.551 3.669
   483   1383655_at   8.171 8.09 8.209 8.216 8.644 6.551 6.43 6.333 6.221 6.08
   484   1367786_at   11.841 11.837 11.784 11.888 11.967 8.991 7.874 7.664 7.759 7.707
   485   1377635_at   5.996 6.05 5.928 5.521 5.965 7.76 7.576 7.546 7.945 7.726
   486   1374284_at   8.022 8.085 7.98 8.332 7.967 5.857 5.762 5.34 5.357 4.988
   487   1382283_at   8.633 8.573 8.708 8.72 8.763 7.46 7.311 7.413 7.466 7.031
   488   1387232_at   6.857 6.93 7.183 6.96 6.751 8.287 8.193 8.487 8.36 8.519
   489   1390849_at   6.899 7.001 7.225 7.159 7.403 3.372 3.513 2.941 4.29 3.612
   490   1388596_at   10.38 10.354 10.484 10.436 10.36 9.558 9.368 9.444 9.681 9.513
   491   1369979_at   10.565 10.587 10.536 10.368 10.546 9.613 9.348 9.609 9.42 9.39
   492   1367974_at   11.027 10.817 11.217 11.22 11.156 9.123 8.83 8.756 8.535 8.32
   493   1387856_at   12.148 12.122 12.083 12.045 12.233 11.385 11.415 11.296 11.325 11.172
   494   1373912_at   8.903 8.992 8.912 8.965 9.245 7.82 7.491 7.697 7.513 7.721
   495   1369031_at   10.858 10.888 10.954 11.014 10.989 7.725 6.239 6.614 6.173 6.507
   496   1388160_a_at   10.592 10.542 10.636 10.585 10.504 10.956 10.969 10.937 10.992 11.016
   497   1388347_at   12.303 12.31 12.171 12.105 12.233 11.018 10.469 10.692 10.822 10.696
   498   1371925_at   8.878 8.943 9.079 8.916 8.925 8.259 8.169 8.208 8.341 8.167
   499   1388175_at   10.155 10.167 10.283 10.008 10.031 11.159 11.098 11.154 11.361 11.323
   500   1368482_at   10.761 10.738 10.922 10.864 10.794 7.637 6.748 6.345 6.484 6.026
   501   1398303_s_at   10.694 10.82 10.918 10.764 10.865 9.781 9.863 9.571 9.811 9.582
   502   1371948_at   9.74 9.673 9.725 9.503 9.803 10.566 10.703 10.715 10.844 10.755
   503   1376775_at   6.645 6.316 6.556 6.454 6.294 5.111 5.229 4.84 5.044 5.054
   504   1369737_at   6.835 6.657 6.788 6.952 6.903 5.277 5.236 5.186 4.799 5.358
   505   1387946_at   11.427 11.407 11.36 11.316 11.411 10.158 9.651 9.557 9.715 9.632
   506   1375955_at   9.496 9.593 9.579 9.748 9.637 8.29 7.765 8.084 7.966 7.76
   507   1395697_at   7.582 7.728 7.635 7.613 7.484 6.507 6.359 6.279 6.181 6.039
   508   1374478_at   8.869 8.777 8.91 9.119 9.004 10.071 10.043 10.228 10.339 10.297
   509   1374627_at   8.175 8.397 8.307 8.309 8.324 7.021 6.689 6.419 6.526 6.556
   510   1389222_at   7.729 7.603 7.628 7.618 7.671 6.924 6.937 7.072 6.812 6.894
   511   1392958_at   8.889 8.781 8.737 8.899 8.976 7.665 7.477 7.76 7.52 7.329
   512   1385088_at   6.985 6.95 7.088 7.116 7.083 7.759 8.05 7.923 7.874 7.904
   513   1373897_at   9.902 9.765 9.511 9.85 9.891 8.269 8.02 8.117 7.785 7.789
   514   1387502_at   8.751 8.679 8.958 8.76 8.61 7.496 7.518 7.511 7.174 7.328
   515   1379613_at   9.722 9.665 9.545 9.795 9.778 7.269 6.907 6.307 6.231 6.419
   516   1367908_at   10.07 10.059 9.906 10.194 9.965 11.117 11.228 11.241 11.324 10.991
   517   1389553_at   10.673 10.724 11.048 11.015 11.138 8.768 8.305 8.498 8.17 7.912
   518   1393187_at   9.12 9.31 9.111 9.233 9.285 5.977 5.241 4.668 4.913 4.379
   519   1370621_at   7.806 7.762 8.105 7.949 7.581 5.373 4.217 4.525 4.584 4.707
   520   1381124_at   7.613 7.655 7.4 7.633 7.598 8.599 8.601 8.85 8.887 8.694
   521   1374129_at   8.076 8.199 8.243 8.103 8.186 9.093 8.866 9.099 8.966 8.931
   522   1393987_s_at   10.292 10.284 10.352 10.307 10.248 9.163 8.927 9.331 9.05 8.897
   523   1370249_at   11.531 11.554 11.565 11.345 11.566 10.214 10.04 9.924 9.807 9.647
   524   1374730_at   9.971 10.348 10.682 10.273 10.584 7.958 7.765 7.559 7.623 7.141
   525   1388212_a_at   11.806 11.667 11.722 11.548 11.841 9.496 8.409 8.645 8.86 8.608
   526   1387878_at   10.861 10.85 10.805 10.869 10.727 11.27 11.364 11.377 11.34 11.285
   527   1388537_at   8.65 8.692 8.828 8.914 8.669 9.676 9.874 9.929 9.998 9.847
   528   1392496_at   8.989 8.856 9.253 9.025 8.863 5.157 3.788 4.051 3.306 3.434
   529   1379452_at   7.009 7.11 7.009 6.881 6.57 8.908 9.143 9.032 9.327 8.705
   530   1387134_at   10.857 10.714 10.928 10.441 10.719 7.63 6.898 6.153 6.556 7.011
   531   1389867_at   8.483 8.425 8.688 8.523 8.479 7.45 7.152 7.516 7.327 7.4
   532   1381268_at   7.225 7.285 7.267 7.373 7.382 8.03 7.932 8.116 8.17 8.094
   533   1368291_at   8.73 8.797 8.869 8.948 9.04 7.919 7.652 7.719 7.718 7.594
   534   1372009_at   9.872 9.862 9.815 10.125 9.998 8.833 8.539 8.796 8.54 8.6
   535   1373065_at   9.915 9.824 9.894 9.816 10.118 8.901 8.741 8.599 8.557 8.676
   536   1382311_at   10.002 10.105 10.044 10.04 10.126 7.59 7.113 6.934 6.448 6.51
   537   1389997_at   8.957 8.896 9.525 9.078 8.7 5.953 5.009 4.952 5.046 4.609
   538   1390249_at   6.663 6.74 6.6 6.905 6.726 5.794 5.915 5.807 5.718 5.713
   539   1375856_at   7.603 7.685 7.835 7.554 7.411 8.908 9.043 8.795 9.058 8.838
   540   1385434_at   7.927 7.887 8.009 8.146 8 5.496 4.615 5.179 4.483 5.267
   541   1392334_at   12.034 12.113 11.897 11.939 11.89 10.893 10.797 10.494 10.757 10.571
   542   1392935_at   6.065 5.871 5.883 5.854 6.226 7.482 7.252 7.299 7.255 7.184
   543   1384778_at   6.812 6.909 7.054 7.02 7.427 4.791 4.731 4.54 4.205 4.912
   544   1398987_at   9.235 9.154 9.176 9.256 9.244 8.802 8.844 8.83 8.784 8.735
   545   1370436_at   11.237 11.325 11.488 11.515 11.373 12.18 12.363 12.293 12.248 12.301
   546   1380905_at   7.369 7.444 7.853 7.622 7.503 8.905 9.114 9.034 8.974 8.864
   547   1376944_at   7.116 7.159 7.231 7.483 7.003 9.044 9.145 9.571 9.172 8.948
   548   1386927_at   8.402 8.417 8.329 8.287 8.283 8.88 8.806 8.833 8.865 8.788
   549   1396163_at   11.577 11.493 11.66 11.336 11.363 8.262 7.431 6.872 6.972 6.725
   550   1377375_at   10.371 10.369 10.557 10.495 10.403 11.081 11.097 11.166 11.236 11.103
   551   1369726_at   10.491 10.478 10.155 10.193 10.405 8.272 8.406 8.126 8.051 7.619
   552   1382902_at   8.114 8.441 8.337 8.613 8.29 5.376 5.166 5.049 4.488 5.696
   553   1377016_at   9.416 9.492 9.647 9.71 9.832 8.116 8.257 8.329 7.971 8.214
   554   1372004_at   8.985 8.987 9.14 9.108 9.12 7.374 7.382 6.941 7 6.715
   555   1375716_at   8.449 8.129 8.319 8.292 8.344 6.648 6.531 6.448 5.953 6.379
   556   1380021_at   8.525 8.407 8.534 8.685 8.614 7.678 7.598 7.519 7.402 7.429
   557   1372303_at   9.983 10.12 10.332 10.277 10.365 8.593 8.312 8.47 8.125 8.005
   558   1388337_at   12.571 12.462 12.417 12.209 12.384 11.189 10.72 10.772 10.804 10.724
   559   1371466_at   7.361 7.525 7.375 7.386 7.374 8.012 8.036 8.098 8.062 7.925
   560   1387835_at   6.531 6.69 6.819 6.724 6.276 2.227 1.787 0.714 1.239 0.287
   561   1374329_at   5.815 5.683 5.575 5.599 5.573 6.953 7.067 7.283 7.224 7.507
   562   1367973_at   11.683 11.652 11.553 11.494 11.558 9.059 8.294 7.986 7.797 7.939
   563   1374512_at   8.157 8.193 8.312 8.291 8.121 9.203 9.051 9.373 9.37 9.268
   564   1389140_at   9.988 10.014 10.07 10.082 10.102 9.609 9.49 9.546 9.44 9.541
   565   1377750_at   8.715 8.624 8.705 8.82 8.881 7.873 7.875 7.922 7.686 7.761
   566   1386908_at   10.015 10.012 10.177 10.095 10.189 8.205 7.879 8.034 7.714 7.333
   567   1387354_at   12.674 12.614 12.572 12.378 12.498 10.217 9.33 9.102 9.229 9.127
   568   1387495_at   7.065 7.245 7.186 7.216 7.357 6.304 5.946 6.052 6.003 6.091
   569   1389034_at   9.6 9.697 9.55 9.673 9.793 6.797 5.296 5.67 5.913 6.082
   570   1398390_at   10.642 10.651 10.364 10.055 9.861 7.608 7.453 7.367 7.085 6.856
   571   1392737_at   9.505 9.495 9.57 9.565 9.602 6.727 5.959 5.803 6.056 5.202
   572   1368066_at   8.103 8.032 8.37 8.481 8.275 6.523 6.321 6.079 6.648 6.351
   573   1377286_at   8.48 8.467 8.912 8.619 8.524 7.214 6.865 6.943 7.126 6.865
   574   1374387_at   10.714 10.694 10.767 10.794 10.868 10 9.986 10.099 9.879 9.845
   575   1372256_at   10.169 10.163 10.317 10.251 10.33 8.713 8.771 8.287 8.25 8.264
   576   1369520_a_at   8.602 8.514 8.691 8.856 8.541 6.215 5.934 5.478 5.235 6.069
   577   1378520_at   3.436 3.449 4.241 3.285 3.643 0.411 0 0.816 0 0.322
   578   1378914_a_at   7.156 7.368 7.701 7.383 7.227 5.348 5.748 5.471 5.248 5.223
   579   1388784_at   9.886 9.905 9.849 9.909 9.739 8.671 8.473 8.481 8.792 8.31
   580   1372276_at   7.442 7.205 7.284 7.236 7.124 8.19 8.188 8.335 8.226 8.101
   581   1372013_at   11.461 11.515 11.565 11.577 11.621 10.282 9.871 10.409 10.203 10.16
   582   1389084_at   5.697 5.678 6.027 5.927 6.006 7.336 7.28 7.588 7.597 7.713
   583   1370855_at   11.008 11.037 10.984 10.946 11.072 11.576 11.563 11.504 11.7 11.552
   584   1368894_at   1.104 1.506 1.036 2.098 1.339 4.364 4.458 4.615 4.43 4.933
   585   1385889_at   8.922 8.834 9.017 8.998 8.835 9.681 9.617 9.53 9.591 9.676
   586   1369873_at   6.666 6.644 6.821 6.9 6.89 7.984 7.695 7.798 7.818 7.839
   587   1389147_at   9.474 9.551 9.741 9.706 9.646 10.386 10.5 10.522 10.389 10.459
   588   1372513_at   11.001 11.162 11.061 10.977 11.088 10.439 10.359 10.276 10.213 10.274
   589   1387672_at   6.367 6.536 6.675 6.666 6.06 8.382 8.404 8.469 8.837 8.644
   590   1381228_at   9.679 9.715 9.451 9.697 10.012 7.411 6.592 6.503 6.809 6.362
   591   1383783_at   4.147 4.186 3.757 4.45 3.776 6.808 6.452 7.098 7.322 7.025
   592   1371511_at   11.93 11.863 11.877 11.777 11.908 11.341 11.333 11.356 11.292 11.195
   593   1367850_at   11.503 11.459 11.567 11.389 11.672 9.043 8.375 8.294 8.04 7.744
   594   1370539_at   8.161 8.084 8.323 8.315 8.424 7.091 6.748 6.851 6.633 6.713
   595   1388822_at   10.064 10.043 10.072 10.068 10.154 10.477 10.453 10.499 10.441 10.397
   596   1382314_at   11.473 11.455 11.093 11.489 11.618 8.325 7.036 6.771 7.15 7.036
   597   1383516_at   8.299 8.396 8.681 8.479 8.669 6.911 6.437 6.527 6.381 6.212
   598   1370790_at   9.202 9.063 8.904 8.687 8.674 4.696 3.653 3.245 2.548 3.02
   599   1368188_at   11.623 11.613 11.657 11.648 11.619 12.47 12.816 12.581 12.482 12.507
   600   1382043_at   10.764 10.796 10.695 10.729 10.823 9.027 8.854 8.217 8.642 8.423
   601   1398246_s_at   11.965 11.89 11.856 11.709 11.992 9.56 9.206 8.852 8.822 8.404
   602   1379496_at   8.593 8.652 8.604 8.744 8.662 6.884 5.932 6.121 6.15 6.237
   603   1398756_at   8.809 8.898 8.975 8.885 9.087 7.883 7.555 7.781 7.517 7.535
   604   1371605_at   10.997 11.02 10.989 11.037 11.062 11.305 11.33 11.323 11.285 11.382
   605   1369936_at   12.03 12.049 11.859 11.906 11.936 11.16 11.099 10.95 11.199 10.984
   606   1368490_at   10.396 10.459 10.433 10.352 10.537 7.956 7.814 7.649 7.285 6.847
   607   1367790_at   9.721 9.634 9.764 9.844 9.865 8.946 8.695 8.853 8.749 8.924
   608   1370367_at   8.744 8.644 8.836 8.947 8.465 10.008 10.11 10.247 10.287 10.07
   609   1392531_at   7.326 7.161 7.157 7.294 7.215 6.402 6.542 6.293 6.436 6.295
   610   1387280_a_at   6.811 6.731 6.87 7.107 6.926 5.803 5.679 5.775 5.913 5.865
   611   1372323_at   7.153 7.385 7.442 7.574 7.005 8.782 8.808 8.849 8.903 8.793
   612   1374657_at   7.743 7.583 7.746 7.656 7.632 8.467 8.494 8.746 8.714 8.599
   613   1383420_at   6.983 7.05 6.992 7.082 7.099 7.868 8.213 8.13 8.002 7.919
   614   1376006_at   9.97 9.869 9.928 10.038 9.945 8.641 8.445 8.411 8.111 8.06
   615   1369425_at   3.666 3.557 3.24 3.459 3.458 5.119 5.128 5.087 5.606 5.132
   616   1371922_at   10.676 10.701 11.035 10.846 10.754 11.734 11.727 11.777 11.763 11.735
   617   1389093_at   9.152 9.221 9.154 9.291 9.291 8.406 8.107 8.199 8.018 8.099
   618   1374337_at   9.76 9.705 9.622 9.876 9.895 7.461 6.741 6.483 6.39 6.307
   619   1398644_at   6.54 6.702 6.635 6.705 6.656 4.097 3.566 4.43 3.338 3.807
   620   1371483_at   10.484 10.399 10.518 10.433 10.311 11.144 11.085 11.286 11.108 11.133
   621   1369794_a_at   7.487 7.661 7.946 7.936 7.373 5.896 5.461 5.509 5.53 5.755
   622   1394819_at   8.44 8.229 8.61 8.487 8.89 6.425 5.973 5.845 6.42 6.325
   623   1397652_at   6.836 6.987 6.906 7.002 7.089 7.711 7.678 7.866 7.762 7.709
   624   1383478_at   11.024 11.007 10.991 11.127 10.981 10.463 10.364 10.379 10.532 10.369
   625   1382601_at   8.813 8.855 8.856 8.951 9.223 6.444 5.878 5.93 5.309 5.378
   626   1392528_at   8.013 8.075 7.988 8.246 8.252 6.95 6.628 6.783 6.489 6.563
   627   1389645_at   8.495 8.354 8.409 8.618 8.438 9.45 9.782 9.868 9.781 9.629
   628   1379698_at   9.372 9.283 9.434 9.595 9.524 6.543 5.721 5.212 5.443 5.045
   629   1390226_at   8.248 8.235 8.331 8.465 8.481 6.593 6.434 5.88 6.015 5.937
   630   1370314_at   9.689 9.73 9.697 9.81 9.886 8.818 8.525 8.804 8.588 8.502
   631   1389686_at   8.485 8.538 8.415 8.534 8.485 7.769 7.789 7.726 7.505 7.711
   632   1389379_at   6.494 6.757 6.819 6.564 6.709 5.479 5.433 5.194 5.497 5.189
   633   1370463_x_at   9.931 9.993 10.288 9.953 9.764 8.758 8.568 8.448 8.555 8.535
   634   1379563_at   8.094 7.954 7.986 7.757 7.587 6.2 5.927 5.722 6.232 6.014
   635   1384603_at   5.58 5.558 5.617 5.391 5.542 6.768 6.911 7.223 7.023 7.303
   636   1367745_at   8.438 8.473 8.336 8.334 8.402 7.645 7.331 7.542 7.605 7.483
   637   1382622_at   10.245 10.195 10.391 10.151 10.031 7.108 6.092 5.736 5.654 5.519
   638   1389432_at   8.461 8.391 8.421 8.376 8.398 8.068 7.98 8.062 8.103 8.051
   639   1382546_at   9.646 9.615 9.483 9.717 9.737 6.936 5.8 5.458 5.976 5.728
   640   1395268_at   7.214 7.246 7.481 7.417 7.139 8.432 8.278 8.558 8.538 8.476
   641   1388361_at   10.169 10.2 10.16 10.183 10.26 10.687 10.673 10.6 10.784 10.674
   642   1369070_at   6.945 6.952 7.067 7.056 6.946 7.583 7.678 7.68 7.817 7.629
   643   1367919_at   7.705 7.791 7.662 7.988 7.513 6.532 6.211 6.321 6.42 6.419
   644   1377950_at   11.438 11.413 11.288 11.224 11.48 8.538 7.232 7.228 7.134 7.147
   645   1378080_at   8.394 8.255 8.305 8.38 8.407 7.881 7.76 7.785 7.843 7.854
   646   1396262_at   9.501 9.556 9.455 9.301 9.803 6.868 6.336 5.919 5.82 5.58
   647   1373757_at   9.041 9.119 9.143 9.267 9.408 7.734 7.091 7.17 7.424 7.244
   648   1382074_at   8.66 8.82 8.925 9.02 8.956 7.363 6.679 6.774 6.871 6.701
   649   1398491_at   7.03 7.266 7.172 7.162 7.016 7.942 7.821 7.919 8.012 7.95
   650   1370138_at   5.101 5.198 5.389 5.586 5.644 1.208 1.856 0.31 0.322 1.124
   651   1391629_at   5.851 5.917 6.217 6.182 5.896 7.285 7.613 7.387 7.54 7.664
   652   1388309_at   8.273 8.371 8.416 8.521 8.34 7.342 7.251 7.414 7.363 7.041
   653   1374749_at   7.6 7.738 7.623 7.688 7.608 8.453 8.297 8.562 8.476 8.34
   654   1381054_at   4.204 4.355 4.335 4.31 4.361 4.951 5.022 4.936 4.937 5.14
   655   1370339_at   11.712 11.712 11.796 11.728 11.864 11.062 10.819 10.841 10.805 10.717
   656   1385926_at   8.692 8.827 8.63 8.847 8.91 7.328 6.962 7.049 6.696 6.693
   657   1370869_at   9.467 9.542 9.508 9.617 9.677 6.898 6.09 5.626 5.484 6.052
   658   1379381_at   9.772 9.659 9.697 9.812 9.928 7.008 5.938 5.436 5.951 5.861
   659   1370960_at   10.054 9.973 10.287 10.018 10.167 11.301 11.059 11.316 11.399 11.405
   660   1370047_at   6.169 6.004 6.34 6.192 6.018 7.151 7.193 7.087 7.193 7.028
   661   1368379_at   7.921 8.032 7.85 8.08 8.188 7.074 6.955 6.854 6.909 6.772
   662   1372254_at   12.455 12.337 12.452 12.187 12.273 11.306 10.986 11.183 11.122 10.904
   663   1371976_at   8.843 8.855 9.072 9.052 8.9 9.774 9.842 9.866 10.047 9.889
   664   1377576_at   9.528 9.528 9.623 9.679 9.609 10.284 10.347 10.521 10.289 10.294
   665   1383131_at   9.417 9.267 9.558 9.421 9.133 7.835 7.516 7.593 7.164 7.413
   666   1377916_at   10.922 10.84 10.714 10.663 10.888 8.518 7.966 7.581 7.436 7.411
   667   1368038_at   8.451 8.506 8.606 8.628 8.324 9.444 9.584 9.511 9.75 9.539
   668   1368566_a_at   10.139 10.049 10.191 10.159 10.033 10.887 10.976 10.987 11.195 10.913
   669   1373262_at   8.657 8.594 8.606 8.759 8.435 7.005 6.731 7.018 6.942 6.402
   670   1395007_at   4.567 4.68 4.53 4.387 4.746 5.712 5.587 5.557 5.682 5.816
   671   1376487_at   8.416 8.379 8.386 8.321 8.321 9.18 9.183 9.141 9.47 9.323
   672   1395426_at   10.677 10.742 10.673 10.714 10.76 10.175 10.028 10.065 10.033 9.893
   673   1376652_at   11.6 11.588 11.723 11.507 11.507 9.874 9.585 9.324 9.445 8.974
   674   1398482_at   8.115 7.84 7.896 8.384 8.227 6.465 6.408 6.307 6.072 6.072
   675   1368073_at   12.186 12.125 12.057 12.037 12.094 9.446 8.508 8.3 8.27 7.922
   676   1376573_at   8.703 8.692 8.515 8.46 8.498 9.459 9.545 9.535 9.623 9.371
   677   1369630_at   10.664 10.722 10.674 10.596 10.471 11.356 11.336 11.344 11.401 11.24
   678   1378377_at   7.732 7.472 7.624 7.471 7.466 4.48 3.444 4.03 4.85 4.151
   679   1374667_at   6.585 6.63 6.614 6.791 6.817 7.769 8.082 8.115 7.903 7.764
   680   1369497_at   7.876 7.904 7.446 7.976 8.02 5.463 4.783 4.643 4.26 4.763
   681   1373807_at   8.351 8.435 8.587 8.504 8.313 9.184 9.184 9.155 9.113 9.146
   682   1384787_at   9.583 9.553 9.919 9.58 9.276 6.527 6.023 5.009 5.947 5.627
   683   1379418_at   6.414 6.851 6.48 6.652 6.77 7.893 7.922 7.999 8.141 7.882
   684   1367660_at   9.758 9.815 10.036 10.019 9.655 11.192 11.048 11.095 11.201 11.398
   685   1370810_at   7.178 7.392 7.445 7.563 7.232 5.801 5.296 5.854 5.621 5.751
   686   1395744_at   7.691 7.659 7.626 7.709 7.747 6.33 6.553 6.443 6.539 6.054
   687   1377744_at   7.468 7.423 7.33 7.371 7.525 6.253 5.893 5.681 5.801 6.076
   688   1399130_at   8.567 8.473 8.332 8.496 8.585 9.162 9.36 9.377 9.338 9.326
   689   1368045_at   8.592 8.627 8.845 8.741 8.521 9.553 9.607 9.506 9.5 9.467
   690   1375901_at   8.828 9.052 8.815 9.005 9.142 7.841 7.878 8.049 7.771 7.869
   691   1389114_at   9.223 9.061 9.052 9.146 9.103 9.816 10.015 10.087 10.15 10.097
   692   1385832_s_at   8.524 8.632 8.838 8.84 8.405 6.591 6.803 6.662 6.28 6.089
   693   1368385_a_at   9.638 9.753 9.85 9.964 9.823 8.928 8.823 8.948 8.967 8.767
   694   1376785_at   8.161 8.275 8.114 8.149 8.017 9.077 9.014 9.332 9.308 9.159
   695   1367672_at   10.268 10.453 10.301 10.355 10.107 11.126 11.098 11.116 11.213 11.221
   696   1388463_at   9.45 9.47 9.562 9.598 9.282 10.259 10.341 10.439 10.459 10.406
   697   1368134_a_at   8.556 8.57 8.653 8.714 8.632 7.151 6.581 6.923 7.061 7.24
   698   1382189_at   8.86 8.872 9.069 9.151 8.59 10.425 10.444 10.599 10.375 10.283
   699   1383392_at   10.827 10.804 10.781 10.741 10.739 7.909 6.97 6.369 7.11 6.45
   700   1389041_at   8.611 8.445 8.604 8.529 8.564 7.884 7.833 7.926 7.712 7.709
   701   1372064_at   11.265 11.249 11.034 11.243 11.161 10.283 10.202 10.292 10.26 9.983
   702   1393373_at   7.788 7.852 7.716 7.894 8.099 6.63 6.644 6.424 6.526 6.228
   703   1382861_at   10.425 10.343 10.294 10.183 10.171 9.531 9.365 9.383 9.293 9.27
   704   1384302_at   7.209 7.423 7.39 7.554 6.977 8.973 8.88 9.07 9.344 9.007
   705   1373043_at   9.963 9.953 9.906 10.041 10.294 8.98 8.706 8.771 8.625 8.659
   706   1387918_at   10.974 10.904 10.891 11.018 11.07 9.894 9.667 9.536 9.364 9.407
   707   1374393_at   9.014 8.913 8.91 9.029 9.085 8.142 7.864 8.136 7.97 7.842
   708   1387776_at   10.511 10.542 10.288 10.502 10.588 9.12 8.906 8.822 9 8.494
   709   1387076_at   11.991 11.853 11.935 11.706 11.668 10.696 10.441 10.509 10.365 10.218
   710   1368059_at   9.978 9.907 10.021 10.109 9.92 10.991 10.834 11.07 11.158 11.231
   711   1389154_at   8.505 8.501 8.572 8.506 8.421 8.958 8.97 9.056 9.095 8.963
   712   1382803_at   7.34 7.328 7.296 7.405 6.975 5.585 5.08 4.911 5.005 5.437
   713   1367485_at   10.608 10.501 10.602 10.54 10.547 9.836 9.78 9.893 9.633 9.611
   714   1373218_at   6.479 5.967 6.473 6.319 6.319 7.851 7.803 7.694 8.091 7.91
   715   1374165_at   7.433 7.631 7.633 7.75 7.819 5.177 5.821 5.466 5.581 4.962
   716   1370180_at   11.337 11.354 11.397 11.301 11.295 11.963 11.838 11.847 12.006 12.026
   717   1370358_at   8.991 8.887 8.956 9.11 8.804 9.866 9.78 9.822 9.851 9.671
   718   1367905_at   8.987 9.27 9.219 9.439 9.544 7.89 7.697 7.882 7.617 7.621
   719   1376920_at   8.898 8.844 8.623 8.978 8.962 7.351 6.931 7.236 6.688 6.977
   720   1378700_at   7.56 7.62 7.556 7.679 7.748 6.049 6.004 5.964 6.517 5.951
   721   1377018_at   6.076 5.76 5.197 5.639 6.066 8.036 8.081 8.256 7.973 7.983
   722   1370890_at   11.102 11.16 11.199 11.109 11.129 10.36 10.01 10.282 10.125 10.024
   723   1376700_at   9.613 9.58 9.49 9.639 9.798 8.701 8.581 8.486 8.702 8.407
   724   1381504_at   3.146 3.602 2.93 3.278 3.546 5.198 5.181 5.113 5.341 4.999
   725   1389498_at   7.642 7.754 7.691 7.785 7.584 8.552 8.546 8.711 8.601 8.387
   726   1394673_at   8.185 8.344 8.355 8.373 8.542 6.047 5.361 5.909 5.671 4.963
   727   1380071_at   9.597 9.691 9.703 9.869 10.138 8.154 7.563 7.621 7.657 7.439
   728   1372620_at   9.307 9.151 9.245 9.359 9.323 8.685 8.532 8.511 8.576 8.452
   729   1371631_at   8.707 8.565 8.833 8.821 8.672 7.82 7.936 8 7.885 7.813
   730   1371073_at   8.79 8.682 8.633 8.675 8.742 7.381 7.12 6.765 7.057 6.72
   731   1382603_at   9.724 9.768 9.798 9.743 9.718 6.566 5.273 5.348 5.486 4.611
   732   1388948_at   9.27 9.232 9.235 9.264 9.079 10.227 10.28 10.25 10.616 10.506
   733   1368575_at   9.398 9.371 9.597 9.521 9.402 10.552 10.252 10.602 10.573 10.536
   734   1388026_at   6.8 6.709 7.181 7.002 6.79 4.885 4.392 4.656 4.631 5.18
   735   1392107_at   8.657 8.621 8.694 8.862 8.85 7.451 7.146 7.093 6.912 6.8
   736   1382293_at   9.005 8.964 9.031 8.747 8.995 9.994 9.877 10.171 10.098 10.251
   737   1389166_at   6.028 6.221 6.412 6.154 6.116 7.676 7.365 7.757 7.577 7.385
   738   1383422_at   6.208 6.364 6.585 6.56 6.324 7.617 7.715 7.923 7.817 7.559
   739   1397772_at   7.716 7.894 7.936 8.016 7.708 6.345 6.139 5.811 5.636 5.847
   740   1383158_at   9.241 9.208 9.166 9.134 9.3 8.058 8.083 8.075 7.812 7.634
   741   1373777_at   7.771 7.738 7.866 8 7.861 5.992 6.043 5.638 5.397 5.268
   742   1383654_a_at   6.179 6.332 6.805 6.285 6.23 7.905 8.166 8.139 8.141 7.947
   743   1394228_at   8.391 8.344 8.499 8.643 8.248 9.463 9.46 9.732 9.56 9.575
   744   1389227_at   9.84 9.794 9.71 10.03 9.992 8.854 8.668 8.963 8.791 8.712
   745   1381282_at   5.474 5.666 5.42 5.61 5.415 6.323 6.341 6.29 6.4 6.503
   746   1386968_at   9.006 8.875 9.048 8.936 8.574 10.486 10.429 10.722 10.899 10.946
   747   1385590_at   7.053 7.021 7.559 7.334 7.079 3.157 4.087 3.7 2.566 3.623
   748   1371078_at   9.937 9.778 9.875 9.892 9.803 7.84 6.901 6.614 7.054 7.035
   749   1391131_at   5.241 5.247 5.263 5.2 5.345 6.46 6.164 6.649 6.619 6.587
   750   1374196_at   7.948 7.978 7.941 8.053 8.006 8.524 8.746 8.623 8.66 8.525
   751   1390325_at   9.364 9.182 9.221 9.68 9.635 6.934 5.936 5.882 5.717 5.699
   752   1379791_at   9.563 9.586 10.027 9.621 9.216 5.691 4.709 3.998 4.168 3.584
   753   1371967_at   9.483 9.466 9.474 9.49 9.371 10.056 10.317 10.278 10.237 10.103
   754   1383731_at   7.985 7.995 8.09 8.05 8.086 8.703 8.671 8.902 8.757 8.635
   755   1370080_at   9.929 9.968 10.614 10.591 10.268 7.691 7.063 6.69 7.45 7.336
   756   1377666_at   8.567 8.499 8.46 8.609 8.394 9.278 9.304 9.341 9.535 9.263
   757   1370682_at   8.102 8.26 8.59 8.352 8.264 6.678 6.491 6.582 6.355 6.011
   758   1393831_at   8.893 8.817 9.094 9.197 8.744 10.34 10.517 10.513 10.352 10.171
   759   1374594_at   7.369 7.456 7.65 7.526 7.59 8.447 8.736 8.876 8.616 8.686
   760   1370200_at   10.106 10.231 10.269 10.384 10.197 10.893 10.894 10.996 10.999 10.895
   761   1389448_at   7.677 7.728 7.451 7.883 8.074 9.537 9.462 9.984 9.576 9.563
   762   1370990_at   8.08 8.146 8.115 8.053 8.375 6.829 6.731 7.12 7.049 6.858
   763   1381174_at   7.372 7.325 7.366 7.42 7.401 7.774 7.813 7.918 7.927 7.937
   764   1370090_at   9.027 9.098 9.129 9.151 9.002 6.218 5.483 5.401 6.118 4.807
   765   1370897_at   8.224 8.12 8.281 8.267 8.331 9.097 9.089 9.332 9.42 9.223
   766   1370918_a_at   12.085 12.051 12.043 11.966 12.021 12.474 12.573 12.431 12.494 12.589
   767   1370197_a_at   7.402 7.552 7.784 7.632 7.456 8.597 8.544 8.619 8.452 8.523
   768   1371977_at   11.524 11.5 11.464 11.472 11.622 10.772 10.788 10.845 10.629 10.566
   769   1382227_at   6.874 6.95 7.049 7.128 6.791 8.09 7.907 8.041 7.881 7.872
   770   1398849_at   11.692 11.606 11.635 11.678 11.688 11.219 11.169 11.102 10.999 11.119
   771   1373611_at   8.65 8.607 8.798 8.72 8.864 7.65 7.16 7.31 7.548 7.334
   772   1373216_at   7.3 7.47 7.18 7.531 7.293 8.321 8.206 8.244 8.239 8.219
   773   1371696_at   9.857 9.863 9.848 10.025 9.724 10.531 10.554 10.627 10.621 10.516
   774   1393000_at   6.199 6.137 6.126 6.307 6.43 7.576 7.176 7.484 7.38 7.441
   775   1399043_at   10.493 10.515 10.498 10.474 10.608 9.994 9.799 9.919 9.827 9.751
   776   1379344_at   12.018 12.03 12.033 11.665 11.813 10.068 9.509 9.631 9.496 9.062
   777   1369262_at   8.346 8.503 8.315 8.432 8.622 7.047 6.973 6.838 6.919 6.438
   778   1389403_at   8.414 8.352 8.411 8.668 8.354 9.402 9.538 9.46 9.542 9.285
   779   1376908_at   9.624 9.611 9.233 9.809 9.75 6.569 4.814 5.277 5.517 5.262
   780   1370166_at   8.93 8.981 9.184 8.943 8.449 10.566 10.576 10.613 10.467 10.498
   781   1388255_x_at   10.322 10.472 10.609 10.391 10.412 8.462 7.555 7.265 7.631 7.668
   782   1375732_at   7.277 7.446 7.583 7.615 7.35 6.407 6.531 6.468 6.377 6.564
   783   1375016_at   6.017 6.06 6.088 5.922 5.797 6.82 7.029 7.026 7.062 6.843
   784   1367472_at   10.662 10.769 10.649 10.845 10.795 10.21 10.154 10.158 10.146 10.061
   785   1374190_at   7.978 7.755 7.838 7.715 7.929 8.831 8.893 8.937 8.703 8.666
   786   1369877_at   8.899 8.842 9.53 9.565 9.046 5.759 4.233 4.252 4.716 4.071
   787   1370460_at   7.806 7.768 7.798 7.884 7.849 7.276 7.343 7.239 7.11 7.262
   788   1368375_a_at   7.936 7.851 7.613 7.75 7.877 6.339 6.006 5.815 6.038 6.43
   789   1368092_at   10.015 10.024 10.152 10.127 9.899 10.803 10.88 10.937 11.027 11.09
   790   1388408_at   10.9 10.936 10.821 10.786 10.881 10.226 9.995 10.091 9.993 9.894
   791   1387130_at   8.128 8.303 8.211 8.183 8.1 9.111 9.222 9.273 9.055 8.93
   792   1392910_at   7.911 8.016 7.967 8.164 7.982 7.409 7.231 7.232 7.313 7.247
   793   1384525_at   8.776 8.874 8.879 8.94 8.995 7.243 6.788 7.189 6.942 6.449
   794   1372776_at   9.918 10.043 10.259 10.093 10.233 9.022 9.012 9.136 8.972 8.783
   795   1371184_x_at   7.902 7.89 8.021 8.086 8.138 6.4 6.821 6.467 6.769 6.348
   796   1378633_at   8.602 8.583 8.811 8.624 8.252 7.292 7.184 7.188 7.033 6.945
   797   1374307_at   7.936 7.909 7.985 7.728 7.596 9.001 9.205 9.218 9.432 9.416
   798   1386904_a_at   10.666 10.667 10.674 10.738 10.609 11.142 11.2 11.256 11.358 11.322
   799   1375635_at   9.496 9.633 9.522 9.583 9.616 8.707 8.667 8.894 8.847 8.587
   800   1387806_at   11.498 11.498 11.471 11.288 11.505 10.299 10.177 10.239 10.097 9.773
   801   1373693_at   8.541 8.554 8.705 8.627 8.281 9.456 9.598 9.558 9.515 9.569
   802   1367958_at   8.228 8.249 8.337 8.413 8.297 7.589 7.546 7.629 7.428 7.36
   803   1377603_at   8.237 8.309 8.314 8.273 8.366 7.459 7.056 7.417 7.195 7.305
   804   1391808_at   7.667 7.709 7.593 7.828 7.978 6.043 5.765 5.873 5.25 5.473
   805   1373709_at   7.274 7.194 7.464 7.36 7.368 8.029 8.046 7.982 7.958 7.939
   806   1387027_a_at   9.4 9.413 8.984 9.115 9.348 7.706 7.018 7.291 7.461 7.19
   807   1374897_at   9.45 9.66 9.513 9.708 9.759 8.779 8.69 8.707 8.627 8.528
   808   1368809_at   11.381 11.334 11.18 11.098 11.321 10.314 10.293 10.204 10.012 10.032
   809   1368528_at   7.128 7.039 7.155 7.177 7.286 8.198 8.093 8.503 8.357 8.162
   810   1373253_at   7.937 7.622 7.848 8.032 7.807 8.905 8.824 9.014 9.117 9.046
   811   1375006_at   9.586 9.717 9.309 9.568 9.594 8.35 8.162 8.278 8.151 7.868
   812   1372907_at   9.532 9.514 9.761 9.616 9.502 10.362 10.309 10.342 10.461 10.514
   813   1379568_at   9.491 9.594 9.337 9.589 9.812 7.577 6.67 6.68 6.892 6.479
   814   1367720_at   9.076 9.067 9.277 9.183 9.111 10.005 9.95 10.204 9.906 9.952
   815   1387656_at   6.469 6.298 6.824 6.798 6.644 8 7.985 7.915 8.001 7.877
   816   1368266_at   7.898 7.948 8.146 8.694 8.353 5.738 5.954 5.51 5.611 5.129
   817   1389363_at   9.087 9.031 9.224 9.238 9.287 9.83 9.879 9.867 9.842 9.791
   818   1367763_at   10.85 10.721 10.775 10.777 10.756 11.163 11.264 11.296 11.164 11.216
   819   1369969_at   10.046 10.032 10.012 10.128 10.253 9.168 9.005 9.067 8.959 8.735
   820   1372461_at   10.365 10.243 10.298 10.445 10.437 9.661 9.581 9.743 9.574 9.502
   821   1398373_at   10.006 10.257 10.06 10.186 10.364 8.621 8.564 8.559 8.566 8.014
   822   1369909_s_at   8.253 8.191 8.376 8.56 8.205 9.569 9.61 9.735 10.045 9.851
   823   1382464_at   8.433 8.476 8.56 8.773 8.447 9.379 9.493 9.557 9.595 9.457
   824   1390454_at   9.685 9.528 9.685 9.666 9.596 10.258 10.54 10.481 10.569 10.546
   825   1387867_at   9.807 9.772 9.8 10.024 9.735 10.609 10.635 10.72 10.779 10.858
   826   1398516_at   6.173 6.327 6.397 6.135 5.845 7.542 7.545 7.463 7.528 7.378
   827   1392888_at   9.513 9.69 9.617 9.779 9.636 10.222 10.274 10.342 10.354 10.33
   828   1372255_at   10.42 10.252 10.031 10.405 10.521 9.24 9.07 9.009 9.016 9.1
   829   1387818_at   9.164 9.069 8.892 9.07 9.137 7.232 6.923 7.074 6.874 6.284
   830   1372107_at   8.424 8.302 8.275 8.346 8.582 9.613 9.444 9.685 9.378 9.362
   831   1395574_at   8.557 8.632 8.582 8.749 8.822 7.938 7.698 7.835 7.844 7.864
   832   1388066_a_at   7.005 6.9 7.059 7.062 6.872 6.053 5.923 5.802 5.736 5.587
   833   1374169_at   7.481 7.649 7.631 7.732 7.532 8.321 8.364 8.584 8.433 8.425
   834   1372350_at   8.239 8.277 8.245 8.246 8.275 8.788 8.651 8.713 8.666 8.6
   835   1370265_at   8.491 8.663 8.674 8.435 8.513 6.981 6.519 6.547 6.675 6.079
   836   1398426_at   4.807 4.664 4.825 4.989 4.82 5.802 5.737 6.016 5.849 5.686
   837   1372232_at   8.016 8.206 8.141 8.151 8.294 8.941 8.787 8.874 8.812 8.833
   838   1370036_at   7.757 7.72 7.478 7.738 7.465 8.612 8.808 8.973 8.968 8.86
   839   1380079_at   8.316 8.143 8.187 8.212 7.626 6.385 6.431 6.068 6.064 6.181
   840   1367866_at   8.545 8.561 8.373 8.526 8.986 10.415 9.973 10.312 10.335 10.245
   841   1372097_at   10.287 10.332 10.195 10.225 10.322 7.667 6.552 6.409 6.897 6.066
   842   1392914_at   6.082 6.382 6.567 6.45 6.298 7.607 7.398 7.639 7.627 7.67
   843   1388374_at   8.886 8.837 8.784 8.966 8.907 9.457 9.461 9.442 9.562 9.639
   844   1374057_at   9.785 9.795 9.879 9.832 10.003 10.746 10.57 10.836 10.839 10.913
   845   1367925_at   10.022 9.936 9.977 10.091 10.266 9.205 9.008 9.091 9.052 8.889
   846   1370422_at   9.784 9.839 9.678 9.765 9.897 7.565 7.059 6.784 6.373 6.371
   847   1386928_at   8.436 8.387 8.473 8.41 8.311 9.006 8.827 8.937 8.897 8.937
   848   1388196_at   10.209 10.244 10.273 10.156 10.076 10.81 10.818 10.705 10.756 10.692
   849   1389256_at   6.298 6.27 6.075 6.298 6.526 7.581 7.322 7.666 7.603 7.438
   850   1370628_at   11.153 11.098 11.632 11.534 11.095 7.049 5.515 5.192 5.409 4.392
   851   1367810_at   9.114 9.155 9.226 9.213 9.049 9.857 9.677 9.836 9.788 9.91
   852   1398282_at   7.968 7.959 8.049 8.13 7.799 9.037 9.165 9.463 9.186 9.072
   853   1383906_at   11.401 11.349 10.72 11.141 11.622 8.948 8.099 8.399 8.298 8.013
   854   1379406_at   7.158 7.27 7.324 7.376 7.371 6.53 6.512 6.658 6.702 6.58
   855   1393342_at   8.003 8.024 8.314 8.376 7.966 9.295 9.253 9.442 9.406 9.307
   856   1382003_at   7.369 7.019 7.416 7.378 7.062 8.414 8.41 8.477 8.585 8.64
   857   1389386_at   8.636 8.709 8.755 8.783 8.745 9.205 9.107 9.137 9.178 9.242
   858   1372825_at   8.392 8.273 8.468 8.512 8.503 7.435 7.144 6.978 6.982 6.919
   859   1383391_a_at   11.008 11.04 11.111 11.001 11.051 8.191 7.735 6.76 6.99 6.73
   860   1393085_at   7.992 7.98 7.954 8.164 8.369 6.576 5.786 6.167 6.202 6.044
   861   1370493_a_at   8.439 8.405 8.62 8.435 8.299 6.565 6.232 5.821 6.029 5.505
   862   1379847_at   5.46 5.475 5.752 5.629 5.612 6.808 6.626 6.89 6.844 6.514
   863   1370186_at   12.604 12.519 12.466 12.27 12.466 9.937 8.939 8.753 8.743 8.33
   864   1379229_at   7.584 7.577 7.928 7.878 7.794 5.671 4.507 5.189 5.293 5.09
   865   1377844_at   8.476 8.361 8.442 8.542 8.626 9.152 9.273 9.395 9.275 9.376
   866   1367832_at   10.246 10.217 10.39 10.231 10.232 10.81 10.84 10.869 10.778 10.713
   867   1384164_at   7.243 7.212 7.204 7.162 6.931 8.557 8.789 9.054 8.909 8.45
   868   1387807_at   9.377 9.381 9.36 9.468 9.503 9.023 8.897 8.877 8.919 8.89
   869   1369839_at   4.957 4.995 4.787 4.919 4.935 6.048 5.743 5.998 6.074 5.776
   870   1382050_at   8.421 8.556 8.567 8.57 8.025 9.964 10.079 10.259 10.092 9.837
   871   1388673_at   8.559 8.558 8.687 8.353 8.395 6.234 6.111 5.936 5.368 6.458
   872   1387434_at   6.403 6.325 6.426 6.307 6.219 7.074 7.286 7.47 7.377 7.31
   873   1394359_at   6.681 6.678 6.781 6.901 6.839 5.345 5.017 4.941 4.501 4.986
   874   1393049_at   8.241 8.388 8.641 8.507 8.006 6.294 6.029 5.481 5.51 5.474
   875   1392637_at   11.34 11.412 11.277 11.374 11.434 8.104 6.646 6.476 6.652 5.956
   876   1376102_at   9.279 9.45 9.203 9.41 9.518 8.548 8.206 8.325 8.314 8.34
   877   1386980_at   10.718 10.616 10.742 10.733 10.425 11.467 11.746 11.597 11.704 11.662
   878   1388509_at   8.627 8.702 8.523 8.557 8.574 7.628 7.534 7.91 7.735 7.648
   879   1389185_at   6.398 6.699 6.517 6.566 6.46 7.54 7.744 7.451 7.809 7.8
   880   1376110_at   5.574 5.743 5.567 6.044 5.435 7.061 7.304 7.269 7.501 7.381
   881   1368610_at   7.613 7.553 7.408 7.497 7.585 5.319 4.82 5.215 4.747 4.152
   882   1371998_at   10.019 10.02 10.002 9.986 9.9 9.446 9.237 9.37 9.225 9.184
   883   1370820_at   9.943 10.069 10.078 10.035 10.056 9.573 9.559 9.534 9.677 9.533
   884   1390389_at   7.788 8.195 8.05 7.895 7.836 6.676 6.823 6.736 6.78 6.458
   885   1373462_at   8.603 8.617 8.492 8.652 8.723 7.732 7.825 7.802 7.653 7.486
   886   1389181_at   9.035 8.973 9.258 9.094 8.947 7.791 7.774 7.842 7.676 7.326
   887   1386888_at   8.874 8.801 8.848 9.098 9.2 7.851 7.531 7.425 7.455 7.61
   888   1371795_at   8.767 8.733 9.034 8.759 8.716 9.931 9.655 9.929 10.023 9.943
   889   1372734_at   9.712 9.694 9.731 9.862 9.652 10.269 10.341 10.393 10.488 10.42
   890   1368192_at   8.371 8.158 8.649 8.15 8.029 5.775 5.648 5.307 5.332 4.605
   891   1397628_at   8.622 8.58 8.408 8.825 8.808 7.559 7.143 7.301 7.31 7.386
   892   1376537_at   6.077 5.956 5.951 6.227 6.016 6.782 6.878 6.927 6.83 7.03
   893   1387976_at   6.507 6.543 6.573 6.708 6.595 7.75 7.348 7.464 7.531 7.578
   894   1375895_at   8.794 8.939 8.762 9.035 8.964 7.999 7.757 7.915 7.705 7.621
   895   1387242_at   8.343 8.528 8.093 8.484 8.587 6.987 6.781 7.027 7.202 6.995
   896   1387603_at   4.743 5.249 5.72 5.338 4.454 0.918 0.604 0 1.59 0.546
   897   1368091_at   9.48 9.245 9.437 9.631 9.415 10.633 10.404 10.55 10.488 10.777
   898   1376693_at   10.627 10.579 10.301 10.418 10.739 7.562 5.958 6.167 5.893 5.819
   899   1389592_at   7.3 7.367 7.49 7.689 7.39 8.507 8.443 8.595 8.556 8.334
   900   1371381_at   11.895 11.858 11.928 11.819 11.897 12.328 12.401 12.253 12.328 12.435
   901   1387061_at   8.007 8.032 8.176 8.341 8.296 9.222 9.004 9.179 9.168 9.164
   902   1375920_at   7.519 7.84 7.761 7.897 7.652 6.121 5.401 5.555 6.047 5.66
   903   1377497_at   8.757 8.124 8.559 8.408 8.126 6.409 5.905 5.61 6.209 6.137
   904   1373629_at   8.299 8.014 8.005 7.916 8.307 6.473 6.805 6.28 6.692 6.449
   905   1368003_at   6.268 6.415 6.399 6.463 6.723 7.483 7.436 7.565 7.467 7.65
   906   1389744_at   6.664 6.913 7.142 7.231 6.944 8.232 8.333 8.503 8.415 8.515
   907   1399089_at   7.295 7.342 7.304 7.474 7.079 6.271 6.36 6.156 6.208 6.008
   908   1367834_at   8.333 8.302 8.434 8.405 8.423 7.979 8.021 7.957 8.037 8.009
   909   1368100_at   8.195 8.331 8.219 8.422 8.43 9.342 9.073 9.243 9.403 9.344
   910   1386865_at   6.417 6.419 6.463 6.661 6.951 8.046 8.145 8.171 8.333 7.937
   911   1369044_a_at   7.148 6.887 7.45 7.402 7.422 5.598 5.226 5.718 5.536 5.322
   912   1391489_at   11.73 11.741 11.584 11.594 11.751 8.653 7.471 7.108 7.224 6.658
   913   1389151_at   8.048 8.269 8.353 8.376 8.367 7.082 6.419 6.584 6.554 6.552
   914   1377654_at   7.681 7.742 7.571 7.789 7.731 8.365 8.577 8.38 8.426 8.336
   915   1387566_at   9.54 9.551 9.419 9.49 9.825 8.341 7.904 8.362 8.193 8.075
   916   1370014_at   9.602 9.625 9.501 9.707 9.771 8.94 8.652 8.822 8.822 8.724
   917   1388330_at   9.283 9.33 9.507 9.575 9.559 10.419 10.288 10.585 10.492 10.395
   918   1398805_at   10.34 10.453 10.552 10.506 10.562 9.542 9.195 9.585 9.299 9.268
   919   1371671_at   5.552 5.512 5.802 5.416 5.417 6.61 6.429 6.563 6.507 6.474
   920   1376026_at   7.069 7.032 6.848 6.851 6.782 5.822 5.556 5.356 5.797 5.628
   921   1380617_at   8.347 8.464 8.89 8.79 8.519 7.13 6.875 7.074 7.094 6.716
   922   1392894_at   9.931 9.992 10.011 10.113 9.996 8.312 8.426 8.113 7.925 7.568
   923   1393329_at   8.124 7.918 8.618 8.598 8.043 6.077 5.203 5.759 5.804 5.673
   924   1383457_at   7.813 7.676 8.055 7.767 7.664 9.185 8.868 9.226 9.039 9.321
   925   1391347_at   9.154 9.245 9.65 9.508 9.702 7.713 7.522 7.902 7.648 7.962
   926   1385440_at   7.579 7.102 7.816 7.549 7.069 5.459 4.866 5.025 4.757 5.132
   927   1392145_at   7.061 7.225 7.358 7.483 7.431 8.515 8.291 8.595 8.616 8.535
   928   1390956_at   10.314 10.195 9.975 10.349 10.295 6.754 5.301 5.274 4.455 4.954
   929   1389866_at   10.33 10.423 10.493 10.548 10.458 11.032 10.98 11.094 11.181 11.112
   930   1372655_at   8.808 8.836 9.054 8.995 8.828 9.6 9.558 9.691 9.733 9.642
   931   1376749_at   8.487 8.537 8.368 8.44 8.81 9.925 9.832 10.03 10.053 9.622
   932   1393245_at   10.489 10.408 10.346 10.495 10.442 10.972 11.08 11.083 11.203 10.982
   933   1368220_at   9.254 9.284 9.216 9.083 9.339 8.627 8.64 8.536 8.502 8.635
   934   1373074_at   7.936 7.604 7.641 7.741 7.789 8.634 8.545 8.743 8.546 8.583
   935   1370062_at   10.609 10.598 10.71 10.807 10.693 10.193 10.1 10.178 10.087 10.149
   936   1372762_at   8.109 7.951 8.198 8.05 8.183 8.877 9.14 8.906 9.155 9.096
   937   1386882_at   9.88 9.871 9.994 10.045 10.188 8.998 9.094 9.114 8.918 8.814
   938   1371379_at   11.041 11.053 11.285 11.173 11.074 11.75 11.862 11.721 11.78 11.841
   939   1392486_at   8.606 8.68 8.931 8.689 8.66 9.48 9.668 9.667 9.511 9.54
   940   1368006_at   9.931 9.776 10.139 9.969 9.927 8.164 7.848 7.915 7.899 7.195
   941   1382115_at   6.487 6.563 6.447 6.684 6.595 7.201 7.116 7.213 7.093 7.216
   942   1376038_at   8.216 8.121 8.203 8.223 8.095 8.881 8.694 8.919 8.944 8.776
   943   1387739_at   7.451 7.503 8.221 7.699 7.177 3.939 4.46 3.452 4.438 3.309
   944   1390350_at   6.855 7.169 6.985 7.216 6.926 6.054 5.83 5.927 5.96 5.709
   945   1386586_at   8.068 8.18 8.236 8.247 8.539 6.768 6.216 6.794 6.34 6.55
   946   1395886_at   9.762 9.873 9.929 9.834 9.849 8.552 8.472 8.515 8.264 7.916
   947   1377060_at   9.205 9.457 9.474 9.56 9.292 10.303 10.17 10.291 10.307 10.346
   948   1374838_at   8.984 9.194 9.12 9.126 9.177 7.596 6.998 7.12 6.671 7.318
   949   1381525_at   7.593 7.467 7.418 7.706 7.636 6.76 6.672 6.546 6.797 6.741
   950   1372492_at   9.967 10.084 10.228 10.204 9.928 11.139 10.897 11.084 11.136 11.225
   951   1388275_at   9.991 9.923 10.457 10.106 9.738 6.589 5.704 4.826 5.828 4.751
   952   1387083_at   6.264 6.145 6.331 6.351 5.981 7.403 7.25 7.437 7.392 7.683
   953   1368856_at   7.919 8.232 7.846 8.023 8.22 6.413 6.18 5.98 5.618 5.706
   954   1384275_at   5.34 4.741 4.745 4.709 4.808 6.523 6.382 6.581 6.713 6.799
   955   1388521_at   8.23 7.991 8.327 8.205 8.261 7.204 7.25 7.183 6.881 7.139
   956   1387413_at   7.695 7.717 7.748 7.544 7.253 5.703 4.95 5.599 5.022 5.514
   957   1373439_at   9.494 9.629 9.538 9.55 9.695 8.747 8.449 8.64 8.363 8.351
   958   1371171_at   8.033 7.835 7.882 7.661 7.763 6.232 5.703 5.514 5.945 6.22
   959   1379651_at   6.974 6.979 6.822 7.054 6.788 5.655 5.899 5.576 6 5.759
   960   1382995_at   7.995 7.856 7.745 8.068 8.194 6.744 6.385 6.588 6.256 6.276
   961   1372654_at   9.56 9.627 9.557 9.603 9.437 10.412 10.221 10.549 10.279 10.41
   962   1387794_at   6.295 6.349 6.219 6.532 6.454 4.246 4.401 4.53 4.805 3.895
   963   1384507_at   7.327 7.329 7.445 7.582 7.685 6.108 5.508 6.076 5.774 5.812
   964   1367695_at   10.539 10.634 10.699 10.663 10.422 11.256 11.206 11.331 11.296 11.351
   965   1390942_at   6.53 6.37 6.598 6.768 6.492 7.399 7.627 7.653 7.624 7.501
   966   1371941_at   9.429 9.449 9.594 9.506 9.525 9.998 10.001 10.14 10.058 9.96
   967   1368927_at   7.624 7.664 7.747 7.968 7.726 6.976 6.918 6.798 6.956 6.92
   968   1374592_at   7.895 8.023 8.022 8.045 8.028 8.77 8.654 8.789 9.018 8.865
   969   1388815_at   9.329 9.244 9.304 9.434 9.243 8.751 8.583 8.785 8.702 8.661
   970   1374693_at   6.805 6.677 6.615 6.693 6.757 7.2 7.31 7.421 7.357 7.389
   971   1367989_at   4.771 4.639 4.754 4.904 4.243 6.639 6.439 6.454 6.552 7.051
   972   1390541_at   7.849 7.978 8.095 8.159 8.063 8.683 8.774 8.824 8.863 8.798
   973   1367663_at   12.744 12.644 12.598 12.345 12.504 11.529 11.448 11.251 11.268 11.108
   974   1371640_at   9.849 9.776 9.831 9.686 9.891 10.284 10.328 10.231 10.325 10.316
   975   1377646_at   9.97 9.952 10.209 9.845 9.63 11.211 11.264 11.162 11.314 11.058
   976   1378168_at   6.064 6.518 6.038 6.643 6.397 7.923 8.199 8.399 8.476 8.2
   977   1395034_at   9.791 9.758 9.916 9.695 9.92 6.658 4.911 5.009 4.814 4.59
   978   1390917_at   6.524 6.822 6.689 6.875 6.846 5.04 5.175 5.408 5.023 5.551
   979   1382181_at   9.319 9.404 8.991 9.114 9.341 7.688 7.503 7.394 7.5 6.935
   980   1377751_at   7.462 7.537 7.587 7.392 7.464 8.61 8.63 8.705 8.713 8.265
   981   1367911_at   9.61 9.668 9.678 9.617 9.69 10.078 9.992 10.089 10.155 10.174
   982   1379818_at   8.55 8.614 8.537 8.655 8.624 5.983 4.933 5.447 5.57 4.43
   983   1375028_at   7.389 7.37 7.032 7.163 7.106 5.242 5.18 4.439 5.119 5.328
   984   1378171_at   7.945 7.744 7.88 8.214 8.201 6.482 6.342 5.897 6.333 5.99
   985   1376151_a_at   12.281 12.194 12.192 12.078 12.219 10.147 9.242 9.308 8.975 8.785
   986   1392647_at   10.327 10.268 10.901 10.411 10.104 6.789 6.004 5.618 5.018 4.743
   987   1372884_at   6.597 6.51 6.32 6.632 6.52 7.487 7.544 7.479 7.587 7.262
   988   1393050_at   7.953 8.057 7.702 8.023 8.229 6.511 6.337 6.668 6.249 6.093
   989   1379957_at   7.932 8.093 7.747 7.83 8.067 6.678 6.446 6.336 6.174 6.024
   990   1384344_at   5.113 5.228 5.2 5.394 5.276 6.076 6.24 6.044 6.168 5.939
   991   1373830_at   8.959 8.897 8.874 9.002 9.214 7.916 7.61 7.543 7.46 7.326
   992   1377995_at   9.176 9.144 8.992 9.001 9.215 9.973 9.811 9.999 10.073 9.834
   993   1388497_at   7.398 7.448 7.616 7.828 7.571 8.656 8.505 8.674 8.774 8.777
   994   1384529_at   4.725 5.11 5.107 5.359 5.019 6.411 6.791 6.908 6.804 6.618
   995   1388149_at   10.894 10.853 10.738 10.898 11.033 8.334 7.191 6.77 6.973 6.696
   996   1367769_at   10.075 9.973 10.009 9.963 10.02 9.58 9.507 9.601 9.436 9.428
   997   1370382_at   10.053 10.405 10.099 10.429 10.241 8.021 7.395 6.665 7.283 7.588
   998   1373136_at   8.105 8.206 8.094 8.15 8.246 7.251 7.119 7.342 7.273 6.92
   999   1397304_at   12.298 12.241 12.328 12.082 12.187 9.651 8.469 8.353 8.245 7.87
   1000   1374884_at   6.923 6.923 6.706 6.792 6.693 7.507 7.397 7.471 7.493 7.446
   1001   1376810_at   7.309 7.383 7.479 7.563 7.535 6.855 6.818 6.895 6.807 6.844
   1002   1374122_at   5.357 5.757 5.578 5.76 5.354 6.739 6.68 6.69 6.633 6.779
   1003   1372790_at   8.891 9.066 9.134 9.12 8.759 10.082 9.951 10.11 10.101 10.295
   1004   1369491_at   10.965 10.923 11.271 11.099 10.863 11.984 11.944 11.927 12.036 12.108
   1005   1385571_at   9.592 9.868 9.588 9.47 9.795 6.958 5.382 5.303 5.464 5.928
   1006   1374300_at   8.303 8.179 8.374 8.291 8.45 8.959 8.914 8.892 9.041 8.959
   1007   1368081_at   6.93 6.942 6.98 7.229 6.857 8.199 8.172 8.114 8.171 8.565
   1008   1372847_at   7.846 7.537 7.775 7.801 7.527 8.623 8.649 8.916 8.755 8.776
   1009   1399125_at   9.015 8.999 8.898 9.198 9.24 8.171 7.938 8.176 8.171 8.09
   1010   1375297_at   9.655 9.668 9.639 9.742 9.533 10.277 10.471 10.253 10.5 10.38
   1011   1379732_at   6.97 6.863 7.266 7.172 7.378 4.893 4.419 5.288 4.333 4.52
   1012   1375504_at   7.075 6.923 7.226 7.05 6.805 8.167 8.191 8.366 8.041 8.026
   1013   1382531_at   6.752 6.835 6.926 6.495 6.901 5.15 4.748 4.172 4.538 4.58
   1014   1379285_at   10.139 10.155 10.229 10.43 10.53 8.122 6.896 6.798 7.163 6.872
   1015   1367619_at   10.879 10.862 10.925 10.819 10.685 11.43 11.578 11.455 11.455 11.389
   1016   1375917_at   9.467 9.554 9.97 9.097 9.418 7.115 6.346 5.878 6.63 6.615
   1017   1368754_at   8.496 8.213 8.538 8.472 8.459 6.998 6.677 6.494 6.662 6.133
   1018   1371781_at   10.217 10.021 9.964 10.023 9.954 8.984 8.313 8.537 8.482 8.532
   1019   1368043_at   9.731 9.789 9.813 9.815 9.73 9.191 9.065 9.127 9.156 8.887
   1020   1368050_at   7.878 7.819 8.069 7.941 8 7.364 7.254 7.309 7.393 7.268
   1021   1376390_at   10.861 10.897 10.838 10.714 10.934 7.462 6.227 5.758 5.695 5.116
   1022   1387053_at   9.889 10.026 10.089 9.955 9.388 11.389 11.51 11.353 11.439 11.391
   1023   1374785_at   4.97 5.301 5.331 5.299 5.461 6.496 6.398 6.409 6.533 6.252
   1024   1376546_at   6.409 6.507 6.473 6.215 6.088 7.69 7.361 7.628 7.705 7.522
   1025   1391935_at   7.27 7.354 7.665 7.407 8.125 5.488 5.446 5.543 5.594 5.122
   1026   1374738_at   6.921 7.021 6.817 7.074 6.86 7.784 7.835 7.972 7.639 7.808
   1027   1372406_at   8.436 8.465 8.388 8.458 8.354 7.428 6.991 7.008 6.733 6.912
   1028   1372341_at   8.658 8.734 8.846 8.607 8.551 9.336 9.369 9.293 9.343 9.424
   1029   1368334_at   6.264 6.352 6.292 6.648 6.276 7.476 7.408 7.375 7.708 7.598
   1030   1394352_at   7.005 7.016 6.994 7.37 7.153 5.623 5.369 5.182 4.792 5.293
   1031   1371850_at   9.243 9.245 9.421 9.377 9.332 10.055 9.852 10.034 10.102 10.106
   1032   1383986_at   11.224 11.241 11.113 11.207 11.332 10.406 10.211 10.519 10.246 10.156
   1033   1390287_at   8.671 8.653 8.781 8.898 8.6 7.82 7.829 7.797 7.951 7.613
   1034   1367883_at   8.525 8.44 8.347 8.486 8.807 7.526 7.607 7.551 7.481 7.43
   1035   1388761_at   9.153 9.255 9.17 9.186 9.232 8.592 8.35 8.409 8.289 8.197
   1036   1375796_at   9.642 9.69 10.012 9.744 10.328 7.172 6.328 6.522 5.895 5.628
   1037   1369006_at   6.732 6.863 6.947 7.367 7.196 4.874 4.412 4.871 4.376 3.941
   1038   1386901_at   7.688 7.801 7.424 7.498 7.667 8.852 8.774 9.075 9.146 8.725
   1039   1389654_at   8.392 8.38 8.416 8.616 8.601 9.42 9.476 9.565 9.229 9.359
   1040   1385051_at   10.004 9.897 9.623 9.854 9.992 6.203 5.473 5.017 4.835 3.814
   1041   1372743_at   10.834 10.849 10.991 10.886 11.012 10.208 10.049 10.113 9.995 9.833
   1042   1368645_at   7.822 8.093 8.246 8.165 8.174 6.566 6.362 6.591 5.986 6.613
   1043   1390312_at   8.793 8.94 8.68 9.164 9.084 7.859 7.593 7.515 7.611 7.526
   1044   1375978_at   4.183 4.32 4.977 4.92 4.708 1.956 1.299 2.214 1.824 1.31
   1045   1390406_at   8.196 8.199 8.25 8.332 8.347 8.954 9.095 9.318 9.097 9.021
   1046   1370927_at   8.083 8.21 8.069 8.349 8.578 9.541 9.734 9.845 9.604 9.487
   1047   1385658_at   9.209 9.239 9.173 9.631 9.571 7.931 7.776 8.008 7.66 7.517
   1048   1391417_at   10.883 10.924 10.982 10.992 10.784 11.486 11.725 11.734 11.745 11.78
   1049   1398597_at   5.577 5.803 5.433 5.75 5.904 6.988 6.84 6.885 7.01 6.716
   1050   1392612_at   6.627 6.784 6.561 6.647 5.986 8.35 8.462 8.459 8.484 8.12
   1051   1398828_at   10.907 10.897 10.965 10.966 10.919 10.492 10.296 10.399 10.26 10.243
   1052   1369670_at   8.427 8.373 8.429 8.44 8.324 7.412 7.643 7.773 7.515 7.452
   1053   1386975_at   8.494 8.35 8.607 8.616 8.32 9.259 9.353 9.236 9.414 9.321
   1054   1392731_at   7.275 7.02 7.144 7.055 7.366 4.642 3.54 3.516 4.03 2.991
   1055   1382926_s_at   8.754 8.868 8.938 8.907 8.968 6.504 5.221 4.933 5.068 5.012
   1056   1369636_at   10.703 10.587 10.717 10.628 10.281 11.509 11.672 11.565 11.583 11.595
   1057   1370237_at   10.248 10.243 10.307 10.426 10.273 11.128 10.953 11.326 11.272 11.208
   1058   1373346_at   9.347 9.43 9.238 9.331 9.357 9.898 10.032 10.088 9.95 9.859
   1059   1387271_at   10.81 10.862 10.855 10.931 10.913 11.303 11.265 11.284 11.382 11.431
   1060   1379248_at   7.903 7.738 7.918 7.901 7.893 6.67 6.799 6.501 6.478 6.161
   1061   1393906_at   6.933 6.675 6.925 7.06 6.559 8.031 8.05 8.221 7.937 8.087
   1062   1391690_at   7.004 6.786 6.909 6.53 6.513 4.692 4.013 4.94 4.252 4.474
   1063   1381357_at   7.673 7.397 8.096 7.697 7.475 4.361 4.002 3.748 3.55 2.496
   1064   1369677_at   3.944 3.628 3.595 3.824 3.747 5.209 4.788 4.795 4.955 4.996
   1065   1384842_s_at   7.739 7.832 7.56 7.736 7.895 6.115 5.558 5.384 5.509 5.009
   1066   1398511_at   8.608 8.584 8.653 8.717 8.164 9.889 9.813 10.228 10.015 10.08
   1067   1380577_at   9.172 9.072 9.265 9.364 9.089 9.992 10.044 9.997 10.056 9.845
   1068   1376160_at   7.481 7.718 7.828 7.83 7.792 6.818 6.431 6.479 6.41 6.585
   1069   1386879_at   11.72 11.693 11.954 11.646 11.526 10.448 10.576 10.114 10.226 10.082
   1070   1383148_at   6.724 6.875 6.88 6.948 6.682 7.86 7.961 8.131 7.843 7.678
   1071   1393935_at   8.152 8.02 8.234 8.12 7.939 9.11 9.171 9.548 9.179 9.145
   1072   1392554_a_at   7.137 7.278 7.646 7.443 6.998 5.311 5.756 5.742 5.699 5.635
   1073   1370429_at   7.48 7.535 7.776 8.019 8.093 4.502 5.122 3.526 4.011 4.32
   1074   1388304_at   10.978 10.911 11.034 11.005 11.102 11.497 11.452 11.56 11.563 11.641
   1075   1392670_at   7.765 7.957 7.875 7.918 7.647 9.06 9.033 9.365 8.996 8.836
   1076   1388118_at   11.357 11.331 11.389 11.227 11.171 11.814 11.78 11.854 11.824 11.884
   1077   1383397_at   5.3 5.209 5.686 5.246 5.236 6.515 6.461 6.347 6.499 6.438
   1078   1372090_at   9.802 9.785 10.057 10.119 10.07 8.907 8.618 8.913 8.965 8.786
   1079   1372157_at   7.965 7.949 8.005 8.094 7.922 8.505 8.441 8.571 8.611 8.65
   1080   1398255_at   8.02 8.102 8.216 8.318 7.683 9.566 9.402 9.802 9.694 9.527
   1081   1368437_at   8.323 8.519 8.294 8.67 8.583 9.486 9.538 9.35 9.376 9.472
   1082   1369319_at   10.451 10.536 10.434 10.411 10.611 9.632 9.724 9.581 9.417 9.337
   1083   1369290_at   9.422 9.494 9.536 9.712 9.901 6.72 5.281 5.104 5.519 4.731
   1084   1388977_at   9.755 9.895 9.826 9.789 9.977 9.211 9.265 9.318 9.108 9.2
   1085   1375940_a_at   6.552 6.476 6.369 6.612 6.552 7.257 7.239 7.388 7.514 7.237
   1086   1392990_at   5.245 5.647 5.999 5.607 5.698 6.996 7.16 7.163 7.204 7.251
   1087   1372248_at   7.016 7.242 7.128 7.131 7.148 7.908 7.733 7.961 8.078 7.916
   1088   1369629_at   9.316 9.257 9.377 9.291 9.044 9.94 9.954 9.931 9.942 10.021
   1089   1393832_at   7.454 6.762 7.18 6.881 7.164 5.113 4.9 5.044 5.377 4.612
   1090   1388921_at   5.66 5.956 5.943 5.821 5.654 7.194 6.902 7.087 6.906 7.349
   1091   1378085_at   6.022 6.221 6.35 6.231 6.137 7.343 7.146 7.399 7.556 7.655
   1092   1387558_at   7.078 7.224 7.198 7.56 7.207 8.449 8.227 8.413 8.48 8.287
   1093   1376226_at   5.478 5.589 5.365 5.373 5.754 6.886 6.896 6.9 6.849 6.481
   1094   1389063_at   9.365 9.47 9.602 9.643 9.61 8.802 8.686 8.892 8.801 8.832
   1095   1381441_at   6.945 7.057 7.279 7.095 6.553 4.679 3.316 3.939 3.479 3.801
   1096   1389833_at   8.594 8.635 8.82 8.841 8.541 9.469 9.578 9.623 9.756 9.722
   1097   1383564_at   11.243 11.238 11.277 11.268 11.258 8.999 7.719 7.56 8.049 8.475
   1098   1389688_at   7.635 7.77 7.77 7.954 7.743 8.771 8.776 8.923 8.731 8.525
   1099   1375020_at   8.651 8.546 8.841 8.862 8.868 7.521 7.213 7.636 7.266 7.106
   1100   1370609_a_at   8.574 8.567 8.883 8.611 8.392 9.955 9.676 9.938 10.043 10.181
   1101   1387793_at   10.937 10.972 11.125 11.073 10.905 11.501 11.492 11.491 11.539 11.54
   1102   1376675_at   7.734 7.725 7.758 7.707 8.005 6.093 6.002 5.796 5.613 5.167
   1103   1369672_at   8.211 8.319 8.119 8.346 8.745 6.736 7.016 6.966 6.647 6.722
   1104   1382177_at   7.577 7.742 7.551 7.633 7.622 6.459 6.082 6.084 6.37 5.781
   1105   1388617_at   9.206 9.252 9.495 9.372 9.137 10.014 10.187 10.104 10.138 10.115
   1106   1372546_at   7.582 7.239 7.664 7.408 7.515 6.599 6.535 6.58 6.445 6.424
   1107   1368507_at   11.236 11.333 11.425 11.506 11.428 10.771 10.569 10.743 10.659 10.598
   1108   1392655_at   9.11 9.057 8.835 8.947 9.064 7.516 7.254 7.343 7.556 6.796
   1109   1370832_at   8.709 8.462 9.315 9.389 8.609 6.444 5.675 5.814 5.393 5.962
   1110   1371701_at   11.76 11.755 11.745 11.726 11.673 12.06 11.999 12.063 12.075 12.149
   1111   1390786_at   8.53 8.517 8.437 8.484 8.366 7.829 7.56 7.677 7.765 7.858
   1112   1368609_at   3.091 3.136 2.687 3.124 2.888 5.123 4.788 4.5 5.022 5.326
   1113   1372621_at   8.079 7.901 8.047 7.848 8.382 9.224 9.182 9.117 9.21 9.148
   1114   1371849_at   5.668 5.733 5.935 5.553 5.947 7.646 7.241 7.284 7.013 7.207
   1115   1388998_at   8.366 8.248 8.457 8.563 8.164 9.715 9.379 9.499 9.797 9.756
   1116   1375872_at   6.125 6.422 6.294 6.277 6.216 7.143 6.984 7.284 7.104 7.024
   1117   1390412_at   7.625 7.406 7.83 7.644 7.67 8.804 8.912 9.066 8.891 8.589
   1118   1376084_a_at   6.262 6.303 6.337 6.42 6.314 5.769 5.532 5.543 5.574 5.399
   1119   1368976_at   8.304 8.231 8.61 8.622 8.223 5.385 4.029 3.849 4.66 3.45
   1120   1378802_at   8.883 8.86 9.139 9.102 8.898 9.909 9.858 10.11 10.23 10.058
   1121   1391262_at   7.071 7.418 7.091 7.488 7.311 6.03 5.965 5.875 5.709 5.53
   1122   1387608_at   7.28 7.431 7.72 7.706 7.465 3.842 4.424 4.37 2.738 3.7
   1123   1386954_at   10.716 10.697 10.738 10.881 10.878 10.197 10.226 10.1 10.095 10.01
   1124   1367975_at   7.373 7.573 7.663 7.367 7.586 4.786 3.139 3.939 4.2 4.522
   1125   1389413_at   7.847 8.151 8.133 7.853 7.752 6.85 6.941 6.887 6.717 6.665
   1126   1373852_at   8.205 8.364 8.461 8.444 8.576 7.501 7.396 7.622 7.339 7.538
   1127   1373533_at   7.338 7.292 6.931 7.05 7.128 8.42 8.37 8.564 8.288 8.139
   1128   1371152_a_at   9.206 9.265 8.832 9.216 9.377 6.496 5.639 4.991 6.116 6.232
   1129   1388738_at   8.752 9.095 8.845 8.783 8.81 10.11 10.075 10.447 10.124 9.891
   1130   1393830_at   7.002 7.34 7.759 7.637 6.863 4.311 2.809 2.836 2.916 2.478
   1131   1378245_at   6.134 5.843 5.91 6.008 6.294 7.452 7.345 7.711 7.61 7.971
   1132   1383795_at   7.656 8.038 7.804 8.116 7.864 8.938 9.208 9.402 9.22 9.299
   1133   1376845_at   11.333 11.338 11.61 11.301 11.573 9.613 8.623 8.338 8.796 8.807
   1134   1387018_at   6.734 6.805 6.872 6.531 6.943 8.031 7.849 8.367 8.05 8.013
   1135   1373422_at   9.919 9.911 9.822 9.989 9.96 9.271 9.103 9.178 9.15 8.894
   1136   1383964_at   6.104 6.166 5.921 6.22 5.874 7.246 6.988 7.297 7.069 7.03
   1137   1369700_at   7.044 7.073 7.267 7.154 7.148 6.147 6.255 6.285 5.873 6.226
   1138   1377945_at   8.34 8.368 8.204 8.296 8.178 7.542 7.636 7.683 7.397 7.544
   1139   1372457_at   7.838 7.88 7.898 7.898 7.806 8.635 8.443 8.847 8.682 8.622
   1140   1383633_at   9.333 9.347 9.53 9.391 9.246 9.979 9.933 10.064 9.938 10.04
   1141   1398853_at   11.336 11.314 11.267 11.143 11.145 10.767 10.76 10.748 10.718 10.699
   1142   1390797_at   8.005 7.931 7.923 7.975 7.892 4.056 4.912 5.273 4.176 5.368
   1143   1374892_at   8.088 8.224 8.406 8.38 7.977 9.266 9.173 9.313 9.351 9.446
   1144   1388324_at   10.428 10.442 10.511 10.463 10.261 10.945 11.111 11.16 11.141 11.161
   1145   1389640_at   8.288 8.087 8.193 8.289 8.021 9.014 8.823 9.095 8.984 9.02
   1146   1382590_at   7.79 7.696 7.904 7.987 7.759 6.831 6.943 6.762 7.099 6.964
   1147   1385432_at   6.355 6.52 6.845 6.651 6.752 7.827 7.815 7.936 7.732 8.114
   1148   1371933_at   6.439 6.549 6.623 6.423 6.101 7.933 7.782 8.101 7.846 7.592
   1149   1394968_at   7.007 7.041 7.039 6.954 6.903 6.482 6.412 6.388 6.352 6.201
   1150   1372485_at   9.692 9.786 10.069 10.059 9.75 10.792 10.867 10.876 11.015 10.936
   1151   1397549_at   4.825 4.641 4.531 4.713 4.497 2.789 2.147 2.709 1.84 2.602
   1152   1388641_at   8.099 8.226 8.087 8.191 8.144 7.705 7.748 7.622 7.675 7.56
   1153   1382748_at   8.533 8.497 8.519 8.53 8.536 9.077 9.139 9.379 9.099 9.128
   1154   1379293_at   9.42 9.354 10.516 10.138 9.384 5.93 4.912 3.986 4.109 3.998
   1155   1368428_at   8.425 8.326 8.68 8.718 8.337 9.662 9.64 10.007 9.865 9.691
   1156   1379801_at   5.089 5.503 5.409 5.113 5.159 6.557 6.58 6.743 6.444 6.322
   1157   1392819_at   8.983 9.107 9.02 8.827 9.003 6.099 4.895 5.397 4.064 4.879
   1158   1377048_at   8.917 8.872 9.052 9.079 8.747 10.087 9.961 10.098 10.366 10.431
   1159   1396152_s_at   8.153 8.061 8.282 7.958 7.976 9.226 9.034 9.202 9.466 9.469
   1160   1388364_at   10.614 10.553 10.679 10.501 10.459 11.157 11.036 11.076 11.191 11.129
   1161   1374499_at   9.302 9.439 9.283 9.513 9.689 7.922 7.69 7.978 7.49 7.257
   1162   1373032_at   5.11 5.076 5.229 5.213 5.411 6.211 5.948 6.273 6.276 6.214
   1163   1384964_at   8.44 8.496 8.495 8.781 8.685 9.518 9.737 9.891 9.754 9.54
   1164   1379676_a_at   8.864 9.051 9.084 8.918 9.137 8.253 7.895 8.087 8.162 8.123
   1165   1370002_at   8.659 8.67 8.734 8.844 8.563 8.131 8.01 8.003 8.114 7.979
   1166   1370978_at   9.28 9.257 9.233 9.299 9.222 8.682 8.7 8.512 8.44 8.419
   1167   1384292_at   7.584 7.542 7.501 7.629 7.778 5.807 5.881 5.89 5.461 5.031
   1168   1370891_at   10.493 10.49 10.629 10.387 10.32 9.426 9.356 9.643 9.553 9.241
   1169   1389066_at   6.991 6.749 6.944 6.834 6.345 8.165 8.231 8.548 8.454 8.281
   1170   1380726_at   3.693 3.741 4.163 3.315 3.234 5.617 5.601 5.665 5.766 5.486
   1171   1384251_at   5.22 5.261 5.104 5.361 5.064 6.189 5.951 5.936 6.128 6.021
   1172   1388148_a_at   10.221 10.311 10.265 10.392 10.309 10.98 11.145 11.144 10.963 10.86
   1173   1375026_at   9.862 9.825 9.95 10.06 10.099 10.763 11.137 10.932 10.931 10.826
   1174   1372109_at   8.863 8.863 8.921 8.901 8.804 9.373 9.459 9.388 9.398 9.227
   1175   1382034_at   4.317 4.606 4.73 5.1 4.841 6.673 6.237 6.353 6.612 6.584
   1176   1386916_at   9.33 9.356 9.504 9.524 9.367 10.266 10.337 10.506 10.711 10.626
   1177   1376796_at   8.751 8.765 8.869 8.783 8.817 8.38 8.307 8.383 8.446 8.463
   1178   1387889_at   10.954 10.875 10.917 10.902 10.884 11.477 11.792 11.647 11.68 11.485
   1179   1380772_at   6.239 6.208 6.201 6.547 6.098 5.052 4.707 4.37 4.719 4.801
   1180   1368391_at   7.786 7.759 7.863 8.086 8.007 6.903 6.717 6.828 6.423 6.649
   1181   1372671_at   10.121 10.238 10.039 10.222 10.06 10.865 10.84 10.929 10.742 10.692
   1182   1384934_at   7.531 7.61 7.107 7.406 7.577 5.75 6.23 6.109 5.876 5.801
   1183   1393119_at   6.534 6.561 7.016 6.884 6.464 5.146 4.917 5.206 5.197 4.721
   1184   1375529_at   7.787 7.764 7.791 7.756 7.947 8.421 8.76 8.766 8.618 8.613
   1185   1373841_at   8.493 8.568 8.494 8.478 8.34 7.647 7.475 7.481 7.606 7.199
   1186   1371844_at   9.846 9.843 9.794 9.924 9.901 10.278 10.256 10.385 10.27 10.213
   1187   1383465_at   7.836 7.716 7.566 7.81 7.718 7.183 7.003 7.053 7.101 7.015
   1188   1387788_at   8.681 8.636 8.473 8.668 8.613 7.665 7.257 7.224 7.252 6.974
   1189   1379891_at   10.014 10.057 10.229 10.164 10.026 9.427 9.141 9.274 9.256 9.43
   1190   1383058_at   8.163 8.067 8.37 8.421 8.129 9.775 9.374 9.699 10.05 9.919
   1191   1385696_at   8.311 8.392 8.662 8.978 8.357 6.002 4.647 5.111 5.72 4.851
   1192   1392037_at   6.699 7.072 6.434 6.822 7.064 5.087 5.254 5.48 5.108 5.244
   1193   1368783_at   8.493 8.478 8.747 8.746 8.1 5.664 3.931 4.993 4.183 4.908
   1194   1382661_at   7.541 7.605 7.596 7.349 7.403 6.897 6.851 6.875 6.918 6.814
   1195   1383015_at   7.115 6.939 7.026 7.065 6.741 4.652 3.705 3.713 4.356 3.189
   1196   1372438_at   10.603 10.637 10.781 10.484 10.45 11.403 11.49 11.417 11.348 11.268
   1197   1379885_at   8.497 8.664 8.898 8.822 8.193 10.061 10.035 10.247 10.308 10.211
   1198   1368079_at   7.41 7.334 7.182 7.242 7.501 5.854 6.296 6.157 6.349 6.121
   1199   1370952_at   7.521 7.466 7.707 7.773 7.927 9.269 8.851 9.115 9.2 9.474
   1200   1380393_at   9.715 9.721 9.85 9.85 9.523 10.534 10.651 10.902 10.8 10.803
   1201   1371916_at   10.833 10.804 11.023 10.867 10.642 11.553 11.526 11.594 11.669 11.663
   1202   1387952_a_at   10.009 10.033 9.692 9.83 9.772 7.881 8.089 7.639 7.191 7.12
   1203   1374222_at   10.023 10.073 9.982 10.191 10.087 10.768 10.636 10.819 10.934 10.926
   1204   1382010_at   6.75 6.818 6.831 6.824 6.813 7.896 7.826 7.901 7.577 7.522
   1205   1373751_at   5.771 5.99 6.227 6.002 5.716 4.826 4.457 4.348 4.594 4.43
   1206   1379710_at   4.825 4.741 4.841 5.002 4.851 5.963 5.706 6.002 5.738 6.146
   1207   1397437_at   6.289 6.217 6.62 6.538 6.428 4.664 4.343 4.066 3.752 3.621
   1208   1369688_s_at   7.956 7.988 8.181 8.163 7.885 6.877 6.185 6.252 6.303 6.109
   1209   1373273_at   8.003 8.156 8.236 8.119 8.277 7.515 7.445 7.607 7.549 7.446
   1210   1398877_at   10.029 10.096 10.036 10.139 10.19 9.623 9.392 9.551 9.404 9.453
   1211   1386895_at   8.926 8.934 8.799 8.837 9.055 9.777 9.637 9.627 9.568 9.509
   1212   1374034_at   7.811 7.75 7.832 7.998 7.857 7.052 6.945 7.178 7.173 7.218
   1213   1391803_at   7.166 7.154 7.117 6.885 7.07 5.045 5.595 5.477 4.855 4.802
   1214   1386770_x_at   0 0.057 0 0 0.566 2.014 2.011 2.597 2.822 2.257
   1215   1388436_at   8.233 8.14 8.169 8.414 8.37 7.302 7.292 7.282 6.993 6.933
   1216   1382490_at   4.351 4.595 4.771 4.629 4.338 5.837 5.773 5.575 5.807 5.548
   1217   1399012_at   11.336 11.392 11.39 11.396 11.387 11.673 11.588 11.729 11.674 11.662
   1218   1391509_at   6.951 6.916 7.187 7.252 6.936 8.278 7.927 8.189 8.039 8.073
   1219   1377092_at   8.921 9.148 8.984 9.43 9.363 7.343 7.278 6.88 6.744 6.425
   1220   1376869_at   9.054 8.779 8.893 8.916 8.945 8.133 8.01 8.273 8.211 7.994
   1221   1379598_at   7.176 7.609 7.463 7.509 7.324 5.414 5.384 4.855 4.447 4.495
   1222   1368965_at   7.971 8.3 8.02 8.548 8.547 6.39 5.759 5.755 5.243 5.538
   1223   1373324_at   6.211 6.096 6.053 6.455 6.33 7.565 7.293 7.501 7.174 7.369
   1224   1393219_at   11.202 11.201 11.192 10.843 11.075 7.928 7.326 6.632 6.914 5.728
   1225   1374265_at   8.999 8.736 8.576 8.768 8.912 7.905 7.785 7.843 7.668 7.601
   1226   1393863_at   7.706 7.823 8.375 7.867 7.889 5.326 4.057 4.818 3.952 5.013
   1227   1382447_at   6.573 6.391 6.812 6.539 6.332 7.672 7.649 8.013 7.825 8.026
   1228   1372626_at   6.325 6.299 6.419 6.365 6.447 7.269 7.229 7.549 7.725 7.514
   1229   1388794_at   9.422 9.382 9.349 9.355 9.572 8.719 8.694 8.599 8.436 8.42
   1230   1371447_at   12.791 12.704 12.582 12.33 12.57 11.164 10.646 10.754 10.524 10.227
   1231   1368158_at   9.372 9.466 9.531 9.365 9.495 8.939 8.966 8.803 8.884 8.763
   1232   1386978_at   9.61 9.589 9.519 9.58 9.67 10.113 10.189 10.199 10.152 9.975
   1233   1372615_at   4.858 4.776 5.111 5.601 5.039 6.808 7.218 7.536 7.314 7.081
   1234   1382270_at   6.051 5.821 5.747 6.139 5.918 6.826 6.986 7.122 6.853 6.906
   1235   1383290_at   7.918 7.878 7.891 7.882 8.013 8.521 8.505 8.68 8.491 8.385
   1236   1387659_at   10.275 10.265 10.502 10.402 10.998 8.587 8.409 8.774 8.327 8.025
   1237   1368420_at   10.779 10.78 10.193 10.447 10.687 9.222 8.766 8.935 8.694 8.652
   1238   1393067_at   7.875 7.92 7.701 7.888 8.126 8.796 8.956 9.211 8.952 8.93
   1239   1382158_at   6.984 7.155 6.69 6.817 7.069 5.95 5.801 6.015 5.916 5.93
   1240   1373992_at   11.983 12.011 11.944 11.785 11.954 8.893 7.507 6.734 7.332 6.664
   1241   1386953_at   11.504 11.538 11.541 11.41 11.363 11.865 11.995 11.937 11.933 11.939
   1242   1367988_at   11.929 11.901 12.045 11.765 11.874 12.506 12.612 12.617 12.427 12.561
   1243   1384151_at   6.212 6.262 6.287 6.321 6.193 5.353 5.344 5.659 5.479 5.245
   1244   1368504_at   11.472 11.506 11.456 11.436 11.577 11.866 11.926 11.815 11.948 11.846
   1245   1389310_at   6.963 6.746 6.835 6.844 6.767 8.062 7.731 8.295 7.933 7.871
   1246   1385019_at   5.967 6.21 6.29 5.736 5.814 4.241 4.077 4.326 4.479 4.663
   1247   1373502_at   8.185 8.13 8.164 8.236 8.18 8.556 8.443 8.58 8.591 8.51
   1248   1371613_at   8.943 8.926 9.009 8.783 8.864 8.262 8.045 8.215 8.054 7.927
   1249   1369801_at   8.42 8.46 8.56 8.398 8.508 5.326 3.402 5.133 4.083 4.664
   1250   1380410_at   9.265 9.28 9.081 9.162 9.464 7.711 7.516 7.354 6.993 6.82
   1251   1379356_at   8.496 8.578 8.528 8.615 8.732 7.548 7.341 7.417 7.083 6.94
   1252   1385072_at   9.448 9.425 9.302 9.334 9.076 10.348 10.119 10.419 10.514 10.354
   1253   1369208_at   7.09 7.14 7.244 7.19 7.056 5.948 5.701 5.568 5.391 5.124
   1254   1371388_at   10.215 10.309 10.37 10.402 10.275 11.036 10.78 10.96 10.914 11.009
   1255   1391331_at   6.69 6.59 6.759 6.697 6.551 7.716 7.601 8.156 8.061 7.969
   1256   1398366_at   6.082 5.93 6.037 6.043 6.039 6.714 6.819 6.675 6.627 6.504
   1257   1383448_at   8.991 8.96 8.984 9.084 9.179 7.712 6.927 6.893 7.221 6.77
   1258   1381776_at   6.674 7.009 6.35 7.127 7.394 9.211 9.25 9.509 9.072 8.936
   1259   1388555_at   10.044 10.028 10.335 10.252 10.027 10.93 11.058 11.073 11.208 10.955
   1260   1378150_at   7.989 7.902 7.92 8.062 8.069 5.92 5.563 4.747 5.676 5.912
   1261   1387932_at   7.646 7.723 8.095 8.122 7.677 9.118 9.143 9.451 9.481 9.268
   1262   1387926_at   8.164 8.201 8.328 8.063 7.893 9.052 9.009 8.953 9.069 8.916
   1263   1373736_at   5.327 5.328 4.914 5.314 5.224 6.389 6.669 6.915 6.918 6.524
   1264   1397830_at   7.256 7.155 7.475 7.207 7.242 8.3 8.232 8.239 8.664 8.505
   1265   1387925_at   8.375 8.466 8.467 8.551 8.545 7.568 7.705 7.682 7.412 7.292
   1266   1372030_at   8.745 8.689 8.961 8.814 8.806 9.537 9.514 9.537 9.463 9.325
   1267   1370469_at   8.076 7.95 8.292 7.952 7.879 6.045 5.62 4.788 4.983 4.985
   1268   1370207_at   10.938 10.938 10.979 11.112 11.114 11.66 11.583 11.609 11.694 11.834
   1269   1368509_at   7.362 7.09 7.17 7.235 6.668 8.427 8.438 8.658 8.632 8.729
   1270   1375926_at   7.565 7.402 7.757 7.657 7.201 8.907 8.63 8.669 8.71 8.838
   1271   1373585_at   9.833 9.864 9.986 9.984 9.83 10.458 10.357 10.445 10.439 10.314
   1272   1382384_at   5.894 6.158 5.934 6.035 5.815 6.804 6.751 6.853 6.682 6.963
   1273   1373181_at   5.881 5.843 6.003 5.986 5.991 6.712 6.588 6.728 6.82 6.992
   1274   1372199_at   7.119 7.158 7.314 7.533 7.149 8.423 8.188 8.568 8.624 8.545
   1275   1368085_at   8.269 8.414 8.52 8.72 8.386 9.512 9.327 9.539 9.671 9.639
   1276   1374857_at   7.921 7.999 8.006 8.228 8.181 7.221 7.085 7.162 6.832 6.952
   1277   1388694_at   10.031 9.898 9.616 9.776 10.016 8.019 7.479 6.96 7.363 7.932
   1278   1373557_at   8.05 8.241 8.008 8.096 8.045 7.104 7.107 7.002 6.633 7.091
   1279   1373515_at   8.019 7.998 8.145 8.074 7.92 8.703 9.011 8.832 8.814 8.677
   1280   1390738_at   11.653 11.625 11.69 11.762 11.843 10.221 9.234 9.307 9.531 9.867
   1281   1373854_at   8.324 8.517 8.456 8.572 8.575 9.257 9.221 9.547 9.43 9.271
   1282   1368371_at   6.825 6.843 6.984 6.628 6.72 7.542 7.774 7.788 7.918 7.873
   1283   1383826_at   5.08 5.683 5.622 5.692 5.502 6.934 6.733 7.112 7.01 6.988
   1284   1372604_at   8.869 8.587 8.597 8.826 9.042 6.808 5.355 6.033 5.983 5.743
   1285   1372643_at   9.085 9.193 9.396 9.515 9.18 8.299 7.882 7.967 8.052 7.818
   1286   1374800_at   8.396 8.501 8.461 8.806 8.407 9.459 9.469 9.767 9.523 9.543
   1287   1373434_at   7.291 7.379 7.189 7.461 7.233 7.855 7.996 7.936 7.884 7.915
   1288   1374725_at   8.007 7.979 7.742 8.095 8.183 6.734 6.28 6.646 6.235 6.084
   1289   1389907_at   9.402 9.436 9.388 9.46 9.452 9.755 9.667 9.813 9.726 9.793
   1290   1388397_at   9.106 9.051 8.962 9.167 9.353 8.295 8.334 8.242 8.105 8.077
   1291   1390020_at   10.638 10.707 10.73 10.74 10.601 11.428 11.482 11.201 11.514 11.559
   1292   1391228_at   4.09 3.406 3.466 3.654 3.754 5.702 5.141 5.232 5.458 5.455
   1293   1368548_at   9.829 9.899 10.003 9.982 10.292 10.942 10.912 11.057 10.866 11.04
   1294   1395248_at   7.972 8.192 8.174 8.159 8.102 7.235 7.274 7.123 6.976 6.815
   1295   1368178_at   10.465 10.509 10.729 10.712 10.446 11.258 11.27 11.302 11.235 11.245
   1296   1391411_at   10.112 10.233 10.252 10.228 10.136 9.508 9.366 9.488 9.466 9.147
   1297   1370769_a_at   7.282 7.178 7.819 7.335 7.107 4.524 4.987 4.011 3.856 4.916
   1298   1387763_at   6.321 6.359 6.728 6.49 6.448 4.979 4.93 4.455 4.537 4.165
   1299   1370974_at   8.477 8.489 8.315 8.621 8.543 7.81 7.892 7.87 7.883 7.951
   1300   1372058_at   9.018 9.064 8.982 9.091 8.981 8.733 8.596 8.584 8.669 8.612
   1301   1385656_at   10.116 10.255 10.396 10.375 10.22 11.017 11.011 11.043 10.945 10.839
   1302   1378197_at   6.611 6.628 6.891 6.687 6.255 7.877 7.968 8.125 8.39 8.162
   1303   1370384_a_at   7.597 7.306 7.757 8.097 7.188 9.558 9.877 10.448 10.154 9.972
   1304   1378307_at   6.45 6.669 7.033 6.915 6.567 7.939 8.154 8.364 8.298 8.109
   1305   1371417_at   11.58 11.607 11.651 11.389 11.553 12.099 12.054 12.033 12.074 12.059
   1306   1375989_a_at   8.148 8.226 7.691 7.932 8.152 6.559 6.813 6.436 6.342 6.212
   1307   1368882_at   7.698 7.653 7.641 7.574 7.333 8.443 8.695 8.771 8.757 8.478
   1308   1383601_at   7.376 7.578 7.482 7.511 7.479 6.091 5.646 6.04 5.273 5.476
   1309   1379419_at   7.132 7.293 7.328 7.095 7.493 8.468 8.864 8.582 8.613 8.292
   1310   1376784_at   6.84 6.455 6.783 6.733 6.623 5.106 4.938 5.119 4.962 5.616
   1311   1375585_at   8.251 8.327 8.201 8.469 8.526 7.413 6.942 7.351 7.24 7.154
   1312   1398325_at   10.065 10.038 9.999 10.147 10.203 10.789 10.59 10.701 10.629 10.607
   1313   1393729_at   6.089 6.009 5.65 6.046 5.777 7.028 6.888 7.124 6.916 6.907
   1314   1382325_at   7.343 7.436 7.234 7.51 7.394 8.406 8.093 8.254 8.328 8.519
   1315   1398500_at   6.92 6.962 6.825 7.375 7.02 8.031 8.099 8.207 8.067 8.106
   1316   1368467_at   9.775 9.857 9.839 10.146 9.503 11.043 11.17 11.048 11.412 11.157
   1317   1387667_at   7.823 7.781 8.249 8.104 7.556 1.144 3.493 1.692 0 1.59
   1318   1374601_at   9.555 9.749 9.644 9.608 9.68 8.489 8.289 8.277 8.14 7.712
   1319   1387742_at   8.082 8.22 7.884 7.858 8.053 5.194 4.407 4.071 3.063 4.277
   1320   1374397_at   10.891 10.833 10.887 10.818 10.901 10.19 10.392 10.192 10.034 10.104
   1321   1399118_at   7.328 7.125 7.164 7.383 7.185 6.403 6.217 5.995 6.085 6.371
   1322   1370130_at   10.43 10.446 10.528 10.56 10.429 10.127 9.992 9.985 10.077 9.971
   1323   1383368_at   9.538 9.463 9.399 9.423 9.396 8.984 8.887 8.978 8.777 8.793
   1324   1387866_at   8 7.697 7.843 7.958 7.933 6.863 6.825 7.028 6.996 6.595
   1325   1372398_at   6.654 6.77 6.682 6.514 6.57 7.407 7.427 7.674 7.678 7.785
   1326   1372104_at   8.489 8.476 8.347 8.631 8.481 9.413 9.382 9.767 9.529 9.316
   1327   1393116_at   5.775 5.931 5.724 5.727 5.768 6.457 6.492 6.714 6.479 6.395
   1328   1369306_at   6.207 6.358 6.584 6.189 5.798 4.347 3.745 3.835 4.108 4.507
   1329   1367752_at   7.675 7.639 7.757 7.946 7.531 8.582 8.427 8.537 8.598 8.621
   1330   1368103_at   9.064 9.095 9.022 9.464 9.212 7.859 7.561 8.01 7.858 7.455
   1331   1379311_at   7.463 7.539 7.451 7.659 7.71 5.579 4.963 4.089 4.418 4.605
   1332   1371803_at   9.655 9.54 9.651 9.535 9.587 8.95 9.07 9.03 8.899 8.763
   1333   1368187_at   11.443 11.524 11.739 11.636 11.558 9.54 9.337 9.349 9.093 8.289
   1334   1372502_at   7.71 7.522 7.766 7.886 7.552 8.64 8.563 8.877 8.555 8.666
   1335   1379719_at   6.693 6.755 6.602 6.648 6.578 7.272 7.3 7.484 7.25 7.522
   1336   1374508_at   8.624 8.562 8.773 8.676 8.68 9.169 9.142 9.327 9.144 9.191
   1337   1389456_at   6.488 6.398 6.561 6.553 6.393 7.088 7.12 7.08 7.361 7.185
   1338   1369471_at   6.529 6.284 6.558 6.453 6.26 5.498 5.012 5.047 5.249 5.328
   1339   1382730_at   6.815 7.049 6.654 6.841 6.71 5.402 5.47 5.695 5.82 5.302
   1340   1394407_at   7.145 7.128 7.015 7.277 7.172 7.717 7.695 7.811 7.651 7.651
   1341   1396267_at   7.644 7.838 7.818 7.805 7.683 6.977 6.58 6.91 6.74 6.568
   1342   1371494_at   7.298 7.384 7.146 7.407 7.361 8.06 8.05 8.294 8.313 8.055
   1343   1392032_at   6.665 6.679 6.897 6.816 6.549 5.677 5.189 5.286 5.665 5.49
   1344   1386277_at   8.527 8.494 8.421 8.577 8.747 6.996 6.313 5.793 6.157 6.431
   1345   1389477_at   7.52 7.542 7.564 7.869 7.589 6.616 6.633 6.357 6.25 6.201
   1346   1376051_at   9.891 9.887 10.053 9.973 9.584 10.885 10.753 10.949 11.076 11.032
   1347   1368653_a_at   10.919 10.898 10.942 11.001 11.031 11.253 11.348 11.346 11.285 11.278
   1348   1390918_at   8.48 8.426 8.623 8.701 8.46 9.193 9.195 9.412 9.358 9.262
   1349   1368552_at   9.706 9.648 9.759 9.796 9.774 10.109 10.173 10.225 10.273 10.12
   1350   1388994_at   6.208 6.555 6.042 6.273 6.424 7.439 7.48 7.547 7.24 7.368
   1351   1379587_at   5.756 5.831 5.694 6.014 5.417 6.912 6.826 6.971 7.087 7.175
   1352   1398818_at   5.939 6.11 6.171 5.995 6.127 6.817 6.785 6.718 6.639 6.606
   1353   1367622_at   11.838 11.776 11.76 11.694 11.828 12.065 12.112 12.122 12.079 12.15
   1354   1378244_at   3.121 2.793 4.039 3.257 3.609 0 0.227 0 1.007 0.084
   1355   1382221_at   6.034 6.205 6.209 6.519 6.035 7.373 7.373 7.582 7.53 7.843
   1356   1390508_at   7.452 7.604 7.515 7.566 7.419 8.163 8.151 8.313 8.001 8.185
   1357   1373803_a_at   7.22 7.182 7.401 7.34 6.94 8.099 8.272 8.249 8.351 8.122
   1358   1368000_at   11.62 11.643 10.641 10.98 11.521 8.746 8.059 7.54 7.622 7.41
   1359   1371027_at   7.477 7.548 7.794 7.923 7.764 6.184 6.099 6.679 6.185 6.194
   1360   1378482_at   7.839 7.831 7.87 7.751 7.905 7.178 6.942 7.077 6.852 6.744
   1361   1386946_at   9.91 9.973 9.798 9.747 9.936 10.458 10.436 10.668 10.515 10.492
   1362   1375961_at   7.479 7.456 7.19 7.392 7.788 8.641 8.823 8.913 9.071 8.651
   1363   1382660_at   7.118 7.239 6.918 7.313 7.201 8.009 8.214 8.411 8.22 8.097
   1364   1388809_at   10.543 10.443 10.939 10.572 10.51 9.391 9.32 9.328 9.662 9.493
   1365   1375436_at   10.315 10.336 10.575 10.624 10.356 11.249 11.48 11.29 11.485 11.256
   1366   1373465_at   7.792 8.014 7.923 8.079 7.777 8.741 8.67 8.973 8.749 8.92
   1367   1379464_at   8.985 9.091 9.266 8.776 8.771 10.246 10.293 10.337 10.748 10.328
   1368   1388603_a_at   9.45 9.488 9.519 9.541 9.549 9.788 9.879 9.892 9.948 9.824
   1369   1388150_at   9.532 9.54 9.273 9.426 9.404 8.725 8.734 8.723 8.587 8.454
   1370   1379450_at   9.191 9.203 9.227 9.333 9.262 8.714 8.421 8.596 8.365 8.482
   1371   1371363_at   8.927 8.811 9.357 9.176 8.69 10.441 10.62 10.306 10.708 10.456
   1372   1385382_at   6.342 6.428 6.19 6.464 6.106 4.86 5.131 4.878 5.251 4.659
   1373   1388353_at   9.42 9.229 9.553 9.61 9.471 8.516 8.406 8.577 8.355 8.685
   1374   1382792_at   5.69 5.629 5.984 5.966 5.785 4.855 4.588 4.305 4.465 4.375
   1375   1374089_at   5.919 6.059 6.06 6.26 6.109 7.167 6.99 6.915 6.785 6.992
   1376   1388753_at   9.999 10.016 9.737 9.792 10.078 9.097 8.845 9.045 9.098 9.092
   1377   1373198_at   7.848 7.889 7.805 8.107 7.901 8.552 8.596 8.689 8.75 8.532
   1378   1392149_at   5.277 5.73 5.289 5.553 5.371 6.466 6.414 6.46 6.318 6.477
   1379   1372460_at   10.087 10.168 10.062 10.169 10.156 9.675 9.7 9.625 9.456 9.587
   1380   1380867_a_at   7.995 8.145 8.202 8.08 8.003 7.311 6.964 7.226 7.366 7.098
   1381   1372055_at   8.604 8.711 8.931 8.935 8.735 7.238 6.557 7.049 7.072 6.363
   1382   1391526_at   6.891 7.04 7.069 6.91 6.832 7.517 7.541 7.751 7.628 7.59
   1383   1369625_at   11.151 11.075 11.32 11.147 10.953 11.91 11.849 11.887 11.953 12.119
   1384   1394881_at   5.713 5.547 6.011 6.354 5.651 7.68 7.37 7.756 7.905 7.717
   1385   1387851_at   10.222 10.216 10.259 10.342 10.367 11.023 11.097 11.16 11.172 10.816
   1386   1380357_a_at   8.135 8.634 8.343 8.578 8.376 7.229 6.706 7.083 6.787 6.678
   1387   1383794_at   5.771 6.024 6.054 6.101 6.073 4.728 4.775 4.68 4.123 4.619
   1388   1395183_at   6.773 6.906 7.119 7.045 7.069 5.855 5.708 6.087 5.539 5.836
   1389   1384147_at   7.491 7.378 7.456 7.58 7.518 6.725 6.614 6.462 6.274 6.289
   1390   1371665_at   6.398 6.558 6.175 6.414 6.116 7.384 7.252 7.385 7.567 7.59
   1391   1372011_at   11.079 11.142 11.113 10.874 11.196 9.14 9.283 8.961 8.906 8.141
   1392   1386881_at   7.603 7.542 7.329 7.273 7.336 8.477 8.359 8.457 8.843 8.569
   1393   1384970_at   4.125 4.409 4.821 4.671 4.678 6.396 6.233 6.267 5.885 6.482
   1394   1387880_at   5.602 5.422 6.066 5.178 5.696 2.975 1.614 2.795 1.731 2.266
   1395   1388618_at   8.317 8.236 8.114 8.538 8.465 9.477 9.338 9.592 9.307 9.245
   1396   1368179_at   8.345 8.376 8.502 8.609 8.519 7.424 7.587 7.771 7.435 7.347
   1397   1373933_at   9.692 9.728 9.725 9.786 9.728 8.904 8.868 9.152 8.779 8.683
   1398   1387084_at   9.75 9.538 9.356 9.831 9.67 10.579 10.81 10.787 10.564 10.643
   1399   1386891_at   11.508 11.504 11.562 11.466 11.502 11.973 11.914 11.883 11.987 12.121
   1400   1367767_at   9.221 9.283 9.275 9.227 9.213 9.557 9.566 9.533 9.516 9.443
   1401   1369085_s_at   6.677 6.663 6.553 6.719 6.9 7.682 7.62 7.778 7.58 7.393
   1402   1367806_at   6.479 7.007 6.452 6.575 6.575 8.373 8.408 8.39 8.091 7.837
   1403   1372827_at   9.615 9.716 9.694 9.812 9.637 9.339 9.31 9.334 9.301 9.31
   1404   1390348_at   8.783 9 9.578 9.327 9.149 7.61 7.302 7.25 7.077 6.877
   1405   1377244_at   6.374 6.519 6.357 6.542 6.519 6.947 7.001 6.915 7.092 7.014
   1406   1382843_at   7.769 7.652 7.824 7.914 7.69 6.763 6.456 6.256 6.809 6.429
   1407   1374400_at   8.958 8.959 8.858 8.951 8.917 8.594 8.563 8.663 8.614 8.511
   1408   1373625_at   9.378 9.382 9.441 9.402 9.152 10.005 10.031 9.978 9.961 9.868
   1409   1386009_at   7.931 7.881 7.837 8.027 8.105 5.731 4.724 5.078 3.951 4.737
   1410   1368136_at   9.205 9.273 9.212 9.271 9.176 8.661 8.418 8.508 8.301 8.259
   1411   1376513_a_at   6.601 6.567 7.004 6.916 6.687 5.776 5.547 5.677 5.383 5.598
   1412   1388195_at   7.74 7.567 8.186 8.104 7.962 6.695 6.145 6.33 6.329 6.307
   1413   1375267_at   9.152 9.231 9.159 9.5 9.215 10.224 10.123 10.565 10.319 10.468
   1414   1377993_at   5.82 5.972 5.93 6.328 5.692 7.225 7.411 7.521 7.502 7.84
   1415   1368512_a_at   10.442 10.393 10.576 10.578 10.494 11.039 11.225 11.332 11.319 11.127
   1416   1394020_at   8.182 8.223 8.176 7.935 7.817 9.303 9.112 9.432 9.579 9.669
   1417   1382039_at   6.662 6.661 6.561 6.775 6.763 7.369 7.214 7.477 7.389 7.252
   1418   1371694_at   10.31 10.321 10.332 10.325 10.543 9.73 9.391 9.482 9.415 9.301
   1419   1370399_at   6.479 6.265 6.074 5.974 6.19 7.861 7.552 7.957 8.151 7.49
   1420   1368236_at   10.342 10.393 10.553 10.419 9.921 11.407 11.488 11.471 11.501 11.501
   1421   1390674_at   6.913 7.2 7.381 7.225 7.01 5.515 4.363 4.926 5.035 4.609
   1422   1390292_at   8.499 8.474 8.547 8.553 8.507 9.136 8.928 9.163 9.23 9.035
   1423   1395694_at   7.691 7.929 7.806 7.66 7.86 8.788 8.492 8.876 8.836 8.619
   1424   1370020_at   9.292 9.17 9.24 9.479 9.021 10.222 10.234 10.519 10.632 10.511
   1425   1369552_at   6.96 7.326 7.701 7.611 7.593 4.766 4.667 5.439 4.951 5.548
   1426   1367843_at   10.526 10.497 10.891 10.655 10.272 11.68 11.734 11.635 11.888 11.833
   1427   1371079_at   8.897 8.903 8.927 8.775 9.089 6.397 6.261 5.187 5.576 4.807
   1428   1392915_at   5.385 4.966 4.572 5.345 5.739 7.624 7.269 7.786 7.562 7.374
   1429   1367892_at   9.572 9.654 9.81 9.861 9.552 10.573 10.445 10.749 10.823 10.691
   1430   1374708_at   6.446 6.533 6.723 6.485 6.649 7.407 7.177 7.442 7.247 7.365
   1431   1372422_at   7.426 7.319 7.456 7.648 7.245 8.278 8.328 8.166 8.248 8.141
   1432   1390602_a_at   8.058 8.088 8.238 8.179 7.948 8.778 8.887 8.982 9.038 8.758
   1433   1385502_at   6.755 7.123 6.693 6.796 6.696 5.265 4.615 5.041 4.978 5.47
   1434   1394612_at   6.637 6.681 6.796 6.703 6.377 7.426 7.404 7.438 7.344 7.399
   1435   1382683_a_at   6.068 6.473 6.342 6.365 5.801 8.284 8.125 8.344 8.888 9.072
   1436   1393666_at   6.277 6.392 6.232 6.242 6.384 7.102 7.03 7.414 7.169 7.02
   1437   1374432_at   6.876 7.077 6.622 6.777 7.087 8.107 8.054 8.427 8.4 8.025
   1438   1393531_at   6.53 6.286 6.744 6.827 6.512 7.607 7.711 7.872 7.966 7.934
   1439   1398326_at   11.963 11.899 11.995 11.934 12.014 12.378 12.478 12.37 12.455 12.604
   1440   1368973_at   7.82 8.083 7.879 8.203 8.153 6.988 6.307 6.608 6.662 6.611
   1441   1388416_at   9.449 9.658 9.469 9.563 9.559 8.315 7.9 8.229 8.351 7.672
   1442   1397584_at   7.264 7.198 7.544 7.621 7.431 8.351 8.318 8.368 8.551 8.578
   1443   1373508_at   10.169 10.171 10.32 10.233 10.195 9.842 9.853 9.763 9.824 9.701
   1444   1368555_at   7.595 7.613 7.892 7.869 7.704 6.633 6.28 6.216 6.544 6.74
   1445   1371609_at   9.207 9.133 9.305 9.338 9.339 9.687 9.739 9.75 9.738 9.82
   1446   1388992_at   7.972 8.047 7.784 8.157 8.042 7.15 6.995 7.265 7.265 7.097
   1447   1388345_at   9.82 9.943 9.82 9.892 9.946 9.2 9.025 9.342 9.011 8.998
   1448   1383603_at   8.628 8.503 8.607 8.68 8.589 7.963 7.997 8.155 7.847 7.903
   1449   1398812_at   11.529 11.588 11.546 11.489 11.683 11.149 11.139 11.171 11.108 11.016
   1450   1370613_s_at   11.088 11.021 11.077 10.999 10.989 11.452 11.55 11.592 11.744 11.67
   1451   1389111_at   7.12 7.179 7.265 7.313 7.051 8.018 7.962 8.103 7.848 8.235
   1452   1393209_at   7.546 7.601 7.87 7.591 7.54 8.619 8.594 8.871 8.938 9.093
   1453   1382950_at   12.36 12.362 12.345 11.979 12.25 9.891 8.719 8.43 8.37 7.897
   1454   1386914_at   5.836 5.607 5.781 5.815 5.716 6.695 6.627 7.172 6.837 6.824
   1455   1393745_at   7.418 7.558 7.599 7.493 7.479 6.314 6.353 6.49 6.263 5.806
   1456   1372976_at   7.181 7.004 7.398 7.409 6.915 8.678 8.334 8.852 8.913 8.903
   1457   1396565_at   4.705 4.487 4.654 4.248 4.491 5.33 5.409 5.339 5.428 5.423
   1458   1373371_a_at   8.489 8.495 8.439 8.667 8.489 9.078 9.172 9.294 9.299 9.069
   1459   1393060_at   4.884 5.221 5.14 5.084 5.482 7.425 6.626 7.265 7.669 7.455
   1460   1377869_at   7.675 7.719 7.759 8.195 7.9 6.434 6.079 6.5 6.12 6.623
   1461   1382999_at   4.491 5.183 5.126 5.227 5.164 3.441 2.996 2.646 2.648 2.934
   1462   1382331_at   9.597 9.345 9.37 9.671 9.352 10.372 10.387 10.219 10.235 10.236
   1463   1387803_at   5.407 5.231 5.185 5.383 5.174 6.298 6.252 6.573 6.81 6.397
   1464   1368573_at   8.367 8.522 8.312 8.542 8.511 7.397 7.182 7.217 6.692 6.957
   1465   1383248_at   8.03 7.885 7.888 7.987 7.791 8.651 8.677 8.851 9.04 8.721
   1466   1393673_at   4.033 3.597 4.024 3.944 3.343 5.381 5.729 6.041 6.209 5.954
   1467   1373970_at   9.388 9.325 9.176 9.305 8.91 10.192 10.31 10.335 10.252 10.078
   1468   1370290_at   10.895 10.869 10.798 10.898 10.933 10.098 10.071 9.836 9.618 9.737
   1469   1387121_a_at   7.884 8.038 8.112 8.26 8.019 9.344 8.893 9.4 9.41 9.402
   1470   1393070_at   6.266 5.897 6.287 6.378 6.094 7.293 7.065 7.328 7.434 7.393
   1471   1393061_at   8.1 8.14 8.302 8.369 7.747 9.298 9.321 9.523 9.644 9.556
   1472   1393281_at   7.111 7.036 7.383 7.312 6.949 8.178 8.1 8.141 8.281 8.004
   1473   1391955_at   6.695 6.415 6.524 6.46 6.527 5.35 4.453 4.757 4.898 5.027
   1474   1383137_at   8.278 8.285 7.941 8.34 8.554 9.589 9.382 9.577 9.526 9.294
   1475   1389650_at   6.235 6.271 6.347 6.518 6.425 7.049 7.246 7.195 7.299 7.466
   1476   1371015_at   10.642 10.638 10.172 10.341 10.666 7.812 6.528 5.575 6.54 6.704
   1477   1397655_at   6.579 7.007 6.696 7.132 6.785 5.459 5.309 4.704 4.999 4.957
   1478   1368267_at   6.575 6.627 6.728 6.745 6.701 7.127 7.224 7.18 7.238 7.052
   1479   1373679_at   7.52 7.419 7.699 7.605 7.457 6.662 6.441 6.639 6.589 6.204
   1480   1393217_at   8.568 8.711 8.515 8.546 8.824 7.568 7.609 7.364 7.295 7.021
   1481   1370082_at   8.602 8.351 8.795 8.684 8.622 7.705 7.505 7.47 7.409 7.189
   1482   1372110_at   7.384 7.355 7.432 7.605 7.194 8.361 8.302 8.675 8.669 8.385
   1483   1385831_at   5.444 5.105 5.681 5.786 5.739 3.941 3.566 3.99 3.569 3.306
   1484   1396492_at   7.075 7.551 7.638 7.542 7.077 4.441 2.909 3.704 2.526 2.452
   1485   1391669_at   5.947 6.229 6.156 6.066 6.038 7.105 6.95 7.421 7.091 6.964
   1486   1372395_at   9.132 9.144 9.332 9.293 9.169 9.874 10.011 10.053 9.982 9.746
   1487   1367864_at   7.601 7.7 7.66 7.948 7.877 8.646 8.553 8.773 8.941 8.832
   1488   1373011_at   9.244 9.231 9.367 9.33 9.224 9.959 10.061 10.149 10.177 9.816
   1489   1371984_at   4.258 4.465 4.116 4.695 4.589 5.833 5.66 5.64 5.763 5.5
   1490   1372872_at   6.128 6.11 6.208 6.227 5.929 7.302 7.326 7.795 7.493 7.133
   1491   1372475_at   8.714 8.804 8.755 8.72 8.393 9.654 9.563 9.767 9.962 9.919
   1492   1384056_at   6.208 6.332 6.313 6.45 6.568 7.83 7.746 7.861 7.445 7.344
   1493   1368877_at   7.78 7.777 8.229 7.789 7.662 8.806 8.963 9.061 9.045 9.003
   1494   1368545_at   7.775 7.882 7.715 7.91 7.586 7.074 6.986 7.177 7.04 6.962
   1495   1371821_at   10.09 10.14 10.091 10.244 10.193 9.759 9.676 9.743 9.782 9.622
   1496   1383945_at   6.952 7.433 7.13 7.177 7.505 6.205 6.123 6.176 6.076 5.998
   1497   1372190_at   7.483 7.522 7.421 7.339 7.72 8.492 8.202 8.483 8.598 8.5
   1498   1387859_at   10.168 10.081 10.012 10.018 9.958 10.456 10.502 10.581 10.448 10.546
   1499   1374217_at   6.911 7.009 7.015 7.239 6.612 8.135 8.055 8.197 8.206 8.437
   1500   1375307_at   7.326 7.497 7.413 7.618 7.534 8.476 8.198 8.639 8.646 8.432
   1501   1388470_at   7.891 7.855 8.023 8.051 7.675 9.025 8.878 8.932 8.729 8.693
   1502   1373615_at   7.637 7.622 7.703 7.499 8.05 8.707 8.782 8.859 9.033 8.769
   1503   1368975_at   9.474 9.525 9.606 9.557 9.562 6.576 4.71 4.346 4.225 4.102
   1504   1388734_at   8.029 8.192 8.146 8.223 8.125 9.084 8.829 9.341 9.089 8.999
   1505   1371903_at   7.01 7.179 7.448 7.182 7.1 8.033 7.987 8.03 8.236 8.046
   1506   1391637_at   8.115 8.159 8.181 8.254 8.422 6.927 6.414 6.413 6.678 5.968
   1507   1388142_at   7.192 7.141 6.828 7.393 7.688 5.511 5.227 5.357 4.762 5.392
   1508   1383453_at   8.17 8.352 8.11 8.249 8.238 8.972 9.247 9.327 9.174 8.928
   1509   1384301_at   6.339 6.068 5.873 5.932 6.055 7.272 7.364 7.897 7.712 7.859
   1510   1373345_at   7.798 7.825 7.617 7.787 8.352 6.522 6.519 6.636 6.546 6.391
   1511   1369930_at   11.827 11.775 11.686 11.59 11.701 11.074 11.143 11.186 10.988 10.923
   1512   1368329_at   9.273 9.15 9.511 9.289 8.828 10.496 10.678 10.887 11.125 10.81
   1513   1387947_at   7.757 7.69 8.17 8.281 8.26 6.605 5.946 6.231 5.937 6.266
   1514   1371482_at   10.373 10.354 10.322 10.466 10.513 10.799 10.789 10.846 10.817 10.766
   1515   1374221_at   7.95 7.972 7.943 8.277 7.924 8.805 8.716 9.003 8.902 8.877
   1516   1392978_at   8.825 8.78 8.743 8.88 9.016 8.118 7.759 7.877 8.11 8.02
   1517   1382013_at   8.138 8.258 8.246 8.018 8.046 8.858 8.74 8.742 8.973 8.794
   1518   1384936_at   4.498 4.494 4.274 4.223 4.737 5.387 5.583 5.544 5.711 5.489
   1519   1373519_at   7 6.925 7.149 7.171 6.946 8.063 7.758 8.164 7.908 7.866
   1520   1367926_at   7.608 7.89 7.774 7.72 7.798 8.36 8.39 8.28 8.249 8.414
   1521   1369948_at   9.011 9.037 9.083 9.248 9.2 9.959 9.774 10.088 10.212 10.109
   1522   1387975_at   6.919 6.983 7.248 7.148 7.028 5.854 5.339 5.726 5.065 5.504
   1523   1383199_at   7.355 7.452 7.435 7.628 7.37 6.611 6.325 6.297 6.652 6.232
   1524   1393114_at   8.556 8.6 8.407 8.526 8.654 7.645 7.739 7.948 7.825 7.576
   1525   1388558_at   10.638 10.615 10.721 10.741 10.718 11.22 11.063 11.23 11.256 11.088
   1526   1387800_at   8.187 8.088 8.034 8.128 7.877 6.66 5.906 6.702 6.266 6.577
   1527   1398272_at   8.322 8.31 8.603 8.454 8.233 7.659 7.468 7.576 7.674 7.65
   1528   1371484_at   8.981 8.896 8.8 8.986 8.974 8.136 8.107 8.209 8.43 8.105
   1529   1375346_at   8.221 8.269 8.29 8.435 8.138 8.854 8.899 8.997 8.893 9.096
   1530   1388476_at   8.881 8.792 8.881 8.938 8.959 8.398 8.349 8.46 8.455 8.244
   1531   1372904_at   8.402 8.374 8.563 8.751 8.727 7.448 6.741 7.069 6.935 6.739
   1532   1372016_at   7.986 8.091 7.882 8.188 8.042 6.437 6.524 6.755 5.964 5.965
   1533   1382624_at   5.868 6.106 6.151 5.914 6.334 5.038 4.442 4.768 4.714 4.823
   1534   1380507_at   5.374 5.051 5.215 5.224 4.819 6.086 6.335 6.469 6.292 6.314
   1535   1390049_at   6.456 6.296 6.327 5.976 6.04 7.559 7.361 7.351 7.35 7.162
   1536   1386716_at   6.793 6.899 6.643 6.405 6.354 5.355 5.099 4.812 5.037 5.317
   1537   1373280_at   9.291 9.229 9.046 9.237 9.134 8.341 8.363 8.407 8.308 7.98
   1538   1387995_a_at   11.555 11.565 11.437 11.587 11.701 10.984 10.623 10.756 10.723 10.876
   1539   1377523_at   6.337 6.011 6.245 6.282 5.928 6.988 7.183 7.077 6.996 7.12
   1540   1389658_at   9.021 9.1 9.123 9.213 9.407 8.424 8.317 8.41 8.15 8.336
   1541   1371646_at   10.039 10.078 10.076 10.207 10.378 9.499 9.179 9.279 9.22 9.195
   1542   1376055_at   8.366 8.253 8.311 8.228 8.31 7.306 7.286 7.573 6.978 7.315
   1543   1369617_at   9.264 9.152 9.238 9.123 8.891 8.329 8.017 7.924 7.835 7.872
   1544   1380110_at   9.031 9.026 9.058 8.968 9.135 7.74 7.538 7.914 7.613 7.078
   1545   1374200_at   8.436 8.543 8.75 8.698 8.408 9.444 9.251 9.479 9.339 9.413
   1546   1388465_at   7.59 7.577 7.468 7.594 7.489 8.31 8.134 8.516 8.404 8.189
   1547   1394275_at   8.345 8.185 8.041 8.049 8.406 7.346 7.318 7.482 7.35 7.406
   1548   1386120_at   4.227 4.087 5.015 4.134 3.932 6.763 7.012 6.475 6.938 6.282
   1549   1373207_at   8.082 8.305 8.002 8.012 8.262 7.532 7.425 7.411 7.413 7.415
   1550   1382689_at   9.109 8.953 9.142 9.074 9.149 9.54 9.635 9.641 9.604 9.472
   1551   1390127_at   4.14 4.999 4.458 4.681 4.622 5.95 6.14 6.095 6.03 6.127
   1552   1389520_at   10.748 10.696 10.887 10.888 10.783 10.383 10.265 10.249 10.171 10.185
   1553   1385276_a_at   9.034 9.138 8.747 8.876 9.168 6.119 3.622 4.469 4.652 4.07
   1554   1373876_at   9.479 9.492 9.482 9.55 9.535 8.874 8.73 8.981 8.938 8.641
   1555   1367706_at   11.674 11.63 11.71 11.532 11.49 12.14 12.098 12.049 12.048 12.166
   1556   1367948_a_at   8.282 8.347 7.901 8.141 8.126 9.102 8.955 9.277 9.158 9.081
   1557   1388134_at   10.726 10.708 10.634 10.743 10.847 10.332 10.303 10.309 10.297 10.178
   1558   1380828_at   2.918 2.791 2.816 3.079 2.406 4.472 5.003 4.901 4.251 4.532
   1559   1368332_at   12.834 12.743 12.595 12.593 12.714 10.025 8.604 8.27 8.193 7.624
   1560   1380321_at   6.668 6.59 6.845 6.708 6.502 7.291 7.435 7.249 7.344 7.366
   1561   1397548_at   7.162 6.894 6.745 6.986 6.843 5.917 5.85 5.364 5.677 5.726
   1562   1371505_at   10.903 10.893 10.751 10.897 10.963 10.429 10.363 10.419 10.261 10.239
   1563   1384457_at   7.876 7.924 7.684 7.962 8.036 6.671 6.606 6.605 5.982 6.291
   1564   1373991_at   9.964 9.99 10.389 10.215 9.746 11.163 11.332 11.197 11.369 11.245
   1565   1382100_at   7.566 7.717 7.707 7.84 7.705 8.43 8.23 8.513 8.311 8.425
   1566   1375922_at   8.176 8.15 8.229 8.272 8.208 8.502 8.623 8.566 8.498 8.504
   1567   1385605_at   6.525 7.016 6.399 6.502 6.751 3.149 2.104 3.838 3.747 3.432
   1568   1398909_at   10.481 10.37 10.421 10.37 10.49 10.92 11.136 10.883 11.03 10.911
   1569   1388799_at   7.477 7.556 7.308 7.518 7.656 8.345 8.27 8.607 8.393 8.255
   1570   1386379_at   7.395 7.463 7.293 7.331 7.496 6.428 6.472 6.198 6.391 5.919
   1571   1398354_at   6.71 6.71 6.719 6.754 6.878 7.816 7.525 7.776 7.647 7.386
   1572   1371912_at   10.952 10.916 11.02 10.83 10.83 11.331 11.322 11.271 11.392 11.356
   1573   1391652_at   9.683 9.458 9.954 9.66 9.274 10.721 10.813 10.929 10.885 10.988
   1574   1386926_at   9.849 9.472 9.879 9.903 9.877 9.015 8.807 8.855 8.79 8.881
   1575   1371567_at   11.375 11.273 11.381 11.464 11.24 11.793 11.813 11.842 11.859 11.961
   1576   1371763_at   9.85 9.788 10.061 10.023 9.355 11.11 11.092 11.314 11.358 11.39
   1577   1371641_at   10.447 10.439 10.388 10.555 10.568 10.044 9.947 10.104 9.943 9.997
   1578   1377250_at   7.901 7.811 7.681 7.956 7.626 7.072 7.111 7.124 7.158 7.178
   1579   1385252_at   8.522 8.631 8.621 8.355 8.753 7.229 7.074 7.202 7.105 6.478
   1580   1384060_at   9.202 9.325 9.454 9.484 9.326 8.759 8.686 8.55 8.641 8.462
   1581   1398806_at   9.706 9.679 9.769 9.916 9.887 9.297 9.33 9.256 9.228 9.235
   1582   1388340_at   9.767 9.739 9.878 9.688 9.619 8.652 8.229 8.349 7.86 7.923
   1583   1375756_at   7.746 7.987 8.17 7.938 7.994 9.007 8.776 8.818 8.995 9.156
   1584   1394070_at   6.368 6.435 6.824 6.618 6.555 7.928 7.633 8.246 8.042 7.75
   1585   1390289_at   6.91 7.131 7.147 7.159 7.004 7.782 7.682 7.573 7.716 7.644
   1586   1377877_at   5.005 5.317 4.932 5.093 5.649 6.8 6.447 6.936 6.808 6.813
   1587   1372536_at   7 6.896 6.544 7.248 6.709 8.385 8.283 8.137 8.487 8.147
   1588   1396112_at   7.263 7.279 7.41 7.453 7.238 8.026 7.996 8.085 8.02 7.801
   1589   1370818_at   9.685 9.658 9.866 9.577 9.27 10.546 10.673 10.778 10.813 10.756
   1590   1368271_a_at   5.288 4.954 4.544 5.133 4.891 6.908 6.485 7.419 7.199 6.98
   1591   1390704_at   6.452 6.426 6.497 6.432 6.507 5.381 5.681 5.728 5.257 5.601
   1592   1377116_at   8.123 8.388 8.09 8.596 8.56 6.963 6.424 6.971 6.793 6.982
   1593   1383063_a_at   7.381 7.442 7.675 7.447 7.786 8.976 8.7 9.178 9.446 8.928
   1594   1380619_at   7.62 7.77 7.829 7.579 7.643 6.424 6.063 6.423 6.532 5.824
   1595   1376858_at   6.818 6.545 6.391 6.481 6.777 7.853 7.766 8.32 8.337 8.026
   1596   1367507_at   8.624 8.464 8.454 8.42 8.331 7.688 7.638 7.497 7.88 7.635
   1597   1372170_at   11.612 11.587 11.774 11.716 11.524 12.263 12.322 12.274 12.478 12.54
   1598   1368476_at   6.35 6.237 6.222 6.263 6.388 7.16 6.841 7.296 7.165 7.156
   1599   1387036_at   6.879 6.794 6.922 6.783 6.543 8.169 7.929 8.6 7.984 8.373
   1600   1367933_at   9.099 8.963 9.147 9.108 9.059 9.516 9.503 9.583 9.47 9.416
   1601   1368471_at   5.973 6.159 6.073 5.83 5.757 7.115 7.005 6.99 6.901 7.375
   1602   1386973_a_at   7.596 7.868 8.111 8.182 7.885 9.052 8.997 9.141 9.332 9.278
   1603   1374199_at   6.52 6.479 6.5 6.424 6.715 7.511 7.281 7.395 7.185 7.214
   1604   1367838_at   8.663 8.641 9.033 8.888 8.213 10.206 10.043 10.256 10.253 10.449
   1605   1389196_at   7.766 7.909 8.118 7.992 7.811 8.549 8.663 8.658 8.553 8.606
   1606   1371409_at   9.822 9.795 9.891 9.894 9.906 10.253 10.151 10.342 10.258 10.318
   1607   1378315_at   4.986 4.918 4.503 4.475 4.328 6.11 6.676 6.977 6.616 6.512
   1608   1374150_at   7.887 7.903 7.99 7.982 7.994 7.621 7.488 7.497 7.378 7.443
   1609   1388483_at   9.284 9.215 9.308 9.405 9.195 10.056 9.934 9.94 9.911 9.741
   1610   1372358_at   6.979 6.619 6.819 7.064 6.818 7.939 7.732 8.009 7.871 7.688
   1611   1377819_at   7.01 6.945 7.028 7.112 7.1 6.395 6.343 6.444 6.372 6.082
   1612   1374628_at   10.093 10.12 10.241 10.283 9.876 10.878 10.971 11.041 10.96 11.181
   1613   1368132_at   9.601 9.613 9.59 9.522 9.444 10.238 10.469 10.423 10.333 10.09
   1614   1384393_at   6.508 6.825 6.814 6.792 6.953 4.684 5.126 4.849 4.316 3.96
   1615   1388528_at   9.874 9.907 9.876 10.006 10.14 8.976 8.886 9.098 8.606 8.603
   1616   1370907_at   9.642 9.601 9.819 9.778 9.395 10.835 10.425 10.733 10.818 10.766
   1617   1370258_at   8.408 8.424 8.364 8.456 8.552 7.654 7.583 7.635 7.475 7.179
   1618   1372828_at   8.202 8.307 8.21 8.456 8.331 8.8 8.848 8.74 8.814 8.844
   1619   1368834_at   8.738 8.765 8.769 8.818 8.764 8.417 8.509 8.54 8.403 8.413
   1620   1377969_at   6.12 5.991 6.508 6.484 5.802 7.735 7.502 7.729 7.67 7.875
   1621   1377299_at   8.327 8.3 8.244 8.512 8.487 7.607 7.311 7.506 7.125 7.339
   1622   1381014_at   8.098 8.067 7.891 8.381 8.48 6.686 5.773 6.125 6.419 6.402
   1623   1373017_at   11.301 11.281 11.166 11.263 11.264 11.65 11.639 11.689 11.732 11.854
   1624   1368774_a_at   7.61 7.478 7.639 7.731 7.35 8.581 8.625 8.914 9.096 9.087
   1625   1367676_at   10.85 10.853 10.921 10.791 10.825 9.701 9.285 9.278 8.86 8.802
   1626   1396000_at   7.263 7.076 7.331 7.314 6.753 8.281 8.408 8.282 8.591 8.35
   1627   1393587_a_at   8.256 8.421 8.5 8.561 8.498 9.107 9.094 9.181 9.137 8.961
   1628   1367732_at   9.692 9.703 9.781 9.799 9.919 9.108 9.11 9.069 8.921 8.769
   1629   1371899_at   8.186 8.122 8.29 8.179 8.158 8.822 8.76 8.913 8.84 8.6
   1630   1372882_at   7.664 7.565 7.81 7.536 7.766 8.232 8.297 8.396 8.473 8.419
   1631   1370247_a_at   7.514 7.813 7.75 7.464 7.892 8.893 8.732 8.628 8.634 8.591
   1632   1368137_at   7.6 7.546 7.783 7.959 7.541 8.652 8.446 8.6 8.679 8.621
   1633   1398249_at   8.546 8.607 8.658 8.652 8.703 9.116 9.007 9.167 9.14 8.989
   1634   1398769_at   10.14 9.943 9.861 10.066 10.255 9.239 8.857 9.14 8.936 8.863
   1635   1391463_at   9.232 9.282 9.313 9.46 9.509 8.339 7.728 8.233 8.011 7.757
   1636   1373850_at   8.12 8.08 8.323 8.177 8.047 7.4 7.249 6.875 7.124 7.102
   1637   1377634_at   8.402 8.21 8.333 8.442 8.495 7.46 7.237 7.452 7.19 6.912
   1638   1370963_at   7.638 7.535 7.446 7.505 7.783 6.481 5.795 6.124 5.846 5.64
   1639   1378138_at   6.239 6.043 6.134 6.63 6.263 7.293 7.424 7.544 7.444 7.262
   1640   1398783_at   9.954 9.919 9.983 9.866 9.78 10.318 10.329 10.255 10.374 10.287
   1641   1390782_at   5.078 5.182 5.096 4.933 5.119 5.992 5.853 6.322 6.351 6.102
   1642   1395862_at   5.791 5.862 5.678 6.119 5.858 4.806 4.941 4.937 4.568 4.932
   1643   1387015_at   7.981 7.696 7.81 7.824 8.024 8.823 8.534 8.737 8.794 8.936
   1644   1381593_x_at   12.123 12.094 11.905 12.021 11.915 10.734 10.691 10.168 10.803 10.896
   1645   1386876_at   5.471 5.391 5.503 5.693 5.497 6.629 6.167 6.387 6.507 6.429
   1646   1387913_at   4.56 4.728 4.964 4.715 4.322 5.792 5.684 5.914 5.843 5.997
   1647   1371888_at   10.048 10.119 9.873 9.93 10.186 9.388 9.481 9.405 9.432 9.356
   1648   1389015_at   8.172 8.316 8.557 8.364 8.203 9.039 9.049 9.201 9.102 9.019
   1649   1385869_at   8.591 8.672 8.635 8.652 8.613 7.758 7.541 7.592 7.135 7.221
   1650   1368314_at   8.017 7.965 7.746 7.958 8.14 8.907 8.853 8.795 8.739 8.612
   1651   1376613_at   8.608 8.679 8.804 8.776 8.704 8.222 7.969 8.144 7.951 7.953
   1652   1375106_at   8.825 8.709 8.939 8.943 8.563 9.835 9.618 9.633 9.671 9.549
   1653   1385361_at   6.748 6.693 6.234 6.493 6.702 4.89 4.534 4.969 4.9 5.372
   1654   1378389_at   6.566 6.813 6.622 6.856 6.716 5.517 5.71 5.839 5.933 5.505
   1655   1371729_at   8.827 8.811 8.927 8.908 8.895 9.289 9.401 9.571 9.497 9.36
   1656   1372396_at   6.864 6.948 6.775 7.012 6.91 7.501 7.316 7.52 7.549 7.482
   1657   1370313_at   8.676 8.788 8.78 8.909 8.847 7.727 7.719 8.1 7.503 7.642
   1658   1379445_at   4.816 4.52 5.216 5.458 4.311 7.128 6.958 7.557 7.597 7.543
   1659   1398333_at   10.116 10.109 10.024 10.167 10.314 10.668 10.642 10.718 10.678 10.603
   1660   1368574_at   5.547 5.433 4.934 5.539 5.579 6.8 6.546 6.916 6.729 6.765
   1661   1389170_at   9.383 9.407 9.857 9.633 9.477 8.101 8.132 8.234 8.416 8.58
   1662   1393887_at   10.364 10.217 10.432 10.371 10.003 11.034 11.145 11.272 11.319 11.198
   1663   1372433_at   8.055 8.11 8.059 8.222 8.103 7.402 7.285 7.416 7.451 7.653
   1664   1387984_at   8.459 8.5 8.719 8.493 8.734 7.31 6.802 7.316 6.699 6.607
   1665   1392627_x_at   9.012 9.096 9.169 9.123 9.297 9.97 9.959 10.323 10.131 9.883
   1666   1373264_at   6.887 7 6.715 6.903 6.812 7.923 7.591 8.127 7.94 7.781
   1667   1368077_at   11.819 11.746 11.735 11.634 11.388 12.436 12.589 12.388 12.525 12.548
   1668   1378546_at   7.072 6.935 7.303 7.252 7.032 6.171 5.675 6.002 6.157 5.954
   1669   1391478_at   6.025 5.817 5.876 6.125 5.737 6.612 6.701 6.66 6.665 6.807
   1670   1389085_at   7.707 7.609 7.896 8.039 7.379 9.059 8.969 9.516 9.25 9.187
   1671   1374113_at   8.195 8.165 7.996 7.763 8.1 7.001 6.702 6.789 6.402 6.408
   1672   1367871_at   9.486 9.597 9.774 9.745 9.024 10.959 11.577 11.364 11.222 11.098
   1673   1367679_at   13.217 13.118 13.079 12.8 12.924 11.985 11.765 11.323 11.739 11.792
   1674   1370427_at   8.255 8.181 8.333 8.322 8.285 7.541 7.086 7.473 7.433 7.511
   1675   1391661_at   8.934 8.851 9.143 8.894 8.458 9.976 10.092 10.191 10.066 9.935
   1676   1380182_at   7.05 7.052 6.588 7.133 7.286 8.306 8.384 8.558 8.378 8.15
   1677   1377424_at   6.541 6.454 6.717 6.579 6.383 7.449 7.149 7.509 7.423 7.341
   1678   1371074_a_at   8.755 8.795 8.606 8.688 8.776 8.052 7.73 7.861 7.573 7.937
   1679   1367999_at   10.319 10.25 10.247 10.521 10.188 10.923 10.966 11.07 11.188 11.047
   1680   1379250_at   8.503 8.59 8.89 8.912 8.506 9.825 9.607 9.865 9.973 10.058
   1681   1375709_at   8.175 7.994 8.436 8.133 8.079 7.081 6.768 6.767 7.005 7.253
   1682   1377698_at   9.164 9.116 8.818 9.069 9.247 6.431 4.036 5.345 4.888 4.761
   1683   1390172_at   8.721 8.745 8.78 8.856 8.197 9.771 9.864 9.878 9.856 9.851
   1684   1386052_at   4.727 4.693 4.683 4.846 4.616 2.932 2.653 3.297 2.478 2.121
   1685   1385400_at   7.029 7.311 7.371 7.597 7.246 8.419 8.259 8.306 8.612 8.465
   1686   1387916_at   6.744 7.218 6.93 6.909 6.908 7.724 7.78 7.684 7.691 7.793
   1687   1377098_at   7.745 7.66 8.116 7.846 7.273 9.301 9.325 9.346 9.244 8.982
   1688   1372676_at   9.417 9.369 9.675 9.667 9.443 10.564 10.236 10.68 10.671 10.624
   1689   1392777_at   6.601 6.871 6.918 6.972 6.912 4.489 4.712 4.311 4.302 3.213
   1690   1371575_at   11.581 11.58 11.513 11.544 11.551 11.207 11.196 11.138 10.996 11.034
   1691   1375684_at   6.343 6.461 6.616 6.439 6.405 7.211 7.011 7.264 7.246 7.408
   1692   1379932_at   7.365 7.193 7.22 7.285 6.916 8.059 8.021 8.21 8.206 7.966
   1693   1387038_at   8.269 8.272 8.416 8.482 8.447 8.92 8.928 8.972 8.814 9.012
   1694   1371633_at   8.627 8.574 8.415 8.689 8.608 7.994 7.861 7.951 7.807 8.067
   1695   1369635_at   10.383 10.44 10.574 10.467 10.143 11.151 11.204 11.203 11.102 11.089
   1696   1388915_at   8.388 8.466 8.235 8.645 8.576 7.519 7.098 7.492 7.513 7.378
   1697   1368016_at   11 10.97 10.979 11.025 11.147 11.611 11.654 11.56 11.663 11.411
   1698   1373122_at   7.628 7.443 7.648 7.989 7.448 8.793 8.915 8.857 8.558 8.803
   1699   1379318_at   7.766 7.63 7.738 8.039 7.805 8.774 8.571 8.98 8.69 8.915
   1700   1383352_at   6.397 6.516 6.482 6.422 6.615 6.015 5.867 6.005 5.941 5.797
   1701   1372301_at   8.374 8.26 8.267 8.381 8.49 8.822 8.809 8.863 8.959 8.957
   1702   1372747_at   6.781 6.844 6.667 6.834 6.637 7.298 7.421 7.269 7.217 7.267
   1703   1383548_at   7.751 7.6 7.684 7.735 7.917 7.079 7.071 7.143 6.871 6.93
   1704   1367604_at   9.123 8.991 9.28 9.245 9.332 10.18 9.843 10.066 10.179 10.017
   1705   1375030_at   6.464 6.438 6.233 6.506 5.888 7.693 7.425 7.947 7.863 7.781
   1706   1387765_at   6.238 6.143 6.419 6.125 6.242 7.181 7.033 7.386 7.122 7.513
   1707   1367957_at   8.352 8.215 8.4 8.551 8.379 7.87 7.802 7.832 7.756 7.744
   1708   1370369_at   7.344 7.067 7.429 7.5 6.811 4.254 2.06 1.766 1.727 2.104
   1709   1390530_at   10.281 10.402 10.409 10.293 10.068 11.443 11.46 11.258 11.01 11.316
   1710   1391201_at   5.902 5.512 5.528 5.578 5.664 4.677 4.482 4.336 4.161 4.636
   1711   1368858_at   4.703 4.918 5.259 4.642 4.58 6.207 6.017 6.223 6.408 6.124
   1712   1388916_at   8.459 8.612 8.532 8.81 8.403 9.649 9.58 9.741 9.548 9.306
   1713   1373851_at   7.363 7.196 7.388 7.454 7.21 7.939 7.858 8.101 8.08 7.98
   1714   1390133_at   8.531 8.543 8.584 8.608 8.768 7.994 7.762 7.974 7.83 7.65
   1715   1393029_at   8.495 8.57 8.66 8.49 8.64 7.761 7.449 7.664 7.587 7.187
   1716   1377135_at   6.01 5.729 6.405 6.615 5.575 7.887 8.117 8.384 8.202 8.261
   1717   1374171_at   5.887 6.051 5.829 5.79 6.416 7.628 7.832 8.138 7.763 7.312
   1718   1375645_at   9.3 9.253 9.212 9.402 9.452 8.821 8.873 8.769 8.815 8.886
   1719   1390447_at   8.183 8.185 8.271 8.351 8.127 7.797 7.577 7.682 7.738 7.683
   1720   1375040_at   6.002 6.227 6.084 6.051 6.354 4.336 4.159 3.245 3.17 3.394
   1721   1390383_at   10.921 10.856 10.889 10.967 10.986 10.205 10.115 10.245 9.863 9.842
   1722   1390255_at   7.189 7.428 7.306 7.35 7.166 7.965 8.071 8.215 7.916 7.931
   1723   1384015_at   7.007 7.277 6.939 7.236 7.121 6.269 6.437 6.264 6.094 6.118
   1724   1370638_at   7.408 7.522 7.902 7.888 7.495 8.658 8.767 9.038 8.886 8.884
   1725   1382031_at   3.95 4.197 4.989 4.883 5.472 7.543 7.376 7.866 8.165 7.669
   1726   1381556_at   7.646 7.838 7.473 7.756 8.118 5.412 3.877 4.053 5.132 4.122
   1727   1387078_at   8.122 8.102 8.1 7.921 7.835 7.374 7.319 7.174 7.408 7.224
   1728   1388874_at   9.198 9.269 9.52 9.591 9.206 10.183 10.215 10.215 10.173 10.131
   1729   1378470_at   8.694 8.697 8.971 8.938 8.448 9.712 9.672 9.831 9.942 9.813
   1730   1368240_a_at   7.036 7.036 7.022 7.262 6.686 4.015 4.921 3.129 3.638 4.142
   1731   1387270_at   7.035 6.79 7.154 7.309 7.374 5.99 5.499 5.513 5.87 5.789
   1732   1377966_at   8.408 8.267 8.296 8.334 7.875 9.217 9.379 9.509 9.718 9.595
   1733   1382663_at   7.989 8.094 7.947 8.419 8.351 5.406 5.054 5.191 3.539 4.461
   1734   1398891_at   10.351 10.187 10.349 10.333 10.284 10.669 10.786 10.733 10.895 10.795
   1735   1371727_at   10.4 10.47 10.647 10.424 10.226 11.251 11.318 11.227 11.318 11.065
   1736   1377456_at   5.367 5.23 5.595 4.968 5.606 6.591 6.56 6.91 6.67 6.64
   1737   1373152_at   10.393 10.317 10.31 10.251 10.259 10.727 10.906 11.008 10.846 10.772
   1738   1382452_at   5.492 5.634 5.214 5.667 5.703 7.149 6.526 7.117 7.036 6.934
   1739   1392411_at   8.07 8.279 8.656 8.422 8.135 9.649 9.891 10.124 9.602 9.576
   1740   1370066_at   8.057 8.033 8.036 8.247 8.312 7.306 7.384 7.272 7.45 7.536
   1741   1398572_at   8.667 8.676 8.671 8.765 9.001 7.964 7.773 7.853 7.809 7.503
   1742   1377680_at   6.723 6.725 7.354 7.114 6.693 8.264 8.209 8.437 8.285 8.514
   1743   1380728_at   7.529 7.466 7.323 7.659 7.703 6.615 6.24 6.017 6.399 6.443
   1744   1388776_at   10.629 10.758 10.919 10.85 10.856 10.205 10.03 10.171 10.133 9.919
   1745   1368294_at   6.598 6.875 6.12 6.351 6.356 4.831 5.036 4.625 4.305 4.812
   1746   1391224_at   5.85 5.702 5.701 5.863 5.779 6.329 6.499 6.537 6.762 6.591
   1747   1375932_at   9.22 9.306 9.115 9.138 9.095 9.695 9.756 10.009 9.806 9.815
   1748   1379499_at   8.316 8.023 7.804 7.897 7.832 5.78 5.729 4.929 4.411 5.473
   1749   1384028_at   9.096 9.096 9.257 9.39 9.391 10.107 9.882 10.074 10.129 10.209
   1750   1386898_at   11.675 11.685 11.64 11.645 11.732 11.883 11.977 11.902 11.99 11.979
   1751   1375197_at   11.06 10.983 11.166 11.19 11.197 11.652 11.662 11.639 11.754 11.875
   1752   1387950_at   8.141 8.179 8.09 8.149 8.368 7.547 7.615 7.535 7.364 7.339
   1753   1385143_at   7.886 8.119 7.733 8.006 8 6.908 6.664 6.72 6.253 6.31
   1754   1396300_at   7.191 7.189 7.099 7.326 6.976 8.047 7.895 8.339 8.312 8.284
   1755   1367759_at   9.202 9.161 9.034 9.274 9.197 9.66 9.571 9.685 9.659 9.564
   1756   1392975_at   6.872 6.765 7.12 7.006 6.88 7.89 7.575 7.767 7.759 7.643
   1757   1374298_at   8.401 8.321 8.115 7.929 7.836 6.606 6.567 6.661 5.953 6.16
   1758   1374496_at   6.618 6.635 6.381 6.749 7.395 8.853 8.369 8.684 8.972 8.616
   1759   1381819_at   7.068 7.236 6.667 7.134 7.216 5.831 5.706 6.048 5.827 5.574
   1760   1380975_at   7.551 7.198 7.417 7.671 7.506 8.426 8.231 8.392 8.262 8.294
   1761   1390518_at   4.365 4.401 3.48 4.288 3.839 6.175 5.725 6.377 6.352 6.357
   1762   1388836_at   8.369 8.253 8.884 8.715 8.212 7.165 6.784 7.173 6.903 6.896
   1763   1372367_at   8.005 7.834 7.648 7.902 7.775 8.378 8.49 8.513 8.485 8.56
   1764   1376601_at   5.78 5.897 5.716 5.74 5.556 6.491 6.419 6.434 6.382 6.682
   1765   1395534_at   5.023 5.274 5.31 5.287 5.123 6.138 5.825 5.891 6.038 6.084
   1766   1377662_at   5.332 5.341 5.669 5.371 5.524 6.456 6.257 6.16 6.197 6.398
   1767   1373740_at   8.549 8.652 9.019 9.042 8.721 9.931 9.754 10.058 9.907 9.804
   1768   1387314_at   8.999 9.108 9.293 9.286 9.152 9.871 10.015 10.309 10.262 10.13
   1769   1391273_at   10.191 10.196 10.451 10.367 10.463 10.919 11.088 11.068 11.183 11.006
   1770   1383578_at   8.085 8.238 7.981 8.014 7.914 7.161 6.781 6.953 6.56 7.033
   1771   1387343_at   8.974 8.761 8.888 8.89 9.209 7.382 7.067 6.737 6.33 6.276
   1772   1383928_a_at   6.295 6.127 6.031 6.018 5.891 6.693 6.924 6.983 6.93 6.943
   1773   1374624_at   9.042 9.051 9.342 9.091 8.52 10.447 10.249 10.315 10.398 10.354
   1774   1372557_at   7.846 7.759 7.738 7.797 7.91 8.5 8.633 8.765 8.475 8.364
   1775   1389556_at   7.451 7.732 7.562 7.658 7.293 8.501 8.58 8.942 8.799 8.518
   1776   1384173_at   7.14 7.158 7.109 7.226 7.039 5.558 6.126 6.075 6.137 5.695
   1777   1372003_at   9.009 9.086 8.807 8.855 8.893 8.122 8.148 8.285 8.218 7.909
   1778   1383962_at   8.284 8.22 8.512 8.154 8.267 7.708 7.64 7.595 7.649 7.513
   1779   1382997_at   5.709 5.966 5.394 6.371 5.434 7.957 7.508 8.14 7.92 8.314
   1780   1371807_at   8.291 8.346 8.311 8.303 8.463 9.149 9.17 9.191 9.068 8.816
   1781   1388323_at   10.778 10.721 10.806 10.714 10.75 10.962 10.953 11.051 11.022 11.005
   1782   1379794_at   9.781 9.846 10.447 10.303 9.753 6.131 4.51 3.912 4.231 2.543
   1783   1387759_s_at   10.516 10.443 10.654 10.535 10.406 11.027 11.062 11.007 11.123 11.24
   1784   1383648_x_at   7.727 7.679 7.723 7.698 7.763 8.07 8.132 8.12 8.021 7.958
   1785   1372158_at   7.585 7.804 7.163 7.751 7.312 8.697 8.688 8.845 8.861 8.86
   1786   1379550_a_at   7.234 7.266 7.093 7.329 7.357 7.995 7.894 8.154 8.146 7.852
   1787   1386956_at   6.861 6.962 6.971 6.829 7.049 6.253 6.323 6.25 6.14 5.942
   1788   1374191_at   7.31 7.16 7.277 7.591 7.38 8.127 7.986 8.16 8.106 8.082
   1789   1388158_at   10.497 10.526 10.528 10.446 10.531 10.133 9.809 9.83 9.901 9.825
   1790   1376595_at   7.221 7.294 7.228 7.157 7.141 7.76 7.597 7.856 7.789 7.656
   1791   1372423_at   8.806 8.952 8.874 8.99 9.057 9.675 9.647 9.821 9.619 9.456
   1792   1377626_at   12.948 12.901 12.992 12.783 12.676 10.001 8.644 7.771 7.762 7.432
   1793   1379298_at   7.743 7.929 7.628 7.756 7.781 6.962 6.941 7.09 6.9 6.634
   1794   1374293_at   8.339 8.407 8.521 8.646 8.38 9.082 8.971 9.114 9.116 9.026
   1795   1371954_at   10.03 10.044 10.115 10.309 10.213 10.904 10.892 11.319 11.069 11.048
   1796   1367642_at   10.609 10.598 10.604 10.652 10.765 10.958 10.93 10.97 10.98 10.958
   1797   1368781_at   5.131 4.983 5.282 5.386 5.251 3.778 3.935 3.448 3.053 3.681
   1798   1369158_at   7.273 7.288 7.468 7.322 7.127 8.199 8.103 8.579 8.577 8.447
   1799   1376661_at   7.438 7.099 7.288 7.355 7.074 8.233 8.11 8.215 7.979 8.001
   1800   1378505_at   4.748 3.97 4.317 4.647 4.573 6.365 5.947 5.984 6.135 5.851
   1801   1394082_at   9.518 9.509 9.606 9.552 9.717 8.953 8.829 8.725 8.574 8.514
   1802   1379679_at   6.009 5.994 5.913 6.165 5.695 6.857 7.336 7.486 7.478 7.286
   1803   1368981_at   5.037 5.112 5.162 4.905 5.093 5.919 5.706 6.049 6.007 6.221
   1804   1383844_at   5.949 5.88 5.365 5.963 5.866 7.067 7.021 7.436 7.113 7.001
   1805   1370699_a_at   10.561 10.592 10.681 10.647 10.371 11.113 11.193 11.14 11.233 11.318
   1806   1387971_a_at   7.272 7.279 7.745 7.562 7.571 8.709 8.375 8.575 8.823 8.685
   1807   1384007_at   3.828 3.497 3.942 3.726 3.64 4.504 4.869 4.781 4.782 4.601
   1808   1392203_at   7.538 7.639 7.864 7.862 7.859 8.459 8.848 8.628 8.78 8.679
   1809   1387605_at   8.009 8.046 7.927 7.989 8.449 6.353 6.458 5.978 6.132 5.39
   1810   1376263_at   6.54 6.109 6.655 6.784 6.283 7.734 7.785 8.044 8.238 8.111
   1811   1372568_at   8.109 8.008 8.093 7.808 7.816 8.896 8.796 9.211 9.201 8.91
   1812   1380611_at   8.374 8.404 7.857 7.838 8.064 6.448 6.817 6.907 6.596 6.508
   1813   1389362_at   7.319 7.256 7.248 7.217 7.061 7.649 7.75 7.722 7.849 7.816
   1814   1378287_at   7.842 7.932 8.005 8.042 7.927 8.418 8.299 8.355 8.3 8.356
   1815   1398398_at   7.909 8.113 7.899 8.219 8.214 8.788 8.727 8.879 8.782 8.883
   1816   1376691_at   7.967 8.002 8.184 8.098 7.688 8.866 9.032 8.992 8.814 9.13
   1817   1376737_at   8.684 8.664 8.681 8.497 8.64 7.941 7.855 7.884 7.718 7.483
   1818   1373134_at   8.5 8.391 8.441 8.61 8.461 9.04 9.111 9.402 9.211 9.336
   1819   1383665_at   8.225 8.222 7.84 8.274 8.25 7.321 7.245 7.302 7.356 7.425
   1820   1367793_at   10.455 10.427 10.739 10.714 10.521 11.308 11.221 11.381 11.501 11.484
   1821   1387691_at   5.5 6.425 6.272 6.168 6.093 3.845 2.832 2.84 2.392 2.025
   1822   1388341_at   9.132 9.186 9.162 9.444 9.302 8.411 8.378 8.664 8.303 8.449
   1823   1372506_at   9.403 9.46 9.362 9.631 9.613 8.93 8.976 8.826 8.849 8.793
   1824   1381976_at   7.116 7.423 7.344 7.499 7.303 8.225 8.107 8.424 8.157 8.076
   1825   1369983_at   8.879 8.909 8.432 9.313 7.905 6.097 5.541 6.163 6.297 5.59
   1826   1373767_at   7.123 7.241 7.507 7.342 7.376 6.492 6.289 6.047 6.052 5.927
   1827   1368233_at   7.614 7.794 7.703 7.747 7.882 7.143 7.125 6.986 6.801 7.035
   1828   1373544_at   12.534 12.475 12.331 12.113 12.431 9.312 7.781 7.268 6.995 6.362
   1829   1387844_at   8.024 7.933 8.066 8.094 8.172 7.704 7.609 7.571 7.571 7.579
   1830   1398925_at   9.643 9.614 9.631 9.903 9.733 9.157 8.921 9.05 8.931 9.087
   1831   1394527_at   5.251 5.684 5.865 5.569 5.961 4.29 4.207 4.375 4.006 3.832
   1832   1383194_a_at   5.153 5.633 5.611 5.08 5.708 6.639 6.901 7.004 6.773 6.919
   1833   1370030_at   10.716 10.731 10.857 10.733 10.507 11.406 11.267 11.369 11.354 11.526
   1834   1367586_at   12.105 12.034 12.103 12.026 12.003 11.486 11.54 11.412 11.16 11.332
   1835   1388703_at   8.269 8.141 8.037 8.272 8.097 8.74 8.676 8.942 8.881 8.902
   1836   1373921_at   7.327 7.543 7.162 7.451 7.417 8.293 8.346 8.206 8.01 8.334
   1837   1371932_at   8.802 8.676 8.685 8.633 8.94 8.157 8.134 7.922 8.014 8.113
   1838   1371990_at   8.193 8.284 8.38 8.397 8.316 8.937 8.795 9.131 9.109 9.043
   1839   1394259_at   4.89 5.021 4.85 4.76 4.713 5.86 5.559 5.957 5.596 5.868
   1840   1371986_at   8.878 9.036 9.079 8.963 9.062 9.691 9.64 9.673 9.501 9.449
   1841   1381080_at   6.468 6.516 7.193 6.72 5.874 8.857 8.554 9.315 9.21 8.911
   1842   1370379_at   9.01 9.025 9.246 9.169 8.726 9.893 10 10.013 10.224 10.152
   1843   1390765_at   9.468 9.33 9.424 9.387 9.464 9.795 9.796 10.006 9.909 9.962
   1844   1369862_at   6.698 6.678 6.622 6.897 7.169 4.627 4.883 3.782 4.908 4.077
   1845   1382228_at   6.416 6.599 6.577 6.459 6.362 7.306 7.043 7.23 7.252 7.037
   1846   1383922_a_at   6.874 6.696 6.654 6.825 6.723 6.153 6.315 6.316 6.166 6.242
   1847   1373831_at   8.672 9.266 8.934 8.736 9.173 7.885 7.72 7.568 7.546 7.477
   1848   1398507_at   4.927 5.093 5.385 4.977 4.827 6.261 6.147 6.76 6.361 6.495
   1849   1398591_at   6.816 6.973 7.215 6.993 7 5.439 4.938 4.659 5.49 5.542
   1850   1390394_at   7.732 7.995 7.775 7.72 7.83 6.923 6.929 7.132 6.742 6.645
   1851   1395875_at   7.657 7.756 7.597 7.826 7.752 7.079 7.104 6.918 7.246 7.008
   1852   1384907_at   7.556 7.555 7.426 7.477 7.53 8.522 8.723 9.099 8.737 8.38
   1853   1384243_at   5.351 5.306 5.495 5.851 5.639 6.63 6.512 6.814 6.862 6.577
   1854   1374550_at   7.664 7.72 7.9 8.158 8.157 6.963 6.652 6.824 6.846 6.752
   1855   1388763_at   10.759 10.749 10.783 10.542 10.545 9.985 9.869 9.918 9.647 9.626
   1856   1370848_at   9.06 8.973 9 9.259 8.99 8.432 8.299 8.519 8.248 8.385
   1857   1368643_at   7.288 7.189 6.935 7.188 7.138 7.987 8.092 8.073 8.029 7.731
   1858   1382266_at   6.144 6.269 6.396 6.226 6.169 5.658 5.661 5.353 5.455 5.539
   1859   1369971_a_at   9.529 9.627 9.336 9.628 9.732 8.759 8.49 8.825 8.489 8.479
   1860   1388711_at   9.581 9.53 9.291 9.54 9.718 8.699 8.625 8.672 8.543 8.286
   1861   1387711_at   6.86 7.05 7.511 7.416 7.086 3.349 0.444 2.007 1 0
   1862   1390411_at   7.451 7.381 7.507 7.859 7.869 8.734 8.556 8.805 8.703 8.89
   1863   1374197_at   8.585 8.516 8.497 8.47 8.408 8.943 8.874 8.934 9.023 8.822
   1864   1372585_at   13.401 13.256 13.221 12.877 13.043 11.838 10.869 10.78 10.891 10.746
   1865   1384968_at   7.283 7.352 7.514 7.637 7.557 6.288 6.467 6.131 5.887 5.774
   1866   1370165_at   5.002 6.095 5.202 5.754 5.81 7.552 7.518 7.993 8.106 7.995
   1867   1370566_at   6.471 6.983 7.187 7.045 6.689 8.103 8.163 8.485 8.253 8.397
   1868   1388194_at   8.812 8.807 8.761 8.823 8.79 9.309 9.353 9.46 9.258 9.144
   1869   1370844_at   10.347 10.289 10.283 10.492 10.469 9.881 9.865 9.718 9.641 9.713
   1870   1371728_at   7.477 7.451 7.575 7.718 7.467 8.089 8.007 8.217 8.121 8.16
   1871   1375553_at   5.686 5.711 5.159 5.72 5.416 6.717 6.547 6.783 6.768 6.947
   1872   1377979_at   5.468 5.523 5.276 5.426 5.229 6.185 6.496 6.813 6.744 6.569
   1873   1383191_at   7.209 7.187 7.209 6.845 6.581 8.58 8.248 8.773 8.875 8.91
   1874   1376605_at   7.902 7.956 8.163 8.117 7.746 8.921 8.747 8.852 9.114 8.852
   1875   1368361_a_at   8.94 8.789 8.7 9.047 9.217 7.805 7.993 7.986 7.632 7.704
   1876   1398897_at   10.824 10.734 10.729 10.908 10.947 10.272 10.137 10.352 10.32 10.173
   1877   1388909_at   7.791 7.915 7.705 7.941 7.547 8.518 8.424 8.517 8.506 8.596
   1878   1383917_at   7.876 7.737 7.865 8.324 8.215 6.95 6.596 6.868 6.702 6.622
   1879   1370060_at   10.237 10.187 10.373 10.342 10.317 10.795 10.65 10.725 10.744 10.854
   1880   1376861_at   7.356 7.104 7.363 7.178 7.008 7.904 7.89 7.88 8.04 7.896
   1881   1373249_at   6.332 6.072 6.323 6.503 6.366 7.19 7.142 7.038 6.96 7.083
   1882   1370017_at   7.779 7.916 8.095 8.08 7.874 7.211 6.905 7.11 6.874 6.752
   1883   1390863_at   7.573 7.709 7.698 7.591 7.409 8.254 8.142 8.38 8.233 8.181
   1884   1372028_at   9.228 9.359 9.23 9.365 9.508 8.731 8.841 8.797 8.797 8.668
   1885   1371467_at   9.803 9.712 9.641 9.772 9.715 9.23 9.159 9.315 9.079 9.014
   1886   1384988_at   6.576 6.363 6.308 6.135 6.357 5.643 5.505 5.372 5.412 5.254
   1887   1367873_at   10.399 10.36 10.438 10.433 10.415 10.602 10.614 10.621 10.544 10.619
   1888   1367504_at   8.069 8.175 8.081 7.983 7.931 8.658 8.522 8.646 8.733 8.512
   1889   1393508_at   8.035 7.934 8.152 7.608 6.923 9.901 10.381 10.252 10.03 9.784
   1890   1376043_at   9.031 9.034 8.854 8.781 8.677 8.114 8.061 8.187 8.247 8.109
   1891   1373267_at   7.041 7.184 7.236 7.099 7.015 7.859 7.891 7.947 7.851 7.586
   1892   1388176_at   7.255 7.343 7.525 7.423 6.9 8.327 8.911 8.933 8.839 8.736
   1893   1371938_at   11.404 11.397 11.301 11.336 11.269 11.018 10.885 10.894 10.956 10.795
   1894   1397892_at   8.868 8.687 8.896 8.882 8.96 8.175 7.98 8.013 7.656 7.879
   1895   1384211_at   2.897 3.5 2.021 3.155 3.933 6.386 5.944 6.693 6.53 6.271
   1896   1369962_at   9.409 9.454 9.257 9.432 9.512 8.638 8.639 8.908 8.851 8.754
   1897   1372455_at   8.438 8.424 8.161 8.228 8.53 9.261 9.185 9.585 9.482 9.224
   1898   1387178_a_at   8.212 8.194 8.405 8.483 7.801 9.452 9.246 9.426 9.433 9.512
   1899   1368245_at   8.903 8.869 8.92 9.06 8.429 10.073 10.036 10.335 10.389 10.644
   1900   1383165_at   3.742 4.396 4.46 4.538 4.43 6.07 5.704 5.998 5.848 5.686
   1901   1375987_at   7.806 8.011 8.067 8.108 8.075 7.274 6.961 7.291 6.895 7.038
   1902   1386454_at   6.733 7 7.109 7.332 6.863 8.399 8.072 8.024 8.362 8.364
   1903   1383143_at   4.875 4.816 4.937 5.119 5.306 5.974 6.181 6.123 5.921 5.887
   1904   1398880_at   11.453 11.424 11.535 11.522 11.466 11.224 11.06 11.175 11.169 11.163
   1905   1379538_at   6.731 7.066 6.91 7.069 7.186 5.97 5.535 5.851 5.739 5.332
   1906   1375964_at   7.614 7.702 7.999 7.852 7.55 8.752 8.804 8.539 8.833 8.576
   1907   1368934_at   8.549 8.635 8.94 8.953 8.883 9.772 9.982 10.211 9.905 9.731
   1908   1388315_at   11.701 11.726 11.793 11.662 11.654 12.076 11.963 12.005 12.097 12.053
   1909   1367627_at   11.636 11.626 11.536 11.311 11.285 12.501 12.658 12.376 12.25 12.591
   1910   1368013_at   5.075 5.269 4.984 5.077 5.875 7.072 6.844 6.922 6.701 7.09
   1911   1382211_at   7.931 7.8 8.054 8.205 7.81 9.453 9.254 10.007 9.572 9.2
   1912   1374626_at   7.237 7.268 7.472 7.294 7.389 6.693 6.577 6.875 6.759 6.693
   1913   1373837_at   8.458 8.32 8.391 8.535 8.542 9.117 8.871 9.07 9.046 9.139
   1914   1372437_at   11.736 11.731 11.806 11.689 11.773 12.065 11.962 12.029 11.976 11.994
   1915   1380187_at   7.424 7.188 7.128 7.651 7.494 6.216 6.316 6.443 6.486 6.305
   1916   1377738_a_at   7.024 7.17 7.168 7.29 7.371 8.061 7.952 8.319 8.386 8.081
   1917   1392971_at   7.588 7.782 7.62 7.985 8.079 9.168 9.312 9.517 8.988 8.922
   1918   1373804_at   7.26 7.311 7.599 7.481 7.536 6.754 6.795 6.838 6.65 6.733
   1919   1384022_at   8.804 9.037 9.162 9.093 9.078 8.227 8.155 8.241 7.944 7.837
   1920   1374437_at   9.484 9.529 9.6 9.691 9.595 9.148 9.04 8.999 8.86 8.887
   1921   1384658_at   7.507 7.682 7.828 7.662 7.708 3.531 3.529 2.202 1.316 0.687
   1922   1373480_at   7.905 7.816 7.876 7.946 7.725 8.419 8.298 8.424 8.349 8.238
   1923   1369007_at   5.96 6.142 6.271 6.44 5.945 5.029 4.508 5.04 4.952 4.816
   1924   1370330_at   6.785 6.672 6.865 6.621 6.671 7.271 7.436 7.173 7.362 7.321
   1925   1399152_at   8.701 8.691 8.831 8.627 8.917 8.019 8.071 8.065 8.062 7.752
   1926   1391662_at   5.938 6.019 6.202 5.954 5.988 4.919 5.005 4.502 4.755 4.288
   1927   1379631_at   8.309 8.332 7.703 8.178 8.127 6.954 6.342 6.181 6.171 6.419
   1928   1388414_at   10.744 10.697 10.866 10.939 10.936 11.302 11.411 11.337 11.471 11.374
   1929   1368921_a_at   9.587 9.684 9.362 9.406 9.353 7.684 7.802 7.702 6.94 6.799
   1930   1374180_at   9.345 9.431 9.393 9.413 9.398 8.455 8.444 8.5 8.265 7.877
   1931   1387300_at   7.59 7.651 7.464 7.351 7.564 7.065 6.924 6.899 6.88 6.874
   1932   1368412_a_at   8.589 8.693 8.576 8.475 8.359 7.829 7.63 7.747 7.798 7.97
   1933   1383855_at   10.321 10.27 10.618 10.324 10.496 11.125 10.965 11.153 11.159 11.132
   1934   1368098_a_at   7.223 7.279 7.359 7.39 7.329 8.144 7.83 8.099 8.319 8.122
   1935   1389528_s_at   10.422 10.339 10.271 10.617 10.508 9.454 9.341 9.33 9.058 8.814
   1936   1389496_at   8.071 8.182 8.397 8.483 8.413 7.349 7.098 7.376 7.498 7.137
   1937   1382592_at   7.009 7.042 7.131 7.226 7.051 6.34 6.241 5.9 5.848 5.847
   1938   1385124_at   6.211 6.295 5.954 6.383 6.27 6.928 7.142 7.028 7.27 7.211
   1939   1372152_at   7.58 7.763 7.861 7.805 7.842 8.234 8.287 8.293 8.402 8.345
   1940   1389230_at   8.772 8.813 9.003 9.017 8.993 9.868 9.537 10.021 9.848 9.768
   1941   1371539_at   9.816 9.664 9.754 9.819 9.877 9.193 9.215 8.962 8.877 8.907
   1942   1374485_at   7.551 7.538 7.44 7.729 7.71 8.11 8.158 8.223 8.145 8.1
   1943   1389964_at   10.81 10.803 10.949 10.723 10.844 11.21 11.322 11.176 11.301 11.258
   1944   1375767_at   7.215 7.276 7.341 7.326 6.752 8.315 8.145 8.301 8.271 8.217
   1945   1398430_at   7.528 7.662 7.624 7.852 7.473 8.645 8.366 8.906 8.647 8.609
   1946   1389193_at   4.792 4.864 4.936 5.082 4.903 5.615 5.707 5.711 6.077 5.86
   1947   1368470_at   10.53 10.544 10.643 10.71 10.724 11.217 11.425 11.626 11.331 11.309
   1948   1382868_at   6.105 6.251 6.344 6.468 6.466 7.147 7.142 7.569 7.426 7.385
   1949   1376180_at   5.918 6.194 6.149 6.378 6.319 7.207 6.929 7.212 7.127 7.016
   1950   1376579_at   9.565 9.523 9.853 9.715 9.663 9.01 8.93 8.9 8.652 8.909
   1951   1393165_at   7.036 7.136 7.292 6.936 7.061 7.698 7.699 7.651 7.641 7.63
   1952   1395659_at   5.306 5.569 5.464 5.695 5.084 6.404 6.488 6.704 6.501 6.681
   1953   1393066_at   4.799 4.897 4.796 5.076 4.849 5.461 5.5 5.488 5.734 5.618
   1954   1370814_at   9.419 9.364 9.505 9.585 9.583 9.996 9.931 10.083 10.071 10.169
   1955   1384051_at   4.365 4.727 4.71 5.245 5.016 6.321 6.117 6.384 6.328 6.224
   1956   1380250_at   6.236 6.402 6.163 6.051 6.442 7.549 7.129 7.493 7.709 7.28
   1957   1372670_at   7.619 7.643 7.553 7.575 7.359 8.133 8.013 8.146 8.246 8.211
   1958   1370878_at   9.316 9.498 9.332 9.296 9.418 9.981 9.887 9.888 9.8 9.771
   1959   1390566_a_at   8.89 9.087 8.875 9.014 8.95 9.453 9.619 9.778 9.518 9.599
   1960   1368924_at   6.413 6.486 6.48 5.946 6.042 7.423 7.464 7.555 7.319 7.346
   1961   1387071_a_at   7.788 7.735 8.038 7.874 7.459 8.758 8.731 9.017 9.073 8.813
   1962   1372543_at   8.459 8.509 8.641 8.695 8.706 7.745 7.267 7.768 7.368 7.333
   1963   1375558_at   6.496 6.596 6.571 6.544 6.75 7.196 7.029 7.081 7.116 7.023
   1964   1373697_at   5.702 5.666 5.935 5.724 5.48 6.552 6.499 6.439 6.779 6.552
   1965   1373161_at   9.129 9.046 8.798 8.984 9.033 9.796 9.626 9.903 9.696 9.607
   1966   1373191_at   8.245 8.274 8.233 8.335 8.049 8.805 8.658 8.742 8.792 8.708
   1967   1387654_at   7.844 7.866 7.827 7.847 7.788 8.42 8.558 8.263 8.285 8.527
   1968   1387821_at   7.567 7.427 7.649 7.803 7.618 8.337 8.246 8.555 8.462 8.308
   1969   1383064_at   7.954 7.791 8.128 8.237 8.324 9.167 8.976 9.43 9.281 9.261
   1970   1396065_at   3.967 4.212 3.346 4.261 4.681 1.856 0.422 0.556 0.595 0.251
   1971   1370883_at   12.927 12.844 12.777 12.455 12.703 11.428 11.154 10.375 10.941 10.9
   1972   1385627_at   7.351 7.438 6.889 7.671 7.491 5.939 5.425 5.695 5.602 5.064
   1973   1369799_at   7.797 7.657 7.923 7.94 7.527 8.584 8.448 8.549 8.564 8.626
   1974   1387819_at   8.944 8.798 9.286 9.103 9.089 10.162 9.849 10.306 10.032 10.052
   1975   1376317_at   6.955 6.858 6.684 6.81 6.972 6.107 5.864 6.255 5.908 6.063
   1976   1373290_at   7.496 7.674 7.697 7.566 7.554 6.821 6.435 6.472 6.097 6.261
   1977   1387770_at   12.28 12.263 12.245 12.165 12.365 11.252 10.483 10.37 10.813 10.92
   1978   1388786_at   8.189 8.122 8.15 8.404 8.275 8.929 8.824 9.201 9.125 8.991
   1979   1382924_at   8.38 8.598 8.534 8.404 8.005 9.541 9.267 9.634 9.582 9.517
   1980   1383255_at   7.996 8.264 8.039 8.158 8.326 7.202 7.152 7.42 7.406 7.028
   1981   1387306_a_at   7.737 7.897 7.978 8.137 7.983 6.555 6.038 6.514 5.608 5.825
   1982   1371106_at   7.094 7.167 7.461 7.235 7.505 5.144 4.596 4.677 3.437 4.761
   1983   1384925_at   5.097 5.396 5.533 5.71 5.385 6.761 6.319 6.617 6.627 6.746
   1984   1391543_at   7.495 7.343 7.245 7.376 7.162 6.213 6.093 5.659 6.338 6.23
   1985   1382873_at   7.499 7.577 7.592 7.779 7.631 7.004 6.898 7.15 7.079 7.054
   1986   1384303_s_at   5.2 4.635 4.999 5.246 5.193 6.564 6.049 6.46 6.534 6.508
   1987   1368847_at   9.701 9.908 9.499 9.762 9.447 8.859 8.814 8.719 8.849 8.623
   1988   1374434_at   5.785 6.19 5.952 6.159 5.989 6.941 6.9 7.328 6.908 7.118
   1989   1369531_at   8.242 8.413 8.935 8.852 7.925 10.22 10.342 10.143 10.406 10.261
   1990   1380345_at   4.707 5.033 4.676 4.984 4.757 5.968 5.874 5.805 5.575 6.06
   1991   1385132_at   7.418 7.307 7.725 7.413 6.888 8.718 8.541 8.72 8.679 8.574
   1992   1389082_at   6.911 7.024 7.043 6.995 6.902 7.654 7.586 7.741 7.819 7.431
   1993   1370259_a_at   8.923 8.73 9.045 9.361 8.641 10.167 10.15 10.579 10.457 10.343
   1994   1389251_at   7.648 7.779 8.022 7.959 7.919 8.774 8.496 8.86 8.706 8.675
   1995   1387870_at   10 10.129 10.206 10.1 10.044 8.972 8.32 8.429 8.359 7.827
   1996   1369200_at   8.022 8.071 8.356 8.326 7.927 9.032 9.128 9.182 9.235 8.905
   1997   1369289_at   8.079 8.068 8.367 8.231 7.913 8.878 8.957 8.849 9.007 9.095
   1998   1394565_at   9.476 9.479 9.529 9.541 9.514 9.347 9.339 9.266 9.279 9.26
   1999   1368563_at   9.166 9.102 9.612 9.331 9.038 10.303 10.727 10.504 10.467 10.281
   2000   1394694_at   4.646 4.985 4.819 4.866 4.715 5.857 5.898 5.775 5.435 5.873
   2001   1398349_at   11.499 11.466 11.456 11.602 11.634 11.131 11.023 11.19 11.1 11.05
   2002   1398856_at   11.305 11.199 11.194 11.191 11.288 10.46 10.748 10.451 10.255 10.423
   2003   1383679_at   7.85 7.606 7.797 7.922 8.033 7.026 6.99 7.191 7.028 6.851
   2004   1371407_at   11.065 11.072 10.916 11.105 11.085 11.509 11.56 11.599 11.39 11.471
   2005   1372023_at   7.41 7.612 7.316 7.307 7.415 8.065 7.991 8.302 8.23 8.101
   2006   1369347_s_at   6.099 6.233 6.111 6.147 6.334 7.36 6.866 7.35 7.255 7.514
   2007   1374903_at   8.665 8.732 8.671 8.708 8.5 9.069 9.285 9.34 9.224 9.19
   2008   1383625_a_at   9.568 9.533 9.477 9.576 9.569 8.912 8.701 8.863 8.567 8.428
   2009   1381461_at   8.957 8.933 8.759 8.974 9.149 8.331 8.113 8.319 8.31 8.167
   2010   1367484_at   10.157 10.114 10.052 9.896 10.196 9.492 9.563 9.503 9.493 9.324
   2011   1377064_at   8.734 8.713 8.58 8.664 8.715 8.33 8.311 8.41 8.277 8.239
   2012   1383102_at   7.972 7.666 7.947 7.757 7.836 6.989 7.073 7.269 6.884 6.988
   2013   1386181_at   5.746 5.663 5.607 5.411 5.435 4.93 4.703 4.811 4.948 4.853
   2014   1382378_at   6.7 6.887 7.221 6.887 6.927 4.401 1.945 3.048 2.167 2.508
   2015   1371033_at   12.782 12.68 12.72 12.472 12.504 11.566 10.899 10.499 10.906 10.842
   2016   1392510_at   5.272 5.094 5.598 4.796 4.471 6.901 6.814 6.972 7.32 6.986
   2017   1393629_at   6.261 6.783 6.717 6.702 6.687 4.332 4.84 5.279 4.413 4.202
   2018   1369197_at   7.885 7.813 7.791 7.762 8.201 6.737 6.436 6.406 6.369 5.832
   2019   1383753_at   4.704 4.64 4.903 4.862 4.768 5.426 5.864 5.595 5.639 5.487
   2020   1398561_at   8.194 8.44 8.145 7.884 8.087 6.894 7.039 6.363 6.921 6.771
   2021   1384350_at   7.029 6.819 6.779 6.669 6.541 5.659 4.957 5.156 5.534 5.481
   2022   1375867_at   7.651 7.585 7.704 7.664 7.809 8.253 8.305 8.365 8.534 8.181
   2023   1370499_at   6.564 6.454 6.829 6.123 6.171 4.632 4.109 3.953 3.265 4.254
   2024   1369754_a_at   7.885 7.867 7.932 7.787 7.843 7.134 7.278 7.142 6.96 7.375
   2025   1374414_at   7.755 7.657 7.607 7.922 7.891 8.422 8.306 8.518 8.464 8.401
   2026   1393947_at   8.372 8.271 8.469 8.544 8.026 9.372 9.338 9.486 9.269 9.136
   2027   1367599_at   11.572 11.604 11.6 11.584 11.616 11.992 12.011 11.881 12.047 12.163
   2028   1372986_at   7.486 7.606 7.49 7.284 7.302 6.629 6.763 6.788 6.874 6.761
   2029   1383742_at   8.667 8.663 8.621 8.685 8.523 9.344 9.2 9.62 9.392 9.241
   2030   1372890_at   9.15 9.133 9.396 9.571 9.428 8.514 8.022 8.33 8.225 8.171
   2031   1367998_at   11.895 11.793 11.786 11.624 12.012 8.819 7.219 6.012 6.728 5.907
   2032   1390474_at   7.198 6.854 7.062 6.902 7.169 7.803 7.713 7.786 8.013 7.797
   2033   1384791_at   7.043 7.189 7.245 7.193 7.133 6.641 6.634 6.738 6.771 6.535
   2034   1389911_at   8.851 8.875 8.895 9.066 9.229 8.352 8.165 8.265 8.106 8.185
   2035   1389649_at   10.025 10.023 9.97 9.91 10.026 10.731 11.036 10.825 10.857 10.519
   2036   1388644_at   8.812 8.751 8.56 8.785 8.442 10.112 9.785 10.203 10.734 10.278
   2037   1387412_at   6.994 7.039 7.252 7.081 7.23 6.486 6.236 6.47 6.15 6.4
   2038   1394842_at   8.076 8.076 8.021 8.238 8.396 7.489 7.49 7.309 7.347 7.228
   2039   1373222_at   10.311 10.206 10.255 10.373 10.418 10.836 10.75 10.771 10.688 10.689
   2040   1387092_at   8.208 8.267 8.637 8.263 8.564 9.622 9.232 9.428 9.307 9.52
   2041   1371942_at   11.524 11.603 11.691 11.627 11.667 12.227 12.129 12.132 12.264 12.464
   2042   1388151_at   8.193 8.17 8.284 8.324 8.102 7.759 7.523 7.607 7.761 7.643
   2043   1382692_at   8.604 8.62 8.832 8.808 8.26 6.725 6.892 7.149 7.262 6.354
   2044   1371794_at   8.167 8.154 7.898 8.038 8.1 8.723 8.592 8.933 8.716 8.769
   2045   1393244_at   8.315 8.31 8.346 8.425 8.295 8.032 8.074 8.082 8.129 8.035
   2046   1373865_at   4.738 4.448 5.174 4.955 4.316 6.897 6.715 6.96 6.366 6.279
   2047   1370433_at   8.863 8.91 8.765 8.92 9.068 7.927 7.906 8.072 7.872 7.449
   2048   1367605_at   12.588 12.498 12.64 12.358 12.469 11.768 11.844 11.392 11.487 11.601
   2049   1371372_at   11.389 11.392 11.368 11.31 11.416 11.173 11.147 11.156 11.064 11.145
   2050   1392530_at   10.402 10.244 10.339 10.397 10.466 10.874 10.758 10.879 11.024 10.942
   2051   1372389_at   8.105 8.208 8.394 8.587 8.554 7.457 7.31 7.272 7.221 6.975
   2052   1372728_at   7.474 7.481 7.481 7.761 7.767 8.568 8.302 8.75 8.656 8.792
   2053   1398966_at   8.825 8.886 8.962 8.973 8.951 9.202 9.341 9.394 9.346 9.285
   2054   1371126_x_at   3.89 4.241 4.522 4.411 3.487 1.807 0 0 0 0.536
   2055   1396217_at   6.59 6.661 7.055 6.462 6.636 4.561 4.499 3.165 4.038 4.426
   2056   1388352_at   10.274 10.218 10.187 10.11 10.234 9.67 9.665 9.567 9.471 9.308
   2057   1394051_at   6.061 6.11 6.25 6.201 5.778 2.976 2.558 2.305 0.651 1.057
   2058   1384244_at   9.918 9.985 9.921 10.105 9.889 10.329 10.486 10.527 10.577 10.507
   2059   1387085_at   8.839 8.808 8.684 8.856 8.794 8.255 8.249 8.297 7.988 8.054
   2060   1388541_at   9.105 9.041 8.892 9.016 8.904 9.612 9.415 9.551 9.623 9.447
   2061   1382111_at   8.677 8.807 9.205 8.897 8.559 9.989 9.909 9.869 10.086 9.804
   2062   1388870_at   9.332 9.198 9.335 9.271 9.477 9.951 9.861 10.124 10.121 9.886
   2063   1393757_at   7.036 6.93 7.092 7.143 6.826 5.115 3.891 5.287 4.633 4.446
   2064   1392116_at   7.244 7.072 7.275 6.944 7.014 6.394 6.519 6.448 6.404 6.26
   2065   1374431_at   10.813 10.851 10.802 10.812 10.762 11.104 10.986 11.051 11.053 11.002
   2066   1383086_at   6.871 6.726 7.081 6.84 6.743 7.919 7.591 8.062 8.121 8.181
   2067   1380009_at   7.967 7.998 7.57 7.83 7.79 7.024 6.659 7.023 6.861 6.937
   2068   1370071_at   8.599 8.39 8.598 8.807 8.596 7.79 7.701 8.017 7.843 7.782
   2069   1375516_at   10.665 10.636 10.742 10.789 10.669 11.076 11.054 11.207 11.103 11.254
   2070   1392322_at   6.683 6.921 7.312 7.119 6.674 4.168 3.067 2.669 4.109 4.345
   2071   1373546_at   10.159 10.172 9.951 10.062 10.054 10.563 10.554 10.715 10.618 10.484
   2072   1380336_at   7.016 6.661 6.662 6.707 7.034 5.767 5.49 5.657 5.212 5.137
   2073   1368659_at   11.306 11.258 11.185 11.271 11.285 11.687 12.005 11.973 11.841 11.752
   2074   1368041_at   9.064 9.112 9.265 9.373 9.335 9.91 9.78 9.924 9.93 9.787
   2075   1370547_at   8.815 8.716 8.777 8.873 8.306 9.598 9.985 10.126 10.047 9.925
   2076   1386376_at   7.807 7.708 7.891 7.933 7.462 8.924 8.642 9.131 9.179 9.256
   2077   1383171_at   7.857 7.735 7.94 7.931 7.676 8.323 8.354 8.516 8.507 8.43
   2078   1370001_at   9.653 9.781 9.752 9.737 9.708 9.403 9.231 9.353 9.428 9.288
   2079   1394306_at   7.639 8.137 8.267 7.876 7.393 5.22 3.329 2.63 2.98 3.027
   2080   1382255_at   10.833 10.417 10.406 10.166 11.013 8.717 7.352 7.932 8.093 7.42
   2081   1398424_at   8.659 8.649 8.795 8.985 8.75 9.351 9.28 9.402 9.395 9.353
   2082   1372850_at   8.218 8.467 8.295 8.303 8.429 7.721 7.346 7.681 7.486 7.367
   2083   1395147_at   7.005 7.054 7.241 7.136 7.457 6.284 6.324 6.283 6.329 6.517
   2084   1386915_at   10.346 10.364 10.278 10.487 10.523 9.828 9.496 9.772 9.498 9.541
   2085   1385580_at   5.718 5.569 5.484 5.382 5.452 4.633 4.261 4.741 4.71 4.52
   2086   1371596_at   10.216 10.128 10.143 10.115 10.144 9.693 9.69 9.598 9.515 9.368
   2087   1367775_at   9.691 9.675 10.017 9.947 9.11 11.111 11.09 11.201 11.329 11.224
   2088   1397411_at   6.617 6.378 6.386 6.77 6.248 4.411 3.55 4.687 4.208 4.769
   2089   1367657_at   11.583 11.696 11.754 11.597 11.642 11.283 11.062 11.18 11.251 11.105
   2090   1393777_at   8.132 8.144 8.375 8.511 8.182 9.022 8.983 9.243 9.051 9.026
   2091   1388547_at   8.631 8.474 8.151 8.467 8.584 7.698 7.742 7.642 7.574 7.614
   2092   1389584_at   3.449 2.299 3.189 3.118 3.428 5.198 5.079 5.658 5.251 5.04
   2093   1377727_at   7.216 7.164 7.436 7.383 7.942 5.914 5.844 6.255 6.088 5.896
   2094   1368095_at   10.342 10.359 10.399 10.224 10.148 10.794 10.933 10.835 11 10.779
   2095   1388688_at   7.64 7.526 7.815 7.805 7.567 8.464 8.281 8.651 8.509 8.372
   2096   1373283_at   9.005 8.683 8.699 8.712 8.844 8.107 7.902 7.966 7.738 7.658
   2097   1391384_at   7.838 7.555 7.236 7.475 7.313 4.434 2.824 4.151 5.017 3.829
   2098   1392786_at   5.996 5.547 6.141 6.027 6.271 7.267 7.262 7.656 7.38 7.163
   2099   1391547_at   4.931 5.009 5.167 4.963 5.369 6.323 6.192 6.743 6.905 6.873
   2100   1392908_at   8.772 8.948 8.968 8.949 8.962 8.212 8.271 8.27 7.861 8.14
   2101   1367687_a_at   9.484 9.409 9.26 9.493 9.569 10.005 10.022 10.176 9.986 9.957
   2102   1371398_at   11.752 11.643 11.682 11.569 11.71 12.105 12.019 12.066 12.071 12.228
   2103   1367808_at   10.407 10.381 10.636 10.68 10.598 11.136 11.046 11.18 11.198 11.177
   2104   1371793_at   6.339 6.225 6.465 6.56 6.747 7.374 7.548 7.427 7.801 7.477
   2105   1387969_at   12.744 12.629 12.425 12.383 12.612 9.938 8.305 7.748 7.695 7.128
   2106   1382571_at   7.583 7.108 7.348 7.286 7.135 8.169 8.055 8.38 8.23 8.355
   2107   1374254_a_at   8.821 8.747 9.074 9.129 9.004 9.949 9.727 9.962 10.15 10.249
   2108   1398923_at   10.367 10.429 10.516 10.558 10.631 11.055 11.016 11.066 11.293 11.127
   2109   1369929_at   12.272 12.262 12.323 12.104 12.095 11.64 11.601 11.337 11.605 11.409
   2110   1370991_at   7.66 7.636 8.028 7.417 6.821 9.138 9.502 9.461 9.503 9.497
   2111   1376575_at   9.247 9.291 9.013 9.117 9.221 10.014 9.946 10.24 10.104 9.773
   2112   1381470_at   6.487 6.539 6.66 6.726 6.875 7.497 7.436 7.841 7.492 7.527
   2113   1367726_at   5.822 6.004 6.182 6.005 6.192 7.296 6.901 7.221 7.628 7.446
   2114   1370209_at   9.498 9.643 9.641 9.754 9.542 10.24 10.115 10.379 10.317 10.125
   2115   1383565_at   5.674 5.692 5.78 5.908 5.483 4.73 4.395 4.902 4.374 4.621
   2116   1398879_at   10.12 10.206 10.171 10.198 10.339 9.673 9.718 9.849 9.827 9.75
   2117   1370948_a_at   8.783 8.709 8.882 8.502 8.952 7.841 7.425 7.537 7.693 7.191
   2118   1373335_at   8.43 8.148 8.165 8.203 8.182 8.768 8.87 9.108 8.961 8.886
   2119   1375633_at   12.264 12.258 12.226 12.179 12.298 11.83 11.413 11.476 11.558 11.603
   2120   1369816_at   5.927 6.056 5.721 5.438 5.742 6.936 7.088 6.825 6.744 7.155
   2121   1375879_at   8.44 8.508 8.541 8.552 8.596 9.225 9.198 9.394 9.268 8.958
   2122   1388868_at   10.285 10.286 10.23 10.22 10.336 9.754 9.598 9.774 9.804 9.479
   2123   1370880_at   9.998 10.034 10.203 10.322 10.203 9.62 9.517 9.362 9.386 9.408
   2124   1379371_at   10.633 10.656 10.355 10.644 10.638 9.788 9.575 9.986 9.847 9.754
   2125   1378915_x_at   6.277 6.525 6.742 6.359 6.518 4.944 4.255 3.648 4.364 4.69
   2126   1375034_at   8.665 8.667 8.893 9.054 8.713 8.166 7.87 7.961 7.919 7.916
   2127   1373110_at   7.515 7.589 7.689 7.618 7.438 8.444 8.281 8.886 8.69 8.689
   2128   1371412_a_at   8.721 8.766 8.449 8.944 9.324 10.576 10.083 10.779 10.71 10.551
   2129   1392668_at   7.588 7.419 7.591 7.492 7.331 6.781 6.351 6.325 6.105 6.212
   2130   1376733_at   8.502 8.586 8.749 8.897 8.664 9.744 9.462 10.023 10.035 9.78
   2131   1392464_at   8.323 8.225 8.313 8.404 8.291 7.888 7.842 7.913 7.675 7.893
   2132   1392681_at   10.662 10.602 10.701 10.716 10.685 11.06 10.919 11.126 11 11.048
   2133   1367812_at   6.236 6.35 6.44 6.794 6.551 7.543 7.289 7.603 7.69 7.708
   2134   1370187_at   10.366 10.269 10.438 10.414 10.268 10.862 10.872 10.843 10.994 11.134
   2135   1394789_at   5.351 5.619 5.615 5.909 5.611 6.449 6.377 6.53 6.565 6.631
   2136   1379669_at   7.09 7.075 7.212 7.467 7.287 8.005 7.822 8.021 8.021 7.925
   2137   1389213_at   7.535 7.491 7.492 7.762 7.626 6.576 6.82 6.864 6.794 6.999
   2138   1378241_at   7.06 7.068 7.165 7.296 7.408 7.919 7.783 7.977 7.929 8.07
   2139   1372592_at   8.248 8.269 8.561 8.681 8.197 9.433 9.189 9.551 9.444 9.507
   2140   1391563_at   3.349 3.136 3.152 3.853 2.935 5.126 4.772 5.013 4.974 4.627
   2141   1387253_at   3.834 4.144 4.452 4.059 4.231 5.184 5.392 5.183 5.129 5.532
   2142   1383813_at   7.86 7.801 8.109 8.068 8.006 8.663 8.547 8.677 8.634 8.481
   2143   1398245_at   5.889 6.277 6.387 6.421 5.828 7.22 7.356 7.407 7.289 7.399
   2144   1368527_at   6.852 7.023 6.843 7.018 7.028 5.702 5.07 5.637 5.396 4.757
   2145   1372258_at   9.2 9.25 9.259 9.303 9.181 9.524 9.45 9.562 9.528 9.47
   2146   1393997_at   7.84 7.651 7.496 7.52 7.319 8.397 8.762 8.76 8.477 8.547
   2147   1389402_at   7.671 7.64 7.45 7.872 7.733 6.319 6.238 6.592 5.985 5.686
   2148   1391527_at   8.64 8.688 8.639 8.858 8.846 8.083 7.62 7.716 7.742 7.993
   2149   1367629_at   12.185 12.116 12.219 12.073 12.115 12.503 12.545 12.422 12.456 12.593
   2150   1372794_at   7.596 7.68 8.078 7.545 7.54 8.853 8.603 9.064 8.861 8.732
   2151   1372162_at   9.904 9.906 10.14 10.205 9.813 10.661 10.656 10.773 10.814 10.722
   2152   1370105_at   6.516 6.728 7.012 7.004 7.027 5.068 4.558 5.392 5.014 5.51
   2153   1379287_at   8.245 8.425 8.237 8.469 8.384 7.227 7.111 7.331 6.533 7.015
   2154   1384837_at   8.625 8.669 8.859 8.947 8.447 4.982 2.17 1.459 0.848 1.876
   2155   1370280_at   10.647 10.682 10.823 10.757 10.79 10.211 10.011 10.221 9.945 9.883
   2156   1387872_at   10.031 10.076 9.985 10.21 10.346 9.353 9.137 8.991 8.786 8.813
   2157   1372876_at   9.741 9.79 9.941 9.795 9.586 10.32 10.407 10.331 10.377 10.246
   2158   1390001_at   4.611 4.791 4.609 4.593 4.366 5.452 5.654 5.722 5.326 5.388
   2159   1367953_at   5.877 6.012 5.888 5.717 6.292 6.999 6.837 7.295 7.25 7.092
   2160   1390923_a_at   7.677 7.76 8.112 7.88 7.856 8.535 8.475 8.588 8.59 8.666
   2161   1373528_at   8.291 8.359 8.307 8.267 8.16 6.127 4.7 6.029 6.023 6.115
   2162   1390731_at   8.912 8.837 8.501 8.739 8.995 7.46 6.633 7.067 7.492 7.299
   2163   1367880_at   6.935 6.993 6.996 7.226 7.422 8.104 7.853 8.129 8.054 8.1
   2164   1367901_at   8.914 8.95 9.024 9.119 9.194 8.264 8.069 8.17 8.271 7.782
   2165   1390124_at   7.895 8.017 7.679 7.72 7.936 7.248 7.148 7.188 6.998 6.998
   2166   1398903_at   10.435 10.523 10.543 10.655 10.622 10.97 11.171 11.064 11.192 11.003
   2167   1376465_at   6.054 6.045 5.883 6.182 6.158 7.047 6.899 7.34 7.044 6.783
   2168   1392579_at   9.513 9.464 9.568 9.504 9.319 8.496 8.539 8.485 8.566 7.969
   2169   1376868_at   8.696 8.814 9.103 9.009 8.691 9.727 9.765 9.987 9.776 9.623
   2170   1375272_at   8.72 8.973 8.858 8.83 8.692 8.376 8.221 8.344 8.251 8.242
   2171   1390386_at   8.746 8.678 8.73 8.649 8.678 7.89 7.758 7.749 7.692 7.223
   2172   1371965_at   9.186 9.09 9.179 9.187 9.079 9.594 9.677 9.825 9.577 9.545
   2173   1388764_at   10.376 10.398 10.29 10.329 10.263 10.01 9.932 9.942 9.848 9.773
   2174   1379352_at   6.036 5.952 5.786 6.006 5.99 6.395 6.49 6.453 6.643 6.475
   2175   1397526_at   9.005 8.939 9.059 9.072 8.693 9.517 9.637 9.682 9.705 9.633
   2176   1373715_at   7.115 7.179 6.969 7.086 7.092 6.609 6.542 6.278 6.546 6.511
   2177   1388892_at   8.099 8.074 8.25 8.272 8.188 8.649 8.555 8.774 8.616 8.691
   2178   1393197_at   6.568 6.483 6.288 6.222 6.657 7.273 7.168 7.414 7.439 7.249
   2179   1371863_at   8.56 8.78 8.368 8.518 8.613 7.818 7.55 7.637 7.313 7.328
   2180   1380616_at   6.332 6.235 6.116 5.814 5.903 3.57 3.434 2.428 1.609 2.866
   2181   1397729_x_at   6.886 6.785 7.364 7.289 7.107 8.431 8.333 8.575 8.037 8.425
   2182   1379935_at   10.305 10.173 9.893 10.189 10.278 6.123 5.111 4.083 2.202 4.27
   2183   1383519_at   8.007 8.274 7.927 8.352 8.248 6.151 6.353 6.405 5.144 5.816
   2184   1372343_at   7.732 7.934 7.937 7.653 7.884 7.102 6.906 7.212 6.828 7.078
   2185   1390822_at   10.698 10.561 10.425 10.392 10.671 11.38 11.538 11.386 11.343 11.128
   2186   1389778_a_at   8.154 8.354 8.583 8.442 8.443 7.81 7.672 7.68 7.745 7.63
   2187   1388622_at   9.208 9.173 9.017 9.345 9.333 8.464 8.723 8.517 8.5 8.509
   2188   1386410_at   7.236 7.264 7.446 7.636 7.445 6.21 6.126 6.455 6.116 5.679
   2189   1396305_at   4.492 4.419 4.114 4.204 4.555 5.763 5.766 5.761 6.271 5.408
   2190   1374505_at   10.302 10.348 10.434 10.188 10.407 9.799 9.595 9.73 9.815 9.52
   2191   1376870_at   5.468 5.23 5.535 5.933 5.573 6.877 6.544 7.094 7.12 6.908
   2192   1394786_at   8.924 8.941 9.238 9.412 9.125 10.048 9.941 10.27 10.085 10.036
   2193   1370728_at   8.559 8.711 8.482 8.366 8.428 7.517 7.85 7.4 7.34 7.3
   2194   1367955_at   8.045 7.938 7.979 8.148 8.118 7.446 7.383 7.01 7.102 7.239
   2195   1374443_at   8.831 8.938 8.911 8.998 8.958 9.257 9.206 9.192 9.32 9.261
   2196   1387651_at   10.676 10.631 11.061 10.868 10.584 11.62 11.629 11.528 11.733 11.814
   2197   1387135_at   8.766 8.918 8.9 8.959 9.09 8.166 8.228 8.324 8.35 8.012
   2198   1385128_at   8.202 8.155 8.048 8.207 8.313 7.54 7.448 7.327 7.323 7.032
   2199   1374959_at   8.877 8.901 8.984 8.906 8.601 9.384 9.596 9.614 9.594 9.544
   2200   1398732_at   5.598 5.781 5.442 5.603 6.142 6.734 6.928 7.189 7.177 7.081
   2201   1393335_at   5.307 4.892 4.977 5.119 5.229 6.424 5.886 6.448 6.271 6.167
   2202   1381969_at   9.273 9.413 9.475 9.545 9.5 8.733 8.355 8.775 8.732 8.57
   2203   1378345_at   6.624 6.598 6.66 6.372 6.565 5.496 5.707 5.58 5.995 5.659
   2204   1372493_at   8.113 8.247 8.014 8.288 8.517 7.314 7.236 7.5 7.392 7.178
   2205   1374756_at   6.828 6.637 6.381 6.726 6.659 5.464 5.414 5.483 4.923 4.833
   2206   1396127_at   3.892 5.13 5.045 4.446 4.368 6.66 6.605 6.649 6.853 6.547
   2207   1372758_at   9.463 9.399 9.431 9.407 9.406 10.041 10.055 10.231 9.934 9.834
   2208   1376860_at   6.702 6.862 7.155 6.891 6.452 7.796 8.188 8.115 8.26 7.985
   2209   1388294_at   11.129 11.126 11.27 11.121 11.104 11.601 11.466 11.581 11.542 11.696
   2210   1384941_at   5.457 5.333 5.586 5.219 5.485 5.974 6.164 6.002 6.097 6.04
   2211   1392603_at   8.149 8.057 8.079 8.257 8.088 8.588 8.445 8.653 8.613 8.645
   2212   1385414_at   9.421 9.397 9.837 9.624 9.318 5.583 2.787 3.222 2.858 1.111
   2213   1370320_at   10.206 10.14 10.165 10.126 10.076 10.552 10.66 10.636 10.887 10.796
   2214   1385486_at   7.038 7.386 7.326 7.486 7.537 8.351 8.516 8.966 8.691 8.434
   2215   1373016_at   9.331 9.49 9.585 9.73 9.635 10.232 10.213 10.139 10.29 10.149
   2216   1382287_at   8.713 8.725 8.628 8.537 8.639 8.041 8.018 7.653 7.641 7.813
   2217   1368418_a_at   10.829 10.877 10.651 10.776 10.899 10.043 9.605 9.985 9.871 9.515
   2218   1389286_at   10.291 10.244 10.344 10.543 10.452 10.884 10.844 11.006 10.905 10.918
   2219   1387839_at   6.615 6.283 6.006 6.121 6.333 5.015 4.106 4.474 4.19 4.753
   2220   1379326_at   7.319 7.636 8.112 7.229 7.467 8.833 9.059 9.204 9.191 9.002
   2221   1371371_at   11.458 11.309 11.589 11.49 11.593 12.041 11.984 11.931 11.998 11.943
   2222   1367835_at   6.175 6.31 6.475 6.69 6.229 7.497 7.245 7.405 7.754 7.494
   2223   1369393_at   6.279 6.428 6.092 6.325 6.628 4.923 4.982 4.323 4.006 4.366
   2224   1374833_at   5.273 5.799 5.528 5.695 5.263 6.589 6.713 7.06 7.072 6.68
   2225   1391840_at   6.787 6.978 6.887 6.847 6.962 7.723 8.277 8.026 8.181 7.695
   2226   1389466_at   10.273 10.328 10.552 10.619 10.266 11.2 11.048 11.385 11.32 11.228
   2227   1370806_at   10.714 10.664 10.662 10.863 10.557 11.202 11.245 11.491 11.429 11.432
   2228   1370170_at   10.692 10.69 10.609 10.809 10.799 10.185 10.356 10.133 10.148 10.067
   2229   1398932_at   11.299 11.252 11.228 11.25 11.324 11.499 11.628 11.577 11.507 11.634
   2230   1368414_at   8.745 8.589 8.767 9.018 8.496 9.7 9.662 9.666 9.483 9.509
   2231   1378181_at   7.955 8.167 7.955 8.13 8.258 6.954 7.179 7.399 7.028 6.83
   2232   1385690_at   8.11 8.03 8.142 8.055 7.81 8.535 8.577 8.543 8.526 8.576
   2233   1368032_at   8.41 8.484 8.359 8.604 8.763 7.809 7.71 7.772 7.538 7.476
   2234   1378169_at   7.033 6.954 6.983 6.714 7.112 7.632 7.62 7.797 7.554 7.636
   2235   1392755_at   7.985 7.535 7.826 7.86 7.093 6.086 5.892 5.449 5.149 5.403
   2236   1371251_at   8.18 8.391 8.382 8.297 8.511 9.145 8.882 9.077 9.238 9.256
   2237   1375368_at   8.701 8.791 8.657 8.805 8.703 9.091 9.047 9.133 9.03 9.2
   2238   1398784_at   11.188 11.141 11.192 11.182 11.302 10.669 10.648 10.68 10.895 10.61
   2239   1382277_at   8.378 8.421 8.375 8.576 8.782 7.492 7.079 7.285 7.042 6.667
   2240   1369451_a_at   8.839 9.017 9.165 8.946 9.071 10.019 9.711 9.68 10.025 10.077
   2241   1375247_at   9.299 9.29 9.588 9.4 9.188 10.725 10.322 10.473 11.095 10.836
   2242   1377923_at   8.417 8.424 8.458 8.663 8.48 8.953 8.934 9.04 8.935 9.116
   2243   1387287_a_at   3.869 4.151 3.508 3.432 3.926 5.547 5.126 5.744 5.096 5.536
   2244   1377879_at   5.987 6.165 5.959 6.365 5.919 6.881 6.84 7.152 7.135 6.988
   2245   1373659_at   4.041 4.151 4.345 4.331 4.613 5.633 5.207 5.727 5.526 5.856
   2246   1368304_at   9.691 9.639 9.73 9.809 8.882 11.07 10.94 11.228 11.284 11.324
   2247   1389392_at   7.09 7.157 7.117 7.271 7.128 7.941 7.819 8.409 8.173 8.217
   2248   1369122_at   8.77 8.619 8.65 8.812 9.059 8.086 8.064 8.061 8.101 7.939
   2249   1374025_at   6.642 6.789 6.763 6.901 6.668 7.311 7.481 7.508 7.413 7.212
   2250   1382137_at   6.298 6.672 6.191 6.596 6.324 7.559 7.46 7.706 7.215 7.52
   2251   1379900_at   5.529 5.888 5.789 5.763 5.745 6.561 6.592 6.381 6.844 6.529
   2252   1372373_at   7.327 7.306 7.289 7.378 7.347 7.947 7.709 8.063 7.991 7.813
   2253   1389548_at   8.545 8.664 8.898 8.619 8.239 9.658 9.436 9.727 9.643 9.723
   2254   1387905_at   7.953 8.143 8.206 8.232 8.031 8.669 8.643 8.879 8.748 8.897
   2255   1391502_at   6.173 6.415 6.151 6.462 6.625 4.922 4.295 4.22 5.111 4.344
   2256   1387391_at   7.068 7.69 7.418 7.443 7.295 5.725 5.2 4.19 4.618 4.931
   2257   1397287_at   5.821 5.841 5.948 6.145 5.782 6.524 6.796 6.84 6.935 6.68
   2258   1375270_at   6.951 6.743 6.772 6.965 6.844 7.647 7.422 7.809 7.799 7.935
   2259   1383863_at   8.757 8.649 8.683 8.689 8.954 8.014 7.589 7.83 7.994 7.596
   2260   1388791_at   8.493 8.477 8.477 8.842 8.653 7.798 7.364 7.77 7.702 7.52
   2261   1389521_at   10.012 9.959 10.224 10.216 10.356 10.984 10.824 11.059 10.813 10.911
   2262   1371559_at   10.042 10.147 10.108 10.065 10.168 9.83 9.636 9.729 9.793 9.76
   2263   1392969_at   10.425 10.337 10.631 10.666 10.357 11.118 11.188 11.334 11.43 11.414
   2264   1392973_at   6.325 6.419 6.695 6.662 6.641 7.21 7.386 7.377 7.183 7.346
   2265   1391073_at   4.599 4.5 4.721 4.76 4.598 5.654 5.424 5.508 5.448 5.964
   2266   1371137_at   4.752 4.83 4.883 5.527 4.886 6.377 6.12 6.221 6.417 6.317
   2267   1371805_at   8.213 8.228 8.099 8.266 8.083 8.621 8.669 8.895 8.891 8.869
   2268   1368253_at   8.261 8.495 8.638 8.541 8.149 9.417 9.24 9.661 9.711 9.635
   2269   1390195_at   7.011 6.863 6.866 6.97 6.787 5.569 4.833 5.057 5.245 4.298
   2270   1392913_at   7.691 7.664 7.742 7.642 7.834 6.993 6.642 7.039 7.104 6.81
   2271   1374724_at   5.935 6.165 6.228 6.257 5.925 6.722 7.038 7.145 7.011 7.08
   2272   1379315_at   7.343 7.561 7.392 7.557 7.362 8.121 7.951 8.015 7.874 8.047
   2273   1388516_at   10.201 10.069 10.029 10.201 10.107 9.644 9.471 9.732 9.678 9.636
   2274   1390937_at   8.044 8.054 8.039 8.319 8.212 8.821 8.659 8.988 8.765 8.846
   2275   1373686_at   1.546 0 1.189 0 0 3.95 4.457 3.946 4.928 3.568
   2276   1379073_at   7.283 7.42 7.195 7.692 6.737 9.061 8.533 9.04 9.034 9.235
   2277   1367694_at   11.182 11.189 11.214 11.089 11.147 11.426 11.425 11.417 11.549 11.506
   2278   1377015_at   6.563 6.596 6.423 6.734 6.889 7.41 7.335 7.508 7.351 7.347
   2279   1382138_at   6.636 6.96 6.688 6.665 6.914 5.897 5.575 5.938 5.708 5.389
   2280   1383542_at   8.528 8.317 8.344 8.327 8.352 7.36 7.035 7.061 6.397 6.947
   2281   1376571_at   7.555 7.536 7.623 7.665 7.765 8.08 8.314 8.352 8.496 8.363
   2282   1371529_at   8.391 8.337 8.43 8.676 8.589 7.806 7.36 7.712 7.644 7.526
   2283   1384345_at   5.911 5.814 5.935 5.884 5.878 6.736 6.794 6.439 6.423 6.514
   2284   1389468_at   8.897 8.849 8.905 9.186 9.185 8.38 8.152 8.213 8.034 8.163
   2285   1387914_at   8.988 8.755 8.442 8.677 8.893 7.608 7.359 7.218 7.335 6.736
   2286   1393956_at   8.869 8.917 9.113 8.891 8.754 8.35 8.185 8.183 8.069 7.965
   2287   1387060_at   9.127 9.011 9.113 9.346 9.558 8.083 7.777 8.026 7.616 7.346
   2288   1383080_at   11.01 11.027 10.909 10.777 10.9 10.369 10.192 10.348 10.157 9.998
   2289   1376230_at   6.731 6.618 6.456 6.563 6.816 7.544 7.871 7.633 7.623 7.31
   2290   1388674_at   8.375 8.518 8.767 8.871 8.587 7.703 7.158 7.19 6.867 7.123
   2291   1370243_a_at   12.323 12.224 12.223 12.216 12.382 11.882 11.906 11.8 11.678 11.741
   2292   1383073_at   10.39 10.422 10.401 10.528 10.425 10.042 9.877 10.115 10.005 9.895
   2293   1387472_at   8.392 8.373 9.065 8.78 8.224 5.355 4.29 4.616 4.618 2.561
   2294   1368647_at   8.159 8.076 8.164 8.261 7.919 7.315 7.266 7.602 7.445 7.323
   2295   1377672_at   9.437 9.331 9.912 9.505 8.922 10.91 10.778 10.985 10.9 10.737
   2296   1383786_at   9.52 9.56 9.524 9.309 9.885 8.542 8.632 8.494 8.13 8.256
   2297   1377404_at   6.664 6.477 6.548 6.808 6.817 7.5 7.842 8.003 7.484 7.795
   2298   1375900_at   7.059 7.7 7.66 8.165 7.417 5.788 5.649 5.908 6.044 5.419
   2299   1369986_at   9.398 9.391 9.668 9.833 9.365 10.346 10.331 10.569 10.566 10.52
   2300   1374861_at   4.938 4.631 5.378 5.451 5.567 7.116 6.641 6.911 7.061 6.796
   2301   1383495_at   6.722 6.583 6.679 6.676 6.679 6.981 7.049 7.081 7.141 6.939
   2302   1383443_at   6.763 6.964 6.933 7.069 6.902 7.501 7.81 7.772 7.797 7.51
   2303   1373680_at   8.432 8.379 8.387 8.746 8.173 9.193 9.235 9.412 9.42 9.34
   2304   1383220_at   5.892 5.429 5.451 5.638 5.545 6.766 6.412 6.834 6.91 6.529
   2305   1398264_at   6.276 5.938 6.165 6.298 5.831 6.869 6.994 7.107 7.213 7.115
   2306   1373607_at   7.222 7.077 6.986 7.193 6.836 7.664 7.721 7.689 7.842 7.801
   2307   1389275_at   7.536 7.382 7.386 7.626 7.155 4.623 5.352 3.417 3.517 4.149
   2308   1371342_at   11.381 11.305 11.295 11.358 11.49 11.813 11.777 11.744 11.675 11.698
   2309   1395887_at   6.288 5.855 6.487 6.413 5.765 7.547 7.526 7.386 7.412 7.517
   2310   1376281_at   7.003 6.754 6.986 7.023 7.009 5.252 5.8 5.159 5.476 5.971
   2311   1377940_at   6.557 6.821 7.009 7.105 6.463 8.206 7.893 8.598 8.344 8.246
   2312   1373521_at   6.139 6.495 6.372 6.626 6.247 7.587 7.333 7.924 7.367 7.643
   2313   1368214_at   8.447 8.569 8.553 8.444 8.538 8.176 8.016 8.034 7.866 8.03
   2314   1377408_at   6.807 6.618 6.651 6.878 6.471 8.017 7.79 7.73 8.077 8.499
   2315   1387432_at   6.919 7.317 7.233 7.289 6.778 6.159 5.793 6.086 5.972 6.068
   2316   1369318_at   6.168 6.037 5.716 6.005 5.992 7.009 7.455 7.578 7.256 6.927
   2317   1388332_at   11.318 11.304 11.214 11.304 11.377 10.949 10.685 10.904 10.807 10.735
   2318   1372744_at   8.915 8.931 8.905 9.133 8.773 9.511 9.545 9.849 9.708 9.702
   2319   1376336_at   7.517 7.717 7.606 7.608 7.843 7.04 6.921 7.137 6.83 6.849
   2320   1390767_at   8.145 8.091 8.169 8.125 8.288 7.756 7.675 7.652 7.5 7.472
   2321   1394479_at   7.704 7.76 7.868 7.918 7.559 8.496 8.455 8.761 8.808 8.867
   2322   1374208_at   8.389 8.325 8.233 8.426 8.606 7.35 7.385 6.883 7.554 7.282
   2323   1378605_at   8.164 8.018 7.947 7.964 7.83 8.509 8.506 8.416 8.575 8.533
   2324   1383107_at   7.99 7.851 7.934 7.9 7.964 7.313 6.876 7.297 6.912 7.105
   2325   1386967_at   9.161 9.156 9.134 9.236 9.282 8.587 8.524 8.526 8.626 8.198
   2326   1384695_at   5.496 5.569 5.372 6.115 5.398 7.262 6.763 6.877 6.947 7.094
   2327   1369683_at   8.235 8.204 8.413 8.38 8.368 6.892 6.809 5.71 6.423 6.516
   2328   1381216_at   6.268 6.321 6.876 6.651 6.764 4.404 4.483 4.276 4.334 3.104
   2329   1397168_at   5.967 5.885 5.959 5.504 6.051 6.989 7.106 7.416 6.853 6.94
   2330   1388486_at   7.75 7.655 7.72 7.985 7.628 8.398 8.377 8.552 8.33 8.334
   2331   1389510_at   8.36 8.37 8.195 8.406 8.548 7.496 7.053 7.031 6.627 6.765
   2332   1372449_at   8.118 8.29 8.513 8.4 8.411 9.198 8.951 9.168 9.298 9.375
   2333   1373749_at   6.702 6.924 7.361 7.164 6.616 8.157 8.167 8.406 8.57 8.439
   2334   1370481_at   7.539 7.391 7.538 7.5 7.358 7.976 8.28 7.939 8.139 8.149
   2335   1382454_at   12.69 12.605 12.256 12.304 12.548 9.33 7.615 6.823 6.7 5.759
   2336   1390838_at   6.141 6.27 6.508 6.524 6.276 7.384 7.268 7.966 7.73 7.646
   2337   1388102_at   7.895 8 7.736 7.943 8.168 8.581 8.52 8.619 8.606 8.557
   2338   1392794_at   7.35 7.644 6.955 7.143 7.208 6.002 5.615 4.978 5.355 5.183
   2339   1387072_at   7.869 8.054 7.704 7.757 8.143 8.802 8.607 8.935 8.976 8.983
   2340   1388384_at   5.037 5.002 4.525 4.663 4.95 6.062 5.794 6.091 6.097 5.728
   2341   1373947_at   11.175 11.109 10.927 10.928 11.195 10.542 10.312 10.353 10.503 10.309
   2342   1390967_a_at   7.582 7.45 7.757 7.703 7.541 7.084 7.098 7.09 7.109 7.167
   2343   1387223_at   11.987 11.896 12.111 11.89 11.712 12.672 12.554 12.468 12.58 12.711
   2344   1377755_at   7.741 7.957 8.01 7.859 7.804 7.328 7.413 7.211 7.114 7.194
   2345   1388129_at   9.345 9.343 9.286 9.499 9.467 8.794 8.602 8.839 8.833 8.515
   2346   1389425_at   7.67 7.641 7.822 7.936 8.134 6.711 6.731 6.511 6.915 6.289
   2347   1391109_x_at   5.805 5.329 5.795 5.825 5.428 7.045 6.964 7.096 7.571 7.761
   2348   1367951_at   1.7 2.457 1.828 0.604 0 5.899 5.157 6.375 5.633 5.151
   2349   1388333_at   11.295 11.301 11.244 11.059 11.276 10.802 10.757 10.624 10.572 10.571
   2350   1374709_at   5.448 5.748 5.834 5.736 5.344 6.71 7.104 7.188 6.828 6.609
   2351   1384239_at   8.134 7.919 8.073 8.081 8.048 7.366 7.432 7.522 7.265 7.086
   2352   1370789_a_at   7.36 7.339 7.632 7.662 6.957 8.59 8.613 9.174 8.831 8.912
   2353   1389549_at   9.679 9.714 9.886 9.833 9.598 10.275 10.354 10.311 10.312 10.154
   2354   1396455_at   4.531 4.434 4.279 4.462 4.166 6.158 5.795 5.254 6.005 5.905
   2355   1368071_at   9.209 9.348 9.386 9.418 9.354 10.062 9.816 10.187 10.001 9.88
   2356   1398831_at   12.02 11.952 11.945 11.787 11.958 11.477 11.491 11.49 11.337 11.269
   2357   1378166_at   7.36 7.207 7.315 7.415 7.345 6.932 7.01 6.965 6.837 6.856
   2358   1374524_at   9.655 9.602 9.81 9.779 9.608 10.431 10.151 10.479 10.538 10.568
   2359   1392922_at   8.103 8.264 8.363 8.267 8.179 7.739 7.444 7.547 7.619 7.348
   2360   1385066_a_at   6.828 6.681 6.669 7.002 6.99 6.07 5.821 6.187 5.902 5.837
   2361   1393028_at   7.145 7.037 6.884 7.029 6.975 7.383 7.581 7.593 7.498 7.477
   2362   1383943_at   6.643 6.835 7.034 7.168 6.595 8.185 8.455 8.4 8.199 7.814
   2363   1392454_at   7.566 7.711 7.545 7.759 8.02 6.851 6.632 6.966 6.688 6.545
   2364   1377725_at   8.058 8.17 8.275 8.211 8.171 8.908 8.619 9.075 8.974 8.913
   2365   1371493_at   8.547 8.491 8.485 8.678 8.582 9.199 9.007 9.317 9.335 9.081
   2366   1383407_at   7.878 7.833 7.942 7.768 7.762 5.428 5.338 5.238 3.57 4.703
   2367   1387588_at   7.101 6.996 7.172 7.27 6.952 6.575 6.481 6.562 6.4 6.578
   2368   1367678_at   11.24 11.294 11.402 11.431 11.351 11.715 11.633 11.752 11.662 11.733
   2369   1383936_at   7.612 7.546 7.771 7.894 8.161 5.78 5.768 5.838 5.645 4.561
   2370   1372810_at   9.643 9.599 9.749 9.633 9.532 9.13 8.755 8.939 8.649 8.791
   2371   1392477_at   7.778 7.796 8.209 7.687 8.128 8.743 8.897 8.997 9.084 8.984
   2372   1373473_a_at   8.87 8.891 8.631 8.916 9.075 8.183 8.059 8.19 7.846 7.995
   2373   1391856_at   9.25 9.126 9.091 9.377 9.121 9.804 9.717 9.931 9.977 10.069
   2374   1367715_at   10.022 10.045 10.169 10.2 10.341 9.49 9.014 9.11 9.061 8.828
   2375   1368460_at   7.552 7.895 7.797 8.119 7.114 9.294 9.06 9.491 9.301 9.257
   2376   1373631_at   7.426 7.372 7.483 7.463 7.427 8.173 8.082 8.39 8.5 8.667
   2377   1370714_a_at   7.932 7.836 8.053 7.944 7.312 9.191 8.991 8.961 9.233 9.44
   2378   1381445_at   6.751 6.715 6.779 6.954 6.975 7.641 7.773 8.106 8.081 7.625
   2379   1374771_at   7.882 8.097 7.845 8.156 8.162 9.245 8.681 9.187 9.138 9.171
   2380   1385058_at   9.861 10.058 10.161 9.989 10.098 10.59 10.5 10.686 10.613 10.488
   2381   1376804_at   8.809 8.691 8.594 9.02 8.984 9.575 9.667 9.7 9.639 9.465
   2382   1398341_at   8.745 8.738 8.932 8.967 8.443 9.804 9.56 9.868 10.051 9.78
   2383   1383121_at   8.559 8.69 8.664 8.899 8.663 9.291 9.436 9.564 9.631 9.307
   2384   1377058_at   6.092 6.507 6.645 6.567 6.598 5.569 5.633 5.28 5.398 5.476
   2385   1382134_at   7.756 7.648 7.787 7.511 7.942 8.373 8.591 8.482 8.466 8.343
   2386   1388552_at   9.626 9.685 9.839 9.703 9.711 10.207 10.036 10.142 10.263 10.253
   2387   1378043_at   7.555 7.464 7.549 7.217 7.315 4.491 3.359 4.141 2.637 1.903
   2388   1372815_at   9.961 9.896 9.896 9.894 10.064 9.397 9.311 9.44 9.149 9.073
   2389   1389633_at   8.624 8.722 8.877 8.832 8.661 8.118 7.954 7.911 8.243 7.879
   2390   1374433_at   8.814 8.823 8.709 8.818 8.636 8.099 7.923 8.21 8.108 7.774
   2391   1387941_s_at   6.311 6.542 6.088 6.3 6.5 7.616 7.284 7.597 7.918 8.051
   2392   1382763_at   6.919 6.91 7.025 7.16 6.899 7.436 7.44 7.374 7.433 7.478
   2393   1372481_at   6.695 7.242 6.802 6.998 7.371 8.138 8.089 8.193 8.363 8.151
   2394   1389469_at   8.435 8.589 8.486 8.813 8.399 9.29 9.336 9.728 9.541 9.445
   2395   1367747_at   8.051 7.934 8.099 8.086 8.349 8.676 8.851 8.941 8.992 8.803
   2396   1374247_at   4.063 4.56 3.731 4.313 4.854 6.546 6.187 6.648 6.464 5.884
   2397   1385933_at   9.27 9.332 9.522 9.253 9.286 8.611 8.569 8.568 8.366 8.127
   2398   1397356_at   5.319 5.294 5.573 5.63 5.618 6.433 6.357 6.191 6.538 6.199
   2399   1387157_at   5.454 5.433 5.36 5.379 5.614 6.25 6.475 6.832 6.246 6.634
   2400   1399019_at   9.403 9.43 9.465 9.531 9.495 9.868 9.7 9.847 9.83 9.751
   2401   1396820_at   9.19 9.213 9.329 9.301 9.42 8.86 8.774 8.591 8.633 8.558
   2402   1374110_at   6.15 6.249 6.304 6.068 6.423 7.002 6.877 6.963 6.773 7.093
   2403   1398358_a_at   8.51 8.289 8.212 8.32 8.123 9.263 8.969 9.111 9.49 9.233
   2404   1394470_at   6.859 6.642 6.673 6.581 6.959 4.598 4.162 2.943 4.016 4.615
   2405   1384228_at   6.694 7.142 7.195 7.135 6.919 5.303 4.369 4.434 3.683 4.92
   2406   1391212_at   6.562 6.63 6.488 6.839 6.808 7.489 7.552 7.851 7.653 7.361
   2407   1368811_at   7.549 7.559 7.568 7.935 7.809 6.644 6.269 6.348 6.006 5.736
   2408   1392514_at   7.318 7.234 6.993 7.31 7.645 6.42 6.272 6.317 6.028 6.172
   2409   1388487_at   10.283 10.295 10.35 10.362 10.248 10.722 10.531 10.689 10.674 10.619
   2410   1370201_at   10.535 10.489 10.627 10.141 10.285 11.454 11.281 11.216 11.632 11.547
   2411   1379493_at   6.271 6.283 6.121 6.013 6.46 5.589 5.394 5.406 5.541 5.316
   2412   1377153_a_at   7.376 7.413 7.567 7.467 7.185 6.588 6.234 5.807 6.139 5.958
   2413   1373895_at   7.232 7.34 7.426 6.817 7.055 8.265 8.121 8.206 8.373 8.562
   2414   1386866_at   10.714 10.714 10.699 10.937 10.936 10.189 10.137 10.237 10.049 9.898
   2415   1372886_at   7.845 7.905 7.814 7.855 7.899 7.134 6.637 6.465 6.279 6.41
   2416   1389274_at   6.932 7 6.816 6.985 7.238 7.745 7.942 8.123 7.738 7.769
   2417   1395381_at   6.816 6.756 6.814 6.483 6.917 7.544 7.392 7.757 7.533 7.673
   2418   1383205_at   7.502 7.6 7.343 7.348 7.336 8.257 8.13 8.606 8.514 8.194
   2419   1384614_at   5.848 6.172 6.312 6.385 6.1 8.009 7.394 7.711 8.269 8.453
   2420   1390783_at   7.366 7.497 6.793 7.196 7.782 8.912 8.724 8.864 8.882 8.625
   2421   1399107_at   7.759 7.633 7.801 7.703 7.747 8.225 8.228 8.309 8.311 8.034
   2422   1372684_at   6.569 5.979 5.773 6.618 6.598 7.834 7.764 8.067 8.108 7.899
   2423   1367970_at   6.259 6.145 6.092 6.219 6.517 7.279 6.901 7.013 7.074 7.044
   2424   1371936_at   11.636 11.641 11.617 11.751 11.869 11.101 11.083 11.064 10.743 10.857
   2425   1368118_at   9.609 9.498 9.69 9.809 9.701 9.11 8.913 9.072 8.928 8.744
   2426   1378569_at   3.944 4.307 4.756 3.756 4.262 6.041 6.062 6.168 5.744 5.601
   2427   1394761_at   7.156 7.397 7.223 7.489 7.326 7.914 7.816 7.843 7.975 8.002
   2428   1384852_at   7.374 7.351 7.588 7.549 7.385 6.05 5.475 5.822 4.858 4.978
   2429   1373810_at   6.808 6.857 6.983 6.907 6.679 7.472 7.432 7.382 7.299 7.292
   2430   1372490_at   8.321 8.547 8.542 8.552 8.524 9.117 9.229 9.593 9.487 9.252
   2431   1390576_at   8.305 8.425 8.213 8.384 8.321 7.777 7.646 7.899 7.674 7.518
   2432   1398743_at   1.84 0.454 1.189 1.411 0.632 3.632 3.541 3.529 3.964 3.108
   2433   1386660_at   5.945 6.086 6.134 6.202 6.386 5.493 4.989 5.251 5.081 5.144
   2434   1373529_at   9.693 9.634 9.697 9.755 9.773 10.331 10.153 10.464 10.318 10.127
   2435   1390415_at   6.949 6.95 6.744 6.834 7.062 6.169 5.754 5.746 5.431 5.88
   2436   1367743_at   8.952 8.982 9.038 9.188 9.035 8.354 8.272 8.409 7.981 8.399
   2437   1384035_at   6.332 6.297 6.218 6.516 6.648 7.369 7.836 7.636 7.574 7.221
   2438   1387382_at   8.455 8.368 8.493 8.698 7.557 10.015 9.952 10.27 10.256 10.54
   2439   1388527_at   5.882 5.923 5.592 5.861 6.007 7.461 7.18 7.141 7.443 6.686
   2440   1371155_at   5.189 5.157 6.657 5.107 4.394 2.189 1.345 1.637 2.081 1.021
   2441   1386252_at   7.582 7.736 7.446 7.778 7.927 6.248 6.405 6.726 6.808 6.291
   2442   1371796_at   9.703 9.731 9.664 9.636 9.695 10.096 10.18 10.148 9.986 9.965
   2443   1383260_at   8.959 9.007 9.031 9.013 8.922 9.338 9.274 9.507 9.34 9.336
   2444   1373196_at   5.548 5.64 5.909 6.19 5.845 6.846 7.238 7.494 7.126 7.031
   2445   1387948_at   6.925 6.711 6.753 6.788 6.824 7.282 7.164 7.316 7.286 7.134
   2446   1390843_at   6.938 6.556 6.535 6.895 6.796 6.01 5.775 5.524 5.796 5.645
   2447   1373373_at   9.141 9.199 9.2 9.392 9.368 8.493 8.201 8.698 8.294 8.321
   2448   1369650_at   8.055 8.094 7.899 8.061 7.667 7.044 7.145 6.957 7.223 7.266
   2449   1394456_at   8.08 8.13 8.402 8.422 8.142 9.015 8.891 9.293 9.256 9.194
   2450   1377190_at   6.601 6.532 6.705 6.46 6.646 5.5 4.64 5.263 4.519 5.008
   2451   1367656_at   10.73 10.639 10.716 10.837 10.772 10.363 10.178 10.345 10.251 10.389
   2452   1388071_x_at   12.95 12.847 12.825 12.594 12.81 12.281 12.148 11.913 12.008 12.04
   2453   1370141_at   9.733 9.81 9.835 10.001 9.856 9.034 9.066 8.942 8.747 8.478
   2454   1368102_at   10.739 10.747 10.683 10.537 10.8 11.263 11.148 11.29 11.483 11.298
   2455   1379277_at   7.771 7.573 7.638 7.878 7.869 8.45 8.424 8.858 8.644 8.559
   2456   1385354_at   3.784 4.341 4.529 4.486 4.428 5.504 5.779 5.803 5.498 5.992
   2457   1369187_a_at   7.706 7.657 7.58 7.919 7.659 8.505 8.622 8.223 8.396 8.468
   2458   1378034_at   5.742 6.008 6.344 6.237 6.222 7.475 7.36 7.123 7.129 7.074
   2459   1379495_at   6.559 6.586 7.086 6.992 6.876 7.841 8.088 7.954 7.889 7.671
   2460   1376321_at   7.355 7.439 7.401 7.727 7.696 6.874 6.57 6.692 6.728 6.512
   2461   1387029_at   8.788 8.887 8.708 8.685 9.181 9.934 9.849 10.024 10.024 9.578
   2462   1376624_at   7.436 7.765 7.823 7.5 7.392 6.241 5.81 6.135 6.487 5.592
   2463   1382175_at   8.932 8.847 8.846 8.843 8.998 8.454 8.341 8.545 8.328 8.251
   2464   1391572_at   7.151 7.234 7.124 7.252 7.037 6.612 6.759 6.443 6.664 6.562
   2465   1373226_at   7.513 7.331 7.231 7.289 7.38 6.396 6.224 6.026 6.69 6.15
   2466   1372297_at   10.656 10.524 10.84 10.855 10.761 11.354 11.26 11.372 11.464 11.556
   2467   1368099_at   8.176 8.155 8.123 8.063 8.112 7.719 7.68 7.853 7.842 7.667
   2468   1392060_at   0 0.379 0 0 1.367 3.43 2.595 3.326 2.705 3.521
   2469   1378679_at   10.763 10.81 10.582 10.571 10.722 9.562 9.654 9.659 9.069 8.996
   2470   1379469_at   4.722 5.002 4.307 4.814 5.148 3.051 3.659 3.26 2.962 3.123
   2471   1384584_at   7.54 7.631 7.617 7.726 7.873 6.96 6.88 6.933 6.869 6.505
   2472   1390598_at   4.569 4.798 5.172 5.261 4.816 6.501 6.028 6.882 6.541 6.641
   2473   1380655_at   5.209 5.395 5.373 5.631 5.318 6.002 5.989 6.158 6.192 6.255
   2474   1370208_at   9.5 9.418 9.553 9.532 9.456 9.173 9.218 9.091 9.238 9.098
   2475   1381919_at   6.651 6.803 6.444 6.632 6.782 5.937 5.715 5.659 5.525 5.306
   2476   1370680_at   6.297 6.234 6.277 6.305 6.213 6.969 6.681 6.796 6.961 6.686
   2477   1374890_at   7.978 8.03 8.046 8.143 8.242 8.608 8.562 8.807 8.682 8.559
   2478   1390801_at   9.712 9.614 9.468 9.658 9.972 8.603 8.836 8.783 8.607 8.291
   2479   1368488_at   7.417 7.057 7.283 7.693 7.569 6.357 6.403 6.391 6.009 6.273
   2480   1372470_at   5.99 6.132 6.306 6.171 6.647 7.115 7.338 7.319 7.507 7.339
   2481   1377607_at   7.555 7.42 7.411 7.445 7.401 7.138 6.955 7.02 6.953 6.849
   2482   1389725_at   6.81 6.532 6.65 6.289 6.999 7.842 7.94 7.621 7.732 8.006
   2483   1383963_at   7.904 7.712 7.87 7.939 7.966 8.363 8.411 8.543 8.639 8.374
   2484   1388788_at   10.701 10.652 10.658 10.654 10.311 11.222 11.373 11.49 11.624 11.589
   2485   1371266_at   7.578 7.576 7.782 7.74 7.047 8.796 8.573 8.749 8.975 8.948
   2486   1390698_at   8.199 8.106 8.086 8.437 8.396 7.348 7.362 7.59 7.233 7.099
   2487   1383852_at   7.45 7.585 7.836 7.974 7.83 8.742 8.51 8.712 8.7 8.521
   2488   1389789_at   8.14 8.182 8.058 8.322 8.331 6.977 5.895 6.591 6.853 6.395
   2489   1373504_at   8.677 8.628 8.974 8.638 8.793 7.989 7.857 7.583 7.514 7.4
   2490   1389857_at   9.693 9.514 9.582 9.861 9.851 10.256 10.308 10.352 10.275 10.353
   2491   1388506_at   8.806 8.862 8.713 8.883 8.663 9.317 9.239 9.535 9.282 9.344
   2492   1389780_at   5.562 5.383 5.391 5.411 5.484 6.234 6.03 6.464 6.169 6.538
   2493   1389729_at   9.478 9.528 9.687 9.369 9.43 8.96 8.967 8.901 8.919 8.704
   2494   1377334_at   5.227 5.047 5.209 5.501 5.888 3.091 3.9 3.534 3.898 3.637
   2495   1382398_at   2.83 2.746 2.342 2.536 3.214 4.326 5.033 5.3 4.346 4.776
   2496   1393184_at   7.017 7.203 7.04 6.983 7.087 6.601 6.51 6.307 6.319 6.221
   2497   1367584_at   11.735 11.664 11.646 11.688 11.712 11.233 11.264 11.035 11.009 10.897
   2498   1387378_at   10.322 10.24 9.787 9.911 10.136 6.482 5.581 4.814 2.791 4.775
   2499   1374874_at   5.414 5.897 5.18 5.763 5.878 6.914 6.912 7.108 6.998 6.717
   2500   1378443_at   8.307 8.247 8.41 8.6 8.285 7.306 6.881 6.199 6.637 6.824
   2501   1391067_at   5.293 4.977 5.213 5.614 5.678 6.856 6.423 7.19 7.013 6.982
   2502   1398931_at   9.236 9.404 9.381 9.195 9.254 9.743 9.807 9.795 9.836 9.625
   2503   1368356_a_at   11.144 11.281 11.215 11.234 11.395 9.819 9.831 10.33 9.72 9.269
   2504   1390707_at   8.682 8.647 8.984 8.836 8.573 7.763 7.91 7.755 7.91 7.4
   2505   1389572_at   6.712 6.817 6.494 6.851 6.722 7.375 7.323 7.692 7.477 7.456
   2506   1396993_at   5.772 5.637 5.787 6.093 6.166 4.636 3.895 3.733 4.419 3.687
   2507   1387151_at   6.571 6.704 7.133 7.13 7.16 5.748 5.829 5.896 5.745 5.954
   2508   1392940_at   6.587 6.947 6.665 6.96 6.596 7.858 7.58 8.087 8.117 8.274
   2509   1374686_at   6.907 7.098 6.886 6.855 7.166 6.016 6.056 6.39 5.84 5.972
   2510   1386893_at   11.593 11.651 11.633 11.678 11.899 11.167 10.736 10.992 10.897 10.964
   2511   1388178_at   7.35 6.966 7.201 7.44 7.316 6.39 6.294 6.076 6.467 6.012
   2512   1393414_at   7.062 7.215 7.539 7.016 7.055 8.059 8.155 7.978 8.248 8.002
   2513   1369313_at   7.626 7.786 7.869 7.978 7.955 8.878 8.725 8.712 8.813 8.406
   2514   1370610_at   7.619 7.65 8.374 7.451 6.974 9.699 9.37 9.478 9.745 9.756
   2515   1381323_at   7.522 7.664 7.709 7.73 7.702 7.201 7.193 6.969 7.05 6.884
   2516   1374243_at   6.275 6.55 6.317 6.384 6.553 7.095 7.205 7.481 7.132 7.084
   2517   1388054_a_at   7.607 7.174 7.623 7.536 8.489 5.893 5.793 5.456 5.135 5.354
   2518   1370859_at   11.291 11.25 11.142 11.159 11.422 10.789 10.694 10.642 10.52 10.491
   2519   1392090_at   9.527 9.495 9.64 9.595 9.363 10.109 10.348 10.343 10.225 10.007
   2520   1374527_at   6.981 7.003 7.289 7.351 7.094 7.945 7.838 8.283 8.188 8.206
   2521   1375215_x_at   10.005 10.129 10.58 10.363 10.469 11.144 11.447 11.592 11.339 11.434
   2522   1376722_at   8.964 8.96 8.913 8.979 9.031 8.345 8.171 8.281 7.822 7.925
   2523   1381567_at   5.48 5.795 5.501 5.561 5.926 4.573 4.837 4.922 4.604 4.645
   2524   1378216_at   4.007 3.579 3.502 3.959 4.223 4.943 5.276 5.573 5.537 5.563
   2525   1392527_at   9.826 9.839 9.915 9.961 9.928 10.359 10.392 10.581 10.554 10.273
   2526   1377970_at   7.492 7.453 7.496 7.492 7.946 6.54 5.879 6.39 6.472 6.09
   2527   1370870_at   7.733 7.711 7.867 8.188 7.5 9.282 8.957 9.291 9.654 9.842
   2528   1371581_at   10.42 10.402 10.217 10.377 10.432 9.967 9.996 10.031 9.896 9.862
   2529   1371346_at   10.844 10.723 10.916 10.636 10.742 11.309 11.261 11.172 11.216 11.357
   2530   1369738_s_at   7.714 7.678 7.857 7.906 7.828 7.33 7.184 7.067 6.971 7.238
   2531   1389215_at   7.532 7.661 7.862 7.853 7.64 8.264 8.271 8.339 8.27 8.213
   2532   1373233_at   8.326 8.327 8.543 8.205 8.358 7.804 7.656 7.8 7.526 7.522
   2533   1372056_at   10.293 10.339 10.138 10.287 10.534 9.415 9.23 9.316 9.128 8.702
   2534   1386880_at   9.432 9.534 9.481 9.579 9.569 10.193 10.588 10.923 10.606 10.42
   2535   1372667_at   8.83 8.823 8.617 8.716 8.854 8.31 8.371 8.362 8.238 8.19
   2536   1369169_at   8.211 8.275 8.567 8.497 7.856 9.58 9.211 9.582 9.625 9.683
   2537   1377898_at   2.403 2.428 2.556 2.85 1.227 5.904 5.782 4.505 5.601 6.08
   2538   1382709_at   6.498 6.961 7.192 6.836 6.54 5.347 5.642 4.941 5.318 5.485
   2539   1388569_at   10.862 10.915 10.689 10.997 10.893 11.555 11.794 11.595 11.515 11.385
   2540   1393380_at   6.993 7.133 6.986 7.075 6.899 7.642 7.65 7.9 7.815 7.493
   2541   1382153_at   7.87 7.92 7.898 7.966 7.829 6.864 6.321 6.752 6.724 5.998
   2542   1387344_at   11.143 11.103 11.253 11.012 11.001 11.669 11.765 11.638 11.484 11.652
   2543   1371579_at   10.698 10.679 10.54 10.701 10.676 10.185 10.109 10.314 10.169 9.991
   2544   1377874_at   6.206 6.288 6.414 6.551 6.549 7.151 6.934 7.171 7.272 7.192
   2545   1373337_at   10.799 10.753 10.974 10.876 10.32 11.647 11.751 11.66 11.82 11.711
   2546   1368308_at   8.44 8.574 8.064 8.443 8.284 7.254 6.535 7.225 6.644 6.612
   2547   1379308_at   7.209 7.328 7.237 7.261 6.837 8.407 7.886 8.419 8.399 8.396
   2548   1374303_at   6.833 6.884 6.71 6.981 7.056 7.442 7.495 7.656 7.512 7.736
   2549   1398719_at   5.665 5.118 5.549 5.889 5.638 4.174 4.314 4.467 4.576 4.256
   2550   1386695_at   7.196 7.46 7.294 7.732 7.787 4.914 3.738 2.18 2.398 2.975
   2551   1382105_at   6.171 6.157 6.503 6.425 6.408 7.267 7.168 7.725 7.217 7.457
   2552   1369291_at   8.173 8.17 8.152 7.994 8.265 8.829 8.718 8.89 9.032 8.642
   2553   1396187_at   7.944 7.775 7.903 8.157 7.923 7.119 7.332 6.871 6.834 6.972
   2554   1379805_at   6.005 5.933 6.108 6.527 6.754 4.955 4.798 4.562 4.372 4.336
   2555   1378668_at   8.095 8.029 8.035 8.045 7.979 8.475 8.428 8.557 8.402 8.291
   2556   1369998_at   9.873 9.979 10.161 9.957 9.954 9.495 9.255 9.017 9.181 9.204
   2557   1383879_at   8.473 8.454 8.73 8.586 8.578 8.971 9.209 9.085 9.228 9.157
   2558   1380544_at   6.801 7.026 7.238 6.986 7.001 6.043 5.816 6.38 5.929 5.951
   2559   1383056_a_at   7.58 7.455 7.921 7.706 7.525 8.483 8.274 8.487 8.378 8.564
   2560   1389176_at   7.565 7.721 7.687 7.718 7.4 8.269 8.189 8.349 8.445 8.168
   2561   1389062_at   9.102 9.125 9.005 9.199 9.45 8.552 8.466 8.551 8.358 8.362
   2562   1370167_at   8.828 8.717 9.129 8.875 8.035 10.224 10.275 10.238 10.21 10.269
   2563   1376501_at   7.816 7.921 7.776 7.764 7.992 7.196 6.767 7.163 6.791 6.739
   2564   1383395_at   9.173 9.145 9.232 9.464 8.95 9.987 9.856 10.201 10.187 10.152
   2565   1398295_at   5.763 5.631 5.89 5.535 5.431 7.44 6.59 7.483 7.533 7.563
   2566   1370402_at   6.759 6.543 6.627 6.824 7.03 7.625 7.374 7.666 7.629 7.627
   2567   1377737_at   9.491 9.536 9.395 9.4 9.525 8.703 8.832 8.671 8.72 8.276
   2568   1373200_at   9.48 9.485 9.431 9.485 9.659 9.027 9.04 9.098 8.975 8.813
   2569   1375335_at   11.729 11.7 11.709 11.648 12.007 11.139 11 11.248 11.146 11.152
   2570   1388505_at   8.589 8.815 8.917 8.759 8.579 7.985 7.756 7.912 7.693 7.422
   2571   1371252_at   8.57 8.521 8.668 8.899 8.894 5.33 2.511 3.931 3.436 1.546
   2572   1398327_at   9.822 9.825 10.003 9.965 9.947 10.397 10.379 10.484 10.333 10.24
   2573   1388152_at   4.974 4.457 4.612 5.001 4.961 5.829 5.812 6.31 6.013 6.181
   2574   1381922_at   7.756 7.999 8.145 8.198 7.925 8.632 8.693 8.696 8.655 8.667
   2575   1392933_a_at   6.807 6.808 6.76 6.842 6.636 7.803 7.394 7.866 7.471 7.512
   2576   1393364_at   6.972 6.901 6.853 6.981 6.535 7.528 7.93 7.97 8.022 7.793
   2577   1375663_at   9.288 9.21 9.281 9.315 9.255 8.992 9.023 8.979 8.934 8.827
   2578   1367750_at   8.396 8.25 8.246 8.413 8.444 8.875 8.692 8.806 8.882 8.773
   2579   1380297_at   8.199 8.205 7.836 8.176 8.408 6.981 6.939 6.472 6.597 6.098
   2580   1380383_at   6.768 6.831 6.603 6.591 6.84 7.553 7.274 7.543 7.329 7.647
   2581   1377090_at   5.567 5.726 5.319 5.45 5.711 6.4 6.358 6.618 6.348 6.769
   2582   1379507_at   6.476 6.664 7.444 7.015 6.418 2.718 0.678 0.888 3.614 1.907
   2583   1373488_at   9.158 9.145 8.985 9.213 9.224 8.579 8.469 8.609 8.321 8.228
   2584   1374484_at   7.319 7.153 7.169 7.203 7.328 6.89 6.917 6.93 6.904 6.833
   2585   1389269_at   7.495 7.423 7.479 7.164 7.331 6.84 6.705 6.705 6.9 6.774
   2586   1380471_at   7.259 7.66 7.479 8.028 7.653 8.688 8.68 9.083 8.836 8.991
   2587   1381161_a_at   6.935 6.992 6.956 7.085 6.782 4.754 3.926 5.332 4.991 3.961
   2588   1371403_at   10.781 10.738 10.682 10.839 10.91 10.425 10.357 10.432 10.245 10.328
   2589   1370861_at   11.983 11.887 11.901 11.83 11.918 12.228 12.271 12.11 12.241 12.271
   2590   1375424_at   10.866 10.876 10.903 10.899 11.076 10.373 10.149 10.498 10.158 10.265
   2591   1390404_at   6.207 6.221 6.105 6.346 6.949 8.011 7.722 8.069 8.063 7.551
   2592   1375566_at   8.917 8.902 8.798 9.005 8.974 9.224 9.371 9.377 9.467 9.345
   2593   1367836_at   8.908 8.975 8.927 8.792 8.769 9.278 9.259 9.362 9.485 9.322
   2594   1380962_at   6.11 6.354 6.329 6.449 5.822 7.254 7.199 7.664 7.68 7.606
   2595   1374124_at   6.992 6.853 7.007 7.267 7.222 7.777 7.715 7.918 7.851 7.698
   2596   1391611_at   8.237 8.406 8.203 8.107 8.356 8.974 9.007 8.897 8.934 8.69
   2597   1389367_at   6.323 6.235 6.511 6.703 6.322 7.293 7.135 7.23 7.11 7.387
   2598   1378958_at   7.036 7.057 7.077 7.224 6.924 6.5 6.237 6.366 5.994 6.215
   2599   1384112_at   7.175 7.147 7.624 7.568 7.435 8.235 8.287 8.443 8.306 8.163
   2600   1367811_at   8.636 8.658 8.981 8.96 8.767 9.403 9.41 9.485 9.392 9.403
   2601   1379278_at   8.048 8.094 8.094 8.195 8.239 7.57 7.456 7.631 7.707 7.358
   2602   1372293_at   5.135 4.701 5.375 4.993 5.155 5.973 5.951 6.127 6.005 6.08
   2603   1387631_at   6.591 6.726 6.339 6.584 5.93 8.547 8.698 8.872 8.619 7.693
   2604   1368080_at   8.168 8.201 7.995 8.033 7.65 8.876 9.014 9.315 8.923 9.034
   2605   1372362_at   8.428 8.439 8.549 8.501 8.688 8.935 8.963 9.101 9.127 8.987
   2606   1368056_at   7.629 7.669 7.738 7.728 7.564 8.227 7.967 8.136 8.227 8.138
   2607   1368863_at   7.943 7.902 7.959 8.15 8.309 8.757 8.738 8.815 8.667 8.667
   2608   1390021_at   9.872 9.832 9.891 10.042 9.858 10.216 10.214 10.2 10.291 10.276
   2609   1383733_at   8.734 8.786 8.795 8.623 8.478 9.372 9.268 9.254 9.349 9.143
   2610   1377392_at   4.957 5.379 5.693 5.44 4.927 6.59 6.38 6.739 6.79 6.637
   2611   1367783_at   10.774 10.773 10.666 10.668 10.821 11.02 10.98 11.047 10.998 11.042
   2612   1382769_at   8.93 8.603 8.757 8.75 8.857 8.061 7.838 8.279 8.02 7.989
   2613   1376789_at   2.868 3.621 3.259 3.22 1.491 6.009 6.395 6.202 6.156 5.681
   2614   1383893_at   9.703 9.573 9.845 9.837 9.613 10.457 10.333 10.463 10.549 10.817
   2615   1373144_at   6.761 6.991 6.849 6.999 6.845 7.483 7.243 7.437 7.491 7.465
   2616   1376076_at   7.123 7.065 7.13 7.341 7.728 6.102 5.733 5.993 5.81 6.284
   2617   1379592_at   8.61 8.434 8.379 8.441 8.11 9.15 9.033 9.192 9.037 9.168
   2618   1388772_at   8.293 8.513 8.455 8.345 8.495 7.538 7.826 7.64 7.559 7.238
   2619   1374137_at   9.143 9.188 9.068 8.898 9.017 8.428 8.301 8.477 8.294 8.034
   2620   1378415_at   7.113 7.298 7.165 7.224 7.039 7.707 7.599 7.926 7.87 7.751
   2621   1372987_at   7.451 7.69 7.794 7.817 7.845 7.131 7.009 7.151 7.108 7.068
   2622   1372106_at   8.136 8.167 8.013 8.114 8.139 7.549 7.399 7.774 7.596 7.535
   2623   1385248_a_at   7.699 7.611 7.581 7.624 8.147 8.938 8.863 8.85 9.151 8.583
   2624   1390114_at   7.062 7.154 7.039 7.175 7.249 7.951 7.568 7.849 8.026 7.844
   2625   1384265_at   6.995 7.13 7.149 6.999 6.92 6.388 6.313 6.6 6.31 6.543
   2626   1388614_at   9.438 9.395 9.385 9.615 9.567 9.068 8.867 8.968 9.045 9.027
   2627   1396077_at   7.415 7.068 7.259 7.483 7.67 6.488 6.231 6.364 6.034 5.96
   2628   1390925_a_at   5.217 5.466 5.281 4.969 5.235 6.252 5.959 5.899 6.132 6.17
   2629   1387183_at   7.949 8.031 7.841 7.896 7.821 8.269 8.452 8.255 8.352 8.338
   2630   1370808_at   9.168 9.218 8.999 9.371 9.38 9.948 9.767 10.104 9.981 10.01
   2631   1372721_at   9.798 9.876 9.921 9.916 10.092 9.525 9.447 9.506 9.385 9.361
   2632   1390207_at   8.914 8.948 8.723 8.993 8.939 9.563 9.596 9.633 9.427 9.328
   2633   1397176_at   6.974 6.934 6.962 6.858 7.109 6.513 6.411 6.267 6.558 6.348
   2634   1377808_at   4.584 5.023 5.062 4.618 4.863 5.595 5.881 5.752 5.961 5.767
   2635   1376440_at   8.759 8.748 8.595 8.779 8.984 8.074 8.123 8.294 8.082 7.977
   2636   1392810_at   6.246 6.308 6.359 6.074 6.531 5.256 4.523 5.021 5.3 4.87
   2637   1371636_at   10.508 10.559 10.522 10.367 10.327 10.802 10.853 10.917 10.87 10.868
   2638   1371519_at   9.017 9.132 9.183 9.192 9.041 9.402 9.535 9.536 9.565 9.625
   2639   1373909_at   6.731 6.972 7.079 6.677 6.543 7.644 7.918 7.697 7.654 7.949
   2640   1377748_at   6.26 6.193 5.991 6.184 6.499 6.941 7.092 7.003 6.958 6.85
   2641   1377524_at   8.327 8.304 8.254 8.166 8.305 8.656 8.696 8.806 8.651 8.908
   2642   1370899_at   8.564 8.477 8.214 8.593 8.488 7.839 7.79 7.966 7.745 7.718
   2643   1368607_at   10.766 10.804 10.977 10.993 10.762 11.342 11.381 11.29 11.372 11.515
   2644   1376282_at   4.848 5.302 5.403 5.361 5.609 6.599 6.326 6.334 6.656 6.701
   2645   1380809_at   0.722 0 1.599 0 0.176 3.905 3.018 3.909 4.183 3.144
   2646   1388982_at   7.774 7.715 7.687 7.797 7.908 8.141 8.085 8.252 8.217 8.164
   2647   1388751_at   9.05 9.056 9.055 8.819 8.708 9.657 9.673 9.76 9.581 9.503
   2648   1377818_at   5.601 5.98 5.406 5.861 5.671 6.628 6.768 7.197 6.743 6.901
   2649   1374777_at   6.615 6.717 6.569 6.608 6.716 6.148 6.034 5.827 5.681 5.977
   2650   1388391_at   11.601 11.566 11.681 11.66 11.61 11.968 11.941 11.937 12.082 12.154
   2651   1387782_at   8.387 8.437 8.573 8.794 8.567 9.245 9.159 9.324 9.531 9.329
   2652   1375908_at   7.543 7.612 7.702 7.632 7.351 8.488 8.144 8.439 8.278 8.187
   2653   1387377_a_at   7.732 7.95 7.237 7.511 7.793 6.185 6.038 6.233 5.687 5.296
   2654   1388140_at   8.78 8.739 8.707 8.854 9.028 7.982 7.836 7.934 7.847 7.353
   2655   1389511_s_at   7.577 7.645 7.854 7.887 7.693 8.384 8.188 8.432 8.456 8.5
   2656   1368374_a_at   11.657 11.813 11.796 11.891 11.707 12.424 12.283 12.166 12.358 12.495
   2657   1385806_at   7.992 8.181 7.933 8.088 8.181 7.382 6.727 7.121 6.875 7.166
   2658   1389805_at   5.643 5.334 5.918 5.894 6.135 6.887 7.162 7.073 6.885 6.938
   2659   1375996_at   7.448 7.1 7.464 7.608 6.967 5.924 6.363 6.03 5.749 6.091
   2660   1391878_at   5.024 5.246 6.049 5.938 5.362 3.939 3.338 3.02 2.934 3.458
   2661   1374766_at   8.536 8.64 8.707 8.677 8.57 9.254 9.086 9.387 9.168 9.046
   2662   1397958_at   8.213 8.123 8.134 8.298 8.429 4.349 4.598 1.718 3.513 1.766
   2663   1368762_at   12.733 12.623 12.216 12.235 12.365 9.574 7.574 7.184 6.992 5.869
   2664   1390789_at   7.149 7.424 7.195 7.534 7.251 7.998 8.118 8.314 7.995 8.006
   2665   1367912_at   6.117 6.005 6.305 6.321 6.846 7.437 7.625 7.67 7.489 7.518
   2666   1389551_at   10.269 10.28 10.726 10.364 9.923 11.428 11.381 11.419 11.415 11.271
   2667   1397227_at   3.227 3.144 3.434 3.011 3.109 4.692 4.385 4.278 5.226 4.646
   2668   1391871_at   6.158 6.094 6.427 6.354 6.227 7.224 6.882 7.172 7.236 6.883
   2669   1398300_at   10.518 10.473 10.678 10.572 10.71 10.113 9.689 9.953 9.958 9.866
   2670   1372095_at   6.453 6.607 6.257 6.531 6.712 7.163 7.282 7.303 7.194 7.482
   2671   1381947_at   4.472 4.216 4.759 4.655 3.897 5.849 5.721 5.626 6.118 6.004
   2672   1395364_at   8.006 7.96 7.809 7.708 7.94 7.395 7.347 7.425 7.245 7.214
   2673   1373225_at   10.751 10.673 10.794 10.806 10.749 10.175 10.023 10.235 9.997 9.744
   2674   1393708_at   3.811 4.096 3.592 3.466 4.249 5.803 5.417 5.565 5.213 5.095
   2675   1390437_at   6.617 6.597 6.256 6.301 6.811 7.534 7.492 7.517 7.261 7.561
   2676   1369682_at   8.341 8.233 8.103 8.605 8.443 9.225 9.086 9.501 9.571 9.472
   2677   1373494_at   8.597 8.524 8.546 8.496 8.533 7.968 7.613 8.064 7.97 7.832
   2678   1373389_at   7.29 7.477 7.587 7.48 7.482 7.85 7.922 7.926 7.929 7.827
   2679   1369057_at   8.431 8.424 8.438 8.62 8.458 8.033 8.011 8.002 7.857 7.742
   2680   1370067_at   7.621 7.698 7.742 7.851 7.225 9.079 8.63 9.069 9.35 9.515
   2681   1372497_at   9.056 9.12 9.035 9.193 9.22 9.534 9.63 9.643 9.656 9.432
   2682   1377884_at   7.076 7.044 6.924 7.095 7.076 7.602 7.432 7.689 7.6 7.409
   2683   1397611_at   6.679 7.028 6.636 6.9 6.933 5.552 5.755 5.589 4.968 5.733
   2684   1373400_at   9.181 9.174 9.02 9.305 9.119 9.687 9.649 9.924 9.745 9.636
   2685   1367455_at   11.148 11.108 11.095 11.241 11.271 10.912 10.801 10.799 10.846 10.839
   2686   1372363_at   8.041 8.151 8.37 8.322 8.514 7.455 7.405 7.614 7.419 7.202
   2687   1370196_at   6.664 6.555 6.577 6.638 6.671 7.091 6.895 7.139 6.962 7.038
   2688   1388064_a_at   6.718 6.732 6.76 6.757 6.232 4.316 4.796 3.566 4.551 5.016
   2689   1369370_s_at   6.914 7.057 7.174 7.308 6.87 7.973 8.126 7.895 7.894 7.711
   2690   1383998_at   9.375 9.423 9.429 9.261 9.427 8.854 8.791 8.886 8.787 8.496
   2691   1388358_at   10.057 9.889 9.97 10.302 10.209 10.743 10.731 10.928 10.853 11.039
   2692   1389774_at   5.253 5.836 5.341 5.602 5.176 6.413 6.458 6.494 6.43 6.4
   2693   1375640_at   8.608 8.79 8.579 8.953 9.174 9.915 9.669 10.053 9.785 9.976
   2694   1382780_at   6.547 6.413 6.958 6.453 6.55 7.454 7.516 7.804 7.692 7.414
   2695   1372326_at   5.509 5.153 5.58 5.671 5.658 4.379 4.695 4.692 4.305 4.454
   2696   1375074_at   6.719 6.938 6.987 6.869 6.623 6.015 5.54 5.23 5.664 5.276
   2697   1381971_at   6.926 6.824 6.78 7.043 7.252 7.683 7.801 7.733 8.04 7.955
   2698   1391538_at   7.483 7.185 7.079 7.677 7.219 8.186 8.341 8.317 8.192 8.232
   2699   1392918_at   10.211 10.294 10.009 10.353 10.253 9.648 9.779 9.514 9.637 9.596
   2700   1388986_at   8.938 9.214 8.647 8.746 9.099 7.855 7.809 7.771 7.44 7.23
   2701   1386679_at   5.324 4.874 4.478 4.995 5.174 6.202 6.3 6.825 6.415 6.724
   2702   1371366_at   11.016 11.024 10.984 11.001 11.094 10.439 10.08 10.315 10.369 10.569
   2703   1393431_at   9.74 9.606 9.374 9.468 9.929 8.73 8.637 8.843 8.473 8.46
   2704   1383262_at   8.944 8.844 8.87 9.014 8.98 8.499 8.522 8.597 8.553 8.351
   2705   1379849_at   7.461 7.249 7.409 7.455 7.417 6.869 6.424 6.674 6.643 6.829
   2706   1383041_x_at   7.607 7.588 7.537 7.596 7.871 8.248 8.285 8.369 8.37 8.09
   2707   1369177_at   7.261 7.205 7.564 7.23 7.282 6.488 6.371 6.74 6.545 6.284
   2708   1369895_s_at   7.981 7.918 7.97 8.159 7.917 8.566 8.459 8.756 8.66 8.86
   2709   1390185_at   8.448 8.474 8.474 8.407 8.555 7.881 7.85 8.111 7.804 7.673
   2710   1370979_at   7.265 7.149 7.141 7.228 7.113 6.579 6.556 6.816 6.609 6.4
   2711   1374295_at   6.67 6.594 6.659 6.853 6.785 7.181 7.101 7.144 7.297 7.281
   2712   1392174_at   6.153 6.035 5.949 6.465 6.666 4.976 5.239 5.151 5.066 4.937
   2713   1372352_at   10.412 10.481 10.348 10.537 10.958 9.632 9.55 9.678 9.359 9.333
   2714   1388884_at   10.482 10.372 10.504 10.582 10.278 10.865 11.023 11.118 11.157 11.156
   2715   1370691_a_at   4.484 4.233 5.207 4.576 4.767 6.275 5.898 6.437 6.66 6.622
   2716   1390664_at   5.573 6.021 5.847 5.767 5.639 6.969 6.587 6.596 6.721 7.072
   2717   1379665_at   7.221 7.085 7.01 6.989 7.138 6.542 6.48 6.35 6.083 6.243
   2718   1376830_at   4.288 4.551 4.584 4.549 4.393 5.018 5.069 5.319 5.22 5.384
   2719   1372372_at   10.806 10.842 10.915 10.802 10.259 11.592 11.76 11.722 11.714 11.774
   2720   1381199_at   6.58 6.783 6.508 6.487 6.935 5.311 4.722 5.046 5.637 5.381
   2721   1371489_at   9.596 9.577 9.64 9.668 9.764 9.243 9.116 9.091 8.883 8.917
   2722   1371755_at   9.948 10.022 9.858 9.929 9.907 10.463 10.524 10.554 10.414 10.233
   2723   1373786_at   6.107 5.826 6.382 6.122 5.855 7.075 7.071 7.29 7.636 7.143
   2724   1393123_at   6.109 5.88 5.975 6.227 6 6.732 7.001 7.114 7.415 7.301
   2725   1393309_at   6.483 6.563 6.412 6.683 6.448 7.306 7.185 7.423 7.005 7.139
   2726   1388417_at   10.538 10.526 10.338 10.409 10.398 10.787 10.794 10.753 10.732 10.772
   2727   1387550_a_at   5.783 5.42 5.933 6.296 6.422 7.6 7.261 7.656 7.569 7.758
   2728   1389446_at   8.219 8.395 8.155 8.283 8.587 7.605 7.157 7.084 7.37 7.473
   2729   1394112_at   4.927 4.906 3.932 5.196 4.53 6.76 7.224 7.417 6.892 6.431
   2730   1368201_at   6.768 6.956 6.958 7.041 7.067 6.249 6.474 6.48 6.457 6.341
   2731   1382387_at   4.465 3.914 4.247 4.51 4.851 6.302 5.68 5.937 5.99 5.728
   2732   1390685_at   5.912 5.989 5.571 6.016 6.121 4.908 4.839 5.073 4.529 4.532
   2733   1392944_at   5.529 5.411 5.717 5.876 5.531 7.058 6.43 6.742 6.538 6.715
   2734   1389412_at   9.039 8.894 8.949 9.088 8.711 9.623 9.636 9.914 9.671 9.53
   2735   1393330_at   6.175 6.537 6.277 6.495 6.543 7.303 7.365 7.434 7.788 7.195
   2736   1373967_at   7.528 8.003 7.981 7.762 7.894 8.518 8.552 8.779 8.707 8.623
   2737   1368215_at   9.828 9.743 9.887 9.894 9.669 10.227 10.229 10.316 10.248 10.127
   2738   1384971_at   7.441 7.426 7.703 7.262 7.202 8.11 8.265 8.499 8.473 8.255
   2739   1393516_at   8.855 8.804 8.949 8.703 8.502 9.782 10.08 9.888 9.815 9.438
   2740   1395856_at   6.222 5.922 6.059 5.502 5.957 6.843 6.929 6.97 6.976 6.864
   2741   1380897_at   3.646 3.526 3.472 3.081 3.081 4.478 4.69 4.926 5.327 5.161
   2742   1374954_at   5.538 5.8 5.903 5.934 5.775 6.665 6.584 6.631 6.721 7.118
   2743   1377189_at   6.222 6.17 6.125 6.07 6.39 6.873 6.811 6.744 6.811 7.111
   2744   1387885_at   8.956 8.924 8.889 9.306 9.167 9.873 9.613 9.89 9.871 9.84
   2745   1369942_at   10.561 10.51 10.623 10.811 10.542 11.092 11.007 11.11 11.079 11.176
   2746   1388488_at   9.474 9.564 9.451 9.608 9.48 9.163 9.147 9.244 9.069 9.225
   2747   1393148_at   8.403 8.476 8.802 8.821 8.462 9.34 9.309 9.371 9.297 9.307
   2748   1395410_at   7.785 7.843 7.707 7.96 7.925 8.307 8.575 8.649 8.563 8.361
   2749   1376640_at   8.938 8.954 8.998 9.261 9.451 9.975 9.898 10.15 10.196 10.01
   2750   1387265_at   5.424 4.707 6.262 5.871 5.404 7.514 7.637 7.936 8.233 8.043
   2751   1371092_at   6.869 7.14 7.03 7.44 6.838 5.245 3.629 4.936 3.762 4.029
   2752   1377302_a_at   5.573 5.598 5.715 5.749 5.479 6.104 6.047 6.324 6.201 6.162
   2753   1381364_at   7.619 7.602 7.325 7.523 7.534 6.759 7.013 6.995 6.952 6.744
   2754   1370332_at   4.633 5.224 5.035 4.307 4.395 0.864 2.293 1.922 2.558 2.198
   2755   1372841_at   7.119 6.921 7.326 7.522 6.959 8.351 8.129 8.576 8.818 8.713
   2756   1372123_at   11.502 11.484 11.47 11.421 11.522 11.778 11.635 11.75 11.747 11.791
   2757   1373469_at   8.227 8.264 8.086 8.289 8.365 8.81 8.658 8.762 8.686 8.619
   2758   1376663_at   7.014 6.866 7.078 7.21 7.165 6.468 6.214 6.519 6.283 6.46
   2759   1373201_at   8.069 8.339 8.485 8.524 8.475 8.96 9.151 9.197 9.207 9.187
   2760   1374666_at   5.945 6.304 6.397 6.231 6.323 6.995 6.975 6.998 7.301 7.297
   2761   1383301_at   6.927 7.018 6.847 6.799 7.174 6.371 6.402 6.454 6.43 6.375
   2762   1384838_at   8.95 8.77 9.103 8.967 8.776 9.666 9.756 9.849 9.98 9.488
   2763   1383278_at   7.032 7.101 7.272 7.295 7.216 7.902 7.888 8.079 7.725 7.707
   2764   1373151_at   7.375 7.389 7.493 7.846 7.859 8.724 8.422 8.795 8.735 8.501
   2765   1391241_at   5.265 5.218 4.797 5.079 5.695 6.309 6.951 6.772 6.737 6.558
   2766   1376169_at   7.061 7.017 7.006 7.094 7.11 7.559 7.853 7.578 7.571 7.463
   2767   1386555_at   6.112 6.119 5.798 6.504 6.397 4.899 4.021 4.273 4.791 4.678
   2768   1375414_at   10.02 10.093 10.09 10.003 10.027 9.56 9.437 9.737 9.421 9.379
   2769   1389803_at   4.685 4.941 4.993 5.537 4.525 6.372 6.395 6.795 6.454 6.329
   2770   1388762_at   9.589 9.597 9.518 9.573 9.554 9.244 8.981 9.018 8.992 8.817
   2771   1389526_at   9.444 9.524 9.535 9.51 9.489 8.971 8.721 9.136 8.828 8.963
   2772   1388166_at   8.7 8.789 8.681 8.604 8.449 7.555 7.58 7.148 7.687 6.909
   2773   1372062_at   7.699 7.816 7.705 7.601 7.878 7.125 7.267 7.221 6.968 6.967
   2774   1376211_a_at   8.137 8.036 8.317 8.242 7.968 8.962 8.873 8.965 8.906 8.617
   2775   1395157_at   7.843 7.912 7.835 8.01 8.103 8.574 8.61 8.608 8.642 8.329
   2776   1382928_at   6.944 6.945 7.448 7.479 7.062 8.061 8.241 8.5 8.491 8.408
   2777   1387127_at   6.748 6.716 6.627 6.885 6.783 7.28 7.339 7.537 7.437 7.175
   2778   1370188_at   10.475 10.507 10.474 10.488 10.604 9.953 9.959 9.935 9.754 9.538
   2779   1395519_at   5.555 6.206 6.186 5.953 5.809 7.089 6.861 7.336 7.088 7.319
   2780   1374262_at   4.492 4.299 4.741 5.106 5.187 6.08 6.465 6.463 6.152 6.208
   2781   1388459_at   8.93 8.835 8.848 9.129 9.134 9.804 9.465 9.788 9.727 9.675
   2782   1372452_at   7.835 7.928 7.984 8.227 7.794 8.788 8.559 8.684 8.631 8.568
   2783   1383514_s_at   8.03 8.046 7.77 7.835 7.839 7.398 7.273 7.465 7.219 7.257
   2784   1385336_at   5.587 5.493 5.728 6.012 6.193 4.628 3.452 3.84 3.609 3.568
   2785   1372172_at   6.34 6.743 6.565 6.322 6.332 7.203 7.103 7.436 7.317 7.401
   2786   1377594_at   9.154 9.213 9.245 9.474 9.4 8.685 8.601 8.648 8.391 8.298
   2787   1380582_at   9.005 9.069 8.953 8.954 8.781 8.411 8.237 8.414 8.343 8.554
   2788   1369927_at   10.957 10.906 10.873 11.033 11.009 11.252 11.198 11.191 11.245 11.288
   2789   1379467_at   3.845 4.987 5.317 4.591 4.545 6.491 6.752 7.005 6.864 6.627
   2790   1378421_at   3.924 4.142 4.333 4.302 4.202 4.972 5.36 5.569 5.197 5.031
   2791   1385029_at   8.422 8.429 8.402 8.681 8.866 7.156 6.948 6.978 6.397 6
   2792   1388588_at   8.932 8.935 8.992 8.906 8.902 8.668 8.477 8.582 8.486 8.376
   2793   1389832_at   10.849 10.855 11.028 11.083 10.997 11.433 11.426 11.568 11.463 11.332
   2794   1374272_at   7.362 7.426 7.473 7.63 7.341 7.954 7.859 8.144 8.011 8.039
   2795   1376570_at   11.499 11.456 11.452 11.433 11.576 11.172 10.972 11.012 10.828 10.895
   2796   1368355_at   7.682 7.649 7.822 7.998 7.788 8.854 8.88 8.853 8.553 8.399
   2797   1377642_at   8.093 8.073 8.239 8.092 8.157 8.834 9.285 9.135 9.352 8.786
   2798   1376007_at   6.936 7.113 7.109 7.097 7.206 7.613 7.943 8.021 8.231 8.065
   2799   1372764_at   8.855 8.734 8.749 8.909 8.797 8.416 8.359 8.398 8.287 8.129
   2800   1379557_at   6.527 6.747 6.846 6.986 6.955 4.789 4.163 4.811 3.517 5.082
   2801   1373363_at   6.277 6.332 6.264 6.15 6.595 7.427 7.306 7.464 7.293 6.926
   2802   1368300_at   7.133 7.339 6.722 7.573 7.148 6.059 5.846 6.091 5.979 5.716
   2803   1393794_at   7.4 7.313 7.243 7.71 7.653 8.467 8.239 8.836 8.605 8.74
   2804   1384877_at   7.023 7.123 7.202 7.366 6.796 8.072 7.884 8.079 7.905 7.836
   2805   1389128_at   9.194 9.237 9.203 9.397 9.34 9.762 9.758 9.895 9.695 9.629
   2806   1368021_at   8.976 8.888 9.193 8.827 7.858 10.441 10.858 10.893 10.822 10.567
   2807   1379497_at   9.787 9.97 9.608 10.054 9.985 9.129 9.265 9.252 9.205 9.146
   2808   1378282_at   7.37 7.326 7.556 7.578 7.837 6.743 6.581 6.795 6.404 6.485
   2809   1379581_at   7.637 7.596 7.37 7.621 7.675 7.065 6.982 6.973 6.699 6.926
   2810   1387703_a_at   6.668 6.731 6.981 6.548 6.416 7.487 7.349 7.599 7.607 7.654
   2811   1393048_at   5.383 5.184 4.838 5.246 5.853 6.849 6.524 6.941 7.023 6.654
   2812   1373094_at   8.174 7.942 7.889 8.125 8.052 7.498 7.053 7.32 7.377 7.168
   2813   1373447_at   9.119 9.045 9.011 9.094 9.146 8.614 8.6 8.709 8.484 8.345
   2814   1374083_at   5.943 5.793 5.58 6.027 6.058 7.009 6.624 6.99 6.663 6.962
   2815   1381173_at   5.83 6.111 5.638 5.834 5.689 4.093 3.633 4.67 3.811 3.336
   2816   1388493_at   8.36 8.504 8.782 8.488 8.566 7.671 7.594 7.765 7.373 7.158
   2817   1388365_at   11.084 11.086 11.177 11.123 10.962 11.531 11.379 11.516 11.641 11.527
   2818   1380688_at   6.449 5.839 6.084 6.334 5.877 7.454 6.989 7.555 7.382 7.323
   2819   1390815_at   3.784 4.036 3.846 3.568 3.648 4.77 4.718 5.429 5.276 5.251
   2820   1389561_at   6.899 6.482 6.838 6.883 6.78 7.49 7.386 7.603 7.451 7.381
   2821   1389162_at   8.602 8.716 8.576 8.793 8.992 8.007 8.112 7.968 7.722 7.811
   2822   1390333_at   8.274 8.062 8.491 8.56 8.094 7.433 7.507 7.359 7.347 7.134
   2823   1390113_a_at   10.273 10.438 10.166 10.522 10.501 9.597 9.515 9.77 9.691 9.348
   2824   1392289_at   8.031 7.697 7.955 8.073 8.095 7.369 7.326 7.306 7.11 7.34
   2825   1373583_at   6.957 7.086 7.239 7.333 7.312 7.959 7.715 8.069 7.905 8.031
   2826   1373678_at   10.208 10.261 10.112 10.299 10.201 9.84 9.821 9.594 9.599 9.579
   2827   1368378_at   9.15 9.229 9.348 9.457 9.282 9.983 9.799 10.036 10.169 10.268
   2828   1376078_at   7.526 7.329 8.039 7.883 7.008 9.015 9.14 9.592 9.106 9.325
   2829   1369382_at   7.082 6.926 7.25 7.313 6.95 6.143 5.12 5.58 5.805 5.619
   2830   1373293_at   7.405 7.42 7.037 7.366 7.445 6.693 6.613 6.796 6.58 6.573
   2831   1387108_at   10.649 10.693 10.714 10.663 10.631 10.475 10.471 10.451 10.453 10.338
   2832   1390706_at   8.464 8.457 8.559 8.656 8.527 8.962 8.949 9.158 8.915 8.935
   2833   1376291_at   5.853 6.321 6.366 6.416 6.503 5.421 4.957 5.181 4.838 5.091
   2834   1370361_at   6.15 6.069 5.944 5.582 5.942 6.864 6.753 7.293 7.099 7.179
   2835   1377686_at   7.048 7.033 7.113 6.633 6.919 5.766 6.155 5.793 5.622 5.374
   2836   1384515_at   4.743 4.653 5.072 4.825 4.67 5.802 6.065 5.793 5.759 5.447
   2837   1386960_at   9.156 9.036 9.496 9.394 8.761 10.157 10.186 10.446 10.437 10.299
   2838   1371427_at   11.532 11.522 11.556 11.551 11.618 11.424 11.39 11.416 11.397 11.429
   2839   1387145_at   5.785 6.144 6.594 5.588 5.629 7.383 7.554 7.742 7.379 7.616
   2840   1394122_at   8.078 8.336 8.346 8.668 8.697 5.953 3.23 4.777 4.647 3.429
   2841   1374651_at   6.98 6.746 6.98 6.783 6.998 5.869 5.85 5.424 6.138 6.006
   2842   1375432_at   7.856 8.139 7.89 7.9 8.067 7.525 7.537 7.5 7.417 7.519
   2843   1375517_at   9.106 9.203 9.104 9.082 8.985 9.775 9.545 9.586 9.94 9.757
   2844   1382151_at   5.756 5.752 5.607 5.594 5.841 6.149 6.128 6.2 6.143 6.046
   2845   1383719_at   7.078 7.118 7.355 7.356 6.939 7.847 8.127 7.815 8.06 8.065
   2846   1387871_at   8.055 8.295 8.352 8.219 8.497 7.67 7.408 7.433 7.193 7.198
   2847   1372287_at   8.191 8.438 8.582 8.533 8.563 9.231 9.252 9.543 9.181 9.125
   2848   1373848_at   7.847 7.917 7.687 7.805 7.704 8.341 8.461 8.617 8.311 8.262
   2849   1390134_at   8.198 8.366 8.313 8.419 8.406 7.58 6.845 7.361 7.456 7.288
   2850   1376703_at   8.26 8.21 8.166 8.225 8.239 7.829 7.91 7.913 7.852 7.658
   2851   1376030_at   6.533 6.811 7.034 6.781 6.567 2.563 4.655 3.919 4.268 3.256
   2852   1369299_at   7.078 7.387 6.898 7.006 6.261 5.498 5.09 4.748 5.169 4.63
   2853   1381811_at   5.603 5.954 5.918 5.768 5.739 6.345 6.454 6.541 6.737 6.478
   2854   1390160_at   5.445 5.652 5.744 5.575 5.67 6.216 6.116 6.039 6.145 6.017
   2855   1392542_at   8.481 8.418 8.644 8.426 8.364 8.102 7.962 7.963 7.84 7.878
   2856   1369190_at   7.611 7.414 7.617 7.6 7.334 4.456 1.748 3.744 3.937 4.313
   2857   1386867_at   11.337 11.326 11.459 11.198 11.125 11.917 11.826 11.73 11.841 11.988
   2858   1399161_a_at   10.675 10.612 10.598 10.597 10.583 9.913 9.694 9.511 9.842 9.23
   2859   1368026_at   7.706 7.834 8.015 7.897 7.826 7.085 6.762 7.188 7.127 6.699
   2860   1373127_at   9.247 9.276 9.372 9.178 9.143 9.674 9.753 9.649 9.542 9.655
   2861   1383694_at   5.351 5.693 5.194 5.573 5.392 6.715 6.149 6.844 6.733 6.787
   2862   1389246_at   8.54 8.688 8.854 8.934 8.894 8.01 7.61 8.078 7.692 7.926
   2863   1375981_a_at   8.232 8.267 8.179 8.405 8.424 7.503 7.233 7.21 6.648 7.13
   2864   1370184_at   11.873 11.838 11.916 11.731 11.839 11.518 11.528 11.337 11.333 11.347
   2865   1386900_at   9.819 9.8 9.996 9.914 9.998 9.442 9.541 9.338 9.289 9.246
   2866   1382404_at   7.476 7.702 7.581 7.758 7.569 7.025 6.869 6.794 7.179 6.872
   2867   1368543_at   7.181 7.091 7.128 6.777 6.056 8.427 8.898 8.937 8.666 8.483
   2868   1373975_at   7.886 7.74 8.166 7.84 6.822 9.759 9.458 9.521 9.9 10.199
   2869   1393896_at   6.592 6.503 6.487 6.755 7.034 8.071 7.494 8.161 8.244 7.934
   2870   1368317_at   10.347 10.285 10.339 10.407 10.165 10.712 10.63 10.699 10.615 10.651
   2871   1370219_at   11.695 11.752 11.82 11.576 11.588 11.084 11.145 10.752 10.913 10.721
   2872   1378247_at   4.143 4.22 3.782 4.617 3.911 5.842 5.154 5.652 5.754 5.587
   2873   1378056_at   7.596 7.208 7.758 7.487 7.613 6.727 6.424 6.122 6.143 6.508
   2874   1388395_at   9.114 9.086 9.295 9.273 9.029 9.88 9.971 9.949 9.871 9.578
   2875   1369081_at   8.193 8.015 8.097 8.522 8.278 8.822 9.132 9.119 9.205 9.157
   2876   1396035_at   6.797 6.624 6.754 6.496 5.847 7.783 8.049 8.111 7.989 8.407
   2877   1378783_at   4.271 4.255 3.808 4.155 4.153 5.046 5.122 5.325 5.15 4.779
   2878   1387023_at   4.996 5.42 5.395 5.192 5.776 6.337 6.402 6.561 6.749 6.53
   2879   1385887_at   7.383 7.697 7.589 7.756 7.535 8.442 8.122 8.511 8.525 8.288
   2880   1370973_at   2.844 2.503 2.098 1.989 2.325 4.064 3.7 4.067 4.744 4.51
   2881   1398386_at   8.094 8.262 8.17 8.049 8.075 7.806 7.735 7.74 7.588 7.723
   2882   1380334_at   9.44 9.423 9.753 9.677 9.567 10.206 10.255 10.54 10.198 10.227
   2883   1380513_at   8.362 8.273 8.122 8.31 8.458 7.573 7.751 7.528 7.368 7.237
   2884   1367680_at   10.324 10.363 10.442 10.383 9.945 11.038 11.056 11.13 11.335 11.394
   2885   1370909_at   8.479 8.576 8.364 8.686 8.739 8.06 7.664 7.822 7.851 7.779
   2886   1374036_at   8.724 8.825 8.99 8.72 8.696 8.118 8.235 8.247 7.878 8.123
   2887   1372469_at   7.213 7.17 7.122 7.554 7.435 8.147 7.939 8.36 8.366 8.17
   2888   1397674_at   9.069 9.076 9.258 9.304 9.368 8.665 8.763 8.586 8.593 8.727
   2889   1373217_at   6.462 6.311 6.223 6.331 6.583 7.148 6.891 7.168 7.148 6.939
   2890   1371939_at   10.971 11.003 10.933 11.005 10.945 10.655 10.621 10.733 10.692 10.499
   2891   1373859_at   6.984 6.974 7.344 7.166 7.103 7.647 7.841 7.938 7.83 7.687
   2892   1373392_at   8.524 8.667 8.715 8.76 8.746 8.31 8.237 8.339 8.267 8.147
   2893   1388112_at   11.31 11.284 11.25 11.271 11.347 11.741 11.549 11.577 11.565 11.701
   2894   1389471_at   7.725 7.745 7.769 8.097 7.893 7.077 6.799 7.198 7.193 7.046
   2895   1393152_at   8.309 8.297 8.453 8.486 8.235 9.001 9.059 9.331 9.533 9.126
   2896   1387587_at   6.096 6.535 7.293 6.454 6.187 4.911 4.181 4.776 4.322 4.195
   2897   1371336_at   10.354 10.346 10.354 10.509 10.566 9.634 9.048 9.631 9.311 9.033
   2898   1369361_at   3.874 3.851 3.79 3.916 3.432 5.802 5.133 4.956 5.325 4.89
   2899   1372696_at   8.023 8.203 7.743 8.052 8.13 8.843 8.924 9.089 8.861 8.631
   2900   1368431_at   9.425 9.402 9.422 9.537 9.253 10.048 9.841 10.023 10.274 10.131
   2901   1375341_at   7.936 7.726 7.851 7.866 7.891 7.392 7.007 7.307 7.301 7.077
   2902   1371553_at   9.253 9.28 9.361 9.304 9.25 9.499 9.55 9.679 9.658 9.658
   2903   1389297_at   7.931 8.051 8.05 8.255 7.996 7.439 6.972 7.329 7.029 7.336
   2904   1372612_at   9.801 9.807 10.045 10.055 10.075 10.54 10.542 10.86 10.593 10.772
   2905   1367754_s_at   9.505 9.616 9.71 9.39 9.287 10.226 10.138 10.08 10.296 10.107
   2906   1386872_at   10.429 10.391 10.765 10.671 10.157 11.247 11.385 11.491 11.437 11.35
   2907   1398970_at   9.457 9.444 9.366 9.368 9.303 9.836 9.895 10.155 9.963 9.794
   2908   1395847_at   4.715 5.056 4.823 4.949 5.087 5.448 5.739 5.71 5.553 5.732
   2909   1379462_at   6.458 6.175 6.14 6.127 6.313 5.592 5.483 5.71 5.644 5.365
   2910   1389039_at   8.412 8.305 8.082 8.305 8.596 7.586 7.023 7.338 7.191 6.914
   2911   1385334_at   7.502 7.638 7.548 7.473 7.648 8.542 8.542 8.491 8.325 8.019
   2912   1385716_at   7.369 7.099 7.678 7.546 7.541 8.376 8.147 8.267 8.437 8.37
   2913   1367935_at   9.277 9.229 9.294 9.374 9.424 8.893 8.888 9.005 8.73 8.788
   2914   1383001_at   6.399 6.578 6.564 6.995 6.796 5.78 5.355 5.522 5.437 5.034
   2915   1373164_at   9.294 9.166 9.616 9.458 9.311 8.47 8.311 8.469 7.95 7.882
   2916   1383833_at   6.806 6.853 7.209 6.899 6.874 7.644 7.544 7.971 7.74 7.683
   2917   1371254_at   11.843 11.764 11.863 11.743 11.722 12.115 12.063 11.986 12.114 12.104
   2918   1374395_at   9.12 9.067 8.968 9.144 9.23 8.645 8.172 8.455 8.331 8.234
   2919   1391274_at   6.371 6.468 6.66 6.585 6.801 7.711 7.243 7.56 7.665 7.329
   2920   1388327_at   11.893 11.848 12.011 11.836 11.742 12.295 12.203 12.264 12.293 12.406
   2921   1372069_at   8.058 8.032 8.286 8.222 7.904 8.829 8.67 9.081 8.785 8.89
   2922   1374417_at   5.698 5.674 5.497 5.628 6.011 6.761 6.488 6.612 6.755 6.36
   2923   1376808_at   6.819 6.577 6.567 6.795 6.392 7.931 7.462 8.343 8.207 8.318
   2924   1370823_at   7.87 7.785 8.015 8.048 7.655 8.48 8.546 8.495 8.692 8.448
   2925   1388908_at   9.291 9.273 9.545 9.261 9.011 9.907 10.036 10.215 10.283 10.12
   2926   1387013_at   11.796 11.727 11.854 11.549 11.702 12.438 12.6 12.325 12.255 12.254
   2927   1389324_at   6.152 6.565 6.441 6.446 6.614 7.225 7.056 7.252 7.181 7.436
   2928   1368173_at   9.371 9.267 9.16 9.338 9.339 8.562 8.847 8.75 8.526 8.416
   2929   1367670_at   10.782 10.759 10.746 10.905 10.829 11.044 11.117 11.091 11.015 11.095
   2930   1397647_at   7.887 7.969 8.174 8.123 7.617 9.057 9.01 8.851 8.654 8.92
   2931   1367718_at   8.194 8.306 8.279 8.15 8.366 8.77 8.622 8.81 8.796 8.602
   2932   1368186_a_at   7.22 6.757 7.113 6.993 6.863 5.779 5.029 5.724 5.566 5.945
   2933   1386986_at   8.826 8.347 8.449 8.81 8.925 7.378 7.254 7.606 7.761 7.159
   2934   1392965_a_at   8.087 8.137 7.697 8.632 8.598 9.918 9.575 10.152 9.784 9.625
   2935   1393917_at   6.438 6.61 7.492 7.095 7.07 4.388 1.787 1.971 3.305 1.782
   2936   1378825_a_at   7.452 7.19 7.297 7.244 7.106 6.592 6.25 6.12 6.445 5.958
   2937   1375525_at   9.393 9.287 9.421 9.516 9.414 9.114 9.025 9.086 8.977 8.946
   2938   1371071_at   7.663 7.729 7.781 7.9 8.245 6.992 6.701 6.916 6.931 6.518
   2939   1377336_at   6.713 6.103 6.428 6.549 6.237 7.567 7.149 7.507 7.573 7.645
   2940   1384453_at   5.474 5.408 5.422 5.539 5.527 5.943 6.343 5.973 6.184 6.021
   2941   1370865_at   11.244 11.233 11.279 11.018 11.025 11.719 11.696 11.561 11.628 11.649
   2942   1389919_at   6.648 6.514 6.485 6.725 6.431 6.001 6.028 6.106 5.927 6.145
   2943   1379633_a_at   8.383 8.287 8.072 8.315 8.644 5.73 2.918 2.811 4.377 4.469
   2944   1376683_at   8.061 8.215 8.362 8.276 8.142 8.709 8.701 9.004 8.912 8.786
   2945   1367881_at   8.512 8.679 8.481 8.812 8.773 8.17 8.059 8.063 7.996 7.922
   2946   1369381_a_at   5.516 5.438 5.852 5.202 5.099 6.786 7.138 6.519 7.239 7.377
   2947   1374448_at   7.649 7.878 8.094 8.19 8.054 9.021 8.654 9.073 8.966 8.85
   2948   1390866_at   10.621 10.616 10.475 10.468 10.541 9.666 9.714 9.768 9.385 9.088
   2949   1389013_at   9.551 9.553 9.533 9.658 9.6 9.362 9.186 9.215 9.114 9.225
   2950   1390807_at   9.433 9.485 9.582 9.655 9.329 10.09 10.18 10.365 10.088 10.005
   2951   1387111_at   10.356 10.387 11.02 10.477 10.406 11.507 11.435 11.659 11.683 11.801
   2952   1375336_at   12.412 12.362 12.089 12.224 12.329 11.857 11.835 11.743 11.816 11.817
   2953   1388643_at   7.908 7.981 7.737 7.788 7.791 8.344 8.385 8.464 8.478 8.19
   2954   1374501_at   9.452 9.342 9.275 9.369 9.332 9.024 8.832 9.004 8.826 8.76
   2955   1368464_at   8.695 8.631 8.677 8.842 9.499 7.584 7.04 6.817 7.079 6.494
   2956   1372661_at   7.579 7.505 7.549 7.71 7.696 7.321 7.134 7.192 7.123 7.187
   2957   1388636_at   7.913 7.925 7.996 8.038 7.867 8.415 8.276 8.273 8.497 8.328
   2958   1382135_at   8.63 8.609 8.563 8.64 8.482 8.326 8.174 8.207 8.192 8.065
   2959   1376202_at   7.459 7.462 7.42 7.78 7.748 8.227 8.231 8.491 8.337 8.221
   2960   1392111_at   8.076 8.074 7.844 8.058 8.132 7.512 7.518 7.631 7.466 7.656
   2961   1370792_at   9.258 9.216 9.355 9.355 9.225 8.922 8.715 8.735 8.792 8.534
   2962   1375073_at   3.329 4.018 3.898 3.918 4.099 4.848 4.973 5.339 5.112 5.194
   2963   1374276_at   5.319 5.846 5.737 5.771 5.645 6.932 6.938 6.807 6.742 6.354
   2964   1388774_at   10.995 10.959 11.282 11.119 11.092 10.656 10.508 10.61 10.411 10.475
   2965   1395126_at   2.47 2.703 0.714 3.06 2.39 5.378 6.016 5.733 5.882 5.161
   2966   1385505_at   6.915 6.922 7.035 7.005 7.065 7.556 7.391 7.615 7.55 7.313
   2967   1390972_at   6.001 6.093 6.079 6.057 6.26 6.768 6.888 6.811 7.039 6.542
   2968   1373258_at   7.511 7.423 7.458 7.47 7.592 8.213 8.116 8.47 8.325 7.948
   2969   1396301_x_at   7.219 7.468 7.647 7.738 7.507 8.39 8.285 8.686 8.736 8.844
   2970   1373452_at   7.754 7.902 7.97 8.065 7.899 7.509 7.338 7.39 7.273 7.483
   2971   1395058_at   7.001 6.819 7.117 6.832 6.936 6.331 6.507 6.358 6.218 6.168
   2972   1389581_at   8.152 8.12 8.377 8.14 8.145 9.002 9.357 9.431 9.117 8.808
   2973   1387809_at   4.895 5.478 4.955 5.36 5.445 6.626 6.093 6.539 6.375 6.507
   2974   1381979_at   6.94 7.095 7.078 7.061 7.116 7.524 7.496 7.458 7.452 7.296
   2975   1373428_at   8.474 8.28 8.347 8.5 8.489 7.969 7.842 7.887 7.999 7.691
   2976   1371338_at   9.33 9.251 9.545 9.573 9.436 10.008 9.895 10.187 10.039 10.171
   2977   1389199_at   7.832 7.945 8.005 8.127 8.057 8.518 8.442 8.655 8.502 8.401
   2978   1381221_at   6.274 5.9 6.1 6.097 6.161 5.119 4.991 4.473 4.449 4.156
   2979   1390163_at   6.999 6.775 6.83 6.89 6.993 6.539 6.49 6.536 6.375 6.407
   2980   1370446_at   7.913 7.761 8.044 8.081 7.555 8.73 8.686 8.815 8.642 9.02
   2981   1382925_at   7.185 7.347 7.332 7.419 7.441 4.283 4.409 2.053 4.407 4.064
   2982   1391559_at   6.274 6.325 6.037 6.451 6.684 7.268 7.069 7.356 7.286 7.311
   2983   1388856_at   7.501 7.496 7.683 7.491 7.371 8.13 8.18 8.395 8.263 7.959
   2984   1370929_at   8.757 8.712 9.216 9.251 8.644 9.988 10.2 10.4 10.084 9.917
   2985   1383162_at   9.832 9.868 10.146 10.023 9.696 10.478 10.627 10.521 10.587 10.692
   2986   1374664_at   7.542 7.581 7.65 7.771 7.713 8.021 8.088 8.181 8.153 7.982
   2987   1382041_at   7.156 7.496 7.764 7.487 7.201 8.603 8.483 8.317 8.24 8.383
   2988   1388757_at   7.067 7.425 7.676 7.553 7.446 5.958 5.948 6.396 6.37 6.507
   2989   1386961_at   6.775 6.798 6.842 7.135 7.153 8.009 7.603 7.778 7.687 7.905
   2990   1374526_at   9.339 9.382 9.189 9.237 9.292 8.982 8.818 8.889 8.791 8.7
   2991   1382921_at   7.145 7.383 7.103 7.24 7.41 5.943 5.598 6.327 5.404 5.274
   2992   1393388_at   8.902 8.586 8.457 8.622 8.723 9.347 9.495 9.555 9.442 9.197
   2993   1373089_at   5.814 5.719 5.95 6.081 6.107 6.759 6.799 6.532 6.531 6.795
   2994   1393018_at   7.584 7.844 8.346 8.029 7.434 9.178 9.014 9.329 9.086 9.371
   2995   1374830_at   6.061 6.172 5.823 6.211 6.051 7.286 6.683 6.991 7.26 7.248
   2996   1391989_at   3.101 4.025 4.54 3.945 4.407 1.356 0.333 1.58 1.496 1.705
   2997   1384591_at   5.969 5.845 5.411 5.864 5.894 6.624 6.489 6.763 6.798 6.647
   2998   1391302_at   5.806 6.023 5.391 6.059 5.919 7.249 6.718 7.076 6.906 7.184
   2999   1379321_at   5.224 5.375 5.347 5.098 4.782 6.092 6.015 6.172 5.98 5.961
   3000   1370177_at   8.479 8.428 8.311 8.66 8.372 7.377 7.084 6.916 6.68 6.193
   3001   1398602_at   7.965 7.892 7.901 8.081 7.856 7.044 7.158 7.116 6.494 6.725
   3002   1393551_at   8.266 8.392 8.133 8.421 8.667 8.966 9.199 9.259 9.279 9.224
   3003   1389565_at   6.69 6.922 7.369 7.083 6.772 5.926 5.865 6.122 5.958 6.031
   3004   1374815_at   8.675 8.596 8.798 8.744 8.587 8.244 8.022 8.239 7.915 8.047
   3005   1388423_at   8.512 8.436 8.373 8.584 8.4 8.899 8.778 8.933 9.058 9.013
   3006   1388455_at   9.985 10.085 10.016 10.056 10.275 9.451 9.114 9.502 9.074 9.055
   3007   1373209_at   8.186 8.315 8.255 8.378 8.258 7.931 7.585 7.797 7.642 7.601
   3008   1373748_at   7.3 7.226 7.05 7.14 7.598 7.966 8.075 8.082 7.929 8.12
   3009   1375431_at   7.564 7.384 7.248 7.363 7.476 7.833 7.83 8.031 7.914 7.912
   3010   1383557_at   7.875 8.161 8.097 8.066 7.863 8.779 8.975 9.151 8.696 8.669
   3011   1369949_at   8.484 8.355 8.7 8.713 8.589 9.295 9.086 9.368 9.284 9.513
   3012   1393338_at   5.246 5.629 5.076 5.562 5.494 6.269 6.454 6.59 6.26 6.727
   3013   1374235_at   5.738 6.189 5.957 6.197 6.104 7.16 6.874 7.13 7.198 7.647
   3014   1367897_at   9.25 9.365 9.126 9.359 9.356 9.867 9.67 10.028 9.992 9.882
   3015   1392013_at   4.566 5.112 4.507 4.373 4.906 6.274 7.186 6.798 7.32 6.18
   3016   1371700_at   7.541 7.602 6.995 7.386 8.155 9.67 9.051 9.246 10.04 9.419
   3017   1385005_at   5.72 5.244 6.004 5.687 4.936 6.933 7.47 7.438 7.406 6.96
   3018   1379076_at   2.018 2.602 2.793 3.172 2.784 5.04 4.806 4.553 4.014 4.764
   3019   1373412_at   8.806 8.728 8.63 8.758 8.907 8.311 8.179 8.268 8.054 8.348
   3020   1383715_at   7.519 7.449 7.567 7.6 7.428 7.889 8.193 7.936 8.186 8.127
   3021   1372252_at   9.59 9.738 9.681 9.792 9.893 10.233 10.175 10.291 10.36 10.131
   3022   1376401_at   7.799 7.988 7.854 8.076 7.978 8.391 8.319 8.378 8.523 8.38
   3023   1390543_at   7.792 7.784 8.188 8.204 7.402 8.891 9.08 9.127 9.102 9.119
   3024   1372637_at   4.451 4.192 3.905 4.323 3.977 5.327 5.085 5.53 4.984 5.308
   3025   1371775_at   9.848 10.067 10.187 9.85 9.77 10.661 10.506 10.541 10.704 10.699
   3026   1393142_at   6.434 6.273 5.865 6.318 6.326 6.997 7.137 7.049 7.043 6.943
   3027   1374491_at   6.119 6.206 5.623 6.231 5.547 6.968 7.119 7.236 7.242 7.161
   3028   1367698_a_at   9.647 9.65 9.8 9.848 9.746 9.283 8.931 9.144 9.281 9.153
   3029   1396215_at   5.632 5.629 5.579 5.734 5.045 6.517 6.55 6.648 6.483 6.383
   3030   1399002_at   9.76 9.853 9.906 9.784 9.727 10.263 10.355 10.387 10.17 10.132
   3031   1375289_at   8.986 8.959 9.111 9.177 8.984 9.671 9.499 9.455 9.525 9.748
   3032   1381915_at   7.746 7.923 8.02 8.083 7.856 8.417 8.359 8.588 8.559 8.522
   3033   1384628_at   8.623 8.748 8.889 8.895 8.373 9.439 9.486 9.761 9.796 9.738
   3034   1383939_at   6.057 6.471 6.535 6.572 6.002 7.362 7.659 7.702 7.358 7.287
   3035   1393550_at   6.13 6.086 5.85 6.12 6.299 5.583 5.507 5.33 5.387 5.449
   3036   1378013_at   6.566 6.762 6.722 6.593 5.264 8.598 8.4 8.649 8.884 8.556
   3037   1387101_at   10.028 10.046 9.775 9.967 10.216 9.484 9.234 9.365 9.292 9.137
   3038   1372853_at   8.516 8.399 8.631 8.584 8.556 8.135 7.977 8.142 8.095 7.881
   3039   1389012_at   10.895 10.878 10.872 10.722 10.94 11.194 11.253 11.123 11.167 11.193
   3040   1389288_at   10.023 10.034 10.062 10.202 10.157 10.54 10.435 10.69 10.745 10.723
   3041   1390246_at   6.047 6.316 6.488 6.252 6.069 7.192 7.167 7.383 7.422 6.872
   3042   1372752_at   9.744 9.727 9.792 9.787 9.759 9.184 9.321 9.387 9.149 8.973
   3043   1372813_at   9.156 9.141 9.289 9.211 9.201 9.571 9.459 9.591 9.696 9.508
   3044   1383845_at   7.276 7.358 7.301 7.339 7.173 7.795 7.724 7.924 7.661 7.646
   3045   1388722_at   9.312 9.246 9.265 9.439 9.53 8.966 8.841 8.932 8.795 8.814
   3046   1371738_at   7.201 6.886 7.063 7.282 7.12 7.88 7.737 7.92 7.634 8.014
   3047   1372402_at   8.867 9 8.884 9.005 9.203 8.537 8.536 8.485 8.378 8.48
   3048   1391855_at   5.225 5.081 5.174 4.983 5.063 5.977 5.812 6.385 6.343 5.871
   3049   1382711_at   5.702 5.491 5.45 5.65 5.463 6.428 6.31 6.205 6.096 6.044
   3050   1370011_at   5.674 5.405 5.663 6.072 5.942 7.181 6.778 6.699 6.816 7.149
   3051   1388778_at   8.301 8.223 8.142 8.32 7.755 9.07 8.903 9.38 9.143 9.264
   3052   1398369_at   8.94 8.955 9.025 9.232 9.069 8.286 8.043 8.463 8.172 7.861
   3053   1386274_at   5.677 5.123 5.859 5.613 5.089 6.842 6.603 6.956 6.605 6.864
   3054   1373719_at   8.859 8.718 8.917 8.741 8.932 9.32 9.201 9.234 9.17 9.199
   3055   1388803_at   8.284 8.229 8.197 8.04 8.17 7.754 7.451 7.771 7.638 7.589
   3056   1377037_at   7.528 7.328 7.94 7.465 7.214 8.638 8.964 8.733 9.007 8.416
   3057   1396383_at   7.015 6.488 6.962 7.129 6.903 5.89 5.639 5.903 5.745 5.274
   3058   1373917_at   9.417 9.502 9.337 9.45 9.492 9.199 9.083 9.136 9.02 9.019
   3059   1369957_at   5.893 5.743 5.689 5.958 6.53 7.137 7.36 7.167 7.122 7.061
   3060   1376165_at   3.93 4.337 4.279 4.663 4.141 5.73 5.328 5.795 5.938 6.351
   3061   1373843_at   11.125 11.132 11.123 11.154 11.178 10.766 10.938 10.934 10.858 10.783
   3062   1382569_at   5.146 5.659 5.822 5.618 5.583 6.65 6.404 6.959 6.873 7.016
   3063   1372329_at   8.337 8.432 8.474 8.584 8.617 8.058 7.859 8.056 7.892 7.807
   3064   1387823_at   9.176 9.308 9.266 9.394 9.332 8.886 8.953 9.046 8.903 8.944
   3065   1381474_at   5.386 5.19 5.311 5.713 5.901 3.65 4.049 2.935 3.65 2.772
   3066   1371712_at   8.413 8.549 8.589 8.672 8.536 8.172 8.168 7.961 8.157 8.097
   3067   1382350_at   6.678 6.515 6.831 6.804 6.816 6.115 6.037 6.092 5.678 5.908
   3068   1387831_at   9.947 9.949 10.03 9.932 9.665 6.499 4.697 4.249 4.534 1.975
   3069   1380379_at   6.655 6.582 6.623 6.608 6.512 7.53 7.175 7.573 7.868 7.889
   3070   1371089_at   6.265 6.388 6.268 6.531 6.661 7.132 7.046 7.496 7.325 7.35
   3071   1390387_at   8.422 8.284 8.435 8.393 8.331 8.927 8.929 9.053 8.945 8.667
   3072   1375869_at   6.593 6.665 6.531 6.97 6.597 7.416 7.202 7.487 7.354 7.351
   3073   1367494_at   10.377 10.415 10.434 10.382 10.406 10.074 10.151 10.125 10.021 9.9
   3074   1377185_at   7.833 7.932 7.933 8.116 8.271 5.559 3.63 5.389 5.597 5.403
   3075   1372535_at   8.249 7.938 8.08 7.899 8.243 6.839 7.066 6.639 7.099 7.356
   3076   1371513_at   8.229 8.088 7.934 8.191 8.201 8.683 8.517 8.774 8.743 8.621
   3077   1371782_at   8.463 8.451 8.236 8.438 8.614 7.625 7.483 7.724 7.141 7.161
   3078   1383104_at   6.921 6.895 6.961 6.691 6.597 7.372 7.428 7.668 7.659 7.704
   3079   1371415_at   11.35 11.267 11.383 11.295 11.493 11.729 11.718 11.763 11.686 11.886
   3080   1394745_at   5.981 5.869 5.681 5.621 5.775 4.747 4.262 4.154 3.573 3.644
   3081   1374576_at   7.193 7.084 7.397 6.97 6.944 6.182 6.549 6.281 6.128 6.149
   3082   1374638_at   9.094 9.111 9.266 9.138 9.17 9.463 9.386 9.52 9.469 9.408
   3083   1382363_at   6.605 6.203 6.675 6.695 6.472 7.315 7.569 7.452 7.397 7.175
   3084   1371618_s_at   6.45 6.442 6.681 6.856 7.161 5.511 4.969 5.06 4.522 4.404
   3085   1370802_at   6.019 6.424 6.336 6.413 6.435 7.308 6.893 7.388 7.416 7.395
   3086   1379642_at   6.505 6.829 7.026 6.826 6.601 4.95 4.405 4.537 4.603 3.159
   3087   1372270_at   9.193 9.231 8.979 9.319 9.581 8.604 8.472 8.453 8.372 8.407
   3088   1368339_at   4.511 4.253 3.817 4.043 3.668 5.144 5.875 5.642 6.04 5.957
   3089   1374444_at   5.692 5.546 4.995 5.759 5.574 6.57 6.728 6.72 6.692 6.426
   3090   1370984_at   6.846 6.85 6.775 7.044 7 7.494 7.468 7.679 7.324 7.447
   3091   1396114_at   0 0 0 0.632 0.934 2.766 1.96 2.414 2.22 3.168
   3092   1395953_at   5.33 5.439 4.997 5.244 5.276 5.766 5.99 6.043 5.865 5.947
   3093   1373053_at   7.258 7.109 7.163 6.841 6.907 7.824 7.764 8.204 8.165 7.873
   3094   1390485_at   9.566 9.566 9.574 9.712 9.723 9.253 9.008 9.242 9.244 9.14
   3095   1372076_at   10.739 10.733 10.779 10.823 10.824 10.968 11.122 11.176 11.137 11.059
   3096   1382475_at   4.884 4.665 5.098 5.413 4.89 5.871 6.303 6.148 6.391 6.097
   3097   1372292_at   7.978 7.912 8.183 8.121 7.954 8.456 8.413 8.445 8.427 8.536
   3098   1371964_at   9.793 9.707 9.797 9.891 9.807 10.161 10.126 10.253 10.256 10.05
   3099   1371764_at   9.175 9.156 9.163 9.308 9.334 8.897 8.606 8.77 8.788 8.665
   3100   1388111_at   4.899 4.582 4.807 5.657 5.491 7.063 6.467 6.953 7.303 7.081
   3101   1367760_at   9.197 9.31 9.483 9.499 9.41 8.897 8.892 8.924 8.704 8.743
   3102   1375029_at   7.72 7.676 7.7 7.83 7.882 7.464 7.308 7.378 7.255 7.25
   3103   1379724_at   7.381 7.404 7.757 7.845 7.833 8.659 8.58 9.268 9.036 8.837
   3104   1380210_at   6.379 6.161 6.645 6.66 5.962 5.094 5.364 5.296 4.873 5.073
   3105   1367874_at   8.456 8.438 8.303 8.529 8.43 7.981 7.692 7.774 7.721 7.442
   3106   1370853_at   5.875 5.86 6.279 6.532 5.888 7.16 7.084 7.345 7.323 7.571
   3107   1370309_a_at   11.205 11.207 11.241 11.278 11.29 10.899 10.881 10.986 10.984 10.759
   3108   1376071_at   6.687 6.855 6.483 6.675 6.5 7.341 7.492 7.315 7.78 7.389
   3109   1395761_at   5.471 5.868 5.782 5.978 5.695 6.526 6.78 6.816 6.821 6.41
   3110   1388410_at   10.046 10.05 9.986 10.004 10.103 10.194 10.236 10.271 10.24 10.195
   3111   1393032_x_at   5.279 5.399 5.451 5.528 5.636 5.966 6.111 6.041 5.91 6.18
   3112   1388960_at   10.073 10.052 9.963 10.113 10.179 9.685 9.375 9.615 9.39 9.594
   3113   1396039_at   8.078 8.037 8.679 8.67 7.768 9.652 9.727 9.723 10.176 10.197
   3114   1382190_at   4.536 4.379 4.122 4.219 4.925 5.903 5.859 6.084 6.412 5.533
   3115   1374947_at   5.147 5.326 5.022 5.008 5.104 6.059 5.796 5.846 5.984 5.604
   3116   1372390_at   5.619 5.642 5.359 5.315 6.198 7.486 6.785 7.544 7.301 7.031
   3117   1374537_at   8.66 8.575 8.808 8.817 8.857 7.985 7.924 7.959 7.749 7.378
   3118   1387114_at   8.615 8.685 8.644 8.979 8.953 8.196 8.078 8.164 8.202 8.06
   3119   1376828_at   5.236 5.37 5.231 5.241 4.992 5.804 5.811 5.837 5.889 6.164
   3120   1377232_at   7.249 6.954 7.349 7.422 7.317 8.022 7.895 8.274 8.09 7.948
   3121   1390790_a_at   6.568 6.759 6.825 7.194 6.715 5.736 5.329 5.341 5.764 5.917
   3122   1380245_at   7.636 7.62 7.851 7.678 7.423 8.232 8.406 8.515 8.297 8.184
   3123   1370519_at   6.541 6.484 6.567 6.733 6.653 5.396 5.613 5.432 5.732 6.05
   3124   1373406_at   7.832 7.972 7.718 7.843 8.014 7.338 7.23 7.37 7.135 6.985
   3125   1368244_at   7.026 7.31 7.051 7.142 7.341 7.793 7.868 8.19 7.834 7.843
   3126   1371479_at   8.101 8.063 8.005 8.131 8.165 8.448 8.487 8.5 8.756 8.584
   3127   1390777_at   8.137 8.235 8.053 7.697 7.677 8.835 8.887 8.963 8.752 8.987
   3128   1367712_at   10.475 10.54 10.517 10.544 10.506 9.334 9.58 9.078 8.728 8.507
   3129   1371405_at   10.103 10.164 10.147 10.184 10.195 10.803 10.728 10.962 11.101 11.336
   3130   1383231_at   7.86 7.987 7.716 7.903 8.089 7.486 7.395 7.418 7.324 7.378
   3131   1388027_a_at   10.247 10.325 10.201 10.208 10.313 9.975 9.892 9.915 9.938 9.734
   3132   1393331_at   7.247 7.457 7.479 7.467 7.464 6.823 6.994 6.816 7.073 6.906
   3133   1374060_at   6.723 6.775 6.189 6.686 6.756 7.661 7.567 7.817 7.799 7.388
   3134   1382210_at   6.077 5.984 6.149 6.223 6.485 4.97 4.84 4.814 5.492 4.709
   3135   1373840_at   8.185 7.876 7.879 8.292 8.286 7.304 7.246 6.855 7.19 7.258
   3136   1376585_at   8.956 8.776 9.02 8.727 8.852 9.293 9.356 9.353 9.34 9.228
   3137   1374731_at   9.457 9.786 9.646 10.074 9.985 8.932 8.687 8.951 8.802 8.571
   3138   1386897_at   10.165 10.115 10.04 10.104 10.252 9.62 9.687 9.576 9.389 9.286
   3139   1371329_at   11.529 11.494 11.578 11.558 11.564 11.31 11.161 11.239 11.04 11.197
   3140   1375785_at   7.228 7.342 7.805 7.518 7.363 8.287 8.243 8.292 8.279 8.111
   3141   1384389_at   3.652 4.676 3.611 2.753 3.686 1.077 1.485 0.88 0.322 1.118
   3142   1383324_at   7.86 7.965 7.985 8.1 7.83 8.557 8.308 8.642 8.667 8.559
   3143   1391534_at   6.676 6.574 6.602 6.46 6.227 7.43 7.509 7.514 7.502 7.048
   3144   1370127_at   7.203 7.162 7.055 6.869 6.982 6.595 6.347 6.317 6.232 6.478
   3145   1367624_at   10.722 10.715 10.919 11.157 10.884 10.27 10.305 10.252 10.17 10.255
   3146   1375182_at   6.858 6.559 6.721 6.925 6.604 7.465 7.204 7.561 7.537 7.555
   3147   1379573_at   4.741 5.385 5.04 4.526 4.713 5.998 6.195 6.379 6.005 6.077
   3148   1386574_at   6.202 6.014 6.055 6.106 6.08 6.569 6.436 6.616 6.823 6.691
   3149   1374819_at   5.25 5.114 5.238 5.5 5.394 6.024 6.005 6.385 5.92 6.067
   3150   1396202_at   6.002 6.081 5.741 5.981 5.817 6.435 6.667 6.776 6.467 6.656
   3151   1385585_at   6.878 6.721 6.608 6.621 6.282 7.355 7.524 7.835 7.875 7.788
   3152   1367567_at   12.267 12.256 12.277 12.189 12.304 12.079 12.114 12.007 12.037 12.021
   3153   1371800_at   9.202 9.083 9.101 9.427 9.379 8.57 8.232 8.445 8.607 8.191
   3154   1379205_at   5.362 5.848 5.338 5.63 5.619 4.483 4.808 4.547 4.539 4.158
   3155   1386886_at   11.786 11.685 11.73 11.624 11.656 12.01 11.916 11.978 11.904 11.987
   3156   1389611_at   7.538 7.817 7.885 7.742 7.537 8.383 8.535 8.45 8.441 8.183
   3157   1382611_at   6.426 7.049 6.777 6.777 5.85 8.168 8.258 8.309 8.567 8.02
   3158   1373247_at   6.218 6.222 6 5.981 6.25 7.106 7.06 7.336 7.313 6.698
   3159   1398710_at   5.259 6.029 5.513 5.741 6.06 7.05 7.076 7.113 7.293 6.73
   3160   1386577_at   3.949 3.841 3.93 4.424 4.168 4.963 4.878 5.246 4.876 5.068
   3161   1389634_at   8.532 8.32 8.342 8.464 8.339 7.961 7.883 7.783 7.937 7.618
   3162   1372205_at   6.813 6.821 6.679 6.859 6.826 7.364 7.184 7.56 7.603 7.36
   3163   1386935_at   7.559 7.5 7.762 7.369 7.163 6.448 6.641 6.323 6.31 6.734
   3164   1374694_at   6.793 7.036 7.011 6.779 7.013 7.653 7.517 7.601 7.575 7.322
   3165   1372894_at   8.409 8.475 8.462 8.615 8.519 8.841 8.76 8.929 8.977 8.872
   3166   1380057_at   6.578 6.268 6.978 6.744 6.412 8.042 7.453 8.179 7.986 7.878
   3167   1398107_at   5.34 5.398 5.782 5.431 5.437 6.962 6.289 6.326 6.64 6.724
   3168   1398838_at   10.317 10.242 10.222 10.191 10.181 9.855 10.017 9.771 9.808 9.772
   3169   1375882_at   8.386 8.725 8.456 8.697 8.737 7.873 7.979 8.028 7.778 7.679
   3170   1386671_at   6.945 7.094 7.412 7.113 7.076 8.314 8.584 8.197 8.182 7.815
   3171   1371776_at   9.01 8.991 8.822 9.025 8.898 8.181 8.392 8.226 8.002 7.78
   3172   1372032_at   9.065 9.249 9.555 9.63 9.436 8.2 7.722 8.388 7.572 7.536
   3173   1373847_at   9.282 9.241 9.472 9.603 9.545 8.911 8.79 8.776 8.573 8.737
   3174   1388624_at   7.732 7.916 7.908 8.097 7.966 8.586 8.483 8.857 8.926 8.96
   3175   1370969_at   7.039 6.984 6.767 6.532 6.678 7.636 7.598 7.734 7.779 7.434
   3176   1373868_at   7.322 7.262 7.271 7.238 7.322 6.622 6.67 6.388 6.496 6.046
   3177   1389717_at   8.367 8.38 8.457 8.552 8.365 8.103 8.041 8.092 7.992 7.883
   3178   1395167_at   7.44 7.359 7.527 7.506 7.225 8.046 8.098 8.579 8.458 8.226
   3179   1387911_at   9.85 9.815 9.712 9.999 10.011 9.344 9.332 9.267 9.22 9.493
   3180   1374611_at   8.249 8.212 8.444 8.384 8.271 7.844 7.721 7.82 7.706 7.461
   3181   1371041_at   10.889 10.779 10.942 11.086 11.116 11.456 11.411 11.586 11.443 11.537
   3182   1374094_at   7.446 7.476 7.567 7.406 7.566 8.05 7.772 8.045 7.907 7.942
   3183   1367857_at   8.91 8.886 8.531 8.745 8.942 9.608 9.647 9.391 9.907 9.809
   3184   1372553_at   7.491 7.36 7.423 7.332 7.37 7.998 7.871 7.706 7.832 8.042
   3185   1383785_at   6.648 5.984 6.643 6.279 6.412 4.155 4.575 5.073 5.02 5.033
   3186   1383686_at   5.624 5.289 5.95 6.027 6.142 7.143 7.318 6.938 7.196 6.908
   3187   1387373_at   5.443 5.665 5.695 5.022 5.302 6.785 6.508 6.536 6.7 6.273
   3188   1377643_at   8.096 8.005 8.019 7.886 8.097 9.084 8.478 9.105 9.055 9.015
   3189   1377192_a_at   9.024 9.097 9.309 9.275 8.832 9.742 9.728 9.914 9.971 9.917
   3190   1385791_at   6.808 6.82 7.01 7.159 6.428 7.902 7.795 8.169 8.335 8.413
   3191   1397672_at   3.775 4.28 3.848 4.114 3.612 4.951 5.047 5.102 5.527 5.579
   3192   1367651_at   11.594 11.672 11.595 11.512 11.759 12.052 11.901 11.998 12.059 12.076
   3193   1389788_at   6.31 6.527 6.115 6.453 6.379 7.029 7.245 7.641 7.189 7.253
   3194   1380108_at   6.097 5.756 6.187 6.138 6.015 7.24 6.966 7.127 6.82 6.693
   3195   1378260_at   4.806 4.512 5.496 4.517 4.55 6.329 6.519 6.37 6.461 5.991
   3196   1373943_at   8.189 8.222 8.439 8.486 8.143 7.612 7.178 7.601 7.405 7.604
   3197   1367777_at   10.132 10.124 10.156 10.251 10.22 10.573 10.723 11.021 10.883 10.709
   3198   1388130_at   9.541 9.474 9.722 9.945 9.758 9.016 8.718 8.998 8.607 8.688
   3199   1398634_at   6.766 6.751 7.066 6.935 6.534 7.504 7.837 7.86 7.672 7.528
   3200   1370831_at   8.97 8.89 9.381 9.184 9.395 10.387 10.123 10.75 11.087 10.684
   3201   1374430_at   9.393 9.412 9.411 9.249 9.605 8.824 8.843 8.836 8.661 8.466
   3202   1396280_at   6.478 6.745 6.844 6.845 6.993 5.891 5.963 6.032 5.508 5.874
   3203   1369268_at   8.673 8.468 8.692 8.424 8.549 6.704 6.466 6.073 5.101 4.909
   3204   1382161_at   7.166 7.179 7.016 7.202 7.28 6.615 6.502 6.833 6.617 6.491
   3205   1388018_at   5.192 5.446 4.658 5.007 5.115 2.373 2.973 2.707 1.245 3.044
   3206   1378753_at   8.895 8.982 8.995 9.072 8.995 9.448 9.463 9.753 9.48 9.384
   3207   1370968_at   9.392 9.394 9.295 9.494 9.452 8.316 8.703 8.654 8.114 8.038
   3208   1373399_at   7.734 7.464 7.497 7.845 7.768 8.336 8.2 8.524 8.307 8.373
   3209   1384431_at   3.864 4.541 4.095 3.843 3.467 5.562 5.375 5.549 5.253 5.188
   3210   1375674_at   7.109 7.416 7.208 7.27 7.076 8.231 7.83 7.951 8.2 8.434
   3211   1382005_at   10.538 10.559 10.559 10.398 10.517 9.889 10.082 10.087 10.005 9.721
   3212   1380850_at   6.006 5.97 5.945 5.63 5.594 6.47 6.747 6.954 6.68 6.91
   3213   1397949_at   5.91 6.164 6.039 6.011 5.978 6.662 6.738 6.953 6.621 6.466
   3214   1397300_at   8.124 8.183 8.258 8.29 7.974 8.671 8.567 8.802 8.861 8.738
   3215   1393357_at   6.659 6.675 6.677 6.735 6.874 5.958 5.827 5.219 5.866 5.696
   3216   1392449_at   5.938 6.611 6.494 6.687 6.426 5.288 5.155 5.401 4.784 5.268
   3217   1375948_at   7.671 7.769 7.637 7.793 7.76 7.381 7.343 7.453 7.305 7.192
   3218   1398750_at   11.938 11.92 11.893 11.836 11.935 11.605 11.511 11.443 11.247 11.402
   3219   1388792_at   8.479 8.131 8.357 8.668 8.587 7.519 7.416 7.322 6.821 6.915
   3220   1398342_at   7.216 7.406 7.329 7.229 7.271 7.699 7.872 7.766 7.626 7.63
   3221   1371500_at   8.242 8.559 7.98 8.499 8.675 9.92 9.256 9.855 9.844 9.577
   3222   1368301_at   6.704 6.238 6.308 6.204 6.534 4.275 2.632 3.231 4.616 3.583
   3223   1396847_at   5.009 4.806 5.106 4.961 4.659 5.524 5.72 5.783 5.663 6.011
   3224   1387505_at   6.979 7.19 7.332 7.222 6.747 8.068 7.907 8.463 8.284 8.043
   3225   1368813_at   8.454 8.407 8.489 8.741 8.686 7.415 6.66 6.331 6.071 5.832
   3226   1388355_at   8.89 8.854 8.808 9.085 9.13 8.33 7.962 8.39 8.027 8.044
   3227   1368370_at   6.623 6.344 6.522 6.937 6.686 5.847 5.584 5.648 5.446 5.881
   3228   1388917_at   8.801 8.581 8.76 8.877 8.807 9.512 9.177 9.531 9.306 9.357
   3229   1367940_at   7.679 7.823 7.999 8.17 8.081 8.745 8.822 9.037 8.767 9.256
   3230   1367574_at   12.078 12.005 12.008 11.869 12.046 11.151 11.199 10.853 10.789 10.374
   3231   1371012_at   7.781 7.549 7.782 7.53 7.09 8.51 8.42 8.719 8.586 8.517
   3232   1371719_at   8.389 8.462 8.253 8.479 8.298 8.087 8.017 7.944 7.961 7.987
   3233   1390164_at   4.823 5.916 5.297 5.633 5.137 6.723 7.379 7.413 7.498 6.89
   3234   1391500_at   8.11 8.062 8.191 8.197 8.22 8.505 8.391 8.475 8.428 8.57
   3235   1381903_at   8.167 8.079 8.182 8.037 8.202 7.706 7.582 7.565 7.308 7.3
   3236   1392053_at   4.337 4.664 4.161 3.948 3.995 5.796 5.135 5.448 5.782 5.801
   3237   1382154_at   9.961 9.887 9.903 9.892 9.868 9.433 9.49 9.522 9.382 9.144
   3238   1387348_at   7.388 7.774 8.224 7.77 7.991 9.078 9.058 9.901 9.767 9.667
   3239   1389786_at   5.686 5.383 5.776 5.606 5.109 6.637 6.387 7.06 6.727 6.697
   3240   1376609_at   8.666 8.597 8.45 8.515 8.55 8.155 8.191 8.281 8.155 8.042
   3241   1367994_at   6.722 6.884 6.71 6.733 6.48 7.404 7.148 7.418 7.367 7.248
   3242   1369119_a_at   5.838 5.655 5.405 6.024 5.991 4.029 4.586 4.24 4.652 3.719
   3243   1383263_at   8.197 8.179 7.844 7.644 7.903 9.141 9.158 9.109 9.612 8.821
   3244   1389208_at   4.686 4.114 4.719 4.984 4.948 6.048 6.12 5.67 6.033 6.034
   3245   1374836_at   7.993 7.653 7.894 7.797 7.963 7.335 7.082 7.105 7.149 6.818
   3246   1390032_at   8.13 8.135 8.294 8.453 8.328 7.69 7.568 7.726 7.833 7.553
   3247   1391390_at   8.003 7.553 7.738 8.167 7.511 8.687 8.794 9.1 8.826 8.906
   3248   1388974_at   8.893 8.868 8.96 8.955 8.912 8.472 8.355 8.578 8.215 8.202
   3249   1367977_at   4.654 4.637 3.846 4.353 3.374 6.48 5.896 6.557 7.066 7.102
   3250   1382802_x_at   6.33 6.384 6.609 6.805 6.786 7.336 7.397 7.253 7.326 7.33
   3251   1372868_at   6.806 7.261 7.263 7.427 7.334 6.424 6.38 6.367 6.249 6.073
   3252   1373513_at   6.495 6.002 6.222 6.262 6.83 5.365 4.463 4.738 4.839 4.912
   3253   1388902_at   8.747 8.673 8.48 8.115 8.537 9.75 9.757 10.005 9.558 9.319
   3254   1373436_at   6.359 6.344 6.463 6.548 6.317 7.01 7.372 7.628 7.208 7.111
   3255   1387028_a_at   8.699 8.572 8.634 8.726 8.878 9.068 9.09 9.165 9.124 9.074
   3256   1373419_at   7.169 7.48 7.2 7.168 7.359 8.198 8.073 8.268 8.02 7.763
   3257   1372392_at   7.067 7.202 7.141 7.568 7.32 8.056 7.963 8.434 8.143 8.075
   3258   1368268_at   8.08 8.242 8.557 8.284 8.198 7.767 7.534 7.464 7.515 7.417
   3259   1382629_at   7.677 7.765 7.718 7.51 8.051 6.32 6.69 6.995 6.831 6.617
   3260   1371358_at   9.703 9.635 9.653 9.698 9.921 10.218 10.097 10.146 10.116 10.27
   3261   1393474_at   6.36 6.35 6.555 6.546 5.546 7.606 7.625 7.61 7.739 7.741
   3262   1386892_at   9.6 9.47 9.54 9.684 9.465 10.253 9.914 10.008 10.121 10.05
   3263   1382021_at   7.959 8.057 8.342 8.266 7.964 8.927 8.91 9.08 8.882 8.649
   3264   1371920_at   8.91 8.888 8.892 8.781 8.768 9.285 9.09 9.24 9.342 9.199
   3265   1388615_at   10.771 10.751 10.82 10.667 10.586 10.256 10.105 10.203 10.365 10.029
   3266   1385704_at   7.814 7.724 7.379 7.811 7.766 8.567 8.861 9.155 8.561 8.585
   3267   1393314_at   8.837 8.9 8.92 9.113 8.996 9.3 9.42 9.494 9.45 9.321
   3268   1382682_at   6.997 7.295 6.927 7.226 7.338 6.36 6.256 6.541 6.009 6.209
   3269   1375406_s_at   5.894 6.176 6.073 6.592 6.27 5.233 5.083 5.448 5.236 5.085
   3270   1378457_at   7.052 7.166 7.253 7.308 7.009 7.972 7.684 8.258 8.09 7.892
   3271   1398356_at   10.071 10.044 10.06 9.888 10.034 9.484 9.33 9.408 9.146 8.944
   3272   1397766_at   10.504 10.569 10.311 10.341 10.741 9.692 9.787 9.957 9.768 9.578
   3273   1373397_at   10.133 10.055 10.05 10.142 10.166 9.764 9.8 9.823 9.609 9.604
   3274   1370684_s_at   8.556 8.492 8.572 8.716 8.631 8.98 9.041 8.861 9 8.909
   3275   1392082_a_at   7.307 7.562 7.96 7.579 6.817 5.955 5.678 5.728 6.204 5.933
   3276   1373080_at   9.334 9.403 9.584 9.573 9.395 8.821 9.025 9.006 8.973 8.759
   3277   1378047_at   7.344 7.277 7.433 7.238 7.24 3.87 0.401 3.558 0.888 2.325
   3278   1384841_at   7.18 7.186 7.416 7.24 7.258 6.213 6.105 6.398 6.543 5.74
   3279   1382185_at   5.109 5.761 5.208 6.045 6.213 7.672 7.121 7.705 7.695 7.331
   3280   1371949_at   10.116 10.201 10.063 10.125 10.291 9.761 9.85 9.666 9.642 9.57
   3281   1368588_at   7.2 7.336 7.363 7.572 7.442 6.725 6.885 6.876 6.808 6.586
   3282   1372381_at   5.94 6.621 6.117 6.731 6.327 7.42 7.387 7.815 7.68 7.653
   3283   1371248_at   5.848 5.51 5.424 6.324 6.103 7.881 7.072 7.285 7.521 7.432
   3284   1374038_at   7.535 7.776 7.42 7.649 7.728 8.364 8.333 8.752 8.429 8.237
   3285   1378365_at   4.105 4.492 3.517 3.679 3.797 5.454 5.31 5.563 5.583 5.03
   3286   1372579_at   8.175 8 8.09 8.346 8.427 8.966 8.742 8.921 9.127 9.067
   3287   1371347_at   8.981 9.028 8.987 8.994 8.948 9.284 9.122 9.296 9.311 9.292
   3288   1369120_a_at   5.384 5.531 5.375 5.335 5.15 5.916 5.909 5.896 5.779 6.064
   3289   1375266_at   9.099 9.105 9.126 9.702 9.266 8.004 7.571 8.312 8.178 8.016
   3290   1399098_at   9.436 9.423 9.397 9.642 9.73 10.066 10.039 10.161 10.041 9.992
   3291   1367742_at   6.16 6.242 6.092 6.356 6.241 7.041 6.826 7.555 7.394 7.305
   3292   1395960_at   4.933 4.24 5.119 5.773 4.524 2.84 2.198 3.101 2.293 2.081
   3293   1378860_at   7.483 7.555 7.605 7.797 7.655 8.156 8.216 8.38 8.031 8.154
   3294   1371627_at   7.138 7.04 7.214 7.287 7.321 7.931 7.66 7.915 8.09 7.741
   3295   1381129_at   4.203 5.218 5.22 5.26 5.613 3.445 2.868 3.173 3.411 2.954
   3296   1387408_at   6.151 6.151 5.736 6.16 6.173 4.688 5.24 4.367 4.989 4.887
   3297   1382084_at   6.795 6.738 6.888 6.862 6.637 7.797 7.389 7.832 8.064 8.209
   3298   1370871_at   11.464 11.506 11.602 11.514 11.587 11.213 11.083 10.891 10.842 11.022
   3299   1397610_at   4.171 3.645 3.879 4.159 4.34 5.328 4.885 5.266 5.337 5.005
   3300   1380370_at   4.302 4.218 3.831 4.493 3.617 5.437 5.608 5.535 5.129 5.355
   3301   1384887_at   6.446 6.505 6.533 6.5 6.096 7.168 7.303 7.418 7.853 7.513
   3302   1389902_at   9.975 10.009 9.827 9.965 10.104 9.561 9.565 9.632 9.501 9.382
   3303   1367917_at   9.833 9.94 9.788 10.158 9.3 10.679 10.999 11.051 11.025 11.092
   3304   1367995_at   11.314 11.325 11.375 11.375 10.974 11.847 11.872 11.945 11.998 12.167
   3305   1369654_at   7.276 7.038 7.666 7.412 7.348 8.324 8.262 8.935 8.592 8.408
   3306   1372208_at   5.561 5.821 5.996 5.831 5.614 7.14 6.62 7.286 7.527 7.723
   3307   1395192_at   7.451 7.227 6.818 6.873 7.031 6.176 6.206 5.907 5.788 5.718
   3308   1372820_at   7.02 7.217 6.711 6.776 7.083 8.004 7.787 7.949 7.903 7.583
   3309   1376774_at   7.642 7.614 7.388 7.61 7.47 8.004 7.974 8.189 8.103 7.932
   3310   1371465_at   9.236 9.288 9.218 9.42 9.412 9.899 9.743 9.84 9.646 9.717
   3311   1372720_at   9.082 9.218 9.004 9.184 9.277 9.571 9.533 9.734 9.657 9.543
   3312   1388471_at   7.383 7.549 7.76 7.732 7.863 8.381 8.274 8.544 8.443 8.277
   3313   1388864_at   8.034 7.924 7.627 8.118 8.108 7.305 7.22 7.326 7.107 7.257
   3314   1390857_at   8.976 8.979 9.194 9.51 9.211 9.899 9.952 10.229 10.347 10.238
   3315   1372380_at   8.433 8.347 8.564 8.685 8.529 8.885 9.129 9.02 9.053 9.019
   3316   1374509_at   6.524 6.558 6.579 6.74 6.662 7.083 7.009 7.398 7.294 7.21
   3317   1389562_at   6.943 7.03 6.675 6.775 6.957 7.686 7.482 7.593 7.95 7.524
   3318   1388857_at   9.516 9.587 9.695 9.782 9.595 9.132 8.991 9.277 8.931 8.983
   3319   1387531_at   8.024 8.203 8.63 8.25 7.699 9.206 9.37 9.283 9.292 9.514
   3320   1379243_at   10.118 10.231 10.504 10.616 10.459 11.063 11.018 11.137 11.071 11.221
   3321   1373578_at   7.556 7.557 7.8 7.862 7.604 8.276 8.174 8.527 8.272 8.305
   3322   1374073_at   5.111 4.571 4.775 4.992 5.236 5.875 6.267 5.842 5.912 5.944
   3323   1386987_at   6.43 6.204 6.488 6.208 6.804 5.382 5.648 5.261 5.12 4.999
   3324   1389123_at   8.23 8.146 8.087 7.788 7.75 8.887 9.013 9.135 9.249 8.698
   3325   1375951_at   6.351 6.822 6.778 6.55 6.564 7.389 7.298 7.396 7.642 7.268
   3326   1398781_at   10.782 10.791 10.901 10.805 10.822 11.025 11.15 11.117 11.025 11.028
   3327   1387812_at   5.82 5.713 5.703 5.986 5.193 6.877 6.727 6.776 6.872 7.317
   3328   1373925_at   7.417 7.566 7.618 7.63 7.637 6.84 7.051 7.139 7.156 7.106
   3329   1370854_at   4.457 4.563 4.062 4.635 4.846 5.951 5.831 5.45 6.237 5.742
   3330   1396231_at   5.997 6.111 6.275 5.689 5.15 3.154 2.07 2.803 0.496 1.111
   3331   1385632_at   5.77 5.491 5.553 5.674 5.563 6.168 6.146 5.946 6.175 6.041
   3332   1385853_at   5.938 6.419 6.538 6.472 6.794 5.328 4.59 5.228 4.976 4.66
   3333   1389820_at   11.484 11.377 11.471 11.427 11.502 11.755 11.821 11.677 11.65 11.782
   3334   1370335_at   7.068 7.056 7.225 7.069 7.397 8.084 7.653 7.916 7.982 7.803
   3335   1390240_at   6.72 6.761 6.708 6.655 6.449 5.656 4.999 5.301 5.539 4.608
   3336   1385098_at   6.872 6.989 6.898 7.015 6.862 7.844 7.925 8.096 7.763 7.394
   3337   1368533_at   5.59 5.86 5.012 5.852 5.94 6.982 7.138 7.25 7.352 6.766
   3338   1372098_at   7.61 7.598 7.617 7.635 7.53 7.78 7.867 7.97 7.828 7.912
   3339   1375630_at   10.146 10.251 10.343 10.43 10.433 9.815 9.912 9.879 9.665 9.803
   3340   1389735_at   5.538 5.045 5.061 5.27 5.558 6.103 6.275 6.565 6.501 6.192
   3341   1385426_at   6.655 6.904 7.13 7.299 6.865 5.371 4.812 3.717 4.963 3.971
   3342   1370397_at   10.794 10.744 11.249 10.976 10.587 11.649 11.662 11.763 11.781 11.875
   3343   1385087_at   5.261 5.445 5.26 5.655 5.138 6.217 6.268 6.282 6.141 5.919
   3344   1393385_at   4.262 4.213 4.318 3.982 4.428 5.105 5.02 4.747 5.073 4.876
   3345   1371487_at   10.107 10.157 10.305 10.369 10.423 9.796 9.773 9.812 9.713 9.58
   3346   1368915_at   8.89 8.856 9.009 9.07 8.398 9.748 9.794 9.727 10.127 10.024
   3347   1395251_at   6.04 6.269 6.381 6.271 6.415 6.713 6.987 7.081 6.902 6.943
   3348   1380538_at   6.601 6.761 6.536 6.627 6.164 5.892 5.66 5.448 5.494 5.503
   3349   1370224_at   10.195 10.288 10.682 10.533 10.772 9.416 8.945 9.682 9.164 9.18
   3350   1374279_at   8.629 8.71 8.568 8.855 8.855 8.265 8.174 8.341 8.123 8.172
   3351   1399055_at   8.741 8.797 8.595 8.866 8.779 8.062 7.761 8.239 8.19 7.904
   3352   1392961_at   8.526 8.35 8.233 8.148 8.273 7.61 7.546 7.406 7.234 6.976
   3353   1379972_at   6.085 5.931 5.979 5.812 6.383 5.229 4.964 5.012 5.382 5.154
   3354   1376912_at   5.229 5.446 5.303 5.251 5.392 5.786 5.927 5.877 5.837 5.621
   3355   1383416_at   7.197 6.603 7.018 7.325 6.868 7.887 7.9 7.9 8.019 8.001
   3356   1371704_at   9.181 9.08 8.879 9.196 9.16 8.677 8.549 8.704 8.59 8.546
   3357   1383669_at   5.005 4.981 4.737 5.052 4.663 5.866 5.672 5.43 5.631 5.633
   3358   1372442_at   9.887 9.988 9.797 10.033 10.086 9.452 9.096 9.432 9.231 9.057
   3359   1377002_at   6.333 6.344 6.449 6.812 6.548 5.677 5.295 4.919 5.438 5.519
   3360   1371773_at   8.566 9.072 8.754 8.779 8.524 9.482 9.509 9.672 9.434 9.446
   3361   1368901_at   4.98 5.247 5.207 4.868 5.076 6.242 5.686 6.508 6.303 6.19
   3362   1382396_at   7.982 8.003 7.888 8.126 8.217 7.595 7.597 7.392 7.582 7.45
   3363   1378191_at   7.9 7.964 8.151 8.167 7.39 8.854 9.117 9.308 9.251 8.981
   3364   1381012_at   6.215 6.367 6.284 6.623 6.299 7.043 7.273 7.366 7.475 6.963
   3365   1374420_at   10.457 10.407 10.779 10.683 10.341 11.147 11.187 11.364 11.17 11.168
   3366   1387956_s_at   7.437 7.517 7.773 7.692 7.908 6.092 6.395 6.156 5.648 5.081
   3367   1397372_at   6.866 6.798 6.842 6.805 6.801 5.674 6.161 6.246 6.2 5.929
   3368   1381973_at   7.601 7.693 7.928 7.911 7.319 8.769 8.506 8.613 8.951 9.038
   3369   1367591_at   10.639 10.66 10.744 10.617 10.643 10.932 10.843 10.966 10.862 10.984
   3370   1373312_at   8.462 8.455 8.608 8.908 8.66 9.471 9.158 9.569 9.59 9.645
   3371   1369494_a_at   3.684 4.465 4.391 4.309 4.468 5.198 5.523 5.767 5.635 5.773
   3372   1374703_at   7.735 7.778 7.484 8.024 7.924 8.797 8.531 9.133 9.104 8.72
   3373   1388581_at   9.58 9.648 9.639 9.804 9.923 9.227 9.043 9.126 9.054 8.822
   3374   1380366_at   7.287 7.445 7.359 7.211 6.92 6.161 5.301 6.173 6.047 5.747
   3375   1383401_at   9.799 9.875 9.666 9.756 9.823 9.214 9.342 9.245 9.031 8.89
   3376   1387879_a_at   6.555 7.507 7.471 7.054 7.075 5.935 5.292 5.522 5.6 5.044
   3377   1380019_at   8.295 8.295 8.393 8.016 8.431 7.181 6.348 6.927 6.643 5.915
   3378   1387945_at   6.537 6.196 6.427 6.247 6.489 4.256 2.465 4.034 3.367 2.05
   3379   1373041_at   11.192 11.149 11.186 11.216 11.359 11.596 11.517 11.651 11.728 11.792
   3380   1383146_at   6.237 6.221 6.285 6.394 6.565 7.122 7.463 7.749 7.512 7.055
   3381   1390455_at   8.029 7.844 7.554 7.677 7.748 7.06 7.256 6.997 7.105 6.937
   3382   1379375_at   7.249 7.356 7.113 7.634 7.547 6.153 6.28 6.682 6.424 6.543
   3383   1387646_a_at   7.47 7.655 8.145 7.977 7.713 6.793 7.028 6.754 6.855 6.879
   3384   1396247_at   5.153 5.338 5.483 5.463 5.091 6.225 6.753 6.237 6.057 6.375
   3385   1370851_a_at   4.611 4.321 4.932 4.663 4.278 6.001 5.722 5.424 5.587 5.611
   3386   1379361_at   7.432 7.278 7.309 7.119 7.052 7.803 7.916 8.242 7.884 8.037
   3387   1375628_at   8.257 8.086 8.364 8.058 8.297 8.874 8.657 8.663 8.847 8.736
   3388   1379571_at   6.522 6.459 6.958 6.843 6.776 7.388 7.359 7.511 7.617 7.564
   3389   1379322_at   8.857 8.95 8.848 8.963 9.044 8.636 8.411 8.272 8.299 8.375
   3390   1390638_at   5.244 5.392 5.172 5.766 5.362 6.049 6.584 6.66 6.578 6.674
   3391   1398463_at   8.715 8.597 9.025 8.565 8.051 10.182 10.035 9.626 9.96 9.799
   3392   1389537_at   6.874 6.708 6.68 6.909 6.576 5.844 5.776 5.662 6.254 5.945
   3393   1392929_at   8.062 7.929 8.022 8.154 7.727 8.823 8.821 8.874 8.927 8.453
   3394   1390659_at   9.868 9.914 9.542 9.837 9.855 8.246 8.53 8.372 7.758 7.233
   3395   1379312_at   6.912 6.951 6.68 7.004 7.205 6.224 5.987 5.927 5.484 5.818
   3396   1370967_at   6.394 6.535 6.214 6.343 6.463 5.223 5.433 4.532 5.24 4.593
   3397   1374679_at   2.227 3.69 2.449 1.422 1.609 4.99 5.517 5.676 5.651 5.011
   3398   1395190_at   5.273 5.244 5.845 5.684 5.787 6.637 6.8 6.583 7.054 7.247
   3399   1374144_at   8.195 8.215 8.256 8.43 8.196 8.803 8.866 8.847 8.849 8.556
   3400   1370076_at   9.735 9.781 10.069 9.804 9.418 10.637 11.024 10.684 10.624 10.576
   3401   1369900_at   3.713 2.996 4.071 3.983 3.76 2.128 1.091 1.903 0.864 1.58
   3402   1389569_at   8.75 8.911 8.601 8.81 8.874 8.181 8.369 8.283 8.091 8.018
   3403   1372246_at   10.268 10.264 10.279 10.253 10.564 9.727 9.672 9.948 9.641 9.748
   3404   1385243_at   9.807 9.826 10.085 10.074 9.835 10.65 10.646 10.792 10.781 10.361
   3405   1393079_at   6.884 6.917 6.765 6.978 6.598 7.509 7.483 7.391 7.416 7.252
   3406   1393321_at   5.03 4.877 4.915 5.003 5.081 5.548 5.33 5.417 5.697 5.554
   3407   1378029_at   5.555 5.863 6.199 5.739 5.648 6.703 6.882 7.181 6.984 6.628
   3408   1372682_at   5.749 5.946 5.823 6.006 6.241 6.697 6.537 6.843 6.999 6.879
   3409   1387992_at   7.457 6.545 7.9 7.427 7.666 5.633 5.4 4.955 4.577 4.311
   3410   1367746_a_at   9.644 9.658 9.483 9.835 9.878 9.134 8.861 9.181 9.054 8.974
   3411   1390154_at   8.312 8.258 8.034 8.371 8.291 7.549 7.268 7.362 7.682 7.08
   3412   1372021_at   7.556 7.523 7.353 7.467 7.51 7.122 6.81 6.997 6.987 6.75
   3413   1377994_at   6.771 6.795 6.734 6.814 6.821 5.76 5.944 5.754 4.99 5.474
   3414   1377223_at   5.921 5.616 5.703 5.892 5.776 6.176 6.355 6.441 6.556 6.459
   3415   1371838_at   11.001 10.932 10.856 10.806 10.886 10.571 10.126 10.346 10.232 10.277
   3416   1371963_at   8.494 8.658 8.87 8.83 8.445 9.373 9.187 9.493 9.461 9.425
   3417   1376239_at   6.627 6.311 6.134 5.777 6.404 7.697 7.549 7.145 7.638 7.432
   3418   1367683_at   10.216 10.119 10.102 10.109 10.107 9.785 9.549 9.555 9.312 9.37
   3419   1372681_at   7.199 7.23 6.999 6.983 6.996 7.936 7.775 8.017 7.696 7.553
   3420   1398378_at   8.623 8.539 8.505 8.52 8.883 9.156 9.232 9.451 9.585 9.387
   3421   1393910_at   4.886 5.124 5.058 5.472 5.428 6.053 6.214 6.086 5.975 6.37
   3422   1375461_at   8.065 8.059 8.044 8.17 8.137 8.6 8.704 8.539 9.006 8.662
   3423   1378179_a_at   7.577 7.828 7.631 7.752 7.839 6.953 6.771 6.453 6.507 6.037
   3424   1396512_at   4.369 5.024 4.685 4.426 4.619 5.555 5.482 5.451 5.407 5.646
   3425   1390024_at   11.681 11.707 11.811 11.498 11.652 11.174 11.245 11.143 11.085 10.893
   3426   1397942_at   3.459 3.489 3.568 3.834 3.346 4.235 4.145 4.197 4.403 4.498
   3427   1370283_at   11.87 11.761 11.695 11.62 12.141 11.156 11.054 11.192 10.891 11.03
   3428   1371389_at   10.517 10.369 10.593 10.477 9.775 11.405 11.306 11.463 11.559 11.504
   3429   1382945_at   7.119 6.778 7.016 7.037 7.136 7.677 7.6 7.547 7.806 7.953
   3430   1393230_s_at   7.123 7.128 7.235 7.375 7.33 7.981 7.791 8.285 8.271 7.894
   3431   1388373_at   10.256 10.362 10.308 10.472 10.349 10.639 10.612 10.771 10.775 10.704
   3432   1390144_at   7.854 7.677 7.567 7.281 7.551 6.696 6.608 6.769 6.906 6.388
   3433   1395375_at   8.846 8.977 9.096 8.841 8.891 8.455 8.102 8.333 8.179 7.94
   3434   1389165_at   9.658 9.637 9.731 9.569 9.592 9.345 9.018 9.216 9.023 9.042
   3435   1367664_at   3.341 3.721 3.445 3.623 3.146 4.694 4.932 4.959 4.315 5.313
   3436   1368916_at   9.198 9.167 9.242 9.305 9.058 9.661 9.57 9.535 9.681 9.491
   3437   1373211_at   8.211 8.096 8.545 8.322 8.048 7.433 7.409 7.012 6.8 7.209
   3438   1393105_at   7.469 7.197 7.14 7.205 7.69 6.063 6.12 6.602 5.956 5.777
   3439   1371985_a_at   9.24 9.127 9.002 9.131 9.27 8.561 8.39 8.73 8.498 8.254
   3440   1373633_at   6.531 6.275 6.241 6.432 6.527 5.803 5.302 5.779 5.411 5.435
   3441   1398997_at   9.204 9.147 9.144 9.297 9.417 9.834 9.759 9.959 9.738 9.627
   3442   1379772_at   3.574 2.639 2.036 3.325 4.048 5.551 5.537 5.762 5.858 5.871
   3443   1385314_at   7.5 7.618 7.397 7.558 7.843 6.63 6.642 6.664 6.229 6.931
   3444   1393730_at   6.702 6.76 6.796 6.814 6.989 6.313 5.848 5.931 6.07 5.673
   3445   1368283_at   8.98 8.873 8.983 9.042 8.468 9.671 9.761 10.122 10.191 10.258
   3446   1372925_at   7.237 7.266 7.303 7.345 7.536 7.91 8.237 7.915 8.004 7.813
   3447   1370181_at   6.969 6.982 7.008 7.482 7.325 7.821 7.933 8.054 8.11 8.05
   3448   1377121_at   7.227 7.306 7.281 7.287 7.478 8.081 8.012 8.066 7.847 7.684
   3449   1372889_at   7.351 7.414 7.287 7.517 7.072 7.976 8.162 8.046 7.869 7.884
   3450   1384467_at   8.636 8.693 8.861 8.777 8.868 8.404 8.054 8.274 8.241 8.271
   3451   1373924_at   8.19 8.494 8.378 8.325 8.362 8.783 8.902 8.674 8.917 8.826
   3452   1392791_at   5.852 5.751 5.807 5.823 5.64 3.944 3.525 2.604 1.623 2.795
   3453   1378328_at   3.608 1.618 1.043 1.632 1.651 4.702 5.686 5.684 5.096 5.727
   3454   1383111_at   9.986 10.005 10.14 10.05 9.03 11.252 11.392 11.206 11.452 11.472
   3455   1379206_at   6.034 6.556 6.195 5.953 6.222 7.137 6.907 7.227 6.928 7.208
   3456   1390450_a_at   6.179 6.622 6.504 6.435 7.102 7.985 7.919 8.305 8.407 7.624
   3457   1388940_at   6.495 6.604 6.746 7.112 6.912 5.038 3.975 5.035 3.709 4.985
   3458   1385729_at   7.038 7.165 7.404 7.342 6.694 8.21 8.233 8.685 8.167 8.132
   3459   1373758_at   8.018 8.232 8.118 8.271 8.184 7.744 7.752 7.633 7.795 7.851
   3460   1391086_at   7.124 7.21 7.157 7.328 7.411 6.856 6.611 6.715 6.683 6.494
   3461   1381391_at   4.32 4.133 4.138 4.15 4.737 5.325 5.258 5.081 5.57 5.255
   3462   1388679_at   8.995 9.006 9.034 9.007 8.865 9.352 9.315 9.317 9.279 9.173
   3463   1376727_at   8.572 8.608 8.829 8.793 8.79 9.244 9.223 9.389 9.286 9.076
   3464   1390529_at   8.114 8.222 7.719 8.067 7.835 7.233 6.758 6.721 6.965 7.206
   3465   1375944_at   10.713 10.664 10.869 10.736 10.16 11.511 11.417 11.61 11.531 11.63
   3466   1374462_at   7.4 7.479 7.416 7.597 7.577 8.189 7.922 8.329 8.125 7.955
   3467   1368284_at   8.688 8.648 9.069 8.827 9.242 10.011 9.626 9.978 10.258 10.076
   3468   1374405_at   8.506 8.43 8.588 8.646 8.573 9.053 8.848 8.868 8.925 8.895
   3469   1373503_at   5.713 5.937 5.817 6.122 6.083 6.686 6.482 6.832 6.537 6.757
   3470   1368251_at   7.571 7.339 7.668 7.649 7.609 5.735 5.772 6.397 6.248 6.708
   3471   1389332_at   7.202 7.267 7.34 7.702 7.804 8.37 8.415 8.914 8.629 8.523
   3472   1367992_at   4.725 5.166 4.587 4.501 4.869 5.921 5.985 5.665 5.642 5.624
   3473   1385574_at   7.416 7.631 7.597 7.814 7.33 8.112 8.331 8.493 8.54 8.551
   3474   1393167_at   4.074 4.238 3.87 3.64 3.862 4.998 5.601 4.803 5.464 5.172
   3475   1383716_at   7.052 7.291 7.107 6.897 7.188 6.539 6.691 6.612 6.574 6.598
   3476   1373940_at   9.014 9.111 9.158 9.181 9.194 8.55 8.658 8.688 8.661 8.301
   3477   1373734_at   7.607 7.676 7.389 7.442 7.547 7.925 7.986 8.132 7.949 7.954
   3478   1379339_at   8.038 7.953 8.194 8.007 7.806 8.467 8.434 8.595 8.451 8.517
   3479   1384743_at   8.563 8.559 8.702 8.728 8.806 9.382 9.563 9.933 9.468 9.297
   3480   1373733_at   7.203 7.386 7.269 7.585 7.836 8.674 8.18 8.707 8.847 8.832
   3481   1377522_a_at   5.454 5.433 5.793 5.747 5.583 6.263 6.182 6.546 6.352 6.197
   3482   1369891_at   6.876 7.031 6.643 6.686 6.963 4.896 4.799 3.59 4.602 3.069
   3483   1385856_at   7.424 7.243 7.245 7.566 7.479 6.871 6.294 6.517 6.381 6.632
   3484   1393894_at   9.017 9.025 9.372 9.574 9.541 10.176 10.522 10.356 10.606 10.182
   3485   1372814_at   8.894 8.954 8.87 9.064 9.056 9.572 9.392 9.652 9.467 9.319
   3486   1384273_at   6.876 7.142 6.652 6.485 6.785 7.521 7.825 7.609 7.924 7.766
   3487   1389102_at   7.937 8.243 7.886 7.999 8.068 8.718 8.616 9.016 8.687 8.591
   3488   1392916_at   8.698 8.691 8.961 9.168 8.763 9.541 9.434 9.601 9.672 9.676
   3489   1368163_at   11.126 11.107 10.997 10.86 10.888 11.618 11.763 11.612 11.532 11.383
   3490   1387283_at   9.072 8.7 8.336 9.23 9.43 7.131 4.446 5.315 5.154 5.524
   3491   1371350_at   10.56 10.537 10.569 10.312 10.211 11.151 10.926 11.135 10.968 11.133
   3492   1387498_a_at   6.91 7.041 6.92 7.237 7.102 7.73 7.489 7.774 7.768 7.528
   3493   1372044_at   7.413 7.829 7.77 7.28 7.383 8.777 8.565 8.565 8.519 8.235
   3494   1378819_at   7.093 6.918 7.675 7.492 6.838 8.276 8.381 8.365 8.476 8.555
   3495   1384499_at   7.778 7.833 7.765 7.994 8.272 8.721 8.943 9.239 8.719 8.827
   3496   1370385_at   6.318 6.396 5.904 6.67 6.318 7.358 7.177 7.674 7.758 7.815
   3497   1376098_a_at   7.549 7.489 7.549 7.508 7.414 6.548 6.823 5.985 6.076 5.997
   3498   1375806_at   7.122 7.286 6.825 6.799 6.803 6.222 5.46 5.509 5.413 5.235
   3499   1388376_at   10.005 10.012 10.112 10.165 10.123 9.806 9.647 9.826 9.668 9.639
   3500   1368676_at   6.604 6.5 6.1 6.344 6.395 7.377 6.98 7.462 7.134 7.275
   3501   1389336_at   7.564 7.572 7.794 7.499 7.466 8.075 8.084 8.042 8.141 7.93
   3502   1368961_at   6.16 6.162 6.277 6.55 6.76 7.318 7.285 7.31 7.133 7.225
   3503   1382426_at   6.937 6.979 6.877 6.943 6.798 7.289 7.304 7.135 7.293 7.403
   3504   1388137_a_at   9.181 9.177 9.286 9.318 9.447 8.876 8.775 8.97 8.821 8.776
   3505   1376848_at   7.604 7.353 7.908 7.804 7.596 8.299 8.545 8.469 8.413 8.314
   3506   1379905_at   6.586 6.579 6.441 6.605 6.594 6.085 6.143 5.685 5.96 5.926
   3507   1371592_at   9.012 9.099 9.154 9.359 9.295 8.582 8.117 8.549 8.345 8.52
   3508   1374685_at   5.666 5.709 5.59 5.524 5.988 6.445 6.648 6.457 6.41 6.222
   3509   1389343_at   10.762 10.711 10.813 10.788 10.902 11.126 10.984 11.106 11.114 11.092
   3510   1378331_at   4.336 5.044 3.352 4.978 4.824 2.242 1.536 1.816 0.714 1.868
   3511   1370505_at   7.784 7.858 7.89 7.79 7.955 7.252 6.766 7.308 7.181 6.918
   3512   1369621_s_at   10.017 10.057 10.187 10.176 10.324 9.692 9.671 9.552 9.468 9.331
   3513   1372607_at   10.02 10.045 9.979 10.141 10.183 9.719 9.676 9.814 9.611 9.593
   3514   1385103_at   8.335 8.254 8.296 8.54 8.715 9.177 9.006 9.416 9.393 9.441
   3515   1367456_at   12.2 12.151 12.114 11.866 11.931 11.506 11.506 11.42 11.338 11.136
   3516   1388713_at   8.109 8.181 8.158 8.31 8.35 8.719 8.628 8.884 8.763 8.587
   3517   1384898_at   6.259 6.259 5.678 6.219 6.373 4.878 3.566 4.151 3.783 4.633
   3518   1371619_at   9.629 9.686 9.747 9.803 9.873 10.218 10.07 10.259 10.113 10.088
   3519   1388230_at   5.881 5.87 6.218 6.119 5.798 7.018 6.844 6.912 6.507 6.783
   3520   1376728_at   9.63 9.799 9.8 9.902 9.728 10.341 10.193 10.593 10.371 10.253
   3521   1372780_at   7.039 7.048 7.179 7.065 6.983 7.544 7.333 7.438 7.532 7.644
   3522   1373224_at   8.6 8.443 8.348 8.487 8.726 5.729 4.701 4.883 4.1 2.205
   3523   1371956_at   8.841 8.931 8.843 9.054 9.156 9.51 9.449 9.694 9.841 9.602
   3524   1370943_at   11.814 11.769 11.919 11.576 11.33 12.519 12.701 12.424 12.438 12.436
   3525   1378049_at   8.16 8.336 8.574 8.578 8.407 9.2 8.945 9.376 9.294 9.092
   3526   1374029_at   6.093 6.146 5.918 5.943 6.296 7.305 6.888 7.354 7.271 6.717
   3527   1369029_at   9.256 9.242 8.932 9.096 9.329 8.435 8.425 8.474 8.164 7.938
   3528   1394290_at   5.731 5.569 5.617 5.554 5.32 4.719 4.53 4.244 3.776 3.981
   3529   1368089_at   6.87 7.167 7.344 7.483 7.258 8.196 7.881 8.257 8.437 8.168
   3530   1383371_at   6.592 6.253 6.62 6.24 6.079 7.03 7.239 7.212 7.108 7.187
   3531   1388568_at   9.932 9.928 9.918 10.182 10.11 9.451 9.481 9.671 9.377 9.386
   3532   1384944_at   5.733 5.657 6.001 6.075 6.34 3.349 4.609 4.478 3.978 4.322
   3533   1371572_at   11.105 11.051 10.944 11.087 11.211 11.442 11.409 11.359 11.413 11.378
   3534   1398974_at   9.351 9.264 9.206 9.12 9.142 8.896 8.618 8.715 8.529 8.71
   3535   1371886_at   9.027 9.072 9.235 8.971 8.577 9.835 9.731 9.748 10.009 9.79
   3536   1396942_at   4.976 5.018 5.18 5.304 5.048 6.026 5.666 6.029 6.371 6.296
   3537   1377789_at   5.687 5.963 5.991 6.247 5.647 6.655 6.867 7.227 7.074 6.889
   3538   1380116_at   8.648 8.698 8.747 8.642 8.477 8.287 8.159 8.214 8.03 7.992
   3539   1384638_at   8.816 8.882 9.039 9.063 8.385 9.652 9.756 10.073 9.982 10.138
   3540   1388466_at   8.845 8.718 8.752 8.898 8.862 8.42 8.396 8.552 8.541 8.516
   3541   1398863_at   10.612 10.599 10.673 10.671 10.794 10.385 10.171 10.338 10.207 10.128
   3542   1386615_at   6.614 6.924 7.069 7.203 6.742 7.927 7.638 7.79 7.748 7.644
   3543   1388859_at   7.065 7.005 6.808 7.128 7.435 7.79 8.299 7.959 7.964 8.114
   3544   1369830_at   7.237 7.033 7.978 7.701 7.255 5.994 4.864 4.771 5.214 5.796
   3545   1377616_at   7.553 7.458 7.59 7.572 7.754 8.207 8.188 8.631 8.464 8.149
   3546   1372959_at   6.123 5.92 6.265 6.12 5.481 6.914 7.02 7.186 7.314 7.49
   3547   1371253_at   10.39 10.36 10.521 10.464 10.4 10.738 10.79 10.776 10.733 10.611
   3548   1378094_at   6.989 7.409 6.812 7.436 7.559 8.309 8.355 8.624 8.366 8.195
   3549   1389113_at   5.993 6.255 6.456 6.388 5.902 7.167 7.08 7.249 7.393 7.754
   3550   1383624_at   6.804 6.915 7.101 7.234 7.046 7.757 7.524 7.631 7.833 7.61
   3551   1373440_at   8.438 8.447 8.449 8.377 8.4 8.693 8.892 8.879 8.975 8.702
   3552   1387375_at   8.969 8.805 9.439 9.502 8.642 10.343 10.214 10.449 10.577 10.82
   3553   1382128_at   7.414 7.337 7.361 7.486 7.315 6.831 6.798 6.988 6.959 6.582
   3554   1398344_at   7.938 7.897 7.938 7.798 7.957 7.621 7.443 7.516 7.631 7.398
   3555   1392308_at   8.264 8.312 8.686 8.542 7.703 2.744 4.313 4.297 2.254 0.138
   3556   1383251_at   7.874 7.807 7.552 7.741 7.824 7.334 7.202 7.245 7.115 6.971
   3557   1368409_at   7.101 7.096 7.307 7.378 7.154 7.749 7.548 7.804 7.819 7.8
   3558   1388199_at   11.808 11.798 11.744 11.802 11.918 12.041 12.04 12.096 12.018 12.139
   3559   1383862_at   7.854 7.831 7.98 8.028 7.802 7.381 6.997 7.335 6.997 6.866
   3560   1389373_at   9.033 9.139 8.889 9.127 9.329 8.515 8.499 8.558 8.528 8.262
   3561   1383850_at   7.07 7.329 7.155 7.045 7.193 7.688 7.927 8.162 8.057 7.705
   3562   1376672_at   5.577 5.876 5.865 5.934 6.033 6.955 6.413 6.98 6.718 7.012
   3563   1384008_at   7.402 7.426 7.739 7.769 7.246 8.28 8.322 8.688 8.781 8.49
   3564   1369421_at   7.486 7.433 7.293 7.532 7.505 6.918 6.707 7.068 6.81 6.625
   3565   1390776_at   7.902 8.049 8.02 8.276 8.31 8.861 8.767 9.26 8.951 8.828
   3566   1389504_at   7.411 7.412 7.343 7.391 7.162 8.366 7.858 8.534 8.475 8.142
   3567   1371959_at   7.465 7.445 7.443 7.517 7.587 8.121 8.074 8.62 8.483 8.677
   3568   1377183_at   5.975 5.919 5.933 5.867 5.66 6.336 6.674 6.916 6.929 6.76
   3569   1389171_at   5.65 5.708 6.208 5.654 6.132 6.992 6.657 6.94 7.212 7.23
   3570   1384900_at   6.393 6.614 6.883 6.695 6.512 5.854 5.458 5.01 5.368 5.647
   3571   1389333_at   8.66 8.809 8.868 8.948 8.938 9.187 9.279 9.361 9.24 9.254
   3572   1371314_at   10.37 10.369 10.102 10.031 10.382 11.148 11.389 11.151 11.013 10.811
   3573   1397185_at   8.819 8.587 8.887 8.715 8.128 9.534 9.726 9.703 9.634 9.506
   3574   1380559_at   5.378 5.56 5.116 5.711 5.356 3.91 2.428 3.561 2.373 3.396
   3575   1388441_at   9.794 9.62 9.94 10.165 10.001 10.678 10.582 11.068 10.86 11.054
   3576   1370282_at   9.308 9.327 9.533 9.47 9.238 9.717 9.89 10.018 9.961 9.898
   3577   1374126_at   7.901 7.953 7.891 8.013 7.886 8.419 8.499 8.89 8.673 8.424
   3578   1376839_at   8.216 7.998 8.159 8.333 8.34 7.276 7.111 7.567 7.652 7.281
   3579   1384368_at   7.358 7.415 7.541 7.661 7.482 7.946 7.986 8.173 7.946 7.896
   3580   1379541_at   6.679 6.942 6.906 7.091 7.253 7.783 7.754 7.895 7.634 7.615
   3581   1391338_at   5.135 5.196 5.025 5.252 4.976 5.815 5.715 6.374 6.008 6.118
   3582   1369409_at   8.335 8.454 8.369 8.382 7.907 7.521 7.327 7.602 7.135 7.422
   3583   1374186_at   7.189 7.265 7.156 7.184 7.482 6.715 6.761 6.882 6.794 6.82
   3584   1382962_at   3.677 3.372 3.851 3.957 4.093 5.034 5.42 5.133 5.061 4.609
   3585   1389177_at   8.452 8.434 8.622 8.411 8.669 9.09 9.247 9.143 9.027 8.887
   3586   1387791_at   5.892 5.854 5.802 6.013 5.686 6.244 6.274 6.507 6.463 6.358
   3587   1383472_at   5.237 5.183 4.515 4.795 4.636 5.795 6.011 6.377 6.252 6.087
   3588   1396302_at   5.181 5.356 5.421 5.577 4.991 6.222 6.082 5.962 6.141 6.363
   3589   1383632_at   9.613 9.521 9.434 9.597 9.695 9.081 8.962 9.237 8.976 8.852
   3590   1369560_at   6.852 7.174 7.624 7.098 6.76 8.563 8.25 8.079 8.353 8.24
   3591   1392460_at   8.14 7.807 8.102 8.093 8.167 8.574 8.491 8.734 8.711 8.579
   3592   1386909_a_at   10.526 10.509 10.851 10.785 10.609 11.257 11.242 11.064 11.249 11.192
   3593   1389757_at   6.94 7 6.895 7.149 6.613 7.666 7.533 7.897 7.901 7.624
   3594   1367914_at   8.993 9.021 9.21 9.231 9.414 8.647 8.584 8.43 8.33 8.316
   3595   1397916_s_at   7.711 7.773 8.201 7.774 7.803 7.259 6.768 7.03 6.733 6.92
   3596   1395173_at   8.697 8.672 8.783 8.72 8.716 8.244 8.376 8.004 8.3 8.044
   3597   1383869_at   3.996 4.088 4.365 3.803 3.898 5.014 5.104 4.656 5.292 5.215
   3598   1368109_at   8.148 7.934 8.055 8.092 7.986 8.977 9.111 9.187 8.725 8.558
   3599   1388918_at   8.449 8.392 8.572 8.802 8.539 9.072 9.023 9.296 9.184 9.268
   3600   1379528_at   8.669 8.765 8.565 8.842 8.64 9.075 9.161 9.175 9.233 9.012
   3601   1395165_at   6.148 6.492 6.325 6.584 6.369 5.463 5.797 5.718 5.75 5.797
   3602   1373997_at   7.792 7.886 7.884 7.797 7.73 7.21 7.035 6.964 6.746 6.452
   3603   1379542_at   9.821 9.834 10.088 10.406 10.298 9.199 9.283 9.264 9.062 8.956
   3604   1370838_s_at   9.991 10.173 10.388 10.458 10.237 10.786 10.828 10.82 10.888 10.886
   3605   1367585_a_at   12.322 12.296 12.29 12.116 12.234 12.63 12.559 12.499 12.515 12.59
   3606   1391227_at   6.965 7.152 6.701 6.89 6.975 4.404 3.256 2.053 3.106 4.753
   3607   1377812_a_at   7.93 7.828 7.92 7.769 7.434 8.457 8.421 8.367 8.505 8.405
   3608   1386860_at   7.982 8.226 8.149 8.206 8.423 9.071 8.643 9.06 8.914 8.97
   3609   1393559_at   6.143 6.368 6.146 6.703 6.563 7.217 7.49 7.813 7.27 7.485
   3610   1384096_at   4.584 3.829 4.123 4.222 4.04 5.457 4.967 5.548 5.506 5.167
   3611   1389906_at   7.028 6.94 7.365 7.267 7.228 8.053 7.783 7.806 7.741 8.036
   3612   1378032_at   8.227 8.158 7.601 7.821 8.532 6.505 6.306 6.485 5.566 5.379
   3613   1371473_at   9.373 9.439 9.399 9.606 9.249 9.863 9.82 9.824 10.008 9.96
   3614   1372310_at   8.781 8.934 9.056 9.138 8.815 9.703 9.364 9.755 9.799 9.757
   3615   1397406_at   5.762 6.113 6.013 5.574 5.649 6.774 6.827 6.725 6.767 6.425
   3616   1384505_at   5.154 5.868 5.826 5.793 5.588 6.534 6.515 6.742 6.775 6.868
   3617   1367652_at   8.127 8.052 8.359 8.231 8.136 9.334 8.885 9.314 9.894 9.602
   3618   1368040_at   7.414 7.613 7.727 7.786 7.766 8.4 8.18 8.688 8.682 8.452
   3619   1387623_at   4.31 4.08 3.403 3.374 3.91 4.943 5.743 5.576 5.336 5.486
   3620   1374187_at   4.395 4.205 3.886 4.111 4.153 5.351 5.977 6.168 5.681 5.078
   3621   1368204_at   7.804 7.813 7.819 8.014 7.88 7.455 7.147 7.396 7.129 7.41
   3622   1395986_at   5.44 5.386 5.402 5.662 6.024 6.731 6.429 7.048 6.774 6.543
   3623   1372119_at   8.494 8.44 8.37 8.564 8.734 7.982 8.079 8.11 8.014 7.903
   3624   1385800_at   7.277 7.239 7.816 7.785 7.512 6.57 6.465 6.016 6.137 6.579
   3625   1371321_at   11.628 11.598 11.687 11.668 11.789 11.979 11.868 12.022 11.992 12.027
   3626   1389337_at   8.589 8.512 8.368 8.423 8.727 8.081 7.944 7.804 7.926 7.698
   3627   1395427_at   7.435 7.185 7.307 7.235 7.466 8.211 8.043 8.173 7.886 7.776
   3628   1391544_at   4.198 3.899 3.863 4.945 4.346 5.69 5.781 5.882 6.125 6.583
   3629   1384294_at   6.152 6.422 6.357 6.662 6.477 5.288 4.073 4.355 4.31 5.131
   3630   1385465_at   0 1.96 1.864 2.585 1.975 4.836 5.382 4.886 5.059 4.422
   3631   1388298_at   8.344 8.295 8.643 8.65 8.96 9.626 9.523 9.721 9.365 9.423
   3632   1392928_at   8.7 8.455 8.567 8.343 8.117 9.266 9.69 9.546 9.307 9.174
   3633   1377893_at   6.995 7.137 6.84 7.053 6.942 6.408 6.412 6.497 6.527 6.133
   3634   1373904_at   7.117 7.308 7.257 7.343 7.603 6.799 6.3 6.363 6.186 6.428
   3635   1399085_at   8.935 9.056 9.173 9.179 9.083 9.376 9.532 9.611 9.68 9.608
   3636   1372117_at   7.688 7.569 7.714 7.771 7.745 8.226 8.29 8.768 8.398 8.338
   3637   1372809_at   5.652 5.598 4.896 5.953 5.904 3.692 4.39 4.082 3.674 4.076
   3638   1377084_at   5.99 6.231 6.326 6.06 6.404 6.983 6.696 6.899 6.878 7.088
   3639   1384101_at   7.583 7.661 7.725 7.751 7.603 8.078 8.036 8.109 8.022 7.877
   3640   1398811_at   9.192 9.302 9.224 9.423 9.483 8.877 8.407 8.71 8.646 8.704
   3641   1380813_at   7.69 7.839 7.848 7.672 7.759 8.192 8.165 8.335 8.093 8.074
   3642   1388571_at   9.894 9.846 9.876 9.936 9.882 9.592 9.652 9.656 9.721 9.517
   3643   1391936_a_at   9.224 9.265 9.139 8.876 9.309 9.682 9.9 9.844 9.758 10.035
   3644   1376770_at   9.001 8.994 8.876 8.88 8.728 9.579 9.406 9.741 10.04 9.794
   3645   1372414_at   7.479 7.263 7.606 7.571 7.494 7.933 7.931 8.152 8.046 7.932
   3646   1377851_at   5.978 5.591 5.946 5.382 5.623 4.633 4.613 4.907 4.176 4.471
   3647   1387966_at   8.37 8.38 8.418 8.923 8.52 9.354 9.162 9.624 9.484 9.494
   3648   1369743_a_at   8.314 8.44 8.57 8.784 8.803 7.972 7.658 7.834 7.607 7.543
   3649   1372521_at   5.417 5.943 5.558 5.621 5.426 6.428 6.362 6.427 6.759 6.304
   3650   1392989_at   7.573 7.583 7.69 7.644 7.58 8.16 8.358 8.444 8.284 7.945
   3651   1379960_at   5.19 4.913 5.727 5.274 5.349 6.223 6.297 6.677 6.709 6.874
   3652   1383471_at   5.624 5.609 5.825 5.727 5.747 6.412 6.123 6.097 6.125 6.242
   3653   1376496_at   7.371 7.275 6.861 7.407 7.579 4.53 4.46 1.993 4.089 2.459
   3654   1370592_at   6.465 6.095 6.685 6.77 6.308 7.552 7.618 8.33 7.714 7.639
   3655   1376082_at   8.607 8.868 8.58 8.751 8.904 9.473 9.311 9.905 9.781 9.574
   3656   1389020_at   8.117 8.437 8.106 8.746 8.91 9.987 9.491 9.87 10.159 9.709
   3657   1373149_at   7.586 7.704 7.712 7.975 7.904 8.384 8.284 8.465 8.7 8.481
   3658   1384981_at   7.315 6.967 7.038 7.344 6.547 5.169 3.501 4.813 4.802 3.463
   3659   1370098_at   9.207 9.137 9.399 9.412 9.277 9.67 9.713 9.674 9.625 9.757
   3660   1370218_at   11.894 11.922 11.971 11.899 11.973 12.375 12.249 12.178 12.335 12.495
   3661   1391255_at   6.68 6.703 6.291 7.021 6.8 5.824 5.555 5.902 5.844 5.604
   3662   1372260_at   8.888 9.055 8.867 9.05 9.105 9.642 9.416 9.914 9.921 9.734
   3663   1375277_at   7.117 7.001 7.216 7.219 7.099 6.556 6.038 6.211 5.995 6.524
   3664   1395894_at   8.351 8.559 8.571 8.462 8.425 8.17 8.104 8.058 7.93 7.938
   3665   1373015_at   8.383 8.478 8.691 8.641 8.439 9.241 8.898 9.253 9.227 9.116
   3666   1392992_at   6.652 6.45 6.537 6.579 6.441 7.199 6.943 7.395 7.236 7.014
   3667   1389180_at   7.615 7.42 7.232 7.11 7.14 7.892 8.196 8.064 8.048 8.01
   3668   1370007_at   9.595 9.76 9.524 9.894 10.002 9.108 9.197 8.959 8.888 8.934
   3669   1377720_x_at   10.554 10.681 11 10.951 10.644 11.365 11.494 11.369 11.511 11.649
   3670   1371331_at   9.077 9.027 9.054 9.232 9.542 10.136 9.787 10.025 10.106 9.898
   3671   1378925_at   8.983 8.961 8.789 9.352 8.868 8.177 7.765 8.281 7.894 8.132
   3672   1392595_at   5.402 5.297 5.576 5.662 5.747 6.5 6.381 6.785 6.535 6.136
   3673   1376670_at   5.961 6.095 6.263 5.971 6.073 6.727 6.805 7.045 6.614 6.588
   3674   1376005_at   7.177 7.078 7.255 7.359 7.312 6.795 6.613 6.782 6.429 6.506
   3675   1382403_at   4.817 4.873 4.474 4.424 5.198 5.825 5.867 5.985 5.661 5.73
   3676   1374251_at   9.994 10.17 10.338 10.428 9.58 11.108 11.194 11.326 11.169 11.1
   3677   1376692_at   6.866 6.881 7.01 7.29 7.029 7.964 7.623 8.397 8.182 8.24
   3678   1390321_at   6.571 6.564 6.297 6.497 6.358 6.027 5.631 5.952 5.73 5.789
   3679   1368680_a_at   12.141 12.075 12.128 11.983 11.697 12.622 12.687 12.505 12.593 12.695
   3680   1388427_at   7.813 7.895 7.737 7.757 8.407 9.335 8.831 9.142 9.221 8.788
   3681   1387655_at   9.092 9.077 8.951 8.797 9.096 9.531 9.37 9.531 9.692 9.607
   3682   1381396_s_at   7.13 7 7.564 7.289 6.625 8.099 8.269 8.371 8.215 8.46
   3683   1368695_at   6.609 6.569 5.552 5.999 5.767 2.305 3.631 3.266 2.376 4.223
   3684   1368604_at   7.145 7.561 7.301 6.673 6.766 5.666 5.778 4.945 5.762 5.429
   3685   1370341_at   6.286 6.232 6.476 6.28 6.595 5.381 5.861 5.455 5.514 5.198
   3686   1374306_at   7.325 7.375 6.929 7.599 7.757 6.474 6.466 6.332 6.178 6.32
   3687   1395027_at   6.061 6.083 6.505 6.519 5.926 7.28 6.988 7.172 7.15 7.423
   3688   1394022_at   8.561 8.042 8.476 8.525 8.077 9.39 9.545 10.103 9.316 9.917
   3689   1372079_at   10.837 10.851 10.938 10.94 10.871 10.498 10.468 10.504 10.292 10.151
   3690   1379693_at   5.208 5.225 4.588 5.173 5.656 6.381 6.425 6.612 6.427 6.237
   3691   1374063_at   10.818 10.859 10.68 10.745 10.918 10.305 10.332 10.053 9.849 10.043
   3692   1374425_at   4.539 4.117 4.1 4.223 4.277 4.994 5.307 5.554 5.709 5.092
   3693   1367550_a_at   8.67 8.55 8.603 8.638 8.687 9.065 8.897 9.09 9.053 8.865
   3694   1374570_at   7.114 7.034 7.272 7.329 7.459 7.767 7.969 8.05 7.924 7.764
   3695   1397255_at   7.128 6.967 6.821 6.89 6.929 5.992 6.391 6.455 6.096 6.292
   3696   1369527_at   4.928 5.472 5.362 5.076 4.777 6.447 6.147 6.155 6.059 6.01
   3697   1390152_at   7.674 7.393 7.627 7.374 7.511 7.082 6.994 6.976 6.74 6.847
   3698   1374883_at   5.873 5.935 5.921 5.957 6.261 6.641 6.53 6.816 6.489 6.794
   3699   1380254_at   4.407 4.639 4.603 4.626 4.032 5.65 5.32 5.981 5.45 5.492
   3700   1374547_at   8.07 8.022 7.855 8.162 8.143 8.462 8.505 8.698 8.485 8.501
   3701   1369584_at   7.641 7.903 8.013 7.947 7.722 6.772 6.326 5.574 5.328 5.622
   3702   1372071_at   8.419 8.363 8.392 8.095 8.443 9.078 8.898 9.448 9.315 9.036
   3703   1386718_at   7.438 7.166 7.016 7.127 7.118 8.097 8.002 8.57 8.275 7.847
   3704   1374844_at   9.315 9.146 8.789 8.966 9.277 8.515 8.369 8.237 8.241 8.255
   3705   1379365_at   13.175 13.021 12.632 12.626 12.892 11.041 9.439 9.251 8.899 7.932
   3706   1372308_at   7.246 7.159 7.425 7.396 7.318 6.9 6.57 6.823 6.866 6.761
   3707   1376955_at   4.979 4.791 5.584 5.009 4.849 6.225 5.978 6.62 6.672 6.31
   3708   1373996_at   7.305 7.177 7.394 7.309 7.127 8.087 7.734 8.104 8.121 7.751
   3709   1374625_at   7.821 8.091 8.43 8.417 8.311 9.47 9.014 9.617 9.85 9.897
   3710   1376997_at   8.071 8.048 7.986 8.195 8.164 8.545 8.543 8.983 8.746 8.781
   3711   1371657_at   9.648 9.654 9.701 9.81 9.881 9.228 9.211 9.4 9.136 9.009
   3712   1379516_at   7.339 7.187 7.455 7.624 7.43 8.084 7.855 8.041 8.141 7.945
   3713   1373315_at   7.827 7.819 7.964 7.917 7.798 8.227 8.305 8.357 8.094 8.34
   3714   1371588_at   8.304 8.363 8.438 8.556 8.649 9.078 9.099 9.187 8.968 8.867
   3715   1387621_at   6.453 6.376 6.226 6.552 6.553 5.273 5.441 4.934 4.252 4.916
   3716   1381836_at   7.859 7.855 7.92 8.077 8.083 7.448 7.518 7.49 7.164 7.37
   3717   1398626_s_at   11.917 11.764 11.821 11.797 11.871 11.537 11.136 11.372 11.251 11.376
   3718   1368075_at   9.078 8.974 8.915 9.022 8.991 9.394 9.443 9.515 9.302 9.242
   3719   1372173_at   7.95 7.857 8.035 8.186 8.162 7.17 7.366 7.375 6.76 7.095
   3720   1388507_at   10.699 10.813 10.774 10.951 10.809 10.525 10.506 10.514 10.389 10.454
   3721   1375945_at   7.029 7.121 7.25 7.241 7.416 7.799 7.701 7.992 7.9 7.688
   3722   1384310_at   6.272 6.23 6.022 6.278 6.51 7.075 7.11 7.506 6.908 7.062
   3723   1374419_at   6.363 6.324 6.209 6.209 6.158 7.034 6.672 6.906 7.269 7.129
   3724   1377437_at   5.054 4.841 5.661 4.861 5.377 6.524 6.116 6.553 6.48 6.83
   3725   1376191_at   6.471 6.946 7.074 7.154 7.102 8.784 8.623 9 8.723 7.814
   3726   1389678_at   6.882 6.867 7.043 7.226 6.528 8.069 7.606 8.26 8.295 8.238
   3727   1374475_at   7.311 6.947 7.275 7.385 7.536 8.133 7.893 8.173 7.993 8.016
   3728   1368231_at   7.479 7.265 7.555 7.507 7.365 6.4 6.549 6.731 6.857 6.906
   3729   1372150_at   9.304 9.353 9.291 9.295 9.419 9.518 9.535 9.618 9.577 9.508
   3730   1383147_at   7.543 7.633 7.918 7.927 7.779 8.662 8.37 8.547 8.454 8.273
   3731   1395557_at   8.085 8.091 7.695 7.906 7.93 6.849 7.115 6.521 6.387 6.056
   3732   1376766_at   8.433 8.426 8.618 8.594 8.271 7.648 7.318 7.481 7.801 7.895
   3733   1372149_at   8.407 8.846 8.438 8.741 8.729 9.376 9.439 9.767 9.606 9.263
   3734   1389759_at   5.492 5.393 5.936 5.788 5.615 7.032 6.456 6.54 6.644 6.486
   3735   1370363_at   6.218 6.325 6.572 6.367 5.371 7.808 7.666 7.5 7.74 7.544
   3736   1371356_at   6.485 6.74 6.73 6.661 6.258 7.601 7.169 7.552 7.851 7.602
   3737   1376255_at   7.198 7.177 7.345 7.429 7.179 6.197 4.235 4.846 4.825 5.051
   3738   1391414_at   7.174 7.171 7.213 7.343 7.163 6.917 6.794 6.64 6.558 6.614
   3739   1396337_at   4.034 4.24 3.849 3.711 3.555 5.646 5.494 5.117 4.72 5.295
   3740   1372482_at   8.126 8.164 8.069 7.973 8.168 8.633 8.958 8.918 8.568 8.585
   3741   1372333_at   10.914 11.031 11.062 11.042 11.105 10.569 10.748 10.665 10.59 10.421
   3742   1371503_at   9.553 9.507 9.631 9.606 9.569 9.109 9.299 9.145 8.895 9.082
   3743   1371108_a_at   11.99 11.938 11.998 11.766 11.911 12.455 12.492 12.219 12.296 12.38
   3744   1387429_at   7.492 7.629 7.932 8.16 7.924 7.022 6.839 6.419 6.803 6.542
   3745   1392858_at   8.762 8.744 8.743 8.837 8.934 8.464 8.492 8.477 8.515 8.312
   3746   1377625_at   6.453 6.306 6.193 6.459 6.46 5.723 5.563 5.775 5.986 5.829
   3747   1373539_at   7.945 7.597 7.578 7.786 7.776 8.365 8.213 8.582 8.362 8.294
   3748   1378547_at   5.171 5.187 5.099 5.594 5.596 6.366 6.074 6.349 6.352 6.035
   3749   1385679_at   8.913 8.923 9.072 9.07 9.254 9.77 9.829 9.957 9.699 9.469
   3750   1379943_at   4.061 3.885 3.559 4.089 4.184 4.833 5.045 4.654 4.754 4.887
   3751   1369198_at   6.142 6.196 6.465 6.399 6.042 5.077 4.977 5.464 4.872 5.538
   3752   1378386_at   6.19 6.179 6.747 6.493 6.779 3.198 4.424 4.867 4.715 3.966
   3753   1386166_at   7.496 7.474 7.571 7.817 7.513 7.955 8.315 8.247 8.364 8.349
   3754   1385512_at   7.673 7.411 7.586 7.732 7.573 8.599 8.411 8.021 8.444 8.617
   3755   1391811_at   7.121 7.269 7.295 7.323 7.252 6.98 7.002 6.957 6.939 6.837
   3756   1379323_at   7.235 7.399 7.431 7.53 7.628 8.133 7.861 8.259 8.131 8.034
   3757   1387624_at   8.428 8.325 8.158 8.518 8.523 7.865 7.774 7.704 7.438 7.475
   3758   1391849_at   6.606 6.957 6.665 6.668 6.514 6.134 6.049 5.928 6.212 5.947
   3759   1383553_at   6.617 6.47 6.715 6.628 6.676 7.286 7.014 7.433 7.592 7.486
   3760   1379909_at   7.076 6.775 6.562 6.56 6.832 7.513 7.525 7.813 7.485 7.444
   3761   1399084_at   8.198 8.262 8.322 8.309 8.231 7.674 7.739 7.897 7.945 7.572
   3762   1389408_at   10.422 10.531 10.462 10.404 10.238 9.727 9.53 9.373 8.921 9.005
   3763   1372519_at   8.447 8.504 8.365 8.589 8.743 7.904 7.401 7.887 7.562 7.827
   3764   1367829_at   9.983 9.913 9.953 9.794 9.677 10.403 10.231 10.267 10.431 10.443
   3765   1376867_at   6.669 6.988 7.09 6.755 6.378 7.748 7.853 7.709 7.589 7.589
   3766   1383369_at   8.243 8.225 8.172 8.235 8.3 7.131 7.205 6.735 6.358 7.387
   3767   1389467_at   5.017 5.122 4.649 4.753 4.717 5.684 6.007 5.564 5.953 5.566
   3768   1372504_at   8.744 8.802 8.685 8.918 8.926 9.116 9.25 9.401 9.265 9.316
   3769   1382521_at   9.889 9.929 10.056 9.899 10.015 9.541 9.41 9.418 9.118 9.107
   3770   1389080_at   9.061 9.178 8.964 8.999 9.072 8.734 8.735 8.656 8.636 8.478
   3771   1376567_at   6.646 6.864 6.925 6.977 6.928 6.473 6.295 6.272 6.07 6.151
   3772   1376990_at   7.288 7.298 7.29 7.633 7.71 6.842 6.44 6.596 6.672 6.367
   3773   1376222_at   5.442 4.807 5.922 5.811 5.38 7.01 6.688 7.293 6.868 7.258
   3774   1384145_at   9.48 9.399 9.514 9.263 9.392 9.985 9.963 9.944 10.354 9.925
   3775   1371551_at   7.941 7.951 7.961 8.212 8.093 8.518 8.378 8.434 8.416 8.565
   3776   1378078_at   5.011 4.617 4.779 5.09 4.999 5.448 5.527 5.553 5.708 5.687
   3777   1393443_a_at   2.832 1.986 1.718 3.094 1.104 5.41 4.856 5.689 5.064 4.373
   3778   1373427_at   6.807 6.793 6.976 7.251 6.956 7.734 7.415 7.689 7.691 7.713
   3779   1372672_at   9.095 9.018 9.33 9.294 8.896 9.735 9.733 10.025 9.904 10.137
   3780   1372550_at   9 8.95 9.064 9.277 9.227 8.471 8.117 8.53 8.196 7.966
   3781   1370828_at   6.383 6.32 6.308 6.415 6.8 7.408 7.378 7.485 7.409 6.966
   3782   1367929_at   10.426 10.432 10.635 10.458 10.545 10.82 10.896 11.128 11.123 11.06
   3783   1373922_at   8.121 8.067 8.169 8.267 8.194 9.035 8.64 9.274 8.89 8.809
   3784   1386275_at   5.458 5.981 5.909 5.996 6.104 3.911 0.433 1.014 1.989 1.384
   3785   1369222_at   5.278 5.198 5.455 5.26 5.235 6.145 5.898 5.843 6.161 6.468
   3786   1374920_at   7.744 7.794 7.695 7.687 7.483 8.058 8.138 8.096 8.021 8.21
   3787   1379043_at   6.047 6.071 6.339 6.598 6.282 7.096 6.853 7.145 7.149 7.004
   3788   1384238_at   7.901 7.821 7.744 7.961 7.794 8.379 8.372 8.654 8.508 8.201
   3789   1378033_at   6.739 6.772 6.657 7.004 7.047 4.861 5.955 4.838 5.558 5.317
   3790   1375349_at   8.083 8.102 8.206 8.483 8.1 8.742 8.95 9.229 9.074 8.848
   3791   1398794_at   10.564 10.593 10.478 10.622 10.625 10.257 10.267 10.404 10.344 10.264
   3792   1373825_at   8.676 8.549 8.497 8.53 8.309 8.975 8.948 8.917 9.071 8.902
   3793   1389125_at   8.598 8.624 8.532 8.542 8.55 8.206 8.146 8.367 8.052 8.08
   3794   1369184_at   7.304 6.776 7.103 6.91 6.869 8.161 7.618 8.378 8.368 8.127
   3795   1369852_at   6.62 6.339 6.203 6.397 6.204 3.986 4.581 2.278 2.558 2.563
   3796   1369651_at   6.242 6.105 6.273 5.899 7.023 8.044 7.685 7.648 7.609 7.613
   3797   1388684_at   7.768 7.778 7.775 7.722 7.483 7.38 7.166 7.172 7.076 7.159
   3798   1395316_at   6.674 6.871 6.835 7.012 7.237 8.006 7.645 8.314 8.02 7.753
   3799   1374584_at   8.794 8.826 9.023 9.117 8.879 9.29 9.44 9.481 9.525 9.398
   3800   1371024_at   7.312 7.331 7.454 7.757 7.557 8.247 8.16 8.63 8.327 8.143
   3801   1393955_at   7.07 7.205 6.926 6.858 6.928 6.428 6.488 6.013 6.199 6.12
   3802   1374313_at   6.929 7.075 6.88 7.021 6.666 6.248 6.234 5.945 5.646 6.038
   3803   1370159_at   7.719 7.883 7.866 7.782 7.881 7.49 7.172 7.306 7.256 7.012
   3804   1385733_at   5.776 6.203 5.914 5.906 5.856 4.54 5.046 5.075 4.209 4.421
   3805   1370253_at   12.142 12.082 12.087 12.065 12.113 11.939 11.953 11.945 11.961 11.847
   3806   1374629_at   6.987 7.111 6.86 7.017 7.349 6.401 6.373 5.954 6.372 6.111
   3807   1371020_at   5.362 5.275 5.192 5.304 5.495 6.148 5.956 6.085 6.662 6.413
   3808   1377197_at   5.6 5.432 5.644 5.73 5.65 6.343 6.366 6.538 6.059 6.131
   3809   1370573_at   6.395 6.167 6.632 6.321 6.214 7.833 7.062 7.223 7.393 7.413
   3810   1388634_at   8.572 8.74 8.667 8.748 8.612 9.056 8.99 9.069 9.307 9.09
   3811   1376086_at   8.907 8.885 8.831 8.916 9.016 9.283 9.275 9.288 9.294 9.091
   3812   1384330_at   7.138 7.354 7.112 7.255 7.211 6.309 6.591 6.672 6.659 6.193
   3813   1368209_at   10.485 10.208 10.564 10.526 10.426 11.117 11.471 11.259 11.003 11.016
   3814   1394056_at   6.185 6.05 5.901 5.903 5.871 6.57 6.866 6.634 6.407 6.665
   3815   1388069_at   6.162 6.138 6.106 5.9 6.087 2.118 0.864 3.913 2.932 1.057
   3816   1368589_at   8.184 8.062 8.087 8.33 8.466 7.576 7.216 7.547 7.197 6.989
   3817   1388771_at   9.482 9.539 9.463 9.508 9.513 9.129 9.069 9.218 8.94 8.844
   3818   1383129_at   7.915 7.844 7.755 7.86 7.876 8.442 8.349 8.789 8.36 8.339
   3819   1377959_at   8.403 8.226 8.283 8.548 8.618 7.887 7.967 7.786 7.864 7.797
   3820   1392218_at   1.664 2.025 1.718 1.986 1.669 2.776 4.123 3.946 3.968 4.527
   3821   1390585_at   6.783 6.526 6.713 6.475 6.712 5.888 6.195 5.781 5.873 5.594
   3822   1373772_at   8.006 8.239 7.998 8.075 8.223 7.49 7.518 7.553 7.061 7.409
   3823   1372019_at   8.857 8.736 8.753 8.971 9.135 8.38 8.041 8.298 8.054 7.968
   3824   1369952_at   12.386 12.32 12.336 12.217 12.379 11.86 11.959 11.923 11.833 11.58
   3825   1367644_at   7.254 7.011 7.265 7.394 7.638 8.307 7.885 8.238 8.24 8.349
   3826   1383838_at   6.54 6.933 7.072 7.185 6.563 7.687 7.716 7.832 7.811 7.829
   3827   1380794_at   7.571 7.779 7.565 7.859 7.974 6.934 7.18 7.228 6.915 6.965
   3828   1368199_at   9.1 9.104 8.95 9.041 9.142 8.411 8.662 8.429 8.342 8.068
   3829   1387783_a_at   9.426 9.513 9.247 9.535 8.805 10.217 10.302 10.467 10.6 10.833
   3830   1374159_at   5.553 5.822 5.506 5.317 5.496 6.305 5.995 6.27 6.225 6.216
   3831   1375597_at   4.172 3.977 4.349 4.207 4.15 5.06 4.803 4.731 4.672 4.704
   3832   1379463_at   4.443 4.701 4.874 4.646 4.891 5.582 5.351 6.087 5.887 5.926
   3833   1398062_at   6.178 6.397 6.434 6.375 6.38 5.764 5.012 5.184 5.534 5.033
   3834   1388592_at   7.485 7.005 7.561 7.817 7.421 8.599 8.5 8.816 8.473 8.274
   3835   1390161_at   5.819 6.101 5.883 6.246 6.171 6.594 6.925 6.947 7.169 6.815
   3836   1384935_at   8.409 8.313 8.317 8.464 8.433 7.523 7.913 7.92 7.739 7.47
   3837   1395840_at   8.265 8.416 8.386 8.554 8.605 7.541 7.816 7.542 7.108 7.108
   3838   1383065_at   7.054 7.194 7.167 7.41 6.924 7.72 7.723 7.686 7.74 7.657
   3839   1371744_at   7.19 7.064 7.05 7.096 7.283 7.482 7.663 7.581 7.464 7.477
   3840   1380610_at   6.41 6.598 6.604 6.722 6.274 7.189 7.132 7.533 7.412 7.654
   3841   1374747_at   7.218 7.096 6.843 7.424 7.28 8.087 7.964 7.733 8.047 7.943
   3842   1373024_at   9.215 9.249 9.408 9.446 9.457 8.871 8.725 8.86 8.699 8.434
   3843   1379395_a_at   10.155 10.121 10.17 10.32 10.144 10.485 10.511 10.641 10.662 10.776
   3844   1370133_at   7.676 7.446 7.512 7.523 7.631 4.606 5.719 5.969 5.645 4.247
   3845   1368514_at   5.989 6.088 5.912 5.938 6.391 6.601 6.838 6.852 6.675 6.722
   3846   1382267_at   5.017 4.869 5.115 5.325 4.598 6.269 5.688 6.042 6.047 6.107
   3847   1372839_at   8.24 8.292 8.409 8.371 8.431 8.621 8.643 8.698 8.829 8.683
   3848   1384295_at   8.538 8.459 8.388 8.464 8.808 8.049 7.97 7.914 7.836 7.703
   3849   1369165_at   5.232 4.744 4.667 4.87 4.736 5.659 5.494 5.791 5.792 5.533
   3850   1374241_at   7.937 8.013 8.044 8.642 8.144 9.077 8.991 9.312 9.115 8.99
   3851   1387059_at   7.851 7.829 7.945 8.067 7.847 8.511 8.277 8.693 8.685 8.79
   3852   1376205_at   7.43 7.67 7.553 7.762 7.761 6.75 6.337 6.937 6.628 6.992
   3853   1397808_at   10.166 10.269 10.116 10.232 10.288 9.092 8.63 9.54 9.203 8.645
   3854   1371709_at   10.225 10.215 10.233 10.167 10.191 10.115 10.001 10.014 10.002 9.971
   3855   1384427_at   8.503 8.624 8.601 8.6 8.559 8.047 7.806 7.845 7.687 7.308
   3856   1383814_at   5.483 5.862 5.746 5.65 5.168 6.498 6.613 6.691 7.016 7.229
   3857   1398367_at   8.76 8.667 8.693 8.871 8.906 8.059 7.711 8.247 8.032 8.226
   3858   1370921_at   9.127 9.104 9.262 9.36 9.337 9.804 9.581 9.913 9.974 9.966
   3859   1376852_at   8.833 8.888 9.093 9.167 8.601 9.627 9.636 9.672 9.732 9.987
   3860   1398383_at   8.037 8.068 7.841 8.061 7.951 8.464 8.315 8.652 8.457 8.634
   3861   1373193_at   10.532 10.408 10.539 10.559 10.726 11.014 10.876 11.108 11.204 11.081
   3862   1389644_at   5.842 5.771 5.78 6.237 5.997 7.004 6.784 6.821 6.474 6.7
   3863   1390948_at   5.802 5.433 5.871 5.592 5.64 4.561 4.318 3.496 3.807 3.186
   3864   1397835_at   4.662 4.355 4.609 4.614 3.961 5.648 5.327 5.495 5.266 5.656
   3865   1388271_at   12.613 12.567 12.311 12.418 12.346 11.987 12.065 12.046 11.965 12.061
   3866   1397535_at   8.027 8.041 8.303 8.402 8.259 8.843 8.755 9.195 9.055 8.863
   3867   1394390_at   6.057 6.158 5.916 5.686 6.074 5.351 4.606 4.989 5.081 4.954
   3868   1367813_at   6.09 5.638 5.942 5.9 5.863 6.608 6.424 6.793 7.02 6.74
   3869   1375056_at   9.046 9.09 8.998 8.961 9.067 8.531 8.599 8.63 8.501 8.199
   3870   1384392_at   4.09 3.964 3.577 4.211 4.703 5.325 6.065 6.801 6.511 6.534
   3871   1378556_at   6.767 7.061 6.41 6.774 6.338 7.645 7.89 7.573 7.565 7.59
   3872   1397326_at   3.558 3.955 4.581 3.64 4.138 5.365 5.271 5.155 5.445 5.152
   3873   1367590_at   12.107 12.02 12.085 11.89 12.003 11.675 11.634 11.478 11.333 11.386
   3874   1368884_at   6.911 6.651 6.852 6.849 6.859 5.639 5.688 5.412 5.204 4.447
   3875   1370070_at   9.146 8.842 9.344 9.137 9.154 8.489 8.58 8.594 8.591 8.424
   3876   1374244_at   6.947 6.977 7.277 7.268 5.91 8.461 8.472 8.818 8.944 8.747
   3877   1388848_at   7.106 6.805 7.428 7.351 6.973 6.067 5.218 6.113 5.619 5.331
   3878   1382477_at   6.274 6.56 6.75 6.436 6.663 6.007 5.603 5.632 5.376 5.478
   3879   1373027_at   8.627 8.686 8.782 8.974 8.71 9.413 9.321 9.489 9.251 9.149
   3880   1369024_at   7.091 6.993 7.024 7.041 7.027 6.796 6.681 6.51 6.766 6.644
   3881   1395723_at   4.221 4.829 4.492 4.383 4.34 5.283 5.166 5.24 5.112 5.447
   3882   1373078_at   8.921 9.02 9.474 9.11 8.989 9.809 9.751 9.812 10.06 9.83
   3883   1371739_at   8.96 8.844 9.015 9.03 9.06 9.57 9.29 9.708 9.757 9.603
   3884   1386641_at   5.463 5.999 6.565 6.007 5.546 7.043 7.451 7.721 7.819 7.775
   3885   1372875_at   8.238 8.264 8.324 8.477 8.205 7.862 7.777 7.831 7.507 7.541
   3886   1371839_at   9.524 9.471 9.532 9.426 9.367 9.037 8.924 8.937 8.539 8.745
   3887   1384556_at   4.447 3.648 3.797 3.497 4.815 2.211 2.147 2.516 2.342 2.23
   3888   1374910_at   6.342 6.444 5.926 6.51 6.989 7.714 7.443 7.732 7.641 7.868
   3889   1376394_at   5.859 5.557 6.066 5.194 5.398 0.926 1.021 1.007 3.411 2.893
   3890   1391199_at   7.317 7.667 7.345 7.413 7.542 6.765 6.714 6.925 6.822 6.41
   3891   1374982_at   7.056 6.633 6.865 6.532 6.697 7.585 7.564 7.397 7.303 7.546
   3892   1375966_at   6.263 6.207 6.433 6.593 6.569 6.948 7.11 7.325 7.479 7.225
   3893   1372200_at   7.442 7.45 7.9 8.13 7.862 6.78 6.558 6.333 6.892 6.659
   3894   1388698_at   7.207 7.629 7.447 7.434 7.792 8.233 8.135 8.258 8.53 8.375
   3895   1371531_at   8.97 8.855 9.139 9.333 9.205 9.659 9.648 9.742 9.686 9.829
   3896   1389481_at   9.44 9.486 9.408 9.433 9.425 9.804 9.68 9.756 9.844 9.607
   3897   1392548_at   6.637 6.21 6.367 6.487 6.667 5.692 5.771 5.993 5.812 5.783
   3898   1391570_at   6.151 6.268 6.064 6.416 6.378 7.108 7.027 7.115 6.704 6.838
   3899   1397851_at   6.263 6.542 6.272 6.435 6.386 5.712 5.52 4.904 5.365 5.506
   3900   1395523_at   6.021 6.045 6.148 6.083 6.346 7.255 6.785 7.128 7.192 6.669
   3901   1375353_at   6.115 6.112 6.116 6.441 6.115 7.032 6.653 6.895 7.104 6.763
   3902   1393803_at   6.66 6.864 6.563 6.861 6.846 5.787 5.615 6.123 5.194 5.489
   3903   1368047_at   10.94 10.927 11.25 11.125 10.762 11.516 11.606 11.714 11.761 11.843
   3904   1393990_at   8.515 8.358 8.171 8.281 8.351 8.837 9.12 9.225 9.131 8.798
   3905   1375647_at   6.561 6.569 6.417 7.034 6.786 7.567 7.337 7.862 7.617 7.558
   3906   1374101_at   7.974 8.089 8.378 8.319 8.139 8.701 8.668 8.644 8.722 8.697
   3907   1384440_at   5.349 5.395 4.893 5.333 5.886 7.043 6.427 6.85 6.539 6.538
   3908   1374691_at   6.649 6.31 6.292 6.29 6.45 7.117 6.881 7.024 6.958 6.85
   3909   1398316_at   8.676 8.649 8.546 8.758 8.708 8.874 8.986 9.044 9.034 8.962
   3910   1371731_at   2.414 4.405 3.349 3.425 3.704 5.66 5.662 5.411 5.746 5.956
   3911   1369732_a_at   7.577 7.433 7.575 7.596 7.563 8.284 8.241 8.577 8.207 7.945
   3912   1374195_at   7.226 7.619 7.51 7.966 8.02 8.73 8.64 8.754 8.78 9.133
   3913   1393992_at   5.957 5.305 5.276 5.62 5.44 6.866 6.216 6.875 6.728 6.872
   3914   1370420_at   5.943 6.25 6.185 6.353 6.548 7.106 7.26 6.955 7.031 6.889
   3915   1396460_at   3.978 4.604 4.452 4.851 4.27 5.505 5.459 5.335 5.689 5.474
   3916   1372277_at   11.107 10.98 11.128 11.207 11.032 10.73 10.474 10.633 10.692 10.766
   3917   1392886_a_at   6.585 6.341 6.464 6.266 6.271 4.921 5.367 5.661 5.343 5.657
   3918   1387209_at   5.007 4.467 4.536 4.239 4.625 5.515 5.508 6.05 5.563 5.634
   3919   1389809_at   7.583 7.716 7.96 8.179 7.752 8.576 8.685 8.835 8.741 9.156
   3920   1394459_at   5.938 5.99 5.856 5.851 6.055 6.724 6.423 6.848 6.451 6.442
   3921   1395073_at   5.597 5.664 5.869 6.025 6.413 6.884 6.798 7.22 7.158 7.199
   3922   1373782_a_at   6.787 6.877 6.337 6.263 6.706 5.767 5.721 5.577 5.301 5.305
   3923   1398265_at   4.144 4.781 4.89 4.271 5.601 6.485 6.752 7.144 6.599 6.4
   3924   1374385_at   11.332 11.374 11.274 11.139 11.248 11.043 10.927 10.94 10.967 10.884
   3925   1395725_at   8.281 8.268 8.316 8.282 8.313 8.573 8.653 8.816 8.614 8.517
   3926   1371928_at   6.879 7.131 6.994 7.273 7.319 6.429 6.194 6.41 5.82 6.104
   3927   1370112_at   8.866 8.81 8.645 8.61 8.675 8.307 8.422 8.187 8.291 8.181
   3928   1392583_at   3.688 3.341 3.471 3.242 2.838 4.341 4.646 4.206 4.707 4.854
   3929   1379025_at   7.131 6.859 6.705 7.075 7.031 6.416 6.39 6.379 6.142 6.142
   3930   1379781_at   5.609 5.389 5.809 5.677 5.295 6.074 6.277 6.378 6.336 6.36
   3931   1368129_at   7.337 7.466 7.084 7.31 7.376 6.604 6.319 6.827 6.55 6.262
   3932   1372863_at   8.971 9.133 8.974 9.17 9.187 8.542 8.248 8.696 8.34 8.24
   3933   1388707_at   7.989 7.895 7.563 7.767 7.962 7.152 7.218 7.253 6.928 6.837
   3934   1388363_at   8.729 8.688 8.457 8.818 8.775 8.19 7.99 7.945 7.723 8.148
   3935   1398317_at   9.07 9.052 9.101 9.158 9.156 9.49 9.427 9.7 9.434 9.411
   3936   1383089_at   9.098 9.103 9.356 9.252 9.407 8.875 8.702 8.729 8.809 8.746
   3937   1380088_at   5.273 5.157 4.393 5.108 5.626 6.742 6.304 6.644 6.88 6.476
   3938   1376697_at   7.109 7.32 7.683 7.429 7.534 6.408 6.292 6.122 6.708 5.871
   3939   1383349_at   8.572 8.687 8.651 8.706 8.937 8.264 7.763 8.072 8.002 7.815
   3940   1394850_at   5.895 5.954 5.842 5.19 5.514 6.976 6.708 6.648 6.804 6.577
   3941   1385739_at   9.046 9.054 9.06 9.25 9.101 9.667 9.708 10.162 9.72 9.665
   3942   1383240_at   9.203 9.268 9.361 9.398 9.185 9.871 9.974 10.132 9.766 9.675
   3943   1390416_at   9.341 9.432 9.367 9.527 9.08 10.081 9.786 10.24 10.188 10.256
   3944   1384361_at   5.817 6.178 6.537 6.473 6.613 7.243 7.247 7.387 7.376 7.353
   3945   1371380_at   10.972 10.916 11.014 11.182 10.958 11.391 11.318 11.512 11.44 11.596
   3946   1392503_at   7.764 7.749 7.733 7.836 7.92 7.465 7.229 7.289 7.273 6.997
   3947   1387004_at   6.394 6.672 6.778 6.713 6.775 7.496 7.113 7.401 7.64 7.602
   3948   1395654_at   6.336 6.254 6.128 6.455 5.99 5.179 5.443 5.68 5.483 5.165
   3949   1375554_at   8.723 8.724 8.499 8.554 8.78 8.266 8.067 8.156 7.906 7.895
   3950   1387875_at   7.965 8.111 8.26 8.216 8.074 8.575 8.501 8.566 8.701 8.47
   3951   1398514_at   10.435 10.126 10.604 10.195 9.806 11.08 11.131 11.156 11.233 11.188
   3952   1398432_at   7.585 7.449 7.731 7.77 7.476 8.083 8.146 8.24 8.117 7.985
   3953   1373789_at   5.706 5.757 5.579 6.116 6.017 6.847 6.431 7.136 7.002 7
   3954   1382127_at   9.009 9.191 9.168 9.206 8.466 9.923 10.032 9.959 10.022 9.864
   3955   1371715_at   9.045 9.149 9.098 9.012 9.095 9.525 9.343 9.522 9.326 9.357
   3956   1386308_at   0.263 0.824 0 2.186 0.696 3.194 3.252 3.587 3.988 3.374
   3957   1397669_at   5.846 5.785 5.73 6.011 5.882 4.928 4.889 4.221 5.07 4.376
   3958   1374355_at   6.663 6.925 6.913 7.203 7.386 5.913 5.947 6.277 6.08 6.154
   3959   1376498_at   6.681 6.509 6.677 6.931 7.079 7.617 7.477 7.861 7.77 7.447
   3960   1368399_a_at   10.066 9.896 10.224 10.146 10.052 10.69 10.441 10.569 10.461 10.618
   3961   1393015_at   8.367 8.331 8.332 8.775 8.535 9.224 9.057 9.522 9.379 9.135
   3962   1388378_at   11.083 11.01 11.025 11.129 11.107 10.875 10.843 10.86 10.708 10.766
   3963   1369450_at   7.641 7.828 7.676 7.746 8.288 8.871 8.648 9.217 8.892 8.685
   3964   1372209_at   9.675 9.662 9.67 9.669 9.746 9.543 9.383 9.468 9.37 9.349
   3965   1398757_at   11.755 11.707 11.632 11.639 11.845 11.195 11.053 11.264 10.893 10.745
   3966   1389443_at   6.395 6.549 6.458 6.516 6.512 6.216 6.214 6.286 6.093 6.1
   3967   1398355_at   7.744 8.018 7.997 8.073 8.117 8.595 8.716 8.934 8.7 8.44
   3968   1378433_at   5.901 6.093 5.988 6.209 6.178 6.504 6.743 6.671 6.74 6.46
   3969   1389253_at   8.387 8.289 8.46 8.26 8.534 9.015 9.771 9.761 9.623 9.19
   3970   1388787_at   8.885 8.843 8.748 8.95 9.068 8.555 8.279 8.489 8.343 8.414
   3971   1370043_at   7.524 7.345 7.425 7.562 7.773 8.345 8.139 8.798 8.668 8.332
   3972   1387336_at   9.116 9.044 9.654 9.486 8.463 10.533 10.503 10.953 10.993 11.332
   3973   1393655_at   6.129 6.327 5.848 6.342 6.044 5.305 4.738 5.281 4.5 4.798
   3974   1388145_at   6.007 5.73 6.018 6.351 5.77 6.816 6.637 6.928 7.03 7.144
   3975   1367541_at   7.84 8.022 7.976 7.842 8.097 7.348 7.546 7.555 7.548 7.586
   3976   1378293_at   7.487 7.573 7.612 7.816 7.698 7.188 6.811 6.837 6.887 7.166
   3977   1383581_at   6.728 6.832 6.727 7.182 7.065 6.25 5.809 5.752 5.352 5.752
   3978   1384765_at   7.264 7.403 7.694 7.673 7.58 5.904 4.436 4.73 6.152 4.985
   3979   1398869_at   10.243 10.251 10.252 10.373 10.415 10.05 10.071 10.017 10.086 10.004
   3980   1370190_at   11.918 11.804 11.717 11.681 11.763 11.371 11.262 11.427 11.2 11.052
   3981   1398408_at   6.388 6.327 6.356 6.132 6.268 4.639 3.392 4.515 4.931 4.795
   3982   1382918_at   4.48 4.725 4.825 4.892 3.963 5.526 5.858 6.146 6.213 6.062
   3983   1382537_at   9.454 9.317 9.547 9.574 9.508 9.166 9.07 9.141 9.109 8.947
   3984   1391240_at   4.833 4.706 4.238 4.457 4.265 5.687 5.294 5.765 5.463 5.314
   3985   1386917_at   8.443 8.383 8.692 8.79 7.962 9.482 9.461 9.731 9.478 9.336
   3986   1383047_at   8.053 8.034 8.329 8.371 8.725 9.119 9.132 9.367 9.14 9.188
   3987   1387039_at   6.295 6.73 6.679 6.522 6.537 7.184 7.025 7.216 7.153 7.403
   3988   1388155_at   7.752 7.715 7.796 8.026 8.014 8.614 8.457 8.556 8.36 8.285
   3989   1391765_at   4.264 4.482 5.029 5.031 4.569 5.681 5.718 6.087 5.768 5.735
   3990   1398402_at   4.236 5.084 4.587 4.586 4.401 2.534 3.429 2.832 3.329 3.338
   3991   1390561_at   5.949 6.25 6.111 6.041 5.988 6.621 6.533 6.694 6.423 6.466
   3992   1367839_at   8.361 8.293 8.644 8.606 8.309 9.387 9.073 9.016 9.2 9.514
   3993   1385423_at   7.739 7.688 7.623 7.784 7.878 8.294 8.39 8.038 8.169 8.321
   3994   1392079_at   6.262 6.319 6.546 6.735 6.629 4.86 5.347 5.731 4.951 5.468
   3995   1376353_at   6.651 6.41 6.442 6.491 6.327 5.676 6.077 5.893 5.636 5.626
   3996   1373778_at   5.19 5.76 4.223 4.924 5.022 6.602 6.605 6.763 6.727 6.645
   3997   1388831_at   6.187 6.065 6.274 6.497 6.563 7.244 6.974 7.101 7.531 7.515
   3998   1393279_at   4.637 4.123 4.792 4.688 4.153 5.469 5.681 6.161 5.531 6.014
   3999   1381937_at   6.207 6.002 6.368 6.104 6.794 7.487 7.068 7.613 7.389 7.376
   4000   1398894_at   10.458 10.428 10.548 10.578 10.566 10.782 10.752 10.948 10.853 10.781
   4001   1368272_at   9.946 9.94 10.1 9.722 9.903 10.441 10.434 10.322 10.379 10.279
   4002   1371724_at   9.973 9.827 9.959 10.036 10.16 9.433 9.569 9.622 9.346 9.316
   4003   1377007_at   6.498 6.563 6.478 6.644 6.763 7.174 6.956 7.37 7.093 7.272
   4004   1390650_at   7.097 7.385 7.163 7.452 7.412 6.796 6.755 6.86 6.589 6.697
   4005   1389790_at   7.122 7.338 7.129 7.149 7.163 6.631 6.619 6.813 6.645 6.335
   4006   1377905_at   7.913 7.95 8.048 8.258 8.078 7.606 7.397 7.564 7.665 7.463
   4007   1370195_at   9.106 9.204 9.195 9.322 9.313 8.949 8.906 8.901 8.848 8.735
   4008   1377340_at   6.391 6.347 6.83 6.639 6.352 5.213 4.931 4.894 5.195 3.923
   4009   1373333_at   2.032 3.383 2.101 3.209 3.205 4.909 4.749 5.601 4.846 4.829
   4010   1384087_at   6.034 5.767 6.032 5.93 5.869 4.789 4.754 5.389 4.476 4.98
   4011   1374156_at   8.167 8.123 8.062 8.085 8.079 8.616 8.668 9.002 8.863 8.478
   4012   1384027_a_at   9.086 8.903 8.963 9.279 9.342 9.752 9.905 9.825 9.802 9.591
   4013   1377753_at   5.699 5.886 6.127 5.87 6.542 7.235 7.054 7.09 7.065 6.864
   4014   1372450_at   7.298 7.528 7.67 7.604 7.582 8.16 7.905 8.271 8.079 8.172
   4015   1374828_at   6.32 6.527 6.461 6.995 7.015 7.717 7.572 7.712 8.041 7.663
   4016   1371771_at   6.466 6.529 6.694 6.888 6.749 7.305 7.187 7.409 7.197 7.534
   4017   1388958_a_at   4.846 4.58 5.015 4.826 4.258 5.804 5.841 5.688 6.135 6.493
   4018   1370136_at   7.629 7.62 7.849 7.887 7.614 7.171 7.196 7.097 6.939 6.733
   4019   1390851_at   10.521 10.523 10.913 10.679 9.858 11.631 11.582 11.678 11.694 11.758
   4020   1380199_at   4.734 4.624 4.569 4.418 4.322 5.227 5.017 5.363 5.291 5.564
   4021   1375562_at   5.427 5.235 5.433 5.794 5.651 6.518 6.179 6.781 6.584 6.271
   4022   1390845_at   8.699 8.811 8.682 8.925 8.993 9.332 9.2 9.469 9.565 9.401
   4023   1373257_at   4.625 5.276 5.333 5.189 4.952 1.832 2.597 0 2.128 2.563
   4024   1368801_at   1.766 0 0.322 0.949 2.39 4.418 3.654 4.304 4.188 4.066
   4025   1393810_at   3.68 2.905 3.758 3.004 3.029 4.4 4.92 4.517 4.741 5.085
   4026   1389325_at   10.4 10.335 10.358 10.237 10.435 9.623 9.861 9.947 9.617 9.417
   4027   1374821_at   4.577 5.012 4.722 4.73 5.157 6.339 5.681 6.06 5.748 5.747
   4028   1385269_s_at   6.485 6.285 6.149 6.331 6.44 5.705 4.954 5.208 5.465 4.865
   4029   1393822_at   6.787 6.423 5.975 6.624 6.743 5.546 5.2 5.623 5.402 5.304
   4030   1386979_at   6.385 6.256 6.252 6.172 5.973 7.002 6.916 6.781 6.868 6.607
   4031   1379488_at   6.234 6.141 6.112 6.121 6.479 5.531 5.682 5.775 5.397 5.438
   4032   1373118_at   6.168 5.877 6.19 6.164 6.073 6.752 6.722 7.179 6.623 6.876
   4033   1375997_at   7.724 7.47 7.96 7.867 7.144 8.641 8.505 8.631 8.853 8.725
   4034   1388645_at   10.329 10.313 10.332 10.478 10.562 9.961 9.991 10.126 9.888 9.898
   4035   1380546_at   8.587 8.519 8.515 8.559 8.739 9.065 8.911 9.258 9.038 8.979
   4036   1368614_at   6.045 6.038 5.979 5.662 5.854 6.743 6.376 6.486 6.581 6.491
   4037   1398954_at   10.974 10.994 10.875 10.779 10.868 10.648 10.489 10.633 10.577 10.557
   4038   1371125_at   7.639 7.714 7.637 7.783 7.544 7.221 7.266 7.252 7.448 7.252
   4039   1381296_at   5.47 4.974 5.319 5.6 5.085 6.272 6.174 6.221 6.273 5.911
   4040   1393732_at   6.026 6.354 6.065 6.412 6.335 7.06 7.331 7.643 6.961 7.051
   4041   1372829_at   9.774 9.762 9.621 9.879 10.001 9.217 9.09 9.369 8.871 9.012
   4042   1395730_at   7.921 7.84 7.717 7.947 7.852 7.616 7.39 7.291 7.321 7.332
   4043   1367855_at   7.116 7.034 7.269 7.18 7.17 6.806 6.544 6.312 6.446 6.266
   4044   1384254_at   8.409 8.286 8.443 8.324 8.144 7.667 7.735 7.889 7.662 7.361
   4045   1370232_at   9.631 9.775 9.878 9.837 9.82 10.315 10.07 10.385 10.492 10.405
   4046   1368194_at   7.51 7.663 7.289 7.713 8.018 6.401 6.873 6.151 6.671 6.286
   4047   1371518_at   10.207 10.338 9.881 10.213 10.55 10.952 10.967 11.186 11.039 10.924
   4048   1392893_a_at   6.93 7.015 7.061 7.224 7.05 7.496 7.857 8.042 7.947 7.618
   4049   1398796_at   10.353 10.354 10.351 10.379 10.45 10.225 10.136 10.247 10.179 10.136
   4050   1397317_at   6.04 6.143 6.11 6.609 6.569 4.871 4.905 5.377 4.273 5.005
   4051   1368159_at   5.564 5.384 5.536 6.324 6.16 6.973 6.895 7.233 7.207 7.266
   4052   1371558_at   8.675 8.611 8.677 8.883 8.867 9.467 9.068 9.352 9.342 9.287
   4053   1388850_at   11.912 11.886 12.023 11.814 12.074 11.541 11.231 11.462 11.453 11.561
   4054   1392809_at   4.138 3.955 4.797 4.434 4.688 5.68 6.059 6.467 5.466 5.97
   4055   1390440_at   7.272 7.382 6.986 7.23 7.289 7.75 7.755 7.971 7.793 7.65
   4056   1395928_at   7.016 6.866 7.248 7.316 7.42 8.051 7.744 7.922 8.175 8.088
   4057   1385277_x_at   8.815 8.517 8.372 8.65 8.905 6.484 4.841 3.686 4.722 2.716
   4058   1399034_at   7.699 7.405 7.653 7.808 7.714 8.054 8.089 8.193 8.156 8.228
   4059   1384215_at   5.573 5.377 5.382 4.959 5.26 6.506 5.983 6.453 6.716 6.896
   4060   1397419_at   6.866 6.989 6.816 7.148 7.072 7.652 7.753 7.76 7.413 7.451
   4061   1375569_at   6.23 6.02 6.036 6.027 6.586 6.778 6.964 6.956 7.019 7.037
   4062   1398449_at   7.451 7.293 7.586 7.61 7.089 4.661 3.306 4.207 1.761 1.293
   4063   1372910_at   7.269 7.395 7.471 7.596 7.557 6.943 6.676 7.082 6.789 6.753
   4064   1371848_at   10.29 10.299 10.177 10.418 10.423 10.019 9.913 10.049 9.962 9.905
   4065   1370189_at   10.303 10.413 10.345 10.297 10.414 10.013 10.107 9.981 9.806 9.828
   4066   1377085_at   6.177 6.346 6.136 6.38 6.32 6.627 6.974 7.129 7.191 7.136
   4067   1389315_at   7.926 8.021 8.043 8.018 7.934 7.529 7.477 7.442 7.271 7.002
   4068   1392168_at   5.763 5.708 5.74 5.623 5.696 5.241 5.399 5.153 5.002 4.943
   4069   1378733_at   6.605 6.494 5.902 6.63 6.324 5.534 4.849 4.385 4.953 4.693
   4070   1370414_at   6.518 6.588 6.668 6.845 7.033 6.123 5.751 6.118 5.942 5.854
   4071   1390141_at   5.561 6.268 6.07 6.369 6.094 4.632 4.954 4.645 4.477 3.766
   4072   1385873_at   7.578 7.86 7.675 7.666 7.822 7.235 7.318 7.054 6.95 6.895
   4073   1398633_at   0 0.411 0.791 0.623 1.491 3.58 2.31 2.628 3.741 3.004
   4074   1371944_at   10.852 10.813 10.944 10.795 10.827 10.482 10.663 10.463 10.456 10.365
   4075   1367982_at   9.096 9.094 9.181 9.137 9.036 9.427 9.604 9.323 9.434 9.428
   4076   1390097_at   5.377 5.92 6.144 6.252 6.047 7.008 6.825 7.314 7.398 7.316
   4077   1376645_at   7.084 7.14 7.33 7.117 7.586 8.264 8.487 8.55 7.978 7.977
   4078   1383502_at   7.766 7.645 7.517 7.739 7.61 7.043 6.937 7.158 6.543 6.744
   4079   1393776_at   7.432 7.519 7.366 7.584 7.891 6.487 5.155 6.249 5.828 6.077
   4080   1398991_at   7.891 8.066 7.83 8.004 7.96 8.523 8.313 8.852 8.718 8.598
   4081   1379659_at   5.997 6.031 6.226 6.348 6.461 5.332 5.434 5.506 4.882 5.028
   4082   1385816_at   5.935 5.369 5.5 5.407 5.442 4.699 3.745 4.359 4.345 4.422
   4083   1374370_at   5.249 5.534 5.707 5.371 5.404 6.233 5.958 6.403 6.435 6.603
   4084   1380229_at   6.706 6.514 6.651 6.808 6.637 5.53 5.647 5.618 5.348 4.587
   4085   1370386_at   8.082 8.009 7.836 8.161 8.135 7.267 7.584 7.586 7.291 7.196
   4086   1381043_at   6.568 6.565 6.611 6.652 6.812 7.094 7.139 7.309 7.166 6.939
   4087   1388705_at   8.246 8.16 8.353 8.492 8.757 9.144 9.272 9.364 9.149 9.01
   4088   1373892_at   8.897 9.242 9.149 9.166 9.267 9.823 9.662 10.133 9.953 9.694
   4089   1393433_at   4.149 5.078 4.008 5.207 4.702 6.35 6.087 6.675 6.497 6.134
   4090   1369148_at   6.575 6.554 6.45 6.588 6.398 7.058 7.469 7.713 7.98 7.462
   4091   1391107_at   6.046 6.066 6.288 5.899 6.49 5.059 5.079 5.331 5.184 5.572
   4092   1374632_at   7.265 7.571 7.158 7.255 7.43 6.459 6.704 5.883 6.211 6.382
   4093   1370727_at   4.71 4.417 4.026 4.644 4.413 5.723 5.516 5.825 5.207 5.339
   4094   1371737_at   8.899 8.784 9.008 9.089 9.006 8.459 8.498 8.641 8.354 8.325
   4095   1369292_at   4.964 4.885 4.678 4.762 4.587 5.609 5.485 5.655 6.128 6.193
   4096   1374466_at   6.856 6.811 6.861 6.985 6.855 7.495 7.528 8.13 7.679 7.521
   4097   1377137_at   7.342 7.67 7.451 7.656 7.726 7.005 6.92 6.884 6.641 6.496
   4098   1388295_s_at   5.169 5.09 4.899 5.22 5.066 5.713 5.736 5.472 5.467 5.668
   4099   1390208_at   7.993 8.103 8.366 8.138 8.191 7.691 7.4 7.634 7.438 7.716
   4100   1370932_at   5.597 5.243 5.435 6.059 5.651 6.814 6.352 6.875 6.784 6.567
   4101   1390142_at   8.471 8.142 8.442 8.367 8.291 7.622 7.106 7.217 6.811 6.495
   4102   1368046_at   7.889 7.983 8.223 7.806 7.537 8.702 8.798 8.635 8.718 8.521
   4103   1370509_at   7.985 8.028 8.567 8.321 8.264 9.099 8.952 9.413 9.04 9.001
   4104   1389430_at   5.011 5.081 5.477 5.371 5.293 6.358 5.789 6.044 6.22 6.376
   4105   1372082_at   9.449 9.498 9.436 9.632 9.579 9.131 8.892 9.143 9.142 8.86
   4106   1378302_at   5.678 5.893 5.86 5.358 5.616 6.625 6.593 6.243 6.41 6.399
   4107   1389200_at   7.444 7.523 7.669 7.705 7.614 7.232 7.237 7.147 6.978 7.183
   4108   1367570_at   8.9 8.683 9.413 9.421 9.235 10.089 10.243 10.243 9.951 10.211
   4109   1380930_at   6.076 5.904 5.615 6.105 6.256 4.175 4.736 3.978 3.032 4.27
   4110   1383117_at   7.871 7.786 8.232 8.227 7.998 8.73 8.643 9.055 9.219 9.028
   4111   1382515_at   7.134 7.271 7.285 7.523 7.414 6.572 6.738 6.879 6.505 6.385
   4112   1376688_a_at   7.415 7.612 7.441 7.309 7.635 6.509 6.181 6.378 6.046 5.328
   4113   1367468_at   9.483 9.737 9.756 9.835 9.604 10.03 10.317 10.227 10.323 10.182
   4114   1397698_at   7.001 7.194 7.408 7.193 7.095 7.964 8.083 8.139 8.323 7.643
   4115   1391397_at   6.338 6.117 6.602 6.287 6.3 7.215 6.811 7.239 7.185 7.475
   4116   1391434_at   5.749 5.343 6.006 6.051 6.372 7.36 7.25 6.809 7.414 7.509
   4117   1372357_at   8.714 8.662 8.689 8.668 8.73 8.208 7.928 8.393 8.246 8.05
   4118   1391506_at   5.394 4.97 5.064 5.683 5.488 6.308 6.294 6.11 6.162 6.2
   4119   1388697_at   8.008 7.881 7.879 8.143 7.954 8.419 8.244 8.418 8.525 8.5
   4120   1382184_at   6.358 6.202 5.643 6.064 6.332 7.034 7.051 6.978 6.856 7.072
   4121   1375726_at   7.572 7.554 7.765 8.133 7.763 8.451 8.356 8.681 8.534 8.568
   4122   1376842_at   6.58 6.678 6.71 6.818 6.897 6.121 5.801 6.27 5.936 5.679
   4123   1390818_at   6.995 6.98 6.447 6.974 6.812 7.715 7.517 7.753 7.757 7.443
   4124   1370298_at   10.606 10.601 10.622 10.466 10.637 10.819 10.806 10.83 10.923 10.795
   4125   1395555_at   7.252 7.426 7.381 7.364 7.336 7.038 6.962 7.024 7.044 6.767
   4126   1380355_s_at   3.817 3.167 3.553 3.449 3.184 4.677 4.136 4.984 4.752 4.589
   4127   1387503_at   6.15 6.454 6.657 6.736 6.49 7.307 7.265 7.55 7.272 7.103
   4128   1393140_at   5.949 6.378 5.955 6.152 6.344 4.469 5.381 5.024 5.203 4.903
   4129   1387772_at   11.312 11.287 11.082 11.261 11.3 10.912 10.561 10.691 10.869 10.641
   4130   1395508_at   10.366 10.366 10.326 10.365 10.539 9.894 10.063 9.802 9.641 9.611
   4131   1390374_at   7.566 7.72 8.168 7.788 7.787 7.07 7.013 7.081 6.715 6.595
   4132   1396057_at   4.129 3.944 4.594 4.631 4.57 5.993 5.47 5.876 5.294 6.148
   4133   1380017_at   6.689 6.644 6.893 6.956 6.907 7.391 7.381 7.657 7.276 7.326
   4134   1389103_at   7.419 7.326 7.566 7.265 7.477 6.795 6.772 6.889 6.339 6.481
   4135   1385753_at   7.517 7.187 7.554 7.387 7.337 7.946 7.925 7.997 7.872 7.752
   4136   1398892_at   11.009 11.09 11.051 11.092 11.113 10.901 10.746 10.843 10.672 10.723
   4137   1370278_at   11.574 11.53 11.627 11.371 11.371 11.995 11.95 11.787 12.094 12.036
   4138   1372401_at   9.494 9.438 9.434 9.491 9.573 9.032 9.217 9.245 9.067 9.223
   4139   1387690_at   8.057 7.945 8.179 8.157 7.885 7.198 7.195 6.497 6.267 6.469
   4140   1390717_at   7.985 7.944 8.233 8.337 8.051 8.761 8.929 9.039 8.743 8.585
   4141   1374048_at   5.123 5.027 5.381 5.513 4.737 6.665 5.969 6.168 6.515 6.542
   4142   1383004_at   8.349 8.566 8.63 8.342 8.441 8.888 8.907 9.106 8.996 8.837
   4143   1379672_at   4.454 4.047 5.101 4.586 4.795 5.841 5.582 6.193 6.016 6.268
   4144   1380852_at   4.372 4.912 4.757 4.819 4.73 5.539 6.384 5.66 6.331 6.342
   4145   1368292_at   7.414 7.564 7.633 7.79 7.707 8.241 8.068 8.512 8.375 8.6
   4146   1383084_at   3.951 4.421 3.994 4.013 3.693 5.619 4.975 5.466 5.413 4.816
   4147   1382008_at   8.684 8.691 8.778 8.576 8.602 9.014 9.038 9.015 9.29 9.034
   4148   1371730_at   9.475 9.581 9.456 9.786 9.718 9.985 10.056 10.263 10.211 10.17
   4149   1383988_at   5.568 5.866 5.442 5.648 5.511 6.218 6.284 6.009 6.39 6.221
   4150   1380640_at   4.08 4.042 4.025 4.615 4.388 5.42 5.202 5.172 4.985 5.658
   4151   1399158_a_at   11.652 11.736 11.483 11.565 11.723 10.806 11.094 10.624 10.444 10.247
   4152   1369944_at   10.036 10.12 9.941 10.209 10.204 9.746 9.302 9.488 9.325 9.218
   4153   1373305_at   8.394 8.342 8.154 8.287 8.482 7.931 7.804 7.966 7.662 7.727
   4154   1374868_at   6.184 6.043 5.788 6.153 6.141 6.599 6.912 6.868 6.521 6.828
   4155   1392551_at   5.581 5.313 5.757 5.615 6.138 6.673 6.524 6.719 6.736 6.454
   4156   1394627_at   7.009 7.047 7.098 7.157 7.018 7.46 7.513 7.409 7.388 7.247
   4157   1374037_at   6.771 6.809 6.999 6.873 6.878 7.39 7.17 7.488 7.335 7.207
   4158   1399065_at   8.364 8.537 8.597 8.883 8.659 9.425 9.197 9.679 9.282 9.294
   4159   1387158_at   7.784 7.791 8.188 8.482 7.156 9.163 9.371 9.718 9.909 9.937
   4160   1398647_at   5.648 5.784 5.782 5.98 5.841 6.527 6.415 6.733 7.017 7.146
   4161   1380045_at   6.849 7.361 7.68 7.833 7.443 8.523 8.437 8.8 8.69 8.525
   4162   1379314_at   7.803 7.888 7.889 7.986 7.956 8.294 8.395 8.562 8.234 8.245
   4163   1369492_at   5.208 5.536 5.357 5.272 4.525 6.562 7.113 7.06 7.159 6.203
   4164   1382727_at   7.335 7.516 7.416 7.375 7.677 6.162 6.688 6.577 5.715 6.324
   4165   1390145_at   7.675 7.749 7.555 7.81 7.748 8.153 8.137 8.391 8.165 8.034
   4166   1388863_at   8.918 8.928 9.214 8.915 9.215 8.537 8.625 8.354 8.431 8.494
   4167   1385830_at   4.782 4.228 3.831 4.474 4.115 5.22 5.727 5.507 5.367 5.451
   4168   1371988_at   9.281 9.342 9.397 9.328 9.273 8.891 8.52 8.622 8.552 8.149
   4169   1387098_at   7.143 7.104 7.024 7.362 6.958 6.567 6.709 6.415 6.491 6.638
   4170   1393100_at   6.624 6.663 6.38 6.55 6.609 7.015 6.909 7.238 7.179 7.298
   4171   1373842_at   8.821 8.91 9.009 8.945 8.784 9.601 9.653 9.811 9.492 9.238
   4172   1398431_at   5.266 5.076 6.073 5.642 5.862 6.721 6.648 7.024 7.087 6.99
   4173   1378592_at   8.169 8.269 8.213 8.22 8.412 7.859 7.621 7.834 7.425 7.549
   4174   1369788_s_at   9.283 9.409 8.94 9.25 9.115 8.637 8.631 8.55 8.288 8.295
   4175   1392001_at   5.388 5.243 4.995 4.968 4.903 5.765 5.736 5.873 6.177 6.134
   4176   1373497_at   7.286 7.367 7.413 7.674 7.254 8.336 8.142 9.039 8.787 8.413
   4177   1383361_at   9.747 9.744 9.664 9.653 9.756 9.263 9.364 9.44 9.464 9.183
   4178   1391981_at   5.699 5.816 6.256 5.943 6.067 6.922 6.523 6.915 6.664 6.715
   4179   1374565_at   8.343 8.33 8.188 8.214 8.422 7.593 7.77 7.545 7.274 7.06
   4180   1388136_at   8.318 8.156 8.358 8.354 8.327 8.55 8.678 8.692 8.659 8.537
   4181   1379044_at   5.795 5.525 5.79 6.305 5.794 6.705 6.709 7.119 7.085 6.71
   4182   1372103_at   6.937 6.872 6.972 7.157 6.915 7.323 7.52 7.791 7.552 7.532
   4183   1398304_at   5.123 5.153 5.71 5.475 5.909 6.547 6.525 6.339 6.559 6.487
   4184   1390449_at   5.722 6.254 6.382 6.503 6.756 7.568 7.383 7.645 7.438 7.364
   4185   1390727_at   6.532 6.313 6.697 7.013 6.838 7.569 7.362 7.849 7.786 7.593
   4186   1371635_at   7.131 6.919 7.089 7.411 7.184 7.65 7.628 7.876 7.775 7.733
   4187   1377033_at   9.381 9.267 9.302 9.609 9.151 9.766 9.878 10.045 10.06 10.04
   4188   1376388_at   0.214 1.526 1.824 1.124 1.287 4.745 4.567 2.971 3.572 3.766
   4189   1367996_a_at   5.098 4.57 5.57 5.421 5.368 6.866 6.149 6.81 7.109 6.704
   4190   1372014_at   7.6 7.481 7.673 7.949 7.942 7.076 6.816 7.196 6.925 6.838
   4191   1371323_at   11.945 11.871 11.97 11.911 11.846 12.324 12.25 12.107 12.318 12.441
   4192   1368180_s_at   11.168 11.089 11.513 11.295 10.498 12.135 12.217 12.224 12.191 12.284
   4193   1382463_at   8.014 8.414 8.488 8.142 8.026 7.622 7.424 7.487 7.529 7.234
   4194   1377235_a_at   6.943 7.012 7.33 7.005 6.842 7.576 7.786 7.489 7.757 7.706
   4195   1397292_at   6.519 6.383 7.208 6.721 6.762 5.198 2.014 2.422 2.759 2.736
   4196   1393808_at   5.847 6.532 6.356 6.51 6.312 7.508 6.989 7.477 7.517 7.784
   4197   1383221_at   9.007 8.922 8.966 9.375 9.301 9.678 9.675 9.908 9.852 9.937
   4198   1367648_at   7.6 7.586 7.597 7.693 7.476 8.706 8.329 8.642 8.429 8.002
   4199   1382972_at   6.883 6.897 7.097 7.166 6.765 6.564 6.372 6.291 6.376 6.227
   4200   1389540_at   8.82 8.837 8.936 9.078 8.279 9.939 9.505 9.763 9.938 9.978
   4201   1370336_at   8.494 8.39 8.799 8.786 7.455 10.162 9.767 9.943 10.141 10.221
   4202   1378003_at   6.713 6.691 7.049 6.908 6.77 7.376 7.413 7.836 7.719 7.443
   4203   1386861_at   12.295 12.234 12.257 12.127 12.37 11.95 11.793 11.722 11.512 11.65
   4204   1371043_a_at   8.294 8.255 8.022 8.448 8.532 8.98 8.834 9.219 9.055 8.941
   4205   1389440_at   7.298 7.572 7.317 7.449 7.238 6.064 6.762 6.119 5.684 6.305
   4206   1372708_at   5.126 5.081 5.056 5.135 5.697 6.263 5.852 6.48 6.382 6.475
   4207   1393212_at   4.92 4.594 4.99 4.842 4.584 6.095 5.323 5.787 5.72 5.771
   4208   1393755_at   5.741 6.223 5.661 5.963 6.113 5.095 4.624 5.217 4.711 5.044
   4209   1377756_at   8.165 8.153 8.358 8.477 8.52 7.645 7.757 7.933 7.699 7.626
   4210   1388062_at   1.091 3.209 2.091 1.705 1.384 4.842 4.311 4.368 4.127 4.236
   4211   1388901_at   8.009 7.831 7.547 7.834 7.713 7.028 7.217 7.367 7.164 7.158
   4212   1386687_at   5.581 5.881 5.685 5.616 5.696 6.243 6.011 6.246 6.198 6.052
   4213   1370176_at   7.586 7.687 7.846 7.907 7.727 8.321 8.273 8.63 8.347 8.187
   4214   1388754_at   9.9 9.83 9.805 9.851 9.99 9.544 9.482 9.376 9.329 9.128
   4215   1370966_at   6.292 6.715 6.423 6.141 6.38 7.115 6.953 7.212 6.982 7.249
   4216   1377508_at   3.483 3.669 3.618 3.581 3.288 4.855 4.459 5.149 4.175 4.759
   4217   1399035_at   7.863 7.847 7.983 8.1 7.894 7.367 7.281 7.52 7.191 6.95
   4218   1387364_at   5.397 5.795 5.791 4.963 4.819 6.797 6.961 6.776 6.592 6.548
   4219   1392461_at   8.526 8.541 8.39 8.585 8.433 8.207 7.947 8.183 7.931 8.018
   4220   1392044_at   8.236 8.248 8.068 8.554 8.501 9.025 8.79 9.131 9.044 9.016
   4221   1382940_at   6.111 5.862 6.274 5.853 5.964 6.935 6.462 6.925 6.703 6.851
   4222   1398902_at   9.352 9.506 9.699 9.638 9.535 10.168 9.85 10.138 10.148 10.19
   4223   1387669_a_at   9.532 9.542 9.642 9.698 9.569 9.976 9.937 10.35 10.229 10.167
   4224   1375080_at   8.168 8.534 8.475 8.395 8.358 8.951 8.71 8.869 8.876 8.891
   4225   1368325_at   10.044 10.113 10.374 10.235 9.045 11.457 11.505 11.52 11.449 11.922
   4226   1374896_at   5.411 5.947 5.503 5.79 5.412 6.295 6.432 6.381 6.226 6.386
   4227   1388024_s_at   9.209 9.119 9.384 9.247 9.411 8.526 8.261 8.845 8.659 8.579
   4228   1376765_at   9.343 9.386 9.671 9.496 8.8 10.144 10.285 10.403 10.479 10.402
   4229   1374636_at   7.936 7.911 8.18 8.291 8.067 8.814 8.906 9.175 8.931 8.542
   4230   1378883_at   5.337 5.617 6.129 5.885 5.717 2.874 3.18 1.322 3.94 3.076
   4231   1385828_at   5.824 5.719 5.83 6.155 5.935 6.643 6.713 6.488 6.358 6.841
   4232   1390205_at   8.055 8.129 8.453 8.287 8.298 7.687 7.775 7.76 7.673 7.456
   4233   1395628_at   4.839 4.377 4.677 4.236 4.091 5.552 5.456 5.331 5.421 5.913
   4234   1374860_at   7.249 7.145 6.993 7.131 7.216 7.524 7.533 7.807 7.62 7.509
   4235   1391571_at   5.182 5.064 5.306 5.47 5.18 6.119 5.718 6.276 5.858 6.048
   4236   1388122_at   11.129 11.106 10.877 11.118 11.231 11.667 11.47 11.645 11.746 11.924
   4237   1393929_at   6.405 5.777 7.112 6.777 6.169 3.885 1.104 1.345 3.709 1.646
   4238   1388354_at   8.983 8.845 8.748 8.797 8.772 8.387 8.492 8.447 8.168 8.212
   4239   1373731_at   7.402 6.929 7.577 7.279 7.386 8.129 8.107 8.169 8.198 7.868
   4240   1374957_at   5.268 5.456 5.327 5.593 5.422 5.832 5.904 5.811 5.745 5.764
   4241   1389238_at   8.141 7.858 7.928 8.32 8.355 7.446 7.151 7.514 7.086 7.281
   4242   1378163_at   5.983 6.364 6.039 6.016 6.628 7.115 6.994 7.387 7.331 7.675
   4243   1398928_at   9.784 9.753 9.783 10.012 10.056 10.294 10.25 10.31 10.367 10.302
   4244   1388135_at   8.067 8.071 8.285 8.213 7.949 7.179 7.301 7.551 6.799 7.359
   4245   1368141_at   11.693 11.628 11.475 11.46 11.564 11.247 11.278 11.25 11.125 11.102
   4246   1380884_at   4.006 4.388 4.593 4.358 3.601 5.536 5.239 5.431 5.341 5.241
   4247   1376159_at   7.176 6.932 7.069 6.934 7.017 7.503 7.374 7.609 7.437 7.331
   4248   1370147_at   8.623 8.468 9.002 8.474 7.612 9.906 10.269 9.721 10.086 9.989
   4249   1371752_at   8.176 8.063 8.405 8.355 8.41 8.746 8.966 8.749 8.765 8.764
   4250   1387357_at   8.636 8.773 8.933 8.888 8.097 9.661 9.512 9.677 9.851 9.871
   4251   1374519_at   7.864 8.069 7.697 7.777 7.917 7.154 7.227 7.365 6.864 6.824
   4252   1387658_at   6.219 6.243 5.556 6.161 6.31 4.278 4.787 4.937 5.172 4.901
   4253   1384752_at   5.591 5.532 5.978 5.905 5.686 3.575 3.493 1.124 2.687 1.449
   4254   1379683_at   6.355 6.14 5.863 5.844 6.144 6.922 6.782 7.152 6.678 6.804
   4255   1373790_at   7.746 7.843 7.879 7.503 7.756 8.806 9.208 8.833 8.999 8.266
   4256   1367677_at   11.702 11.715 11.746 11.759 11.862 11.384 11.261 11.373 11.45 11.552
   4257   1368453_at   6.829 6.973 6.977 7.228 6.996 7.644 7.534 7.781 8.134 8.021
   4258   1368785_a_at   6.291 6.179 6.46 6.522 6.401 7.074 7.272 7.265 6.842 6.882
   4259   1392134_x_at   6.737 6.751 6.825 6.546 6.772 6.186 5.391 5.289 5.725 5.785
   4260   1382234_at   6.756 6.651 6.504 6.782 6.559 7.03 7.022 7.217 7.014 7.187
   4261   1391485_at   3.172 4.411 4.773 4.868 4.66 6.357 6.084 6.884 6.936 6.886
   4262   1372617_at   7.674 7.881 7.86 8.14 8.05 8.653 8.333 8.542 8.585 8.571
   4263   1373978_at   9.372 9.409 9.732 9.524 9.09 10.144 10.445 10.248 10.179 10.046
   4264   1373338_at   7.546 7.618 7.645 7.568 7.382 8.456 7.956 8.452 8.721 8.649
   4265   1387483_at   6.189 6.309 5.794 6.158 6.439 5.134 4.045 4.099 4.261 3.251
   4266   1373634_at   8.755 8.831 8.523 8.781 8.855 9.244 9.419 9.513 9.256 9.139
   4267   1374376_at   7.043 6.848 6.844 7.3 7.163 7.762 7.86 7.982 7.969 7.518
   4268   1369989_at   8.399 8.376 8.457 8.334 8.315 8.722 8.607 8.894 8.902 8.95
   4269   1391653_at   3.444 2.287 2.941 2.897 2.889 0.88 0 1.104 0 1.459
   4270   1391206_at   5.914 6.296 6.205 6.128 6.237 6.725 6.594 6.684 6.634 6.52
   4271   1372772_at   7.827 8.012 8.181 8.132 7.961 8.718 8.44 8.731 8.518 8.483
   4272   1389307_at   5.903 5.622 5.912 5.838 5.793 6.442 6.252 6.254 6.627 6.632
   4273   1374160_at   5.288 5.168 5.488 5.499 5.943 6.476 6.484 6.244 6.494 6.842
   4274   1380346_at   9.303 9.082 9.65 9.228 9.282 8.724 8.356 8.589 8.651 8.653
   4275   1388312_at   8.081 7.518 7.919 8.083 8.313 9.252 8.738 9.255 9.367 8.917
   4276   1372273_at   8.433 8.508 8.687 8.673 8.664 7.87 7.547 7.879 7.777 7.126
   4277   1393162_at   7.06 7.159 6.863 7.202 7.335 6.315 6.493 6.547 6.02 6.088
   4278   1382291_at   5.188 5.233 5.051 4.961 4.913 4.238 4.163 4.662 3.966 4.22
   4279   1384889_at   6.45 6.274 6.506 6.653 6.578 6.09 5.71 6.008 5.899 5.871
   4280   1391091_at   6.938 6.958 7.052 7.186 6.901 6.368 5.851 5.604 5.568 5.156
   4281   1376285_at   5.979 6.81 6.519 6.83 6.711 7.839 7.578 8.032 7.479 7.783
   4282   1389576_at   8.535 8.282 8.389 8.435 8.417 7.799 8.066 7.633 7.929 7.613
   4283   1378092_at   7.784 7.786 7.79 7.881 8.035 7.537 7.357 7.477 7.272 7.229
   4284   1370220_at   11.816 11.77 11.823 11.772 11.871 12.039 12.206 12.071 12.14 11.978
   4285   1388700_at   8.278 8.36 7.965 8.219 8.285 7.726 7.778 7.566 7.575 7.377
   4286   1374688_at   7.272 7.233 7.332 7.245 6.908 7.902 7.625 7.791 7.678 7.821
   4287   1368078_at   7.304 7.325 7.709 7.563 7.199 8.308 8.058 8.234 8.029 7.999
   4288   1395561_at   10.444 10.37 10.587 10.643 10.643 10.256 9.909 9.959 9.948 9.913
   4289   1372507_at   8.797 8.795 9.069 9.074 8.705 9.372 9.341 9.586 9.433 9.499
   4290   1391453_at   6.194 6.055 6.663 6.575 6.155 5.475 5.333 5.263 5.514 5.653
   4291   1372787_at   8.912 9.164 8.656 9.079 9.185 8.355 8.283 8.47 8.255 8.316
   4292   1398860_at   11.559 11.555 11.507 11.653 11.68 11.346 11.383 11.306 11.389 11.389
   4293   1389396_at   6.185 6.231 6.67 6.362 6.014 7.035 6.965 6.974 7.29 7.161
   4294   1382313_at   5.436 5.53 5.572 5.005 5.928 6.364 6.714 6.472 6.574 6.408
   4295   1377162_at   6.511 6.444 6.245 6.465 6.415 7.021 6.966 7.243 6.975 6.746
   4296   1388742_at   7.542 7.466 8.001 7.995 7.426 6.916 6.403 6.723 6.656 6.258
   4297   1384438_at   6.487 6.575 7.28 7.369 7.102 8.077 8.08 8.623 8.261 8.299
   4298   1380170_at   7.662 7.826 7.582 7.881 7.567 8.152 8.038 8.166 8.236 8.181
   4299   1378088_at   6.704 6.641 6.25 6.375 6.819 7.349 7.14 7.257 7.268 7.409
   4300   1379264_at   9.443 9.287 9.331 9.618 9.239 9.733 9.945 10.091 9.987 10.042
   4301   1370083_at   7.127 7.201 7.129 7.054 7.881 4.711 4.603 3.396 2.573 1.551
   4302   1372719_at   8.617 8.754 8.559 8.91 8.929 8.226 7.996 8.123 8.289 7.91
   4303   1385783_at   7.576 7.689 7.799 8.091 7.462 8.499 8.339 8.46 8.47 8.373
   4304   1382869_at   4.31 4.323 4.098 3.654 4.485 5.12 5.051 5.335 5.11 5.061
   4305   1390221_at   7.035 7.315 7.564 7.492 6.898 5.683 4.13 5.611 4.779 5.618
   4306   1383824_at   5.973 5.353 5.132 5.212 5.879 6.881 6.751 6.834 7.486 6.689
   4307   1395781_at   5.462 5.147 5.472 5.167 5.006 5.936 6.044 5.789 5.871 6.155
   4308   1389806_at   3.981 3.738 3.379 4.475 3.506 5.109 5.197 5.093 5.006 4.939
   4309   1394957_at   7.308 7.461 7.568 7.589 7.898 8.146 8.365 8.357 8.418 8.129
   4310   1372902_at   7.357 7.63 7.574 7.665 7.632 6.968 6.793 7.206 7.103 6.895
   4311   1373955_at   9.034 9.142 9.212 9.277 9.233 8.646 8.896 8.703 8.687 8.477
   4312   1384943_at   5.588 5.57 6.335 5.893 5.37 4.678 4.474 4.002 3.688 4.297
   4313   1370879_at   9.837 9.887 10.028 10.134 10.059 10.524 10.3 10.611 10.677 10.492
   4314   1388533_at   9.183 9.232 9.316 9.414 9.159 9.754 9.529 9.794 9.915 9.853
   4315   1375119_at   10.271 10.215 10.084 10.237 10.457 10.877 10.788 10.931 10.725 10.593
   4316   1371474_at   10.226 10.182 10.285 10.257 10.311 10.546 10.399 10.557 10.646 10.545
   4317   1370238_at   12.068 12.014 11.971 11.864 11.931 12.297 12.187 12.182 12.284 12.322
   4318   1388745_at   9.773 9.883 9.679 10.082 9.927 9.442 9.446 9.411 9.289 9.251
   4319   1371524_at   8.338 8.483 8.485 8.442 8.583 7.984 8.13 7.965 7.722 7.716
   4320   1367537_at   8.008 8.04 8.059 8.074 8.08 7.722 7.308 7.686 7.635 7.498
   4321   1376214_at   8.106 8.115 8.169 7.657 7.876 8.587 8.943 8.691 8.983 8.651
   4322   1376989_at   6.142 6.255 5.936 6.462 6.58 7.073 6.901 7.375 7.322 7.38
   4323   1376188_at   5.228 6.155 6.484 5.747 5.86 4.687 4.386 4.669 4.357 4.365
   4324   1395518_at   8.271 8.445 8.476 8.369 8.261 9.011 8.932 9.231 9.475 8.882
   4325   1378002_at   6.201 6.378 5.883 6.3 6.304 6.783 6.708 6.842 6.751 6.812
   4326   1379959_at   6.788 6.611 6.548 6.602 6.86 7.096 7.079 7.24 7.142 7.034
   4327   1388113_at   12.375 12.293 12.411 12.24 12.253 12.676 12.569 12.58 12.654 12.809
   4328   1382438_at   3.516 4.089 3.888 3.081 3.335 5.185 4.527 5.108 5.455 5.069
   4329   1373137_at   6.91 7.021 7.095 7.394 7.037 7.771 7.498 7.863 7.848 7.904
   4330   1376878_at   8.484 8.349 8.579 8.934 8.778 9.755 9.214 9.685 9.462 9.446
   4331   1374871_at   8.952 8.922 9.094 9.236 8.483 10.03 9.552 10.072 10.123 10.097
   4332   1380923_at   3.811 3.69 4.222 3.617 4.52 5.257 4.91 5.037 5.314 5.286
   4333   1377141_at   8.016 8.087 8.013 8.192 8.165 8.588 8.993 8.957 8.859 8.481
   4334   1371420_at   7.581 7.492 7.683 7.926 7.642 8.452 8.083 8.539 8.428 8.69
   4335   1393183_at   8.161 8.342 8.202 8.312 8.419 7.856 7.871 7.448 7.66 7.76
   4336   1369301_at   2.561 1.989 3.934 2.26 2.31 4.979 4.51 4.966 4.734 4.99
   4337   1393320_at   7.579 7.781 7.542 7.869 7.757 7.224 7.153 7.364 7.02 7.125
   4338   1372365_at   9.948 9.942 10.198 10.184 10.169 9.554 9.408 9.741 9.584 9.365
   4339   1398360_at   9.8 9.668 9.981 10.011 9.871 10.341 10.175 10.348 10.391 10.345
   4340   1375027_at   8.553 8.619 8.583 8.609 8.779 9.049 9.006 9.366 9.076 9.038
   4341   1372650_at   7.622 7.35 7.729 7.453 7.531 7.953 7.915 7.939 7.991 8.023
   4342   1397278_at   5.031 5.003 4.74 5.131 4.848 5.915 5.556 5.612 5.402 5.676
   4343   1395532_at   8.57 8.602 8.45 8.762 8.448 8.994 8.874 9.029 9.023 8.939
   4344   1393647_at   9.725 9.761 9.87 10.037 9.917 9.319 9.169 9.541 9.195 9.363
   4345   1377765_at   9.777 9.679 9.688 9.838 9.669 9.329 9.389 8.961 9.195 9.053
   4346   1399033_at   9.972 10.128 10.171 9.875 10.001 9.54 9.317 9.528 9.21 9.011
   4347   1370574_a_at   7.141 6.948 7.033 7.309 7.224 6.674 6.658 6.516 6.567 6.776
   4348   1385912_at   6.055 6.314 6.973 6.467 5.823 7.87 7.658 7.772 7.868 7.328
   4349   1389662_at   6.559 6.629 6.017 6.737 6.799 7.383 7.629 7.817 7.465 7.442
   4350   1387420_at   9.219 9.259 9.49 9.624 9.293 8.918 8.914 8.67 8.79 8.756
   4351   1371521_at   9.315 9.283 9.396 9.503 9.638 8.964 8.972 9.054 8.816 8.968
   4352   1376847_at   6.079 6.317 6.776 6.437 5.805 7.453 7.98 8.22 7.616 7.358
   4353   1398615_at   8.325 8.193 7.843 8.307 8.423 7.65 7.47 7.281 7.477 7.551
   4354   1372789_at   8.086 8.148 7.856 8.085 8.135 8.339 8.479 8.482 8.525 8.42
   4355   1368814_at   9.804 9.88 10.104 9.851 9.342 10.589 10.791 10.542 10.547 10.66
   4356   1388628_at   8.844 8.807 8.62 8.578 8.901 8.285 8.047 8.225 8.263 7.906
   4357   1370217_at   8.119 8.257 7.978 8.489 8.26 8.825 8.903 9.032 8.769 8.721
   4358   1383914_at   6.064 6.192 5.936 6.202 6.391 6.848 6.659 7.272 7.118 6.955
   4359   1390149_at   8.592 8.706 8.958 9.181 8.643 9.568 9.447 9.597 9.472 9.577
   4360   1368910_at   7.267 7.286 7.41 7.19 7.43 7.009 6.868 6.752 6.728 6.581
   4361   1372043_at   8.21 8.375 7.885 8.384 8.28 7.709 7.621 7.634 7.437 7.496
   4362   1397363_at   6.672 6.856 6.85 6.866 6.787 7.243 7.36 7.582 7.601 7.174
   4363   1375696_at   7.428 7.774 7.75 7.486 7.377 6.972 6.928 6.435 6.753 6.528
   4364   1374061_at   9.448 9.509 9.521 9.332 9.637 8.929 8.891 9.205 9.087 8.966
   4365   1388602_at   7.343 7.14 7.463 7.516 7.376 6.196 5.203 6.162 6.394 5.512
   4366   1378214_at   6.079 6.182 5.844 6.369 6.371 7.01 6.653 7.05 7.057 6.939
   4367   1373300_at   6.393 6.314 6.626 6.87 6.539 7.445 7.033 7.546 7.38 7.524
   4368   1370063_at   7.289 7.186 7.456 7.935 7.748 8.537 8.283 8.512 8.678 8.421
   4369   1387372_at   6.678 6.556 6.946 6.831 5.569 7.925 8.066 8.122 8.048 8.213
   4370   1387876_at   6.889 7.045 7.223 6.952 7.041 6.055 5.702 5.873 6.447 6.355
   4371   1389301_at   9.878 9.946 9.839 9.864 9.799 10.275 10.53 10.46 10.228 10.158
   4372   1399166_a_at   7.939 7.905 8.024 8.097 8.082 8.227 8.334 8.434 8.481 8.371
   4373   1394097_at   4.799 4.951 4.587 5.221 4.345 5.666 5.97 6.157 6.031 5.669
   4374   1371811_at   9.069 9.009 9.321 9.195 9.176 9.615 9.59 9.674 9.937 9.594
   4375   1369073_at   8.133 7.964 8.064 7.831 7.607 8.697 8.838 8.808 8.704 8.383
   4376   1374984_at   5.987 6.119 6.208 5.922 5.753 6.915 7.19 7.201 6.78 6.535
   4377   1376100_at   9.396 9.118 9.132 9.213 9.523 8.285 7.932 7.333 6.798 7.005
   4378   1367671_at   11.382 11.308 11.308 11.31 11.201 11.012 10.8 10.952 10.64 10.894
   4379   1389457_at   5.968 6.139 5.695 5.964 6.103 5.333 4.823 5.28 4.615 4.845
   4380   1371915_at   7.089 7.342 7.186 7.678 7.277 7.916 7.895 8.02 8.036 7.942
   4381   1388132_at   8.787 8.829 8.913 9.14 9.217 8.371 8.432 8.469 8.16 8.245
   4382   1393360_at   7.976 7.955 7.624 7.963 7.911 8.375 8.469 8.544 8.296 8.297
   4383   1372911_at   6.579 6.344 6.669 6.411 5.951 7.65 7.136 7.741 8.014 8.179
   4384   1398950_at   8.641 8.931 9.09 9.144 8.76 9.49 9.452 9.543 9.563 9.567
   4385   1367815_at   7.98 8.086 8.31 8.411 7.681 9.038 8.764 8.942 8.983 9.069
   4386   1375491_at   0.595 1.091 0.714 2.585 1.275 3.998 3.325 4.528 4.112 3.391
   4387   1373927_at   6.804 6.574 6.539 6.584 6.594 6.191 5.758 6.036 6.075 5.685
   4388   1398467_at   4.48 5.216 4.784 4.626 5.362 6.509 5.971 6.23 6.388 5.862
   4389   1383398_at   5.284 4.368 4.483 5.336 4.385 6.737 6.502 6.661 5.975 6.177
   4390   1369129_at   5.532 5.708 5.482 5.383 4.967 4.489 3.745 3.762 4.411 3.522
   4391   1388046_at   7.827 7.817 8.107 7.913 7.71 5.535 4.681 3.187 4.109 1.782
   4392   1377998_at   8.348 8.47 8.064 8.209 8.291 7.773 7.747 7.841 7.767 7.509
   4393   1391146_at   3.604 3.566 3.363 3.795 3.878 4.6 4.539 4.924 5.447 4.626
   4394   1372306_at   10.068 10.131 10.232 10.32 10.16 10.663 10.524 10.914 10.926 11.004
   4395   1396449_at   6.526 6.618 6.896 6.778 6.561 6.246 5.735 6.079 5.998 5.93
   4396   1382072_at   5.558 5.159 5.179 5.288 5.355 6.369 5.756 6.593 6.355 6.327
   4397   1370936_at   7.894 8.009 7.865 8.08 7.412 8.445 8.925 9.113 8.826 8.951
   4398   1376936_at   7.532 7.493 7.589 7.639 7.721 7.148 6.873 7.234 6.869 7.157
   4399   1373194_at   7.977 8.139 8.149 7.841 8.072 8.556 8.678 8.671 8.545 8.337
   4400   1388647_at   8.774 8.738 8.824 8.959 9.047 8.358 8.168 8.548 8.339 8.233
   4401   1379742_at   7.316 7.839 7.59 7.625 7.629 6.702 5.418 6.301 6.382 6.078
   4402   1387797_at   11.307 11.255 11.222 11.238 11.372 11.041 11.08 10.969 11.004 10.86
   4403   1375146_at   10.745 10.685 10.862 10.721 10.587 11.342 11.107 11.12 11.304 11.535
   4404   1393544_at   3.67 3.172 3.534 4.044 3.425 4.529 4.734 4.398 4.424 4.663
   4405   1383476_at   6.994 6.988 7.18 7.237 7.24 6.546 6.177 6.694 6.477 6.212
   4406   1385171_s_at   8.033 7.758 8.001 8.028 7.981 8.485 8.721 8.721 8.308 8.791
   4407   1387715_at   2.963 4.24 4.17 3.563 3.497 1.96 0 0.993 0 1.422
   4408   1391328_at   4.695 5.353 5.535 3.259 5.429 2.302 2.157 1.257 2.192 1.183
   4409   1370371_a_at   6.586 6.769 7.068 6.805 6.918 6.016 5.201 5.873 6.023 5.731
   4410   1388172_at   9.301 9.104 9.502 9.19 7.988 10.499 10.78 10.76 10.716 10.691
   4411   1372830_at   8.535 8.556 8.143 8.113 8.379 7.694 7.568 7.66 7.38 7.148
   4412   1387778_at   10.627 10.627 10.672 10.741 10.735 10.481 10.376 10.408 10.213 10.367
   4413   1371085_at   5.194 4.988 5.123 4.806 4.832 6.033 5.725 5.942 5.496 5.624
   4414   1378288_at   7.18 7.13 7.46 7.467 7.339 8.033 7.835 7.714 7.919 7.8
   4415   1371436_at   6.388 5.957 5.869 6.366 6.76 7.414 7.129 7.437 7.416 7.26
   4416   1368600_at   8.637 8.969 9.042 9.331 8.668 9.755 9.698 9.92 10.03 9.702
   4417   1384639_at   6.903 6.865 7.137 7.231 6.56 7.894 7.603 8.027 8.34 8.23
   4418   1382269_at   6.198 6.427 6.297 6.826 6.659 7.23 7.081 7.441 7.382 7.395
   4419   1387296_at   6.416 6.482 6.079 6.507 6.255 7.184 6.839 7.567 7.365 7.234
   4420   1371196_at   5.798 6.069 6.086 5.86 5.818 4.914 2.793 3.917 3.599 3.73
   4421   1395351_at   5.135 4.497 5.249 4.545 5.124 5.987 5.808 5.942 5.954 5.903
   4422   1374572_at   7.323 7.291 7.291 7.524 7.449 7.85 8.052 7.659 7.873 7.992
   4423   1375729_at   5.101 4.794 5.636 5.377 5.1 6.06 6.105 6.557 6.594 6.691
   4424   1376885_at   8.243 8.218 8.57 8.666 8.463 9.211 8.888 9.443 9.245 9.315
   4425   1380033_at   6.447 6.673 6.621 6.659 6.748 6.939 6.931 7.125 7.04 7.011
   4426   1398377_at   7.314 7.217 7.054 7.084 7.066 7.871 7.531 7.511 7.691 7.675
   4427   1372344_at   8.46 8.719 8.707 8.787 8.614 8.975 9.053 9.032 9.107 9.188
   4428   1372138_at   8.76 9.196 9.309 8.956 9.08 9.724 9.661 9.881 9.587 9.843
   4429   1368766_at   5.219 3.849 4.548 4.335 4.383 0.614 0 0.604 2.85 1.511
   4430   1376824_at   6.83 6.833 6.798 7.026 7.059 7.351 7.26 7.583 7.525 7.347
   4431   1377158_at   5.024 5.058 4.455 4.883 4.724 5.712 5.486 5.583 5.635 5.446
   4432   1370351_at   8.596 8.581 8.148 8.563 8.598 7.793 7.96 8.009 7.7 7.791
   4433   1385258_at   6.127 6.196 6.053 6.248 6.017 5.656 5.5 5.166 5.049 4.968
   4434   1387284_at   7.652 7.607 8.073 8.078 7.205 8.644 8.663 8.895 8.89 8.986
   4435   1370561_at   7.861 7.878 7.795 8.12 8.076 7.306 7.121 6.956 7.265 6.581
   4436   1389616_at   6.367 6.311 6.559 6.54 6.634 7.14 7.164 7.048 6.863 6.888
   4437   1386466_at   6.85 6.899 6.781 6.674 6.749 7.428 7.527 7.89 7.359 7.27
   4438   1391503_at   6.068 6.495 6.372 6.715 6.646 7.102 7.234 7.335 7.193 7.478
   4439   1393035_at   7.444 7.705 7.284 7.456 7.412 6.938 6.387 6.683 6.891 6.742
   4440   1380542_at   5.027 5.417 5.18 5.509 5.654 1.696 3.279 3.783 2.846 1.664
   4441   1382318_at   6.708 6.683 6.992 7.022 6.888 7.62 7.239 7.386 7.525 7.464
   4442   1370284_at   12.071 11.933 11.879 11.798 11.896 12.152 12.249 12.239 12.282 12.352
   4443   1391754_at   6.716 6.767 6.953 7.19 7.321 5.901 5.218 4.18 4.595 5.249
   4444   1394414_at   7.941 8.198 8.076 8.28 8.364 8.672 8.863 8.945 8.811 8.593
   4445   1372566_at   8.406 8.288 8.441 8.223 8.355 9.006 8.879 8.879 8.675 8.657
   4446   1374590_at   5.992 6.299 6.434 6.508 6.351 5.624 5.655 5.867 5.52 5.47
   4447   1380450_at   6.286 6.726 7.423 6.888 6.647 5.329 5.593 5.595 5.799 5.371
   4448   1394371_at   7.198 7.114 7.194 7.327 7.005 6.724 6.748 6.758 6.492 6.436
   4449   1377169_at   7.017 7.096 7.197 7.3 7.316 6.829 6.664 6.787 6.649 6.507
   4450   1396984_at   8.182 8.366 8.293 8.293 8.3 7.947 7.399 7.399 7.148 7.328
   4451   1375892_at   9.922 9.905 9.988 9.944 9.871 9.697 9.757 9.775 9.693 9.602
   4452   1374345_at   5.898 6.051 6.473 5.716 5.476 7.235 6.992 7.48 6.848 7.192
   4453   1374631_at   8.043 7.99 8.099 7.884 8.015 8.383 8.41 8.336 8.284 8.2
   4454   1383224_at   8.962 8.903 8.639 8.822 8.77 9.31 9.431 9.734 9.39 9.271
   4455   1389836_a_at   10.851 10.795 11.083 11.037 10.587 11.393 11.502 11.379 11.463 11.577
   4456   1398286_at   7.469 7.544 7.609 8.013 7.343 8.59 8.311 9.115 8.708 9.082
   4457   1371491_at   7.605 7.611 7.678 7.886 7.92 7.366 7.148 7.097 6.932 6.939
   4458   1387009_at   8.488 8.537 8.674 8.732 8.594 9.106 8.838 9.171 9.197 9.178
   4459   1386573_a_at   6.579 6.723 6.547 6.352 6.763 7.068 7.037 7.169 7.046 7.023
   4460   1371091_at   6.199 6.21 6.525 6.432 6.317 6.809 6.88 7.176 6.997 6.778
   4461   1388768_at   6.147 6.447 6.659 6.183 6.262 5.791 5.455 5.763 5.453 5.524
   4462   1385025_a_at   7.577 7.615 7.884 7.899 7.617 8.27 8.392 8.185 8.39 8.115
   4463   1390557_at   7.949 7.625 7.606 7.533 7.628 8.416 8.201 8.384 8.266 8.056
   4464   1372092_at   7.938 8.243 8.098 8.337 8.111 8.725 8.729 8.896 8.692 8.508
   4465   1379382_at   5.877 5.967 5.808 6.009 5.813 6.342 6.448 6.819 6.657 6.344
   4466   1373499_at   8.892 8.888 8.982 8.952 9.265 8.474 8.326 8.089 8.059 7.775
   4467   1393051_at   5.569 5.989 6.055 5.842 6.285 6.968 6.861 7.09 6.634 6.679
   4468   1369947_at   6.892 6.35 6.079 6.288 6.538 7.549 7.34 7.793 7.632 7.181
   4469   1373088_at   9.715 9.562 9.907 9.99 9.936 10.424 10.219 10.451 10.366 10.482
   4470   1397836_at   4.508 5.001 5.054 5.422 4.844 6.409 5.83 6.095 5.93 6.029
   4471   1369634_at   5.273 6.003 6.086 6.028 5.624 6.785 6.728 7.16 7.185 6.806
   4472   1373106_at   9.964 9.941 9.803 9.952 9.892 9.535 9.575 9.731 9.62 9.469
   4473   1380553_at   6.931 7.157 7.152 7.038 6.926 6.569 6.726 6.309 6.348 6.444
   4474   1385553_at   8.864 8.975 8.974 8.957 9.389 8.431 8.332 8.346 8.191 7.917
   4475   1372951_at   7.675 7.361 7.523 7.337 7.331 6.813 6.456 6.916 6.808 6.915
   4476   1395363_at   6.964 6.825 6.971 7.222 7.011 6.414 6.242 6.504 6.471 6.622
   4477   1388883_at   8.053 8.034 8.54 8.378 8.246 8.876 8.901 8.816 9.172 9.069
   4478   1374411_at   9.096 9.016 9.065 9.078 9.257 8.756 8.912 8.76 8.761 8.76
   4479   1389353_at   6.92 6.965 6.495 6.948 6.785 7.602 7.334 7.946 7.938 7.944
   4480   1369526_at   6.699 6.566 7.065 6.541 6.448 7.374 7.356 7.227 7.479 7.453
   4481   1388342_at   8.1 8.037 7.926 8.042 7.862 7.53 7.296 7.383 7.214 6.859
   4482   1389816_at   7.023 6.862 7.236 6.859 7.025 7.8 7.576 7.775 7.417 7.932
   4483   1368395_at   7.294 7.063 6.586 6.993 7.632 8.219 8.204 8.259 8.115 8.126
   4484   1386887_at   12.017 11.972 11.914 11.83 11.987 12.178 12.201 12.129 12.131 12.192
   4485   1388928_at   6.795 6.831 6.897 6.956 6.988 7.814 7.466 7.529 7.398 7.263
   4486   1388456_at   8.11 7.992 8.339 8.074 7.91 8.844 8.506 8.88 8.893 9.153
   4487   1393427_s_at   6.013 6.089 6.441 6.29 6.872 7.444 7.127 7.447 7.726 7.721
   4488   1388453_at   9.247 9.169 9.413 9.566 9.216 8.954 8.786 8.783 8.741 8.657
   4489   1395788_at   6.663 6.583 6.344 5.893 6.549 3.023 0 3.062 2.969 0
   4490   1371695_at   8.488 8.426 8.204 8.59 8.553 7.996 7.896 7.986 7.623 7.808
   4491   1372080_at   10.757 10.703 10.798 10.785 10.857 11.062 11.039 11.367 11.184 11.205
   4492   1390832_at   8.757 8.636 8.259 8.933 9.036 7.763 7.772 8.013 7.813 7.542
   4493   1367787_at   6.17 6.397 5.911 6.263 6.477 6.824 6.979 7.008 6.889 6.79
   4494   1378250_at   4.705 4.801 4.994 4.755 4.319 5.685 5.415 5.468 5.885 5.453
   4495   1375083_at   6.801 6.795 6.463 7.114 6.645 7.582 7.348 7.517 7.435 7.428
   4496   1382642_at   8.104 8.1 8.098 8.361 8.405 7.391 7.402 7.788 7.46 7.11
   4497   1368150_at   11.133 11.061 11.175 11.068 10.51 11.657 11.737 11.746 11.837 11.79
   4498   1390380_at   8.285 8.276 8.106 8.515 8.466 8.859 8.682 8.919 8.944 8.892
   4499   1370009_at   8.183 7.929 8.273 8.215 7.909 8.84 9.313 9.234 8.798 8.715
   4500   1398447_at   7.163 7.135 6.946 7.466 7.241 7.99 7.733 8.004 7.977 7.657
   4501   1387513_at   5.241 5.091 4.716 5.219 5.26 6.196 5.774 5.926 6.151 5.728
   4502   1368177_at   7.003 6.898 7.182 7.3 6.989 7.734 7.612 8.056 8.162 8.281
   4503   1393404_at   5.005 5.475 5.287 5.529 5.23 5.931 6.075 6.358 6.279 5.891
   4504   1389585_at   6.401 6.546 6.902 6.786 6.54 7.294 7.351 7.153 7.544 7.619
   4505   1374722_at   6.929 7.048 6.407 6.742 7.33 5.22 5.355 6.086 5.532 5.7
   4506   1373618_at   6.718 6.625 6.395 6.48 6.481 7.291 6.909 7.513 7.203 7.218
   4507   1388669_at   8.469 8.627 8.366 8.659 9.032 8.032 7.936 7.813 7.706 7.794
   4508   1385773_at   5.767 5.679 6.344 5.939 6.051 7.013 7.286 7.116 7.501 6.612
   4509   1367846_at   9.534 9.84 9.929 9.833 10.101 9.28 8.888 8.995 9.295 8.952
   4510   1375303_at   3.248 2.801 3.895 2.398 3.025 4.722 4.621 5.063 4.52 4.566
   4511   1372808_at   7.554 7.619 7.696 8.064 7.575 3 5.373 3.961 5.69 5.063
   4512   1383706_at   5.009 5.222 5.609 5.307 5.108 5.948 6.055 6.218 6.553 6.018
   4513   1388326_at   10.647 10.634 10.632 10.538 10.64 11.041 11.111 10.775 11.024 10.973
   4514   1398792_at   9.846 9.8 9.75 9.83 9.934 9.452 9.325 9.593 9.58 9.421
   4515   1367969_at   10.077 10.009 10.268 10.514 10.38 10.773 10.81 10.953 10.838 10.865
   4516   1367795_at   8.855 8.963 9.087 9.254 8.882 8.339 8.195 8.129 7.739 7.53
   4517   1393743_at   8.025 7.956 7.926 8.065 8.054 8.572 8.506 9.017 8.582 8.471
   4518   1380373_at   7.484 7.448 7.441 7.595 7.636 7.003 7.101 7.192 6.776 7.078
   4519   1398573_at   7.376 7.418 7.396 7.489 7.307 7.915 8.065 8.014 7.775 7.661
   4520   1369167_at   4.78 4.408 4.442 3.402 4.584 6.149 6.204 5.637 5.803 5.814
   4521   1380174_at   5.51 5.381 5.569 5.67 5.695 6.136 5.993 6.556 6.268 6.188
   4522   1395816_at   3.555 3.881 3.384 3.684 3.175 4.162 4.483 4.346 4.516 4.323
   4523   1369286_at   7.269 6.945 7.311 7.395 6.728 7.956 7.902 8.528 8.135 8.26
   4524   1369500_at   6.479 6.776 6.795 6.588 6.65 7.543 7.407 7.382 7.02 7.208
   4525   1390122_at   7.459 7.423 7.343 7.377 7.839 6.958 6.766 6.917 6.624 6.559
   4526   1369795_at   4.394 4.777 4.626 4.9 4.499 5.887 5.23 5.745 5.513 5.377
   4527   1389244_x_at   7.113 7.043 7.289 7.514 7.264 6.014 5.705 6.489 5.145 5.477
   4528   1390609_at   2.313 2.621 1.888 2.628 3.074 3.626 4.574 3.977 4.053 3.883
   4529   1389023_at   9.812 9.833 10.11 9.989 10.027 10.383 10.406 10.673 10.413 10.358
   4530   1368067_at   7.998 8.218 8.334 8.302 8.169 8.821 8.598 8.716 8.801 8.54
   4531   1373407_at   6.809 6.739 7.036 7.095 6.938 7.476 7.662 7.34 7.405 7.385
   4532   1382776_at   7.899 7.976 7.898 8.045 8.353 7.3 7.142 7.53 7.257 6.898
   4533   1398353_at   9.904 10.081 10.184 10.197 10.183 9.749 9.764 9.819 9.709 9.728
   4534   1379636_at   5.829 5.422 5.778 6.185 5.903 6.54 6.524 6.891 6.82 6.958
   4535   1383429_at   9.864 10.008 10.149 10.003 10.174 9.535 9.527 9.546 9.125 9.208
   4536   1382419_at   7.259 7.349 7.033 7.329 7.111 6.593 6.597 6.82 6.286 6.375
   4537   1379396_at   6.772 6.576 7.1 7.211 7.249 5.806 5.479 5.804 6.159 6.14
   4538   1381982_at   8.987 8.916 9.007 9.196 9.083 9.349 9.423 9.426 9.297 9.332
   4539   1388143_at   8.886 8.84 8.697 9.064 9.133 9.605 9.379 9.574 9.604 9.364
   4540   1389734_x_at   8.196 8.265 8.109 8.218 8.457 7.808 7.349 7.593 7.687 7.269
   4541   1387888_at   12.115 12.071 12.076 12.124 12.247 11.955 11.921 11.877 11.857 11.824
   4542   1392338_at   3.44 1.531 1.687 1.996 1.709 5.084 3.926 4.455 5.299 4.518
   4543   1379027_at   6.484 6.767 7.102 7.392 7.059 8.054 8.066 8.264 7.824 7.864
   4544   1398774_at   12.121 12.162 12.157 12.085 12.165 11.965 11.986 11.837 11.958 11.832
   4545   1389163_at   8.08 8.032 8.111 8.252 8.288 8.608 8.538 8.71 8.607 8.414
   4546   1372360_at   9.489 9.65 9.573 9.45 9.454 8.678 9.026 8.822 8.95 8.353
   4547   1388880_at   9.386 9.161 9.271 9.41 9.469 8.953 8.645 8.472 8.779 8.411
   4548   1386938_at   10.523 10.45 10.841 10.898 10.42 11.179 11.2 11.321 11.331 11.329
   4549   1368174_at   5.386 5.872 5.635 5.793 5.205 6.488 6.375 6.769 6.577 7.108
   4550   1390993_at   7.758 7.74 7.836 7.736 7.668 8.21 8.099 8.377 8.598 8.21
   4551   1396309_at   5.666 5.324 5.927 5.704 5.839 6.394 6.44 6.27 6.277 6.512
   4552   1391897_at   6.903 6.709 6.704 6.831 6.606 5.986 6.159 6.244 6.03 5.594
   4553   1376809_at   6.187 6.34 6.285 6.314 6.402 6.797 6.512 6.914 6.86 6.848
   4554   1391837_at   7.148 7.186 7.202 7.178 7.241 6.9 6.696 6.331 6.62 6.518
   4555   1381350_at   5.942 6.138 5.935 6.384 5.54 7.035 6.727 7.448 7.044 7.262
   4556   1371602_at   8.255 8.267 8.131 8.545 8.685 9.298 8.897 9.301 9.169 9.074
   4557   1388631_at   8.141 7.929 8.052 8.171 8.237 7.694 7.637 7.514 7.736 7.369
   4558   1393163_at   4.814 4.716 4.677 4.868 5.036 5.614 5.582 5.174 5.626 5.808
   4559   1376831_at   3.999 4.674 4.082 4.335 4.787 6.098 5.364 5.339 5.698 5.693
   4560   1372428_at   6.486 6.273 6.466 6.391 6.162 6.951 6.797 7.048 7.018 6.7
   4561   1370365_at   10.311 9.787 9.865 10.119 9.544 10.871 10.584 10.884 11.041 11.096
   4562   1390483_at   6.647 6.11 6.785 6.604 6.55 7.195 7.354 7.451 7.224 7.225
   4563   1389739_at   6.058 5.956 6.15 6.382 5.356 7.42 7.058 7.924 8.179 7.356
   4564   1370504_a_at   6.926 6.616 6.906 7.051 6.908 7.532 7.457 7.591 7.257 7.373
   4565   1367506_at   9.468 9.423 9.363 9.656 9.505 9.826 9.797 9.91 10.06 9.866
   4566   1385904_at   6.468 6.852 6.693 6.857 6.777 7.775 7.92 7.462 7.903 7.226
   4567   1373903_at   7.6 7.621 7.686 7.638 7.645 7.341 6.968 7.157 7.315 7.283
   4568   1389941_at   8.859 8.951 9.306 9.3 9.188 9.888 9.568 9.995 9.836 9.813
   4569   1390814_at   8.366 8.615 8.724 8.803 8.744 8.241 7.87 8.127 7.929 7.955
   4570   1396317_at   6.819 6.911 6.525 7.132 6.966 7.658 7.465 8.016 7.713 7.6
   4571   1388579_at   5.891 6.236 6.127 5.641 5.581 7.031 6.576 7.046 7.092 7.445
   4572   1367597_at   12.497 12.448 12.449 12.457 12.6 12.349 12.236 12.177 12.195 12.171
   4573   1371754_at   7.329 6.849 7.176 7.117 7.112 6.407 6.346 6.371 5.946 5.713
   4574   1381508_at   6.131 6.39 6.369 6.423 6.534 7.112 6.988 6.909 7.464 6.974
   4575   1393234_at   7.853 7.99 7.92 8.048 8.093 7.613 7.505 7.602 7.481 7.226
   4576   1367520_at   9.033 9.313 9.247 9.311 9.337 9.587 9.61 9.603 9.74 9.594
   4577   1382934_at   6.935 6.75 6.397 6.608 6.713 6.241 6.035 5.879 6.059 5.965
   4578   1368208_at   8.236 8.326 8.925 8.839 7.349 9.951 9.874 10.219 10.488 10.495
   4579   1372574_at   5.382 4.659 4.985 5.731 5.718 6.601 6.558 6.993 6.773 6.466
   4580   1370785_s_at   10.28 10.33 10.403 10.355 10.253 10.649 10.649 10.64 10.469 10.555
   4581   1391440_at   7.221 7.18 7.39 7.249 7.525 6.787 6.36 6.5 6.429 5.939
   4582   1384352_at   7.419 7.685 7.39 7.404 7.562 6.608 7.055 6.872 6.691 7.033
   4583   1372900_at   9.226 9.234 9.09 9.34 9.069 9.617 9.51 9.639 9.484 9.505
   4584   1390825_at   8.451 8.5 8.365 8.351 8.617 8.985 9.198 9.161 9.388 8.819
   4585   1399081_at   8.205 8.24 8.359 8.284 8.131 7.8 7.707 7.878 7.936 7.549
   4586   1384892_at   6.192 6.609 6.457 6.683 6.69 4.662 5.79 5.266 5.383 5.372
   4587   1370277_at   12.35 12.294 12.342 12.228 12.264 12.649 12.579 12.487 12.517 12.725
   4588   1368278_at   5.341 4.795 4.833 4.159 5.493 6.638 6.295 6.382 6.325 6.318
   4589   1388626_at   8.71 8.624 8.713 8.822 8.662 8.984 8.939 9.2 9.124 9.029
   4590   1370005_at   8.938 8.907 9.07 9.022 8.786 9.509 9.321 9.631 9.505 9.874
   4591   1374399_at   7.847 7.901 7.36 7.56 7.803 7.054 6.59 6.892 7.065 6.716
   4592   1398250_at   9.051 9.119 9.652 9.278 9.164 9.954 10.019 10.092 10.143 9.78
   4593   1374834_at   9.465 9.408 9.424 9.571 9.427 9.241 9.135 9.292 9.218 9.236
   4594   1371232_a_at   7.328 7.261 7.335 7.732 8.531 6.062 6.08 5.836 5.157 6.095
   4595   1379284_at   6.544 6.687 6.843 6.729 6.773 6.29 6.076 6.372 6.055 6.314
   4596   1371509_at   10.506 10.445 10.309 10.278 10.372 9.971 9.938 9.787 9.659 9.462
   4597   1388590_at   8.47 8.37 8.976 8.87 8.743 7.934 7.939 8.081 7.847 7.724
   4598   1375117_at   7.184 7.312 7.495 7.381 7.299 7.876 7.726 7.846 7.6 7.751
   4599   1377433_at   6.151 5.836 6.086 5.946 6.165 6.595 6.416 6.926 6.679 6.7
   4600   1369982_at   9.775 9.738 9.712 9.765 9.793 9.958 10.024 10.029 10.058 9.86
   4601   1394136_at   7.456 7.252 7.239 7.165 7.257 7.008 6.94 6.992 6.926 6.971
   4602   1369493_at   6.942 7.1 7.252 7.578 6.403 8.247 8.044 8.593 8.495 8.41
   4603   1370482_at   7.801 7.765 7.882 7.883 7.835 8.415 8.069 8.453 8.634 8.505
   4604   1367824_at   9.533 9.548 9.406 9.59 9.725 9.166 9.042 9.281 9.134 8.978
   4605   1368648_at   3.546 3.94 4.394 4.128 3.65 4.869 5.202 5.446 5.61 6.036
   4606   1372471_at   9.116 9.214 9.026 9.281 9.229 8.914 8.614 8.744 8.526 8.597
   4607   1392497_at   7.887 7.899 7.763 7.648 8.017 7.364 7.285 7.31 7.017 6.899
   4608   1383712_at   7.98 7.927 7.963 8.027 8.107 7.616 7.204 7.659 7.282 7.338
   4609   1391551_at   4.833 5.213 4.945 5.504 4.756 6.197 6.013 6.778 6.106 6.839
   4610   1382006_at   8.443 8.267 8.419 8.314 8.325 8.744 8.765 8.913 8.756 8.558
   4611   1385668_at   5.793 5.577 5.973 5.469 5.374 6.251 6.363 6.69 6.384 6.444
   4612   1368942_at   1.233 1.799 2.483 2.676 1.864 4.589 4.643 4.565 3.24 5.027
   4613   1383642_at   5.189 5.018 5.474 5.45 5.381 6.037 5.973 6.04 6.614 6.227
   4614   1370675_at   7.174 7.26 7.509 7.703 7.214 8.097 8.038 8.337 8.057 7.948
   4615   1373072_at   9.981 10.05 10.02 9.947 10.018 9.67 9.394 9.51 9.24 9.082
   4616   1374885_at   7.365 7.573 7.105 7.439 7.286 7.826 7.89 8.201 8.275 8.075
   4617   1385702_at   8.347 8.654 8.501 8.247 8.758 7.489 6.425 5.426 5.925 5.48
   4618   1391282_at   7.787 7.899 7.71 7.841 7.748 8.474 8.144 8.752 8.558 8.32
   4619   1378872_at   0.214 1.485 0.506 0.696 0.774 4.785 3.869 4.056 4.78 2.121
   4620   1378572_at   7.8 7.808 8.032 7.819 7.734 7.416 7.452 7.409 7.579 7.525
   4621   1398496_at   6.293 6.233 6.173 6.302 6.191 6.818 6.559 6.713 6.466 6.619
   4622   1377173_at   7.229 6.873 7.273 7.18 7.272 6.297 5.976 6.625 6.434 6.489
   4623   1379687_at   9.259 9.211 9.037 9.102 8.85 8.4 8.351 8.287 7.934 7.647
   4624   1396408_at   5.296 5.791 5.84 5.619 5.364 6.781 6.56 6.672 6.917 6.14
   4625   1376109_at   8.813 8.83 8.654 8.967 9.107 8.129 7.834 8.004 7.386 7.208
   4626   1371478_at   10.956 10.978 11.148 11.098 11.01 10.777 10.51 10.764 10.575 10.548
   4627   1392219_at   4.081 3.536 4.216 3.872 3.959 5.102 4.759 4.888 4.657 4.61
   4628   1398904_at   10.575 10.504 10.414 10.471 10.566 10.285 10.143 10.316 10.178 10.115
   4629   1374323_at   9.456 9.336 8.966 9.328 9.451 8.765 8.459 8.791 8.612 8.446
   4630   1383164_at   4.191 3.758 2.84 3.882 3.658 4.776 5.16 5.267 5.568 5.398
   4631   1388692_at   8.708 8.635 8.836 8.575 8.616 9.036 9.041 9.157 9.232 8.945
   4632   1368992_a_at   9.686 9.573 9.628 9.687 9.43 9.97 9.966 10.19 9.904 10.015
   4633   1393271_at   5.2 4.953 5.108 5.758 4.793 6.63 6.82 6.811 6.392 5.977
   4634   1379363_at   4.088 3.545 3.724 4.128 4.051 4.784 5.659 5.034 4.986 5.716
   4635   1394450_at   5.908 5.789 5.441 6.029 5.568 6.649 6.398 6.466 6.343 6.461
   4636   1380152_at   7.043 7.181 6.898 7.088 6.378 7.576 8.137 8.136 8.025 7.984
   4637   1382355_at   6.26 6.381 6.239 6.547 6.63 5.806 5.656 5.718 5.271 5.817
   4638   1398409_at   8.081 8.301 8.225 8.318 8.365 8.751 8.689 9.036 8.685 8.67
   4639   1384277_at   6.534 6.849 6.624 6.803 6.525 7.135 7.511 7.362 7.407 7.087
   4640   1376194_at   8.539 8.461 8.22 8.833 8.706 9.356 9.284 9.651 9.4 9.127
   4641   1398380_at   6.554 6.789 6.908 7.088 7.177 7.69 7.773 7.617 7.523 7.524
   4642   1383350_at   6.943 7.043 7.153 7.26 6.975 7.684 7.457 7.759 7.669 7.433
   4643   1388539_at   6.799 6.752 6.676 6.75 6.782 7.172 7.471 7.356 7.512 7.034
   4644   1372083_at   9.177 9.197 8.968 9.221 9.285 8.867 8.702 8.809 8.599 8.607
   4645   1381816_at   6.613 6.599 6.811 6.458 6.568 7.075 6.943 7.13 7.03 6.929
   4646   1372108_at   9.35 9.364 9.334 9.252 9.23 9.666 9.644 9.521 9.628 9.471
   4647   1398773_at   10.326 10.27 10.255 10.323 10.374 10.12 10.028 10.118 10.014 9.9
   4648   1390282_at   5.745 5.949 6.185 6.205 5.727 6.957 6.66 7.204 6.832 6.609
   4649   1373860_at   6.749 6.885 7.116 7.324 7.495 8.288 7.83 8.436 8.267 7.993
   4650   1377967_at   7.623 7.816 7.267 7.707 7.805 6.668 6.974 6.707 6.13 6.652
   4651   1374458_at   8.901 8.634 8.309 8.36 8.355 7.585 7.706 7.968 7.75 7.639
   4652   1398943_at   9.432 9.457 9.67 9.607 9.505 9.961 10.065 10.075 9.897 9.785
   4653   1390290_at   7.508 7.64 7.593 7.924 8.083 7.052 7.012 6.993 6.567 6.939
   4654   1382830_at   5.295 5.262 5.155 5.608 4.904 4.145 4.636 4.026 4.304 4.438
   4655   1391539_at   7.696 7.919 8.422 8.02 7.406 8.778 9.087 9.279 9.038 8.922
   4656   1391271_at   5.837 5.541 5.782 5.939 5.676 6.154 6.424 6.687 6.767 6.569
   4657   1387822_at   6.527 6.499 6.845 6.766 6.762 7.129 7.111 7.078 7.196 7.073
   4658   1394397_at   8.776 8.722 8.567 8.639 8.9 8.141 8.401 8.333 8.103 8.121
   4659   1391438_at   7.157 7.461 7.559 7.292 7.56 7.879 7.96 8.38 8.208 8.219
   4660   1383028_at   6.768 6.363 6.998 6.713 6.918 5.928 5.769 6.192 5.742 6.02
   4661   1392726_at   4.675 4.599 4.785 4.848 4.601 5.493 5.309 5.087 5.101 5.209
   4662   1370595_a_at   3.489 4.056 3.91 3.734 4.007 4.589 4.751 4.66 4.355 4.874
   4663   1382146_at   7.174 7.343 7.151 7.111 7.568 8.078 7.914 8.108 8.414 7.809
   4664   1391741_a_at   5.894 5.747 6.381 6.244 5.41 3.722 4.726 4.647 4.626 4.027
   4665   1368824_at   7.653 7.592 7.985 8.132 8.099 8.812 8.46 8.728 8.98 8.697
   4666   1382217_at   8.516 8.438 8.418 8.405 8.573 8.259 8.21 8.316 8.211 8.226
   4667   1399131_at   7.913 7.966 7.836 7.991 8.064 8.184 8.298 8.392 8.247 8.437
   4668   1390407_at   9.762 9.722 9.79 9.765 9.805 9.351 9.41 9.253 9.101 8.887
   4669   1376550_at   0 0 0.774 0.379 0.614 1.428 1.157 1.683 1.795 1.444
   4670   1390101_at   6.15 6.082 6.296 6.528 6.407 6.692 6.83 6.911 6.969 6.862
   4671   1395065_at   6.7 6.631 6.667 7.011 7.024 6.101 6.015 6.148 5.978 6.397
   4672   1382388_at   6.596 6.197 6.604 6.638 6.496 7.059 7.251 6.937 7.093 7.04
   4673   1387910_at   7.211 7.046 7.411 7.547 7.332 8.207 7.712 8.339 8.301 8.281
   4674   1368498_a_at   8.882 8.812 9.24 9.175 8.148 9.994 9.893 10.141 10.259 10.043
   4675   1395944_at   7.044 6.599 7.056 6.927 6.923 6.168 6.335 6.481 6.07 6.261
   4676   1372558_at   9.041 9.122 8.984 9.077 9.076 8.665 8.752 8.749 8.69 8.403
   4677   1367653_a_at   12.043 12.059 12.09 11.96 11.954 12.465 12.354 12.236 12.316 12.541
   4678   1371864_at   9.83 9.779 9.901 9.925 9.676 10.253 10.332 10.348 10.34 10.037
   4679   1384621_at   6.055 6.192 5.835 6.161 5.961 6.595 6.366 6.517 6.466 6.507
   4680   1398798_at   10.336 10.46 10.314 10.324 10.338 9.987 9.995 10.092 9.996 9.73
   4681   1388084_at   6.161 6.162 6.766 5.886 6.224 3.414 1.275 1.722 4.5 2.362
   4682   1383593_at   6.011 6.607 6.607 6.332 6.228 7.015 7.044 7.258 7.024 7.278
   4683   1387340_at   10.144 10.259 10.217 10.217 10.159 10.441 10.315 10.364 10.384 10.441
   4684   1367659_s_at   10.001 10.005 10.017 9.999 10.007 10.309 10.418 10.632 10.815 10.764
   4685   1372517_at   8.342 8.3 8.371 8.852 8.772 7.46 7.637 7.43 6.745 7.52
   4686   1382118_at   4.924 5.334 4.768 5.18 5.335 6.158 6.028 6.809 5.931 6.479
   4687   1380106_at   7.462 7.447 7.415 7.739 7.496 7.122 6.857 6.811 6.67 7.068
   4688   1388895_at   8.294 8.287 8.389 8.437 8.316 7.952 8.043 7.903 7.886 7.61
   4689   1389008_at   8.326 8.278 8.436 8.342 8.53 8.792 8.794 8.983 8.877 8.633
   4690   1384200_at   8.19 7.744 8.361 8.465 8.422 7.351 7.551 7.418 7.413 7.216
   4691   1369518_at   6.198 6.222 6.029 6.212 6.758 7.035 6.937 7.139 7.143 7.02
   4692   1383690_at   6.188 6.813 6.233 6.372 6.795 5.699 5.27 5.2 4.907 5.556
   4693   1398844_at   10.025 10.04 10.131 10.063 10.014 10.434 10.199 10.365 10.464 10.494
   4694   1379783_at   5.79 6.341 5.966 5.93 6.168 6.888 6.752 6.829 6.48 6.828
   4695   1370256_at   9.22 9.25 9.229 9.147 9.409 9.849 9.849 9.89 9.931 9.478
   4696   1390366_at   8.638 8.572 8.525 8.738 8.563 8.088 8.172 8.406 8.249 8.14
   4697   1393806_at   5.748 6.439 6.363 6.62 6.727 7.444 7.466 7.653 7.293 7.395
   4698   1374606_at   5.736 5.636 5.696 5.674 5.63 6.462 6.237 5.943 6.1 6.262
   4699   1379831_at   6.802 7.029 6.959 7.12 7.022 6.442 6.6 6.714 6.498 6.415
   4700   1397507_at   5.876 5.287 5.304 5.444 5.281 3.011 2.924 0.757 2.644 3.48
   4701   1383182_at   6.998 7.312 7.307 7.377 7.247 6.888 6.777 6.757 6.811 6.889
   4702   1374953_at   8.473 8.521 8.249 8.307 8.576 7.883 7.851 7.999 7.982 7.605
   4703   1372012_at   7.828 8.027 8.299 8.256 8.018 9.319 8.694 8.809 9.253 9.447
   4704   1390596_at   0.367 0 0.832 1.384 0 3.542 2.493 3.176 4.682 5.147
   4705   1379653_a_at   6.04 6.172 6.297 6.552 5.628 3.566 2.029 0 3.312 2.475
   4706   1369631_at   9.256 9.275 9.438 9.468 9.093 9.81 9.625 9.753 9.822 9.821
   4707   1384742_at   6.403 7.005 6.144 6.233 6.713 5.4 5.226 5.477 5.562 4.869
   4708   1391388_at   5.545 5.446 5.339 5.401 5.375 4.275 4.646 4.493 3.48 4.152
   4709   1398779_at   9.945 9.906 10.036 10.099 10.02 10.256 10.224 10.188 10.338 10.254
   4710   1395460_at   6.995 6.939 6.743 6.904 6.851 7.822 7.639 7.383 7.444 8.066
   4711   1374783_at   6.69 6.728 7.313 6.987 6.715 7.747 7.765 8.166 7.814 7.552
   4712   1374188_at   9.545 9.539 9.611 9.771 9.753 9.985 9.957 10.28 10.153 10.156
   4713   1382027_at   5.504 6.146 6.694 6.5 6.456 5.07 4.801 4.961 4.434 4.21
   4714   1370506_at   6.945 7.268 7.218 7.12 7.639 8.179 7.877 8.173 8.243 7.867
   4715   1374736_at   7.43 7.473 7.469 7.519 7.333 7.064 7.248 7.196 7.069 7
   4716   1368812_at   5.264 5.22 5.257 5.958 5.375 4.291 3.111 3.687 3.208 4.275
   4717   1384046_at   7.624 7.731 7.781 7.662 7.634 7.998 8.047 7.939 8.212 7.951
   4718   1382330_at   7.121 7.234 7.038 7.29 7.496 7.939 7.719 8.148 7.821 7.778
   4719   1370919_at   9.209 9.132 9.078 9.36 9.42 8.836 8.742 8.749 8.509 8.468
   4720   1373159_at   5.485 5.071 4.876 5.301 5.164 6.072 5.965 6.444 5.994 5.804
   4721   1394310_at   5.422 5.172 5.708 5.69 5.728 6.75 6.139 6.335 6.794 6.615
   4722   1376190_at   7.224 7.33 7.301 7.357 7.433 7.675 7.764 7.781 7.676 8.057
   4723   1377719_a_at   9.531 9.669 9.97 10.089 9.568 10.348 10.455 10.631 10.546 10.76
   4724   1383061_at   8.824 8.814 8.737 8.718 8.619 8.481 8.481 8.378 8.427 8.245
   4725   1369305_at   6.707 6.854 7.026 7.041 7.123 6.457 6.275 5.98 6.06 6.415
   4726   1389550_at   6.436 6.759 6.698 7.017 6.77 7.342 7.297 7.461 7.288 7.245
   4727   1370228_at   10.192 10.176 9.517 10.05 9.523 8.804 8.76 8.906 9.048 8.406
   4728   1383981_at   6.533 6.313 6.657 6.563 6.786 7.394 7.275 7.813 7.291 7.152
   4729   1382381_at   7.757 7.845 7.931 7.964 7.532 8.375 8.199 8.584 8.378 8.402
   4730   1368683_at   6.996 7.68 8.387 8.326 7.258 5.618 6.336 4.678 5.212 4.977
   4731   1388595_at   8.019 7.977 7.902 7.984 8.052 8.341 8.154 8.34 8.471 8.45
   4732   1383161_a_at   9.993 10.021 10.578 10.479 10.136 10.848 11.053 11 11.075 11.224
   4733   1379866_at   5.326 5.203 5.597 5.849 5.351 6.435 6.135 6.759 6.396 6.239
   4734   1372262_at   9.259 9.195 9.478 9.224 9.3 9.889 9.975 10.049 9.861 9.56
   4735   1380100_at   5.404 5.32 5.127 5.479 5.101 5.96 5.92 5.813 5.808 5.652
   4736   1394705_at   8.638 8.776 8.819 8.832 8.506 9.302 9.12 9.235 9.545 9.195
   4737   1371433_at   9.34 9.275 9.214 9.477 9.519 9.73 9.681 9.811 9.722 9.716
   4738   1398254_at   8.041 7.985 8.151 8.292 8.142 8.765 8.423 8.999 8.773 8.851
   4739   1372113_at   9.107 9.005 8.829 9.13 9.229 8.667 8.604 8.514 8.452 8.293
   4740   1367711_at   11.013 11.064 11.049 11.186 11.177 10.801 10.802 10.935 10.851 10.835
   4741   1371164_at   3.747 3.223 3.546 2.757 3.636 0.151 0 0 2.31 0
   4742   1374564_at   6.586 6.699 6.952 6.989 6.799 6.342 5.524 5.705 5.888 5.509
   4743   1393248_at   8.582 8.565 8.283 8.537 8.663 7.906 7.923 8.237 8.005 7.962
   4744   1378954_at   4.918 4.642 4.729 5.087 4.351 5.427 5.49 5.5 5.5 5.56
   4745   1388782_at   8.119 8.041 7.714 8.429 8.426 9.441 8.816 9.471 9.769 9.293
   4746   1383287_at   8.584 8.67 8.84 9.032 8.93 8.408 7.901 7.754 8.009 7.935
   4747   1392636_at   5.646 5.309 5.801 5.631 5.807 6.18 6.419 6.4 6.633 6.194
   4748   1374013_at   6.188 6.311 6.034 6.158 7.323 7.938 7.693 8.134 8.008 7.659
   4749   1379031_at   7.104 7.413 7.325 7.27 7.537 8.054 7.768 8.195 8.088 7.802
   4750   1383439_at   5.279 4.938 5.071 5.163 4.806 6.062 5.664 5.706 5.854 5.548
   4751   1382200_at   5.89 6.075 5.62 6.175 6.017 6.501 6.619 6.601 6.45 6.676
   4752   1374011_at   8.559 8.747 8.575 8.711 8.65 8.246 8.303 8.295 8.298 7.98
   4753   1368427_at   7.424 7.483 7.364 7.596 7.468 7.989 7.717 8.216 8.011 8.119
   4754   1393316_at   7.231 7.345 7.208 7.489 7.51 8.005 7.643 7.961 7.876 7.924
   4755   1381336_at   6.529 6.757 6.829 7.14 7.096 7.526 7.437 7.694 7.648 7.684
   4756   1389804_at   6.813 7.047 7.215 7.103 7.176 6.425 6.504 6.626 6.089 6.303
   4757   1367823_at   6.459 6.572 7.262 6.859 6.9 7.941 7.522 7.88 7.923 7.627
   4758   1393382_at   3.97 3.954 4.087 4.349 3.963 4.783 4.604 5.211 4.859 4.678
   4759   1387390_at   6.011 6.299 6.755 6.824 6.429 5.444 5.422 5.221 5.526 5.713
   4760   1393249_at   7.8 7.985 8.084 8.07 7.954 7.627 7.392 7.231 7.463 7.101
   4761   1389697_at   4.872 5.024 5.146 5.611 5.606 6.401 6.205 6.303 6.118 6.06
   4762   1374748_at   8.402 8.372 8.704 8.71 8.615 9.025 9.15 9.228 9.019 8.963
   4763   1374454_at   7.055 7.204 7.212 7.413 7.488 7.893 7.873 8.164 7.977 7.691
   4764   1390123_at   5.746 6.175 6.723 6.27 6.254 7.111 7.066 7.461 7.237 7.342
   4765   1375428_at   8.89 8.699 9.391 9.195 9.269 8.212 8.089 8.358 8.343 8.365
   4766   1373279_at   6.265 6.68 6.764 6.548 6.789 7.138 7.188 7.231 7.166 7.18
   4767   1371681_at   8.2 8.276 8.448 8.385 8.334 7.965 7.882 8.13 7.929 7.871
   4768   1387188_at   9.32 9.401 9.769 9.567 9.241 10.138 10.011 10.038 10.182 9.957
   4769   1382071_at   6.338 6.375 6.569 6.469 6.413 5.794 5.747 6.212 5.75 5.778
   4770   1389971_at   7.094 7.113 7.072 6.69 7.226 8.005 7.711 7.81 7.492 7.809
   4771   1371132_a_at   6.611 6.311 6.286 5.971 6.438 7.085 6.871 7.002 7.066 7.294
   4772   1372386_at   7.669 7.47 7.424 7.565 7.628 7.021 7.311 7.125 6.948 6.969
   4773   1381489_at   5.767 5.149 5.658 5.583 5.717 6.277 6.138 6.605 6.412 6.451
   4774   1386930_at   9.145 9.09 9.22 9.268 9.372 8.876 8.935 8.903 8.708 8.806
   4775   1386394_at   8.486 8.407 8.609 8.839 8.615 7.937 8.026 8.239 7.824 7.845
   4776   1395404_at   7.855 8.056 8.062 8.244 8.091 8.521 8.45 8.876 8.686 8.602
   4777   1388995_at   9.059 9.238 9.232 9.221 9.303 9.513 9.502 9.704 9.549 9.474
   4778   1388240_a_at   4.55 3.911 3.812 3.534 4.369 5.602 4.978 5.406 5.172 5.655
   4779   1383912_at   8.677 8.776 8.889 8.741 9.014 8.21 8.313 8.341 8.102 7.81
   4780   1388560_at   9.048 9.052 8.977 9.344 9.266 8.637 8.509 8.802 8.457 8.504
   4781   1374994_at   4.934 4.574 4.708 5.071 4.729 5.876 5.902 6.563 6.136 5.455
   4782   1382935_at   7.772 7.386 7.75 7.828 7.641 8.342 8.072 8.564 8.577 8.367
   4783   1373661_a_at   6.859 6.886 7.058 7.038 6.901 5.739 5.763 5.868 5.807 4.637
   4784   1367844_at   9.489 9.511 9.819 9.842 9.836 9.07 8.556 8.969 8.668 9.133
   4785   1372439_at   10.55 10.547 10.591 10.648 10.793 11.371 10.99 11.145 11.322 10.996
   4786   1370331_at   6.789 6.828 6.833 7.196 7.232 7.5 7.609 7.943 7.806 7.676
   4787   1388611_at   7.138 6.545 7.374 7.1 7.059 7.81 7.741 7.994 8.08 8.07
   4788   1382857_at   9.274 9.277 9.328 9.485 9.25 9.682 9.673 9.744 9.869 9.565
   4789   1371324_at   10.079 10.053 10.083 10.091 10.345 9.819 9.848 9.788 9.719 9.755
   4790   1387799_at   11.999 11.941 12.001 11.789 11.723 12.366 12.452 12.216 12.328 12.597
   4791   1392852_at   3.788 4.078 4.256 3.622 3.16 4.872 4.892 5.508 5.659 5.242
   4792   1387820_at   9.146 9.069 9.393 8.735 7.244 10.816 10.772 11.203 11.206 11.47
   4793   1394668_at   0.275 0.978 1.057 0.345 0.856 4.601 1.891 3.645 4.369 4.372
   4794   1373367_at   9.361 9.277 9.445 9.543 9.461 9.793 9.692 10.062 9.947 9.881
   4795   1387903_at   8.274 8.54 8.275 8.374 8.209 8.732 8.691 8.992 8.77 8.769
   4796   1384184_at   5.697 6.036 6.141 5.786 6.584 6.904 7.516 7.188 7.029 7.095
   4797   1373301_at   6.329 6.35 6.495 6.636 6.099 7.251 6.806 7.175 7.015 7.179
   4798   1377011_at   5.693 5.417 6.035 5.841 5.825 6.669 6.38 6.687 6.302 6.503
   4799   1382510_at   4.558 4.441 4.716 4.511 4.452 5.096 4.832 5.373 5.292 5.183
   4800   1392391_at   7.08 6.543 7.128 7.042 6.826 7.694 7.468 7.645 7.561 7.622
   4801   1384148_at   9.156 9.069 9.11 9.485 9.316 8.605 8.432 8.807 8.736 8.635
   4802   1385561_at   7.028 7.077 7.343 6.866 6.66 7.82 7.551 7.767 7.701 7.711
   4803   1370299_at   12.796 12.714 12.676 12.524 12.502 13.074 13.194 12.94 13.006 13.174
   4804   1371814_at   7.923 7.93 7.977 8.041 8.07 7.615 7.075 7.406 7.559 7.403
   4805   1393958_at   6.944 7.135 7.787 6.791 7.573 8.819 8.503 8.356 8.567 8.229
   4806   1393791_at   4.53 4.276 5.057 4.798 4.722 2.558 1.628 0 0 2.39
   4807   1373366_at   8.497 8.529 8.523 8.519 8.499 8.754 8.689 8.863 8.8 8.638
   4808   1384876_at   3.878 4.05 3.835 3.984 3.877 4.386 4.485 4.804 4.282 4.674
   4809   1372509_at   7.375 7.624 7.624 7.454 7.458 8.245 7.927 8.323 7.926 7.984
   4810   1376107_at   5.297 5.365 5.164 4.983 4.854 5.743 5.867 6.023 6.392 5.91
   4811   1390928_at   6.415 6.659 6.535 6.578 6.699 6.038 5.727 6.086 6.057 5.559
   4812   1372985_at   6.972 6.893 6.759 6.903 6.634 7.519 7.21 7.191 7.388 7.451
   4813   1375916_at   7.619 7.5 7.951 7.88 7.966 8.39 8.284 8.516 8.492 8.312
   4814   1371639_at   10.412 10.567 10.516 10.406 10.428 10.904 10.8 11.02 10.862 10.671
   4815   1374720_at   6.311 6.294 6.043 6.567 6.32 5.52 4.863 5.217 5.553 5.615
   4816   1389687_at   6.488 6.277 6.537 6.575 6.416 7.024 6.828 7.058 6.957 6.744
   4817   1371683_at   9.153 9.153 9.192 9.375 9.405 8.701 8.509 8.979 8.665 8.671
   4818   1369697_at   4.385 4.077 3.053 2.104 4.162 0.651 1.257 1.189 0.057 0.444
   4819   1389632_at   7.971 8.003 8.175 8.491 8.179 8.912 8.757 8.933 8.824 8.604
   4820   1392909_at   4.967 5.411 5.239 5.035 5.282 5.917 5.927 5.797 5.551 5.904
   4821   1373703_at   8.265 8.178 7.543 8.104 8.286 6.981 7.004 7.122 6.552 6.169
   4822   1390121_at   8.692 8.844 9.043 8.939 8.703 9.448 9.198 9.235 9.411 9.413
   4823   1392725_at   5.96 6.495 6.487 6.436 6.348 7.283 7.388 7.099 6.817 7.308
   4824   1392818_at   6.891 7.016 7.422 6.697 7.1 5.8 6.407 6.085 6.074 6.21
   4825   1372531_at   7.479 7.419 7.485 7.444 7.306 7.856 8.191 7.881 7.874 7.736
   4826   1370975_at   7.71 7.643 7.689 7.628 7.704 7.196 7.117 7.491 7.107 7.007
   4827   1385451_at   5.553 5.197 5.06 5.336 5.092 3.362 3.783 3.216 4.09 2.245
   4828   1387190_at   8.097 8.325 8.236 8.471 8.129 7.591 7.475 7.842 7.636 7.251
   4829   1390856_at   4.998 4.709 4.559 4.91 4.871 5.527 5.586 5.912 5.465 5.293
   4830   1368698_at   3.817 4.243 3.864 4.094 4.532 4.68 5.311 5.422 5.351 5.237
   4831   1381217_at   6.506 6.563 6.462 6.527 6.66 6.179 6.043 6.054 5.921 5.629
   4832   1389201_at   6.617 6.567 6.441 6.667 6.84 7.101 7.268 7.004 7.361 7.09
   4833   1398523_a_at   6.577 6.656 6.524 6.636 6.408 6.15 5.492 5.775 5.713 5.301
   4834   1387861_at   8.91 8.717 8.845 9.016 9.14 9.719 9.325 9.468 9.76 9.816
   4835   1374014_at   8.175 8.023 8.399 8.572 8.377 7.709 7.517 7.816 7.416 7.384
   4836   1373857_at   6.403 6.465 6.303 6.952 6.307 7.206 7.17 7.208 7.151 7.355
   4837   1367643_at   12.307 12.266 12.165 11.983 12.193 12.619 12.784 12.518 12.573 12.548
   4838   1392480_at   5.828 6.092 6.391 5.921 6.366 7.306 6.629 7.238 7.219 7.072
   4839   1376042_at   5.75 5.821 5.748 5.787 5.972 6.212 6.541 6.585 6.294 6.18
   4840   1396246_at   4.3 4.705 4.635 4.836 4.349 5.412 5.5 5.595 5.899 5.134
   4841   1384474_at   4.089 4.68 5.068 4.836 3.976 5.934 5.696 6.701 6.18 6.71
   4842   1373055_at   7.819 7.704 7.855 8.004 7.821 8.262 8.119 8.527 8.478 8.411
   4843   1373530_at   6.133 6.29 5.983 6.574 6.634 5.328 5.096 5.626 5.488 5.536
   4844   1389271_at   6.307 6.584 6.345 6.755 6.623 7.01 7.023 7.039 7.141 7.27
   4845   1371446_at   9.888 9.9 9.552 10.016 9.814 9.393 9.371 9.379 9.402 9.281
   4846   1373915_at   5.857 5.797 6.049 6.401 6.552 7.119 6.918 7.493 7.064 7.388
   4847   1377459_at   4.322 4.787 5.147 4.942 4.38 5.951 5.846 5.692 5.582 5.598
   4848   1390656_at   6.006 5.773 5.677 5.855 4.851 6.674 7.162 7.555 7.107 6.92
   4849   1392534_at   7.913 7.932 8.355 8.461 8.207 8.916 8.713 9.025 9.019 9.232
   4850   1367452_at   11.095 11.185 11.336 11.213 11.225 11.012 10.925 10.981 10.868 10.879
   4851   1378536_at   7.658 7.894 7.904 7.809 7.621 8.36 8.41 8.765 8.961 8.362
   4852   1388825_at   7.716 7.699 7.875 7.873 7.827 8.448 8.403 8.853 8.422 8.188
   4853   1372765_a_at   10.099 10.016 10.209 10.023 9.791 10.426 10.42 10.501 10.413 10.425
   4854   1391045_at   7.212 7.351 7.406 7.269 6.844 7.902 7.706 7.887 7.897 8.109
   4855   1374995_at   7.29 7.375 7.324 7.193 7.488 7.992 7.7 7.623 7.876 7.853
   4856   1370542_a_at   8.188 8.053 8.048 7.766 7.74 7.299 7.183 7.013 7.059 6.496
   4857   1369626_at   8.024 8.024 8.217 7.938 8.062 8.744 8.592 8.691 8.616 8.287
   4858   1379671_at   10.164 10.172 10.161 10.22 10.175 9.957 9.684 9.535 9.459 9.426
   4859   1398281_at   7.533 7.68 7.797 7.569 7.785 8.004 8.002 8.06 8.081 7.941
   4860   1369463_at   3.858 4.576 4.256 4.318 3.628 5.073 5.251 5.046 5.107 5.301
   4861   1374870_at   5.657 5.919 5.301 6.168 5.877 6.81 6.45 6.862 6.79 6.731
   4862   1391157_at   6.35 6.5 6.46 6.377 6.587 7.028 6.862 7.059 6.699 7.087
   4863   1376088_at   5.98 6.11 5.84 6.45 6.14 6.744 6.695 6.881 6.882 6.622
   4864   1383796_at   5.849 5.918 5.923 6.191 5.635 7.03 7.047 7.821 6.763 6.861
   4865   1387267_at   6.123 6.064 6.575 5.989 5.089 7.69 7.736 7.788 7.473 7.093
   4866   1388301_at   11.484 11.528 11.44 11.322 11.342 11.733 11.726 11.596 11.75 11.765
   4867   1371448_at   9.189 9.236 9.419 9.505 9.135 9.8 9.64 9.826 9.913 10
   4868   1384138_at   5.513 5.41 5.19 5.354 5.679 6.131 5.833 6.139 6.301 6.401
   4869   1390592_at   7.101 7.113 7.364 7.467 7.327 7.767 7.732 8.217 8.137 8.041
   4870   1388814_at   9.591 9.547 9.851 9.835 9.278 10.213 10.248 10.448 10.504 10.225
   4871   1398123_at   0 0.651 0.111 0 0 1.952 2.195 1.328 1.151 1.239
   4872   1367885_at   8.429 8.417 8.597 8.67 8.325 8.883 8.878 9.007 9.049 9.227
   4873   1389558_at   8.403 8.315 8.619 8.264 8.485 7.809 7.825 7.81 7.833 7.304
   4874   1371365_at   10.233 10.274 10.261 10.395 10.34 10.141 9.863 9.913 9.852 9.795
   4875   1398540_at   6.874 6.858 6.973 6.895 6.603 3.921 3.118 3.5 3.948 0.39
   4876   1388871_at   8.652 8.733 8.65 8.894 9.029 8.384 8.123 8.364 8.303 8.219
   4877   1379778_at   7.843 7.793 7.642 7.93 7.673 7.15 7.127 7.43 6.961 6.803
   4878   1374019_at   7.995 8.117 8.027 8.113 8.194 8.549 8.36 8.82 8.669 8.513
   4879   1371905_at   9.918 9.866 9.986 10.097 10.047 10.213 10.247 10.267 10.289 10.199
   4880   1379330_s_at   8.935 9.146 9.051 9.373 9.284 9.725 9.618 10.161 10.035 9.907
   4881   1371951_at   8.091 8.223 8.785 8.595 8.755 9.631 9.199 9.613 9.651 9.294
   4882   1371818_at   9.404 9.45 9.495 9.512 9.215 9.71 9.747 9.974 9.862 9.832
   4883   1373021_at   8.975 8.879 8.881 8.534 8.598 9.239 9.37 9.238 9.34 9.31
   4884   1374758_at   8.371 8.5 8.307 8.616 8.57 8.789 8.888 8.908 8.809 8.803
   4885   1382953_at   4.215 4.32 4.882 4.574 4.565 5.239 5.13 5.571 5.591 5.733
   4886   1388556_at   8.41 8.348 8.234 8.409 8.445 7.975 7.646 7.736 7.953 7.434
   4887   1372625_at   7.299 7.168 7.38 7.321 7.554 6.989 6.625 6.715 6.921 6.829
   4888   1377664_at   7.398 7.276 7.305 7.441 7.764 6.774 6.686 6.63 6.701 6.11
   4889   1387361_s_at   11.775 11.768 11.91 11.801 11.873 11.638 11.499 11.33 11.353 11.495
   4890   1367577_at   8.027 7.97 8.045 8.123 8.371 8.629 8.542 8.576 8.48 8.494
   4891   1387462_at   4.376 4.846 5.068 5.275 5.022 5.688 5.83 5.842 5.938 5.821
   4892   1384098_at   6.774 7.072 6.981 6.931 6.961 6.371 6.03 6.337 6.516 6.522
   4893   1370579_at   6.367 6.114 6.405 6.534 6.434 6.994 6.837 6.767 6.851 7.113
   4894   1381832_at   7.637 7.6 7.585 8.007 7.862 8.183 8.368 8.337 8.481 8.208
   4895   1382840_at   9.326 9.296 9.536 9.062 8.966 10.113 10.533 10.287 10.101 9.777
   4896   1399088_at   7.544 7.654 7.661 7.806 7.764 7.219 7.406 7.37 7.122 7.122
   4897   1390112_at   6.832 6.848 6.671 6.433 7.033 7.331 7.515 7.522 7.305 7.353
   4898   1379370_at   2.31 1.888 1.333 2.838 3.678 4.77 5.193 4.602 4.949 4.599
   4899   1388445_at   10.301 10.289 10.333 10.317 10.12 10.553 10.473 10.639 10.557 10.511
   4900   1389209_at   7.824 7.829 7.776 7.921 7.858 8.165 8.109 8.235 7.997 8.066
   4901   1368432_a_at   4.711 5.093 5.048 5.674 4.469 5.941 6.408 6.761 6.47 6.418
   4902   1373501_at   9.608 9.632 9.567 9.676 9.49 9.807 9.949 9.897 9.949 9.771
   4903   1388189_at   0.506 0.263 0 0.287 0.227 0.84 0.926 0.642 0.926 1.091
   4904   1377311_at   7.084 7.318 7.099 7.207 7.53 7.75 7.903 8.117 7.884 7.716
   4905   1367900_at   9.819 9.82 9.826 9.721 9.907 9.477 9.388 9.475 9.349 9.066
   4906   1373415_at   8.035 7.886 7.841 8.097 7.984 8.401 8.238 8.534 8.609 8.371
   4907   1384958_at   8.935 8.915 8.875 9.083 8.987 8.605 8.33 8.723 8.458 8.481
   4908   1370289_x_at   4.964 4.658 4.728 4.656 4.047 5.699 6.622 5.82 5.63 6.229
   4909   1390708_at   5.742 6.018 5.931 5.834 6.224 4.94 4.809 4.996 5.513 5.213
   4910   1379258_at   8.826 8.703 8.617 8.831 8.88 8.243 7.888 8.267 8.046 7.603
   4911   1373421_at   9.811 9.909 9.522 9.875 9.883 8.905 9.036 8.788 8.496 7.979
   4912   1382032_at   7.081 7.397 7.072 7.33 7.246 7.678 7.526 7.808 7.847 7.805
   4913   1388370_at   11 10.992 10.922 10.896 11.112 11.308 11.236 11.345 11.217 11.188
   4914   1367638_at   8.215 8.183 8.48 8.284 8.142 8.636 8.611 8.746 8.841 8.936
   4915   1377900_at   9.805 9.849 10.234 10.031 9.323 10.671 10.7 10.906 11.203 10.935
   4916   1390457_at   6.323 6.556 6.39 6.648 6.68 7.053 6.82 7.105 7.054 7.101
   4917   1390173_at   7.61 7.745 7.941 8.053 7.824 8.373 8.22 8.535 8.628 8.412
   4918   1387957_a_at   7.919 7.892 8.374 8.149 8.196 7.584 6.946 7.452 7.259 7.205
   4919   1374817_at   4.805 5.477 4.952 5.307 5.649 6.107 6.146 6.255 6.189 6.119
   4920   1375425_at   9.053 9.079 9.015 9.259 9.254 8.844 8.708 8.823 8.803 8.83
   4921   1376642_at   5.936 6.005 5.623 6.249 6.126 4.47 4.835 4.959 5.425 4.618
   4922   1383621_at   7.648 7.964 7.549 7.829 7.834 7.096 7.15 7.237 7.302 6.814
   4923   1390403_at   7.605 7.298 7.695 7.712 7.441 8.143 7.924 8.404 8.295 8.267
   4924   1374285_at   8.278 8.289 8.07 8.336 8.314 8.628 8.777 8.506 8.627 8.776
   4925   1379057_at   5.313 5.676 5.506 6.06 5.653 6.722 6.204 6.503 6.497 6.591
   4926   1372565_at   6.381 5.897 6.188 6.284 6.747 7.451 7.178 7.307 7.671 7.016
   4927   1375540_at   8.385 8.394 8.138 8.342 8.226 7.797 7.868 7.467 7.813 7.403
   4928   1368031_at   7.879 7.893 7.794 7.927 8.008 7.42 7.491 7.692 7.603 7.396
   4929   1383576_at   8.084 7.795 8.12 7.959 7.865 7.274 7.014 7.13 7.22 6.426
   4930   1370538_at   5.136 4.115 3.574 5.11 4.837 6.386 6.548 6.76 6.762 6.013
   4931   1388575_at   9.071 9.054 9.094 9.057 9.133 9.618 9.564 9.633 9.408 9.264
   4932   1392196_at   2.101 1.433 0 0 2.118 4.411 3.307 4.148 4.713 4.606
   4933   1382265_at   6.974 6.904 7.338 7.294 7.191 7.877 7.557 8.165 8.117 8.143
   4934   1394438_at   8.533 8.539 8.43 8.608 8.63 8.313 8.293 8.247 8.19 8.368
   4935   1373980_at   7.318 7.412 7.41 7.363 7.478 7.093 7.098 7.114 6.872 7.162
   4936   1382047_at   5.662 6.044 6.143 6.086 6.128 6.483 6.584 6.51 6.675 6.552
   4937   1373461_at   8.426 8.488 8.646 8.825 8.719 8.222 8.117 8.229 8.004 8.113
   4938   1392296_at   5.339 5.5 6.015 5.584 5.03 4.334 4.27 3.044 3.786 3.94
   4939   1370398_at   6.537 6.693 6.338 6.686 6.873 7.267 7.1 7.285 7.395 7.112
   4940   1382230_at   6.692 6.327 6.322 6.942 6.787 7.814 7.202 7.506 7.51 7.612
   4941   1374152_at   7.428 7.186 7.253 7.394 7.245 6.807 6.818 6.809 6.397 6.863
   4942   1378543_at   7.485 7.513 7.475 7.531 7.447 8.103 7.883 8.289 7.857 7.849
   4943   1386096_at   7.375 7.489 7.697 7.613 7.808 7.123 7.123 7.101 7.171 6.904
   4944   1371658_at   8.849 8.691 8.493 8.604 8.69 8.259 8.313 8.324 8.098 8.28
   4945   1370830_at   6.358 6.045 6.247 6.325 6.403 5.822 5.913 5.87 5.969 5.815
   4946   1373321_at   6.982 6.862 6.905 7.066 7.216 7.509 7.305 7.453 7.531 7.392
   4947   1367877_at   8.916 8.881 8.689 9.072 9.059 9.37 9.39 9.441 9.385 9.244
   4948   1387347_at   6.722 6.557 7.29 6.548 6.393 7.599 7.64 7.809 8.172 8.28
   4949   1371309_at   11.999 11.979 11.883 11.862 11.889 12.245 12.196 12.116 12.118 12.324
   4950   1393201_at   6.98 7.085 7.141 7.077 6.84 6.29 5.661 6.105 5.787 5.073
   4951   1389490_at   5.277 5.112 5.13 5.475 5.811 6.284 6.166 6.847 6.39 6.228
   4952   1387591_at   7.144 6.4 6.913 6.677 6.331 4.731 0.74 3.583 2.248 2.367
   4953   1389374_at   8.041 8.084 8.317 8.285 8.131 8.599 8.617 8.747 8.595 8.432
   4954   1381075_at   5.931 5.892 5.703 6.068 6.06 6.422 6.388 6.67 6.328 6.4
   4955   1387037_at   9.775 9.897 9.732 10.253 10.178 10.765 11.038 11.138 10.619 10.612
   4956   1397663_at   5.771 5.301 5.364 5.789 5.019 1.748 0 1.541 3.715 2.401
   4957   1371058_at   6.226 5.924 7 6.131 5.657 4.42 2.852 2.736 4.59 3.569
   4958   1388401_at   8.705 8.736 8.546 8.876 8.826 9.115 9.223 9.257 9.351 9.03
   4959   1384182_at   6.73 6.883 6.791 6.68 6.727 7.02 7.451 7.287 7.226 7.134
   4960   1374098_at   7.226 7.452 7.322 7.067 7.61 7.969 7.815 8.203 7.972 7.935
   4961   1379584_at   5.674 5.679 6.052 5.555 5.692 4.678 3.782 4.849 4.79 4.169
   4962   1368700_at   6.914 7.589 7.296 7.321 7.039 8.074 8.076 8.625 8.258 7.935
   4963   1368303_at   6.645 6.804 6.417 6.586 6.783 7.122 7.587 7.324 7.329 7.103
   4964   1388117_at   10.731 10.727 10.871 10.697 10.759 10.609 10.503 10.441 10.383 10.418
   4965   1389109_at   8.998 9.022 8.995 9.116 9.023 8.644 8.652 8.851 8.465 8.513
   4966   1383832_at   7.518 7.626 8.084 7.615 7.03 8.745 9.026 8.599 8.692 8.412
   4967   1377480_at   7.154 7.149 7.061 7.059 7.288 6.944 6.621 6.729 6.661 6.757
   4968   1375600_at   5.663 6.061 5.799 5.691 5.877 6.389 6.305 6.312 6.415 6.141
   4969   1390709_at   7.629 7.632 7.413 7.724 7.789 8.165 8.316 8.231 8.094 7.945
   4970   1370285_at   6.728 6.782 6.951 7.184 7.158 7.687 7.378 7.758 7.923 7.781
   4971   1373994_at   5.677 5.956 5.998 5.954 5.91 6.337 6.183 6.248 6.326 6.428
   4972   1369517_at   6.564 6.801 6.827 6.72 6.736 6.459 6.103 6.312 6.283 6.077
   4973   1371919_at   7.936 7.651 7.713 7.734 7.499 8.169 8.251 8.273 8.29 8.02
   4974   1368571_at   8.825 8.499 8.196 8.529 8.313 7.95 7.199 7.527 7.334 7.12
   4975   1373580_at   5.029 5.318 5.644 5.781 5.375 6.161 6.53 6.681 6.141 6.467
   4976   1390418_at   7.982 8.184 7.899 8.405 8.205 8.756 8.614 8.685 8.703 8.601
   4977   1367950_at   9.823 9.463 9.919 9.569 9.385 10.169 10.515 10.367 10.443 10.195
   4978   1383492_at   7.62 7.529 7.605 7.969 8.007 8.302 8.319 8.677 8.765 8.647
   4979   1384312_at   7.442 7.264 6.996 7.566 7.221 8.111 7.759 8.406 8.411 8.388
   4980   1368784_at   4.783 4.957 5.831 5.181 5.259 6.558 6.007 6.881 6.84 6.51
   4981   1375685_at   8.16 8.399 8.584 8.748 8.631 7.786 7.549 8.041 7.708 7.612
   4982   1393799_at   6.24 5.941 6.35 6.374 6.3 7.022 6.684 6.835 6.699 6.805
   4983   1374085_at   7.512 7.375 7.639 7.588 7.913 8.308 8.118 8.563 8.434 8.134
   4984   1377774_at   6.021 5.922 6.099 5.822 6.232 6.519 6.88 7.433 7.384 7.121
   4985   1377726_at   9.401 9.144 9.156 9.438 9.698 8.701 8.317 8.843 8.478 8.593
   4986   1372431_at   9.966 9.917 9.77 9.899 10.138 10.288 10.334 10.304 10.227 10.36
   4987   1367502_at   8.643 8.71 8.819 8.858 8.837 8.481 8.474 8.579 8.532 8.459
   4988   1371461_at   9.957 9.885 9.951 9.901 9.869 10.069 10.122 10.101 10.085 9.994
   4989   1370328_at   6.143 6.409 6.029 6.321 6.911 7.721 7.542 7.608 7.518 7.01
   4990   1388366_at   9.672 9.67 9.693 9.665 9.627 9.959 9.804 9.869 9.9 9.778
   4991   1372196_at   8.161 8.442 8.419 8.622 8.378 8.817 8.783 8.905 8.923 8.832
   4992   1378319_at   6.254 6.568 6.671 6.791 6.009 5.454 5.065 5.418 5.73 5.512
   4993   1369764_at   8.383 8.618 7.294 8.097 7.183 4.396 4.1 0 2.599 0
   4994   1387917_at   9.691 9.866 9.861 9.898 9.861 9.33 9.25 9.653 9.362 9.295
   4995   1388888_at   7.076 7.13 7.21 7.356 7.064 7.542 7.532 7.658 7.472 7.762
   4996   1385728_at   5.019 5.126 5.335 5.377 5.29 4.905 4.681 4.593 4.459 4.457
   4997   1379770_at   9.778 9.841 10.077 9.958 9.475 10.353 10.428 10.465 10.616 10.421
   4998   1380327_at   7.175 7.07 7.135 7.406 7.276 6.641 6.148 6.455 6.455 5.793
   4999   1387773_at   12.241 12.13 12.242 11.986 12.021 12.581 12.577 12.404 12.414 12.581
   5000   AFFX_Rat_beta-actin_5_at   12.915 12.776 12.868 12.615 12.683 12.414 12.366 12.004 12.104 12.253
   5001   1371736_at   9.849 9.769 9.803 9.593 9.784 10.427 10.486 10.365 10.498 9.987
   5002   1371772_at   8.708 8.858 8.977 8.898 9.026 8.523 8.099 8.253 8.22 8.511
   5003   1398936_at   8.703 8.7 8.8 8.786 8.745 8.216 8.361 8.54 8.504 8.328
   5004   1385924_at   5.406 5.796 5.416 5.386 5.473 5.943 6.342 6.697 6.364 6.225
   5005   1389053_at   8.816 8.852 8.877 8.958 8.869 8.52 8.687 8.396 8.565 8.346
   5006   1371873_at   9.301 9.26 9.424 9.406 9.198 8.756 8.715 8.969 8.722 8.355
   5007   1382167_at   5.747 5.816 6.353 6.043 5.573 3.586 4.261 4.671 4.893 4.592
   5008   1373424_at   6.81 6.557 6.94 7.201 7.043 7.618 7.559 7.694 7.633 7.42
   5009   1375536_at   8.201 8.349 8.308 8.582 8.357 8.744 8.877 8.956 8.851 8.664
   5010   1389522_at   7.502 7.888 7.811 7.561 7.463 8.254 8.258 8.522 8.617 8.132
   5011   1371544_at   10.892 10.802 10.911 10.796 10.936 10.548 10.451 10.555 10.228 10.191
   5012   1385001_at   7.486 7.476 7.101 7.393 7.606 6.99 6.417 6.15 6.16 6.181
   5013   1376443_at   5.107 5.285 5.048 5.385 5.512 5.828 6.236 6.174 5.798 6.372
   5014   1399160_a_at   11.639 11.581 11.76 11.346 11.391 11.07 11.123 10.819 10.884 10.708
   5015   1367621_at   9.273 9.194 9.495 9.634 9.648 8.957 8.876 8.897 8.88 8.951
   5016   1377013_at   6.126 5.333 5.662 5.365 5.15 6.575 6.713 6.494 6.618 6.368
   5017   1393204_at   4.729 5.149 5.065 5.209 5.48 5.823 6.113 5.818 5.78 6.028
   5018   1375011_at   7.365 7.124 7.12 7.352 7.425 7.783 7.84 8.279 7.937 7.777
   5019   1374641_at   8.685 8.767 8.852 8.958 8.769 9.266 9.121 9.546 9.425 9.211
   5020   1390914_at   7.634 7.522 7.78 7.862 7.781 7.329 6.752 7.112 7.162 7.001
   5021   1376918_at   4.891 4.752 4.929 5.254 5.041 6.379 5.52 6.321 5.773 6.207
   5022   1368202_a_at   11.327 11.267 11.308 11.375 11.371 11.656 11.701 11.846 11.648 11.495
   5023   1372736_at   7.129 7.119 7.029 7.377 7.215 7.571 7.761 7.941 7.74 7.531
   5024   1388605_at   3.366 3.925 3.522 4 2.478 5.578 4.747 5.106 5.566 5.207
   5025   1379848_at   1.39 3.279 1.795 3.838 2.511 5.03 5.148 5.265 5.667 5.014
   5026   1380037_at   5.887 6.196 5.992 6.042 6.272 5.094 4.313 4.521 3.35 4.661
   5027   1382132_at   3.67 0.799 0.978 2.365 0.774 5.081 4.797 5.266 5.133 4.754
   5028   1368138_at   7.031 7.283 7.376 7.597 7.085 8.001 7.836 8.245 8.458 8.502
   5029   1373270_at   7.115 7.274 7.318 7.343 7.615 7.965 8.006 8.033 7.852 7.697
   5030   1399030_at   8.144 8.017 8.043 8.272 8.098 8.412 8.637 8.495 8.581 8.367
   5031   1368546_at   7.295 7.249 7.314 7.73 7.291 6.292 6.623 6.825 6.156 6.004
   5032   1367842_at   6.007 6.196 5.509 6.104 5.975 6.741 6.696 6.684 6.605 6.56
   5033   1371659_at   8.72 8.751 8.75 8.981 9.027 9.252 9.192 9.351 9.334 9.188
   5034   1377310_at   8.026 8.042 7.946 8.198 8.249 7.654 7.551 7.716 7.6 7.283
   5035   1368133_at   6.492 6.536 6.295 6.59 6.813 7.085 7.381 7.466 7.091 7.071
   5036   1367803_at   8.173 8.138 8.048 7.911 8.372 7.603 7.642 7.329 7.26 7.068
   5037   1387148_at   6.819 6.823 6.65 6.982 7.063 7.368 7.193 7.36 7.386 7.298
   5038   1389943_at   2.768 1.74 2.78 2.516 2.176 5.348 3.392 5.763 5.144 5.198
   5039   1369960_at   6.179 6.068 6.046 6.439 6.568 7.191 6.968 6.879 6.849 6.865
   5040   1385563_at   3.055 3.348 3.32 3.315 3.305 4.306 4.009 3.589 4.163 4.005
   5041   1388429_at   8.034 8.118 8.314 7.937 8.154 7.663 7.668 7.603 7.541 7.247
   5042   1376564_at   3.769 3.004 3.475 4.013 3.249 4.386 4.465 4.777 4.84 4.856
   5043   1392465_at   10.443 10.444 10.402 10.401 10.514 10.314 10.289 10.297 10.287 10.197
   5044   1372029_at   9.279 9.185 9.298 9.216 9.281 9.358 9.447 9.439 9.432 9.38
   5045   1373597_at   8.499 8.61 8.645 8.811 8.791 8.263 8.358 8.303 8.103 8.283
   5046   1394472_at   8.19 8.201 8.544 8.323 8.385 8.9 8.674 9.143 9.123 8.878
   5047   1388566_at   10.861 10.795 10.616 10.906 10.836 10.541 10.391 10.504 10.315 10.445
   5048   1373064_at   9.352 9.396 9.675 9.494 9.626 9.918 9.799 9.891 9.924 9.901
   5049   1389728_at   6.746 6.778 6.79 6.81 7.351 7.727 7.659 7.984 7.987 7.454
   5050   1388887_at   9.149 8.93 9.111 8.96 8.599 9.544 9.436 9.593 9.547 9.49
   5051   1386868_at   12.041 11.947 11.874 11.779 11.966 11.686 11.627 11.59 11.697 11.585
   5052   1372972_at   4.504 3.668 3.99 3.986 3.624 5.124 5.085 4.745 5.292 4.819
   5053   1395026_at   6.653 6.816 7.216 6.589 5.71 7.832 8.365 8.072 8.125 8
   5054   1370131_at   5.595 5.981 6.377 5.834 5.548 7.25 7.221 6.499 7.045 7.032
   5055   1373035_at   8.72 8.665 8.362 8.739 8.866 7.961 7.855 7.745 7.407 7.032
   5056   1375870_a_at   8.545 8.73 8.736 8.71 9.004 8.266 8.047 8.09 7.766 7.605
   5057   1379682_at   0.678 0.74 1.124 0.791 0.74 2.036 2.763 2.287 1.48 1.911
   5058   1386906_a_at   9.298 9.24 9.488 9.27 9.121 8.555 8.308 8.681 8.671 8.922
   5059   1386346_at   7.696 7.75 7.733 7.847 7.882 7.48 7.179 7.189 6.938 7.352
   5060   1378021_at   5.872 5.54 5.688 5.507 5.431 6.193 6.463 6.622 6.08 6.232
   5061   1371953_at   8.923 8.909 9.279 8.963 9.269 8.441 8.021 8.485 8.256 7.811
   5062   1372995_at   6.918 6.684 6.613 7.037 6.897 6.425 6.157 6.394 6.151 6.272
   5063   1387268_at   6.356 6.193 6.336 6.532 6.693 5.745 5.647 5.473 5.96 5.319
   5064   1392924_at   5.889 6.21 6.483 6.334 6.076 6.685 6.811 6.893 6.811 6.853
   5065   1383805_at   5.007 4.98 5.229 4.75 5.221 5.543 5.508 5.657 5.632 5.839
   5066   1380070_at   5.671 5.783 5.347 5.503 5.551 6.334 6.122 6.538 6.202 6.001
   5067   1370156_at   8.296 8.431 8.306 8.463 8.642 9.009 8.862 9.092 8.884 8.747
   5068   1373875_at   7.552 7.77 7.724 7.717 7.826 6.994 7.335 6.804 7.222 6.745
   5069   1375886_at   8.602 8.592 8.867 8.719 8.608 8.973 9.214 9.188 9.266 8.996
   5070   1372466_at   10.929 10.967 10.731 10.87 10.923 10.473 10.567 10.591 10.532 10.276
   5071   1389546_at   8.448 8.581 8.693 8.979 8.53 9.498 9.268 9.661 9.245 9.186
   5072   1369515_s_at   1.787 1.872 1.007 3.002 1.782 4.45 3.335 4.204 4.072 4.868
   5073   1371472_at   6.81 6.577 6.408 6.867 7.33 7.917 8.225 8.646 8.093 7.621
   5074   1399049_at   9.014 8.978 9.087 9.196 9.04 9.393 9.311 9.508 9.35 9.286
   5075   1371326_at   8.413 8.443 8.637 8.701 8.399 8.189 8.109 8.168 8.181 8.092
   5076   1392265_s_at   6.706 6.517 6.098 6.575 7.096 7.616 7.655 7.653 7.76 7.313
   5077   1371451_at   9.034 8.822 8.797 8.913 9.079 8.324 8.315 8.538 8.513 8.06
   5078   1391421_at   7.331 7.367 7.309 7.389 7.43 7.938 7.568 8.06 7.841 7.798
   5079   1384570_at   4.764 5.055 4.401 4.723 4.586 3.037 2.367 3.118 2.86 4.014
   5080   1390531_at   7.613 7.582 7.509 7.978 7.873 6.751 5.405 6.512 6.489 6.35
   5081   1385268_at   6.309 6.728 6.241 6.046 6.645 5.571 5.674 5.487 5.639 5.159
   5082   1398535_at   6.282 6.469 6.586 6.602 6.577 7.256 6.812 7.065 6.963 7.133
   5083   1376041_at   5.335 5.41 5.57 5.836 5.384 6.666 6.062 6.464 6.456 7.035
   5084   1377743_at   8.234 8.402 8.357 8.238 8.134 7.739 7.464 7.674 7.618 7.026
   5085   1394651_at   4.756 5.001 4.916 5.107 5.46 6.148 5.578 6.269 5.971 5.952
   5086   1375205_at   9.697 9.799 9.75 9.82 9.85 9.487 9.334 9.545 9.201 9.198
   5087   1370154_at   13.223 13.131 12.849 12.705 12.825 12.435 12.366 11.94 12.075 12.113
   5088   1384394_at   8.134 8.054 8.485 8.56 8.439 8.969 8.846 9.336 9.199 9.047
   5089   1390478_at   7.302 7.055 6.924 7.094 6.992 7.588 7.653 7.771 7.688 7.352
   5090   1387458_at   9.389 9.367 9.544 9.442 9.385 9.133 9.028 8.797 8.852 8.613
   5091   1388987_at   6.495 6.669 6.577 6.65 6.799 5.615 5.924 6.133 6.176 5.596
   5092   1381536_at   4.768 4.737 3.708 4.752 4.461 2.818 1.417 2.43 0.31 1.926
   5093   1393563_at   6.907 6.602 6.824 6.802 6.928 6.199 6.467 6.272 6.154 5.892
   5094   1373408_at   10.633 10.694 10.696 10.801 10.839 10.965 10.96 10.976 11.049 10.925
   5095   1373581_at   4.738 5.008 4.884 5.197 4.834 5.334 5.641 5.769 5.461 5.516
   5096   1398371_at   8.301 8.183 8.218 8.331 8.309 7.862 7.974 8.044 8.044 7.795
   5097   1374511_at   5.144 4.805 5.531 5.109 4.918 5.743 5.972 5.904 5.888 5.732
   5098   1370376_a_at   9.387 9.447 9.897 9.688 9.531 8.683 9.087 8.77 8.592 8.26
   5099   1390957_at   6.036 6.095 5.904 6.246 6.347 6.846 6.446 6.827 6.779 6.723
   5100   1382131_at   7.365 7.512 7.105 7.247 7.26 7.847 7.747 8.116 7.839 7.705
   5101   1385114_at   3.617 3.804 4.319 4.351 3.972 4.859 4.854 4.995 4.74 4.804
   5102   1371468_at   8.544 8.428 8.287 8.581 8.502 8.91 8.74 8.926 8.922 8.745
   5103   1388545_at   4.119 3.798 3.926 5.113 2.891 5.79 6.021 6.159 5.975 6.014
   5104   1391893_at   6.544 6.552 6.605 6.78 6.892 6.356 6.101 5.873 5.987 5.877
   5105   1372278_at   4.389 4.955 5.559 4.848 5.064 4.074 2.895 2.744 2.457 3.223
   5106   1369452_a_at   9.836 9.797 9.843 9.744 9.656 9.449 9.473 9.2 9.353 9.08
   5107   1372572_at   5.708 5.992 5.657 5.736 5.436 6.129 6.7 6.365 6.66 6.546
   5108   1393401_at   7.932 8.005 7.894 8.101 8.115 7.16 7.299 7.308 7.296 6.512
   5109   1378263_at   4.653 3.816 4.239 4.101 3.834 4.995 5.174 4.939 5.033 4.965
   5110   1376250_at   8.886 9.064 8.811 8.963 9.001 8.254 8.137 8.434 8.259 7.634
   5111   1392274_at   7.959 8.088 7.967 8.076 8.205 8.776 8.872 8.863 8.754 8.293
   5112   1379342_at   5.529 5.715 5.381 5.442 5.688 6.138 5.921 5.958 6.033 6.269
   5113   1382049_at   6.291 5.891 6.21 6.056 6.02 6.905 6.664 6.712 6.735 6.418
   5114   1391415_at   8.375 8.759 8.143 8.351 8.96 7.678 7.762 7.659 7.387 7.385
   5115   1370642_s_at   6.904 6.774 7.217 7.341 7.314 7.873 7.74 7.794 7.898 7.665
   5116   1378213_at   4.887 5.33 5.293 4.969 5.565 3.332 0.888 1.782 3.403 1.057
   5117   1371587_at   9.519 9.403 9.648 9.445 9.651 9.265 8.99 9.121 8.845 8.931
   5118   1370158_at   7.674 7.744 7.841 8.232 8.248 9.064 8.629 8.988 8.655 8.685
   5119   1378506_at   7.719 7.659 7.387 7.451 7.513 8.091 8.186 8.354 8.123 7.85
   5120   1370942_at   7.445 7.523 7.514 7.373 7.776 7.227 6.765 6.671 6.776 6.614
   5121   1377867_at   6 6.421 6.084 5.871 5.79 5.092 4.083 4.245 4.389 3.165
   5122   1370132_at   4.065 4.743 3.699 4.668 5.064 5.891 5.72 6.386 6.153 5.808
   5123   1371496_at   9.406 9.297 9.536 9.516 9.006 10.282 9.855 10.335 10.48 10.768
   5124   1367636_at   10.443 10.321 10.392 10.367 9.764 11.065 11.072 10.945 10.911 10.925
   5125   1384821_at   7.294 7.206 7.107 7.335 7.322 6.927 6.83 7.06 6.94 6.846
   5126   1375338_at   10.157 10.165 10.382 10.391 10.21 9.988 9.718 9.948 9.852 9.834
   5127   1382150_at   9.204 9.053 8.835 9.194 9.034 9.512 9.512 9.598 9.378 9.453
   5128   1373596_at   6.34 6.258 5.786 6.452 6.465 7.402 6.973 7.547 7.583 6.962
   5129   1370004_at   9.215 9.134 8.875 9.374 9.436 8.576 8.473 8.704 8.684 8.424
   5130   1374354_at   6.067 6.03 6.117 6.34 6.058 5.719 5.531 5.438 5.831 5.492
   5131   1383886_at   5.68 5.648 5.823 5.821 5.238 6.294 6.39 6.62 6.154 6.503
   5132   1399059_at   8.31 8.387 8.285 8.523 8.297 8.637 8.579 8.78 8.666 8.684
   5133   1384939_at   5.988 6.271 6.287 6.3 6.483 4.336 4.899 4.716 3.54 2.84
   5134   1395495_at   0 0.485 0 1.029 0 3.858 4.505 4.057 1.465 4.523
   5135   1383331_at   4.823 4.93 5.1 4.733 4.614 5.4 5.521 6.076 5.818 5.529
   5136   1384430_at   5.544 5.582 5.637 5.542 5.548 6.329 5.859 6.243 5.923 6.068
   5137   1378037_at   7.648 7.768 7.778 7.827 7.714 8.371 7.963 8.309 8.521 8.269
   5138   1371560_at   8.078 8.001 8.297 8.234 8.326 8.727 8.538 8.501 8.733 8.741
   5139   1383099_at   7.296 7.536 7.356 7.47 7.609 7.008 7.171 7.026 6.892 6.805
   5140   1391194_at   7.401 7.489 7.303 7.248 7.509 7.682 7.86 8.058 7.939 7.752
   5141   1372538_at   8.548 8.525 8.705 8.696 8.628 9.132 8.956 9.209 8.934 8.888
   5142   1376638_at   5.852 6.05 5.73 5.943 6.248 6.368 6.526 6.558 6.71 6.718
   5143   1380465_at   8.176 8.017 8.563 8.341 7.735 8.872 9.054 9.029 9.112 8.901
   5144   1379336_at   7.072 7.143 7.092 7.408 7.126 7.598 7.704 8.146 8.209 7.926
   5145   1399095_at   10.365 10.33 10.36 10.409 10.287 10.187 9.923 10.142 9.981 10.042
   5146   1373645_at   7.441 7.299 7.698 7.586 7.317 8.061 8.023 8.41 7.901 8.255
   5147   1388756_at   8.111 8.245 8.031 8.282 8.087 8.648 8.466 8.972 9.004 8.78
   5148   1371004_at   5.379 5.398 5.123 5.524 5.755 6.261 6.01 6.635 6.419 6.099
   5149   1374678_at   9.072 9.344 9.064 9.293 9.457 8.31 8.518 8.786 8.707 8.243
   5150   1390501_at   7.84 7.964 7.933 7.797 7.704 8.208 8.362 8.326 8.446 8.085
   5151   1390819_at   7.265 7.3 7.283 7.54 7.203 7.782 7.773 8.28 7.879 7.924
   5152   1372443_at   8.125 7.992 7.998 8.132 8.261 8.685 8.794 8.88 8.94 8.363
   5153   1383640_at   7.184 7.016 7.035 7.225 7.458 7.737 7.769 8.061 7.9 7.59
   5154   1396101_at   6.36 5.93 6.11 6.375 6.248 6.99 7.272 7.294 6.595 7.103
   5155   1389380_at   7.039 7.332 7.016 6.757 6.994 7.6 7.561 7.478 7.58 7.532
   5156   1367496_at   10.531 10.54 10.594 10.638 10.71 10.939 10.877 10.993 10.76 10.868
   5157   1372520_at   10.746 10.732 10.822 10.686 10.489 10.227 10.017 10.243 9.74 9.714
   5158   1377052_at   6.573 6.942 6.895 7.094 6.801 6.29 6.372 5.946 6.062 5.787
   5159   1391847_at   3.497 4.057 4.264 3.821 3.599 2.411 2.901 2.801 2.511 3.16
   5160   1370235_at   11.511 11.476 11.619 11.588 11.681 11.911 11.822 11.813 11.769 11.953
   5161   1378327_at   5.525 5.23 5.493 5.616 6.068 6.42 6.272 6.621 6.971 6.703
   5162   1398826_s_at   8.347 8.465 8.428 8.589 8.588 9.125 8.715 8.989 9.209 9.118
   5163   1371997_at   8.7 8.885 8.645 9.127 9.028 9.47 9.477 9.679 9.459 9.32
   5164   1387041_at   9.51 9.534 9.59 9.659 9.669 9.364 9.387 9.231 9.228 9.12
   5165   1370554_at   9.534 9.529 9.328 9.324 9.521 8.946 9.021 9.14 8.879 8.669
   5166   1370148_at   5.308 5.482 5.503 5.601 5.516 3.814 2.83 4.533 4.255 3.825
   5167   1388851_at   10.431 10.516 10.37 10.63 10.415 10.712 10.741 10.746 10.769 10.864
   5168   1389050_at   9.321 9.166 9.344 9.488 9.245 9.716 9.581 9.882 9.916 9.728
   5169   1382616_at   7.538 7.538 7.676 7.385 7.377 6.907 6.725 6.488 5.986 6.008
   5170   1390665_at   5.409 5.515 6.062 5.509 5.511 6.234 6.246 6.2 6.379 6.319
   5171   1372419_at   9.322 9.183 9.324 9.129 9.203 8.992 8.932 8.914 8.936 8.727
   5172   1374413_at   9.898 10.069 10.126 10.021 9.972 10.275 10.24 10.327 10.365 10.214
   5173   1385667_x_at   4.824 5.22 5.214 5.524 5.882 6.572 6.265 6.676 6.274 6.32
   5174   1371668_at   8.156 8.06 8.202 8.316 8.384 8.752 8.454 8.665 8.714 8.745
   5175   1374969_at   6.378 6.322 6.639 6.526 6.629 7.307 6.811 7.305 7.391 7.097
   5176   1367702_at   11.08 11.076 11.166 11.271 11.07 11.514 11.396 11.61 11.713 11.848
   5177   1392125_at   6.155 6.629 6.893 6.446 6.512 7.199 7.302 7.284 7.373 7.087
   5178   1383675_at   6.627 6.143 6.514 6.881 5.909 5.147 5.343 5.579 5.48 5.223
   5179   1373826_at   9.265 9.108 9.319 9.327 9.123 9.545 9.522 9.479 9.574 9.461
   5180   1392705_at   5.726 5.311 5.513 5.632 5.617 6.274 6.057 5.972 6.437 6.098
   5181   1368914_at   7.711 7.604 7.487 7.804 8.235 6.909 6.557 6.789 6.005 5.907
   5182   1378440_at   7.207 7.411 7.438 7.413 7.51 6.93 6.947 6.938 6.718 6.458
   5183   1381190_at   8.709 8.57 8.512 9.048 9.011 9.315 9.635 9.781 9.461 9.46
   5184   1393761_at   2.893 3.163 2.685 3.013 2.68 3.493 3.651 4.285 4.198 4.353
   5185   1385638_at   7.113 7.07 6.79 6.935 7.205 7.684 7.427 7.801 7.865 7.475
   5186   1372175_at   9.832 9.812 9.579 9.553 9.475 10.071 10.069 10.176 10.228 10.003
   5187   1368358_a_at   6.589 6.954 6.722 6.95 6.748 7.168 7.308 7.476 7.706 7.534
   5188   1384757_at   5.822 5.49 5.845 5.439 5.698 5.295 4.889 4.969 4.805 5.052
   5189   1380115_at   4.666 3.902 4.008 3.873 3.635 5.004 5.064 5.752 6.033 5.522
   5190   1368128_at   9.552 9.645 9.036 8.97 9.451 6.095 5.505 4.762 4.061 0.934
   5191   1371879_at   8.442 8.237 7.995 8.109 8.248 7.592 7.742 7.696 7.536 7.235
   5192   1374712_at   9.871 9.78 10.084 10.036 10.051 9.539 9.4 9.716 9.408 9.419
   5193   1389418_at   7.125 6.809 6.839 6.94 6.904 7.409 7.397 7.744 7.55 7.26
   5194   1369935_at   8.939 8.794 8.948 8.984 8.979 8.785 8.509 8.57 8.553 8.514
   5195   1370474_at   5.264 5.252 5.956 5.629 5.949 6.516 6.425 6.935 6.63 6.507
   5196   1392644_s_at   7.017 7.466 7.36 7.583 7.318 7.852 7.956 7.939 8.015 7.806
   5197   1392713_a_at   7.927 7.748 7.8 7.888 7.471 8.2 8.388 8.388 8.217 8.194
   5198   1374404_at   8.718 8.801 8.573 8.841 8.885 7.858 7.809 6.877 7.037 6.427
   5199   1388530_at   9.147 9.573 9.498 9.435 9.633 9.036 9.01 8.925 8.876 8.786
   5200   1376013_at   5.931 5.848 5.774 6.019 6.04 6.515 6.732 6.549 6.368 6.22
   5201   1394195_at   4.353 4.775 4.314 5.067 4.672 5.794 5.296 5.463 5.727 5.505
   5202   1376974_at   8.521 8.512 8.582 8.735 8.695 8.285 8.095 8.391 8.169 8.229
   5203   1386999_at   10.513 10.557 10.225 10.066 10.33 9.758 9.7 9.892 9.739 9.481
   5204   1370437_at   7.594 7.777 7.662 7.616 7.636 7.412 7.421 7.47 7.428 7.273
   5205   1383258_at   4.936 4.839 4.869 5.202 5.223 5.78 5.815 5.876 5.625 6.435
   5206   1368126_at   6.656 6.808 6.756 6.795 6.926 6.048 5.825 5.731 5.147 6.162
   5207   1387863_at   10.24 10.183 10.045 10.193 10.215 10.391 10.369 10.409 10.504 10.371
   5208   1373142_at   9.942 9.966 9.861 9.987 10.004 9.681 9.707 9.746 9.787 9.556
   5209   1376776_at   6.553 6.179 6.794 6.732 6.552 7.105 7.193 7.162 7.204 7.145
   5210   1399159_a_at   9.812 10.054 10.047 10.056 10.214 9.697 9.293 9.601 9.537 9.424
   5211   1376751_at   6.947 7.392 7.218 7.287 7.581 6.518 6.582 6.732 6.181 6.577
   5212   1390357_at   6.812 6.648 6.49 6.682 6.856 7.066 7.032 7.243 7.135 7.076
   5213   1375915_at   6.983 6.917 6.581 6.967 6.828 7.43 7.819 7.606 7.776 7.245
   5214   1374012_at   3.898 4.331 5.448 5.253 5.125 6.255 6.72 6.642 6.484 6.455
   5215   1367655_at   11.424 11.554 11.822 10.865 11.248 10.425 10.382 10.179 9.483 9.838
   5216   1374613_at   7.35 7.028 7.129 7.116 7.102 7.574 7.875 8.182 7.833 7.617
   5217   1391672_at   6.218 6.166 5.612 5.807 6.109 6.888 6.629 6.57 6.716 6.668
   5218   1377196_at   5.718 5.828 6.287 6.127 6.067 6.566 6.56 7.004 6.838 6.996
   5219   1371745_at   9.147 9.118 9.175 9.236 9.303 8.93 8.722 8.834 8.906 8.998
   5220   1376656_at   7.52 7.579 7.706 8.053 7.501 8.28 8.261 8.315 8.202 8.189
   5221   1372282_at   8.145 8.408 8.123 8.359 8.559 9.144 8.84 8.872 9.005 8.735
   5222   1376705_a_at   7.785 7.722 7.94 8.064 7.964 8.253 8.204 8.449 8.506 8.432
   5223   1377380_at   6.554 6.701 6.876 6.944 6.932 6.163 6.306 6.174 6.158 5.71
   5224   1391161_at   7.215 7.05 6.864 6.707 6.918 6.409 6.415 6.261 5.877 6.057
   5225   1371249_at   10.621 10.613 10.465 10.649 10.717 10.281 10.268 10.398 10.275 10.41
   5226   1390890_at   5.259 5.789 5.657 6.051 4.897 2.245 4.295 3.275 2.521 3.629
   5227   1377675_at   8.255 8.403 8.516 8.607 8.331 7.84 7.743 7.978 8.055 7.575
   5228   1379578_at   8.528 8.536 8.759 8.928 9.023 9.546 9.268 9.889 9.766 9.439
   5229   1381363_at   3.949 3.725 3.899 4.396 3.599 4.487 4.869 5.07 4.824 5.053
   5230   1389148_at   8.24 8.017 8.08 8.177 8.299 8.486 8.735 8.757 8.583 8.46
   5231   1388518_at   8.816 8.95 8.942 8.827 8.777 9.217 9.049 9.364 9.265 9.16
   5232   1398262_at   8.727 8.637 8.73 8.71 8.641 9.091 8.947 9.406 9.096 9.349
   5233   1373632_at   7.822 8.108 7.852 7.983 7.804 7.242 7.368 7.107 7.143 7.635
   5234   1397676_at   7.201 7.228 7.885 7.112 7.742 6.785 5.789 6.047 6.153 5.858
   5235   1381940_at   4.757 4.537 5.582 5.708 5.518 6.509 6.484 6.554 6.827 6.523
   5236   1391068_at   4.394 4.928 4.993 5.108 4.868 5.924 5.977 5.534 6.438 5.75
   5237   1379545_at   4.816 5.303 5.24 5.791 6.027 6.723 6.611 6.942 6.648 6.49
   5238   1380531_at   5.086 5.202 4.808 5.486 5.138 3.982 3.053 3.506 1.807 2.958
   5239   1372774_at   9.368 9.405 9.602 9.479 8.921 10.073 9.881 10.297 10.458 10.38
   5240   1387892_at   11.149 11.121 11.105 11.137 11.152 10.865 10.48 10.36 10.078 10.284
   5241   1373759_at   4.634 4.939 5.203 5.551 4.661 3.126 4.079 3.733 3.202 3.855
   5242   1371059_at   6.179 6.116 6.102 5.852 5.722 6.976 6.41 6.633 6.962 6.793
   5243   1388925_at   8.158 7.967 7.967 8.072 8.278 8.395 8.473 8.585 8.446 8.415
   5244   1373401_at   8.342 8.408 8.578 8.632 8.412 7.822 7.748 7.629 6.93 7.606
   5245   1388383_at   8.553 8.586 8.422 8.4 8.204 8.934 8.926 9.072 8.992 8.714
   5246   1379780_at   5.702 5.731 6.045 5.686 5.389 4.548 4.674 4.129 4.868 5.081
   5247   1398762_at   12.267 12.138 12.263 12.007 12.156 11.922 11.873 11.866 11.71 11.776
   5248   1370302_at   7.254 7.087 7.434 7.499 7.394 7.988 7.87 8.575 8.111 8.027
   5249   1381264_at   4.024 4.376 4.826 4.505 4.398 5.078 5.235 5.169 5.591 5.482
   5250   1392128_at   5.65 5.967 5.843 5.837 5.783 5.425 5.216 4.903 5.408 5.14
   5251   1375071_at   6.525 6.589 6.676 6.328 6.433 6.067 5.359 5.126 5.055 5.62
   5252   1373052_at   9.062 9.052 9.085 9.253 9.204 8.87 8.918 8.811 8.679 8.807
   5253   1382004_at   5.842 6.283 5.901 5.988 6.003 6.727 6.731 6.794 6.397 6.442
   5254   1372840_at   7.477 7.567 7.624 7.715 7.725 7.065 7.255 7.218 7.319 6.929
   5255   1388932_at   7.153 7.243 7.636 7.719 7.349 8.103 7.959 8.24 7.988 8.177
   5256   1390872_at   6.164 6.381 6.257 6.285 6.62 7.12 6.867 6.81 6.935 7.325
   5257   1367477_at   9.716 9.808 9.802 9.995 9.869 10.205 10.237 10.095 10.23 10.054
   5258   1368260_at   8.153 8.207 8.062 8.195 7.884 7.59 7.091 6.894 6.444 6.501
   5259   1371354_at   6.588 6.665 6.88 6.488 6.227 7.161 7.185 7.649 7.277 7.397
   5260   1380979_a_at   7.821 8.077 8.203 8.11 8.133 8.417 8.447 8.569 8.707 8.491
   5261   1372733_at   8.153 8.324 8.269 8.36 8.269 8.656 8.682 8.995 8.778 8.546
   5262   1397788_at   7.037 6.969 7.063 7.152 7.553 7.772 7.757 8.043 8.388 8.179
   5263   1375973_at   7.557 7.571 7.598 7.681 7.675 7.221 7.482 7.131 7.127 7.192
   5264   1372465_at   6.857 7.028 6.953 7.027 6.714 7.294 7.239 7.242 7.284 7.207
   5265   1381403_at   7.875 7.799 7.715 8.068 7.536 6.617 7.367 6.954 6.801 7.049
   5266   1391822_at   4.719 4.951 4.728 4.595 5.217 5.466 5.548 5.917 6.109 6.198
   5267   1399102_at   8.087 8.057 8.323 8.333 8.379 8.713 8.788 9.012 8.565 8.864
   5268   1376630_at   5.422 5.536 5.771 5.871 5.431 6.337 6.032 6.135 6.296 6.161
   5269   1373162_at   7.758 7.786 7.722 8.119 8.11 8.586 8.316 8.738 8.5 8.451
   5270   1394160_at   7.01 6.985 6.799 6.871 7.215 6.267 6.191 6.126 5.805 5.315
   5271   1371862_at   8.815 8.876 8.624 8.789 8.804 8.525 8.4 8.407 8.171 8.181
   5272   1390390_at   6.905 6.992 6.721 7.122 6.898 6.407 6.227 6.532 6.117 6.507
   5273   1371615_at   7.487 7.575 7.749 7.794 7.271 8.21 8.11 8.224 8.443 8.672
   5274   1394467_at   6.111 5.916 6.238 6.286 5.674 6.634 6.71 6.592 6.831 6.776
   5275   1380201_at   7.692 7.545 7.769 7.993 7.691 7.341 6.985 7.333 7.155 7.18
   5276   1377166_at   7.622 7.87 7.664 8.081 7.935 8.329 8.209 8.517 8.52 8.495
   5277   1384233_at   4.517 5.19 4.975 5.329 5.029 3.839 4.22 4.142 3.372 3.488
   5278   1383616_at   9.021 9.181 8.884 9.03 9.355 8.367 8.485 8.686 8.552 8.188
   5279   1374118_at   8.989 8.914 9.001 9.111 9.007 8.483 8.581 8.611 8.243 8.054
   5280   1383567_at   3.186 4.84 4.355 4.169 4.037 5.955 5.609 5.527 5.59 5.586
   5281   1388532_at   8.111 8.139 8.073 8.157 8.211 7.941 7.951 7.963 7.818 7.772
   5282   1377760_at   7.783 7.551 7.745 7.635 7.806 7.271 7.336 7.318 7.171 6.911
   5283   1380120_at   4.439 4.893 5.421 4.512 4.881 6.352 5.824 5.959 5.682 6.289
   5284   1371962_at   10.074 9.996 10.232 10.214 10.197 10.515 10.338 10.423 10.484 10.524
   5285   1387638_a_at   6.034 6.116 6.988 6.351 6.025 3.786 4.818 2.392 4.06 2.362
   5286   1396977_at   3.472 3.722 4.375 3.938 3.471 4.727 4.929 4.637 4.703 5.021
   5287   1392487_at   7.469 7.645 7.592 7.983 7.794 7.257 6.993 7.244 6.983 7.098
   5288   1383250_at   7.514 7.402 7.69 7.704 7.649 7.228 6.968 7.278 7.116 6.943
   5289   1397307_at   5.82 5.534 5.818 5.841 5.18 6.478 6.405 6.588 6.565 6.183
   5290   1371711_at   8.63 8.63 8.729 8.744 8.902 9.19 9.025 9.097 9.309 9.433
   5291   1377079_a_at   6.686 6.813 7.059 6.966 6.919 7.4 7.514 7.904 7.406 7.403
   5292   1377735_at   9.294 9.287 9.18 9.294 9.169 8.866 9.003 9.108 8.902 8.908
   5293   1398985_at   7.653 7.835 7.681 7.875 7.823 7.46 6.795 7.066 6.815 6.649
   5294   1375243_at   4.895 5.015 5.19 4.181 4.822 5.682 5.788 5.746 6.004 5.801
   5295   1371824_at   9.841 9.76 9.998 10.201 9.375 10.592 10.509 10.943 10.691 10.691
   5296   1371099_at   10.165 9.992 10.062 10.162 9.757 9.569 9.513 9.647 9.368 9.603
   5297   1373069_at   8.395 8.524 8.55 8.66 8.57 8.84 8.883 8.79 8.971 8.761
   5298   1389313_at   7.362 7.533 7.408 7.552 7.421 7.828 7.81 8.101 7.797 7.74
   5299   1384487_at   5.564 4.804 4.556 4.708 4.327 6.107 5.845 6.121 6.073 5.812
   5300   1374630_at   5.924 6.167 6.118 6.133 5.168 7.073 6.731 7.05 7.191 7.396
   5301   1374305_at   7.119 7.293 7.477 7.253 7.12 6.994 6.685 6.617 6.627 6.731
   5302   1396109_at   3.727 4.109 4.351 3.78 4.424 5.243 5.266 4.686 5.674 5.434
   5303   1392706_at   6.013 6.063 6.087 6.178 6.368 5.773 5.26 5.593 5.476 5.665
   5304   1394216_at   5.338 5.938 5.64 4.984 5.807 6.6 6.61 6.481 6.336 6.56
   5305   1374615_at   7.722 7.74 7.682 7.819 7.727 7.013 7.032 7.492 6.956 6.719
   5306   1368967_at   7.177 7.397 7.475 7.49 7.442 6.986 7.036 6.711 6.974 7.023
   5307   1392316_at   3.178 2.147 3.077 3.42 3.172 4.274 4.872 4.376 4.256 4.146
   5308   1370906_at   10.02 9.717 9.931 10.124 9.868 10.514 10.219 10.665 10.463 10.528
   5309   1392947_at   5.195 5.319 5.104 5.287 5.421 6.641 5.631 6.74 6.493 6.37
   5310   1383299_at   6.601 6.59 6.22 6.418 6.643 6.928 7.074 7.334 7.262 6.955
   5311   1385470_at   6.059 6.025 5.809 5.936 6.004 6.494 6.45 6.543 6.151 6.415
   5312   1391443_at   7.358 7.462 7.67 7.566 7.474 7.86 7.94 8.104 7.855 7.802
   5313   1373448_at   8.039 7.971 8.25 8.221 7.941 8.484 8.445 8.795 8.528 8.586
   5314   1398801_at   10.735 10.327 10.319 10.399 10.445 10.031 9.918 10.01 10.07 9.847
   5315   1367722_at   10.593 10.536 10.787 10.637 10.717 11.115 11.142 10.956 11.152 10.87
   5316   1385921_at   9.305 9.42 9.234 9.484 9.539 8.833 8.928 9.128 9.06 8.956
   5317   1382400_at   6.602 6.359 6.107 6.498 6.709 6.054 5.599 5.533 5.545 5.67
   5318   1392498_at   8.155 8.194 8.401 8.408 8.147 7.836 7.945 7.781 7.536 7.777
   5319   1370006_at   10.831 10.819 10.784 10.96 10.974 11.228 11.041 11.326 11.3 11.368
   5320   1372432_at   7.963 7.904 7.703 8.105 8.03 7.638 7.249 7.323 7.371 7.107
   5321   1368143_at   9.703 9.782 9.671 9.86 9.875 9.29 9.53 9.392 9.26 9.07
   5322   1371973_at   11.409 11.38 11.396 11.219 11.385 10.946 10.892 10.825 10.747 10.351
   5323   1385929_at   5.724 6.324 5.895 6.17 5.694 6.544 6.629 6.819 6.743 6.679
   5324   1384824_at   6.099 6.266 6.199 6.418 6.639 7.671 7.142 7.351 7.216 6.816
   5325   1373689_at   5.39 6.149 6.211 6.072 5.62 7.217 6.572 7.319 7.258 6.905
   5326   1383497_at   6.434 6.266 6.236 6.949 6.265 7.077 7.152 7.49 7.636 7.439
   5327   1389038_at   9.11 9.195 9.276 9.508 9.39 8.996 8.823 8.883 8.792 8.848
   5328   1376576_at   8.528 8.363 8.543 8.597 8.64 7.899 8.156 8.124 8.159 7.751
   5329   1395465_at   8.085 7.98 8.003 7.813 8.048 7.474 7.645 7.167 7.595 7.305
   5330   1375057_at   4.38 3.123 4.739 5.025 4.71 6.133 6.077 6.257 6.314 6.499
   5331   1380763_at   1.727 2.956 3.063 3.239 2.032 3.856 4.7 5.267 4.84 4.674
   5332   1389281_at   5.674 6.028 5.829 6.059 6.324 6.741 6.682 6.622 6.509 6.561
   5333   1374807_at   7.252 7.192 7.473 7.322 7.464 7.764 7.586 7.754 7.685 7.671
   5334   1372296_at   4.262 4.403 4.489 4.692 4.547 4.787 4.981 5.084 5.18 4.993
   5335   1376673_at   7.842 7.84 8.182 8.144 8.179 8.625 8.429 8.586 8.65 8.434
   5336   1386045_at   4.403 3.942 4.133 3.2 2.832 5.361 5.377 5.503 5.247 5.179
   5337   1389428_at   9.271 9.149 9.083 9.349 9.202 8.948 8.899 9.004 8.864 8.928
   5338   1391656_at   6.509 6.492 6.44 6.531 6.18 7.496 6.78 7.593 7.184 7.45
   5339   1367693_at   10.7 10.613 10.57 10.739 10.759 10.298 10.285 10.246 9.991 10.416
   5340   1399138_at   7.139 7.218 7.04 7.251 7.089 7.441 7.427 7.788 7.679 7.759
   5341   1381974_at   6.595 6.956 6.549 6.772 6.731 7.572 7.681 7.595 7.379 7.041
   5342   1396521_at   6.449 6.471 6.637 6.629 6.625 6.018 5.978 5.565 6.029 6.185
   5343   1389265_at   9.394 9.584 9.444 9.363 9.351 9.709 9.681 9.991 9.816 9.812
   5344   1385838_a_at   8.372 8.385 8.488 8.538 8.594 8.823 8.691 8.961 8.963 8.782
   5345   1382219_at   6.846 7.06 7.156 7.299 6.932 7.505 7.522 7.725 7.5 7.512
   5346   1384831_at   11.022 11.25 11.243 11.517 10.421 12.051 12.108 12.102 12.09 12.4
   5347   1370419_a_at   7.259 7.06 7.272 7.911 7.498 6.691 6.205 6.344 6.575 6.573
   5348   1370910_at   8.172 8.293 8.36 8.387 8.254 7.136 7.713 7.637 7.474 7.892
   5349   1373595_at   9.153 9.208 9.286 9.428 9.51 8.893 8.866 9.009 8.894 8.72
   5350   1368674_at   9.173 9.127 9.062 9.281 9.249 8.664 8.769 8.904 8.806 8.468
   5351   1376518_at   5.93 6.418 6.877 6.858 6.539 7.341 7.666 7.709 7.715 7.388
   5352   1397468_at   8.96 9.086 9.171 9.126 9.117 9.344 9.351 9.6 9.636 9.653
   5353   1370134_at   8.154 8.222 8.222 8.206 8.22 8.526 8.492 8.626 8.382 8.362
   5354   1387184_at   4.723 4.941 4.747 5.289 5.253 5.821 5.648 6.36 5.904 5.824
   5355   1385344_at   6.32 6.397 6.638 6.628 6.495 5.772 5.678 6.172 6.04 5.84
   5356   1377673_at   7.969 7.894 8.029 8.132 7.91 8.395 8.631 8.875 8.544 8.333
   5357   1374441_at   6.964 7.047 7.199 7.061 6.929 7.391 7.42 7.29 7.267 7.328
   5358   1397821_at   5.353 5.382 5.855 5.783 5.547 6.324 6.211 6.263 6.423 6.85
   5359   1370588_a_at   4.744 4.365 5.366 4.823 4.23 5.783 5.805 5.776 5.973 5.708
   5360   1370817_at   8.72 8.842 8.731 8.854 9.279 8.251 8.28 8.387 8.372 8.265
   5361   1367845_at   6.039 5.846 6.037 6.118 6.502 7.105 6.621 7.254 7.35 6.857
   5362   1369278_at   7.815 7.723 7.662 7.693 7.634 8.004 8.199 8.068 8.257 7.91
   5363   1376170_at   4.542 5.443 4.944 4.831 5.189 6.205 6.139 6.288 6.205 5.568
   5364   1383376_at   7.896 7.974 8.001 7.643 7.52 8.224 8.327 8.477 8.487 8.441
   5365   1383074_at   8.377 8.366 8.571 8.517 8.439 8.225 7.998 8.145 8.056 7.867
   5366   1398765_at   11.641 11.584 11.519 11.663 11.677 11.335 11.414 11.4 11.163 11.223
   5367   1381650_at   3.418 3.152 3.717 3.306 2.667 4.917 4.588 5.037 4.043 4.405
   5368   1398980_at   8.422 8.458 8.544 8.718 8.587 8.289 7.89 8.103 7.827 7.999
   5369   1380600_at   6.212 6.17 5.84 6.468 6.432 6.931 6.76 6.814 7.122 7.015
   5370   1374680_at   6.731 6.792 6.691 6.769 6.924 7.056 7.21 7.502 7.151 7.305
   5371   1390409_at   8.08 8.001 8.367 8.305 8.195 7.913 7.517 7.508 7.683 7.512
   5372   1367641_at   12.219 12.068 12.08 12.095 12.029 12.297 12.393 12.301 12.428 12.53
   5373   1376334_at   4.709 4.895 4.265 4.883 4.895 5.57 5.643 5.574 5.51 5.212
   5374   1388128_at   7.89 7.808 7.814 7.875 7.876 7.204 6.83 7.02 7.433 6.517
   5375   1372027_at   8.624 8.683 8.455 8.735 8.635 8.083 7.728 8.328 8.098 7.967
   5376   1389263_at   8.531 8.699 8.471 8.7 8.588 8.369 7.916 8.03 7.88 7.773
   5377   1396113_at   6.478 6.85 6.652 7.017 6.221 7.497 7.267 7.452 7.772 7.663
   5378   1375246_at   11.184 11.177 11.121 11.105 11.177 10.933 10.903 10.99 11.011 10.791
   5379   1373138_at   7.569 7.419 7.67 7.667 7.943 7.075 6.772 6.842 6.843 6.208
   5380   1375894_at   8.507 8.678 8.858 8.617 8.811 9.099 9.171 9.154 9.426 9.062
   5381   1390108_at   7.657 7.421 7.609 7.836 7.756 8.049 7.972 8.138 8.149 8.065
   5382   1380089_at   5.398 4.769 5.195 5.016 4.351 5.907 5.935 6.234 6.232 5.828
   5383   1379402_at   7.712 7.681 7.918 8.168 7.865 8.649 8.196 8.717 8.621 8.578
   5384   1379832_at   9.901 9.973 9.809 9.762 9.682 9.201 9.532 9.392 9.221 9.009
   5385   1374104_at   6.369 6.479 6.238 6.753 6.815 7.454 6.94 7.488 7.396 7.419
   5386   1374112_at   6.806 6.629 6.979 6.808 6.879 7.268 7.035 7.249 7.231 7.273
   5387   1376091_at   8.252 8.325 8.109 8.303 8.358 7.968 8.003 8.046 7.829 7.844
   5388   1378896_at   5.845 5.936 6.121 5.95 5.259 6.57 6.524 6.868 6.983 6.796
   5389   1368501_s_at   6.615 6.679 6.708 6.084 6.691 4.548 4.318 4.844 3.547 2.16
   5390   1368473_at   5.362 5.777 5.546 5.993 5.709 6.295 6.229 6.315 6.185 6.344
   5391   1367870_at   10.346 10.324 10.216 10.209 10.36 10.015 10.166 10.049 10.007 9.985
   5392   1396014_at   4.676 4.728 3.956 4.32 3.569 5.549 5.411 6.218 5.447 5.76
   5393   1394953_at   6.005 5.621 5.919 6.279 6.317 5.101 5.239 5.306 4.541 4.88
   5394   1375954_at   6.933 7.091 6.963 7.368 7.584 8.034 7.789 8.007 8.013 7.791
   5395   1376872_at   6.293 6.21 6.192 6.441 6.232 4.456 3.744 3.632 3.754 1.322
   5396   1397579_x_at   5.242 5.243 4.808 5.697 5.826 6.79 6.128 7.124 7.419 6.949
   5397   1374039_at   6.563 6.857 6.905 6.498 6.74 7.454 7.626 7.219 7.624 7.13
   5398   1389450_at   8.551 8.48 8.29 8.184 8.189 7.997 7.91 7.887 7.8 7.738
   5399   1384439_at   6.828 6.716 6.77 6.823 6.612 6.011 6.434 6.247 5.848 6.248
   5400   1391317_at   6.685 6.945 6.462 6.658 6.817 6.046 5.711 5.422 4.71 5.282
   5401   1370174_at   7.391 7.556 7.848 7.954 7.592 6.943 6.89 6.708 6.631 6.042
   5402   1368224_at   7.973 8.063 7.577 8.061 7.861 7.091 7.233 6.633 6.314 6.182
   5403   1394948_at   7.206 7.207 7.19 7.127 7.061 7.996 7.551 8.107 7.715 7.564
   5404   1384451_at   7.39 7.593 7.346 7.577 7.572 6.951 6.55 7.171 6.92 6.774
   5405   1367849_at   7.896 7.937 7.984 7.904 7.848 7.487 7.445 7.627 7.625 7.202
   5406   1376619_at   8.189 8.185 8.368 8.371 8.315 8.84 8.832 9.397 8.921 8.746
   5407   1385614_at   6.216 6.205 6.711 6.62 6.338 7.097 6.988 7.041 7.373 7.506
   5408   1374194_at   8.242 8.202 8.185 8.317 8.424 7.822 7.741 8.014 7.767 7.495
   5409   1395533_at   7.986 8.07 8.016 8.206 8.352 7.747 7.758 7.619 7.436 7.442
   5410   1379039_at   6.232 4.771 5.675 6.057 5.681 4.22 3.356 3.454 4.534 3.77
   5411   1369586_at   4.08 4.012 3.342 3.838 4.005 0.322 0 2.96 0 0.575
   5412   1370230_at   11.067 11.121 11.312 11.256 11.296 11.577 11.451 11.528 11.664 11.748
   5413   1378292_at   6.326 6.568 6.533 6.719 6.443 7.039 6.957 7.614 7.507 7.489
   5414   1372417_at   8.474 8.463 8.504 8.659 8.661 8.052 8.204 8.228 7.857 7.808
   5415   1370813_at   5.785 6.175 6.09 6.174 6.709 7.249 7.221 7.172 7.061 6.798
   5416   1395265_at   10.046 10.031 10.183 10.154 10.03 10.238 10.472 10.331 10.407 10.374
   5417   1376483_at   8.432 8.578 8.523 8.555 8.817 8.071 7.854 8.243 8.09 7.766
   5418   1388138_at   3.956 4.024 4.043 4.561 4.268 5.142 5.478 5.349 4.806 4.811
   5419   1374384_at   2.945 3.374 2.868 3.43 1.057 4.849 5.162 5.367 5.452 4.926
   5420   1386344_at   6.255 6.088 6.384 6.34 5.977 6.695 6.608 6.73 6.62 6.586
   5421   1368735_a_at   6.297 6.344 6.244 6.326 6.052 4.372 4.374 2.263 4.284 4.488
   5422   1370057_at   10.276 10.291 10.2 10.426 10.571 10.024 9.731 10.001 9.894 9.822
   5423   1368672_at   7.79 7.648 8.004 7.715 7.263 8.426 8.25 8.488 8.725 8.835
   5424   1370697_a_at   4.647 4.61 4.445 4.864 5.008 5.554 5.833 5.259 5.269 5.609
   5425   1368953_at   7.753 7.679 7.683 7.664 7.973 7.526 7.096 7.156 7.083 7.219
   5426   1374735_at   6.541 6.89 7.069 6.337 6.233 5.455 5.052 4.723 4.515 3.489
   5427   1373000_at   5.032 4.548 4.762 5.208 6.134 6.528 6.438 6.816 6.774 6.722
   5428   1372332_at   6.422 6.387 6.166 6.328 6.696 7.244 6.836 7.155 7.115 6.851
   5429   1372870_at   7.136 7.104 6.978 7.031 7.505 7.905 7.993 7.787 7.701 7.565
   5430   1398829_at   10.608 10.594 10.698 10.595 10.742 10.46 10.404 10.246 10.151 10.277
   5431   1369652_at   6.548 6.455 6.112 6.465 7.354 7.835 7.91 7.778 7.855 7.464
   5432   1368660_at   5.858 6.177 5.998 5.851 6.008 6.678 6.314 6.491 6.335 6.632
   5433   1377702_at   7.216 6.812 6.9 6.727 6.831 7.498 7.821 7.401 7.409 7.442
   5434   1377041_at   6.818 6.561 6.818 6.806 6.914 6.055 5.953 6.227 6.342 5.61
   5435   1388997_at   10.184 10.227 10.142 10.422 10.437 9.842 9.666 10.008 9.817 9.634
   5436   1387547_a_at   8.156 8.273 8.376 8.353 8.424 8.817 8.676 8.887 8.921 8.543
   5437   1374133_at   7.39 7.528 7.266 7.454 7.835 6.849 6.816 6.888 6.529 7.028
   5438   1370143_at   4.866 4.985 4.271 5.197 5.17 6.499 5.942 6.02 6.034 5.692
   5439   1369182_at   9.407 9.419 9.611 9.451 9.36 8.936 8.947 8.908 8.792 8.327
   5440   1389169_at   7.444 7.55 7.411 7.59 7.397 7.853 7.899 8.258 8.213 7.838
   5441   1375530_at   9.005 9.176 9.15 9.171 9.091 9.6 9.548 9.49 9.388 9.291
   5442   1389033_at   9.013 8.918 8.934 9.14 8.921 9.252 9.375 9.25 9.428 9.21
   5443   1371422_at   11.263 11.254 11.137 11.154 11.362 11.072 10.91 10.858 10.799 10.81
   5444   1373599_at   6.5 6.766 6.792 7.035 7.019 7.896 7.247 7.94 7.684 7.584
   5445   1374605_at   7.044 7 7.171 7.32 6.831 7.617 7.494 8.059 7.842 7.75
   5446   1382371_at   6.951 6.746 7.169 7.06 7.109 7.646 7.948 8.008 7.623 7.402
   5447   1375745_at   8.548 8.389 8.313 8.49 8.633 8.839 8.835 8.717 8.845 8.785
   5448   1390944_at   6.46 6.871 7.023 7.207 6.5 7.6 7.55 7.602 7.598 7.622
   5449   1395621_at   7.826 7.798 8.354 8.3 7.961 8.841 8.548 8.79 8.748 8.721
   5450   1371367_at   9.583 9.644 9.478 9.803 9.823 9.34 9.013 9.295 9.153 9.028
   5451   1374403_at   6.996 6.863 7.158 7.146 7.06 7.839 7.607 7.61 7.334 7.484
   5452   1389459_at   8.334 8.258 8.38 8.259 8.141 8.652 8.867 8.592 8.616 8.528
   5453   1376734_at   4.743 4.728 5.314 4.12 4.957 5.635 5.923 5.916 5.857 5.935
   5454   1391944_at   5.477 5.233 5.05 5.57 5.598 6.14 6.226 6.035 6.4 5.856
   5455   1385211_at   6.403 6.224 6.732 6.367 6.397 7.026 7.087 7.185 6.795 6.906
   5456   1384334_at   3.225 3.459 3.494 4.44 3.887 6.136 6.481 6.242 4.983 4.992
   5457   1387228_at   9.152 9.241 9.126 9.215 8.896 10.167 10.518 10.356 9.891 9.554
   5458   1397865_at   4.204 3.629 4.129 3.619 3.889 4.807 4.685 4.488 4.519 4.58
   5459   1389338_at   8.771 8.729 8.823 8.77 8.944 9.167 9.287 9.263 9.272 8.96
   5460   1389559_at   9.273 9.171 9.253 9.351 9.427 9.003 8.924 8.953 8.924 8.65
   5461   1389345_at   7.87 7.471 7.679 7.845 7.669 7.369 7.194 7.235 7.241 6.999
   5462   1370317_at   6.695 6.882 6.464 6.856 6.726 7.665 7.472 7.435 7.118 7.21
   5463   1377076_at   7.753 7.805 7.793 8.016 7.692 8.339 8.397 8.618 8.343 8.078
   5464   1392835_at   7.422 7.459 7.39 7.457 7.627 7.942 7.816 7.99 7.956 7.649
   5465   1372701_at   12.397 12.372 12.261 12.071 12.18 11.921 11.972 11.781 11.732 11.878
   5466   1368632_at   2.578 2.37 1.757 3.128 2.986 4.001 4.714 3.948 4.049 3.821
   5467   1374315_at   4.828 4.624 5.038 4.881 4.572 5.319 5.793 5.923 5.699 5.294
   5468   1391812_at   5.492 5.403 5.518 5.432 5.845 5.923 6.049 5.946 6.125 5.981
   5469   1380002_at   6.504 6.493 6.677 6.692 6.536 6.813 6.943 6.987 7.213 7.016
   5470   1370752_a_at   8.013 7.687 7.874 8.074 7.824 7.598 7.181 7.38 7.163 7.356
   5471   1369061_at   8.15 8.287 8.639 8.47 8.254 8.988 8.764 8.796 8.88 8.936
   5472   1397365_at   5.004 4.541 5.07 5.464 5.071 5.82 5.708 5.987 5.82 5.943
   5473   1367785_at   5.062 4.476 4.713 4.902 4.787 5.486 5.429 5.322 5.405 5.844
   5474   1388969_at   8.465 8.318 8.524 8.707 8.377 8.842 8.808 9.104 8.929 8.979
   5475   1391628_at   8.299 8.317 8.287 8.415 8.445 8.068 7.973 8.212 7.978 7.92
   5476   1367774_at   10.736 10.687 10.904 10.768 10.698 11.195 11.266 11.287 11.655 11.817
   5477   1371495_at   10.823 10.762 10.861 10.779 10.851 10.601 10.475 10.638 10.49 10.328
   5478   1368366_at   8.638 8.637 9.15 9.002 7.619 9.96 9.84 10.187 10.417 10.461
   5479   1388930_at   10.949 10.95 10.827 10.801 10.999 10.541 10.624 10.683 10.54 10.361
   5480   1373619_at   7.204 6.872 7.204 6.76 6.866 7.692 7.495 7.653 7.708 7.374
   5481   1371431_at   7.826 7.66 7.823 8.351 7.755 8.451 8.495 8.689 8.756 8.582
   5482   1368386_at   9.569 9.536 9.777 9.916 9.738 9.27 9.165 9.426 9.24 9.259
   5483   1383756_at   3.643 3.488 3.64 4.718 2.872 5.123 5.696 5.299 5.724 5.155
   5484   1369207_at   6.015 6.339 6.16 6.08 6.036 5.462 4.689 4.097 4.169 3.57
   5485   1389280_at   1.618 1.48 0.614 2.872 1.803 3.963 3.096 4.065 4.284 4.034
   5486   1382679_at   6.965 7.144 6.823 6.89 6.956 6.15 6.536 6.346 5.708 6.052
   5487   1392987_at   7.65 7.751 7.682 7.873 7.79 7.907 7.985 8.012 8.026 8.02
   5488   1371564_at   10.262 10.327 10.247 10.481 10.483 10.7 10.555 10.745 10.762 10.724
   5489   1370360_at   8.559 8.432 8.57 8.618 8.671 8.886 8.811 9.078 8.929 8.817
   5490   1371030_at   10.953 10.848 10.894 11.056 9.861 12.062 12.102 11.789 11.805 11.898
   5491   1368424_at   7.922 7.63 7.936 8.057 7.816 7.562 7.31 7.481 7.283 7.301
   5492   1380437_at   5.177 4.571 4.515 5.146 4.928 5.572 5.717 6.114 6.227 5.716
   5493   1374095_at   6.183 6.25 6.219 6.54 5.488 5.306 4.822 4.538 4.433 5.149
   5494   1387728_at   5.722 5.17 5.305 5.55 5.412 1.506 3.461 3.548 3.932 2.16
   5495   1368826_at   11.714 11.692 11.807 11.583 11.408 12.071 12.364 12.122 12.074 12.011
   5496   1372298_at   8.738 8.747 8.65 8.825 8.895 9.004 9.198 9.291 9.066 9.067
   5497   1374653_at   7.503 7.214 7.707 7.088 7.462 8.135 7.895 8.24 8.108 7.951
   5498   1376996_at   7.508 7.371 7.212 7.605 7.605 7.015 6.862 7.139 6.924 7.008
   5499   1380130_at   5.995 6.404 5.613 6.175 6.227 5.023 4.658 5.268 4.531 5.351
   5500   1392926_at   4.944 5.235 5.405 5.81 5.204 6.126 5.98 6.183 6.252 6.052
   5501   1390109_at   7.959 7.908 8.181 8.111 8.201 7.6 7.41 7.647 7.737 7.289
   5502   1367649_at   7.413 7.669 7.635 7.998 7.534 8.503 8.251 8.05 8.399 8.507
   5503   1390523_at   5.393 5.442 5.705 6.047 5.624 6.652 6.287 6.955 6.479 6.318
   5504   1381017_at   7.022 7.01 7.001 7.019 7.131 6.47 6.75 6.505 6.595 6.143
   5505   1369514_at   4.23 2.84 2.566 2.039 1.17 5.185 5.239 5.13 5.303 5.369
   5506   1391280_at   6.845 6.737 6.723 7.174 7.06 7.546 7.316 7.713 7.41 7.689
   5507   1370003_at   11.738 11.615 11.651 11.613 11.712 11.534 11.411 11.431 11.361 11.272
   5508   1383432_at   5.433 5.209 4.598 5.087 4.961 5.594 6.043 6.143 6.353 6.332
   5509   1368440_at   12.61 12.543 12.631 12.461 12.135 13.024 13.06 12.914 12.929 13.192
   5510   1391173_at   6.001 6.339 6.254 6.505 6.146 6.991 7.028 7.222 6.881 6.611
   5511   1387012_at   8.282 8.405 8.112 8.42 8.363 7.924 7.925 8.106 7.936 7.918
   5512   1398553_at   6.57 6.388 7.225 6.872 7.334 5.872 5.821 5.954 5.941 5.934
   5513   1383685_at   6.559 6.445 6.682 7.007 7.272 5.767 5.997 6.14 5.684 5.743
   5514   1372145_at   8.243 8.341 8.349 8.671 8.502 8.068 7.854 8.051 7.811 7.801
   5515   1376446_at   7.789 7.747 8.038 7.906 7.833 8.277 8.052 8.311 8.261 8.225
   5516   1371255_at   8.9 8.866 8.855 9.027 8.92 9.267 9.109 9.254 9.511 9.361
   5517   1374438_at   8.671 8.917 8.825 9.055 8.991 8.38 8.128 8.617 8.197 8.208
   5518   1384904_at   4.943 4.599 4.928 4.719 5.375 5.506 5.648 5.677 5.923 5.756
   5519   1389484_at   6.16 6.373 6.463 6.667 6.161 7.056 6.985 7.071 6.774 6.882
   5520   1390119_at   6.319 5.984 5.147 5.439 5.751 6.755 6.833 6.914 7.208 6.769
   5521   1383543_at   6.931 7.104 7.277 7.166 7.088 6.211 6.851 6.48 6.326 6.475
   5522   1384073_at   7.24 7.452 8.055 7.158 6.351 8.974 8.536 8.782 8.622 9.036
   5523   1392754_at   4.659 5.426 5.718 5.179 4.93 4.172 3.625 4.052 3.697 2.975
   5524   1380835_at   5.558 5.736 6.062 5.721 5.541 4.758 3.608 4.802 3.579 4.369
   5525   1398610_at   6.323 6.422 7.048 6.758 6.895 7.267 7.582 7.532 7.449 7.603
   5526   1395373_at   7.132 7.424 7.193 7.204 6.62 7.745 7.752 8.056 7.996 7.921
   5527   1385868_at   4.169 4.568 4.36 3.927 4.553 5.626 5.153 5.755 5.119 5.011
   5528   1393110_at   8.628 8.906 8.709 9.003 8.961 9.27 9.384 9.374 9.314 9.136
   5529   1371653_at   11.394 11.393 11.318 11.369 11.466 11.204 11.132 11.053 10.869 10.932
   5530   1396297_at   5.025 5.291 5.669 5.462 4.711 3.021 2.39 2.648 3.568 0.614
   5531   1388749_at   8.285 8.341 8.566 8.588 8.238 8.858 8.782 9.139 8.826 8.989
   5532   1377736_at   5.363 4.972 4.545 5.116 5.069 6.62 6.315 6.842 6.253 5.603
   5533   1398233_at   3.748 4.673 4.649 4.719 4.651 5.662 5.259 5.573 5.556 5.683
   5534   1388517_at   7.962 8.224 8.115 7.999 8.135 8.297 8.372 8.453 8.466 8.354
   5535   1373036_at   7.847 7.954 7.846 8.264 7.94 8.371 8.398 8.544 8.571 8.343
   5536   1383152_at   7.756 7.727 7.656 7.897 8.114 7.273 7.4 7.223 6.845 6.97
   5537   1396219_at   5.412 4.885 5.202 5.11 5.365 6.027 5.839 5.642 5.707 6.029
   5538   1373019_at   9.445 9.494 9.466 9.54 9.628 9.283 9.032 9.172 9.032 8.817
   5539   1372259_at   10.618 10.616 10.469 10.51 10.496 10.314 10.216 10.381 10.29 10.142
   5540   1381850_at   6.011 6.091 6.384 6.16 6.125 5.591 5.519 5.182 5.605 5.786
   5541   1383150_at   7.477 7.34 7.482 7.623 7.371 7.054 6.718 6.9 7.165 7.039
   5542   1386162_at   5.984 6.098 6.31 6.308 5.869 5.211 5.42 5.401 5.692 4.989
   5543   1383705_at   8.581 8.567 8.711 8.572 8.672 8.289 8.454 8.24 8.224 8.061
   5544   1384724_at   5.639 5.553 5.138 5.174 5.834 6.261 6.255 6.651 6.497 6.093
   5545   1370223_at   8.298 8.225 8.367 8.537 8.53 8.121 7.911 8.018 7.808 7.811
   5546   1387508_at   4.056 4.19 4.427 4.6 3.539 5.868 5.379 5.038 5.646 5.153
   5547   1371642_at   11.284 11.214 11.396 11.323 11.324 11.479 11.48 11.55 11.484 11.447
   5548   1384542_at   5.09 5.705 5.53 5.707 5.536 6.113 6.36 6.502 6.609 6.066
   5549   1380389_at   7.226 7.502 6.935 7.343 7.226 8.014 7.602 8.036 8.03 8.25
   5550   1398832_at   10.584 10.683 10.739 10.708 10.851 10.327 10.148 10.533 10.209 10.19
   5551   1384127_at   7.991 7.996 8.139 8.167 8.157 7.882 7.864 7.774 7.901 7.797
   5552   1371014_at   8.22 8.246 8.341 8.414 8.107 8.786 8.906 9.049 8.823 8.503
   5553   1370862_at   11.163 11.122 11.343 11.302 11.197 11.747 11.57 11.464 11.486 11.574
   5554   1382866_at   7.081 7.133 7.416 7.256 7.008 7.579 7.533 7.795 7.916 7.714
   5555   1368039_at   5.887 6.05 6.35 6.015 5.382 7.014 7.35 6.879 6.606 7.133
   5556   1378093_at   2.83 3.655 3.465 2.529 4.196 5.688 4.993 5.157 4.637 5.212
   5557   1387675_at   11.617 11.54 11.67 11.658 11.541 11.973 11.831 11.922 11.763 11.799
   5558   1371040_at   8.081 8.548 8.42 8.34 8.282 7.1 7.077 7.826 7.026 7.695
   5559   1392743_at   6.885 6.914 7.061 7.18 7.307 6.184 6.373 6.565 6.304 5.742
   5560   1371302_at   11.82 11.724 11.834 11.632 11.774 12.032 12.076 11.948 11.959 11.95
   5561   1377327_at   7.309 7.554 7.623 7.344 7.471 7.101 7.05 6.675 6.739 6.581
   5562   1387664_at   10.864 10.82 10.842 10.705 10.596 10.387 10.545 10.355 10.326 10.179
   5563   1375412_at   9.404 9.5 9.3 9.485 9.599 9.988 10.046 10.126 10.013 9.649
   5564   1393336_at   8.802 8.778 8.877 9.061 8.842 8.573 8.327 8.46 8.459 8.122
   5565   1376747_at   5.452 5.386 5.178 5.22 4.789 5.776 5.897 6.174 5.846 5.868
   5566   1368661_at   7.722 7.756 8.246 7.985 7.137 8.602 8.682 8.922 8.931 8.843
   5567   1372129_at   7.56 7.85 7.733 7.961 7.729 8.238 8.103 8.325 8.376 8.096
   5568   1383533_at   6.811 6.474 6.581 6.498 6.474 7.137 7.066 7.233 7.102 6.808
   5569   1372348_at   6.507 6.293 6.289 6.505 6.427 6.98 7.007 6.866 6.653 6.729
   5570   1370881_at   10.966 10.948 11.174 10.949 10.714 11.32 11.298 11.433 11.445 11.33
   5571   1387295_at   5.66 5.627 5.285 5.029 5.506 6.246 6.016 6.522 6.63 6.96
   5572   1383597_at   5.953 5.982 6.199 5.814 6.001 4.758 3.826 5.109 4.553 5.158
   5573   1385369_at   5.964 5.695 5.548 5.819 5.977 6.346 6.245 6.672 6.84 6.462
   5574   1369401_at   8.115 8.199 8.733 8.622 6.58 10.027 9.882 10.259 10.14 10.653
   5575   1378028_at   6.406 6.242 6.764 6.767 6.626 5.949 6.045 5.645 5.596 5.379
   5576   1388413_at   8.538 8.607 8.592 8.95 8.923 8.282 8.156 8.231 8.311 8.185
   5577   1370321_at   9.681 9.743 9.717 9.859 9.926 10.032 9.98 10.126 10.084 10.085
   5578   1380133_at   4.34 4.527 4.351 4.18 4.374 5.273 5.395 4.665 5.582 5.159
   5579   1391564_at   5.479 6.046 5.785 5.454 5.695 6.431 6.792 6.438 6.655 6.162
   5580   1383088_at   6.257 6.174 6.185 6.201 6.375 6.555 6.568 6.644 6.565 6.92
   5581   1371498_at   9.44 9.473 9.497 9.414 9.509 9.318 9.297 9.251 9.124 9.11
   5582   1386890_at   11.482 11.368 11.204 11.354 11.503 10.742 10.968 10.815 10.438 10.253
   5583   1367518_at   8.135 8.019 7.989 8.226 8.191 8.564 8.297 8.595 8.452 8.595
   5584   1391744_at   6.077 6.149 5.996 5.939 6.248 6.5 6.519 6.602 6.359 6.35
   5585   1398335_at   7.921 7.891 8.06 8.351 8.125 8.722 8.355 8.786 8.764 8.759
   5586   1399080_at   9.48 9.589 9.517 9.544 9.27 9.864 9.928 9.92 9.74 9.75
   5587   1396755_at   6.996 7.12 6.85 7.199 7.067 5.839 6.649 6.229 6.415 6.292
   5588   1371612_at   10.133 10.156 10.082 10.138 10.189 10.44 10.231 10.471 10.371 10.363
   5589   1387046_at   6.388 6.472 6.463 6.123 6.346 6.836 6.921 6.83 6.857 6.587
   5590   1381073_at   5.27 4.885 5.404 5.594 5.253 6.146 5.827 5.921 5.98 5.895
   5591   1389047_at   7.198 7.386 7.564 7.612 7.224 7.935 8.075 7.928 7.739 7.937
   5592   1392993_at   7.085 7.224 7.224 7.298 7.1 7.527 7.362 7.518 7.424 7.582
   5593   1372704_at   10.108 10.121 9.901 9.969 10.156 9.737 9.705 9.802 9.585 9.787
   5594   1383991_at   6.106 4.764 5.38 5.687 6.136 7.049 6.942 7.089 7.071 6.772
   5595   1374062_x_at   4.722 4.887 5.245 5.363 5.227 5.715 6.125 5.781 6.251 6.468
   5596   1371418_at   10.299 10.385 10.277 10.526 10.505 10.153 10.144 10.08 10.131 10.062
   5597   1385090_at   7.714 7.294 7.162 7.39 7.558 7.003 6.754 6.898 6.71 6.601
   5598   1395547_at   6.639 6.612 6.892 6.811 6.591 7.12 7.019 7.306 7.027 7.173
   5599   1379519_at   7.182 6.991 6.896 7.397 6.998 7.684 7.456 7.901 7.767 7.667
   5600   1388121_at   9.945 9.616 9.72 9.805 9.78 10.395 10.023 10.244 10.126 10.243
   5601   1374331_at   7.539 7.664 7.761 8.013 7.934 7.32 7.248 7.409 7.292 7.227
   5602   1385359_at   5.638 6 5.925 5.905 5.695 5.401 5.206 4.887 4.986 5.391
   5603   1383573_at   7.987 8.127 7.804 8.083 7.797 8.523 8.469 8.663 8.528 8.223
   5604   1374727_at   6.125 6.353 6.238 6.071 6.174 6.363 6.696 6.662 6.61 6.644
   5605   1378644_at   3.183 3.956 3.756 3.322 3.622 4.47 4.591 4.131 4.369 4.532
   5606   1374482_at   6.722 6.722 6.623 6.78 6.653 7.149 7.078 7.486 6.977 7.162
   5607   1372640_at   6.235 6.307 6.454 6.331 6.342 5.693 5.14 5.93 5.445 5.053
   5608   1374131_at   5.67 5.836 5.734 6.172 5.922 6.901 6.491 7.099 6.979 6.339
   5609   1381070_at   3.995 2.907 4.031 3.422 3.583 4.868 4.469 4.787 4.693 4.969
   5610   1398800_at   10.806 10.777 10.664 10.737 10.73 10.63 10.573 10.627 10.551 10.549
   5611   1389351_at   9.088 9.026 8.902 9.174 9.046 8.565 8.432 8.77 8.745 8.398
   5612   1388099_a_at   6.757 6.557 6.922 6.412 6.906 5.901 6.132 6.089 5.62 6.141
   5613   1390384_at   8.128 8.512 8.086 8.332 8.291 7.926 7.497 7.703 7.413 7.311
   5614   1397383_at   6.053 6.595 6.524 6.324 6.248 6.972 6.877 6.993 6.829 7.249
   5615   1372788_at   6.927 7.216 7.08 7.131 6.928 6.759 6.527 6.615 6.688 6.774
   5616   1368270_at   7.579 7.523 8.186 7.74 7.584 6.977 6.911 6.659 6.692 6.051
   5617   1373126_at   6.933 6.929 6.863 6.873 6.783 7.004 7.209 7.177 7.157 7.062
   5618   1388990_at   8.891 8.878 8.882 8.922 8.745 8.476 8.29 8.671 8.358 8.563
   5619   1380448_at   7.944 7.724 8.12 8.072 7.856 7.607 7.164 7.397 7.498 7.283
   5620   1387671_at   4.524 4.299 5.132 4.771 3.977 5.563 5.394 5.769 5.86 5.69
   5621   1376213_at   4.643 3.898 4.774 4.397 4.069 5.163 5.122 5.289 5.396 5.39
   5622   1372286_at   8.669 8.518 8.426 8.537 8.991 9.567 9.286 9.484 9.426 9.008
   5623   1389217_at   8.039 7.837 8.149 7.922 7.813 8.366 8.204 8.383 8.493 8.358
   5624   1389036_at   8.518 8.607 8.467 8.405 8.562 8.174 8.226 8.375 8.148 8.102
   5625   1379810_at   8.705 8.859 8.799 8.962 8.901 8.485 8.341 8.662 8.368 8.493
   5626   1388422_at   8.067 8.216 8.416 8.152 7.902 8.578 8.791 8.847 8.717 8.534
   5627   1390202_at   6.481 6.372 6.693 6.753 6.48 6.977 7.038 6.92 6.966 7.234
   5628   1367540_at   8.369 8.385 7.913 8.259 8.24 7.65 7.589 7.852 7.555 7.292
   5629   1385551_at   4.71 5.603 5.758 5.271 5.803 4.202 3.16 4.019 2.336 2.897
   5630   1388038_at   6.649 7.287 7.3 7.202 7.502 8.053 7.92 8.022 7.999 7.832
   5631   1376687_at   8.724 8.759 8.722 8.543 8.639 8.419 8.274 8.445 8.205 8.112
   5632   1387008_at   8.017 7.832 8.166 8.103 8.044 8.329 8.517 8.62 8.548 8.314
   5633   1391730_at   4.828 4.442 4.535 5.179 5.068 5.527 5.445 5.78 5.888 5.729
   5634   1379235_x_at   7.467 7.344 7.369 7.599 7.439 7.048 6.451 7.03 6.768 6.859
   5635   1367464_at   9.44 9.38 9.313 9.513 9.565 9.135 9.028 9.221 9.114 8.913
   5636   1374999_at   4.241 4.437 4.195 4.479 4.014 4.97 4.736 5.267 5.581 5.121
   5637   1379945_at   5.439 5.341 5.317 5.229 5.595 4.807 4.916 4.815 4.351 4.887
   5638   1387730_at   8.086 8.129 8.069 8.294 8.075 8.506 8.667 8.529 8.35 8.404
   5639   1370097_a_at   6.869 6.813 6.969 7.008 6.506 5.749 5.339 6.097 5.522 4.616
   5640   1368885_at   7.473 7.488 7.289 7.743 7.672 6.669 7.036 6.915 6.802 6.286
   5641   1391581_at   7.258 7.368 7.379 7.369 7.211 7.65 7.638 7.978 7.586 7.778
   5642   1376519_at   7.184 7.373 7.239 7.533 7.355 7.032 6.972 6.897 6.9 6.683
   5643   1376464_at   5.162 4.999 5.452 5.091 4.826 5.588 5.756 5.879 5.767 5.595
   5644   1386691_at   4.713 4.566 5.294 5.497 4.496 6.373 5.963 6.378 6.145 5.783
   5645   1382832_at   7.876 7.52 7.682 7.998 8.057 7.172 7.047 7.433 7.113 7.243
   5646   1376209_at   10.479 10.416 10.835 10.804 10.61 11.097 11.02 11.144 11.316 11.385
   5647   1368279_at   6.497 6.375 6.046 6.211 6.298 5.349 4.93 5.511 5.711 4.707
   5648   1393793_at   8.37 8.364 8.692 8.664 8.16 9.218 9.007 9.356 9.011 8.951
   5649   1379716_at   5.239 6 6.662 6.649 6.741 5.204 4.347 4.326 3.981 4.127
   5650   1394797_at   6.054 6.177 6.152 6.315 6.552 5.56 5.645 5.763 5.355 5.082
   5651   1384707_at   3.525 4.097 1.561 3.016 3.789 6.144 5.303 5.815 5.792 5.294
   5652   1388942_at   8.414 8.669 8.628 8.684 8.616 9.105 8.815 9.128 9.344 9.192
   5653   1399083_at   7.883 7.79 7.929 7.898 8.038 8.209 8.072 8.237 8.181 8.321
   5654   1389101_at   5.574 6.04 5.728 5.725 6.063 5.243 4.796 5.257 5.059 4.598
   5655   1387745_at   5.249 5.365 5.589 5.135 5.369 4.19 3.459 3.914 4.442 4.721
   5656   1383067_a_at   8.78 8.791 8.742 8.836 8.997 8.572 8.477 8.56 8.265 8.406
   5657   1376316_at   5.767 5.293 5.629 5.616 5.964 4.976 5.11 4.566 4.622 4.984
   5658   1393589_at   2.58 3.065 1.566 3.325 2.729 4.101 4.059 4.455 4.854 4.487
   5659   1389321_at   6.634 6.721 6.523 7.198 6.935 7.385 7.389 7.527 7.418 7.49
   5660   1378129_a_at   4.348 4.758 3.747 3.69 4.254 5.137 5.224 5.592 5.187 5.241
   5661   1390946_at   7.017 7.598 7.855 7.872 7.814 6.066 5.431 4.172 3.246 5.405
   5662   1388104_at   6.787 6.717 6.422 6.843 6.892 7.605 7.529 7.609 7.405 7.034
   5663   1377832_at   7.307 7.381 7.465 7.315 7.144 6.852 6.582 6.803 6.417 6.087
   5664   1387855_at   9.326 9.373 9.348 9.458 9.526 9.844 9.575 9.849 9.83 9.672
   5665   1371889_at   6.615 6.166 6.57 6.609 6.943 7.319 7.404 7.613 7.093 7.345
   5666   1380865_at   5.197 5.402 5.925 5.944 4.903 6.691 6.769 6.768 6.724 6.268
   5667   1369632_a_at   4.415 4.452 4.498 4.356 4.08 5.071 4.892 4.727 4.776 4.809
   5668   1398480_at   4.786 3.395 4.5 4.883 4.94 6.096 5.838 5.994 6.347 5.986
   5669   1370925_at   6.776 7.113 6.744 7.421 7.078 7.699 7.647 7.717 7.607 7.792
   5670   1373464_at   7.809 7.841 7.781 7.893 7.66 7.612 7.4 7.581 7.48 7.427
   5671   1369990_at   9.131 9.138 9.142 9.072 9.033 9.393 9.404 9.51 9.365 9.212
   5672   1387234_at   5.701 5.907 5.743 5.966 5.308 6.663 6.263 6.515 6.948 7.076
   5673   1373195_at   10.227 10.208 10.278 10.426 10.447 9.991 9.628 10.002 9.608 9.802
   5674   1398889_at   10.33 10.371 10.574 10.577 10.455 10.805 10.719 10.752 10.838 10.689
   5675   1382048_at   9.082 9.222 9.441 9.365 9.363 9.77 9.619 9.84 9.763 9.577
   5676   1394458_at   6.181 6.138 5.854 6.084 6.185 6.796 6.96 7.561 7.022 6.592
   5677   1393069_at   2.628 1.986 3.219 3.149 2.568 3.988 3.61 4.425 4.265 4.579
   5678   1378785_at   5.988 5.739 5.686 5.578 5.364 6.372 6.171 6.358 6.25 6.131
   5679   1377785_at   7.486 7.608 7.457 7.729 7.621 8.009 8.022 8.223 7.906 7.845
   5680   1375790_at   4.229 4.647 4.413 4.272 4.848 5.53 5.133 5.332 5.429 4.979
   5681   1394625_at   6.16 6.46 6.263 6.145 5.829 6.983 7.179 6.952 6.547 6.969
   5682   1397953_at   7.533 7.709 7.255 7.623 7.739 6.711 6.064 6.968 6.862 6.59
   5683   1373269_at   7.417 7.239 7.416 7.616 7.6 8.018 7.904 8.022 7.973 7.712
   5684   1374023_at   5.603 5.922 5.751 6.201 5.676 6.327 6.354 6.777 6.645 6.66
   5685   1376158_at   4.973 5.195 5.154 5.36 6.047 6.624 6.031 6.695 6.547 6.528
   5686   1386478_at   8.288 8.144 8.667 8.685 8.428 9.224 8.895 9.465 9.129 9.126
   5687   1370261_at   7.869 7.821 7.601 7.964 7.826 7.462 7.014 7.421 7.308 7.08
   5688   1398933_at   9.455 9.482 9.609 9.889 9.826 10.028 10.118 10.206 10.265 10.158
   5689   1381145_at   6.998 7.123 7.036 7.309 7.403 6.798 6.658 6.669 6.729 6.432
   5690   1378887_at   5.437 5.207 5.111 4.979 5.418 4.044 3.867 3.552 2.42 2.214
   5691   1367713_at   9.838 9.851 9.728 9.65 10.054 9.421 9.25 9.356 9.222 8.883
   5692   1383332_at   8.075 8.121 8.608 8.453 8.204 9.171 8.676 9.325 9.136 9.02
   5693   1382496_at   7.394 7.347 7.883 7.626 7.307 8.028 8.085 8.106 8.413 8.307
   5694   1374739_at   8.192 8.345 8.402 8.666 8.348 7.946 7.254 7.821 7.582 7.696
   5695   1379075_at   5.345 5.1 5.382 5.631 5.028 6.939 6.007 6.102 6.418 6.171
   5696   1374791_at   5.458 5.532 5.623 5.027 5.492 6.228 5.883 6.384 6.717 6.305
   5697   1382160_at   6.177 6.452 6.032 6.38 6.556 5.915 5.463 5.539 5.307 5.103
   5698   1375140_at   7.749 7.603 7.724 7.616 7.44 8.162 8.119 7.978 8.051 7.846
   5699   1374540_at   8.077 7.807 7.869 7.892 7.92 6.589 6.454 6.154 4.406 5.68
   5700   1385659_at   4.807 4.492 5.211 5.456 5.621 6.258 6.139 6.428 6.058 6.487
   5701   1373728_at   6.05 6.436 6.019 6.457 6.06 6.88 6.914 6.744 6.888 6.595
   5702   1385038_at   4.529 3.777 5.187 5.219 4.432 6.004 6 6.625 5.922 6.301
   5703   1388512_at   8.093 7.99 8.164 7.976 8.001 8.22 8.344 8.209 8.314 8.304
   5704   1375003_at   8.022 7.84 8.104 8.113 8.407 8.638 8.831 8.785 8.502 8.584
   5705   1377262_at   7.552 7.704 7.777 7.69 7.723 7.408 7.416 7.256 7.328 7.049
   5706   1378746_at   6.816 6.675 6.801 6.577 6.663 6.221 5.652 5.465 6.15 5.373
   5707   1379327_at   8.329 8.488 8.427 8.697 8.257 7.775 7.902 7.978 8.103 8.016
   5708   1380090_at   4.835 4.434 5.018 4.889 5.213 6.295 5.808 5.596 5.708 6.438
   5709   1388409_at   8.517 8.584 8.306 8.619 8.638 7.99 7.458 8.128 7.91 7.605
   5710   1391905_at   7.544 7.636 7.522 7.637 7.744 7.08 6.673 7.215 6.409 6.859
   5711   1388544_at   8.713 8.739 8.884 9.019 9.127 8.473 8.402 8.485 8.512 8.492
   5712   1376494_at   6.991 6.536 6.878 6.956 6.453 7.293 7.53 7.517 7.345 7.312
   5713   1397049_at   4.545 4.152 4.648 4.891 4.013 5.744 5.067 5.565 5.57 5.963
   5714   1385517_at   6.778 6.891 6.924 6.918 6.657 7.71 7.513 7.117 7.425 7.3
   5715   1388759_at   11.656 11.583 11.549 11.584 11.585 11.413 11.48 11.425 11.314 11.285
   5716   1389191_at   7.738 7.846 7.838 8.086 8.091 7.573 7.162 7.501 7.253 7.41
   5717   1374117_at   6.087 6.437 6.477 6.605 6.422 7.221 7.375 7.366 6.758 7.034
   5718   1370937_a_at   4.962 5.722 5.409 5.751 4.916 6.351 6.133 6.311 6.55 6.35
   5719   1368419_at   10.491 10.575 10.232 10.321 10.556 9.815 9.558 10.039 10.001 9.552
   5720   1373685_at   5.864 5.686 6.15 6.466 5.597 6.615 6.877 6.88 6.841 6.866
   5721   1378207_at   1.367 1.623 1.57 1.333 1.202 1.868 2.462 2.316 2.004 2.637
   5722   1376272_s_at   9.477 9.521 9.048 9.376 9.728 8.622 8.85 8.855 8.918 8.689
   5723   1398771_at   10.651 10.635 10.813 10.747 10.61 10.957 10.988 10.948 10.911 10.84
   5724   1371644_at   10.172 10.116 10.251 10.399 10.366 10.708 10.709 10.773 10.615 10.466
   5725   1398627_at   3.684 4.346 3.329 4.323 4.46 4.984 5.37 5.353 5.316 5.146
   5726   1382818_at   7.926 8.014 7.937 8.085 8.144 8.445 8.365 8.683 8.721 8.309
   5727   1398413_at   6.491 6.616 6.606 6.486 6.494 7.236 7.154 6.811 6.878 6.9
   5728   1389720_at   3.699 4.305 4.305 4.864 4.192 5.69 5.265 5.142 5.679 5.239
   5729   1381997_at   5.189 5.671 4.839 5.236 4.75 6.162 6.334 6.171 6.458 5.76
   5730   1396238_at   5.255 4.853 6.367 5.878 5.851 7.114 7.127 6.829 7.338 6.96
   5731   1374030_at   7.672 7.745 7.742 7.876 7.712 8.038 8.105 8.268 7.928 8.084
   5732   1378243_at   5.97 5.85 5.853 5.851 6.095 6.447 6.297 6.797 6.286 6.486
   5733   1374074_at   6.945 7.022 6.988 7.053 6.874 7.279 7.408 7.678 7.236 7.435
   5734   1373628_at   4.741 4.172 4.481 4.331 5.947 6.494 6.533 6.284 6.188 6.581
   5735   1390991_at   3.184 4.371 4.104 4.667 4.405 5.425 5.881 5.431 5.411 5.586
   5736   1383798_at   10.085 10.093 10.433 10.161 9.81 10.807 11.136 10.851 10.945 10.511
   5737   1371361_at   8.681 8.753 9.056 8.668 8.887 9.475 9.261 10.141 9.683 9.688
   5738   1367896_at   8.04 8.156 6.959 7.571 8.178 9.153 8.816 9.48 9.319 8.92
   5739   1370392_at   5.234 4.964 5.135 5.557 5.57 5.888 6.231 5.813 6.277 6.141
   5740   1396058_at   5.756 6.291 6.309 6.486 6.078 6.751 7.134 7.077 6.785 6.943
   5741   1371693_at   9.574 9.608 9.308 9.871 9.877 9.111 9.072 9.125 9.051 9.12
   5742   1390785_at   8.929 8.787 8.818 8.858 8.531 9.048 9.235 9.177 9.186 9.202
   5743   1384405_at   7.155 7.058 7.072 6.997 7.654 7.813 7.756 8.005 8.053 7.773
   5744   1370286_at   8.948 9.095 9.18 9.547 9.281 9.96 9.819 10.086 9.779 9.646
   5745   1370688_at   12.034 11.902 12.139 12.01 11.312 12.766 12.525 12.592 12.758 12.874
   5746   1373450_at   7.791 7.674 7.822 7.905 7.655 7.514 7.285 7.52 7.449 7.301
   5747   1384304_at   5.854 5.415 5.903 5.571 4.951 6.307 6.391 7.015 6.894 6.839
   5748   1398585_at   5.534 5.822 5.845 6.026 5.427 6.353 6.215 6.4 6.332 6.284
   5749   1399062_at   9.174 9.151 9.182 9.178 9.191 8.991 8.891 8.988 8.836 8.679
   5750   1371656_at   11.252 11.149 11.148 11.068 11.297 10.956 10.606 10.847 10.777 10.562
   5751   1370553_at   4.102 4.614 4.283 4.545 4.452 5.207 4.856 5.71 5.281 5.677
   5752   1397838_at   4.775 5.12 4.939 5.313 5.47 6.118 5.854 6.196 5.65 6.354
   5753   1398935_at   8.644 8.442 8.63 8.82 8.522 9.065 8.887 9.326 9.08 9.177
   5754   1376206_at   7.491 7.61 7.539 7.537 7.385 7.222 7.187 7.088 6.894 6.78
   5755   1399103_at   7.001 6.937 7.167 7.163 7.307 7.57 7.412 7.642 7.893 7.753
   5756   1394710_at   5.886 6.182 6.25 6.156 6.051 5.565 5.814 5.296 5.351 5.601
   5757   1379431_at   6.291 6.446 6.656 6.144 6.116 6.794 6.8 6.972 7.103 7.091
   5758   1386191_a_at   6.545 6.374 6.772 6.883 6.615 5.907 5.889 6.067 5.895 5.287
   5759   1383181_at   9.046 9.079 9.094 9.234 9.221 8.914 8.691 8.913 8.64 8.753
   5760   1398576_at   6.087 6.195 6.267 6.077 6.08 4.361 4.108 3.111 1.176 1.761
   5761   1377806_at   7.832 7.588 7.794 8.013 7.86 8.394 8.181 8.527 8.246 8.192
   5762   1380030_at   6.526 6.812 6.801 6.511 6.925 6.215 6.002 6.315 5.83 5.781
   5763   1377209_at   7.683 7.745 8.176 7.98 7.632 8.438 8.548 8.667 8.571 8.234
   5764   1387427_at   6.146 6.403 6.222 6.464 6.018 5.783 5.863 5.712 5.496 5.76
   5765   1385974_at   3.989 4.151 3.834 3.661 4.253 5.04 4.874 4.578 5.137 4.494
   5766   1367491_at   10.157 10.185 10.191 10.143 10.063 9.904 9.943 9.922 9.689 9.691
   5767   1388919_at   7.252 7.291 7.218 7.334 7.423 7.586 7.529 7.552 7.801 7.593
   5768   1370892_at   11.958 11.846 12.054 11.886 11.785 10.961 11.079 11.111 10.476 9.916
   5769   1395072_at   1.449 1.506 2.17 2.803 3.383 4.091 3.87 4.67 4.545 4.928
   5770   1395763_at   1.956 0.124 1.899 0.971 0.748 3.041 3.547 2.897 3.074 2.759
   5771   1388105_at   8.539 8.646 8.573 8.504 8.5 8.374 8.203 8.395 8.309 8.187
   5772   1384240_at   9.051 8.879 8.83 8.486 8.756 9.586 9.753 9.798 9.668 9.119
   5773   1371202_a_at   8.109 7.98 7.949 8.271 8.191 8.797 8.473 8.936 8.763 8.446
   5774   1371437_at   10.277 10.321 10.31 10.511 10.435 10.157 10.154 10.152 10.126 10.093
   5775   1377761_at   6.649 6.654 6.891 6.874 6.775 6.566 6.357 6.275 6.405 6.225
   5776   1373420_at   7.902 7.996 8.156 8.25 7.928 8.49 8.446 8.319 8.513 8.635
   5777   1388920_at   5.932 6.255 6.049 6.112 6.573 7.034 6.577 6.916 6.976 6.899
   5778   1383228_at   7.103 7.279 7.361 7.643 7.483 7.763 7.984 7.942 8.005 7.816
   5779   1397221_s_at   5.151 5.075 5.065 5.043 5.512 4.245 4.56 4.421 3.884 3.567
   5780   1393621_at   7.113 7.044 7.35 6.935 7.08 6.668 6.841 6.697 6.567 6.562
   5781   1373966_at   7.206 7.105 7.123 7.055 7.278 7.521 7.324 7.63 7.468 7.451
   5782   1387636_a_at   9.802 9.848 9.998 10.053 10.177 9.668 9.391 9.574 9.236 9.368
   5783   1389704_at   5.095 4.704 4.513 5.201 4.789 5.725 5.442 6.341 6.001 6.346
   5784   1398268_at   9.055 8.974 9.196 9.243 9.076 9.428 9.325 9.572 9.398 9.545
   5785   1392792_at   6.993 7.199 6.951 7.04 7.122 6.823 6.615 6.786 6.473 6.593
   5786   1375440_at   8.444 8.417 8.628 8.762 8.608 8.296 8.25 8.227 8.192 8.164
   5787   1377890_at   5.984 6.194 6.332 6.183 5.921 6.927 6.428 7.199 7.187 6.928
   5788   1388356_at   9.282 9.297 9.291 9.399 9.415 9.827 9.493 9.76 9.676 9.601
   5789   1372777_at   9.143 9.128 9.207 9.308 9.127 9.592 9.451 9.567 9.345 9.426
   5790   1372317_at   8.968 8.982 9.174 9.133 9.088 9.277 9.354 9.512 9.335 9.327
   5791   1393620_at   7.058 6.742 7.45 7.457 7.137 6.348 6.269 6.684 6.255 6.167
   5792   1391864_at   8.575 8.456 8.782 8.712 8.221 9.393 9.595 9.143 9.01 9.234
   5793   1372879_at   8.478 8.524 8.762 8.704 8.686 9.135 9.088 9.097 9.395 8.902
   5794   1368909_a_at   7.362 7.293 7.119 7.79 7.588 6.815 6.588 6.892 6.805 6.501
   5795   1382215_at   7.104 7.142 7.15 7.193 7.072 7.619 7.345 7.613 7.624 7.323
   5796   1395809_at   5.91 5.785 6.023 5.698 6.34 6.505 6.446 6.54 6.654 6.651
   5797   1378240_at   6.354 6.365 6.435 6.193 6.183 6.049 5.223 5.171 5.416 5.116
   5798   1371421_at   11.297 11.128 11.38 11.143 10.944 11.665 11.711 11.493 11.664 11.9
   5799   1388489_at   10.946 11.044 11.151 11.128 11.134 11.384 11.232 11.464 11.553 11.464
   5800   1369773_at   5.281 5.265 4.714 5.233 4.858 5.594 5.741 5.827 6.115 6.136
   5801   1370260_at   8.209 8.199 8.698 8.565 8.746 9.048 9.076 9.467 9.238 9.159
   5802   1373516_at   7.881 7.799 7.86 8.032 7.859 7.65 7.501 7.716 7.585 7.49
   5803   1383714_at   7.626 7.778 7.741 8.073 7.89 8.364 8.143 8.491 8.516 8.247
   5804   1371969_at   9.177 9.292 9.636 9.7 9.764 10.373 10.367 10.358 10.136 9.971
   5805   1397855_at   0.651 0 3.728 3.005 0 5.946 6.13 5.422 5.024 5.606
   5806   1374573_at   7.735 7.661 7.707 7.836 7.945 8.093 8.233 8.327 8.148 8.005
   5807   1373560_at   8.68 8.359 8.739 8.616 8.785 9.408 9.001 9.104 9.076 9.236
   5808   1377155_at   6.744 6.418 6.397 7.065 5.755 7.553 7.437 7.724 7.961 7.696
   5809   1371296_at   10.644 10.53 10.775 10.66 10.565 11.006 10.836 10.901 10.889 11.066
   5810   1390989_at   9.58 9.736 9.809 9.456 9.649 9.169 8.774 9.326 9.032 8.797
   5811   1380747_at   3.725 4.533 4.226 3.994 5.286 5.368 5.895 6.153 5.977 6.093
   5812   1368055_a_at   8.905 8.848 8.988 8.999 9.034 8.713 8.61 8.608 8.355 8.316
   5813   1378399_at   6.679 6.82 7.031 7.108 6.95 6.229 6.516 6.583 6.263 6.374
   5814   1392842_at   8.808 8.951 8.86 9.127 9.388 8.316 7.491 8.226 8.219 8.194
   5815   1370577_at   6.745 6.709 7.056 6.603 7.063 6.01 6.329 6.227 6.278 5.777
   5816   1370356_at   8.136 8.255 7.938 8.389 8.407 7.757 7.681 7.781 7.797 7.505
   5817   1367482_at   9.036 9.135 9.059 9.173 9.285 8.949 8.748 8.847 8.844 8.889
   5818   1378800_at   6.627 7.07 6.57 6.836 6.627 6.221 6.047 6.215 6.063 6.371
   5819   1377911_at   8.882 8.952 9.001 8.996 8.558 9.415 9.494 9.599 9.416 9.188
   5820   1377042_at   8.443 8.345 8.76 8.756 8.59 8.159 7.887 8.165 8.099 8.106
   5821   1374585_at   8.139 8.176 7.998 8.305 8.435 8.681 8.48 8.815 8.762 8.701
   5822   1368515_at   8.545 8.607 8.897 9.008 8.53 9.177 9.473 9.585 9.411 9.184
   5823   1384926_at   6.823 7.126 6.819 7.206 7.175 6.32 6.229 6.734 6.214 6.094
   5824   1374610_at   8.515 8.317 8.493 8.642 8.299 8.816 9.055 9.114 8.801 8.836
   5825   1388715_at   10.218 10.09 10.03 10.344 10.193 9.796 9.322 9.775 9.74 9.619
   5826   1395710_at   6.06 6.066 5.846 6.043 5.647 5.081 5.222 5.646 5.161 4.996
   5827   1376002_at   5.838 5.943 6.057 6.194 6.652 5.47 4.577 4.353 5.134 4.947
   5828   1398598_at   9.132 9.157 9.063 9.248 9.017 9.594 9.383 9.614 9.4 9.358
   5829   1393277_at   5.553 5.237 5.254 5.417 5.432 5.765 5.939 6.131 5.958 5.662
   5830   1369263_at   4.509 4.872 3.932 4.792 4.521 5.106 5.835 5.976 5.751 5.624
   5831   1391050_at   7.195 7.026 7.311 7.437 7.458 6.917 6.447 6.832 6.458 6.552
   5832   1381528_at   6.717 6.1 6.273 6.865 6.687 7.297 7.26 7.389 7.288 7.191
   5833   1393409_at   5.628 6.109 5.488 5.327 5.548 6.602 6.905 6.438 6.221 6.394
   5834   1395246_at   7.179 7.01 6.885 6.892 6.923 7.437 7.32 7.406 7.773 7.772
   5835   1373003_at   7.807 7.726 7.538 7.98 7.888 7.127 6.887 7.439 6.925 7.281
   5836   1374103_at   6.144 6.089 6.299 6.507 6.357 7.135 6.778 7.051 7.021 6.594
   5837   1385941_at   7.922 7.902 7.838 7.833 8.02 7.57 7.367 7.609 7.614 7.699
   5838   1379784_at   7.838 8.086 7.937 7.846 7.98 8.143 8.306 8.178 8.306 8.202
   5839   1389733_at   8.629 8.664 8.57 8.804 8.695 8.472 8.402 8.35 8.153 8.3
   5840   1376136_at   6.654 6.39 6.74 6.713 6.534 7.165 6.904 7.201 7.41 7.047
   5841   1374622_at   7.382 7.362 7.54 7.611 7.456 8.171 7.858 8.591 8.232 7.954
   5842   1389681_at   6.609 6.343 6.615 6.49 6.696 6.282 5.919 6.168 5.958 6.114
   5843   1382781_at   6.521 6.555 6.555 6.339 6.573 6.753 6.958 7.113 6.797 7.091
   5844   1390434_at   7.654 7.347 7.557 7.498 7.643 7.138 6.992 7.27 6.938 6.775
   5845   1369871_at   6.738 6.533 5.169 6.576 6.725 4.073 4.387 5.25 3.663 4.315
   5846   1373174_at   8.495 8.441 8.56 8.648 8.684 8.756 8.959 9.117 8.898 9.015
   5847   1368519_at   6.873 7.011 8.349 8.06 6.384 5.064 5.687 4.859 3.55 4.458
   5848   1377400_at   7.948 8.022 7.975 8.159 8.197 7.568 7.784 7.693 7.636 7.342
   5849   1376679_at   6.248 5.984 6.286 6.234 6.178 6.496 6.5 6.767 6.874 6.889
   5850   1388910_at   7.391 7.371 7.539 7.265 7.087 7.639 7.8 7.824 7.876 7.707
   5851   1370503_s_at   8.082 7.877 8.122 8.194 7.914 8.341 8.702 8.741 8.693 8.388
   5852   1372845_at   9.733 9.831 9.699 9.421 9.533 9.135 9.045 8.921 9.182 8.565
   5853   1384616_at   3.294 2.609 2.722 2.854 2.508 4.323 3.985 4.01 3.268 4.157
   5854   1384242_at   7.001 7.684 7.342 7.521 7.42 6.46 6.966 6.346 6.336 6.678
   5855   1372366_at   8.522 8.523 8.445 8.504 8.481 8.358 8.421 8.409 8.341 8.317
   5856   1372542_at   9.286 9.219 8.825 8.899 9.379 8.398 8.417 8.425 8.016 7.686
   5857   1381113_at   0.322 0.623 1.868 1.444 0.422 2.316 2.578 2.965 3.35 2.519
   5858   1388797_at   9.589 9.599 9.462 9.864 9.843 9.212 9.241 9.308 9.156 9.316
   5859   1373723_at   1.795 1.674 2.299 2.511 1.506 3.731 5.443 4.977 4.715 3.036
   5860   1384866_at   4.747 4.482 4.223 4.504 4.249 5.257 5.033 4.858 5.234 4.957
   5861   1397119_at   4.01 4.319 4.398 5.168 4.584 6.008 5.779 5.523 5.404 5.438
   5862   1376121_at   4.539 4.693 4.538 5.114 4.935 5.453 5.662 5.597 5.228 5.374
   5863   1382811_at   6.262 5.957 5.774 5.853 5.599 6.5 6.81 6.913 6.744 6.34
   5864   1395747_at   5.067 5.111 5.122 5.114 5.396 4.863 4.309 3.928 4.359 4.252
   5865   1392638_at   6.886 6.69 7.086 6.757 6.623 6.131 6.028 6.339 5.503 5.699
   5866   1398277_at   6.431 6.051 6.317 6.533 5.95 6.97 7.002 6.962 6.734 6.77
   5867   1383477_at   8.999 8.683 8.755 9.008 8.973 8.274 8.083 8.572 8.4 8.402
   5868   1389302_at   7.63 7.814 8.004 7.882 7.899 7.306 7.524 7.494 7.547 7.448
   5869   1382457_at   4.148 3.41 2.725 3.466 3.096 4.784 4.608 5.086 4.36 5.281
   5870   1376610_a_at   5.426 5.58 5.815 6.033 5.541 6.79 6.283 6.534 6.264 6.298
   5871   1383750_at   3.454 3.727 2.459 3.767 2.842 4.448 4.399 4.94 4.953 4.679
   5872   1388866_at   5.181 5.048 4.033 4.979 5.527 6.349 6.104 6.333 6.196 6.196
   5873   1394729_at   3.638 3.859 4.191 3.706 3.987 4.43 4.9 4.518 4.362 4.698
   5874   1371861_at   9.737 9.697 9.681 9.912 10.154 10.485 10.321 10.626 10.348 10.243
   5875   1371743_at   8.959 8.911 9.008 9.083 8.976 9.465 9.117 9.387 9.276 9.35
   5876   1386948_at   5.308 5.784 6.122 6.095 5.504 6.961 6.331 7.094 6.793 6.842
   5877   1369956_at   10.154 10.015 10.2 10.132 9.964 9.778 9.737 9.739 9.712 9.394
   5878   1374700_at   8.435 8.077 8.107 8.225 8.425 7.781 7.687 7.944 7.868 7.722
   5879   1375120_at   6.845 7.209 7.169 7.23 7.209 7.611 7.469 8.055 7.85 7.882
   5880   1391014_at   6.741 6.553 6.602 6.841 6.941 6.353 6.151 6.427 6.301 6.154
   5881   1374139_at   8.001 7.894 8.074 8.303 7.913 8.927 9.007 8.951 8.285 8.84
   5882   1382094_at   9.426 9.523 9.495 9.566 9.681 8.965 8.87 9.307 8.663 8.64
   5883   1395132_at   5.876 5.344 5.34 5.749 5.506 6.412 6.405 6.705 6.142 6.079
   5884   1368324_at   6.328 6.536 6.368 6.357 6.654 5.176 2.379 4.596 3.699 2.648
   5885   1369590_a_at   7.767 7.746 8.061 8.295 7.781 7.378 7.269 7.126 7.395 7.424
   5886   1379580_at   5.815 6.19 5.57 5.889 5.752 6.41 6.629 6.268 6.389 6.573
   5887   1390225_at   2.114 1.541 0.098 0 1.551 3.387 3.573 2.986 4.27 3.203
   5888   1380948_at   4.114 4.133 4.076 3.895 3.303 4.911 4.561 4.801 5.291 5.282
   5889   1392952_at   7.265 7.375 7.578 7.726 7.158 8.121 7.939 7.924 8.417 8.328
   5890   1388619_at   8.565 8.773 8.792 8.696 8.757 9.076 9.174 9.39 9.149 8.935
   5891   1390545_at   7.923 7.997 7.696 8.028 8.214 8.382 8.459 8.871 8.616 8.532
   5892   1394743_at   6.618 6.516 6.154 6.897 6.218 5.705 5.531 5.471 4.919 5.729
   5893   1383469_at   6.757 6.529 7.462 6.095 6.8 8.197 7.524 8.139 8.087 8.105
   5894   1375673_at   8.94 8.903 8.616 9.063 8.941 8.426 8.216 8.387 7.753 8.077
   5895   1387143_at   7.016 7.045 6.981 7.221 7.241 6.681 6.332 6.714 6.445 6.778
   5896   1390272_at   6.64 6.753 6.739 6.881 6.927 6.29 6.576 6.403 6.154 6.2
   5897   1395635_at   6.203 6.081 6.585 6.727 6.762 5.608 4.918 5.417 4.104 4.658
   5898   1367865_at   7.932 7.824 7.655 7.933 7.837 7.468 7.221 7.583 7.491 7.436
   5899   1386745_at   7.232 7.346 7.244 7.14 7.228 6.374 6.849 6.51 6.343 5.811
   5900   1381871_at   6.564 6.385 6.55 6.852 7.013 7.688 7.16 8.039 7.509 7.556
   5901   1382078_at   7.21 6.979 7.147 7.025 6.893 6.438 6.682 6.016 6.52 6.104
   5902   1380117_at   6.482 5.971 6.017 6.392 5.848 7.069 6.55 7.042 7.118 7.211
   5903   1381439_at   3.474 1.683 0.782 1.807 1.864 4.524 3.852 3.921 5.044 5.082
   5904   1378692_at   9.045 9.09 8.953 9.338 9.11 9.635 9.561 9.777 9.43 9.434
   5905   1388632_at   3.548 3.796 4.007 2.814 3.784 4.793 4.486 4.771 4.585 4.753
   5906   1369752_a_at   4.731 4.55 4.494 4.814 4.663 3.764 1.406 0.566 1.899 2.227
   5907   1369173_at   5.609 5.611 6.068 5.985 5.874 4.769 5.306 4.857 4.845 5.37
   5908   1399048_at   7.398 7.301 7.131 7.344 7.52 7.003 6.737 6.935 6.744 7.027
   5909   1385238_at   8.881 8.796 9.032 9.069 9.05 9.382 9.184 9.405 9.246 9.423
   5910   1384010_at   5.107 5.392 5.699 5.489 5.539 5.859 5.997 6.287 6.176 6.011
   5911   1383336_at   7.834 8.037 8.029 8.179 8.415 7.526 7.484 7.624 7.649 7.281
   5912   1398885_at   12.43 12.397 12.338 12.284 12.392 12.245 12.24 12.191 12.159 12.117
   5913   1384111_at   6.291 6.203 6.305 6.097 5.542 3.989 3.366 4.33 4.327 1.753
   5914   1382093_at   6.074 5.793 6.164 6.121 6.128 6.322 6.839 6.617 6.635 6.557
   5915   1368096_at   9.647 9.695 9.775 9.849 9.319 10.303 10.025 10.68 10.607 10.396
   5916   1390219_at   6.096 5.989 6.013 6.055 5.633 6.699 6.499 6.797 6.405 6.363
   5917   1372403_at   6.672 6.57 6.582 7.036 6.323 7.631 7.346 7.585 7.408 7.066
   5918   1374081_at   6.209 5.979 5.666 6.121 6.607 7.036 7.054 7.388 7.109 6.712
   5919   1394790_at   5.664 5.354 5.82 5.978 5.495 6.208 6.231 6.303 6.654 6.372
   5920   1376163_at   8.349 8.414 8.563 8.538 8.211 8.903 8.954 9.031 8.715 8.729
   5921   1372990_at   8.325 8.401 8.452 8.713 8.661 8.042 7.926 8.175 7.884 7.664
   5922   1377632_at   2.926 4.223 4.295 4.053 4.619 5.681 5.768 5.177 5.592 5.612
   5923   1378601_at   5.505 5.472 5.752 4.953 5.709 6.456 6.203 6.568 6.48 6.038
   5924   1382476_x_at   5.002 5.035 5.167 5.143 4.418 5.425 6.266 5.927 6.112 6.26
   5925   1384863_at   6.047 6.25 6.686 6.314 6.471 7.068 6.929 7.178 7.652 7.074
   5926   1380186_at   7.074 7.236 7.124 7.5 7.323 6.802 6.732 6.883 6.679 6.423
   5927   1392545_at   7.248 7.32 7.16 6.961 7.465 7.66 7.653 8.164 7.999 7.986
   5928   1379283_at   6.752 6.978 6.803 6.849 6.854 7.254 7.115 7.389 7.086 7.456
   5929   1381618_at   4.53 4.614 4.324 5.307 4.864 5.523 5.509 5.987 5.69 5.688
   5930   1371109_at   4.194 3.406 4.172 4.163 3.955 4.983 5.283 5.063 4.505 4.98
   5931   1388404_at   10.021 10.035 10.139 10.118 10.108 9.827 9.749 9.915 9.822 9.581
   5932   1371624_at   8.332 8.5 8.027 8.382 8.365 7.893 7.78 7.983 7.917 7.715
   5933   1393017_at   6.639 6.755 7.42 7.492 6.987 8.155 8.19 7.676 8.469 8.496
   5934   1371517_at   7.906 7.748 7.641 7.926 7.933 8.531 8.233 8.385 8.409 8.07
   5935   1372169_at   8.227 8.326 8.311 8.552 8.501 8.129 7.943 8.07 8.015 7.899
   5936   1389295_at   6.884 7.154 7.05 7.207 7.672 8.053 7.866 7.963 8.154 7.726
   5937   1372797_at   8.34 8.23 8.337 8.254 8.509 7.804 8.026 8 8.072 8.048
   5938   1368839_at   6.497 6.352 6.535 6.356 6.439 6.759 6.815 7.018 6.994 7.35
   5939   1373829_at   5.729 5.97 6.245 6.302 6.433 6.939 6.83 7.154 6.794 6.697
   5940   1378027_at   7.408 7.537 7.745 7.749 7.929 8.32 8.278 8.554 8.422 8.009
   5941   1389642_at   8.335 8.301 8.487 8.626 8.574 8.786 8.746 9.052 9.032 9.005
   5942   1398807_at   9.368 9.487 9.562 9.619 9.429 9.726 9.751 10.016 9.884 9.816
   5943   1389417_at   6.836 6.888 6.654 6.862 6.734 6.348 6.349 6.041 5.759 5.638
   5944   1389035_at   6.158 6.075 5.904 5.966 6.267 6.346 6.583 6.524 6.812 6.747
   5945   1392803_at   4.063 1.941 4.256 3.903 2.496 5.618 5.55 5.662 5.748 5.842
   5946   1378259_at   7.211 7.141 7.316 7.292 7.399 7.117 6.796 6.716 6.65 6.775
   5947   1367681_at   10.109 10.079 10.133 10.228 10.34 10.67 10.423 10.64 10.523 10.416
   5948   1387054_at   6.711 6.626 6.543 6.785 6.529 6.05 5.914 5.556 4.879 5.211
   5949   1398847_at   9.574 9.791 9.821 9.865 9.729 10.346 10.306 10.32 10.635 11.072
   5950   1379755_at   6.654 6.722 7.191 7.084 7.041 7.925 7.418 8.297 8.62 8.105
   5951   1382263_at   5.546 6.004 5.751 5.919 5.825 6.258 6.329 6.229 6.178 6.156
   5952   1387201_at   7.235 7.222 7.513 7.454 7.532 6.522 6.657 6.685 6.842 5.924
   5953   1376641_at   7.961 8.103 8.134 8.166 8.104 7.6 7.837 7.565 7.353 7.185
   5954   1384390_at   6.958 7.106 7.06 7.408 7.228 6.74 6.614 6.706 6.854 6.626
   5955   1379465_at   3.136 3.603 3.263 3.767 3.374 4.32 4.129 4.501 4.449 3.855
   5956   1398397_at   9.123 9.063 8.979 9.081 8.916 9.379 9.241 9.297 9.261 9.196
   5957   1369814_at   7.019 6.994 5.65 6.66 6.79 4.953 5.103 5.457 4.011 4.888
   5958   1391270_at   7.617 7.332 7.856 7.93 7.44 8.463 8.011 8.538 8.464 8.621
   5959   1377630_at   8.193 8.234 8.513 8.436 8.332 7.802 7.631 8.127 7.792 7.839
   5960   1367800_at   9.425 9.366 9.448 9.515 9.466 9.933 10.034 9.804 9.654 9.691
   5961   1372665_at   10.754 10.791 11.1 11.083 10.668 11.35 11.26 11.49 11.375 11.603
   5962   1387042_at   4.894 4.661 4.649 5.193 5.072 5.742 5.25 5.701 5.647 5.626
   5963   1393523_at   3.51 2.905 1.465 3.39 2.884 1.007 0.516 0 0.934 1.17
   5964   1376068_at   5.697 5.767 5.998 6.121 5.743 6.584 6.137 6.557 6.622 6.757
   5965   1371267_at   6.356 6.478 6.443 6.389 6.435 5.486 3.704 5.26 4.684 3.796
   5966   1367899_at   9.821 9.833 10.158 9.999 10.118 10.429 10.388 10.482 10.31 10.289
   5967   1395965_at   5.967 6.248 6.156 5.712 6.011 6.567 6.479 6.714 6.458 6.469
   5968   1395445_at   6.893 6.985 7.119 7.152 7.235 6.791 6.722 6.399 6.391 6.518
   5969   1389291_at   9.686 9.759 9.68 9.784 9.893 9.956 9.92 9.976 9.98 9.982
   5970   1374109_at   7.686 7.763 7.851 7.856 7.731 8.214 8.084 8.189 8.059 7.914
   5971   1372279_at   7.62 7.573 7.51 7.604 7.8 7.257 6.751 7.134 6.98 7.27
   5972   1376970_at   4.312 3.815 4.408 4.624 4.492 5.307 5.275 4.918 5.206 4.962
   5973   1384645_at   5.348 5.207 5.548 5.707 5.166 5.807 6.035 6.442 6.47 6.404
   5974   1377593_at   8.992 8.947 8.907 9.141 9.283 9.369 9.443 9.496 9.445 9.363
   5975   1389904_at   11.661 11.583 11.617 11.392 11.473 11.265 11.264 11.058 10.966 10.888
   5976   1398362_at   8.629 8.589 8.596 8.784 8.846 8.39 8.048 8.239 7.99 7.754
   5977   1386621_at   5.281 5.207 5.686 5.649 5.707 4.498 4.463 5.18 4.545 4.487
   5978   1382046_at   8.561 8.721 8.701 8.982 8.763 8.369 8.375 8.419 8.308 8.129
   5979   1377840_at   4.408 4.584 4.262 4.388 4.195 4.851 5.128 4.737 5.409 5.076
   5980   1376124_at   4.372 3.94 4.365 4.861 3.22 5.486 5.71 5.523 5.435 5.565
   5981   1376303_a_at   7.747 7.581 7.763 7.845 7.586 7.975 8.088 7.985 8.267 8.057
   5982   1396344_at   6.496 6.401 6.418 6.424 6.536 6.784 6.644 6.581 6.733 6.643
   5983   1377703_at   7.307 6.964 7.311 7.254 7.282 7.54 7.506 7.8 7.725 7.717
   5984   1374682_at   7.02 6.99 6.922 7.344 6.774 7.35 7.679 7.703 7.803 7.554
   5985   1398886_at   10.264 10.204 10.319 10.324 10.464 9.979 9.903 9.922 9.683 9.464
   5986   1372338_at   7.061 7.077 7.492 7.362 7.437 7.991 7.846 8.333 7.859 7.77
   5987   1374733_at   7.331 7.197 7.097 7.264 7.367 6.682 6.708 6.853 7.047 6.562
   5988   1380421_s_at   3.323 3.335 4.533 4.015 3.697 2.526 0.057 0 1.511 1.496
   5989   1390107_at   7.934 7.659 7.438 7.85 7.741 8.594 8.168 8.696 8.211 8.328
   5990   1371113_a_at   7.454 7.38 7.768 7.802 7.054 8.439 8.345 8.207 8.015 8.481
   5991   1397500_x_at   5.993 6.361 6.312 6.187 6.037 6.761 6.529 7.033 6.984 7.298
   5992   1385052_at   7.13 7.086 7.184 6.777 7.295 6.504 6.63 6.239 6.689 6.515
   5993   1379897_at   4.291 4.702 3.978 3.542 3.916 5.488 5.009 5.417 4.873 5.486
   5994   1379254_at   9.636 9.751 9.388 9.555 9.333 9.975 10.027 9.952 9.886 9.876
   5995   1388515_at   7.854 8.116 8.086 8.375 8.175 8.561 8.684 9.123 8.726 8.685
   5996   1397313_at   8.626 8.643 8.54 8.401 8.571 8.093 8.364 8.092 7.964 7.836
   5997   1373215_at   6.805 6.806 6.781 6.961 7.381 5.614 6.167 6.263 6.073 6.349
   5998   1389554_at   10.092 10.179 9.989 9.997 10.332 10.658 10.4 10.625 10.587 10.411
   5999   1380256_at   7.909 7.705 7.748 7.898 8.035 7.153 7.669 7.25 7.172 7.118
   6000   1398628_at   8.796 8.833 9.367 8.818 8.373 9.608 9.641 9.514 9.795 9.796
   6001   1369212_s_at   7.237 7.105 7.383 7.5 7.407 7.905 7.673 8.052 8.332 7.89
   6002   1382964_at   7.152 6.779 6.875 7.012 7.143 7.504 7.392 7.995 7.738 7.545
   6003   1379525_at   8.261 8.461 8.57 8.817 8.397 8.954 9.313 9.47 9.304 8.954
   6004   1370821_at   9.781 9.911 9.997 10.216 9.371 10.546 10.476 10.793 11.064 11.065
   6005   1371430_at   8.152 8.129 8.484 8.559 8.551 8.96 8.748 9.128 9.083 8.896
   6006   1383713_at   5.544 5.142 5.709 6.054 5.775 6.566 6.295 6.413 6.394 6.701
   6007   1393143_at   7.833 7.793 8.228 8.26 7.875 8.487 8.411 8.638 8.674 8.578
   6008   1387182_at   4.44 3.919 4.307 4.986 5.047 5.59 5.589 6.092 6.087 5.527
   6009   1393368_at   6.426 6.855 6.694 7.196 7.06 7.507 7.467 7.631 7.505 7.765
   6010   1371680_at   10.096 10.128 10.071 10.282 10.07 10.452 10.371 10.824 10.635 10.549
   6011   1378089_at   0.705 1.098 0.39 0.485 0.705 1.465 2.16 2.266 1.696 2.976
   6012   1388691_at   8.229 8.265 8.267 8.565 8.612 8.961 8.847 8.757 8.827 8.784
   6013   1383938_at   9.927 10.081 9.904 10.103 9.926 10.303 10.524 10.599 10.303 10.247
   6014   1382593_at   6.445 6.76 6.672 6.544 6.753 6.981 7.294 7.032 7.507 7.172
   6015   1396013_at   7.662 7.95 7.933 8.375 7.897 8.907 8.55 8.559 8.866 8.51
   6016   1370135_at   6.829 7.166 7.431 6.629 6.971 7.9 7.988 7.636 7.743 7.628
   6017   1375411_at   10.228 10.17 10.374 10.187 10.191 10.692 11.137 10.788 10.607 10.587
   6018   1371477_at   7.872 7.881 7.864 7.788 7.955 7.73 7.436 7.644 7.607 7.576
   6019   1376790_at   7.956 8.076 8.228 8.017 7.883 8.502 8.506 8.708 8.506 8.253
   6020   1388561_at   5.218 6.43 5.158 6.365 6.292 7.431 7.303 7.177 7.418 7.42
   6021   1378019_at   5.751 5.407 5.607 5.414 5.504 6.235 6.04 5.982 5.904 5.846
   6022   1375187_at   6.539 6.714 6.404 6.39 6.489 6.098 6.208 6.115 6.033 5.784
   6023   1370334_at   4.564 5.523 5.511 5.215 5.762 6.807 6.048 6.709 6.466 6.769
   6024   1395170_at   2.915 3.677 3.128 2.918 2.651 3.867 4.522 4.643 4.007 5.01
   6025   1375723_at   5.368 5.052 5.006 5.658 5.194 5.712 6.08 5.92 5.892 6.061
   6026   1374534_at   7.323 7.599 7.524 7.64 7.631 7.955 7.751 7.98 7.907 7.888
   6027   1373879_at   8.03 7.95 8.045 8.066 7.856 7.781 7.794 7.719 7.608 7.575
   6028   1371428_at   11.294 11.402 11.236 11.184 11.287 11.111 11.081 11.013 10.924 10.899
   6029   1375526_at   9.284 9.191 9.453 9.36 9.5 9.698 9.567 9.735 9.856 9.845
   6030   1377802_at   5.159 4.725 5.055 5.634 4.974 5.88 5.89 6.29 6.495 5.887
   6031   1377088_at   5.109 5.477 5.454 5.699 5.073 6.318 5.824 6.271 6.675 6.21
   6032   1382225_at   6.533 6.699 6.748 6.547 6.182 5.977 5.624 6.105 5.545 5.455
   6033   1373882_at   7.455 7.552 6.77 7.208 7.647 8.263 7.989 8.178 8.374 8.079
   6034   1379517_at   0 2.011 1.384 3.055 1.88 4.512 3.915 5.517 5.037 3.939
   6035   1378929_at   4.423 4.402 3.983 4.823 3.888 5.402 4.95 6.011 5.526 5.839
   6036   1388274_at   5.185 5.249 4.71 5.482 5.698 6.377 5.999 6.83 6.115 6.574
   6037   1371295_at   12.355 12.259 12.368 12.274 12.384 12.057 12.214 12.061 11.91 11.968
   6038   1387007_at   5.563 5.694 5.963 5.852 5.81 6.495 6.039 6.587 6.444 6.775
   6039   1376050_at   6.734 6.897 6.699 6.807 6.772 6.31 6.632 6.186 6.258 6.093
   6040   1386669_at   5.76 5.73 6.003 6.031 6.109 5.476 4.874 4.278 4.714 5.049
   6041   1390513_at   6.856 7.043 7.043 6.781 6.508 5.925 6.289 6.183 6.209 6.427
   6042   1384762_at   6.355 6.116 6.188 6.249 6.161 5.868 5.519 5.477 4.917 5.319
   6043   1378854_at   5.623 6.007 5.91 5.395 5.736 6.149 6.497 6.76 6.418 6.453
   6044   1371150_at   8.303 8.325 8.676 8.56 8.291 8.886 8.962 9.341 8.87 9.29
   6045   1374563_at   5.72 6.18 6.4 6.508 6.26 4.946 5.57 5.531 5.123 4.803
   6046   1368743_a_at   3.077 3.266 2.86 3.205 3.683 3.734 4.214 4.547 4.057 4.235
   6047   1382086_at   7.697 7.697 7.138 7.334 7.474 6.602 6.503 6.472 6.574 5.543
   6048   1397495_at   5.423 5.565 5.127 4.87 4.403 6.151 5.875 6.516 6.5 6.751
   6049   1381808_at   4.576 3.672 4.163 4.672 4.192 5.162 5.394 5.297 5.388 4.93
   6050   1393927_at   3.246 2.836 3.718 2.541 3.818 4.692 4.114 4.804 4.705 4.668
   6051   1374268_at   6.755 6.666 6.471 6.867 6.549 7.11 7.148 6.913 7.195 7.065
   6052   1383605_at   7.807 8.045 8.09 7.991 7.124 8.787 8.447 8.982 9.013 9.023
   6053   1389117_at   6.934 6.862 7.074 7.061 6.916 6.533 6.398 6.495 6.679 6.088
   6054   1398872_at   12.302 12.236 12.302 12.066 12.231 12.007 11.889 11.559 11.772 11.783
   6055   1385790_at   8.366 8.409 8.465 8.569 8.224 7.986 8.206 8.09 7.987 8.003
   6056   1381993_at   9.438 9.537 9.269 9.313 9.571 8.96 8.841 9.166 9.096 8.82
   6057   1390806_at   7.329 7.224 7.306 7.593 7.614 7.861 7.765 7.955 7.981 7.771
   6058   1389913_at   5.211 5.369 5.621 5.634 5.657 6.218 6.091 5.99 5.947 5.885
   6059   1383830_a_at   7.74 7.63 7.768 7.82 7.52 8.083 8.056 7.889 8.089 8.213
   6060   1382746_s_at   6.301 6.178 6.086 6.556 6.568 5.692 5.439 5.886 5.867 5.518
   6061   1372415_at   7.294 7.181 7.159 7.278 7.264 6.267 7.001 6.647 6.57 6.694
   6062   1392563_at   6.912 6.809 7.013 7.3 6.777 7.563 7.345 7.756 7.958 7.984
   6063   1379160_at   5.319 5.195 4.849 5.205 4.745 5.831 5.772 5.703 5.713 6.339
   6064   1383741_at   6.451 6.311 6.269 6.501 6.284 6.024 5.545 5.751 5.284 5.845
   6065   1382126_at   8.188 8.203 8.112 8.227 8.058 8.628 8.573 8.821 8.699 8.31
   6066   1382913_at   4.982 4.932 4.46 5.381 5.143 5.594 6.34 6.56 6.068 5.921
   6067   1367961_at   9.833 9.779 10.131 9.226 8.338 10.802 10.984 11.303 11.116 11.517
   6068   1396175_at   4.711 4.683 4.542 4.825 4.684 5.124 5.318 4.916 4.989 5.197
   6069   1388924_at   8.746 8.828 8.316 8.981 9.002 7.281 7.506 7.79 6.467 6.004
   6070   1376112_a_at   7.693 7.69 7.49 7.687 7.587 7.778 8.058 8.007 7.997 7.893
   6071   1378763_at   4.537 5.126 5.353 4.995 4.488 5.966 5.469 5.998 6.012 5.928
   6072   1379826_at   6.198 6.535 6.28 5.784 6.201 6.655 6.906 6.904 6.884 6.858
   6073   1393192_at   6.887 6.741 6.969 6.928 6.954 7.339 7.455 7.618 7.104 7.301
   6074   1382778_at   7.13 7.453 7.403 7.915 6.944 6.468 6.403 6.698 6.508 6.512
   6075   1397622_at   5.32 4.989 5.188 5.339 5.474 5.926 5.65 6.421 6.41 6.065
   6076   1369213_at   5.906 6.359 6.045 6.741 6.111 6.959 6.852 7.573 7.398 7.468
   6077   1395704_at   6.721 6.816 6.551 6.548 6.631 6.466 6.006 6.191 6.218 6.114
   6078   1397615_at   4.569 4.433 4.77 4.974 4.708 6.039 5.636 5.596 5.578 5.061
   6079   1393132_at   6.059 5.737 6.235 6.387 6.327 6.741 6.746 6.692 6.976 6.733
   6080   1394568_at   6.888 7.186 6.69 7.22 7.339 6.542 6.393 6.403 6.455 6.498
   6081   1390509_a_at   8.723 8.603 8.614 8.855 8.74 8.929 8.91 9.129 9.075 9.038
   6082   1388430_at   8.503 8.288 8.117 7.933 8.234 8.674 8.721 8.699 8.846 8.63
   6083   1371798_at   10.313 10.32 10.163 10.207 10.325 10.57 10.507 10.618 10.63 10.386
   6084   1373254_at   5.148 5.259 4.758 5.353 5.489 5.889 5.776 6.361 5.9 6.269
   6085   1399078_at   7.917 8.015 7.888 8.221 8.093 8.334 8.271 8.546 8.533 8.467
   6086   1373475_at   8.472 8.575 8.611 8.719 8.753 8.337 8.299 8.38 8.297 8.103
   6087   1371044_at   7.176 7.477 7.238 7.039 7.162 6.5 5.804 6.807 6.273 6.282
   6088   1389464_at   5.344 4.591 5.374 5.193 5.552 6.105 5.831 6.567 6.383 6.595
   6089   1390743_at   3.942 3.796 3.517 3.761 3.684 4.178 4.806 4.208 4.341 4.448
   6090   1380203_at   5.955 5.919 6.03 6.335 6.367 6.754 6.716 7.485 6.91 6.895
   6091   1396815_at   3.987 4.448 3.95 3.53 4.25 5.129 4.601 5.622 5.235 5.132
   6092   1371021_at   8.81 8.92 8.845 8.818 8.818 9.25 9.338 9.28 9.212 8.934
   6093   1382186_a_at   5.315 5.588 6.27 5.914 5.969 4.896 4.538 4.264 5.135 4.195
   6094   1368465_at   4.298 3.442 2.616 4.186 5.063 6.037 5.985 6.445 5.778 5.947
   6095   1372454_at   8.02 8.118 8.142 8.191 8.051 8.36 8.343 8.38 8.684 8.517
   6096   1383976_at   6.005 6.18 5.904 6.077 6.231 6.467 6.717 6.939 6.633 6.402
   6097   1380561_at   7.177 7.001 6.984 7.239 7.079 7.436 7.264 7.465 7.395 7.504
   6098   1384774_at   6.496 6.626 6.949 6.912 5.58 7.716 7.548 7.846 7.882 7.895
   6099   1384327_at   8.533 8.435 8.574 8.697 8.572 9.171 8.749 9.405 9.122 9.296
   6100   1374252_at   6.643 6.452 6.808 6.737 6.813 6.278 6.12 6.336 5.962 5.797
   6101   1371926_at   9.562 9.711 9.292 9.58 9.465 9.25 8.986 9.178 9.029 8.963
   6102   1372537_at   6.365 7.025 6.838 7.109 7.166 7.725 7.486 7.871 7.65 7.655
   6103   1376710_at   4.233 3.57 4.491 4.505 4.093 5.017 5.03 5.511 4.995 5.517
   6104   1373431_at   9.406 9.599 9.803 9.581 9.144 10.026 10.012 10.183 10.288 10.044
   6105   1368413_at   7.345 7.278 6.151 6.752 7.396 5.735 5.234 3.961 5.341 5.235
   6106   1389218_at   8.788 8.765 8.688 8.866 8.939 8.581 8.36 8.519 8.605 8.468
   6107   1388662_at   7.466 7.64 7.638 7.705 7.449 7.997 8.212 8.179 8.006 7.781
   6108   1373605_at   7.89 7.977 7.865 8.038 7.948 8.159 8.348 8.168 8.524 8.347
   6109   1373100_at   9.769 9.733 9.875 9.914 9.825 9.683 9.458 9.608 9.564 9.594
   6110   1377961_at   5.365 5.68 5.828 5.834 6.116 6.289 6.467 6.918 6.563 6.517
   6111   1372257_at   8.576 8.477 8.484 8.797 8.609 8.927 8.841 8.99 9.056 8.861
   6112   1368341_at   7.708 7.722 7.753 7.74 7.513 7.447 7.493 7.224 7.232 7.268
   6113   1377254_a_at   6.742 6.937 6.74 7.113 6.947 7.449 7.519 7.838 7.567 7.191
   6114   1392483_at   6.176 6.155 6.925 6.165 6.459 7.124 7.178 7.877 7.296 7.329
   6115   1379286_at   5.716 5.186 5.227 5.202 5.531 5.861 6.083 6.583 6.67 6.226
   6116   1387262_at   9.694 9.594 9.492 9.706 9.905 9.259 9.305 9.402 9.216 9.082
   6117   1395145_at   8.256 8.138 8.38 8.384 8.186 8.538 8.545 8.767 8.928 8.778
   6118   1389179_at   5.979 6.557 6.188 6.175 6.286 6.886 6.576 6.868 6.932 6.734
   6119   1375550_at   9.266 9.174 9.028 9.314 9.335 8.895 8.803 8.99 8.946 8.906
   6120   1393298_at   5.886 5.744 5.704 5.575 5.818 4.88 4.941 5.55 4.745 4.968
   6121   1394093_at   7.278 7.773 7.534 7.932 7.486 7.182 6.953 6.849 6.694 6.909
   6122   1390458_at   5.664 5.4 5.677 5.616 5.463 5.795 6.113 6.155 6.054 6.376
   6123   1390684_at   7.695 7.832 7.71 7.758 7.864 8.114 8.15 8.391 8.098 7.972
   6124   1374178_at   9.364 9.19 9.019 9.28 9.088 8.841 8.705 8.958 8.627 8.68
   6125   1380932_at   4.446 4.84 4.82 4.907 4.615 6.156 5.469 5.398 5.871 6.603
   6126   1388542_at   9.31 9.163 9.108 9.424 9.296 9.481 9.569 9.619 9.614 9.537
   6127   1368167_at   6.65 6.532 6.82 6.605 6.247 6.252 5.885 5.831 6.014 5.953
   6128   1379374_at   3.331 4.011 3 3.711 2.986 4.736 5.054 5.001 4.09 4.644
   6129   1368060_at   11.98 11.961 12.084 11.796 11.766 12.256 12.298 12.18 12.226 12.433
   6130   1398411_at   4.13 2.539 4.543 4.374 4.483 6.557 6.04 5.491 5.896 6.341
   6131   1390556_at   6.398 6.359 6.461 6.664 6.227 6.815 6.942 7.109 7.395 7.502
   6132   1389612_at   6.402 6.563 6.23 6.142 6.441 6.693 6.809 6.954 6.838 6.693
   6133   1370295_at   9.111 9.208 9.191 9.291 9.458 9.017 8.868 8.963 8.867 8.934
   6134   1375707_at   4.189 5.6 4.079 4.455 6.274 7.409 6.8 7.764 7.575 6.775
   6135   1393421_at   6.683 6.75 7.457 7.091 6.273 7.888 7.592 8.009 8.084 8.035
   6136   1380526_at   7.28 7.184 7.385 6.931 6.927 7.57 7.508 7.805 7.729 7.773
   6137   1395396_at   7.321 7.274 6.832 7.213 7.172 6.803 6.739 6.382 6.432 6.474
   6138   1389586_at   9.451 9.584 9.187 9.513 9.823 10.013 10.039 10.392 10.211 10.015
   6139   1369991_at   10.069 10.098 10.138 10.246 10.355 9.839 9.713 9.894 9.549 9.438
   6140   1393765_at   2.72 1.852 2.483 2.094 3.943 4.592 4.565 4.622 4.448 4.092
   6141   1384634_at   3.876 4.307 3.779 4.529 3.814 5.456 5.233 4.973 4.671 5.684
   6142   1379300_at   5.515 5.845 6.447 6.452 5.972 6.923 6.861 7.773 7.353 7.328
   6143   1388244_s_at   11.764 11.689 11.763 11.86 11.881 11.653 11.462 11.563 11.54 11.589
   6144   1385126_at   4.391 4.78 4.989 4.759 4.428 5.297 5.527 5.508 5.166 5.198
   6145   1384309_at   4.436 4.621 4.703 3.684 4.505 5.318 5.352 5.14 5.32 5.508
   6146   1368900_at   4.998 5.175 5.365 5.327 5.381 5.979 5.939 5.527 6.259 5.912
   6147   1376793_at   9.593 9.633 9.574 9.665 9.679 9.353 9.315 9.441 9.251 9.032
   6148   1382016_at   4.103 4.163 4.177 4.459 4.288 5.081 5.297 4.595 5.366 4.783
   6149   1368333_at   12.183 12.105 12.311 12.086 11.47 12.77 12.796 12.672 12.758 13.046
   6150   1390711_at   6.289 5.984 6.513 6.413 6.114 6.941 7.191 7.295 6.864 6.664
   6151   1379971_at   4.51 5.223 4.938 4.532 4.664 5.445 5.752 6.01 5.471 5.471
   6152   1395466_at   3.422 4.159 4.11 3.703 4.51 4.857 4.842 5.249 5.595 5.108
   6153   1388656_at   9.358 9.375 9.422 9.321 9.414 9.236 9.088 9.279 9.11 9.088
   6154   1379491_at   5.076 5.409 5.329 5.259 3.644 6.416 6.34 6.816 6.796 7.099
   6155   1372261_at   11.44 11.346 11.469 11.156 10.783 11.849 11.935 11.837 11.802 12.052
   6156   1376649_at   8.037 8.122 8.024 8.206 8.139 8.463 8.436 8.829 8.731 8.385
   6157   1369777_a_at   6.869 6.846 6.87 7.105 7.061 7.323 7.192 7.646 7.543 7.415
   6158   1389219_at   6.893 6.765 6.639 7.14 7.067 7.318 7.508 7.515 7.367 7.312
   6159   1393263_at   4.305 3.611 3.734 4.268 4.448 5.213 4.69 5.748 5.889 5.428
   6160   1376156_at   5.198 5.706 5.489 5.747 6.046 6.442 6.387 6.492 6.528 6.151
   6161   1368169_at   6.309 6.246 6.485 6.671 6.527 6.862 7.1 6.746 7.069 6.925
   6162   1372947_at   9.424 9.184 9.707 9.428 9.432 9.898 9.773 10.091 9.926 9.872
   6163   1376569_at   8.372 8.411 8.744 8.519 8.68 8.019 7.811 8.179 8.215 7.99
   6164   1392864_at   6.117 6.285 6.192 6.05 6.654 6.949 6.924 7.599 7.289 6.849
   6165   1373165_at   5.995 6.211 6.22 6.712 6.076 6.739 6.858 7.283 7.117 7.265
   6166   1380431_at   6.89 7.106 6.701 6.835 6.571 7.145 7.366 7.599 7.52 7.38
   6167   1375982_at   2.714 3.233 3.441 3.178 3.476 4.889 3.706 4.316 4.701 4.944
   6168   1377407_at   4.979 4.645 5.436 5.71 4.758 6.059 6.26 6.056 6.432 6.028
   6169   1375933_at   10.005 10.035 9.846 10.148 9.831 10.448 10.482 10.399 10.283 10.2
   6170   1371634_at   9.446 9.596 9.501 9.4 9.44 9.711 9.852 9.815 9.777 9.598
   6171   1368082_at   7.727 7.94 7.681 7.806 7.825 8.336 7.934 8.426 8.325 8.304
   6172   1372421_at   8.934 8.723 8.713 8.973 9.073 9.343 9.422 9.396 9.374 9.106
   6173   1391222_at   6.547 6.639 7.196 6.991 7.532 6.063 5.82 6.298 5.552 5.705
   6174   1395629_at   4.79 3.97 4.925 4.24 4.879 5.473 5.397 5.819 5.495 5.65
   6175   1397376_at   2.973 0 1.832 1.459 1.444 4.705 3.718 4.424 4.251 3.469
   6176   1398901_at   8.081 8.243 8.005 8.139 8.274 7.973 7.784 7.857 7.778 7.703
   6177   1372858_at   4.431 3.635 4.453 4.578 4.566 5.589 5.451 5.282 4.99 5.789
   6178   1367669_a_at   9.689 9.7 9.989 9.83 9.649 10.112 10.179 10.033 10.105 10.062
   6179   1377723_at   8.423 8.364 8.489 8.583 8.54 8.819 8.808 9.114 8.787 8.746
   6180   1381651_at   3.563 3.307 3.704 3.438 3.605 3.858 4.007 4.265 4.539 4.336
   6181   1379782_a_at   6.251 6.379 6.309 6.642 7.134 7.226 7.34 7.467 7.637 7.873
   6182   1383202_at   9.324 9.336 9.407 9.565 9.526 9.2 9.047 9.24 9.082 9.081
   6183   1380333_at   5.989 6.302 6.928 6.248 5.31 3.839 3.498 1 4.063 3.449
   6184   1390351_at   6.391 6.633 6.61 6.614 6.513 6.322 6.096 6.245 5.891 6.006
   6185   1371305_at   12.245 12.275 12.124 12.101 12.307 12.001 12.013 11.946 11.942 12.041
   6186   1390521_at   2.433 0.705 1.189 2.011 2.59 3.899 3.771 4.111 4.331 3.076
   6187   1368838_at   10.11 10.05 10.377 10.298 10.291 9.869 9.967 9.645 9.53 9.643
   6188   1392324_at   6.648 6.807 7.094 6.617 6.135 7.509 7.297 7.404 7.569 7.743
   6189   1389785_at   10.511 10.511 10.864 10.846 10.316 11.275 10.983 11.365 11.505 11.613
   6190   1374205_at   7.424 7.547 7.501 7.357 7.411 7.107 6.974 6.825 7.275 7.07
   6191   1395331_at   8.354 8.416 8.425 8.55 8.604 8.647 9.001 9.027 9.189 8.924
   6192   1386988_at   6.867 7.002 6.998 7.086 6.973 7.325 7.128 7.2 7.333 7.39
   6193   1372500_at   9.078 9.064 9.155 9.264 9.56 8.697 8.65 8.876 8.397 8.468
   6194   1374633_at   6.8 6.885 6.924 6.871 6.435 7.206 7.691 7.597 7.443 7.211
   6195   1373495_at   8.072 8.123 8.174 8.096 8.017 7.775 7.929 7.613 7.486 7.483
   6196   1394871_at   8.091 8.493 8.231 8.488 7.954 8.834 8.825 8.707 8.86 8.734
   6197   1391079_at   6.106 6.146 5.997 6.752 6.531 7.049 6.816 7.338 7.374 7.271
   6198   1389032_at   8.699 8.582 8.584 8.855 8.589 9.112 9.057 8.887 9.312 9.046
   6199   1382294_at   6.124 5.842 5.803 5.787 5.971 6.639 6.763 6.389 6.27 6.297
   6200   1395551_at   6.954 6.893 7.297 7.033 6.847 7.544 7.785 7.941 7.308 7.707
   6201   1398874_at   9.198 9.386 9.408 9.361 9.333 9.693 9.669 9.597 9.546 9.483
   6202   1387974_a_at   9.395 9.369 9.752 9.437 8.306 10.477 10.259 10.494 10.615 10.586
   6203   1382838_at   5.448 5.515 4.528 4.888 5.305 6.424 5.991 6.366 6.268 5.876
   6204   1389234_at   6.834 6.815 6.847 7.173 5.938 7.704 7.949 8.297 7.655 7.767
   6205   1377963_at   4.098 4.682 4.489 4.255 4.67 5.22 4.866 5.241 5.176 4.996
   6206   1382063_at   8.48 8.581 8.398 8.532 8.512 8.236 8.251 8.401 8.154 8.172
   6207   1384357_at   6.362 6.478 6.495 6.841 6.783 6.082 5.586 6.025 5.564 6.057
   6208   1368313_a_at   6.552 6.135 6.56 6.696 6.278 7.32 7.689 7.135 7.041 6.992
   6209   1380407_at   9.483 9.424 9.607 9.576 9.535 9.804 9.633 9.878 9.766 9.8
   6210   1377899_at   9.112 9.195 9.079 9.199 9.198 9.346 9.762 10.016 9.673 9.667
   6211   1391044_s_at   5.96 5.461 5.462 5.891 5.562 4.929 5.07 4.788 4.603 5.232
   6212   1388448_at   8.957 9.026 8.943 9.002 9.021 8.726 8.377 8.802 8.514 8.395
   6213   1390690_at   4.55 3.662 4.098 4.547 4.752 5.469 5.385 5.411 5.033 5.57
   6214   1373119_at   7.174 6.676 6.687 6.895 7.018 7.798 7.21 7.788 7.519 7.653
   6215   1394510_at   6.26 6.278 6.104 6.526 6.24 6.627 6.744 7.088 6.902 6.676
   6216   1389027_at   7.614 7.525 7.645 7.728 7.91 8.321 7.997 8.132 8.384 8.034
   6217   1371563_at   8.363 8.469 8.266 8.44 8.529 7.941 7.805 7.822 7.825 7.175
   6218   1372779_at   8.25 8.344 8.405 8.66 8.464 8.079 7.961 8.02 8.057 8.16
   6219   1379270_at   4.725 5.02 4.532 4.608 4.75 5.137 5.312 5.694 5.319 5.783
   6220   1384720_at   3.263 3.982 4.551 3.639 3.851 5.038 5.21 5.107 4.652 4.917
   6221   1376801_at   7.49 7.826 7.56 7.738 7.75 6.907 7.111 7.402 7.068 6.714
   6222   1379814_at   5.15 5.46 5.724 6.065 5.862 6.707 6.6 7.297 6.726 6.344
   6223   1373549_at   7.096 6.959 7.067 7.319 7.262 6.613 6.274 6.799 6.22 6.014
   6224   1388119_at   10.98 11.035 11.051 11.072 11.114 10.887 10.811 10.925 10.699 10.713
   6225   1388979_at   8.837 8.833 8.878 8.979 8.964 8.722 8.629 8.656 8.527 8.4
   6226   1368843_at   10.208 9.954 10.042 9.973 10.045 9.795 9.68 9.722 9.589 9.375
   6227   1372877_at   9.276 9.4 9.229 9.466 9.512 9.091 9.01 9.014 8.718 8.721
   6228   1387417_a_at   6.771 7.117 6.985 7.003 6.791 7.233 7.274 7.312 7.547 7.454
   6229   1394486_at   6.201 6.563 6.447 6.528 6.1 5.664 5.389 4.76 5.463 5.763
   6230   1383776_at   7.919 7.745 7.751 7.982 7.99 8.416 8.224 8.543 8.332 8.112
   6231   1381923_at   6.463 6.486 6.77 6.737 6.723 7.034 7.019 6.902 7.029 6.935
   6232   1383558_at   7.914 7.726 7.419 7.946 7.638 8.073 8.258 8.247 8.339 8.33
   6233   1372166_at   8.782 8.764 8.813 8.998 9.033 8.567 8.274 8.608 8.513 8.367
   6234   1372214_at   9.365 9.382 9.716 9.627 9.601 9.897 9.921 9.873 9.917 9.827
   6235   1372318_at   6.715 6.549 6.347 6.652 6.762 6.887 7.122 7.313 7.125 7.418
   6236   1394741_at   6.786 6.788 6.832 6.85 6.687 7.188 7.216 6.995 7.081 6.936
   6237   1367847_at   7.525 7.693 7.966 7.842 7.776 8.253 8.145 8.535 8.295 8.742
   6238   1387530_a_at   5.91 5.973 6.028 6.143 5.647 5.351 5.428 5.391 5.542 4.999
   6239   1383188_at   5.278 5.923 6.24 6.194 5.172 6.797 6.811 6.845 7.01 6.897
   6240   1391383_at   1.516 3.69 1.084 0.604 1.501 4.732 4.542 5.476 4.779 3.608
   6241   1382462_at   5.844 5.91 6.032 6.545 5.548 6.667 7.187 7.252 7.281 6.683
   6242   1398839_at   13.41 13.307 13.159 12.915 13.069 12.828 12.752 12.552 12.579 12.696
   6243   1393654_at   6.202 6.38 6.787 6.382 6.516 7.082 6.958 7.05 7.26 6.832
   6244   1387269_s_at   6.237 5.609 6.738 6.498 6.222 5.522 5.206 4.707 4.657 4.323
   6245   1369153_at   5.708 5.429 5.252 5.788 5.712 6.102 6.082 6.171 6.084 6.457
   6246   1379456_at   9.315 9.24 9.798 9.659 9.411 10.081 9.886 10.316 10.129 10.062
   6247   1384770_at   4.951 4.834 4.498 3.984 2.322 6.392 6.098 6.598 6.646 6.932
   6248   1387900_at   9.45 9.374 9.41 9.68 9.647 9.801 9.825 9.975 9.851 9.816
   6249   1398837_at   10.819 10.727 10.902 10.91 11.005 11.093 11.08 11.122 11.106 11.105
   6250   1393261_at   5.05 5.382 5.446 4.952 5.518 6.005 5.616 6.163 6.057 6.131
   6251   1373838_at   6.589 7.016 6.979 6.902 6.852 6.518 6.35 6.564 6.42 6.291
   6252   1382685_at   5.742 5.789 5.715 5.253 5.227 6.84 6.751 7.021 6.167 6.179
   6253   1377658_at   4.382 3.884 3.64 4.643 3.745 5.056 5.291 5.279 4.806 5.027
   6254   1373869_at   7.671 7.926 7.887 8.132 8.004 7.297 7.591 7.479 7.554 7.494
   6255   1374637_at   7.468 7.464 7.366 7.684 7.76 7.041 6.858 7.307 6.803 6.929
   6256   1377020_at   7.705 7.796 7.922 7.568 7.848 7.548 7.446 7.319 7.408 7.336
   6257   1393128_at   6.329 6.31 6.041 5.939 5.802 3.797 5.252 5.038 4.867 3.998
   6258   1371599_at   9.673 9.738 9.858 9.911 9.76 10.193 9.926 10.231 10.232 10.353
   6259   1369980_s_at   9.08 9.17 9.264 9.351 9.387 9.692 9.42 9.762 9.722 9.658
   6260   1395274_at   7.048 6.966 6.979 7.203 6.898 7.472 7.248 7.499 7.274 7.354
   6261   1390498_at   7.691 7.577 7.632 7.938 7.532 7.952 8.434 8.209 8.201 8.123
   6262   1373491_at   8.674 8.669 8.625 8.627 8.764 8.419 8.393 8.366 8.014 8.273
   6263   1382897_at   4.112 3.847 3.694 3.871 4.564 4.769 5.003 4.846 4.705 4.692
   6264   1397508_at   6.55 6.415 6.714 6.632 7.015 6.195 5.871 5.689 6.222 5.831
   6265   1373793_at   7.064 6.443 6.677 7.072 6.985 5.953 6.235 6.117 5.431 6.012
   6266   1375194_at   6.257 6.367 6.132 6.236 6.122 6.676 6.902 6.403 6.698 6.644
   6267   1376058_at   6.542 6.481 6.77 6.821 6.551 7.519 7.131 6.991 7.067 7.205
   6268   1388966_at   6.728 6.713 6.693 6.741 6.506 5.719 5.712 4.971 6.19 5.857
   6269   1386966_a_at   8.509 8.301 8.452 8.231 8.361 7.789 7.625 7.866 8.162 7.542
   6270   1392515_at   8.412 7.944 8.465 7.078 8.073 6.983 6.692 5.701 6.029 5.564
   6271   1373228_at   8.149 8.187 8.209 8.132 8.404 8.476 8.449 8.625 8.465 8.48
   6272   1371335_at   11.858 11.84 11.81 11.631 11.867 12.12 12.011 11.997 12.016 12.04
   6273   1374489_at   7.795 7.967 7.909 7.97 8.107 7.633 7.357 7.452 7.268 6.914
   6274   1383921_at   3.977 2.637 3.195 3.751 3.645 1.884 1.118 0.575 2.457 0.623
   6275   1377696_at   7.853 8.109 7.857 7.877 7.591 6.749 6.656 7.148 7.098 7.496
   6276   1382943_at   5.427 5.474 5.422 5.921 5.938 6.452 6.339 6.025 6.41 6.518
   6277   1395579_at   7.106 7.061 7.017 7.093 6.999 7.264 7.448 7.171 7.335 7.45
   6278   1383566_at   4.6 4.614 3.278 4.808 4.804 5.893 5.538 6.367 6.063 5.93
   6279   1388076_at   11.559 11.506 11.299 11.49 11.748 11.077 11.075 11.235 11.197 11.117
   6280   1376907_at   6.64 6.657 6.557 6.905 6.768 6.195 5.856 6.38 6.18 6.308
   6281   1371885_at   9.376 9.394 9.374 9.291 9.612 9.205 9.09 9.134 9.086 8.996
   6282   1379918_at   6.799 6.179 7.216 7.187 6.777 7.825 7.796 8.009 7.545 7.85
   6283   1388767_at   10.383 10.377 10.359 10.527 10.493 10.212 10.031 10.237 10.235 10.06
   6284   1394392_at   6.489 6.37 7.062 7.228 6.948 7.618 7.473 7.857 7.674 7.837
   6285   1385556_at   6.017 6.013 6.539 6.621 6.539 7.266 6.911 6.995 7.008 7.206
   6286   1393848_at   8.295 8.307 8.557 8.04 8.27 7.746 7.331 6.787 6.524 6.252
   6287   1388750_at   9.648 9.659 9.883 9.904 9.1 10.516 10.577 10.237 10.254 10.616
   6288   1395153_at   3.824 5.074 3.379 4.139 4.875 5.731 6.236 5.809 5.643 6.504
   6289   1370867_at   9.224 9.131 9.359 9.398 9.545 9.038 8.863 9.027 8.898 8.865
   6290   1374650_at   8.285 8.294 8.578 8.471 8.173 8.658 8.716 8.901 8.76 8.76
   6291   1379774_at   5.521 5.5 5.367 5.391 5.35 4.74 5.104 5.117 4.676 5.052
   6292   1373984_at   8.433 8.271 7.935 8.195 8.002 7.666 7.588 7.66 7.487 7.103
   6293   1369439_at   5.223 5.531 5.195 5.188 5.755 6.455 5.766 6.679 6.44 6.193
   6294   1391948_at   5.767 5.133 5.978 6.262 5.727 1.422 4.82 3.316 2.111 2.23
   6295   1385246_at   5.468 5.935 5.807 6.365 6.317 6.751 6.678 6.809 7.102 7.089
   6296   1368521_at   11.457 11.398 11.4 11.444 11.488 11.602 11.688 11.636 11.623 11.498
   6297   1379608_at   6.233 7.005 6.664 6.238 6.227 5.919 5.421 5.684 5.357 5.329
   6298   1388399_at   7.108 6.957 6.731 7.265 7.181 7.487 7.48 7.479 7.553 7.541
   6299   1378566_at   3.18 2.846 3.654 3.987 3.392 4.624 4.264 4.242 4.964 4.781
   6300   1371765_at   6.77 6.895 6.799 7.144 7.418 7.777 7.638 7.862 7.746 7.435
   6301   1387512_at   5.506 5.749 5.856 5.837 5.285 6.166 6.297 6.166 6.116 6.235
   6302   1380741_at   4.778 5.193 5.808 5.539 5.018 6.033 6.625 6.443 6.254 6.109
   6303   1374538_at   7.971 8.07 7.959 7.864 8.17 8.184 8.383 8.388 8.331 8.292
   6304   1368348_at   5.128 4.026 4.284 4.96 4.456 5.434 5.781 5.697 5.486 5.675
   6305   1382102_at   4.146 3.573 4.519 4.408 3.625 4.901 5.64 5.033 5.151 5.131
   6306   1378629_at   6.721 6.658 7.012 6.894 6.537 5.595 4.444 5.839 5.928 5.089
   6307   1369270_at   5.646 6.059 6.193 6.064 5.815 6.528 6.27 6.626 6.682 6.62
   6308   1378416_at   6.013 6.19 6.226 6.207 6.49 7.015 7.215 7.181 7.059 6.452
   6309   1384602_at   6.057 6.122 6.013 6.4 6.139 5.811 5.638 5.535 5.266 5.715
   6310   1370264_at   7.051 6.772 7.169 7.062 6.992 7.65 7.464 7.603 7.305 7.329
   6311   1399110_at   6.449 6.529 6.413 6.625 6.264 6.954 6.822 7.003 6.778 6.699
   6312   1374100_at   6.44 6.661 6.399 6.461 6.482 6.974 6.843 7.2 7.305 6.781
   6313   1368537_at   7.101 6.951 7.067 7.166 7.04 6.754 6.82 6.676 6.939 6.679
   6314   1390854_at   6.931 6.82 7.183 7.154 6.744 7.339 7.549 7.34 7.521 7.441
   6315   1373377_at   7.224 6.725 7.414 7.285 7.037 7.599 7.706 7.997 8.028 8.139
   6316   1379768_at   7.561 7.668 7.621 7.57 7.517 7.127 7.243 7.192 7.128 6.661
   6317   1374326_at   8.028 7.979 7.898 8.077 8.298 7.56 7.782 7.642 7.633 7.367
   6318   1371868_at   9.222 9.202 9.208 9.363 9.434 8.793 8.638 8.972 8.964 8.467
   6319   1398416_at   4.35 4.118 4.762 3.958 4.698 5.459 5.272 4.962 5.529 5.166
   6320   1387964_a_at   6.38 6.102 6.63 6.287 6.715 5.815 5.125 5.826 5.449 4.999
   6321   1374546_at   5.341 5.368 4.827 5.711 5.652 6.109 6.51 6.65 6.123 6.147
   6322   1385529_at   3.51 3.704 3.726 3.678 3.546 4.41 3.782 4.459 4.376 4.274
   6323   1386257_at   4.093 3.612 3.863 3.786 3.958 4.547 4.425 4.263 4.874 4.981
   6324   1371908_at   8.337 8.36 8.282 8.422 8.232 7.628 8.006 8.007 8.067 7.779
   6325   1378867_at   4.916 5.529 5.85 5.082 5.08 4.388 3.237 4.37 3.147 3.703
   6326   1370683_at   4.033 3.202 3.748 3.867 3.133 4.409 4.398 4.602 4.48 4.542
   6327   1373809_at   8.804 8.755 8.427 8.702 8.504 9.151 9.104 9.244 9.543 8.994
   6328   1391584_at   8.134 8.177 8.106 8.349 8.275 8.031 7.869 8 7.864 7.844
   6329   1388733_at   8.025 8.047 7.905 7.935 8.098 7.638 7.599 7.459 7.753 7.225
   6330   1371825_at   7.729 7.59 7.765 7.843 7.839 8.191 8.126 7.986 8.176 7.939
   6331   1374745_at   5.596 5.296 5.207 5.619 5.803 6.252 5.961 6.136 6.664 6.452
   6332   1375634_at   9.758 9.713 10.097 9.997 9.956 10.267 10.402 10.328 10.372 10.166
   6333   1378423_at   2.694 3.552 3.358 3.121 3.831 4.461 4.22 4.092 4.477 4.263
   6334   1388380_at   10.03 10.018 10.008 10.303 10.145 10.564 10.258 10.59 10.533 10.537
   6335   1383168_at   7.176 7.619 7.271 7.518 7.527 7.104 6.935 6.68 6.937 6.842
   6336   1391197_at   4.299 3.852 3.706 3.706 4.383 4.723 5.055 4.927 4.66 4.621
   6337   1381558_at   5.405 5.489 5.827 5.642 5.922 6.495 6.427 6.471 6.125 6.038
   6338   1373811_at   8.229 8.369 8.324 8.252 8.165 7.833 7.954 7.868 8.132 7.737
   6339   1372480_at   5.628 5.557 5.856 6.188 5.879 6.278 6.588 6.572 6.413 6.899
   6340   1387339_at   5.441 5.414 6.031 6.149 5.46 6.803 6.504 6.278 6.63 6.803
   6341   1371774_at   11.451 11.404 11.62 11.372 11.601 11.174 10.98 11.264 11.217 11.042
   6342   1383159_at   7.242 7.4 7.509 7.722 7.496 8.078 7.849 8.485 8.054 8.028
   6343   1382969_at   2.287 2.281 3.055 2.501 1.642 3.59 3.45 3.526 4.461 4.435
   6344   1379899_at   1.511 0.202 2.95 0.687 2.422 4.71 3.59 4.644 4.434 3.827
   6345   1396149_at   5.728 6.089 5.791 5.451 5.415 6.411 6.396 6.273 6.345 6.176
   6346   1373091_at   6.621 7.008 7.275 7.368 7.261 6.508 6.121 6.469 6.39 6.419
   6347   1392746_x_at   9.081 8.917 8.989 9.344 9.101 8.418 8.133 8.758 8.351 8.634
   6348   1374840_at   8.15 7.938 7.907 7.977 7.938 7.621 7.76 7.562 7.287 7.356
   6349   1397015_at   4.745 5.068 4.668 5.605 5.715 6.255 6.005 6.699 6.763 6.277
   6350   1382320_at   4.82 4.869 4.436 4.494 5.182 5.861 5.312 5.734 5.376 5.617
   6351   1381220_at   6.573 6.259 6.396 6.759 6.664 7.038 6.971 7.014 7.087 6.872
   6352   1376242_at   5.963 5.867 6.119 6.248 6.243 7.265 6.766 7.592 7.152 6.521
   6353   1379449_at   7.628 7.525 7.696 7.793 7.603 8 7.936 8.194 8.104 7.841
   6354   1399004_at   9.458 9.591 9.578 9.722 9.757 9.94 9.995 9.99 9.932 9.796
   6355   1396028_at   5.707 5.818 6.206 6.308 5.912 6.989 6.399 6.594 6.915 6.859
   6356   1397744_at   4.348 5.363 5.061 4.599 4.959 5.821 5.594 6.629 6.069 6.403
   6357   1374473_at   8.227 8.106 8.146 8.334 8.36 7.914 7.62 7.909 7.944 7.601
   6358   1396885_at   4.771 4.995 5.543 5.503 4.795 6.14 5.848 5.957 5.925 6.396
   6359   1370578_at   5.827 6.167 6.14 5.998 5.888 6.368 6.495 6.527 6.65 6.256
   6360   1368948_at   9.522 9.654 9.681 9.657 9.432 9.153 9.332 8.991 8.848 8.685
   6361   1397385_at   7.167 7.289 6.927 7.433 7.442 6.782 6.712 6.794 6.364 6.405
   6362   1375442_at   6.601 6.542 6.372 6.686 6.983 6.159 6.106 5.937 6.134 5.708
   6363   1381972_at   8.828 9.076 9.33 9.073 9.007 8.631 8.537 8.611 8.298 8.089
   6364   1383790_at   6.422 6.519 6.417 6.212 6.514 7.08 7.198 7.305 6.839 6.688
   6365   1391422_at   7.029 7.149 6.635 7.282 7.127 6.485 6.289 6.574 6.35 6.565
   6366   1386985_at   7.722 8.435 8.275 8.3 8.559 8.942 8.864 9.081 8.928 9.017
   6367   1398905_at   9.759 9.936 9.778 9.854 10.029 9.518 9.243 9.136 9.292 9.642
   6368   1398085_at   0.043 0.214 0.31 0 0 2.205 2.597 1.599 1.903 0.575
   6369   1398267_at   7.114 7.247 7.291 7.681 7.148 7.871 7.677 7.949 7.798 8.06
   6370   1386128_at   6.673 6.99 6.545 7.189 7.791 8.537 7.969 8.528 8.36 7.929
   6371   1389388_at   7.464 7.304 7.522 7.64 7.493 7.687 7.821 7.836 7.775 7.748
   6372   1395739_at   8.19 8.305 8.248 8.305 8.335 7.969 7.409 7.923 7.801 7.487
   6373   1372769_at   8.719 8.469 8.7 8.732 8.646 8.334 8.401 8.323 8.322 8.057
   6374   1383404_at   6.404 6.505 5.871 6.446 6.973 7.631 7.182 7.667 7.365 7.2
   6375   1373049_at   8.87 9.039 9.114 9.276 9.165 8.64 8.656 8.874 8.597 8.484
   6376   1374806_at   5.965 6.185 6.187 6.103 6.291 7.013 6.793 6.622 6.379 6.719
   6377   1380248_at   4.834 5.187 4.696 5.065 5.971 6.526 6.163 6.504 5.985 6.486
   6378   1387779_at   8.92 8.827 8.493 8.872 8.742 8.209 8.315 8.221 8.272 8.539
   6379   1382262_at   4.058 4.082 4.552 3.887 3.815 4.635 4.786 5.439 5.011 4.978
   6380   1375068_at   6.521 6.312 6.744 6.757 6.9 7.233 7.186 7.352 7.11 7.077
   6381   1399091_at   11.08 11.17 11.287 11.277 11.324 11.048 10.826 10.981 10.978 10.901
   6382   1368330_at   7.171 7.113 7.293 7.404 7.569 6.922 6.972 6.847 6.672 6.66
   6383   1386911_at   4.948 4.87 4.491 4.925 5.373 6.035 5.613 6.141 6.11 5.451
   6384   1372314_at   8.287 8.355 8.177 8.348 8.434 8.567 8.571 8.698 8.61 8.479
   6385   1388435_at   3.154 2.711 3.915 2.616 3.909 4.794 4.374 4.643 5.474 5.082
   6386   1370380_s_at   7.101 7.305 7.35 7.456 7.638 8.074 7.822 7.816 8.005 7.733
   6387   1382423_at   7.127 7.08 7.036 7.4 6.957 7.545 7.487 7.651 7.446 7.469
   6388   1371083_at   2.011 1.214 1.975 0.856 0 4.553 3.016 2.797 3.611 4.45
   6389   1372143_at   8.416 8.326 8.359 8.25 8.525 7.963 8.155 8.165 7.98 7.879
   6390   1391370_at   5.934 6.157 6.307 6.307 5.952 6.609 6.587 6.91 6.646 6.483
   6391   1393168_at   5.011 5.314 5.579 5.367 5.173 6.099 6.159 6.025 6.048 5.552
   6392   1373263_at   7.81 7.883 7.553 7.788 7.9 7.396 6.925 7.45 7.309 7.159
   6393   1389682_at   6.332 6.51 5.906 6.711 6.862 7.35 7.302 7.411 7.317 7.092
   6394   1382923_at   7.776 8.191 8.048 8.184 8.323 7.598 7.602 7.676 7.23 7.533
   6395   1368019_at   7.463 7.733 7.475 7.682 7.61 7.868 7.823 7.838 7.869 7.935
   6396   1380563_at   2.916 4.165 3.524 3.783 4.127 5.479 4.731 4.761 5.472 4.868
   6397   1384276_at   3.348 3.757 3.45 3.666 2.814 5.098 4.463 4.697 4.123 4.293
   6398   1373564_at   7.98 8.043 8.032 8.17 8.294 8.33 8.521 8.687 8.637 8.42
   6399   1383960_at   7.131 7.384 7.311 7.587 7.48 7.678 7.777 7.95 7.98 8.122
   6400   1368390_at   7.843 7.809 8.152 8.064 7.82 8.309 8.27 8.316 8.227 8.305
   6401   1384195_at   4.559 5.02 5.155 5.04 5.992 6.678 6.263 6.652 6.678 5.979
   6402   1386894_at   11.982 11.945 11.833 11.938 11.91 12.058 12.049 12.031 12.036 12.038
   6403   1393270_at   4.648 4.699 5.058 4.23 4.409 5.71 5.785 5.37 5.34 5.174
   6404   1394916_at   2.711 3.548 3.243 3.165 3.472 4.858 3.748 5.272 4.475 4.73
   6405   1372663_at   8.271 8.315 8.236 8.526 8.548 8.639 8.671 8.864 8.833 8.769
   6406   1389326_at   7.307 7.427 7.299 7.558 7.68 7.068 6.897 7.159 6.706 6.87
   6407   1371702_at   8.639 8.495 8.41 8.482 8.816 9.125 8.781 9.274 9.161 9.102
   6408   1368794_at   8.951 8.832 9.228 9.192 8.736 9.584 9.386 9.684 10.012 10.034
   6409   1396211_at   3.405 2.787 2.575 2.077 2.441 4.274 3.584 3.964 3.567 4.206
   6410   1368122_at   8.154 8.103 8.079 8.282 8.159 8.573 8.556 8.637 8.345 8.344
   6411   1392724_at   5.684 5.379 5.972 5.594 5.717 6.804 6.572 6.485 5.988 6.4
   6412   1391356_at   8.349 8.666 8.765 8.8 8.788 8.217 7.883 8.159 8.306 7.853
   6413   1382124_at   7.261 7.252 7.358 7.16 7.042 7.706 7.364 7.671 7.698 7.796
   6414   1388578_at   6.719 6.764 6.813 6.683 6.532 6.294 5.763 6.308 6.304 6.099
   6415   1388680_at   8.694 8.707 8.654 8.617 8.846 8.99 9.064 9.121 9.194 8.844
   6416   1367509_at   9.166 9.067 9.215 9.275 9.405 9.448 9.577 9.737 9.54 9.561
   6417   1372927_at   7.367 7.382 7.543 7.721 7.702 7.965 7.853 8.158 7.93 7.96
   6418   1398384_at   6.726 7.06 7.033 7.176 6.843 6.387 6.156 6.617 6.561 6.195
   6419   1383253_at   9.605 9.69 9.785 9.994 9.788 9.319 9.219 9.44 9.474 9.467
   6420   1374706_at   4.935 5.058 5.285 5.241 5.812 5.895 6.005 6.188 5.967 6.023
   6421   1385220_at   6.438 6.724 6.404 6.538 6.442 6.239 6.088 6.157 5.848 5.84
   6422   1395214_at   6.519 6.173 6.236 6.142 6.39 6.71 6.762 6.629 6.851 6.566
   6423   1383319_at   6.713 6.316 6.659 7.056 6.214 7.926 7.313 7.485 7.256 7.902
   6424   1371970_at   7.807 7.662 7.474 7.708 7.74 6.97 7.177 7.055 6.479 6.246
   6425   1397693_at   8.409 8.448 8.192 8.101 8.318 7.709 8.1 7.744 7.673 7.539
   6426   1369176_at   4.345 4.751 5.822 6.196 5.045 3.68 3.754 3.605 3.055 3.452
   6427   1369191_at   5.815 5.769 5.676 5.246 5.067 3.991 3.167 2.922 2.733 0.496
   6428   1398361_at   6.98 6.796 7.196 6.872 6.678 7.208 7.408 7.441 7.453 7.35
   6429   1390947_at   7.728 7.765 7.55 7.585 7.737 7.381 7.192 7.485 7.31 7.112
   6430   1389671_at   6.462 6.724 6.101 6.657 6.683 5.89 5.285 5.872 5.954 5.617
   6431   1373221_at   8.312 8.221 8.346 8.171 8.21 8.04 7.761 7.971 8.024 8.014
   6432   1392330_at   7.565 7.668 7.777 7.877 7.853 8.15 7.974 8.124 8.137 7.999
   6433   1370347_at   7.767 7.734 7.997 8.056 8.027 7.534 7.688 7.483 7.541 7.466
   6434   1392824_at   3.403 3.121 3.616 3.713 4.009 4.873 4.723 4.115 4.438 4.421
   6435   1371660_at   9.354 9.412 9.235 9.485 9.652 9.034 9.181 9.071 9.041 9.04
   6436   1393389_at   5.84 5.214 5.502 6.31 5.678 3.969 5.164 3.557 3.281 3.726
   6437   1382239_at   2.639 0.782 3.595 3.497 3.343 5.275 5.029 5.713 5.402 5.158
   6438   1390356_at   10.431 10.335 10.357 10.402 10.503 10.129 10.108 10.277 9.936 9.971
   6439   1379107_at   3.847 3.934 3.708 3.604 3.721 4.223 4.128 4.746 4.233 4.524
   6440   1370905_at   7.307 7.311 7.162 7.255 7.195 7.839 7.658 7.978 7.85 7.396
   6441   1375649_at   5.009 5.631 5.708 5.075 5.044 3.811 2.956 3.821 3.183 4.684
   6442   1383871_at   7.348 7.313 7.577 7.477 7.627 7.29 7.006 6.959 7.073 6.983
   6443   1383931_at   6.546 6.292 6.641 6.769 6.824 7.239 7.266 7.322 6.936 7.088
   6444   1379662_a_at   8.37 8.21 8.344 8.681 8.646 8.008 7.862 7.857 7.825 7.38
   6445   1389340_at   6.238 5.821 6.148 6.186 6.239 6.634 6.513 6.413 6.766 6.642
   6446   1375123_at   6.072 5.775 6.077 6.514 6.779 7.464 6.822 7.488 7.44 7.182
   6447   1374938_at   7.697 7.715 7.891 7.904 7.98 7.573 7.461 7.555 7.481 7.622
   6448   1387491_at   7.28 7.696 8.517 6.617 7.076 9.479 9.692 9.837 9.643 8.372
   6449   1375551_at   7.351 7.042 7.152 7.278 7.283 7.036 6.7 6.851 6.799 6.641
   6450   1369679_a_at   7.247 8.009 7.892 7.614 7.644 8.2 8.446 9.116 8.662 8.827
   6451   1373776_at   7.314 7.342 7.187 7.351 7.447 6.717 6.338 7.121 6.356 6.367
   6452   1388511_at   7.761 7.813 7.753 7.989 8.106 7.558 7.23 7.357 7.537 7.11
   6453   1373958_at   7.44 7.361 7.68 7.559 7.684 7.874 7.888 7.809 7.973 7.837
   6454   1385109_at   6.217 6.037 6.465 6.236 6.392 5.869 5.688 5.881 5.879 5.965
   6455   1383280_at   5.947 6.275 5.802 5.604 5.889 6.771 6.295 7.175 7.074 6.694
   6456   1380387_at   3.449 4.033 4.628 4.344 3.121 5.311 5.116 6.052 5.281 5.428
   6457   1384002_at   7.603 7.558 7.568 7.59 7.376 7.173 7.026 7.379 6.896 6.959
   6458   1367902_at   7.848 7.897 7.549 7.832 8.411 8.883 8.542 8.699 8.929 8.452
   6459   1381630_at   5.04 5.152 5.393 5.975 4.964 1.098 1.395 1.761 4.11 3.436
   6460   1392672_at   6.407 6.267 6.216 6.426 7.376 7.966 7.536 7.605 7.744 7.387
   6461   1390136_at   8.511 8.305 8.418 8.581 8.634 7.838 8.075 8.286 7.955 7.803
   6462   1367831_at   9.201 9.283 9.049 9.449 9.4 8.993 8.769 8.687 8.593 8.36
   6463   1387542_at   8.551 8.77 8.724 8.306 7.839 9.31 9.534 9.379 9.458 9.028
   6464   1368851_at   9.166 9.374 9.287 9.313 8.95 8.848 8.789 8.85 8.661 8.462
   6465   1384522_at   5.895 5.884 6.208 6.077 5.229 4.249 4.618 4.603 5.099 5.129
   6466   1381196_at   5.858 5.735 6.341 6.536 6.413 7.251 6.916 6.976 6.833 7.021
   6467   1396009_at   4.137 4.519 4.79 4.667 5.688 5.885 6.022 5.915 6.017 6.081
   6468   1399041_at   7.742 7.508 7.743 8.035 7.409 8.3 8.293 8.493 8.974 8.347
   6469   1376660_at   8.079 7.985 8.03 7.96 7.966 8.44 8.528 8.736 8.569 8.137
   6470   1388359_at   10.139 10.203 10.195 10.578 10.354 10.807 10.784 10.999 10.597 10.715
   6471   1378378_at   7.625 7.552 7.559 7.612 7.808 7.28 6.989 7.438 7.072 7.219
   6472   1388681_at   9.929 9.974 10.034 10.098 9.907 10.1 10.393 10.264 10.329 10.269
   6473   1372754_at   6.821 6.181 6.896 6.56 6.655 7.385 7.139 7.132 7.338 7.327
   6474   1378681_at   3.266 4.049 3.445 3.781 2.926 4.124 4.904 5.416 5.271 4.791
   6475   1395441_at   6.32 6.416 6.856 6.672 6.283 7.142 6.925 7.115 7.006 7.172
   6476   1381951_at   3.566 3.333 3.667 4.205 2.956 4.236 4.881 5.018 5.648 5.322
   6477   1383437_at   8.247 8.288 8.461 8.846 8.473 9.001 8.872 8.973 9.181 9.22
   6478   1368259_at   8.015 7.853 8.171 7.876 7.955 7.715 7.206 7.591 7.275 7.472
   6479   1387010_s_at   6.507 7.06 6.825 6.917 6.322 7.739 7.144 7.814 7.72 8.116
   6480   1387363_at   6.177 6.288 5.908 5.928 4.474 7.557 7.724 7.607 7.25 6.991
   6481   1368899_at   7.566 7.352 7.385 7.573 7.487 8.007 7.982 8.199 8.115 7.622
   6482   1390855_at   6.322 6.5 6.536 6.593 6.429 7.038 6.724 7.322 7.163 7.539
   6483   1382835_at   7.289 7.135 7.718 7.75 7.344 8.322 7.808 8.348 8.465 8.213
   6484   1388725_at   9.572 9.632 9.667 9.794 9.89 9.367 8.907 9.325 9.208 8.828
   6485   1374182_at   7.206 7.182 7.238 7.372 7.268 6.917 6.984 6.995 6.819 6.578
   6486   1397826_at   6.023 6.599 6.295 6.461 5.998 7.185 7.197 6.622 7.108 7.006
   6487   1395116_at   6.815 6.382 7.32 7.05 7.014 4.748 4.091 0.757 2.376 4.464
   6488   1398389_at   8.95 8.881 8.783 8.805 8.816 9.268 9.089 9.325 9.178 8.984
   6489   1376168_at   8.553 8.516 8.5 8.266 8.349 8.855 8.809 8.925 8.677 8.669
   6490   1388504_at   10.235 10.194 10.078 10.224 10.181 10.438 10.354 10.505 10.378 10.299
   6491   1390316_at   5.715 5.974 5.732 6.155 5.703 6.608 6.849 6.364 6.263 6.937
   6492   1391442_at   8.074 8.017 8.285 8.243 8.335 7.635 7.709 7.984 7.563 7.415
   6493   1376115_at   7.608 7.711 7.847 7.732 7.368 8.343 8.011 8.13 8.202 7.999
   6494   1392025_x_at   6.18 5.837 6.679 6.026 6.712 7.458 6.9 7.445 7.852 7.272
   6495   1374586_at   8.177 8.237 8.307 8.379 8.427 8.651 8.448 8.757 8.698 8.603
   6496   1371355_at   10.566 10.541 10.472 10.474 10.568 10.662 10.789 10.625 10.664 10.73
   6497   1388852_at   8.916 8.82 8.828 9.151 8.849 9.451 9.107 9.293 9.349 9.324
   6498   1374847_at   7.679 7.741 7.742 7.723 7.566 7.906 7.937 7.98 8.033 7.8
   6499   1386923_at   9.834 9.812 9.792 9.996 9.979 9.638 9.624 9.506 9.352 9.255
   6500   1399146_at   8.489 8.545 8.298 8.725 8.714 8.876 9.028 9.112 8.99 8.888
   6501   1377671_at   11.048 11.071 11.076 10.924 10.745 10.57 10.627 10.571 10.753 10.658
   6502   1389453_at   6.322 6.281 5.953 6.161 6.294 6.802 6.536 7.161 6.716 6.708
   6503   1390563_at   8.054 7.954 8.182 8.268 8.371 8.834 8.786 8.98 8.739 8.385
   6504   1370418_s_at   6.894 7.072 7.079 7.313 7.346 7.613 7.406 7.748 7.84 7.753
   6505   1371614_at   8.489 8.497 8.589 8.648 8.487 8.766 8.719 8.975 8.859 8.74
   6506   1395743_at   4.138 4.473 4.836 4.333 4.606 4.81 5.4 5.347 5.252 5.441
   6507   1384157_at   9.066 9.157 9.25 8.947 8.886 8.596 8.8 8.526 8.652 8.326
   6508   1385352_at   4.804 3.087 5.073 4.928 4.182 5.978 5.935 6.392 6.287 6.321
   6509   1375719_s_at   2.392 3.558 3.183 3.746 3.979 4.849 4.497 5.993 5.897 5.09
   6510   1398312_s_at   5.076 5.377 5.396 5.079 5.593 6.601 5.686 6.067 6.113 6.405
   6511   1369239_at   3.981 4.561 4.545 4.421 3.9 5.027 4.912 4.831 5.079 5.173
   6512   1384553_at   6.978 7.334 7.219 7.302 7.38 6.96 5.86 6.059 6.369 6.295
   6513   1368583_a_at   6.077 6.331 6.754 6.129 5.138 7.091 7.403 7.526 7.438 7.433
   6514   1390649_at   4.753 5.618 5.411 5.667 5.331 6.336 5.966 6.119 6.185 6.309
   6515   1388437_at   6.977 6.853 7.338 7.557 7.403 8.063 7.747 7.925 7.901 7.841
   6516   1368463_at   7.394 7.202 7.379 7.339 7.624 7.867 7.651 7.741 7.876 7.709
   6517   1368237_at   4.574 4.467 4.208 4.953 4.434 5.022 5.277 5.19 5.274 5.027
   6518   1398606_at   8.042 7.907 7.838 7.877 7.934 8.094 8.127 8.352 8.312 8.164
   6519   1386041_a_at   6.484 6.549 6.858 6.784 6.833 6.405 6.17 6.163 6.28 6.355
   6520   1396055_at   6.849 6.84 7.032 7.073 7.136 7.694 7.355 7.405 7.57 7.245
   6521   1388936_at   8.324 8.374 8.089 8.725 9.147 9.789 9.334 9.791 9.707 9.214
   6522   1382461_at   6.997 7.071 7.38 7.315 7.07 6.873 6.711 6.768 6.803 6.6
   6523   1383721_at   5.956 6.311 5.679 6.369 6.109 6.845 6.538 6.706 6.784 6.72
   6524   1374620_at   7.462 7.491 7.25 7.468 7.07 6.851 6.722 7.064 6.915 6.682
   6525   1379249_at   7.668 7.942 7.527 7.706 7.559 7.336 7.308 7.232 7.204 6.938
   6526   1378304_at   4.886 5.14 5.7 5.532 5.328 6.23 5.814 6.141 6.106 6.026
   6527   1371463_at   9.389 9.481 9.284 9.464 9.493 9.138 9.184 9.168 9.193 8.918
   6528   1392244_at   6.108 5.883 5.632 5.883 5.785 6.104 6.653 6.945 7.017 6.758
   6529   1386321_s_at   5.684 5.582 6.02 5.913 5.757 4.782 4.556 5.014 3.37 3.861
   6530   1389306_at   7.128 7.359 6.832 7.37 7.622 7.99 7.76 8.03 8.08 7.853
   6531   1384910_at   0.669 0.757 1.766 1.982 1.744 3.903 3.54 3.725 2.192 4.446
   6532   1372528_at   8.129 7.915 8.019 8.192 8.008 7.806 7.296 7.58 7.645 7.665
   6533   1397845_at   4.508 3.867 4.672 4.406 3.915 2.58 0.978 3.072 0.014 0.941
   6534   1367804_at   8.597 8.443 8.844 8.543 8.036 9.138 9.283 9.521 9.551 8.98
   6535   1386648_at   4.668 3.64 3.921 4.006 3.345 5.304 5.013 5.709 5.419 4.698
   6536   1387804_at   3.45 3.23 1.628 2.31 2.548 4.96 3.872 4.157 4.355 4.736
   6537   1390027_at   9.031 9.037 9.322 9.181 9.29 9.477 9.456 9.761 9.607 9.504
   6538   1378814_at   3.079 3.297 4.038 3.699 3.356 4.854 3.973 4.716 4.554 4.789
   6539   1372578_at   7.404 7.832 7.628 7.676 7.786 7.213 6.814 6.705 5.995 6.686
   6540   1382844_at   4.554 4.963 4.745 4.745 4.589 5.245 5.113 5.642 5.253 5.136
   6541   1373819_at   9.974 10.128 10.291 10.289 10.249 10.675 10.44 10.455 10.579 10.522
   6542   1382783_at   6.208 6.389 6.344 6.341 6.442 5.777 5.59 6.211 5.812 5.792
   6543   1389202_at   8.212 8.613 8.576 8.682 8.505 8.033 7.799 8.169 7.671 7.49
   6544   1378449_at   2.687 3.417 2.621 3.257 3.686 3.891 4.262 4.279 4.747 4.865
   6545   1391919_at   5.204 5.179 5.463 4.515 4.598 5.952 6.235 6.017 5.942 5.652
   6546   1374683_at   1.978 2.403 2.114 1.82 2.06 2.446 4.957 4.887 4.817 4.786
   6547   1373643_at   6.914 6.763 6.849 7.383 7.186 7.937 7.936 8.1 7.722 7.374
   6548   1378396_at   6.083 5.584 5.683 5.822 5.763 6.11 6.588 6.445 6.256 6.283
   6549   1372561_at   8.157 8.164 8.223 8.35 8.287 8.049 7.441 7.834 7.692 7.597
   6550   1377493_at   7.067 6.711 6.956 7.067 7.099 7.261 7.533 7.464 7.384 7.3
   6551   1390193_at   5.585 5.503 5.896 5.905 6.101 6.158 6.521 6.707 6.4 6.635
   6552   1372271_at   7.2 7.248 7.536 7.602 7.209 6.882 7.021 6.853 6.92 6.974
   6553   1390118_at   7.296 7.205 7.37 7.534 7.398 7.033 7.051 7.192 7.013 7.01
   6554   1376016_at   7.647 7.645 7.943 8.107 7.888 8.117 8.431 8.377 8.45 8.357
   6555   1388689_at   5.243 5.406 5.995 6.304 5.862 6.825 6.975 6.963 6.536 6.555
   6556   1373304_at   7.282 7.145 7.458 7.456 7.162 7.7 7.632 8.039 8.024 7.699
   6557   1381970_at   10.157 10.168 10.083 10.174 10.169 9.915 9.777 10.053 9.799 9.689
   6558   1385706_at   6.34 6.151 6.051 6.248 6.072 5.84 5.596 5.972 5.689 5.555
   6559   1372412_at   7.778 7.837 7.861 8.083 7.942 8.097 8.246 8.387 8.201 8.213
   6560   1396742_at   6.507 6.338 6.523 6.092 6.213 5.801 6.016 5.453 5.304 5.381
   6561   1390610_at   3.363 3.513 3.619 4.785 3.558 5.135 4.951 5.076 4.924 4.866
   6562   1385086_at   7.105 7.193 7.178 7.194 7.148 6.686 6.43 6.422 5.54 5.922
   6563   1377809_at   8.153 8.066 8.131 8.371 8.4 8.576 8.594 8.717 8.961 8.59
   6564   1372844_at   5.875 5.864 6.16 6.508 6.211 6.866 6.882 7.098 6.726 6.548
   6565   1393196_at   4.482 4.693 4.755 4.793 4.632 5.081 5.556 5.285 5.275 4.929
   6566   1380401_at   3.815 4.031 4.532 3.517 4.463 4.812 5.221 5.463 4.888 5.328
   6567   1398374_at   6.134 6.351 6.477 6.163 6.04 6.572 6.69 6.639 6.58 6.596
   6568   1398350_at   9.922 9.89 9.807 10.038 10.179 10.28 10.399 10.598 10.449 10.235
   6569   1398876_at   8.947 8.849 8.929 9.019 9.012 8.694 8.747 8.795 8.591 8.504
   6570   1386540_at   5.835 6.118 5.601 5.909 6.096 6.793 6.398 6.501 6.278 6.579
   6571   1397632_at   3.827 4.702 4.799 4.404 4.119 5.315 5.038 5.322 5.299 5.262
   6572   1383523_at   7.76 8.062 7.933 8.345 7.911 8.318 8.619 8.588 8.693 8.517
   6573   1392045_at   4.22 4.148 4.07 4.518 4.235 4.896 4.877 4.561 5.198 5.375
   6574   1373391_at   9.646 9.628 9.834 9.83 9.934 10.077 10.11 10.165 10.038 10.003
   6575   1383193_at   6.253 6.313 6.678 6.488 6.457 6.882 6.689 7.005 7.076 7.201
   6576   1375643_at   6.568 6.42 6.04 6.394 6.579 7.085 6.989 7.094 6.718 6.899
   6577   1382193_at   6.25 6.622 6.665 6.621 6.963 7.101 7.37 7.237 7.07 7.277
   6578   1388801_at   8.052 8.073 7.941 8.152 8.184 7.817 7.679 7.292 7.59 7.718
   6579   1371443_at   8.081 7.982 8.275 8.335 8.054 8.443 8.505 8.51 8.416 8.485
   6580   1384382_at   6.152 6.561 6.756 6.625 6.335 7.059 7.152 7.01 7.059 6.872
   6581   1372139_at   5.928 5.726 6.254 6.602 6.214 7.109 6.899 7.037 6.693 6.855
   6582   1367539_at   8.577 8.636 8.698 8.564 8.622 8.476 8.455 8.491 8.542 8.488
   6583   1376237_at   6.677 6.477 6.398 6.401 7.099 5.531 5.852 5.561 6.114 5.954
   6584   1383421_at   7.152 7.23 7.27 7.474 7.201 8.134 7.86 7.823 7.478 8.135
   6585   1367668_a_at   8.571 8.585 8.924 9.234 8.721 9.745 9.558 9.291 10.004 10.232
   6586   1390370_at   3.388 3.841 3.445 3.989 4.05 4.644 4.865 4.123 4.744 4.88
   6587   1392980_at   5.488 5.284 5.606 5.752 5.525 5.085 4.523 4.238 4.958 4.848
   6588   1373020_at   9.216 9.154 9.338 9.343 9.38 9.621 9.666 9.44 9.683 9.506
   6589   1381163_at   8.305 8.438 8.672 8.58 8.428 8.946 9.141 8.995 8.922 8.686
   6590   1390681_at   5.271 5.471 5.292 5.603 5.375 5.163 4.777 5.039 4.772 4.663
   6591   1378652_at   5.562 5.406 5.856 5.537 5.48 5.826 5.957 6.037 6.071 6.155
   6592   1372832_at   6.242 6.397 6.533 6.452 7.008 7.146 7.343 7.342 7.505 6.976
   6593   1377849_at   6.256 6.46 6.032 6.241 6.567 5.665 5.606 6.01 5.697 5.377
   6594   1388454_at   9.085 8.933 8.863 9.306 9.231 9.712 9.307 9.795 9.707 9.697
   6595   1369101_at   5.553 5.369 5.64 5.446 4.616 6.212 6.177 6.291 6.039 6.286
   6596   1370211_at   4.83 5.421 5.583 5.427 4.537 6.342 5.864 6.184 6.17 6.419
   6597   1388320_at   11.418 11.308 11.122 11.316 11.287 11.651 11.416 11.646 11.621 11.672
   6598   1388661_at   6.9 6.683 6.864 6.646 6.278 7.208 7.193 7.185 7.604 7.351
   6599   1397878_at   5.368 4.862 5.925 5.573 5.43 6.179 6.733 6.41 6.12 6.437
   6600   1370824_at   6.806 6.858 7.082 7.039 6.189 8.05 7.272 8.055 8.505 8.509
   6601   1386864_at   12.466 12.426 12.339 12.201 12.255 12.143 12.109 11.938 11.993 12.057
   6602   1391995_at   6.031 6.438 5.771 5.941 6.187 6.983 6.622 6.644 6.632 6.6
   6603   1376962_at   6.316 6.447 6.775 6.607 5.755 7.559 7.47 7.218 7.073 7.161
   6604   1376057_at   7.321 7.19 7.099 7.021 7.437 7.749 7.657 8.029 7.71 7.522
   6605   1372770_at   10.117 10.053 10.004 10.042 10.247 9.904 9.86 9.838 9.65 9.689
   6606   1391260_at   5.149 5.388 5.396 5.444 5.209 3.556 4.233 4.537 2.746 4.094
   6607   1394737_at   8.71 8.712 8.764 9.049 8.981 9.239 9.095 9.374 9.389 9.21
   6608   1376767_at   5.852 5.936 6.126 6.34 6.324 6.519 6.615 7.123 6.898 7.119
   6609   1376235_at   7.217 6.975 6.993 7.331 7.125 7.345 7.461 7.599 7.598 7.579
   6610   1379384_at   6.537 6.451 6.531 6.749 6.625 6.254 6.068 5.921 6.375 6.117
   6611   1398977_at   9.397 9.478 9.646 9.591 9.492 9.826 9.734 9.892 9.748 9.701
   6612   1373746_at   7.287 7.58 7.592 7.424 7.53 7.243 7.104 7.149 7.237 7.213
   6613   1374495_at   8.022 8.308 8.123 8.244 8.006 8.325 8.662 8.545 8.531 8.505
   6614   1374356_at   5.848 6.066 6.225 6.163 5.95 6.507 6.288 6.717 6.546 6.803
   6615   1398287_at   10.854 10.838 10.911 11.002 10.815 11.169 11.304 11.255 11.078 11.032
   6616   1376128_at   8.301 7.948 8.165 8.326 8.076 8.523 8.518 8.45 8.769 8.645
   6617   1383272_at   5.017 4.898 5.142 4.723 5.113 1.111 4.496 1.189 1.718 1.872
   6618   1371547_at   10.727 10.734 10.556 10.64 10.828 10.87 10.915 10.989 10.959 10.919
   6619   1374931_at   6.699 6.648 6.868 6.923 6.817 7.166 7.193 6.966 7.036 7.153
   6620   1395852_at   7.186 7.238 7.237 7.446 7.155 7.816 7.592 8.146 8.145 7.615
   6621   1379733_at   2.984 3.614 3.479 4.336 4.037 5.313 4.485 5.533 5.306 4.804
   6622   1391512_at   5.806 5.527 5.266 5.506 5.666 4.804 5.102 4.88 5.153 4.543
   6623   1374146_at   7.942 7.774 7.911 7.964 7.835 7.66 7.358 7.329 7.407 7.695
   6624   1399010_at   8.758 8.72 8.769 8.954 8.88 9.06 8.961 9.156 9.165 9.066
   6625   1379816_at   6.029 6.24 6.497 6.541 6.378 6.857 6.953 6.978 6.959 6.61
   6626   1370073_at   8.703 8.64 8.486 8.704 8.944 8.231 8.272 8.411 8.393 8.214
   6627   1372136_at   9.483 9.416 9.379 9.624 9.58 9.248 9.009 9.277 9.239 9.142
   6628   1371573_at   11.274 11.274 11.258 11.303 11.51 11.113 11.093 11.143 11.054 11.03
   6629   1368772_at   4.844 4.927 4.248 4.803 4.543 5.177 5.377 5.176 5.493 5.34
   6630   1373914_at   5.306 5.848 5.537 5.8 6.41 7.101 6.986 6.946 6.796 6.341
   6631   1375255_at   0.575 0 0 0 0.322 1.669 1.183 1.118 1.963 0.714
   6632   1377273_at   5.027 5.221 5.361 5.25 5.283 5.51 5.589 5.835 5.483 5.686
   6633   1367888_at   6.065 6.35 5.702 5.702 5.756 4.156 3.603 4.439 2.018 4.439
   6634   1372124_at   8.605 8.621 8.656 8.886 8.753 9.013 9.083 8.97 9.079 8.875
   6635   1387877_at   5.427 5.652 5.351 5.773 4.688 6.291 6.059 6.381 6.632 6.781
   6636   1380001_at   6.955 6.811 6.928 6.803 6.89 6.656 6.384 6.188 6.134 6.565
   6637   1385093_at   5.611 5.39 5.53 5.305 5.203 6.228 6.39 6.1 6.719 5.732
   6638   1398481_at   8.426 8.191 7.985 8.363 8.235 7.8 7.654 7.865 7.595 7.253
   6639   1369847_at   5.885 5.838 5.558 5.745 5.607 5.315 5.227 5.434 4.941 5.305
   6640   1376187_at   7.878 8.09 8.053 8.255 8.011 8.443 8.344 8.57 8.291 8.432
   6641   1376219_at   7.224 7.544 7.547 7.4 7.759 7.008 7.089 7.086 7.093 7.11
   6642   1378612_at   2.348 2.414 1.373 0.766 1.131 2.868 3.733 3.385 3.004 3.677
   6643   1372846_at   7.772 7.654 7.615 7.524 7.791 7.463 7.046 7.367 7.205 7.075
   6644   1392662_at   8.557 8.597 8.556 8.526 8.573 8.318 8.271 8.345 8.277 7.959
   6645   1382580_at   3.805 4.213 4.155 4.955 5.918 6.587 6.039 6.67 6.548 6.303
   6646   1392816_at   7.337 7.252 7.368 7.603 7.346 7.565 7.751 7.892 7.762 7.754
   6647   1373185_at   9.883 9.878 9.901 9.91 10.025 9.759 9.668 9.652 9.49 9.398
   6648   1376560_at   7.367 7.323 7.087 7.278 7.414 6.923 6.784 7.137 6.818 6.782
   6649   1368442_at   7.75 7.755 8.068 8.309 7.44 8.515 8.475 8.707 8.663 8.55
   6650   1367937_at   12.521 12.494 12.654 12.36 12.014 12.988 13.089 12.801 12.901 13.078
   6651   1368637_at   5.84 5.591 5.148 4.989 5.376 3.383 2.281 4.294 2.189 0.84
   6652   1389801_at   7.146 7.055 7.196 6.896 6.925 7.867 7.205 7.953 7.758 7.75
   6653   1371311_at   11.737 11.716 11.843 11.669 11.653 11.959 11.98 11.884 11.879 12.087
   6654   1369970_at   10.541 10.476 10.486 10.542 10.585 10.239 10.319 10.469 10.305 10.306
   6655   1395607_at   3.916 4.098 4.285 4.008 4.04 4.31 4.355 4.432 4.586 4.462
   6656   1388307_at   10.391 10.567 10.775 10.532 10.529 10.852 10.97 10.93 10.833 10.804
   6657   1383781_at   6.225 6.42 6.891 6.192 6.29 7.179 7.079 7.042 7.032 6.864
   6658   1385649_at   6.782 6.592 7.026 6.936 7.015 6.556 6.388 6.071 6.276 6.409
   6659   1395978_at   4.786 4.43 5.049 4.862 4.325 5.465 5.353 5.574 5.389 5.167
   6660   1374472_at   7.723 7.687 7.955 7.661 7.604 7.298 7.526 7.268 7.316 7.084
   6661   1374916_at   6.349 6.611 6.482 6.493 6.634 5.993 6.106 6.372 5.943 6.07
   6662   1383120_at   6.652 6.091 6.612 6.257 6.38 7.11 6.966 7.276 7 6.764
   6663   1383924_at   4.191 4.472 4.493 4.388 4.826 4.796 5.085 5.013 5.159 5.193
   6664   1377716_at   6.496 6.941 6.525 6.825 6.842 7.364 6.991 7.687 7.674 7.532
   6665   1394524_at   8.895 8.967 9.066 9.046 9.093 9.379 9.319 9.459 9.178 9.205
   6666   1376022_at   7.859 7.836 8.087 7.899 7.85 7.556 6.906 7.385 7.491 7.027
   6667   1372816_at   7.255 7.261 7.062 7.002 7.274 7.626 7.589 7.864 7.616 7.377
   6668   1392652_at   3.363 4.256 4.168 3.279 4.153 4.644 4.949 5.036 5.632 5.606
   6669   1383396_at   7.601 7.881 7.686 7.772 7.872 7.347 7.459 7.575 7.408 7.244
   6670   1368249_at   8.474 8.797 9.214 8.794 7.993 9.593 9.52 9.905 9.657 9.537
   6671   1376419_at   6.204 6.331 6.732 6.47 6.538 6.064 5.495 5.642 6.043 5.702
   6672   1376486_at   7.186 7.257 7.007 7.308 7.209 7.713 7.568 7.671 7.415 7.428
   6673   1383891_a_at   6.551 6.305 5.731 6.16 6.388 7.041 6.872 7.216 7.142 6.707
   6674   1391476_at   7.081 7.269 7.481 7.297 7.05 7.507 8.004 7.947 8.082 7.694
   6675   1385626_at   4.39 5.657 5.335 5.127 5.067 6.405 5.931 6.068 6.071 6.466
   6676   1388962_at   5.163 5.716 5.702 5.794 5.199 6.213 6.1 6.06 6.234 6.245
   6677   1391279_at   7.479 7.323 7.514 7.807 7.473 8.275 7.943 8.524 7.914 8.535
   6678   1389775_at   5.604 5.908 5.401 5.822 5.671 6.145 6.112 6.421 6.11 6.095
   6679   1371762_at   7.075 6.998 6.964 7.454 5.744 8.404 7.857 8.489 9.081 9.298
   6680   1371140_a_at   1.722 1.546 3.603 3.711 1.642 4.75 4.162 5.282 5.383 5.168
   6681   1389740_at   5.185 4.434 4.037 3.539 4.768 5.419 5.733 5.945 5.993 5.837
   6682   1386732_at   4.271 4.095 3.685 5.366 3.7 5.913 5.774 5.5 5.484 5.777
   6683   1391428_at   7.476 7.828 7.193 7.442 7.774 8.275 8.266 8.605 8.272 7.93
   6684   1395045_at   6.427 6.063 6.548 6.025 6.253 7.085 6.563 7.023 6.947 6.841
   6685   1367499_at   8.407 8.398 8.492 8.672 8.53 8.192 8.219 8.332 8.249 8.13
   6686   1385994_at   5.491 4.517 3.491 4.86 4.949 6.016 6.377 6.197 6.417 6.123
   6687   1389541_at   7.123 7.425 7.15 7.099 7.271 7.726 7.604 7.954 7.689 7.448
   6688   1387299_at   8.133 8.167 8.265 8.29 8.187 8.342 8.397 8.335 8.347 8.349
   6689   1386701_at   6.398 6.229 6.222 6.562 5.931 4.887 4.726 5.607 5.424 5.668
   6690   1392865_at   7.085 7.182 7.108 7.597 7.118 7.621 7.609 7.971 7.88 7.755
   6691   1370780_at   6.343 6.505 6.528 6.893 6.67 6.147 6.034 6.166 6.232 5.955
   6692   1376953_at   7.829 8.169 8.228 8.235 8.042 8.482 8.439 8.4 8.653 8.599
   6693   1394419_at   8.484 8.48 8.337 8.615 8.614 8.189 7.618 7.801 7.078 7.497
   6694   1387977_at   8.301 8.487 8.458 8.551 8.582 8.149 8.248 8.209 8.088 7.906
   6695   1394348_at   5.958 6.049 5.865 6.448 6.264 6.795 6.491 6.999 6.643 6.763
   6696   1370456_at   4.698 4.401 4.581 4.844 4.119 5.206 4.899 5.366 5.393 5.354
   6697   1392043_at   6.018 5.967 6.171 6.142 5.744 6.391 6.915 6.788 6.346 6.716
   6698   1383999_at   8.64 8.331 8.379 8.803 8.656 8.132 7.896 8.238 7.92 7.749
   6699   1398778_at   10.56 10.733 10.65 10.491 10.626 10.05 10.49 9.871 9.98 10.072
   6700   1376792_at   7.771 7.727 8.095 7.848 7.625 8.087 8.219 8.212 8.363 8.281
   6701   1368306_at   4.508 4.91 4.812 4.739 4.939 5.209 5.578 5.06 5.424 5.57
   6702   1381056_at   5.995 5.578 5.109 5.891 5.677 6.36 6.772 6.234 6.858 6.5
   6703   1391236_at   7.502 7.265 7.143 7.12 7.022 6.638 6.988 6.65 6.552 6.498
   6704   1377422_at   3.777 3.242 3.922 4.362 4.774 5.704 4.755 5.828 5.531 5.724
   6705   1394594_at   6.511 6.593 6.619 6.637 6.336 6.996 7.346 7.595 7.099 6.877
   6706   1377452_at   5.745 6.129 5.861 6.091 6.739 7.348 6.732 7.059 7.395 6.922
   6707   1391181_at   5.309 5.911 5.631 5.032 5.699 6.253 6.229 6.12 6.413 6.286
   6708   1380579_at   5.727 5.817 4.269 5.006 5.118 6.647 6.316 6.311 6.769 6.972
   6709   1387549_at   6.493 6.152 6.728 6.687 6.284 7.167 6.895 7.058 7.432 7.542
   6710   1367547_at   7.913 7.999 7.875 8.081 7.991 8.301 8.168 8.365 8.207 8.125
   6711   1392557_at   9.436 9.601 9.072 9.619 9.82 10.296 10.332 10.409 10.003 10
   6712   1368265_at   6.37 6.299 5.917 6.397 6.249 6.778 6.958 6.961 6.757 6.513
   6713   1373908_at   8.38 8.438 8.25 8.495 8.92 7.614 7.967 7.463 7.398 6.73
   6714   1377786_at   3.5 4.274 4.057 4.197 3.855 4.918 5.098 5.672 4.783 4.629
   6715   1373153_at   5.18 5.366 5.553 5.579 5.331 5.813 5.877 5.747 5.659 5.814
   6716   1392282_at   0 0 0.275 1.787 1.696 2.82 3.063 2.558 2.263 2.98
   6717   1393136_at   7.072 7.402 7.103 7.205 6.926 7.703 7.884 7.927 7.707 7.371
   6718   1380452_at   6.179 6.446 6.347 6.357 6.255 6.541 6.622 6.961 6.71 6.933
   6719   1370454_at   3.556 3.615 3.013 4.164 3.72 4.558 4.246 4.583 4.758 4.55
   6720   1367717_at   12.55 12.483 12.455 12.363 12.56 12.302 12.294 12.36 12.283 12.297
   6721   1373256_at   6.497 6.245 6.363 6.92 6.283 7.038 7.045 7.15 7.515 7.055
   6722   1383618_at   7.534 7.6 7.705 7.676 7.537 7.89 7.726 7.896 7.844 8.005
   6723   1368550_at   5.935 5.681 6.088 6.201 6.143 6.657 6.398 6.812 6.471 6.466
   6724   1384797_at   7.591 7.503 7.678 7.593 7.787 7.326 6.695 7.232 7.131 7.056
   6725   1393672_at   1.941 3.736 3.5 3.913 4.396 5.653 5.476 5.735 5.241 5.207
   6726   1382070_at   7.705 7.569 7.84 7.771 7.906 7.522 7.294 7.494 7.236 7.177
   6727   1389463_at   5.933 6.226 5.918 6.273 6.548 6.64 6.774 6.953 6.691 7.029
   6728   1394535_at   6.096 6.175 6.697 6.509 6.273 6.843 6.884 7.053 6.771 7.007
   6729   1395821_at   7.246 7.391 7.737 7.775 7.633 6.745 7.246 6.831 6.663 6.418
   6730   1377904_at   5.051 5.274 4.997 4.814 5.406 5.915 6.143 6.239 5.622 5.553
   6731   1368189_at   8.168 8.103 8.202 8.498 8.157 8.944 8.625 8.492 8.661 8.836
   6732   1369678_a_at   6.455 6.361 6.119 6.773 6.819 7.416 7.236 7.414 7.536 6.887
   6733   1388550_at   10.628 10.689 10.58 10.629 10.691 10.33 10.242 10.562 10.318 10.164
   6734   1376760_at   7.092 7.225 6.862 6.929 7.289 6.487 6.656 6.827 6.517 6.441
   6735   1371940_at   8.427 8.47 8.361 8.773 8.511 8.938 8.891 9.227 8.876 8.86
   6736   1372132_at   11.593 11.61 11.554 11.728 11.607 11.387 11.319 11.333 11.475 11.503
   6737   1393915_at   9.72 9.719 9.762 9.761 9.657 9.996 9.962 9.826 10.049 10.182
   6738   1373951_at   10.253 10.254 10.079 10.172 10.301 9.976 9.787 10.052 9.917 9.75
   6739   1376755_at   7.625 7.603 7.844 7.755 8.198 8.444 8.191 8.442 8.454 8.26
   6740   1393891_at   4.817 4.827 4.996 4.48 5.358 6.261 5.749 5.979 5.715 5.4
   6741   1387987_at   8.271 8.12 8.904 8.642 7.651 9.352 9.206 9.562 9.189 9.562
   6742   1384246_at   7.781 8.115 7.435 7.801 7.876 7.129 7.005 7.39 7.356 7.063
   6743   1368644_at   6.085 5.807 5.528 5.875 5.759 6.424 6.226 6.705 6.517 6.185
   6744   1391647_at   3.907 4.515 4.854 4.496 4.27 5.126 5.975 5.324 5.236 5.315
   6745   1374542_at   2.878 4.045 3.681 3.919 3.773 4.519 4.587 4.898 4.599 5.189
   6746   1376635_at   5.399 5.824 5.574 5.85 5.489 6.341 6.047 6.29 6.364 5.936
   6747   1390547_at   5.895 5.91 5.585 5.901 6.155 6.602 6.165 6.537 6.579 6.362
   6748   1390111_at   9.113 9.182 9.2 9.182 9.212 9.492 9.326 9.453 9.396 9.26
   6749   1391162_at   5.896 6.227 6.142 6.54 6.819 7.229 7.28 7.534 6.95 7.01
   6750   1392884_at   5.611 5.636 5.833 5.159 6.226 6.787 6.252 6.746 6.544 6.65
   6751   1394222_at   7.16 7.362 7.264 7.256 7.171 7.493 7.588 7.339 7.548 7.556
   6752   1385215_at   0 0 0.014 0.151 1.609 3.257 1.401 2.299 2.254 2.406
   6753   1379410_at   7.795 7.861 7.406 7.718 7.548 8.206 8.111 8.244 7.929 8.11
   6754   1395998_at   6.971 6.766 6.422 6.665 6.795 5.967 6.111 5.597 6.403 5.813
   6755   1393613_at   5.152 5.706 5.744 5.788 5.896 6.368 6.536 6.728 6.48 6.063
   6756   1378404_at   6.135 5.934 6.25 6.322 6.221 6.893 6.65 6.553 6.431 6.642
   6757   1387125_at   7.643 7.739 6.981 7.868 8.725 5.585 5.245 6.967 5.068 4.333
   6758   1384171_x_at   5.446 5.769 5.365 5.926 5.888 6.559 6.072 6.382 6.214 6.455
   6759   1370982_at   3.535 1.566 3.134 2.551 3.852 4.961 4.423 5.683 5.054 4.836
   6760   1387972_at   9.938 9.842 9.844 10.069 9.822 10.302 10.208 10.368 10.442 10.055
   6761   1383375_at   8.281 8.138 8.291 8.249 8.478 8.562 8.818 8.661 8.56 8.548
   6762   1386073_at   5.801 5.583 5.235 5.291 5.15 5.048 4.598 4.711 4.642 4.755
   6763   1377266_at   6.369 6.344 6.176 6.563 6.437 6.965 6.731 7.29 6.853 6.765
   6764   1383493_at   6.253 6.367 6.354 6.433 6.354 3.675 5.436 5.493 4.89 4.234
   6765   1395984_at   6.787 6.488 6.741 6.582 6.361 6.894 7.05 6.993 6.949 6.965
   6766   1397631_at   4.309 4.627 3.904 4.464 4.074 5.345 5.199 5.245 5.279 4.599
   6767   1369323_at   8.044 8.253 8.232 8.366 8.375 8.047 7.765 7.964 7.795 7.715
   6768   1384283_at   7.014 6.897 7.054 7.142 6.598 7.793 7.764 7.856 7.378 7.305
   6769   1393933_at   8.237 8.319 8.529 8.645 8.298 8.98 8.642 9.137 8.863 9.085
   6770   1374786_at   3.666 3.583 3.948 3.091 3.448 4.446 4.061 4.55 4.198 4.251
   6771   1382289_at   6.96 7.11 7.341 6.955 7.123 7.507 7.806 7.64 7.539 7.35
   6772   1384676_at   6.112 6.086 6.053 6.458 6.277 5.844 5.589 5.535 5.654 5.927
   6773   1382244_at   4.271 4.539 5.189 5.468 4.692 5.727 5.69 6.081 6.256 5.937
   6774   1389409_at   8.376 8.233 8.556 8.566 8.842 8.21 7.657 7.626 7.877 7.87
   6775   1392424_at   7.255 7.765 7.558 7.252 7.543 7.872 7.989 8.135 8.131 7.865
   6776   1372539_at   7.522 7.526 6.999 7.479 7.314 8.067 8.006 8.134 7.927 7.658
   6777   1389167_at   8.051 7.883 8.057 8.211 7.936 8.255 8.318 8.293 8.294 8.292
   6778   1383305_at   7.214 7.245 7.231 7.467 7.113 7.795 7.685 8.014 7.542 7.579
   6779   1378338_at   6.808 6.794 6.72 6.7 6.583 6.516 6.457 6.244 6.067 6.164
   6780   1371617_at   8.835 8.894 8.801 8.964 9.167 8.591 8.551 8.621 8.577 8.713
   6781   1369919_at   4.711 4.8 5.161 5.29 4.956 5.782 5.664 5.719 5.823 5.271
   6782   1384340_a_at   7.242 7.473 7.183 6.841 7.422 6.623 6.484 6.785 6.763 6.36
   6783   1383644_at   4.963 5.375 4.8 4.751 5.557 6.355 5.614 6.428 6.01 5.98
   6784   1372831_at   6.456 5.725 6.121 6.187 6.161 6.81 6.567 6.941 6.64 6.748
   6785   1386902_at   11.316 11.165 11.111 11.208 11.209 11.395 11.415 11.452 11.309 11.505
   6786   1385814_at   6.278 6.021 6.191 5.973 6.154 5.668 5.629 5.965 5.748 5.547
   6787   1398698_at   5.181 5.16 5.385 5.089 5.591 5.674 5.777 5.988 5.65 5.815
   6788   1376248_at   6.106 5.833 5.521 5.655 4.262 7.1 6.703 7.008 6.96 7.027
   6789   1390901_at   6.927 7 6.371 6.95 7.579 8.28 7.987 8.381 8.282 7.523
   6790   1388328_at   10.052 9.855 10.116 9.889 10.075 9.683 9.834 9.641 9.617 9.729
   6791   1385559_at   4.977 5.156 5.13 5.195 5.122 5.912 5.725 5.424 5.36 5.864
   6792   1374123_at   4.267 4.72 4.869 5.097 4.381 5.51 5.117 5.609 5.723 5.594
   6793   1369568_at   6.442 6.662 6.317 6.898 6.821 6.002 5.201 5.332 6.002 5.909
   6794   1371679_at   5.353 5.858 5.334 5.956 5.508 6.407 6.191 6.397 6.178 6.098
   6795   1390976_at   5.021 5.123 4.214 5.363 5.237 5.878 6.236 6.096 5.868 6.735
   6796   1374189_at   7.556 7.445 7.372 7.555 7.925 8.223 7.874 8.403 8.296 8.071
   6797   1393569_at   6.798 7.034 6.963 7.09 7.082 6.644 6.593 6.549 6.854 6.592
   6798   1390423_at   8.357 8.332 8.284 8.48 8.562 8.059 7.891 8.261 7.921 7.898
   6799   1382492_a_at   8.161 8.407 7.944 8.291 8.212 8.713 8.97 8.905 8.868 8.414
   6800   1383994_at   4.326 4.891 4.379 5.357 5.01 5.921 5.408 6.105 5.759 6.063
   6801   1376827_at   6.905 6.727 6.926 6.848 7.233 6.534 6.258 6.443 6.499 6.595
   6802   1373582_at   3.826 4.191 4.379 4.647 4.941 5.525 5.096 5.404 5.432 5.215
   6803   1373235_at   8.348 8.372 8.529 8.309 8.341 8.881 8.713 8.898 8.771 8.498
   6804   1380831_at   4.703 4.448 5.133 4.868 5.202 5.616 5.472 5.892 5.541 5.462
   6805   1376268_at   9.328 9.359 9.481 9.665 9.819 9.054 8.741 8.986 8.881 8.309
   6806   1376225_at   1.566 2.417 2.962 2.989 2.59 5.154 4.068 3.54 5.828 4.799
   6807   1371170_a_at   6.017 5.41 5.12 5.197 5.621 3.768 3.004 4.408 4.32 4.507
   6808   1385805_at   7.849 7.909 8.009 8.245 8.098 8.598 8.29 8.904 8.554 8.947
   6809   1397233_at   4.823 4.596 4.712 3.995 5.206 5.732 5.944 5.369 6.393 5.756
   6810   1367931_a_at   10.485 10.548 10.541 10.548 10.558 10.378 9.991 10.25 10.195 9.915
   6811   1386858_at   12.234 12.182 12.053 12.036 12.218 11.921 11.968 11.71 11.816 11.657
   6812   1397541_at   7.555 7.641 7.488 7.481 7.561 8.235 7.735 8.186 8.034 7.836
   6813   1389157_at   7.517 7.696 7.064 7.778 7.876 8.32 8.169 8.353 8.239 8.199
   6814   1373484_at   7.109 7.082 6.763 6.718 7.175 7.753 7.886 7.794 7.255 7.524
   6815   1368154_at   8.498 8.491 8.116 8.239 8.815 9.339 8.919 9.478 9.538 8.942
   6816   1371115_at   6.278 5.785 6.125 6.358 6.181 4.674 3.612 1.934 4.904 3.147
   6817   1380118_at   5.983 6.002 6.063 6.072 6.042 6.284 6.184 6.537 6.43 6.244
   6818   1368057_at   9.173 8.447 8.683 8.983 9.027 9.435 9.494 10.016 9.73 9.494
   6819   1388817_at   8.146 8.099 8.113 8.216 8.332 8.596 8.352 8.727 8.508 8.458
   6820   1368397_at   9.771 9.916 10.407 9.87 9.253 10.52 10.655 10.876 10.8 10.776
   6821   1371194_at   6.337 5.933 6.088 5.754 6.327 5.6 5.325 5.293 5.153 4.631
   6822   1393361_at   7.2 7.105 7.088 7.392 7.37 7.029 6.484 6.68 6.603 6.797
   6823   1373378_at   7.492 7.242 7.182 7.502 7.144 7.748 7.63 8.146 7.941 7.776
   6824   1396286_at   6.473 6.745 6.818 7.022 6.924 6.192 5.684 6.384 6.08 6.23
   6825   1389054_at   8.474 8.512 8.254 8.194 8.727 8.871 9.207 8.96 9.177 8.798
   6826   1371101_at   7.424 7.618 7.308 7.204 7.585 7.834 7.857 7.823 7.866 7.691
   6827   1370310_at   6.193 6.155 5.387 5.338 5.577 6.858 6.704 8.113 8.169 7.084
   6828   1374760_at   6.063 5.956 5.772 5.904 5.802 6.495 6.21 6.506 6.292 6.117
   6829   1382878_at   6.91 7.066 6.858 6.994 7.056 7.884 7.749 7.404 7.285 7.371
   6830   1389242_at   6.926 7.061 6.846 6.97 6.547 7.366 7.113 7.463 7.512 7.491
   6831   1382343_at   6.52 6.414 6.529 6.663 6.589 5.6 5.976 5.059 5.649 6.133
   6832   1369253_at   2.826 2.774 3.133 3.961 3.698 4.387 4.377 4.485 4.23 4.943
   6833   1380013_at   5.256 5.336 5.583 5.895 4.969 6.397 5.925 6.616 6.285 6.197
   6834   1373699_at   5.261 5.286 5.073 5.136 5.723 6.269 5.642 6.439 6.747 6.215
   6835   1375357_at   8.071 8.164 8.155 8.16 8.227 8.063 7.986 7.965 8.05 7.953
   6836   1371460_at   10.458 10.397 10.399 10.574 10.289 10.703 10.711 10.595 10.834 10.654
   6837   1376580_at   8.123 8.216 8.137 8.088 8.123 8.405 8.722 8.389 8.583 8.927
   6838   1396766_at   3.739 4.205 4.25 4.064 4.101 4.7 4.626 4.515 4.568 4.36
   6839   1371610_at   8.211 8.025 8.166 8.211 8.255 8.63 8.476 8.734 8.534 8.319
   6840   1372299_at   8.295 8.359 8.091 8.546 9.316 9.522 9.386 10.116 9.928 9.448
   6841   1372116_at   8.919 8.916 8.961 9.097 8.91 9.279 9.118 9.407 9.255 9.151
   6842   1390569_at   8.274 8.315 8.255 8.269 8.013 8.669 8.7 9.11 9.354 9.611
   6843   1372409_at   7.41 7.713 7.624 7.853 7.75 7.286 7.018 7.295 7.377 7.073
   6844   1372430_at   8.336 8.089 8.29 8.039 8.193 7.826 7.489 7.985 7.502 7.614
   6845   1377790_at   6.412 6.493 6.316 6.638 6.522 6.742 6.824 6.787 6.755 6.653
   6846   1395601_at   6.059 6.596 5.866 6.848 6.872 7.233 7.287 8.026 8.111 7.848
   6847   1386870_at   12.243 12.132 12.305 12.226 12.057 12.424 12.481 12.374 12.47 12.355
   6848   1395412_at   9.932 9.816 9.905 9.814 9.768 9.359 9.718 9.148 9.447 9.377
   6849   1384521_at   3.622 4.451 3.889 4.834 4.728 5.53 5.115 5.468 5.73 6.036
   6850   1395442_at   5.653 6.142 5.749 6.056 5.961 5.302 4.525 4.745 3.491 4.55
   6851   1375523_at   9.988 10.03 9.716 9.657 10.094 9.38 8.986 9.349 9.441 8.771
   6852   1388563_at   9.341 9.402 9.126 9.278 9.381 8.961 8.891 9.165 8.979 8.827
   6853   1372753_at   7.728 7.634 7.588 7.394 7.508 8.02 8.048 7.764 7.977 7.832
   6854   1380313_at   5.135 5.603 5.804 5.394 4.756 2.807 0.516 2.991 3.596 0
   6855   1395414_at   5.412 5.626 5.589 5.319 5.565 6.364 6.127 6.235 6.331 5.653
   6856   1375049_at   6.477 6.611 6.591 6.377 6.924 7.331 7.392 6.838 7.623 7.366
   6857   1389339_at   8.005 7.999 8.083 8.202 8.095 8.407 8.272 8.571 8.831 8.688
   6858   1373004_at   7.477 7.541 7.305 7.514 7.551 7.161 7.106 7.341 7.059 6.953
   6859   1387808_at   10.24 10.256 10.384 10.365 10.133 10.635 10.969 10.735 10.733 10.454
   6860   1382480_at   6.38 6.317 6.857 6.336 6.208 6.957 6.986 6.971 7.203 6.846
   6861   1398002_at   4.238 3.417 3.88 4.105 3.88 5.32 4.42 4.725 4.84 5.365
   6862   1395970_at   4.044 4.164 3.812 4.213 4.547 4.609 5.149 4.727 4.845 4.824
   6863   1373050_at   8.415 8.559 8.218 8.509 8.378 8.747 8.836 9.041 8.765 8.66
   6864   1393172_at   8.411 8.254 8.502 8.408 8.472 8.111 7.856 7.969 8.195 8.171
   6865   1370954_at   9.098 8.941 8.926 9.159 9.069 8.694 8.799 8.659 8.322 8.377
   6866   1374108_at   6.315 6.389 6.547 6.443 6.317 6.814 6.621 6.923 6.602 6.723
   6867   1372658_at   6.554 5.806 5.808 5.901 5.823 6.969 6.407 7.049 6.746 6.848
   6868   1390572_at   6.7 6.419 6.893 6.743 6.444 6.136 5.993 5.881 6.374 6.033
   6869   1389146_at   9.951 9.925 10.206 10.135 9.631 10.623 10.748 10.752 10.491 10.271
   6870   1390628_at   7.325 7.361 7.405 7.324 7.326 7.718 7.69 8.034 7.758 7.502
   6871   1394785_at   5.584 5.732 5.803 5.731 5.898 6.023 6.143 6.312 5.956 6.296
   6872   1373030_at   9.128 9.212 9.118 9.312 9.31 8.839 8.702 9.056 8.713 8.498
   6873   1393585_at   6.521 6.702 6.881 7.228 7.076 7.772 7.619 8.261 7.722 7.373
   6874   1373983_at   9.044 9.015 9.005 8.992 9.139 8.73 8.732 8.871 8.75 8.453
   6875   1373141_at   6.565 6.275 6.578 6.637 6.653 7.078 6.716 7.202 7.023 7.124
   6876   1378947_at   4.45 4.026 4.454 4.896 4.601 5.285 5.471 5.451 5.268 4.892
   6877   1368426_at   9.385 9.299 9.211 9.227 9.533 9.665 9.77 9.927 9.591 9.619
   6878   1382007_at   8.016 8.06 8.182 8.196 8.126 7.885 7.802 7.651 7.352 7.42
   6879   1377469_at   5.738 5.672 5.457 6.148 6.258 6.561 6.448 6.811 6.565 6.54
   6880   1391553_at   5.409 4.665 5.157 5.264 4.935 5.837 5.553 5.775 5.686 5.709
   6881   1389245_at   9.692 9.824 9.65 9.839 9.877 9.447 9.2 9.606 9.465 9.337
   6882   1370352_at   10.555 10.546 10.603 10.22 10.039 11.055 11.093 11.225 11.054 10.707
   6883   1397392_at   2.814 2 0 0.566 1.281 3.615 3.197 3.944 4.849 3.873
   6884   1387450_at   4.155 4.285 4.167 4.872 4.151 4.886 5.304 4.848 5.088 5.235
   6885   1372253_at   7.72 7.923 7.993 8.184 8.325 7.618 7.439 7.515 7.477 7.147
   6886   1368984_at   8.767 8.647 8.583 8.578 8.735 8.305 8.487 7.989 8.098 7.918
   6887   1387898_at   6.888 6.873 6.667 6.677 6.477 7.716 6.915 7.655 7.486 7.838
   6888   1372161_at   2.618 3.537 3.846 4.512 4.498 5.322 5.271 5.955 6.107 5.268
   6889   1385379_at   6.013 6.211 5.936 6.161 6.054 6.354 6.494 6.786 6.408 6.404
   6890   1369174_at   6.82 7.187 6.385 6.999 6.968 6.317 6.207 6.239 6.323 6.101
   6891   1388546_at   8.352 8.235 8.377 8.461 8.066 8.672 8.491 8.772 8.757 8.712
   6892   1395531_at   3.507 1.888 3.093 2.678 3.091 0.722 1.333 1.485 1.595 1.761
   6893   1373791_at   7.79 7.873 7.805 8.154 8.015 8.239 8.17 8.372 8.276 8.311
   6894   1373813_at   8.715 8.666 8.775 8.819 8.785 9.008 8.813 9.07 9.02 9.027
   6895   1377647_at   6.756 7.091 6.779 7.166 6.893 7.348 7.194 7.464 7.422 7.383
   6896   1370266_at   7.194 6.779 6.656 7.035 6.889 7.694 7.447 7.515 7.595 7.182
   6897   1375144_at   8.282 8.483 8.996 8.696 9.112 9.915 9.123 9.68 9.762 9.699
   6898   1385320_at   2.511 4.009 3.921 4.268 3.833 4.673 5.354 5.274 5.411 5.503
   6899   1372929_at   4.732 4.58 4.134 4.569 4.229 4.79 5.202 5.285 5.041 5.117
   6900   1393966_at   4.726 4.81 4.766 4.733 4.964 5.624 5.123 5.674 5.326 5.112
   6901   1384468_at   4.299 3.434 4.982 4.457 4.38 5.426 5.426 5.905 5.703 5.26
   6902   1378685_at   5.992 6.07 6.548 6.494 6.572 5.134 5.318 5.747 5.867 5.125
   6903   1374343_at   4.969 5.574 4.994 5.098 4.817 5.74 5.588 5.942 6.067 5.647
   6904   1367630_at   12.533 12.455 12.354 12.262 12.409 12.196 12.253 12.126 12.078 12.112
   6905   1393170_at   5.793 6.366 5.798 5.734 6.25 6.749 6.841 6.974 6.736 6.369
   6906   1389618_at   5.956 6.021 5.835 5.474 5.709 6.211 6.419 6.787 6.431 6.274
   6907   1382204_at   1.465 0.345 0 2.319 0 3.274 2.683 3.222 2.72 4.159
   6908   1389190_at   6.508 6.617 6.671 6.734 6.992 7.352 7.024 7.17 7.098 7.088
   6909   1378172_at   7.091 7.046 6.701 7.387 7.417 6.537 6.205 6.587 6.27 6.582
   6910   1381203_at   7.617 7.559 7.448 7.717 7.782 7.321 7.037 7.421 7.204 7.137
   6911   1375662_at   8.502 8.434 8.547 8.703 8.762 8.91 8.915 9.139 9.055 8.818
   6912   1373986_at   9.573 9.62 9.77 9.88 9.505 10.009 10.123 10.241 10.064 9.9
   6913   1375093_at   8.215 7.917 8.157 8.212 8.312 8.454 8.661 8.488 8.564 8.42
   6914   1379313_at   5.573 5.192 5.408 5.347 4.911 4.111 4.071 5.023 3.999 4.336
   6915   1368402_at   8.828 8.874 8.786 8.981 8.95 9.239 9.057 9.448 9.238 9.128
   6916   1385665_at   3.592 3.819 4.191 2.128 3.736 1.674 0.411 2.403 0.903 0.832
   6917   1395479_at   7.111 7.094 6.987 6.989 7.159 6.72 6.756 6.612 6.229 6.192
   6918   1377740_at   6.666 6.748 6.397 6.751 6.767 6.965 7.018 7.217 6.963 7.041
   6919   1393139_at   4.509 4.935 5.233 4.761 3.31 6.13 6.046 5.81 6.487 6.969
   6920   1368446_at   8.265 8.369 8.119 8.084 7.985 8.336 8.808 8.876 8.884 8.668
   6921   1387019_at   9.799 10.283 10.179 10.404 10.254 10.954 10.923 10.438 10.847 10.884
   6922   1377577_at   8.904 8.945 8.749 9.04 8.95 8.697 8.566 8.74 8.686 8.567
   6923   1395686_at   6.504 6.235 5.971 6.015 5.949 5.516 5.557 5.796 5.618 5.595
   6924   1388712_at   7.046 7.47 7.34 7.668 7.852 8.419 7.944 8.73 8.456 8.15
   6925   1394702_at   4.487 4.881 5.066 4.549 4.807 5.527 5.01 5.528 5.583 5.523
   6926   1370615_at   4.154 4.323 4.279 4.305 4.845 5.491 4.722 5.329 5.881 5.469
   6927   1377140_at   5.247 6.044 5.512 5.358 5.706 6.631 6.107 6.248 6.282 6.731
   6928   1380404_at   5.756 5.679 5.815 5.841 5.52 6.347 5.943 6.233 6.14 6.02
   6929   1388668_at   7.509 7.493 7.523 7.609 7.662 7.132 7.3 7.367 6.907 6.98
   6930   1387052_at   4.747 5.183 5.003 4.444 5.228 6.426 6.259 5.323 6.103 5.881
   6931   1372791_at   10.047 10.156 9.969 10.044 10.014 10.317 10.229 10.631 10.489 10.319
   6932   1387000_at   6.482 6.489 6.591 6.936 6.715 7.063 7.111 7.229 6.974 7.433
   6933   1372549_at   6.824 6.828 7.051 7.339 7.298 7.67 7.403 7.961 7.801 7.713
   6934   1388502_at   9.27 9.203 8.853 9.664 9.263 8.481 7.686 8.679 8.445 8.128
   6935   1387652_at   7.421 7.522 7.624 7.668 7.551 7.787 7.958 7.947 7.819 7.709
   6936   1368033_at   6.766 6.552 6.404 6.338 6.634 6.078 6.1 5.783 5.972 5.403
   6937   1371732_at   10.916 10.894 10.798 10.813 11.003 10.605 10.275 10.627 10.658 10.481
   6938   1392072_at   6.461 6.104 6.013 6.307 5.768 6.756 6.495 7.097 7.198 7.012
   6939   1378429_at   5.265 5.447 5.45 6.077 5.759 6.046 6.468 6.354 6.461 6.302
   6940   1398708_at   5.142 5.325 5.392 5.865 5.776 6.047 6.017 6.387 6.586 6.434
   6941   1398981_at   8.534 8.708 8.681 8.929 8.625 8.928 9.1 9.016 9.118 8.983
   6942   1389131_at   7.869 7.597 7.492 7.723 7.636 7.973 8.137 8.219 8.102 7.869
   6943   1375781_at   4.496 4.89 5.413 4.117 4.888 1.637 3.372 3.521 1.774 3.173
   6944   1369522_a_at   5.104 5.699 6.074 6.441 5.752 3.363 0 3.717 3.245 2.903
   6945   1397238_at   3.694 3.3 4.013 3.29 3.463 4.894 4.446 4.974 4.819 3.925
   6946   1378674_at   4.926 5.439 5.085 5.549 5.041 5.802 5.574 6.073 6.029 5.924
   6947   1391106_at   9.327 9.424 9.083 9.141 9.371 8.628 8.764 8.632 8.296 7.777
   6948   1397666_at   4.853 5.546 5.63 5.178 5.149 4.36 4.255 4.828 4.6 4.438
   6949   1379439_at   6.574 6.58 6.355 6.508 6.376 5.699 6.016 6.322 5.803 5.697
   6950   1391946_at   5.848 5.901 5.652 5.731 6.667 3.97 3.651 3.767 0.934 3.908
   6951   1371923_at   7.152 7.511 7.077 7.389 7.481 6.884 6.815 6.424 6.405 5.992
   6952   1388913_at   8.765 8.813 9.002 9.047 9.022 9.422 9.248 9.624 9.403 9.159
   6953   1391412_at   9.026 8.926 9.052 9.131 9.052 8.68 8.743 8.924 8.779 8.57
   6954   1373509_at   7.158 7.023 7.06 7.185 7.18 7.487 7.37 7.52 7.349 7.236
   6955   1372571_at   9.982 10.029 10.027 9.955 10.069 10.486 10.413 10.406 10.371 10.067
   6956   1397402_at   4.69 3.669 4.268 4.081 3.609 5.071 5.117 4.688 5.333 5.132
   6957   1393134_at   5.14 4.53 5.36 4.61 4.54 6.131 5.434 6.077 6.109 5.551
   6958   1368963_at   6.997 7.016 7.186 7.069 6.223 7.612 7.962 7.7 7.547 7.905
   6959   1369353_at   2.384 3.383 2.884 2.459 2.793 3.948 3.775 4.204 4.42 3.322
   6960   1371533_at   8.698 8.619 9.004 8.959 8.69 9.183 9.258 9.062 9.368 9.49
   6961   1367744_at   7.37 7.333 6.852 7.3 8.055 8.316 8.313 8.361 8.354 8.047
   6962   1375017_at   4.673 5.096 5.82 5.646 4.822 6.283 5.907 6.478 6.734 6.631
   6963   1369067_at   6.051 6.181 5.508 6.203 5.297 5.009 3.16 3.996 4.101 2.465
   6964   1375946_at   5.737 6.619 6.307 6.507 6.32 6.995 7.239 6.83 7.057 7.048
   6965   1367768_at   8.31 8.324 8.105 8.356 8.656 7.792 7.797 7.89 7.342 7.161
   6966   1367895_at   11.523 11.515 11.523 11.494 11.652 11.367 11.438 11.277 11.221 11.178
   6967   1389342_at   4.18 4.75 4.719 4.388 5.132 5.126 5.849 5.53 5.955 5.563
   6968   1387436_at   8.649 8.512 8.702 8.634 8.573 8.374 8.47 8.232 8.118 8.087
   6969   1372050_at   8.066 8.252 8.288 8.402 8.561 8.015 7.768 7.955 7.83 7.927
   6970   1368627_at   9.128 9.165 8.974 9.528 7.97 10.018 9.872 10.436 10.693 10.716
   6971   1389385_at   8.036 8.088 8.035 8.116 8.217 8.334 8.47 8.745 8.544 8.308
   6972   1379736_at   8.375 8.358 8.768 8.673 8.372 8.984 8.803 8.901 8.961 8.989
   6973   1380485_at   4.868 4.574 4.816 5.121 5.143 5.768 5.679 5.292 5.392 5.456
   6974   1382520_at   3.465 3.738 3.521 3.411 3.806 4.333 4.276 4.487 3.842 4.051
   6975   1376195_at   8.397 8.431 8.445 8.492 8.296 8.119 8.303 7.993 7.871 7.996
   6976   1389258_at   6.95 7.072 6.838 7.028 7.312 6.576 6.028 6.645 6.412 5.955
   6977   1392505_at   8.306 8.378 8.383 8.371 8.353 8.063 8.164 8.269 8.148 7.961
   6978   1368508_at   8.508 8.539 8.625 8.531 8.656 8.304 8.181 8.501 8.251 8.215
   6979   1395362_at   7.994 8.044 7.93 8.065 7.808 8.25 8.191 8.444 8.259 8.175
   6980   1378397_at   5.349 5.827 5.702 6.023 5.79 6.232 6.671 6.717 6.828 6.15
   6981   1375904_at   9.771 9.815 9.733 9.668 9.844 9.397 9.606 9.532 9.33 9.188
   6982   1384347_at   3.458 4.337 3.839 4.363 4.558 5.047 4.891 5.148 4.955 5.111
   6983   1377637_at   4.529 5.226 4.789 4.949 5.021 1.744 3.479 2.872 3.806 1.183
   6984   1372920_at   6.942 7.045 7.095 6.807 6.605 7.657 7.504 7.787 7.125 7.796
   6985   1373248_at   6.561 6.654 6.49 6.768 6.855 7.425 6.952 7.224 7.175 6.977
   6986   1373329_at   8.395 8.557 8.159 8.604 8.798 9.213 8.804 9.347 9.292 9.067
   6987   1370276_at   12.107 12.044 11.945 11.678 11.831 12.33 12.416 12.188 12.256 12.312
   6988   1370665_at   6.973 7.106 7.027 7.229 7.06 6.962 6.485 6.263 6.359 6.237
   6989   1382218_at   7.074 7.045 7.016 7.124 6.935 6.478 6.387 6.92 6.169 6.249
   6990   1372860_at   8.115 8.373 8.229 8.508 8.29 8.537 8.561 8.744 8.708 8.73
   6991   1388847_at   7.725 7.777 7.918 7.681 7.74 7.229 7.401 7.32 7.641 7.132
   6992   1372996_at   8.942 8.766 8.622 8.812 8.959 9.255 8.983 9.341 9.183 9.261
   6993   1383723_at   7.075 7.193 7.001 7.309 7.27 7.474 7.459 7.771 7.708 7.444
   6994   1387057_at   7.469 7.635 7.634 7.972 7.468 7.986 8.111 8.208 8.248 8.558
