# Supplementary material for: Activation of counter-regulatory mechanisms in a rat renal acute rejection model
Source: BMC Genomics. 2008 Feb 8;9:71. doi: 10.1186/1471-2164-9-71 (PMC2262896; doi:10.1186/1471-2164-9-71)
Supplement: Additional file 1 — Gene lists from the class comparison between the groups analyzed with BRB ArrayTools. [file 1471-2164-9-71-S1.zip › syngeneicvsallogeneic/syngeneicvsallogeneic.html]

 

 
 
 Class Comparison Results 
 
P {font-size: 9pt}
TABLE {font-size: 9pt}
 
 
 
 
function popUp(text) {
   var prop =
   "location=no,scrollbars=yes,menubars=no,toolbars=no,resizable=yes";
   popup = window.open("","mywin",prop);
   popup.document.open();
   popup.document.write(text);
   popup.document.close();
   popup.focus();
   }
    
 HELP   
   Description of the problem:   
 
Number of classes: 2
 
Number of genes that passed filtering criteria: 31099
 
Type of univariate test used: Two-sample T-test (with random variance model)
 
 
Column of the Experiment Descriptors sheet that defines class variable : syngeneic vs allogeneic
 
 
WARNING: Distribution assumptions underlying the univariate test random variance model are not satisfied. Random variance model was not used
 
 
Exact Multivariate Permutations test was computed based on 126 available permutations
 
 
 
Nominal significance level of each univariate test: 0.001
 
 
Confidence level of false discovery rate assessment: 90 %
 
Maximum allowed number of false-positive genes: 10
 
Maximum allowed proportion of false-positive genes: 0.1
 
 
   Summary of Results:   
 
 
Number of genes significant at 0.001 level of the univariate test: 5902
 
 
Probability of getting at least 5902 genes significant by chance (at the 0.001 level) if there are no real differences between the classes: 0.00794
 
 
   Genes which discriminate among classes:   
  Table 1  - Sorted by p-value of the univariate test.
 
The first 5902 genes are significant at the nominal 0.001 level of the univariate test
 
With probability of 90 % the first 4035 genes contain no more than 10 false discoveries.
 
 
With probability of 90% the first 6994 genes contain no more than 10% of false discoveries. 
Further extension of the list was halted because the list would contain more than 100 false discoveries
 
 
  
    Parametric  p-value   Geom mean of  intensities  in class  1 :  allogeneic  Geom mean of  intensities  in class  2 :  syngeneic  Fold difference of geom means   Probe set Description Anno- tations Gene symbol
   1 p  511.5 63.4 8.068  1391737_at  similar to p40-phox  Info   LOC500904 
   2 p  502.9 113.9 4.415  1388600_at  Similar to putative membrane steroid receptor (predicted)  Info    
   3 p  877 178.7 4.908  1376795_at  phosphoinositide-3-kinase adaptor protein 1 (predicted)  Info   Pik3ap1_predicted 
   4 p  263.2 25.7 10.241  1384191_at  Transcribed locus  Info    
   5 p  543.6 109.2 4.978  1392785_at  potassium channel tetramerisation domain containing 12 (predicted)  Info   Kctd12_predicted 
   6 p  750.6 209.1 3.59  1377759_at  BH3 interacting domain death agonist  Info   Bid 
   7 p  443.8 37.6 11.803  1385439_x_at  Transcribed locus, strongly similar to XP_220978.3 PREDICTED: similar to Probable ATP-dependent helicase LGP2 (Protein D11Lgp2) [Rattus norvegicus]  Info    
   8 p  412.6 121.3 3.401  1373949_at  similar to RIKEN cDNA 1110012E06 (predicted)  Info   RGD1307977_predicted 
   9 p  1552.9 2215.5 0.701  1371577_at  NADH dehydrogenase (ubiquinone) Fe-S protein 1, 75kDa  Info   RGD:1359670 
   10 p  1535.7 395.4 3.884  1373672_at    Info    
   11 p  643.7 50.5 12.747  1379766_at  Thyroglobulin  Info   Tg 
   12 p  300.8 945.7 0.318  1368064_a_at  dopa decarboxylase  Info   Ddc 
   13 p  1023.8 293.9 3.483  1378428_at  Transcribed locus  Info    
   14 p  264.4 135.2 1.956  1373115_at  similar to 2310061I09Rik protein (predicted)  Info   RGD1304653_predicted 
   15 p  224.1 23.4 9.577  1391593_at  Ras association (RalGDS/AF-6) domain family 4 (predicted)  Info   Rassf4_predicted 
   16 p  831.5 146.7 5.668  1368010_at  protein tyrosine phosphatase, non-receptor type 6  Info   Ptpn6 
   17 p  265.3 43.1 6.155  1369387_at  vav 1 oncogene  Info   Vav1 
   18 p  161.3 367.8 0.439  1390050_at  golgi phosphoprotein 2 (predicted)  Info   Golph2_predicted 
   19 p  601.8 142.9 4.211  1368742_at  complement component 5, receptor 1  Info   C5r1 
   20 p  600.3 282.1 2.128  1383574_at  mannosidase 1, alpha (predicted)  Info   Man1a_predicted 
   21 p  694.9 1459.8 0.476  1371847_at    Info    
   22 p  241.2 111.8 2.157  1368311_at  O-6-methylguanine-DNA methyltransferase  Info   Mgmt 
   23 p  575.1 65.8 8.74  1388740_at  similar to cDNA sequence BC032204 (predicted)  Info   RGD1310168_predicted 
   24 p  2358.1 850.8 2.772  1373881_at  Rho, GDP dissociation inhibitor (GDI) beta (predicted)  Info   Arhgdib_predicted 
   25 p  287 50.2 5.717  1378321_at  Ras association (RalGDS/AF-6) domain family 4 (predicted)  Info   Rassf4_predicted 
   26 p  494.9 115 4.303  1379604_at  apolipoprotein L, 3 (predicted) /// similar to RIKEN cDNA 2210421G13  Info   Apol3_predicted /// LOC500903 
   27 p  2905.3 500.9 5.8  1388574_at  tryptophanyl-tRNA synthetase (predicted)  Info   Wars_predicted 
   28 p  349.3 39.6 8.821  1375041_at  Similar to F-box only protein 27  Info    
   29 p  440.2 188.3 2.338  1373961_at  similar to 4930453N24Rik protein  Info   RGD:1359320 
   30 p  3145.1 1449.5 2.17  1369931_at  similar to pyruvate kinase (EC 2.7.1.40) isozyme M2 - rat  Info   LOC498661 
   31 p  502.9 117.9 4.265  1372236_at  Similar to Caspase recruitment domain protein 4  Info    
   32 p  503.5 942 0.535  1372093_at  Max interacting protein 1  Info   Mxi1 
   33 p  406.5 183.9 2.21  1392544_at  rcd1 (required for cell differentiation) homolog 1 (S. pombe) (predicted)  Info   Rqcd1_predicted 
   34 p  1886.2 575.8 3.276  1390042_at  similar to 1110007F12Rik protein  Info   MGC109491 
   35 p  1632 182.9 8.923  1370913_at  Best5 protein  Info   RGD:620495 
   36 p  713.3 153.6 4.644  1368679_a_at  Yamaguchi sarcoma viral (v-yes-1) oncogene homolog  Info   Lyn 
   37 p  2403.4 582.7 4.125  1371913_at  transforming growth factor, beta induced  Info   Tgfbi 
   38 p  198.2 405.9 0.488  1373754_at  Transcribed locus  Info    
   39 p  1406.8 418.8 3.359  1388460_at  capping protein (actin filament), gelsolin-like (predicted)  Info   Capg_predicted 
   40 p  470.4 906.4 0.519  1367673_at  selenium binding protein 2  Info   Selenbp1 
   41 p  530.1 177.9 2.98  1383372_at  platelet-activating factor receptor  Info   Ptafr 
   42 p  200.4 37.7 5.316  1380377_at  Bruton agammaglobulinemia tyrosine kinase  Info   RGD:1359683 
   43 p  765.5 423.2 1.809  1388492_at  TNFAIP3 interacting protein 1 (predicted)  Info   Tnip1_predicted 
   44 p  521.6 99.8 5.226  1390391_at  Transcribed locus  Info    
   45 p  3295.5 653.4 5.044  1370882_at  major histocompatibility complex, class II, DM beta  Info   RGD:735096 
   46 p  256.8 43.6 5.89  1380621_at  Similar to tyrosine kinase Fps/Fes  Info    
   47 p  1164.8 188.1 6.192  1373932_at  Cytochrome b-245, beta polypeptide  Info   Cybb 
   48 p  294 101.3 2.902  1369557_at  caspase 7  Info   Casp7 
   49 p  302.4 77.3 3.912  1384483_at  Similar to RIKEN cDNA E030034P13 (predicted)  Info    
   50 p  138.2 277.1 0.499  1380123_at    Info    
   51 p  748.9 128.8 5.814  1373037_at  ubiquitin-conjugating enzyme E2L 6 (predicted)  Info   Ube2l6_predicted 
   52 p  226.1 36.6 6.178  1376197_at  transcription factor 7, T-cell specific (predicted)  Info   Tcf7_predicted 
   53 p  1302.3 410.5 3.172  1368274_at  drebrin-like  Info   Dbnl 
   54 p  288.1 76.7 3.756  1381376_at  Similar to [Mouse primary response gene B94 mRNA, 3end.], gene product  Info    
   55 p  929.1 156.4 5.941  1369186_at  caspase 1  Info   Casp1 
   56 p  1022.2 109.1 9.369  1390798_at    Info    
   57 p  594.2 34.5 17.223  1393400_at  zeta-chain (TCR) associated protein kinase 70kDa  Info   Zap70 
   58 p  301.1 56.3 5.348  1379425_at  similar to RIKEN cDNA 3110037I16 (predicted)  Info   RGD1309802_predicted 
   59 p  965.2 329.6 2.928  1372478_at  similar to LNV  Info   LOC501065 
   60 p  1124 193.7 5.803  1378257_at  three prime repair exonuclease 1 (predicted)  Info   Trex1_predicted 
   61 p  314.4 148.8 2.113  1379302_at  Transcribed locus  Info    
   62 p  406.8 120.4 3.379  1369669_at  neurolysin (metallopeptidase M3 family)  Info   Nln 
   63 p  4028.6 837.3 4.811  1376029_at  RAB2, member RAS oncogene family-like  Info   RGD:1302985 
   64 p  1070.8 120.7 8.872  1368732_at  transporter 2, ATP-binding cassette, sub-family B (MDR/TAP)  Info   Tap2 
   65 p  1240.2 237.6 5.22  1387087_at  CCAAT/enhancer binding protein (C/EBP), beta  Info   Cebpb 
   66 p  571.2 58.4 9.781  1398256_at  interleukin 1 beta  Info   Il1b 
   67 p  1096.2 259.3 4.228  1376976_at  secreted and transmembrane 1  Info   Sectm1 
   68 p  1811.2 174.4 10.385  1391925_at  chemokine (C-C motif) ligand 19 (predicted)  Info   Ccl19_predicted 
   69 p  2476.5 547.4 4.524  1370281_at  fatty acid binding protein 5, epidermal  Info   Fabp5 
   70 p  682.4 151.2 4.513  1383658_at  lysosomal-associated protein transmembrane 5  Info   Laptm5 
   71 p  470.7 139.8 3.367  1372468_at  CD97 antigen (predicted)  Info   Cd97_predicted 
   72 p  5116.3 1404.9 3.642  1373575_at  similar to NADH dehydrogenase (ubiquinone) Fe-S protein 2  Info   LOC498279 
   73 p  284.1 80.3 3.538  1387198_at  inositol polyphosphate-5-phosphatase D  Info   Inpp5d 
   74 p  1371.4 539.3 2.543  1372182_at  phosphofructokinase, platelet  Info   Pfkp 
   75 p  207.4 56.4 3.677  1394678_at  FYVE, RhoGEF and PH domain containing 2 (predicted)  Info    
   76 p  4732 906.2 5.222  1393108_at  RT1 class I, A3  Info   RT1-A3 
   77 p  444.2 760.1 0.584  1375560_at  similar to RIKEN cDNA 0610010D20 (predicted)  Info   RGD1310475_predicted 
   78 p  1907.7 356.9 5.345  1372404_at  RAS-related C3 botulinum substrate 2 (predicted)  Info   Rac2_predicted 
   79 p  209.1 32.4 6.454  1385213_at  Similar to epithelial stromal interaction 1 isoform a  Info    
   80 p  426 110.8 3.845  1372135_at  protein tyrosine kinase 9-like (A6-related protein) (predicted)  Info   Ptk9l_predicted 
   81 p  797.5 1833.4 0.435  1379741_at  ATPase, H+ transporting, lysosomal V0 subunit A isoform 4 (predicted)  Info   Atp6v0a4_predicted 
   82 p  608.4 127.2 4.783  1377826_at  Transcribed locus, weakly similar to NP_036457.1 microtubule-associated protein, RP/EB family, member 1; adenomatous polyposis coli-binding protein EB1 [Homo sapiens]  Info    
   83 p  817.9 157.7 5.186  1380063_at  cholesterol 25-hydroxylase (predicted)  Info   Ch25h_predicted 
   84 p  695.5 153.1 4.543  1393144_at  N-myc (and STAT) interactor (predicted)  Info    
   85 p  4422.3 1222.7 3.617  1369973_at  xanthine dehydrogenase /// hypothetical gene supported by NM_017154  Info   Xdh /// LOC497811 
   86 p  1309.6 135.3 9.679  1373514_at  similar to chromosome 17 open reading frame 27 (predicted)  Info   RGD1308168_predicted 
   87 p  505 80.1 6.305  1380129_at  Transcribed locus  Info    
   88 p  105 405.6 0.259  1395810_at  Transcribed locus  Info    
   89 p  560 327.1 1.712  1398473_at  biogenesis of lysosome-related organelles complex-1, subunit 2 (predicted)  Info   Bloc1s2_predicted 
   90 p  68.6 183.4 0.374  1377125_at  DnaJ (Hsp40) homolog, subfamily C, member 6 (predicted)  Info   Dnajc6_predicted 
   91 p  647.5 32.4 19.985  1370096_at  perforin 1 (pore forming protein)  Info   Prf1 
   92 p  1232 208.6 5.906  1369181_at  cytochrome b-245, beta polypeptide  Info   Cybb 
   93 p  914.5 369.8 2.473  1387227_at  Wiskott-Aldrich syndrome protein interacting protein  Info   Waspip 
   94 p  2973 1528.6 1.945  1398786_at  proteasome (prosome, macropain) subunit, beta type 2  Info   Psmb2 
   95 p  3461.3 494.7 6.997  1389006_at  Macrophage expressed gene 1  Info   Mpeg1 
   96 p  184.4 512.3 0.36  1368281_at  dipeptidase 1 (renal)  Info   Dpep1 
   97 p  1240.8 197.5 6.283  1379748_at  Transcribed locus  Info    
   98 p  736.2 111.8 6.585  1370516_at  peptide/histidine transporter PHT2  Info   Slc15a3 
   99 p  966.5 573.7 1.685  1388565_at  acid cluster protein 33  Info   RGD:1359141 
   100 p  3159.2 1151.7 2.743  1386925_at  actin related protein 2/3 complex, subunit 1B  Info   Arpc1b 
   101 p  371.9 78.3 4.75  1368144_at  regulator of G-protein signaling 2  Info   Rgs2 
   102 p  1068.5 521.9 2.047  1379561_at  ATPase family, AAA domain containing 1 (predicted)  Info   Atad1_predicted 
   103 p  557.2 132.3 4.212  1370987_at  sialophorin  Info   Spn 
   104 p  345 52.6 6.559  1382680_at    Info    
   105 p  892.5 63.8 13.989  1370119_at  leucocyte specific transcript 1  Info   Lst1 
   106 p  1582.5 286.5 5.524  1370904_at  major histocompatibility complex, class II, DM alpha  Info   RGD:735053 
   107 p  1203.3 311 3.869  1373490_at  glia maturation factor, gamma  Info   Gmfg 
   108 p  131 357.9 0.366  1375638_at  serum deprivation response protein  Info   RGD:1359345 
   109 p  108.5 657.1 0.165  1369113_at  gremlin 1 homolog, cysteine knot superfamily (Xenopus laevis)  Info   Grem1 
   110 p  501.4 778.4 0.644  1369412_a_at  solute carrier family 19, member 1  Info   Slc19a1 
   111 p  130.4 228 0.572  1393256_at  selenophosphate synthetase 1 (predicted)  Info   Sephs1_predicted 
   112 p  3933.1 654.4 6.01  1370202_at  HRAS like suppressor  Info   Hrasls3 
   113 p  134.2 30.7 4.371  1387277_at  Yamaguchi sarcoma viral (v-yes-1) oncogene homolog  Info   Lyn 
   114 p  1181.7 2007.7 0.589  1389488_at  hypothetical protein LOC498544  Info   LOC498544 
   115 p  4413.7 969.2 4.554  1370056_at  Ly6-C antigen  Info   Ly6c 
   116 p  303.3 70.9 4.278  1379688_at  Transcribed locus  Info    
   117 p  357.8 152.5 2.346  1391817_at  Transcribed locus  Info    
   118 p  331.5 89.7 3.696  1370623_at  fibrinogen-like 2  Info   Fgl2 
   119 p  1407.1 631.6 2.228  1381968_at  cellular repressor of E1A-stimulated genes (predicted)  Info   Creg_predicted 
   120 p  259.1 73.9 3.506  1370064_at  presenilin 2  Info   Psen2 
   121 p  1223.1 258.5 4.732  1374006_at  kynurenine aminotransferase III  Info   RGD:1359262 
   122 p  477.8 1103.8 0.433  1386943_at  transmembrane 4 superfamily member 11  Info   Tm4sf11 
   123 p  662.3 153.9 4.303  1388773_at  similar to [Mouse primary response gene B94 mRNA, 3end.], gene product  Info   LOC299339 
   124 p  239.6 110.8 2.162  1367600_at  desmin  Info   Des 
   125 p  908.1 87.6 10.366  1368693_at  Gardner-Rasheed feline sarcoma viral (Fgr) oncogene homolog  Info   Fgr 
   126 p  1362.5 346.5 3.932  1373592_at  similar to SPI6  Info   MGC94010 
   127 p  141.2 35 4.034  1371017_at  T-cell receptor gamma chain  Info   Tcrg 
   128 p  1770.7 152.5 11.611  1369110_x_at  RT1 class Ib, locus Aw2  Info   RT1-Aw2 
   129 p  608.8 91.6 6.646  1392407_at  Similar to RIKEN cDNA 6030405P05 gene  Info    
   130 p  1131.1 2320 0.488  1392590_at  Rho GTPase activating protein 24 (predicted)  Info   Arhgap24_predicted 
   131 p  645.7 100.3 6.438  1369665_a_at  interleukin 18  Info   Il18 
   132 p  187.7 16.2 11.586  1368930_at  potassium intermediate/small conductance calcium-activated channel, subfamily N, member 4  Info   Kcnn4 
   133 p  426.8 145.6 2.931  1370440_at  peptide/histidine transporter  Info   Slc15a4 
   134 p  439 137.8 3.186  1369716_s_at  lectin, galactose binding, soluble 5 /// lectin, galactose binding, soluble 9  Info   Lgals5 /// Lgals9 
   135 p  2702.6 395.5 6.833  1369964_at  coronin, actin binding protein 1A  Info   Coro1a 
   136 p  287.7 81.8 3.517  1382275_at  similar to PAK/PLC-interacting protein 1  Info   LOC361232 
   137 p  1632.2 440.6 3.704  1389732_at  Transcribed locus, strongly similar to NP_082154.2 hypothetical protein LOC71712 [Mus musculus]  Info    
   138 p  143 24.4 5.861  1384580_at  complement component 6  Info   C6 
   139 p  339.5 111.5 3.045  1370415_at  Ras association (RalGDS/AF-6) domain family 5  Info   Rassf5 
   140 p  345.1 40.9 8.438  1378418_at  similar to MGC37193 protein (predicted)  Info   RGD1311681_predicted 
   141 p  2414.9 338.9 7.126  1368518_at  CD53 antigen  Info   Cd53 
   142 p  1205.9 2612.1 0.462  1398348_at  Transcribed locus  Info    
   143 p  892.6 253.6 3.52  1389885_at  similar to RIKEN cDNA 0610025L06 (predicted)  Info   RGD1309967_predicted 
   144 p  417.8 90.1 4.637  1368227_at  solute carrier family 28 (sodium-coupled nucleoside transporter), member 2  Info   Slc28a2 
   145 p  543.9 78.5 6.929  1369294_at  bone marrow stromal cell antigen 1  Info   Bst1 
   146 p  217.6 78 2.79  1371111_at  RT1 class I, A3  Info   RT1-A3 
   147 p  598.1 41.1 14.552  1389470_at  B-factor, properdin  Info   Bf 
   148 p  789.1 1515.4 0.521  1387040_at  myelin and lymphocyte protein  Info   Mal 
   149 p  167.1 295.2 0.566  1375423_at    Info    
   150 p  493.7 49.6 9.954  1379357_at  Transcribed locus  Info    
   151 p  1422.1 189.2 7.516  1372691_at  uridine phosphorylase 1 (predicted)  Info   Upp1_predicted 
   152 p  51.8 143 0.362  1386529_at  Transcribed locus  Info    
   153 p  411.5 93.2 4.415  1385925_at  Transcribed locus  Info    
   154 p  1424.5 582.2 2.447  1372459_at  vasodilator-stimulated phosphoprotein (predicted)  Info   Vasp_predicted 
   155 p  737 162.3 4.541  1387074_at  regulator of G-protein signaling 2  Info   Rgs2 
   156 p  833.2 1855.5 0.449  1367765_at  transcobalamin 2  Info   Tcn2 
   157 p  548.1 137.5 3.986  1389873_at  apoptosis-associated speck-like protein containing a CARD  Info   Pycard 
   158 p  1212.4 102.7 11.805  1369204_at  hemopoietic cell kinase  Info   Hck 
   159 p  5997.3 1968.3 3.047  1371440_at  Beta-2 microglobulin  Info   B2m 
   160 p  758 181.9 4.167  1387897_at  cyclic nucleotide phosphodiesterase 1  Info   Cnp1 
   161 p  1048.2 221.4 4.734  1367816_at  homeobox only domain  Info   Hod 
   162 p  109.5 253.4 0.432  1369100_at  angiotensin/vasopressin receptor  Info   Nalp6 
   163 p  1331.6 187.7 7.094  1395124_at  tryptophanyl-tRNA synthetase (predicted)  Info   Wars_predicted 
   164 p  100.9 25 4.036  1393297_at  Transcribed locus  Info    
   165 p  525.1 65.5 8.017  1387687_at  immunoglobulin superfamily, member 6  Info   Igsf6 
   166 p  285.5 871.8 0.327  1372646_at  similar to RIKEN cDNA 1500015O10 (predicted)  Info   RGD1305645_predicted 
   167 p  249.7 40.7 6.135  1385572_at  Transcribed locus  Info    
   168 p  267.7 76.4 3.504  1382535_at  Potassium channel tetramerisation domain containing 12 (predicted)  Info    
   169 p  376.3 29.9 12.585  1391214_at  hypothetical protein LOC294430  Info   RGD1304835_predicted 
   170 p  1143.9 329.4 3.473  1389538_at  nuclear factor of kappa light chain gene enhancer in B-cells inhibitor, alpha  Info   Nfkbia 
   171 p  466.1 74.8 6.231  1392280_at  toll-like receptor 2  Info   Tlr2 
   172 p  974.2 115.3 8.449  1371210_s_at  RT1 class Ib, locus Aw2 /// RT1 class I, CE5  Info   RT1-Aw2 /// RT1-CE5 
   173 p  526.3 168.5 3.123  1374157_at    Info    
   174 p  3927.4 454.6 8.639  1370428_x_at  RT1 class Ib, locus Aw2 /// RT1 class Ia, locus A2 /// RT1 class I, A3  Info   RT1-Aw2 /// RT1-A2 /// RGD:1359610 
   175 p  631.4 45.4 13.907  1369427_at  macrophage expressed gene 1  Info   Mpeg1 
   176 p  1283.8 307.2 4.179  1374551_at  interferon-induced protein 35 (predicted)  Info   Ifi35_predicted 
   177 p  923.6 127.9 7.221  1383320_at  lymphocyte protein tyrosine kinase  Info   Lck 
   178 p  3935.4 1346 2.924  1370172_at  superoxide dismutase 2, mitochondrial  Info   Sod2 
   179 p  398.3 185.1 2.152  1383711_at  N-myristoyltransferase 1  Info   Nmt1 
   180 p  2625.9 341.9 7.68  1389014_at  pre-B-cell colony enhancing factor 1  Info   Pbef1 
   181 p  311.8 692.9 0.45  1373240_at  dehydrogenase/reductase (SDR family) member 3 (predicted)  Info   Dhrs3_predicted 
   182 p  325.9 216.6 1.505  1389344_at  ubiquitin specific protease 39 (predicted)  Info   Usp39_predicted 
   183 p  2267.9 771.1 2.941  1371662_at  lysyl-tRNA synthetase  Info   RGD:1359653 
   184 p  632 177.5 3.561  1374578_at  Transcribed locus  Info    
   185 p  462.3 308.2 1.5  1393317_at  similar to THUMP domain containing 3  Info   LOC500288 
   186 p  428 754.6 0.567  1376723_a_at    Info    
   187 p  4730.6 962.7 4.914  1367710_at  protease (prosome, macropain) 28 subunit, beta  Info   Psme2 
   188 p  288.9 50.8 5.687  1383426_at  proline-serine-threonine phosphatase-interacting protein 1 (predicted)  Info   Pstpip1_predicted 
   189 p  2053.4 179.5 11.44  1389092_at  interleukin 2 receptor, gamma (severe combined immunodeficiency)  Info   Il2rg 
   190 1e-07 433.8 36.3 11.95  1368723_at  linker for activation of T cells  Info   Lat 
   191 1e-07 2532.5 3906.7 0.648  1386871_at  glutathione peroxidase 4  Info   Gpx4 
   192 1e-07 489.8 53.7 9.121  1382442_at  Similar to NF-kappa-B-repressing factor (NFKB-repressing factor) (Transcription factor NRF)  Info    
   193 1e-07 43.1 116.6 0.37  1392863_at  Similar to fibronectin leucine rich transmembrane protein 3  Info    
   194 1e-07 4308.4 1935.3 2.226  1375211_at  ribonuclease T2 (predicted)  Info   Rnaset2_predicted 
   195 1e-07 515.3 35.6 14.475  1389696_at  Transcribed locus  Info    
   196 1e-07 317.3 625.4 0.507  1376977_at  Prostaglandin E receptor 3 (subtype EP3)  Info   Ptger3 
   197 1e-07 557.1 90.8 6.135  1382026_at  Rho GTPase activating protein 9 (predicted)  Info   Arhgap9_predicted 
   198 1e-07 2946.4 650.8 4.527  1387005_at  cathepsin S  Info   Ctss 
   199 1e-07 450.3 74.7 6.028  1392881_at  alpha-2,3-sialyltransferase ST3Gal IV  Info   RGD:1303068 
   200 1e-07 390 140.1 2.784  1373785_at  similar to expressed sequence AI132321  Info   LOC362792 
   201 1e-07 560.2 1183.6 0.473  1371629_at  CXXC finger 5  Info   RGD:1359466 
   202 1e-07 464 42.6 10.892  1371070_at  tumor stroma and activated macrophage protein DLM-1  Info   Zbp1 
   203 1e-07 107.9 325.5 0.331  1390126_at    Info    
   204 1e-07 3793 1984.5 1.911  1370928_at  LPS-induced TN factor  Info   Litaf 
   205 1e-07 53.1 101.6 0.523  1373642_at    Info    
   206 1e-07 466.1 146.7 3.177  1376835_at  Solute carrier family 35, member B2  Info   Slc35b2 
   207 1e-07 1457.9 638.6 2.283  1369559_a_at  CD47 antigen (Rh-related antigen, integrin-associated signal transducer)  Info   Cd47 
   208 1e-07 171.4 24.3 7.053  1368377_at  granzyme C  Info   Gzmc 
   209 1e-07 201.4 367.2 0.548  1377830_a_at  similar to RIKEN cDNA 1810014F10 gene (predicted)  Info   RGD1311186_predicted 
   210 1e-07 141.4 19.2 7.365  1393957_at  Transcribed locus  Info    
   211 1e-07 1068.7 168 6.361  1387205_at  RT1 class Ib, locus M3  Info   RT1-M3 
   212 1e-07 305.5 73.8 4.14  1382346_at  Transcribed locus  Info    
   213 1e-07 290.8 593.8 0.49  1394577_at  Transcribed locus  Info    
   214 1e-07 2789.4 485.5 5.745  1389210_at  lymphocyte cytosolic protein 1 (predicted)  Info   Lcp1_predicted 
   215 1e-07 157.9 68.5 2.305  1379482_at  transmembrane 6 superfamily member 1 (predicted)  Info   Tm6sf1_predicted 
   216 1e-07 1394.9 387.7 3.598  1374388_at  EF hand domain containing 2 (predicted)  Info   Efhd2_predicted 
   217 1e-07 368.6 682.4 0.54  1371931_at  general transcription factor II I  Info   Gtf2i 
   218 1e-07 2864.2 234.1 12.235  1370885_at  cathepsin Z  Info   Ctsz 
   219 1e-07 635.3 278.7 2.28  1372302_at  similar to hypothetical protein FLJ10579 (predicted)  Info   RGD1308697_predicted 
   220 1e-07 414.8 700.2 0.592  1367716_at  T-cell immunomodulatory protein  Info   Cda08 
   221 1e-07 773.6 469.5 1.648  1375522_at  N-myristoyltransferase 1  Info   Nmt1 
   222 1e-07 405 214.7 1.886  1394724_at  transcription elongation factor A (SII) 1 (predicted)  Info   Tcea1_predicted 
   223 1e-07 794.8 143.7 5.531  1398616_at  similar to Adapter-related protein complex 1 sigma 1B subunit (Sigma-adaptin 1B) (Adaptor protein complex AP-1 sigma-1B subunit) (Golgi adaptor HA1/AP1 adaptin sigma-1B subunit) (Clathrin assembly protein complex 1 sigma-1B small chain) (Sigma 1B s...  Info   LOC302671 
   224 1e-07 424.7 1009.3 0.421  1369663_at  epoxide hydrolase 2, cytoplasmic  Info   Ephx2 
   225 1e-07 379 72.2 5.249  1370400_at  RT1 class Ib gene, H2-TL-like, grc region (N1) /// RT1 class Ib gene, H2-TL-like, grc region (N3) /// RT1 class Ib gene, H2-TL-like, grc region(N2)  Info   RT1-N1 /// RT1-N3 /// RT1-N2 
   226 1e-07 502.1 1066.3 0.471  1372587_at  endomucin  Info   Emcn 
   227 1e-07 137.1 315.6 0.434  1389159_at  Transcribed locus  Info    
   228 1e-07 75.8 313.3 0.242  1377086_at  C1q and tumor necrosis factor related protein 3 (predicted)  Info   C1qtnf3_predicted 
   229 1e-07 746.4 190.5 3.918  1376327_at  tumor necrosis factor receptor superfamily, member 14 (herpesvirus entry mediator) (predicted)  Info   Tnfrsf14_predicted 
   230 1e-07 244.1 85.4 2.858  1389071_at  tangerin  Info   LOC309169 
   231 1e-07 1130.1 660.7 1.71  1367856_at  glucose-6-phosphate dehydrogenase  Info   G6pdx 
   232 1e-07 322.9 71.8 4.497  1376943_at    Info    
   233 1e-07 1478.7 229.8 6.435  1368558_s_at  allograft inflammatory factor 1  Info   Aif1 
   234 1e-07 138.6 44.4 3.122  1368940_at  purinergic receptor P2Y, G-protein coupled 2  Info   P2ry2 
   235 1e-07 206.3 338.3 0.61  1388109_at  G protein-coupled receptor 116  Info   Gpr116 
   236 1e-07 293.1 31 9.455  1397167_at    Info    
   237 1e-07 163.7 15.8 10.361  1381771_at  Transcribed locus  Info    
   238 1e-07 542.6 1181.3 0.459  1373900_at  keratin complex 2, basic, gene 7 (predicted)  Info   Krt2-7_predicted 
   239 1e-07 252.3 64 3.942  1370693_a_at  cyclic nucleotide phosphodiesterase 1  Info   Cnp1 
   240 1e-07 2608.2 523.5 4.982  1373025_at  complement component 1, q subcomponent, gamma polypeptide (predicted)  Info   C1qg_predicted 
   241 1e-07 2346.2 1055.2 2.223  1387884_at  proteasome (prosome, macropain) subunit, alpha type 5  Info   Psma5 
   242 1e-07 147.2 982.9 0.15  1367805_at  glutaminase  Info   Gls 
   243 1e-07 1583.5 178 8.896  1388164_at  RT1 class Ib, locus S3  Info   RT1-S3 
   244 1e-07 3153.5 1543.6 2.043  1372070_at  interferon gamma inducible protein 30 (predicted)  Info   Ifi30_predicted 
   245 1e-07 1970 902.1 2.184  1368808_at  CAP, adenylate cyclase-associated protein 1 (yeast)  Info   Cap1 
   246 1e-07 3589.3 567.9 6.32  1372757_at  Signal transducer and activator of transcription 1  Info   Stat1 
   247 1e-07 907.7 165.5 5.485  1393038_at  Fc receptor, IgG, high affinity I (predicted)  Info   Fcgr1_predicted 
   248 1e-07 267.5 41.4 6.461  1374429_at  Transcribed locus, weakly similar to XP_518443.1 PREDICTED: pim-1 oncogene [Pan troglodytes]  Info    
   249 1e-07 898.6 2406.4 0.373  1367909_at  dicarbonyl L-xylulose reductase  Info   Dcxr 
   250 1e-07 366.3 69.2 5.293  1373066_at  Transcribed locus  Info    
   251 1e-07 1974 987.7 1.999  1375137_at  actin related protein 2/3 complex, subunit 2 (predicted)  Info   Arpc2_predicted 
   252 1e-07 611.1 43.1 14.179  1368455_at  natural killer cell group 7 sequence  Info   Nkg7 
   253 1e-07 188.6 91.1 2.07  1371122_at  TRAF family member-associated Nf-kappa B activator /// hypothetical gene supported by NM_145788  Info   Tank /// LOC497768 
   254 1e-07 178 341.1 0.522  1382014_at  similar to CGI-121 protein (predicted)  Info   RGD1309786_predicted 
   255 1e-07 1785.8 925.8 1.929  1377034_at  serine (or cysteine) proteinase inhibitor, clade B, member 1a (predicted)  Info   Serpinb1a_predicted 
   256 1e-07 333.5 77.7 4.292  1389250_at  Transcribed locus  Info    
   257 1e-07 325.3 71.6 4.543  1379760_at  similar to RIKEN cDNA 1810019A08 (predicted)  Info   RGD1310519_predicted 
   258 1e-07 821.8 316.4 2.597  1367922_at  a disintegrin and metalloproteinase domain 17 (tumor necrosis factor, alpha, converting enzyme)  Info   Adam17 
   259 1e-07 205.4 45.6 4.504  1385557_at  Transcribed locus  Info    
   260 1e-07 770.2 2413 0.319  1379281_at  uterine sensitization-associated gene 1 protein  Info   Sostdc1 
   261 1e-07 1095.1 419.2 2.612  1388236_x_at  RT1 class I, CE12  Info   RGD:1359103 
   262 1e-07 1042.2 1977.3 0.527  1391409_at  amnionless (predicted)  Info   Amn_predicted 
   263 1e-07 59.6 121.7 0.49  1386293_at    Info    
   264 1e-07 601.7 128.4 4.686  1383662_at  hypothetical protein LOC500956  Info   LOC500956 
   265 1e-07 1233.4 57.3 21.525  1370924_at  similar to T-cell receptor beta-2 chain C region  Info   LOC362350 
   266 1e-07 532 64.6 8.235  1390909_at  Transcribed locus  Info    
   267 1e-07 370 99.5 3.719  1374544_at  CDNA clone MGC:112715 IMAGE:7377326  Info    
   268 1e-07 94 350 0.269  1376623_at  similar to hypothetical protein FLJ20898  Info   RGD:1359314 
   269 1e-07 580.6 1189.3 0.488  1368008_at  prominin 1  Info   Prom1 
   270 1e-07 1460.3 761.3 1.918  1392900_at  Similar to mKIAA1631 protein  Info    
   271 1e-07 593.3 136 4.362  1370449_at  G protein-coupled receptor 105  Info   Gpr105 
   272 1e-07 2168.7 4618 0.47  1369954_at  isocitrate dehydrogenase 1 (NADP+), soluble  Info   Idh1 
   273 1e-07 3509.1 714 4.915  1370383_s_at  RT1 class II, locus Db1  Info   RT1-Db1 
   274 1e-07 557.3 122.4 4.553  1373670_at  Signal transducer and activator of transcription 2 (predicted)  Info    
   275 1e-07 272.1 64.2 4.238  1387982_at  toll-like receptor 4  Info   Tlr4 
   276 1e-07 314.7 721.2 0.436  1375231_a_at  CXXC finger 5  Info   RGD:1359466 
   277 1e-07 128.7 386.6 0.333  1367689_a_at  cd36 antigen /// similar to fatty acid translocase/CD36  Info   Cd36 /// LOC499984 
   278 1e-07 944.3 241.2 3.915  1372930_at  SP110 nuclear body protein (predicted)  Info    
   279 1e-07 1068.7 257.9 4.144  1367705_at  glutaredoxin 1 (thioltransferase)  Info   Glrx1 
   280 1e-07 2316.6 1693.5 1.368  1369940_at  transaldolase 1  Info   Taldo1 
   281 1e-07 265.1 147.4 1.799  1391167_at  Transcribed locus  Info    
   282 1e-07 1015.3 1962.6 0.517  1386934_at  choline transporter  Info   Slc6a8 
   283 1e-07 1290.6 220.4 5.856  1379717_at  similar to ATP-binding cassette, subfamily G, member 3  Info   LOC498435 
   284 1e-07 364.4 105.9 3.441  1380077_at  Transcribed locus  Info    
   285 1e-07 663.1 74.1 8.949  1370113_at  inhibitor of apoptosis protein 1  Info   Birc3 
   286 1e-07 402.3 67.9 5.925  1381279_at  receptor (TNFRSF)-interacting serine-threonine kinase 2 (predicted)  Info   Ripk2_predicted 
   287 1e-07 2435.3 1026.5 2.372  1371968_at  CGI-119 protein  Info   Cgi119 
   288 1e-07 2868.2 920.8 3.115  1387893_at  complement component 1, s subcomponent /// similar to complement component 1, s subcomponent  Info   C1s /// LOC500313 
   289 1e-07 917.2 454.1 2.02  1370173_at  superoxide dismutase 2, mitochondrial  Info   Sod2 
   290 1e-07 1558.4 337.6 4.616  1387202_at  intercellular adhesion molecule 1  Info   Icam1 
   291 1e-07 418.7 34.6 12.101  1372852_at  similar to protein tyrosine phosphatase, receptor type, C polypeptide-associated protein  Info   LOC499300 
   292 1e-07 1136 3603.8 0.315  1367952_at  low density lipoprotein receptor-related protein 2  Info   Lrp2 
   293 1e-07 871.6 142.5 6.116  1380521_at  hematopoietic cell specific Lyn substrate 1 (predicted)  Info   Hcls1_predicted 
   294 1e-07 427.4 167 2.559  1384413_at  Similar to solute carrier family 35, member A5  Info    
   295 1e-07 139.3 70.7 1.97  1382777_at  Neurolysin (metallopeptidase M3 family)  Info   Nln 
   296 1e-07 1280.8 280.2 4.571  1370972_x_at    Info   Multiple Gene Symbols 
   297 1e-07 1583.4 4009.8 0.395  1368568_at  aquaporin 2  Info   Aqp2 
   298 1e-07 490.5 103.8 4.725  1393460_at  similar to GARP protein precursor (Garpin) (Glycoprotein A repetitions predominant) (predicted)  Info   RGD1310771_predicted 
   299 1e-07 155.6 369.6 0.421  1370930_at  kinesin family member 1C  Info   Kif1c 
   300 1e-07 198.4 73.9 2.685  1372896_at  Similar to CDNA sequence BC063749  Info    
   301 1e-07 693.2 175.5 3.95  1373197_at  Similar to Ab1-133 (predicted)  Info    
   302 1e-07 938 1549.4 0.605  1371557_at  THAP domain containing 4  Info   RGD:1359473 
   303 1e-07 172.6 58.5 2.95  1376077_at    Info    
   304 1e-07 741.8 127.4 5.823  1384187_at  similar to Adapter-related protein complex 1 sigma 1B subunit (Sigma-adaptin 1B) (Adaptor protein complex AP-1 sigma-1B subunit) (Golgi adaptor HA1/AP1 adaptin sigma-1B subunit) (Clathrin assembly protein complex 1 sigma-1B small chain) (Sigma 1B s...  Info   LOC302671 
   305 1e-07 1070.8 216.5 4.946  1393280_at  lymphocyte antigen 86 (predicted)  Info   Ly86_predicted 
   306 1e-07 1154.7 346.7 3.331  1372112_at  similar to 9230105E10Rik protein (predicted)  Info   RGD1304579_predicted 
   307 1e-07 1017.8 283.4 3.591  1373775_at  Hypothetical LOC311956  Info    
   308 1e-07 3299.5 1716.8 1.922  1371869_at  proteasome (prosome, macropain) subunit, alpha type 7  Info   Psma7 
   309 1e-07 101.7 63 1.614  1392508_at  similar to RIKEN cDNA 3110010F15 (predicted)  Info   RGD1308378_predicted 
   310 1e-07 1192.9 2145.9 0.556  1390421_at  Similar to RIKEN cDNA 0610010D20 (predicted)  Info    
   311 1e-07 465.6 129.3 3.601  1379295_at    Info    
   312 1e-07 495 162.8 3.041  1392960_at  similar to RIKEN cDNA 2310004I24 gene (predicted)  Info   RGD1309906_predicted 
   313 1e-07 1397.5 329.6 4.24  1382274_at  retinoic acid receptor responder (tazarotene induced) 1 (predicted)  Info   Rarres1_predicted 
   314 1e-07 931.4 1746.7 0.533  1386885_at  enoyl coenzyme A hydratase 1, peroxisomal  Info   Ech1 
   315 1e-07 277.4 83.5 3.322  1390396_at  Similar to eukaryotic translation initiation factor 4E member 3  Info    
   316 1e-07 532.5 1624.1 0.328  1387100_at  aquaporin 3  Info   Aqp3 
   317 1e-07 484.5 764 0.634  1372755_at  mal, T-cell differentiation protein 2  Info   Mal2 
   318 1e-07 1456.1 241.1 6.039  1375010_at  CD68 antigen (predicted)  Info   Cd68_predicted 
   319 1e-07 1939.6 426.5 4.548  1368474_at  vascular cell adhesion molecule 1  Info   Vcam1 
   320 1e-07 635.8 80.2 7.928  1383585_s_at  Sorting nexin 10 (predicted)  Info    
   321 1e-07 1244.2 149.1 8.345  1371016_at  similar to T-cell receptor alpha chain precursor V and C regions (TRA29) - rat (fragment)  Info   RGD:1359684 
   322 1e-07 587.1 128.7 4.562  1377239_at  amyloid beta (A4) precursor protein-binding, family B, member 1 interacting protein (predicted)  Info   Apbb1ip_predicted 
   323 1e-07 559.4 40 13.985  1369214_a_at  MHC class II transactivator  Info   Mhc2ta 
   324 1e-07 5370.3 2885.7 1.861  1369937_at  calmodulin 1  Info   Calm1 
   325 1e-07 274 72.1 3.8  1377669_at  low Mr GTP-binding protein  Info   Rab27a 
   326 2e-07 374.4 128.2 2.92  1367936_at  serine/threonine kinase 10  Info   Stk10 
   327 2e-07 1736.6 3750.1 0.463  1387022_at  aldehyde dehydrogenase family 1, member A1  Info   Aldh1a1 
   328 2e-07 227.9 55.5 4.106  1395313_s_at    Info    
   329 2e-07 3778.5 2711.2 1.394  1398788_at  glucose regulated protein, 58 kDa  Info   Grp58 
   330 2e-07 161.1 55.4 2.908  1390579_at  similar to RIKEN cDNA 1810029B16 (predicted)  Info   RGD1305222_predicted 
   331 2e-07 1118.7 520.2 2.151  1388629_at  inosine 5-monophosphate dehydrogenase 2  Info   Impdh2 
   332 2e-07 282.8 131.1 2.157  1394340_at  inositol polyphosphate-1-phosphatase (predicted)  Info   Inpp1_predicted 
   333 2e-07 296.4 32.6 9.092  1387221_at  GTP cyclohydrolase 1  Info   Gch 
   334 2e-07 1501.3 761.1 1.973  1372577_at  actin related protein 2/3 complex, subunit 4 (predicted)  Info   Arpc4_predicted 
   335 2e-07 234 791.2 0.296  1377145_at  similar to very large G-protein coupled receptor 1  Info   LOC294614 
   336 2e-07 249.8 124.6 2.005  1380583_s_at  colony stimulating factor 1 (macrophage)  Info   Csf1 
   337 2e-07 233.5 48.5 4.814  1371213_at  RT1 class I, A3  Info   RGD:1359610 
   338 2e-07 1069.2 3078.5 0.347  1368461_at  solute carrier family 22 (organic anion transporter), member 8  Info   Slc22a8 
   339 2e-07 393.2 682.1 0.576  1372562_at  similar to RIKEN cDNA C030006K11  Info   RGD:1359663 
   340 2e-07 3055.9 309.9 9.861  1371209_at  RT1 class I, CE5  Info   RT1-CE5 
   341 2e-07 1192.5 1699.8 0.702  1371328_at  Cleavage and polyadenylation specific factor 5 (predicted)  Info    
   342 2e-07 1508.1 422.6 3.569  1373818_at  Potassium channel tetramerisation domain containing 12 (predicted)  Info    
   343 2e-07 184.2 33.6 5.482  1380537_at  hematopoietic protein 1 (predicted)  Info   Hem1_predicted 
   344 2e-07 922.3 108.8 8.477  1373889_at  similar to immunoglobulin superfamily, member 7  Info   LOC498022 
   345 2e-07 438.6 866.7 0.506  1388976_at  similar to BolA domain-containing protein like (11.4 kD) (1P25) (predicted)  Info   RGD1305975_predicted 
   346 2e-07 678.4 99.3 6.832  1391979_at  Transcribed locus  Info    
   347 2e-07 1674.9 2751.5 0.609  1367662_at  hydroxysteroid (17-beta) dehydrogenase 10  Info   Hsd17b10 
   348 2e-07 356 847.4 0.42  1370248_at  FXYD domain-containing ion transport regulator 6  Info   Fxyd6 
   349 2e-07 1822.6 174.7 10.433  1390510_at  membrane-spanning 4-domains, subfamily A, member 6B  Info   RGD:1359381 
   350 2e-07 237.3 34.3 6.918  1397214_at  Transcribed locus  Info    
   351 2e-07 1230.5 2718.7 0.453  1388988_at  similar to RIKEN cDNA 1810013B01  Info   RGD:1359649 
   352 2e-07 483.8 123.4 3.921  1395003_at  dendritic cell inhibitory receptor 4  Info   RGD:1359109 
   353 2e-07 276.2 24.2 11.413  1369025_at  CD5 antigen  Info   Cd5 
   354 2e-07 1979.4 4666.2 0.424  1369660_at  defensin beta 1  Info   Defb1 
   355 2e-07 863.7 208.3 4.146  1371537_at  Transcribed locus  Info    
   356 2e-07 1364.8 572.8 2.383  1372865_at  zinc finger protein 364 (predicted)  Info   Zfp364_predicted 
   357 2e-07 1002.6 75.2 13.332  1378927_at    Info    
   358 2e-07 2018.3 363.8 5.548  1370215_at  complement component 1, q subcomponent, beta polypeptide  Info   C1qb 
   359 2e-07 681.8 1456.1 0.468  1368191_a_at  solute carrier family 22 (organic cation transporter), member 1  Info   Slc22a1 
   360 2e-07 256.8 105.3 2.439  1380445_at  Transcribed locus, strongly similar to XP_515868.1 PREDICTED: sodium channel, voltage-gated, type III, alpha [Pan troglodytes]  Info    
   361 2e-07 305.1 99.9 3.054  1374539_at  Similar to type IV putative aminophospholipid transporting ATPase  Info    
   362 2e-07 33 136.8 0.241  1384874_at  Transcribed locus  Info    
   363 2e-07 641.2 1825.8 0.351  1370072_at  membrane metallo endopeptidase  Info   Mme 
   364 2e-07 368.4 126.4 2.915  1376379_a_at  similar to RIKEN cDNA 2310004I24 gene (predicted)  Info   RGD1309906_predicted 
   365 2e-07 727.6 287.7 2.529  1368207_at  FXYD domain-containing ion transport regulator 5  Info   Fxyd5 
   366 2e-07 233.7 31.9 7.326  1392722_at  Transcribed locus  Info    
   367 2e-07 956.5 346.3 2.762  1374468_at  myeloid differentiation primary response gene 88  Info   Myd88 
   368 2e-07 518.7 79.5 6.525  1369549_at  NKR-P2, ortholog of human NKG2D  Info   Nkrp2 
   369 2e-07 2713.7 3918.7 0.693  1388403_at  similar to NADP+-specific isocitrate dehydrogenase  Info   LOC361596 
   370 2e-07 878.3 261.1 3.364  1382108_at  V-maf musculoaponeurotic fibrosarcoma oncogene family, protein B (avian)  Info   Mafb 
   371 2e-07 1004.1 130.8 7.677  1388202_at  RT1 class Ib, locus Aw2  Info   RT1-Aw2 
   372 2e-07 555.4 47.3 11.742  1370603_a_at  protein tyrosine phosphatase, receptor type, C  Info   Ptprc 
   373 2e-07 1486.8 107.6 13.818  1390687_at  pleckstrin (predicted)  Info   Plek_predicted 
   374 2e-07 357.2 47.6 7.504  1393044_at  thymidylate kinase family LPS-inducible member (predicted)  Info   Tyki_predicted 
   375 2e-07 174.5 17.2 10.145  1370483_at  CD244 natural killer cell receptor 2B4  Info   Cd244 
   376 2e-07 302.1 70.5 4.285  1380425_at  Ribonuclease L (2',5'-oligoisoadenylate synthetase-dependent)  Info   Rnasel 
   377 2e-07 635 1204.3 0.527  1388534_at  Solute carrier family 31 (copper transporters), member 1  Info   Slc31a1 
   378 2e-07 427.7 46.8 9.139  1387394_at  interleukin 2 receptor, beta chain  Info   Il2rb 
   379 2e-07 374.7 228.1 1.643  1367861_at  Ena-vasodilator stimulated phosphoprotein  Info   Evl 
   380 2e-07 1958.5 848.1 2.309  1371239_s_at  tropomyosin 3, gamma  Info   Tpm3 
   381 2e-07 1120.6 623.5 1.797  1386884_at  protease, serine, 11 (Igf binding)  Info   Prss11 
   382 2e-07 241.1 55.7 4.329  1386637_at  fibrinogen-like 2  Info   Fgl2 
   383 2e-07 1234 59.5 20.739  1392547_at  hypothetical LOC302884  Info   RGD:1359583 
   384 2e-07 2276.1 647.5 3.515  1368655_at  proteoglycan peptide core protein  Info   RGD:619969 
   385 2e-07 738.4 1020.6 0.723  1374846_at  cardiac lineage protein 1  Info   Clp1 
   386 2e-07 1564.3 2251.9 0.695  1389903_at  pituitary tumor-transforming 1 interacting protein (predicted)  Info   Pttg1ip_predicted 
   387 2e-07 439 98.8 4.443  1394940_at  similar to hypothetical protein FLJ20037 (predicted)  Info   RGD1311381_predicted 
   388 2e-07 3498.8 1528.9 2.288  1369943_at  transglutaminase 2, C polypeptide  Info   Tgm2 
   389 2e-07 970.3 185.9 5.219  1389571_at  signal transducer and activator of transcription 2 (predicted)  Info   Stat2_predicted 
   390 2e-07 304.9 108.7 2.805  1379316_at  Transcribed locus  Info    
   391 2e-07 4552.8 440.3 10.34  1373523_at  Fc fragment of IgG, low affinity IIIa, receptor for (CD16)  Info   Fcgr3a 
   392 2e-07 208.8 35.1 5.949  1369815_at  chemokine (C-C motif) ligand 3  Info   Ccl3 
   393 2e-07 1111.8 2106.7 0.528  1390835_at  similar to 1300013J15Rik protein (predicted)  Info   RGD1311123_predicted 
   394 2e-07 1788.8 239.1 7.481  1372034_at  Similar to hypothetical protein MGC29390 (predicted)  Info    
   395 2e-07 275.7 113.3 2.433  1387678_at  neuroblastoma RAS viral (v-ras) oncogene homolog  Info   Nras 
   396 2e-07 401.7 175.4 2.29  1382533_at  similar to HIV-1 Rev binding protein  Info   LOC363266 
   397 2e-07 74.3 2.5 29.72  1369766_at  prostaglandin E receptor 2, subtype EP2  Info   Ptger2 
   398 2e-07 269.7 70 3.853  1369103_at  fyn proto-oncogene  Info   Fyn 
   399 2e-07 52.2 113.9 0.458  1374574_at  Similar to hypothetical protein MGC38960 (predicted)  Info    
   400 2e-07 2013.2 834.4 2.413  1370340_x_at  tropomyosin 3, gamma  Info   Tpm3 
   401 2e-07 372 76.6 4.856  1371146_at  transcription factor EC  Info   Tfec 
   402 2e-07 516.7 91.3 5.659  1395184_at    Info    
   403 2e-07 814.1 148.3 5.49  1370445_at  phosphatidylserine-specific phospholipase A1  Info   RGD:621261 
   404 2e-07 343 59.6 5.755  1379275_at  sorting nexin 10 (predicted)  Info   Snx10_predicted 
   405 2e-07 2262.5 4751.3 0.476  1387034_at  phenylalanine hydroxylase  Info   Pah 
   406 2e-07 354.9 73.5 4.829  1381875_at  N-myc (and STAT) interactor (predicted)  Info   Nmi_predicted 
   407 2e-07 2721.2 318.8 8.536  1374033_at  proteasome (prosome, macropain) subunit, beta type 10 (predicted)  Info   Psmb10_predicted 
   408 2e-07 595.4 105.3 5.654  1385641_at  Transcribed locus, strongly similar to NP_780521.1 hypothetical protein LOC101148 [Mus musculus]  Info    
   409 2e-07 124.4 224.6 0.554  1372953_at  neurocalcin delta  Info   Ncald 
   410 2e-07 384.1 114.4 3.358  1396268_at  SP110 nuclear body protein (predicted)  Info    
   411 2e-07 201.4 53.3 3.779  1397866_at  serine (or cysteine) proteinase inhibitor, clade B, member 6b (predicted)  Info   Serpinb6b_predicted 
   412 2e-07 611 34.9 17.507  1392171_at  chitinase 3-like 1  Info   Chi3l1 
   413 2e-07 161.3 427.1 0.378  1368765_at  chloride channel K1  Info   Clcnk1 
   414 2e-07 341.6 718.5 0.475  1372328_at  kinesin-like 8 (predicted)  Info   Knsl8_predicted 
   415 2e-07 204.3 406.6 0.502  1373524_at  BCL2-associated transcription factor 1 (predicted)  Info    
   416 2e-07 374.7 111.7 3.355  1370585_a_at  protein kinase C, beta 1  Info   Prkcb1 
   417 2e-07 135 531.6 0.254  1374172_at  Procollagen, type VIII, alpha 2 (predicted)  Info    
   418 2e-07 1421.1 842.9 1.686  1371445_at  similar to ribosome-binding protein p34 - rat (predicted)  Info   RGD1305092_predicted 
   419 2e-07 315.6 97.5 3.237  1368027_at  thromboxane A synthase 1  Info   Tbxas1 
   420 2e-07 3288.8 367.7 8.944  1371123_x_at  RT1 class Ib, locus S3  Info   RT1-S3 
   421 2e-07 335.1 47.1 7.115  1377023_at  dual specificity phosphatase 2 (predicted)  Info   Dusp2_predicted 
   422 2e-07 626.4 1143.5 0.548  1389622_at  similar to citrin  Info   LOC362322 
   423 2e-07 401.1 65.4 6.133  1384298_at  myosin IF (predicted)  Info   Myo1f_predicted 
   424 2e-07 1132.3 1628.1 0.695  1375220_at  RE70703p-like  Info   RGD:1303292 
   425 3e-07 330.1 158.4 2.084  1376130_a_at  dystrobrevin, beta (predicted)  Info   Dtnb_predicted 
   426 3e-07 427.2 45.3 9.43  1368565_at  solute carrier family 1 (glial high affinity glutamate transporter), member 3  Info   Slc1a3 
   427 3e-07 1285.5 2277 0.565  1398296_at  membrane interacting protein of RGS16  Info   Mir16 
   428 3e-07 224.9 517.8 0.434  1372111_at    Info    
   429 3e-07 295.6 592.6 0.499  1386773_at  biotinidase (predicted)  Info   Btd_predicted 
   430 3e-07 1177.3 191.2 6.157  1388203_x_at  RT1 class Ib, locus Aw2 /// RT1 class I, CE5 /// RT1 class I, CE2 /// RT1 class I, CE15 /// RT1 class I, CE10  Info   RT1-Aw2 /// RT1-CE5 /// RT1-CE2 /// RGD:1359630 /// RGD:1359292 
   431 3e-07 237.9 106 2.244  1376275_at  Transcribed locus  Info    
   432 3e-07 708.2 101.5 6.977  1383424_at  thymidylate kinase family LPS-inducible member (predicted)  Info   Tyki_predicted 
   433 3e-07 255.3 74.5 3.427  1373683_at  fyn proto-oncogene  Info   Fyn 
   434 3e-07 540.6 277.9 1.945  1376880_at  similar to T2K protein kinase homolog  Info   LOC299827 
   435 3e-07 1064.8 142.8 7.457  1384180_at  interferon-induced protein with tetratricopeptide repeats 2 (predicted)  Info   Ifit2_predicted 
   436 3e-07 1091.1 1637.9 0.666  1371576_at  mitochondrial ribosomal protein S36 (predicted)  Info   Mrps36_predicted 
   437 3e-07 1617.9 3058.2 0.529  1392597_at    Info    
   438 3e-07 85.7 316 0.271  1390285_at  similar to BC026645 protein  Info   LOC309112 
   439 3e-07 5182.1 1099.4 4.714  1370822_at  RT1 class II, locus Ba  Info   RT1-Ba 
   440 3e-07 280.1 643.9 0.435  1388526_at  Transcribed locus  Info    
   441 3e-07 426.4 221.1 1.929  1367943_at  nuclear factor of kappa light chain gene enhancer in B-cells inhibitor, beta  Info   Nfkbib 
   442 3e-07 2922.1 5174.5 0.565  1388300_at  microsomal glutathione S-transferase 3 (predicted)  Info   Mgst3_predicted 
   443 3e-07 1947.1 260.8 7.466  1376144_at  similar to B aggressive lymphoma (predicted)  Info   RGD1307534_predicted 
   444 3e-07 3482.4 1754.2 1.985  1390022_at  actin related protein 2/3 complex, subunit 5 (predicted)  Info   Arpc5_predicted 
   445 3e-07 949.9 263.9 3.599  1387048_at  nuclear RNA helicase, DECD variant of DEAD box family  Info   Ddx39 
   446 3e-07 1774.2 181.4 9.781  1374718_at  Similar to deltex 3-like  Info    
   447 3e-07 422.9 797 0.531  1395411_at  nicotinate phosphoribosyltransferase-like protein  Info   RGD:1302945 
   448 3e-07 1173.9 624.5 1.88  1367837_at  proteasome (prosome, macropain) subunit, alpha type 4  Info   Psma4 
   449 3e-07 158 46.5 3.398  1383709_at  Transcribed locus  Info    
   450 3e-07 138.9 79.5 1.747  1379987_at  similar to RIKEN cDNA 6330416G13 gene (predicted)  Info   RGD1304595_predicted 
   451 3e-07 328.8 137.5 2.391  1367738_at  UNC-119 homolog (C. elegans)  Info   Unc119 
   452 3e-07 255.6 112 2.282  1370443_at  deoxyribonuclease II  Info   Dnase2 
   453 3e-07 2370.9 243.2 9.749  1388213_a_at  RT1 class Ib, locus S3  Info   RT1-S3 
   454 3e-07 634.3 93.7 6.769  1384946_at    Info    
   455 3e-07 1084.6 485.4 2.234  1389387_at  CDNA clone MGC:93913 IMAGE:7112877  Info    
   456 3e-07 1650.1 208.5 7.914  1383189_at  Transcribed locus  Info    
   457 3e-07 224.2 481 0.466  1373057_at  ProSAPiP1 protein  Info   ProSAPiP1 
   458 3e-07 1437 80 17.962  1378193_at  membrane-spanning 4-domains, subfamily A, member 7 (predicted)  Info   Ms4a7_predicted 
   459 3e-07 928.8 2016.4 0.461  1389107_at  Transcribed locus, weakly similar to XP_236385.3 PREDICTED: similar to junction-associated coiled-coil protein [Rattus norvegicus]  Info    
   460 3e-07 339.8 592.5 0.574  1371604_at  mitochondrial ribosomal protein L34  Info   RGD:1359397 
   461 3e-07 5358.6 771.6 6.945  1368835_at  signal transducer and activator of transcription 1  Info   Stat1 
   462 3e-07 386.7 86.5 4.471  1367825_at  ral guanine nucleotide dissociation stimulator  Info   Ralgds 
   463 3e-07 533.1 1188.9 0.448  1374705_at  similar to type IV collagen alpha 5 chain  Info   LOC363457 
   464 3e-07 529.1 168.9 3.133  1376056_at  similar to poly (ADP-ribose) polymerase family, member 10  Info   LOC300043 
   465 3e-07 596.7 303.8 1.964  1378137_at  similar to ribosome-binding protein p34 - rat (predicted)  Info   RGD1305092_predicted 
   466 3e-07 166.5 76.5 2.176  1397904_at  Similar to osteoclast inhibitory lectin  Info    
   467 3e-07 2399.2 3834.9 0.626  1367589_at  aconitase 2, mitochondrial  Info   Aco2 
   468 3e-07 418.4 147.1 2.844  1374670_at  similar to hypothetical protein MGC20700 (predicted)  Info   RGD1307722_predicted 
   469 3e-07 3324.8 2089.6 1.591  1388351_at  similar to chromosome 13 open reading frame 12 (predicted)  Info   RGD1305831_predicted 
   470 3e-07 758.2 1335.1 0.568  1388570_at  similar to RIKEN cDNA 2310005O14  Info   LOC498909 
   471 3e-07 672.2 1188.9 0.565  1372741_at  similar to RIKEN cDNA C330023F11 (predicted)  Info   RGD1311440_predicted 
   472 3e-07 82.6 218.9 0.377  1368282_at  dipeptidase 1 (renal)  Info   Dpep1 
   473 3e-07 621.1 181.4 3.424  1371256_at  protein tyrosine phosphatase, non-receptor type 18 (predicted)  Info   Ptpn18_predicted 
   474 3e-07 625.7 1370.5 0.457  1372613_at  dehydrogenase/reductase (SDR family) member 6 (predicted)  Info   Dhrs6_predicted 
   475 3e-07 3122.1 895.4 3.487  1383241_at  complement component 1, r subcomponent (predicted)  Info   C1r_predicted 
   476 3e-07 139.3 30.4 4.582  1390507_at  interferon-stimulated protein (predicted)  Info   Isg20_predicted 
   477 3e-07 595.8 223.4 2.667  1389365_at  Similar to RIKEN cDNA 1600014C10  Info    
   478 3e-07 116.6 36.8 3.168  1383684_at  ASF1 anti-silencing function 1 homolog B (S. cerevisiae) (predicted)  Info   Asf1b_predicted 
   479 3e-07 463.3 38.7 11.972  1374469_at  Similar to C20orf118  Info    
   480 3e-07 551.5 236.7 2.33  1389355_at  immediate early response 5  Info   Ier5 
   481 3e-07 304.6 176.1 1.73  1383748_at  Unknown (protein for MGC:72598)  Info   RGD:735063 
   482 3e-07 156.1 12.4 12.589  1377110_at  similar to ADP-ribosylation-like factor 12 protein  Info   LOC497990 
   483 3e-07 307.9 80.1 3.844  1383655_at  TNFAIP3 interacting protein 2 (predicted)  Info   Tnip2_predicted 
   484 3e-07 3726.4 255.8 14.568  1367786_at  proteosome (prosome, macropain) subunit, beta type 8  Info   Psmb8 
   485 3e-07 59.4 209.5 0.284  1377635_at  flavin containing monooxygenase 2  Info   Fmo2 
   486 3e-07 270 44 6.136  1374284_at  Ras association (RalGDS/AF-6) domain family 4 (predicted)  Info   Rassf4_predicted 
   487 3e-07 410.1 161.6 2.538  1382283_at  Wiskott-Aldrich syndrome protein interacting protein  Info   Waspip 
   488 3e-07 122.5 330.6 0.371  1387232_at  bone morphogenetic protein 4  Info   Bmp4 
   489 3e-07 140.8 11.7 12.034  1390849_at  Transcribed locus  Info    
   490 3e-07 1353.8 730.5 1.853  1388596_at  coactosin-like 1 (Dictyostelium) (predicted)  Info   Cotl1_predicted 
   491 3e-07 1468.8 712 2.063  1369979_at  src family associated phosphoprotein 2  Info   Scap2 
   492 3e-07 2176 419.6 5.186  1367974_at  annexin A3  Info   Anxa3 
   493 3e-07 4470.8 2554.1 1.75  1387856_at  calponin 3, acidic  Info   Cnn3 
   494 3e-07 513.2 200.6 2.558  1373912_at  Transcribed locus, weakly similar to XP_488820.1 PREDICTED: hypothetical protein XP_488820 [Mus musculus]  Info    
   495 3e-07 1965.4 100.5 19.556  1369031_at  interferon gamma inducing factor binding protein  Info   Il18bp 
   496 4e-07 1522 2011.6 0.757  1388160_a_at  isocitrate dehydrogenase 3 (NAD+) beta  Info   Idh3B 
   497 4e-07 4785.5 1709.4 2.8  1388347_at  similar to lymphocyte antigen 6 complex, locus E  Info   LOC362934 
   498 4e-07 493.9 300.1 1.646  1371925_at  ATPase type 13A (predicted)  Info   Atp13a_predicted 
   499 4e-07 1119.5 2383.9 0.47  1388175_at  chloride channel Kb  Info   Clcnkb 
   500 4e-07 1802.4 100.3 17.97  1368482_at  B-cell leukemia/lymphoma 2 related protein A1  Info   Bcl2a1 
   501 4e-07 1798 844.2 2.13  1398303_s_at  tropomyosin 3, gamma  Info   Tpm3 
   502 4e-07 825.4 1682.7 0.491  1371948_at  Down syndrome critical region gene 5 (predicted)  Info   Dscr5_predicted 
   503 4e-07 87.6 33.3 2.631  1376775_at  similar to RIKEN cDNA 2600010E01 (predicted)  Info   RGD1309969_predicted 
   504 4e-07 113.5 36 3.153  1369737_at  cAMP responsive element modulator  Info   Crem 
   505 4e-07 2672.5 856.7 3.12  1387946_at  lectin, galactoside-binding, soluble, 3 binding protein  Info   Lgals3bp 
   506 4e-07 781.8 251.3 3.111  1375955_at  zinc finger protein 313  Info   RGD:1303139 
   507 4e-07 195.1 77.3 2.524  1395697_at  similar to Ezh2 protein  Info   LOC312299 
   508 4e-07 489.7 1172.7 0.418  1374478_at  similar to RIKEN cDNA 2610528J11 (predicted)  Info   RGD1305347_predicted 
   509 4e-07 315.6 99.9 3.159  1374627_at  Transcribed locus  Info    
   510 4e-07 200.8 121.8 1.649  1389222_at  Tryptophan hydroxylase  Info   Tph 
   511 4e-07 463.5 187.4 2.473  1392958_at  similar to cylindromatosis (turban tumor syndrome)  Info   LOC498035 
   512 4e-07 132 239.2 0.552  1385088_at  hypothetical LOC304650 (predicted)  Info   RGD1310262_predicted 
   513 4e-07 881.5 255.3 3.453  1373897_at    Info    
   514 4e-07 431 169.6 2.541  1387502_at  serine/threonine kinase 17b (apoptosis-inducing)  Info   Stk17b 
   515 4e-07 832.2 98.8 8.423  1379613_at  Similar to hypothetical protein MGC29390 (predicted)  Info    
   516 4e-07 1051.8 2320.1 0.453  1367908_at  glycine cleavage system protein H (aminomethyl carrier)  Info   Gcsh 
   517 4e-07 1936.9 321.9 6.017  1389553_at  dendritic cell inhibitory receptor 3  Info   RGD:1359528 
   518 4e-07 592.9 32.8 18.076  1393187_at  Similar to Ifi204 protein  Info    
   519 4e-07 229.2 25.7 8.918  1370621_at  CD3 antigen, zeta polypeptide  Info   Cd3z 
   520 4e-07 191.3 423.5 0.452  1381124_at  Solute carrier family 25 (mitochondrial carrier; ornithine transporter) member 15 (predicted)  Info    
   521 4e-07 286.3 508.9 0.563  1374129_at  similar to product is unknown~seizure-related gene  Info   LOC501454 
   522 4e-07 1257.7 538.8 2.334  1393987_s_at  G protein-coupled receptor kinase 6  Info   Gprk6 
   523 4e-07 2920.7 973 3.002  1370249_at  benzodiazepin receptor  Info   Bzrp 
   524 4e-07 1324.8 195.2 6.787  1374730_at  Tyro protein tyrosine kinase binding protein  Info   Tyrobp 
   525 4e-07 3365.3 446.8 7.532  1388212_a_at  RT1 class Ib, locus S3  Info   RT1-S3 
   526 4e-07 1810.5 2569.6 0.705  1387878_at  glutamate dehydrogenase 1  Info   Glud1 
   527 4e-07 430.7 932.4 0.462  1388537_at  4-nitrophenylphosphatase domain and non-neuronal SNAP25-like protein homolog 1 (C. elegans) (predicted)  Info   Nipsnap1_predicted 
   528 4e-07 511.1 15.4 33.188  1392496_at  Trypsin V-A  Info   LOC312273 
   529 4e-07 120.7 520.3 0.232  1379452_at  similar to growth arrest-specific protein 2 - mouse  Info   LOC499156 
   530 4e-07 1700.9 115.3 14.752  1387134_at  schlafen 3  Info   Slfn3 
   531 4e-07 367 165.3 2.22  1389867_at  CUG triplet repeat,RNA-binding protein 2  Info   Cugbp2 
   532 4e-07 158.3 268.5 0.59  1381268_at  Forkhead box I1 (predicted)  Info    
   533 4e-07 470.1 210.9 2.229  1368291_at  baculoviral IAP repeat-containing 2  Info   Birc2 
   534 4e-07 978.4 405 2.416  1372009_at  tyrosyl-tRNA synthetase (predicted)  Info   Yars_predicted 
   535 4e-07 964.3 414.3 2.328  1373065_at  protein tyrosine phosphatase, non-receptor type 18 (predicted)  Info   Ptpn18_predicted 
   536 4e-07 1069.9 121 8.842  1382311_at  TRAF2 binding protein  Info   RGD:1359151 
   537 4e-07 523.2 34.6 15.121  1389997_at  CD3 antigen, epsilon polypeptide (predicted)  Info   Cd3e_predicted 
   538 4e-07 105.9 55.3 1.915  1390249_at  similar to DKFZP434H132 protein (predicted)  Info   RGD1305464_predicted 
   539 4e-07 196.4 487.2 0.403  1375856_at  Transcribed locus  Info    
   540 4e-07 254.9 32.2 7.916  1385434_at  Prostaglandin E receptor 2, subtype EP2  Info   Ptger2 
   541 4e-07 4024.4 1666.3 2.415  1392334_at  RT1 class II, locus Ba  Info   RT1-Ba 
   542 4e-07 63.1 157 0.402  1392935_at  Transcribed locus  Info    
   543 4e-07 132 24.9 5.301  1384778_at  chymotrypsin C (caldecrin) (predicted)  Info   Ctrc_predicted 
   544 4e-07 593.4 445.4 1.332  1398987_at  suppressor of Ty 4 homolog 2 (S. cerevisiae) (predicted)  Info   Supt4h2_predicted 
   545 4e-07 2679.2 4963.4 0.54  1370436_at  kidney-specific protein (KS)  Info   RGD:708383 
   546 4e-07 188.5 504.3 0.374  1380905_at  Transcribed locus, weakly similar to NP_083358.1 hypothetical protein LOC74742 [Mus musculus]  Info    
   547 4e-07 146.9 578.5 0.254  1376944_at  Prolactin receptor  Info   Prlr 
   548 4e-07 324.9 456.5 0.712  1386927_at  carnitine palmitoyltransferase 2  Info   Cpt2 
   549 4e-07 2868.1 152.5 18.807  1396163_at  interferon gamma induced GTPase (predicted)  Info   Igtp_predicted 
   550 4e-07 1388.1 2251.2 0.617  1377375_at  aminoadipate-semialdehyde synthase (predicted)  Info   Aass_predicted 
   551 4e-07 1300 273.4 4.755  1369726_at  TAP binding protein  Info   Tapbp 
   552 4e-07 328.3 35.6 9.222  1382902_at  potential ubiquitin ligase  Info   Herc6 
   553 4e-07 786.6 289.5 2.717  1377016_at  similar to RIKEN cDNA 5730592L21 (predicted)  Info   RGD1310614_predicted 
   554 4e-07 536.8 135.5 3.962  1372004_at  heme binding protein 1 (predicted)  Info   Hebp1_predicted 
   555 4e-07 316.7 84 3.77  1375716_at  interferon gamma receptor 2 (predicted)  Info   Ifngr2_predicted 
   556 4e-07 375.6 184.2 2.039  1380021_at  similar to 2010308M01Rik protein (predicted)  Info   RGD1305120_predicted 
   557 4e-07 1188.6 315.4 3.769  1372303_at  similar to 0910001A06Rik protein (predicted)  Info   RGD1307697_predicted 
   558 4e-07 5437.3 1835.1 2.963  1388337_at  nucleoside phosphorylase  Info   Np 
   559 4e-07 169.4 260.7 0.65  1371466_at  similar to RIKEN cDNA 0610009B22 (predicted) /// similar to Sedlin (Trafficking protein particle complex protein 2)  Info   RGD1306925_predicted /// LOC501550 
   560 4e-07 97.5 2.4 40.625  1387835_at  interleukin 1 receptor antagonist  Info   Il1rn 
   561 4e-07 50.2 147.7 0.34  1374329_at  Mcf.2 transforming sequence-like  Info   Mcf2l 
   562 4e-07 3079 297.2 10.36  1367973_at  chemokine (C-C motif) ligand 2  Info   Ccl2 
   563 4e-07 297.1 610.1 0.487  1374512_at  Transcribed locus, strongly similar to XP_214823.2 PREDICTED: similar to Blvrb protein [Rattus norvegicus]  Info    
   564 4e-07 1061 736.8 1.44  1389140_at  similar to RIKEN cDNA 5730406I15 (predicted)  Info   RGD1311253_predicted 
   565 4e-07 430.3 226.5 1.9  1377750_at  Rho guanine nucleotide exchange factor (GEF) 3 (predicted)  Info    
   566 4e-07 1095.5 228 4.805  1386908_at  glutaredoxin 1 (thioltransferase)  Info   Glrx1 
   567 5e-07 5985.4 676 8.854  1387354_at  signal transducer and activator of transcription 1  Info   Stat1 
   568 5e-07 148.5 67.6 2.197  1387495_at  transducin-like enhancer of split 4, E(spl) homolog (Drosophila)  Info   Tle4 
   569 5e-07 810.3 61.9 13.09  1389034_at  similar to ubiquitin specific protease UBP43  Info   RGD:1359153 
   570 5e-07 1273.5 154.7 8.232  1398390_at  similar to Small inducible cytokine B13 precursor (CXCL13) (B lymphocyte chemoattractant) (CXC chemokine BLC)  Info   LOC498335 
   571 5e-07 748.3 61.8 12.108  1392737_at  Transcribed locus  Info    
   572 5e-07 304.9 83.5 3.651  1368066_at  BCL2-antagonist/killer 1  Info   Bak1 
   573 5e-07 388.1 128.2 3.027  1377286_at  GTPase, IMAP family member 4  Info   Gimap4 
   574 5e-07 1742.8 997.2 1.748  1374387_at  ADP-ribosylation factor-like 6 interacting protein 5  Info   Arl6ip5 
   575 5e-07 1214.4 351.5 3.455  1372256_at    Info    
   576 5e-07 399.2 55.2 7.232  1369520_a_at  branched chain aminotransferase 1, cytosolic  Info   Bcat1 
   577 5e-07 12.2 1.2 10.167  1378520_at  B-cell leukemia/lymphoma 11B (predicted)  Info   Bcl11b_predicted 
   578 5e-07 165.1 42.4 3.894  1378914_a_at  hypothetical protein LOC308990  Info   LOC308990 
   579 5e-07 927.8 373.6 2.483  1388784_at  colony stimulating factor 1 receptor  Info   Csf1r 
   580 5e-07 153.1 295.6 0.518  1372276_at  Transcribed locus  Info    
   581 5e-07 2994 1164.1 2.572  1372013_at  interferon induced transmembrane protein 1 (predicted)  Info   Ifitm1_predicted 
   582 5e-07 58.4 181.4 0.322  1389084_at    Info    
   583 5e-07 2061.4 3059.4 0.674  1370855_at  cystatin C  Info   Cst3 
   584 5e-07 2.7 23.6 0.114  1368894_at  CAP, adenylate cyclase-associated protein, 2 (yeast)  Info   Cap2 
   585 5e-07 484.8 786.3 0.617  1385889_at  Similar to hypothetical protein MGC52110  Info    
   586 5e-07 110.2 227.1 0.485  1369873_at  arginine vasopressin receptor 2  Info   Avpr2 
   587 5e-07 788.8 1399.8 0.564  1389147_at  Transcribed locus  Info    
   588 5e-07 2131.9 1271.2 1.677  1372513_at  ras-related C3 botulinum toxin substrate 1  Info   Rac1 
   589 5e-07 88.1 374.1 0.235  1387672_at  glycine N-methyltransferase  Info   Gnmt 
   590 5e-07 838 106.5 7.869  1381228_at  F-box protein 39 (predicted)  Info    
   591 5e-07 16.7 122.8 0.136  1383783_at  protocadherin 9 (predicted)  Info   Pcdh9_predicted 
   592 5e-07 3745.7 2527.5 1.482  1371511_at  actin related protein 2/3 complex, subunit 2 (predicted)  Info   Arpc2_predicted 
   593 5e-07 2932.5 315 9.31  1367850_at  Fc gamma receptor II beta  Info   LOC498276 
   594 5e-07 306.8 112 2.739  1370539_at  RAB8B, member RAS oncogene family  Info   Rab8b 
   595 5e-07 1082.6 1402.3 0.772  1388822_at  short coiled-coil protein (predicted) /// similar to short coiled-coil protein  Info   Scoc_predicted /// LOC498931 
   596 5e-07 2750.8 153.7 17.897  1382314_at  interferon, alpha-inducible protein (clone IFI-15K) (predicted)  Info   G1p2_predicted 
   597 5e-07 363.3 90.1 4.032  1383516_at  fibrinogen-like 2  Info   Fgl2 
   598 5e-07 479.6 10.8 44.407  1370790_at  interferon gamma  Info   Ifng 
   599 5e-07 3173.8 6086.1 0.521  1368188_at  4-hydroxyphenylpyruvic acid dioxygenase  Info   Hpd 
   600 5e-07 1735.7 396.9 4.373  1382043_at  unc-93 homolog B (C. elegans) (predicted)  Info   Unc93b_predicted 
   601 5e-07 3775.2 501 7.535  1398246_s_at  Fc receptor, IgG, low affinity III /// Fc gamma receptor II beta  Info   Fcgr3 /// LOC498276 
   602 5e-07 402 76.9 5.228  1379496_at  Transcribed locus  Info    
   603 5e-07 488 201.4 2.423  1398756_at  nucleophosmin 1 /// similar to Nucleophosmin (NPM) (Nucleolar phosphoprotein B23) (Numatrin) (Nucleolar protein NO38)  Info   Npm1 /// LOC300303 
   604 5e-07 2078.5 2565.3 0.81  1371605_at  similar to RIKEN cDNA 2410011G03 (predicted)  Info   RGD1311462_predicted 
   605 5e-07 3973.3 2162.4 1.837  1369936_at  calmodulin 1  Info   Calm1 
   606 5e-07 1384.8 182.3 7.596  1368490_at  CD14 antigen  Info   Cd14 
   607 5e-07 870.4 456.2 1.908  1367790_at  staphylococcal nuclease domain containing 1  Info   Snd1 
   608 5e-07 423.8 1131.9 0.374  1370367_at  solute carrier family 1 (neuronal/epithelial high affinity glutamate transporter, system Xag), member 1  Info   Slc1a1 
   609 5e-07 150.2 84.1 1.786  1392531_at  Transcribed locus  Info    
   610 5e-07 118.5 56 2.116  1387280_a_at  tumor-associated protein 1  Info   Slc7a5 
   611 5e-07 158.9 454.1 0.35  1372323_at  sarcosine dehydrogenase  Info   Sardh 
   612 5e-07 203.9 389.1 0.524  1374657_at  Transcribed locus  Info    
   613 5e-07 131.7 260.8 0.505  1383420_at  Similar to lactation elevated 1  Info    
   614 5e-07 989.2 322.6 3.066  1376006_at  Transcribed locus  Info    
   615 5e-07 11.1 37.1 0.299  1369425_at  cadherin 13  Info   Cdh13 
   616 5e-07 1785.7 3438 0.519  1371922_at  Transcribed locus  Info    
   617 5e-07 597.1 287.2 2.079  1389093_at    Info    
   618 5e-07 874 102.3 8.543  1374337_at  Similar to chromosome 17 open reading frame 27 (predicted)  Info    
   619 5e-07 100.3 14.4 6.965  1398644_at  Transcribed locus  Info    
   620 5e-07 1378.8 2274.1 0.606  1371483_at  nicotinamide nucleotide transhydrogenase  Info   Nnt 
   621 5e-07 205.2 49.5 4.145  1369794_a_at  6-phosphofructo-2-kinase/fructose-2,6-biphosphatase 3  Info   Pfkfb3 
   622 5e-07 369.9 73.4 5.04  1394819_at  SP110 nuclear body protein (predicted)  Info    
   623 5e-07 124.9 214.6 0.582  1397652_at  Transcribed locus  Info    
   624 5e-07 2085.5 1371.3 1.521  1383478_at  Janus kinase 1  Info   Jak1 
   625 5e-07 491 55.2 8.895  1382601_at  Similar to scavenger receptor type A SR-A  Info    
   626 6e-07 277.2 102.7 2.699  1392528_at  Granulocyte-macrophage colony stimulating receptor alpha (CSFR alpha gene)  Info    
   627 6e-07 352.8 832.9 0.424  1389645_at  proline dehydrogenase (oxidase) 2 (predicted)  Info   Prodh2_predicted 
   628 6e-07 695.4 48.3 14.398  1379698_at  Transcribed locus  Info    
   629 6e-07 326.7 72.1 4.531  1390226_at  similar to hypothetical protein LOC340061  Info   LOC498840 
   630 6e-07 868.4 401 2.166  1370314_at  solute carrier family 20 (phosphate transporter), member 1  Info   Slc20a1 
   631 6e-07 359.9 207.9 1.731  1389686_at  Hypothetical LOC363470  Info    
   632 6e-07 101.7 41 2.48  1389379_at  DNA cross-link repair 1C, PSO2 homolog (S. cerevisiae)  Info   Mt1a 
   633 6e-07 1014 380.8 2.663  1370463_x_at  RT1 class I, CE16  Info   RGD:1359403 
   634 6e-07 234.8 64.9 3.618  1379563_at  interferon regulatory factor 5 (predicted)  Info   Irf5_predicted 
   635 6e-07 46.5 132.1 0.352  1384603_at  ATP-binding cassette, sub-family A (ABC1), member 4 (predicted)  Info   Abca4_predicted 
   636 6e-07 337 183.7 1.835  1367745_at  diacylglycerol kinase zeta  Info   Dgkz 
   637 6e-07 1178.5 65 18.131  1382622_at  cystatin F (leukocystatin) (predicted)  Info   Cst7_predicted 
   638 6e-07 340 265.5 1.281  1389432_at  pre-B-cell leukemia transcription factor 2  Info   RGD:1303084 
   639 6e-07 797.6 63.1 12.64  1382546_at  PHD finger protein 11 (predicted)  Info   Phf11_predicted 
   640 6e-07 157.5 351.2 0.448  1395268_at  WW domain containing E3 ubiquitin protein ligase 1 (predicted)  Info   Wwp1_predicted 
   641 6e-07 1171.8 1644.8 0.712  1388361_at    Info    
   642 6e-07 127.4 204.7 0.622  1369070_at  peroxisomal biogenesis factor 12  Info   Pex12 
   643 6e-07 212.6 83.3 2.552  1367919_at  nuclear pore membrane glycoprotein 210  Info   Pom210 
   644 6e-07 2644.1 175.6 15.058  1377950_at  similar to interferon-inducible GTPase (predicted)  Info   RGD1309362_predicted 
   645 6e-07 325.9 226.7 1.438  1378080_at  similar to SCO cytochrome oxidase deficient homolog 1 (yeast)  Info   LOC497930 
   646 6e-07 735.8 68.8 10.695  1396262_at  pre-B-cell colony enhancing factor 1  Info   Pbef1 
   647 6e-07 586.3 161.2 3.637  1373757_at  FLN29 gene product  Info   Fln29 
   648 6e-07 469.9 117.6 3.996  1382074_at  IBR domain containing 3 (predicted)  Info   Ibrdc3_predicted 
   649 6e-07 140 243.7 0.574  1398491_at  Transcribed locus, strongly similar to NP_598870.1 immunoglobulin-like domain containing receptor 1 [Mus musculus]  Info    
   650 6e-07 41.7 2 20.85  1370138_at  lymphoid enhancer binding factor 1  Info   Lef1 
   651 6e-07 64.6 180.7 0.357  1391629_at  hypothetical LOC362677  Info   LOC362677 
   652 6e-07 334.1 155.7 2.146  1388309_at  high mobility group AT-hook 1  Info   Hmga1 
   653 6e-07 201 343.8 0.585  1374749_at    Info    
   654 6e-07 19.9 31.9 0.624  1381054_at  similar to KIAA0980 protein (predicted)  Info   RGD1306152_predicted 
   655 6e-07 3474 1844.1 1.884  1370339_at  Tropomyosin 3, splice variant 1  Info   Tpm3 
   656 6e-07 440 123.3 3.569  1385926_at  Transcribed locus  Info    
   657 6e-07 756 65.4 11.56  1370869_at  branched chain aminotransferase 1, cytosolic  Info   Bcat1 
   658 6e-07 875.2 65.7 13.321  1379381_at  MHC class II transactivator  Info   C2ta 
   659 6e-07 1097.4 2514.9 0.436  1370960_at  insulin-like growth factor binding protein 5  Info   Igfbp5 
   660 6e-07 70.7 140.1 0.505  1370047_at  ectonucleotide pyrophosphatase/phosphodiesterase 1  Info   Enpp1 
   661 6e-07 258.5 120.5 2.145  1368379_at  CD36 antigen (collagen type I receptor, thrombospondin receptor)-like 2  Info   Scarb2 
   662 6e-07 5187.2 2195.4 2.363  1372254_at  serine (or cysteine) peptidase inhibitor, clade G, member 1  Info   Serping1 
   663 6e-07 492.6 944.7 0.521  1371976_at  similar to RIKEN cDNA 9530058B02  Info   LOC497875 
   664 6e-07 772.6 1302.4 0.593  1377576_at  similar to RIKEN cDNA 1110013G13 (predicted)  Info   RGD1307965_predicted 
   665 6e-07 656.8 181.6 3.617  1383131_at  integrin beta 2 (predicted)  Info   Itgb2_predicted 
   666 6e-07 1789.9 220.1 8.132  1377916_at  schlafen 2 (predicted)  Info   Slfn2_predicted 
   667 6e-07 362.8 757.8 0.479  1368038_at  synaptojanin 2 binding protein  Info   Synj2bp 
   668 6e-07 1108.1 2036.3 0.544  1368566_a_at  MIPP65 protein  Info   RGD:621020 
   669 6e-07 390.8 113 3.458  1373262_at  Similar to 2310014H01Rik protein (predicted)  Info    
   670 6e-07 24 50.9 0.472  1395007_at  Transcribed locus  Info    
   671 7e-07 329.6 612.9 0.538  1376487_at  Rhesus blood group-associated C glycoprotein  Info   Rhcg 
   672 7e-07 1678.7 1051.7 1.596  1395426_at  similar to ubiquitin-conjugating enzyme E2 variant 1  Info   LOC499945 
   673 7e-07 3071.3 694.7 4.421  1376652_at  complement component 1, q subcomponent, alpha polypeptide (predicted)  Info   C1qa_predicted 
   674 7e-07 272.9 76.9 3.549  1398482_at  B-cell leukemia/lymphoma 3 (predicted)  Info   Bcl3_predicted 
   675 7e-07 4389.9 359.3 12.218  1368073_at  interferon regulatory factor 1  Info   Irf1 
   676 7e-07 381 727.5 0.524  1376573_at  RAB34, member of RAS oncogene family (predicted)  Info   Rab34_predicted 
   677 7e-07 1580 2583.9 0.611  1369630_at  adenosine kinase  Info   Adk 
   678 7e-07 187.8 18.3 10.262  1378377_at  dual adaptor for phosphotyrosine and 3-phosphoinositides 1 (predicted)  Info   Dapp1_predicted 
   679 7e-07 103.1 243.3 0.424  1374667_at  CDNA clone IMAGE:7302289  Info    
   680 7e-07 229.8 27.5 8.356  1369497_at  Robo-1  Info   LOC24906 
   681 7e-07 346.8 570.6 0.608  1373807_at  Vascular endothelial growth factor A  Info   Vegf 
   682 7e-07 766.5 56.8 13.495  1384787_at  CD3 antigen, gamma polypeptide (predicted)  Info   Cd3g_predicted 
   683 7e-07 99.3 250.2 0.397  1379418_at  LOC501008  Info   LOC501008 
   684 7e-07 927.3 2331.3 0.398  1367660_at  fatty acid binding protein 3  Info   Fabp3 
   685 7e-07 164.5 50.7 3.245  1370810_at  cyclin D2  Info   Ccnd2 
   686 7e-07 206 83.5 2.467  1395744_at  Transcribed locus, moderately similar to XP_579465.1 PREDICTED: DNA binding protein N5 [Rattus norvegicus]  Info    
   687 7e-07 171.7 61.4 2.796  1377744_at  Transcribed locus, moderately similar to XP_576460.1 PREDICTED: similar to hypothetical protein PB402898.00.0 [Rattus norvegicus]  Info    
   688 7e-07 359.7 635.8 0.566  1399130_at  similar to RIKEN cDNA 2610022G08  Info   LOC502782 
   689 7e-07 406 737.5 0.551  1368045_at  solute carrier family 31 (copper transporters), member 1  Info   RGD:620059 
   690 7e-07 500.9 235.8 2.124  1375901_at  DEAD (Asp-Glu-Ala-Asp) box polypeptide 21b (predicted)  Info   Ddx21b_predicted 
   691 7e-07 555.3 1047.5 0.53  1389114_at  similar to Hypothetical protein MGC59076 (predicted)  Info   RGD1309144_predicted 
   692 7e-07 401.1 89.6 4.477  1385832_s_at  Similar to RIKEN cDNA 1200013B08  Info    
   693 7e-07 894.9 473.3 1.891  1368385_a_at  growth factor receptor bound protein 2  Info   Grb2 
   694 7e-07 282.7 579.2 0.488  1376785_at  Synaptonemal complex protein 3  Info   Sycp3 
   695 7e-07 1258 2280.2 0.552  1367672_at  hydroxysteroid (17-beta) dehydrogenase 4  Info   Hsd17b4 
   696 7e-07 710.3 1333.4 0.533  1388463_at  putative secreted protein ZSIG11  Info   RGD:1359353 
   697 8e-07 394.8 127.2 3.104  1368134_a_at  interleukin 4 receptor  Info   Il4r 
   698 8e-07 480.5 1374.8 0.35  1382189_at  syndecan 2  Info   Sdc2 
   699 8e-07 1756.1 124.6 14.094  1383392_at  complement component 2  Info   C2 
   700 8e-07 375 224.9 1.667  1389041_at  vacuole 14 protein (S. cerevisiae)  Info   Vac14 
   701 8e-07 2337.1 1179.7 1.981  1372064_at  similar to chemokine (C-X-C motif) ligand 16  Info   Cxcl16 
   702 8e-07 233.9 89.9 2.602  1393373_at  similar to encephalopsin  Info   LOC498289 
   703 8e-07 1246 660.9 1.885  1382861_at  similar to transcription elongation factor IIS - mouse  Info   LOC498453 
   704 8e-07 158.8 531.8 0.299  1384302_at  solute carrier family 6 (neurotransmitter transporter), member 17  Info   Slc6a17 
   705 8e-07 1046.6 430 2.434  1373043_at    Info    
   706 8e-07 2007.8 761.9 2.635  1387918_at  tropomyosin 3, gamma  Info   Tpm3 
   707 8e-07 508.5 254.3 2  1374393_at  protein tyrosine phosphatase-like (proline instead of catalytic arginine), member b (predicted)  Info   Ptplb_predicted 
   708 8e-07 1434.3 467.3 3.069  1387776_at  transglutaminase 2, C polypeptide  Info   Tgm2 
   709 8e-07 3642.4 1394.9 2.611  1387076_at  hypoxia inducible factor 1, alpha subunit  Info   Hif1a 
   710 8e-07 1015 2130.2 0.476  1368059_at  crystallin, mu  Info   Crym 
   711 8e-07 362.3 515 0.703  1389154_at  lipoic acid synthetase (predicted)  Info   Lias_predicted 
   712 8e-07 154.2 36.8 4.19  1382803_at  mitogen activated protein kinase kinase kinase kinase 1 (predicted)  Info   Map4k1_predicted 
   713 8e-07 1509 861.3 1.752  1367485_at  transcription elongation factor A (SII) 1 (predicted) /// similar to transcription elongation factor IIS - mouse  Info   Tcea1_predicted /// LOC498453 
   714 8e-07 79.4 233.9 0.339  1373218_at  Similar to 6430514L14Rik protein (predicted)  Info    
   715 8e-07 201.3 42.3 4.759  1374165_at  similar to Rasa4 protein  Info   LOC288589 
   716 8e-07 2586.9 3918.3 0.66  1370180_at  nudix (nucleoside diphosphate linked moiety X)-type motif 4  Info   Nudt4 
   717 8e-07 494.4 890.1 0.555  1370358_at  two pore channel 1  Info   Tpcn1 
   718 8e-07 626.8 214 2.929  1367905_at  ectonucleotide pyrophosphatase/phosphodiesterase 3  Info   Enpp3 
   719 8e-07 465 131.3 3.542  1376920_at  similar to mKIAA2005 protein /// similar to mKIAA2005 protein /// similar to mKIAA2005 protein  Info   LOC500012 /// LOC500014 /// LOC500015 
   720 8e-07 198.5 68.5 2.898  1378700_at  Transcribed locus  Info    
   721 8e-07 53.7 268 0.2  1377018_at  Similar to E430002G05Rik protein (predicted)  Info    
   722 8e-07 2256.3 1144.3 1.972  1370890_at  ARP3 actin-related protein 3 homolog (yeast)  Info   Actr3 
   723 8e-07 789 381.5 2.068  1376700_at  Transcribed locus, moderately similar to XP_580018.1 PREDICTED: hypothetical protein XP_580018 [Rattus norvegicus]  Info    
   724 8e-07 9.9 35.9 0.276  1381504_at  similar to asporin precursor  Info   LOC306805 
   725 8e-07 206.6 377.3 0.548  1389498_at    Info    
   726 8e-07 328.5 48.2 6.815  1394673_at  similar to m33-A isoform  Info   LOC499142 
   727 8e-07 891.2 206.1 4.324  1380071_at  Transcribed locus  Info    
   728 8e-07 620.5 375.1 1.654  1372620_at  acidic (leucine-rich) nuclear phosphoprotein 32 family, member E (predicted)  Info   Anp32e_predicted 
   729 8e-07 421.6 237.3 1.777  1371631_at  similar to 2010003J03Rik protein (predicted)  Info   RGD1304686_predicted 
   730 8e-07 417.2 128.8 3.239  1371073_at  UDP-Gal:betaGlcNAc beta 1,4- galactosyltransferase, polypeptide 1  Info   B4galt1 
   731 8e-07 861.2 43.9 19.617  1382603_at  Programmed cell death 1 ligand 2 (predicted)  Info    
   732 8e-07 594.8 1328.6 0.448  1388948_at  START domain containing 10 (predicted)  Info   Stard10_predicted 
   733 8e-07 703.1 1451.2 0.484  1368575_at  solute carrier family 6 (neurotransmitter transporter), member 18  Info   Slc6a18 
   734 8e-07 119.1 26.9 4.428  1388026_at  CD3 antigen, zeta polypeptide  Info   Cd3z 
   735 9e-07 426.6 135.3 3.153  1392107_at  similar to KIAA0963 protein (predicted)  Info   RGD1307194_predicted 
   736 9e-07 494 1081.1 0.457  1382293_at    Info    
   737 9e-07 72.8 187.7 0.388  1389166_at  calcium and integrin binding family member 2 (predicted)  Info   Cib2_predicted 
   738 9e-07 84.9 211.7 0.401  1383422_at  similar to RIKEN cDNA 2310026E23  Info   LOC362564 
   739 9e-07 231.4 62.1 3.726  1397772_at  Similar to putative membrane steroid receptor (predicted)  Info    
   740 9e-07 592.1 244.3 2.424  1383158_at  similar to HIV-1 Rev binding protein  Info   LOC363266 
   741 9e-07 230.3 50.8 4.533  1373777_at  Transcribed locus  Info    
   742 9e-07 82.5 266.8 0.309  1383654_a_at  similar to fructosamine-3-kinase  Info   Fnsk 
   743 9e-07 343.7 753.8 0.456  1394228_at  Transcribed locus  Info    
   744 9e-07 937.8 444.9 2.108  1389227_at  ras homolog gene family, member G (predicted)  Info   Rhog_predicted 
   745 9e-07 45.8 82.8 0.553  1381282_at  similar to cDNA sequence BC026432 (predicted)  Info   RGD1308345_predicted 
   746 9e-07 473.7 1659.1 0.286  1386968_at  protein phosphatase 1, regulatory (inhibitor) subunit 1A  Info   Ppp1r1a 
   747 9e-07 148 10.8 13.704  1385590_at  Transcribed locus  Info    
   748 9e-07 927.4 136.1 6.814  1371078_at  RT1 class Ib, locus Aw2  Info   RT1-Aw2 
   749 9e-07 38.3 90.2 0.425  1391131_at  Transcribed locus  Info    
   750 9e-07 253.4 392.2 0.646  1374196_at  lanC (bacterial lantibiotic synthetase component C)-like 1  Info   Lancl1 
   751 9e-07 683.4 65.5 10.434  1390325_at  CD38 antigen  Info   Cd38 
   752 9e-07 777.5 21.6 35.995  1379791_at  CD3 antigen, epsilon polypeptide (predicted)  Info    
   753 9e-07 702.8 1174.7 0.598  1371967_at  mitochondrial ribosomal protein L16  Info   Mrpl16 
   754 9e-07 263.4 425.7 0.619  1383731_at  Similar to RIKEN cDNA 2310036D22  Info   MGC94135 
   755 9e-07 1238.3 151.8 8.157  1370080_at  heme oxygenase (decycling) 1  Info   Hmox1 
   756 9e-07 363.5 650 0.559  1377666_at  choline dehydrogenase  Info   Chdh 
   757 9e-07 318.1 85.8 3.707  1370682_at  paired-Ig-like receptor A2 (predicted) /// similar to PIRA5  Info   Pira2_predicted /// LOC365169 
   758 9e-07 494.2 1331.2 0.371  1393831_at  Transcribed locus  Info    
   759 9e-07 183.3 407.9 0.449  1374594_at  similar to RIKEN cDNA 1600029D21  Info   RGD:1359217 
   760 9e-07 1207.1 1958.4 0.616  1370200_at  glutamate dehydrogenase 1  Info   Glud1 
   761 9e-07 217.1 789.3 0.275  1389448_at  peanut-like 2 (Drosophila) (predicted)  Info   Pnutl2_predicted 
   762 9e-07 284.8 120.9 2.356  1370990_at  similar to cytokine receptor related protein 4  Info   RGD:621517 
   763 9e-07 166.2 234.6 0.708  1381174_at  similar to Hypothetical protein KIAA0141  Info   RGD:735029 
   764 9e-07 541.8 48.7 11.125  1370090_at  lymphocyte cytosolic protein 2  Info   Lcp2 
   765 9e-07 303.3 601.4 0.504  1370897_at  Branched chain ketoacid dehydrogenase E1, alpha polypeptide  Info   Bckdha 
   766 9e-07 4191.2 5842.8 0.717  1370918_a_at  ATP synthase, H+ transporting, mitochondrial F1 complex, gamma polypeptide 1  Info   Atp5c1 
   767 9e-07 189.4 374.1 0.506  1370197_a_at  protein kinase C, zeta  Info   Prkcz 
   768 9e-07 2929.2 1686.9 1.736  1371977_at  actin related protein 2/3 complex, subunit 3 (predicted)  Info   Arpc3_predicted 
   769 1e-06 124.4 248.7 0.5  1382227_at  1-acylglycerol-3-phosphate O-acyltransferase 3 (predicted)  Info    
   770 1e-06 3235.3 2227.9 1.452  1398849_at  H3 histone, family 3B  Info   RGD:621095 
   771 1e-06 424 168.9 2.51  1373611_at  interleukin 17 receptor (predicted)  Info   Il17r_predicted 
   772 1e-06 163.7 303.6 0.539  1373216_at    Info    
   773 1e-06 931.4 1519.9 0.613  1371696_at  G protein-coupled receptor 56  Info   Gpr56 
   774 1e-06 75.6 170.3 0.444  1393000_at    Info    
   775 1e-06 1466 928 1.58  1399043_at  capping protein (actin filament) muscle Z-line, alpha 2  Info   Capza2 
   776 1e-06 3853.1 751.3 5.129  1379344_at  Cytochrome b-245, beta polypeptide  Info   Cybb 
   777 1e-06 348.1 114.8 3.032  1369262_at  caspase 8  Info   Casp8 
   778 1e-06 347.3 697.2 0.498  1389403_at  bone morphogenetic protein 7  Info   Bmp7 
   779 1e-06 779 44.9 17.35  1376908_at  similar to This ORF is capable of encoding 404aa which is homologous to two human interferon-inducible proteins, 54 kDa and 56 kDa proteins; ORF  Info   RGD:1359681 
   780 1e-06 476.8 1492.8 0.319  1370166_at  syndecan 2  Info   Sdc2 
   781 1e-06 1390.3 210.3 6.611  1388255_x_at  RT1 class I, CE5  Info   RT1-CE5 
   782 1e-06 175.4 88.6 1.98  1375732_at  Transcribed locus  Info    
   783 1e-06 63 124.2 0.507  1375016_at  Transcribed locus  Info    
   784 1e-06 1714.8 1132.9 1.514  1367472_at  similar to ubiquitin-protein ligase (EC 6.3.2.19) E1 - mouse  Info   RGD:1359327 
   785 1e-06 229.6 447.5 0.513  1374190_at  citrate lyase beta like (predicted)  Info   Clybl_predicted 
   786 1e-06 578.7 24.4 23.717  1369877_at  CD8 antigen, alpha chain  Info   Cd8a 
   787 1e-06 226.1 151.8 1.489  1370460_at  ubiquitin specific protease 15  Info   Usp15 
   788 1e-06 223.7 69.8 3.205  1368375_a_at  interleukin 15  Info   Il15 
   789 1e-06 1055.2 1975 0.534  1368092_at  fumarylacetoacetate hydrolase  Info   Fah 
   790 1e-06 1864.5 1052.5 1.771  1388408_at  similar to RIKEN cDNA 1110020C13 (predicted)  Info   RGD1307129_predicted 
   791 1e-06 291.1 555.8 0.524  1387130_at  solute carrier family 39 (iron-regulated transporter), member 1  Info   Slc40a1 
   792 1e-06 257.4 156.1 1.649  1392910_at  block of proliferation 1 (predicted)  Info   Bop1_predicted 
   793 1e-06 475.4 121.3 3.919  1384525_at  similar to spleen specific 238 kDa protein with PH domain  Info   LOC313438 
   794 1e-06 1104.5 506.7 2.18  1372776_at  F-box and leucine-rich repeat protein 5 (predicted)  Info   Fbxl5_predicted 
   795 1e-06 257.3 94.4 2.726  1371184_x_at  Tropomyosin 3, splice variant 1  Info   Tpm3 
   796 1e-06 381.3 139.9 2.726  1378633_at  leupaxin (predicted)  Info   Lpxn_predicted 
   797 1e-06 227.7 610.8 0.373  1374307_at  CaM-kinase II inhibitor alpha  Info   LOC287005 
   798 1e-06 1630 2445.2 0.667  1386904_a_at  cytochrome b-5  Info   Cyb5 
   799 1e-06 760.1 427.7 1.777  1375635_at  Transcribed locus  Info    
   800 1e-06 2801.5 1110.5 2.523  1387806_at  RAS related protein 1b  Info   Rap1b 
   801 1e-06 372.6 743.9 0.501  1373693_at  G protein-coupled receptor, family C, group 5, member C (predicted)  Info   Gprc5c_predicted 
   802 1e-06 316.2 182.3 1.735  1367958_at  abl-interactor 1  Info   Abi1 
   803 1e-06 315.2 156.1 2.019  1377603_at  sorting nexing 24 (predicted)  Info   Snx24_predicted 
   804 1e-06 216 51.3 4.211  1391808_at  Transcribed locus  Info    
   805 1e-06 161.1 254.4 0.633  1373709_at  hypothetical LOC289900  Info   RGD:1359592 
   806 1e-06 609.7 161.3 3.78  1387027_a_at  lectin, galactose binding, soluble 9  Info   Lgals9 
   807 1.1e-06 785.8 406.2 1.935  1374897_at    Info    
   808 1.1e-06 2456.9 1153.1 2.131  1368809_at  CAP, adenylate cyclase-associated protein 1 (yeast)  Info   Cap1 
   809 1.1e-06 142.7 307.1 0.465  1368528_at  MIC2 like 1  Info   RGD:620896 
   810 1.1e-06 230.6 505.4 0.456  1373253_at  acyl-Coenzyme A binding domain containing 4 (predicted)  Info   Acbd4_predicted 
   811 1.1e-06 752.1 286.4 2.626  1375006_at  Similar to RIKEN cDNA B130055L09 (predicted)  Info    
   812 1.1e-06 768.2 1348.9 0.57  1372907_at  ATPase, H+ transporting, V0 subunit E isoform 2  Info   Atp6v0e2 
   813 1.1e-06 757.1 116.2 6.515  1379568_at  interferon-induced protein with tetratricopeptide repeats 2 (predicted)  Info   Ifit2_predicted 
   814 1.1e-06 565.3 1026.5 0.551  1367720_at  aminolevulinate, delta-, dehydratase  Info   Alad 
   815 1.1e-06 97.4 248.2 0.392  1387656_at  solute carrier family 4, member 1  Info   Slc4a1 
   816 1.1e-06 295.7 48.1 6.148  1368266_at  arginase 1  Info   Arg1 
   817 1.1e-06 577.3 917.7 0.629  1389363_at  aci-reductone dioxygenase (ARD)-like protein 1  Info   Alp1 
   818 1.1e-06 1753.1 2386.4 0.735  1367763_at  acetyl-coenzyme A acetyltransferase 1  Info   Acat1 
   819 1.1e-06 1093 507.3 2.155  1369969_at  ADP-ribosyltransferase 1  Info   Adprt 
   820 1.1e-06 1311.9 782.7 1.676  1372461_at  SET translocation (predicted) /// LOC499767  Info   Set_predicted /// LOC499767 
   821 1.1e-06 1155.9 353.3 3.272  1398373_at  UDP-Gal:betaGlcNAc beta 1,3-galactosyltransferase, polypeptide 3 (predicted)  Info   B3galt3_predicted 
   822 1.1e-06 318.9 868.2 0.367  1369909_s_at  fasting-inducible integral membrane protein TM6P1  Info   Tm6p1 
   823 1.1e-06 371.6 722.1 0.515  1382464_at  similar to ENSANGP00000021391 (predicted)  Info   RGD1309779_predicted 
   824 1.1e-06 793.4 1427 0.556  1390454_at  4-nitrophenylphosphatase domain and non-neuronal SNAP25-like protein homolog 1 (C. elegans) (predicted)  Info   Nipsnap1_predicted 
   825 1.1e-06 908.7 1686.9 0.539  1387867_at  aldehyde dehydrogenase family 9, subfamily A1  Info   Aldh9a1 
   826 1.1e-06 72.3 179.9 0.402  1398516_at  similar to RIKEN cDNA 0610012C01  Info   LOC498230 
   827 1.1e-06 801.7 1264.8 0.634  1392888_at  similar to glypican 4  Info   RGD:1359399 
   828 1.1e-06 1283.6 543.8 2.36  1372255_at  arginyl-tRNA synthetase (predicted)  Info   Rars_predicted 
   829 1.1e-06 536.1 117.6 4.559  1387818_at  caspase 11  Info   Casp11 
   830 1.1e-06 334.5 722.3 0.463  1372107_at    Info    
   831 1.1e-06 406.8 228.5 1.78  1395574_at  ROD1 regulator of differentiation 1 (S. pombe)  Info   Rod1 
   832 1.1e-06 126.2 56.5 2.234  1388066_a_at  G protein-coupled receptor kinase 6  Info   Gprk6 
   833 1.1e-06 194.7 343.8 0.566  1374169_at  similar to chromosome 16 open reading frame 5 (predicted)  Info   RGD1310686_predicted 
   834 1.1e-06 305.8 411.2 0.744  1372350_at  suppressor of var1, 3-like 1 (S. cerevisiae) (predicted)  Info   Supv3l1_predicted 
   835 1.1e-06 376.2 94.4 3.985  1370265_at  arrestin, beta 2  Info   Arrb2 
   836 1.1e-06 28.3 56.4 0.502  1398426_at  similar to RIKEN cDNA B130052G07  Info   LOC498309 
   837 1.1e-06 286.3 461.3 0.621  1372232_at  WW domain binding protein 1 (predicted)  Info   Wbp1_predicted 
   838 1.2e-06 198.3 459.6 0.431  1370036_at  sulfite oxidase  Info   Suox 
   839 1.2e-06 273.8 74.8 3.66  1380079_at  similar to mKIAA2005 protein  Info   LOC500015 
   840 1.2e-06 387.5 1222.9 0.317  1367866_at  fibulin 5  Info   Fbln5 
   841 1.2e-06 1236.6 105.3 11.744  1372097_at  Interferon consensus sequence binding protein 1 (predicted)  Info   Icsbp1 
   842 1.2e-06 81.9 192.4 0.426  1392914_at    Info    
   843 1.2e-06 469.8 730.3 0.643  1388374_at    Info    
   844 1.2e-06 928.6 1759.4 0.528  1374057_at  Similar to Homeobox protein Hox-D8 (Hox-4.3) (Hox-5.4)  Info    
   845 1.2e-06 1066.2 529.7 2.013  1367925_at  major vault protein  Info   Mvp 
   846 1.2e-06 886.9 113.8 7.793  1370422_at  receptor-interacting serine-threonine kinase 3  Info   Ripk3 
   847 1.2e-06 338.6 484.6 0.699  1386928_at  branched chain aminotransferase 2, mitochondrial  Info   Bcat2 
   848 1.2e-06 1169.4 1729.4 0.676  1388196_at  NCK-associated protein 1  Info   Nckap1 
   849 1.2e-06 78.4 183.8 0.427  1389256_at  Transcribed locus  Info    
   850 1.2e-06 2526.1 45.6 55.397  1370628_at  granzyme B  Info   Gzmb 
   851 1.2e-06 568.6 899.8 0.632  1367810_at  choline transporter  Info   Slc6a8 
   852 1.2e-06 252.7 581.9 0.434  1398282_at  kynureninase (L-kynurenine hydrolase)  Info   Kynu 
   853 1.2e-06 2429.9 326.6 7.44  1383906_at  similar to lung inducible neuralized-related C3HC4 RING finger protein  Info   RGD:1359633 
   854 1.2e-06 157.5 96.8 1.627  1379406_at  similar to chromosome 14 open reading frame 21 (predicted)  Info   RGD1308396_predicted 
   855 1.2e-06 281.4 648.3 0.434  1393342_at  Solute carrier family 25 (mitochondrial carrier; ornithine transporter) member 15 (predicted)  Info    
   856 1.2e-06 152.1 363.3 0.419  1382003_at  solute carrier family 16 (monocarboxylic acid transporters), member 13  Info   RGD:1359138 
   857 1.2e-06 423.3 577.6 0.733  1389386_at  similar to hypothetical protein DKFZp313N0621  Info   LOC363169 
   858 1.2e-06 344.8 136.4 2.528  1372825_at  LOC499775  Info   LOC499775 
   859 1.2e-06 2108.6 155.6 13.551  1383391_a_at  complement component 2  Info   C2 
   860 1.2e-06 272.8 71.3 3.826  1393085_at  similar to hypothetical protein BC018453 (predicted)  Info   RGD1307700_predicted 
   861 1.2e-06 347.2 65.4 5.309  1370493_a_at  leukocyte immunoglobulin-like receptor, subfamily B (with TM and ITIM domains), member 3 (predicted)  Info   Lilrb3_predicted 
   862 1.3e-06 48 106.6 0.45  1379847_at  dysferlin (predicted)  Info   Dysf_predicted 
   863 1.3e-06 5654.3 491.3 11.509  1370186_at  proteosome (prosome, macropain) subunit, beta type 9  Info   Psmb9 
   864 1.3e-06 215.6 35.5 6.073  1379229_at    Info    
   865 1.3e-06 359.4 627.9 0.572  1377844_at  zinc binding alcohol dehydrogenase, domain containing 1 (predicted)  Info   Zadh1_predicted 
   866 1.3e-06 1229.1 1785.3 0.688  1367832_at  lysophospholipase 1  Info   Lypla1 
   867 1.3e-06 142.1 431.1 0.33  1384164_at  Transcribed locus  Info    
   868 1.3e-06 683.9 484.8 1.411  1387807_at  platelet-activating factor acetylhydrolase, isoform Ib, alpha subunit 45kDa  Info   Pafah1b1 
   869 1.3e-06 30.3 60.9 0.498  1369839_at  amphiphysin 1  Info   Amph1 
   870 1.3e-06 344.4 1057.1 0.326  1382050_at  Transcribed locus  Info    
   871 1.3e-06 364.6 65 5.609  1388673_at  lymphocyte specific 1 (predicted)  Info   Lsp1_predicted 
   872 1.3e-06 80.8 157.9 0.512  1387434_at  solute carrier family 22 (organic cation transporter), member 4  Info   Slc22a4 
   873 1.3e-06 109.6 31.1 3.524  1394359_at  similar to DUF729 domain containing 1  Info   LOC364500 
   874 1.3e-06 327.8 54.1 6.059  1393049_at  myosin IG (predicted)  Info   Myo1g_predicted 
   875 1.3e-06 2642.1 108.9 24.262  1392637_at  Similar to interferon-inducible GTPase (predicted)  Info    
   876 1.3e-06 662.6 325.5 2.036  1376102_at  similar to RECS1  Info   RGD:1359409 
   877 1.3e-06 1603.1 3181.5 0.504  1386980_at  apolipoprotein M  Info   Apom 
   878 1.3e-06 387 206.7 1.872  1388509_at    Info    
   879 1.3e-06 92.3 203.5 0.454  1389185_at    Info    
   880 1.3e-06 51 157.9 0.323  1376110_at  ribonuclease P 25 subunit (human) (predicted)  Info   Rpp25_predicted 
   881 1.3e-06 185 28.9 6.401  1368610_at  mast cell antigen 32  Info   Mca32 
   882 1.3e-06 1013.7 627.1 1.616  1371998_at  Similar to Mob4B protein  Info    
   883 1.3e-06 1050 762.8 1.377  1370820_at  F-box only protein 6b  Info   Fbxo6b 
   884 1.3e-06 247.8 103.6 2.392  1390389_at    Info    
   885 1.3e-06 392.7 207.9 1.889  1373462_at  embryonic ectoderm development (predicted)  Info   Eed_predicted 
   886 1.3e-06 534.3 205.3 2.603  1389181_at  Rap guanine nucleotide exchange factor (GEF) 6 (predicted)  Info   Rapgef6_predicted 
   887 1.4e-06 499.5 190.6 2.621  1386888_at  eukaryotic translation initiation factor 4E binding protein 1  Info   Eif4ebp1 
   888 1.4e-06 446.2 953 0.468  1371795_at    Info    
   889 1.4e-06 849.4 1334.8 0.636  1372734_at  small cell adhesion glycoprotein  Info   Smagp 
   890 1.4e-06 309 40.3 7.667  1368192_at  chemokine (C-X-C motif) receptor 3  Info   Cxcr3 
   891 1.4e-06 401.4 162 2.478  1397628_at  similar to RIKEN cDNA 2900092E17 (predicted)  Info   RGD1305592_predicted 
   892 1.4e-06 66.1 118.5 0.558  1376537_at  Similar to Tyrosine-protein phosphatase, non-receptor type 3 (Protein-tyrosine phosphatase H1) (PTP-H1)  Info    
   893 1.4e-06 96 185.4 0.518  1387976_at  solute carrier family 9 (sodium/hydrogen exchanger), isoform 3 regulator 2  Info   Slc9a3r2 
   894 1.4e-06 477.3 222.7 2.143  1375895_at  Serine racemase  Info   Srr 
   895 1.4e-06 339.4 127.9 2.654  1387242_at  Protein kinase, interferon-inducible double stranded RNA dependent  Info   Prkr 
   896 1.4e-06 34.3 1.7 20.176  1387603_at  killer cell lectin-like receptor subfamily C, member 2  Info   Klrc2 
   897 1.4e-06 695.3 1520.3 0.457  1368091_at  5-oxoprolinase (ATP-hydrolysing)  Info   Oplah 
   898 1.4e-06 1481.6 77.7 19.068  1376693_at  similar to OEF2  Info   LOC500011 
   899 1.4e-06 174.5 358.8 0.486  1389592_at  Transcribed locus  Info    
   900 1.4e-06 3767.7 5216.6 0.722  1371381_at  similar to TBC1 domain family member 4  Info   LOC306117 
   901 1.4e-06 288.1 567.2 0.508  1387061_at  junction plakoglobin  Info   Jup 
   902 1.4e-06 212.9 54.1 3.935  1375920_at  similar to TNF intracellular domain-interacting protein (predicted)  Info   RGD1311487_predicted 
   903 1.4e-06 336.5 66.4 5.068  1377497_at  2'-5' oligoadenylate synthetase-like 1 (predicted)  Info   Oasl1_predicted 
   904 1.4e-06 276 93.1 2.965  1373629_at  solute carrier family 7 (cationic amino acid transporter, y+ system), member 6 (predicted)  Info   Slc7a6_predicted 
   905 1.4e-06 87.7 183.6 0.478  1368003_at  aldehyde dehydrogenase family 1, subfamily A2  Info   Aldh1a2 
   906 1.4e-06 126.1 337.8 0.373  1389744_at  LOC499898  Info   LOC499898 
   907 1.4e-06 157.4 73.6 2.139  1399089_at  solute carrier family 38, member 6 (predicted)  Info   Slc38a6_predicted 
   908 1.4e-06 333 256.1 1.3  1367834_at  spermidine synthase  Info   Srm 
   909 1.4e-06 319.4 622.2 0.513  1368100_at  phosphate cytidylyltransferase 2, ethanolamine  Info   Pcyt2 
   910 1.4e-06 95.8 279.4 0.343  1386865_at  SPARC-like 1 (mast9, hevin)  Info   Sparcl1 
   911 1.4e-06 153.5 44.6 3.442  1369044_a_at  phosphodiesterase 4B  Info   Pde4b 
   912 1.4e-06 3281 171.6 19.12  1391489_at  interferon inducible protein 1 (predicted)  Info   Ifi1_predicted 
   913 1.4e-06 311.3 99.6 3.126  1389151_at  Transcribed locus  Info    
   914 1.4e-06 208.4 341.8 0.61  1377654_at  similar to RIKEN cDNA 1810037C20  Info   LOC501664 
   915 1.4e-06 757.5 289 2.621  1387566_at  phospholipase A2, group IVA (cytosolic, calcium-dependent)  Info   Pla2g4a 
   916 1.5e-06 798.5 443.2 1.802  1370014_at  syntaxin 4A (placental)  Info   Stx4a 
   917 1.5e-06 699.8 1385.1 0.505  1388330_at  vitamin K epoxide reductase complex, subunit 1  Info   RGD:1303107 
   918 1.5e-06 1430.8 665.3 2.151  1398805_at  APG3 autophagy 3-like (S. cerevisiae)  Info   Apg3l 
   919 1.5e-06 46.5 91.5 0.508  1371671_at  G protein-coupled receptor, family C, group 5, member B (predicted)  Info   Gprc5b_predicted 
   920 1.5e-06 120.8 49.6 2.435  1376026_at  downstream neighbor of SON (predicted)  Info   Donson_predicted 
   921 1.5e-06 388.6 126 3.084  1380617_at  GTPase, IMAP family member 5  Info   Gimap5 
   922 1.5e-06 1030.1 268.5 3.836  1392894_at  fibrinogen-like 2  Info   Fgl2 
   923 1.5e-06 306.6 52.1 5.885  1393329_at  Inducible T-cell co-stimulator  Info   Ailim 
   924 1.5e-06 222.1 559.5 0.397  1383457_at  similar to hypothetical protein DKFZp761N1114  Info   LOC498228 
   925 1.5e-06 700.3 215.2 3.254  1391347_at  RAB8B, member RAS oncogene family  Info   Rab8b 
   926 1.5e-06 171.6 33.1 5.184  1385440_at  hematopoietic cell signal transducer  Info   RGD:1359238 
   927 1.5e-06 158.9 364.7 0.436  1392145_at    Info    
   928 1.5e-06 1197.3 40.7 29.418  1390956_at  Transcribed locus  Info    
   929 1.5e-06 1399.3 2164.7 0.646  1389866_at    Info    
   930 1.5e-06 479.1 800.6 0.598  1372655_at    Info    
   931 1.6e-06 369.3 950.4 0.389  1376749_at  osteoglycin (predicted)  Info   Ogn_predicted 
   932 1.6e-06 1385.4 2140.9 0.647  1393245_at  Phytanoyl-CoA hydroxylase  Info   Phyh 
   933 1.6e-06 602.8 384.8 1.567  1368220_at  general transcription factor IIB  Info   Gtf2b 
   934 1.6e-06 214.1 390.8 0.548  1373074_at  similar to RIKEN cDNA 2700002I20 (predicted)  Info   RGD1307279_predicted 
   935 1.6e-06 1644.6 1129.3 1.456  1370062_at  hypoxia induced gene 1  Info   RGD:620215 
   936 1.6e-06 274.1 524.4 0.523  1372762_at  similar to RIKEN cDNA 1110033O09 (predicted)  Info   RGD1311839_predicted 
   937 1.6e-06 1020.8 507.6 2.011  1386882_at  t-complex testis expressed 1  Info   Tctex1 
   938 1.6e-06 2234.1 3543.3 0.631  1371379_at  similar to Brain protein 44  Info   LOC289182 
   939 1.6e-06 419.7 761.8 0.551  1392486_at  similar to RIKEN cDNA 2310036D22  Info   RGD:1303037 
   940 1.6e-06 987.9 223.5 4.42  1368006_at  lysosomal-associated protein transmembrane 5  Info   Laptm5 
   941 1.6e-06 94 143.8 0.654  1382115_at  similar to RIKEN cDNA 4930521E07 (predicted)  Info   RGD1305166_predicted 
   942 1.6e-06 288.3 459.1 0.628  1376038_at  testis expressed gene 2 (predicted)  Info   Tex2_predicted 
   943 1.6e-06 195.4 15.1 12.94  1387739_at  CD8 antigen, beta chain  Info   Cd8b 
   944 1.6e-06 130.7 59.5 2.197  1390350_at  similar to hypothetical protein AL133206 (predicted)  Info   RGD1308418_predicted 
   945 1.6e-06 305.2 92.6 3.296  1386586_at  similar to PAK/PLC-interacting protein 1  Info   LOC361232 
   946 1.6e-06 922.3 324.8 2.84  1395886_at  ARP3 actin-related protein 3 homolog (yeast)  Info   Actr3 
   947 1.6e-06 674.4 1246.2 0.541  1377060_at  methylcrotonoyl-Coenzyme A carboxylase 2 (beta) (predicted)  Info   Mccc2_predicted 
   948 1.6e-06 556.5 141.1 3.944  1374838_at  SP140 nuclear body protein (predicted)  Info   Sp140_predicted 
   949 1.6e-06 189.2 104.2 1.816  1381525_at    Info    
   950 1.6e-06 1084 2189.3 0.495  1372492_at  claudin 10 (predicted)  Info   Cldn10_predicted 
   951 1.6e-06 1055 46.5 22.688  1388275_at  T-cell receptor beta chain  Info   Tcrb 
   952 1.6e-06 74.3 172.8 0.43  1387083_at  cardiotrophin 1  Info   Ctf1 
   953 1.7e-06 264.7 63.1 4.195  1368856_at  Janus kinase 2  Info   Jak2 
   954 1.7e-06 29.2 97 0.301  1384275_at  Transcribed locus  Info    
   955 1.7e-06 294.6 140.2 2.101  1388521_at  pyrroline-5-carboxylate synthetase (glutamate gamma-semialdehyde synthetase) (predicted)  Info   Pycs_predicted 
   956 1.7e-06 192.8 41 4.702  1387413_at  neutrophil cytosolic factor 1  Info   Ncf1 
   957 1.7e-06 765.9 364.6 2.101  1373439_at  Lamin B receptor  Info   Lbr 
   958 1.7e-06 228.3 60.7 3.761  1371171_at  RT1 class Ib, locus Aw2  Info   RT1-Aw2 
   959 1.7e-06 121.4 54.9 2.211  1379651_at  Forkhead box P1 (predicted)  Info    
   960 1.7e-06 251 87.4 2.872  1382995_at  Neuropilin 2  Info   Nrp2 
   961 1.7e-06 753.2 1327.1 0.568  1372654_at  EPS8-like 2 (predicted)  Info   Eps8l2_predicted 
   962 1.7e-06 82.7 20.8 3.976  1387794_at  ficolin A  Info   Fcna 
   963 1.7e-06 177.7 57.9 3.069  1384507_at  Similar to hypothetical protein FLJ11127  Info    
   964 1.7e-06 1543 2500.6 0.617  1367695_at  quinoid dihydropteridine reductase  Info   Qdpr 
   965 1.7e-06 93.8 188.8 0.497  1390942_at  Transcribed locus  Info    
   966 1.7e-06 724.3 1046.3 0.692  1371941_at  similar to RIKEN cDNA 5230400G24 (predicted)  Info   RGD1310230_predicted 
   967 1.7e-06 214.7 120.5 1.782  1368927_at  membrane bound C2 domain containing protein  Info   Mbc2 
   968 1.7e-06 256.5 451.7 0.568  1374592_at    Info    
   969 1.7e-06 635.2 414.8 1.531  1388815_at  similar to B430201G11Rik protein (predicted)  Info   RGD1311409_predicted 
   970 1.7e-06 104.7 161.5 0.648  1374693_at  hypothetical LOC315760 (predicted)  Info   RGD1306243_predicted 
   971 1.7e-06 25.3 98.8 0.256  1367989_at  solute carrier family 2 (facilitated glucose transporter), member 4  Info   Slc2a4 
   972 1.7e-06 261.2 442.2 0.591  1390541_at  harmonin a1  Info   RGD:1303329 
   973 1.7e-06 6068.1 2558 2.372  1367663_at  protease (prosome, macropain) 28 subunit, alpha  Info   Psme1 
   974 1.7e-06 895.5 1257.9 0.712  1371640_at  hypothetical protein LOC500855  Info   LOC500855 
   975 1.7e-06 969.6 2355.6 0.412  1377646_at  Transcribed locus  Info    
   976 1.7e-06 80.5 302.1 0.266  1378168_at  hypothetical LOC287534  Info   RGD:1359691 
   977 1.7e-06 901.3 36.7 24.559  1395034_at  similar to RIKEN cDNA 4933430F08 (predicted)  Info   RGD1311481_predicted 
   978 1.8e-06 107.7 37.8 2.849  1390917_at    Info    
   979 1.8e-06 602.1 169.4 3.554  1382181_at  similar to RIKEN cDNA A530088I07 gene  Info   LOC311984 
   980 1.8e-06 179.6 383.9 0.468  1377751_at  Transcribed locus  Info    
   981 1.8e-06 804.9 1095.8 0.735  1367911_at  isocitrate dehydrogenase 3 (NAD+) alpha  Info   Idh3a 
   982 1.8e-06 387 38.7 10  1379818_at    Info    
   983 1.8e-06 148.3 33.4 4.44  1375028_at  Transcribed locus  Info    
   984 1.8e-06 255.4 74 3.451  1378171_at  Neuropilin 2  Info   Nrp2 
   985 1.8e-06 4681.9 626.7 7.471  1376151_a_at    Info    
   986 1.8e-06 1353.3 49.7 27.229  1392647_at  Tryptophan hydroxylase  Info   Tph 
   987 1.8e-06 91.5 177.5 0.515  1372884_at  Transcribed locus  Info    
   988 1.8e-06 254.7 82.8 3.076  1393050_at  Strain Wistar Rap2a mRNA, 5' UTR  Info    
   989 1.8e-06 244.5 80.5 3.037  1379957_at  similar to schlafen 8  Info   RGD:1359139 
   990 1.8e-06 37.8 68.3 0.553  1384344_at  FK506 binding protein 7 (predicted)  Info   Fkbp7_predicted 
   991 1.8e-06 508.2 190.2 2.672  1373830_at  hypothetical protein LOC619574  Info   LOC619574 
   992 1.8e-06 551 980.9 0.562  1377995_at  similar to hypothetical protein DKFZp761D0211 (predicted)  Info   RGD1311484_predicted 
   993 1.8e-06 190.3 409.3 0.465  1388497_at  thioesterase superfamily member 2 (predicted)  Info   Them2_predicted 
   994 1.9e-06 33.5 104.5 0.321  1384529_at  Transcribed locus  Info    
   995 1.9e-06 1888.4 146.3 12.908  1388149_at  transporter 1, ATP-binding cassette, sub-family B (MDR/TAP)  Info   Tap1 
   996 1.9e-06 1029.6 729.3 1.412  1367769_at  polymerase (RNA) II (DNA directed) polypeptide G  Info   Polr2g 
   997 1.9e-06 1213.9 167.8 7.234  1370382_at  RT1 class II, locus Db1  Info   RT1-Db1 
   998 1.9e-06 286.1 145.1 1.972  1373136_at  hypothetical LOC294390 (predicted)  Info   RGD1307672_predicted 
   999 1.9e-06 4795.7 366.5 13.085  1397304_at  interferon gamma induced GTPase (predicted)  Info   Igtp_predicted 
   1000 1.9e-06 112 176.4 0.635  1374884_at  protein phosphatase 1D magnesium-dependent, delta isoform (predicted)  Info   Ppm1d_predicted 
   1001 1.9e-06 175.3 114.9 1.526  1376810_at  ADP-ribosyltransferase (NAD+; poly (ADP-ribose) polymerase)-like 1 (predicted)  Info   Adprtl1_predicted 
   1002 1.9e-06 47.2 104.3 0.453  1374122_at  Transcribed locus  Info    
   1003 1.9e-06 509.8 1103.4 0.462  1372790_at  Malate dehydrogenase 1, NAD (soluble)  Info   Mdh1 
   1004 1.9e-06 2082.7 4095.7 0.509  1369491_at  D-amino acid oxidase  Info   Dao1 
   1005 1.9e-06 810.5 56 14.473  1385571_at  Similar to SP140 nuclear body protein isoform 1  Info    
   1006 1.9e-06 319.4 495.6 0.644  1374300_at    Info    
   1007 1.9e-06 126.9 303.2 0.419  1368081_at  ATP-binding cassette, sub-family A (ABC1), member 2  Info   Abca2 
   1008 1.9e-06 207.5 428.7 0.484  1372847_at  ACN9 homolog (S. cerevisiae) (predicted)  Info   Acn9_predicted 
   1009 1.9e-06 537.4 276.1 1.946  1399125_at  inositol polyphosphate-1-phosphatase (predicted)  Info   Inpp1_predicted 
   1010 2e-06 801.9 1328.9 0.603  1375297_at  similar to RIKEN cDNA 0610008C08  Info   LOC363474 
   1011 2e-06 140.1 25.8 5.43  1379732_at  syntaxin 11 (predicted)  Info   Stx11_predicted 
   1012 2e-06 129.4 285.7 0.453  1375504_at  Polymerase (DNA directed), gamma 2, accessory subunit (predicted)  Info    
   1013 2e-06 110 24.9 4.418  1382531_at  similar to toll-like receptor 7  Info   LOC317468 
   1014 2e-06 1257.9 144 8.735  1379285_at  similar to 5830458K16Rik protein (predicted)  Info   RGD1306974_predicted 
   1015 2e-06 1825.3 2819.8 0.647  1367619_at  progesterone receptor membrane component 1  Info   Pgrmc1 
   1016 2e-06 724.7 91.6 7.912  1375917_at  glycoprotein 49b /// similar to GP49B1  Info   RGD:1359090 /// LOC499078 
   1017 2e-06 346.2 96.5 3.588  1368754_at  pyrimidinergic receptor P2Y, G-protein coupled, 6  Info   P2ry6 
   1018 2e-06 1049.7 380 2.762  1371781_at  signal transducer and activator of transcription 3  Info   Stat3 
   1019 2e-06 876.5 543.1 1.614  1368043_at  sorting nexin 1  Info   Snx1 
   1020 2e-06 245.8 159.5 1.541  1368050_at  cyclin L1  Info   Ccnl1 
   1021 2e-06 1844.3 66.3 27.817  1376390_at  membrane-spanning 4-domains, subfamily A, member 11 (predicted)  Info   Ms4a11_predicted 
   1022 2e-06 935.3 2733.4 0.342  1387053_at  flavin containing monooxygenase 1  Info   Fmo1 
   1023 2e-06 38.6 85.5 0.451  1374785_at  similar to CD69 antigen (p60, early T-cell activation antigen)  Info   LOC296985 
   1024 2e-06 80.9 191.5 0.422  1376546_at  similar to hypothetical protein  Info   LOC499157 
   1025 2e-06 189.3 43.4 4.362  1391935_at  similar to eukaryotic translation initiation factor 4E member 3  Info   LOC297481 
   1026 2e-06 122.7 224 0.548  1374738_at  serologically defined colon cancer antigen 10 (predicted)  Info   Sdccag10_predicted 
   1027 2e-06 342.5 129.3 2.649  1372406_at  minichromosome maintenance deficient 3 (S. cerevisiae) (predicted) /// similar to DNA replication licensing factor MCM3 (DNA polymerase alpha holoenzyme-associated protein P1) (P1-MCM3)  Info   Mcm3_predicted /// LOC367976 
   1028 2e-06 409.9 653.9 0.627  1372341_at  Transcribed locus, strongly similar to XP_484968.2 PREDICTED: similar to RIKEN cDNA C330005L02 [Mus musculus]  Info    
   1029 2e-06 82.5 182.7 0.452  1368334_at  growth factor receptor bound protein 7  Info   Grb7 
   1030 2e-06 137.9 38.1 3.619  1394352_at    Info    
   1031 2e-06 640.8 1045.3 0.613  1371850_at  3'-phosphoadenosine 5'-phosphosulfate synthase 1 (predicted)  Info   Papss1_predicted 
   1032 2e-06 2391 1267.4 1.887  1383986_at  tropomyosin 3, gamma  Info   Tpm3 
   1033 2.1e-06 421.8 223.2 1.89  1390287_at  bridging integrator 2 (predicted)  Info   Bin2_predicted 
   1034 2.1e-06 367.4 183.4 2.003  1367883_at  survival of motor neuron 1, telomeric  Info   SMN1 
   1035 2.1e-06 587.8 330.3 1.78  1388761_at  histone deacetylase 1 (predicted)  Info   Hdac1_predicted 
   1036 2.1e-06 944.3 79.3 11.908  1375796_at  interferon gamma induced GTPase (predicted) /// similar to Ac2-233  Info   Igtp_predicted /// LOC501695 
   1037 2.1e-06 129.9 22.5 5.773  1369006_at  hexokinase 2  Info   Hk2 
   1038 2.1e-06 196.1 482.6 0.406  1386901_at  similar to fatty acid translocase/CD36  Info   LOC499984 
   1039 2.1e-06 357.3 680.1 0.525  1389654_at  Transcribed locus  Info    
   1040 2.1e-06 938.2 33.6 27.923  1385051_at  Transcribed locus  Info    
   1041 2.1e-06 1929.8 1052.5 1.834  1372743_at  sorting nexin 5 (predicted)  Info   Snx5_predicted 
   1042 2.1e-06 274.4 85.9 3.194  1368645_at  protein tyrosine phosphatase, non-receptor type 1  Info   Ptpn1 
   1043 2.1e-06 488.5 196.8 2.482  1390312_at  similar to mKIAA2005 protein /// similar to mKIAA2005 protein /// similar to mKIAA2005 protein  Info   LOC500012 /// LOC500014 /// LOC500015 
   1044 2.1e-06 24.6 3.3 7.455  1375978_at  FCH domain only 1 (predicted)  Info   Fcho1_predicted 
   1045 2.1e-06 307.6 547.7 0.562  1390406_at    Info    
   1046 2.1e-06 306.1 799 0.383  1370927_at  procollagen, type XII, alpha 1  Info   Col12a1 
   1047 2.1e-06 659.2 219.5 3.003  1385658_at  zinc finger protein 313  Info   RGD:1303139 
   1048 2.1e-06 1928 3312.6 0.582  1391417_at  similar to betaine-homocysteine methyltransferase 2  Info   LOC365972 
   1049 2.1e-06 51.7 118.4 0.437  1398597_at  Transcribed locus  Info    
   1050 2.1e-06 91.8 332 0.277  1392612_at  Prolactin receptor  Info   Prlr 
   1051 2.1e-06 1951.9 1294.4 1.508  1398828_at  FK506 binding protein 1a  Info   Fkbp1a 
   1052 2.1e-06 337.5 188.6 1.79  1369670_at  antigen identified by monoclonal antibody MRC OX-2  Info   Mox2 
   1053 2.2e-06 356.4 637.6 0.559  1386975_at  pyruvate dehydrogenase kinase, isoenzyme 2  Info   Pdk2 
   1054 2.2e-06 144.2 13.4 10.761  1392731_at  hypothetical gene supported by NM_130426  Info   LOC497808 
   1055 2.2e-06 473.5 40.7 11.634  1382926_s_at  LOC499533  Info   LOC499533 
   1056 2.2e-06 1534.1 3071.8 0.499  1369636_at  sorbitol dehydrogenase  Info   Sord 
   1057 2.2e-06 1260.2 2316.1 0.544  1370237_at  L-3-hydroxyacyl-Coenzyme A dehydrogenase, short chain  Info   Hadhsc 
   1058 2.2e-06 648.4 999.9 0.648  1373346_at  similar to hypothetical protein CL25084 (predicted)  Info   RGD1306508_predicted 
   1059 2.2e-06 1876.9 2579.9 0.728  1387271_at  phytanoyl-CoA hydroxylase  Info   Phyh 
   1060 2.2e-06 234 91.9 2.546  1379248_at  Prolylcarboxypeptidase (angiotensinase C) (predicted)  Info    
   1061 2.2e-06 113.8 267.8 0.425  1393906_at  cytochrome P450, family 4, subfamily v, polypeptide 3 (predicted)  Info   Cyp4v3_predicted 
   1062 2.2e-06 107.5 22.2 4.842  1391690_at  Transcribed locus  Info    
   1063 2.2e-06 203.3 12.4 16.395  1381357_at  protein tyrosine phosphatase, non-receptor type 8 (predicted)  Info   Ptpn8_predicted 
   1064 2.2e-06 13.4 30.9 0.434  1369677_at  cannabinoid receptor 1 (brain)  Info   Cnr1 
   1065 2.2e-06 215.6 45.7 4.718  1384842_s_at  Tumor necrosis factor receptor superfamily, member 6  Info   RGD:619831 
   1066 2.2e-06 373.6 1027.6 0.364  1398511_at  sushi domain containing 2 (predicted)  Info   Susd2_predicted 
   1067 2.2e-06 585.1 1014.7 0.577  1380577_at  ATP-binding cassette, sub-family G (WHITE), member 2  Info   Abcg2 
   1068 2.2e-06 212.3 93.4 2.273  1376160_at  similar to Avian reticuloendotheliosis viral (v-rel) oncogene related B  Info   LOC365510 
   1069 2.2e-06 3345.1 1251.2 2.674  1386879_at  lectin, galactose binding, soluble 3  Info   Lgals3 
   1070 2.2e-06 113.1 238 0.475  1383148_at  Transcribed locus  Info    
   1071 2.2e-06 273 600.8 0.454  1393935_at  Transcribed locus  Info    
   1072 2.2e-06 157.6 49.5 3.184  1392554_a_at    Info    
   1073 2.2e-06 219.9 19.6 11.219  1370429_at  RT1 class Ib, locus Aw2  Info   RT1-Aw2 
   1074 2.2e-06 2056.5 2983.4 0.689  1388304_at  NADH dehydrogenase (ubiquinone) 1 beta subcomplex, 5 (predicted)  Info   Ndufb5_predicted 
   1075 2.2e-06 227.9 533 0.428  1392670_at  Transcribed locus  Info    
   1076 2.3e-06 2512.7 3643.9 0.69  1388118_at  3-hydroxyisobutyrate dehydrogenase  Info   Hibadh 
   1077 2.3e-06 40.4 87.6 0.461  1383397_at  receptor-associated protein of the synapse (predicted)  Info   Rapsn_predicted 
   1078 2.3e-06 1000.6 457.5 2.187  1372090_at  similar to RIKEN cDNA 1700108M19  Info   LOC298963 
   1079 2.3e-06 253.7 376.3 0.674  1372157_at  similar to CGI-143 protein (predicted)  Info   RGD1310586_predicted 
   1080 2.3e-06 268.4 775.1 0.346  1398255_at  solute carrier family 15 (H+/peptide transporter), member 2  Info   Slc15a2 
   1081 2.3e-06 356.5 696.8 0.512  1368437_at  carbonic anhydrase 4  Info   Ca4 
   1082 2.3e-06 1436.7 743.6 1.932  1369319_at  ADP-ribosylation factor-like 6 interacting protein 5  Info   Arl6ip5 
   1083 2.3e-06 783 44.4 17.635  1369290_at  chemokine (C-C) receptor 5  Info   Ccr5 
   1084 2.3e-06 921.9 596.6 1.545  1388977_at  similar to RIKEN cDNA 9930118K05  Info   RGD:1359727 
   1085 2.3e-06 91.3 160.6 0.568  1375940_a_at  similar to hypothetical protein FLJ12242 (predicted)  Info   RGD1311154_predicted 
   1086 2.3e-06 49.8 142.5 0.349  1392990_at  SRY-box containing gene 17 (predicted)  Info   Sox17_predicted 
   1087 2.3e-06 140.4 242 0.58  1372248_at  sestrin 1 (predicted)  Info   Sesn1_predicted 
   1088 2.3e-06 611.8 994.3 0.615  1369629_at  adenosine kinase  Info   Adk 
   1089 2.3e-06 136 32.2 4.224  1393832_at  Similar to RIKEN cDNA 0610033I05 (predicted)  Info    
   1090 2.3e-06 56 136 0.412  1388921_at  Transcribed locus  Info    
   1091 2.4e-06 73.1 171.2 0.427  1378085_at  epsin 3 (predicted)  Info   Epn3_predicted 
   1092 2.4e-06 152.6 331.1 0.461  1387558_at  transient receptor potential cation channel, subfamily V, member 4  Info   Trpv4 
   1093 2.4e-06 45.6 111.6 0.409  1376226_at  Similar to Ab2-076  Info    
   1094 2.4e-06 743.5 446.5 1.665  1389063_at  exportin 6 (predicted)  Info   Xpo6_predicted 
   1095 2.4e-06 126.7 14.3 8.86  1381441_at    Info    
   1096 2.4e-06 411.9 792.1 0.52  1389833_at  Sulfatase modifying factor 1 (predicted)  Info    
   1097 2.4e-06 2446.9 286.1 8.553  1383564_at    Info    
   1098 2.4e-06 218.9 429 0.51  1389688_at  Transcribed locus  Info    
   1099 2.4e-06 431.6 163 2.648  1375020_at  Transcribed locus  Info    
   1100 2.4e-06 389.4 995.1 0.391  1370609_a_at  solute carrier family 16 (monocarboxylic acid transporters), member 7 /// hypothetical gene supported by NM_017302  Info   Slc16a7 /// LOC497820 
   1101 2.4e-06 2051.6 2921.8 0.702  1387793_at  ERM-binding phosphoprotein  Info   Slc9a3r1 
   1102 2.4e-06 220.7 53.2 4.148  1376675_at  similar to mast cell-derived paired immunoglobulin-like receptor1  Info   LOC501736 
   1103 2.4e-06 325.8 112.8 2.888  1369672_at  arachidonate 5-lipoxygenase activating protein  Info   Alox5ap 
   1104 2.4e-06 197.4 71.3 2.769  1382177_at  Transcribed locus  Info    
   1105 2.4e-06 627 1106.4 0.567  1388617_at  biphenyl hydrolase-like (serine hydrolase, breast epithelial mucin-associated antigen) (predicted)  Info   Bphl_predicted 
   1106 2.4e-06 178.7 91.6 1.951  1372546_at  mitogen-activated protein kinase-activated protein kinase 3 (predicted)  Info   Mapkapk3_predicted 
   1107 2.4e-06 2675.7 1627 1.645  1368507_at  proteasome (prosome, macropain) subunit, alpha type 3  Info   Psma3 
   1108 2.4e-06 513 156.8 3.272  1392655_at  Similar to SP140 nuclear body protein isoform 1  Info    
   1109 2.5e-06 476.6 58 8.217  1370832_at  small inducible cytokine A4  Info   Ccl4 
   1110 2.5e-06 3401.5 4297.5 0.792  1371701_at  NADH dehydrogenase (ubiquinone) 1 beta subcomplex, 9 (predicted)  Info   Ndufb9_predicted 
   1111 2.5e-06 353.8 213.4 1.658  1390786_at  rho/rac guanine nucleotide exchange factor (GEF) 2 (predicted)  Info   Arhgef2_predicted 
   1112 2.5e-06 7.9 30.9 0.256  1368609_at  solute carrier family 10 (sodium/bile acid cotransporter family), member 1  Info   Slc10a1 
   1113 2.5e-06 265.3 578.5 0.459  1372621_at  similar to 2610027C15Rik protein (predicted)  Info   RGD1308876_predicted 
   1114 2.5e-06 54.5 155.2 0.351  1371849_at  similar to hypothetical protein FLJ12442 (predicted)  Info   RGD1305524_predicted 
   1115 2.5e-06 328.5 792 0.415  1388998_at  erythrocyte protein band 4.9 (predicted)  Info   Epb4.9_predicted 
   1116 2.5e-06 77 137.9 0.558  1375872_at  Similar to hypothetical protein D4Ertd89e (predicted)  Info    
   1117 2.5e-06 198.8 462.2 0.43  1390412_at  Solute carrier family 39 (iron-regulated transporter), member 1  Info   Slc40a1 
   1118 2.5e-06 80.3 47.3 1.698  1376084_a_at  extra spindle poles like 1 (S. cerevisiae) (predicted)  Info   Espl1_predicted 
   1119 2.5e-06 337.3 19.4 17.387  1368976_at  CD38 antigen  Info   Cd38 
   1120 2.5e-06 503.7 1047.7 0.481  1378802_at  similar to hypothetical protein MGC13047  Info   RGD:1303232 
   1121 2.5e-06 155 56.6 2.739  1391262_at  SUMO/sentrin specific protease 5 (predicted) /// similar to SUMO/sentrin specific protease 5  Info   Senp5_predicted /// LOC499574 
   1122 2.6e-06 183.6 14.1 13.021  1387608_at  indoleamine 2,3-dioxygenase  Info   Indo 
   1123 2.6e-06 1760.8 1117.1 1.576  1386954_at  adenylate kinase 2  Info   Ak2 
   1124 2.6e-06 182.6 17.4 10.494  1367975_at  annexin A3  Info   Anxa3 
   1125 2.6e-06 246.8 112.4 2.196  1389413_at  Oligodendrocyte-myelin glycoprotein  Info   Omg 
   1126 2.6e-06 340.1 178.4 1.906  1373852_at  similar to SRD5A2L (predicted)  Info   RGD1308828_predicted 
   1127 2.6e-06 141.8 327.7 0.433  1373533_at    Info    
   1128 2.6e-06 579.7 59.5 9.743  1371152_a_at  2',5'-oligoadenylate synthetase 1, 40/46kDa  Info   Oas1 
   1129 2.6e-06 463.7 1120 0.414  1388738_at  Similar to mKIAA0534 protein  Info    
   1130 2.6e-06 159.8 8.4 19.024  1393830_at  SH2 domain protein 2A  Info   RGD:1303076 
   1131 2.6e-06 65.7 196.4 0.335  1378245_at  similar to 6430514L14Rik protein (predicted)  Info   RGD1311958_predicted 
   1132 2.6e-06 238.1 593.7 0.401  1383795_at  CDNA clone IMAGE:7379585  Info    
   1133 2.6e-06 2761.1 456.8 6.044  1376845_at  putative ISG12(b) protein  Info   isg12(b) 
   1134 2.6e-06 109.7 267.2 0.411  1387018_at  Arg/Abl-interacting protein ArgBP2  Info   Argbp2 
   1135 2.6e-06 968.9 556.1 1.742  1373422_at    Info    
   1136 2.6e-06 66.6 139.7 0.477  1383964_at  Transcribed locus  Info    
   1137 2.7e-06 140.8 71.4 1.972  1369700_at  chloride channel 7  Info   Clcn7 
   1138 2.7e-06 310.3 188.7 1.644  1377945_at  similar to RIKEN cDNA 2310005B10  Info   MGC95311 
   1139 2.7e-06 232.9 400.5 0.582  1372457_at  mitochondrial tumor suppressor 1  Info   Mtus1 
   1140 2.7e-06 661.3 1017.6 0.65  1383633_at  Transcribed locus  Info    
   1141 2.7e-06 2420.3 1708.4 1.417  1398853_at  proteasome (prosome, macropain) subunit, beta type 3  Info   Psmb3 
   1142 2.7e-06 246.5 27 9.13  1390797_at  lymphocyte cytosolic protein 2  Info   Lcp2 
   1143 2.7e-06 297.2 634.7 0.468  1374892_at  spermidine/spermine N1-acetyl transferase 2 (predicted)  Info   Sat2_predicted 
   1144 2.7e-06 1371 2200.4 0.623  1388324_at  nitrilase 1  Info   Nit1 
   1145 2.8e-06 289.1 507.5 0.57  1389640_at  Similar to SH3 domain binding glutamic acid-rich protein like 2  Info    
   1146 2.8e-06 227.1 121.1 1.875  1382590_at  Similar to RIKEN cDNA 2310015N21  Info    
   1147 2.8e-06 98.7 236.3 0.418  1385432_at  CDNA clone MGC:95146 IMAGE:7129361  Info    
   1148 2.8e-06 86.1 230.8 0.373  1371933_at    Info    
   1149 2.8e-06 127 82.5 1.539  1394968_at  Transcribed locus  Info    
   1150 2.8e-06 936.6 1907.2 0.491  1372485_at  pterin 4 alpha carbinolamine dehydratase/dimerization cofactor of hepatocyte nuclear factor 1 alpha (TCF1) 1  Info   Pcbd1 
   1151 2.8e-06 25 5.3 4.717  1397549_at  centaurin, gamma 1  Info   Centg1 
   1152 2.8e-06 284 202.5 1.402  1388641_at  phosphoribosylglycinamide formyltransferase (predicted)  Info   Gart_predicted 
   1153 2.8e-06 367.8 573.8 0.641  1382748_at    Info    
   1154 2.8e-06 868.5 24 36.188  1379293_at  granzyme A  Info   Gzma 
   1155 2.8e-06 361.3 874.9 0.413  1368428_at  X-prolyl aminopeptidase (aminopeptidase P) 2, membrane-bound  Info   Xpnpep2 
   1156 2.8e-06 38.2 92.3 0.414  1379801_at  LOC499790  Info    
   1157 2.8e-06 507.7 33.5 15.155  1392819_at  membrane-spanning 4-domains, subfamily A, member 11 (predicted)  Info   Ms4a11_predicted 
   1158 2.8e-06 488.9 1166.9 0.419  1377048_at  similar to cDNA sequence BC021917 (predicted)  Info   RGD1311026_predicted 
   1159 2.8e-06 271.7 621.5 0.437  1396152_s_at  insulin-like growth factor binding protein 5  Info   Igfbp5 
   1160 2.8e-06 1510.8 2222.2 0.68  1388364_at  NADH dehydrogenase (ubiquinone) Fe-S protein 3 (predicted)  Info   Ndufs3_predicted 
   1161 2.8e-06 697 203.3 3.428  1374499_at  TATA box binding protein (Tbp)-associated factor, RNA polymerase I, A (predicted)  Info    
   1162 2.8e-06 37 72.7 0.509  1373032_at  musculoskeletal, embryonic nuclear protein 1  Info   Mustn1 
   1163 2.8e-06 382.5 824.8 0.464  1384964_at  similar to Solute carrier family 6 (neurotransmitter transporter), member 20  Info   LOC301083 
   1164 2.8e-06 515.9 275.1 1.875  1379676_a_at  similar to RIKEN cDNA 2310005K03  Info   RGD:1359588 
   1165 2.8e-06 414.1 264.6 1.565  1370002_at  Rho guanine nucleotide exchange factor (GEF) 1  Info   Arhgef1 
   1166 2.8e-06 612.3 374.9 1.633  1370978_at  secretory carrier membrane protein 1  Info   Scamp1 
   1167 2.8e-06 194.9 49 3.978  1384292_at  docking protein 1 (predicted)  Info   Dok1_predicted 
   1168 2.9e-06 1412.6 696.4 2.028  1370891_at  CD48 antigen  Info   Cd48 
   1169 2.9e-06 109.3 323.1 0.338  1389066_at  Down syndrome critical region gene 1-like 1  Info   Dscr1l1 
   1170 2.9e-06 12.4 49.4 0.251  1380726_at  Similar to asporin precursor  Info    
   1171 2.9e-06 36.8 66 0.558  1384251_at  similar to leucine rich repeat containing 27  Info   LOC499281 
   1172 2.9e-06 1260.4 2074.3 0.608  1388148_a_at  low density lipoprotein receptor-related protein associated protein 1  Info   Lrpap1 
   1173 2.9e-06 995.3 1934.4 0.515  1375026_at  ceroid-lipofuscinosis, neuronal 6 (predicted)  Info   Cln6_predicted 
   1174 2.9e-06 468 661.2 0.708  1372109_at    Info    
   1175 2.9e-06 26.3 90 0.292  1382034_at    Info    
   1176 2.9e-06 683.2 1437.3 0.475  1386916_at  aconitase 1  Info   Aco1 
   1177 2.9e-06 444.7 336.9 1.32  1376796_at  Transcribed locus  Info    
   1178 3e-06 1919.2 3138.9 0.611  1387889_at  folate receptor 1 (adult)  Info   Folr1 
   1179 3e-06 76.6 26.5 2.891  1380772_at  Transcribed locus  Info    
   1180 3e-06 238.9 104.3 2.291  1368391_at  solute carrier family 7 (cationic amino acid transporter, y+ system), member 1  Info   Slc7a1 
   1181 3e-06 1125.3 1799.7 0.625  1372671_at  similar to RIKEN cDNA 0610038L10 gene /// similar to riboflavin kinase /// similar to riboflavin kinase  Info   LOC317214 /// LOC499328 /// LOC501601 
   1182 3e-06 174.4 62 2.813  1384934_at  Transcribed locus  Info    
   1183 3e-06 103.4 32.8 3.152  1393119_at  Transcribed locus  Info    
   1184 3e-06 224.3 397.7 0.564  1375529_at  carbonic reductase 4  Info   Cbr4 
   1185 3e-06 355.7 178.7 1.99  1373841_at  Rac/cdc42 guanine nucleotide exchange factor 6  Info   arhgef6 
   1186 3.1e-06 930.3 1243.8 0.748  1371844_at    Info    
   1187 3.1e-06 212.2 134.4 1.579  1383465_at  F-box and leucine-rich repeat protein 14 (predicted)  Info   Fbxl14_predicted 
   1188 3.1e-06 391.8 154.8 2.531  1387788_at  Jun-B oncogene  Info   Junb 
   1189 3.1e-06 1095.9 632.7 1.732  1379891_at  scotin  Info   RGD:1359661 
   1190 3.1e-06 300.3 869.1 0.346  1383058_at  Transcribed locus  Info    
   1191 3.1e-06 372.2 38.5 9.668  1385696_at    Info    
   1192 3.1e-06 112.9 37.7 2.995  1392037_at  Transcribed locus  Info    
   1193 3.1e-06 365.3 26.6 13.733  1368783_at  inducible T-cell co-stimulator  Info   Icos 
   1194 3.2e-06 180.9 117.1 1.545  1382661_at  nucleoporin 160 (predicted)  Info   Nup160_predicted 
   1195 3.2e-06 126 15.2 8.289  1383015_at  Similar to DKFZP434P1750 protein (predicted)  Info    
   1196 3.2e-06 1542.3 2674.8 0.577  1372438_at  similar to Nit protein 2 (predicted)  Info   RGD1310494_predicted 
   1197 3.2e-06 392 1154 0.34  1379885_at  flavin containing monooxygenase 4  Info   Fmo4 
   1198 3.2e-06 161.3 71.3 2.262  1368079_at  pyruvate dehydrogenase kinase 1  Info   Pdk1 
   1199 3.2e-06 204.9 580.8 0.353  1370952_at  glutathione S-transferase, mu 2  Info   Gstm2 
   1200 3.2e-06 850.2 1708 0.498  1380393_at  crystallin, zeta (predicted)  Info   Cryz_predicted 
   1201 3.2e-06 1825 3106.8 0.587  1371916_at  CDNA clone MGC:105753 IMAGE:7316479  Info    
   1202 3.2e-06 934.1 191.8 4.87  1387952_a_at  CD44 antigen  Info   Cd44 
   1203 3.2e-06 1075.9 1803.3 0.597  1374222_at  tumor suppressing subtransferable candidate 5  Info   RGD:1303323 
   1204 3.2e-06 112 214.4 0.522  1382010_at    Info    
   1205 3.2e-06 61.4 23.1 2.658  1373751_at  Transcribed locus  Info    
   1206 3.2e-06 28.9 60.2 0.48  1379710_at  Transcribed locus, weakly similar to NP_081541.1 hypothetical protein LOC69933 [Mus musculus]  Info    
   1207 3.3e-06 85.5 17 5.029  1397437_at  AT rich interactive domain 5A (Mrf1 like) (predicted)  Info    
   1208 3.3e-06 262.2 81.3 3.225  1369688_s_at  protein tyrosine kinase 2 beta  Info   Ptk2b 
   1209 3.3e-06 285.7 182.6 1.565  1373273_at  similar to hypothetical protein FLJ14936 (predicted)  Info   RGD1311129_predicted 
   1210 3.3e-06 1096 716.3 1.53  1398877_at  stress-induced-phosphoprotein 1  Info   Stip1 
   1211 3.3e-06 481.2 788.9 0.61  1386895_at  melanoma antigen, family D, 1  Info   Maged1 
   1212 3.3e-06 230.6 138.5 1.665  1374034_at  cysteinyl-tRNA synthetase (predicted)  Info   Cars_predicted 
   1213 3.3e-06 135.1 35.6 3.795  1391803_at  Transcribed locus  Info    
   1214 3.3e-06 1.1 5.1 0.216  1386770_x_at  potassium voltage-gated channel, Isk-related family, member 2  Info   Kcne2 
   1215 3.4e-06 307.7 143.1 2.15  1388436_at  small nuclear ribonucleoprotein polypeptide A (predicted)  Info   Snrpa_predicted 
   1216 3.4e-06 23.2 52.3 0.444  1382490_at    Info    
   1217 3.4e-06 2665.1 3247.4 0.821  1399012_at  similar to RIKEN cDNA 1110001M20 (predicted)  Info   RGD1310313_predicted 
   1218 3.4e-06 132.4 274.6 0.482  1391509_at  tumor-associated calcium signal transducer 2  Info   RGD:1359498 
   1219 3.4e-06 575.7 122.3 4.707  1377092_at  Transcribed locus  Info    
   1220 3.4e-06 483.6 279.1 1.733  1376869_at  Transducin-like enhancer of split 4, E(spl) homolog (Drosophila)  Info   Tle4 
   1221 3.5e-06 170.8 30.3 5.637  1379598_at  Adenylate cyclase 7  Info   Adcy7 
   1222 3.5e-06 310.2 53.3 5.82  1368965_at  monocarboxylate transporter  Info   Slc16a3 
   1223 3.5e-06 75 166.6 0.45  1373324_at  dual specificity phosphatase 14 (predicted) /// similar to Dual specificity phosphatase 14  Info   Dusp14_predicted /// LOC499736 
   1224 3.5e-06 2199 119.9 18.34  1393219_at  complement component 2  Info   C2 
   1225 3.5e-06 445.1 216.9 2.052  1374265_at  Similar to arylacetamide deacetylase (esterase) (predicted)  Info    
   1226 3.5e-06 244.2 24.8 9.847  1393863_at  CDNA clone MGC:124654 IMAGE:5622000  Info    
   1227 3.5e-06 92.4 228.6 0.404  1382447_at    Info    
   1228 3.5e-06 82.8 175.7 0.471  1372626_at  Similar to tumor protein D53  Info    
   1229 3.5e-06 683.2 381 1.793  1388794_at  RNA binding motif protein, X chromosome retrogene (predicted)  Info   Rbmxrt_predicted 
   1230 3.5e-06 6188.1 1621.7 3.816  1371447_at  placenta-specific 8 (predicted)  Info   Plac8_predicted 
   1231 3.5e-06 697.3 468.2 1.489  1368158_at  sec1 family domain containing 1  Info   Scfd1 
   1232 3.5e-06 772.7 1117.1 0.692  1386978_at  BCL2/adenovirus E1B 19 kDa-interacting protein 3-like  Info   Bnip3l 
   1233 3.6e-06 33.8 146.1 0.231  1372615_at  amine oxidase, copper containing 3  Info   Aoc3 
   1234 3.6e-06 61.2 122.7 0.499  1382270_at  Transcribed locus  Info    
   1235 3.6e-06 241.6 366.2 0.66  1383290_at  hepatocyte growth factor activator inhibitor 1  Info   RGD:1303138 
   1236 3.6e-06 1436.6 343.6 4.181  1387659_at  guanine deaminase  Info   Gda 
   1237 3.7e-06 1528 462.7 3.302  1368420_at  ceruloplasmin  Info   Cp 
   1238 3.7e-06 239.2 501.2 0.477  1393067_at    Info    
   1239 3.7e-06 123 60.6 2.03  1382158_at  Transcribed locus  Info    
   1240 3.7e-06 3916 172 22.767  1373992_at  similar to interferon-inducible GTPase /// similar to MGC108823 protein /// similar to MGC108823 protein  Info   RGD:1359421 /// LOC498871 /// LOC498872 
   1241 3.7e-06 2839 3912.8 0.726  1386953_at  hydroxysteroid 11-beta dehydrogenase 1  Info   Hsd11b1 
   1242 3.7e-06 3829.3 5974.2 0.641  1367988_at  cytochrome P450, family 2, subfamily c, polypeptide 23  Info   Cyp2c23 
   1243 3.7e-06 76.4 42.7 1.789  1384151_at  Transcribed locus  Info    
   1244 3.7e-06 2875.1 3770.3 0.763  1368504_at  lysosomal membrane glycoprotein 1  Info   Lamp1 
   1245 3.7e-06 113.8 252.2 0.451  1389310_at  Transcribed locus  Info    
   1246 3.7e-06 64.1 20.5 3.127  1385019_at    Info    
   1247 3.7e-06 289.8 371.2 0.781  1373502_at  Similar to RIKEN cDNA 4933427L07 (predicted)  Info    
   1248 3.7e-06 479.4 274.5 1.746  1371613_at  engulfment and cell motility 2, ced-12 homolog (C. elegans) (predicted)  Info   Elmo2_predicted 
   1249 3.7e-06 354.4 23 15.409  1369801_at  selectin, lymphocyte  Info   Sell 
   1250 3.7e-06 609.1 155.3 3.922  1380410_at  Transcribed locus  Info    
   1251 3.7e-06 385.3 153.9 2.504  1379356_at  Similar to RIKEN cDNA C230093N12 (predicted)  Info    
   1252 3.7e-06 637.8 1305.9 0.488  1385072_at  galactose mutarotase (aldose 1-epimerase)  Info   RGD:1359459 
   1253 3.7e-06 141.4 46.7 3.028  1369208_at  interleukin 7  Info   Il7 
   1254 3.8e-06 1273.2 1964.5 0.648  1371388_at  pyruvate dehydrogenase (lipoamide) beta  Info   RGD:1359146 
   1255 3.8e-06 100.9 239 0.422  1391331_at  Transcribed locus  Info    
   1256 3.8e-06 65.2 101.7 0.641  1398366_at  LOC497968  Info   LOC497968 
   1257 3.8e-06 526.2 137.6 3.824  1383448_at  interferon dependent positive acting transcription factor 3 gamma (predicted)  Info   Isgf3g_predicted 
   1258 3.8e-06 120.3 586.4 0.205  1381776_at  similar to RIKEN cDNA 1810015P03 (predicted)  Info   RGD1305260_predicted 
   1259 3.8e-06 1126.3 2113 0.533  1388555_at  thioredoxin-like 5 (predicted)  Info   Txnl5_predicted 
   1260 3.8e-06 253.9 47.3 5.368  1378150_at  caspase recruitment domain family, member 11 (predicted)  Info   Card11_predicted 
   1261 3.8e-06 231.1 626.9 0.369  1387932_at  solute carrier family 1 (neuronal/epithelial high affinity glutamate transporter, system Xag), member 1  Info   Slc1a1 
   1262 3.9e-06 280.1 512 0.547  1387926_at  sterol-C5-desaturase (fungal ERG3, delta-5-desaturase) homolog (S. cerevisae)  Info   Sc5d 
   1263 3.9e-06 37.3 102.7 0.363  1373736_at  Transcribed locus, weakly similar to NP_082606.1 hypothetical protein LOC72704 [Mus musculus]  Info    
   1264 3.9e-06 154 335 0.46  1397830_at  Insulin-like growth factor binding protein 5  Info   Igfbp5 
   1265 3.9e-06 357.3 185.1 1.93  1387925_at  asparagine synthetase  Info   Asns 
   1266 3.9e-06 446.7 711.8 0.628  1372030_at  zinc finger, FYVE domain containing 21 (predicted)  Info   Zfyve21_predicted 
   1267 3.9e-06 261.3 39 6.7  1370469_at  protein tyrosine phosphatase, non-receptor type 7  Info   Ptpn7 
   1268 3.9e-06 2071 3272.1 0.633  1370207_at  ATP synthase, H+ transporting, mitochondrial F0 complex, subunit c (subunit 9), isoform 2 /// similar to ATP synthase, H+ transporting, mitochondrial F0 complex, subunit c (subunit 9), isoform 2  Info   Atp5g2 /// LOC499512 
   1269 3.9e-06 137.7 381.8 0.361  1368509_at  Bardet-Biedl syndrome 2 (human)  Info   Bbs2 
   1270 3.9e-06 183.1 430.8 0.425  1375926_at  Transcribed locus  Info    
   1271 3.9e-06 955 1353.7 0.705  1373585_at  similar to RIKEN cDNA 1810020G14 (predicted)  Info   RGD1310781_predicted 
   1272 3.9e-06 62.6 112.2 0.558  1382384_at  similar to Ras association (RalGDS/AF-6) domain family 6  Info   MGC114410 
   1273 3.9e-06 61.4 109 0.563  1373181_at  Similar to actin filament associated protein; actin filament-associated protein, 110 kDa (predicted)  Info    
   1274 3.9e-06 152.7 354.5 0.431  1372199_at  PTEN induced putative kinase 1 (predicted)  Info   Pink1_predicted 
   1275 3.9e-06 352.6 743.2 0.474  1368085_at  GTP cyclohydrolase I feedback regulator  Info   Gchfr 
   1276 4e-06 268.2 132.6 2.023  1374857_at  similar to nucleolar protein family A, member 1  Info   LOC499709 
   1277 4e-06 934 187.5 4.981  1388694_at  RT1-149 protein  Info   RT1-149 
   1278 4e-06 272.1 126.9 2.144  1373557_at  mini chromosome maintenance deficient 4 homolog (S. cerevisiae) /// similar to mcdc21 protein  Info   Mcmd4 /// LOC310429 
   1279 4e-06 261.6 448 0.584  1373515_at  Similar to RIKEN cDNA 1110067D22 (predicted)  Info    
   1280 4e-06 3360.3 793.4 4.235  1390738_at  bone marrow stromal cell antigen 2  Info   Bst2 
   1281 4e-06 359.3 650.4 0.552  1373854_at  CDNA clone IMAGE:7324675  Info    
   1282 4e-06 111.4 219.6 0.507  1368371_at  potassium voltage-gated channel, subfamily Q, member 1  Info   Kcnq1 
   1283 4e-06 45.8 124.1 0.369  1383826_at  Rab40b, member RAS oncogene family (predicted)  Info   Rab40b_predicted 
   1284 4e-06 440.9 63.3 6.965  1372604_at  similar to RIKEN cDNA 2210421G13  Info   LOC315106 
   1285 4e-06 619 256.6 2.412  1372643_at  similar to protein 4.1G  Info   LOC309557 
   1286 4e-06 365.6 750.7 0.487  1374800_at  Solute carrier family 25 (mitochondrial carrier; ornithine transporter) member 15 (predicted)  Info    
   1287 4e-06 158.8 241.7 0.657  1373434_at  Transcribed locus  Info    
   1288 4e-06 256.2 84.2 3.043  1374725_at  similar to mKIAA1631 protein  Info   LOC310756 
   1289 4e-06 688.5 861.5 0.799  1389907_at  similar to archease  Info   LOC297885 
   1290 4e-06 559.5 296.2 1.889  1388397_at  EBNA1 binding protein 2 (predicted)  Info   Ebna1bp2_predicted 
   1291 4e-06 1644.1 2771.9 0.593  1390020_at    Info    
   1292 4e-06 12.8 42.1 0.304  1391228_at  testis specific gene A2 (predicted)  Info   Tsga2_predicted 
   1293 4e-06 1024.7 1996.7 0.513  1368548_at  solute carrier family 12, member 1  Info   Slc12a1 
   1294 4.1e-06 278.2 135.7 2.05  1395248_at  similar to mKIAA0212 protein  Info   LOC297504 
   1295 4.1e-06 1522.4 2455.8 0.62  1368178_at  PDZ domain containing 1  Info   Pdzk1 
   1296 4.1e-06 1169.9 673.3 1.738  1391411_at  Formin-like 2 (predicted)  Info    
   1297 4.1e-06 162.5 22 7.386  1370769_a_at  inducible T-cell co-stimulator  Info   Icos 
   1298 4.1e-06 88.6 24.5 3.616  1387763_at  low Mr GTP-binding protein  Info   Rab27a 
   1299 4.1e-06 359.3 235.7 1.524  1370974_at  Vps54-like  Info   Vps54 
   1300 4.1e-06 521.8 398.6 1.309  1372058_at  Transcribed locus, strongly similar to NP_776109.1 NudC domain containing 3 [Mus musculus]  Info    
   1301 4.1e-06 1236.7 2007.4 0.616  1385656_at  similar to hypothetical protein MGC13034  Info   LOC499516 
   1302 4.1e-06 98 275.2 0.356  1378197_at  kinesin family member C2  Info   KIFC2 
   1303 4.2e-06 192.6 1025.2 0.188  1370384_a_at  prolactin receptor  Info   Prlr 
   1304 4.2e-06 105.9 288.5 0.367  1378307_at  Pleckstrin homology domain containing, family H (with MyTH4 domain) member 1 (predicted)  Info    
   1305 4.2e-06 3011.1 4281.2 0.703  1371417_at  low molecular mass ubiquinone-binding protein  Info   Qpc 
   1306 4.2e-06 261.3 88.8 2.943  1375989_a_at  similar to nuclear factor kappa B subunit p100 (predicted)  Info   RGD1307189_predicted 
   1307 4.2e-06 191.3 395.9 0.483  1368882_at  sialyltransferase 7c  Info   Siat7c 
   1308 4.2e-06 179.2 52.2 3.433  1383601_at  DUF729 domain containing 1 (predicted)  Info    
   1309 4.2e-06 154.1 378.4 0.407  1379419_at  DNA sequence AY228474  Info   AY228474 
   1310 4.2e-06 103 35.5 2.901  1376784_at  LOC499775  Info   LOC499775 
   1311 4.2e-06 327.4 149.1 2.196  1375585_at  Transcribed locus  Info    
   1312 4.2e-06 1090.4 1621.5 0.672  1398325_at  transmembrane 4 superfamily member 8  Info   RGD:1359444 
   1313 4.2e-06 60.3 125.6 0.48  1393729_at  neuropilin (NRP) and tolloid (TLL)-like 2 (predicted)  Info   Neto2_predicted 
   1314 4.2e-06 167 319.5 0.523  1382325_at  glycine C-acetyltransferase (2-amino-3-ketobutyrate-coenzyme A ligase) (predicted)  Info   Gcat_predicted 
   1315 4.3e-06 129.8 274.8 0.472  1398500_at  G protein-coupled receptor 116  Info   Gpr116 
   1316 4.3e-06 906.4 2297.5 0.395  1368467_at  cytochrome P450, family 4, subfamily F, polypeptide 2  Info   Cyp4f2 
   1317 4.3e-06 239.3 3 79.767  1387667_at  nitric oxide synthase 2, inducible  Info   Nos2 
   1318 4.3e-06 801.9 290.3 2.762  1374601_at  interferon gamma receptor 2 (predicted)  Info   Ifngr2_predicted 
   1319 4.3e-06 259.5 18.4 14.103  1387742_at  chemokine (C-C motif) receptor 2  Info   Ccr2 
   1320 4.3e-06 1866.3 1162.1 1.606  1374397_at  eukaryotic translation initiation factor 2, subunit 2 (beta)  Info   Eif2s2 
   1321 4.3e-06 150.9 74.3 2.031  1399118_at  similar to RIKEN cDNA 4121402D02  Info   LOC303514 
   1322 4.3e-06 1426.9 1045.8 1.364  1370130_at  ras homolog gene family, member A  Info   RGD:619921 
   1323 4.3e-06 696.5 472.4 1.474  1383368_at  Unknown (protein for MGC:72610)  Info   MGC72610 
   1324 4.3e-06 236.6 116.3 2.034  1387866_at  myosin IXb  Info   Myo9b 
   1325 4.3e-06 99.6 193.2 0.516  1372398_at  similar to Potassium channel tetramerisation domain containing 15  Info   LOC499129 
   1326 4.4e-06 358.3 714.8 0.501  1372104_at    Info    
   1327 4.4e-06 55.1 91 0.605  1393116_at  Transcribed locus  Info    
   1328 4.4e-06 74.9 17.2 4.355  1369306_at  killer cell lectin-like receptor, subfamily D, member 1  Info   Klrd1 
   1329 4.4e-06 209.3 375.6 0.557  1367752_at  breast cancer anti-estrogen resistance 1  Info   Bcar1 
   1330 4.4e-06 576.6 215 2.682  1368103_at  ATP-binding cassette, sub-family G (WHITE), member 1  Info   Abcg1 
   1331 4.5e-06 189.3 26.6 7.117  1379311_at  AT rich interactive domain 5A (Mrf1 like) (predicted)  Info    
   1332 4.5e-06 772.6 491.9 1.571  1371803_at  GM2 ganglioside activator protein  Info   Gm2a 
   1333 4.5e-06 3061 557.1 5.495  1368187_at  glycoprotein (transmembrane) nmb  Info   Gpnmb 
   1334 4.5e-06 206.1 404.6 0.509  1372502_at  Transcribed locus, moderately similar to NP_035028.2 neurofibromatosis 2 [Mus musculus]  Info    
   1335 4.5e-06 100.8 164.9 0.611  1379719_at  Transcribed locus  Info    
   1336 4.5e-06 405.3 585.9 0.692  1374508_at  nephronophthisis 1 (juvenile) (predicted)  Info   Nphp1_predicted 
   1337 4.5e-06 89.2 143.7 0.621  1389456_at  Transcribed locus  Info    
   1338 4.5e-06 85.4 37.4 2.283  1369471_at  rapostlin  Info   Fnbp1 
   1339 4.5e-06 112.5 46.5 2.419  1382730_at  Similar to osteoclast inhibitory lectin  Info    
   1340 4.5e-06 141.8 208.7 0.679  1394407_at  Transcribed locus  Info    
   1341 4.5e-06 216.4 108 2.004  1396267_at    Info    
   1342 4.5e-06 159.7 284.9 0.561  1371494_at  Transcribed locus  Info    
   1343 4.5e-06 105.5 44.1 2.392  1392032_at  GRB2-related adaptor protein (predicted)  Info   Grap_predicted 
   1344 4.6e-06 375.7 80.9 4.644  1386277_at  ubiquitin-activating enzyme E1-like (predicted)  Info   Ube1l_predicted 
   1345 4.6e-06 196.3 85.1 2.307  1389477_at  Transcribed locus  Info    
   1346 4.6e-06 940.7 1963.3 0.479  1376051_at  crystallin, lamda 1  Info   Cryl1 
   1347 4.6e-06 1989.4 2524.9 0.788  1368653_a_at  fertility protein SP22  Info   Park7 
   1348 4.6e-06 371.7 623.4 0.596  1390918_at  GH regulated TBC protein 1 (predicted)  Info   Grtp1_predicted 
   1349 4.6e-06 853.1 1160.1 0.735  1368552_at  GrpE-like 1, mitochondrial  Info   Grpel1 
   1350 4.6e-06 78.8 170.6 0.462  1388994_at  Transcribed locus  Info    
   1351 4.6e-06 53.5 127.5 0.42  1379587_at    Info    
   1352 4.6e-06 67.1 104.9 0.64  1398818_at  guanine nucleotide binding protein (G protein), gamma 5 subunit  Info   Gng5 
   1353 4.7e-06 3514.9 4406.5 0.798  1367622_at  ATP synthase, H+ transporting, mitochondrial F0 complex, subunit d  Info   Atp5h 
   1354 4.7e-06 10.3 1.2 8.583  1378244_at  Transcribed locus  Info    
   1355 4.7e-06 73.5 186.1 0.395  1382221_at  Transcribed locus  Info    
   1356 4.7e-06 182.4 286.6 0.636  1390508_at  Similar to hypothetical protein MGC27648 (predicted)  Info    
   1357 4.8e-06 148.7 297.9 0.499  1373803_a_at  growth hormone receptor  Info   Ghr 
   1358 4.8e-06 2488.5 234.8 10.598  1368000_at  complement component 3  Info   C3 
   1359 4.8e-06 208.1 77.1 2.699  1371027_at  Casitas B-lineage lymphoma b  Info   Cblb 
   1360 4.8e-06 229 124.4 1.841  1378482_at  kidney cell line derived transcript 1 (predicted)  Info   Kdt1_predicted 
   1361 4.8e-06 937.7 1462 0.641  1386946_at  carnitine palmitoyltransferase 1, liver  Info   Cpt1a 
   1362 4.8e-06 176.2 451.9 0.39  1375961_at  frizzled-related protein (predicted)  Info   Frzb_predicted 
   1363 4.8e-06 142.8 292 0.489  1382660_at  Transcribed locus  Info    
   1364 4.9e-06 1553.6 694 2.239  1388809_at  acid sphingomyelinase-like phosphodiesterase 3A  Info   RGD:1359277 
   1365 4.9e-06 1390.3 2613.8 0.532  1375436_at  nicotinate phosphoribosyltransferase-like protein  Info   RGD:1302945 
   1366 4.9e-06 241.7 449.1 0.538  1373465_at  PQ loop repeat containing 1 (predicted)  Info   Pqlc1_predicted 
   1367 4.9e-06 504.2 1342.1 0.376  1379464_at  Transcribed locus  Info    
   1368 4.9e-06 728.7 933.3 0.781  1388603_a_at  HesB protein  Info   Hbld2 
   1369 4.9e-06 692 400.2 1.729  1388150_at  exportin 1, CRM1 homolog (yeast)  Info   Xpo1 
   1370 4.9e-06 606.1 366 1.656  1379450_at  similar to cDNA sequence BC003236 (predicted)  Info   RGD1310822_predicted 
   1371 4.9e-06 509.2 1454.2 0.35  1371363_at  glycerol-3-phosphate dehydrogenase 1 (soluble)  Info   Gpd1 
   1372 5e-06 79.1 31 2.552  1385382_at  Transcribed locus  Info    
   1373 5e-06 702.5 364 1.93  1388353_at  proliferation-associated 2G4, 38kDa  Info   RGD:1302994 
   1374 5e-06 56.1 22.9 2.45  1382792_at  Transcribed locus  Info    
   1375 5e-06 67.7 125.3 0.54  1374089_at  Transcribed locus  Info    
   1376 5e-06 971.8 524.7 1.852  1388753_at  sulfatase 2 (predicted)  Info   Sulf2_predicted 
   1377 5e-06 240.5 394.5 0.61  1373198_at  similar to RIKEN cDNA 2810451A06 (predicted)  Info   RGD1311098_predicted 
   1378 5e-06 43.5 86 0.506  1392149_at  Eukaryotic translation initiation factor 4B (predicted)  Info   MGC94923 
   1379 5e-06 1119.3 780.7 1.434  1372460_at  LOC499767  Info   LOC499767 
   1380 5e-06 271.5 146.3 1.856  1380867_a_at  pleckstrin homology domain containing, family M (with RUN domain) member 1 (predicted)  Info   Plekhm1_predicted 
   1381 5.1e-06 440.6 115.8 3.805  1372055_at  Similar to P-Rex1 (predicted)  Info    
   1382 5.1e-06 123.5 194.7 0.634  1391526_at  Kinesin family member 16B (predicted)  Info    
   1383 5.1e-06 2239.9 3938.9 0.569  1369625_at  aquaporin 1  Info   Aqp1 
   1384 5.1e-06 57.9 205.9 0.281  1394881_at  similar to 6430514L14Rik protein (predicted)  Info   RGD1311958_predicted 
   1385 5.1e-06 1244.4 2125.5 0.585  1387851_at  phosphotriesterase related  Info   Pter 
   1386 5.1e-06 340.9 119.1 2.862  1380357_a_at  Transcribed locus  Info    
   1387 5.1e-06 64.2 24 2.675  1383794_at    Info    
   1388 5.2e-06 126.4 55.9 2.261  1395183_at  Transcribed locus  Info    
   1389 5.2e-06 179.1 88.8 2.017  1384147_at  eukaryotic translation initiation factor 1A (predicted)  Info   Eif1a_predicted 
   1390 5.2e-06 80.6 173.1 0.466  1371665_at  SWI/SNF related, matrix associated, actin dependent regulator of chromatin, subfamily d, member 3 (predicted)  Info   Smarcd3_predicted 
   1391 5.2e-06 2166.1 473.1 4.579  1372011_at  Guanine deaminase  Info   Gda 
   1392 5.2e-06 170.9 372.5 0.459  1386881_at  insulin-like growth factor binding protein 3  Info   Igfbp3 
   1393 5.2e-06 23.3 76.2 0.306  1384970_at  similar to RIKEN cDNA 5330437I02 gene  Info   LOC361340 
   1394 5.2e-06 48.3 4.8 10.062  1387880_at  CUG triplet repeat,RNA-binding protein 2  Info   Cugbp2 
   1395 5.2e-06 322.6 671.6 0.48  1388618_at  nidogen 2 (predicted)  Info   Nid2_predicted 
   1396 5.2e-06 354.6 182.6 1.942  1368179_at  thymic stromal-derived lymphopoietin, receptor  Info   Tslpr 
   1397 5.2e-06 850.4 470.2 1.809  1373933_at  Rap guanine nucleotide exchange factor (GEF) 2 (predicted)  Info   Rapgef2_predicted 
   1398 5.2e-06 791.7 1636.8 0.484  1387084_at  dipeptidylpeptidase 4  Info   Dpp4 
   1399 5.3e-06 2913.4 4027.4 0.723  1386891_at  phosphatidylethanolamine binding protein  Info   Pbp 
   1400 5.3e-06 606.3 735.8 0.824  1367767_at  3-hydroxy-3-methylglutaryl-Coenzyme A lyase  Info   Hmgcl 
   1401 5.3e-06 104.1 195.4 0.533  1369085_s_at  small nuclear ribonucleoprotein N /// SNRPN upstream reading frame  Info   Snrpn /// Snurf 
   1402 5.3e-06 98.2 298.1 0.329  1367806_at  glutaminase  Info   Gls 
   1403 5.3e-06 828.7 638.6 1.298  1372827_at  peptidylprolyl isomerase D  Info   RGD:1303174 
   1404 5.3e-06 575 149.4 3.849  1390348_at  folate receptor 2 (fetal) (predicted)  Info   Folr2_predicted 
   1405 5.3e-06 88.2 127.5 0.692  1377244_at  Zinc finger protein 95 (predicted)  Info    
   1406 5.3e-06 218.2 93.2 2.341  1382843_at  sphingosine phosphate lyase 1  Info   Sgpl1 
   1407 5.3e-06 487.3 385.1 1.265  1374400_at  similar to RIKEN cDNA C920006C10 (predicted)  Info   RGD1305976_predicted 
   1408 5.3e-06 653.1 1002 0.652  1373625_at  serine hydroxymethyl transferase 1 (soluble) (predicted)  Info   Shmt1_predicted 
   1409 5.3e-06 248.3 28.7 8.652  1386009_at  Transcribed locus  Info    
   1410 5.4e-06 599.4 344.7 1.739  1368136_at  thymopoietin  Info   Tmpo 
   1411 5.4e-06 108 48.4 2.231  1376513_a_at    Info    
   1412 5.4e-06 240.8 82.2 2.929  1388195_at  CUG triplet repeat,RNA-binding protein 2  Info   Cugbp2 
   1413 5.4e-06 609.5 1296 0.47  1375267_at  peptidylprolyl isomerase C  Info   RGD:1303221 
   1414 5.4e-06 61.8 181 0.341  1377993_at  similar to hypothetical protein FLJ12681 (predicted)  Info   RGD1310180_predicted 
   1415 5.4e-06 1444.7 2365.9 0.611  1368512_a_at  glutamyl aminopeptidase  Info   Enpep 
   1416 5.5e-06 268.1 684.6 0.392  1394020_at  Transcribed locus  Info    
   1417 5.5e-06 102.9 162 0.635  1382039_at  RAB, member of RAS oncogene family-like 2A (predicted)  Info   Rabl2a_predicted 
   1418 5.5e-06 1319.7 706.2 1.869  1371694_at    Info    
   1419 5.5e-06 73.3 223.2 0.328  1370399_at  cytochrome P450, family 4, subfamily b, polypeptide 1  Info   Cyp4b1 
   1420 5.5e-06 1283 2843.8 0.451  1368236_at  meprin 1 alpha  Info   Mep1a 
   1421 5.5e-06 141.6 29.6 4.784  1390674_at  Transcribed locus  Info    
   1422 5.5e-06 366.1 548.1 0.668  1390292_at  transmembrane protein 8 (five membrane-spanning domains) (predicted)  Info   Tmem8_predicted 
   1423 5.5e-06 221.2 422.3 0.524  1395694_at  f-box only protein 9 (predicted)  Info   Fbxo9_predicted 
   1424 5.5e-06 604.9 1373.4 0.44  1370020_at  solute carrier family 25 (mitochondrial carrier; dicarboxylate transporter), member 10  Info   RGD:621430 
   1425 5.5e-06 173.4 33.7 5.145  1369552_at  SAM domain, SH3 domain and nuclear localisation signals, 1  Info   Samsn1 
   1426 5.5e-06 1518.4 3453.7 0.44  1367843_at  aldo-keto reductase family 7, member A2 (aflatoxin aldehyde reductase)  Info   RGD:620311 
   1427 5.5e-06 483.7 50.1 9.655  1371079_at  Fc receptor, IgG, low affinity IIb  Info   Fcgr2b 
   1428 5.6e-06 36.8 183.9 0.2  1392915_at  procollagen, type XI, alpha 1  Info   Col11a1 
   1429 5.6e-06 825.9 1613.9 0.512  1367892_at  pyruvate dehydrogenase kinase, isoenzyme 2  Info   Pdk2 
   1430 5.6e-06 94.8 160.6 0.59  1374708_at  similar to Rho guanine nucleotide exchange factor (GEF) 10  Info   LOC306618 
   1431 5.6e-06 171.1 300.7 0.569  1372422_at  Sulfatase modifying factor 1 (predicted)  Info    
   1432 5.6e-06 274.8 473.9 0.58  1390602_a_at  similar to RIKEN cDNA C430004E15  Info   LOC499749 
   1433 5.6e-06 112.4 33.7 3.335  1385502_at  Tripartite motif protein 21 (predicted)  Info    
   1434 5.7e-06 99.6 169.2 0.589  1394612_at    Info    
   1435 5.7e-06 74 373 0.198  1382683_a_at    Info    
   1436 5.7e-06 79.1 141.7 0.558  1393666_at  Transcribed locus  Info    
   1437 5.7e-06 118.4 294.6 0.402  1374432_at    Info    
   1438 5.7e-06 95.7 225.7 0.424  1393531_at  Transcribed locus  Info    
   1439 5.7e-06 3987.4 5623.1 0.709  1398326_at  similar to Nur77 downstream protein 2  Info   RGD:1359417 
   1440 5.7e-06 260.9 99.4 2.625  1368973_at  adenosine deaminase, RNA-specific  Info   Adar 
   1441 5.7e-06 744.2 273.1 2.725  1388416_at  low density lipoprotein receptor-related protein 1 (predicted)  Info   Lrp1_predicted 
   1442 5.7e-06 170.2 345.7 0.492  1397584_at  Transcribed locus  Info    
   1443 5.8e-06 1190.9 889.3 1.339  1373508_at  similar to Selenoprotein T precursor  Info   LOC499625 
   1444 5.8e-06 213 89.4 2.383  1368555_at  CD37 antigen  Info   Cd37 
   1445 5.8e-06 614.9 859.1 0.716  1371609_at  homolog of zebrafish ES1  Info   RGD:1303003 
   1446 5.8e-06 256.1 142.4 1.798  1388992_at  E1A binding protein p400 (predicted)  Info   Ep400_predicted 
   1447 5.8e-06 945.1 554.5 1.704  1388345_at    Info    
   1448 5.8e-06 388.4 251.3 1.546  1383603_at  Transcribed locus, moderately similar to XP_579465.1 PREDICTED: DNA binding protein N5 [Rattus norvegicus]  Info    
   1449 5.8e-06 3033.9 2220.5 1.366  1398812_at  proteasome (prosome, macropain) subunit, beta type 1  Info   Psmb1 
   1450 5.8e-06 2098.4 3107.5 0.675  1370613_s_at    Info   Ugt1a1 /// Ugt1a6 /// Ugt1a7 /// Ugt1a8 /// Ugt1a2 /// Ugt1a4 /// Ugt1a11 /// Ugt1a5 
   1451 5.9e-06 145.6 262 0.556  1389111_at  Transcribed locus  Info    
   1452 5.9e-06 198 452.8 0.437  1393209_at  Bartter syndrome, infantile, with sensorineural deafness (Barttin)  Info   bsnd 
   1453 5.9e-06 4902.4 404.9 12.108  1382950_at  Guanylate nucleotide binding protein 3 (predicted)  Info    
   1454 5.9e-06 53.9 113.9 0.473  1386914_at  guanosine monophosphate reductase  Info   Gmpr 
   1455 5.9e-06 182.2 75.8 2.404  1393745_at  origin recognition complex, subunit 3-like (S. cerevisiae) (predicted)  Info   Orc3l_predicted 
   1456 5.9e-06 145.1 426.4 0.34  1372976_at  similar to Dorz1 (predicted)  Info   RGD1309721_predicted 
   1457 5.9e-06 22.9 41.8 0.548  1396565_at  LOC499891  Info   LOC499891 
   1458 5.9e-06 366 581 0.63  1373371_a_at  similar to RIKEN cDNA 1110001J03  Info   MGC112899 
   1459 5.9e-06 35.8 156.3 0.229  1393060_at  similar to KIAA0605 gene product (predicted)  Info   RGD1305459_predicted 
   1460 5.9e-06 230.7 81.6 2.827  1377869_at  similar to carbon catabolite repression 4 protein homolog  Info   LOC310395 
   1461 5.9e-06 32.9 7.6 4.329  1382999_at  ADP-ribosyltransferase 2b  Info   Art2b 
   1462 5.9e-06 707.7 1252 0.565  1382331_at  similar to RIKEN cDNA 0610038L10 gene /// similar to RIKEN cDNA 0610038L10 gene /// similar to riboflavin kinase /// similar to riboflavin kinase  Info   LOC311241 /// LOC317214 /// LOC499328 /// LOC501601 
   1463 6e-06 38.7 88.4 0.438  1387803_at  protein phosphatase 2 (formerly 2A), regulatory subunit B (PR 52), beta isoform  Info   Ppp2r2b 
   1464 6e-06 349.9 136.2 2.569  1368573_at  karyopherin (importin) beta 1  Info   Kpnb1 
   1465 6e-06 241.6 442.1 0.546  1383248_at  flavin containing monooxygenase 5  Info   Fmo5 
   1466 6e-06 13.8 58.2 0.237  1393673_at  similar to HCDI protein (predicted)  Info   RGD1309307_predicted 
   1467 6e-06 596.7 1203.7 0.496  1373970_at  similar to RIKEN cDNA 9230117N10 (predicted)  Info   RGD1311155_predicted 
   1468 6e-06 1882.7 937 2.009  1370290_at  tubulin, beta 5  Info   Tubb5 
   1469 6e-06 267.4 625.9 0.427  1387121_a_at  N-myc downstream regulated gene 2  Info   Ndrg2 
   1470 6e-06 72.7 157.9 0.46  1393070_at  secernin 2 (predicted)  Info   Scrn2_predicted 
   1471 6.1e-06 280.4 708.4 0.396  1393061_at  similar to cDNA sequence BC021608  Info   RGD:1359544 
   1472 6.1e-06 142.8 282.3 0.506  1393281_at  caveolin  Info   Cav 
   1473 6.1e-06 92 29.8 3.087  1391955_at  similar to RAB19, member RAS oncogene family  Info   LOC500088 
   1474 6.1e-06 310.8 710.9 0.437  1383137_at  SRY-box containing gene 4 (predicted)  Info    
   1475 6.1e-06 82.1 152.3 0.539  1389650_at  Similar to zinc finger protein 366 (predicted)  Info    
   1476 6.1e-06 1440.2 99.2 14.518  1371015_at  myxovirus (influenza virus) resistance 1  Info   Mx1 
   1477 6.1e-06 114.6 34 3.371  1397655_at    Info    
   1478 6.1e-06 102.2 143.4 0.713  1368267_at  protein-O-mannosyltransferase 1  Info   Pomt1 
   1479 6.2e-06 186.1 91 2.045  1373679_at  Transcribed locus  Info    
   1480 6.2e-06 397 165.6 2.397  1393217_at  ATP-binding cassette, sub-family G (WHITE), member 3  Info   RGD:1303294 
   1481 6.2e-06 391 175.5 2.228  1370082_at  transforming growth factor, beta 1  Info   Tgfb1 
   1482 6.2e-06 168.2 356.7 0.472  1372110_at  Similar to hypothetical protein (predicted)  Info    
   1483 6.2e-06 46.9 12.8 3.664  1385831_at  Similar to RIKEN cDNA 1200013B08  Info    
   1484 6.2e-06 166.2 9.2 18.065  1396492_at  similar to NFAT activation molecule 1 (predicted)  Info   RGD1308316_predicted 
   1485 6.2e-06 68 137.8 0.493  1391669_at  protein tyrosine phosphatase, receptor type, B (predicted)  Info   Ptprb_predicted 
   1486 6.2e-06 593.9 977.6 0.608  1372395_at  Similar to KIAA0597 protein  Info    
   1487 6.3e-06 216.3 430.2 0.503  1367864_at  phosphofructokinase, muscle  Info   Pfkm 
   1488 6.3e-06 621.4 1047.3 0.593  1373011_at  hypothetical protein LOC619558  Info   LOC619558 
   1489 6.3e-06 21.5 51.2 0.42  1371984_at  Similar to mKIAA0613 protein  Info    
   1490 6.3e-06 69.6 170 0.409  1372872_at  Similar to adenylate kinase isozyme 5  Info    
   1491 6.3e-06 409.4 874.7 0.468  1372475_at  PTEN induced putative kinase 1 (predicted)  Info   Pink1_predicted 
   1492 6.3e-06 83 200.2 0.415  1384056_at  Fibroblast growth factor receptor 3  Info   Fgfr3 
   1493 6.3e-06 230.3 503.4 0.457  1368877_at  zinc finger protein 354A  Info   Znf354a 
   1494 6.3e-06 218.9 132.3 1.655  1368545_at  CASP8 and FADD-like apoptosis regulator  Info   Cflar 
   1495 6.3e-06 1137.5 841.2 1.352  1371821_at  WAS protein family, member 2 (predicted)  Info    
   1496 6.3e-06 151.1 69.3 2.18  1383945_at  uridine monophosphate kinase (predicted)  Info   Umpk_predicted 
   1497 6.3e-06 180.6 351 0.515  1372190_at  Aquaporin 4  Info   Aqp4 
   1498 6.3e-06 1058.4 1454.7 0.728  1387859_at  nitrogen fixation gene 1 (S. cerevisiae)  Info   Nfs1 
   1499 6.4e-06 124.3 295.3 0.421  1374217_at  similar to chromosome 16 open reading frame 5 (predicted)  Info   RGD1310686_predicted 
   1500 6.4e-06 178.3 356.6 0.5  1375307_at  chromobox homolog 6 (predicted)  Info   Cbx6_predicted 
   1501 6.4e-06 238.7 462 0.517  1388470_at  f-box only protein 9 (predicted)  Info   Fbxo9_predicted 
   1502 6.4e-06 208.3 455.1 0.458  1373615_at  frizzled-related protein (predicted)  Info   Frzb_predicted 
   1503 6.4e-06 746.9 27.7 26.964  1368975_at  CD38 antigen  Info   Cd38 
   1504 6.5e-06 282.7 536.8 0.527  1388734_at  Transcribed locus  Info    
   1505 6.5e-06 145.4 268 0.543  1371903_at  STIP1 homology and U-Box containing protein 1 (predicted)  Info    
   1506 6.5e-06 299.5 89.3 3.354  1391637_at  similar to RIKEN cDNA 5830480G12  Info   RGD:1359304 
   1507 6.5e-06 152 38 4  1388142_at  chondroitin sulfate proteoglycan 2  Info   Cspg2 
   1508 6.5e-06 298.9 560.1 0.534  1383453_at  similar to olfactomedin-like 1 /// olfactomedin-like 1 (predicted)  Info   LOC308917 /// Olfml1_predicted 
   1509 6.5e-06 66.4 196.9 0.337  1384301_at  Similar to Kelch-like protein 3  Info    
   1510 6.5e-06 234.9 91.9 2.556  1373345_at  amphoterin induced gene 2  Info   Amigo2 
   1511 6.5e-06 3364 2139.1 1.573  1369930_at    Info    
   1512 6.5e-06 592.3 1781.8 0.332  1368329_at  solute carrier family 22 (organic anion transporter), member 6  Info   Slc22a6 
   1513 6.6e-06 261.7 73.4 3.565  1387947_at  v-maf musculoaponeurotic fibrosarcoma oncogene family, protein B (avian)  Info   Mafb 
   1514 6.6e-06 1356.3 1787.1 0.759  1371482_at  NADH dehydrogenase (ubiquinone) Fe-S protein 2 (predicted)  Info   Ndufs2_predicted 
   1515 6.6e-06 258.4 464.8 0.556  1374221_at  Solute carrier family 29 (nucleoside transporters), member 3  Info   Slc29a3 
   1516 6.6e-06 461 251.9 1.83  1392978_at    Info    
   1517 6.6e-06 282.3 452.4 0.624  1382013_at  RNA binding motif protein 24 (predicted)  Info   Rbm24_predicted 
   1518 6.7e-06 21.8 46.6 0.468  1384936_at    Info    
   1519 6.7e-06 131.4 247.6 0.531  1373519_at  Transcribed locus  Info    
   1520 6.7e-06 216.5 323.7 0.669  1367926_at  prohibitin  Info   Phb 
   1521 6.7e-06 554.8 1044.3 0.531  1369948_at  nerve growth factor receptor (TNFRSF16) associated protein 1  Info   Ngfrap1 
   1522 6.7e-06 133.9 45.2 2.962  1387975_at  UDP-glucose ceramide glucosyltransferase  Info   Ugcg 
   1523 6.7e-06 174.6 85.8 2.035  1383199_at  Friend leukemia integration 1 (predicted)  Info    
   1524 6.7e-06 374.4 214.8 1.743  1393114_at  Transcribed locus  Info    
   1525 6.7e-06 1648.2 2306.5 0.715  1388558_at  Adenylate kinase 3  Info   Ak3 
   1526 6.7e-06 267.4 85.8 3.117  1387800_at  Fas death domain-associated protein  Info   Daxx 
   1527 6.7e-06 334.2 194.7 1.716  1398272_at  UDP-N-acetyl-alpha-D-galactosamine:(N-acetylneuraminyl)-galactosylglucosylceramide-beta-1, 4-N-acetylgalactosaminyltransferase  Info   Galgt1 
   1528 6.7e-06 486.9 293.6 1.658  1371484_at    Info    
   1529 6.7e-06 308.8 493.7 0.625  1375346_at  similar to hypothetical protein FLJ20010  Info   LOC500993 
   1530 6.7e-06 474.5 333.4 1.423  1388476_at  Tial1 cytotoxic granule-associated RNA binding protein-like 1  Info   Tial1 
   1531 6.7e-06 378.3 126.8 2.983  1372904_at  MOB1, Mps One Binder kinase activator-like 2B (yeast) (predicted)  Info   Mobkl2b_predicted 
   1532 6.8e-06 262.7 80.4 3.267  1372016_at  growth arrest and DNA-damage-inducible 45 beta (predicted)  Info   Gadd45b_predicted 
   1533 6.8e-06 67.4 27 2.496  1382624_at    Info    
   1534 6.8e-06 35.2 78.7 0.447  1380507_at  Similar to hypothetical protein MGC33630  Info    
   1535 6.8e-06 74.5 163.9 0.455  1390049_at  four and a half LIM domains 1  Info   Fhl1 
   1536 6.8e-06 98.3 34.9 2.817  1386716_at  Similar to putative membrane steroid receptor (predicted)  Info    
   1537 6.8e-06 583 310.8 1.876  1373280_at  RuvB-like 2 (E. coli) (predicted)  Info   Ruvbl2_predicted 
   1538 6.8e-06 3038.8 1773.2 1.714  1387995_a_at  interferon induced transmembrane protein 3  Info   Ifitm3 
   1539 6.8e-06 71.5 134.6 0.531  1377523_at  Transcribed locus  Info    
   1540 6.8e-06 577.2 321.2 1.797  1389658_at  similar to CG6133-PA (predicted)  Info   RGD1311954_predicted 
   1541 6.9e-06 1140.7 619.3 1.842  1371646_at  phosphogluconate dehydrogenase  Info   Pgd 
   1542 6.9e-06 313.8 156.7 2.003  1376055_at  Transcribed locus  Info    
   1543 6.9e-06 561.6 255.2 2.201  1369617_at  ubiquitin-conjugating enzyme E2N  Info   Ube2n 
   1544 6.9e-06 527.7 190.9 2.764  1380110_at    Info    
   1545 6.9e-06 379.2 668.7 0.567  1374200_at  solute carrier family 29 (nucleoside transporters), member 3  Info   Slc29a3 
   1546 6.9e-06 186.5 317.5 0.587  1388465_at  Transcribed locus  Info    
   1547 6.9e-06 295.1 166.6 1.771  1394275_at    Info    
   1548 7e-06 19.4 103.5 0.187  1386120_at  LOC500416  Info   LOC500416 
   1549 7e-06 280.6 173.5 1.617  1373207_at  Transcribed locus  Info    
   1550 7e-06 543.2 764.5 0.711  1382689_at  similar to RGD1310870_predicted protein  Info   LOC499242 
   1551 7e-06 23.9 67.1 0.356  1390127_at  Transcribed locus  Info    
   1552 7e-06 1783.3 1218.2 1.464  1389520_at  similar to Wdr1 protein  Info   LOC360950 
   1553 7e-06 509.4 24 21.225  1385276_a_at  similar to hypothetical protein FLJ11354 (predicted)  Info   RGD1310093_predicted 
   1554 7e-06 727.8 456 1.596  1373876_at    Info    
   1555 7.1e-06 3119.9 4390.5 0.711  1367706_at  voltage-dependent anion channel 1  Info   Vdac1 
   1556 7.1e-06 285.9 554.4 0.516  1367948_a_at  kinase insert domain protein receptor  Info   Kdr 
   1557 7.1e-06 1700.5 1246.3 1.364  1388134_at  eukaryotic translation elongation factor 1 delta (guanine nucleotide exchange protein)  Info   Eef1d 
   1558 7.1e-06 7 24.8 0.282  1380828_at  gamma-aminobutyric acid A receptor, alpha 1  Info   Gabra1 
   1559 7.1e-06 6633.9 373 17.785  1368332_at  guanylate nucleotide binding protein 2  Info   Gbp2 
   1560 7.1e-06 101.3 161.7 0.626  1380321_at  mitochondrial tumor suppressor 1  Info   Mtus1 
   1561 7.1e-06 121.6 52.2 2.33  1397548_at  similar to PTPL1-associated RhoGAP 1 (predicted)  Info   RGD1308662_predicted 
   1562 7.1e-06 1886.4 1298 1.453  1371505_at  heterogeneous nuclear ribonucleoprotein C (predicted)  Info   Hnrpc_predicted 
   1563 7.1e-06 238.2 86.3 2.76  1384457_at  similar to Fbxw17 protein  Info   LOC498713 
   1564 7.2e-06 1068.1 2454.5 0.435  1373991_at  Transcribed locus  Info    
   1565 7.2e-06 208.9 333.6 0.626  1382100_at  similar to RIKEN cDNA 2310047O13 (predicted)  Info   RGD1309605_predicted 
   1566 7.2e-06 295.5 371.8 0.795  1375922_at    Info    
   1567 7.2e-06 99.7 9.5 10.495  1385605_at    Info    
   1568 7.2e-06 1376.1 2014.2 0.683  1398909_at  similar to QIL1  Info   LOC301124 
   1569 7.2e-06 181.4 331.8 0.547  1388799_at  kelch-like 7 (Drosophila) (predicted)  Info   Klhl7_predicted 
   1570 7.2e-06 168.4 77.8 2.165  1386379_at  Similar to MGC68837 protein  Info    
   1571 7.2e-06 108 198.1 0.545  1398354_at  catenin alpha-like 1 (predicted)  Info   Catnal1_predicted 
   1572 7.3e-06 1923.8 2581.9 0.745  1371912_at  NADH dehydrogenase (ubiquinone) Fe-S protein 7 (predicted)  Info   Ndufs7_predicted 
   1573 7.3e-06 779.3 1868.1 0.417  1391652_at  glycine-N-acyltransferase (predicted)  Info   Glyat_predicted 
   1574 7.3e-06 888.9 467.8 1.9  1386926_at  acyl-CoA synthetase long-chain family member 5  Info   Acsl5 
   1575 7.3e-06 2603.8 3700.6 0.704  1371567_at  aldehyde dehydrogenase family 7, member A1 (predicted)  Info   Aldh7a1_predicted 
   1576 7.3e-06 901.1 2440 0.369  1371763_at  similar to RIKEN cDNA 4931406C07 (predicted)  Info   RGD1309534_predicted 
   1577 7.4e-06 1427.6 1029 1.387  1371641_at  chaperonin subunit 7 (eta) (predicted)  Info   Cct7_predicted 
   1578 7.4e-06 222.1 139.9 1.588  1377250_at  Transcribed locus  Info    
   1579 7.4e-06 381.7 129.6 2.945  1385252_at  Tripartite motif protein 34 (predicted)  Info    
   1580 7.5e-06 656.3 393.3 1.669  1384060_at  Janus kinase 1  Info   Jak1 
   1581 7.5e-06 886 617.1 1.436  1398806_at  phosphatidylinositol transfer protein  Info   Pitpn 
   1582 7.5e-06 854.1 294.6 2.899  1388340_at  Ns5atp9 protein  Info   RGD:1303041 
   1583 7.5e-06 250.2 494.8 0.506  1375756_at  Transcribed locus  Info    
   1584 7.5e-06 94.4 242.2 0.39  1394070_at  Transcribed locus  Info    
   1585 7.5e-06 134.4 205 0.656  1390289_at  similar to RIKEN cDNA A530089I17 (predicted)  Info   RGD1311375_predicted 
   1586 7.5e-06 36.7 108.4 0.339  1377877_at    Info    
   1587 7.5e-06 117.7 312.5 0.377  1372536_at  chaperone, ABC1 activity of bc1 complex like (S. pombe) (predicted)  Info   Cabc1_predicted 
   1588 7.6e-06 160.7 253.5 0.634  1396112_at  similar to BB128963 protein (predicted)  Info   RGD1305958_predicted 
   1589 7.6e-06 782 1678.8 0.466  1370818_at  2-4-dienoyl-Coenzyme A reductase 2, peroxisomal  Info   Decr2 
   1590 7.6e-06 31.2 127.8 0.244  1368271_a_at  fatty acid binding protein 4, adipocyte  Info   Fabp4 
   1591 7.6e-06 88.2 46.2 1.909  1390704_at  SDA1 domain containing 1  Info   RGD:1359112 
   1592 7.6e-06 326.6 113.5 2.878  1377116_at  Ribonuclease L (2',5'-oligoisoadenylate synthetase-dependent)  Info   Rnasel 
   1593 7.7e-06 186.9 528.4 0.354  1383063_a_at  CDNA clone IMAGE:7379585  Info    
   1594 7.7e-06 206.3 76.3 2.704  1380619_at  similar to RIKEN cDNA 3110001I22 /// similar to RIKEN cDNA 3110001I22  Info   LOC293819 /// LOC361116 
   1595 7.7e-06 97.2 267 0.364  1376858_at    Info    
   1596 7.7e-06 351.8 203.3 1.73  1367507_at  Unknown (protein for MGC:72981)  Info   RGD:735224 
   1597 7.7e-06 3196.8 5312.7 0.602  1372170_at  aminoacylase 1  Info   Acy1 
   1598 7.7e-06 78.4 139.5 0.562  1368476_at  nuclear receptor subfamily 3, group C, member 2  Info   Nr3c2 
   1599 7.7e-06 110.2 296.3 0.372  1387036_at  hairy and enhancer of split 1 (Drosophila)  Info   Hes1 
   1600 7.7e-06 539.4 722.7 0.746  1367933_at  S-adenosylmethionine decarboxylase 1  Info   Amd1 
   1601 7.7e-06 62.2 135 0.461  1368471_at  guanylate cyclase activator 2A  Info   Guca2a 
   1602 7.7e-06 243.6 572.1 0.426  1386973_a_at  mitogen activated protein kinase 8 interacting protein  Info   Mapk8ip 
   1603 7.8e-06 92.3 159.5 0.579  1374199_at  similar to Disco-interacting protein 2 homolog  Info   LOC309692 
   1604 7.8e-06 412.3 1210.5 0.341  1367838_at  CTL target antigen  Info   Cth 
   1605 7.8e-06 242 389.6 0.621  1389196_at    Info    
   1606 7.8e-06 930.3 1229.9 0.756  1371409_at  similar to cDNA sequence BC031181  Info   LOC498886 
   1607 7.8e-06 25 95.6 0.262  1378315_at  Transcribed locus  Info    
   1608 7.8e-06 247.5 179.2 1.381  1374150_at  Similar to EWS/FLI1 activated transcript 2 (EAT-2)  Info    
   1609 7.9e-06 622.3 966.3 0.644  1388483_at  cofilin 2, muscle (predicted)  Info   Cfl2_predicted 
   1610 7.9e-06 116.1 230.4 0.504  1372358_at  TG interacting factor (predicted)  Info   Tfig 
   1611 7.9e-06 131.5 80.3 1.638  1377819_at  Similar to mKIAA1111 protein  Info    
   1612 7.9e-06 1114.9 2057 0.542  1374628_at  crystallin, zeta (predicted)  Info   Cryz_predicted 
   1613 7.9e-06 751.7 1270 0.592  1368132_at  transducer of ERBB2, 1  Info   Tob1 
   1614 7.9e-06 109.8 24 4.575  1384393_at  Transcribed locus  Info    
   1615 7.9e-06 996.5 456.2 2.184  1388528_at  fibrillarin (predicted)  Info   Fbl_predicted 
   1616 7.9e-06 801.7 1681.4 0.477  1370907_at  sialyltransferase 1  Info   Siat1 
   1617 8e-06 347.4 181.7 1.912  1370258_at  basic leucine zipper and W2 domains 2  Info   Bzw2 
   1618 8e-06 315.5 448.6 0.703  1372828_at  methionine sulfoxide reductase B (predicted)  Info   Mrsb_predicted 
   1619 8e-06 436.8 351.3 1.243  1368834_at  calcium/calmodulin-dependent protein kinase II, delta  Info   Camk2d 
   1620 8e-06 72.5 208.2 0.348  1377969_at    Info    
   1621 8e-06 331.8 166.3 1.995  1377299_at  nuclear autoantigenic sperm protein  Info   RGD:1359609 
   1622 8e-06 290.7 77.8 3.737  1381014_at  interferon-induced protein 44 (predicted)  Info   Ifi44_predicted 
   1623 8e-06 2443.8 3356.5 0.728  1373017_at  succinate-Coenzyme A ligase, GDP-forming, beta subunit (predicted)  Info   Suclg2_predicted 
   1624 8e-06 188.9 464.9 0.406  1368774_a_at  espin  Info   Espn 
   1625 8.1e-06 1843.1 582.2 3.166  1367676_at  high mobility group box 2 /// similar to High mobility group protein 2 (HMG-2) /// similar to High mobility group protein 2 (HMG-2) /// similar to High mobility group protein 2 (HMG-2)  Info   Hmgb2 /// LOC498072 /// LOC498388 /// LOC498988 
   1626 8.1e-06 141.8 333.7 0.425  1396000_at  Transcribed locus  Info    
   1627 8.1e-06 349.1 547.2 0.638  1393587_a_at    Info    
   1628 8.1e-06 878.5 510.4 1.721  1367732_at  guanine nucleotide binding protein, beta 1  Info   Gnb1 
   1629 8.1e-06 291.4 441.7 0.66  1371899_at  protein kinase, interferon inducible double stranded RNA dependent activator (predicted)  Info   Prkra_predicted 
   1630 8.1e-06 203.4 329.3 0.618  1372882_at  similar to CG12379-PA (predicted)  Info   RGD1308635_predicted 
   1631 8.1e-06 206 414.6 0.497  1370247_a_at  peripheral myelin protein 22  Info   Pmp22 
   1632 8.1e-06 205.9 387.9 0.531  1368137_at  microtubule-associated protein tau /// hypothetical gene supported by NM_017212  Info   Mapt /// LOC497674 
   1633 8.2e-06 397 542.6 0.732  1398249_at  solute carrier family 25 (carnitine/acylcarnitine translocase), member 20  Info   Slc25a20 
   1634 8.2e-06 1062.4 514.5 2.065  1398769_at  coronin, actin-binding protein, 1B  Info   Coro1b 
   1635 8.2e-06 656.8 258.4 2.542  1391463_at  DEAD (Asp-Glu-Ala-Asp) box polypeptide 58 (predicted)  Info    
   1636 8.2e-06 283.9 142 1.999  1373850_at  sphingomyelin phosphodiesterase, acid-like 3B (predicted)  Info   Smpdl3b_predicted 
   1637 8.2e-06 332.3 152.2 2.183  1377634_at  BCL2/adenovirus E1B 19kDa-interacting protein 1, NIP2 (predicted)  Info   Bnip2_predicted 
   1638 8.2e-06 191.5 63 3.04  1370963_at  growth arrest specific 7  Info   Gas7 
   1639 8.2e-06 76.7 168.1 0.456  1378138_at  kinesin family member 16B (predicted)  Info   Kif16b_predicted 
   1640 8.2e-06 955.7 1271.6 0.752  1398783_at  G protein pathway suppressor 1  Info   Gps1 
   1641 8.2e-06 33.9 69.7 0.486  1390782_at  similar to RIKEN cDNA 1200003C23 (predicted)  Info   RGD1306232_predicted 
   1642 8.2e-06 58.1 28.6 2.031  1395862_at  Transcribed locus  Info    
   1643 8.3e-06 233.5 435 0.537  1387015_at  profilin 2  Info   Pfn2 
   1644 8.3e-06 4129.9 1616.2 2.555  1381593_x_at  RT1 class II, locus Ba  Info   RT1-Ba 
   1645 8.3e-06 45.6 85.8 0.531  1386876_at  adenylate cyclase 6  Info   Adcy6 
   1646 8.3e-06 25.2 57.5 0.438  1387913_at  cytochrome P450, family 2, subfamily d, polypeptide 22  Info   Cyp2d22 
   1647 8.4e-06 1046.4 681.5 1.535  1371888_at  mitochondrial ribosomal protein L24  Info   RGD:1359289 
   1648 8.4e-06 320.1 541.9 0.591  1389015_at  similar to FUN14 domain containing 2  Info   LOC361288 
   1649 8.4e-06 396.9 174.8 2.271  1385869_at  Zinc finger protein 281 (predicted)  Info    
   1650 8.4e-06 249.8 439.9 0.568  1368314_at  gamma-glutamyl carboxylase  Info   Ggcx 
   1651 8.5e-06 420 264.6 1.587  1376613_at  similar to hypothetical protein  Info   MGC73022 
   1652 8.5e-06 444.4 809.7 0.549  1375106_at  amnionless (predicted)  Info   Amn_predicted 
   1653 8.5e-06 95.3 30.5 3.125  1385361_at  ATPase, class V, type 10A (predicted)  Info   Atp10a_predicted 
   1654 8.5e-06 105 52 2.019  1378389_at  Similar to nuclear factor of activated T-cells, cytoplasmic, calcineurin-dependent 1  Info    
   1655 8.6e-06 469.1 686.6 0.683  1371729_at  Yippee-like 5 (Drosophila) (predicted)  Info    
   1656 8.6e-06 119.6 177.8 0.673  1372396_at  Similar to 2310047B19Rik protein (predicted)  Info    
   1657 8.6e-06 445.7 213.5 2.088  1370313_at  brain acyl-CoA hydrolase  Info   Bach 
   1658 8.6e-06 29.1 163.9 0.178  1379445_at  LOC500609  Info    
   1659 8.6e-06 1133.1 1619.9 0.699  1398333_at  Endothelial PAS domain protein 1  Info   Epas1 
   1660 8.6e-06 42.4 107.7 0.394  1368574_at  adrenergic receptor, alpha 1b  Info   Adra1b 
   1661 8.6e-06 750.5 313.5 2.394  1389170_at  caspase 7  Info   Casp7 
   1662 8.6e-06 1241.1 2342 0.53  1393887_at  similar to choline phosphotransferase 1; cholinephosphotransferase 1 alpha; cholinephosphotransferase 1  Info   RGD:1359283 
   1663 8.6e-06 276.2 173.8 1.589  1372433_at  similar to CG11030-PA (predicted)  Info   RGD1310211_predicted 
   1664 8.7e-06 383 123.4 3.104  1387984_at  chemokine-like factor 1  Info   Cklf1 
   1665 8.7e-06 564 1062.5 0.531  1392627_x_at    Info    
   1666 8.7e-06 116.4 234.3 0.497  1373264_at  zinc finger, BED domain containing 3 (predicted)  Info   Zbed3_predicted 
   1667 8.7e-06 3245.7 5781.2 0.561  1368077_at  fructose-1,6- biphosphatase 1  Info   Fbp1 
   1668 8.8e-06 139 63.6 2.186  1378546_at  Transcribed locus  Info    
   1669 8.8e-06 60.4 103.2 0.585  1391478_at  zinc finger protein 532 (predicted)  Info   Znf532_predicted 
   1670 8.8e-06 211.7 586.6 0.361  1389085_at  Transcribed locus  Info    
   1671 8.8e-06 263.9 101.1 2.61  1374113_at  Bromodomain adjacent to zinc finger domain, 1A (predicted)  Info    
   1672 8.8e-06 736.8 2425.3 0.304  1367871_at  cytochrome P450, family 2, subfamily e, polypeptide 1  Info   Cyp2e1 
   1673 8.8e-06 8350.3 3375.2 2.474  1367679_at  CD74 antigen (invariant polpypeptide of major histocompatibility class II antigen-associated)  Info   Cd74 
   1674 8.8e-06 309.9 169.9 1.824  1370427_at  platelet derived growth factor, alpha  Info   Pdgfa 
   1675 8.8e-06 463.4 1061.6 0.437  1391661_at  Hypothetical gene supported by NM_024381  Info   Gyk 
   1676 8.8e-06 130 327.5 0.397  1380182_at  similar to KIAA1217  Info   LOC291356 
   1677 8.9e-06 92.7 165.9 0.559  1377424_at  Transcribed locus  Info    
   1678 8.9e-06 422.9 227.6 1.858  1371074_a_at  mini chromosome maintenance deficient 6 (S. cerevisiae)  Info   Mcmd6 
   1679 8.9e-06 1265.1 2103.5 0.601  1367999_at  aldehyde dehydrogenase 2  Info   Aldh2 
   1680 8.9e-06 410.2 932.8 0.44  1379250_at  galactose mutarotase (aldose 1-epimerase)  Info   RGD:1359459 
   1681 8.9e-06 286.7 125.8 2.279  1375709_at  signal-induced proliferation-associated gene 1  Info   RGD:1303184 
   1682 8.9e-06 542.2 34.1 15.9  1377698_at  tumor necrosis factor receptor superfamily, member 5  Info   RGD:619830 
   1683 9e-06 404.4 919.1 0.44  1390172_at  dehydrogenase E1 and transketolase domain containing 1 (predicted)  Info   Dhtkd1_predicted 
   1684 9e-06 26.2 6.5 4.031  1386052_at  Transcribed locus  Info    
   1685 9e-06 158.8 340.6 0.466  1385400_at  ATPase, H+ transporting, V1 subunit B, isoform 1 (predicted)  Info   Atp6v1b1_predicted 
   1686 9e-06 122.9 213 0.577  1387916_at  cytochrome P450 4F6  Info   RGD:708365 
   1687 9.1e-06 212 604.4 0.351  1377098_at  Transcribed locus  Info    
   1688 9.1e-06 731.3 1504.2 0.486  1372676_at  similar to RIKEN cDNA 1110025H10 (predicted)  Info   RGD1304560_predicted 
   1689 9.1e-06 115.7 18.4 6.288  1392777_at  Transcribed locus  Info    
   1690 9.1e-06 3006.4 2216.5 1.356  1371575_at  Moesin  Info   Msn 
   1691 9.1e-06 87.6 149.9 0.584  1375684_at  neuraminidase 1  Info   Neu1 
   1692 9.1e-06 146.6 272.9 0.537  1379932_at  Putative chloride channel (similar to Mm Clcn4-2)  Info   Clcn4-2 
   1693 9.1e-06 332.5 487.4 0.682  1387038_at  copper chaperone for superoxide dismutase  Info   Ccs 
   1694 9.1e-06 383.3 244.9 1.565  1371633_at  beta catenin-like 1  Info   Ctnnbl1 
   1695 9.2e-06 1352.5 2272.2 0.595  1369635_at  sorbitol dehydrogenase  Info   Sord 
   1696 9.2e-06 352.7 168.9 2.088  1388915_at  Transcribed locus  Info    
   1697 9.2e-06 2082.8 3060.5 0.681  1368016_at  peroxisomal trans-2-enoyl-CoA reductase  Info   Pecr 
   1698 9.2e-06 198.3 441.1 0.45  1373122_at  ajuba homolog (Xenopus laevis)  Info   Jub 
   1699 9.2e-06 222.2 441.4 0.503  1379318_at  Transcribed locus  Info    
   1700 9.2e-06 89.7 60.8 1.475  1383352_at  Transcribed locus  Info    
   1701 9.2e-06 327.3 471.8 0.694  1372301_at  AE binding protein 1 (predicted)  Info   Aebp1_predicted 
   1702 9.2e-06 107.8 157 0.687  1372747_at  solute carrier family 20, member 2  Info   Slc20a2 
   1703 9.3e-06 213.4 129.7 1.645  1383548_at  Similar to hypothetical protein FLJ20189 (predicted)  Info    
   1704 9.3e-06 585.8 1065.3 0.55  1367604_at  cysteine-rich protein 2  Info   RGD:1302959 
   1705 9.3e-06 79.1 214 0.37  1375030_at  Transcribed locus  Info    
   1706 9.3e-06 75.2 151.9 0.495  1387765_at  mannose binding lectin 1, protein A  Info   Mbl1 
   1707 9.3e-06 333 223 1.493  1367957_at  regulator of G-protein signalling 3  Info   Rgs3 
   1708 9.3e-06 150.2 5.2 28.885  1370369_at  granzyme M (lymphocyte met-ase 1)  Info   Gzmm 
   1709 9.4e-06 1252.4 2517.1 0.498  1390530_at  Transcribed locus  Info    
   1710 9.4e-06 49.8 22 2.264  1391201_at  WD repeat and HMG-box DNA binding protein 1 (predicted)  Info    
   1711 9.4e-06 28.3 73.3 0.386  1368858_at  UDP-glucuronosyltransferase 8  Info   Ugt8 
   1712 9.4e-06 378.3 757.4 0.499  1388916_at  Transcribed locus  Info    
   1713 9.4e-06 160 254.5 0.629  1373851_at  Transcribed locus  Info    
   1714 9.5e-06 389.8 229.4 1.699  1390133_at  BCL2/adenovirus E1B 19kDa-interacting protein 1, NIP2 (predicted)  Info   Bnip2_predicted 
   1715 9.5e-06 380.3 184.8 2.058  1393029_at  CUG triplet repeat, RNA binding protein 1 (predicted)  Info    
   1716 9.5e-06 67 288 0.233  1377135_at    Info    
   1717 9.5e-06 63.8 213 0.3  1374171_at  Similar to SRY (sex determining region Y)-box 5 isoform a  Info   Abcc9 
   1718 9.5e-06 640.8 456 1.405  1375645_at  similar to DNA segment, Chr 4, ERATO Doi 22, expressed  Info   LOC500575 
   1719 9.5e-06 298.8 207.3 1.441  1390447_at    Info    
   1720 9.6e-06 70.7 12.6 5.611  1375040_at  nuclear factor, erythroid derived 2 (predicted)  Info   Nfe2_predicted 
   1721 9.6e-06 1942.4 1063.2 1.827  1390383_at  adipose differentiation-related protein  Info   RGD:1359288 
   1722 9.6e-06 156.3 259.5 0.602  1390255_at  similar to mKIAA0843 protein  Info   LOC307395 
   1723 9.6e-06 138.7 75.4 1.84  1384015_at  cytoplasmic linker associated protein 1 (predicted)  Info   Clasp1_predicted 
   1724 9.7e-06 199.9 460.3 0.434  1370638_at  ankyrin 3, epithelial  Info   RGD:620157 
   1725 9.8e-06 26 211.4 0.123  1382031_at  Transcribed locus  Info    
   1726 9.8e-06 217.7 22.9 9.507  1381556_at  Similar to BC013672 protein  Info    
   1727 9.8e-06 258.9 157.6 1.643  1387078_at  inositol polyphosphate-4-phosphatase, type 1  Info   Inpp4a 
   1728 9.8e-06 655.6 1162.8 0.564  1388874_at  Transcribed locus  Info    
   1729 9.8e-06 430.4 887.6 0.485  1378470_at  similar to i-45 protein (49.8 kD) (4H318) (predicted)  Info   RGD1311259_predicted 
   1730 9.8e-06 128.7 15.7 8.197  1368240_a_at  protein kinase C, beta 1  Info   Prkcb1 
   1731 9.9e-06 140.3 53.2 2.637  1387270_at  hematopoietically expressed homeobox  Info   Hhex 
   1732 9.9e-06 301.5 715.9 0.421  1377966_at  Transcribed locus, weakly similar to XP_579465.1 PREDICTED: DNA binding protein N5 [Rattus norvegicus]  Info    
   1733 9.9e-06 286 26.5 10.792  1382663_at  basic leucine zipper transcription factor, ATF-like (predicted)  Info   Batf_predicted 
   1734 9.9e-06 1261.4 1752.9 0.72  1398891_at  mitochondrial ribosomal protein L15 (predicted)  Info   Mrpl15_predicted 
   1735 9.9e-06 1383 2411.5 0.574  1371727_at    Info    
   1736 9.9e-06 40.9 102.1 0.401  1377456_at  Transcribed locus  Info    
   1737 9.9e-06 1265.9 1848.3 0.685  1373152_at  protease, serine, 23  Info   RGD:1359545 
   1738 9.9e-06 46.6 123.9 0.376  1382452_at  serum deprivation response protein  Info   RGD:1359345 
   1739 9.9e-06 317.9 872.1 0.365  1392411_at  tubulointerstitial nephritis antigen  Info   RGD:1359482 
   1740 9.9e-06 281.5 167.7 1.679  1370066_at  Kelch-like ECH-associated protein 1  Info   Keap1 
   1741 9.9e-06 432.4 219.8 1.967  1398572_at  RNA (guanine-7-) methyltransferase (predicted)  Info   Rnmt_predicted 
   1742 9.9e-06 121.2 324.4 0.374  1377680_at  glial cell line derived neurotrophic factor family receptor alpha 3  Info   Gfra3 
   1743 9.9e-06 185.6 81.2 2.286  1380728_at  Similar to collapsin response mediator protein-2A  Info    
   1744 1e-05 1785.6 1091.1 1.637  1388776_at  scotin  Info   RGD:1359661 
   1745 1e-05 88 26.4 3.333  1368294_at  deoxyribonuclease I-like 3  Info   Dnase1l3 
   1746 1e-05 54.9 93.3 0.588  1391224_at  similar to 4921517L17Rik protein (predicted)  Info   RGD1311678_predicted 
   1747 1.01e-05 577.9 901.4 0.641  1375932_at  Phosphoribosyl pyrophosphate synthetase 2  Info   Prps2 
   1748 1.01e-05 251.5 38.4 6.549  1379499_at  lymphotoxin B  Info   RGD:1303162 
   1749 1.01e-05 607.2 1082.5 0.561  1384028_at  Transcribed locus  Info    
   1750 1.02e-05 3270.9 3945.4 0.829  1386898_at  heat shock 10 kDa protein 1  Info   Hspe1 
   1751 1.02e-05 2224.4 3364.7 0.661  1375197_at    Info    
   1752 1.02e-05 291.2 178.5 1.631  1387950_at  Saccharomyces cerevisiae Nip7p homolog  Info   RGD:620069 
   1753 1.03e-05 247 95.1 2.597  1385143_at    Info    
   1754 1.03e-05 142.6 289.1 0.493  1396300_at  Transcribed locus  Info    
   1755 1.03e-05 577.4 791.1 0.73  1367759_at  H1 histone family, member 0  Info   H1f0 
   1756 1.03e-05 121.8 211.8 0.575  1392975_at  Transcribed locus, strongly similar to XP_525786.1 PREDICTED: similar to chromosome 2 open reading frame 7 [Pan troglodytes]  Info    
   1757 1.03e-05 278.3 83.8 3.321  1374298_at  Transcribed locus, moderately similar to XP_580018.1 PREDICTED: hypothetical protein XP_580018 [Rattus norvegicus]  Info    
   1758 1.03e-05 108 415.5 0.26  1374496_at  similar to RIKEN cDNA 1200009O22; EST AI316813 (predicted)  Info   RGD1310827_predicted 
   1759 1.04e-05 133.8 55.6 2.406  1381819_at  Transcribed locus  Info    
   1760 1.04e-05 177.2 319.8 0.554  1380975_at  Transcribed locus  Info    
   1761 1.04e-05 16.9 73.4 0.23  1390518_at  similar to putative emu1 protein  Info   LOC498403 
   1762 1.04e-05 358.7 126.6 2.833  1388836_at  protein kinase C, eta  Info   Prkch 
   1763 1.04e-05 228 358.4 0.636  1372367_at    Info    
   1764 1.04e-05 53.4 89.4 0.597  1376601_at  Sema domain, transmembrane domain (TM), and cytoplasmic domain, (semaphorin) 6A (predicted)  Info    
   1765 1.04e-05 36.8 63.8 0.577  1395534_at  Transcribed locus  Info    
   1766 1.04e-05 43.6 78.4 0.556  1377662_at  pirin  Info   RGD:1359212 
   1767 1.04e-05 444.7 949.5 0.468  1373740_at  Transcribed locus, weakly similar to XP_418630.1 PREDICTED: similar to ORF2 [Gallus gallus]  Info    
   1768 1.04e-05 575.1 1110.9 0.518  1387314_at  sulfotransferase family 1B, member 1  Info   Sult1b1 
   1769 1.04e-05 1290.6 2124.7 0.607  1391273_at  Na+ dependent glucose transporter 1  Info   RGD:631438 
   1770 1.05e-05 264.4 119.2 2.218  1383578_at  Transcribed locus  Info    
   1771 1.05e-05 492.7 108.3 4.549  1387343_at  CCAAT/enhancer binding protein (C/EBP), delta  Info   Cebpd 
   1772 1.05e-05 67.3 119 0.566  1383928_a_at  Transcribed locus  Info    
   1773 1.05e-05 515.3 1307.7 0.394  1374624_at  UDP-N-acetyl-alpha-D-galactosamine:polypeptide N-acetylgalactosaminyltransferase 11 (GalNAc-T11)  Info   Galnt11 
   1774 1.05e-05 224.4 374.1 0.6  1372557_at  ADP-ribosylation factor-like 6 (predicted)  Info   Arl6_predicted 
   1775 1.05e-05 186 406.8 0.457  1389556_at  kinesin-associated protein 3 (predicted)  Info   Kifap3_predicted 
   1776 1.05e-05 140.5 60.5 2.322  1384173_at  src family associated phosphoprotein 1  Info   Scap1 
   1777 1.06e-05 487.7 281.4 1.733  1372003_at  Transcribed locus, strongly similar to NP_056312.2 DKFZP564G2022 protein [Homo sapiens]  Info    
   1778 1.06e-05 312.5 196.9 1.587  1383962_at  Cd27 binding protein (Hindu God of destruction)  Info   Siva 
   1779 1.06e-05 54.7 250.3 0.219  1382997_at  solute carrier family 22 (organic cation transporter), member 13 (predicted)  Info   Slc22a13_predicted 
   1780 1.06e-05 324.7 540.8 0.6  1371807_at  similar to apoptosis related protein APR-3; p18 protein (predicted)  Info   RGD1311605_predicted 
   1781 1.06e-05 1726.6 2046.2 0.844  1388323_at  NADH dehydrogenase (ubiquinone) 1 alpha subcomplex, 9 (predicted)  Info   Ndufa9_predicted 
   1782 1.06e-05 1042.7 19.2 54.307  1379794_at  granzyme B  Info   Gzmb 
   1783 1.07e-05 1459 2182.3 0.669  1387759_s_at    Info   Ugt1a1 /// Ugt1a6 /// Ugt1a7 /// Ugt1a8 /// Ugt1a2 /// Ugt1a4 /// Ugt1a11 /// Ugt1a5 
   1784 1.07e-05 210.5 266.9 0.789  1383648_x_at  similar to Crumbs homolog 3  Info   LOC301112 
   1785 1.07e-05 183.9 442.7 0.415  1372158_at  LRP16 protein  Info   Lrp16 
   1786 1.07e-05 152.8 257.5 0.593  1379550_a_at  general transcription factor II I repeat domain-containing 1  Info   Gtf2ird1 
   1787 1.07e-05 122.3 72.6 1.685  1386956_at  scavenger receptor class B, member 1  Info   Scarb1 
   1788 1.07e-05 162.4 272.9 0.595  1374191_at  rhomboid family 1 (Drosophila) (predicted)  Info   Rhbdf1_predicted 
   1789 1.07e-05 1453.7 955.2 1.522  1388158_at  HLA-B-associated transcript 1A  Info   Bat1a 
   1790 1.08e-05 147.9 212.6 0.696  1376595_at  similar to peroxisome biogenesis factor 1  Info   LOC500006 
   1791 1.08e-05 489.8 800 0.612  1372423_at  PERP, TP53 apoptosis effector (predicted)  Info   Perp_predicted 
   1792 1.09e-05 7434.8 320 23.234  1377626_at    Info    
   1793 1.09e-05 217.9 119.9 1.817  1379298_at  interferon (alpha and beta) receptor 1 (predicted)  Info   Ifnar1_predicted 
   1794 1.09e-05 351.8 534.4 0.658  1374293_at    Info    
   1795 1.09e-05 1130.1 2114.9 0.534  1371954_at    Info    
   1796 1.09e-05 1601.7 1990.8 0.805  1367642_at  succinate-CoA ligase, GDP-forming, alpha subunit  Info   Suclg1 
   1797 1.09e-05 36.9 12 3.075  1368781_at  RAS guanyl releasing protein 4  Info   Rasgrp4 
   1798 1.09e-05 157.1 333.4 0.471  1369158_at  calcium-sensing receptor  Info   Casr 
   1799 1.09e-05 152.3 275.9 0.552  1376661_at  similar to CG11206-PA /// similar to kazrin isoform A  Info   RGD:1359147 /// LOC500579 
   1800 1.1e-05 21.9 66.5 0.329  1378505_at    Info    
   1801 1.1e-05 765.6 421.5 1.816  1394082_at  similar to T-Brain-1  Info   LOC311078 
   1802 1.1e-05 62 156.3 0.397  1379679_at  Transcribed locus  Info    
   1803 1.1e-05 33.4 63.1 0.529  1368981_at  aquaporin 4  Info   Aqp4 
   1804 1.1e-05 55.9 139.8 0.4  1383844_at  Transcribed locus  Info    
   1805 1.1e-05 1520.6 2351.7 0.647  1370699_a_at  epidermal growth factor receptor /// peptidase D  Info   Egfr /// Pepd 
   1806 1.11e-05 179.2 397.2 0.451  1387971_a_at  mitogen activated protein kinase 8 interacting protein  Info   Mapk8ip 
   1807 1.11e-05 13.2 26.1 0.506  1384007_at  atlastin-like  Info   RGD:1359232 
   1808 1.11e-05 215.6 409.8 0.526  1392203_at  homeo box B2 (predicted)  Info   Hoxb2_predicted 
   1809 1.11e-05 271.4 66.8 4.063  1387605_at  caspase 12  Info   Casp12 
   1810 1.11e-05 88.9 252.9 0.352  1376263_at  similar to 1810034B16Rik protein (predicted)  Info   RGD1306222_predicted 
   1811 1.11e-05 250.2 513 0.488  1372568_at  EPM2A (laforin) interacting protein 1 (predicted)  Info    
   1812 1.12e-05 275.8 100.8 2.736  1380611_at  FK506 binding protein 5 (predicted)  Info   Fkbp5_predicted 
   1813 1.12e-05 149.1 216.3 0.689  1389362_at  protein tyrosine phosphatase, non-receptor type 3 (predicted)  Info   Ptpn3_predicted 
   1814 1.12e-05 247.2 325.3 0.76  1378287_at  radixin  Info   RGD:1359472 
   1815 1.12e-05 268.9 449.3 0.598  1398398_at  similar to Homeobox protein A10  Info   LOC368057 
   1816 1.13e-05 253.9 500.3 0.507  1376691_at  Transcribed locus  Info    
   1817 1.13e-05 397 219.2 1.811  1376737_at  similar to SUMO/sentrin specific protease 5  Info   LOC499312 
   1818 1.13e-05 357.2 596.4 0.599  1373134_at  similar to RIKEN cDNA B430104H02  Info   LOC296131 
   1819 1.13e-05 286.5 160.9 1.781  1383665_at  Transcribed locus  Info    
   1820 1.13e-05 1521.5 2663.5 0.571  1367793_at  D-dopachrome tautomerase  Info   Ddt 
   1821 1.13e-05 68.2 6.9 9.884  1387691_at  tumor necrosis factor superfamily, member 2  Info   Tnf 
   1822 1.13e-05 606.8 347.5 1.746  1388341_at  RAN GTPase activating protein 1 (predicted)  Info   Rangap1_predicted 
   1823 1.13e-05 720.9 469.5 1.535  1372506_at  proteaseome (prosome, macropain) 28 subunit, 3 (predicted)  Info   Psme3_predicted 
   1824 1.13e-05 161.7 293.6 0.551  1381976_at  kinesin family member 21A (predicted)  Info   Kif21a_predicted 
   1825 1.14e-05 412.3 61.3 6.726  1369983_at  chemokine (C-C motif) ligand 5  Info   Ccl5 
   1826 1.14e-05 159.5 71.6 2.228  1373767_at  similar to arsenite inducible RNA associated protein (predicted)  Info   RGD1310991_predicted 
   1827 1.14e-05 214.9 129.6 1.658  1368233_at  general transcription factor IIF, polypeptide 2  Info   Gtf2f2 
   1828 1.14e-05 5318.8 186.6 28.504  1373544_at  chemokine (C-X-C motif) ligand 9  Info   Cxcl9 
   1829 1.15e-05 266.4 195 1.366  1387844_at  LIM and SH3 protein 1  Info   Lasp1 
   1830 1.15e-05 834.4 522.5 1.597  1398925_at  similar to RIKEN cDNA 1300018I05 (predicted)  Info   RGD1307801_predicted 
   1831 1.15e-05 50.8 17.7 2.87  1394527_at  Transcribed locus  Info    
   1832 1.15e-05 43.3 115.1 0.376  1383194_a_at  CDNA clone IMAGE:7379585  Info    
   1833 1.15e-05 1673.6 2673.1 0.626  1370030_at  glutamate cysteine ligase, modifier subunit  Info   Gclm 
   1834 1.16e-05 4252.5 2676.5 1.589  1367586_at  lactate dehydrogenase A  Info   Ldha 
   1835 1.17e-05 286.7 454.5 0.631  1388703_at  endothelial cell adhesion molecule  Info   RGD:1303286 
   1836 1.17e-05 166.6 301.9 0.552  1373921_at  CDNA clone MGC:124810 IMAGE:7932594  Info    
   1837 1.17e-05 429.7 268.4 1.601  1371932_at  similar to hypothetical protein FLJ38482 (predicted)  Info   RGD1309341_predicted 
   1838 1.17e-05 318.2 513.1 0.62  1371990_at  CDNA clone IMAGE:7930963  Info    
   1839 1.17e-05 28.8 54.5 0.528  1394259_at  Claudin 15 (predicted)  Info    
   1840 1.17e-05 513.2 771.1 0.666  1371986_at  Transcribed locus  Info    
   1841 1.18e-05 94 501.3 0.188  1381080_at  Transcribed locus  Info    
   1842 1.18e-05 524.5 1064.9 0.493  1370379_at  protease, serine, 8 (prostasin)  Info   Prss8 
   1843 1.18e-05 682.4 951.4 0.717  1390765_at  WAP four-disulfide core domain 2  Info   Wfdc2 
   1844 1.18e-05 112.4 21.9 5.132  1369862_at  proviral integration site 1  Info   Pim1 
   1845 1.19e-05 89.4 144.4 0.619  1382228_at  similar to Zinc finger protein 431  Info   LOC499597 
   1846 1.19e-05 108 75.5 1.43  1383922_a_at  similar to N-acetyltransferase ARD1  Info   LOC363518 
   1847 1.19e-05 496.6 199.4 2.49  1373831_at  hypothetical protein LOC619574  Info   LOC619574 
   1848 1.19e-05 32.9 84.7 0.388  1398507_at    Info    
   1849 1.19e-05 128 37.1 3.45  1398591_at  chemokine (C-C motif) receptor-like 2 (predicted)  Info   Ccrl2_predicted 
   1850 1.19e-05 224.5 117.3 1.914  1390394_at  Transcribed locus  Info    
   1851 1.2e-05 210.5 134.4 1.566  1395875_at  similar to KIAA0372 gene product (predicted)  Info   RGD1306062_predicted 
   1852 1.21e-05 182.1 413.6 0.44  1384907_at    Info    
   1853 1.21e-05 46.2 102.5 0.451  1384243_at  Transcribed locus  Info    
   1854 1.21e-05 242.2 112 2.163  1374550_at  similar to map kinase interacting kinase  Info   MGC112775 
   1855 1.21e-05 1635.3 897.1 1.823  1388763_at  Transcribed locus, strongly similar to NP_031751.1 calponin 2 [Mus musculus]  Info    
   1856 1.22e-05 532.4 332.4 1.602  1370848_at  solute carrier family 2 (facilitated glucose transporter), member 1  Info   Slc2a1 
   1857 1.22e-05 141.8 252.9 0.561  1368643_at  spermatogenesis associated 6  Info   Spata6 
   1858 1.22e-05 75.6 46.3 1.633  1382266_at  similar to G protein-coupled receptor 146  Info   LOC498153 
   1859 1.22e-05 760.3 390.3 1.948  1369971_a_at  heterogeneous nuclear ribonucleoprotein D  Info   Hnrpd 
   1860 1.22e-05 740.3 378.7 1.955  1388711_at  interleukin 13 receptor, alpha 1  Info   Il13ra1 
   1861 1.22e-05 145.5 2.6 55.962  1387711_at  interleukin 10  Info   Il10 
   1862 1.23e-05 195.8 426.8 0.459  1390411_at  claudin 19 (predicted)  Info   Cldn19_predicted 
   1863 1.23e-05 360.8 484.1 0.745  1374197_at  Similar to expressed sequence AA415817  Info    
   1864 1.23e-05 9150.7 2083.8 4.391  1372585_at  LOC499318  Info   LOC499318 
   1865 1.23e-05 177.1 69 2.567  1384968_at  SH3-domain binding protein 2 (predicted)  Info   Sh3bp2_predicted 
   1866 1.23e-05 47.6 228 0.209  1370165_at  small muscle protein, X-linked  Info   Smpx 
   1867 1.23e-05 117.4 310.9 0.378  1370566_at  retinol dehydrogenase 2  Info   Rdh2 
   1868 1.23e-05 445.2 632.5 0.704  1388194_at  dihydrolipoamide S-acetyltransferase (E2 component of pyruvate dehydrogenase complex)  Info   Dlat 
   1869 1.23e-05 1328.9 869.3 1.529  1370844_at  heterogeneous nuclear ribonucleoprotein F  Info   Hnrpf 
   1870 1.23e-05 185.8 278 0.668  1371728_at  Tripartite motif-containing 35  Info    
   1871 1.24e-05 46.5 107.8 0.431  1375553_at  similar to RIKEN cDNA 5133400G04 (predicted)  Info   RGD1311742_predicted 
   1872 1.24e-05 41.8 94.4 0.443  1377979_at  Similar to RIKEN cDNA 2610022G08  Info    
   1873 1.24e-05 128.5 409.3 0.314  1383191_at  protein phosphatase 1, regulatory (inhibitor) subunit 1A  Info   Ppp1r1a 
   1874 1.24e-05 251.9 476.8 0.528  1376605_at  solute carrier family 17 (anion/sugar transporter), member 5 (predicted)  Info   Slc17a5_predicted 
   1875 1.24e-05 490.6 226.6 2.165  1368361_a_at  protein tyrosine phosphatase, non-receptor type 2  Info   Ptpn2 
   1876 1.24e-05 1817.9 1218.3 1.492  1398897_at  ubiquitin-conjugating enzyme E2 variant 1 (predicted)  Info   Ube2v1_predicted 
   1877 1.25e-05 219.8 365.1 0.602  1388909_at  similar to cDNA sequence BC019806 (predicted)  Info   RGD1311534_predicted 
   1878 1.25e-05 256.6 107.5 2.387  1383917_at  ABI gene family, member 3 (predicted)  Info   Abi3_predicted 
   1879 1.25e-05 1253.2 1726.7 0.726  1370060_at  solute carrier family 25 (mitochondrial carrier; oxoglutarate carrier), member 11  Info   Slc25a11 
   1880 1.26e-05 147.2 242.5 0.607  1376861_at  similar to RIKEN cDNA 1810018L05 /// similar to motile sperm domain containing 1  Info   RGD:1359486 /// LOC501648 
   1881 1.26e-05 79.8 135.6 0.588  1373249_at  Similar to Ubiquitin-like protein 4 (Ubiquitin-like protein GDX)  Info    
   1882 1.26e-05 247.1 125.4 1.97  1370017_at  emerin  Info   Emd 
   1883 1.26e-05 193.5 301.9 0.641  1390863_at  solute carrier family 19 (thiamine transporter), member 2 (predicted)  Info   Slc19a2_predicted 
   1884 1.26e-05 647.2 435.5 1.486  1372028_at  similar to Protein CGI-117 (Protein HSPC111) (predicted)  Info   RGD1305727_predicted 
   1885 1.26e-05 848.4 571.8 1.484  1371467_at  similar to RIKEN cDNA 0610007P06  Info   RGD:1359455 
   1886 1.27e-05 81.5 43.3 1.882  1384988_at  F-box only protein 5 (predicted)  Info   Fbxo5_predicted 
   1887 1.27e-05 1359.5 1552.1 0.876  1367873_at  ATPase, H+ transporting, lysosomal (vacuolar proton pump), subunit 1  Info   Atp6ap1 
   1888 1.27e-05 264.6 391.9 0.675  1367504_at    Info    
   1889 1.27e-05 212.4 1074.7 0.198  1393508_at  Transcribed locus  Info    
   1890 1.28e-05 469.6 282.8 1.661  1376043_at  similar to PHD zinc finger containing protein JUNE1  Info   RGD:1302969 
   1891 1.28e-05 138.6 227 0.611  1373267_at  Sh3 domain YSC-like 1 (predicted)  Info   Sh3yl1_predicted 
   1892 1.28e-05 156.4 430.3 0.363  1388176_at  camello-like 5  Info   Cml5 
   1893 1.28e-05 2594.6 1923.6 1.349  1371938_at  GPI-anchored membrane protein 1 (predicted)  Info    
   1894 1.28e-05 464.2 245.7 1.889  1397892_at  eukaryotic translation initiation factor 1A (predicted)  Info   Eif1a_predicted 
   1895 1.28e-05 8.6 82.4 0.104  1384211_at  procollagen, type XI, alpha 1  Info   Col11a1 
   1896 1.28e-05 681.7 432.9 1.575  1369962_at  5-aminoimidazole-4-carboxamide ribonucleotide formyltransferase/IMP cyclohydrolase  Info   Atic 
   1897 1.29e-05 327.7 651.5 0.503  1372455_at  transmembrane 4 superfamily member 12 (predicted)  Info   Tm4sf12_predicted 
   1898 1.29e-05 298 682 0.437  1387178_a_at  cystathionine beta synthase  Info   Cbs 
   1899 1.29e-05 457.1 1256.8 0.364  1368245_at  ureidopropionase, beta  Info   Upb1 
   1900 1.29e-05 19.9 58.1 0.343  1383165_at  similar to KIAA1324 protein (predicted)  Info   RGD1310209_predicted 
   1901 1.29e-05 258.4 136.4 1.894  1375987_at  ceramide kinase (predicted)  Info   Cerk_predicted 
   1902 1.29e-05 128.7 303.2 0.424  1386454_at  solute carrier family 23 (nucleobase transporters), member 3 (predicted)  Info   Slc23a3_predicted 
   1903 1.3e-05 32.2 64.8 0.497  1383143_at  similar to Ephb2 protein  Info   LOC313633 
   1904 1.3e-05 2856.9 2285.5 1.25  1398880_at  RNA polymerase II transcriptional coactivator  Info   Rpo2tc1 
   1905 1.3e-05 127.3 51.5 2.472  1379538_at  centromere protein J (predicted)  Info   Cenpj_predicted 
   1906 1.3e-05 214.3 416.1 0.515  1375964_at  phosphoserine phosphatase (predicted)  Info   Psph_predicted 
   1907 1.3e-05 443.2 969 0.457  1368934_at  cytochrome P450, family 4, subfamily a, polypeptide 10 /// cytochrome P450, family 4, subfamily A, polypeptide 22  Info   Cyp4a10 /// Cyp4a22 
   1908 1.3e-05 3343.8 4207.4 0.795  1388315_at  similar to neuronal protein 15.6  Info   LOC299310 
   1909 1.32e-05 2854.6 5693 0.501  1367627_at  glycine amidinotransferase (L-arginine:glycine amidinotransferase)  Info   Gatm 
   1910 1.32e-05 38.2 121.6 0.314  1368013_at  DNA-damage-inducible transcript 4-like  Info   Ddit4l 
   1911 1.32e-05 249 722.7 0.345  1382211_at  Transcribed locus  Info    
   1912 1.32e-05 161.1 105.4 1.528  1374626_at  similar to leucine-rich alpha-2-glycoprotein  Info   RGD:1359464 
   1913 1.32e-05 349.5 529.6 0.66  1373837_at  fused toes (predicted)  Info   Fts_predicted 
   1914 1.33e-05 3437.5 4111.2 0.836  1372437_at  S-phase kinase-associated protein 1A  Info   RGD:1359648 
   1915 1.33e-05 166.2 81.8 2.032  1380187_at  similar to TNF intracellular domain-interacting protein (predicted)  Info   RGD1311487_predicted 
   1916 1.33e-05 147.5 286 0.516  1377738_a_at  cysteine and histidine rich 1  Info   Cyhr1 
   1917 1.33e-05 224.5 580.6 0.387  1392971_at  Transcribed locus  Info    
   1918 1.33e-05 173.3 107.9 1.606  1373804_at  Forkhead box P1 (predicted)  Info    
   1919 1.33e-05 524.5 270.7 1.938  1384022_at    Info    
   1920 1.33e-05 765.3 507.2 1.509  1374437_at  asparaginyl-tRNA synthetase (predicted)  Info   Nars_predicted 
   1921 1.33e-05 204.7 4.8 42.646  1384658_at  Transcribed locus  Info    
   1922 1.34e-05 231.3 325.3 0.711  1373480_at  Transcribed locus  Info    
   1923 1.34e-05 71.1 29.2 2.435  1369007_at  nuclear receptor subfamily 4, group A, member 2  Info   Nr4a2 
   1924 1.34e-05 105.6 159 0.664  1370330_at  signal-induced proliferation-associated 1 like 1  Info   Sipa1l1 
   1925 1.34e-05 431.6 254.9 1.693  1399152_at  epidermal growth factor receptor pathway substrate 15 (predicted)  Info   Eps15_predicted 
   1926 1.35e-05 64.9 25.9 2.506  1391662_at  similar to RIKEN cDNA 9430015G10 (predicted) /// similar to RIKEN cDNA 9430015G10  Info   RGD1311517_predicted /// LOC500599 
   1927 1.35e-05 280.1 85.2 3.288  1379631_at  colony stimulating factor 1 (macrophage)  Info   Csf1 
   1928 1.35e-05 1828.4 2663.4 0.686  1388414_at  NADH dehydrogenase (ubiquinone) Fe-S protein 5b, 15kDa (NADH-coenzyme Q reductase) (predicted)  Info   Ndufs5b_predicted 
   1929 1.35e-05 713.3 167.2 4.266  1368921_a_at  CD44 antigen  Info   Cd44 
   1930 1.35e-05 673.7 316.9 2.126  1374180_at  Transcribed locus  Info    
   1931 1.35e-05 184.1 121.8 1.511  1387300_at  crooked neck protein  Info   Crnkl1 
   1932 1.36e-05 371.8 222.1 1.674  1368412_a_at  protein tyrosine phosphatase, receptor type, O  Info   Ptpro 
   1933 1.36e-05 1356.8 2205.4 0.615  1383855_at    Info    
   1934 1.37e-05 159.3 274.9 0.579  1368098_a_at  small nuclear ribonucleoprotein N  Info   Snrpn 
   1935 1.37e-05 1380.8 588 2.348  1389528_s_at  v-jun sarcoma virus 17 oncogene homolog (avian)  Info   Jun 
   1936 1.37e-05 317.1 156.7 2.024  1389496_at  A kinase (PRKA) anchor protein 7  Info   RGD:1303071 
   1937 1.38e-05 136.4 65.6 2.079  1382592_at    Info    
   1938 1.39e-05 74.7 138.7 0.539  1385124_at  similar to Tribbles homolog 2  Info   LOC313974 
   1939 1.39e-05 218.3 317.8 0.687  1372152_at  similar to RIKEN cDNA 5430428G01 (predicted) /// similar to RIKEN cDNA 5430428G01  Info   RGD1312003_predicted /// LOC499699 
   1940 1.39e-05 484.3 896.6 0.54  1389230_at  Arrestin domain containing 3  Info   Arrdc3 
   1941 1.39e-05 882.9 523.1 1.688  1371539_at  nucleolar protein family A, member 2 (predicted)  Info   Nola2_predicted 
   1942 1.39e-05 193.1 283.5 0.681  1374485_at  Transcribed locus  Info    
   1943 1.39e-05 1814.8 2441.1 0.743  1389964_at  NADH dehydrogenase (ubiquinone) 1, alpha/beta subcomplex, 1 (predicted)  Info   Ndufab1_predicted 
   1944 1.4e-05 145.2 304.4 0.477  1375767_at  similar to common-site lymphoma leukemia guanine nucleotide exchange factor like (5N754) (predicted)  Info   RGD1308089_predicted 
   1945 1.4e-05 197.8 397.4 0.498  1398430_at  Transcribed locus  Info    
   1946 1.4e-05 30.2 55.5 0.544  1389193_at  Transcribed locus  Info    
   1947 1.4e-05 1585.1 2667.8 0.594  1368470_at  gamma-glutamyl hydrolase  Info   Ggh 
   1948 1.41e-05 80.3 161.3 0.498  1382868_at  sema domain, transmembrane domain (TM), and cytoplasmic domain, (semaphorin) 6A (predicted)  Info   Sema6a_predicted 
   1949 1.41e-05 73.1 137 0.534  1376180_at    Info    
   1950 1.41e-05 811.1 471.3 1.721  1376579_at  leucine aminopeptidase 3 (predicted)  Info   Lap3_predicted 
   1951 1.41e-05 136.4 202.8 0.673  1393165_at  similar to RIKEN cDNA 2310028N02  Info   RGD:1359339 
   1952 1.41e-05 42.9 94.1 0.456  1395659_at    Info    
   1953 1.41e-05 29.5 47.2 0.625  1393066_at  Similar to pregnancy-specific beta 1-glycoprotein  Info    
   1954 1.41e-05 719.6 1060 0.679  1370814_at  dehydrogenase/reductase (SDR family) member 4  Info   Dhrs4 
   1955 1.41e-05 28.1 77.4 0.363  1384051_at  Transcribed locus  Info    
   1956 1.42e-05 76.6 172.7 0.444  1380250_at  sushi, nidogen and EGF-like domains 1  Info   Sned1 
   1957 1.42e-05 187.4 284 0.66  1372670_at  similar to hypothetical protein FLJ21827 (predicted)  Info   RGD1307682_predicted 
   1958 1.43e-05 662.5 932.8 0.71  1370878_at  uroporphyrinogen decarboxylase  Info   Urod 
   1959 1.43e-05 499 772.4 0.646  1390566_a_at  creatine kinase, mitochondrial 1, ubiquitous  Info   Ckmt1 
   1960 1.43e-05 77.4 171.4 0.452  1368924_at  growth hormone receptor  Info   Ghr 
   1961 1.44e-05 219.6 470.6 0.467  1387071_a_at  microtubule-associated protein tau /// hypothetical gene supported by NM_017212  Info   Mapt /// LOC497674 
   1962 1.44e-05 388.6 180.5 2.153  1372543_at  similar to RIKEN cDNA 2610029G23  Info   LOC363485 
   1963 1.44e-05 96.4 136.1 0.708  1375558_at  PREDICTED: Rattus norvegicus RAB34, member of RAS oncogene family (predicted) (Rab34_predicted), mRNA  Info    
   1964 1.44e-05 52 94.6 0.55  1373697_at  myosin binding protein C, fast-type (predicted)  Info   Mybpc2_predicted 
   1965 1.44e-05 511.3 846.6 0.604  1373161_at  similar to RIKEN cDNA 6530411B15  Info   RGD:1359736 
   1966 1.44e-05 299.7 427.8 0.701  1373191_at  similar to hypothetical protein  Info   LOC500897 
   1967 1.44e-05 228.2 340.3 0.671  1387654_at  unconventional myosin Myr2 I heavy chain  Info   RGD:620443 
   1968 1.44e-05 195.8 333.5 0.587  1387821_at  RAB3A interacting protein  Info   Rab3ip 
   1969 1.44e-05 271.9 597.6 0.455  1383064_at  CDNA clone IMAGE:7379585  Info    
   1970 1.45e-05 17.1 1.7 10.059  1396065_at  Transcribed locus  Info    
   1971 1.45e-05 6846.9 1991.6 3.438  1370883_at  RT1 class II, locus Da  Info   RT1-Da 
   1972 1.45e-05 165.2 46.7 3.537  1385627_at  B-cell leukemia/lymphoma 3 (predicted)  Info   Bcl3_predicted 
   1973 1.46e-05 218.1 375.9 0.58  1369799_at  4-aminobutyrate aminotransferase  Info   Abat 
   1974 1.46e-05 527.9 1082.5 0.488  1387819_at  elastase 1, pancreatic  Info   Ela1 
   1975 1.46e-05 115.8 65.8 1.76  1376317_at    Info    
   1976 1.47e-05 193.6 85.5 2.264  1373290_at  similar to Ezh2 protein  Info   LOC312299 
   1977 1.47e-05 4916.5 1743.4 2.82  1387770_at  putative ISG12(a) protein  Info   RGD:1303168 
   1978 1.47e-05 299.9 517 0.58  1388786_at  Transcribed locus  Info    
   1979 1.47e-05 334.1 728.3 0.459  1382924_at  pantothenate kinase 1 (predicted)  Info   Pank1_predicted 
   1980 1.48e-05 285.3 151.4 1.884  1383255_at  Transcribed locus  Info    
   1981 1.48e-05 246.6 69 3.574  1387306_a_at  early growth response 2  Info   Egr2 
   1982 1.49e-05 156.8 23 6.817  1371106_at  integrin, beta 7  Info   Itgb7 
   1983 1.49e-05 42.9 97.9 0.438  1384925_at  Similar to IL-17D  Info    
   1984 1.49e-05 160.3 68.9 2.327  1391543_at  receptor (TNFRSF)-interacting serine-threonine kinase 1 (predicted)  Info   Ripk1_predicted 
   1985 1.49e-05 196.2 131.3 1.494  1382873_at  similar to cDNA sequence BC003236 (predicted)  Info   RGD1310822_predicted 
   1986 1.5e-05 33.2 85.8 0.387  1384303_s_at  solute carrier family 6 (neurotransmitter transporter), member 17  Info   Slc6a17 
   1987 1.5e-05 810.9 437.5 1.853  1368847_at  RAB10, member RAS oncogene family  Info   Rab10 
   1988 1.5e-05 64.7 131.5 0.492  1374434_at  similar to specifically androgen-regulated protein  Info   LOC498222 
   1989 1.5e-05 355.4 1238.7 0.287  1369531_at  sulfotransferase family, cytosolic, 1C, member 2  Info   Sult1c2 
   1990 1.5e-05 28.5 57.9 0.492  1380345_at  Transcribed locus  Info    
   1991 1.51e-05 163.2 400.8 0.407  1385132_at  myeloblastosis oncogene-like 1 (predicted)  Info   Mybl1_predicted 
   1992 1.52e-05 125.8 200.4 0.628  1389082_at    Info    
   1993 1.52e-05 491.2 1295.5 0.379  1370259_a_at  parathyroid hormone receptor 1  Info   Pthr1 
   1994 1.52e-05 233.2 416.5 0.56  1389251_at  nudix (nucleoside diphosphate linked moiety X)-type motif 7 (predicted)  Info   Nudt7_predicted 
   1995 1.53e-05 1094.3 333.5 3.281  1387870_at  zinc finger protein 36  Info   Zfp36 
   1996 1.53e-05 282.2 547.3 0.516  1369200_at  5 nucleotidase  Info   Nt5 
   1997 1.53e-05 280.5 497 0.564  1369289_at  hepatocyte nuclear factor 4, alpha  Info   Hnf4a 
   1998 1.53e-05 728 629.6 1.156  1394565_at  coatomer protein complex, subunit zeta 1 (predicted)  Info   Copz1_predicted 
   1999 1.53e-05 608.7 1405.1 0.433  1368563_at  aspartoacylase  Info   Aspa 
   2000 1.53e-05 28 54.5 0.514  1394694_at    Info    
   2001 1.54e-05 2960.1 2193.2 1.35  1398349_at  adenylate kinase 2  Info   Ak2 
   2002 1.54e-05 2411 1415.5 1.703  1398856_at  proteasome (prosome, macropain) subunit, alpha type 2  Info   Psma2 
   2003 1.54e-05 229.4 129.5 1.771  1383679_at  ring finger protein 31 (predicted)  Info   Rnf31_predicted 
   2004 1.54e-05 2118.5 2907.5 0.729  1371407_at  NCK-associated protein 1  Info   Nckap1 
   2005 1.55e-05 170.3 281.7 0.605  1372023_at  LOC498674  Info   LOC498674 
   2006 1.55e-05 72.8 154.2 0.472  1369347_s_at  prominin 2  Info   Prom2 
   2007 1.55e-05 403.2 597 0.675  1374903_at  glucosaminyl (N-acetyl) transferase 2, I-branching enzyme /// hypothetical gene supported by NM_001001511  Info   Gcnt2 /// LOC497712 
   2008 1.55e-05 746.7 414.2 1.803  1383625_a_at  similar to zinc finger protein  Info   LOC500989 
   2009 1.55e-05 496.1 304 1.632  1381461_at  Transcribed locus  Info    
   2010 1.55e-05 1084.5 711.6 1.524  1367484_at  ubiquitin-conjugating enzyme E2E 2 (UBC4/5 homolog, yeast) (predicted) /// similar to Ubiquitin-conjugating enzyme E2 E1 (Ubiquitin-protein ligase E1) (Ubiquitin carrier protein E1) (UbcM3)  Info   Ube2e2_predicted /// LOC498449 
   2011 1.55e-05 410.5 318.1 1.29  1377064_at  dual specificity phosphatase 6  Info   Dusp6 
   2012 1.56e-05 228.4 131.7 1.734  1383102_at  Transcribed locus  Info    
   2013 1.56e-05 47.6 28.8 1.653  1386181_at  Transcribed locus  Info    
   2014 1.56e-05 121.5 7 17.357  1382378_at  cathepsin W (predicted)  Info   Ctsw_predicted 
   2015 1.56e-05 6345.7 1967.8 3.225  1371033_at  RT1 class II, locus Bb  Info   RT1-Bb 
   2016 1.57e-05 33 127.9 0.258  1392510_at  similar to hypothetical protein B230314O19 (predicted)  Info   RGD1308013_predicted 
   2017 1.57e-05 99 24.5 4.041  1393629_at  H2.0-like homeo box 1 (Drosophila) (predicted)  Info   Hlx1_predicted 
   2018 1.58e-05 237.3 81.9 2.897  1369197_at  apoptotic peptidase activating factor 1  Info   Apaf1 
   2019 1.58e-05 27.4 48.6 0.564  1383753_at    Info    
   2020 1.59e-05 284 111.2 2.554  1398561_at  Transcribed locus  Info    
   2021 1.59e-05 108.9 41 2.656  1384350_at  Transcribed locus  Info    
   2022 1.59e-05 205.4 321.3 0.639  1375867_at  zinc finger and BTB domain containing 4 (predicted)  Info   Zbtb4_predicted 
   2023 1.59e-05 86.1 16.5 5.218  1370499_at  killer cell lectin-like receptor subfamily B member 1A  Info   Klrb1a 
   2024 1.59e-05 232.8 144.8 1.608  1369754_a_at  calpastatin  Info   Cast 
   2025 1.59e-05 217.7 343.1 0.635  1374414_at  similar to RIKEN cDNA 1300010M03 (predicted)  Info   RGD1306844_predicted 
   2026 1.59e-05 323.2 639.2 0.506  1393947_at  solute carrier family 25 (mitochondrial carrier; ornithine transporter) member 15 (predicted)  Info   Slc25a15_predicted 
   2027 1.6e-05 3093.7 4149.6 0.746  1367599_at  ATP synthase, H+ transporting, mitochondrial F0 complex, subunit c (subunit 9), isoform 1  Info   Atp5g1 
   2028 1.6e-05 172.8 108.6 1.591  1372986_at    Info    
   2029 1.6e-05 396.7 656.8 0.604  1383742_at  sorting nexin 7 (predicted)  Info   Snx7_predicted 
   2030 1.6e-05 646.1 305 2.118  1372890_at  Sphingosine phosphate lyase 1  Info   Spgl1 
   2031 1.6e-05 3621.2 122.5 29.561  1367998_at  secretory leukocyte peptidase inhibitor /// similar to secretory leukocyte protease inhibitor  Info   Slpi /// LOC296356 
   2032 1.61e-05 131.3 226.3 0.58  1390474_at  similar to RIKEN cDNA 9330161F08  Info   LOC362899 
   2033 1.61e-05 143.1 101.4 1.411  1384791_at  UDP-GlcNAc:betaGal beta-1,3-N-acetylglucosaminyltransferase 1 (predicted)  Info   B3gnt1_predicted 
   2034 1.61e-05 506.1 297.1 1.703  1389911_at  similar to cDNA sequence BC019776  Info   RGD:1359271 
   2035 1.62e-05 1017.5 1775.1 0.573  1389649_at  Transcribed locus  Info    
   2036 1.62e-05 407.3 1194.6 0.341  1388644_at  Monoglyceride lipase  Info   Mgll 
   2037 1.62e-05 139 81.5 1.706  1387412_at  phosphatidylinositol-4-phosphate 5-kinase, type II, alpha  Info   Pip5k2a 
   2038 1.62e-05 286.3 165.7 1.728  1394842_at  Unknown (protein for MGC:72591)  Info   Tmem19 
   2039 1.63e-05 1271.7 1718.2 0.74  1373222_at  hexosaminidase A  Info   Hexa 
   2040 1.63e-05 334.9 686 0.488  1387092_at  FXYD domain-containing ion transport regulator 4  Info   Fxyd4 
   2041 1.63e-05 3153 4848.1 0.65  1371942_at  similar to Glutathione S-transferase, theta 3  Info   LOC499422 
   2042 1.63e-05 297.1 202 1.471  1388151_at  coronin 7  Info   Coro7 
   2043 1.63e-05 394.8 117.5 3.36  1382692_at  Transcribed locus  Info    
   2044 1.63e-05 269 429.5 0.626  1371794_at  Transcribed locus, strongly similar to NP_035873.1 SH3-domain binding protein 3 [Mus musculus]  Info    
   2045 1.63e-05 323.7 268.8 1.204  1393244_at  HGF-regulated tyrosine kinase substrate  Info   Hgs 
   2046 1.63e-05 26.5 100 0.265  1373865_at  synaptosomal-associated protein, 91kDa homolog (mouse)  Info   Snap91 
   2047 1.63e-05 479.4 229.9 2.085  1370433_at  CCA2 protein  Info   Hsd3b7 
   2048 1.64e-05 5835 3144 1.856  1367605_at  profilin 1  Info   Pfn1 
   2049 1.64e-05 2656 2251.9 1.179  1371372_at  similar to Sid3177p  Info   LOC362809 
   2050 1.64e-05 1323.2 1904.5 0.695  1392530_at  similar to NADH dehydrogenase (ubiquinone) 1, subcomplex unknown, 1  Info   LOC499612 
   2051 1.64e-05 330.7 151.9 2.177  1372389_at  similar to immediate early response 2  Info   RGD:1359581 
   2052 1.64e-05 193 391.6 0.493  1372728_at  Sortilin 1  Info   Sort1 
   2053 1.64e-05 484.2 636.3 0.761  1398966_at  Transcribed locus, strongly similar to NP_081070.1 differentially expressed in B16F10 1 [Mus musculus]  Info    
   2054 1.64e-05 17.3 1.4 12.357  1371126_x_at  granzyme B  Info   Gzmb 
   2055 1.65e-05 102.6 17.6 5.83  1396217_at  Similar to Adapter-related protein complex 1 sigma 1B subunit (Sigma-adaptin 1B) (Adaptor protein complex AP-1 sigma-1B subunit) (Golgi adaptor HA1/AP1 adaptin sigma-1B subunit) (Clathrin assembly protein complex 1 sigma-1B small chain) (Sigma 1B s...  Info    
   2056 1.65e-05 1180.3 742.4 1.59  1388352_at  N-acetyltransferase 5 (ARD1 homolog, S. cerevisiae) (predicted)  Info   Nat5_predicted 
   2057 1.65e-05 67.7 3.8 17.816  1394051_at  Transcribed locus  Info    
   2058 1.65e-05 998.5 1433.2 0.697  1384244_at  similar to RIKEN cDNA 2610207I16 (predicted)  Info   RGD1305387_predicted 
   2059 1.65e-05 444.6 287.7 1.545  1387085_at  phosphoribosyl pyrophosphate synthetase 1  Info   Prps1 
   2060 1.65e-05 509.1 739.1 0.689  1388541_at  Similar to Hypothetical protein KIAA0152 (predicted)  Info    
   2061 1.65e-05 454.8 976.3 0.466  1382111_at  similar to RIKEN cDNA 2010001H14  Info   LOC302554 
   2062 1.66e-05 640.2 1015.9 0.63  1388870_at  Transcribed locus, strongly similar to NP_620412.1 musashi 2 isoform a [Homo sapiens]  Info    
   2063 1.66e-05 128.5 25.5 5.039  1393757_at  Similar to OTTHUMP00000018227  Info    
   2064 1.66e-05 138.1 84.7 1.63  1392116_at  similar to RIKEN cDNA 5730470L24 (predicted)  Info   RGD1311316_predicted 
   2065 1.67e-05 1792.9 2104.5 0.852  1374431_at  ATPase, H+ transporting, V1 subunit A, isoform 1 (predicted)  Info    
   2066 1.67e-05 115.6 251.5 0.46  1383086_at  Transcribed locus  Info    
   2067 1.67e-05 227.7 119.5 1.905  1380009_at  vaccinia related kinase 1 (predicted)  Info   Vrk1_predicted 
   2068 1.67e-05 387.5 227 1.707  1370071_at  adenosine deaminase  Info   Ada 
   2069 1.67e-05 1663.6 2254.8 0.738  1375516_at  NADH dehydrogenase (ubiquinone) 1, subcomplex unknown, 2 (predicted)  Info   Ndufc2_predicted 
   2070 1.67e-05 122.9 12.7 9.677  1392322_at  GTPase, IMAP family member 7  Info   Gimap7 
   2071 1.68e-05 1082.1 1537.9 0.704  1373546_at  ATPase, class VI, type 11A (predicted)  Info    
   2072 1.68e-05 112.7 43.8 2.573  1380336_at  Transcribed locus  Info    
   2073 1.68e-05 2454.3 3695.7 0.664  1368659_at  alanine-glyoxylate aminotransferase 2  Info   Agxt2 
   2074 1.69e-05 600.5 933.2 0.643  1368041_at  synaptojanin 2 binding protein  Info   Synj2bp 
   2075 1.69e-05 415.1 979.7 0.424  1370547_at  pregnancy-zone protein  Info   Pzp 
   2076 1.69e-05 216.8 521.5 0.416  1386376_at  Transcribed locus  Info    
   2077 1.69e-05 227.2 344 0.66  1383171_at  transcription factor B2, mitochondrial (predicted)  Info   Tfb2m_predicted 
   2078 1.7e-05 846.9 648.2 1.307  1370001_at  RAB8A, member RAS oncogene family  Info   Rab8a 
   2079 1.7e-05 232.7 10.8 21.546  1394306_at  zeta-chain (TCR) associated protein kinase 70kDa  Info   Zap70 
   2080 1.71e-05 1517 239.3 6.339  1382255_at    Info    
   2081 1.71e-05 435.9 655.3 0.665  1398424_at  WD SOCS-box protein 2  Info   Wsb2 
   2082 1.71e-05 324.6 183.5 1.769  1372850_at  Similar to Expressed sequence AU019823  Info    
   2083 1.71e-05 144.9 81.4 1.78  1395147_at  Excision repair cross-complementing rodent repair deficiency, complementation group 3 (predicted)  Info    
   2084 1.72e-05 1350.8 790.8 1.708  1386915_at  acidic nuclear phosphoprotein 32 family, member B  Info   Anp32b 
   2085 1.72e-05 45.9 23.8 1.929  1385580_at  Transcribed locus  Info    
   2086 1.72e-05 1135.6 761.6 1.491  1371596_at  ribonucleic acid binding protein S1 (predicted)  Info   Rnps1_predicted 
   2087 1.73e-05 824.9 2338 0.353  1367775_at  alpha-methylacyl-CoA racemase  Info   Amacr 
   2088 1.74e-05 89.2 20 4.46  1397411_at  FYVE, RhoGEF and PH domain containing 2 (predicted)  Info   Fgd2_predicted 
   2089 1.74e-05 3223.3 2314.1 1.393  1367657_at  B-cell translocation gene 1, anti-proliferative  Info   Btg1 
   2090 1.74e-05 308.4 535.6 0.576  1393777_at  Transcribed locus  Info    
   2091 1.74e-05 352.5 201.4 1.75  1388547_at  claudin 4 (predicted)  Info   Cldn4_predicted 
   2092 1.74e-05 8.6 37.9 0.227  1389584_at  Transcribed locus  Info    
   2093 1.75e-05 172.2 64 2.691  1377727_at  bromodomain adjacent to zinc finger domain, 1A (predicted)  Info   Baz1a_predicted 
   2094 1.75e-05 1255.7 1869.2 0.672  1368095_at  adenylate kinase 3  Info   Ak3 
   2095 1.75e-05 203.7 351 0.58  1388688_at  Transcribed locus  Info    
   2096 1.76e-05 442.2 234.6 1.885  1373283_at  Thymopoietin  Info   Tmpo 
   2097 1.76e-05 179 16.6 10.783  1391384_at  G4 protein  Info   Tnf 
   2098 1.76e-05 63.8 162.7 0.392  1392786_at    Info    
   2099 1.77e-05 34 97.5 0.349  1391547_at  CDNA clone IMAGE:7320555  Info    
   2100 1.77e-05 484.3 284.2 1.704  1392908_at  hypothetical protein LOC595134  Info   LOC595134 
   2101 1.77e-05 696.1 1044.9 0.666  1367687_a_at  peptidylglycine alpha-amidating monooxygenase  Info   Pam 
   2102 1.77e-05 3261.2 4383.8 0.744  1371398_at    Info    
   2103 1.78e-05 1489.3 2268.3 0.657  1367808_at  translocase of inner mitochondrial membrane 8 homolog b (yeast)  Info   Timm8b 
   2104 1.79e-05 88.5 184.2 0.48  1371793_at  Transcribed locus, strongly similar to NP_081632.1 hypothetical protein LOC70240 [Mus musculus]  Info    
   2105 1.79e-05 6033.3 286.6 21.051  1387969_at  chemokine (C-X-C motif) ligand 10  Info   Cxcl10 
   2106 1.79e-05 156.7 301.8 0.519  1382571_at  Transcribed locus  Info    
   2107 1.79e-05 496.3 1029.4 0.482  1374254_a_at  hypothetical LOC300441  Info   LOC300441 
   2108 1.79e-05 1448.5 2212.2 0.655  1398923_at  similar to D11Bwg0434e protein (predicted)  Info   RGD1305687_predicted 
   2109 1.79e-05 4742 2933.7 1.616  1369929_at  prosaposin  Info   Psap 
   2110 1.79e-05 182.6 685.1 0.267  1370991_at  camello-like 3  Info   Cml3 
   2111 1.79e-05 579.2 1034.9 0.56  1376575_at  Transcribed locus  Info    
   2112 1.79e-05 100.9 188.5 0.535  1381470_at  V-kit Hardy-Zuckerman 4 feline sarcoma viral oncogene homolog  Info   Kit 
   2113 1.8e-05 65.8 157.4 0.418  1367726_at  thyroid hormone receptor alpha  Info   Thra 
   2114 1.8e-05 784.4 1205.2 0.651  1370209_at  basic transcription element binding protein 1  Info   Bteb1 
   2115 1.81e-05 52.3 24.3 2.152  1383565_at  Transcribed locus  Info    
   2116 1.81e-05 1181.8 869.1 1.36  1398879_at  similar to hypothetical protein MGC8721  Info   RGD:1303011 
   2117 1.81e-05 435.2 185.8 2.342  1370948_a_at  myristoylated alanine rich protein kinase C substrate  Info   Marcks 
   2118 1.81e-05 299.3 483.8 0.619  1373335_at  similar to zinc finger, DHHC domain containing 9  Info   LOC302808 
   2119 1.83e-05 4853.6 3052.8 1.59  1375633_at  chloride intracellular channel 1  Info   RGD:1303043 
   2120 1.83e-05 54.8 123.6 0.443  1369816_at  RAB3A, member RAS oncogene family  Info   Rab3a 
   2121 1.84e-05 369 591.7 0.624  1375879_at  G protein-coupled receptor 48  Info   Gpr48 
   2122 1.84e-05 1235.8 821.1 1.505  1388868_at  zinc finger protein 216 (predicted)  Info   Zfp216_predicted 
   2123 1.84e-05 1137.8 703.6 1.617  1370880_at  ribonuclease/angiogenin inhibitor 1  Info   Rnh1 
   2124 1.85e-05 1536.3 885.4 1.735  1379371_at  Transcribed locus  Info    
   2125 1.85e-05 89.5 20.8 4.303  1378915_x_at  hypothetical protein LOC308990  Info   LOC308990 
   2126 1.86e-05 445.2 250.1 1.78  1375034_at  lysosomal phospholipase A2  Info   RGD:1302982 
   2127 1.86e-05 190 387.6 0.49  1373110_at  Transcribed locus  Info    
   2128 1.86e-05 458.5 1488.6 0.308  1371412_a_at  Neuronal regeneration related protein  Info   Nrep 
   2129 1.86e-05 179 81.8 2.188  1392668_at  Transcribed locus  Info    
   2130 1.86e-05 410 896.9 0.457  1376733_at  immunoglobulin superfamily, member 11 (predicted)  Info   Igsf11_predicted 
   2131 1.86e-05 317.6 229.5 1.384  1392464_at    Info    
   2132 1.86e-05 1632.9 2091.6 0.781  1392681_at  RAB2, member RAS oncogene family  Info   Rab2 
   2133 1.87e-05 88.9 189.6 0.469  1367812_at  beta-spectrin 3  Info   Spnb3 
   2134 1.87e-05 1305.9 1965.6 0.664  1370187_at  propionyl coenzyme A carboxylase, beta polypeptide  Info   Pccb 
   2135 1.87e-05 49.2 91.2 0.539  1394789_at  LOC499812  Info   LOC499812 
   2136 1.87e-05 149.7 248.8 0.602  1379669_at    Info    
   2137 1.87e-05 191.5 112.3 1.705  1389213_at  Transcribed locus  Info    
   2138 1.88e-05 147 244.8 0.6  1378241_at  Transcribed locus  Info    
   2139 1.88e-05 335.7 687.2 0.489  1372592_at  histone deacetylase 6  Info   Hdac6 
   2140 1.89e-05 9.7 29.9 0.324  1391563_at  Transcribed locus  Info    
   2141 1.89e-05 17.7 39 0.454  1387253_at  guanylate cyclase activator 2b  Info   Guca2b 
   2142 1.89e-05 250.6 388.1 0.646  1383813_at  ADP-ribosylation factor-like 2 binding protein  Info   Arl2bp 
   2143 1.89e-05 71.5 161.4 0.443  1398245_at  synuclein, gamma  Info   Sncg 
   2144 1.9e-05 123.9 39.7 3.121  1368527_at  prostaglandin-endoperoxide synthase 2  Info   Ptgs2 
   2145 1.9e-05 604.1 727.5 0.83  1372258_at  Transcribed locus, moderately similar to XP_529507.1 PREDICTED: hypothetical protein XP_529507 [Pan troglodytes]  Info    
   2146 1.9e-05 189.4 385 0.492  1393997_at  similar to hypothetical protein FLJ32825  Info   LOC500595 
   2147 1.9e-05 204.1 71.7 2.847  1389402_at  Transcribed locus  Info    
   2148 1.9e-05 425.8 227.7 1.87  1391527_at  Ngfi-A binding protein 2 (predicted)  Info   Nab2 
   2149 1.91e-05 4518.4 5808.8 0.778  1367629_at  cytochrome c oxidase, subunit 7a 3  Info   Cox7a3 
   2150 1.91e-05 206.2 452.8 0.455  1372794_at  death-associated kinase 2 (predicted)  Info   Dapk2_predicted 
   2151 1.92e-05 1019.4 1692.9 0.602  1372162_at  acetyl-Coenzyme A synthetase 2 (AMP forming)-like (predicted)  Info   Acas2l_predicted 
   2152 1.92e-05 116 34.5 3.362  1370105_at  lunatic fringe gene homolog (Drosophila)  Info   Lfng 
   2153 1.93e-05 326.7 131.9 2.477  1379287_at  retinitis pigmentosa 1 (autosomal dominant) (predicted) /// similar to cylindromatosis (turban tumor syndrome) /// similar to cylindromatosis (turban tumor syndrome)  Info   Rp1_predicted /// LOC361373 /// LOC498035 
   2154 1.93e-05 418.5 4.8 87.188  1384837_at  CD69 antigen  Info   Cd69 
   2155 1.93e-05 1709.7 1063.3 1.608  1370280_at  hypoxanthine guanine phosphoribosyl transferase  Info   Hprt 
   2156 1.93e-05 1120.3 517.7 2.164  1387872_at  heterogeneous nuclear ribonucleoprotein A1  Info   Hnrpa1 
   2157 1.94e-05 873.5 1292.8 0.676  1372876_at  selenophosphate synthetase 2 (predicted)  Info   Sephs2_predicted 
   2158 1.94e-05 24.1 45.5 0.53  1390001_at  Rho-related BTB domain containing 2 (predicted)  Info   Rhobtb2_predicted 
   2159 1.95e-05 62.1 136.7 0.454  1367953_at  TYRO3 protein tyrosine kinase 3  Info   Tyro3 
   2160 1.95e-05 231.8 380.3 0.61  1390923_a_at  oxysterol binding protein-like 1A  Info   Osbpl1a 
   2161 1.95e-05 310.1 55.7 5.567  1373528_at  Phosphatidylinositol 3-kinase catalytic delta polypeptide (predicted)  Info    
   2162 1.95e-05 444.7 146 3.046  1390731_at  Transcribed locus  Info    
   2163 1.96e-05 138.6 264.6 0.524  1367880_at  laminin, beta 2  Info   Lamb2 
   2164 1.96e-05 526.5 276.5 1.904  1367901_at  glucuronidase, beta  Info   Gusb 
   2165 1.96e-05 230.6 138.7 1.663  1390124_at  similar to hypothetical protein FLJ38426  Info   LOC499866 
   2166 1.97e-05 1505 2164.6 0.695  1398903_at  Esterase D/formylglutathione hydrolase  Info   Esd 
   2167 1.97e-05 66.9 130 0.515  1376465_at  Transcribed locus  Info    
   2168 1.98e-05 711.1 340.4 2.089  1392579_at  similar to 5830411E10Rik protein (predicted)  Info   RGD1306658_predicted 
   2169 1.98e-05 465.5 876.5 0.531  1376868_at    Info    
   2170 1.98e-05 450.3 312.2 1.442  1375272_at  Phosphatidylinositol 4-kinase type 2 alpha  Info   Pi4KII 
   2171 1.99e-05 414.8 202.6 2.047  1390386_at  caspase 3  Info   Casp3 
   2172 1.99e-05 565.9 799.9 0.707  1371965_at  similar to RIKEN cDNA 2010311D03  Info   RGD:1303272 
   2173 1.99e-05 1288.2 956.1 1.347  1388764_at  IQ motif containing GTPase activating protein 1 (predicted)  Info    
   2174 1.99e-05 62 90 0.689  1379352_at  phosphatidylinositol-4-phosphate 5-kinase, type 1 alpha (predicted)  Info   Pip5k1a_predicted 
   2175 2e-05 495.7 795 0.624  1397526_at  glutaryl-Coenzyme A dehydrogenase (predicted)  Info   Gcdh_predicted 
   2176 2e-05 136.1 90.3 1.507  1373715_at  similar to hypothetical protein DKFZp434K1815  Info   RGD:1359442 
   2177 2.01e-05 289.3 403.6 0.717  1388892_at  RAB2B, member RAS oncogene family (predicted)  Info   Rab2b_predicted 
   2178 2.01e-05 87.1 158.5 0.55  1393197_at  abhydrolase domain containing 8 (predicted)  Info   Abhd8_predicted 
   2179 2.01e-05 379.4 184.7 2.054  1371863_at  similar to phosphatidylglycerophosphate synthase (predicted)  Info   RGD1305052_predicted 
   2180 2.01e-05 67.6 6.9 9.797  1380616_at  sialyltransferase 8 (alpha-2, 8-sialytransferase) D  Info   Siat8d 
   2181 2.01e-05 135.9 328.6 0.414  1397729_x_at  similar to RIKEN cDNA 1600029D21  Info   RGD:1359217 
   2182 2.01e-05 1150.3 20.5 56.112  1379935_at  chemokine (C-C motif) ligand 7  Info   RGD:1359152 
   2183 2.01e-05 286.3 62.9 4.552  1383519_at  Hexokinase 2  Info   Hk2 
   2184 2.02e-05 227.2 130.3 1.744  1372343_at  exosome component 8 (predicted)  Info   Exosc8_predicted 
   2185 2.02e-05 1498.6 2619.4 0.572  1390822_at  cadherin 16 (predicted)  Info   Cdh16_predicted 
   2186 2.02e-05 336.7 209 1.611  1389778_a_at  transcription elongation factor B (SIII), polypeptide 3  Info   Tceb3 
   2187 2.02e-05 594.3 372.9 1.594  1388622_at  nucleolar protein 5A (predicted)  Info   Nol5a_predicted 
   2188 2.03e-05 169.5 69.4 2.442  1386410_at  Transcribed locus  Info    
   2189 2.03e-05 20.5 55.5 0.369  1396305_at  Transcribed locus  Info    
   2190 2.03e-05 1292.4 827 1.563  1374505_at  similar to growth hormone-inducible soluble protein  Info   LOC502922 
   2191 2.04e-05 46.8 120.1 0.39  1376870_at  zinc finger protein 278 (predicted)  Info   Znf278_predicted 
   2192 2.04e-05 559.4 1079.4 0.518  1394786_at  Transcribed locus  Info    
   2193 2.04e-05 364.4 178.7 2.039  1370728_at  interleukin 13 receptor, alpha 1  Info   Il13ra1 
   2194 2.04e-05 264.2 150.8 1.752  1367955_at  RAB4B, member RAS oncogene family  Info   Rab4b 
   2195 2.04e-05 486.8 607.7 0.801  1374443_at  immature colon carcinoma transcript 1 (predicted)  Info   Ict1_predicted 
   2196 2.04e-05 1739.2 3247.3 0.536  1387651_at  aquaporin 1  Info   Aqp1 
   2197 2.05e-05 486.6 297.3 1.637  1387135_at  a disintegrin and metalloproteinase domain 15 (metargidin)  Info   Adam15 
   2198 2.05e-05 291.1 161.4 1.804  1385128_at  Similar to MGC68837 protein  Info    
   2199 2.05e-05 462.6 747.8 0.619  1374959_at  NAD(P)H dehydrogenase, quinone 2  Info   RGD:1303320 
   2200 2.05e-05 52.5 129.9 0.404  1398732_at  Transcribed locus  Info    
   2201 2.05e-05 34.4 75.6 0.455  1393335_at  epidermal growth factor-like protein 6  Info   Egfl6 
   2202 2.05e-05 695.1 397 1.751  1381969_at  Transcribed locus  Info    
   2203 2.06e-05 94.6 51.5 1.837  1378345_at  Transcribed locus  Info    
   2204 2.06e-05 301.4 160.2 1.881  1372493_at  similar to RIKEN cDNA 2410005K17 (predicted)  Info   RGD1311232_predicted 
   2205 2.07e-05 100.1 37.4 2.676  1374756_at  similar to 1500031N24Rik protein (predicted) /// similar to 1500031N24Rik protein  Info   RGD1311188_predicted /// LOC362936 
   2206 2.07e-05 23.9 101.3 0.236  1396127_at    Info    
   2207 2.07e-05 685.6 1037.6 0.661  1372758_at  Transcribed locus  Info    
   2208 2.07e-05 112.4 268.5 0.419  1376860_at  Transcribed locus  Info    
   2209 2.08e-05 2272.5 3055.9 0.744  1388294_at  succinate dehydrogenase complex, subunit D, integral membrane protein  Info   Sdhd 
   2210 2.08e-05 42.7 66.5 0.642  1384941_at  similar to Shb-like adapter protein, Shf - human  Info   LOC362205 
   2211 2.08e-05 279.4 385 0.726  1392603_at  EGL nine homolog 1 (C. elegans)  Info   Egln1 
   2212 2.09e-05 733.9 8.6 85.337  1385414_at  CD8 antigen, alpha chain  Info   Cd8a 
   2213 2.09e-05 1130.2 1670.6 0.677  1370320_at  MAWD binding protein  Info   Mawbp 
   2214 2.09e-05 163.7 385.8 0.424  1385486_at  Basonuclin 2 (predicted)  Info   LOC298189 
   2215 2.09e-05 751.8 1180 0.637  1373016_at  dihydrolipoamide dehydrogenase  Info   Dld 
   2216 2.1e-05 401.2 228 1.76  1382287_at  ribonucleic acid binding protein S1 (predicted)  Info   Rnps1_predicted 
   2217 2.1e-05 1790.8 893.8 2.004  1368418_a_at  ceruloplasmin  Info   Cp 
   2218 2.1e-05 1327.9 1926 0.689  1389286_at  similar to 2900070E19Rik protein (predicted)  Info   RGD1308383_predicted 
   2219 2.11e-05 77.3 22.7 3.405  1387839_at  RT1 class Ib gene, H2-TL-like, grc region (N1) /// RT1 class Ib gene, H2-TL-like, grc region (N3) /// RT1 class Ib gene, H2-TL-like, grc region(N2)  Info   RT1-N1 /// RT1-N3 /// RT1-N2 
   2220 2.11e-05 187.8 533 0.352  1379326_at  similar to 9530008L14Rik protein (predicted)  Info   RGD1305679_predicted 
   2221 2.11e-05 2871.8 4038 0.711  1371371_at  NADH dehydrogenase (ubiquinone) 1 beta subcomplex, 4, 15kDa (predicted)  Info   Ndufb4_predicted 
   2222 2.12e-05 83.1 178.4 0.466  1367835_at  proprotein convertase subtilisin/kexin type 1 inhibitor  Info   Pcsk1n 
   2223 2.12e-05 81.6 22.9 3.563  1369393_at  mitogen-activated protein kinase kinase kinase 8  Info   Map3k8 
   2224 2.12e-05 45.6 113.2 0.403  1374833_at  Transcribed locus  Info    
   2225 2.13e-05 118.8 252.5 0.47  1391840_at  similar to RIKEN cDNA 1700027N10 (predicted)  Info   RGD1310693_predicted 
   2226 2.13e-05 1358.3 2411.9 0.563  1389466_at  Ankyrin 3, epithelial isoform g  Info    
   2227 2.14e-05 1654 2628.3 0.629  1370806_at  all-trans-13,14-dihydroretinol saturase  Info   Retsat 
   2228 2.14e-05 1686.4 1158.2 1.456  1370170_at  heterogeneous nuclear ribonucleoprotein U  Info   Hnrpu 
   2229 2.14e-05 2470.6 3038.2 0.813  1398932_at  histidine triad nucleotide binding protein 1 (predicted)  Info   Hint1_predicted 
   2230 2.14e-05 422.6 778.3 0.543  1368414_at  solute carrier family 5 (sodium/glucose cotransporter), member 2  Info   Slc5a2 
   2231 2.15e-05 273 135.1 2.021  1378181_at  ribonuclease P 40 subunit (human) (predicted)  Info   Rpp40_predicted 
   2232 2.15e-05 261.3 375.2 0.696  1385690_at  similar to Mut protein /// similar to Mut protein  Info   LOC301276 /// LOC363201 
   2233 2.15e-05 368.1 202.4 1.819  1368032_at  nucleolar and coiled-body phosphoprotein 1  Info   Nolc1 
   2234 2.15e-05 124.4 200.5 0.62  1378169_at  similar to peroxisomal long chain acyl-CoA thioesterase Ia  Info   LOC314304 
   2235 2.16e-05 202.2 48.4 4.178  1392755_at  similar to hypothetical protein AD158 (predicted)  Info   RGD1306585_predicted 
   2236 2.16e-05 326.8 556.3 0.587  1371251_at  galactose-1-phosphate uridyl transferase (predicted)  Info   Galt_predicted 
   2237 2.16e-05 425 548.8 0.774  1375368_at  similar to RIKEN cDNA 1700012G19 gene (predicted)  Info   RGD1307773_predicted 
   2238 2.17e-05 2353.9 1663.9 1.415  1398784_at  complement component 1, q subcomponent binding protein  Info   C1qbp 
   2239 2.17e-05 363.7 138.4 2.628  1382277_at    Info    
   2240 2.17e-05 514.7 956.9 0.538  1369451_a_at  potassium inwardly-rectifying channel, subfamily J, member 1  Info   Kcnj1 
   2241 2.18e-05 653.9 1652.2 0.396  1375247_at  Monoglyceride lipase  Info   Mgll 
   2242 2.18e-05 359.2 510.4 0.704  1377923_at  Transcribed locus, weakly similar to XP_576460.1 PREDICTED: similar to hypothetical protein PB402898.00.0 [Rattus norvegicus]  Info    
   2243 2.18e-05 13.7 42.5 0.322  1387287_a_at  ATP-binding cassette, sub-family C (CFTR/MRP), member 9  Info   Abcc9 
   2244 2.18e-05 67.6 127.9 0.529  1377879_at  Transcribed locus  Info    
   2245 2.18e-05 19.6 48.2 0.407  1373659_at    Info    
   2246 2.18e-05 749.7 2302.7 0.326  1368304_at  Flavin containing monooxygenase 3  Info   Fmo3 
   2247 2.19e-05 142.3 276.6 0.514  1389392_at  Transcribed locus  Info    
   2248 2.19e-05 440.2 265.1 1.661  1369122_at  Bcl2-associated X protein  Info   Bax 
   2249 2.2e-05 107.8 167.1 0.645  1374025_at  nicotinamide nucleotide adenylyltransferase 3 (predicted) /// similar to Nicotinamide nucleotide adenylyltransferase 3  Info   Nmnat3_predicted /// LOC501040 
   2250 2.2e-05 85.4 180 0.474  1382137_at  abhydrolase domain containing 3 (predicted)  Info   Abhd3_predicted 
   2251 2.2e-05 53.6 95.8 0.559  1379900_at  Transcribed locus  Info    
   2252 2.21e-05 160.8 239.6 0.671  1372373_at  Similar to putative WD-repeat protein  Info    
   2253 2.21e-05 386.1 796.4 0.485  1389548_at  alcohol dehydrogenase, iron containing, 1 (predicted)  Info   Adhfe1_predicted 
   2254 2.21e-05 276.9 435.8 0.635  1387905_at  J domain protein 1  Info   LOC619393 
   2255 2.22e-05 82.5 23.9 3.452  1391502_at  Transcribed locus  Info    
   2256 2.22e-05 166.9 30.5 5.472  1387391_at  cyclin-dependent kinase inhibitor 1A  Info   Cdkn1a 
   2257 2.22e-05 60 108 0.556  1397287_at  similar to 4931417G12Rik protein  Info   LOC500294 
   2258 2.22e-05 115.8 211.2 0.548  1375270_at  Transcribed locus  Info    
   2259 2.22e-05 429.4 223.6 1.92  1383863_at    Info    
   2260 2.23e-05 384.9 198.2 1.942  1388791_at  similar to 2810022L02Rik protein (predicted)  Info   RGD1309930_predicted 
   2261 2.23e-05 1138.9 1935 0.589  1389521_at  influenza virus NS1A binding protein (predicted)  Info   Ivns1abp_predicted 
   2262 2.23e-05 1102.1 860.6 1.281  1371559_at  CDNA clone MGC:109551 IMAGE:7325365  Info    
   2263 2.24e-05 1431.3 2515.7 0.569  1392969_at  Transcribed locus  Info    
   2264 2.24e-05 93.6 157.6 0.594  1392973_at  LOC499153  Info   LOC499153 
   2265 2.24e-05 24.9 48.5 0.513  1391073_at  Transcribed locus  Info    
   2266 2.25e-05 31.5 78.3 0.402  1371137_at  acyl-Coenzyme A oxidase 2, branched chain  Info   Acox2 
   2267 2.26e-05 289.6 442.3 0.655  1371805_at  similar to RIKEN cDNA C730048E16 (predicted)  Info   RGD1309382_predicted 
   2268 2.26e-05 341.8 740.6 0.462  1368253_at  guanidinoacetate methyltransferase  Info   Gamt 
   2269 2.26e-05 119.4 32 3.731  1390195_at  Pleckstrin and Sec7 domain containing 4 (predicted)  Info    
   2270 2.27e-05 210.1 120.9 1.738  1392913_at  similar to zinc finger protein 366 (predicted)  Info   RGD1306491_predicted 
   2271 2.27e-05 68.7 127.9 0.537  1374724_at  Transcribed locus  Info    
   2272 2.27e-05 174 256.3 0.679  1379315_at  similar to RIKEN cDNA 2400009B11 gene (predicted)  Info   RGD1306244_predicted 
   2273 2.27e-05 1113.7 793.6 1.403  1388516_at  Similar to CDV-3B  Info    
   2274 2.27e-05 280.8 450.6 0.623  1390937_at  similar to chromosome 14 open reading frame 50 (predicted)  Info   RGD1309051_predicted 
   2275 2.27e-05 1.5 18 0.083  1373686_at  serine (or cysteine) proteinase inhibitor, clade A (alpha-1 antiproteinase, antitrypsin), member 6  Info   Serpina6 
   2276 2.28e-05 153.9 505.2 0.305  1379073_at  Similar to RIKEN cDNA 2310067G05  Info    
   2277 2.28e-05 2295.2 2825.9 0.812  1367694_at  hydroxyacyl-Coenzyme A dehydrogenase/3-ketoacyl-Coenzyme A thiolase/enoyl-Coenzyme A hydratase (trifunctional protein), beta subunit  Info   Hadhb 
   2278 2.29e-05 99.8 167.8 0.595  1377015_at  Malic enzyme 3, NADP(+)-dependent, mitochondrial (predicted)  Info    
   2279 2.29e-05 109.3 52 2.102  1382138_at  similar to hypothetical protein FLJ20433  Info   LOC499745 
   2280 2.3e-05 331.6 124.5 2.663  1383542_at  RAS guanyl releasing protein 1  Info   Rasgrp1 
   2281 2.3e-05 197.9 319.8 0.619  1376571_at  similar to RIKEN cDNA 0610027O18 (predicted)  Info   RGD1307118_predicted 
   2282 2.3e-05 358.2 195.3 1.834  1371529_at  Receptor (TNFRSF)-interacting serine-threonine kinase 1 (predicted)  Info    
   2283 2.3e-05 59.1 95.7 0.618  1384345_at  zinc binding alcohol dehydrogenase, domain containing 1 (predicted)  Info   Zadh1_predicted 
   2284 2.31e-05 513.5 291.7 1.76  1389468_at  ribose 5-phosphate isomerase A (predicted)  Info   Rpia_predicted 
   2285 2.31e-05 430.8 152.4 2.827  1387914_at  cytochrome P450, family 27, subfamily a, polypeptide 1  Info   Cyp27a1 
   2286 2.31e-05 480.6 284.2 1.691  1393956_at  serine/threonine kinase 17b (apoptosis-inducing)  Info   Stk17b 
   2287 2.31e-05 600.8 218.2 2.753  1387060_at  core promoter element binding protein  Info   Copeb 
   2288 2.32e-05 1943.6 1186.7 1.638  1383080_at  Lysosomal membrane glycoprotein 2  Info   Lamp2 
   2289 2.32e-05 99.5 193.5 0.514  1376230_at    Info    
   2290 2.33e-05 394.4 147.9 2.667  1388674_at  cyclin-dependent kinase inhibitor 1A  Info   Cdkn1a 
   2291 2.33e-05 4950.6 3568.9 1.387  1370243_a_at  prothymosin alpha  Info   Ptma 
   2292 2.34e-05 1382.6 1014.7 1.363  1383073_at  ubiquitin specific protease 14 (predicted)  Info   Usp14_predicted 
   2293 2.34e-05 379.2 19.5 19.446  1387472_at  CD3 antigen delta polypeptide  Info   Cd3d 
   2294 2.35e-05 277.4 167.7 1.654  1368647_at  G protein-coupled receptor kinase 6  Info   Gprk6 
   2295 2.35e-05 685.7 1861.4 0.368  1377672_at  sulfotransferase family, cytosolic, 1C, member 2  Info   Sult1c2 
   2296 2.35e-05 754.6 340.3 2.217  1383786_at  Transcribed locus  Info    
   2297 2.35e-05 101.3 211.6 0.479  1377404_at  stanniocalcin 1  Info   Stc1 
   2298 2.35e-05 194 54.3 3.573  1375900_at  similar to T-cell antigen 4-1BB precursor - mouse  Info   LOC500590 
   2299 2.36e-05 739.8 1414.9 0.523  1369986_at  hydroxyacyl glutathione hydrolase  Info   Hagh 
   2300 2.36e-05 36.6 119.8 0.306  1374861_at  transducin-like enhancer of split 2, homolog of Drosophila E(spl) (predicted)  Info   Tle2_predicted 
   2301 2.36e-05 101.7 131.4 0.774  1383495_at  DNA cross-link repair 1A, PSO2 homolog (S. cerevisiae) (predicted)  Info   Dclre1a_predicted 
   2302 2.36e-05 121.6 204.8 0.594  1383443_at  similar to RIKEN cDNA 1110018J18  Info   LOC498685 
   2303 2.36e-05 343.3 639.2 0.537  1373680_at  Transcribed locus  Info    
   2304 2.37e-05 48.2 103.3 0.467  1383220_at  Transcribed locus  Info    
   2305 2.38e-05 68.7 133.4 0.515  1398264_at  solute carrier family 30 (zinc transporter), member 2  Info   Slc30a2 
   2306 2.38e-05 133.7 214.3 0.624  1373607_at  Transcribed locus  Info    
   2307 2.38e-05 170.9 18.5 9.238  1389275_at    Info    
   2308 2.39e-05 2639.1 3423.6 0.771  1371342_at  cytochrome c-1 (predicted)  Info   Cyc1_predicted 
   2309 2.39e-05 71.6 178.2 0.402  1395887_at  similar to RIKEN cDNA 1600029D21  Info   RGD:1359217 
   2310 2.39e-05 124.1 46.3 2.68  1376281_at  Similar to hypothetical protein FLJ22349  Info    
   2311 2.39e-05 110.8 306 0.362  1377940_at  hypothetical LOC287534  Info   RGD:1359691 
   2312 2.39e-05 83.1 190.1 0.437  1373521_at  similar to RIKEN cDNA D430044G18 (predicted)  Info   RGD1309038_predicted 
   2313 2.4e-05 364.6 260.4 1.4  1368214_at  MAD homolog 2 (Drosophila)  Info   Madh2 
   2314 2.4e-05 102.9 260 0.396  1377408_at  phospholipase A2, group VI  Info   Pla2g6 
   2315 2.4e-05 137.9 64.7 2.131  1387432_at  ROD1 regulator of differentiation 1 (S. pombe)  Info   Rod1 
   2316 2.4e-05 63.3 151.7 0.417  1369318_at  fragile histidine triad gene  Info   Fhit 
   2317 2.4e-05 2527.6 1802.8 1.402  1388332_at  Ras-related C3 botulinum toxin substrate 1  Info   Rac1 
   2318 2.4e-05 488.2 810.7 0.602  1372744_at  plakophilin 4 (predicted)  Info   Pkp4_predicted 
   2319 2.4e-05 202 124.1 1.628  1376336_at  Transcribed locus  Info    
   2320 2.41e-05 286.8 195.5 1.467  1390767_at  signal sequence receptor, alpha (predicted)  Info   Ssr1_predicted 
   2321 2.42e-05 217.1 409.4 0.53  1394479_at  Transcribed locus  Info    
   2322 2.42e-05 336.8 156.6 2.151  1374208_at  zinc finger CCCH type domain containing 7 (predicted)  Info   Zc3hdc7_predicted 
   2323 2.42e-05 253.3 364 0.696  1378605_at  similar to single Ig IL-1 receptor related protein (predicted)  Info   RGD1306732_predicted 
   2324 2.42e-05 243.5 137.3 1.773  1383107_at  small nuclear ribonucleoprotein D1 (predicted)  Info   Snrpd1_predicted 
   2325 2.43e-05 585.7 360.1 1.626  1386967_at  ras homolog gene family, member Q  Info   Rhoq 
   2326 2.43e-05 48.2 127 0.38  1384695_at  uroplakin 3A (predicted)  Info   Upk3a_predicted 
   2327 2.43e-05 319.6 88.7 3.603  1369683_at  BH3 interacting domain death agonist  Info   Bid 
   2328 2.44e-05 95.4 17.4 5.483  1381216_at    Info    
   2329 2.44e-05 58.6 133.5 0.439  1397168_at  Similar to CD2-associated protein (predicted)  Info    
   2330 2.44e-05 214.9 337.4 0.637  1388486_at  Dipeptidylpeptidase 8 (predicted)  Info    
   2331 2.44e-05 332.1 127.5 2.605  1389510_at  Ly1 antibody reactive clone (predicted)  Info   Lyar_predicted 
   2332 2.44e-05 325.5 587.4 0.554  1372449_at  Transcribed locus  Info    
   2333 2.45e-05 123.9 325.8 0.38  1373749_at  syntrophin, acidic 1 (predicted)  Info   Snta1_predicted 
   2334 2.46e-05 176.7 273.7 0.646  1370481_at  sodium channel, nonvoltage-gated 1 gamma  Info   Scnn1g 
   2335 2.46e-05 5715.5 151.7 37.676  1382454_at  chemokine (C-X-C motif) ligand 9  Info   Cxcl9 
   2336 2.47e-05 81.2 193.8 0.419  1390838_at  similar to KIAA1034-like DNA binding protein  Info   LOC501145 
   2337 2.47e-05 247.1 381.7 0.647  1388102_at  leukotriene B4 12-hydroxydehydrogenase  Info   Ltb4dh 
   2338 2.47e-05 153.3 43 3.565  1392794_at  Transcribed locus  Info    
   2339 2.47e-05 239.8 464.8 0.516  1387072_at  protein kinase, lysine deficient 1  Info   Prkwnk1 
   2340 2.48e-05 28.5 62 0.46  1388384_at  Dynein, cytoplasmic, light chain 1  Info   Dnclc1 
   2341 2.48e-05 2145.3 1354.6 1.584  1373947_at  dermatopontin (predicted)  Info   Dpt_predicted 
   2342 2.49e-05 194.9 138.1 1.411  1390967_a_at  Similar to RIKEN cDNA 2410006F12 (predicted)  Info    
   2343 2.5e-05 3873.2 6195.7 0.625  1387223_at  aminoadipate aminotransferase  Info   Aadat 
   2344 2.51e-05 234.6 152.4 1.539  1377755_at  helicase, ATP binding 1 (predicted)  Info   Helic1_predicted 
   2345 2.52e-05 670.1 420.7 1.593  1388129_at  structure specific recognition protein 1  Info   Ssrp1 
   2346 2.52e-05 229.2 99.1 2.313  1389425_at  similar to 5(3)-deoxyribonucleotidase  Info   LOC501746 
   2347 2.52e-05 49.7 156.2 0.318  1391109_x_at    Info    
   2348 2.53e-05 2.5 50 0.05  1367951_at  phosphoglycerate mutase 2  Info   Pgam2 
   2349 2.53e-05 2410.3 1623.8 1.484  1388333_at  Ring-box 1 (predicted)  Info    
   2350 2.53e-05 49.2 118.4 0.416  1374709_at  Transcribed locus  Info    
   2351 2.53e-05 265.2 161.4 1.643  1384239_at  Ribosomal protein L31  Info   Rpl31 
   2352 2.53e-05 167.7 453.1 0.37  1370789_a_at  prolactin receptor  Info   Prlr 
   2353 2.54e-05 856.3 1244.3 0.688  1389549_at  proline synthetase co-transcribed (predicted)  Info   Prosc_predicted 
   2354 2.54e-05 20.7 56.6 0.366  1396455_at  cytochrome c oxidase, subunit VIb (predicted)  Info   Cox6b_predicted 
   2355 2.55e-05 649.5 1016.2 0.639  1368071_at  Mg87 protein  Info   Mg87 
   2356 2.56e-05 3908.7 2726.2 1.434  1398831_at  proteasome (prosome, macropain) subunit, beta type 4  Info   Psmb4 
   2357 2.56e-05 160.7 121.1 1.327  1378166_at  Gene trap locus 7 (predicted)  Info    
   2358 2.56e-05 826.5 1382.7 0.598  1374524_at  selenocysteine lyase  Info   RGD:1359514 
   2359 2.56e-05 301.3 186 1.62  1392922_at  Transcribed locus  Info    
   2360 2.57e-05 114.1 62.4 1.829  1385066_a_at  Similar to hypothetical protein FLJ13089 (predicted)  Info    
   2361 2.57e-05 129.2 181.8 0.711  1393028_at  Transcribed locus  Info    
   2362 2.58e-05 115.8 296.2 0.391  1383943_at  dynein, axonemal, heavy polypeptide 7  Info   Dnah7 
   2363 2.58e-05 210.8 106.6 1.977  1392454_at  similar to hypothetical protein FLJ20729 (predicted)  Info   RGD1308723_predicted 
   2364 2.58e-05 289.4 477 0.607  1377725_at  Transcribed locus  Info    
   2365 2.58e-05 376.5 583.2 0.646  1371493_at  Transcribed locus  Info    
   2366 2.59e-05 228.6 28.9 7.91  1383407_at  Similar to RIKEN cDNA 1200013B08  Info    
   2367 2.59e-05 137 91.7 1.494  1387588_at  EH-domain containing 3  Info   Ehd3 
   2368 2.59e-05 2598.4 3324.5 0.782  1367678_at  succinate dehydrogenase complex, subunit A, flavoprotein (Fp)  Info   Sdha 
   2369 2.6e-05 222.4 45.8 4.856  1383936_at  Elastin microfibril interfacer 2 (predicted)  Info    
   2370 2.61e-05 793 462.4 1.715  1372810_at    Info    
   2371 2.61e-05 242.1 491.5 0.493  1392477_at  ets variant gene 1 (predicted)  Info   Etv1_predicted 
   2372 2.62e-05 470 265.9 1.768  1373473_a_at  nucleosome assembly protein 1-like 1  Info   Nap1l1 
   2373 2.62e-05 585.2 955.1 0.613  1391856_at  similar to semaphorin sem2 (predicted)  Info   RGD1306079_predicted 
   2374 2.62e-05 1140.3 549 2.077  1367715_at  tumor necrosis factor receptor superfamily, member 1a  Info   Tnfrsf1a 
   2375 2.63e-05 207.3 621.9 0.333  1368460_at  solute carrier family 2, member 5  Info   Slc2a5 
   2376 2.63e-05 173 329.1 0.526  1373631_at  RAP1, GTPase activating protein 1 (predicted)  Info   Rap1ga1_predicted 
   2377 2.63e-05 225.2 573.3 0.393  1370714_a_at  sialyltransferase 1  Info   Siat1 
   2378 2.63e-05 114.2 229.9 0.497  1381445_at  Transcribed locus  Info    
   2379 2.63e-05 261.1 542.8 0.481  1374771_at    Info    
   2380 2.64e-05 1048.1 1525.8 0.687  1385058_at  Claudin 8 (predicted)  Info    
   2381 2.64e-05 451.8 780.9 0.579  1376804_at  similar to Myosin VI  Info   LOC315840 
   2382 2.65e-05 435.1 899.2 0.484  1398341_at  hypothetical LOC287661  Info   LOC287661 
   2383 2.65e-05 414.5 697.4 0.594  1383121_at  Ceroid-lipofuscinosis, neuronal 2  Info   Cln2 
   2384 2.65e-05 89.4 44.4 2.014  1377058_at  similar to solute carrier family 37 (glycerol-3-phosphate transporter), member 2  Info   LOC500973 
   2385 2.65e-05 212.1 349.9 0.606  1382134_at  similar to olfactomedin-like 1 /// olfactomedin-like 1 (predicted)  Info   LOC308917 /// Olfml1_predicted 
   2386 2.65e-05 839.2 1160.1 0.723  1388552_at  sphingomyelin phosphodiesterase 1, acid lysosomal  Info   Smpd1 
   2387 2.65e-05 171.3 9.9 17.303  1378043_at  similar to OEF2  Info   LOC500011 
   2388 2.66e-05 983.7 619 1.589  1372815_at  mago-nashi homolog, proliferation-associated (Drosophila) (predicted)  Info   Magoh_predicted 
   2389 2.67e-05 428.5 259.7 1.65  1389633_at  E2F transcription factor 4 (predicted)  Info    
   2390 2.67e-05 433.5 260 1.667  1374433_at  Similar to Ferritin light chain (Ferritin L subunit)  Info    
   2391 2.67e-05 81.5 206.9 0.394  1387941_s_at  phospholipase A2, group VI  Info   Pla2g6 
   2392 2.67e-05 126.5 172.7 0.732  1382763_at    Info    
   2393 2.67e-05 129.9 291.4 0.446  1372481_at  Transcribed locus, moderately similar to NP_598415.1 CD34 antigen [Mus musculus]  Info    
   2394 2.67e-05 373.4 708.2 0.527  1389469_at  chromodomain helicase DNA binding protein 1-like (predicted)  Info   Chd1l_predicted 
   2395 2.68e-05 275.1 462.3 0.595  1367747_at  ADP-ribosylation factor-like 3  Info   Arl3 
   2396 2.68e-05 19.8 81.3 0.244  1374247_at  stabilin 1 (predicted)  Info   Stab1_predicted 
   2397 2.68e-05 644.7 349.3 1.846  1385933_at  Transcribed locus  Info    
   2398 2.68e-05 44.8 81.2 0.552  1397356_at  Rho GTPase activating protein 1 (predicted)  Info    
   2399 2.68e-05 43.6 89.7 0.486  1387157_at  polyamine modulated factor 1 binding protein 1  Info   Pmfbp1 
   2400 2.68e-05 706.5 890.9 0.793  1399019_at  abhydrolase domain containing 4 (predicted)  Info   Abhd4_predicted 
   2401 2.69e-05 626.3 411.1 1.523  1396820_at  histone deacetylase 1 (predicted)  Info   Hdac1_predicted 
   2402 2.69e-05 75.5 122.9 0.614  1374110_at  Mannosidase 2, alpha 2 (predicted)  Info    
   2403 2.69e-05 313.2 593.5 0.528  1398358_a_at  similar to integrin beta-5  Info   LOC498091 
   2404 2.69e-05 107.1 16.8 6.375  1394470_at  similar to hypothetical protein DKFZp566A1524 (predicted)  Info   RGD1305961_predicted 
   2405 2.7e-05 129.5 23.3 5.558  1384228_at  similar to nuclear factor of activated T-cells, cytoplasmic, calcineurin-dependent 1  Info   LOC307231 
   2406 2.7e-05 101.5 191.5 0.53  1391212_at  transcription elongation factor A (SII)-like 1  Info   Tceal1 
   2407 2.7e-05 205.6 73.6 2.793  1368811_at  lamin B1  Info   Lmnb1 
   2408 2.72e-05 157.6 75.7 2.082  1392514_at  brix domain containing 1 (predicted)  Info   Bxdc1_predicted 
   2409 2.72e-05 1267.2 1603.5 0.79  1388487_at  Adducin 1 (alpha)  Info   Add1 
   2410 2.72e-05 1365.7 2751.5 0.496  1370201_at  calbindin 1  Info   Calb1 
   2411 2.73e-05 75 43.7 1.716  1379493_at  Transcribed locus  Info    
   2412 2.73e-05 169.1 70.8 2.388  1377153_a_at  kelch-like 6 (Drosophila) (predicted)  Info   Klhl6_predicted 
   2413 2.73e-05 144.4 316.4 0.456  1373895_at    Info    
   2414 2.74e-05 1783 1099 1.622  1386866_at  tyrosine 3-monooxgenase/tryptophan 5-monooxgenase activation protein, gamma polypeptide  Info   Ywhag 
   2415 2.75e-05 232.9 96 2.426  1372886_at  transforming acidic coiled coil 3  Info   RGD:1302948 
   2416 2.75e-05 127.5 232.8 0.548  1389274_at  similar to RIKEN cDNA 6720485C15  Info   RGD:1359211 
   2417 2.75e-05 108.2 191.3 0.566  1395381_at    Info    
   2418 2.76e-05 172 324.1 0.531  1383205_at  dapper homolog 2, antagonist of beta-catenin (xenopus) (predicted)  Info   Dact2_predicted 
   2419 2.78e-05 71.7 250.2 0.287  1384614_at    Info    
   2420 2.78e-05 160.5 446.1 0.36  1390783_at  ATP-binding cassette, sub-family A (ABC1), member 8a (predicted)  Info   Abca8a_predicted 
   2421 2.78e-05 212.1 298.5 0.711  1399107_at  similar to RIKEN cDNA 1810030N24 (predicted)  Info   RGD1305158_predicted 
   2422 2.79e-05 79.2 244.6 0.324  1372684_at  smoothelin (predicted)  Info   Smtn_predicted 
   2423 2.8e-05 75.9 133.6 0.568  1367970_at  profilin 2  Info   Pfn2 
   2424 2.8e-05 3333.9 2005.3 1.663  1371936_at  eukaryotic translation initiation factor 4A1  Info   Eif4a1 
   2425 2.8e-05 809.7 495.7 1.633  1368118_at  B-cell CLL/lymphoma 10  Info   Bcl10 
   2426 2.8e-05 18.4 60.7 0.303  1378569_at  Transcribed locus  Info    
   2427 2.8e-05 159.6 240.5 0.664  1394761_at  similar to Rho-GTPase-activating protein 26 (Oligophrenin-1 like protein) (GTPase regulator associated with focal adhesion kinase)  Info   LOC300350 
   2428 2.81e-05 174.8 43.3 4.037  1384852_at  low Mr GTP-binding protein  Info   Rab27a 
   2429 2.82e-05 115.1 166 0.693  1373810_at  phospholipase A2, group XIIA (predicted)  Info   Pla2g12a_predicted 
   2430 2.82e-05 361.3 646.1 0.559  1372490_at  Similar to mKIAA0089 protein  Info    
   2431 2.83e-05 321.7 208.4 1.544  1390576_at  Transcribed locus  Info    
   2432 2.83e-05 2.2 11.8 0.186  1398743_at  Transcribed locus  Info    
   2433 2.83e-05 71 36.5 1.945  1386660_at  Transcribed locus  Info    
   2434 2.83e-05 837.9 1242.2 0.675  1373529_at  Transcribed locus  Info    
   2435 2.84e-05 120.1 55.6 2.16  1390415_at  Thyroid hormone receptor interactor 13 (predicted)  Info    
   2436 2.85e-05 526 311.5 1.689  1367743_at  phosphofructokinase, liver, B-type  Info   Pfkl 
   2437 2.85e-05 84.6 184.5 0.459  1384035_at  Similar to SPT3-associated factor 42 (predicted)  Info    
   2438 2.85e-05 318.2 1181.6 0.269  1387382_at  histamine N-methyltransferase  Info   Hnmt 
   2439 2.86e-05 57.8 145.2 0.398  1388527_at  Transcribed locus  Info    
   2440 2.86e-05 39.4 3.1 12.71  1371155_at  killer cell lectin-like receptor subfamily C, member 1  Info   Klrc1 
   2441 2.87e-05 207 90.2 2.295  1386252_at  Transcribed locus  Info    
   2442 2.87e-05 823.6 1078.7 0.764  1371796_at  Thioredoxin-related transmembrane protein 2  Info   MGC94529 
   2443 2.87e-05 507.3 656.7 0.772  1383260_at  similar to RIKEN cDNA G630024C07 gene (predicted)  Info   RGD1305903_predicted 
   2444 2.87e-05 56.7 141.7 0.4  1373196_at  EF hand domain family, member A2  Info   Efha2 
   2445 2.87e-05 111.4 150.8 0.739  1387948_at  intestinal cell kinase  Info   Ick 
   2446 2.87e-05 107.2 53.8 1.993  1390843_at  similar to hypothetical protein LOC92922 (predicted)  Info   RGD1308684_predicted 
   2447 2.88e-05 613.1 338.2 1.813  1373373_at  Glutamine fructose-6-phosphate transaminase 1  Info   MGC95214 
   2448 2.89e-05 248.2 139.8 1.775  1369650_at  p21 (CDKN1A)-activated kinase 2  Info   Pak2 
   2449 2.89e-05 301.4 560.3 0.538  1394456_at  Transcribed locus  Info    
   2450 2.9e-05 96.2 31.7 3.035  1377190_at  Similar to mKIAA0386 protein (predicted)  Info    
   2451 2.9e-05 1708.8 1265.2 1.351  1367656_at  proteasome (prosome, macropain) subunit, beta type 7  Info   Psmb7 
   2452 2.91e-05 7156.7 4323.2 1.655  1388071_x_at  RT1 class Ib, locus Aw2  Info   RT1-Aw2 
   2453 2.92e-05 921 462.5 1.991  1370141_at  myeloid cell leukemia sequence 1  Info   Mcl1 
   2454 2.93e-05 1665.1 2514.9 0.662  1368102_at  hydroxysteroid 11-beta dehydrogenase 2  Info   Hsd11b2 
   2455 2.93e-05 214.7 384.6 0.558  1379277_at  Utrophin  Info   Utrn 
   2456 2.93e-05 19.9 52.5 0.379  1385354_at  similar to integrin alpha 8  Info   LOC364786 
   2457 2.93e-05 208.5 348 0.599  1369187_a_at  prostaglandin E receptor 3 (subtype EP3)  Info   Ptger3 
   2458 2.94e-05 69.1 150.3 0.46  1378034_at  similar to transcription termination factor-like protein (predicted)  Info   RGD1311836_predicted 
   2459 2.94e-05 113 237 0.477  1379495_at  Transcribed locus  Info    
   2460 2.94e-05 184 102.2 1.8  1376321_at  family with sequence similarity 38, member A (predicted)  Info   Fam38a_predicted 
   2461 2.95e-05 461.4 943.4 0.489  1387029_at  complement component factor H  Info   Cfh 
   2462 2.95e-05 191.8 66.4 2.889  1376624_at  Transcribed locus  Info    
   2463 2.95e-05 475.5 334 1.424  1382175_at  Similar to Wilms tumor 1-associating protein (WT1-associated protein) (Putative pre-mRNA splicing regulator female-lethal(2D) homolog)  Info    
   2464 2.96e-05 143 97.5 1.467  1391572_at  cysteinyl-tRNA synthetase (predicted)  Info   Cars_predicted 
   2465 2.96e-05 163 78.6 2.074  1373226_at  Similar to 60S ribosomal protein L23  Info    
   2466 2.97e-05 1695.2 2704.2 0.627  1372297_at  glutathione S-transferase, alpha 4 (predicted)  Info   Gsta4_predicted 
   2467 2.98e-05 279.3 215.6 1.295  1368099_at  CLIP associating protein 2  Info   Clasp2 
   2468 2.98e-05 1.3 8.7 0.149  1392060_at    Info    
   2469 2.98e-05 1651.3 669.9 2.465  1378679_at  Transcribed locus  Info    
   2470 2.98e-05 27.8 9.3 2.989  1379469_at  similar to transducin (beta)-like 1 X-linked  Info   LOC302711 
   2471 2.99e-05 204.7 113.7 1.8  1384584_at  Transcribed locus  Info    
   2472 3.01e-05 30.3 91.7 0.33  1390598_at  Transcribed locus  Info    
   2473 3.01e-05 41.8 69.5 0.601  1380655_at  renal tumor antigen (predicted)  Info   Rage_predicted 
   2474 3.01e-05 719.9 573.4 1.255  1370208_at  nuclear receptor subfamily 1, group H, member 2  Info   Nr1h2 
   2475 3.01e-05 101.3 49.5 2.046  1381919_at  Hermansky-Pudlak syndrome 6  Info   Hps6 
   2476 3.01e-05 76.9 112.9 0.681  1370680_at  staufen, RNA binding protein, homolog 2 (Drosophila)  Info   Stau2 
   2477 3.02e-05 272.1 400 0.68  1374890_at  Transcribed locus, strongly similar to NP_031990.1 ecotropic viral integration site 5 [Mus musculus]  Info    
   2478 3.02e-05 823.1 394.5 2.086  1390801_at  similar to hypothetical protein MGC34760  Info   RGD:1359349 
   2479 3.03e-05 169.3 78.1 2.168  1368488_at  nuclear factor, interleukin 3 regulated  Info   Nfil3 
   2480 3.03e-05 76.1 160.2 0.475  1372470_at  armadillo repeat gene deleted in velo-cardio-facial syndrome (predicted)  Info   Arvcf_predicted 
   2481 3.03e-05 174.4 126.5 1.379  1377607_at  mitogen activated protein kinase kinase kinase 3 (predicted)  Info   Map3k3_predicted 
   2482 3.03e-05 100.8 227.2 0.444  1389725_at  transmembrane 7 superfamily member 2 (predicted)  Info   Tm7sf2_predicted 
   2483 3.04e-05 235.3 353.7 0.665  1383963_at  Transcribed locus  Info    
   2484 3.04e-05 1546.9 2816.6 0.549  1388788_at  glutaryl-Coenzyme A dehydrogenase (predicted)  Info   Gcdh_predicted 
   2485 3.04e-05 186.7 448.2 0.417  1371266_at  afamin  Info   Afm 
   2486 3.04e-05 303.3 160.5 1.89  1390698_at  Transcribed locus, weakly similar to XP_579465.1 PREDICTED: DNA binding protein N5 [Rattus norvegicus]  Info    
   2487 3.04e-05 213 398.1 0.535  1383852_at  Transcribed locus  Info    
   2488 3.05e-05 295.4 93.2 3.17  1389789_at  Transcribed locus  Info    
   2489 3.05e-05 428.2 203.4 2.105  1373504_at  GLI pathogenesis-related 1 (glioma) (predicted)  Info   Glipr1_predicted 
   2490 3.05e-05 831.8 1268.4 0.656  1389857_at  similar to WW domain binding protein 5  Info   LOC294067 
   2491 3.05e-05 441.3 649.5 0.679  1388506_at  desmoplakin (predicted)  Info   Dsp_predicted 
   2492 3.05e-05 43.6 78.1 0.558  1389780_at  tissue factor pathway inhibitor  Info   Tfpi 
   2493 3.06e-05 723.4 474.5 1.525  1389729_at  similar to cDNA sequence BC003324  Info   RGD:1303140 
   2494 3.06e-05 41.5 12.2 3.402  1377334_at  RT1 class II, locus Ba  Info   RT1-Ba 
   2495 3.06e-05 6.7 27 0.248  1382398_at  similar to MGC68837 protein  Info   LOC498356 
   2496 3.07e-05 134 84 1.595  1393184_at  P21 (CDKN1A)-activated kinase 2  Info   Pak2 
   2497 3.07e-05 3301.4 2176.4 1.517  1367584_at  annexin A2  Info   Anxa2 
   2498 3.07e-05 1081.9 29.6 36.551  1387378_at  ficolin B  Info   Fcnb 
   2499 3.07e-05 49.4 121.9 0.405  1374874_at  similar to RIKEN cDNA 5730509K17 gene  Info   LOC498386 
   2500 3.08e-05 330.8 109.1 3.032  1378443_at  SLAM family member 9 (predicted)  Info   Slamf9_predicted 
   2501 3.09e-05 40.9 118.8 0.344  1391067_at    Info    
   2502 3.09e-05 627.8 867.8 0.723  1398931_at    Info    
   2503 3.09e-05 2441.5 887.7 2.75  1368356_a_at  type 1 tumor necrosis factor receptor shedding aminopeptidase regulator  Info   Arts1 
   2504 3.09e-05 428.8 214.9 1.995  1390707_at  regulator of G-protein signalling 10  Info   Rgs10 
   2505 3.1e-05 105.4 176.6 0.597  1389572_at  malic enzyme 3, NADP(+)-dependent, mitochondrial (predicted)  Info   Me3_predicted 
   2506 3.1e-05 59.3 16.8 3.53  1396993_at  Transcribed locus  Info    
   2507 3.1e-05 122.7 57.1 2.149  1387151_at  nucleoporin 107  Info   Nup107 
   2508 3.12e-05 107.7 253 0.426  1392940_at  Transcribed locus  Info    
   2509 3.12e-05 126.5 66.5 1.902  1374686_at  solute carrier family 30 (zinc transporter), member 7 (predicted)  Info   Slc30a7_predicted 
   2510 3.12e-05 3306.3 1979.9 1.67  1386893_at  granulin  Info   Grn 
   2511 3.12e-05 152.7 76 2.009  1388178_at  nuclear receptor coactivator 3  Info   Ncoa3 
   2512 3.14e-05 144.8 272.2 0.532  1393414_at  Transcribed locus  Info    
   2513 3.14e-05 229.6 417.9 0.549  1369313_at  four and a half LIM domains 2  Info   Fhl2 
   2514 3.14e-05 195.8 781.3 0.251  1370610_at  Solute carrier family 34 (sodium phosphate), member 1  Info   Slc34a1 
   2515 3.15e-05 203 133.4 1.522  1381323_at  retinoblastoma binding protein 5 (predicted)  Info   Rbbp5_predicted 
   2516 3.16e-05 85.4 147 0.581  1374243_at  Transcribed locus  Info    
   2517 3.17e-05 205.9 46.1 4.466  1388054_a_at  chondroitin sulfate proteoglycan 2  Info   Cspg2 
   2518 3.17e-05 2439.7 1581.6 1.543  1370859_at  thioredoxin domain containing 7  Info   Txndc7 
   2519 3.18e-05 736.1 1181.6 0.623  1392090_at  serine (or cysteine) proteinase inhibitor, clade C (antithrombin), member 1 (predicted)  Info   Serpinc1_predicted 
   2520 3.18e-05 141.4 272.9 0.518  1374527_at  similar to hypothetical protein D4Ertd765e (predicted)  Info   RGD1308525_predicted 
   2521 3.19e-05 1268.6 2686.2 0.472  1375215_x_at  pyroglutamyl-peptidase I  Info   RGD:1303133 
   2522 3.19e-05 501.3 276.1 1.816  1376722_at  nucleoporin 205kDa (predicted)  Info   Nup205_predicted 
   2523 3.2e-05 50.3 26.3 1.913  1381567_at  hypothetical LOC294390 (predicted)  Info   RGD1307672_predicted 
   2524 3.21e-05 14.5 41.6 0.349  1378216_at  Transcribed locus, weakly similar to NP_032201.1 granulin [Mus musculus]  Info    
   2525 3.21e-05 951.4 1381.2 0.689  1392527_at  similar to homolog of yeast TIM14 isoform c  Info   LOC502525 
   2526 3.21e-05 190.8 77.4 2.465  1377970_at  similar to poly (ADP-ribose) polymerase family, member 8  Info   LOC294762 
   2527 3.21e-05 222.8 677.9 0.329  1370870_at  malic enzyme 1  Info   Me1 
   2528 3.21e-05 1323.2 989.4 1.337  1371581_at  Vascular endothelial zinc finger 1 (predicted)  Info    
   2529 3.21e-05 1749 2457.5 0.712  1371346_at  NADH dehydrogenase (ubiquinone) 1 beta subcomplex, 6, 17kDa (predicted)  Info   Ndufb6_predicted 
   2530 3.21e-05 222.3 142.8 1.557  1369738_s_at  cAMP responsive element modulator  Info   Crem 
   2531 3.22e-05 209.3 309 0.677  1389215_at  selenophosphate synthetase 1 (predicted)  Info   Sephs1_predicted 
   2532 3.22e-05 326.7 202.5 1.613  1373233_at  Transcribed locus  Info    
   2533 3.22e-05 1276.9 571.4 2.235  1372056_at  Chemokine-like factor super family 6 (predicted)  Info   Cklfsf6 
   2534 3.23e-05 733.6 1494.9 0.491  1386880_at  acetyl-Coenzyme A acyltransferase 2 (mitochondrial 3-oxoacyl-Coenzyme A thiolase)  Info   Acaa2 
   2535 3.23e-05 435.9 313.9 1.389  1372667_at  similar to RIKEN cDNA 1110059E24  Info   RGD:1359158 
   2536 3.24e-05 311.1 742.5 0.419  1369169_at  solute carrier family 23 (nucleobase transporters), member 1  Info   Slc23a1 
   2537 3.24e-05 4.9 47.7 0.103  1377898_at  solute carrier family 39 (metal ion transporter), member 5 (predicted)  Info   Slc39a5_predicted 
   2538 3.24e-05 111.8 40.7 2.747  1382709_at    Info    
   2539 3.25e-05 1873.1 3037.9 0.617  1388569_at  serine (or cysteine) proteinase inhibitor, clade F), member 1  Info   Serpinf1 
   2540 3.25e-05 129.6 208 0.623  1393380_at  Transcribed locus  Info    
   2541 3.25e-05 238.3 92.5 2.576  1382153_at  C-type (calcium dependent, carbohydrate recognition domain) lectin, superfamily member 6  Info   RGD:1359662 
   2542 3.26e-05 2199 3195 0.688  1387344_at  aldehyde dehydrogenase family 6, subfamily A1  Info   Aldh6a1 
   2543 3.26e-05 1616.4 1138.9 1.419  1371579_at  Clone UI-R-FJ0-cpu-m-15-0-UI unknown mRNA  Info    
   2544 3.26e-05 84.5 141.4 0.598  1377874_at  T-box 2 (predicted)  Info   Tbx2 
   2545 3.26e-05 1715.7 3368.3 0.509  1373337_at  glyoxylate reductase/hydroxypyruvate reductase (predicted)  Info   Grhpr_predicted 
   2546 3.26e-05 328.8 115.7 2.842  1368308_at  myelocytomatosis viral oncogene homolog (avian)  Info   Myc 
   2547 3.27e-05 144.5 315.5 0.458  1379308_at  Transcribed locus, strongly similar to NP_031761.1 procollagen, type IV, alpha 4 [Mus musculus]  Info    
   2548 3.27e-05 118.8 189.8 0.626  1374303_at  similar to prostate cancer antigen-1 (predicted)  Info   RGD1306377_predicted 
   2549 3.28e-05 47.6 20.5 2.322  1398719_at  chromodomain protein, Y chromosome-like 2 (predicted)  Info   Cdyl2_predicted 
   2550 3.29e-05 180.3 9.5 18.979  1386695_at  Transcribed locus  Info    
   2551 3.3e-05 80.6 165.1 0.488  1382105_at  guanine nucleotide binding protein, beta 5  Info   Gnb5 
   2552 3.3e-05 284.2 452.7 0.628  1369291_at  angiotensin II receptor, type 1 (AT1A)  Info   Agtr1a 
   2553 3.3e-05 245.7 130.3 1.886  1396187_at  Hypothetical protein LOC606294  Info    
   2554 3.3e-05 76.9 24.3 3.165  1379805_at  solute carrier family 41, member 2 (predicted)  Info   Slc41a2_predicted 
   2555 3.31e-05 262.6 345 0.761  1378668_at  CDNA clone IMAGE:7317308  Info    
   2556 3.31e-05 1013.3 600.7 1.687  1369998_at  ADP-ribosylation factor 6  Info   Arf6 
   2557 3.31e-05 378.5 560.3 0.676  1383879_at  Transcribed locus  Info    
   2558 3.31e-05 128.9 65.1 1.98  1380544_at  Transcribed locus  Info    
   2559 3.31e-05 199.1 346.6 0.574  1383056_a_at  LOC499072  Info   LOC499072 
   2560 3.32e-05 196.5 311.7 0.63  1389176_at  Inositol polyphosphate-5-phosphatase F (predicted)  Info    
   2561 3.32e-05 578.6 351.6 1.646  1389062_at  RED protein  Info   RGD:1359352 
   2562 3.32e-05 420.7 1212.2 0.347  1370167_at  syndecan 2  Info   Sdc2 
   2563 3.32e-05 231.3 122 1.896  1376501_at  Rho GTPase activating protein 8  Info   RGD:1303143 
   2564 3.32e-05 585.1 1079.8 0.542  1383395_at  agmatine ureohydrolase (agmatinase) (predicted)  Info   Agmat_predicted 
   2565 3.32e-05 50.2 160 0.314  1398295_at  solute carrier family 29 (nucleoside transporters), member 1  Info   Slc29a1 
   2566 3.33e-05 108.1 191.9 0.563  1370402_at  EGF-like domain 7  Info   Egfl7 
   2567 3.33e-05 708.9 399 1.777  1377737_at  Similar to hypothetical protein MGC5466 (predicted)  Info    
   2568 3.34e-05 728.2 508.8 1.431  1373200_at  eukaryotic translation elongation factor 1 epsilon 1 (predicted)  Info   Eef1e1_predicted 
   2569 3.34e-05 3464.9 2251.9 1.539  1375335_at  heat shock 90kDa protein 1, beta  Info   RGD:1303075 
   2570 3.36e-05 425.2 215.8 1.97  1388505_at  Transcribed locus  Info    
   2571 3.36e-05 418.9 10.2 41.069  1371252_at  suppressor of cytokine signaling 1  Info   Socs1 
   2572 3.37e-05 963.8 1320.2 0.73  1398327_at  pleckstrin homology domain containing, family C (with FERM domain) member 1 (predicted)  Info   Plekhc1_predicted 
   2573 3.37e-05 27.9 65.3 0.427  1388152_at  microtubule-associated protein 2  Info   Mtap2 
   2574 3.37e-05 256.9 406.9 0.631  1381922_at  solute carrier family 5 (sodium/glucose cotransporter), member 11  Info   Slc5a11 
   2575 3.38e-05 109.2 195.3 0.559  1392933_a_at  B-cell CLL/lymphoma 7A (predicted)  Info    
   2576 3.38e-05 115.2 230.5 0.5  1393364_at  cation-chloride cotransporter 9  Info   Slc12a8 
   2577 3.39e-05 617.3 495 1.247  1375663_at  similar to RIKEN cDNA 2510010F15 (predicted)  Info   RGD1307608_predicted 
   2578 3.39e-05 326.3 447.5 0.729  1367750_at  phosphoribosyl pyrophosphate synthetase-associated protein 1  Info   Prpsap1 
   2579 3.39e-05 287 98.2 2.923  1380297_at    Info    
   2580 3.39e-05 105.9 177.2 0.598  1380383_at  ADP-ribosylation factor 4-like (predicted)  Info    
   2581 3.4e-05 47 90.4 0.52  1377090_at  dedicator of cytokinesis 6 (predicted)  Info   Dock6_predicted 
   2582 3.4e-05 111.7 3.9 28.641  1379507_at  Transcribed locus  Info    
   2583 3.4e-05 566.1 347.5 1.629  1373488_at  Transcribed locus  Info    
   2584 3.41e-05 150.6 119 1.266  1374484_at  similar to RIKEN cDNA 2610033C09 (predicted)  Info   RGD1306421_predicted 
   2585 3.41e-05 166.4 110.3 1.509  1389269_at  similar to RIKEN cDNA 4933433P14 gene (predicted)  Info   RGD1308470_predicted 
   2586 3.41e-05 196.2 463.2 0.424  1380471_at  Transcribed locus  Info    
   2587 3.43e-05 123.6 24.1 5.129  1381161_a_at  Similar to hypothetical protein FLJ21127  Info    
   2588 3.43e-05 1770.5 1311.8 1.35  1371403_at  chaperonin subunit 3 (gamma)  Info   Cct3 
   2589 3.44e-05 3831.6 4784.2 0.801  1370861_at  Cytochrome c oxidase, subunit VIa, polypeptide 1  Info   Cox6a1 
   2590 3.45e-05 1943 1250.9 1.553  1375424_at  ARP2 actin-related protein 2 homolog (yeast) (predicted)  Info   Actr2_predicted 
   2591 3.45e-05 82.5 236.1 0.349  1390404_at  laminin, alpha 2 (predicted)  Info   Lama2_predicted 
   2592 3.45e-05 484.1 655.7 0.738  1375566_at  angiotensin II receptor-associated protein  Info   RGD:1359346 
   2593 3.47e-05 469.2 648.7 0.723  1367836_at  carnitine palmitoyltransferase 1, liver  Info   Cpt1a 
   2594 3.48e-05 74.2 178.6 0.415  1380962_at  Angiotensin I converting enzyme (peptidyl-dipeptidase A) 2  Info   Ace2 
   2595 3.48e-05 134.2 221.6 0.606  1374124_at  similar to RIKEN cDNA 2610003J06 (predicted)  Info   RGD1307381_predicted 
   2596 3.48e-05 306.9 477.8 0.642  1391611_at  kinesin family member 12 (predicted)  Info   Kif12_predicted 
   2597 3.49e-05 85.6 150.2 0.57  1389367_at  schwannomin interacting protein 1 (predicted)  Info   Schip1_predicted 
   2598 3.49e-05 133.8 76.8 1.742  1378958_at    Info    
   2599 3.5e-05 167.7 312.3 0.537  1384112_at  5 nucleotidase  Info   Nt5 
   2600 3.5e-05 445.8 684.4 0.651  1367811_at  3-phosphoglycerate dehydrogenase  Info   Phgdh 
   2601 3.51e-05 280.9 186.6 1.505  1379278_at  similar to hypothetical protein FLJ10871 (predicted)  Info   RGD1311539_predicted 
   2602 3.51e-05 33.6 65.2 0.515  1372293_at  Toll-like receptor 5  Info   Tlr5 
   2603 3.52e-05 86.5 358.5 0.241  1387631_at  15-hydroxyprostaglandin dehydrogenase  Info   Hpgd 
   2604 3.52e-05 257.6 523.6 0.492  1368080_at  response gene to complement 32  Info   Rgc32 
   2605 3.53e-05 367.3 520.1 0.706  1372362_at  similar to N-acetylgalactosamine kinase (GalNAc kinase) (Galactokinase 2)  Info   LOC296117 
   2606 3.54e-05 203.1 281.9 0.72  1368056_at  tuberous sclerosis 2  Info   Tsc2 
   2607 3.54e-05 265.5 424.3 0.626  1368863_at  non-metastatic cells 3, protein expressed in  Info   Nme3 
   2608 3.54e-05 954.8 1209 0.79  1390021_at  histone 1, H2bh (predicted)  Info   Hist1h2bh_predicted 
   2609 3.54e-05 411 620.5 0.662  1383733_at  Adenylate kinase 3  Info   Ak3 
   2610 3.55e-05 38.8 98.8 0.393  1377392_at  Transcribed locus, weakly similar to XP_236909.3 PREDICTED: similar to dishevelled associated activator of morphogenesis 2 [Rattus norvegicus]  Info    
   2611 3.55e-05 1710.7 2072.8 0.825  1367783_at  GABA(A) receptor-associated protein like 2  Info   Gabarapl2 
   2612 3.55e-05 439.4 262.7 1.673  1382769_at  interferon (alpha and beta) receptor 1 (predicted)  Info   Ifnar1_predicted 
   2613 3.57e-05 7.4 68.1 0.109  1376789_at  Similar to Myosin light chain kinase 2, skeletal/cardiac muscle (MLCK2) (predicted)  Info    
   2614 3.58e-05 840 1472.5 0.57  1383893_at  ATPase, H+ transporting, V1 subunit G, isoform 3 (predicted)  Info   Atp6v1g3_predicted 
   2615 3.58e-05 118.5 171.7 0.69  1373144_at  similar to Ac2-125  Info   LOC499654 
   2616 3.59e-05 155.1 63.3 2.45  1376076_at    Info    
   2617 3.59e-05 336.6 554.9 0.607  1379592_at  similar to citrin  Info   LOC362322 
   2618 3.59e-05 342.6 188.7 1.816  1388772_at  LSM8 homolog, U6 small nuclear RNA associated (S. cerevisiae) (predicted)  Info   Lsm8_predicted 
   2619 3.6e-05 534.8 316.7 1.689  1374137_at  E74-like factor 1  Info   Elf1 
   2620 3.61e-05 143.8 218.4 0.658  1378415_at  Protein kinase, cAMP-dependent, regulatory, type 2, alpha  Info   Prkar2a 
   2621 3.61e-05 210.7 136.6 1.542  1372987_at  Transcribed locus, strongly similar to XP_233741.1 PREDICTED: similar to Non-receptor tyrosine-protein kinase TYK2 [Rattus norvegicus]  Info    
   2622 3.62e-05 277 190.1 1.457  1372106_at  EH-domain containing 4  Info   Ehd4 
   2623 3.62e-05 212.7 470.1 0.452  1385248_a_at  osteoglycin (predicted)  Info   Ogn_predicted 
   2624 3.63e-05 140.7 230.4 0.611  1390114_at  myelin protein zero-like 1  Info   RGD:1359140 
   2625 3.63e-05 131.5 86.3 1.524  1384265_at  polymerase (DNA-directed), delta 3, accessory subunit (predicted)  Info   Pold3_predicted 
   2626 3.63e-05 714.1 510.3 1.399  1388614_at  splicing factor 3b, subunit 2 (predicted)  Info   Sf3b2_predicted 
   2627 3.63e-05 166.4 74.3 2.24  1396077_at  Transcribed locus  Info    
   2628 3.64e-05 37.6 67.8 0.555  1390925_a_at  Transcribed locus  Info    
   2629 3.64e-05 240.1 322.5 0.744  1387183_at  carnitine O-octanoyltransferase  Info   Crot 
   2630 3.65e-05 599.3 997.5 0.601  1370808_at  diaphorase 1  Info   Dia1 
   2631 3.66e-05 969 696.8 1.391  1372721_at    Info    
   2632 3.66e-05 478.8 728.8 0.657  1390207_at  Large tumor suppressor 2 (predicted)  Info    
   2633 3.66e-05 125.1 85.6 1.461  1397176_at  similar to RIKEN cDNA 1600013P15  Info   RGD:1359565 
   2634 3.68e-05 28.4 55.4 0.513  1377808_at  Similar to IQ motif and WD repeats 1  Info    
   2635 3.68e-05 437.5 276.3 1.583  1376440_at  ring finger protein 139 (predicted)  Info   Rnf139_predicted 
   2636 3.68e-05 79 31.9 2.476  1392810_at    Info    
   2637 3.69e-05 1405 1861 0.755  1371636_at  Similar to novel protein  Info    
   2638 3.69e-05 553.7 740.7 0.748  1371519_at  electron-transferring-flavoprotein dehydrogenase  Info   Etfdh 
   2639 3.69e-05 111.5 218.7 0.51  1373909_at    Info    
   2640 3.7e-05 74.8 125.3 0.597  1377748_at  Transcribed locus  Info    
   2641 3.7e-05 308.9 428.5 0.721  1377524_at  similar to CG18661-PA (predicted)  Info   RGD1307155_predicted 
   2642 3.7e-05 353.9 224.7 1.575  1370899_at  NonO/p54nrb homolog  Info   Sfpq 
   2643 3.7e-05 1858.9 2665.2 0.697  1368607_at  cytochrome P450, 4a12  Info   RGD:628846 
   2644 3.71e-05 39.5 92 0.429  1376282_at  Transcribed locus  Info    
   2645 3.72e-05 1.4 12.4 0.113  1380809_at    Info    
   2646 3.73e-05 219.2 288.4 0.76  1388982_at    Info    
   2647 3.73e-05 490.4 795 0.617  1388751_at  RNA binding motif protein 24 (predicted)  Info   Rbm24_predicted 
   2648 3.74e-05 52.1 115.1 0.453  1377818_at  Similar to junction-mediating and regulatory protein  Info    
   2649 3.75e-05 100.1 61.1 1.638  1374777_at  hypothetical protein LOC502894  Info   LOC502894 
   2650 3.75e-05 3155.2 4142.7 0.762  1388391_at  similar to NADH dehydrogenase  Info   LOC363441 
   2651 3.76e-05 375.2 638.1 0.588  1387782_at  dynein light chain-2  Info   Dlc2 
   2652 3.77e-05 189.8 316.7 0.599  1375908_at  epithelial V-like antigen (predicted)  Info   Eva_predicted 
   2653 3.79e-05 200.1 59.2 3.38  1387377_a_at  p21 (CDKN1A)-activated kinase 1  Info   Pak1 
   2654 3.79e-05 452.5 221.4 2.044  1388140_at  RAB13, member RAS oncogene family  Info   Rab13 
   2655 3.8e-05 212.5 335.9 0.633  1389511_s_at    Info    
   2656 3.8e-05 3499.5 5203.1 0.673  1368374_a_at  gamma-glutamyltransferase 1  Info   Ggt1 
   2657 3.81e-05 269.7 132.9 2.029  1385806_at  similar to PAP-1  Info   LOC363032 
   2658 3.83e-05 55.1 127 0.434  1389805_at  Transcribed locus  Info    
   2659 3.83e-05 159.5 65.4 2.439  1375996_at  septin 1 (predicted)  Info   Sept1_predicted 
   2660 3.83e-05 46 10.1 4.554  1391878_at  similar to Myb proto-oncogene protein (C-myb)  Info   LOC498982 
   2661 3.83e-05 395.1 583.4 0.677  1374766_at  Similar to mKIAA0493 protein  Info    
   2662 3.84e-05 302.2 9.1 33.209  1397958_at  Transcribed locus  Info    
   2663 3.84e-05 5535.1 173.5 31.903  1368762_at  ubiquitin D  Info   Ubd 
   2664 3.84e-05 158.8 271.7 0.584  1390789_at  similar to 5730439E10Rik protein (predicted)  Info   RGD1306270_predicted 
   2665 3.84e-05 79.8 187.1 0.427  1367912_at  latent transforming growth factor beta binding protein 1  Info   Ltbp1 
   2666 3.85e-05 1271.6 2670.3 0.476  1389551_at  lactamase, beta 2 (predicted)  Info   Lactb2_predicted 
   2667 3.85e-05 9.1 25 0.364  1397227_at  spinal cord expression protein 4  Info   RGD:1303093 
   2668 3.85e-05 76.2 135.2 0.564  1391871_at  Transcribed locus  Info    
   2669 3.86e-05 1541.6 966 1.596  1398300_at  ATPase, Na+/K+ transporting, beta 3 polypeptide  Info   Atp1b3 
   2670 3.86e-05 91.3 155.9 0.586  1372095_at  Similar to KIAA1161 protein (predicted)  Info    
   2671 3.86e-05 21.1 58.2 0.363  1381947_at  hypothetical LOC295622  Info   LOC295622 
   2672 3.86e-05 236.3 160.4 1.473  1395364_at  interferon (alpha and beta) receptor 1 (predicted)  Info   Ifnar1_predicted 
   2673 3.87e-05 1727.7 1049.1 1.647  1373225_at  Myeloid cell leukemia sequence 1  Info   Mcl1 
   2674 3.88e-05 14.3 42.8 0.334  1393708_at  basic helix-loop-helix domain containing, class B5 (predicted)  Info   Bhlhb5_predicted 
   2675 3.88e-05 91.5 177.7 0.515  1390437_at  Transcribed locus  Info    
   2676 3.89e-05 325.2 662.1 0.491  1369682_at  transcription factor 2  Info   Tcf2 
   2677 3.93e-05 372 237.1 1.569  1373494_at  breakpoint cluster region (predicted)  Info   Bcr_predicted 
   2678 3.93e-05 176.5 237.3 0.744  1373389_at  acyl-Coenzyme A dehydrogenase family, member 9  Info   Acad9 
   2679 3.93e-05 355.6 243.7 1.459  1369057_at  syntaxin binding protein 2  Info   Stxbp2 
   2680 3.93e-05 197.7 559.7 0.353  1370067_at  malic enzyme 1  Info   Me1 
   2681 3.94e-05 558.3 764.9 0.73  1372497_at  similar to Nbr1 (predicted)  Info   RGD1311421_predicted 
   2682 3.94e-05 131.9 187 0.705  1377884_at  Transcribed locus  Info    
   2683 3.94e-05 114.2 45.9 2.488  1397611_at  LOC501124  Info   LOC501124 
   2684 3.94e-05 572 848.2 0.674  1373400_at  Protein kinase, cAMP-dependent, regulatory, type 2, alpha  Info   Prkar2a 
   2685 3.94e-05 2308.1 1832.3 1.26  1367455_at  valosin-containing protein  Info   Vcp 
   2686 3.95e-05 310.7 171.1 1.816  1372363_at  similar to polymerase (RNA) II (DNA directed) polypeptide H /// similar to polymerase (RNA) II (DNA directed) polypeptide H  Info   LOC291795 /// LOC498109 
   2687 3.95e-05 98.4 130.2 0.756  1370196_at  protein inhibitor of activated STAT 3  Info   Pias3 
   2688 3.96e-05 99.7 21.8 4.573  1388064_a_at  solute carrier family 1 (glial high affinity glutamate transporter), member 3  Info   Slc1a3 
   2689 3.96e-05 133.9 242.2 0.553  1369370_s_at  transient receptor potential cation channel, subfamily V, member 4 /// transient receptor potential cation channel, subfamily V, member 1  Info   Trpv4 /// Trpv1 
   2690 3.97e-05 667.7 434.4 1.537  1383998_at  Transcribed locus, moderately similar to XP_421321.1 PREDICTED: similar to hypothetical protein FLJ20707 isoform 1 [Gallus gallus]  Info    
   2691 3.97e-05 1086.5 1857.1 0.585  1388358_at  electron-transfer-flavoprotein, beta polypeptide  Info   RGD:1303312 
   2692 3.97e-05 43.5 86.8 0.501  1389774_at  Transcribed locus  Info    
   2693 3.97e-05 452.2 942 0.48  1375640_at  FK506 binding protein 9  Info   Fkbp9 
   2694 3.97e-05 96 190.8 0.503  1382780_at  Transcribed locus  Info    
   2695 3.98e-05 45.7 22.7 2.013  1372326_at  Solute carrier family 2 (facilitated glucose transporter), member 3  Info   Slc2a3 
   2696 3.98e-05 113.6 46.7 2.433  1375074_at  ProSAPiP2 protein  Info   Prosapip2 
   2697 3.98e-05 124.9 229.5 0.544  1381971_at  SRY-box containing gene 18 (predicted)  Info   Sox18_predicted 
   2698 3.98e-05 160.7 305.2 0.527  1391538_at  cell death-inducing DNA fragmentation factor, alpha subunit-like effector B (predicted)  Info   Cideb_predicted 
   2699 3.99e-05 1195.9 795 1.504  1392918_at  chaperonin subunit 8 (theta) (predicted)  Info   Cct8_predicted 
   2700 3.99e-05 487.4 196.9 2.475  1388986_at  Core promoter element binding protein  Info   Copeb 
   2701 4.02e-05 31.3 90.1 0.347  1386679_at    Info    
   2702 4.03e-05 2082.1 1309.1 1.59  1371366_at  Rho GDP dissociation inhibitor (GDI) alpha  Info   RGD:1359547 
   2703 4.03e-05 788.7 395.8 1.993  1393431_at  Transcribed locus  Info    
   2704 4.03e-05 487.8 363.2 1.343  1383262_at  Unknown (protein for MGC:72591)  Info   Tmem19 
   2705 4.06e-05 168.7 103.1 1.636  1379849_at  THO complex 3 (predicted)  Info    
   2706 4.06e-05 199.4 309.2 0.645  1383041_x_at    Info    
   2707 4.06e-05 158.5 89.6 1.769  1369177_at  phosphatidylinositol 4-kinase type 2 alpha  Info   Pi4k2a 
   2708 4.06e-05 254.1 404.6 0.628  1369895_s_at  podocalyxin-like  Info   Podxl 
   2709 4.07e-05 355 232.9 1.524  1390185_at  mRNA decapping enzyme  Info   Dcps 
   2710 4.08e-05 144.9 96.5 1.502  1370979_at  DEAD/H (Asp-Glu-Ala-Asp/His) box polypeptide 20, 103kD  Info   RGD:621744 
   2711 4.09e-05 104.9 147.1 0.713  1374295_at  polymerase (RNA) mitochondrial (DNA directed) (predicted)  Info   Polrmt_predicted 
   2712 4.09e-05 76.3 33.7 2.264  1392174_at  carbohydrate sulfotransferase 12 (predicted)  Info   Chst12_predicted 
   2713 4.1e-05 1496.4 729.3 2.052  1372352_at  arginine-rich, mutated in early stage tumors (predicted)  Info   Armet_predicted 
   2714 4.1e-05 1392.6 2140.4 0.651  1388884_at  similar to RIKEN cDNA 1810022C23 (predicted)  Info   RGD1310224_predicted 
   2715 4.1e-05 25.2 83.2 0.303  1370691_a_at  thyroid hormone receptor alpha  Info   Thra 
   2716 4.11e-05 54.5 110.6 0.493  1390664_at    Info    
   2717 4.12e-05 136.1 81 1.68  1379665_at  similar to RIKEN cDNA 4632422M10 (predicted)  Info   RGD1310204_predicted 
   2718 4.12e-05 22.2 36.8 0.603  1376830_at  similar to RIKEN cDNA E430013E20 gene (predicted)  Info   RGD1311132_predicted 
   2719 4.12e-05 1692.3 3355.6 0.504  1372372_at  similar to Ab2-225 (predicted)  Info   RGD1306952_predicted 
   2720 4.13e-05 101 37.3 2.708  1381199_at    Info    
   2721 4.13e-05 802.9 530 1.515  1371489_at  Ring finger protein 4  Info   Rnf4 
   2722 4.14e-05 977.3 1386.6 0.705  1371755_at  Similar to RIKEN cDNA B230219D22  Info    
   2723 4.14e-05 66.6 151.5 0.44  1373786_at  Transcribed locus  Info    
   2724 4.15e-05 65.7 138.4 0.475  1393123_at  complement component 8, gamma polypeptide (predicted)  Info   C8g_predicted 
   2725 4.16e-05 91.6 148.2 0.618  1393309_at  Transcribed locus  Info    
   2726 4.18e-05 1390.8 1743.3 0.798  1388417_at  anaphase-promoting complex subunit 5 (predicted)  Info   Anapc5_predicted 
   2727 4.2e-05 62.7 189.9 0.33  1387550_a_at  solute carrier family 14 (urea transporter), member 2  Info   Slc14a2 
   2728 4.2e-05 321.4 161.8 1.986  1389446_at  small nuclear ribonucleoprotein polypeptide A' (predicted)  Info   Snrpa1_predicted 
   2729 4.21e-05 26 123.2 0.211  1394112_at  Hydroxyacid oxidase 1  Info   Hao1 
   2730 4.21e-05 124.3 84.5 1.471  1368201_at  natriuretic peptide receptor 1  Info   Npr1 
   2731 4.21e-05 21.1 60.9 0.346  1382387_at  Transmembrane protein 16A (predicted)  Info    
   2732 4.23e-05 60.6 27.4 2.212  1390685_at  Scm-like with four mbt domains 1  Info   Sfmbt1 
   2733 4.23e-05 48.9 103.7 0.472  1392944_at  similar to talin 2  Info   LOC315776 
   2734 4.23e-05 489.9 817.4 0.599  1389412_at    Info    
   2735 4.24e-05 84.8 170.9 0.496  1393330_at    Info    
   2736 4.25e-05 228.2 397.8 0.574  1373967_at  Transcribed locus  Info    
   2737 4.25e-05 894.1 1200.6 0.745  1368215_at  ceroid-lipofuscinosis, neuronal 2  Info   Cln2 
   2738 4.26e-05 169.7 319.7 0.531  1384971_at  similar to Hypothetical protein MGC19163 (predicted)  Info   RGD1309720_predicted 
   2739 4.26e-05 434.3 891.8 0.487  1393516_at  Transcribed locus  Info    
   2740 4.26e-05 61.1 120.8 0.506  1395856_at  Hypothetical LOC308765 (predicted)  Info    
   2741 4.27e-05 10.3 30.2 0.341  1380897_at  Transcribed locus  Info    
   2742 4.27e-05 55.3 107.2 0.516  1374954_at  histone deacetylase 11 (predicted)  Info   Hdac11_predicted 
   2743 4.27e-05 73.3 117 0.626  1377189_at  similar to BC282485_1 (predicted)  Info   RGD1307390_predicted 
   2744 4.27e-05 529.6 902.2 0.587  1387885_at  Fc receptor, IgG, alpha chain transporter  Info   Fcgrt 
   2745 4.27e-05 1562.4 2183.9 0.715  1369942_at  actinin alpha 4  Info   Actn4 
   2746 4.3e-05 731.9 575.9 1.271  1388488_at  LSM3 homolog, U6 small nuclear RNA associated (S. cerevisiae) (predicted)  Info   Lsm3_predicted 
   2747 4.31e-05 386.1 641.3 0.602  1393148_at  transmembrane 7 superfamily member 1 (predicted)  Info   Tm7sf1_predicted 
   2748 4.31e-05 229.8 359.8 0.639  1395410_at  protein phosphatase 2 (formerly 2A), regulatory subunit B'', alpha (predicted)  Info   Ppp2r3a_predicted 
   2749 4.31e-05 556.6 1056.9 0.527  1376640_at    Info    
   2750 4.31e-05 46.3 234.4 0.198  1387265_at  diacylglycerol kinase, gamma  Info   Dgkg 
   2751 4.33e-05 133.7 20 6.685  1371092_at  preprotrypsinogen IV  Info   RGD:708585 
   2752 4.36e-05 49.3 71.9 0.686  1377302_a_at  methylmalonic aciduria (cobalamin deficiency) type A (predicted)  Info   Mmaa_predicted 
   2753 4.36e-05 183.6 118.8 1.545  1381364_at  transportin 1 (predicted)  Info   Tnpo1_predicted 
   2754 4.36e-05 26.3 3.9 6.744  1370332_at  Munc13-4 protein  Info   Unc13h4 
   2755 4.36e-05 143.9 366.4 0.393  1372841_at  deleted in polyposis 1-like 1 (predicted)  Info   Dp1l1_predicted 
   2756 4.36e-05 2856.2 3421.1 0.835  1372123_at  succinate dehydrogenase complex, subunit B, iron sulfur (Ip) (predicted)  Info   Sdhb_predicted 
   2757 4.36e-05 303.6 417.9 0.726  1373469_at  Transcribed locus  Info    
   2758 4.36e-05 134 83.8 1.599  1376663_at  similar to hypothetical protein 6720484B16 (predicted)  Info   RGD1304881_predicted 
   2759 4.38e-05 332.8 564.3 0.59  1373201_at  Dihydrolipoamide branched chain transacylase E2  Info   Dbt 
   2760 4.39e-05 75.6 138.4 0.546  1374666_at  Similar to mKIAA0824 protein  Info    
   2761 4.4e-05 123.9 84.8 1.461  1383301_at  hypothetical protein LOC498154  Info   LOC498154 
   2762 4.4e-05 482.2 859.9 0.561  1384838_at  solute carrier family 34 (sodium phosphate), member 3  Info   Slc34a3 
   2763 4.4e-05 145.3 232.4 0.625  1383278_at  centaurin, gamma 2 (predicted)  Info   Centg2_predicted 
   2764 4.4e-05 193 397.7 0.485  1373151_at  similar to RIKEN cDNA 2810489O06  Info   MGC109397 
   2765 4.4e-05 37 101.5 0.365  1391241_at  Transcribed locus  Info    
   2766 4.41e-05 133.2 194.6 0.684  1376169_at  Transcribed locus  Info    
   2767 4.41e-05 72.8 23.1 3.152  1386555_at  casein kinase II, alpha 2, polypeptide (predicted)  Info   Csnk2a2_predicted 
   2768 4.42e-05 1057.6 727.5 1.454  1375414_at  TAF9 RNA polymerase II, TATA box binding protein (TBP)-associated factor  Info   Taf9 
   2769 4.42e-05 30.6 88.6 0.345  1389803_at  Transcribed locus  Info    
   2770 4.43e-05 758 515.7 1.47  1388762_at    Info    
   2771 4.44e-05 724.3 485.7 1.491  1389526_at  similar to RIKEN cDNA C920006C10 (predicted)  Info   RGD1305976_predicted 
   2772 4.45e-05 400.2 166.1 2.409  1388166_at  similar to Igh-6 protein /// similar to Ig H-chain V-region precursor /// similar to single chain Fv antibody fragment scFv 7-10A /// similar to Ig heavy chain V region MC101 precursor  Info   RGD:1359202 /// LOC299458 /// LOC314509 /// LOC366747 
   2773 4.45e-05 213.7 138.1 1.547  1372062_at  similar to cyclin-dependent kinase 2-interacting protein  Info   LOC299334 
   2774 4.46e-05 282.1 466.1 0.605  1376211_a_at  potassium channel tetramerisation domain containing 6 (predicted)  Info   Kctd6_predicted 
   2775 4.46e-05 245.7 375.5 0.654  1395157_at  neural precursor cell expressed, developmentally down-regulated gene 4A  Info   Nedd4a 
   2776 4.46e-05 144.6 324.1 0.446  1382928_at  Transcribed locus  Info    
   2777 4.46e-05 107.8 163.6 0.659  1387127_at  attractin  Info   Atrn 
   2778 4.47e-05 1457.8 908.8 1.604  1370188_at  splicing factor, arginine/serine-rich 10 (transformer 2 homolog, Drosophila)  Info   Sfrs10 
   2779 4.47e-05 61.5 140.9 0.436  1395519_at  similar to B230212L03Rik protein  Info   MGC95152 
   2780 4.47e-05 27.2 77.4 0.351  1374262_at  Transcribed locus  Info    
   2781 4.48e-05 503.3 827 0.609  1388459_at  collagen, type XVIII, alpha 1  Info   Col18a1 
   2782 4.5e-05 247.9 400.6 0.619  1372452_at  Glycerol-3-phosphate acyltransferase, mitochondrial  Info   Gpam 
   2783 4.5e-05 239.5 160.1 1.496  1383514_s_at  NMDA receptor-regulated gene 1 (predicted)  Info   Narg1_predicted 
   2784 4.5e-05 55.8 14.1 3.957  1385336_at  docking protein 2 (predicted)  Info   Dok2_predicted 
   2785 4.51e-05 88 156.7 0.562  1372172_at  nephronophthisis 1 (juvenile) (predicted)  Info   Nphp1_predicted 
   2786 4.51e-05 629.1 368.2 1.709  1377594_at  Src homology 2 domain-containing transforming protein C1  Info   Shc1 
   2787 4.51e-05 495.3 335.9 1.475  1380582_at  colony stimulating factor 1 (macrophage)  Info   Csf1 
   2788 4.51e-05 1985.8 2409.7 0.824  1369927_at  malate dehydrogenase, mitochondrial  Info   Mor1 
   2789 4.53e-05 25.2 107.5 0.234  1379467_at    Info    
   2790 4.53e-05 18.1 37.4 0.484  1378421_at  Transcribed locus  Info    
   2791 4.54e-05 377.4 103.7 3.639  1385029_at  Transcribed locus  Info    
   2792 4.54e-05 488.9 366.5 1.334  1388588_at  mammary tumor virus receptor 2 (predicted)  Info   Mtvr2_predicted 
   2793 4.54e-05 1995.5 2786.6 0.716  1389832_at  glutathione S-transferase omega 1  Info   Gsto1 
   2794 4.54e-05 174.4 256.2 0.681  1374272_at  Transcribed locus  Info    
   2795 4.55e-05 2862.7 2013.9 1.421  1376570_at  chaperonin subunit 5 (epsilon)  Info   Cct5 
   2796 4.55e-05 221 418.1 0.529  1368355_at  myosin 5B  Info   Myo5b 
   2797 4.56e-05 280.3 540.6 0.518  1377642_at  caveolin 2  Info   Cav2 
   2798 4.56e-05 136.4 251.5 0.542  1376007_at  Transcribed locus, moderately similar to XP_286747.1 PREDICTED: hypothetical protein XP_286747 [Mus musculus]  Info    
   2799 4.56e-05 448.4 319.1 1.405  1372764_at  similar to density regulated protein drp1  Info   LOC498180 
   2800 4.57e-05 112.4 22.2 5.063  1379557_at  Similar to Gamma-parvin  Info    
   2801 4.57e-05 80.1 155.8 0.514  1373363_at  microtubule-associated protein 1b  Info   Map1b 
   2802 4.57e-05 145.3 61.3 2.37  1368300_at  adenosine A2a receptor  Info   Adora2a 
   2803 4.57e-05 176.5 382 0.462  1393794_at  Similar to tetratricopeptide repeat domain 21B  Info    
   2804 4.58e-05 137.4 248.2 0.554  1384877_at  aquaporin 11  Info   Aqp11 
   2805 4.58e-05 619.2 859.8 0.72  1389128_at  WD repeat and FYVE domain containing 3 (predicted)  Info    
   2806 4.59e-05 430.1 1682.2 0.256  1368021_at  alcohol dehydrogenase 1  Info   Adh1 
   2807 4.59e-05 942.8 588 1.603  1379497_at    Info    
   2808 4.6e-05 185.2 97.1 1.907  1378282_at  casein kinase II, alpha 2, polypeptide (predicted)  Info   Csnk2a2_predicted 
   2809 4.6e-05 191.3 121.9 1.569  1379581_at  Transcribed locus, weakly similar to XP_579465.1 PREDICTED: DNA binding protein N5 [Rattus norvegicus]  Info    
   2810 4.6e-05 101.7 186 0.547  1387703_a_at  ubiquitin specific protease 2  Info   Usp2 
   2811 4.61e-05 39.4 111.3 0.354  1393048_at  Adrenergic receptor, alpha 2a  Info   Adra2a 
   2812 4.63e-05 262.5 155.8 1.685  1373094_at  general transcription factor II H, polypeptide 1 (predicted)  Info   Gtf2h1_predicted 
   2813 4.63e-05 542.3 374.9 1.447  1373447_at  similar to HN1-like protein (predicted)  Info   RGD1305117_predicted 
   2814 4.64e-05 58.9 115.3 0.511  1374083_at  Transcribed locus  Info    
   2815 4.65e-05 56.5 15 3.767  1381173_at  Transcribed locus  Info    
   2816 4.66e-05 372.3 182.6 2.039  1388493_at  Similar to vomeronasal V1r-type receptor V1rc41  Info    
   2817 4.67e-05 2174.6 2934.6 0.741  1388365_at  ATPase, H+ transporting, V0 subunit D isoform 1 (predicted)  Info   Atp6v0d1_predicted 
   2818 4.67e-05 69.4 162.1 0.428  1380688_at  hypothetical LOC293181 (predicted)  Info   RGD1309623_predicted 
   2819 4.67e-05 13.7 34 0.403  1390815_at  Cyclin M1 (predicted)  Info    
   2820 4.67e-05 109.6 176.3 0.622  1389561_at  similar to RIKEN cDNA 1810021J13 (predicted)  Info   RGD1311364_predicted 
   2821 4.68e-05 426.3 242.8 1.756  1389162_at  histone cell cycle regulation defective interacting protein 5 (predicted)  Info   Hirip5_predicted 
   2822 4.68e-05 314.4 163.8 1.919  1390333_at  protein phosphatase 4 (formerly X), catalytic subunit  Info   RGD:621225 
   2823 4.69e-05 1332.5 767.5 1.736  1390113_a_at  Similar to KIAA0731 protein (predicted)  Info    
   2824 4.7e-05 250.8 156.5 1.603  1392289_at    Info    
   2825 4.73e-05 145.6 244.9 0.595  1373583_at  Transcribed locus  Info    
   2826 4.73e-05 1189.5 824.1 1.443  1373678_at  Chloride intracellular channel 4  Info   Clic4 
   2827 4.74e-05 627.4 1060.8 0.591  1368378_at  formyltetrahydrofolate dehydrogenase  Info   Fthfd 
   2828 4.76e-05 188.3 602.9 0.312  1376078_at  Transcribed locus  Info    
   2829 4.76e-05 137.6 50.3 2.736  1369382_at  c-mer proto-oncogene tyrosine kinase  Info   Mertk 
   2830 4.77e-05 161.4 100.5 1.606  1373293_at  similar to DNA segment, Chr 16, Brigham & Womens Genetics 1547 expressed (predicted)  Info   RGD1309089_predicted 
   2831 4.77e-05 1629.5 1386.8 1.175  1387108_at  casein kinase 2, beta subunit  Info   Csnk2b 
   2832 4.8e-05 370.3 506.3 0.731  1390706_at  spectrin beta 2  Info   Spnb2 
   2833 4.81e-05 78.4 34.2 2.292  1376291_at  similar to WD repeat domain 4  Info   LOC499412 
   2834 4.81e-05 61.3 131.4 0.467  1370361_at  cell growth regulator with EF hand domain 1  Info   Cgref1 
   2835 4.82e-05 123.6 53.5 2.31  1377686_at  Transcribed locus  Info    
   2836 4.84e-05 27.7 54.7 0.506  1384515_at  Transcribed locus  Info    
   2837 4.84e-05 575.6 1265 0.455  1386960_at  solute carrier family 37 (glycerol-6-phosphate transporter), member 4  Info   Slc37a4 
   2838 4.84e-05 3010.8 2723.1 1.106  1371427_at  similar to signal peptidase 12kDa (predicted)  Info   RGD1309125_predicted 
   2839 4.85e-05 61.7 185.5 0.333  1387145_at  gap junction membrane channel protein beta 1  Info   Gjb1 
   2840 4.85e-05 343.7 21.2 16.212  1394122_at  ras homolog gene family, member H (predicted)  Info   Rhoh_predicted 
   2841 4.85e-05 119.2 58 2.055  1374651_at  similar to KIAA0933 protein (predicted)  Info   RGD1306097_predicted 
   2842 4.85e-05 250.8 181 1.386  1375432_at  DD6A4-12(7)-2 mRNA, partial sequence  Info    
   2843 4.85e-05 547.2 843.7 0.649  1375517_at  Tumor protein p53 inducible nuclear protein 2  Info   LOC362246 
   2844 4.86e-05 52.3 70.2 0.745  1382151_at  TruB pseudouridine (psi) synthase homolog 1 (E. coli) (predicted)  Info   Trub1_predicted 
   2845 4.87e-05 143.9 252.9 0.569  1383719_at  microtubule-associated protein 7 (predicted)  Info   Mtap7_predicted 
   2846 4.87e-05 311.6 166.6 1.87  1387871_at  cofilin 1  Info   Cfl1 
   2847 4.91e-05 352.5 615.7 0.573  1372287_at  Myotubularin related protein 6 (predicted)  Info    
   2848 4.91e-05 221.6 337.4 0.657  1373848_at  similar to RIKEN cDNA 5730449L18 (predicted)  Info   RGD1308584_predicted 
   2849 4.92e-05 324.2 158.3 2.048  1390134_at  Transcribed locus  Info    
   2850 4.92e-05 298.2 227.9 1.308  1376703_at  nucleoporin 37 (predicted)  Info   Nup37_predicted 
   2851 4.93e-05 107.3 13.3 8.068  1376030_at  centaurin, beta 1 (predicted)  Info   Centb1_predicted 
   2852 4.94e-05 121.6 32.6 3.73  1369299_at  platelet-activating factor receptor  Info   Ptafr 
   2853 4.94e-05 55.6 91.2 0.61  1381811_at  Transcribed locus  Info    
   2854 4.95e-05 49.1 68.9 0.713  1390160_at  Transcribed locus  Info    
   2855 4.95e-05 353.8 247.1 1.432  1392542_at  similar to CDC42 small effector 2 /// similar to CDC42 small effector 2  Info   LOC501691 /// LOC502327 
   2856 4.97e-05 182.9 12.5 14.632  1369190_at  CD2 antigen /// hypothetical gene supported by NM_012830  Info   Cd2 /// LOC497761 
   2857 4.97e-05 2502.3 3718.1 0.673  1386867_at  brain protein 44-like  Info   Brp44l 
   2858 4.98e-05 1565.9 796.8 1.965  1399161_a_at  type 1 tumor necrosis factor receptor shedding aminopeptidase regulator  Info   Arts1 
   2859 4.98e-05 231.6 125.5 1.845  1368026_at  hepatoma-derived growth factor, related protein 2  Info   Hdgfrp2 
   2860 4.99e-05 606 805.9 0.752  1373127_at  Similar to Tripartite motif protein 44 (DIPB protein)  Info    
   2861 4.99e-05 43.4 100.1 0.434  1383694_at  similar to RIKEN cDNA 1110061N23  Info   LOC499748 
   2862 5e-05 440.2 232.9 1.89  1389246_at  Transcribed locus  Info    
   2863 5.01e-05 315.5 141.5 2.23  1375981_a_at  similar to Su(var)3-9 homolog  Info   LOC302553 
   2864 5.02e-05 3664.4 2725.8 1.344  1370184_at  cofilin 1  Info   Cfl1 
   2865 5.02e-05 959 662.2 1.448  1386900_at  ribosome associated membrane protein 4  Info   RAMP4 
   2866 5.05e-05 196.3 123.5 1.589  1382404_at  Transcribed locus  Info    
   2867 5.05e-05 115.1 410.8 0.28  1368543_at  NADPH oxidase 4  Info   Nox4 
   2868 5.07e-05 206.6 871.5 0.237  1373975_at  similar to thioether S-methyltransferase  Info   LOC368066 
   2869 5.07e-05 102.1 252.6 0.404  1393896_at  similar to ionized calcium binding adapter molecule 2 (Iba2) (predicted)  Info   RGD1305081_predicted 
   2870 5.08e-05 1268.2 1619.9 0.783  1368317_at  aquaporin 7  Info   Aqp7 
   2871 5.08e-05 3295.3 1941.7 1.697  1370219_at  cytochrome b-245, alpha polypeptide  Info   Cyba 
   2872 5.1e-05 17.6 48.4 0.364  1378247_at  ELL associated factor 2  Info   Eaf2 
   2873 5.1e-05 185.1 83.6 2.214  1378056_at  geminin (predicted)  Info   Gmnn_predicted 
   2874 5.12e-05 571.8 922.8 0.62  1388395_at  G0/G1 switch gene 2 (predicted)  Info   G0s2_predicted 
   2875 5.12e-05 298.4 543.9 0.549  1369081_at  neuraminidase 1  Info   Neu1 
   2876 5.13e-05 90.7 268.3 0.338  1396035_at  similar to RPE-spondin  Info   LOC297757 
   2877 5.13e-05 17.5 33.9 0.516  1378783_at  Transcribed locus  Info    
   2878 5.14e-05 40.9 91.5 0.447  1387023_at  glutathione S-transferase, mu type 3  Info   Gstm3 
   2879 5.14e-05 192.9 332.6 0.58  1385887_at  Transcribed locus  Info    
   2880 5.14e-05 5.1 18.6 0.274  1370973_at  sodium channel, voltage-gated, type 6, alpha polypeptide  Info   RGD:61922 
   2881 5.15e-05 280.1 210.6 1.33  1398386_at  Transcribed locus, strongly similar to NP_705766.2 zinc finger, CCHC domain containing 6 [Mus musculus]  Info    
   2882 5.16e-05 761.2 1247.8 0.61  1380334_at  hypothetical RNA binding protein  Info   RGD:1359713 
   2883 5.16e-05 316.3 179.9 1.758  1380513_at  similar to Expressed sequence AW060207 (predicted)  Info   RGD1309034_predicted 
   2884 5.16e-05 1253.1 2337.6 0.536  1367680_at  acyl-Coenzyme A oxidase 1, palmitoyl  Info   Acox1 
   2885 5.16e-05 379.7 228.3 1.663  1370909_at  nucleoporin 62  Info   Nup62 
   2886 5.16e-05 442.9 278.3 1.591  1374036_at  minichromosome maintenance deficient 2 mitotin (S. cerevisiae) (predicted)  Info   Mcm2_predicted 
   2887 5.17e-05 157.5 293.3 0.537  1372469_at  Transcribed locus  Info    
   2888 5.17e-05 594.2 406.4 1.462  1397674_at  eukaryotic translation initiation factor 3, subunit 8, 110kDa (predicted)  Info   Eif3s8_predicted 
   2889 5.18e-05 83.4 133.3 0.626  1373217_at  Transcribed locus  Info    
   2890 5.18e-05 2008 1595.8 1.258  1371939_at  GPI-anchored membrane protein 1 (predicted)  Info   Gpiap1_predicted 
   2891 5.18e-05 138.5 221.1 0.626  1373859_at  Transcribed locus  Info    
   2892 5.19e-05 410.9 306.6 1.34  1373392_at  TPA regulated locus (predicted)  Info   Tparl_predicted 
   2893 5.19e-05 2508.4 3162.2 0.793  1388112_at  solute carrier family 25 (mitochondrial adenine nucleotide translocator) member 4  Info   Slc25a4 
   2894 5.2e-05 230 133.7 1.72  1389471_at  Translocase of outer mitochondrial membrane 34 (predicted)  Info    
   2895 5.21e-05 327.6 592.2 0.553  1393152_at    Info    
   2896 5.21e-05 91.3 22.3 4.094  1387587_at  tumor necrosis factor (ligand) superfamily, member 6  Info   Tnfsf6 
   2897 5.22e-05 1375.8 644.2 2.136  1371336_at  hematological and neurological expressed sequence 1  Info   RGD:1359325 
   2898 5.23e-05 13.7 37.3 0.367  1369361_at  Klotho  Info   Kl 
   2899 5.26e-05 261.4 467.6 0.559  1372696_at  Transcribed locus  Info    
   2900 5.27e-05 679.4 1070.1 0.635  1368431_at  hepsin  Info   Hpn 
   2901 5.27e-05 231.4 148.8 1.555  1375341_at  Kua homolog (predicted)  Info   Kua_predicted 
   2902 5.27e-05 625.9 780.8 0.802  1371553_at  mitochondrial ribosomal protein L36 (predicted)  Info   Mrpl36_predicted 
   2903 5.27e-05 266.2 149.2 1.784  1389297_at  ERO1-like (S. cerevisiae)  Info   Ero1l 
   2904 5.28e-05 993.8 1619.6 0.614  1372612_at  dynein light chain-2  Info   Dlc2 
   2905 5.28e-05 724.9 1151.5 0.63  1367754_s_at  argininosuccinate lyase /// heterogeneous nuclear ribonucleoprotein A/B  Info   Asl /// Hnrpab 
   2906 5.28e-05 1430.7 2668.4 0.536  1386872_at  insulin-like growth factor 2 receptor  Info   Igf2r 
   2907 5.28e-05 669.9 974.4 0.688  1398970_at  Translocation protein 1 (predicted)  Info    
   2908 5.28e-05 30.4 49.7 0.612  1395847_at    Info    
   2909 5.28e-05 75.7 47.1 1.607  1379462_at  Transcribed locus, moderately similar to XP_580018.1 PREDICTED: hypothetical protein XP_580018 [Rattus norvegicus]  Info    
   2910 5.28e-05 324 148.1 2.188  1389039_at  Transcribed locus  Info    
   2911 5.29e-05 189 334 0.566  1385334_at  Cytochrome P450, family 4, subfamily v, polypeptide 3 (predicted)  Info   Loc266761 
   2912 5.29e-05 174.4 319.4 0.546  1385716_at  Transcribed locus  Info    
   2913 5.3e-05 639.1 464.8 1.375  1367935_at  smu-1 suppressor of mec-8 and unc-52 homolog (C. elegans)  Info   Smu1 
   2914 5.32e-05 101.6 43 2.363  1383001_at  Transcribed locus  Info    
   2915 5.32e-05 661.4 297.4 2.224  1373164_at  Serine/threonine kinase 17b (apoptosis-inducing)  Info   Stk17b 
   2916 5.32e-05 121.8 210.3 0.579  1383833_at  hypothetical protein LOC500991  Info   LOC500991 
   2917 5.32e-05 3532.9 4318.7 0.818  1371254_at  ubiquinol-cytochrome c reductase, Rieske iron-sulfur polypeptide 1  Info   Uqcrfs1 
   2918 5.32e-05 550.9 330.3 1.668  1374395_at  Transcribed locus  Info    
   2919 5.32e-05 95.5 181.2 0.527  1391274_at  Transcribed locus  Info    
   2920 5.33e-05 3732.4 5015.9 0.744  1388327_at  similar to DNA segment, Chr 10, ERATO Doi 214, expressed (predicted)  Info   RGD1309529_predicted 
   2921 5.34e-05 274.5 461.8 0.594  1372069_at  ankyrin repeat domain 15 (predicted)  Info   Ankrd15_predicted 
   2922 5.34e-05 52 96.7 0.538  1374417_at  Siah binding protein 1; FBP interacting repressor; pyrimidine tract binding splicing factor; Ro ribonucleoprotein-binding protein 1  Info   Siahbp1 
   2923 5.34e-05 99 265.4 0.373  1376808_at  Transcribed locus  Info    
   2924 5.34e-05 234.7 370.2 0.634  1370823_at  BMP and activin membrane-bound inhibitor, homolog (Xenopus laevis)  Info   Bambi 
   2925 5.35e-05 620 1106.8 0.56  1388908_at  similar to Peci protein  Info   RGD:1359427 
   2926 5.35e-05 3385.9 5309.7 0.638  1387013_at  kidney-specific membrane protein  Info   Tmem27 
   2927 5.35e-05 87 150.2 0.579  1389324_at  tyrosine kinase receptor 1  Info   Tie1 
   2928 5.35e-05 628.1 393.4 1.597  1368173_at  nucleolar protein 5  Info   Nol5 
   2929 5.37e-05 1788.4 2153.3 0.831  1367670_at  fumarate hydratase 1  Info   Fh1 
   2930 5.37e-05 248 477.2 0.52  1397647_at  solute carrier family 25 (mitochondrial carrier; ornithine transporter) member 15 (predicted)  Info   Slc25a15_predicted 
   2931 5.38e-05 306.3 421.6 0.727  1367718_at  choline kinase beta  Info   Chkb 
   2932 5.39e-05 127 48.8 2.602  1368186_a_at  spleen tyrosine kinase  Info   Syk 
   2933 5.4e-05 407.8 172.7 2.361  1386986_at  opioid growth factor receptor  Info   Ogfr 
   2934 5.4e-05 300.3 898.1 0.334  1392965_a_at  SPARC related modular calcium binding 2 (predicted)  Info   Smoc2_predicted 
   2935 5.41e-05 122.9 6.3 19.508  1393917_at  CD163 antigen (predicted)  Info   Cd163_predicted 
   2936 5.41e-05 153 77.3 1.979  1378825_a_at  Similar to brain specific protein 4  Info    
   2937 5.41e-05 678.5 522.7 1.298  1375525_at  Microtubule-associated protein, RP/EB family, member 1  Info   Mapre1 
   2938 5.42e-05 232.9 112.3 2.074  1371071_at  guanine nucleotide binding protein beta 4 subunit  Info   RGD:1359321 
   2939 5.43e-05 84.8 179.6 0.472  1377336_at  sema domain, immunoglobulin domain (Ig), short basic domain, secreted, (semaphorin) 3B (predicted)  Info   Sema3b_predicted 
   2940 5.43e-05 44.5 68.2 0.652  1384453_at  CDNA clone IMAGE:7303447  Info    
   2941 5.44e-05 2287.9 3215.1 0.712  1370865_at  isocitrate dehydrogenase 3, gamma  Info   Idh3g 
   2942 5.44e-05 94.4 65.9 1.432  1389919_at  parvin, beta (predicted)  Info   Parvb_predicted 
   2943 5.45e-05 324.1 16.7 19.407  1379633_a_at  ubiquitin-activating enzyme E1-like (predicted)  Info   Ube1l_predicted 
   2944 5.45e-05 296.3 452.7 0.655  1376683_at  Transcribed locus  Info    
   2945 5.46e-05 402.1 263.6 1.525  1367881_at  protein tyrosine phosphatase, non-receptor type substrate 1  Info   Ptpns1 
   2946 5.46e-05 42.9 129.1 0.332  1369381_a_at  solute carrier family 15 (oligopeptide transporter), member 1  Info   Slc15a1 
   2947 5.46e-05 251.3 482 0.521  1374448_at  reversion-inducing-cysteine-rich protein with kazal motifs (predicted)  Info   Reck_predicted 
   2948 5.47e-05 1493.1 736.3 2.028  1390866_at  Transcribed locus  Info    
   2949 5.47e-05 764.7 596.6 1.282  1389013_at  similar to RIKEN cDNA 1500016H10 (predicted)  Info   RGD1308469_predicted 
   2950 5.47e-05 722.5 1132.8 0.638  1390807_at  carbonic anhydrase VB, mitochondrial  Info   Ca5b 
   2951 5.48e-05 1477.8 3141.1 0.47  1387111_at  dimethylarginine dimethylaminohydrolase 1  Info   Ddah1 
   2952 5.48e-05 4984.1 3600 1.384  1375336_at  heat shock 90kDa protein 1, beta  Info   RGD:1303075 
   2953 5.48e-05 229.3 331.3 0.692  1388643_at  fucosyltransferase 8 (alpha (1,6) fucosyltransferase)  Info   RGD:1303096 
   2954 5.49e-05 654.5 474.2 1.38  1374501_at    Info    
   2955 5.5e-05 467.5 128.3 3.644  1368464_at  macrophage galactose N-acetyl-galactosamine specific lectin 1  Info   Mgl1 
   2956 5.51e-05 195.1 146.2 1.334  1372661_at  transducin (beta)-like 3 (predicted)  Info   Tbl3_predicted 
   2957 5.51e-05 246.9 328.1 0.753  1388636_at  similar to RIKEN cDNA 5730408C10 (predicted)  Info   RGD1305972_predicted 
   2958 5.52e-05 384 292.6 1.312  1382135_at  similar to CG9346-PA (predicted)  Info   RGD1307882_predicted 
   2959 5.52e-05 190.5 315.4 0.604  1376202_at  UDP-GlcNAc:betaGal beta-1,3-N-acetylglucosaminyltransferase 6 (predicted)  Info   B3gnt6_predicted 
   2960 5.53e-05 262.6 188.3 1.395  1392111_at  exosome component 3 (predicted)  Info   Exosc3_predicted 
   2961 5.53e-05 622.5 427.4 1.456  1370792_at  microtubule-associated protein, RP/EB family, member 1  Info   Mapre1 
   2962 5.56e-05 14.4 34.1 0.422  1375073_at  similar to sterile alpha motif domain containing 11  Info   LOC500601 
   2963 5.58e-05 50.7 108 0.469  1374276_at  Similar to SPT3-associated factor 42 (predicted)  Info    
   2964 5.59e-05 2179.2 1480.5 1.472  1388774_at    Info    
   2965 5.59e-05 4.8 49.7 0.097  1395126_at    Info    
   2966 5.59e-05 127 179.1 0.709  1385505_at  Transcribed locus  Info    
   2967 5.6e-05 68.5 112.2 0.611  1390972_at  similar to CGI-121 protein (predicted)  Info   RGD1309786_predicted 
   2968 5.61e-05 179.9 297 0.606  1373258_at  cathepsin F (predicted)  Info   Ctsf_predicted 
   2969 5.62e-05 183 384.9 0.475  1396301_x_at  Transcribed locus  Info    
   2970 5.62e-05 241.8 168.7 1.433  1373452_at  RNA terminal phosphate cyclase-like 1 (predicted)  Info   Rcl1_predicted 
   2971 5.62e-05 122.9 79.7 1.542  1395058_at  similar to CG12050-PA  Info   LOC314545 
   2972 5.64e-05 291.4 565.4 0.515  1389581_at  similar to RIKEN cDNA 9230117N10 (predicted)  Info   RGD1311155_predicted 
   2973 5.65e-05 37.4 86.1 0.434  1387809_at  mitogen-activated protein kinase kinase 6  Info   Map2k6 
   2974 5.67e-05 133.3 174.3 0.765  1381979_at  sulfatase modifying factor 2  Info   Sumf2 
   2975 5.68e-05 342.1 235.2 1.455  1373428_at  breast cancer metastasis-suppressor 1 (predicted)  Info   Brms1_predicted 
   2976 5.68e-05 688.5 1067.7 0.645  1371338_at  similar to RIKEN cDNA 1700023M09 (predicted)  Info   RGD1312037_predicted 
   2977 5.68e-05 254.8 362.9 0.702  1389199_at  Similar to Ab2-095 (predicted)  Info    
   2978 5.68e-05 68.9 24.9 2.767  1381221_at  similar to T cell receptor V delta 6 (predicted)  Info   RGD1311300_predicted 
   2979 5.69e-05 119.2 88.6 1.345  1390163_at    Info    
   2980 5.69e-05 234 439.2 0.533  1370446_at  non-metastatic cells 7, protein expressed in  Info   Nme7 
   2981 5.7e-05 162.5 14.4 11.285  1382925_at    Info    
   2982 5.7e-05 81.8 153 0.535  1391559_at  similar to RIKEN cDNA 0610030G03  Info   RGD:1359195 
   2983 5.73e-05 182.1 291.1 0.626  1388856_at  kit ligand  Info   Kitl 
   2984 5.73e-05 483 1111.1 0.435  1370929_at  low density lipoprotein receptor-related protein associated protein 1  Info   Lrpap1 
   2985 5.74e-05 964.1 1531.9 0.629  1383162_at    Info    
   2986 5.74e-05 201 271.6 0.74  1374664_at  Similar to TTF-I interacting peptide 20; TIP20; Transcription Termination Factor I Interacting Peptide 20 (predicted)  Info    
   2987 5.74e-05 171.3 339 0.505  1382041_at  1-acylglycerol-3-phosphate O-acyltransferase 3 (predicted)  Info   Agpat3_predicted 
   2988 5.75e-05 172.8 75.4 2.292  1388757_at  Transcribed locus  Info    
   2989 5.75e-05 122.8 222.3 0.552  1386961_at  phosphofructokinase, muscle  Info   Pfkm 
   2990 5.75e-05 625 457 1.368  1374526_at    Info    
   2991 5.77e-05 152.9 52.3 2.924  1382921_at  SDA1 domain containing 1  Info   RGD:1359112 
   2992 5.77e-05 404 678.9 0.595  1393388_at  Zinc finger, DHHC domain containing 3 (predicted)  Info   Zdhhc3 
   2993 5.8e-05 61.1 102.8 0.594  1373089_at  cadherin 3, type 1, P-cadherin (placental)  Info   Cdh3 
   2994 5.82e-05 230.3 586.3 0.393  1393018_at  Similar to RING finger protein 33  Info    
   2995 5.83e-05 66.9 136.6 0.49  1374830_at  similar to Coiled-coil domain containing protein 3 precursor  Info   LOC498795 
   2996 5.83e-05 16 2.5 6.4  1391989_at    Info    
   2997 5.83e-05 55.6 101.4 0.548  1384591_at  Similar to hypothetical protein FLJ32884 (predicted)  Info    
   2998 5.85e-05 57.3 130.4 0.439  1391302_at    Info    
   2999 5.85e-05 35.9 66 0.544  1379321_at  Hypothetical LOC305960 (predicted)  Info    
   3000 5.86e-05 349.7 115.4 3.03  1370177_at  poliovirus receptor  Info   PVR 
   3001 5.86e-05 245.4 120.1 2.043  1398602_at  MAD2 (mitotic arrest deficient, homolog)-like 1 (yeast) (predicted)  Info    
   3002 5.88e-05 332.2 582.1 0.571  1393551_at    Info    
   3003 5.9e-05 125.1 63.1 1.983  1389565_at  Transcribed locus  Info    
   3004 5.9e-05 410.1 273.1 1.502  1374815_at  STARD3 N-terminal like (predicted)  Info   Stard3nl_predicted 
   3005 5.91e-05 352.3 489.8 0.719  1388423_at  hypothetical protein MGC15854  Info   RGD:1302996 
   3006 5.91e-05 1085.2 604.2 1.796  1388455_at  guanine nucleotide binding protein (G protein), gamma 10  Info   Gng10 
   3007 5.92e-05 310.5 209.5 1.482  1373209_at  Transcribed locus  Info    
   3008 5.92e-05 153.6 262.1 0.586  1373748_at  PDZ domain containing RING finger 3 (predicted)  Info   Pdzrn3_predicted 
   3009 5.93e-05 169.7 239.5 0.709  1375431_at  Transcribed locus  Info    
   3010 5.93e-05 258.2 462.8 0.558  1383557_at  Transcribed locus  Info    
   3011 5.94e-05 379.6 634.4 0.598  1369949_at  Lutheran blood group (Auberger b antigen included)  Info   Lu 
   3012 5.94e-05 42.3 88 0.481  1393338_at    Info    
   3013 5.94e-05 65.7 147.2 0.446  1374235_at  Down syndrome critical region gene 1-like 1  Info   Dscr1l1 
   3014 5.95e-05 626.5 947.4 0.661  1367897_at  acyl-Coenzyme A dehydrogenase, very long chain  Info   Acadvl 
   3015 5.95e-05 25.9 107.7 0.24  1392013_at  DEP domain containing 2 (predicted)  Info    
   3016 5.95e-05 185.6 716.6 0.259  1371700_at  Microfibrillar-associated protein 4 (predicted)  Info    
   3017 5.95e-05 45.8 151.3 0.303  1385005_at    Info    
   3018 5.95e-05 6.4 24.9 0.257  1379076_at  Transcribed locus  Info    
   3019 5.95e-05 435.3 300.6 1.448  1373412_at  5'-nucleotidase, cytosolic III (predicted)  Info   Nt5c3_predicted 
   3020 5.96e-05 182.6 268 0.681  1383715_at  similar to hypothetical protein D4Ertd89e (predicted)  Info   RGD1305703_predicted 
   3021 5.97e-05 854.3 1207.8 0.707  1372252_at  trafficking protein particle complex 1 (predicted)  Info   Trappc1_predicted 
   3022 5.98e-05 245.4 337.4 0.727  1376401_at  Transcribed locus  Info    
   3023 5.98e-05 234.6 535.1 0.438  1390543_at  Transcribed locus  Info    
   3024 5.98e-05 18 38 0.474  1372637_at    Info    
   3025 5.99e-05 985.3 1576.2 0.625  1371775_at  Acyl-Coenzyme A dehydrogenase, short/branched chain  Info   Acadsb 
   3026 6e-05 75.8 131 0.579  1393142_at  similar to p10-binding protein  Info   LOC367153 
   3027 6e-05 61.6 141.6 0.435  1374491_at  chemokine-like factor super family 8  Info   Cklfsf8 
   3028 6e-05 854.1 571.5 1.494  1367698_a_at  septin 9  Info   Sept9 
   3029 6e-05 46 91.5 0.503  1396215_at  Similar to RIKEN cDNA 2610022G08  Info    
   3030 6.01e-05 895.2 1227.3 0.729  1399002_at  mitochondrial ribosomal protein S17 (predicted)  Info   Mrps17_predicted 
   3031 6.02e-05 527.7 765.1 0.69  1375289_at    Info    
   3032 6.04e-05 243.2 359.3 0.677  1381915_at  Transcribed locus  Info    
   3033 6.05e-05 417.5 800 0.522  1384628_at  similar to RIKEN cDNA 0610009A07 (predicted)  Info   RGD1309288_predicted 
   3034 6.05e-05 80.3 177.7 0.452  1383939_at  Transcribed locus, moderately similar to XP_580018.1 PREDICTED: hypothetical protein XP_580018 [Rattus norvegicus]  Info    
   3035 6.06e-05 68.5 43.7 1.568  1393550_at  Transcribed locus  Info    
   3036 6.06e-05 83.4 392.8 0.212  1378013_at  hypothetical LOC361199  Info   LOC361199 
   3037 6.07e-05 1028.8 631.4 1.629  1387101_at  acyl-CoA synthetase long-chain family member 4  Info   Acsl4 
   3038 6.07e-05 371.5 264.3 1.406  1372853_at  v-rel reticuloendotheliosis viral oncogene homolog A (avian)  Info   Rela 
   3039 6.07e-05 1860.9 2329.7 0.799  1389012_at  NADH dehydrogenase (ubiquinone) 1 beta subcomplex, 2 (predicted)  Info   Ndufb2_predicted 
   3040 6.08e-05 1094.2 1581 0.692  1389288_at  NADH dehydrogenase (ubiquinone) 1 alpha subcomplex, 2 (predicted)  Info   Ndufa2_predicted 
   3041 6.08e-05 75.3 147.8 0.509  1390246_at  Transcribed locus, moderately similar to NP_796093.1 hypothetical protein LOC320264 [Mus musculus]  Info    
   3042 6.1e-05 868.1 589.2 1.473  1372752_at  transmembrane 4 superfamily member 7 (predicted)  Info   Tm4sf7_predicted 
   3043 6.1e-05 587.9 757.4 0.776  1372813_at  similar to RIKEN cDNA 1810046J19 (predicted)  Info   RGD1306682_predicted 
   3044 6.11e-05 156.4 215.3 0.726  1383845_at  Transcribed locus  Info    
   3045 6.11e-05 656.4 467.7 1.403  1388722_at  DnaJ (Hsp40) homolog, subfamily B, member 1 (predicted)  Info   Dnajb1_predicted 
   3046 6.11e-05 138.2 228.7 0.604  1371738_at  similar to hypothetical protein FLJ10375 (predicted)  Info   RGD1308260_predicted 
   3047 6.12e-05 509.1 357.9 1.422  1372402_at  N-acetylneuraminic acid synthase (sialic acid synthase) (predicted)  Info   Nans_predicted 
   3048 6.12e-05 34.4 67.5 0.51  1391855_at  B-cell CLL/lymphoma 7A (predicted)  Info    
   3049 6.13e-05 46.9 74.4 0.63  1382711_at  zinc finger protein 36 (KOX 18)  Info   MGC116363 
   3050 6.15e-05 53.9 121.5 0.444  1370011_at  adenylate kinase 1  Info   Ak1 
   3051 6.16e-05 283.7 568.9 0.499  1388778_at  Transcribed locus  Info    
   3052 6.16e-05 528 287 1.84  1398369_at  Transcribed locus  Info    
   3053 6.16e-05 44.4 109.4 0.406  1386274_at  solute carrier family 17 (sodium phosphate), member 2 (predicted)  Info   Slc17a2_predicted 
   3054 6.16e-05 456.1 598.4 0.762  1373719_at  mitogen-activated protein kinase kinase kinase kinase 3  Info   Map4k3 
   3055 6.17e-05 290.8 199.5 1.458  1388803_at  deoxyhypusine synthase  Info   RGD:1303326 
   3056 6.17e-05 180.4 431 0.419  1377037_at  similar to peroxisomal long chain acyl-CoA thioesterase Ia  Info   LOC314304 
   3057 6.17e-05 119.4 51.6 2.314  1396383_at  fatty aldehyde dehydrogenase-like  Info   RGD:1359546 
   3058 6.19e-05 694.5 545.5 1.273  1373917_at  Transcribed locus  Info    
   3059 6.19e-05 62.4 143.9 0.434  1369957_at  regulator of G-protein signaling 5  Info   Rgs5 
   3060 6.2e-05 19.3 56.8 0.34  1376165_at  Transcribed locus  Info    
   3061 6.2e-05 2260.5 1853.1 1.22  1373843_at  Transcribed locus  Info    
   3062 6.21e-05 47.4 109.9 0.431  1382569_at  Transcribed locus  Info    
   3063 6.22e-05 359.2 244.6 1.469  1372329_at  similar to hypothetical protein PRO0971 (predicted)  Info   RGD1311435_predicted 
   3064 6.23e-05 628.2 493.4 1.273  1387823_at  pleiotropic regulator 1 homolog (Arabidopsis)  Info   Plrg1 
   3065 6.23e-05 45.3 10.6 4.274  1381474_at  similar to mCHCR  Info   LOC302492 
   3066 6.24e-05 375.3 276.5 1.357  1371712_at  similar to snRNP core protein SMX5d  Info   LOC292030 
   3067 6.24e-05 106.1 62.5 1.698  1382350_at  signal transducer and activator of transcription 6 (predicted)  Info   Stat6_predicted 
   3068 6.24e-05 958.4 21 45.638  1387831_at  small inducible cytokine subfamily C, member 1 (lymphotactin)  Info   Xcl1 
   3069 6.28e-05 96.7 194.9 0.496  1380379_at  Similar to : F-box protein 44  Info    
   3070 6.28e-05 85.8 154.3 0.556  1371089_at  glutathione S-transferase Yc2 subunit  Info   Yc2 
   3071 6.28e-05 331.5 479.1 0.692  1390387_at  Similar to SH3 domain protein D19 (predicted)  Info    
   3072 6.29e-05 101.9 164.5 0.619  1375869_at  Unc-51 like kinase 1 (C. elegans)  Info   Ulk1 
   3073 6.3e-05 1353.6 1063.4 1.273  1367494_at  similar to CGI-35 protein (predicted)  Info   RGD1310899_predicted 
   3074 6.31e-05 259 34.7 7.464  1377185_at    Info    
   3075 6.31e-05 270.9 128 2.116  1372535_at  similar to RIKEN cDNA 2210012G02  Info   LOC366431 
   3076 6.32e-05 279.8 406.6 0.688  1371513_at  LOC500067  Info   LOC500067 
   3077 6.32e-05 347.4 172.1 2.019  1371782_at  NIPSNAP-related protein  Info   RGD:1359688 
   3078 6.33e-05 112.4 189.5 0.593  1383104_at  similar to RIKEN cDNA 1810020D17  Info   LOC361606 
   3079 6.34e-05 2624.1 3459.2 0.759  1371415_at  ubiquinol-cytochrome c reductase hinge protein (predicted)  Info   Uqcrh_predicted 
   3080 6.35e-05 55.2 16.9 3.266  1394745_at  Similar to hypothetical protein FLJ20037 (predicted)  Info    
   3081 6.35e-05 138.9 76.5 1.816  1374576_at  similar to mKIAA0841 protein  Info   LOC361546 
   3082 6.36e-05 570.4 698.9 0.816  1374638_at  peroxisomal biogenesis factor 13 (predicted)  Info   Pex13_predicted 
   3083 6.37e-05 92.4 166.7 0.554  1382363_at  membrane protein, palmitoylated 5 (MAGUK p55 subfamily member 5) (predicted)  Info   Mpp5_predicted 
   3084 6.37e-05 105.3 29.7 3.545  1371618_s_at  tubulin, beta 3  Info   Tubb3 
   3085 6.38e-05 80.2 155.4 0.516  1370802_at  similar to integrin beta-5  Info   LOC498091 
   3086 6.4e-05 108.2 20.1 5.383  1379642_at  Transcribed locus  Info    
   3087 6.41e-05 613.3 352.5 1.74  1372270_at  dolichyl-phosphate (UDP-N-acetylglucosamine) N-acetylglucosaminephosphotransferase 1 (GlcNAc-1-P transferase)  Info   H2afx 
   3088 6.42e-05 16.7 53.1 0.315  1368339_at  calbindin 3, (vitamin D-dependent calcium binding protein)  Info   Calb3 
   3089 6.44e-05 45.7 98.9 0.462  1374444_at  Plexin B1 (predicted)  Info   LOC316009 
   3090 6.45e-05 119.7 178.8 0.669  1370984_at  Zinc finger protein 46 (predicted)  Info    
   3091 6.45e-05 1.2 5.7 0.211  1396114_at  F-box only protein 33 (predicted)  Info    
   3092 6.45e-05 38.2 60.6 0.63  1395953_at  Transcribed locus  Info    
   3093 6.46e-05 133 250 0.532  1373053_at    Info    
   3094 6.47e-05 791.4 578.8 1.367  1390485_at  ring finger protein 153 (predicted)  Info   Rnf153_predicted 
   3095 6.49e-05 1757.8 2183.5 0.805  1372076_at  hepatitis B virus x interacting protein (predicted)  Info   Hbxip_predicted 
   3096 6.49e-05 31.8 71.6 0.444  1382475_at  Similar to pellino 3 alpha (predicted)  Info    
   3097 6.49e-05 261.3 351 0.744  1372292_at  Similar to Serine/threonine protein kinase 24  Info    
   3098 6.5e-05 890.8 1151.3 0.774  1371964_at  G-rich RNA sequence binding factor 1 (predicted)  Info   Grsf1_predicted 
   3099 6.5e-05 599.3 429.1 1.397  1371764_at  similar to Ubc6p homolog  Info   RGD:1359523 
   3100 6.51e-05 34 125.6 0.271  1388111_at  elastin  Info   Eln 
   3101 6.51e-05 666.3 455.7 1.462  1367760_at  mitogen activated protein kinase kinase 1  Info   Map2k1 
   3102 6.53e-05 217 161 1.348  1375029_at    Info    
   3103 6.55e-05 200 469.9 0.426  1379724_at    Info    
   3104 6.55e-05 82.2 35.3 2.329  1380210_at    Info    
   3105 6.55e-05 345.2 211.1 1.635  1367874_at  ras homolog gene family, member Q  Info   Rhoq 
   3106 6.56e-05 68 157.2 0.433  1370853_at  CaM-kinase II inhibitor alpha  Info   RGD:708430 
   3107 6.56e-05 2425.7 1913.3 1.268  1370309_a_at  heterogeneous nuclear ribonucleoprotein A/B  Info   Hnrpab 
   3108 6.57e-05 99.7 176.5 0.565  1376071_at  Transcribed locus  Info    
   3109 6.59e-05 54.1 101.9 0.531  1395761_at  Sema domain, immunoglobulin domain (Ig), short basic domain, secreted, (semaphorin) 3B (predicted)  Info    
   3110 6.59e-05 1051.2 1198.9 0.877  1388410_at  UDP-glucose pyrophosphorylase 2 (predicted)  Info   Ugp2_predicted 
   3111 6.61e-05 44 65.9 0.668  1393032_x_at  RAB, member of RAS oncogene family-like 5 (predicted)  Info   Rabl5_predicted 
   3112 6.61e-05 1079.4 740.1 1.458  1388960_at  pyrophosphatase  Info   Pp 
   3113 6.61e-05 303.7 952.1 0.319  1396039_at  solute carrier family 22 (organic anion/cation transporter), member 12  Info   Slc22a12 
   3114 6.61e-05 21.6 62.2 0.347  1382190_at  MAS-related GPR, member F  Info   Mrgprf 
   3115 6.62e-05 34.8 58 0.6  1374947_at  breast cancer anti-estrogen resistance 3 (predicted)  Info   Bcar3_predicted 
   3116 6.63e-05 49.4 150.1 0.329  1372390_at  Transcribed locus  Info    
   3117 6.63e-05 428.6 222.7 1.925  1374537_at  Carbohydrate (chondroitin) synthase 1 (predicted)  Info    
   3118 6.64e-05 438.1 282.1 1.553  1387114_at  protein kinase C, delta  Info   Prkcd 
   3119 6.65e-05 37.1 59.8 0.62  1376828_at  retinoic acid induced 3 (predicted)  Info   Rai3_predicted 
   3120 6.66e-05 153.1 264.3 0.579  1377232_at  similar to hypothetical protein (predicted)  Info   RGD1305269_predicted 
   3121 6.66e-05 112.4 49.1 2.289  1390790_a_at  hypothetical LOC361349  Info   LOC361349 
   3122 6.67e-05 199.7 321.1 0.622  1380245_at    Info    
   3123 6.68e-05 96.7 50 1.934  1370519_at  syntaxin binding protein 1  Info   Stxbp1 
   3124 6.69e-05 234.9 148.2 1.585  1373406_at  Torsin family 1, member B (predicted)  Info    
   3125 6.71e-05 144.4 239.8 0.602  1368244_at  arsenic (+3 oxidation state) methyltransferase  Info   As3mt 
   3126 6.71e-05 273 376.2 0.726  1371479_at  LOC500939  Info   LOC500939 
   3127 6.72e-05 249 472.7 0.527  1390777_at  sterol-C5-desaturase (fungal ERG3, delta-5-desaturase) homolog (S. cerevisae)  Info   Sc5d 
   3128 6.73e-05 1464.8 528.4 2.772  1367712_at  tissue inhibitor of metalloproteinase 1  Info   Timp1 
   3129 6.74e-05 1142.8 2028.3 0.563  1371405_at  Similar to hypothetical protein MGC52110  Info    
   3130 6.75e-05 240.7 168.9 1.425  1383231_at  similar to Smad nuclear interacting protein  Info   RGD:1359268 
   3131 6.77e-05 1225.2 949.3 1.291  1388027_a_at  reticulon 4  Info   Rtn4 
   3132 6.78e-05 171.6 121.3 1.415  1393331_at  Transcribed locus  Info    
   3133 6.83e-05 98.8 200.4 0.493  1374060_at  Transcribed locus  Info    
   3134 6.84e-05 72.7 31.2 2.33  1382210_at  Strain Wistar Rap2a mRNA, 5' UTR  Info    
   3135 6.84e-05 275 144.1 1.908  1373840_at  nudix -type motif 9  Info   RGD:1359522 
   3136 6.84e-05 466.7 636.6 0.733  1376585_at  mitochondrial ribosomal protein L50 (predicted)  Info   Mrpl50_predicted 
   3137 6.85e-05 884.9 442.2 2.001  1374731_at  Tripartite motif protein 25  Info   Trim25 
   3138 6.86e-05 1124.5 729.9 1.541  1386897_at  heterogeneous nuclear ribonucleoproteins methyltransferase-like 2 (S. cerevisiae)  Info   Hrmt1l2 
   3139 6.86e-05 2987 2335.2 1.279  1371329_at    Info    
   3140 6.86e-05 175 302.9 0.578  1375785_at  Similar to hypothetical protein D4Ertd89e (predicted)  Info    
   3141 6.87e-05 12.8 2 6.4  1384389_at  Reticulon 4 receptor-like 2  Info   Rtn4rl2 
   3142 6.88e-05 246.9 374 0.66  1383324_at  Similar to PALS2-alpha splice variant  Info    
   3143 6.88e-05 91 169 0.538  1391534_at  elongation of very long chain fatty acids (FEN1/Elo2, SUR4/Elo3, yeast)-like 2 (predicted)  Info   Elovl2_predicted 
   3144 6.88e-05 132.9 84.1 1.58  1370127_at  polymerase (DNA directed), delta 1, catalytic subunit  Info   Pold1 
   3145 6.89e-05 1883.5 1218.1 1.546  1367624_at  activating transcription factor 4  Info   Atf4 
   3146 6.92e-05 106.4 176.6 0.602  1375182_at  Similar to RIKEN cDNA D030028O16 (predicted)  Info   MGC105586 
   3147 6.93e-05 29.5 70.1 0.421  1379573_at    Info    
   3148 6.94e-05 68.2 98.8 0.69  1386574_at  Transcribed locus  Info    
   3149 6.94e-05 39.4 67.7 0.582  1374819_at  Transcribed locus  Info    
   3150 6.94e-05 60.7 97 0.626  1396202_at  Transcribed locus  Info    
   3151 6.96e-05 98.5 204.4 0.482  1385585_at  Transcribed locus  Info    
   3152 6.96e-05 4899.5 4245.9 1.154  1367567_at  ribosomal protein L6  Info   Rpl6 
   3153 6.97e-05 604 339.9 1.777  1371800_at  polyhomeotic-like 2 (Drosophila) (predicted)  Info   Phc2_predicted 
   3154 6.97e-05 47.2 22.7 2.079  1379205_at  Transcribed locus  Info    
   3155 6.99e-05 3318.1 3981.7 0.833  1386886_at  CD164 antigen  Info   Cd164 
   3156 6.99e-05 208.5 337.4 0.618  1389611_at  very low density lipoprotein receptor  Info   Vldlr 
   3157 7e-05 95.4 307.5 0.31  1382611_at  Transcribed locus  Info    
   3158 7.02e-05 70.2 137.4 0.511  1373247_at  Beta-site APP cleaving enzyme  Info   Bace 
   3159 7.02e-05 52.7 132.7 0.397  1398710_at  cytochrome P450, family 2, subfamily u, polypeptide 1 (predicted)  Info   Cyp2u1_predicted 
   3160 7.03e-05 16.7 32.1 0.52  1386577_at  Similar to RIKEN cDNA 4930429H24 (predicted)  Info    
   3161 7.03e-05 337.6 228.6 1.477  1389634_at  methionyl aminopeptidase 1 (predicted)  Info   Metap1_predicted 
   3162 7.04e-05 111.4 170.6 0.653  1372205_at  zinc finger protein 278 (predicted)  Info   Znf278_predicted 
   3163 7.07e-05 177.4 90 1.971  1386935_at  nuclear receptor subfamily 4, group A, member 1  Info   Nr4a1 
   3164 7.07e-05 121.6 185.3 0.656  1374694_at  Transcribed locus  Info    
   3165 7.08e-05 361 469.8 0.768  1372894_at  similar to PL6 protein (predicted)  Info   RGD1310540_predicted 
   3166 7.09e-05 96.7 240.1 0.403  1380057_at  Transcribed locus  Info    
   3167 7.1e-05 44.6 96.2 0.464  1398107_at  similar to RIKEN cDNA A030007L17; EST AA673177 (predicted) /// similar to RIKEN cDNA A030007L17  Info   RGD1304876_predicted /// LOC362370 
   3168 7.11e-05 1201.5 919.3 1.307  1398838_at  RAB7, member RAS oncogene family  Info   Rab7 
   3169 7.11e-05 388.1 233.5 1.662  1375882_at  protein phosphatase 3, catalytic subunit, beta isoform  Info   Ppp3cb 
   3170 7.12e-05 139.9 297.9 0.47  1386671_at  Coiled-coil domain containing 2  Info   Ccdc2 
   3171 7.13e-05 494.3 277.5 1.781  1371776_at  Phosphatidylinositol 3-kinase, regulatory subunit, polypeptide 1  Info   Pik3r1 
   3172 7.14e-05 669.5 236.1 2.836  1372032_at    Info    
   3173 7.17e-05 689.2 432.8 1.592  1373847_at  transmembrane 4 superfamily member 1 (predicted)  Info   Tm4sf1_predicted 
   3174 7.18e-05 242.8 434.3 0.559  1388624_at  similar to Hypothetical UPF0184 protein C9orf16 homolog  Info   LOC499780 
   3175 7.19e-05 111.4 198.9 0.56  1370969_at  homeo box A5  Info   Hoxa5 
   3176 7.2e-05 155.7 87.1 1.788  1373868_at  BCL2-associated transcription factor 1 (predicted)  Info   Bclaf1_predicted 
   3177 7.22e-05 343.5 260 1.321  1389717_at  similar to KIAA0157 gene product is novel. (predicted)  Info   RGD1308918_predicted 
   3178 7.23e-05 170.2 311.2 0.547  1395167_at  LOC362424 (predicted)  Info   RGD1308850_predicted 
   3179 7.23e-05 940.4 644.1 1.46  1387911_at  Rab geranylgeranyl transferase componenet, subunit beta  Info   Rabggtb 
   3180 7.23e-05 317.8 209.4 1.518  1374611_at    Info    
   3181 7.25e-05 1995.2 2869.7 0.695  1371041_at  NADH dehydrogenase (ubiquinone) flavoprotein 2  Info   Ndufv2 
   3182 7.25e-05 180 246.1 0.731  1374094_at  Calcium binding protein 39-like (predicted)  Info    
   3183 7.27e-05 446.6 815.9 0.547  1367857_at  fatty acid desaturase 1  Info   Fads1 
   3184 7.27e-05 168.3 237.2 0.71  1372553_at  similar to RIKEN cDNA 2610301B20; EST AI428449  Info   MGC94199 
   3185 7.27e-05 84 27.3 3.077  1383785_at  Lymphoid enhancer binding factor 1  Info   Lef1 
   3186 7.28e-05 56 137.2 0.408  1383686_at  synaptogyrin 1  Info   Syngr1 
   3187 7.29e-05 43 94.4 0.456  1387373_at  phosphodiesterase 4D interacting protein (myomegalin)  Info   Pde4dip 
   3188 7.3e-05 259.7 493.7 0.526  1377643_at  homeo box D10 (predicted)  Info   Hoxd10_predicted 
   3189 7.3e-05 551.6 925.6 0.596  1377192_a_at  caseinolytic protease X (E.coli) (predicted)  Info   Clpx_predicted 
   3190 7.31e-05 115 278.7 0.413  1385791_at  similar to villin-like protein (predicted)  Info   RGD1304843_predicted 
   3191 7.33e-05 15.2 37.8 0.402  1397672_at  Transcribed locus  Info    
   3192 7.34e-05 3161 4144.9 0.763  1367651_at  cathepsin D  Info   Ctsd 
   3193 7.34e-05 82 154.5 0.531  1389788_at  hypothetical protein LOC293950  Info   RGD1307336_predicted 
   3194 7.34e-05 65.7 125.3 0.524  1380108_at  WW domain binding protein 1 (predicted)  Info    
   3195 7.35e-05 27.4 80.7 0.34  1378260_at  Alcohol dehydrogenase 1  Info   Adh1 
   3196 7.36e-05 314.3 178.5 1.761  1373943_at  CDNA clone IMAGE:7365681  Info    
   3197 7.37e-05 1157.3 1760.4 0.657  1367777_at  2,4-dienoyl CoA reductase 1, mitochondrial  Info   Decr1 
   3198 7.38e-05 824.7 447.5 1.843  1388130_at  zyxin  Info   Zyx 
   3199 7.38e-05 112.2 205.1 0.547  1398634_at    Info    
   3200 7.39e-05 573.6 1558.6 0.368  1370831_at  monoglyceride lipase  Info   Mgll 
   3201 7.4e-05 682.2 423.5 1.611  1374430_at  similar to RIKEN cDNA 2700085E05 (predicted)  Info   RGD1307010_predicted 
   3202 7.4e-05 110 57.8 1.903  1396280_at  similar to G patch domain and KOW motifs  Info   LOC302560 
   3203 7.42e-05 377.7 57.7 6.546  1369268_at  activating transcription factor 3  Info   Atf3 
   3204 7.44e-05 143.9 97.8 1.471  1382161_at  M-phase phosphoprotein 10 (U3 small nucleolar ribonucleoprotein) (predicted)  Info   Mphosph10_predicted 
   3205 7.44e-05 33.9 5.5 6.164  1388018_at  selectin, endothelial cell  Info   Sele 
   3206 7.44e-05 507.7 727 0.698  1378753_at  thiopurine methyltransferase  Info   Tpmt 
   3207 7.46e-05 678.1 329.7 2.057  1370968_at  nuclear factor of kappa light chain gene enhancer in B-cells 1, p105  Info   Nfkb1 
   3208 7.47e-05 202.4 325.8 0.621  1373399_at  WD repeat domain 6 (predicted)  Info   Wdr6_predicted 
   3209 7.48e-05 15.6 41.8 0.373  1384431_at  Similar to RIKEN cDNA 4930431B11 gene (predicted)  Info    
   3210 7.5e-05 148.7 279.9 0.531  1375674_at  similar to chromosome 16 open reading frame 5 (predicted)  Info   RGD1310686_predicted 
   3211 7.54e-05 1462.6 993.8 1.472  1382005_at  ROD1 regulator of differentiation 1 (S. pombe)  Info   Rod1 
   3212 7.54e-05 56.8 107.8 0.527  1380850_at  Transcribed locus  Info    
   3213 7.55e-05 64.9 103.1 0.629  1397949_at    Info    
   3214 7.55e-05 287.1 423.9 0.677  1397300_at  Transcribed locus  Info    
   3215 7.56e-05 105.7 52.5 2.013  1393357_at  GLE1 RNA export mediator-like (yeast (predicted)  Info   Gle1l_predicted 
   3216 7.57e-05 86.3 36.2 2.384  1392449_at  RAD18 homolog (S. cerevisiae) (predicted)  Info   Rad18_predicted 
   3217 7.57e-05 211.7 161.4 1.312  1375948_at  Similar to hypothetical protein FLJ20308 (predicted)  Info    
   3218 7.58e-05 3833.4 2781.5 1.378  1398750_at  calreticulin  Info   Calr 
   3219 7.59e-05 348.3 146.9 2.371  1388792_at  growth arrest and DNA-damage-inducible 45 gamma (predicted)  Info   Gadd45g_predicted 
   3220 7.59e-05 156.5 210.7 0.743  1398342_at  similar to Fus1 protein  Info   LOC501052 
   3221 7.6e-05 335.7 826.3 0.406  1371500_at  latent transforming growth factor beta binding protein 4 (predicted)  Info   Ltbp4_predicted 
   3222 7.63e-05 84.3 12.7 6.638  1368301_at  adenosine A2a receptor  Info   Adora2a 
   3223 7.64e-05 30 53.5 0.561  1396847_at  Transcribed locus  Info    
   3224 7.65e-05 136.6 284.6 0.48  1387505_at  guanine nucleotide binding protein, alpha inhibiting 1  Info   Gnai1 
   3225 7.65e-05 376.2 88.2 4.265  1368813_at  CCAAT/enhancer binding protein (C/EBP), delta  Info   Cebpd 
   3226 7.66e-05 495.7 284.1 1.745  1388355_at  RNA binding motif protein 17 (predicted)  Info   Rbm17_predicted 
   3227 7.66e-05 98.5 51.3 1.92  1368370_at  adenylate cyclase 4  Info   Adcy4 
   3228 7.66e-05 435.1 664.8 0.654  1388917_at  myosin IC  Info   Myo1c 
   3229 7.68e-05 247.4 486.2 0.509  1367940_at  chemokine orphan receptor 1  Info   Cmkor1 
   3230 7.69e-05 4099.3 1875.9 2.185  1367574_at  vimentin  Info   Vim 
   3231 7.71e-05 186.9 374.9 0.499  1371012_at  2-hydroxyphytanoyl-Coenzyme A lyase  Info   Hpcl2 
   3232 7.71e-05 332.3 255.9 1.299  1371719_at  bromodomain-containing 2  Info   RGD:1303324 
   3233 7.72e-05 41.1 145.1 0.283  1390164_at  Transcribed locus  Info    
   3234 7.73e-05 285.2 355.5 0.802  1391500_at  Transcribed locus  Info    
   3235 7.73e-05 280.8 180 1.56  1381903_at  F-box only protein 33 (predicted)  Info   Fbxo33_predicted 
   3236 7.74e-05 18.6 48.3 0.385  1392053_at    Info    
   3237 7.74e-05 957 672.7 1.423  1382154_at  Transcribed locus, strongly similar to NP_035333.2 protein tyrosine phosphatase, non-receptor type 12 [Mus musculus]  Info    
   3238 7.74e-05 227.4 721.2 0.315  1387348_at  insulin-like growth factor binding protein 5  Info   Igfbp5 
   3239 7.76e-05 45.6 104.1 0.438  1389786_at  Similar to RIKEN cDNA 2410005O16  Info    
   3240 7.77e-05 376.3 287 1.311  1376609_at  similar to TRS85 homolog (predicted)  Info   RGD1309102_predicted 
   3241 7.77e-05 104.4 159.5 0.655  1367994_at  dihydropyrimidine dehydrogenase  Info   Dpyd 
   3242 7.78e-05 55 19 2.895  1369119_a_at  5-hydroxytryptamine (serotonin) receptor 7  Info   Htr7 
   3243 7.78e-05 247.9 575.4 0.431  1383263_at  osteoglycin (predicted)  Info   Ogn_predicted 
   3244 7.78e-05 25.8 63.2 0.408  1389208_at  similar to AI448196 protein  Info   LOC363495 
   3245 7.8e-05 232.3 137 1.696  1374836_at  RNA, U3 small nucleolar interacting protein 2 (predicted)  Info   Rnu3ip2_predicted 
   3246 7.8e-05 308.2 204.3 1.509  1390032_at  RNA binding motif, single stranded interacting protein 2 (predicted)  Info   Rbms2_predicted 
   3247 7.82e-05 222 465.4 0.477  1391390_at    Info    
   3248 7.82e-05 483.5 329.6 1.467  1388974_at  signal recognition particle 19 (predicted)  Info   Srp19_predicted 
   3249 7.83e-05 18 98.4 0.183  1367977_at  synuclein, alpha  Info   Snca 
   3250 7.83e-05 95.8 160.7 0.596  1382802_x_at    Info    
   3251 7.84e-05 148.9 78.7 1.892  1372868_at  torsin family 3, member A (predicted)  Info   Tor3a_predicted 
   3252 7.85e-05 82.3 29.1 2.828  1373513_at  GTP cyclohydrolase 1  Info   Gch 
   3253 7.85e-05 364.7 819 0.445  1388902_at  lysyl oxidase-like 1 (predicted)  Info   Loxl1_predicted 
   3254 7.85e-05 84.8 153.9 0.551  1373436_at  similar to RIKEN cDNA 1700040L02 (predicted)  Info   RGD1306739_predicted 
   3255 7.86e-05 416.4 550.3 0.757  1387028_a_at  Inhibitor of DNA binding 1, helix-loop-helix protein (splice variation)  Info   Id1 
   3256 7.86e-05 154.9 267.7 0.579  1373419_at    Info    
   3257 7.87e-05 153.2 281 0.545  1372392_at  Transcribed locus  Info    
   3258 7.89e-05 309.2 186 1.662  1368268_at  thymine-DNA glycosylase  Info   Tdg 
   3259 7.91e-05 214.4 103.3 2.076  1382629_at  similar to male sterility domain containing 2  Info   LOC499247 
   3260 7.92e-05 844.5 1151.6 0.733  1371358_at  glycoprotein, synaptic 2  Info   Gpsn2 
   3261 7.93e-05 77.3 202.9 0.381  1393474_at  sulfotransferase family, cytosolic, 2B, member 1 (predicted)  Info   Sult2b1_predicted 
   3262 7.94e-05 750.6 1074.3 0.699  1386892_at  parathymosin  Info   Ptms 
   3263 7.94e-05 277.7 474.3 0.585  1382021_at  Similar to polycystic kidney disease 2  Info    
   3264 7.95e-05 460.7 601 0.767  1371920_at  polymerase (DNA-directed), delta interacting protein 2 (predicted)  Info   Poldip2_predicted 
   3265 7.97e-05 1685.3 1169.4 1.441  1388615_at  Ras-related protein RAP-1A  Info   RGD:1359694 
   3266 7.97e-05 207.7 429.3 0.484  1385704_at    Info    
   3267 7.99e-05 495.7 674.2 0.735  1393314_at  Fibroblast growth factor 1  Info   Fgf1 
   3268 7.99e-05 142.7 77.4 1.844  1382682_at  Similar to hypothetical protein PRO0971 (predicted)  Info    
   3269 8.01e-05 73.6 37.2 1.978  1375406_s_at  rapostlin  Info   Fnbp1 
   3270 8.01e-05 142.8 252.3 0.566  1378457_at  Transcribed locus  Info    
   3271 8.01e-05 1037.8 614.1 1.69  1398356_at  cleavage and polyadenylation specific factor 5 (predicted)  Info   Cpsf5_predicted 
   3272 8.02e-05 1441.4 865 1.666  1397766_at  Similar to RIKEN cDNA 4933405A16 (predicted)  Info    
   3273 8.02e-05 1104.5 843.5 1.309  1373397_at    Info    
   3274 8.02e-05 386.3 497.3 0.777  1370684_s_at  hypothetical protein LOC501546  Info   LOC501546 
   3275 8.03e-05 174.3 59.7 2.92  1392082_a_at  CD7 antigen (predicted)  Info   Cd7_predicted 
   3276 8.03e-05 703.2 483.3 1.455  1373080_at  poly (A) polymerase alpha (predicted)  Info   Papola_predicted 
   3277 8.04e-05 158.3 4.6 34.413  1378047_at  Spi-C transcription factor (Spi-1/PU.1 related) (predicted)  Info   Spic_predicted 
   3278 8.04e-05 152.8 73.5 2.079  1384841_at  Cathepsin C  Info   Ctsc 
   3279 8.05e-05 50.8 181.6 0.28  1382185_at  similar to C1q and tumor necrosis factor related protein 2  Info   LOC497886 
   3280 8.06e-05 1143.6 830.3 1.377  1371949_at  basic leucine zipper and W2 domains 1  Info   Bzw1 
   3281 8.06e-05 166.9 109.6 1.523  1368588_at  ATP-dependent, RNA helicase  Info   Ddx52 
   3282 8.06e-05 81.4 192.8 0.422  1372381_at  TSPY-like 4 (predicted)  Info    
   3283 8.07e-05 57.3 173.4 0.33  1371248_at  similar to Cornifin alpha (Small proline-rich protein 1) (SPRR1)  Info   LOC499660 
   3284 8.11e-05 196.9 343.2 0.574  1374038_at  Transcribed locus  Info    
   3285 8.14e-05 15.1 41.9 0.36  1378365_at  Transcribed locus  Info    
   3286 8.14e-05 295.6 499.6 0.592  1372579_at  Endoglin  Info    
   3287 8.14e-05 507.6 613.5 0.827  1371347_at  similar to RIKEN cDNA B230114J08 (predicted)  Info   RGD1307561_predicted 
   3288 8.14e-05 40.9 60.3 0.678  1369120_a_at  luteinizing hormone beta  Info   Lhb 
   3289 8.14e-05 613.1 258.9 2.368  1375266_at    Info    
   3290 8.15e-05 737.1 1067.3 0.691  1399098_at  glyoxylase 1  Info   Glo1 
   3291 8.16e-05 74.5 149.5 0.498  1367742_at  carnitine palmitoyltransferase 1b  Info   Cpt1b 
   3292 8.18e-05 30.2 5.7 5.298  1395960_at  Transcribed locus, moderately similar to NP_862904.1 epidermodysplasia verruciformis 2 [Mus musculus]  Info    
   3293 8.2e-05 196.6 291.5 0.674  1378860_at  Similar to hypothetical protein D930020E02  Info    
   3294 8.2e-05 147 233.5 0.63  1371627_at  Angiomotin-like 1 (predicted)  Info    
   3295 8.21e-05 34.4 9 3.822  1381129_at    Info    
   3296 8.22e-05 67.4 28.5 2.365  1387408_at  seven in absentia 2  Info   RGD:620778 
   3297 8.22e-05 110.2 232 0.475  1382084_at  Similar to EHM2  Info    
   3298 8.24e-05 2966.6 2062.6 1.438  1370871_at  hypothetical gene supported by Y16641; Y16641  Info   LOC288146 
   3299 8.27e-05 16.4 35.9 0.457  1397610_at  Transcribed locus  Info    
   3300 8.27e-05 17.1 42.6 0.401  1380370_at    Info    
   3301 8.27e-05 85.4 175 0.488  1384887_at    Info    
   3302 8.28e-05 1007.2 738.3 1.364  1389902_at  histocompatibility 13 (predicted)  Info   H13_predicted 
   3303 8.29e-05 893.7 2004.8 0.446  1367917_at  cytochrome P450, family 2, subfamily d, polypeptide 26  Info   Cyp2d26 
   3304 8.3e-05 2473.9 3999.7 0.619  1367995_at  catalase  Info   Cat 
   3305 8.3e-05 162.9 363.1 0.449  1369654_at  protein kinase, AMP-activated, alpha 2 catalytic subunit  Info   Prkaa2 
   3306 8.3e-05 54.4 153.2 0.355  1372208_at  protein phosphatase 1, regulatory (inhibitor) subunit 1B  Info   Ppp1r1b 
   3307 8.31e-05 135.3 62.2 2.175  1395192_at  origin recognition complex, subunit 3-like (S. cerevisiae) (predicted)  Info   Orc3l_predicted 
   3308 8.31e-05 124.6 230 0.542  1372820_at  Transcribed locus  Info    
   3309 8.32e-05 186.7 263.3 0.709  1376774_at  Putative membrane protein Re9 (member 1)  Info    
   3310 8.36e-05 636.8 872.5 0.73  1371465_at    Info    
   3311 8.37e-05 569.3 780 0.73  1372720_at  BTB (POZ) domain containing 1 (predicted)  Info   Btbd1_predicted 
   3312 8.37e-05 201.9 334 0.604  1388471_at  similar to RIKEN cDNA E430026E19 (predicted)  Info   RGD1307494_predicted 
   3313 8.38e-05 249.4 151.5 1.646  1388864_at  similar to ATP-dependent RNA-helicase  Info   LOC360815 
   3314 8.4e-05 577.7 1123.1 0.514  1390857_at    Info    
   3315 8.41e-05 364.9 519.6 0.702  1372380_at  similar to RNA binding protein gene with multiple splicing  Info   LOC498642 
   3316 8.44e-05 97.9 146.9 0.666  1374509_at  Similar to RIKEN cDNA 1110018O08  Info   MGC94226 
   3317 8.45e-05 117.4 200.4 0.586  1389562_at  Transcribed locus  Info    
   3318 8.46e-05 795.1 534.9 1.486  1388857_at  SEC23B (S. cerevisiae) (predicted)  Info   Sec23b_predicted 
   3319 8.47e-05 286.2 644.9 0.444  1387531_at  methionine sulfoxide reductase A  Info   Msra 
   3320 8.47e-05 1337.7 2198.1 0.609  1379243_at  NADH dehydrogenase (ubiquinone) 1 alpha subcomplex, 6 (B14) (predicted)  Info   Ndufa6_predicted 
   3321 8.48e-05 204.5 317.5 0.644  1373578_at  Transcribed locus, strongly similar to NP_032717.1 neurofilament 3, medium [Mus musculus]  Info    
   3322 8.51e-05 30.6 62.6 0.489  1374073_at  similar to DNA segment, Chr 11, ERATO Doi 18, expressed (predicted)  Info   RGD1309472_predicted 
   3323 8.52e-05 86 38.9 2.211  1386987_at  interleukin 6 receptor  Info   Il6r 
   3324 8.52e-05 256.1 510.8 0.501  1389123_at  chemokine (C-C motif) ligand 6  Info   RGD:1303200 
   3325 8.53e-05 97.9 168.7 0.58  1375951_at  thrombomodulin  Info   Thbd 
   3326 8.53e-05 1808.3 2148.3 0.842  1398781_at  ATPase, H+ transporting, V1 subunit F  Info   Atp6v1f 
   3327 8.53e-05 51.4 120.6 0.426  1387812_at  Subtilisin - like endoprotease  Info   Pace4 
   3328 8.53e-05 190.5 133.3 1.429  1373925_at    Info    
   3329 8.54e-05 22.8 57.4 0.397  1370854_at  nexilin  Info   Nexn 
   3330 8.56e-05 57.5 3.8 15.132  1396231_at  similar to Ras GTPase-activating protein nGAP (RAS protein activator like 1) (predicted)  Info   RGD1309019_predicted 
   3331 8.57e-05 48.8 68.4 0.713  1385632_at  similar to RIKEN cDNA A430093J20 gene  Info   LOC367113 
   3332 8.58e-05 86.3 31 2.784  1385853_at  bromodomain adjacent to zinc finger domain, 1A (predicted)  Info   Baz1a_predicted 
   3333 8.58e-05 2801.9 3412.8 0.821  1389820_at    Info    
   3334 8.61e-05 143.3 236.8 0.605  1370335_at  disabled homolog 2 (Drosophila) interacting protein  Info   Dab2ip 
   3335 8.61e-05 101 37.3 2.708  1390240_at  Transcribed locus  Info    
   3336 8.62e-05 121.7 223.6 0.544  1385098_at  Transcribed locus  Info    
   3337 8.64e-05 50.2 137 0.366  1368533_at  hephaestin  Info   Heph 
   3338 8.67e-05 193.8 234.2 0.827  1372098_at    Info    
   3339 8.67e-05 1279 900.7 1.42  1375630_at  similar to NHP2-like protein 1 (High mobility group-like nuclear protein 2 homolog 1) ([U4/U6.U5] tri-snRNP 15.5 kDa protein) (OTK27)  Info   RGD:1303103 
   3340 8.68e-05 39.2 80.3 0.488  1389735_at  Transcribed locus  Info    
   3341 8.68e-05 125.4 23.7 5.291  1385426_at  similar to hypothetical protein FLJ20647 (predicted)  Info   RGD1305326_predicted 
   3342 8.68e-05 1871.6 3434.6 0.545  1370397_at  cytochrome P450, family 4, subfamily a, polypeptide 14  Info   Cyp4a14 
   3343 8.69e-05 40.8 71.8 0.568  1385087_at  Transcribed locus  Info    
   3344 8.71e-05 18.9 31.2 0.606  1393385_at  LOC498405  Info   LOC498405 
   3345 8.71e-05 1236.6 852 1.451  1371487_at  SH3 domain binding glutamic acid-rich protein-like 3 (predicted)  Info   Sh3bgrl3_predicted 
   3346 8.72e-05 459.7 944.9 0.487  1368915_at  kynurenine 3-monooxygenase (kynurenine 3-hydroxylase)  Info   Kmo 
   3347 8.72e-05 77.4 121.5 0.637  1395251_at    Info    
   3348 8.73e-05 92.9 48.5 1.915  1380538_at  Similar to cytochrome c oxidase subunit VIc  Info    
   3349 8.73e-05 1442.2 620.5 2.324  1370224_at  signal transducer and activator of transcription 3  Info   Stat3 
   3350 8.74e-05 422.7 297.1 1.423  1374279_at  small inducible cytokine subfamily E, member 1  Info   Scye1 
   3351 8.75e-05 432.3 261.6 1.653  1399055_at  bromodomain containing 7 (predicted)  Info   Brd7_predicted 
   3352 8.76e-05 316.5 163.6 1.935  1392961_at  Similar to Mob4B protein  Info    
   3353 8.77e-05 65.7 35.5 1.851  1379972_at  similar to 1700060H10Rik protein (predicted)  Info   RGD1308699_predicted 
   3354 8.78e-05 40.1 56.1 0.715  1376912_at  hypothetical LOC287388 (predicted)  Info   RGD1310324_predicted 
   3355 8.79e-05 128.2 245.8 0.522  1383416_at  Acetyl-coenzyme A acetyltransferase 1  Info   Acat1 
   3356 8.81e-05 548.4 391.6 1.4  1371704_at  Rho family GTPase 1 (predicted)  Info   Rnd1 
   3357 8.82e-05 29.6 50.1 0.591  1383669_at  Transcribed locus  Info    
   3358 8.82e-05 994.6 610.3 1.63  1372442_at  Transcribed locus  Info    
   3359 8.84e-05 90.3 41.3 2.186  1377002_at  Transcribed locus  Info    
   3360 8.85e-05 427.3 728.4 0.587  1371773_at  Transcribed locus  Info    
   3361 8.86e-05 33.7 72.8 0.463  1368901_at  thrombomodulin  Info   Thbd 
   3362 8.87e-05 263.7 183.9 1.434  1382396_at  Similar to D7Wsu128e protein (predicted)  Info    
   3363 8.88e-05 241.3 549.6 0.439  1378191_at  nuclear cap binding protein subunit 1, 80kDa (predicted)  Info   Ncbp1_predicted 
   3364 8.89e-05 82 149.5 0.548  1381012_at  Serine (or cysteine) proteinase inhibitor, clade F), member 1  Info   Serpinf1 
   3365 8.89e-05 1482.1 2364.5 0.627  1374420_at  similar to RIKEN cDNA 2310001A20 (predicted)  Info   RGD1308874_predicted 
   3366 8.91e-05 203 58.7 3.458  1387956_s_at  chemokine-like factor 1  Info   Cklf1 
   3367 8.91e-05 113.2 65.9 1.718  1397372_at  similar to Mob4B protein /// MOB1, Mps One Binder kinase activator-like 1B (yeast) (predicted)  Info   LOC308302 /// Mobk1b_predicted 
   3368 8.92e-05 206.6 438.2 0.471  1381973_at  solute carrier family 25, member 30  Info   RGD:1359702 
   3369 8.92e-05 1618.6 1933.9 0.837  1367591_at  peroxiredoxin 3  Info   Prdx3 
   3370 8.93e-05 393.1 717.4 0.548  1373312_at  Transcribed locus, strongly similar to NP_064383.1 brain protein 17 [Mus musculus]  Info    
   3371 8.93e-05 19.2 47.8 0.402  1369494_a_at  growth hormone releasing hormone receptor  Info   Ghrhr 
   3372 8.93e-05 221.2 463.7 0.477  1374703_at  Chromobox homolog 6 (predicted)  Info    
   3373 8.93e-05 842.7 531.7 1.585  1388581_at  hematological and neurological expressed sequence 1  Info   RGD:1359325 
   3374 8.97e-05 151.6 59.1 2.565  1380366_at  similar to single chain Fv antibody fragment scFv 7-10A  Info   LOC314509 
   3375 8.98e-05 881.5 565.9 1.558  1383401_at  similar to Testis derived transcript  Info   LOC500040 
   3376 8.98e-05 140.3 44.6 3.146  1387879_a_at  CUG triplet repeat,RNA-binding protein 2  Info   Cugbp2 
   3377 8.99e-05 312.1 97.2 3.211  1380019_at  T-cell, immune regulator 1  Info   Tcirg1 
   3378 9.01e-05 83.2 9.4 8.851  1387945_at  Similar to TRGV9 protein  Info    
   3379 9.03e-05 2386.3 3228.6 0.739  1373041_at  NADH dehydrogenase (ubiquinone) 1 beta subcomplex 3 (predicted)  Info   Ndufb3_predicted 
   3380 9.05e-05 81 166.6 0.486  1383146_at  similar to neurobeachin  Info   LOC361948 
   3381 9.06e-05 218.3 134.4 1.624  1390455_at  abhydrolase domain containing 2 (predicted)  Info   Abhd2_predicted 
   3382 9.06e-05 166.6 85.4 1.951  1379375_at  Platelet derived growth factor, alpha  Info   Pdgfa 
   3383 9.06e-05 221.7 116.3 1.906  1387646_a_at  Max protein  Info   Max 
   3384 9.07e-05 39.6 80.4 0.493  1396247_at  Transcribed locus, strongly similar to NP_079543.1 hypothetical protein MGC2744 [Homo sapiens]  Info    
   3385 9.08e-05 23.6 50.9 0.464  1370851_a_at  kalirin, RhoGEF kinase  Info   Kalrn 
   3386 9.08e-05 150.9 251.8 0.599  1379361_at  peroxisomal biogenesis factor 11A  Info   Pex11a 
   3387 9.08e-05 296.6 432.1 0.686  1375628_at  NADH dehydrogenase (ubiquinone) 1 beta subcomplex 8 (predicted)  Info   Ndufb8_predicted 
   3388 9.1e-05 104.8 179.5 0.584  1379571_at  plakophilin 4 (predicted)  Info   Pkp4_predicted 
   3389 9.1e-05 488.6 337.4 1.448  1379322_at  proteasome (prosome, macropain) 26S subunit, non-ATPase, 7 (predicted)  Info   Psmd7_predicted 
   3390 9.11e-05 41.9 91.1 0.46  1390638_at  Similar to Eph receptor A4  Info    
   3391 9.12e-05 385.5 969.1 0.398  1398463_at  Transcribed locus  Info    
   3392 9.12e-05 107.6 59.5 1.808  1389537_at  Treacher Collins Franceschetti syndrome 1, homolog (predicted)  Info   Tcof1_predicted 
   3393 9.13e-05 252.2 439.4 0.574  1392929_at  Solute carrier family 4, sodium bicarbonate transporter-like, member 11 (predicted)  Info    
   3394 9.14e-05 893.5 261 3.423  1390659_at  CD44 antigen  Info   Cd44 
   3395 9.16e-05 123.7 59.2 2.09  1379312_at  peroxisome proliferative activated receptor, gamma, coactivator-related 1 (predicted)  Info   Pprc1_predicted 
   3396 9.16e-05 83.8 32.1 2.611  1370967_at  Ig active lambda2-like chain mRNA, 3' end  Info    
   3397 9.17e-05 4.9 41.3 0.119  1374679_at  similar to RIKEN cDNA 2310056K19 (predicted)  Info   RGD1306907_predicted 
   3398 9.18e-05 47.4 116.5 0.407  1395190_at  aldo-keto reductase family 1, member B10 (aldose reductase) (predicted)  Info   Akr1b10_predicted 
   3399 9.18e-05 306.2 440.8 0.695  1374144_at  Transcribed locus  Info    
   3400 9.2e-05 867.9 1673.9 0.518  1370076_at  potassium inwardly-rectifying channel, subfamily J, member 16  Info   Kcnj16 
   3401 9.2e-05 13 2.9 4.483  1369900_at  Tumor necrosis factor receptor superfamily, member 6  Info   RGD:619831 
   3402 9.2e-05 442.5 291.7 1.517  1389569_at  similar to BRIX (predicted)  Info   RGD1308508_predicted 
   3403 9.21e-05 1283.3 859.3 1.493  1372246_at  osteoclast stimulating factor 1  Info   Ostf1 
   3404 9.22e-05 972.4 1602.4 0.607  1385243_at  V-maf musculoaponeurotic fibrosarcoma (avian) oncogene homolog (c-maf)  Info   Maf 
   3405 9.23e-05 113.6 170.1 0.668  1393079_at  similar to sialic-acid O-acetylesterase (predicted)  Info   RGD1310431_predicted 
   3406 9.23e-05 31.6 45.5 0.695  1393321_at  kelch domain containing 1 (predicted)  Info   Klhdc1_predicted 
   3407 9.26e-05 55.8 117.4 0.475  1378029_at  Transcribed locus  Info    
   3408 9.26e-05 61.9 110.8 0.559  1372682_at  similar to RIKEN cDNA 2810432L12 (predicted)  Info   RGD1307218_predicted 
   3409 9.27e-05 168.8 31.5 5.359  1387992_at  Leukocyte immunoglobulin-like receptor, subfamily B (with TM and ITIM domains), member 3 (predicted)  Info    
   3410 9.28e-05 831.4 526.7 1.579  1367746_a_at  flotillin 2  Info   Flot2 
   3411 9.28e-05 305.1 167.5 1.821  1390154_at  Transcribed locus  Info    
   3412 9.29e-05 178.7 122.2 1.462  1372021_at  nucleoporin 188 (predicted)  Info   Nup188_predicted 
   3413 9.3e-05 110.4 48 2.3  1377994_at  Transcribed locus  Info    
   3414 9.32e-05 55 84.3 0.652  1377223_at  Transcribed locus  Info    
   3415 9.34e-05 1905.8 1269.8 1.501  1371838_at  similar to splicing factor, arginine/serine-rich 2  Info   RGD:1359422 
   3416 9.36e-05 404.3 669.9 0.604  1371963_at  propionyl-coenzyme A carboxylase, alpha polypeptide  Info   Pcca 
   3417 9.37e-05 76.1 180 0.423  1376239_at  similar to RIKEN cDNA 1110038G14  Info   RGD:1359430 
   3418 9.38e-05 1121.1 731.2 1.533  1367683_at  karyopherin (importin) alpha 2  Info   Kpna2 
   3419 9.4e-05 135.4 222.2 0.609  1372681_at  Transcribed locus  Info    
   3420 9.4e-05 391.9 658.2 0.595  1398378_at  glutathione S-transferase kappa 1  Info   Gstk1 
   3421 9.41e-05 36.6 70.5 0.519  1393910_at  similar to Fam13a1 protein (predicted)  Info   RGD1309807_predicted 
   3422 9.42e-05 273.4 416.5 0.656  1375461_at  similar to hypothetical protein FLJ10241  Info   MGC109149 
   3423 9.43e-05 211.6 93.3 2.268  1378179_a_at  Transcribed locus  Info    
   3424 9.47e-05 24.7 45.5 0.543  1396512_at  Transcribed locus  Info    
   3425 9.49e-05 3257.7 2207.4 1.476  1390024_at  Similar to osteoclast inhibitory lectin  Info    
   3426 9.49e-05 11.6 19.6 0.592  1397942_at  Cell division cycle 37 homolog (S. cerevisiae)-like (predicted)  Info    
   3427 9.5e-05 3608.9 2142 1.685  1370283_at  heat shock 70kD protein 5  Info   Hspa5 
   3428 9.5e-05 1301.9 2792.9 0.466  1371389_at  hypothetical LOC306766  Info   RGD:1359256 
   3429 9.52e-05 129.5 210.3 0.616  1382945_at  Similar to cDNA sequence BC006662 (predicted)  Info    
   3430 9.53e-05 151 264 0.572  1393230_s_at  Dead end homolog 1 (zebrafish) (predicted)  Info    
   3431 9.53e-05 1304.7 1663.7 0.784  1388373_at  adiponectin receptor 1  Info   RGD:1303151 
   3432 9.54e-05 192.1 102.1 1.881  1390144_at  similar to RIKEN cDNA 3110043O21 gene  Info   RGD:1359108 
   3433 9.56e-05 487.9 294.5 1.657  1395375_at  similar to hypothetical protein  Info   MGC73022 
   3434 9.57e-05 796.5 559.8 1.423  1389165_at  similar to myocardial ischemic preconditioning upregulated protein 2  Info   LOC498301 
   3435 9.58e-05 11 28.7 0.383  1367664_at  ankyrin repeat domain 1 (cardiac muscle)  Info   Ankrd1 
   3436 9.58e-05 585.7 769.4 0.761  1368916_at  argininosuccinate lyase  Info   Asl 
   3437 9.58e-05 303.3 144.3 2.102  1373211_at  F-box and WD-40 domain protein 4 (predicted)  Info   Fbxw4_predicted 
   3438 9.6e-05 162 68.8 2.355  1393105_at  similar to zinc finger protein  Info   LOC500989 
   3439 9.61e-05 569.7 358.7 1.588  1371985_a_at  HLA-B associated transcript 5  Info   RGD:1303164 
   3440 9.62e-05 84.5 46.7 1.809  1373633_at  Transcribed locus  Info    
   3441 9.63e-05 605.5 881.2 0.687  1398997_at  phosphohistidine phosphatase 1 (predicted)  Info   Phpt1_predicted 
   3442 9.63e-05 8.7 52.6 0.165  1379772_at  angiotensin receptor-like 1  Info   Agtrl1 
   3443 9.65e-05 191.8 98.3 1.951  1385314_at  Transcribed locus  Info    
   3444 9.65e-05 112.4 62.6 1.796  1393730_at  Transcribed locus  Info    
   3445 9.66e-05 467.7 1024.3 0.457  1368283_at  enoyl-Coenzyme A, hydratase/3-hydroxyacyl Coenzyme A dehydrogenase  Info   Ehhadh 
   3446 9.66e-05 161.7 251.7 0.642  1372925_at    Info    
   3447 9.66e-05 142.4 254.8 0.559  1370181_at  RAB4A, member RAS oncogene family  Info   Rab4a 
   3448 9.66e-05 159.3 245.2 0.65  1377121_at  Discs, large homolog 5 (Drosophila) (predicted)  Info    
   3449 9.68e-05 160.7 253.8 0.633  1372889_at  Solute carrier organic anion transporter family, member 2a1  Info   Slco2a1 
   3450 9.71e-05 435.7 304.2 1.432  1384467_at  CDNA clone MGC:109551 IMAGE:7325365  Info    
   3451 9.72e-05 326.3 452 0.722  1373924_at  similar to C530044N13Rik protein (predicted)  Info   RGD1306568_predicted 
   3452 9.72e-05 54.7 7.5 7.293  1392791_at  Transcribed locus  Info    
   3453 9.73e-05 3.8 41.6 0.091  1378328_at  Transcribed locus  Info    
   3454 9.74e-05 917.9 2618.9 0.35  1383111_at  2-amino-3-carboxymuconate-6-semialdehyde decarboxylase  Info   Acmsd 
   3455 9.74e-05 73.1 135.4 0.54  1379206_at  similar to RIKEN cDNA 1110001E17 (predicted)  Info   RGD1307901_predicted 
   3456 9.75e-05 94.9 264.6 0.359  1390450_a_at  osteoglycin (predicted)  Info   Ogn_predicted 
   3457 9.76e-05 109.4 23.4 4.675  1388940_at  similar to Rho-GTPase-activating protein 25  Info   LOC500246 
   3458 9.77e-05 139.9 312 0.448  1385729_at  Transcribed locus  Info    
   3459 9.77e-05 286.9 216 1.328  1373758_at    Info    
   3460 9.78e-05 151.8 102 1.488  1391086_at  katanin p60 subunit A 1  Info   RGD:1303062 
   3461 9.78e-05 19.6 39.3 0.499  1381391_at  hypothetical LOC287798  Info   RGD:1359207 
   3462 9.78e-05 505.4 624.8 0.809  1388679_at  TBC1 domain family, member 14 (predicted)  Info   Tbc1d14_predicted 
   3463 9.79e-05 421.2 606 0.695  1376727_at  similar to RIKEN cDNA 2310034L04 (predicted)  Info   RGD1310157_predicted 
   3464 9.8e-05 254.5 125.9 2.021  1390529_at  CD83 antigen (predicted)  Info   Cd83_predicted 
   3465 9.85e-05 1583.1 2977.1 0.532  1375944_at    Info    
   3466 9.86e-05 180.3 275.1 0.655  1374462_at  kinesin-associated protein 3 (predicted)  Info   Kifap3_predicted 
   3467 9.87e-05 476 1016.7 0.468  1368284_at  plasmalemma vesicle associated protein  Info   Plvap 
   3468 9.89e-05 374.4 483.6 0.774  1374405_at  heterogeneous nuclear ribonucleoprotein A3  Info   Hnrpa3 
   3469 9.9e-05 61.2 101 0.606  1373503_at  leucine-rich repeats and calponin homology (CH) domain containing 4 (predicted)  Info   Lrch4_predicted 
   3470 9.9e-05 189.6 72.1 2.63  1368251_at  Janus kinase 3  Info   Jak3 
   3471 9.94e-05 176.4 380.1 0.464  1389332_at  Transcribed locus  Info    
   3472 9.94e-05 27.3 54.5 0.501  1367992_at  secretory granule neuroendocrine protein 1  Info   Sgne1 
   3473 9.96e-05 188.4 339.1 0.556  1385574_at  similar to cDNA sequence AF397014 (predicted)  Info   RGD1308114_predicted 
   3474 9.97e-05 15.3 36.9 0.415  1393167_at    Info    
   3475 9.98e-05 137.9 97.2 1.419  1383716_at  Transcribed locus, strongly similar to NP_777569.1 hypothetical protein MGC23909 [Homo sapiens]  Info    
   3476 9.98e-05 560.8 380.4 1.474  1373940_at  non-catalytic region of tyrosine kinase adaptor protein 1 (predicted)  Info   Nck1_predicted 
   3477 0.0001001 185.1 254.1 0.728  1373734_at  Solute carrier organic anion transporter family, member 3a1  Info   Slco3a1 
   3478 0.0001001 255.9 360.2 0.71  1379339_at  similar to tumor-related protein (predicted)  Info   RGD1307632_predicted 
   3479 0.0001003 407.8 738.5 0.552  1384743_at  Transcribed locus  Info    
   3480 0.0001004 175.6 401.2 0.438  1373733_at  Bcl-2-related ovarian killer protein  Info   Bok 
   3481 0.0001004 48.6 79.2 0.614  1377522_a_at  similar to KIAA1086 protein (predicted)  Info   RGD1304737_predicted 
   3482 0.0001005 114.6 18.3 6.262  1369891_at  Jun dimerization protein 1  Info   Jdp1 
   3483 0.0001006 167.9 93 1.805  1385856_at  RIO kinase 1 (yeast) (predicted)  Info   Riok1_predicted 
   3484 0.0001006 633 1322 0.479  1393894_at  cytochrome P450, 4a12  Info   RGD:628846 
   3485 0.0001007 500.6 714.2 0.701  1372814_at  similar to RIKEN cDNA 2010005O13 (predicted)  Info   RGD1310623_predicted 
   3486 0.0001007 110.5 212.2 0.521  1384273_at  Carbohydrate kinase-like (predicted)  Info    
   3487 0.0001008 260.8 423.3 0.616  1389102_at  similar to Dock1 protein  Info   LOC309081 
   3488 0.0001012 463.5 768 0.604  1392916_at  microtubule-associated protein 7 (predicted)  Info   Mtap7_predicted 
   3489 0.0001013 2041.7 3064.9 0.666  1368163_at  dipeptidylpeptidase 4  Info   Dpp4 
   3490 0.0001015 495.8 45.7 10.849  1387283_at  myxovirus (influenza virus) resistance 2  Info   Mx2 
   3491 0.0001015 1387 2138.8 0.648  1371350_at  similar to S-adenosylmethionine synthetase gamma form (Methionine adenosyltransferase) (AdoMet synthetase) (MAT-II)  Info   LOC498211 
   3492 0.0001018 131.8 202 0.652  1387498_a_at  Fibroblast growth factor receptor 1  Info   Fgfr1 
   3493 0.0001019 185.4 370.2 0.501  1372044_at  similar to Ser/Thr-rich protein T10 in DGCR region (predicted)  Info   RGD1310348_predicted 
   3494 0.0001024 147.3 340.3 0.433  1378819_at  similar to RIKEN cDNA 0610012D14 (predicted)  Info   RGD1310111_predicted 
   3495 0.0001024 243.6 474.4 0.513  1384499_at  Butyryl Coenzyme A synthetase 1 (predicted)  Info    
   3496 0.0001025 80 188.3 0.425  1370385_at  phospholipase A2, group VI  Info   Pla2g6 
   3497 0.0001027 181.2 78 2.323  1376098_a_at  myosin IG (predicted)  Info   Myo1g_predicted 
   3498 0.0001028 125.1 47.4 2.639  1375806_at  similar to [Mouse primary response gene B94 mRNA, 3end.], gene product  Info   LOC299339 
   3499 0.0001029 1085.1 841.5 1.289  1388376_at  similar to RIKEN cDNA 0610038D11 (predicted)  Info   RGD1309710_predicted 
   3500 0.000103 83.8 151.8 0.552  1368676_at  dynein, cytoplasmic, heavy polypeptide 2 /// hypothetical gene supported by NM_023024  Info   Dnch2 /// LOC497827 
   3501 0.000103 191.2 265.9 0.719  1389336_at  processing of precursor 5, ribonuclease P/MRP family (S. cerevisiae) (predicted)  Info   Pop5_predicted 
   3502 0.000103 83.4 152.7 0.546  1368961_at  Matrix metalloproteinase 23  Info   Mmp23 
   3503 0.0001032 120 155.9 0.77  1382426_at    Info    
   3504 0.0001034 622.5 459.3 1.355  1388137_a_at  zygin-related protein  Info   RGD:628711 
   3505 0.0001034 201.3 339.7 0.593  1376848_at  Transcribed locus  Info    
   3506 0.0001035 94.4 62.2 1.518  1379905_at  GTP binding protein 1 (predicted)  Info   Gtpbp1_predicted 
   3507 0.0001035 581.6 343.2 1.695  1371592_at  c-src tyrosine kinase (predicted)  Info   Csk_predicted 
   3508 0.0001037 51.8 86.6 0.598  1374685_at  Similar to ALEX3 protein  Info    
   3509 0.0001037 1777 2171.4 0.818  1389343_at  similar to Hypothetical UPF0327 protein  Info   LOC362641 
   3510 0.0001038 22.7 3.1 7.323  1378331_at  Mesoderm development candiate 2 (predicted)  Info   Mesdc2 
   3511 0.0001039 231.6 135.8 1.705  1370505_at  ADP-ribosylarginine hydrolase  Info   Adprh 
   3512 0.000104 1138 745.8 1.526  1369621_s_at  FK506 binding protein 1a  Info   Fkbp1a 
   3513 0.000104 1077.5 821.9 1.311  1372607_at  nucleotide binding protein 2 (predicted)  Info   Nubp2_predicted 
   3514 0.000104 344.4 624.5 0.551  1385103_at  fused toes (predicted)  Info   Fts_predicted 
   3515 0.000104 4247.2 2667.4 1.592  1367456_at  ubiquitin-conjugating enzyme E2D 3 (UBC4/5 homolog, yeast)  Info   Ube2d3 
   3516 0.0001043 298.5 420.6 0.71  1388713_at  thiamine triphosphatase  Info   Thtpa 
   3517 0.0001044 71.4 18.4 3.88  1384898_at  Transcribed locus  Info    
   3518 0.0001044 859.6 1136.1 0.757  1371619_at  similar to HTGN29 protein; keratinocytes associated transmembrane protein 2 (predicted)  Info   RGD1310352_predicted 
   3519 0.0001044 63 112.4 0.56  1388230_at  ajuba homolog (Xenopus laevis)  Info   Jub 
   3520 0.0001048 874.1 1305.4 0.67  1376728_at  Transcribed locus  Info    
   3521 0.000105 133.7 180.8 0.739  1372780_at  similar to RIKEN cDNA 1110038M16 (predicted)  Info   RGD1307066_predicted 
   3522 0.0001054 367.3 20 18.365  1373224_at  alpha-2,3-sialyltransferase ST3Gal IV  Info   RGD:1303068 
   3523 0.0001055 499.8 786.4 0.636  1371956_at  CDNA clone MGC:93760 IMAGE:7108525  Info    
   3524 0.0001056 3284.8 5807.8 0.566  1370943_at  sulfotransferase family, cytosolic, 1C, member 1 (predicted)  Info   Sult1c1_predicted 
   3525 0.0001057 340.4 580.7 0.586  1378049_at  similar to Cas-associated zinc finger protein (predicted)  Info   RGD1306498_predicted 
   3526 0.0001057 67.6 137.8 0.491  1374029_at    Info    
   3527 0.0001058 576.5 312.4 1.845  1369029_at  phospholipid scramblase 1  Info   Plscr1 
   3528 0.0001058 47.1 19 2.479  1394290_at  Transcribed locus  Info    
   3529 0.0001059 149.6 291.6 0.513  1368089_at  phosphodiesterase 2A, cGMP-stimulated  Info   Pde2a 
   3530 0.000106 81.9 142.6 0.574  1383371_at  Transcribed locus  Info    
   3531 0.000106 1034 710.7 1.455  1388568_at  eukaryotic translation initiation factor 3 subunit 7  Info   RGD:1303255 
   3532 0.0001064 62.3 17.7 3.52  1384944_at  B-cell leukemia/lymphoma 11B (predicted)  Info   Bcl11b_predicted 
   3533 0.0001066 2164 2702.7 0.801  1371572_at  amyloid beta (A4) precursor protein  Info   App 
   3534 0.0001067 594.9 414 1.437  1398974_at  chromosome segregation 1-like (S. cerevisiae) (predicted)  Info   Cse1l_predicted 
   3535 0.0001069 503.8 905.5 0.556  1371886_at  carnitine acetyltransferase  Info   RGD:1303031 
   3536 0.0001072 34.4 67.5 0.51  1396942_at  hypothetical LOC301267  Info   LOC301267 
   3537 0.0001073 60 123 0.488  1377789_at  Transcribed locus  Info    
   3538 0.0001074 399.5 281.4 1.42  1380116_at  Transcribed locus  Info    
   3539 0.0001075 457.3 968.8 0.472  1384638_at  Similar to RIKEN cDNA 1700013H19 (predicted)  Info    
   3540 0.0001076 450.4 358.3 1.257  1388466_at  proteasome (prosome, macropain) 26S subunit, non-ATPase, 3 (predicted)  Info   Psmd3_predicted 
   3541 0.0001077 1629 1214.3 1.342  1398863_at  guanine nucleotide binding protein, beta polypeptide 2  Info   Gnb2 
   3542 0.0001077 120.3 215.2 0.559  1386615_at  carbohydrate sulfotransferase 2 (predicted)  Info   Chst2_predicted 
   3543 0.0001078 136 260.5 0.522  1388859_at  PKD2 interactor, golgi and endoplasmic reticulum associated 1  Info   Pgea1 
   3544 0.0001078 173.7 40.2 4.321  1369830_at  protein kinase C, eta  Info   Prkch 
   3545 0.0001079 192 321.3 0.598  1377616_at  similar to RIKEN cDNA 6720467C03 (predicted)  Info   RGD1310681_predicted 
   3546 0.000108 63.2 145.5 0.434  1372959_at  similar to mKIAA0665 protein (predicted)  Info   RGD1308952_predicted 
   3547 0.000108 1376.7 1698.1 0.811  1371253_at  electron transferring flavoprotein, alpha polypeptide  Info   Etfa 
   3548 0.0001084 151.3 330.8 0.457  1378094_at  Similar to hypothetical protein FLJ11342 (predicted)  Info    
   3549 0.0001084 73.4 160.8 0.456  1389113_at  Similar to Hypothetical protein MGC32471 (predicted)  Info    
   3550 0.0001084 129.8 203.8 0.637  1383624_at  similar to hypothetical protein LOC284018 isoform b  Info   LOC498014 
   3551 0.0001089 343 454.5 0.755  1373440_at  caspase 8 associated protein 2 (predicted)  Info   Casp8ap2_predicted 
   3552 0.0001089 538 1428.8 0.377  1387375_at  ketohexokinase  Info   Khk 
   3553 0.0001092 166.9 113.9 1.465  1382128_at  Transcribed locus  Info    
   3554 0.0001092 239.8 183.8 1.305  1398344_at  cofactor required for Sp1 transcriptional activation, subunit 6 (predicted)  Info   Crsp6_predicted 
   3555 0.0001093 315.5 6.7 47.09  1392308_at  phospholipase A2, group IID (predicted)  Info   Pla2g2d_predicted 
   3556 0.0001093 216.7 144.3 1.502  1383251_at  ADP-ribosyltransferase (NAD+, poly(ADP-ribose) polymerase)-like 2 (predicted)  Info   Adprtl2_predicted 
   3557 0.0001094 147.8 214.4 0.689  1368409_at  glutathione S-transferase, theta 2  Info   Gstt2 
   3558 0.0001095 3600.6 4290.3 0.839  1388199_at  tumor-associated calcium signal transducer 1  Info   Tacstd1 
   3559 0.0001096 238.7 138.6 1.722  1383862_at  Similar to osteoclast inhibitory lectin  Info    
   3560 0.0001096 550 355.2 1.548  1389373_at  SMAD, mothers against DPP homolog 1 (Drosophila)  Info   Smad1 
   3561 0.0001097 142.9 240.2 0.595  1383850_at  Similar to RIKEN cDNA 1600014C10  Info    
   3562 0.0001097 58 112.6 0.515  1376672_at  Transcribed locus  Info    
   3563 0.0001099 183.1 365.1 0.502  1384008_at  Similar to hypothetical protein FLJ40283 (predicted)  Info    
   3564 0.0001099 174.8 113.4 1.541  1369421_at  topoisomerase (DNA) I  Info   Top1 
   3565 0.0001101 276.6 488.9 0.566  1390776_at  Iroquois related homeobox 3 (Drosophila) (predicted)  Info   Irx3_predicted 
   3566 0.0001101 162.4 309.8 0.524  1389504_at  Similar to Deltex3  Info    
   3567 0.0001102 179.9 336.6 0.534  1371959_at  histone 2, H2aa (predicted) /// similar to Hist2h2aa1 protein  Info   Hist2h2aa_predicted /// LOC499676 
   3568 0.0001103 58.5 105.6 0.554  1377183_at  Transcribed locus  Info    
   3569 0.0001105 58.5 128.6 0.455  1389171_at  similar to RIKEN cDNA 1110001E17 (predicted)  Info   RGD1307901_predicted 
   3570 0.0001107 98.3 44.2 2.224  1384900_at  MOB1, Mps One Binder kinase activator-like 2B (yeast) (predicted)  Info   Mobkl2b_predicted 
   3571 0.0001107 459.7 614.9 0.748  1389333_at    Info    
   3572 0.0001108 1218.4 2198.7 0.554  1371314_at  neural precursor cell expressed, developmentally down-regulated gene 4A  Info   Nedd4a 
   3573 0.0001111 395.4 787.2 0.502  1397185_at  Transcribed locus  Info    
   3574 0.0001112 42.9 8.8 4.875  1380559_at  Transcribed locus  Info    
   3575 0.0001113 958 1843.7 0.52  1388441_at    Info    
   3576 0.0001113 664 953.3 0.697  1370282_at  cysteine and glycine-rich protein 2  Info   RGD:61950 
   3577 0.0001113 243.7 382.9 0.636  1374126_at  Transcribed locus  Info    
   3578 0.0001114 296 166.3 1.78  1376839_at  Transcribed locus  Info    
   3579 0.0001119 179.9 254.1 0.708  1384368_at  DNA2 DNA replication helicase 2-like (yeast) (predicted)  Info    
   3580 0.0001119 125.7 213.2 0.59  1379541_at    Info    
   3581 0.000112 34.7 64.3 0.54  1391338_at  Transcribed locus  Info    
   3582 0.0001121 312.9 169.1 1.85  1369409_at  Ngfi-A binding protein 1  Info   Nab1 
   3583 0.0001122 152.8 111 1.377  1374186_at  Similar to KIAA1185 protein (predicted)  Info    
   3584 0.0001124 13.8 33.2 0.416  1382962_at  Transcribed locus  Info    
   3585 0.0001125 366.4 540.7 0.678  1389177_at  PERP, TP53 apoptosis effector (predicted)  Info   Perp_predicted 
   3586 0.0001128 57.7 82.7 0.698  1387791_at  angiotensin 1 converting enzyme  Info   Ace 
   3587 0.0001129 29.3 68.8 0.426  1383472_at  aldehyde dehydrogenase 1 family, member B1 (predicted)  Info   Aldh1b1_predicted 
   3588 0.000113 39.5 71.2 0.555  1396302_at  Transcribed locus  Info    
   3589 0.000113 761.1 519.7 1.464  1383632_at  Solute carrier family 30 (zinc transporter), member 1  Info   Slc30a1 
   3590 0.0001131 137.3 314.5 0.437  1369560_at  glycerol-3-phosphate dehydrogenase 1 (soluble)  Info   Gpd1 
   3591 0.0001131 267.2 392.8 0.68  1392460_at  Similar to hypothetical protein MGC20460 (predicted)  Info    
   3592 0.0001132 1613.6 2353.9 0.686  1386909_a_at  voltage-dependent anion channel 1  Info   Vdac1 
   3593 0.0001132 121 211.4 0.572  1389757_at  similar to acetyl-coA dehydrogenase -related (111.6 kD) (5G231) (predicted)  Info   RGD1310159_predicted 
   3594 0.0001136 577.5 352.5 1.638  1367914_at  epithelial membrane protein 3  Info   Emp3 
   3595 0.0001136 231.1 122.9 1.88  1397916_s_at  protein tyrosine phosphatase, non-receptor type 1  Info   Ptpn1 
   3596 0.0001137 421 292.8 1.438  1395173_at  GPI-anchored membrane protein 1 (predicted)  Info   Gpiap1_predicted 
   3597 0.0001138 16.3 33.3 0.489  1383869_at  Transcribed locus  Info    
   3598 0.0001138 263.7 481.6 0.548  1368109_at  sialyltransferase 9 (CMP-NeuAc:lactosylceramide alpha-2,3-sialyltransferase)  Info   Siat9 
   3599 0.0001139 375 575.5 0.652  1388918_at  High density lipoprotein binding protein  Info   Hdlbp 
   3600 0.0001141 414.8 560.7 0.74  1379528_at  Transcribed locus  Info    
   3601 0.0001141 83.5 52.2 1.6  1395165_at  PC4 and SFRS1 interacting protein 1  Info   Psip1 
   3602 0.0001142 225.6 117.9 1.913  1373997_at  Transcribed locus  Info    
   3603 0.0001142 1089.4 569.3 1.914  1379542_at  similar to Small nuclear ribonucleoprotein F (snRNP-F) (Sm protein F) (Sm-F) (SmF) /// small nuclear ribonucleoprotein polypeptide F (predicted)  Info   LOC296377 /// Snrpf_predicted 
   3604 0.0001142 1217.3 1835.2 0.663  1370838_s_at  alpha-spectrin 2 /// hypothetical gene supported by NM_171983  Info   Spna2 /// LOC497766 
   3605 0.0001144 4877.2 6033 0.808  1367585_a_at  ATPase, Na+/K+ transporting, alpha 1 polypeptide  Info   Atp1a1 
   3606 0.0001144 122.5 11.4 10.746  1391227_at  Transcribed locus  Info    
   3607 0.0001145 219.2 345.1 0.635  1377812_a_at  transmembrane protein 24 (predicted)  Info   Tmem24_predicted 
   3608 0.0001147 293.5 488.3 0.601  1386860_at  milk fat globule-EGF factor 8 protein  Info   Mfge8 
   3609 0.0001147 83.6 175.4 0.477  1393559_at  Transcribed locus  Info    
   3610 0.0001149 17.9 40.2 0.445  1384096_at  Transcribed locus  Info    
   3611 0.0001149 143.6 236.2 0.608  1389906_at  Farnesyl diphosphate farnesyl transferase 1  Info   Fdft1 
   3612 0.000115 268.3 66.2 4.053  1378032_at    Info    
   3613 0.000115 681.8 952.1 0.716  1371473_at  cleft lip and palate associated transmembrane protein 1 (predicted)  Info   Clptm1_predicted 
   3614 0.0001153 492.7 817.7 0.603  1372310_at  similar to tumor-related protein (predicted)  Info   RGD1307632_predicted 
   3615 0.0001154 56.6 104.2 0.543  1397406_at  UDP-N-acteylglucosamine pyrophosphorylase 1-like 1 (predicted)  Info    
   3616 0.0001154 50.1 103 0.486  1384505_at  Transcribed locus  Info    
   3617 0.0001159 290.2 678.4 0.428  1367652_at  insulin-like growth factor binding protein 3  Info   Igfbp3 
   3618 0.000116 202.4 357.1 0.567  1368040_at  synaptojanin 2 binding protein  Info   Synj2bp 
   3619 0.0001161 14.1 42.7 0.33  1387623_at  stanniocalcin 1  Info   Stc1 
   3620 0.0001162 17.8 50.2 0.355  1374187_at  Transcribed locus  Info    
   3621 0.0001162 233.2 158.4 1.472  1368204_at  ligase I, DNA, ATP-dependent /// ligase I, DNA, ATP-dependent (predicted)  Info   Lig1 /// Lig1_predicted 
   3622 0.0001162 47.9 104.3 0.459  1395986_at  slit homolog 2 (Drosophila)  Info   Slit2 
   3623 0.0001162 367.2 259.1 1.417  1372119_at  ubiquitin-activating enzyme E1-domain containing 1 (predicted)  Info   Ube1dc1_predicted 
   3624 0.0001163 184.3 81.8 2.253  1385800_at    Info    
   3625 0.0001163 3267.4 4033 0.81  1371321_at    Info    
   3626 0.0001165 368.1 237.3 1.551  1389337_at  Transcribed locus  Info    
   3627 0.0001165 160.4 259.2 0.619  1395427_at  Transcribed locus  Info    
   3628 0.0001166 19 64.5 0.295  1391544_at  Similar to adiponutrin  Info    
   3629 0.0001167 85.3 24.8 3.44  1384294_at  Transcribed locus  Info    
   3630 0.0001167 3.2 30.2 0.106  1385465_at  Transcribed locus  Info    
   3631 0.0001168 382.3 740.2 0.516  1388298_at  myosin, light polypeptide 9, regulatory (predicted)  Info   Myl9_predicted 
   3632 0.0001169 346.4 674.1 0.514  1392928_at  similar to RIKEN cDNA 2810055F11 (predicted)  Info   RGD1305721_predicted 
   3633 0.0001169 127.4 84.2 1.513  1377893_at  Transcribed locus  Info    
   3634 0.0001169 160.4 85.3 1.88  1373904_at  similar to RIKEN cDNA 2210402C18 (predicted)  Info   RGD1304585_predicted 
   3635 0.000117 543.1 755.6 0.719  1399085_at  Zinc metalloproteinase, STE24 homolog (S. cerevisiae) (predicted)  Info    
   3636 0.0001171 207.6 338.7 0.613  1372117_at  similar to BB128963 protein (predicted)  Info   RGD1305958_predicted 
   3637 0.0001171 48.5 15.8 3.07  1372809_at  hypothetical gene supported by AF152002  Info   LOC290595 
   3638 0.0001171 73.6 120.2 0.612  1377084_at  Similar to hypothetical protein (predicted)  Info    
   3639 0.0001173 202.9 260.4 0.779  1384101_at  Type II keratin Kb1  Info   Kb1 
   3640 0.0001177 641.3 406.9 1.576  1398811_at  jumping translocation breakpoint  Info   Jtb 
   3641 0.0001179 217 288.4 0.752  1380813_at    Info    
   3642 0.0001179 946.7 791.1 1.197  1388571_at  Transcribed locus  Info    
   3643 0.0001181 573.1 919 0.624  1391936_a_at  Transcribed locus  Info    
   3644 0.0001181 476.2 838.6 0.568  1376770_at  similar to EF hand domain containing 1  Info   LOC501181 
   3645 0.0001182 178.9 255.8 0.699  1372414_at  Transcribed locus  Info    
   3646 0.0001186 52.1 23.6 2.208  1377851_at  Transcribed locus, weakly similar to NP_997348.1 FLJ45337 protein [Homo sapiens]  Info    
   3647 0.0001189 367.6 686.8 0.535  1387966_at  asparaginase-like sperm autoantigen  Info   Asrgl1 
   3648 0.0001189 383.3 211.3 1.814  1369743_a_at  purinergic receptor P2X, ligand-gated ion channel 4  Info   P2rx4 
   3649 0.000119 48.3 87.8 0.55  1372521_at  ras homolog gene family, member N (predicted)  Info   Rohn_predicted 
   3650 0.000119 195.9 301.9 0.649  1392989_at  Transcribed locus  Info    
   3651 0.0001192 39.1 94.1 0.416  1379960_at  sushi domain containing 3 (predicted)  Info   Susd3_predicted 
   3652 0.0001192 52.2 73.5 0.71  1383471_at  similar to RIKEN cDNA 1200006F02 (predicted)  Info   RGD1307594_predicted 
   3653 0.0001195 157.4 11.4 13.807  1376496_at  hypothetical protein LOC503164  Info   LOC503164 
   3654 0.0001195 88.3 218.3 0.404  1370592_at  kidney expressed gene 1  Info   Keg1 
   3655 0.0001196 428.1 780.9 0.548  1376082_at  ecotropic viral integration site 1 (predicted)  Info   Evi1_predicted 
   3656 0.0001199 352.9 918.5 0.384  1389020_at  Transcribed locus  Info    
   3657 0.0001199 219.2 352.9 0.621  1373149_at  similar to yippee-like 3  Info   LOC293491 
   3658 0.00012 131.8 20.4 6.461  1384981_at  CD6 antigen  Info   Cd6 
   3659 0.00012 624.4 824.8 0.757  1370098_at  synaptobrevin-like 1  Info   Sybl1 
   3660 0.00012 3907 5135.9 0.761  1370218_at  lactate dehydrogenase B  Info   Ldhb 
   3661 0.00012 103.9 53.7 1.935  1391255_at  zinc finger, MYND domain containing 15 (predicted)  Info   Zmynd15_predicted 
   3662 0.0001203 509.5 846.5 0.602  1372260_at  leucine zipper domain protein  Info   MGC116147 
   3663 0.0001204 140.1 76.9 1.822  1375277_at  similar to hypothetical protein FLJ20433  Info   LOC499745 
   3664 0.0001205 355.4 263.2 1.35  1395894_at  apoptosis inhibitor 5 (predicted)  Info   Api5_predicted 
   3665 0.0001206 368.7 567 0.65  1373015_at  ring finger protein 11 (predicted)  Info   Rnf11_predicted 
   3666 0.0001207 92.5 142.7 0.648  1392992_at  Transcribed locus  Info    
   3667 0.0001208 158 263.6 0.599  1389180_at  Similar to phosphorylase kinase (EC 2.7.1.38) beta chain, non-muscle - rabbit  Info    
   3668 0.000121 864 518.2 1.667  1370007_at  protein disulfide isomerase related protein (calcium-binding protein, intestinal-related)  Info   Erp70 
   3669 0.000121 1741.5 2852 0.611  1377720_x_at    Info    
   3670 0.000121 582.7 1017.2 0.573  1371331_at  follistatin-like 1  Info   Fstl1 
   3671 0.000121 508.6 265 1.919  1378925_at    Info    
   3672 0.0001211 46.4 88.5 0.524  1392595_at  zinc finger protein 618 (predicted)  Info   Znf618_predicted 
   3673 0.0001213 67.3 108.1 0.623  1376670_at  similar to RIKEN cDNA 1300010K09 (predicted)  Info   RGD1307512_predicted 
   3674 0.0001213 150.8 98.7 1.528  1376005_at  Kinesin family member 1B  Info   Kif1b 
   3675 0.0001214 27 56.2 0.48  1382403_at  similar to RIKEN cDNA 2610019F03  Info   LOC498662 
   3676 0.0001214 1099.1 2318.9 0.474  1374251_at  Transcribed locus  Info    
   3677 0.0001215 129.3 270.8 0.477  1376692_at  homeodomain interacting protein kinase 2 (predicted)  Info   Hipk2_predicted 
   3678 0.0001216 87.9 56.7 1.55  1390321_at  similar to F730047E07Rik protein  Info   LOC499392 
   3679 0.0001217 4110.5 6296.6 0.653  1368680_a_at  solute carrier family 34 (sodium phosphate), member 1  Info   Slc34a1 
   3680 0.0001218 242.5 535 0.453  1388427_at  limitrin  Info   RGD:1359356 
   3681 0.0001221 512.9 747.6 0.686  1387655_at  chemokine (C-X-C motif) ligand 12  Info   Cxcl12 
   3682 0.0001221 139.2 311.5 0.447  1381396_s_at  Kruppel-like factor 15  Info   Klf15 
   3683 0.0001223 68.6 8.9 7.708  1368695_at  complement component 4 binding protein, beta  Info   C4bpb 
   3684 0.0001225 136.1 45.8 2.972  1368604_at  Mediterranean fever  Info   Mefv 
   3685 0.0001225 82.9 44.7 1.855  1370341_at  enolase 2, gamma  Info   Eno2 
   3686 0.0001226 168.6 81.8 2.061  1374306_at  Transcribed locus  Info    
   3687 0.0001227 74.5 147.3 0.506  1395027_at  Transcribed locus  Info    
   3688 0.0001228 323.2 805.8 0.401  1394022_at  inhibitor of DNA binding 4  Info   Idb4 
   3689 0.0001229 1894 1334.8 1.419  1372079_at  Transcribed locus  Info    
   3690 0.0001234 36 85.4 0.422  1379693_at  Roundabout 2 (Drosophila)  Info   Robo2 
   3691 0.0001236 1787.8 1110.1 1.61  1374063_at  splicing factor, arginine/serine-rich 3 (SRp20) (predicted)  Info   Sfrs3_predicted 
   3692 0.0001237 19 40.3 0.471  1374425_at  transducin-like enhancer of split 1, homolog of Drosophila E(spl) (predicted)  Info   Tle1_predicted 
   3693 0.0001237 396 509.9 0.777  1367550_a_at  beta-amyloid binding protein precursor (predicted)  Info   Bbp_predicted 
   3694 0.0001238 151.3 238 0.636  1374570_at  1-acylglycerol-3-phosphate O-acyltransferase 2 (lysophosphatidic acid acyltransferase, beta) (predicted)  Info   Agpat2_predicted 
   3695 0.0001239 123.4 75.9 1.626  1397255_at  similar to hypothetical protein MGC2574  Info   RGD:1359280 
   3696 0.0001239 34.9 71.7 0.487  1369527_at  chemokine (C-X3-C) receptor 1  Info   Cx3cr1 
   3697 0.0001239 183 121.8 1.502  1390152_at  toll-like receptor adaptor molecule 2 (predicted)  Info   Ticam2_predicted 
   3698 0.000124 63.5 100.7 0.631  1374883_at  myotubularin related protein 7 (predicted)  Info   Mtmr7_predicted 
   3699 0.000124 22 47.8 0.46  1380254_at  Transcribed locus, moderately similar to NP_079928.1 general transcription factor III A [Mus musculus]  Info    
   3700 0.0001242 265.1 369.7 0.717  1374547_at  Similar to RIKEN cDNA A130038L21  Info    
   3701 0.0001242 230 60.7 3.789  1369584_at  suppressor of cytokine signaling 3  Info   Socs3 
   3702 0.0001243 324.5 570 0.569  1372071_at  similar to 8D6 antigen (predicted)  Info   RGD1305860_predicted 
   3703 0.0001245 144.3 285.7 0.505  1386718_at  similar to aldo-keto reductase family 1, member C12  Info   LOC307096 
   3704 0.0001246 548.2 320.4 1.711  1374844_at  Transcribed locus, strongly similar to NP_006319.1 RNA binding motif protein 14; coactivator activator; SYT interacting protein [Homo sapiens]  Info    
   3705 0.0001247 7481.8 635.8 11.768  1379365_at  chemokine (C-X-C motif) ligand 11  Info   Cxcl11 
   3706 0.0001247 158.5 110.2 1.438  1372308_at  CDNA clone IMAGE:7366335  Info    
   3707 0.0001248 33 82.2 0.401  1376955_at  procollagen, type IV, alpha 4 (predicted)  Info   Col4a4_predicted 
   3708 0.0001248 153.5 248.9 0.617  1373996_at  Transcribed locus  Info    
   3709 0.000125 296.9 759.9 0.391  1374625_at  hairy and enhancer of split 6 (Drosophila) (predicted)  Info   Hes6_predicted 
   3710 0.0001251 273 421.5 0.648  1376997_at  Transcribed locus  Info    
   3711 0.0001253 854.4 586.8 1.456  1371657_at  ubiquitin-like 1 (sentrin) activating enzyme E1B (predicted)  Info   Uble1b_predicted 
   3712 0.0001253 169.7 258.3 0.657  1379516_at  similar to RIKEN cDNA 2310022B05  Info   LOC498967 
   3713 0.0001253 233.1 307.5 0.758  1373315_at  Similar to RIKEN cDNA 2210412D01 (predicted)  Info    
   3714 0.0001254 352.6 526.4 0.67  1371588_at  Parvin, alpha  Info   Parva 
   3715 0.0001257 86.3 31.2 2.766  1387621_at  tumor necrosis factor receptor superfamily, member 4  Info   Tnfrsf4 
   3716 0.0001258 248.8 168.7 1.475  1381836_at  similar to GTP-binding protein RAB20  Info   LOC502099 
   3717 0.0001259 3650.7 2582.3 1.414  1398626_s_at  ARP2 actin-related protein 2 homolog (yeast) (predicted)  Info   Actr2_predicted 
   3718 0.000126 510.6 665.9 0.767  1368075_at  lipase A, lysosomal acid  Info   Lipa 
   3719 0.0001261 262.8 142.3 1.847  1372173_at  similar to U5 snRNP-specific protein (Prp8-binding) (predicted)  Info   RGD1309198_predicted 
   3720 0.0001261 1794.3 1426.1 1.258  1388507_at  integrin beta 4 binding protein (predicted)  Info   Itgb4bp_predicted 
   3721 0.0001262 148.2 225.3 0.658  1375945_at  plexin domain containing 2 (predicted)  Info   Plxdc2_predicted 
   3722 0.0001265 76.8 140.3 0.547  1384310_at  Transcribed locus  Info    
   3723 0.0001267 76.3 128.2 0.595  1374419_at  Adenylate cyclase 9 (predicted)  Info    
   3724 0.0001272 35.7 90.5 0.394  1377437_at  similar to Veph-A (predicted)  Info   RGD1308217_predicted 
   3725 0.0001272 123.6 385 0.321  1376191_at  15-hydroxyprostaglandin dehydrogenase  Info   Hpgd 
   3726 0.0001273 120.2 273.2 0.44  1389678_at  Transcribed locus  Info    
   3727 0.0001279 156.6 263.5 0.594  1374475_at  abhydrolase domain containing 1 (predicted)  Info   Abhd1_predicted 
   3728 0.000128 172.9 103.2 1.675  1368231_at  signal transducer and activator of transcription 5A  Info   Stat5a 
   3729 0.0001284 644.7 750.3 0.859  1372150_at  Similar to mKIAA0190 protein  Info    
   3730 0.0001285 216.8 352.4 0.615  1383147_at  Transcribed locus  Info    
   3731 0.0001285 245.8 96 2.56  1395557_at  Core promoter element binding protein  Info   Copeb 
   3732 0.000129 354.2 197.9 1.79  1376766_at  formin-like 1 (predicted)  Info   Fmnl1_predicted 
   3733 0.0001292 396.7 719.1 0.552  1372149_at  AU RNA binding protein/enoyl-coenzyme A hydratase (predicted)  Info   Auh_predicted 
   3734 0.0001293 50 99.1 0.505  1389759_at    Info    
   3735 0.0001295 72 201.1 0.358  1370363_at  carboxylesterase 3  Info   Ces3 
   3736 0.0001295 95.3 188.1 0.507  1371356_at  tensin like C1 domain containing phosphatase (predicted)  Info   Tenc1_predicted 
   3737 0.0001295 153.9 32.7 4.706  1376255_at  mitogen activated protein kinase kinase kinase kinase 1 (predicted)  Info   Map4k1_predicted 
   3738 0.0001297 148.3 104.3 1.422  1391414_at    Info    
   3739 0.00013 14.7 38.2 0.385  1396337_at    Info    
   3740 0.00013 274.3 425.4 0.645  1372482_at  solute carrier family 39 (zinc transporter), member 13 (predicted)  Info   Slc39a13_predicted 
   3741 0.0001302 2092.1 1550.4 1.349  1372333_at  small nuclear ribonucleoprotein E (predicted) /// similar to small nuclear ribonucleoprotein E  Info   Snrpe_predicted /// LOC501075 
   3742 0.0001305 761.9 551 1.383  1371503_at  nucleotide binding protein 1 (predicted)  Info   Nubp1_predicted 
   3743 0.0001306 3876.6 5287.7 0.733  1371108_a_at  ATPase, Na+/K+ transporting, alpha 1 polypeptide  Info   Atp1a1 
   3744 0.0001307 227.1 105.8 2.147  1387429_at  adrenergic receptor kinase, beta 1  Info   Adrbk1 
   3745 0.0001309 447 350.2 1.276  1392858_at  nuclear cap binding protein subunit 2 (predicted)  Info   Ncbp2_predicted 
   3746 0.000131 83 54.8 1.515  1377625_at  similar to CGI-09 protein (predicted)  Info   RGD1308877_predicted 
   3747 0.000131 213.3 329.3 0.648  1373539_at  Transcribed locus  Info    
   3748 0.0001311 40.2 75.3 0.534  1378547_at  Transcribed locus  Info    
   3749 0.0001311 528.8 857.9 0.616  1385679_at  Transcribed locus  Info    
   3750 0.0001311 15.5 28.5 0.544  1379943_at  Transcribed locus  Info    
   3751 0.0001312 76 36.4 2.088  1369198_at  apoptotic peptidase activating factor 1  Info   Apaf1 
   3752 0.0001315 89.1 18.8 4.739  1378386_at  solute carrier family 2 (facilitated glucose transporter), member 6 (predicted)  Info   Slc2a6_predicted 
   3753 0.0001316 190.6 303.6 0.628  1386166_at  ATPase, H+ transporting, V1 subunit G, isoform 3 (predicted)  Info   Atp6v1g3_predicted 
   3754 0.0001316 193.3 342.1 0.565  1385512_at  Transcribed locus, strongly similar to NP_705811.8 APG4-D protein [Mus musculus]  Info    
   3755 0.0001318 152.4 123 1.239  1391811_at  Transcribed locus  Info    
   3756 0.0001318 174.2 271.3 0.642  1379323_at  similar to hypothetical protein MGC14327 (predicted)  Info   RGD1306304_predicted 
   3757 0.0001321 335.5 201 1.669  1387624_at  upstream transcription factor 1  Info   Usf1 
   3758 0.0001324 102.7 66.4 1.547  1391849_at  Similar to MBD2 (methyl-CpG-binding protein)-interacting zinc finger protein (predicted)  Info    
   3759 0.0001325 98.4 164.6 0.598  1383553_at  similar to CDNA sequence BC034204 (predicted)  Info   RGD1311293_predicted 
   3760 0.0001326 108.5 188.2 0.577  1379909_at  G kinase anchoring protein 1 (predicted)  Info   Gkap1_predicted 
   3761 0.0001326 307.6 217.6 1.414  1399084_at  DEAH (Asp-Glu-Ala-His) box polypeptide 16  Info   RGD:1302963 
   3762 0.0001327 1362 635.3 2.144  1389408_at  ribonucleotide reductase M2  Info   Rrm2 
   3763 0.0001328 369.6 210.3 1.757  1372519_at  nucleoporin 93 (predicted)  Info   Nup93_predicted 
   3764 0.0001331 931.8 1309.5 0.712  1367829_at  enoyl Coenzyme A hydratase, short chain, 1, mitochondrial  Info   Echs1 
   3765 0.0001331 109.6 207.6 0.528  1376867_at  similar to RIKEN cDNA 1110067D22 (predicted)  Info   RGD1307414_predicted 
   3766 0.0001333 301.3 124.8 2.414  1383369_at  tripartite motif protein 26  Info   RGD:1359126 
   3767 0.0001333 28.9 54 0.535  1389467_at  similar to RIKEN cDNA 1810057C19  Info   MGC108778 
   3768 0.0001334 450.3 617.2 0.73  1372504_at    Info   LOC502017 /// LOC502540 
   3769 0.0001334 994.3 638.7 1.557  1382521_at  glutaminase  Info   Gls 
   3770 0.0001337 531.8 401.1 1.326  1389080_at  zinc responsive protein ZD7  Info   RGD:1359343 
   3771 0.000134 116.8 76.2 1.533  1376567_at  FtsJ homolog 2 (E. coli) (predicted)  Info    
   3772 0.0001342 174.1 95.9 1.815  1376990_at  Similar to HCDI protein (predicted)  Info    
   3773 0.0001344 44.4 130.1 0.341  1376222_at  similar to HCDI protein (predicted)  Info   RGD1309307_predicted 
   3774 0.0001344 680.1 1048.5 0.649  1384145_at  Similar to KIAA1919 protein (predicted)  Info    
   3775 0.0001346 261.7 352.7 0.742  1371551_at  Tnf receptor associated factor 4 (predicted)  Info   Traf4_predicted 
   3776 0.0001347 29.8 48 0.621  1378078_at  Neuronal PAS domain protein 1 (predicted)  Info    
   3777 0.0001348 4.4 33.8 0.13  1393443_a_at  similar to CGI-112 protein (predicted)  Info   RGD1308113_predicted 
   3778 0.0001349 124.2 200.6 0.619  1373427_at  Ras-related GTP binding D (predicted)  Info   Rragd_predicted 
   3779 0.000135 559 959.9 0.582  1372672_at  quinolinate phosphoribosyltransferase (predicted)  Info   Qprt_predicted 
   3780 0.0001351 550.1 305.7 1.799  1372550_at    Info    
   3781 0.0001351 87.1 160.8 0.542  1370828_at  zinc finger, DHHC domain containing 2  Info   Zdhhc2 
   3782 0.0001352 1447.4 2055.8 0.704  1367929_at  CD59 antigen  Info   Cd59 
   3783 0.0001354 286.8 487.6 0.588  1373922_at  TPR domain, ankyrin-repeat and coiled-coil-containing  Info   Tanc 
   3784 0.0001354 59.3 3.4 17.441  1386275_at  Transcribed locus  Info    
   3785 0.0001356 39 68.7 0.568  1369222_at  potassium channel subunit (Slack)  Info   RGD:621106 
   3786 0.0001359 205.2 275.3 0.745  1374920_at  Transcribed locus  Info    
   3787 0.000136 77 132.5 0.581  1379043_at  GH regulated TBC protein 1 (predicted)  Info    
   3788 0.0001362 229.8 343.2 0.67  1384238_at  Similar to Tweety homolog 2  Info    
   3789 0.0001362 114.9 39.6 2.902  1378033_at    Info    
   3790 0.0001367 293 500.9 0.585  1375349_at  Transcribed locus, strongly similar to NP_033192.1 sorbin and SH3 domain containing 1 [Mus musculus]  Info    
   3791 0.0001368 1527.1 1267 1.205  1398794_at  transcription elongation factor B (SIII), polypeptide 1  Info   Tceb1 
   3792 0.000137 365.1 499 0.732  1373825_at  Hypothetical LOC314717 (predicted)  Info    
   3793 0.0001371 379.8 288 1.319  1389125_at  mitochondrial ribosomal protein L1 (predicted)  Info   Mrpl1_predicted 
   3794 0.0001371 127.3 280.2 0.454  1369184_at  claudin 16  Info   Cldn16 
   3795 0.0001371 81.7 9.1 8.978  1369852_at  coagulation factor X  Info   F10 
   3796 0.0001371 79.3 210.8 0.376  1369651_at  thymus cell antigen 1, theta  Info   Thy1 
   3797 0.0001372 208.7 146.1 1.428  1388684_at  formin binding protein 4 (predicted)  Info   Fnbp4_predicted 
   3798 0.0001373 121.6 246.9 0.493  1395316_at  DEAD (Asp-Glu-Ala-Asp) box polypeptide 21a (predicted) /// melanoma antigen, family H, 1  Info   Ddx21a_predicted /// RGD:1359316 
   3799 0.0001375 486.9 688.2 0.707  1374584_at  similar to Serine/threonine protein kinase 24  Info   LOC361092 
   3800 0.0001375 178.8 315.5 0.567  1371024_at  cut-like 1 (Drosophila)  Info   Cutl1 
   3801 0.0001377 127.8 76.1 1.679  1393955_at  WD repeat domain 44  Info   Wdr44 
   3802 0.0001379 120.6 65 1.855  1374313_at  BPY2 interacting protein 1 (predicted)  Info   Bpy2ip1_predicted 
   3803 0.0001379 227 151.9 1.494  1370159_at  SWI/SNF related, matrix associated, actin dependent regulator of chromatin, subfamily d, member 2  Info   Smarcd2 
   3804 0.0001379 61 25.2 2.421  1385733_at  exonuclease 1 (predicted)  Info   Exo1_predicted 
   3805 0.0001379 4383.4 3899.2 1.124  1370253_at  ribosomal protein L22  Info   Rpl22 
   3806 0.000138 133.9 75.7 1.769  1374629_at  mediator of RNA polymerase II transcription, subunit 8 homolog (yeast) (predicted)  Info   Med8_predicted 
   3807 0.000138 40.1 76.2 0.526  1371020_at  RIM binding protein 2  Info   Rimbp2 
   3808 0.000138 48.9 78.1 0.626  1377197_at  Transcribed locus  Info    
   3809 0.0001381 81.4 167.1 0.487  1370573_at  sarcosine dehydrogenase  Info   Sardh 
   3810 0.0001382 406.6 549.7 0.74  1388634_at  phosphoglucomutase 1  Info   Pgm1 
   3811 0.0001383 481.3 607.3 0.793  1376086_at  similar to RIKEN cDNA 1810047C23 (predicted)  Info   RGD1311161_predicted 
   3812 0.0001387 148.5 89.6 1.657  1384330_at  similar to hypothetical protein FLJ20546  Info   RGD:1359513 
   3813 0.0001388 1390.9 2309.3 0.602  1368209_at  membrane-associated protein 17  Info   Map17 
   3814 0.0001393 63.2 98.9 0.639  1394056_at  CDNA clone MGC:124727 IMAGE:7936966  Info    
   3815 0.0001394 67.6 4.5 15.022  1388069_at  killer cell lectin-like receptor subfamily B member 1B  Info   Klrb1b 
   3816 0.0001394 299.4 158.1 1.894  1368589_at  protein tyrosine phosphatase, receptor type, J  Info   Ptprj 
   3817 0.0001395 724.5 526.4 1.376  1388771_at  CGG triplet repeat binding protein 1 (predicted)  Info    
   3818 0.0001395 230.7 351.1 0.657  1383129_at  CG6210-like  Info   RGD:735139 
   3819 0.0001396 341.5 232.4 1.469  1377959_at  similar to RIKEN cDNA 2510049I19  Info   LOC498600 
   3820 0.0001396 3.5 14.6 0.24  1392218_at  Similar to C14orf25 protein  Info    
   3821 0.0001397 99.8 58.3 1.712  1390585_at  mannan-binding lectin serine peptidase 1  Info   Masp1 
   3822 0.00014 275.9 169.6 1.627  1373772_at  DNA (cytosine-5-)-methyltransferase 1  Info   Dnmt1 
   3823 0.00014 474.5 283.7 1.673  1372019_at  similar to DNA segment, Chr 13, Wayne State University 50, expressed (predicted)  Info   RGD1310128_predicted 
   3824 0.00014 5140.4 3642.9 1.411  1369952_at  poly(A) binding protein, cytoplasmic 1  Info   Pabpc1 
   3825 0.00014 158.9 294.8 0.539  1367644_at    Info    
   3826 0.0001402 116.1 219 0.53  1383838_at    Info    
   3827 0.0001404 215.2 132 1.63  1380794_at  Aquarius (predicted)  Info    
   3828 0.0001404 536.5 333.8 1.607  1368199_at  nucleoporin 88  Info   Nup88 
   3829 0.0001407 632.7 1432 0.442  1387783_a_at  acetyl-Coenzyme A acyltransferase 1 (peroxisomal 3-oxoacyl-Coenzyme A thiolase)  Info   Acaa1 
   3830 0.0001407 46.5 73.6 0.632  1374159_at  Similar to RN49018 (predicted)  Info    
   3831 0.0001408 18 27.7 0.65  1375597_at  Inner membrane protein, mitochondrial (predicted)  Info    
   3832 0.0001409 26.2 54.4 0.482  1379463_at  filamin A interacting protein 1  Info   Filip1 
   3833 0.000141 81.7 39.5 2.068  1398062_at  similar to hypothetical protein AL133206 (predicted)  Info   RGD1308418_predicted 
   3834 0.000141 175.8 370.3 0.475  1388592_at  Transcribed locus  Info    
   3835 0.0001412 66 118.6 0.556  1390161_at  similar to cysteine and tyrosine-rich protein 1  Info   RGD:1359578 
   3836 0.0001413 334.8 209.8 1.596  1384935_at  Cleavage stimulation factor, 3' pre-RNA, subunit 3, 77kDa (predicted)  Info    
   3837 0.0001414 348.5 171.6 2.031  1395840_at  eukaryotic translation initiation factor 1A (predicted)  Info   Eif1a_predicted 
   3838 0.0001414 142 208.7 0.68  1383065_at  CDNA clone MGC:124727 IMAGE:7936966  Info    
   3839 0.0001415 140.7 185.2 0.76  1371744_at  Transcribed locus, strongly similar to NP_057481.2 TH1-like protein; TH1 drosophila homolog; negative elongation factor proteins C and D; TH1-like (Drosophila homolog) [Homo sapiens]  Info    
   3840 0.0001416 91.9 167 0.55  1380610_at  Transcribed locus  Info    
   3841 0.0001416 144.2 248.1 0.581  1374747_at  PFTAIRE protein kinase 1 (predicted)  Info    
   3842 0.0001417 654.8 421 1.555  1373024_at  adaptor-related protein complex 3, sigma 1 subunit (predicted)  Info   Ap3s1_predicted 
   3843 0.0001417 1161.7 1568.3 0.741  1379395_a_at  histidine triad nucleotide binding protein 2 (predicted)  Info   Hint2_predicted 
   3844 0.0001418 188.4 37.7 4.997  1370133_at  regulator of G-protein signaling 19  Info   Rgs19 
   3845 0.0001421 66.9 106.7 0.627  1368514_at  monoamine oxidase B  Info   Maob 
   3846 0.0001422 31.7 65.4 0.485  1382267_at  four jointed box 1 (Drosophila) (predicted)  Info   Fjx1_predicted 
   3847 0.0001425 326 414.4 0.787  1372839_at  mitochondrial ribosomal protein S35 (predicted)  Info   Mrps35_predicted 
   3848 0.0001425 370.1 237.9 1.556  1384295_at  heat shock protein hsp70-related protein  Info   RGD:1303296 
   3849 0.0001427 28.8 50.3 0.573  1369165_at  transient receptor potential cation channel, subfamily C, member 3  Info   Trpc3 
   3850 0.0001428 285.3 547.6 0.521  1374241_at  Transcribed locus  Info    
   3851 0.0001429 240.1 385.6 0.623  1387059_at  serine/threonine kinase 39, STE20/SPS1 homolog (yeast)  Info   Stk39 
   3852 0.000143 198.8 106.1 1.874  1376205_at  Transcribed locus  Info    
   3853 0.0001431 1188.1 519.8 2.286  1397808_at  Cathepsin C  Info   Ctsc 
   3854 0.0001432 1181.4 1038.7 1.137  1371709_at  mitochondrial ribosomal protein L3 (predicted)  Info   Mrpl3_predicted 
   3855 0.0001432 381.9 213.6 1.788  1384427_at  transformed mouse 3T3 cell double minute 2 (predicted)  Info   Mdm2_predicted 
   3856 0.0001435 47.9 112.2 0.427  1383814_at    Info    
   3857 0.0001437 439.4 265.9 1.653  1398367_at  speckle-type POZ protein (predicted)  Info   Spop_predicted 
   3858 0.0001439 603.8 921.5 0.655  1370921_at  secretory carrier membrane protein 3  Info   Scamp3 
   3859 0.0001441 483.2 849.7 0.569  1376852_at  methylcrotonoyl-Coenzyme A carboxylase 1 (alpha) (predicted)  Info   Mccc1_predicted 
   3860 0.0001442 254.5 363.1 0.701  1398383_at  cytochrome b-561 (predicted)  Info   Cyb561_predicted 
   3861 0.0001444 1502.1 2130.1 0.705  1373193_at    Info    
   3862 0.0001447 60.8 108.1 0.562  1389644_at  similar to CG16896-PA  Info   LOC299949 
   3863 0.0001448 50.8 14.7 3.456  1390948_at  pan hematopoietic expression (predicted)  Info   Phemx_predicted 
   3864 0.000145 21.7 44.6 0.487  1397835_at    Info    
   3865 0.0001452 5599.2 4167.3 1.344  1388271_at    Info    
   3866 0.0001453 295.4 491.9 0.601  1397535_at  membrane protein, palmitoylated 5 (MAGUK p55 subfamily member 5) (predicted)  Info   Mpp5_predicted 
   3867 0.0001454 63.1 31.9 1.978  1394390_at  Transcribed locus  Info    
   3868 0.0001454 59.2 105.2 0.563  1367813_at  protein phosphatase 1, regulatory (inhibitor) subunit 14A  Info   Ppp1r14a 
   3869 0.0001455 523.7 360 1.455  1375056_at  similar to OCIA domain containing 2  Info   LOC289589 
   3870 0.0001456 17.3 76 0.228  1384392_at  cytochrome P450, family 26, subfamily b, polypeptide 1  Info   Cyp26b1 
   3871 0.0001461 101.8 201.3 0.506  1378556_at  Ectonucleotide pyrophosphatase/phosphodiesterase 1  Info   Enpp1 
   3872 0.0001462 15.7 38.8 0.405  1397326_at  Catechol-O-methyltransferase  Info   Comt 
   3873 0.0001464 4156.5 2898.3 1.434  1367590_at  RAN, member RAS oncogene family  Info   Ran 
   3874 0.0001465 113.3 38.8 2.92  1368884_at  ectonucleoside triphosphate diphosphohydrolase 1  Info   Entpd1 
   3875 0.0001465 558.1 371.1 1.504  1370070_at  synaptojanin 1  Info   Synj1 
   3876 0.0001467 117.4 412.6 0.285  1374244_at  Ab2-060  Info    
   3877 0.0001471 140.3 50.9 2.756  1388848_at  similar to hypothetical protein MGC13251 (predicted)  Info   RGD1308350_predicted 
   3878 0.0001471 92.8 49.1 1.89  1382477_at  SUMO/sentrin specific protease 5 (predicted)  Info    
   3879 0.0001475 432.3 641.2 0.674  1373027_at    Info    
   3880 0.0001483 131.1 102.5 1.279  1369024_at  rabaptin, RAB GTPase binding effector protein 2  Info   Rabep2 
   3881 0.0001484 21.9 38 0.576  1395723_at  Transcribed locus, weakly similar to XP_621938.1 PREDICTED: hypothetical protein XP_621938 [Mus musculus]  Info    
   3882 0.0001485 549.8 924.5 0.595  1373078_at  Transcribed locus  Info    
   3883 0.0001485 505.6 768.2 0.658  1371739_at  Leucine zipper-EF-hand containing transmembrane protein 1  Info   Letm1 
   3884 0.0001487 60.4 189 0.32  1386641_at  Transcribed locus  Info    
   3885 0.0001489 315.5 208.5 1.513  1372875_at  Transcribed locus  Info    
   3886 0.0001489 706.3 457.1 1.545  1371839_at  similar to splicing factor, arginine/serine-rich 2  Info   RGD:1359422 
   3887 0.0001491 16.5 4.9 3.367  1384556_at  ATPase, class V, type 10A (predicted)  Info   Atp10a_predicted 
   3888 0.0001492 87 205 0.424  1374910_at    Info    
   3889 0.0001493 49 3.6 13.611  1376394_at  Transcribed locus  Info    
   3890 0.0001494 175.7 106 1.658  1391199_at  Fas (TNFRSF6)-associated via death domain  Info   Fadd 
   3891 0.0001494 108.1 178.4 0.606  1374982_at  protein phosphatase 1, regulatory (inhibitor) subunit 16A (predicted)  Info   Ppp1r16a_predicted 
   3892 0.0001495 85.2 148.8 0.573  1375966_at  Multimerin 2 (predicted)  Info    
   3893 0.0001495 216.3 100 2.163  1372200_at  G-protein signalling modulator 3 (AGS3-like, C. elegans)  Info   RGD:1303014 
   3894 0.0001497 181.3 316.5 0.573  1388698_at  extracellular matrix protein 1  Info   Ecm1 
   3895 0.0001498 548.8 839.2 0.654  1371531_at  Hypothetical LOC299944 (predicted)  Info    
   3896 0.0001499 693.8 854.1 0.812  1389481_at  similar to GI:13385412-like protein splice form I  Info   RGD:735065 
   3897 0.0001501 88.9 56.1 1.585  1392548_at  similar to mKIAA1604 protein  Info   LOC362153 
   3898 0.0001505 76.4 124.4 0.614  1391570_at  similar to mKIAA0843 protein  Info   LOC307395 
   3899 0.0001507 83.3 42.3 1.969  1397851_at  Similar to QUAKING isoform 6  Info   Qki 
   3900 0.0001511 70 128.5 0.545  1395523_at  Transcribed locus  Info    
   3901 0.0001513 72.5 118.6 0.611  1375353_at  Cdk5 and Abl enzyme substrate 1 (predicted)  Info    
   3902 0.0001516 108.3 49.9 2.17  1393803_at  caspase 8 associated protein 2 (predicted)  Info   Casp8ap2_predicted 
   3903 0.000152 2049.3 3299.2 0.621  1368047_at  solute carrier family 13 (sodium-dependent dicarboxylate transporter), member 3  Info   Slc13a3 
   3904 0.000152 322.9 520 0.621  1393990_at  zinc finger protein 503 (predicted)  Info   Zfp503_predicted 
   3905 0.000152 102.1 192.4 0.531  1375647_at  Transcribed locus  Info    
   3906 0.000152 290 412 0.704  1374101_at  TBC1 domain family, member 10 (predicted)  Info   Tbc1d10_predicted 
   3907 0.0001521 41.4 102.5 0.404  1384440_at  Similar to LRRGT00021  Info    
   3908 0.0001531 84.3 125 0.674  1374691_at  sulfotransferase family 5A, member 1 (predicted)  Info   Sult5a1_predicted 
   3909 0.0001532 406.5 505 0.805  1398316_at  similar to LEYDIG CELL TUMOR 10 KD PROTEIN  Info   RGD:735128 
   3910 0.0001535 11 51.5 0.214  1371731_at  Phosphofructokinase, liver, B-type  Info   Pfkl 
   3911 0.0001535 187.2 304.6 0.615  1369732_a_at  sialyltransferase 4B (beta-galactoside alpha-2,3-sialyltransferase)  Info   Siat4b 
   3912 0.0001536 203.4 448 0.454  1374195_at  ladinin (predicted)  Info   Lad1_predicted 
   3913 0.0001538 45.9 104.8 0.438  1393992_at  homeo box A7  Info   Hoxa7 
   3914 0.0001541 76.4 132.3 0.577  1370420_at  steroid 5 alpha-reductase 1  Info   Srd5a1 
   3915 0.0001542 21.6 45 0.48  1396460_at  Transcribed locus, strongly similar to NP_780301.1 ras homolog gene family, member f [Mus musculus]  Info    
   3916 0.0001544 2180.9 1616.9 1.349  1372277_at  Similar to intermediate filament-like protein MGC:2625 isoform 2; HOM-TES-103 tumor antigen-like (predicted)  Info   Tas1r2 
   3917 0.0001544 83.6 41.9 1.995  1392886_a_at  similar to FCRL (predicted)  Info   RGD1306176_predicted 
   3918 0.0001549 23.8 50.3 0.473  1387209_at  regucalcin gene promotor region related protein  Info   Rgpr 
   3919 0.0001555 228.8 445.3 0.514  1389809_at  Transcribed locus  Info    
   3920 0.0001555 61.3 95.5 0.642  1394459_at  Similar to Protein CXorf17 homolog  Info    
   3921 0.0001555 60.3 132.7 0.454  1395073_at  ecotropic viral integration site 1 (predicted)  Info   Evi1_predicted 
   3922 0.0001559 96.6 46.3 2.086  1373782_a_at  similar to LOC495800 protein  Info   LOC499770 
   3923 0.0001559 26.7 102.3 0.261  1398265_at  ATP-binding cassette, sub-family C (CFTR/MRP), member 9  Info   Abcc9 
   3924 0.0001563 2475.3 1981.2 1.249  1374385_at  similar to Poly(rC)-binding protein 1 (Alpha-CP1) (hnRNP-E1)  Info   LOC500242 
   3925 0.0001575 313.4 397.5 0.788  1395725_at  similar to intracellular membrane-associated calcium-independent phospholipase A2 gamma (predicted)  Info   RGD1311444_predicted 
   3926 0.0001576 139 73.1 1.902  1371928_at  cell division cycle associated 8  Info   Cdca8 
   3927 0.0001576 422.1 310.3 1.36  1370112_at  phosphatase and tensin homolog  Info   Pten 
   3928 0.0001576 10 23.4 0.427  1392583_at  Transcribed locus  Info    
   3929 0.0001578 124.5 78.4 1.588  1379025_at  retinoblastoma-associated factor 600  Info   Rbaf600 
   3930 0.0001579 47 78 0.603  1379781_at  Similar to BAF53a (predicted)  Info    
   3931 0.0001579 159.2 91.3 1.744  1368129_at  Scm-like with four mbt domains 1  Info   Sfmbt1 
   3932 0.0001581 543.9 340.9 1.595  1372863_at  pam, highwire, rpm 1 (predicted)  Info   Phr1_predicted 
   3933 0.0001587 228.3 135.1 1.69  1388707_at  Transcribed locus  Info    
   3934 0.0001591 413.9 255.9 1.617  1388363_at  hnRNP-associated with lethal yellow (predicted)  Info   Raly_predicted 
   3935 0.0001591 551.6 720.3 0.766  1398317_at  3'(2'),5'-bisphosphate nucleotidase  Info   Bpnt1 
   3936 0.0001592 606 437.2 1.386  1383089_at  RAB21, member RAS oncogene family  Info   RGD:1303150 
   3937 0.0001595 34.6 97.6 0.355  1380088_at  Transcribed locus  Info    
   3938 0.0001595 170.7 77.7 2.197  1376697_at  carbohydrate sulfotransferase 12 (predicted)  Info   Chst12_predicted 
   3939 0.0001595 418.9 253.1 1.655  1383349_at  CDNA clone IMAGE:7304849  Info    
   3940 0.0001601 51.2 107.1 0.478  1394850_at    Info    
   3941 0.0001601 549.5 881.8 0.623  1385739_at  Transcribed locus  Info    
   3942 0.0001601 623 944.5 0.66  1383240_at  Integrin, alpha 6  Info   Itga6 
   3943 0.0001603 652.4 1105.2 0.59  1390416_at  solute carrier family 25, member 30  Info   RGD:1359702 
   3944 0.0001604 80.1 159.9 0.501  1384361_at  mal, T-cell differentiation protein 2  Info   Mal2 
   3945 0.0001605 2060.1 2800.8 0.736  1371380_at  pyruvate dehydrogenase E1 alpha 1  Info   Pdha1 
   3946 0.0001605 222.9 152.3 1.464  1392503_at  RNA binding motif protein 22 (predicted)  Info   Rbm22_predicted 
   3947 0.0001605 101.6 174.9 0.581  1387004_at  neuroblastoma, suppression of tumorigenicity 1  Info   Nbl1 
   3948 0.0001607 75.2 41.9 1.795  1395654_at  Similar to RIKEN cDNA 2610528H13 (predicted)  Info    
   3949 0.0001609 403.4 266.5 1.514  1375554_at  DEAD (Asp-Glu-Ala-Asp) box polypeptide 47 (predicted)  Info   Ddx47_predicted 
   3950 0.0001609 279.2 378.1 0.738  1387875_at  PTK2 protein tyrosine kinase 2  Info   Ptk2 
   3951 0.0001615 1203.5 2284.5 0.527  1398514_at  homogentisate 1, 2-dioxygenase (predicted)  Info   Hgd_predicted 
   3952 0.0001617 194.3 277.1 0.701  1398432_at  similar to Ankyrin repeat domain protein 28  Info   LOC306264 
   3953 0.0001619 57.1 118 0.484  1373789_at  Similar to zinc finger protein 651  Info    
   3954 0.0001627 514.9 996.1 0.517  1382127_at    Info    
   3955 0.000163 541.2 682.5 0.793  1371715_at  LOC500651  Info   MGC112883 
   3956 0.0001631 1.7 11.2 0.152  1386308_at  Transcribed locus  Info    
   3957 0.0001631 57.7 25.9 2.228  1397669_at  Chemokine (C-C motif) receptor 6 (predicted)  Info    
   3958 0.0001633 129.6 67.4 1.923  1374355_at  similar to MGC47262 protein  Info   LOC315283 
   3959 0.0001636 109.5 198.7 0.551  1376498_at  similar to 2900002H16Rik protein (predicted)  Info   RGD1307973_predicted 
   3960 0.0001639 1080 1505.1 0.718  1368399_a_at  plasma glutamate carboxypeptidase  Info   Pgcp 
   3961 0.000164 354.1 614.5 0.576  1393015_at  similar to hypothetical protein FLJ14146 (predicted)  Info   RGD1310587_predicted 
   3962 0.0001643 2151.3 1796.1 1.198  1388378_at  eukaryotic translation initiation factor 3, subunit 8, 110kDa (predicted)  Info   Eif3s8_predicted 
   3963 0.0001643 228.5 465.5 0.491  1369450_at  integral membrane transport protein UST5r  Info   UST5r 
   3964 0.0001644 822.7 686.2 1.199  1372209_at  COP9 (constitutive photomorphogenic) homolog, subunit 5 (Arabidopsis thaliana) (predicted)  Info   Cops5_predicted 
   3965 0.0001646 3363.4 2091.5 1.608  1398757_at  nucleophosmin 1  Info   Npm1 
   3966 0.0001646 89.6 72.6 1.234  1389443_at    Info    
   3967 0.000165 254.2 409.3 0.621  1398355_at  Similar to transient receptor potential phospholipase C interacting kinase  Info   Trpm7 
   3968 0.000165 67.4 98.6 0.684  1378433_at  similar to RIKEN cDNA 5730557B15  Info   LOC310200 
   3969 0.0001653 334.6 710.1 0.471  1389253_at  vanin 1 (predicted)  Info   Vnn1_predicted 
   3970 0.0001659 477.3 341.5 1.398  1388787_at  Similar to hypothetical protein FLJ13855 (predicted)  Info    
   3971 0.0001662 184.3 351.3 0.525  1370043_at  activated leukocyte cell adhesion molecule  Info   Alcam 
   3972 0.0001662 569.1 1862 0.306  1387336_at  N-acetyltransferase 8 (camello like)  Info   Nat8 
   3973 0.0001662 70.4 30.4 2.316  1393655_at  RAD54 like (S. cerevisiae) (predicted)  Info   Rad54l_predicted 
   3974 0.0001663 62.9 120.3 0.523  1388145_at  tenascin XA  Info   Tnxa 
   3975 0.0001663 248.2 183.1 1.356  1367541_at  similar to 2810410A08Rik protein  Info   LOC502632 
   3976 0.0001667 199.1 126 1.58  1378293_at  tripartite motif protein 26  Info   RGD:1359126 
   3977 0.000167 120 55.1 2.178  1383581_at    Info    
   3978 0.0001673 183.9 37.8 4.865  1384765_at    Info    
   3979 0.0001673 1266.6 1056.8 1.199  1398869_at  proteasome (prosome, macropain) 26S subunit, ATPase, 4  Info   Psmc4 
   3980 0.0001675 3508.4 2456.5 1.428  1370190_at  H3 histone, family 3B  Info   RGD:621095 
   3981 0.0001679 78.5 21.9 3.584  1398408_at    Info    
   3982 0.0001679 23.9 62.3 0.384  1382918_at  Transcribed locus  Info    
   3983 0.0001679 714.2 543.7 1.314  1382537_at  Ras-related GTP binding C (predicted)  Info   Rragc_predicted 
   3984 0.000168 22.6 45.4 0.498  1391240_at    Info    
   3985 0.0001683 350.6 722.9 0.485  1386917_at  Pyruvate carboxylase  Info   Pc 
   3986 0.0001683 315.7 583.7 0.541  1383047_at  growth arrest specific 6  Info   Gas6 
   3987 0.0001684 93.9 146.7 0.64  1387039_at  glypican 1  Info   Gpc1 
   3988 0.0001685 232.4 350.7 0.663  1388155_at  keratin complex 1, acidic, gene 18  Info   Krt1-18 
   3989 0.0001687 25.5 55.6 0.459  1391765_at  similar to RIKEN cDNA 4930449E07 (predicted)  Info   RGD1309150_predicted 
   3990 0.0001689 23.9 8.5 2.812  1398402_at  forkhead box P4 (predicted)  Info   Foxp4_predicted 
   3991 0.000169 67.1 93.5 0.718  1390561_at  Transcribed locus  Info    
   3992 0.0001693 347.9 603.8 0.576  1367839_at  farnesyl diphosphate farnesyl transferase 1  Info   Fdft1 
   3993 0.0001695 214.1 302.8 0.707  1385423_at  inner membrane protein, mitochondrial (predicted)  Info   Immt_predicted 
   3994 0.0001696 90.4 38.6 2.342  1392079_at  A kinase (PRKA) anchor protein 7  Info   RGD:1303071 
   3995 0.0001696 88.3 55 1.605  1376353_at  ARP5 actin-related protein 5 homolog (yeast) (predicted)  Info   Actr5_predicted 
   3996 0.0001697 32.5 101.7 0.32  1373778_at  Acetyl-Coenzyme A carboxylase beta  Info   Acacb 
   3997 0.0001697 79.7 154.7 0.515  1388831_at  Solute carrier family 9 (sodium/hydrogen exchanger), isoform 3 regulator 2  Info   Slc9a3r2 
   3998 0.0001702 22.3 54.6 0.408  1393279_at    Info    
   3999 0.0001704 78.5 167.3 0.469  1381937_at  similar to 60S ribosomal protein L7a  Info   LOC499344 
   4000 0.0001705 1464 1811.7 0.808  1398894_at  Unknown (protein for MGC:72959)  Info   Commd3 
   4001 0.0001708 970.3 1324.3 0.733  1368272_at  glutamate oxaloacetate transaminase 1  Info   Got1 
   4002 0.0001709 1017.6 702.9 1.448  1371724_at  small fragment nuclease (predicted)  Info   Smfn_predicted 
   4003 0.0001711 96.3 144.3 0.667  1377007_at    Info    
   4004 0.0001712 157.8 106.8 1.478  1390650_at  pericentrin 1 (predicted)  Info   Pcnt1_predicted 
   4005 0.0001713 145 97.6 1.486  1389790_at  Transcribed locus  Info    
   4006 0.0001713 264.9 186 1.424  1377905_at  similar to RIKEN cDNA 2700023B17 (predicted)  Info   RGD1305695_predicted 
   4007 0.0001716 599.6 467.1 1.284  1370195_at    Info    
   4008 0.0001716 91.3 28.5 3.204  1377340_at  tissue factor pathway inhibitor 2  Info   Tfpi2 
   4009 0.0001717 6.9 31.7 0.218  1373333_at  similar to Microsomal signal peptidase 23 kDa subunit (SPase 22 kDa subunit) (SPC22/23)  Info   MGC109340 
   4010 0.000173 60.8 29.4 2.068  1384087_at    Info    
   4011 0.0001736 275 423.2 0.65  1374156_at  membrane protein, palmitoylated 5 (MAGUK p55 subfamily member 5) (predicted)  Info   Mpp5_predicted 
   4012 0.0001736 554.3 876.2 0.633  1384027_a_at  Transcribed locus  Info    
   4013 0.0001741 65.1 133.6 0.487  1377753_at  Transcribed locus  Info    
   4014 0.0001742 185.7 277.7 0.669  1372450_at    Info    
   4015 0.0001742 101.4 213.9 0.474  1374828_at  protein disulfide isomerase-associated 5  Info   RGD:1359236 
   4016 0.0001743 101.5 160.5 0.632  1371771_at  Similar to novel protein  Info    
   4017 0.0001744 26.1 63.7 0.41  1388958_a_at  solute carrier family 2 (facilitated glucose transporter), member 4  Info   Slc2a4 
   4018 0.0001746 210.8 130.4 1.617  1370136_at  lamin B receptor  Info   Lbr 
   4019 0.0001747 1446.9 3255 0.445  1390851_at    Info    
   4020 0.0001747 23.2 39.2 0.592  1380199_at  WD repeat domain 22 (predicted)  Info   Wdr22 
   4021 0.0001748 45.5 88.4 0.515  1375562_at  Transcribed locus  Info    
   4022 0.000175 452.6 672.4 0.673  1390845_at  Transcribed locus  Info    
   4023 0.0001752 33.7 3.5 9.629  1373257_at  similar to protein phosphatase 1, regulatory (inhibitory) subunit 1C; thymocyte ARPP; DNA segment, Chr 9, Brigham & Womens Genetics 1012 expressed (predicted)  Info   RGD1307215_predicted 
   4024 0.0001755 2.1 17.5 0.12  1368801_at  CXXC finger 4  Info   Cxxc4 
   4025 0.0001756 9.7 26.6 0.365  1393810_at  similar to RIKEN cDNA 1810073M12 (predicted)  Info   RGD1309181_predicted 
   4026 0.000176 1307.9 827.6 1.58  1389325_at  similar to programmed cell death 10  Info   RGD:1359329 
   4027 0.0001763 28.6 60.4 0.474  1374821_at  RAB3A interacting protein  Info   RABIN3 
   4028 0.0001764 80.9 37.8 2.14  1385269_s_at  hypothetical protein LOC304743  Info   LOC304743 
   4029 0.0001765 91.2 42.7 2.136  1393822_at  Transcribed locus  Info    
   4030 0.0001767 73.9 114.2 0.647  1386979_at  developmentally regulated protein TPO1  Info   Tpo1 
   4031 0.0001768 74.4 47.3 1.573  1379488_at  TP53 regulating kinase (predicted)  Info   Trp53rk_predicted 
   4032 0.000177 68.3 113.8 0.6  1373118_at    Info    
   4033 0.0001773 198.5 407.6 0.487  1375997_at  Transcribed locus  Info    
   4034 0.0001775 1353.8 1004.8 1.347  1388645_at    Info    
   4035 0.0001776 383.7 530.2 0.724  1380546_at  similar to hypothetical protein FLJ10986 /// similar to hypothetical protein FLJ10986  Info   LOC298250 /// LOC298251 
   4036 0.0001785 60.4 92.8 0.651  1368614_at  Williams-Beuren syndrome chromosome region 14 homolog (human)  Info   Wbscr14 
   4037 0.0001789 1908.3 1531.6 1.246  1398954_at  aspartyl aminopeptidase  Info   Dnpep 
   4038 0.0001792 202.7 156.3 1.297  1371125_at  kinesin heavy chain member 2  Info   Kif2 
   4039 0.0001793 39.1 72 0.543  1381296_at  Arginase 2  Info   Arg2 
   4040 0.0001796 75.5 148 0.51  1393732_at  Transcribed locus  Info    
   4041 0.0001799 895.9 553.2 1.619  1372829_at  RNA binding motif protein 8 (predicted)  Info   Rbm8_predicted 
   4042 0.00018 231.6 167.7 1.381  1395730_at  glycyl-tRNA synthetase (predicted) /// similar to Glycyl-tRNA synthetase  Info   Gars_predicted /// LOC500135 
   4043 0.00018 142.4 88.9 1.602  1367855_at  scavenger receptor class B, member 1  Info   Scarb1 
   4044 0.0001803 319.8 202.6 1.578  1384254_at  similar to OTU domain containing 1  Info   LOC498803 
   4045 0.0001804 884.3 1290.2 0.685  1370232_at  isovaleryl coenzyme A dehydrogenase  Info   Ivd 
   4046 0.0001805 199.3 89 2.239  1368194_at  1-acylglycerol-3-phosphate O-acyltransferase 4  Info   Agpat4 
   4047 0.0001805 1207.5 2066.9 0.584  1371518_at  Nidogen (entactin)  Info   Nid 
   4048 0.0001807 133 221.6 0.6  1392893_a_at  Transcribed locus  Info    
   4049 0.0001807 1330 1163.9 1.143  1398796_at  transmembrane trafficking protein 21  Info   Tmp21 
   4050 0.0001809 78.5 29.6 2.652  1397317_at  Transcribed locus  Info    
   4051 0.000181 55.5 138.6 0.4  1368159_at  ATP-binding cassette, sub-family B (MDR/TAP), member 6  Info   Abcb6 
   4052 0.0001813 428.3 631.8 0.678  1371558_at  nischarin (predicted)  Info   Nisch_predicted 
   4053 0.0001818 3934 2797 1.407  1388850_at  heat shock protein 1, alpha  Info   Hspca 
   4054 0.0001822 21.1 60.9 0.346  1392809_at  Transcribed locus  Info    
   4055 0.0001824 150.3 220.4 0.682  1390440_at  similar to mKIAA0267 protein  Info   LOC302863 
   4056 0.0001828 144.3 255.3 0.565  1395928_at  Nuclear receptor subfamily 1, group D, member 1  Info   Nr1d1 
   4057 0.000183 402.2 22.5 17.876  1385277_x_at  similar to hypothetical protein FLJ11354 (predicted)  Info   RGD1310093_predicted 
   4058 0.0001831 201.7 282.9 0.713  1399034_at  pecanex homolog (Drosophila) (predicted)  Info   Pcnx_predicted 
   4059 0.0001831 39.7 91.2 0.435  1384215_at    Info    
   4060 0.0001837 126.1 194.8 0.647  1397419_at  Similar to PALS2-alpha splice variant  Info    
   4061 0.0001838 72.5 123.7 0.586  1375569_at  glutamine synthetase 1  Info   Glul 
   4062 0.0001838 169.6 8.3 20.434  1398449_at  Transcribed locus  Info    
   4063 0.0001841 175.8 115.3 1.525  1372910_at  Villin (predicted)  Info    
   4064 0.0001842 1279.5 1002.7 1.276  1371848_at    Info    
   4065 0.0001845 1309.1 987 1.326  1370189_at  splicing factor, arginine/serine-rich 10 (transformer 2 homolog, Drosophila)  Info   Sfrs10 
   4066 0.0001845 77.3 129 0.599  1377085_at  carbonic anhydrase 7 (predicted)  Info   Car7_predicted 
   4067 0.0001846 254 162.5 1.563  1389315_at  similar to Git2 protein  Info   RGD:1359708 
   4068 0.0001847 52.2 35.4 1.475  1392168_at  vaccinia related kinase 1 (predicted)  Info   Vrk1_predicted 
   4069 0.0001849 83.9 29.5 2.844  1378733_at  Transcribed locus, moderately similar to XP_580018.1 PREDICTED: hypothetical protein XP_580018 [Rattus norvegicus]  Info    
   4070 0.0001849 106.2 62.2 1.707  1370414_at  Rab38, member of RAS oncogene family  Info   Rab38 
   4071 0.0001853 67.3 22.5 2.991  1390141_at  formyltetrahydrofolate synthetase domain containing 1 (predicted)  Info   Fthfsdc1_predicted 
   4072 0.0001855 210.9 136.3 1.547  1385873_at  Transcribed locus  Info    
   4073 0.0001855 1.6 8.3 0.193  1398633_at  Transcribed locus, strongly similar to XP_341142.2 PREDICTED: similar to KIAA1096 protein [Rattus norvegicus]  Info    
   4074 0.0001856 1841.2 1434.1 1.284  1371944_at  ubiquitin-conjugating enzyme E2L 3 (predicted)  Info   Ube2l3_predicted 
   4075 0.0001856 552.2 696.2 0.793  1367982_at  aminolevulinic acid synthase 1  Info   Alas1 
   4076 0.0001856 61.7 144.2 0.428  1390097_at  TSPY-like 4 (predicted)  Info   Tspyl4_predicted 
   4077 0.0001859 152.4 304.7 0.5  1376645_at  Similar to RIKEN cDNA 6330406I15 (predicted)  Info    
   4078 0.0001859 201.6 118.2 1.706  1383502_at  similar to mKIAA0259 protein  Info   LOC315969 
   4079 0.000186 188.5 62.2 3.031  1393776_at  Similar to lymphocyte antigen 6 complex, locus E  Info    
   4080 0.0001864 247.3 388.3 0.637  1398991_at  Similar to expressed sequence AA408877 (predicted)  Info    
   4081 0.0001864 74.2 37.7 1.968  1379659_at  BMP-2 inducible kinase  Info    
   4082 0.0001869 46.2 19.9 2.322  1385816_at  Transcribed locus, strongly similar to NP_780591.1 hypothetical protein LOC108900 [Mus musculus]  Info    
   4083 0.0001869 43.8 80.3 0.545  1374370_at    Info    
   4084 0.0001871 101.4 40.7 2.491  1380229_at  Transcribed locus  Info    
   4085 0.0001872 264 167.1 1.58  1370386_at  RuvB-like protein 1  Info   Ruvbl1 
   4086 0.0001872 99.8 140 0.713  1381043_at  Spermatogenesis associated, serine-rich 1  Info   Spats1 
   4087 0.0001874 338.1 583.2 0.58  1388705_at  similar to selenoprotein SelM  Info   LOC498398 
   4088 0.0001878 565.9 924.8 0.612  1373892_at  Transcribed locus  Info    
   4089 0.0001878 24.7 81.5 0.303  1393433_at  similar to Agrin (predicted)  Info   RGD1306592_predicted 
   4090 0.0001882 91.3 185.6 0.492  1369148_at  transcription factor 1  Info   Tcf1 
   4091 0.0001884 71.4 37.9 1.884  1391107_at  similar to 5-3 exonuclease (predicted)  Info   RGD1309713_predicted 
   4092 0.0001885 161.6 80.3 2.012  1374632_at  phosphatidylserine receptor (predicted)  Info   Ptdsr_predicted 
   4093 0.0001886 21.7 45.9 0.473  1370727_at  platelet-derived growth factor, D polypeptide  Info   Pdgfd 
   4094 0.0001887 497.1 351 1.416  1371737_at  tripartite motif protein 27 (predicted)  Info   Trim27_predicted 
   4095 0.0001888 27.4 56.3 0.487  1369292_at  hydroxysteroid (17-beta) dehydrogenase 1  Info   Hsd17b1 
   4096 0.0001889 117.3 203.8 0.576  1374466_at  Protein kinase C, epsilon  Info   Prkce 
   4097 0.0001893 189.9 110.6 1.717  1377137_at  tyrosyl-DNA phosphodiesterase 1 (predicted)  Info   Tdp1_predicted 
   4098 0.0001895 34 48.9 0.695  1388295_s_at  fibroblast growth factor receptor substrate 3 (predicted)  Info   Frs3_predicted 
   4099 0.0001895 285.7 190.8 1.497  1390208_at  HIV-1 Tat interactive protein 2 (predicted)  Info   Htatip2_predicted 
   4100 0.0001899 48.4 102.4 0.473  1370932_at  low density lipoprotein receptor-related protein 4  Info   Lrp4 
   4101 0.00019 324.6 132.5 2.45  1390142_at  Zinc finger, CW-type with coiled-coil domain 3 (predicted)  Info    
   4102 0.0001901 236.8 408.6 0.58  1368046_at  solute carrier family 31 (copper transporters), member 1  Info   RGD:620059 
   4103 0.0001904 300.9 549.1 0.548  1370509_at  pyruvate dehydrogenase phosphatase isoenzyme 2  Info   Pdp2 
   4104 0.0001906 38 71.4 0.532  1389430_at  Transcribed locus  Info    
   4105 0.0001907 733.6 524 1.4  1372082_at  Unknown (protein for MGC:72598)  Info   RGD:735063 
   4106 0.0001909 51.3 87.7 0.585  1378302_at    Info    
   4107 0.000191 192.8 142.5 1.353  1389200_at  bystin-like  Info   Bysl 
   4108 0.0001912 560.3 1134.3 0.494  1367570_at  transgelin  Info   Tagln 
   4109 0.0001914 63.6 16.4 3.878  1380930_at  similar to KIAA1607 protein  Info   LOC290569 
   4110 0.0001919 260.1 489.4 0.531  1383117_at  peroxisomal membrane protein 4  Info   Pxmp4 
   4111 0.0001919 160.4 98.1 1.635  1382515_at  cleavage and polyadenylation specific factor 2 (predicted)  Info   Cpsf2_predicted 
   4112 0.0001919 178.9 68 2.631  1376688_a_at  Transcribed locus, moderately similar to NP_705730.1 paired immunoglobin-like type 2 receptor alpha [Mus musculus]  Info    
   4113 0.0001921 821.9 1189.4 0.691  1367468_at  SCAN domain-containing 1 (predicted)  Info   Scand1_predicted 
   4114 0.0001929 144.8 261.5 0.554  1397698_at  Transcribed locus  Info    
   4115 0.000194 80.4 145.5 0.553  1391397_at  Transcribed locus  Info    
   4116 0.0001943 59.9 154.2 0.388  1391434_at  RAB3A, member RAS oncogene family  Info   Rab3a 
   4117 0.0001943 413.7 287 1.441  1372357_at  Transcribed locus  Info    
   4118 0.0001945 39.9 74.3 0.537  1391506_at  PHD finger protein 17 (predicted)  Info    
   4119 0.0001946 251.2 342.8 0.733  1388697_at  inositol polyphosphate-5-phosphatase A (predicted)  Info   Inpp5a_predicted 
   4120 0.0001952 69.5 127.8 0.544  1382184_at  similar to regulator of Fas-induced apoptosis /// Fas apoptotic inhibitory molecule  Info   LOC498224 /// RGD:1359282 
   4121 0.0001953 216.4 366.6 0.59  1375726_at  LIM domain only protein 7  Info   RGD:1302984 
   4122 0.0001957 106.6 62.3 1.711  1376842_at  Transcribed locus  Info    
   4123 0.0001959 114.7 199.1 0.576  1390818_at  ATPase, Na+/K+ transporting, beta 3 polypeptide  Info   Atp1b3 
   4124 0.000196 1537.6 1826.3 0.842  1370298_at  similar to hypothetical protein D11Ertd99e  Info   LOC498000 
   4125 0.0001965 163.3 125.1 1.305  1395555_at  golgi SNAP receptor complex member 1  Info   Gosr1 
   4126 0.0001965 10.8 24.7 0.437  1380355_s_at  solute carrier family 6 (neurotransmitter transporter), member 17  Info   Slc6a17 
   4127 0.0001965 90.4 157.5 0.574  1387503_at  carboxypeptidase N, polypeptide 1, 50kD  Info   Cpn1 
   4128 0.0001971 71.3 31.9 2.235  1393140_at    Info    
   4129 0.0001972 2432.8 1704.1 1.428  1387772_at  calmodulin 1  Info   Calm1 
   4130 0.0001979 1343.9 892.8 1.505  1395508_at  chaperonin subunit 5 (epsilon)  Info   Cct5 
   4131 0.000198 223.7 119 1.88  1390374_at  fibroblast growth factor receptor-like 1  Info   Fgfrl1 
   4132 0.0001981 20.7 54 0.383  1396057_at  Similar to actin monomer-binding protein (predicted)  Info    
   4133 0.0001982 112.8 169.6 0.665  1380017_at  Transcribed locus  Info    
   4134 0.000199 170.1 100.8 1.688  1389103_at  similar to RIKEN cDNA 2810037C03 (predicted)  Info   RGD1307397_predicted 
   4135 0.0001991 168.5 238.6 0.706  1385753_at  Similar to mKIAA0089 protein  Info    
   4136 0.0001992 2151.4 1754.9 1.226  1398892_at  Niemann Pick type C2  Info   Npc2 
   4137 0.0001992 2885.5 4018.4 0.718  1370278_at  ATP synthase, H+ transporting, mitochondrial F1 complex, delta subunit  Info   Atp5d 
   4138 0.0001994 717.1 570.8 1.256  1372401_at  N-acetylneuraminic acid synthase (sialic acid synthase) (predicted)  Info   Nans_predicted 
   4139 0.0001994 264 105.8 2.495  1387690_at  caspase 3  Info   Casp3 
   4140 2e-04 276.2 449.3 0.615  1390717_at  similar to chromosome 20 open reading frame 155 (predicted)  Info   RGD1311037_predicted 
   4141 0.0002001 35.7 82.8 0.431  1374048_at  neurturin  Info   Nrtn 
   4142 0.0002006 353.5 493.5 0.716  1383004_at    Info    
   4143 0.0002008 24.2 63.1 0.384  1379672_at  Transcribed locus  Info    
   4144 0.000201 26.3 66.3 0.397  1380852_at  hypothetical LOC294883 (predicted)  Info   RGD1305844_predicted 
   4145 0.000201 196.9 328.4 0.6  1368292_at  dynamin 1  Info   Dnm1 
   4146 0.0002013 16.2 38.3 0.423  1383084_at  Similar to hypothetical protein FLJ23033  Info    
   4147 0.0002014 406.2 540.5 0.752  1382008_at  similar to hypothetical protein FLJ11218 (predicted)  Info   RGD1309804_predicted 
   4148 0.0002014 777.9 1125.9 0.691  1371730_at  similar to RIKEN cDNA 1300002A08 (predicted)  Info   RGD1305466_predicted 
   4149 0.0002014 48.7 74.8 0.651  1383988_at    Info    
   4150 0.0002015 18.8 39.1 0.481  1380640_at  Transcribed locus  Info    
   4151 0.0002015 3173.8 1598.8 1.985  1399158_a_at  nucleophosmin 1  Info   Npm1 
   4152 0.0002016 1098.8 683 1.609  1369944_at  MARCKS-like protein  Info   Mlp 
   4153 0.0002016 322.2 225.6 1.428  1373305_at  sorting nexin 4 (predicted)  Info   Snx4_predicted 
   4154 0.0002017 66.8 107.3 0.623  1374868_at  Transcribed locus  Info    
   4155 0.0002019 51.3 98.5 0.521  1392551_at  Transcribed locus  Info    
   4156 0.0002021 134 169.3 0.791  1394627_at    Info    
   4157 0.0002022 116.6 159.6 0.731  1374037_at  Similar to 1110008L16Rik protein (predicted)  Info    
   4158 0.0002023 390.2 664.2 0.587  1399065_at    Info    
   4159 0.0002025 235.6 786.7 0.299  1387158_at  meprin 1 beta  Info   Mep1b 
   4160 0.0002027 56 109 0.514  1398647_at  Similar to hypothetical protein FLJ40283 (predicted)  Info    
   4161 0.0002029 172.8 386.7 0.447  1380045_at  pyruvate dehydrogenase phosphatase isoenzyme 2  Info   Pdp2 
   4162 0.0002029 239.6 325.4 0.736  1379314_at    Info    
   4163 0.0002033 36.2 112.9 0.321  1369492_at  arylacetamide deacetylase (esterase)  Info   Aadac 
   4164 0.0002034 176.5 78.4 2.251  1382727_at    Info    
   4165 0.0002036 209 289.3 0.722  1390145_at  Transcribed locus, strongly similar to XP_510406.1 PREDICTED: similar to rabconnectin-3 [Pan troglodytes]  Info    
   4166 0.0002038 525.7 359.1 1.464  1388863_at  similar to ubiquitin-conjugating enzyme HR6A  Info   RGD:1359534 
   4167 0.0002039 19.5 43.8 0.445  1385830_at  similar to hypothetical protein FLJ31606  Info   LOC292078 
   4168 0.0002042 641 374 1.714  1371988_at  mannosidase 1, alpha (predicted)  Info   Man1a_predicted 
   4169 0.0002044 138.9 94.6 1.468  1387098_at  RNA polymerase 1-4  Info   Rpo1-4 
   4170 0.0002044 94.7 139.8 0.677  1393100_at  similar to 1110008L16Rik protein (predicted)  Info   RGD1305089_predicted 
   4171 0.0002045 475.6 754.3 0.631  1373842_at  Type II keratin Kb1  Info   Kb1 
   4172 0.0002046 48 118.9 0.404  1398431_at  carbonic anhydrase 8 (predicted)  Info   Car8_predicted 
   4173 0.0002048 305.9 201.9 1.515  1378592_at  Similar to RIKEN cDNA 2310035M22 (predicted)  Info    
   4174 0.0002051 588 357.1 1.647  1369788_s_at  v-jun sarcoma virus 17 oncogene homolog (avian)  Info   Jun 
   4175 0.0002052 34.3 61.3 0.56  1392001_at  Pannexin 1  Info   Panx1 
   4176 0.0002053 168.7 373.1 0.452  1373497_at  Homeodomain interacting protein kinase 2 (predicted)  Info    
   4177 0.0002053 839.1 649.3 1.292  1383361_at    Info    
   4178 0.0002055 62.1 107.5 0.578  1391981_at  Transcribed locus  Info    
   4179 0.0002055 315 174.6 1.804  1374565_at  NIMA (never in mitosis gene a)-related expressed kinase 6  Info   Nek6 
   4180 0.0002056 315.8 394.2 0.801  1388136_at  translocase of inner mitochondrial membrane 9 homolog (yeast)  Info   Timm9 
   4181 0.0002057 57.4 116.6 0.492  1379044_at  Similar to RIKEN cDNA 1700108L22 (predicted)  Info    
   4182 0.0002058 125.4 186.6 0.672  1372103_at  DnaJ (Hsp40) homolog, subfamily A, member 4 (predicted)  Info    
   4183 0.0002059 44.4 90 0.493  1398304_at  frizzled homolog 2 (Drosophila)  Info   Fzd2 
   4184 0.0002062 80.1 178.5 0.449  1390449_at  CDNA clone IMAGE:7309127  Info    
   4185 0.0002064 102.4 198.3 0.516  1390727_at  Transcribed locus  Info    
   4186 0.0002067 141.7 212.7 0.666  1371635_at  transmembrane domain protein regulated in adipocytes  Info   Tpra40 
   4187 0.0002069 649 994.5 0.653  1377033_at  serine (or cysteine) proteinase inhibitor, clade F, member 2 (predicted)  Info   Serpinf2_predicted 
   4188 0.000207 2.3 15.2 0.151  1376388_at  Transcribed locus  Info    
   4189 0.0002074 36.9 106 0.348  1367996_a_at  latrophilin 1  Info   Lphn1 
   4190 0.0002077 212.1 125.4 1.691  1372014_at  similar to cofactor required for Sp1 transcriptional activation subunit 8  Info   LOC296612 
   4191 0.0002078 3844.4 5001.3 0.769  1371323_at    Info    
   4192 0.000208 2214.5 4738.7 0.467  1368180_s_at  glutathione-S-transferase, alpha type2  Info   Gsta2 
   4193 0.0002081 297.5 175.9 1.691  1382463_at  Transcribed locus  Info    
   4194 0.0002083 130.4 202.7 0.643  1377235_a_at  protein phosphatase 2, regulatory subunit B (B56), beta isoform  Info   Ppp2r5b 
   4195 0.0002086 105.3 8.1 13  1397292_at  LOC500333  Info   LOC500333 
   4196 0.0002087 79.4 175.5 0.452  1393808_at  Transcribed locus, strongly similar to NP_835187.1 fatty acid 2-hydroxylase [Mus musculus]  Info    
   4197 0.0002089 554.2 897.7 0.617  1383221_at  hypothetical LOC360478  Info   LOC360478 
   4198 0.0002096 192.7 342.9 0.562  1367648_at  insulin-like growth factor binding protein 2  Info   Igfbp2 
   4199 0.0002097 124.7 82.5 1.512  1382972_at  Transcribed locus, strongly similar to XP_226713.2 PREDICTED: similar to Src-associated protein SAW [Rattus norvegicus]  Info    
   4200 0.0002098 442.6 906.9 0.488  1389540_at  TG interacting factor (predicted)  Info   Tfig 
   4201 0.0002098 334.3 1057.8 0.316  1370336_at  pregnancy-induced growth inhibitor  Info   Okl38 
   4202 0.0002099 113.5 188.3 0.603  1378003_at  Transcribed locus  Info    
   4203 0.00021 4892.6 3385.7 1.445  1386861_at  H2A histone family, member Z  Info   H2afz 
   4204 0.0002101 317.4 514.1 0.617  1371043_a_at  POU domain, class 3, transcription factor 3  Info   Pou3f3 
   4205 0.0002103 166 72.9 2.277  1389440_at  Transcribed locus  Info    
   4206 0.0002104 37.2 78.3 0.475  1372708_at  Transcribed locus  Info    
   4207 0.0002107 27.6 53.4 0.517  1393212_at  Transcribed locus  Info    
   4208 0.0002108 61.4 30.7 2  1393755_at  phosphatidylinositol 3-kinase catalytic delta polypeptide (predicted)  Info   Pik3cd_predicted 
   4209 0.000211 322.9 212.6 1.519  1377756_at  similar to RIKEN cDNA 5830446M03 (predicted)  Info   RGD1307688_predicted 
   4210 0.0002111 3.7 20.8 0.178  1388062_at  extracellular signal-regulated kinase 7  Info   RGD:628675 
   4211 0.0002112 220.8 145.7 1.515  1388901_at  FK506 binding protein 5 (predicted)  Info   Fkbp5_predicted 
   4212 0.0002115 51.7 71 0.728  1386687_at  Transcribed locus, strongly similar to NP_666072.2 mitochondrial translational release factor 1 [Mus musculus]  Info    
   4213 0.0002117 215.4 326.7 0.659  1370176_at  amyotrophic lateral sclerosis 2 (juvenile) chromosome region, candidate 3 homolog (human)  Info   Als2cr3 
   4214 0.0002125 939 662.4 1.418  1388754_at    Info    
   4215 0.0002129 83.9 137.4 0.611  1370966_at  hyperpolarization activated cyclic nucleotide-gated potassium channel 2  Info   Hcn2 
   4216 0.0002132 11.5 25.6 0.449  1377508_at  Immunoglobulin superfamily, member 11 (predicted)  Info    
   4217 0.0002139 245.1 153.5 1.597  1399035_at  polymerase (RNA) III (DNA directed) polypeptide H (predicted)  Info   Polr3h_predicted 
   4218 0.000214 40.9 106.5 0.384  1387364_at  folate hydrolase  Info   Folh1 
   4219 0.0002141 360.8 266.4 1.354  1392461_at  PRP4 pre-mRNA processing factor 4 homolog (yeast) (predicted)  Info   Prpf4_predicted 
   4220 0.0002142 319.9 512.5 0.624  1392044_at  Transcribed locus  Info    
   4221 0.0002143 64.6 109.5 0.59  1382940_at    Info    
   4222 0.0002143 747.6 1096.5 0.682  1398902_at  Similar to mKIAA0664 protein (predicted)  Info    
   4223 0.0002145 774.2 1121.8 0.69  1387669_a_at  epoxide hydrolase 1  Info   Ephx1 
   4224 0.0002145 334.5 464.5 0.72  1375080_at  Similar to expressed sequence AW491445 (predicted)  Info    
   4225 0.0002146 997.4 3042 0.328  1368325_at  epidermal growth factor  Info   Egf 
   4226 0.0002148 48.9 81.2 0.602  1374896_at  Transcribed locus  Info    
   4227 0.0002148 619.1 381.1 1.625  1388024_s_at  lamina-associated polypeptide 1B  Info   Lap1b 
   4228 0.0002158 647.6 1298.4 0.499  1376765_at  similar to maestro  Info   LOC361348 
   4229 0.000216 270 469.1 0.576  1374636_at  PHD finger protein 17 (predicted)  Info    
   4230 0.0002162 53.3 7.4 7.203  1378883_at  Transcribed locus  Info    
   4231 0.0002165 59.4 97.6 0.609  1385828_at  Transcribed locus, strongly similar to XP_525786.1 PREDICTED: similar to chromosome 2 open reading frame 7 [Pan troglodytes]  Info    
   4232 0.0002165 303.3 203.7 1.489  1390205_at  zinc finger, SWIM domain containing 6 (predicted)  Info   Zswim6_predicted 
   4233 0.0002165 21.8 46.3 0.471  1395628_at  Transcribed locus  Info    
   4234 0.0002166 141.7 193.8 0.731  1374860_at    Info    
   4235 0.0002167 37.8 64.2 0.589  1391571_at  Transcribed locus  Info    
   4236 0.0002169 2183.1 3305.2 0.661  1388122_at  glutathione-S-transferase, pi 1 /// glutathione S-transferase, pi 2  Info   Gstp1 /// Gstp2 
   4237 0.000217 87.3 5.1 17.118  1393929_at  chemokine (C-C motif) receptor 6 (predicted)  Info   Ccr6_predicted 
   4238 0.0002175 454.7 324.3 1.402  1388354_at  similar to U5 snRNP-specific protein, 200 kDa  Info   LOC296126 
   4239 0.0002176 159.2 273.3 0.583  1373731_at  similar to phosphoseryl-tRNA kinase  Info   LOC361661 
   4240 0.000218 42.6 56.1 0.759  1374957_at  similar to ribosomal protein L27a  Info   LOC292474 
   4241 0.0002184 278.3 157.1 1.771  1389238_at  similar to PES1 protein  Info   LOC289740 
   4242 0.0002186 73.8 157.6 0.468  1378163_at  Transcribed locus  Info    
   4243 0.0002189 940.6 1264.7 0.744  1398928_at  divalent cation tolerant protein CUTA  Info   RGD:1303306 
   4244 0.0002196 277.6 150.9 1.84  1388135_at  replication protein A2  Info   Rpa2 
   4245 0.0002197 3027.8 2353.1 1.287  1368141_at  cellular nucleic acid binding protein 1  Info   Cnbp1 
   4246 0.0002199 18.2 41 0.444  1380884_at  Transcribed locus  Info    
   4247 0.00022 130.3 175 0.745  1376159_at  Transcribed locus  Info    
   4248 0.0002201 346.3 1019.9 0.34  1370147_at  2-amino-3-carboxymuconate-6-semialdehyde decarboxylase  Info   Acmsd 
   4249 0.0002201 311.2 445.1 0.699  1371752_at  similar to Ran-interacting protein MOG1  Info   LOC287419 
   4250 0.0002202 406.1 840 0.483  1387357_at  trimethyllysine hydroxylase, epsilon  Info   Tmlhe 
   4251 0.0002206 233.1 135.9 1.715  1374519_at    Info    
   4252 0.0002216 68.5 28.1 2.438  1387658_at  eukaryotic elongation factor-2 kinase  Info   Eef2k 
   4253 0.0002218 53.4 5.5 9.709  1384752_at  similar to osteoclast inhibitory lectin  Info   LOC362446 
   4254 0.0002222 67.1 116.8 0.574  1379683_at  Similar to hypothetical protein MGC17839  Info    
   4255 0.0002222 214.6 452.6 0.474  1373790_at  carbonic anhydrase 14 (predicted)  Info   Car14_predicted 
   4256 0.0002225 3460.4 2710.3 1.277  1367677_at  peroxiredoxin 5  Info   Prdx5 
   4257 0.0002225 128 226.4 0.565  1368453_at  fatty acid desaturase 2  Info   Fads2 
   4258 0.0002231 82.7 134.1 0.617  1368785_a_at  paired-like homeodomain transcription factor 2  Info   Pitx2 
   4259 0.000224 105.9 51.1 2.072  1392134_x_at  similar to KIAA0456 protein  Info   LOC360840 
   4260 0.0002242 100.5 136.6 0.736  1382234_at  Hypothetical protein LOC311254  Info    
   4261 0.0002245 20.8 99 0.21  1391485_at    Info    
   4262 0.0002248 242.3 371.4 0.652  1372617_at  similar to BTB (POZ) domain containing 2  Info   LOC500793 
   4263 0.0002248 687.6 1186.4 0.58  1373978_at  nuclear cap binding protein subunit 1, 80kDa (predicted)  Info   Ncbp1_predicted 
   4264 0.0002249 187.6 349 0.538  1373338_at  Transcribed locus  Info    
   4265 0.0002251 72.4 17.9 4.045  1387483_at  phospholipase C, gamma 2  Info   Plcg2 
   4266 0.0002251 430.2 636.6 0.676  1373634_at  Transcribed locus  Info    
   4267 0.0002256 131.6 225.7 0.583  1374376_at  Rho-guanine nucleotide exchange factor (predicted)  Info   Rgnef_predicted 
   4268 0.0002259 332.2 450.4 0.738  1369989_at  pyridoxine 5'-phosphate oxidase  Info   Pnpo 
   4269 0.0002262 7.4 1.6 4.625  1391653_at  gamma-aminobutyric acid A receptor, gamma 2  Info   Gabrg2 
   4270 0.0002263 71.3 99.1 0.719  1391206_at  Transcribed locus  Info    
   4271 0.0002264 260.1 382.1 0.681  1372772_at    Info    
   4272 0.0002264 56.2 86.9 0.647  1389307_at  similar to Amyloid beta (A4) precursor-like protein 1  Info   LOC502317 
   4273 0.0002267 44.5 91 0.489  1374160_at  Similar to RIKEN cDNA 1110063G11 (predicted)  Info    
   4274 0.0002269 634.3 386.6 1.641  1380346_at  serine (or cysteine) proteinase inhibitor, clade B, member 1a (predicted)  Info   Serpinb1a_predicted 
   4275 0.0002269 252.9 550.9 0.459  1388312_at  Transcribed locus  Info    
   4276 0.0002274 386.2 199.4 1.937  1372273_at  glycophorin C (Gerbich blood group) (predicted) /// similar to glycophorin C  Info   Gypc_predicted /// LOC498835 
   4277 0.0002274 139.5 78.4 1.779  1393162_at  solute carrier family 39 (metal ion transporter), member 6 (predicted)  Info   Slc39a6_predicted 
   4278 0.0002278 33.6 19 1.768  1382291_at  Similar to SRY sex determining region Y-box 9 protein  Info    
   4279 0.0002281 90 60.4 1.49  1384889_at  Transcribed locus  Info    
   4280 0.0002282 128.6 52.3 2.459  1391091_at  retinitis pigmentosa 1 (autosomal dominant) (predicted) /// similar to cylindromatosis (turban tumor syndrome)  Info   Rp1_predicted /// LOC361373 
   4281 0.0002289 95 214.1 0.444  1376285_at  GULP, engulfment adaptor PTB domain containing 1 (predicted)  Info   Gulp1_predicted 
   4282 0.0002291 340.6 224.1 1.52  1389576_at  U2 small nuclear ribonucleoprotein B (predicted)  Info   Snrpb2_predicted 
   4283 0.0002294 231.6 165.9 1.396  1378092_at  similar to magnesium-dependent phosphatase-1 (predicted)  Info   RGD1311147_predicted 
   4284 0.0002295 3591.4 4349.9 0.826  1370220_at  serine carboxypeptidase 1  Info   Scpep1 
   4285 0.0002304 298.5 194.6 1.534  1388700_at  CDNA clone IMAGE:7317367  Info    
   4286 0.0002305 146.8 217.2 0.676  1374688_at  Tial1 cytotoxic granule-associated RNA binding protein-like 1  Info   Tial1 
   4287 0.000231 171.2 279.3 0.613  1368078_at  endothelial cell-specific molecule 1  Info   Esm1 
   4288 0.0002319 1486.4 1021.8 1.455  1395561_at  heterogeneous nuclear ribonucleoprotein A2/B1 (predicted)  Info   Hnrpa2b1_predicted 
   4289 0.0002319 473.8 697.6 0.679  1372507_at  similar to RIKEN cDNA 9130410M22  Info   RGD:1359333 
   4290 0.000232 80.4 43.6 1.844  1391453_at  Transcribed locus, strongly similar to XP_576700.1 PREDICTED: similar to RIKEN cDNA 2900016D05 [Rattus norvegicus]  Info    
   4291 0.0002322 511.7 323.1 1.584  1372787_at  charged amino acid rich leucine zipper 1 (predicted)  Info   Crlz1_predicted 
   4292 0.0002324 3084.5 2632.9 1.172  1398860_at  neural precursor cell expressed, developmentally down-regulated gene 8  Info   Nedd8 
   4293 0.0002331 78.4 135.8 0.577  1389396_at    Info    
   4294 0.0002333 45.1 90.9 0.496  1382313_at  CDNA clone IMAGE:7379585  Info    
   4295 0.0002334 85.4 127.1 0.672  1377162_at  Transcribed locus  Info    
   4296 0.0002337 205.9 96.4 2.136  1388742_at    Info    
   4297 0.0002338 124.7 308.2 0.405  1384438_at  Transcribed locus, moderately similar to NP_080232.2 hypothetical protein LOC67082 [Mus musculus]  Info    
   4298 0.0002339 208.5 285 0.732  1380170_at  GABA(A) receptor-associated protein like 2  Info   Gabarapl2 
   4299 0.000234 94.2 155.9 0.604  1378088_at  similar to Zdhhc1 protein  Info   LOC291967 
   4300 0.0002344 667.9 995.7 0.671  1379264_at  zinc and ring finger 1 (predicted)  Info   Znrf1_predicted 
   4301 0.0002347 155.2 10.3 15.068  1370083_at  macrophage inflammatory protein-1 alpha receptor gene  Info   Ccr1 
   4302 0.000235 431.7 276.1 1.564  1372719_at  Transcribed locus  Info    
   4303 0.0002351 211.3 344.5 0.613  1385783_at  similar to BH3-only member B protein  Info   LOC498630 
   4304 0.0002352 18.1 35.1 0.516  1382869_at  Similar to hypothetical protein FLJ14547 (predicted)  Info    
   4305 0.0002356 153.4 35.9 4.273  1390221_at  Transcribed locus  Info    
   4306 0.0002358 45.6 121.8 0.374  1383824_at  LIM domain binding 2 (predicted)  Info   Ldb2_predicted 
   4307 0.0002361 38.1 62.2 0.613  1395781_at  v-myc myelocytomatosis viral oncogene homolog 1, lung carcinoma derived (avian)  Info   Mycl1 
   4308 0.0002361 14.1 33.6 0.42  1389806_at  Transcribed locus  Info    
   4309 0.0002365 189.3 311.5 0.608  1394957_at  nischarin (predicted)  Info   Nisch_predicted 
   4310 0.0002365 190.3 127.4 1.494  1372902_at  asparagine-linked glycosylation 12 homolog (yeast, alpha-1,6-mannosyltransferase) (predicted)  Info   Alg12_predicted 
   4311 0.0002365 579.8 410.7 1.412  1373955_at  karyopherin (importin) beta 3 (predicted)  Info   Kpnb3_predicted 
   4312 0.0002369 53.9 18.7 2.882  1384943_at  similar to DNA segment, Chr 16, ERATO Doi 472, expressed  Info   LOC360692 
   4313 0.0002369 1016.1 1469.3 0.692  1370879_at  dihydrolipoamide S-succinyltransferase (E2 component of 2-oxo-glutarate complex)  Info   RGD:1359615 
   4314 0.0002373 613.4 872.4 0.703  1388533_at  CTD (carboxy-terminal domain, RNA polymerase II, polypeptide A) small phosphatase 1 (predicted)  Info   Ctdsp1_predicted 
   4315 0.0002375 1220.2 1761.8 0.693  1375119_at  neural precursor cell expressed, developmentally down-regulated gene 4A  Info   Nedd4a 
   4316 0.0002376 1219.7 1487.6 0.82  1371474_at  mitochondrial carrier homolog 1 (C. elegans) (predicted)  Info   Mtch1_predicted 
   4317 0.0002379 4010.6 4886.4 0.821  1370238_at    Info    
   4318 0.0002384 935 660.6 1.415  1388745_at  sema domain, immunoglobulin domain (Ig), transmembrane domain (TM) and short cytoplasmic domain, (semaphorin) 4A (predicted)  Info   Sema4a_predicted 
   4319 0.0002387 353.7 239.4 1.477  1371524_at  gene trap locus 3 (predicted)  Info   Gtl3_predicted 
   4320 0.0002388 265.4 190 1.397  1367537_at  similar to Clast4 protein  Info   LOC305468 
   4321 0.000239 253.3 436.9 0.58  1376214_at  Similar to RIKEN cDNA 1810037K07 (predicted)  Info    
   4322 0.000239 77.5 148.1 0.523  1376989_at  Transcribed locus  Info    
   4323 0.0002392 59.5 22.5 2.644  1376188_at  Transcribed locus, strongly similar to NP_780541.1 hypothetical protein LOC103551 [Mus musculus]  Info    
   4324 0.0002394 329.5 551.1 0.598  1395518_at  Transcribed locus  Info    
   4325 0.0002394 74.2 109.9 0.675  1378002_at  osmotic stress protein 94 kDa (predicted)  Info   OSP94_predicted 
   4326 0.0002397 102.7 138.9 0.739  1379959_at  tryptophan rich basic protein  Info   Wrb 
   4327 0.0002404 5093.6 6461 0.788  1388113_at  cytochrome c oxidase, subunit VIIIa  Info   Cox8a 
   4328 0.0002406 12 33.6 0.357  1382438_at  Transcribed locus, moderately similar to NP_787088.2 hypothetical protein FLJ37266 [Homo sapiens]  Info    
   4329 0.0002407 136.4 219.3 0.622  1373137_at  Transcribed locus  Info    
   4330 0.0002411 394.8 730.3 0.541  1376878_at  similar to RIKEN cDNA 2310022B05  Info   LOC498967 
   4331 0.0002412 490.3 1006.3 0.487  1374871_at  asparaginase-like sperm autoantigen  Info   Asrgl1 
   4332 0.0002416 15.7 35.8 0.439  1380923_at  N-acylsphingosine amidohydrolase 3-like (predicted)  Info    
   4333 0.0002419 273.3 438.3 0.624  1377141_at  Similar to RIKEN cDNA 1110018J18  Info    
   4334 0.0002428 202.9 346.9 0.585  1371420_at  92Aa-Protein  Info   RGD:1359519 
   4335 0.0002432 312.4 210.7 1.483  1393183_at  similar to StAR-related protein 1-4E  Info   LOC502348 
   4336 0.000244 6.1 28.6 0.213  1369301_at  angiotensin receptor-like 1  Info   Agtrl1 
   4337 0.0002444 208.8 144.7 1.443  1393320_at  CDNA clone IMAGE:7375955  Info    
   4338 0.000245 1088.6 739.5 1.472  1372365_at  Transcribed locus  Info    
   4339 0.0002454 933.2 1278.2 0.73  1398360_at  CDNA clone MGC:93791 IMAGE:7109632  Info    
   4340 0.0002457 395.8 551.4 0.718  1375027_at  Cell cycle progression 1 (predicted)  Info    
   4341 0.0002458 185.7 249.7 0.744  1372650_at  similar to Dynamin binding protein (Scaffold protein Tuba)  Info   LOC309362 
   4342 0.0002459 30.9 49.6 0.623  1397278_at  Transcribed locus  Info    
   4343 0.0002459 379.1 502.1 0.755  1395532_at  Transcribed locus, strongly similar to NP_035873.1 SH3-domain binding protein 3 [Mus musculus]  Info    
   4344 0.0002461 930.5 638 1.458  1393647_at  histone aminotransferase 1 (predicted)  Info   Hat1_predicted 
   4345 0.0002465 849.4 582.2 1.459  1377765_at  chloride intracellular channel 4  Info   Clic4 
   4346 0.0002466 1045 639.6 1.634  1399033_at  core binding factor beta  Info   Cbfb 
   4347 0.0002469 140.2 99.6 1.408  1370574_a_at  protein tyrosine phosphatase, non-receptor type substrate 1  Info   Ptpns1 
   4348 0.0002469 80.3 207.8 0.386  1385912_at  similar to interferon regulatory factor 10  Info   LOC502685 
   4349 0.0002474 93.6 187.1 0.5  1389662_at  protein kinase, lysine deficient 4  Info   Prkwnk4 
   4350 0.0002475 664.9 448.7 1.482  1387420_at  chloride intracellular channel 4  Info   Clic4 
   4351 0.0002481 688.3 496.3 1.387  1371521_at  Pyruvate dehydrogenase E1 alpha 2  Info   Pdha2 
   4352 0.0002483 77.9 211.6 0.368  1376847_at  CDNA clone IMAGE:7323916  Info    
   4353 0.0002486 297.8 179.3 1.661  1398615_at  Transcribed locus  Info    
   4354 0.0002486 267.2 349.5 0.765  1372789_at  zinc finger protein 1  Info   AZF1 
   4355 0.0002487 889 1579.9 0.563  1368814_at  aldehyde dehydrogenase family 6, subfamily A1  Info   Aldh6a1 
   4356 0.000249 430.6 283.1 1.521  1388628_at  integral type I protein  Info   RGD:1303327 
   4357 0.0002495 298.3 461.5 0.646  1370217_at  discoidin domain receptor family, member 1  Info   Ddr1 
   4358 0.0002496 71.4 125.4 0.569  1383914_at  Transcribed locus  Info    
   4359 0.00025 450.7 740.4 0.609  1390149_at  transforming acidic coiled coil 2  Info   RGD:1303004 
   4360 0.0002501 159.4 110.5 1.443  1368910_at  protein phosphatase 2C, magnesium dependent, catalytic subunit  Info   Ppm2c 
   4361 0.0002502 299.6 191.3 1.566  1372043_at  similar to ribosomal protein P0-like protein; 60S acidic ribosomal protein PO; ribosomal protein, large, P0-like (predicted)  Info   RGD1311709_predicted 
   4362 0.0002507 111.9 168 0.666  1397363_at  poliovirus receptor-related 3 (predicted)  Info   Pvrl3_predicted 
   4363 0.0002508 189.1 105.7 1.789  1375696_at  interferon (alpha and beta) receptor 1 (predicted)  Info   Ifnar1_predicted 
   4364 0.0002512 718.8 517.5 1.389  1374061_at  similar to RIKEN cDNA 1110055L24 (predicted)  Info   RGD1307606_predicted 
   4365 0.0002514 165.2 59.4 2.781  1388602_at  adipsin  Info   Adn 
   4366 0.0002515 71.9 122.9 0.585  1378214_at    Info    
   4367 0.0002517 93.6 167.2 0.56  1373300_at    Info    
   4368 0.0002519 183.9 358.6 0.513  1370063_at  nuclear receptor subfamily 2, group F, member 2  Info   Nr2f2 
   4369 0.0002519 91.5 269.6 0.339  1387372_at  solute carrier family 6 (neurotransmitter transporter, GABA), member 13  Info   Slc6a13 
   4370 0.0002524 130.7 68 1.922  1387876_at  signal transducer and activator of transcription 5B  Info   Stat5b 
   4371 0.0002525 932.6 1287.4 0.724  1389301_at  Muscleblind-like 2 (predicted)  Info    
   4372 0.0002526 257.6 330.7 0.779  1399166_a_at  Similar to Bcl7b protein  Info    
   4373 0.0002533 27.5 59.7 0.461  1394097_at  Transcribed locus  Info    
   4374 0.0002534 569.7 821.5 0.693  1371811_at  Bernardinelli-Seip congenital lipodystrophy 2 homolog (human)  Info   Bscl2 
   4375 0.0002546 242.1 411.8 0.588  1369073_at  nuclear receptor subfamily 1, group H, member 4  Info   Nr1h4 
   4376 0.0002554 63.9 121.4 0.526  1374984_at  erythrocyte protein band 4.1-like 5 (predicted)  Info   Epb4.1l5_predicted 
   4377 0.0002557 620 177.4 3.495  1376100_at  similar to RIKEN cDNA 2310057H16 (predicted)  Info   RGD1305887_predicted 
   4378 0.0002559 2524.4 1857.9 1.359  1367671_at  proliferating cell nuclear antigen  Info   Pcna 
   4379 0.000256 62.9 31.5 1.997  1389457_at  myeloblastosis oncogene-like 2 (predicted)  Info   Mybl2_predicted 
   4380 0.0002562 159.2 249.3 0.639  1371915_at  poly(rC) binding protein 4 (predicted)  Info   Pcbp4_predicted 
   4381 0.0002562 503.9 323 1.56  1388132_at  NonO/p54nrb homolog  Info   Sfpq 
   4382 0.0002566 236.5 337 0.702  1393360_at  Similar to zinc finger protein 426  Info    
   4383 0.0002566 83.9 214.4 0.391  1372911_at  Transcribed locus, moderately similar to XP_580018.1 PREDICTED: hypothetical protein XP_580018 [Rattus norvegicus]  Info    
   4384 0.0002567 482.1 735.5 0.655  1398950_at    Info    
   4385 0.0002569 273.2 497.7 0.549  1367815_at  solute carrier family 5 (sodium-dependent vitamin transporter), member 6  Info   Slc5a6 
   4386 0.0002573 2.4 14.6 0.164  1375491_at    Info    
   4387 0.0002573 98.3 61.8 1.591  1373927_at  Transcribed locus, strongly similar to XP_226315.3 PREDICTED: similar to High mobility group box 2-like 1 [Rattus norvegicus]  Info    
   4388 0.0002587 29.7 73.1 0.406  1398467_at  hypothetical LOC360644  Info   LOC360644 
   4389 0.0002597 27.3 85.1 0.321  1383398_at  Similar to integrin alpha 8  Info   Itga8 
   4390 0.0002599 42.7 15.8 2.703  1369129_at  RAS guanyl releasing protein 1  Info   Rasgrp1 
   4391 0.0002601 234.7 14.5 16.186  1388046_at  integrin alpha M  Info   Itgam 
   4392 0.0002603 310.1 211.9 1.463  1377998_at  coproporphyrinogen oxidase (predicted)  Info   Cpox_predicted 
   4393 0.0002608 12.5 28.4 0.44  1391146_at  Transcribed locus  Info    
   4394 0.0002613 1162 1790.4 0.649  1372306_at  ethylmalonic encephalopathy 1 (predicted)  Info   Ethe1_predicted 
   4395 0.0002614 102.2 63.9 1.599  1396449_at    Info    
   4396 0.0002615 39.6 77.7 0.51  1382072_at  Similar to cis-Golgi matrix protein GM130  Info    
   4397 0.0002617 231.1 462 0.5  1370936_at  dimethylglycine dehydrogenase precursor  Info   Dmgdh 
   4398 0.0002617 193.3 133.1 1.452  1376936_at  similar to sodium/calcium exchanger protein  Info   LOC498185 
   4399 0.0002622 262.4 376.7 0.697  1373194_at  caspase 8 associated protein 2 (predicted)  Info   Casp8ap2_predicted 
   4400 0.0002625 467.3 321.6 1.453  1388647_at    Info    
   4401 0.0002625 194 72.3 2.683  1379742_at  similar to SP140 nuclear body protein isoform 1  Info   LOC501175 
   4402 0.0002627 2484.3 2034.9 1.221  1387797_at  RAB7, member RAS oncogene family  Info   Rab7 
   4403 0.000263 1686.6 2489.5 0.677  1375146_at  similar to RIKEN cDNA 3010027G13 (predicted)  Info   RGD1306193_predicted 
   4404 0.0002634 11.9 23.4 0.509  1393544_at  Similar to RIKEN cDNA 1110007C24  Info    
   4405 0.0002635 139.9 85.7 1.632  1383476_at  Transcribed locus  Info    
   4406 0.0002637 249 389.4 0.639  1385171_s_at    Info    
   4407 0.0002642 12.9 1.8 7.167  1387715_at  extracellular peptidase inhibitor  Info   Expi 
   4408 0.0002642 28.9 3.5 8.257  1391328_at    Info    
   4409 0.0002643 113.7 54.5 2.086  1370371_a_at  CEA-related cell adhesion molecule 1 /// CEA-related cell adhesion molecule 10  Info   Ceacam1 /// Ceacam10 
   4410 0.0002644 518.1 1650.9 0.314  1388172_at  integral membrane transport UST1r  Info   Ust1r 
   4411 0.0002645 325.2 179.8 1.809  1372830_at  similar to PI-3-kinase-related kinase SMG-1 isoform 2  Info   LOC499250 
   4412 0.0002648 1641 1322.4 1.241  1387778_at  stromal cell derived factor 4  Info   Sdf4 
   4413 0.0002652 31.7 54.3 0.584  1371085_at  achaete-scute complex homolog-like 3 (Drosophila)  Info   Ascl3 
   4414 0.0002661 159.3 232.4 0.685  1378288_at  similar to RIKEN cDNA D630035O19 (predicted)  Info   RGD1311100_predicted 
   4415 0.0002665 77.1 161 0.479  1371436_at  dimethylarginine dimethylaminohydrolase 2  Info   RGD:1302955 
   4416 0.0002665 487.6 904.4 0.539  1368600_at  solute carrier family 26 (sulfate transporter), member 1  Info   Slc26a1 
   4417 0.0002665 122.7 259.4 0.473  1384639_at  deleted in polyposis 1-like 1 (predicted)  Info   Dp1l1_predicted 
   4418 0.0002666 89.4 158.2 0.565  1382269_at  cyclin M2 (predicted)  Info   Cnnm2_predicted 
   4419 0.0002668 81.5 150.9 0.54  1387296_at  cytochrome P450, family 2, subfamily J, polypeptide 4  Info   Cyp2j4 
   4420 0.0002669 60.8 13.8 4.406  1371196_at  MBP13 Vbeta14 gene  Info    
   4421 0.0002671 30.1 60.5 0.498  1395351_at  Transcribed locus, weakly similar to XP_342574.1 PREDICTED: similar to Csr1 [Rattus norvegicus]  Info    
   4422 0.0002674 166.1 236.4 0.703  1374572_at  similar to opposite strand transcription unit to Stag3; Gats protein (predicted)  Info   RGD1304774_predicted 
   4423 0.0002674 36.8 84.5 0.436  1375729_at  similar to Eph receptor A4  Info   LOC316539 
   4424 0.0002676 345.4 596.6 0.579  1376885_at  Similar to chemokine-like factor super family 4  Info    
   4425 0.0002679 99 128.8 0.769  1380033_at  Transcribed locus  Info    
   4426 0.0002681 141.7 201.7 0.703  1398377_at  similar to 4930488P06Rik protein  Info   RGD:1359094 
   4427 0.0002682 403.8 537.8 0.751  1372344_at  Hbs1-like (S. cerevisiae) (predicted)  Info   Hbs1l_predicted 
   4428 0.0002682 533.8 854.7 0.625  1372138_at  Cut-like 1 (Drosophila)  Info   Cutl1 
   4429 0.0002683 22.1 2.2 10.045  1368766_at  matrix metalloproteinase 7  Info   Mmp7 
   4430 0.0002698 120.2 170.5 0.705  1376824_at  Similar to RIKEN cDNA 1700027M01 (predicted)  Info    
   4431 0.0002703 28.4 47.6 0.597  1377158_at  Transcribed locus  Info    
   4432 0.0002705 361.4 230.8 1.566  1370351_at  tudor domain containing 7  Info   Tdrd7 
   4433 0.0002708 69.9 38.5 1.816  1385258_at  UDP-GlcNAc:betaGal beta-1,3-N-acetylglucosaminyltransferase 7 (predicted)  Info   B3gnt7_predicted 
   4434 0.000271 211.3 450.5 0.469  1387284_at  dihydropyrimidinase  Info   Dpys 
   4435 0.0002712 246.6 132.1 1.867  1370561_at  iGb3 synthase  Info   RGD:727913 
   4436 0.0002715 89.4 129.8 0.689  1389616_at  Transcribed locus  Info    
   4437 0.0002715 110.7 180.4 0.614  1386466_at  Transcribed locus  Info    
   4438 0.0002716 88 154.2 0.571  1391503_at  similar to ARF-GAP, RHO-GAP, ankyrin repeat and pleckstrin homology domains-containing protein 3; dual-specificity Rho- and Arf-GTPase activating protein 1 (predicted)  Info   RGD1304938_predicted 
   4439 0.0002718 176.1 106 1.661  1393035_at  Transcribed locus  Info    
   4440 0.0002719 41 6.3 6.508  1380542_at    Info    
   4441 0.000272 116 174.5 0.665  1382318_at    Info    
   4442 0.0002723 3862.4 4887.9 0.79  1370284_at  ATP synthase, H+ transporting, mitochondrial F1 complex, epsilon subunit  Info   Atp5e 
   4443 0.0002728 127.1 32.6 3.899  1391754_at  2 ' -5 ' oligoadenylate synthetase 1I  Info   RGD:1359379 
   4444 0.0002731 288.4 438.5 0.658  1394414_at  similar to RIKEN cDNA 2310034L04 (predicted)  Info   RGD1310157_predicted 
   4445 0.0002735 324.5 451.7 0.718  1372566_at  mitochondrial carrier homolog 2 (C. elegans) (predicted)  Info   Mtch2_predicted 
   4446 0.0002741 79.7 49.4 1.613  1374590_at  phospholipase C-like 2 (predicted)  Info   Plcl2_predicted 
   4447 0.0002742 111 46.4 2.392  1380450_at  benzodiazapine receptor associated protein 1  Info   Bzrap1 
   4448 0.0002742 143.8 99.2 1.45  1394371_at  nuclear fragile X mental retardation protein interacting protein 1  Info   RGD:1359440 
   4449 0.0002746 145.5 103 1.413  1377169_at  Transcribed locus  Info    
   4450 0.0002749 312.3 174.2 1.793  1396984_at  UDP-glucose ceramide glucosyltransferase-like 1  Info   Ugcgl1 
   4451 0.0002754 972.8 834.6 1.166  1375892_at  ELAV (embryonic lethal, abnormal vision, Drosophila)-like 1 (Hu antigen R) (predicted)  Info   Elavl1_predicted 
   4452 0.000276 60.7 142 0.427  1374345_at  Transcribed locus  Info    
   4453 0.0002761 257.1 320.2 0.803  1374631_at  Similar to RIKEN cDNA 2610036N15 (predicted)  Info    
   4454 0.0002763 451.7 688.5 0.656  1383224_at  par-6 (partitioning defective 6) homolog beta (C. elegans) (predicted)  Info   Pard6b_predicted 
   4455 0.0002764 1872.1 2822.7 0.663  1389836_a_at  Synapsin III  Info   Syn3 
   4456 0.0002765 193.4 433.8 0.446  1398286_at  cysteine sulfinic acid decarboxylase  Info   Csad 
   4457 0.0002766 213.8 136.8 1.563  1371491_at  Notch gene homolog 1 (Drosophila)  Info   Notch1 
   4458 0.0002767 389.4 547.9 0.711  1387009_at  calpain 1  Info   Capn1 
   4459 0.000277 96.5 134.2 0.719  1386573_a_at  Transcribed locus  Info    
   4460 0.0002771 80.8 121.8 0.663  1371091_at  unknown protein  Info   RGD:708546 
   4461 0.0002774 81 48.4 1.674  1388768_at  RUN and TBC1 domain containing 1 (predicted)  Info    
   4462 0.0002776 210.7 308.7 0.683  1385025_a_at  transmembrane 7 superfamily member 1 (predicted)  Info   Tm7sf1_predicted 
   4463 0.0002777 203.4 307.6 0.661  1390557_at  Transcribed locus  Info    
   4464 0.0002781 283.1 418.8 0.676  1372092_at  Amyotrophic lateral sclerosis 2 (juvenile) chromosome region, candidate 3 homolog (human)  Info   Als2cr3 
   4465 0.0002781 59.5 91.9 0.647  1379382_at  Transcribed locus  Info    
   4466 0.0002784 510.6 283 1.804  1373499_at  growth arrest specific 5  Info   Gas5 
   4467 0.0002787 61.7 115.1 0.536  1393051_at  similar to 40S ribosomal protein S29  Info   LOC501619 
   4468 0.0002789 86.2 180.9 0.477  1369947_at  cathepsin K  Info   Ctsk 
   4469 0.000279 905.1 1340.4 0.675  1373088_at    Info    
   4470 0.000279 31.3 66.7 0.469  1397836_at  sirtuin (silent mating type information regulation 2 homolog) 4 (S. cerevisiae) (predicted)  Info   Sirt4_predicted 
   4471 0.0002792 55.8 122.2 0.457  1369634_at  solute carrier family 4, member 1  Info   Slc4a1 
   4472 0.0002796 962.3 768.6 1.252  1373106_at  Zinc finger protein 36, C3H type-like 2 (predicted)  Info    
   4473 0.0002797 131.7 89.2 1.476  1380553_at  similar to Protein C8orf1 (hT41) (predicted)  Info   RGD1306323_predicted 
   4474 0.00028 523.5 303 1.728  1385553_at  nuclear cap binding protein subunit 2 (predicted)  Info   Ncbp2_predicted 
   4475 0.0002803 174.3 110 1.585  1372951_at  Transcribed locus  Info    
   4476 0.0002804 127.9 87.5 1.462  1395363_at  methionine-tRNA synthetase (predicted)  Info   Mars_predicted 
   4477 0.0002812 304.5 500.4 0.609  1388883_at  polymerase (DNA-directed), delta 4 (predicted)  Info   Pold4_predicted 
   4478 0.0002819 549.6 442.6 1.242  1374411_at  Mitochondrial ribosomal protein L52 (predicted)  Info    
   4479 0.000282 113.2 215.7 0.525  1389353_at  Sema domain, transmembrane domain (TM), and cytoplasmic domain, (semaphorin) 6D (predicted)  Info    
   4480 0.0002822 101.4 166.3 0.61  1369526_at  acyl-Coenzyme A dehydrogenase, short/branched chain  Info   Acadsb 
   4481 0.0002822 254.9 152.9 1.667  1388342_at  Transcribed locus  Info    
   4482 0.0002825 128.1 207.9 0.616  1389816_at    Info    
   4483 0.0002826 138.5 291 0.476  1368395_at  glypican 3  Info   Gpc3 
   4484 0.0002843 3940.2 4595.8 0.857  1386887_at  cytochrome c oxidase subunit Vb  Info   Cox5b 
   4485 0.0002849 118.9 180.3 0.659  1388928_at  cofilin 2, muscle (predicted)  Info   Cfl2_predicted 
   4486 0.0002853 271.5 463 0.586  1388456_at  S100 calcium binding protein A1  Info   S100a1 
   4487 0.0002858 81.1 180.2 0.45  1393427_s_at  EGF-like domain 7  Info   Egfl7 
   4488 0.0002867 640.2 440.8 1.452  1388453_at  myeloid-associated differentiation marker  Info   Myadm 
   4489 0.0002868 84.8 3.5 24.229  1395788_at  Transcribed locus  Info    
   4490 0.0002874 350.3 232.6 1.506  1371695_at  translocated promoter region (predicted)  Info   Tpr_predicted 
   4491 0.0002884 1758.7 2306.3 0.763  1372080_at  Inner membrane protein, mitochondrial (predicted)  Info    
   4492 0.0002886 423 219.9 1.924  1390832_at  Transcribed locus  Info    
   4493 0.0002888 75.8 119.3 0.635  1367787_at  islet cell autoantigen 1  Info   Ica1 
   4494 0.0002892 26.3 47.9 0.549  1378250_at  Histocompatibility 13 (predicted)  Info    
   4495 0.0002893 108.7 176.3 0.617  1375083_at  Transcribed locus  Info    
   4496 0.0002895 296.9 172.5 1.721  1382642_at  zinc finger protein 281 (predicted)  Info   Zfp281_predicted 
   4497 0.0002896 2032.8 3452.4 0.589  1368150_at  solute carrier family 27 (fatty acid transporter), member 2 /// hypothetical gene supported by NM_031736  Info   Slc27a2 /// LOC497779 
   4498 0.0002899 321.7 464.5 0.693  1390380_at  Similar to Ski protein  Info    
   4499 0.00029 274.8 504.9 0.544  1370009_at  apolipoprotein C-III  Info   Apoc3 
   4500 0.00029 146.1 234.3 0.624  1398447_at  Transcribed locus  Info    
   4501 0.0002901 34.4 62 0.555  1387513_at  pleckstrin homology, Sec7 and coiled-coil domains 3  Info   Pscd3 
   4502 0.0002919 134.8 250.5 0.538  1368177_at  acyl-CoA synthetase long-chain family member 3  Info   Acsl3 
   4503 0.000292 39.5 68.9 0.573  1393404_at  Reticulon 4 receptor-like 1  Info   Rtn4rl1 
   4504 0.0002922 99.4 168 0.592  1389585_at    Info    
   4505 0.0002925 118.7 47.8 2.483  1374722_at  C1q and tumor necrosis factor related protein 1  Info   RGD:1359204 
   4506 0.0002926 93 149.8 0.621  1373618_at  cAMP responsive element binding protein-like 2 (predicted)  Info   Crebl2_predicted 
   4507 0.0002935 396.4 231.7 1.711  1388669_at  splicing factor 3a, subunit 3 (predicted)  Info   Sf3a3_predicted 
   4508 0.0002939 62.1 137.7 0.451  1385773_at  Transcribed locus, weakly similar to XP_576460.1 PREDICTED: similar to hypothetical protein PB402898.00.0 [Rattus norvegicus]  Info    
   4509 0.0002943 921 542 1.699  1367846_at  S100 calcium-binding protein A4  Info   S100a4 
   4510 0.000295 8.4 26 0.323  1375303_at  similar to mKIAA0613 protein  Info   LOC498587 
   4511 0.0002951 208.2 24.5 8.498  1372808_at  similar to methylenetetrahydrofolate dehydrogenase (NAD) (EC 1.5.1.15) / methenyltetrahydrofolate cyclohydrolase (EC 3.5.4.9) precursor - mouse  Info   LOC313410 
   4512 0.0002955 38.1 71.4 0.534  1383706_at  similar to RIKEN cDNA 2900011O08 (predicted)  Info   RGD1305733_predicted 
   4513 0.0002958 1571.9 2026.7 0.776  1388326_at  NADH dehydrogenase (ubiquinone) Fe-S protein 8 (predicted)  Info   Ndufs8_predicted 
   4514 0.0002958 911.5 711.2 1.282  1398792_at  peptidase (prosome, macropain) 26S subunit, ATPase 1  Info   Psmc1 
   4515 0.0002963 1217.5 1842.9 0.661  1367969_at  peroxiredoxin 6  Info   Prdx6 
   4516 0.0002979 514.9 253.6 2.03  1367795_at  interferon-related developmental regulator 1  Info   Ifrd1 
   4517 0.0002982 256.9 396.1 0.649  1393743_at  Transcribed locus  Info    
   4518 0.0002983 183.6 130.7 1.405  1380373_at  guanine monphosphate synthetase (predicted)  Info   Gmps_predicted 
   4519 0.0002983 168.6 236.6 0.713  1398573_at  zinc finger protein 307 (predicted)  Info   Zfp307_predicted 
   4520 0.0002987 20 60.6 0.33  1369167_at  glial cell line derived neurotrophic factor family receptor alpha 2  Info   Gfra2 
   4521 0.0002989 47.3 75 0.631  1380174_at  Transcribed locus  Info    
   4522 0.0002997 11.6 20.6 0.563  1395816_at    Info    
   4523 0.0002997 140 285.3 0.491  1369286_at  protein C  Info   Proc 
   4524 3e-04 101 158.9 0.636  1369500_at  potassium channel, subfamily K, member 1  Info   Kcnk1 
   4525 0.0003003 179.5 108.7 1.651  1390122_at  Similar to triggering receptor expressed on myeloid cells-like 1  Info    
   4526 0.0003008 24.9 46.9 0.531  1369795_at  potassium inwardly-rectifying channel, subfamily J, member 12  Info   Kcnj12 
   4527 0.0003011 151.7 54.4 2.789  1389244_x_at  chemokine (C-X-C motif) receptor 4  Info   Cxcr4 
   4528 0.0003013 5.7 16.3 0.35  1390609_at  Transcribed locus  Info    
   4529 0.0003017 992 1395.3 0.711  1389023_at  Similar to Oligosaccharyl transferase 3 CG7748-PA (predicted)  Info    
   4530 0.0003018 295 414.5 0.712  1368067_at  zinc finger protein 148  Info   Znf148 
   4531 0.0003018 121.4 175.3 0.693  1373407_at  Transcribed locus  Info    
   4532 0.0003019 262.1 149.7 1.751  1382776_at  similar to O-acyltransferase (membrane bound) domain containing 1  Info   LOC498741 
   4533 0.0003019 1104.9 863.3 1.28  1398353_at  SAR1a gene homolog 1  Info   RGD:1359540 
   4534 0.000302 56.6 107.4 0.527  1379636_at  similar to hypothetical protein FLJ32954  Info   LOC313840 
   4535 0.000302 1052.4 670.2 1.57  1383429_at  similar to T-Brain-1  Info   LOC311078 
   4536 0.000302 148.7 92.7 1.604  1382419_at  SoxLZ/Sox6 leucine zipper binding protein in testis (predicted)  Info   Solt_predicted 
   4537 0.000302 126.4 58.8 2.15  1379396_at  engulfment and cell motility 1, ced-12 homolog (C. elegans) (predicted)  Info   Elmo1_predicted 
   4538 0.000302 525.5 659.5 0.797  1381982_at  UDP-N-acteylglucosamine pyrophosphorylase 1-like 1 (predicted)  Info   Uap1l1_predicted 
   4539 0.0003022 485.7 726.7 0.668  1388143_at  collagen, type XVIII, alpha 1  Info   Col18a1 
   4540 0.0003023 304.2 186.3 1.633  1389734_x_at  RT1 class Ib, locus Aw2 /// RT1 class I, CE12 /// RT1 class I, CE1 /// MHC class I RT1.O type 149 processed pseudogene /// RT1 class I, CE7 /// RT1-149 protein /// RT1 class I, CE16  Info   RT1-Aw2 /// RGD:1359103 /// RT1-CE1 /// RGD:727839 /// RT1-CE7 /// RT1-149 /// RGD:1359403 
   4541 0.0003027 4471.7 3786.9 1.181  1387888_at  ribosomal protein S9  Info   Rps9 
   4542 0.0003032 4.2 25.2 0.167  1392338_at  Transcribed locus  Info    
   4543 0.0003032 124.6 258.6 0.482  1379027_at  similar to KIAA0869 protein (predicted)  Info   RGD1308329_predicted 
   4544 0.0003033 4507.7 3863.7 1.167  1398774_at  ribosomal protein L30  Info   Rpl30 
   4545 0.0003033 284.6 381.5 0.746  1389163_at  tripartite motif protein 32 (predicted)  Info   Trim32_predicted 
   4546 0.0003036 735.8 435.3 1.69  1372360_at  Transcribed locus  Info    
   4547 0.0003038 647.8 402.2 1.611  1388880_at  Schlafen 5 (predicted)  Info    
   4548 0.000304 1580.9 2473.1 0.639  1386938_at  alanyl (membrane) aminopeptidase  Info   Anpep 
   4549 0.0003041 47.8 101.4 0.471  1368174_at  hypothetical gene supported by NM_019371  Info   LOC497816 
   4550 0.0003042 214.9 314.9 0.682  1390993_at  MAWD binding protein  Info   Mawbp 
   4551 0.0003044 51.7 83.2 0.621  1396309_at  Similar to RIKEN cDNA 2810453K03 (predicted)  Info    
   4552 0.0003046 107.7 64.1 1.68  1391897_at  similar to rab6 GTPase activating protein (GAP and centrosome-associated) (predicted)  Info   RGD1304620_predicted 
   4553 0.0003046 79.1 110.4 0.716  1376809_at  similar to RIKEN cDNA 2410025L10  Info   LOC304572 
   4554 0.0003047 146.1 97.9 1.492  1391837_at  similar to chr2 synaptotagmin  Info   LOC299488 
   4555 0.0003048 63.5 137.5 0.462  1381350_at  Transcribed locus  Info    
   4556 0.0003048 332.3 567.2 0.586  1371602_at  Transcribed locus  Info    
   4557 0.0003053 275.5 192.6 1.43  1388631_at  5-azacytidine induced gene 2 (predicted)  Info   Azi2_predicted 
   4558 0.0003053 28.3 47.2 0.6  1393163_at  Transcribed locus  Info    
   4559 0.0003056 20.8 49.8 0.418  1376831_at  similar to hypothetical protein FLJ10156 (predicted)  Info   RGD1308747_predicted 
   4560 0.0003057 81.9 119.7 0.684  1372428_at  DnaJ (Hsp40) homolog, subfamily C, member 4 (predicted)  Info   Dnajc4_predicted 
   4561 0.0003059 972.2 1904.5 0.51  1370365_at  glutathione synthetase  Info   Gss 
   4562 0.0003061 93 156.5 0.594  1390483_at  solute carrier family 25 (mitochondrial carrier, palmitoylcarnitine transporter), member 29 (predicted)  Info   Slc25a29_predicted 
   4563 0.0003064 63.1 192.3 0.328  1389739_at  neuralized-like 2 (Drosophila) (predicted)  Info   Neurl2_predicted 
   4564 0.0003064 117.9 173.9 0.678  1370504_a_at  peripheral myelin protein 22  Info   Pmp22 
   4565 0.0003068 715.7 950.1 0.753  1367506_at  mitochondrial ribosomal protein L11  Info   RGD:1359665 
   4566 0.0003076 106.1 201.9 0.526  1385904_at  cold inducible RNA binding protein  Info   Cirbp 
   4567 0.0003077 199.2 148.3 1.343  1373903_at  similar to Hypothetical protein MGC32399 (predicted)  Info   RGD1304715_predicted 
   4568 0.0003078 556.7 903.9 0.616  1389941_at  ADP-ribosylation factor-like 2 binding protein  Info   Arl2bp 
   4569 0.0003084 401.8 260.4 1.543  1390814_at  pellino homolog 1 (Drosophila) (predicted)  Info   Peli1_predicted 
   4570 0.0003087 117 206.6 0.566  1396317_at  cell cycle progression 1 (predicted)  Info   Ccpg1_predicted 
   4571 0.0003087 59.5 131.4 0.453  1388579_at  Similar to hypothetical gene MGC19595 (predicted)  Info    
   4572 0.0003089 5752.9 4789.2 1.201  1367597_at  ribosomal protein S8  Info   Rps8 
   4573 0.0003106 138.8 71.3 1.947  1371754_at  solute carrier family 25 (mitochondrial carrier, phosphate carrier), member 25  Info   Slc25a25 
   4574 0.000311 82.7 136.2 0.607  1381508_at  Transcribed locus  Info    
   4575 0.0003112 252.6 179.2 1.41  1393234_at  Transcribed locus  Info    
   4576 0.0003115 608 790.6 0.769  1367520_at  apolipoprotein A-I binding protein (predicted)  Info   Apoa1bp_predicted 
   4577 0.000312 102.6 65.6 1.564  1382934_at  Pannexin 1  Info   Panx1 
   4578 0.0003121 323 1180.5 0.274  1368208_at  camello-like 1  Info   Cml1 
   4579 0.0003123 39.3 102.4 0.384  1372574_at  TEA domain family member 2  Info   Tead2 
   4580 0.0003124 1281.9 1543.9 0.83  1370785_s_at  translocase of outer mitochondrial membrane 20 homolog (yeast)  Info   Tomm20 
   4581 0.0003128 159 84.6 1.879  1391440_at  testis expressed gene 10 (predicted)  Info   Tex10_predicted 
   4582 0.0003132 180 115.5 1.558  1384352_at  PAP associated domain containing 4 (predicted)  Info   Papd4_predicted 
   4583 0.0003135 584.7 750.1 0.779  1372900_at  phosphatidylinositol glycan, class T (predicted)  Info   Pigt_predicted 
   4584 0.0003138 351.4 552.6 0.636  1390825_at  solute carrier family 35, member B3 (predicted)  Info   Slc35b3_predicted 
   4585 0.0003146 303.2 218.9 1.385  1399081_at  similar to pellino protein  Info   LOC499085 
   4586 0.0003147 92.2 39.2 2.352  1384892_at  Transcribed locus  Info    
   4587 0.0003158 5027.8 6171.3 0.815  1370277_at  solute carrier family 25 (mitochondrial carrier; adenine nucleotide translocator), member 3  Info   Slc25a3 
   4588 0.0003163 30.4 84 0.362  1368278_at  lectin, galactoside-binding, soluble 2  Info   Lgals2 
   4589 0.0003166 417.7 532 0.785  1388626_at  similar to chromosome 20 open reading frame 116 (predicted)  Info   RGD1309979_predicted 
   4590 0.0003168 492.7 759 0.649  1370005_at  cytochrome b5, outer mitochondrial membrane isoform  Info   Cyb5m 
   4591 0.0003175 207.1 116.4 1.779  1374399_at  EH-domain containing 4  Info   Ehd4 
   4592 0.0003178 610.1 1022.4 0.597  1398250_at  cytosolic acyl-CoA thioesterase 1  Info   Cte1 
   4593 0.0003179 703.9 598.3 1.177  1374834_at  Splicing factor 3b, subunit 4 (predicted)  Info    
   4594 0.000318 199.1 57.5 3.463  1371232_a_at  chondroitin sulfate proteoglycan 2  Info   Cspg2 
   4595 0.0003183 105.1 74.6 1.409  1379284_at  similar to RIKEN cDNA 2810457I06 (predicted)  Info   RGD1310357_predicted 
   4596 0.0003183 1334.5 869.1 1.535  1371509_at  transforming growth factor beta regulated gene 1 (predicted)  Info   Tbrg1_predicted 
   4597 0.0003183 411.7 239.7 1.718  1388590_at  zinc ribbon domain containing, 1  Info   RGD:1303114 
   4598 0.0003184 161.4 216.7 0.745  1375117_at  AKT1 substrate 1 (proline-rich) (predicted)  Info   Akt1s1_predicted 
   4599 0.0003185 65.6 101.4 0.647  1377433_at  zinc finger protein 580 (predicted)  Info   Zfp580_predicted 
   4600 0.0003186 865 1014 0.853  1369982_at  adaptor protein complex AP-2, alpha 2 subunit  Info   Ap2a2 
   4601 0.0003187 154.7 125.1 1.237  1394136_at  PAP associated domain containing 1 (predicted)  Info   Papd1_predicted 
   4602 0.0003191 132.9 328.1 0.405  1369493_at  prolactin receptor  Info   Prlr 
   4603 0.0003195 228 341.3 0.668  1370482_at  sodium channel, nonvoltage-gated 1 beta  Info   Scnn1b 
   4604 0.0003198 755.1 556.5 1.357  1367824_at  farnesyltransferase, CAAX box, alpha  Info   Fnta 
   4605 0.0003198 15.3 43.2 0.354  1368648_at  cytochrome c oxidase subunit IV isoform 2  Info   Cox4i2 
   4606 0.0003199 577.4 409.9 1.409  1372471_at  UDP-glucose ceramide glucosyltransferase-like 1  Info   Ugcgl1 
   4607 0.0003199 229.6 144.5 1.589  1392497_at  Transcribed locus  Info    
   4608 0.0003199 256.1 171.2 1.496  1383712_at  U87HG mRNA, complete sequence  Info    
   4609 0.00032 33.1 83.7 0.395  1391551_at    Info    
   4610 0.0003201 327.1 429.7 0.761  1382006_at  SNAP-associated protein  Info   Snapap 
   4611 0.0003202 49.8 86 0.579  1385668_at  Transcribed locus  Info    
   4612 0.0003204 4 21.3 0.188  1368942_at  hairy and enhancer of split 5 (Drosophila)  Info   Hes5 
   4613 0.0003204 39.5 72.4 0.546  1383642_at    Info    
   4614 0.0003206 165.7 273.5 0.606  1370675_at  transient receptor potential cation channel, subfamily V, member 1  Info   Trpv1 
   4615 0.0003209 1026.3 665.9 1.541  1373072_at  Transcribed locus  Info    
   4616 0.0003212 163.5 265.6 0.616  1374885_at  Solute carrier family 12, member 6 (predicted)  Info    
   4617 0.0003215 362.4 71 5.104  1385702_at  myeloid cell nuclear differentiation antigen (predicted)  Info   Mnda_predicted 
   4618 0.0003217 222.4 349.6 0.636  1391282_at  Transcribed locus, moderately similar to XP_484071.1 PREDICTED: similar to Als2 protein [Mus musculus]  Info    
   4619 0.0003219 1.7 15.2 0.112  1378872_at  DEAH (Asp-Glu-Ala-His) box polypeptide 32 (predicted)  Info    
   4620 0.0003219 228.9 178.1 1.285  1378572_at  toll interacting protein (predicted)  Info   Tollip_predicted 
   4621 0.0003233 75.5 99.4 0.76  1398496_at  Transcribed locus  Info    
   4622 0.0003236 143.5 82.4 1.742  1377173_at  Transcribed locus, moderately similar to XP_580018.1 PREDICTED: hypothetical protein XP_580018 [Rattus norvegicus]  Info    
   4623 0.0003236 545.6 279 1.956  1379687_at  ARP2 actin-related protein 2 homolog (yeast) (predicted)  Info   Actr2_predicted 
   4624 0.0003237 47.9 97.9 0.489  1396408_at  Transcribed locus  Info    
   4625 0.0003239 469.2 209.7 2.237  1376109_at  Transcribed locus, weakly similar to NP_062289.1 DNA binding protein with his-thr domain [Mus musculus]  Info    
   4626 0.000324 2102.6 1590.1 1.322  1371478_at  similar to RIKEN cDNA 1110008F13 (predicted)  Info   RGD1307752_predicted 
   4627 0.0003247 15.3 27.9 0.548  1392219_at    Info    
   4628 0.0003248 1454.4 1182.4 1.23  1398904_at  non-POU domain containing, octamer-binding /// similar to Nono protein  Info   Nono /// LOC501589 
   4629 0.0003253 633.5 392 1.616  1374323_at  BRCA2 and CDKN1A interacting protein (predicted)  Info   Bccip_predicted 
   4630 0.0003256 12.7 37.6 0.338  1383164_at  Transcribed locus  Info    
   4631 0.0003264 408.5 542 0.754  1388692_at  Transcribed locus, strongly similar to NP_079814.1 spermatogenesis associated 11 [Mus musculus]  Info    
   4632 0.0003271 776.4 1030.5 0.753  1368992_a_at  splicing factor, arginine/serine-rich 5  Info   Sfrs5 
   4633 0.0003274 35.8 92.2 0.388  1393271_at  Transcribed locus  Info    
   4634 0.0003276 15 37.7 0.398  1379363_at  Maltase-glucoamylase (predicted)  Info    
   4635 0.0003277 53.7 88.2 0.609  1394450_at  Transcribed locus, moderately similar to XP_484812.1 PREDICTED: hypothetical protein XP_484812 [Mus musculus]  Info    
   4636 0.000328 120.9 251 0.482  1380152_at  Similar to RIKEN cDNA 2700059L22 (predicted)  Info    
   4637 0.000328 85.1 50.3 1.692  1382355_at  UDP-N-acetyl-alpha-D-galactosamine:polypeptide N-acetylgalactosaminyltransferase 4 (GalNAc-T4)  Info   Galnt4 
   4638 0.000328 306.1 435.4 0.703  1398409_at  Suppression of tumorigenicity 13  Info   St13 
   4639 0.0003282 101.6 157.6 0.645  1384277_at  Transcribed locus, moderately similar to NP_076356.1 hypothetical protein LOC77547 [Mus musculus]  Info    
   4640 0.0003285 375.3 658.7 0.57  1376194_at  Transcribed locus  Info    
   4641 0.0003287 119.7 197.5 0.606  1398380_at  similar to von Willebrand factor A-domain containing 1 (predicted)  Info   RGD1311476_predicted 
   4642 0.0003295 134.8 194 0.695  1383350_at  solute carrier family 35, member C2 (predicted)  Info   Slc35c2_predicted 
   4643 0.0003295 107.8 158.6 0.68  1388539_at  plakophilin 2 (predicted)  Info   Pkp2_predicted 
   4644 0.00033 575.9 420.8 1.369  1372083_at  polymerase (RNA) II (DNA directed) polypeptide B (predicted)  Info   Polr2b_predicted 
   4645 0.0003301 97.7 129.9 0.752  1381816_at  similar to hypothetical protein FLJ11218 (predicted)  Info   RGD1309804_predicted 
   4646 0.0003301 633 768.6 0.824  1372108_at  similar to RIKEN cDNA 2810422B04  Info   LOC500199 
   4647 0.0003303 1269.2 1049.7 1.209  1398773_at  src associated in mitosis, 68 kDa  Info   Khdrbs1 
   4648 0.0003306 62.3 115.5 0.539  1390282_at  Cytochrome P450, family 2, subfamily s, polypeptide 1 (predicted)  Info    
   4649 0.0003306 138.5 286.6 0.483  1373860_at  SRY-box containing gene 4 (predicted)  Info   Sox4_predicted 
   4650 0.0003308 200 98.8 2.024  1377967_at  Retroviral integration site 2 (predicted)  Info    
   4651 0.0003309 365 212.3 1.719  1374458_at  Transcribed locus, strongly similar to NP_573483.1 serum/glucocorticoid regulated kinase 3 [Mus musculus]  Info    
   4652 0.0003312 741.5 993.6 0.746  1398943_at  similar to Eso3 protein  Info   LOC293863 
   4653 0.0003315 215.2 120.5 1.786  1390290_at  surfeit gene 6 (predicted)  Info   Surf6_predicted 
   4654 0.0003318 37.9 19.8 1.914  1382830_at  Transcribed locus  Info    
   4655 0.0003319 237.6 519.4 0.457  1391539_at  Transcribed locus  Info    
   4656 0.0003322 54 91.8 0.588  1391271_at  LOC499153  Info   LOC499153 
   4657 0.0003323 102.5 138.9 0.738  1387822_at  guanine nucleotide binding protein, alpha 11  Info   Gna11 
   4658 0.0003332 422 298.1 1.416  1394397_at  Ac1158  Info   LOC302898 
   4659 0.0003335 169.6 280 0.606  1391438_at  Transcribed locus, strongly similar to NP_031990.1 ecotropic viral integration site 5 [Mus musculus]  Info    
   4660 0.0003336 107.8 61 1.767  1383028_at  LRRGT00141  Info   LOC499235 
   4661 0.0003339 26 37.8 0.688  1392726_at  similar to pyruvate dehydrogenase phosphatase regulatory subunit (predicted)  Info   RGD1308307_predicted 
   4662 0.000334 14.3 25 0.572  1370595_a_at  potassium channel interacting protein 4  Info   Kcnip4 
   4663 0.0003346 154.3 267.7 0.576  1382146_at  similar to transmembrane 4 superfamily member 6  Info   LOC302313 
   4664 0.0003351 61.2 20.4 3  1391741_a_at  Transcribed locus  Info    
   4665 0.0003358 237.6 426.2 0.557  1368824_at  caldesmon 1  Info   Cald1 
   4666 0.0003361 354.6 303.3 1.169  1382217_at  Transcribed locus  Info    
   4667 0.0003367 247.9 317.7 0.78  1399131_at  Coatomer protein complex, subunit beta 2 (beta prime)  Info   Copb2 
   4668 0.0003372 872.3 588.4 1.482  1390407_at  similar to Protein C3orf4 homolog  Info   RGD:1359273 
   4669 0.0003372 1.3 2.8 0.464  1376550_at  Transcribed locus  Info    
   4670 0.0003377 78.4 115.6 0.678  1390101_at  similar to hypothetical protein MGC31967  Info   LOC313496 
   4671 0.0003377 111.9 69.9 1.601  1395065_at  WD repeat domain 41 (predicted)  Info   Wdr41_predicted 
   4672 0.0003382 90.9 134.9 0.674  1382388_at  Similar to hypothetical protein MGC27699 (predicted)  Info    
   4673 0.0003384 158.6 287.6 0.551  1387910_at  erythrocyte protein band 4.1-like 1  Info   Epb4.1l1 
   4674 0.0003391 461.8 1072 0.431  1368498_a_at  kidney specific organic anion transporter  Info   RGD:621387 
   4675 0.0003392 120.2 76.8 1.565  1395944_at  Transcribed locus  Info    
   4676 0.0003396 533.7 402.3 1.327  1372558_at  NMDA receptor-regulated gene 1 (predicted)  Info   Narg1_predicted 
   4677 0.0003397 4157 5338.9 0.779  1367653_a_at  malate dehydrogenase 1, NAD (soluble)  Info   Mdh1 
   4678 0.0003398 905.3 1228 0.737  1371864_at  Basic transcription element binding protein 1  Info   Bteb1 
   4679 0.0003405 65.8 89.9 0.732  1384621_at  Transcribed locus, weakly similar to XP_519783.1 PREDICTED: similar to hypothetical protein MGC34646 [Pan troglodytes]  Info    
   4680 0.0003405 1309.1 995.9 1.314  1398798_at  methionine aminopeptidase 2  Info   Metap2 
   4681 0.0003406 75.6 6.3 12  1388084_at  killer cell lectin-like receptor subfamily A, member 22  Info   Klra22 
   4682 0.0003406 82 139.5 0.588  1383593_at  Transcribed locus  Info    
   4683 0.0003406 1175.6 1341 0.877  1387340_at  reticulon 3  Info   Rtn3 
   4684 0.0003411 1028.2 1539 0.668  1367659_s_at  dodecenoyl-coenzyme A delta isomerase  Info   Dci 
   4685 0.0003414 369 164.1 2.249  1372517_at  peptidylprolyl isomerase (cyclophilin)-like 1 (predicted)  Info   Ppil1_predicted 
   4686 0.0003429 34.5 77.8 0.443  1382118_at  paternally expressed 3 (predicted)  Info   Peg3_predicted 
   4687 0.0003431 182.5 119.9 1.522  1380106_at  zinc finger protein 162  Info   Zfp162 
   4688 0.0003434 325.1 235.4 1.381  1388895_at    Info    
   4689 0.0003439 333.6 450.7 0.74  1389008_at  Sprouty-related, EVH1 domain containing 2 (predicted)  Info    
   4690 0.0003447 301.6 167.7 1.798  1384200_at    Info    
   4691 0.0003449 77.9 133 0.586  1369518_at  phosphatidylinositol 3 kinase, regulatory subunit, polypeptide 3  Info   Pik3r3 
   4692 0.000345 89.3 40.1 2.227  1383690_at  similar to ornithine decarboxylase-like protein  Info   LOC366473 
   4693 0.0003453 1063.5 1342.9 0.792  1398844_at  thioredoxin 2  Info   Txn2 
   4694 0.000346 65.7 108 0.608  1379783_at    Info    
   4695 0.0003466 609.3 891.1 0.684  1370256_at  frizzled homolog 1 (Drosophila)  Info   Fzd1 
   4696 0.0003468 390 296.3 1.316  1390366_at  similar to PC-LKC gene product  Info   LOC291002 
   4697 0.0003479 83.3 174.9 0.476  1393806_at  Transcribed locus  Info    
   4698 0.0003485 51.1 73.6 0.694  1374606_at  MYST histone acetyltransferase 1 (predicted)  Info   Prss8 
   4699 0.0003487 126.8 92.7 1.368  1379831_at  similar to multiple hat domains (predicted)  Info   RGD1311017_predicted 
   4700 0.0003488 43.4 5.9 7.356  1397507_at  amyloid beta (A4) precursor protein-binding, family B, member 1 interacting protein (predicted)  Info   Apbb1ip_predicted 
   4701 0.0003491 152 113.3 1.342  1383182_at  serine/arginine-rich protein specific kinase 1 (predicted)  Info   Srpk1_predicted 
   4702 0.0003492 343.7 233 1.475  1374953_at  similar to CG12279-PA  Info   LOC500420 
   4703 0.0003494 271.7 550.4 0.494  1372012_at  24-dehydrocholesterol reductase (predicted)  Info   Dhcr24_predicted 
   4704 0.0003501 1.4 14 0.1  1390596_at  melan-A (predicted)  Info   Mlana_predicted 
   4705 0.0003502 70.4 4.8 14.667  1379653_a_at  similar to SH2 domain protein 1A (Signaling lymphocyte activation molecule-associated protein) (SLAM-associated protein) (T cell signal transduction molecule SAP)  Info   LOC501502 
   4706 0.0003504 632.9 871 0.727  1369631_at  unconventional myosin Myr2 I heavy chain  Info   RGD:620443 
   4707 0.0003504 90.5 39.6 2.285  1384742_at  Alpha thalassemia/mental retardation syndrome X-linked homolog (human)  Info   Atrx 
   4708 0.0003506 42.8 18.5 2.314  1391388_at  Transcribed locus  Info    
   4709 0.000351 1025 1219.6 0.84  1398779_at  actin related protein 2/3 complex, subunit 1A  Info   Arpc1a 
   4710 0.0003513 118.3 203.8 0.58  1395460_at  cask-interacting protein 2 (predicted)  Info   Caskin2_predicted 
   4711 0.000352 118.3 224.2 0.528  1374783_at  Similar to suppressor of Ty 3 homolog  Info    
   4712 0.0003524 799.9 1102.2 0.726  1374188_at  Translocation protein 1 (predicted)  Info    
   4713 0.0003526 76.6 25.9 2.958  1382027_at  LOC498010  Info   Itgb3 
   4714 0.0003529 151 268.3 0.563  1370506_at  demethyl-Q 7  Info   Coq7 
   4715 0.0003529 174.2 138.7 1.256  1374736_at  PR domain containing 4  Info   Prdm4 
   4716 0.0003534 42.7 13.1 3.26  1368812_at  hypothetical gene supported by NM_053905  Info   LOC497722 
   4717 0.0003538 206 261.3 0.788  1384046_at    Info    
   4718 0.0003538 150.7 235.7 0.639  1382330_at  Transcribed locus  Info    
   4719 0.0003542 604.6 404.8 1.494  1370919_at  heterogeneous nuclear ribonucleoprotein M  Info   Hnrpm 
   4720 0.0003546 36.2 66.5 0.544  1373159_at  Transcribed locus  Info    
   4721 0.0003553 46.7 92.2 0.507  1394310_at  SPARC related modular calcium binding 2 (predicted)  Info   Smoc2_predicted 
   4722 0.0003554 160.8 221.4 0.726  1376190_at  phosphatidylinositol 3-kinase, regulatory subunit, polypeptide 2  Info   Pik3r2 
   4723 0.0003558 870.3 1497.1 0.581  1377719_a_at    Info    
   4724 0.0003558 428.2 338.3 1.266  1383061_at  Transcribed locus  Info    
   4725 0.0003562 123.7 75.5 1.638  1369305_at  RAB3A interacting protein (rabin3)-like 1  Info   Rab3il1 
   4726 0.0003568 106.6 160.5 0.664  1389550_at  SH3 domain protein 2A  Info   Sh3gl2 
   4727 0.000357 949.8 441.1 2.153  1370228_at  Transferrin /// signal recognition particle receptor, B subunit (predicted)  Info   Tf /// Srprb_predicted 
   4728 0.0003571 95 167.2 0.568  1383981_at  transformation related protein 53 binding protein 2 (predicted)  Info   Trp53bp2_predicted 
   4729 0.0003577 223.8 334.9 0.668  1382381_at  Transcribed locus  Info    
   4730 0.0003578 212.2 41.2 5.15  1368683_at  oxidized low density lipoprotein (lectin-like) receptor 1  Info   Oldlr1 
   4731 0.0003578 253.7 326.5 0.777  1388595_at  DEAH (Asp-Glu-Ala-His) box polypeptide 30 (predicted)  Info   Dhx30_predicted 
   4732 0.0003581 1210.5 2105.6 0.575  1383161_a_at    Info    
   4733 0.0003581 44.2 84 0.526  1379866_at  Septin 6 (predicted)  Info    
   4734 0.0003585 626.5 933.6 0.671  1372262_at  LOC500344  Info    
   4735 0.0003586 39 56.9 0.685  1380100_at  similar to Traf2 and NCK interacting kinase, splice variant 4  Info   LOC294917 
   4736 0.0003587 420 621.6 0.676  1394705_at  Transcribed locus, weakly similar to XP_580018.1 PREDICTED: hypothetical protein XP_580018 [Rattus norvegicus]  Info    
   4737 0.0003588 659.4 850.4 0.775  1371433_at  Transcribed locus  Info    
   4738 0.0003589 278.7 434.2 0.642  1398254_at  renin binding protein  Info   Renbp 
   4739 0.0003591 533.7 363.6 1.468  1372113_at  flightless I homolog (Drosophila) (predicted)  Info   Fliih_predicted 
   4740 0.0003592 2191.9 1839.4 1.192  1367711_at  proteasome (prosome, macropain) 26S subunit, ATPase 2  Info   Psmc2 
   4741 0.0003597 10.4 1.4 7.429  1371164_at  mast cell protease 10  Info   Mcpt10 
   4742 0.0003597 111.8 55.5 2.014  1374564_at  deltex 2 homolog (Drosophila)  Info   Dtx2 
   4743 0.0003599 368.6 257.2 1.433  1393248_at  similar to expressed sequence C87860 (predicted)  Info   RGD1308468_predicted 
   4744 0.0003603 26.8 45.1 0.594  1378954_at    Info    
   4745 0.0003605 283.2 656.3 0.432  1388782_at  transcription factor 21  Info   Tcf21 
   4746 0.0003606 449.2 256.2 1.753  1383287_at  Similar to semaF cytoplasmic domain associated protein 2  Info    
   4747 0.0003607 49.8 82.4 0.604  1392636_at  Transcribed locus  Info    
   4748 0.0003626 84.6 236.6 0.358  1374013_at  C1q and tumor necrosis factor related protein 5 (predicted)  Info   C1qtnf5_predicted 
   4749 0.0003633 160.8 252.7 0.636  1379031_at  Transcribed locus  Info    
   4750 0.0003636 33.2 54.4 0.61  1383439_at  Neuronal PAS domain protein 2 (predicted)  Info    
   4751 0.0003641 62.1 95 0.654  1382200_at  similar to Prr6 protein  Info   LOC501702 
   4752 0.0003645 401.2 299.1 1.341  1374011_at  excision repair cross-complementing rodent repair deficiency, complementation group 3 (predicted)  Info   Ercc3_predicted 
   4753 0.0003649 176.9 257.9 0.686  1368427_at  A kinase (PRKA) anchor protein 11  Info   Akap11 
   4754 0.0003649 163.9 235.9 0.695  1393316_at    Info    
   4755 0.000365 117 193.7 0.604  1381336_at  Transcribed locus  Info    
   4756 0.0003654 134.4 83.8 1.604  1389804_at  Transcribed locus  Info    
   4757 0.0003656 112.2 219.6 0.511  1367823_at  tissue inhibitor of metalloproteinase 2  Info   Timp2 
   4758 0.000366 16.7 28.4 0.588  1393382_at  Similar to Hypothetical protein A430033K04  Info    
   4759 0.0003661 88.3 44.2 1.998  1387390_at  granzyme K  Info   Gzmk 
   4760 0.0003662 252.2 164.6 1.532  1393249_at  Friend leukemia integration 1 (predicted)  Info    
   4761 0.0003662 38.1 74.4 0.512  1389697_at  Transcribed locus  Info    
   4762 0.0003665 377.6 540.1 0.699  1374748_at  serine hydroxymethyl transferase 1 (soluble) (predicted)  Info   Shmt1_predicted 
   4763 0.0003671 154.8 242.1 0.639  1374454_at  Similar to RIKEN cDNA 5330414D10 (predicted)  Info    
   4764 0.0003677 75.2 151.5 0.496  1390123_at  similar to cDNA sequence BC018222 (predicted)  Info   RGD1305586_predicted 
   4765 0.0003683 544.5 309.4 1.76  1375428_at  cellular repressor of E1A-stimulated genes (predicted)  Info   Creg_predicted 
   4766 0.0003685 97.6 145 0.673  1373279_at  similar to beta-catenin-interacting protein ICAT  Info   LOC503000 
   4767 0.0003689 321.5 248.2 1.295  1371681_at  epidermal Langerhans cell protein LCP1  Info   RGD:708449 
   4768 0.000369 703.9 1071.4 0.657  1387188_at  solute carrier family 17 (sodium phosphate), member 1  Info   RGD:620099 
   4769 0.0003692 86.4 57.9 1.492  1382071_at  Nuclear respiratory factor 1 (predicted)  Info    
   4770 0.0003698 131.5 217.6 0.604  1389971_at  sarcoglycan, epsilon  Info   RGD:1303201 
   4771 0.0003699 80.1 133.8 0.599  1371132_a_at  ankyrin 3, epithelial  Info   RGD:620157 
   4772 0.0003701 187.5 134.8 1.391  1372386_at  SUMO/sentrin specific protease 2  Info   Senp2 
   4773 0.0003709 47.7 83.1 0.574  1381489_at  SRY-box containing gene 6 (predicted)  Info   Sox6 
   4774 0.0003712 596 460 1.296  1386930_at  proteasome (prosome, macropain) 26S subunit, non-ATPase, 4  Info   Psmd4 
   4775 0.0003712 385.7 251.5 1.534  1386394_at    Info    
   4776 0.0003718 267.2 395.3 0.676  1395404_at  Transcribed locus  Info    
   4777 0.0003725 592.6 748.7 0.792  1388995_at  Transcribed locus  Info    
   4778 0.0003732 16.4 41.1 0.399  1388240_a_at  integrin alpha 7  Info   Itga7 
   4779 0.0003733 451.8 285.1 1.585  1383912_at  CDNA clone MGC:109201 IMAGE:7306744  Info    
   4780 0.0003739 563.1 383.2 1.469  1388560_at  similar to Ac2-269 (predicted)  Info   RGD1310479_predicted 
   4781 0.0003741 27.9 63.4 0.44  1374994_at  Nei endonuclease VIII-like 1 (E. coli) (predicted)  Info    
   4782 0.0003744 204.4 334.2 0.612  1382935_at  similar to Hypothetical protein KIAA0141  Info   RGD:735029 
   4783 0.0003745 123.5 47.3 2.611  1373661_a_at  chemokine (C-X-C motif) receptor 4  Info   Cxcr4 
   4784 0.0003747 831.4 470.9 1.766  1367844_at  guanine nucleotide binding protein, alpha inhibiting 2  Info   Gnai2 
   4785 0.0003747 1580 2296 0.688  1372439_at  procollagen, type IV, alpha 1 (predicted)  Info   Col4a1_predicted 
   4786 0.0003748 125.8 208.9 0.602  1370331_at  interleukin 11 receptor, alpha chain 1  Info   Il11ra1 
   4787 0.0003749 131.9 245.4 0.537  1388611_at  transcription elongation factor A (SII), 3 (predicted)  Info   Tcea3_predicted 
   4788 0.0003749 640.3 835.6 0.766  1382857_at  Transcribed locus  Info    
   4789 0.0003756 1120.6 882.7 1.27  1371324_at  Transcribed locus, strongly similar to NP_112577.1 SF3b10 [Homo sapiens]  Info    
   4790 0.0003756 3797.1 5373.5 0.707  1387799_at  FXYD domain-containing ion transport regulator 2  Info   Fxyd2 
   4791 0.0003761 13.7 37.6 0.364  1392852_at    Info    
   4792 0.0003762 420.9 2185.2 0.193  1387820_at  kallikrein 7  Info   Klk7 
   4793 0.0003763 1.6 13.7 0.117  1394668_at  immunoglobulin superfamily, member 4B (predicted)  Info   Igsf4b_predicted 
   4794 0.0003775 683.9 939 0.728  1373367_at  Transcribed locus  Info    
   4795 0.0003782 322.8 442.9 0.729  1387903_at  praja 2, RING-H2 motif containing  Info   Pja2 
   4796 0.0003787 66.2 141.7 0.467  1384184_at  Transcribed locus  Info    
   4797 0.0003788 83.4 135.8 0.614  1373301_at    Info    
   4798 0.0003798 54.3 91 0.597  1377011_at  Similar to hypothetical protein CG003 (predicted)  Info    
   4799 0.0003801 23.2 35.6 0.652  1382510_at  Transcribed locus  Info    
   4800 0.0003805 121.4 193.7 0.627  1392391_at  dystroglycan 1  Info   Dag1 
   4801 0.0003807 599.4 399.8 1.499  1384148_at  Procollagen, type IV, alpha 2 (predicted)  Info    
   4802 0.0003811 127.5 209.4 0.609  1385561_at  similar to peroxisome biogenesis factor 1  Info   LOC500006 
   4803 0.0003814 6394.2 8644.5 0.74  1370299_at  aldolase B  Info   Aldob 
   4804 0.000382 253.9 170.3 1.491  1371814_at  ubiquitin-conjugating enzyme E2G 2 (predicted)  Info   Ube2g2_predicted 
   4805 0.000382 151.8 360.8 0.421  1393958_at  Arginine vasopressin receptor 2  Info   Avpr2 
   4806 0.0003822 25.6 2.5 10.24  1393791_at  Transcribed locus  Info    
   4807 0.0003824 365.4 430.1 0.85  1373366_at  alpha glucosidase 2, alpha neutral subunit (predicted)  Info   G2an_predicted 
   4808 0.0003826 15.2 23 0.661  1384876_at  RAD9 homolog B (S. cerevisiae) (predicted)  Info   Rad9b_predicted 
   4809 0.0003833 181.9 270.8 0.672  1372509_at  Transcribed locus  Info    
   4810 0.0003834 35.1 63.4 0.554  1376107_at  Arginase 2  Info   Arg2 
   4811 0.0003837 95.5 59.4 1.608  1390928_at  similar to tigger transposable element derived 2  Info   LOC500150 
   4812 0.0003841 113.9 163.3 0.697  1372985_at  zinc finger protein 444 (predicted)  Info   Znf444_predicted 
   4813 0.0003842 220.3 337.5 0.653  1375916_at  Similar to RIKEN cDNA 5330414D10 (predicted)  Info    
   4814 0.0003851 1414.2 1847.5 0.765  1371639_at  vacuolar protein sorting 29 (S. pombe) (predicted)  Info   Vps29_predicted 
   4815 0.0003855 79.2 40.9 1.936  1374720_at  Transcribed locus  Info    
   4816 0.0003855 87.9 121.3 0.725  1389687_at  Abelson helper integration site 1  Info   Ahi1 
   4817 0.0003862 611.3 417.3 1.465  1371683_at  LSM4 homolog, U6 small nuclear RNA associated (S. cerevisiae) (predicted)  Info   Lsm4_predicted 
   4818 0.0003863 11.8 1.6 7.375  1369697_at  interleukin 8 receptor, beta  Info   Il8rb 
   4819 0.0003867 286.8 447.6 0.641  1389632_at  Transcribed locus, weakly similar to XP_228131.3 PREDICTED: similar to Rho-related BTB domain-containing protein 1 [Rattus norvegicus]  Info    
   4820 0.0003871 36.4 56.5 0.644  1392909_at  Transcribed locus  Info    
   4821 0.0003871 269.7 108.8 2.479  1373703_at  Transcribed locus  Info    
   4822 0.0003872 459.5 648.6 0.708  1390121_at  GLIS family zinc finger 2 (predicted)  Info    
   4823 0.0003872 81.3 144.9 0.561  1392725_at  similar to cDNA sequence BC006662 (predicted)  Info   RGD1304748_predicted 
   4824 0.0003873 130.3 69.3 1.88  1392818_at  growth arrest specific 5  Info   Gas5 
   4825 0.0003873 172 240.1 0.716  1372531_at  protein tyrosine phosphatase, receptor-type, F interacting protein, binding protein 2  Info   Ppfibp2 
   4826 0.000388 204.3 145.4 1.405  1370975_at  jumonji domain containing 1A /// hypothetical gene supported by NM_175764  Info   Jmjd1a /// LOC497786 
   4827 0.0003885 38 10.1 3.762  1385451_at  sialyltransferase 8 (alpha-2, 8-sialytransferase) D  Info   Siat8d 
   4828 0.0003888 304.8 188.6 1.616  1387190_at  diacylglycerol kinase, alpha (80 kDa)  Info   Dgka 
   4829 0.0003895 28 47.1 0.594  1390856_at  Transcribed locus  Info    
   4830 0.0003898 17.3 36.8 0.47  1368698_at  ATPase, Ca++ transporting, plasma membrane 2  Info   Atp2b2 
   4831 0.0003901 93.3 62.5 1.493  1381217_at  similar to CGI-09 protein (predicted)  Info   RGD1308877_predicted 
   4832 0.0003907 98.8 143.5 0.689  1389201_at  PHD finger protein 10 (predicted)  Info    
   4833 0.0003912 94.4 51.5 1.833  1398523_a_at  interferon (alpha and beta) receptor 1 (predicted)  Info   Ifnar1_predicted 
   4834 0.0003912 486.2 785.7 0.619  1387861_at  amino-terminal enhancer of split  Info   Aes 
   4835 0.0003913 317.2 189.8 1.671  1374014_at  neutral sphingomyelinase (N-SMase) activation associated factor /// hypothetical gene supported by NM_181389  Info   Nsmaf /// LOC497801 
   4836 0.0003915 89.6 148.9 0.602  1373857_at  Family with sequence similarity 36, member A (predicted)  Info    
   4837 0.000392 4648.9 6244.8 0.744  1367643_at  basigin  Info   Bsg 
   4838 0.0003925 69.5 136.5 0.509  1392480_at  poly(A) binding protein, nuclear 1  Info   Pabpn1 
   4839 0.0003931 56.3 82.3 0.684  1376042_at  Similar to Faciogenital dysplasia homolog  Info    
   4840 0.0003932 23.7 45.5 0.521  1396246_at  Ubiquitin-conjugating enzyme E2D 1, UBC4/5 homolog (yeast) (predicted)  Info    
   4841 0.0003942 23.1 75.8 0.305  1384474_at  Transcribed locus  Info    
   4842 0.0003955 229.2 328.5 0.698  1373055_at  Transcribed locus  Info    
   4843 0.0003957 80.1 42.7 1.876  1373530_at  cyclin E  Info   Ccne1 
   4844 0.000396 92 136.9 0.672  1389271_at    Info    
   4845 0.0003962 912.7 659.4 1.384  1371446_at  MAP kinase-activated protein kinase 2  Info   Mapkapk2 
   4846 0.0003963 70.1 146.7 0.478  1373915_at  dystrophia myotonica kinase, B15 (predicted)  Info   Dm15_predicted 
   4847 0.0003971 26.3 53.2 0.494  1377459_at  lymphocyte antigen 6 complex, locus G6E  Info   RGD:1303112 
   4848 0.0003971 49.6 135.6 0.366  1390656_at  Transcribed locus  Info    
   4849 0.000398 288.8 505.2 0.572  1392534_at  transmembrane, prostate androgen induced RNA (predicted)  Info   Tmepai_predicted 
   4850 0.0003988 2370.3 1955.4 1.212  1367452_at  SMT3 suppressor of mif two 3 homolog 2 (yeast)  Info   Sumo2 
   4851 0.0003999 219.4 380.5 0.577  1378536_at  Transcribed locus  Info    
   4852 0.0004001 222.5 352.8 0.631  1388825_at  Transcribed locus  Info    
   4853 0.0004008 1043.8 1386.4 0.753  1372765_a_at  similar to Peci protein /// similar to RIKEN cDNA 1810022C23 (predicted)  Info   RGD:1359427 /// RGD1310224_predicted 
   4854 0.0004009 148.7 238.9 0.622  1391045_at  diacylglycerol O-acyltransferase homolog 2 (mouse)  Info   Dgat2 
   4855 0.0004012 161.3 224.3 0.719  1374995_at  engulfment and cell motility 3, ced-12 homolog (C. elegans) (predicted)  Info   Elmo3_predicted 
   4856 0.0004015 248.8 128.9 1.93  1370542_a_at  E74-like factor 1  Info   Elf1 
   4857 0.0004016 265.6 384.3 0.691  1369626_at  insulin degrading enzyme  Info   Ide 
   4858 0.0004017 1158.9 782.7 1.481  1379671_at  similar to RIKEN cDNA 1200003O06 (predicted)  Info   RGD1305751_predicted 
   4859 0.000402 204.1 259.1 0.788  1398281_at  thiopurine methyltransferase  Info   Tpmt 
   4860 0.0004024 17.5 35.6 0.492  1369463_at  5-hydroxytryptamine (serotonin) receptor 5A  Info   Htr5a 
   4861 0.0004024 55.1 106 0.52  1374870_at  Procollagen, type XXVII, alpha 1  Info   Col27a1 
   4862 0.0004024 87.7 123.4 0.711  1391157_at  Similar to CG7744-PA (predicted)  Info    
   4863 0.000403 68.8 108.7 0.633  1376088_at  similar to solute carrier family 25 (mitochondrial deoxynucleotide carrier), member 19  Info   MGC93911 
   4864 0.0004041 59.8 137.6 0.435  1383796_at  CDNA clone IMAGE:7323916  Info    
   4865 0.0004049 62.6 188.2 0.333  1387267_at  neurotrophin 3  Info   Ntf3 
   4866 0.0004049 2746.3 3359 0.818  1388301_at  ubiquinol-cytochrome c reductase core protein I  Info   RGD:1303314 
   4867 0.0004054 629 913.8 0.688  1371448_at  CDNA clone IMAGE:7303896  Info    
   4868 0.0004062 43.1 71.5 0.603  1384138_at  Potassium channel subunit (Slack)  Info   Slack 
   4869 0.0004064 154.8 252.3 0.614  1390592_at  Transcribed locus  Info    
   4870 0.0004067 787.1 1285.1 0.612  1388814_at  DEAD (Asp-Glu-Ala-Asp) box polypeptide 19  Info   Ddx19 
   4871 0.0004068 1.1 3 0.367  1398123_at  Transcribed locus  Info    
   4872 0.0004071 358.9 515.1 0.697  1367885_at  peroxisomal membrane protein 2  Info   Pxmp2 
   4873 0.0004072 341.8 210.3 1.625  1389558_at  Transcribed locus  Info    
   4874 0.0004072 1261.3 963.9 1.309  1371365_at  similar to Ubiquitin-conjugating enzyme E2S  Info   LOC292588 
   4875 0.0004077 114.6 7.9 14.506  1398540_at  regulator of G-protein signaling 1  Info   Rgs1 
   4876 0.0004086 443.1 310.5 1.427  1388871_at  ash2 (absent, small, or homeotic)-like (Drosophila) (predicted)  Info   Ash2l_predicted 
   4877 0.000409 219.2 136.6 1.605  1379778_at  similar to HSPC043 protein (predicted)  Info   RGD1307983_predicted 
   4878 0.0004093 272.3 383.3 0.71  1374019_at  LOC499758  Info    
   4879 0.0004096 1011.8 1212 0.835  1371905_at  similar to 0610007L01Rik protein  Info   RGD:1303319 
   4880 0.0004096 571.1 948.2 0.602  1379330_s_at  CDNA clone IMAGE:7316839  Info    
   4881 0.0004098 359.5 713 0.504  1371951_at  four and a half LIM domains 2  Info   Fhl2 
   4882 0.0004105 682.8 906.9 0.753  1371818_at  Exportin, tRNA (nuclear export receptor for tRNAs) (predicted)  Info    
   4883 0.0004109 437.5 630.1 0.694  1373021_at  Similar to NipSnap2 protein (Glioblastoma amplified sequence)  Info    
   4884 0.000411 355.3 458.1 0.776  1374758_at  Transcribed locus  Info    
   4885 0.0004113 22.8 43.8 0.521  1382953_at  Transcribed locus  Info    
   4886 0.0004114 330.7 215.1 1.537  1388556_at  syntaxin 6  Info   Stx6 
   4887 0.0004116 162.5 112.6 1.443  1372625_at  similar to protein phosphatase 1, regulatory subunit 12C  Info   LOC499076 
   4888 0.0004119 173.2 95.7 1.81  1377664_at  KARP-1 binding protein 1  Info   Kab 
   4889 0.0004119 3628.5 2822.8 1.285  1387361_s_at  phosphoglycerate kinase 1  Info   Pgk1 
   4890 0.0004122 275.8 373.3 0.739  1367577_at  heat shock 27kDa protein 1  Info   Hspb1 
   4891 0.0004125 30.2 56.6 0.534  1387462_at  cholinergic receptor, muscarinic 3  Info   Chrm3 
   4892 0.000414 123.1 81.9 1.503  1384098_at  ring finger protein 125 (predicted)  Info   Rnf125_predicted 
   4893 0.000414 82.8 120.4 0.688  1370579_at  glycine-, glutamate-, thienylcyclohexylpiperidine-binding protein  Info   RGD:708505 
   4894 0.0004143 213.5 318.6 0.67  1381832_at  pyrroline-5-carboxylate reductase-like (predicted)  Info   Pycrl_predicted 
   4895 0.0004145 603.4 1145.8 0.527  1382840_at    Info    
   4896 0.000415 205.9 152 1.355  1399088_at  tousled-like kinase 2 (Arabidopsis) (predicted)  Info   Tlk2_predicted 
   4897 0.0004153 108.6 169.5 0.641  1390112_at  epidermal growth factor-containing fibulin-like extracellular matrix protein 1 (predicted)  Info   Efemp1_predicted 
   4898 0.0004154 5.3 28.3 0.187  1379370_at  Transcribed locus  Info    
   4899 0.0004166 1236.4 1496 0.826  1388445_at  annexin A11 (predicted)  Info   Anxa11_predicted 
   4900 0.0004166 229.4 277.1 0.828  1389209_at  similar to hypothetical protein BC002942 (predicted)  Info   RGD1306274_predicted 
   4901 0.0004172 32 84.4 0.379  1368432_a_at  v-ros UR2 sarcoma virus oncogene homolog 1 (avian)  Info   Ros1 
   4902 0.0004172 773.2 938.7 0.824  1373501_at  Transcribed locus  Info    
   4903 0.0004173 1.2 1.8 0.667  1388189_at  glutamate receptor, metabotropic 3  Info   Grm3 
   4904 0.0004175 152 234.6 0.648  1377311_at  similar to empty spiracles-like protein 2  Info   LOC499380 
   4905 0.0004176 903.1 653 1.383  1367900_at  glycogenin 1  Info   Gyg1 
   4906 0.0004181 250.5 345 0.726  1373415_at  Transcribed locus  Info    
   4907 0.0004186 497.6 367 1.356  1384958_at  Transcribed locus  Info    
   4908 0.0004202 24.4 64 0.381  1370289_x_at  kallikrein, submaxillary gland S3  Info   Klks3 
   4909 0.0004203 61.8 34.2 1.807  1390708_at    Info    
   4910 0.0004205 437 257.7 1.696  1379258_at  kelch-like 5 (Drosophila) (predicted)  Info   Klhl5_predicted 
   4911 0.0004206 891.5 399.1 2.234  1373421_at  TG interacting factor (predicted)  Info   Tgif_predicted 
   4912 0.0004208 149.7 212.7 0.704  1382032_at  Transcribed locus  Info    
   4913 0.000421 2025.9 2450.5 0.827  1388370_at  cyclin I (predicted)  Info   Ccni_predicted 
   4914 0.0004212 306.7 431.7 0.71  1367638_at  malonyl-CoA decarboxylase  Info   Mlycd 
   4915 0.0004213 921.9 1888.4 0.488  1377900_at  similar to RIKEN cDNA 1810013B01  Info   RGD:1359649 
   4916 0.0004214 91.7 130.4 0.703  1390457_at  Transcribed locus  Info    
   4917 0.0004218 228.3 345.7 0.66  1390173_at  Transcribed locus  Info    
   4918 0.0004221 275.5 156.4 1.762  1387957_a_at  SH3-domain kinase binding protein 1  Info   Sh3kbp1 
   4919 0.0004225 37.7 71.7 0.526  1374817_at  Transcribed locus  Info    
   4920 0.0004227 561 446.2 1.257  1375425_at  hypothetical LOC287541 (predicted)  Info   RGD1309400_predicted 
   4921 0.0004228 63.5 29.1 2.182  1376642_at  similar to hypothetical protein FLJ10307 (predicted)  Info   RGD1305649_predicted 
   4922 0.0004228 217.5 139.1 1.564  1383621_at  Transcribed locus  Info    
   4923 0.0004231 187.4 295.4 0.634  1390403_at  similar to CG8312-PA (predicted)  Info   RGD1304790_predicted 
   4924 0.0004235 306 405.3 0.755  1374285_at  Transcribed locus  Info    
   4925 0.0004242 49.9 90.7 0.55  1379057_at  Transcribed locus, strongly similar to NP_659132.1 hypothetical protein LOC227545 [Mus musculus]  Info    
   4926 0.0004242 78.8 160.3 0.492  1372565_at  similar to toll-associated serine protease (predicted)  Info   RGD1308120_predicted 
   4927 0.0004251 314.5 203.6 1.545  1375540_at  similar to hypothetical protein D11Ertd497e (predicted)  Info   RGD1308696_predicted 
   4928 0.0004256 238.9 183.6 1.301  1368031_at  nucleolar and coiled-body phosphoprotein 1  Info   Nolc1 
   4929 0.0004256 249.8 129.2 1.933  1383576_at  Protein phosphatase 1, regulatory (inhibitor) subunit 12A  Info   Ppp1r12a 
   4930 0.0004257 23.5 90.1 0.261  1370538_at  laminin, alpha 3  Info   Lama3 
   4931 0.0004258 541.9 722.7 0.75  1388575_at  Transcribed locus, moderately similar to XP_488956.1 PREDICTED: hypothetical protein LOC66602 [Mus musculus]  Info    
   4932 0.0004266 2.2 18.9 0.116  1392196_at  Discs, large homolog 2 (Drosophila)  Info   Dlgh2 
   4933 0.0004272 141.1 251 0.562  1382265_at  similar to KIAA1128 protein (predicted)  Info   RGD1304626_predicted 
   4934 0.0004272 374.3 311.3 1.202  1394438_at  ARP1 actin-related protein 1 homolog A (yeast) (predicted)  Info   Actr1a_predicted 
   4935 0.0004277 168.5 134.2 1.256  1373980_at  similar to XPA binding protein 1  Info   LOC500619 
   4936 0.000429 64.6 94.4 0.684  1382047_at  similar to AI325464 protein (predicted)  Info   RGD1307976_predicted 
   4937 0.0004299 393.7 281.5 1.399  1373461_at  dermal papilla derived protein 6  Info   RGD:1303303 
   4938 0.0004308 45.1 14.7 3.068  1392296_at  Transcribed locus  Info    
   4939 0.000431 98.7 150.3 0.657  1370398_at  spermatogenesis associated 7  Info   Spata7 
   4940 0.0004322 98 184.7 0.531  1382230_at  similar to RIKEN cDNA A630065K24 (predicted)  Info   RGD1305132_predicted 
   4941 0.0004325 157.7 106.8 1.477  1374152_at  WD repeat domain 46  Info   Wdr46 
   4942 0.0004331 179.8 255.3 0.704  1378543_at  Nuclear factor, erythroid derived 2, like 3 (predicted)  Info    
   4943 0.0004331 193.5 135.7 1.426  1386096_at  similar to Metal-response element-binding transcription factor 2 (Metal-response element DNA-binding protein M96) (predicted)  Info   RGD1304727_predicted 
   4944 0.0004332 406 305.5 1.329  1371658_at  neighbor of Cox4 (predicted)  Info   Noc4_predicted 
   4945 0.0004335 77.5 58.8 1.318  1370830_at  epidermal growth factor receptor  Info   Egfr 
   4946 0.0004336 128.6 173.4 0.742  1373321_at  Transcribed locus, strongly similar to XP_574622.1 PREDICTED: similar to UPF0197 protein C11orf10 homolog [Rattus norvegicus]  Info    
   4947 0.0004337 485.5 659.8 0.736  1367877_at  solute carrier family 11 (proton-coupled divalent metal ion transporters), member 2  Info   Slc11a2 
   4948 0.0004339 104.1 238.9 0.436  1387347_at  insulin-like growth factor binding protein 5  Info   Igfbp5 
   4949 0.0004343 3881.3 4704.5 0.825  1371309_at  testis enhanced gene transcript  Info   Tegt 
   4950 0.0004344 130.2 55.1 2.363  1393201_at  similar to lymphoid-restricted membrane protein  Info   LOC500361 
   4951 0.0004347 41.1 83.5 0.492  1389490_at  CD164 sialomucin-like 1 (predicted)  Info   Cd164l1_predicted 
   4952 0.000435 103.5 6.7 15.448  1387591_at  interleukin 2 receptor, alpha chain  Info   Il2ra 
   4953 0.0004351 288.4 387.6 0.744  1389374_at  kinesin family member C3 (predicted)  Info   Kifc3_predicted 
   4954 0.0004353 61 86.9 0.702  1381075_at  Transcribed locus  Info    
   4955 0.0004356 1000.9 1826.1 0.548  1387037_at  cubilin (intrinsic factor-cobalamin receptor)  Info   Cubn 
   4956 0.0004359 43.7 3.7 11.811  1397663_at  Transcribed locus  Info    
   4957 0.0004371 72.9 12.4 5.879  1371058_at    Info    
   4958 0.0004371 426.9 586.1 0.728  1388401_at    Info    
   4959 0.0004374 108.6 149.4 0.727  1384182_at  pleckstrin homology domain containing, family C (with FERM domain) member 1 (predicted)  Info   Plekhc1_predicted 
   4960 0.0004379 161.5 252.2 0.64  1374098_at  Ng3 protein  Info   Ng3 
   4961 0.000438 53.1 21.9 2.425  1379584_at  C-type (calcium dependent, carbohydrate-recognition domain) lectin, superfamily member 2 (activation-induced) (predicted)  Info    
   4962 0.0004381 150.3 292.8 0.513  1368700_at  phospholipase C-like 1  Info   Plcl1 
   4963 0.0004384 100.2 156.8 0.639  1368303_at  period homolog 2 (Drosophila)  Info   Per2 
   4964 0.0004386 1730.6 1419.1 1.22  1388117_at  Similar to small nuclear ribonucleoprotein B  Info   Snrpb 
   4965 0.0004387 523 394.8 1.325  1389109_at  Phosphatidylinositol-4-phosphate 5-kinase, type II, alpha  Info   Pip5k2a 
   4966 0.0004402 190.6 414.4 0.46  1383832_at  similar to MGC52019 protein (predicted)  Info   RGD1311652_predicted 
   4967 0.0004403 141.3 107.1 1.319  1377480_at  similar to RIKEN cDNA D030060M11 (predicted)  Info   RGD1308076_predicted 
   4968 0.0004406 56.4 79.5 0.709  1375600_at  Phosphatidylinositol glycan, class O (predicted)  Info    
   4969 0.0004407 199.1 284.1 0.701  1390709_at  Transcribed locus  Info    
   4970 0.0004409 124.6 208.7 0.597  1370285_at  KIAA1536 protein  Info   RGD:619812 
   4971 0.0004409 59.7 79 0.756  1373994_at  similar to RIKEN cDNA 2010301N04  Info   LOC500247 
   4972 0.0004411 106.1 75.9 1.398  1369517_at  pleckstrin homology, Sec7 and coiled/coil domains 1  Info   Pscd1 
   4973 0.0004414 208.9 294.2 0.71  1371919_at  similar to RP2 protein, testosterone-regulated - ricefield mouse (Mus caroli)  Info   RGD:1302935 
   4974 0.0004415 355.2 172 2.065  1368571_at  cytoplasmic linker 2  Info   Cyln2 
   4975 0.0004417 43.1 84.2 0.512  1373580_at  Transcribed locus  Info    
   4976 0.0004428 281.1 407.8 0.689  1390418_at  ARP1 actin-related protein 1 homolog B (yeast) (predicted)  Info   Actr1b_predicted 
   4977 0.000443 793.4 1294.2 0.613  1367950_at  solute carrier family 22 (organic cation transporter), member 5  Info   Slc22a5 
   4978 0.0004431 214.7 372.8 0.576  1383492_at  Similar to Homeobox protein Hox-D8 (Hox-4.3) (Hox-5.4)  Info    
   4979 0.0004434 157.3 297.1 0.529  1384312_at  Iroquois related homeobox 1 (Drosophila) (predicted) /// similar to iroquois-class homeobox protein Irx1  Info   Irx1_predicted /// LOC501463 
   4980 0.0004437 36.8 94.3 0.39  1368784_at  apobec-1 complementation factor  Info   Acf 
   4981 0.0004441 363.1 213.7 1.699  1375685_at  cytoplasmic linker associated protein 1 (predicted)  Info   Clasp1_predicted 
   4982 0.0004446 75.6 112.1 0.674  1393799_at  Unc-5 homolog B (C. elegans)  Info   Unc5b 
   4983 0.0004454 194.7 317.7 0.613  1374085_at  Max dimerization protein 4 (predicted)  Info   Mxd4_predicted 
   4984 0.0004459 64.9 134.1 0.484  1377774_at    Info    
   4985 0.000446 660.6 384.4 1.719  1377726_at  tripartite motif protein 25  Info   Trim25 
   4986 0.0004467 981 1262.9 0.777  1372431_at  ribosomal protein, mitochondrial, L12  Info   Mrpl12 
   4987 0.0004476 437.6 363.3 1.205  1367502_at  mitochondrial ribosomal protein L21 (predicted)  Info   Mrpl21_predicted 
   4988 0.0004476 963.8 1078 0.894  1371461_at  similar to RIKEN cDNA 2410166I05 (predicted)  Info   RGD1305453_predicted 
   4989 0.0004477 82.3 178.5 0.461  1370328_at  Dickkopf homolog 3 (Xenopus laevis)  Info   Dkk3 
   4990 0.0004491 812 930.5 0.873  1388366_at  mitochondrial ribosomal protein L4 (predicted)  Info   Mrpl4_predicted 
   4991 0.0004494 338.8 462.1 0.733  1372196_at  nuclear receptor co-repressor 2 (predicted)  Info   Ncor2_predicted 
   4992 0.0004499 88 43.3 2.032  1378319_at  similar to Lck-interacting transmembrane adaptor protein LIME  Info   LOC362289 
   4993 0.0004518 241.3 4.7 51.34  1369764_at  complement component 4 binding protein, alpha  Info   C4bpa 
   4994 0.0004525 913.4 665.3 1.373  1387917_at  lamina-associated polypeptide 1B  Info   Lap1b 
   4995 0.0004533 143.7 193.1 0.744  1388888_at  Transcribed locus  Info    
   4996 0.0004534 37.5 24.6 1.524  1385728_at  AE binding protein 2 (predicted)  Info    
   4997 0.0004536 907.5 1405.1 0.646  1379770_at  similar to choline phosphotransferase 1; cholinephosphotransferase 1 alpha; cholinephosphotransferase 1  Info   RGD:1359283 
   4998 0.0004537 148.3 78.7 1.884  1380327_at  Transcribed locus, moderately similar to NP_536694.1 helicase (DNA) B [Mus musculus]  Info    
   4999 0.0004539 4463.8 5838 0.765  1387773_at  cytochrome c, somatic  Info   Cycs 
   5000 0.000454 6991.6 4797.8 1.457  AFFX_Rat_beta-actin_5_at  actin, beta  Info   Actb 
   5001 0.0004544 866.9 1307.6 0.663  1371736_at  Similar to 1810013D10Rik protein  Info    
   5002 0.0004549 475.5 319.8 1.487  1371772_at  similar to RIKEN cDNA 1810020C19 gene  Info   RGD:1359731 
   5003 0.0004554 429.6 335.4 1.281  1398936_at  eukaryotic translation initiation factor 2, subunit 3, structural gene X-linked  Info   Eif2s3x 
   5004 0.000456 45.1 79.6 0.567  1385924_at  Transcribed locus  Info    
   5005 0.0004563 469.4 362.8 1.294  1389053_at  Similar to scruin like at the midline CG5186-PA (predicted)  Info    
   5006 0.0004565 638.2 416.8 1.531  1371873_at  Transcribed locus  Info    
   5007 0.0004565 60 21.1 2.844  1382167_at  similar to SHP2-interacting transmembrane adaptor protein  Info   LOC500449 
   5008 0.000457 120.3 192 0.627  1373424_at  DiGeorge syndrome critical region gene 6 (predicted)  Info   Dgcr6_predicted 
   5009 0.0004573 328.4 451.5 0.727  1375536_at  Numb gene homolog (Drosophila)  Info   Numb 
   5010 0.0004584 200.2 327.8 0.611  1389522_at  Transcribed locus  Info    
   5011 0.0004585 1868.1 1346.1 1.388  1371544_at  similar to Enhancer of rudimentary homolog  Info   LOC503047 
   5012 0.0004586 170.3 83.3 2.044  1385001_at  gasdermin domain containing 1 (predicted)  Info   Gsdmdc1_predicted 
   5013 0.0004605 38.5 67.7 0.569  1376443_at  Regulator of G-protein signalling 3  Info   Rgs3 
   5014 0.0004617 2984.9 1938.8 1.54  1399160_a_at  ubiquitin-conjugating enzyme E2D 3 (UBC4/5 homolog, yeast)  Info   Ube2d3 
   5015 0.0004617 698.9 481.8 1.451  1367621_at  death-associated protein kinase 3  Info   Dapk3 
   5016 0.0004619 46.1 93.9 0.491  1377013_at  Transcribed locus  Info    
   5017 0.0004619 34.9 60.2 0.58  1393204_at  sideroflexin 4 (predicted)  Info   Sfxn4_predicted 
   5018 0.0004622 155.1 242.7 0.639  1375011_at  Transcribed locus  Info    
   5019 0.0004622 447.7 636.3 0.704  1374641_at  Transcribed locus  Info    
   5020 0.0004624 210.2 134.5 1.563  1390914_at  Friend leukemia integration 1 (predicted)  Info   Fli1_predicted 
   5021 0.0004628 31.4 65.8 0.477  1376918_at  Serine (or cysteine) proteinase inhibitor, clade C (antithrombin), member 1 (predicted)  Info    
   5022 0.0004629 2574 3256.7 0.79  1368202_a_at  disabled homolog 2 (Drosophila)  Info   Dab2 
   5023 0.0004635 144.4 209.2 0.69  1372736_at  Amylo-1, 6-glucosidase, 4-alpha-glucanotransferase (glycogen debranching enzyme, glycogen storage disease type III) (predicted)  Info    
   5024 0.0004635 11 37.8 0.291  1388605_at  WAP four-disulfide core domain 3 (predicted)  Info   Wfdc3_predicted 
   5025 0.0004649 5.9 37.4 0.158  1379848_at    Info    
   5026 0.0004649 67.5 20.9 3.23  1380037_at  Hypothetical LOC292764  Info   MGC94040 
   5027 0.0004656 3.3 32.1 0.103  1382132_at  Transcribed locus  Info    
   5028 0.0004668 154.8 295.8 0.523  1368138_at  hypothetical gene supported by NM_017212  Info   LOC497674 
   5029 0.0004669 161.3 240.6 0.67  1373270_at  similar to D11Ertd498e protein (predicted)  Info   RGD1307754_predicted 
   5030 0.0004673 277.2 361.7 0.766  1399030_at  similar to DNA segment, Chr X, Immunex 38, expressed  Info   RGD:1359718 
   5031 0.0004676 166.1 83.3 1.994  1368546_at  human immunodeficiency virus type I enhancer binding protein 2  Info   Hivep2 
   5032 0.0004683 62.2 100.9 0.616  1367842_at  amyloid beta (A4) precursor protein-binding, family B, member 1  Info   Apbb1 
   5033 0.0004692 460.1 614.5 0.749  1371659_at  ras homolog gene family, member C (predicted)  Info   Rhoc_predicted 
   5034 0.0004703 272.9 188.8 1.445  1377310_at  Transcribed locus, weakly similar to XP_575554.1 PREDICTED: similar to RNA binding motif and ELMO domain 1 [Rattus norvegicus]  Info    
   5035 0.000471 93.4 149 0.627  1368133_at  multiple PDZ domain protein  Info   Mpdz 
   5036 0.0004715 279.8 166.6 1.679  1367803_at  nucleoporin 54  Info   Nup54 
   5037 0.0004715 116.8 159.9 0.73  1387148_at  G protein-coupled receptor associated sorting protein 1  Info   Gprasp1 
   5038 0.0004716 5.3 31.3 0.169  1389943_at  ciliary rootlet coiled-coil, rootletin (predicted)  Info   Crocc_predicted 
   5039 0.0004718 76.6 123.7 0.619  1369960_at  FXYD domain-containing ion transport regulator 1  Info   Fxyd1 
   5040 0.0004722 9.6 16.2 0.593  1385563_at  Similar to Catechol O-methyltransferase  Info    
   5041 0.0004722 276.6 186.7 1.482  1388429_at  similar to gem (nuclear organelle) associated protein 7  Info   LOC499391 
   5042 0.0004724 11.3 25.4 0.445  1376564_at  spectrin beta 4 (predicted)  Info   Spnb4_predicted 
   5043 0.0004727 1389.8 1240.3 1.121  1392465_at  similar to SAP18  Info   LOC498002 
   5044 0.0004733 609.7 680.9 0.895  1372029_at  similar to mannose-6-phosphate receptor binding protein 1  Info   LOC316130 
   5045 0.0004737 407.7 306.9 1.328  1373597_at  processing of precursor 7, ribonuclease P family, (S. cerevisiae) (predicted)  Info   Pop7_predicted 
   5046 0.0004742 321.5 492.4 0.653  1394472_at  Ral guanine nucleotide dissociation stimulator,-like 1 (predicted)  Info    
   5047 0.0004749 1786.5 1388.4 1.287  1388566_at  LIM and SH3 protein 1  Info   Lasp1 
   5048 0.0004749 728.3 946.6 0.769  1373064_at  DnaJ (Hsp40) homolog, subfamily D, member 1 (predicted)  Info   Dnajd1_predicted 
   5049 0.0004751 119 217.1 0.548  1389728_at  Meis1, myeloid ecotropic viral integration site 1 homolog 2 (predicted)  Info   Meis2_predicted 
   5050 0.0004755 494.5 735.2 0.673  1388887_at  Glycoprotein galactosyltransferase alpha 1, 3  Info   Ggta1 
   5051 0.0004766 3878.8 3185 1.218  1386868_at  ribosomal protein S10  Info   Rps10 
   5052 0.0004766 15.5 32.3 0.48  1372972_at  Transcribed locus  Info    
   5053 0.0004768 96.8 270.4 0.358  1395026_at  flavin containing monooxygenase 4  Info   Fmo4 
   5054 0.0004773 58.4 128.8 0.453  1370131_at  caveolin  Info   Cav 
   5055 0.0004775 407.4 194 2.1  1373035_at  Transcribed locus  Info    
   5056 0.0004776 429.1 248.1 1.73  1375870_a_at  RNA binding motif, single stranded interacting protein 1 (predicted)  Info   Rbms1_predicted 
   5057 0.0004776 1.8 4.3 0.419  1379682_at  Similar to RIKEN cDNA 6030446I19 gene (predicted)  Info    
   5058 0.0004776 623.1 395.4 1.576  1386906_a_at  septin 9  Info   Sept9 
   5059 0.0004778 220 149.8 1.469  1386346_at  Unknown (protein for MGC:72591)  Info   Tmem19 
   5060 0.0004781 48.8 79.8 0.612  1378021_at  Transcribed locus  Info    
   5061 0.0004787 536.9 294.6 1.822  1371953_at  Cyclin G2 (predicted)  Info   Ccng2 
   5062 0.000479 113.8 77.7 1.465  1372995_at  similar to protein kinase D2 (predicted)  Info   RGD1308054_predicted 
   5063 0.0004793 85.7 49.5 1.731  1387268_at  RNA polymerase 1-2  Info   Rpo1-2 
   5064 0.0004793 73.4 112.2 0.654  1392924_at  Transcribed locus  Info    
   5065 0.0004803 32.8 49.7 0.66  1383805_at  similar to Traf2 and NCK interacting kinase, splice variant 4  Info   LOC294917 
   5066 0.0004821 47.5 75.5 0.629  1380070_at  ATP synthase, H+ transporting, mitochondrial F1 complex, beta polypeptide  Info   Atp5b 
   5067 0.0004824 344.3 483.9 0.712  1370156_at  prion protein  Info   Prnp 
   5068 0.0004824 210.5 129.8 1.622  1373875_at  similar to RIKEN cDNA 1190005P17 (predicted)  Info   RGD1308261_predicted 
   5069 0.0004837 409.5 559.3 0.732  1375886_at  Transcribed locus  Info    
   5070 0.0004845 1889.7 1436.1 1.316  1372466_at  Transforming growth factor, beta receptor II  Info   Tgfbr2 
   5071 0.0004847 400.7 662.5 0.605  1389546_at    Info    
   5072 0.0004856 3.7 18.2 0.203  1369515_s_at  insulin receptor-related receptor  Info   Insrr 
   5073 0.0004861 111.3 274.4 0.406  1371472_at  Transcribed locus  Info    
   5074 0.0004862 534.8 661.5 0.808  1399049_at  similar to RIKEN cDNA 1200003C05 (predicted)  Info   RGD1308917_predicted 
   5075 0.0004882 366.7 283.7 1.293  1371326_at  casein kinase II, alpha 2, polypeptide (predicted)  Info   Csnk2a2_predicted 
   5076 0.0004882 96.9 193.9 0.5  1392265_s_at  Matrix metalloproteinase 23  Info   Mmp23 
   5077 0.0004884 487.4 326.3 1.494  1371451_at  ribonuclease H2, large subunit (predicted)  Info   Rnaseh2a_predicted 
   5078 0.0004885 164.9 229.3 0.719  1391421_at  similar to Acyl-CoA dehydrogenase family member 8, mitochondrial precursor (ACAD-8) (Isobutyryl-CoA dehydrogenase)  Info   LOC367046 
   5079 0.0004888 26.1 8.5 3.071  1384570_at  pleckstrin homology domain containing, family A (phosphoinositide binding specific) member 4  Info   Plekha4 
   5080 0.00049 209.6 78.9 2.657  1390531_at  Similar to Peroxisomal proliferator-activated receptor A interacting complex 285 kDa protein (PPAR-alpha interacting complex protein 285) (predicted)  Info    
   5081 0.0004904 84.1 45.4 1.852  1385268_at  hypothetical protein LOC304743  Info   LOC304743 
   5082 0.0004905 90.7 132.1 0.687  1398535_at  Hbs1-like (S. cerevisiae) (predicted)  Info   Hbs1l_predicted 
   5083 0.0004913 45.5 92.8 0.49  1376041_at  epsin 3 (predicted)  Info   Epn3_predicted 
   5084 0.0004928 309.3 181.6 1.703  1377743_at  Sprouty protein with EVH-1 domain 1, related sequence (predicted)  Info    
   5085 0.0004932 33.1 63.3 0.523  1394651_at  similar to hypothetical protein FLJ32884 (predicted)  Info   RGD1309453_predicted 
   5086 0.0004939 881.1 653.9 1.347  1375205_at  p300/CBP-associated factor (predicted)  Info   Pcaf_predicted 
   5087 0.000494 7894.1 4659.1 1.694  1370154_at  lysozyme  Info   Lyz 
   5088 0.0004946 322.8 541 0.597  1384394_at    Info    
   5089 0.0004962 134.7 195.4 0.689  1390478_at  origin recognition complex, subunit 4  Info   RGD:619987 
   5090 0.0004974 687.5 472.6 1.455  1387458_at  ring finger protein 4  Info   Rnf4 
   5091 0.0004974 99.6 59.3 1.68  1388987_at  similar to expressed sequence AA960436 (predicted)  Info   RGD1305215_predicted 
   5092 0.0004978 22.4 3.4 6.588  1381536_at  Similar to expressed sequence C79127  Info    
   5093 0.0004982 112.4 73.4 1.531  1393563_at  interleukin 1 receptor accessory protein  Info   Il1rap 
   5094 0.0004983 1701.5 2012.7 0.845  1373408_at  tubulin cofactor a (predicted)  Info   Tbca_predicted 
   5095 0.0004983 30.5 46.7 0.653  1373581_at  similar to KIAA0672 gene product (predicted)  Info   RGD1305664_predicted 
   5096 0.0004987 308.4 246.2 1.253  1398371_at  similar to RIKEN cDNA 1700034M03 gene (predicted)  Info   RGD1304751_predicted 
   5097 0.0004987 34.3 57.6 0.595  1374511_at    Info    
   5098 0.0004989 770.8 409.7 1.881  1370376_a_at  cold shock domain protein A  Info   Csda 
   5099 0.0004993 69.8 105.7 0.66  1390957_at  Similar to Protein C14orf101 homolog  Info    
   5100 0.0004995 157.3 230.9 0.681  1382131_at  Transcribed locus  Info    
   5101 0.0004996 16.1 28.9 0.557  1385114_at  similar to Protein C20orf46  Info   LOC499917 
   5102 5e-04 354.2 461 0.768  1371468_at  Transcribed locus, strongly similar to NP_001003955.1 RAB11 family interacting protein 5 (class I) isoform 1 [Mus musculus]  Info    
   5103 0.0005007 15.7 63.6 0.247  1388545_at  Transcribed locus  Info    
   5104 0.0005009 102.1 65.7 1.554  1391893_at  similar to ATP/GTP-binding protein (predicted)  Info   RGD1307679_predicted 
   5105 0.0005012 31.2 8.4 3.714  1372278_at    Info    
   5106 0.0005013 876.3 635.1 1.38  1369452_a_at  phosphatidylinositol binding clathrin assembly protein  Info   Picalm 
   5107 0.0005025 52.2 89.3 0.585  1372572_at  sialyltransferase 7F  Info   RGD:1303196 
   5108 0.0005039 257.7 138.6 1.859  1393401_at  Similar to 9530046H09Rik protein (predicted)  Info    
   5109 0.0005058 17.5 32.5 0.538  1378263_at  Transcribed locus  Info    
   5110 0.0005059 492.8 282.8 1.743  1376250_at  nuclear fragile X mental retardation protein interacting protein 1  Info   RGD:1359440 
   5111 0.0005062 266.7 419.3 0.636  1392274_at  Transcribed locus  Info    
   5112 0.0005066 46.9 66.9 0.701  1379342_at  similar to hypothetical protein FLJ20531 (predicted)  Info   RGD1306740_predicted 
   5113 0.0005072 68.3 103 0.663  1382049_at  similar to thymus atrophy-related protein (predicted)  Info   RGD1304607_predicted 
   5114 0.0005081 366.5 190.6 1.923  1391415_at  putatative 28 kDa protein  Info   RGD:727882 
   5115 0.0005082 138.1 222 0.622  1370642_s_at  platelet derived growth factor receptor, beta polypeptide /// hypothetical gene supported by NM_031525  Info   Pdgfrb /// LOC497724 
   5116 0.0005082 37 4.3 8.605  1378213_at  Transcribed locus  Info    
   5117 0.0005103 740.9 522.9 1.417  1371587_at  mitogen-activated protein kinase kinase 1 interacting protein 1 (predicted)  Info   Map2k1ip1_predicted 
   5118 0.0005104 246.9 447 0.552  1370158_at  myosin heavy chain 10, non-muscle  Info   Myh10 
   5119 0.0005106 186.9 278.4 0.671  1378506_at  Transcribed locus  Info    
   5120 0.0005112 184.3 112.2 1.643  1370942_at  RAS p21 protein activator 3  Info   Rasa3 
   5121 0.0005115 65.5 18.3 3.579  1377867_at  similar to Glutaminyl-peptide cyclotransferase precursor (QC) (Glutaminyl-tRNA cyclotransferase) (Glutaminyl cyclase)  Info   LOC313837 
   5122 0.0005116 21.8 63.6 0.343  1370132_at  FK506 binding protein 1b  Info   Fkbp1b 
   5123 0.0005117 653.6 1299.7 0.503  1371496_at    Info    
   5124 0.0005122 1224.1 2024.6 0.605  1367636_at  insulin-like growth factor 2 receptor  Info   Igf2r 
   5125 0.0005125 152.5 121.1 1.259  1384821_at  Transcribed locus  Info    
   5126 0.0005136 1227.2 934.4 1.313  1375338_at  RAB10, member RAS oncogene family  Info   Rab10 
   5127 0.0005138 535.2 719.4 0.744  1382150_at  similar to RIKEN cDNA 1200007B05 gene (predicted)  Info   RGD1305072_predicted 
   5128 0.0005143 76.6 156.9 0.488  1373596_at  similar to hypothetical protein FLJ31737 (predicted)  Info   RGD1310423_predicted 
   5129 0.0005147 590.9 380.7 1.552  1370004_at  H2A histone family, member Y  Info   H2afy 
   5130 0.0005153 69.7 48.6 1.434  1374354_at  LOC499785  Info   LOC499785 
   5131 0.0005153 49.9 84 0.594  1383886_at  Transcribed locus  Info    
   5132 0.0005156 328.6 407 0.807  1399059_at  Enhancer of zeste homolog 1 (Drosophila) (predicted)  Info    
   5133 0.0005158 76.9 16.7 4.605  1384939_at  Transcribed locus  Info    
   5134 0.0005165 1.2 12.8 0.094  1395495_at  Transcribed locus  Info    
   5135 0.0005172 28.6 50.9 0.562  1383331_at  Transcribed locus  Info    
   5136 0.0005174 47.5 67.9 0.7  1384430_at  Transcribed locus  Info    
   5137 0.0005182 214.8 312.3 0.688  1378037_at  similar to RIKEN cDNA 0610009J22 (predicted)  Info   RGD1310738_predicted 
   5138 0.0005183 291.5 401.1 0.727  1371560_at  interferon regulatory factor 3  Info   Irf3 
   5139 0.0005198 175.3 126.3 1.388  1383099_at  LOC499526  Info   LOC499526 
   5140 0.0005199 167.7 232 0.723  1391194_at  Sal-like 1 (Drosophila) (predicted)  Info    
   5141 0.0005199 393.5 520.5 0.756  1372538_at  solute carrier family 25 (mitochondrial carrier, peroxisomal membrane protein), member 17 (predicted)  Info   Slc25a17_predicted 
   5142 0.0005202 62.5 95.4 0.655  1376638_at  similar to RIKEN cDNA 2700067D09 (predicted)  Info   RGD1305833_predicted 
   5143 0.0005211 287.3 509.7 0.564  1380465_at  Transcribed locus  Info    
   5144 0.0005228 143.8 241.6 0.595  1379336_at  Transcribed locus  Info    
   5145 0.0005238 1305.5 1063.9 1.227  1399095_at  SMT3 suppressor of mif two 3 homolog 1 (yeast) (predicted)  Info   Sumo1_predicted 
   5146 0.0005247 177.1 280.1 0.632  1373645_at  Transcribed locus  Info    
   5147 0.0005251 284.3 437.7 0.65  1388756_at  similar to RIKEN cDNA 6330579B17 gene (predicted)  Info   RGD1311399_predicted 
   5148 0.0005254 43.3 78 0.555  1371004_at  sortilin 1  Info   Sort1 
   5149 0.000526 607.2 365.3 1.662  1374678_at  Transcribed locus, strongly similar to NP_038687.1 semaphorin 4B precursor [Mus musculus]  Info    
   5150 0.000526 230.3 312 0.738  1390501_at  Transcribed locus  Info    
   5151 0.0005261 159.6 243.5 0.655  1390819_at  Thyrotroph embryonic factor  Info   Tef 
   5152 0.0005264 274.7 425.4 0.646  1372443_at  low density lipoprotein receptor-related protein 11 (predicted)  Info   Lrp11_predicted 
   5153 0.0005265 145.4 224.6 0.647  1383640_at  Phosphatidylinositol glycan, class N (predicted)  Info    
   5154 0.0005266 73.7 132.6 0.556  1396101_at  stanniocalcin 1  Info   Stc1 
   5155 0.0005272 130.5 187.4 0.696  1389380_at    Info    
   5156 0.0005274 1554.9 1894.4 0.821  1367496_at  transmembrane 9 superfamily member 2  Info   RGD:1359230 
   5157 0.0005276 1657.8 1015.7 1.632  1372520_at  myeloid cell leukemia sequence 1  Info   Mcl1 
   5158 0.0005284 116.2 68.2 1.704  1377052_at  Tripartite motif protein 26  Info   Trim26 
   5159 0.0005308 14.4 6.8 2.118  1391847_at  Peroxisome proliferator activated receptor alpha  Info   Ppara 
   5160 0.0005315 3050.9 3700.7 0.824  1370235_at  diazepam binding inhibitor  Info   Dbi 
   5161 0.000532 48 96.8 0.496  1378327_at  doublesex and mab-3 related transcription factor 2 (predicted)  Info   Dmrt2_predicted 
   5162 0.0005322 357.9 523.3 0.684  1398826_s_at  nuclear receptor subfamily 2, group F, member 6  Info   Nr2f6 
   5163 0.0005323 470.1 714.6 0.658  1371997_at  aldo-keto reductase family 1, member E1 (predicted)  Info   Akr1e1_predicted 
   5164 0.0005328 772 615.8 1.254  1387041_at  ubiquilin 1  Info   Ubqln1 
   5165 0.0005333 698 488 1.43  1370554_at  ubiquitin carboxyl-terminal esterase L3 (ubiquitin thiolesterase) /// similar to ubiqutin carboxyl-terminal hydrolase l3  Info   Uchl3 /// LOC498560 
   5166 0.0005333 44.7 14.4 3.104  1370148_at  haptoglobin  Info   Hp 
   5167 0.0005335 1420.8 1741.8 0.816  1388851_at  heat shock protein, A (predicted)  Info   Hspa9a_predicted 
   5168 0.0005337 635.9 869.7 0.731  1389050_at  Similar to Dual specificity protein phosphatase 3 (T-DSP11)  Info    
   5169 0.0005345 181.4 85.8 2.114  1382616_at  glutaminase  Info   Gls 
   5170 0.0005349 48.5 77.5 0.626  1390665_at  LOC362424 (predicted)  Info    
   5171 0.0005359 601.3 477.8 1.258  1372419_at  vaccinia related kinase 3  Info   Vrk3 
   5172 0.0005364 1036.2 1246.9 0.831  1374413_at  ubiquitin-like 3 (predicted)  Info   Ubl3_predicted 
   5173 0.0005368 40.3 85.7 0.47  1385667_x_at  CDNA clone IMAGE:7379585  Info    
   5174 0.000537 298.9 406.1 0.736  1371668_at  Retinoid X receptor alpha  Info   Rxra 
   5175 0.0005372 90.4 145.2 0.623  1374969_at  forkhead box D4  Info   Foxd4 
   5176 0.0005391 2245 3139.1 0.715  1367702_at  acetyl-Coenzyme A dehydrogenase, medium chain  Info   Acadm 
   5177 0.0005398 92.2 152.1 0.606  1392125_at    Info    
   5178 0.0005399 85.3 40.9 2.086  1383675_at  Transcribed locus  Info    
   5179 0.0005404 599.9 732.2 0.819  1373826_at    Info    
   5180 0.0005407 47.2 71.9 0.656  1392705_at  similar to mKIAA0267 protein  Info   LOC302863 
   5181 0.0005409 218 86.4 2.523  1368914_at  runt related transcription factor 1  Info   Runx1 
   5182 0.0005414 168.4 111.3 1.513  1378440_at  Transcribed locus  Info    
   5183 0.0005418 436.5 739.6 0.59  1381190_at    Info    
   5184 0.0005428 7.4 16 0.463  1393761_at  Transcribed locus, moderately similar to XP_221920.3 PREDICTED: similar to RB-associated KRAB repressor [Rattus norvegicus]  Info    
   5185 0.000543 130 200.9 0.647  1385638_at  Transcribed locus  Info    
   5186 0.0005433 803.5 1104.6 0.727  1372175_at  Transcribed locus, weakly similar to XP_342574.1 PREDICTED: similar to Csr1 [Rattus norvegicus]  Info    
   5187 0.0005441 110.9 173.5 0.639  1368358_a_at  protein tyrosine phosphatase, receptor type, R  Info   Ptprr 
   5188 0.0005441 50.5 32 1.578  1384757_at  similar to mKIAA1612 protein (predicted)  Info   RGD1308848_predicted 
   5189 0.0005442 16.2 44.5 0.364  1380115_at    Info    
   5190 0.0005442 644 19.3 33.368  1368128_at  phospholipase A2, group IIA (platelets, synovial fluid)  Info   Pla2g2a 
   5191 0.0005444 295.3 188.8 1.564  1371879_at  similar to RIKEN cDNA A930011F22 gene (predicted)  Info   RGD1308919_predicted 
   5192 0.0005461 999 722.3 1.383  1374712_at  proteasome (prosome, macropain) 26S subunit, non-ATPase, 11 (predicted)  Info   Psmd11_predicted 
   5193 0.0005485 121.4 177.5 0.684  1389418_at    Info    
   5194 0.0005491 487.3 384.4 1.268  1369935_at  cyclin D3  Info   Ccnd3 
   5195 0.0005495 48.8 97.2 0.502  1370474_at  thyroid hormone receptor beta  Info   Thrb 
   5196 0.00055 163 241.1 0.676  1392644_s_at  similar to RIKEN cDNA 2610019F03  Info   LOC498662 
   5197 0.0005502 217.8 310.3 0.702  1392713_a_at    Info    
   5198 0.0005508 434.6 147.2 2.952  1374404_at  v-jun sarcoma virus 17 oncogene homolog (avian)  Info   Jun 
   5199 0.0005509 703 486.6 1.445  1388530_at  Similar to hypothetical protein  Info    
   5200 0.0005514 60.7 89.1 0.681  1376013_at  similar to mKIAA0227 protein  Info   LOC500689 
   5201 0.0005523 24.9 47.1 0.529  1394195_at  Transcribed locus  Info    
   5202 0.0005525 390.5 301 1.297  1376974_at  tetratricopeptide repeat domain 7 (predicted)  Info   Ttc7_predicted 
   5203 0.0005526 1294.6 839.9 1.541  1386999_at  tyrosine 3-monooxygenase/tryptophan 5-monooxygenase activation protein, beta polypeptide  Info   Ywhab 
   5204 0.0005536 201.8 169 1.194  1370437_at  nucleoporin like 1  Info   Nupl1 
   5205 0.0005537 32.3 60 0.538  1383258_at  similar to hypothetical protein FLJ14146 (predicted)  Info   RGD1310587_predicted 
   5206 0.0005541 110.5 55.1 2.005  1368126_at  acetoacetyl-CoA synthetase  Info   Aacs 
   5207 0.0005544 1156.3 1359.3 0.851  1387863_at  upstream of NRAS  Info   Unr 
   5208 0.0005549 990.6 829.1 1.195  1373142_at  Growth hormone inducible transmembrane protein  Info   Ghitm 
   5209 0.000555 94.5 143.2 0.66  1376776_at  similar to hypothetical protein FLJ33084  Info   LOC500528 
   5210 0.0005551 1050.4 729.4 1.44  1399159_a_at  vesicle-associated membrane protein 3  Info   Vamp3 
   5211 0.0005552 155.9 91.6 1.702  1376751_at  serine/threonine kinase 19  Info   RGD:1359601 
   5212 0.0005554 103.8 138.2 0.751  1390357_at  zinc finger protein 574 (predicted)  Info   Znf574_predicted 
   5213 0.0005567 115.8 190.7 0.607  1375915_at  interleukin-1 receptor-associated kinase 1 binding protein 1 (predicted)  Info   Irak1bp1_predicted 
   5214 0.000557 28.1 91.2 0.308  1374012_at  Mitsugumin 29 (predicted)  Info   Mg29 
   5215 0.0005571 2669.7 1068.5 2.499  1367655_at  similar to thymosin, beta 10  Info   LOC502600 
   5216 0.0005575 141.5 225.4 0.628  1374613_at  Transcribed locus  Info    
   5217 0.0005578 63.2 103.6 0.61  1391672_at  Transcribed locus  Info    
   5218 0.000558 64.2 110.9 0.579  1377196_at  Solute carrier family 5 (sodium/glucose cotransporter), member 10 (predicted)  Info    
   5219 0.0005593 586.4 470.4 1.247  1371745_at  hypothetical LOC300361 (predicted)  Info   RGD1310669_predicted 
   5220 0.0005594 203.9 304.3 0.67  1376656_at  ubiquitin specific protease 20 (predicted)  Info   Usp20_predicted 
   5221 0.0005598 319.3 484.1 0.66  1372282_at  similar to RIKEN cDNA 2310016C16 (predicted)  Info   RGD1307506_predicted 
   5222 0.0005599 238.1 330.5 0.72  1376705_a_at  RAB24, member RAS oncogene family (predicted)  Info   Rab24_predicted 
   5223 0.0005605 111.5 68.7 1.623  1377380_at  Transcribed locus  Info    
   5224 0.0005615 123.7 73.7 1.678  1391161_at    Info    
   5225 0.000562 1566.5 1283.9 1.22  1371249_at  X-box binding protein 1  Info   RGD:1303073 
   5226 0.0005625 46.2 9.1 5.077  1390890_at  Transcribed locus  Info    
   5227 0.0005628 343.1 228.8 1.5  1377675_at  LOC498261  Info   LOC498261 
   5228 0.0005642 431.9 766.2 0.564  1379578_at  similar to Zbtb20 protein  Info   LOC288105 
   5229 0.0005644 15.1 29.1 0.519  1381363_at  Low density lipoprotein receptor-related protein 6 (predicted)  Info    
   5230 0.0005649 286.5 389.1 0.736  1389148_at  similar to RIKEN cDNA 9430083G14  Info   LOC317191 
   5231 0.0005655 465.4 592.6 0.785  1388518_at  F-box and WD-40 domain protein 5 (predicted)  Info   Fbxw5_predicted 
   5232 0.0005661 412.7 579.2 0.713  1398262_at  phosphoribosyl pyrophosphate synthetase 2  Info   Prps2 
   5233 0.0005673 241.2 157.5 1.531  1373632_at  TAF9 RNA polymerase II, TATA box binding protein (TBP)-associated factor  Info   Taf9 
   5234 0.0005677 172.9 69.9 2.474  1397676_at  Similar to osteoclast inhibitory lectin  Info    
   5235 0.0005684 37.3 95.6 0.39  1381940_at  Transcribed locus  Info    
   5236 0.0005696 29 60.7 0.478  1391068_at  E030032D13Rik gene  Info   RGD:1303180 
   5237 0.0005713 43.3 102.7 0.422  1379545_at  Transcribed locus, weakly similar to NP_783624.1 hypothetical protein LOC319321 [Mus musculus]  Info    
   5238 0.0005718 35.4 8.3 4.265  1380531_at  solute carrier family 9 (sodium/hydrogen exchanger), isoform 9 (predicted)  Info   Slc9a9_predicted 
   5239 0.0005722 654.9 1190.7 0.55  1372774_at  Coenzyme Q6 homolog (yeast) (predicted)  Info    
   5240 0.0005728 2245.4 1363.9 1.646  1387892_at  tubulin, beta 5  Info   Tubb5 
   5241 0.0005729 31.9 12.1 2.636  1373759_at  Transcribed locus  Info    
   5242 0.000574 63.7 108 0.59  1371059_at  protein kinase, cAMP-dependent, regulatory, type 2, alpha  Info   Prkar2a 
   5243 0.0005742 272.1 352.8 0.771  1388925_at  sirtuin 5  Info   RGD:1303285 
   5244 0.000575 355.7 187 1.902  1373401_at  Transcribed locus  Info    
   5245 0.0005769 345.5 487 0.709  1388383_at  Transcribed locus  Info    
   5246 0.0005772 52.4 25.3 2.071  1379780_at  Similar to mKIAA0386 protein (predicted)  Info    
   5247 0.0005772 4595.5 3639.3 1.263  1398762_at  syndecan binding protein  Info   Sdcbp 
   5248 0.0005774 161.3 277.1 0.582  1370302_at  thyroid hormone-response protein-1  Info   RGD:620882 
   5249 0.0005778 21.5 39.7 0.542  1381264_at  Transcribed locus  Info    
   5250 0.0005783 56.3 37.2 1.513  1392128_at  Transcribed locus  Info    
   5251 0.0005785 91.1 43.6 2.089  1375071_at  nucleoporin 133 (predicted)  Info   Nup133_predicted 
   5252 0.0005789 560.8 451 1.243  1373052_at  phosducin-like 3 (predicted)  Info   Pdcl3_predicted 
   5253 0.00058 64.1 98.2 0.653  1382004_at  Transcribed locus  Info    
   5254 0.0005817 197 142.7 1.381  1372840_at  Similar to Zbed4 protein  Info    
   5255 0.000582 171.2 273.1 0.627  1388932_at  laminin, alpha 5  Info   Lama5 
   5256 0.0005823 81.1 129 0.629  1390872_at  Similar to RIKEN cDNA 4921524P20 (predicted)  Info    
   5257 0.0005827 915.1 1147.5 0.797  1367477_at  mitochondrial ribosomal protein L53 (predicted)  Info   Mrpl53_predicted 
   5258 0.0005834 274.4 119.8 2.29  1368260_at  aurora kinase B  Info   Aurkb 
   5259 0.000584 95 161.3 0.589  1371354_at  Troponin C, cardiac/slow skeletal (predicted)  Info    
   5260 0.0005843 268.5 368.7 0.728  1380979_a_at  similar to KIAA0913 protein (predicted)  Info   RGD1309414_predicted 
   5261 0.0005844 309.7 425.1 0.729  1372733_at  Similar to intracellular membrane-associated calcium-independent phospholipase A2 gamma (predicted)  Info    
   5262 0.0005849 142.5 261 0.546  1397788_at  RIM binding protein 2  Info   LOC266780 
   5263 0.0005849 196.2 150.2 1.306  1375973_at  ariadne ubiquitin-conjugating enzyme E2 binding protein homolog 1 (Drosophila) (predicted)  Info   Arih1_predicted 
   5264 0.000585 120.8 152.5 0.792  1372465_at    Info    
   5265 0.0005859 222.6 124.3 1.791  1381403_at  malic enzyme 2, NAD(+)-dependent, mitochondrial (predicted)  Info   Me2_predicted 
   5266 0.0005859 28.7 57.6 0.498  1391822_at  Transcribed locus  Info    
   5267 0.000586 301.5 442.1 0.682  1399102_at  similar to hypothetical protein MGC15523 (predicted)  Info   RGD1306356_predicted 
   5268 0.0005866 48.7 73.1 0.666  1376630_at    Info    
   5269 0.0005867 238.7 366.7 0.651  1373162_at  NYGGF3  Info    
   5270 0.0005872 125.9 61.4 2.05  1394160_at  Transmembrane protein 2 (predicted)  Info    
   5271 0.0005877 440 323.3 1.361  1371862_at  ribonucleotide reductase M1  Info   Rrm1 
   5272 0.0005878 121.7 82 1.484  1390390_at  transcription factor 20  Info   Tcf20 
   5273 0.0005884 190.7 322.2 0.592  1371615_at  diacylglycerol O-acyltransferase homolog 2 (mouse)  Info   Dgat2 
   5274 0.0005884 66 104.6 0.631  1394467_at  Transcribed locus  Info    
   5275 0.0005888 213.5 146.9 1.453  1380201_at  similar to hypothetical protein FLJ14075  Info   RGD:1359447 
   5276 0.0005889 228.2 341.1 0.669  1377166_at  amyotrophic lateral sclerosis 2 (juvenile) (predicted)  Info   Als2_predicted 
   5277 0.000589 32.2 14 2.3  1384233_at  Transcribed locus  Info    
   5278 0.0005891 546.5 351 1.557  1383616_at  similar to class II cytokine receptor 4  Info   LOC304091 
   5279 0.0005893 513.6 336.4 1.527  1374118_at  leucyl-tRNA synthetase (predicted)  Info   Lars_predicted 
   5280 0.0005898 17.4 50.3 0.346  1383567_at  collectin sub-family member 11 (predicted)  Info   Colec11_predicted 
   5281 0.0005901 281.7 237.1 1.188  1388532_at  similar to hypothetical protein (predicted)  Info   RGD1310571_predicted 
   5282 0.0005909 208.5 147.2 1.416  1377760_at  similar to hypothetical protein MGC28606 (predicted)  Info   RGD1310661_predicted 
   5283 0.0005923 28.4 65 0.437  1380120_at  microtubule-associated protein 7 (predicted)  Info   Mtap7_predicted 
   5284 0.0005923 1130.4 1405.4 0.804  1371962_at  Tu translation elongation factor, mitochondrial (predicted)  Info   Tufm_predicted 
   5285 0.0005927 78.9 11.2 7.045  1387638_a_at  cytotoxic T-lymphocyte-associated protein 4  Info   Ctla4 
   5286 0.0005927 13.9 27.9 0.498  1396977_at  Transcribed locus  Info    
   5287 0.0005934 207.5 138.6 1.497  1392487_at  Transcribed locus  Info    
   5288 0.0005952 192.9 137.8 1.4  1383250_at  similar to KIAA0266 gene product  Info   RGD:1359313 
   5289 0.0005957 49.8 87.1 0.572  1397307_at  Similar to Tescalcin  Info    
   5290 0.0005958 423.7 592.5 0.715  1371711_at  Transcribed locus  Info    
   5291 0.0005964 118.5 184.2 0.643  1377079_a_at  protoporphyrinogen oxidase (predicted)  Info   Ppox_predicted 
   5292 0.0005971 606.7 497.2 1.22  1377735_at  pleckstrin homology domain-containing, family A (phosphoinositide binding specific) member 3 (predicted)  Info   Plekha3_predicted 
   5293 0.0005975 218.8 124.3 1.76  1398985_at  GTP binding protein 1 (predicted)  Info    
   5294 0.0005978 28.3 55.9 0.506  1375243_at  Similar to BTB (POZ) domain containing 2  Info    
   5295 0.0005993 913.4 1646.5 0.555  1371824_at  adenylate kinase 3-like 1  Info   Ak3l1 
   5296 0.0006002 1043.7 744.5 1.402  1371099_at  Polymeric immunoglobulin receptor AATTAA-containing 3'UTR mRNA sequence  Info    
   5297 0.000601 372.2 461.1 0.807  1373069_at  mitochondrial ribosomal protein S30 (predicted)  Info   Mrps30_predicted 
   5298 0.0006015 175.5 231.6 0.758  1389313_at  Transcribed locus, strongly similar to XP_203601.5 PREDICTED: phosphofurin acidic cluster sorting protein 1-like [Mus musculus]  Info    
   5299 0.0006017 27.7 63.6 0.436  1384487_at  doublesex and mab-3 related transcription factor 2 (predicted)  Info   Dmrt2_predicted 
   5300 0.0006017 59.8 136.1 0.439  1374630_at  chloride intracellular channel 3 (predicted)  Info   Clic3_predicted 
   5301 0.0006018 152.5 106.2 1.436  1374305_at  adaptor protein complex AP-1, gamma 2 subunit (predicted)  Info   Ap1g2_predicted 
   5302 0.000602 16.9 38.3 0.441  1396109_at  Transcribed locus  Info    
   5303 0.000604 70.6 47 1.502  1392706_at  Cadherin 23 (otocadherin)  Info   Cdh23 
   5304 0.0006053 46.6 91.6 0.509  1394216_at  similar to RIKEN cDNA 2810055F11 (predicted)  Info   RGD1305721_predicted 
   5305 0.0006059 213.5 131.8 1.62  1374615_at    Info    
   5306 0.0006074 168.5 123.3 1.367  1368967_at  eukaryotic translation initiation factor 2B, subunit 3 gamma  Info   Eif2b3 
   5307 0.000608 8 20.9 0.383  1392316_at  Transcribed locus  Info    
   5308 0.0006087 976.7 1426.1 0.685  1370906_at  branched chain keto acid dehydrogenase E1, beta polypeptide  Info   Bckdhb 
   5309 0.0006088 38.5 83 0.464  1392947_at  CDNA clone IMAGE:7379585  Info    
   5310 0.000609 90.2 138.2 0.653  1383299_at  Similar to KIAA0965 protein  Info    
   5311 0.000609 62.5 85.1 0.734  1385470_at  Transcribed locus  Info    
   5312 0.0006091 181.8 240.9 0.755  1391443_at  similar to chromosome 14 open reading frame 35  Info   LOC498489 
   5313 0.0006091 271.4 379.4 0.715  1373448_at  acylphosphatase 1, erythrocyte (common) type (predicted)  Info   Acyp1_predicted 
   5314 0.0006091 1394.2 1006.5 1.385  1398801_at  CDK105 protein  Info   RGD:621335 
   5315 0.0006099 1611.5 2116 0.762  1367722_at  dipeptidylpeptidase 7  Info   Dpp7 
   5316 0.0006103 674 505.3 1.334  1385921_at  brain zinc finger protein /// similar to brain Zn-finger protein  Info   RGD:1359234 /// LOC498247 
   5317 0.0006123 87.7 51.3 1.71  1382400_at  rearranged L-myc fusion sequence (predicted)  Info   Rlf_predicted 
   5318 0.0006127 306.8 219 1.401  1392498_at  similar to protein phosphatase 2A B56 regulatory subunit gamma 3 isoform  Info   LOC500722 
   5319 0.0006128 1876 2439.8 0.769  1370006_at  NADH dehydrogenase (ubiquinone) Fe-S protein 6  Info   Ndufs6 
   5320 0.0006139 245.8 161.7 1.52  1372432_at  PRP3 pre-mRNA processing factor 3 homolog (yeast) (predicted)  Info   Prpf3_predicted 
   5321 0.0006147 878 634 1.385  1368143_at  annexin A7  Info   Anxa7 
   5322 0.0006148 2624.8 1724.6 1.522  1371973_at  eukaryotic translation initiation factor 3, subunit 6 (predicted)  Info   Eif3s6_predicted 
   5323 0.0006164 62.3 102.7 0.607  1385929_at  Zinc finger protein 11 (predicted)  Info    
   5324 0.0006165 80.1 151.1 0.53  1384824_at  protocadherin 18 (predicted)  Info   Pcdh18_predicted 
   5325 0.0006165 59.2 132.9 0.445  1373689_at  Transcribed locus  Info    
   5326 0.0006168 86.2 164.1 0.525  1383497_at  CDNA clone IMAGE:7300848  Info    
   5327 0.0006169 628.5 467.4 1.345  1389038_at  eukaryotic translation termination factor 1 (predicted)  Info   Etf1_predicted 
   5328 0.0006171 370.7 259.2 1.43  1376576_at  dual specificity phosphatase 11 (RNA/RNP complex 1-interacting) (predicted)  Info   Dusp11_predicted 
   5329 0.0006179 253.5 173.3 1.463  1395465_at    Info    
   5330 0.0006183 21 76.4 0.275  1375057_at  similar to Hypothetical protein 6720430O15  Info   LOC311210 
   5331 0.000619 6.1 25.4 0.24  1380763_at    Info    
   5332 0.0006212 63.2 98.6 0.641  1389281_at  Transcribed locus, strongly similar to XP_238535.3 PREDICTED: similar to ankyrin repeat domain 25 [Rattus norvegicus]  Info    
   5333 0.0006212 162.1 206.8 0.784  1374807_at  similar to RIKEN cDNA 1810063B07 gene (predicted)  Info   RGD1310877_predicted 
   5334 0.0006212 22.3 32.1 0.695  1372296_at  similar to putative SH3BGR protein  Info   LOC498066 
   5335 0.0006229 262.7 373.4 0.704  1376673_at  Transcription factor 8 (represses interleukin 2 expression)  Info   Tcf8 
   5336 0.0006231 13 40.3 0.323  1386045_at  Transcribed locus  Info    
   5337 0.0006238 592.5 487.3 1.216  1389428_at    Info    
   5338 0.0006244 86.2 157.6 0.547  1391656_at    Info    
   5339 0.0006245 1636.4 1215.5 1.346  1367693_at  tyrosine 3-monooxygenase/tryptophan 5-monooxygenase activation protein, eta polypeptide  Info   Ywhah 
   5340 0.0006246 141.8 196.6 0.721  1399138_at  similar to expressed sequence AA408877 (predicted)  Info   RGD1308513_predicted 
   5341 0.0006252 105.5 175.3 0.602  1381974_at  BTB (POZ) domain containing 3 (predicted)  Info    
   5342 0.0006259 94.5 62 1.524  1396521_at  StAR-related lipid transfer (START) domain containing 4 (predicted)  Info   Stard4_predicted 
   5343 0.0006262 688.5 892.5 0.771  1389265_at  glucan (1,4-alpha-), branching enzyme 1 (predicted)  Info   Gbe1_predicted 
   5344 0.0006277 355.9 459.6 0.774  1385838_a_at  beta-amyloid binding protein precursor (predicted)  Info   Bbp_predicted 
   5345 0.0006285 133.3 187.8 0.71  1382219_at  similar to RIKEN cDNA 1700108L22 (predicted)  Info   RGD1307509_predicted 
   5346 0.0006286 2180.6 4545.4 0.48  1384831_at  solute carrier family 7, (cationic amino acid transporter, y+ system) member 13 (predicted)  Info   Slc7a13_predicted 
   5347 0.0006296 168.9 89.1 1.896  1370419_a_at  SH3-domain kinase binding protein 1  Info   Sh3kbp1 
   5348 0.0006297 313.7 190.1 1.65  1370910_at  replication factor C (activator 1) 2  Info   Rfc2 
   5349 0.0006298 637.8 469.9 1.357  1373595_at  similar to RIKEN cDNA 1200015A22  Info   MGC94413 
   5350 0.0006298 579.4 422.3 1.372  1368674_at  liver glycogen phosphorylase  Info   Pygl 
   5351 0.0006298 92 189.2 0.486  1376518_at    Info    
   5352 0.0006305 545.7 732.5 0.745  1397468_at  similar to RIKEN cDNA 2610207I16 (predicted)  Info   RGD1305387_predicted 
   5353 0.0006317 295.1 356.5 0.828  1370134_at  acetyl-CoA transporter  Info   Slc33a1 
   5354 0.0006317 31.8 60.2 0.528  1387184_at  axin2  Info   Axin2 
   5355 0.0006324 90.2 59.7 1.511  1385344_at  AE binding protein 2 (predicted)  Info    
   5356 0.0006335 253.7 376.3 0.674  1377673_at  Similar to cyclin K  Info    
   5357 0.0006345 131.6 161.9 0.813  1374441_at  similar to RIKEN cDNA 4633402N23 gene (predicted)  Info   RGD1306649_predicted 
   5358 0.000635 48 85.3 0.563  1397821_at  Transcribed locus  Info    
   5359 0.0006368 26.1 56.1 0.465  1370588_a_at  solute carrier family 8 (sodium/calcium exchanger), member 1  Info   Slc8a1 
   5360 0.0006372 472.9 317.6 1.489  1370817_at  Sec11-like 3 (S. cerevisiae)  Info   Sec11l3 
   5361 0.0006379 69 131.4 0.525  1367845_at  neurofilament 3, medium  Info   Nef3 
   5362 0.0006383 208.7 272 0.767  1369278_at  guanine nucleotide binding protein, alpha 12  Info   Gna12 
   5363 0.0006385 31.8 67.7 0.47  1376170_at    Info    
   5364 0.0006386 223.9 335.8 0.667  1383376_at  similar to Williams-Beuren syndrome critical region protein 18  Info   LOC368190 
   5365 0.0006389 350.7 266.5 1.316  1383074_at    Info    
   5366 0.0006407 3140.5 2533.5 1.24  1398765_at  adaptor-related protein complex 2, mu 1 subunit  Info   Ap2m1 
   5367 0.0006411 9.5 24.2 0.393  1381650_at  Selenophosphate synthetase 1 (predicted)  Info   Sephs1 
   5368 0.0006414 373.7 259.9 1.438  1398980_at  suppressor of Ty 16 homolog (S. cerevisiae) (predicted)  Info   Supt16h_predicted 
   5369 0.0006416 74.8 121.8 0.614  1380600_at    Info    
   5370 0.0006422 110 151.7 0.725  1374680_at  Transcribed locus  Info    
   5371 0.0006423 291.9 197.6 1.477  1390409_at  LOC361774 (predicted)  Info   RGD1306116_predicted 
   5372 0.0006428 4384 5365.8 0.817  1367641_at  superoxide dismutase 1  Info   Sod1 
   5373 0.0006434 26.5 45.3 0.585  1376334_at  Similar to hypothetical protein FLJ20276 (predicted)  Info    
   5374 0.0006448 231.1 128.1 1.804  1388128_at  ARP3 actin-related protein 3 homolog (yeast)  Info   Actr3 
   5375 0.0006475 395.2 263.4 1.5  1372027_at  Transcribed locus  Info    
   5376 0.0006482 387.5 254.9 1.52  1389263_at  retinoic acid induced 14 (predicted)  Info   Rai14_predicted 
   5377 0.0006484 100 184.9 0.541  1396113_at  similar to Dorz1 (predicted)  Info   RGD1309721_predicted 
   5378 0.0006489 2276.5 1945 1.17  1375246_at    Info    
   5379 0.0006499 201.4 107.5 1.873  1373138_at  nudix-type motif 5  Info   RGD:1359284 
   5380 0.0006509 414.2 581 0.713  1375894_at  leucine zipper transcription factor-like 1 (predicted)  Info   Lztfl1_predicted 
   5381 0.000651 201.7 269.6 0.748  1390108_at  similar to RIKEN cDNA 2310079N02 (predicted)  Info   RGD1306192_predicted 
   5382 0.000651 30.8 65.2 0.472  1380089_at  Transcribed locus  Info    
   5383 0.0006514 233.8 375.4 0.623  1379402_at  ATP-binding cassette, sub-family C (CFTR/MRP), member 4  Info   Abcc4 
   5384 0.0006523 907.3 617.8 1.469  1379832_at  polymerase (RNA) II (DNA directed) polypeptide D (predicted)  Info   Polr2d_predicted 
   5385 0.0006531 92.5 161.9 0.571  1374104_at  claudin 5  Info   Cldn5 
   5386 0.0006533 113 148.2 0.762  1374112_at  similar to MAPK-interacting and spindle-stabilizing protein  Info   LOC499280 
   5387 0.000654 308.6 245.3 1.258  1376091_at  adenylosuccinate lyase (predicted)  Info   Adsl_predicted 
   5388 0.000654 56.6 107.5 0.527  1378896_at    Info    
   5389 0.0006546 94.1 14.8 6.358  1368501_s_at  mast cell protease 8 /// mast cell protease 10 /// mast cell protease 9 /// mast cell protease 8-like 2  Info   Mcpt8 /// Mcpt10 /// Mcpt9 /// RGD:1302938 
   5390 0.000655 51.2 77.4 0.661  1368473_at  gap junction membrane channel protein alpha 5  Info   Gja5 
   5391 0.0006566 1252.7 1056.1 1.186  1367870_at  thioredoxin-like 2  Info   Txnl2 
   5392 0.0006568 19 51.2 0.371  1396014_at  Transcribed locus  Info    
   5393 0.000657 65.3 32.3 2.022  1394953_at  sprouty protein with EVH-1 domain 1, related sequence (predicted)  Info   Spred1_predicted 
   5394 0.0006578 145.8 243.4 0.599  1375954_at  S100 calcium binding protein A13 (predicted)  Info   S100a13_predicted 
   5395 0.0006587 77.4 10.4 7.442  1376872_at  Transcribed locus  Info    
   5396 0.000659 41.2 118 0.349  1397579_x_at  Similar to RIKEN cDNA 2310011J03  Info   MGC94198 
   5397 0.0006592 104.9 170.1 0.617  1374039_at  carbonic anhydrase 14 (predicted)  Info   Car14_predicted 
   5398 0.0006598 323.8 233.4 1.387  1389450_at  similar to Putative methyltransferase HUSSY-03 (Williams-Beuren syndrome chromosome region 22 protein homolog) /// similar to Putative methyltransferase HUSSY-03 (Williams-Beuren syndrome chromosome region 22 protein homolog)  Info   LOC360830 /// LOC368084 
   5399 0.0006599 107.6 71.4 1.507  1384439_at  CDNA clone IMAGE:7379980  Info    
   5400 0.00066 104.9 43.2 2.428  1391317_at  similar to RIKEN cDNA 2810433K01 (predicted)  Info   RGD1310784_predicted 
   5401 0.0006602 203.4 99.9 2.036  1370174_at  myeloid differentiation primary response gene 116  Info   Myd116 
   5402 0.0006603 240 103.3 2.323  1368224_at  Serine protease inhibitor  Info   Spin2c 
   5403 0.0006604 142.8 220.8 0.647  1394948_at  Arrestin domain containing 3  Info   Arrdc3 
   5404 0.0006605 180.4 117.2 1.539  1384451_at  Transcribed locus  Info    
   5405 0.000661 241.2 178.2 1.354  1367849_at  syndecan 1  Info   Sdc1 
   5406 0.0006613 312 493.7 0.632  1376619_at  Similar to protein tyrosine phosphatase, receptor type, D  Info    
   5407 0.0006613 85.5 147.1 0.581  1385614_at  melanophilin (predicted)  Info   Mlph_predicted 
   5408 0.0006626 309.5 218 1.42  1374194_at    Info    
   5409 0.0006637 279.3 194.1 1.439  1395533_at  dead end homolog 1 (zebrafish) (predicted)  Info   Dnd1_predicted 
   5410 0.0006639 51.4 14.6 3.521  1379039_at  Chemokine-like receptor 1  Info   Cmklr1 
   5411 0.0006645 14.5 1.7 8.529  1369586_at  mast cell protease 8  Info   Mcpt8 
   5412 0.0006649 2369.8 3090.9 0.767  1370230_at  ATP synthase, H+ transporting, mitochondrial F0 complex, subunit F6  Info   Atp5j 
   5413 0.0006654 91.6 159.9 0.573  1378292_at  Transcribed locus  Info    
   5414 0.0006666 375.4 261.3 1.437  1372417_at  SERTA domain containing 1  Info   Sertad1 
   5415 0.0006679 72.8 137.2 0.531  1370813_at  glutathione S-transferase, mu 5  Info   Gstm5 
   5416 0.0006681 1089.1 1318.2 0.826  1395265_at  Glucosamine (N-acetyl)-6-sulfatase (predicted)  Info    
   5417 0.0006682 383 256.8 1.491  1376483_at  LSM5 homolog, U6 small nuclear RNA associated (S. cerevisiae) (predicted)  Info   Lsm5_predicted 
   5418 0.0006684 18 34.7 0.519  1388138_at  thrombospondin 4  Info   Thbs4 
   5419 0.0006694 6.7 35.5 0.189  1374384_at  Crystallin, gamma C  Info   Crygc 
   5420 0.0006712 74 100.3 0.738  1386344_at  Progressive ankylosis homolog (mouse)  Info   Ank 
   5421 0.0006715 76.2 15.5 4.916  1368735_a_at  transient receptor potential cation channel, subfamily V, member 2  Info   Trpv2 
   5422 0.0006724 1307.5 951.8 1.374  1370057_at  cysteine and glycine-rich protein 1  Info   Csrp1 
   5423 0.0006728 205.7 373.4 0.551  1368672_at  arginase 2  Info   Arg2 
   5424 0.0006729 26.3 45.4 0.579  1370697_a_at  nexilin  Info   Nexn 
   5425 0.0006733 215.3 148.7 1.448  1368953_at  UDP-glucose ceramide glucosyltransferase-like 1  Info   Ugcgl1 
   5426 0.0006746 98 25.1 3.904  1374735_at  Rho GTPase activating protein 4  Info   Arhgap4 
   5427 0.0006778 35.2 100.8 0.349  1373000_at  similar to Sushi-repeat containing protein  Info   LOC317181 
   5428 0.0006785 84.4 131.6 0.641  1372332_at  Transcribed locus  Info    
   5429 0.0006792 142.1 221.4 0.642  1372870_at  KDEL (Lys-Asp-Glu-Leu) endoplasmic reticulum protein retention receptor 3 (predicted)  Info   Kdelr3_predicted 
   5430 0.0006795 1603.9 1267.4 1.266  1398829_at  FK506 binding protein 1a  Info   Fkbp1a 
   5431 0.0006802 96.1 218 0.441  1369652_at  thymus cell antigen 1, theta  Info   Thy1 
   5432 0.0006814 63.1 89.9 0.702  1368660_at  cAMP-regulated guanine nucleotide exchange factor I (cAMP-GEFI)  Info   Rapgef3 
   5433 0.0006816 119.2 182.8 0.652  1377702_at  Retinoblastoma 1  Info   Rb1 
   5434 0.0006816 110.2 65.7 1.677  1377041_at  manic fringe homolog (Drosophila)  Info   Mfng 
   5435 0.000682 1245.4 887.4 1.403  1388997_at  ADP-ribosylation factor 3  Info   Arf3 
   5436 0.0006823 318.8 436.2 0.731  1387547_a_at  EGF, latrophilin seven transmembrane domain containing 1  Info   Eltd1 
   5437 0.000683 180.3 113.1 1.594  1374133_at  tRNA isopentenyltransferase 1 (predicted)  Info   Trit1_predicted 
   5438 0.0006832 29.8 65.7 0.454  1370143_at  smoothened homolog (Drosophila)  Info   Smo 
   5439 0.0006836 699.1 440.2 1.588  1369182_at  coagulation factor 3  Info   F3 
   5440 0.0006839 178.3 258.2 0.691  1389169_at  progesterone receptor membrane component 2 (predicted)  Info   Pgrmc2_predicted 
   5441 0.000684 555.8 706 0.787  1375530_at  similar to EMeg32 protein  Info   LOC498486 
   5442 0.000684 506.7 631.7 0.802  1389033_at  similar to RIKEN cDNA 2900010M23 (predicted)  Info   RGD1306917_predicted 
   5443 0.0006872 2408.7 1897.5 1.269  1371422_at  MORF-related gene X  Info   RGD:1359471 
   5444 0.0006878 113.1 203.7 0.555  1373599_at  BRF2, subunit of RNA polymerase III transcription initiation factor, BRF1-like (predicted)  Info    
   5445 0.0006882 134.7 215.6 0.625  1374605_at  similar to hypothetical protein MGC33214 (predicted)  Info   RGD1309660_predicted 
   5446 0.0006887 128.6 211.6 0.608  1382371_at  hypothetical protein LOC362011  Info   LOC362011 
   5447 0.000689 355.7 447 0.796  1375745_at  Guanine nucleotide binding protein, alpha q polypeptide  Info   Gnaq 
   5448 0.00069 112.4 193.3 0.581  1390944_at  Snf7 homologue associated with Alix 3  Info   MGC108776 
   5449 0.0006902 264.6 424.5 0.623  1395621_at  similar to Mitochondrial carrier triple repeat 1  Info   LOC305350 
   5450 0.0006911 812.5 574.3 1.415  1371367_at  TAR DNA binding protein (predicted)  Info   Tardbp_predicted 
   5451 0.000692 132 190.6 0.693  1374403_at  ephrin B1  Info   Efnb1 
   5452 0.000692 309.7 402 0.77  1389459_at  Transcribed locus  Info    
   5453 0.0006936 27.3 57.8 0.472  1376734_at  Nephroblastoma overexpressed gene  Info   Nov 
   5454 0.0006943 41.8 70.1 0.596  1391944_at  similar to Hypothetical protein C6orf60 (predicted)  Info   RGD1311294_predicted 
   5455 0.0006944 85.9 128 0.671  1385211_at    Info    
   5456 0.0006957 13 54.4 0.239  1384334_at    Info    
   5457 0.000696 558.7 1095.4 0.51  1387228_at  solute carrier family 2 (facilitated glucose transporter), member 2  Info   Slc2a2 
   5458 0.0006961 14.9 24.5 0.608  1397865_at  Transcribed locus, strongly similar to NP_510964.1 mitochondrial ribosomal protein S21 [Mus musculus]  Info    
   5459 0.0006967 448 584 0.767  1389338_at  hypothetical LOC293114 (predicted)  Info   RGD1308371_predicted 
   5460 0.0006976 628.2 474.6 1.324  1389559_at  similar to Hypothetical protein MGC31278  Info   LOC362919 
   5461 0.0006976 208.9 147.8 1.413  1389345_at  Transcribed locus, strongly similar to NP_997598.1 F-box only protein 3 isoform 1 [Mus musculus]  Info    
   5462 0.0006982 105.8 166.6 0.635  1370317_at  potassium channel regulator 1  Info   RGD:708500 
   5463 0.0006988 224.6 327.5 0.686  1377076_at  Similar to sialic-acid O-acetylesterase (predicted)  Info    
   5464 0.0007003 177.5 234 0.759  1392835_at  zinc finger protein 68 (predicted)  Info   Zfp68_predicted 
   5465 0.0007013 4892.5 3708.7 1.319  1372701_at  heat shock protein 1, alpha  Info   Hspca 
   5466 0.0007016 5.9 17.2 0.343  1368632_at  forkhead box G1  Info   Foxg1 
   5467 0.0007017 27.6 48.7 0.567  1374315_at  Transcribed locus  Info    
   5468 0.0007018 46.5 64.2 0.724  1391812_at  Similar to RIKEN cDNA E330026B02 (predicted)  Info    
   5469 0.0007065 95.7 127.5 0.751  1380002_at  spire homolog 1 (Drosophila) (predicted)  Info   Spire1_predicted 
   5470 0.0007066 237.9 161.6 1.472  1370752_a_at  tumor protein p53  Info   Tp53 
   5471 0.0007067 328.6 468.8 0.701  1369061_at  glutathione reductase  Info   Gsr 
   5472 0.0007077 32.7 57.9 0.565  1397365_at  Similar to ribosomal protein L27a  Info    
   5473 0.0007081 27.6 45.2 0.611  1367785_at  calponin 1  Info   Cnn1 
   5474 0.0007095 356.7 488.6 0.73  1388969_at  similar to hypothetical protein LOC197322  Info   LOC498962 
   5475 0.00071 326.9 261.4 1.251  1391628_at  synovial sarcoma translocation, Chromosome 18 (predicted)  Info   Ss18_predicted 
   5476 0.0007103 1732.3 2785.9 0.622  1367774_at  glutathione S-transferase A5  Info   Gsta5 
   5477 0.0007103 1801.6 1454.7 1.238  1371495_at  cation-dependent mannose-6-phosphate receptor  Info   RGD:1359355 
   5478 0.0007104 390.5 1154.3 0.338  1368366_at  Camello-like 2  Info   Cml2 
   5479 0.0007105 1917.6 1499.1 1.279  1388930_at  similar to RIKEN cDNA 2310075C12 (predicted)  Info   RGD1305625_predicted 
   5480 0.0007109 126.4 191.9 0.659  1373619_at  ankyrin repeat domain 10 (predicted)  Info   Ankrd10_predicted 
   5481 0.000711 236.1 386.6 0.611  1371431_at  Transcribed locus, strongly similar to XP_232343.3 PREDICTED: similar to Pex5 protein [Rattus norvegicus]  Info    
   5482 0.0007118 835.9 618.2 1.352  1368386_at  growth factor receptor bound protein 2  Info   Grb2 
   5483 0.0007127 12.7 42.2 0.301  1383756_at  Transcribed locus  Info    
   5484 0.0007138 69.8 21.1 3.308  1369207_at  interleukin 7  Info   Il7 
   5485 0.0007143 3.2 14.8 0.216  1389280_at  Tubby like protein 1 (predicted)  Info    
   5486 0.0007148 124.1 71.4 1.738  1382679_at  similar to WD-repeat protein 43  Info   LOC362703 
   5487 0.0007151 215.2 254.2 0.847  1392987_at  Similar to hypothetical protein  Info    
   5488 0.0007161 1314.2 1660.4 0.791  1371564_at  ATPase, H+ transporting, V1 subunit E isoform 1  Info   Atp6v1e1 
   5489 0.0007161 380.1 479.1 0.793  1370360_at  testis specific protein  Info   RGD:621352 
   5490 0.0007162 1689.3 3905.6 0.433  1371030_at  secreted phosphoprotein 2  Info   Spp2 
   5491 0.0007163 234.3 167.4 1.4  1368424_at  inhibitor of kappaB kinase beta  Info   Ikbkb 
   5492 0.0007196 29.2 58.5 0.499  1380437_at  Transcribed locus  Info    
   5493 0.0007197 70.3 28.8 2.441  1374095_at  Transcribed locus  Info    
   5494 0.0007198 43.2 7.6 5.684  1387728_at  interleukin 10 receptor, alpha  Info   Il10ra 
   5495 0.0007203 3192.5 4477 0.713  1368826_at  catechol-O-methyltransferase  Info   Comt 
   5496 0.0007213 436.9 558.4 0.782  1372298_at  thioredoxin domain containing 4 (endoplasmic reticulum) (predicted)  Info   Txndc4_predicted 
   5497 0.0007244 168.3 267.9 0.628  1374653_at  Similar to CG12125-PA (predicted)  Info    
   5498 0.0007252 176.1 127.1 1.386  1376996_at  Transcribed locus  Info    
   5499 0.0007257 67.8 31.3 2.166  1380130_at    Info    
   5500 0.000728 39.9 69.5 0.574  1392926_at  laminin, alpha 1 (predicted)  Info   Lama1_predicted 
   5501 0.0007282 269.1 185.7 1.449  1390109_at    Info    
   5502 0.0007282 200.9 324.5 0.619  1367649_at  paralemmin  Info   Palm 
   5503 0.0007282 49.9 92.9 0.537  1390523_at  Transcribed locus  Info    
   5504 0.0007297 131.3 90 1.459  1381017_at  Transcribed locus  Info    
   5505 0.0007306 5.9 37.9 0.156  1369514_at  insulin receptor-related receptor  Info   Insrr 
   5506 0.0007306 120.1 185.4 0.648  1391280_at    Info    
   5507 0.0007307 3248.7 2705.6 1.201  1370003_at  eukaryotic translation elongation factor 2  Info   Eef2 
   5508 0.0007315 33.3 68.3 0.488  1383432_at  LOC362136 (predicted)  Info   RGD1309610_predicted 
   5509 0.0007321 5697.6 8328.2 0.684  1368440_at  solute carrier family 3, member 1  Info   Slc3a1 
   5510 0.0007324 76.1 123.3 0.617  1391173_at  Transcribed locus  Info    
   5511 0.0007325 318.8 249.3 1.279  1387012_at  squamous cell carcinoma antigen recognized by T-cells 1  Info   Sart1 
   5512 0.0007339 117.6 59.9 1.963  1398553_at  similar to CGI-100-like protein  Info   RGD:1359437 
   5513 0.0007341 110.9 58.3 1.902  1383685_at  similar to BAP28 protein  Info   LOC361262 
   5514 0.0007341 342.8 241.6 1.419  1372145_at  threonyl-tRNA synthetase  Info   RGD:1359527 
   5515 0.0007343 232.8 299.2 0.778  1376446_at  similar to RIKEN cDNA D430028G21  Info   RGD:1359371 
   5516 0.0007349 482.2 630.5 0.765  1371255_at  Harvey rat sarcoma viral (v-Ha-ras) oncogene homolog  Info   Hras 
   5517 0.0007362 475 316.4 1.501  1374438_at    Info    
   5518 0.0007364 30.1 52.1 0.578  1384904_at  neuropilin (NRP) and tolloid (TLL)-like 2 (predicted)  Info   Neto2_predicted 
   5519 0.0007365 82.4 123.9 0.665  1389484_at  coenzyme Q4 homolog (yeast) (predicted)  Info   Coq4_predicted 
   5520 0.0007367 53 119.1 0.445  1390119_at  secreted frizzled-related protein 2  Info   Sfrp2 
   5521 0.0007373 138.4 88.6 1.562  1383543_at  similar to CGI-141 protein; EST AA407874 (predicted)  Info   RGD1310691_predicted 
   5522 0.0007383 152.4 442.6 0.344  1384073_at  alcohol dehydrogenase, iron containing, 1 (predicted)  Info   Adhfe1_predicted 
   5523 0.0007384 36.3 13 2.792  1392754_at  Similar to cysteine-rich glycoprotein  Info    
   5524 0.0007391 52.8 18.7 2.824  1380835_at  similar to hypothetical protein FLJ12671  Info   RGD:1359413 
   5525 0.0007409 103.2 179.3 0.576  1398610_at  CDNA clone MGC:124727 IMAGE:7936966  Info    
   5526 0.0007419 138.6 237.9 0.583  1395373_at  Transcribed locus  Info    
   5527 0.000742 19.9 40.3 0.494  1385868_at    Info    
   5528 0.0007428 458.7 628.4 0.73  1393110_at  CDNA clone MGC:108867 IMAGE:7373705  Info    
   5529 0.0007436 2679.6 2102.6 1.274  1371653_at    Info    
   5530 0.0007439 37.6 5.5 6.836  1396297_at  Transcribed locus  Info    
   5531 0.0007441 338.7 484 0.7  1388749_at  Similar to hypothetical protein FLJ13910 (predicted)  Info    
   5532 0.0007441 32.3 80.3 0.402  1377736_at  Transcribed locus  Info    
   5533 0.0007442 22.4 46.7 0.48  1398233_at  Transcribed locus  Info    
   5534 0.0007449 271.9 335.1 0.811  1388517_at  mitochondrial ribosomal protein L40  Info   Mrpl40 
   5535 0.0007453 250.8 348.6 0.719  1373036_at  similar to Ras GTPase-activating-like protein IQGAP2  Info   LOC364885 
   5536 0.0007457 227.5 141.3 1.61  1383152_at  similar to Cc1-9 (predicted)  Info   RGD1308955_predicted 
   5537 0.0007472 36.6 57.6 0.635  1396219_at    Info    
   5538 0.0007473 731.4 536.4 1.364  1373019_at  Epidermal growth factor receptor pathway substrate 15 (predicted)  Info   Eps15 
   5539 0.0007479 1490.9 1233.6 1.209  1372259_at  DEK oncogene (DNA binding)  Info   RGD:1303246 
   5540 0.0007485 71.2 46.4 1.534  1381850_at  protein phosphatase 1, regulatory (inhibitor) subunit 12A  Info   Ppp1r12a 
   5541 0.0007487 175.9 125.8 1.398  1383150_at  Transcribed locus  Info    
   5542 0.0007496 69.3 40.6 1.707  1386162_at  TXK tyrosine kinase (predicted)  Info   Txk_predicted 
   5543 0.0007509 393.7 305.2 1.29  1383705_at  CDNA clone IMAGE:7317308  Info    
   5544 0.0007528 44.3 81.6 0.543  1384724_at    Info    
   5545 0.0007542 335.8 244.5 1.373  1370223_at  ADP-ribosylation factor related protein 1  Info   Arfrp1 
   5546 0.0007548 17.9 42.7 0.419  1387508_at  bile acid-Coenzyme A: amino acid N-acyltransferase  Info   Baat 
   5547 0.0007556 2535.8 2872.2 0.883  1371642_at  eukaryotic translation initiation factor 4A2 (predicted)  Info   Eif4a2_predicted 
   5548 0.0007558 45.7 80.4 0.568  1384542_at  Transcribed locus  Info    
   5549 0.000756 151.9 253.6 0.599  1380389_at  Prostaglandin E receptor 3 (subtype EP3)  Info   Ptger3 
   5550 0.0007579 1678.3 1244.5 1.349  1398832_at  nucleolin  Info   Ncl 
   5551 0.0007582 272.5 229.7 1.186  1384127_at  Transcribed locus  Info    
   5552 0.0007591 307.7 449.9 0.684  1371014_at  phospholipase C, beta 1  Info   Plcb1 
   5553 0.0007591 2394.1 3036.2 0.789  1370862_at  apolipoprotein E  Info   Apoe 
   5554 0.0007594 144.9 209 0.693  1382866_at  similar to Putative protein 15E1.2 (predicted)  Info   RGD1309698_predicted 
   5555 0.0007604 61.2 127.7 0.479  1368039_at  synaptojanin 2 binding protein  Info   Synj2bp 
   5556 0.0007616 10.1 35.2 0.287  1378093_at  Transcribed locus  Info    
   5557 0.0007622 3115.4 3711.5 0.839  1387675_at  Plasminogen activator, urokinase  Info   Plau 
   5558 0.0007636 322.7 162.5 1.986  1371040_at  sodium-dependent neutral amino acid transporter ASCT2  Info   Slc1a5 
   5559 0.0007637 134.3 75.2 1.786  1392743_at  myc induced nuclear antigen  Info   Mina 
   5560 0.0007639 3460.4 4076.5 0.849  1371302_at  Similar to RIKEN cDNA A130038L21  Info    
   5561 0.0007644 176.1 113.7 1.549  1377327_at  Transcribed locus  Info    
   5562 0.0007649 1740.6 1312.8 1.326  1387664_at  ATPase, H+ transporting, V1 subunit B, isoform 2  Info   Atp6v1b2 
   5563 0.0007656 703.1 999 0.704  1375412_at  Arylsulfatase B  Info   Arsb 
   5564 0.0007657 468.5 335 1.399  1393336_at  SWAP complex protein (predicted)  Info   Swap70_predicted 
   5565 0.0007667 36.9 60.2 0.613  1376747_at  Membrane associated guanylate kinase 1 b NT-short isoform  Info    
   5566 0.0007686 218.2 444.4 0.491  1368661_at  solute carrier family 13 (sodium-dependent dicarboxylate transporter), member 2  Info   Slc13a2 
   5567 0.0007686 217.7 299.7 0.726  1372129_at  similar to TBC1 domain family, member 8; BUB2-like protein 1; vascular Rab-GAP/TBC-containing (predicted)  Info   RGD1310147_predicted 
   5568 0.0007688 94.9 134.3 0.707  1383533_at  Transcribed locus  Info    
   5569 0.0007688 84.7 115.1 0.736  1372348_at  Histone cell cycle regulation defective interacting protein 5 (predicted)  Info    
   5570 0.0007691 1978.5 2638.3 0.75  1370881_at  thiosulfate sulfurtransferase  Info   Tst 
   5571 0.0007692 42.9 88.9 0.483  1387295_at  solute carrier family 6 (neurotransmitter transporter, betaine/GABA), member 12  Info   Slc6a12 
   5572 0.0007693 63.5 25.6 2.48  1383597_at  DNA cross-link repair 1C, PSO2 homolog (S. cerevisiae)  Info   Dclre1c 
   5573 0.0007693 55.7 91.3 0.61  1385369_at  similar to hypothetical protein FLJ32844 (predicted)  Info   RGD1311430_predicted 
   5574 0.0007698 265 1169.9 0.227  1369401_at  solute carrier family 21, member 13  Info   Slc21a13 
   5575 0.00077 94.4 52.8 1.788  1378028_at  MAD2 (mitotic arrest deficient, homolog)-like 1 (yeast) (predicted)  Info   Mad2l1_predicted 
   5576 0.0007701 422.3 300.9 1.403  1388413_at  ribosome binding protein 1 homolog 180kDa (dog) (predicted)  Info   Rrbp1_predicted 
   5577 0.0007703 882.2 1068.5 0.826  1370321_at  programmed cell death 8  Info   Pdcd8 
   5578 0.0007705 20.5 37.1 0.553  1380133_at  odd-skipped related 2 (Drosophila) (predicted)  Info   Osr2_predicted 
   5579 0.0007715 51.7 90.2 0.573  1391564_at    Info    
   5580 0.000772 75.5 100.5 0.751  1383088_at  similar to RIKEN cDNA 2610209A20 (predicted)  Info   RGD1305211_predicted 
   5581 0.0007727 707.5 596.4 1.186  1371498_at  similar to Jtv1-pending protein  Info   LOC288480 
   5582 0.0007729 2669.2 1599.1 1.669  1386890_at  S100 calcium binding protein A10 (calpactin)  Info   S100a10 
   5583 0.0007737 276.7 362.2 0.764  1367518_at  similar to fatso (predicted)  Info   RGD1305121_predicted 
   5584 0.0007746 67.7 88.4 0.766  1391744_at  Transcribed locus  Info    
   5585 0.0007749 268.7 409.4 0.656  1398335_at  Tweety homolog 3 (Drosophila) (predicted)  Info    
   5586 0.0007757 714.1 916.7 0.779  1399080_at    Info    
   5587 0.0007765 132.2 78 1.695  1396755_at  Transcribed locus  Info    
   5588 0.0007765 1128 1328.3 0.849  1371612_at  tRNA splicing endonuclease 34 homolog  Info   RGD:1359564 
   5589 0.000778 82 111.9 0.733  1387046_at  selective LIM binding factor, rat homolog  Info   RGD:620744 
   5590 0.0007781 38.9 62 0.627  1381073_at  similar to SPRED-3 (predicted)  Info   RGD1311713_predicted 
   5591 0.0007781 168.5 242.6 0.695  1389047_at  Zinc finger protein 451 (predicted)  Info    
   5592 0.0007784 145.6 178.9 0.814  1392993_at  similar to hypothetical protein MGC37079 (predicted)  Info   RGD1310484_predicted 
   5593 0.0007789 1060.9 845.1 1.255  1372704_at  similar to RIKEN cDNA 2310008M10  Info   LOC362040 
   5594 0.0007804 49 126.6 0.387  1383991_at  Similar to 1810049O03Rik protein  Info    
   5595 0.0007808 34 67.1 0.507  1374062_x_at  microtubule-associated protein, RP/EB family, member 3  Info   RGD:1359297 
   5596 0.0007814 1349.7 1108.2 1.218  1371418_at  chaperonin containing TCP1, subunit 2 (beta)  Info   RGD:1359143 
   5597 0.0007817 171.7 110.9 1.548  1385090_at  RAD17 homolog (S. pombe) (predicted)  Info   Rad17_predicted 
   5598 0.0007823 104.6 140 0.747  1395547_at  Guanine nucleotide binding protein, alpha q polypeptide  Info   Gnaq 
   5599 0.0007824 136.5 207.2 0.659  1379519_at  Transcribed locus, moderately similar to NP_001001306.1 hypothetical protein LOC329695 [Mus musculus]  Info    
   5600 0.0007825 875 1181.5 0.741  1388121_at  amyloid beta (A4) precursor-like protein 2  Info   Aplp2 
   5601 0.0007829 220.1 157.5 1.397  1374331_at  rcd1 (required for cell differentiation) homolog 1 (S. pombe) (predicted)  Info   Rqcd1_predicted 
   5602 0.0007833 57 36.1 1.579  1385359_at  Similar to RIKEN cDNA 4931406C07 (predicted)  Info    
   5603 0.0007856 248.9 357.3 0.697  1383573_at  Serologically defined colon cancer antigen 33 (predicted)  Info    
   5604 0.0007874 73.1 96.7 0.756  1374727_at  similar to DnaJ (Hsp40) homolog, subfamily B, member 12  Info   LOC294513 
   5605 0.0007879 11.9 21.4 0.556  1378644_at  Protein kinase C, epsilon  Info   Prkce 
   5606 0.0007886 104 144.1 0.722  1374482_at    Info    
   5607 0.000789 80.7 43.8 1.842  1372640_at  LOC499410  Info   LOC499410 
   5608 0.0007902 58.4 108.5 0.538  1374131_at  Transcribed locus  Info    
   5609 0.0007903 12 27 0.444  1381070_at    Info    
   5610 0.0007909 1713.6 1537.1 1.115  1398800_at  tyrosine 3-monooxygenase/tryptophan 5-monooxygenase activation protein, beta polypeptide  Info   Ywhab 
   5611 0.0007911 529 383.2 1.38  1389351_at  similar to FLI-LRR associated protein-1  Info   RGD:1359548 
   5612 0.0007932 104.8 63 1.663  1388099_a_at  TCF3 (E2A) fusion partner  Info   Tfpt 
   5613 0.0007941 308.6 190 1.624  1390384_at  similar to Histone H2A.x (H2a/x)  Info   LOC500987 
   5614 0.0007953 81.5 126.6 0.644  1397383_at  similar to empty spiracles homolog 1  Info   LOC500235 
   5615 0.0007956 133.1 102 1.305  1372788_at  ubiquitin-conjugating enzyme E2Q (putative) (predicted)  Info   Ube2q_predicted 
   5616 0.0007956 211.2 101 2.091  1368270_at  apolipoprotein B editing complex 1  Info   Apobec1 
   5617 0.0007958 117.5 139.3 0.844  1373126_at  Transcribed locus, moderately similar to XP_580018.1 PREDICTED: hypothetical protein XP_580018 [Rattus norvegicus]  Info    
   5618 0.0007977 465.8 355 1.312  1388990_at  PREDICTED: Rattus norvegicus spermatogenesis-related protein (Srp) (Mki67ip), mRNA  Info    
   5619 0.0007993 246.1 167.7 1.468  1380448_at  AlkB, alkylation repair homolog (E. coli) (predicted)  Info    
   5620 0.0008002 23.3 50.4 0.462  1387671_at  secretin receptor  Info   Sctr 
   5621 0.0008009 20.5 38.6 0.531  1376213_at  Transcribed locus  Info    
   5622 0.0008018 395.7 654.4 0.605  1372286_at  similar to transmembrane 4 superfamily member 6  Info   LOC302313 
   5623 0.0008024 247.6 328.7 0.753  1389217_at  Radical fringe gene homolog (Drosophila)  Info   Rfng 
   5624 0.0008026 365.1 295 1.238  1389036_at  similar to RIKEN cDNA 9030221M09 gene (predicted)  Info   RGD1305469_predicted 
   5625 0.0008038 459.9 354.5 1.297  1379810_at  small inducible cytokine subfamily E, member 1  Info   Scye1 
   5626 0.0008042 284.2 414 0.686  1388422_at  LIM and senescent cell antigen like domains 2 (predicted)  Info   Lims2_predicted 
   5627 0.0008048 94.1 130.4 0.722  1390202_at  PR domain containing 2, with ZNF domain  Info    
   5628 0.0008049 300.9 192.3 1.565  1367540_at  Transcribed locus  Info    
   5629 0.0008054 43.1 10 4.31  1385551_at  RT1 class Ib, locus H2-Q-like, grc region /// similar to class I histocompatibility antigen alpha chain - cotton-top tamarin  Info   RT1-O /// LOC499402 
   5630 0.0008068 145.8 249.9 0.583  1388038_at  attractin  Info   Atrn 
   5631 0.0008069 409.5 313.1 1.308  1376687_at  ubiquitin specific protease 1 (predicted)  Info   Usp1_predicted 
   5632 0.0008069 261.8 353.6 0.74  1387008_at  sideroflexin 3  Info   Sfxn3 
   5633 0.0008075 28.1 51.1 0.55  1391730_at  Myeloid/lymphoid or mixed-lineage leukemia 2 (predicted)  Info    
   5634 0.0008085 174.1 113.8 1.53  1379235_x_at  similar to CDC45L  Info   LOC287961 
   5635 0.0008087 695.6 542.1 1.283  1367464_at  siah binding protein 1; FBP interacting repressor; pyrimidine tract binding splicing factor; Ro ribonucleoprotein-binding protein 1  Info   RGD:621674 
   5636 0.0008092 19.3 35.1 0.55  1374999_at  Transcribed locus  Info    
   5637 0.0008095 41.8 27 1.548  1379945_at  similar to T-cell activation kelch repeat protein  Info   LOC500262 
   5638 0.0008097 280.2 359.8 0.779  1387730_at  paired box gene 8  Info   Pax8 
   5639 0.0008101 114 44.2 2.579  1370097_a_at  chemokine (C-X-C motif) receptor 4  Info   Cxcr4 
   5640 0.0008107 185.2 107 1.731  1368885_at  ectonucleoside triphosphate diphosphohydrolase 1  Info   Entpd1 
   5641 0.0008118 159.5 211.7 0.753  1391581_at  Rho GTPase activating protein 5 (predicted)  Info    
   5642 0.0008124 161.7 119.2 1.357  1376519_at  brain protein 16  Info   RGD:1359226 
   5643 0.0008126 34.4 52.6 0.654  1376464_at  Thymocyte protein thy28  Info   MGC72984 
   5644 0.0008126 30.1 70 0.43  1386691_at  F-box only protein 36 (predicted)  Info   Fbxo36_predicted 
   5645 0.0008135 227 147.2 1.542  1382832_at  similar to CG9135-PA (predicted)  Info   RGD1309986_predicted 
   5646 0.0008144 1583.4 2340.5 0.677  1376209_at  similar to hypothetical protein supported by AL449243 (predicted)  Info   RGD1311310_predicted 
   5647 0.0008148 78 37.8 2.063  1368279_at  myeloid/lymphoid or mixed-lineage leukemia (trithorax (Drosophila) homolog); translocated to, 3  Info   RGD:620940 
   5648 0.000815 349.7 552 0.634  1393793_at  Transcribed locus  Info    
   5649 0.000818 76.5 21.1 3.626  1379716_at  CD163 antigen (predicted)  Info    
   5650 0.0008182 76.1 44.7 1.702  1394797_at  Transcribed locus  Info    
   5651 0.0008189 9.2 50.9 0.181  1384707_at  Transcribed locus  Info    
   5652 0.00082 388.6 555.1 0.7  1388942_at  Transcribed locus  Info    
   5653 0.0008202 240.2 294.9 0.815  1399083_at  Williams Beuren syndrome chromosome region 21 (predicted) /// similar to Williams-Beuren syndrome critical region protein 21  Info   Wbscr21_predicted /// LOC368083 
   5654 0.0008215 56.7 31.8 1.783  1389101_at  cyclin C  Info   Ccnc 
   5655 0.0008222 40.5 17.7 2.288  1387745_at  antigen identified by monoclonal antibody MRC OX-2 receptor  Info   Mox2r 
   5656 0.0008234 454.9 351.2 1.295  1383067_a_at  deoxythymidylate kinase (predicted)  Info   Dtymk_predicted 
   5657 0.0008243 50.3 28.9 1.74  1376316_at  Origin recognition complex, subunit 6-like (S. cerevisiae) (predicted)  Info    
   5658 0.0008246 6.3 21 0.3  1393589_at  similar to KIAA1687 protein  Info   LOC317196 
   5659 0.0008253 111.6 173.9 0.642  1389321_at  Transcribed locus  Info    
   5660 0.0008258 17.9 38.8 0.461  1378129_a_at  Transcribed locus  Info    
   5661 0.0008261 198.2 29.1 6.811  1390946_at  Runt-related transcription factor 3  Info   Runx3 
   5662 0.0008272 106.3 173.2 0.614  1388104_at  G protein-coupled receptor 48  Info   Gpr48 
   5663 0.0008276 160 93.6 1.709  1377832_at  polo-like kinase 4 (Drosophila) (predicted)  Info   Plk4_predicted 
   5664 0.0008278 678.6 863.5 0.786  1387855_at  guanosine diphosphate dissociation inhibitor 1  Info   Gdi1 
   5665 0.0008283 95.7 163.7 0.585  1371889_at  solute carrier family 22 (organic cation transporter), member 17  Info   Slc22a17 
   5666 0.0008285 44.5 100 0.445  1380865_at  Transcribed locus  Info    
   5667 0.0008292 20.5 28.9 0.709  1369632_a_at  ATP-binding cassette, sub-family C (CFTR/MRP), member 8  Info   Abcc8 
   5668 0.0008294 22.6 66.4 0.34  1398480_at  similar to Peroxidasin CG12002-PA (predicted)  Info   RGD1308720_predicted 
   5669 0.0008295 130.4 206.8 0.631  1370925_at  similar to Potential phospholipid-transporting ATPase IIB  Info   LOC291411 
   5670 0.0008296 222.3 181 1.228  1373464_at  similar to cDNA sequence BC023106 (predicted)  Info   RGD1306588_predicted 
   5671 0.0008299 549.9 664.9 0.827  1369990_at  topoisomerase (DNA) 2 alpha  Info   Top2a 
   5672 0.0008305 52.9 103.5 0.511  1387234_at  alpha-2-glycoprotein 1, zinc  Info   Azgp1 
   5673 0.0008313 1275.7 895.3 1.425  1373195_at  fusion (involved in t(12;16) in malignant liposarcoma) (predicted)  Info   Fus_predicted 
   5674 0.0008315 1409.8 1735 0.813  1398889_at  glutamate receptor, ionotropic, N-methyl D-aspartate-like 1A  Info   Grinl1a 
   5675 0.0008328 627.9 839.6 0.748  1382048_at  ATPase, H+ transporting, V1 subunit A, isoform 1 (predicted)  Info   Atp6v1a1_predicted 
   5676 0.0008332 68 126.8 0.536  1394458_at  SA rat hypertension-associated homolog  Info   Sah 
   5677 0.0008333 6.5 18 0.361  1393069_at  Secreted frizzled-related sequence protein 5 (predicted)  Info    
   5678 0.0008348 50.9 76.5 0.665  1378785_at  Transcribed locus  Info    
   5679 0.0008354 191.4 256.2 0.747  1377785_at  DEAH (Asp-Glu-Ala-His) box polypeptide 40  Info   RGD:1359618 
   5680 0.0008355 22.3 38.9 0.573  1375790_at    Info    
   5681 0.0008367 72.1 121.6 0.593  1394625_at    Info    
   5682 0.0008371 190.3 99.7 1.909  1397953_at  Transcribed locus  Info    
   5683 0.0008373 175.8 243.1 0.723  1373269_at  Transcribed locus  Info    
   5684 0.0008389 56.9 93.9 0.606  1374023_at  Transcribed locus  Info    
   5685 0.0008395 40.7 89.6 0.454  1376158_at    Info    
   5686 0.0008397 347.9 575.2 0.605  1386478_at  mitochondrial carrier triple repeat 1 (predicted)  Info   Mcart1_predicted 
   5687 0.000841 225.4 153 1.473  1370261_at  ribosomal protein S6 kinase polypeptide 1  Info   Rps6ka1 
   5688 0.0008413 804.6 1140 0.706  1398933_at  similar to RIKEN cDNA 2010110M21 (predicted)  Info   RGD1309691_predicted 
   5689 0.0008423 144.4 100.9 1.431  1381145_at  Transcribed locus  Info    
   5690 0.0008436 37.5 9.3 4.032  1378887_at  Mesoderm development candidate 1 (predicted)  Info    
   5691 0.0008459 906.5 599 1.513  1367713_at  eukaryotic translation initiation factor 2, subunit 1 alpha  Info   Eif2s1 
   5692 0.0008466 313.4 535.8 0.585  1383332_at  Transcribed locus, weakly similar to NP_001002778.1 hypothetical protein LOC442814 [Mus musculus]  Info    
   5693 0.0008468 182.5 291.6 0.626  1382496_at    Info    
   5694 0.0008469 335.6 202.2 1.66  1374739_at  similar to hematopoietic progenitor protein  Info   LOC500727 
   5695 0.0008472 39.3 80.3 0.489  1379075_at  O-acyltransferase (membrane bound) domain containing 2 (predicted)  Info   Oact2_predicted 
   5696 0.0008475 43 79 0.544  1374791_at  Similar to sorting nexin 21 isoform a; sorting nexin L; sorting nexin 21 (predicted)  Info    
   5697 0.0008478 79.9 44.2 1.808  1382160_at  Casein kinase II, alpha 2, polypeptide (predicted)  Info    
   5698 0.0008482 197.6 261.6 0.755  1375140_at  muscleblind-like 2 (predicted)  Info   Mbnl2_predicted 
   5699 0.0008483 241 58 4.155  1374540_at  cell division cycle associated 7 (predicted)  Info   Cdca7_predicted 
   5700 0.0008483 34.7 77.4 0.448  1385659_at  N-ethylmaleimide-sensitive factor attachment protein, gamma (predicted)  Info   Napg 
   5701 0.0008499 73.7 111.8 0.659  1373728_at  similar to RIKEN cDNA 1700088E04; DNA segment, Human EST J0827E04; Mus EST J0827E04; DNA segment, EST J0827E04  Info   RGD:1359634 
   5702 0.00085 24.7 72 0.343  1385038_at  similar to hedgehog-interacting protein  Info   LOC291936 
   5703 0.0008512 264.1 310.5 0.851  1388512_at  phosphodiesterase 6D, cGMP-specific, rod, delta (predicted)  Info   Pde6d_predicted 
   5704 0.0008516 273.9 406.7 0.673  1375003_at  serine (or cysteine) peptidase inhibitor, clade B, member 6  Info   Serpinb6 
   5705 0.0008522 206.4 156.6 1.318  1377262_at    Info    
   5706 0.0008522 104.4 54.6 1.912  1378746_at    Info    
   5707 0.0008529 347.2 248.1 1.399  1379327_at  SWI/SNF related, matrix associated, actin dependent regulator of chromatin, subfamily a, member 5 (predicted)  Info   Smarca5_predicted 
   5708 0.000853 29.4 62.6 0.47  1380090_at    Info    
   5709 0.0008531 370.4 225.7 1.641  1388409_at  similar to ubiquitous tetratricopeptide containing protein RoXaN; Rotavirus X associated non-structural protein (predicted)  Info   RGD1307064_predicted 
   5710 0.0008535 196.3 115.2 1.704  1391905_at    Info    
   5711 0.0008539 476.5 355.3 1.341  1388544_at  2,3-bisphosphoglycerate mutase  Info   Bpgm 
   5712 0.0008552 108.6 168.8 0.643  1376494_at  similar to DNA polymerase epsilon p17 subunit (DNA polymerase epsilon subunit 3) (Chromatin accessibility complex 17) (HuCHRAC17) (CHRAC-17)  Info   RGD:1359475 
   5713 0.0008559 21.9 47.9 0.457  1397049_at  Transcribed locus, strongly similar to NP_081963.1 calcium-binding tyrosine-phosphorylation regulated protein [Mus musculus]  Info    
   5714 0.0008562 114.1 170.5 0.669  1385517_at  general transcription factor II I  Info   Gtf2i 
   5715 0.0008563 3086.1 2671.2 1.155  1388759_at  Chloride intracellular channel 4  Info   Clic4 
   5716 0.0008564 242.2 166.5 1.455  1389191_at  similar to RIKEN cDNA 2210415M20 (predicted)  Info   RGD1309754_predicted 
   5717 0.000857 84.8 142.1 0.597  1374117_at  brain-specific angiogenesis inhibitor 1-associated protein 2  Info   Baiap2 
   5718 0.0008589 40.8 80.9 0.504  1370937_a_at  integrin alpha 7  Info   Itga7 
   5719 0.0008593 1384.4 887.1 1.561  1368419_at  ceruloplasmin  Info   Cp 
   5720 0.0008598 61.9 112.7 0.549  1373685_at  similar to low density lipoprotein receptor-related protein binding protein  Info   LOC361149 
   5721 0.0008614 2.7 4.8 0.563  1378207_at  Cartilage acidic protein 1  Info   Crtac1 
   5722 0.0008636 689.8 441.7 1.562  1376272_s_at  AA926063gene  Info   RGD:1303072 
   5723 0.000864 1653.6 1949.3 0.848  1398771_at  solute carrier family 3 (activators of dibasic and neutral amino acid transport), member 2  Info   Slc3a2 
   5724 0.0008651 1227 1611.5 0.761  1371644_at  protein tyrosine kinase 9 (predicted)  Info   Ptk9_predicted 
   5725 0.0008655 16.3 37.6 0.434  1398627_at  Similar to PALS2-alpha splice variant  Info    
   5726 0.0008661 259.8 363.2 0.715  1382818_at  similar to hypothetical protein D630010C10 (predicted)  Info   RGD1309752_predicted 
   5727 0.0008662 93 127.6 0.729  1398413_at  zinc finger protein 444 (predicted)  Info   Znf444_predicted 
   5728 0.0008663 19.3 42.3 0.456  1389720_at  Similar to RIKEN cDNA 1700020L24  Info    
   5729 0.0008663 35.2 72.4 0.486  1381997_at  adipocyte complement related protein of 30 kDa  Info   Acdc 
   5730 0.0008669 49.9 134.7 0.37  1396238_at  UDP-N-acetyl-alpha-D-galactosamine:polypeptide N-acetylgalactosaminyltransferase 14 (predicted)  Info   Galnt14_predicted 
   5731 0.0008679 215.2 271.4 0.793  1374030_at  Transcribed locus  Info    
   5732 0.0008689 60.7 88.2 0.688  1378243_at  transmembrane protein 17 (predicted)  Info   Tmem17_predicted 
   5733 0.0008693 125.9 169.7 0.742  1374074_at  cell division cycle 37 homolog (S. cerevisiae)-like (predicted)  Info   Cdc37l_predicted 
   5734 0.0008693 26.6 85.4 0.311  1373628_at  Transcribed locus  Info    
   5735 0.0008696 17.7 46.7 0.379  1390991_at  hemoglobin, theta 1 (predicted) /// similar to Hemoglobin theta-1 chain  Info   Hbq1_predicted /// LOC501681 
   5736 0.0008699 1110 1845.9 0.601  1383798_at  similar to CDNA sequence BC026682  Info   RGD:1359194 
   5737 0.00087 448.5 803.1 0.558  1371361_at  similar to tensin  Info   LOC301509 
   5738 0.0008704 219.9 563.2 0.39  1367896_at  carbonic anhydrase 3  Info   Ca3 
   5739 0.0008706 39.2 67.2 0.583  1370392_at  transient receptor potential cation channel, subfamily M, member 4  Info   Trpm4 
   5740 0.0008715 72.7 122.6 0.593  1396058_at  Transcribed locus  Info    
   5741 0.000872 802.1 547.2 1.466  1371693_at  Transcribed locus, strongly similar to XP_234523.2 PREDICTED: similar to elk1 [Rattus norvegicus]  Info    
   5742 0.000874 440.9 575.9 0.766  1390785_at  UBX domain containing 4 (predicted)  Info   Ubxd4_predicted 
   5743 0.0008755 145.7 235.5 0.619  1384405_at  Transcribed locus  Info    
   5744 0.000876 592.3 927.9 0.638  1370286_at  solute carrier family 38, member 2  Info   Slc38a2 
   5745 0.0008761 3767.8 6667.9 0.565  1370688_at  glutamate-cysteine ligase, catalytic subunit  Info   Gclc 
   5746 0.0008767 218.2 170.5 1.28  1373450_at    Info    
   5747 0.0008774 46.5 103.2 0.451  1384304_at  LRP16 protein  Info   Lrp16 
   5748 0.0008774 53.1 79.7 0.666  1398585_at  F-box only protein 21 (predicted)  Info   Fbxo21_predicted 
   5749 0.0008779 578.1 470.1 1.23  1399062_at  CDNA clone IMAGE:7317308  Info    
   5750 0.0008786 2325 1721.6 1.35  1371656_at  chaperonin subunit 4 (delta)  Info   Cct4 
   5751 0.0008787 21.1 40.7 0.518  1370553_at  epimorphin  Info   Epim 
   5752 0.0008791 34.9 65.5 0.533  1397838_at  LOC499976  Info    
   5753 0.0008791 391.2 551.3 0.71  1398935_at  Transcribed locus, strongly similar to XP_533460.1 PREDICTED: similar to Sorting nexin-9 [Canis familiaris]  Info    
   5754 0.0008796 182.6 131.1 1.393  1376206_at  Similar to RIKEN cDNA 9030624J02  Info    
   5755 0.0008797 138.6 201.4 0.688  1399103_at    Info    
   5756 0.0008807 68.8 46.1 1.492  1394710_at  5-azacytidine induced gene 2 (predicted)  Info   Azi2_predicted 
   5757 0.0008809 80.5 123.8 0.65  1379431_at  Transcribed locus  Info    
   5758 0.0008813 99.6 56.1 1.775  1386191_a_at  Transcribed locus  Info    
   5759 0.0008817 562.1 440.3 1.277  1383181_at  DnaJ (Hsp40) homolog, subfamily C, member 9 (predicted)  Info   Dnajc9_predicted 
   5760 0.0008844 70.6 7.5 9.413  1398576_at  similar to SIRP beta 1 isoform 1 /// protein tyrosine phosphatase, non-receptor type substrate 1-like 3 (predicted)  Info   LOC310212 /// Ptpns1l3_predicted 
   5761 0.000885 225.6 317 0.712  1377806_at  vascular endothelial zinc finger 1 (predicted)  Info   Vezf1_predicted 
   5762 0.0008866 105 65.3 1.608  1380030_at  zinc finger protein 593 (predicted)  Info   Znf593_predicted 
   5763 0.0008866 229.6 359.9 0.638  1377209_at  similar to hypothetical protein FLJ12587 (predicted)  Info   RGD1310815_predicted 
   5764 0.0008868 76.1 52.8 1.441  1387427_at  RAD50 homolog (S. cerevisiae)  Info   Rad50 
   5765 0.0008874 15.8 28.3 0.558  1385974_at  par-6 partitioning defective 6 homolog gamma (C. elegans) (predicted)  Info   Pard6g_predicted 
   5766 0.0008879 1134.3 910 1.246  1367491_at  apoptosis inhibitor 5 (predicted)  Info   Api5_predicted 
   5767 0.0008897 158 195.7 0.807  1388919_at  zinc finger protein 541 (predicted)  Info   Znf541_predicted 
   5768 0.0008911 3837.6 1673.3 2.293  1370892_at  complement component 4a /// complement component 4, gene 2  Info   C4a /// C4-2 
   5769 0.0008928 4.8 21.4 0.224  1395072_at  Similar to hypothetical protein FLJ10901 (predicted)  Info    
   5770 0.0008938 2.2 8.4 0.262  1395763_at  Transcribed locus  Info    
   5771 0.0008947 375.5 313.8 1.197  1388105_at  D123 gene product  Info   RGD:619766 
   5772 0.0008951 445.8 768 0.58  1384240_at  angiotensin II receptor, type 1 (AT1A)  Info   Agtr1a 
   5773 0.0008973 274.4 411 0.668  1371202_a_at  nuclear factor I/B  Info   Nfib 
   5774 0.0008973 1324.2 1125.5 1.177  1371437_at  SEC13-like 1  Info   RGD:1359555 
   5775 0.0008974 109 82.5 1.321  1377761_at  glutamine-fructose-6-phosphate transaminase 2  Info   RGD:1303097 
   5776 0.0008991 264.4 357.3 0.74  1373420_at  evolutionarily conserved signaling intermediate in Toll pathway  Info   RGD:1359488 
   5777 0.0008999 72.7 117.8 0.617  1388920_at  bone morphogenetic protein 6  Info   Bmp6 
   5778 0.0009011 165.9 239.2 0.694  1383228_at    Info    
   5779 0.0009013 36 17.6 2.045  1397221_s_at  Tumor necrosis factor receptor superfamily, member 6  Info   RGD:619831 
   5780 0.0009014 137.6 101.6 1.354  1393621_at  vitamin K epoxide reductase complex, subunit 1-like 1  Info   RGD:1303249 
   5781 0.0009052 142.4 178.4 0.798  1373966_at  Retinoic acid induced 1 (predicted)  Info    
   5782 0.0009057 1006.9 698.1 1.442  1387636_a_at  P11 protein  Info   RGD:621692 
   5783 0.0009063 29 62.7 0.463  1389704_at  Transcribed locus  Info    
   5784 0.0009079 552.1 701.2 0.787  1398268_at  nuclear transcription factor-Y gamma  Info   Nfyc 
   5785 0.0009081 133.5 101 1.322  1392792_at  Transcribed locus  Info    
   5786 0.0009092 380.5 299.3 1.271  1375440_at  peptidylprolyl isomerase (cyclophilin)-like 2 (predicted)  Info   Ppil2_predicted 
   5787 0.0009097 69.7 122.3 0.57  1377890_at  Transcribed locus  Info    
   5788 0.0009101 646.7 815.3 0.793  1388356_at  S100 calcium binding protein A16 (predicted)  Info   S100a16_predicted 
   5789 0.000911 581.1 712.1 0.816  1372777_at  similar to dJ1033H22.1 (KIAA0554 protein) (predicted)  Info   RGD1305386_predicted 
   5790 0.0009114 537 657.6 0.817  1372317_at  EH-domain containing 1 (predicted)  Info   Ehd1_predicted 
   5791 0.0009116 143.9 81.3 1.77  1393620_at  sestrin 3 (predicted)  Info   Sesn3_predicted 
   5792 0.0009123 374.5 619.5 0.605  1391864_at    Info    
   5793 0.000913 396.4 557.7 0.711  1372879_at  AKT1 substrate 1 (proline-rich) (predicted)  Info   Akt1s1_predicted 
   5794 0.000915 172.5 105.4 1.637  1368909_a_at  GRIP1 associated protein 1  Info   Gripap1 
   5795 0.000915 140.3 181.6 0.773  1382215_at  Similar to Transcription initiation factor TFIID 105 kDa subunit (TAFII-105) (TAFII105)  Info    
   5796 0.0009157 61.9 94.3 0.656  1395809_at  sestrin 1 (predicted)  Info   Sesn1_predicted 
   5797 0.000918 79.1 42.1 1.879  1378240_at  Fanconi anemia D2 protein  Info   RGD:1303172 
   5798 0.0009182 2317.6 3296.5 0.703  1371421_at  Similar to FLJ40243 protein (predicted)  Info    
   5799 0.0009189 2165.6 2738.7 0.791  1388489_at    Info    
   5800 0.0009191 33.6 59 0.569  1369773_at  bone morphogenetic protein 3  Info   Bmp3 
   5801 0.0009194 357.9 587.1 0.61  1370260_at  adducin 3 (gamma)  Info   Add3 
   5802 0.0009203 236.6 192.5 1.229  1373516_at  Similar to RIKEN cDNA 2600005C20 (predicted)  Info    
   5803 0.0009208 226.2 326.8 0.692  1383714_at  Transcribed locus  Info    
   5804 0.000923 730.9 1210.3 0.604  1371969_at  Caldesmon 1  Info   Cald1 
   5805 0.0009244 2.8 49.4 0.057  1397855_at  Similar to cysteine sulfinic acid decarboxylase  Info    
   5806 0.0009263 219.3 286.3 0.766  1374573_at  similar to Dynein 2 light intermediate chain (predicted)  Info   RGD1310286_predicted 
   5807 0.0009265 397.8 574 0.693  1373560_at  similar to N-acetylglucosaminyltrasnferase IVb  Info   LOC303100 
   5808 0.0009267 89 204.3 0.436  1377155_at  Similar to RIKEN cDNA 1700019G17  Info    
   5809 0.0009274 1590.2 1963.8 0.81  1371296_at  succinate dehydrogenase complex, subunit D, integral membrane protein  Info   Sdhd 
   5810 0.0009274 801.1 519 1.544  1390989_at  similar to Mospd2 protein  Info   LOC363463 
   5811 0.0009278 20.4 59.6 0.342  1380747_at  Pre-B-cell leukemia transcription factor 1 (predicted)  Info    
   5812 0.00093 496.2 367.2 1.351  1368055_a_at  lamin A  Info   Lmna 
   5813 0.00093 120.9 84 1.439  1378399_at  similar to RIKEN cDNA 2810453I06  Info   LOC498145 
   5814 0.0009301 521.7 272.3 1.916  1392842_at  Schlafen 5 (predicted)  Info    
   5815 0.0009302 114.2 69.8 1.636  1370577_at  zinc finger protein 455  Info   Zfp455 
   5816 0.0009308 299.2 208.5 1.435  1370356_at  RNA binding motif protein 10  Info   Rbm10 
   5817 0.0009317 563.2 463.1 1.216  1367482_at  similar to anaphase promoting complex subunit 11 homolog  Info   LOC498030 
   5818 0.0009317 107.3 72.7 1.476  1378800_at  Rho GTPase activating protein 8  Info   Arhgap8 
   5819 0.0009319 470.4 686.2 0.686  1377911_at  Transcribed locus  Info    
   5820 0.0009329 382.4 271.2 1.41  1377042_at    Info    
   5821 0.0009338 296.2 412.4 0.718  1374585_at  enoyl Coenzyme A hydratase domain containing 1  Info   RGD:1359654 
   5822 0.0009352 421 659.8 0.638  1368515_at  erythrocyte protein band 4.1-like 3  Info   Epb4.1l3 
   5823 0.0009353 130.7 79.8 1.638  1384926_at  Transcribed locus  Info    
   5824 0.0009367 350.6 485.8 0.722  1374610_at  Similar to hypothetical protein 4933408F15  Info    
   5825 0.0009372 1156.3 803.6 1.439  1388715_at  glycyl-tRNA synthetase (predicted)  Info   Gars_predicted 
   5826 0.0009386 61.1 37.3 1.638  1395710_at  similar to mesoderm induction early response 1 (MI-ER1)  Info   LOC313418 
   5827 0.0009386 70.4 29.8 2.362  1376002_at  Transcribed locus  Info    
   5828 0.0009401 557.7 709 0.787  1398598_at  dystonin (predicted)  Info   Dst_predicted 
   5829 0.0009424 41.6 59.3 0.702  1393277_at    Info    
   5830 0.0009428 23 50.5 0.455  1369263_at  wingless-type MMTV integration site 5A  Info   Wnt5a 
   5831 0.0009434 156 99.8 1.563  1391050_at  similar to Metal-response element-binding transcription factor 2 (Metal-response element DNA-binding protein M96) (predicted)  Info   RGD1304727_predicted 
   5832 0.0009447 92.3 156 0.592  1381528_at    Info    
   5833 0.0009451 49.2 91.3 0.539  1393409_at  Transcribed locus  Info    
   5834 0.0009452 126.1 186.3 0.677  1395246_at  Protein kinase, cAMP-dependent, regulatory, type 2, alpha  Info   Prkar2a 
   5835 0.0009455 221 140.3 1.575  1373003_at  Squamous cell carcinoma antigen recognized by T-cells 3 (predicted)  Info    
   5836 0.0009457 77.7 120.7 0.644  1374103_at  Frequenin homolog (Drosophila)  Info   Freq 
   5837 0.0009463 239.4 190.2 1.259  1385941_at  Wolf-Hirschhorn syndrome candidate 1-like 1 (predicted)  Info    
   5838 0.0009467 245.2 299.6 0.818  1379784_at  Peroxisome biogenesis factor 7 (predicted)  Info    
   5839 0.0009478 408 323 1.263  1389733_at  methionine-tRNA synthetase (predicted)  Info   Mars_predicted 
   5840 0.0009479 97.4 141.5 0.688  1376136_at    Info    
   5841 0.0009491 177.3 286.2 0.619  1374622_at  Transcribed locus  Info    
   5842 0.0009496 93.8 68 1.379  1389681_at  Poliovirus receptor-related 2 (herpesvirus entry mediator B) (predicted)  Info    
   5843 0.0009511 91 123 0.74  1382781_at  Transcribed locus  Info    
   5844 0.0009512 186.1 130 1.432  1390434_at  TNFRSF1A-associated via death domain  Info   Tradd 
   5845 0.0009522 81.5 20.2 4.035  1369871_at  amphiregulin  Info   Areg 
   5846 0.0009522 378.9 494.2 0.767  1373174_at  similar to 1110001K21Rik protein  Info   LOC497867 
   5847 0.0009524 161.5 26.4 6.117  1368519_at  serine (or cysteine) proteinase inhibitor, clade E, member 1  Info   Serpine1 
   5848 0.0009527 266.9 194.6 1.372  1377400_at  HIV-1 Rev binding protein 2 (predicted)  Info   Hrb2_predicted 
   5849 0.0009537 72.8 104.3 0.698  1376679_at  Transcribed locus  Info    
   5850 0.0009537 161 218.1 0.738  1388910_at  mitochondrial ribosomal protein S24 (predicted)  Info   Mrps24_predicted 
   5851 0.0009551 262.8 380.8 0.69  1370503_s_at  erythrocyte protein band 4.1-like 3  Info   Epb4.1l3 
   5852 0.000956 799.7 501.3 1.595  1372845_at  ribonuclease P 21kDa subunit (human)  Info   RGD:1303090 
   5853 0.0009561 7 15.4 0.455  1384616_at  similar to mKIAA0674 protein (predicted)  Info   RGD1304816_predicted 
   5854 0.0009563 168.2 94.2 1.786  1384242_at  Neurolysin (metallopeptidase M3 family)  Info   Nln 
   5855 0.0009573 360.8 330.7 1.091  1372366_at  HIV-1 tat interactive protein, homolog (human)  Info   Htatip 
   5856 0.0009584 557 291.8 1.909  1372542_at  Similar to QUAKING isoform 6  Info   Qki 
   5857 0.0009587 1.9 6.7 0.284  1381113_at  Transcribed locus, weakly similar to XP_342574.1 PREDICTED: similar to Csr1 [Rattus norvegicus]  Info    
   5858 0.0009589 815.4 607.5 1.342  1388797_at  U2 small nuclear ribonucleoprotein auxiliary factor (U2AF) 2 (predicted)  Info   U2af2_predicted 
   5859 0.0009591 3.9 20.8 0.188  1373723_at  similar to high density lipoprotein-binding protein  Info   LOC300027 
   5860 0.0009598 21.7 33.5 0.648  1384866_at  ectonucleoside triphosphate diphosphohydrolase 3  Info   Entpd3 
   5861 0.0009603 22.6 49.5 0.457  1397119_at    Info    
   5862 0.0009613 27.2 44.1 0.617  1376121_at  PHD finger protein 10 (predicted)  Info    
   5863 0.000962 59.3 101.2 0.586  1382811_at  Mitochondrial ribosomal protein L16  Info   Mrpl16 
   5864 0.0009626 35.8 20.3 1.764  1395747_at    Info    
   5865 0.0009627 112.1 61.4 1.826  1392638_at  Similar to myeloid leukemia factor 1 interacting protein  Info    
   5866 0.0009627 76.5 118.4 0.646  1398277_at  activin A receptor, type 1  Info   Acvr1 
   5867 0.0009644 472.4 325.4 1.452  1383477_at  ubiquitin carboxyl-terminal hydrolase L5 (predicted)  Info   Uchl5_predicted 
   5868 0.0009653 230.1 176.6 1.303  1389302_at  similar to RIKEN cDNA D030070L09 (predicted)  Info   RGD1310199_predicted 
   5869 0.0009679 10.3 28.3 0.364  1382457_at    Info    
   5870 0.0009695 51.2 86.4 0.593  1376610_a_at  Similar to OTTHUMP00000028696  Info    
   5871 0.0009696 9.5 25.7 0.37  1383750_at  Transcribed locus  Info    
   5872 0.0009709 31 75.3 0.412  1388866_at  Transcribed locus  Info    
   5873 0.000971 14.7 23.9 0.615  1394729_at  Protein kinase, lysine deficient 1  Info   Prkwnk1 
   5874 0.0009728 914 1355.5 0.674  1371861_at  Transcribed locus  Info    
   5875 0.0009737 507.5 638.7 0.795  1371743_at  guanylate kinase 1 (predicted)  Info   Guk1_predicted 
   5876 0.0009739 54.3 111.7 0.486  1386948_at  nestin  Info   Nes 
   5877 0.0009742 1092.2 815.8 1.339  1369956_at  interferon gamma receptor 1  Info   Ifngr 
   5878 0.0009747 305.3 222.9 1.37  1374700_at  Sprouty protein with EVH-1 domain 1, related sequence (predicted)  Info    
   5879 0.0009749 140.3 218.8 0.641  1375120_at  inhibitor of DNA binding 4  Info   Idb4 
   5880 0.0009798 106.6 77.6 1.374  1391014_at  zinc finger, MYND domain containing 19  Info   Zmynd19 
   5881 0.0009804 262.6 446.4 0.588  1374139_at  similar to cerebellar degeneration-related 2 (predicted)  Info   RGD1310578_predicted 
   5882 0.0009809 743.5 474.1 1.568  1382094_at  Transcribed locus  Info    
   5883 0.000981 47.3 81.5 0.58  1395132_at    Info    
   5884 0.0009813 87.3 13 6.715  1368324_at  breast cancer 2  Info   Brca2 
   5885 0.0009814 243.9 159.6 1.528  1369590_a_at  DNA-damage inducible transcript 3  Info   Ddit3 
   5886 0.0009815 57.4 87.7 0.655  1379580_at  chromodomain helicase DNA binding protein 6 (predicted)  Info   Chd6_predicted 
   5887 0.0009841 2.1 11.2 0.188  1390225_at  NIMA (never in mitosis gene a)- related kinase 9 (predicted)  Info   Nek9_predicted 
   5888 0.0009843 15 31.3 0.479  1380948_at  Transcribed locus  Info    
   5889 0.0009871 171.3 283.2 0.605  1392952_at  hypothetical protein LOC619561  Info   LOC619561 
   5890 0.0009884 420.7 566 0.743  1388619_at  radixin  Info   RGD:1359472 
   5891 0.0009884 251 380.5 0.66  1390545_at  Transcribed locus  Info    
   5892 0.0009886 89.3 44.4 2.011  1394743_at  similar to novel protein  Info   LOC362010 
   5893 0.0009897 106 257.9 0.411  1383469_at  Aldehyde dehydrogenase family 1, subfamily A3  Info   Aldh1a3 
   5894 0.0009909 475.3 288.4 1.648  1375673_at  Mitogen activated protein kinase kinase kinase 1  Info   Map3k1 
   5895 0.0009923 137.3 96.3 1.426  1387143_at  protein phosphatase 1, regulatory subunit 9B  Info   Ppp1r9b 
   5896 0.0009925 110.5 80.2 1.378  1390272_at  similar to RIKEN cDNA 2410012M04 (predicted)  Info   RGD1307867_predicted 
   5897 0.0009926 88.8 30.7 2.893  1395635_at  Tumor necrosis factor receptor superfamily, member 26 (predicted)  Info    
   5898 0.0009929 228.5 173.6 1.316  1367865_at  limkain b1  Info   Lkap 
   5899 0.0009967 151 83.1 1.817  1386745_at  Transcribed locus  Info    
   5900 0.0009969 102 192.7 0.529  1381871_at  BAI1-associated protein 1 (predicted)  Info   Baiap1_predicted 
   5901 0.0009989 132.6 81.7 1.623  1382078_at  Transcribed locus  Info    
   5902 0.0009999 70.6 127.8 0.552  1380117_at  Transcribed locus  Info    
   5903 0.0010007 3.8 22.4 0.17  1381439_at    Info    
   5904 0.001002 551.4 758.8 0.727  1378692_at  Paired box gene 8  Info   Pax8 
   5905 0.0010027 12 25.6 0.469  1388632_at  RAB6B, member RAS oncogene family (predicted)  Info    
   5906 0.0010032 25.1 3.9 6.436  1369752_a_at  calcium/calmodulin-dependent protein kinase IV  Info   Camk4 
   5907 0.0010033 56.9 32.7 1.74  1369173_at  complement component 3a receptor 1  Info   C3ar1 
   5908 0.0010046 161.9 118.5 1.366  1399048_at  coactivator-associated arginine methyltransferase 1 (predicted)  Info   Carm1_predicted 
   5909 0.0010055 499.9 642.8 0.778  1385238_at  Lectin, mannose-binding, 1  Info   Lman1 
   5910 0.0010062 43.6 67 0.651  1384010_at  similar to Neutral alpha-glucosidase C  Info   LOC311345 
   5911 0.0010063 274.1 182.6 1.501  1383336_at  similar to pinin  Info   LOC368070 
   5912 0.0010065 5286.6 4674.4 1.131  1398885_at  ribosomal protein L23  Info   Rpl23 
   5913 0.0010074 68 11.7 5.812  1384111_at  signal transducer and activator of transcription 4 (predicted)  Info   Stat4_predicted 
   5914 0.0010078 66.5 96.6 0.688  1382093_at  Similar to DKFZP434B168 protein (predicted)  Info    
   5915 0.0010087 807.3 1353.4 0.596  1368096_at  Ras-related GTP-binding protein Rab29  Info   Rab7l1 
   5916 0.0010097 62.1 93.9 0.661  1390219_at  similar to WD repeat membrane protein (predicted)  Info   RGD1306997_predicted 
   5917 0.0010101 99.5 169.7 0.586  1372403_at  similar to Nuclear membrane binding protein NUCLING  Info   LOC315732 
   5918 0.0010121 69.4 133.4 0.52  1374081_at  Similar to H63 breast cancer expressed gene isoform a; gene associated with HER-2/neu overexpression (predicted)  Info    
   5919 0.0010127 50.6 81.8 0.619  1394790_at  chloride channel 6 (predicted)  Info   Clcn6_predicted 
   5920 0.0010135 341.4 466.7 0.732  1376163_at  similar to Expressed sequence AI649392 (predicted)  Info   RGD1305043_predicted 
   5921 0.0010152 364.7 245.3 1.487  1372990_at  cAMP responsive element binding protein 3 (predicted)  Info   Creb3_predicted 
   5922 0.0010157 16.3 47.4 0.344  1377632_at    Info    
   5923 0.0010157 44.6 81.5 0.547  1378601_at  Transcribed locus  Info    
   5924 0.0010159 31 63.9 0.485  1382476_x_at  Similar to pellino 3 alpha (predicted)  Info    
   5925 0.0010171 81.8 145 0.564  1384863_at  copine VII (predicted)  Info   Cpne7_predicted 
   5926 0.0010175 152.4 104.2 1.463  1380186_at  Similar to Zinc finger protein 198 (Fused in myeloproliferative disorders protein) (Rearranged in atypical myeloproliferative disorder protein) (predicted)  Info    
   5927 0.0010179 150.2 237.6 0.632  1392545_at  transcription factor AP-2 beta (predicted)  Info   Tcfap2b_predicted 
   5928 0.001018 115.1 153.3 0.751  1379283_at  Transcribed locus  Info    
   5929 0.0010226 26.5 51.3 0.517  1381618_at  Transcribed locus  Info    
   5930 0.001023 15.8 31.2 0.506  1371109_at  complement component 8, beta polypeptide  Info   C8b 
   5931 0.0010239 1085.5 878.4 1.236  1388404_at    Info    
   5932 0.0010249 319.8 231.9 1.379  1371624_at  similar to ubiquitous tetratricopeptide containing protein RoXaN; Rotavirus X associated non-structural protein (predicted)  Info   RGD1307064_predicted 
   5933 0.0010256 133.3 293.5 0.454  1393017_at  rhophilin, Rho GTPase binding protein 1 (predicted)  Info   Rhpn1_predicted 
   5934 0.0010269 227.6 320.8 0.709  1371517_at  Similar to Grb10 protein  Info    
   5935 0.0010269 334 258 1.295  1372169_at  similar to RIKEN cDNA 4121402D02  Info   LOC303514 
   5936 0.0010285 146.4 247.7 0.591  1389295_at  olfactomedin-like 2B (predicted)  Info   Olfml2b_predicted 
   5937 0.0010287 322.7 254.2 1.269  1372797_at  similar to Polyglutamine binding protein 1  Info   LOC302557 
   5938 0.0010295 86.6 126.8 0.683  1368839_at  Wolfram syndrome 1  Info   Wfs1 
   5939 0.0010298 70.3 118 0.596  1373829_at  Fibroblast growth factor receptor 2  Info   Fgfr2 
   5940 0.0010305 204.2 318.8 0.641  1378027_at  poliovirus receptor-related 3 (predicted)  Info   Pvrl3_predicted 
   5941 0.0010309 353.3 485.8 0.727  1389642_at  Transcribed locus  Info    
   5942 0.0010309 720.5 915.6 0.787  1398807_at  protein phosphatase 1B, magnesium dependent, beta isoform  Info   Ppm1b 
   5943 0.0010315 111 65.2 1.702  1389417_at  Transcribed locus  Info    
   5944 0.0010318 67.4 97.2 0.693  1389035_at  similar to DNA segment, Chr 17, Wayne State University 94, expressed (predicted)  Info   RGD1308185_predicted 
   5945 0.0010327 10.1 51.4 0.196  1392803_at    Info    
   5946 0.0010328 154.6 112.3 1.377  1378259_at    Info    
   5947 0.0010339 1158.3 1483.1 0.781  1367681_at  CD151 antigen  Info   Cd151 
   5948 0.0010348 99.6 46 2.165  1387054_at  ATP-binding cassette, sub-family G (WHITE), member 1  Info   Abcg1 
   5949 0.0010364 864.8 1484.5 0.583  1398847_at  nudix (nucleoside diphosphate linked moiety X)-type motif 4  Info   Nudt4 
   5950 0.0010365 122.7 269.3 0.456  1379755_at  Transcribed locus  Info    
   5951 0.0010373 56.1 75.1 0.747  1382263_at  similar to DNA segment, Chr 3, ERATO Doi 250, expressed (predicted)  Info   RGD1308397_predicted 
   5952 0.0010374 167.8 92.1 1.822  1387201_at  ring finger protein 138  Info   Rnf138 
   5953 0.0010379 273.2 182 1.501  1376641_at  THO complex 1 (predicted)  Info   Thoc1_predicted 
   5954 0.0010382 142.2 104.6 1.359  1384390_at  Importin 9 (predicted)  Info    
   5955 0.0010398 10.8 19 0.568  1379465_at  Transcribed locus, moderately similar to XP_421979.1 PREDICTED: similar to connectin/titin [Gallus gallus]  Info    
   5956 0.0010402 523.7 619.4 0.845  1398397_at  Similar to Parg1-pending protein (predicted)  Info   B130017i01rik 
   5957 0.0010414 98.5 29.5 3.339  1369814_at  chemokine (C-C motif) ligand 20  Info   Ccl20 
   5958 0.0010417 198.8 342.3 0.581  1391270_at  Cyclin M3 (predicted)  Info    
   5959 0.0010437 324.4 228.9 1.417  1377630_at  transmembrane 4 superfamily member 13 (predicted) /// similar to transmembrane 4 superfamily member 13  Info   Tm4sf13_predicted /// LOC500643 
   5960 0.0010442 696.5 905.9 0.769  1367800_at  plasminogen activator, tissue  Info   Plat 
   5961 0.0010445 1883.6 2731.6 0.69  1372665_at  phosphoserine aminotransferase 1  Info   Psat1 
   5962 0.0010456 29.7 48.3 0.615  1387042_at  calcium channel, voltage-dependent, beta 3 subunit  Info   Cacnb3 
   5963 0.0010461 7.1 1.7 4.176  1393523_at  Coilin  Info   Coil 
   5964 0.0010463 58.3 92.5 0.63  1376068_at    Info    
   5965 0.0010466 85.6 24 3.567  1371267_at  RT1 class Ib, locus Aw2 /// RT1-149 protein  Info   RT1-Aw2 /// RT1-149 
   5966 0.0010474 1014.1 1332.2 0.761  1367899_at  coagulation factor II (thrombin) receptor  Info   F2r 
   5967 0.0010475 64.8 92.9 0.698  1395965_at  Retinoblastoma binding protein 6 (predicted)  Info    
   5968 0.001048 135 94.6 1.427  1395445_at  similar to regulatory factor X-associated protein  Info   LOC499617 
   5969 0.0010494 867.2 997.9 0.869  1389291_at  coiled-coil-helix-coiled-coil-helix domain containing 3 (predicted)  Info   Chchd3_predicted 
   5970 0.0010498 219.4 272.9 0.804  1374109_at  diacylglycerol kinase, theta (predicted)  Info   Dgkq_predicted 
   5971 0.0010502 196.9 135.2 1.456  1372279_at  BCL2-like 13 (apoptosis facilitator) (predicted)  Info   Bcl2l13_predicted 
   5972 0.0010508 20.1 35.1 0.573  1376970_at  Transcribed locus  Info    
   5973 0.0010535 42.1 75.2 0.56  1384645_at  similar to hypothetical protein DKFZp313N0621  Info   LOC363169 
   5974 0.0010541 531.5 686.7 0.774  1377593_at  dolichol-phosphate (beta-D) mannosyltransferase 1 (predicted)  Info   Dpm1_predicted 
   5975 0.0010548 2988.5 2177.1 1.373  1389904_at    Info    
   5976 0.0010552 412.7 271.4 1.521  1398362_at  Transcribed locus  Info    
   5977 0.0010582 45.5 24.8 1.835  1386621_at    Info    
   5978 0.0010586 429.3 319.6 1.343  1382046_at  mitogen activated protein kinase kinase kinase 3 (predicted)  Info   Map3k3_predicted 
   5979 0.0010595 20.6 32.9 0.626  1377840_at    Info    
   5980 0.0010603 17.8 46.6 0.382  1376124_at  Wiskott-Aldrich syndrome-like (human)  Info   Wasl 
   5981 0.0010609 208.6 269.5 0.774  1376303_a_at  membralin (predicted)  Info   RGD1311136_predicted 
   5982 0.0010611 87.7 102.3 0.857  1396344_at  5'-nucleotidase, cytosolic II-like 1 (predicted)  Info   Col10a1 
   5983 0.0010613 149.4 201.9 0.74  1377703_at  similar to RIKEN cDNA 2900009I07 (predicted)  Info   RGD1310316_predicted 
   5984 0.0010626 128.9 196.5 0.656  1374682_at  Transcribed locus, weakly similar to NP_035569.1 SRY-box 13 [Mus musculus]  Info    
   5985 0.0010627 1273.8 885.4 1.439  1398886_at  similar to Selenoprotein H  Info   LOC502642 
   5986 0.0010641 156.1 248.9 0.627  1372338_at  similar to hypothetical protein FLJ25059  Info   LOC366146 
   5987 0.0010641 152.3 109.2 1.395  1374733_at  symplekin (predicted)  Info   Sympk_predicted 
   5988 0.0010642 13.7 2.2 6.227  1380421_s_at  growth factor independent 1  Info   Gfi1 
   5989 0.0010654 211.5 337.6 0.626  1390107_at  synaptotagmin-like 2 (predicted)  Info   Sytl2_predicted 
   5990 0.001067 180 314.6 0.572  1371113_a_at  Transferrin receptor  Info   Tfrc 
   5991 0.0010678 72.4 121.2 0.597  1397500_x_at  Transcribed locus  Info    
   5992 0.0010687 136.7 91.5 1.494  1385052_at  RAB3A interacting protein (rabin3)-like 1  Info   Rab3il1 
   5993 0.0010691 17 38.2 0.445  1379897_at  Transcribed locus  Info    
   5994 0.00107 740.6 984.5 0.752  1379254_at  similar to RIKEN cDNA 1300007B12; clone MNCb-2755 (predicted)  Info   RGD1309892_predicted 
   5995 0.0010702 278.5 432.2 0.644  1388515_at  Transcribed locus  Info    
   5996 0.0010705 376.4 268.7 1.401  1397313_at  CDNA clone MGC:93828 IMAGE:7110880  Info    
   5997 0.0010713 123.4 68.3 1.807  1373215_at  active BCR-related gene (predicted)  Info   Abr_predicted 
   5998 0.0010719 1111.2 1484.8 0.748  1389554_at  Nuclear receptor subfamily 2, group F, member 2  Info   Nr2f2 
   5999 0.0010729 232.2 154.6 1.502  1380256_at  polymerase (RNA) III (DNA directed) polypeptide C (predicted)  Info   Polr3c_predicted 
   6000 0.0010729 457.4 815.2 0.561  1398628_at    Info    
   6001 0.0010732 160.5 250.8 0.64  1369212_s_at  erythrocyte protein band 4.1-like 1  Info   Epb4.1l1 
   6002 0.0010738 127.3 198.8 0.64  1382964_at    Info    
   6003 0.001074 362.3 587.6 0.617  1379525_at  similar to chromosome 20 open reading frame 155 (predicted)  Info   RGD1311037_predicted 
   6004 0.0010747 926.3 1769.4 0.524  1370821_at  Cb1-812  Info    
   6005 0.0010751 332 499.2 0.665  1371430_at  dystroglycan 1  Info   Dag1 
   6006 0.0010759 50 88.9 0.562  1383713_at  Transcribed locus, moderately similar to XP_132808.1 PREDICTED: hypothetical protein LOC70291 [Mus musculus]  Info    
   6007 0.0010761 255.6 376.8 0.678  1393143_at  Transcribed locus  Info    
   6008 0.0010762 23.3 54.8 0.425  1387182_at  G protein-coupled receptor 37  Info   Gpr37 
   6009 0.0010767 115.1 190.7 0.604  1393368_at  oxysterol binding protein-like 5 (predicted)  Info   Osbpl5_predicted 
   6010 0.0010788 1120.1 1516.1 0.739  1371680_at    Info    
   6011 0.0010788 1.6 4.3 0.372  1378089_at    Info    
   6012 0.0010792 334.9 456.7 0.733  1388691_at  similar to UBIN (predicted)  Info   RGD1308273_predicted 
   6013 0.0010813 1015.8 1346.9 0.754  1383938_at  Similar to nuclear receptor coactivator 7  Info    
   6014 0.0010823 99.4 146.7 0.678  1382593_at  Ets variant gene 1 (predicted)  Info    
   6015 0.0010827 249.6 409.7 0.609  1396013_at  leucine zipper-EF-hand containing transmembrane protein 1  Info   RGD:1359678 
   6016 0.0010828 128.5 219.6 0.585  1370135_at  caveolin 2  Info   Cav2 
   6017 0.0010832 1201 1736.8 0.692  1375411_at  NADH dehydrogenase (ubiquinone) 1 alpha subcomplex, 7 (B14.5a) (predicted)  Info   Ndufa7_predicted 
   6018 0.0010841 234.3 193.8 1.209  1371477_at  Transcribed locus  Info    
   6019 0.001085 261.8 360.8 0.726  1376790_at  Galactosylceramidase  Info   Galc 
   6020 0.0010857 59.4 163.1 0.364  1388561_at  Transcribed locus  Info    
   6021 0.0010861 46.4 64.1 0.724  1378019_at  Transcribed locus  Info    
   6022 0.0010867 91 66.1 1.377  1375187_at  Transcribed locus  Info    
   6023 0.0010869 39.8 94.3 0.422  1370334_at  evectin-1  Info   Plekhb1 
   6024 0.0010873 8.3 21.3 0.39  1395170_at    Info    
   6025 0.0010875 38.2 61.1 0.625  1375723_at  Transcribed locus  Info    
   6026 0.0010897 186.5 238.3 0.783  1374534_at  MIRO2 protein  Info   Rhot2 
   6027 0.0010908 254.1 207.3 1.226  1373879_at  Transcribed locus  Info    
   6028 0.0010914 2487.6 2055.9 1.21  1371428_at    Info    
   6029 0.0010914 656 855.3 0.767  1375526_at  similar to novel protein of unknown function (DUF423) family member  Info   LOC287442 
   6030 0.0010915 34.5 68.1 0.507  1377802_at  Transcribed locus, strongly similar to NP_036462.1 MYST histone acetyltransferase (monocytic leukemia) 4; histone acetyltransferase; monocytic leukemia zinc finger protein-related factor [Homo sapiens]  Info    
   6031 0.0010917 41.1 76.6 0.537  1377088_at  similar to RIKEN cDNA 2310046K01 (predicted)  Info   RGD1304644_predicted 
   6032 0.0010919 93.2 53.5 1.742  1382225_at  Transcribed locus  Info    
   6033 0.001092 160.5 289.3 0.555  1373882_at    Info    
   6034 0.0010924 3.2 24 0.133  1379517_at  regulator of G-protein signaling 6  Info   Rgs6 
   6035 0.0010929 19.7 46.7 0.422  1378929_at  Transcribed locus, weakly similar to XP_580238.1 PREDICTED: hypothetical protein XP_580238 [Rattus norvegicus]  Info    
   6036 0.0010929 38.4 83.2 0.462  1388274_at  brain expressed myelocytomatosis oncogene  Info   Bmyc 
   6037 0.0010934 5141.2 4217.2 1.219  1371295_at  ribosomal protein S20  Info   Rps20 
   6038 0.001094 54.8 88.5 0.619  1387007_at  glial cell line derived neurotrophic factor family receptor alpha 1  Info   Gfra1 
   6039 0.001095 110 78.6 1.399  1376050_at  TAF5 RNA polymerase II, TATA box binding protein (TBP)-associated factor (predicted)  Info   Taf5_predicted 
   6040 0.001095 60.8 29.4 2.068  1386669_at  Transcribed locus  Info    
   6041 0.0010951 115.1 73.9 1.558  1390513_at    Info    
   6042 0.0010986 74.2 42.8 1.734  1384762_at  Transcribed locus  Info    
   6043 0.0010987 53.2 87.8 0.606  1378854_at  5-methyltetrahydrofolate-homocysteine methyltransferase reductase (predicted)  Info   Mtrr_predicted 
   6044 0.0010992 345.1 537.3 0.642  1371150_at  cyclin D1  Info   Ccnd1 
   6045 0.0011 74.2 36.6 2.027  1374563_at  Transcribed locus  Info    
   6046 0.0011018 9.3 17.8 0.522  1368743_a_at  dentin sialophosphoprotein  Info   Dspp 
   6047 0.0011035 177 81 2.185  1382086_at  Transcribed locus  Info    
   6048 0.001106 33.8 82.1 0.412  1397495_at  Similar to Protein C9orf10 (predicted)  Info    
   6049 0.0011077 19.1 37.6 0.508  1381808_at  Heterogeneous nuclear ribonucleoprotein A/B  Info   Hnrpab 
   6050 0.0011083 9.4 24.2 0.388  1393927_at  wingless-related MMTV integration site 2  Info   Wnt2 
   6051 0.0011089 101.3 135.9 0.745  1374268_at  Transcribed locus  Info    
   6052 0.0011095 224.6 461.6 0.487  1383605_at  similar to alpha-fetoprotein  Info   LOC360919 
   6053 0.0011099 125.3 86.7 1.445  1389117_at  O-sialoglycoprotein endopeptidase (predicted)  Info   Osgep_predicted 
   6054 0.0011109 4795.1 3570.7 1.343  1398872_at  ribosomal protein L13 /// ribosomal protein S13  Info   Rpl13 /// Rps13 
   6055 0.0011123 339.3 265.8 1.277  1385790_at  ring finger protein 153 (predicted)  Info   Rnf153_predicted 
   6056 0.0011129 687.7 503.7 1.365  1381993_at  chloride intracellular channel 2 (predicted)  Info   Clic2_predicted 
   6057 0.0011134 170.5 233.4 0.731  1390806_at  similar to RIKEN cDNA B230118H07 (predicted)  Info   RGD1309730_predicted 
   6058 0.0011135 45.2 65.2 0.693  1389913_at    Info    
   6059 0.0011146 207.3 267.9 0.774  1383830_a_at  oxysterol binding protein-like 1A  Info   Osbpl1a 
   6060 0.0011148 80.9 51.3 1.577  1382746_s_at  Similar to lymphocyte alpha-kinase (predicted)  Info    
   6061 0.0011152 150.6 99.4 1.515  1372415_at  general transcription factor IIH, polypeptide 3 (predicted)  Info   Gtf2h3_predicted 
   6062 0.0011153 124.7 211 0.591  1392563_at  Rho GTPase activating protein 8 (predicted)  Info   Arhgap8_predicted 
   6063 0.0011182 33.4 58.6 0.57  1379160_at  Similar to hypothetical protein D930020E02  Info    
   6064 0.0011192 82.3 51.6 1.595  1383741_at  Transcribed locus  Info    
   6065 0.0011196 285.6 389.7 0.733  1382126_at    Info    
   6066 0.0011199 31.5 68.4 0.461  1382913_at  cortactin binding protein 2  Info   Cttnbp2 
   6067 0.0011214 704.9 2263.7 0.311  1367961_at  nerve growth factor, gamma  Info   Ngfg 
   6068 0.0011224 25.8 34.5 0.748  1396175_at  similar to Zinc finger protein 62 homolog (Zfp-62) (ZT3)  Info   LOC497894 
   6069 0.0011227 438 128.9 3.398  1388924_at  angiopoietin-like protein 4  Info   Angptl4 
   6070 0.0011233 198 246.7 0.803  1376112_a_at  nuclear transport factor 2  Info   RGD:1359213 
   6071 0.0011239 29.9 58.7 0.509  1378763_at  hypothetical gene supported by BC088439  Info   LOC500947 
   6072 0.0011257 73.5 114.7 0.641  1379826_at  similar to hypothetical protein MGC31967  Info   LOC313496 
   6073 0.0011263 119.1 164.7 0.723  1393192_at  Transcribed locus  Info    
   6074 0.0011267 165.3 91.6 1.805  1382778_at  Dual specificity phosphatase 6  Info   Dusp6 
   6075 0.0011289 38.4 68.3 0.562  1397622_at    Info    
   6076 0.0011315 75.2 152.2 0.494  1369213_at  neural cell adhesion molecule L1  Info   RGD:619777 
   6077 0.0011323 100.7 73.5 1.37  1395704_at  Transcribed locus  Info    
   6078 0.0011338 25.8 47.9 0.539  1397615_at  Transcribed locus  Info    
   6079 0.0011352 71 109.7 0.647  1393132_at  Similar to inhibitor of MyoD family-a  Info    
   6080 0.0011354 133.8 87.9 1.522  1394568_at  similar to DNA segment, Chr 10, Wayne State University 102, expressed  Info   LOC299700 
   6081 0.0011367 418 517.8 0.807  1390509_a_at  Mitochondrial ribosomal protein L51 (predicted)  Info    
   6082 0.001137 297.1 419.9 0.708  1388430_at  prostate tumor over expressed gene 1 (predicted)  Info   Ptov1_predicted 
   6083 0.0011402 1231 1490.8 0.826  1371798_at  Guanine nucleotide binding protein, alpha 12  Info   Gna12 
   6084 0.0011406 36.8 65.8 0.559  1373254_at  keratin 10  Info   RGD:1359092 
   6085 0.0011416 260.8 344.9 0.756  1399078_at  thyroid hormone receptor associated protein 5 (predicted)  Info   Thrap5_predicted 
   6086 0.0011419 395.1 311.5 1.268  1373475_at  hypothetical LOC288065  Info   LOC288065 
   6087 0.0011437 148.9 80.6 1.847  1371044_at  phosphodiesterase 7A  Info   Pde7a 
   6088 0.0011451 37 78.6 0.471  1389464_at  ligand of numb-protein X 1 (predicted)  Info   Lnx1_predicted 
   6089 0.0011451 13.4 21.1 0.635  1390743_at  Transcribed locus  Info    
   6090 0.0011462 69.6 123.8 0.562  1380203_at    Info    
   6091 0.0011478 16.4 35.3 0.465  1396815_at  LOC501008  Info   LOC501008 
   6092 0.0011488 458.9 589.3 0.779  1371021_at  Arylsulfatase B  Info   Arsb 
   6093 0.0011514 56.2 24.3 2.313  1382186_a_at  similar to RIKEN cDNA 2610029K21 (predicted)  Info   RGD1311086_predicted 
   6094 0.0011522 15.1 65.7 0.23  1368465_at  amiloride-sensitive cation channel 1, neuronal (degenerin)  Info   Accn1 
   6095 0.0011526 275.2 351.4 0.783  1372454_at  similar to RIKEN cDNA 2210016L21 gene (predicted)  Info   RGD1311899_predicted 
   6096 0.0011526 67.6 99.2 0.681  1383976_at  FK506 binding protein 14 (predicted)  Info    
   6097 0.0011535 136.8 170.4 0.803  1380561_at  similar to hypothetical protein FLJ10326 (predicted)  Info   RGD1311857_predicted 
   6098 0.0011549 91.3 219.4 0.416  1384774_at  transmembrane protease, serine 8 (intestinal)  Info   Tmprss8 
   6099 0.0011571 378 567.6 0.666  1384327_at  differentially expressed in FDCP 8 (predicted)  Info   Def8_predicted 
   6100 0.0011578 103.3 68.5 1.508  1374252_at  centrosomal protein 1 (predicted)  Info   Cep1_predicted 
   6101 0.0011581 735.3 541.6 1.358  1371926_at  Interleukin 6 signal transducer  Info   Il6st 
   6102 0.0011587 119.5 204.7 0.584  1372537_at  Echinoderm microtubule associated protein like 1 (predicted)  Info    
   6103 0.0011605 18.1 37.1 0.488  1376710_at  Similar to PYM protein  Info    
   6104 0.0011609 727.3 1105.6 0.658  1373431_at  leucine-rich repeat-containing 5 (predicted)  Info   Lrrc5_predicted 
   6105 0.0011634 126.6 34.3 3.691  1368413_at  amiloride binding protein 1  Info   Abp1 
   6106 0.0011638 448.6 363.7 1.233  1389218_at  UDP-glucose ceramide glucosyltransferase-like 1  Info   Ugcgl1 
   6107 0.0011643 191.3 262.3 0.729  1388662_at  Transcribed locus  Info    
   6108 0.0011656 246.2 317.2 0.776  1373605_at  similar to 106 kDa O-GlcNAc transferase-interacting protein (predicted)  Info   RGD1307844_predicted 
   6109 0.0011676 905.8 766 1.183  1373100_at  similar to RIKEN cDNA 2900010J23  Info   LOC499779 
   6110 0.0011682 54.4 93.7 0.581  1377961_at  similar to AT motif-binding factor  Info   LOC307829 
   6111 0.0011683 385 489.5 0.787  1372257_at  Transcribed locus  Info    
   6112 0.0011688 206.1 161.2 1.279  1368341_at  polymerase (DNA directed), beta  Info   Polb 
   6113 0.0011695 119.1 182.6 0.652  1377254_a_at  Cohen syndrome homolog 1 (predicted)  Info   Cohh1_predicted 
   6114 0.0011696 83.1 164.4 0.505  1392483_at  Similar to choline phosphotransferase 1; cholinephosphotransferase 1 alpha; cholinephosphotransferase 1  Info   MGC95241 
   6115 0.001171 41.4 78 0.531  1379286_at  Transcribed locus  Info    
   6116 0.0011732 819.2 610 1.343  1387262_at  Sjogren syndrome antigen B  Info   Ssb 
   6117 0.0011749 308.4 419.1 0.736  1395145_at  Ral guanine nucleotide dissociation stimulator,-like 1 (predicted)  Info    
   6118 0.0011766 75.4 111.4 0.677  1389179_at  cell death-inducing DNA fragmentation factor, alpha subunit-like effector A (predicted)  Info   Cidea_predicted 
   6119 0.0011777 597.8 480.4 1.244  1375550_at  Bcl2-associated athanogene 1 (predicted)  Info   Bag1_predicted 
   6120 0.0011784 53.6 32.4 1.654  1393298_at  Transcribed locus  Info    
   6121 0.0011799 194.1 120.9 1.605  1394093_at  SWI/SNF related, matrix associated, actin dependent regulator of chromatin, subfamily a, member 5 (predicted)  Info   Smarca5_predicted 
   6122 0.0011801 47.3 68.5 0.691  1390458_at    Info    
   6123 0.0011806 218.6 283.1 0.772  1390684_at  Transcribed locus  Info    
   6124 0.0011807 583.3 434.2 1.343  1374178_at  ADP-ribosylation factor-like 5  Info   Arl5 
   6125 0.0011811 26.5 59.7 0.444  1380932_at    Info    
   6126 0.0011829 613.3 756.9 0.81  1388542_at  similar to RIKEN cDNA 2010315L10 (predicted) /// similar to 2010315L10Rik protein  Info   RGD1306660_predicted /// LOC292282 
   6127 0.0011834 95.1 63.4 1.5  1368167_at  cathepsin E  Info   Ctse 
   6128 0.0011845 10.6 26.1 0.406  1379374_at  plasticity related gene 1  Info   RGD:727806 
   6129 0.0011851 3868.1 4967.9 0.779  1368060_at  heat-responsive protein 12  Info   Hrsp12 
   6130 0.0011862 16.2 66.9 0.242  1398411_at  similar to RIKEN cDNA A430107P09 gene  Info   LOC364357 
   6131 0.0011871 85.7 142.3 0.602  1390556_at    Info    
   6132 0.0011881 81.9 111.2 0.737  1389612_at  Transcribed locus, moderately similar to NP_001001306.1 hypothetical protein LOC329695 [Mus musculus]  Info    
   6133 0.0011882 609.6 487.8 1.25  1370295_at  expressed in non-metastatic cells 1  Info   Nme1 
   6134 0.0011882 30.3 153.8 0.197  1375707_at    Info    
   6135 0.0011887 115.4 242.5 0.476  1393421_at  peroxisomal membrane protein 4  Info   Pxmp4 
   6136 0.0011894 141.2 204.7 0.69  1380526_at  glutaryl-Coenzyme A dehydrogenase (predicted)  Info   Gcdh_predicted 
   6137 0.001191 143.3 94.7 1.513  1395396_at  similar to CG9135-PA (predicted)  Info   RGD1309986_predicted 
   6138 0.0011911 730 1123.7 0.65  1389586_at  Transcribed locus  Info    
   6139 0.0011914 1161 824 1.409  1369991_at  signal peptidase complex 18kD  Info   Spc18 
   6140 0.0011932 6.1 22.1 0.276  1393765_at  Transcribed locus  Info    
   6141 0.0011937 16.7 36.8 0.454  1384634_at    Info    
   6142 0.001194 66.1 152 0.435  1379300_at  carbohydrate sulfotransferase 2 (predicted)  Info   Chst2_predicted 
   6143 0.0011951 3544.4 3022.4 1.173  1388244_s_at  laminin receptor 1 (67kD, ribosomal protein SA)  Info   Lamr1 
   6144 0.0011976 25.4 40.5 0.627  1385126_at  Transcribed locus  Info    
   6145 0.0011981 21 40.2 0.522  1384309_at  Transcribed locus  Info    
   6146 0.0011998 38 60.7 0.626  1368900_at  thrombomodulin  Info   Thbd 
   6147 0.0012023 791.7 620.8 1.275  1376793_at  similar to RIKEN cDNA 5730414C17 (predicted)  Info   RGD1308377_predicted 
   6148 0.0012025 18.9 32.5 0.582  1382016_at  actin related protein 2/3 complex, subunit 5-like (predicted)  Info   Arpc5l_predicted 
   6149 0.0012031 4185.3 7173.4 0.583  1368333_at  uromodulin  Info   Umod 
   6150 0.001208 76.8 127.2 0.604  1390711_at  Transcribed locus  Info    
   6151 0.0012091 27.3 49.5 0.552  1379971_at  zinc finger CCCH type domain containing 6 (predicted)  Info   Zc3hdc6_predicted 
   6152 0.0012091 15.8 35 0.451  1395466_at  Similar to Annexin A9 (Annexin 31) (Annexin XXXI)  Info    
   6153 0.0012099 665.3 572.1 1.163  1388656_at  Transcribed locus, strongly similar to XP_517968.1 PREDICTED: similar to ubiquitin conjugating enzyme [Pan troglodytes]  Info    
   6154 0.0012113 30.8 103.5 0.298  1379491_at  similar to Fat cell-specific low molecular weight protein (Fat tissue-specific low MW protein) (predicted)  Info   RGD1310648_predicted 
   6155 0.0012114 2416.5 3808.3 0.635  1372261_at  Transcribed locus  Info    
   6156 0.0012115 275.4 379.7 0.725  1376649_at  Protein phosphatase 2 (formerly 2A), regulatory subunit A (PR 65), beta isoform (predicted)  Info    
   6157 0.0012122 123.6 171.7 0.72  1369777_a_at  SH3/ankyrin domain gene 2  Info   Shank2 
   6158 0.0012129 119.5 169.4 0.705  1389219_at  Similar to serine/threonine kinase  Info    
   6159 0.0012129 16.8 42 0.4  1393263_at  Kin of IRRE like 3 (Drosophila) (predicted)  Info    
   6160 0.0012164 49.8 84.4 0.59  1376156_at  hypothetical LOC316091 (predicted)  Info   RGD1304827_predicted 
   6161 0.0012171 87.3 122.8 0.711  1368169_at  protein kinase C, alpha binding protein  Info   Prkcabp 
   6162 0.0012173 692.1 963.3 0.718  1372947_at  plastin 3 (T-isoform)  Info   Pls3 
   6163 0.0012176 373.6 263.7 1.417  1376569_at  Kruppel-like factor  Info   RGD:1359220 
   6164 0.001219 76.6 139.3 0.55  1392864_at  Rho GTPase activating protein 5 (predicted)  Info   Arhgap5_predicted 
   6165 0.0012199 75.7 132.7 0.57  1373165_at  Similar to zinc finger, matrin type 1  Info    
   6166 0.0012214 113 169.2 0.668  1380431_at  similar to KIAA0564 protein (predicted)  Info   RGD1308772_predicted 
   6167 0.0012216 9.2 22.8 0.404  1375982_at  Similar to mKIAA0613 protein  Info    
   6168 0.001222 34.4 71.9 0.478  1377407_at  similar to Aa2-174 (predicted)  Info   RGD1309578_predicted 
   6169 0.001223 1005 1316.3 0.764  1375933_at    Info    
   6170 0.0012234 712.5 861.6 0.827  1371634_at  similar to RIKEN cDNA 1810020E01 (predicted)  Info   RGD1305677_predicted 
   6171 0.0012242 222.2 307.7 0.722  1368082_at  solute carrier family 4, member 2  Info   Slc4a2 
   6172 0.0012254 472.2 642.8 0.735  1372421_at  aspartylglucosaminidase (predicted)  Info   Aga_predicted 
   6173 0.001228 126.3 59.2 2.133  1391222_at  similar to Nedd4 binding protein 1 (predicted)  Info   RGD1305179_predicted 
   6174 0.001229 23.6 47.4 0.498  1395629_at  Transcribed locus  Info    
   6175 0.0012296 2.9 17.3 0.168  1397376_at  Transcribed locus  Info    
   6176 0.0012309 283.8 225.8 1.257  1398901_at  nucleoporin 98  Info   Nup98 
   6177 0.0012317 20.1 42.8 0.47  1372858_at    Info    
   6178 0.001232 873.9 1096 0.797  1367669_a_at  microtubule-associated proteins 1A/1B light chain 3  Info   Map1lc3b 
   6179 0.001233 357 462.9 0.771  1377723_at  Transcribed locus  Info    
   6180 0.0012332 11.5 18.4 0.625  1381651_at  Axotrophin (predicted)  Info    
   6181 0.0012337 93.3 182.1 0.512  1379782_a_at    Info    
   6182 0.0012349 690.6 560.3 1.233  1383202_at  similar to U6 snRNA-associated Sm-like protein LSm6 (Sm protein F)  Info   LOC498934 
   6183 0.001235 71.3 9 7.922  1380333_at  protein tyrosine phosphatase, non-receptor type 8 (predicted)  Info   Ptpn8_predicted 
   6184 0.001235 93.8 69.2 1.355  1390351_at  Transcribed locus  Info    
   6185 0.0012351 4738.8 4063.3 1.166  1371305_at  Ribosomal protein L8  Info   Rpl8 
   6186 0.0012362 3.4 14.3 0.238  1390521_at  Transcribed locus, strongly similar to NP_663373.2 O-acetyltransferase [Mus musculus]  Info    
   6187 0.0012415 1197.1 849.6 1.409  1368838_at  tropomyosin 4  Info   Tpm4 
   6188 0.0012452 101.1 181.6 0.557  1392324_at  EPS8-like 2 (predicted)  Info   Eps8l2_predicted 
   6189 0.0012454 1562.5 2606.9 0.599  1389785_at  similar to RIKEN cDNA 0610006H10 gene (predicted)  Info   RGD1305394_predicted 
   6190 0.0012461 174.6 132.5 1.318  1374205_at  transcription factor Pur-beta  Info   pur-beta 
   6191 0.0012478 354.6 497.2 0.713  1395331_at  similar to hypothetical protein CL25084 (predicted)  Info   RGD1306508_predicted 
   6192 0.0012488 126.7 154.9 0.818  1386988_at  deformed epidermal autoregulatory factor 1 (Drosophila)  Info   Deaf1 
   6193 0.0012502 598 392.7 1.523  1372500_at  tropomodulin 3 (predicted)  Info   Tmod3_predicted 
   6194 0.0012515 110.1 172.4 0.639  1374633_at  Transcribed locus  Info    
   6195 0.0012524 273.7 201.9 1.356  1373495_at  ubiquitin-conjugating enzyme E2, J1 (predicted)  Info   Ube2j1_predicted 
   6196 0.0012536 304.8 443.3 0.688  1394871_at  Solute carrier family 31 (copper transporters), member 1  Info   Slc31a1 
   6197 0.001254 79.1 144 0.549  1391079_at  Transcribed locus  Info    
   6198 0.0012551 404.9 542.3 0.747  1389032_at  mucolipin 1 (predicted)  Info   Mcoln1_predicted 
   6199 0.0012566 59.9 88.7 0.675  1382294_at  Activated leukocyte cell adhesion molecule  Info   Alcam 
   6200 0.0012568 128.4 201.8 0.636  1395551_at  Similar to novel protein  Info    
   6201 0.0012583 646.8 774.8 0.835  1398874_at  ataxin 10 homolog (human)  Info   Atxn10 
   6202 0.0012586 609.6 1434.3 0.425  1387974_a_at  kidney specific organic anion transporter  Info   RGD:621387 
   6203 0.0012588 35.2 72.8 0.484  1382838_at    Info    
   6204 0.0012604 105.5 234.7 0.45  1389234_at  von Willebrand factor  Info   RGD:621759 
   6205 0.0012608 21.7 34.3 0.633  1377963_at  Transcribed locus  Info    
   6206 0.0012619 362.2 302.9 1.196  1382063_at  galactosidase, alpha  Info   Gla 
   6207 0.0012626 96.5 58.2 1.658  1384357_at    Info    
   6208 0.001263 87.1 150.7 0.578  1368313_a_at  transient receptor potential cation channel, subfamily V, member 1  Info   Trpv1 
   6209 0.0012632 736.6 876.8 0.84  1380407_at  similar to HTGN29 protein; keratinocytes associated transmembrane protein 2 (predicted)  Info   RGD1310352_predicted 
   6210 0.0012635 570.7 827.6 0.69  1377899_at  similar to RIKEN cDNA 2810025M15 (predicted)  Info   RGD1304982_predicted 
   6211 0.0012635 50.8 30.4 1.671  1391044_s_at  similar to Lck-interacting transmembrane adaptor protein LIME  Info   LOC362289 
   6212 0.0012638 508.4 378.1 1.345  1388448_at  Rap guanine nucleotide exchange factor (GEF) 6 (predicted)  Info    
   6213 0.0012643 20 41.5 0.482  1390690_at  Transcribed locus, moderately similar to XP_507756.1 PREDICTED: similar to ZNF239 protein [Pan troglodytes]  Info    
   6214 0.0012659 118.6 193.2 0.614  1373119_at  potassium channel tetramerisation domain containing 3 (predicted)  Info   Kctd3_predicted 
   6215 0.001267 77.8 112 0.695  1394510_at  Transcribed locus  Info    
   6216 0.0012672 205.7 288.8 0.712  1389027_at  Lectin, mannose-binding 2-like (predicted)  Info    
   6217 0.0012676 341 209.9 1.625  1371563_at  similar to CG9135-PA (predicted)  Info   RGD1309986_predicted 
   6218 0.001268 343.6 266 1.292  1372779_at  UDP-GlcNAc:betaGal beta-1,3-N-acetylglucosaminyltransferase 1 (predicted)  Info    
   6219 0.0012686 26.5 43.7 0.606  1379270_at  Transcribed locus  Info    
   6220 0.0012703 14.5 31.7 0.457  1384720_at    Info    
   6221 0.0012708 204.1 131.6 1.551  1376801_at  hypothetical LOC294291  Info   LOC294291 
   6222 0.0012712 50.3 106.5 0.472  1379814_at  CDNA clone IMAGE:7302159  Info    
   6223 0.0012736 141.1 83.5 1.69  1373549_at  DEAD (Asp-Glu-Ala-Asp) box polypeptide 10 (predicted)  Info   Ddx10_predicted 
   6224 0.0012744 2120.7 1791.3 1.184  1388119_at  hypothetical gene supported by Y16641 /// heterogeneous nuclear ribonucleoprotein A3 /// similar to Hnrpa3 protein /// similar to Hnrpa3 protein  Info   LOC292273 /// Hnrpa3 /// LOC363971 /// LOC499592 
   6225 0.0012748 477.1 384.5 1.241  1388979_at  survival motor neuron domain containing 1 (predicted)  Info   Smndc1_predicted 
   6226 0.001275 1056.1 793.5 1.331  1368843_at  YME1-like 1 (S. cerevisiae)  Info   Yme1l1 
   6227 0.0012756 664.7 481.3 1.381  1372877_at  procollagen-lysine, 2-oxoglutarate 5-dioxygenase 3  Info   Plod3 
   6228 0.0012761 122.2 164.7 0.742  1387417_a_at  SH2-B PH domain containing signaling mediator 1  Info   Sh2bpsm1 
   6229 0.0012769 82.6 42.5 1.944  1394486_at  Ras-related protein RAB43  Info   Rab43 
   6230 0.001277 235.2 320.7 0.733  1383776_at  Transcribed locus  Info    
   6231 0.0012795 99.5 126.6 0.786  1381923_at  similar to LOC387763 protein  Info   LOC499839 
   6232 0.0012798 212.1 304.3 0.697  1383558_at  similar to MJ0495-like protein SelB  Info   LOC500255 
   6233 0.0012799 470.4 353.6 1.33  1372166_at  D4, zinc and double PHD fingers family 2 (predicted)  Info    
   6234 0.00128 743.5 946.7 0.785  1372214_at  mitochondrial ribosomal protein S33 (predicted)  Info   Mrps33_predicted 
   6235 0.0012802 97.3 144.3 0.674  1372318_at  Transcribed locus  Info    
   6236 0.0012807 110.6 135.6 0.816  1394741_at  Heterogeneous nuclear ribonucleoprotein A3  Info   hnRNPA3 
   6237 0.0012815 216.8 336.4 0.644  1367847_at  nuclear protein 1  Info   Nupr1 
   6238 0.001283 61.4 40.6 1.512  1387530_a_at  fos-like antigen 2 /// FBJ osteosarcoma oncogene B (predicted)  Info   Fosl2 /// Fosb_predicted 
   6239 0.0012832 54.2 117.1 0.463  1383188_at  Transcribed locus  Info    
   6240 0.0012848 3.2 24.7 0.13  1391383_at  Cartilage acidic protein 1  Info   Crtac1 
   6241 0.001285 62.9 129.2 0.487  1382462_at  Transcribed locus  Info    
   6242 0.0012852 9229.2 6569.3 1.405  1398839_at  thioredoxin 1  Info   Txn1 
   6243 0.0012861 87.6 131.3 0.667  1393654_at  nei endonuclease VIII-like 1 (E. coli) (predicted)  Info   Neil1_predicted 
   6244 0.0012867 76.7 29.5 2.6  1387269_s_at  plasminogen activator, urokinase receptor  Info   Plaur 
   6245 0.0012871 47.8 72.5 0.659  1369153_at  nephrosis 1 homolog, nephrin (human)  Info   Nphs1 
   6246 0.0012872 716.4 1093.7 0.655  1379456_at  mitochondrial carrier triple repeat 1 (predicted)  Info   Mcart1_predicted 
   6247 0.0012878 17.4 92.6 0.188  1384770_at  Transcribed locus  Info    
   6248 0.0012884 730.3 925.3 0.789  1387900_at  CDP-diacylglycerol--inositol 3-phosphatidyltransferase (phosphatidylinositol synthase)  Info   Cdipt 
   6249 0.0012893 1874.9 2196.6 0.854  1398837_at  transcription elongation factor B (SIII), polypeptide 2  Info   Tceb2 
   6250 0.0012897 38.6 63.7 0.606  1393261_at  Similar to protein phosphatase 2A B56 regulatory subunit gamma 3 isoform  Info    
   6251 0.0012899 116.8 86.1 1.357  1373838_at  fucosyltransferase 4  Info   Fut4 
   6252 0.0012901 46.7 96.4 0.484  1382685_at    Info    
   6253 0.0012912 16.7 34.1 0.49  1377658_at  Similar to actin filament associated protein; actin filament-associated protein, 110 kDa (predicted)  Info    
   6254 0.0012916 242.9 178.9 1.358  1373869_at  sterol O-acyltransferase 1  Info   Soat1 
   6255 0.0012928 187.2 126.9 1.475  1374637_at  similar to hypothetical protein PRO1580 (predicted)  Info   RGD1309263_predicted 
   6256 0.0012956 218 170.2 1.281  1377020_at  CDNA clone IMAGE:7375616  Info    
   6257 0.0012957 67.9 24.1 2.817  1393128_at  similar to RIKEN cDNA 2610029K21 (predicted)  Info   RGD1311086_predicted 
   6258 0.0012958 884 1165.6 0.758  1371599_at  LRRGT00097  Info    
   6259 0.0012958 609.1 803.8 0.758  1369980_s_at  myosin phosphatase-Rho interacting protein  Info   Mrip 
   6260 0.0012969 129.7 165.3 0.785  1395274_at  dystonin (predicted)  Info   Dst_predicted 
   6261 0.0012973 204.2 290.8 0.702  1390498_at  Transcribed locus  Info    
   6262 0.001299 407.8 313.7 1.3  1373491_at  Similar to Metaxin 1, isoform 2  Info    
   6263 0.0012993 16.2 27.9 0.581  1382897_at    Info    
   6264 0.0012996 101.5 62.3 1.629  1397508_at  similar to RIKEN cDNA 2310005B10  Info   MGC95311 
   6265 0.0013004 115.2 61.8 1.864  1373793_at  immunoglobulin superfamily, member 8 (predicted)  Info   Igsf8_predicted 
   6266 0.0013009 74.7 101.4 0.737  1375194_at  Transcribed locus  Info    
   6267 0.0013052 99.2 145.2 0.683  1376058_at  mitochondrial tumor suppressor 1  Info   Mtus1 
   6268 0.0013056 102.3 51.6 1.983  1388966_at  Purinergic receptor P2Y, G-protein coupled 2  Info   P2ry2 
   6269 0.0013088 331 222.4 1.488  1386966_a_at  protein kinase C-binding protein Beta15  Info   Pkcbpb15 
   6270 0.0013093 255 73.2 3.484  1392515_at    Info   RGD:1359643 /// RGD:1359487 /// RGD:1359240 /// RGD:1359669 /// RGD:1359210 /// LOC502904 /// LOC502905 /// LOC502906 /// LOC502907 
   6271 0.0013104 297.4 361.7 0.822  1373228_at  Similar to D8Ertd354e protein  Info    
   6272 0.001311 3568.4 4202.4 0.849  1371335_at  similar to CG6105-PA  Info   RGD:1303259 
   6273 0.0013111 247.2 160.3 1.542  1374489_at  GTP binding protein 2 (predicted)  Info   Gtpbp2_predicted 
   6274 0.0013118 10.9 2.5 4.36  1383921_at  Transcribed locus, strongly similar to XP_579758.1 PREDICTED: hypothetical protein XP_579758 [Rattus norvegicus]  Info    
   6275 0.001313 231.9 130.6 1.776  1377696_at  mesoderm development candidate 1 (predicted)  Info   Mesdc1_predicted 
   6276 0.0013132 49.7 81.5 0.61  1382943_at  protocadherin gamma subfamily A, 10 (predicted)  Info   Pcdhga10_predicted 
   6277 0.001316 133 161.3 0.825  1395579_at  DEAH (Asp-Glu-Ala-His) box polypeptide 32 (predicted)  Info   Dhx32_predicted 
   6278 0.0013162 21.4 62.2 0.344  1383566_at  T-complex-associated testis expressed 1 (predicted)  Info    
   6279 0.0013187 2937.3 2257.1 1.301  1388076_at  PAI-1 mRNA-binding protein  Info   Pairbp1 
   6280 0.0013187 104.4 72.7 1.436  1376907_at  Microtubule-associated protein 6  Info   Mtap6 
   6281 0.0013195 679.9 549.5 1.237  1371885_at  cytoskeleton-associated protein 1 (predicted)  Info   Ckap1_predicted 
   6282 0.0013215 113.9 223.7 0.509  1379918_at    Info    
   6283 0.0013218 1377.5 1140.2 1.208  1388767_at  programmed cell death 6 (predicted)  Info   Pdcd6_predicted 
   6284 0.0013219 112.9 206.8 0.546  1394392_at  similar to dJ881L22.2 (novel protein) (predicted)  Info   RGD1307696_predicted 
   6285 0.001322 81.4 135 0.603  1385556_at    Info    
   6286 0.0013224 313.8 121.8 2.576  1393848_at  ribonucleotide reductase M2  Info   Rrm2 
   6287 0.0013229 797.3 1389.1 0.574  1388750_at  Transferrin receptor  Info   Tfrc 
   6288 0.001323 19.1 63.3 0.302  1395153_at  Claudin 15 (predicted)  Info    
   6289 0.0013236 644.2 490.6 1.313  1370867_at  guanine nucleotide binding protein, beta polypeptide 2  Info   Gnb2 
   6290 0.0013251 328.6 433.3 0.758  1374650_at  neural precursor cell expressed, developmentally down-regulated gene 9 (predicted)  Info   Nedd9_predicted 
   6291 0.0013251 43 30.6 1.405  1379774_at  Transcribed locus  Info    
   6292 0.0013285 287.4 181.1 1.587  1373984_at  Transcribed locus  Info    
   6293 0.0013309 41.6 79.1 0.526  1369439_at  angiotensin II receptor, type-1  Info   RGD:67391 
   6294 0.0013321 54.7 6.9 7.928  1391948_at  B-cell leukemia/lymphoma 11B (predicted)  Info   Bcl11b_predicted 
   6295 0.0013337 63.1 118.3 0.533  1385246_at  Transcribed locus  Info    
   6296 0.0013337 2772.9 3124.5 0.887  1368521_at  napsin A aspartic peptidase  Info   Napsa 
   6297 0.0013348 88.9 46.6 1.908  1379608_at  Transcribed locus  Info    
   6298 0.0013356 132.3 182 0.727  1388399_at  microtubule associated serine/threonine kinase 2 (predicted)  Info   Mast2_predicted 
   6299 0.0013357 10.6 23.8 0.445  1378566_at    Info    
   6300 0.0013373 128.5 206.7 0.622  1371765_at  histone 2a  Info   RGD:621437 
   6301 0.0013378 50.1 73.3 0.683  1387512_at  zinc finger protein 238  Info   Zfp238 
   6302 0.001338 38.5 78.4 0.491  1380741_at  Transcribed locus  Info    
   6303 0.0013391 257.2 318.6 0.807  1374538_at  O-linked mannose beta1,2-N-acetylglucosaminyltransferase  Info   RGD:1359396 
   6304 0.0013393 23.8 49 0.486  1368348_at  solute carrier family 6 (neurotransmitter transporter, serotonin), member 4  Info   Slc6a4 
   6305 0.0013396 16.6 36 0.461  1382102_at  Transcribed locus  Info    
   6306 0.0013411 108.7 41.6 2.613  1378629_at  special AT-rich sequence binding protein 1 (predicted)  Info   Satb1_predicted 
   6307 0.001342 62 93.4 0.664  1369270_at  nuclear receptor subfamily 1, group I, member 2  Info   Nr1i2 
   6308 0.0013421 74.8 126.6 0.591  1378416_at  Tetratricopeptide repeat domain 8 (predicted)  Info    
   6309 0.0013442 70.8 48.3 1.466  1384602_at  G protein-coupled receptor 107 (predicted)  Info   Gpr107_predicted 
   6310 0.0013444 128.8 177.4 0.726  1370264_at  CPG2 protein  Info   RGD:621597 
   6311 0.0013445 87.8 115.5 0.76  1399110_at  zinc finger protein 297  Info   RGD:1359627 
   6312 0.0013452 89.8 129.8 0.692  1374100_at    Info    
   6313 0.0013463 133.9 109.4 1.224  1368537_at  dynactin 4  Info   Dctn4 
   6314 0.0013484 125 173.4 0.721  1390854_at  transmembrane protein 24 (predicted)  Info   Tmem24_predicted 
   6315 0.0013495 140.7 237.8 0.592  1373377_at  sodium channel modifier 1 (predicted)  Info   Scnm1_predicted 
   6316 0.0013502 192.3 134.4 1.431  1379768_at  CDNA clone IMAGE:7374368  Info    
   6317 0.0013507 266.1 193.6 1.374  1374326_at  peter pan homolog (Drosophila) (predicted)  Info   Ppan_predicted 
   6318 0.0013516 624.1 435.6 1.433  1371868_at  breast carcinoma amplified sequence 2 (predicted)  Info   Bcas2_predicted 
   6319 0.0013544 20.8 38.8 0.536  1398416_at    Info    
   6320 0.0013586 85.8 43.5 1.972  1387964_a_at  ERO1-like (S. cerevisiae)  Info   Ero1l 
   6321 0.0013601 41.6 79.2 0.525  1374546_at  Protocadherin 17 (predicted)  Info    
   6322 0.0013602 12.4 19.2 0.646  1385529_at  family with sequence similarity 3, member B (predicted)  Info   Fam3b_predicted 
   6323 0.0013602 14.5 24.6 0.589  1386257_at  Transcribed locus  Info    
   6324 0.0013605 321 238.4 1.346  1371908_at  NTF2-related export protein 1 (predicted)  Info   Nxt1_predicted 
   6325 0.0013611 39.2 13.6 2.882  1378867_at    Info    
   6326 0.0013628 12.1 22.4 0.54  1370683_at  potassium channel, subfamily K, member 15  Info   Kcnk15 
   6327 0.001363 398.6 591.1 0.674  1373809_at  DEAD (Asp-Glu-Ala-Asp) box polypeptide 21b (predicted) /// hypothetical protein LOC606294  Info   Ddx21b_predicted /// LOC606294 
   6328 0.0013637 295.7 242.5 1.219  1391584_at  Transcribed locus  Info    
   6329 0.001364 256.4 185.4 1.383  1388733_at  bifunctional apoptosis regulator (predicted)  Info   Bfar_predicted 
   6330 0.0013652 215.7 271.3 0.795  1371825_at  small nuclear RNA activating complex, polypeptide 2 (predicted)  Info   Snapc2_predicted 
   6331 0.0013662 45.4 78.4 0.579  1374745_at  similar to sorting nexin 21 isoform a; sorting nexin L; sorting nexin 21 (predicted)  Info   RGD1311169_predicted 
   6332 0.0013689 958.2 1266.9 0.756  1375634_at  similar to RIKEN cDNA 2900091E11  Info   LOC299707 
   6333 0.0013697 9.9 19.7 0.503  1378423_at  ataxia, cerebellar, Cayman type (caytaxin) (predicted)  Info   Atcay_predicted 
   6334 0.0013698 1098.1 1444.7 0.76  1388380_at  similar to Protein CGI-51  Info   RGD:1303219 
   6335 0.0013711 171.6 119.4 1.437  1383168_at  Transcribed locus, strongly similar to NP_081141.1 hypothetical protein LOC73635 [Mus musculus]  Info    
   6336 0.0013718 15.9 27.8 0.572  1391197_at  Transcribed locus  Info    
   6337 0.0013726 50.5 79.4 0.636  1381558_at  Similar to 4933427D14Rik protein (predicted)  Info    
   6338 0.0013732 308.2 239.6 1.286  1373811_at  similar to heat shock protein 8  Info   LOC365951 
   6339 0.0013748 56.6 93.7 0.604  1372480_at  zinc finger protein 462 (predicted)  Info   Zfp462_predicted 
   6340 0.0013748 51.9 97.2 0.534  1387339_at  selenoprotein P, plasma, 1  Info   Sepp1 
   6341 0.0013756 2875.7 2249.1 1.279  1371774_at  spermidine/spermine N1-acetyl transferase  Info   Sat 
   6342 0.0013797 177.7 274.2 0.648  1383159_at  Retinoic acid induced 1 (predicted)  Info    
   6343 0.0013798 5.1 14.8 0.345  1382969_at  Transcribed locus  Info    
   6344 0.00138 2.9 18.9 0.153  1379899_at  Transcribed locus  Info    
   6345 0.0013805 51.8 79.9 0.648  1396149_at  Similar to Zdhhc1 protein  Info    
   6346 0.0013809 137.8 83.4 1.652  1373091_at  Transcribed locus  Info    
   6347 0.0013817 543.7 351.9 1.545  1392746_x_at  Similar to KIAA0731 protein (predicted)  Info    
   6348 0.0013829 252.8 183.2 1.38  1374840_at    Info    
   6349 0.0013837 35.8 84.4 0.424  1397015_at  Transcribed locus  Info    
   6350 0.0013838 27.1 47.8 0.567  1382320_at  LOC362793 (predicted)  Info   RGD1307315_predicted 
   6351 0.001389 92.4 127.7 0.724  1381220_at  activin receptor IIA  Info   Acvr2 
   6352 0.0013908 68 133.4 0.51  1376242_at  Transcribed locus  Info    
   6353 0.0013912 200.7 258.7 0.776  1379449_at  RB1-inducible coiled-coil 1 (predicted)  Info   Rb1cc1_predicted 
   6354 0.0013924 787.7 975.9 0.807  1399004_at    Info    
   6355 0.0013934 63.6 107.7 0.591  1396028_at  Transcribed locus  Info    
   6356 0.0013952 29.2 68.7 0.425  1397744_at  Dopamine beta hydroxylase  Info   Dbh 
   6357 0.0013966 301.2 222.5 1.354  1374473_at  Protein phosphatase 1, regulatory (inhibitor) subunit 15b (predicted)  Info    
   6358 0.0013972 34.8 66.4 0.524  1396885_at  smoothelin (predicted)  Info   Smtn_predicted 
   6359 0.0013975 64.2 88 0.73  1370578_at  lin-10 protein homolog (C. elegans)  Info   Lin10 
   6360 0.0013982 770.2 512.6 1.503  1368948_at  moesin  Info   Msn 
   6361 0.0013987 152.4 97.8 1.558  1397385_at  Transcribed locus  Info    
   6362 0.0014006 99.5 64.4 1.545  1375442_at  M-phase phosphoprotein 10 (U3 small nucleolar ribonucleoprotein) (predicted)  Info   Mphosph10_predicted 
   6363 0.0014014 534.8 345.6 1.547  1381972_at  cereblon (predicted)  Info   Crbn_predicted 
   6364 0.0014025 85.4 130 0.657  1383790_at  Transcribed locus  Info    
   6365 0.0014029 132 87.6 1.507  1391422_at  DnaJ (Hsp40) homolog, subfamily B, member 6 (predicted)  Info   Dnajb6_predicted 
   6366 0.001403 306.2 500.2 0.612  1386985_at  glutathione S-transferase, mu 1  Info   Gstm1 
   6367 0.001403 936.6 660 1.419  1398905_at  ATPase, H+ transporting, V1 subunit G isoform 1 (predicted)  Info   Atp6v1g1_predicted 
   6368 0.0014034 1.1 3.4 0.324  1398085_at  Transcribed locus  Info    
   6369 0.0014036 157.2 234.1 0.672  1398267_at  solute carrier family 22 (organic anion transporter), member 7  Info   Slc22a7 
   6370 0.0014049 131.4 307.5 0.427  1386128_at  tetranectin (plasminogen binding protein) (predicted)  Info   Tna_predicted 
   6371 0.001405 179.1 218.8 0.819  1389388_at  Transcribed locus  Info    
   6372 0.001406 310.1 210.5 1.473  1395739_at  similar to RIKEN cDNA C920006C10 (predicted)  Info   RGD1305976_predicted 
   6373 0.0014075 402.5 312.4 1.288  1372769_at  similar to ligatin  Info   LOC498225 
   6374 0.001408 86.8 170 0.511  1383404_at  Transcribed locus  Info    
   6375 0.0014083 546 401.7 1.359  1373049_at  similar to D1Ertd622e protein  Info   LOC501195 
   6376 0.0014096 70.8 104.3 0.679  1374806_at  stratifin (predicted)  Info   Sfn_predicted 
   6377 0.0014102 35.5 80.6 0.44  1380248_at  sema domain, immunoglobulin domain (Ig), transmembrane domain (TM) and short cytoplasmic domain, (semaphorin) 4G (predicted)  Info   Sema4g_predicted 
   6378 0.0014104 436.8 317.6 1.375  1387779_at  MYB binding protein (P160) 1a  Info   Mybbp1a 
   6379 0.0014112 16.9 31.3 0.54  1382262_at  Transcribed locus  Info    
   6380 0.0014133 100.2 146.2 0.685  1375068_at  Similar to surfeit 5 isoform b  Info    
   6381 0.0014139 2398.1 1973.8 1.215  1399091_at  F-actin capping protein beta subunit  Info   RGD:1359099 
   6382 0.0014144 158.7 112.6 1.409  1368330_at  apoptosis antagonizing transcription factor  Info   Aatf 
   6383 0.0014156 30.3 58.5 0.518  1386911_at  ATPase, Na+/K+ transporting, alpha 2 polypeptide  Info   Atp1a2 
   6384 0.0014162 319.6 384 0.832  1372314_at  Transcribed locus  Info    
   6385 0.001417 9.6 29.3 0.328  1388435_at  Crystallin, gamma S (predicted)  Info    
   6386 0.0014171 165.4 237.2 0.697  1370380_s_at  nuclear receptor subfamily 2, group F, member 6  Info   Nr2f6 
   6387 0.0014175 139.1 183.5 0.758  1382423_at  Transcribed locus  Info    
   6388 0.001419 2.3 12.9 0.178  1371083_at  Serine protease inhibitor  Info   Spin2a 
   6389 0.0014207 332 261.1 1.272  1372143_at  ubiquitin-conjugating enzyme E2 variant 2  Info   Ube2v2 
   6390 0.0014208 70.1 100.2 0.7  1391370_at  Transcribed locus  Info    
   6391 0.0014208 39.1 63 0.621  1393168_at  Similar to Mbt domain containing 1  Info    
   6392 0.0014244 220.8 152 1.453  1373263_at  similar to SUMO/sentrin specific protease 5  Info   LOC499312 
   6393 0.0014247 88.3 157 0.562  1389682_at  similar to RIKEN cDNA 2510006C20 (predicted)  Info   RGD1309120_predicted 
   6394 0.0014249 275.2 184.6 1.491  1382923_at  synaptotagmin binding, cytoplasmic RNA interacting protein (predicted)  Info   Syncrip_predicted 
   6395 0.0014259 193 233.4 0.827  1368019_at  FK506 binding protein 12-rapamycin associated protein 1  Info   Frap1 
   6396 0.0014262 13 33.4 0.389  1380563_at  Transcribed locus  Info    
   6397 0.0014281 10.6 23.2 0.457  1384276_at  Transcribed locus  Info    
   6398 0.0014292 275.1 366.9 0.75  1373564_at  3-hydroxyisobutyryl-Coenzyme A hydrolase (predicted)  Info   Hibch_predicted 
   6399 0.0014306 166.4 239.1 0.696  1383960_at  peroxisome biogenesis factor 16 (predicted)  Info   Pex16_predicted 
   6400 0.001435 245.2 312 0.786  1368390_at  v-raf oncogene homolog 1 (murine sarcoma 3611 virus)  Info   Araf1 
   6401 0.0014357 35.6 87.4 0.407  1384195_at    Info    
   6402 0.0014408 3879.9 4217.9 0.92  1386894_at  heat shock protein 1 (chaperonin)  Info   Hspd1 
   6403 0.0014413 24.4 44.5 0.548  1393270_at  Similar to Fbxw17 protein  Info    
   6404 0.001442 9.4 24.5 0.384  1394916_at  Transcribed locus  Info    
   6405 0.0014425 333 432.1 0.771  1372663_at  phosphatidylserine synthase 2 (predicted)  Info   Ptdss2_predicted 
   6406 0.0014437 175.4 122.8 1.428  1389326_at  replication factor C (activator 1) 3 (predicted)  Info   Rfc3_predicted 
   6407 0.0014442 379.6 544.5 0.697  1371702_at  transmembrane 4 superfamily member 2  Info   Tm4sf2 
   6408 0.0014446 507.6 855.1 0.594  1368794_at  3-hydroxyanthranilate 3,4-dioxygenase  Info   Haao 
   6409 0.001446 6.3 15.1 0.417  1396211_at  Similar to RIKEN cDNA 2610207I16 (predicted)  Info    
   6410 0.0014462 285.1 359.8 0.792  1368122_at  ring finger protein 103  Info   Rnf103 
   6411 0.0014483 50.9 87.4 0.582  1392724_at  target of myb1-like 2 (chicken) (predicted)  Info   Tom1l2_predicted 
   6412 0.0014497 408.3 271.3 1.505  1391356_at  Similar to hypothetical protein FLJ10006 (predicted)  Info    
   6413 0.00145 148.5 200.4 0.741  1382124_at  Cyclin M3 (predicted)  Info    
   6414 0.0014517 104.1 71.2 1.462  1388578_at  similar to membrane targeting translocation system protein like (2A562)  Info   LOC289378 
   6415 0.0014528 417 527.4 0.791  1388680_at  similar to contains transmembrane (TM) region and ATP binding region (predicted)  Info   RGD1311230_predicted 
   6416 0.0014532 598.6 761.5 0.786  1367509_at  Similar to general transcription factor IIH, polypeptide 5  Info    
   6417 0.0014544 186.5 251.3 0.742  1372927_at  mitochondrial ribosomal protein L50 (predicted)  Info   Mrpl50_predicted 
   6418 0.0014547 125.2 83.5 1.499  1398384_at  exosome component 9 (predicted)  Info   Exosc9_predicted 
   6419 0.001456 874.6 668.1 1.309  1383253_at  Solute carrier family 30 (zinc transporter), member 1  Info   Slc30a1 
   6420 0.0014572 38.5 64.7 0.595  1374706_at  Growth differentiation factor 11  Info   Gdf11 
   6421 0.0014577 91.1 65.5 1.391  1385220_at    Info    
   6422 0.0014577 78.4 104.2 0.752  1395214_at  LOC497962  Info   LOC497962 
   6423 0.0014586 96.4 190.8 0.505  1383319_at  solute carrier family 4, sodium bicarbonate transporter-like, member 11 (predicted)  Info   Slc4a11_predicted 
   6424 0.0014593 204.8 110.3 1.857  1371970_at  similar to expressed sequence AW413625  Info   LOC499322 
   6425 0.0014595 313.8 215.7 1.455  1397693_at  eukaryotic translation initiation factor 2, subunit 3, structural gene X-linked  Info   Eif2s3x 
   6426 0.0014601 37.6 11.4 3.298  1369176_at  solute carrier family 36 (proton/amino acid symporter), member 1  Info   Slc36a1 
   6427 0.0014617 45.7 6.3 7.254  1369191_at  interleukin 6  Info   Il6 
   6428 0.0014641 119.8 165.7 0.723  1398361_at  similar to Rho-GTPase-activating protein PS-GAP-a  Info   LOC498938 
   6429 0.0014659 204.1 157.2 1.298  1390947_at  Similar to RIKEN cDNA D930036F22 gene  Info    
   6430 0.0014674 92.1 52.8 1.744  1389671_at  transient receptor potential cation channel, subfamily C, member 2  Info   Trpc2 
   6431 0.0014689 304.8 249.3 1.223  1373221_at  Transcribed locus  Info    
   6432 0.0014702 215 270 0.796  1392330_at  similar to N6-DNA-methyltransferase isoform 1 (predicted)  Info   RGD1311843_predicted 
   6433 0.0014712 241.5 186.4 1.296  1370347_at  PDZ and LIM domain 7  Info   Pdlim7 
   6434 0.0014715 11.9 22.8 0.522  1392824_at  BCL2/adenovirus E1B 19kD interacting protein like (predicted)  Info   Bnipl_predicted 
   6435 0.0014737 688.7 538.8 1.278  1371660_at  zinc finger, HIT domain containing 1 (predicted)  Info   Znhit1_predicted 
   6436 0.0014754 52.3 15.3 3.418  1393389_at  Transcribed locus  Info    
   6437 0.0014766 6.8 39.8 0.171  1382239_at  similar to Myosin light chain kinase 2, skeletal/cardiac muscle (MLCK2) (predicted)  Info   RGD1305801_predicted 
   6438 0.0014773 1356.4 1085.7 1.249  1390356_at  Transcribed locus  Info    
   6439 0.0014774 13.6 20.7 0.657  1379107_at  Solute carrier family 4, member 4  Info   Slc4a4 
   6440 0.0014775 151.8 214.4 0.708  1370905_at  dedicator of cytokinesis 9  Info   Dock9 
   6441 0.0014776 39.2 12.9 3.039  1375649_at  Transcribed locus  Info    
   6442 0.0014793 177.1 133.6 1.326  1383871_at  Transcribed locus  Info    
   6443 0.0014797 98 144 0.681  1383931_at  Protein tyrosine phosphatase, receptor type, K, extracellular region  Info    
   6444 0.0014805 349.8 220.8 1.584  1379662_a_at  SNF related kinase  Info   Snrk 
   6445 0.0014805 69.9 96.6 0.724  1389340_at    Info    
   6446 0.0014807 75.8 155.3 0.488  1375123_at  SRY-box containing gene 4 (predicted)  Info   Sox4_predicted 
   6447 0.0014808 228.7 185.9 1.23  1374938_at  ZW10 homolog, centromere/kinetochore protein (Drosophila) (predicted)  Info   Zw10_predicted 
   6448 0.0014812 173.3 677.7 0.256  1387491_at  glycerol kinase  Info   Gyk 
   6449 0.001482 149.2 111.8 1.335  1375551_at  similar to hypothetical protein MGC2574  Info   RGD:1359280 
   6450 0.0014825 205.2 401.7 0.511  1369679_a_at  nuclear factor I/A  Info   Nfia 
   6451 0.0014833 160.7 95.7 1.679  1373776_at  Transcribed locus  Info    
   6452 0.0014858 236.3 164.1 1.44  1388511_at  centaurin, delta 2 (predicted)  Info   Centd2_predicted 
   6453 0.0014864 186.7 235 0.794  1373958_at  similar to ALEX3 protein  Info   LOC367902 
   6454 0.001487 77.1 57.9 1.332  1385109_at  similar to RIKEN cDNA 4930553M18 (predicted)  Info   RGD1309627_predicted 
   6455 0.0014882 59.9 111.6 0.537  1383280_at  Transcribed locus  Info    
   6456 0.001489 15.1 43.3 0.349  1380387_at  Transcribed locus  Info    
   6457 0.0014922 185.9 135.9 1.368  1384002_at  similar to Potential phospholipid-transporting ATPase IF (ATPase class I type 11B) (ATPase IR)  Info   LOC361929 
   6458 0.0014924 240.1 416.2 0.577  1367902_at  guanine nucleotide binding protein (G protein), gamma 11  Info   Gng11 
   6459 0.0014938 39.5 5.1 7.745  1381630_at  similar to RIKEN cDNA A930018P22  Info   LOC362175 
   6460 0.0014938 92.9 200.5 0.463  1392672_at  stem cell growth factor  Info   Scgf 
   6461 0.0014945 359.5 254.5 1.413  1390136_at  Transcribed locus  Info    
   6462 0.0014953 620.1 410.3 1.511  1367831_at  tumor protein p53  Info   Tp53 
   6463 0.0014972 346.8 648.9 0.534  1387542_at  solute carrier family 9, member 3  Info   Slc9a3 
   6464 0.001498 595.5 422.2 1.41  1368851_at  v-ets erythroblastosis virus E26 oncogene homolog 1 (avian)  Info   Ets1 
   6465 0.0014995 58 26.7 2.172  1384522_at  similar to synaptotagmin-like protein 3-a  Info   LOC499017 
   6466 0.0015003 72.3 127.9 0.565  1381196_at  PREDICTED: Rattus norvegicus splicing factor 4 (predicted) (Sf4_predicted), mRNA  Info    
   6467 0.0015006 27.1 63.3 0.428  1396009_at  Transcribed locus  Info    
   6468 0.0015013 206.1 357.4 0.577  1399041_at  similar to OPA3 protein  Info   LOC502305 
   6469 0.0015031 256.7 357.5 0.718  1376660_at  Transcribed locus  Info    
   6470 0.0015035 1255.3 1758.9 0.714  1388359_at  Eukaryotic translation initiation factor 4 gamma, 2  Info   Eif4g2 
   6471 0.001513 198.2 147 1.348  1378378_at  pantothenate kinase 2 (Hallervorden-Spatz syndrome) (predicted)  Info   Pank2_predicted 
   6472 0.0015138 1015.7 1235.8 0.822  1388681_at  SAR1a gene homolog 2 (S. cerevisiae) (predicted)  Info   Sara2_predicted 
   6473 0.001515 98.5 153.7 0.641  1372754_at    Info    
   6474 0.0015159 11.3 29.9 0.378  1378681_at  Transcribed locus  Info    
   6475 0.0015167 91.1 134.5 0.677  1395441_at  poly(rC) binding protein 4 (predicted)  Info   Pcbp4_predicted 
   6476 0.0015171 11.7 32.5 0.36  1381951_at  Transcribed locus  Info    
   6477 0.0015174 352.9 529.9 0.666  1383437_at    Info    
   6478 0.0015183 251.4 175.1 1.436  1368259_at  prostaglandin-endoperoxide synthase 1  Info   Ptgs1 
   6479 0.0015191 105.9 208.9 0.507  1387010_s_at  sodium channel, voltage-gated, type I, beta polypeptide  Info   Scn1b 
   6480 0.0015191 54 171.9 0.314  1387363_at  folate hydrolase  Info   Folh1 
   6481 0.0015197 177.6 253.3 0.701  1368899_at  bone morphogenetic protein receptor, type 1A  Info   Bmpr1a 
   6482 0.0015197 89 142.7 0.624  1390855_at  Prolyl endopeptidase  Info   Prep 
   6483 0.0015219 174.5 300.4 0.581  1382835_at  Transcribed locus  Info    
   6484 0.0015224 838.2 559.1 1.499  1388725_at  leptin receptor overlapping transcript  Info   Leprot 
   6485 0.0015247 152.6 116.1 1.314  1374182_at  SWI/SNF related, matrix associated, actin dependent regulator of chromatin, subfamily c, member 1 (predicted)  Info   Smarcc1_predicted 
   6486 0.0015254 77.4 130.1 0.595  1397826_at  erythrocyte protein band 4.9 (predicted)  Info   Epb4.9_predicted 
   6487 0.0015257 120.8 9.8 12.327  1395116_at  CD8 antigen, alpha chain  Info   Cd8a 
   6488 0.0015263 460.5 575.5 0.8  1398389_at  Platelet-activating factor acetylhydrolase, isoform Ib, alpha subunit 45kDa  Info   Pafah1b1 
   6489 0.0015286 346.5 441.7 0.784  1376168_at  Transcribed locus  Info    
   6490 0.0015341 1162.1 1346.4 0.863  1388504_at  similar to HR21spA  Info   MGC116373 
   6491 0.0015341 57.9 97.3 0.595  1390316_at  Ubiquitin-conjugating enzyme E2Q (putative) (predicted)  Info    
   6492 0.0015392 292.2 202.4 1.444  1391442_at  EH-domain containing 3  Info   Ehd3 
   6493 0.0015394 201.3 281.5 0.715  1376115_at  Transcribed locus  Info    
   6494 0.0015394 78.1 167.2 0.467  1392025_x_at    Info    
   6495 0.0015423 316.4 396.6 0.798  1374586_at  Similar to ADP-ribosylation factor related protein 2  Info    
   6496 0.0015441 1472.7 1656.5 0.889  1371355_at  NADH dehydrogenase (ubiquinone) 1 alpha subcomplex, 8 (predicted)  Info   Ndufa8_predicted 
   6497 0.0015446 481.9 632.5 0.762  1388852_at  similar to pM5 protein; DNA segment, Chr 7, ERATO Doi 156, expressed (predicted)  Info   RGD1305240_predicted 
   6498 0.0015464 206.5 244.1 0.846  1374847_at  leucine-zipper-like transcriptional regulator, 1 (predicted)  Info   Lztr1_predicted 
   6499 0.0015474 944 711.6 1.327  1386923_at  ubiquitin-conjugating enzyme E2B, RAD6 homolog (S. cerevisiae)  Info   Ube2b 
   6500 0.0015486 375.9 504.5 0.745  1399146_at  transmembrane protein 15 (predicted)  Info   Tmem15_predicted 
   6501 0.0015495 2009.8 1591 1.263  1377671_at  Hypothetical gene supported by NM_133555  Info   Csf2rb1 
   6502 0.0015518 73.6 110.3 0.667  1389453_at  RAD52 homolog B (S. cerevisiae) (predicted)  Info   Rad52b_predicted 
   6503 0.001552 287.2 429.1 0.669  1390563_at    Info    
   6504 0.0015588 141.1 203.9 0.692  1370418_s_at  brain and kidney protein /// hypothetical gene supported by NM_138849  Info   Bk /// LOC497736 
   6505 0.0015589 372.7 449.3 0.83  1371614_at  autophagy 12-like (S. cerevisiae) (predicted)  Info   Apg12l_predicted 
   6506 0.001559 22.3 38.1 0.585  1395743_at  Transcribed locus  Info    
   6507 0.0015596 534.1 382.7 1.396  1384157_at  similar to ADP-ribosylation factor-like 10C  Info   LOC500282 
   6508 0.0015609 21.3 72.6 0.293  1385352_at  Solute carrier family 16 (monocarboxylic acid transporters), member 13  Info   MGC93826 
   6509 0.001563 10.4 38.5 0.27  1375719_s_at  cadherin 13  Info   Cdh13 
   6510 0.0015639 39.5 72.2 0.547  1398312_s_at  solute carrier family 14 (urea transporter), member 2  Info   Slc14a2 
   6511 0.0015645 19.4 32.1 0.604  1369239_at  chloride channel 5  Info   Clcn5 
   6512 0.0015649 151.4 79.3 1.909  1384553_at  similar to hypothetical protein FLJ13089 (predicted)  Info   RGD1305685_predicted 
   6513 0.0015651 67.9 166.3 0.408  1368583_a_at  histidine-rich glycoprotein  Info   Hrg 
   6514 0.0015658 41 72.7 0.564  1390649_at  zinc transporter ZnT-3  Info   RGD:1359689 
   6515 0.0015662 149.7 238.1 0.629  1388437_at  Transcribed locus  Info    
   6516 0.0015688 167.4 218.1 0.768  1368463_at  vascular endothelial growth factor C  Info   Vegfc 
   6517 0.0015693 23.1 35.7 0.647  1368237_at  tenomodulin  Info   Tnmd 
   6518 0.0015717 242.1 296.1 0.818  1398606_at  golgi phosphoprotein 4 (predicted)  Info   Golph4_predicted 
   6519 0.001572 104.1 77.4 1.345  1386041_a_at  Kruppel-like factor  Info   RGD:1359220 
   6520 0.001572 126.7 175.3 0.723  1396055_at  procollagen, type VI, alpha 3 (predicted)  Info   Col6a3_predicted 
   6521 0.0015728 370.1 758.4 0.488  1388936_at  cadherin 11  Info   Cdh11 
   6522 0.0015731 143.7 107.7 1.334  1382461_at  similar to arginine N-methyltransferase p82 isoform (predicted)  Info   RGD1304869_predicted 
   6523 0.0015743 67.9 105.3 0.645  1383721_at  similar to transmembrane receptor  Info   MGC112790 
   6524 0.001575 162.9 115.1 1.415  1374620_at  CEA-related cell adhesion molecule 1  Info   Ceacam1 
   6525 0.0015775 205.1 147.4 1.391  1379249_at  Similar to Wilms tumor 1-associating protein (WT1-associated protein) (Putative pre-mRNA splicing regulator female-lethal(2D) homolog)  Info    
   6526 0.001583 39.9 66.9 0.596  1378304_at  Transcribed locus  Info    
   6527 0.0015847 686.1 556.5 1.233  1371463_at  transcription factor INI  Info   Phf5a 
   6528 0.0015854 58 103.6 0.56  1392244_at  LOC498362  Info   LOC498362 
   6529 0.001587 55.4 19.9 2.784  1386321_s_at  tribbles homolog 3 (Drosophila)  Info   Trib3 
   6530 0.0015899 153.5 246 0.624  1389306_at  matrilin 2 (predicted)  Info   Matn2_predicted 
   6531 0.001591 2.6 11.8 0.22  1384910_at  Similar to putative voltage-gated calcium channel alpha(2)delta-4 subunit  Info    
   6532 0.0015932 265.5 193.8 1.37  1372528_at  N-ethylmaleimide sensitive fusion protein  Info   Nsf 
   6533 0.0015935 19.3 2.9 6.655  1397845_at    Info    
   6534 0.0015961 360.2 627.9 0.574  1367804_at  serum amyloid P-component  Info   Sap 
   6535 0.0015966 15.1 37.5 0.403  1386648_at  DnaJ (Hsp40) homolog, subfamily C, member 6 (predicted)  Info   Dnajc6_predicted 
   6536 0.0015969 6.2 21.3 0.291  1387804_at  tripartite motif-containing 63  Info   Trim63 
   6537 0.0015976 576.9 755.3 0.764  1390027_at  ubiquitin specific protease 8 (predicted)  Info   Usp8_predicted 
   6538 0.0015988 11.3 23.9 0.473  1378814_at  Transcribed locus  Info    
   6539 0.0016004 203 102.7 1.977  1372578_at  valyl-tRNA synthetase 2  Info   Vars2 
   6540 0.0016025 26.3 38.8 0.678  1382844_at  Transcribed locus  Info    
   6541 0.0016029 1165.1 1483 0.786  1373819_at  hypothetical protein LOC500855  Info   LOC500855 
   6542 0.0016029 81.3 57.1 1.424  1382783_at  Bloom syndrome (predicted)  Info   Blm_predicted 
   6543 0.0016046 366.5 227.9 1.608  1389202_at    Info    
   6544 0.0016048 8.8 21.2 0.415  1378449_at  keratinocytes proline-rich protein  Info   RGD:1303244 
   6545 0.0016051 31.8 62.2 0.511  1391919_at  similar to transcription elongation regulator 1-like  Info   LOC361669 
   6546 0.0016056 4.2 20.8 0.202  1374683_at  gamma sarcoglycan  Info   RGD:1359577 
   6547 0.0016062 129.7 225 0.576  1373643_at  Transcribed locus  Info    
   6548 0.0016074 55.2 80.8 0.683  1378396_at  Bone specific CMF608  Info   Igsf10 
   6549 0.0016097 301.5 211.2 1.428  1372561_at  similar to hypothetical protein AL133206 (predicted)  Info   RGD1308418_predicted 
   6550 0.0016102 126.3 167.6 0.754  1377493_at  mitochondrial translation optimization 1 homolog (S. cerevisiae) (predicted)  Info   Mto1_predicted 
   6551 0.0016115 55.6 89.5 0.621  1390193_at  Transcribed locus  Info    
   6552 0.001618 164.2 122 1.346  1372271_at  RNA-binding region (RNP1, RRM) containing 1 (predicted)  Info   Rnpc1_predicted 
   6553 0.0016206 164.3 133.4 1.232  1390118_at  similar to ATP/GTP-binding protein (predicted)  Info   RGD1307679_predicted 
   6554 0.001622 230.1 325.5 0.707  1376016_at  similar to RIKEN cDNA 1110007L15 (predicted)  Info   RGD1311660_predicted 
   6555 0.0016225 54.3 109.2 0.497  1388689_at  Transcribed locus, strongly similar to NP_083620.1 acylphosphatase 2, muscle type [Mus musculus]  Info    
   6556 0.0016226 157.6 225.8 0.698  1373304_at  alpha-N-acetylglucosaminidase  Info   Naglu 
   6557 0.0016231 1136.4 920.7 1.234  1381970_at  similar to RIKEN cDNA 2700023B17 (predicted)  Info   RGD1305695_predicted 
   6558 0.0016233 72.1 53.1 1.358  1385706_at  Similar to Testis derived transcript  Info    
   6559 0.0016248 238.9 299.9 0.797  1372412_at  similar to hypothetical protein FLJ10477 (predicted)  Info   RGD1308109_predicted 
   6560 0.0016248 80.7 48.2 1.674  1396742_at  karyopherin (importin) beta 3 (predicted)  Info   Kpnb3_predicted 
   6561 0.0016259 13.6 31.8 0.428  1390610_at    Info    
   6562 0.0016265 143.4 73.5 1.951  1385086_at  budding uninhibited by benzimidazoles 1 homolog (S. cerevisiae) (predicted)  Info   Bub1_predicted 
   6563 0.0016267 299.1 412.2 0.726  1377809_at    Info    
   6564 0.0016286 69.7 113.3 0.615  1372844_at  ephrin A1  Info   Efna1 
   6565 0.0016301 25.5 37.4 0.682  1393196_at  similar to RIKEN cDNA 4930429H24 (predicted)  Info   RGD1307166_predicted 
   6566 0.0016304 16.8 35.3 0.476  1380401_at  Transcribed locus  Info    
   6567 0.0016307 75.2 98.1 0.767  1398374_at  similar to RIKEN cDNA 2810002D13 gene (predicted)  Info   RGD1307128_predicted 
   6568 0.0016357 1001.1 1343.6 0.745  1398350_at  brain abundant, membrane attached signal protein 1  Info   Basp1 
   6569 0.0016366 495 406.2 1.219  1398876_at  ATP-binding cassette, sub-family F (GCN20), member 1  Info   Abcf1 
   6570 0.0016368 60.2 91.1 0.661  1386540_at  V-kit Hardy-Zuckerman 4 feline sarcoma viral oncogene homolog  Info   Kit 
   6571 0.0016385 20.7 38 0.545  1397632_at  SLIT and NTRK-like family, member 1 (predicted)  Info   Slitrk1_predicted 
   6572 0.0016421 256.4 374 0.686  1383523_at  Rho/rac guanine nucleotide exchange factor (GEF) 18 (predicted)  Info    
   6573 0.0016437 18.9 31.6 0.598  1392045_at  transmembrane protein 22 (predicted)  Info   Tmem22_predicted 
   6574 0.0016439 875.7 1081.4 0.81  1373391_at  putative membrane protein  Info   RGD:1359178 
   6575 0.0016444 86.7 125.4 0.691  1383193_at  Transcribed locus  Info    
   6576 0.0016451 84.5 124.2 0.68  1375643_at  cytoskeleton-associated protein 4 (predicted)  Info   Ckap4_predicted 
   6577 0.0016452 98.6 148.1 0.666  1382193_at  Transcribed locus  Info    
   6578 0.0016462 270.7 196.6 1.377  1388801_at  Similar to RIKEN cDNA 9030221M09 gene (predicted)  Info    
   6579 0.0016463 283.1 355 0.797  1371443_at  LOC362671 (predicted)  Info   RGD1304567_predicted 
   6580 0.0016465 89.6 130.7 0.686  1384382_at  O-sialoglycoprotein endopeptidase-like 1 (predicted)  Info   Osgepl1_predicted 
   6581 0.0016465 70.8 121 0.585  1372139_at  similar to RIKEN cDNA 5530601I19 (predicted)  Info   RGD1308399_predicted 
   6582 0.0016487 393.3 359.6 1.094  1367539_at  Transcribed locus  Info    
   6583 0.0016489 97.7 55.8 1.751  1376237_at  Chemokine (C-C motif) receptor 6 (predicted)  Info    
   6584 0.001649 153.9 236.5 0.651  1383421_at  Transcribed locus  Info    
   6585 0.0016519 447.9 870.6 0.514  1367668_a_at  stearoyl-Coenzyme A desaturase 2  Info   Scd2 
   6586 0.0016531 13.4 25.1 0.534  1390370_at  Dehydrogenase/reductase (SDR family) member 4  Info   Dhrs4 
   6587 0.0016534 46.2 26.5 1.743  1392980_at  T-cell lymphoma invasion and metastasis 1 (predicted)  Info   Tiam1_predicted 
   6588 0.0016538 624.2 767.2 0.814  1373020_at  similar to mitochondria-associated granulocyte macrophage CSF signaling molecule  Info   LOC287065 
   6589 0.0016541 358.2 490.4 0.73  1381163_at  Transcribed locus  Info    
   6590 0.0016552 42.3 29.5 1.434  1390681_at  Similar to TGFB-induced factor 2  Info    
   6591 0.0016575 47.4 64.4 0.736  1378652_at  Transcribed locus  Info    
   6592 0.0016596 92.2 153.6 0.6  1372832_at  Transcribed locus  Info    
   6593 0.0016596 79.4 51 1.557  1377849_at  valosin-containing protein (p97)/p47 complex-interacting protein 135  Info   Vcip135 
   6594 0.0016599 542.6 799.8 0.678  1388454_at  Similar to RNA binding protein with multiple splicing 2  Info    
   6595 0.001662 40.1 73.6 0.545  1369101_at  retinoid X receptor alpha  Info   Rxra 
   6596 0.0016656 35.7 73.3 0.487  1370211_at  neurogranin  Info   Nrgn 
   6597 0.0016665 2504.3 3106.2 0.806  1388320_at  serine protease inhibitor, Kunitz type 2  Info   Spint2 
   6598 0.0016671 102.1 158.5 0.644  1388661_at  mitochondrial protein, 18 kDa  Info   RGD:1359705 
   6599 0.0016691 43.2 83 0.52  1397878_at  similar to crystallin, zeta (quinone reductase)-like 1  Info   LOC499978 
   6600 0.0016695 111 270.3 0.411  1370824_at  solute carrier family 38, member 3  Info   Slc38a3 
   6601 0.0016699 5175.7 4235.2 1.222  1386864_at  phosphoglycerate mutase 1  Info   Pgam1 
   6602 0.0016705 67.3 103.7 0.649  1391995_at  Tripartite motif protein 37 (predicted)  Info    
   6603 0.0016707 83.3 157.2 0.53  1376962_at  similar to retinoic acid-responsive protein; STRA6 (predicted)  Info   RGD1307551_predicted 
   6604 0.0016726 148.4 212.8 0.697  1376057_at    Info    
   6605 0.0016772 1091.9 884.2 1.235  1372770_at    Info    
   6606 0.0016777 39.9 14.3 2.79  1391260_at  Transcribed locus  Info    
   6607 0.0016787 459.3 613.6 0.749  1394737_at  similar to CG9752-PA (predicted)  Info   RGD1311345_predicted 
   6608 0.0016797 69.3 115.7 0.599  1376767_at  G protein-coupled receptor 116  Info   Gpr116 
   6609 0.0016798 139.9 183.1 0.764  1376235_at  Transcribed locus  Info    
   6610 0.0016828 95.6 70.9 1.348  1379384_at  Sp1 transcription factor  Info   Sp1 
   6611 0.0016851 734.6 879.3 0.835  1398977_at    Info    
   6612 0.0016854 178.8 146 1.225  1373746_at  similar to HSPC049 protein (predicted)  Info   RGD1306697_predicted 
   6613 0.0016889 282.2 365.5 0.772  1374495_at  LPS-responsive beige-like anchor (predicted)  Info   Lrba_predicted 
   6614 0.0016892 66.3 95.2 0.696  1374356_at  similar to RIKEN cDNA A030007D23 gene  Info   LOC302999 
   6615 0.0016912 1890 2300.3 0.822  1398287_at  plasminogen activator, urokinase  Info   Plau 
   6616 0.001692 286.6 383 0.748  1376128_at  Transcribed locus  Info    
   6617 0.0016925 31.5 4.2 7.5  1383272_at  similar to ankyrin repeat and SOCS box-containing protein 11  Info   LOC302666 
   6618 0.0016937 1660 1951.5 0.851  1371547_at  similar to C10 (predicted)  Info   RGD1309695_predicted 
   6619 0.0016937 110.7 137.5 0.805  1374931_at  mitochondrial ribosomal protein L40  Info   Mrpl40 
   6620 0.0016938 152.4 232.8 0.655  1395852_at  ATP-binding cassette, sub-family A (ABC1), member 3 (predicted)  Info   Abca3_predicted 
   6621 0.0016942 12.9 34 0.379  1379733_at  Transcribed locus  Info    
   6622 0.0016956 47 29.8 1.577  1391512_at  similar to LRRGT00049  Info   LOC502151 
   6623 0.0016957 236.4 179.8 1.315  1374146_at  MAD2 mitotic arrest deficient-like 2 (yeast) (predicted)  Info   Mad2l2_predicted 
   6624 0.001696 450.7 541.8 0.832  1399010_at  COP9 (constitutive photomorphogenic) homolog, subunit 7a (Arabidopsis thaliana) (predicted)  Info   Cops7a_predicted 
   6625 0.0016965 80.8 117.1 0.69  1379816_at  Similar to RIKEN cDNA 2410025L10  Info    
   6626 0.0016965 414.5 316.1 1.311  1370073_at  protein kinase inhibitor p58  Info   Dnajc3 
   6627 0.0016966 722.3 581.2 1.243  1372136_at  similar to tetraspanin similar to TM4SF9 (predicted)  Info   RGD1305714_predicted 
   6628 0.0016977 2563.4 2174.6 1.179  1371573_at  ribosomal protein L36a (predicted)  Info   Rpl36a_predicted 
   6629 0.0016977 25.5 39.7 0.642  1368772_at  solute carrier family 4, member 3  Info   Slc4a3 
   6630 0.0016982 55 114.1 0.482  1373914_at  Stem cell growth factor  Info   Scgf 
   6631 0.0016999 1.1 2.5 0.44  1375255_at  Microtubule associated serine/threonine kinase 2 (predicted)  Info    
   6632 0.0017005 37.5 49.2 0.762  1377273_at  Transcribed locus  Info    
   6633 0.001701 60.3 13.3 4.534  1367888_at  MT-protocadherin  Info   Pcdh21 
   6634 0.0017018 417 513.5 0.812  1372124_at  eukaryotic translation initiation factor 4B (predicted)  Info   Eif4b_predicted 
   6635 0.0017024 41.6 86.2 0.483  1387877_at  formiminotransferase cyclodeaminase  Info   Ftcd 
   6636 0.0017028 117.6 83.6 1.407  1380001_at  similar to pinin  Info   LOC368070 
   6637 0.0017035 42.5 75.3 0.564  1385093_at  similar to hypothetical protein LOC284018 isoform b  Info   LOC498014 
   6638 0.0017052 302.4 198.5 1.523  1398481_at  Transcribed locus  Info    
   6639 0.0017058 53 37.9 1.398  1369847_at  potassium voltage-gated channel, shaker-related subfamily, beta member 1  Info   Kcnab1 
   6640 0.0017085 266.4 341.5 0.78  1376187_at  Transcribed locus  Info    
   6641 0.001709 180.4 135 1.336  1376219_at  Atpase, class I, type 8B, member 2 (predicted)  Info    
   6642 0.0017109 3 10.1 0.297  1378612_at  Similar to RIKEN cDNA 2610207I16 (predicted)  Info    
   6643 0.0017111 203.8 150.3 1.356  1372846_at  similar to hypothetical protein MGC20446 (predicted)  Info   RGD1304670_predicted 
   6644 0.0017116 377.9 301.1 1.255  1392662_at  similar to RIKEN cDNA 1500009M05  Info   LOC295457 
   6645 0.0017119 24.4 86.2 0.283  1382580_at  Transcribed locus  Info    
   6646 0.0017122 166.7 214.5 0.777  1392816_at  similar to DNA segment, Chr 6, Wayne State University 163, expressed (predicted)  Info   RGD1311164_predicted 
   6647 0.0017125 968.5 772.5 1.254  1373185_at  signal sequence receptor, beta (predicted)  Info   Ssr2_predicted 
   6648 0.0017151 156.9 118.5 1.324  1376560_at  Transcribed locus  Info    
   6649 0.0017152 233 383.2 0.608  1368442_at  coagulation factor 2  Info   F2 
   6650 0.0017159 5436.6 8031.6 0.677  1367937_at  myo-inositol oxygenase  Info   Miox 
   6651 0.0017172 41.9 6.1 6.869  1368637_at  caspase recruitment domain family, member 9  Info   Card9 
   6652 0.0017182 131.9 208.9 0.631  1389801_at  fibroblast growth factor receptor 4  Info   Fgfr4 
   6653 0.0017193 3381.8 3977.7 0.85  1371311_at  succinate dehydrogenase complex, subunit C  Info   RGD:1359454 
   6654 0.0017212 1474.6 1285.2 1.147  1369970_at  vesicle-associated membrane protein 8  Info   Vamp8 
   6655 0.0017227 16.8 21.5 0.781  1395607_at  Similar to Ataxin 2-like  Info    
   6656 0.0017231 1508.6 1882.1 0.802  1388307_at  tumor differentially expressed 1, like  Info   Tde2 
   6657 0.0017244 84.7 131.5 0.644  1383781_at    Info    
   6658 0.001726 117 81 1.444  1385649_at  integrin alpha 5  Info   Itga5 
   6659 0.0017262 25.8 41.9 0.616  1395978_at    Info    
   6660 0.0017267 211.7 157.4 1.345  1374472_at  similar to DNA segment, Chr 8, ERATO Doi 531, expressed  Info   MGC116266 
   6661 0.0017285 91.4 68.4 1.336  1374916_at  Transcribed locus  Info    
   6662 0.0017289 84.4 130.1 0.649  1383120_at  similar to RIKEN cDNA 2810429K17 (predicted)  Info   RGD1310182_predicted 
   6663 0.0017321 22.2 33.1 0.671  1383924_at  Transcribed locus  Info    
   6664 0.0017349 105.8 174.8 0.605  1377716_at    Info    
   6665 0.0017383 516.8 633.8 0.815  1394524_at    Info    
   6666 0.0017408 239.9 154.7 1.551  1376022_at  Transcribed locus  Info    
   6667 0.0017421 144.1 196 0.735  1372816_at  Transcribed locus  Info    
   6668 0.0017441 14.4 36.1 0.399  1392652_at  Transcribed locus  Info    
   6669 0.0017444 217.1 169.7 1.279  1383396_at  fibronectin type III domain containing 3 (predicted)  Info   Fndc3_predicted 
   6670 0.001745 403 799.3 0.504  1368249_at  Kruppel-like factor 15  Info   Klf15 
   6671 0.0017451 87.7 55.3 1.586  1376419_at  Transcribed locus  Info    
   6672 0.0017499 146.4 188.6 0.776  1376486_at  SH3-domain GRB2-like endophilin B2 (predicted)  Info   Sh3glb2_predicted 
   6673 0.0017516 74.9 127.6 0.587  1383891_a_at  similar to RIKEN cDNA 2810417J12 gene (predicted)  Info   RGD1311166_predicted 
   6674 0.0017524 150.7 230.2 0.655  1391476_at  Transcribed locus  Info    
   6675 0.0017527 34.7 72.9 0.476  1385626_at  similar to B230212L03Rik protein  Info   MGC95152 
   6676 0.0017532 45.7 72 0.635  1388962_at  ring finger protein 110 (predicted)  Info   Rnf110_predicted 
   6677 0.0017533 183.4 302 0.607  1391279_at    Info    
   6678 0.0017538 51.3 72.3 0.71  1389775_at    Info    
   6679 0.0017553 115.1 395 0.291  1371762_at  retinol binding protein 4, plasma  Info   Rbp4 
   6680 0.0017554 5.4 30.9 0.175  1371140_a_at  amiloride-sensitive cation channel 1, neuronal (degenerin)  Info   Accn1 
   6681 0.0017559 21 55.1 0.381  1389740_at  Transcribed locus, weakly similar to XP_498499.1 PREDICTED: hypothetical protein XP_498499 [Homo sapiens]  Info    
   6682 0.0017563 18.7 51.6 0.362  1386732_at  Transcribed locus  Info    
   6683 0.0017576 186.4 308.6 0.604  1391428_at  Transcribed locus  Info    
   6684 0.0017578 76.8 118.8 0.646  1395045_at  NADH dehydrogenase (ubiquinone) 1 alpha subcomplex, 7 (B14.5a) (predicted)  Info   Ndufa7_predicted 
   6685 0.0017595 362 299.1 1.21  1367499_at  solute carrier family 35, member C1 (predicted)  Info   Slc35c1_predicted 
   6686 0.0017611 25.3 74.9 0.338  1385994_at  sporulation protein, meiosis-specific, SPO11 homolog (S. cerevisiae) (predicted)  Info   Spo11_predicted 
   6687 0.0017625 148.4 205.7 0.721  1389541_at  Transcribed locus  Info    
   6688 0.0017627 295.8 327.2 0.904  1387299_at  Msx-interacting-zinc finger  Info   Miz1 
   6689 0.0017646 77.1 38.4 2.008  1386701_at  Transcribed locus  Info    
   6690 0.0017652 148.9 217.8 0.684  1392865_at  fibroblast growth factor 9  Info   Fgf9 
   6691 0.0017661 96.2 68.9 1.396  1370780_at  RAB31, member RAS oncogene family  Info   Rab31 
   6692 0.0017664 274.5 365.7 0.751  1376953_at  Transcribed locus  Info    
   6693 0.001768 363.6 199 1.827  1394419_at  similar to RIKEN cDNA 6530401L14 gene (predicted)  Info   RGD1309107_predicted 
   6694 0.0017684 356 278.2 1.28  1387977_at  nibrin  Info   Nbn 
   6695 0.0017695 69.4 106.8 0.65  1394348_at  Rho guanine nucleotide exchange factor (GEF) 12 (predicted)  Info   Arhgef12_predicted 
   6696 0.00177 23.1 37.9 0.609  1370456_at  FAT tumor suppressor homolog 3 (Drosophila)  Info   Fat3 
   6697 0.0017709 64.4 99.1 0.65  1392043_at  Transcribed locus  Info    
   6698 0.0017712 377.9 253.8 1.489  1383999_at  Transcribed locus, moderately similar to XP_579465.1 PREDICTED: DNA binding protein N5 [Rattus norvegicus]  Info    
   6699 0.0017717 1565.1 1091.9 1.433  1398778_at  proteasome (prosome, macropain) subunit, alpha type 1  Info   Psma1 
   6700 0.0017725 224.9 300.7 0.748  1376792_at  Transcribed locus  Info    
   6701 0.0017728 27.5 41.3 0.666  1368306_at  glutamate receptor, ionotropic, NMDA2C  Info   Grin2c 
   6702 0.0017729 50.2 93.4 0.537  1381056_at  plakophilin 3 (predicted)  Info   Pkp3_predicted 
   6703 0.0017734 148.1 101.5 1.459  1391236_at  similar to RIKEN cDNA 5730469D23 (predicted)  Info   RGD1308324_predicted 
   6704 0.0017762 16.2 45.5 0.356  1377422_at  myotubularin related protein 1 (predicted)  Info   Mtmr1_predicted 
   6705 0.0017795 93 145.3 0.64  1394594_at  Transcribed locus  Info    
   6706 0.0017795 69.2 136.3 0.508  1377452_at  tetranectin (plasminogen binding protein) (predicted)  Info   Tna_predicted 
   6707 0.00178 45.8 76.6 0.598  1391181_at  Transcribed locus  Info    
   6708 0.0017801 36.4 97.2 0.374  1380579_at  matrilin 1, cartilage matrix protein  Info   RGD:1359410 
   6709 0.0017803 88.6 149 0.595  1387549_at  N-deacetylase/N-sulfotransferase (heparan glucosaminyl) 1  Info   Ndst1 
   6710 0.0017805 251 300.9 0.834  1367547_at  similar to zinc finger protein 198  Info   LOC305913 
   6711 0.0017811 728.9 1182.5 0.616  1392557_at  Transcribed locus  Info    
   6712 0.0017821 75.9 110.9 0.684  1368265_at  cytochrome P450 monooxygenase CYP2T1  Info   RGD:620086 
   6713 0.0017823 361.1 173 2.087  1373908_at  Transcribed locus  Info    
   6714 0.0017879 15.7 32.5 0.483  1377786_at  Transcribed locus  Info    
   6715 0.0017883 42.3 55 0.769  1373153_at  Myelin oligodendrocyte glycoprotein  Info   Mog 
   6716 0.0017889 1.7 6.7 0.254  1392282_at  Similar to brefeldin A-inhibited guanine nucleotide-exchange protein 1  Info    
   6717 0.0017953 141.2 210.7 0.67  1393136_at  similar to RIKEN cDNA 1110007C24  Info   LOC313618 
   6718 0.0017975 79.7 107.9 0.739  1380452_at  Transcribed locus  Info    
   6719 0.0017985 12.2 23.2 0.526  1370454_at  homer homolog 1 (Drosophila)  Info   Homer1 
   6720 0.0017996 5721.1 5067.1 1.129  1367717_at  ribosomal protein S27  Info   Rps27 
   6721 0.0018003 88.1 143.1 0.616  1373256_at  Chromodomain helicase DNA binding protein 3 (predicted)  Info   Chd3 
   6722 0.001801 195.4 234.3 0.834  1383618_at  mitochondrial ribosomal protein L38 (predicted)  Info   Mrpl38_predicted 
   6723 0.0018023 64.4 94.4 0.682  1368550_at  HNF-3/forkhead homolog-1 /// hypothetical gene supported by NM_022858  Info   Foxq1 /// LOC497713 
   6724 0.0018031 198.1 136.1 1.456  1384797_at  similar to RIKEN cDNA 5730596K20 (predicted)  Info   RGD1309871_predicted 
   6725 0.0018035 11.3 44.1 0.256  1393672_at  Similar to OTTHUMP00000065631  Info    
   6726 0.0018039 216.5 162.5 1.332  1382070_at  activating transcription factor 7 interacting protein (predicted)  Info   Atf7ip_predicted 
   6727 0.0018089 72.5 112.8 0.643  1389463_at  protein kinase, cAMP dependent regulatory, type I, beta  Info   Prkar1b 
   6728 0.0018109 81.6 120.4 0.678  1394535_at  kinesin family member 16B (predicted)  Info   Kif16b_predicted 
   6729 0.0018116 188.2 109.9 1.712  1395821_at  core binding factor beta  Info   Cbfb 
   6730 0.0018118 34.5 59.5 0.58  1377904_at  Similar to SPT3-associated factor 42 (predicted)  Info    
   6731 0.0018157 299.4 419.2 0.714  1368189_at  7-dehydrocholesterol reductase  Info   Dhcr7 
   6732 0.0018162 90.9 157.3 0.578  1369678_a_at  nuclear factor I/A  Info   Nfia 
   6733 0.0018183 1599.3 1281.1 1.248  1388550_at  similar to MHR23B  Info   LOC298012 
   6734 0.0018183 135.3 96 1.409  1376760_at    Info    
   6735 0.0018199 364.2 497.4 0.732  1371940_at  similar to microfilament and actin filament cross-linker protein isoform a  Info   LOC362587 
   6736 0.0018215 3144.3 2708.6 1.161  1372132_at  CNDP dipeptidase 2 (metallopeptidase M20 family) (predicted)  Info   Cndp2_predicted 
   6737 0.001823 845.6 1026.1 0.824  1393915_at  gene rich cluster, C3f gene (predicted)  Info   Grcc3f_predicted 
   6738 0.0018232 1185.9 953.2 1.244  1373951_at  Protein kinase, cAMP dependent regulatory, type I, alpha  Info   Prkar1a 
   6739 0.0018248 223.6 328.2 0.681  1376755_at  retinoic acid receptor, beta  Info   Rarb 
   6740 0.0018253 29.8 56.5 0.527  1393891_at  procollagen, type VIII, alpha 1 (predicted)  Info   Col8a1_predicted 
   6741 0.0018258 319 663.6 0.481  1387987_at  solute carrier family 22 (organic anion transporter), member 19  Info   Slc22a19 
   6742 0.0018263 223.1 145.9 1.529  1384246_at  SWI/SNF related, matrix associated, actin dependent regulator of chromatin, subfamily a, member 5 (predicted)  Info   Smarca5_predicted 
   6743 0.0018263 56.1 85.1 0.659  1368644_at  spermatogenesis associated 6  Info   Spata6 
   6744 0.0018287 21.2 42.1 0.504  1391647_at    Info    
   6745 0.0018291 12.6 27.1 0.465  1374542_at  Similar to RIKEN cDNA 1200015N20 (predicted)  Info    
   6746 0.0018297 49.4 73.3 0.674  1376635_at  Transketolase  Info   Tkt 
   6747 0.0018303 59.3 87.4 0.678  1390547_at  Transcribed locus  Info    
   6748 0.0018319 579.2 668.8 0.866  1390111_at    Info    
   6749 0.0018337 80.2 147.1 0.545  1391162_at  Transcribed locus  Info    
   6750 0.0018349 51.7 96.7 0.535  1392884_at    Info    
   6751 0.0018349 151.4 181.6 0.834  1394222_at  similar to RIKEN cDNA G630024C07 gene (predicted)  Info   RGD1305903_predicted 
   6752 0.0018357 1.3 5 0.26  1385215_at  core-binding factor, runt domain, alpha subunit 2; translocated to, 1; cyclin D-related (predicted)  Info   Cbfa2t1_predicted 
   6753 0.0018368 203.1 278.2 0.73  1379410_at  Lin-10 protein homolog (C. elegans)  Info   Lin10 
   6754 0.0018389 105.7 63 1.678  1395998_at  nucleolar protein 5A (predicted)  Info   Nol5a_predicted 
   6755 0.0018397 50.5 86.5 0.584  1393613_at  Transcribed locus  Info    
   6756 0.0018401 72.1 99.3 0.726  1378404_at  Transcribed locus, weakly similar to XP_576460.1 PREDICTED: similar to hypothetical protein PB402898.00.0 [Rattus norvegicus]  Info    
   6757 0.0018424 221.5 43.4 5.104  1387125_at  S100 calcium binding protein A9 (calgranulin B)  Info   S100a9 
   6758 0.0018434 51.2 80.8 0.634  1384171_x_at    Info    
   6759 0.0018444 7.6 31.8 0.239  1370982_at  muscle glycogen phosphorylase  Info   Pygm 
   6760 0.0018458 957.4 1238.8 0.773  1387972_at  mucin and cadherin like  Info   Mucdhl 
   6761 0.001846 312.4 396.1 0.789  1383375_at  similar to RIKEN cDNA 2610319K07 (predicted)  Info   RGD1306225_predicted 
   6762 0.0018468 42.6 26.9 1.584  1386073_at  Similar to T-Brain-1  Info    
   6763 0.0018469 83.2 121.2 0.686  1377266_at  Transcribed locus  Info    
   6764 0.001847 81.7 26.8 3.049  1383493_at  Transcribed locus  Info    
   6765 0.0018473 96.5 125.4 0.77  1395984_at  similar to hypothetical protein FLJ21827 (predicted)  Info   RGD1307682_predicted 
   6766 0.0018485 19.4 35.1 0.553  1397631_at  ubiquitin specific protease 8 (predicted)  Info   Usp8_predicted 
   6767 0.0018489 305.3 231.9 1.317  1369323_at  leptin receptor overlapping transcript  Info   Leprot 
   6768 0.0018493 122.9 196.6 0.625  1384283_at  similar to ankyrin repeat and SOCS box-containing protein 9  Info   LOC367785 
   6769 0.0018499 339.1 491.7 0.69  1393933_at  sortilin-related receptor, L(DLR class) A repeats-containing (predicted)  Info   Sorl1_predicted 
   6770 0.00185 11.7 19.7 0.594  1374786_at  Cytotoxic granule-associated RNA binding protein 1 (predicted)  Info    
   6771 0.0018539 137 189.8 0.722  1382289_at  Transcribed locus  Info    
   6772 0.0018546 73.4 52.3 1.403  1384676_at    Info    
   6773 0.0018563 28.5 61.3 0.465  1382244_at  quinolinate phosphoribosyltransferase (predicted)  Info   Qprt_predicted 
   6774 0.0018572 365.7 230.4 1.587  1389409_at  similar to Testis derived transcript  Info   LOC500040 
   6775 0.0018574 177.9 255.7 0.696  1392424_at  Transcribed locus  Info    
   6776 0.0018578 165.2 248.7 0.664  1372539_at  Transcribed locus  Info    
   6777 0.00186 261 313.1 0.834  1389167_at  mitogen-activated protein kinase associated protein 1 (predicted)  Info   Mapkap1_predicted 
   6778 0.0018609 152.7 211.3 0.723  1383305_at  Transcribed locus  Info    
   6779 0.0018618 105.5 78.2 1.349  1378338_at  similar to hypothetical protein MGC32132 (predicted)  Info   RGD1307915_predicted 
   6780 0.0018622 488.5 390.9 1.25  1371617_at  proteasome (prosome, macropain) 26S subunit, non-ATPase, 13 (predicted)  Info   Psmd13_predicted 
   6781 0.0018631 31.6 50.3 0.628  1369919_at  thyrotroph embryonic factor  Info   Tef 
   6782 0.0018638 150.4 97.2 1.547  1384340_a_at  similar to N-acetyltransferase ARD1  Info   LOC363518 
   6783 0.0018639 34 67.5 0.504  1383644_at  Transcribed locus  Info    
   6784 0.001866 70 107 0.654  1372831_at  Similar to leucine zipper, down-regulated in cancer 1; breast cancer, up-regulated 1 (predicted)  Info    
   6785 0.0018661 2355.7 2730.5 0.863  1386902_at  voltage-dependent anion channel 3  Info   Vdac3 
   6786 0.0018697 69.7 52.4 1.33  1385814_at  mitogen activated protein kinase kinase kinase 3 (predicted)  Info   Map3k3_predicted 
   6787 0.0018702 38.9 55 0.707  1398698_at  Caseinolytic protease X (E.coli) (predicted)  Info   Clpx 
   6788 0.0018704 44.5 124.4 0.358  1376248_at  sulfotransferase family, cytosolic, 2B, member 1 (predicted)  Info   Sult2b1_predicted 
   6789 0.0018709 125 272.6 0.459  1390901_at  bone specific CMF608  Info   Igsf10 
   6790 0.0018721 1022.1 832.2 1.228  1388328_at  similar to Eukaryotic translation initiation factor 3 subunit 2 (eIF-3 beta) (eIF3 p36) (eIF3i) (TGF-beta receptor interacting protein 1) (TRIP-1)  Info   LOC499619 
   6791 0.0018744 34.7 50.5 0.687  1385559_at  Transcribed locus  Info    
   6792 0.0018748 25.4 45.6 0.557  1374123_at  Transcribed locus  Info    
   6793 0.0018768 98.9 51.6 1.917  1369568_at  syntaxin 6  Info   Stx6 
   6794 0.0018769 48.6 76.3 0.637  1371679_at  similar to Synaptopodin-2 (Myopodin)  Info   LOC499702 
   6795 0.0018773 31.8 71.6 0.444  1390976_at  Transcribed locus  Info    
   6796 0.0018774 190.1 288.7 0.658  1374189_at  zinc finger protein 219  Info   RGD:1359259 
   6797 0.0018778 127.4 100.2 1.271  1393569_at  RNA binding motif protein 18 (predicted)  Info   Rbm18_predicted 
   6798 0.0018794 338.5 257 1.317  1390423_at  pam, highwire, rpm 1 (predicted)  Info   Phr1_predicted 
   6799 0.00188 294.7 437.8 0.673  1382492_a_at  hydroxysteroid (17-beta) dehydrogenase 8  Info   RGD:1303158 
   6800 0.0018801 27.7 57.7 0.48  1383994_at    Info    
   6801 0.0018813 121.7 88.4 1.377  1376827_at  Echinoderm microtubule associated protein like 4 (predicted)  Info    
   6802 0.0018824 21.1 40.3 0.524  1373582_at  Similar to CLIP-170-related protein (predicted)  Info    
   6803 0.0018837 333.1 431.2 0.772  1373235_at  SYAP1 protein  Info   Syap1 
   6804 0.0018841 29.3 48.4 0.605  1380831_at  Transcribed locus  Info    
   6805 0.0018845 739.6 443.9 1.666  1376268_at  ADP-ribosylation factor 6  Info   Arf6 
   6806 0.0018854 5.7 25.6 0.223  1376225_at  Calsenilin, presenilin binding protein, EF hand transcription factor  Info   Csen 
   6807 0.0018857 44.4 16 2.775  1371170_a_at  interleukin 1 alpha  Info   Il1a 
   6808 0.0018862 259.9 404.1 0.643  1385805_at    Info    
   6809 0.0018863 25.4 57.2 0.444  1397233_at  similar to hypothetical protein C730036H08 (predicted)  Info   RGD1309232_predicted 
   6810 0.0018863 1485 1132.8 1.311  1367931_a_at  polypyrimidine tract binding protein 1  Info   Ptbp1 
   6811 0.0018871 4528.1 3601.2 1.257  1386858_at  ribosomal protein L13  Info   Rpl13 
   6812 0.0018894 186.8 256.9 0.727  1397541_at  Transcribed locus  Info    
   6813 0.0018916 192.2 305.7 0.629  1389157_at    Info    
   6814 0.0018918 125.3 199.8 0.627  1373484_at  kelch-like 13  Info   Klhl13 
   6815 0.0018928 345.3 606 0.57  1368154_at  guanylate cyclase 1, soluble, alpha 3 /// hypothetical gene supported by NM_017090  Info   Gucy1a3 /// LOC497757 
   6816 0.0018939 70.8 12.6 5.619  1371115_at  protein tyrosine phosphatase, receptor type, E  Info   Ptpre 
   6817 0.0018942 65.4 80.8 0.809  1380118_at  similar to hypothetical gene supported by AK085276  Info   LOC315646 
   6818 0.0018961 465.5 794.5 0.586  1368057_at  ATP-binding cassette, sub-family D (ALD), member 3  Info   Abcd3 
   6819 0.0019012 290.2 369.2 0.786  1388817_at  hypothetical protein LOC310665  Info   LOC310665 
   6820 0.0019063 918.7 1693 0.543  1368397_at  UDP-glucuronosyltransferase 2 family, member 5 /// UDP glycosyltransferase 2 family, polypeptide B4  Info   Ugt2b5 /// Ugt2b4 
   6821 0.0019087 68 36.8 1.848  1371194_at  tumor necrosis factor alpha induced protein 6  Info   Tnfaip6 
   6822 0.0019088 150.2 105.3 1.426  1393361_at  similar to chromosome 16 open reading frame 33; minus -99 protein (predicted)  Info   RGD1310922_predicted 
   6823 0.0019094 159 230.4 0.69  1373378_at  ATP/GTP binding protein 1 (predicted)  Info   Agtpbp1_predicted 
   6824 0.0019098 111.2 69.3 1.605  1396286_at  Transcribed locus  Info    
   6825 0.0019111 345.4 512.9 0.673  1389054_at  similar to RIKEN cDNA 0610040J01  Info   LOC498368 
   6826 0.0019127 172.2 225.1 0.765  1371101_at  receptor-like tyrosine kinase  Info   Ryk 
   6827 0.0019133 53.1 167.2 0.318  1370310_at  3-hydroxy-3-methylglutaryl-Coenzyme A synthase 2  Info   Hmgcs2 
   6828 0.0019135 59.7 80.1 0.745  1374760_at  Transcribed locus  Info    
   6829 0.0019139 126 185.9 0.678  1382878_at  secreted frizzled-related protein 1  Info   Sfrp1 
   6830 0.0019175 117 167.6 0.698  1389242_at  Transcribed locus  Info    
   6831 0.0019186 93.2 51.4 1.813  1382343_at  Transcribed locus  Info    
   6832 0.0019235 9.7 22.4 0.433  1369253_at  kringle containing transmembrane protein  Info   Kremen 
   6833 0.0019261 42.5 77.9 0.546  1380013_at  Similar to adiponutrin  Info    
   6834 0.0019284 39.3 76.8 0.512  1373699_at  Similar to RIKEN cDNA 1810059A23  Info    
   6835 0.00193 285.1 256.6 1.111  1375357_at  dystonia 1  Info   Dyt1 
   6836 0.0019303 1373.2 1662.8 0.826  1371460_at  CDNA clone MGC:105597 IMAGE:7314502  Info    
   6837 0.0019315 281.6 389.4 0.723  1376580_at  similar to frataxin  Info   LOC499335 
   6838 0.0019356 16.8 23.5 0.715  1396766_at  carnitine deficiency-associated gene expressed in ventricle 1  Info   Cdv1 
   6839 0.0019376 288.7 371.8 0.776  1371610_at  Tankyrase, TRF1-interacting ankyrin-related ADP-ribose polymerase (predicted)  Info    
   6840 0.0019382 367.4 820.3 0.448  1372299_at  cyclin-dependent kinase inhibitor 1C (P57)  Info   Cdkn1c 
   6841 0.0019418 498.2 605.5 0.823  1372116_at  Transcribed locus  Info    
   6842 0.001943 299.2 544.4 0.55  1390569_at  similar to carnosinase 1  Info   RGD:1359493 
   6843 0.0019464 203.7 148 1.376  1372409_at  MAD2L1 binding protein (predicted)  Info   Mad2l1bp_predicted 
   6844 0.0019478 291.9 205.5 1.42  1372430_at  similar to RIKEN cDNA 1700037H04 (predicted)  Info   RGD1311739_predicted 
   6845 0.0019502 89 107.8 0.826  1377790_at  Transcribed locus  Info    
   6846 0.0019504 87.3 208.1 0.42  1395601_at  Transcribed locus  Info    
   6847 0.0019508 4680.9 5482.8 0.854  1386870_at  glutamine synthetase 1  Info   Glul 
   6848 0.0019527 921 680.2 1.354  1395412_at  GPI-anchored membrane protein 1 (predicted)  Info   Gpiap1_predicted 
   6849 0.001953 19.8 47.7 0.415  1384521_at  Transcribed locus  Info    
   6850 0.0019555 60.2 23 2.617  1395442_at  Transcribed locus  Info    
   6851 0.0019582 953.4 582.2 1.638  1375523_at  Similar to Myristoylated alanine-rich C-kinase substrate (MARCKS) (ACAMP-81)  Info   Marcks 
   6852 0.0019586 632.8 499.5 1.267  1388563_at  similar to RIKEN cDNA 1700034P14 (predicted)  Info   RGD1305492_predicted 
   6853 0.0019616 190.1 243.6 0.78  1372753_at  coenzyme Q6 homolog (yeast) (predicted)  Info   Coq6_predicted 
   6854 0.0019619 40.5 4 10.125  1380313_at  killer cell lectin-like receptor, family E, member 1  Info   Klre1 
   6855 0.0019646 45.3 70.6 0.642  1395414_at  Amylo-1, 6-glucosidase, 4-alpha-glucanotransferase (glycogen debranching enzyme, glycogen storage disease type III) (predicted)  Info    
   6856 0.0019652 96.7 158.7 0.609  1375049_at    Info    
   6857 0.0019654 270 375.8 0.718  1389339_at  Arylsulfatase A (predicted)  Info    
   6858 0.0019667 178.2 139.5 1.277  1373004_at  DnaJ (Hsp40) homolog, subfamily C, member 2  Info   Dnajc2 
   6859 0.0019667 1239.5 1669.5 0.742  1387808_at  solute carrier family 7 (cationic amino acid transporter, y+ system), member 7  Info   Slc7a7 
   6860 0.0019667 85.6 127.4 0.672  1382480_at  Spinocerebellar ataxia 2 (olivopontocerebellar ataxia 2, autosomal dominant, ataxin 2) (predicted)  Info    
   6861 0.0019673 15 30.6 0.49  1398002_at  Transcribed locus  Info    
   6862 0.0019682 17.8 28.5 0.625  1395970_at  General transcription factor III A  Info   Gtf3a 
   6863 0.0019688 341.5 448.7 0.761  1373050_at  Transcribed locus  Info    
   6864 0.0019705 340 267 1.273  1393172_at  Ngfi-A binding protein 1  Info   Nab1 
   6865 0.0019718 525.8 380 1.384  1370954_at  procollagen-proline, 2-oxoglutarate 4-dioxygenase (proline 4-hydroxylase), alpha 1 polypeptide  Info   P4ha1 
   6866 0.0019726 84.6 106.6 0.794  1374108_at  low density lipoprotein-related protein 12 (predicted)  Info   Lrp12_predicted 
   6867 0.0019731 63 111.7 0.564  1372658_at  desmuslin  Info   Dmn 
   6868 0.0019745 99.7 67.8 1.471  1390572_at  THO complex 3 (predicted)  Info   Thoc3_predicted 
   6869 0.0019787 1002.5 1527.8 0.656  1389146_at  hypothetical protein LOC498796  Info   LOC498796 
   6870 0.0019809 163 213.9 0.762  1390628_at  cytoplasmic polyadenylation element binding protein 2 (predicted)  Info   Cpeb2_predicted 
   6871 0.0019828 53.8 70.8 0.76  1394785_at  Transcribed locus  Info    
   6872 0.0019834 594.6 434 1.37  1373030_at  LOC501594  Info   LOC501594 
   6873 0.0019841 117.9 215.2 0.548  1393585_at  Transcribed locus  Info    
   6874 0.0019843 526 418 1.258  1373983_at  LOC360807  Info   LOC360807 
   6875 0.0019887 93.2 130.6 0.714  1373141_at    Info    
   6876 0.00199 22.4 38.7 0.579  1378947_at  similar to C-terminal tensin-like (predicted)  Info   RGD1310402_predicted 
   6877 0.0019909 643.9 840.2 0.766  1368426_at  carnitine O-octanoyltransferase  Info   Crot 
   6878 0.0019913 277.4 197 1.408  1382007_at  SH3-domain GRB2-like B1 (endophilin) (predicted)  Info   Sh3glb1_predicted 
   6879 0.0019917 57.9 96 0.603  1377469_at  Transcribed locus  Info    
   6880 0.0019918 34 52.4 0.649  1391553_at  Transcribed locus  Info    
   6881 0.0019947 877 680.7 1.288  1389245_at  proteasome (prosome, macropain) 26S subunit, non-ATPase, 7 (predicted)  Info   Psmd7_predicted 
   6882 0.0019956 1344.3 2086.2 0.644  1370352_at  carboxylesterase 1 /// similar to Liver carboxylesterase B-1 precursor (Liver microsomal carboxylesterase)  Info   RGD:621508 /// LOC501232 
   6883 0.0019966 2.5 14.9 0.168  1397392_at    Info    
   6884 0.0019987 20.1 33.6 0.598  1387450_at  transforming growth factor alpha  Info   Tgfa 
   6885 0.0019994 261.2 173.5 1.505  1372253_at  CCR4-NOT transcription complex, subunit 8 (predicted)  Info   Cnot8_predicted 
   6886 0.0019996 405.1 285.9 1.417  1368984_at  septin 2  Info   Sept2 
   6887 0.0020004 105.2 183.8 0.572  1387898_at  heat shock protein, alpha-crystallin-related, B6  Info   Hspb6 
   6888 0.0020045 14 48 0.292  1372161_at  Transcribed locus  Info    
   6889 0.0020047 67.4 89.8 0.751  1385379_at  Transcribed locus  Info    
   6890 0.002006 117.1 75.5 1.551  1369174_at  SMAD, mothers against DPP homolog 1 (Drosophila)  Info   Smad1 
   6891 0.0020076 314.8 410.3 0.767  1388546_at    Info    
   6892 0.0020082 7.2 2.6 2.769  1395531_at  similar to hypothetical protein FLJ12542 (predicted)  Info   RGD1305183_predicted 
   6893 0.0020097 243.4 309.5 0.786  1373791_at  similar to RIKEN cDNA 2310011J03  Info   RGD:1359127 
   6894 0.0020135 431.1 507.6 0.849  1373813_at  DnaJ (Hsp40) homolog, subfamily C, member 10 (predicted)  Info   Dnajc10_predicted 
   6895 0.0020139 122.5 164.5 0.745  1377647_at  sex comb on midleg homolog 1 (predicted)  Info   Scmh1_predicted 
   6896 0.0020193 120.3 179.4 0.671  1370266_at  parvin, alpha  Info   Parva 
   6897 0.0020196 419.9 795.5 0.528  1375144_at  Tissue inhibitor of metalloproteinase 2  Info   Timp2 
   6898 0.0020199 13.1 37.9 0.346  1385320_at  similar to Pdz-containing protein  Info   LOC317379 
   6899 0.0020207 21.8 34 0.641  1372929_at  potassium large conductance calcium-activated channel, subfamily M, alpha member 1  Info   Kcnma1 
   6900 0.0020254 27.9 41.4 0.674  1393966_at  pre-B-cell leukemia transcription factor 1 (predicted)  Info   Pbx1_predicted 
   6901 0.0020296 19.8 46.7 0.424  1384468_at  Transcribed locus, weakly similar to NP_083693.1 hypothetical protein LOC75757 [Mus musculus]  Info    
   6902 0.0020307 80.8 43.4 1.862  1378685_at  Transcribed locus  Info    
   6903 0.0020339 34.1 55.6 0.613  1374343_at  Transcribed locus, weakly similar to XP_580018.1 PREDICTED: hypothetical protein XP_580018 [Rattus norvegicus]  Info    
   6904 0.002034 5414.5 4553.9 1.189  1367630_at  ribosomal protein S11  Info   Rps11 
   6905 0.0020343 63.5 106.4 0.597  1393170_at  similar to homeobox-containing transcription factor  Info   LOC500125 
   6906 0.0020349 55.7 85.9 0.648  1389618_at  Transcribed locus, weakly similar to NP_062289.1 DNA binding protein with his-thr domain [Mus musculus]  Info    
   6907 0.0020392 1.8 9.3 0.194  1382204_at    Info    
   6908 0.0020403 104.3 141.7 0.736  1389190_at  similar to Putative tumor suppressor LAPSER1  Info   RGD:1359249 
   6909 0.0020404 139.9 86.6 1.615  1378172_at  Transcribed locus  Info    
   6910 0.0020411 197.3 149.5 1.32  1381203_at  SH3-domain GRB2-like B1 (endophilin) (predicted)  Info   Sh3glb1_predicted 
   6911 0.0020423 385.3 500.6 0.77  1375662_at  LOC500344  Info   LOC500344 
   6912 0.0020472 814.4 1072.9 0.759  1373986_at  Transcribed locus  Info    
   6913 0.0020489 286.5 366.4 0.782  1375093_at  ribokinase (predicted)  Info   Rbks_predicted 
   6914 0.0020502 39 19.8 1.97  1379313_at    Info    
   6915 0.0020508 472.4 597.2 0.791  1368402_at  dynein, cytoplasmic, light intermediate polypeptide 2  Info   Dncli2 
   6916 0.0020512 11.3 2.4 4.708  1385665_at  SRY-box containing gene 30 (predicted)  Info   Sox30_predicted 
   6917 0.0020531 134.2 90.6 1.481  1395479_at  similar to BRIX (predicted)  Info   RGD1308508_predicted 
   6918 0.0020548 101.5 131.7 0.771  1377740_at  Transcribed locus  Info    
   6919 0.002059 23.4 78.2 0.299  1393139_at  similar to Apolipoprotein C2  Info   LOC292697 
   6920 0.0020601 286.9 420 0.683  1368446_at  serine protease inhibitor, Kazal type 1  Info   Spink1 
   6921 0.0020616 1163.2 1794.5 0.648  1387019_at  ATP synthase, H+ transporting, mitochondrial F0 complex, subunit e  Info   Atp5i 
   6922 0.0020622 483.7 402 1.203  1377577_at  guanine monphosphate synthetase (predicted)  Info   Gmps_predicted 
   6923 0.0020633 70.3 49.1 1.432  1395686_at  Activating transcription factor 7 (predicted)  Info    
   6924 0.002064 177.9 324 0.549  1388712_at    Info    
   6925 0.0020644 27.1 43.2 0.627  1394702_at  Transcribed locus  Info    
   6926 0.0020664 20.8 41.6 0.5  1370615_at  UDP-glucuronosyltransferase  Info   RGD:708417 
   6927 0.0020665 47.6 84.4 0.564  1377140_at    Info    
   6928 0.0020667 52.8 70.4 0.75  1380404_at    Info    
   6929 0.0020698 188.6 140.7 1.34  1388668_at    Info    
   6930 0.0020711 30.3 63.9 0.474  1387052_at  glutamic pyruvic transaminase 1, soluble  Info   Gpt1 
   6931 0.002072 1057.2 1348.4 0.784  1372791_at  Protein kinase, lysine deficient 1  Info   Prkwnk1 
   6932 0.002072 99.9 143.2 0.698  1387000_at  hypothetical gene supported by NM_019385  Info   LOC497835 
   6933 0.0020722 134.2 209.3 0.641  1372549_at  similar to RIKEN cDNA 2600017H02  Info   LOC497936 
   6934 0.0020779 609.1 311.6 1.955  1388502_at  Inositol polyphosphate-5-phosphatase B (predicted)  Info    
   6935 0.0020792 188.4 229.8 0.82  1387652_at  insulin degrading enzyme  Info   Ide 
   6936 0.0020807 93 58.4 1.592  1368033_at  nucleolar and coiled-body phosphoprotein 1  Info   Nolc1 
   6937 0.0020837 1890.9 1477.6 1.28  1371732_at  dermatopontin (predicted)  Info   Dpt_predicted 
   6938 0.0020872 70.1 120.4 0.582  1392072_at  Transcribed locus  Info    
   6939 0.0020895 48.5 80.2 0.605  1378429_at  Transcribed locus  Info    
   6940 0.0020898 45.3 78.5 0.577  1398708_at  LOC499614  Info    
   6941 0.0020907 414.6 522.4 0.794  1398981_at    Info    
   6942 0.0020908 202.7 266.9 0.759  1389131_at  similar to C330027I04Rik protein  Info   RGD:1359229 
   6943 0.002096 27.1 6.5 4.169  1375781_at  Transcribed locus  Info    
   6944 0.0020961 56.3 6.3 8.937  1369522_a_at  CD244 natural killer cell receptor 2B4  Info   Cd244 
   6945 0.0020978 11.7 24.4 0.48  1397238_at  Transcribed locus  Info    
   6946 0.0021023 37 58.9 0.628  1378674_at  Transcribed locus  Info    
   6947 0.0021057 616.9 342.4 1.802  1391106_at  Transcribed locus  Info    
   6948 0.0021088 38.6 22.6 1.708  1397666_at  Transcribed locus  Info    
   6949 0.0021104 89.2 60 1.487  1379439_at  Similar to RIKEN cDNA 6030404E16 (predicted)  Info    
   6950 0.0021105 62.2 9.5 6.547  1391946_at  selectin, platelet  Info   Selp 
   6951 0.002112 160 90.8 1.762  1371923_at  similar to hypothetical protein FLJ20481 (predicted)  Info   RGD1311599_predicted 
   6952 0.002116 487.6 662.3 0.736  1388913_at  phosphatidic acid phosphatase type 2c  Info   Ppap2c 
   6953 0.0021235 525.4 427.3 1.23  1391412_at  5'-3' exoribonuclease 2 (predicted)  Info   Xrn2_predicted 
   6954 0.0021255 139.2 168 0.829  1373509_at  similar to RIKEN cDNA 2810405F18  Info   LOC298426 
   6955 0.002128 1032.9 1303.7 0.792  1372571_at  Similar to 9530046H09Rik protein (predicted)  Info    
   6956 0.0021323 16.7 33.5 0.499  1397402_at  similar to Zinc finger protein 45 (BRC1744)  Info   LOC499096 
   6957 0.0021329 28.6 58.1 0.492  1393134_at  Transcribed locus  Info    
   6958 0.0021339 119.3 214.5 0.556  1368963_at  Max interacting protein 1  Info   Mxi1 
   6959 0.0021367 6.9 15.3 0.451  1369353_at  v-erb-a erythroblastic leukemia viral oncogene homolog 4 (avian)  Info   Erbb4 
   6960 0.0021402 443.9 618.3 0.718  1371533_at  dynactin 6 (predicted)  Info   Dctn6_predicted 
   6961 0.0021417 166.8 310.5 0.537  1367744_at  melanoma antigen, family D, 2  Info   Maged2 
   6962 0.0021424 37.1 84.8 0.438  1375017_at  Transcribed locus  Info    
   6963 0.0021443 57.6 13.4 4.299  1369067_at  nuclear receptor subfamily 4, group A, member 3  Info   Nr4a3 
   6964 0.0021448 78.7 131 0.601  1375946_at  Transcribed locus  Info    
   6965 0.002151 326.3 193.5 1.686  1367768_at  latexin  Info   Lxn 
   6966 0.0021528 2981.1 2514.5 1.186  1367895_at  heterogeneous nuclear ribonucleoprotein K  Info   Hnrpk 
   6967 0.0021532 24.8 48.7 0.509  1389342_at  Transcribed locus  Info    
   6968 0.0021615 391.8 305.8 1.281  1387436_at  septin 7  Info   Sept7 
   6969 0.0021675 318.2 238.7 1.333  1372050_at  Similar to hypothetical protein MGC38524 (predicted)  Info    
   6970 0.002169 495.6 1302.3 0.381  1368627_at  regucalcin  Info   Rgn 
   6971 0.0021699 274.1 357.1 0.768  1389385_at  endothelial precursor protein B9 (predicted)  Info   Eppb9_predicted 
   6972 0.0021706 364.3 486.9 0.748  1379736_at  Similar to hypothetical protein MGC28931 (predicted)  Info    
   6973 0.0021719 29.9 45.8 0.653  1380485_at  Similar to RIKEN cDNA 2600016J21  Info    
   6974 0.0021744 12 18.3 0.656  1382520_at  Transcribed locus  Info    
   6975 0.0021755 340.7 266.2 1.28  1376195_at  spindlin (predicted)  Info   Spin_predicted 
   6976 0.0021762 131.6 80.1 1.643  1389258_at  ring finger protein 138  Info   Rnf138 
   6977 0.0021778 328.1 278.4 1.179  1392505_at    Info    
   6978 0.0021811 380.5 313.1 1.215  1368508_at  proteasome (prosome, macropain) subunit, alpha type 3  Info   Psma3 
   6979 0.0021823 250.4 307.4 0.815  1395362_at  Transcribed locus  Info    
   6980 0.0021829 53.4 91.8 0.582  1378397_at  Transcribed locus  Info    
   6981 0.0021844 870.7 680.6 1.279  1375904_at  Transcribed locus  Info    
   6982 0.0021853 17.3 32.7 0.529  1384347_at  Src homology 2 domain-containing transforming protein C2 (predicted)  Info    
   6983 0.0021858 29.9 6.1 4.902  1377637_at  Transcribed locus  Info    
   6984 0.0021867 119.3 190.5 0.626  1372920_at    Info    
   6985 0.0021867 101.5 142.1 0.714  1373248_at  Similar to hypothetical protein FLJ20674  Info    
   6986 0.0021886 362.7 566 0.641  1373329_at  transmembrane protease, serine 2  Info   Tmprss2 
   6987 0.0021888 3877.9 5044.3 0.769  1370276_at  ATP synthase, H+ transporting, mitochondrial F1 complex, O subunit  Info   Atp5o 
   6988 0.0021895 135.2 88.1 1.535  1370665_at  hypoxia up-regulated 1  Info   Hyou1 
   6989 0.0021921 131.5 86.9 1.513  1382218_at  hypothetical LOC298077 (predicted)  Info   RGD1305807_predicted 
   6990 0.0021936 315.8 403.4 0.783  1372860_at  similar to phospholysine phosphohistidine inorganic pyrophosphate phosphata (5M590)  Info   RGD:1359187 
   6991 0.002197 218 162.5 1.342  1388847_at  Positive cofactor 2, multiprotein complex, glutamine/Q-rich-associated protein (predicted)  Info    
   6992 0.0022006 452 589.9 0.766  1372996_at  Similar to RIKEN cDNA 0610042E07 (predicted)  Info   MGC105521 
   6993 0.0022019 144 190.2 0.757  1383723_at  similar to mKIAA0376 protein (predicted)  Info   RGD1309570_predicted 
   6994 0.0022035 198.9 298.6 0.666  1387057_at  solute carrier family 7 (cationic amino acid transporter, y+ system), member 8 /// synaptic Ras GTPase activating protein 1 homolog (rat)  Info   Slc7a8 /// Syngap1 
 
  
 
    
'Observed v. Expected' table of GO classes and parent classes, in list of 6994 genes shown above:
    
Only GO classes and parent classes with at least 5 observations in the selected subset and with an 'Observed vs. Expected' ratio of at least 2 are shown.  
 Cellular Component 
 
  
  GO id GO classification Observed in selected subset Expected in selected subset Observed/ Expected
  0016942 insulin-like growth factor binding protein complex  5 1.32 3.78
  0005732 small nucleolar ribonucleoprotein complex  7 1.85 3.78
  0042613 MHC class II protein complex  9 2.65 3.4
  0042101 T cell receptor complex  5 1.59 3.15
  0005945 6-phosphofructokinase complex  5 1.59 3.15
  0005664 nuclear origin of replication recognition complex  5 1.59 3.15
  0001772 immunological synapse  39 12.44 3.14
  0042611 MHC protein complex  33 10.58 3.12
  0042612 MHC class I protein complex  23 7.41 3.1
  0005885 Arp2/3 protein complex  9 2.91 3.09
  0005839 proteasome core complex (sensu Eukaryota)  12 4.23 2.83
  0005741 mitochondrial outer membrane  6 2.12 2.83
  0005901 caveola  5 1.85 2.7
  0000808 origin recognition complex  5 1.85 2.7
  0000502 proteasome complex (sensu Eukaryota)  18 8.2 2.19
 
  
 Molecular Function 
 
  
  GO id GO classification Observed in selected subset Expected in selected subset Observed/ Expected
  0000064 L-ornithine transporter activity  5 1.32 3.78
  0042605 peptide antigen binding  5 1.59 3.15
  0045012 MHC class II receptor activity  9 2.91 3.1
  0030106 MHC class I receptor activity  27 9.78 2.76
  0050136 NADH dehydrogenase (quinone) activity  13 4.76 2.73
  0016655 oxidoreductase activity, acting on NADH or NADPH, quinone or similar compound as acceptor  13 4.76 2.73
  0008137 NADH dehydrogenase (ubiquinone) activity  13 4.76 2.73
  0003954 NADH dehydrogenase activity  13 4.76 2.73
  0042287 MHC protein binding  5 1.85 2.7
  0016627 oxidoreductase activity, acting on the CH-CH group of donors  10 3.7 2.7
  0005537 mannose binding  5 1.85 2.7
  0048029 monosaccharide binding  7 2.64 2.65
  0016651 oxidoreductase activity, acting on NADH or NADPH  15 6.08 2.47
  0015081 sodium ion transporter activity  13 5.28 2.46
  0015179 L-amino acid transporter activity  7 2.91 2.41
  0016628 oxidoreductase activity, acting on the CH-CH group of donors, NAD or NADP as acceptor  5 2.11 2.37
  0005044 scavenger receptor activity  5 2.38 2.1
  0008639 small protein conjugating enzyme activity  12 5.81 2.06
  0004177 aminopeptidase activity  6 2.91 2.06
  0008642 ubiquitin-like activating enzyme activity  7 3.43 2.04
 
  
 Biological Process 
 
  
  GO id GO classification Observed in selected subset Expected in selected subset Observed/ Expected
  0042977 tyrosine phosphorylation of JAK2 protein  5 1.36 3.69
  0042976 activation of JAK protein  5 1.36 3.69
  0009618 response to pathogenic bacteria  7 1.9 3.69
  0007595 lactation  6 1.63 3.69
  0051017 actin filament bundle formation  6 1.9 3.16
  0030856 regulation of epithelial cell differentiation  6 1.9 3.16
  0051260 protein homooligomerization  5 1.63 3.07
  0042100 B cell proliferation  5 1.63 3.07
  0030593 neutrophil chemotaxis  5 1.63 3.07
  0008635 caspase activation via cytochrome c  5 1.63 3.07
  0007171 transmembrane receptor protein tyrosine kinase activation (dimerization)  5 1.63 3.07
  0006760 folic acid and derivative metabolism  5 1.63 3.07
  0006108 malate metabolism  5 1.63 3.07
  0042828 response to pathogen  9 2.98 3.02
  0019882 antigen presentation  18 6.24 2.88
  0009065 glutamine family amino acid catabolism  7 2.44 2.87
  0007566 embryo implantation  7 2.44 2.87
  0042773 ATP synthesis coupled electron transport  19 6.78 2.8
  0019363 pyridine nucleotide biosynthesis  6 2.17 2.76
  0009166 nucleotide catabolism  6 2.17 2.76
  0006284 base-excision repair  6 2.17 2.76
  0042775 ATP synthesis coupled electron transport (sensu Eukaryota)  17 6.24 2.72
  0051259 protein oligomerization  8 2.98 2.68
  0006120 mitochondrial electron transport, NADH to ubiquinone  16 5.97 2.68
  0007259 JAK-STAT cascade  21 7.87 2.67
  0009615 response to virus  18 6.78 2.65
  0045736 negative regulation of cyclin dependent protein kinase activity  5 1.9 2.63
  0045672 positive regulation of osteoclast differentiation  5 1.9 2.63
  0045657 positive regulation of monocyte differentiation  5 1.9 2.63
  0019835 cytolysis  10 3.8 2.63
  0015991 ATP hydrolysis coupled proton transport  5 1.9 2.63
  0015988 energy coupled proton transport, against electrochemical gradient  5 1.9 2.63
  0046356 acetyl-CoA catabolism  12 4.61 2.6
  0009617 response to bacteria  12 4.61 2.6
  0009109 coenzyme catabolism  12 4.61 2.6
  0006084 acetyl-CoA metabolism  14 5.43 2.58
  0000079 regulation of cyclin dependent protein kinase activity  7 2.71 2.58
  0006100 tricarboxylic acid cycle intermediate metabolism  9 3.53 2.55
  0009060 aerobic respiration  11 4.34 2.53
  0006099 tricarboxylic acid cycle  11 4.34 2.53
  0045333 cellular respiration  13 5.16 2.52
  0051028 mRNA transport  6 2.44 2.46
  0050900 immune cell migration  8 3.26 2.46
  0048589 developmental growth  6 2.44 2.46
  0045670 regulation of osteoclast differentiation  6 2.44 2.46
  0044242 cellular lipid catabolism  6 2.44 2.46
  0042742 defense response to bacteria  8 3.26 2.46
  0030595 immune cell chemotaxis  6 2.44 2.46
  0030316 osteoclast differentiation  6 2.44 2.46
  0009063 amino acid catabolism  18 7.33 2.46
  0008625 induction of apoptosis via death domain receptors  8 3.26 2.46
  0007569 cell aging  6 2.44 2.46
  0001776 immune cell homeostasis  6 2.44 2.46
  0019221 cytokine and chemokine mediated signaling pathway  11 4.61 2.38
  0050867 positive regulation of cell activation  9 3.8 2.37
  0006119 oxidative phosphorylation  25 10.58 2.36
  0007568 aging  7 2.98 2.35
  0009310 amine catabolism  22 9.5 2.32
  0050871 positive regulation of B cell activation  5 2.17 2.3
  0050727 regulation of inflammatory response  5 2.17 2.3
  0045765 regulation of angiogenesis  5 2.17 2.3
  0042246 tissue regeneration  5 2.17 2.3
  0019362 pyridine nucleotide metabolism  10 4.34 2.3
  0009070 serine family amino acid biosynthesis  5 2.17 2.3
  0007173 epidermal growth factor receptor signaling pathway  5 2.17 2.3
  0006518 peptide metabolism  5 2.17 2.3
  0044270 nitrogen compound catabolism  23 10.04 2.29
  0051251 positive regulation of lymphocyte activation  8 3.53 2.27
  0051240 positive regulation of organismal physiological process  16 7.06 2.27
  0050864 regulation of B cell activation  8 3.53 2.27
  0009066 aspartate family amino acid metabolism  8 3.53 2.27
  0008629 induction of apoptosis by intracellular signals  8 3.53 2.27
  0006733 oxidoreduction coenzyme metabolism  11 4.88 2.25
  0051187 cofactor catabolism  12 5.43 2.21
  0051084 posttranslational protein folding  6 2.71 2.21
  0042402 biogenic amine catabolism  6 2.71 2.21
  0030041 actin filament polymerization  6 2.71 2.21
  0009081 branched chain family amino acid metabolism  6 2.71 2.21
  0015931 nucleobase, nucleoside, nucleotide and nucleic acid transport  13 5.97 2.18
  0051236 establishment of RNA localization  10 4.61 2.17
  0050658 RNA transport  10 4.61 2.17
  0050657 nucleic acid transport  10 4.61 2.17
  0019751 polyol metabolism  7 3.26 2.15
  0001894 tissue homeostasis  7 3.26 2.15
  0043281 regulation of caspase activity  11 5.16 2.13
  0043280 positive regulation of caspase activity  11 5.16 2.13
  0006919 caspase activation  11 5.16 2.13
  0050778 positive regulation of immune response  13 6.24 2.08
  0042108 positive regulation of cytokine biosynthesis  5 2.44 2.05
  0031099 regeneration  5 2.44 2.05
  0007588 excretion  5 2.44 2.05
  0006783 heme biosynthesis  5 2.44 2.05
  0001819 positive regulation of cytokine production  5 2.44 2.05
  0009064 glutamine family amino acid metabolism  11 5.43 2.03
  0046822 regulation of nucleocytoplasmic transport  6 2.98 2.01
  0042219 amino acid derivative catabolism  6 2.98 2.01
  0006405 RNA-nucleus export  6 2.98 2.01
  0000082 G1/S transition of mitotic cell cycle  6 2.98 2.01
 
  
  
 
  
 
  
 
 
 
 
   Date and time of the analysis:   
Name of the project file: Project.xls
 
Time of the analysis: Fri Apr 07 13:50:15 2006
 
BRB-ArrayTools Version: 3.3
 
 
   Filtering parameters:   
   Spot Filters: OFF
    
    
Normalization: OFF
   
   Gene Filters: OFF
   
   Gene Subsets: OFF
   
      View expression of significant genes.
